# Spatial proteomics identifies FXYD6 as a dual-site protein of neuromuscular junction in the diaphragm

**DOI:** 10.1038/s42003-026-10884-8

**Published:** 2026-09-18

**Authors:** Nitin Eapen, Luisa Schmidt, Michael Saynisch, Jan-Wilm Lackmann, Christian Hoegsbjerg, Abigail L. Mackey, Manuel Koch, Bent Brachvogel, Philipp Antczak, Marcus Krüger

**Affiliations:** 1 Institute for Genetics, Cologne Excellence Cluster on Cellular Stress Responses in Aging-Associated Diseases (CECAD), Cologne, Germany; 2 Novo Nordisk Foundation Center for Protein Research, Faculty of Health and Medical Sciences, University of Copenhagen, Copenhagen, Denmark; 3 Department of Orthopaedic Surgery, Institute of Sports Medicine Copenhagen, Copenhagen University Hospital - Bispebjerg and Frederiksberg, Copenhagen, Denmark; 4 Department of Clinical Medicine, Faculty of Health and Medical Sciences, University of Copenhagen, Copenhagen, Denmark; 5 Institute for Dental Research and Oral Musculoskeletal Biology, Faculty of Medicine and University Hospital Cologne, University of Cologne, Cologne, Germany; 6 Department of Pediatrics and Adolescent Medicine, Experimental Neonatology, Medical Faculty, University of Cologne, Cologne, Germany; 7 Center for Biochemistry, Medical Faculty, University of Cologne, Cologne, Germany; 8 Center for Molecular Medicine (CMMC), University of Cologne, Cologne, Germany

**Keywords:** Proteomics, Ion channels in the nervous system

## Abstract

Morphological studies of the diaphragm have provided a detailed view of its architecture, which consists of parallel skeletal muscle fibers and a central ring of neuronal innervation that includes neuromuscular junction (NMJ) units. NMJs are disease-vulnerable synapses; thus, analysis of the NMJ is essential to understand its function in both healthy and disease-related conditions. The diaphragm was analyzed by spatial proteomics and 115 proteins were enriched at the NMJ. Comparison of the protein signatures of the NMJ and myotendinous junction (MTJ) revealed 31 shared proteins, suggesting partially conserved structures between these junctions. Key mediators of synaptic transmission and extracellular matrix organization were observed among the NMJ-enriched components, which indicates the molecular complexity and regulatory potential of the NMJ. A focused study of the uncharacterized NMJ protein FXYD6 demonstrate enhanced FXYD6 expression in type IIa fibers of the diaphragm, which exhibit a unique balance of oxidative and glycolytic capacity. FXYD6 interacts with Na⁺/K⁺-ATPase subunits in the diaphragm, which supports the function of FXYD6 in the ionic homeostasis required for continuous, fatigue-resistant contraction. Overall, the dataset provides a comprehensive molecular atlas of the NMJ in the diaphragm and opens new opportunities to dissect the synaptic mechanisms underlying respiratory function and neuromuscular diseases.

## Abstract (web summary)

Spatial profiling identifies FXYD6 as an NMJ protein in the diaphragm, with further characterization revealing expression in type IIa fibers and Na⁺/K⁺-ATPase interaction supporting ionic homeostasis for fatigue-resistant contraction

## Introduction

The diaphragm is a constantly contracting muscle that is innervated by the bilateral phrenic nerves, and neuromuscular junctions (NMJs) convert motor neuron action potentials into chemical signals that trigger post-synaptic depolarization and muscle contraction^1,2^. In addition, motor neurons instruct the myonuclei at the synapses to express specific genes, and locally regulated protein synthesis may coordinate the assembly of these functional compartments within the muscle. However, a comprehensive proteomic analysis of NMJ structures is essential to elucidate the precise molecular composition of their pre- and post-synaptic components, including the neighboring cells and extracellular matrix (ECM) proteins. This knowledge will provide mechanistic insights into how NMJs regulate synaptic activity, maintain structural integrity, and control skeletal muscle contraction. Synaptic transmission at the NMJ starts when an action potential reaches the presynaptic terminal of a motor neuron, which causes the neurotransmitter acetylcholine (ACh) to be released and diffuse across the synaptic cleft. ACh then binds to nicotinic acetylcholine receptors (nAChRs) on the cell membrane of muscle fibers to induce a post-synaptic action potential. This activation triggers sarcolemmal release of calcium, which binds to troponin C and induces skeletal muscle contraction^3^. Sodium and potassium ATPases (Na⁺/K⁺-ATPase) play crucial roles in maintaining the ion gradients necessary for action potential generation and propagation. These ATPases actively transport sodium ions from outside the plasma membrane into the muscle fiber to restore resting membrane potential after depolarization. The motor endplates of NMJs form specialized nerve-muscle contact sites. The gap between the motor neuron and the muscle fiber is called the synaptic basal lamina. This specialized ECM structure contains a complex assembly of laminins, glycoproteins, and collagens that are crucial for the structural integrity of the NMJ^4^. Schwann cells, the glial cells of the peripheral nervous system, are another integral part of the NMJ. Schwann cells enwrap large nerve axons to form a myelin sheath, and also enwrap smaller axons without forming myelin to establish non-myelinating Schwann cells or terminal Schwann cells (TSCs)^5^. Defects in neuronal innervation or changes in the NMJ can lead to a decrease in muscle strength, which is termed sarcopenia in older individuals^6^. NMJ degeneration is also observed in disorders such as ALS, although the causes of such degeneration are not yet fully understood^7^. Modern mass spectrometers offer greatly improved sensitivity and can be used to analyze small amounts of protein samples in the sub-nanogram range, including single cells or single skeletal and cardiac muscle fibers from mice or human tissues^8–10^. However, to the best of our knowledge, an unbiased LC-MS analysis of NMJ structures has not yet been conducted and is necessary to comprehensively characterize the complex protein networks that characterize NMJ integrity and function. Therefore, to map the spatial protein patterns in the diaphragm, we isolated the diaphragm from the mouse, embedded a strip of the medial diaphragm, and prepared thin cryosections^11^. The sections were assessed using short LC-MS gradients, resulting in ~2500 protein profiles along the longitudinal muscle fiber-tendon axis. By identifying neurofilament, choline acetyltransferase, and a wide range of other protein signatures specific to the NMJ, including the membrane protein FXYD6, our spatial proteomic profiling revealed the molecular landscape along the longitudinal axis of the diaphragm and enabled precise mapping of the NMJ.

## Results

### Dissection of the skeletal muscle in the diaphragm based on thin cryotome sections via short LC-MS gradients

**Fig. 1:**
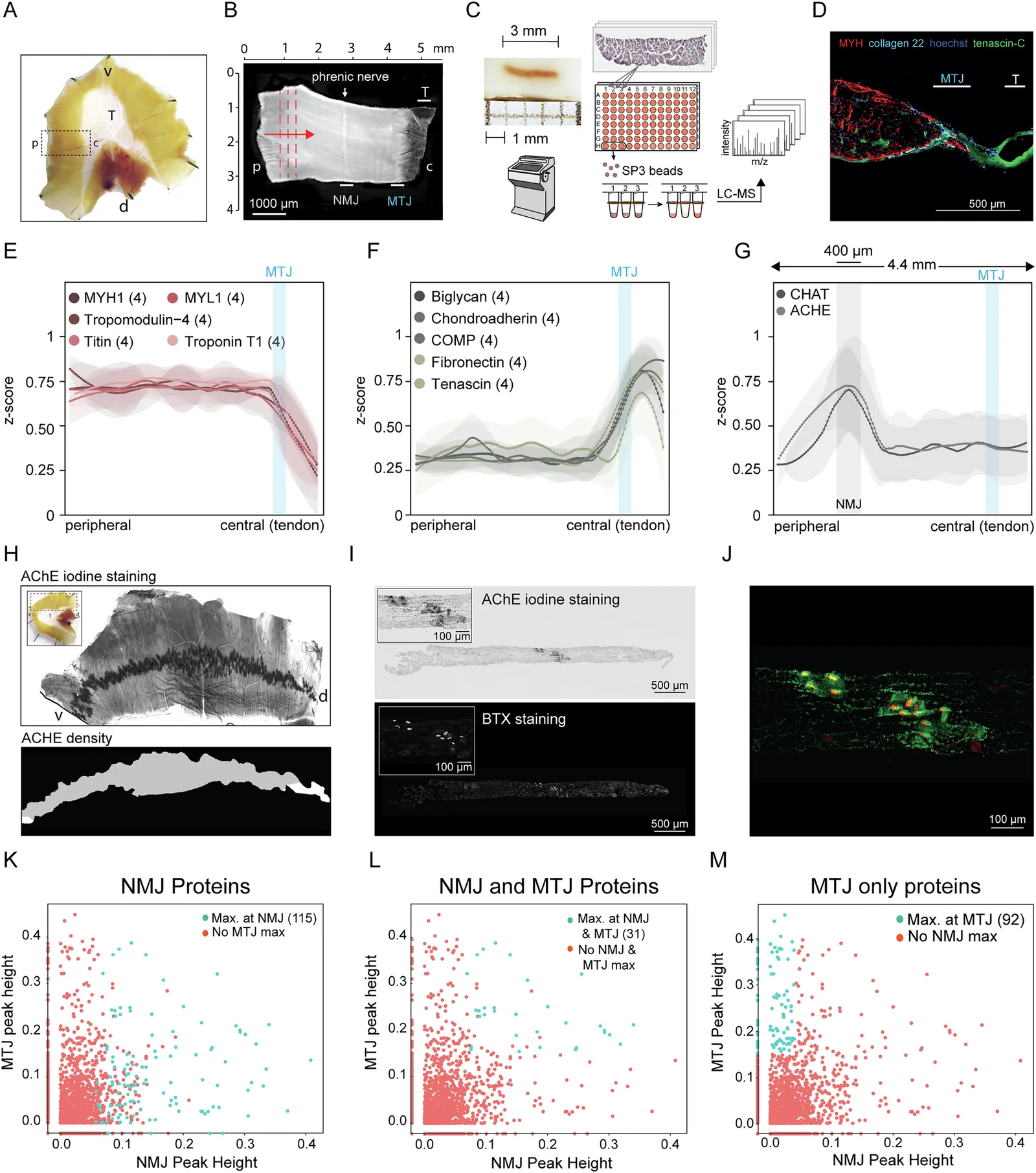
Spatial Proteomics analysis of the dissected area of the diaphragm muscle. **A** Isolated, intact mouse diaphragm. **B** Representative example showing the size and scale of an isolated diaphragm. **C** Experimental workflow. **D** Immunostaining for MYH, tenascin-C, and collagen 22 in a longitudinal mouse diaphragm section. **E**–**G** Selected protein profiles of sarcomeric, extracellular matrix, MTJ, and NMJ marker proteins along the longitudinal axis of the diaphragm. The blue bar represents the MTJ area; v ventral, d dorsal, c central, p peripheral, t tendon. **H** AChE-iodine staining of the whole-mount diaphragm. **I**, **J** AChE and BTX staining of the longitudinal section of the diaphragm. **K**–**M** Scatter plots of differences between the NMJ and MTJ peak heights.

We used the medial costal area of the mouse diaphragm as a model system to identify specific proteins associated with the molecular function of the NMJ. The costal diaphragm muscle merges into a central tendon area, and the junction between the muscle and tendon is called the myotendinous junction (MTJ). The NMJ forms a perpendicular band to the muscle fibers in the central areas of the diaphragm (Fig. 1A, B). To create a spatial protein profile along the longitudinal axis of the diaphragm, we first embedded medial diaphragm strips with a width of 1‒8 mm and diameter of ~1 mm, and created 20 µm-thick cross-sections from the peripheral, rib side (p) to the center (c) of the diaphragm where the central tendon area (t) is located (Fig. 1B; Supplementary Fig. 1A, B)^12^. Each section was transferred individually to a 96-well microplate and digested with trypsin. The peptides were assessed using short LC-MS gradients of 11 min or 21 min, which yielded an average of 8‒10 K peptides, resulting in an average of ~2500 proteins from a single section with a width of ~3 mm (Fig. 1C; Supplementary Fig. 1 C–E; Supplementary Data 2). All samples were analyzed using the DIA-NN software tool^13^. To achieve the following protein profiling of the medial diaphragm, we selected 20 µm-thick slices with a width of ~3 mm and performed 11 min LC-MS gradients on a trapped ion mobility time-of-flight mass spectrometer (timsTOF pro 2). Combined analysis of the 200 RAW files from each experiment resulted in more than 6000 protein hits (*n* = 4; Supplementary Fig. 1F, Supplementary Data 3). Next, we compared our diaphragm proteome with a previously generated soleus proteome^12^. Strikingly, 2408 proteins were found only in the diaphragm, indicating physiological and molecular adaptations in protein expression between these two muscles (Supplementary Fig. 1G). To test the performance and carryover between the diaphragm samples, we analyzed HeLa cell lysates and performed blank runs using the same LC-MS settings as for the skeletal muscle slices (Supplementary Fig. 1H, I). Liquid chromatography performance, including peptide retention times, was controlled for by spiking-in indexed retention time (iRT) peptides to each sample (Supplementary Fig. 1J, K). To generate longitudinal protein expression profiles, we first normalized the data by dividing the individual protein intensities by the sum of the protein intensity per slice. Using a sliding window, data were filtered for at least 70% data completeness over an area of 200 µm (10 sections), followed by linear imputation (Data S1). To compare our protein signatures with known marker proteins for muscle, MTJ, and tendon areas, we prepared longitudinal cryosections of the diaphragm and performed immunostaining with antibodies against pan-MYH for skeletal muscle tissue, tenascin-C (TNC) as a marker for ECM, and collagen 22 (COL22) and thrombospondin-4 (THBS4) to localize the MTJ (Fig. 1D; Supplementary Fig. 2A–C)^14^. In addition, we analyzed the distribution of MYH isoforms in individual cryosections. Previous studies demonstrated that the diaphragm muscle predominantly contains the MYH2 (~40%) and MYH1 (~40%) isoforms, which indicate the presence of type IIa and IIx fibers. The MYH7 isoform, which is mainly expressed in slow type I fibers, is present at significantly lower levels in the diaphragm than the soleus muscle. Similarly, our analysis of single diaphragm fibers revealed fibers positive for MYH2 and MYH1 (Supplementary Fig. 2D, E). Next, we plotted the *z-*score normalized protein intensities of selected sarcomeric, Ca^2+^ transport, and mitochondrial proteins along the longitudinal axis of the diaphragm from the peripheral, muscle side to the central tendon area. This plotting enabled determination of longitudinal protein profiles and characterization of specific areas of the diaphragm, such as the MTJ and NMJ. We observed constant expression of the selected proteins in muscle areas and a steep decrease in their expression at the MTJ, whereas the expression profiles of ECM proteins markedly increased at the MTJ (Fig. 1E, F; Supplementary Fig. 2F–H).

### Specific protein profiles exhibit peak intensities at the NMJ and indicate a complex neuromuscular structure

**Fig. 2:**
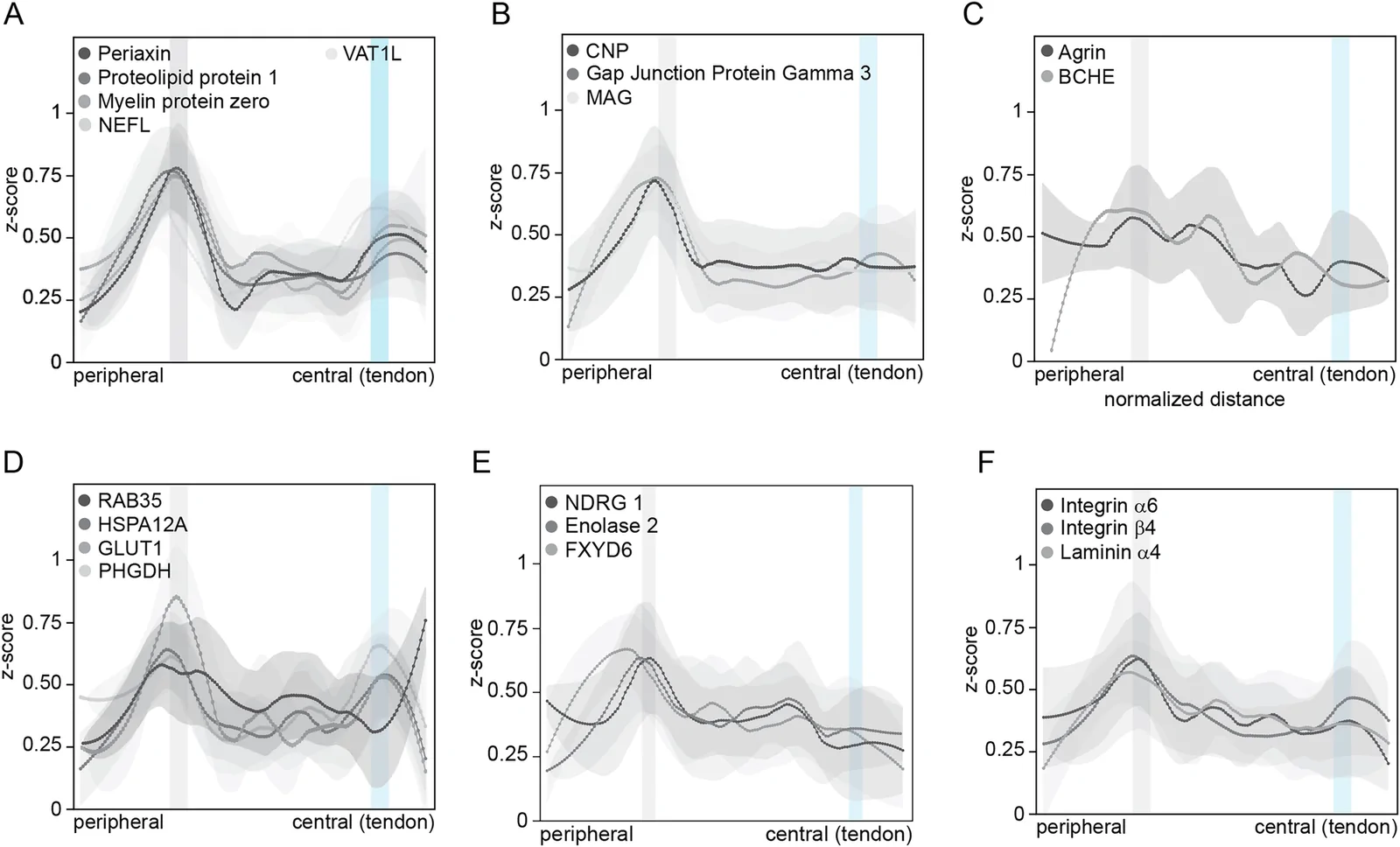
Expression profiles of selected proteins along the longitudinal diaphragm. **A**–**C** Selected neuronal and NMJ marker proteins. **D**–**F** Selected proteins related to signaling, metabolism, and the extracellular matrix that exhibited peak maxima at the NMJ.

**Fig. 3:**
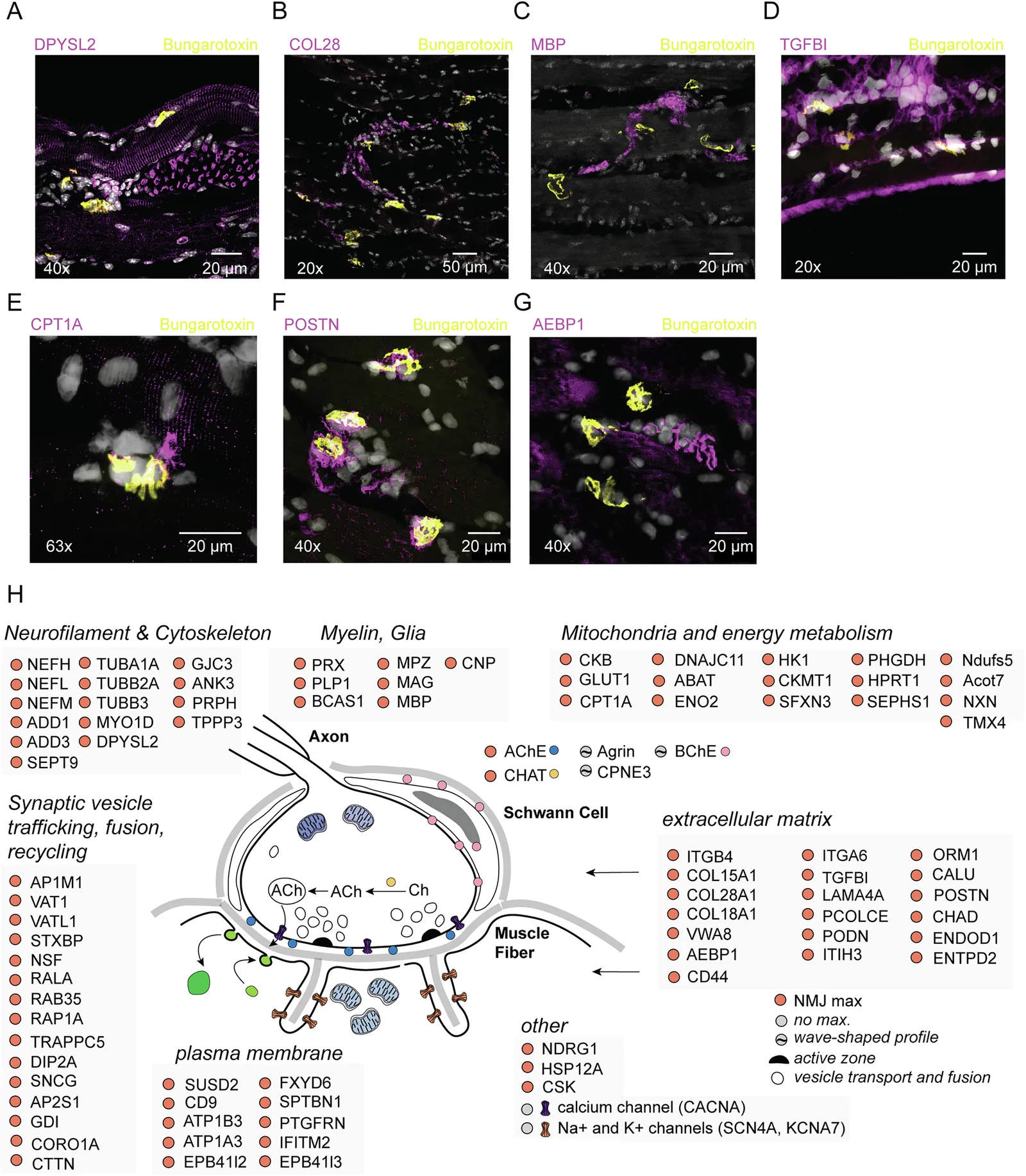
Overview of identified NMJ and MTJ proteins. **A**–**G** Immunostaining of selected proteins with a peak expression at the NMJ on longitudinal mouse diaphragm sections. The NMJ area of the diaphragm is stained with α-bungarotoxin (BTX). **H** Schematic representation of the NMJ and selected proteins with peak maxima in the NMJ (red circle) (Modified from Pratt et al.^50^ and Maselli et al.^51^).

The NMJ, where the phrenic nerve innervates the skeletal muscle along a central ring, is another key structure of the diaphragm^15^. To compare our protein profiling data with the morphometric parameters of the synaptic area of the diaphragm, we first conducted acetylcholinesterase (AChE) staining on whole-mounts and longitudinal sections (Fig. 1H–J). The width of the AChE-positive areas ranged from approximately ~200 µm in the ventral and dorsal regions to ~600 µm in the middle areas of the diaphragm (Supplementary Fig. 3A, B). For our protein profiling, we isolated an intermediate NMJ area with a width of ~400 µm. Analysis of 20 µm-thick cryosections by LC-MS yielded very similar protein profiles and positions of the peak intensities for presynaptic ChAT and the synaptic cleft protein AChE (Fig. 1G) as the morphometric analysis (Fig. 1H–J), further indicating the location of the NMJ. Expression of the acetylcholine receptor (CHRND) was also detected within the same NMJ area, although the profile for this protein was not complete (Supplementary Fig. 3C). To systematically define the NMJ area, we used the protein signature of known NMJ marker proteins, including neurofilaments, myelin proteins, CHAT, and ACHE. Similarly, COL22 was used to localize the MTJ. Based on these protein signatures, we defined the relative NMJ region to span from −0.11 to 0.08 (relative units), and the MTJ region was located beyond 0.55 on the longitudinal axis (Supplementary Fig. 3D). We first calculated the average *z*-score intensity of individual proteins within the non-innervated diaphragm area between the NMJ and MTJ. A valid peak intensity within the NMJ was defined as a minimum difference in the *z*-score intensity of 0.05 between the peak maximum of the protein in the NMJ and its average intensity in the intermediate region. As some profiles showed peak maxima in the region between the NMJ and MTJ, we confirmed that these intermediate peaks did not exceed the NMJ peak to ensure that the NMJ was not a background feature. Thus, we added 0.035 to each peak maximum that occurred between the NMJ and MTJ area, and tested whether this value was still smaller than the maximum in the NMJ or MTJ. Based on this threshold, 115 proteins exhibited maxima at the NMJ, 31 proteins showed dual maxima at both the NMJ and MTJ, and 92 proteins had maxima at the MTJ, including the known MTJ markers COL22 and CILP (Fig. 1K–M; Supplementary Data 4). In confirmation of pre- and post-synaptic activity within the NMJ area, the protein expression profiles of periaxin, 2′,3′-cyclic-nucleotide-3′-phosphodiesterase (CNP), the GAP junction protein gamma 3 (GJP3), and the myelin-associated glycoprotein (MAG) peaked at the NMJ (Fig. 2A, B; Supplementary Fig. 4A, B). Agrin, a protein previously described to be expressed in the NMJ, is secreted by motor neurons and stabilizes acetylcholine receptor (AChR) clusters at the synapse. In addition, the skeletal muscle isoform of agrin contributes to ECM remodeling and interacts with dystroglycans and integrins^16^. LC-MS cannot distinguish between these two isoforms; therefore, we were not able to detect a significant maximum for agrin at the NMJ, and observed the wave-like profile may reflect combined detection of both isoforms (Fig. 2C). A similar pattern was observed for butyrylcholinesterase (BCHE), an enzyme with broad substrate specificity similar to AChE. Other proteins that exhibited peak maxima at the NMJ and MTJ include the GTPase RAB35 and heat shock protein family A12A (HSPA12A) (Fig. 2D). Notably, the peak maxima of the neuron-specific glucose transporter type 1 (GLUT1), the neuronal isoform of creatine kinase, brain (CKB), and enolase 2 (ENO2) at the NMJ (Fig. 2D, E) indicate the presence of neuronal cell metabolism. In contrast, the muscle-specific GLUT4 transporter exhibited a constant longitudinal profile along the diaphragm until the MTJ, followed by a steep decrease in the tendon area (Supplementary Fig. 4C). Further profiles of mitochondrial, vesicle, and membrane proteins with maxima at the NMJ and/or MTJ are shown in Supplementary Fig. 4D, E. Another protein with a maximum peak at the NMJ is the FXYD domain-containing ion transport regulator 6 (FXYD6, also known as phosphohippolin) (Fig. 2E). FXYD6 regulates Na⁺/K⁺-ATPase activity, is primarily expressed in the nervous system, and modulates ion transport kinetics through binding to Na⁺/K⁺ pumps^17^. Integrins and laminins play crucial roles in both structural organization and signaling processes at the NMJ^18^. Our profiling revealed that integrin α6 and integrin β4 exhibited maximum peaks at the NMJ (Fig. 2F), whereas integrin β1, integrin α7, and integrin β5 did not exhibit significant peaks at the NMJ (Supplementary Fig. 4F). The laminin chains α4 and α5 are located in the basal lamina and are the preferred substrates of integrins in skeletal muscle tissue. We observed a maximum for laminin α4 (LAMA4) at the NMJ (Fig. 2F), whereas laminin α5 showed only a modest peak at the NMJ (Data S1). Gene ontology enrichment of the 115 proteins that exhibited maxima at the NMJ revealed terms related to neurofilament bundle assembly, glial cell differentiation, and cellular transport mechanisms at the basement membrane (Supplementary Fig. 4H). To visualize the precise localization of selected NMJ proteins, we performed combined immunostaining for bungarotoxin (BTX), a marker for the  AChR receptor, on longitudinal sections of mouse diaphragm muscles (Fig. 3A–G). Dihydropyrimidinase-related protein 2 (DPYSL2) participates in cellular migration, neurite growth, and guidance to synapse maturation^19^. We observed maximum staining for DPYSL2 at the phrenic nerve, next to BTX-positive areas (Fig. 3A; Supplementary Fig. 4B). Although DPYSL2 is mainly expressed in the developing brain, expression of DPYSL has also been observed during synapse formation in adult stages. DPYSL2 mediates semaphoring-dependent synapse pruning and interacts with cytoplasmic loops of caveolins; thus, DPYSL2 could also be involved in neuromuscular signaling and contribute to neuronal degeneration and regeneration. Carnitine palmitoyltransferase 1a (CPT1A) is a mitochondrial outer membrane enzyme primarily involved in fatty acid β-oxidation^20^. Although CPT1A is mainly expressed in the liver^21^, CPT1A also exhibited a maximum at the NMJ of the diaphragm (Fig. 3E; Supplementary Fig. 4D). Overall, the complex expression patterns of membrane, transport, and matrix proteins observed in our protein profiling indicate the unique structure and cellular landscape within the NMJ (Fig. 3H).

### Dual protein expression of FXYD6 in neuromuscular endplates and skeletal muscle fibers

**Fig. 4:**
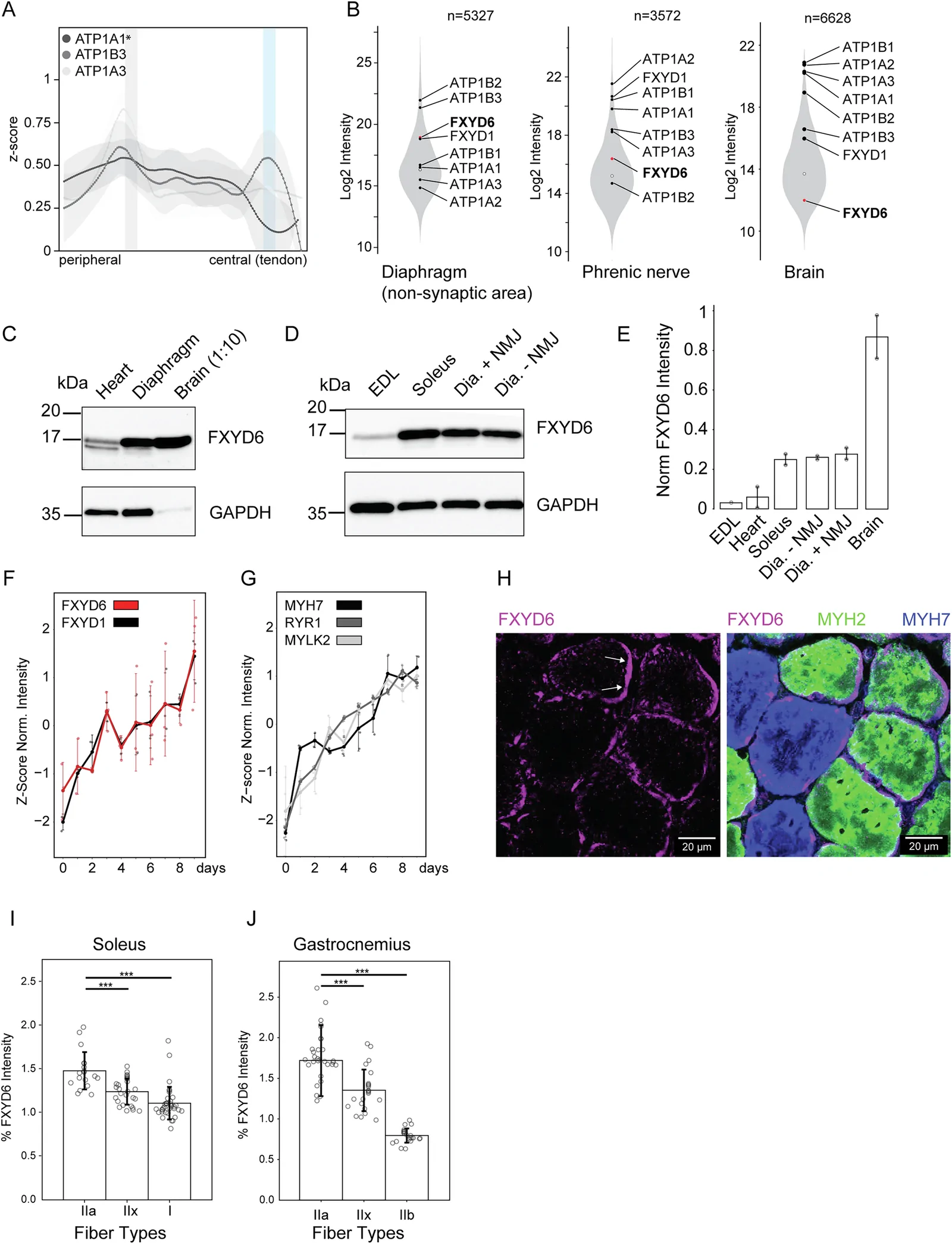
Relative abundance of FXYD6 and other ATPases in neurons, neuromuscular endplates, and skeletal muscle tissue. **A** Protein profile peaks of three selected ATPases. *No peak in the NMJ. **B** Jitter plot of protein intensity in the mouse diaphragm, phrenic nerve, and brain. ATPases and FXYD1/6 are indicated as black and red circles, respectively. **C** Immunoblotting of FXYD6 in the mouse heart, diaphragm, and brain. **D** Immunoblotting of FXYD6 in various mouse skeletal muscles. **E** Quantification of Immunoblot analysis of FXYD6 in various tissues. **F** Normalized protein intensities of the expression of FXYD1 and FXYD6 during myoblast differentiation (*n* = 3; Mean ± SD). **G** Myogenic differentiation factors in primary mouse myoblasts during differentiation. **H** Immunostaining of soleus muscle for FXYD6, MYH2, and MYH7. **I**, **J** Quantification of the corresponding immunohistochemistry (IHC) data for FXYD6 in soleus (**I**) and gastrocnemius (**J**) (*n* = 50 fibers; Mean ± SD; *** adjusted *p* value < 0.001).

**Fig. 5:**
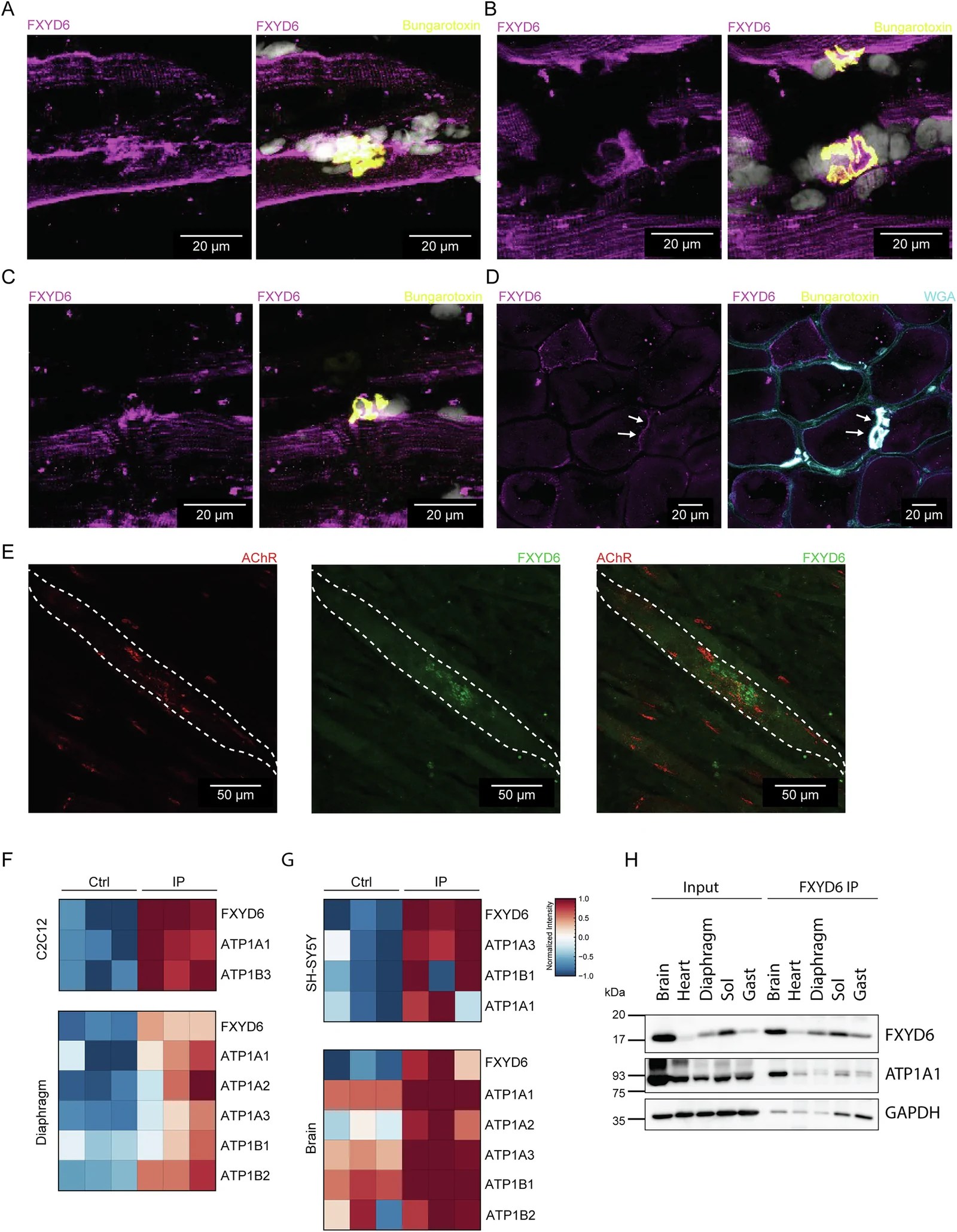
Immunohistochemical analysis of FXYD6 expression in skeletal muscle fibers. **A**–**C** Co-immunostaining for FXYD6 and BTX on longitudinal sections of the diaphragm. **D** Co-immunostaining for FXYD6 and WGA on transverse sections of the tibialis anterior (TA). **E** AChR clustering assay. Differentiated C2C12 myoblasts were transfected with FXYD6 and co-immunostained for AChR and FXYD6. *Z*-score-normalized intensity of the expression of FXYD6 and various ATPases (ATP1A1; A2; A3; B1; B2; B3) based on co-immunoprecipitation using an anti-FLAG antibody in C2C12 myoblasts (**F**) and neuronal SH-SY5Y cells (**G**) and immunoprecipitation with an anti-FXYD6 antibody in diaphragm (**F**) and brain tissue lysates (**G**). **H** Immunoprecipitation with an anti-FXYD6 antibody and immunoblotting of brain and skeletal muscle tissues using anti-FXYD6 and anti-ATP1A1 antibodies.

The transport of small ions, such as sodium (Na⁺) and potassium (K⁺), by specific Na⁺/K⁺-ATPases within the plasma membrane is essential for excitation of both neurons and skeletal muscle fibers. Interestingly, members of the FXYD family are involved in regulating the activity and transport capacity of ATPases in different cell types^22^. We observed maximal expression of FXYD6 at the NMJ; therefore, we investigated whether other specific ATPases also exhibited similar peaks at the NMJ. We plotted the expression profiles of seven ATPase isoforms detected along the longitudinal axis of the diaphragm and observed increased levels of ATP1A3 and ATP1B3 at the NMJ; the other three detected isoforms showed only weak peaks at the NMJ (ATP1A1; Fig. 4A) or a constant protein profile along the longitudinal axis of the diaphragm (ATP1A2 and ATP1B1; Supplementary Fig. 5A). Since FXYD6 has previously been described to be expressed in neuronal tissue^23^, we investigated whether FXYD6 and the ATPase isoforms are also detectable in diaphragm, phrenic nerve, and brain (Fig. 4B; Supplementary Data 5). Immunoblotting of heart, skeletal muscle, and brain tissues with an anti-FXYD6 antibody revealed a ~20 kDa band, with the highest expression observed in brain (Fig. 4C, E). In cardiac tissue, FXYD6 was detected as a doublet with an additional smaller band at ~18 kDa of unspecified origin. Next, we tested skeletal muscles, containing different proportions of slow and fast fibers, including the fast extensor digitorum longus (EDL), slow soleus, and the synaptic and non-synaptic regions of the diaphragm (Supplementary Fig. 5B). FXYD6 expression was similar in the soleus muscle and in both the synaptic and non-synaptic regions of the diaphragm and was expressed at lower levels in the EDL (Fig. 4D). Since FXYD6 was observed in skeletal muscle, we employed an in vitro primary myoblast differentiation system to confirm and characterize its expression during myogenesis. FXYD6 was detected in differentiating primary myoblasts, which indicates FXYD6 plays a role during skeletal muscle development (Fig. 4F, G; Supplementary Fig. 5C). Similarly, expressions of the ATPase subunits ATP1A1 and ATP1A2, as well as ATP1B1 and ATP1B2, increased during myoblast differentiation (Supplementary Fig. 5D). Co-immunostaining of soleus sections further verified that FXYD6 is expressed mainly in MYH2-positive fibers, and to a lesser extent in MYH7-positive fibers, of the soleus and gastrocnemius muscle (GAST) (Fig. 4H–J; Supplementary Fig. 5E, F). Finally, co-staining of FXYD6 with BTX demonstrated that FXYD6 localizes to the plasma membrane, which is in close proximity to the NMJ (Fig. 5A–C). Double staining revealed a significant overlap of FXYD6 and WGA at the NMJ; this is most likely due to the specialized basal lamina of the NMJ, which contains glycoproteins and probably also glycosylated synaptic membranes, and would contribute to WGA binding at these sites (Fig. 5D). The acetylcholine receptor (AChR) clustering assay confirmed the expression and colocalization of FXYD6, using an anti-FXYD6 antibody, with AChR clusters (labeled with BTX) in differentiating myoblasts supplemented with agrin and positively transfected for FXYD6 (Fig. 5E). Moreover, FXYD6 showed higher staining intensity in differentiated myoblasts treated with agrin compared that had not been incubated with agrin (Supplementary Fig. 5G, H). Overall, these results suggest that FXYD6 localizes to the post-synaptic receptor region of the NMJ.

### FXYD6 and ATPase interact in neuronal and muscle tissues

To define the FXYD6 interactome, we performed immunoprecipitation of FLAG-tagged FXYD6 transiently expressed in C2C12 mouse myoblasts. Affinity purification with an  anti-FLAG antibody followed by mass spectrometry identified ATP1A1 and ATP1B3 as FXYD6-interacting proteins in C2C12 myoblasts (Fig. 5F upper heatmap, Supplementary Data 6). Notably, the C-terminally tagged FXYD6 construct yielded stronger enrichment compared to the N-terminally tagged variant, which suggests that N-terminal modification may disrupt proper folding, localization, or binding (Supplementary Fig. 5I, J). In confirmation of the Flag immunoprecipitation assays in C2C12 myoblasts, anti-FXYD6 immunoprecipitation from total diaphragm lysates followed by proteomics identified interactions between FXYD6 and five ATPases (Fig. 5F, lower heatmap). Furthermore, immunoprecipitation assays with the Flag-FXYD6 construct in the human neuroblastoma cell line SH-SY5Y identified several ATPase subunits, including the neuronal ATP1A3 isoform, as interacting partners (Fig. 5G, upper heatmap). In addition, endogenous FXYD6 immunoprecipitated from brain tissue showed a similar interaction with ATPase isoforms compared to the skeletal muscle (Fig. 5G, lower heatmap; Supplementary Fig. 5K–N). The interaction between FXYD6 and ATP1A1 in brain and different skeletal muscles was further substantiated by co-immunoprecipitation using an anti-FXYD6 antibody and immunoblotting with an anti-ATP1A1 antibody (Fig. 5H, Supplementary Data 6). Overall, our results demonstrate FXYD6 localizes to the plasma membrane in the NMJ region, where it associates with different Na⁺/K⁺-ATPase isoforms in muscle and neuronal cells.

## Discussion

Although the NMJ is crucial for muscle activation and is implicated in the progression of neuromuscular diseases, our understanding of its molecular composition is still limited. Previous proteomic analyses of the NMJ have been limited by the difficulty of isolating these structures due to their small size compared to the surrounding muscle tissue. Here, we precisely mapped the NMJ and MTJ of the mouse diaphragm in an unbiased manner via longitudinal protein profiling of single transverse sections. This approach identified 115 proteins that exhibit peak maxima at the NMJ and 92 proteins that exhibit peak maxima at the MTJ. In addition, 31 proteins exhibited dual peaks at both the NMJ and MTJ, suggesting shared structural features, including proteins related to ECM organization and neuronal innervation.

To investigate the correlations between snRNA transcript levels and protein abundance, we overlapped our mapped NMJ protein profiles with a recent snRNA sequencing study in mouse skeletal muscle tissue^24^. However, we only identified overlapping transcriptomic and proteomic profiles for four proteins: ACHE, AChR, ANK3 and FXYD6. Furthermore, our comparison with gene clusters from Schwann cell and TSC nuclei, both of which are components of the NMJ and were also identified in the snRNA seq study, revealed an additional overlap of 10 genes between our protein profiles and the snRNA data (Supplementary Data 7). For example, gene expression of the plasma membrane proteins CD9 and ITGA6 was observed in Schwann cells, which suggests that these proteins contribute to the specialized Schwann cell-muscle interface by regulating cell-cell adhesion, ECM interactions, and stabilization of the synaptic microenvironment; these processes are essential for NMJ maintenance and remodeling. Although RNA persists in the presynaptic terminal, with nuclear RNA absent in the spinal cord, many NMJ structures, including synaptic vesicles, active zones, and post-synaptic scaffolds, contain a specialized protein microenvironment that is missed by transcriptomic analyses. However, proteomics favors detection of abundant proteins, whereas RNA-seq captures actively transcribed genes. Together, these combined biological and methodological factors likely explain the limited overlap between the proteomic and transcriptomic datasets.

Neurotransmission depends on the formation and release of synaptic vesicles. Within the active zone, a specialized region of the presynaptic membrane, synaptic vesicles dock and fuse to release neurotransmitters to muscle fibers^25^. Accordingly, we observed several small GTPases with peak maxima at the NMJ, including RAB35, STXBP1, and VAT1. These findings highlight the sensitivity of our approach to identify synaptic structures from skeletal muscle slices, which mainly contain highly abundant sarcomeric proteins. Cell adhesion is crucial for the formation, maintenance, and function of the NMJ, as it mediates interactions between the pre- and post-synaptic membranes through highly specialized ECM^26^. The number of identified proteins with peak expression at the NMJ further indicates the structural and molecular complexity of the ECM in this area. For example, the prostaglandin F2 receptor negative regulator (PTGFRN, also known as CD9 partner 1, CD9P-1) is a transmembrane glycoprotein that exhibits peak expression at the NMJ. PTGFRN interacts with the tetraspanin and glycoproteins CD9 and CD81, both of which play roles in membrane scaffolding and cell-cell adhesion^27^. The common peak of PTGFRN and CD9 at the NMJ could reflect this interaction and possibly be involved in stabilizing cell-cell contacts within the NMJ (Data S1; Fig. 3H). Interestingly, tetraspanins, like CD9, are often found to be enriched in small extracellular vesicles (sEVs) and tetraspanin-enriched microdomains (TEMs)^28^. Thus, the detection of CD9 at the NMJ might reflect the presence of those vesicles as a further mechanism of cellular communication^29,30^. Laminins bind to integrins, and we identified that the integrin α6β4 heterodimer exhibited a peak maximum at the NMJ (Fig. 2F). The integrin α6β4 dimer is primarily expressed in Schwann cells, where it interacts with synaptic laminins to strengthen Schwann cell-ECM contacts that influence NMJ organization^31^. Another integrin interactor with a peak maximum at the NMJ and MTJ is the matrix protein periostin (PSTN). In accordance with previous studies, we observed that α-BTX-positive areas were closely surrounded by periostin-positive structures (Fig. 3F), which provides confirmation of the accuracy of our protein profiling approach and the localization of specific proteins within the NMJ^24,32^. An additional ECM protein that has not previously been associated with NMJ function, adipocyte enhancer-binding protein 1 (AEBP1), also showed a maximum peak at the NMJ and was located in close proximity to α-BTX-positive regions (Fig. 3G). AEBP1 has been associated with collagens in the ECM and is reported to play several roles in development, tissue repair, and fibrosis^33^. For example, AEBP1 has been described as a transcription repressor that can inhibit the release of cholesterol from macrophages and trigger inflammatory responses^34^. Moreover, AEBP1 also acts as a negative regulator of skeletal muscle differentiation and likely represses myogenesis-related genes and thereby disrupts normal muscle repair processes^35^. Human patients with a mutation in AEBP1 show disease features related to Ehlers-Danlos syndrome (EDS), including joint laxity and hyperextensible skin^36^. Moreover, patients with EDS also experience neuromuscular issues such as muscle weakness and fatigue; in this context, the primary cause is a defect in the formation of collagen that supports connective tissue, which affects muscles and nerve structures. Hence, the expression of AEBP1 within the NMJ might also contribute to poor muscle function and neurological symptoms. Neuronal metabolism within the NMJ was identified by the peak maxima for proteins related to energy production, including GLUT1, CKB, ENO2, and carnitine palmitoyltransferase 1A (CPT1A). Notably, CPT1A is associated with fatty acid metabolism, is located on the outer mitochondrial membrane, and its main function is the conversion of long-chain acyl-CoA into acylcarnitine to support β-oxidation of fatty acids inside mitochondria. However, neurons usually rely on glucose and lactate metabolism, whereas fatty acid oxidation only plays a minor role under physiological conditions^37^. However, astrocytes, glia, and Schwann cells express CPT1A, and β-oxidation can contribute to energy production in these cells, in particular during myelination^38^. Since we observed a peak maximum for CPT1A at the NMJ, we speculate that CPT1A is expressed in Schwann cells and contributes to metabolism in these cells at the NMJ.

Sodium-potassium pumps (Na+/K+ ATPases) are essential for maintaining the electrochemical gradients across the plasma membrane in neurons^39^ and skeletal muscle fibers^40^. Moreover, these ATPases interact with small single-pass membrane proteins of the FXYD family, which modulate the activity of the ATPases in a tissue- and isoform-specific manner^41^. For example, FXYD1 (also known as phospholemman) interacts with and regulates the kinetic properties of the ATPases in contractile tissues, resulting in different muscle contractions^42^. FXYD6, another member of the FXYD family, is predominantly expressed in neuronal tissue, including central and peripheral neurons. FXYD6 modulates the affinity of ATPase dimers to Na+ and K+ ions, which allows cells to restore Na⁺ and K⁺ gradients more efficiently after activity. In addition, overexpression of FXYD6 reduces the number of Na⁺/K⁺ pumps in the plasma membrane in Xenopus oocytes, which suggests that FXYD6 influences the trafficking, assembly, or stability of ATPases^43^.

Since FXYD6 is predominantly expressed in neuronal tissue, the observed peak of this protein in the NMJ likely reflects localization of FXYD6 at neuromuscular endplates (Figs. 2E and 5A–D). Our protein profiling and immunostaining reveal a previously unrecognized localization of FXYD6 at the plasma membrane of diaphragm skeletal muscle fibers. FXYD6 may not only act at the presynaptic site, but may also regulate Na⁺/K⁺-ATPase activity at the post-synaptic membrane of skeletal muscle fibers. Whether this regulation is specific to innervated regions versus non-innervated areas of skeletal muscle fibers should be addressed in future studies. We observed that FXYD6 was mainly expressed in skeletal muscle tissues containing type IIa and type IIx skeletal muscle fibers, which suggests a function in fibers with intermediate oxidative and glycolytic capacities. Type IIa and type IIx fibers are metabolically flexible and capable of adapting to both sustained and rapid contractions, which imposes high demands on ion transport and membrane excitability^44^. Through its interaction with specific ATPase isoforms (Fig. 5F, G), FXYD6 may fine-tune Na⁺/K⁺-ATPase activity to stabilize ion gradients and sustain contractile efficiency. Similarly, FXYD1 has been shown to regulate Na⁺/K⁺-ATPase activity in the heart and modulate excitability, contractility, and Ca²⁺ handling during continuous beating^42^. Therefore, FXYD6 may fulfill a similar role in the diaphragm, where these fibers contribute to the continuous rhythmic contractions required for ventilation.

In addition, we observed enhanced colocalization and staining intensity of FXYD6 at AChR clusters upon agrin stimulation in C2C12 myoblasts (Fig. 5E), which indicates that FXYD6 may play a role in the organization or stabilization of post-synaptic receptor complexes at the NMJ. Given its function as a regulator of ion transport, FXYD6 might contribute to the maturation of the post-synaptic membrane by modulating local ion homeostasis that supports AChR clustering. This suggestion aligns with previous reports that showed that FXYD6 regulates ATPase heterodimers in excitable tissues, including auditory neurons and the stria vascularis^23^, and potentially indicates a broader role for FXYD6 in fine-tuning ion transport mechanisms across different cell types and development. Overall, our proteomic analysis sheds light on the complex molecular architecture of the NMJ, including synaptic vesicle machinery, adhesion proteins, and ion homeostasis. Future studies using single-cell proteomics approaches could further elucidate the cellular environment of the NMJ, including TSCs, neighboring fibroblasts, and the surrounding ECM, and thereby provide a more detailed understanding of NMJ activity.

## Materials and methods

### Experimental model

All animal experiments were performed in accordance with national and institutional guidelines. Wildtype C57BL/6 mice were housed at 22 ± 2 °C, with a humidity of 55 ± 10%, and an air exchange rate of 15 times per hour on a 12:12-h light-dark cycle and had free access to a standard chow diet. All mouse experiments were performed in accordance with the regulations stated in European, national, and institutional guidelines and were approved by the local government authority, Landesamt für Natur, Umwelt und Verbraucherschutz (LANUV) of North Rhine-Westphalia, Germany. We have complied with all relevant ethical regulations for animal use (UniKöln_Anzeige§4.22.007).

### Tissue sectioning and sample preparation

After sacrifice of adult (~3 months) wildtype C57BL/6 mice by cervical dislocation, diaphragm and soleus muscles were fixed using insect needles on cork matrix, embedded in optimal cutting temperature (OCT) resin, and stored at −80 °C until sectioned into 10‒30 µm-thick slices at −25 °C using a cryostat (Leica, Nussloch, Germany) and placed individually into 96-well-plates. As controls, one column on each 96-well-plate was loaded with HEK293T protein lysate. The sections were lysed with 40 µL of 4% SDS in PBS containing 5 mM TCEP and 10 mM CAA at 95 °C for 10 min, followed by Bioruptor sonication at 20 °C with 10 cycles of 30 s on and 30 s off. Samples were digested following the standard SP3 protocol^45^. Briefly, 20 µg of washed SP3 beads were added to each well, followed by one sample volume of acetonitrile (ACN). After incubation for 8 min and 2 min on a magnet, the supernatant was discarded, and the magnetic beads were washed twice with 70% EtOH and once with 100% ACN. Samples were digested with 10 µL of 20 ng LysC and 40 ng trypsin in 50 mM ammonium bicarbonate buffer at 37 °C on a rotating wheel (750 rpm) overnight, acidified by addition of 100 µL 0.1% FA, followed by clean-up with house-made SDB-RPS tips. Peptides were reconstituted in 2% ACN, 5% FA, with iRT peptides.

### Quantitative proteomics analysis of tissue sections

Desalted peptides were loaded onto EvoTips Pure and separated on an EvoSep One system (both Evosep, Denmark) equipped with an in-house packed 8 cm silica emitter column (100 µm inner diameter, packed with 5 μm C18 Poroshell, Agilent) at 30 °C with the pre-programmed 60 (21 min) or 100 (11.4 min) samples per day (SPD) methods. The mobile phases were 0.1% FA as solvent A and 0.1% FA in ACN as solvent B. The HPLC system was coupled to a timsTOF Pro 2 using a CaptiveSpray source (both Bruker). Samples were measured in dia-PASEF mode with ion mobility calibrated after every 50th sample using three ions of Agilent ESI-Low Tuning Mix following the vendor’s specifications. The dia-PASEF window was 1/*k*_0_ 0.7‒1.35, with 24 × 25 Th windows and in dimension *m*/*z* from 350 to 1250.

### Bioinformatic analysis

Data were analyzed with DIA-NN version 1.8 using library-free search against the UniProt mouse database (Sep. 2017) complemented with protein sequences from myosin heavy chain variants and collagens. Mass ranges were set according to the settings of the mass spectrometer; mass deviation was automatically determined from the first data file. Data were further processed using R (V 4.2.2), with the tidyverse, diann, data.table, magrittr, FactoMineR, factoextra, ggplot2, and gprofiler libraries. An in-house modified R script based on the version by V. Demichev was used to calculate MaxLFQ values^13,46^. Data input was filtered for unique precursor, *q*-value < 0.01, Lib.Q.Value < 0.01, PG.Q.Value < 0.01, Global.Q.Value < 0.01, Quantity.Quality > 0.7, Fragment.count ≥ 4. Statistical analysis was performed with R (V 4.2.2) and Perseus (V.1.6.5.0) and visualized with Instant clue (V 0.10.10.20210315). Further calculations were performed within R (version 4.2.2) using the diann, tidyverse, data.table, samr, vsn, ggplot2, and gprofiler libraries. Protein profiles and distance-based network interactions were generated as previously described in the STAR protocols method^11^. The interactions were visualized in Cytoscape and arranged using the Spring Embedded Layout of the delay, leading to a hierarchical arrangement of the proteins over distance. Wilcoxon *t*-tests of the AUC and GOterm enrichment analysis were performed and visualized using R (V 4.2.2) using the packages *gprofiler2* and *ggplot2*.

### Intact tissue sample processing, protein digestion, and desalting

After sacrifice of adult wildtype C57BL/6 mice, the brain, diaphragm muscle, and phrenic nerve were isolated, snap-frozen in liquid nitrogen, and stored at −80 °C. Samples were lyophilized, ground to a fine powder using a mortar and pestle, lysed in 4% SDS in PBS, heated to 95 °C, and sonicated using the Bioruptor sonicator (Diagenode, Belgium) with 10 cycles of 30 s on 30 s off. Cell debris was pelleted by centrifugation at 18,000 × *g* for 10 min and 10 µg of the protein extract was reduced and alkylated for 10 min at 70 °C using 5 mM TCEP and 15 mM CAA. Samples were digested following the standard SP3 protocol, then 20 µg of washed Sera-Mag carboxylate magnetic beads (Cytiva; Cat#2415210505250) were added to each sample, followed by one sample volume of acetonitrile (ACN). After incubation for 8 min, the supernatant was discarded, and the magnetic beads were washed twice with 70% EtOH and once with 100% ACN. Samples were digested with 10 µL of 100 ng LysC (substrate:enzyme ratio 100:1) and 200 ng trypsin (substrate:enzyme ratio 50:1) in 50 mM triethyl ammonium bicarbonate buffer at 37 °C overnight. Peptides were acidified by addition of 100 µL of 0.1% FA the following day, followed by clean-up with house-made SDB-RPS tips.

### Gas phase fractionation of tissue samples

Tryptic peptides from each tissue sample were analyzed using a Vanquish Nano-LC connected to Orbitrap Exploris 480 (Thermo Scientific). From each sample, 1 µg of peptides was injected six times and measured using a 52 min gradient in data-independent acquisition mode. Peptides were separated on a 25 cm Aurora® Ultimate™ 25 × 75 C18 UHPLC column using a binary buffer system consisting of buffer A (0.1% FA) and buffer B (0.1% FA in 80% ACN). Each gas phase fraction was measured in staggered DIA with a first full scan at 30 k in the scan range of 400‒1000 *m*/*z* and a second full scan in a staggered manner at 30 k in the scan range of 398‒1002 *m*/*z*, both in intervals of 100 *m*/*z*. For each run, MS1 and MS2 scans were acquired at 30k resolution with a maximum injection time of 54 ms. AGC target was set to 100% for MS1 and 1000% for MS2 scans. Spectra were acquired in staggered 4 *m*/*z* windows, resulting in nominal 2 *m*/*z* windows after deconvolution using ProteoWizard. Each MS full scan was followed by a first MSMS scan acquired using 25 ×4 Da windows and a second MSMS scan acquired at 26 × 4 Da windows. All scans were acquired in positive ionization mode at 30 k resolution, and DIA scans were acquired after peptide fragmentation at a normalized collision energy (NCE) of 30%. The raw files were analyzed using DIA-NN version 1.8.1 with a library-free search against the UniProt mouse database (2024). Further data processing and statistical analysis were performed with Perseus (V.1.6.5.0) and visualized with Instant clue (V 0.10.10.20210315).

### Histochemistry and immunostaining

Mouse diaphragm muscles were collected, cryo-embedded in OCT, and frozen at −80 °C until further analysis. Longitudinal sections of the diaphragm were cut at 10 µm thickness, collected on glass slides, and stored at −80 °C. Slides were removed from the freezer and dried at room temperature. Sections were fixed in ice-cold acetone for 10 min at −20 °C and stained using primary antibodies against Tenascin-C (1:100; Leica Biosystems Cat# NCL-TENAS-C, RRID:AB_564026), DPYSL2 (1:100; Sigma-Aldrich, Cat# HPA002381, RRID:AB_1847244), MBP (1:25; Abcam Cat# ab218011 RRID: AB_2895537), CPT1A (1:100; Proteintech, Cat# 15184-1-AP, RRID: AB_2084676), FXYD6 (1:200, Proteintech, Cat#15805-1-AP RRID: AB_2108625), α-Bungarotoxin-ATTO Fluor-488 (1:500, Molecular Probes Cat# T1175, RRID:AB_2313931), α-Bungarotoxin-CF 594 (1:500, Biotium, Cat#0007), α-Bungarotoxin-647 (1:500, Invitrogen, Cat#B35450), Collagen 22 (1:500 Gp IgG), COLXXVIII (1:500; KR44 Rb IgG), POSTN (1:200), TGFBI (1:500, KR135, Rb, IgG), or AEBP1 (1:200, Rb, IgG; provided by Manuel Koch, Cologne) overnight at 4 °C. The following day, secondary antibodies (diluted 1:200) and Hoechst (Invitrogen, H1399; 1:100 in TBS) were applied for 60 min at RT. The secondary antibodies were AF568-conjugated anti-rabbit (Molecular Probes Cat# A-11036; RRID:AB_10563566), AF488-conjugated anti-rabbit (Thermo Fisher Scientific Cat# A-11034; RRID:AB_2576217), AF568-conjugated anti-guinea pig (Thermo Fisher Scientific Cat# A-11075, RRID:AB_2534119), AF488-conjugated anti-mouse IgG2b (Thermo Fisher Scientific Cat# A-21141, RRID:AB_2535778), and AF680-conjugated anti-mouse IgG2a (Thermo Fisher Scientific Cat# A31563, RRID:AB_2536177). For myosin heavy chain (MYH) isoform staining, transverse cryosections of the soleus and gastrocnemius muscles were cut at 10 µm, fixed in acetone, and blocked in 10% normal goat serum (Thermo Scientific, Cat# 31872, RRID: AB_2532166). Primary antibodies BA-D5 (MYH7, mouse IgG2b), SC71 (MYH2, mouse IgG1), BF-F3 (MYH4, mouse IgM) (1:100; Developmental Studies Hybridoma Bank, DSHB supernatant), FXYD6 (rb IgG; 1:200; Proteintech, Cat#15805-1-AP RRID:AB_2108625) were applied overnight at 4 °C. Secondary antibodies DyLight 405 labeled goat anti-mouse (IgG, Fcγ 2b subclass specific; 1:200; 115-475-207, Jackson ImmunoResearch Europe), Alexa Fluor-488 labeled goat anti-mouse IgG, Fcγ 1 subclass specific (1:200; 115-545-205, Jackson ImmunoResearch Europe), Alexa Fluor 594 labeled goat anti-mouse IgM, µ chain specific (1:200; 115-585-075 or 115-165-075 Jackson ImmunoResearch Europe), Alexa Fluor 647 labeled goat anti-rabbit IgG (111-607-003, Jackson ImmunoResearch Europe) were diluted in PBS-BSA 1% and incubated for 1 h at room temperature. Sections were washed with TBS between all steps in the protocol and were mounted with coverslips using Prolong Gold Antifade Reagent (Thermo Fisher Scientific). For widefield fluorescence microscopy, images were captured using 10x/0.3NA and 20x/0.5NA objectives on an Olympus BX51 microscope with a 0.5× camera (Olympus DP71, Olympus Deutschland GmBH, Hamburg, Germany) at the ISMC using Olympus cellSens Software (Research Resource Identifier: RRID:SCR_014551). Confocal images were taken with a STELLARIS 5 confocal laser scanning microscope (Leica Microsystems, RRID:SCR_024671) using 40× and 63× objectives (HC PL APO 40×/0.95 CORR, HC PL APO 63x/1.30 GLYC CORR CS2). Two biological replicates were performed for each staining. Images were further processed using ImageJ (version 1.54d; RRID:SCR_003070) and colorblind-friendly pseudo colors were applied to the composite images.

### AChE-iodine histochemical staining

Wholemount and longitudinal cryosections of the diaphragm were fixed in 4% PFA for 2 h and 10 min respectively, incubated with ethopropazine (0.2 mM), acetylthiocholine iodine (4 mM), glycine (10 mM), cupric sulfate (2 mM), and sodium acetate (65 mM, pH 5.5) for 2–4 h at 37 °C, developed for 2–5 min in sodium sulfide (1.25%, pH 6.5), extensively washed with water, and mounted and imaged using a Evos FL Auto2 (Thermo Fisher Scientific) with a 20x objective on brightfield and tile-scan option. Images were further processed using ImageJ (version 1.54d; RRID:SCR_003070).

### Single muscle fiber isolation and digestion

High-throughput proteomics fiber-typing from the diaphragm muscle was done as previously described^47^. Briefly, freshly dissected diaphragm muscles were placed in 0.2% collagenase-P Dulbecco’s Modified Eagle Medium (DMEM) and incubated for 20‒30 min at 37 °C, and digestion was stopped by transferring the muscles to DMEM containing 5 mM EDTA and 10% FBS. Fibers were isolated by flushing the skeletal muscle several times using a glass pipette. Muscle fibers were washed five times with 1x PBS containing 5 mM EDTA. Every single fiber was placed into a 96-well-plate and lysed with 40 µL of 4% SDS in PBS containing 5 mM TCEP and 10 mM CAA. The 96-well-plates were incubated at 95 °C for 10 min, followed by Bioruptor sonication at 20 °C with 10 cycles of 30 s on and 30 s off. Samples were digested, and peptides were purified as described above and reconstituted in 0.1% FA.

### Isolation of primary myoblasts

Myoblasts were isolated as described before^48^. In short, quadriceps muscles were dissected from 4- to 8-week-old wild-type C57BL/6 mice and cultivated as explants on collagen- and Matrigel-coated cell culture dishes. Five days after the explants were established, the cells were collected with trypsin, transferred to a 25 cm^3^ flask, and cultivated for 2‒3 days to reach maximum confluency of 50%. Myoblasts were enriched by 37 °C PBS and gentle shaking of the plate. This procedure was repeated once more to yield a myoblast purity of ~95%.

### Construction of overexpression constructs

The mouse Fxyd6 construct was designed and supplied by VectorBuilder. The construct contains full-length murine *Fxyd6* fused to a 3× GS to a 3× FLAG-tag on the C-terminus or the N-terminus (as shown in Supplementary Fig. 5K). Expression is controlled by the EF1A promoter and a Kozak sequence.

### Co-immunoprecipitation assays

Co-immunoprecipitation experiments were performed using C2C12 (ATCC #CRL-1772) and SHS-Y5Y (ATCC #CRL-2266) cells and mouse brain, diaphragm, heart, soleus, and gastrocnemius tissue lysates. Brain, diaphragm, heart, soleus, and gastrocnemius tissue were isolated from 2- to 4-month-old wild-type C57BL/6 mice, snap-frozen in liquid nitrogen, and stored at −80 °C. Cells were grown to 70% confluency on 6 cm dishes and transfected with FXYD6-FLAG and control vector constructs using lipotransfection following the manufacturer’s protocol (Polyplus #117) for 24 h. Cells and lyophilized tissue samples were lysed in IP buffer (20 mM Tris-HCl, pH 7.5, 150 mM NaCl, 1 mM Na_2_EDTA, 1 mM EGTA, 1% Triton) with protease inhibitors and phosphatase inhibitor cocktail (Absource #B14002). Immunoprecipitation was performed using FLAG-magnetic beads (Sigma #M8823) and Dynabeads Protein A (Invitrogen; Cat#10001D) coupled to FXYD6 Antibody (Proteintech, Cat#15805-1-AP) for cell lysates and tissue lysates, respectively. The lysates were incubated with the respective beads overnight at 4 °C, then the beads were washed three times with IP buffer and digestion buffer (50 mM ammonium bicarbonate), respectively. For western blot analysis, the proteins were eluted from the beads with 1x LDS and DTT and heated to 95 °C. For MS/MS analysis, proteins were eluted with 6 M urea and 2 M thiourea in 50 mM ammonium bicarbonate, reduced and alkylated with TCEP (5 mM) and 2-chloracetamide (15 mM), digested with trypsin (500 ng) and LysC (500 ng) overnight at room temperature, and the peptides were cleaned up with SDB-RPS Stage Tips and reconstituted in 2% ACN and 5% FA.

### Proteomics analysis of immunoprecipitates

Tryptic peptides from enrichment samples were analyzed using an EvoSep One system (Evosep, Denmark) connected to an Orbitrap Eclipse (Thermo Scientific). The peptides were separated using the 30 SPD method with a 15 cm column (Evosep ReproSil-Pur C18, 1.5 µm beads by Dr Maisch, Germany, 15 cm × 150 µm). Spectra for co-immunoprecipitation experiments from in vitro (cell lysate) samples were acquired in DDA with a full scan at 60 k in the scan range of 350‒1400 *m/z* with a normalized AGC target of 100%. MSMS precursor selection was conducted in 1.6 *m/z* window within the cycle time of 1.5 s; the resolution was set to 15 k with a maximum injection time of 118 ms. All methods were operated in positive ionization mode. For the endogenous co-IP from mouse tissue lysates, samples were measured in DIA with a full scan at 30 k over the scan range of 39‒1010 *m/z* with a normalized AGC target of 100% and a maximum injection time of 54 ms. MSMS scans were acquired at 30×20 Da windows from 399.5 to 1000 *m/z* with an overlap of 0.5 Da on each side. All scans were acquired in positive ionization mode at 15 k resolution, and DIA scans were acquired after peptide fragmentation at a normalized collision energy (NCE) of 30%. The DDA raw files were processed with MaxQuant (V 2.6.6.0) and the DIA files were analyzed using DIA-NN 1.8.1 with a library-free search against the UniProt mouse database (2024). Further data processing and statistical analysis were performed with Perseus (V.1.6.5.0) and visualized with Instant clue (V 0.10.10.20210315).

### AChR clustering assay

C2C12 cells (ATCC #CRL-1772) were seeded onto glass coverslips coated with Matrigel (Fisher #11573620) and collagen I (Gibco #A10483) in 6-well-plates (Greiner #657160) in proliferation medium (DMEM, 10% FBS, 1% PSG). When the cells reached ~90% confluence, the medium was switched to differentiation medium (DMEM, 2% horse serum, 1% PSG) and changed every second day. After 5–7 days of differentiation, transient transfections of various plasmids (pRP-mFxyd6-FLAG and pRP-FLAG; VectorBuilder) were performed using lipotransfection following the manufacturer’s protocol (Polyplus #117). To initiate formation of acetylcholine receptor clusters, myotubes were supplemented with 0.1 µg/mL recombinant agrin protein (R&D Systems; Cat#550-AG-100) 48 h after transfection. Cells were fixed 16 h later using 4% PFA in PBS for 10 min at RT. The AChR clusters were stained using Alpha-Bungarotoxin conjugated to Alexa Fluor 647 (Thermo #B35450) and overexpressed protein was detected with either an Fxyd6 antibody (Proteintech #15805-1-AP) or Flag-antibody (Thermo # MA1-142-A488). Confocal images were taken with a STELLARIS 5 confocal laser scanning microscope (Leica Microsystems, RRID:SCR_024671) using a 40× objective (HC PL APO 40×/0.95 CORR, HC PL APO). Images were further processed using ImageJ (version 1.54 d; RRID:SCR_003070)

### Statistics and reproducibility

All experiments were carried out in triplicate, unless otherwise stated. Welch’s *t*-test was calculated using Perseus software. The permutation-based FDR and number of randomizations were set to 0.05 and 500, respectively. Significance is indicated using asterisks (* adjusted *p* value < 0.05. ** adjusted *p* value < 0.01, *** adjusted *p* value < 0.001).

## Supplementary information

Transparent Peer Review file

Supplementary Information

Description of Additional Supplementary Files

Supplementary Data

reporting-summary

## Data Availability

The MS proteomics data have been deposited to the ProteomeXchange Consortium via the PRIDE partner repository with the dataset identifier PXD054352^49^. Original Blots for Figs. 4 and 5 can be obtained from Supplementary Fig. 6. Source data can be obtained from Supplementary Data 1–7 and the PRIDE partner repository.
