# Supplementary material for: Spatial proteomics identifies FXYD6 as a dual-site protein of neuromuscular junction in the diaphragm
Source: Commun Biol. 2026 Sep 18;9:1220. doi: 10.1038/s42003-026-10884-8 (PMC13588806; doi:10.1038/s42003-026-10884-8)
Supplement: Supplementary file 1 [file 42003_2026_10884_MOESM1_ESM.pdf]

nature portfolio

Peer Review File

Spatial proteomics identifies FXYD6 as a dual-site protein of neuromuscular junction in the diaphragm

Corresponding Author: Professor Marcus Krüger

This file contains all reviewer reports in order by version, followed by all author rebuttals in order by version.

Version 0:

Reviewer comments:

Reviewer #1

(Remarks to the Author)
In this paper, Krüger and colleagues are using mass-spectrometry to spatially localise proteins in mouse diaphragm. They created serial sections of the muscle tissue from peripheral to the central (tendon) axis and analysed the sections individually by mass spectrometry. This technique was established by this group before and allows to identify functional regions along the muscle contraction axis by known marker proteins, including the neuromuscular junction (NMJ) and the myotendinous junction (MTJ). Here the authors focus on the NMJ, located in the centre of the diaphragm muscle axis, and first found that the NMJ shares a group of proteins (31 proteins) with the MTJ, despite being functionally very different, while 115 are specific to the NMJ and 92 to the MTJ. A comparison to known marker genes validated the approach, suggesting that also uncharacterised identified proteins are truly enriched at NMJ and MTJ.
Second, the authors focus on one NMJ-enriched protein, FXYD6. Antibody stainings verified that FXYD6 is indeed enriched at NMJs of type IIa fibres of the soleus (MYH2 positive) as well as in the diaphragm, likely including the postsynaptic side. Furthermore, stainings of developing NMJs in differentiating C2C12 cells suggests that FXYD6 may also be involved in NMJ development.
In the nervous system, FXYD6 was known to interact with a Na/K ATPases, suggesting that it may also regulate ion balance in type IIa fibres, potentially at the NMJ. Indeed, Na/K ATPases were also identified here as enriched at the NMJ. To demonstrate binding, the authors performed co-IPs from C2C12 cells transfected with Flag-tagged FXYD6 or co-IPs using an anti-FXYD6 antibody from diaphragm lysates and found binding of FXYD6 to some of the ATPases found enriched at the NMJ in the diaphragm. This suggests that FXYD6 may indeed regulate these ATPases at the NMJ as had been suggested in other systems.

The manuscript is well written and the quality of the data presented is high. Thus, the conclusions are largely supported by the data. Overall, this manuscript provides an interesting atlas of the NMJ-enriched proteins in the diaphragm, a first step towards the functional characterisation of some these not well studied proteins.

Minor points.
1. Typo on page 3" These ATPases actively transport sodium out of potassium into the muscle fiber", please correct.
2. I find the nomenclature in Figure S1 a bit confusing. I would suggest to use "identified peptides" instead of "precursors" in 1A. Showing only 1, 2, 3 etc. mm suggests that the section resolution was only 1mm, but it was 20 µm. This is better visible in Figure 1; hence, I would try to adapt this to Figure S1 as well. What is the difference between S1A, B and S1 C, D?
3. I assume "v" and "d" in Figure 1H mean ventral and dorsal? Please include this in the legend.
4. The comparison to the single nuclei mRNA data could be moved to the results sections and hence displayed in a figure.

Reviewer #2

(Remarks to the Author)
The neuromuscular junction (NMJ) occupies only approximately 0.1–1% of the muscle fiber surface, and synaptic proteins are present at relatively low abundance. Therefore, it is technically challenging to use mass spectrometry to analyze proteins specifically enriched at the NMJ from whole muscle tissue. In this manuscript, the authors applied spatial proteomics to identify NMJ-associated proteins in the diaphragm and identified FXYD6 as an NMJ-enriched protein. The authors further performed immunostaining and immunoprecipitation experiments to validate the localization of FXYD6 and characterize its

interactome. Overall, the manuscript presents a potentially valuable spatial proteomics approach for studying the NMJ. However, several issues should be addressed before the manuscript can be considered for publication.

In Supplementary Figure 3C, the authors show the acetylcholine receptor delta subunit (CHRND). What about the other AChR subunits, such as CHRNA1, CHRNB1, and CHRNG/CHRNE? Were these subunits also detected in the spatial proteomics dataset?
If not, please provide possible explanations.

Rapsyn is known to associate with acetylcholine receptors at approximately a 1:1 stoichiometric ratio. Was rapsyn detected in the mass spectrometry dataset? If so, how abundant was it relative to AChR subunits?
What about other agrin signaling proteins, such as Lrp4, MuSK and Dok7? These proteins are essential for NMJ formation and maintenance. Clarification on this point would help evaluate how well the dataset reflects known NMJ molecular organization.

In Figure 4A–D, the signals are difficult to appreciate. Please provide higher-quality images or present them in a format similar to Figure 4E–G to allow clearer evaluation of the staining patterns.

In Figure 4E and 4G, CPT1A and AEBP1 do not appear to colocalize with AChRs at the NMJ. The authors should clarify this interpretation and provide stronger evidence if these proteins are claimed to be localized at the NMJ.

In Figure 5D, it is unclear why WGA staining was included. Please explain the purpose of this experiment and how WGA staining contributes to the conclusion being drawn.

In Figure 5E, it appears that the authors aim to test whether FXYD6 overexpression affects AChR clustering. However, the colocalization is not clear, and the conclusion is difficult to follow. Does FXYD6 overexpression increase or decrease AChR clustering? Please quantify the effect and clarify the interpretation.

The statement that "FXYD6 showed higher staining intensity at AChR clusters in control-transfected, differentiated myoblasts treated with agrin compared to transfected cells that had not been incubated with agrin" is unclear. Please revise this sentence for clarity and better explain its biological significance.

For Figure 5F–G, would it be possible to provide a volcano plot of the FXYD6 interactome data? This would help readers better assess the specificity and significance of the identified interacting proteins.

In Figure 5H, the co-immunoprecipitation experiment lacks an appropriate negative control. Please include an IgG control, particularly for the brain tissue experiment, to demonstrate the specificity of the interaction.

Reviewer #3

(Remarks to the Author)
In this article, the authors performed unbiased LC-MS analysis of NMJ structures to know the complex protein networks, which characterize NMJ integrity and function. By comparison of the protein and mRNA signatures, which have been analyzed by single-nucleotide RNAseq, they found that 115 proteins at the NMJ. They also revealed that FXYD6 protein is enriched at NMJ and may function in the ionic homeostasis for continuous, fatigue resistant contraction.
Overall, the article presents a novel way to assess the NMJ proteins using LC-MS and the well-analyzed dataset provides a protein atlas of the NMJ in the diaphragm muscle. This is a well-written and interesting manuscript that meets the journal's criteria. I think that the study is suitable for publication after addressing the following minor point.
(1) The article is a bit descriptive and lack of functional validations. In the 115 proteins of NMJs, how many genes are involved in muscle diseases such as CMS or dystrophy, and in neuronal synaptic diseases?

Version 1:

Reviewer comments:

Reviewer #1

(Remarks to the Author)
This revised manuscript by Krüger and colleagues has addressed the few initial suggestions that I had for improvement. As explained in my initial review, this manuscript defines an interesting atlas of the NMJ-enriched proteins in the diaphragm using rigorous mass-spec analysis. This will be of interest to muscle field. Hence, I recommend publication of this revised manuscript.

Reviewer #2

(Remarks to the Author)

The authors have addressed all my concerns, and I have no more questions.

Reviewer #3

(Remarks to the Author)
The authors have adequately addressed the concerns raised during the review process and have provided satisfactory responses to all major comments. The revised manuscript is substantially improved and presents a comprehensive set of data supporting the authors' conclusions.
While no study is without limitations, I find that the remaining issues are minor and do not detract from the overall quality or significance of the work. Importantly, the manuscript satisfactorily addresses the key scientific questions it set out to investigate, and the conclusions are supported by the presented evidence.
I have no further major concerns and believe that the manuscript is suitable for publication in its present form.

Open Access This Peer Review File is licensed under a Creative Commons Attribution 4.0 International License, which permits use, sharing, adaptation, distribution and reproduction in any medium or format, as long as you give appropriate credit to the original author(s) and the source, provide a link to the Creative Commons license, and indicate if changes were made.
In cases where reviewers are anonymous, credit should be given to 'Anonymous Referee' and the source.
The images or other third party material in this Peer Review File are included in the article's Creative Commons license, unless indicated otherwise in a credit line to the material. If material is not included in the article's Creative Commons license and your intended use is not permitted by statutory regulation or exceeds the permitted use, you will need to obtain permission directly from the copyright holder.
To view a copy of this license, visit https://creativecommons.org/licenses/by/4.0/

Point-by-point responses to the reviewer comments

Comments of reviewers are in italic, replies in blue:

Reviewer #1 (Remarks to the Author):

Typo on page 3" These ATPases actively transport sodium out of potassium into the muscle fiber", please correct.

Thanks for the notifying the mistake. The typo has been corrected.

I find the nomenclature in Figure S1 a bit confusing. I would suggest to use "identified peptides" instead of "precursors" in 1A.

- We changed the y axis in S1A and S1C to "identified peptides" instead of identified precursors

Showing only 1, 2, 3 etc. mm suggests that the section resolution was only 1mm, but it was 20 µm. This is better visible in Figure 1; hence, I would try to adapt this to Figure S1 as well.

The values 1, 2, 3, etc. mm correspond to the width of the diaphragm tissue that was embedded prior to cutting. The thickness of the sectioned tissue was always set at 20 µm. To clarify this point, we have added the term "diaphragm width" to the description of the x-axis in SI 1A and SI 1B. In the main Figure 1, we then showed the 3 mm wide section of the diaphragm

What is the difference between S1A, B and S1 C, D?

Figure S1 A and C correspond to the **identified peptides** for different diaphragm width (1-8 mm) of the embedded diaphragm tissue measured at short 11min (100 SPD) and intermediate 21min LC-MS gradients respectively.
Similarly Figure S1 B and D correspond to the **identified proteins** for different width (1-8 mm) of the embedded diaphragm tissue measured at 11min (100 SPD) and 21min LC-MS gradients respectively. The reason for these plots was to determine the size of the isolated diaphragm from which we could obtain sufficient material for proteomic analysis. A diameter of 3 mm yielded sufficient protein hits and was therefore selected for the subsequent experiments.

1. *I assume "v" and "d" in Figure 1H mean ventral and dorsal? Please include this in the legend.*

It is already mentioned in the figure description.

4. The comparison to the single nuclei mRNA data could be moved to the results sections and hence displayed in a figure.

We agree with the reviewer that a comparison of protein and single nuclei mRNA expression could also be of interest for the main figure. However, when we performed this comparison, the overlap was unfortunately very small (**see the attached Figure R1**), which is why we decided

to include it only in the Supplementary Information (**SI Figure X**). Although we consider this comparison to be an important insight, we wish to avoid any confusion and have focused our study on the protein level of the presynaptic and postsynaptic structures/proteins in the main figures.

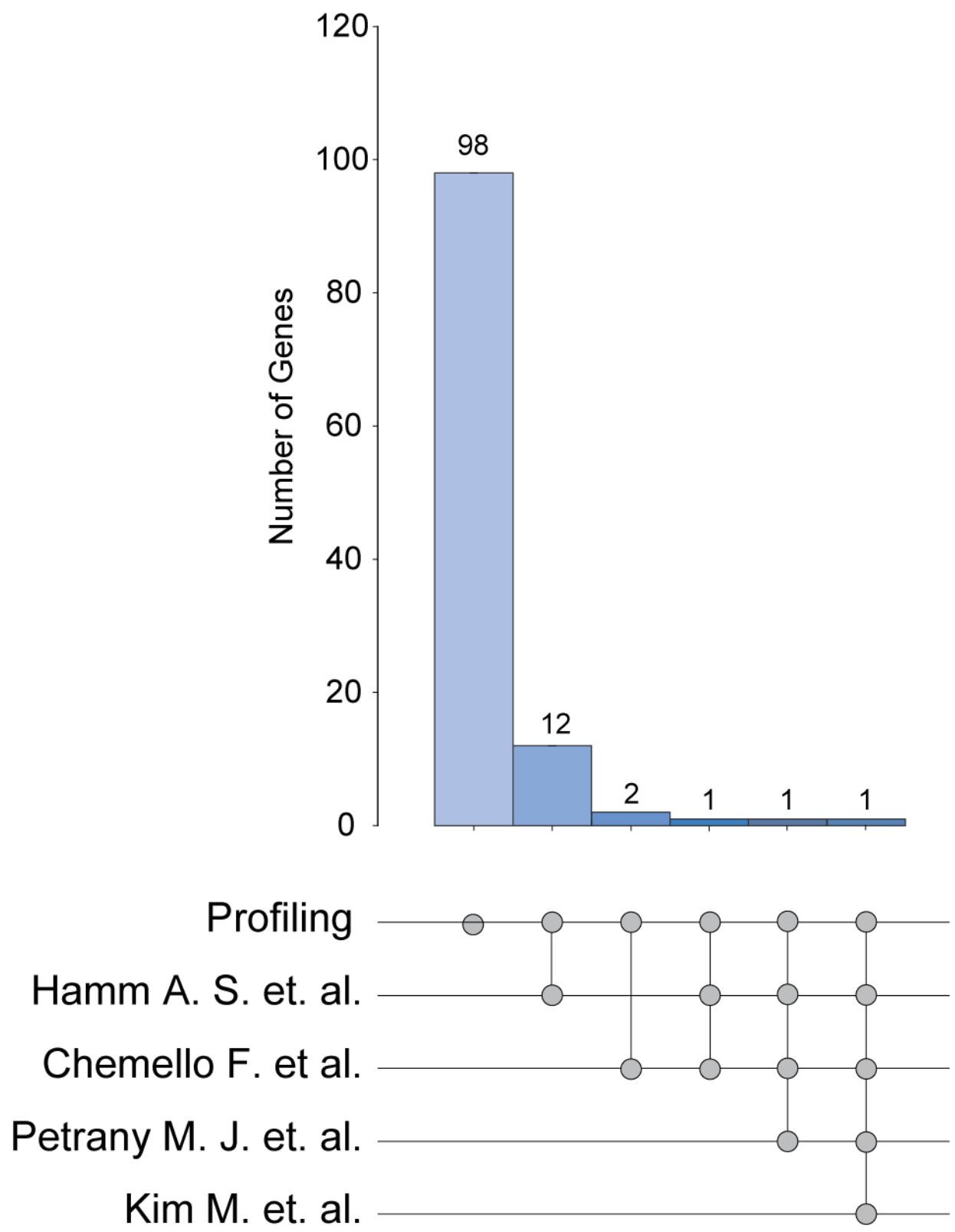

Figure R1: Overview of the comparison of various snRNA seq. data from different publications and our protein profiling. (929 genes from Hamm A. S. et. al.; 347 gene from Chemello F. et. al.; 345 genes from Petrany M. J. et. al.; 51 genes from Kim M. et. al.)

Reviewer #2 (Remarks to the Author):

1. *In Supplementary Figure 3C, the authors show the acetylcholine receptor delta subunit (CHRND). What about the other AChR subunits, such as CHRNA1, CHRNB1, and CHRNG/CHRNE? Were these subunits also detected in the spatial proteomics dataset? If not, please provide possible explanations.*

Unfortunately, only the CHRND subunit was identified with a sufficient q-value for detection, and a protein profile for this gene could not be generated due to the large number of missing values.

Other subunits were detected but did not meet the filter criteria of sufficient global q-value and protein group Q-value (PG.Q.value) which is set at less than 1% for both conditions. Global Q-value is a peptide/precursor level FDR estimated across the entire experiment globally. The PG.Q.Value is the **protein group-level FDR** and reflects the confidence that a given protein group identification is not a false positive, after calculating the precursor/peptide level evidence.

This keeps the data more stringent and robust suitable for downstream analysis. **Figure R2** shows the number of unique peptides, the corresponding intensities, global Q-value and PG. Q value for the different AChR subunits and other NMJ related genes but unfortunately did not meet the 1% FDR cut-offs. Possibly with more sensitive instrumentation or better analysis software this could be possible to detect more peptides for these low abundant proteins with high confidence intervals.

The threshold for both Global Q-value and Protein Group Q-value was set at 1%.

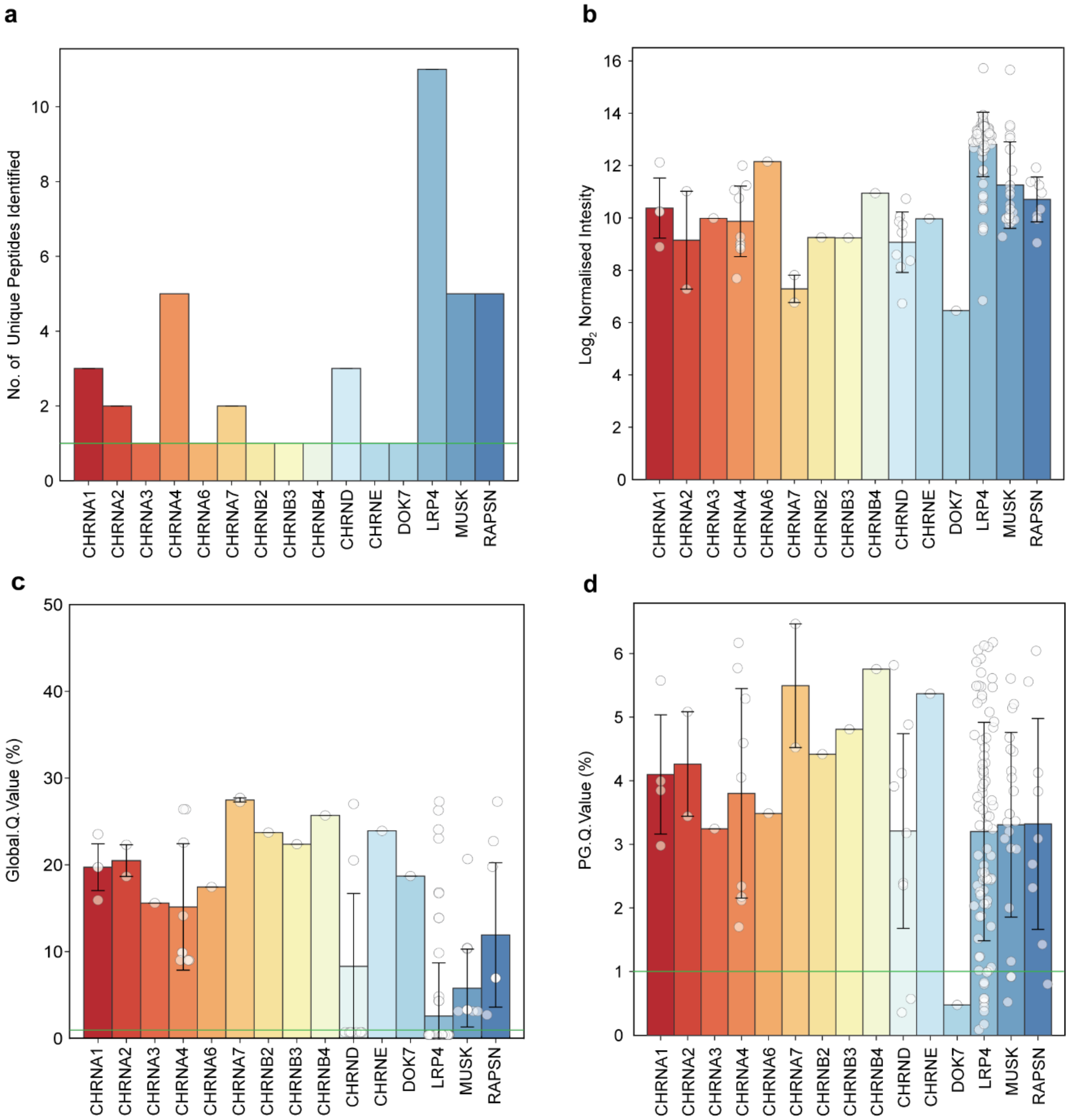

Figure R2. The number of (a) unique peptides, the corresponding (b) log2 normalised intensity, (c) Global Q-value and (d) Protein group Q-value for different AChR subunits (CHRNA1, A2, A3, A4, A6, A7, B2, B3, B4, D, and E), DOK7, LRP4, MUSK and RAPSN. (Green line indicating 1% cutoff for both Global

2. *Rapsyn is known to associate with acetylcholine receptors at approximately a 1:1 stoichiometric ratio. Was rapsyn detected in the mass spectrometry dataset? If so, how abundant was it relative to AChR subunits? What about other agrin signaling proteins,*

such as Lrp4, MuSK and Dok7? These proteins are essential for NMJ formation and maintenance. Clarification on this point would help evaluate how well the dataset reflects known NMJ molecular organization.

As mentioned above, these low abundant proteins are detected but do not meet the recommended FDR cut-offs of 1% therefore are not considered for downstream analysis. With more sensitive instrumentation or improved data analysis software, it may become possible to detect additional peptides from these low-abundance proteins with greater confidence.

3. *In Figure 4A–D, the signals are difficult to appreciate. Please provide higher-quality images or present them in a format similar to Figure 4E–G to allow clearer evaluation of the staining patterns.*

Thanks for the suggestion, the **Figure 4A-D** have been updated to a higher magnification (see below and new **Figure 4A-C**).

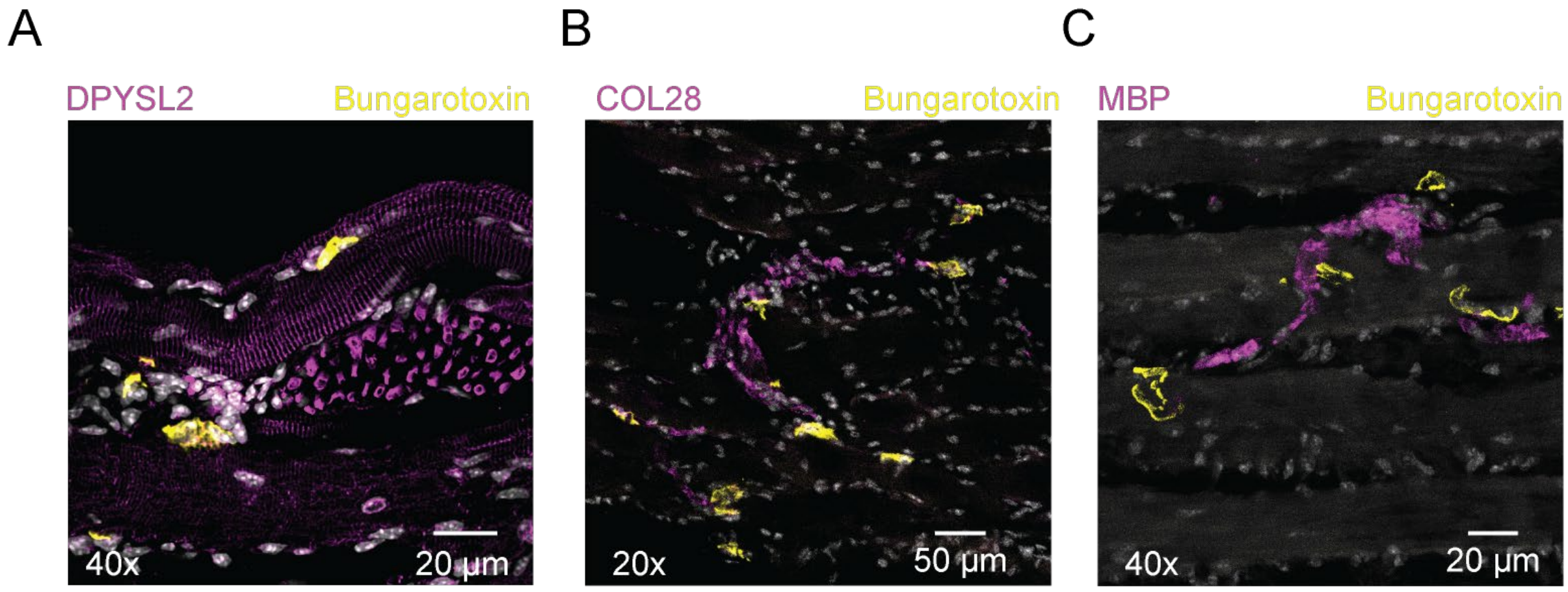

4. *In Figure 4E and 4G, CPT1A and AEBP1 do not appear to colocalize with AChRs at the NMJ. The authors should clarify this interpretation and provide stronger evidence if these proteins are claimed to be localized at the NMJ.*

We speculate CPT1a and AEBP1 could be expressed by glial cells and Schwann cells. Since CPT1A is associated with fatty acid metabolism and supports β-oxidation of fatty acids it can contribute to energy production in these cells, during myelination. So, an exact co-localization might not be the case but rather more localized along the neuronal axons. Future studies should use colocalisation analyses with Schwann cell markers, including SOX10, S100β and GFAP, to determine whether CPT1A also serves as a marker for terminal Schwann cells.
AEBP1, also showed a maximum peak at the NMJ in the protein profiles and was located in proximity to α-BTX-positive regions (**Figure 3G**). AEBP1 has been associated with collagens in the ECM and is reported to play several roles in development, tissue repair, and fibrosis.

5. *In Figure 5D, it is unclear why WGA staining was included. Please explain the purpose of this experiment and how WGA staining contributes to the conclusion being drawn.*

The WGA staining was included to visualize the plasma membrane of each skeletal muscle fiber. In addition to labeling the sarcolemma, WGA also prominently stains the NMJ and T-tubule regions due to the high abundance of extracellular matrix proteins and glycoproteins in these structures, resulting in a staining pattern that partially overlaps with BTX labeling. This helped to better assess the membrane-associated localization of FXYD6 at the NMJ. Furthermore, the WGA co-staining helped in showing the fiber-type specificity of FXYD6 expression, as FXYD6 does not stain all muscle fibers equally, consistent with the observations shown in **Figure 4H**.

6. *In Figure 5E, it appears that the authors aim to test whether FXYD6 overexpression affects AChR clustering. However, the colocalization is not clear, and the conclusion is difficult to follow. Does FXYD6 overexpression increase or decrease AChR clustering? Please quantify the effect and clarify the interpretation.*

We agree with the reviewer that the conclusion from Figure 5E was not sufficiently clear. The experiment was designed as a qualitative assessment to visualize the spatial relationship between FXYD6 expression and AChR cluster formation in differentiating myotubes, rather than to provide a quantitative analysis of clustering changes. Based on the imaging data, FXYD6 overexpression does not show a uniform distribution along the entire myotube. Instead, FXYD6 appears more enriched in regions where AChR clusters are typically located, mainly toward the central regions of the fibers. A similar localization pattern is also observed in Supplementary **Figure 5G** and **H**, with a tendency toward stronger signal in response to Agrin stimulation (see attached **Figure R3**). However, based on the current dataset, it is not possible to conclude whether FXYD6 overexpression increases or decreases AChR clustering. Hence, functional effect on AChR clustering remains speculative, and additional quantitative experiments in isolated myotubes (+/- FXYD6) would be required to determine whether FXYD6 directly modulates AChR cluster formation or stability

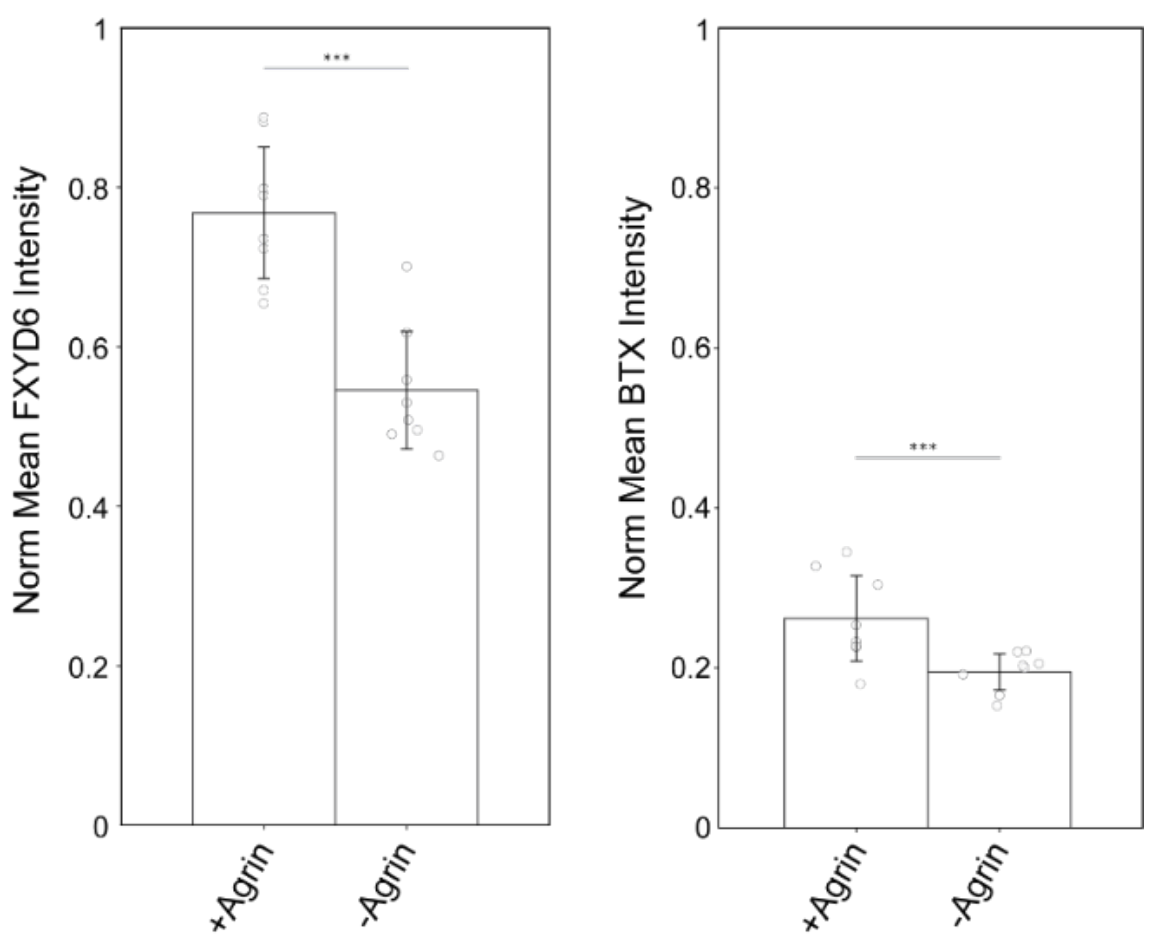

Figure R3. Quantification of the corresponding immunohistochemistry (IHC) data for FXYD6 and BTX in differentiated myofibers (n=10 fibers; Mean ± SD; *** adjusted p-value < 0.001).

7. *The statement that "FXYD6 showed higher staining intensity at AChR clusters in control-transfected, differentiated myoblasts treated with agrin compared to transfected cells that had not been incubated with agrin" is unclear. Please revise this sentence for clarity and better explain its biological significance.*

We thank the reviewer for the suggestion. The statement has been revised in the edited manuscript:

"*FXYD6 showed higher staining intensity in differentiated myoblasts treated with agrin compared that had not been incubated with agrin*."

8. *For Figure 5F–G, would it be possible to provide a volcano plot of the FXYD6 interactome data? This would help readers better assess the specificity and significance of the identified interacting proteins.*

In the edited version of the manuscript, we have now included the volcano plots as **Supplementary Figure 5 K-N**.

9. *In Figure 5H, the co-immunoprecipitation experiment lacks an appropriate negative control. Please include an IgG control, particularly for the brain tissue experiment, to demonstrate the specificity of the interaction.*

We agree that IgG negative controls were not included in the co-immunoprecipitation experiments (**Figure 5H**). If the reviewer's concern relates to antibody specificity, we would like to emphasize that the mass spectrometry–based pulldown performed with the same antibody provides independent support for specificity. In that dataset, FXYD6 and its potential interactors were significantly enriched in the immunoprecipitation, as indicated by both a strong p-value and a robust t-test difference (n=3) (**Supplementary Figure 5K–N**). We assume that using the same antibody and different concentrations of the bait will yield better results than a potential IgG control, which could introduce additional background binders or false-positive candidates.

Reviewer #3 (Remarks to the Author):

(1) The article is a bit descriptive and lack of functional validations. In the 115 proteins of NMJs, how many genes are involved in muscle diseases such as CMS or dystrophy, and in neuronal synaptic diseases?

We agree that our manuscript lacks functional validation of most NMJ candidates. We have done our best to validate several NMJ proteins using immunohistochemistry, including some functional studies on the new NMJ candidate FXYD6.

We are convinced that this work can contribute to the development of an NMJ reference atlas, which will provide a framework for validating genes associated with muscle-related disorders such as CMS. To clarify the relationship between congenital myasthenic syndrome (CMS), muscular dystrophy, neuronal-synaptic diseases and our dataset, we cross-referenced the 115 NMJ proteins with the ClinGen disease-gene database for Congenital Muscular Dystrophy, Myasthenic Syndromes Amyotrophic Lateral Sclerosis Disorders (126 entries *https://search.clinicalgenome.org/kb/gene-validity*) (**Figure R4**). Only three NMJ proteins, neurofilament, CHAT and peripherin are annotated in ClinGen as being directly associated with any of these disease categories. Nevertheless, when the entire set of ~2300 proteins is considered, 61 of them overlap with at least one of the disease-related genes. Since the three disease-associated proteins are also present in several gene ontology terms related to neuronal structure and maintenance, it is premature to conclude that they serve as specific markers for CMS. Their disease relevance is more likely indicative of broader neuronal functions rather than a role specific to CMS. An alternative strategy would be to physically enrich the NMJ niche, for example, by laser-capture microdissection or to apply targeted parallel-reaction-monitoring (PRM) assays for selected candidates. Both approaches could markedly increase the number of CMS-associated protein profiles. These experiments, however, fall outside the scope of the present manuscript and will be addressed in future work. Likewise, proximity-labeling techniques such as TurboID, using an NMJ marker as bait, or photo-activatable biotinylation that labels proteins within pre- or postsynaptic structures constitute promising avenues for future experiments.

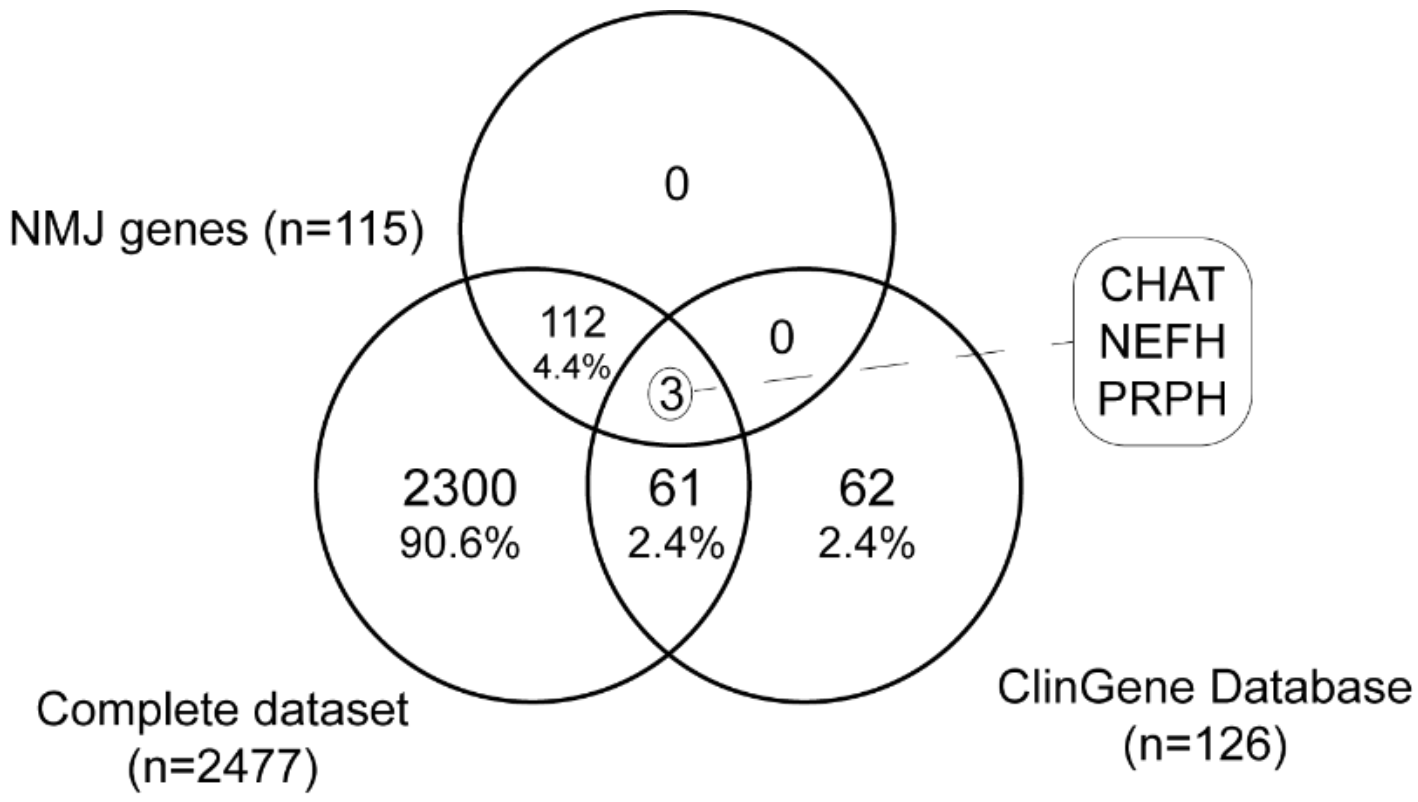

Figure R4. Overlap of genes from the 115 NMJ genes, the complete dataset (n=2477) and ClinGene Database for Congenital Muscular Dystrophy, Myasthenic Syndromes, and Amyotrophic Lateral Sclerosis Disorders (n=126).
