# Supplementary material for: Spatial proteomics identifies FXYD6 as a dual-site protein of neuromuscular junction in the diaphragm
Source: Commun Biol. 2026 Sep 18;9:1220. doi: 10.1038/s42003-026-10884-8 (PMC13588806; doi:10.1038/s42003-026-10884-8)
Supplement: Supplementary file 2 [file 42003_2026_10884_MOESM2_ESM.pdf]

Supplementary Information:

Spatial proteomics identifies FXYD6 as a dual-site protein of neuromuscular junction in the diaphragm

Nitin Eapen[1#,] Luisa Schmidt[2#], Michael Saynisch[1], Jan-Wilm Lackmann[1], Christian Hoegsbjerg[3], Abigail L. Mackey[3], Manuel Koch[4], Bent Brachvogel[1,6,7], Philipp Antczak7, Marcus Krüger[1,7$]

[1] Institute for Genetics, Cologne Excellence Cluster on Cellular Stress Responses in Aging-Associated Diseases (CECAD), Cologne, Germany

[2] Novo Nordisk Foundation Center for Protein Research, Faculty of Health and Medical Sciences, University of Copenhagen, Copenhagen, Denmark

[3] Institute of Sports Medicine Copenhagen, Department of Orthopaedic Surgery, Copenhagen University Hospital - Bispebjerg and Frederiksberg, Copenhagen, Denmark and Department of Clinical Medicine, Faculty of Health and Medical Sciences, University of Copenhagen, Copenhagen, Denmark

[4] Institute for Dental Research and Oral Musculoskeletal Biology, Center for Biochemistry, Medical Faculty, University of Cologne, Cologne, Germany

[5] Department of Pediatrics and Adolescent Medicine, Experimental Neonatology, Faculty of Medicine and University Hospital Cologne, University of Cologne, Cologne, Germany

[6] Department of Pediatrics and Adolescent Medicine, Experimental Neonatology, Medical Faculty, University of Cologne, Cologne, Germany and Center for Biochemistry, Medical Faculty, University of Cologne, Cologne, Germany

[7] Center for Molecular Medicine (CMMC), University of Cologne, 50931 Cologne, Germany
#equal contribution

$ Correspondence: MK Marcus Krüger (marcus.krueger@uni-koeln.de)
Lead Contact: Marcus Krüger, marcus.krueger@uni-koeln.de

Email: marcus.krueger@uni-koeln.de

This PDF file includes:

Supporting text
Figures S1 to S6

Other supporting materials for this manuscript include the following:

Datasets S1 to S7

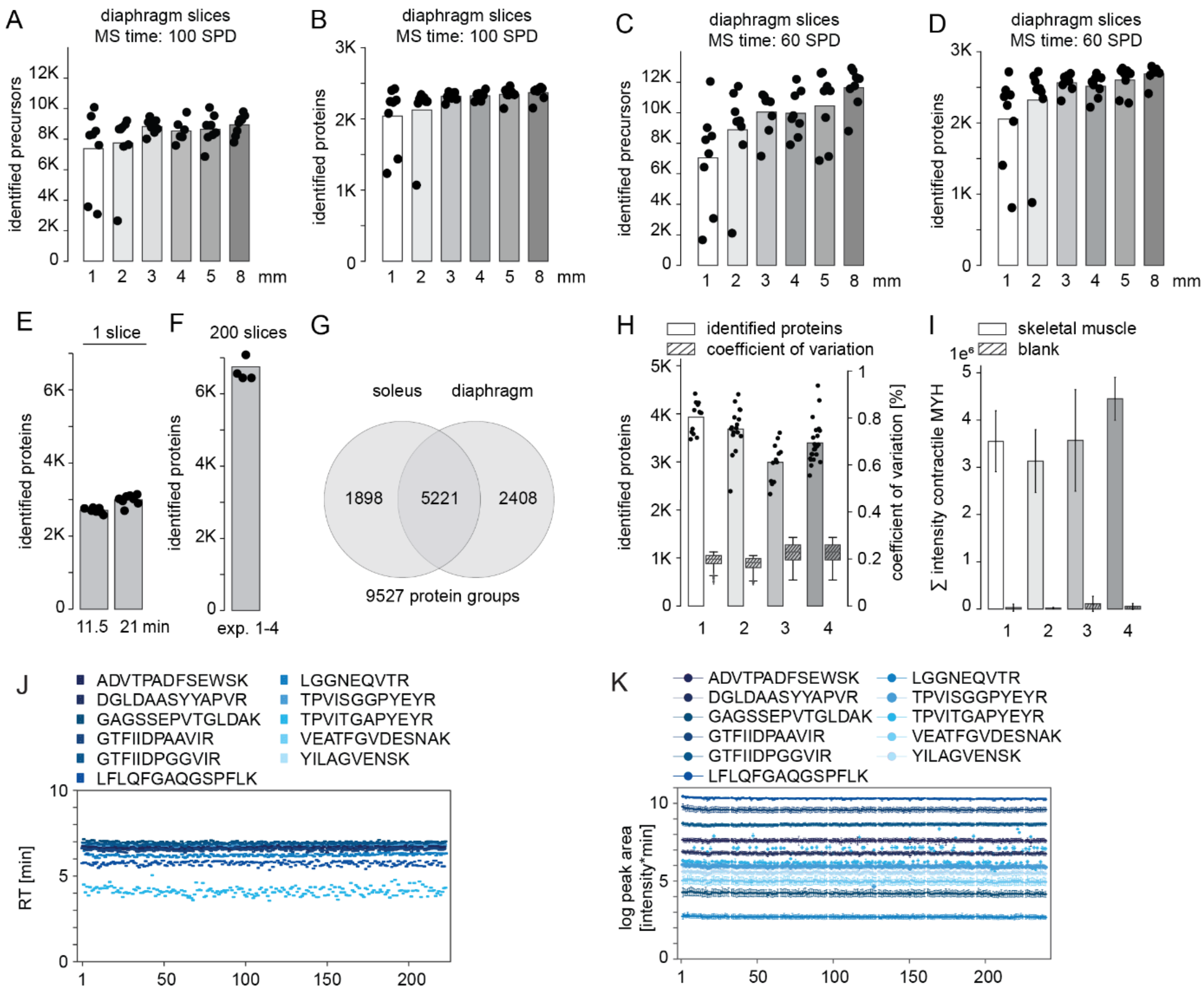

Supplementary Figure S1: Optimization of protein identification rates and quality control. (A-D) Number of identified precursors **(A, C)** and proteins **(B, D)** at different widths of the diaphragm tissue using 1-8 mm, 1 mm diameter, and 20 µm thick transverse sections measured with short LC-MS gradients of 11 min (100 SPD) **(A, B)** or 21 min (60 SPD) **(C, D)**. **(E)** Number of proteins identified in one section using short LC-MS gradients of 11 min (100 SPD) and 21 min (60 SPD). **(F)** Total number of proteins identified in 200 sections from expt. 1–4 **(A-D)**. **(G)** Venn diagram represents the overlap between the number of protein groups identified in soleus and diaphragm skeletal muscle slices. **(H)** Number of protein groups identified from HeLa cell lysates in each 96-well-plate 1-4. **(I)** Summed intensity of contractile myosin heavy chain isoforms (MYH4, MYH7, MYH2, MYH1) in skeletal muscle and blank runs with the same LC-MS settings. **(J)** Liquid

chromatography performance, including peptide retention time and log peak area **(K)** of the spike-in indexed retention time (iRT) peptides to each sample.

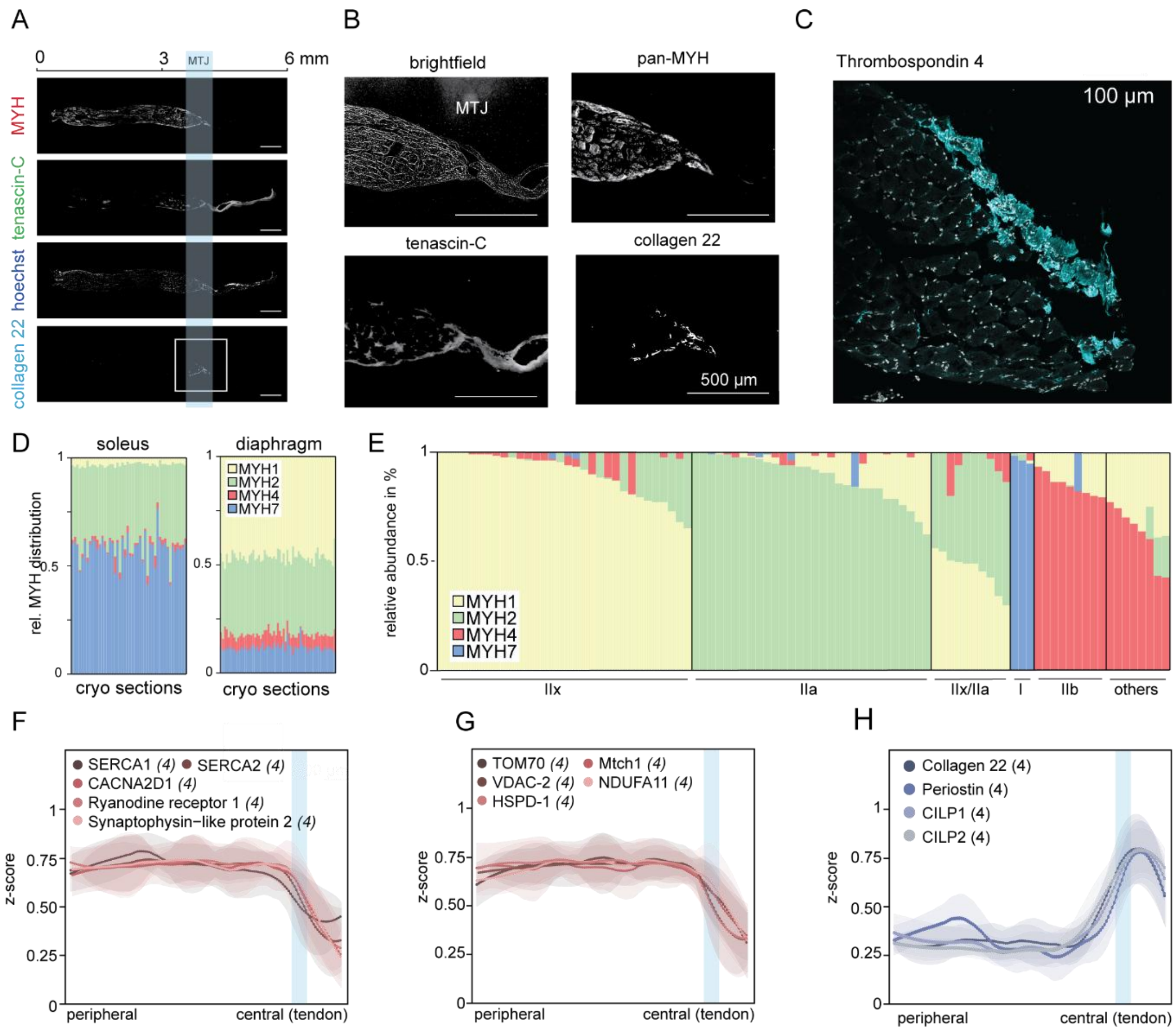

Supplementary Figure S2: Immunostaining with marker proteins and profiling of the MTJ area of the mouse diaphragm. (A-C) Immunohistochemical staining for pan-MYH, tenascin-C (TNC), collagen 22 (COL22), and thombospondin-4 (THBS4) on longitudinal cryosections of the diaphragm. (**D)** Distribution of the MYH isoform in the soleus and diaphragm transversal cryosections (**E**) MYH isoform distribution from single-fiber LC-MS analysis of the diaphragm muscle. (**F**) *Z*-score normalized protein intensity profiles of the sarcomeric proteins SERCA1/2, Ca^{2+} transporter (CACNA2D1), ryanodine receptor 1 (RYR1), and synaptophysin-like protein 2 (SYPL2) and (**G**) the mitochondrial proteins TOM70, VDAC-2, HSPD-1, MTCH1, and NDUFA11 along the longitudinal axis of the diaphragm in 20 µm thick cryo-sections. (**H**) *Z*-score normalized protein intensity profiles of ECM marker proteins that exhibited a peak along the longitudinal axis of the diaphragm at the MTJ region.

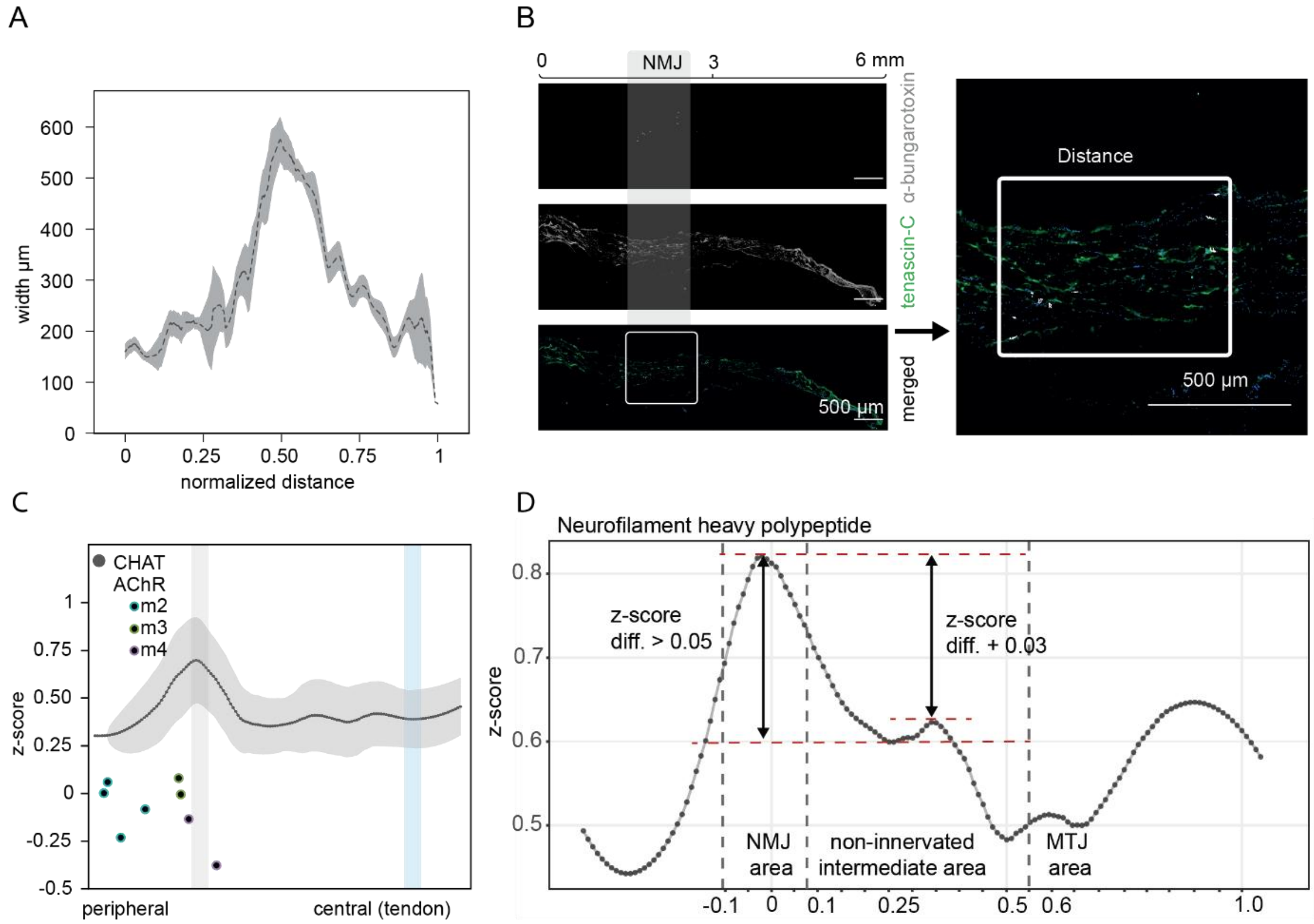

Supplementary Figure S3: Morphometric analysis of the NMJ area of the mouse diaphragm. (**A**) Width of the AChE-stained region along the normalized distance along the dorsal-ventral axis. (**B**) Immunohistochemical staining longitudinal cryosections of the diaphragm for alpha-bungarotoxin and tenascin-C. (**C-D**) *Z*-score normalized protein intensity profiles of choline acetyl transferase (ChAT), acetyl-choline receptor (CHRND), and neurofilament heavy polypeptide (NEFH) (D) along the longitudinal axis of the diaphragm.

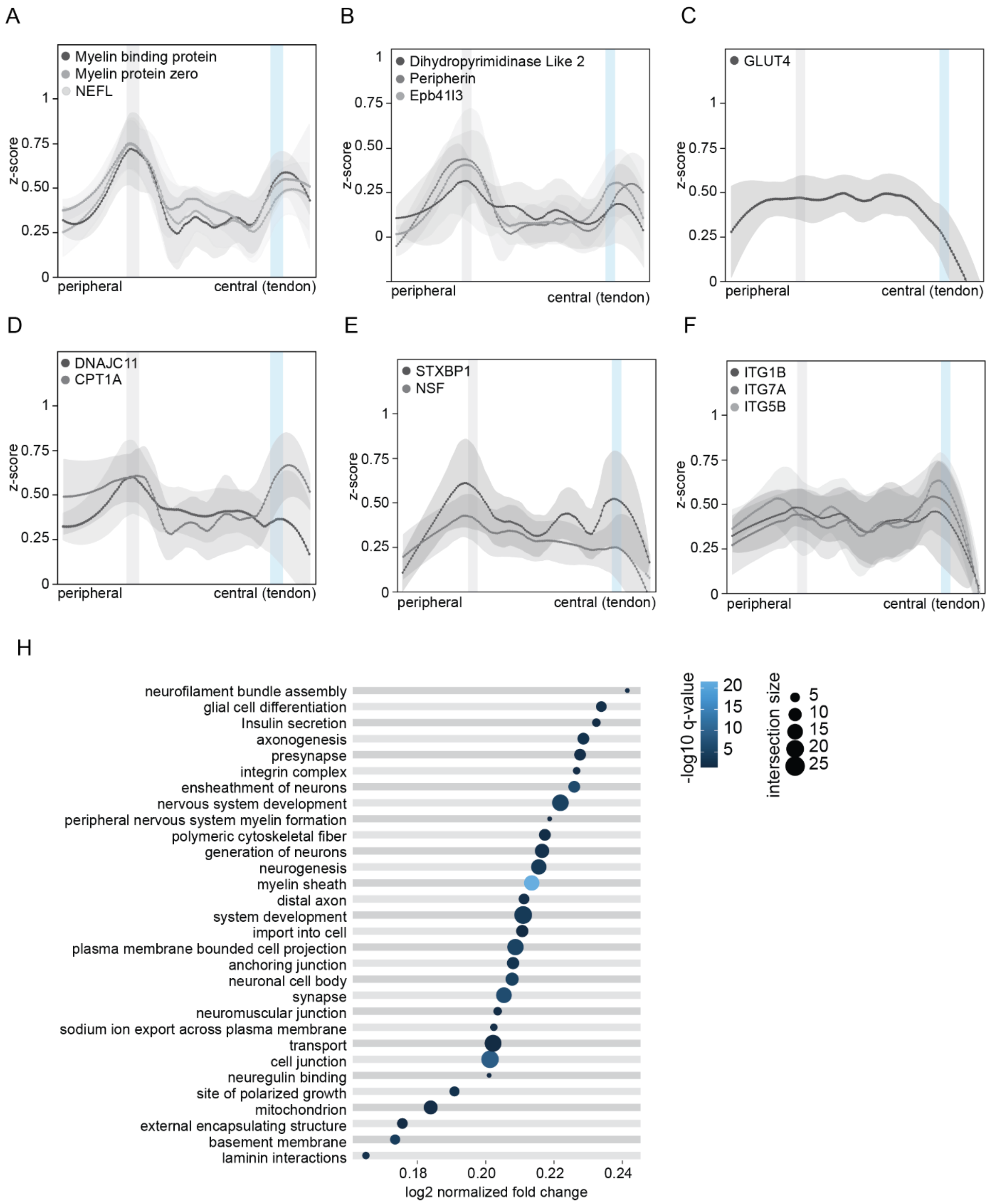

Supplementary Figure S4: Selected profiles with peak maxima at the NMJ and MTJ of the mouse diaphragm. (**A-F**) *Z*-score normalized protein intensity profiles along the longitudinal axis of the diaphragm for the neuronal marker proteins MBP, MPZ, NEFL, DPYSL2, PRPH, and EPB41L3 (**A, B**); muscle-specific marker GLUT4 (**C**); mitochondrial proteins CPT1A and, DNAJC11 (**D**); vesicle and membrane proteins STXBP1 and NSF (**E**); and integrins ITGB1, ITGA7, and ITGB5 (F). (**H**) Gene ontology enrichment of the 115 proteins that showed peak maxima at the NMJ.

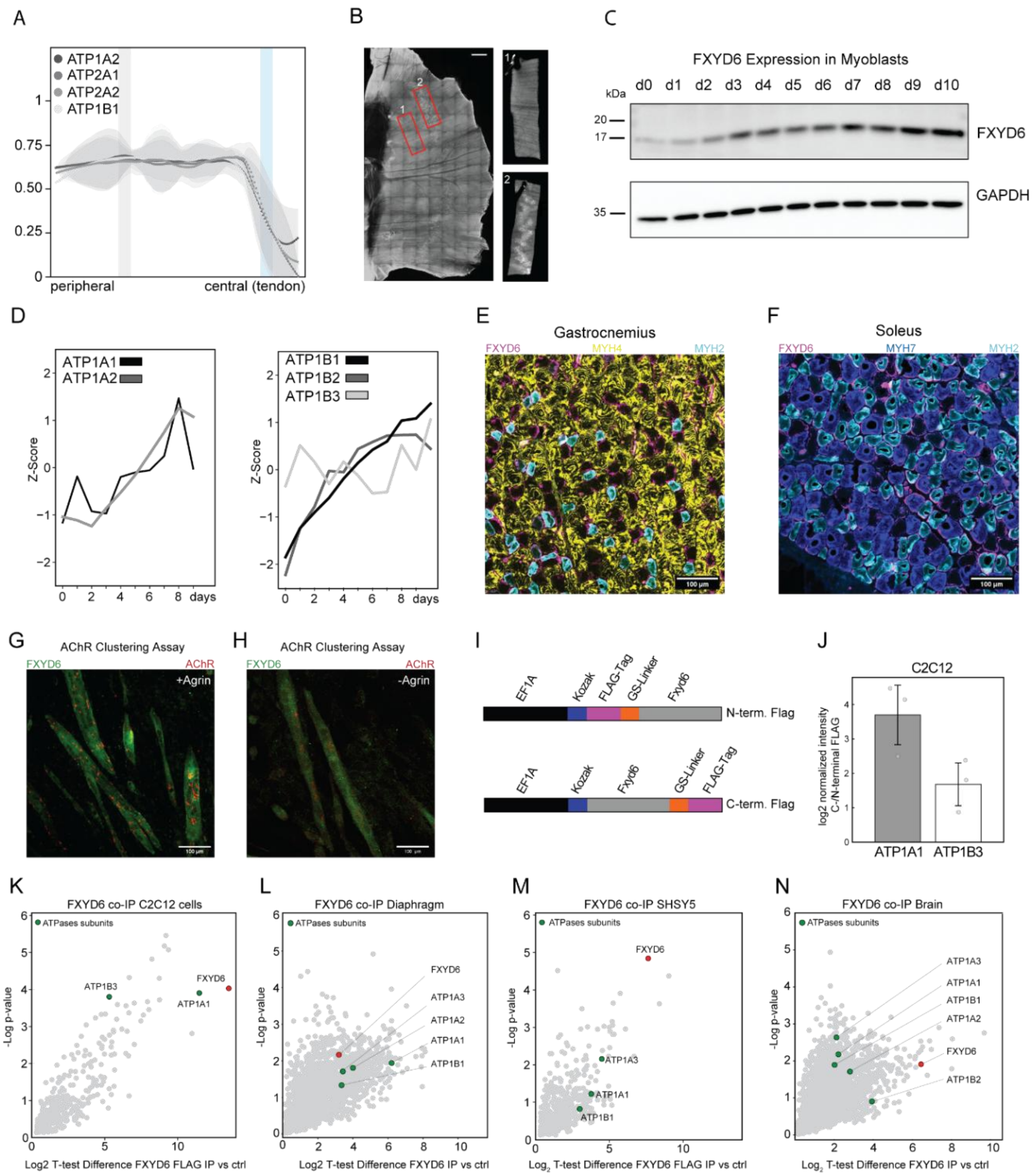

Supplementary Figure S5: ATPase protein profiling and expression in myoblasts and FXYD6 interactome studies. (A) *Z*-score normalized protein intensity profiles along the longitudinal axis of the diaphragm of sodium/potassium-transporting ATPases (ATP1A1, ATP1A1) and SERCA Ca^{2+}-ATPases (ATP2A1, ATP2AA2). (**B**) Immunofluorescent staining of the diaphragm, showing the non-innervated (muscle, top, 1) and innervated (BTX-594, bottom, 2) region of the diaphragm. (**C**) Western blot of FXYD6 expression in differentiating myoblasts from Day 0 to Day 10. (**D**) *Z*-score normalized protein intensity of ATP1a1, ATP1a2, ATP1b1, ATP1b2, and ATP1b3 at different time points (in days) in differentiating primary myoblasts. (**E-F)** Immunohistochemical staining of

FXYD6, MYH2, MYH7, and MYH4 in gastrocnemius (**E**) and soleus (**F**). (**G-H**) AChR clustering assay performed on differentiated C2C12 myoblasts and co-stained for AChR (BTX) and FXYD6 (anti-FXYD6 antibody) with agrin (G) without agrin (**H**). (**I**) Illustration of the C-terminal and N-terminal FLAG-tagged FXYD6 constructs. (**J**) Log_2 normalized intensity ratio of ATP1A1 and ATP1B3 in C2C12 cells overexpressing FXYD6 (C-/N terminal FLAG) from LC-MS analysis. (**K-N**) Volcano plots depicting the proteins that co-immunoprecipitated with an anti-FLAG antibody in FLAG-tagged FXYD6 transfected C2C12 (K) and SHSY5 cells (**M**) and anti-FXYD6 antibody in diaphragm (**L**) and brain tissue lysates (**N**); ATPases are indicated.

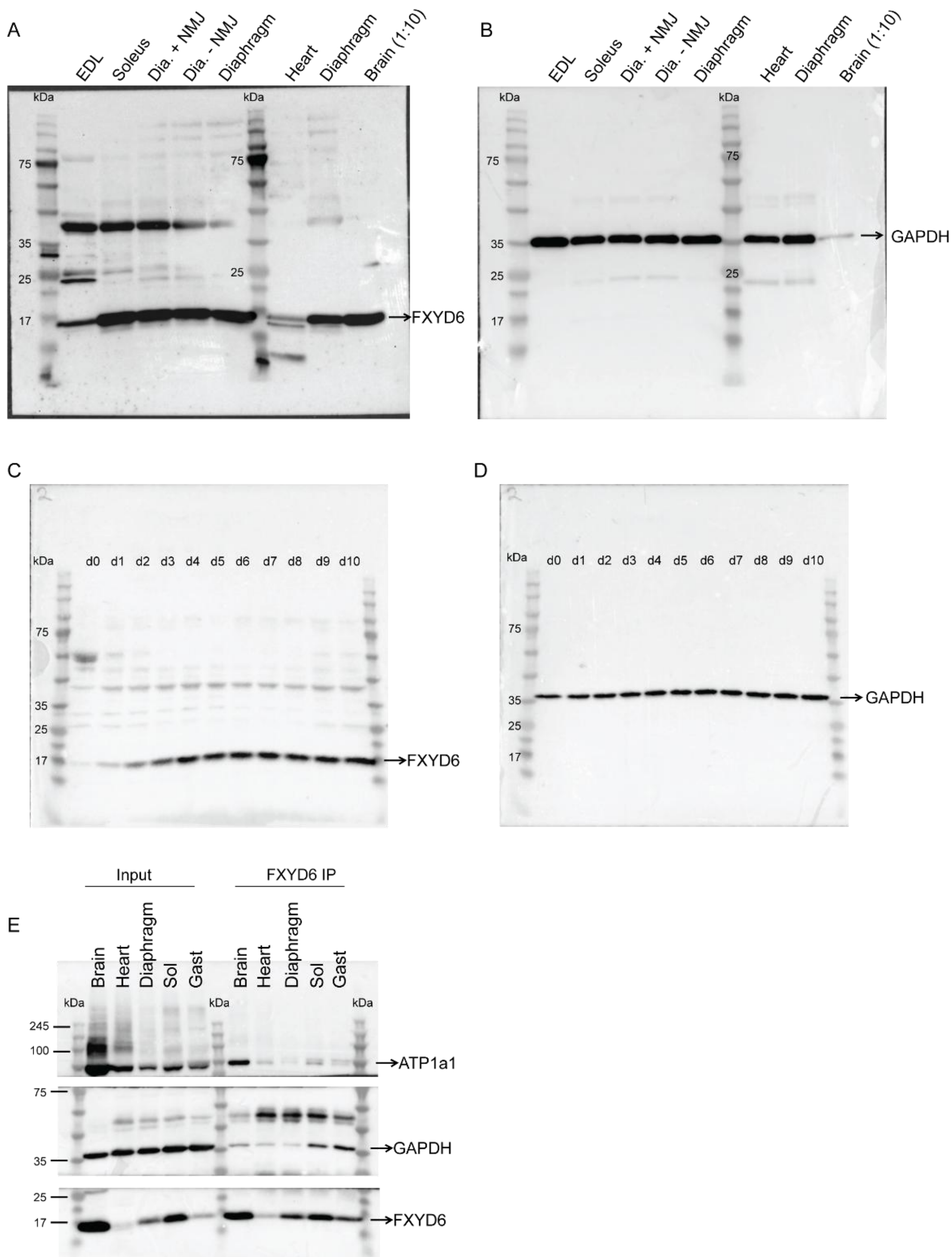

Supplementary Figure S6: Immunoblot analysis of FXYD6. (**A-B**) Immunoblotting of FXYD6 (**A**) and GAPDH (**B**) in various mouse skeletal muscles, heart, and brain. (**C-D**) Western blot of FXYD6 (**C**) and GAPDH (**D**) in differentiating myoblasts from Day 0 to Day 10. (**E)** Immunoprecipitation with an anti-FXYD6 antibody and immunoblotting of brain, heart, and various skeletal muscle tissues using anti-FXYD6 and anti-ATP1A1 antibodies.

Supplementary Data 1: *Z*-score normalized protein profiles of 2490 protein groups along the normalized longitudinal axis of the diaphragm.

Supplementary Data 2: *Z*-score normalized intensity of 2500 protein profiles along the normalized longitudinal axis of the diaphragm, depicting the muscle, pre/post-NMJ, NMJ, MTJ, and tendon region.

Supplementary Data 3: Protein group quantity tables of the raw intensity values for medial diaphragm of 1–8 mm width and 10–20 µm-thick transverse sections measured by short LC-MS gradients of 11 min (100 SPD) and 21 min (60 SPD).

Supplementary Data 4: Summary of protein profiles with a peak maximum at the NMJ, MTJ, and both the NMJ and MTJ.

Supplementary Data 5: Protein expression of FXYD6 in skeletal muscle fibers, phrenic nerve, and brain, measured with a gas-phase fractionation LC-MS method.

Supplementary Data 6: LC-MS data of the interactome of FXYD6 in different cell lines and tissues.

Supplementary Data 7: Overlap between snRNA-seq data and NMJ candidate proteins. Source data for figures 4 E, F, G, I, J,
