# Supplementary material for: Spatial proteomics identifies FXYD6 as a dual-site protein of neuromuscular junction in the diaphragm
Source: Commun Biol. 2026 Sep 18;9:1220. doi: 10.1038/s42003-026-10884-8 (PMC13588806; doi:10.1038/s42003-026-10884-8)
Supplement: Supplementary file 3 [file 42003_2026_10884_MOESM3_ESM.pdf]

Description of Additional Supplementary File

File name: Supplementary data 1
Description: Z-score normalized protein profiles of 2490 protein groups along the normalized longitudinal axis of the diaphragm.

File name: Supplementary data 2
Description: Z-score normalized intensity of 2500 protein profiles along the normalized longitudinal axis of the diaphragm, depicting the muscle, pre/post-NMJ, NMJ, MTJ, and tendon region.

File name: Supplementary data 3
Description: Protein group quantity tables of the raw intensity values for medial diaphragm of 1–8 mm width and 10–20 µm-thick transverse sections measured by short LC-MS gradients of 11 min (100 SPD) and 21 min (60 SPD).

File name: Supplementary data 4
Description: Summary of protein profiles with a peak maximum at the NMJ, MTJ, and both the NMJ and MTJ.

File name: Supplementary data 5
Description: Protein expression of FXYD6 in skeletal muscle fibers, phrenic nerve, and brain, measured with a gas-phase fractionation LC-MS method.

File name: Supplementary data 6
Description: LC-MS data of the interactome of FXYD6 in different cell lines and tissues.

File name: Supplementary data 7
Description: Overlap between snRNA-seq data and NMJ candidate proteins. Source data for figures 4 E, F, G, I, J,
