# Supplementary material for: Spatial proteomics identifies FXYD6 as a dual-site protein of neuromuscular junction in the diaphragm
Source: Commun Biol. 2026 Sep 18;9:1220. doi: 10.1038/s42003-026-10884-8 (PMC13588806; doi:10.1038/s42003-026-10884-8)
Supplement: Supplementary file 5 [file 42003_2026_10884_MOESM4_ESM.zip › Supplementary Data 1.pdf]

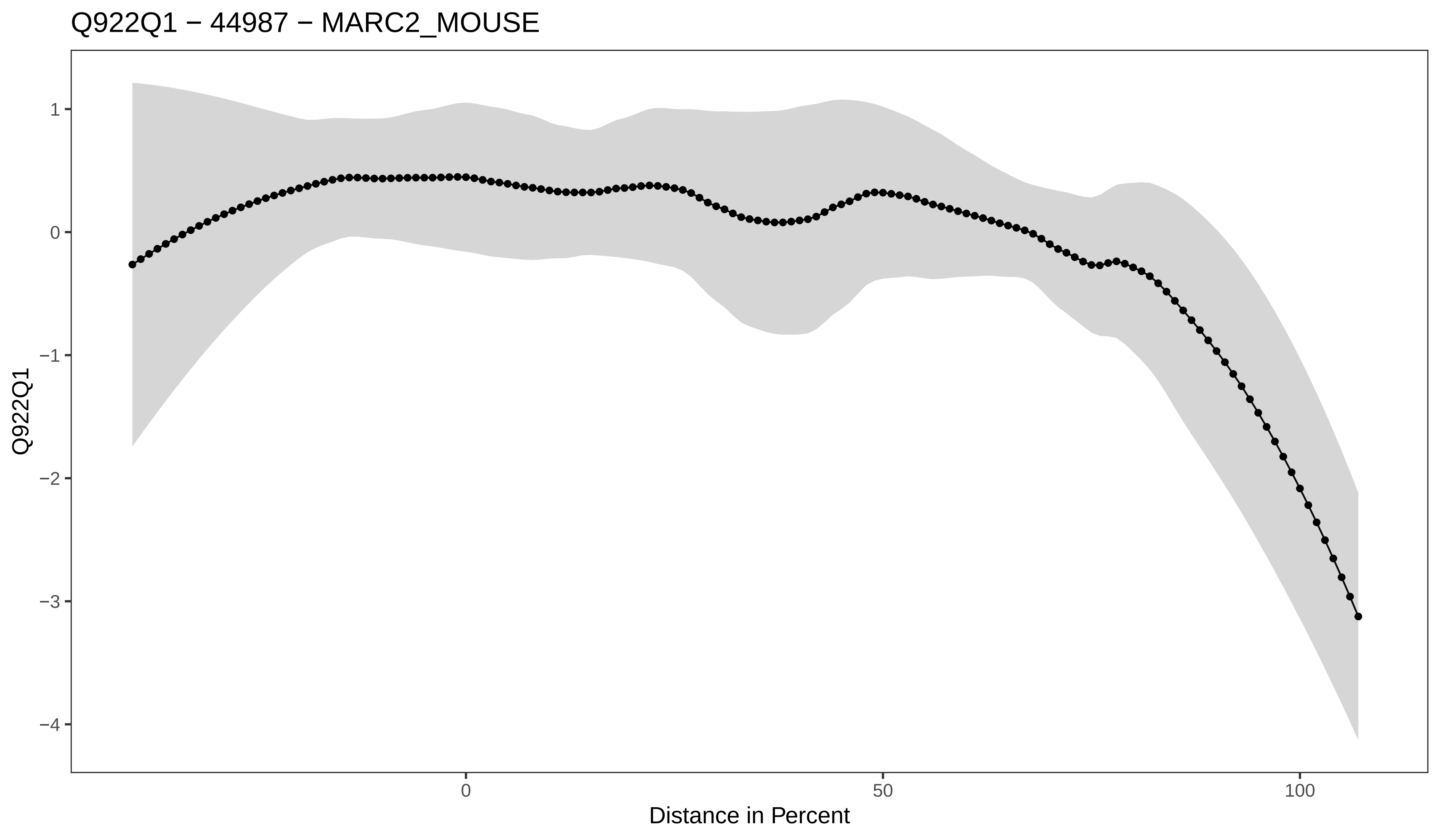

Q922Q1 − 44987 − MARC2_MOUSE
1
0
−1
−2
−3
−4
Q922Q1
0
50
100
Distance in Percent

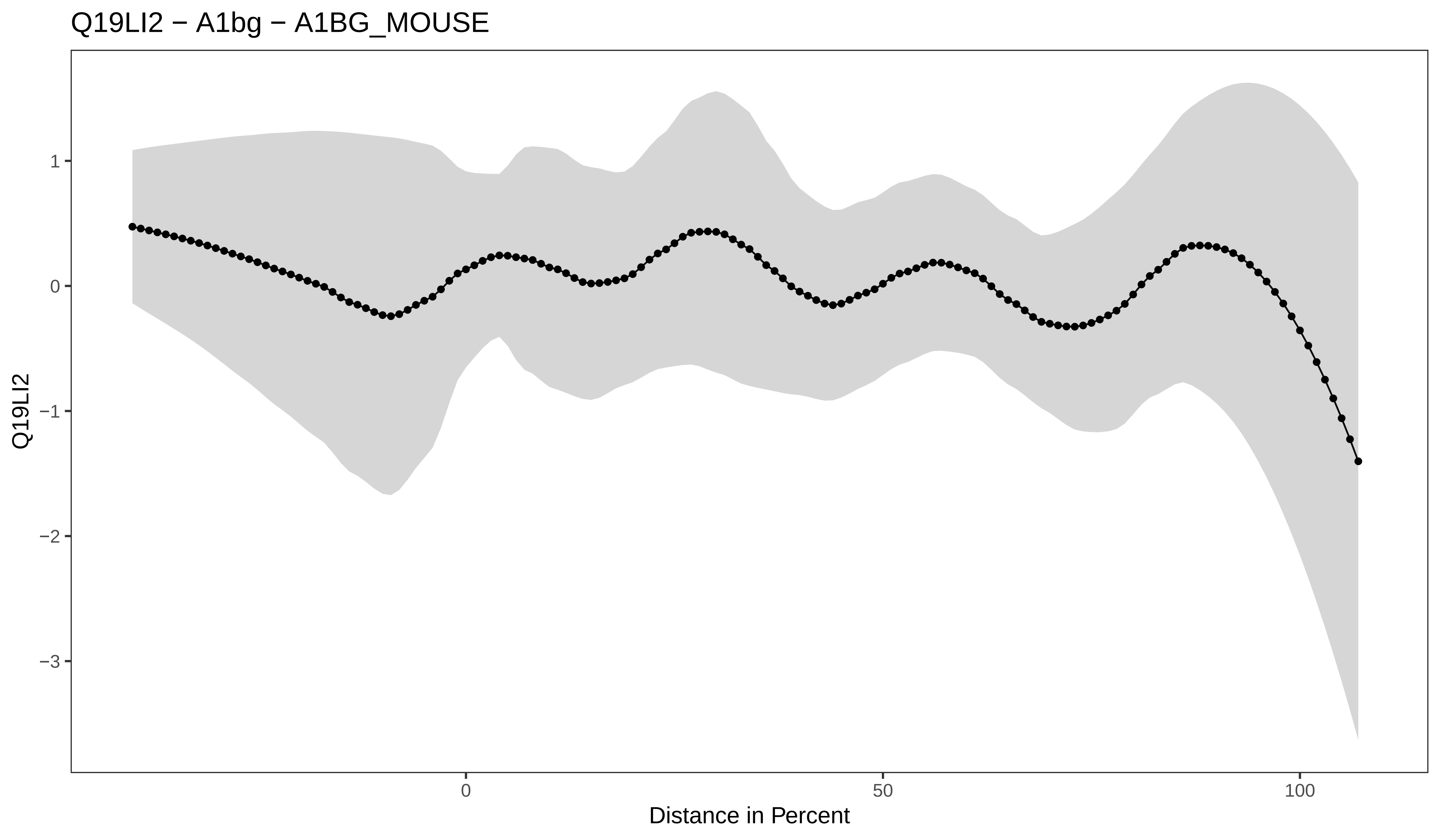

Q19LI2 − A1bg − A1BG_MOUSE
1
0
−1
−2
−3
Q19LI2
0
50
100
Distance in Percent

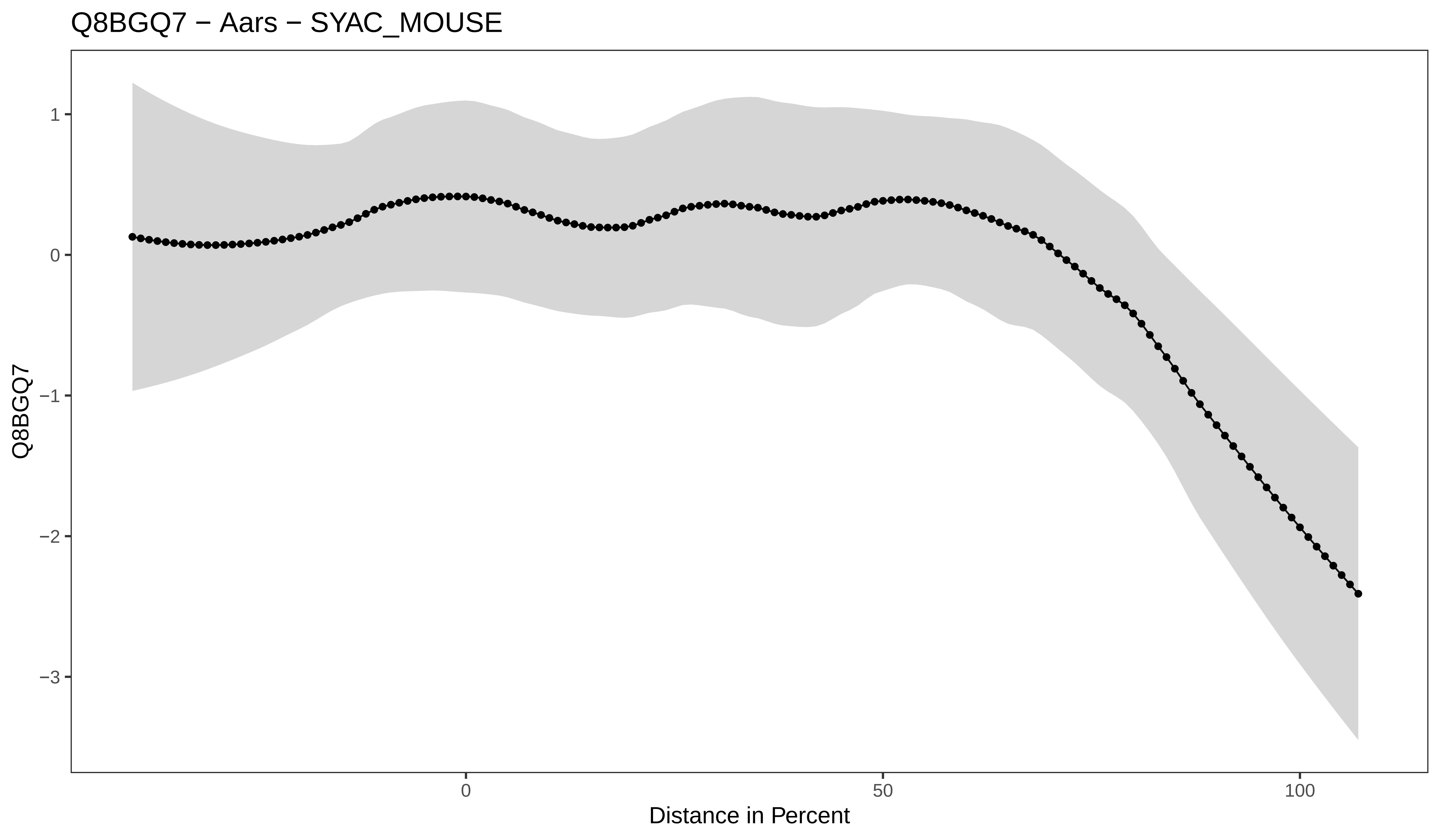
Q8BGQ7 − Aars − SYAC_MOUSE
Q8BGQ7
1
0
−1
−2
−3
0
50
100
Distance in Percent

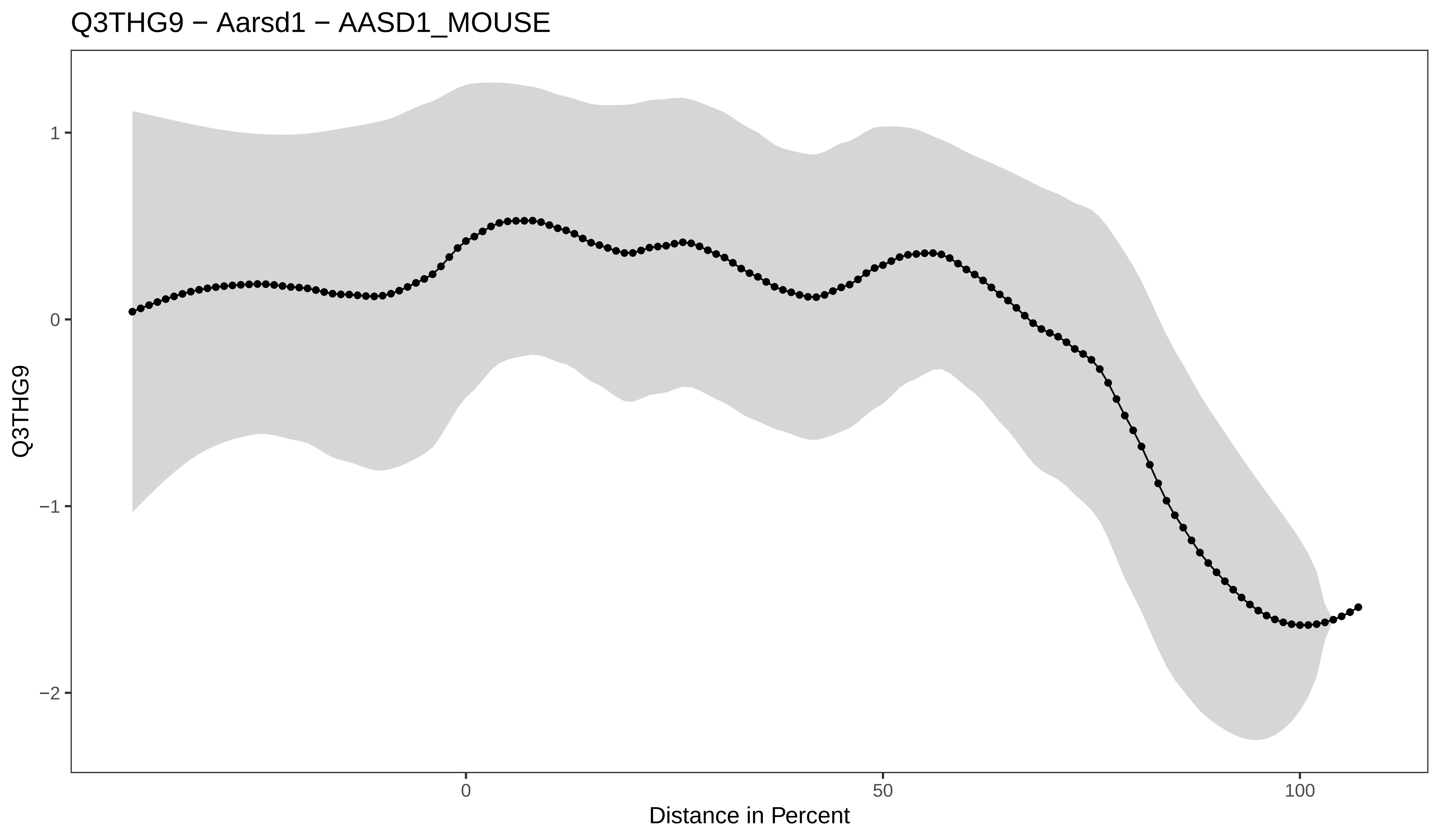

Q3THG9 − Aarsd1 − AASD1_MOUSE
Q3THG9
1
0
−1
−2
0
50
100
Distance in Percent

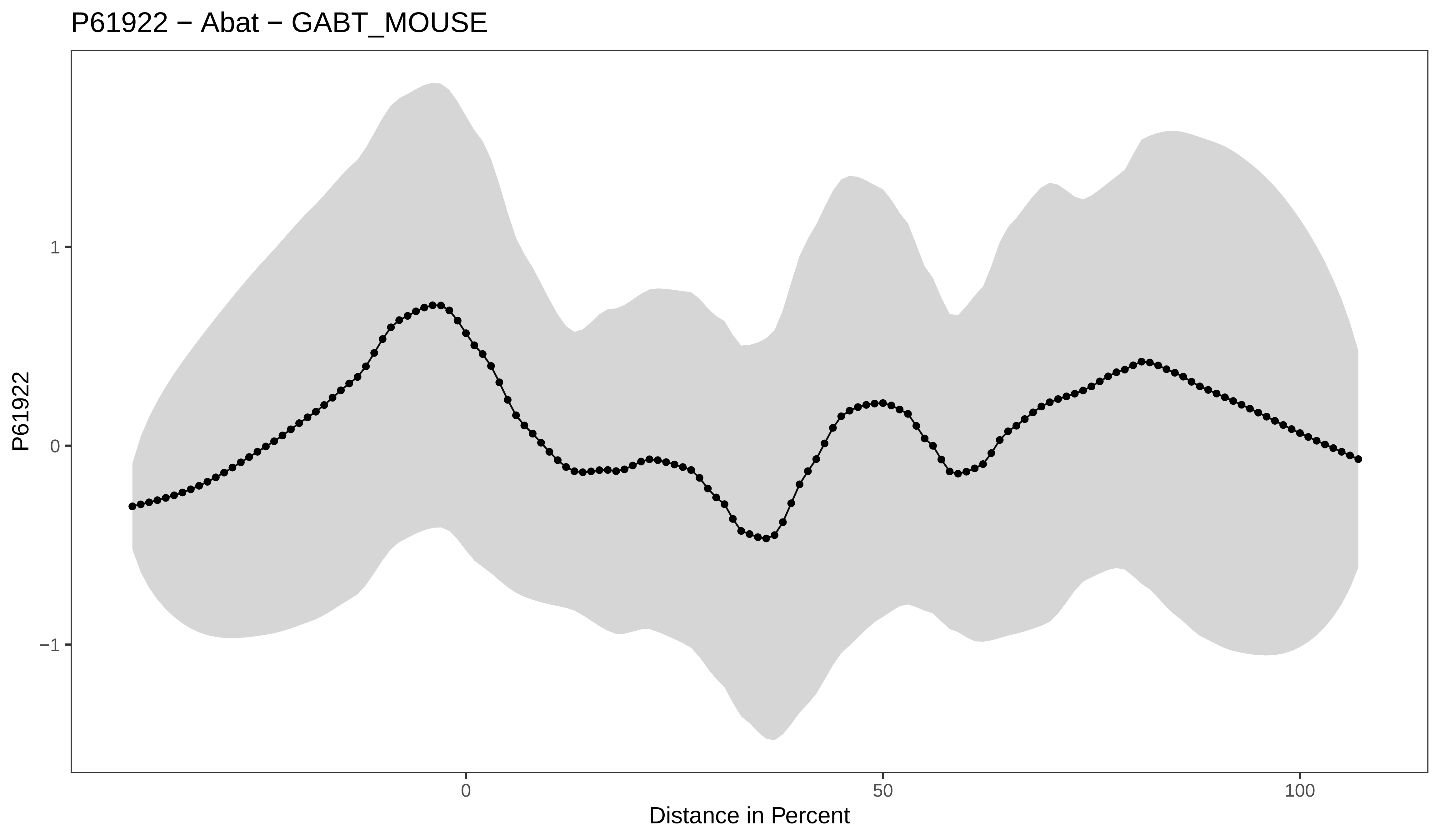

P61922 − Abat − GABT_MOUSE
P61922
1
0
−1
0
50
100
Distance in Percent

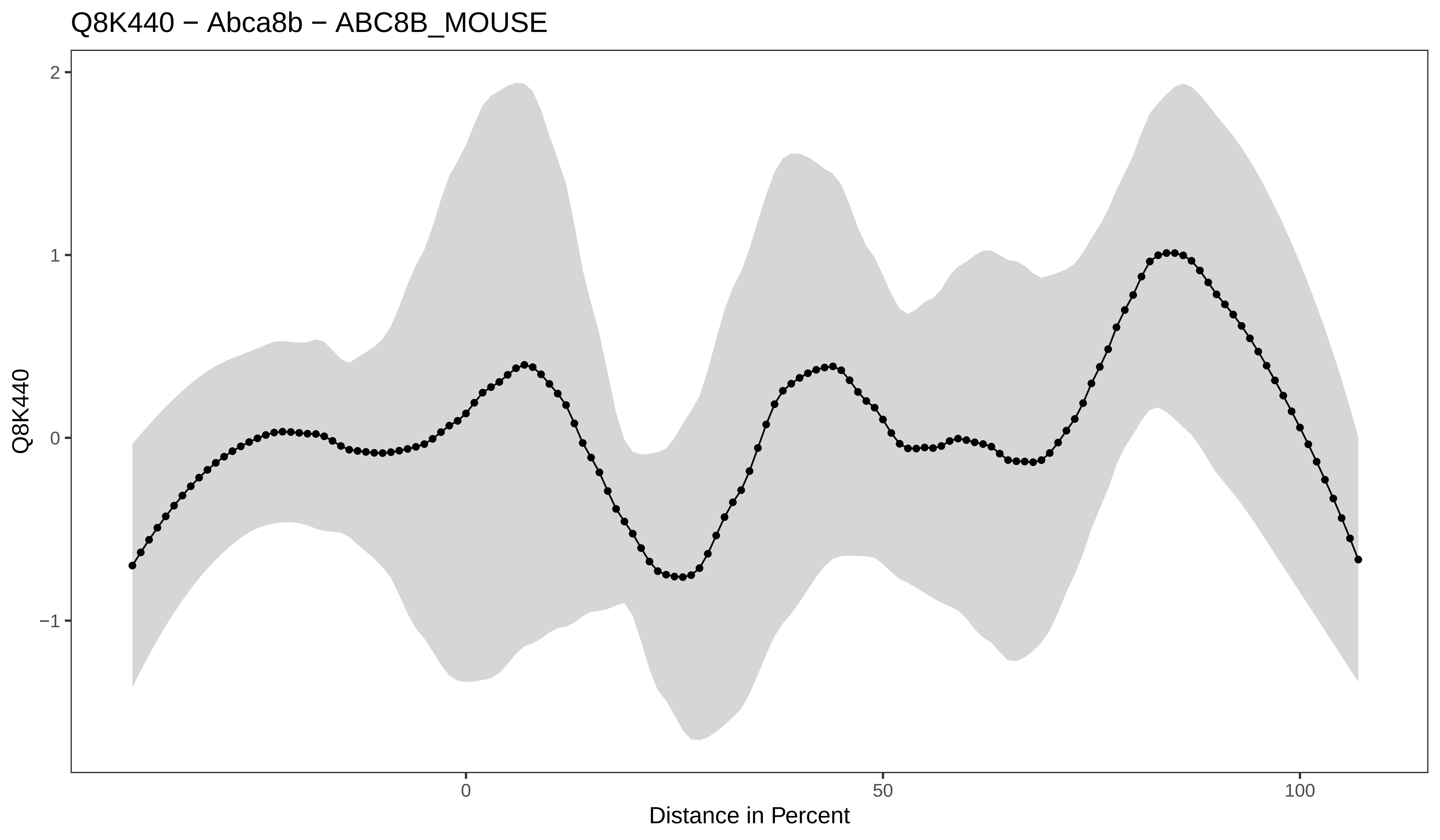
Q8K440 − Abca8b − ABC8B_MOUSE
Q8K440
2
1
0
−1
0
50
100
Distance in Percent

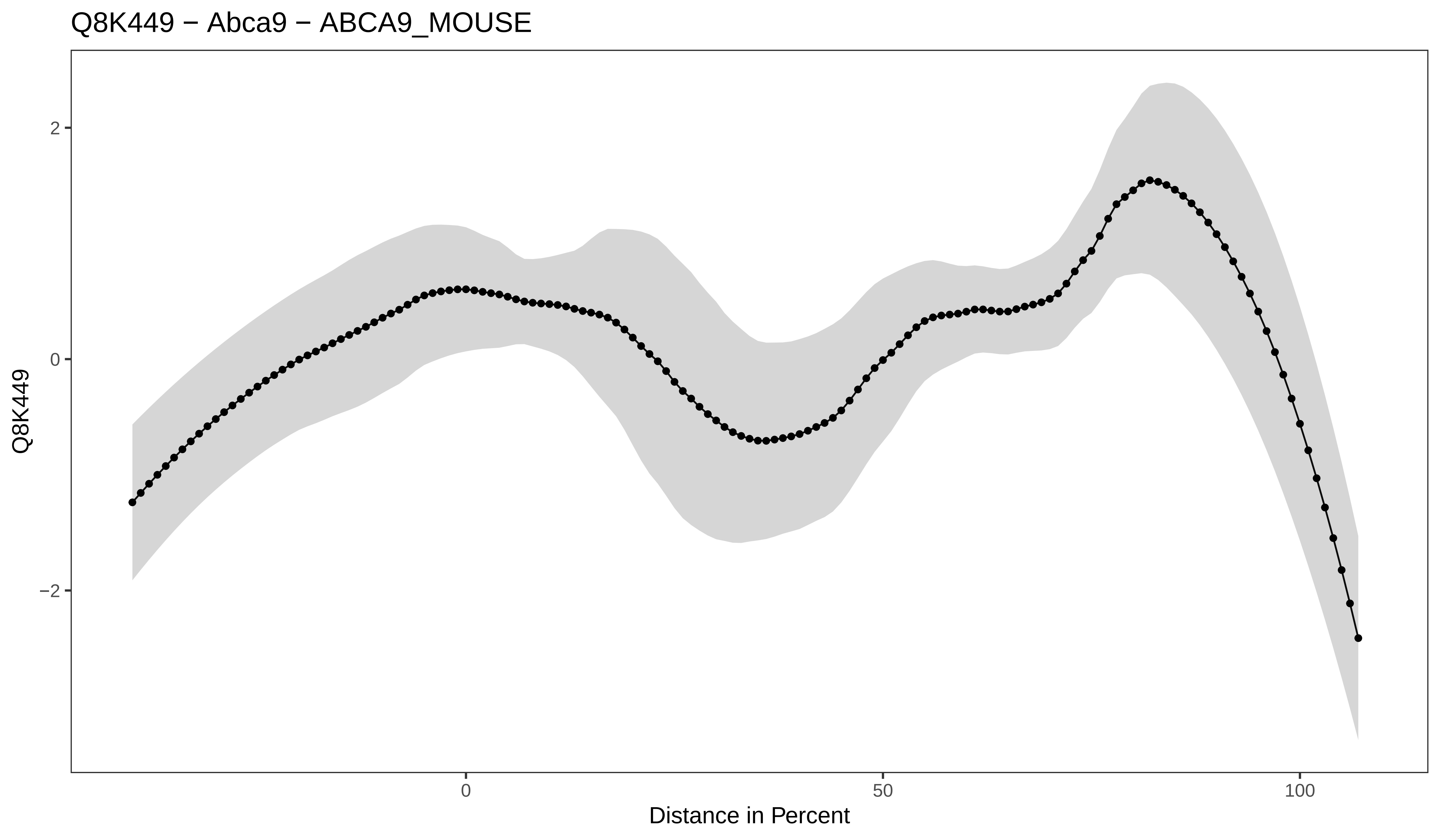

Q8K449 − Abca9 − ABCA9_MOUSE
Q8K449
2
0
−2
0
50
100
Distance in Percent

Q9JI39 − Abcb10 − ABCBA_MOUSE

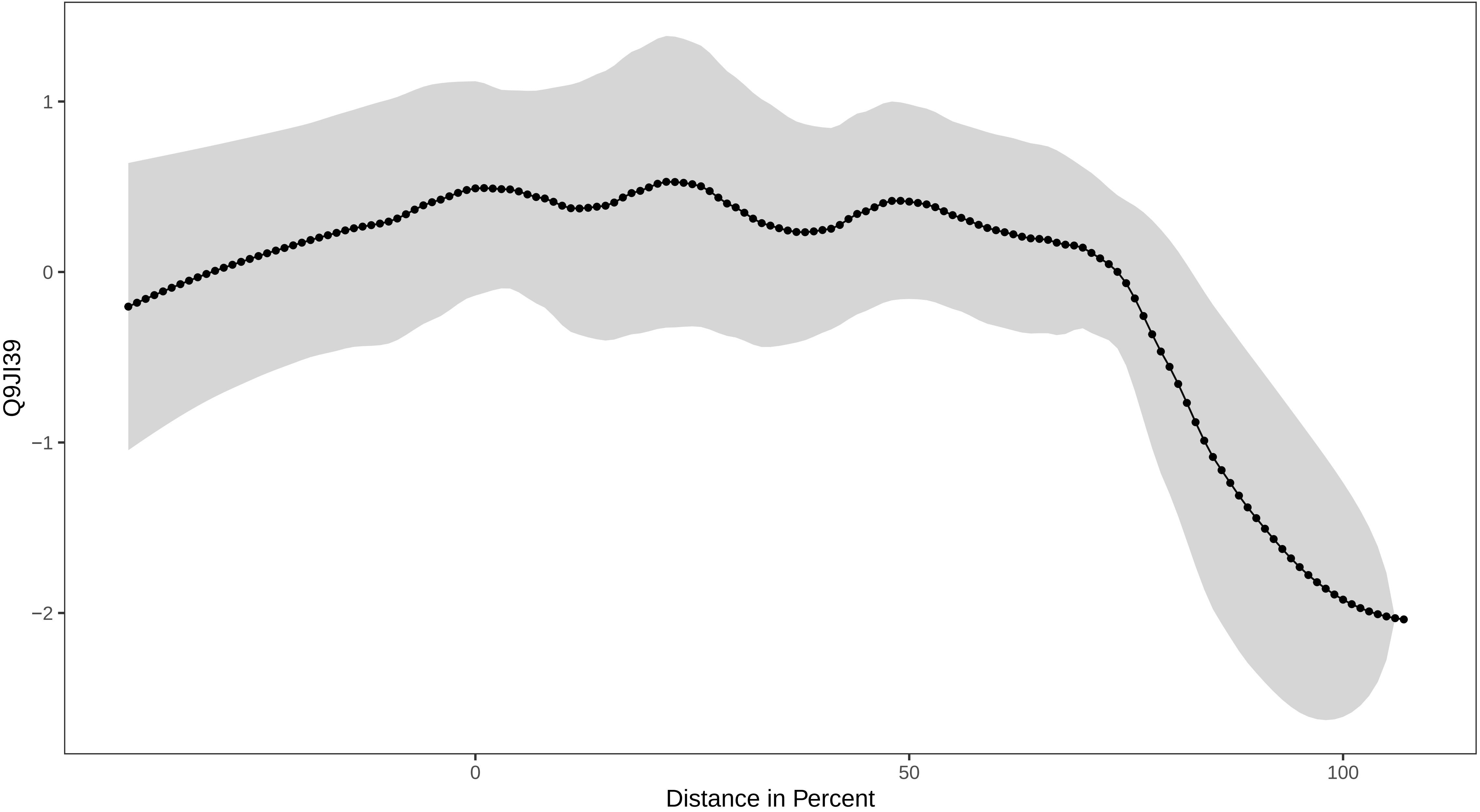

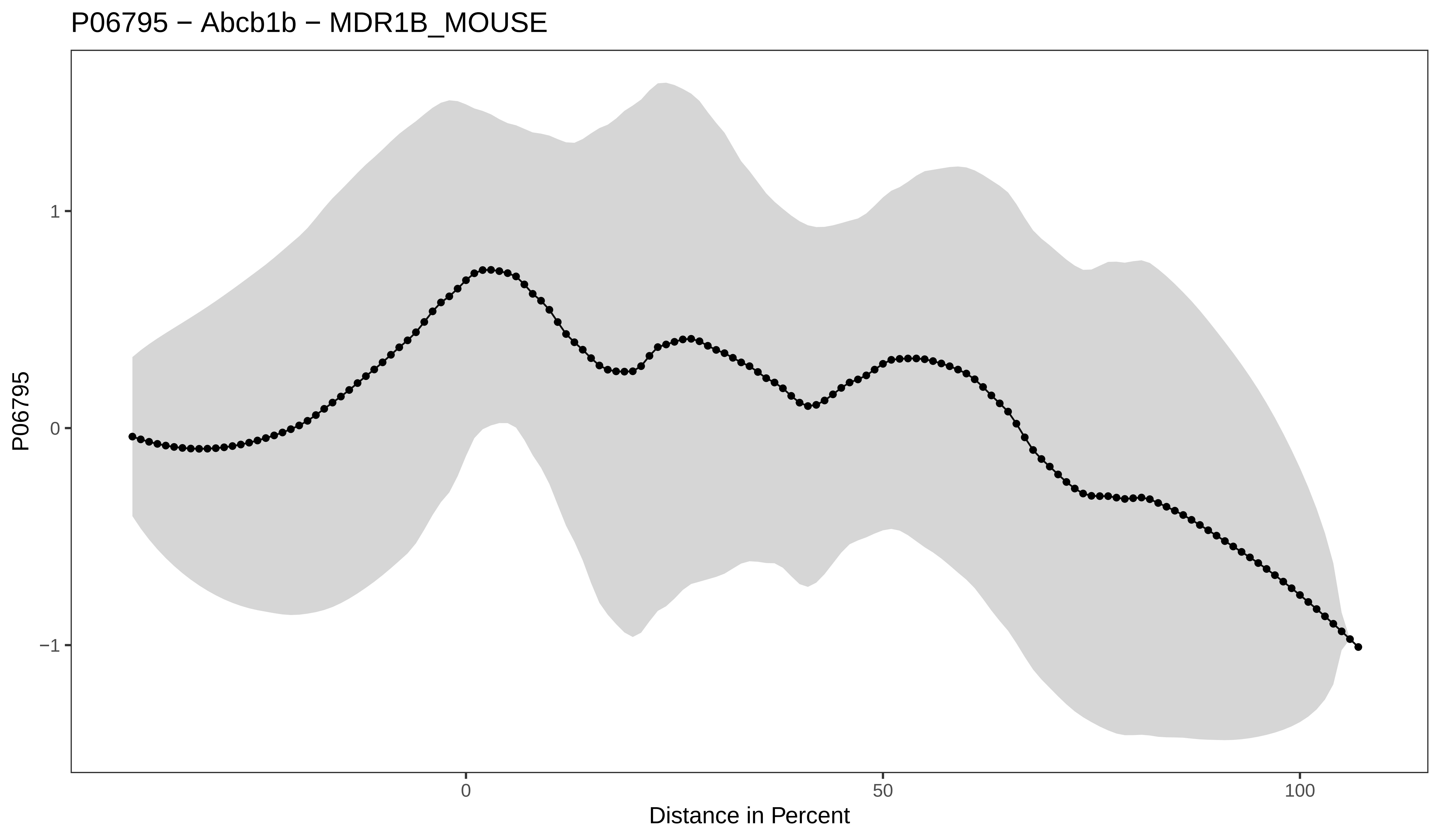

P06795 − Abcb1b − MDR1B_MOUSE
P06795
1
0
−1
0
50
100
Distance in Percent

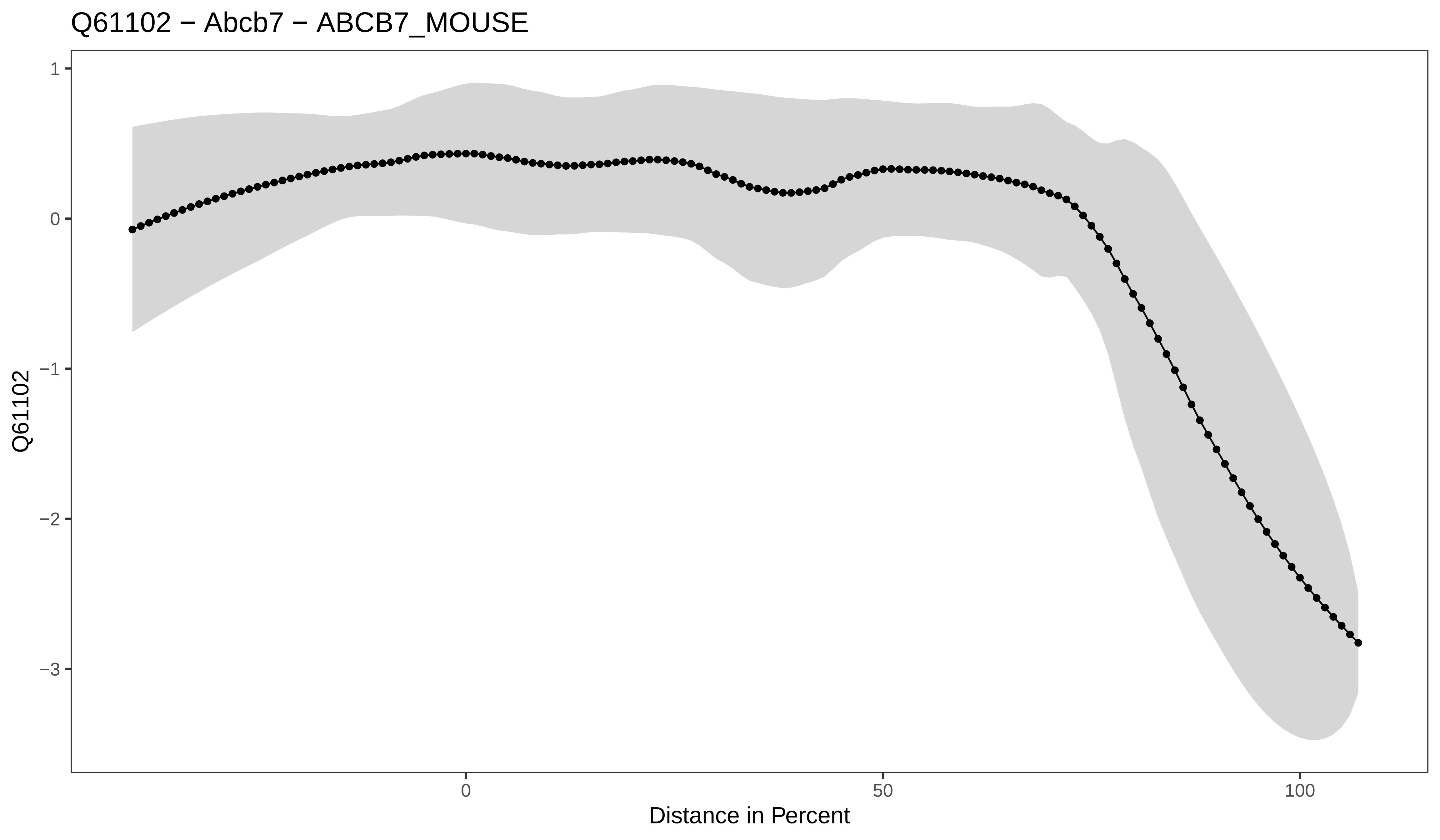

Q61102 − Abcb7 − ABCB7_MOUSE
1
0
−1
−2
−3
Q61102
0
50
100
Distance in Percent

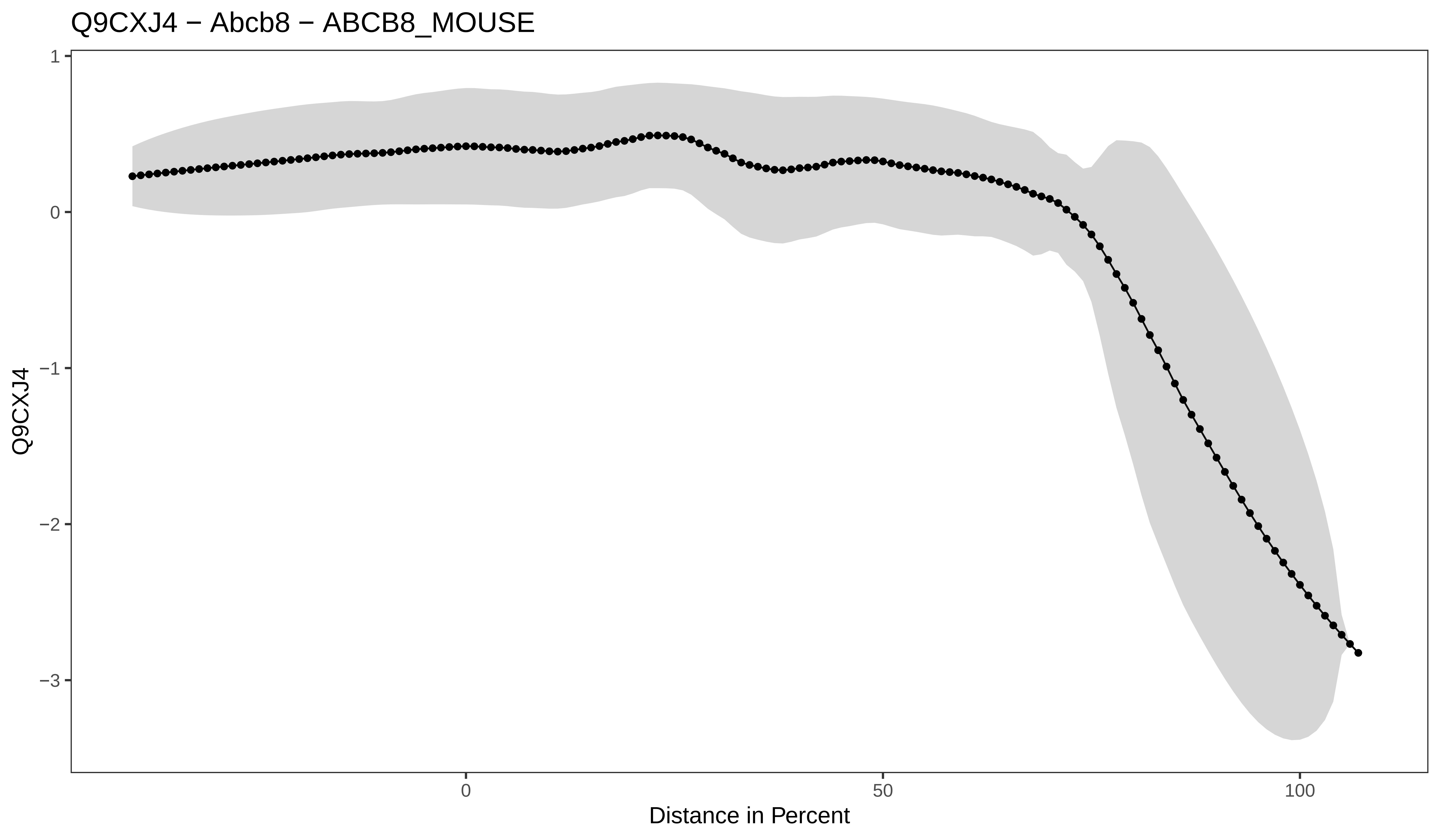
Q9CXJ4 − Abcb8 − ABCB8_MOUSE
1
0
−1
−2
−3
Q9CXJ4
0
50
100
Distance in Percent

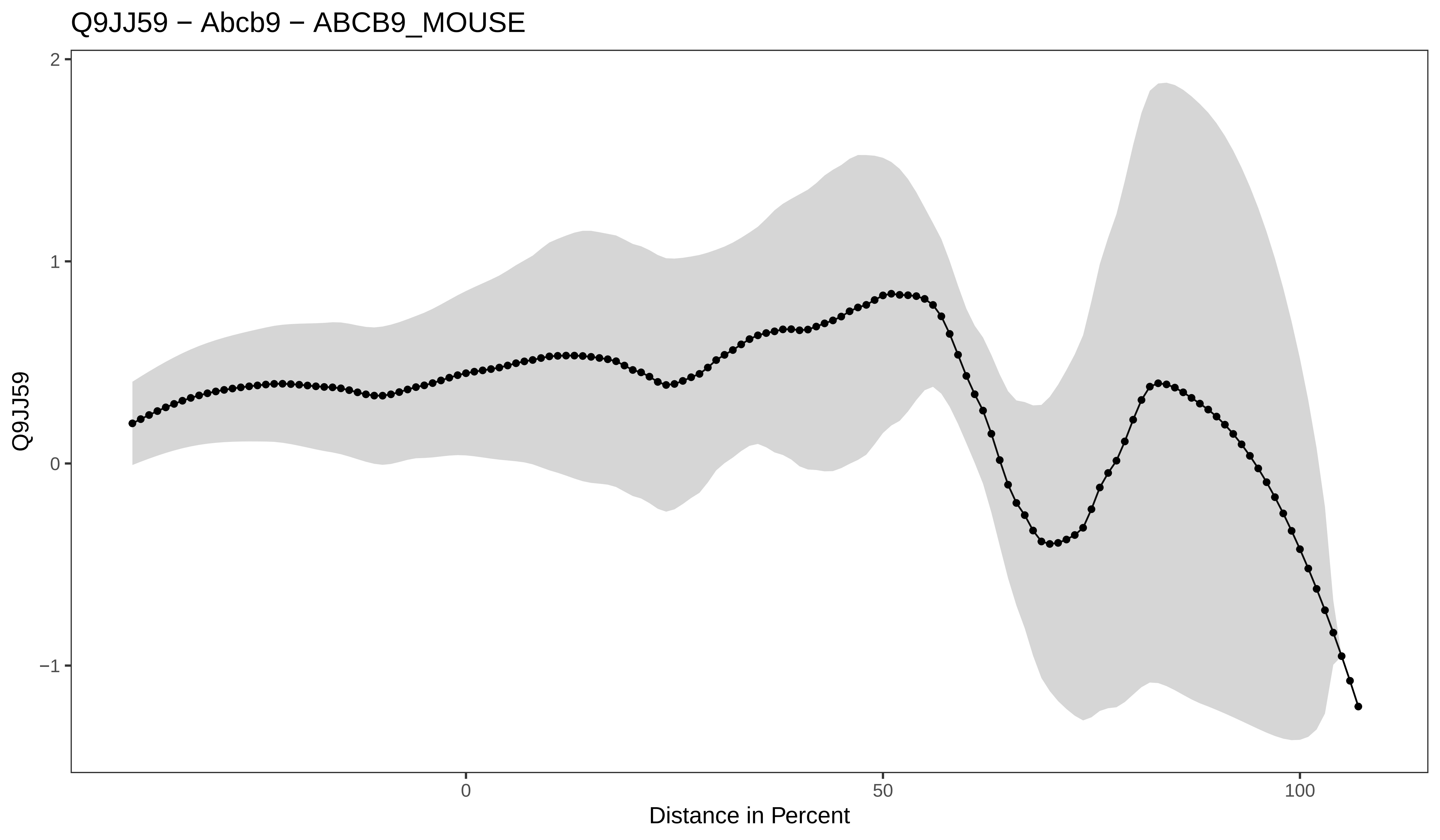

Q9JJ59 − Abcb9 − ABCB9_MOUSE
Q9JJ59
2
1
0
−1
0
50
100
Distance in Percent

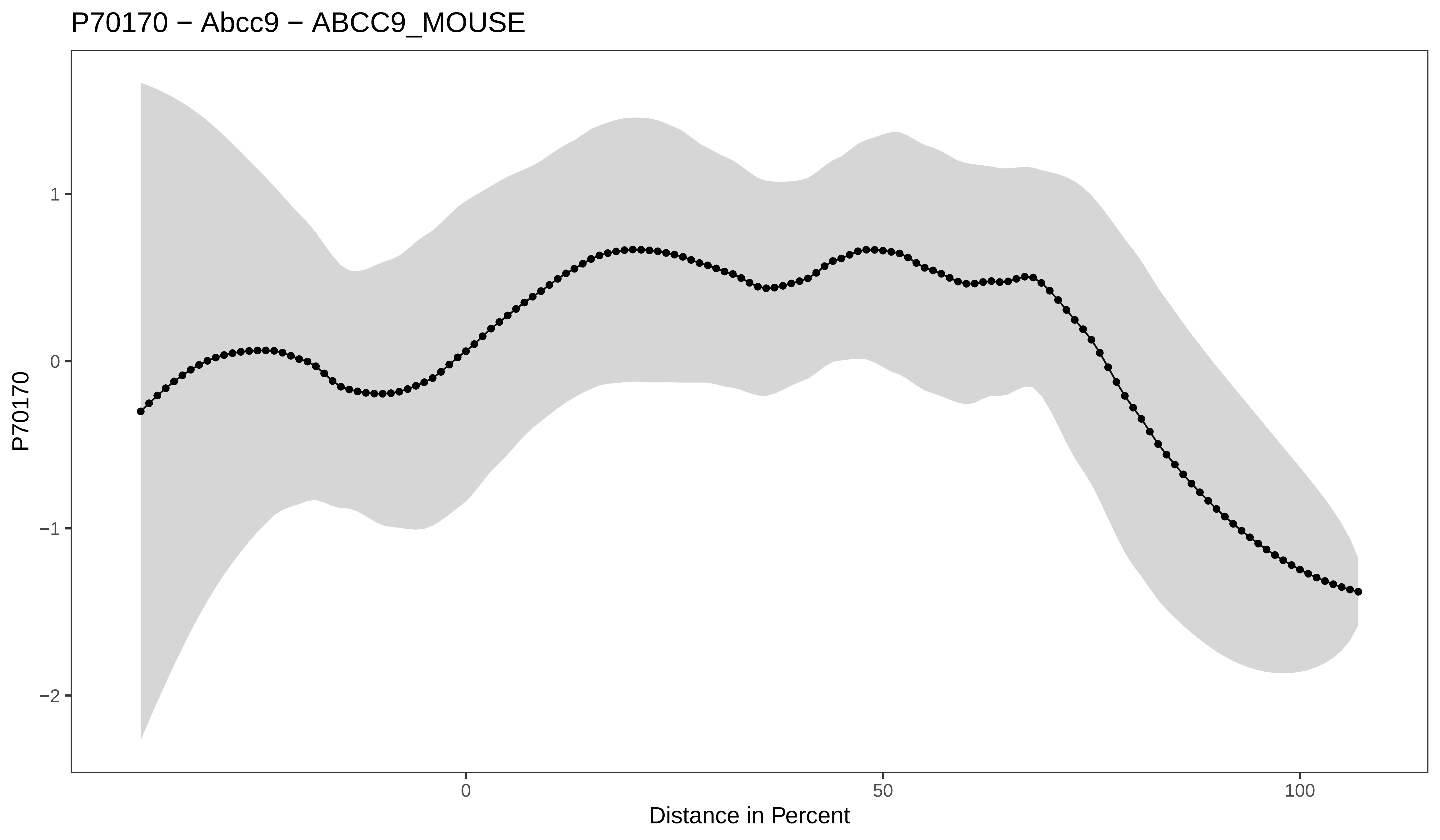
P70170 − Abcc9 − ABCC9_MOUSE
P70170
1
0
−1
−2
0
50
100
Distance in Percent

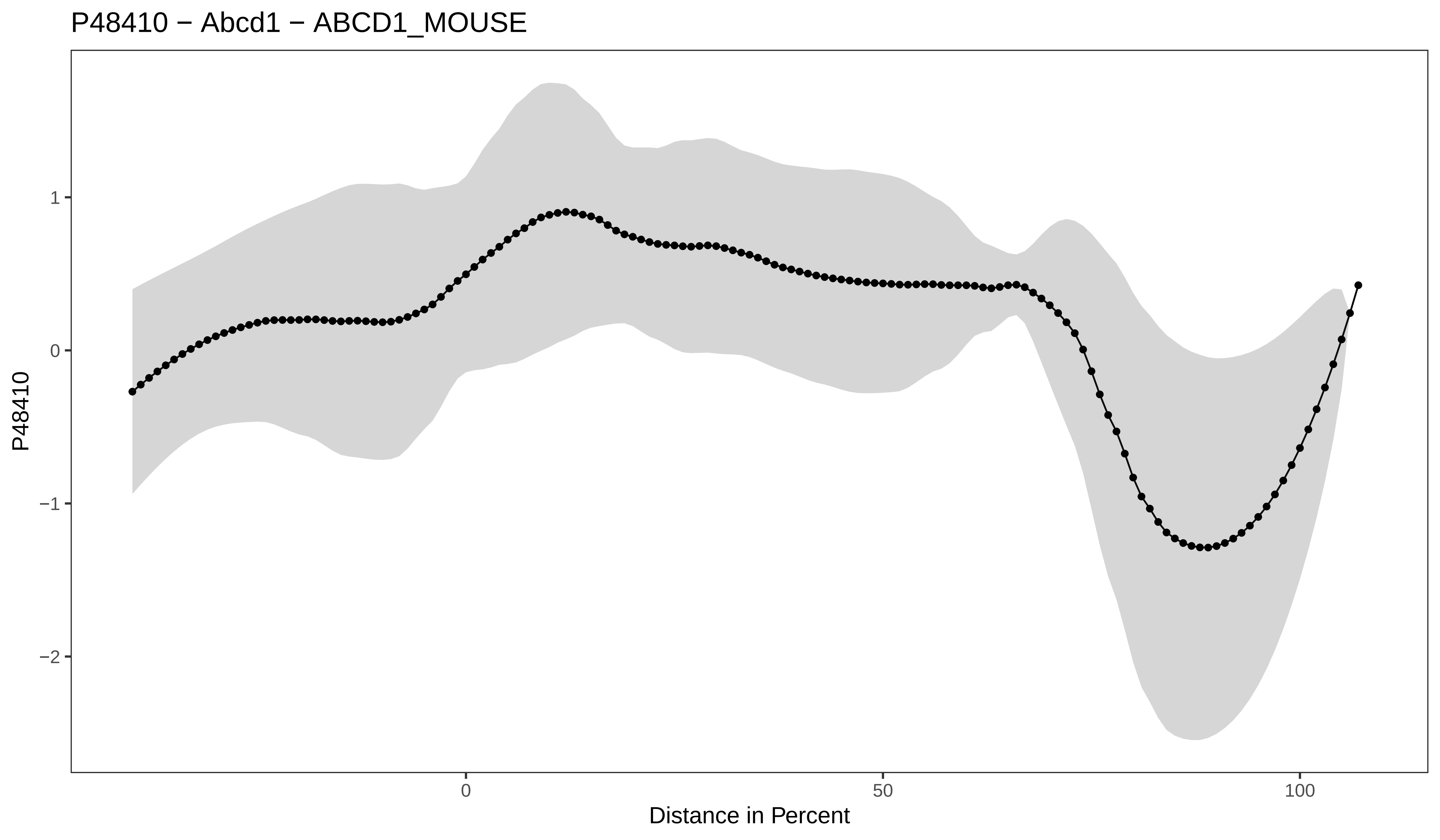
P48410 − Abcd1 − ABCD1_MOUSE
P48410
1
0
−1
−2
0
50
100
Distance in Percent

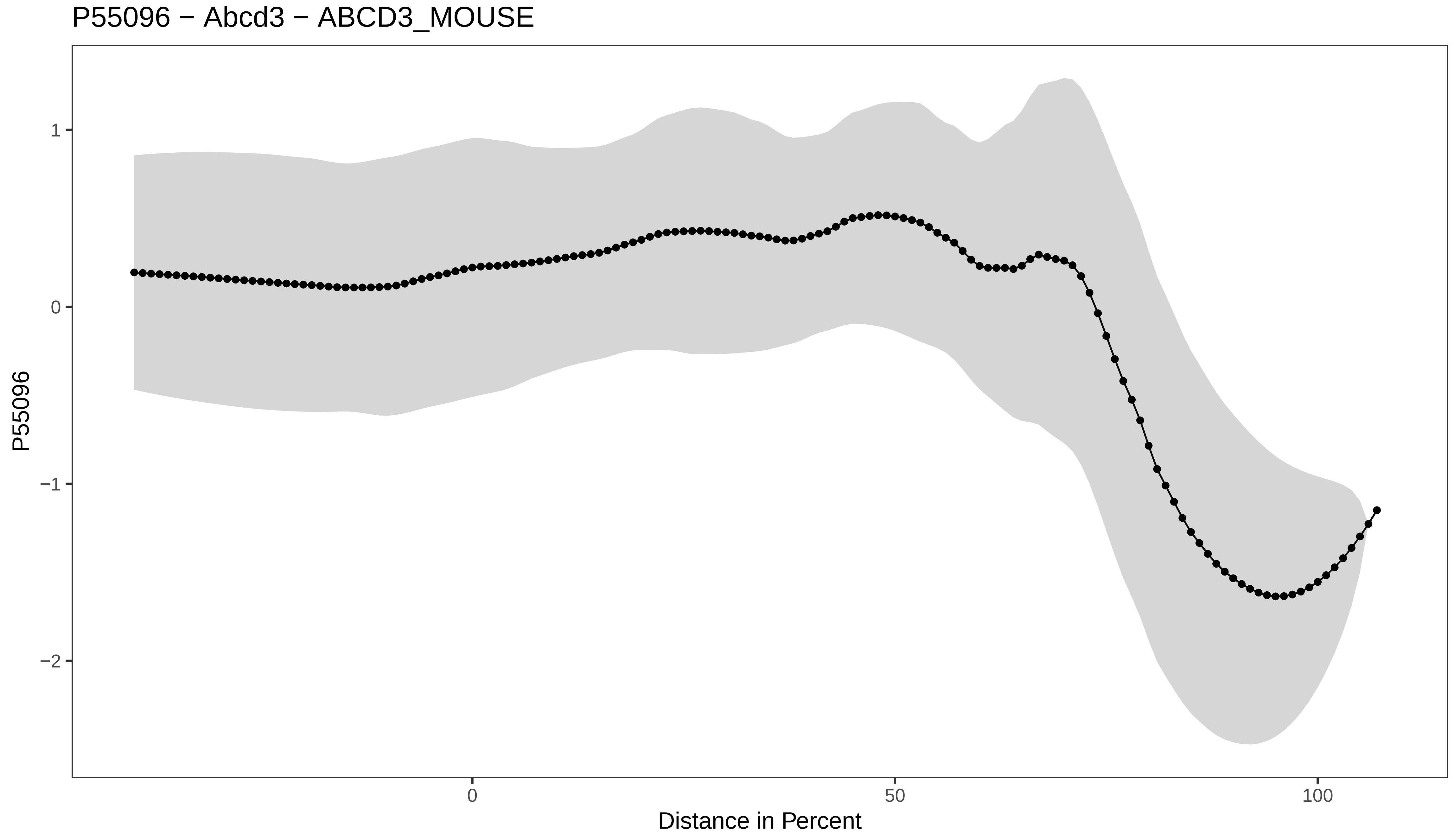
P55096 − Abcd3 − ABCD3_MOUSE
P55096
1
0
−1
−2
0
50
100
Distance in Percent

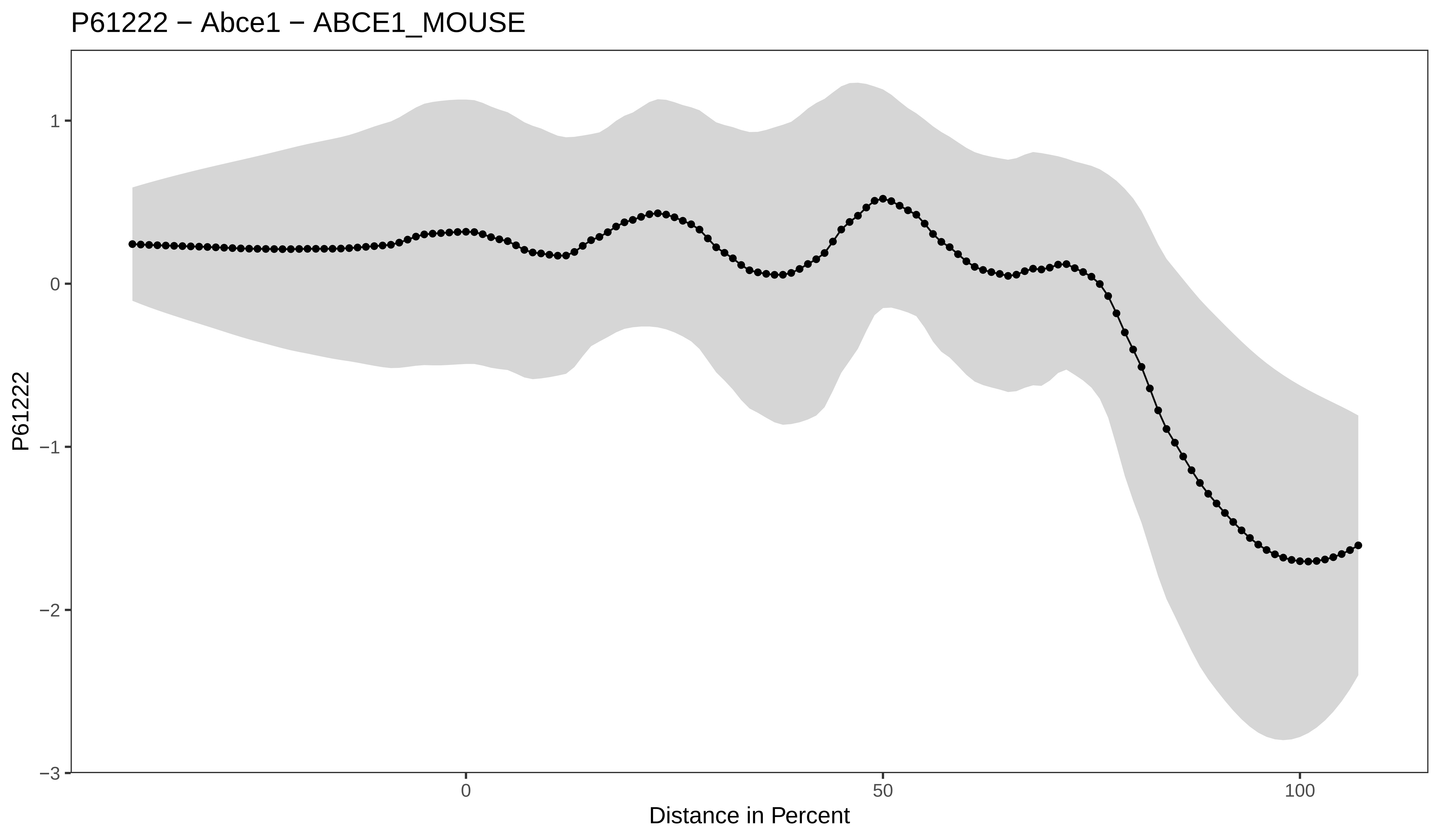
P61222 − Abce1 − ABCE1_MOUSE
P61222
Distance in Percent
1
0
−1
−2
−3
0
50
100

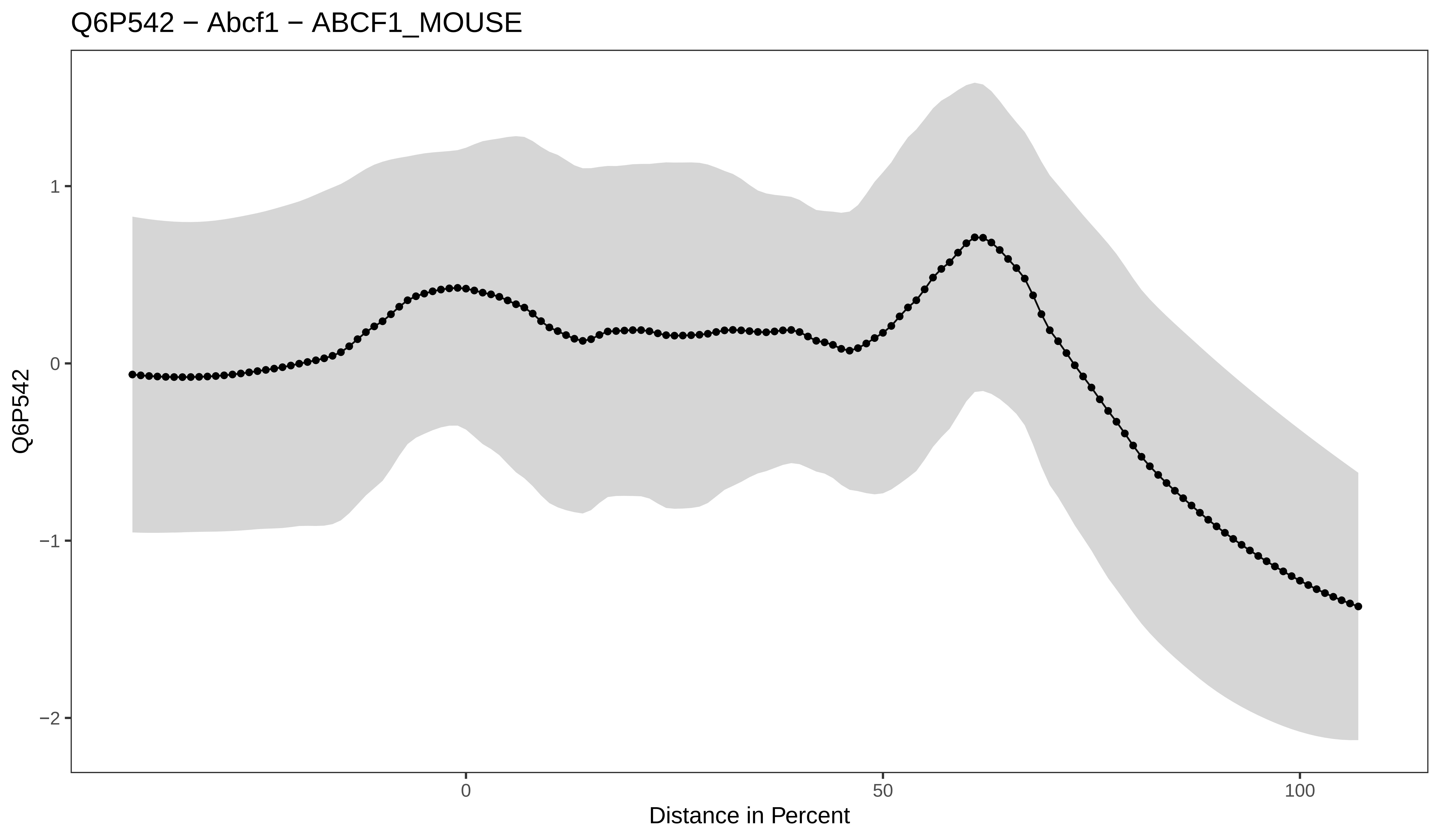
Q6P542 − Abcf1 − ABCF1_MOUSE
1
0
−1
−2
Q6P542
0
50
100
Distance in Percent

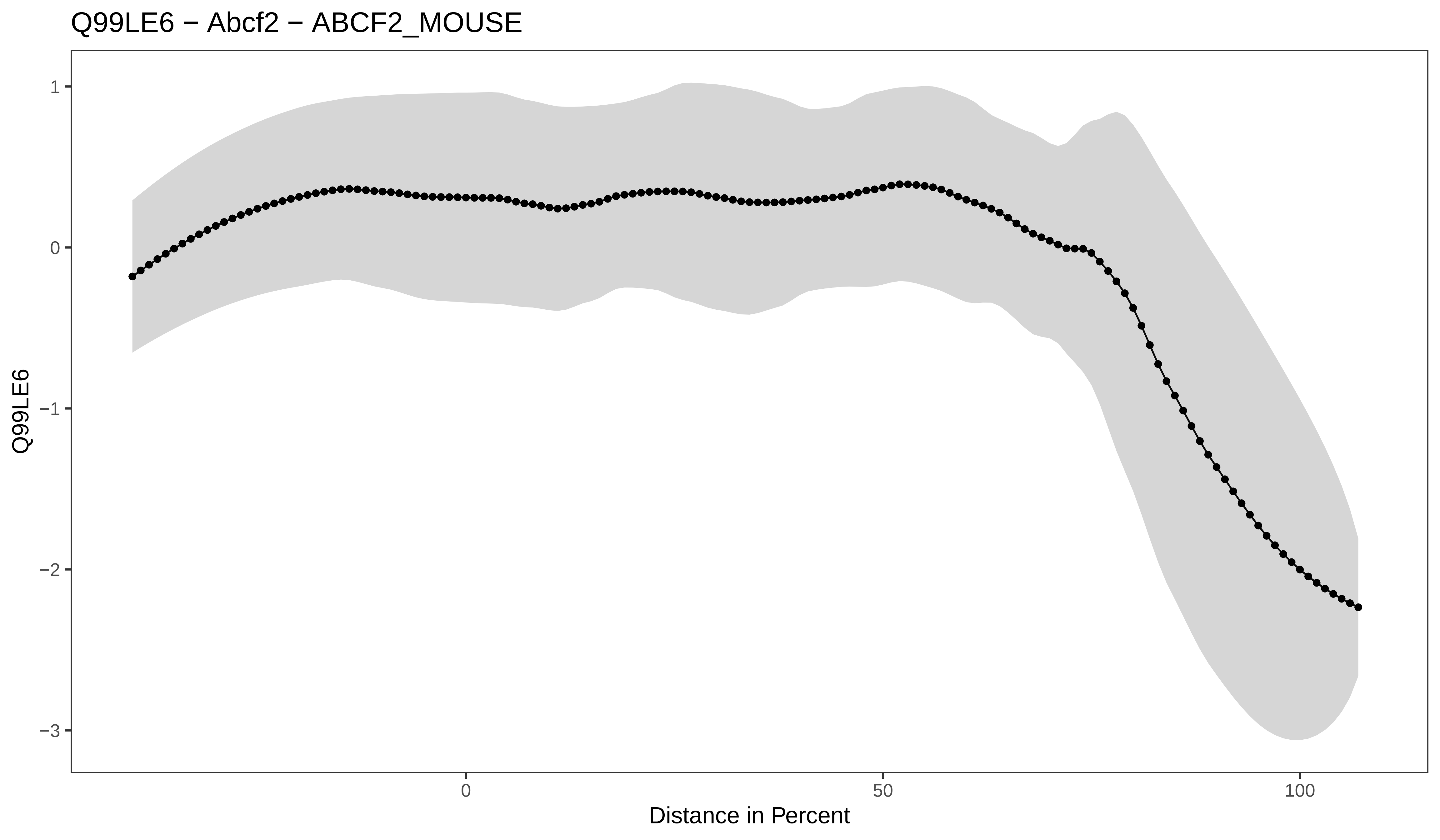

Q99LE6 − Abcf2 − ABCF2_MOUSE
1
0
−1
−2
−3
Q99LE6
0
50
100
Distance in Percent

Q7TMS5 − Abcg2 − ABCG2_MOUSE

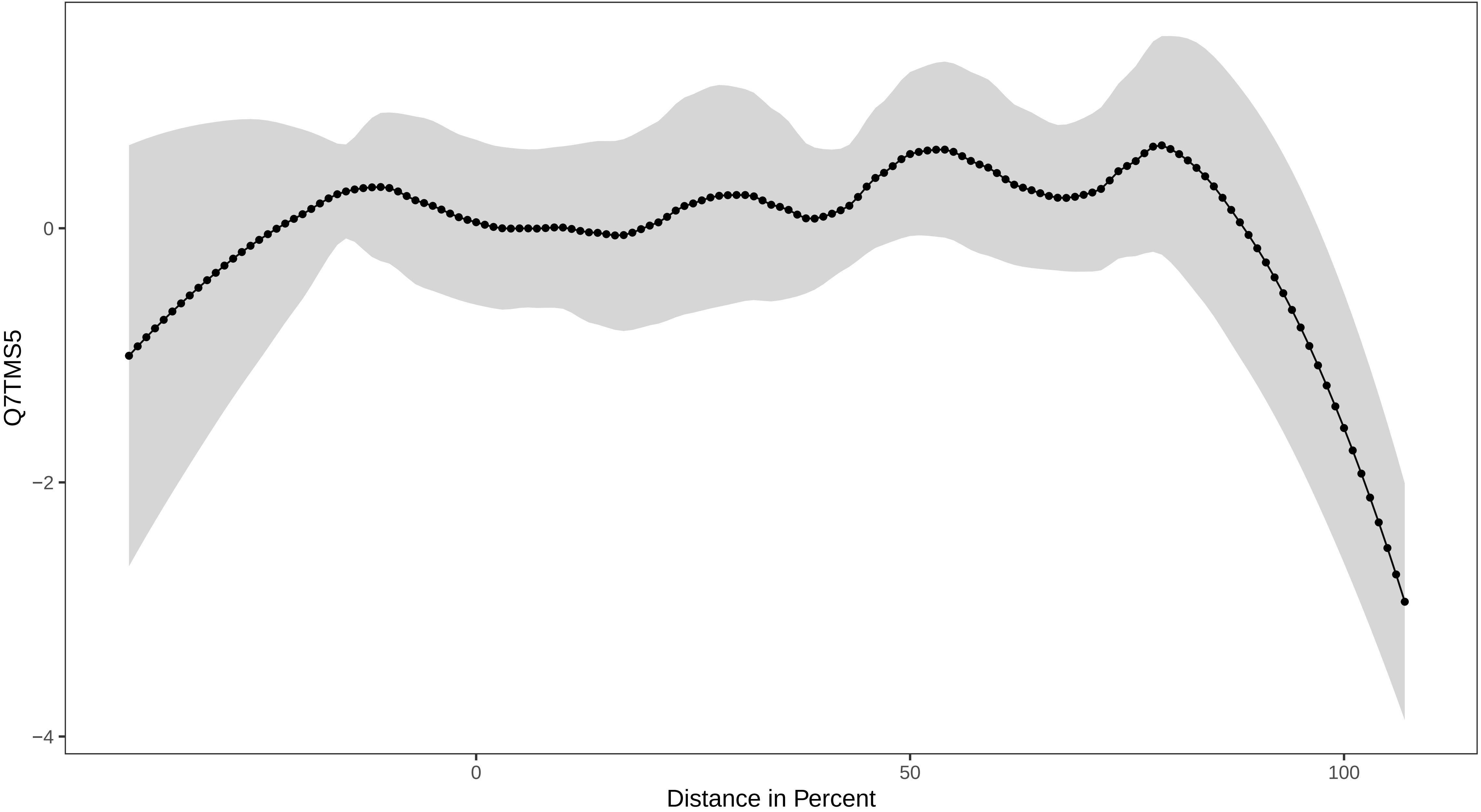

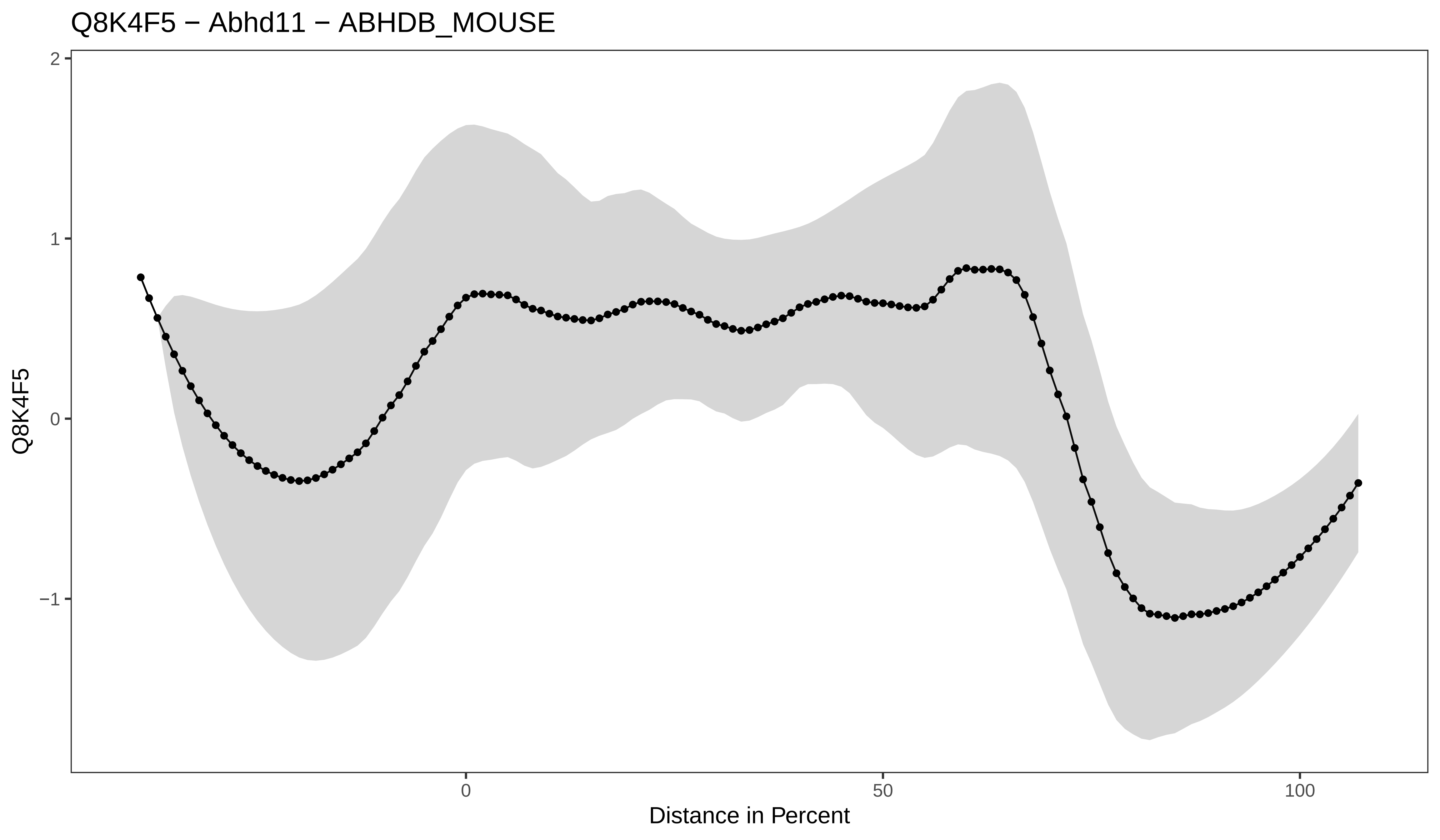
Q8K4F5 − Abhd11 − ABHDB_MOUSE
Q8K4F5
Distance in Percent
2
1
0
−1
0
50
100

Q99LR1 − Abhd12 − ABD12_MOUSE

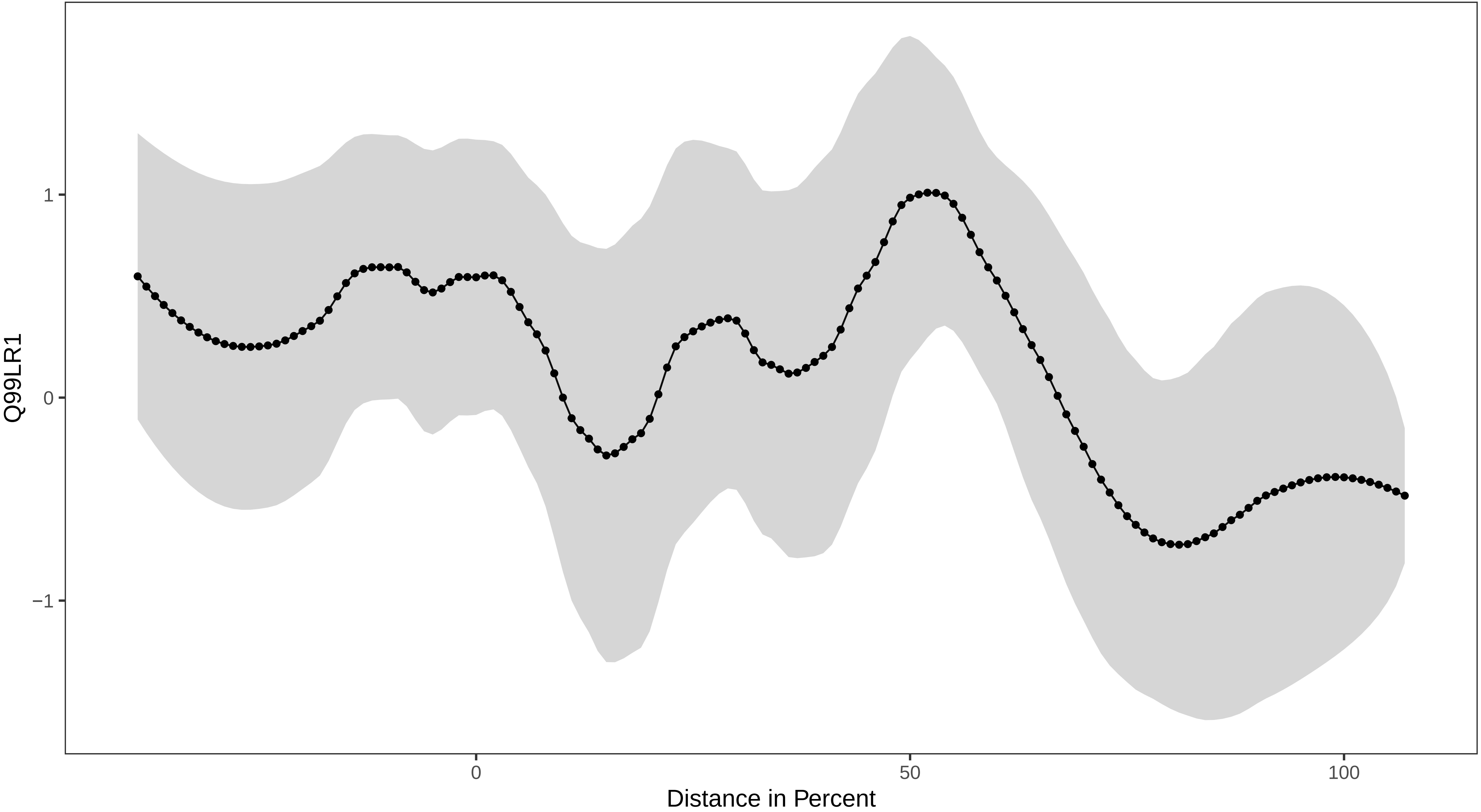

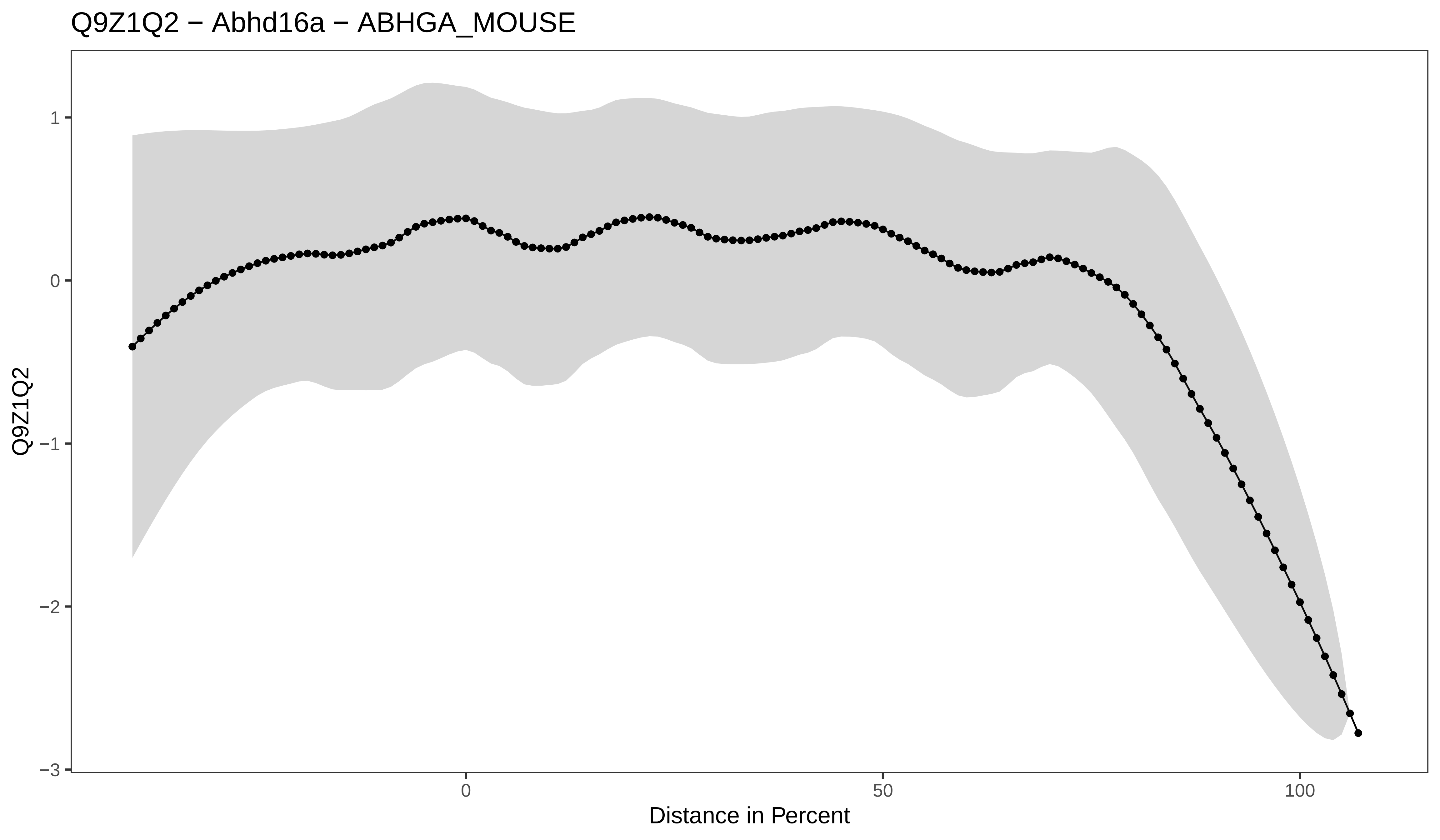

Q9Z1Q2 − Abhd16a − ABHGA_MOUSE
1
0
−1
−2
−3
Q9Z1Q2
0
50
100
Distance in Percent

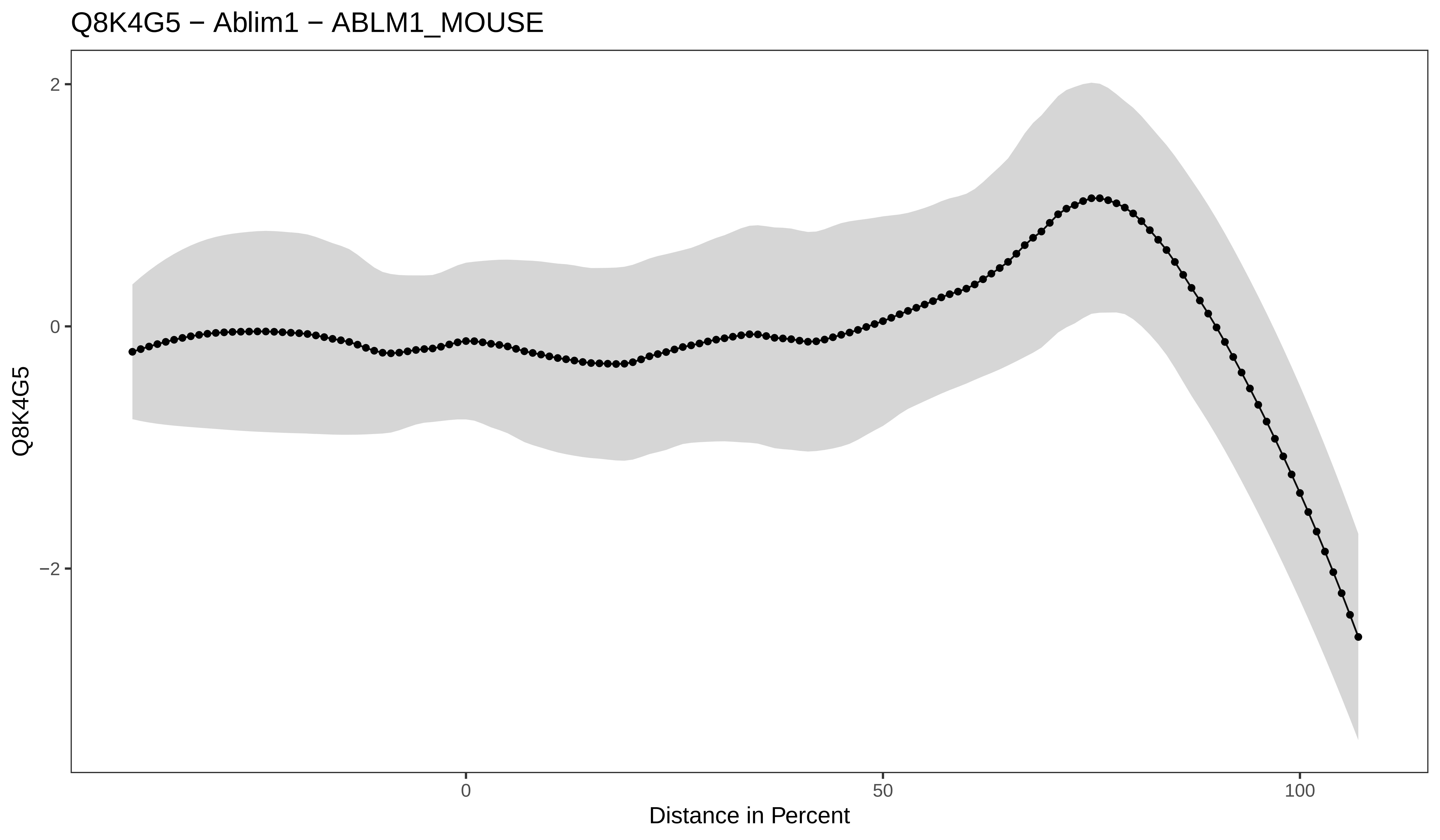
Q8K4G5 − Ablim1 − ABLM1_MOUSE
2
0
−2
Q8K4G5
0
50
100
Distance in Percent

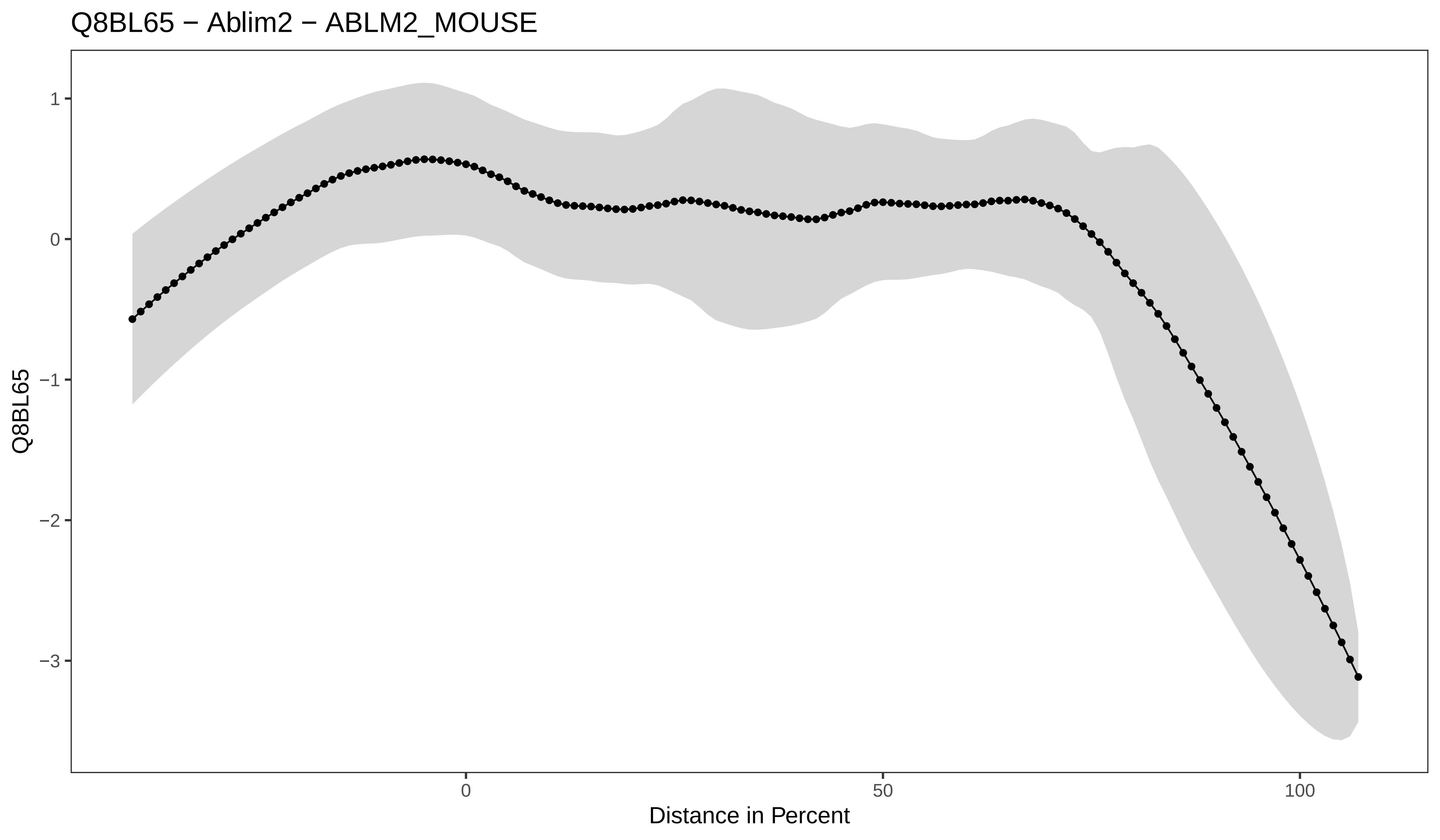
Q8BL65 − Ablim2 − ABLM2_MOUSE
Q8BL65
1
0
−1
−2
−3
0
50
100
Distance in Percent

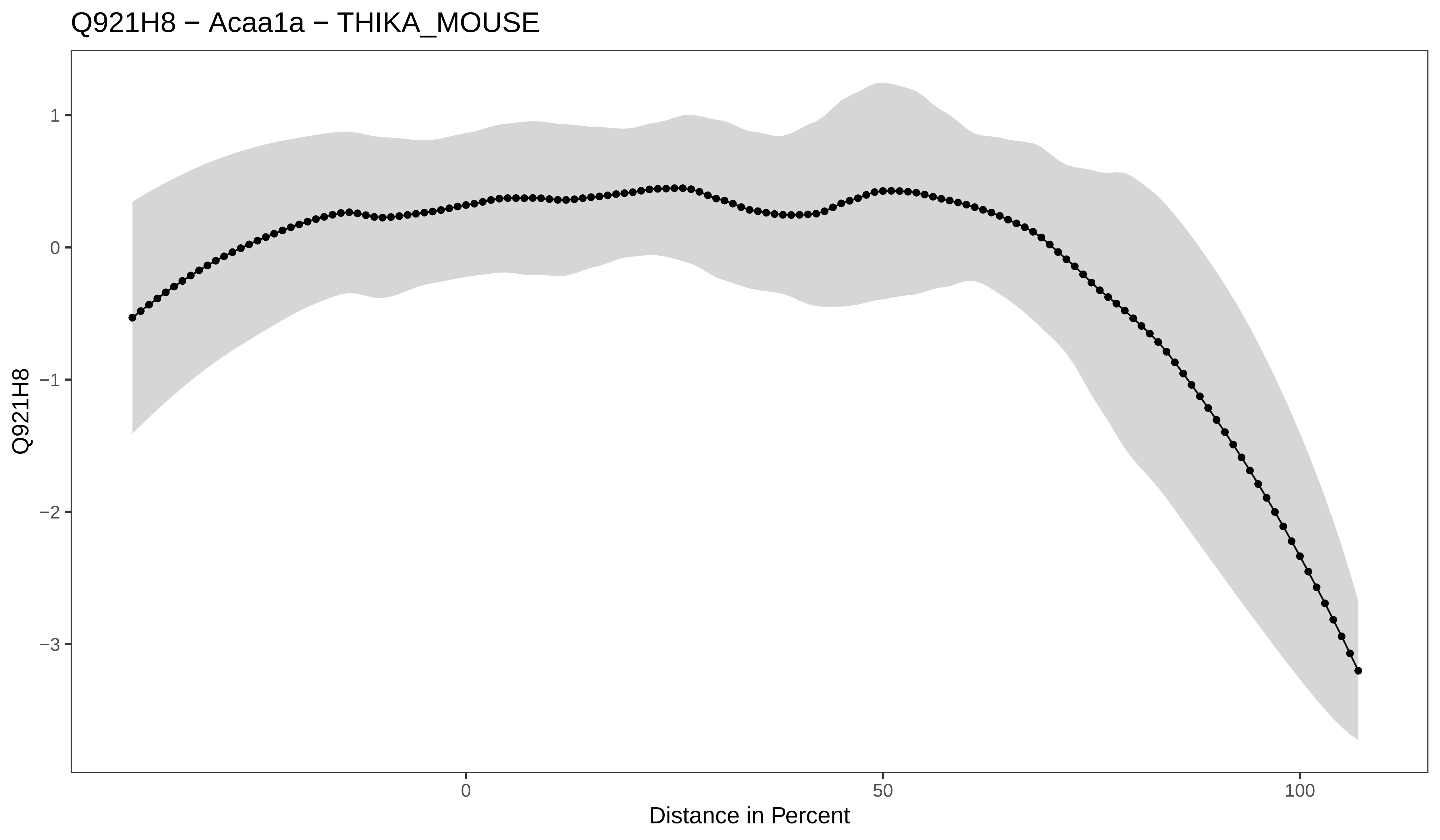

Q921H8 − Acaa1a − THIKA_MOUSE
1
0
−1
−2
−3
Q921H8
0
50
100
Distance in Percent

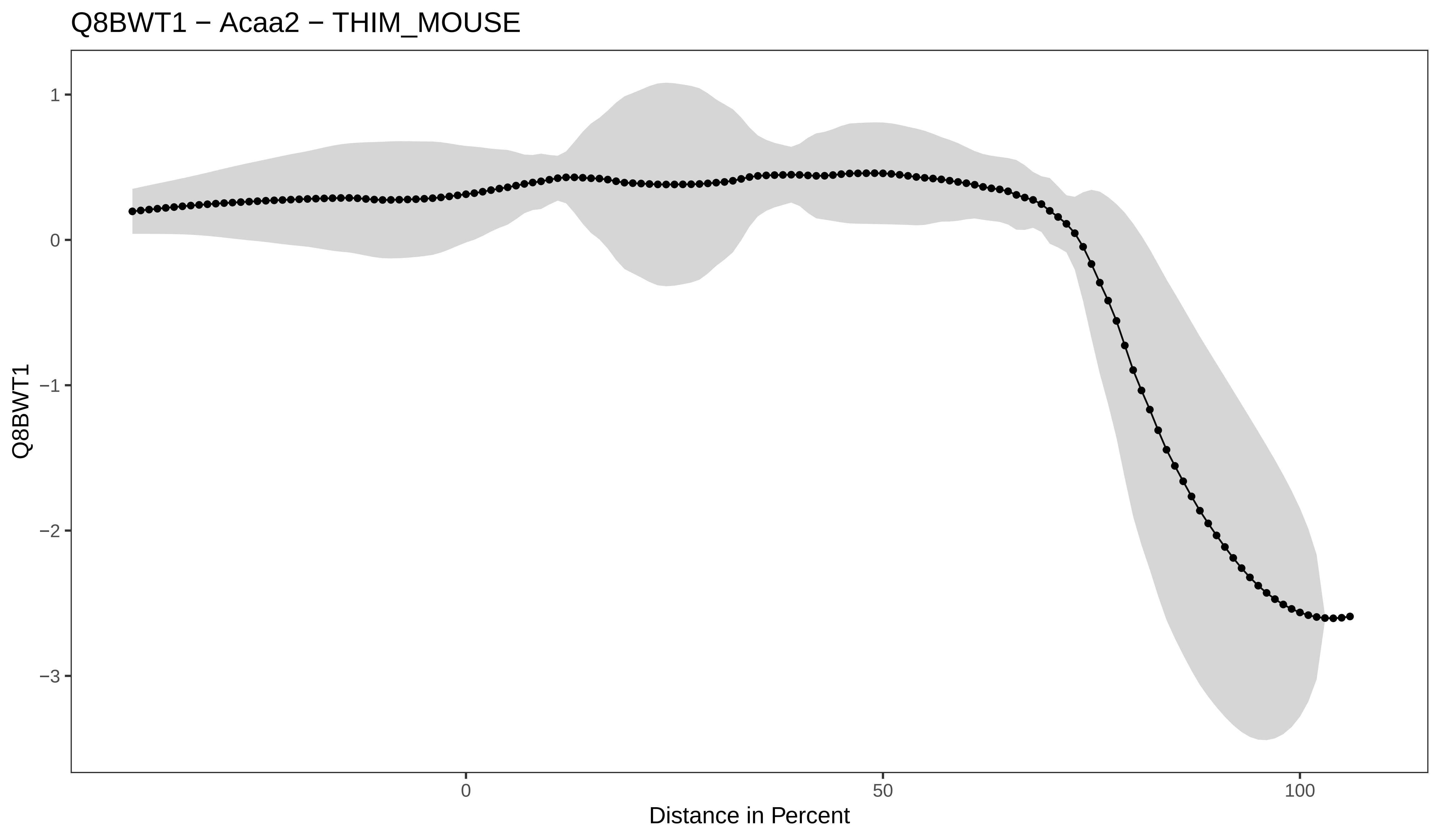

Q8BWT1 − Acaa2 − THIM_MOUSE
1
0
−1
−2
−3
Q8BWT1
0
50
100
Distance in Percent

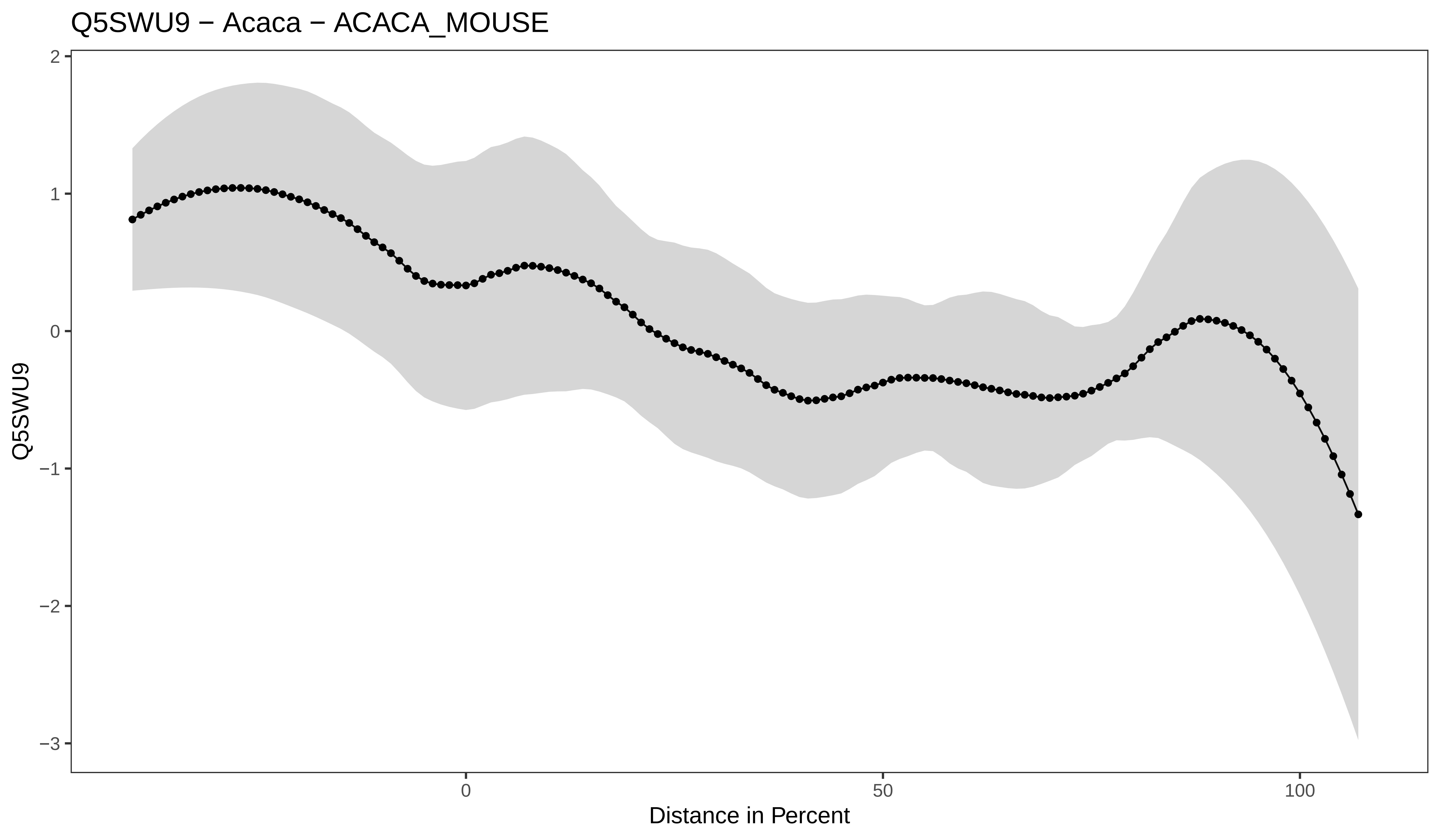
Q5SWU9 − Acaca − ACACA_MOUSE
2
1
0
−1
−2
−3
Q5SWU9
0
50
100
Distance in Percent

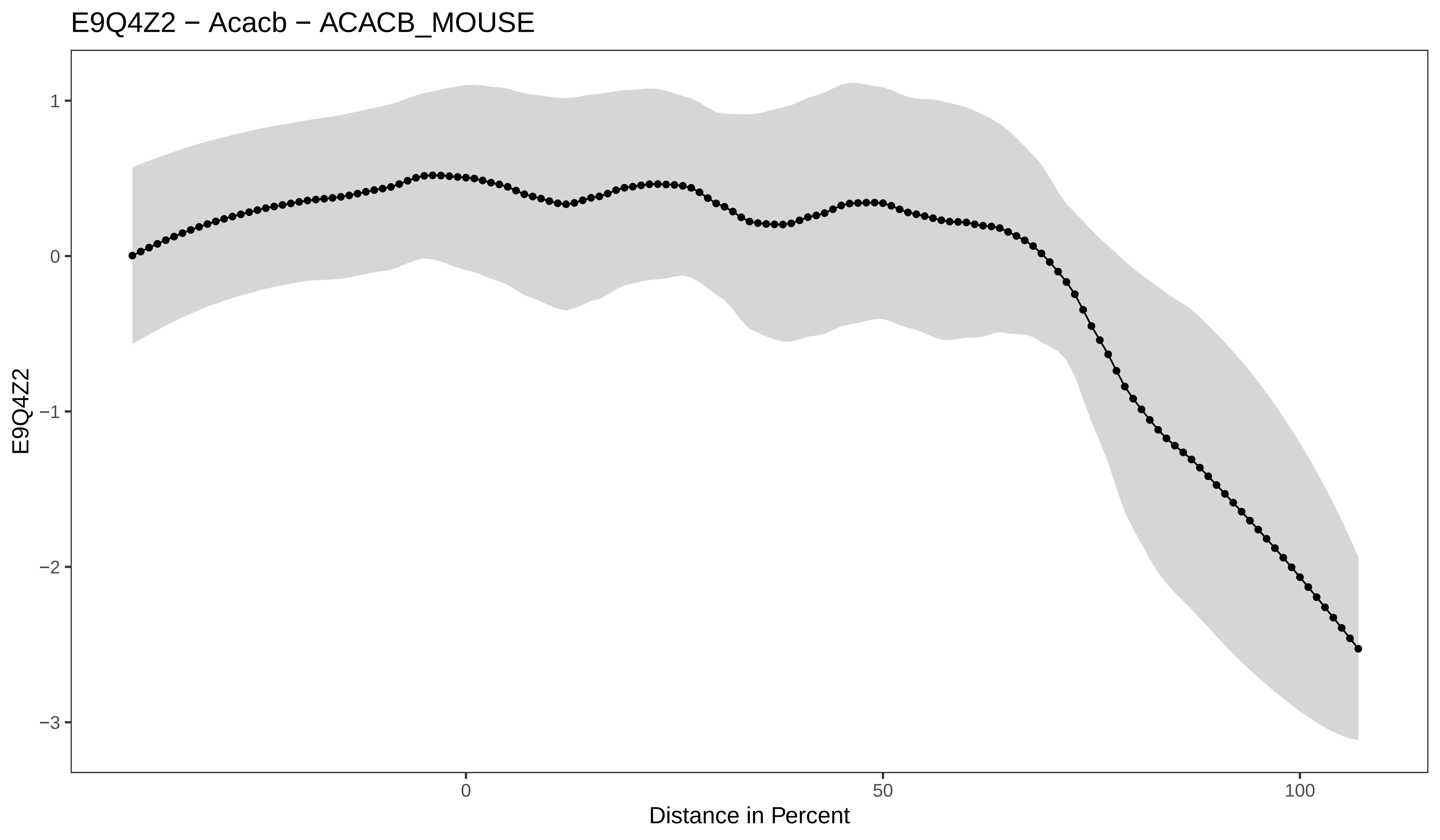

E9Q4Z2 − Acacb − ACACB_MOUSE
E9Q4Z2
1
0
−1
−2
−3
0
50
100
Distance in Percent

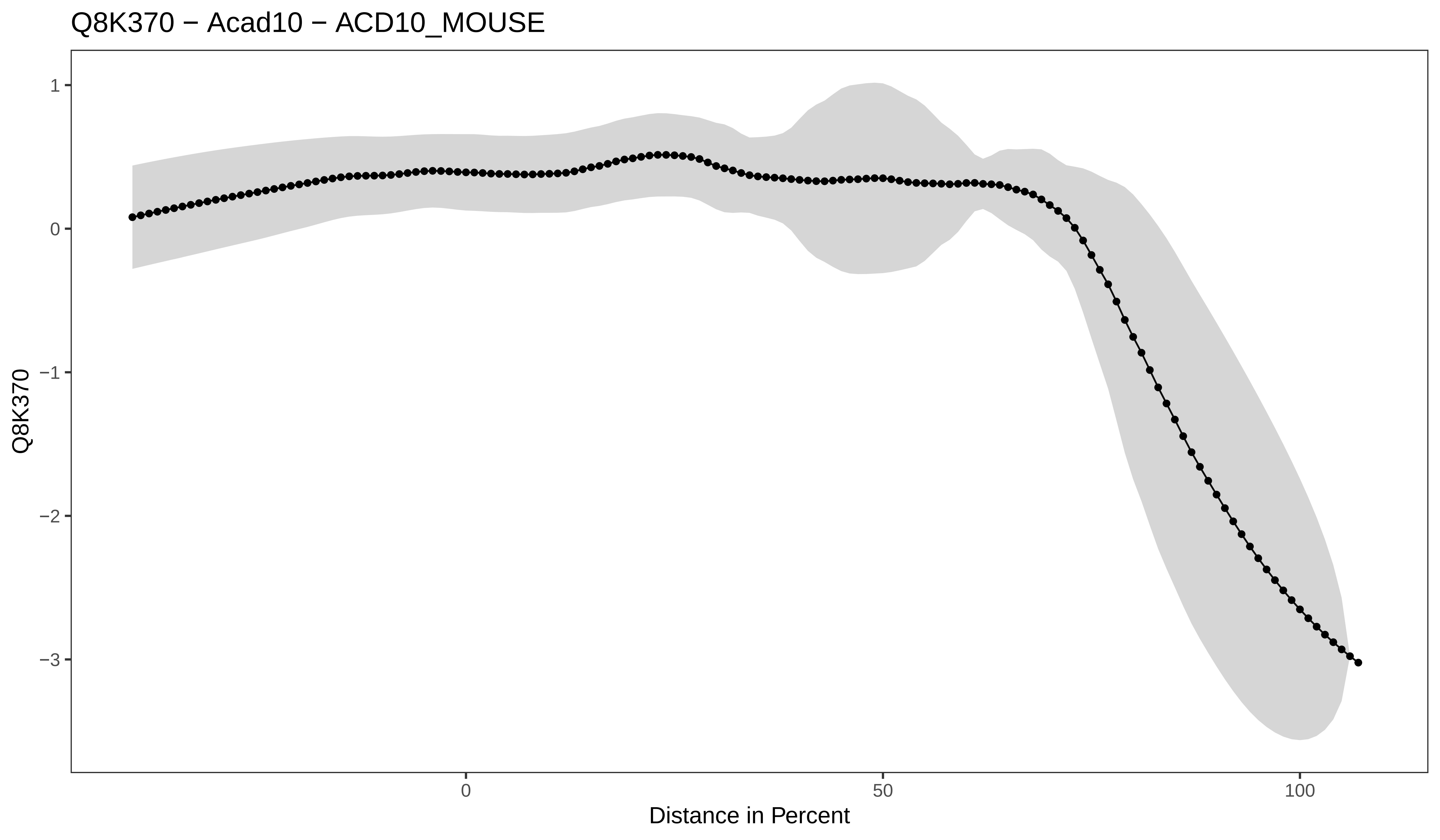

Q8K370 − Acad10 − ACD10_MOUSE
Q8K370
1
0
−1
−2
−3
0
50
100
Distance in Percent

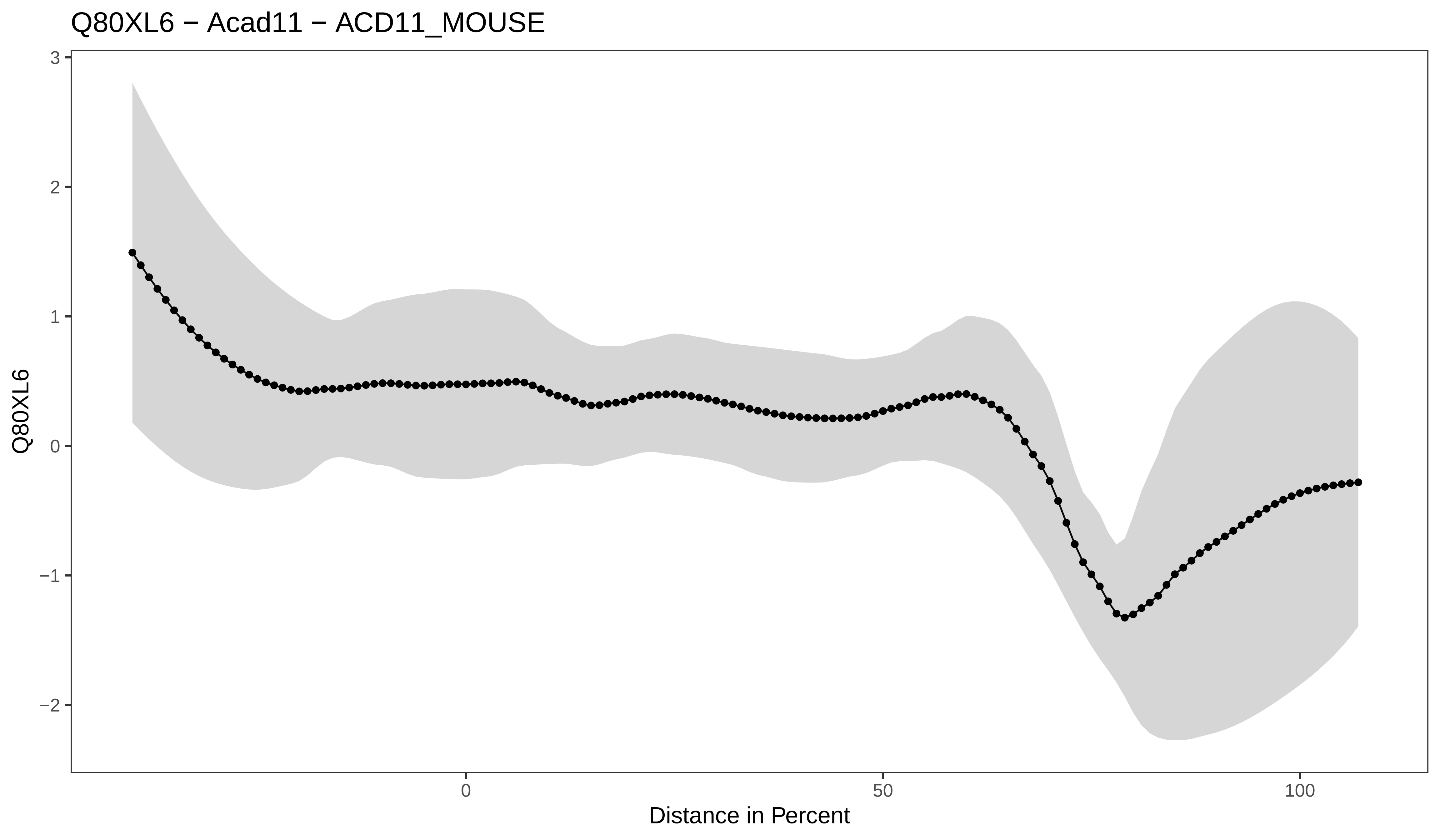

Q80XL6 − Acad11 − ACD11_MOUSE
Q80XL6
Distance in Percent
3
2
1
0
−1
−2
0
50
100

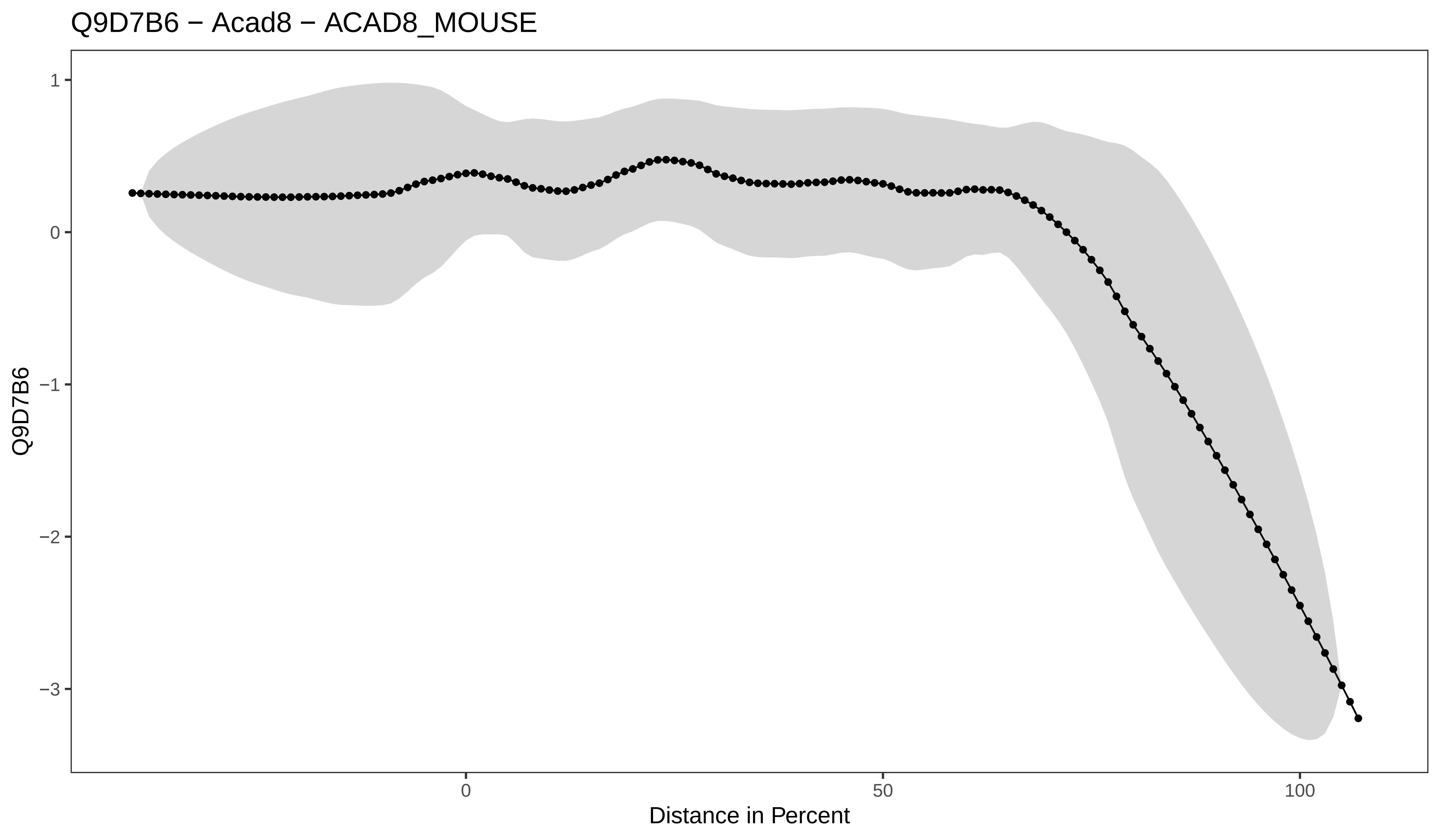
Q9D7B6 − Acad8 − ACAD8_MOUSE
Q9D7B6
1
0
−1
−2
−3
0
50
100
Distance in Percent

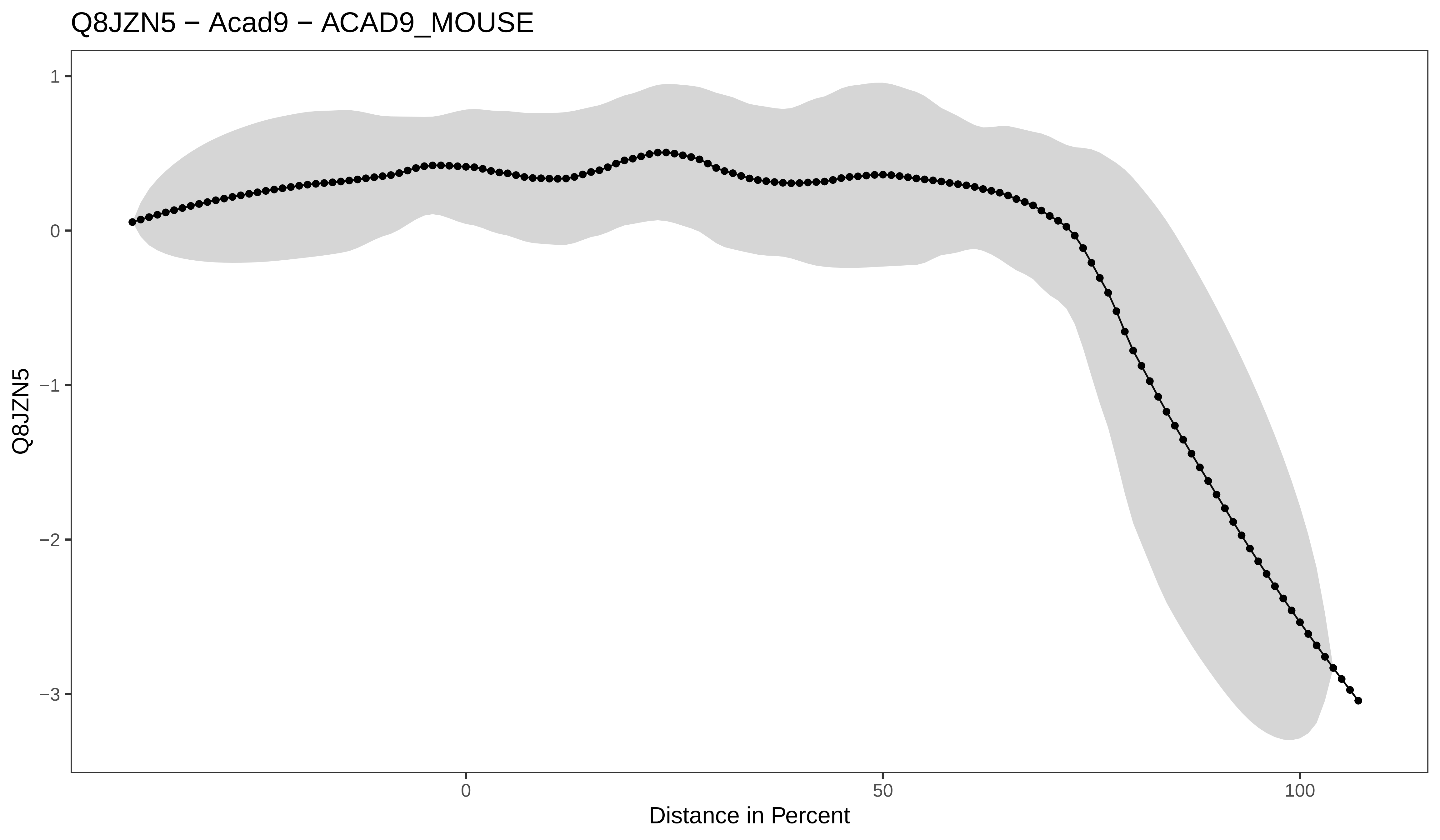
Q8JZN5 − Acad9 − ACAD9_MOUSE
1
0
−1
−2
−3
Q8JZN5
0
50
100
Distance in Percent

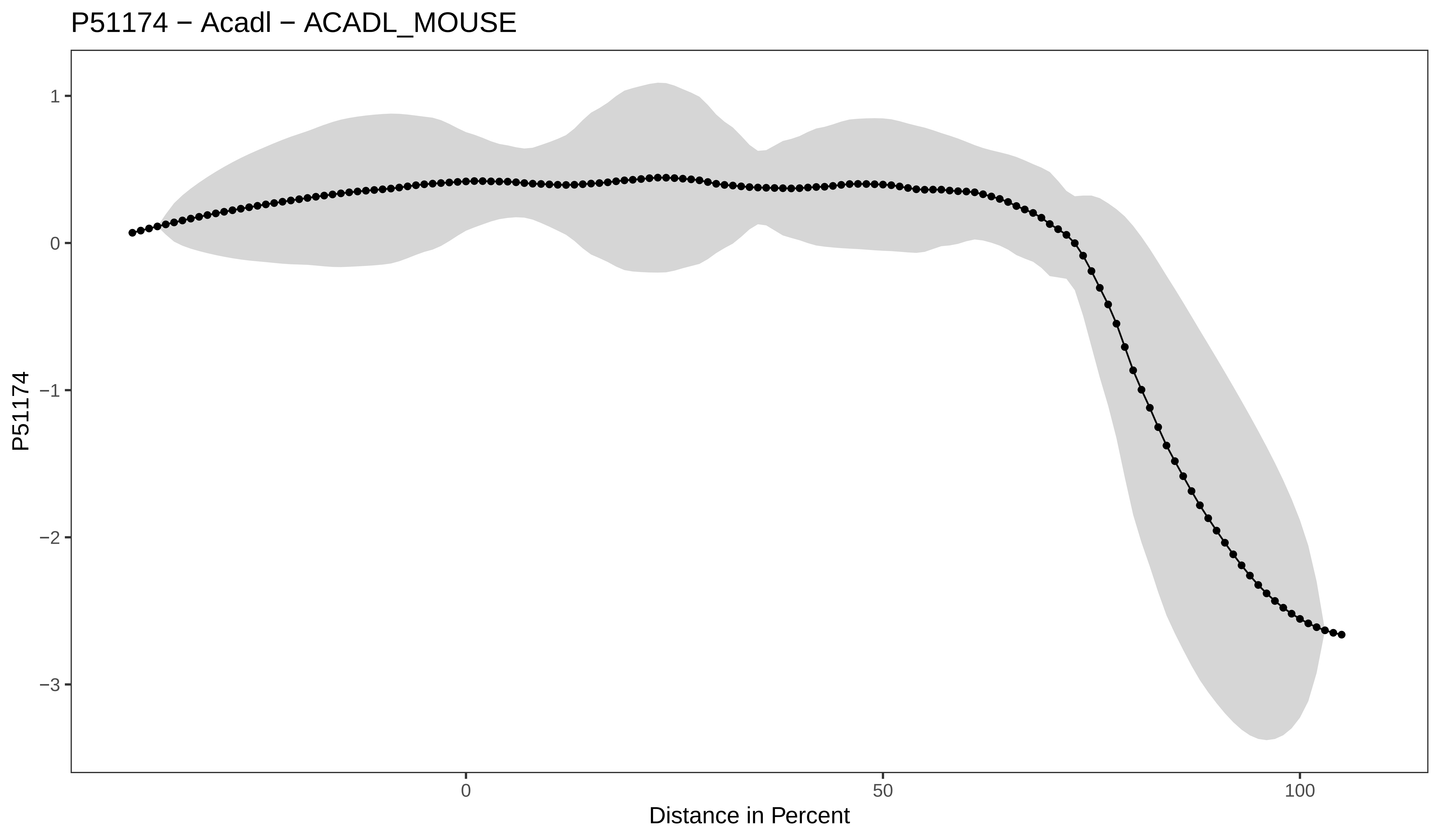

P51174 − Acadl − ACADL_MOUSE
1
0
−1
−2
−3
P51174
0
50
100
Distance in Percent

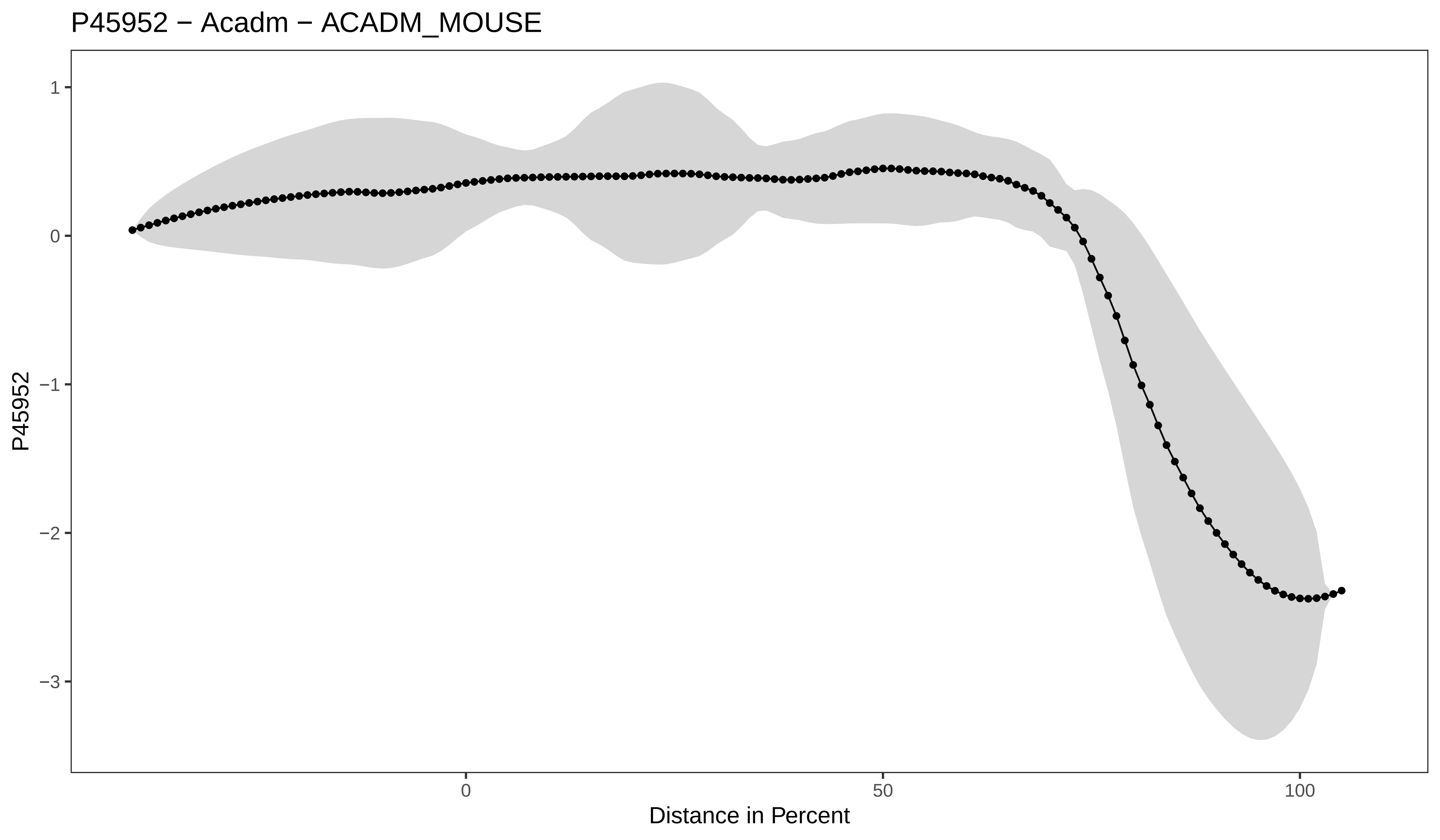
P45952 − Acadm − ACADM_MOUSE
P45952
Distance in Percent
1
0
−1
−2
−3
0
50
100

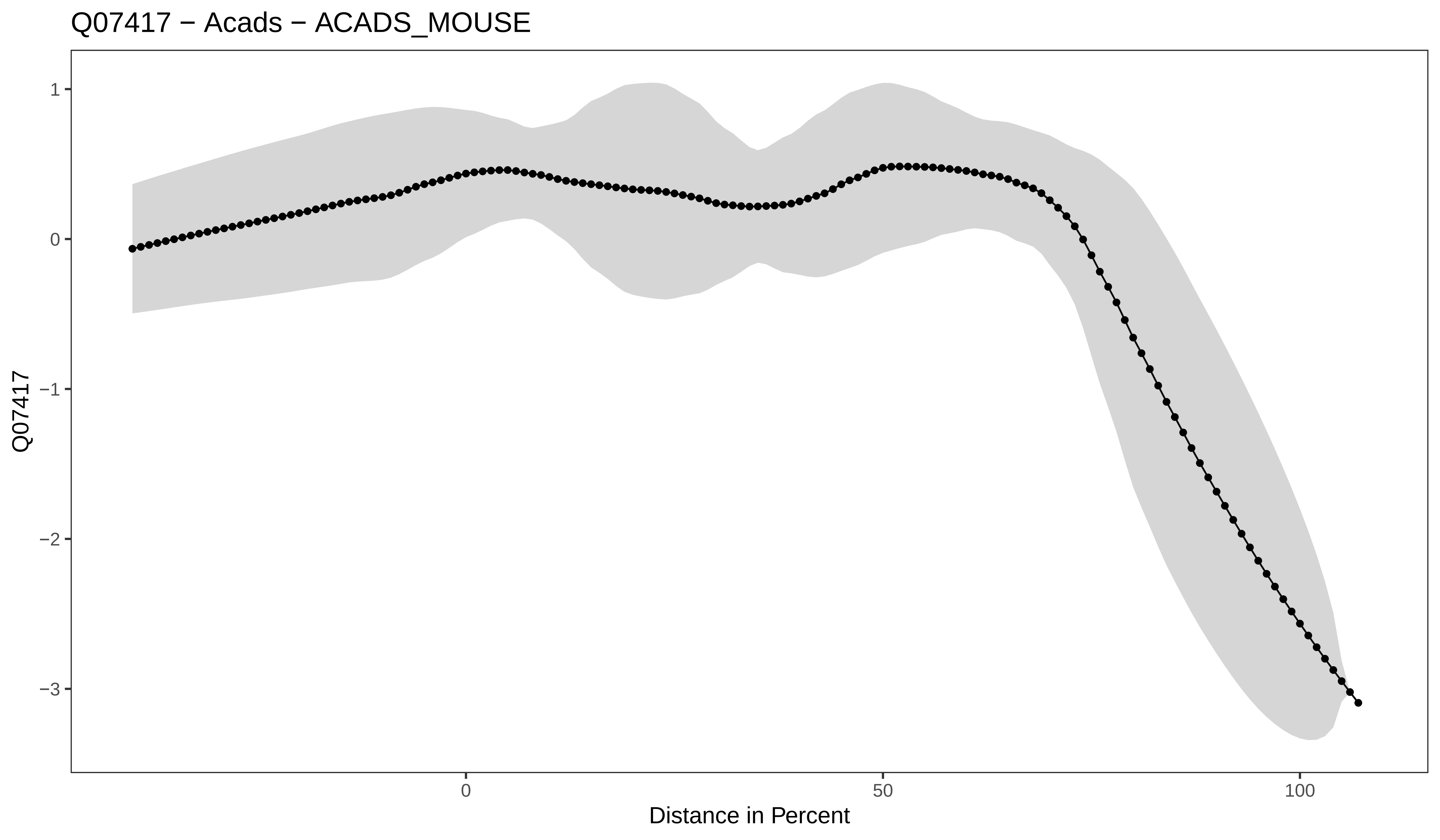

Q07417 − Acads − ACADS_MOUSE
1
0
−1
−2
−3
Q07417
0
50
100
Distance in Percent

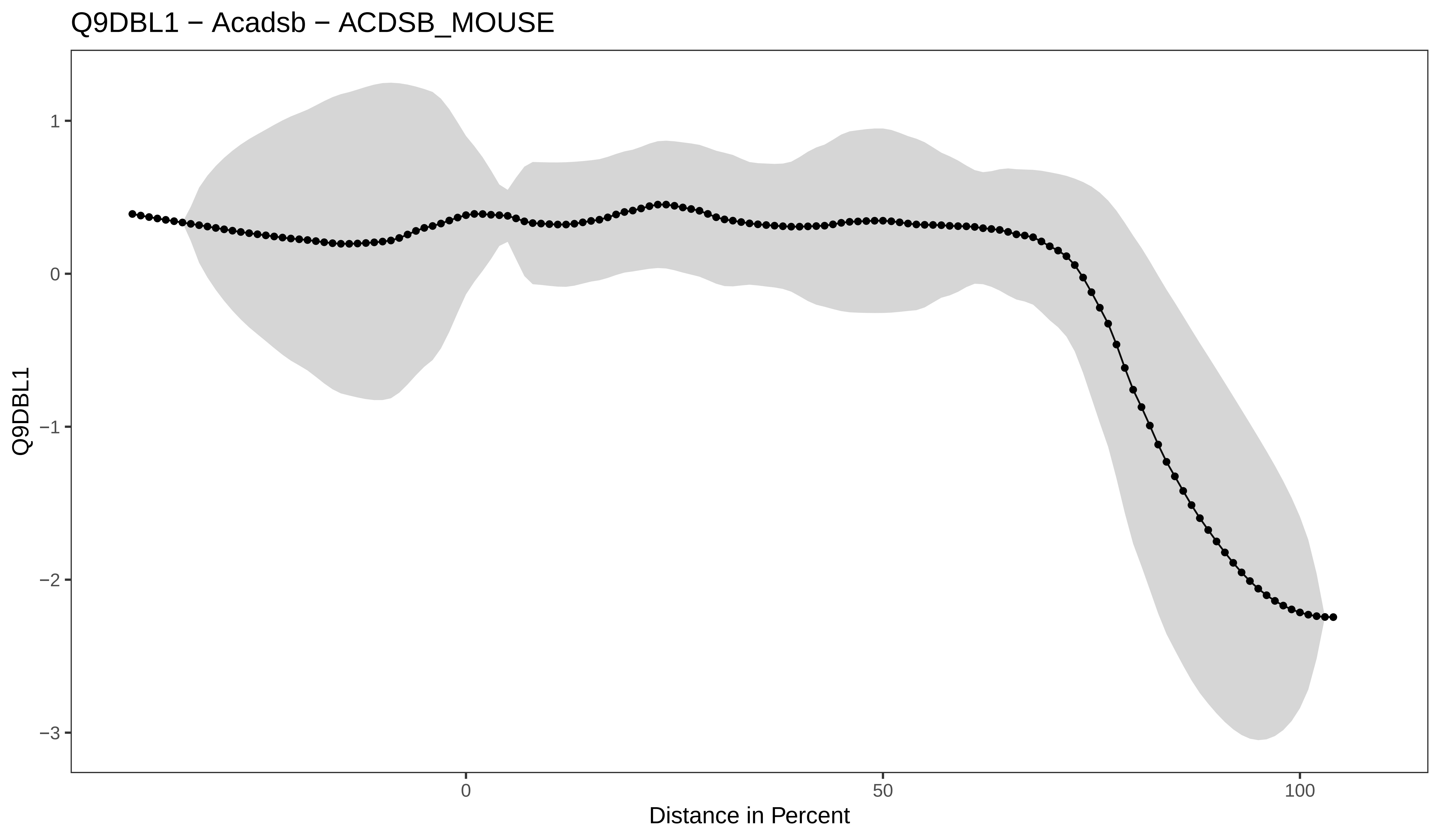

Q9DBL1 − Acadsb − ACDSB_MOUSE
1
0
−1
−2
−3
Q9DBL1
0
50
100
Distance in Percent

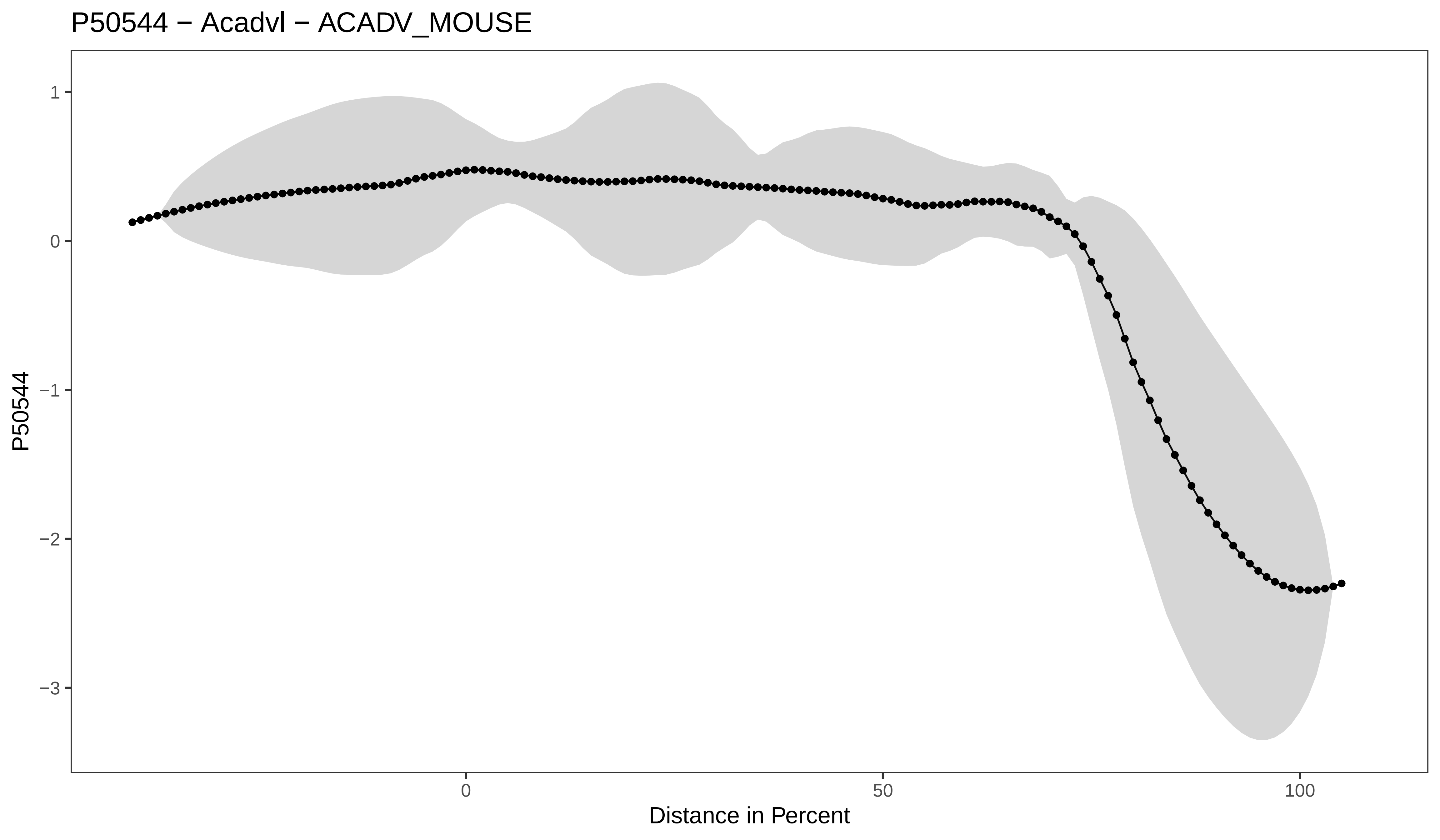
P50544 − Acadvl − ACADV_MOUSE
1
0
−1
−2
−3
0
50
100
Distance in Percent
P50544

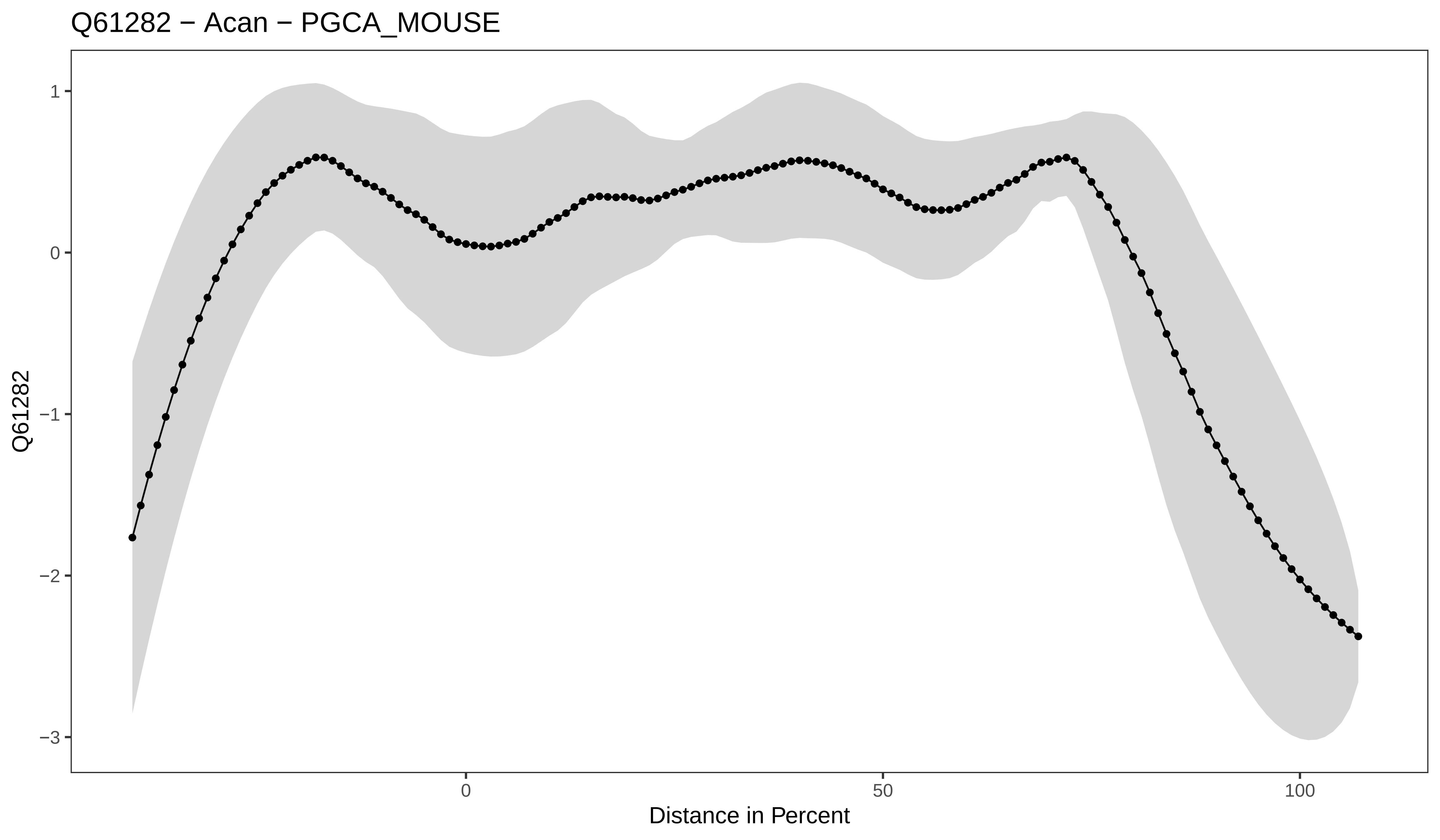

Q61282 − Acan − PGCA_MOUSE
1
0
−1
−2
−3
Q61282
0
50
100
Distance in Percent

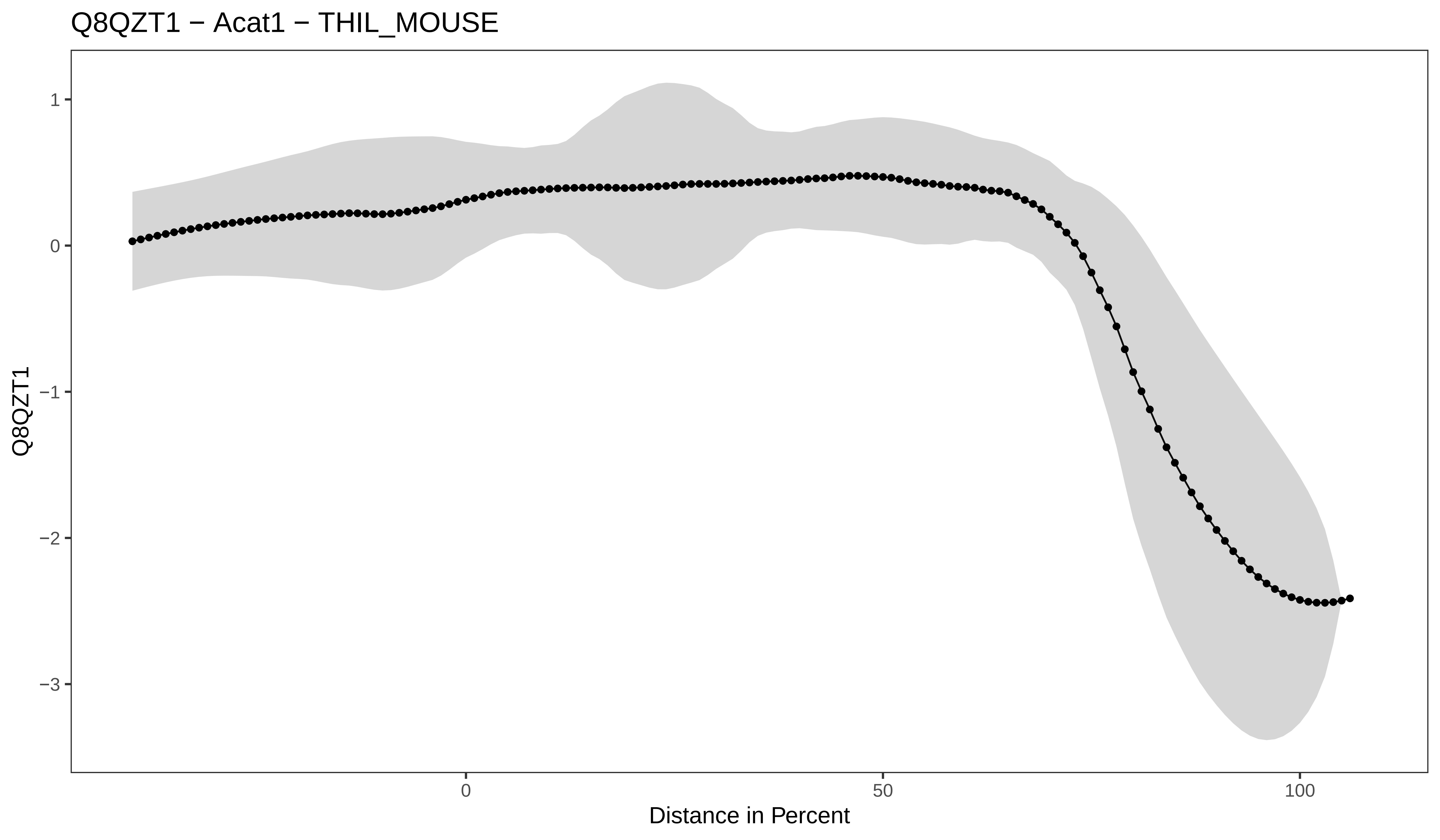

Q8QZT1 − Acat1 − THIL_MOUSE
1
0
−1
−2
−3
Q8QZT1
0
50
100
Distance in Percent

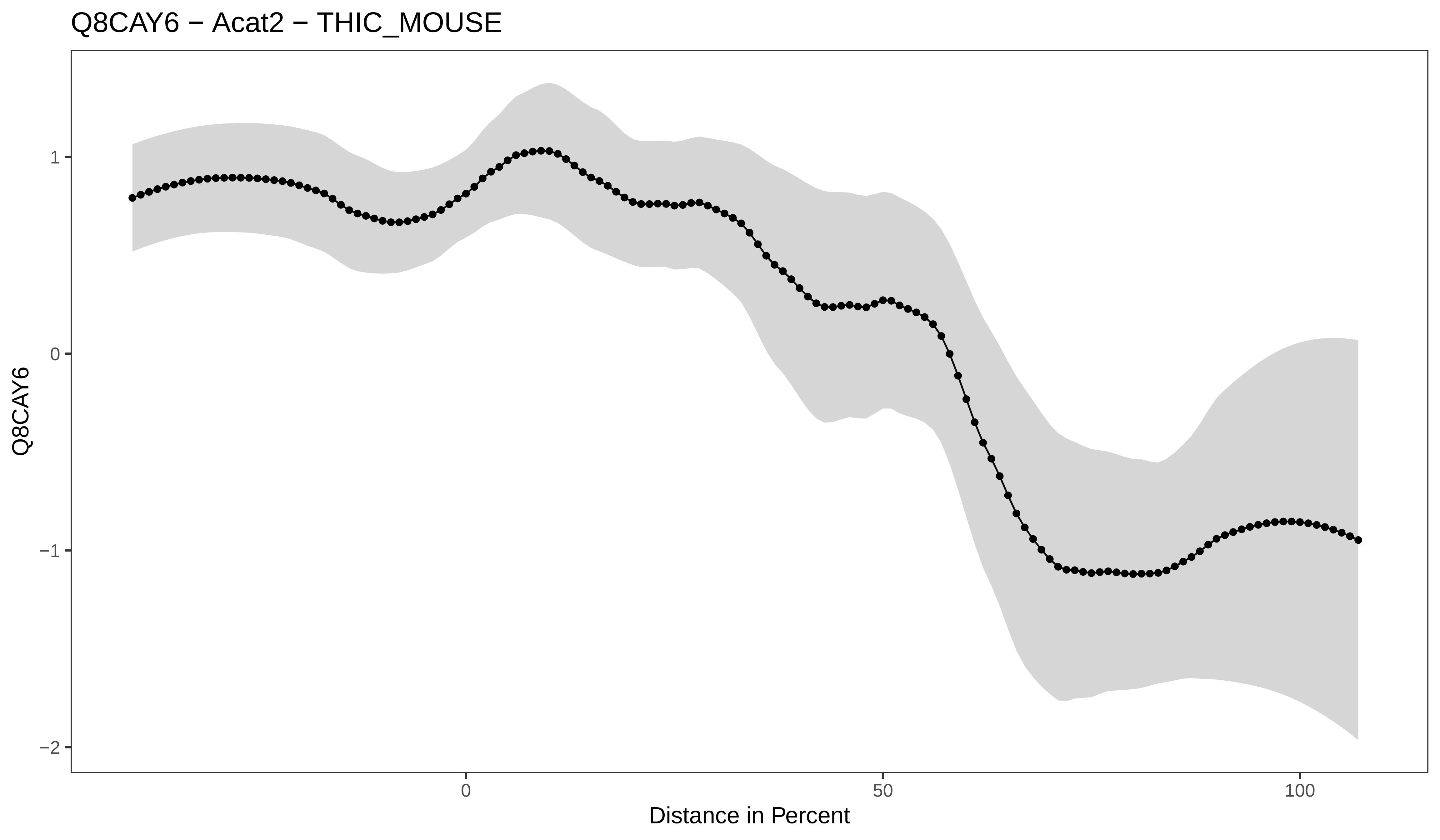
Q8CAY6 − Acat2 − THIC_MOUSE
1
0
−1
−2
Q8CAY6
0
50
100
Distance in Percent

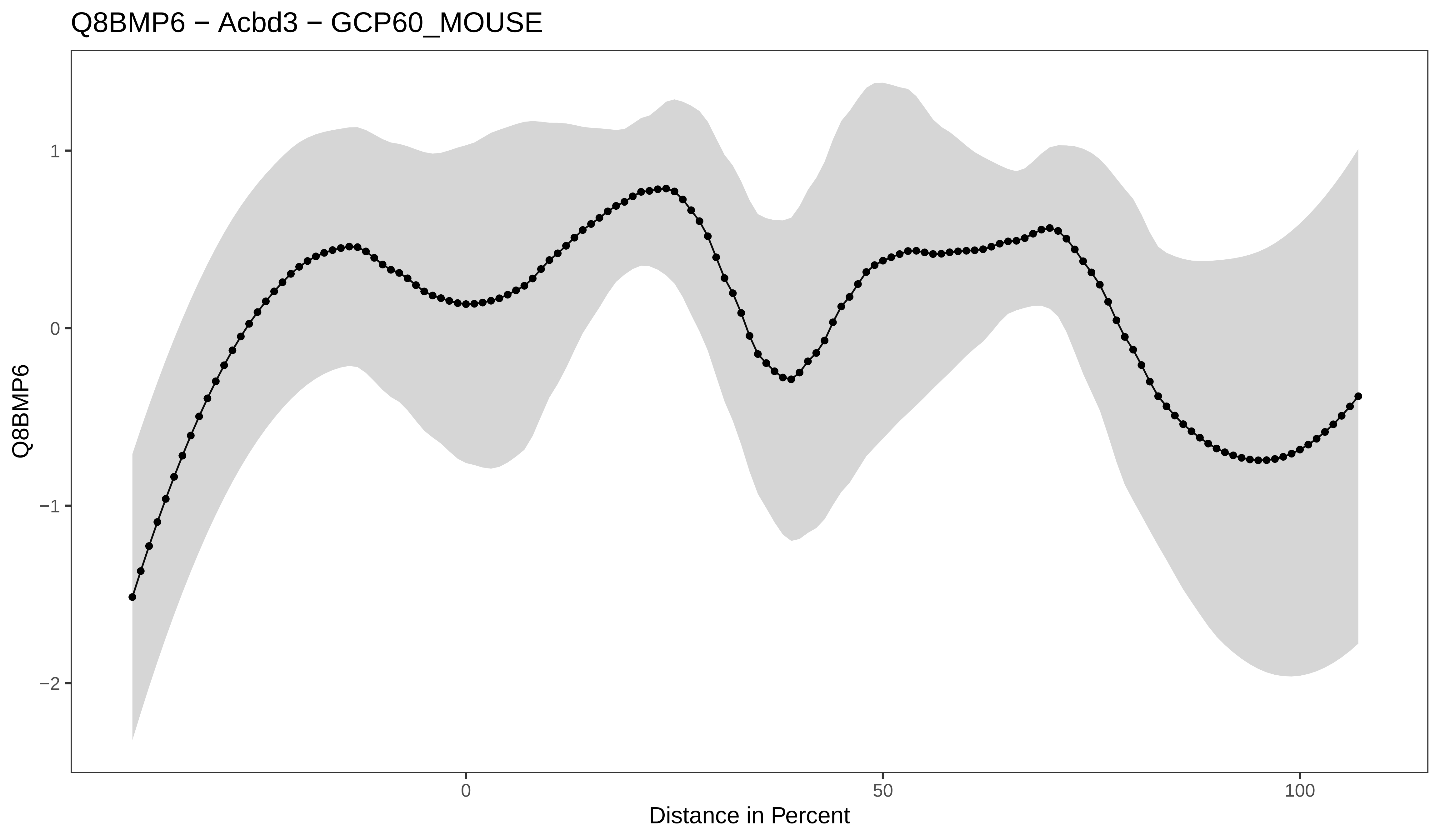

Q8BMP6 − Acbd3 − GCP60_MOUSE
Q8BMP6
1
0
−1
−2
0
50
100
Distance in Percent

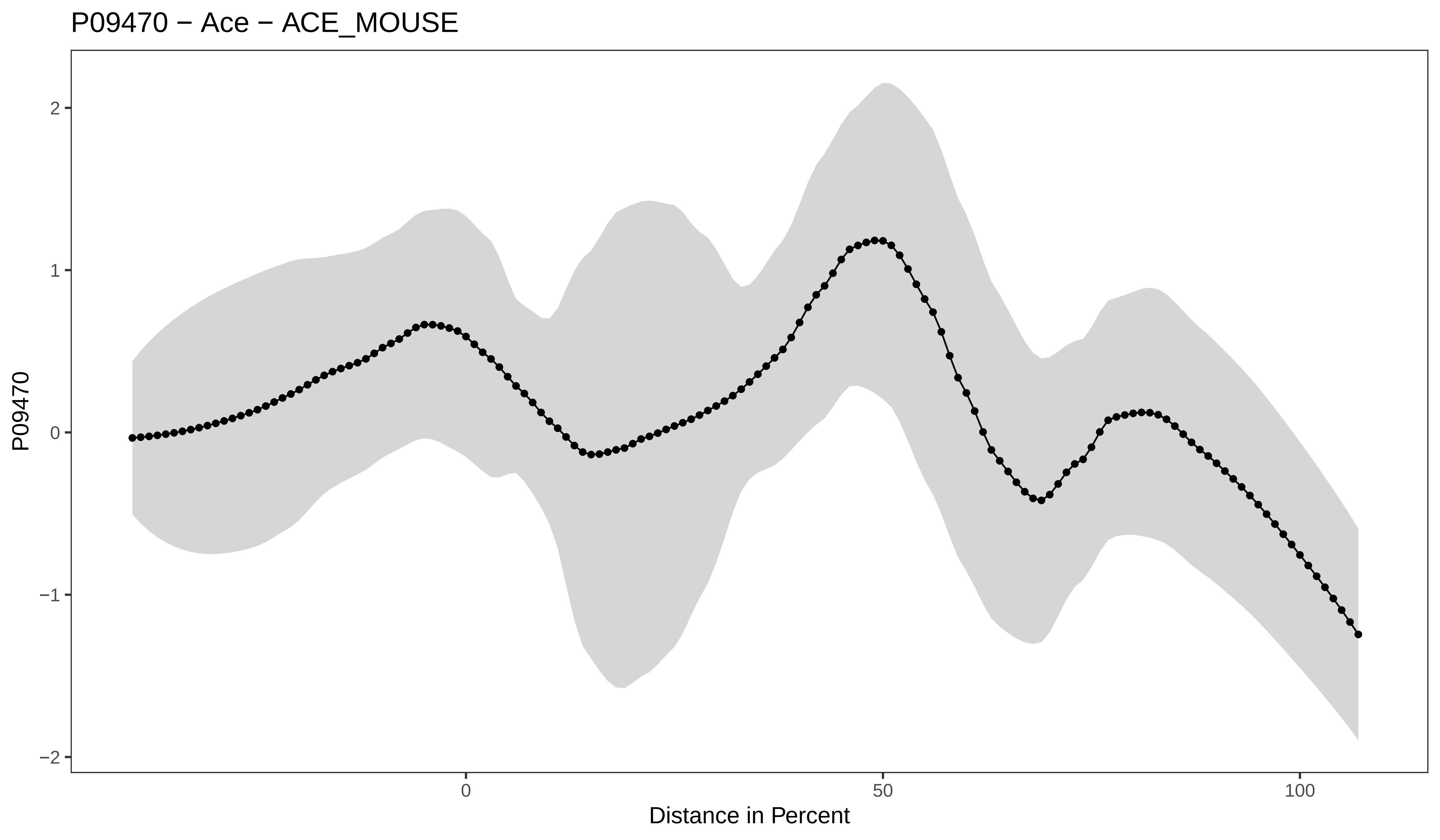

P09470 − Ace − ACE_MOUSE
2
1
0
−1
−2
P09470
0
50
100
Distance in Percent

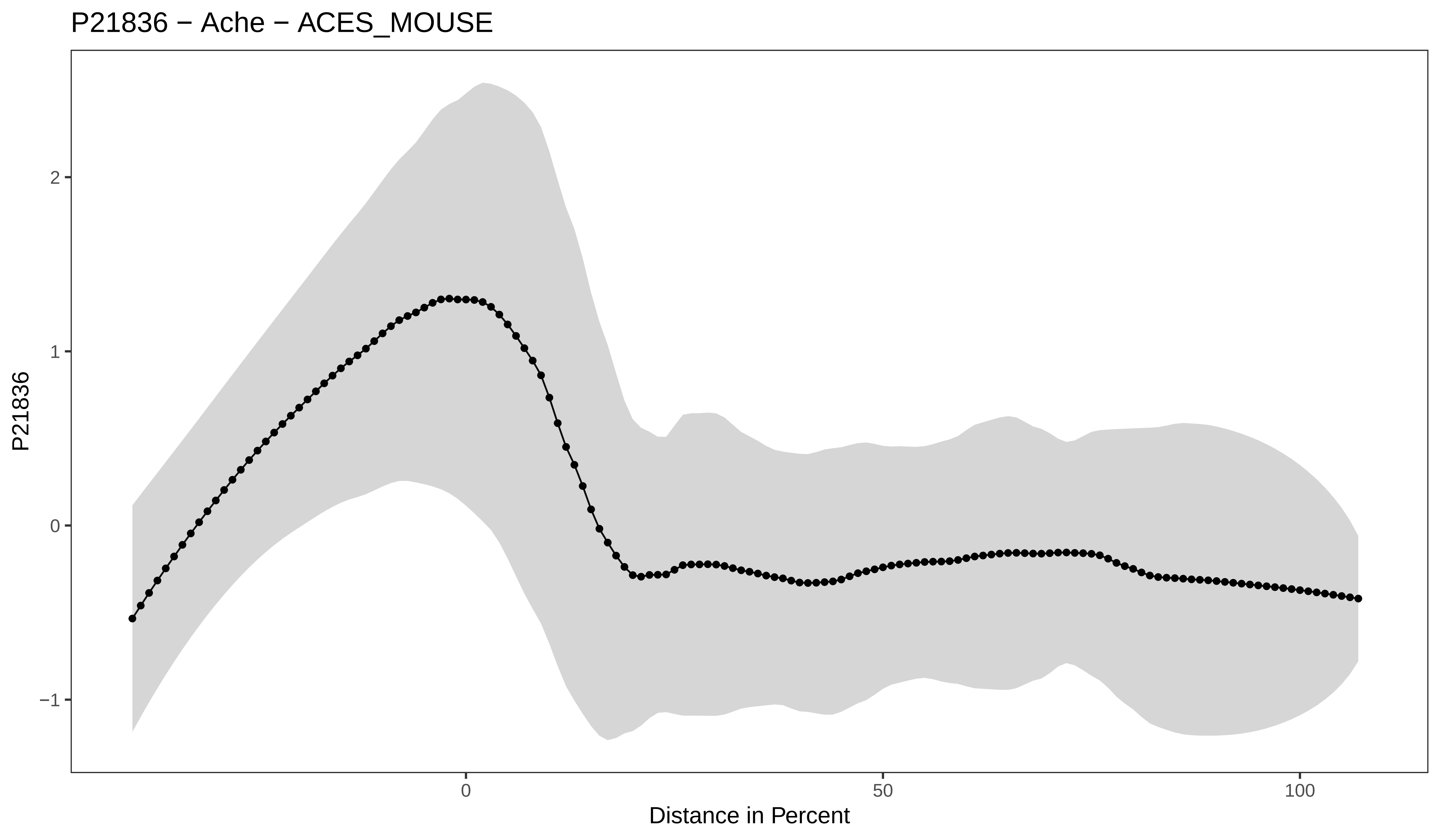
P21836 − Ache − ACES_MOUSE
P21836
2
1
0
−1
0
50
100
Distance in Percent

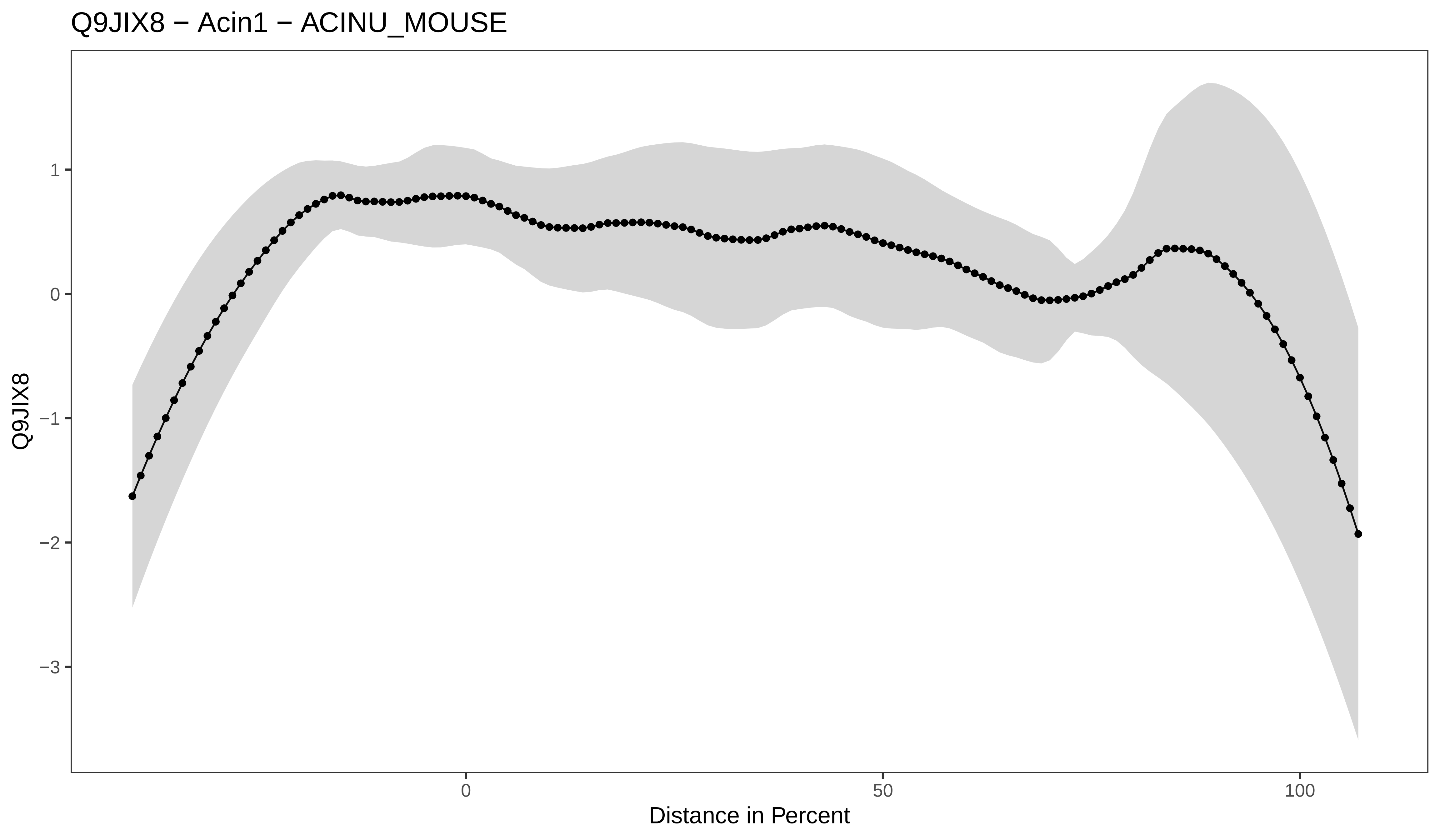

Q9JIX8 − Acin1 − ACINU_MOUSE
Q9JIX8
Distance in Percent
1
0
−1
−2
−3
0
50
100

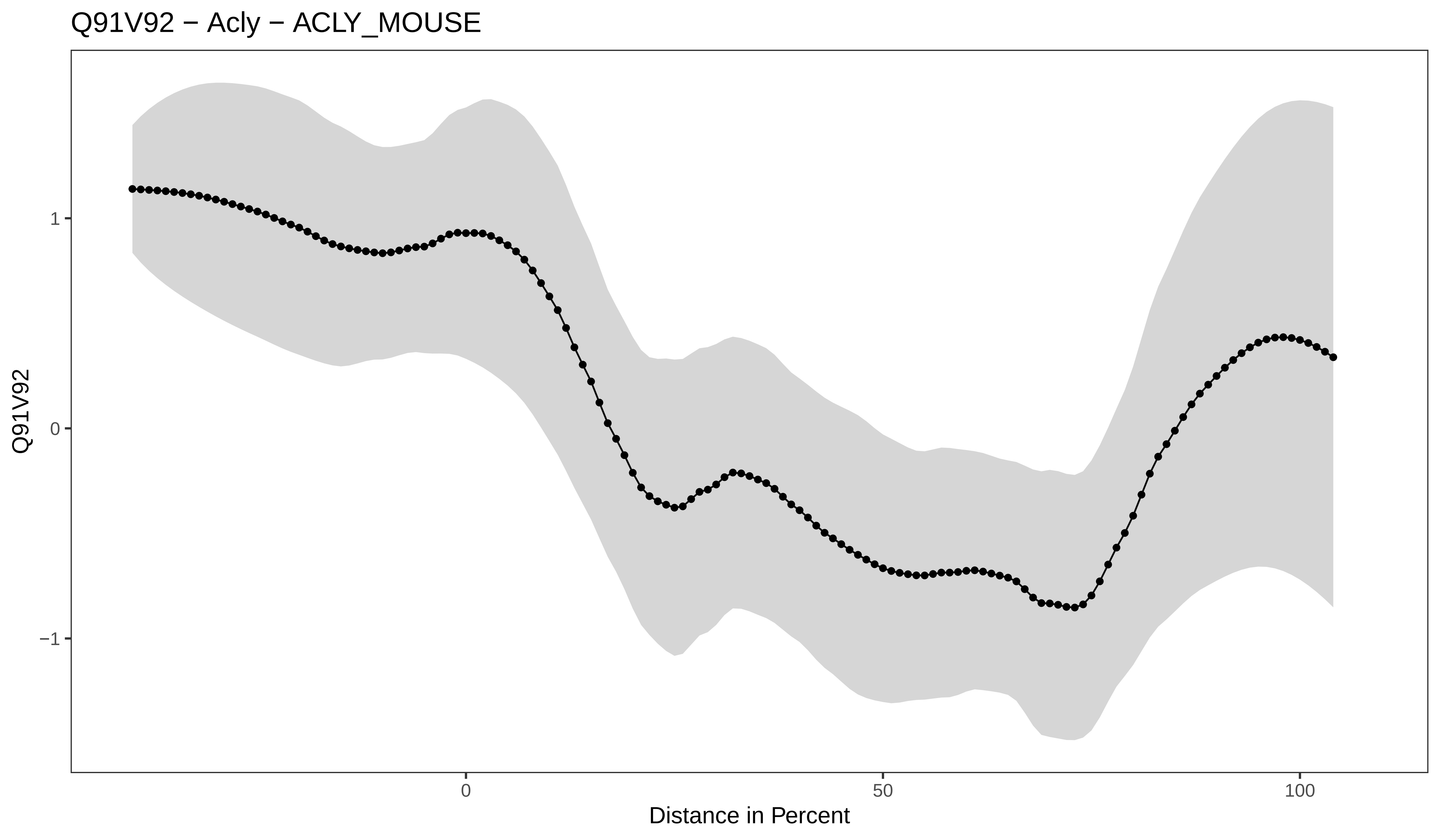
Q91V92 − Acly − ACLY_MOUSE
Q91V92
1
0
−1
0
50
100
Distance in Percent

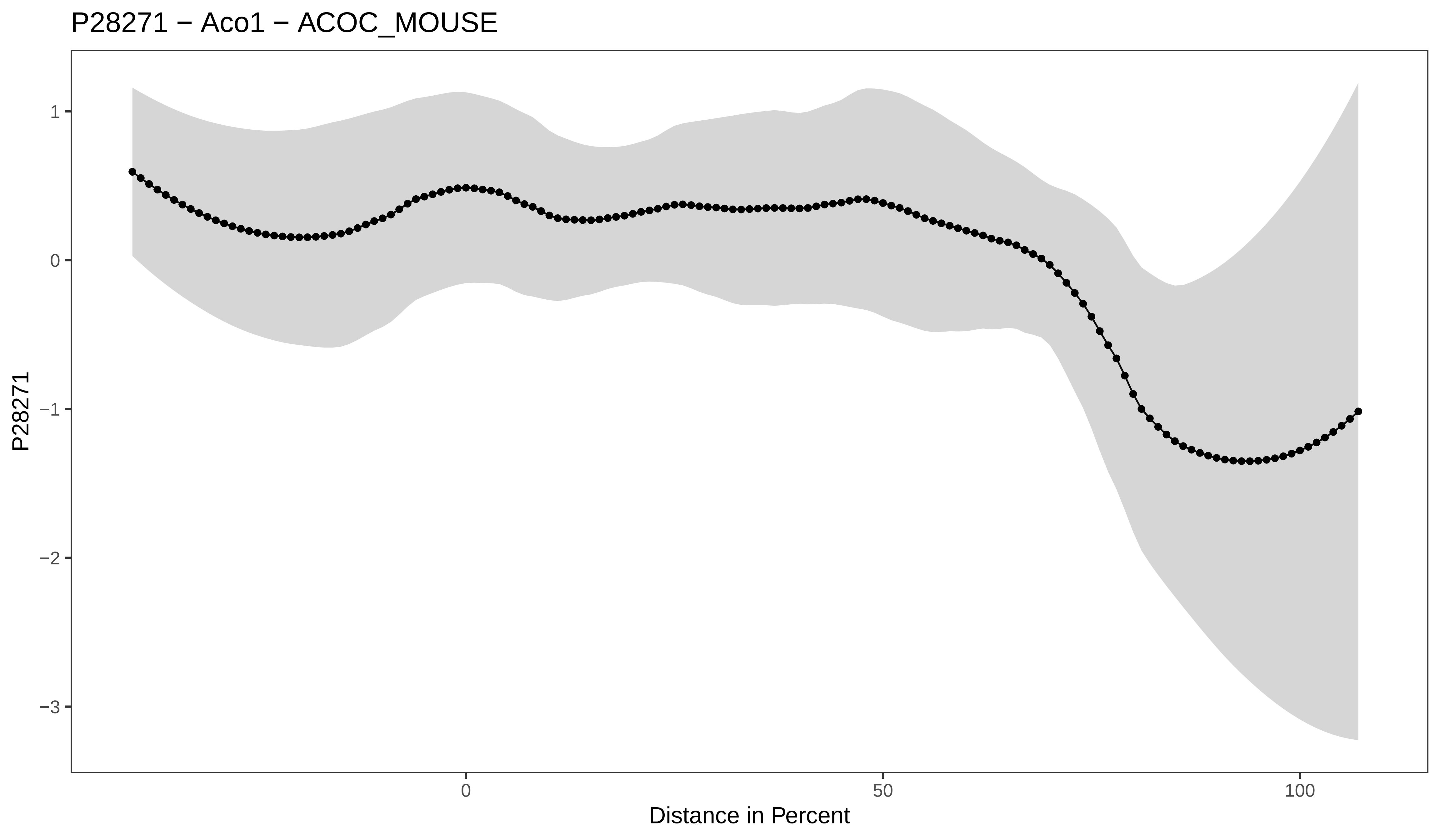
P28271 − Aco1 − ACOC_MOUSE
1
0
−1
−2
−3
P28271
0
50
100
Distance in Percent

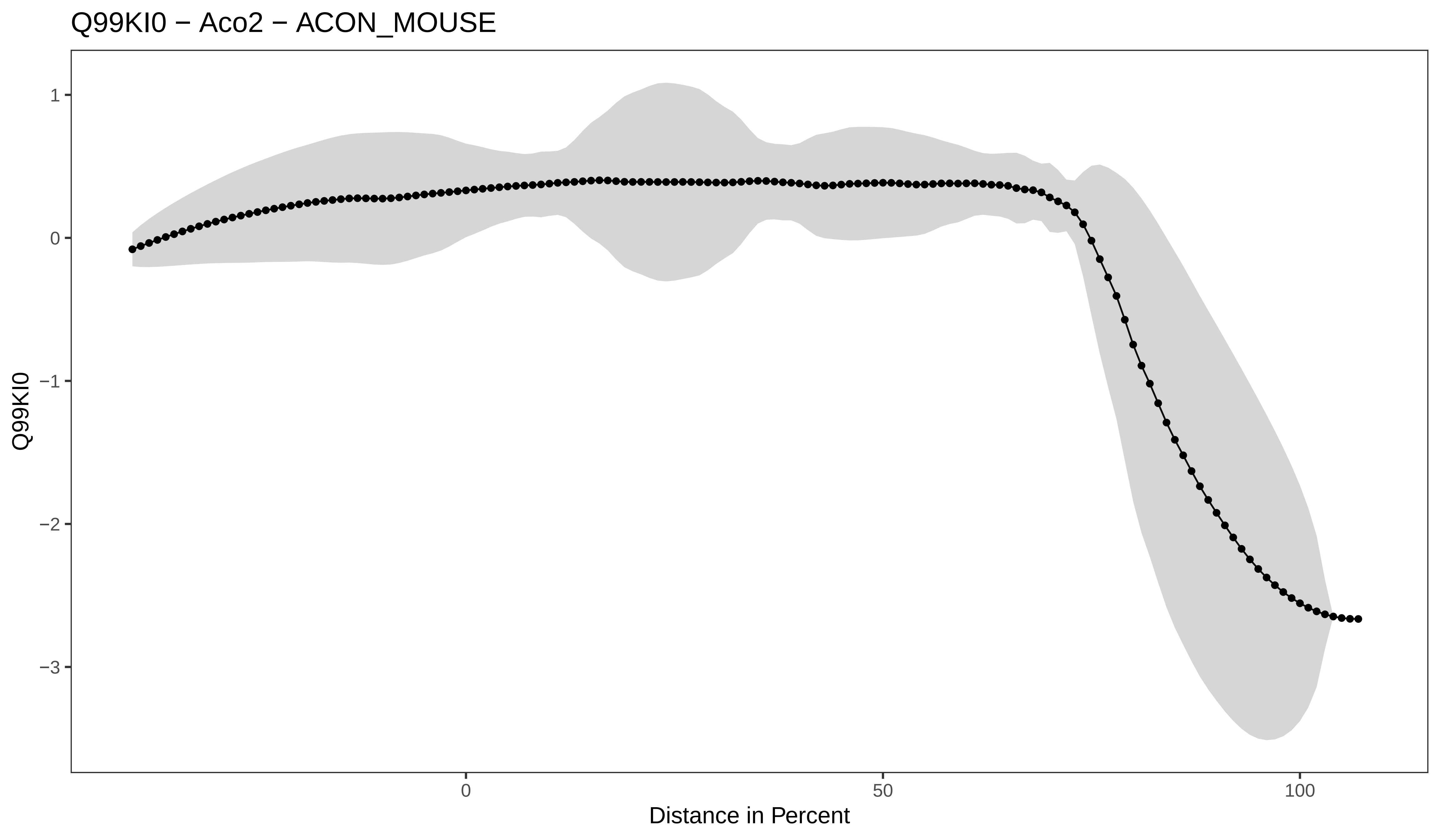

Q99KI0 − Aco2 − ACON_MOUSE
1
0
−1
−2
−3
Q99KI0
0
50
100
Distance in Percent

Q9CQR4 − Acot13 − ACO13_MOUSE

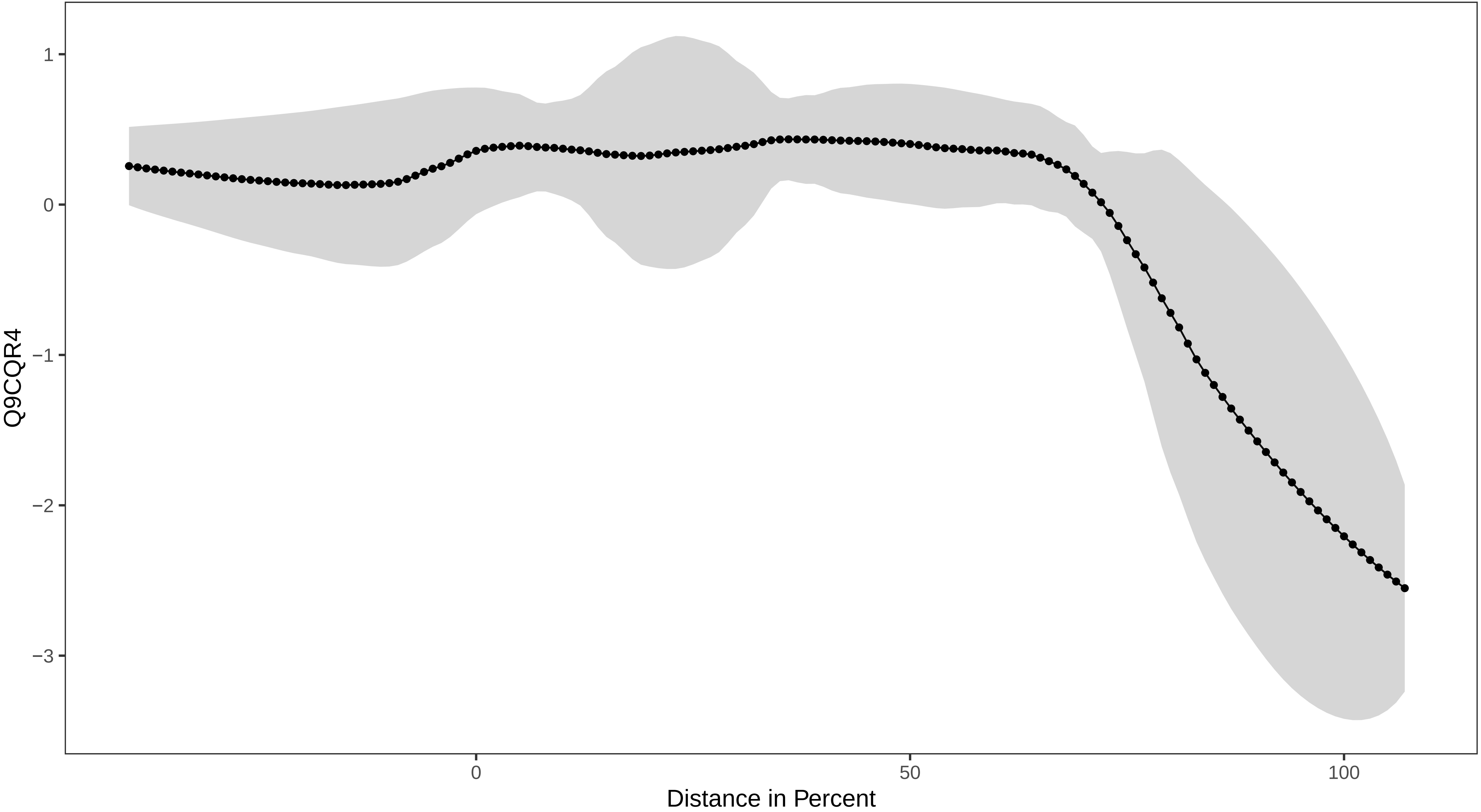

Q9QYR9 − Acot2 − ACOT2_MOUSE

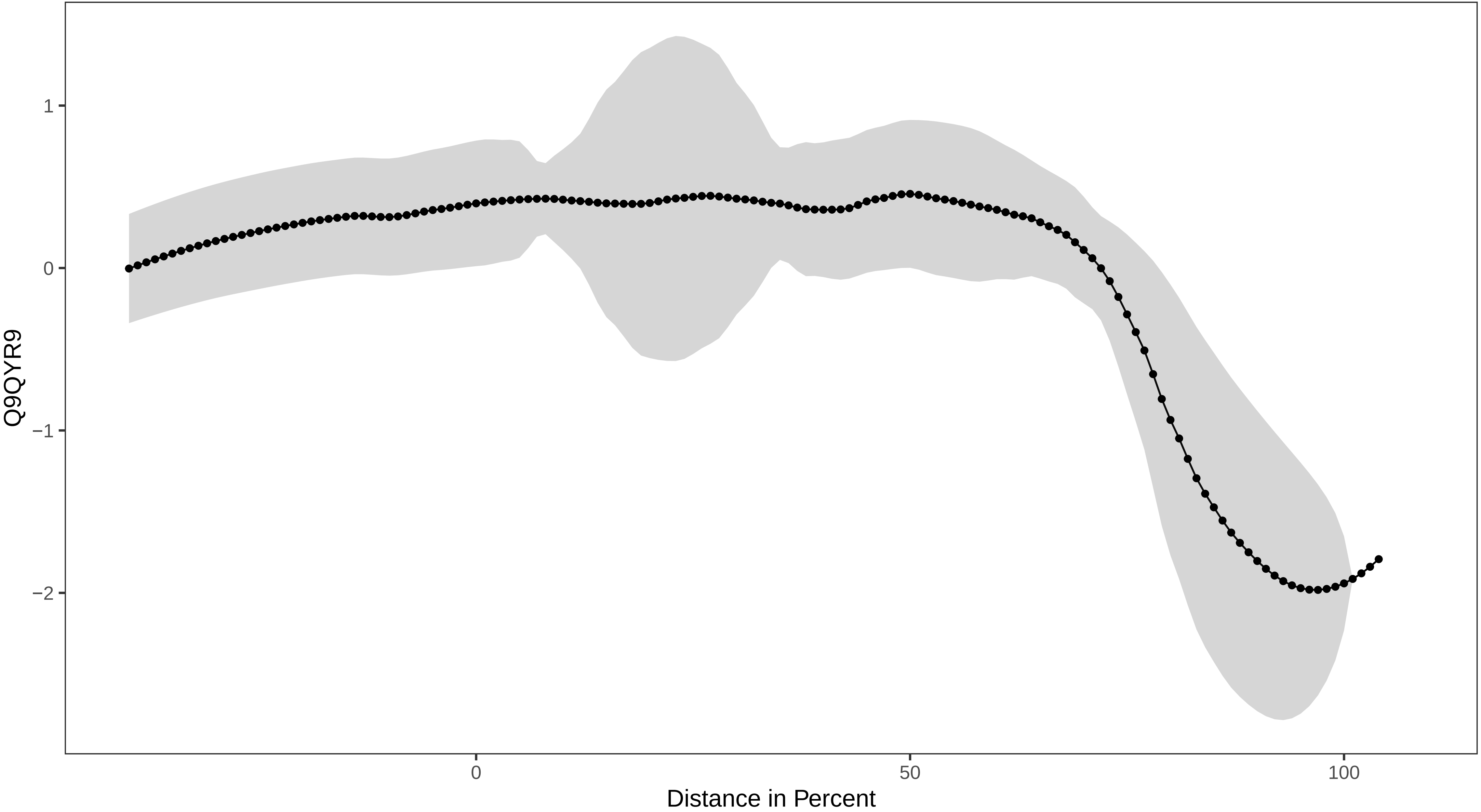

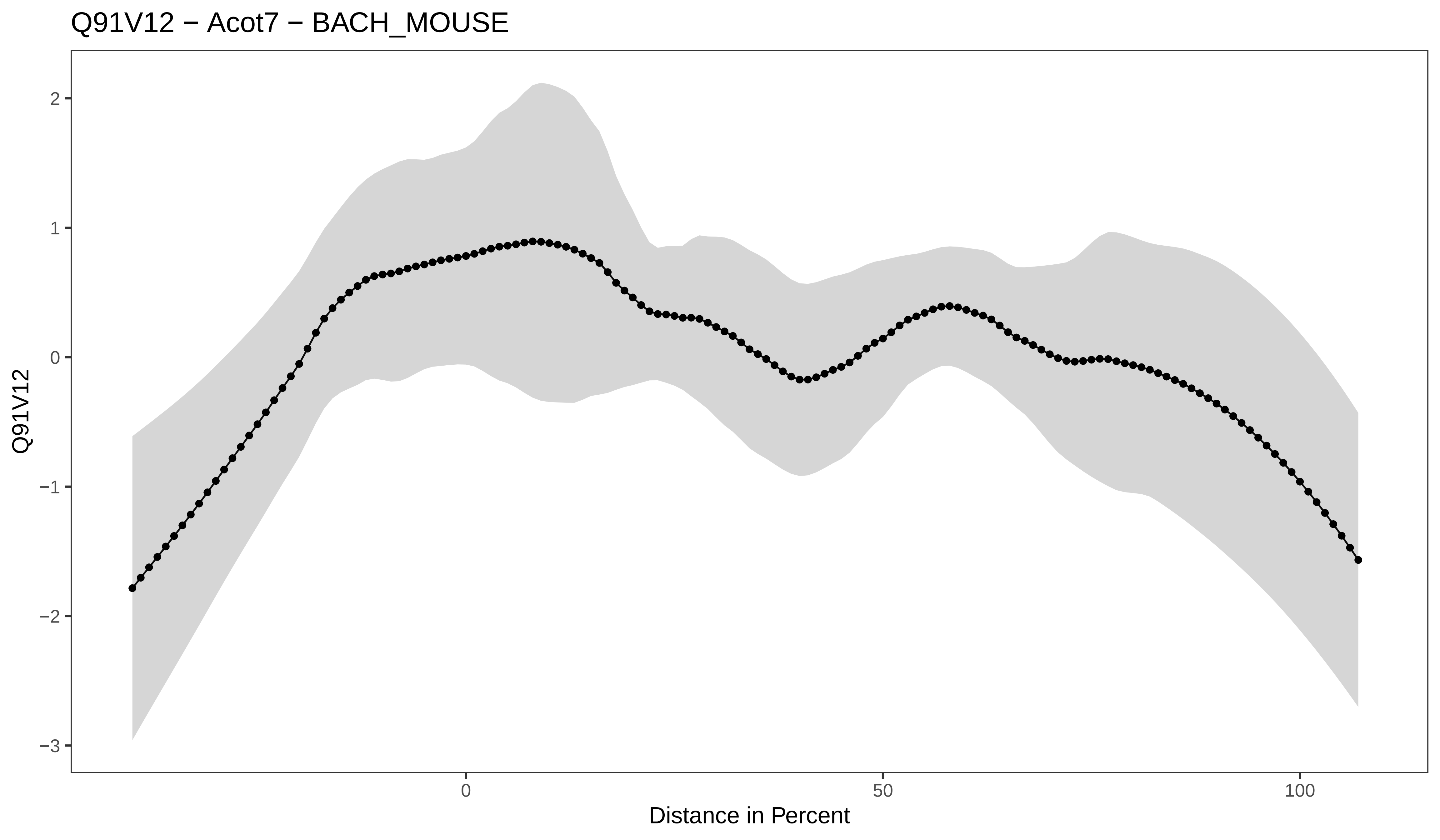
Q91V12 − Acot7 − BACH_MOUSE
Q91V12
Distance in Percent
2
1
0
−1
−2
−3
0
50
100

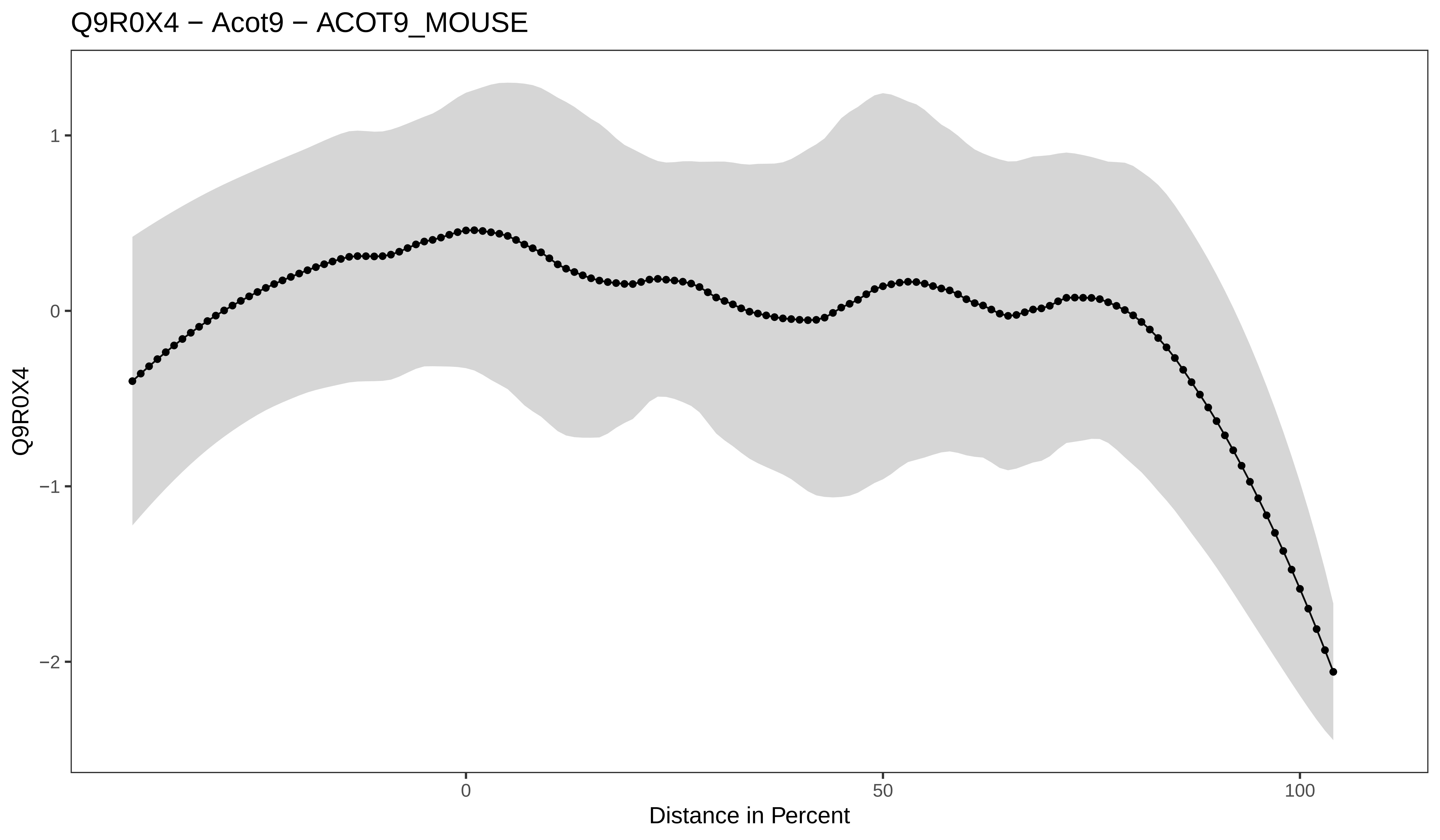
Q9R0X4 − Acot9 − ACOT9_MOUSE
Q9R0X4
Distance in Percent
1
0
−1
−2
0
50
100

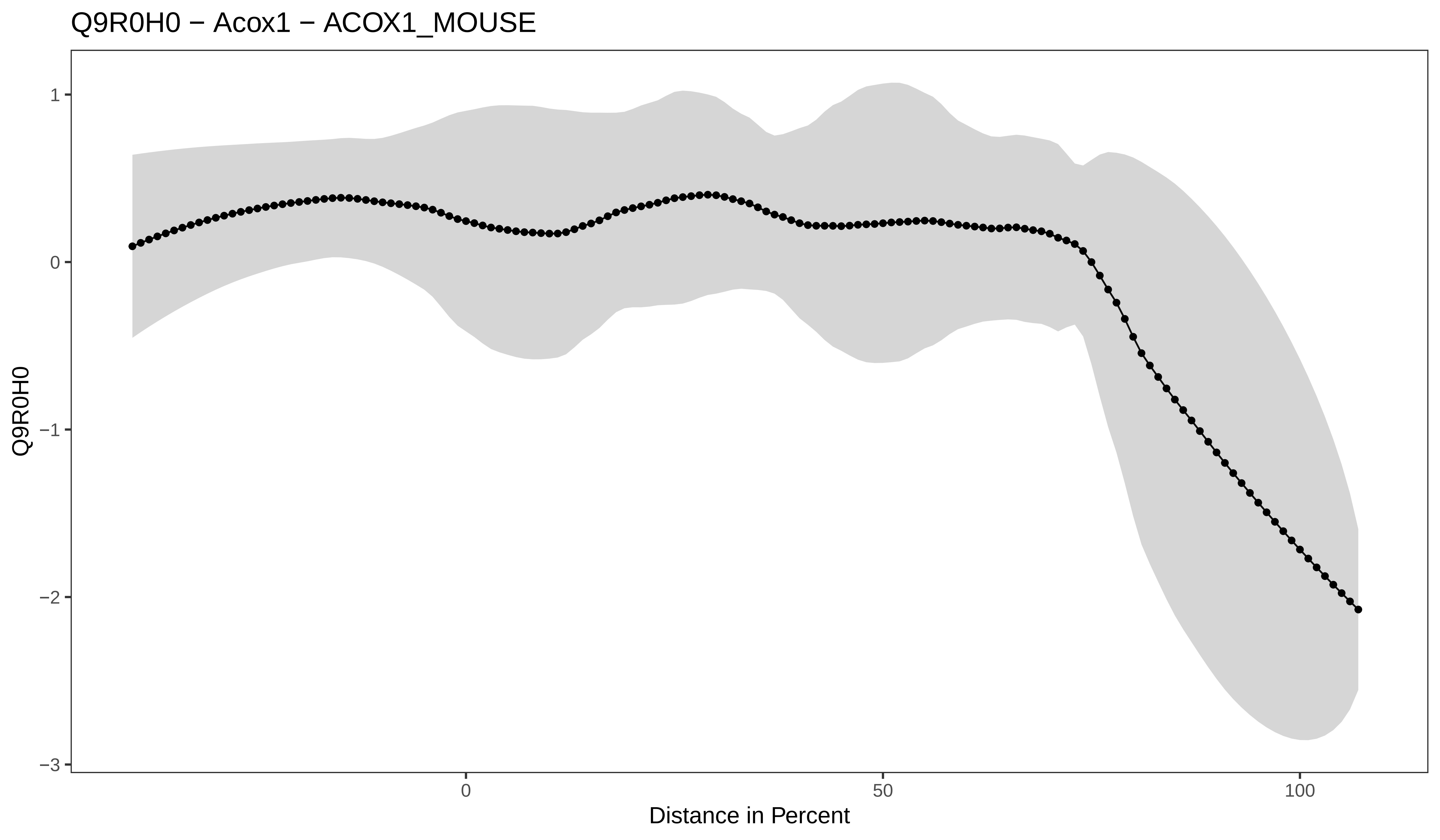

Q9R0H0 − Acox1 − ACOX1_MOUSE
Q9R0H0
1
0
−1
−2
−3
0
50
100
Distance in Percent

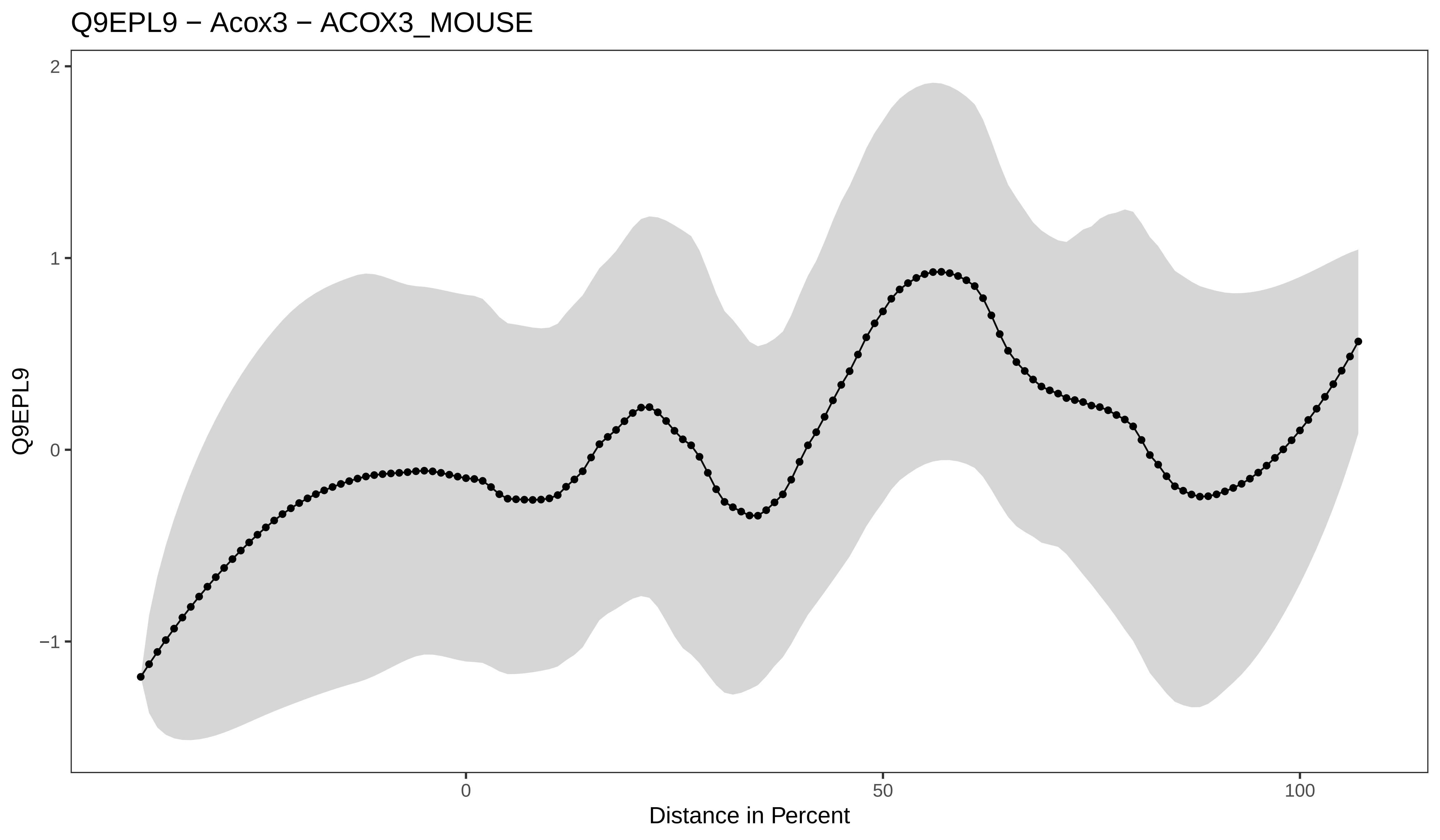

Q9EPL9 − Acox3 − ACOX3_MOUSE
2
1
0
−1
Q9EPL9
0
50
100
Distance in Percent

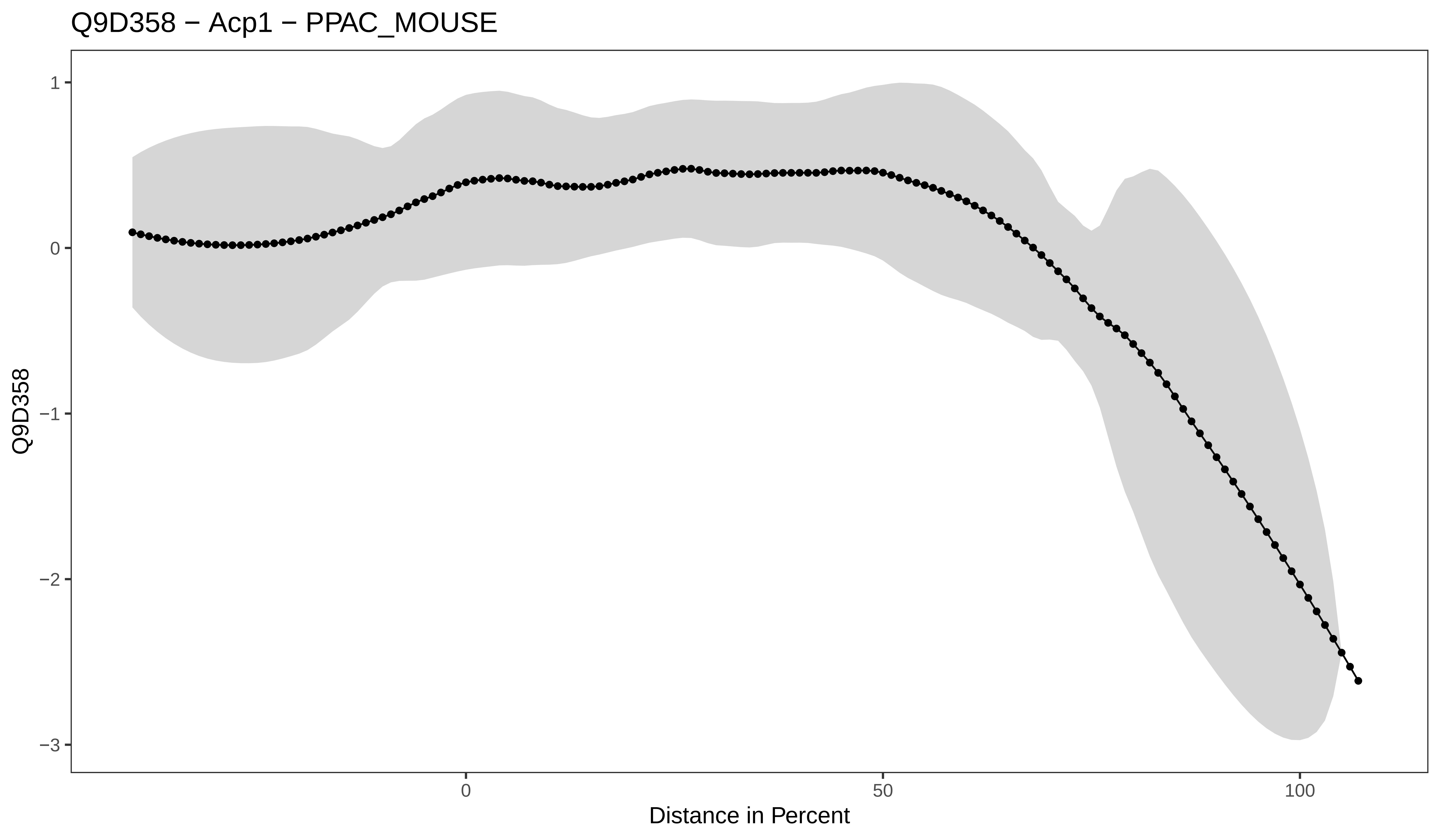

Q9D358 − Acp1 − PPAC_MOUSE
Q9D358
1
0
−1
−2
−3
0
50
100
Distance in Percent

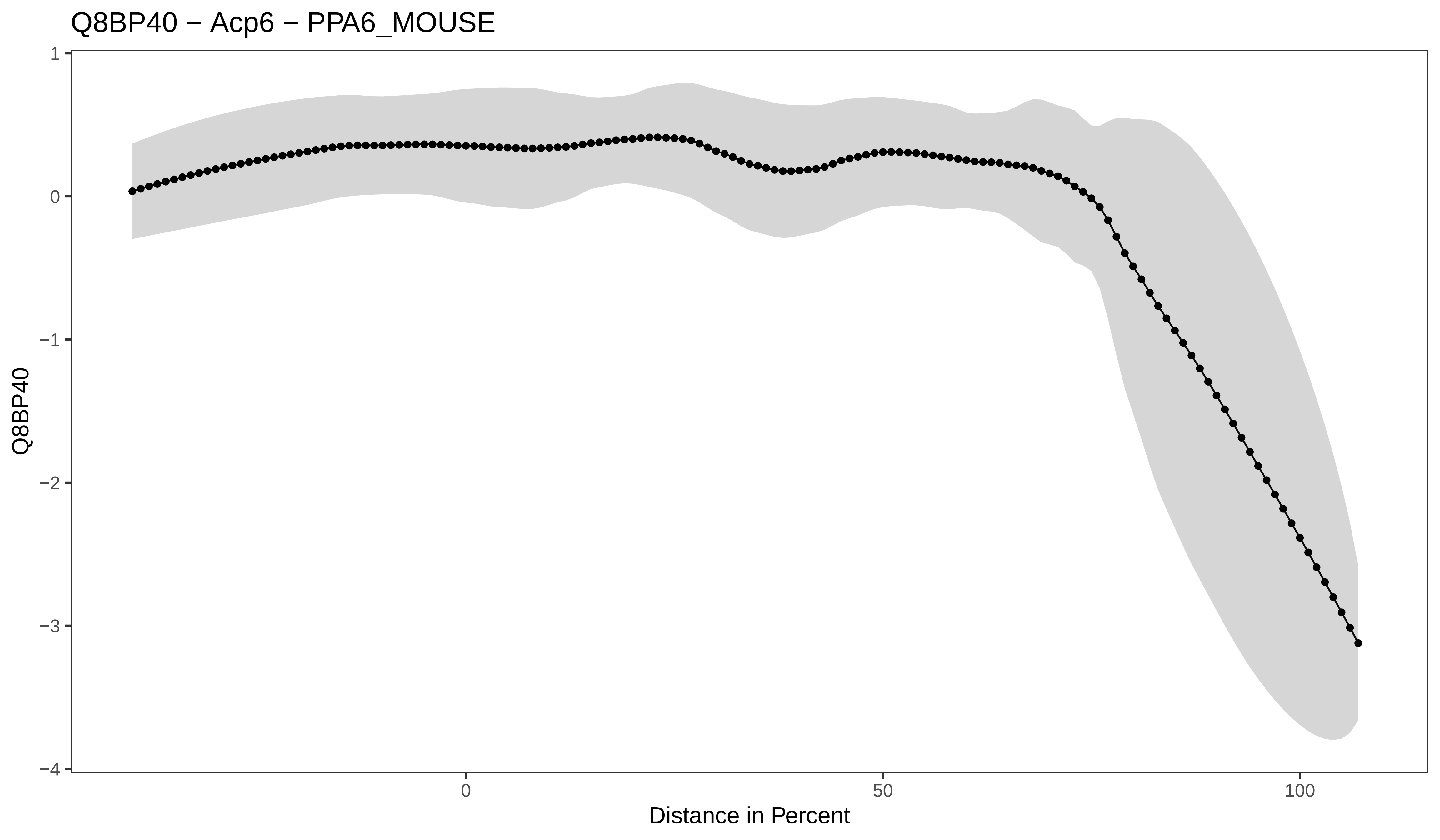
Q8BP40 − Acp6 − PPA6_MOUSE
Q8BP40
Distance in Percent
1
0
−1
−2
−3
−4
0
50
100

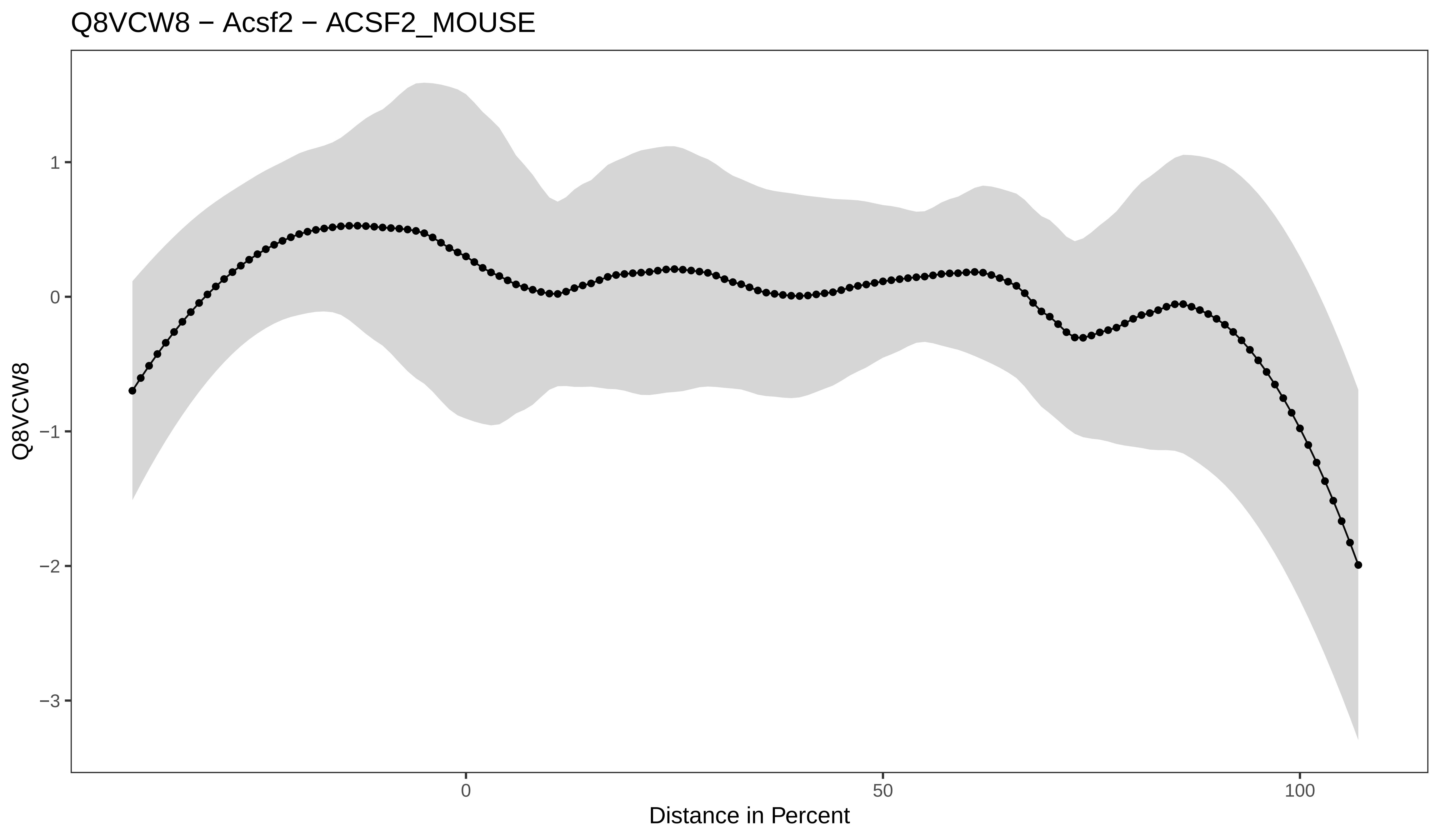

Q8VCW8 − Acsf2 − ACSF2_MOUSE
Q8VCW8
1
0
−1
−2
−3
0
50
100
Distance in Percent

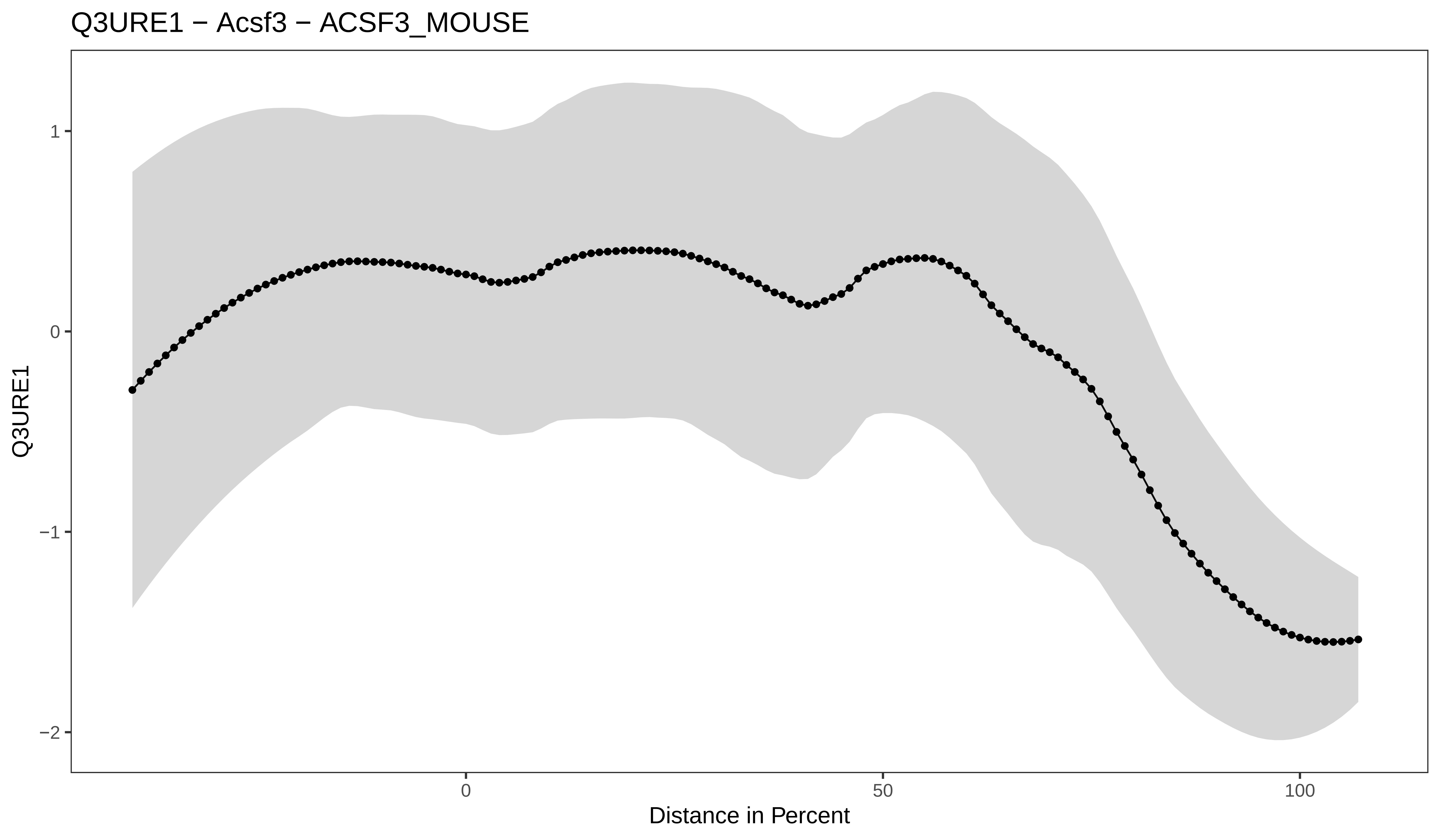
Q3URE1 − Acsf3 − ACSF3_MOUSE
Q3URE1
1
0
−1
−2
0
50
100
Distance in Percent

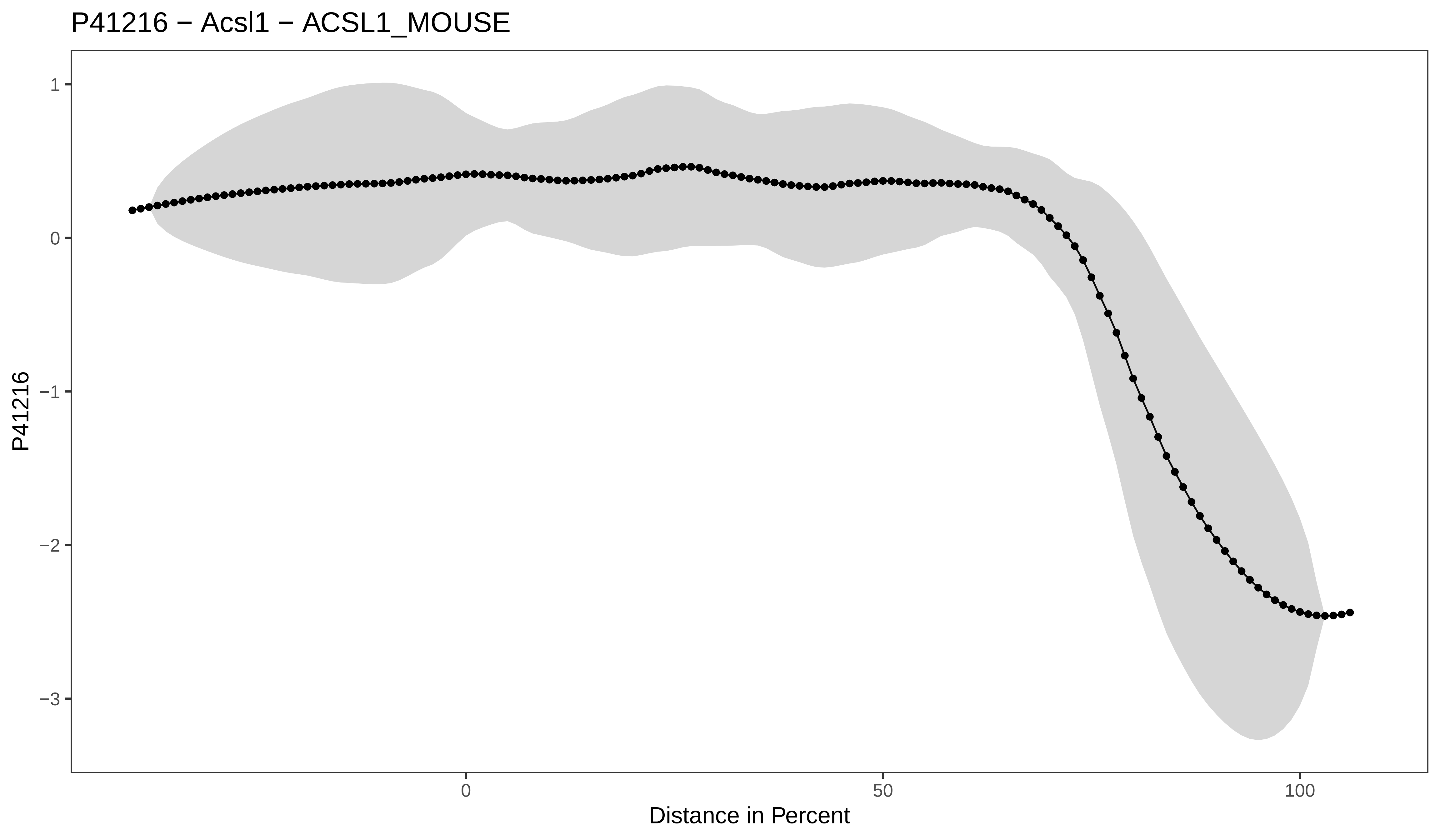

P41216 − Acsl1 − ACSL1_MOUSE
1
0
−1
−2
−3
P41216
0
50
100
Distance in Percent

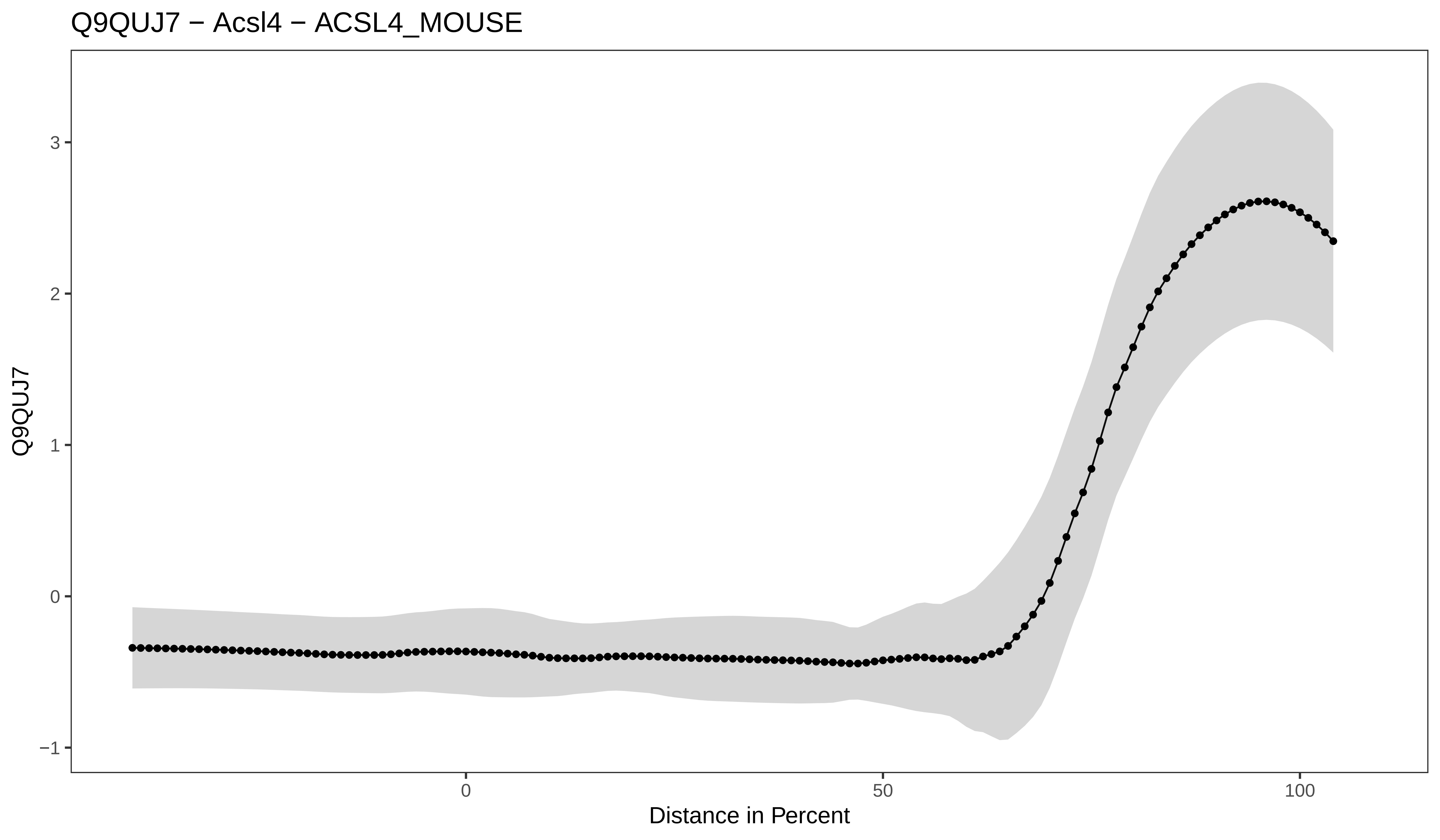
Q9QUJ7 − Acsl4 − ACSL4_MOUSE
Q9QUJ7
3
2
1
0
−1
0
50
100
Distance in Percent

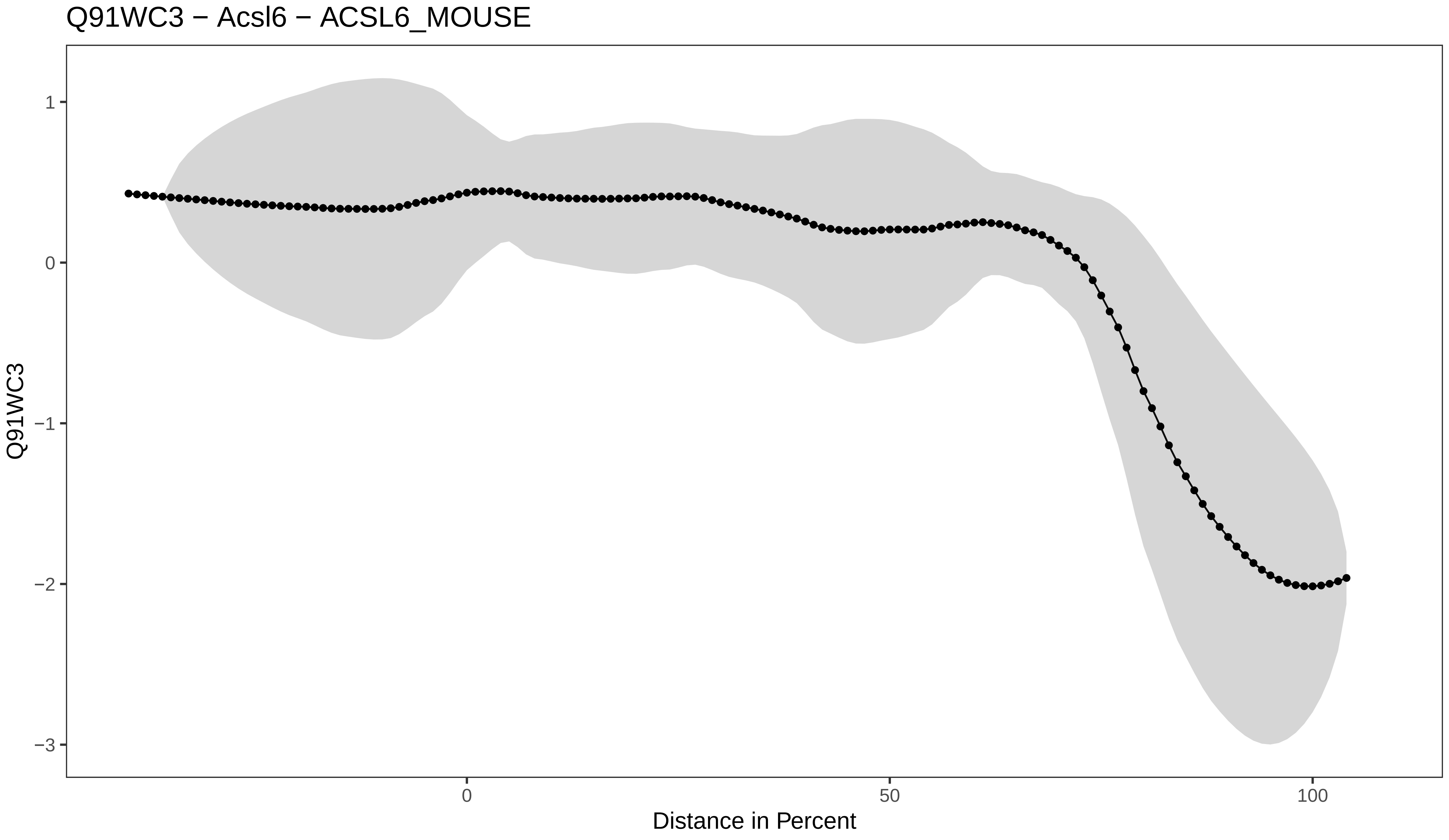

Q91WC3 − Acsl6 − ACSL6_MOUSE
1
0
−1
−2
−3
Q91WC3
0
50
100
Distance in Percent

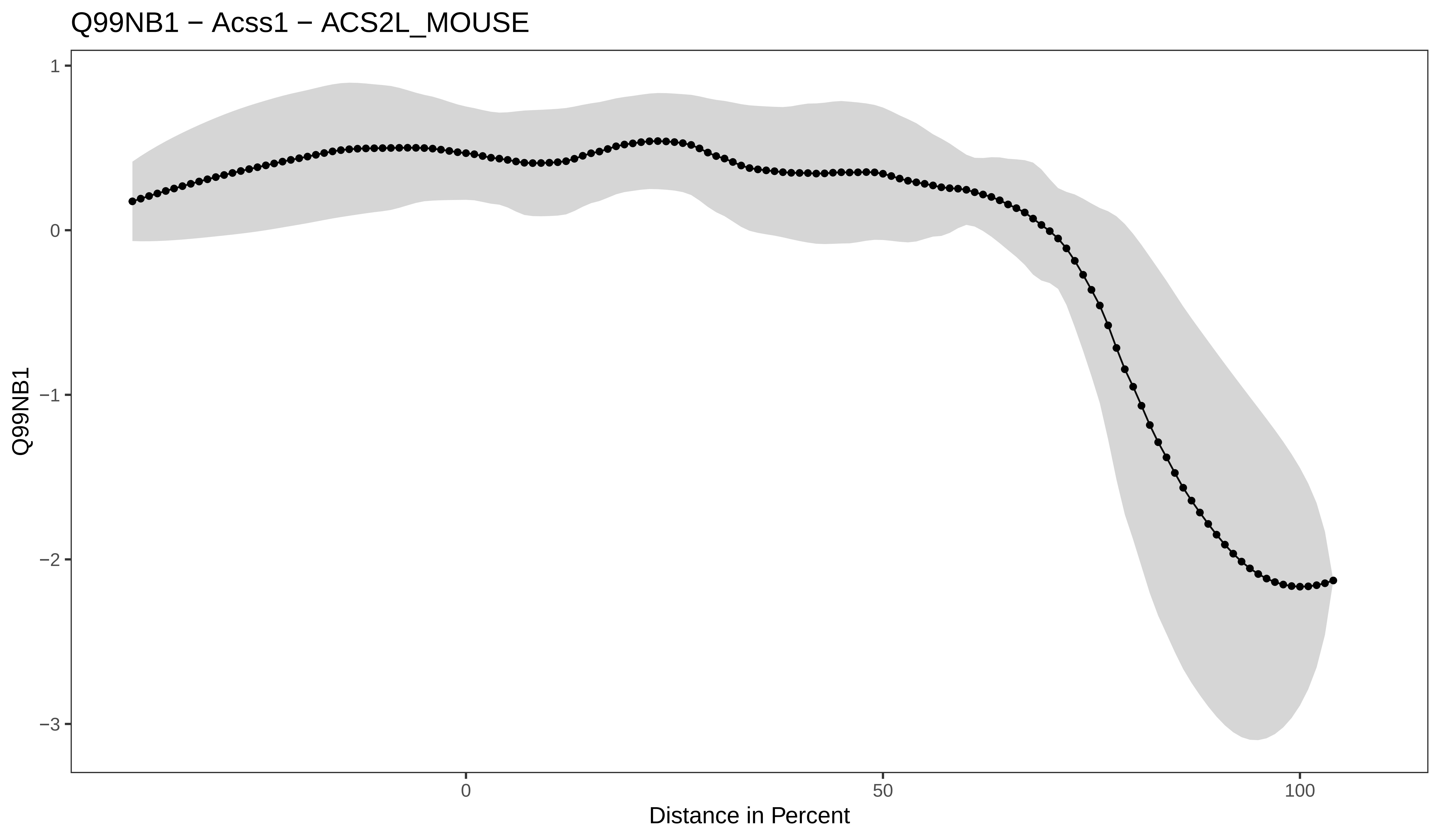

Q99NB1 − Acss1 − ACS2L_MOUSE
Q99NB1
1
0
−1
−2
−3
0
50
100
Distance in Percent

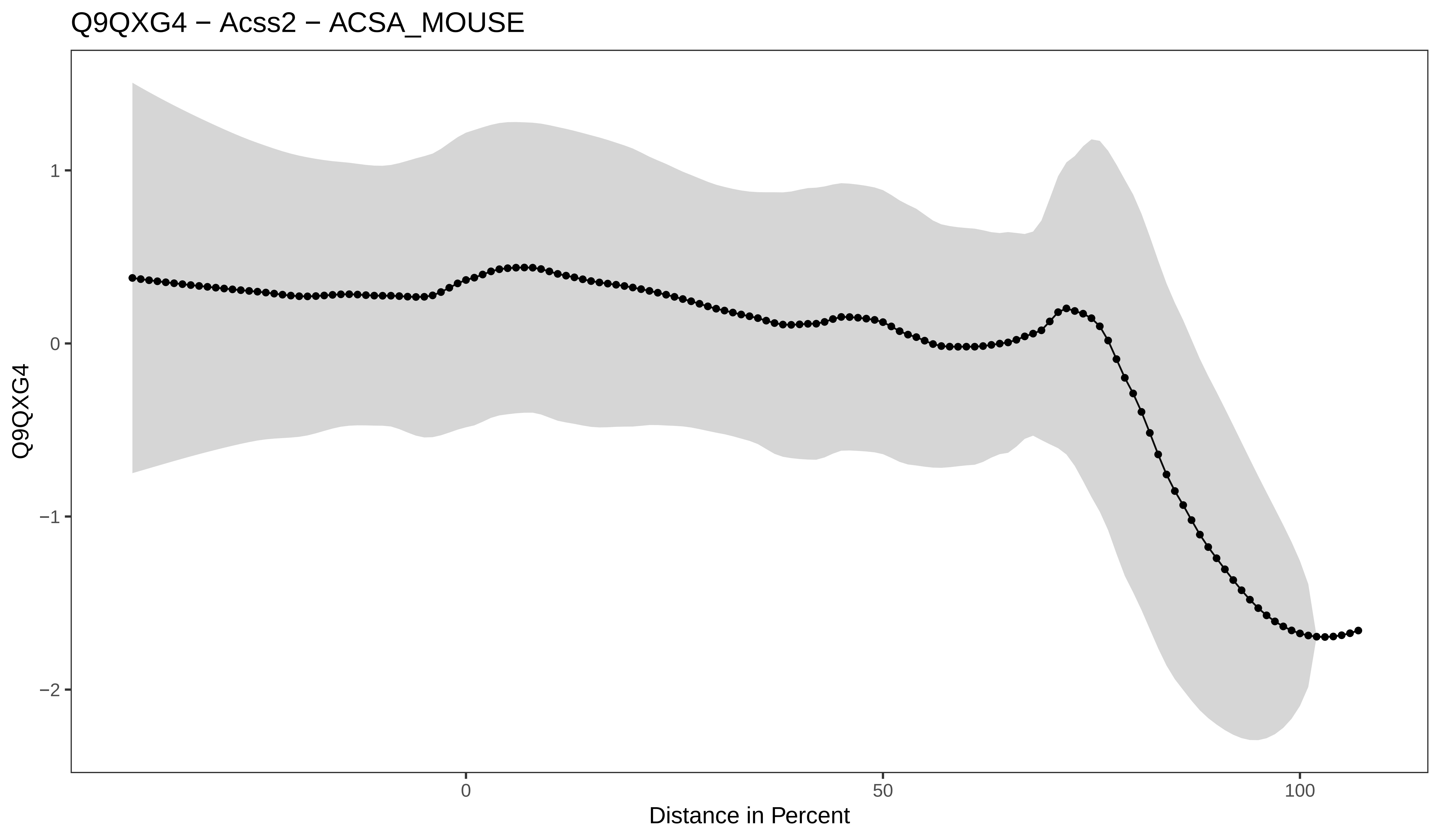

Q9QXG4 − Acss2 − ACSA_MOUSE
1
0
−1
−2
Q9QXG4
0
50
100
Distance in Percent

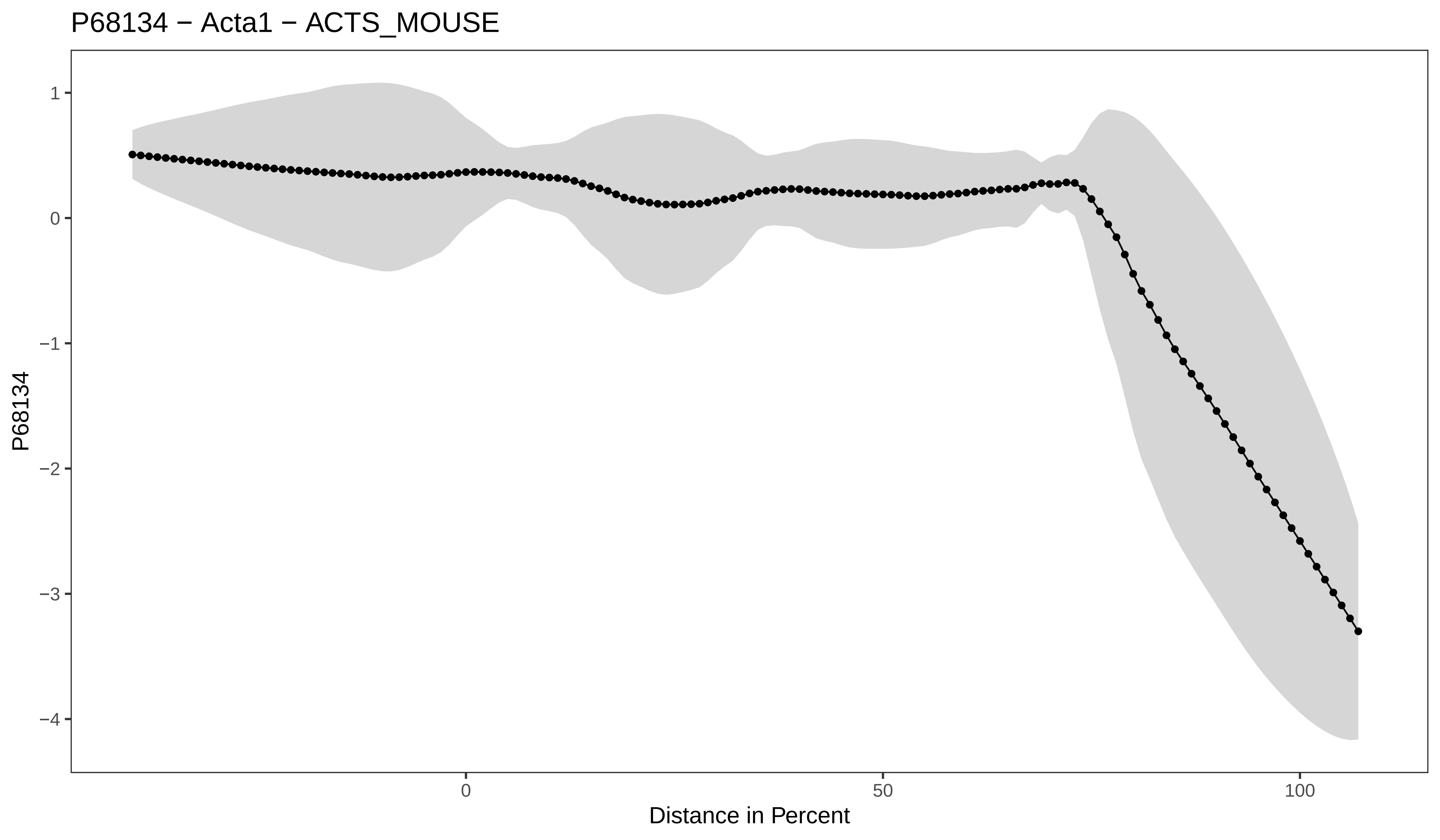
P68134 − Acta1 − ACTS_MOUSE
P68134
Distance in Percent
1
0
−1
−2
−3
−4
0
50
100

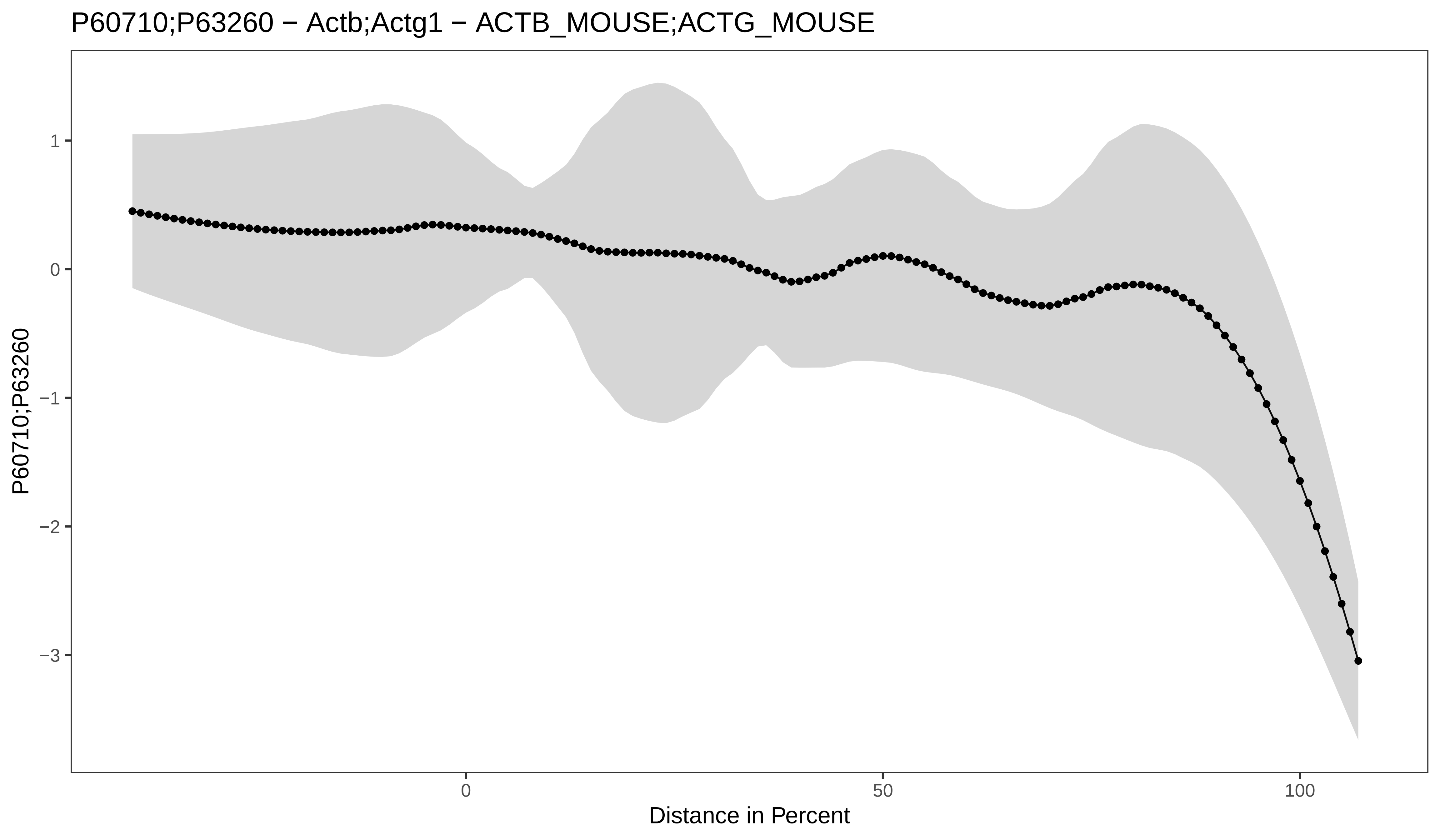
P60710;P63260 − Actb;Actg1 − ACTB_MOUSE;ACTG_MOUSE
P60710;P63260
Distance in Percent
1
0
−1
−2
−3
0
50
100

Q8BFZ3 − Actbl2 − ACTBL_MOUSE

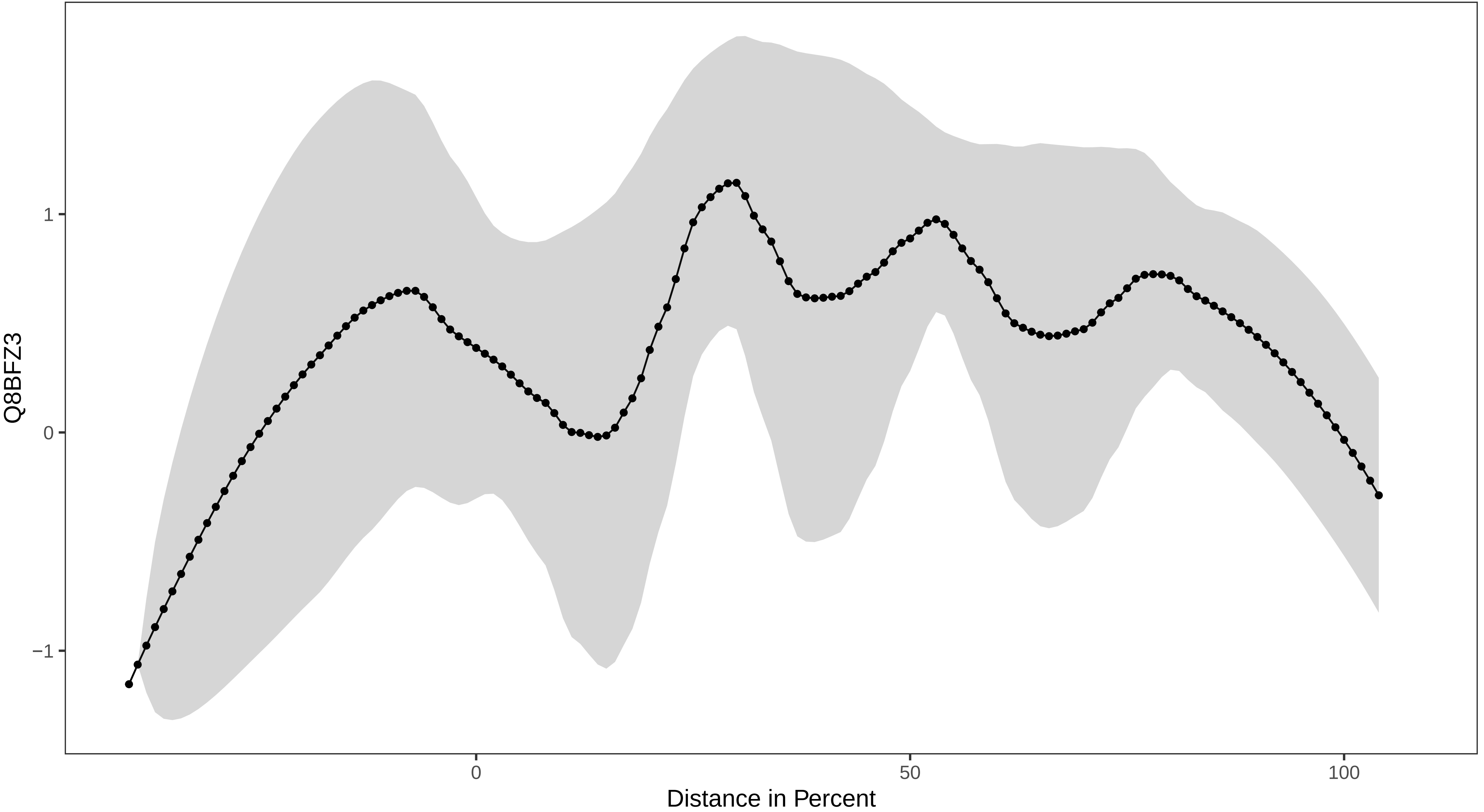

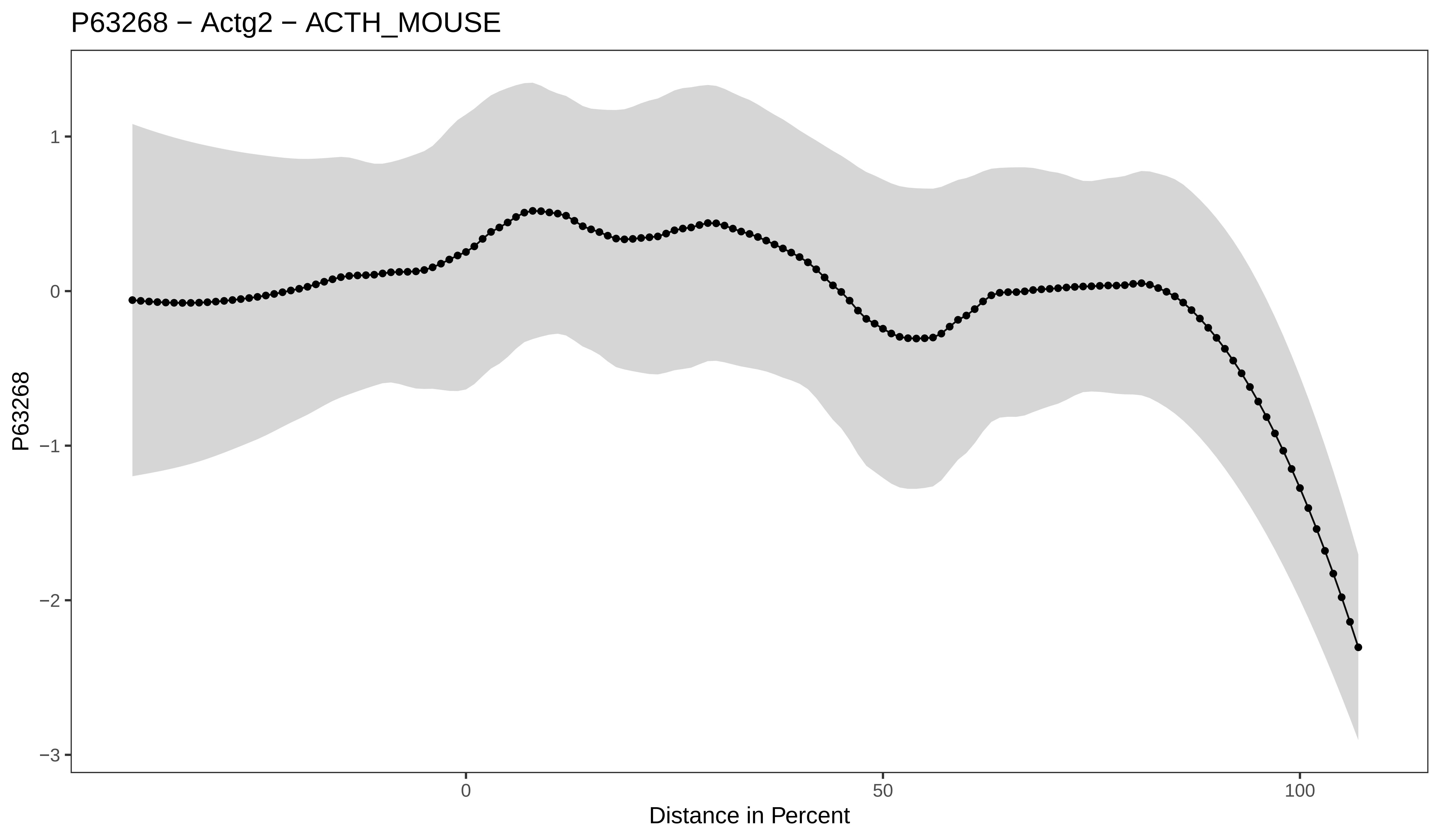
P63268 − Actg2 − ACTH_MOUSE
P63268
1
0
−1
−2
−3
0
50
100
Distance in Percent

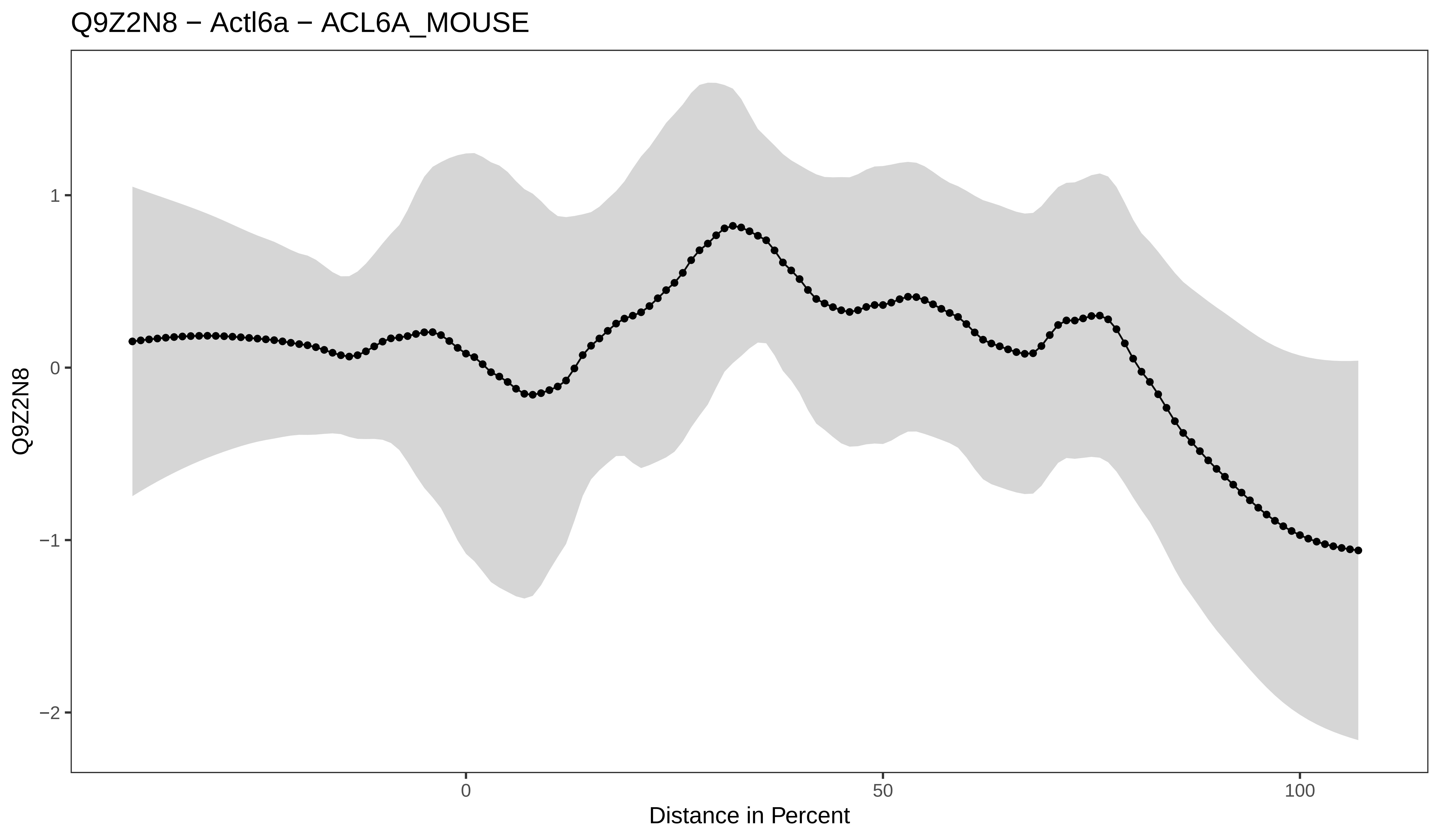

Q9Z2N8 − Actl6a − ACL6A_MOUSE
Q9Z2N8
1
0
−1
−2
0
50
100
Distance in Percent

Q7TPR4 − Actn1 − ACTN1_MOUSE

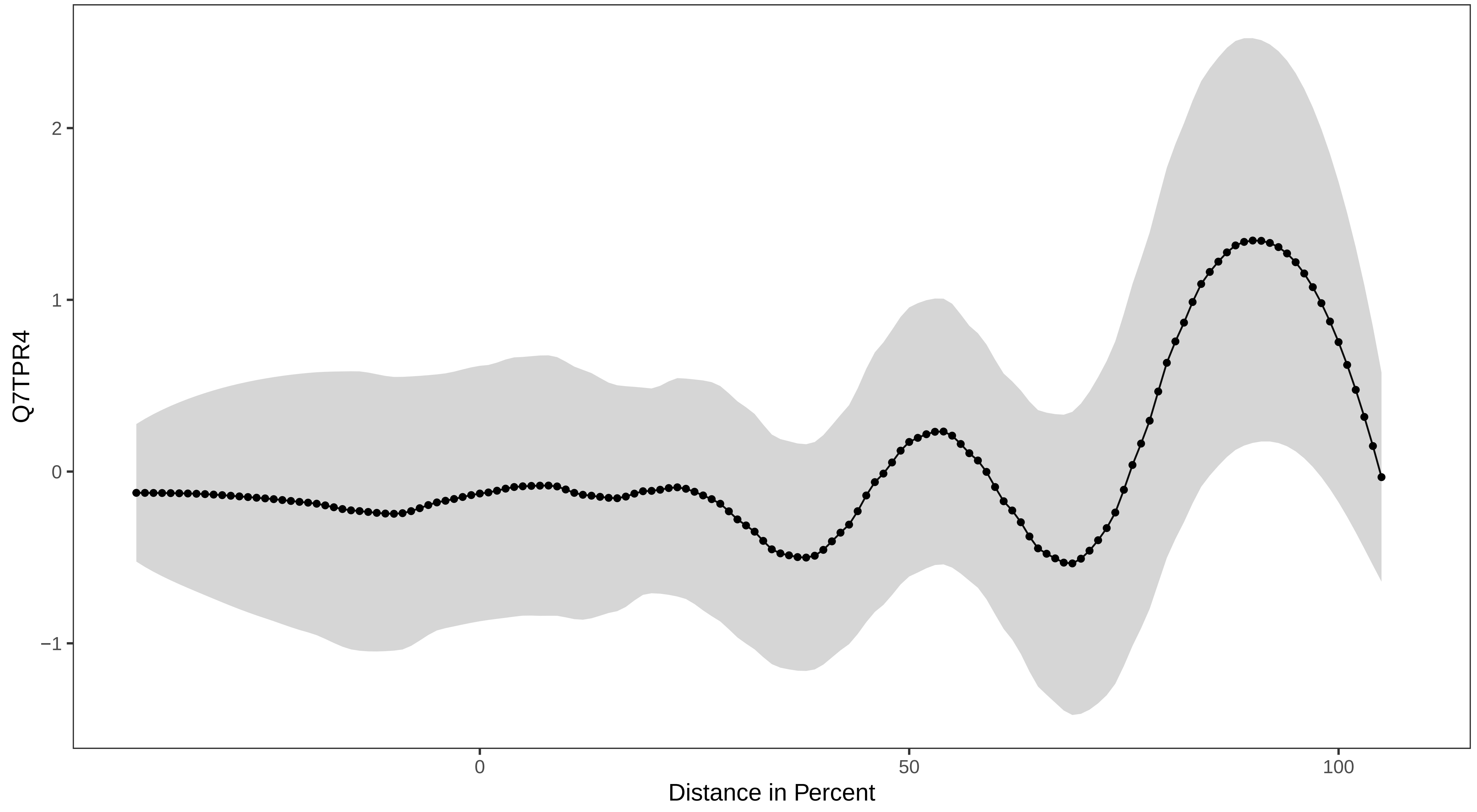

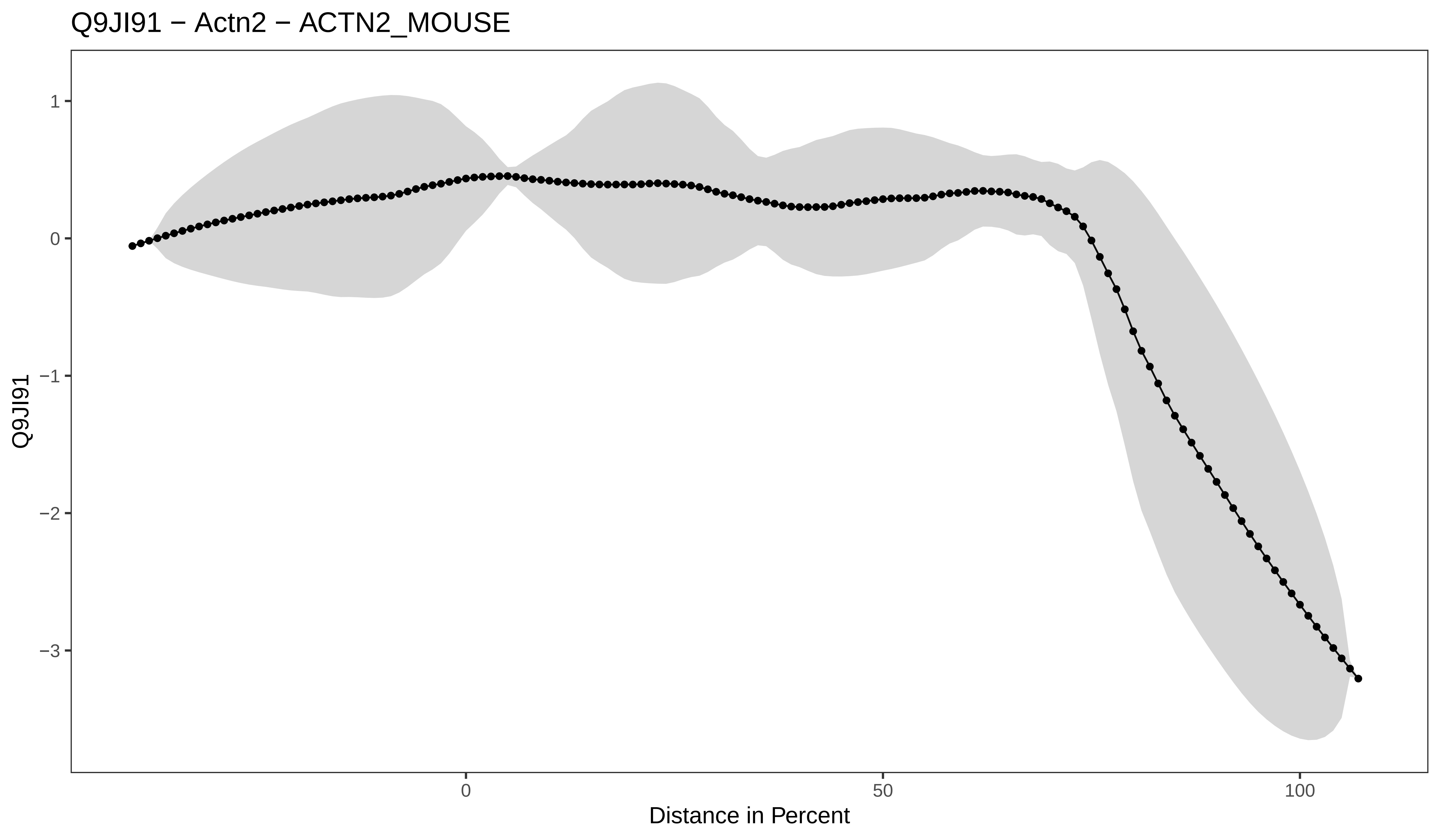

Q9JI91 − Actn2 − ACTN2_MOUSE
1
0
−1
−2
−3
Q9JI91
0
50
100
Distance in Percent

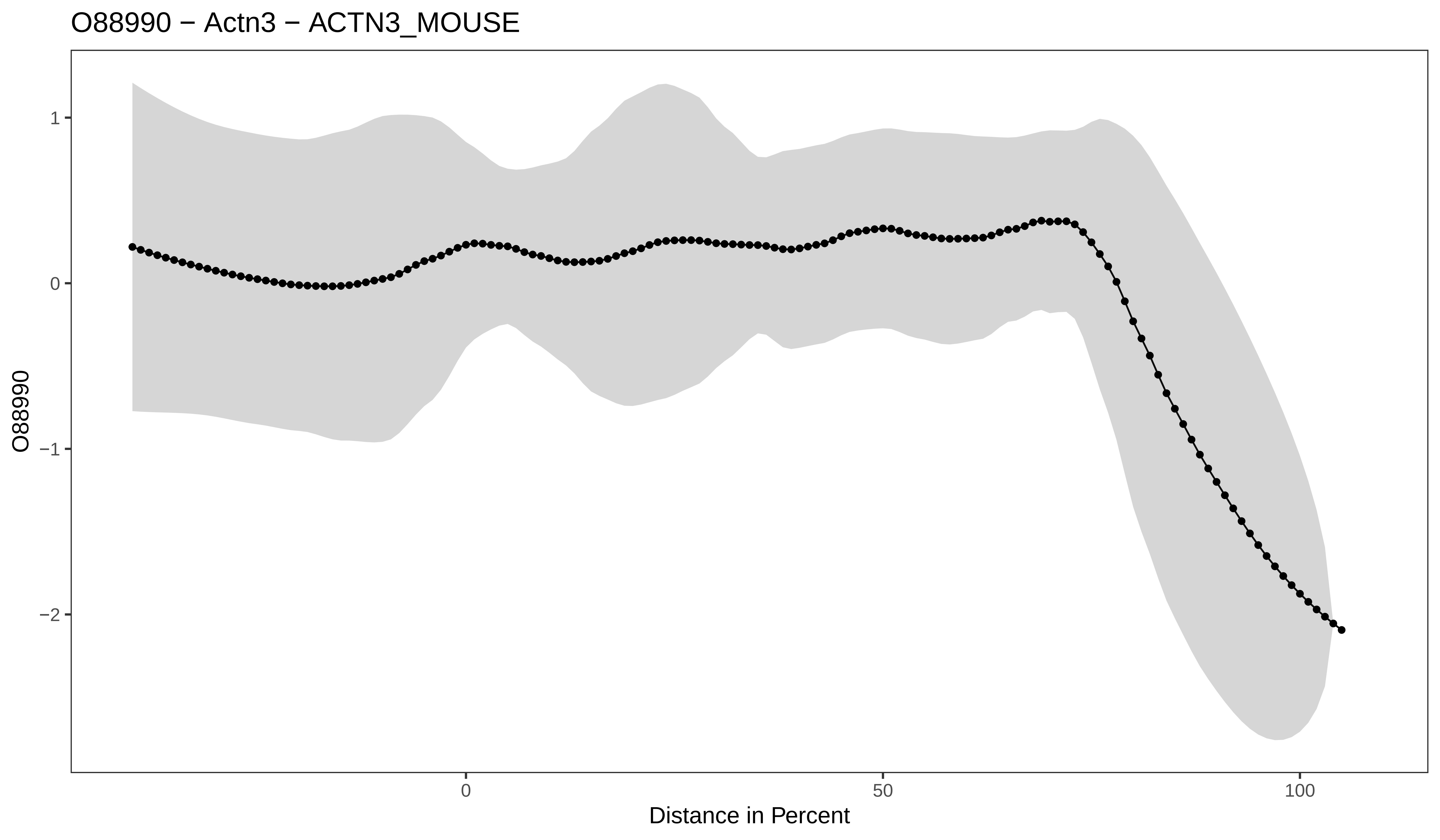
O88990 − Actn3 − ACTN3_MOUSE
O88990
1
0
−1
−2
0
50
100
Distance in Percent

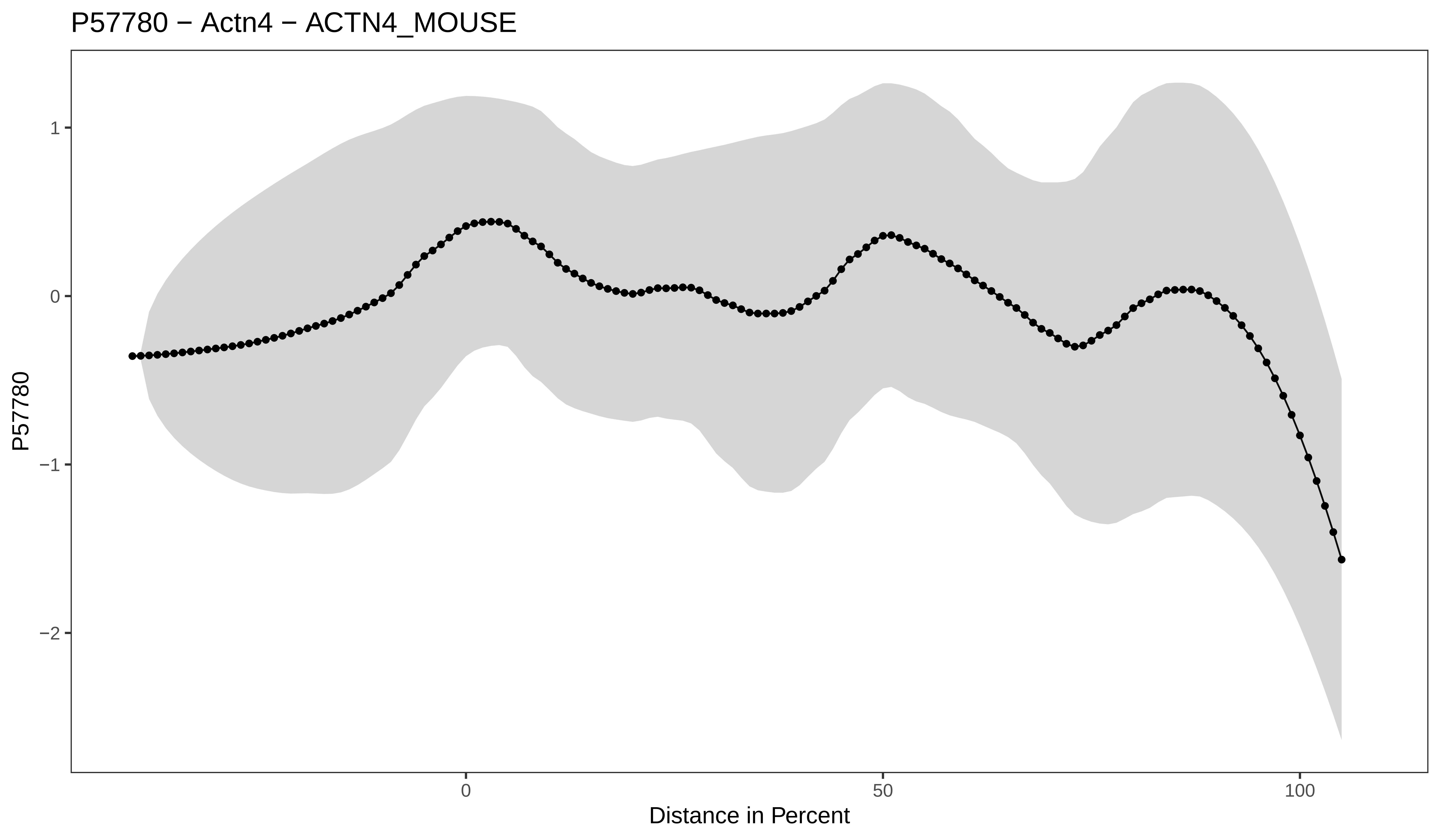
P57780 − Actn4 − ACTN4_MOUSE
P57780
1
0
−1
−2
0
50
100
Distance in Percent

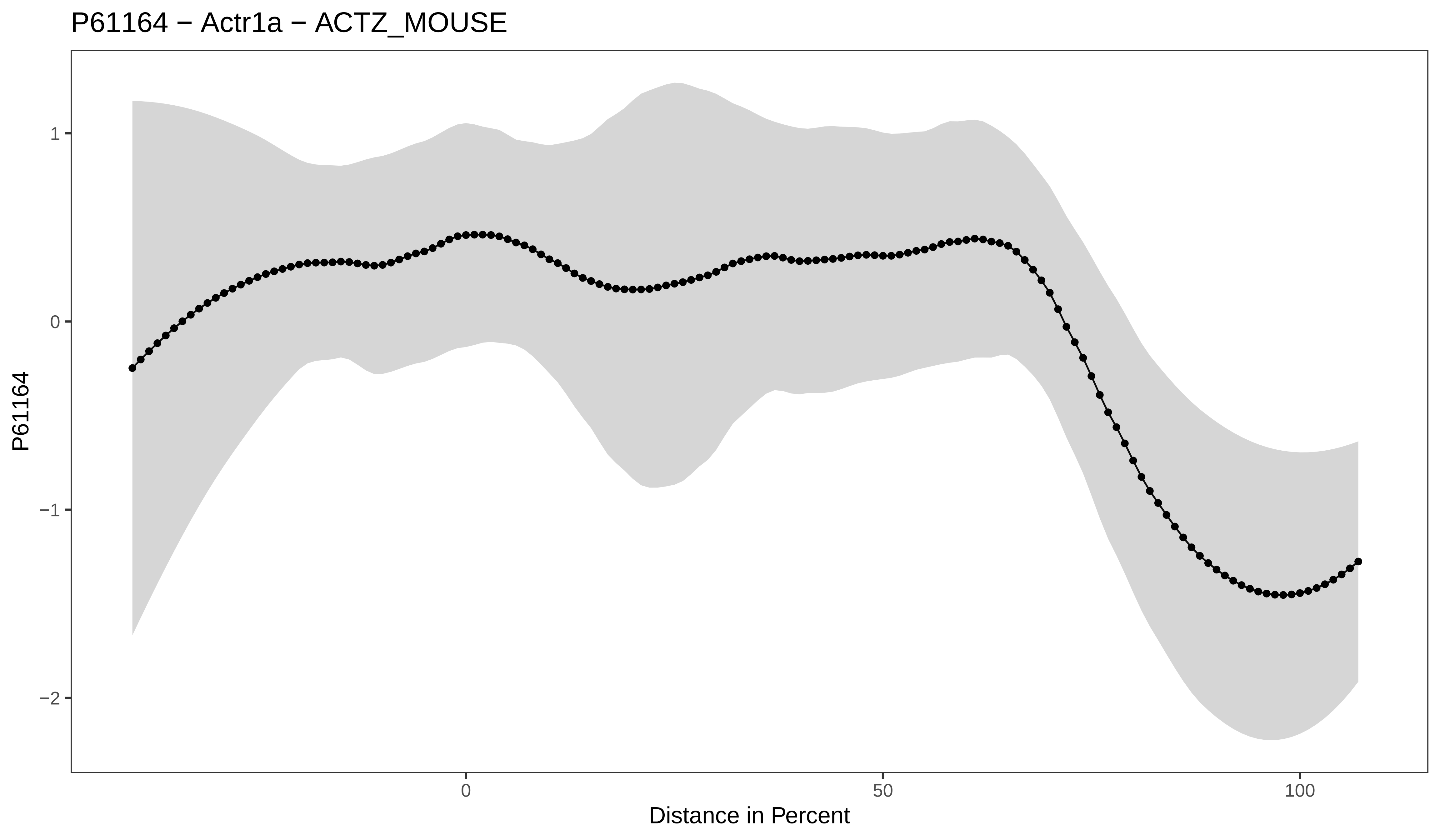

P61164 − Actr1a − ACTZ_MOUSE
P61164
Distance in Percent
1
0
−1
−2
0
50
100

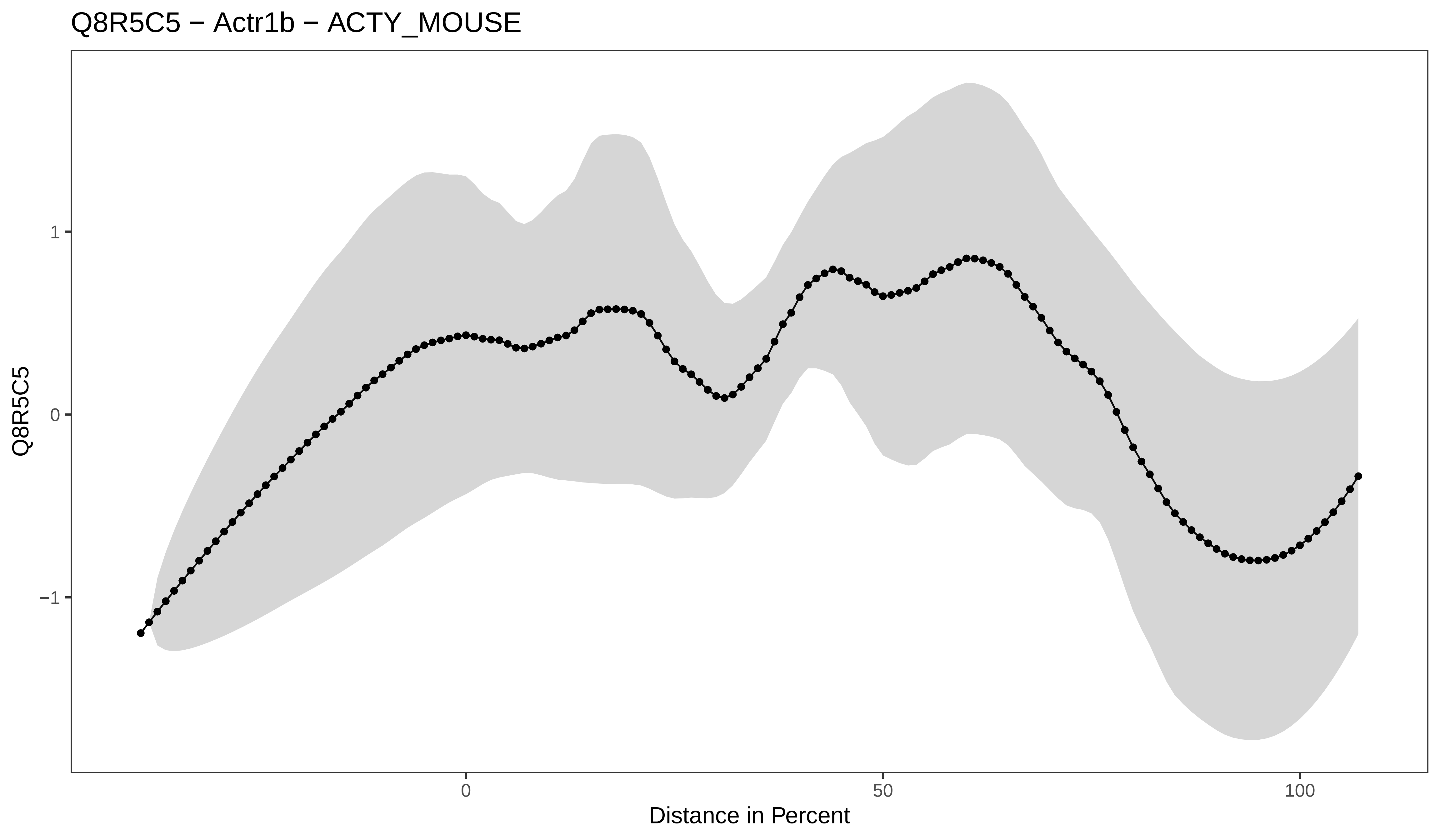
Q8R5C5 − Actr1b − ACTY_MOUSE
Q8R5C5
1
0
−1
0
50
100
Distance in Percent

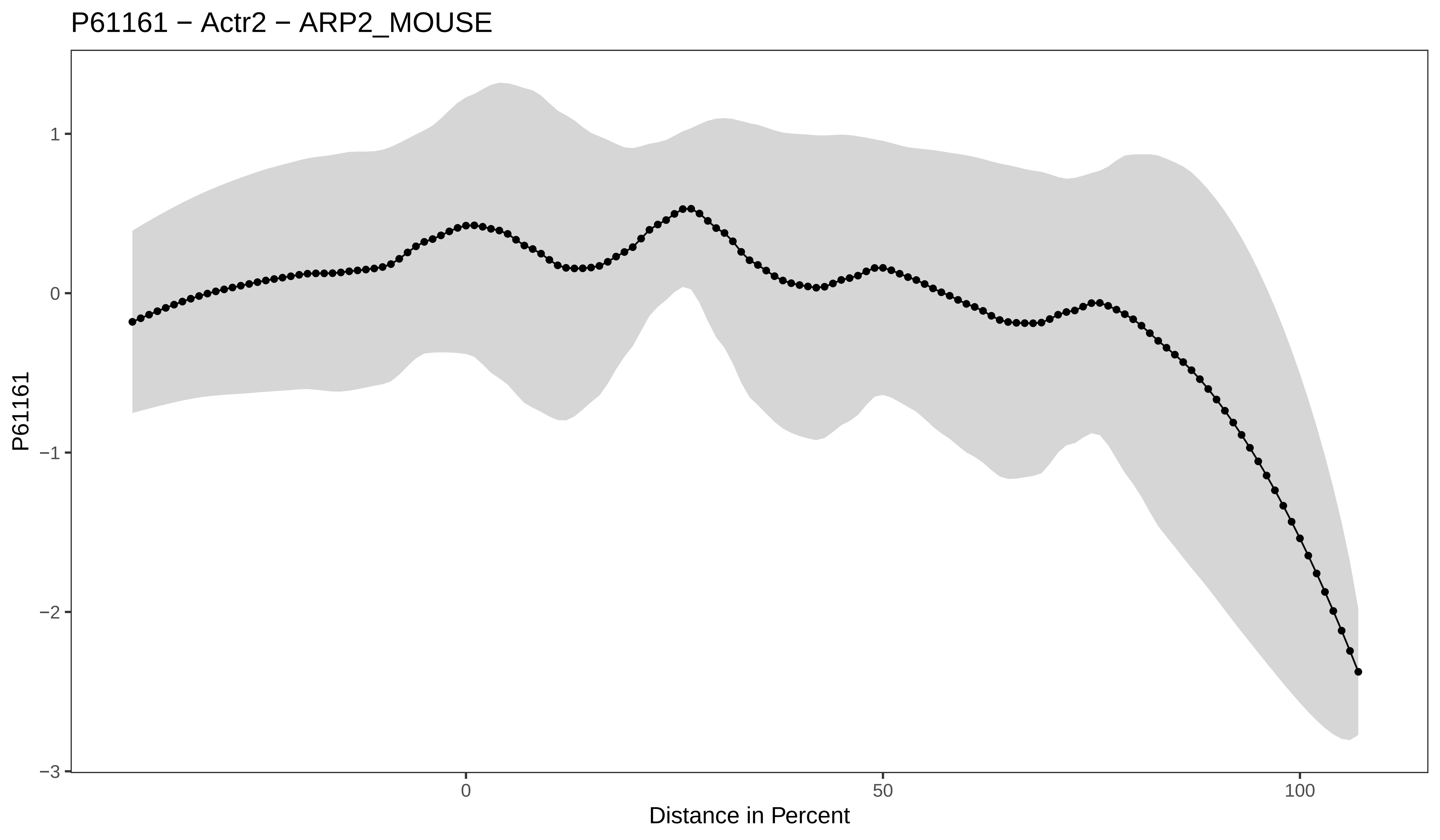
P61161 − Actr2 − ARP2_MOUSE
1
0
−1
−2
−3
P61161
0
50
100
Distance in Percent

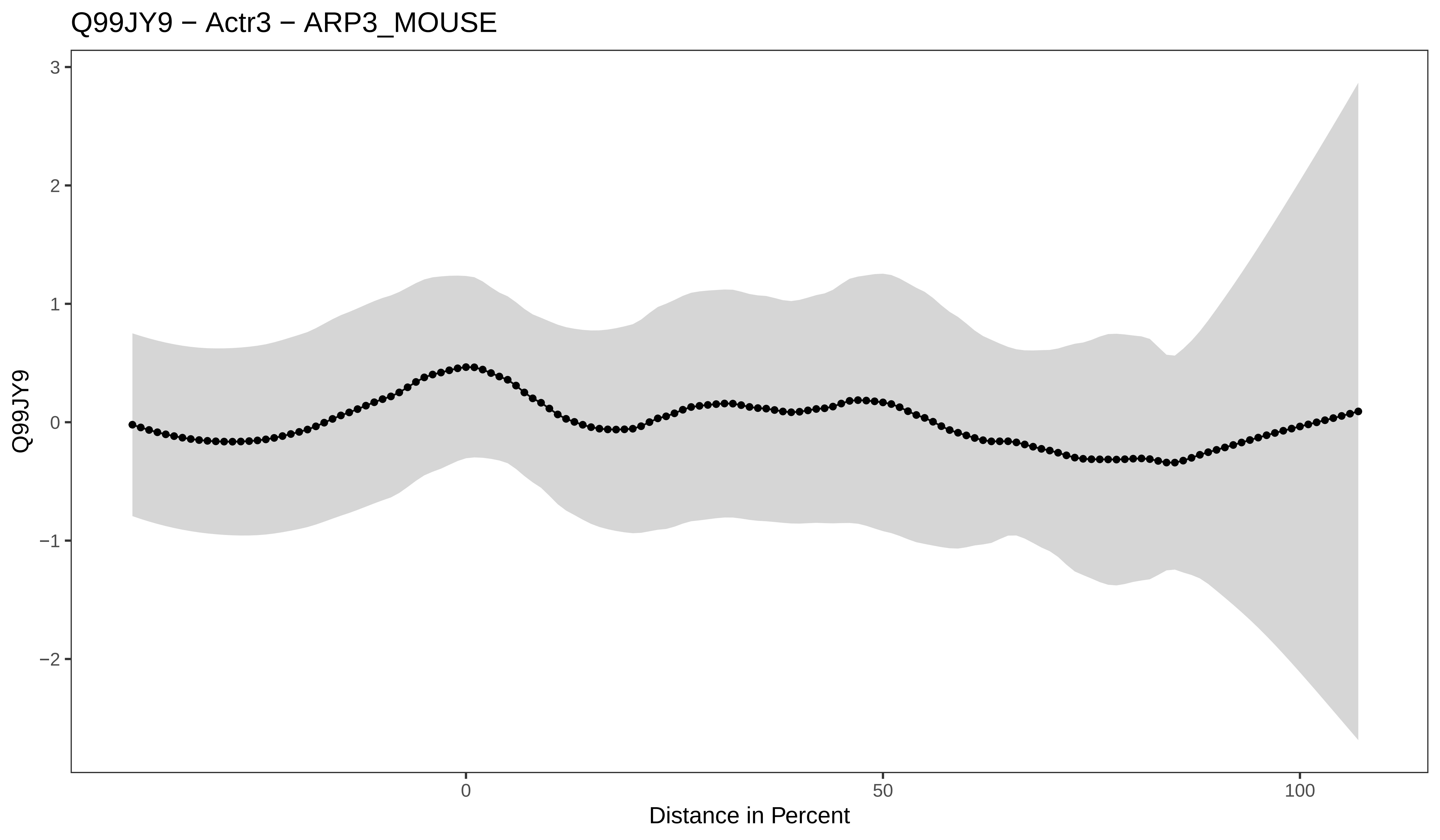
Q99JY9 − Actr3 − ARP3_MOUSE
3
2
1
0
−1
−2
Q99JY9
0
50
100
Distance in Percent

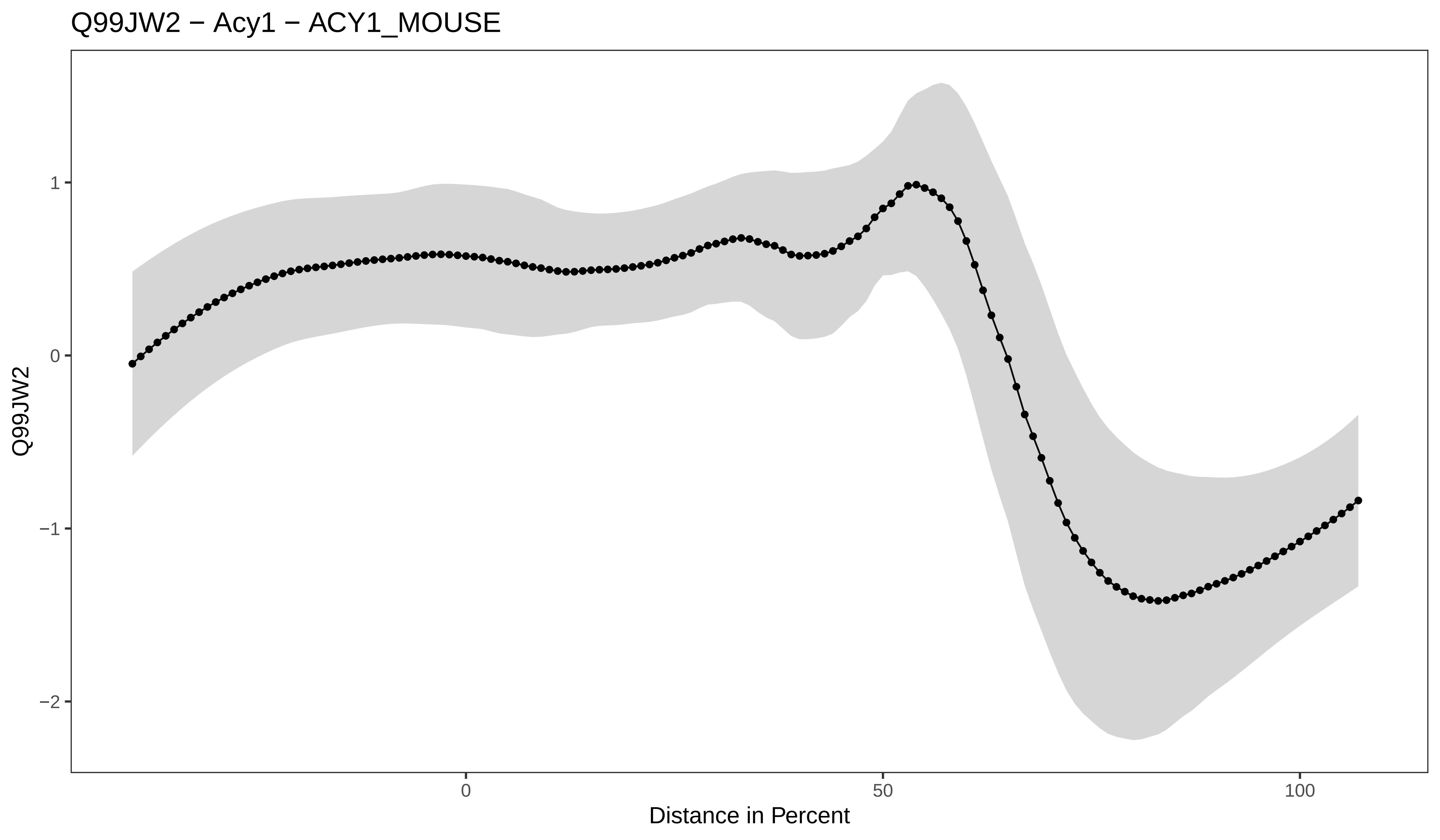
Q99JW2 − Acy1 − ACY1_MOUSE
Q99JW2
1
0
−1
−2
0
50
100
Distance in Percent

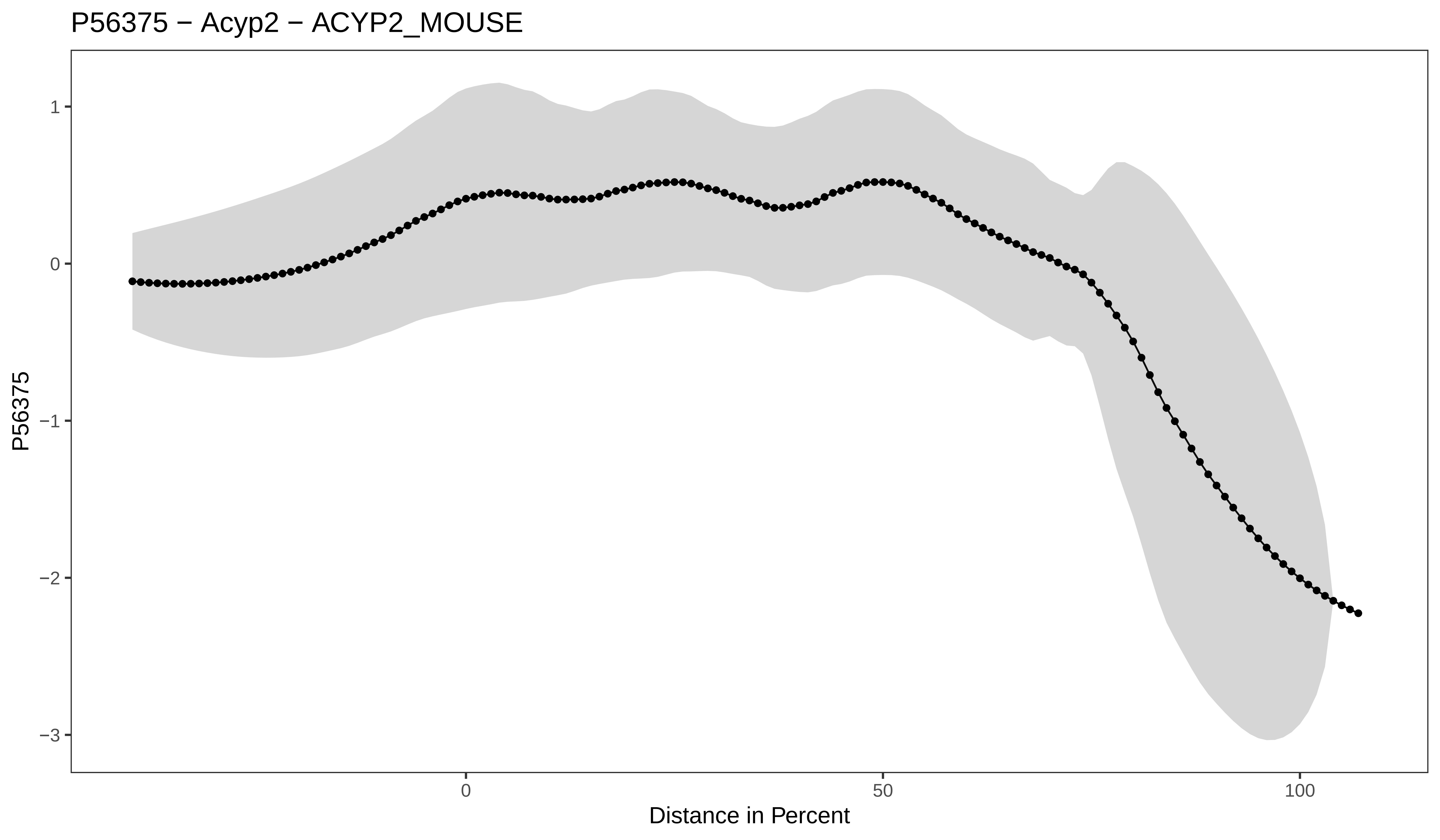
P56375 − Acyp2 − ACYP2_MOUSE
P56375
1
0
−1
−2
−3
0
50
100
Distance in Percent

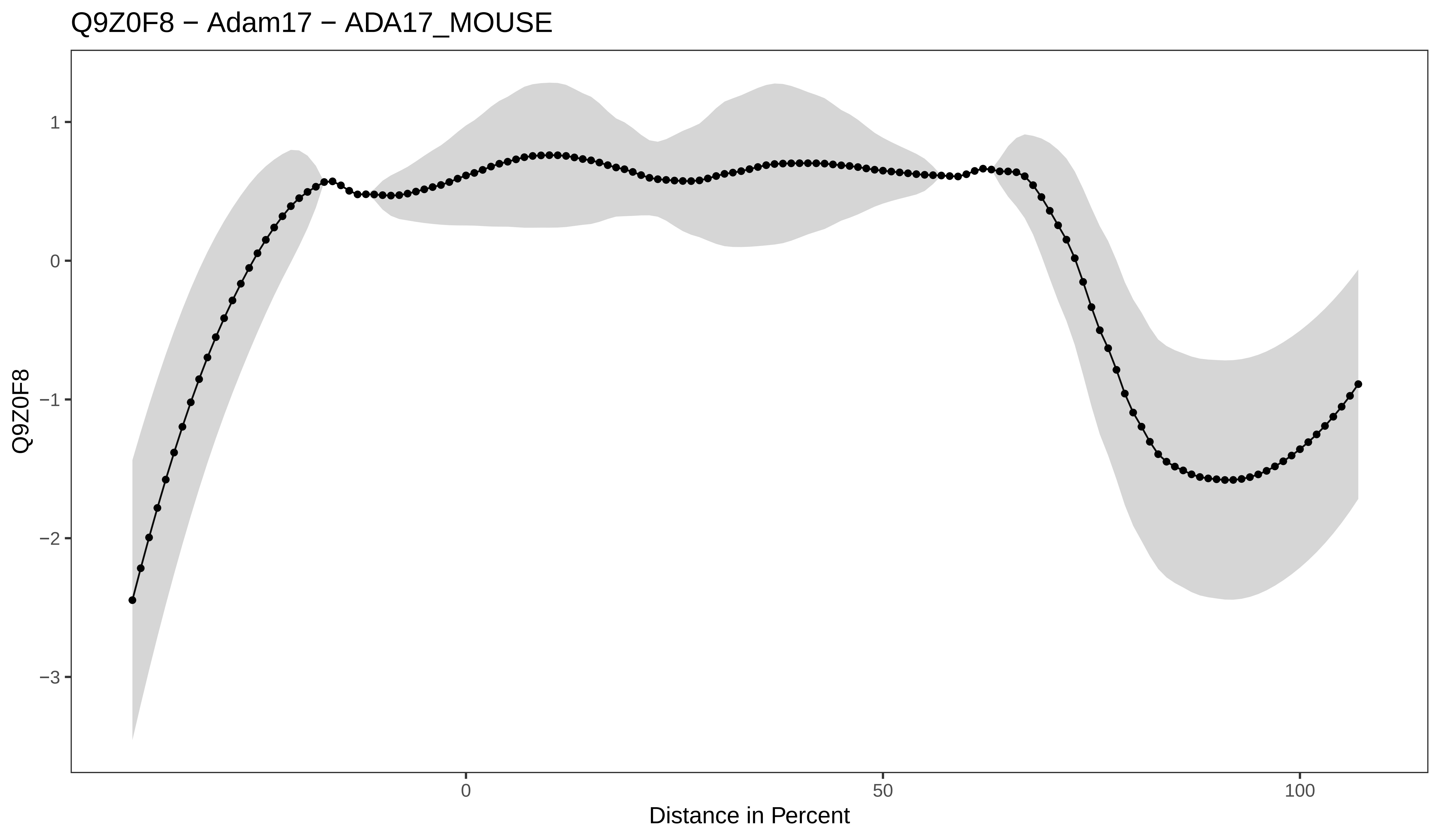

Q9Z0F8 − Adam17 − ADA17_MOUSE
Q9Z0F8
1
0
−1
−2
−3
0
50
100
Distance in Percent

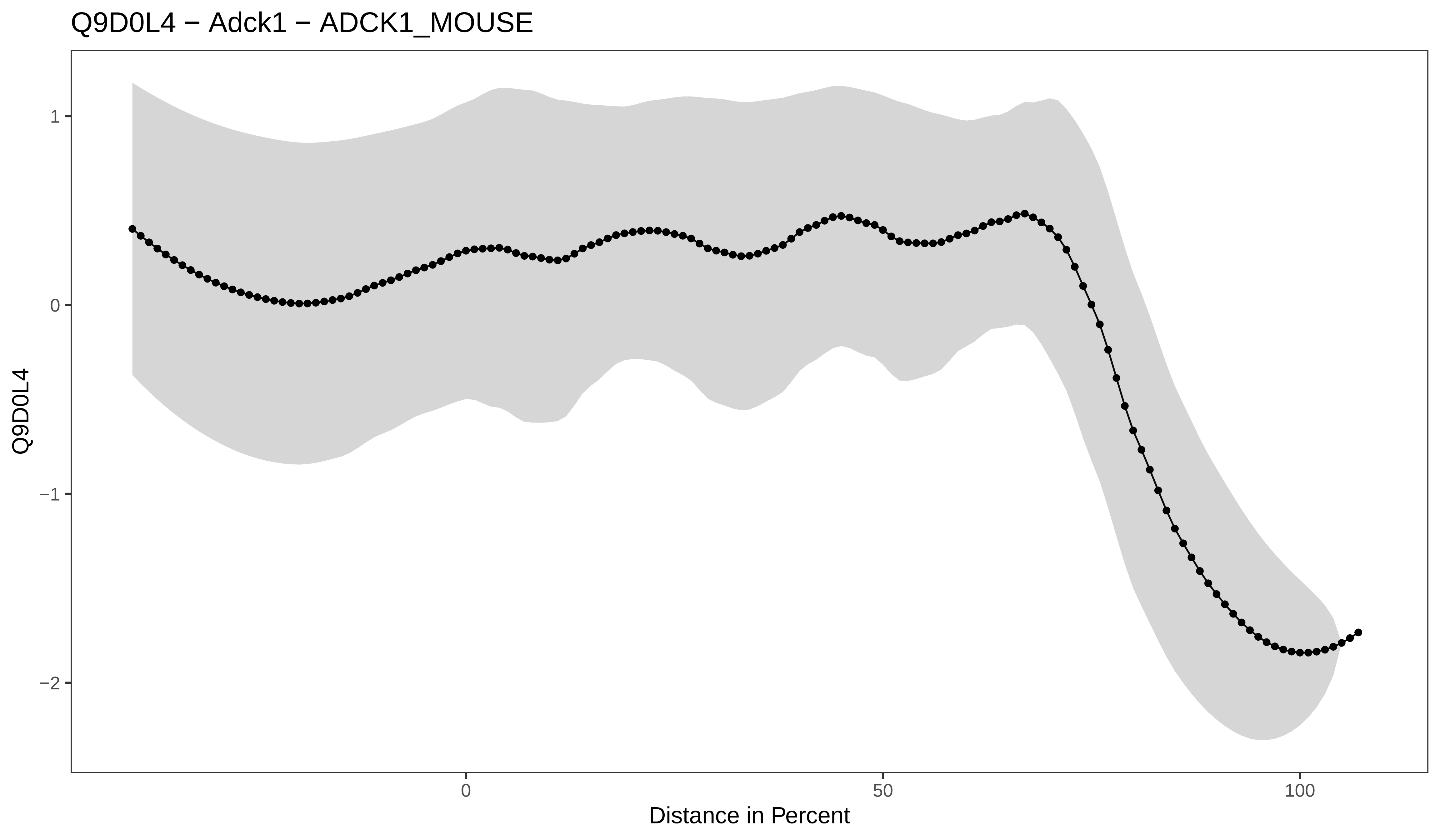

Q9D0L4 − Adck1 − ADCK1_MOUSE
1
0
−1
−2
Q9D0L4
0
50
100
Distance in Percent

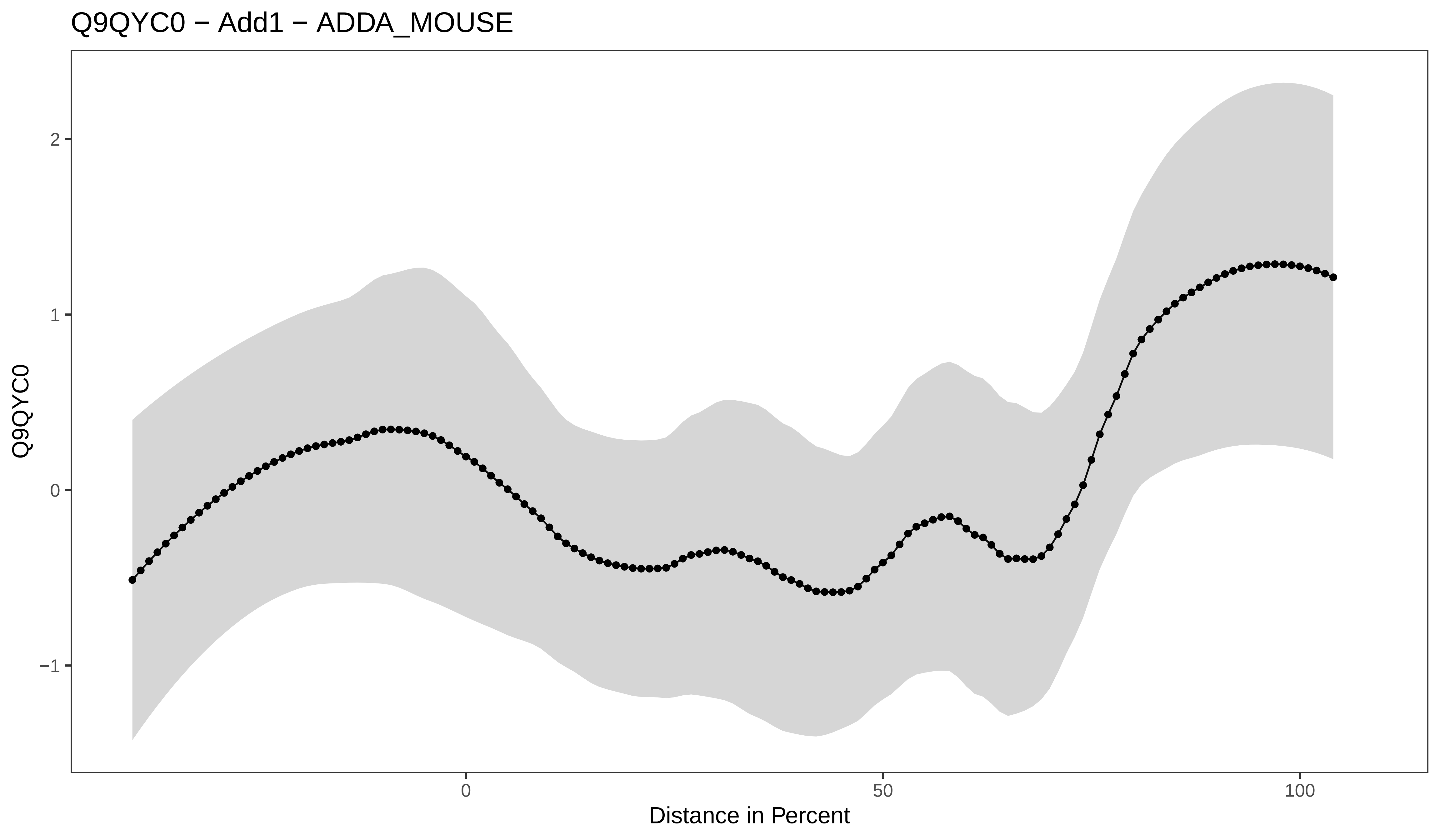

Q9QYC0 − Add1 − ADDA_MOUSE
Q9QYC0
2
1
0
−1
0
50
100
Distance in Percent

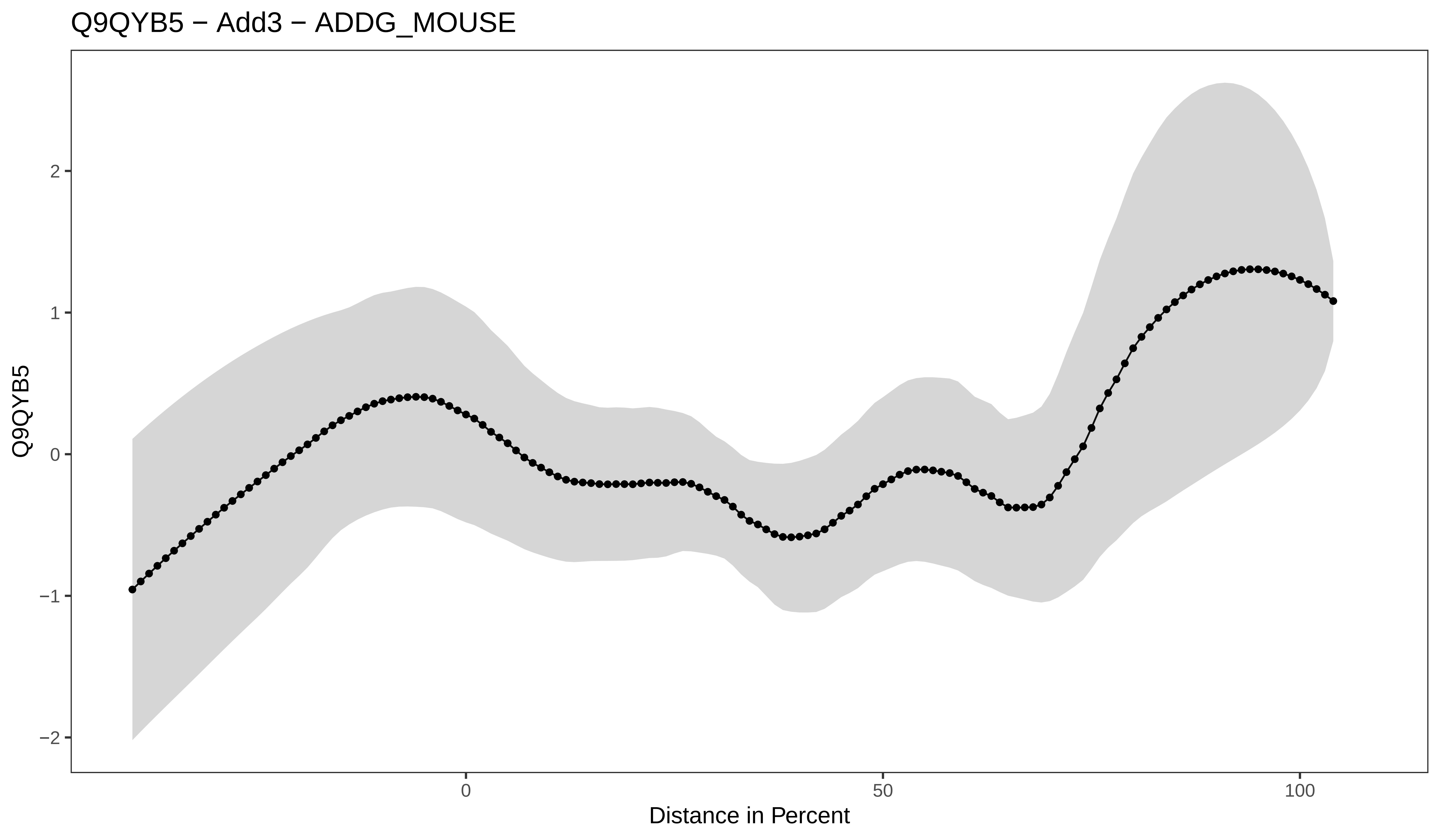
Q9QYB5 − Add3 − ADDG_MOUSE
Q9QYB5
2
1
0
−1
−2
0
50
100
Distance in Percent

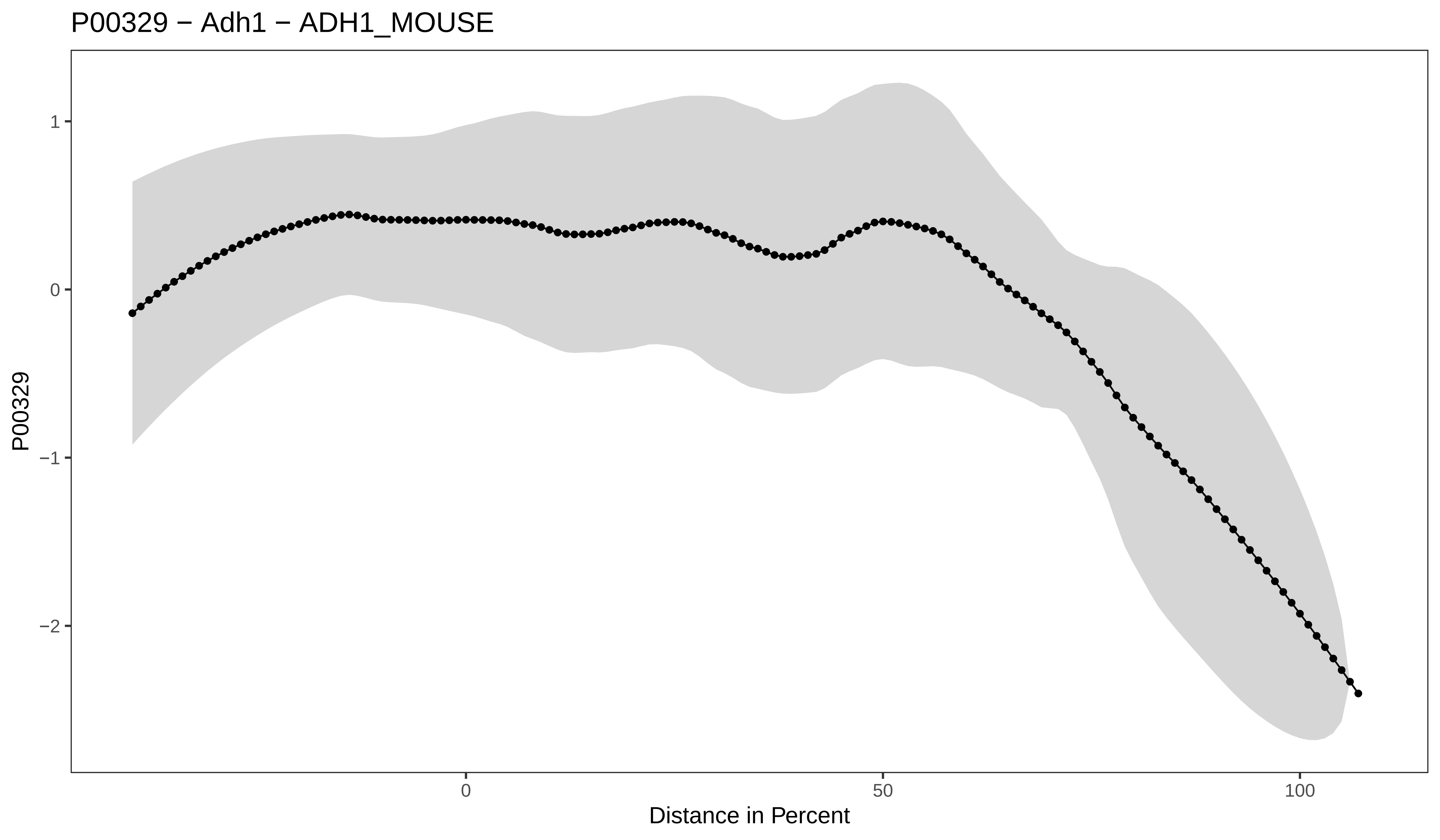
P00329 − Adh1 − ADH1_MOUSE
1
0
−1
−2
P00329
0
50
100
Distance in Percent

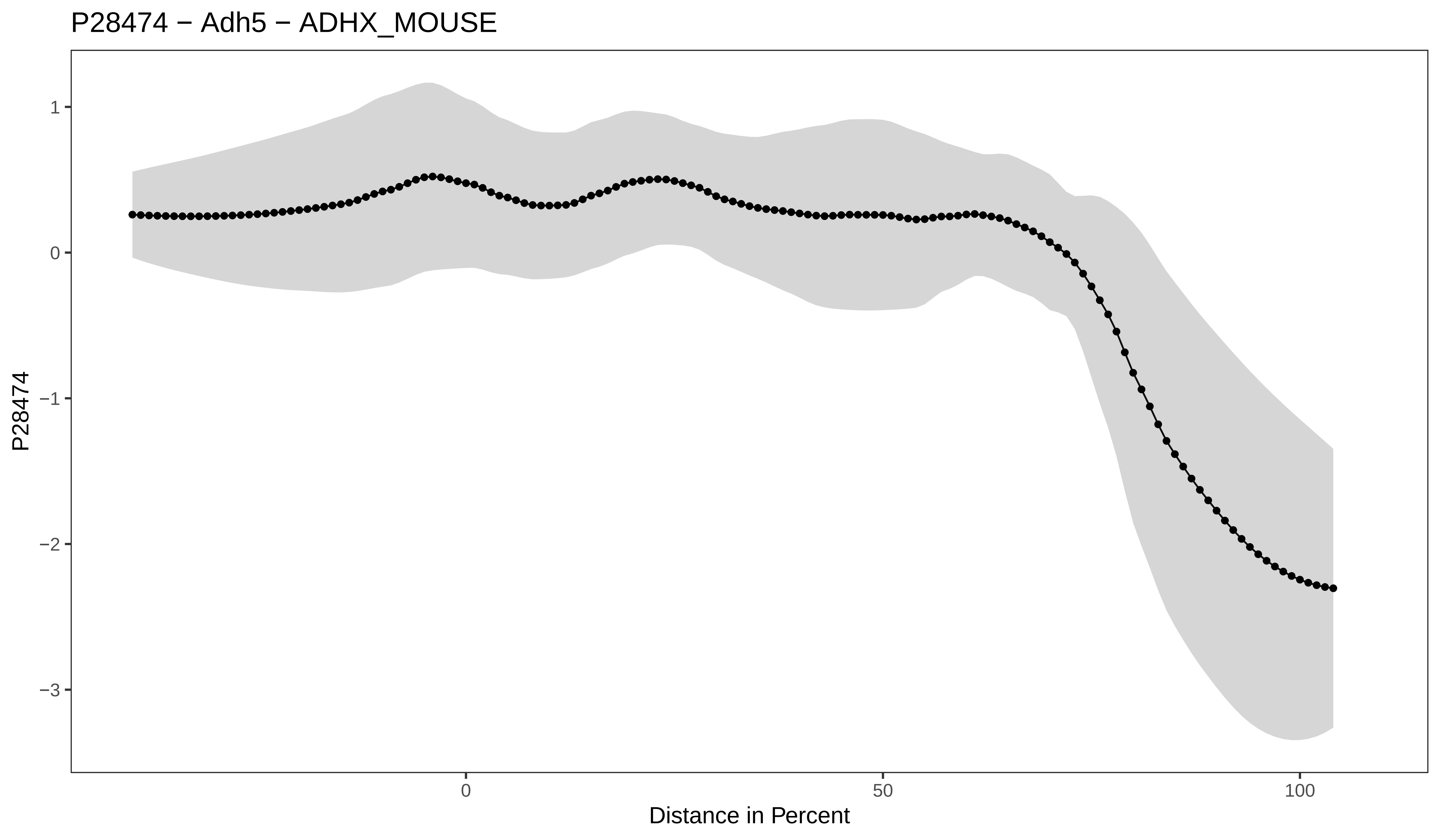
P28474 − Adh5 − ADHX_MOUSE
1
0
−1
−2
−3
P28474
0
50
100
Distance in Percent

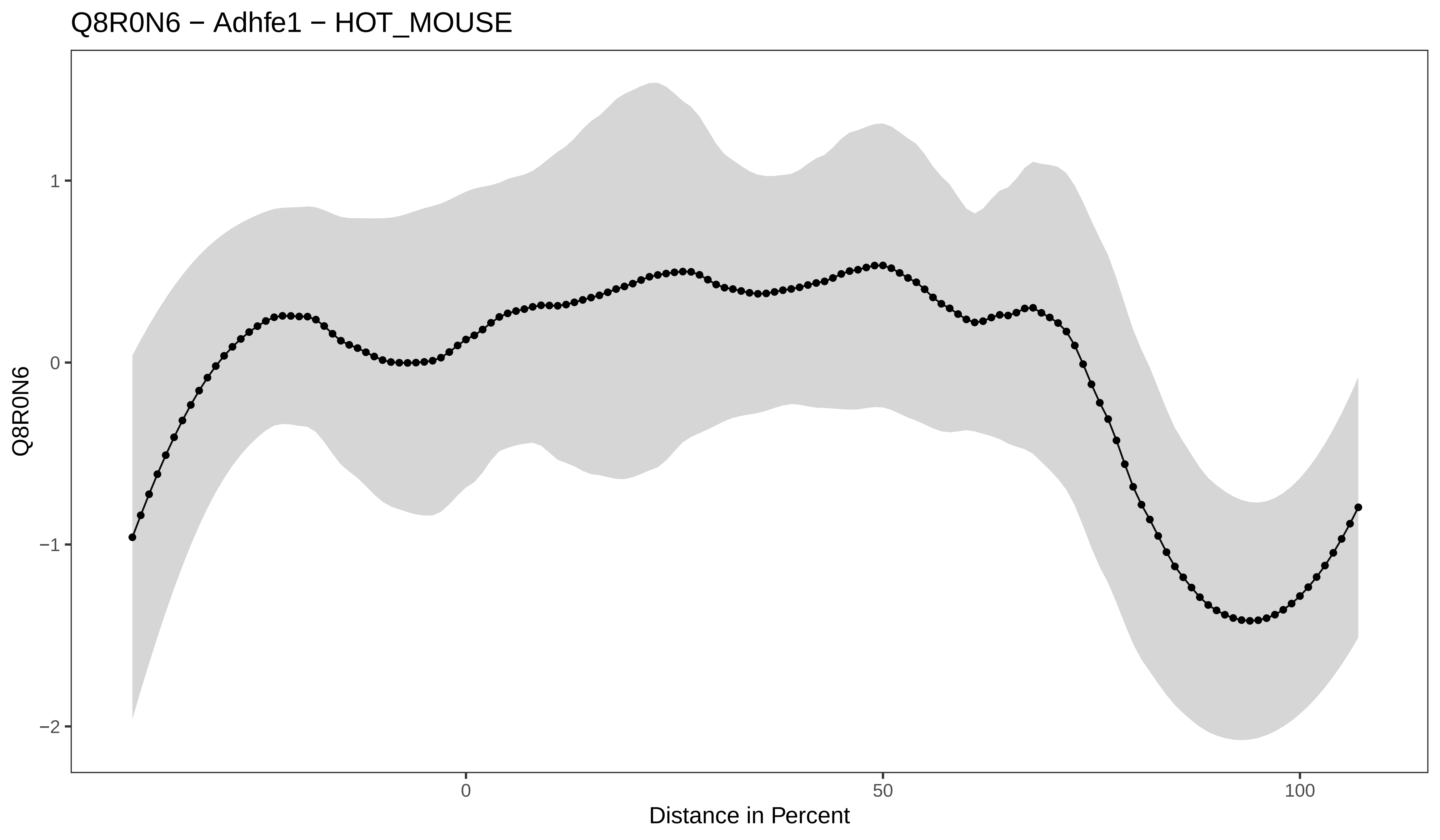
Q8R0N6 − Adhfe1 − HOT_MOUSE
Q8R0N6
Distance in Percent
1
0
−1
−2
0
50
100

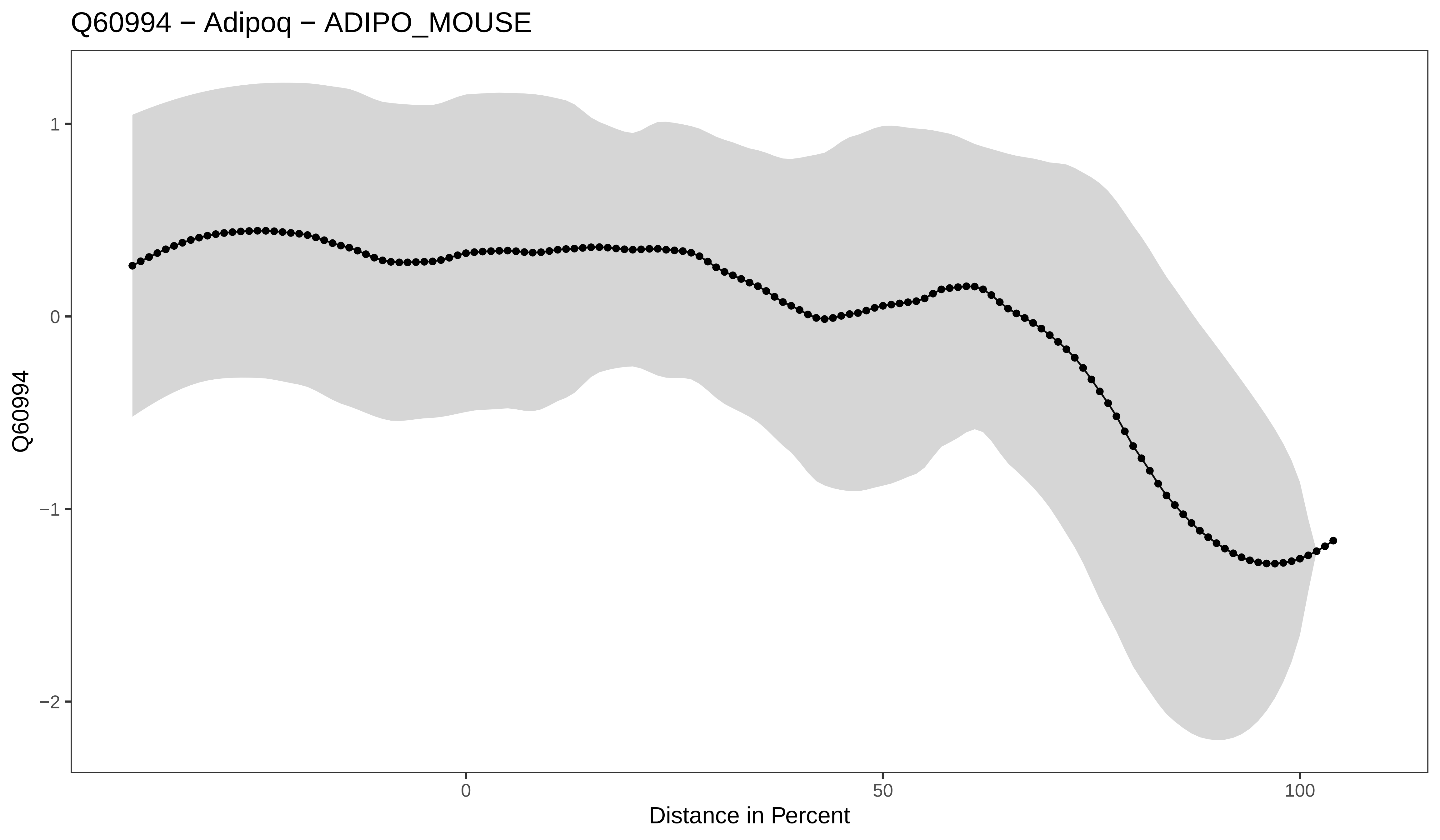

Q60994 − Adipoq − ADIPO_MOUSE
1
0
−1
−2
Q60994
0
50
100
Distance in Percent

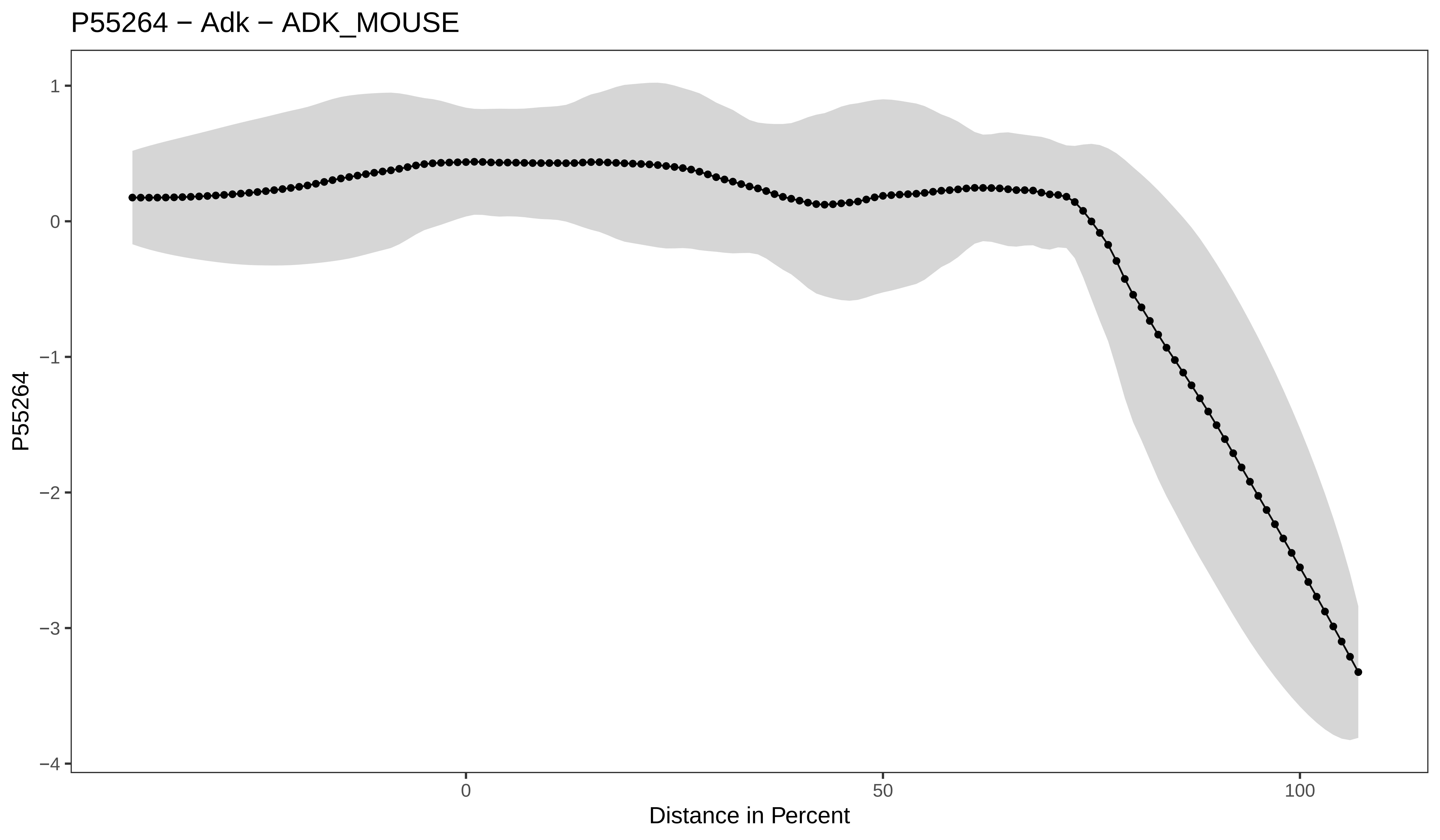

P55264 − Adk − ADK_MOUSE
P55264
1
0
−1
−2
−3
−4
0
50
100
Distance in Percent

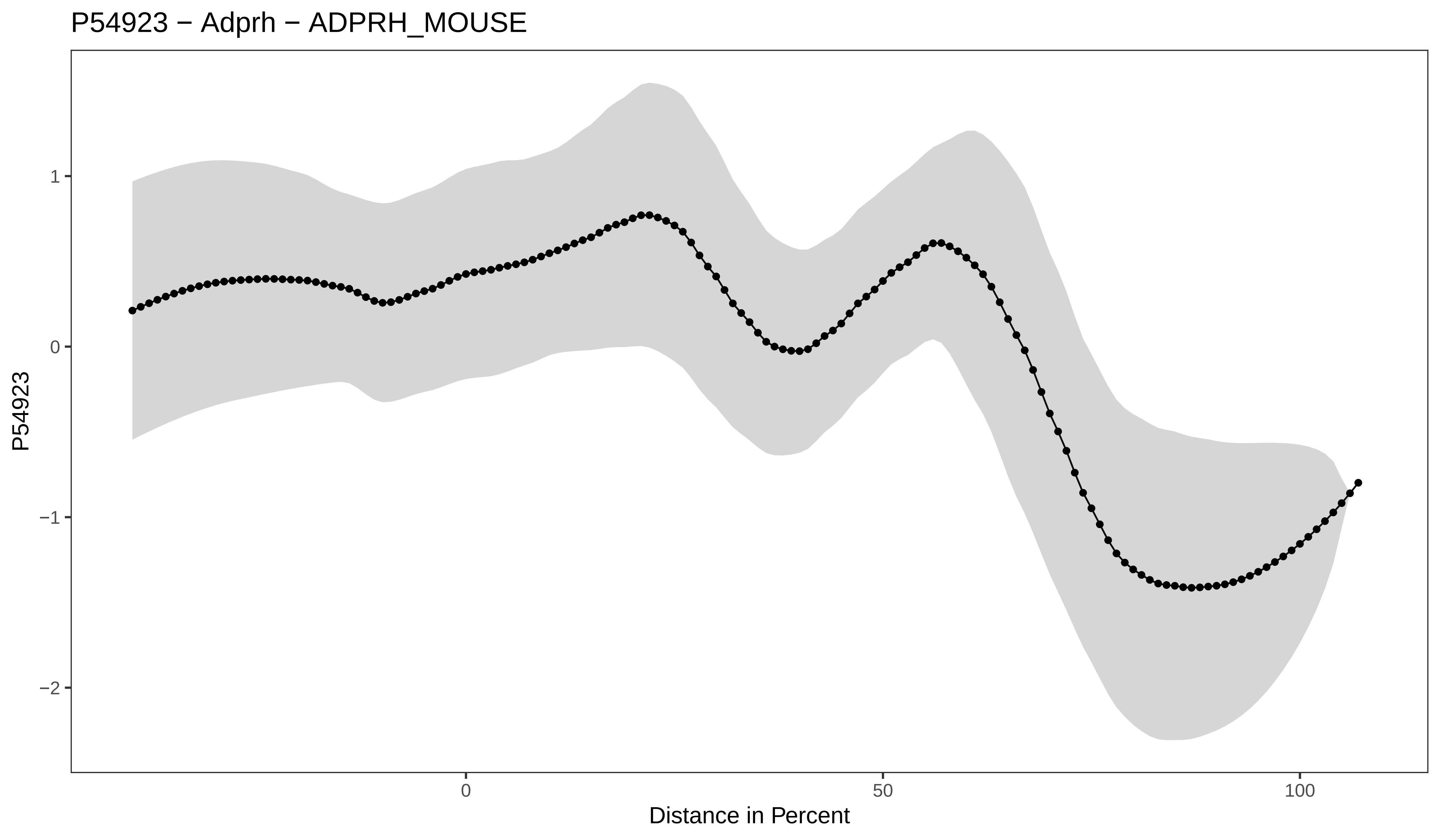

P54923 − Adprh − ADPRH_MOUSE
P54923
1
0
−1
−2
0
50
100
Distance in Percent

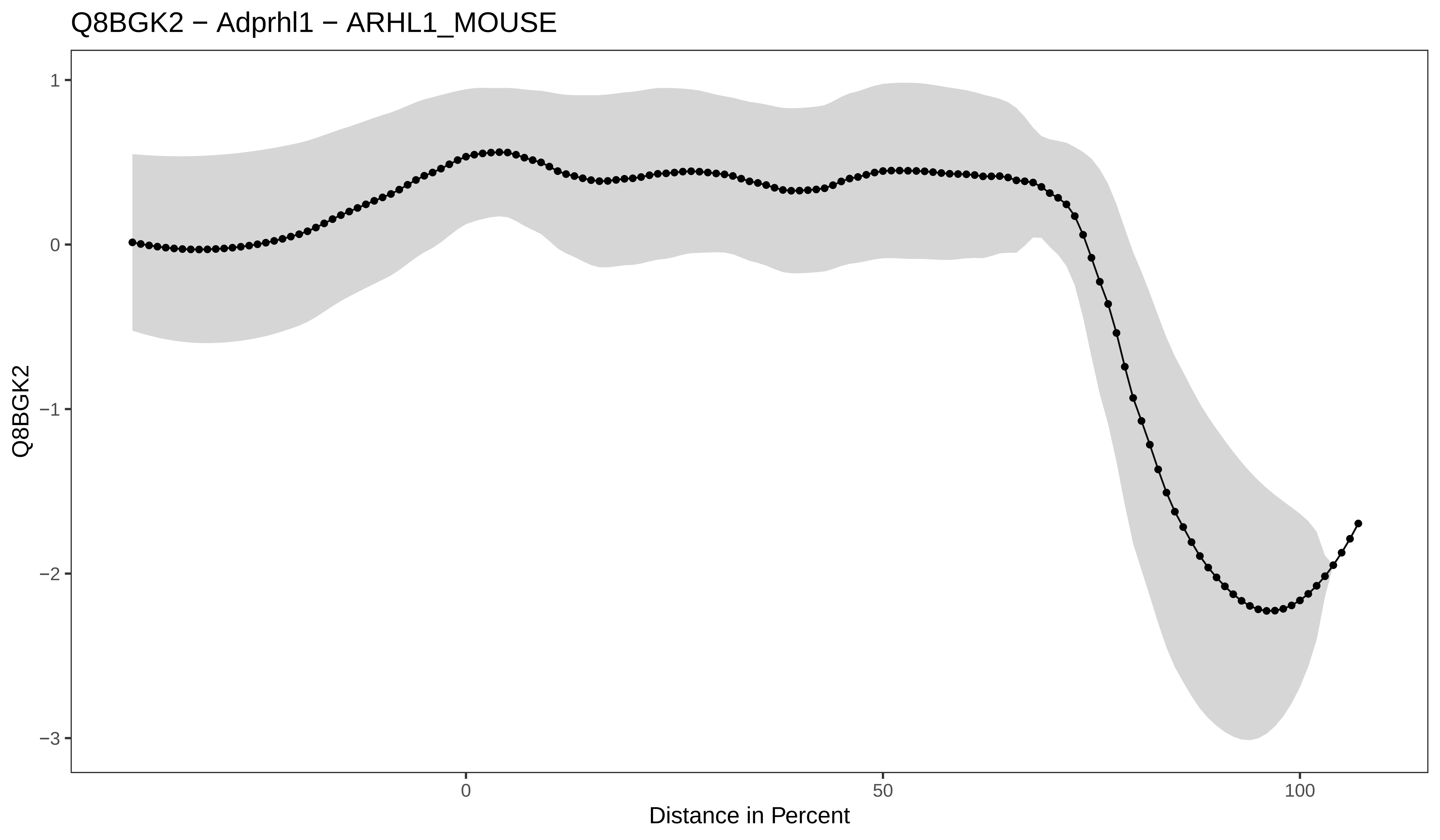
Q8BGK2 − Adprhl1 − ARHL1_MOUSE
Q8BGK2
1
0
−1
−2
−3
0
50
100
Distance in Percent

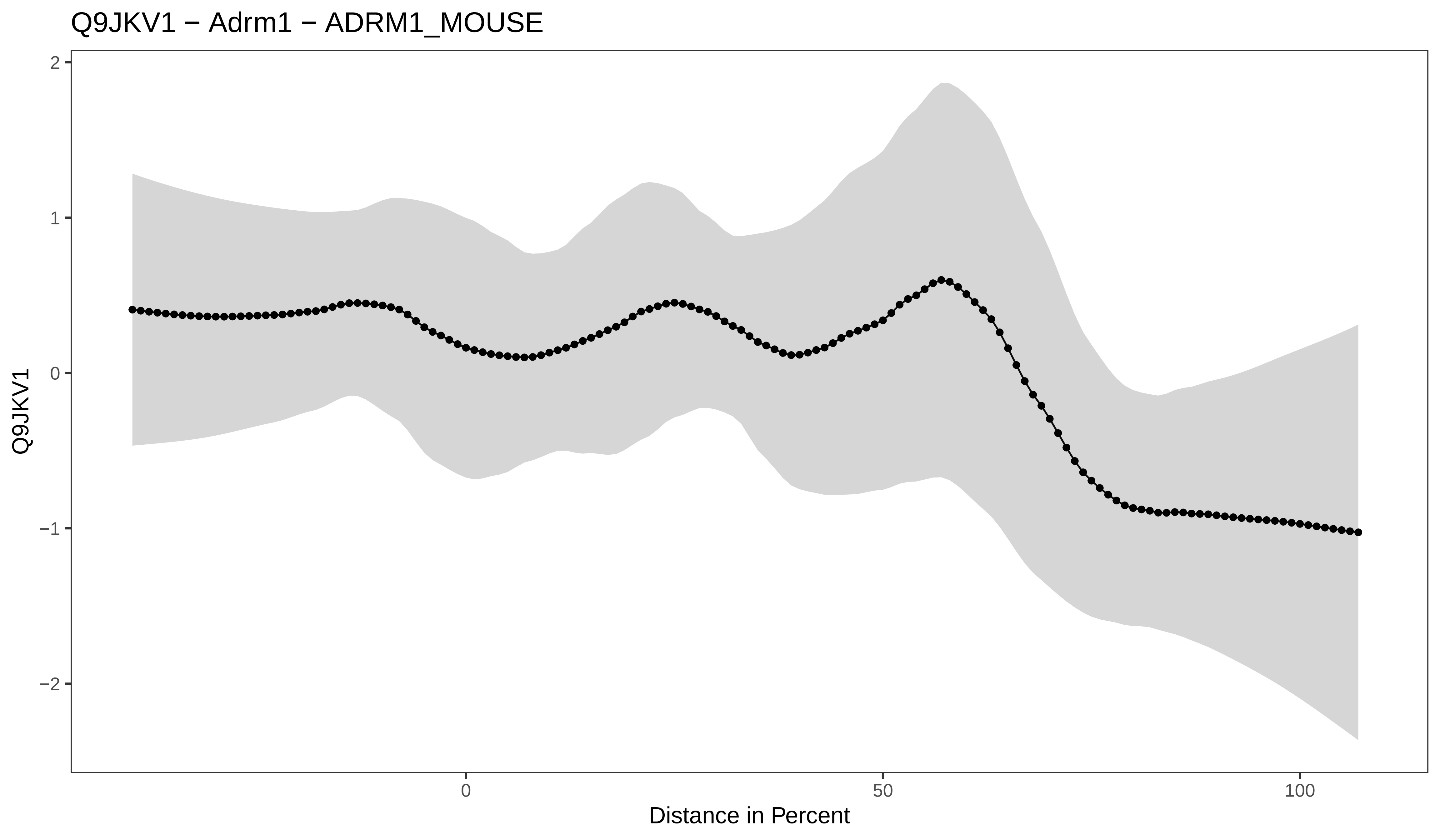
Q9JKV1 − Adrm1 − ADRM1_MOUSE
Q9JKV1
Distance in Percent
2
1
0
−1
−2
0
50
100

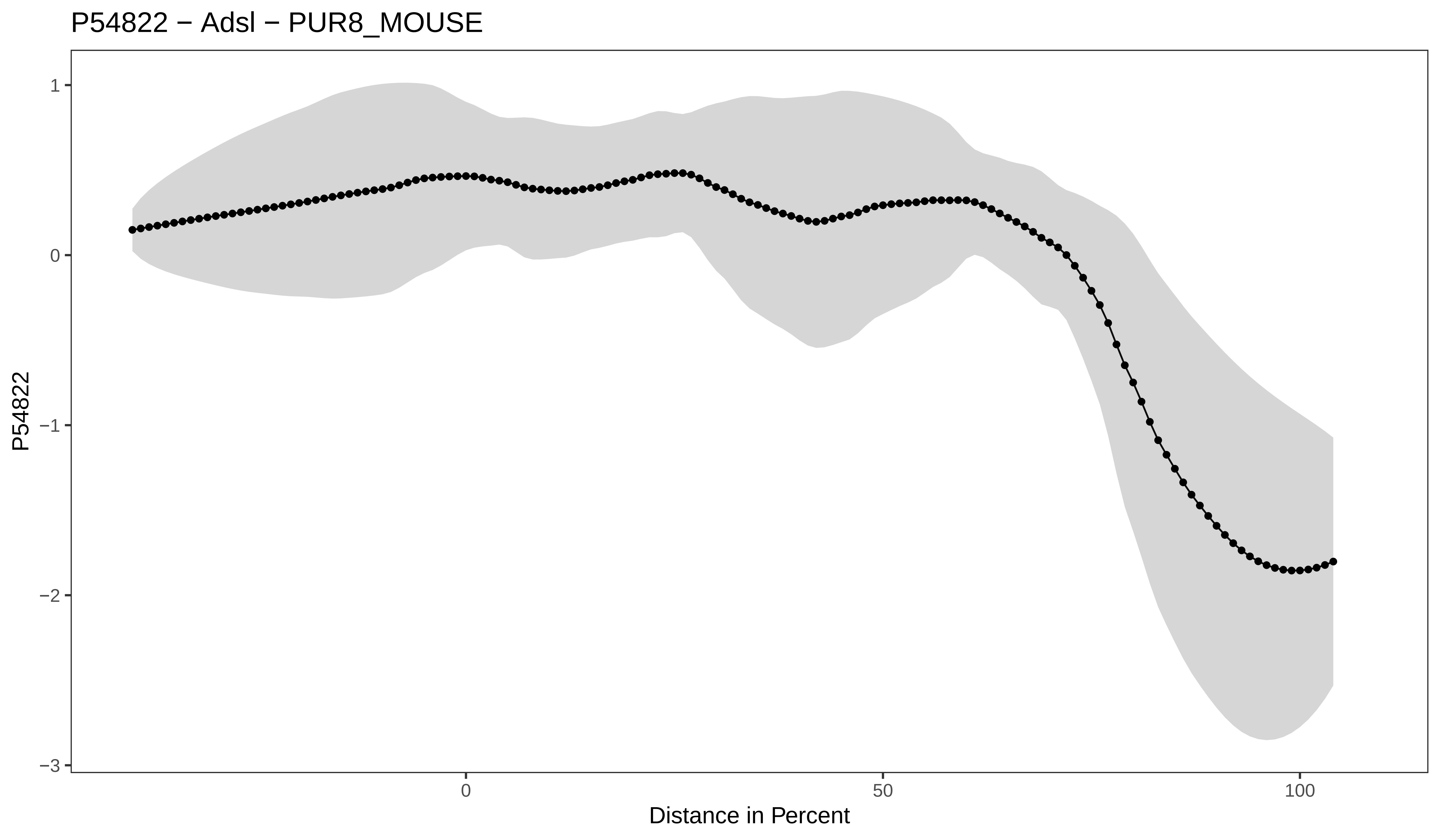
P54822 − Adsl − PUR8_MOUSE
P54822
1
0
−1
−2
−3
0
50
100
Distance in Percent

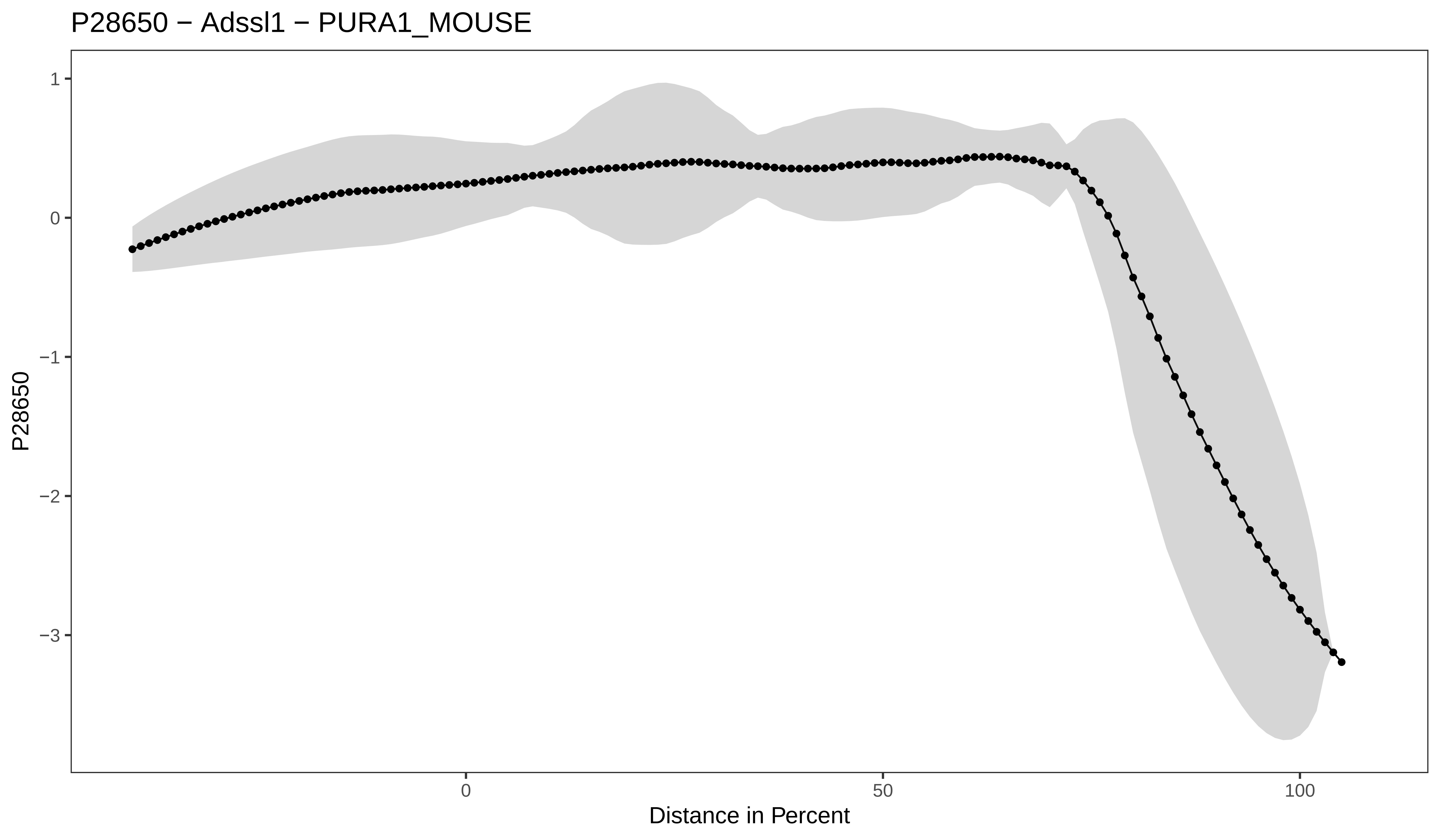

P28650 − Adssl1 − PURA1_MOUSE
1
0
−1
−2
−3
P28650
0
50
100
Distance in Percent

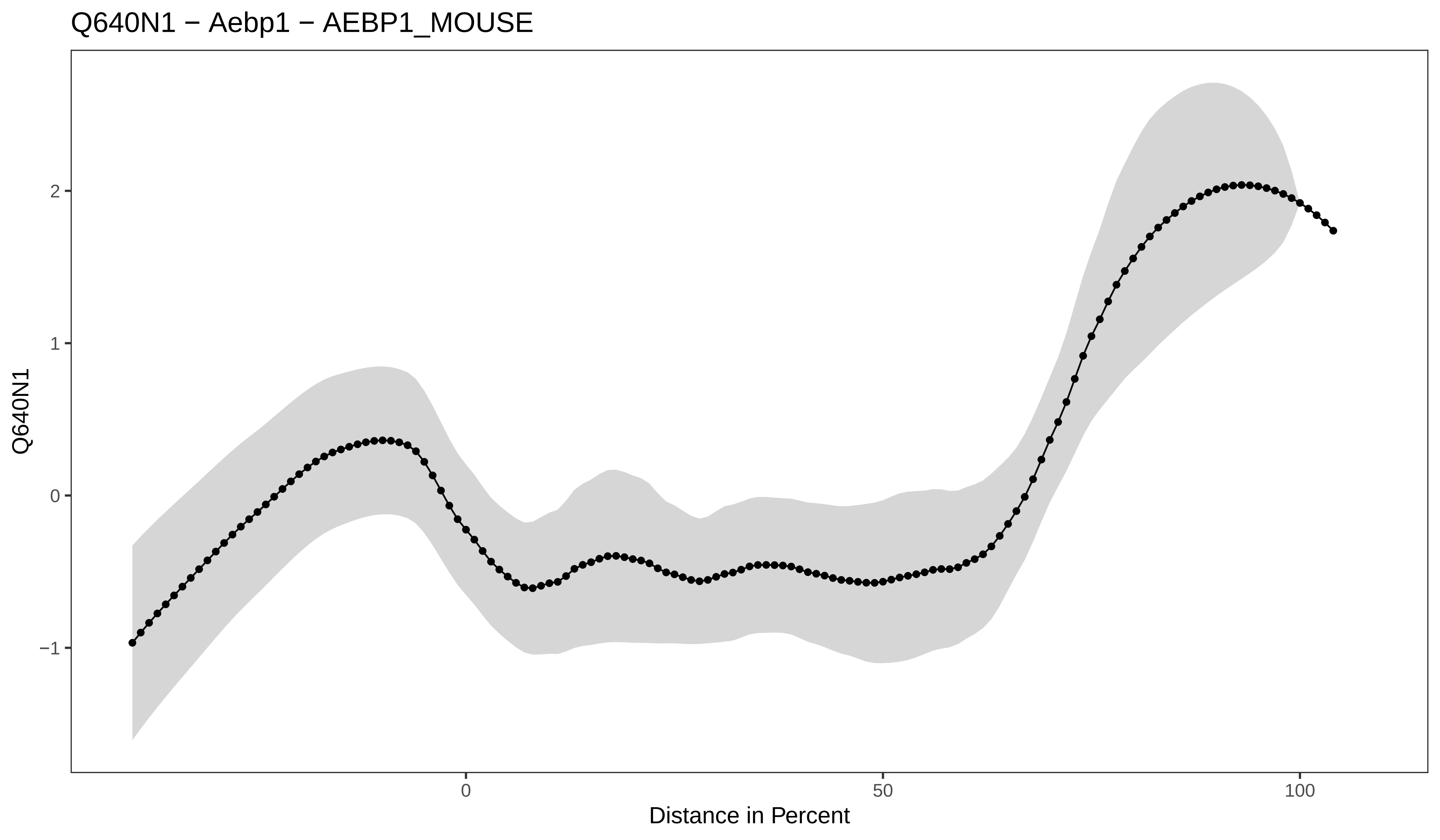
Q640N1 − Aebp1 − AEBP1_MOUSE
Q640N1
2
1
0
−1
0
50
100
Distance in Percent

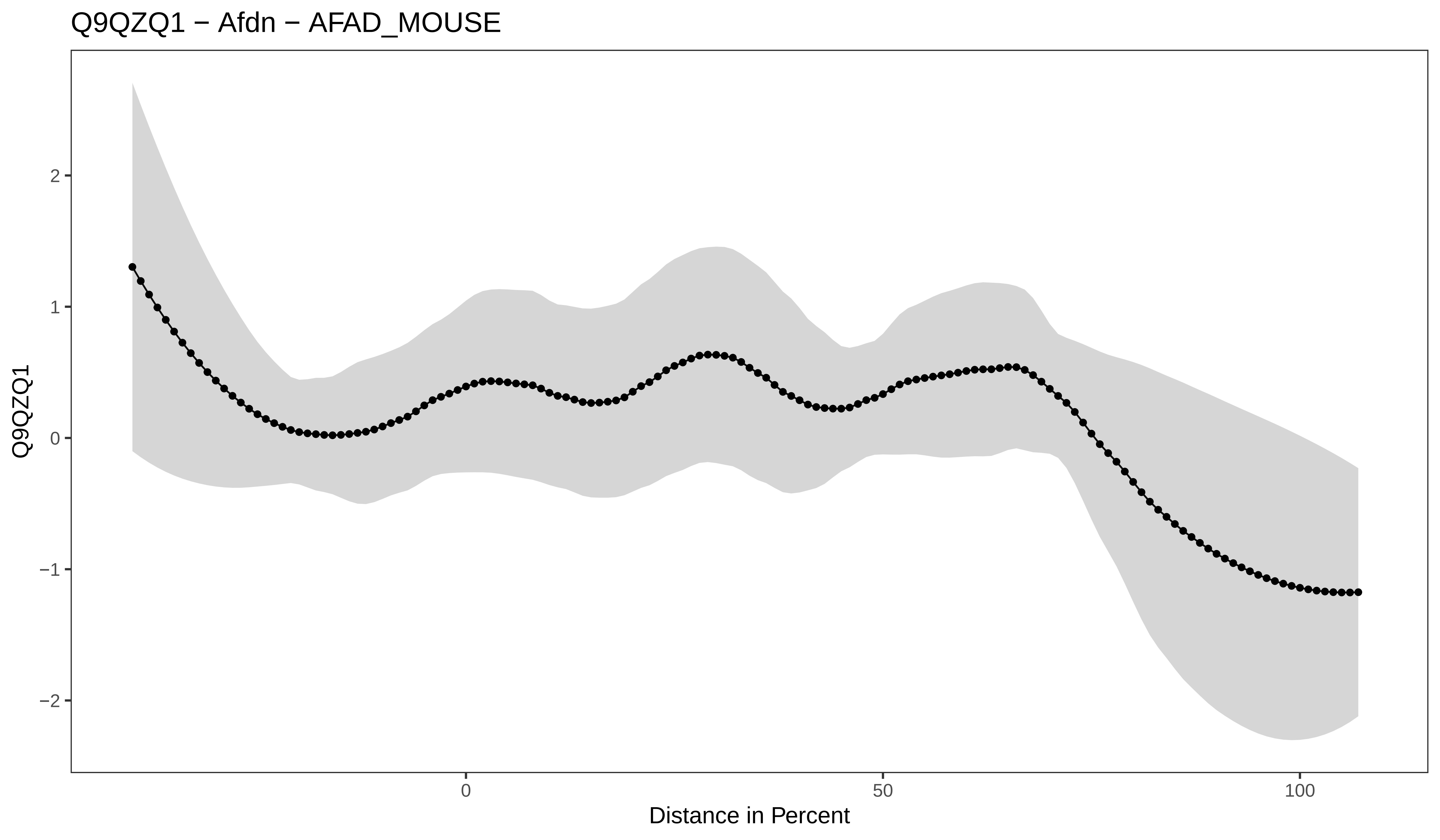
Q9QZQ1 − Afdn − AFAD_MOUSE
Q9QZQ1
2
1
0
−1
−2
0
50
100
Distance in Percent

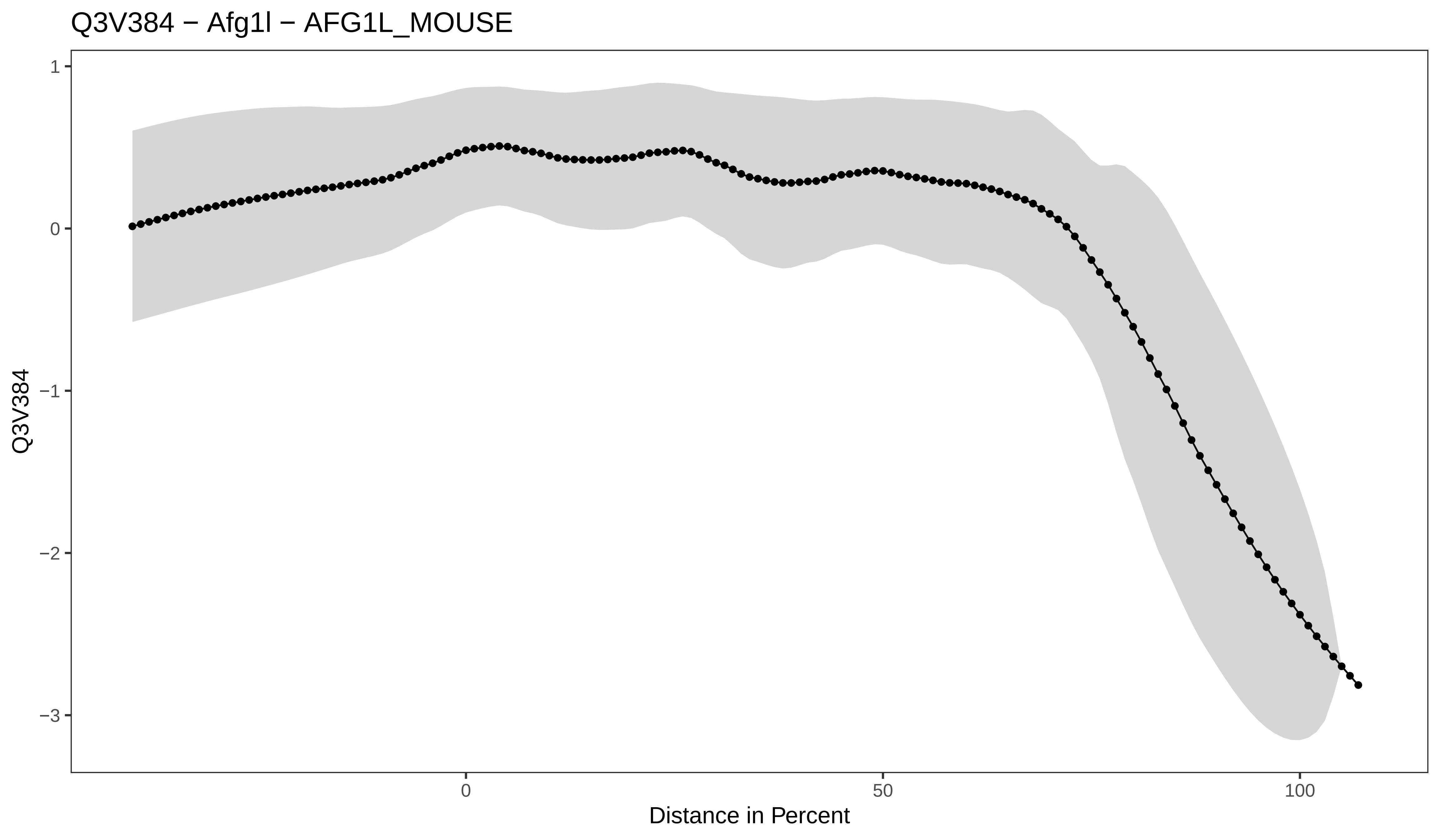
Q3V384 − Afg1l − AFG1L_MOUSE
1
0
−1
−2
−3
Q3V384
0
50
100
Distance in Percent

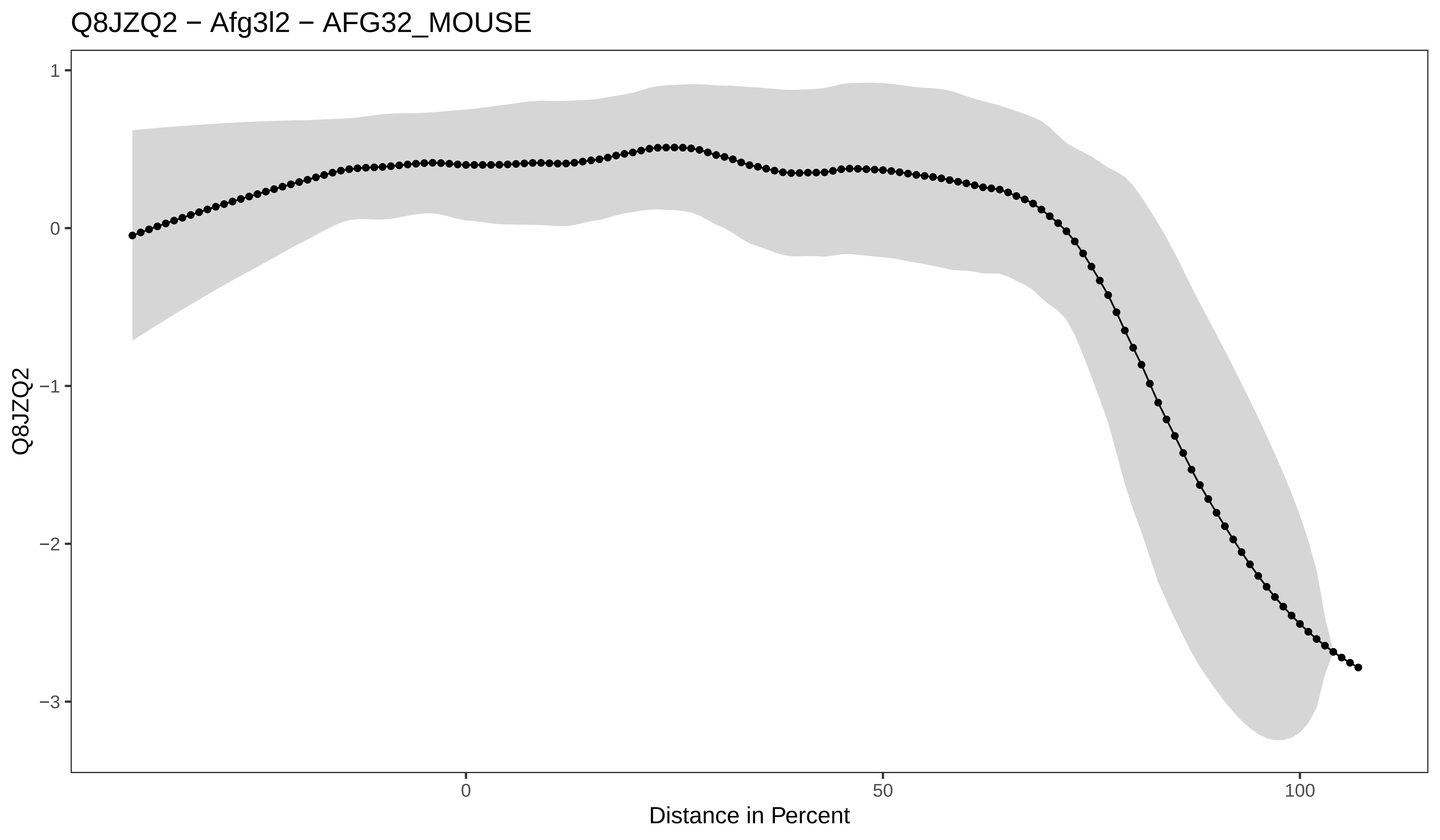

Q8JZQ2 − Afg3l2 − AFG32_MOUSE
1
0
−1
−2
−3
Q8JZQ2
0
50
100
Distance in Percent

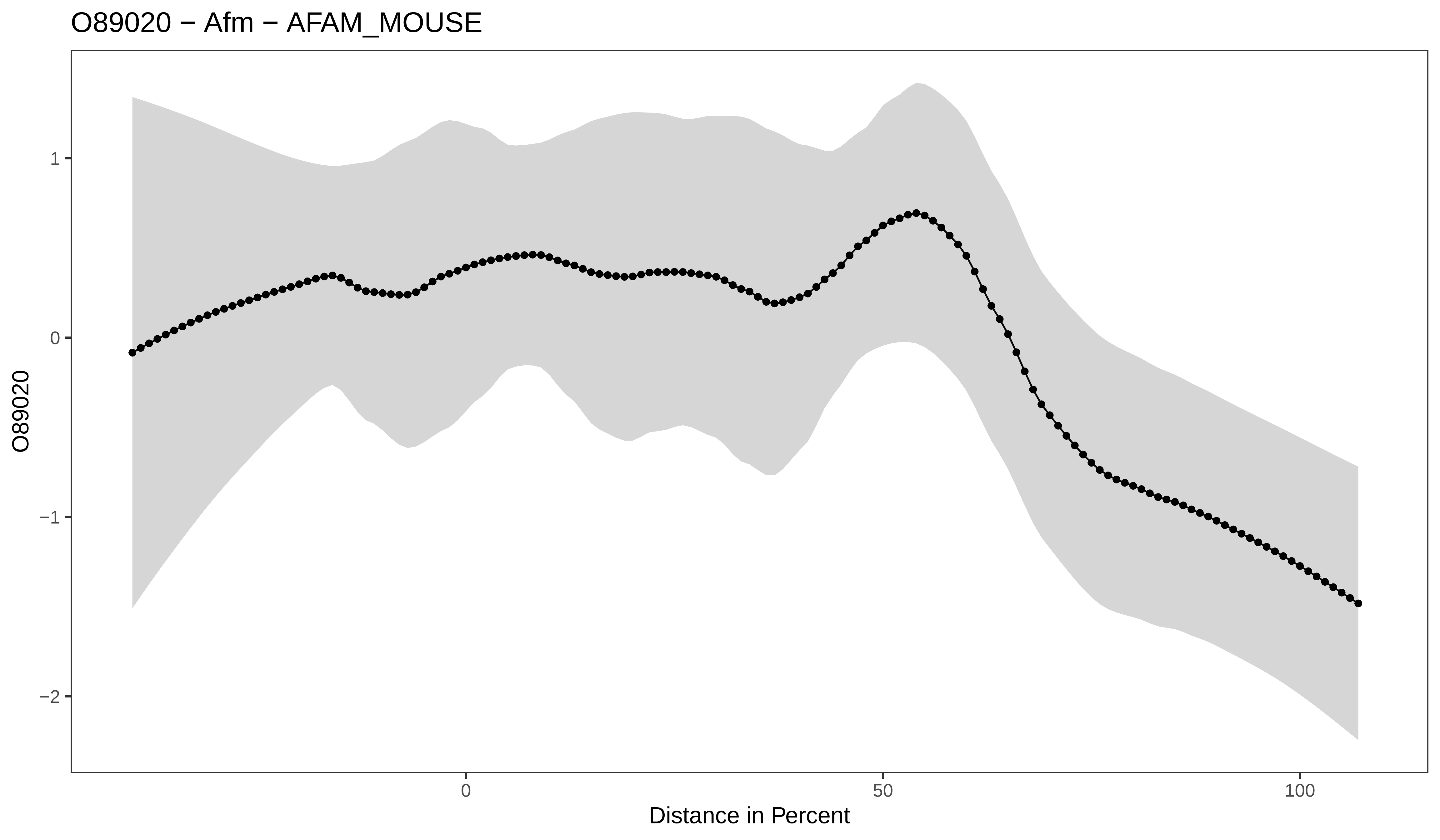
O89020 − Afm − AFAM_MOUSE
O89020
Distance in Percent
1
0
−1
−2
0
50
100

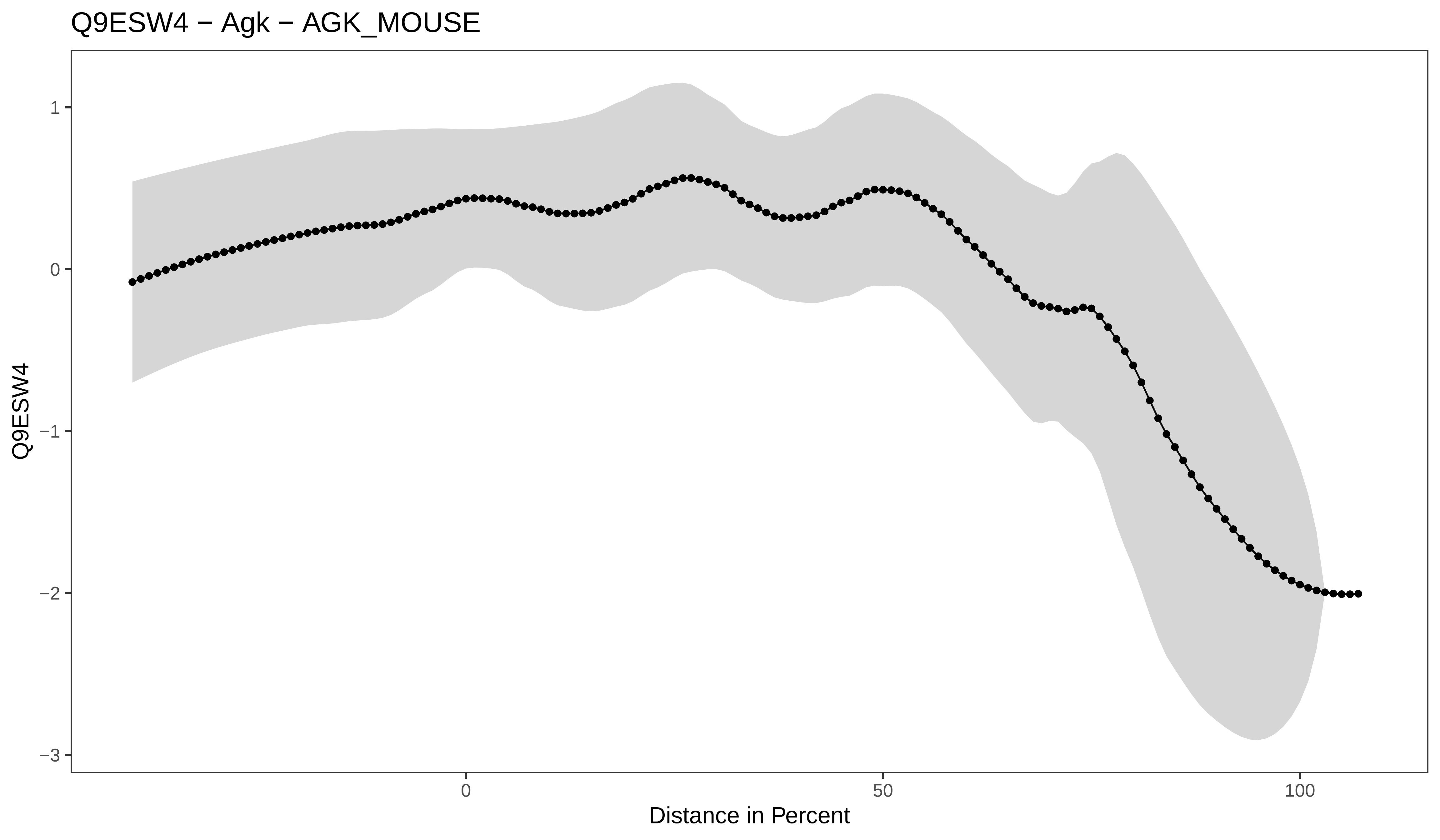
Q9ESW4 − Agk − AGK_MOUSE
Q9ESW4
1
0
−1
−2
−3
0
50
100
Distance in Percent

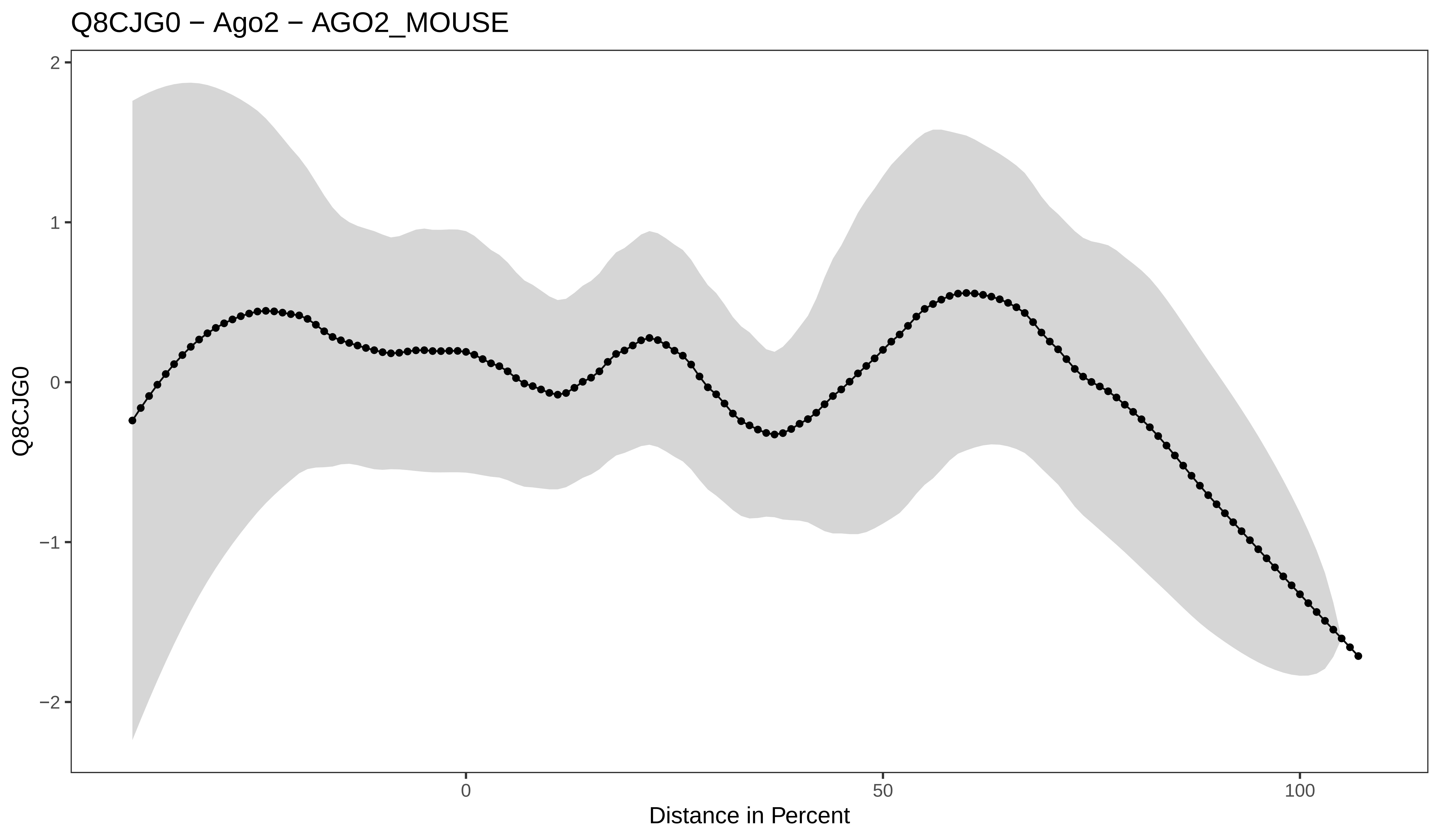

Q8CJG0 − Ago2 − AGO2_MOUSE
Q8CJG0
Distance in Percent
2
1
0
−1
−2
0
50
100

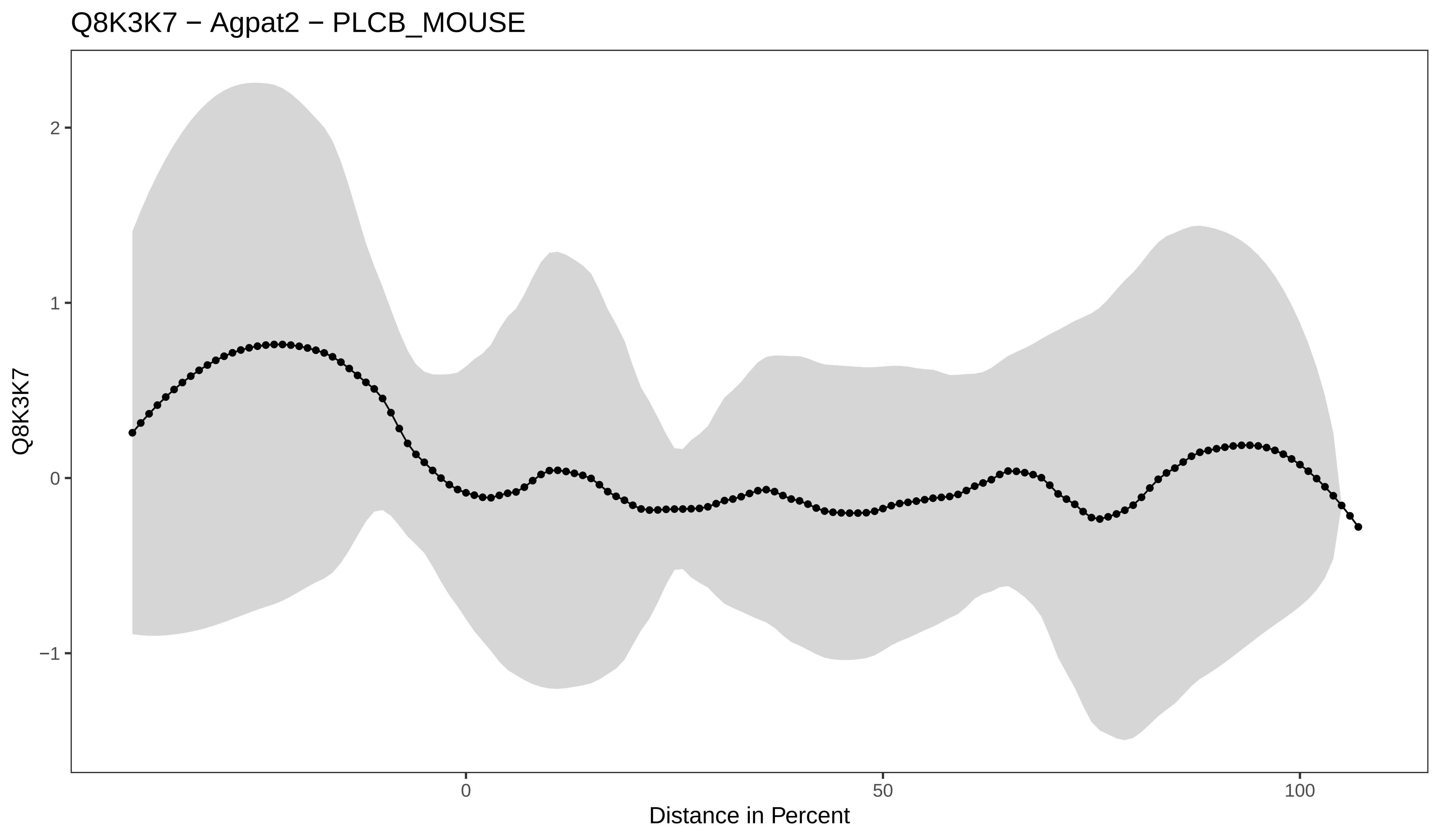
Q8K3K7 − Agpat2 − PLCB_MOUSE
Q8K3K7
2
1
0
−1
0
50
100
Distance in Percent

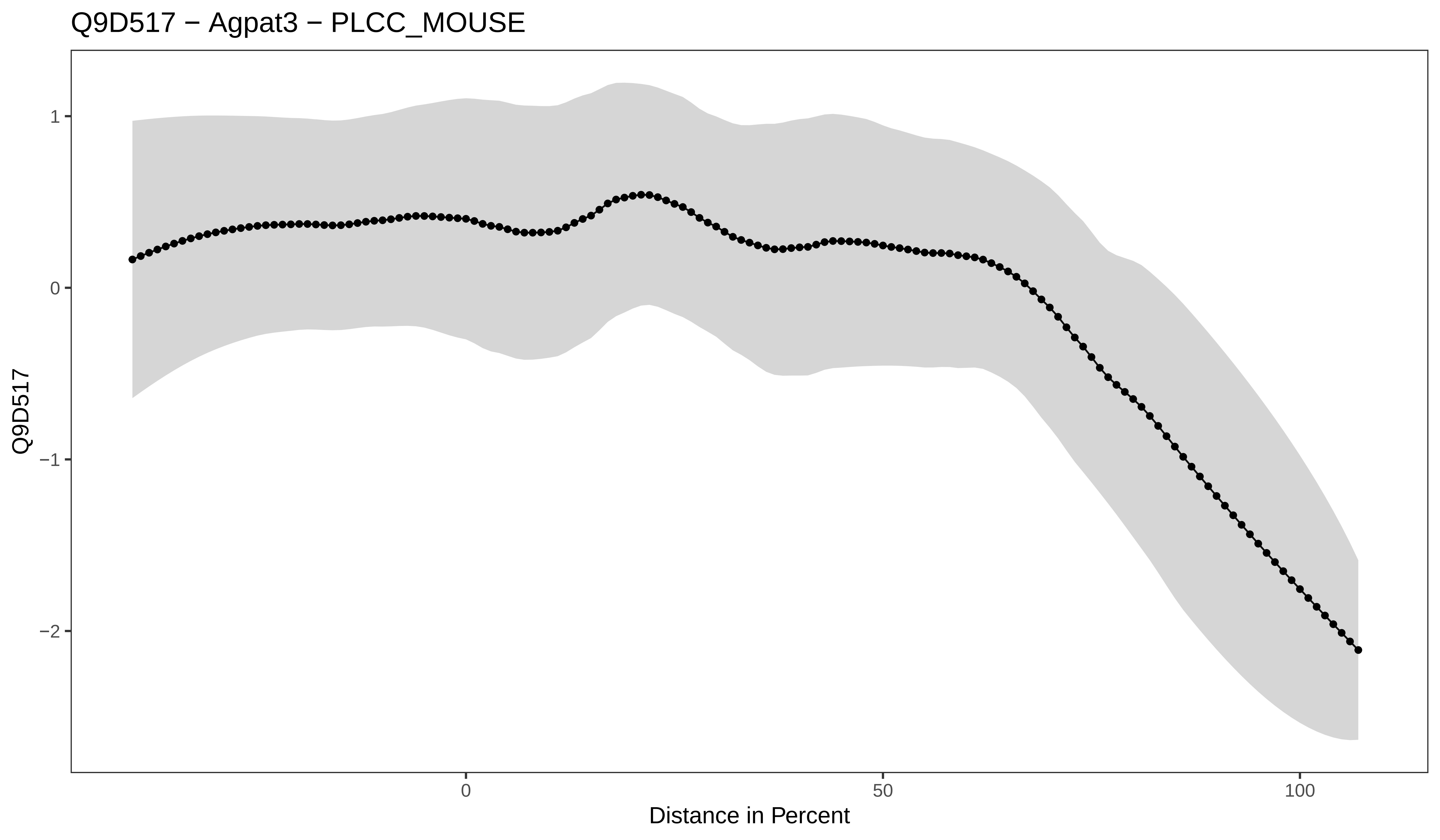

Q9D517 − Agpat3 − PLCC_MOUSE
1
0
−1
−2
Q9D517
0
50
100
Distance in Percent

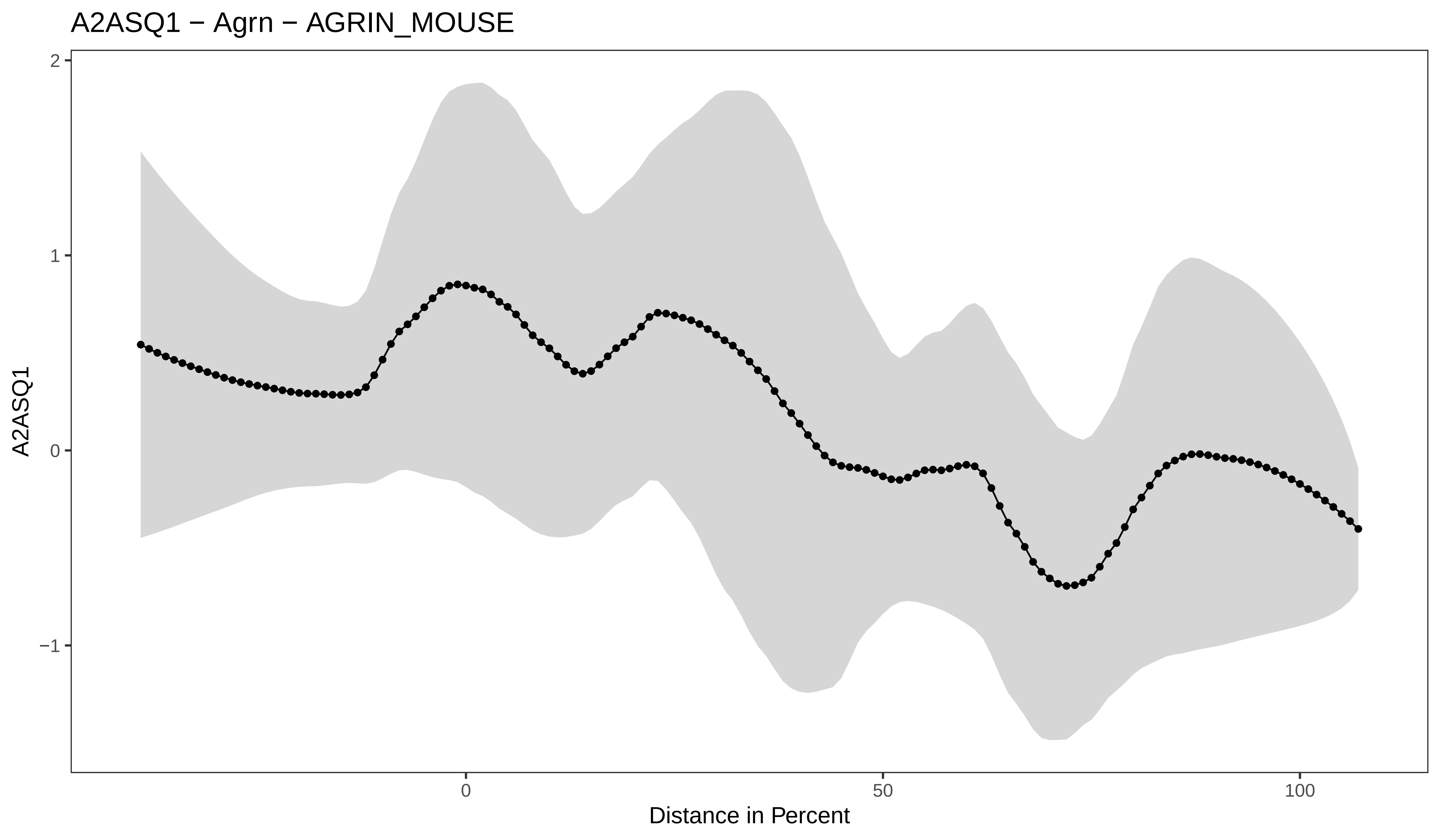

A2ASQ1 − Agrn − AGRIN_MOUSE
2
1
0
−1
A2ASQ1
0
50
100
Distance in Percent

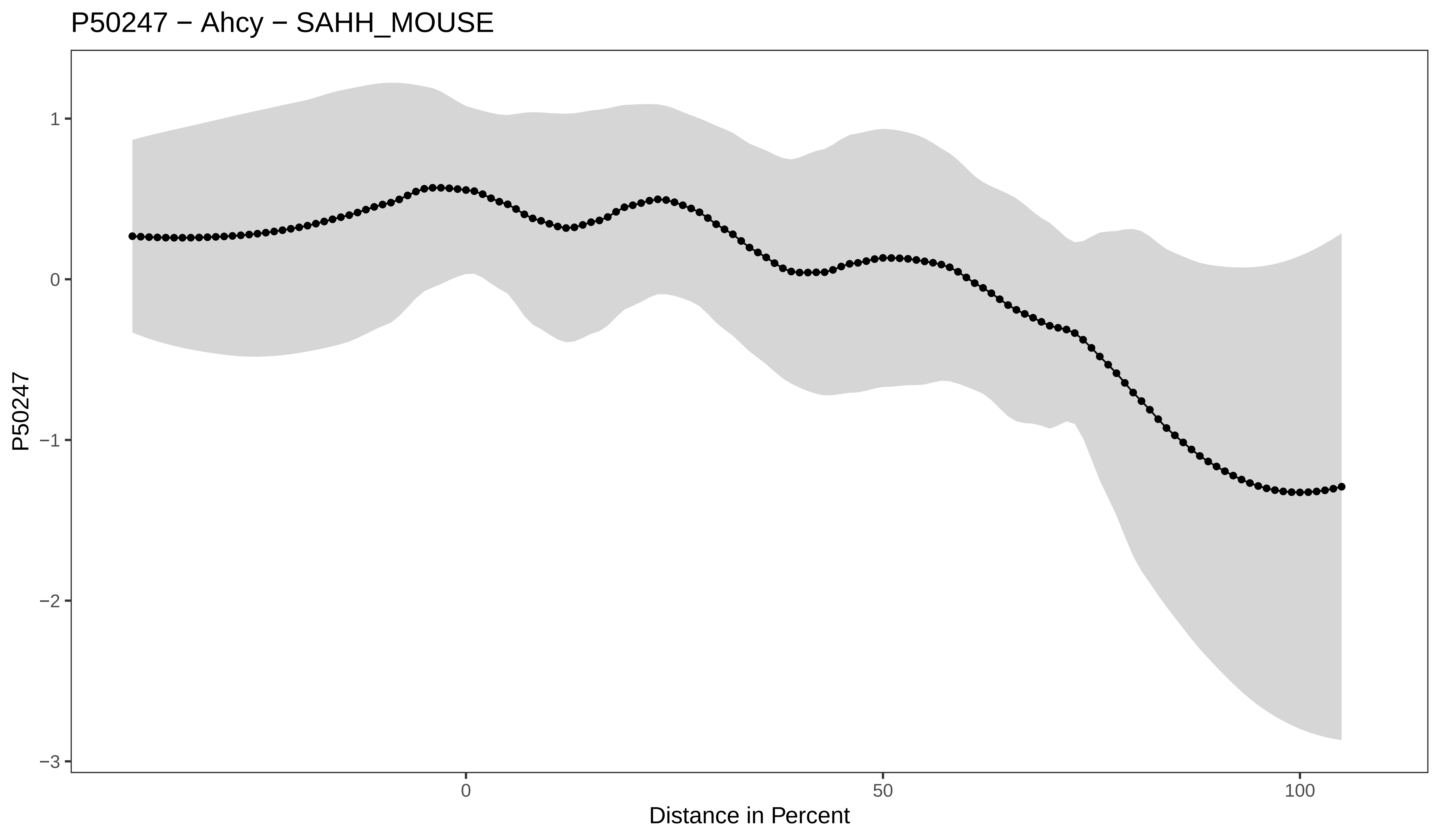
P50247 − Ahcy − SAHH_MOUSE
P50247
Distance in Percent
1
0
−1
−2
−3
0
50
100

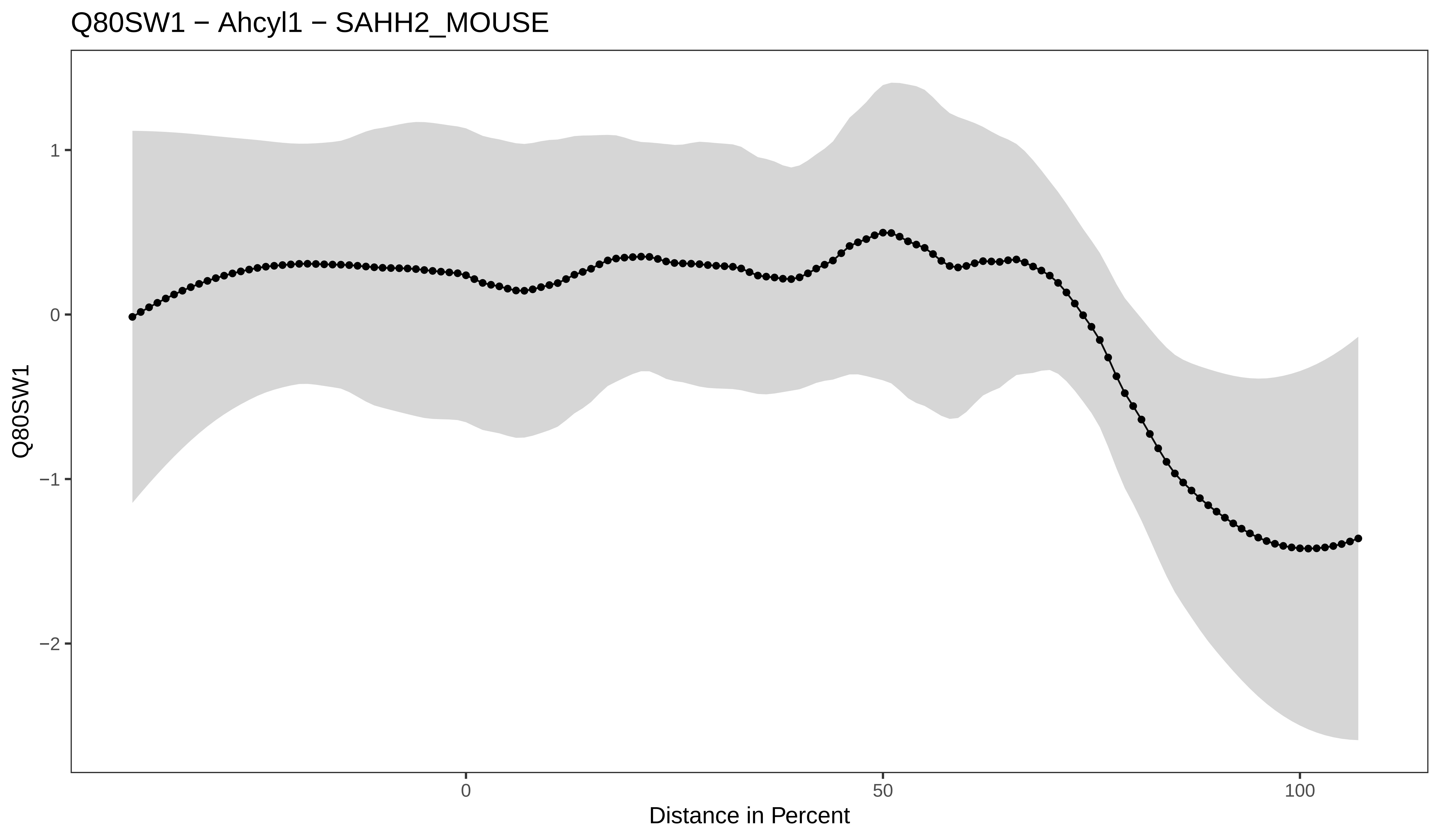

Q80SW1 − Ahcyl1 − SAHH2_MOUSE
Q80SW1
1
0
−1
−2
0
50
100
Distance in Percent

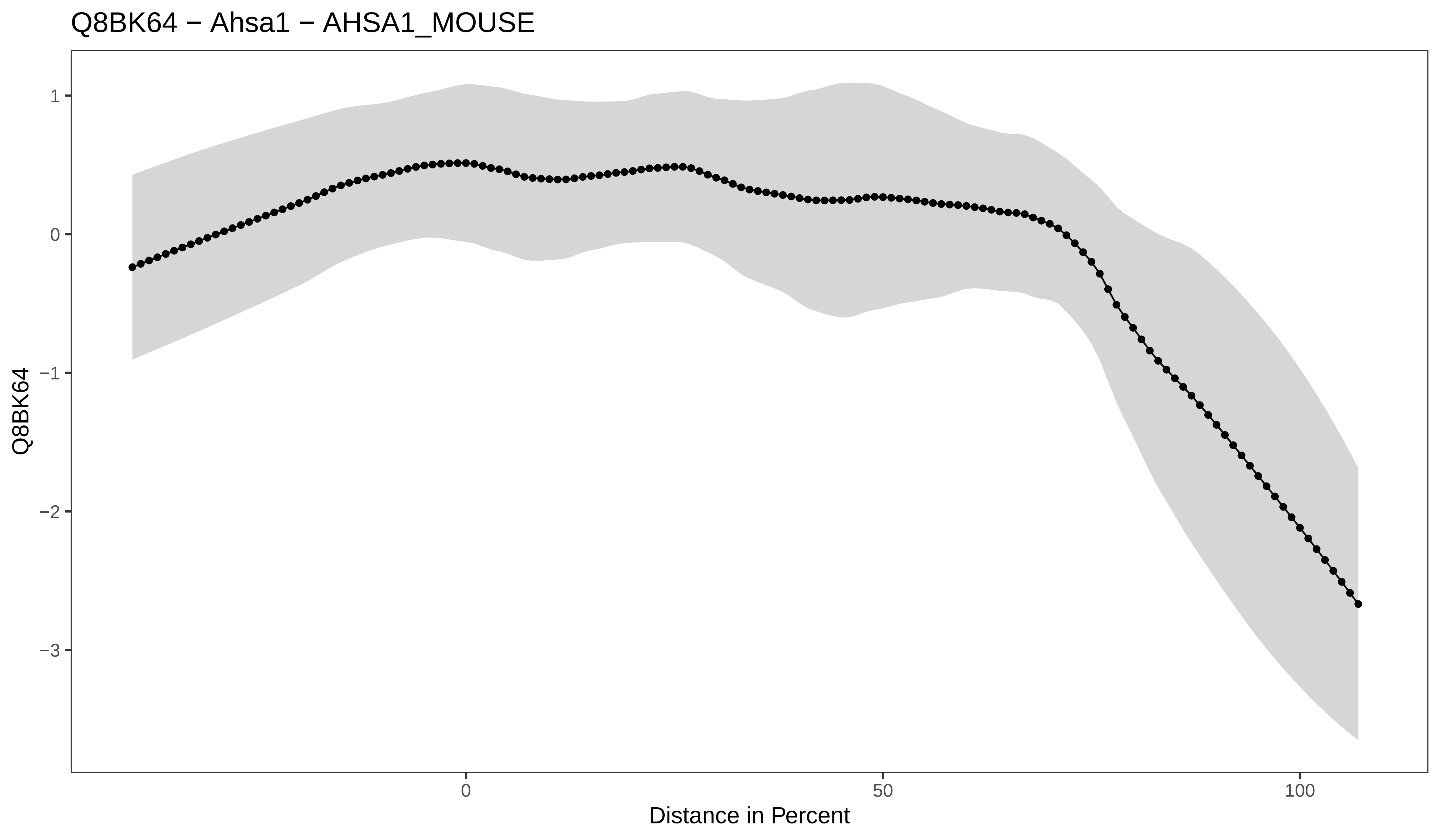

Q8BK64 − Ahsa1 − AHSA1_MOUSE
Q8BK64
1
0
−1
−2
−3
0
50
100
Distance in Percent

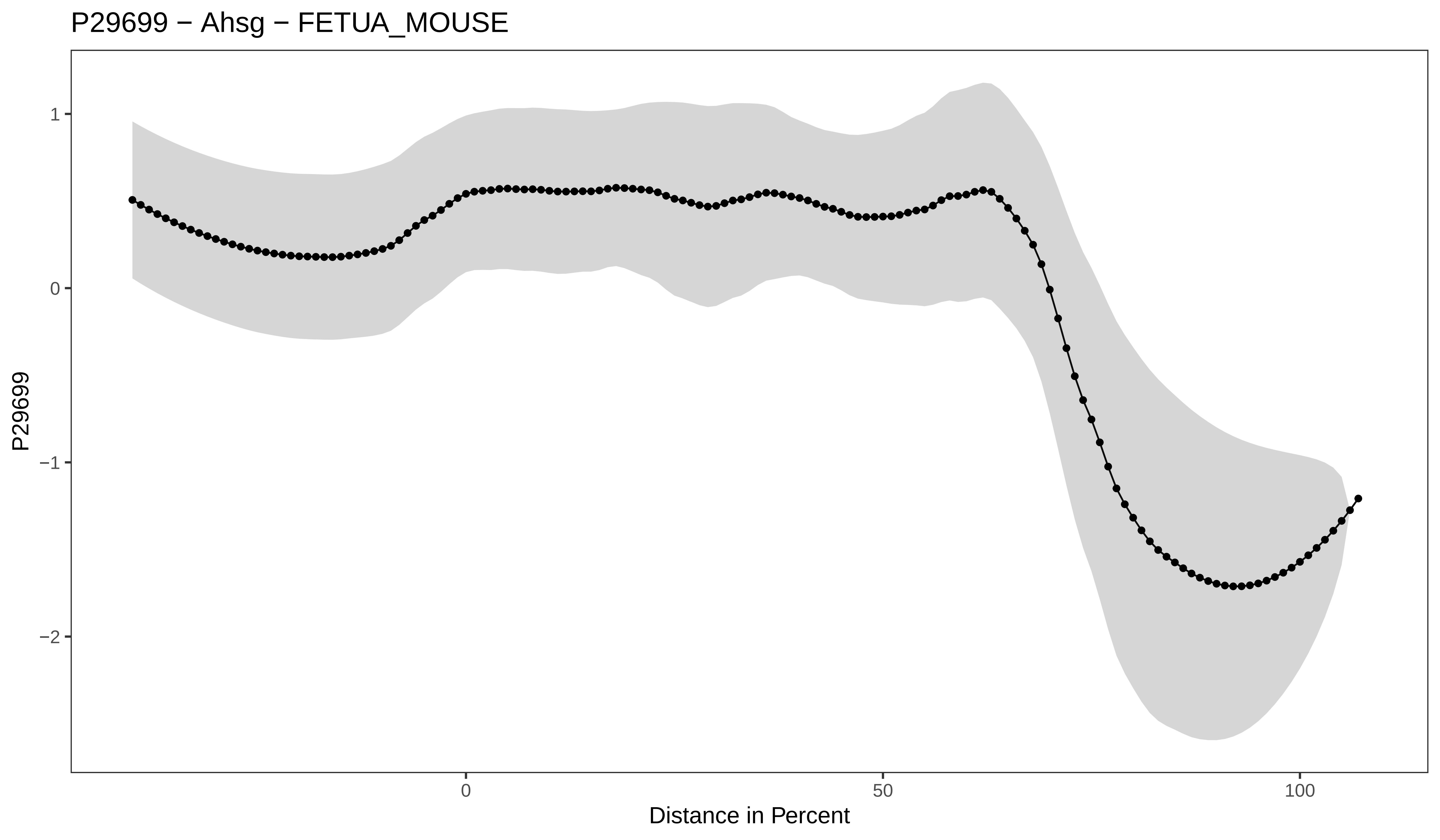

P29699 − Ahsg − FETUA_MOUSE
1
0
−1
−2
P29699
0
50
100
Distance in Percent

Q9Z0X1 − Aifm1 − AIFM1_MOUSE

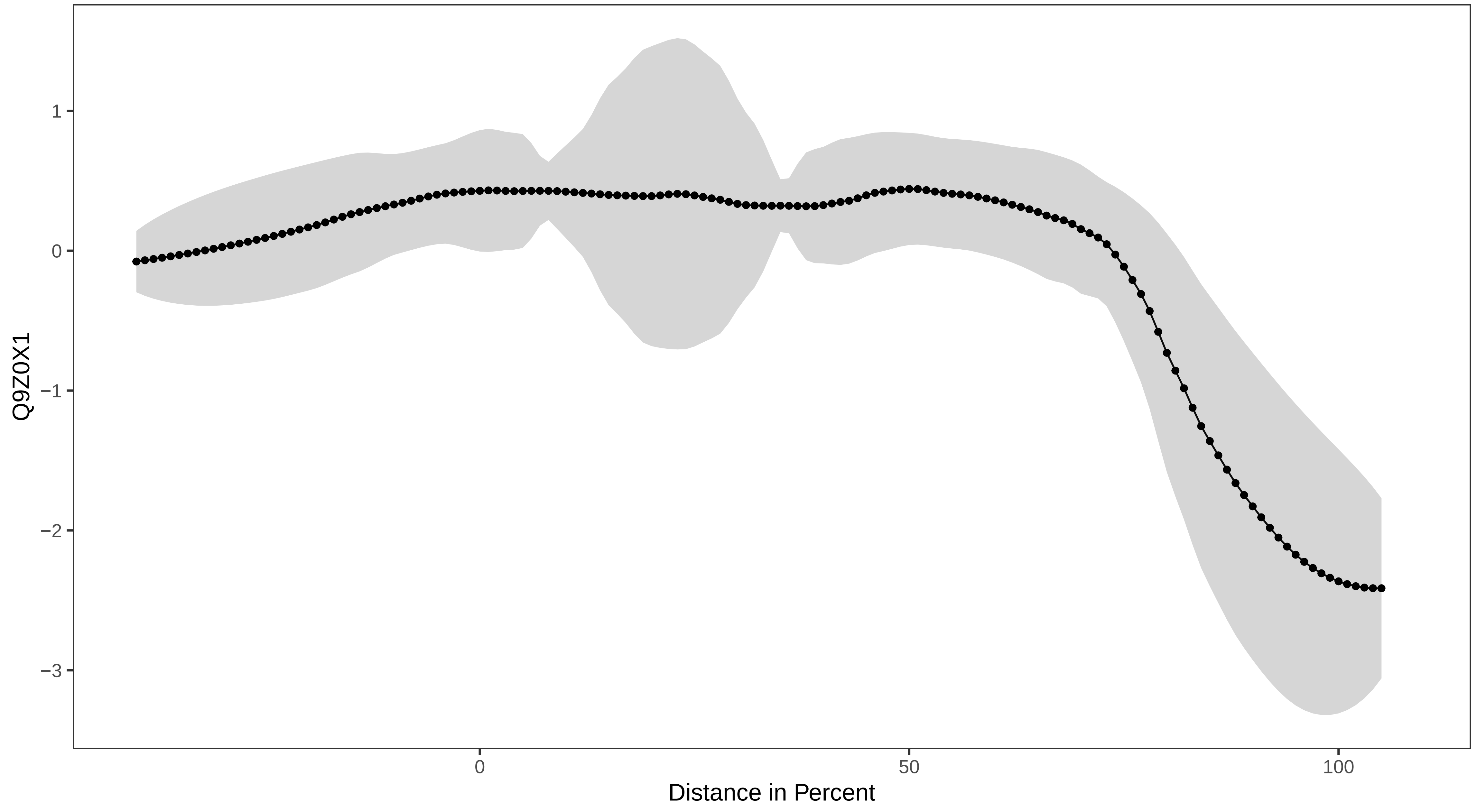

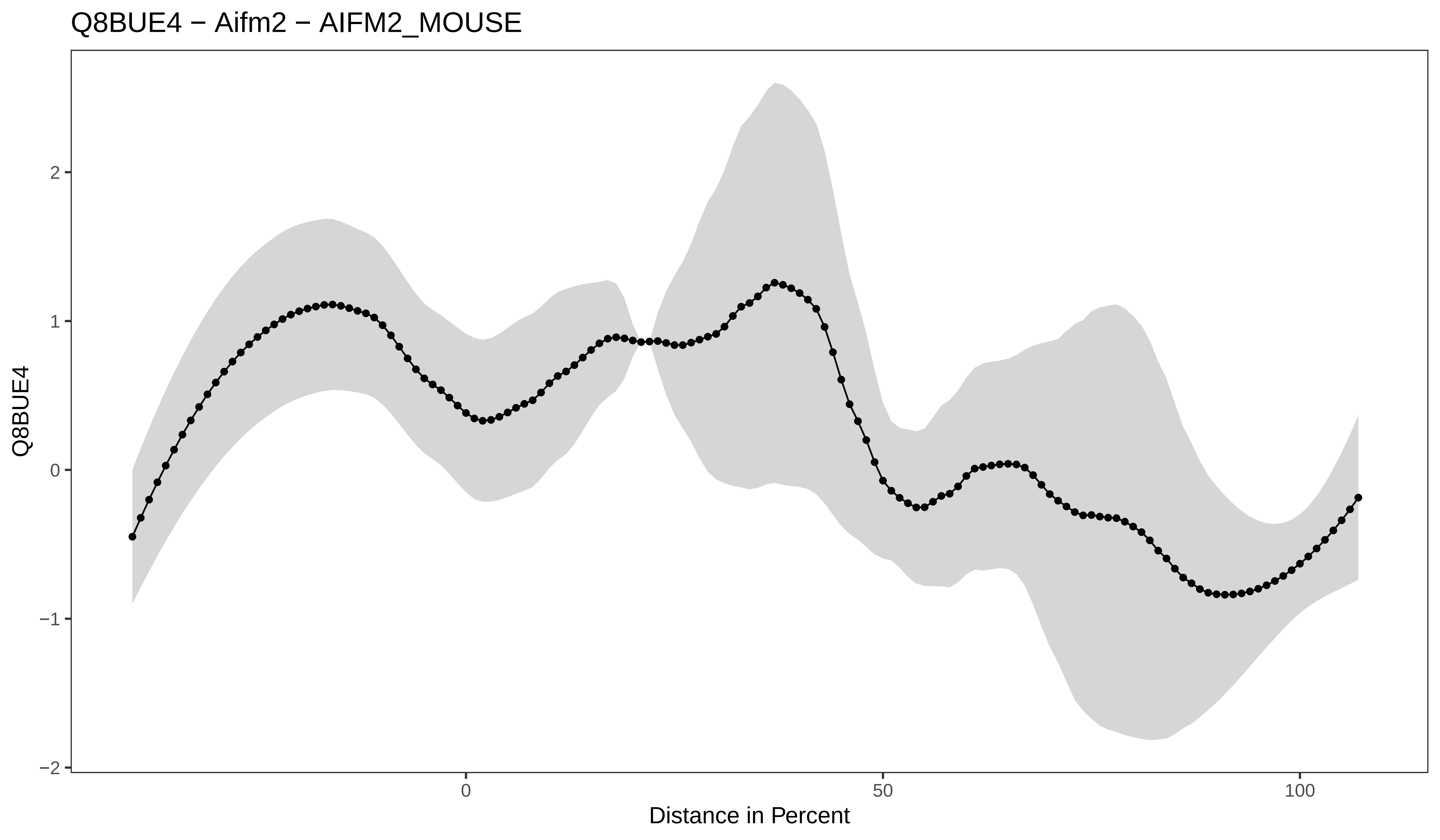
Q8BUE4 − Aifm2 − AIFM2_MOUSE
Q8BUE4
Distance in Percent
2
1
0
−1
−2
0
50
100

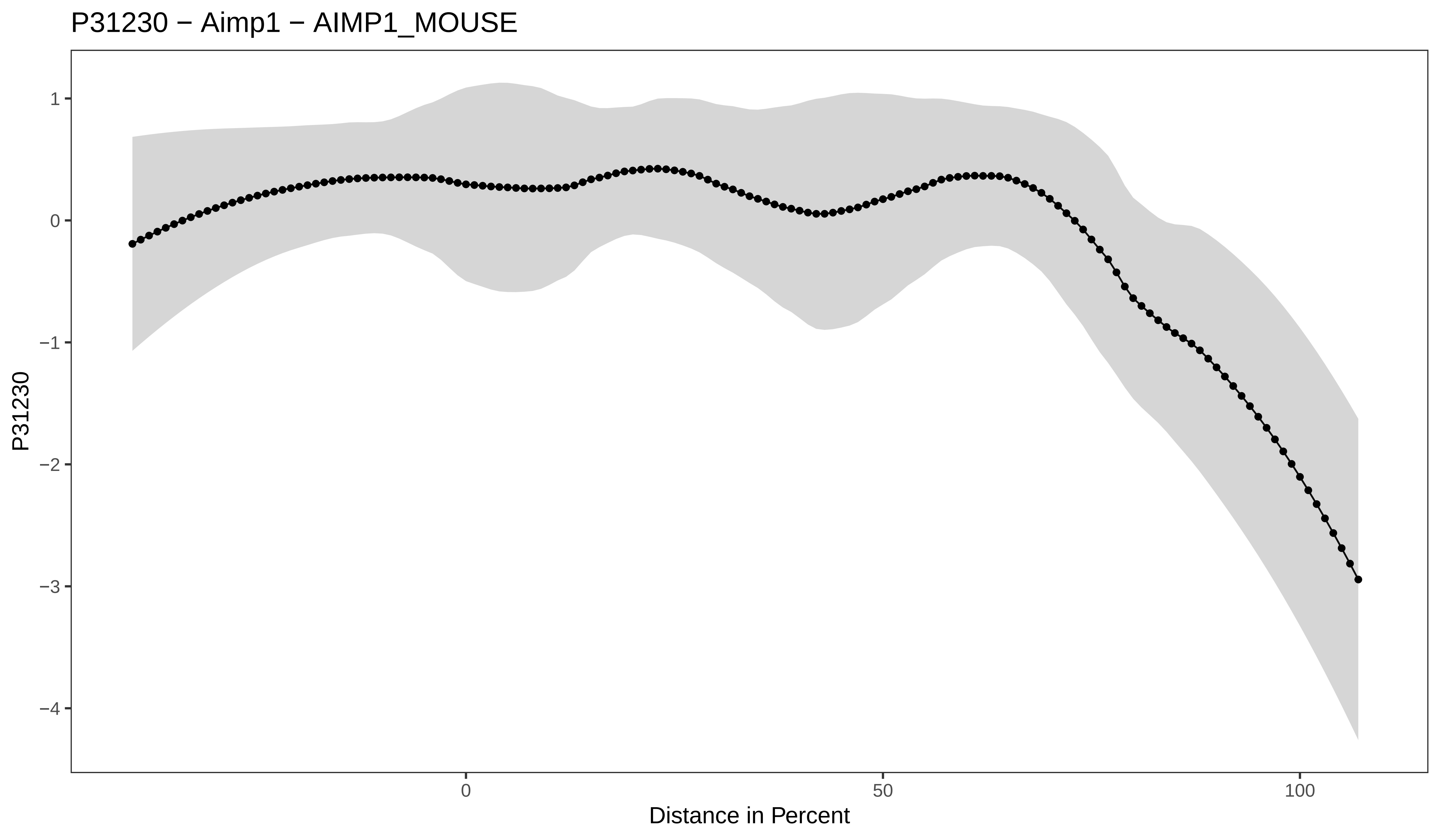
P31230 − Aimp1 − AIMP1_MOUSE
1
0
−1
−2
−3
−4
P31230
0
50
100
Distance in Percent

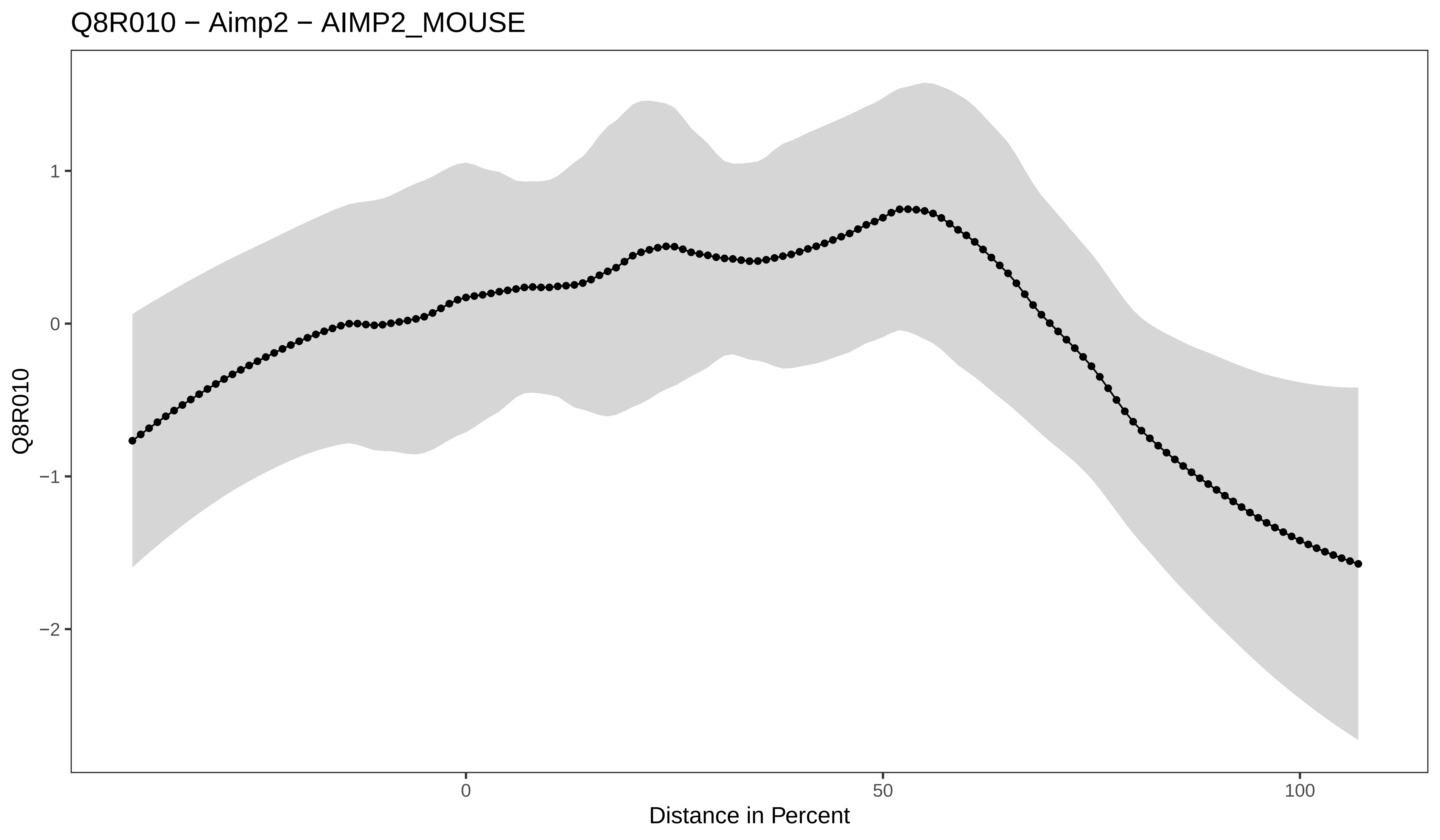

Q8R010 − Aimp2 − AIMP2_MOUSE
1
0
−1
−2
Q8R010
0
50
100
Distance in Percent

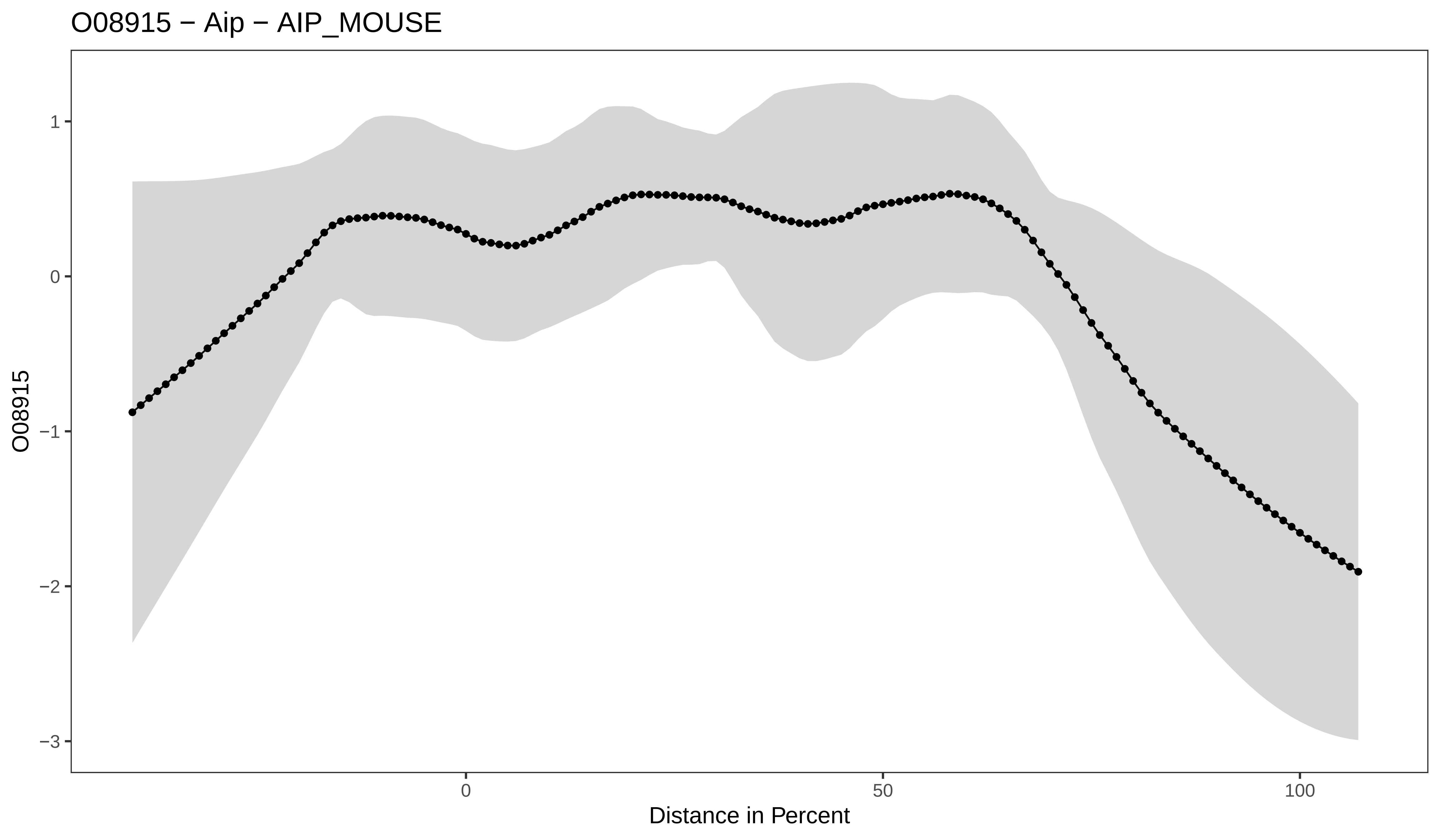
O08915 − Aip − AIP_MOUSE
O08915
Distance in Percent
1
0
−1
−2
−3
0
50
100

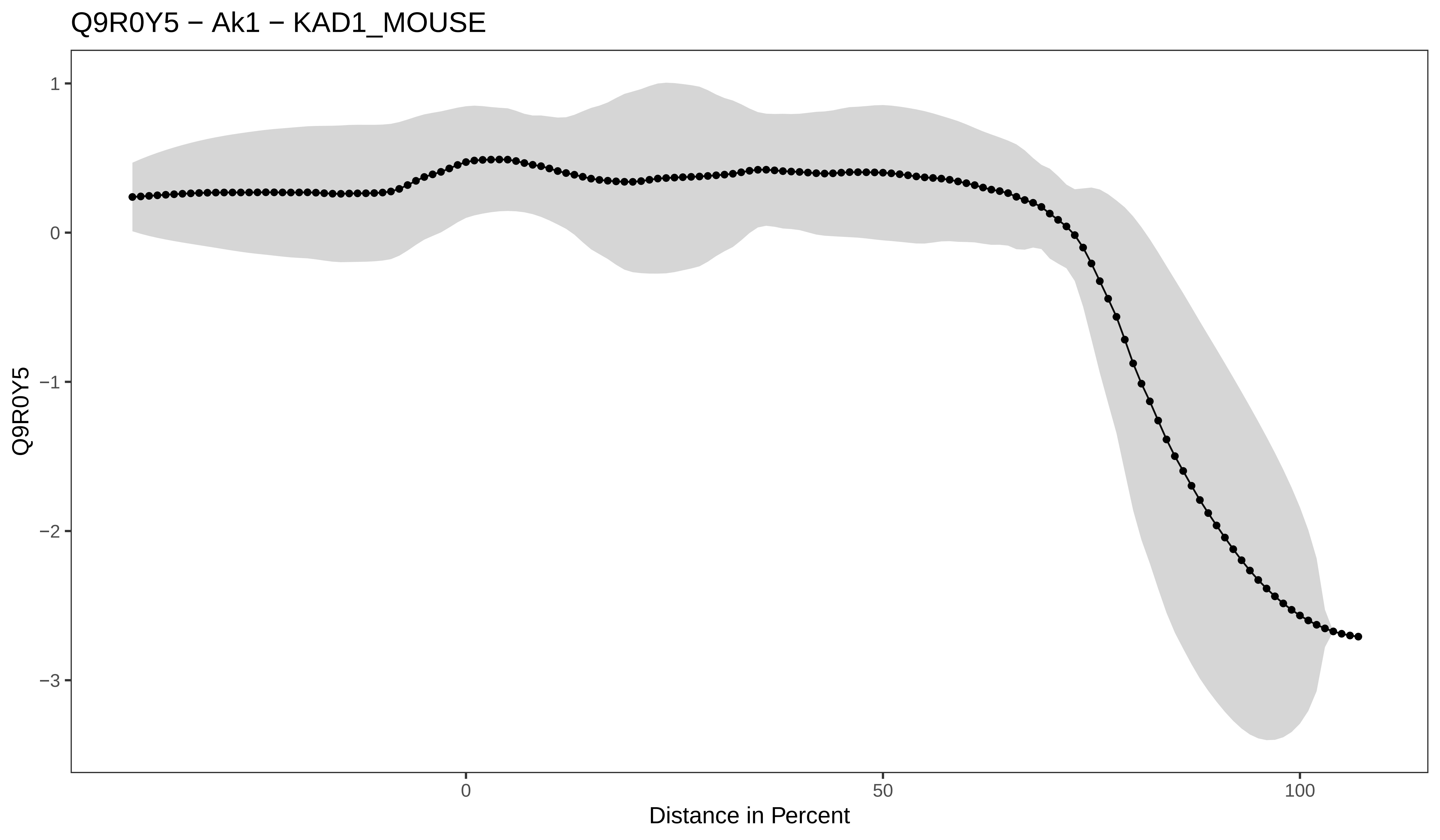
Q9R0Y5 − Ak1 − KAD1_MOUSE
1
0
−1
−2
−3
Q9R0Y5
0
50
100
Distance in Percent

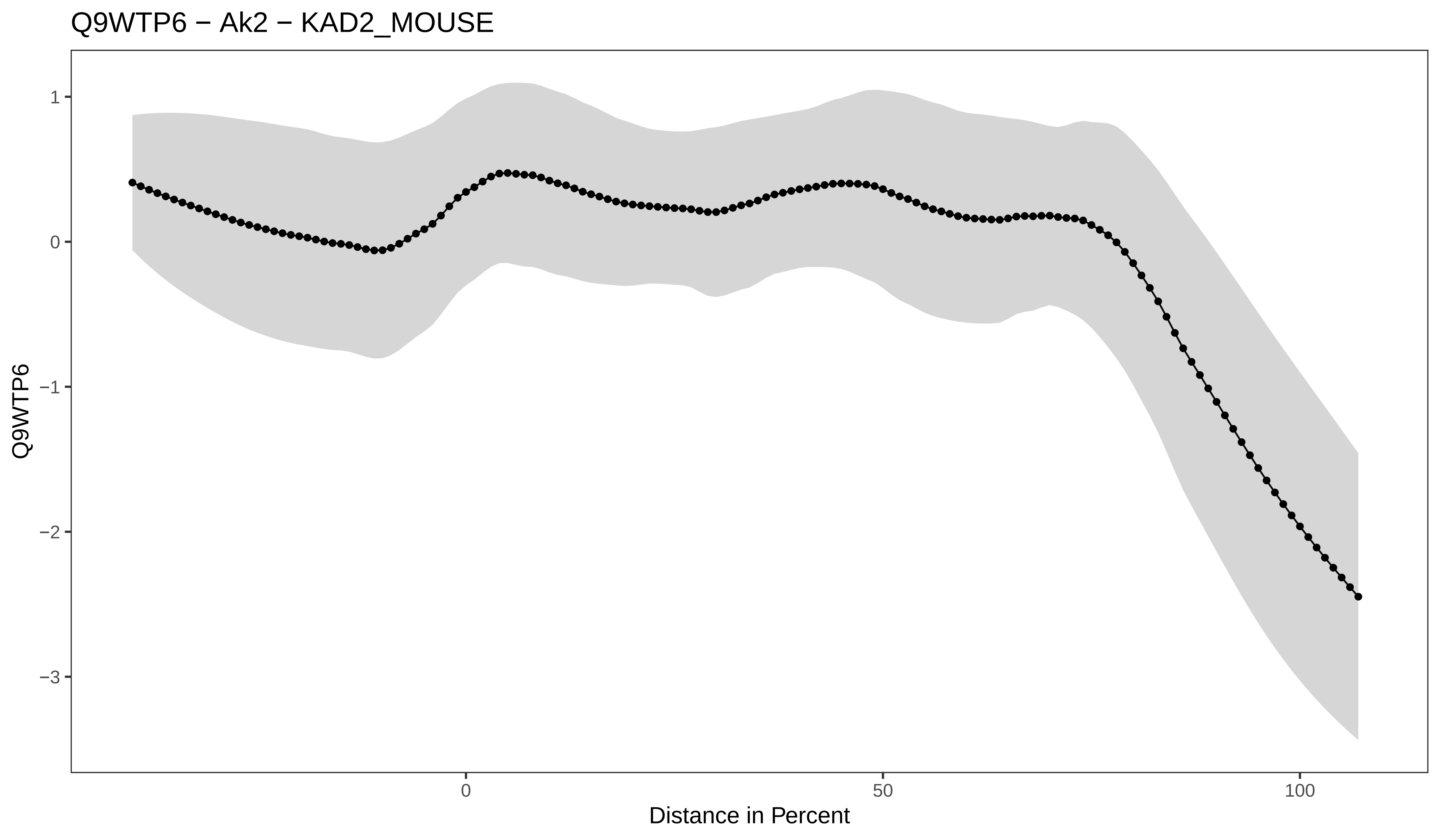
Q9WTP6 − Ak2 − KAD2_MOUSE
Q9WTP6
Distance in Percent
1
0
−1
−2
−3
0
50
100

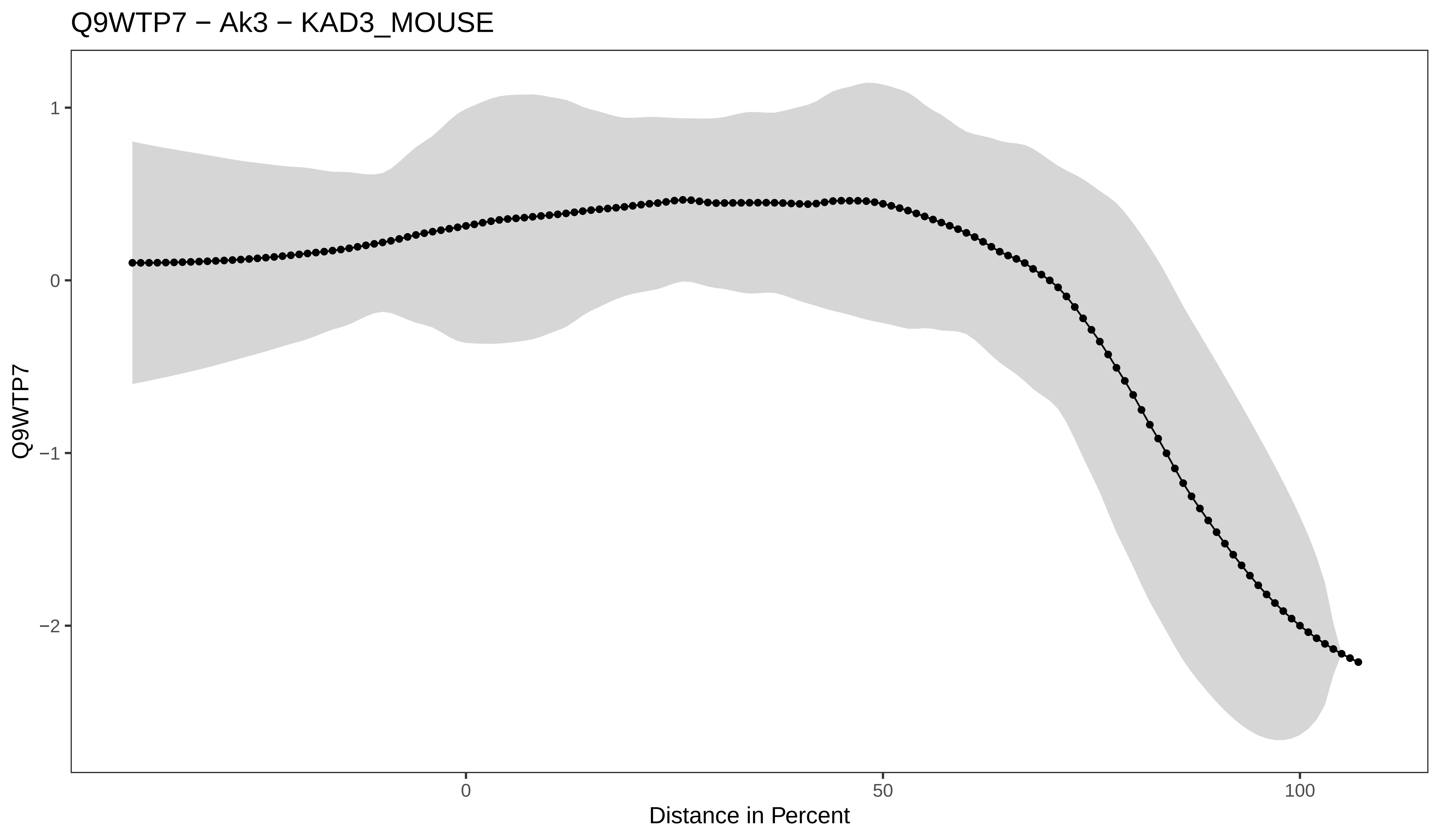

Q9WTP7 − Ak3 − KAD3_MOUSE
Q9WTP7
1
0
−1
−2
0
50
100
Distance in Percent

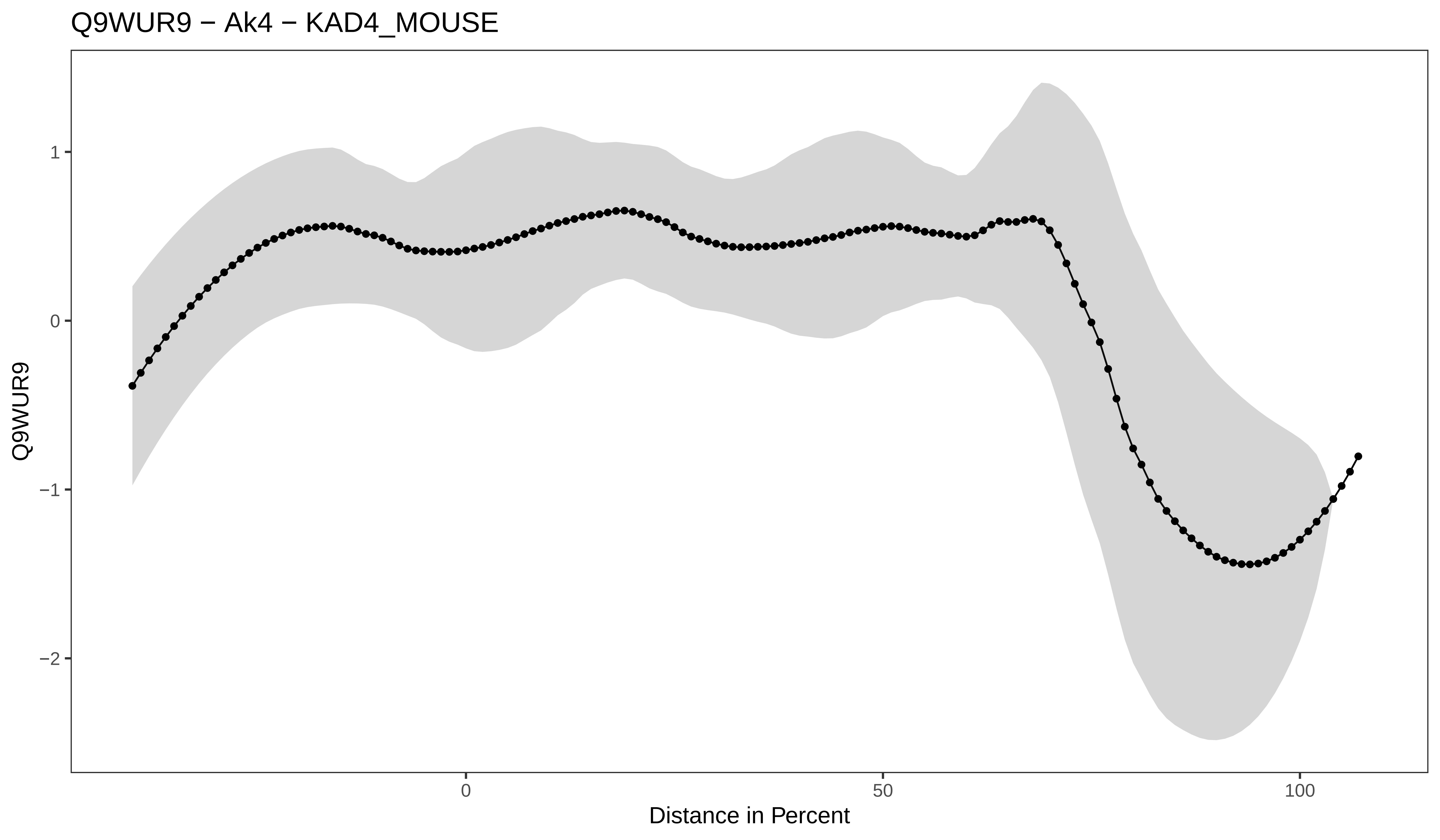

Q9WUR9 − Ak4 − KAD4_MOUSE
1
0
−1
−2
Q9WUR9
0
50
100
Distance in Percent

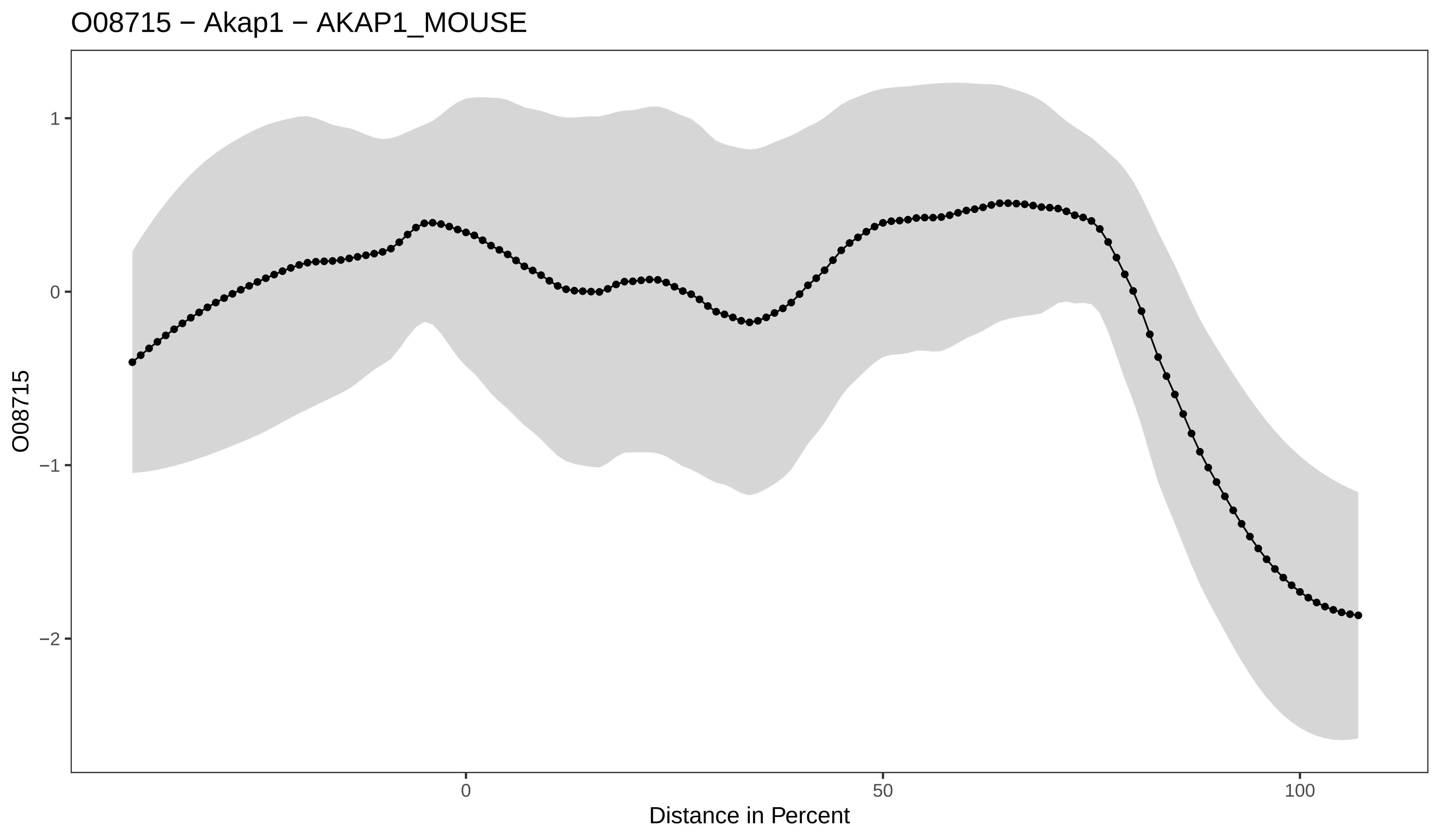

O08715 − Akap1 − AKAP1_MOUSE
O08715
Distance in Percent
1
0
−1
−2
0
50
100

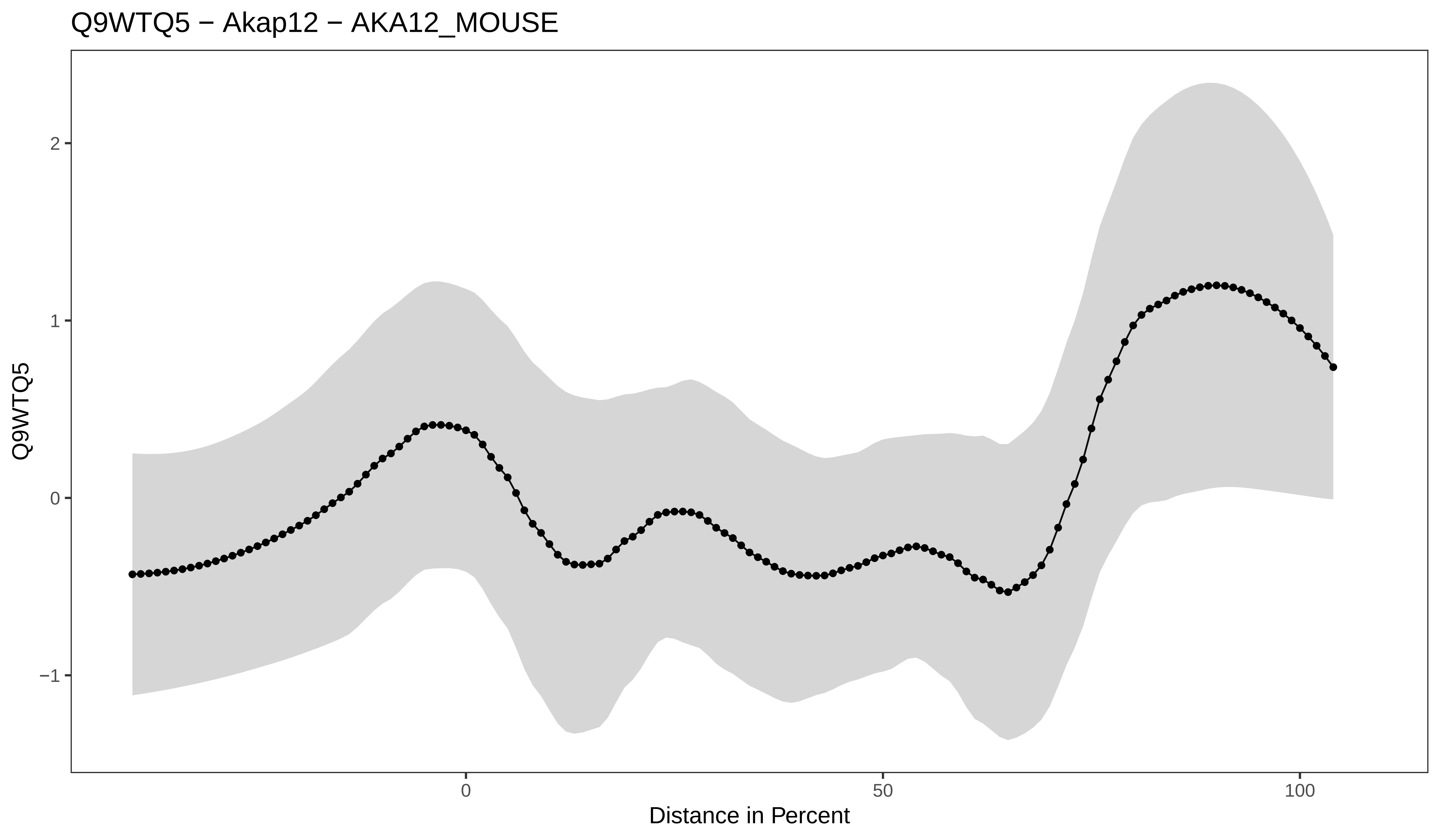
Q9WTQ5 − Akap12 − AKA12_MOUSE
Q9WTQ5
Distance in Percent
2
1
0
−1
0
50
100

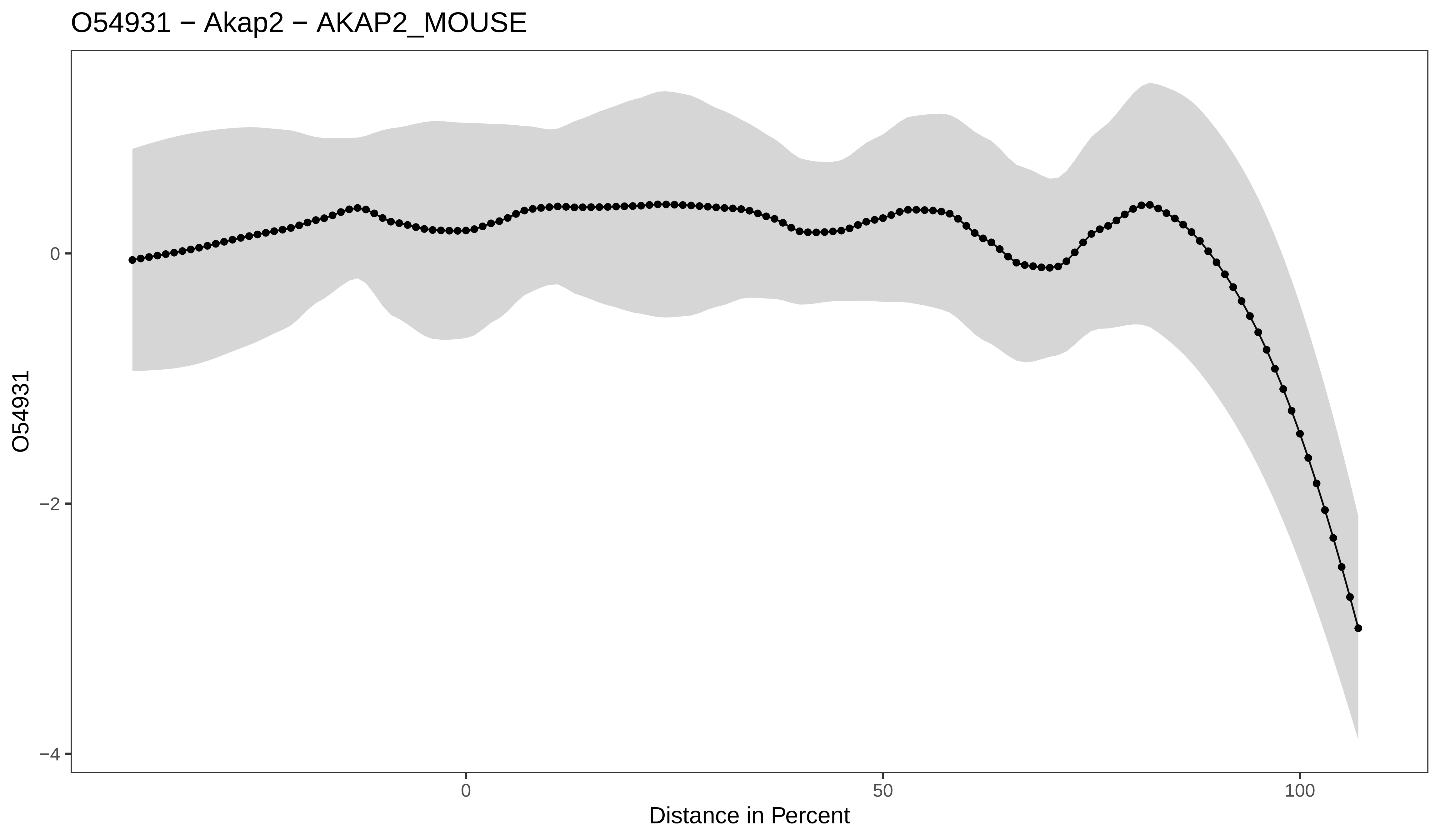
O54931 − Akap2 − AKAP2_MOUSE
O54931
0
−2
−4
0
50
100
Distance in Percent

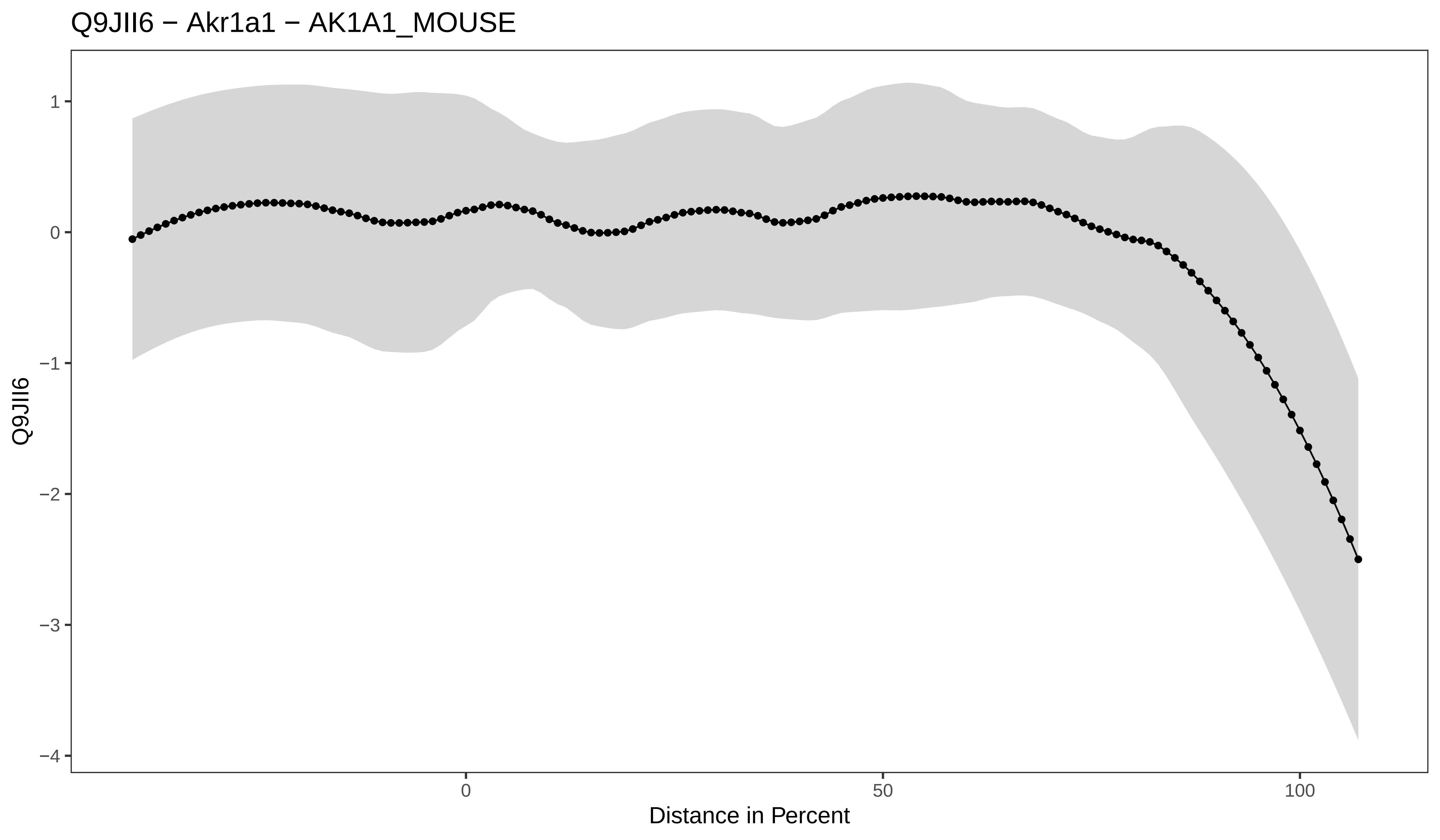

Q9JII6 − Akr1a1 − AK1A1_MOUSE
1
0
−1
−2
−3
−4
Q9JII6
0
50
100
Distance in Percent

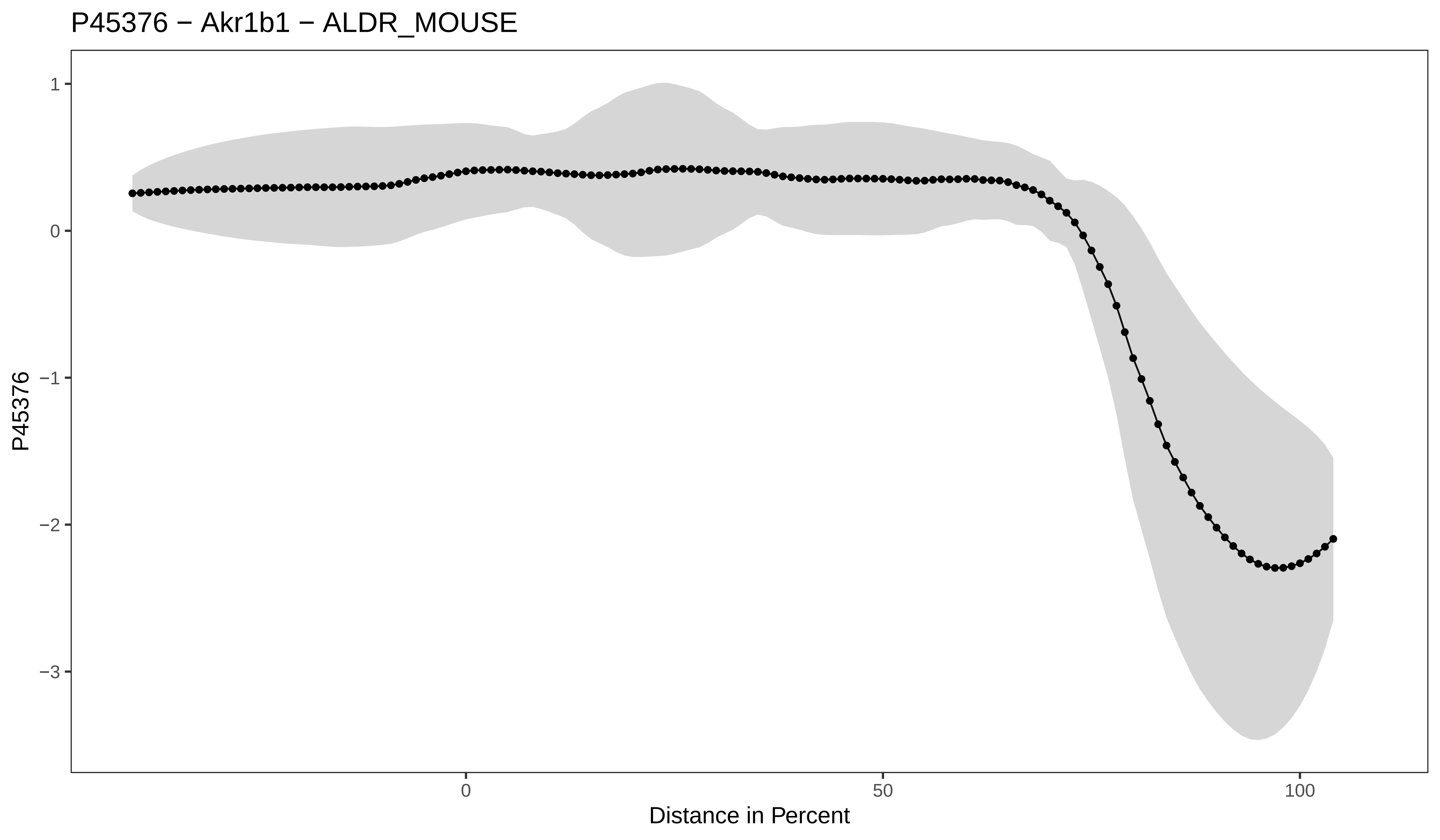

P45376 − Akr1b1 − ALDR_MOUSE
1
0
−1
−2
−3
P45376
0
50
100
Distance in Percent

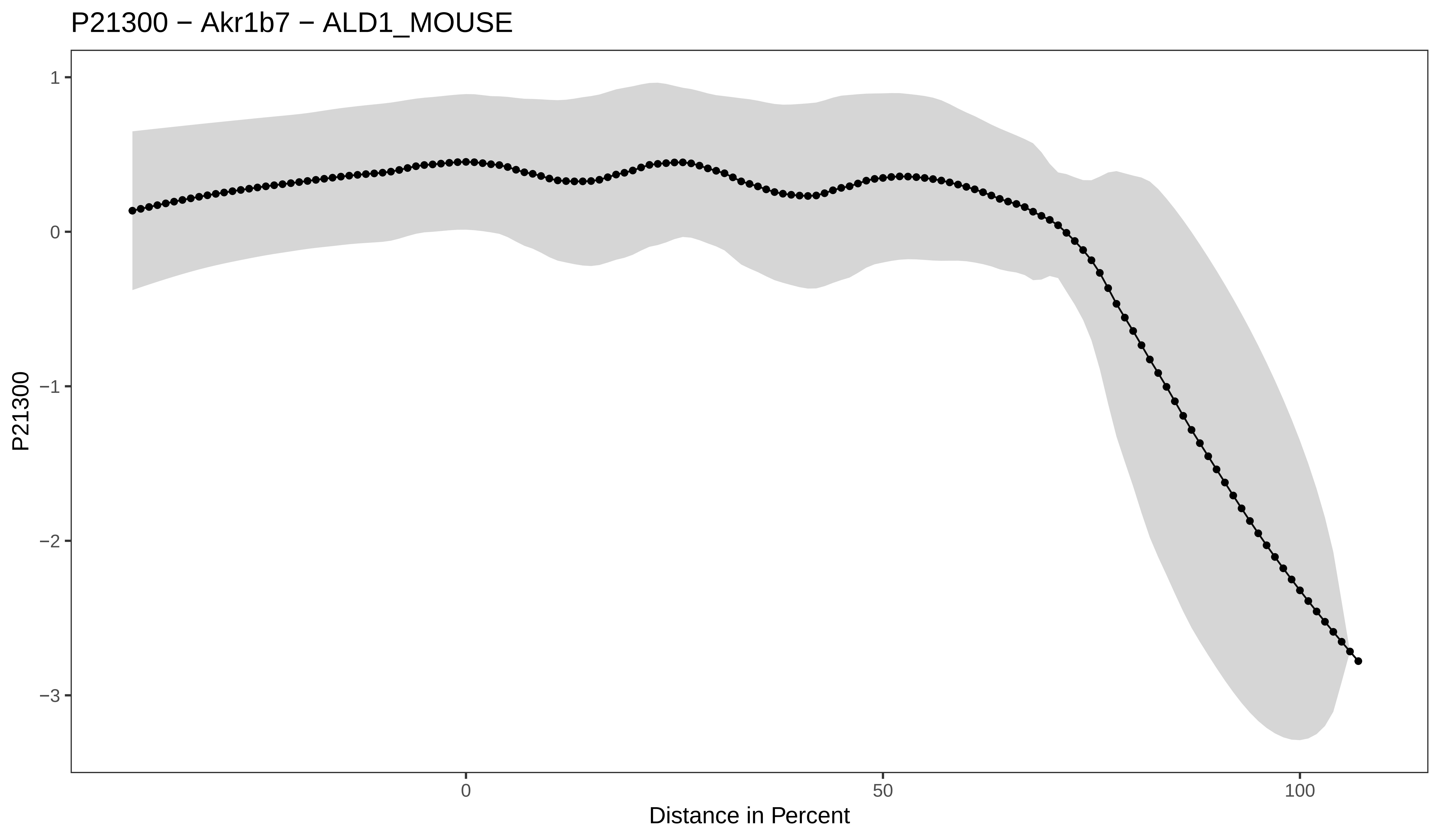

P21300 − Akr1b7 − ALD1_MOUSE
1
0
−1
−2
−3
P21300
0
50
100
Distance in Percent

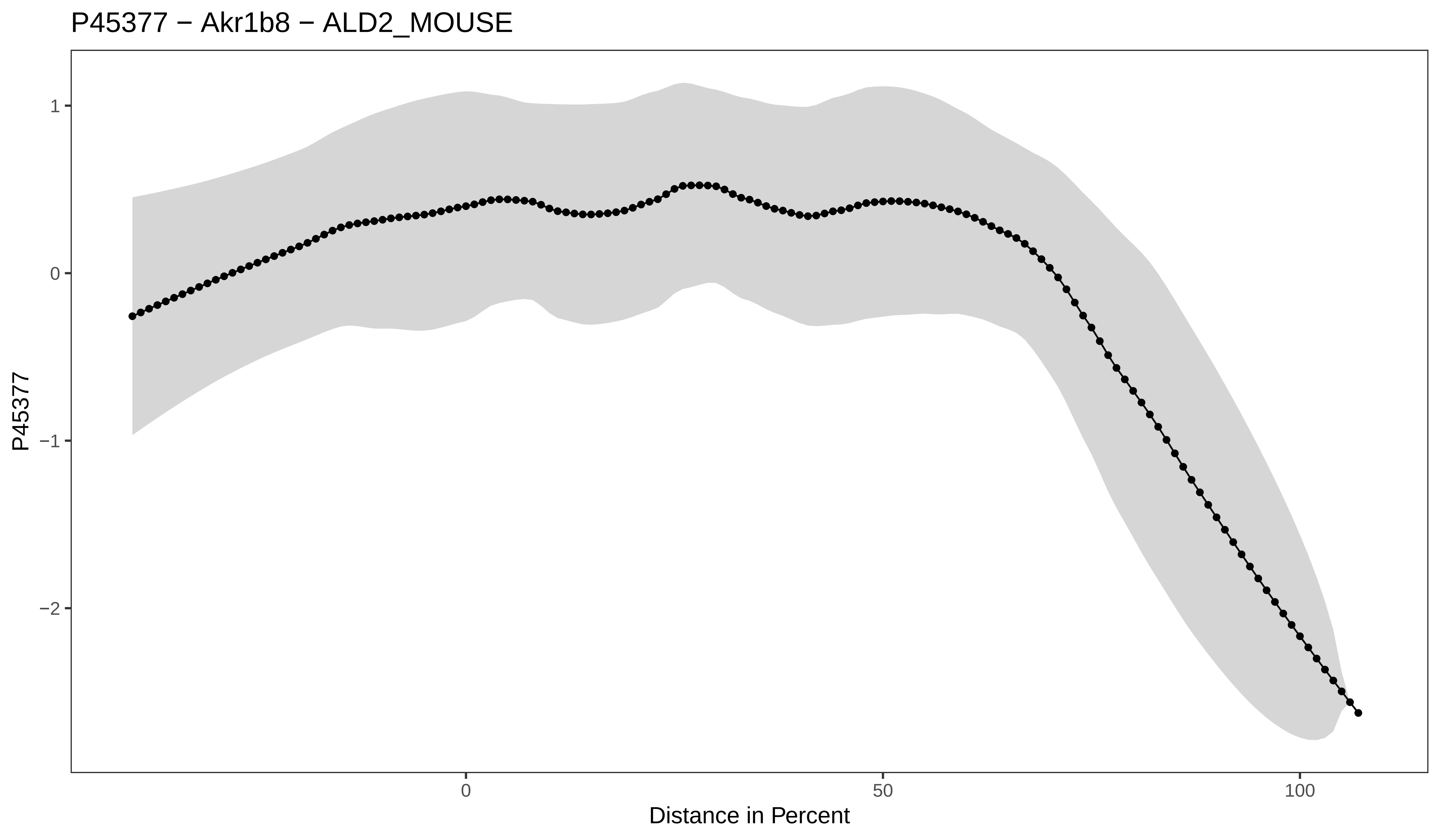
P45377 − Akr1b8 − ALD2_MOUSE
P45377
1
0
−1
−2
0
50
100
Distance in Percent

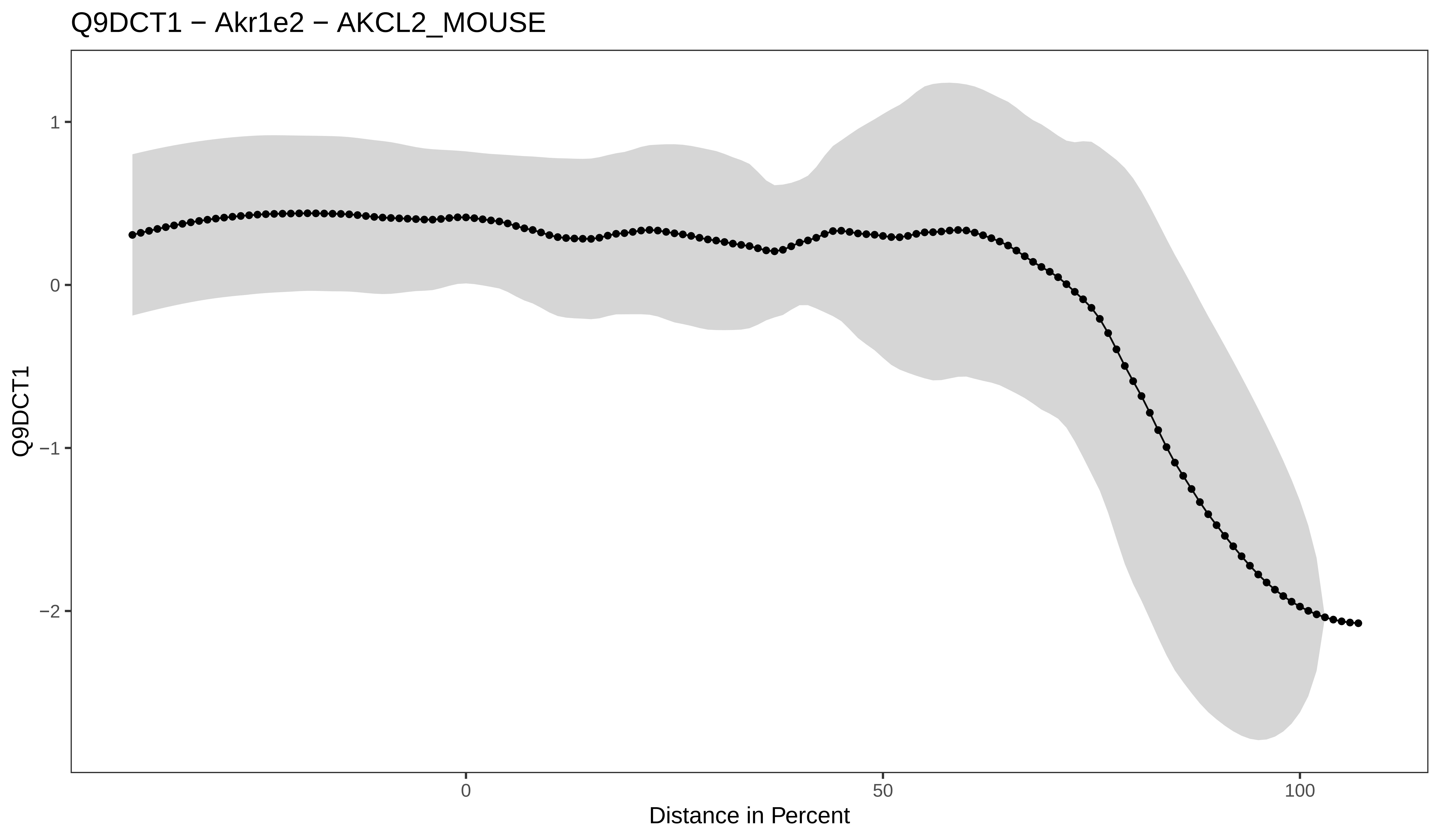

Q9DCT1 − Akr1e2 − AKCL2_MOUSE
1
0
−1
−2
Q9DCT1
0
50
100
Distance in Percent

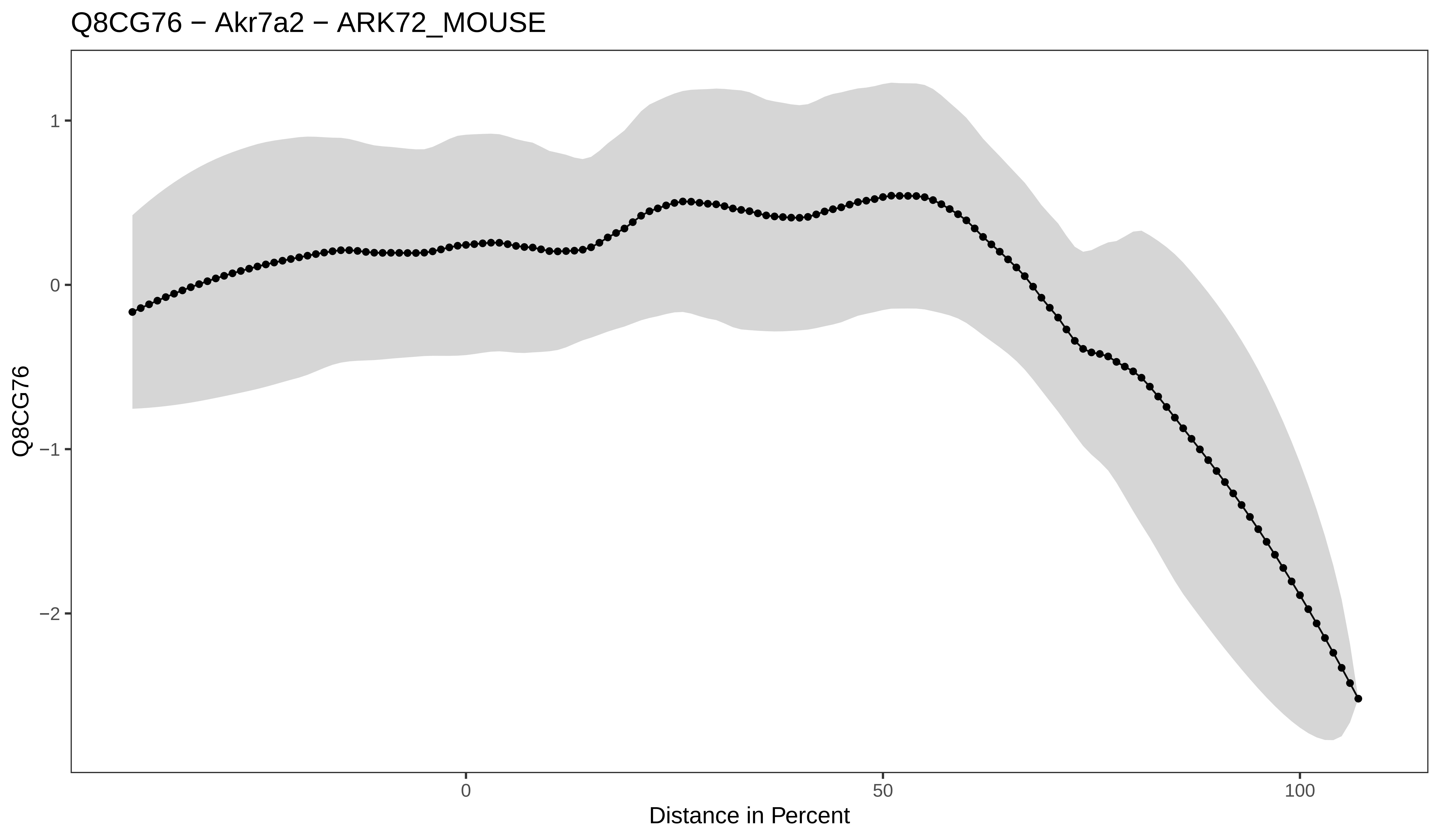

Q8CG76 − Akr7a2 − ARK72_MOUSE
1
0
−1
−2
Q8CG76
0
50
100
Distance in Percent

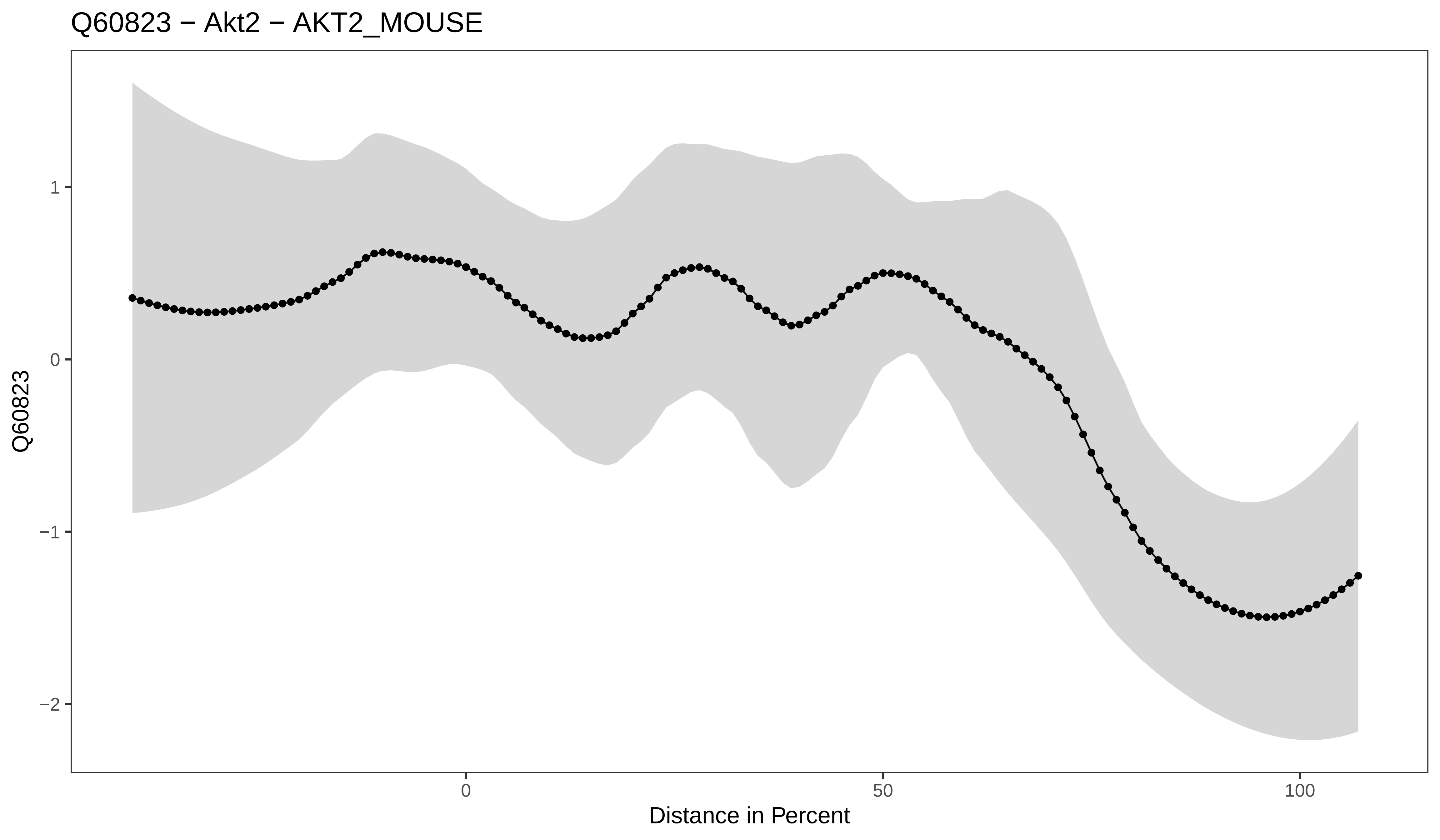

Q60823 − Akt2 − AKT2_MOUSE
Q60823
1
0
−1
−2
0
50
100
Distance in Percent

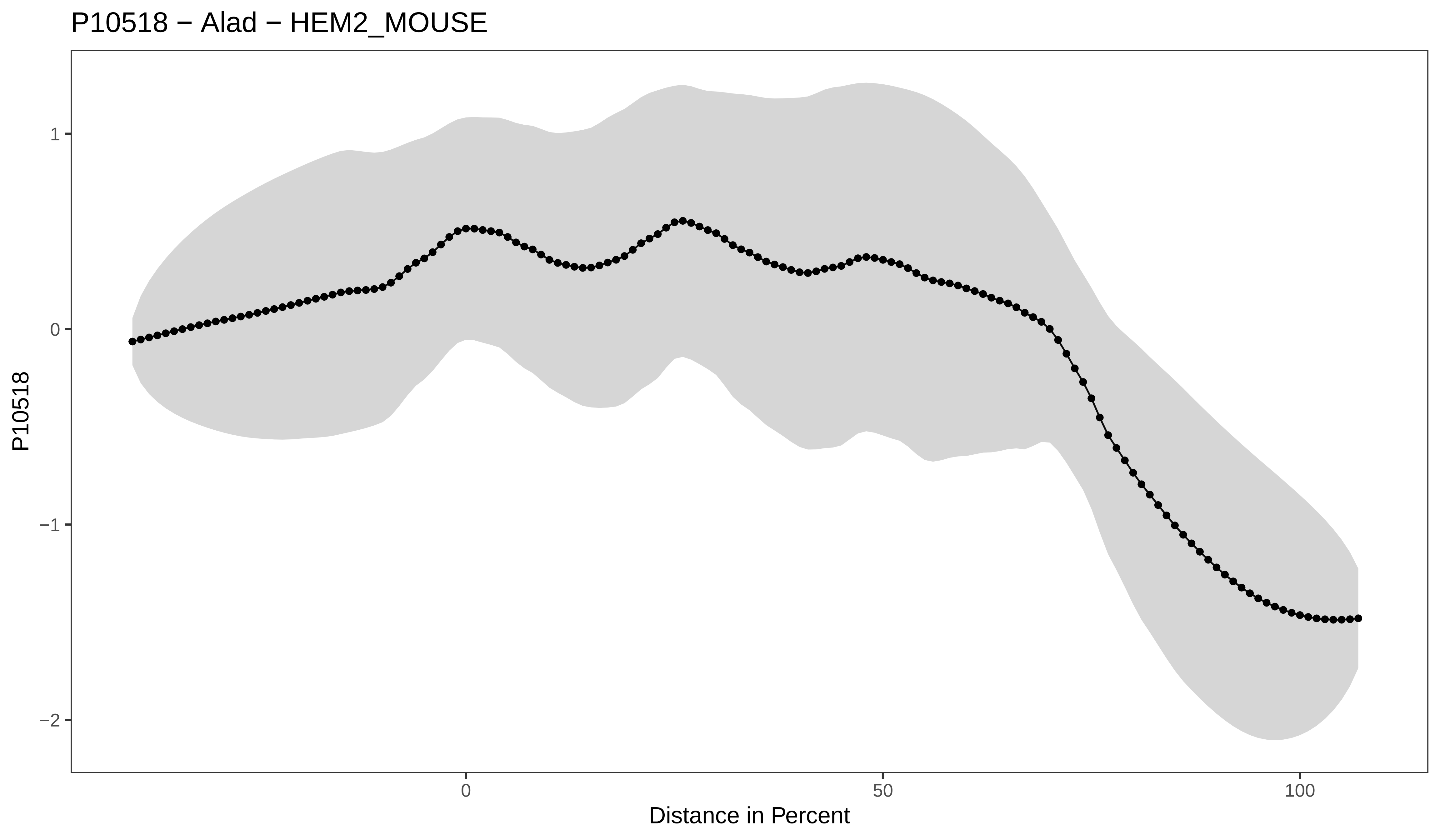

P10518 − Alad − HEM2_MOUSE
P10518
1
0
−1
−2
0
50
100
Distance in Percent

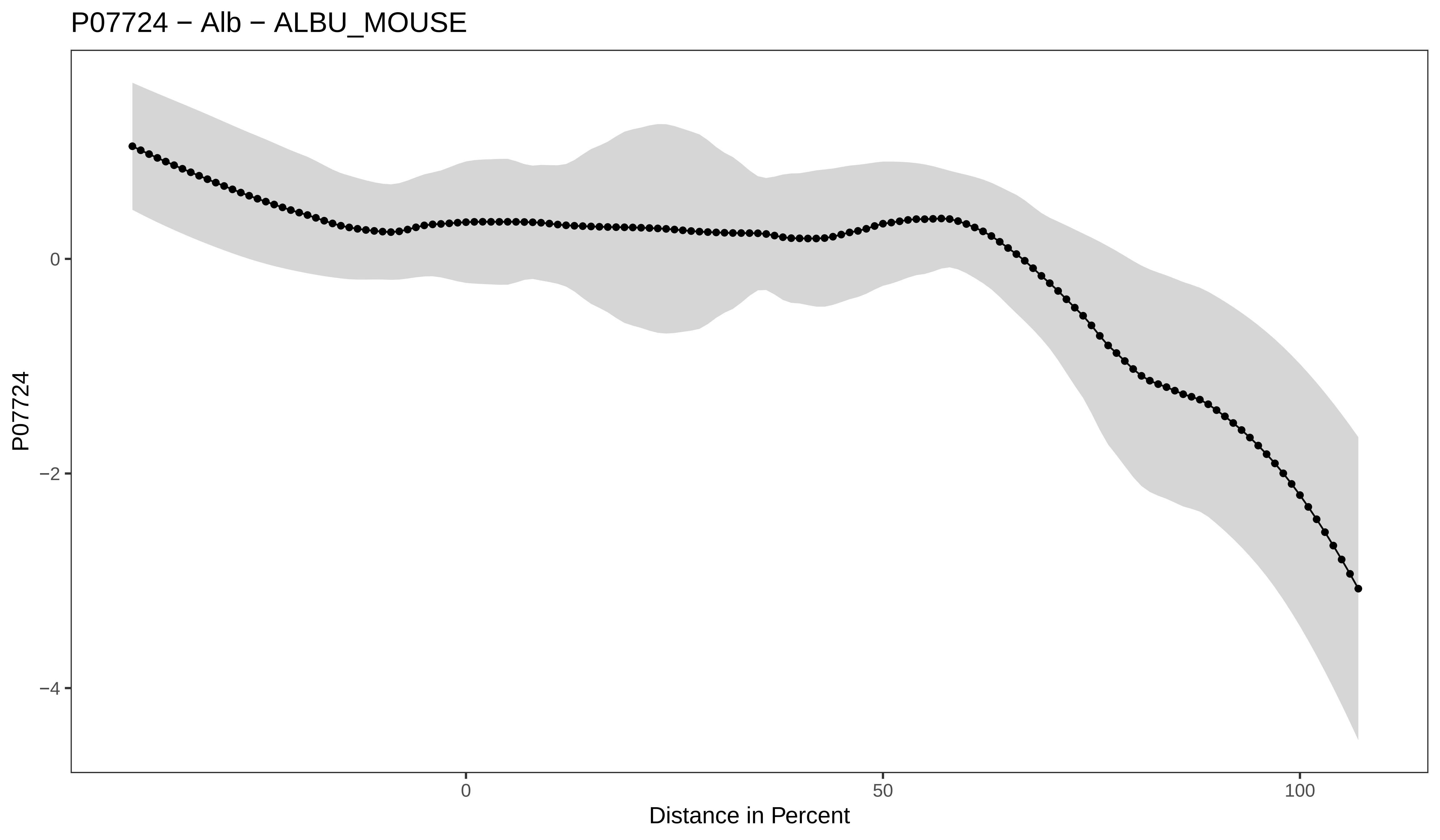

P07724 − Alb − ALBU_MOUSE
P07724
0
−2
−4
0
50
100
Distance in Percent

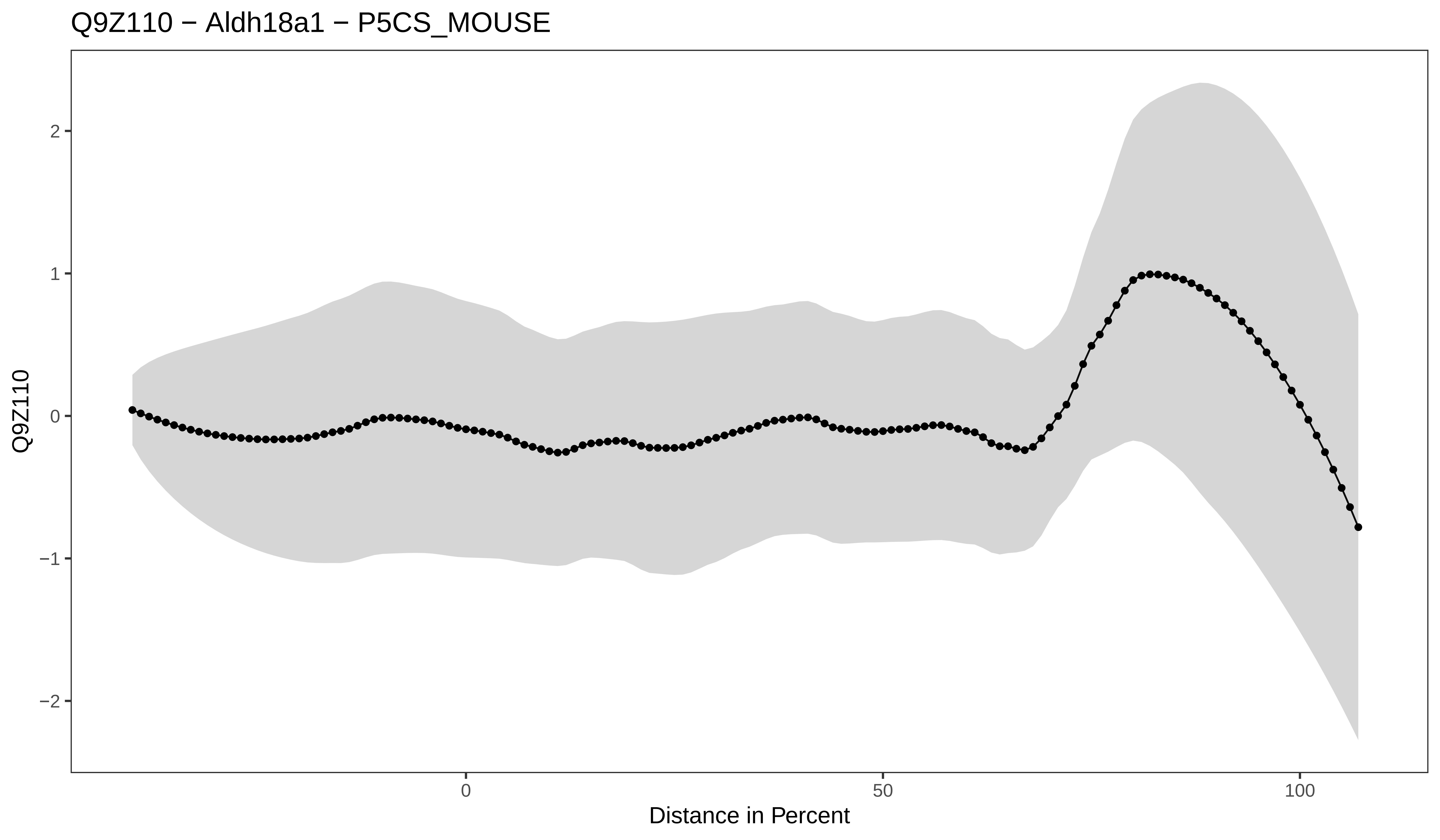

Q9Z110 − Aldh18a1 − P5CS_MOUSE
Q9Z110
2
1
0
−1
−2
0
50
100
Distance in Percent

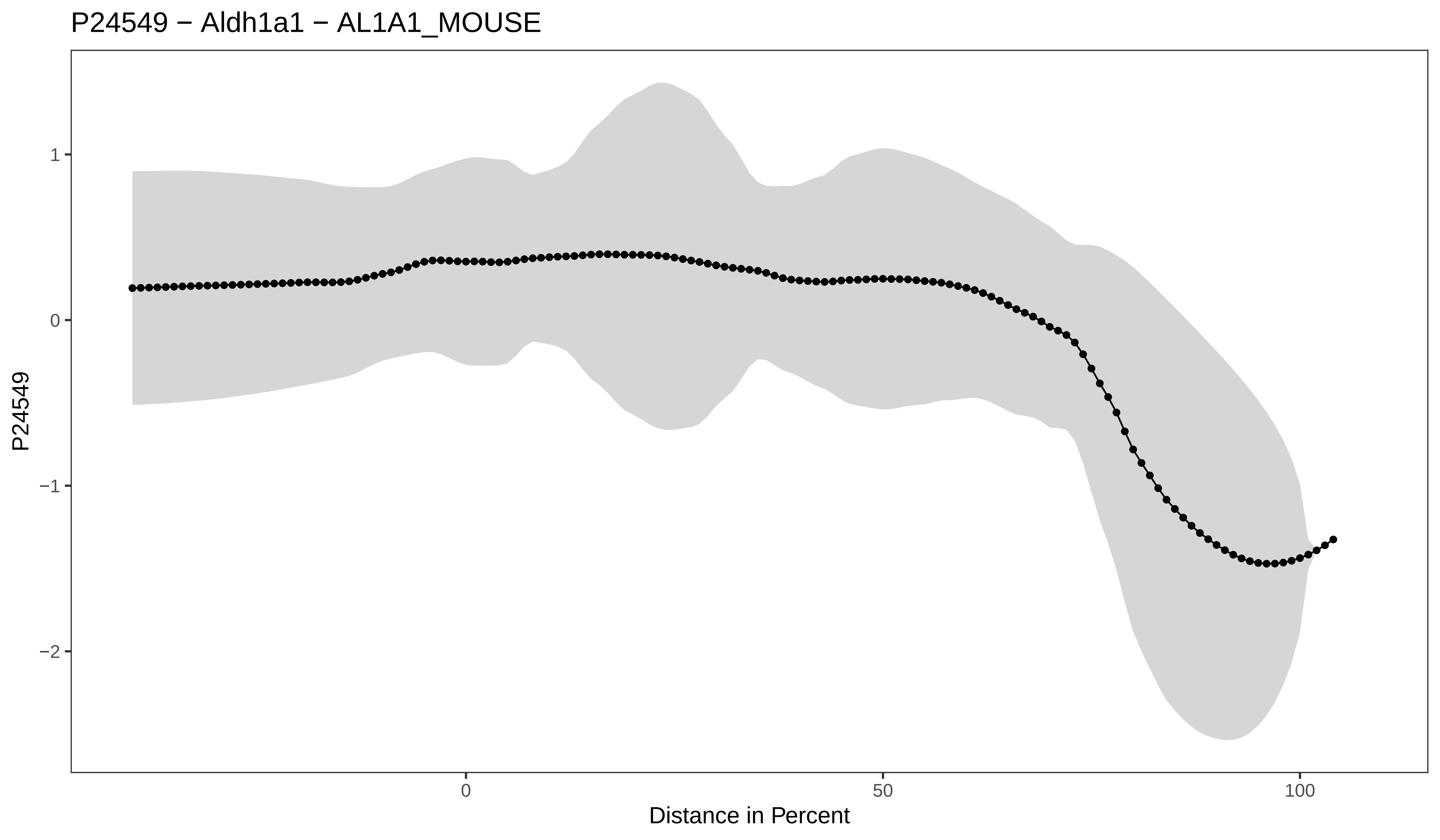
P24549 − Aldh1a1 − AL1A1_MOUSE
P24549
1
0
−1
−2
0
50
100
Distance in Percent

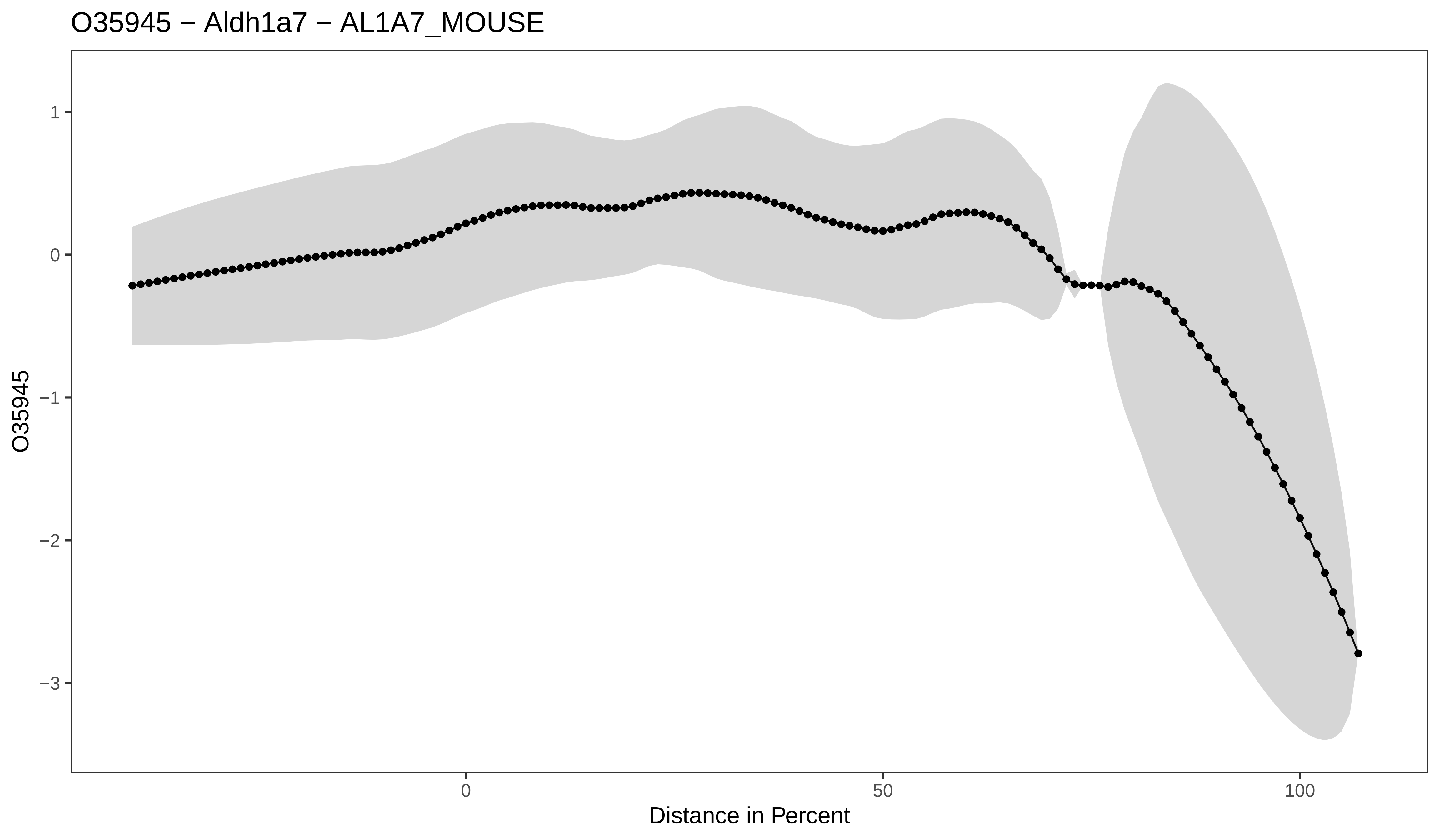
O35945 − Aldh1a7 − AL1A7_MOUSE
O35945
1
0
−1
−2
−3
0
50
100
Distance in Percent

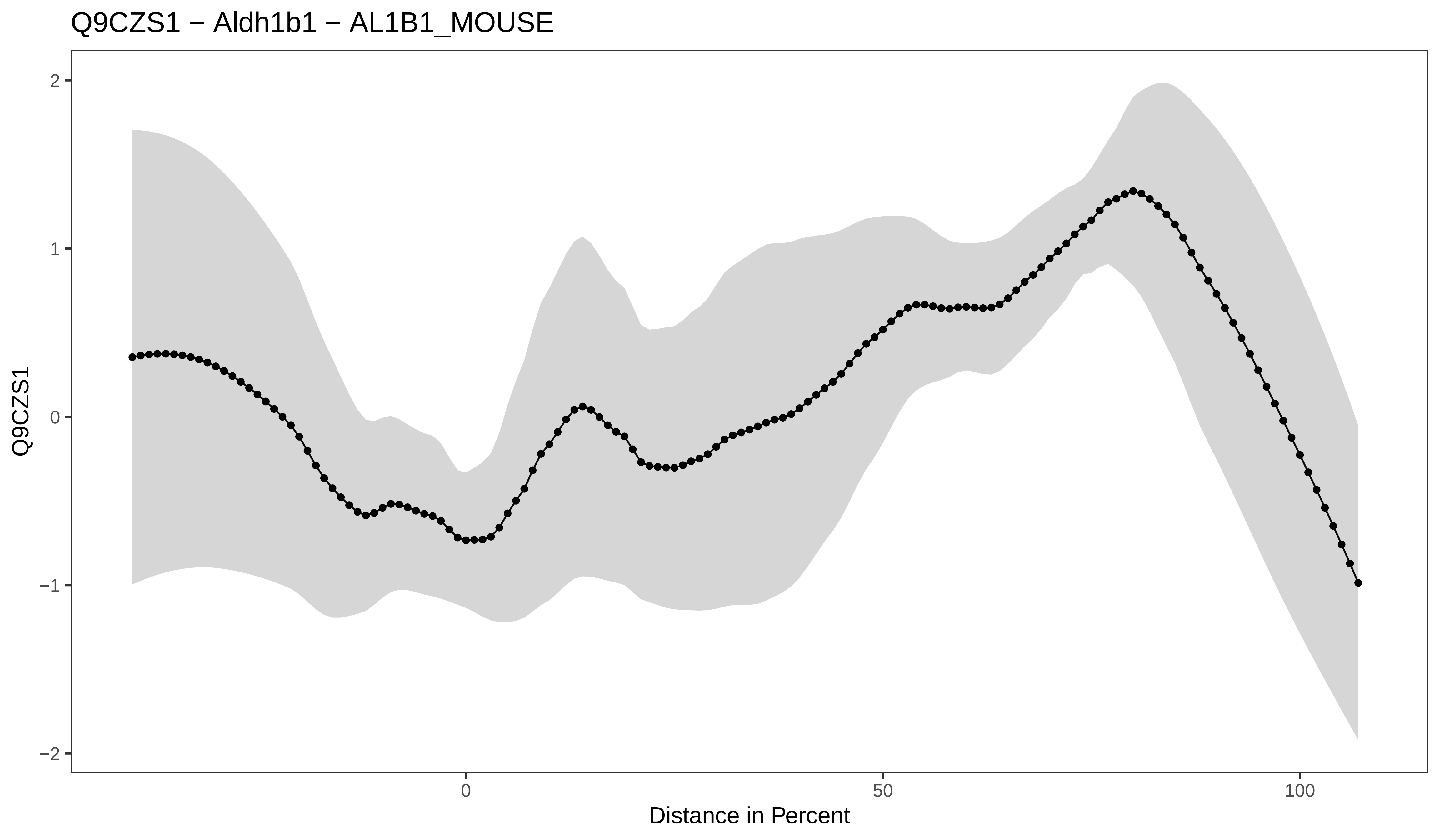
Q9CZS1 − Aldh1b1 − AL1B1_MOUSE
Q9CZS1
2
1
0
−1
−2
0
50
100
Distance in Percent

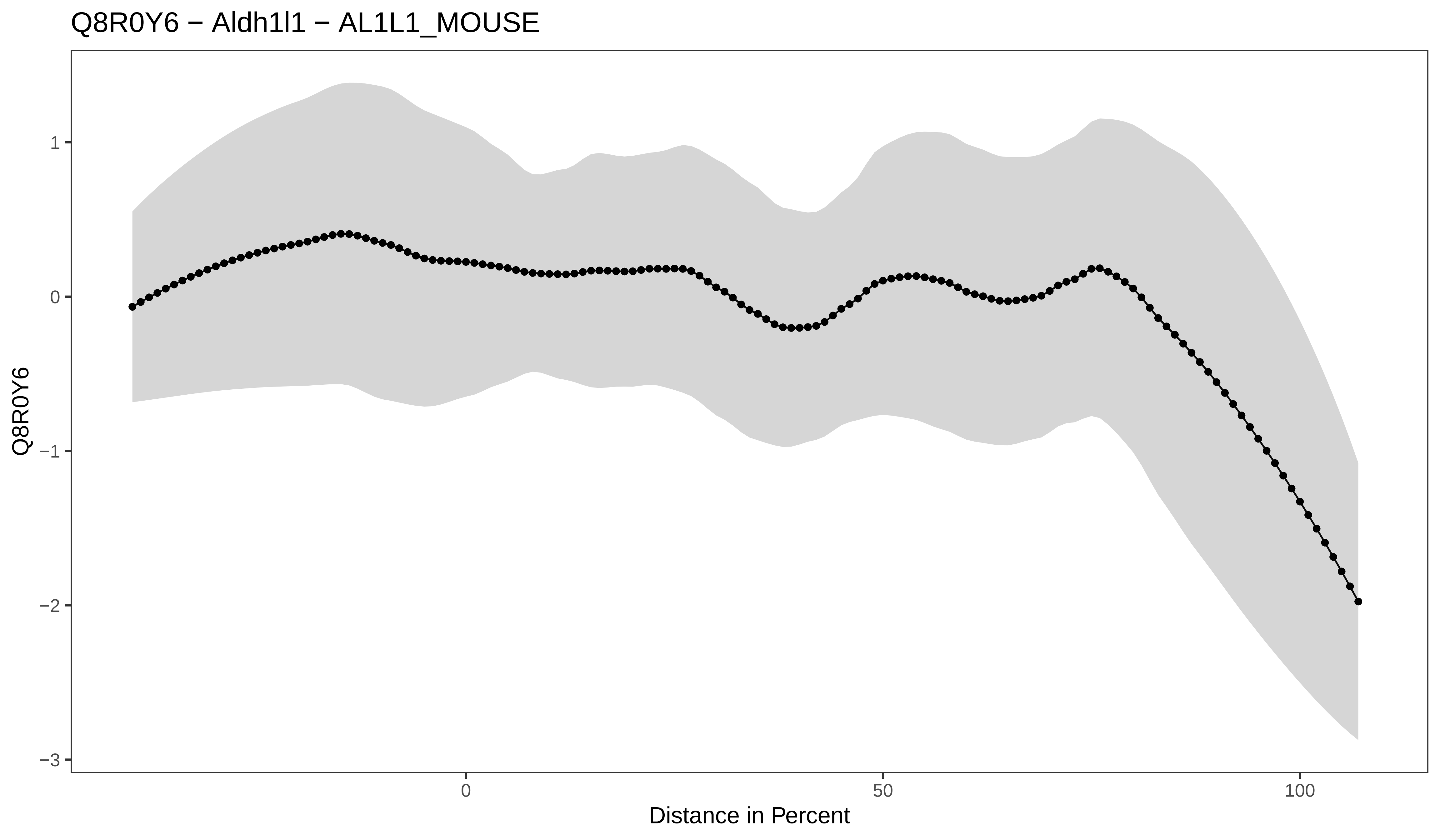

Q8R0Y6 − Aldh1l1 − AL1L1_MOUSE
Q8R0Y6
Distance in Percent
1
0
−1
−2
−3
0
50
100

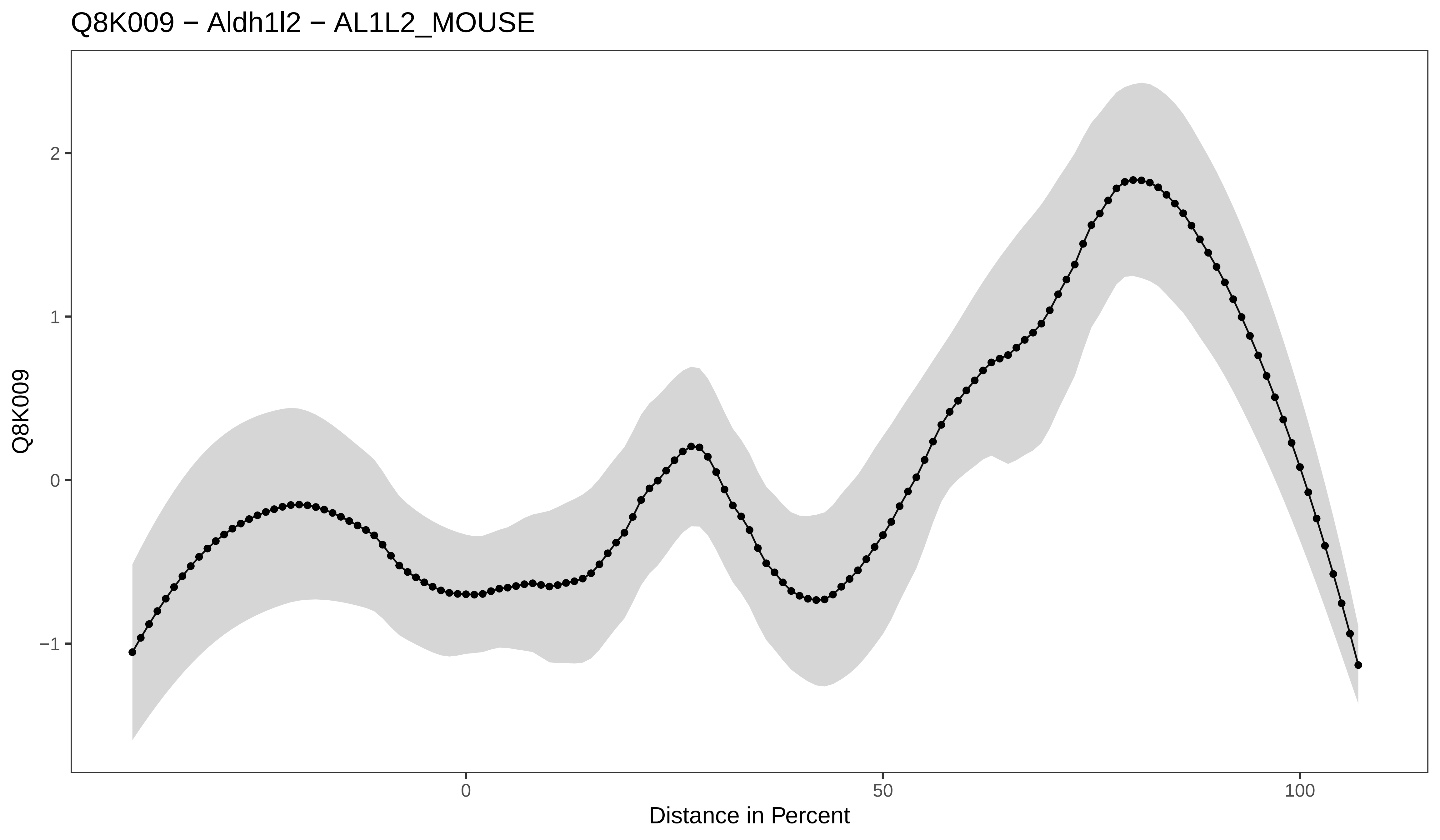

Q8K009 − Aldh1l2 − AL1L2_MOUSE
Q8K009
2
1
0
−1
0
50
100
Distance in Percent

P47738 − Aldh2 − ALDH2_MOUSE

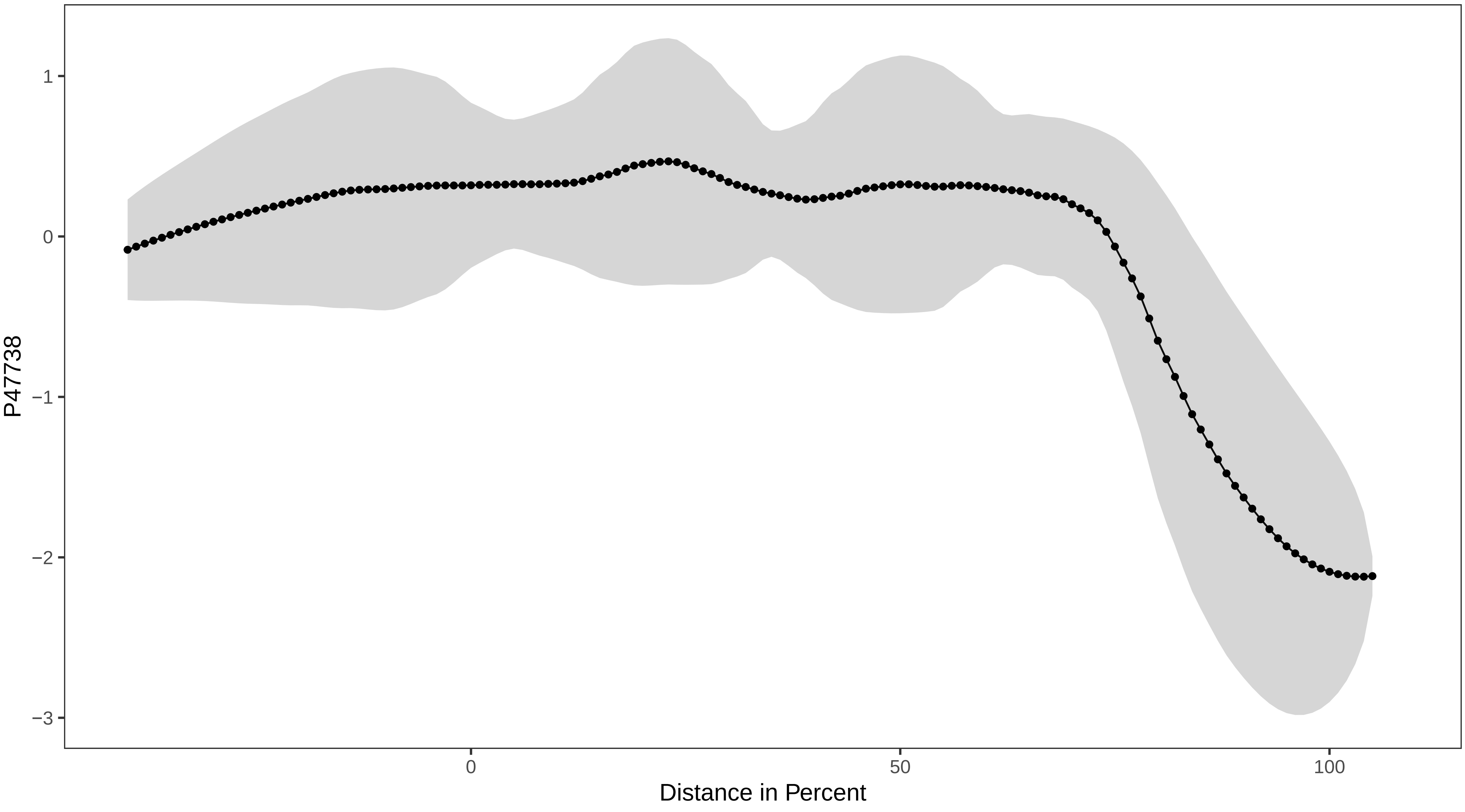

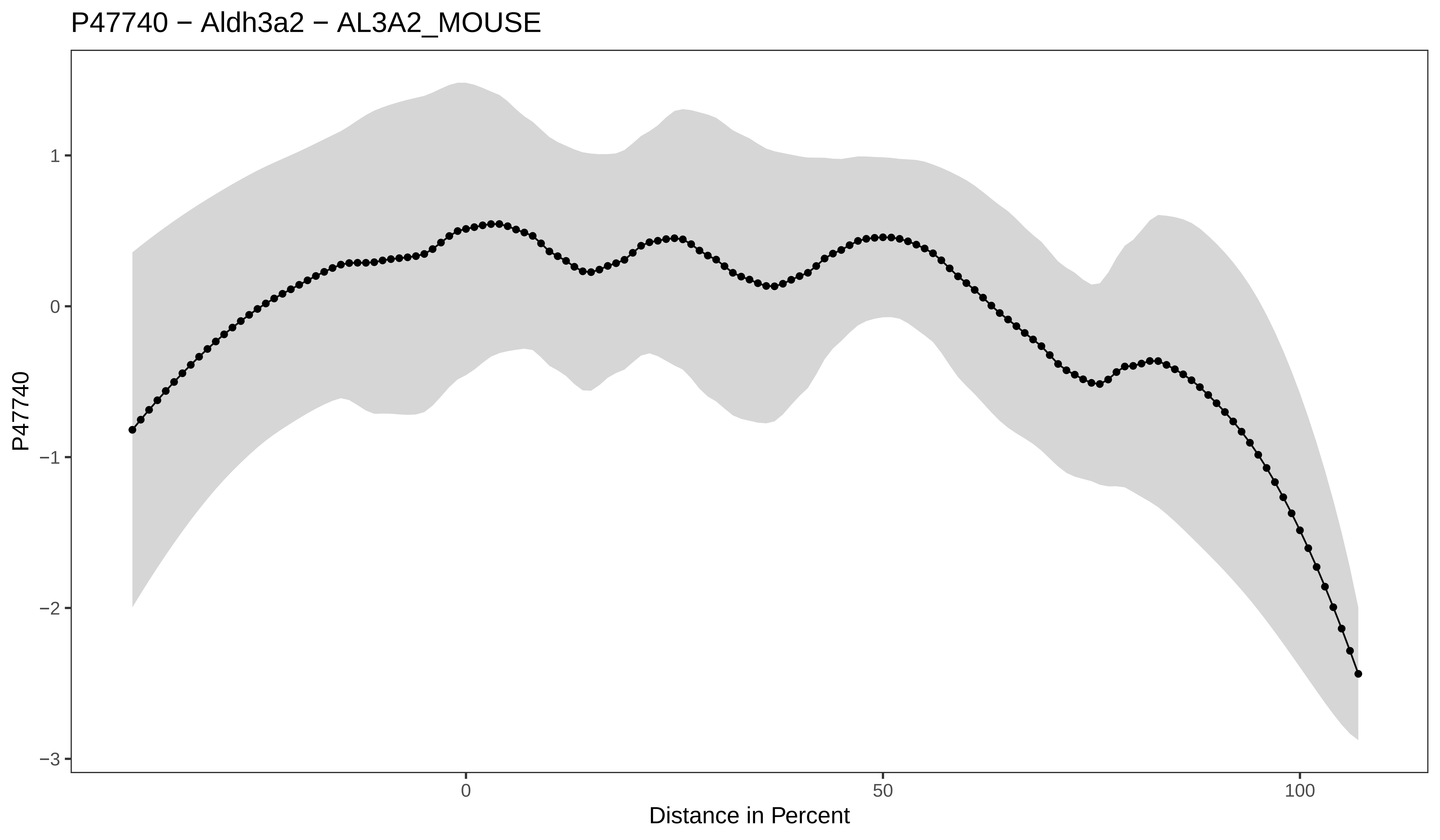
P47740 − Aldh3a2 − AL3A2_MOUSE
1
0
−1
−2
−3
P47740
0
50
100
Distance in Percent

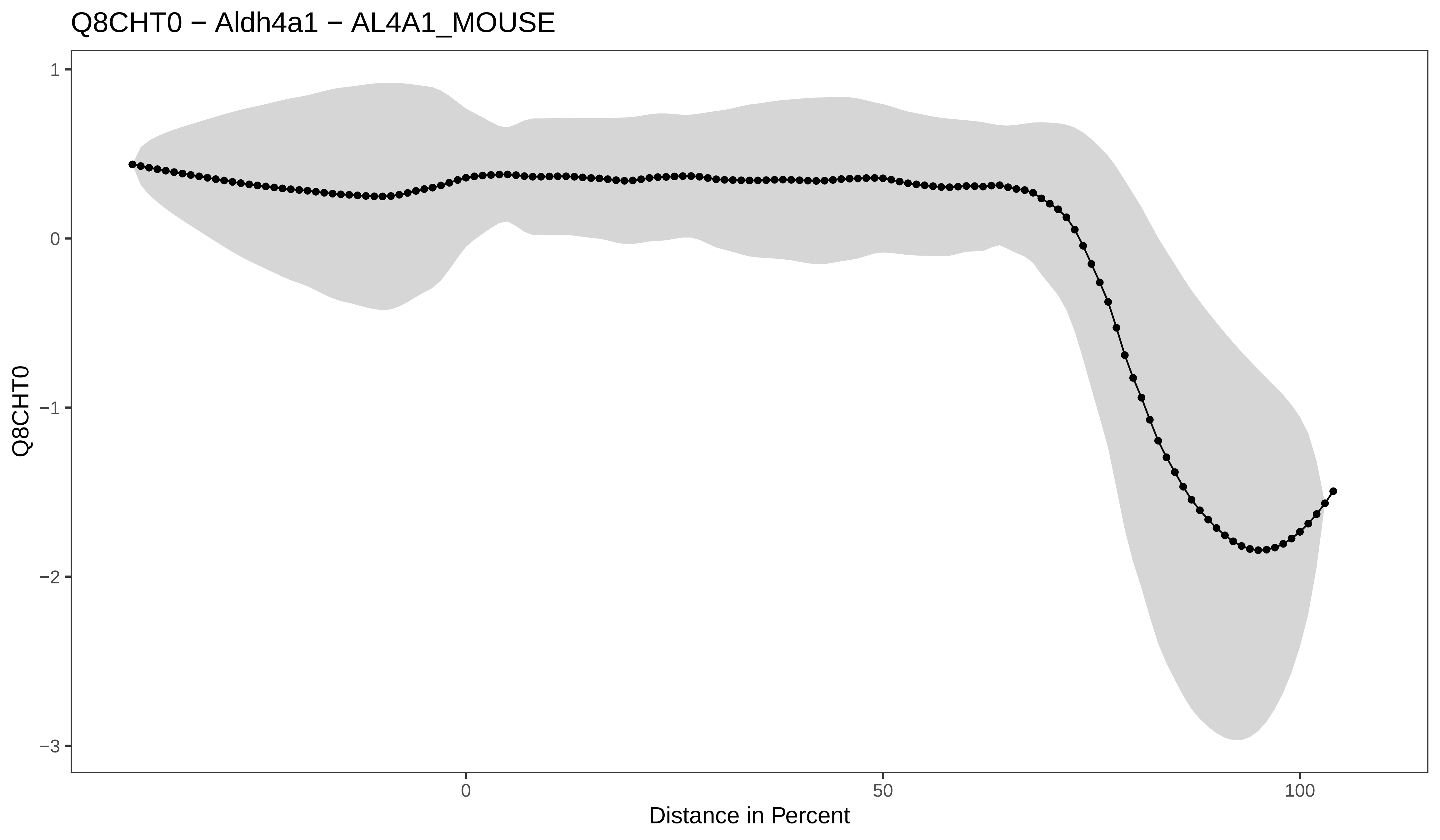
Q8CHT0 − Aldh4a1 − AL4A1_MOUSE
1
0
−1
−2
−3
Q8CHT0
0
50
100
Distance in Percent

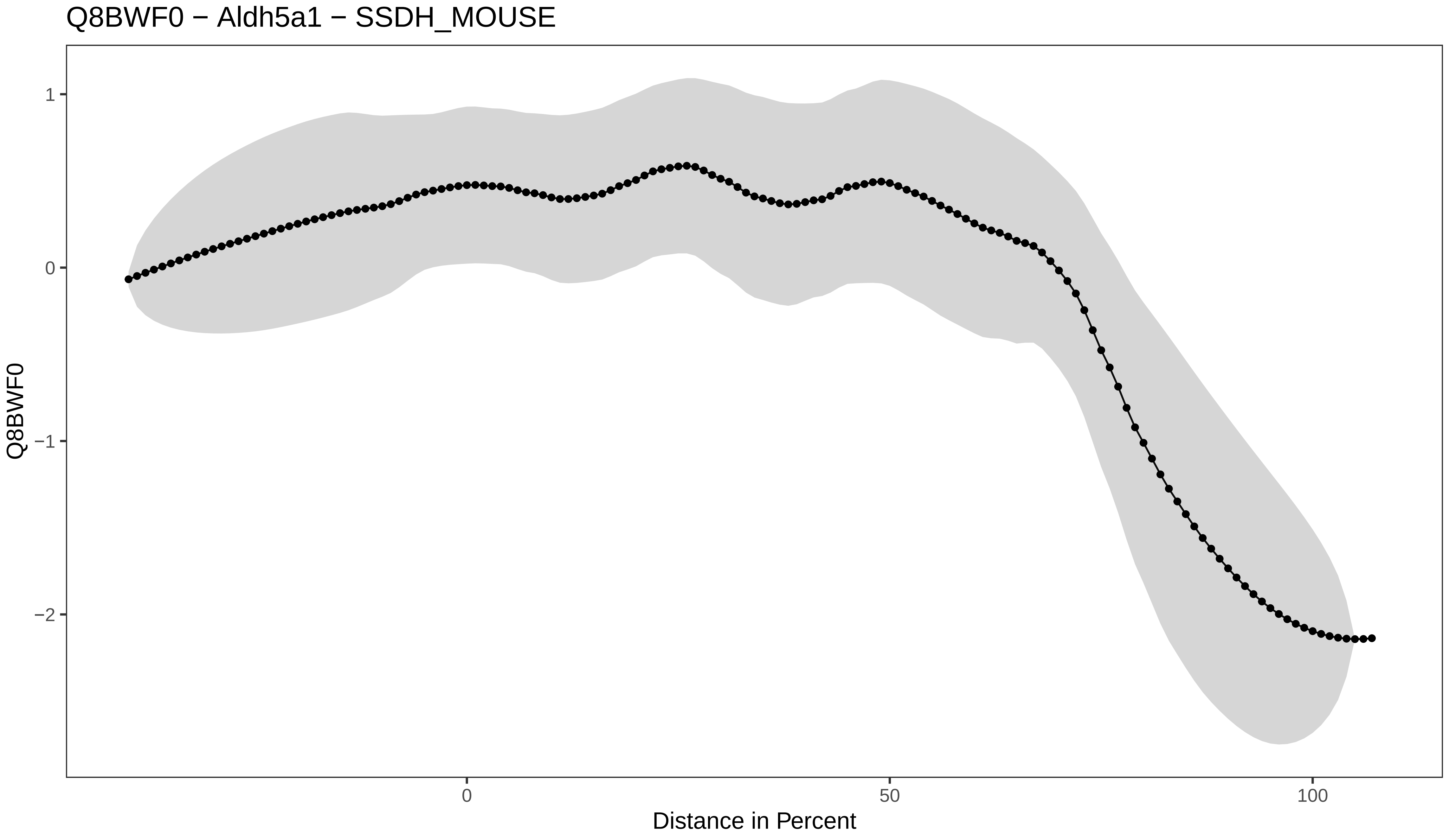
Q8BWF0 − Aldh5a1 − SSDH_MOUSE
1
0
−1
−2
0
50
100
Q8BWF0
Distance in Percent

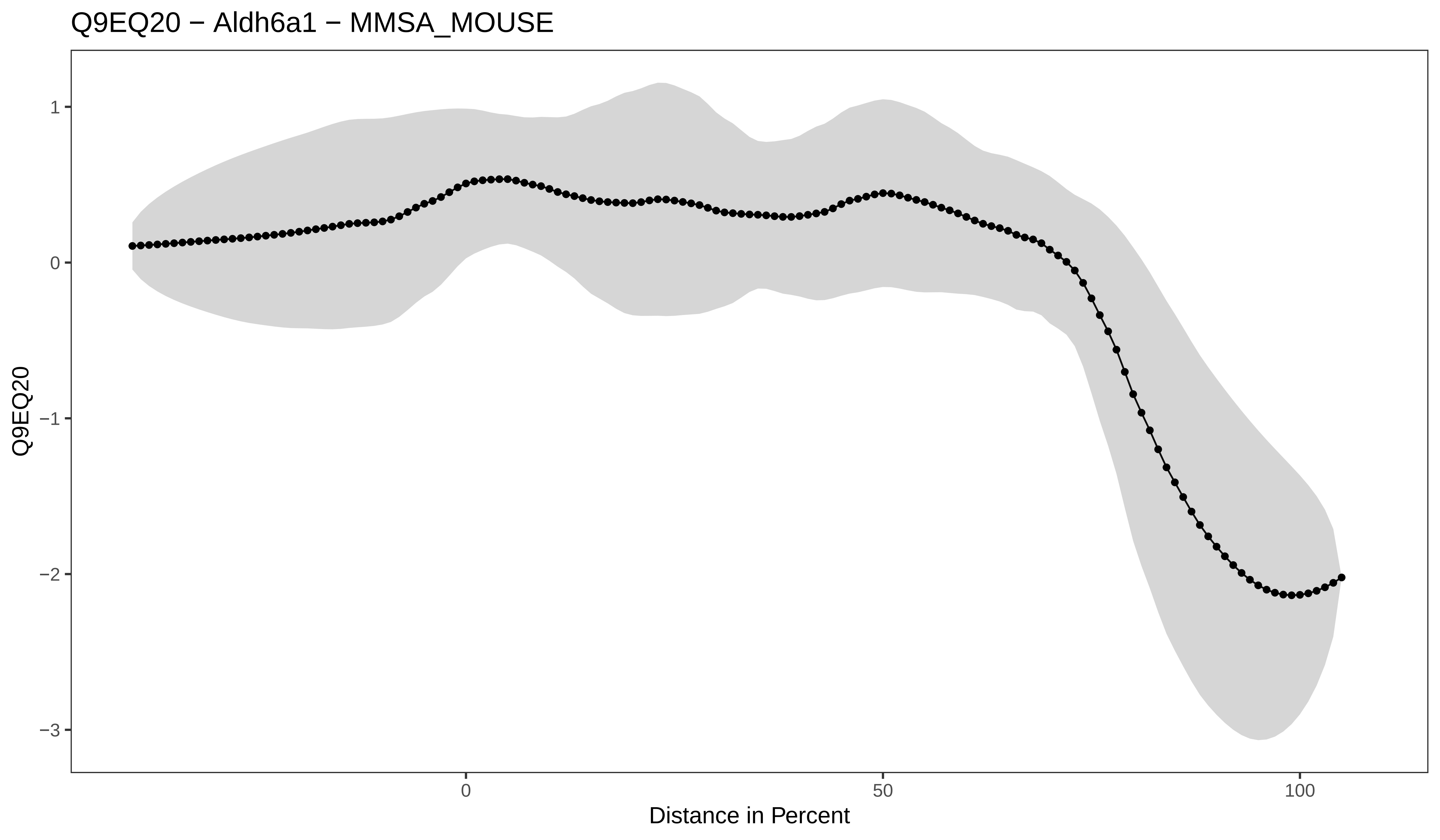
Q9EQ20 − Aldh6a1 − MMSA_MOUSE
Q9EQ20
1
0
−1
−2
−3
0
50
100
Distance in Percent

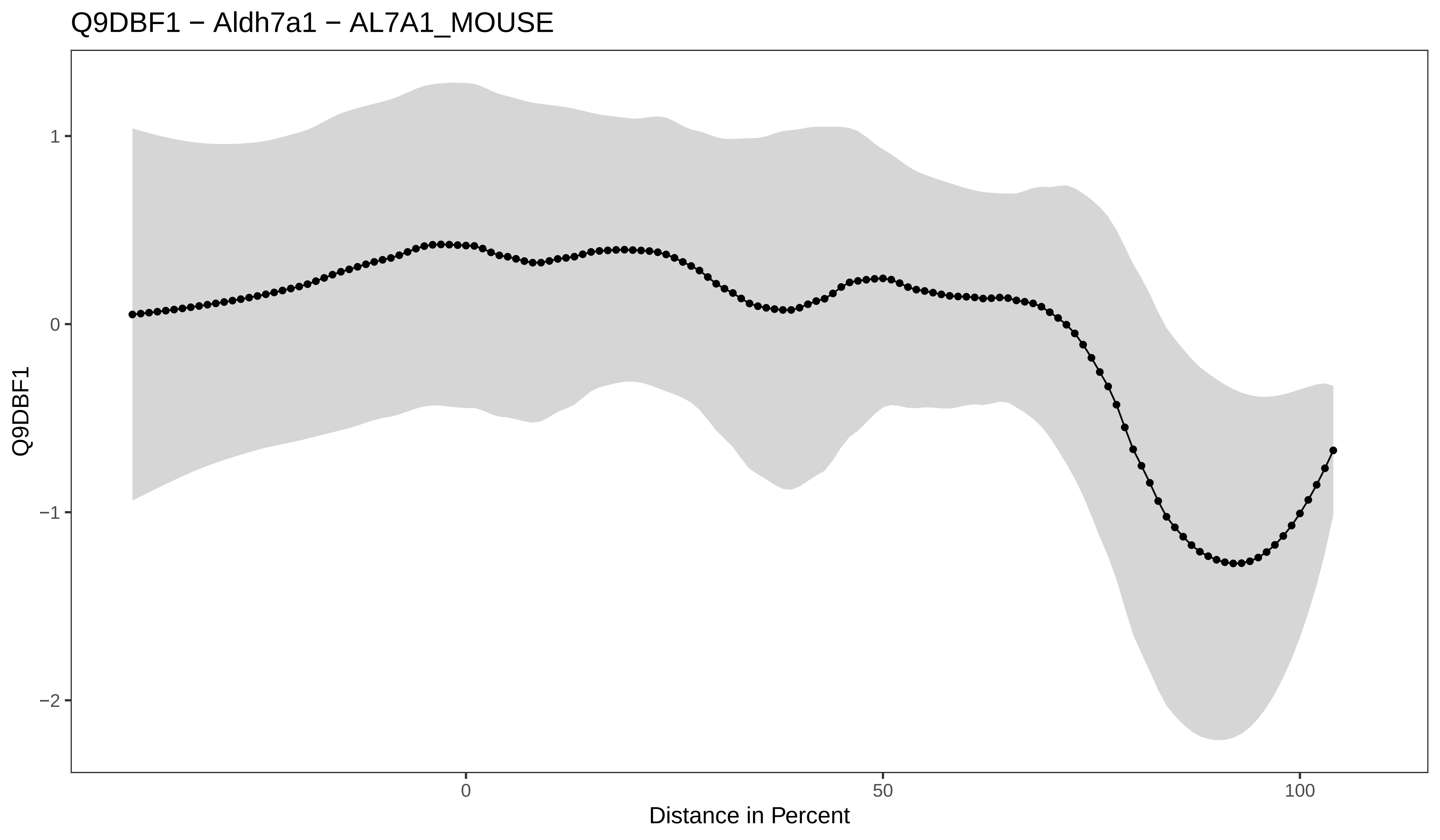

Q9DBF1 − Aldh7a1 − AL7A1_MOUSE
1
0
−1
−2
Q9DBF1
0
50
100
Distance in Percent

Q9JLJ2 − Aldh9a1 − AL9A1_MOUSE

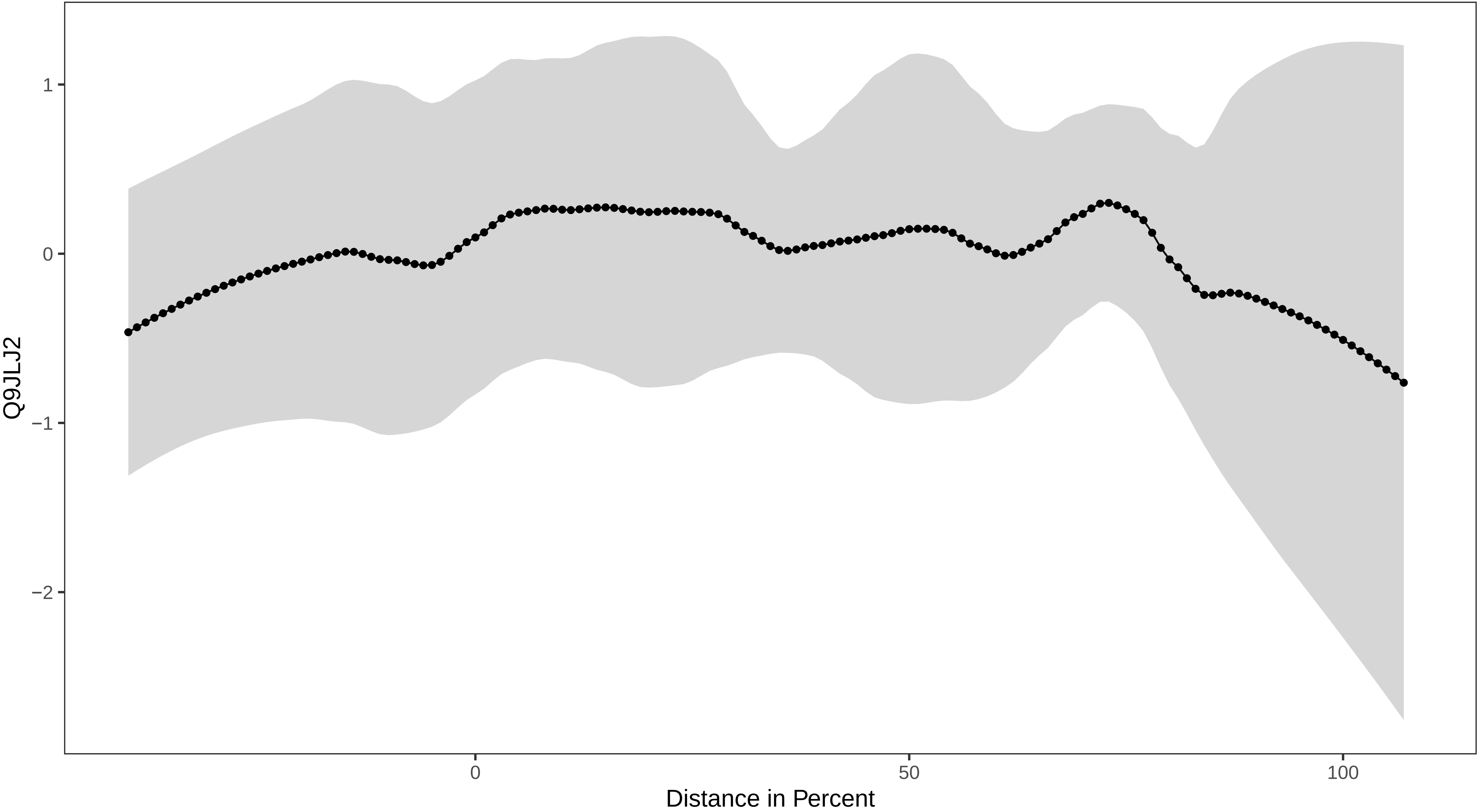

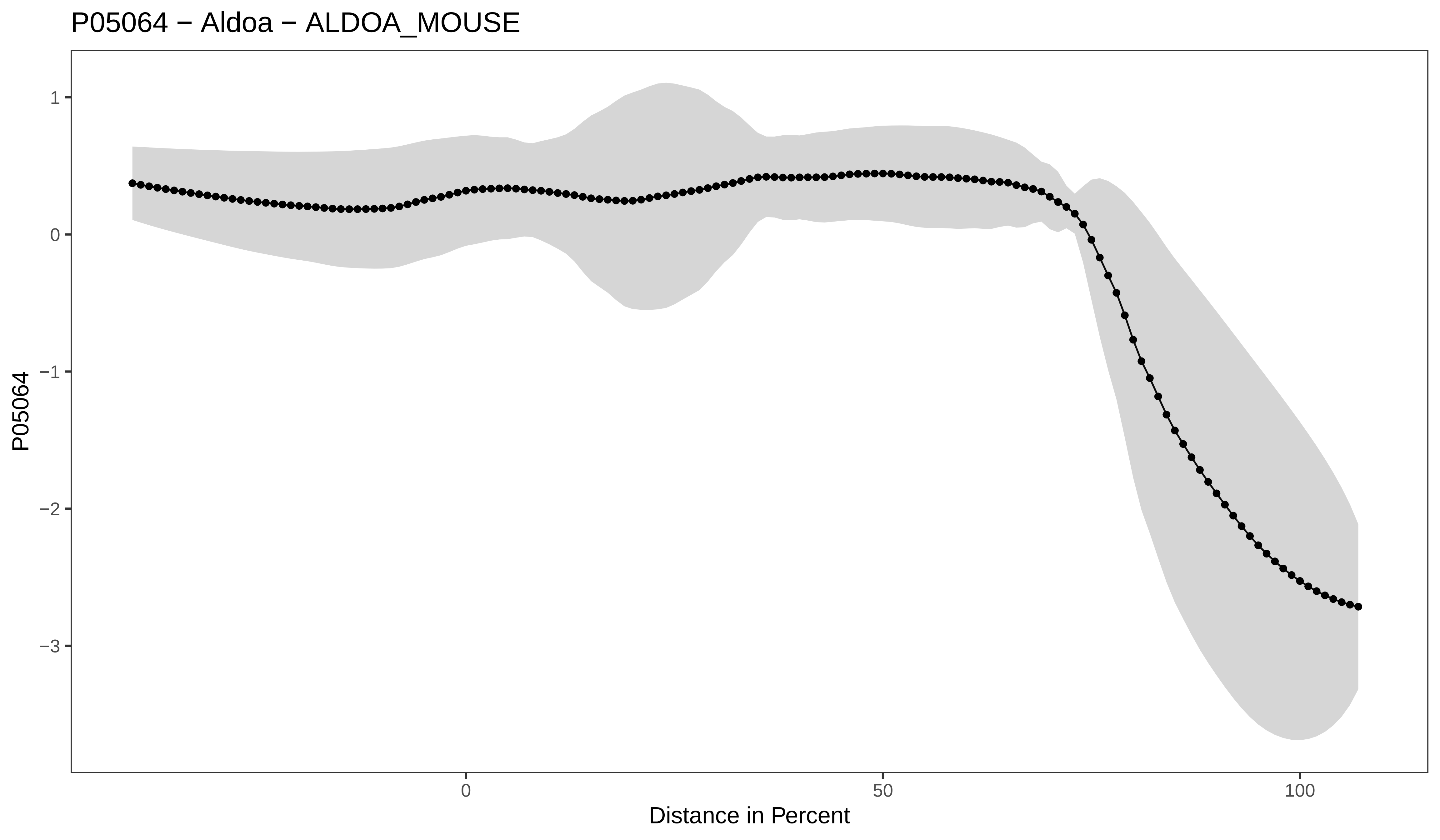

P05064 − Aldoa − ALDOA_MOUSE
1
0
−1
−2
−3
P05064
0
50
100
Distance in Percent

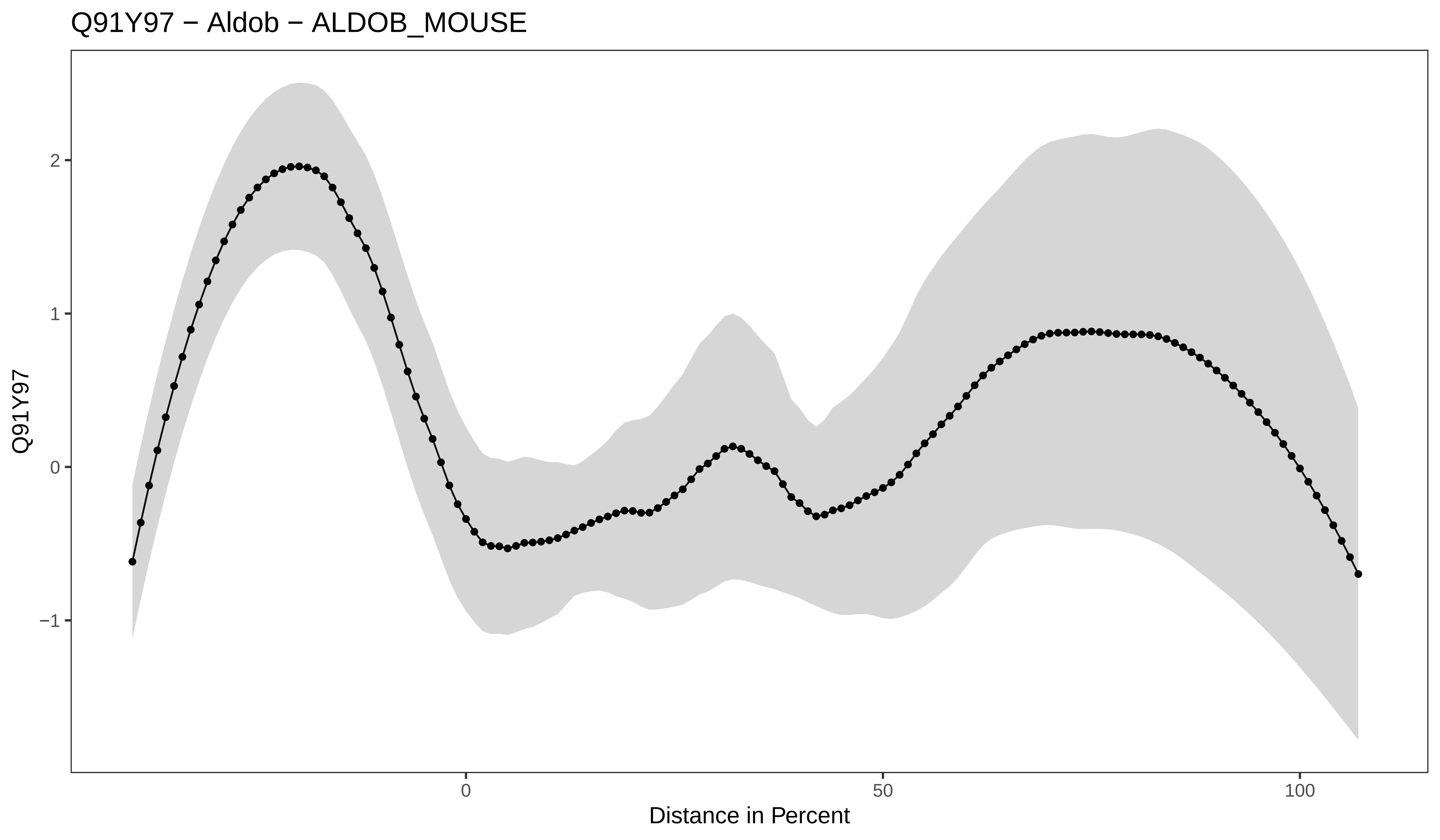
Q91Y97 − Aldob − ALDOB_MOUSE
Q91Y97
2
1
0
−1
0
50
100
Distance in Percent

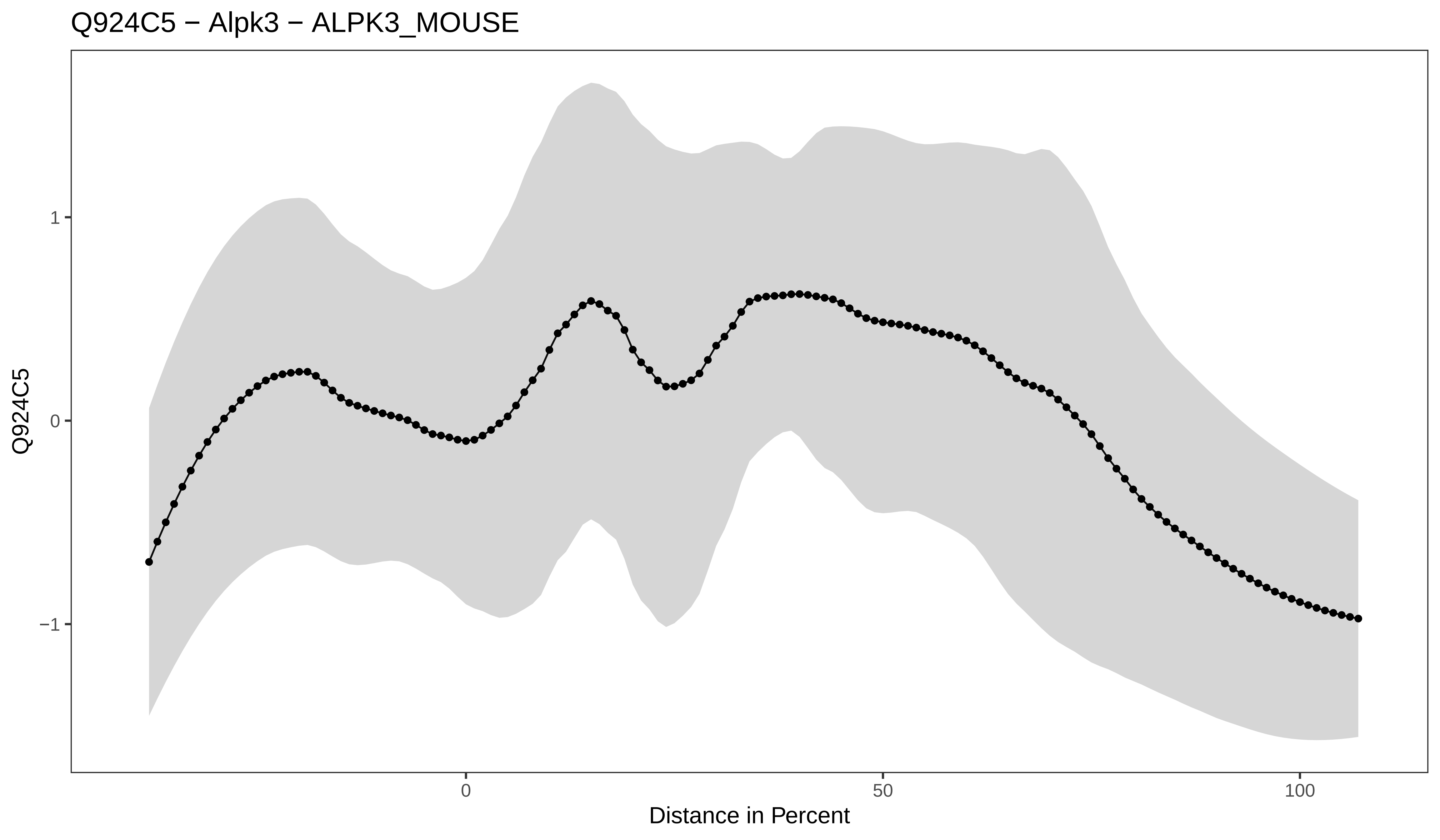

Q924C5 − Alpk3 − ALPK3_MOUSE
1
0
−1
Q924C5
0
50
100
Distance in Percent

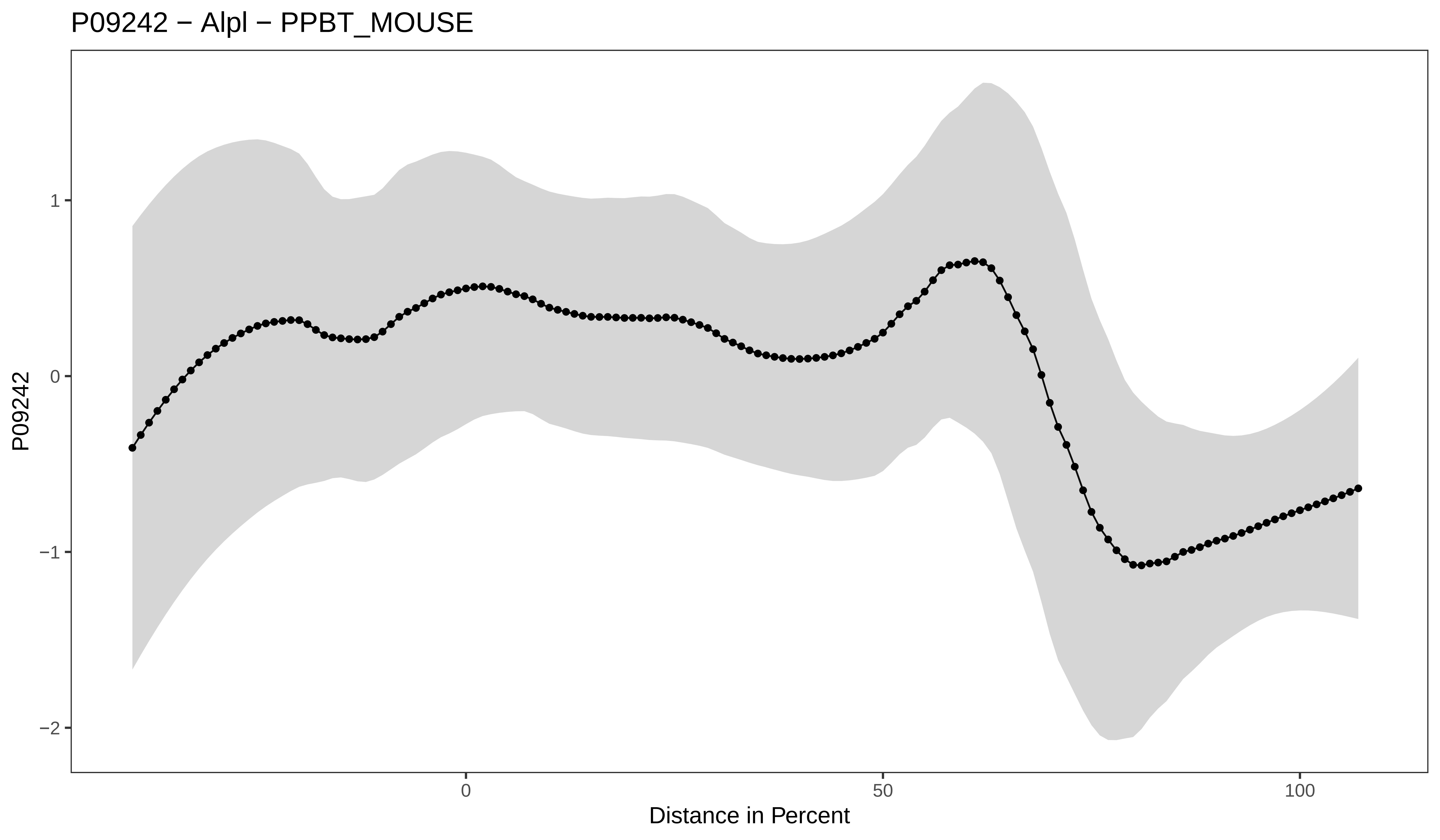
P09242 − Alpl − PPBT_MOUSE
1
0
−1
−2
P09242
0
50
100
Distance in Percent

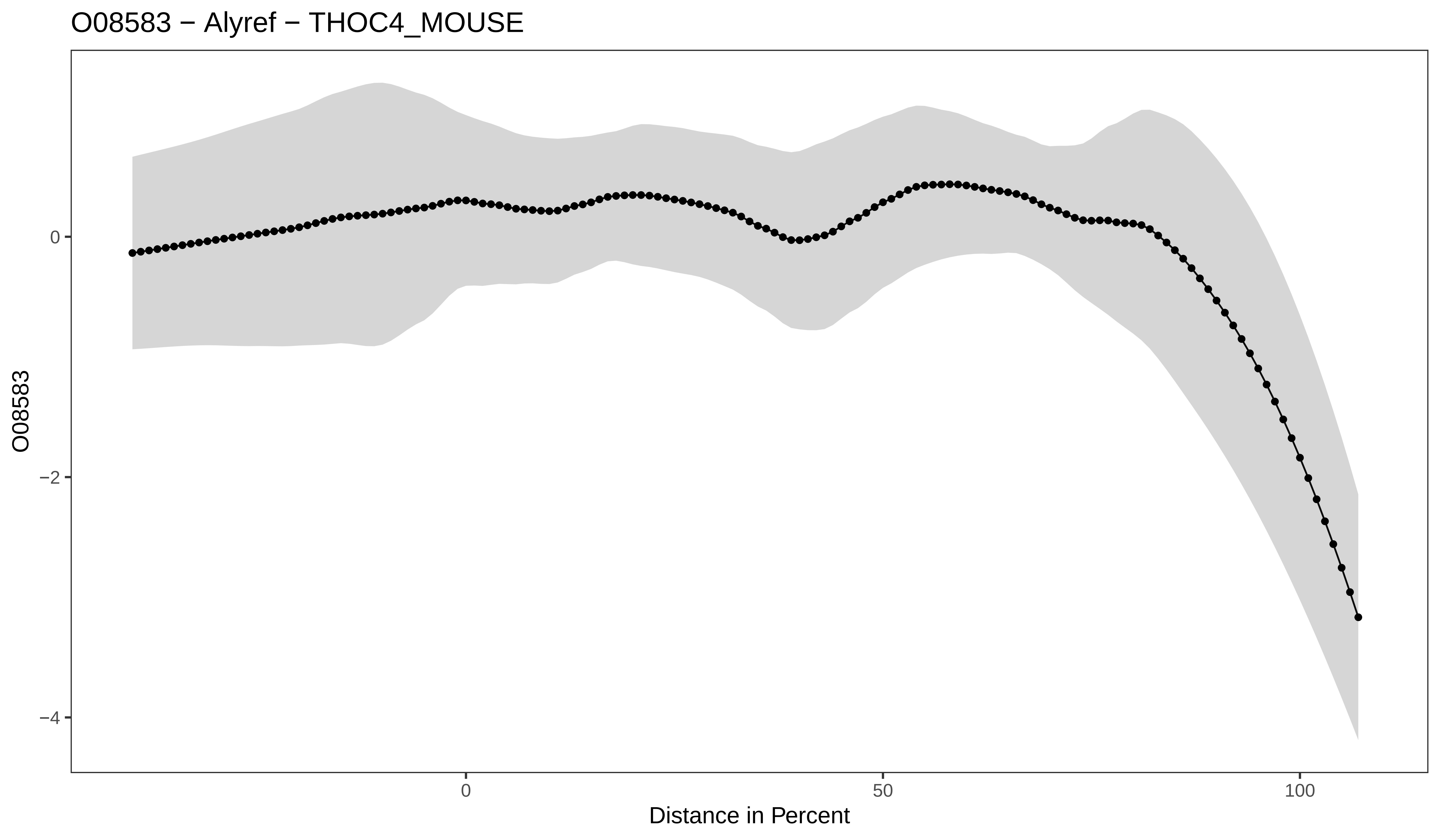
O08583 − Alyref − THOC4_MOUSE
O08583
0
−2
−4
0
50
100
Distance in Percent

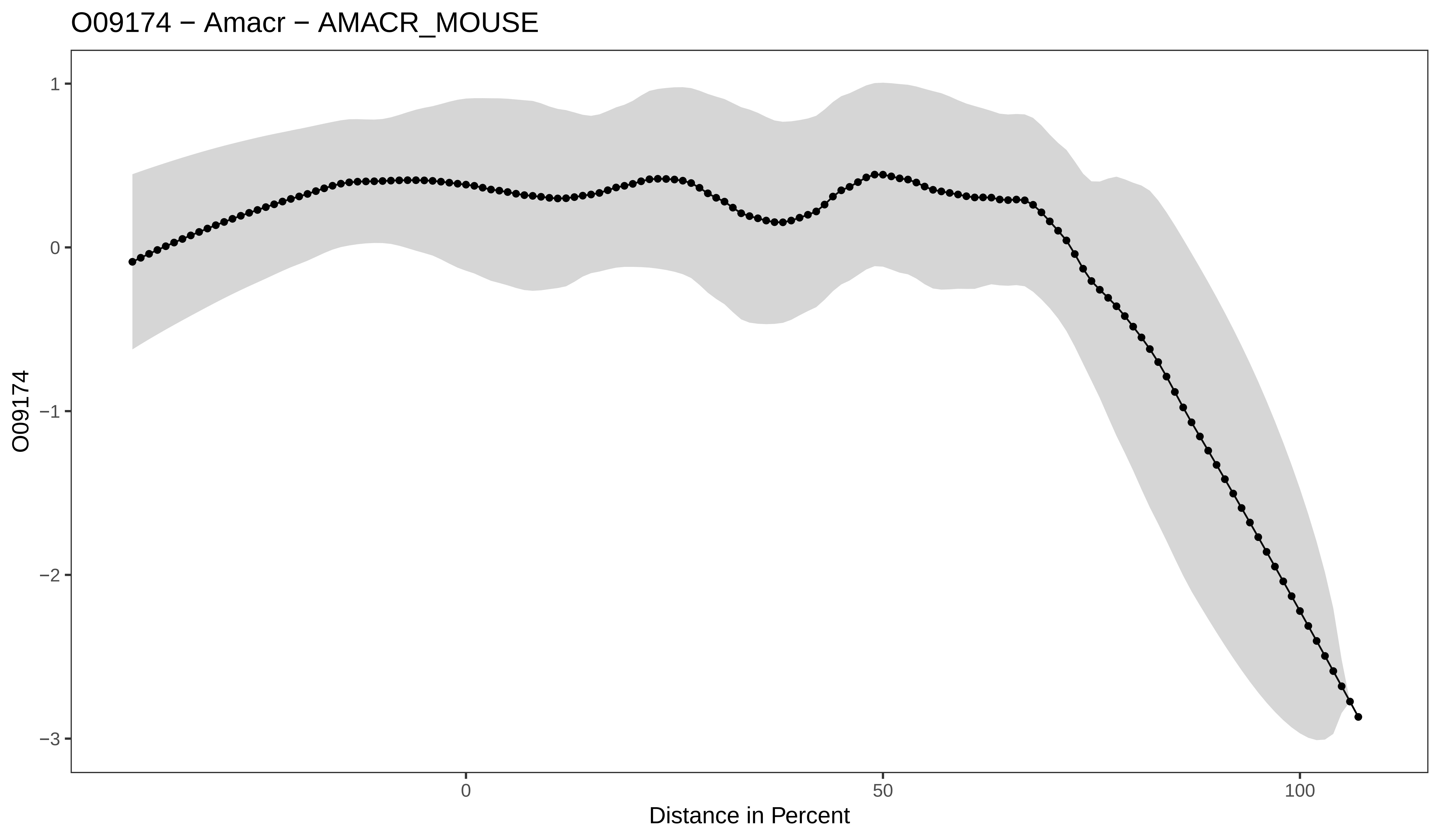

O09174 − Amacr − AMACR_MOUSE
1
0
−1
−2
−3
O09174
0
50
100
Distance in Percent

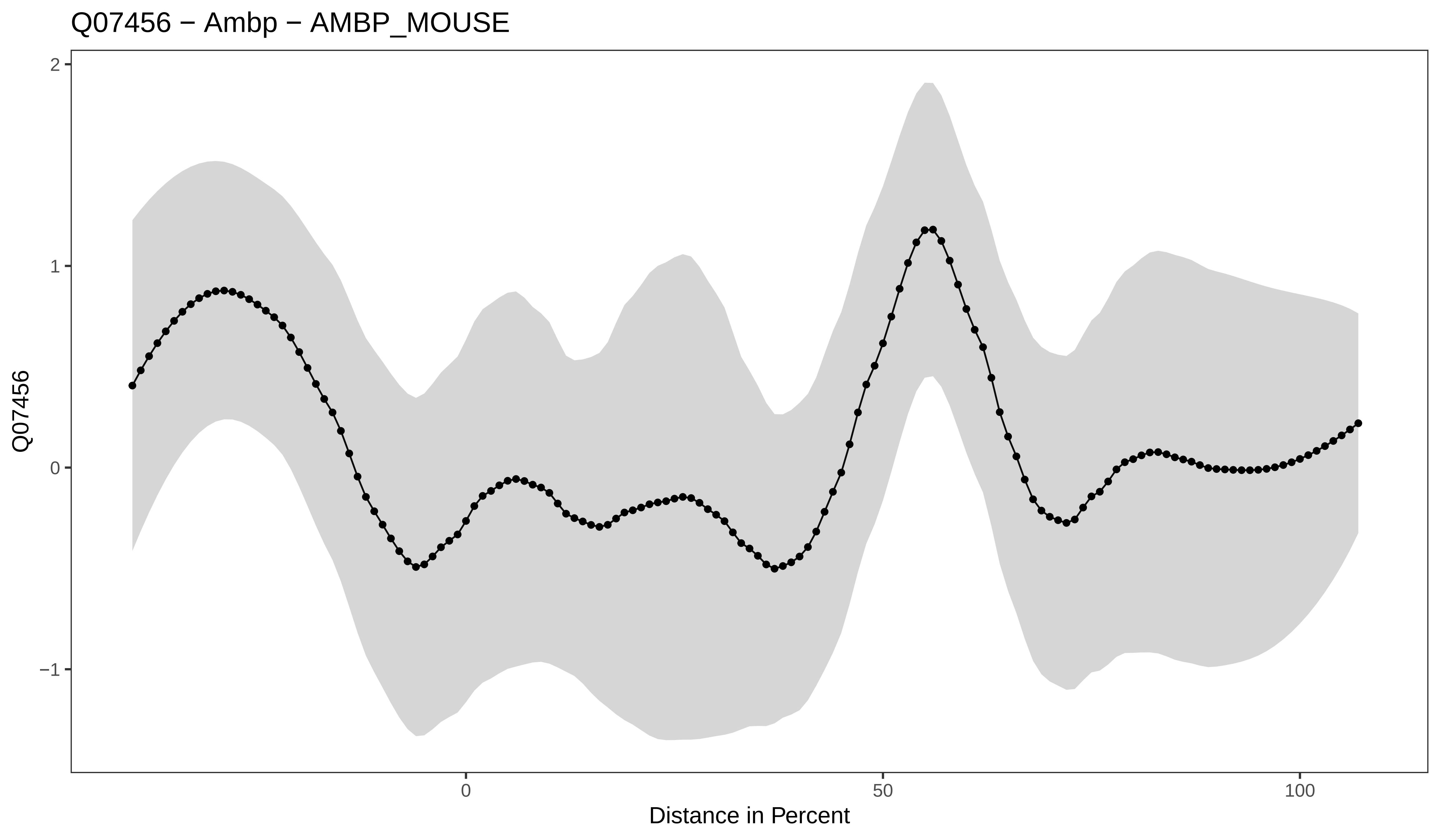

Q07456 − Ambp − AMBP_MOUSE
2
1
0
−1
Q07456
0
50
100
Distance in Percent

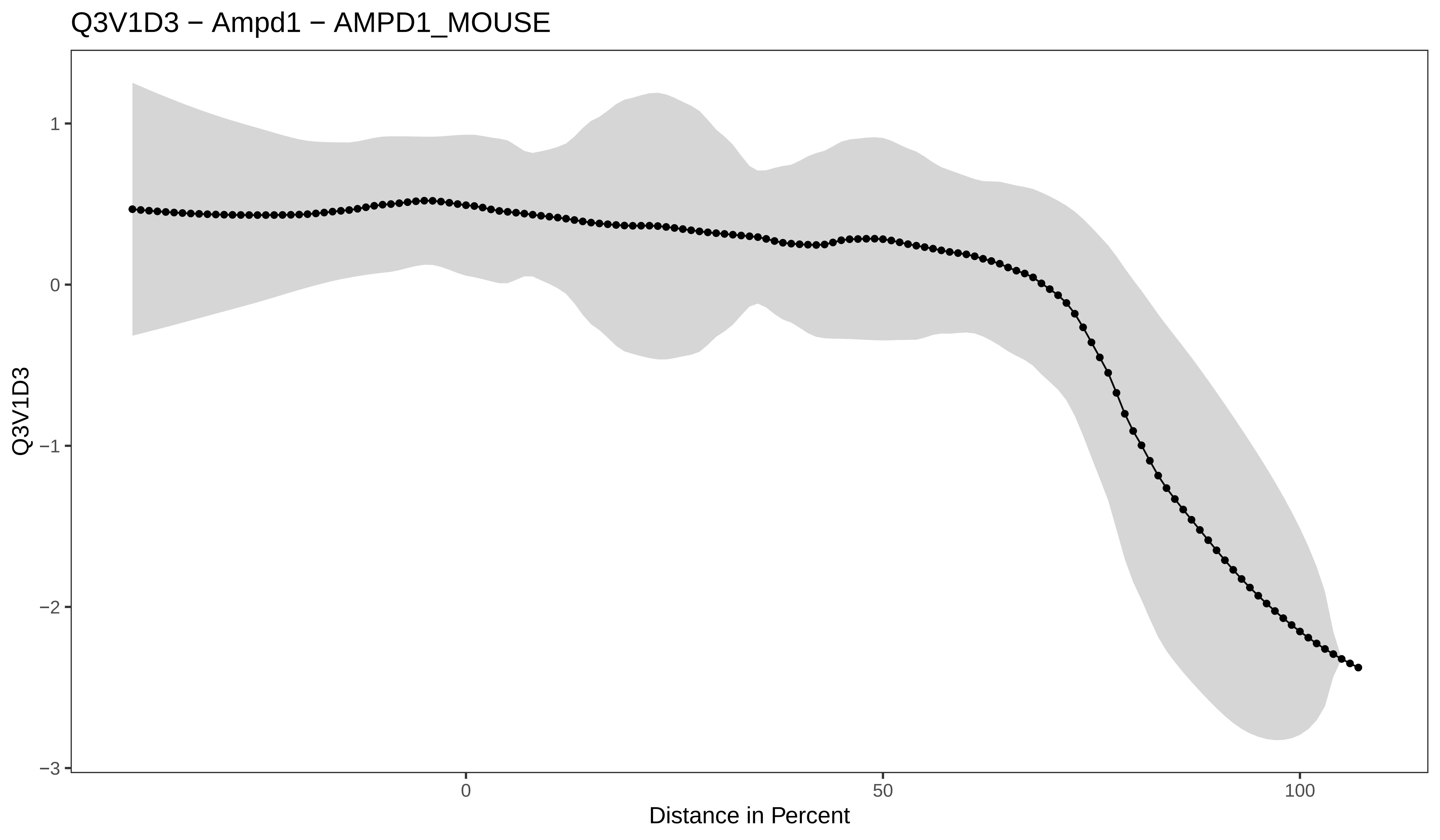

Q3V1D3 − Ampd1 − AMPD1_MOUSE
Q3V1D3
1
0
−1
−2
−3
0
50
100
Distance in Percent

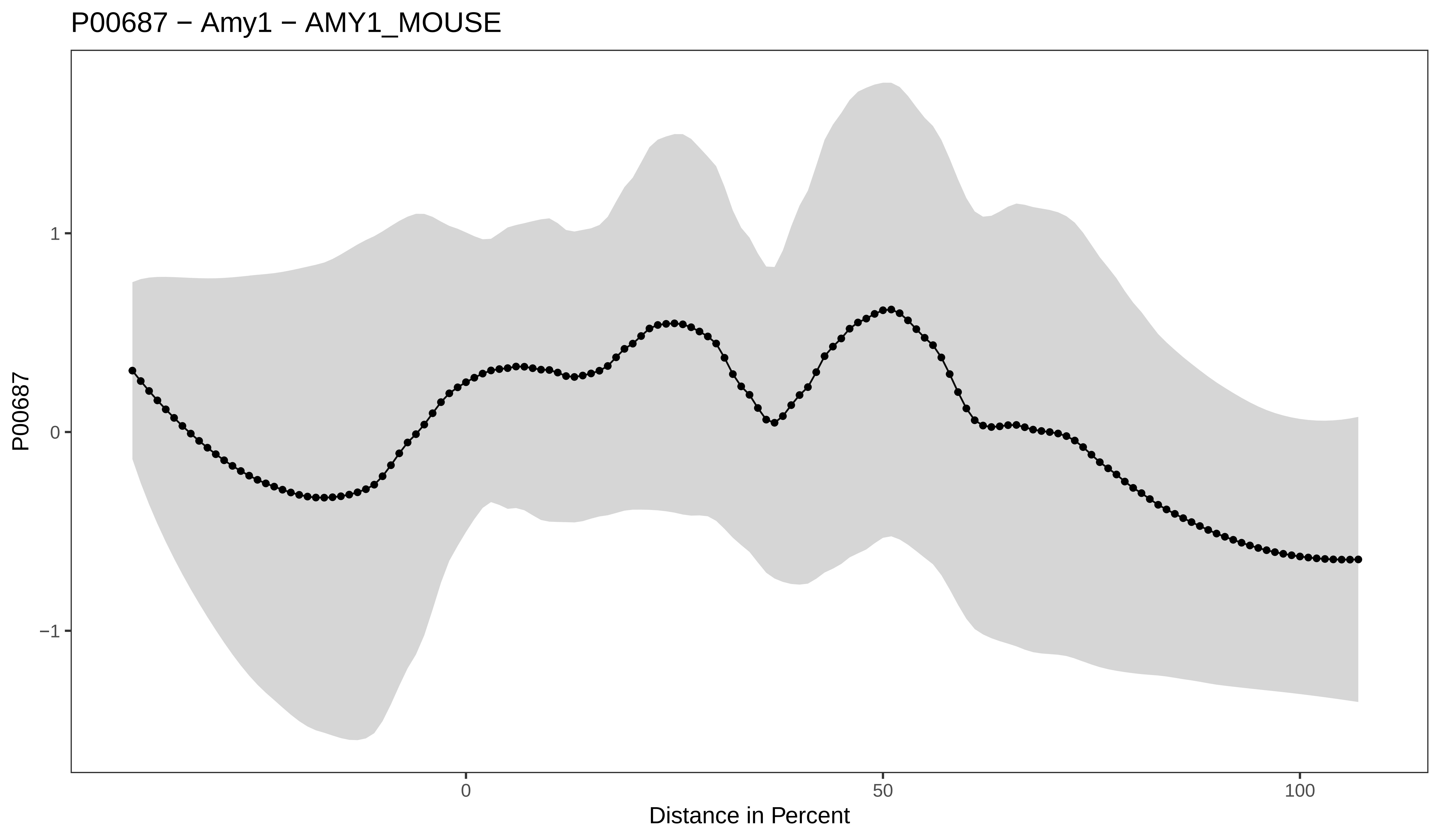
P00687 − Amy1 − AMY1_MOUSE
P00687
1
0
−1
0
50
100
Distance in Percent

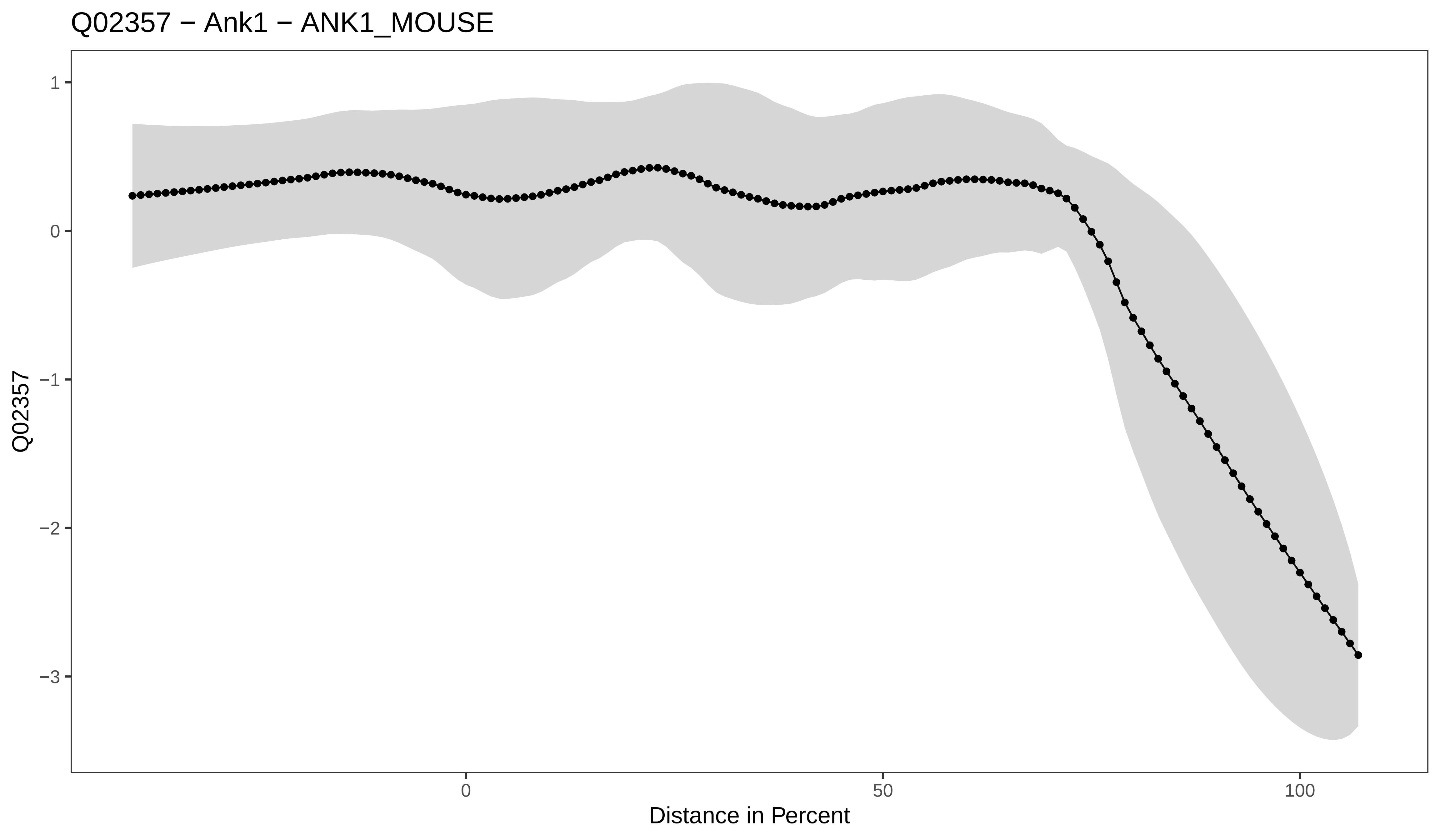

Q02357 − Ank1 − ANK1_MOUSE
1
0
−1
−2
−3
Q02357
0
50
100
Distance in Percent

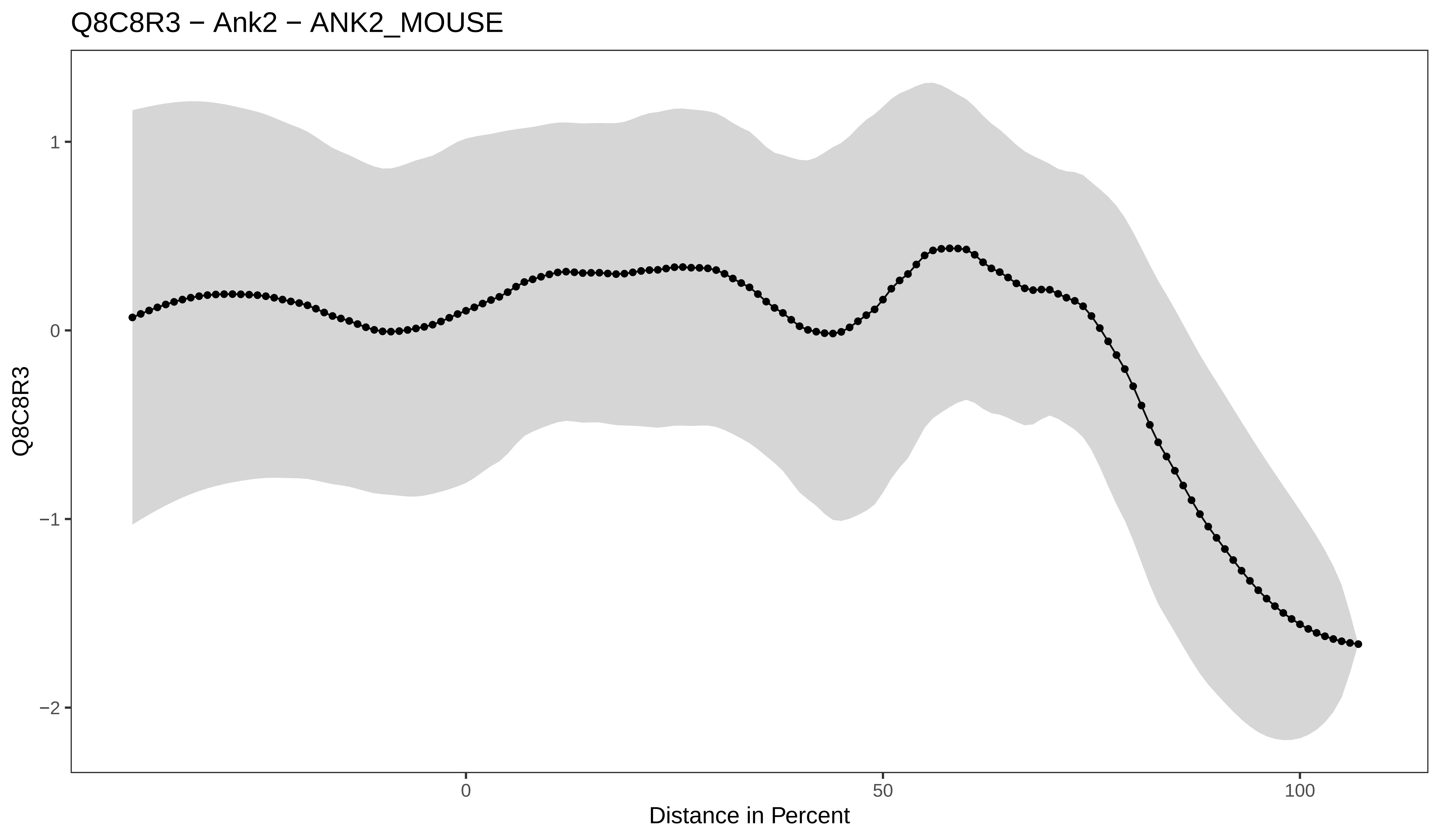

Q8C8R3 − Ank2 − ANK2_MOUSE
1
0
−1
−2
Q8C8R3
0
50
100
Distance in Percent

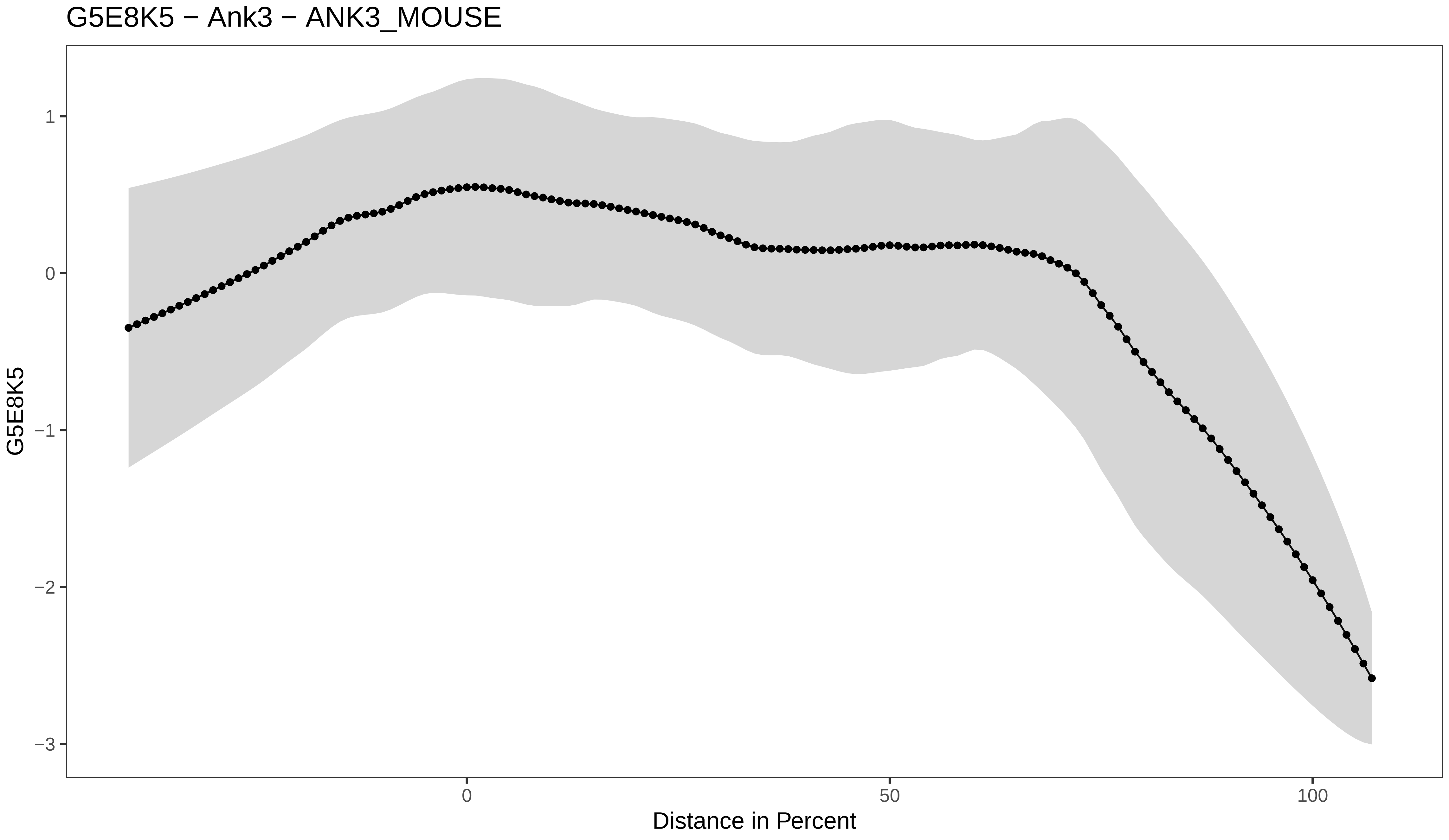

G5E8K5 − Ank3 − ANK3_MOUSE
G5E8K5
Distance in Percent
1
0
−1
−2
−3
0
50
100

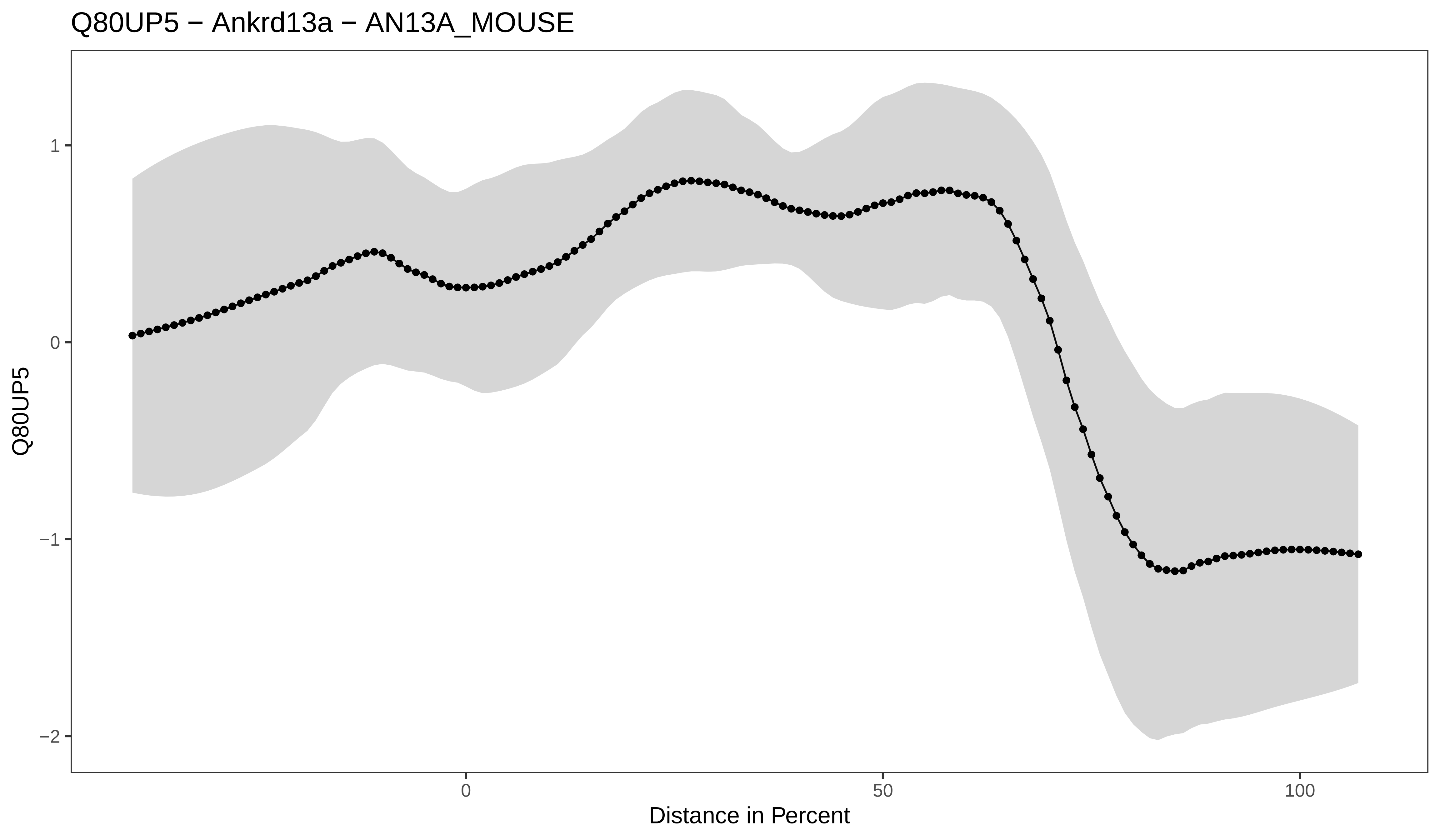

Q80UP5 − Ankrd13a − AN13A_MOUSE
Q80UP5
1
0
−1
−2
0
50
100
Distance in Percent

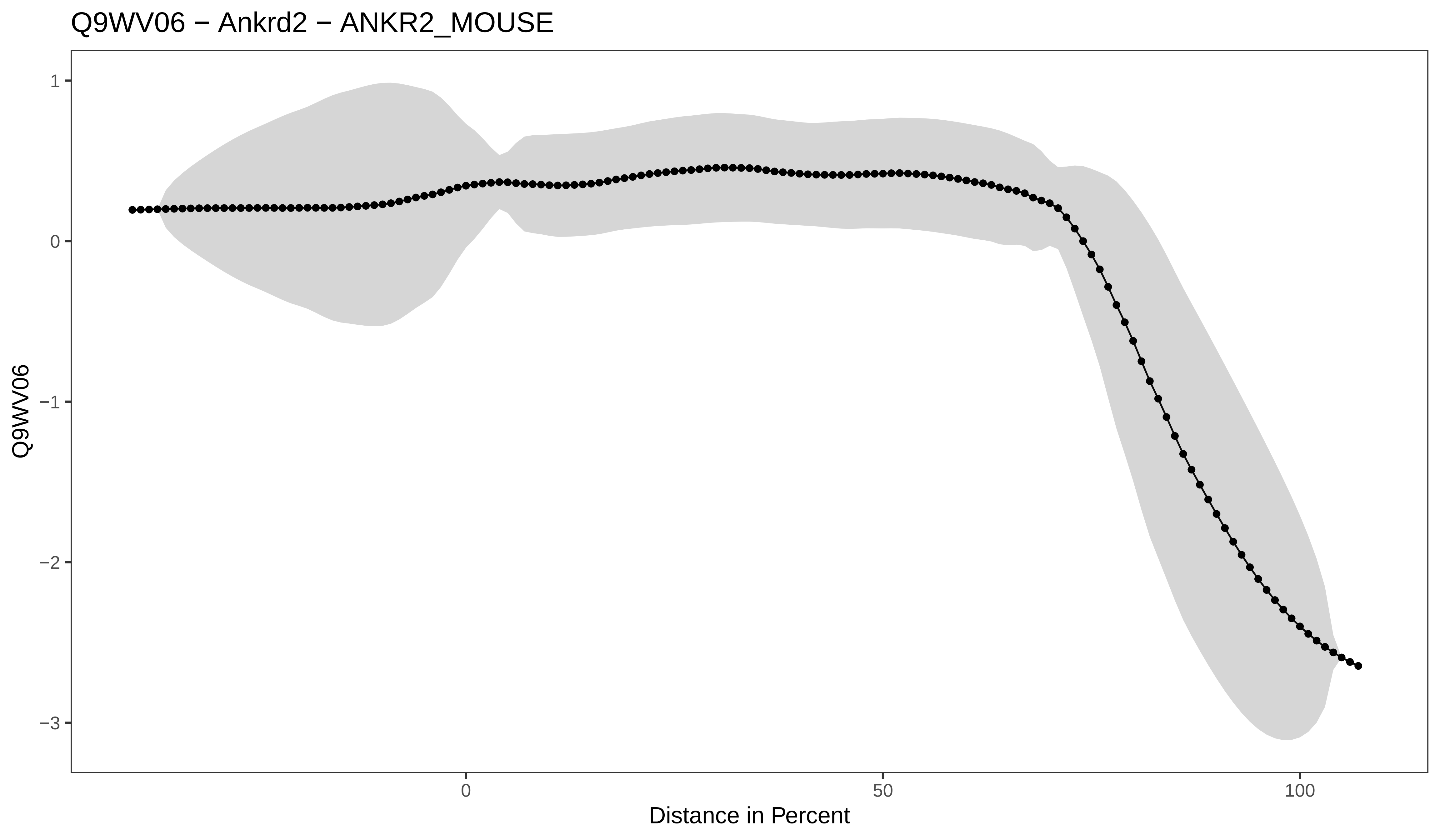
Q9WV06 − Ankrd2 − ANKR2_MOUSE
1
0
−1
−2
−3
Q9WV06
0
50
100
Distance in Percent

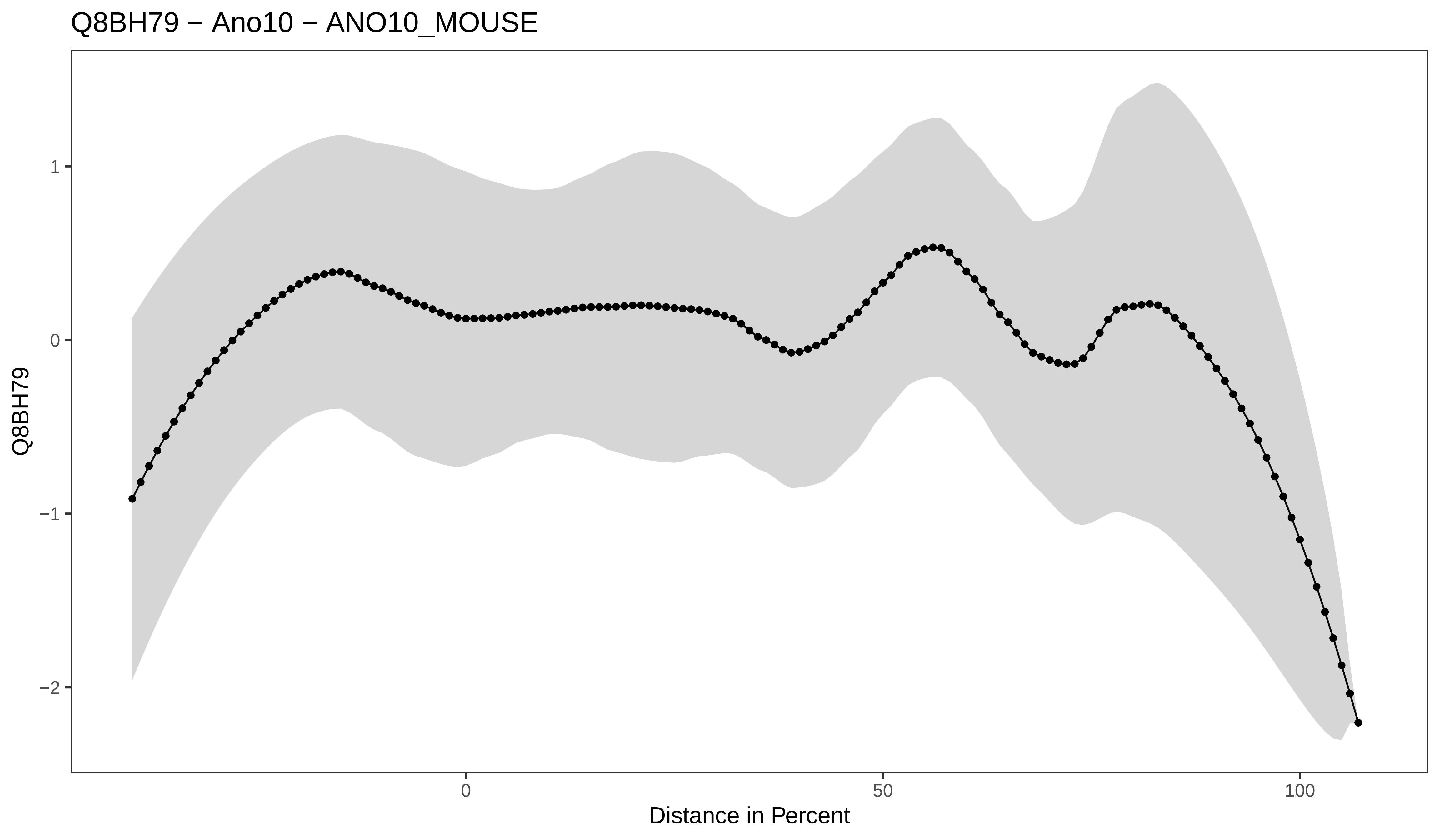
Q8BH79 − Ano10 − ANO10_MOUSE
Q8BH79
1
0
−1
−2
0
50
100
Distance in Percent

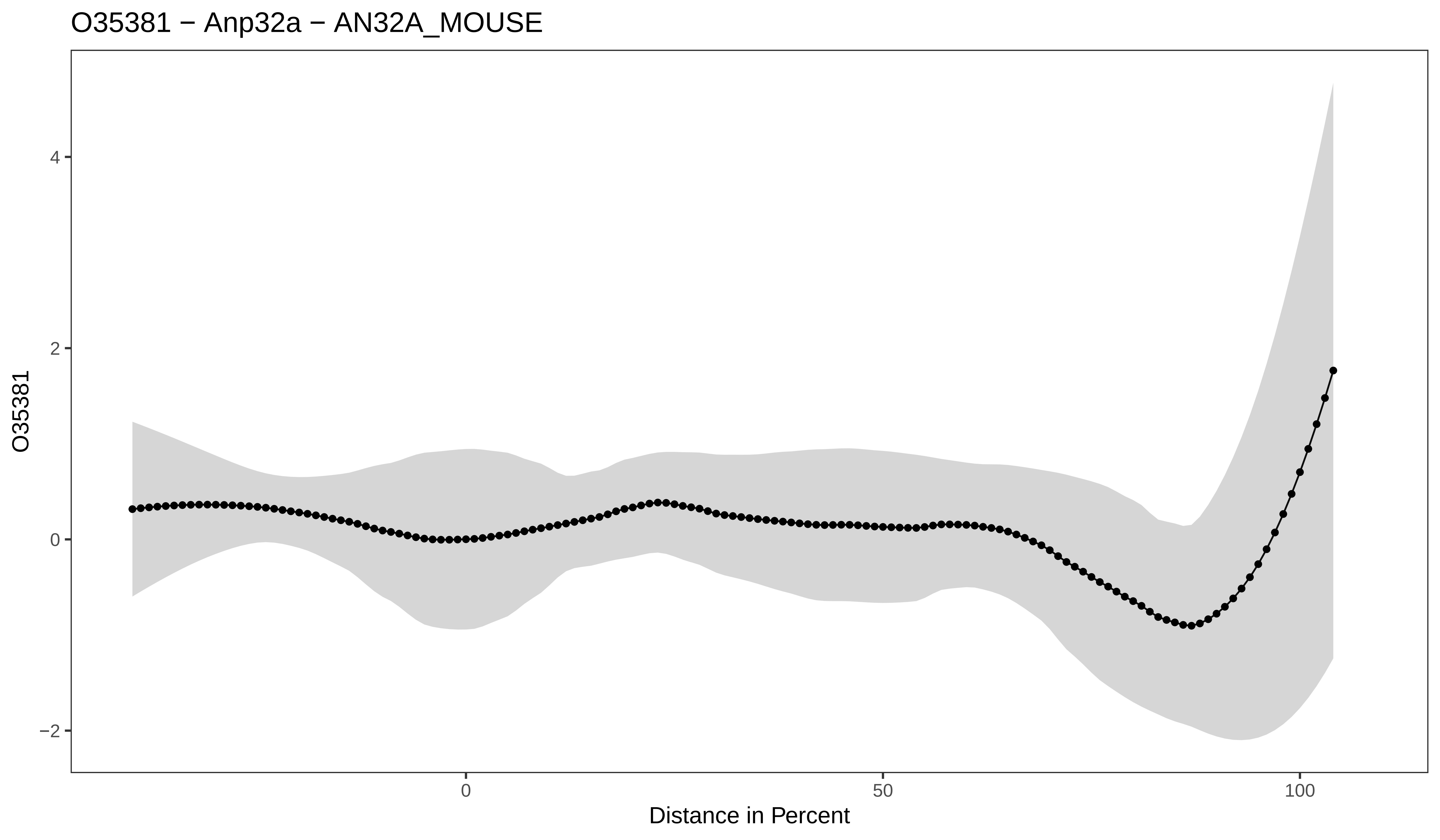

O35381 − Anp32a − AN32A_MOUSE
O35381
Distance in Percent
4
2
0
−2
0
50
100

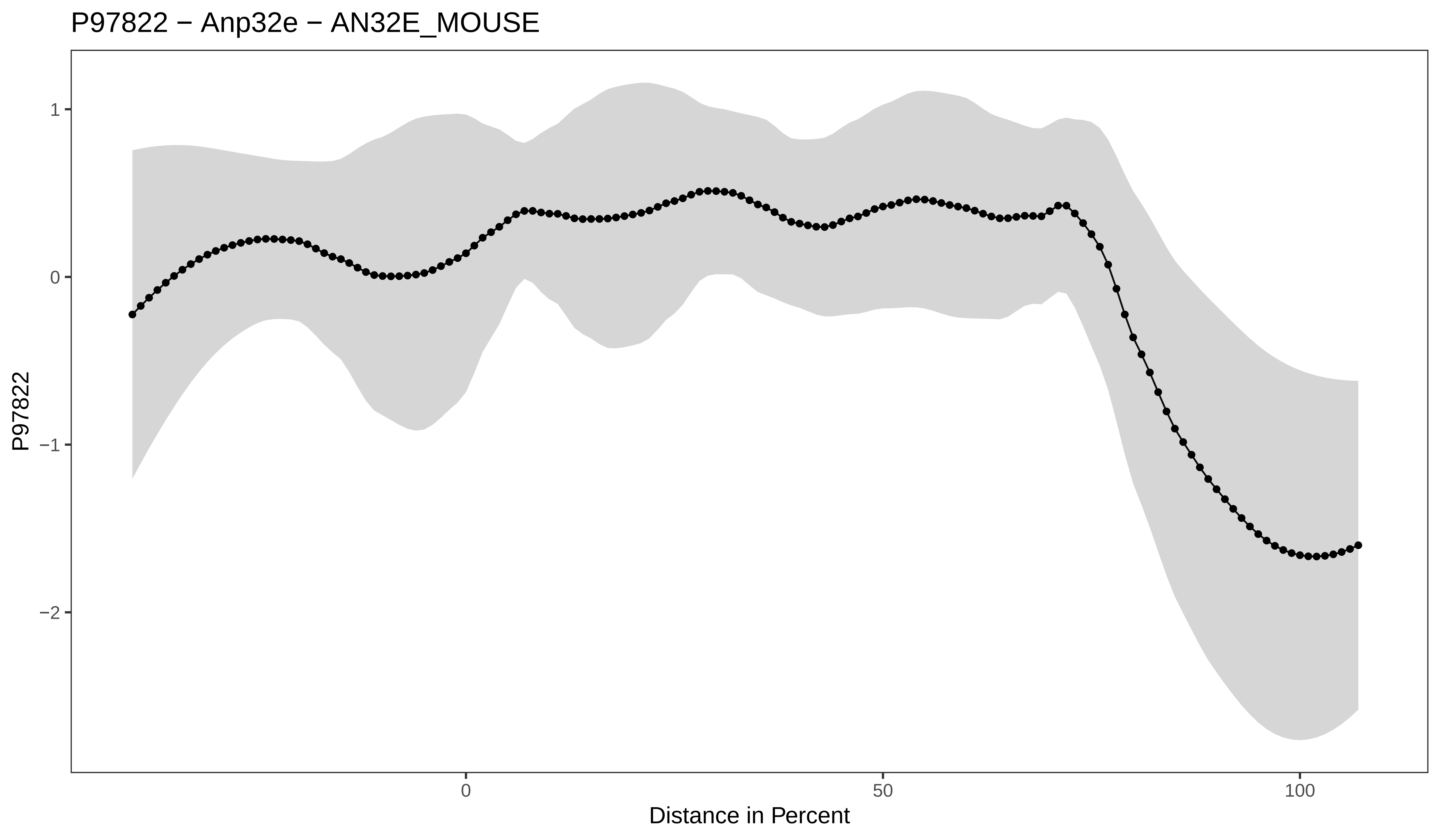
P97822 − Anp32e − AN32E_MOUSE
1
0
−1
−2
P97822
0
50
100
Distance in Percent

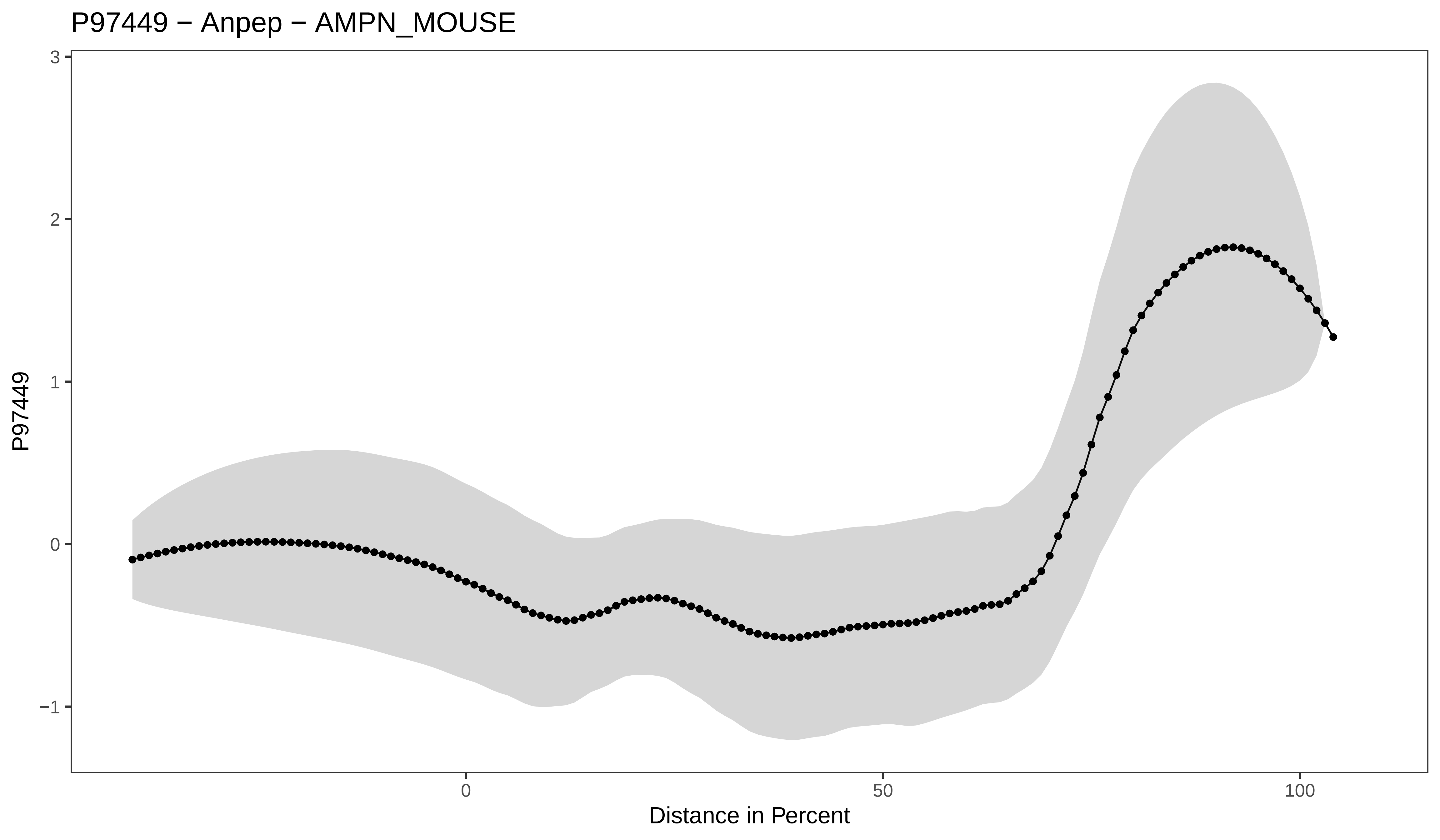

P97449 − Anpep − AMPN_MOUSE
3
2
1
0
−1
P97449
0
50
100
Distance in Percent

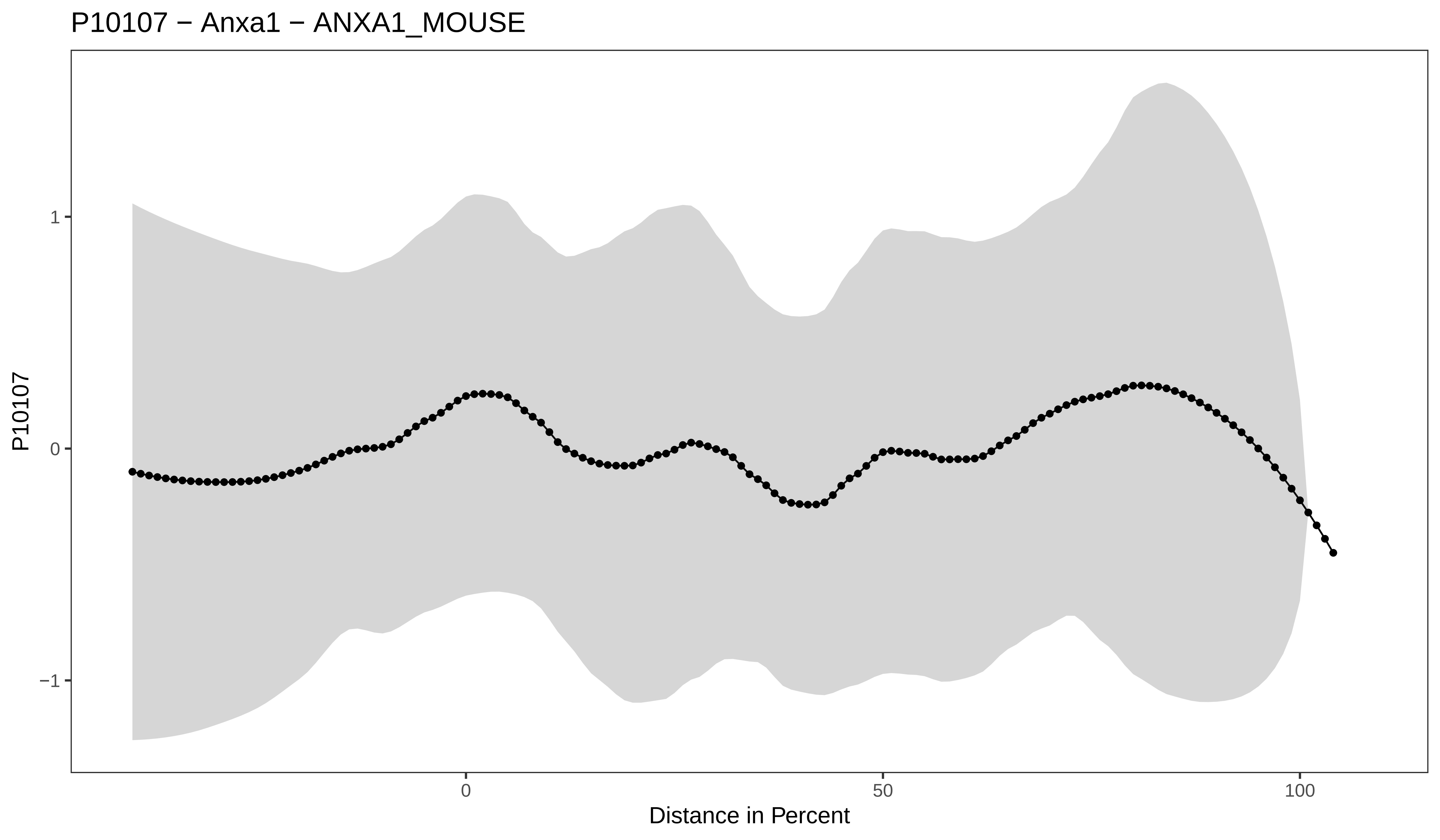

P10107 − Anxa1 − ANXA1_MOUSE
P10107
1
0
−1
0
50
100
Distance in Percent

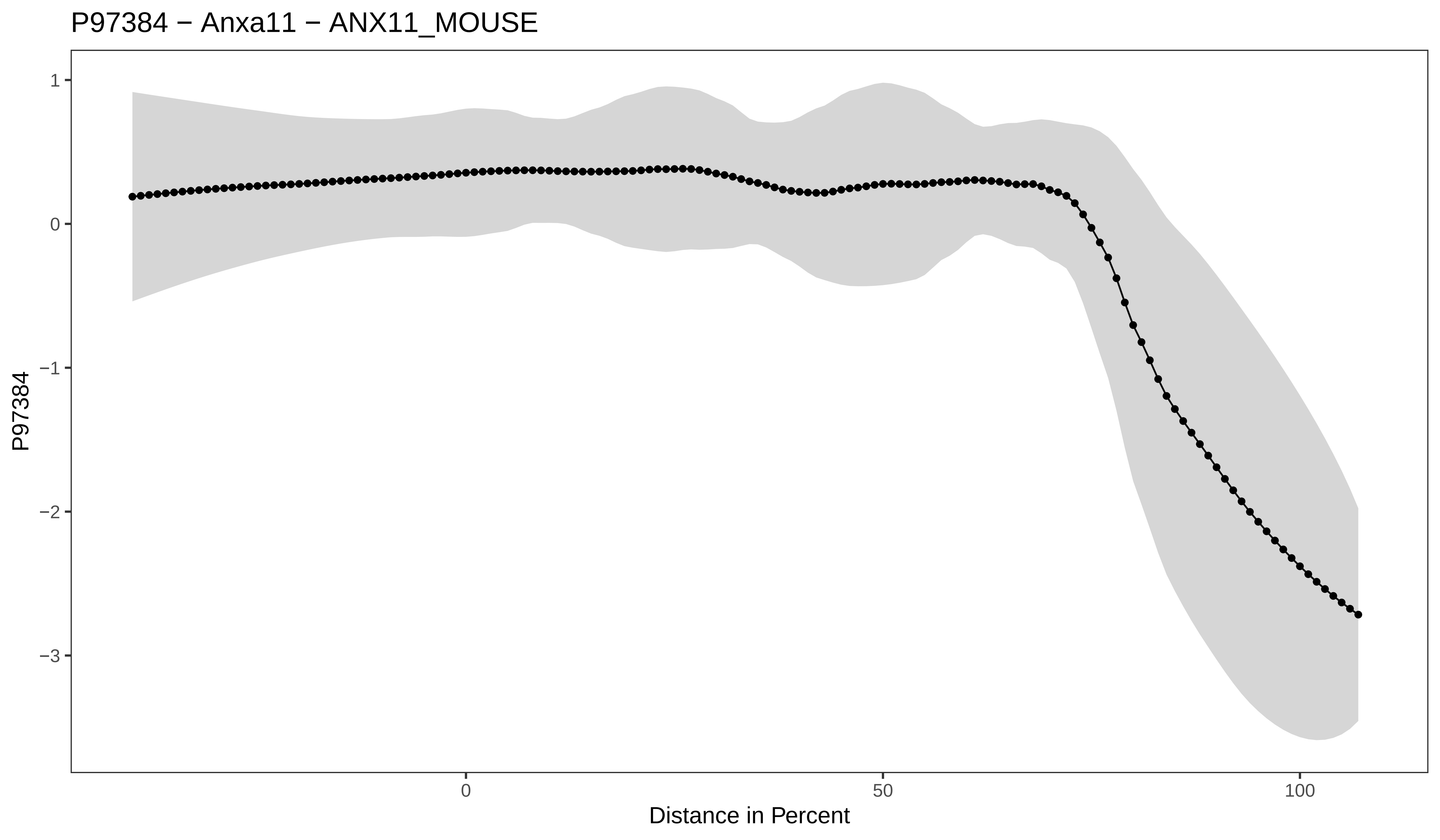

P97384 − Anxa11 − ANX11_MOUSE
1
0
−1
−2
−3
P97384
0
50
100
Distance in Percent

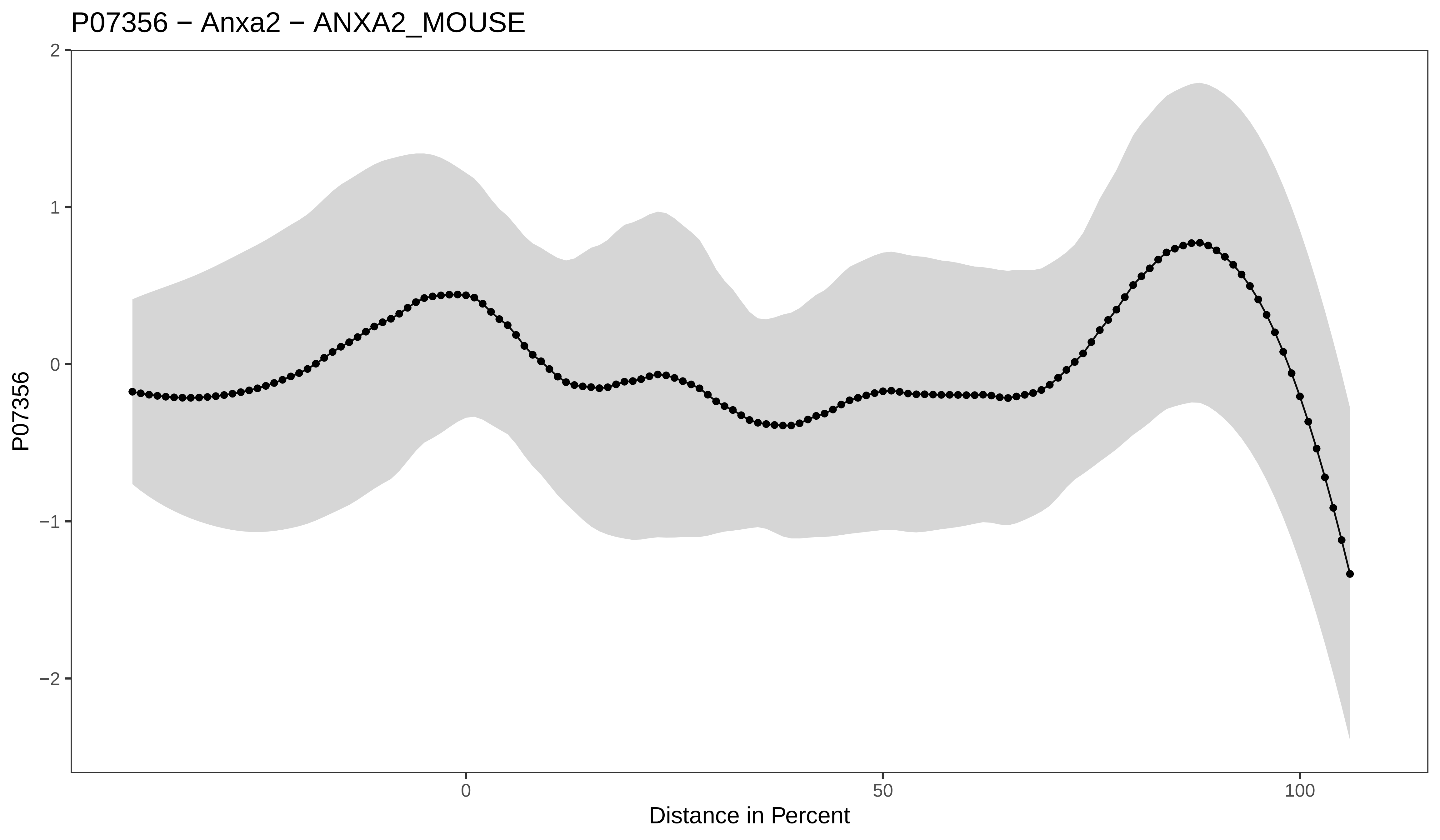
P07356 − Anxa2 − ANXA2_MOUSE
2
1
0
−1
−2
P07356
0
50
100
Distance in Percent

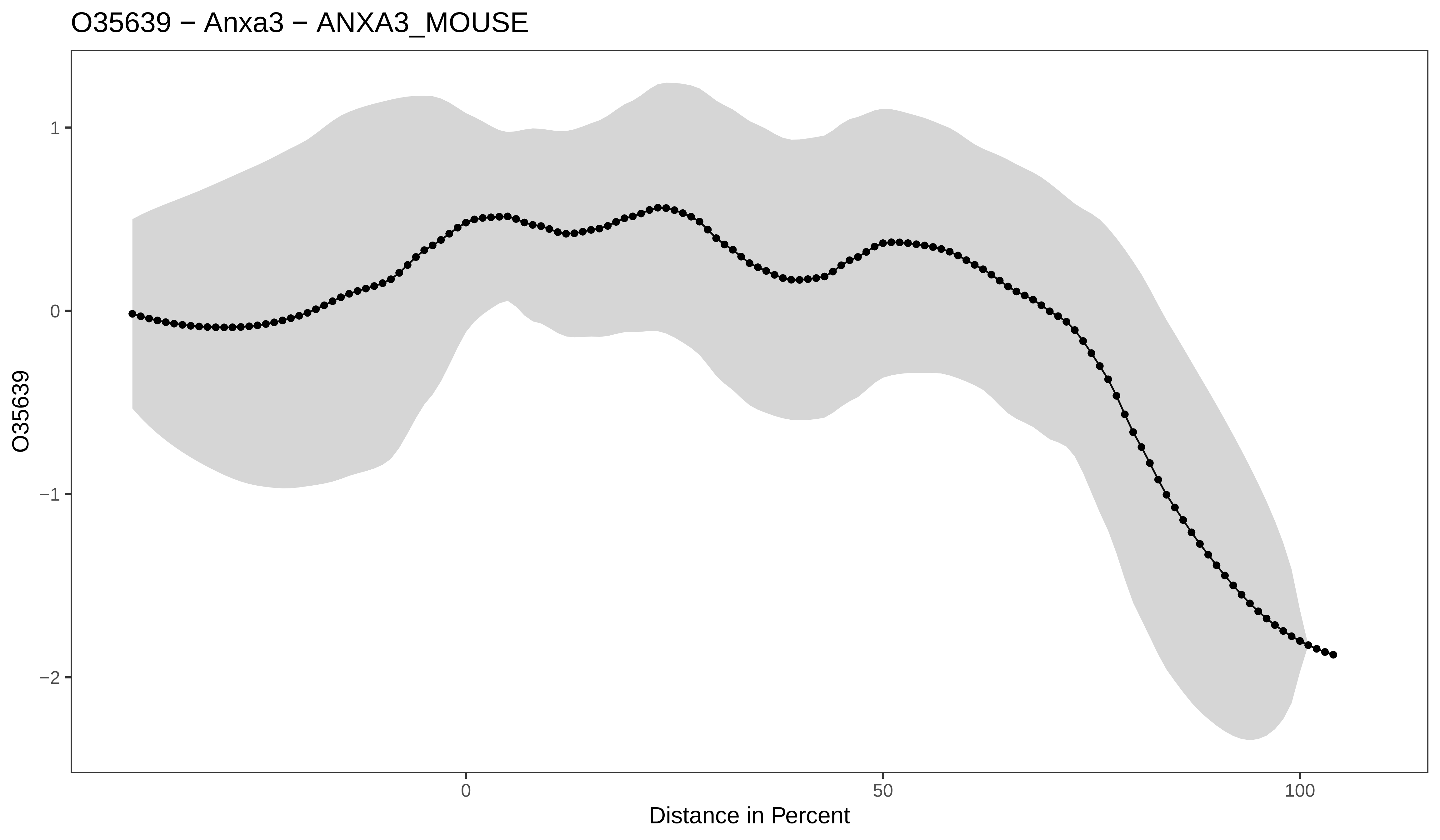
O35639 − Anxa3 − ANXA3_MOUSE
1
0
−1
−2
O35639
0
50
100
Distance in Percent

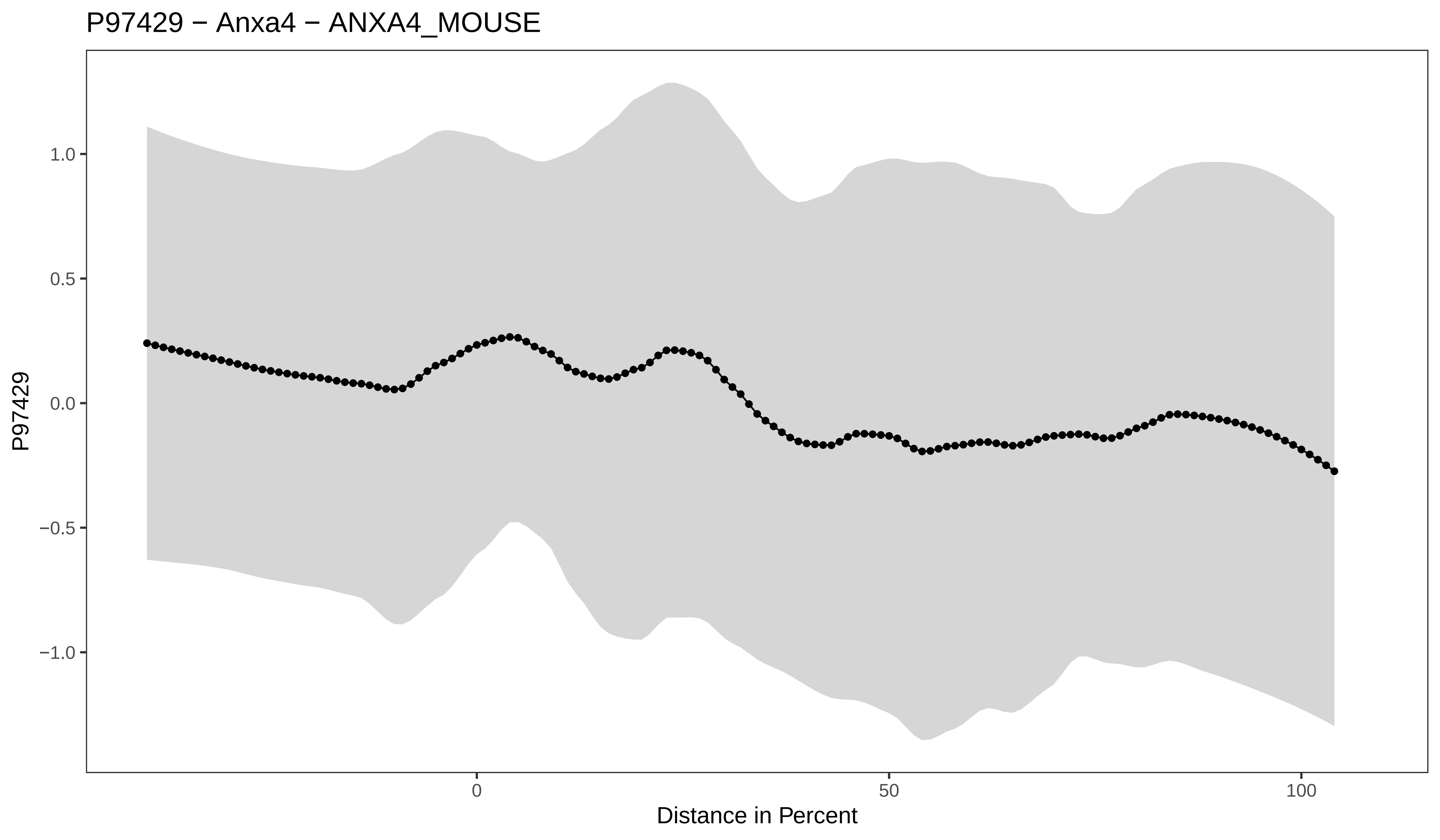
P97429 − Anxa4 − ANXA4_MOUSE
P97429
1.0
0.5
0.0
−0.5
−1.0
0
50
100
Distance in Percent

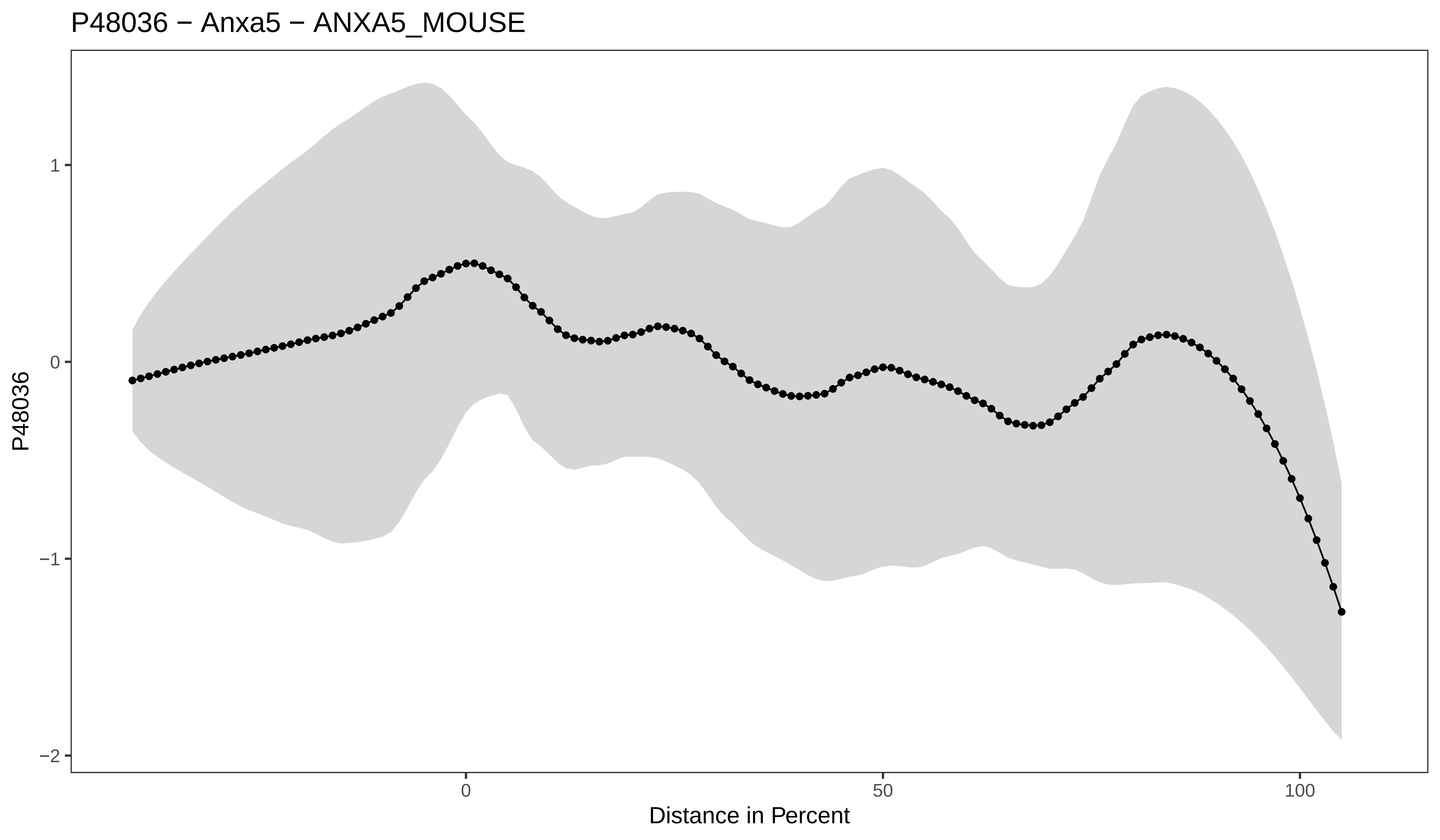
P48036 − Anxa5 − ANXA5_MOUSE
P48036
1
0
−1
−2
0
50
100
Distance in Percent

P14824 − Anxa6 − ANXA6_MOUSE

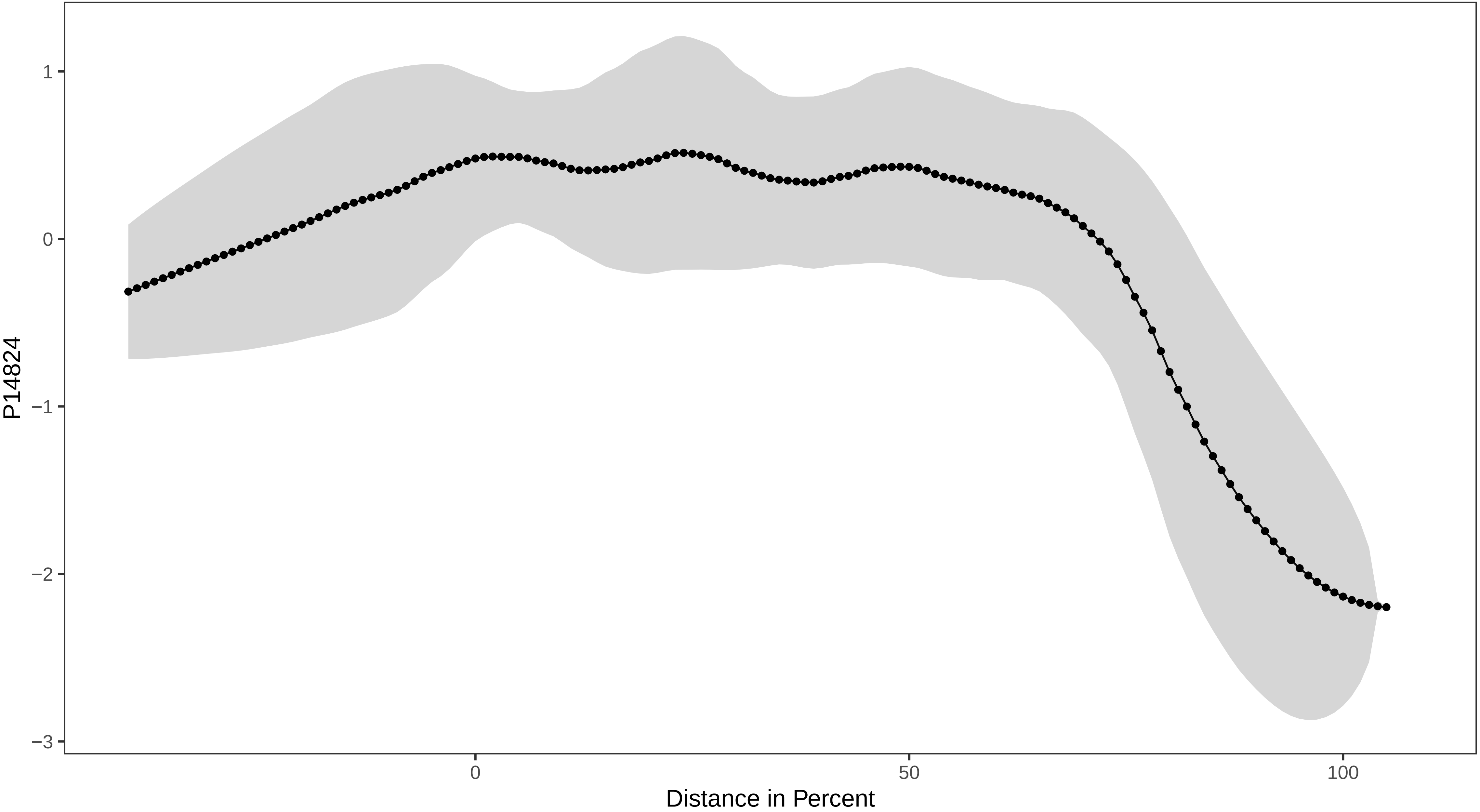

Q07076 − Anxa7 − ANXA7_MOUSE

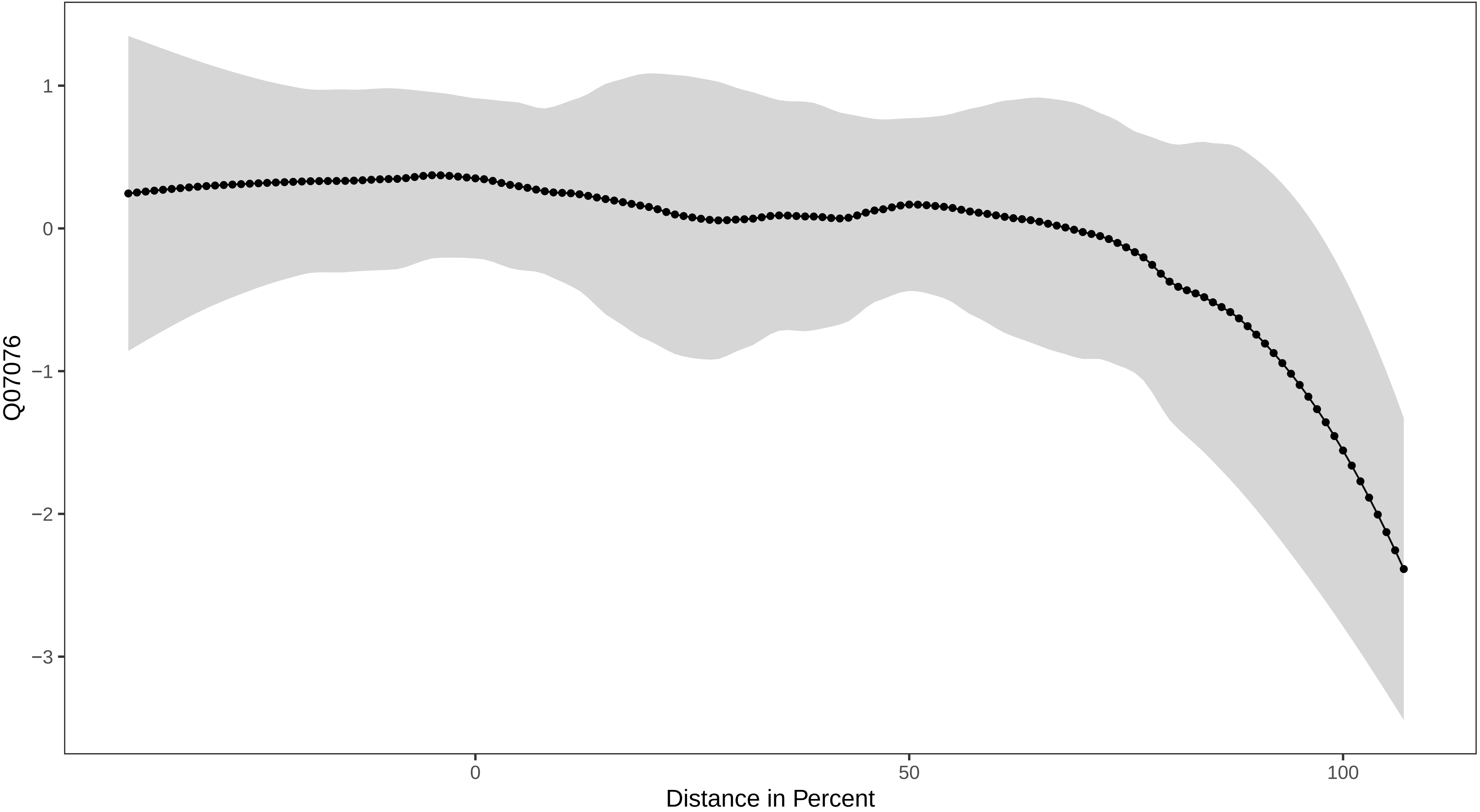

O70423 − Aoc3 − AOC3_MOUSE

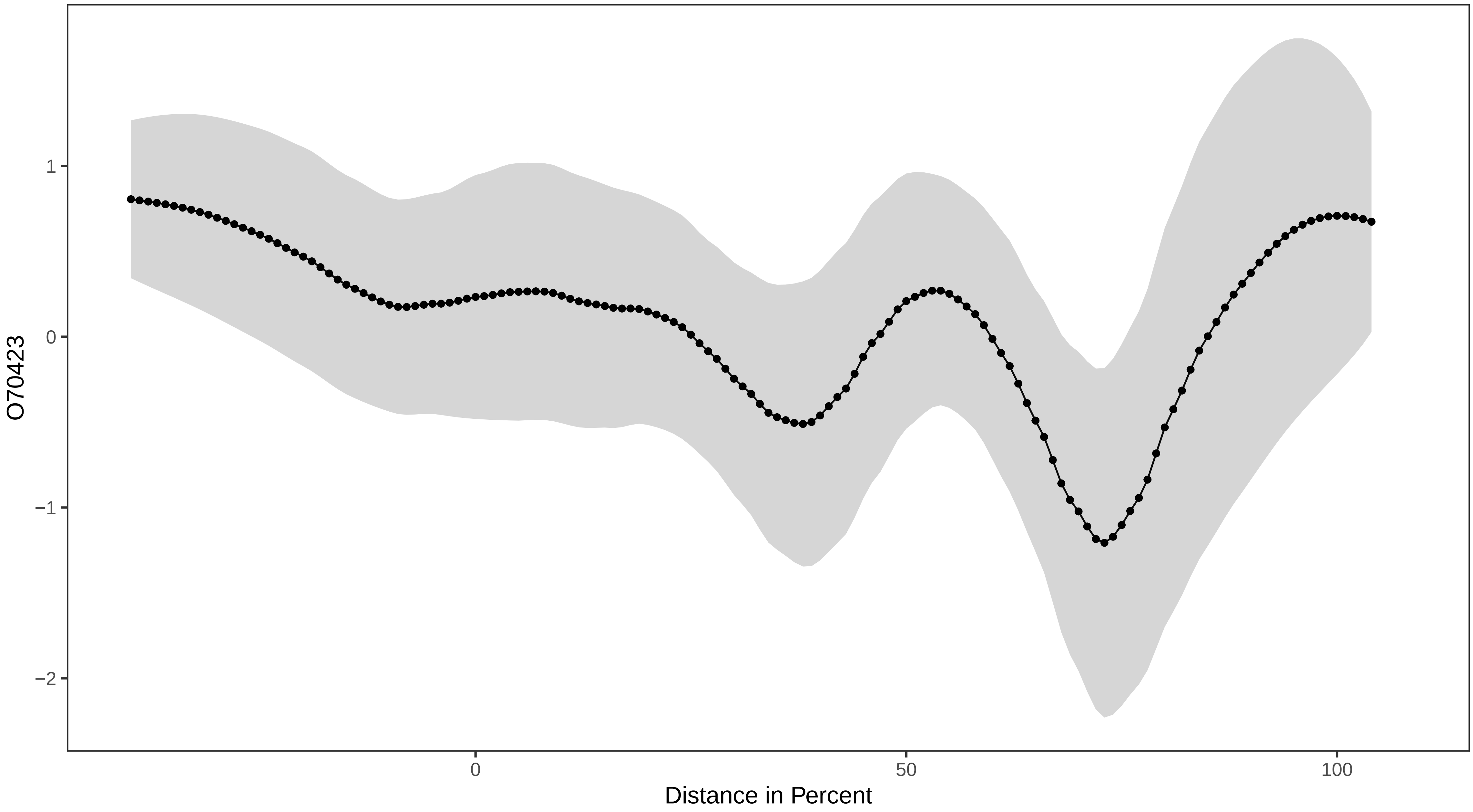

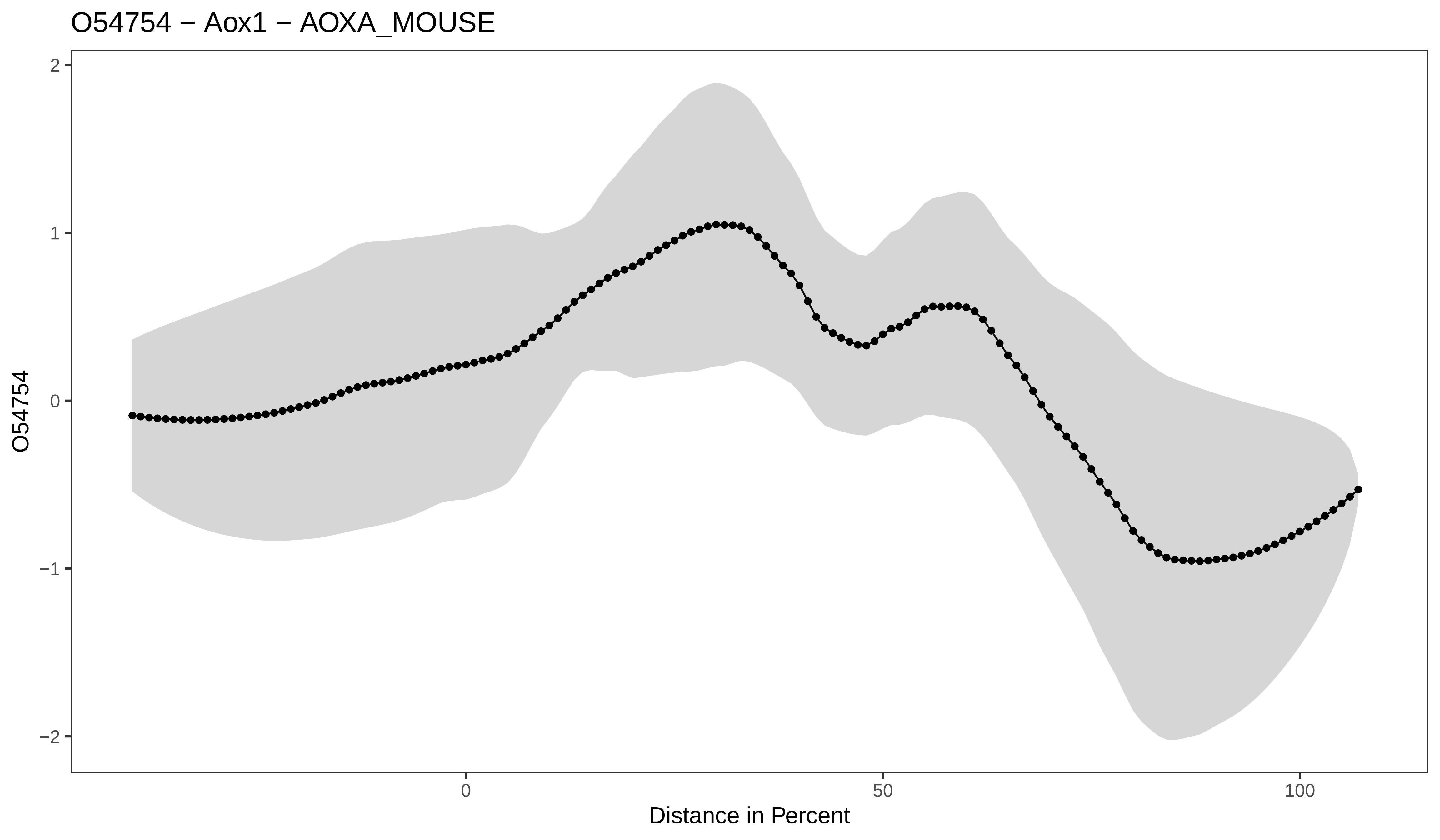
O54754 − Aox1 − AOXA_MOUSE
O54754
Distance in Percent
2
1
0
−1
−2
0
50
100

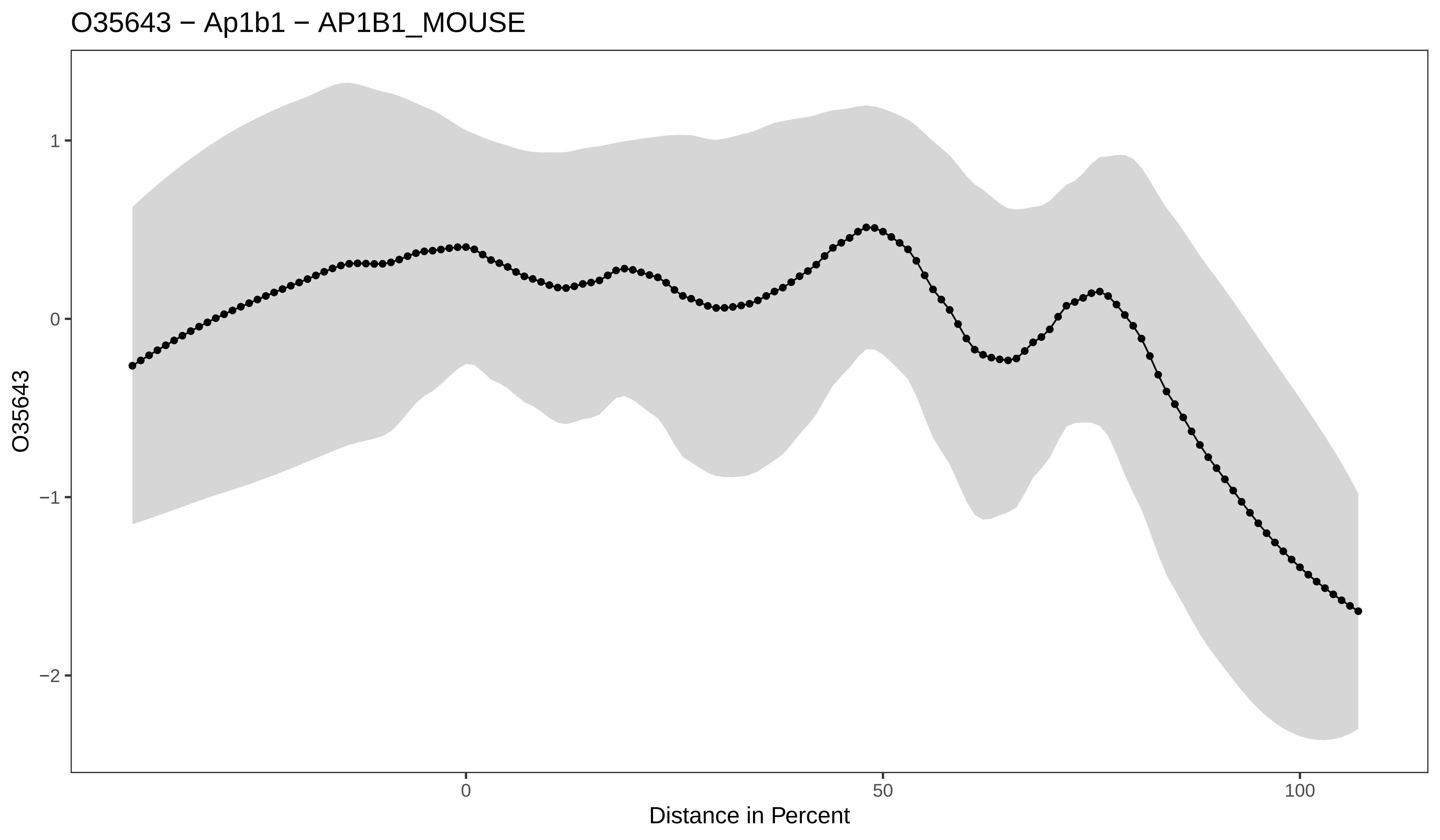
O35643 − Ap1b1 − AP1B1_MOUSE
1
0
−1
−2
O35643
0
50
100
Distance in Percent

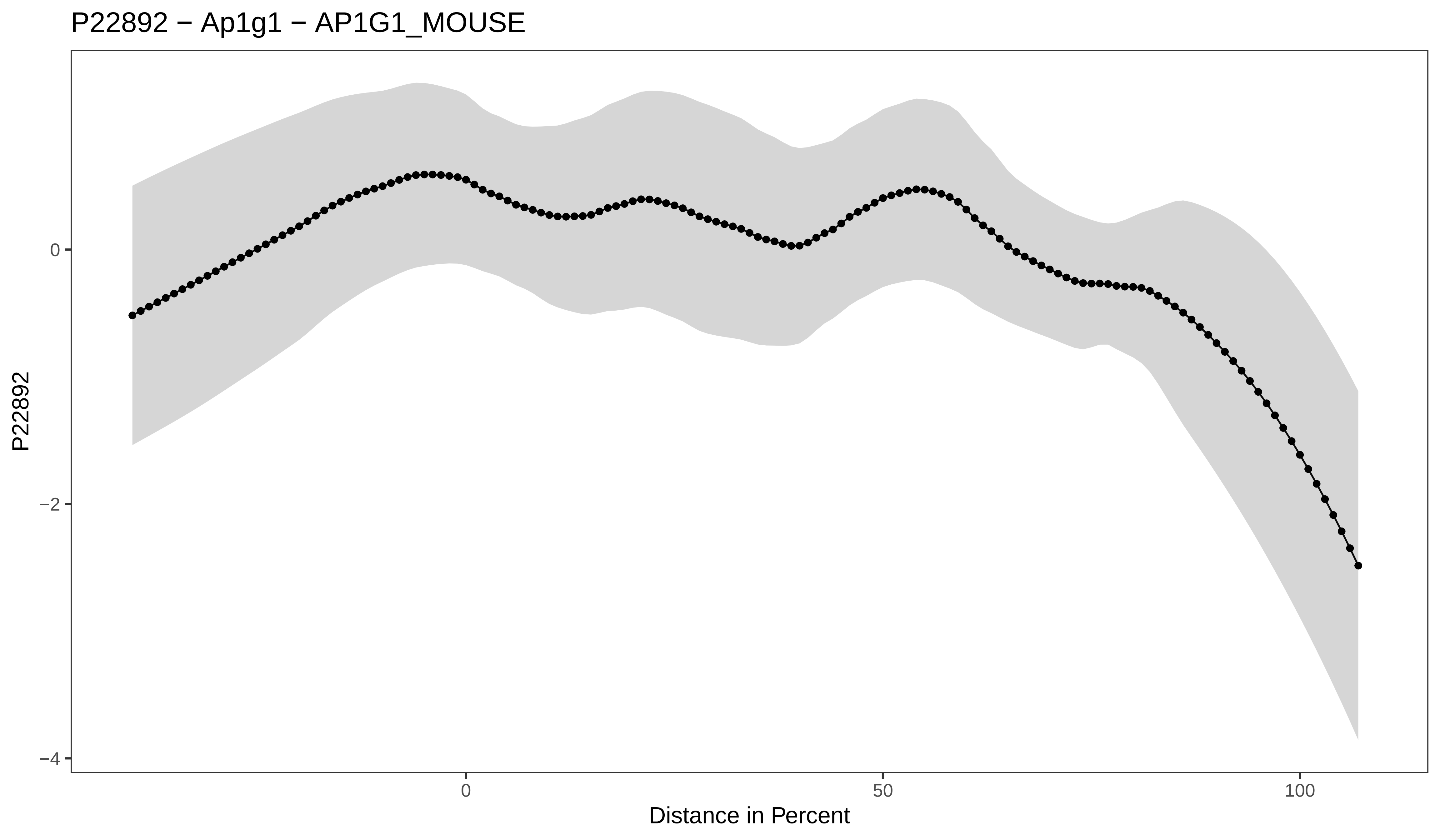
P22892 − Ap1g1 − AP1G1_MOUSE
P22892
0
−2
−4
0
50
100
Distance in Percent

P35585 − Ap1m1 − AP1M1_MOUSE

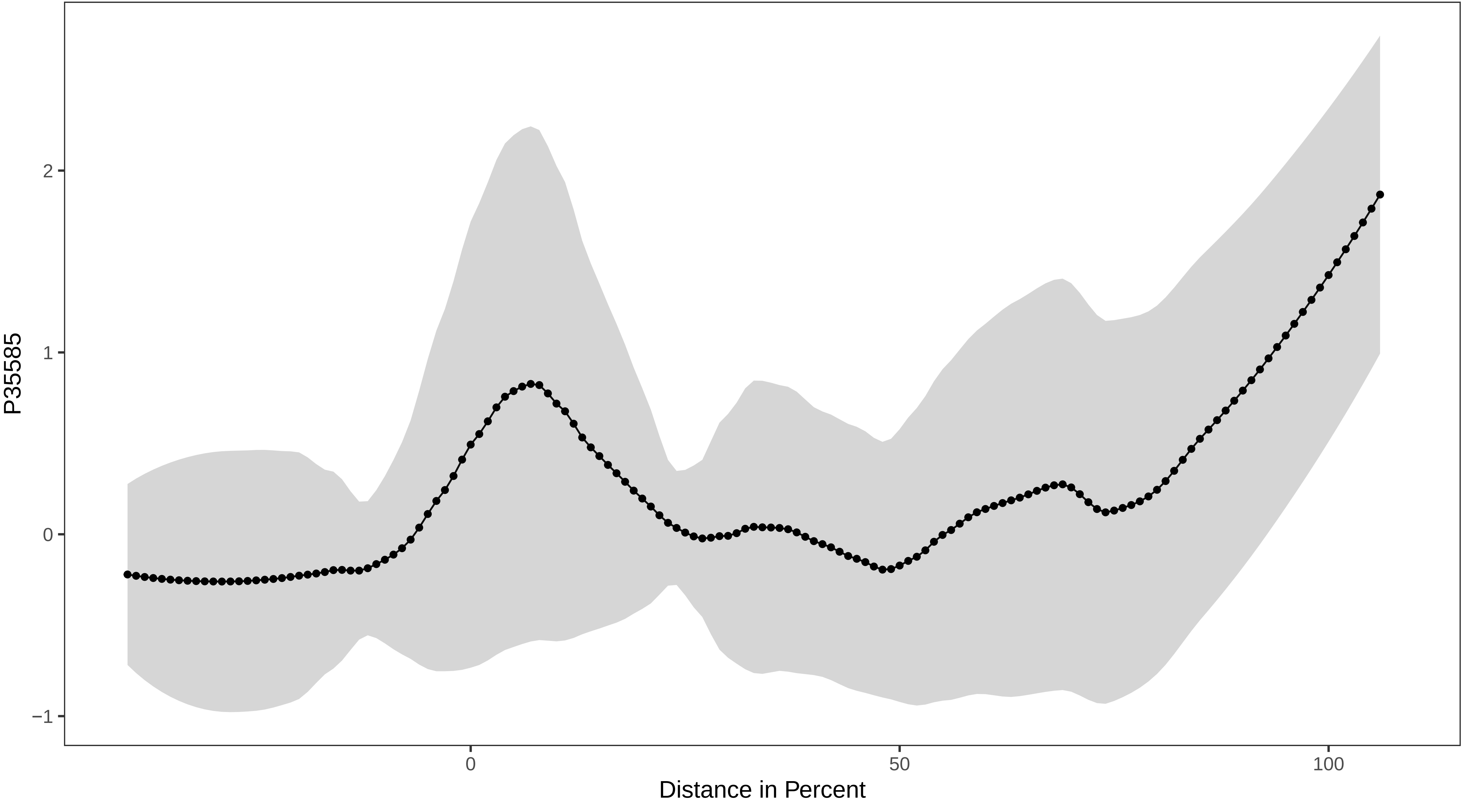

P17426 − Ap2a1 − AP2A1_MOUSE

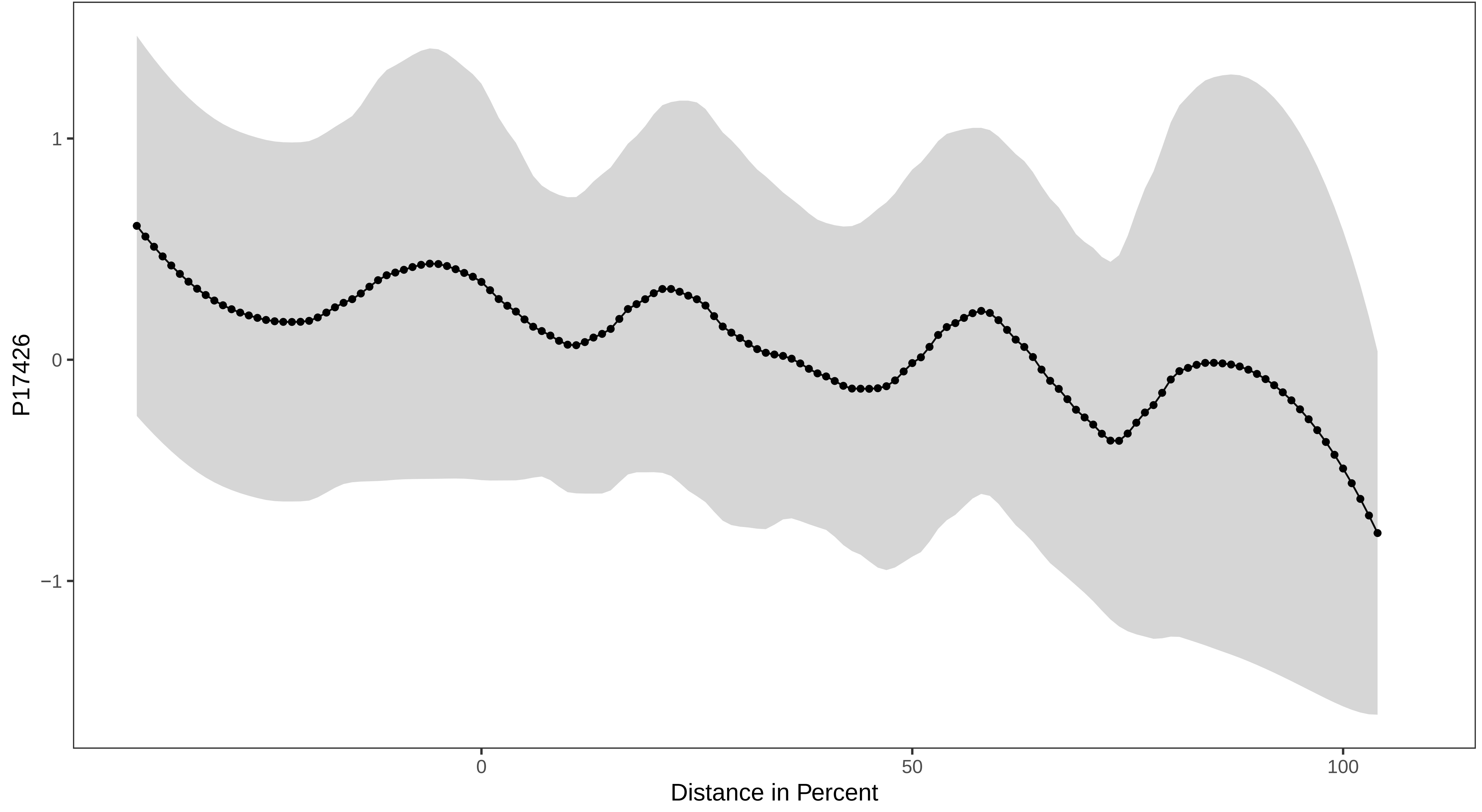

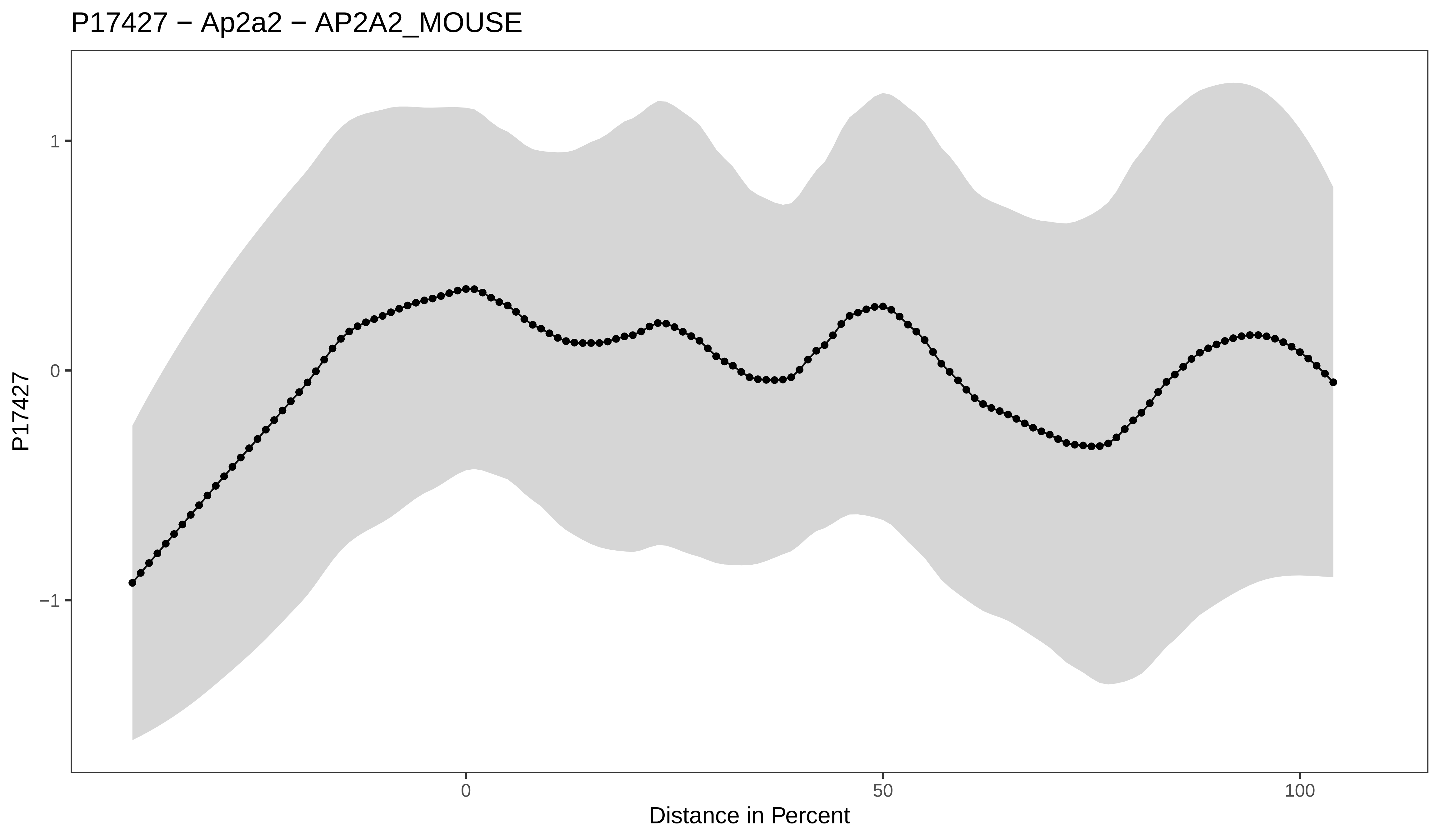

P17427 − Ap2a2 − AP2A2_MOUSE
1
0
−1
P17427
0
50
100
Distance in Percent

Q9DBG3 − Ap2b1 − AP2B1_MOUSE

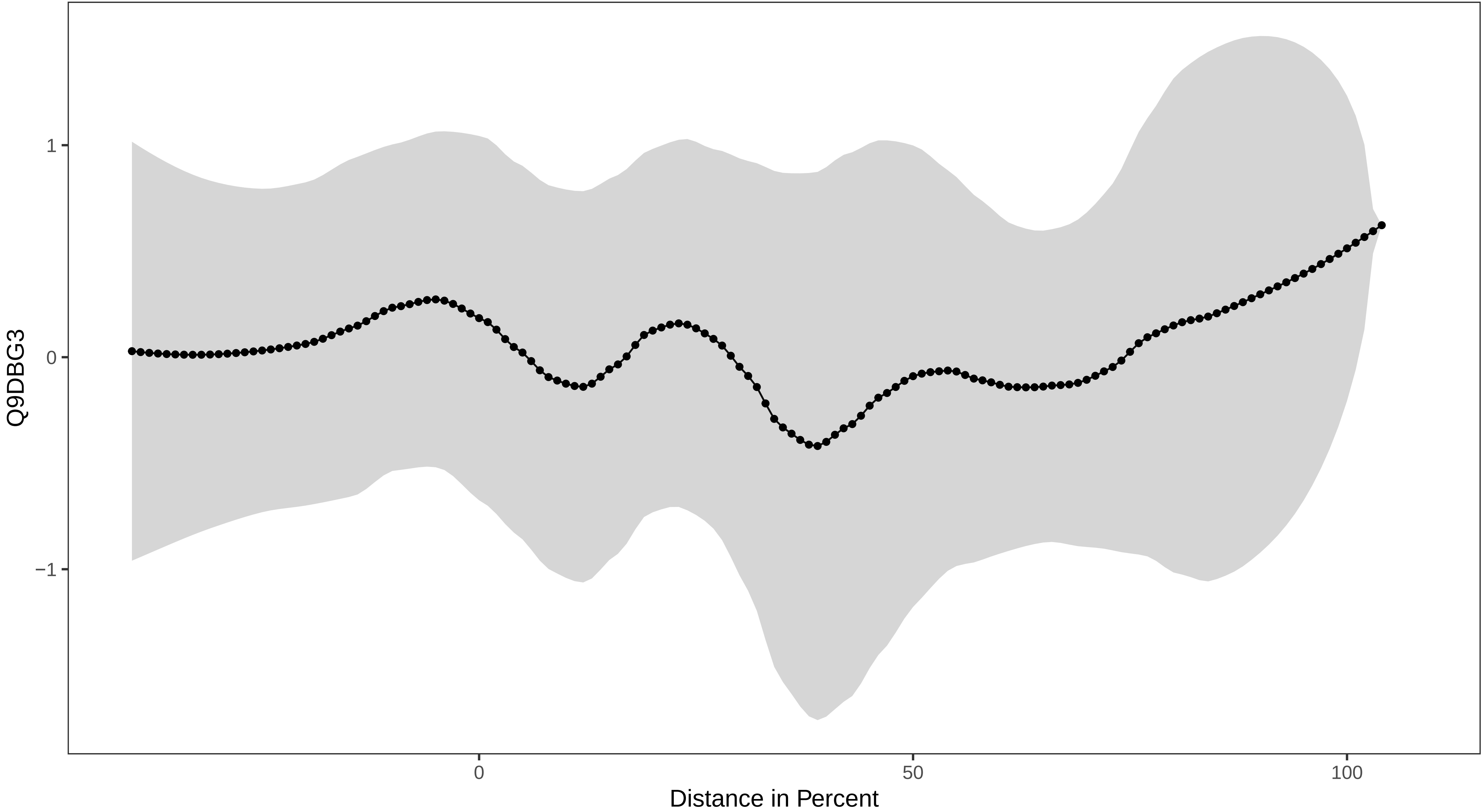

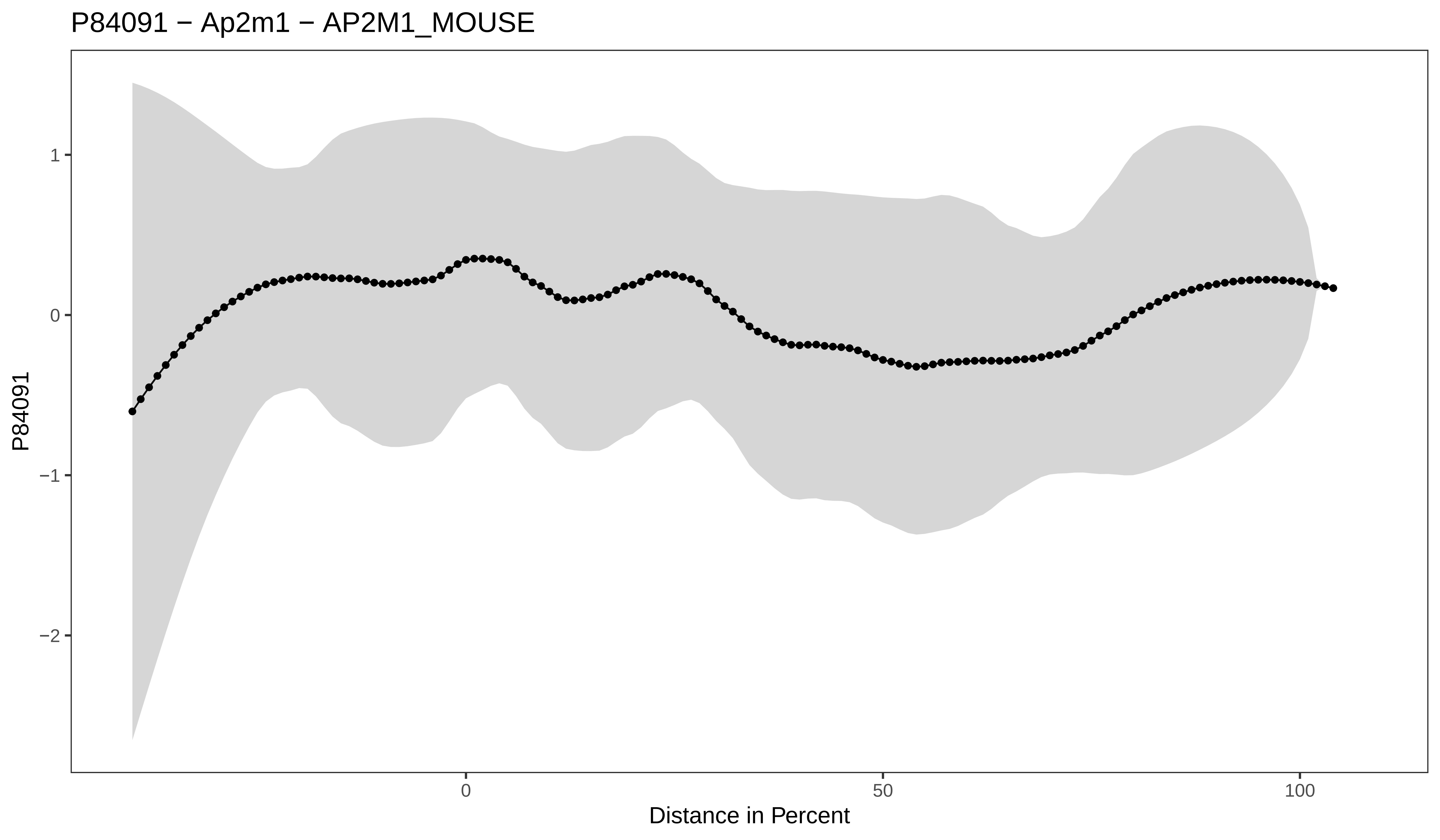
P84091 − Ap2m1 − AP2M1_MOUSE
P84091
1
0
−1
−2
0
50
100
Distance in Percent

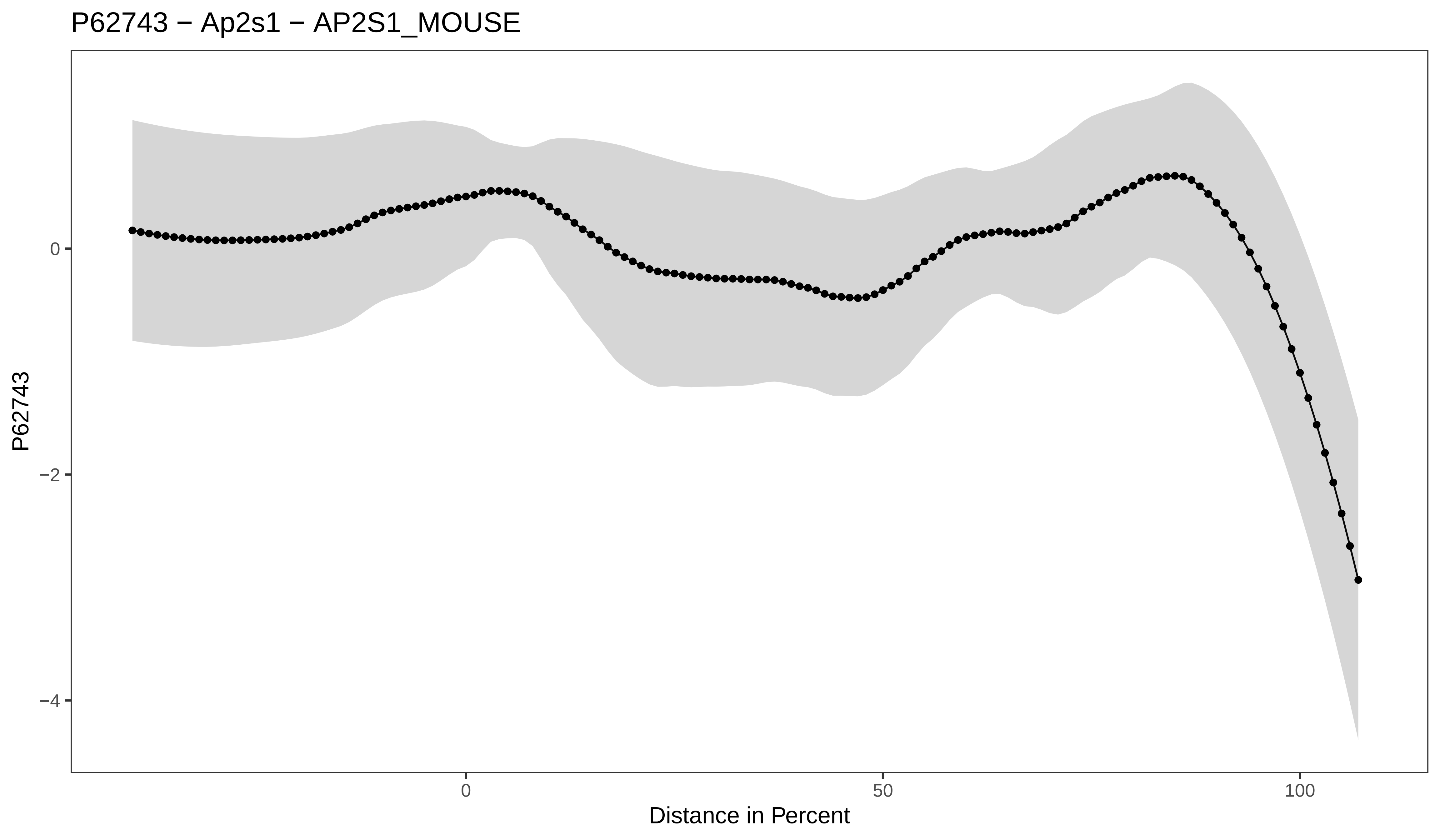

P62743 − Ap2s1 − AP2S1_MOUSE
P62743
0
−2
−4
0
50
100
Distance in Percent

Q9Z1T1 − Ap3b1 − AP3B1_MOUSE

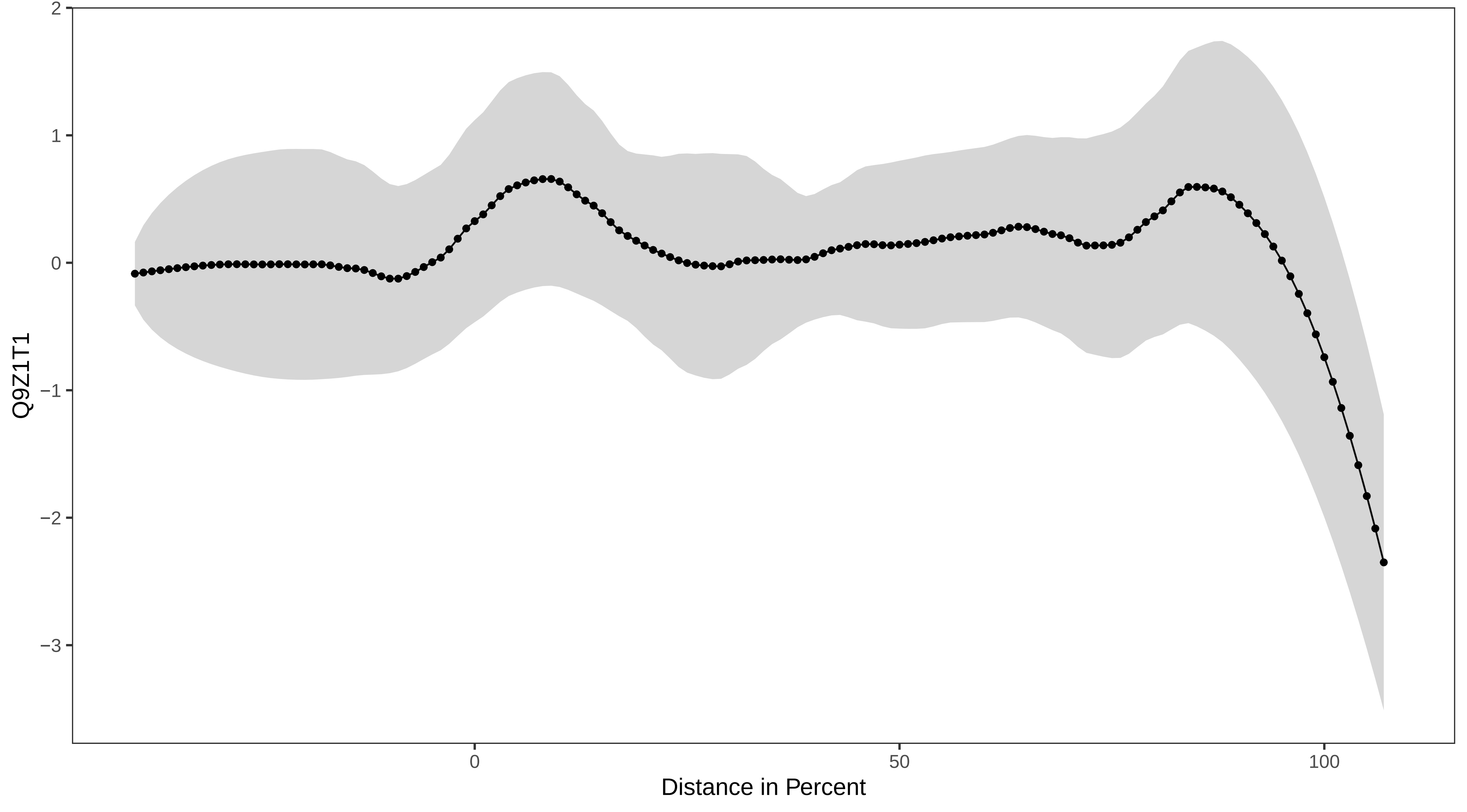

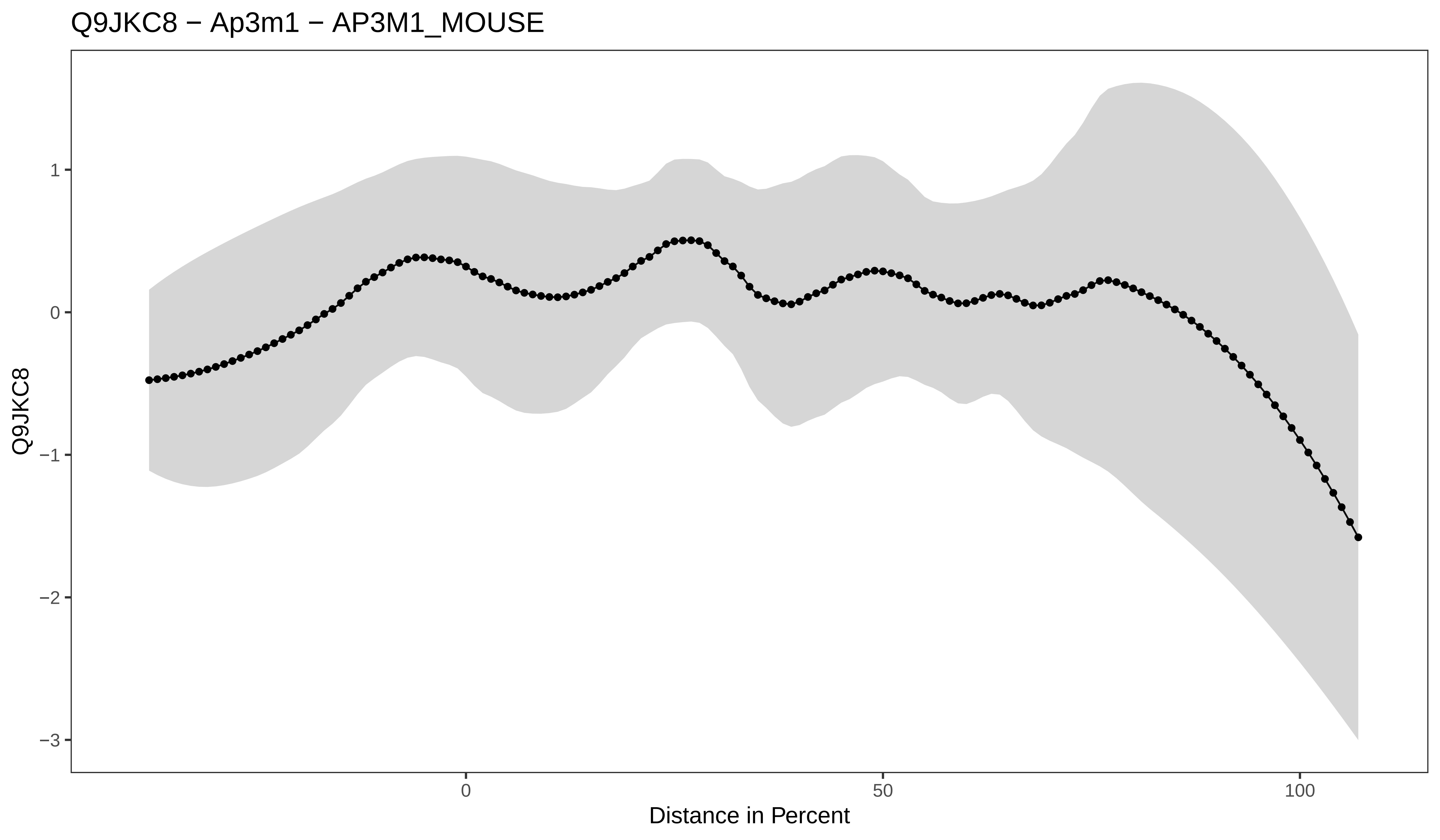

Q9JKC8 − Ap3m1 − AP3M1_MOUSE
1
0
−1
−2
−3
Q9JKC8
0
50
100
Distance in Percent

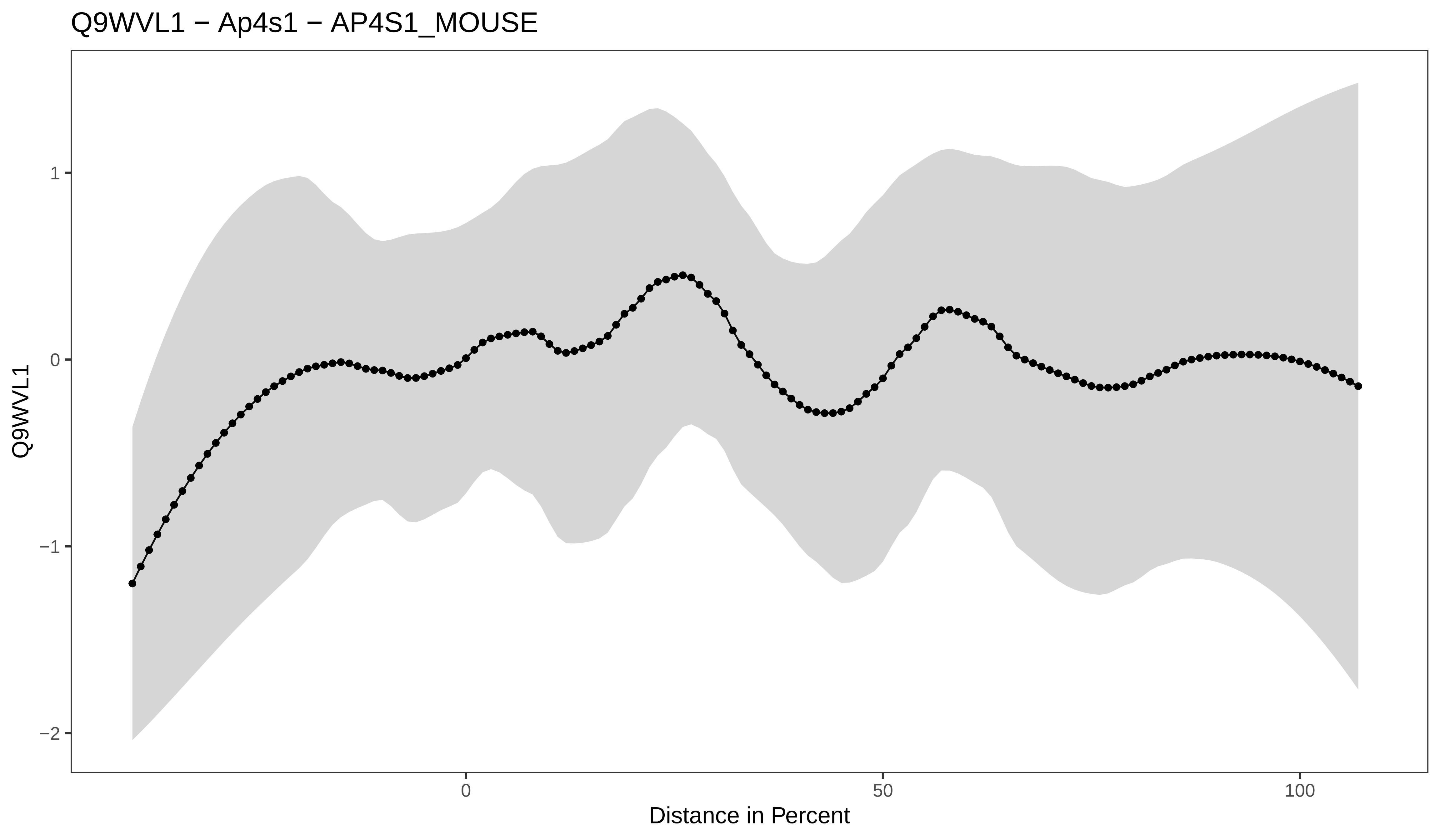

Q9WVL1 − Ap4s1 − AP4S1_MOUSE
1
0
−1
−2
Q9WVL1
0
50
100
Distance in Percent

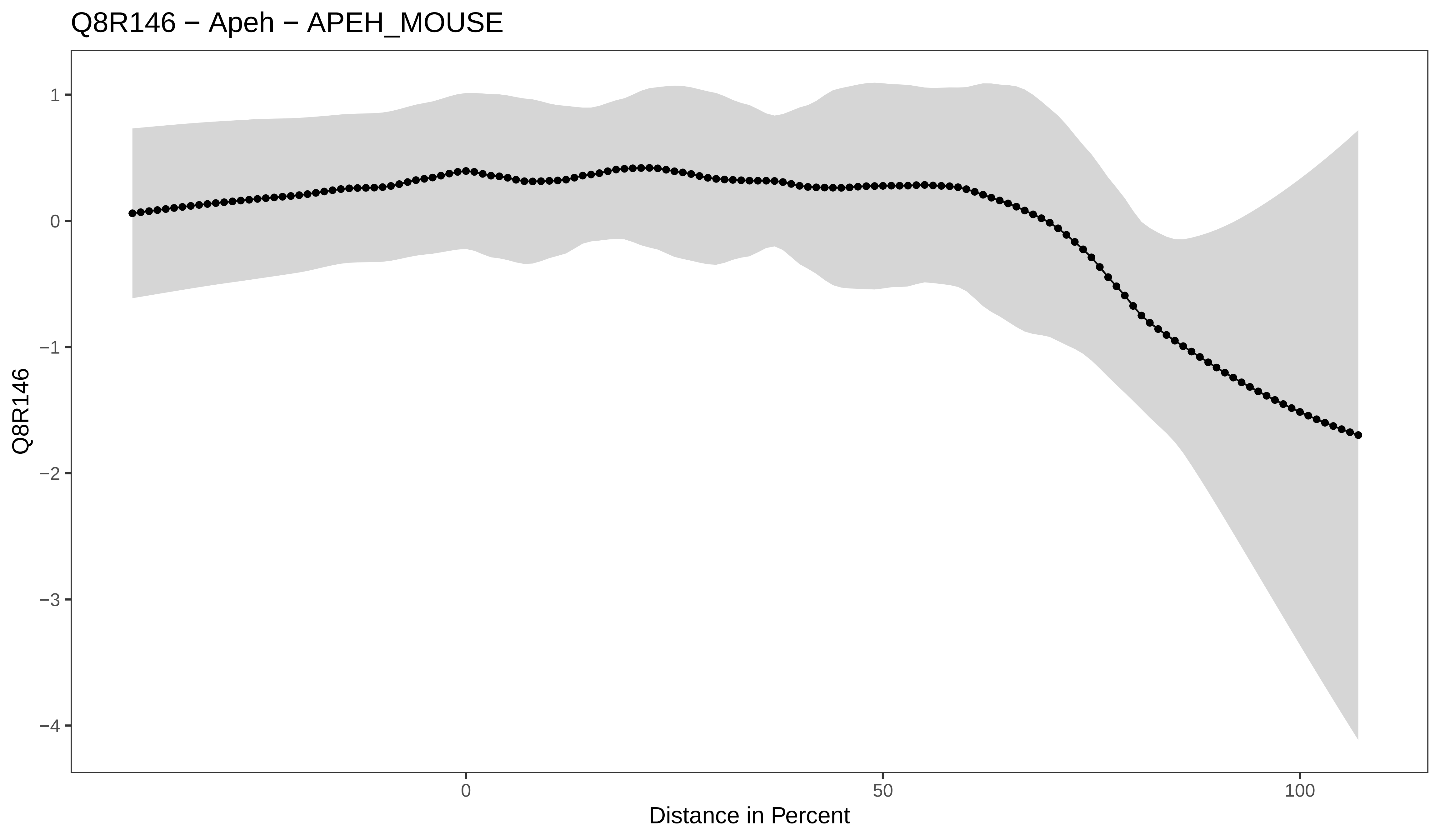
Q8R146 − Apeh − APEH_MOUSE
Q8R146
Distance in Percent
1
0
−1
−2
−3
−4
0
50
100

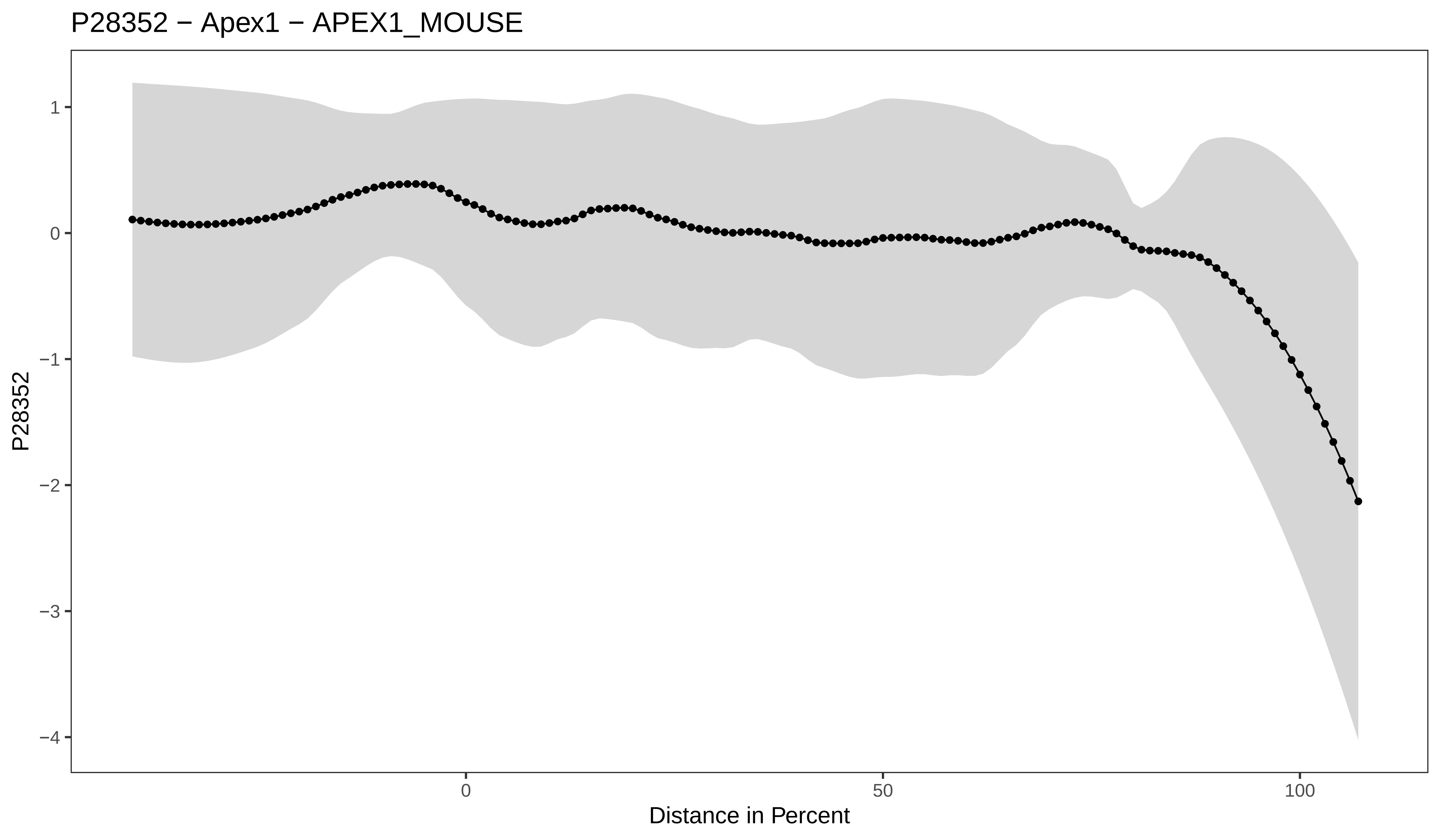

P28352 − Apex1 − APEX1_MOUSE
1
0
−1
−2
−3
−4
P28352
0
50
100
Distance in Percent

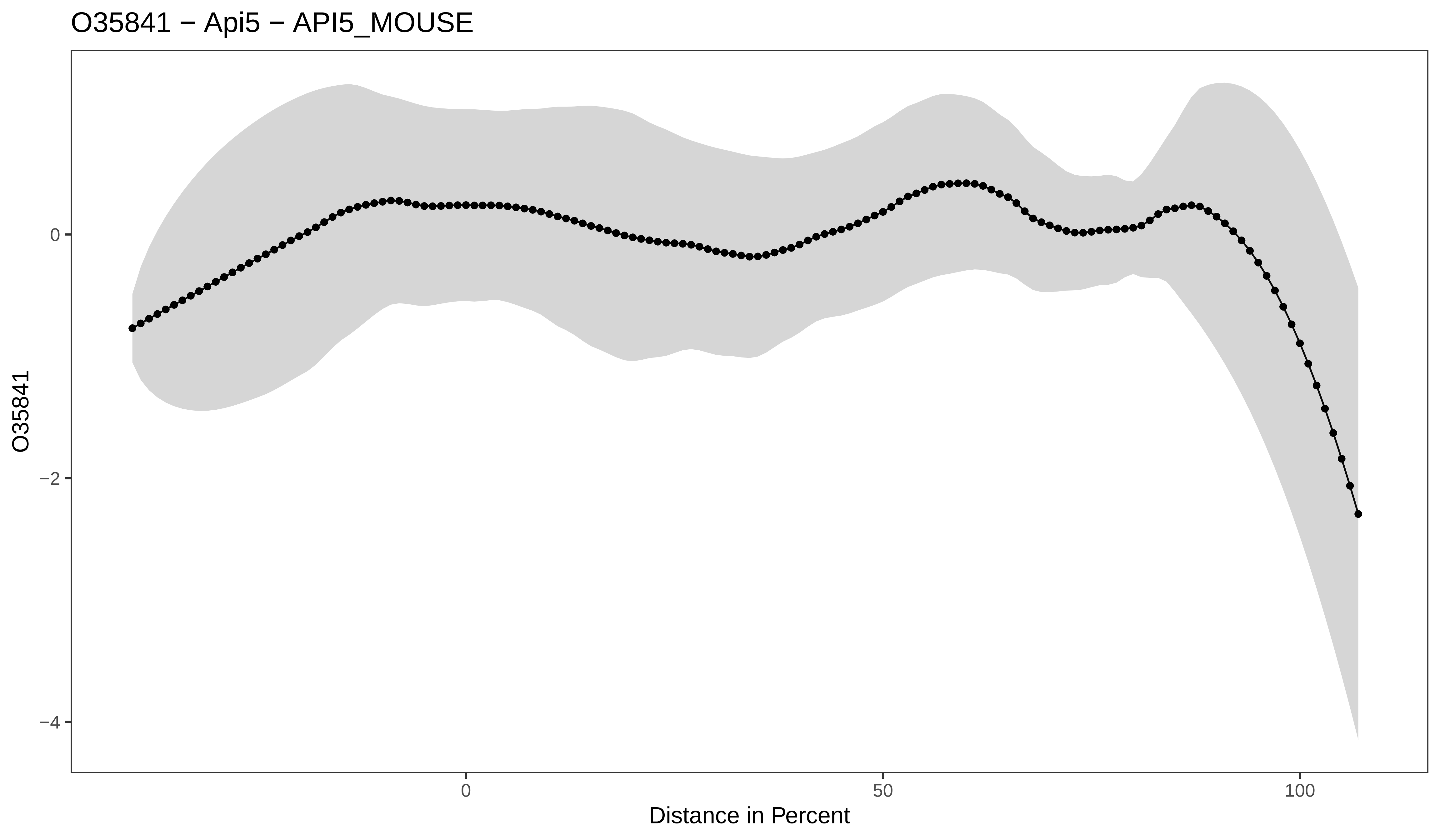

O35841 − Api5 − API5_MOUSE
O35841
0
−2
−4
0
50
100
Distance in Percent

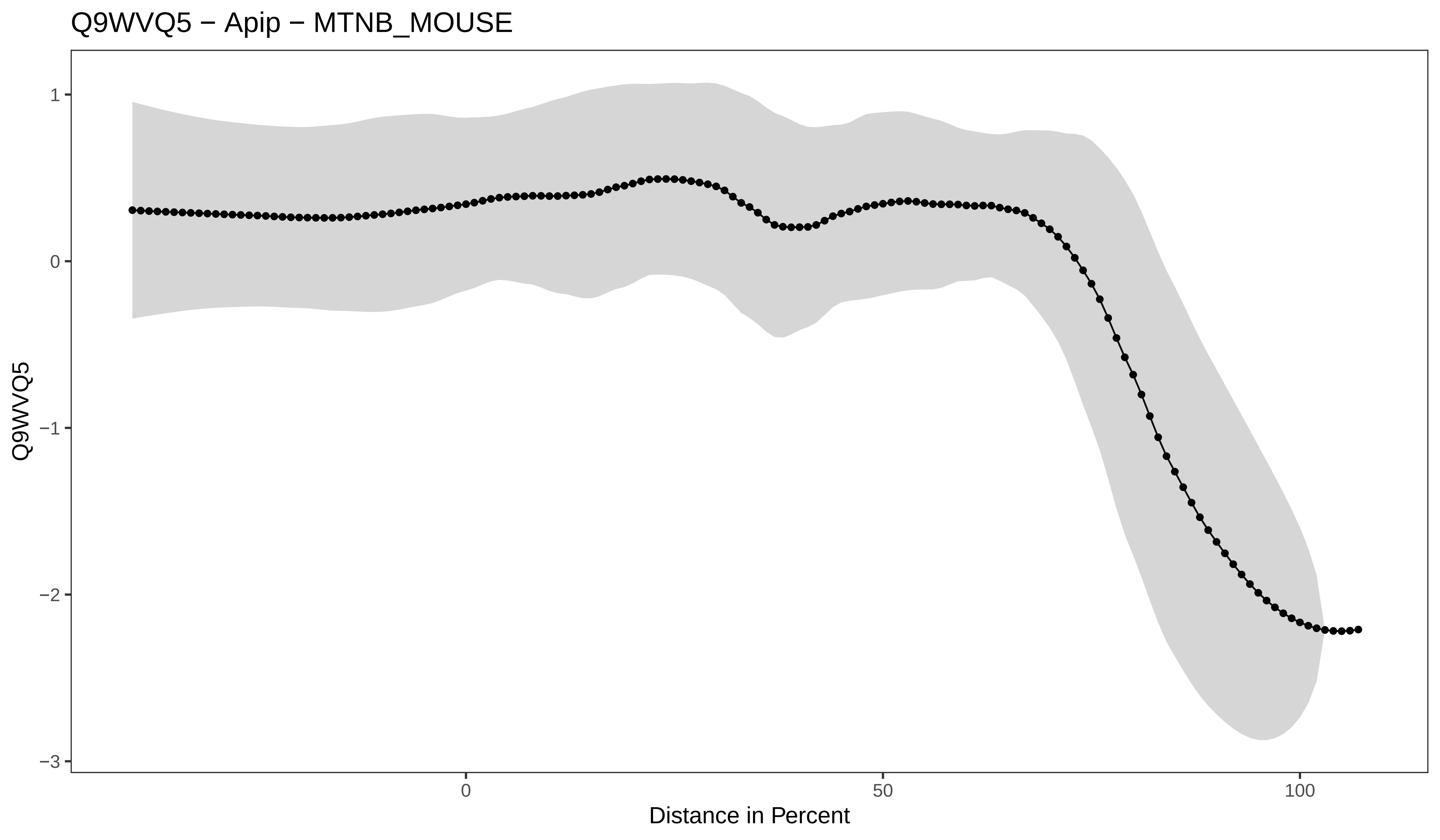

Q9WVQ5 − Apip − MTNB_MOUSE
Q9WVQ5
Distance in Percent
1
0
−1
−2
−3
0
50
100

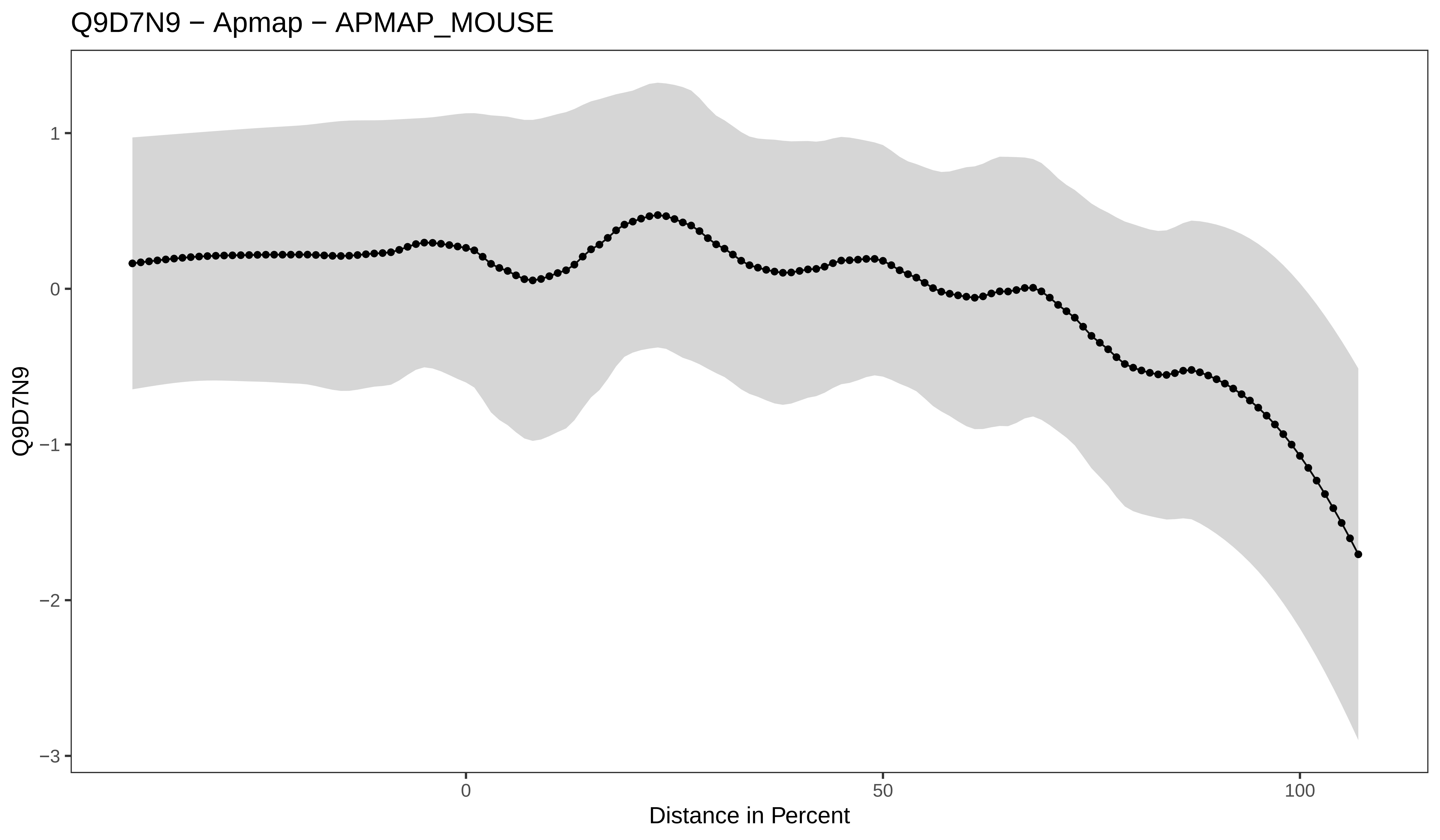

Q9D7N9 − Apmap − APMAP_MOUSE
Q9D7N9
Distance in Percent
1
0
−1
−2
−3
0
50
100

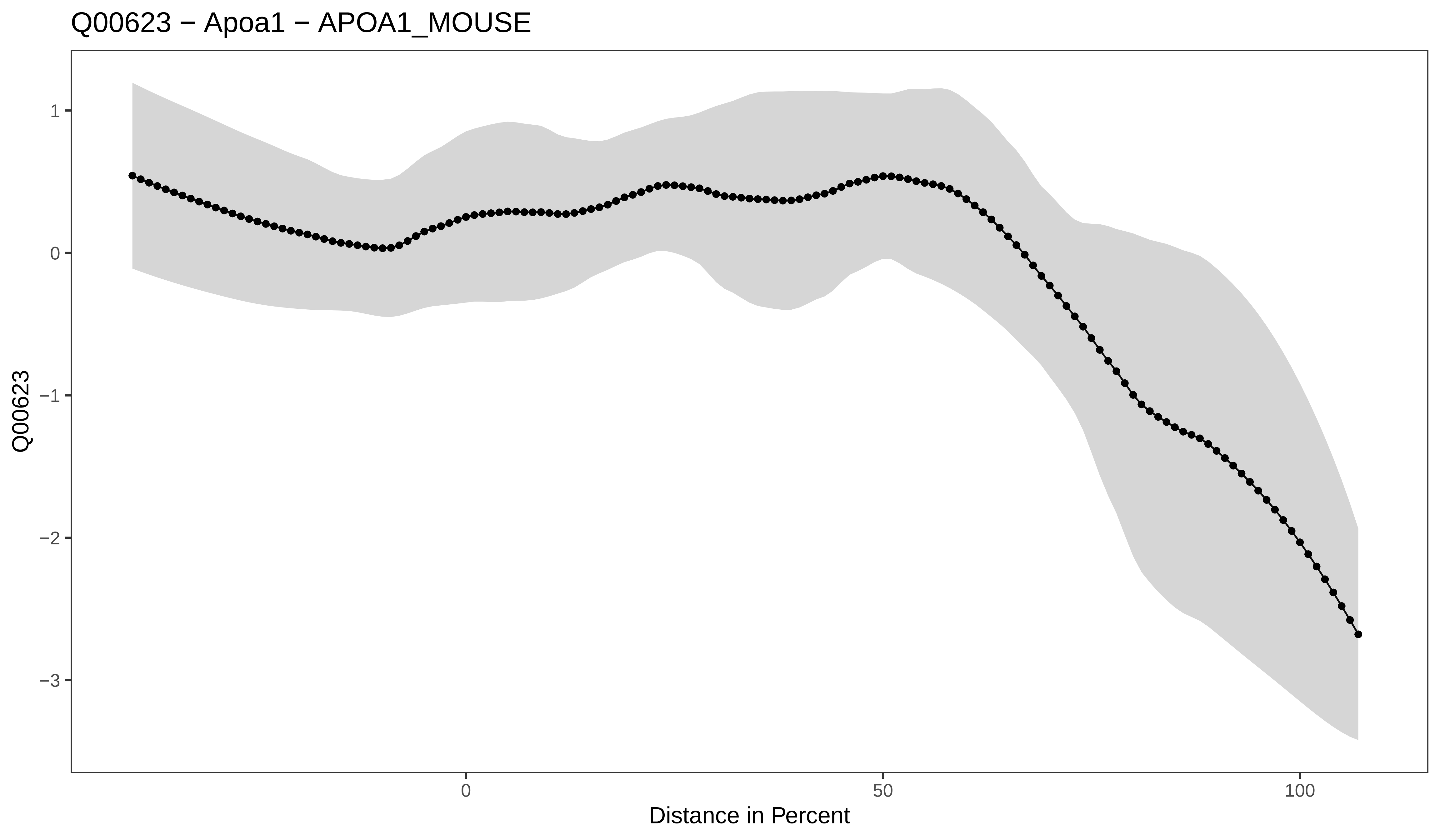
Q00623 − Apoa1 − APOA1_MOUSE
1
0
−1
−2
−3
Q00623
0
50
100
Distance in Percent

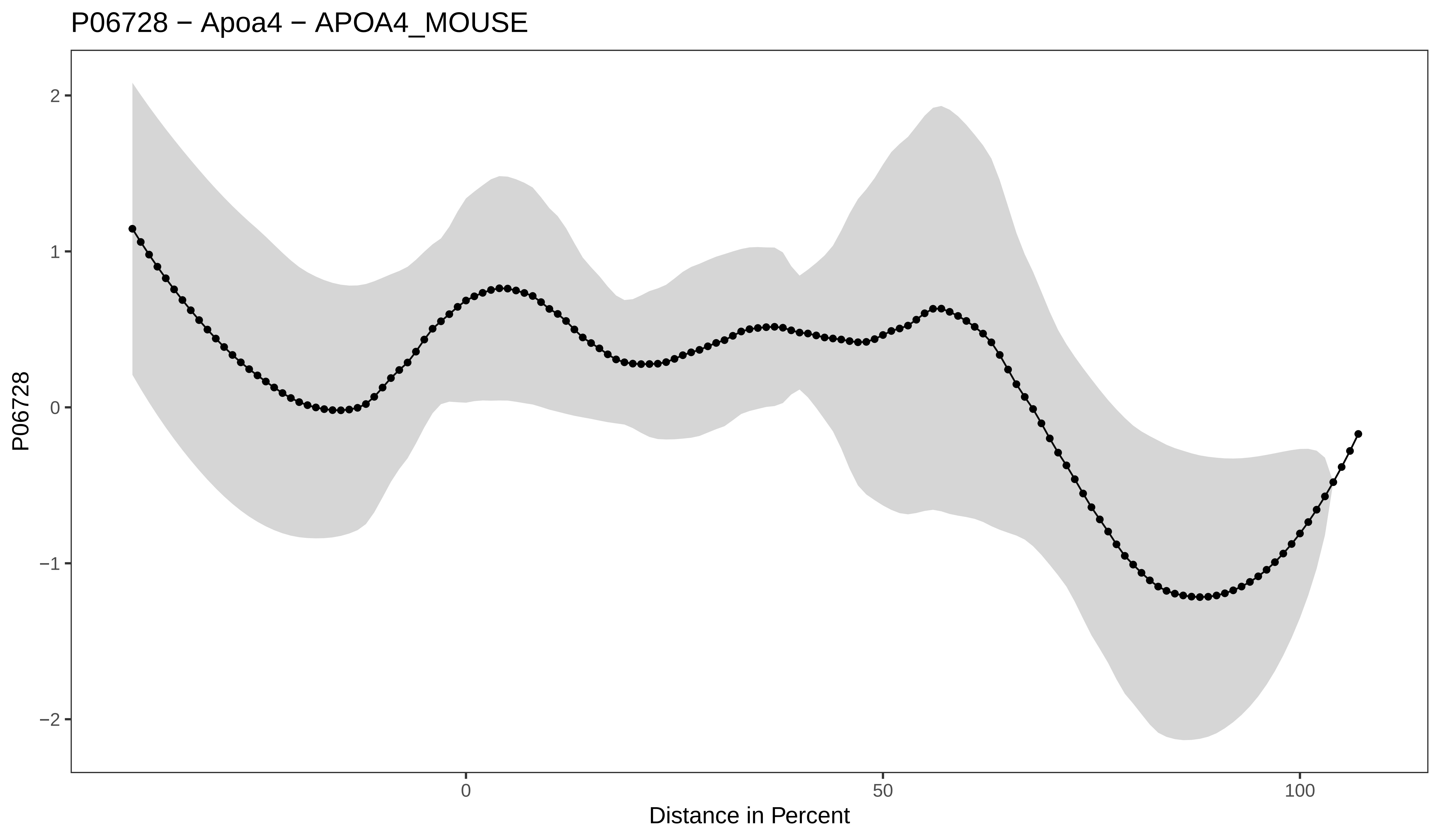

P06728 − Apoa4 − APOA4_MOUSE
P06728
Distance in Percent
2
1
0
−1
−2
0
50
100

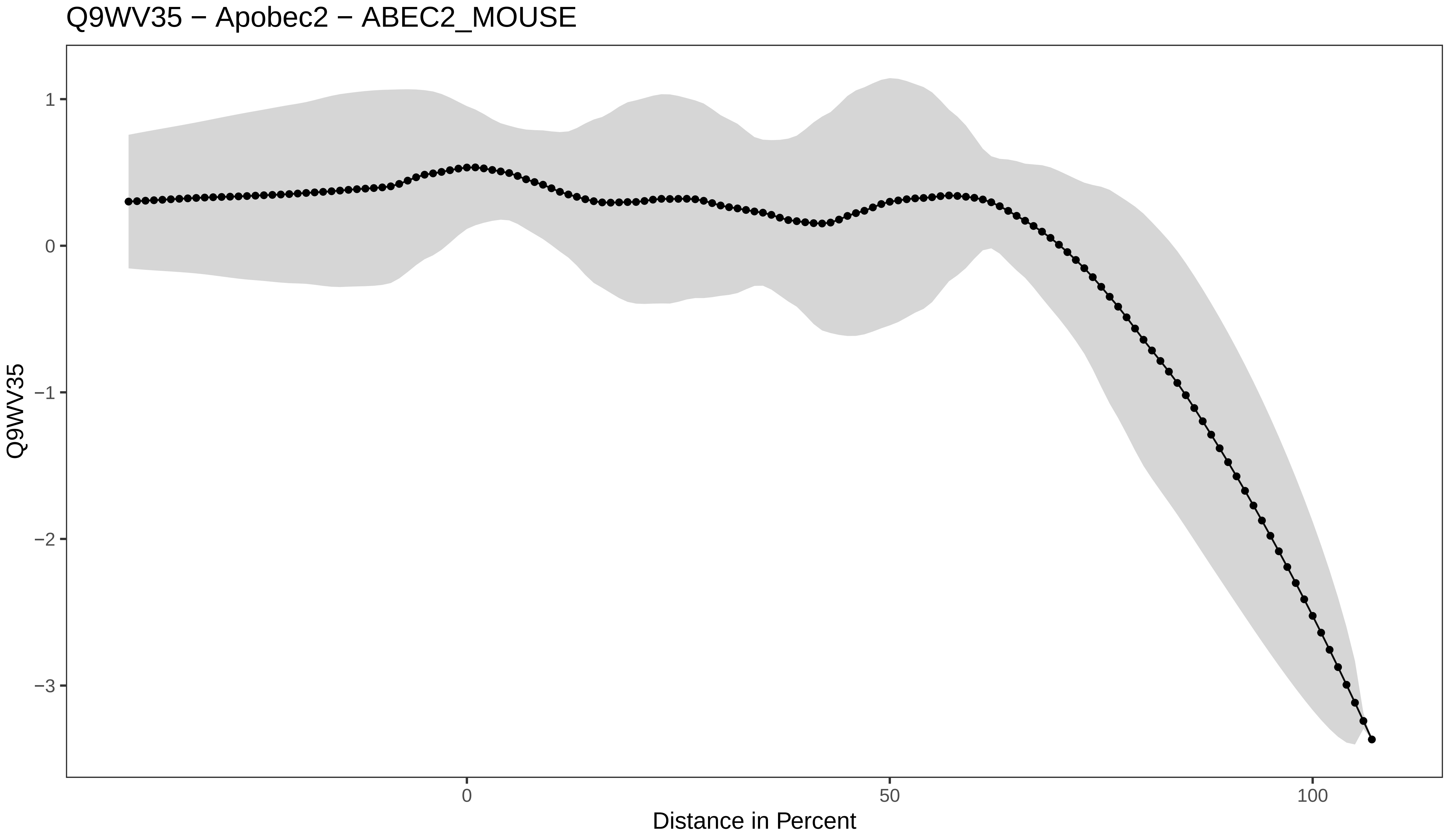

Q9WV35 − Apobec2 − ABEC2_MOUSE
Q9WV35
1
0
−1
−2
−3
0
50
100
Distance in Percent

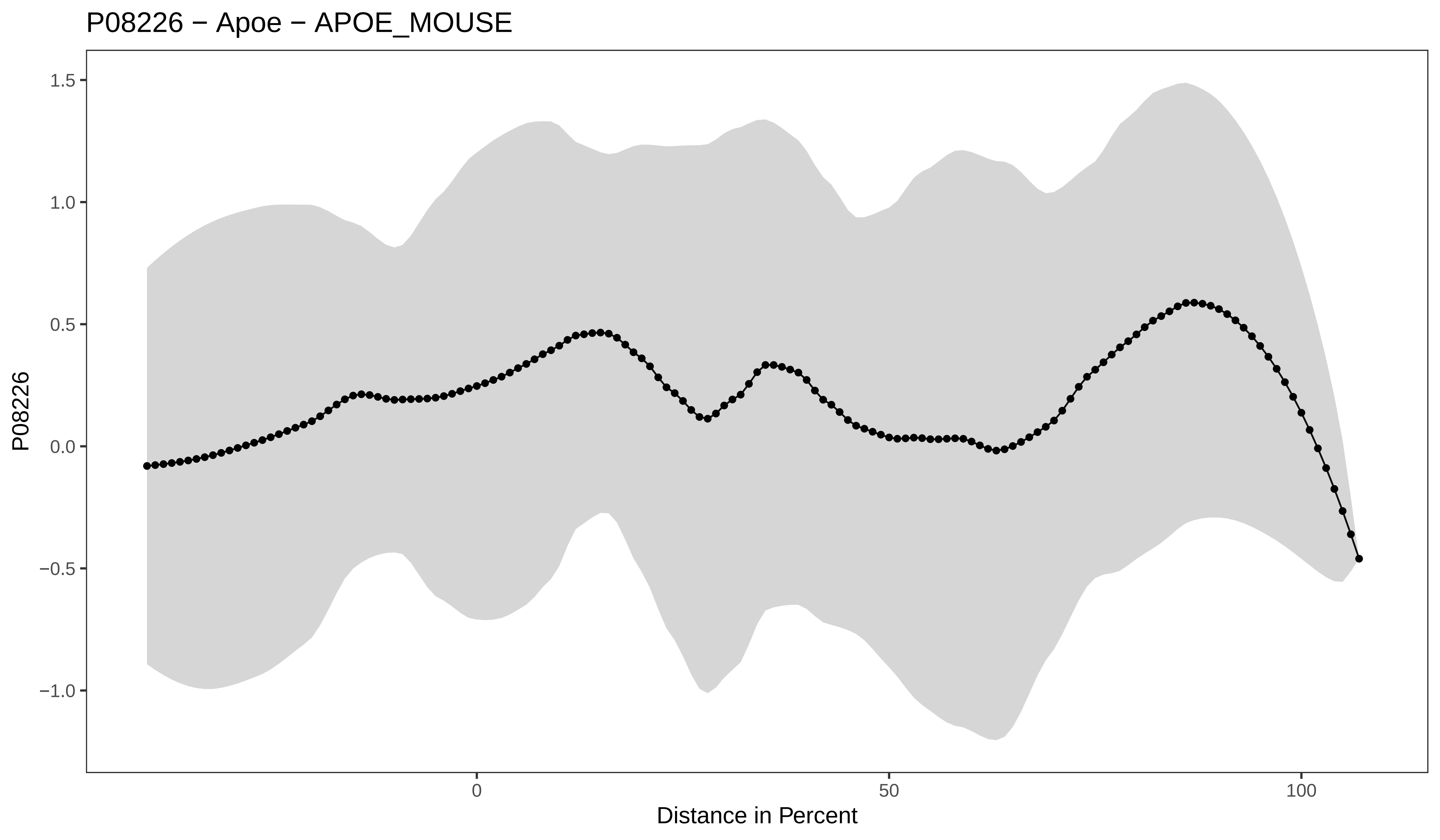
P08226 − Apoe − APOE_MOUSE
1.5
1.0
0.5
0.0
−0.5
−1.0
P08226
0
50
100
Distance in Percent

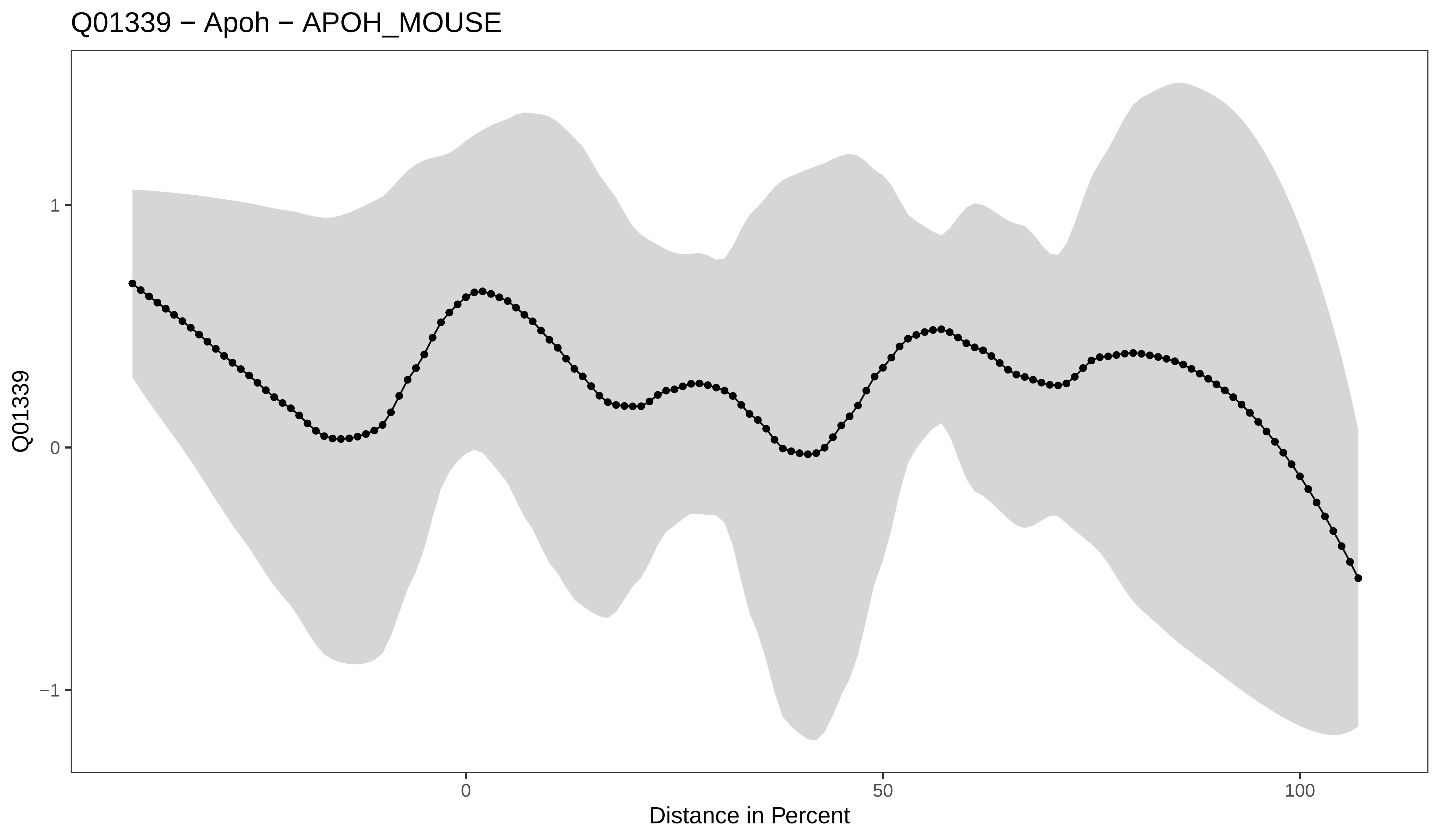
Q01339 − Apoh − APOH_MOUSE
Q01339
1
0
−1
0
50
100
Distance in Percent

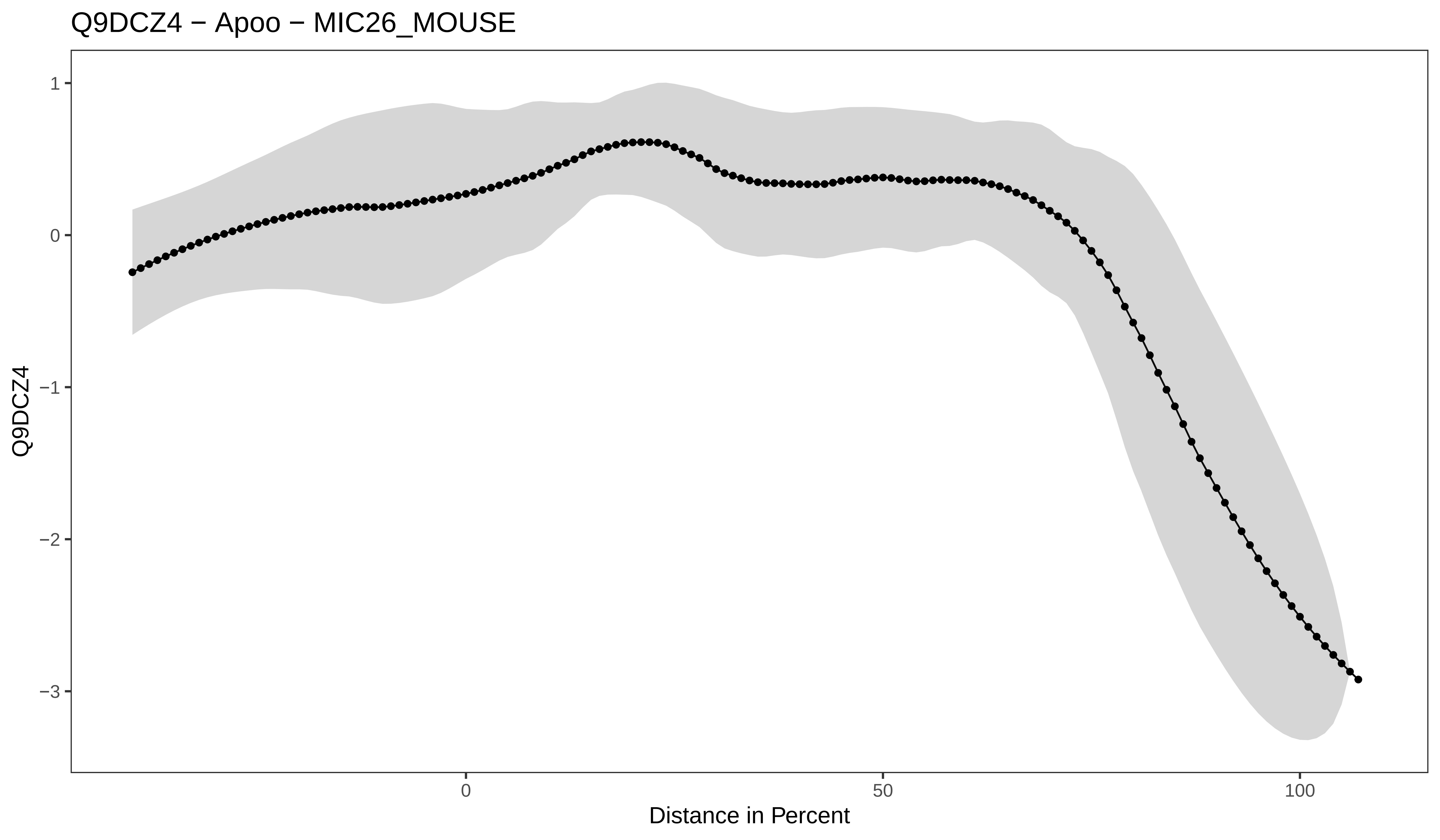

Q9DCZ4 − Apoo − MIC26_MOUSE
Q9DCZ4
1
0
−1
−2
−3
0
50
100
Distance in Percent

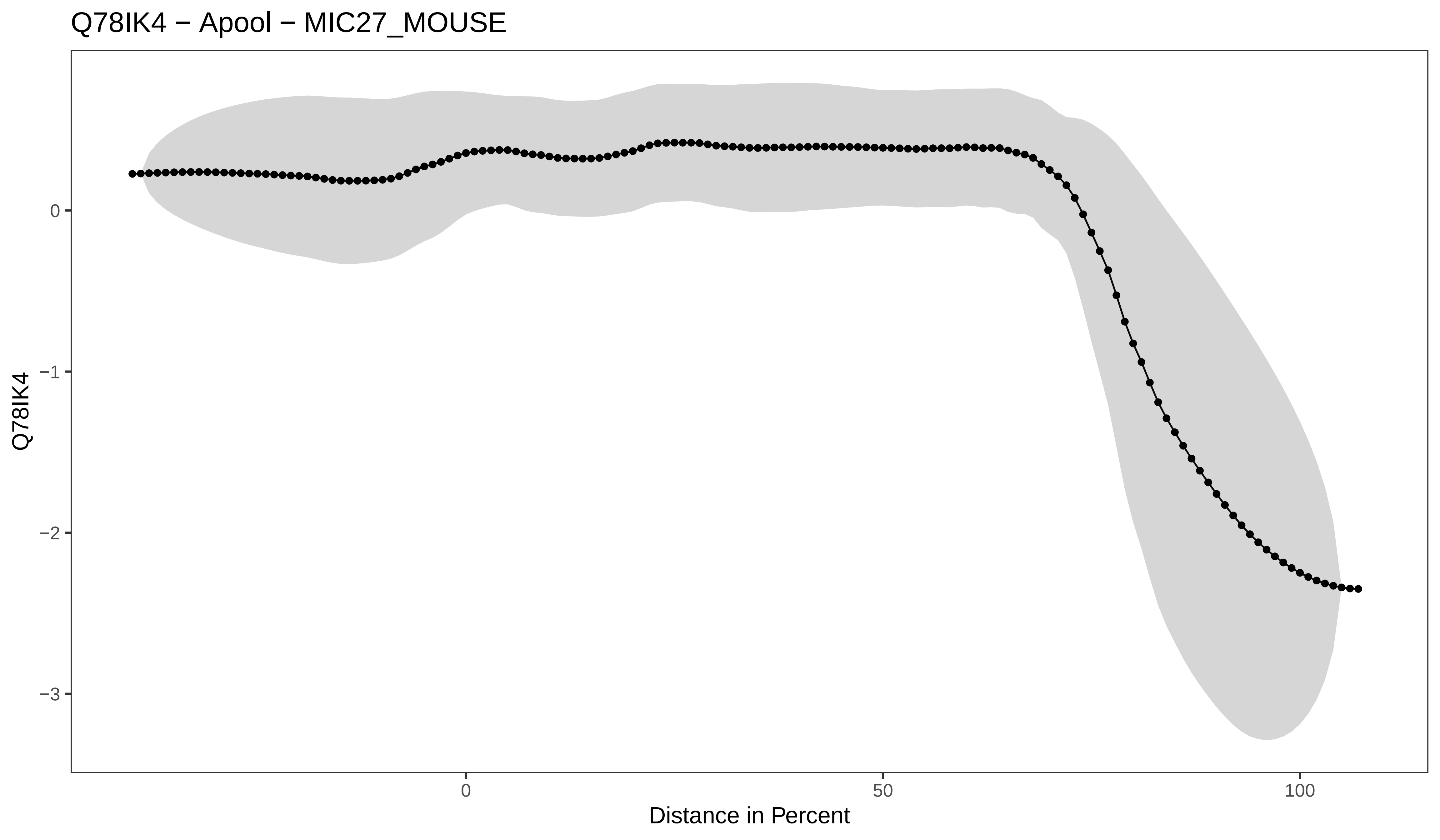

Q78IK4 − Apool − MIC27_MOUSE
Q78IK4
0
−1
−2
−3
0
50
100
Distance in Percent

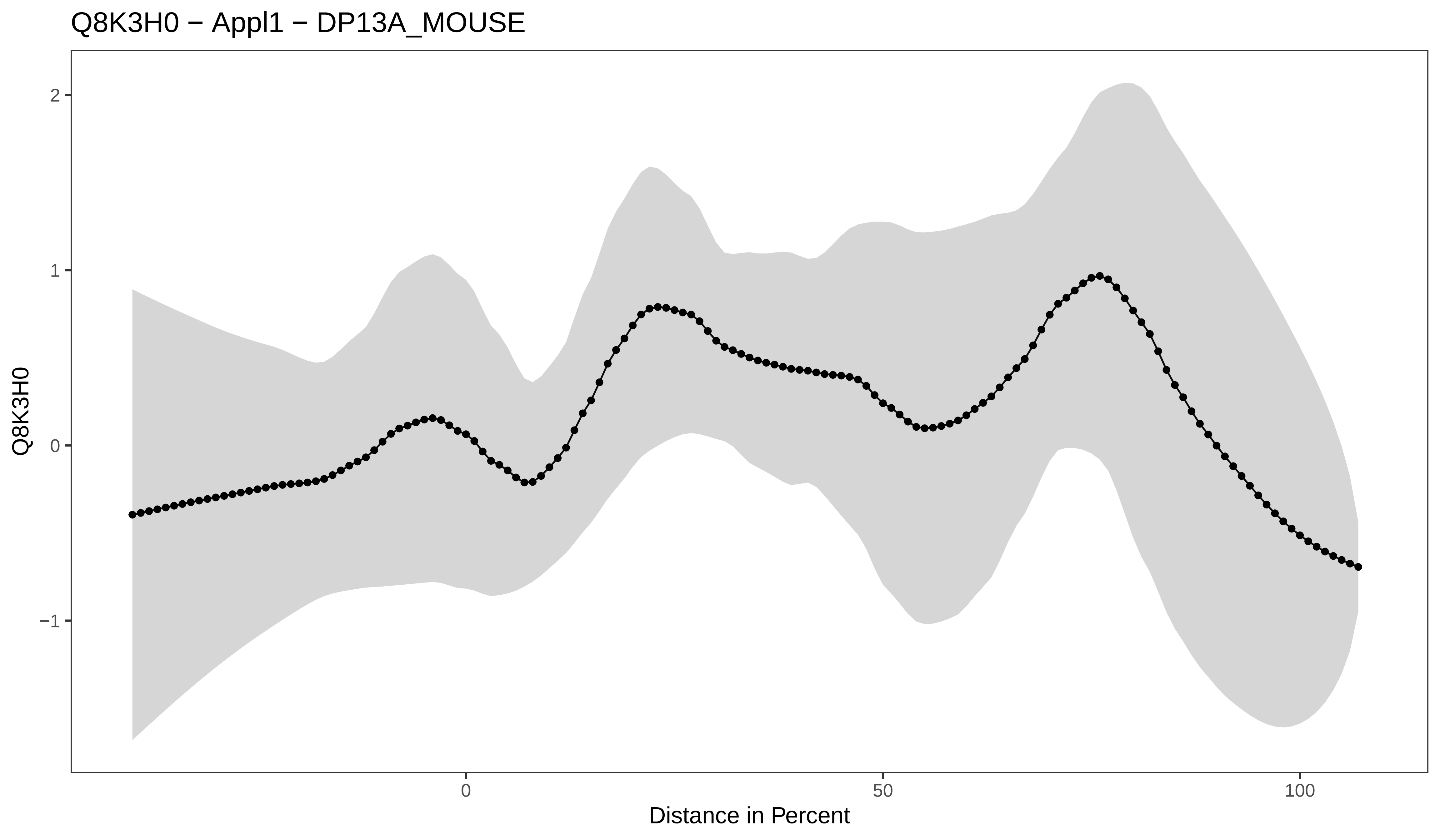

Q8K3H0 − Appl1 − DP13A_MOUSE
Q8K3H0
2
1
0
−1
0
50
100
Distance in Percent

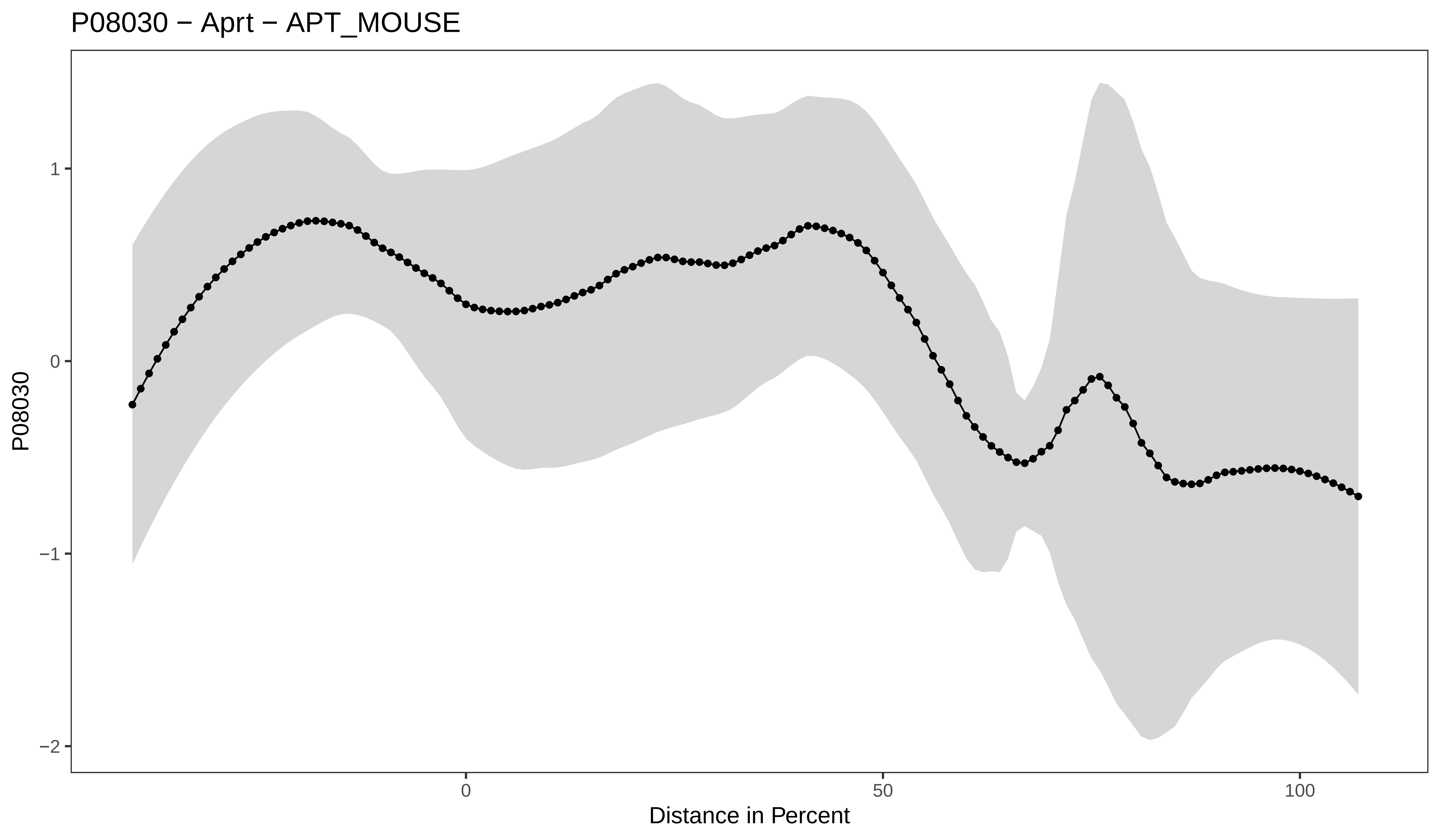
P08030 − Aprt − APT_MOUSE
P08030
1
0
−1
−2
0
50
100
Distance in Percent

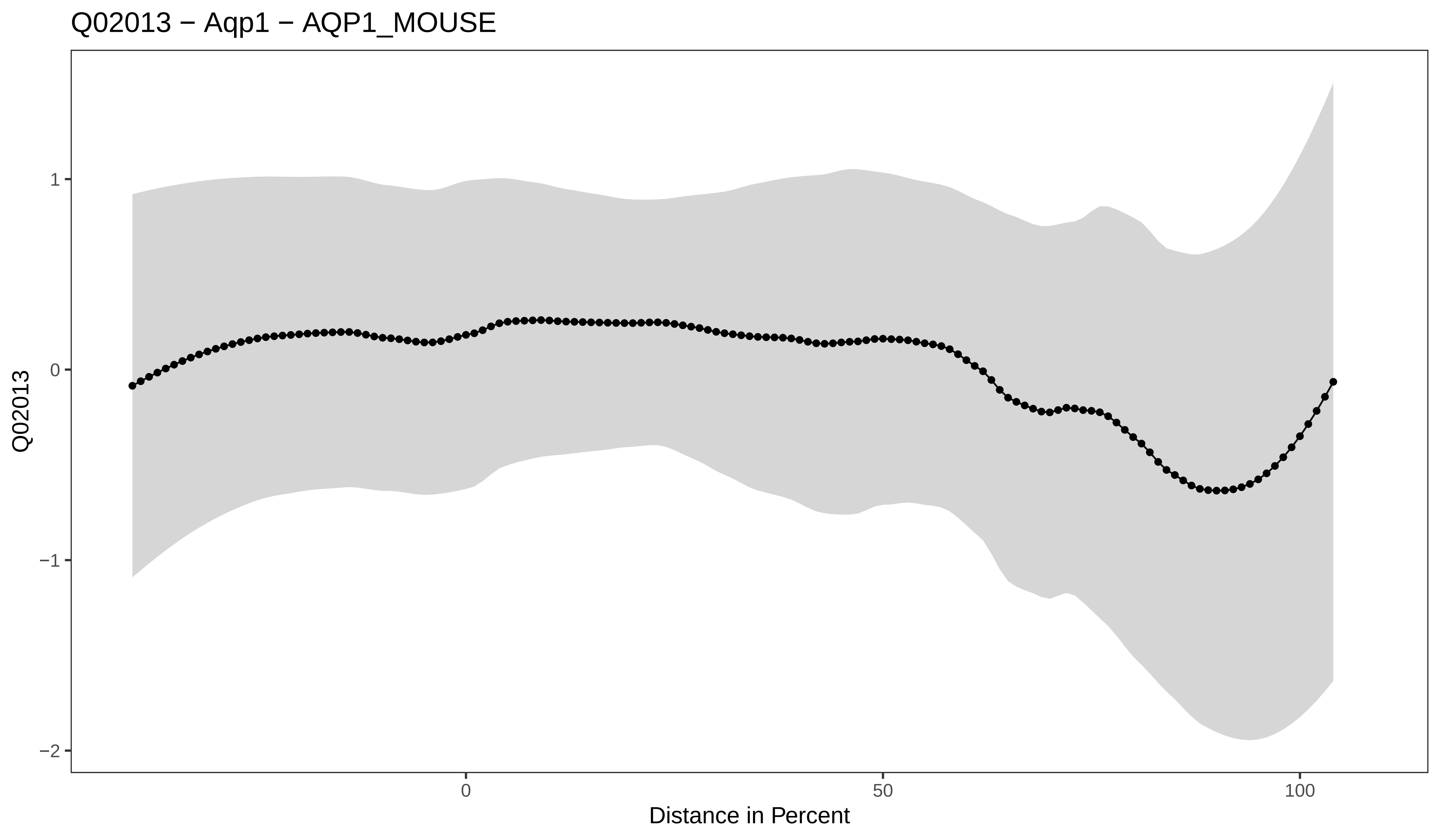
Q02013 − Aqp1 − AQP1_MOUSE
Q02013
1
0
−1
−2
0
50
100
Distance in Percent

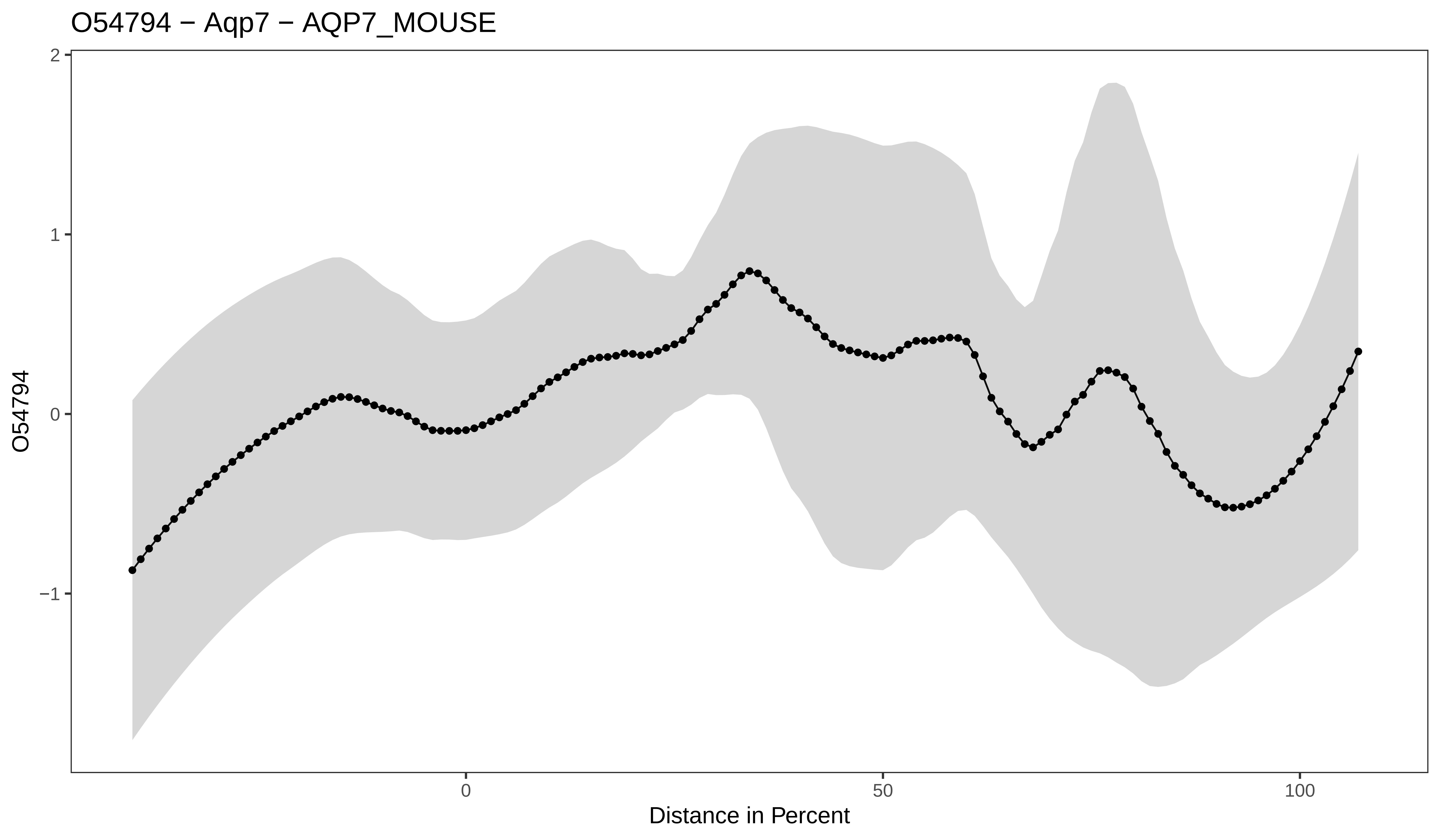

O54794 − Aqp7 − AQP7_MOUSE
O54794
Distance in Percent
2
1
0
−1
0
50
100

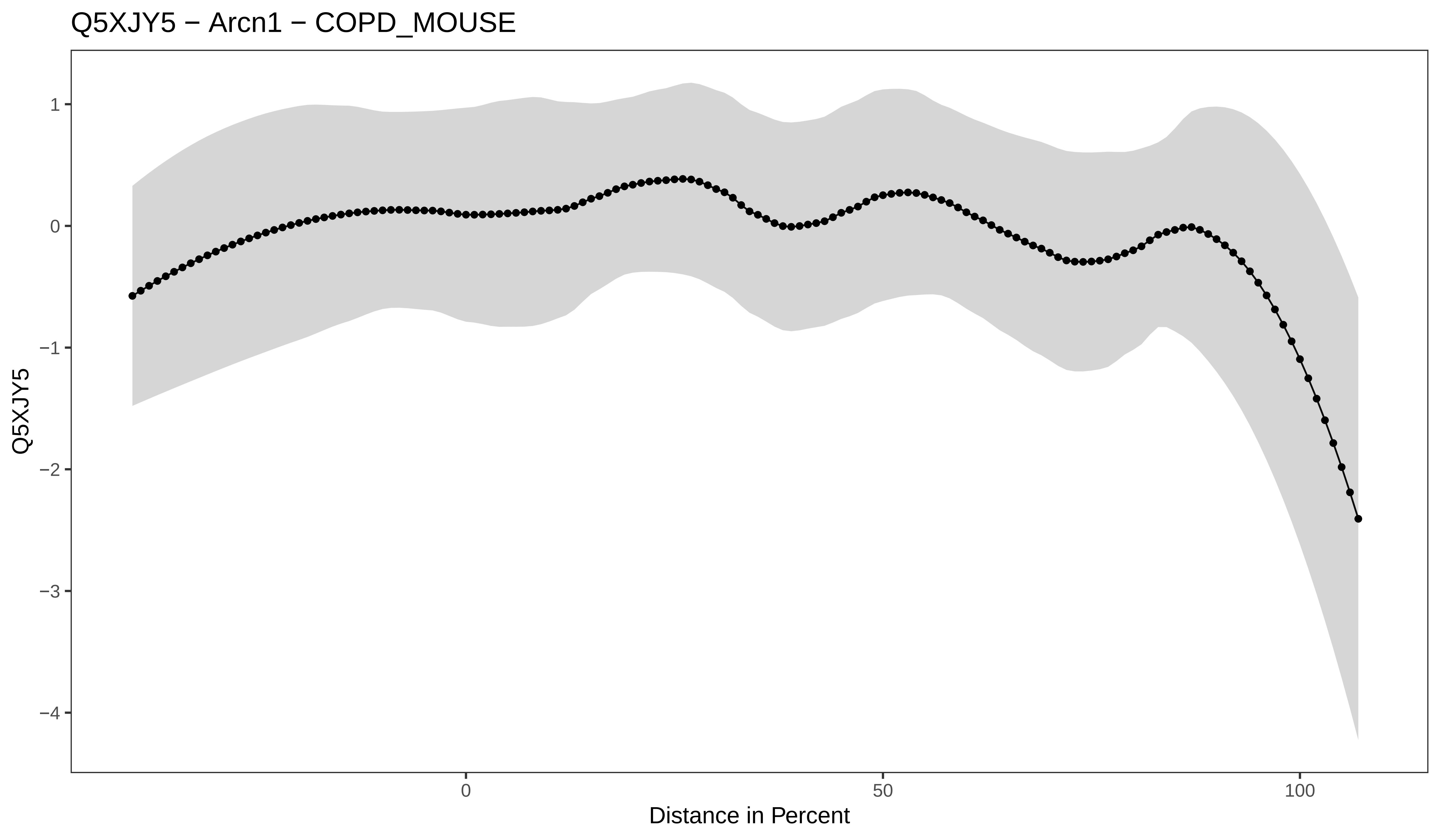

Q5XJY5 − Arcn1 − COPD_MOUSE
1
0
−1
−2
−3
−4
Q5XJY5
0
50
100
Distance in Percent

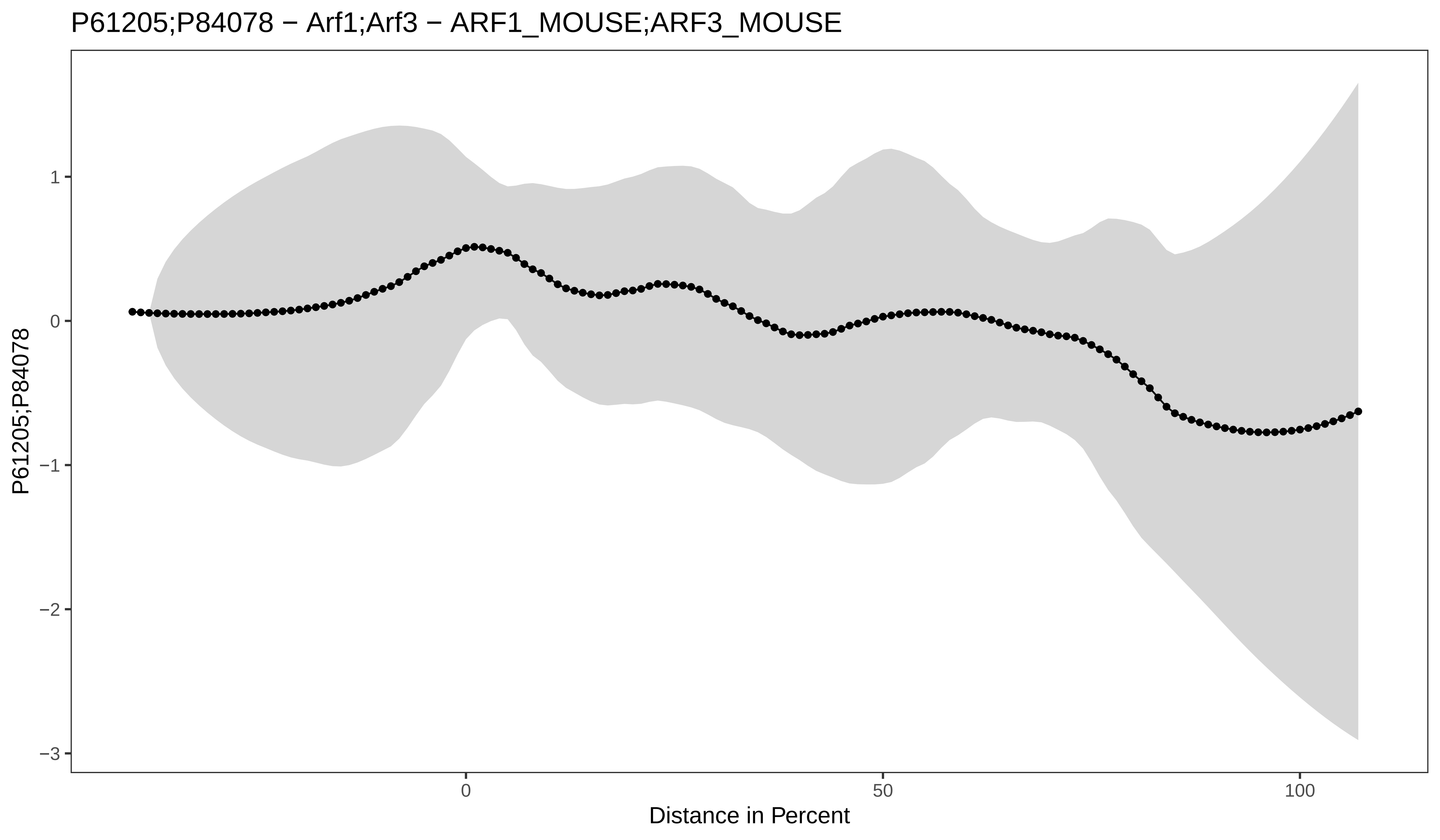
P61205;P84078 − Arf1;Arf3 − ARF1_MOUSE;ARF3_MOUSE
P61205;P84078
1
0
−1
−2
−3
0
50
100
Distance in Percent

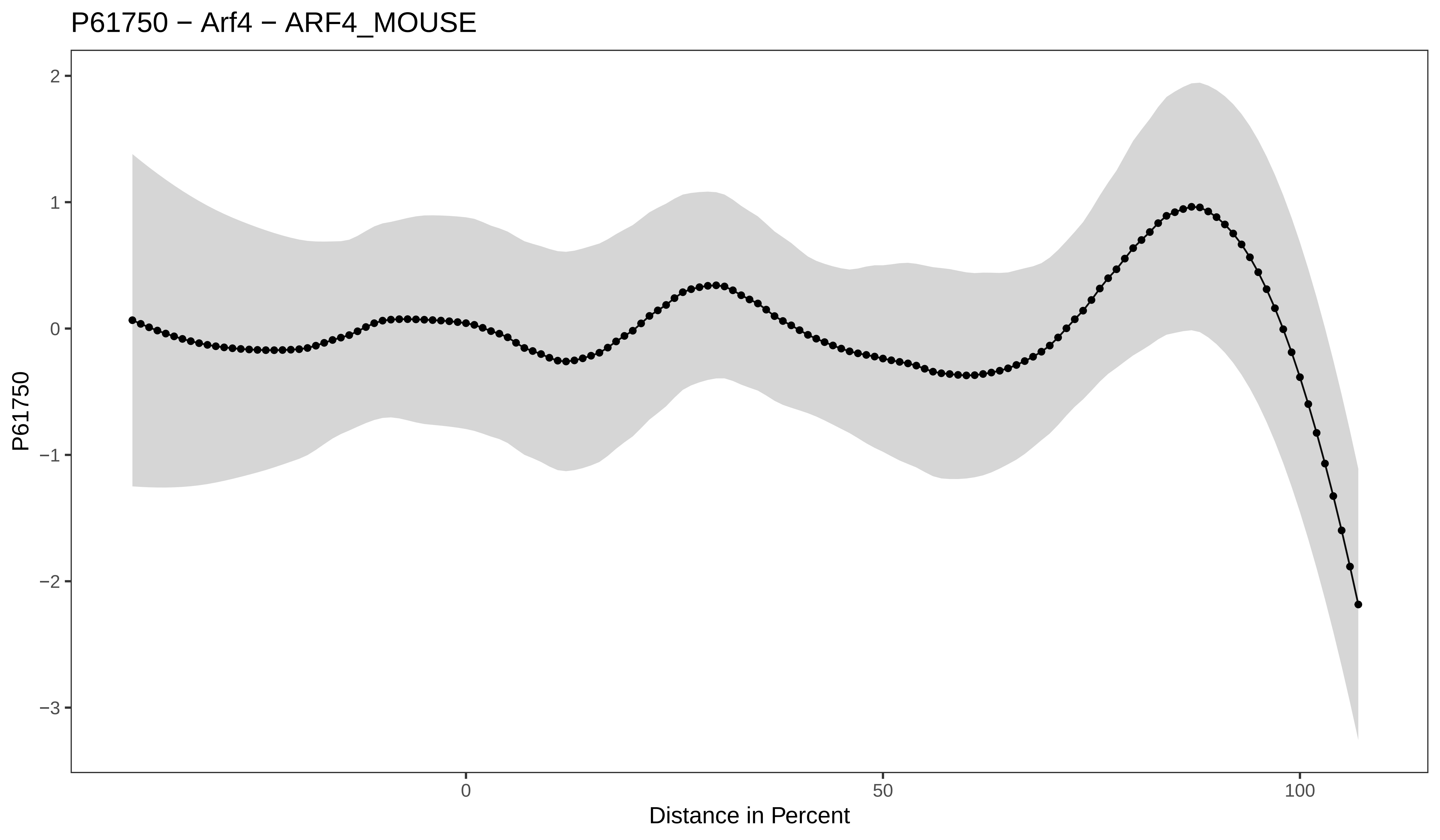

P61750 − Arf4 − ARF4_MOUSE
2
1
0
−1
−2
−3
P61750
0
50
100
Distance in Percent

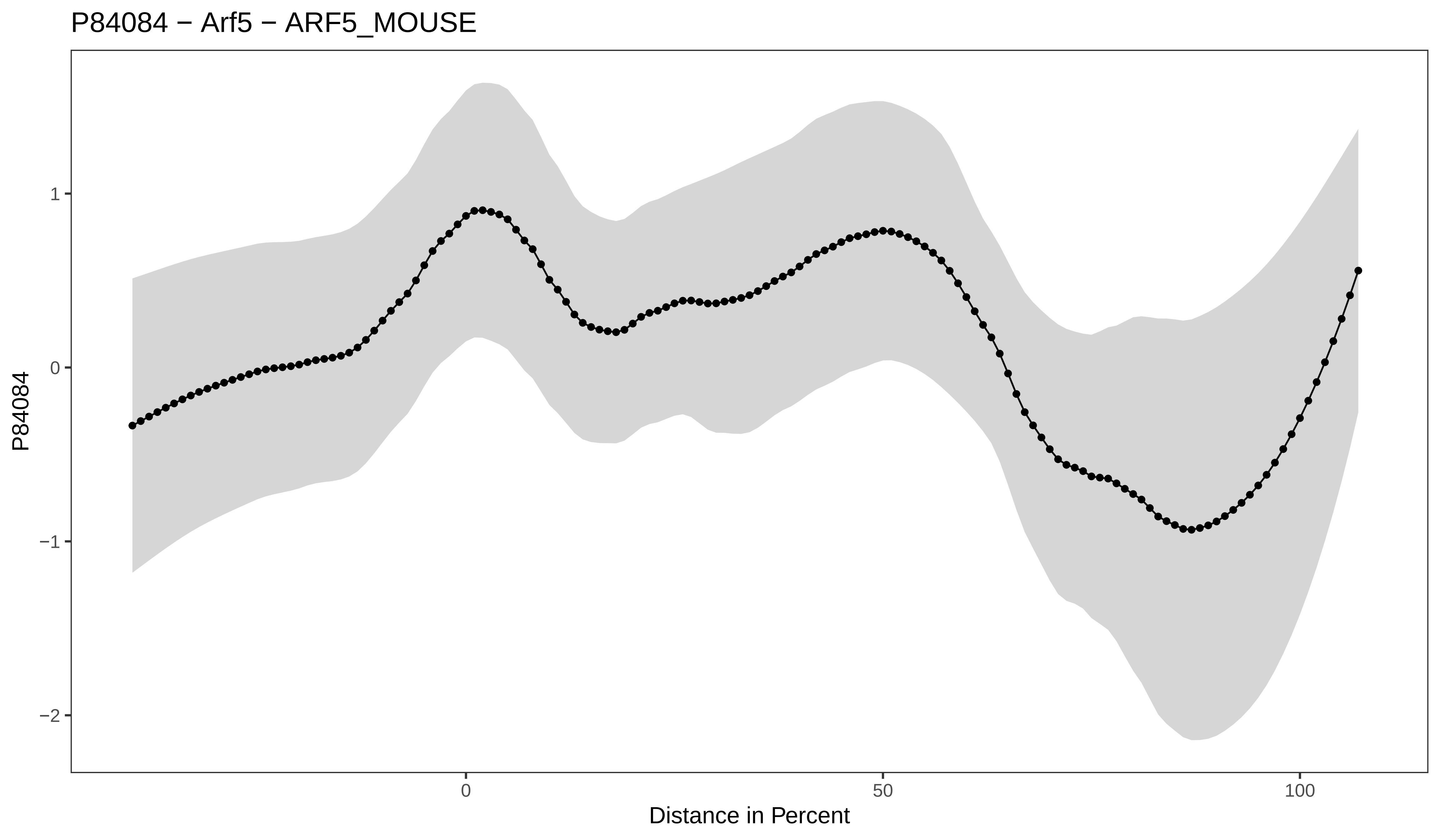
P84084 − Arf5 − ARF5_MOUSE
P84084
1
0
−1
−2
0
50
100
Distance in Percent

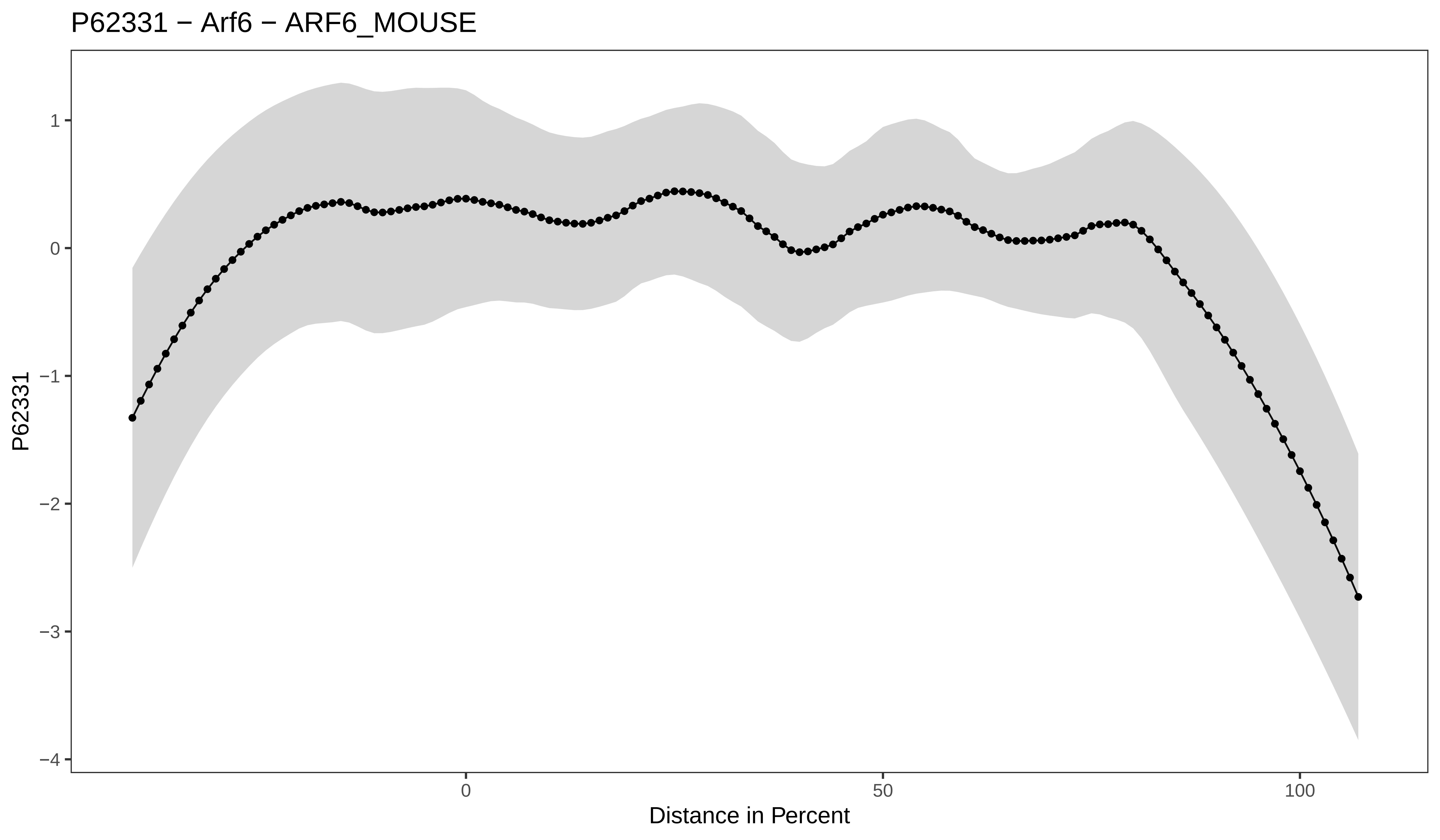

P62331 − Arf6 − ARF6_MOUSE
P62331
1
0
−1
−2
−3
−4
0
50
100
Distance in Percent

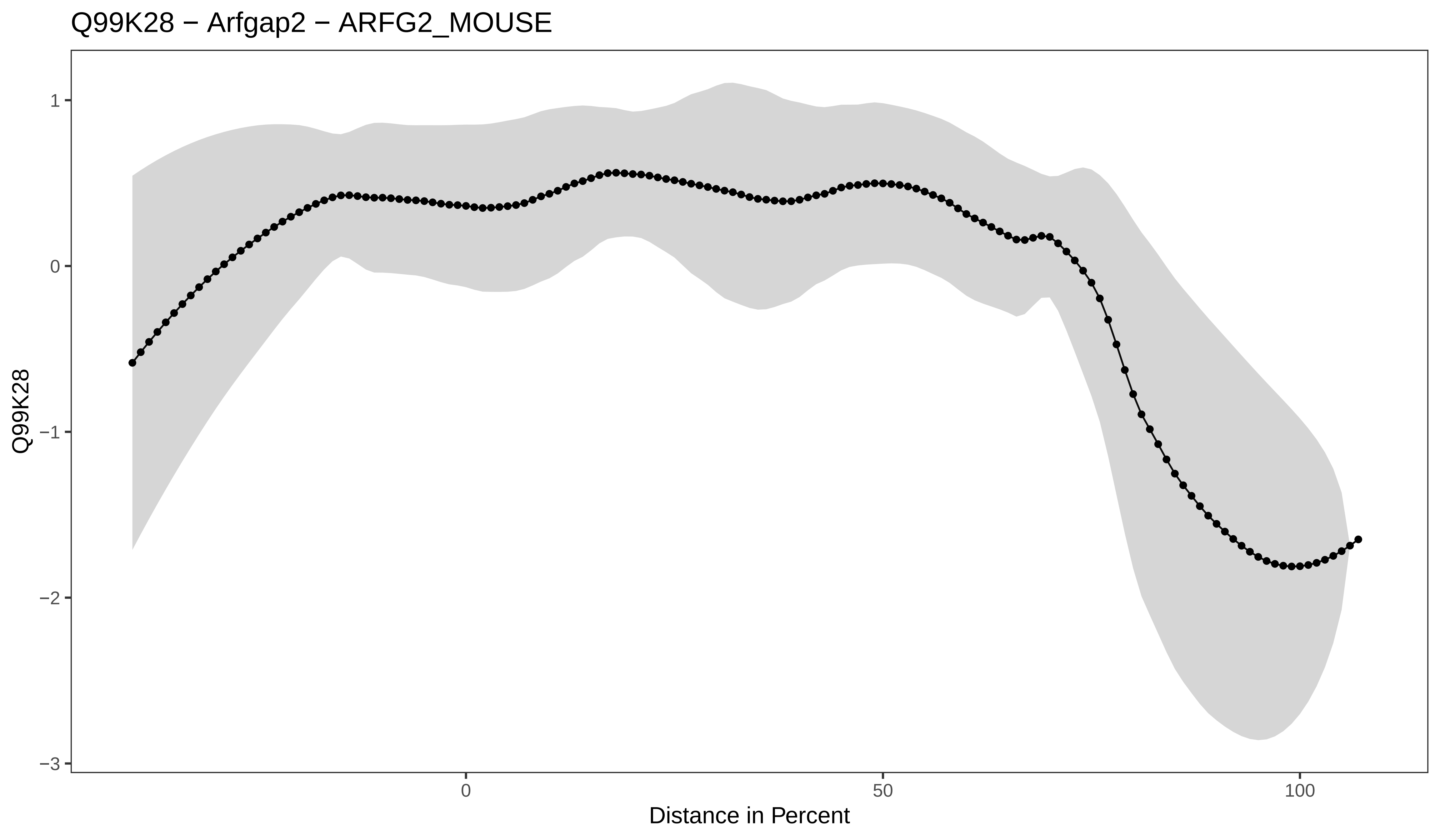
Q99K28 − Arfgap2 − ARFG2_MOUSE
Q99K28
1
0
−1
−2
−3
0
50
100
Distance in Percent

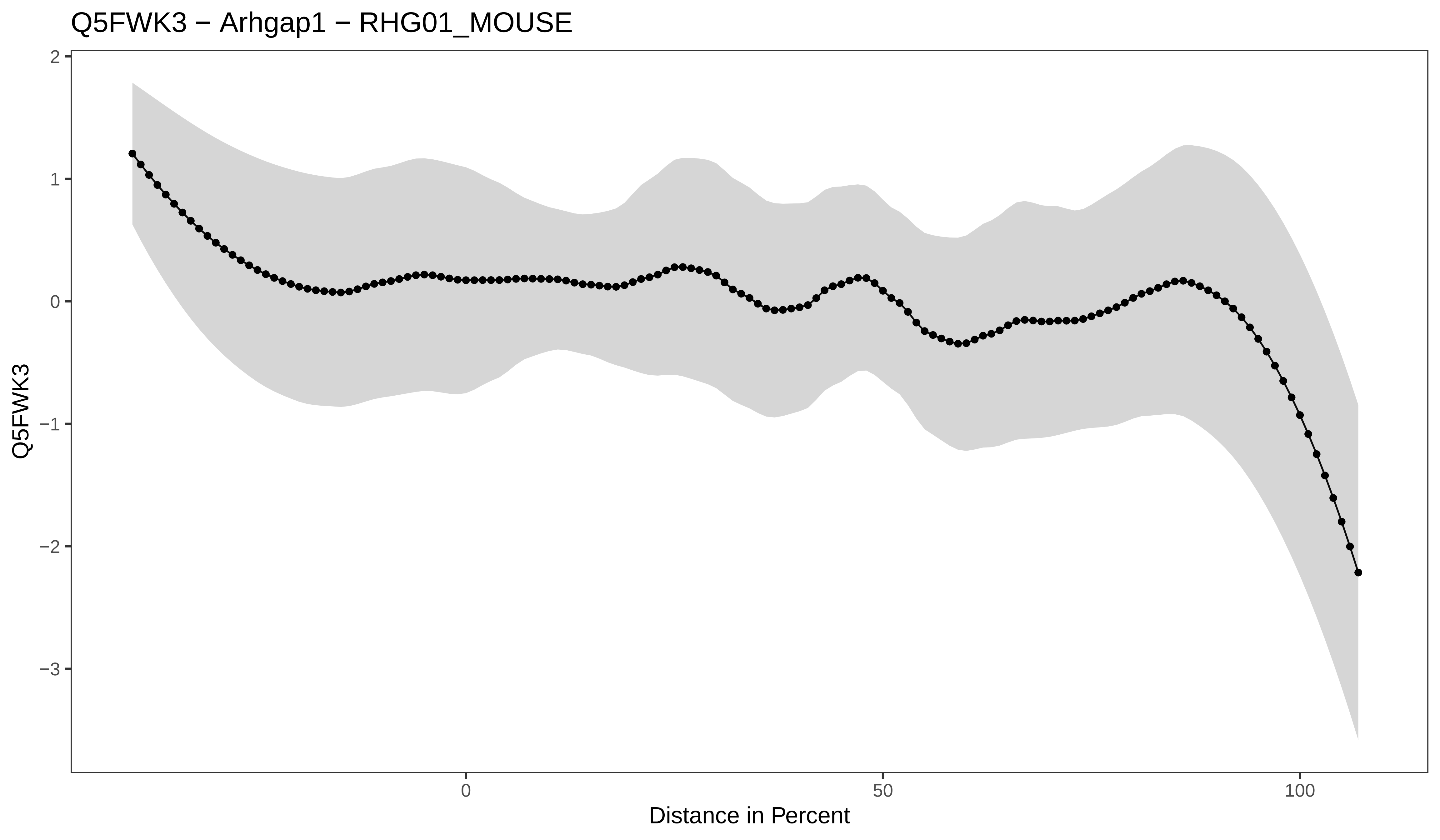

Q5FWK3 − Arhgap1 − RHG01_MOUSE
Q5FWK3
Distance in Percent
2
1
0
−1
−2
−3
0
50
100

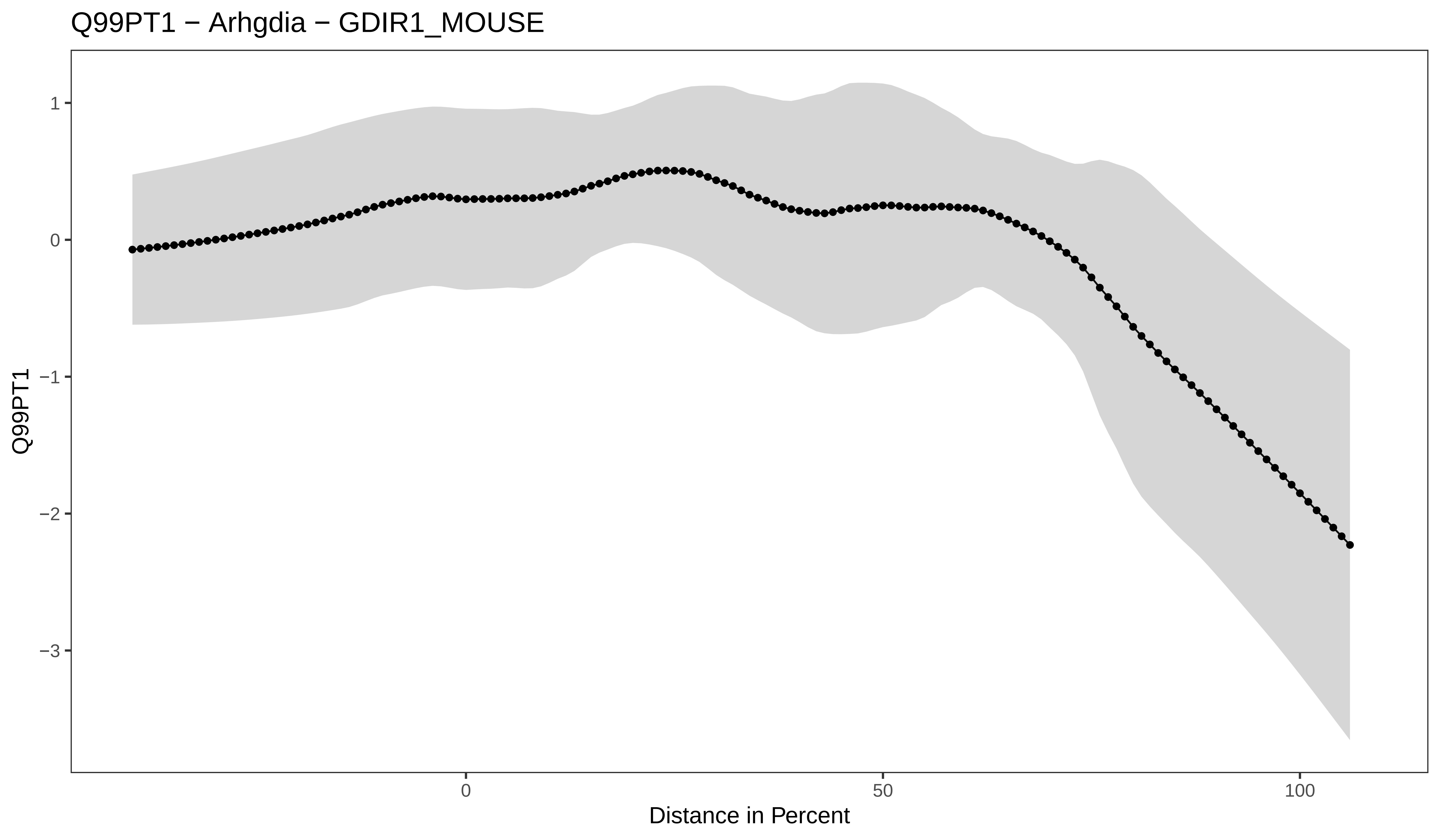
Q99PT1 − Arhgdia − GDIR1_MOUSE
Q99PT1
1
0
−1
−2
−3
0
50
100
Distance in Percent

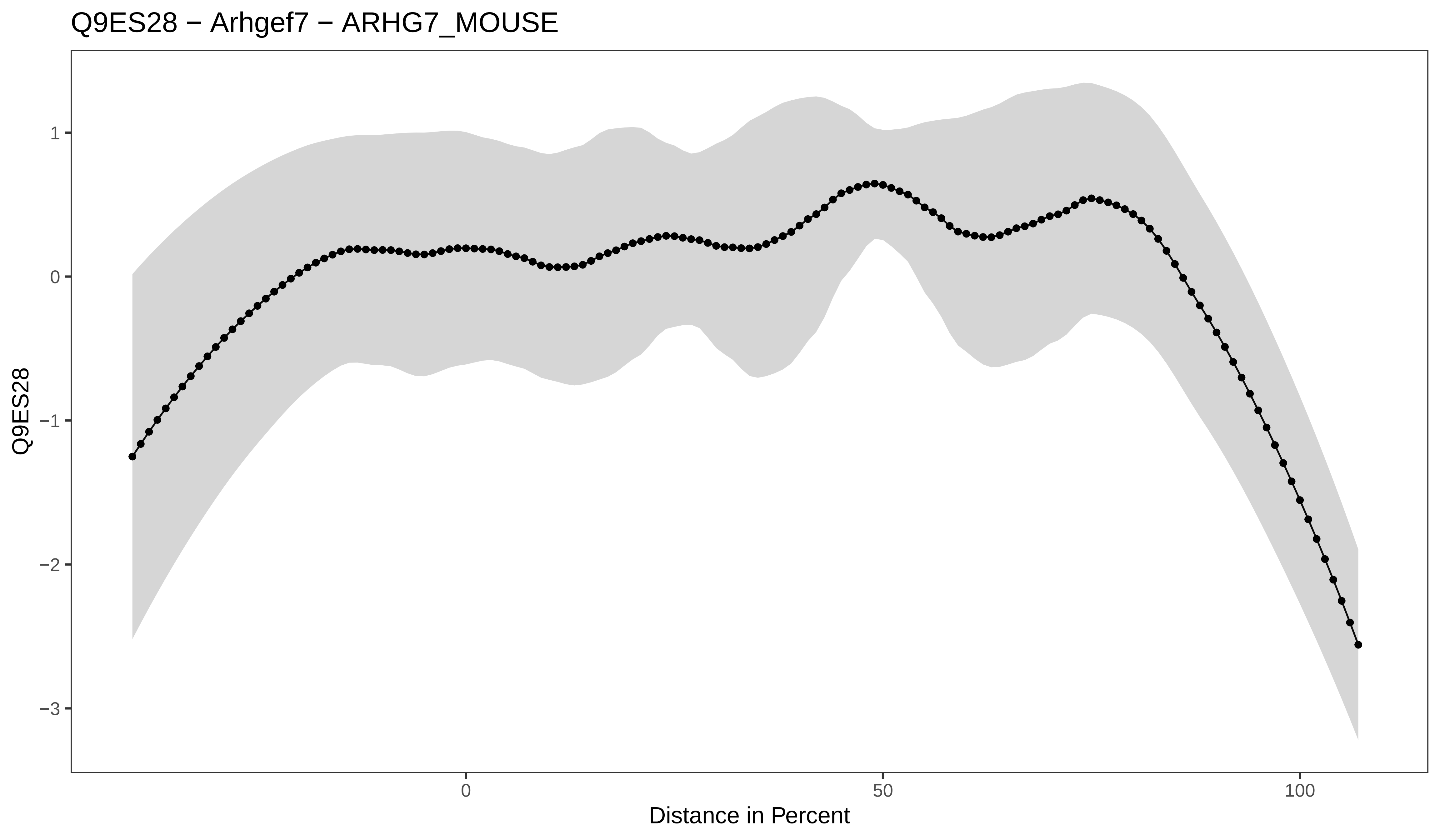

Q9ES28 − Arhgef7 − ARHG7_MOUSE
Q9ES28
Distance in Percent
1
0
−1
−2
−3
0
50
100

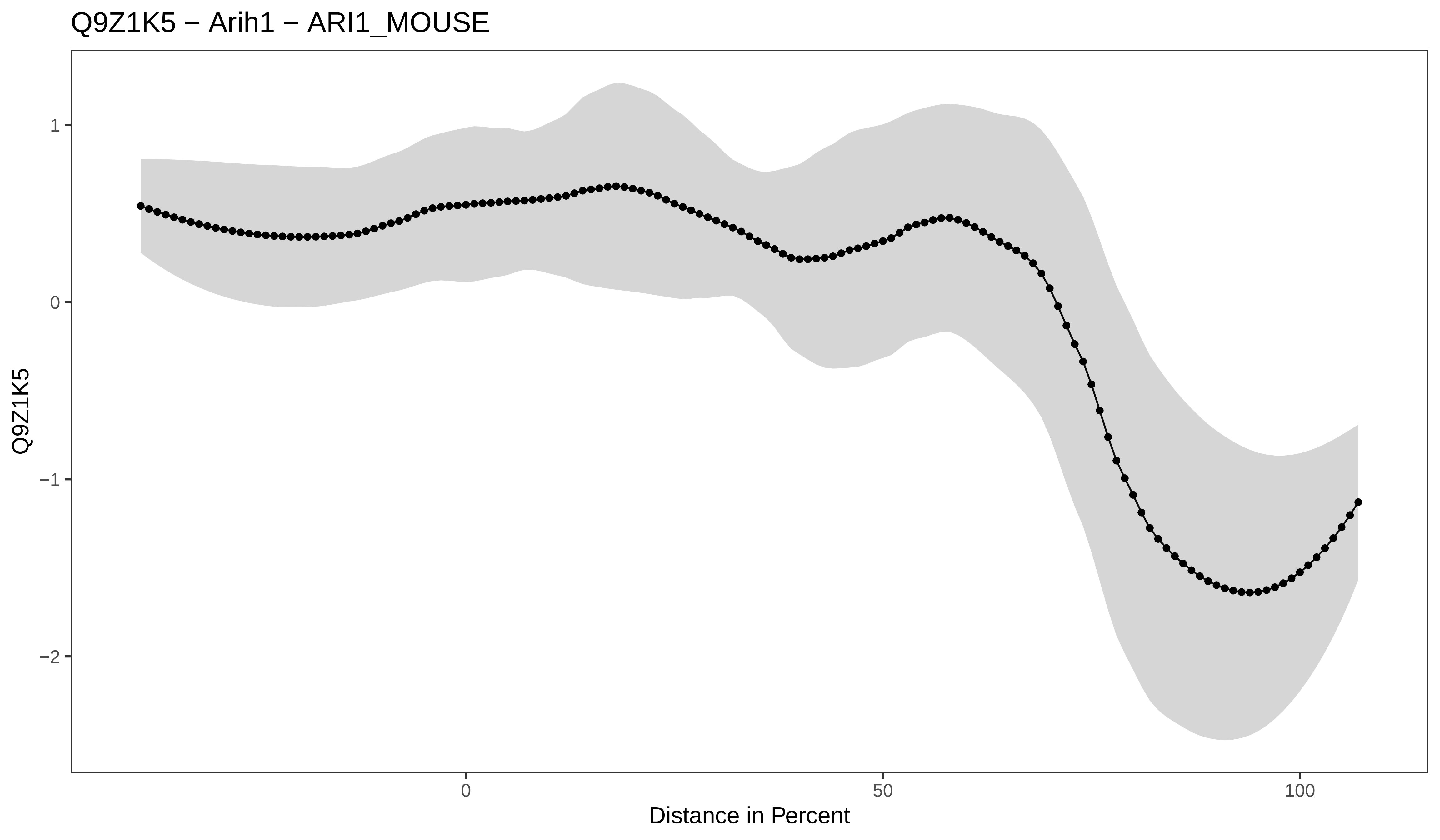
Q9Z1K5 − Arih1 − ARI1_MOUSE
Q9Z1K5
Distance in Percent
1
0
−1
−2
0
50
100

P61211 − Arl1 − ARL1_MOUSE

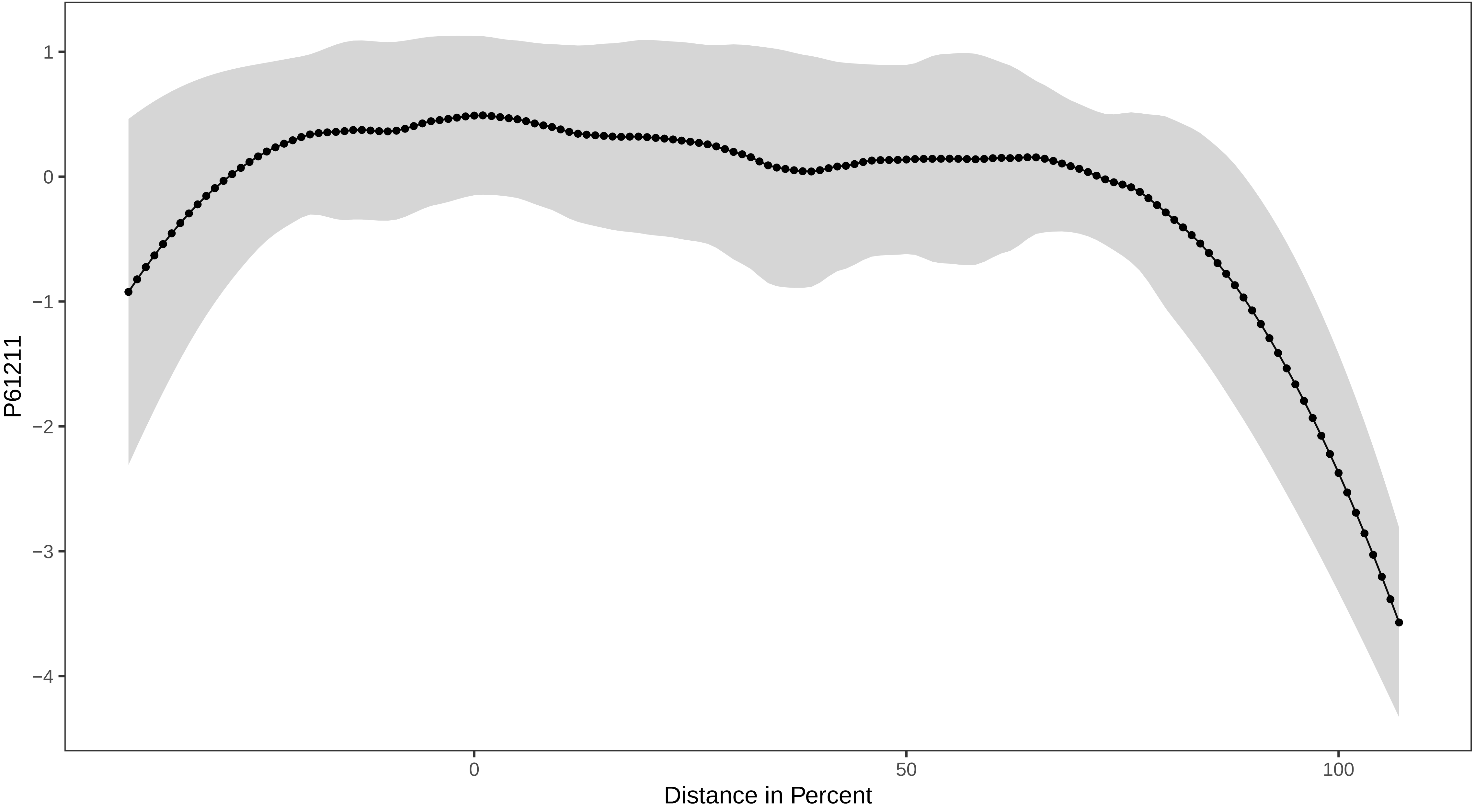

Q9D0J4 − Arl2 − ARL2_MOUSE

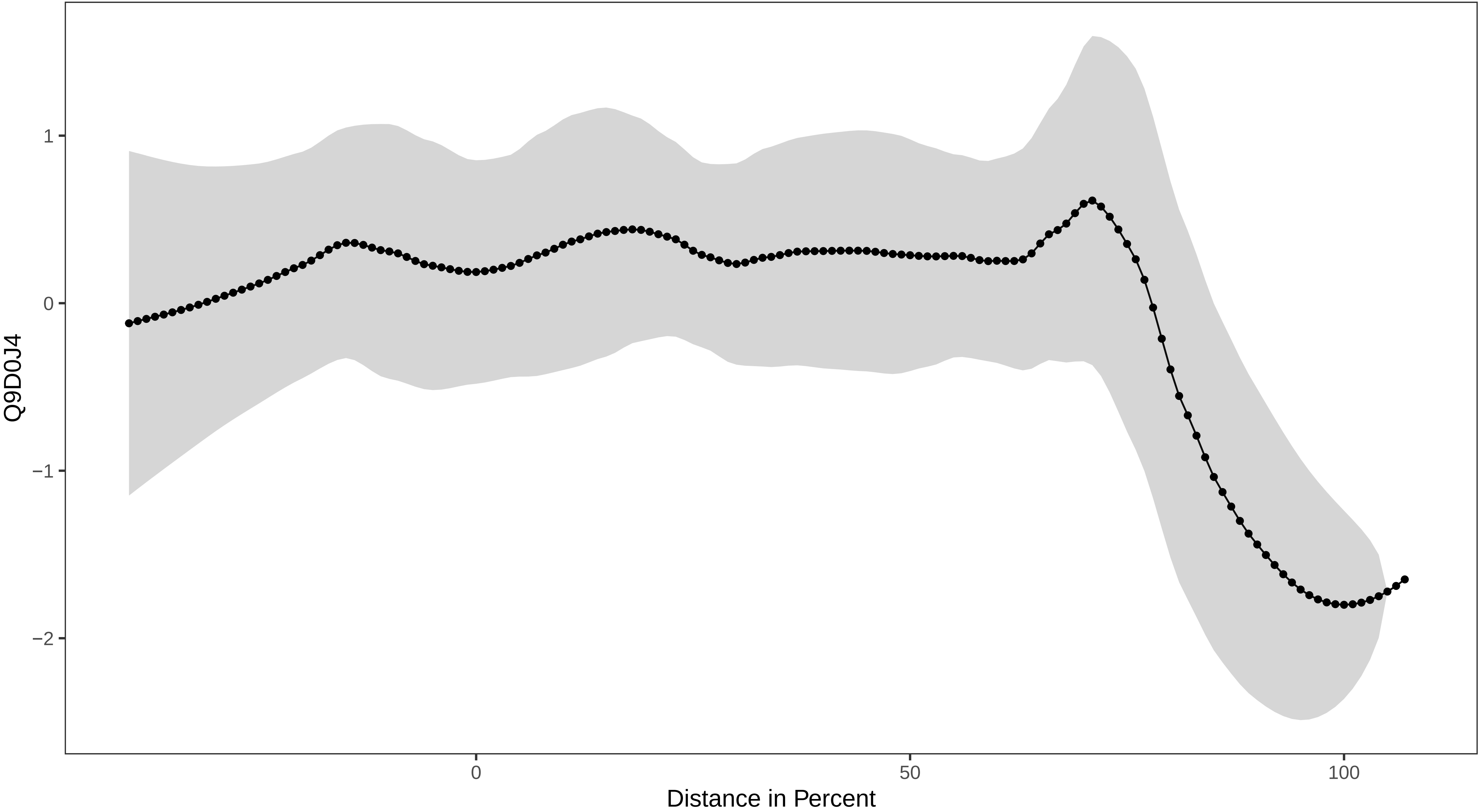

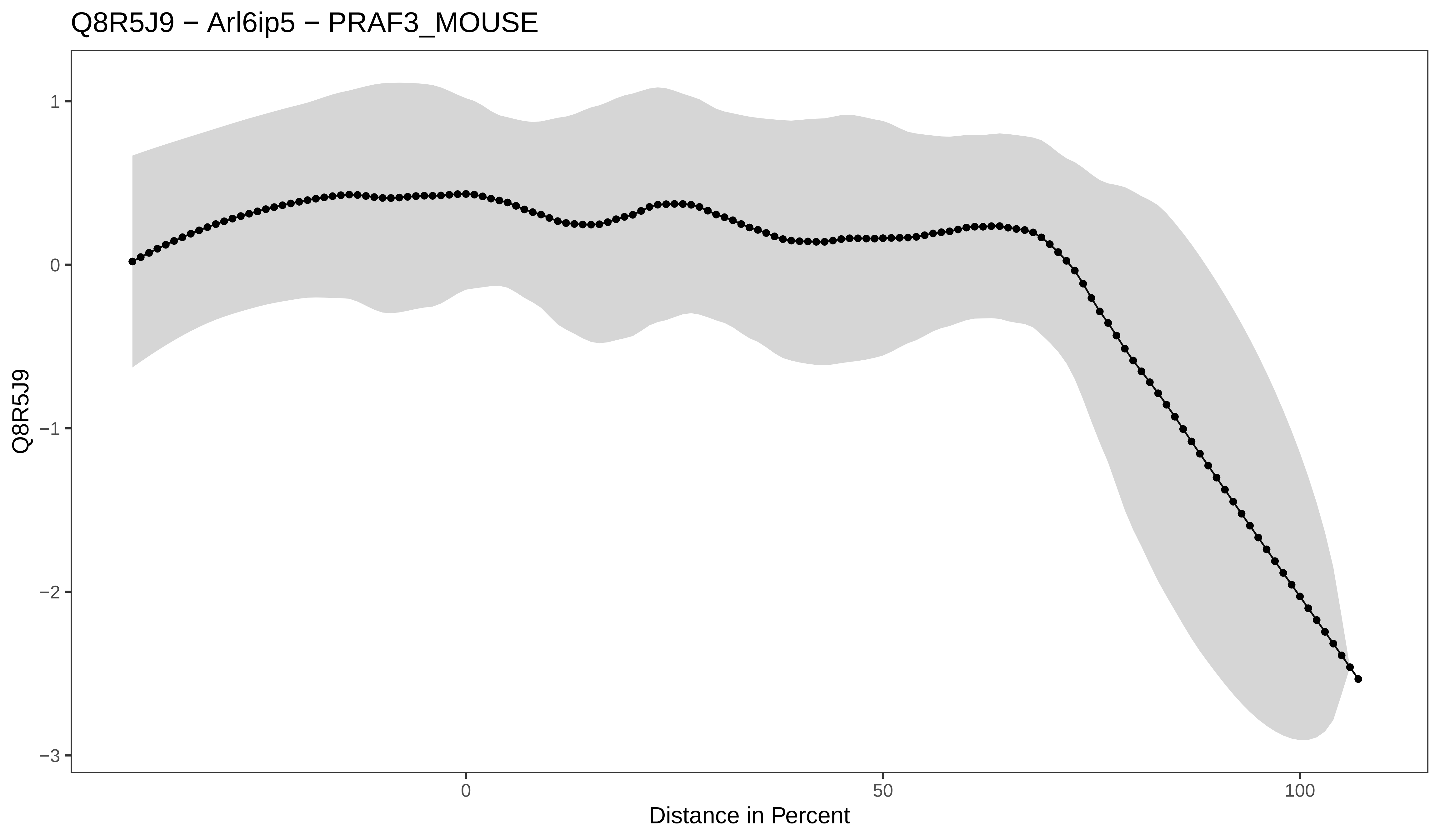

Q8R5J9 − Arl6ip5 − PRAF3_MOUSE
1
0
−1
−2
−3
Q8R5J9
0
50
100
Distance in Percent

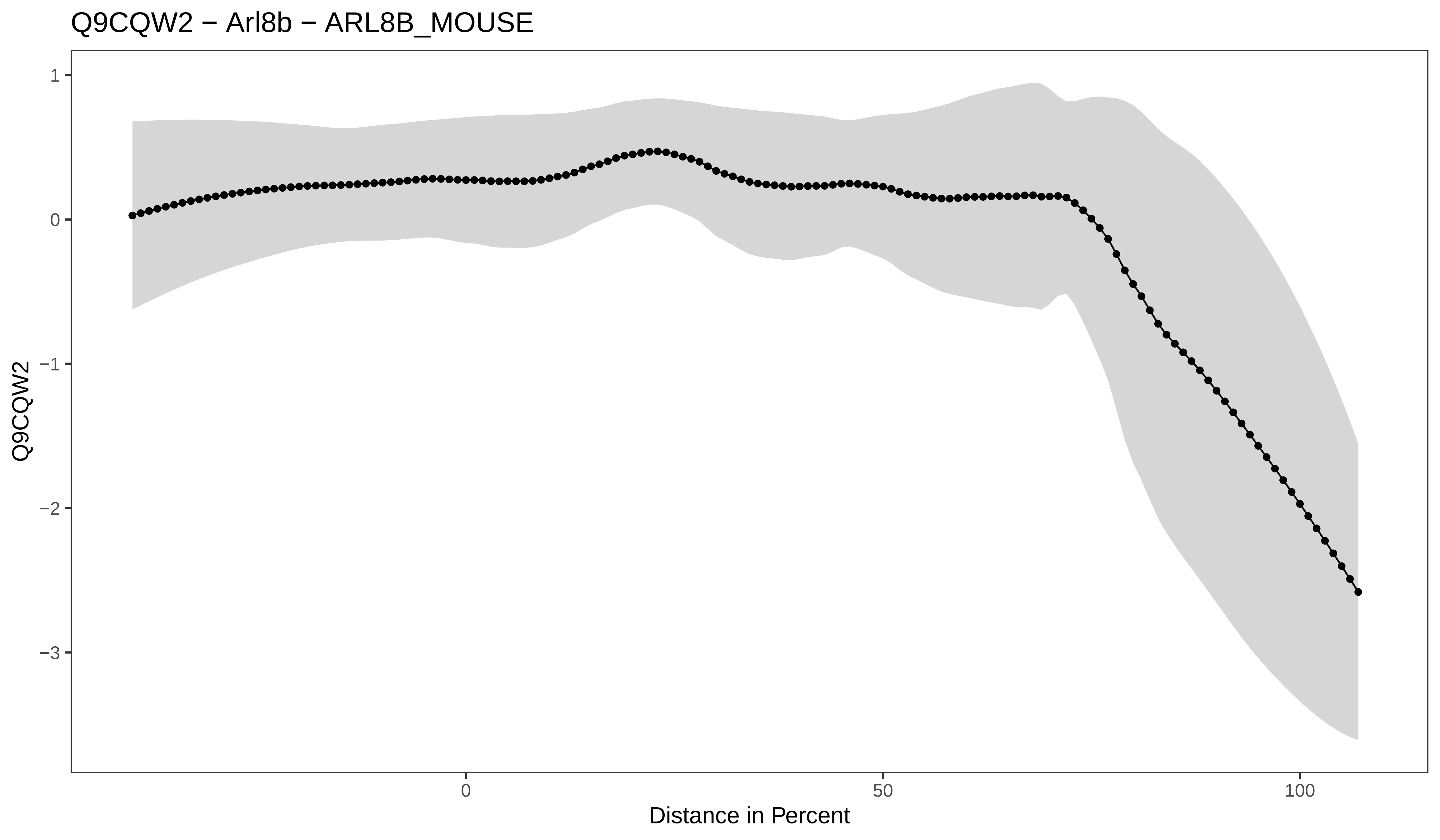

Q9CQW2 − Arl8b − ARL8B_MOUSE
1
0
−1
−2
−3
Q9CQW2
0
50
100
Distance in Percent

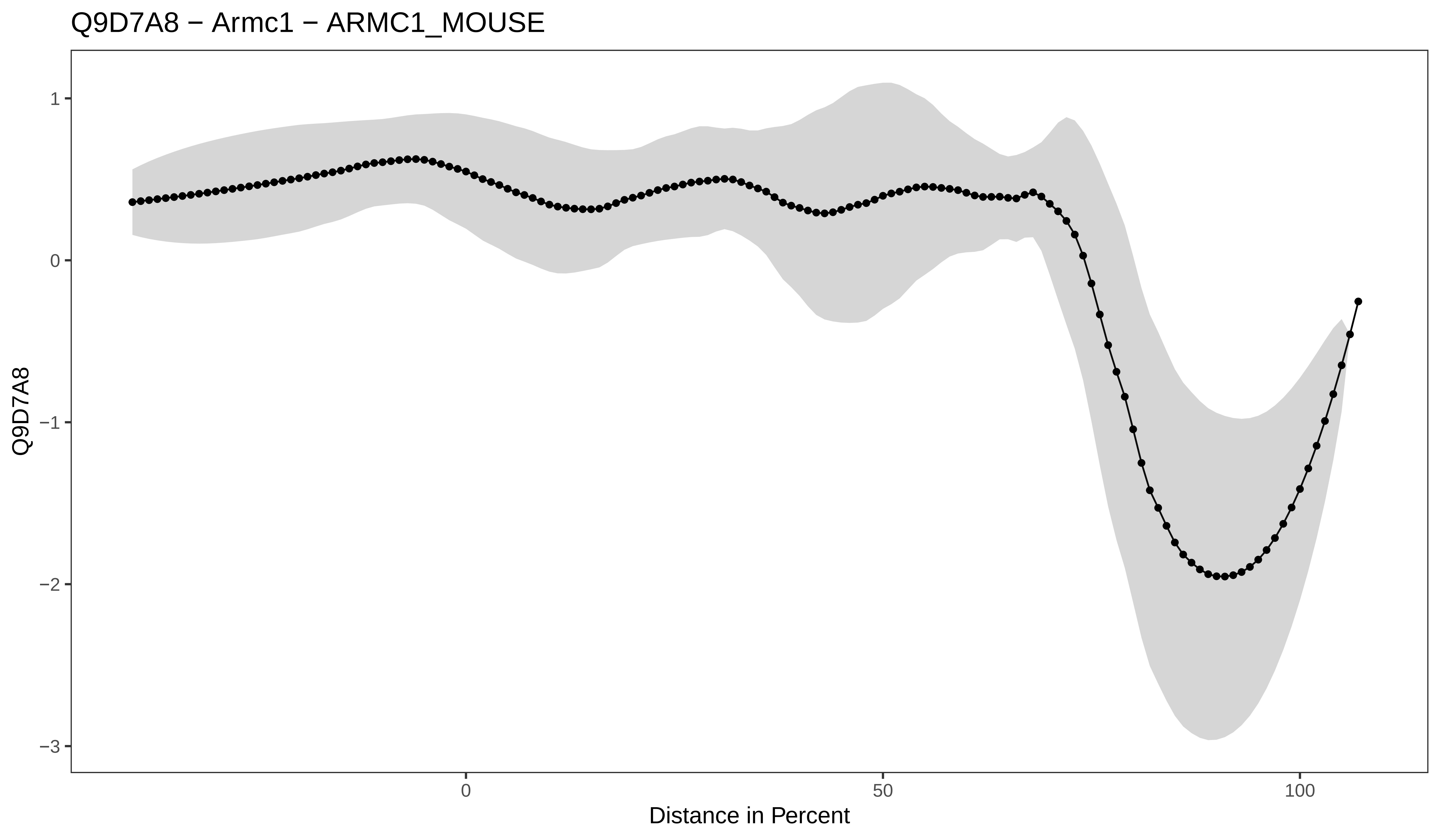

Q9D7A8 − Armc1 − ARMC1_MOUSE
1
0
−1
−2
−3
Q9D7A8
0
50
100
Distance in Percent

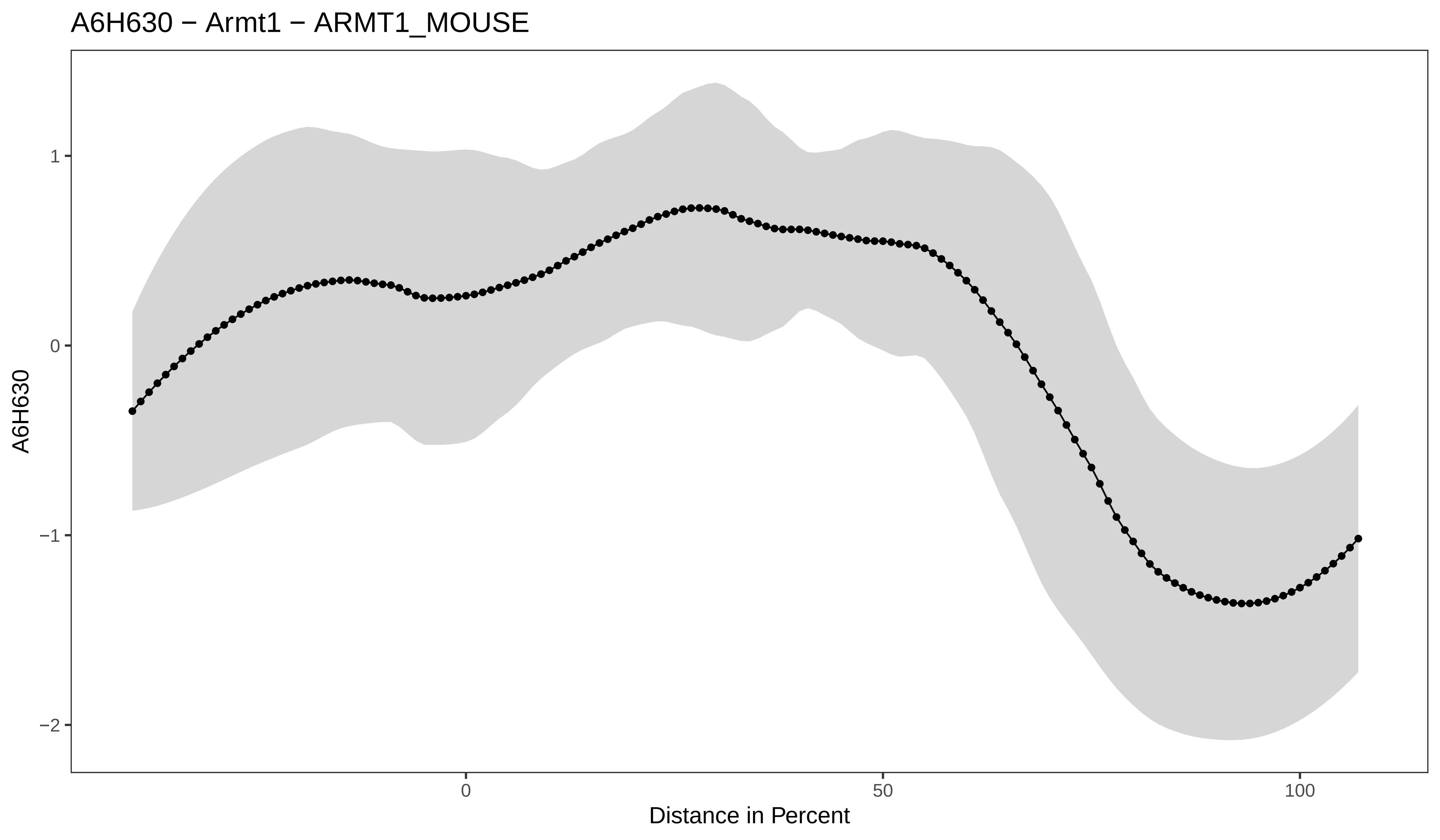

A6H630 − Armt1 − ARMT1_MOUSE
1
0
−1
−2
A6H630
0
50
100
Distance in Percent

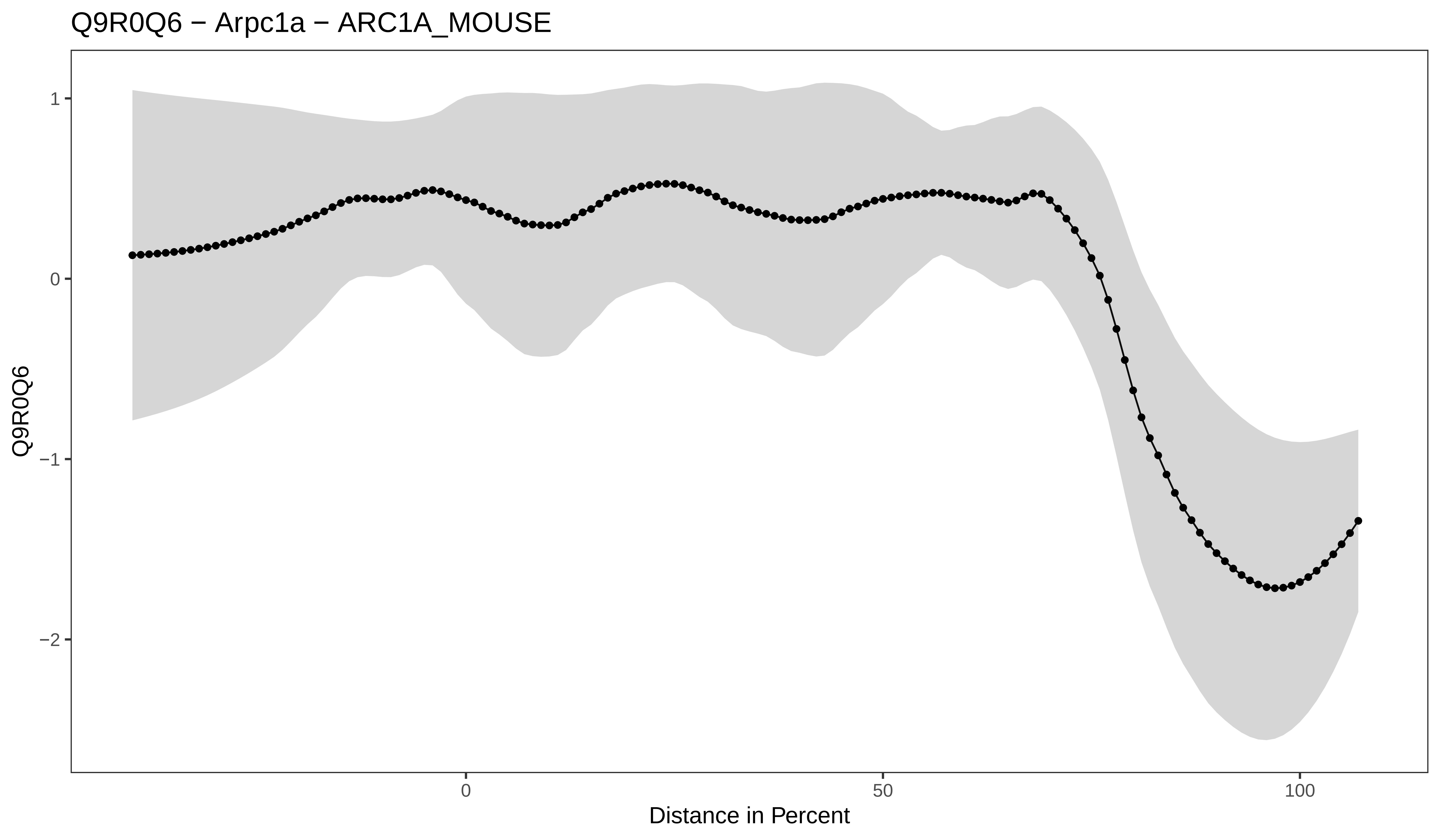

Q9R0Q6 − Arpc1a − ARC1A_MOUSE
Q9R0Q6
1
0
−1
−2
0
50
100
Distance in Percent

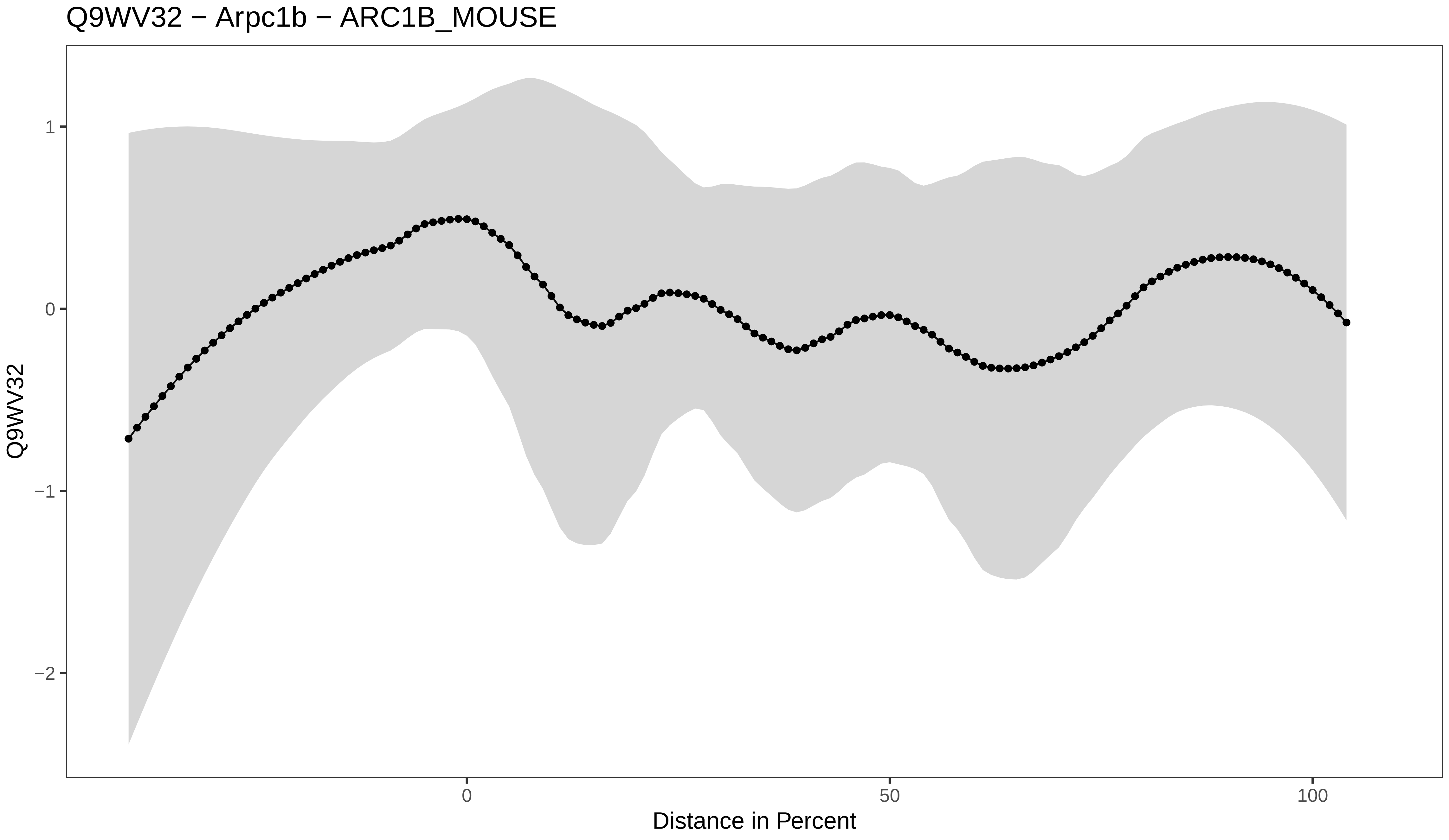

Q9WV32 − Arpc1b − ARC1B_MOUSE
1
0
−1
−2
Q9WV32
0
50
100
Distance in Percent

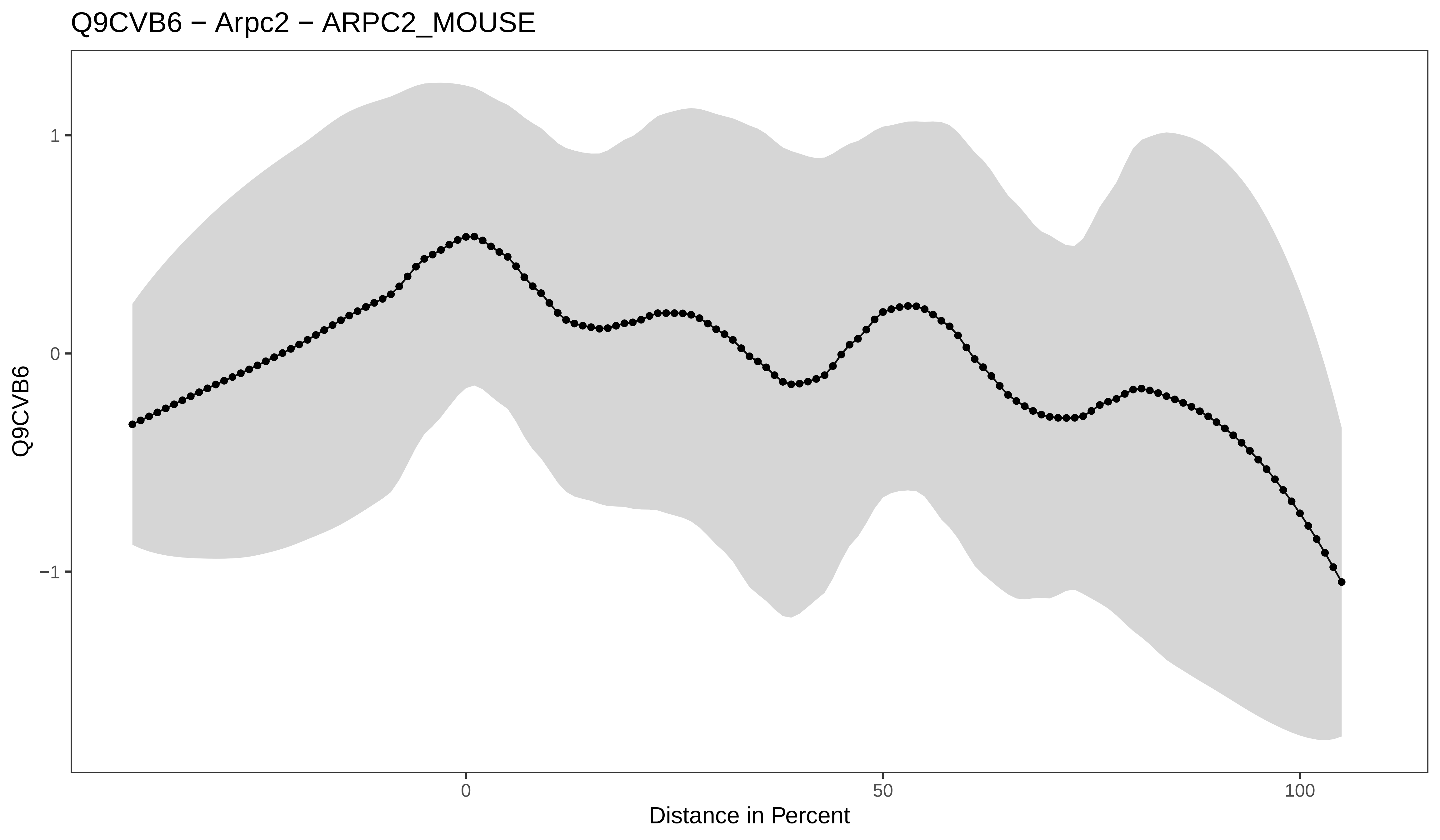

Q9CVB6 − Arpc2 − ARPC2_MOUSE
1
0
−1
Q9CVB6
0
50
100
Distance in Percent

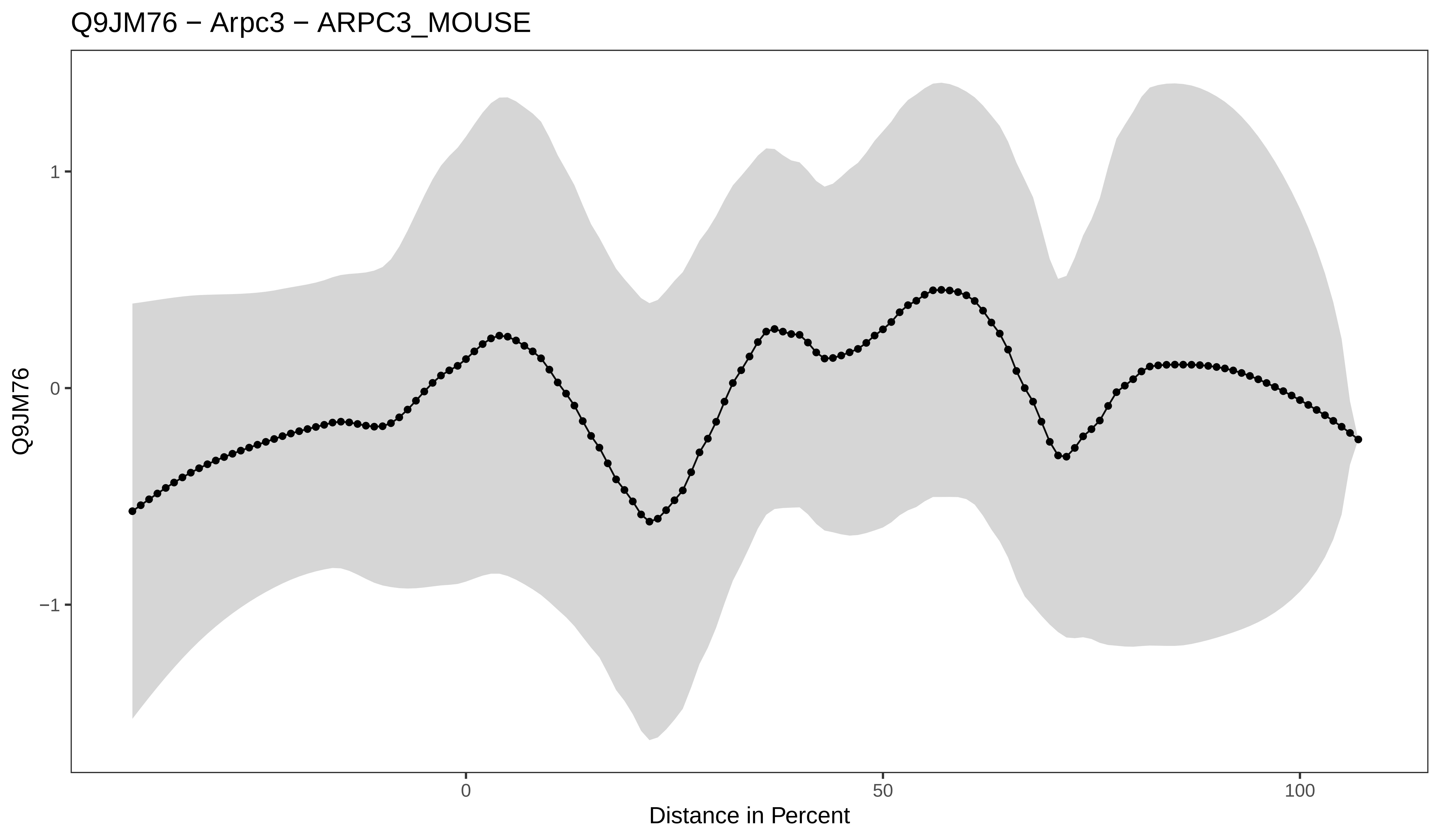

Q9JM76 − Arpc3 − ARPC3_MOUSE
Q9JM76
1
0
−1
0
50
100
Distance in Percent

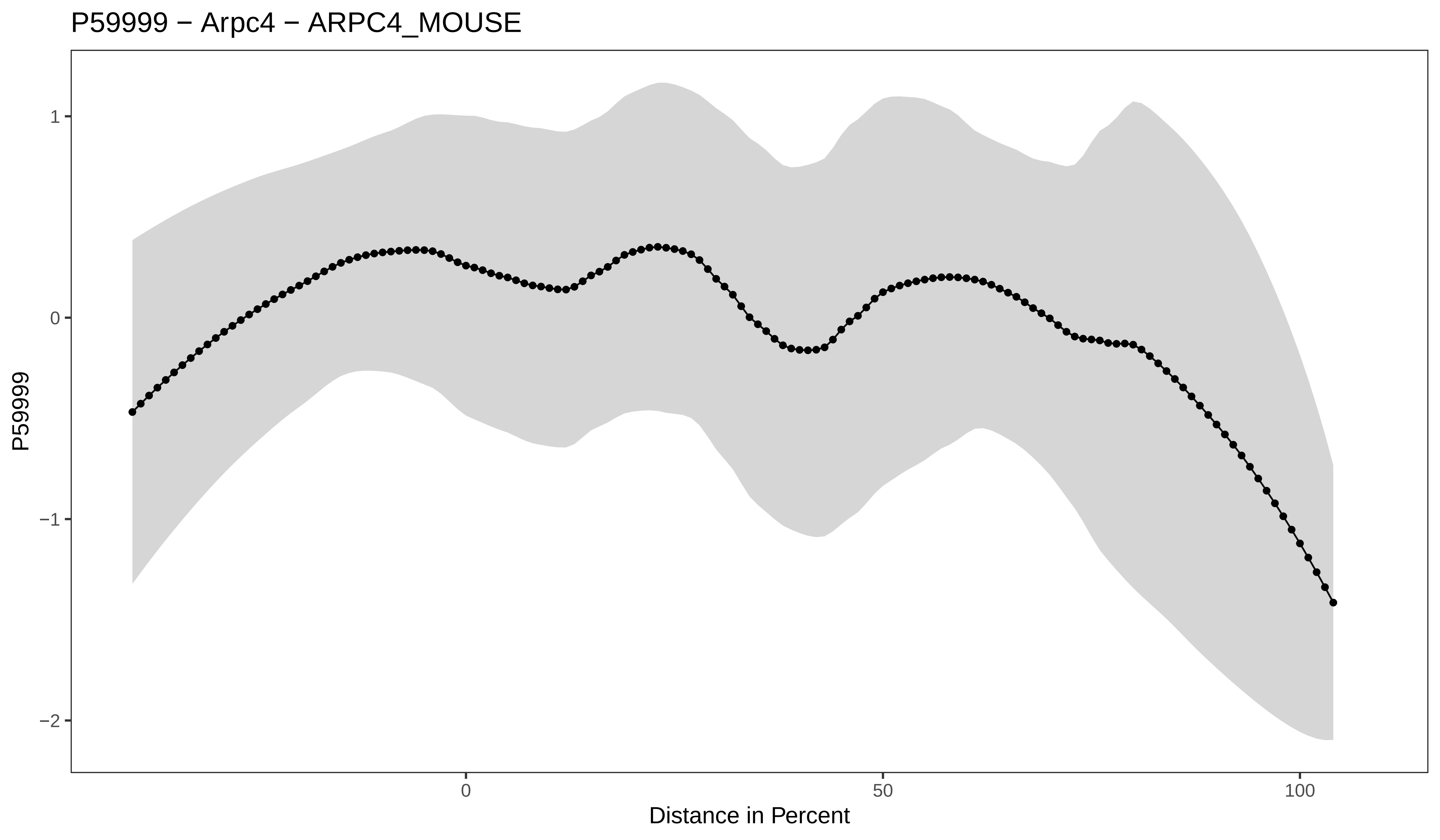
P59999 − Arpc4 − ARPC4_MOUSE
P59999
Distance in Percent
1
0
−1
−2
0
50
100

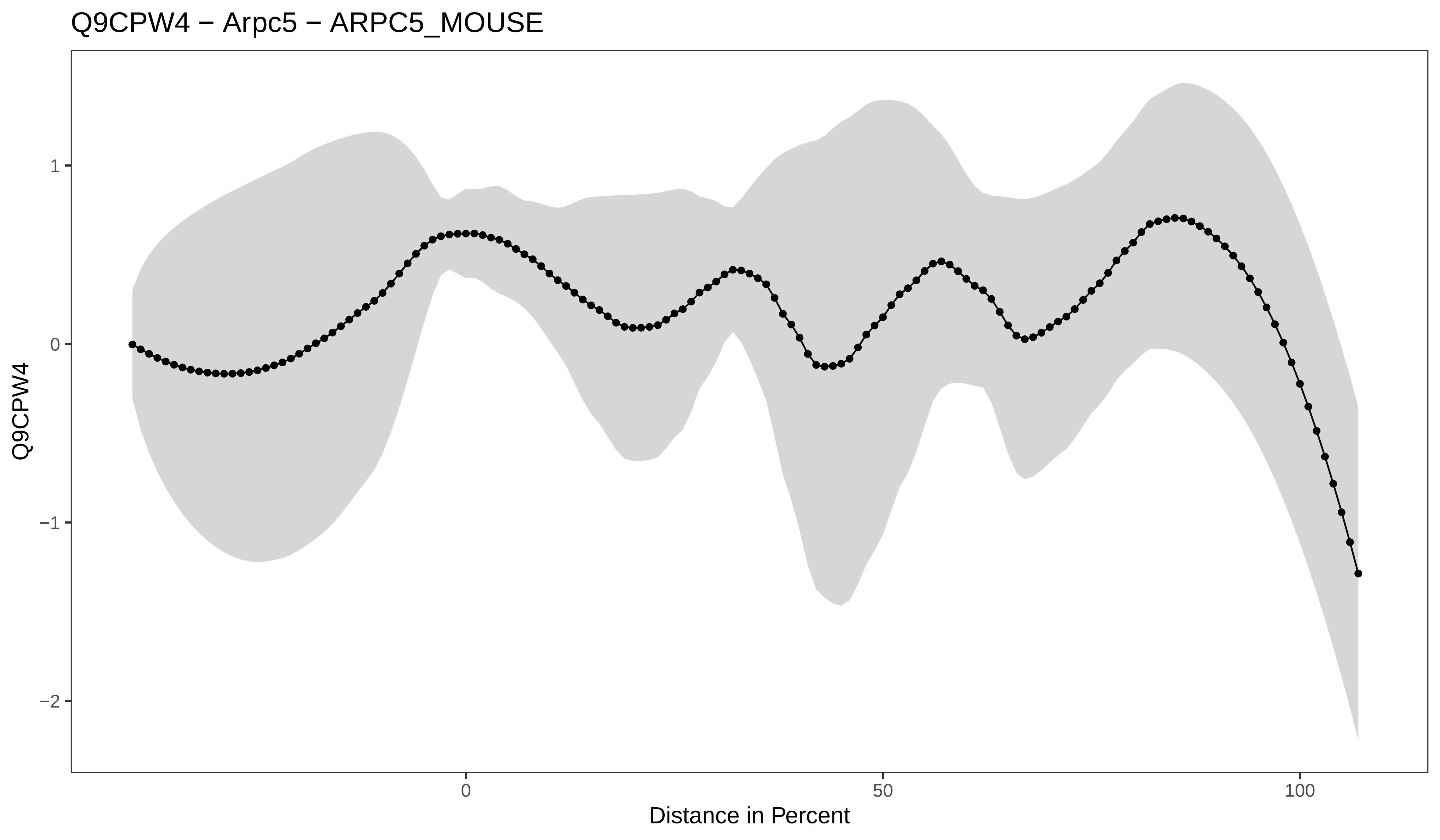

Q9CPW4 − Arpc5 − ARPC5_MOUSE
1
0
−1
−2
Q9CPW4
0
50
100
Distance in Percent

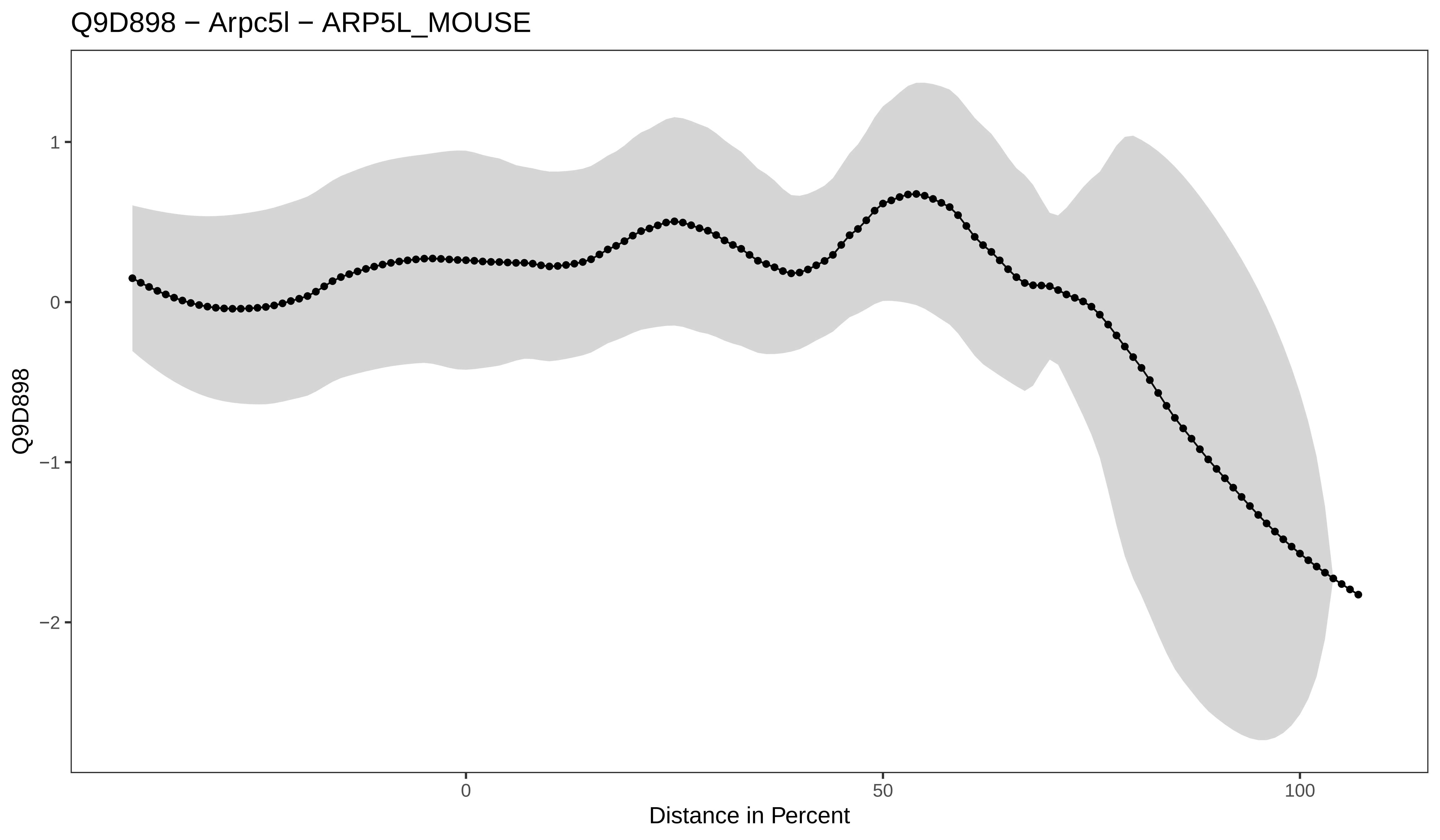
Q9D898 − Arpc5l − ARP5L_MOUSE
Q9D898
1
0
−1
−2
0
50
100
Distance in Percent

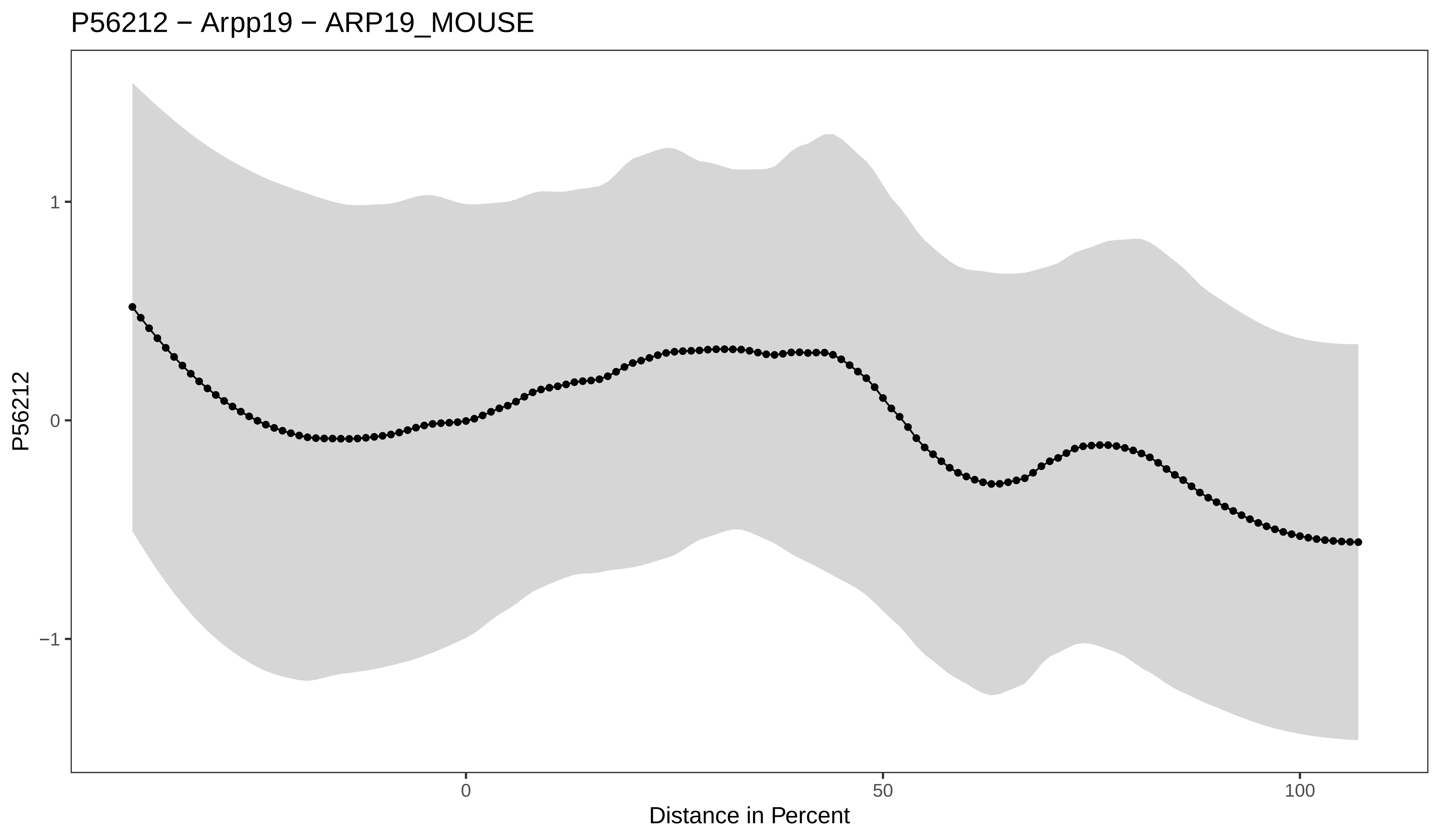
P56212 − Arpp19 − ARP19_MOUSE
P56212
1
0
−1
0
50
100
Distance in Percent

Q60935 − Art1 − NAR1_MOUSE

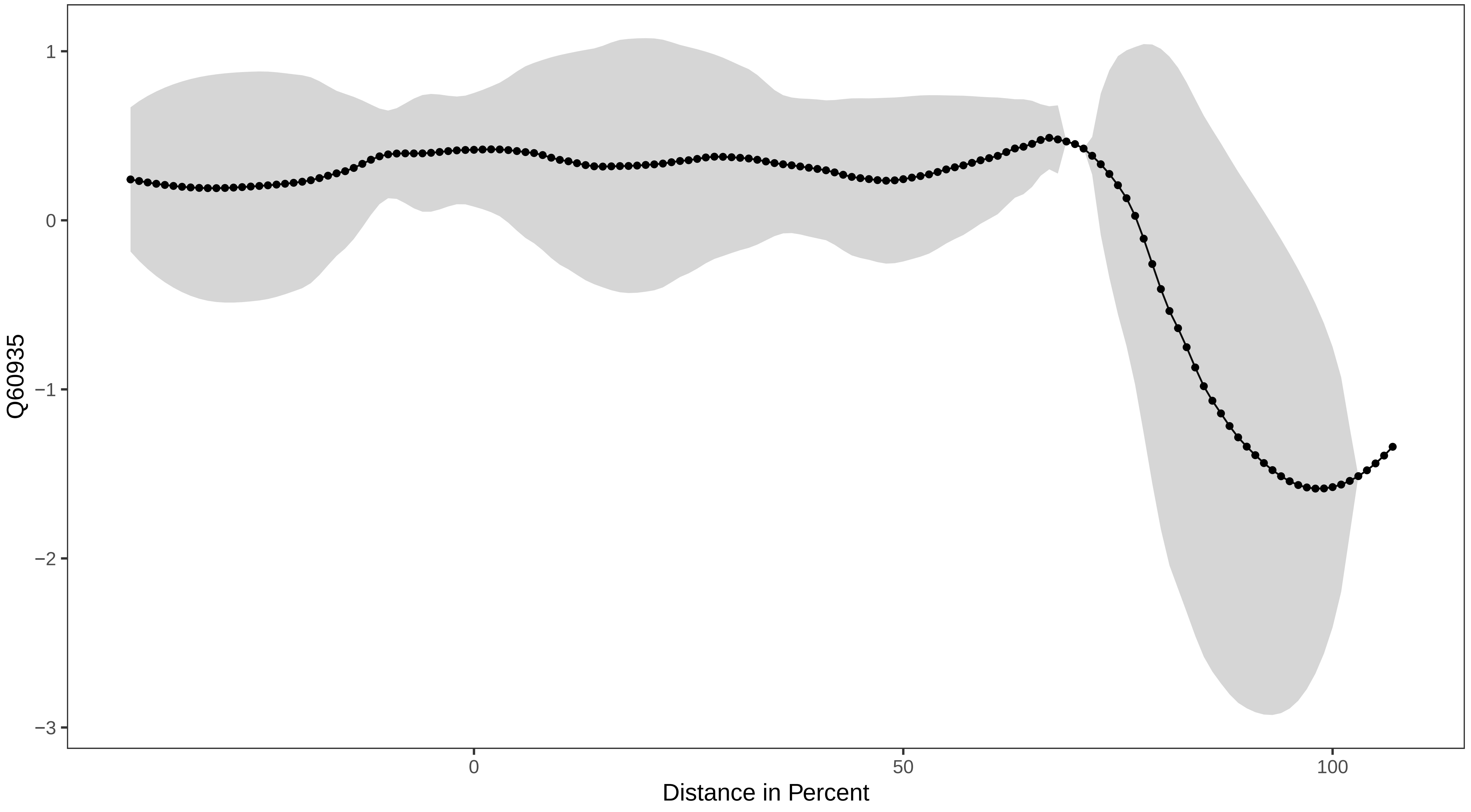

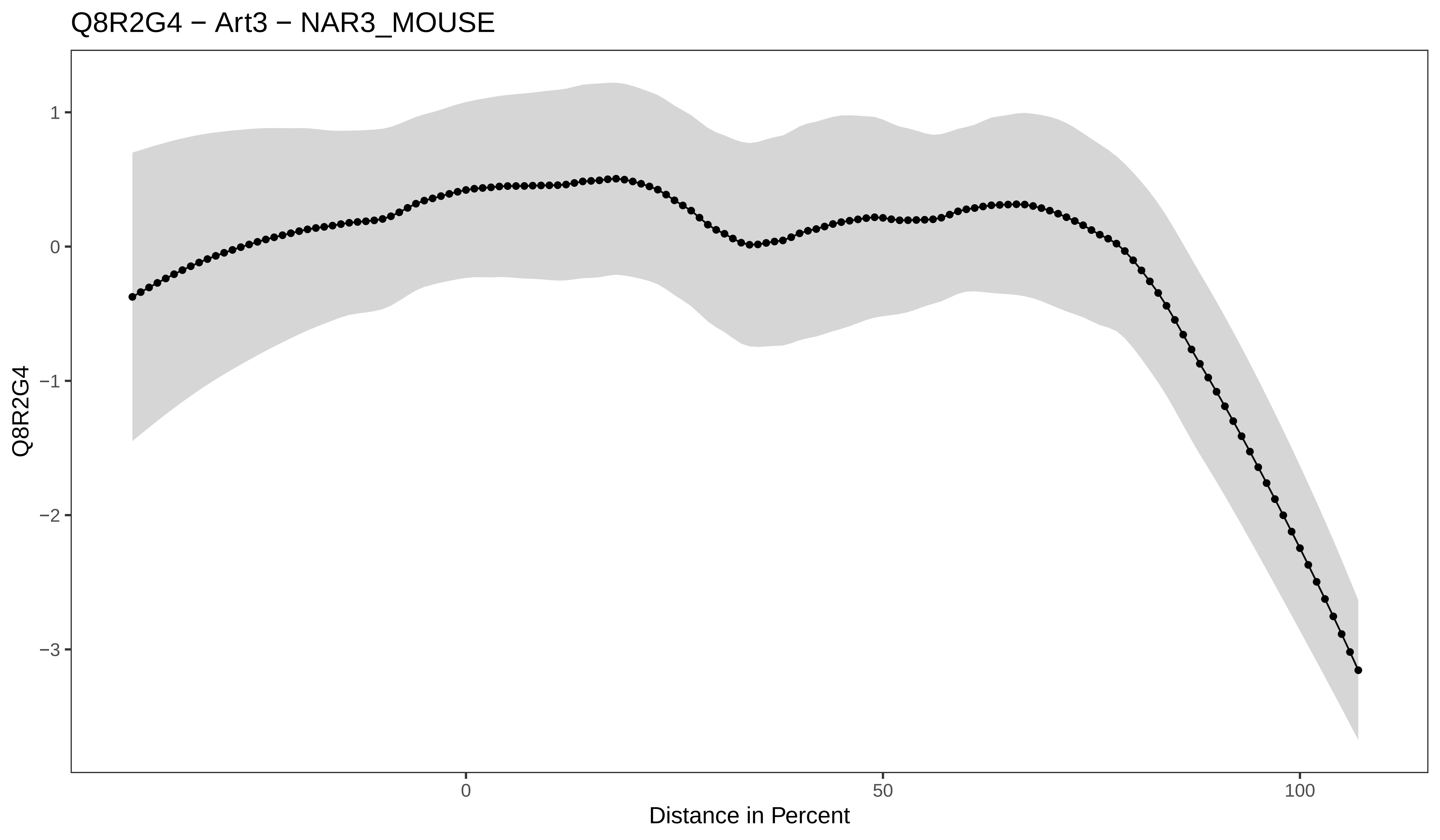

Q8R2G4 − Art3 − NAR3_MOUSE
Q8R2G4
Distance in Percent
1
0
−1
−2
−3
0
50
100

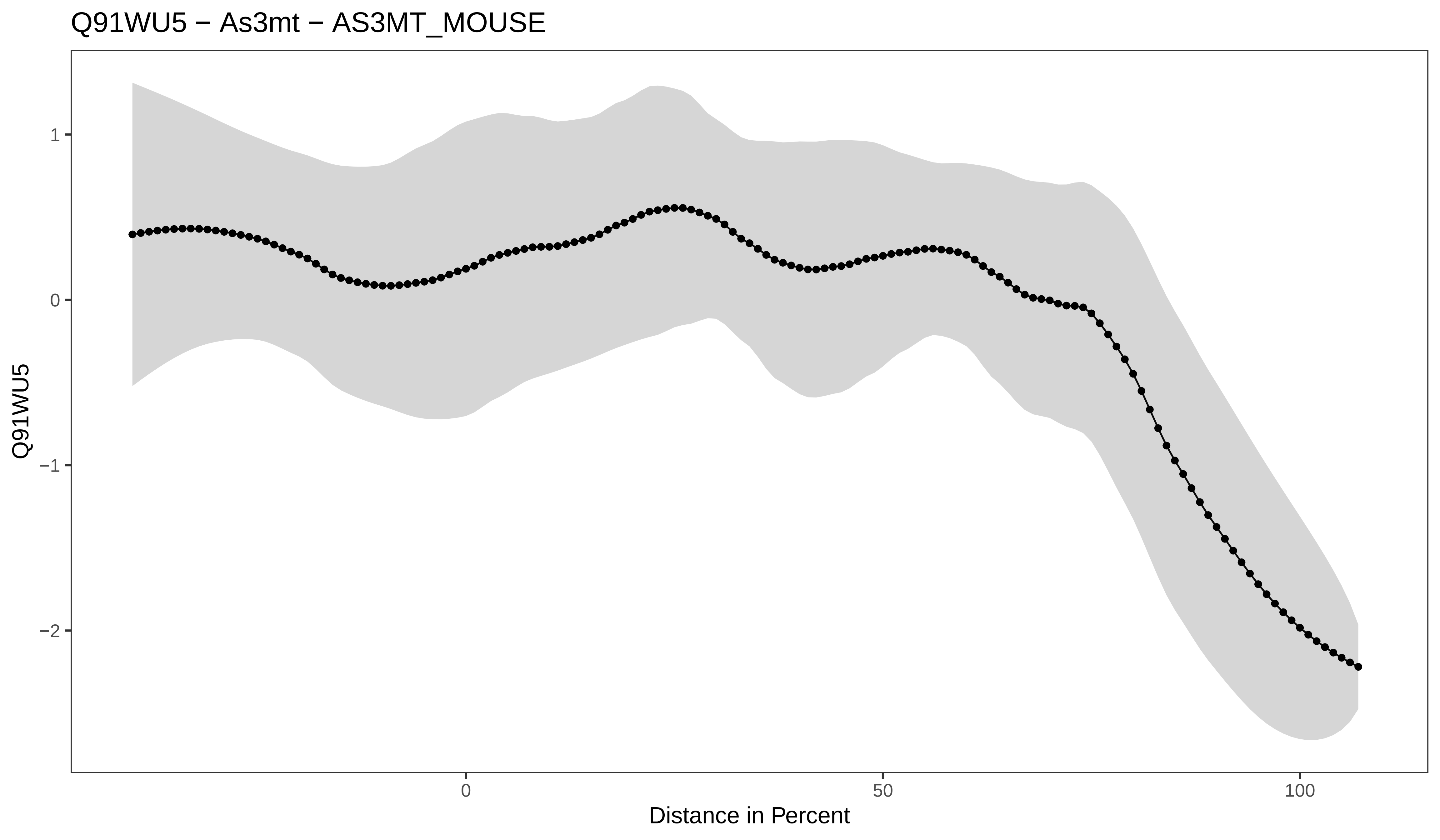

Q91WU5 − As3mt − AS3MT_MOUSE
1
0
−1
−2
Q91WU5
0
50
100
Distance in Percent

Q9WV54 − Asah1 − ASAH1_MOUSE

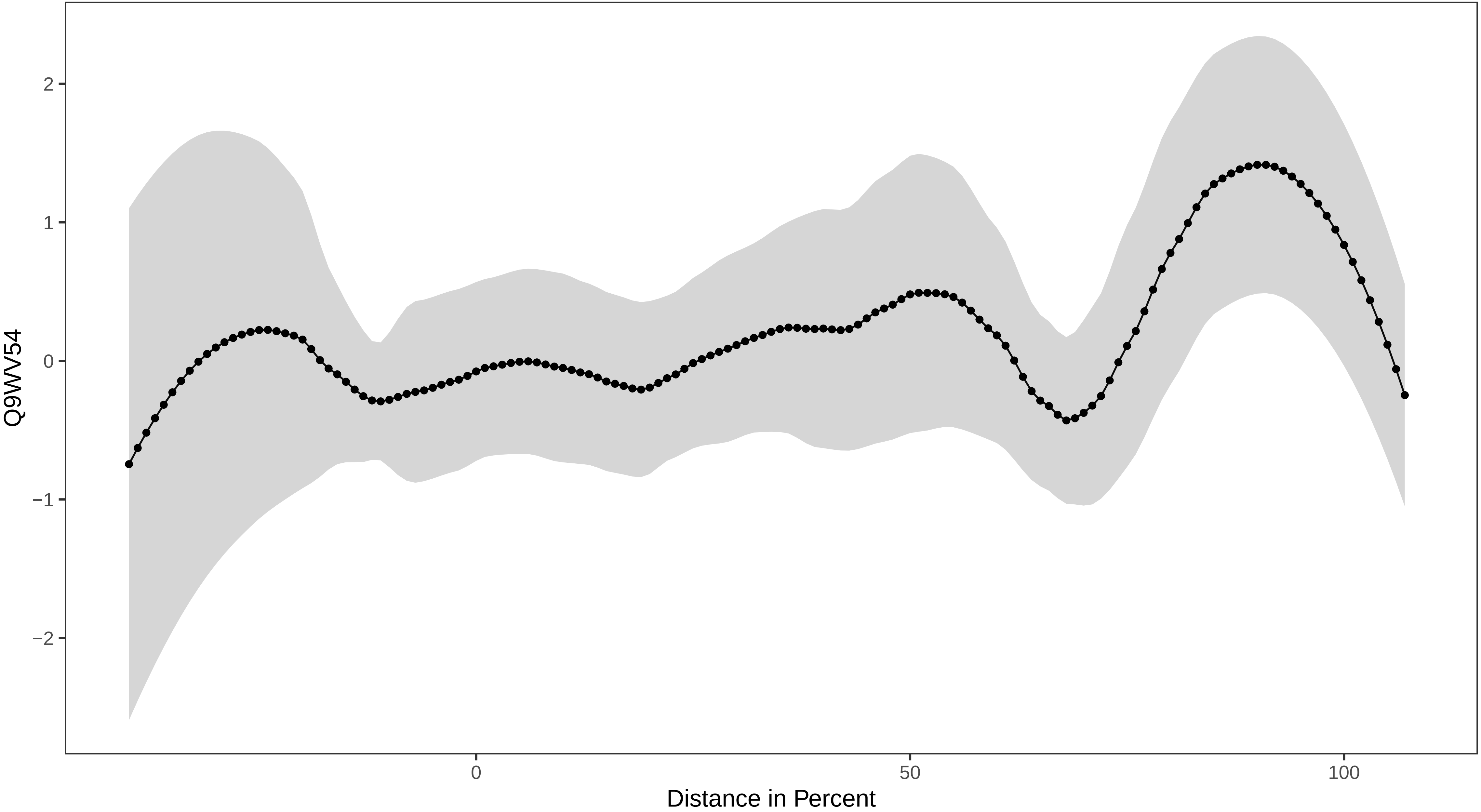

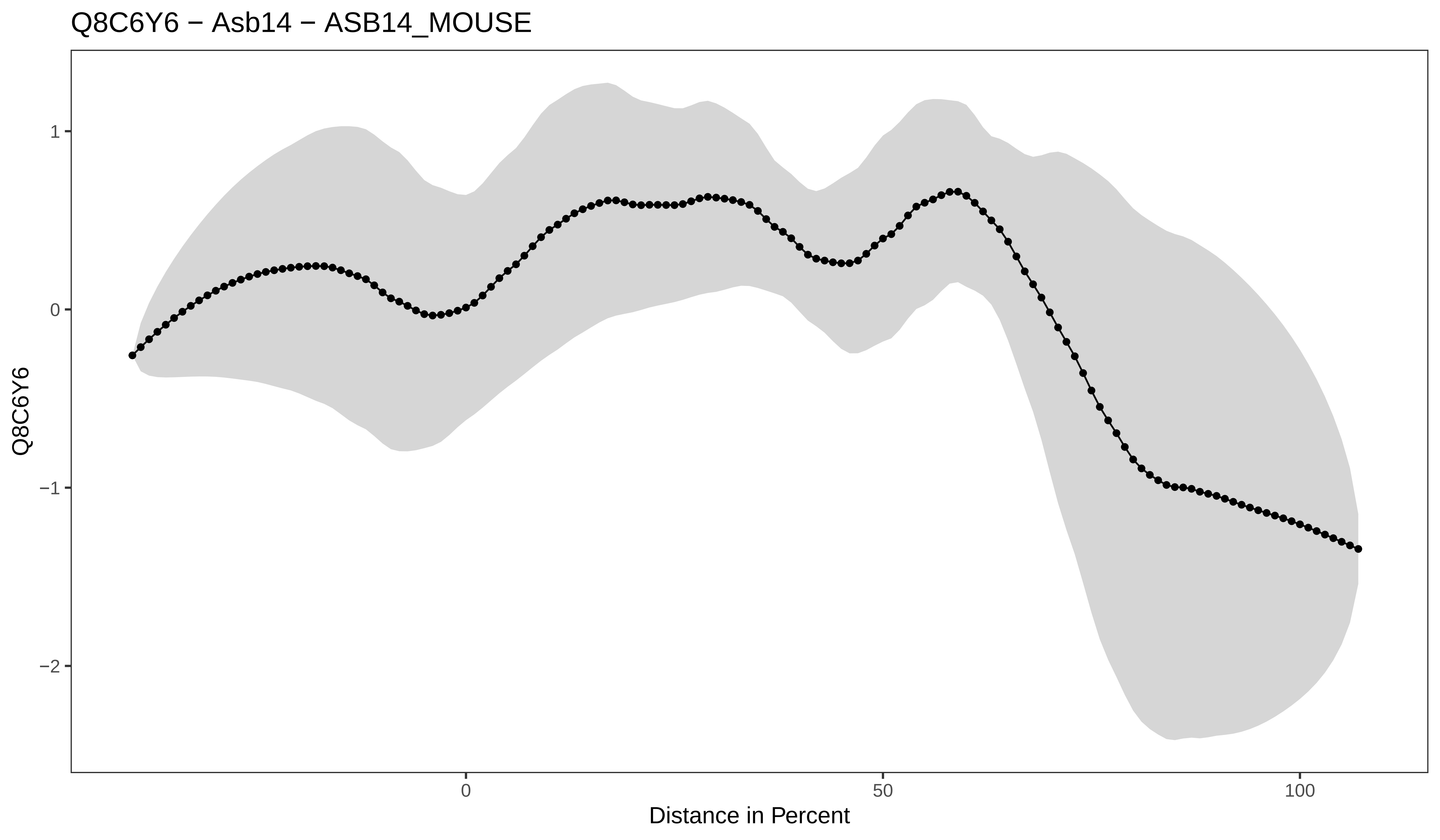

Q8C6Y6 − Asb14 − ASB14_MOUSE
Q8C6Y6
Distance in Percent
1
0
−1
−2
0
50
100

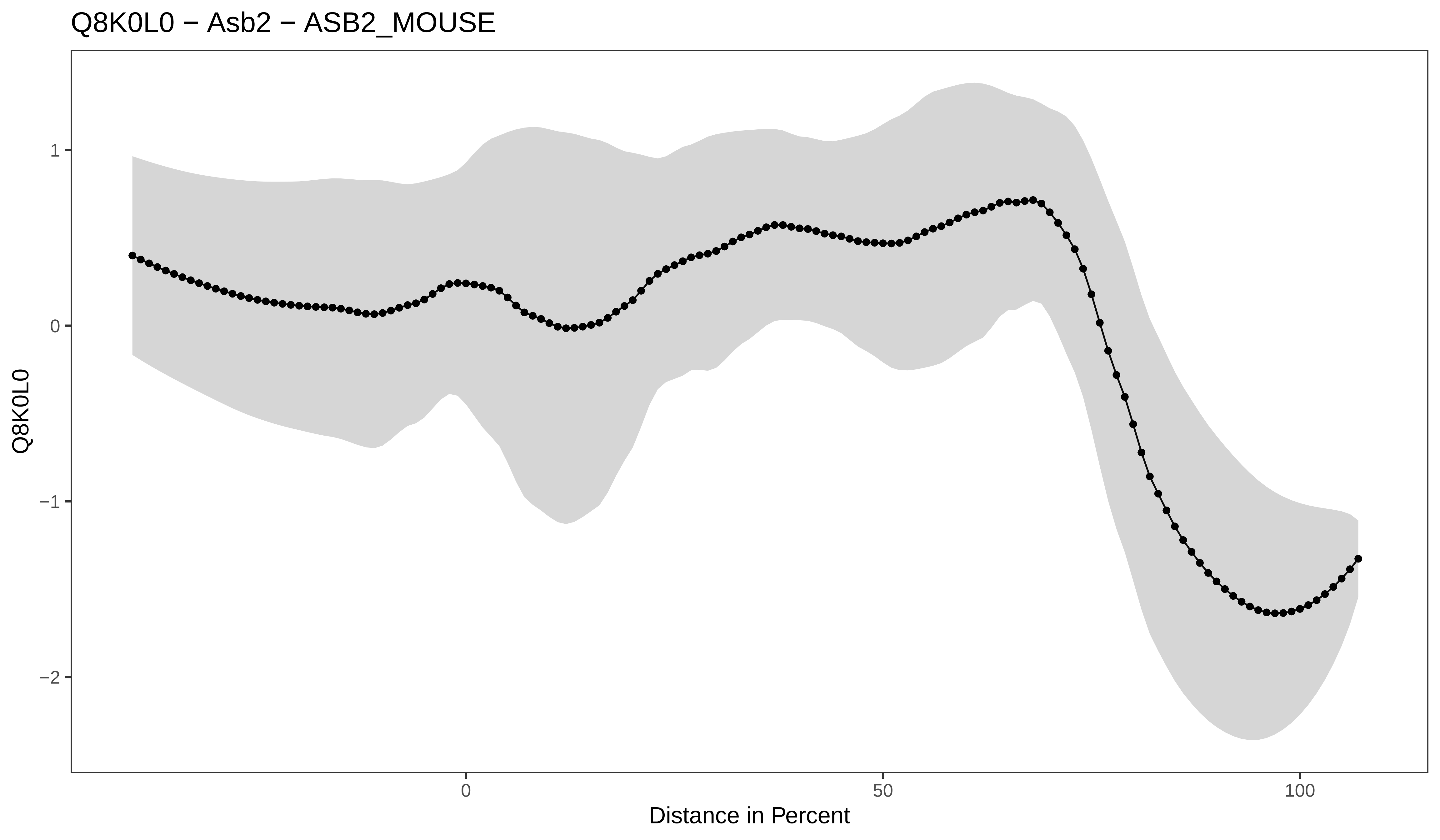

Q8K0L0 − Asb2 − ASB2_MOUSE
Q8K0L0
Distance in Percent
1
0
−1
−2
0
50
100

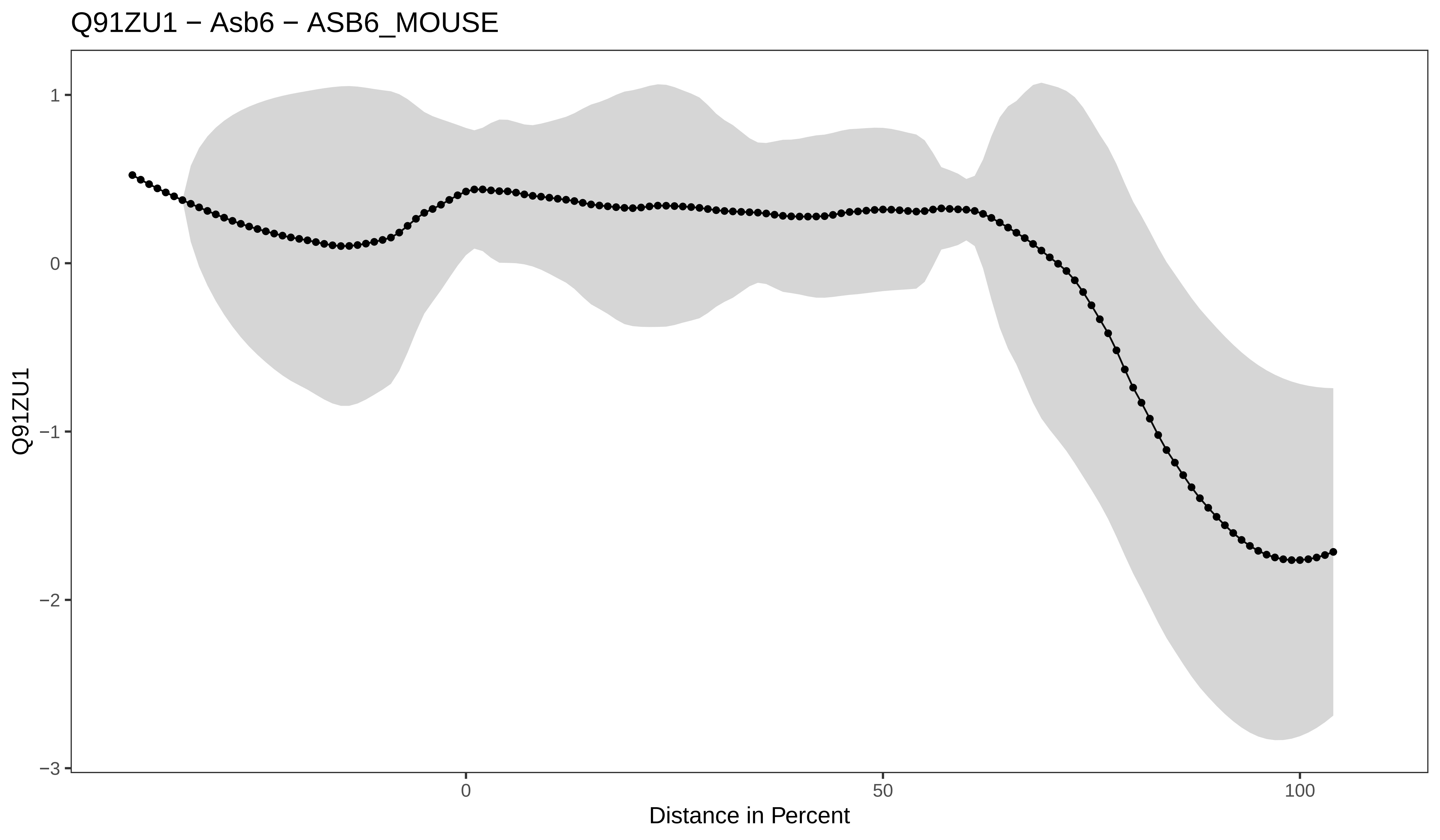

Q91ZU1 − Asb6 − ASB6_MOUSE
1
0
−1
−2
−3
Q91ZU1
0
50
100
Distance in Percent

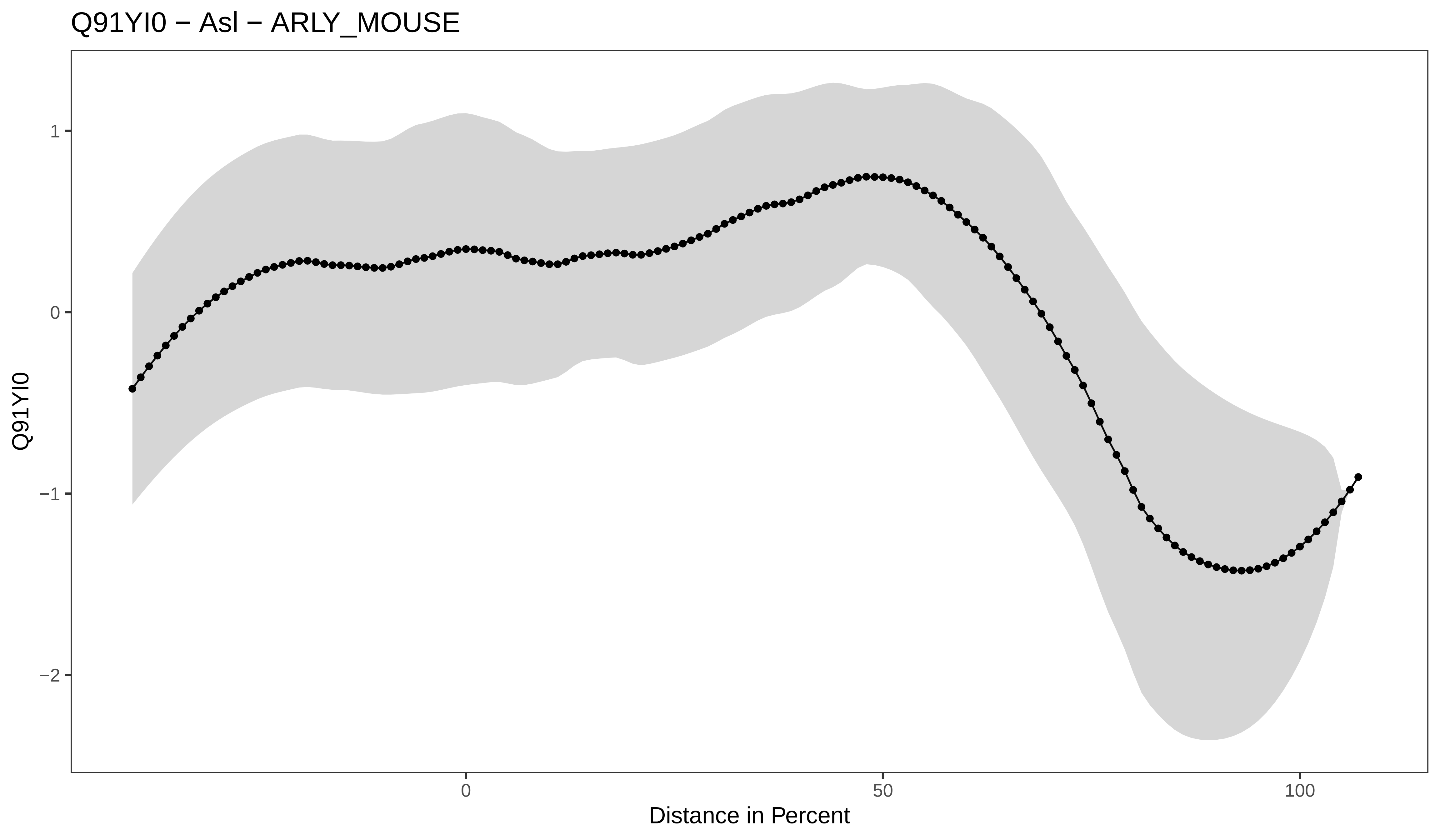

Q91YI0 − Asl − ARLY_MOUSE
Q91YI0
1
0
−1
−2
0
50
100
Distance in Percent

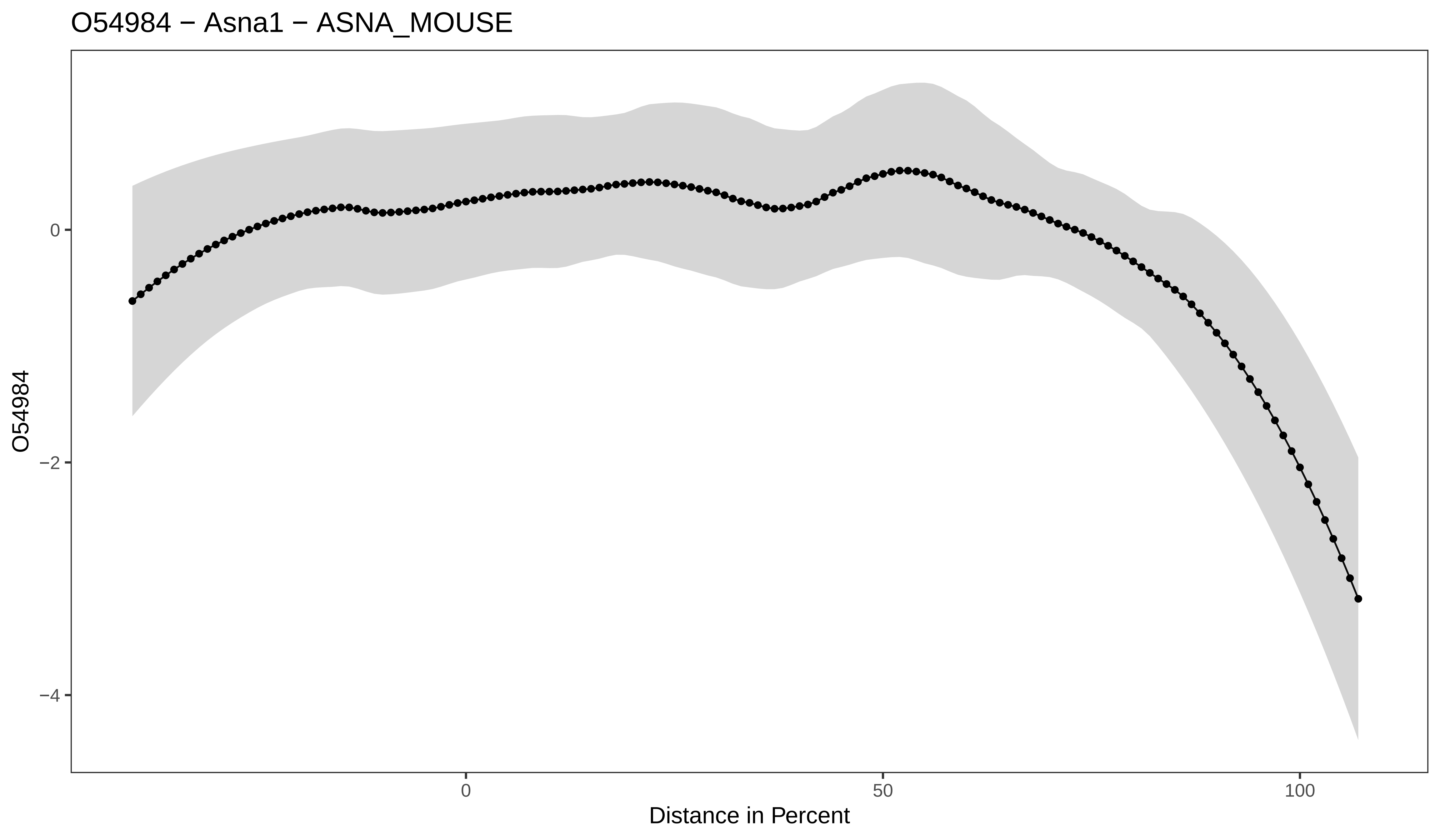

O54984 − Asna1 − ASNA_MOUSE
0
−2
−4
O54984
0
50
100
Distance in Percent

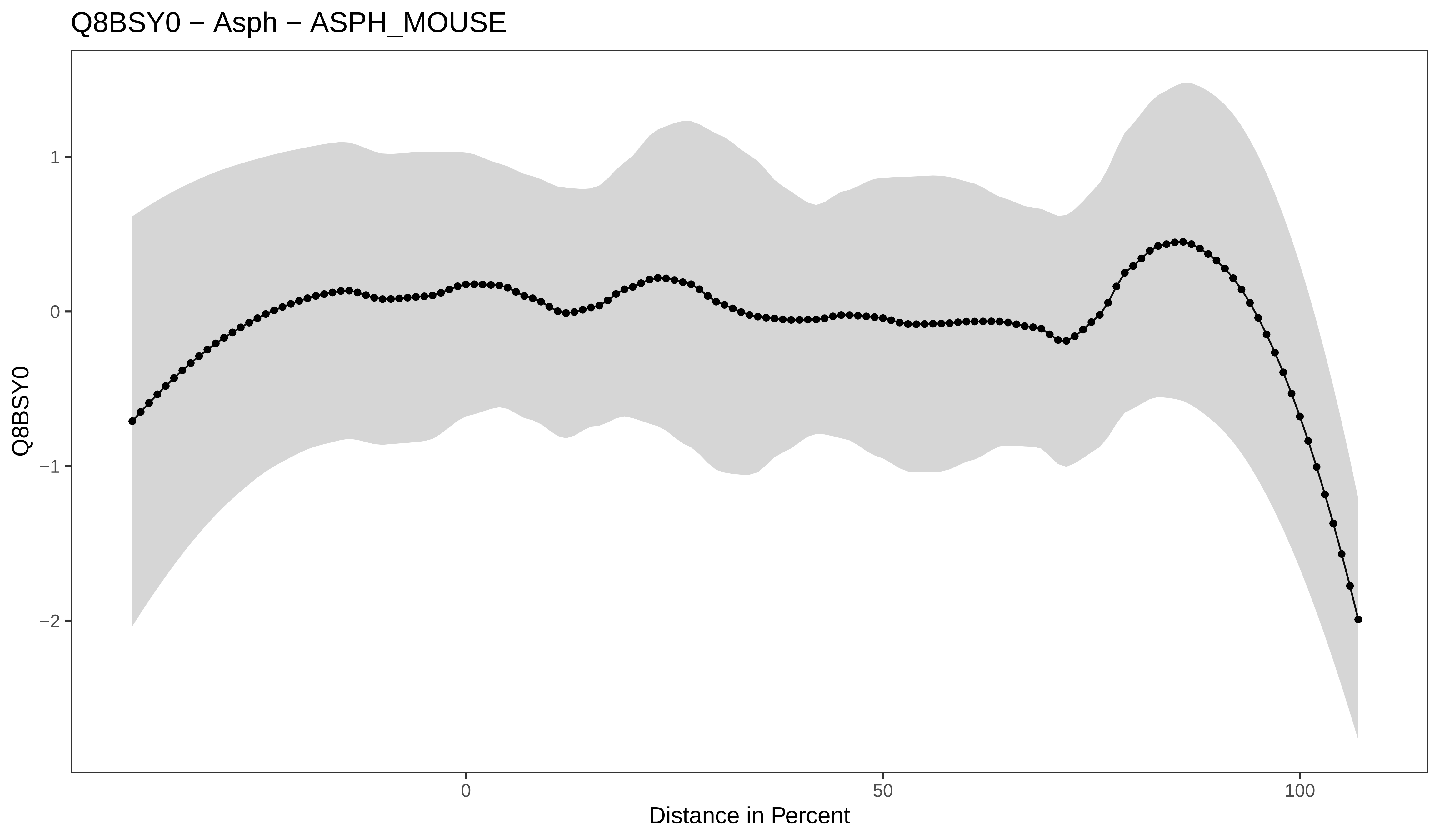

Q8BSY0 − Asph − ASPH_MOUSE
Q8BSY0
1
0
−1
−2
0
50
100
Distance in Percent

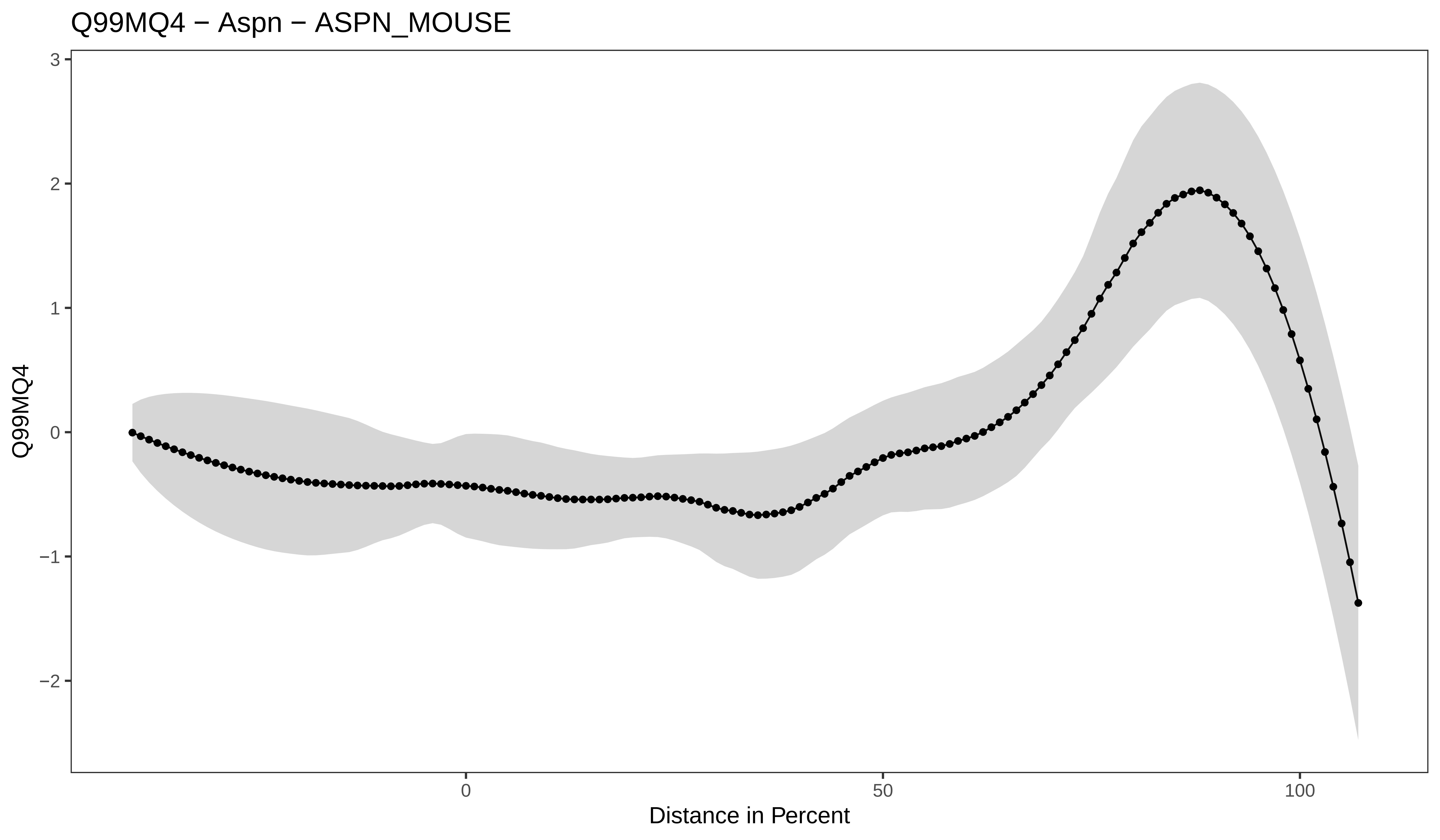

Q99MQ4 − Aspn − ASPN_MOUSE
3
2
1
0
−1
−2
Q99MQ4
0
50
100
Distance in Percent

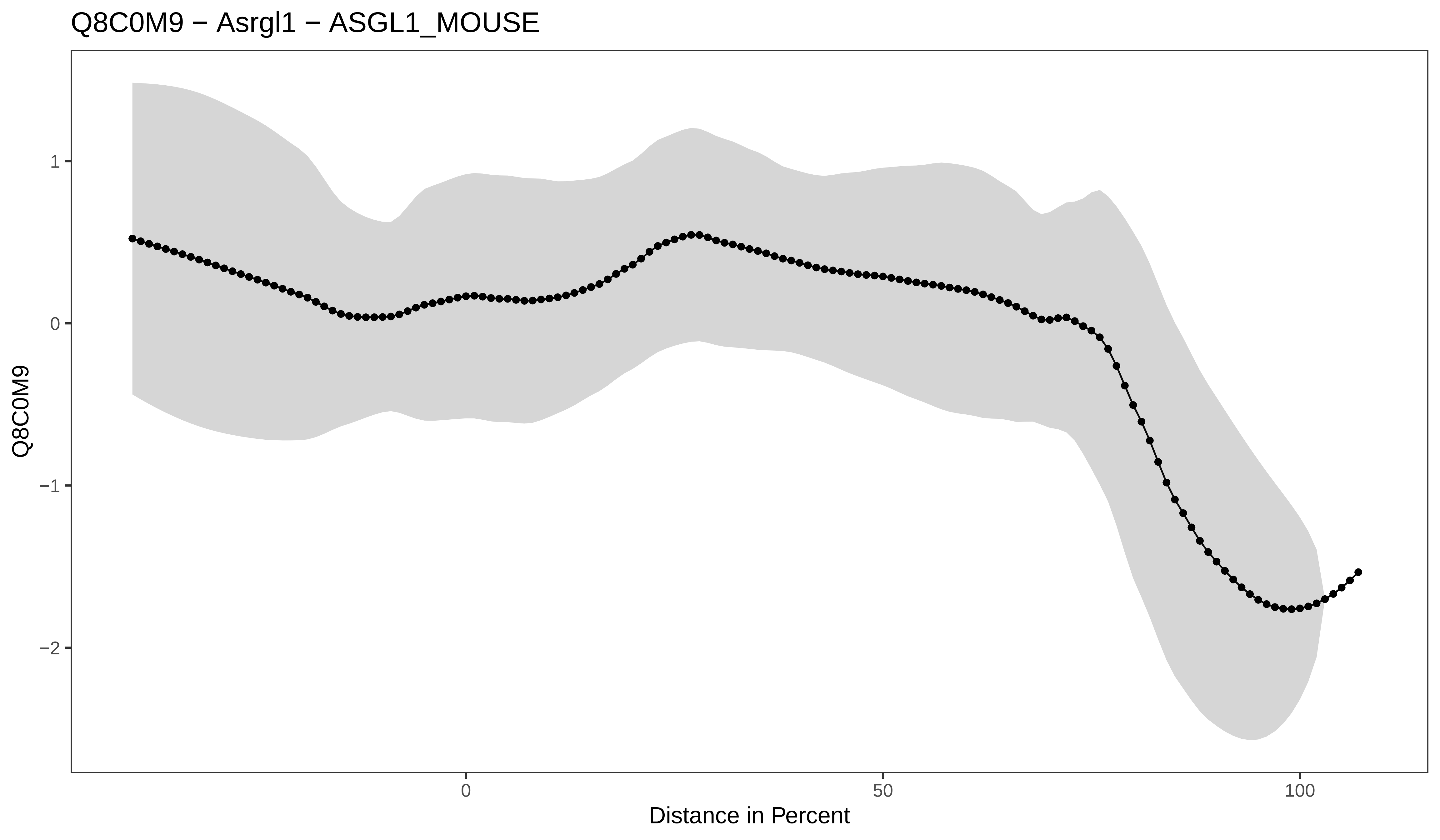

Q8C0M9 − Asrgl1 − ASGL1_MOUSE
Q8C0M9
1
0
−1
−2
0
50
100
Distance in Percent

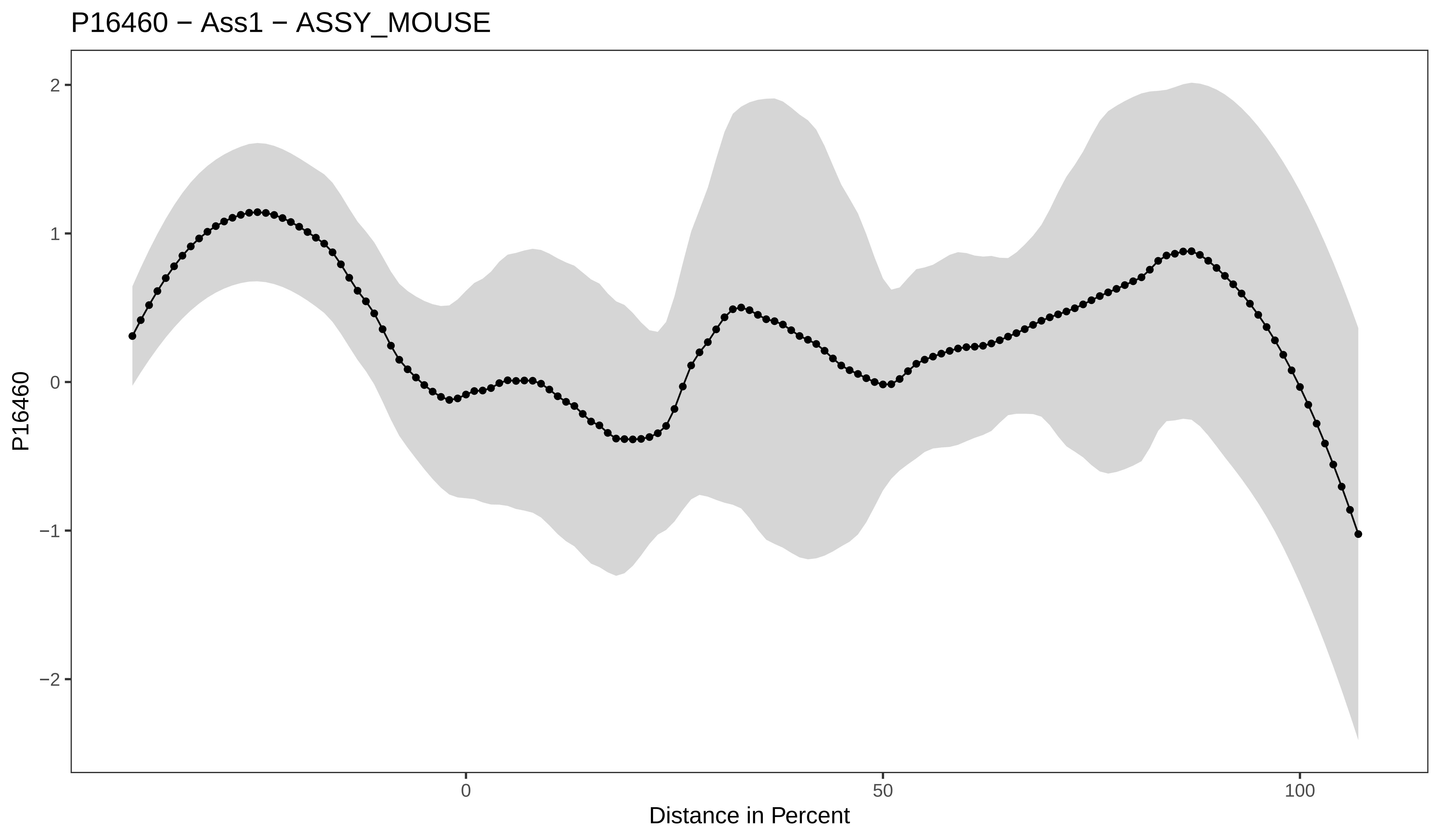
P16460 − Ass1 − ASSY_MOUSE
P16460
Distance in Percent
2
1
0
−1
−2
0
50
100

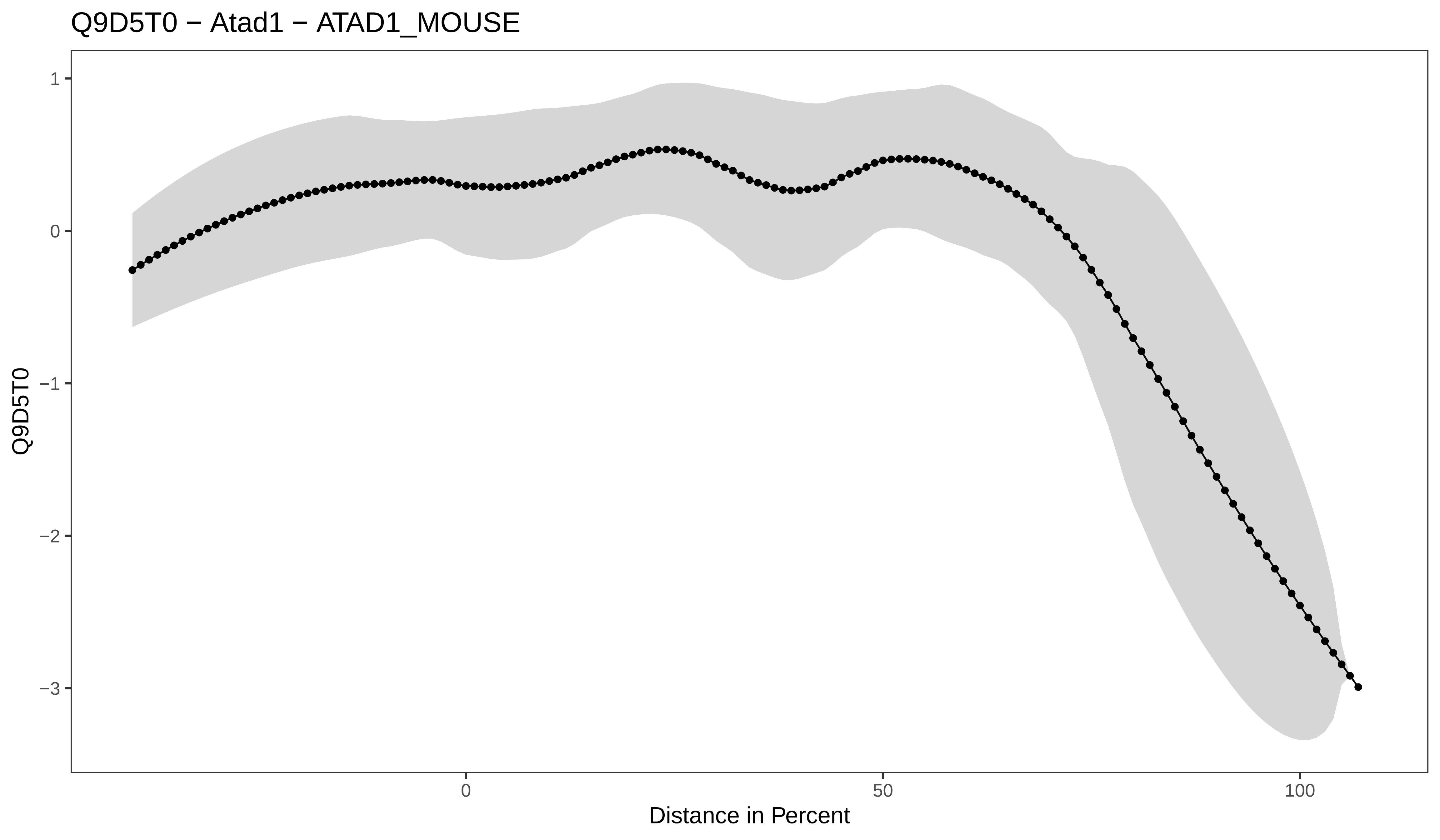

Q9D5T0 − Atad1 − ATAD1_MOUSE
Q9D5T0
1
0
−1
−2
−3
0
50
100
Distance in Percent

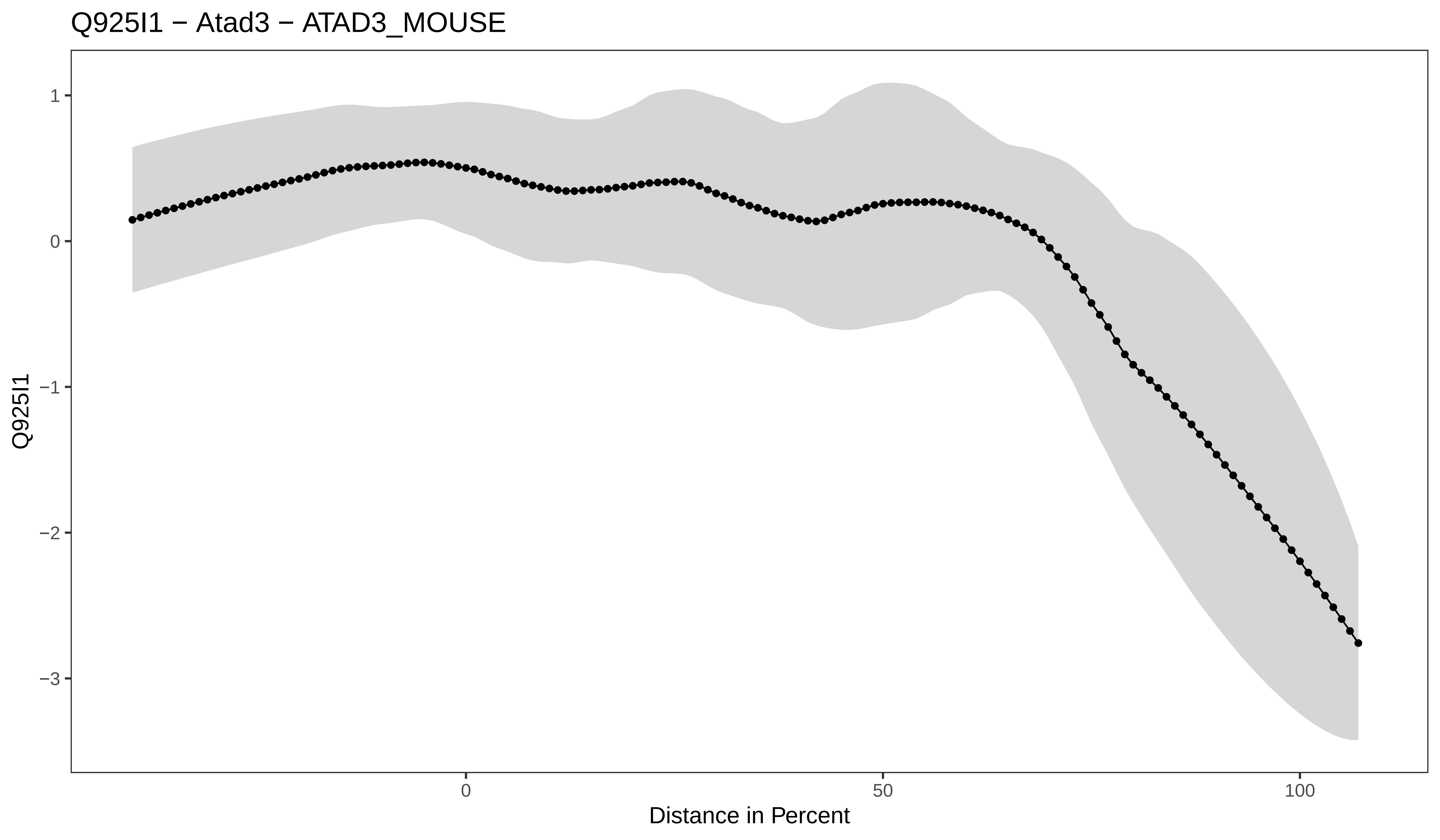
Q925I1 − Atad3 − ATAD3_MOUSE
Q925I1
1
0
−1
−2
−3
0
50
100
Distance in Percent

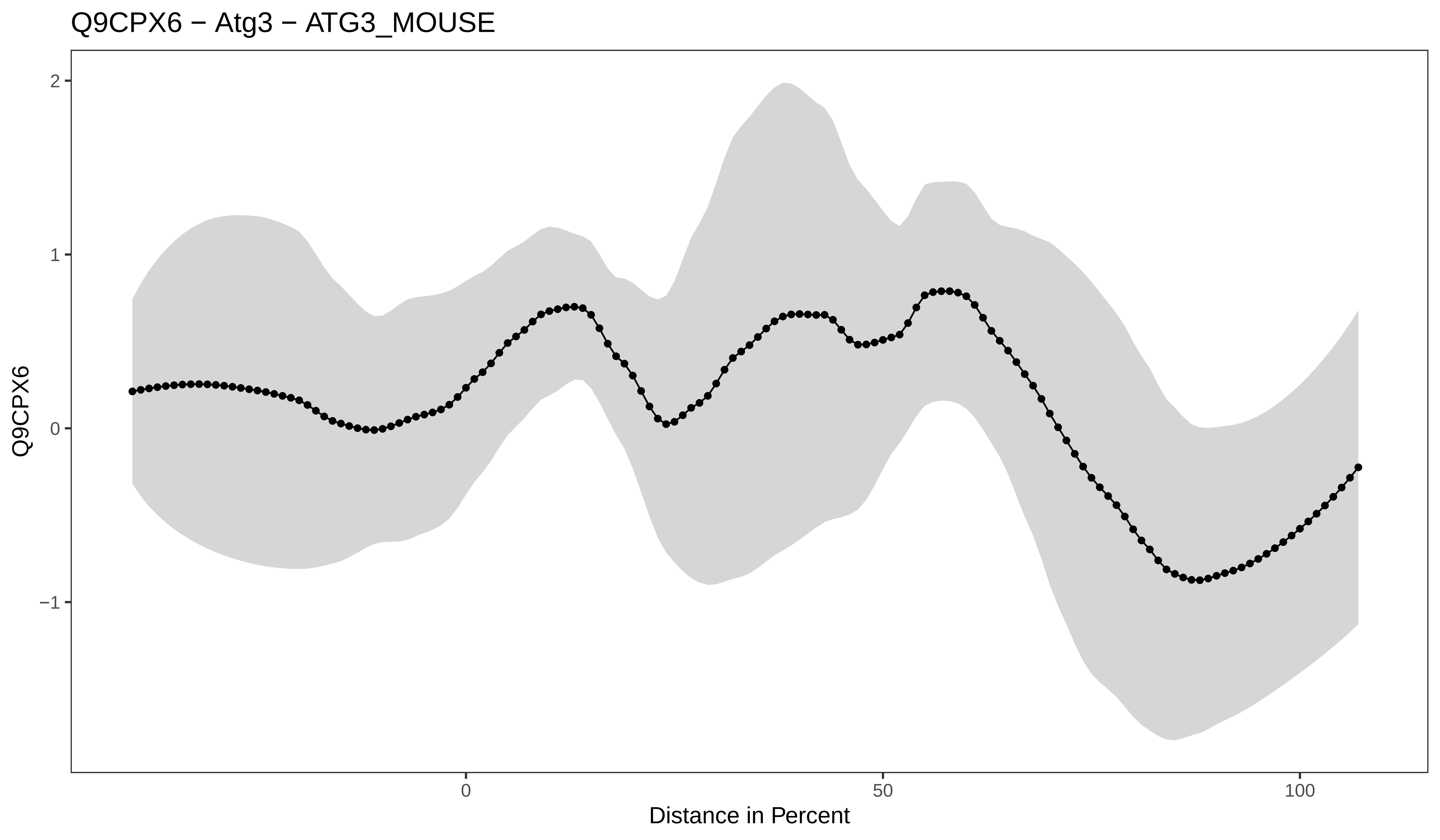
Q9CPX6 − Atg3 − ATG3_MOUSE
Q9CPX6
2
1
0
−1
0
50
100
Distance in Percent

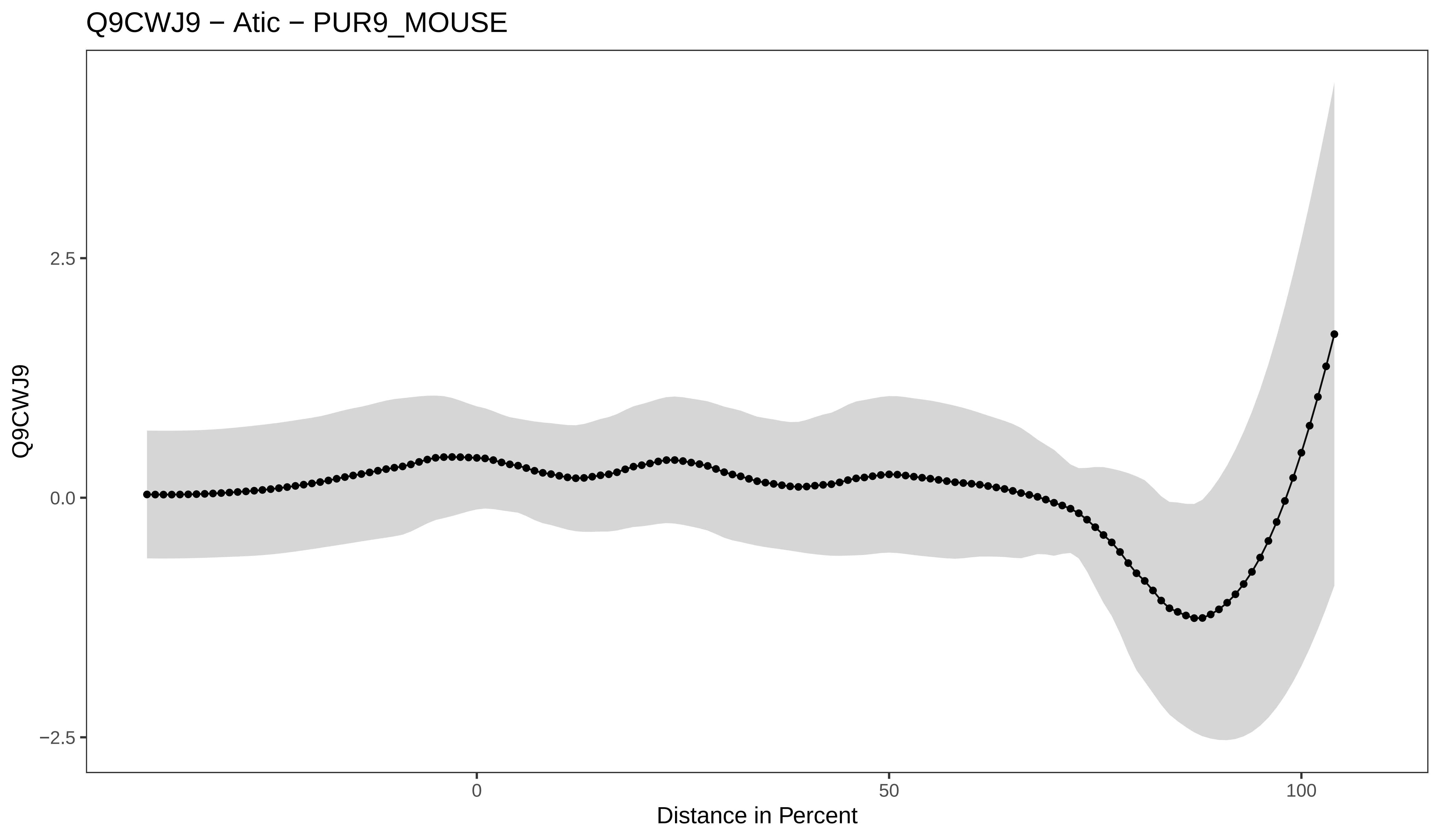

Q9CWJ9 − Atic − PUR9_MOUSE
Q9CWJ9
2.5
0.0
−2.5
0
50
100
Distance in Percent

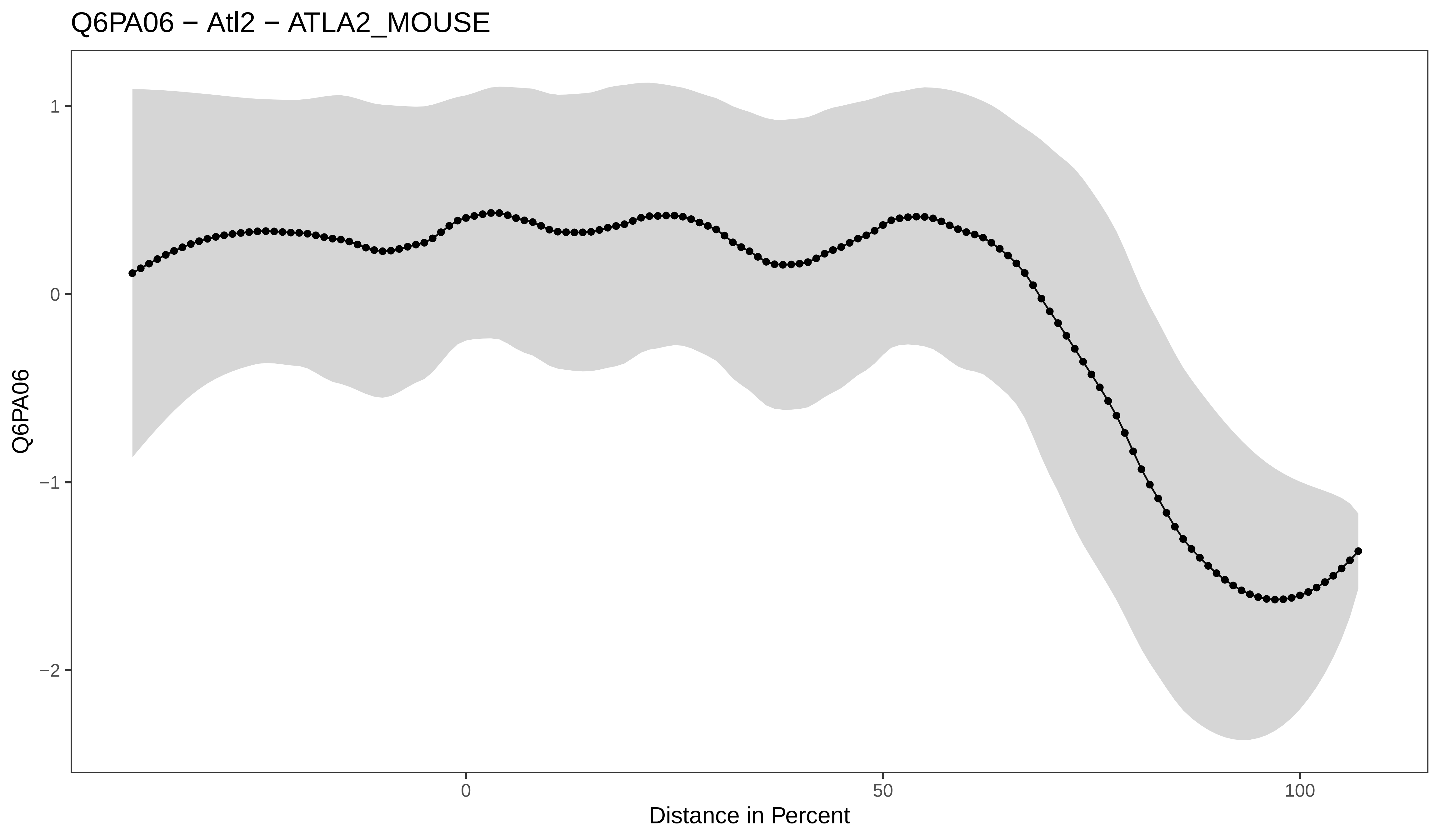

Q6PA06 − Atl2 − ATLA2_MOUSE
1
0
−1
−2
Q6PA06
0
50
100
Distance in Percent

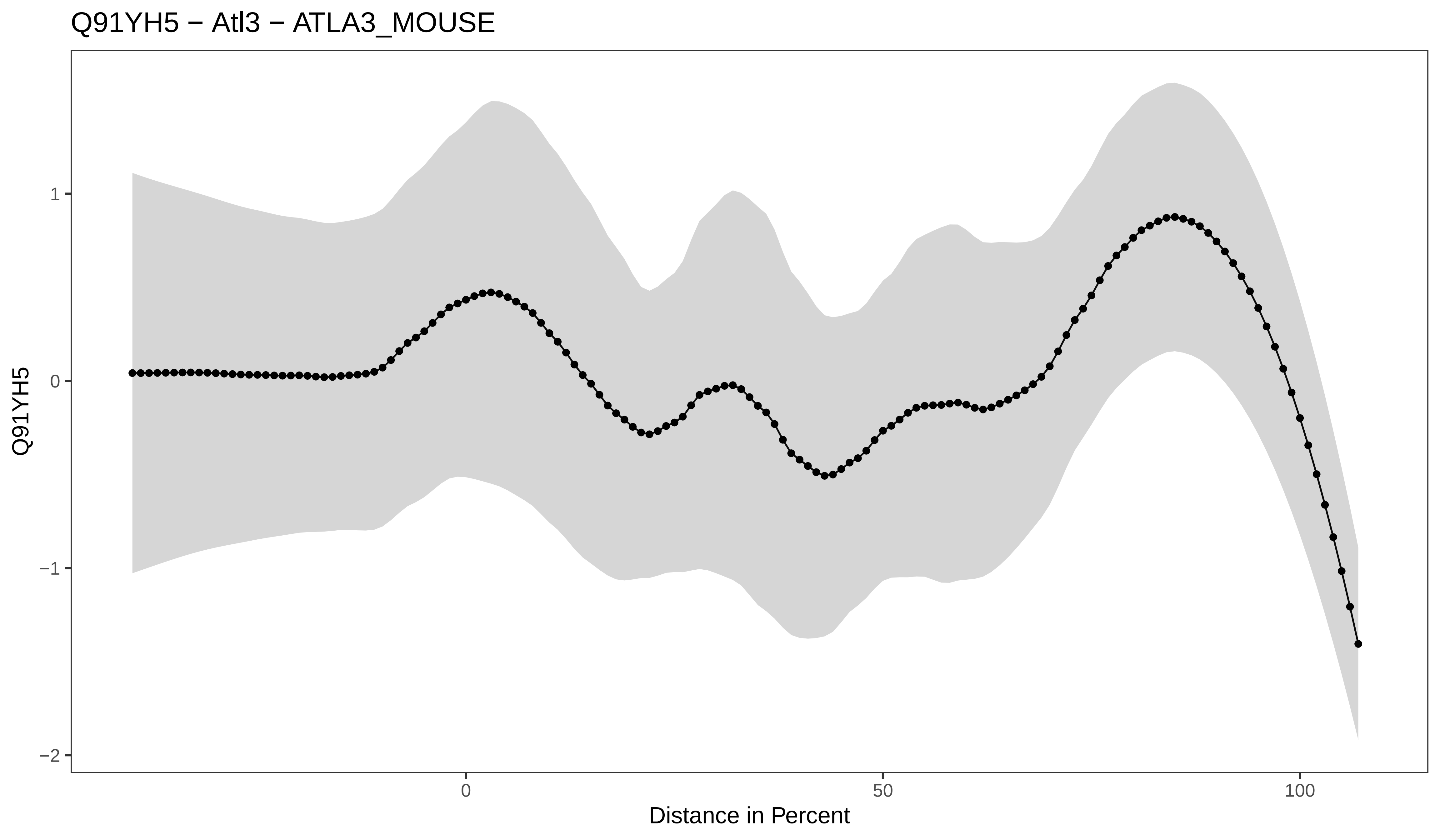

Q91YH5 − Atl3 − ATLA3_MOUSE
Q91YH5
1
0
−1
−2
0
50
100
Distance in Percent

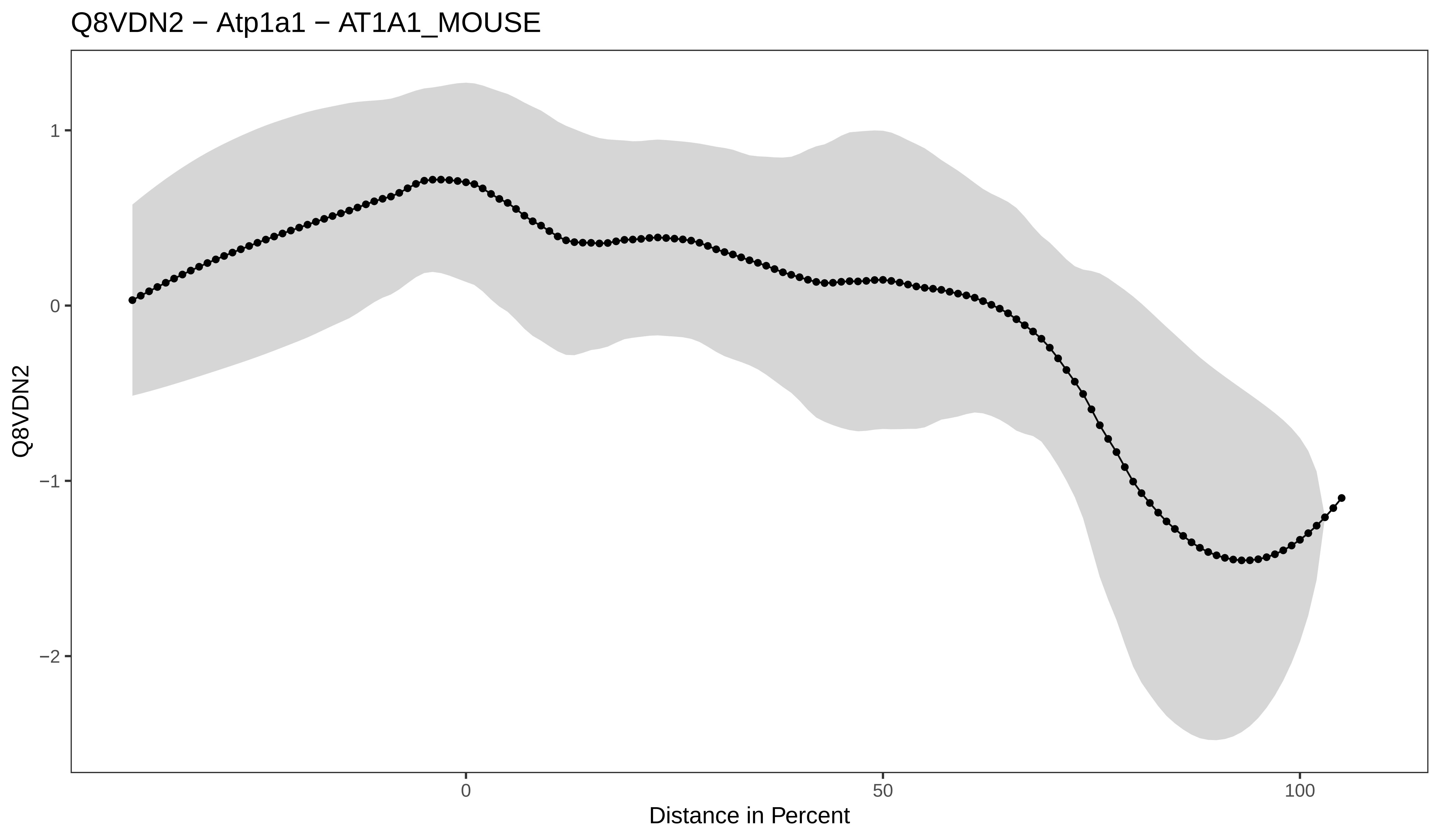
Q8VDN2 − Atp1a1 − AT1A1_MOUSE
Q8VDN2
1
0
−1
−2
0
50
100
Distance in Percent

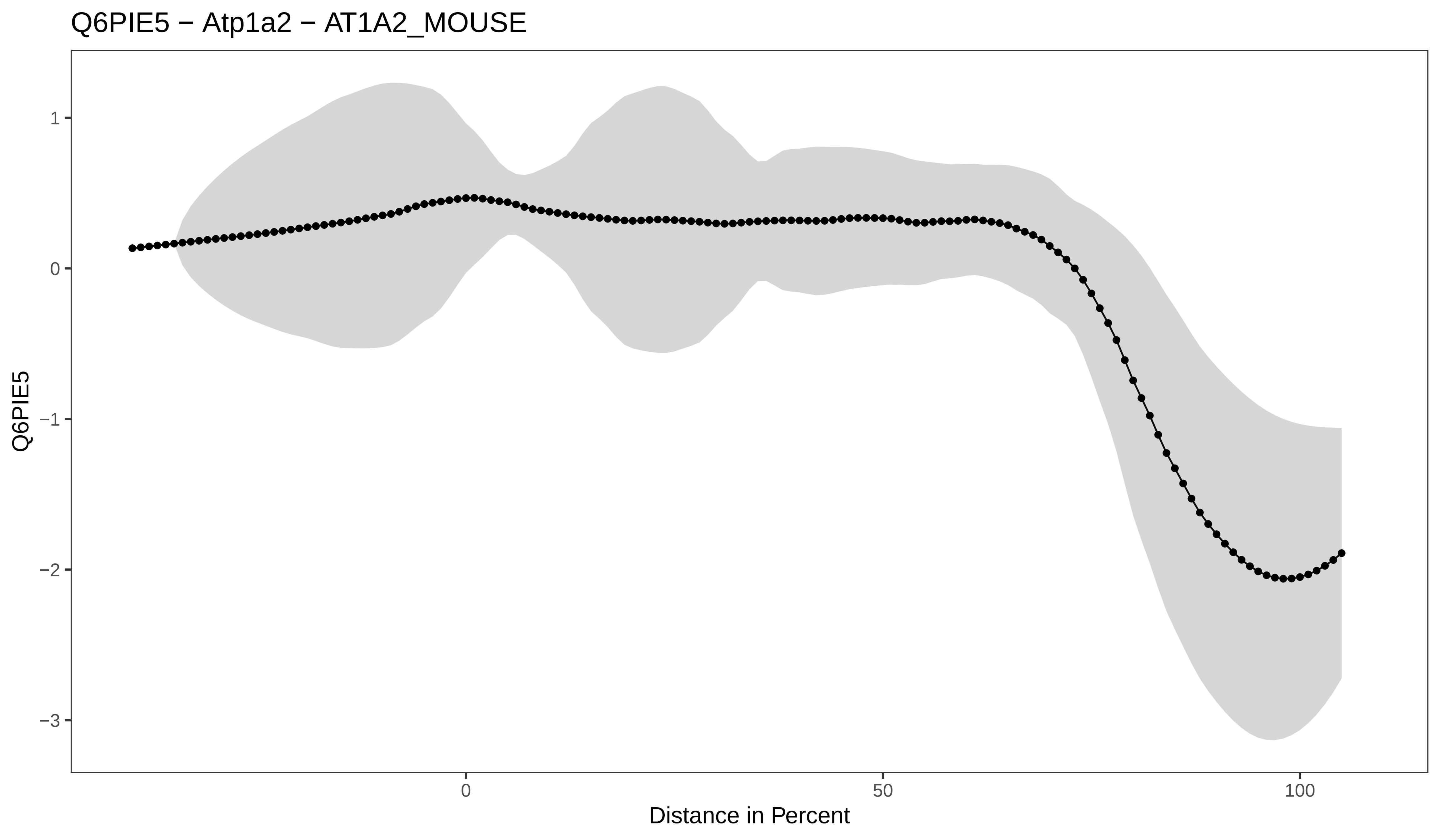

Q6PIE5 − Atp1a2 − AT1A2_MOUSE
1
0
−1
−2
−3
Q6PIE5
0
50
100
Distance in Percent

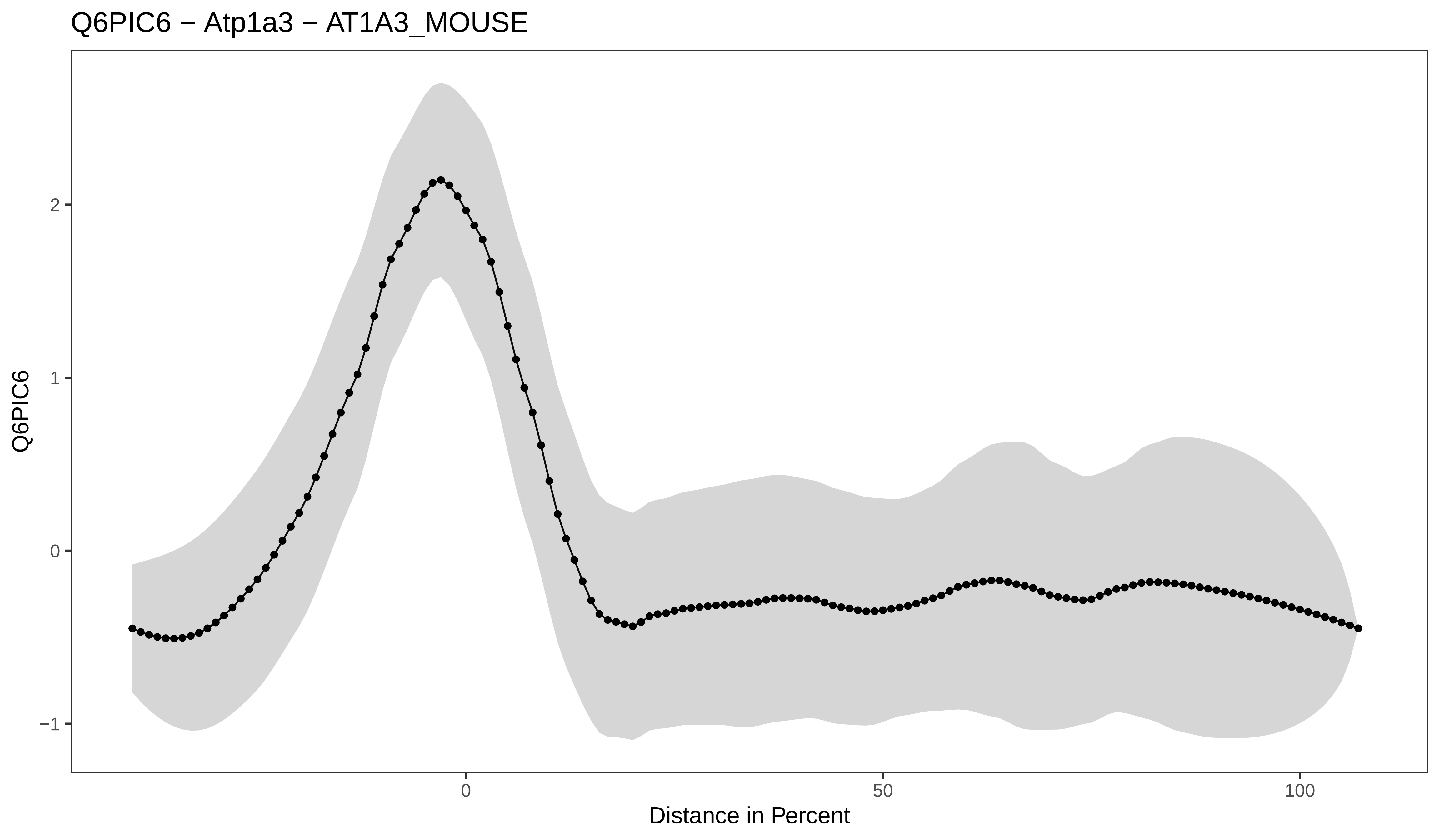

Q6PIC6 − Atp1a3 − AT1A3_MOUSE
Q6PIC6
2
1
0
−1
0
50
100
Distance in Percent

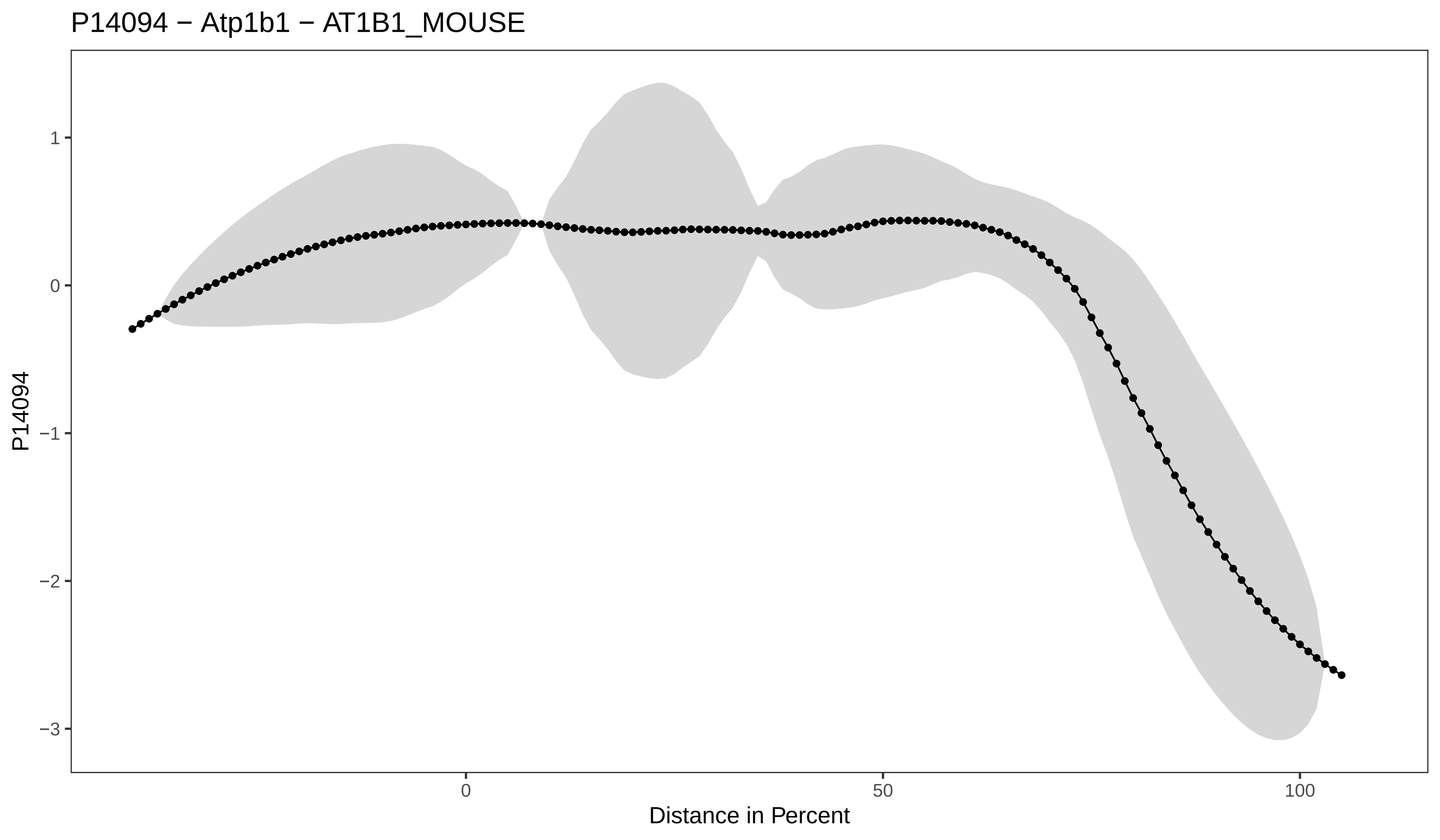
P14094 − Atp1b1 − AT1B1_MOUSE
1
0
−1
−2
−3
P14094
0
50
100
Distance in Percent

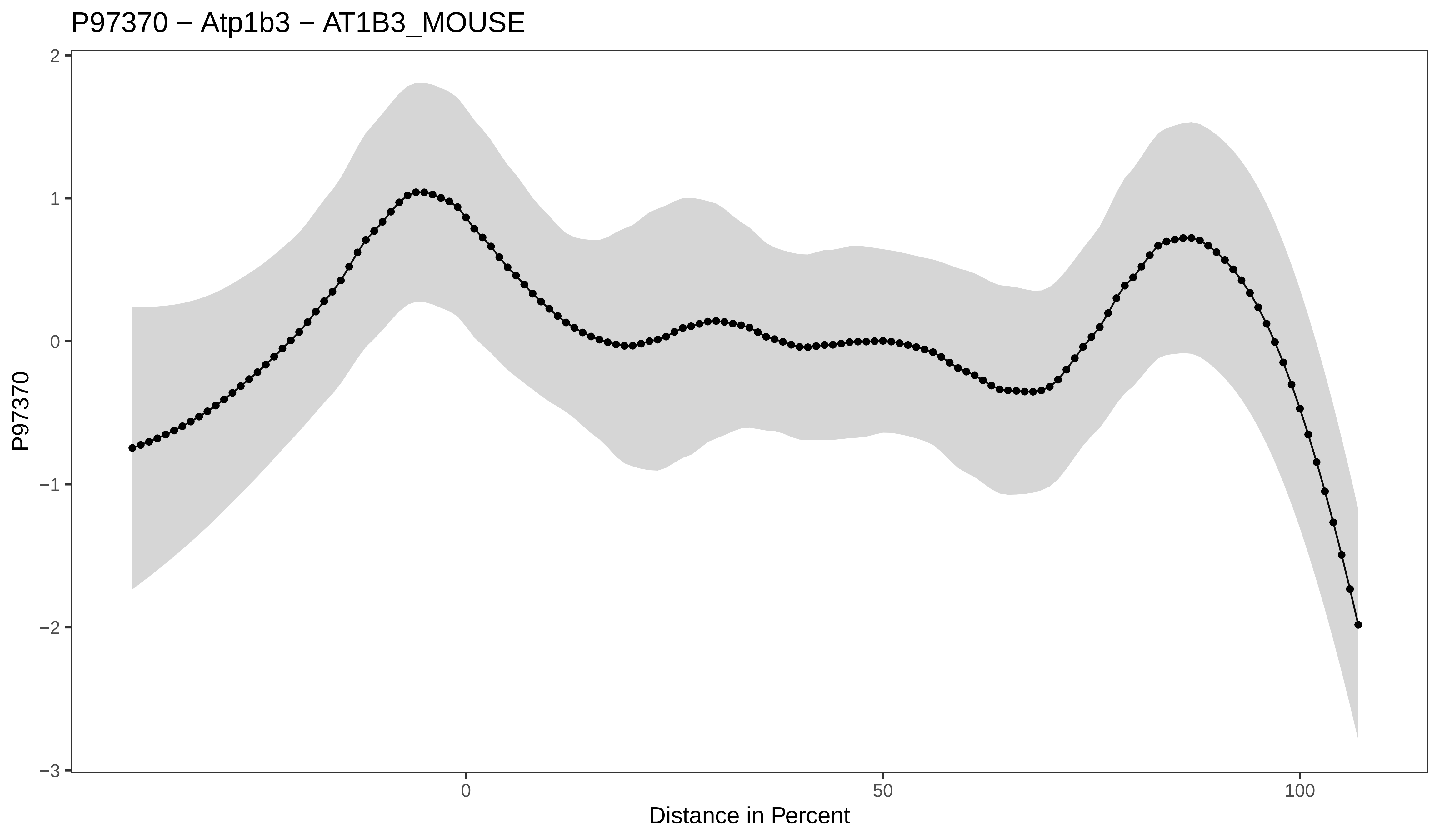

P97370 − Atp1b3 − AT1B3_MOUSE
P97370
Distance in Percent
2
1
0
−1
−2
−3
0
50
100

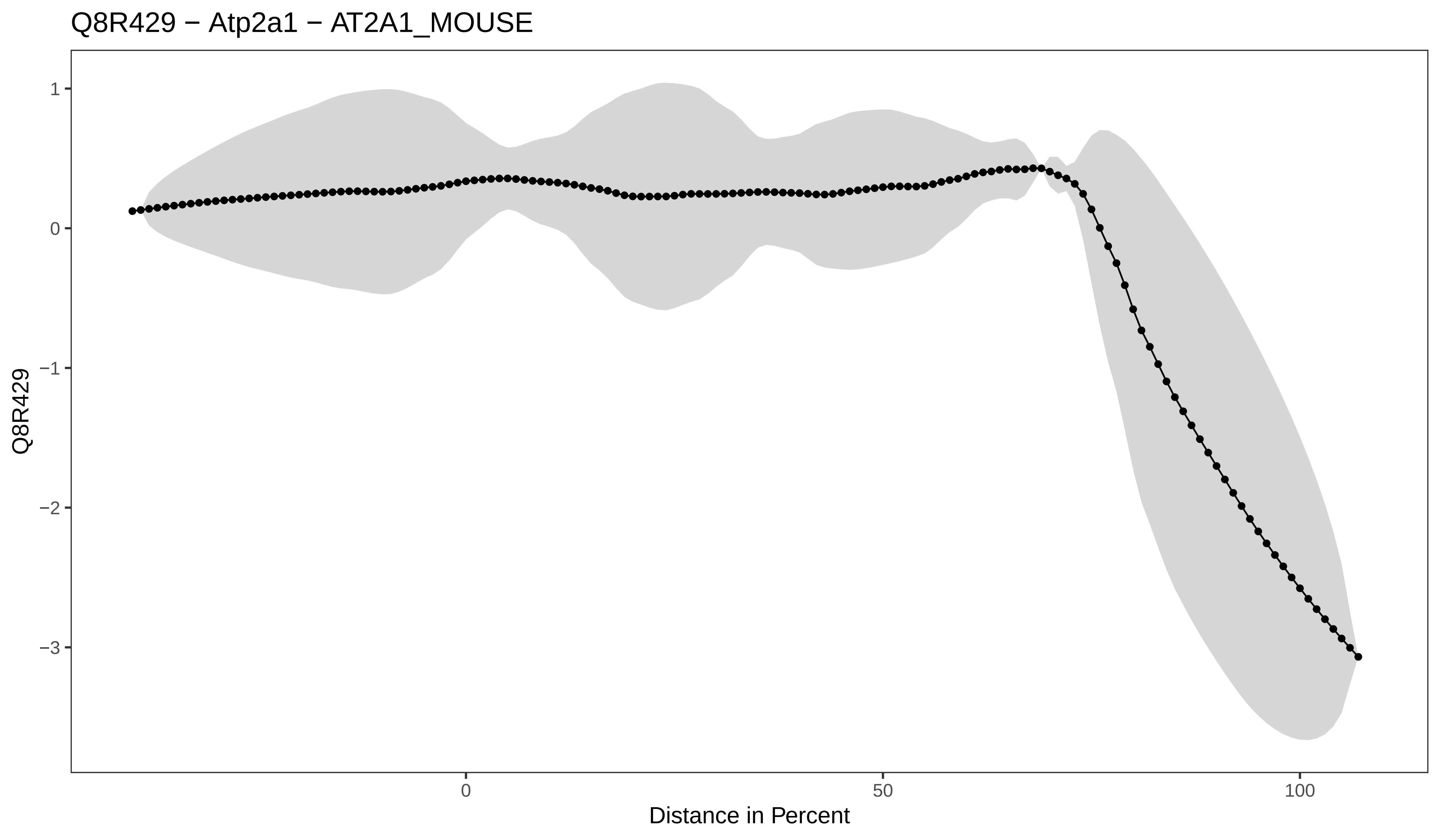
Q8R429 − Atp2a1 − AT2A1_MOUSE
1
0
−1
−2
−3
Q8R429
0
50
100
Distance in Percent

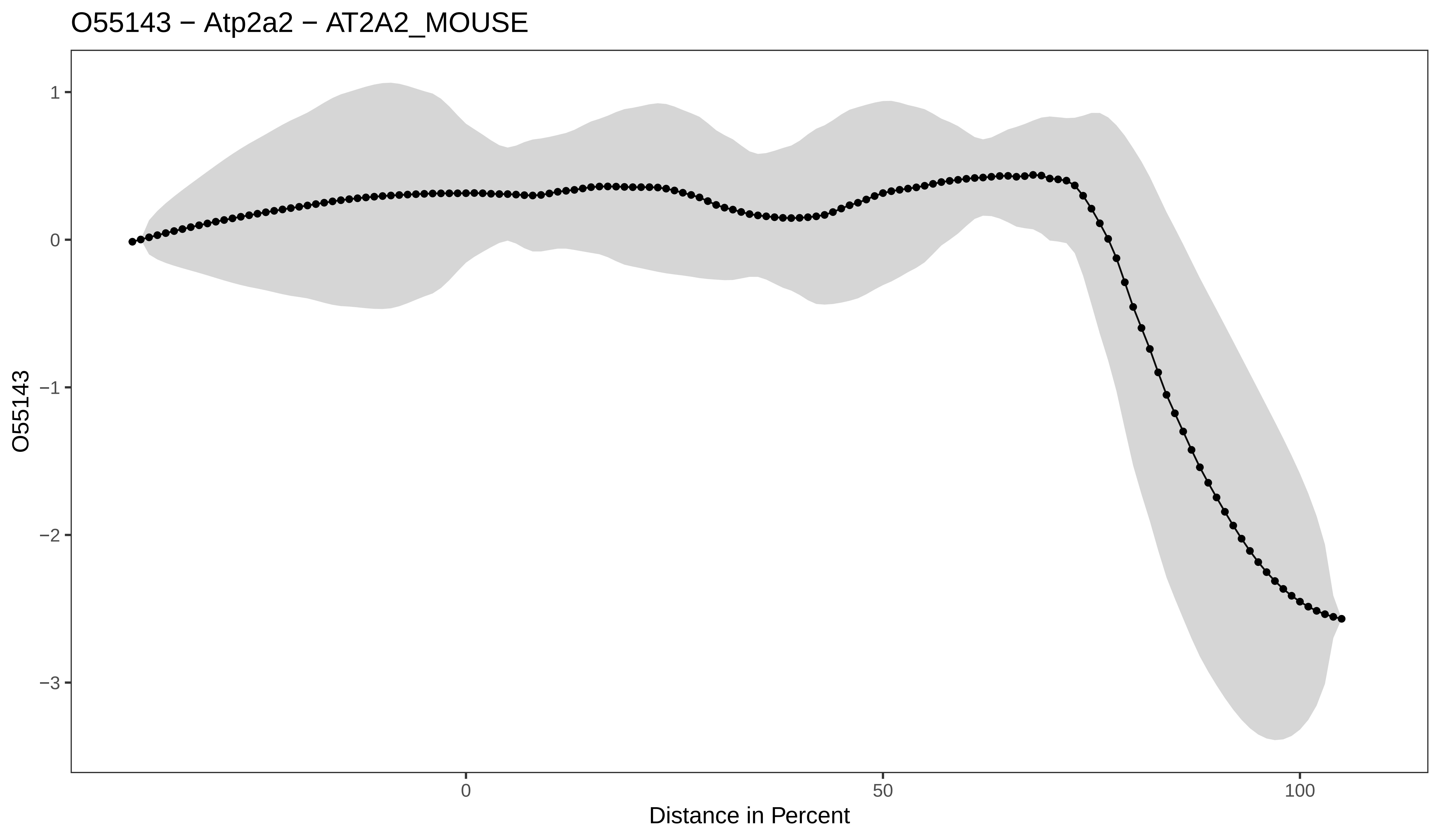

O55143 − Atp2a2 − AT2A2_MOUSE
1
0
−1
−2
−3
O55143
0
50
100
Distance in Percent

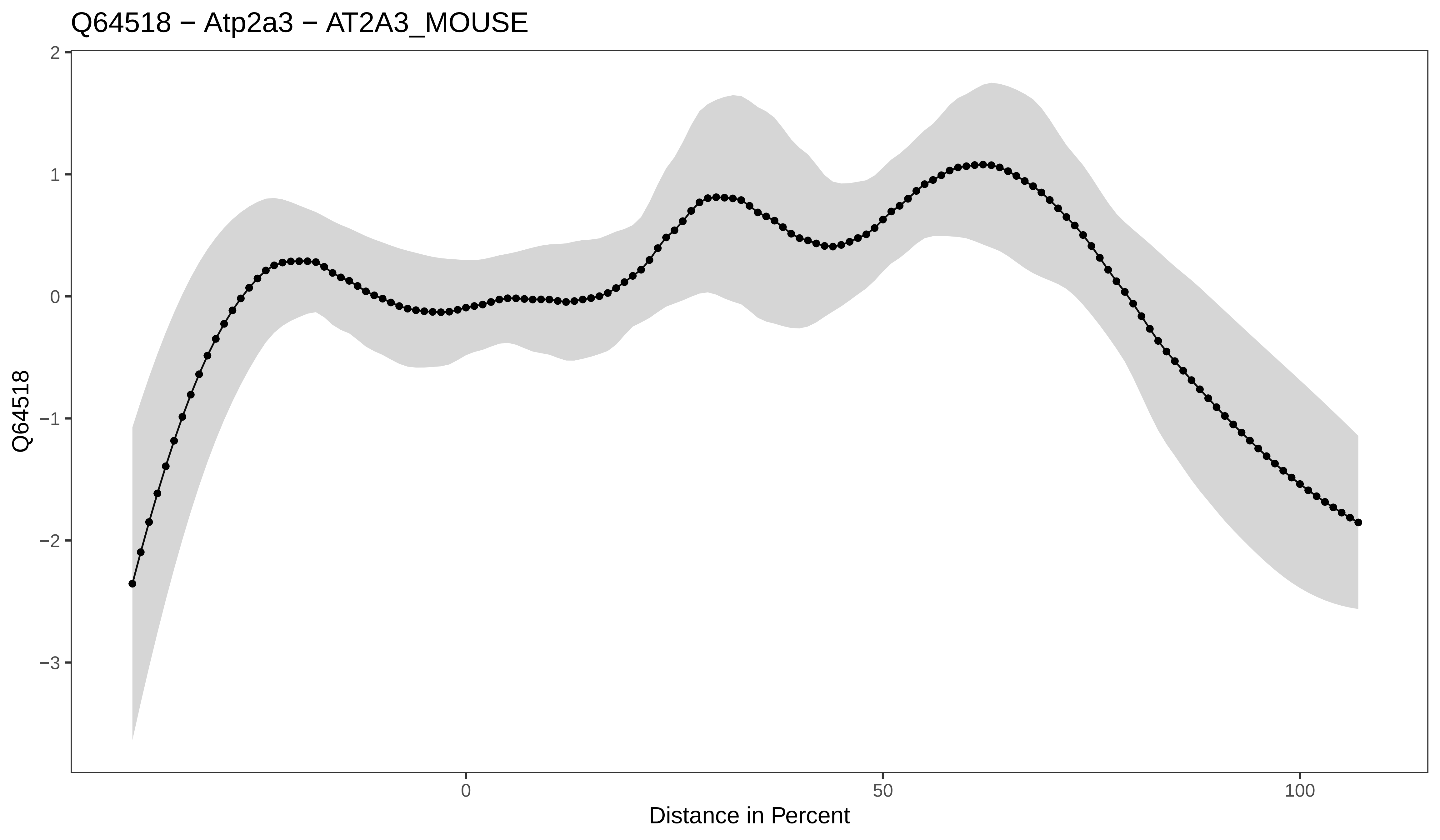
Q64518 − Atp2a3 − AT2A3_MOUSE
Q64518
Distance in Percent
2
1
0
−1
−2
−3
0
50
100

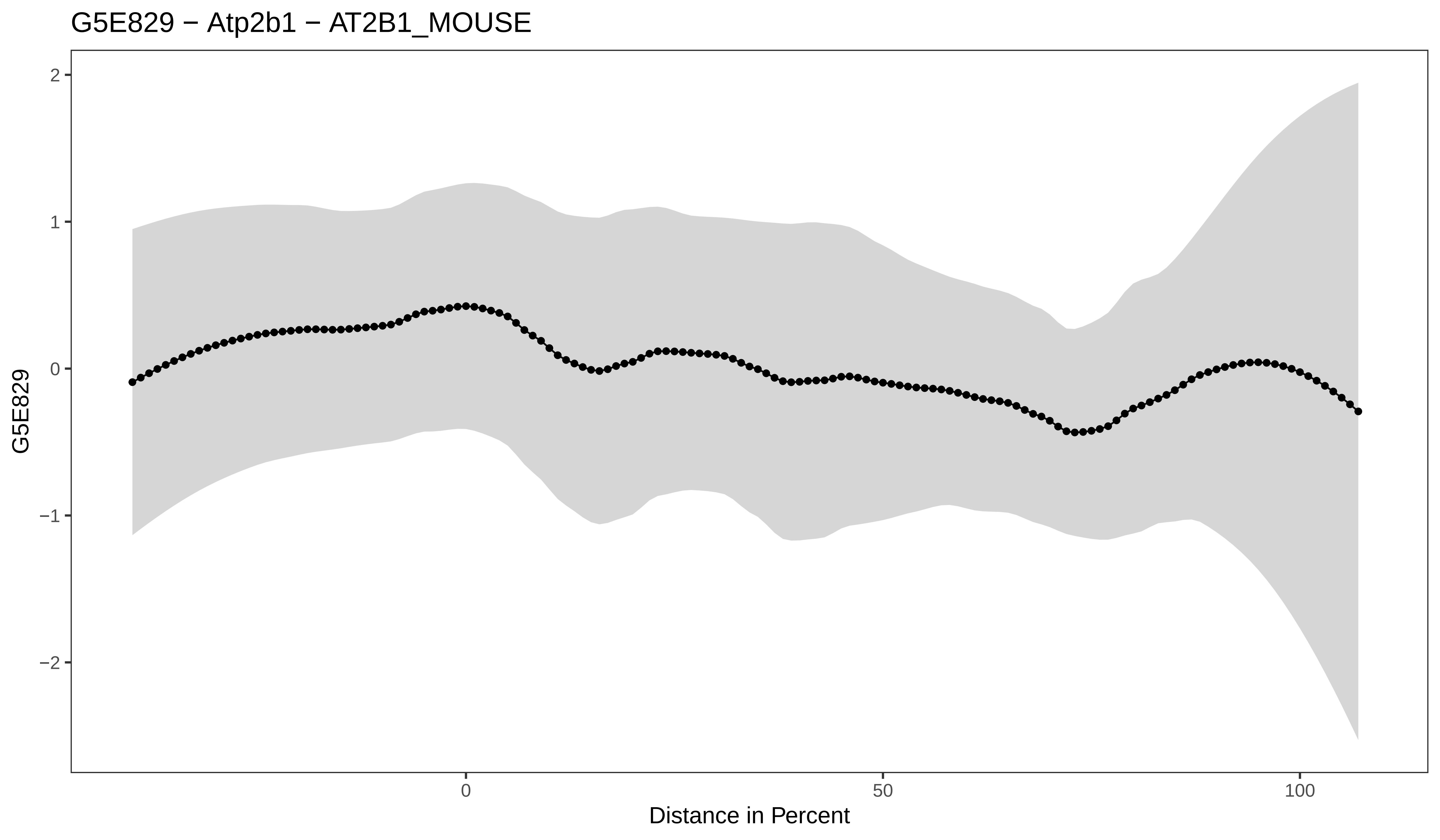

G5E829 − Atp2b1 − AT2B1_MOUSE
2
1
0
−1
−2
G5E829
0
50
100
Distance in Percent

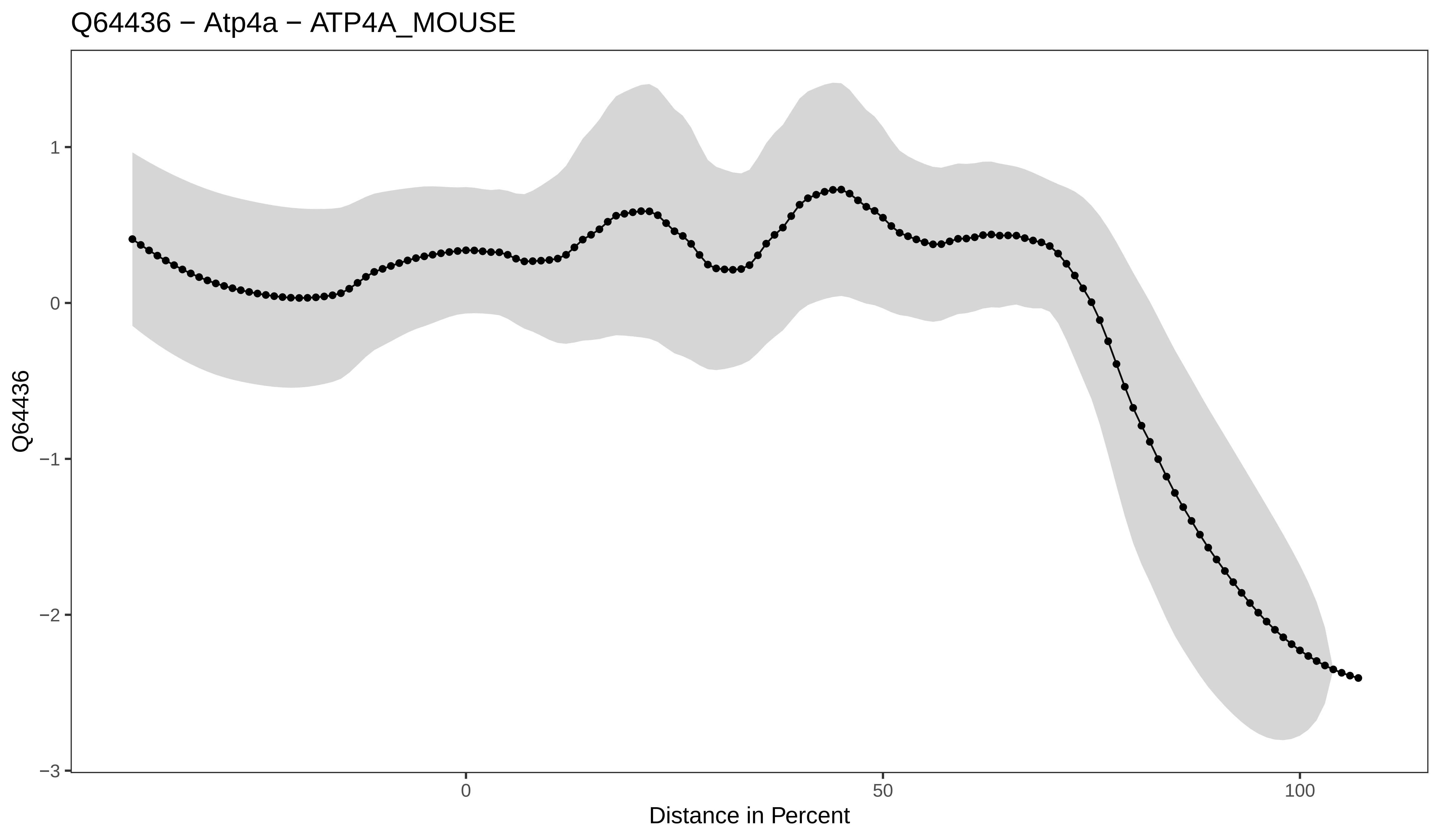

Q64436 − Atp4a − ATP4A_MOUSE
1
0
−1
−2
−3
Q64436
0
50
100
Distance in Percent

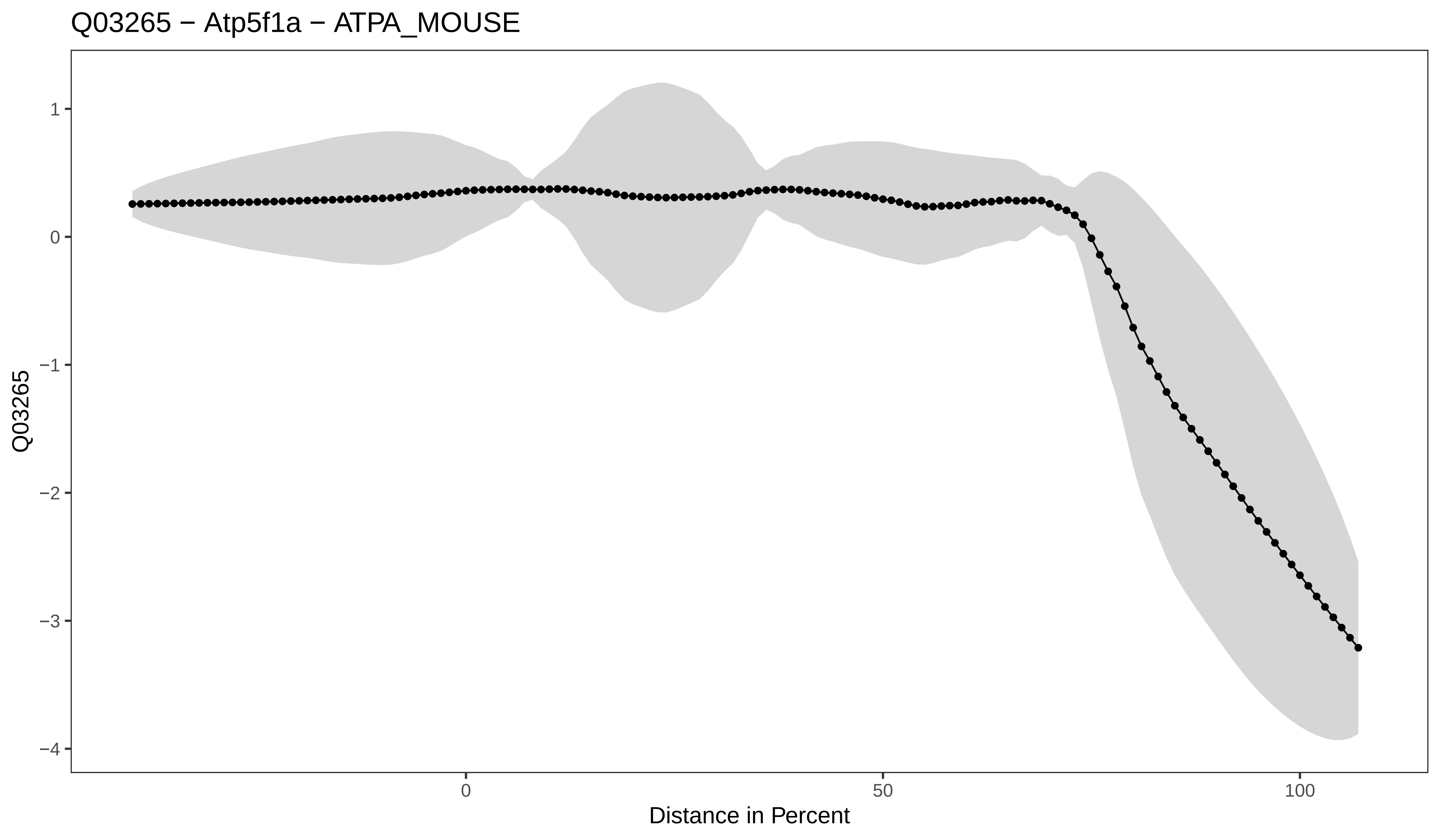

Q03265 − Atp5f1a − ATPA_MOUSE
Q03265
1
0
−1
−2
−3
−4
0
50
100
Distance in Percent

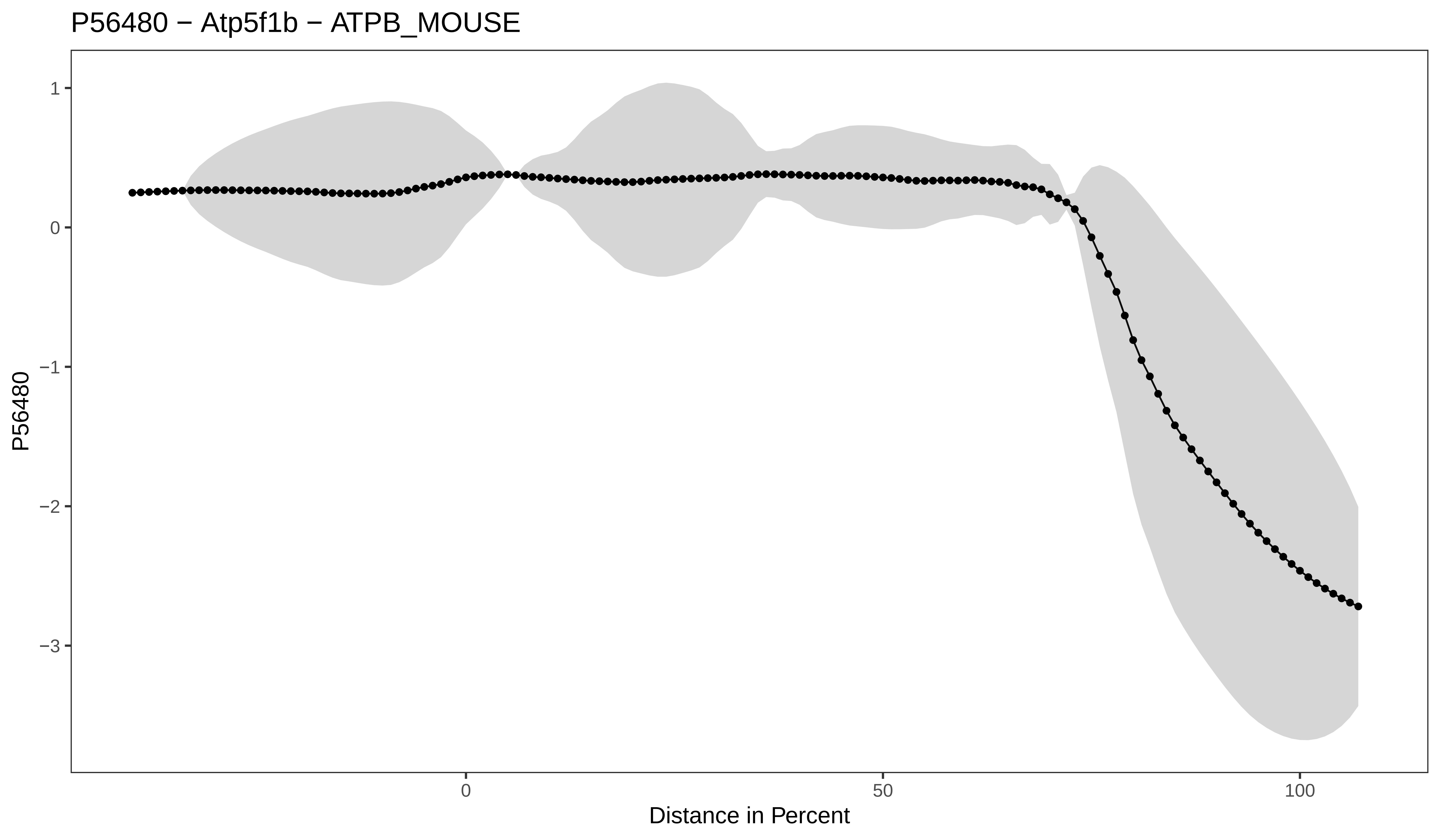

P56480 − Atp5f1b − ATPB_MOUSE
P56480
Distance in Percent
1
0
−1
−2
−3
0
50
100

Q91VR2 − Atp5f1c − ATPG_MOUSE

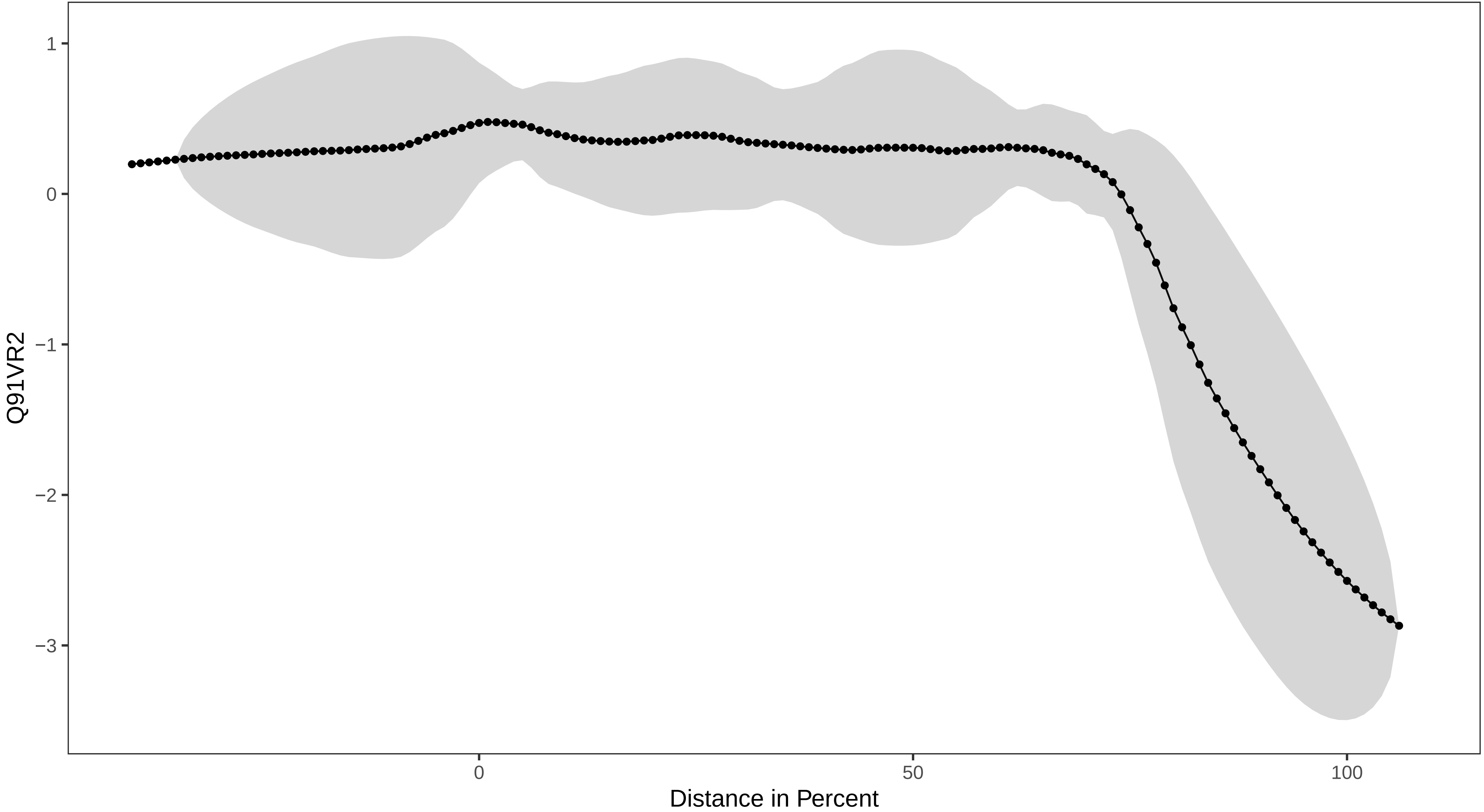

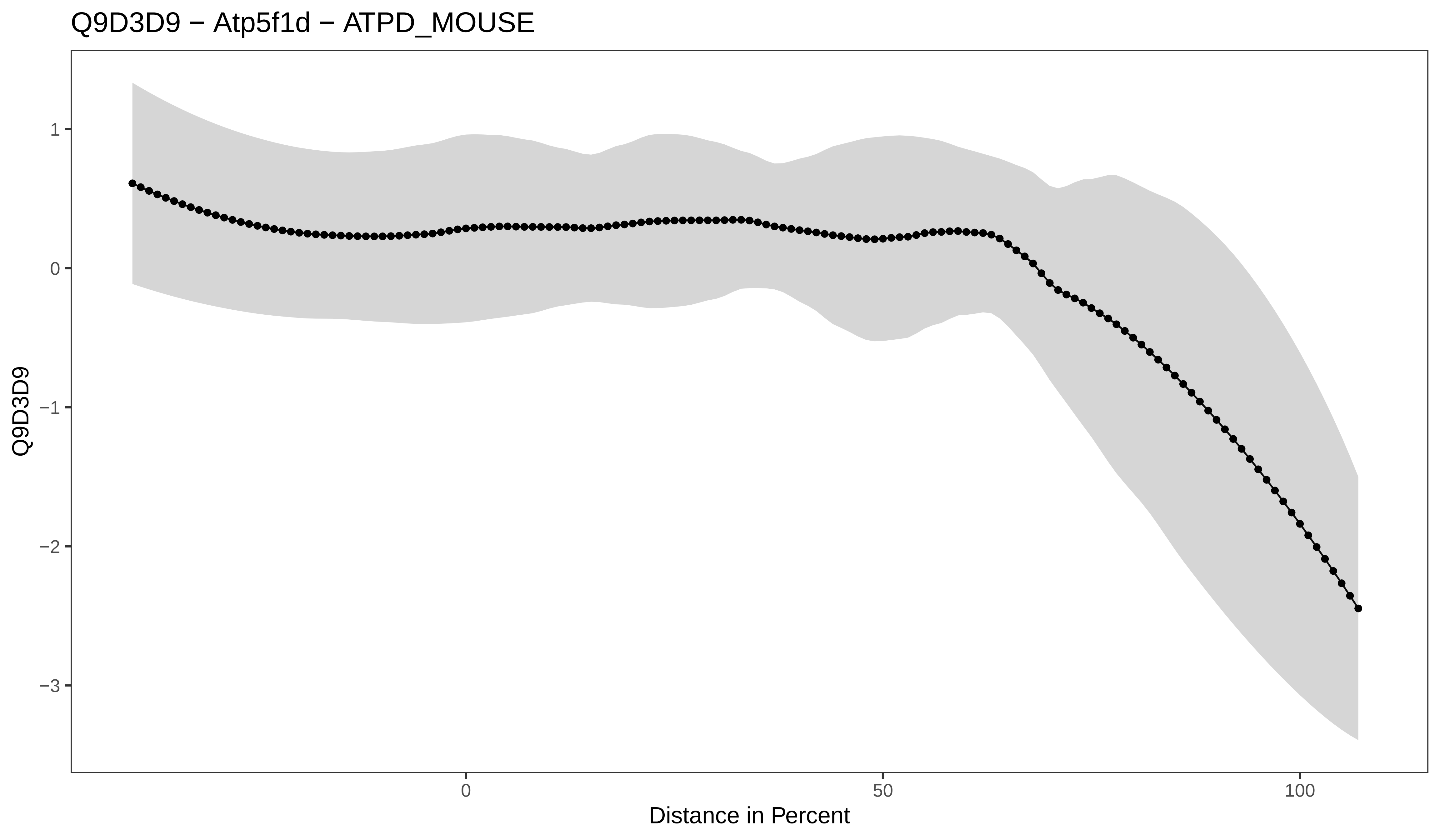
Q9D3D9 − Atp5f1d − ATPD_MOUSE
Q9D3D9
1
0
−1
−2
−3
0
50
100
Distance in Percent

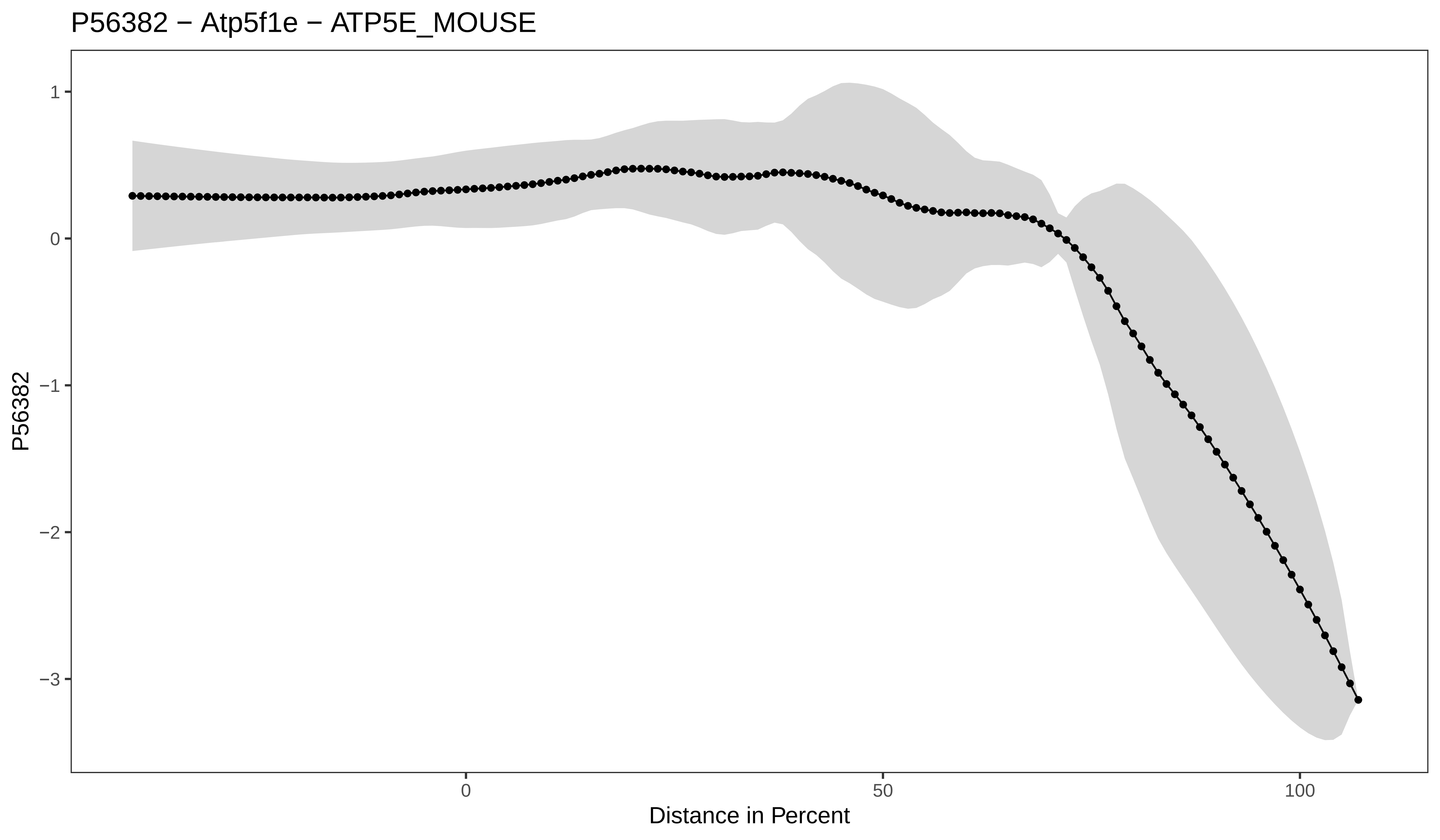

P56382 − Atp5f1e − ATP5E_MOUSE
1
0
−1
−2
−3
P56382
0
50
100
Distance in Percent

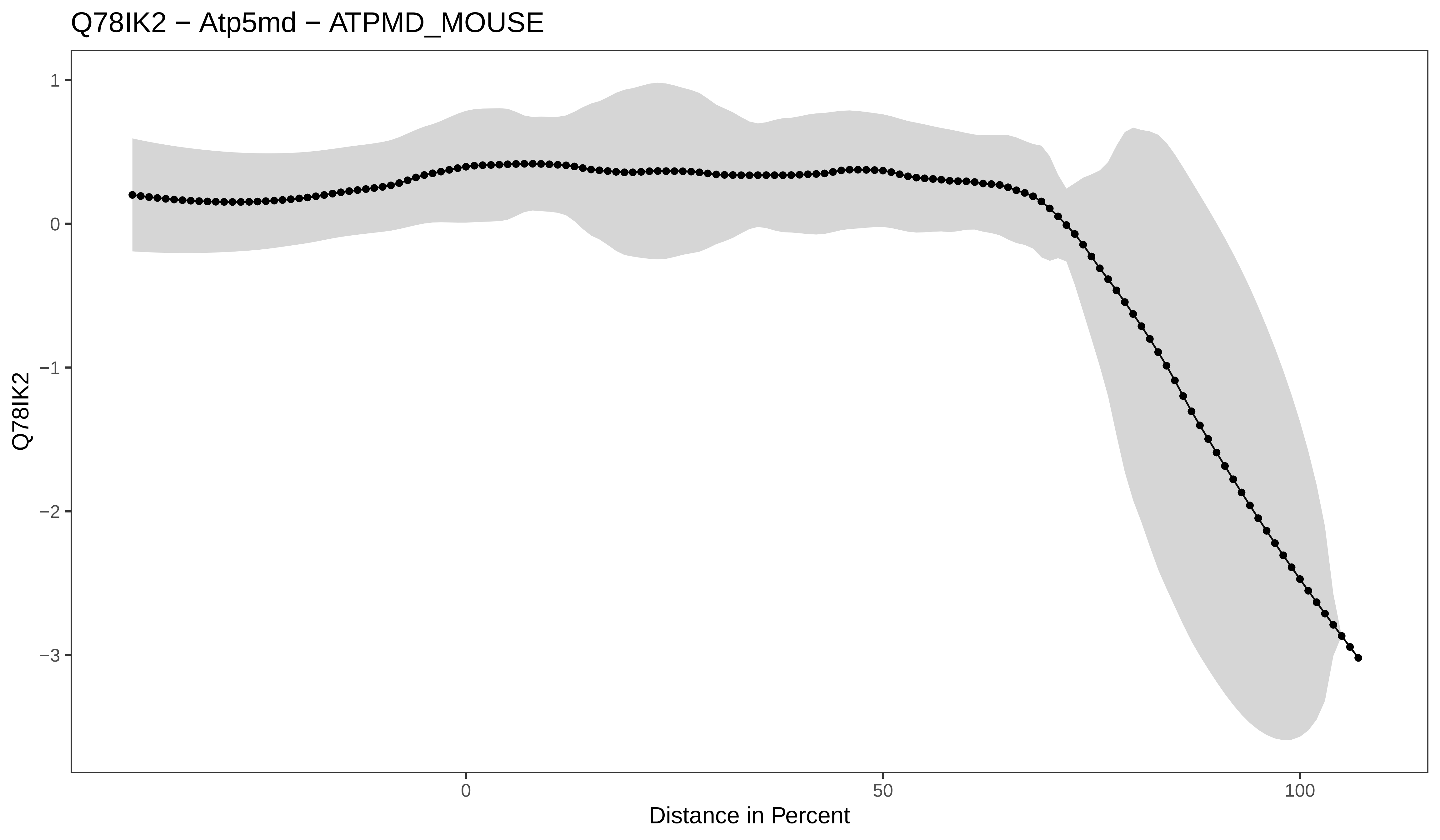

Q78IK2 − Atp5md − ATPMD_MOUSE
1
0
−1
−2
−3
0
50
100
Distance in Percent
Q78IK2

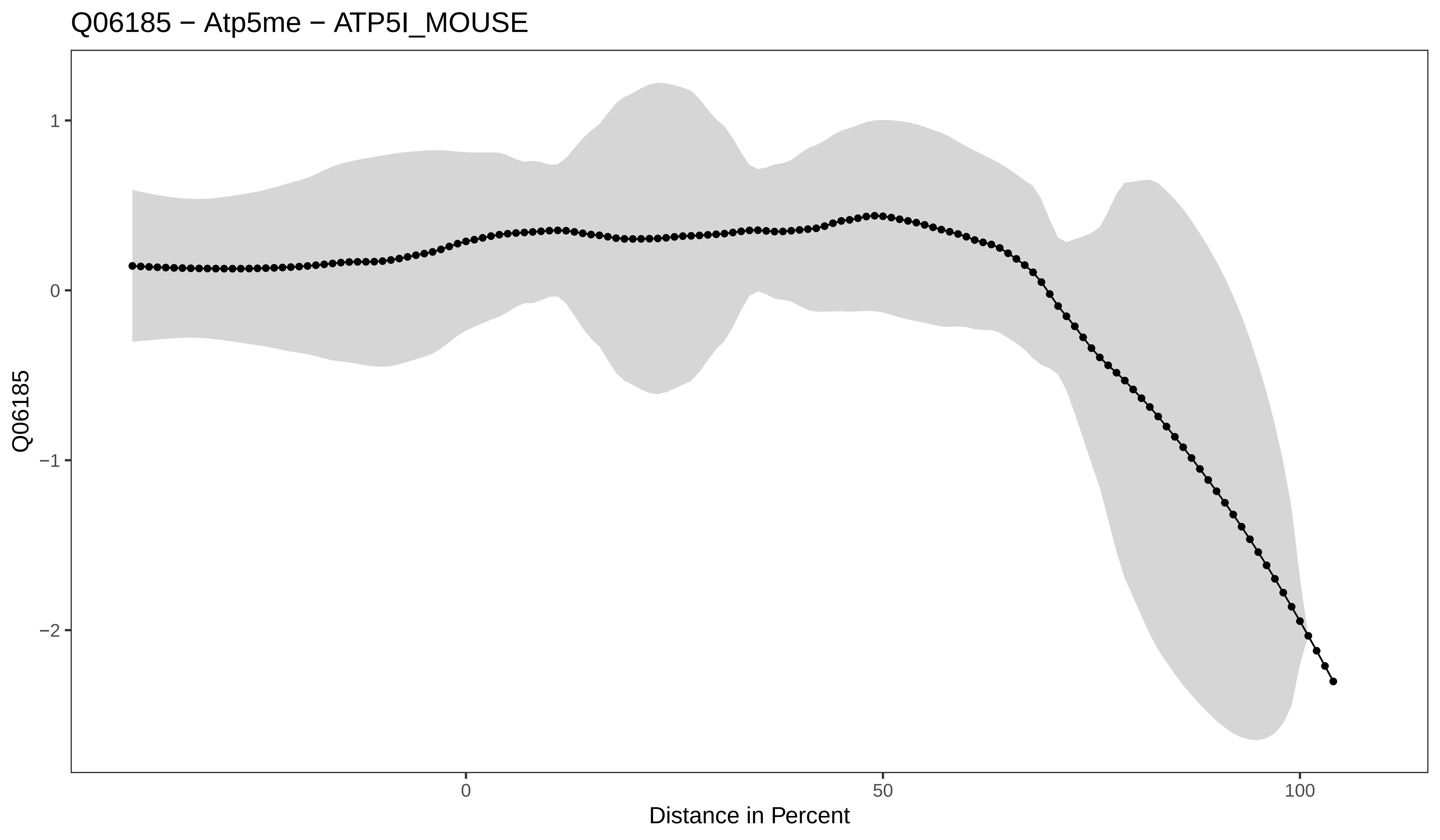

Q06185 − Atp5me − ATP5I_MOUSE
1
0
−1
−2
Q06185
0
50
100
Distance in Percent

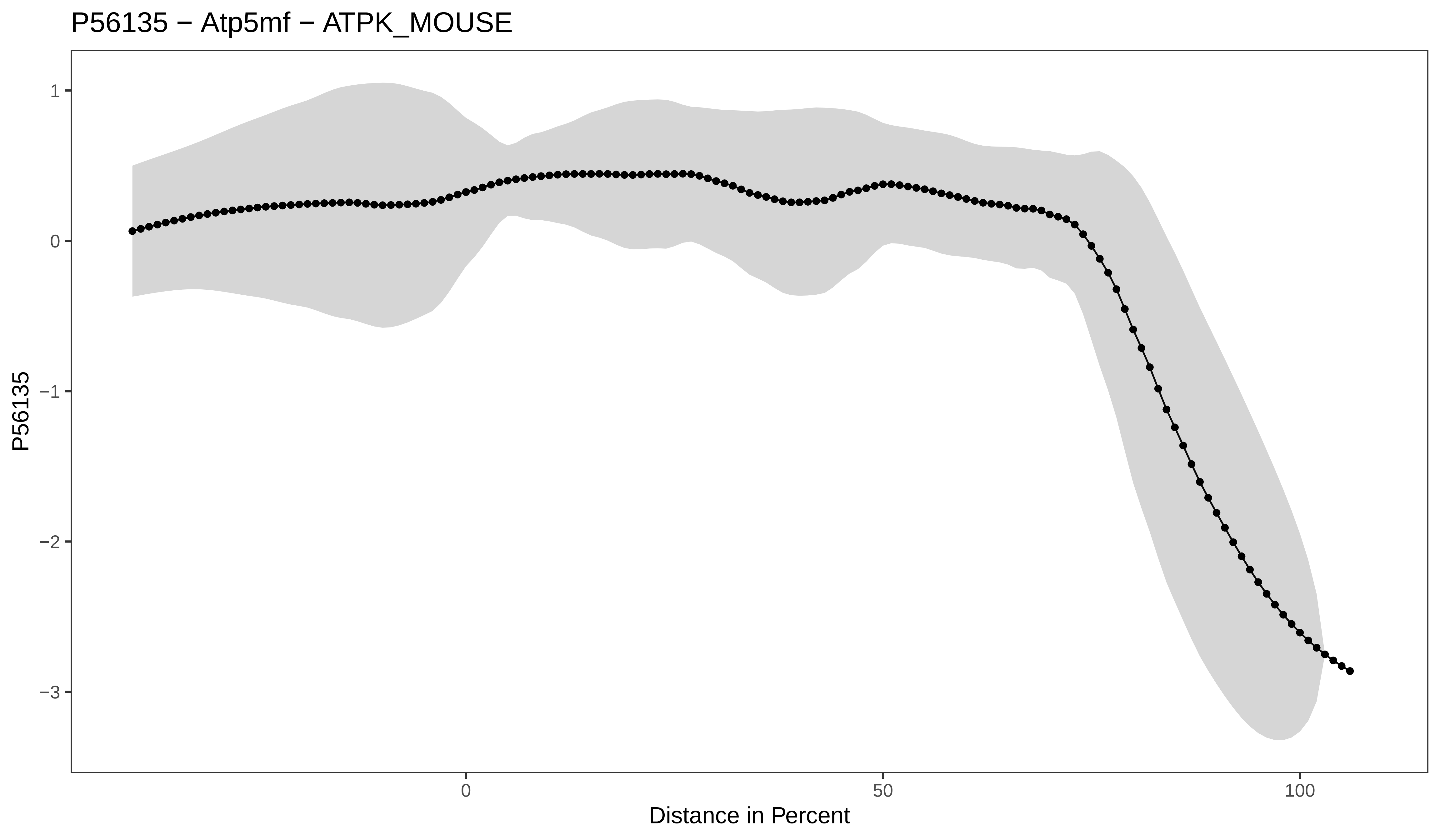

P56135 − Atp5mf − ATPK_MOUSE
1
0
−1
−2
−3
P56135
0
50
100
Distance in Percent

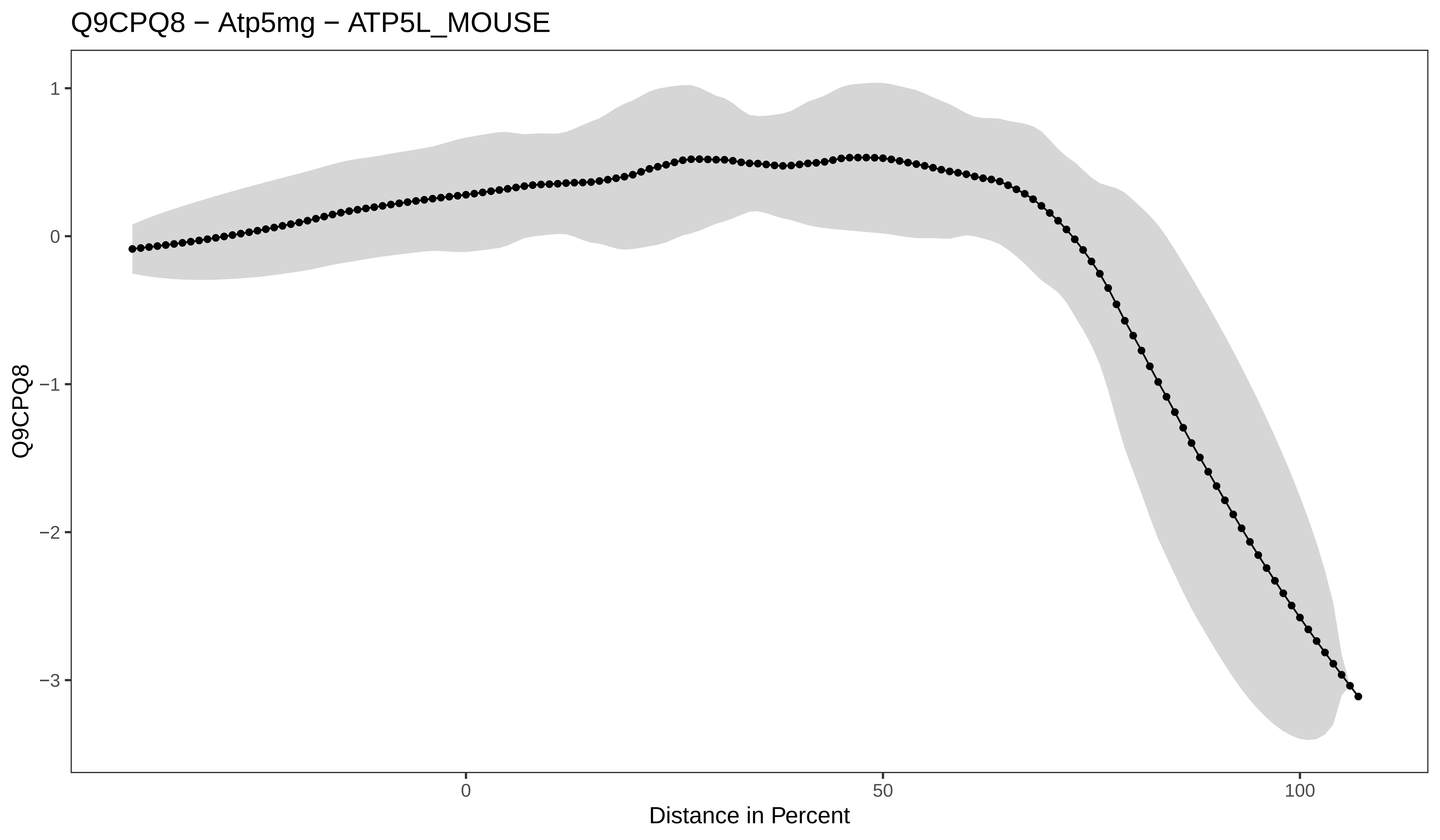
Q9CPQ8 − Atp5mg − ATP5L_MOUSE
1
0
−1
−2
−3
Q9CPQ8
0
50
100
Distance in Percent

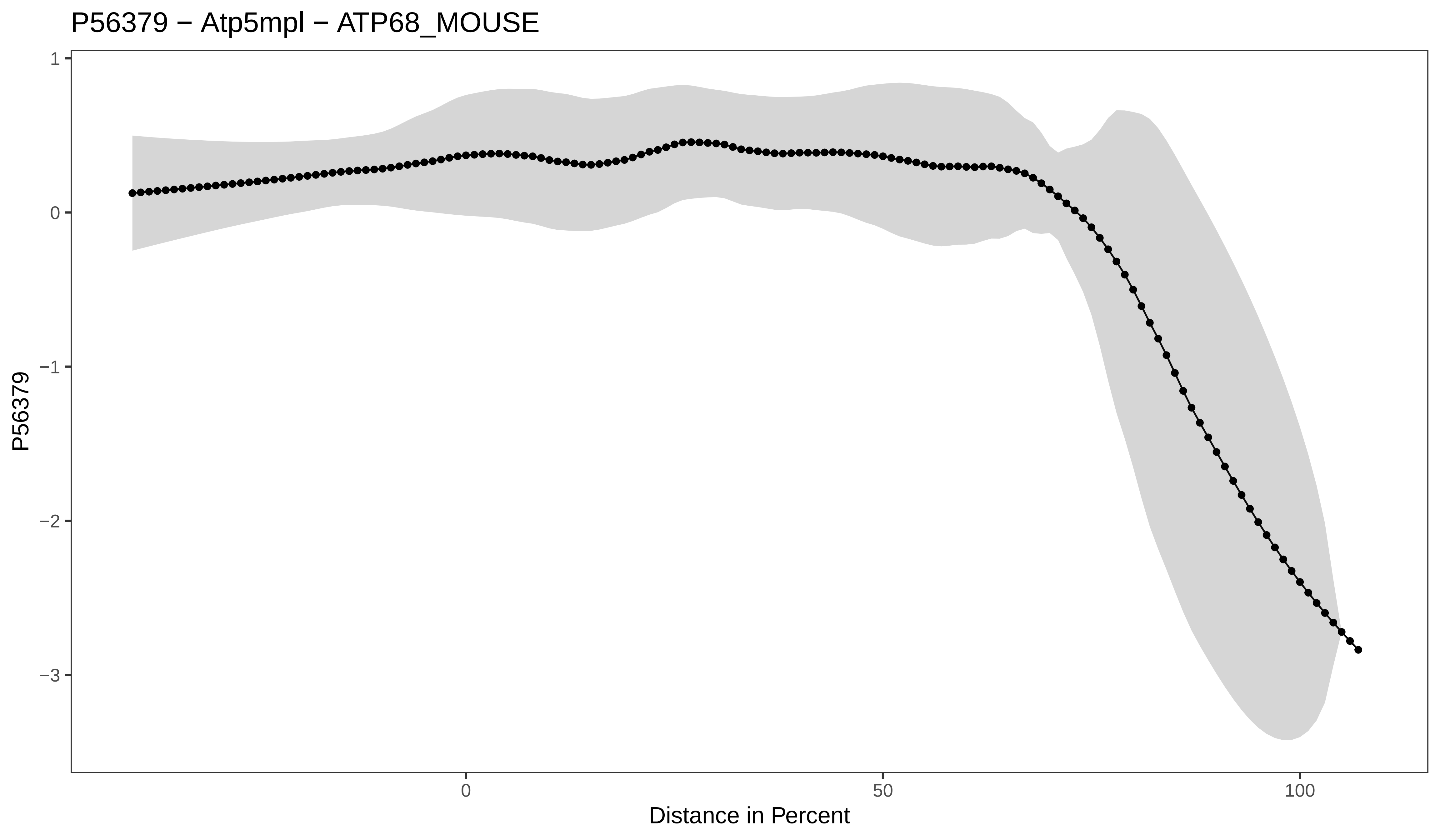

P56379 − Atp5mpl − ATP68_MOUSE
P56379
1
0
−1
−2
−3
0
50
100
Distance in Percent

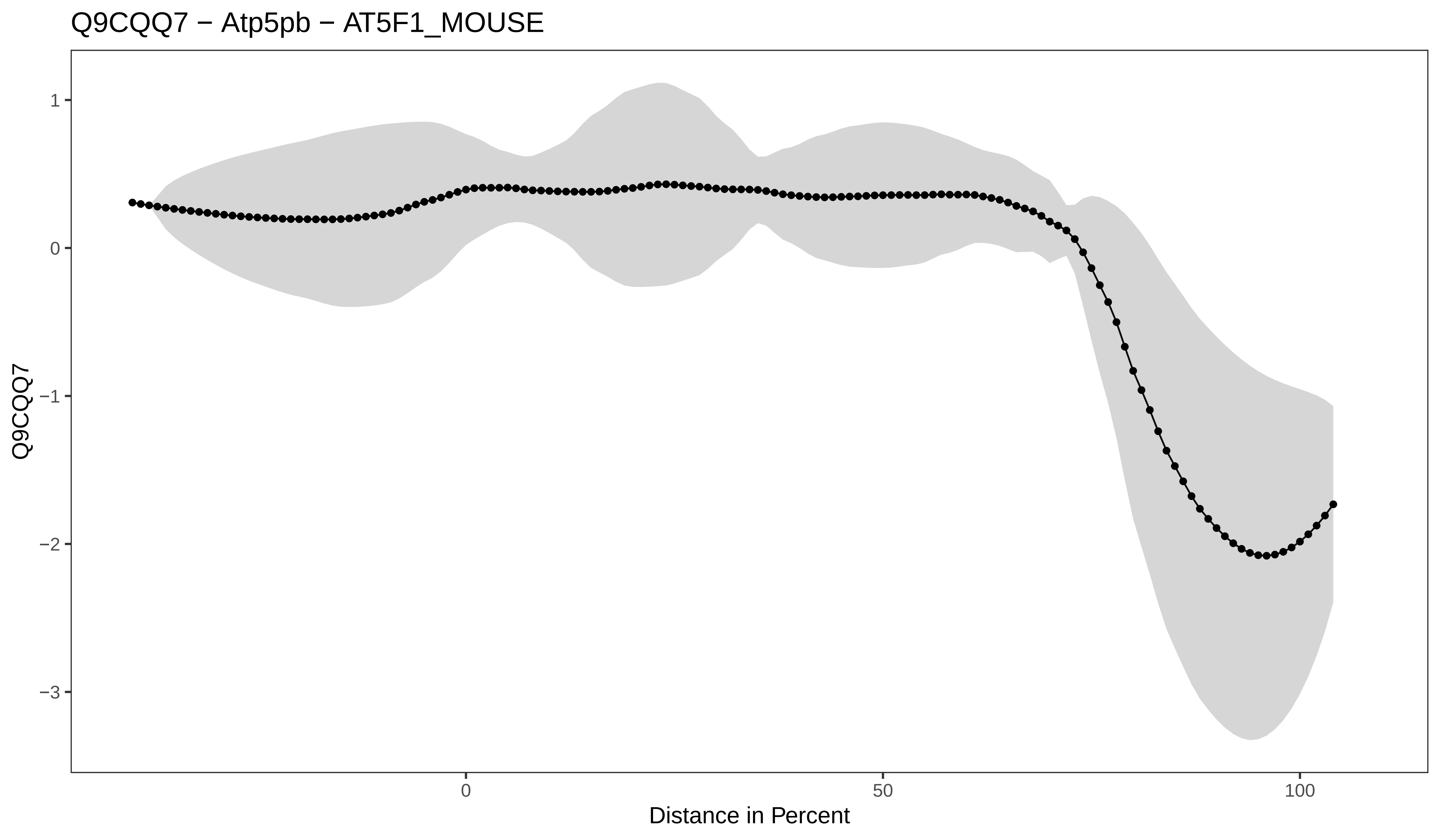

Q9CQQ7 − Atp5pb − AT5F1_MOUSE
1
0
−1
−2
−3
Q9CQQ7
0
50
100
Distance in Percent

Q9DCX2 − Atp5pd − ATP5H_MOUSE

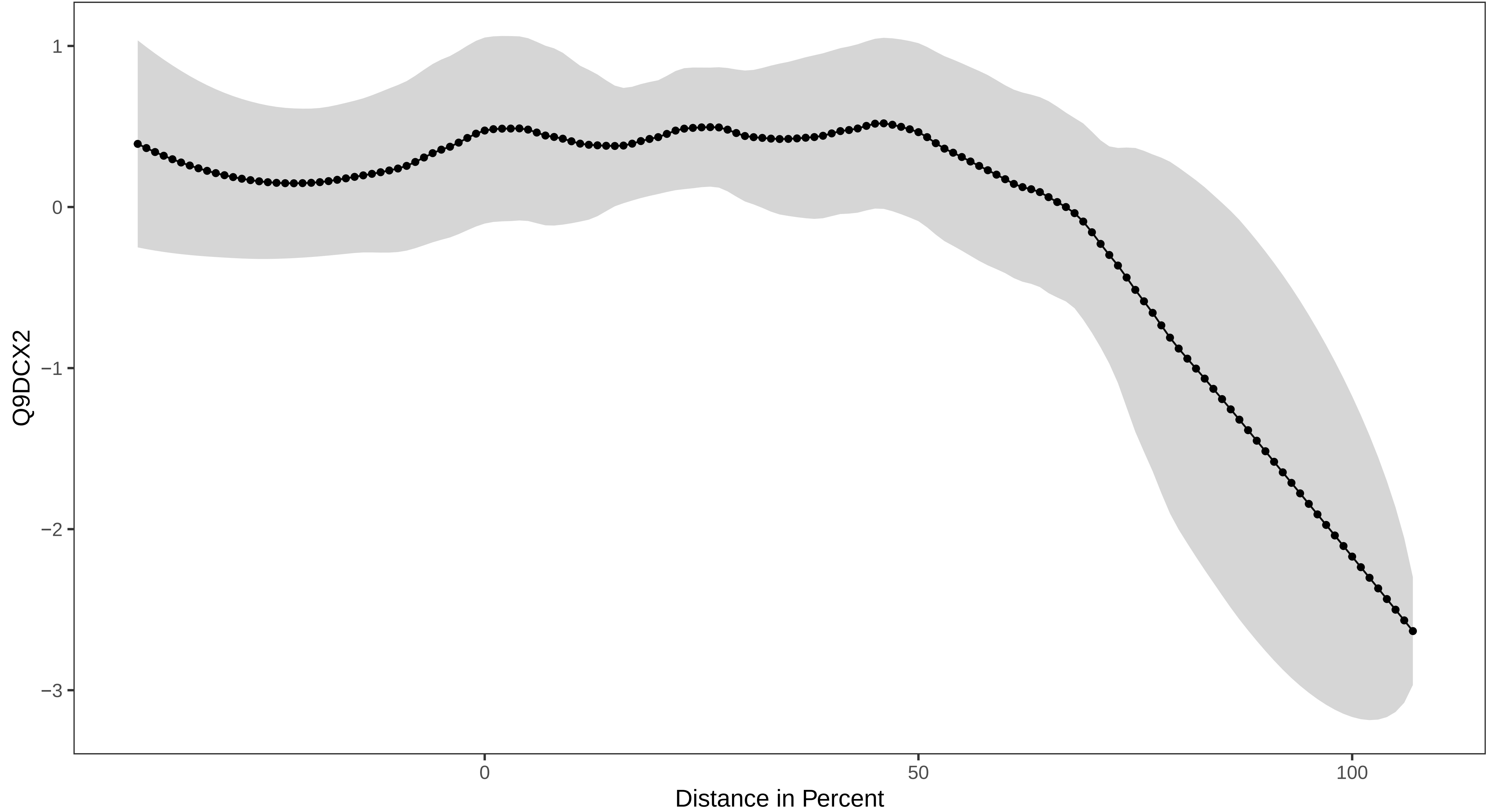

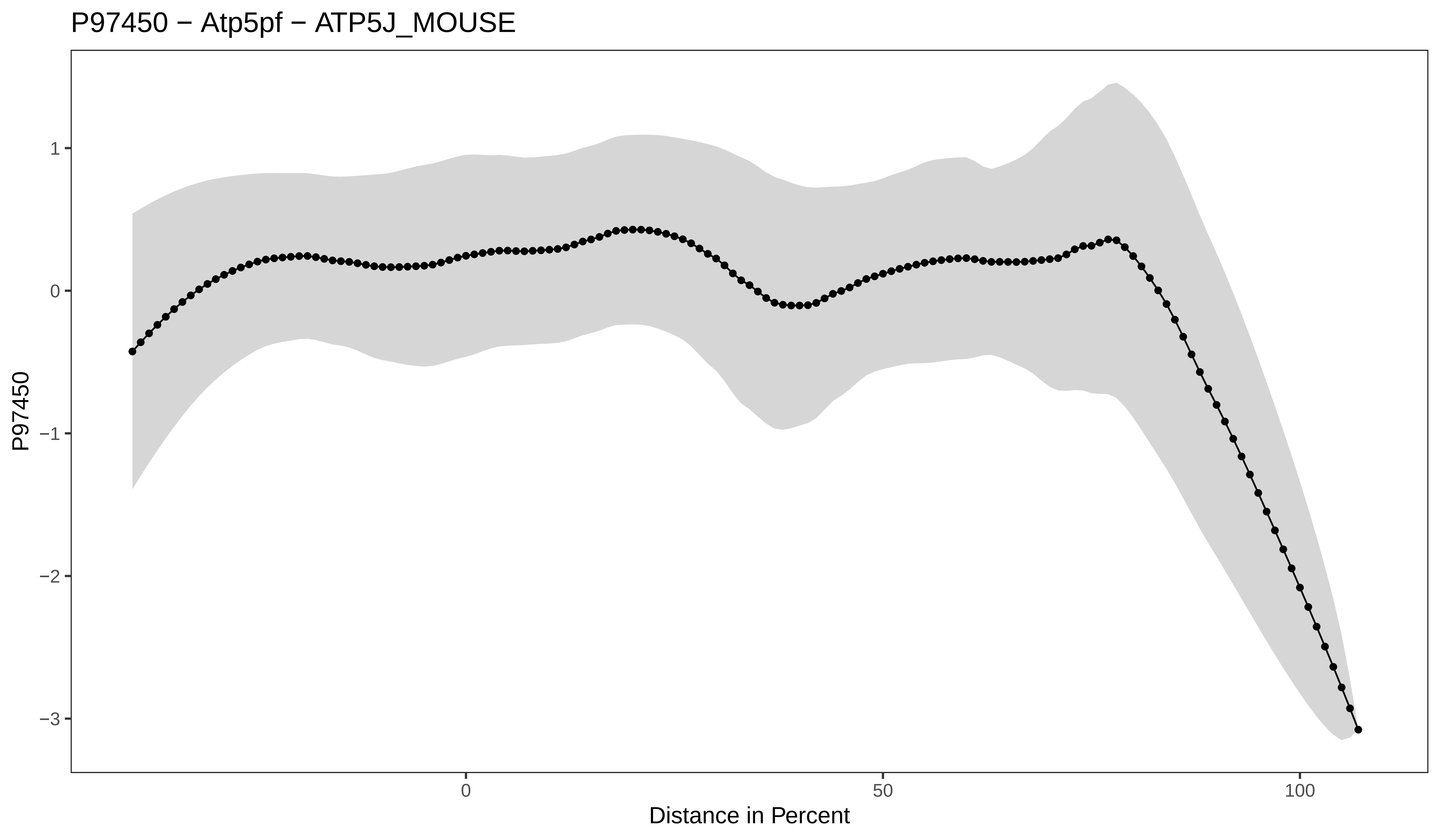

P97450 − Atp5pf − ATP5J_MOUSE
P97450
1
0
−1
−2
−3
0
50
100
Distance in Percent

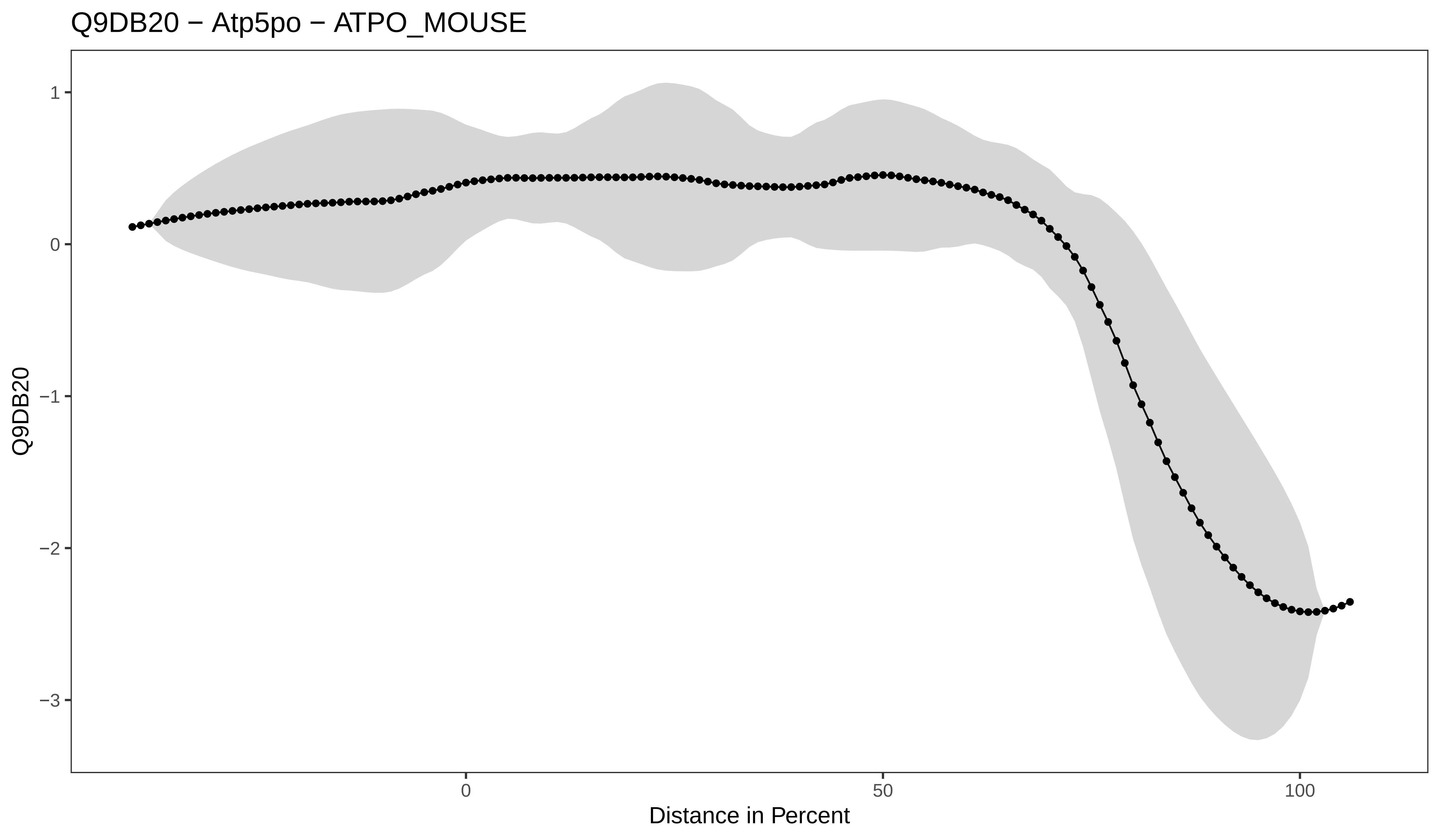

Q9DB20 − Atp5po − ATPO_MOUSE
1
0
−1
−2
−3
Q9DB20
0
50
100
Distance in Percent

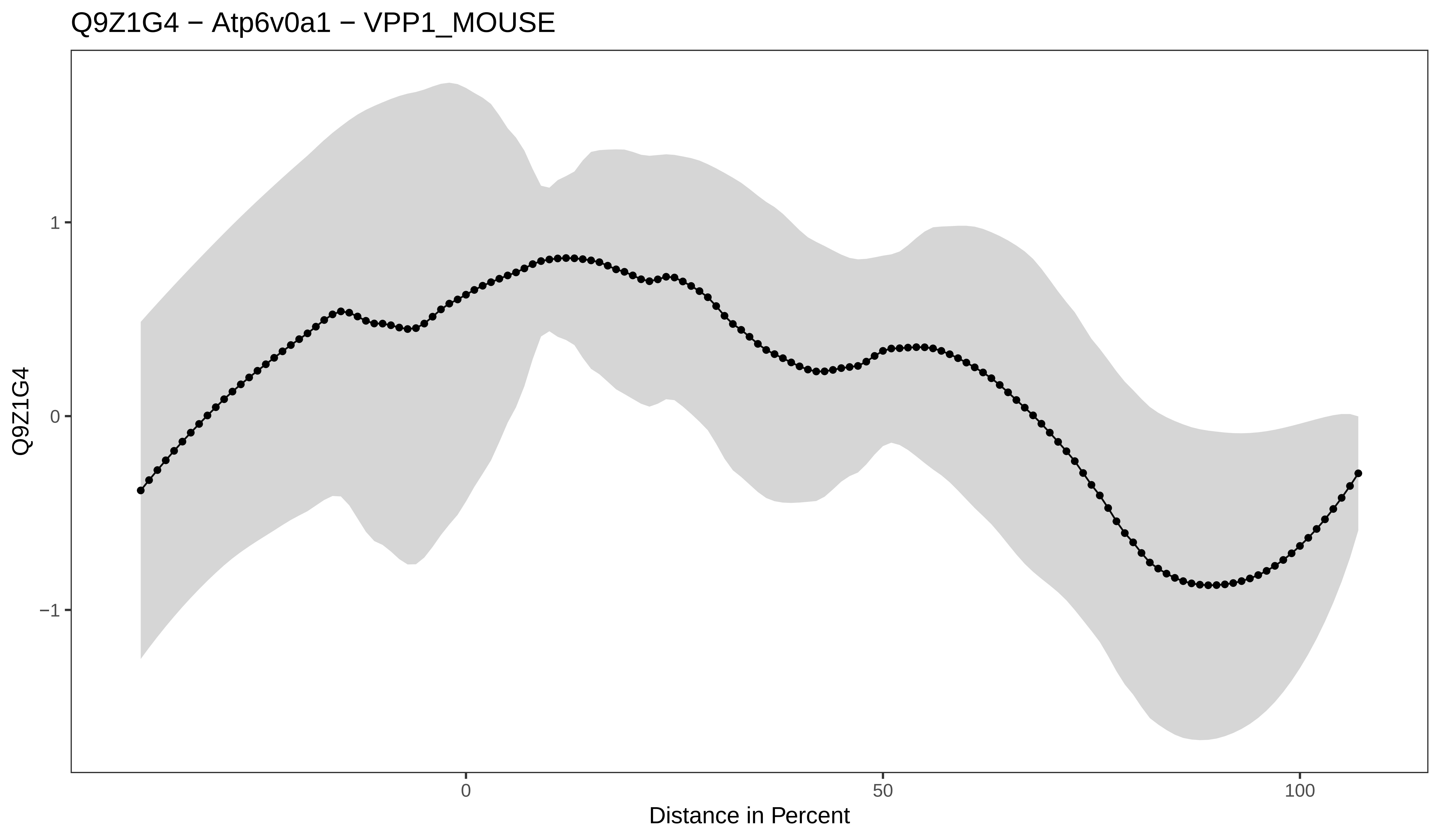

Q9Z1G4 − Atp6v0a1 − VPP1_MOUSE
Q9Z1G4
1
0
−1
0
50
100
Distance in Percent

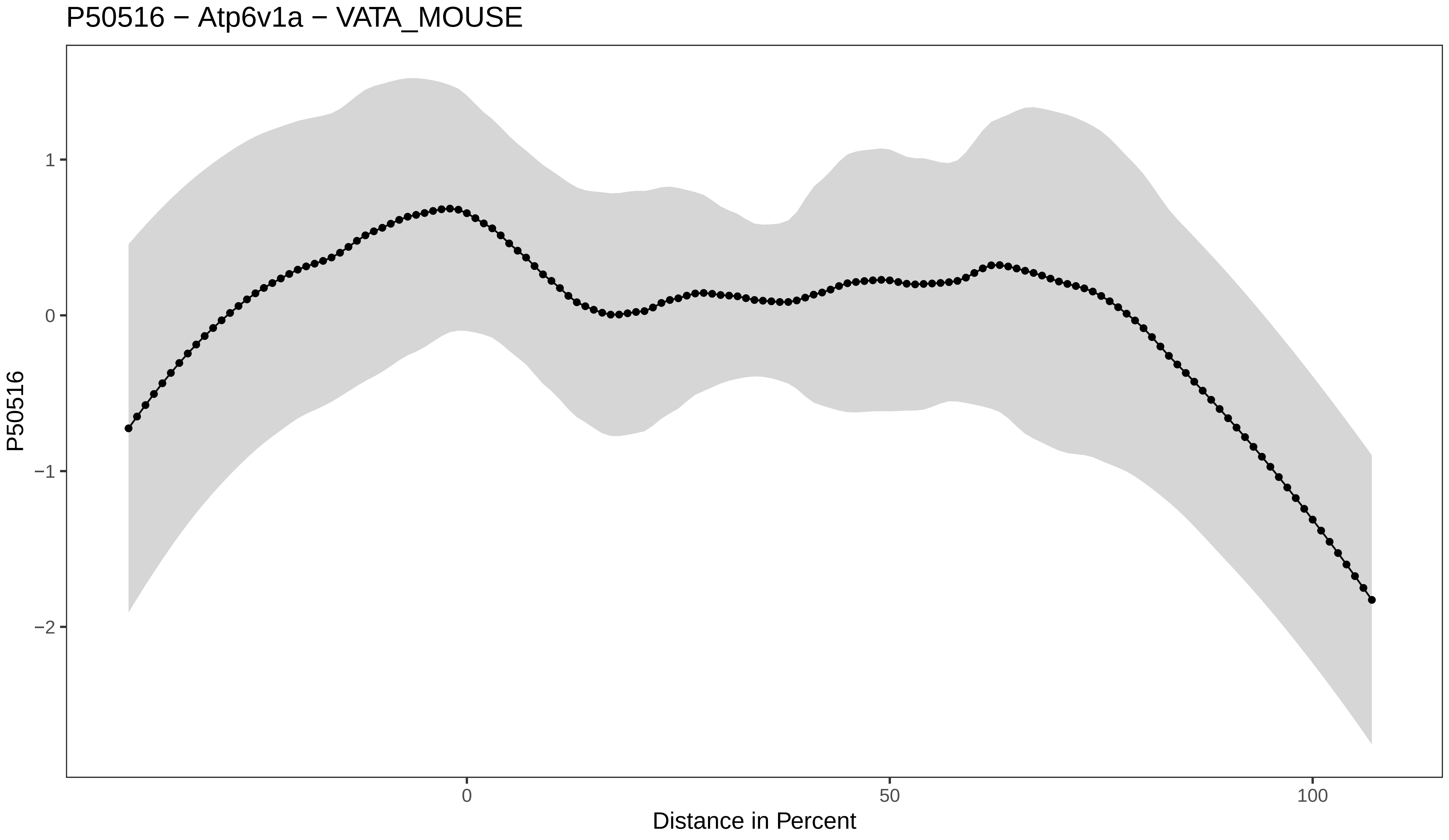
P50516 − Atp6v1a − VATA_MOUSE
P50516
1
0
−1
−2
0
50
100
Distance in Percent

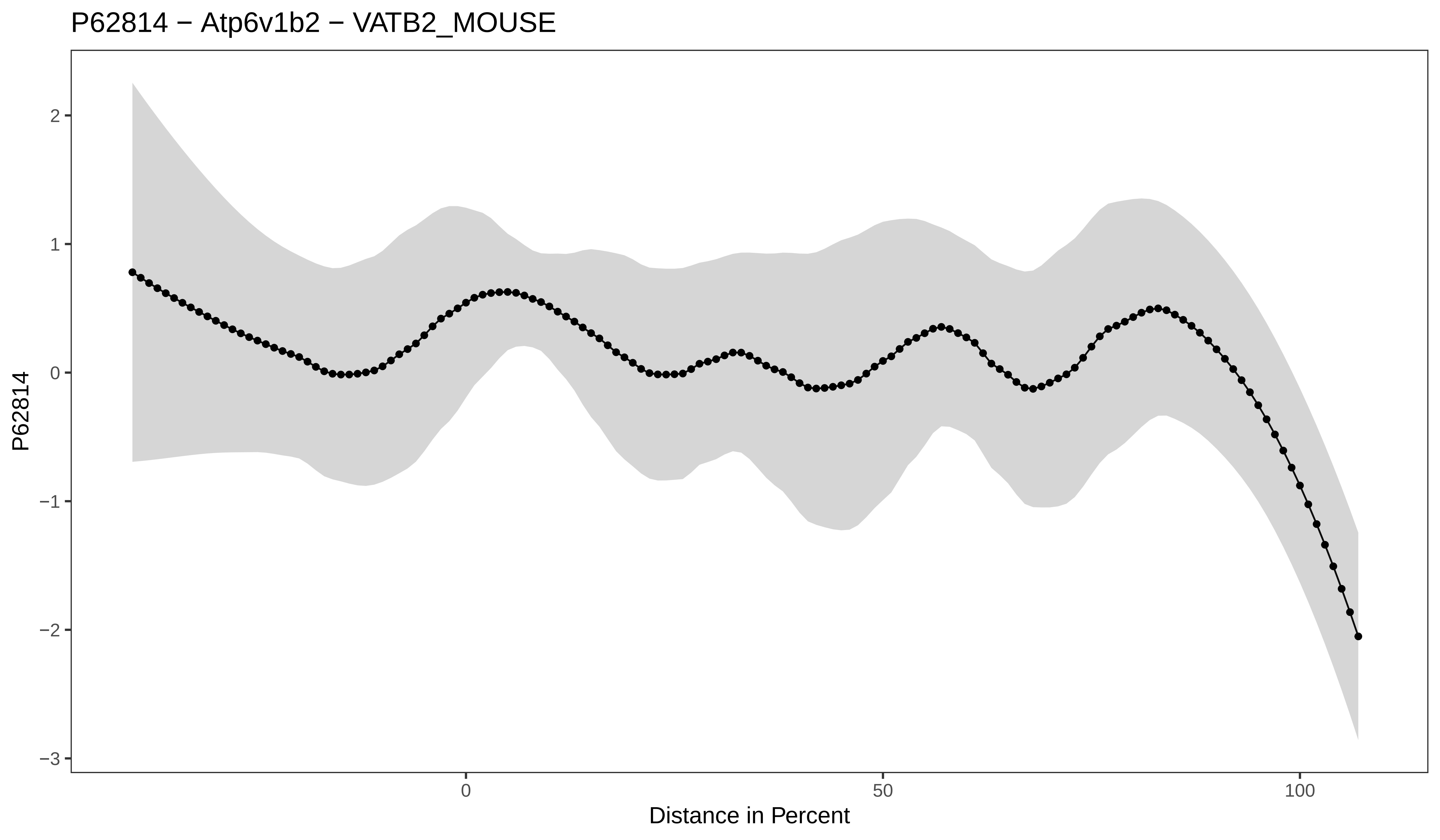

P62814 − Atp6v1b2 − VATB2_MOUSE
P62814
Distance in Percent
2
1
0
−1
−2
−3
0
50
100

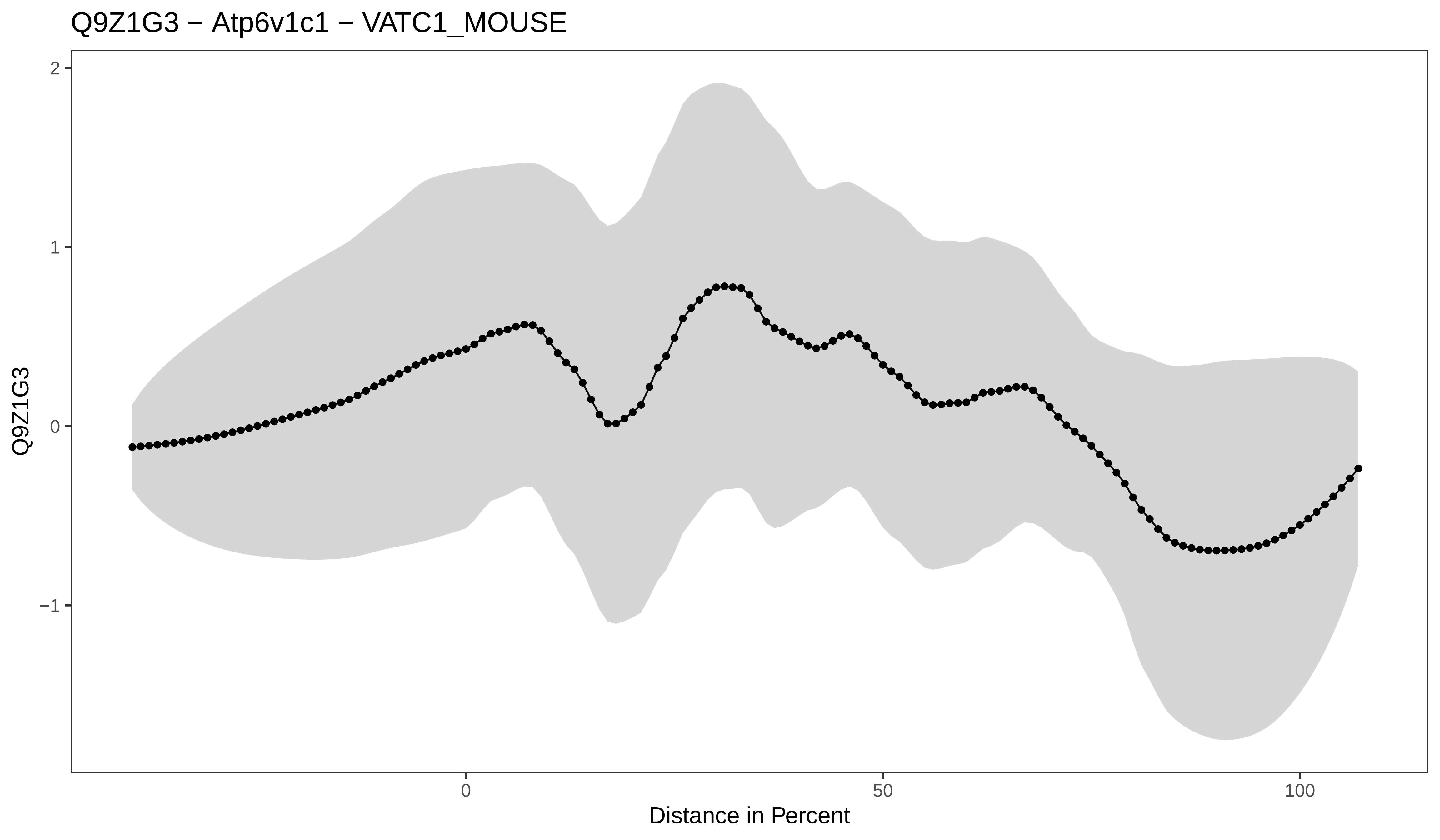
Q9Z1G3 − Atp6v1c1 − VATC1_MOUSE
Q9Z1G3
2
1
0
−1
0
50
100
Distance in Percent

Q8BVE3 − Atp6v1h − VATH_MOUSE

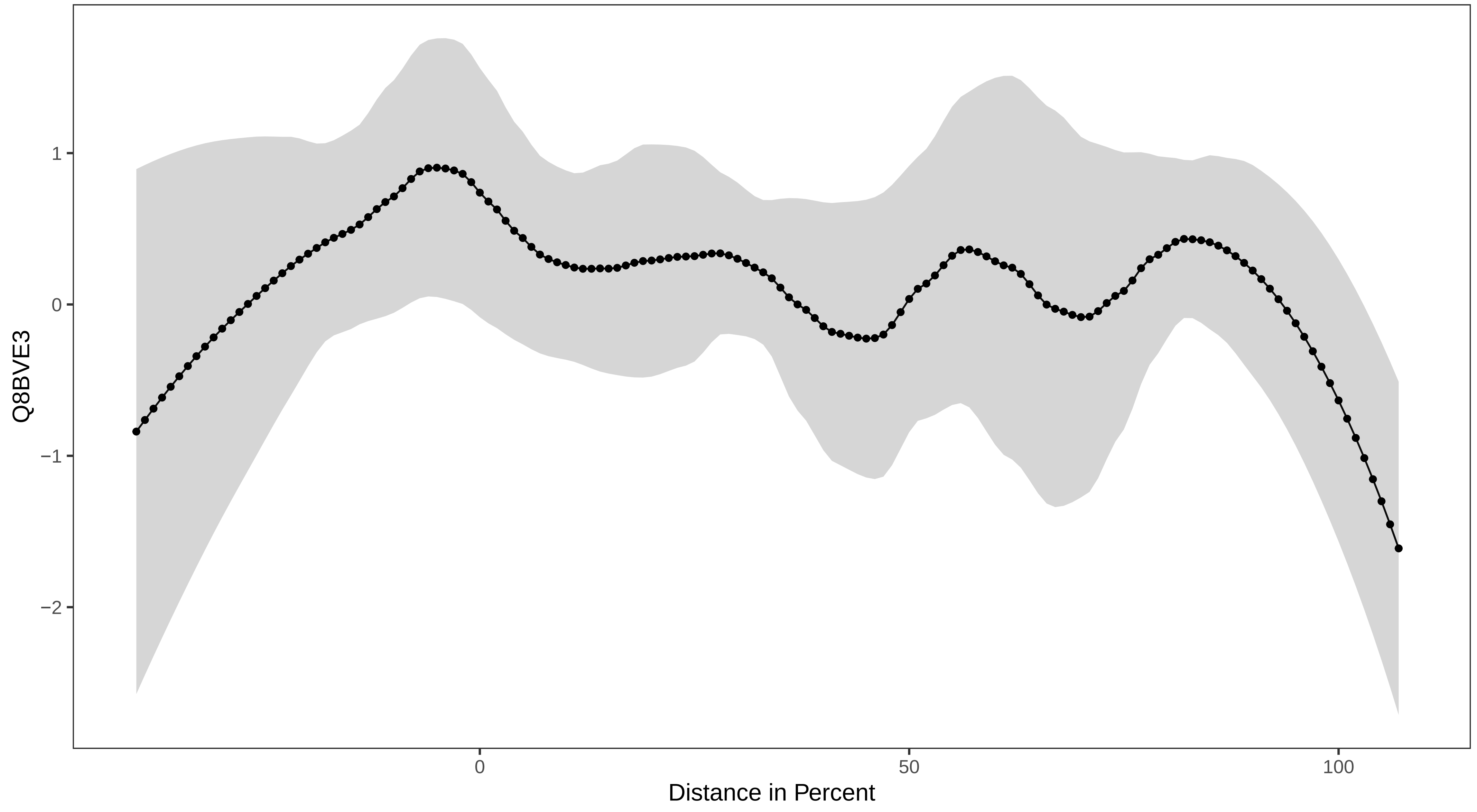

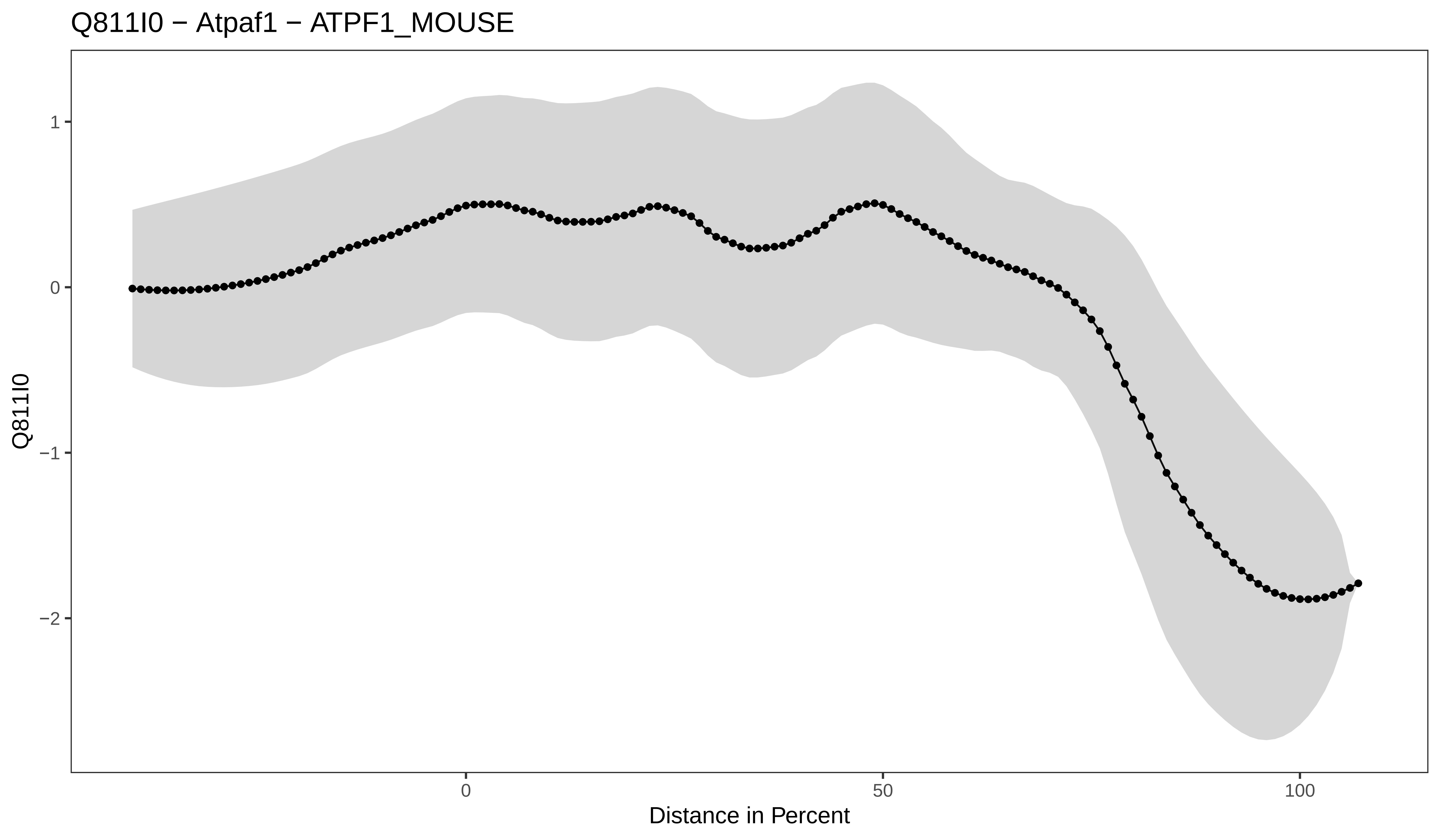
Q811I0 − Atpaf1 − ATPF1_MOUSE
1
0
−1
−2
Q811I0
0
50
100
Distance in Percent

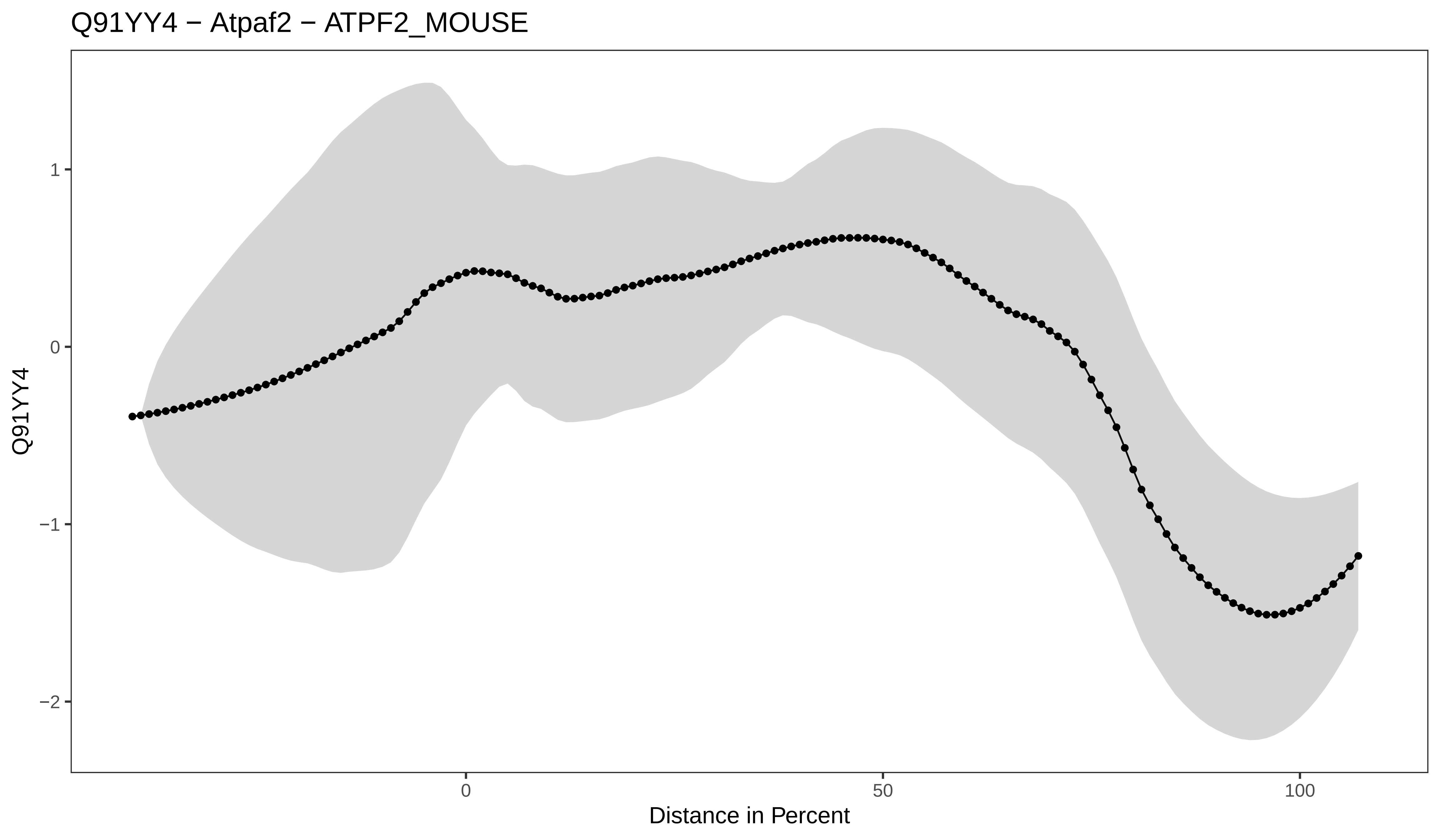

Q91YY4 − Atpaf2 − ATPF2_MOUSE
1
0
−1
−2
Q91YY4
0
50
100
Distance in Percent

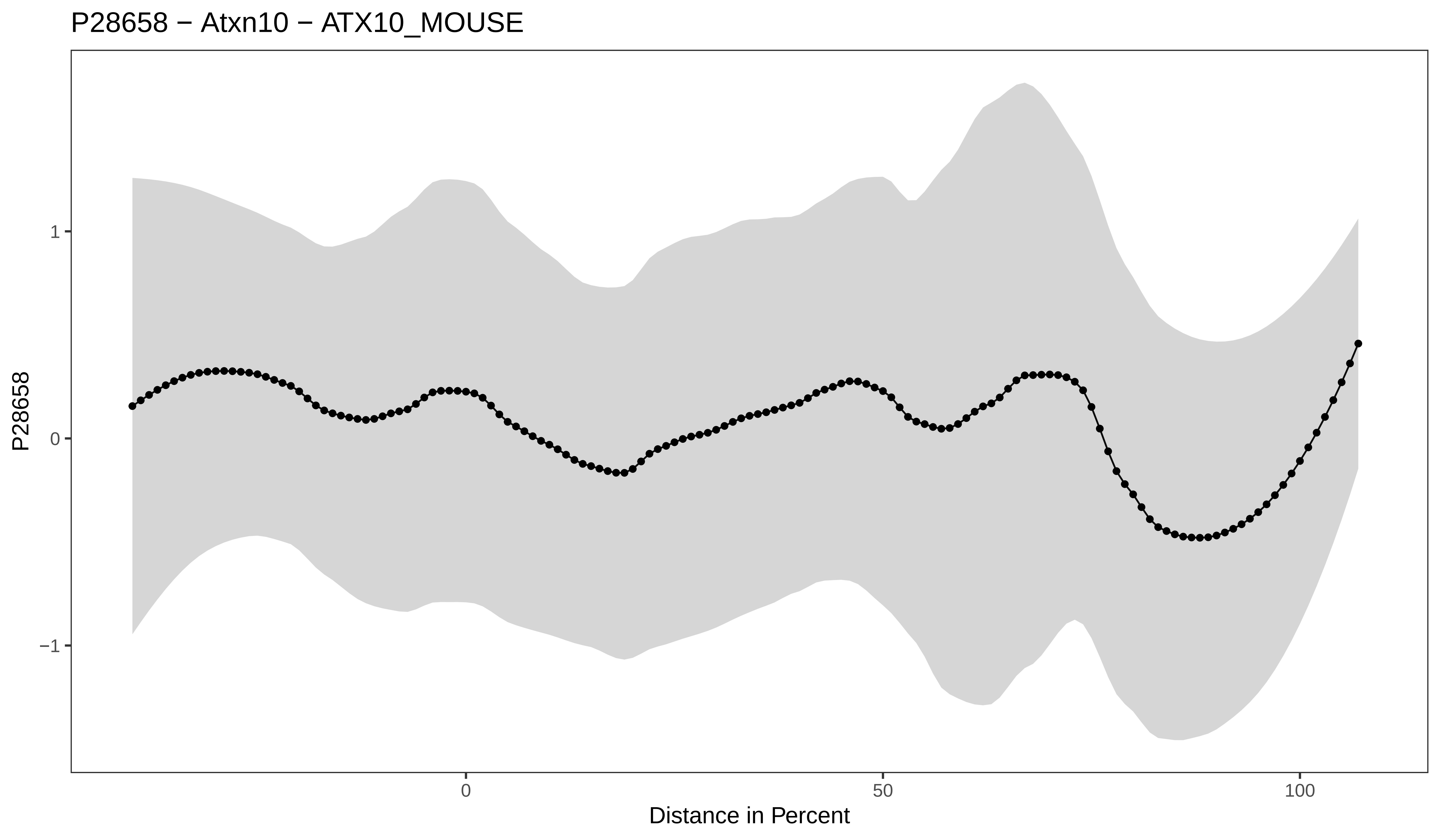

P28658 − Atxn10 − ATX10_MOUSE
1
0
−1
P28658
0
50
100
Distance in Percent

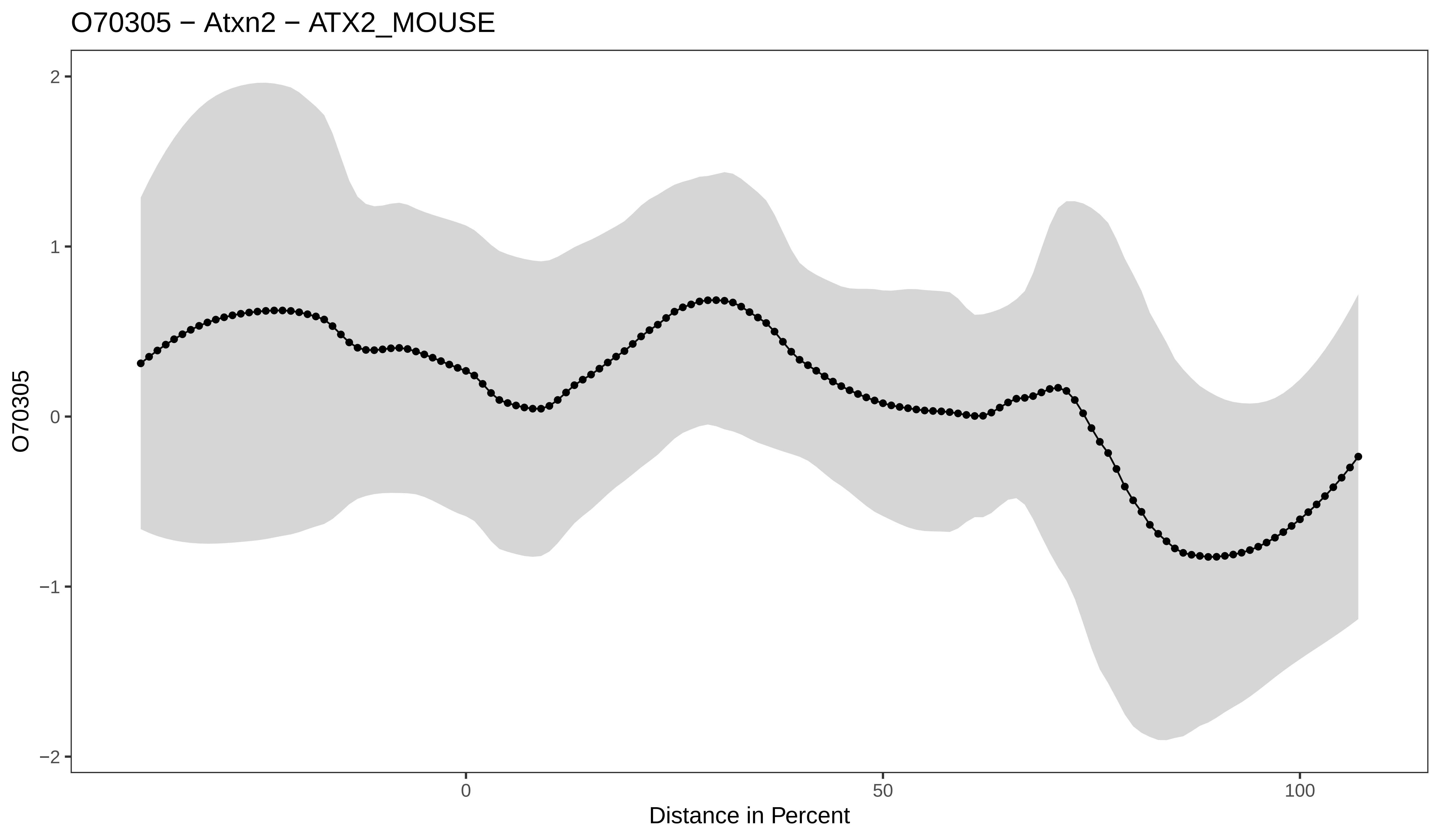
O70305 − Atxn2 − ATX2_MOUSE
O70305
Distance in Percent
2
1
0
−1
−2
0
50
100

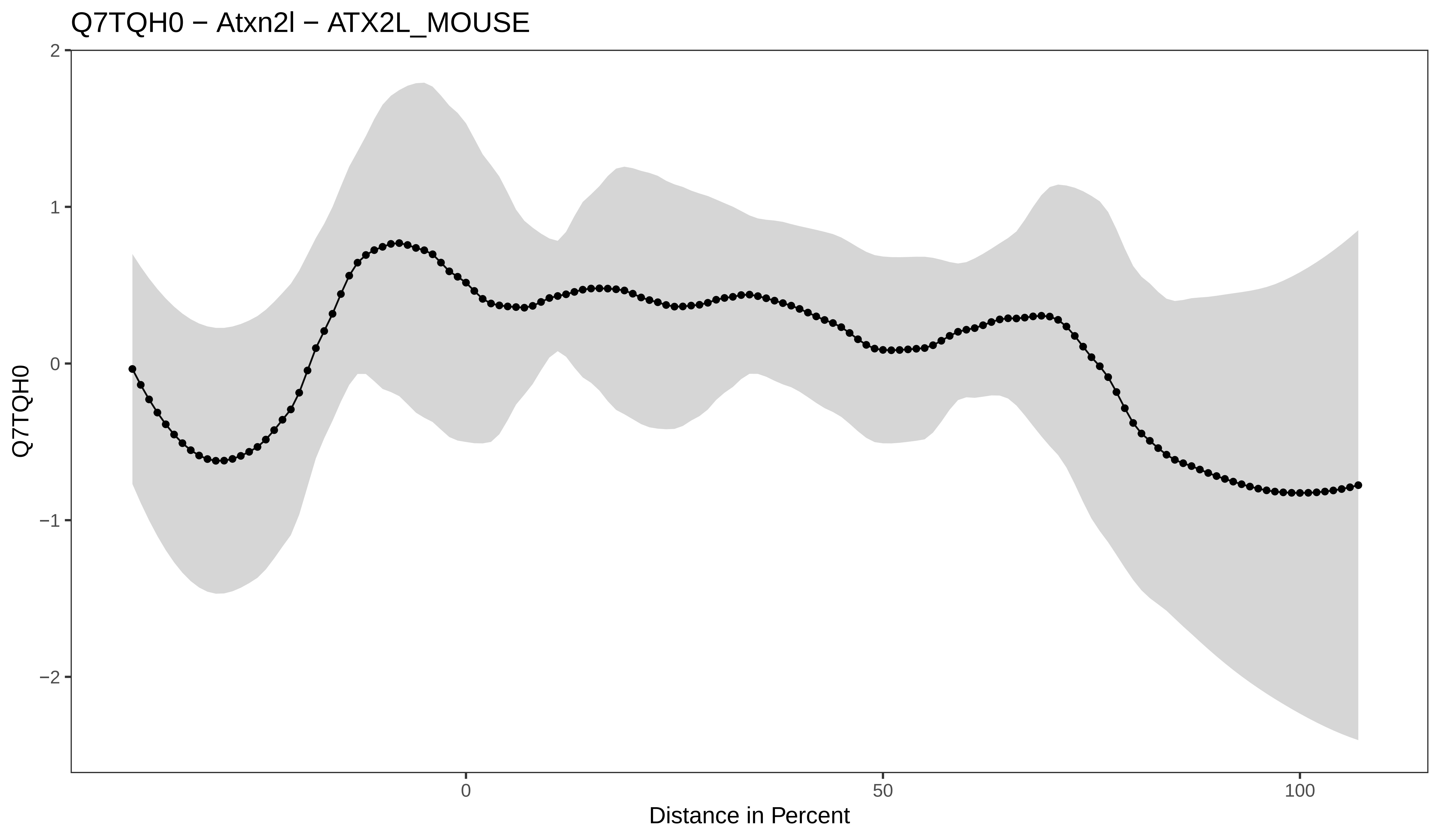

Q7TQH0 − Atxn2l − ATX2L_MOUSE
2
1
0
−1
−2
Q7TQH0
0
50
100
Distance in Percent

Q9JLZ3 − Auh − AUHM_MOUSE

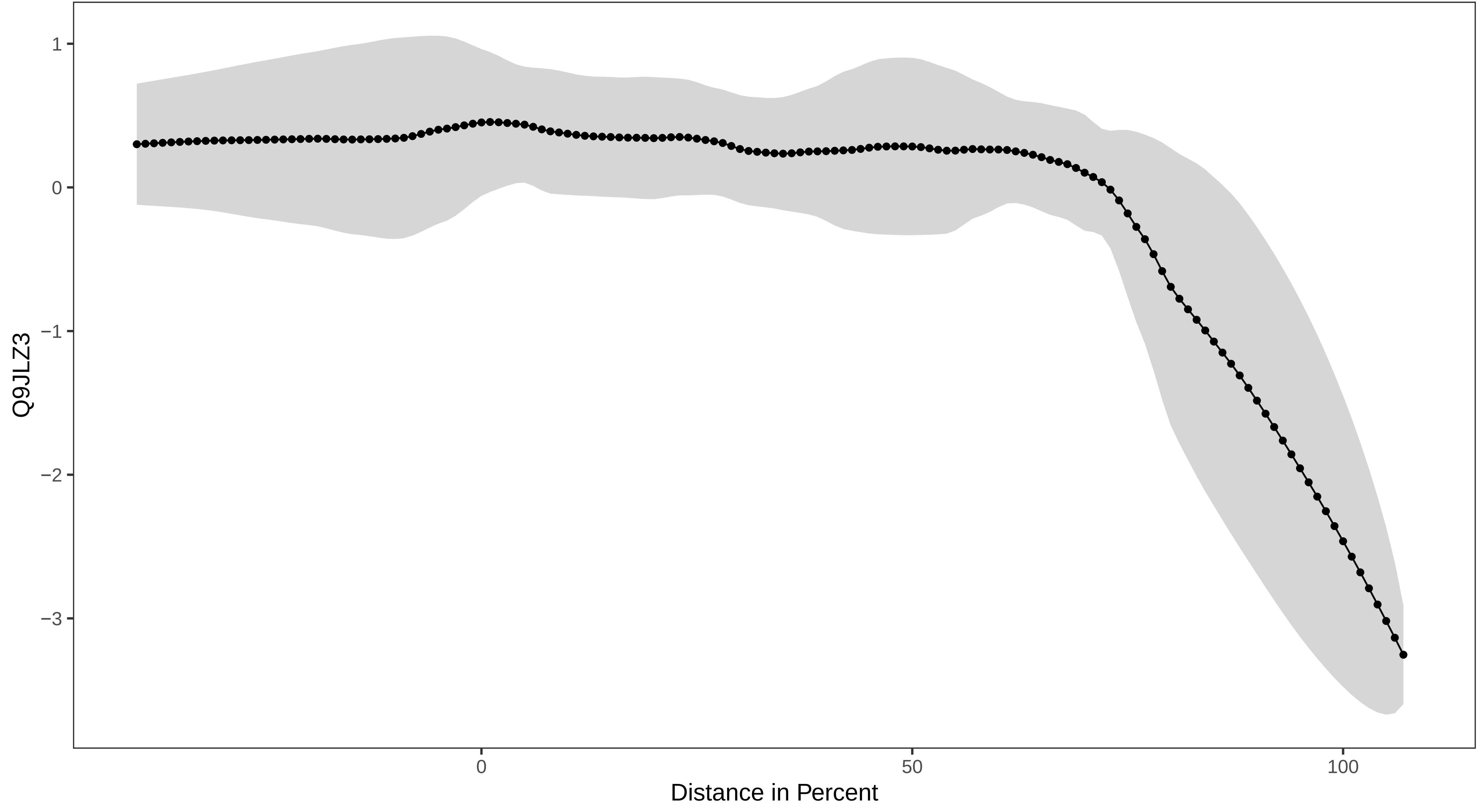

Q91YN9 − Bag2 − BAG2_MOUSE

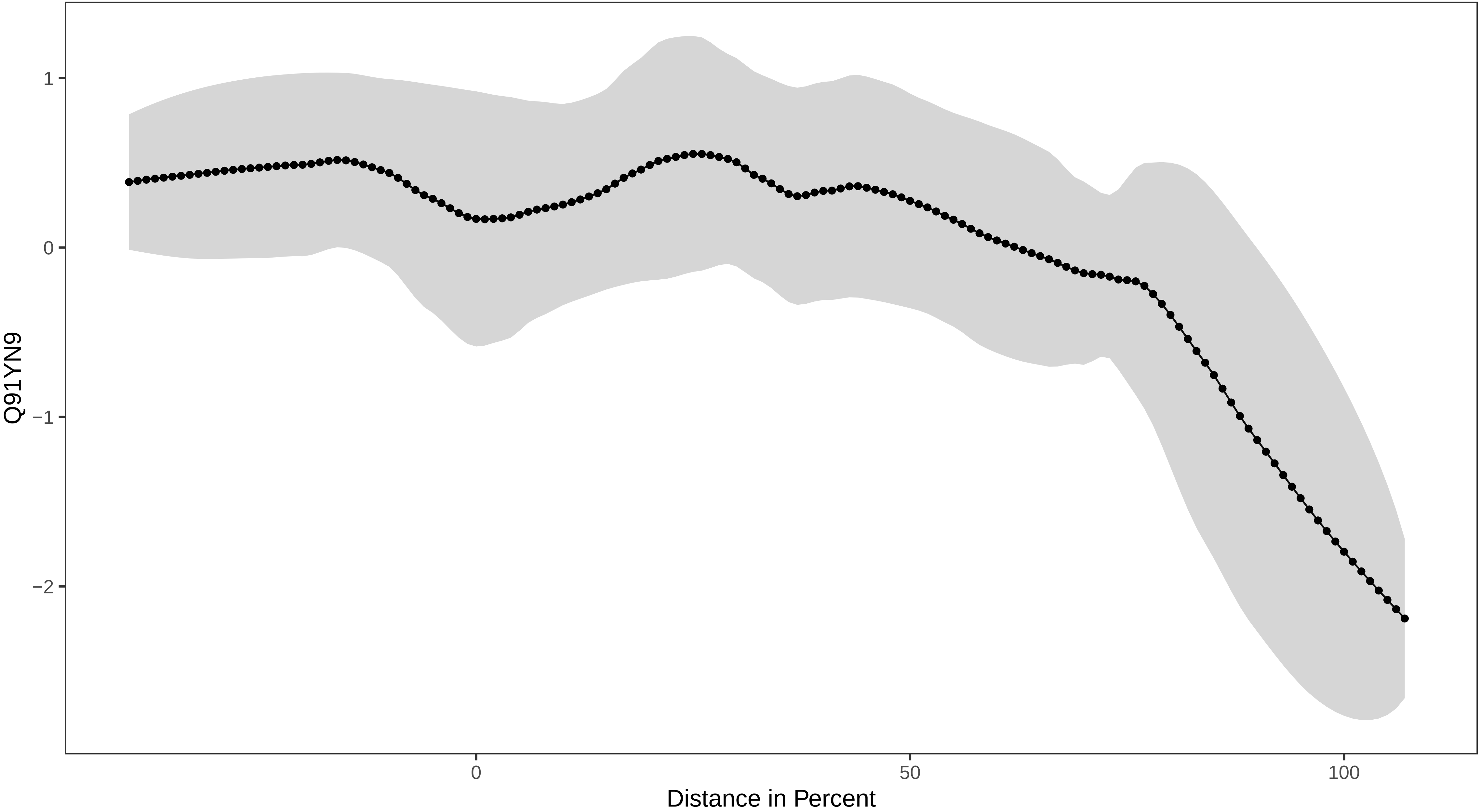

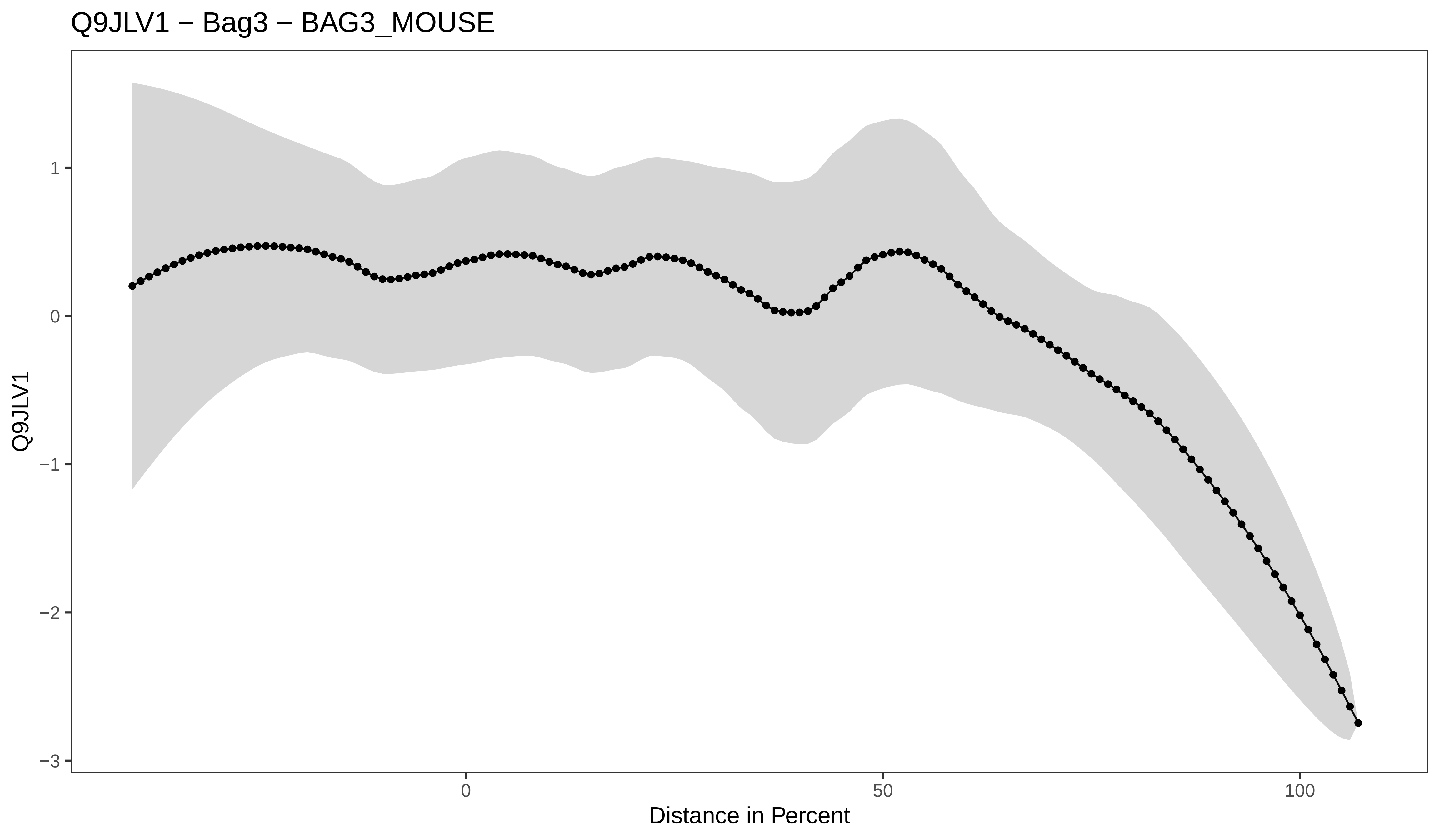
Q9JLV1 − Bag3 − BAG3_MOUSE
1
0
−1
−2
−3
Q9JLV1
0
50
100
Distance in Percent

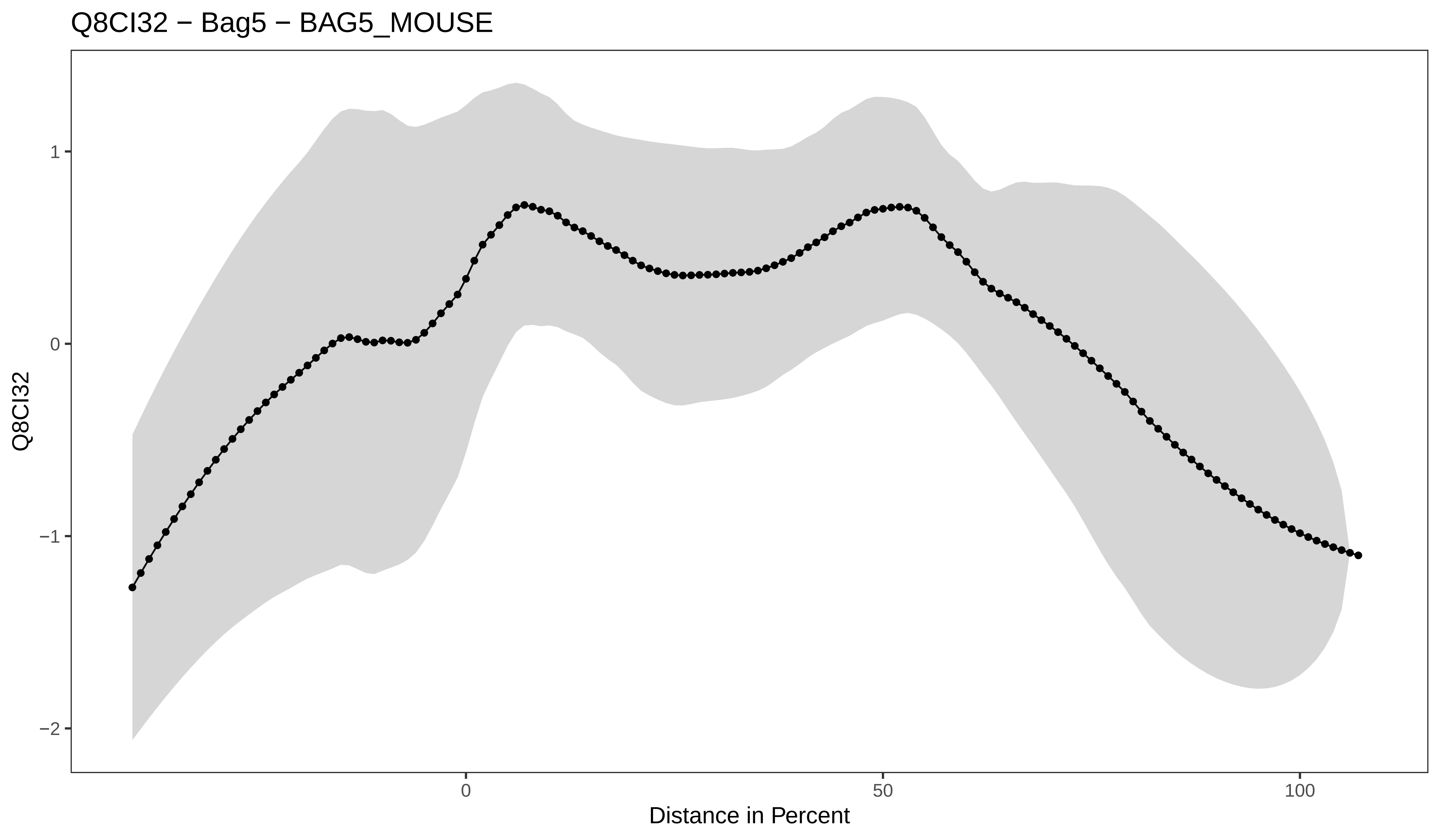

Q8CI32 − Bag5 − BAG5_MOUSE
1
0
−1
−2
Q8CI32
0
50
100
Distance in Percent

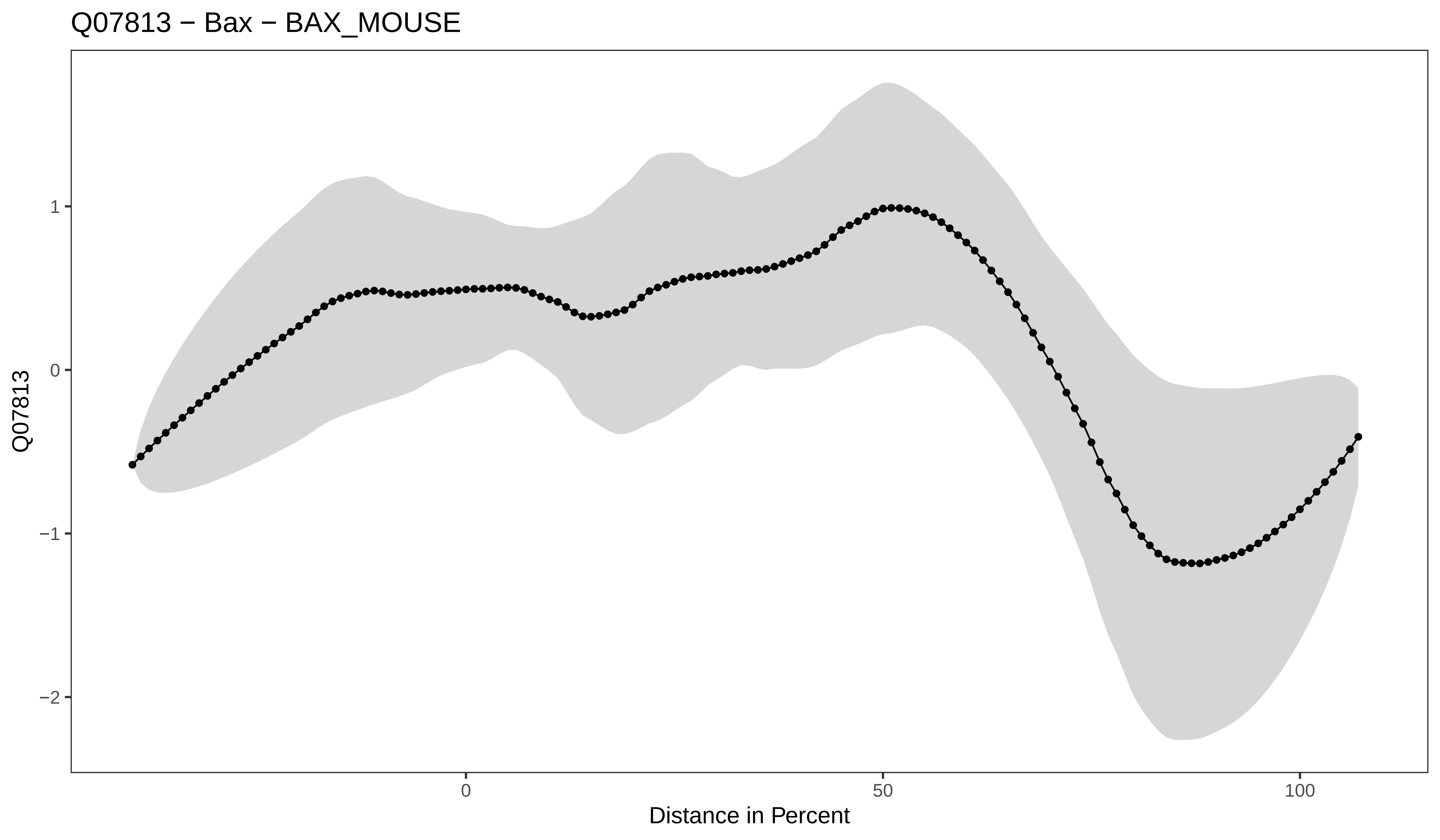

Q07813 − Bax − BAX_MOUSE
Q07813
1
0
−1
−2
0
50
100
Distance in Percent

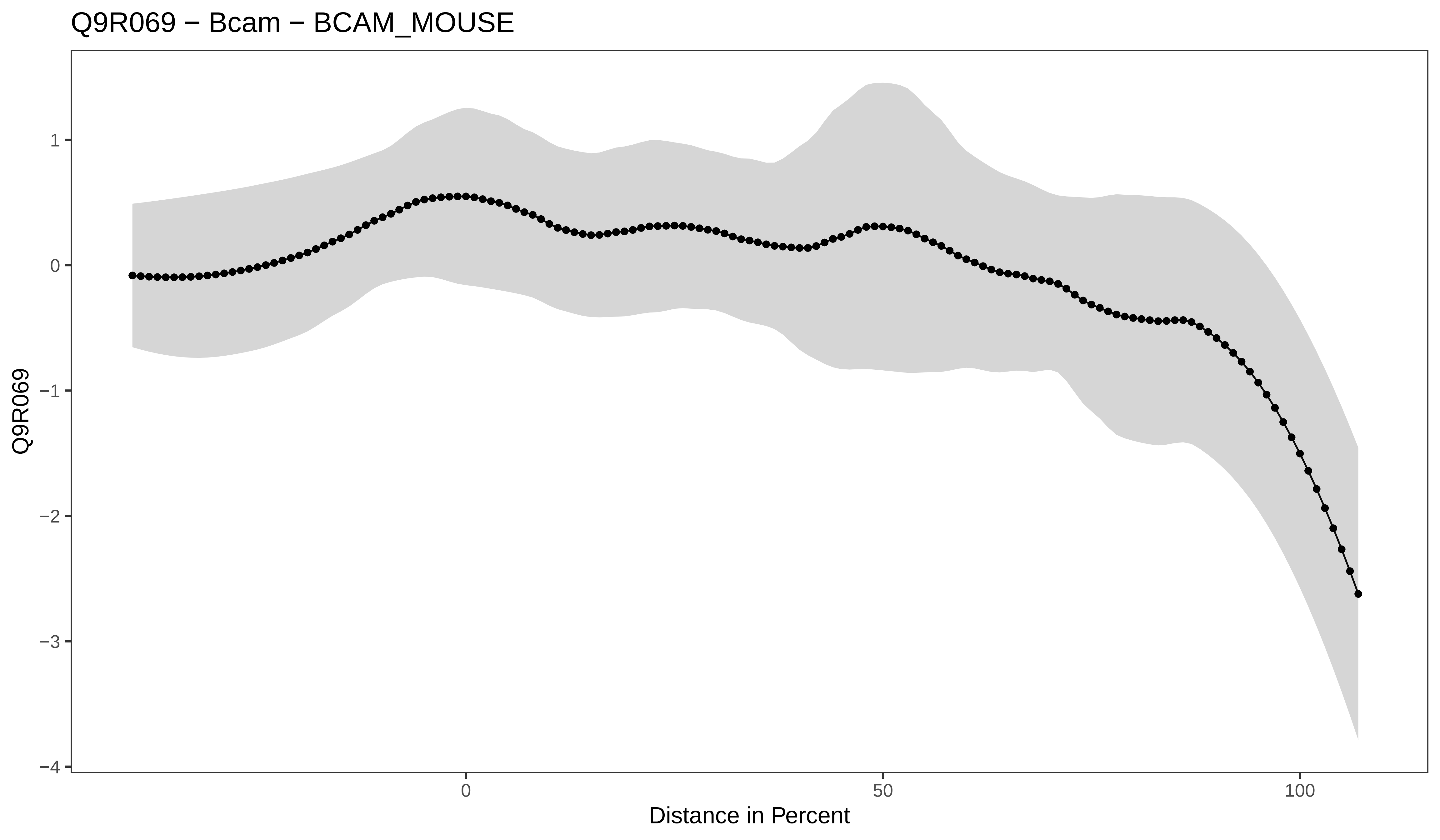
Q9R069 − Bcam − BCAM_MOUSE
Q9R069
Distance in Percent
1
0
−1
−2
−3
−4
0
50
100

Q61335 − Bcap31 − BAP31_MOUSE

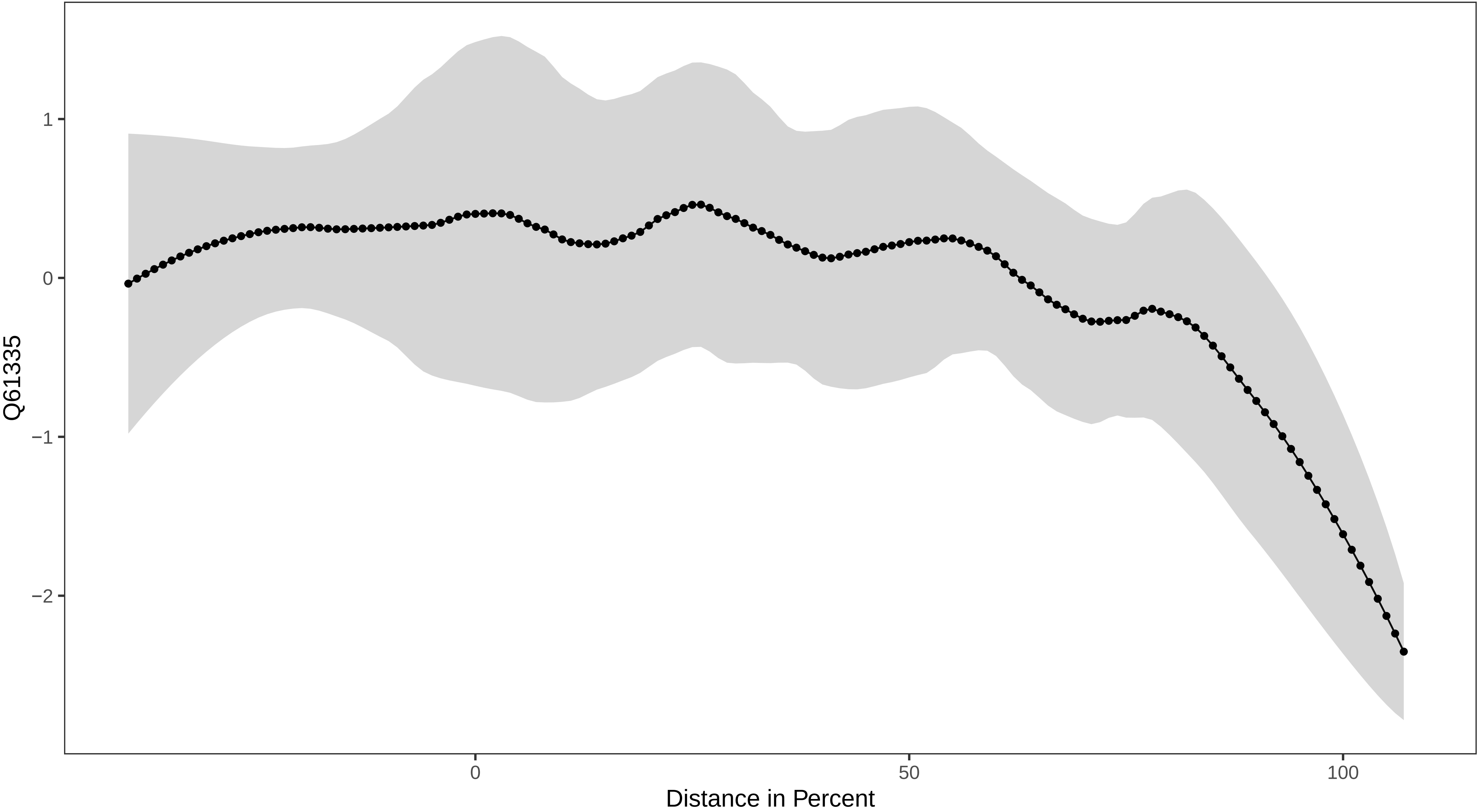

Q80YN3 − Bcas1 − BCAS1_MOUSE

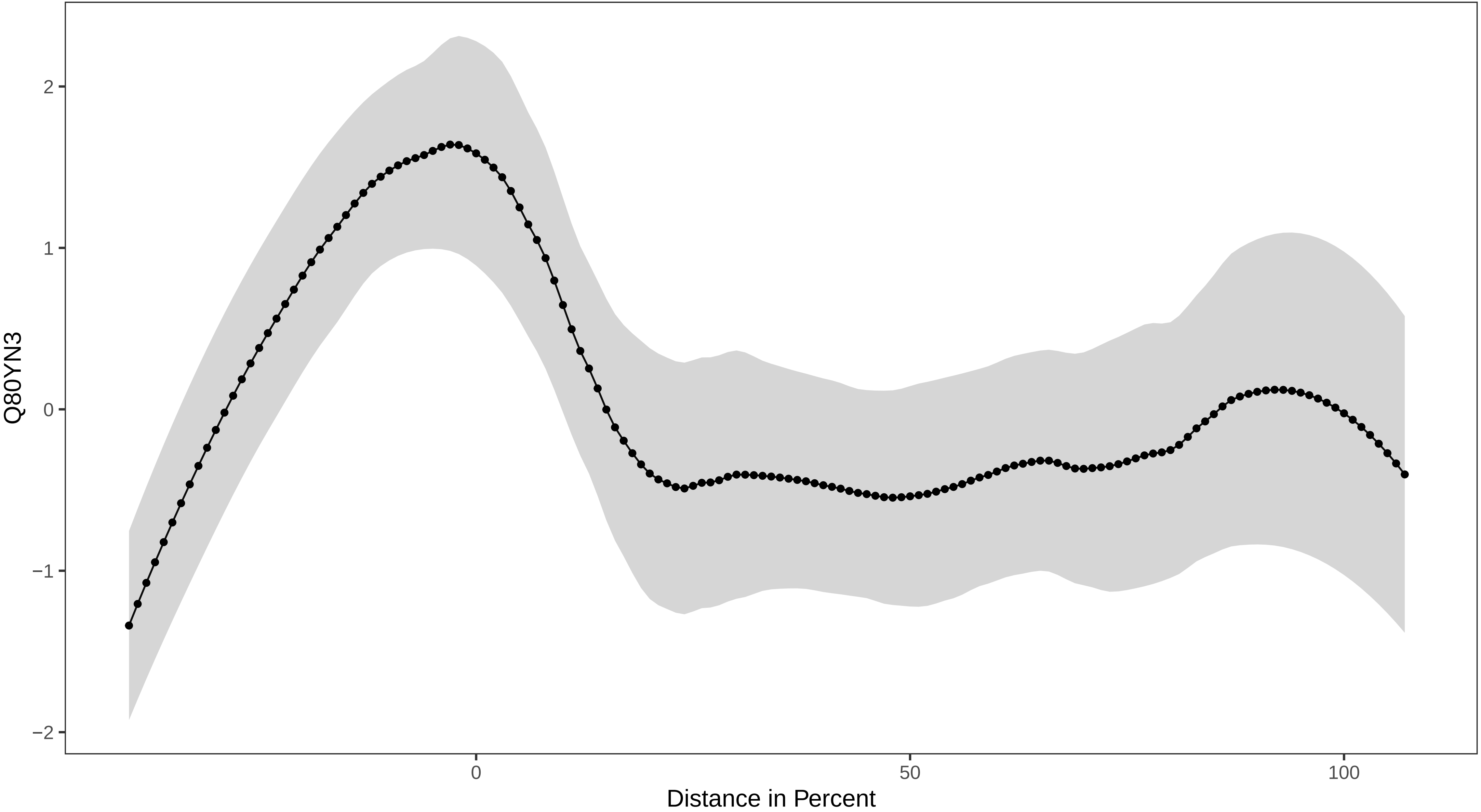

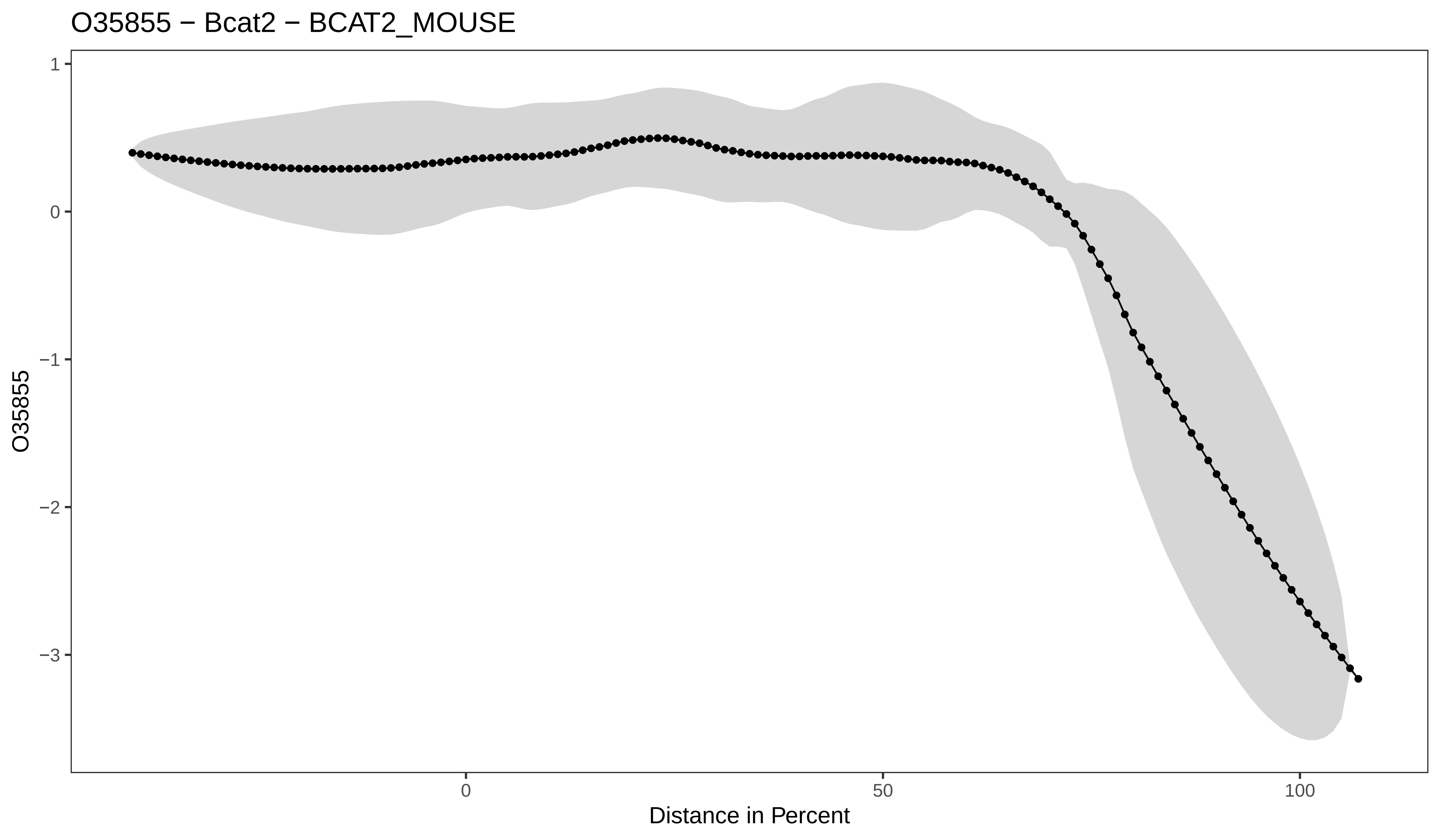
O35855 − Bcat2 − BCAT2_MOUSE
O35855
Distance in Percent
1
0
−1
−2
−3
0
50
100

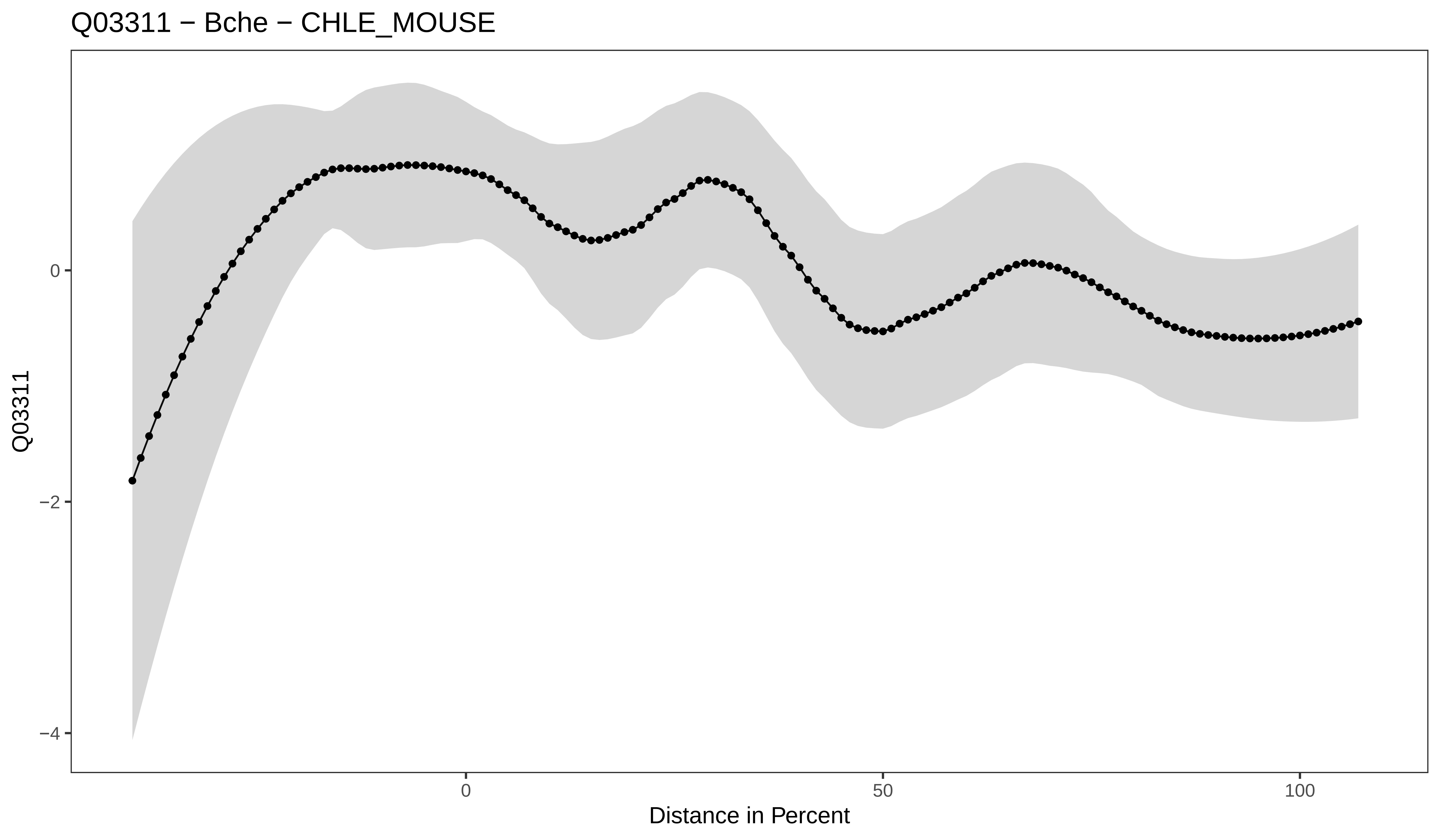

Q03311 − Bche − CHLE_MOUSE
0
−2
−4
Q03311
0
50
100
Distance in Percent

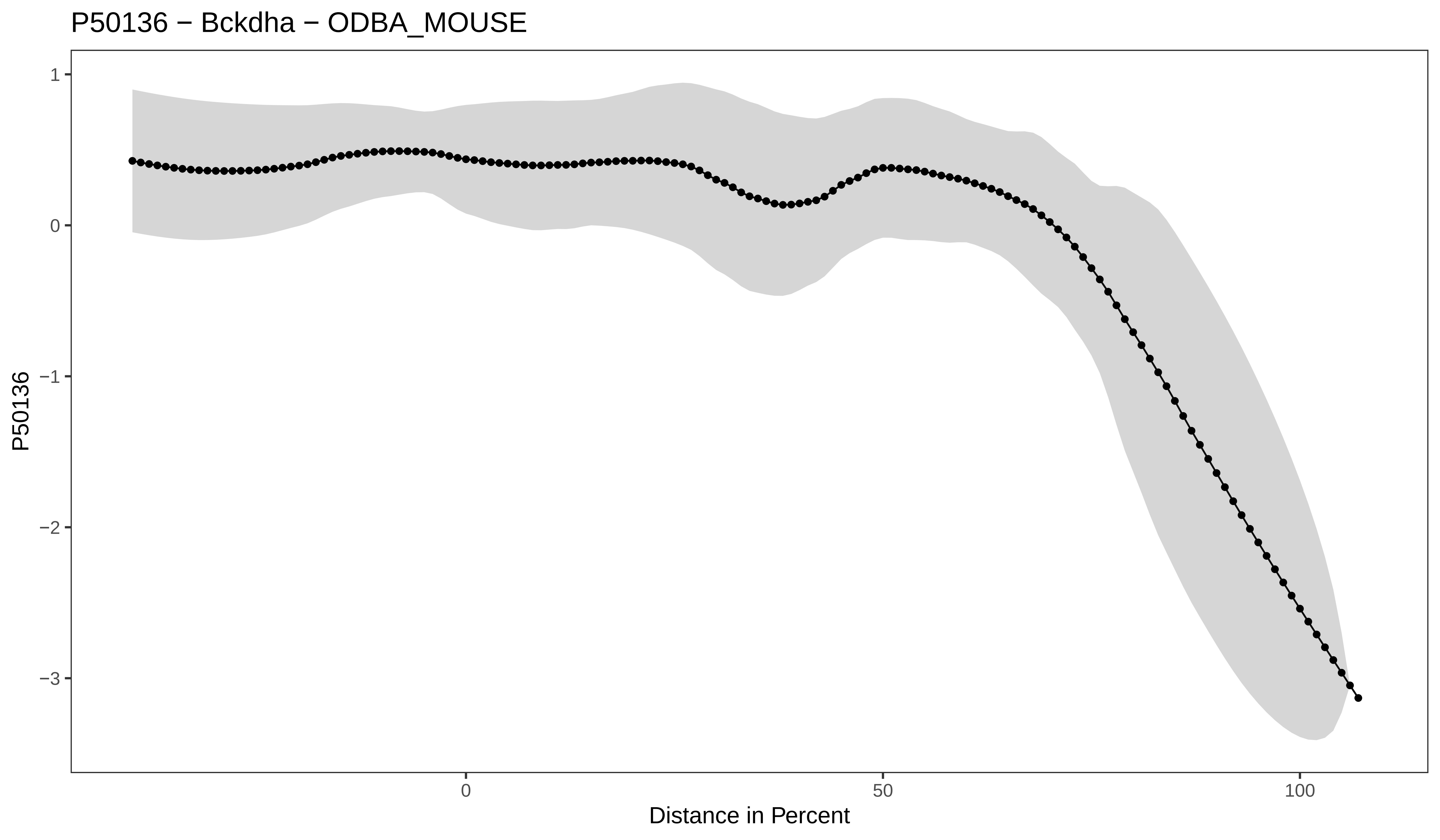

P50136 − Bckdha − ODBA_MOUSE
P50136
1
0
−1
−2
−3
0
50
100
Distance in Percent

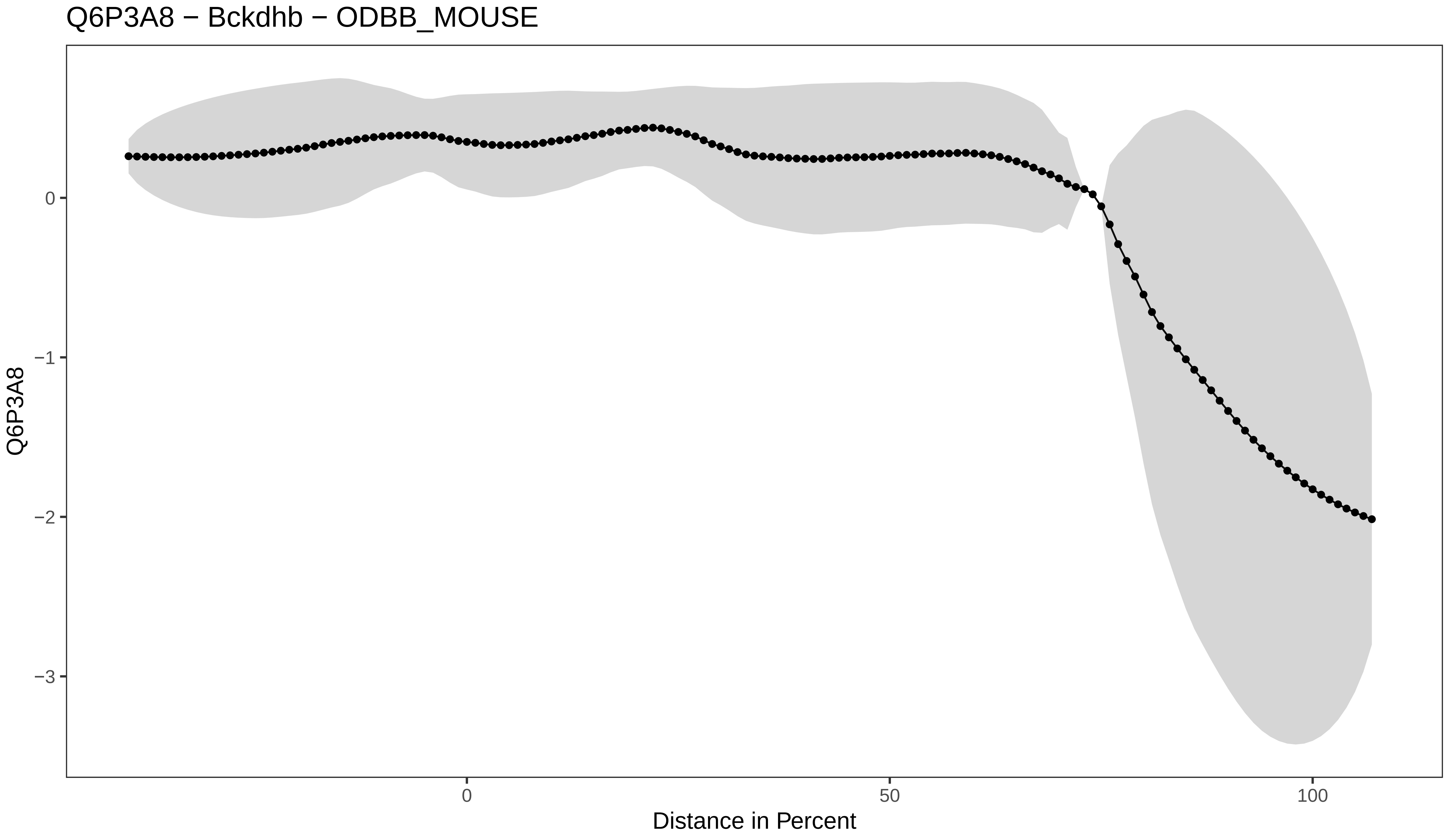

Q6P3A8 − Bckdhb − ODBB_MOUSE
Q6P3A8
0
−1
−2
−3
0
50
100
Distance in Percent

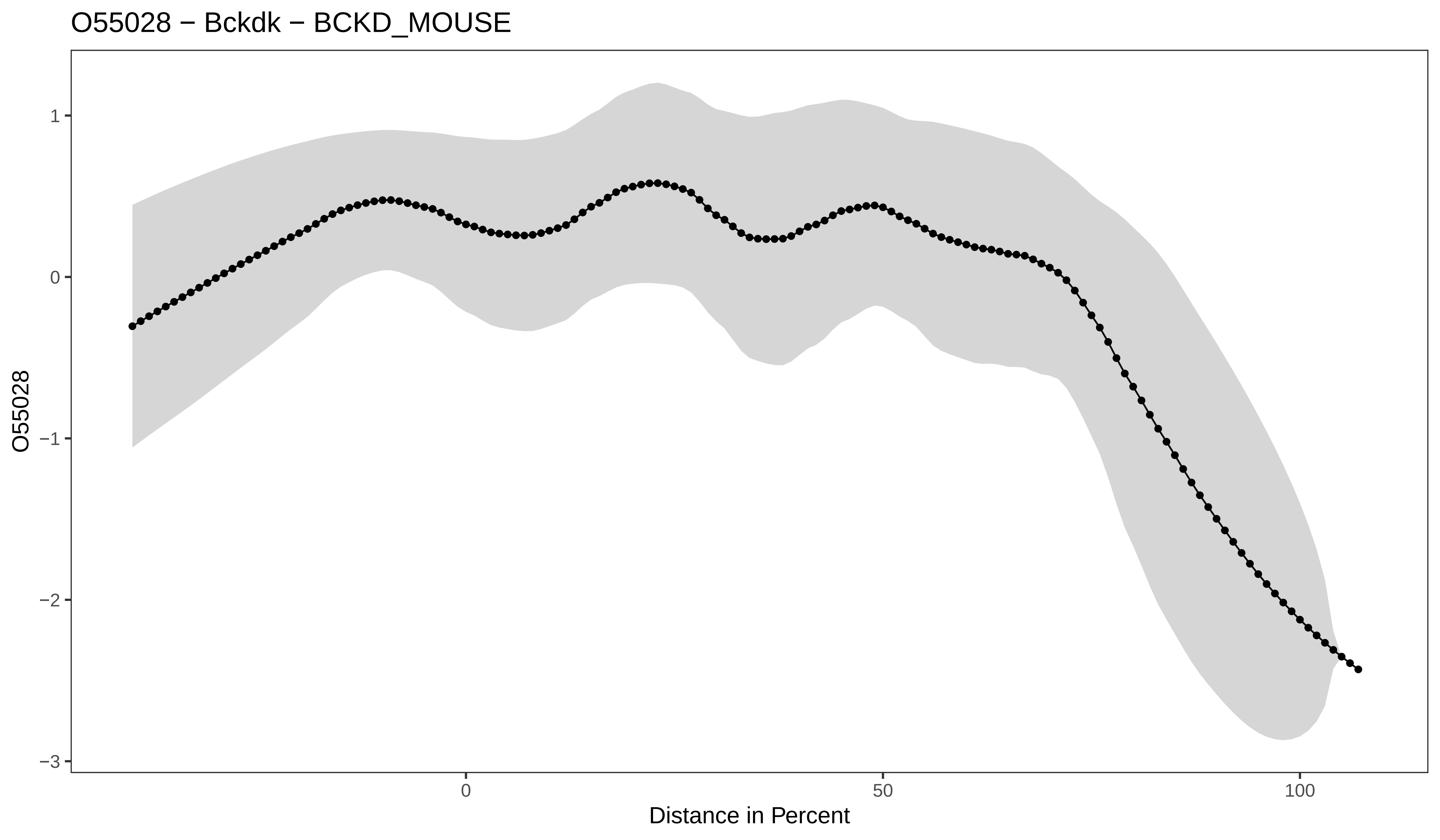
O55028 − Bckdk − BCKD_MOUSE
1
0
−1
−2
−3
O55028
0
50
100
Distance in Percent

P59017 − Bcl2l13 − B2L13_MOUSE

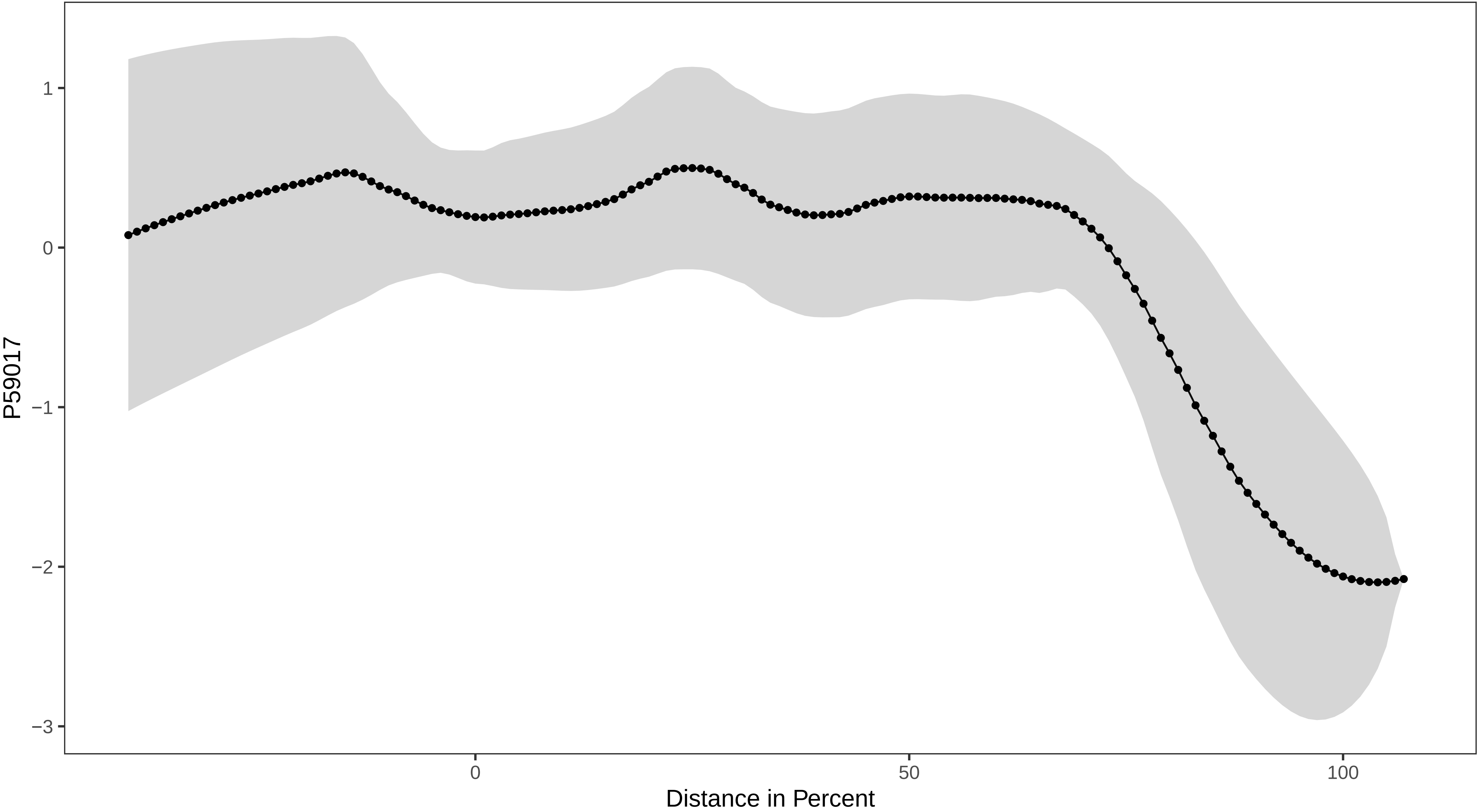

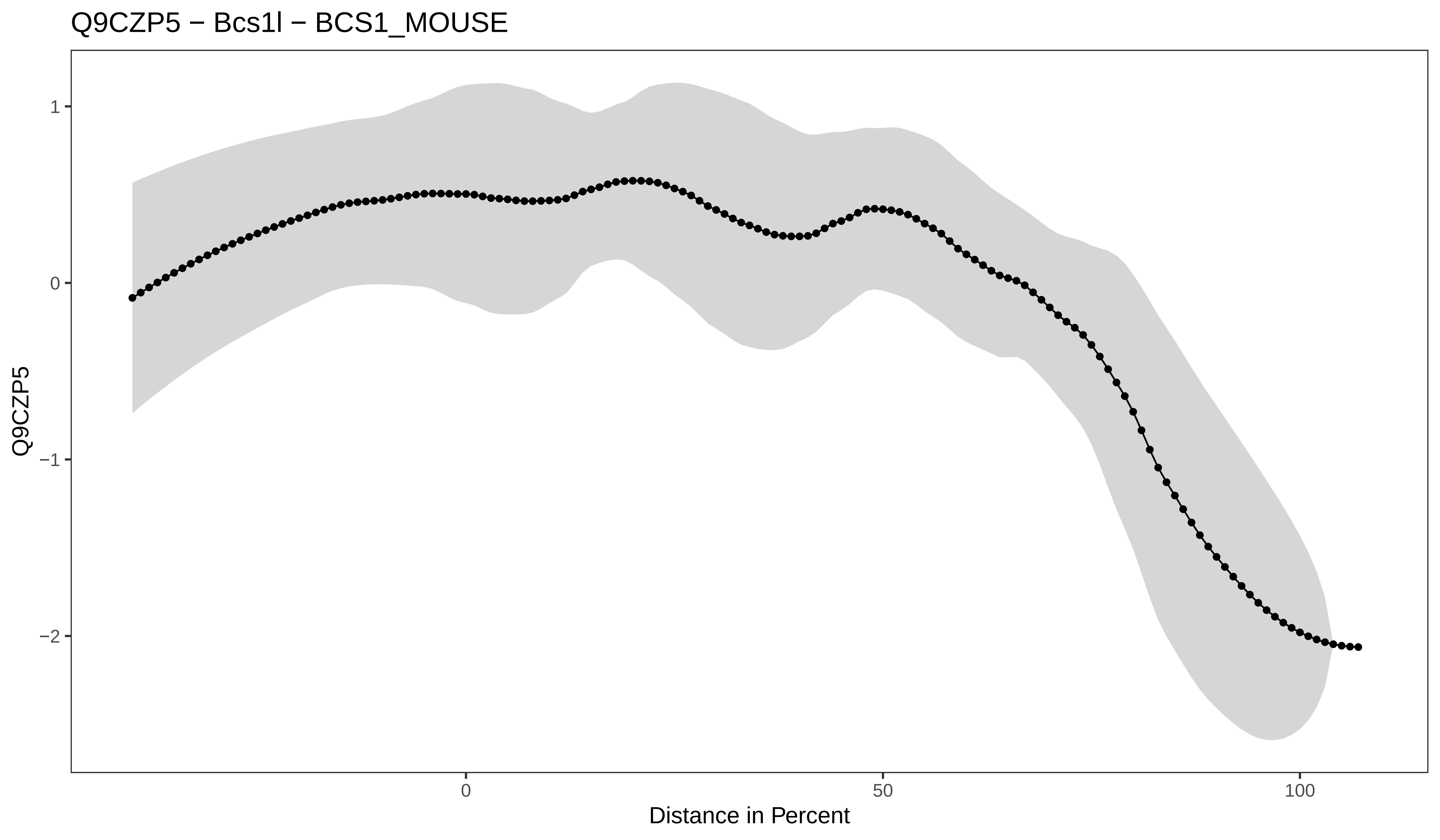

Q9CZP5 − Bcs1l − BCS1_MOUSE
1
0
−1
−2
Q9CZP5
0
50
100
Distance in Percent

Q80XN0 − Bdh1 − BDH_MOUSE

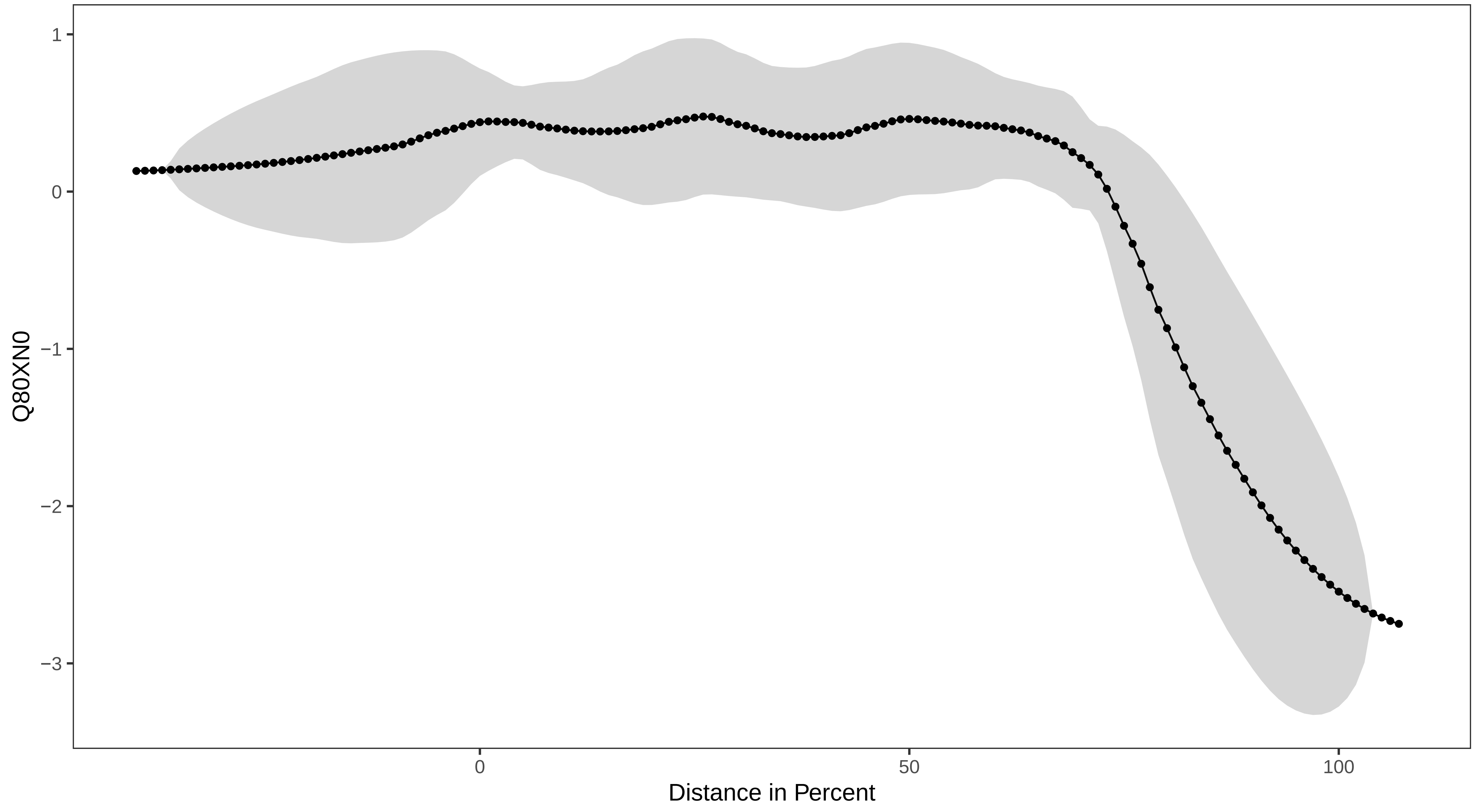

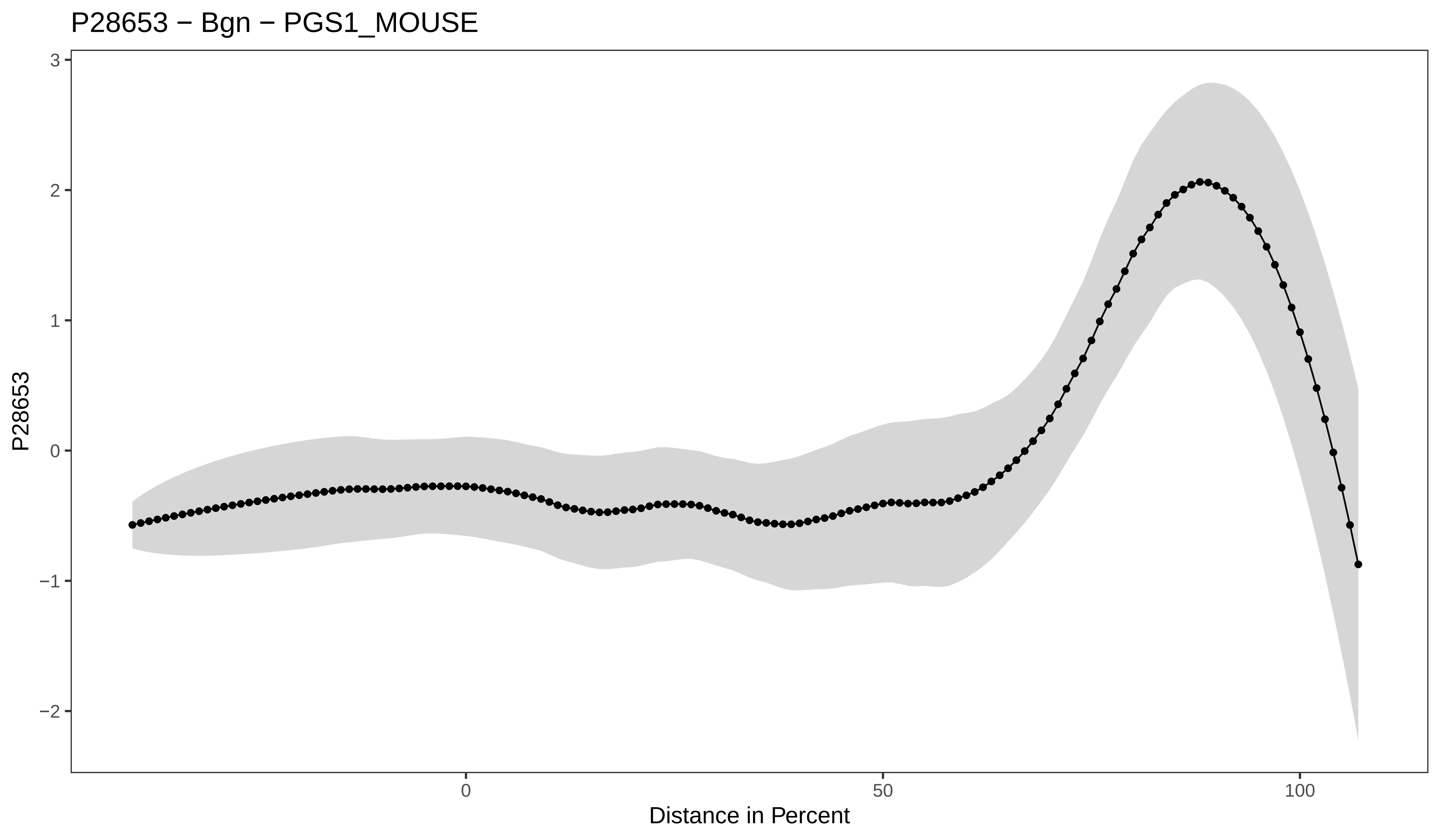
P28653 − Bgn − PGS1_MOUSE
3
2
1
0
−1
−2
P28653
0
50
100
Distance in Percent

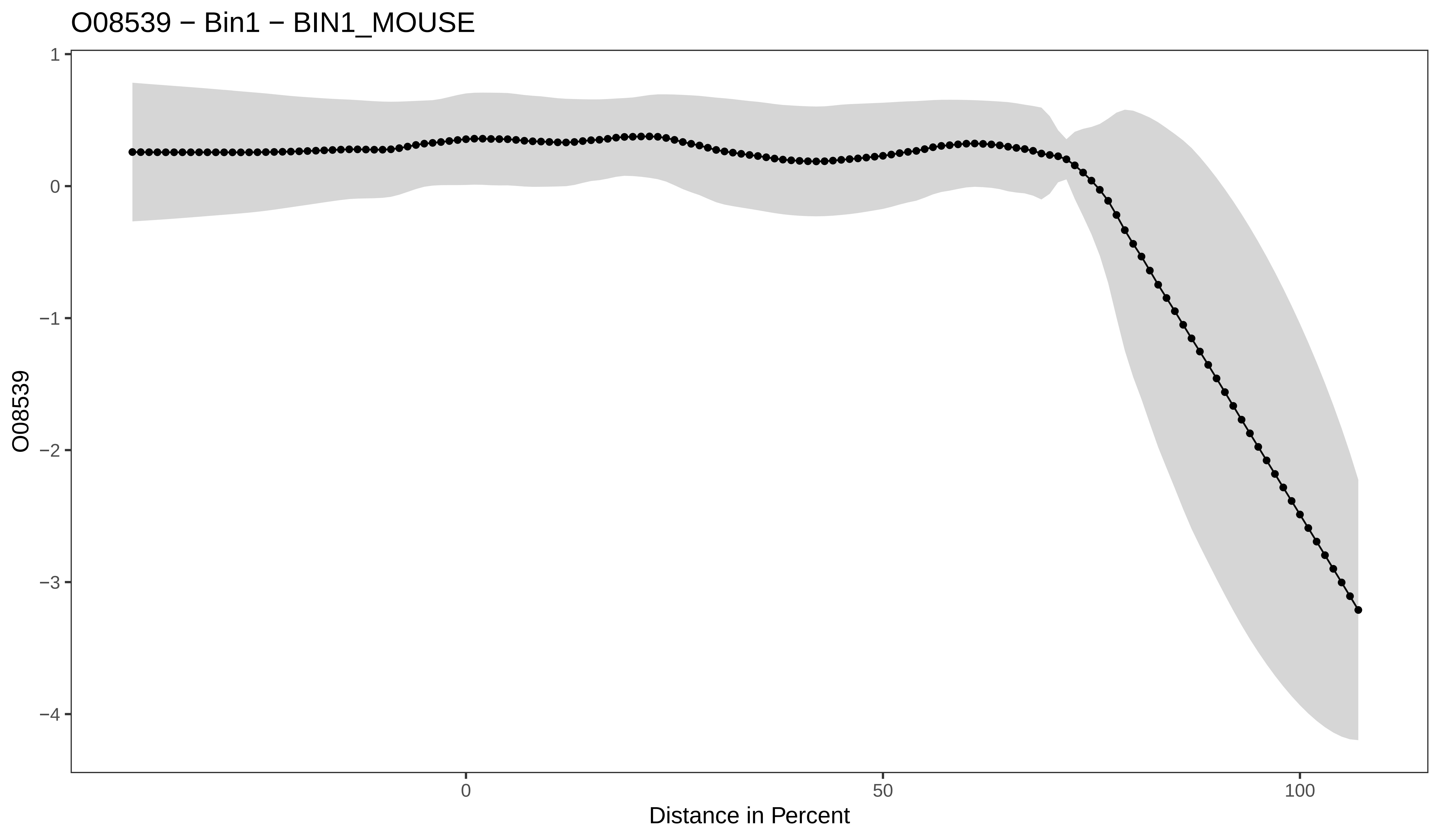

O08539 − Bin1 − BIN1_MOUSE
O08539
1
0
−1
−2
−3
−4
0
50
100
Distance in Percent

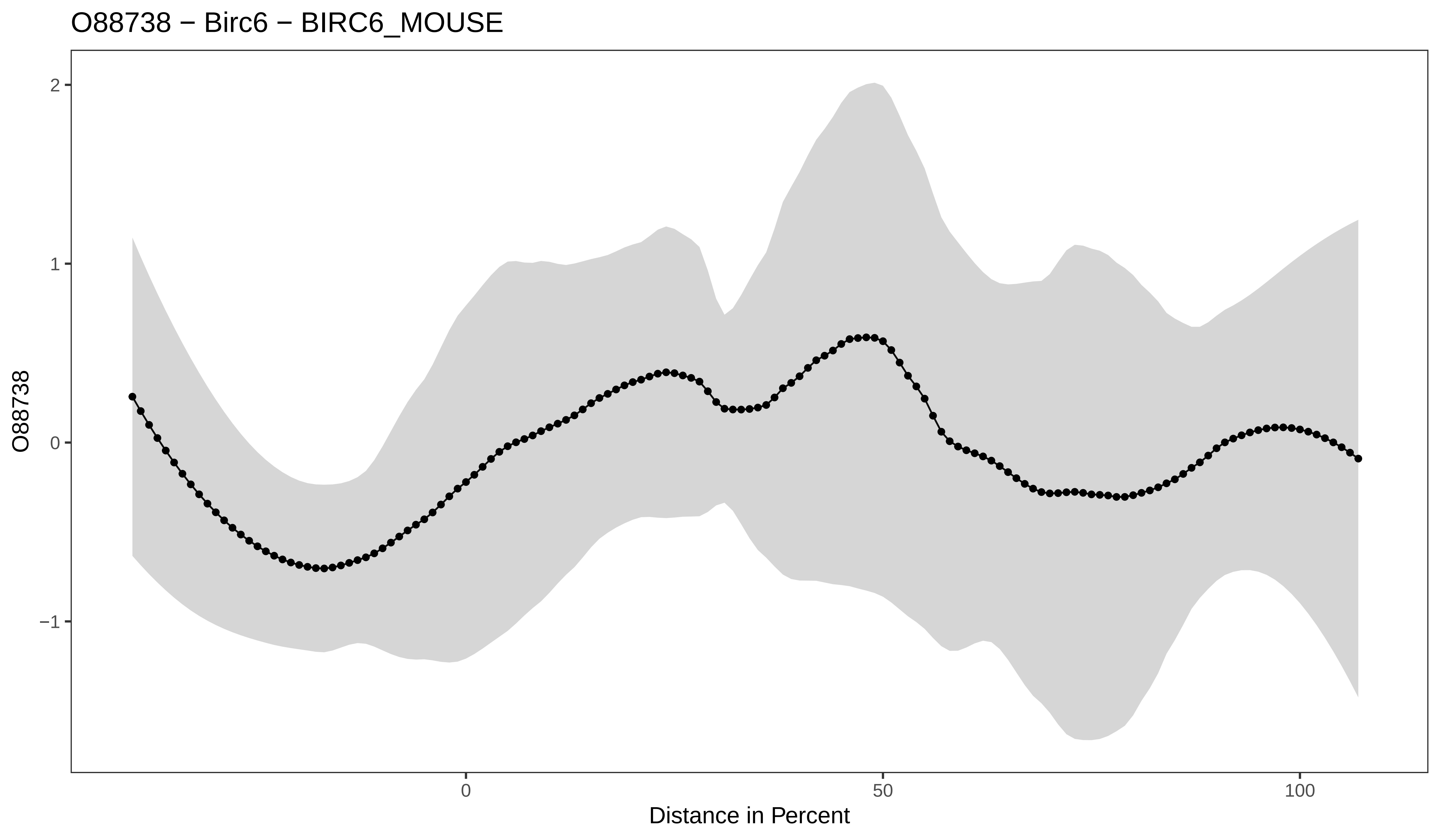
O88738 − Birc6 − BIRC6_MOUSE
O88738
2
1
0
−1
0
50
100
Distance in Percent

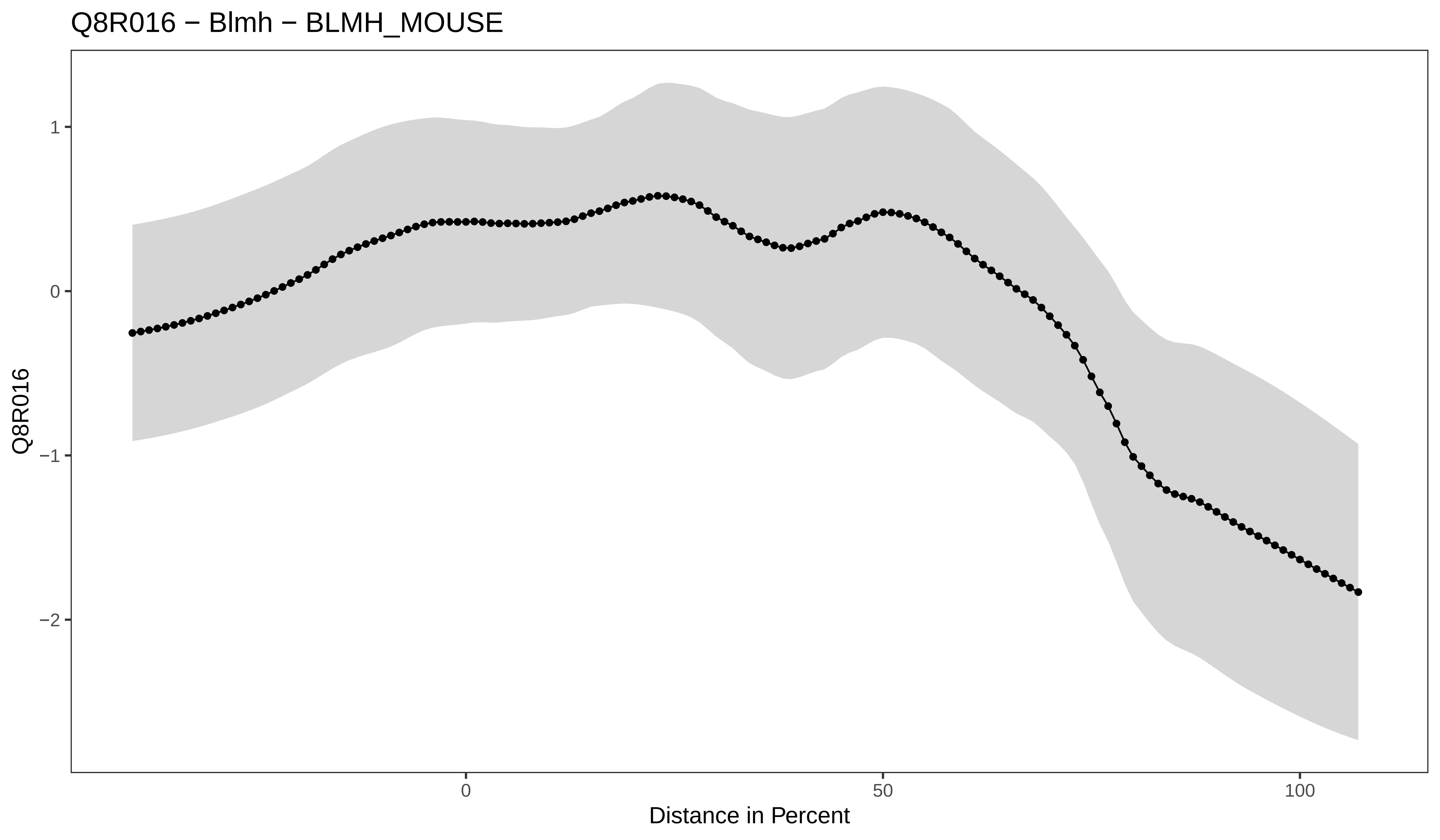
Q8R016 − Blmh − BLMH_MOUSE
1
0
−1
−2
Q8R016
0
50
100
Distance in Percent

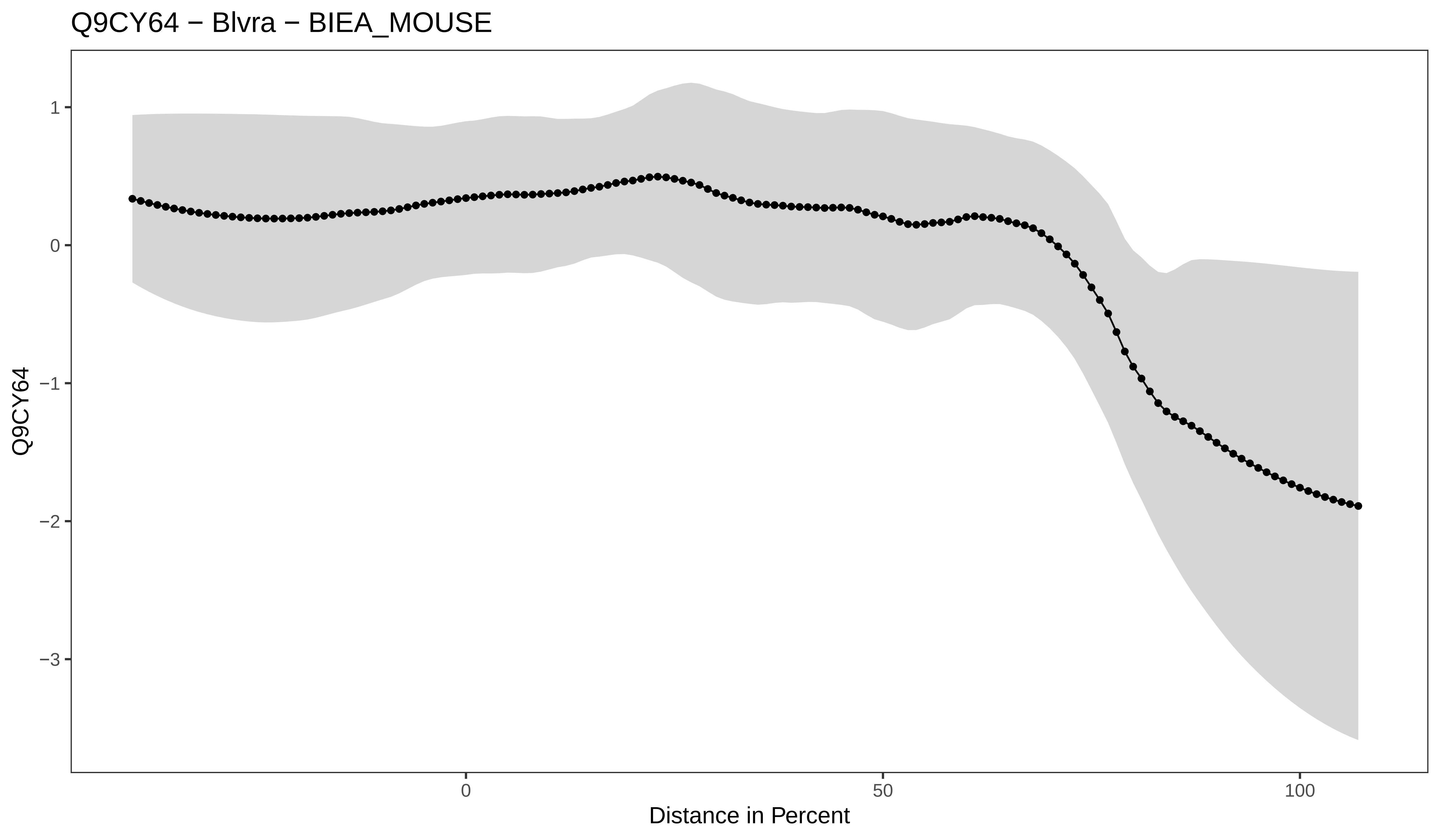
Q9CY64 − Blvra − BIEA_MOUSE
1
0
−1
−2
−3
Q9CY64
0
50
100
Distance in Percent

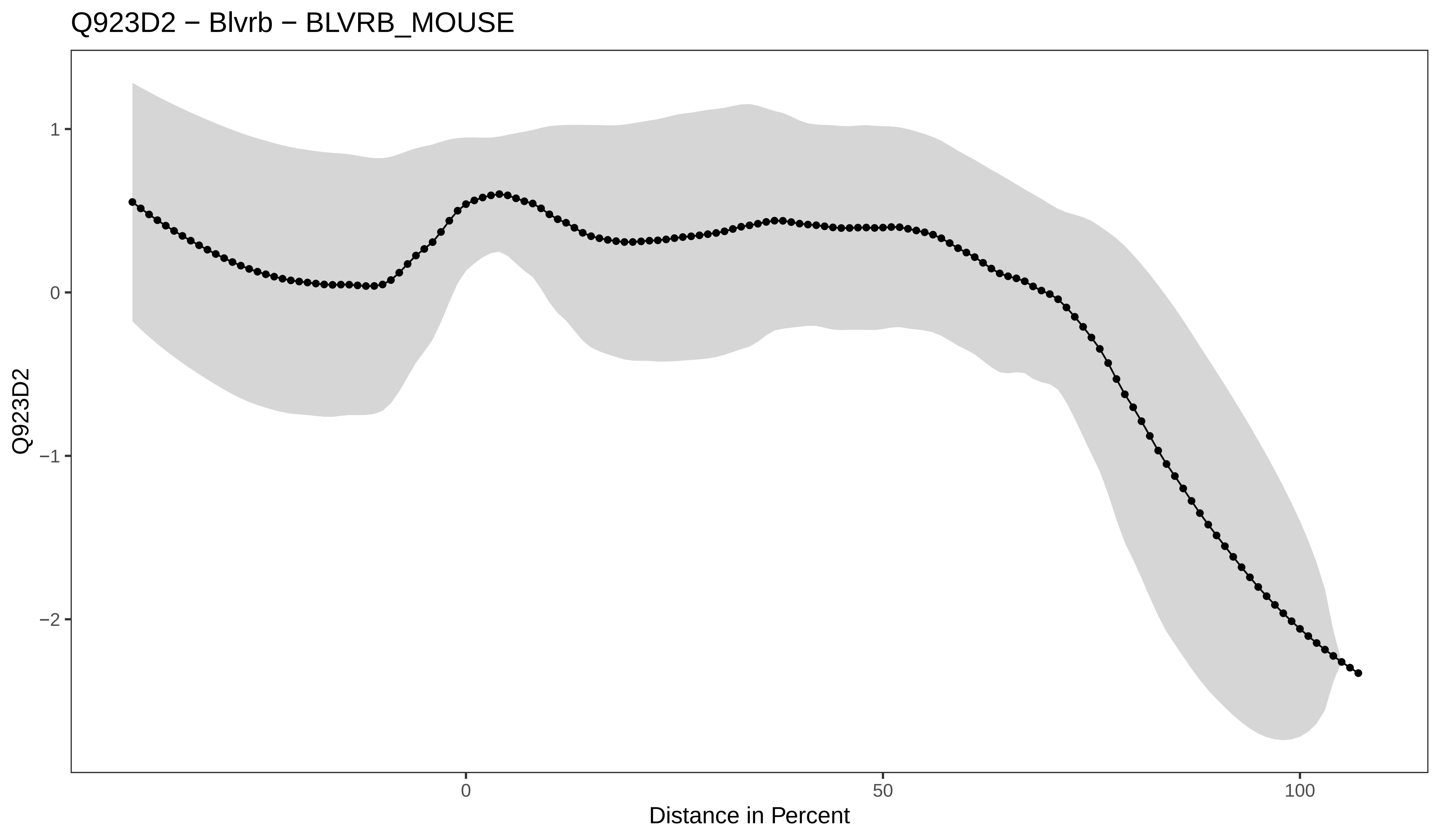
Q923D2 − Blvrb − BLVRB_MOUSE
1
0
−1
−2
Q923D2
0
50
100
Distance in Percent

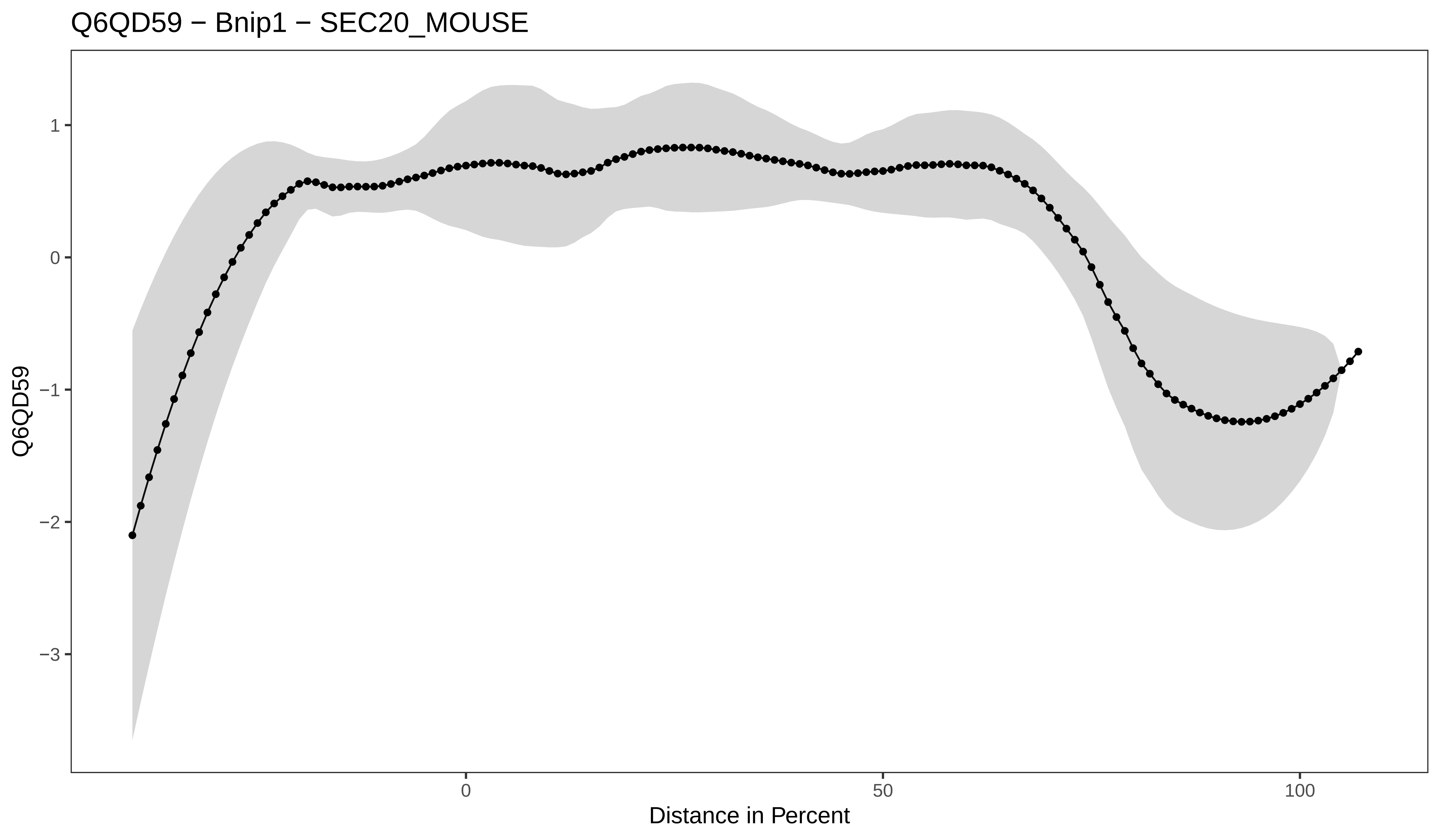

Q6QD59 − Bnip1 − SEC20_MOUSE
Q6QD59
1
0
−1
−2
−3
0
50
100
Distance in Percent

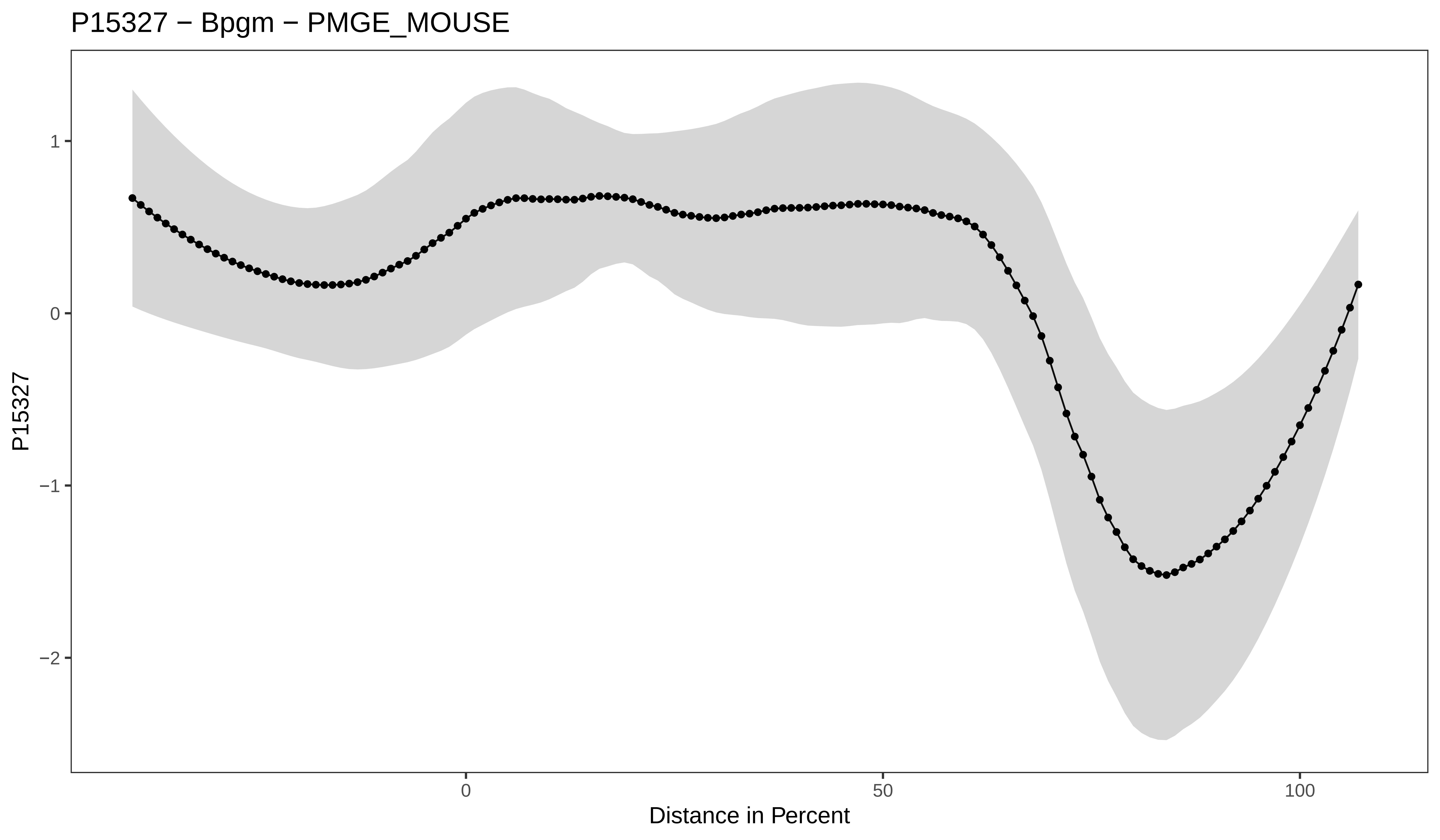

P15327 − Bpgm − PMGE_MOUSE
P15327
Distance in Percent
1
0
−1
−2
0
50
100

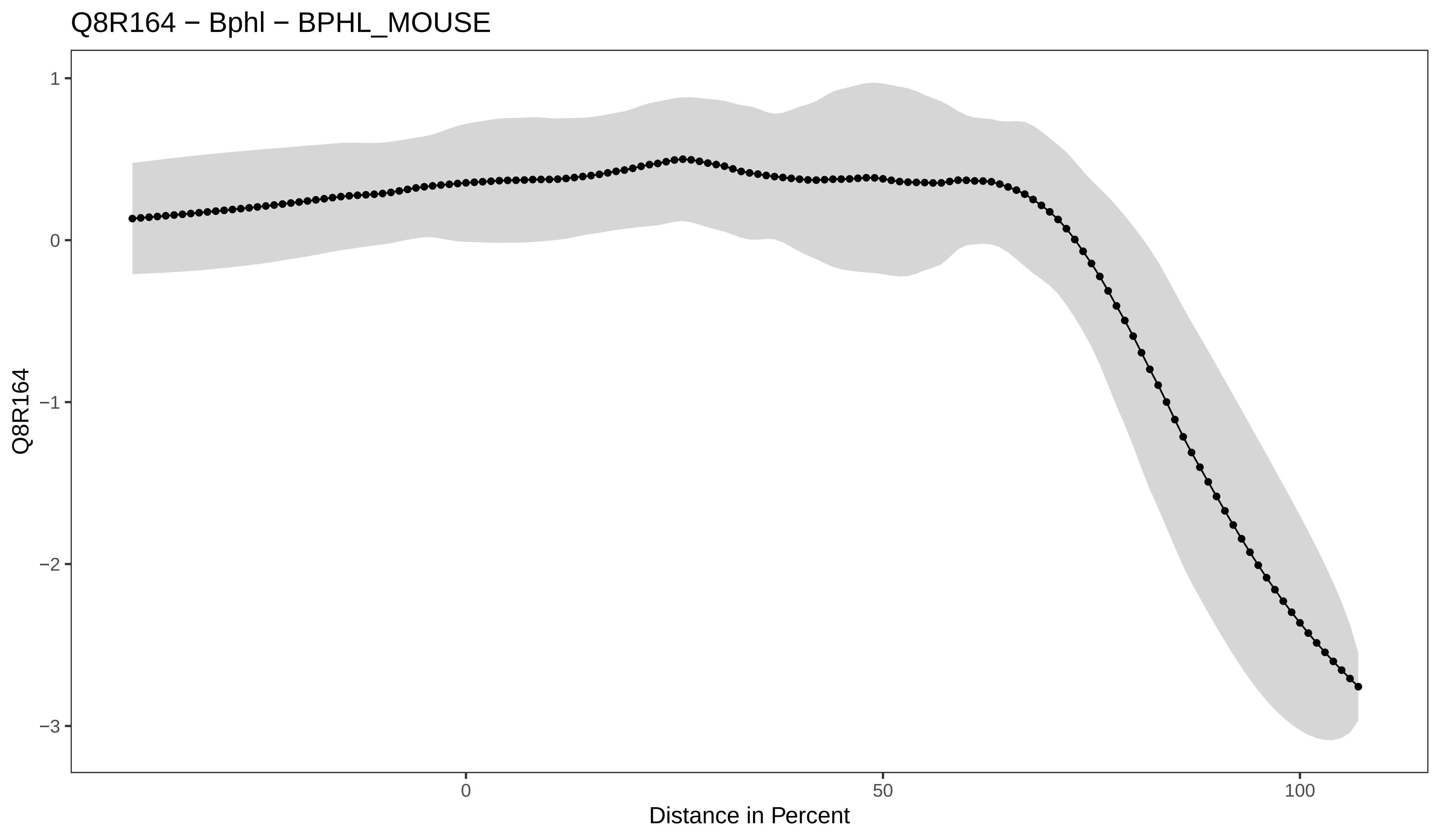
Q8R164 − Bphl − BPHL_MOUSE
1
0
−1
−2
−3
Q8R164
0
50
100
Distance in Percent

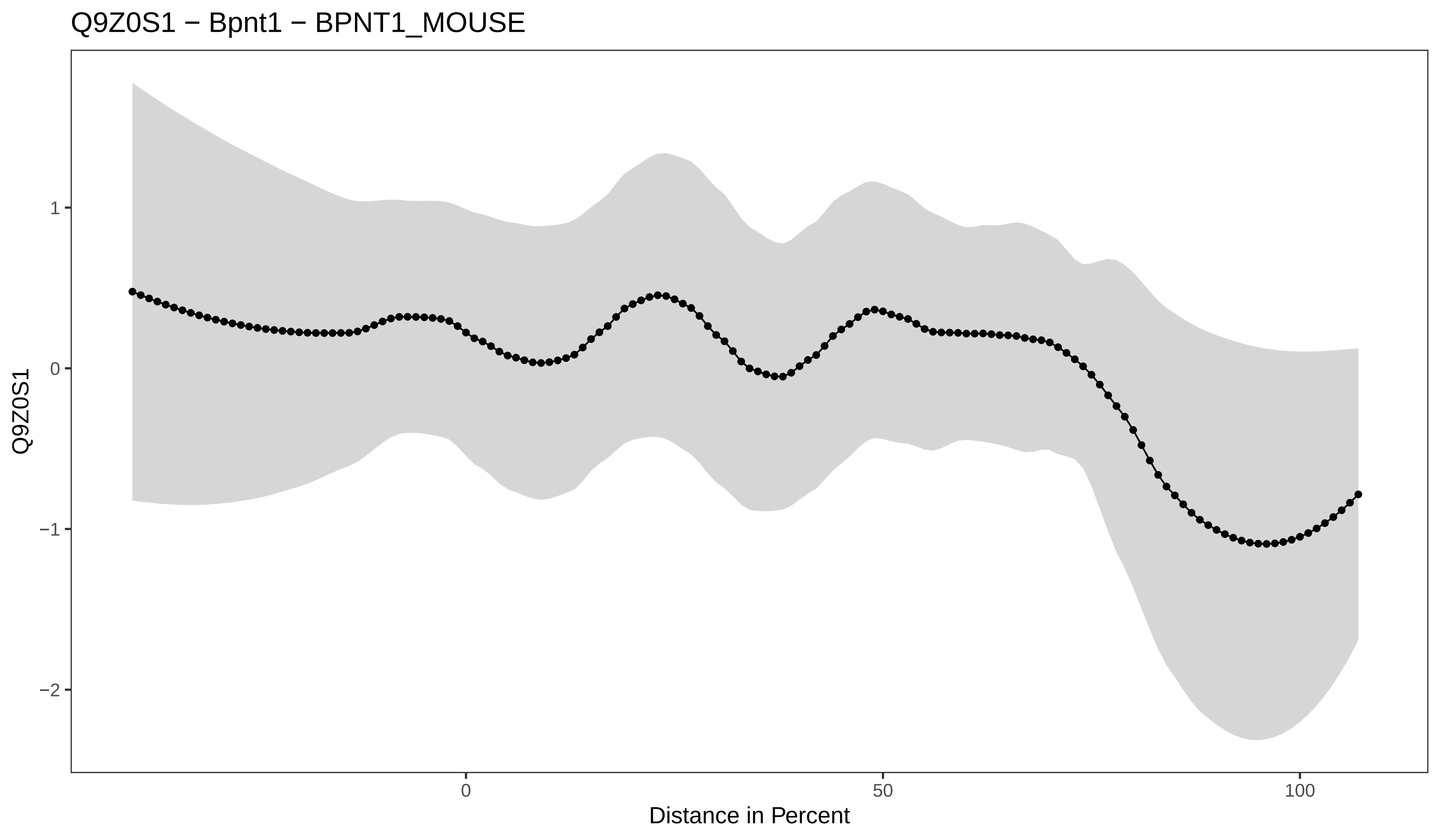
Q9Z0S1 − Bpnt1 − BPNT1_MOUSE
Q9Z0S1
1
0
−1
−2
0
50
100
Distance in Percent

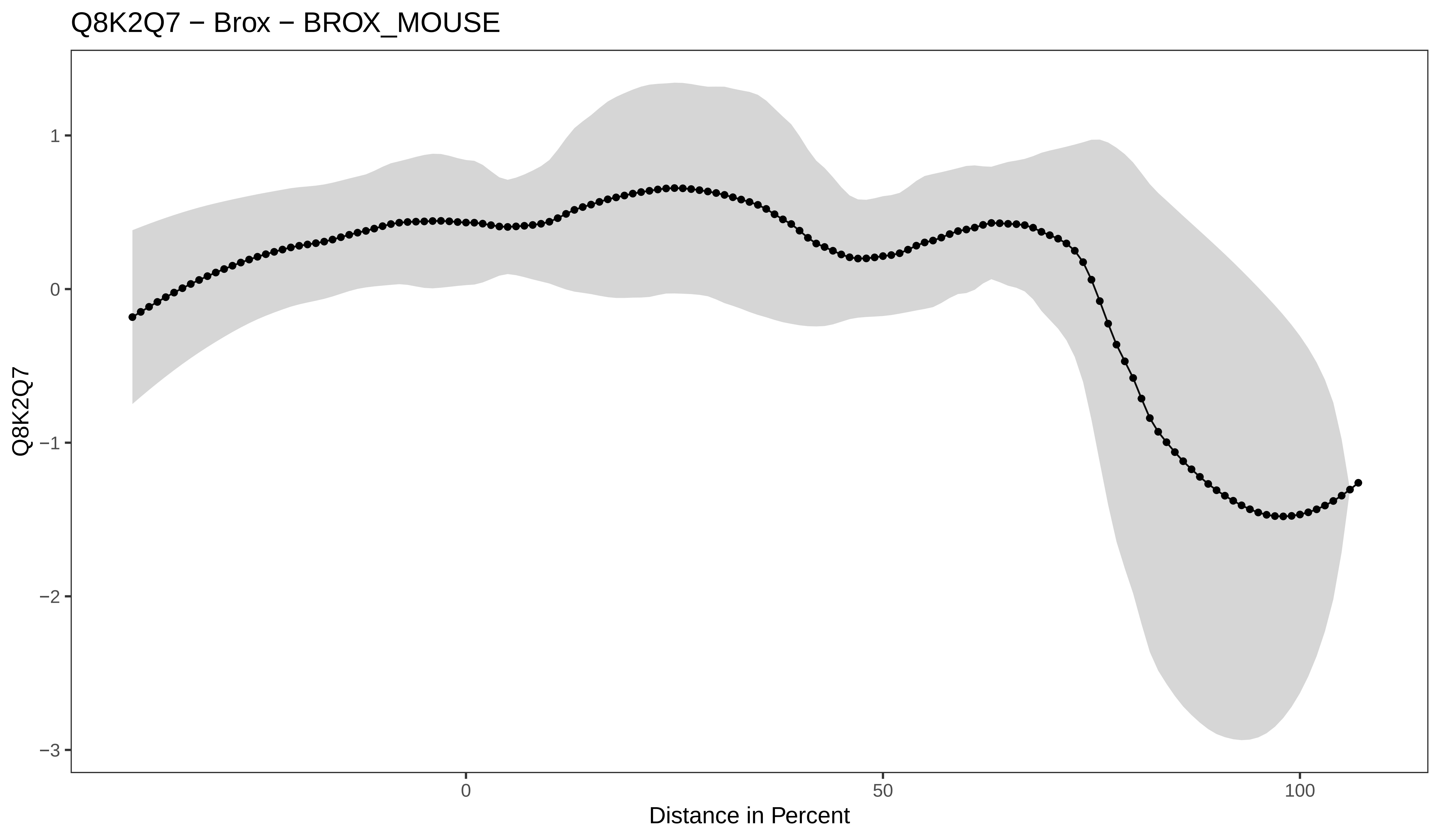

Q8K2Q7 − Brox − BROX_MOUSE
1
0
−1
−2
−3
Q8K2Q7
0
50
100
Distance in Percent

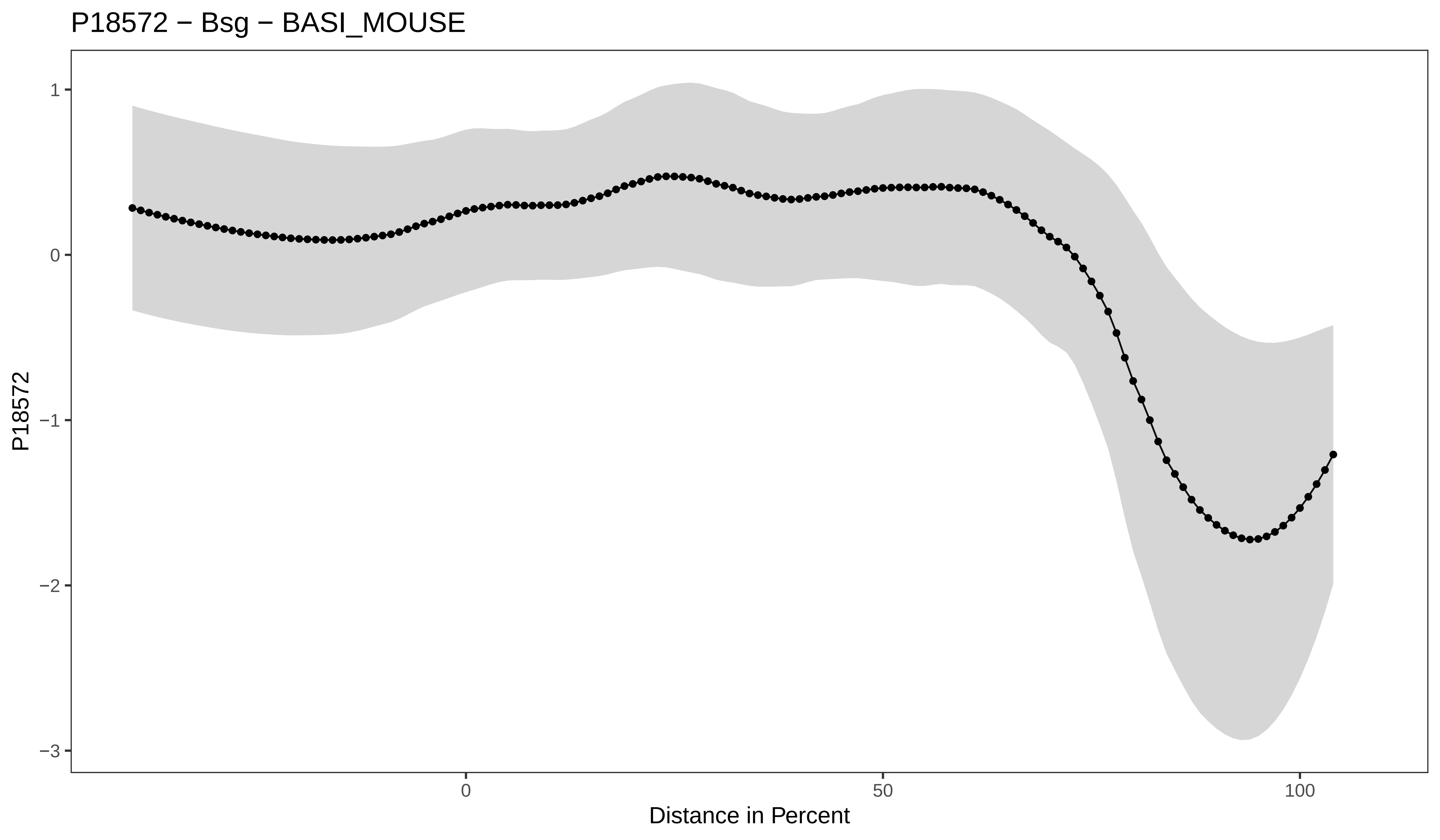

P18572 − Bsg − BASI_MOUSE
P18572
1
0
−1
−2
−3
0
50
100
Distance in Percent

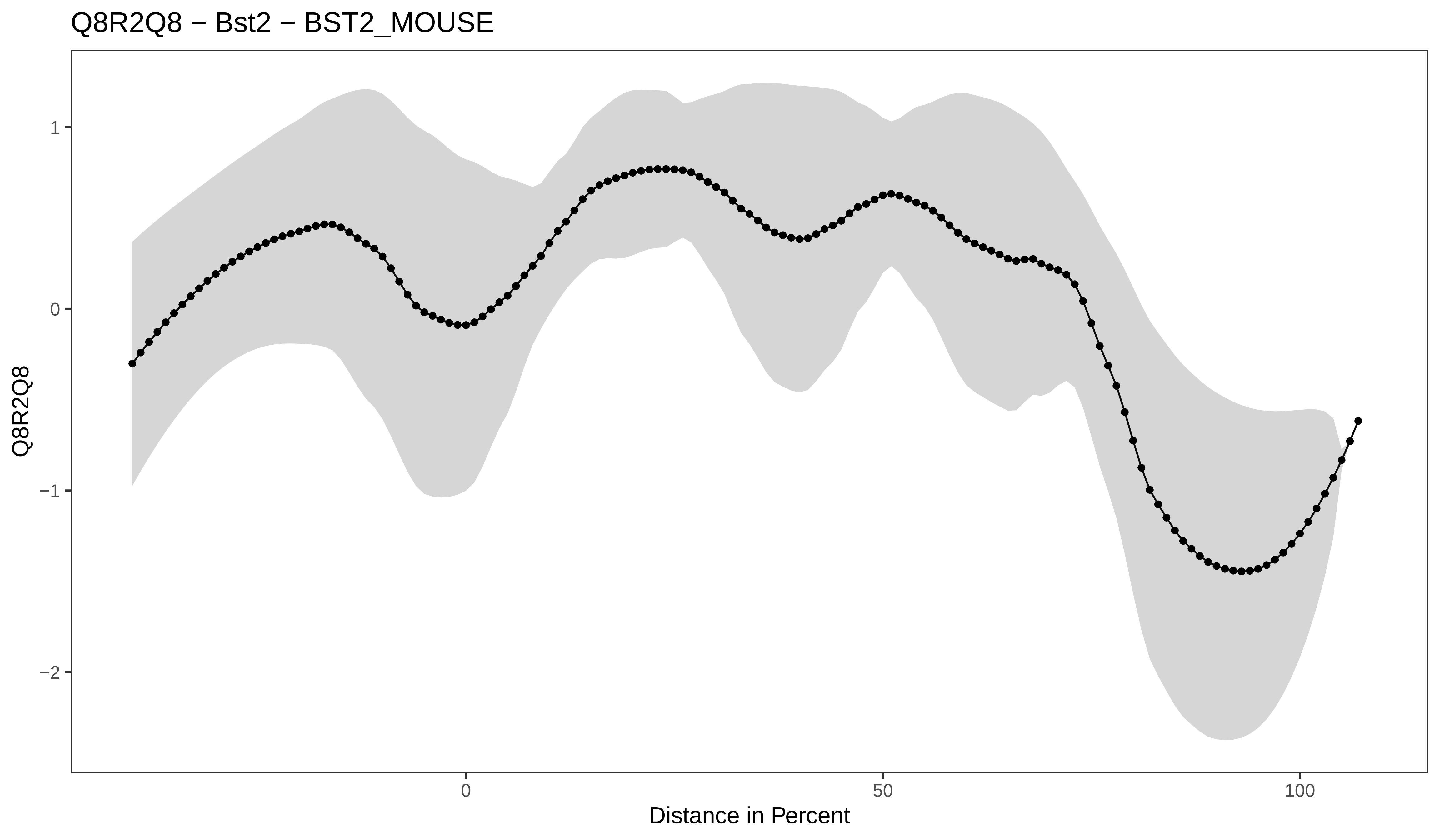
Q8R2Q8 − Bst2 − BST2_MOUSE
Q8R2Q8
Distance in Percent
1
0
−1
−2
0
50
100

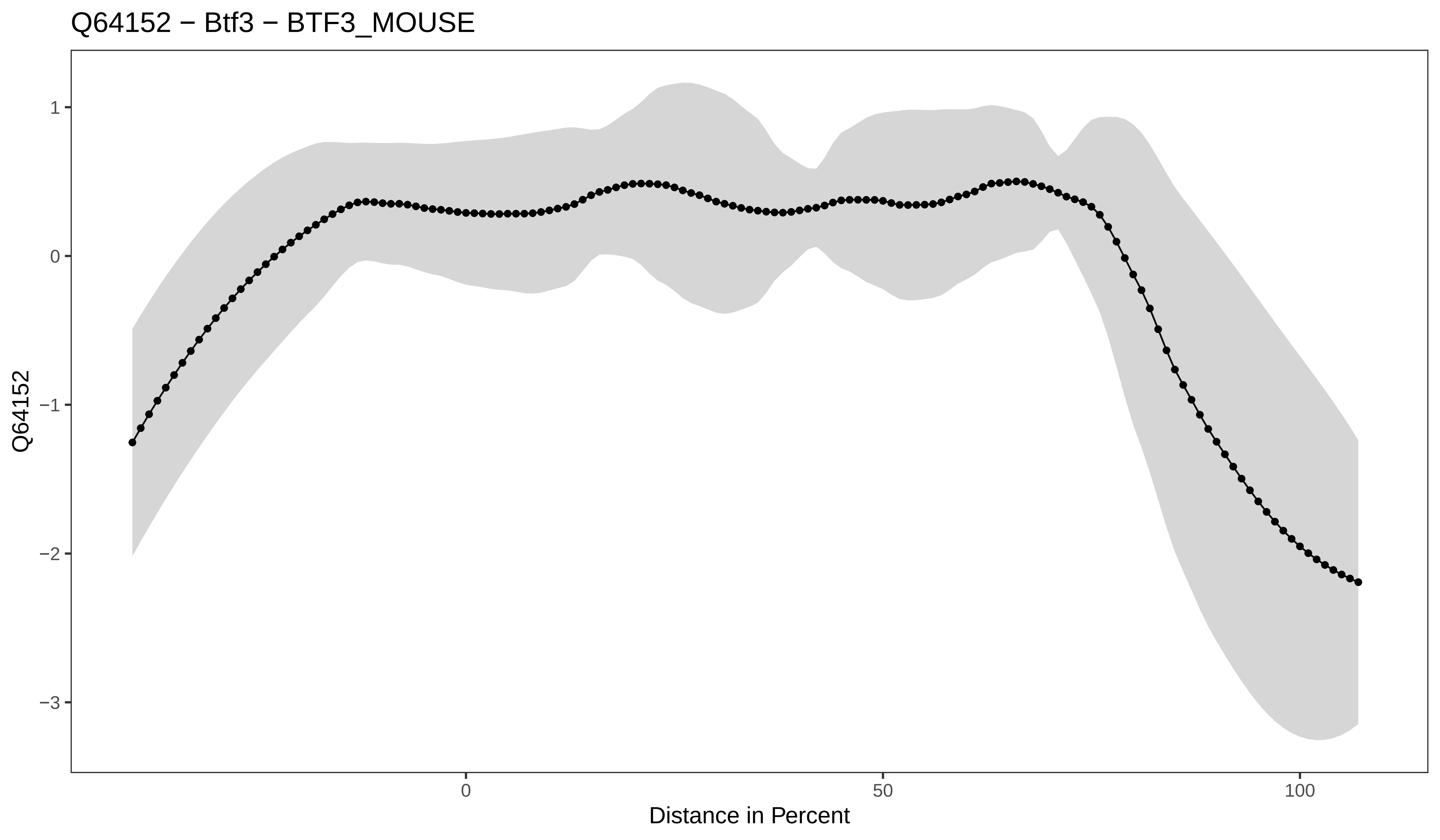

Q64152 − Btf3 − BTF3_MOUSE
Q64152
1
0
−1
−2
−3
0
50
100
Distance in Percent

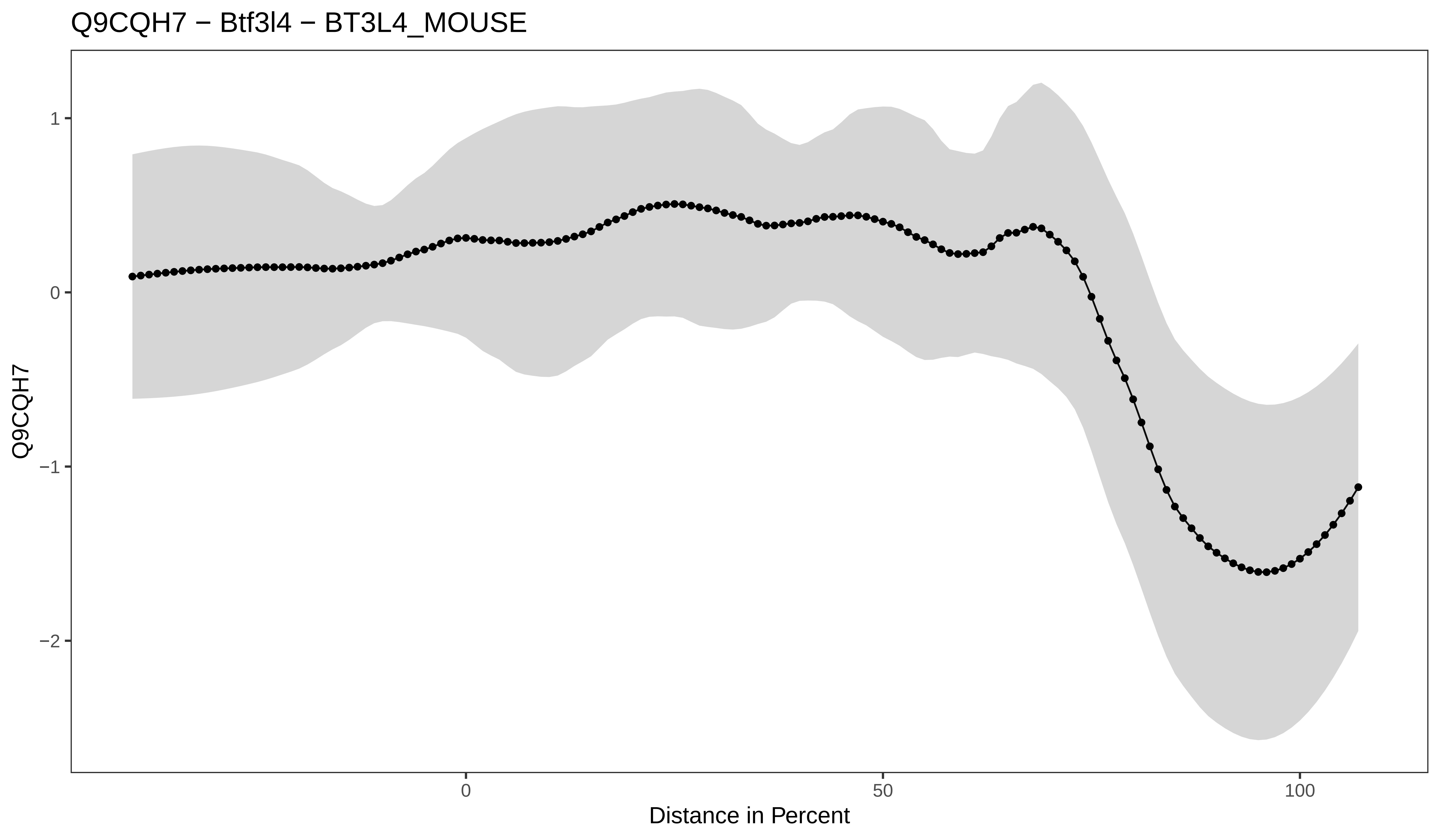
Q9CQH7 − Btf3l4 − BT3L4_MOUSE
1
0
−1
−2
Q9CQH7
0
50
100
Distance in Percent

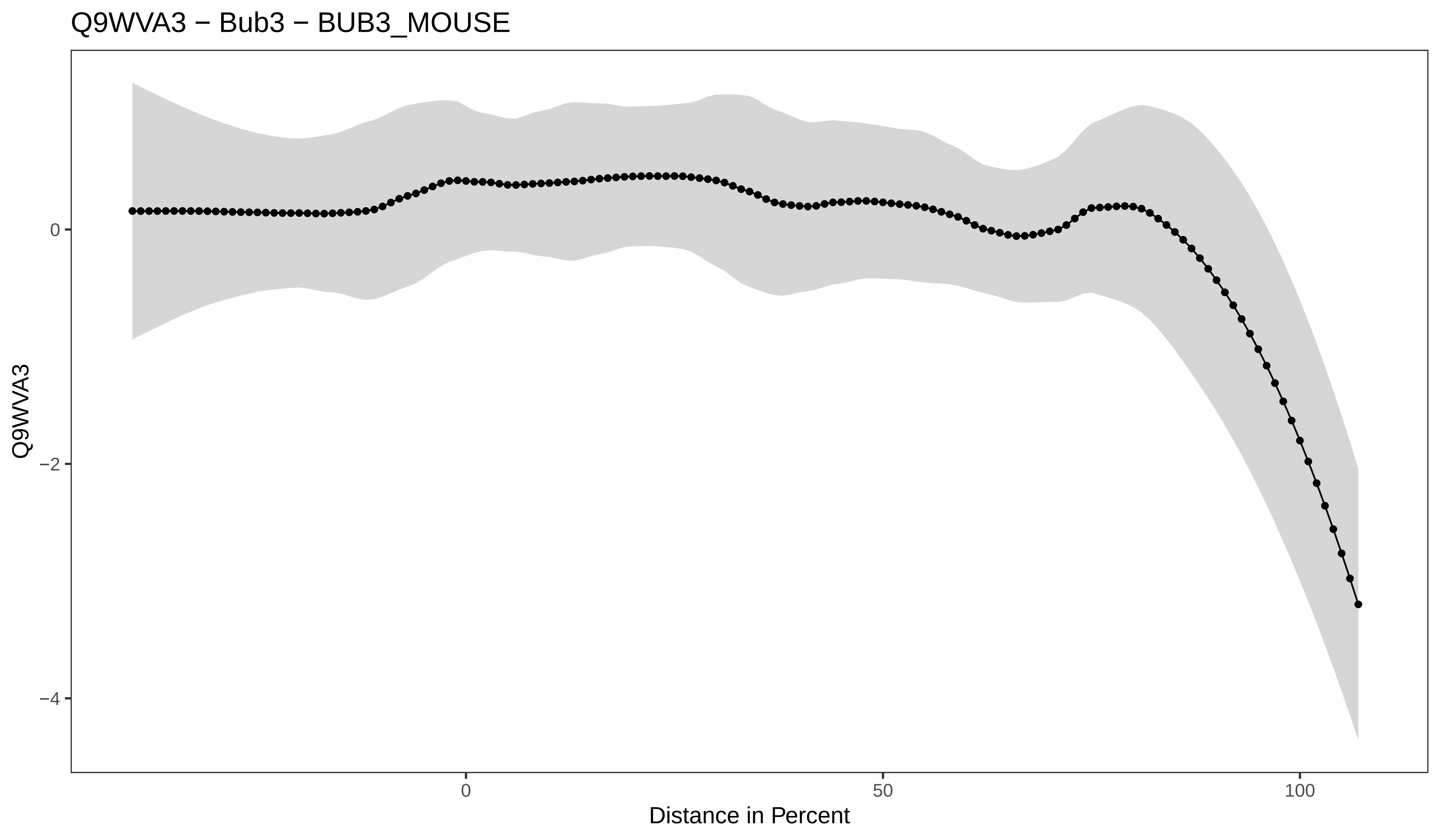

Q9WVA3 − Bub3 − BUB3_MOUSE
Q9WVA3
0
−2
−4
0
50
100
Distance in Percent

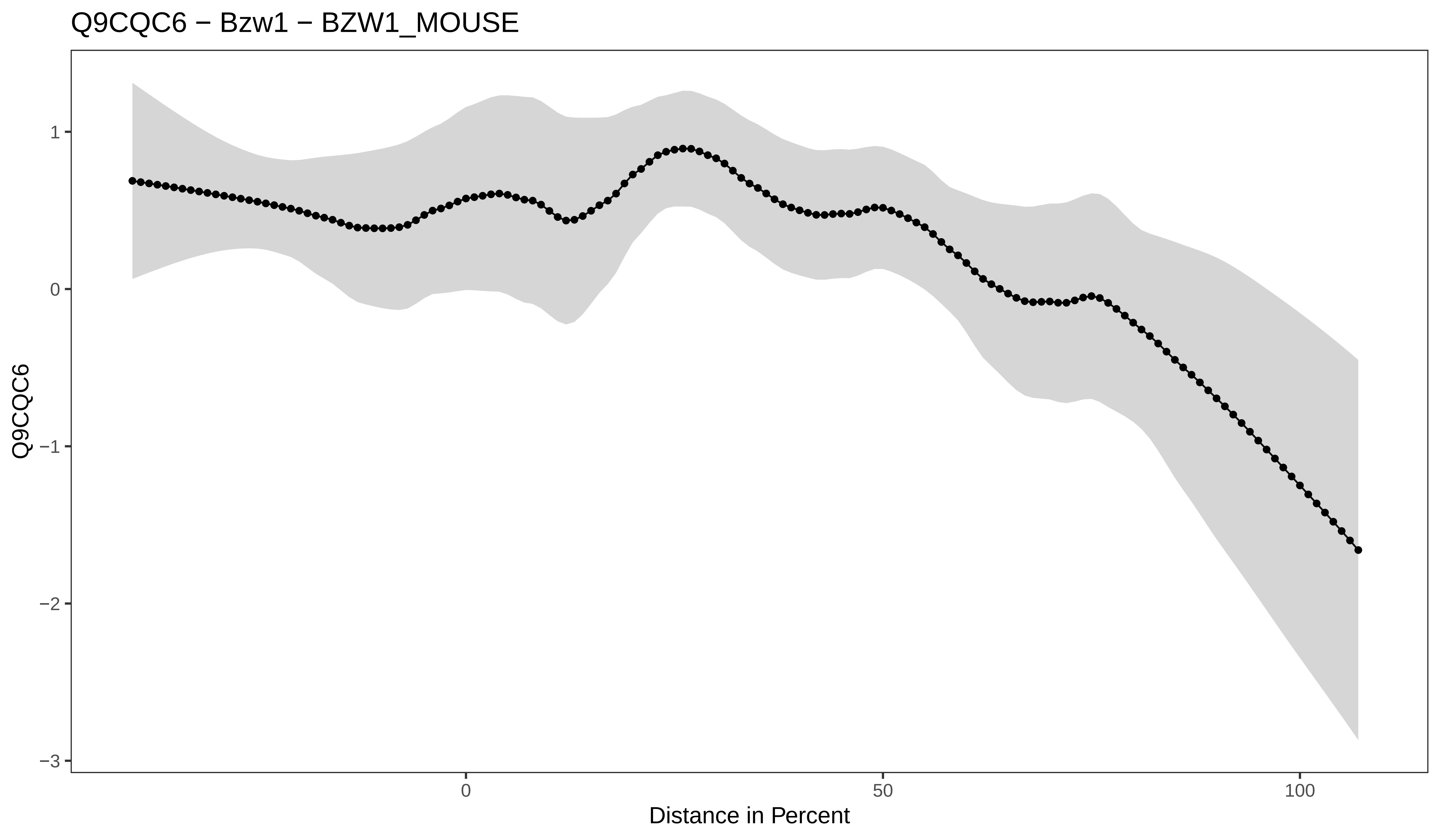
Q9CQC6 − Bzw1 − BZW1_MOUSE
1
0
−1
−2
−3
Q9CQC6
0
50
100
Distance in Percent

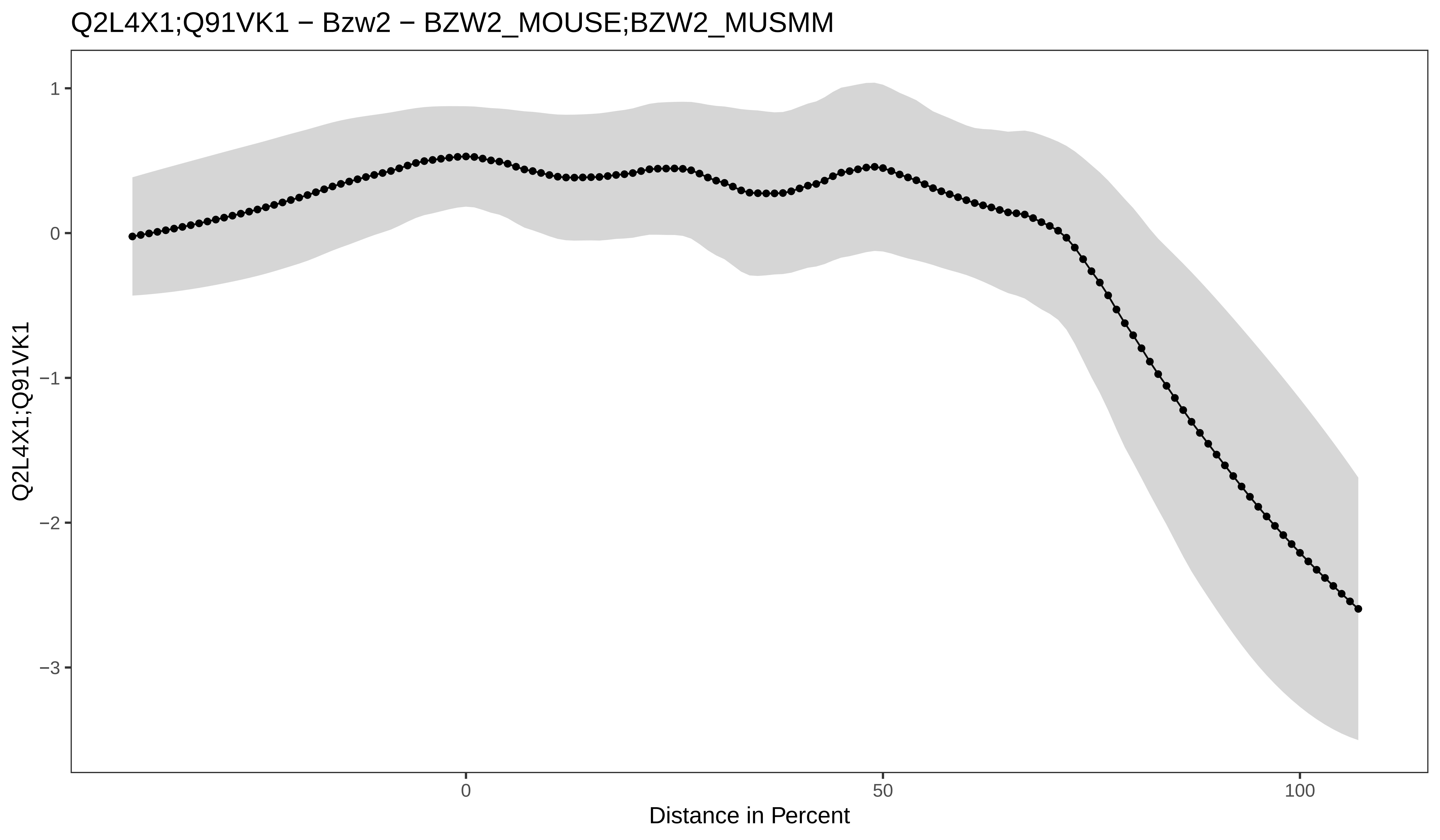

Q2L4X1;Q91VK1 − Bzw2 − BZW2_MOUSE;BZW2_MUSMM
1
0
−1
−2
−3
Q2L4X1;Q91VK1
0
50
100
Distance in Percent

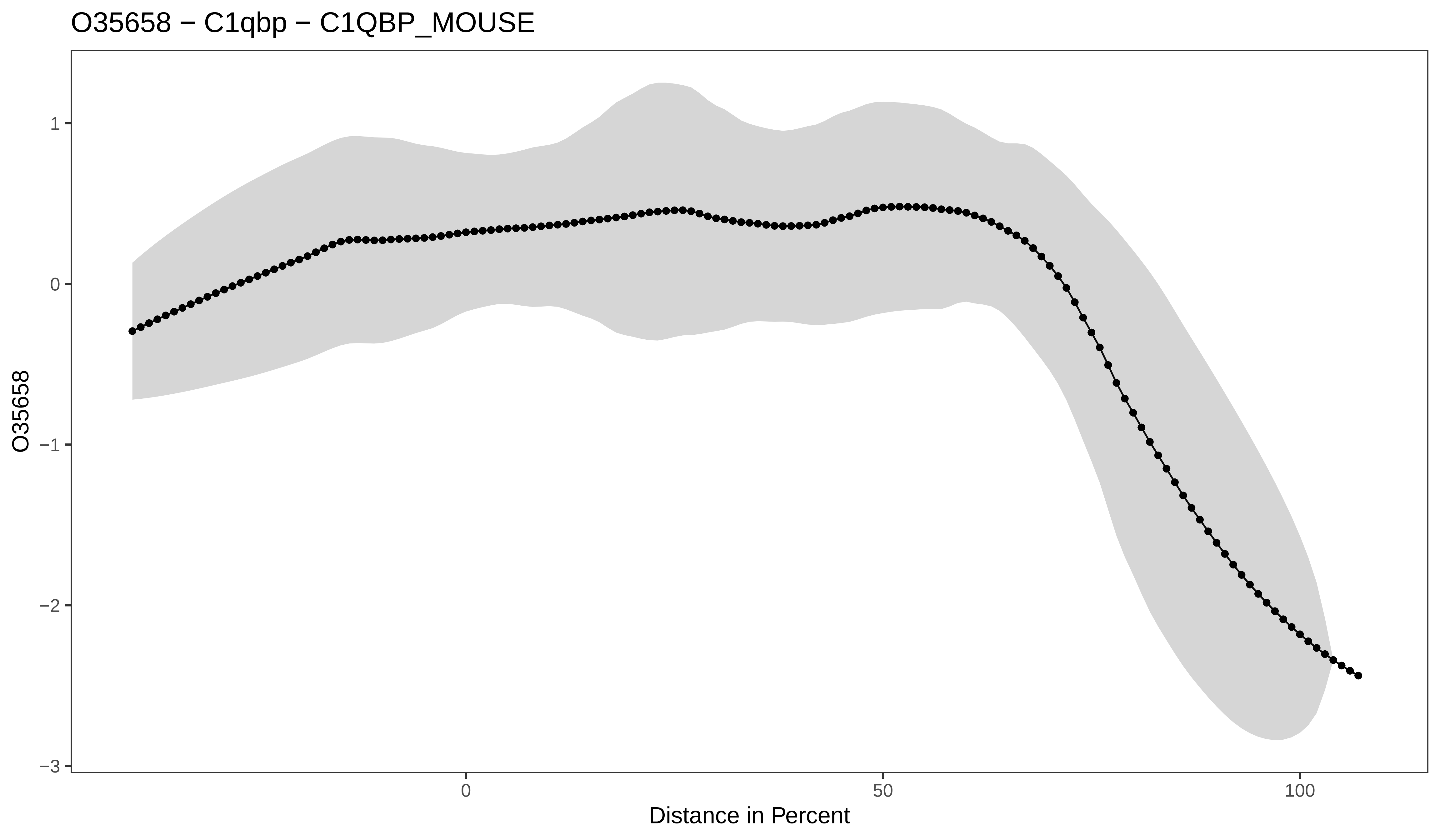
O35658 − C1qbp − C1QBP_MOUSE
O35658
1
0
−1
−2
−3
0
50
100
Distance in Percent

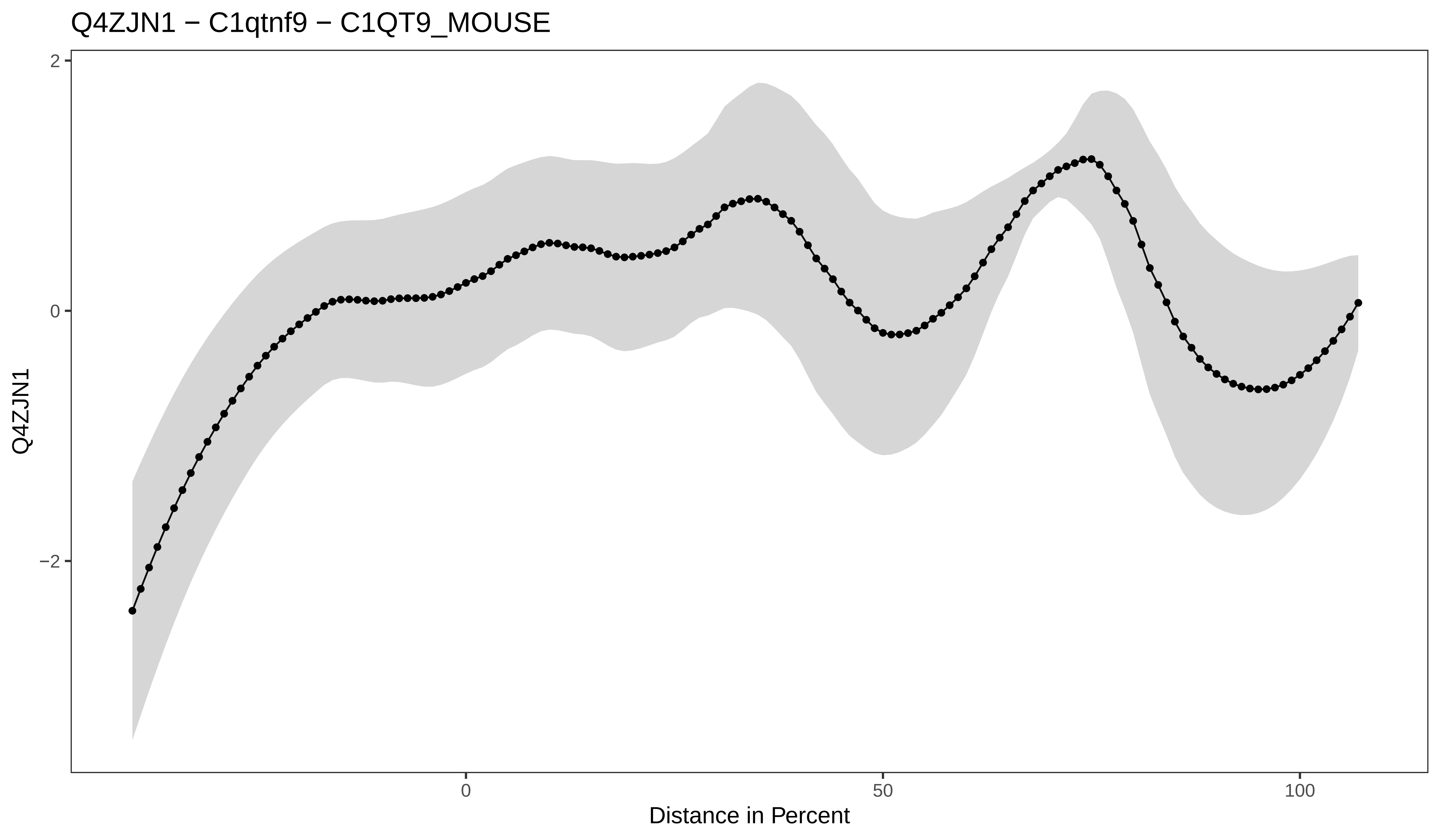
Q4ZJN1 − C1qtnf9 − C1QT9_MOUSE
2
0
−2
Q4ZJN1
0
50
100
Distance in Percent

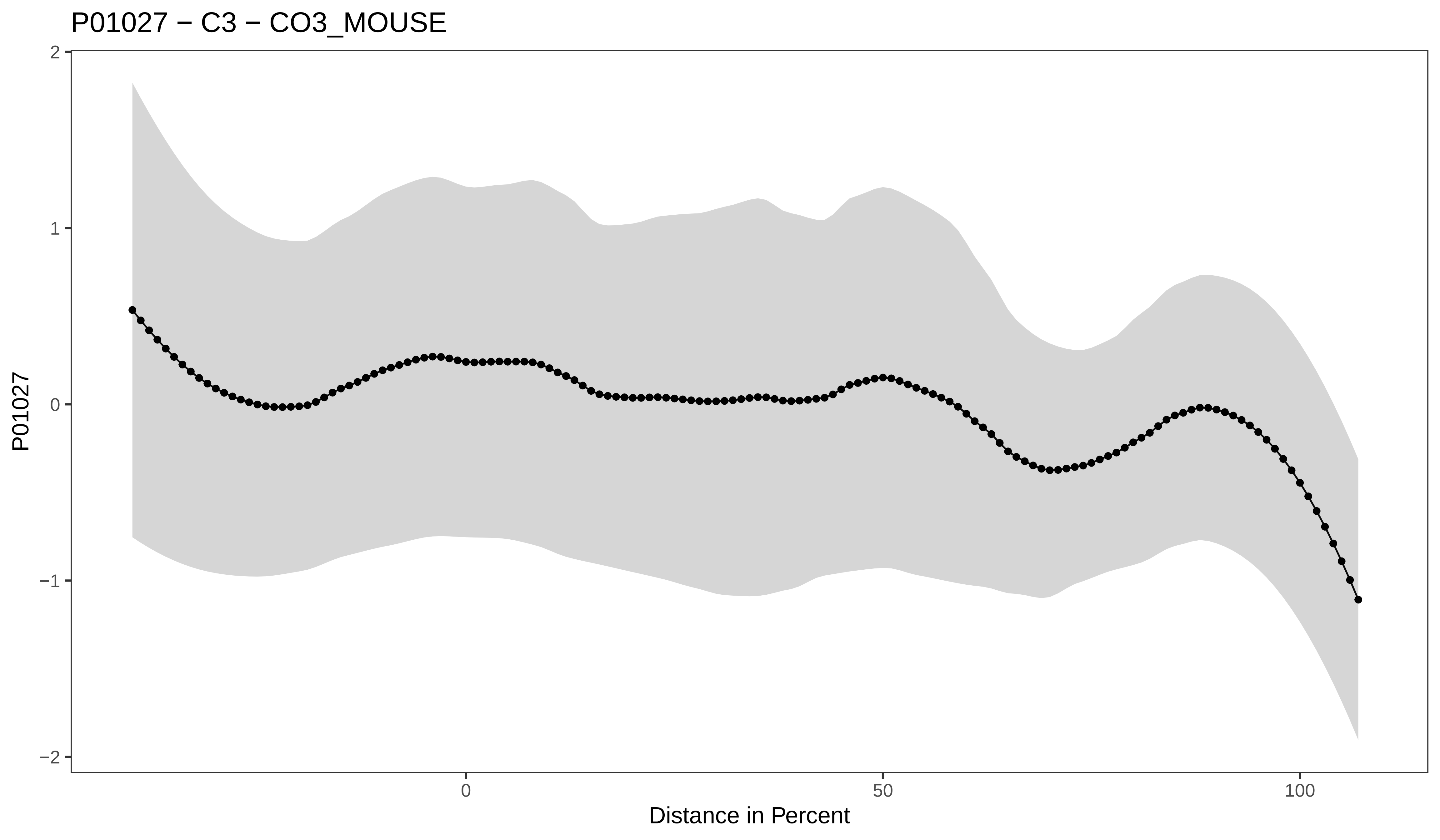
P01027 − C3 − CO3_MOUSE
2
1
0
−1
−2
P01027
0
50
100
Distance in Percent

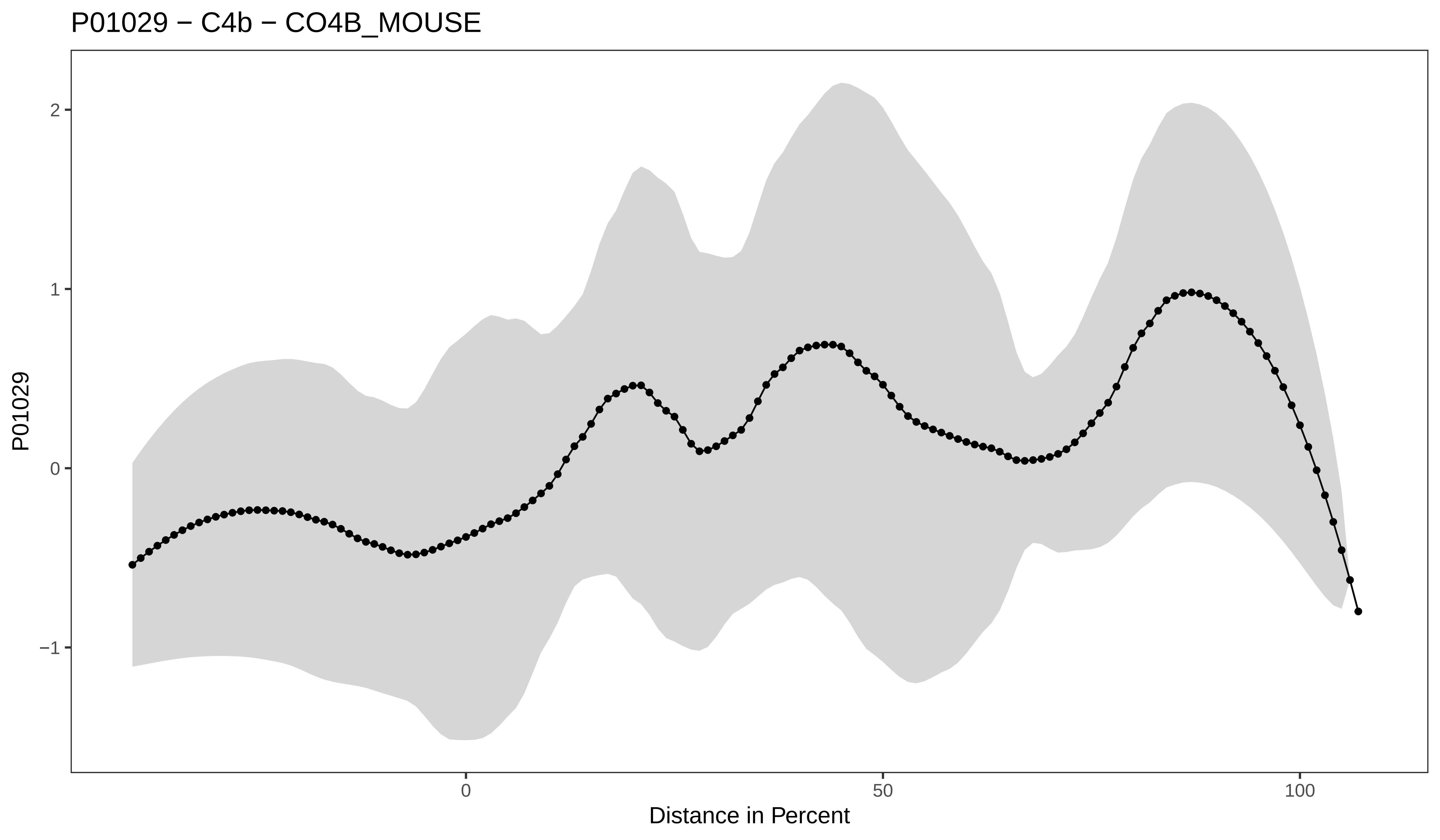
P01029 − C4b − CO4B_MOUSE
P01029
2
1
0
−1
0
50
100
Distance in Percent

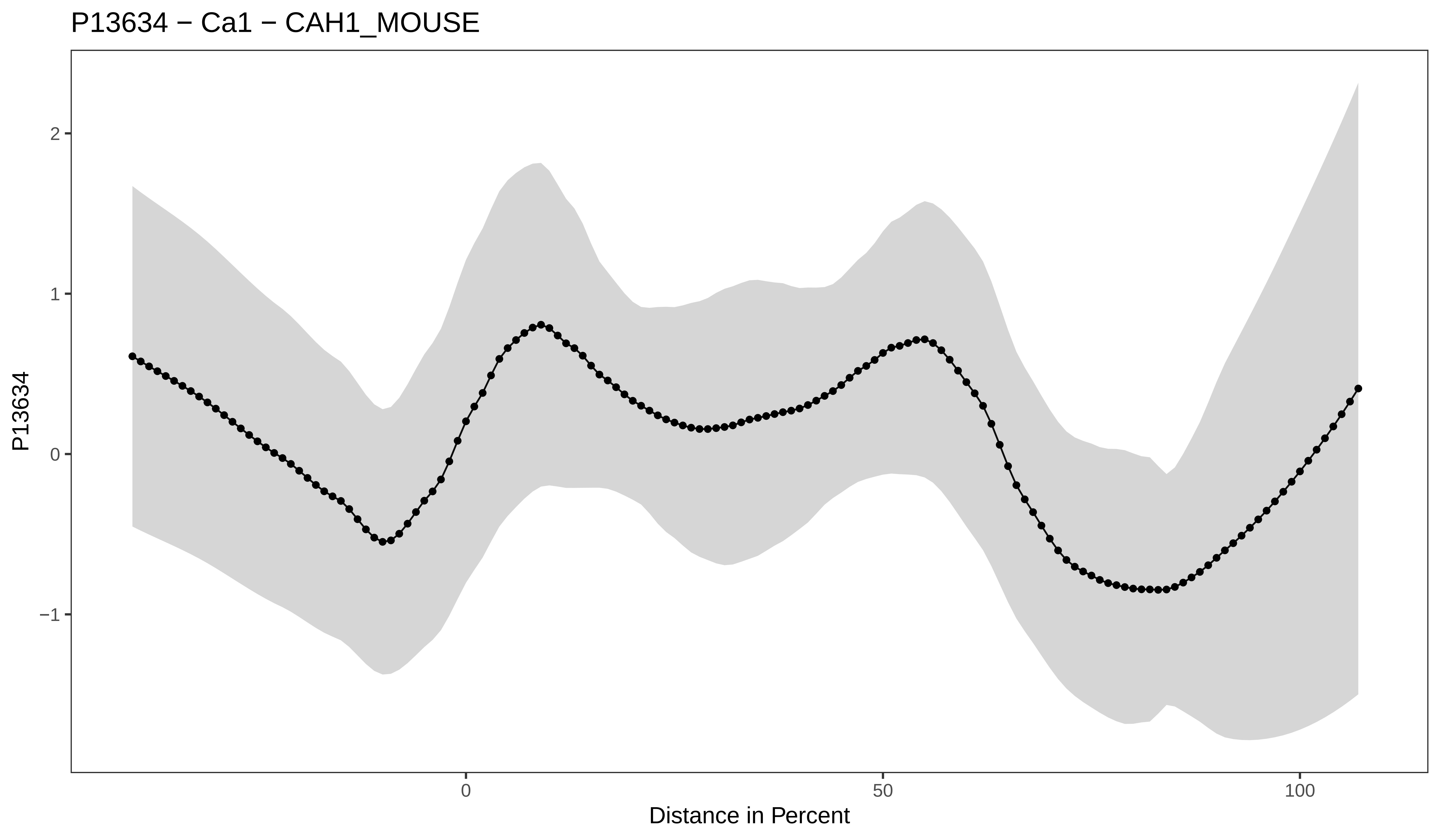

P13634 − Ca1 − CAH1_MOUSE
P13634
2
1
0
−1
0
50
100
Distance in Percent

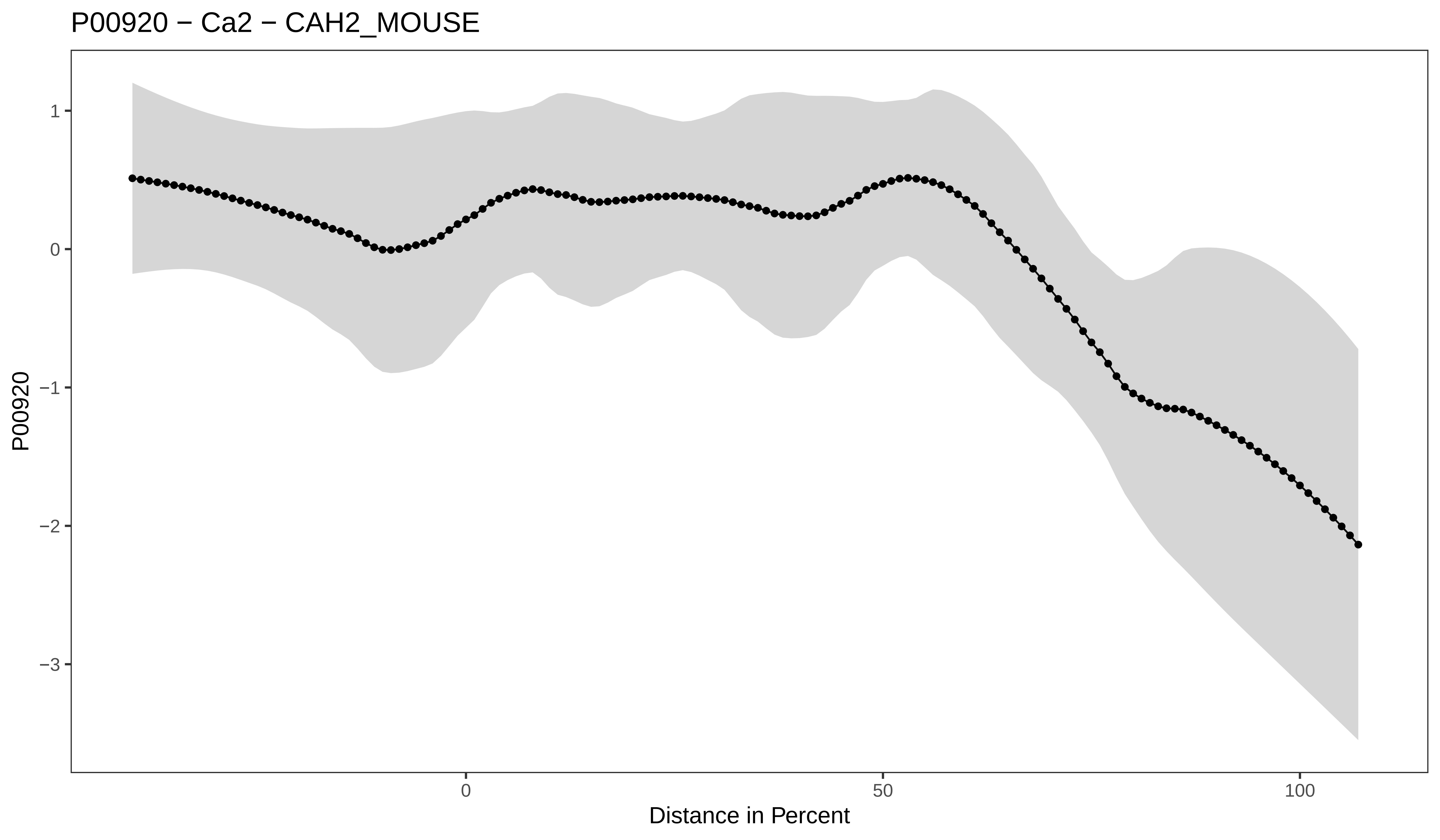

P00920 − Ca2 − CAH2_MOUSE
1
0
−1
−2
−3
P00920
0
50
100
Distance in Percent

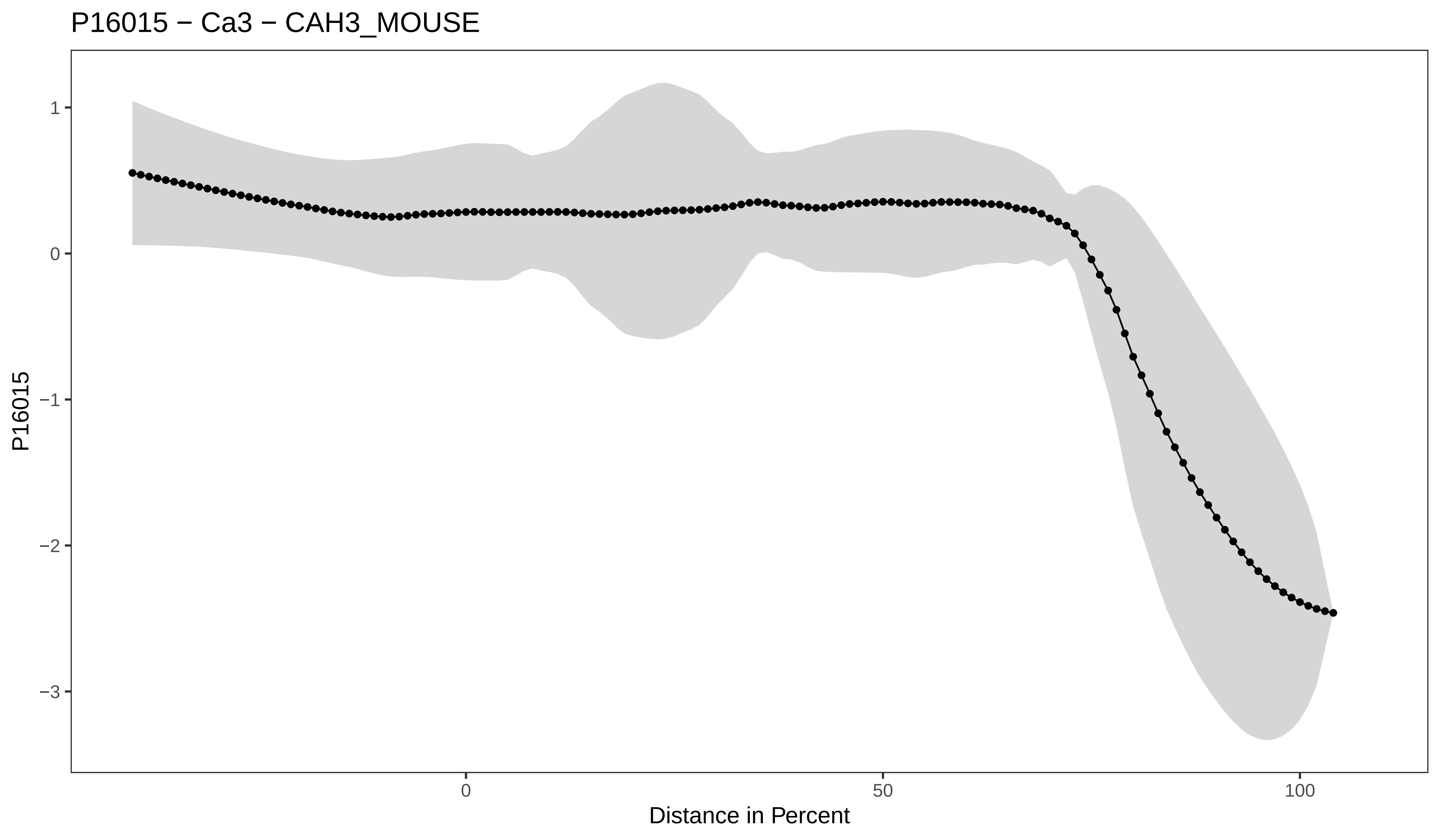

P16015 − Ca3 − CAH3_MOUSE
1
0
−1
−2
−3
P16015
0
50
100
Distance in Percent

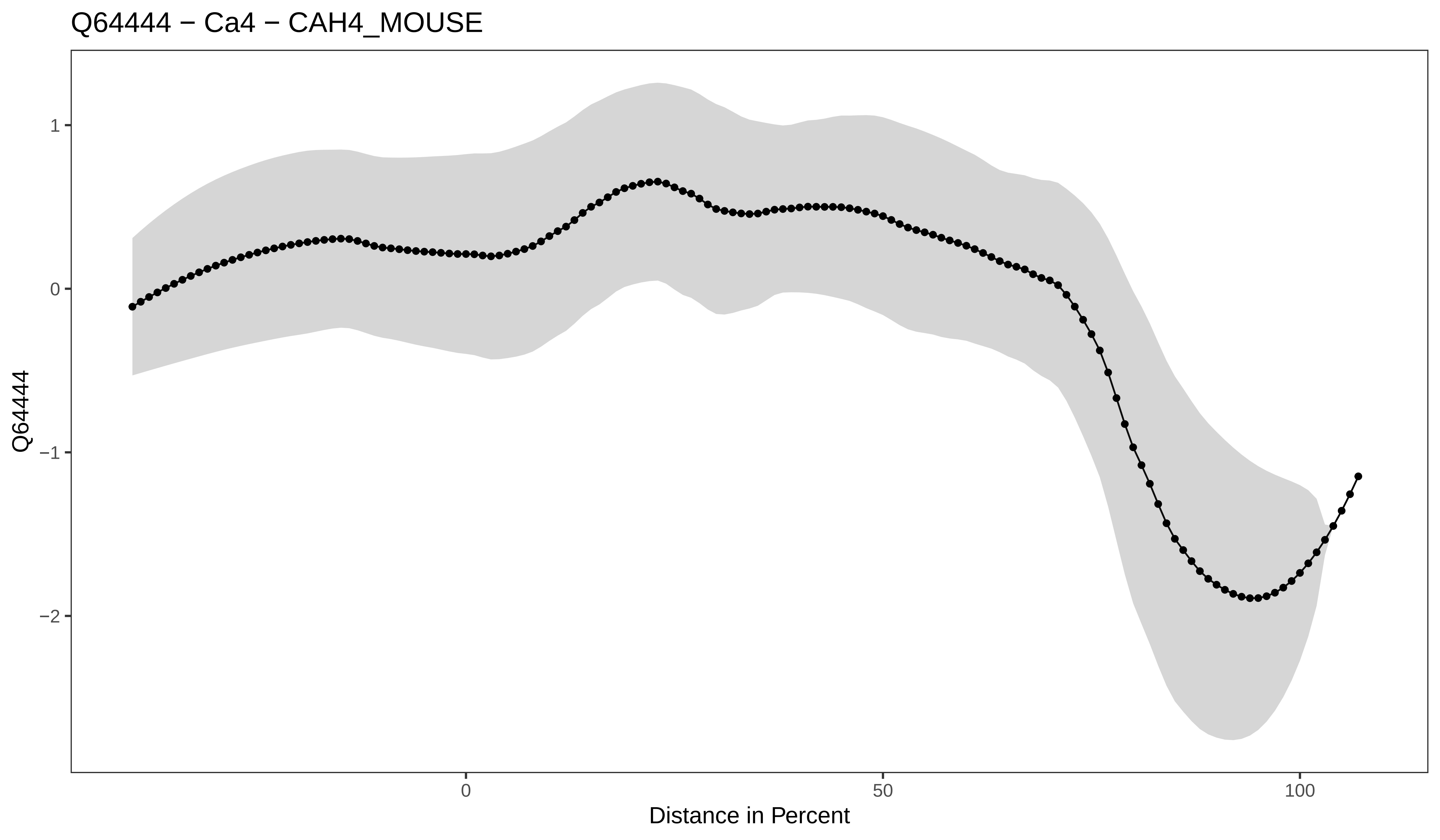

Q64444 − Ca4 − CAH4_MOUSE
Q64444
1
0
−1
−2
0
50
100
Distance in Percent

Q06138 − Cab39 − CAB39_MOUSE

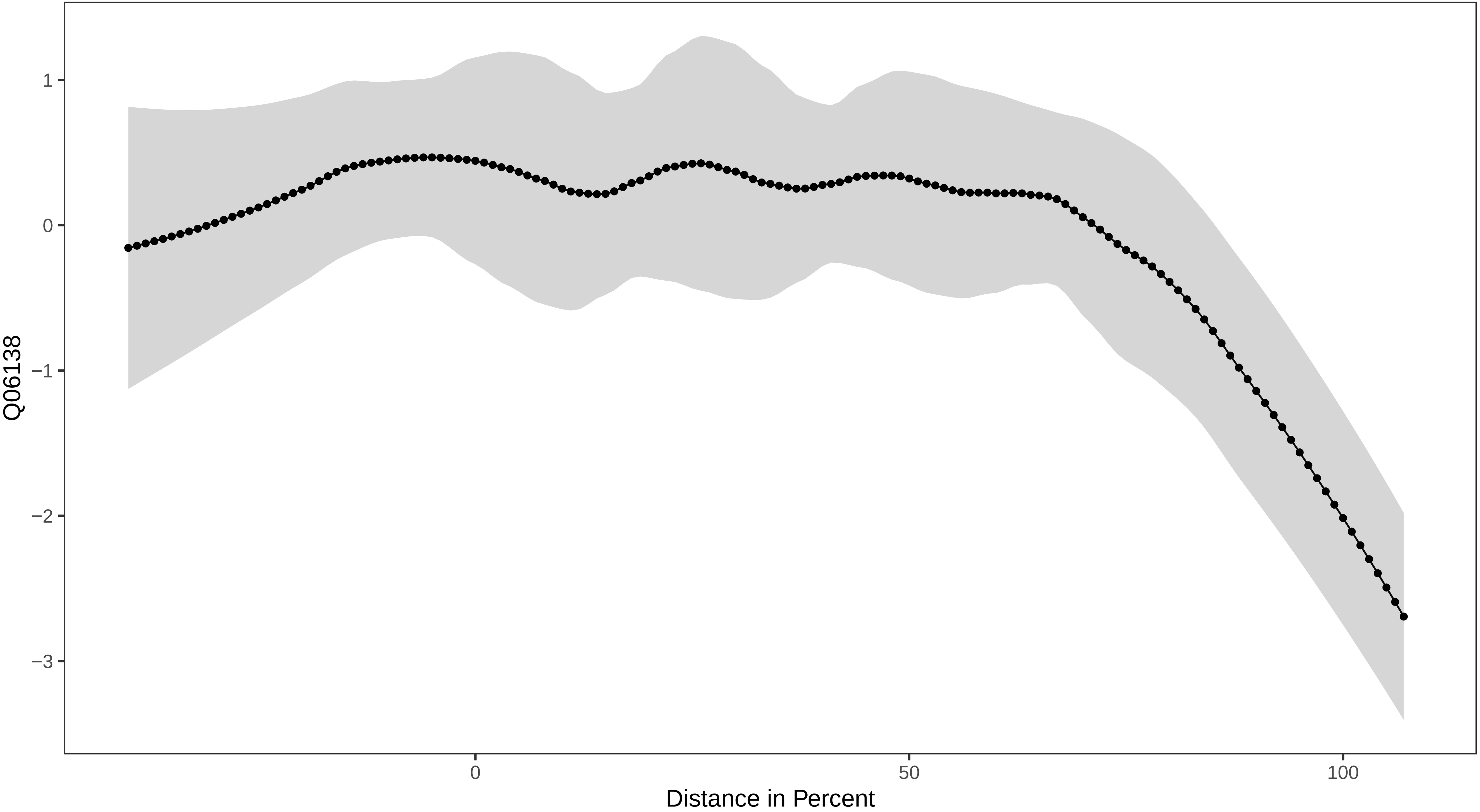

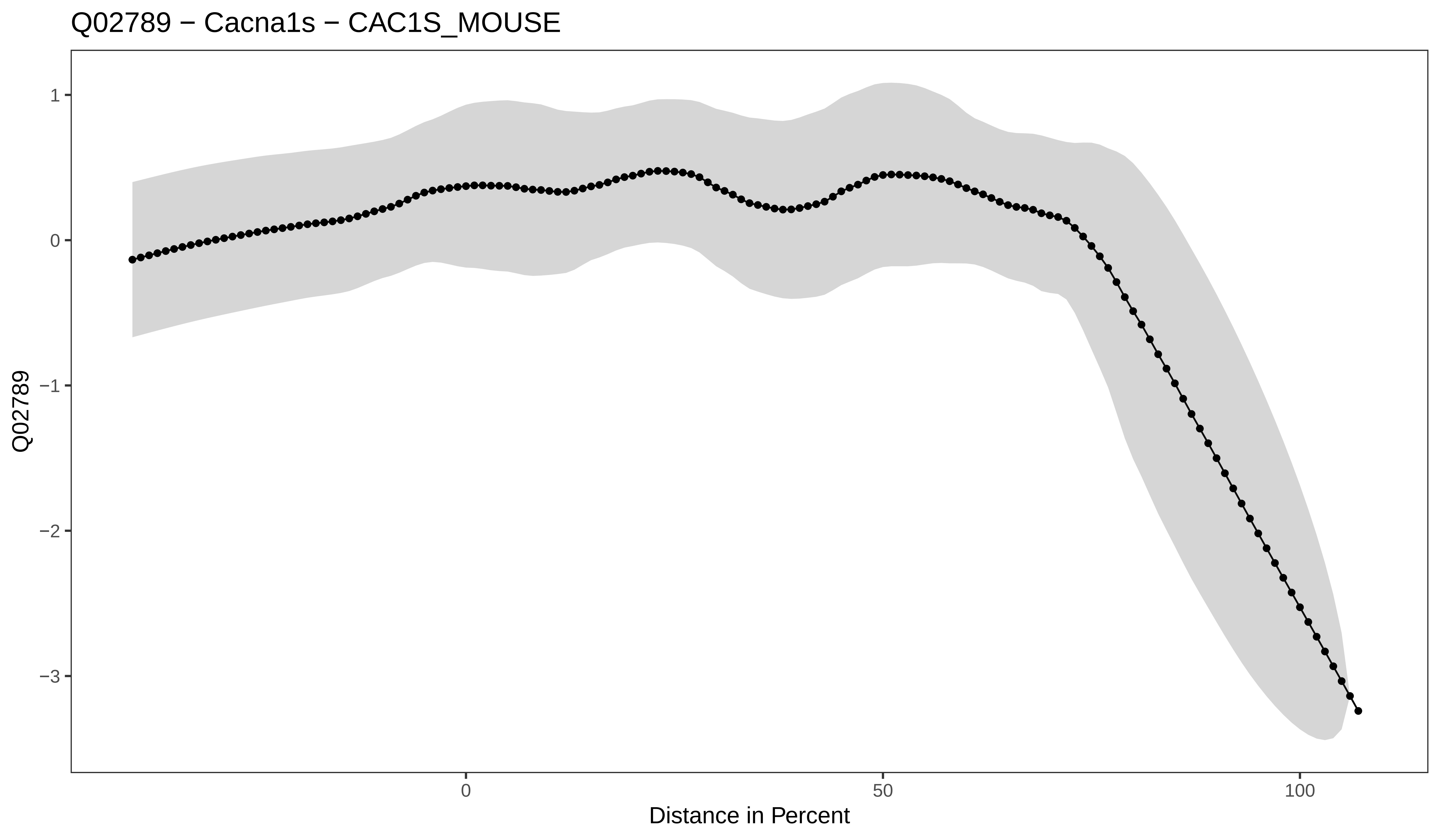

Q02789 − Cacna1s − CAC1S_MOUSE
Q02789
1
0
−1
−2
−3
0
50
100
Distance in Percent

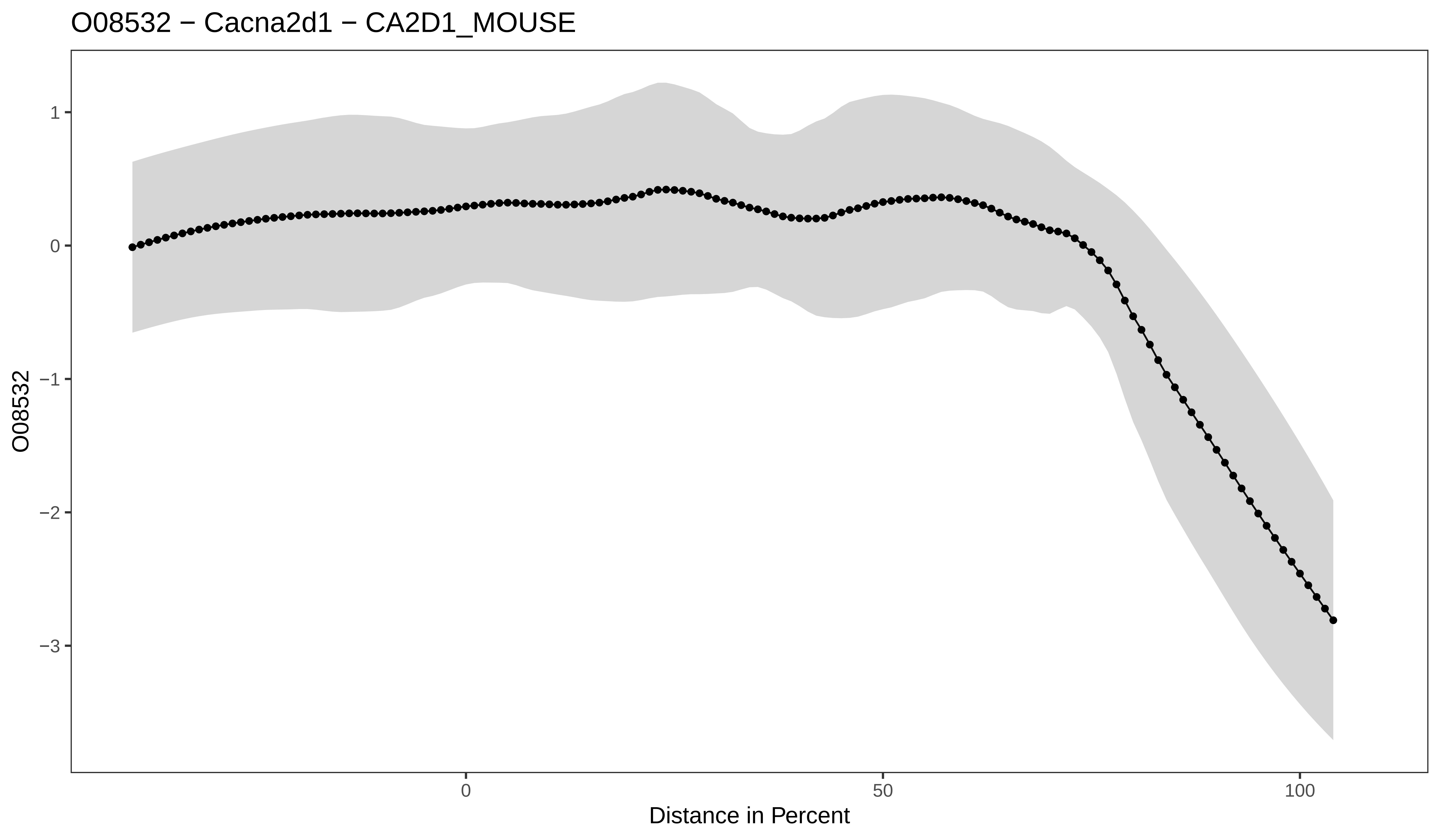

O08532 − Cacna2d1 − CA2D1_MOUSE
1
0
−1
−2
−3
O08532
0
50
100
Distance in Percent

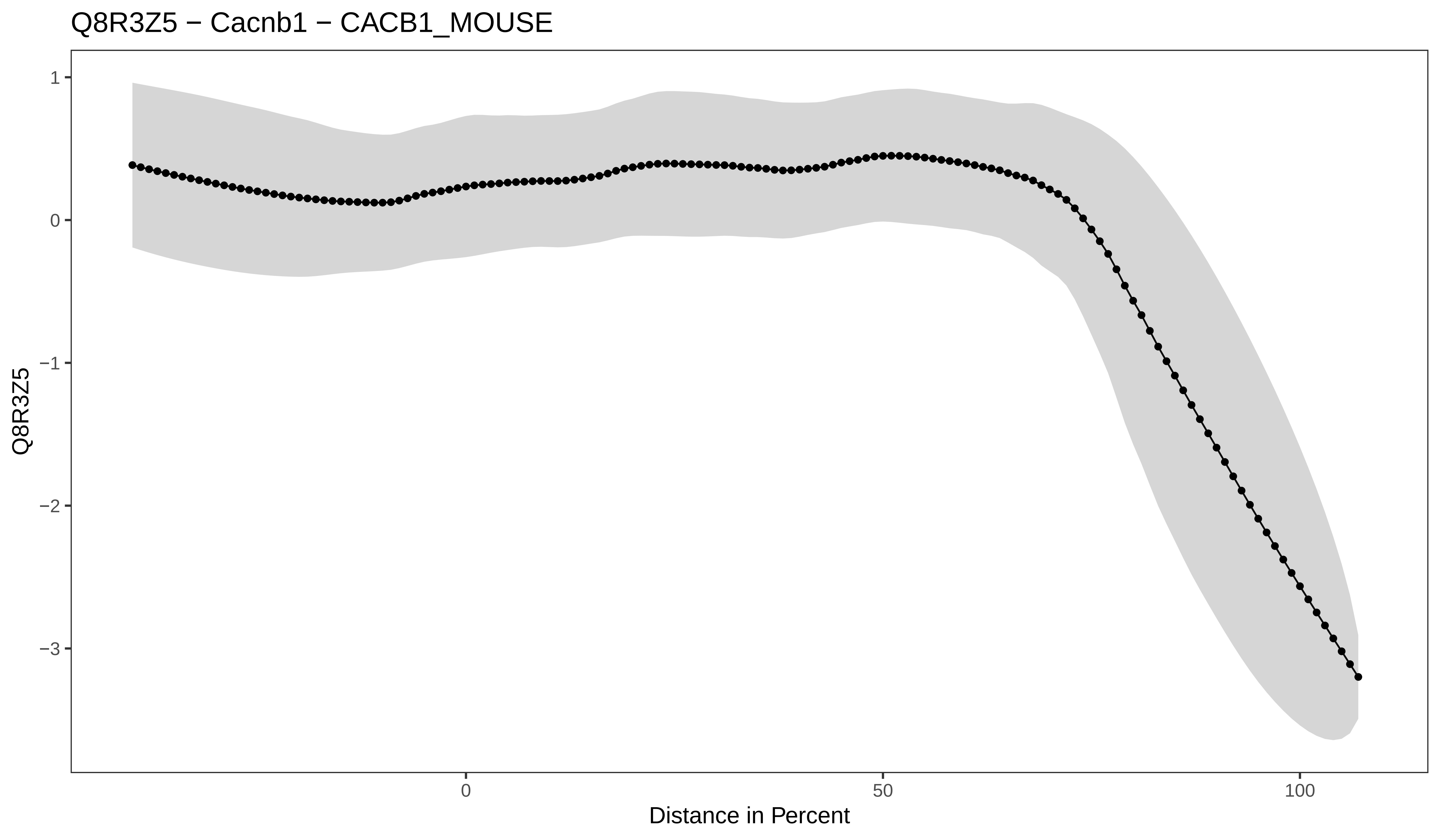

Q8R3Z5 − Cacnb1 − CACB1_MOUSE
1
0
−1
−2
−3
Q8R3Z5
0
50
100
Distance in Percent

Q8VHW3 − Cacng6 − CCG6_MOUSE

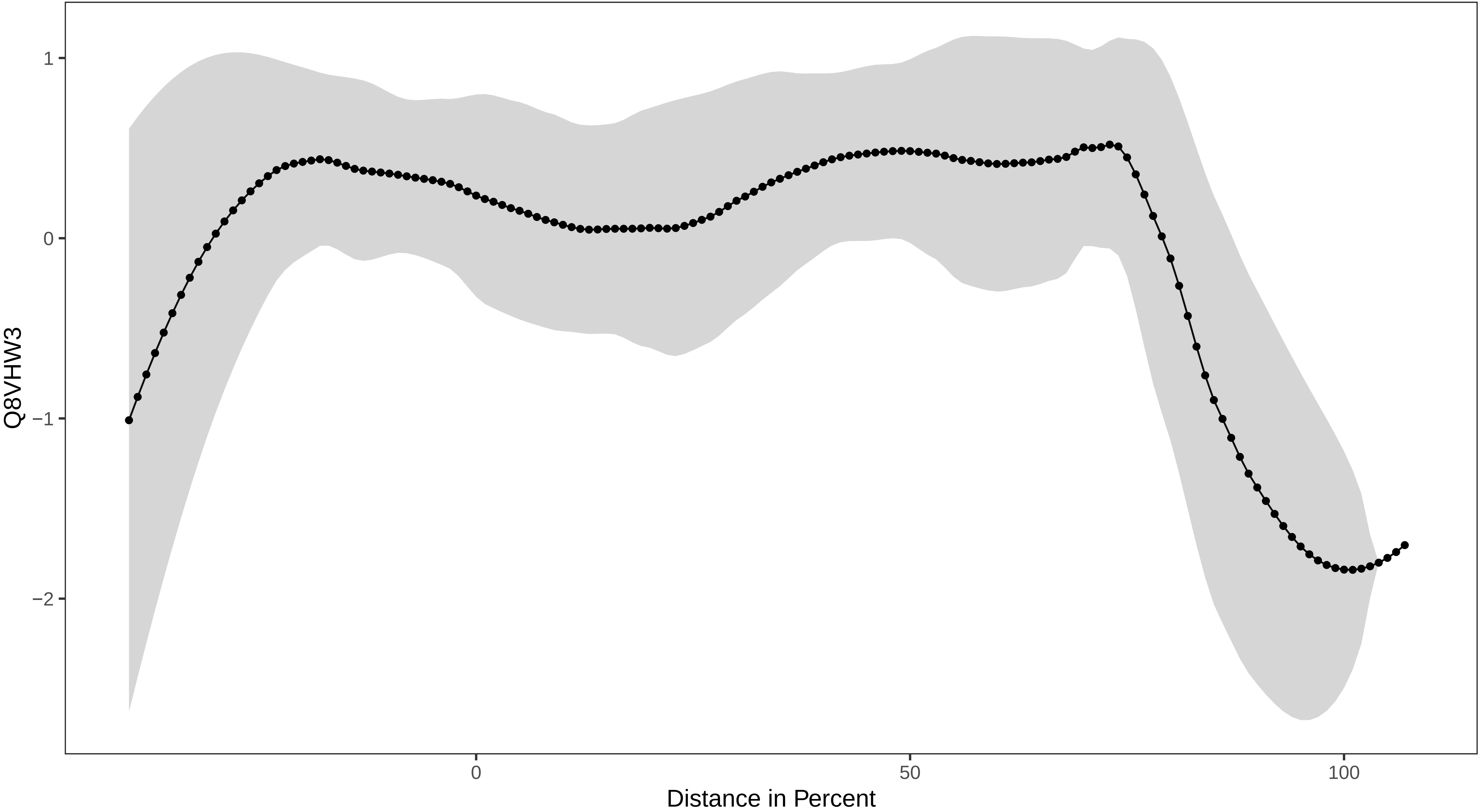

Q9CXW3 − Cacybp − CYBP_MOUSE

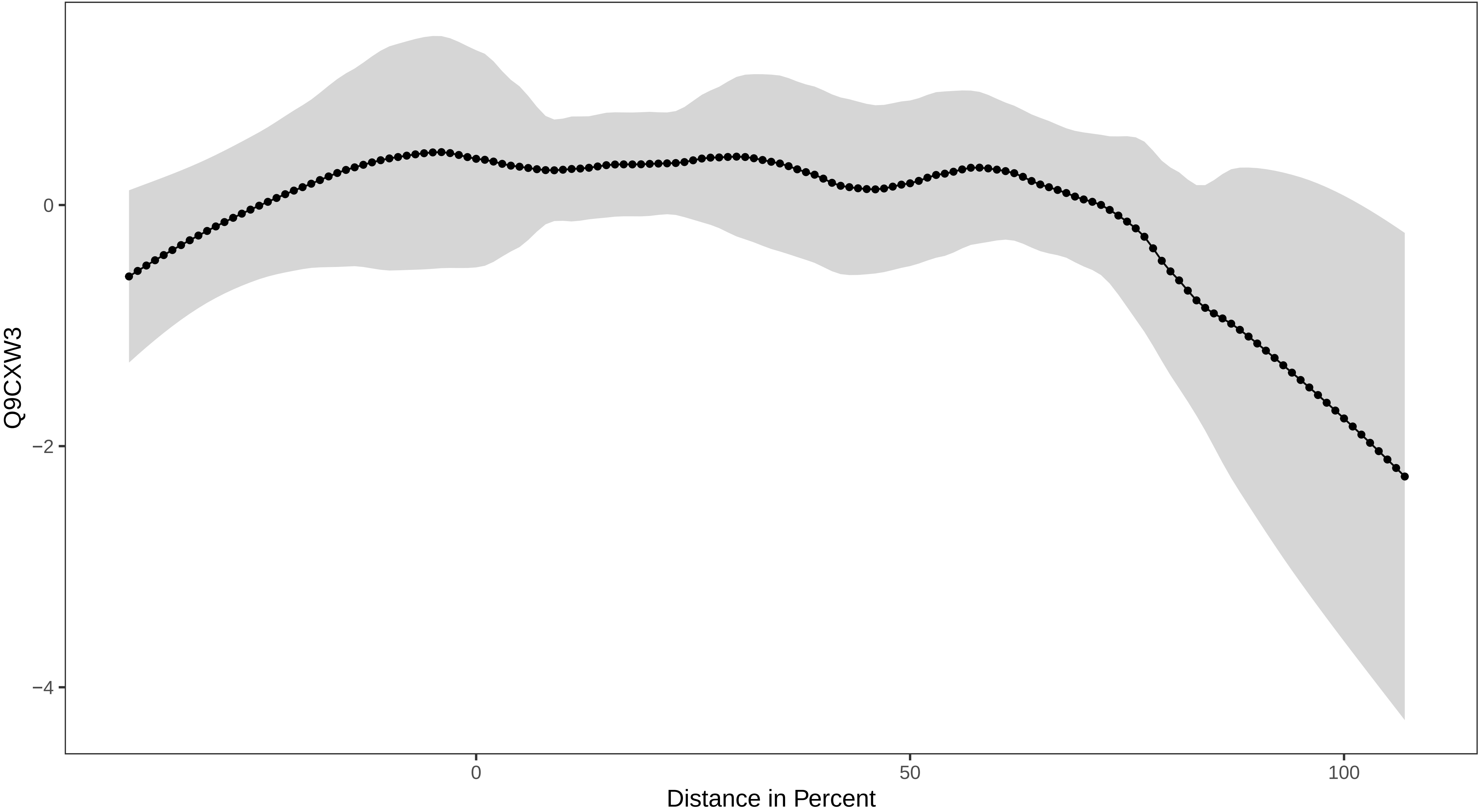

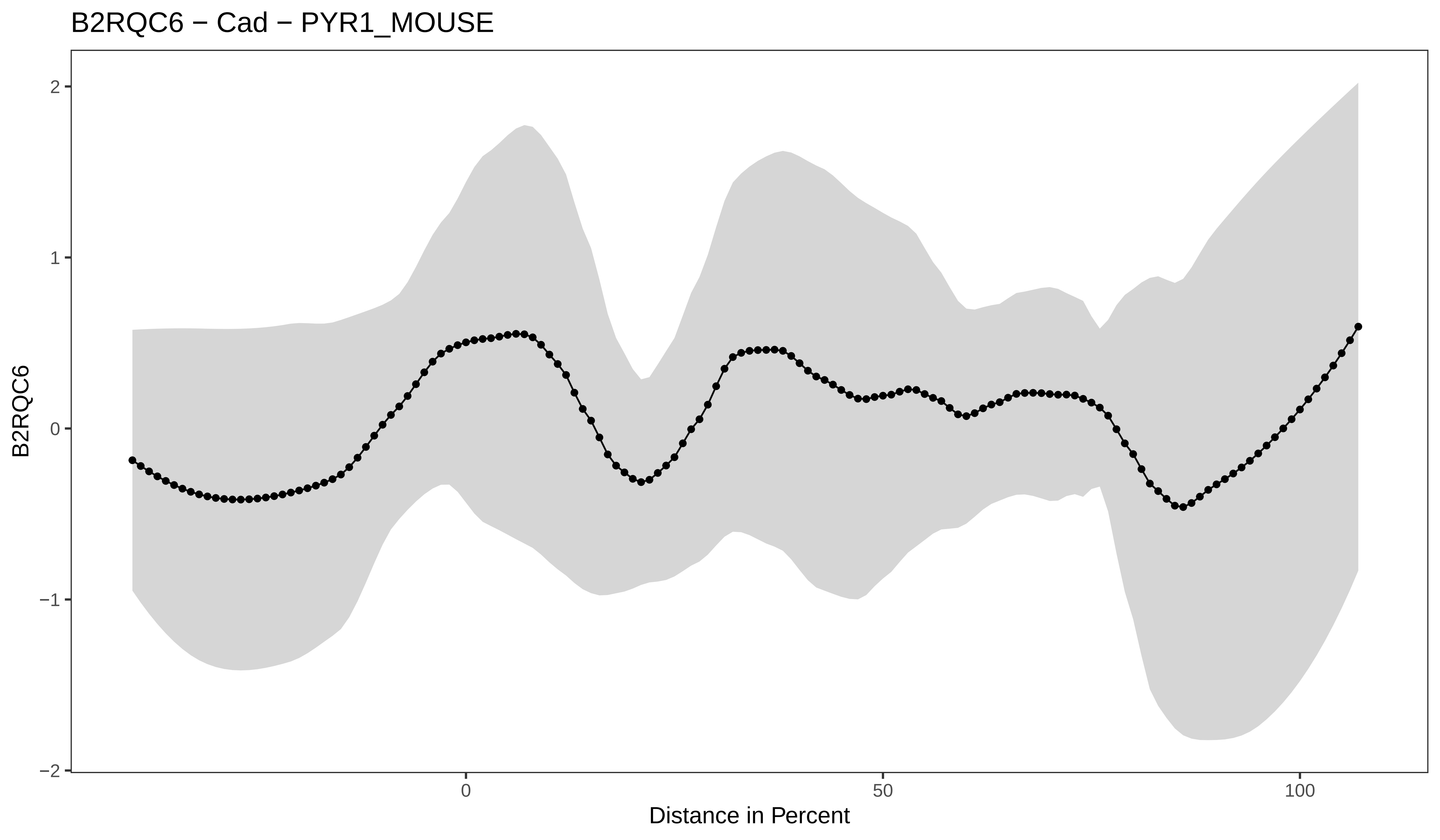

B2RQC6 − Cad − PYR1_MOUSE
B2RQC6
2
1
0
−1
−2
0
50
100
Distance in Percent

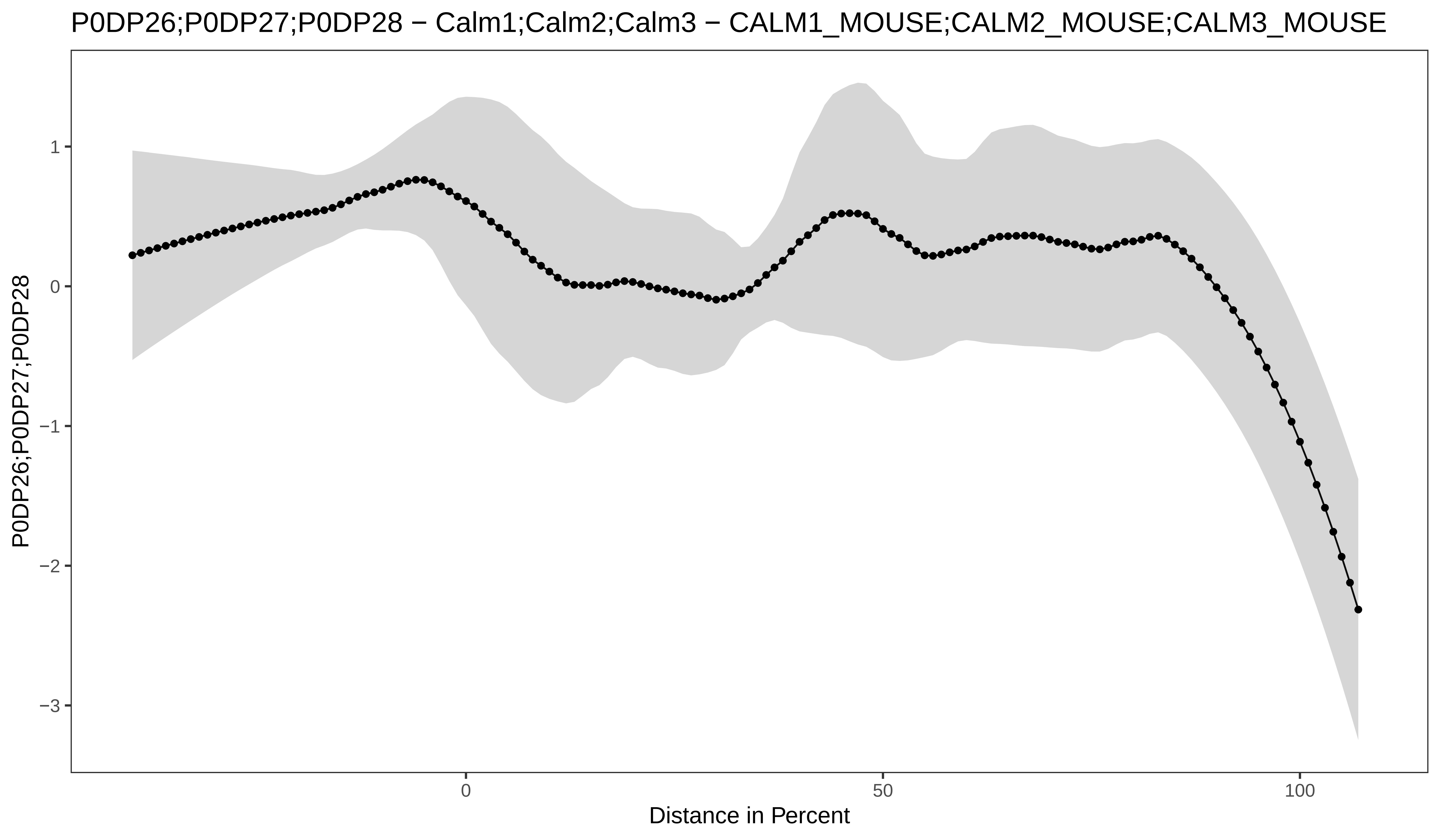
P0DP26;P0DP27;P0DP28 − Calm1;Calm2;Calm3 − CALM1_MOUSE;CALM2_MOUSE;CALM3_MOUSE
1
0
−1
−2
−3
P0DP26;P0DP27;P0DP28
0
50
100
Distance in Percent

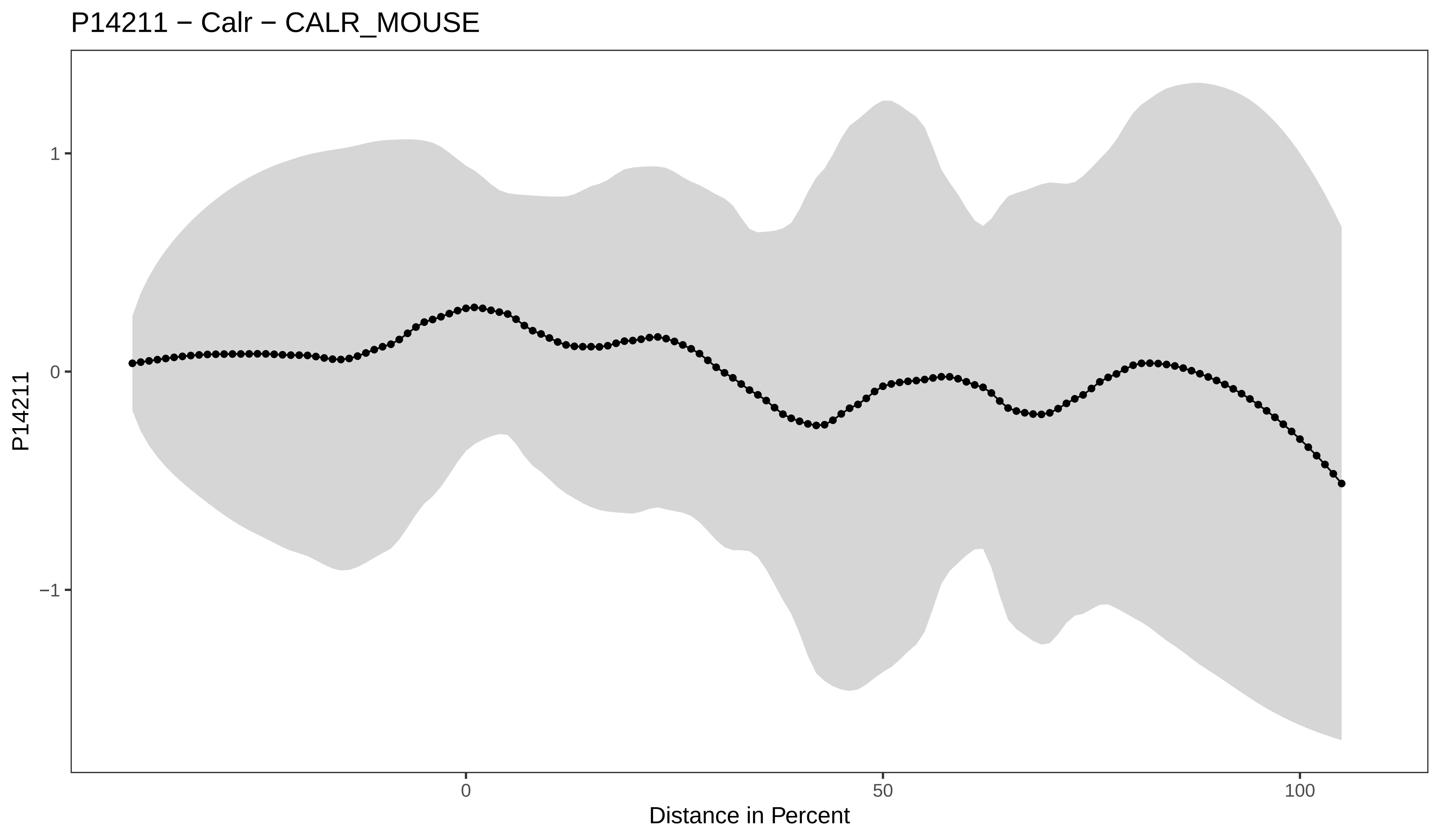
P14211 − Calr − CALR_MOUSE
P14211
1
0
−1
0
50
100
Distance in Percent

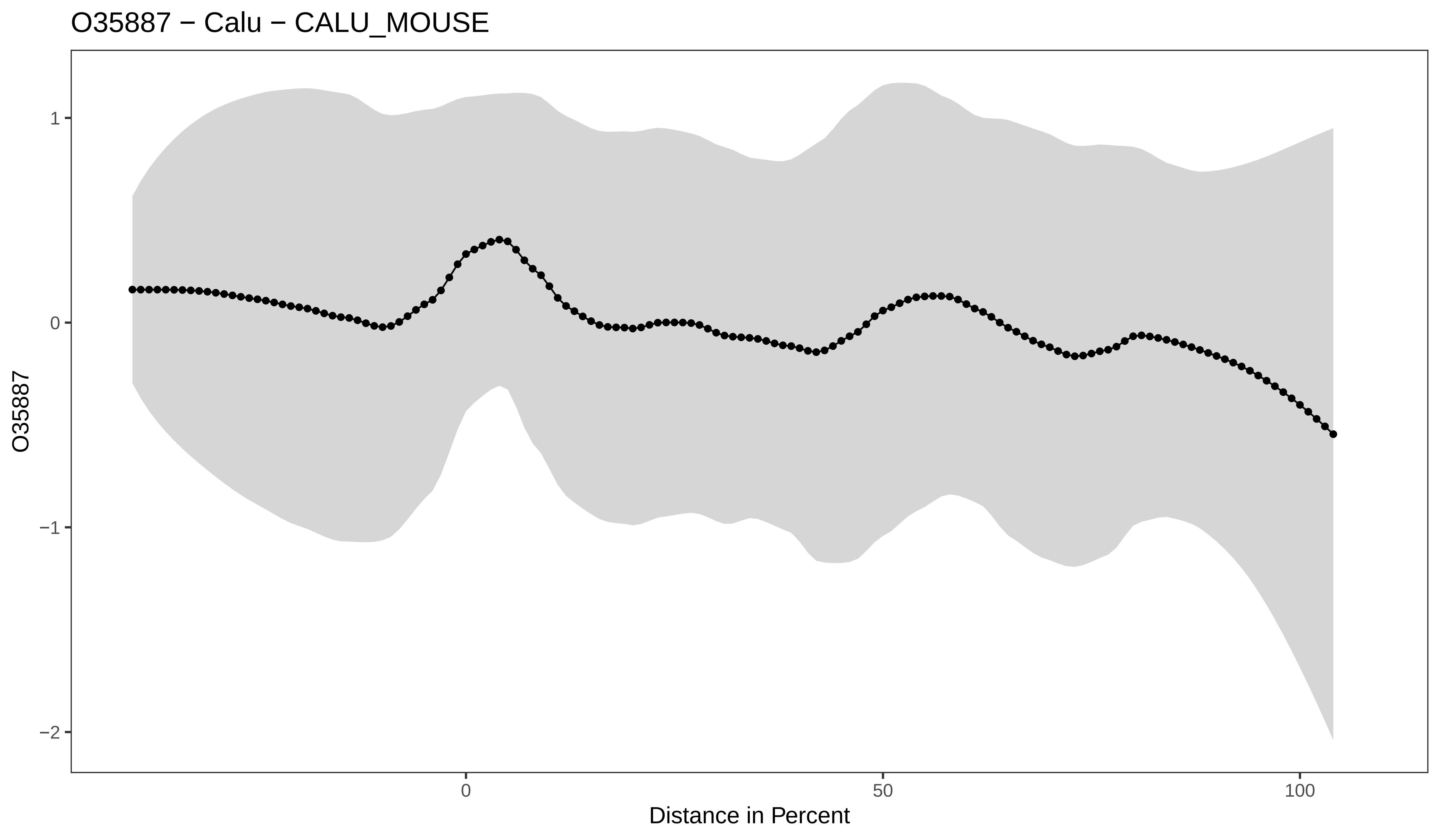

O35887 − Calu − CALU_MOUSE
O35887
1
0
−1
−2
0
50
100
Distance in Percent

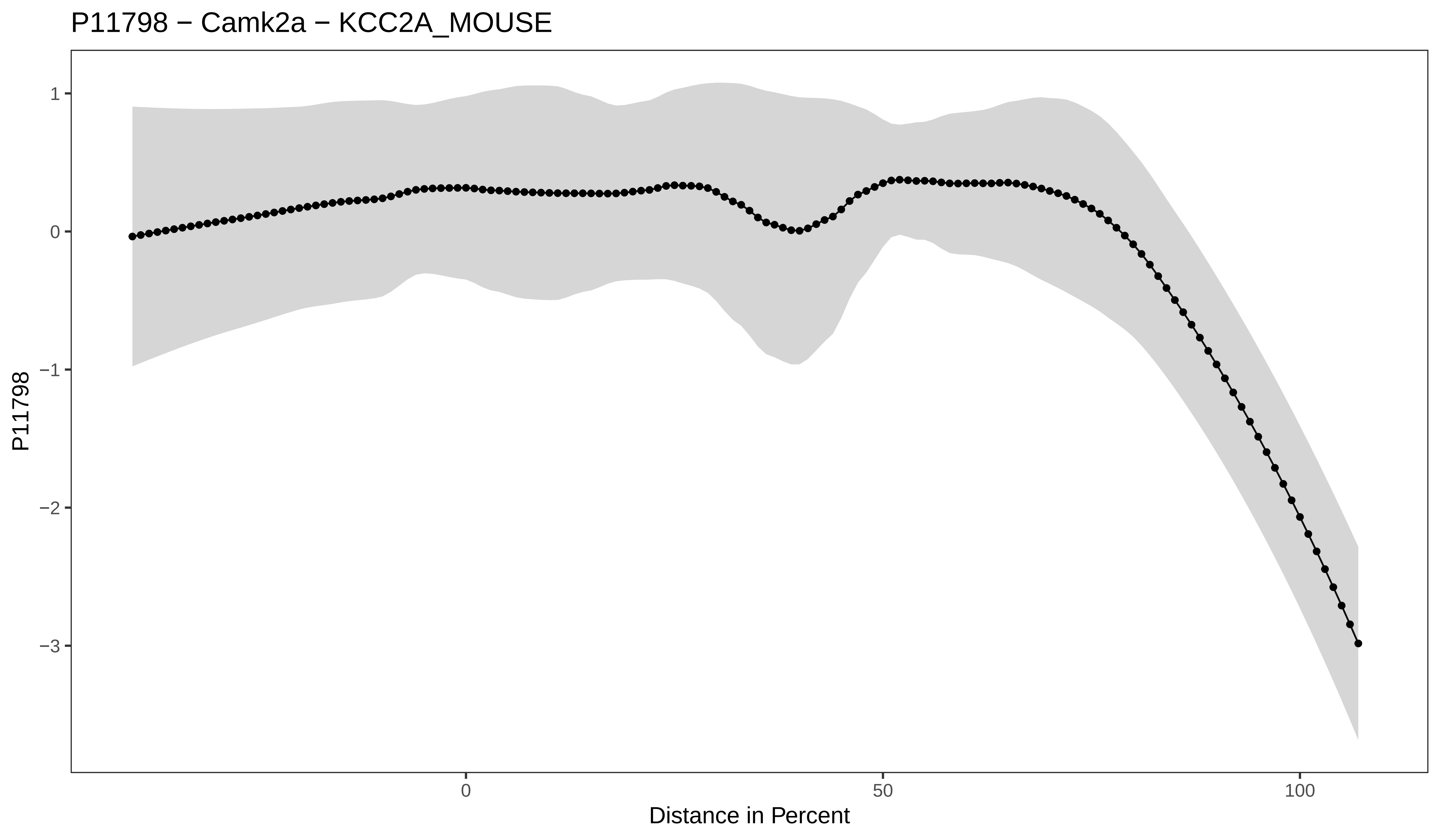

P11798 − Camk2a − KCC2A_MOUSE
1
0
−1
−2
−3
P11798
0
50
100
Distance in Percent

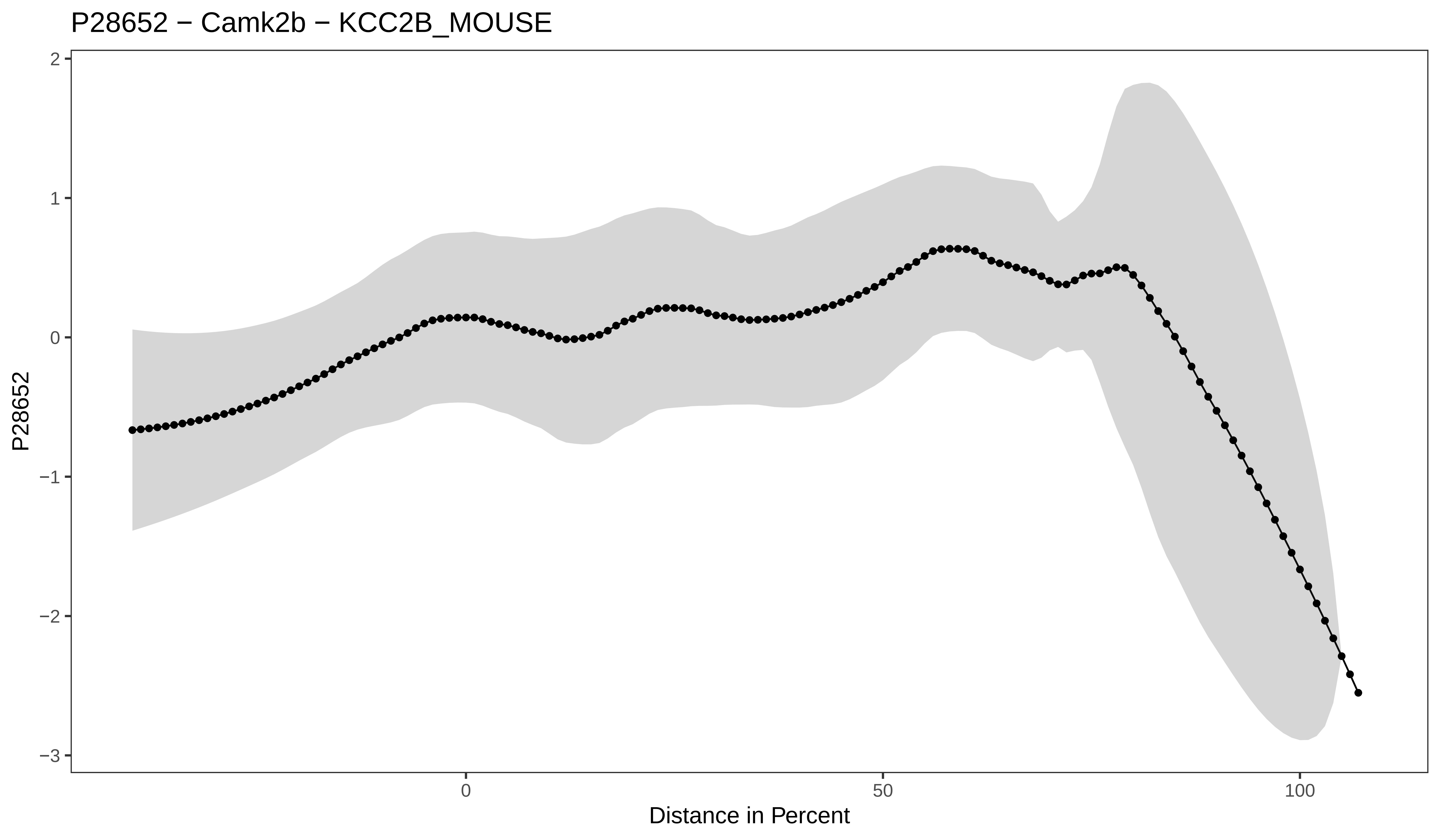
P28652 − Camk2b − KCC2B_MOUSE
P28652
Distance in Percent
2
1
0
−1
−2
−3
0
50
100

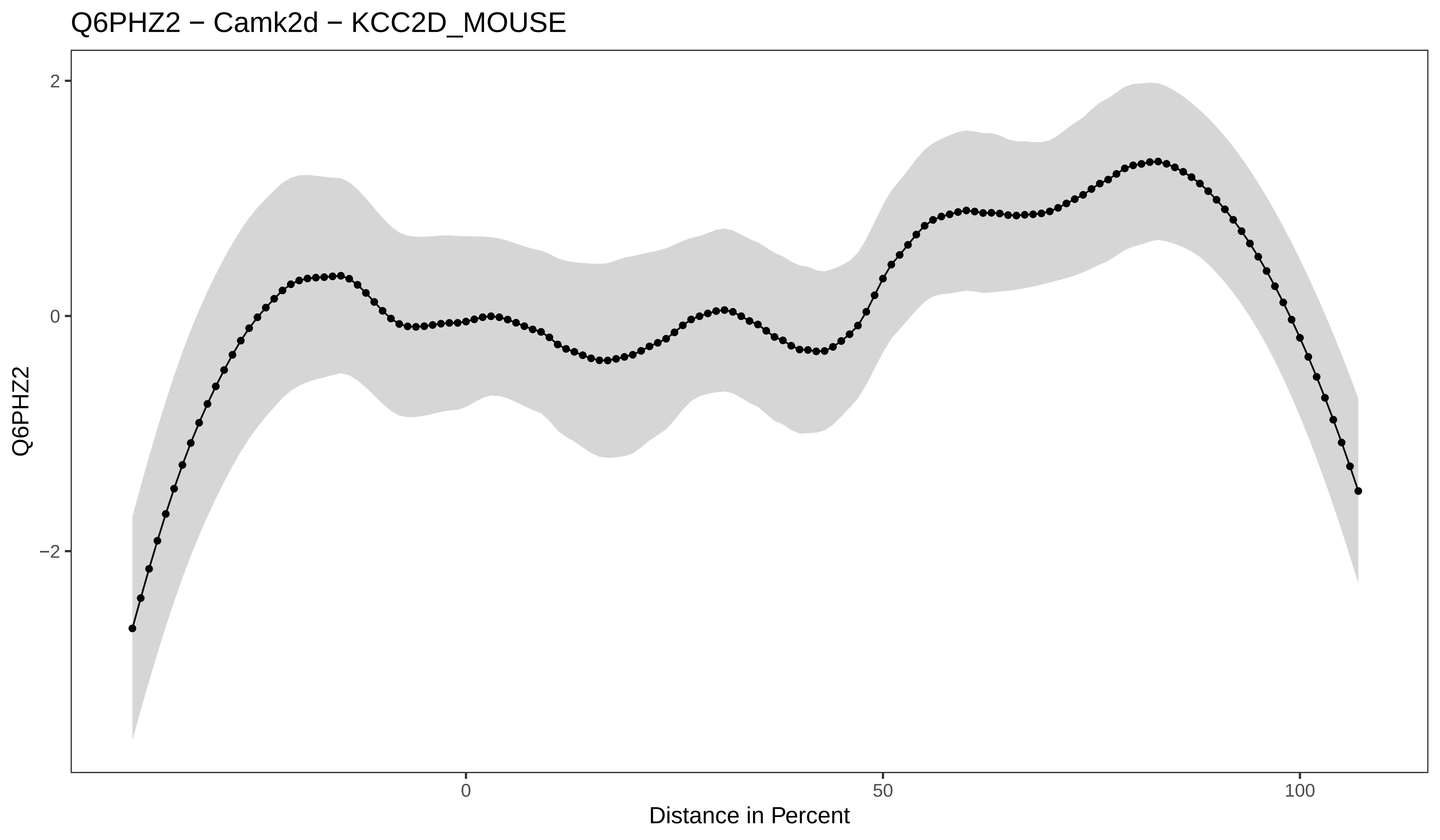

Q6PHZ2 − Camk2d − KCC2D_MOUSE
2
0
−2
Q6PHZ2
0
50
100
Distance in Percent

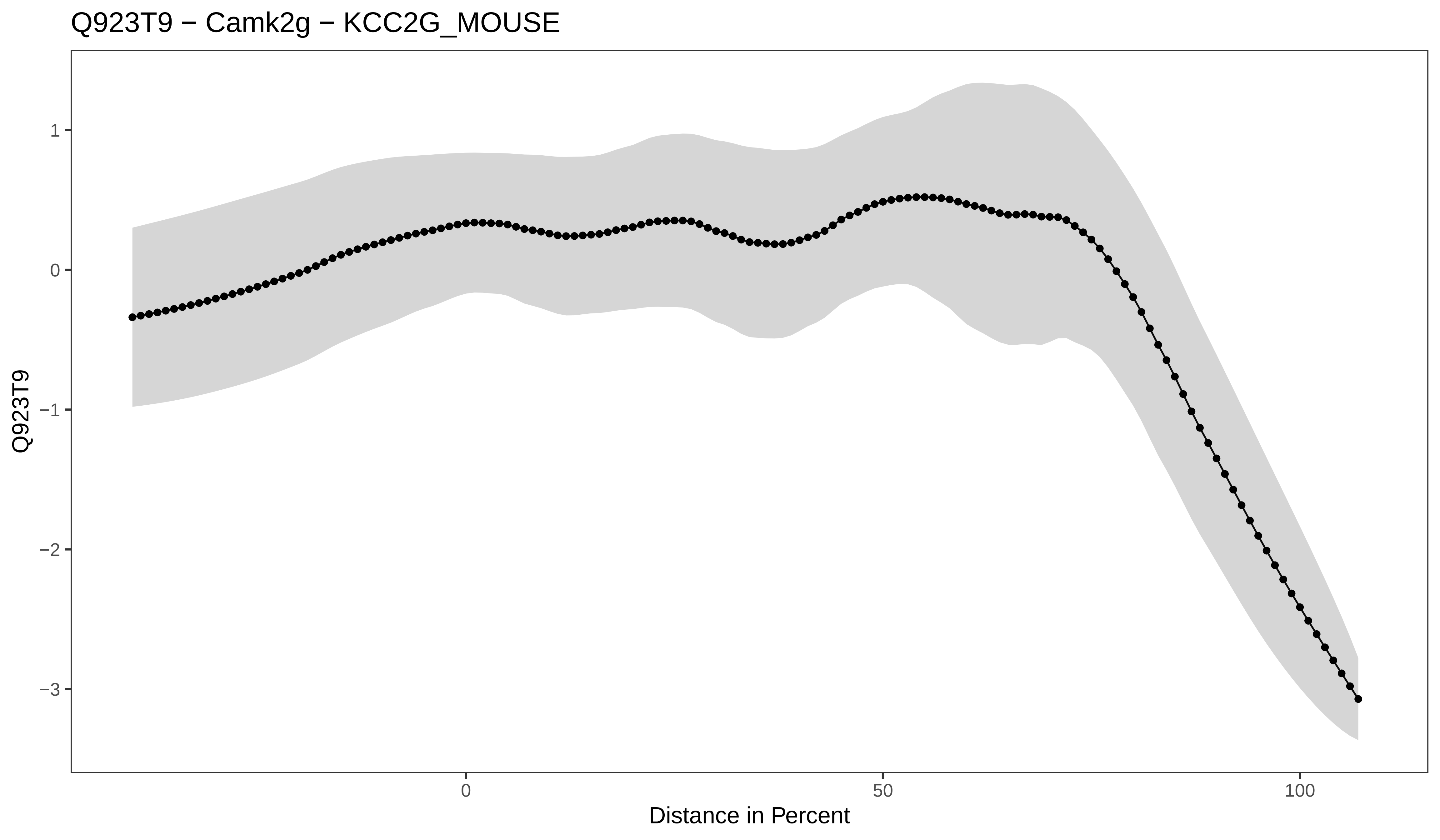

Q923T9 − Camk2g − KCC2G_MOUSE
1
0
−1
−2
−3
Q923T9
0
50
100
Distance in Percent

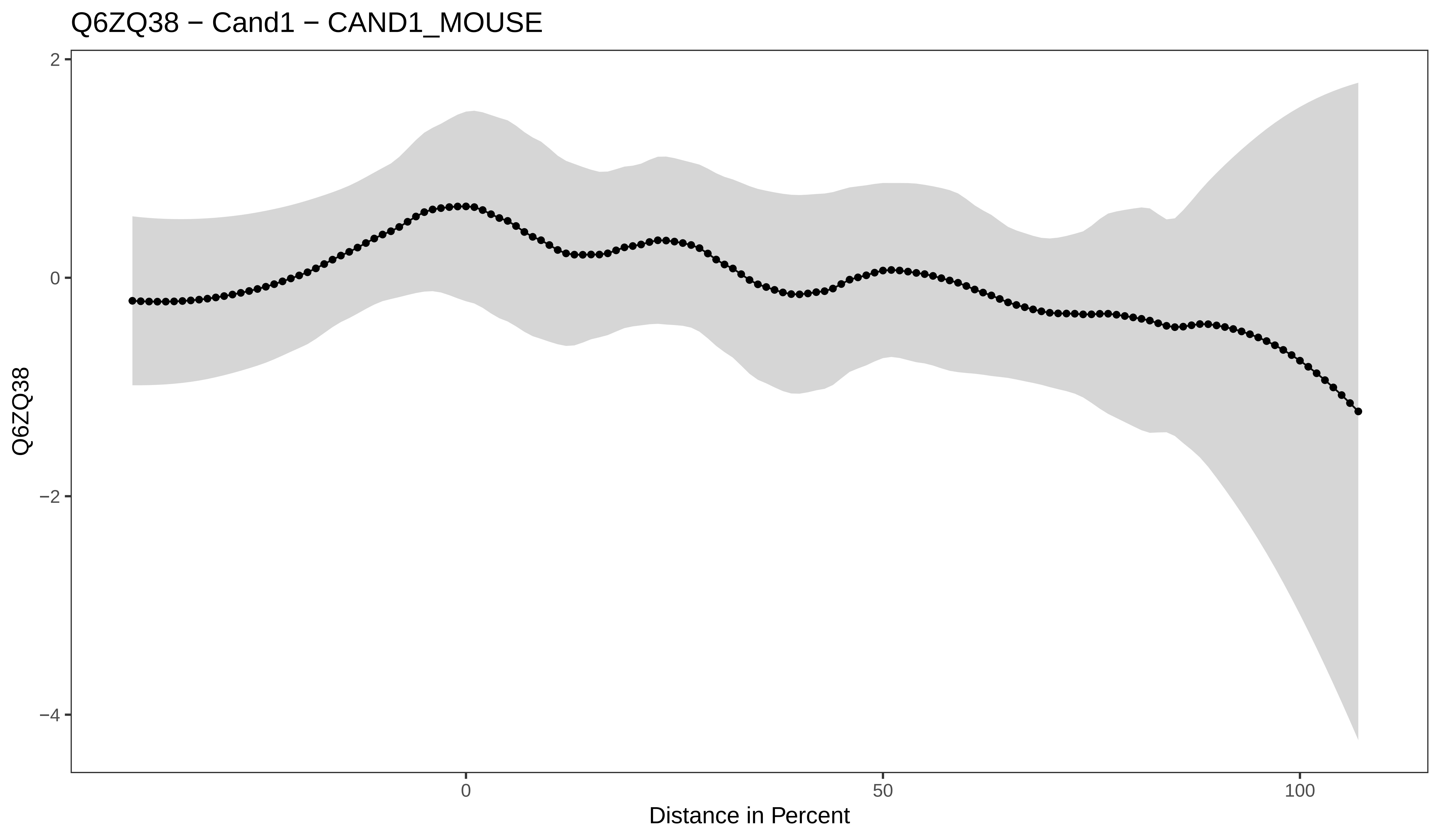

Q6ZQ38 − Cand1 − CAND1_MOUSE
2
0
−2
−4
Q6ZQ38
0
50
100
Distance in Percent

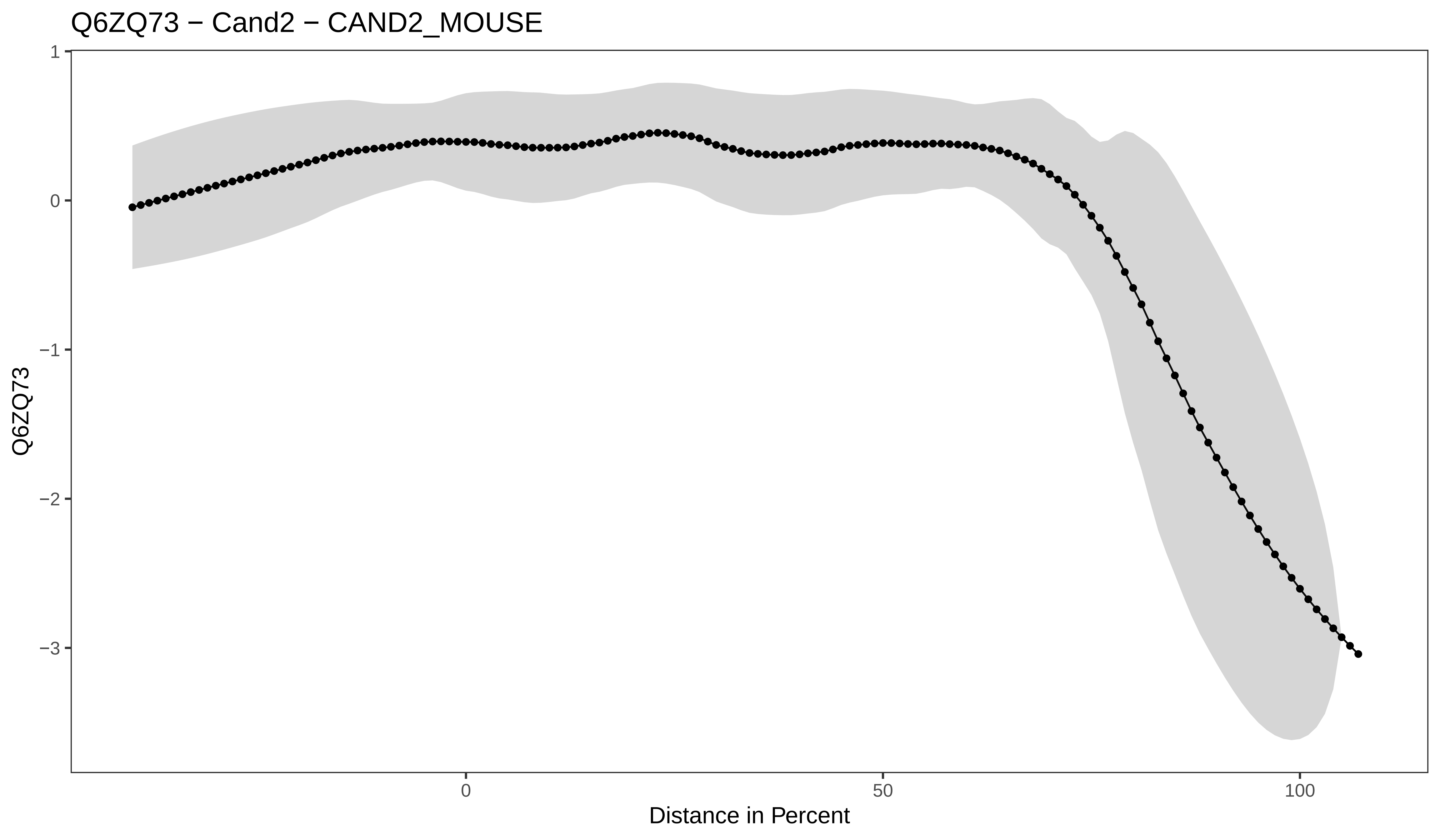

Q6ZQ73 − Cand2 − CAND2_MOUSE
1
0
−1
−2
−3
Q6ZQ73
0
50
100
Distance in Percent

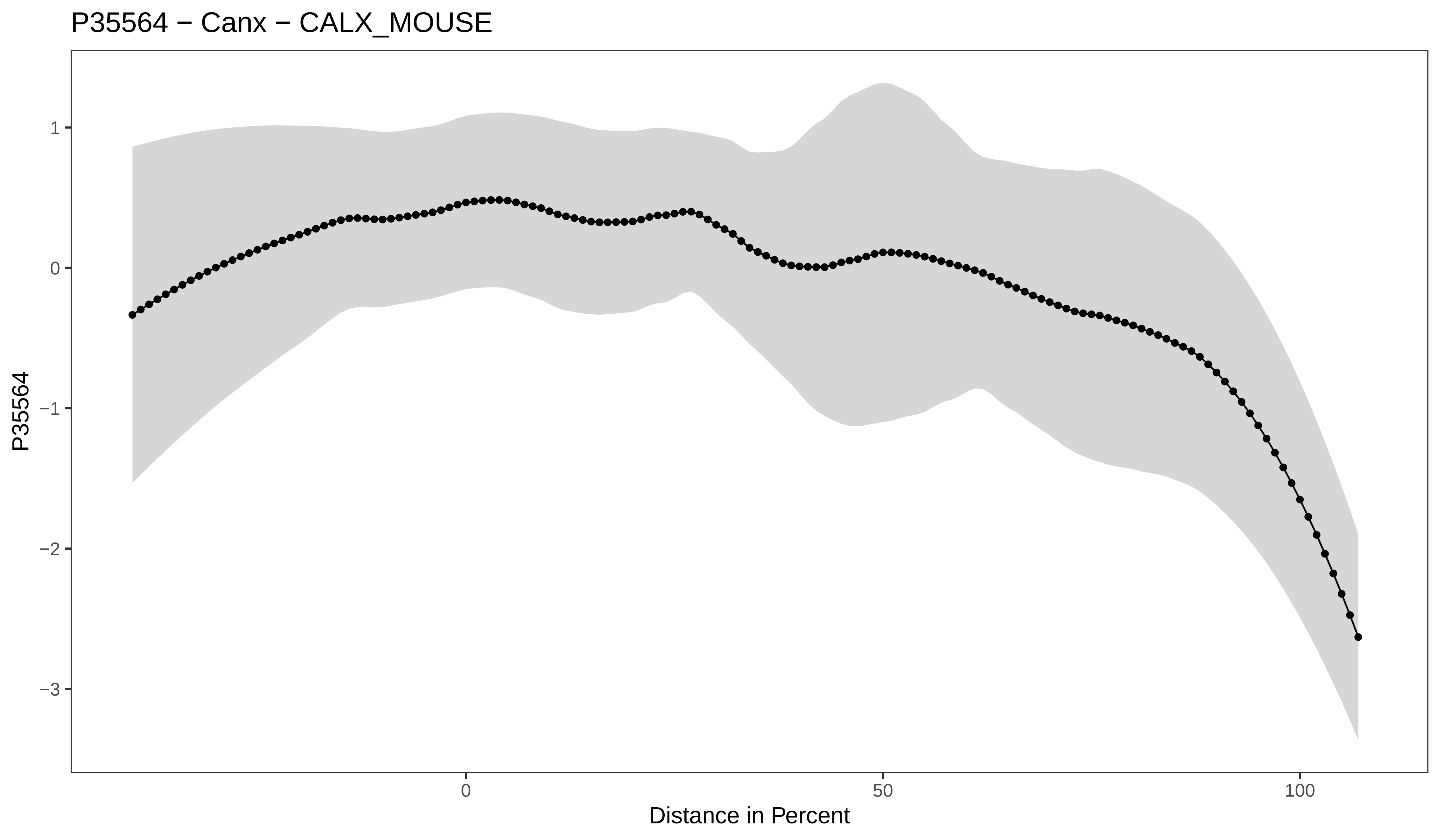
P35564 − Canx − CALX_MOUSE
P35564
Distance in Percent
1
0
−1
−2
−3
0
50
100

P40124 − Cap1 − CAP1_MOUSE

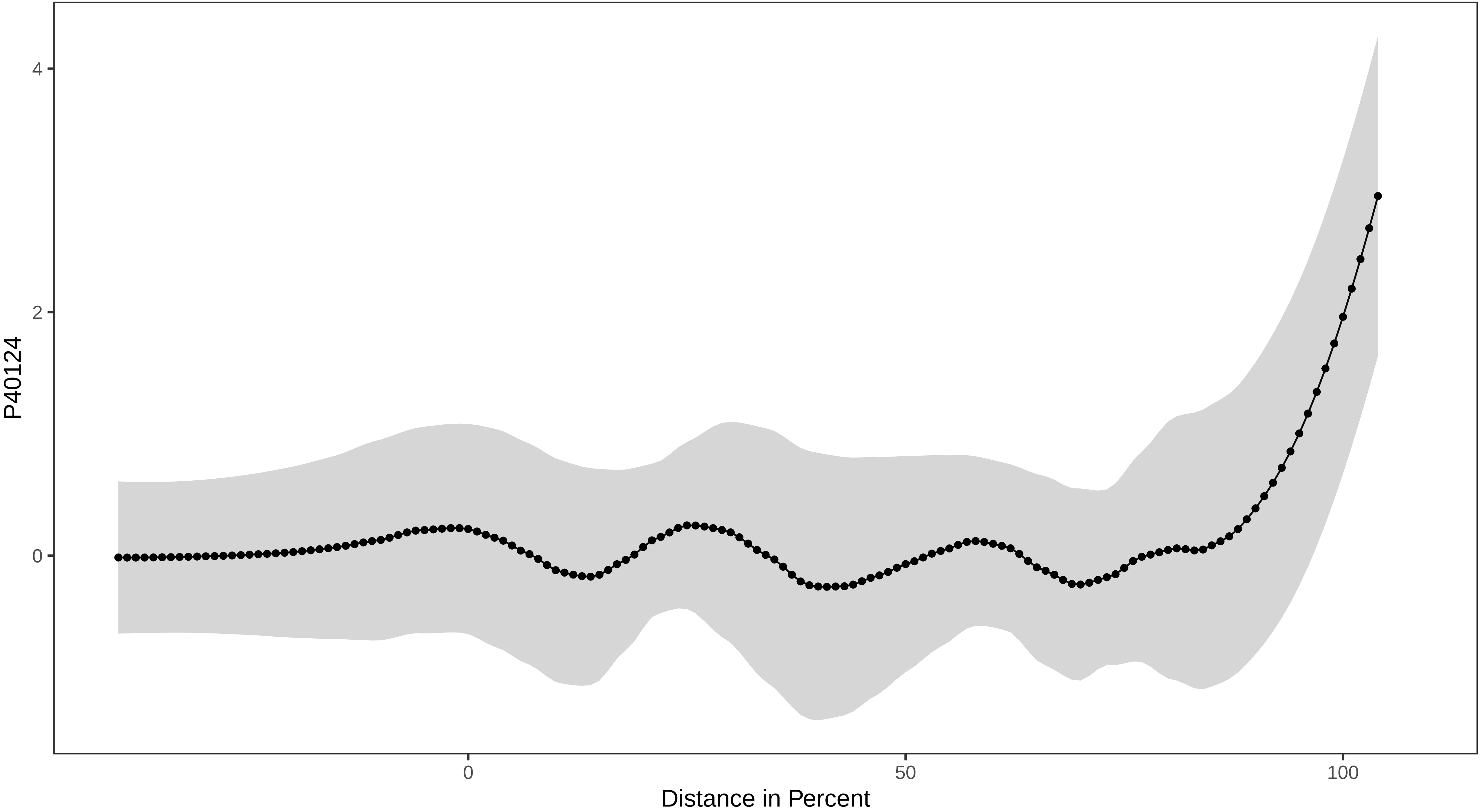

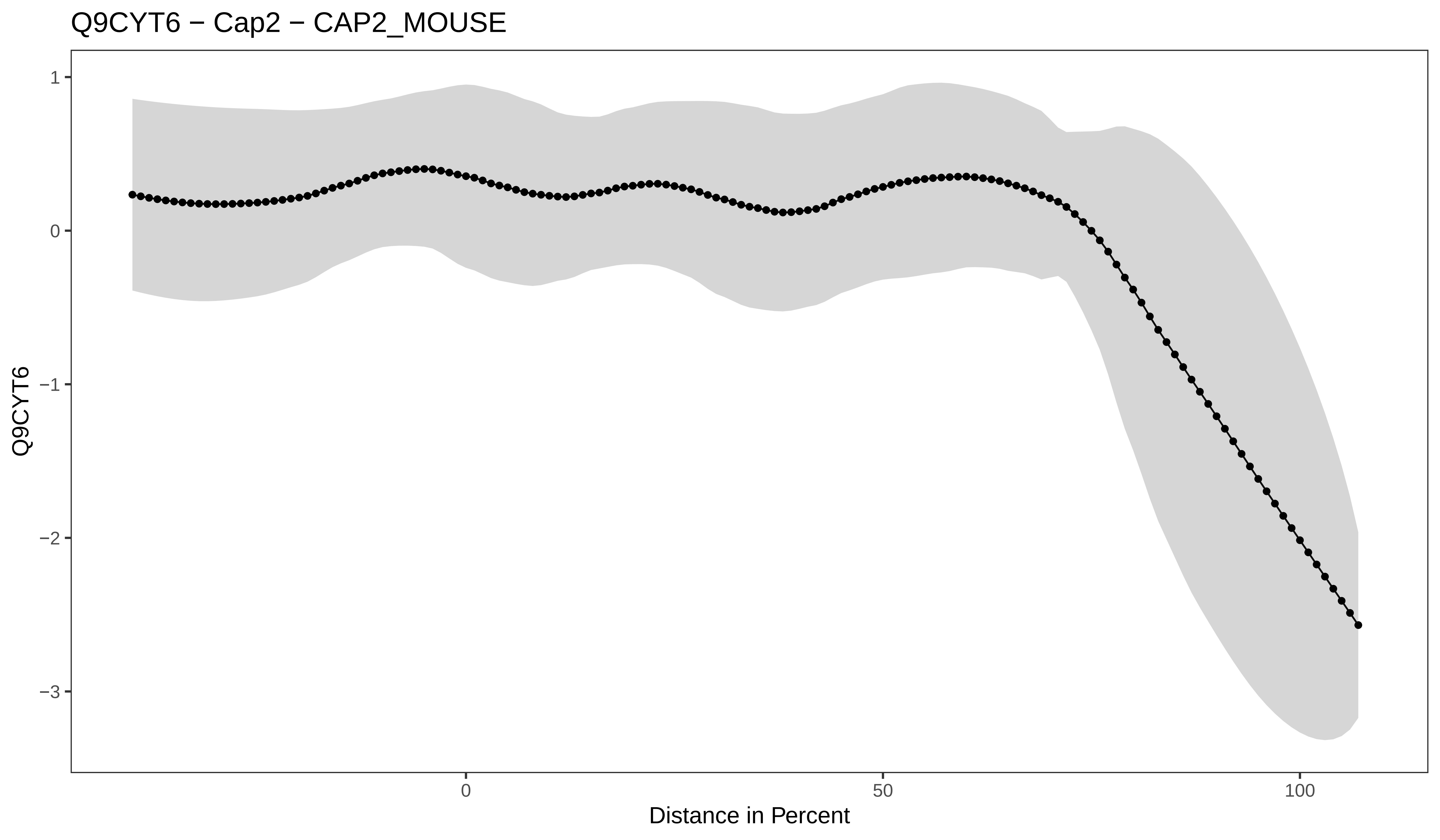

Q9CYT6 − Cap2 − CAP2_MOUSE
1
0
−1
−2
−3
Q9CYT6
0
50
100
Distance in Percent

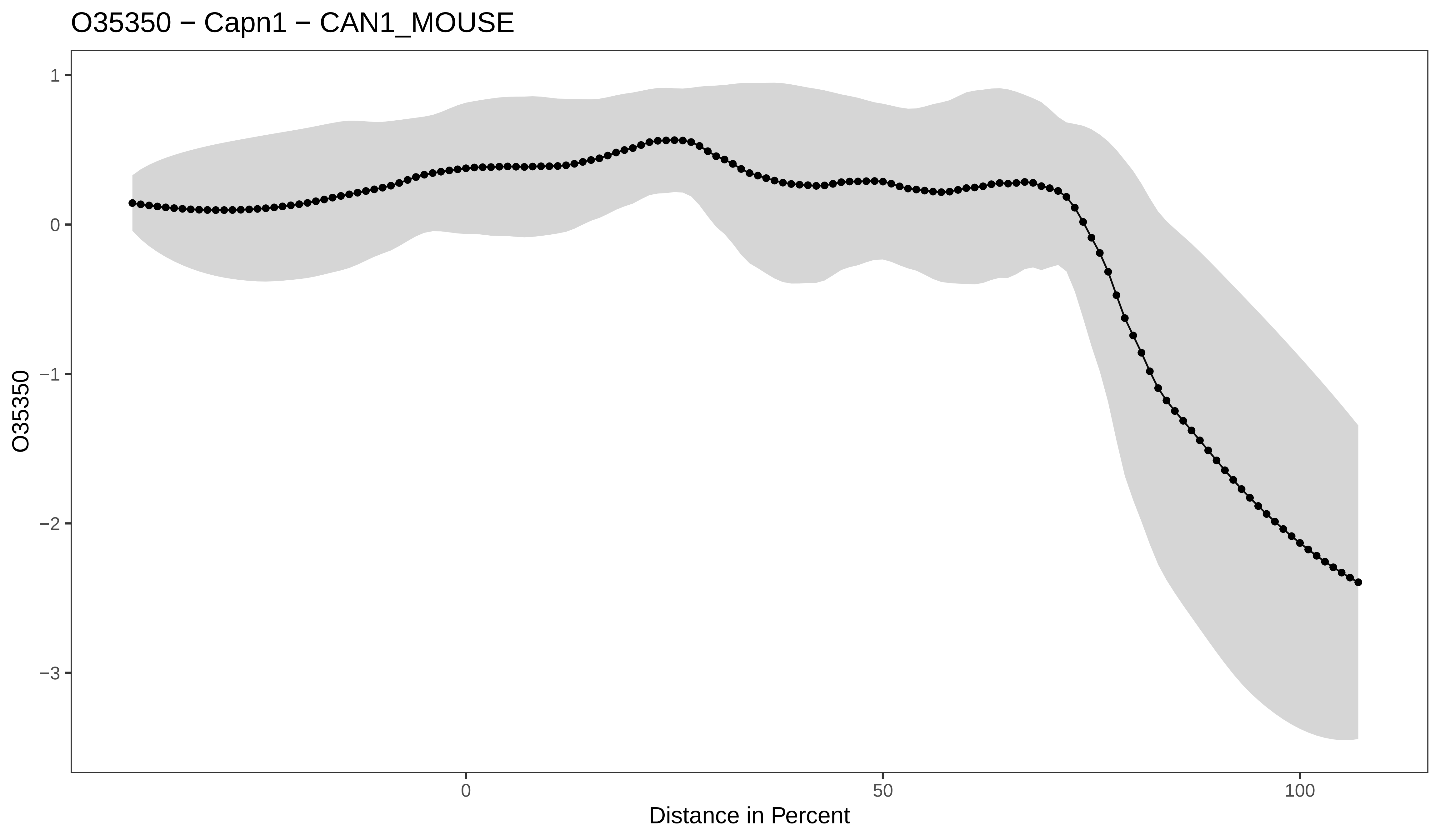
O35350 − Capn1 − CAN1_MOUSE
O35350
1
0
−1
−2
−3
0
50
100
Distance in Percent

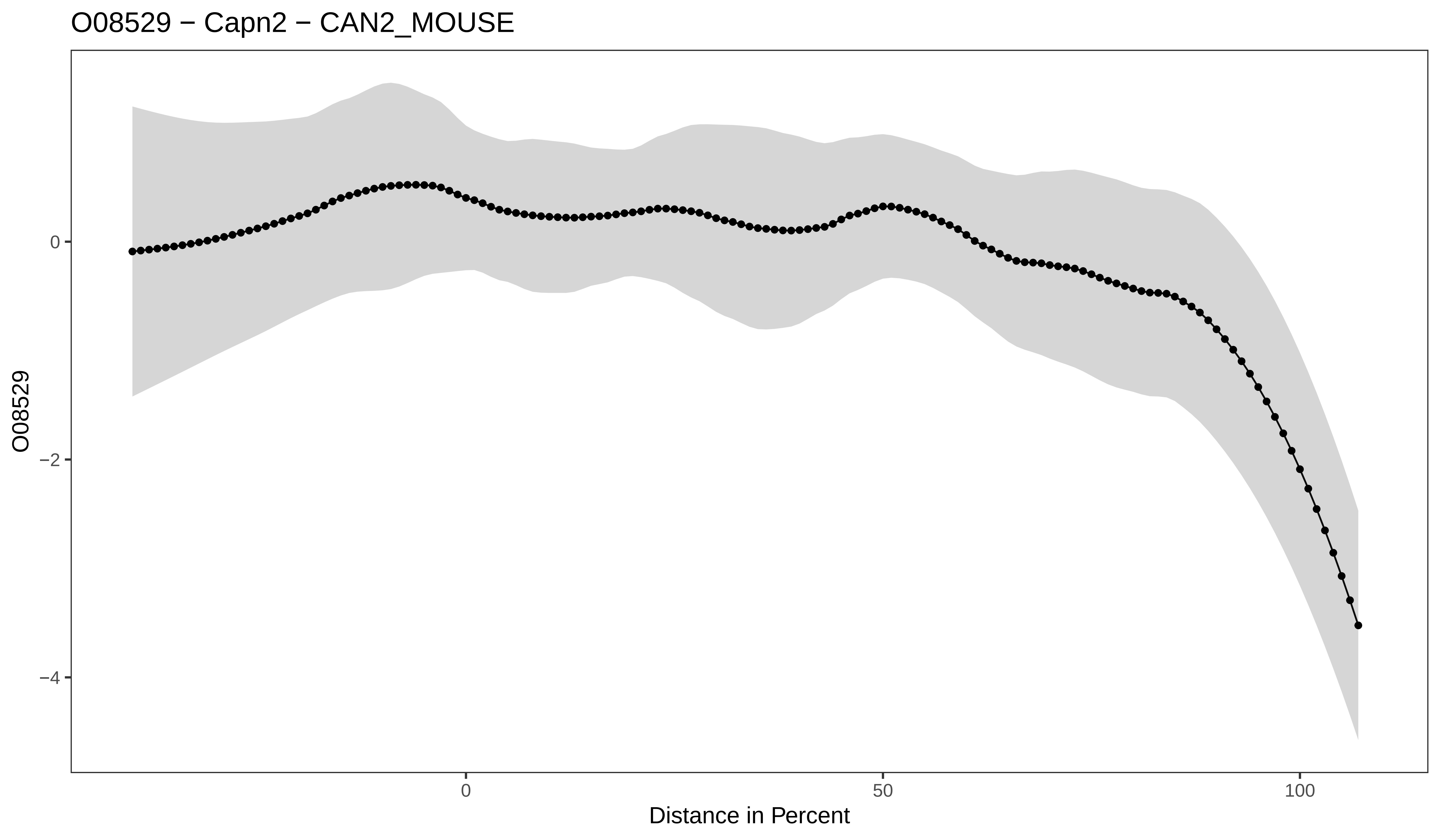
O08529 − Capn2 − CAN2_MOUSE
O08529
0
−2
−4
0
50
100
Distance in Percent

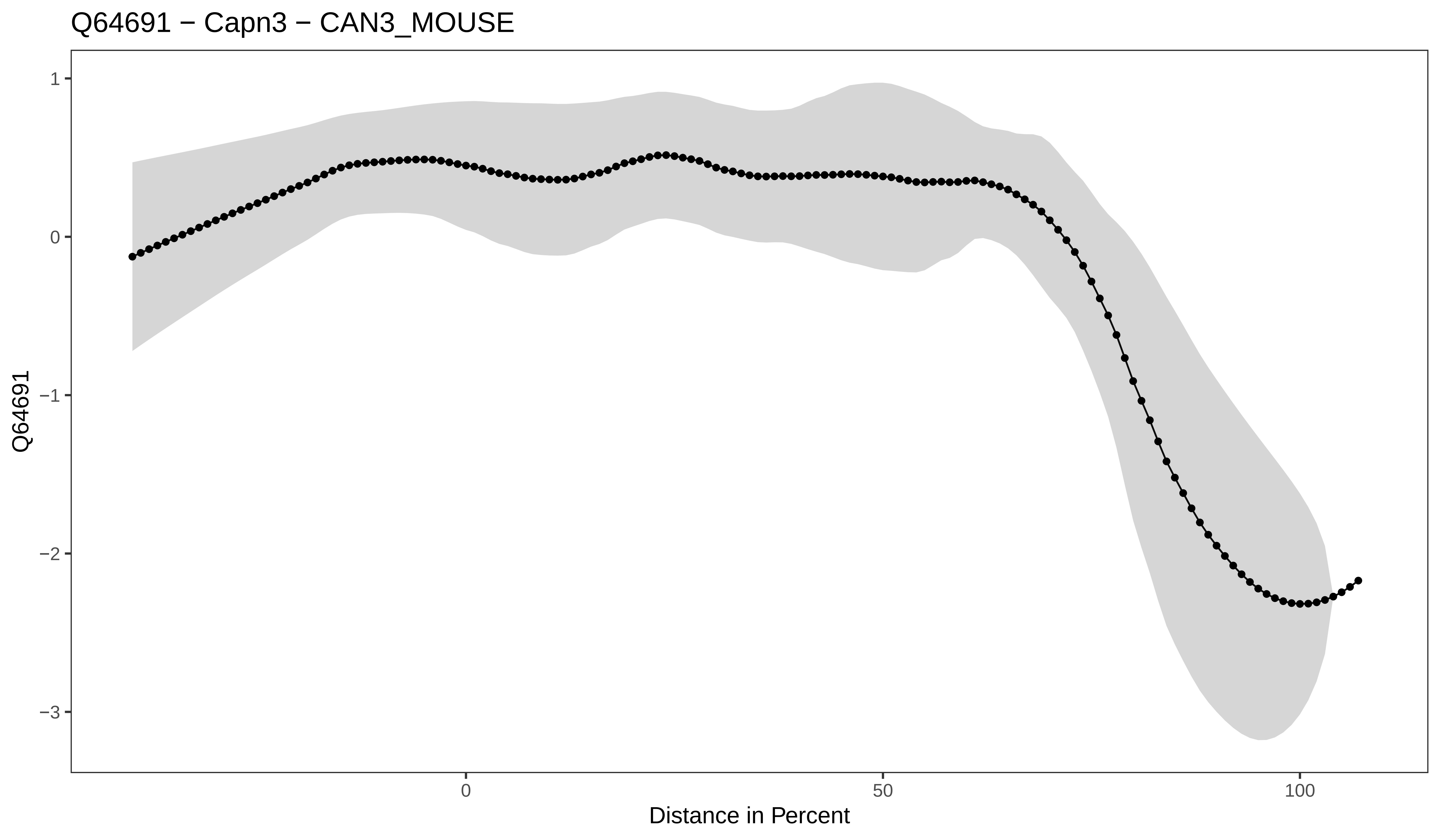

Q64691 − Capn3 − CAN3_MOUSE
Q64691
Distance in Percent
1
0
−1
−2
−3
0
50
100

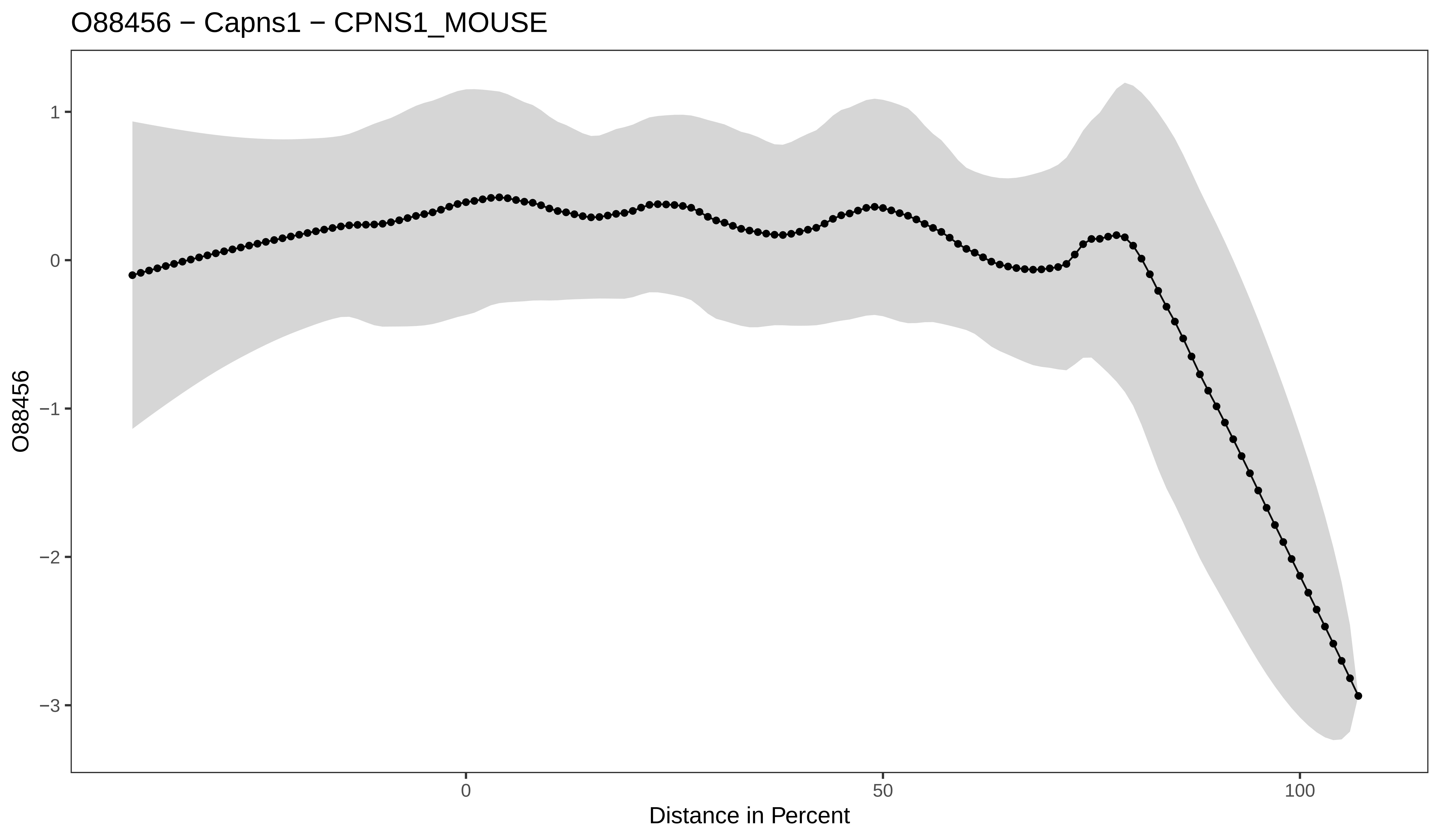

O88456 − Capns1 − CPNS1_MOUSE
O88456
1
0
−1
−2
−3
0
50
100
Distance in Percent

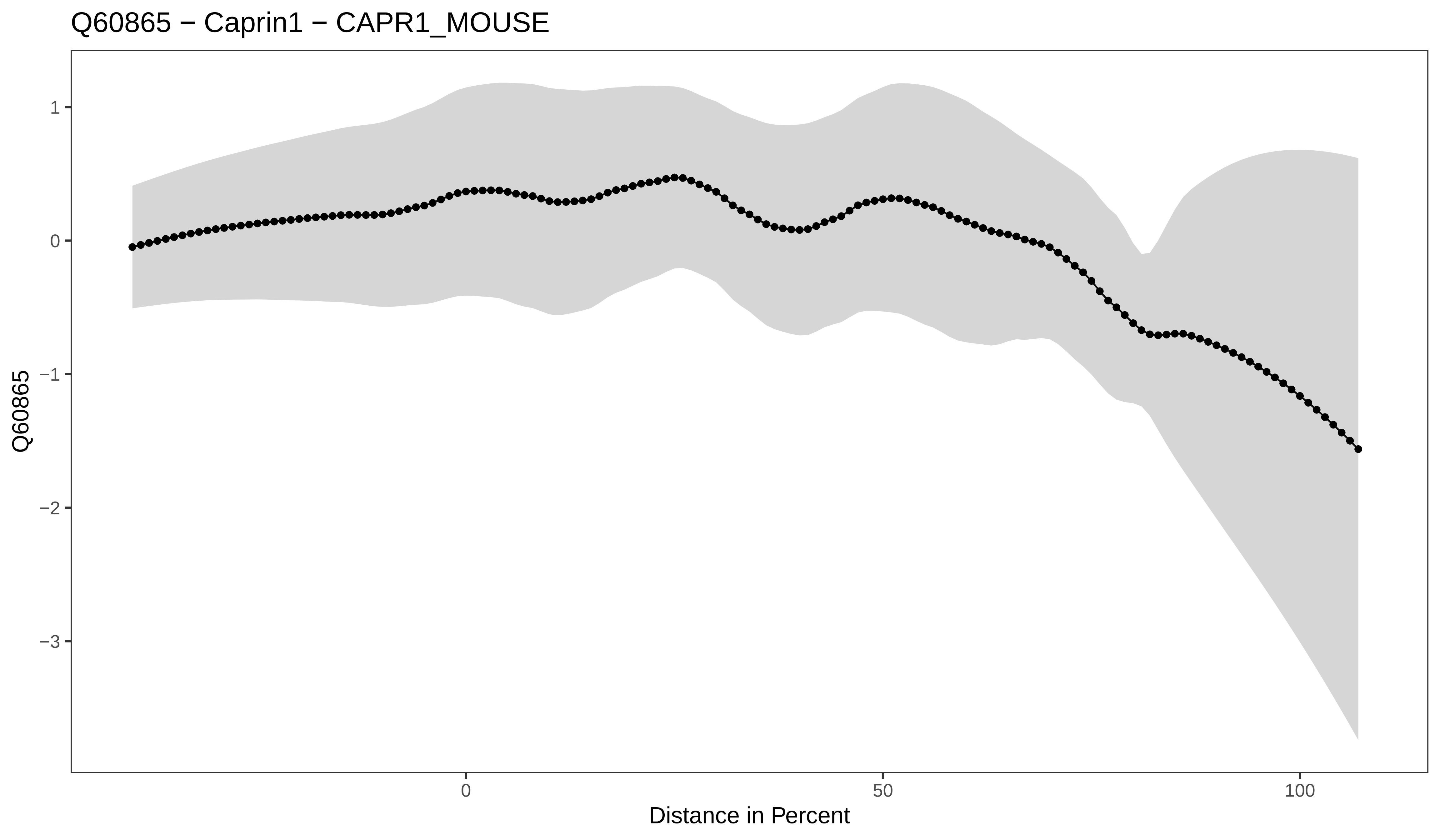

Q60865 − Caprin1 − CAPR1_MOUSE
1
0
−1
−2
−3
Q60865
0
50
100
Distance in Percent

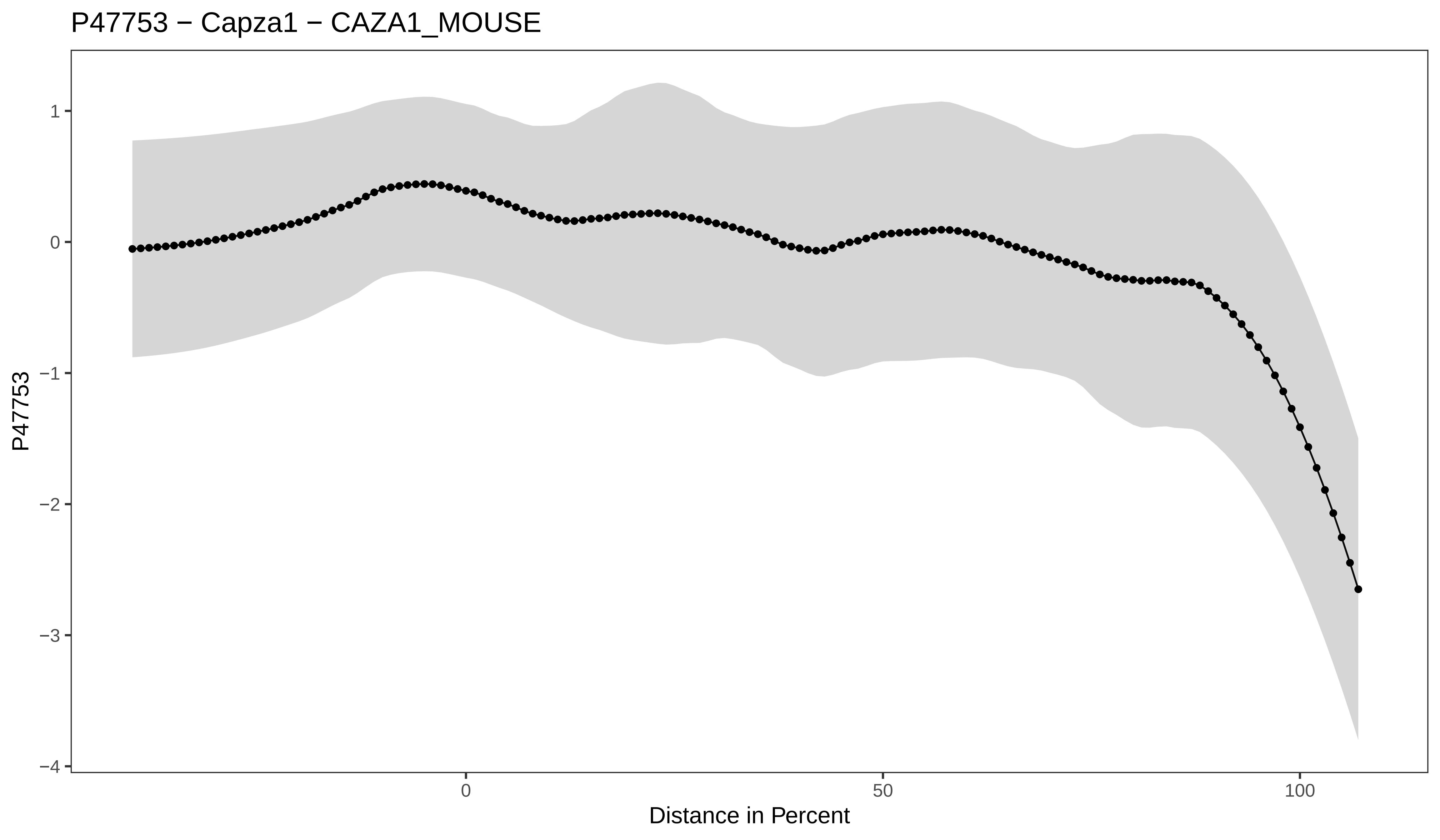
P47753 − Capza1 − CAZA1_MOUSE
P47753
1
0
−1
−2
−3
−4
0
50
100
Distance in Percent

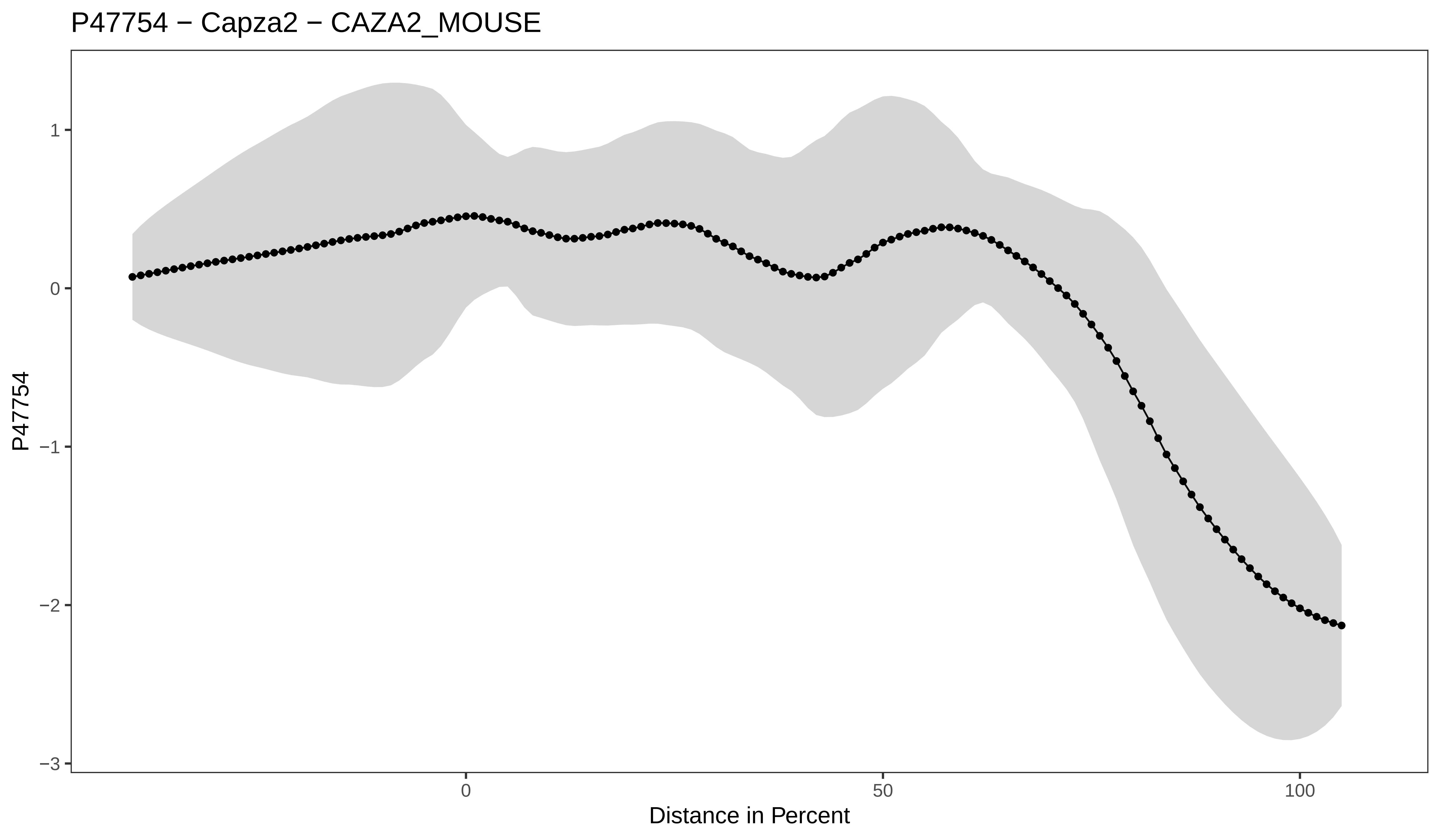
P47754 − Capza2 − CAZA2_MOUSE
P47754
1
0
−1
−2
−3
0
50
100
Distance in Percent

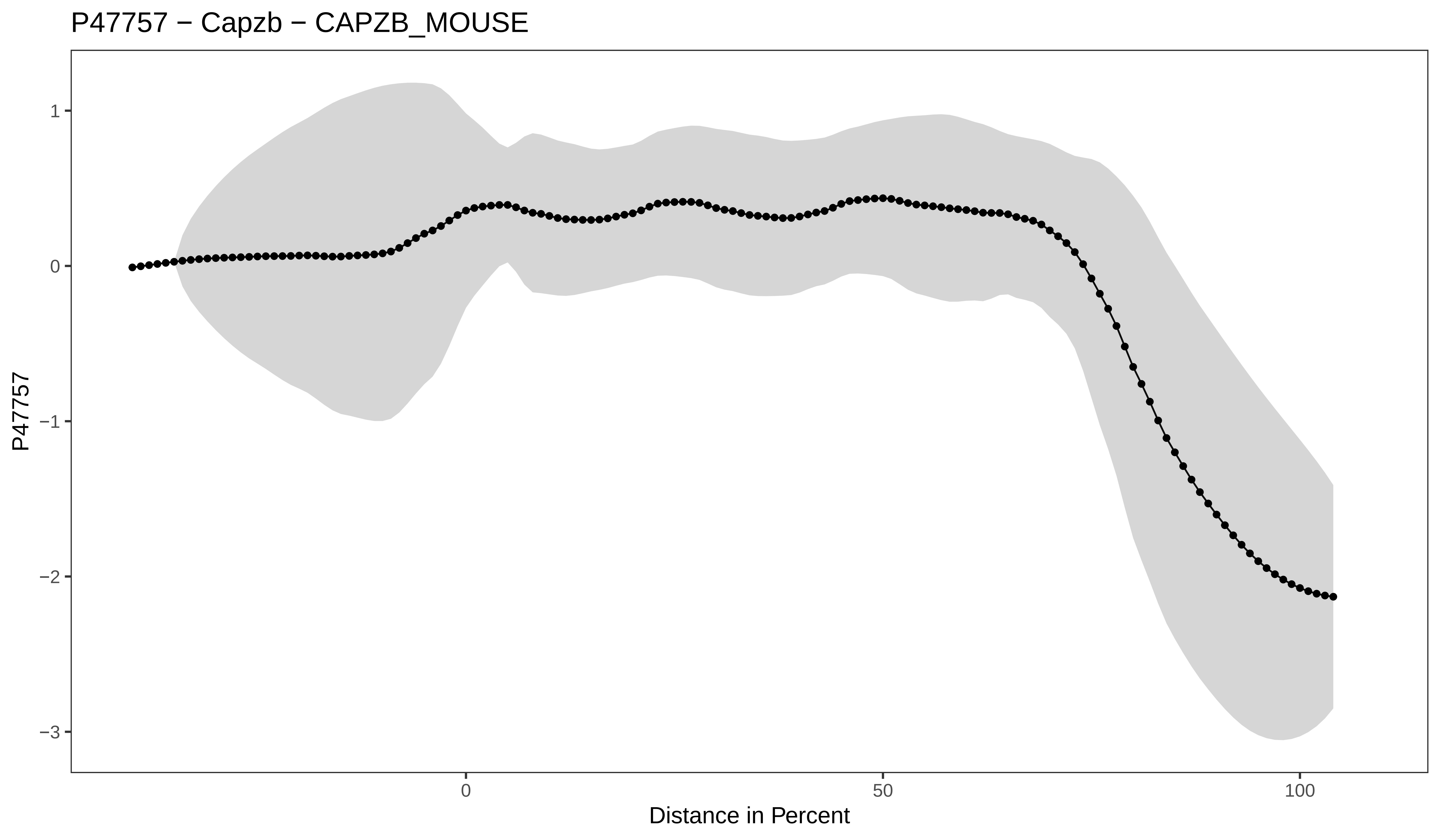

P47757 − Capzb − CAPZB_MOUSE
1
0
−1
−2
−3
P47757
0
50
100
Distance in Percent

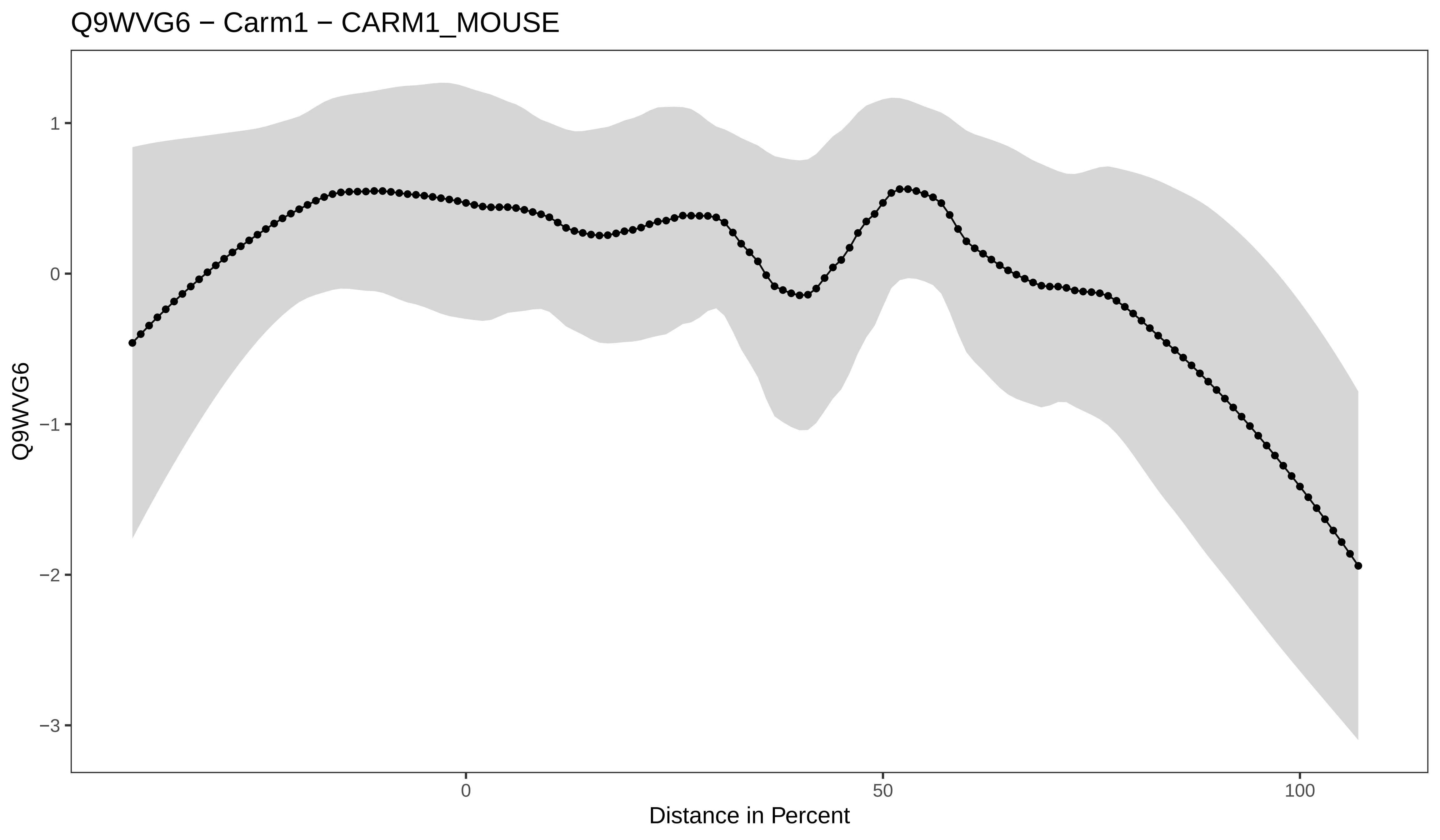
Q9WVG6 − Carm1 − CARM1_MOUSE
Q9WVG6
1
0
−1
−2
−3
0
50
100
Distance in Percent

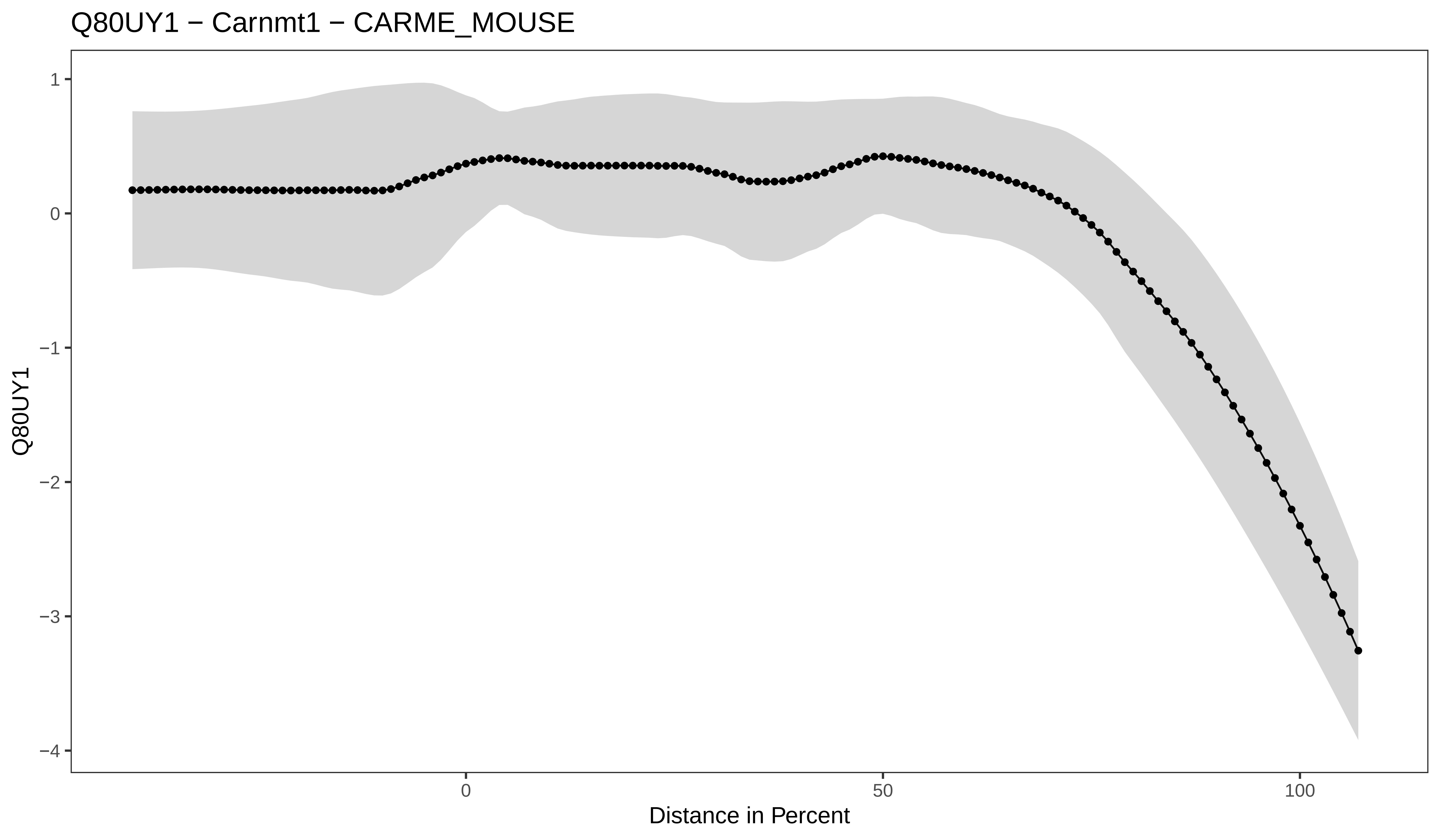

Q80UY1 − Carnmt1 − CARME_MOUSE
1
0
−1
−2
−3
−4
Q80UY1
0
50
100
Distance in Percent

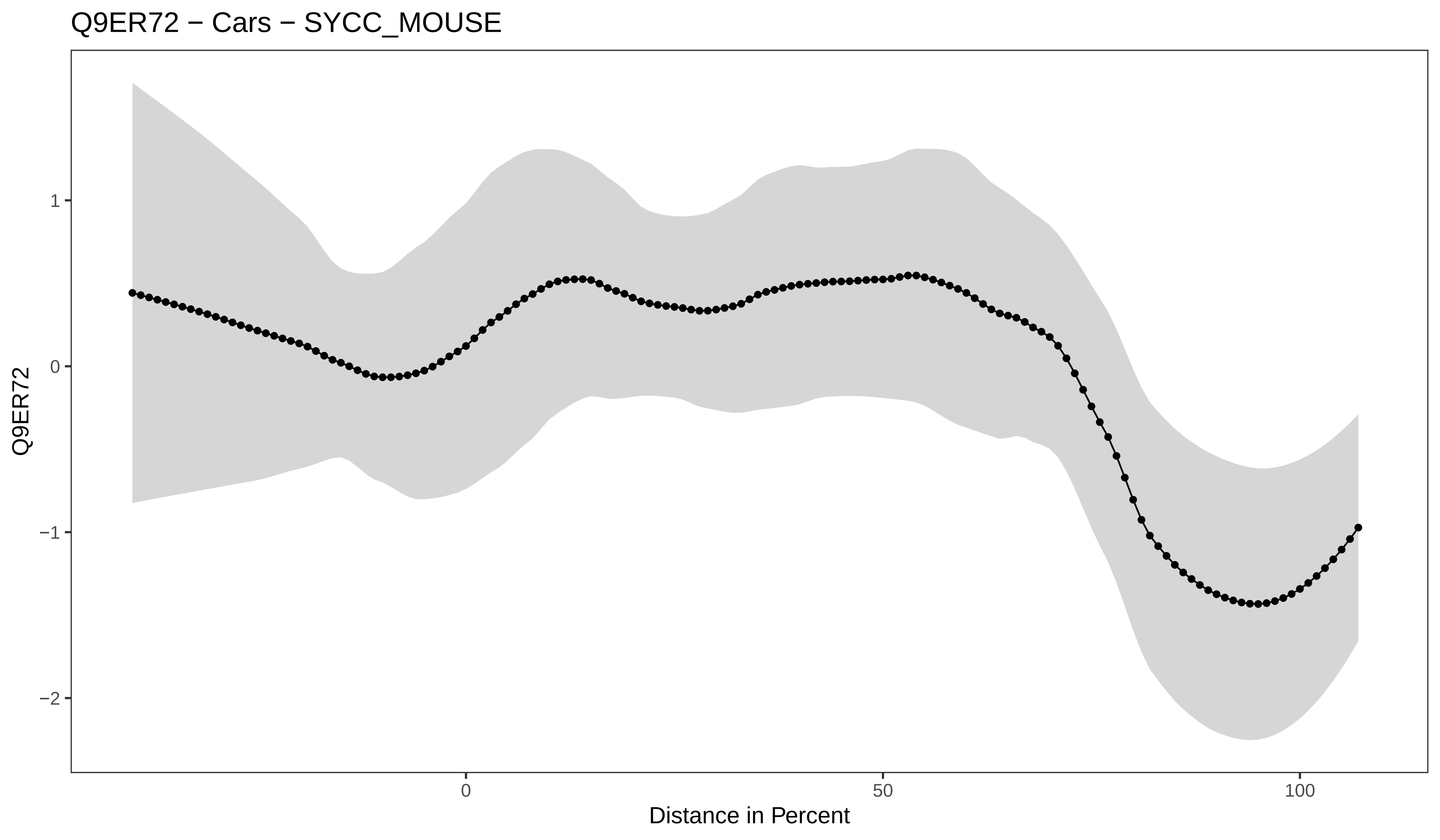

Q9ER72 − Cars − SYCC_MOUSE
Q9ER72
1
0
−1
−2
0
50
100
Distance in Percent

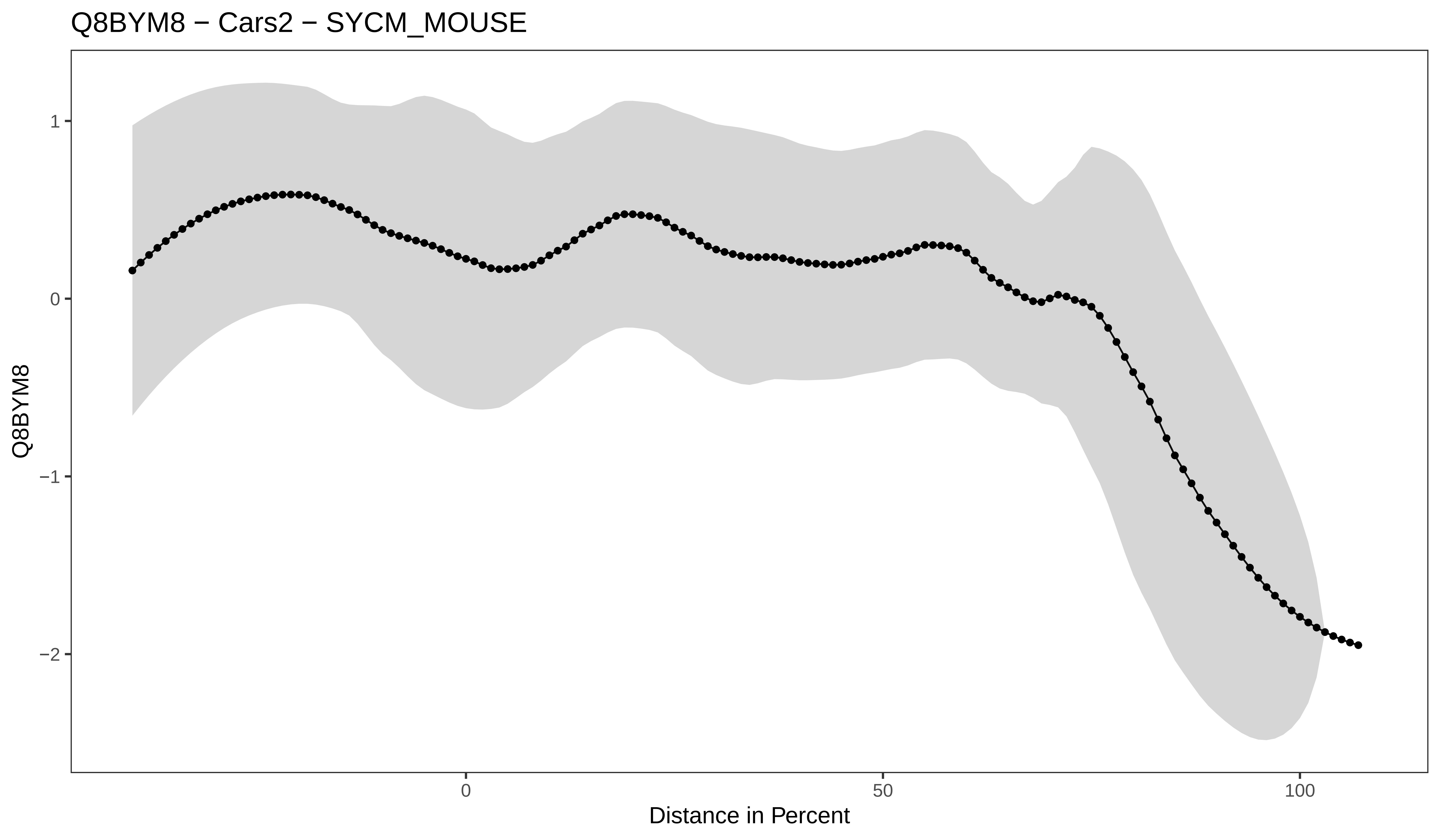
Q8BYM8 − Cars2 − SYCM_MOUSE
Q8BYM8
1
0
−1
−2
0
50
100
Distance in Percent

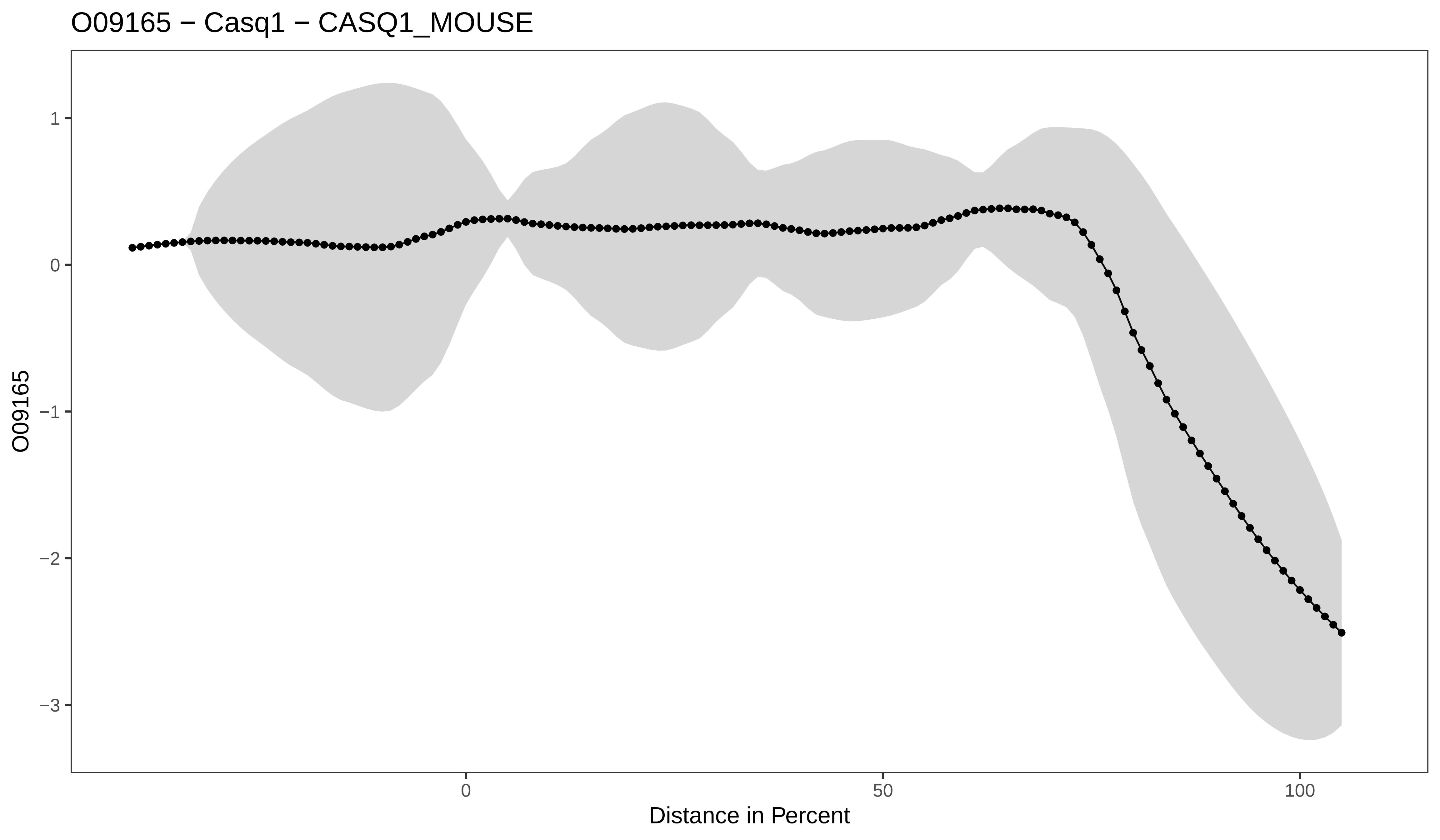
O09165 − Casq1 − CASQ1_MOUSE
1
0
−1
−2
−3
O09165
0
50
100
Distance in Percent

O09161 − Casq2 − CASQ2_MOUSE

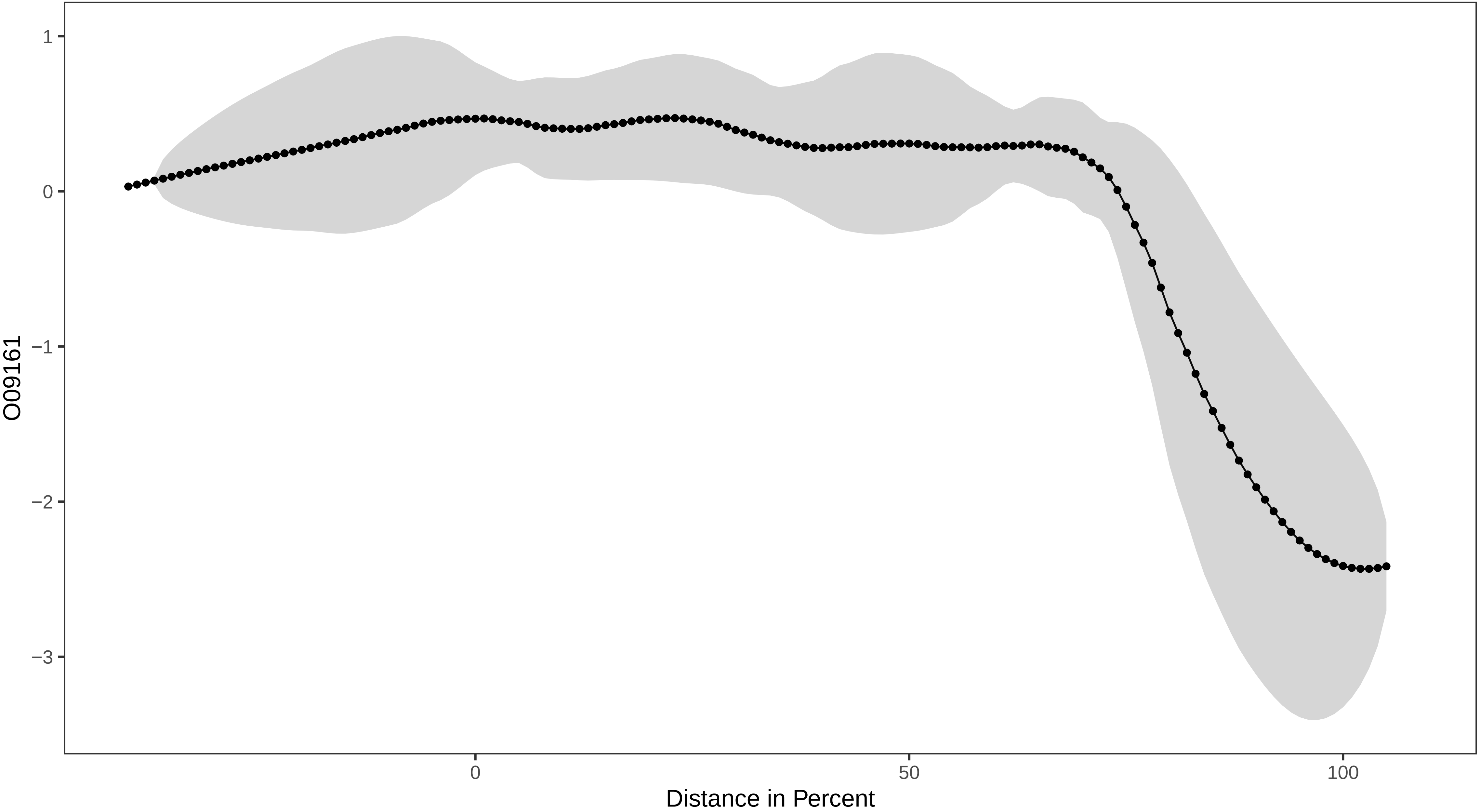

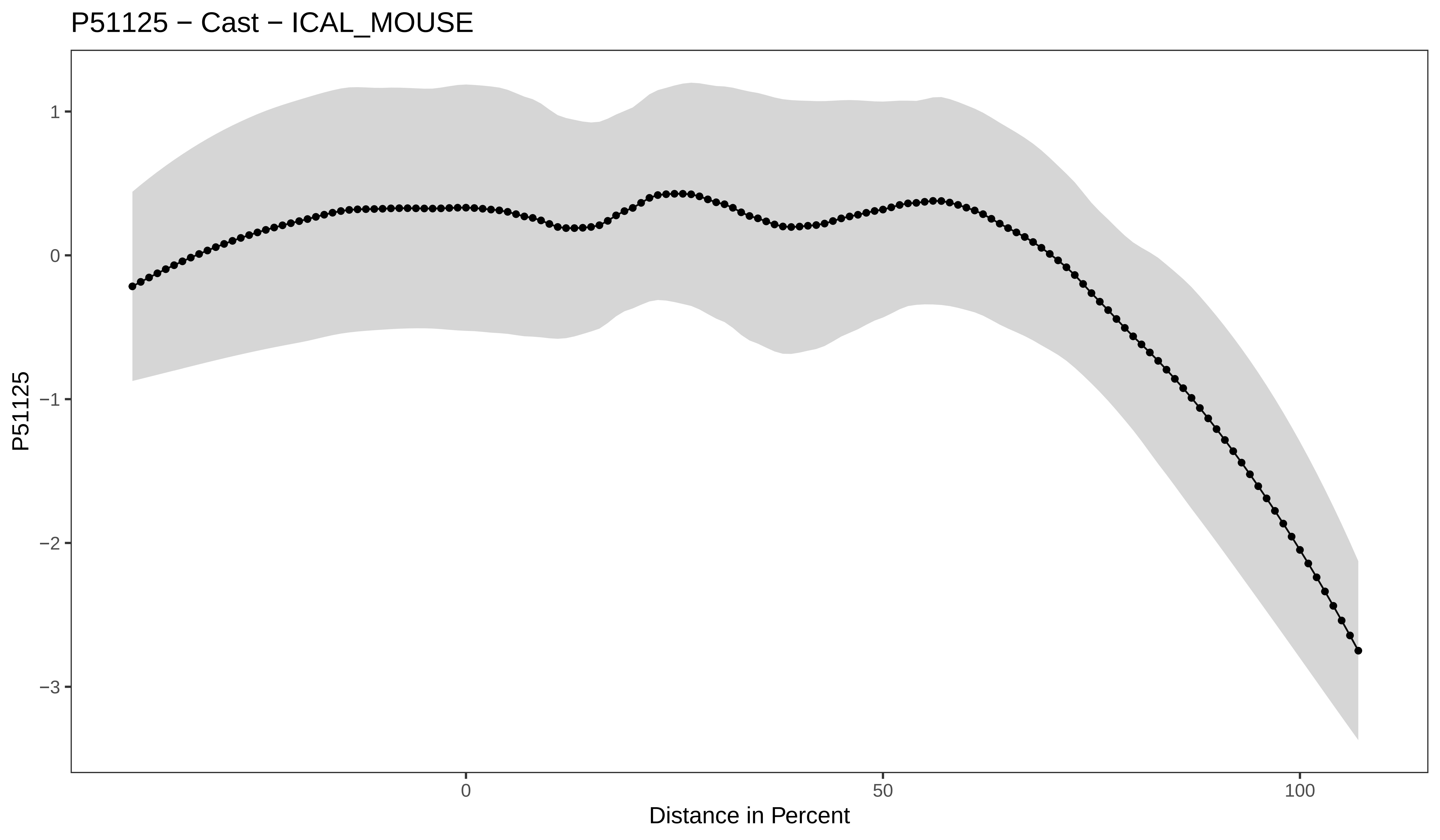
P51125 − Cast − ICAL_MOUSE
P51125
Distance in Percent
1
0
−1
−2
−3
0
50
100

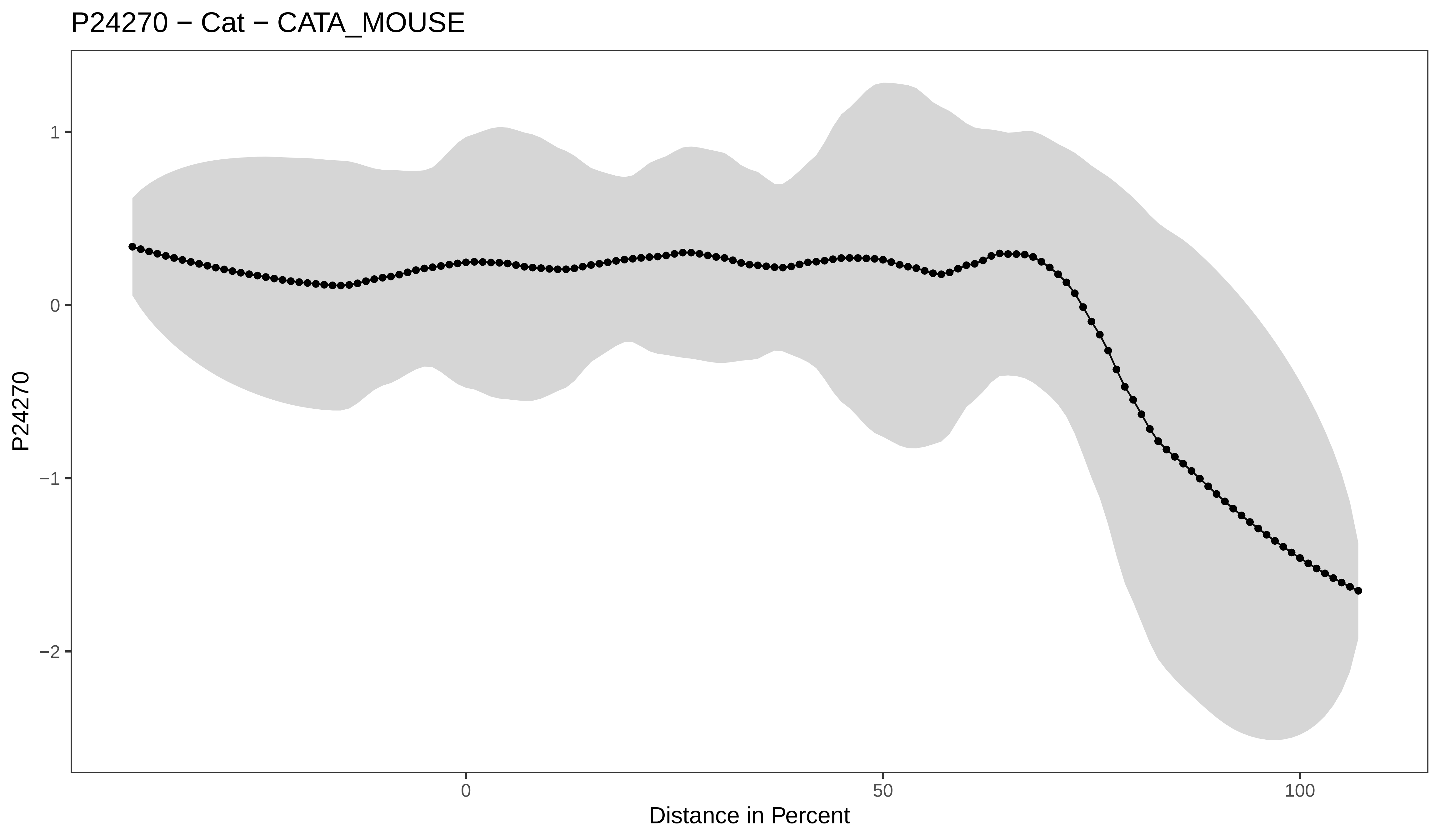

P24270 − Cat − CATA_MOUSE
P24270
1
0
−1
−2
0
50
100
Distance in Percent

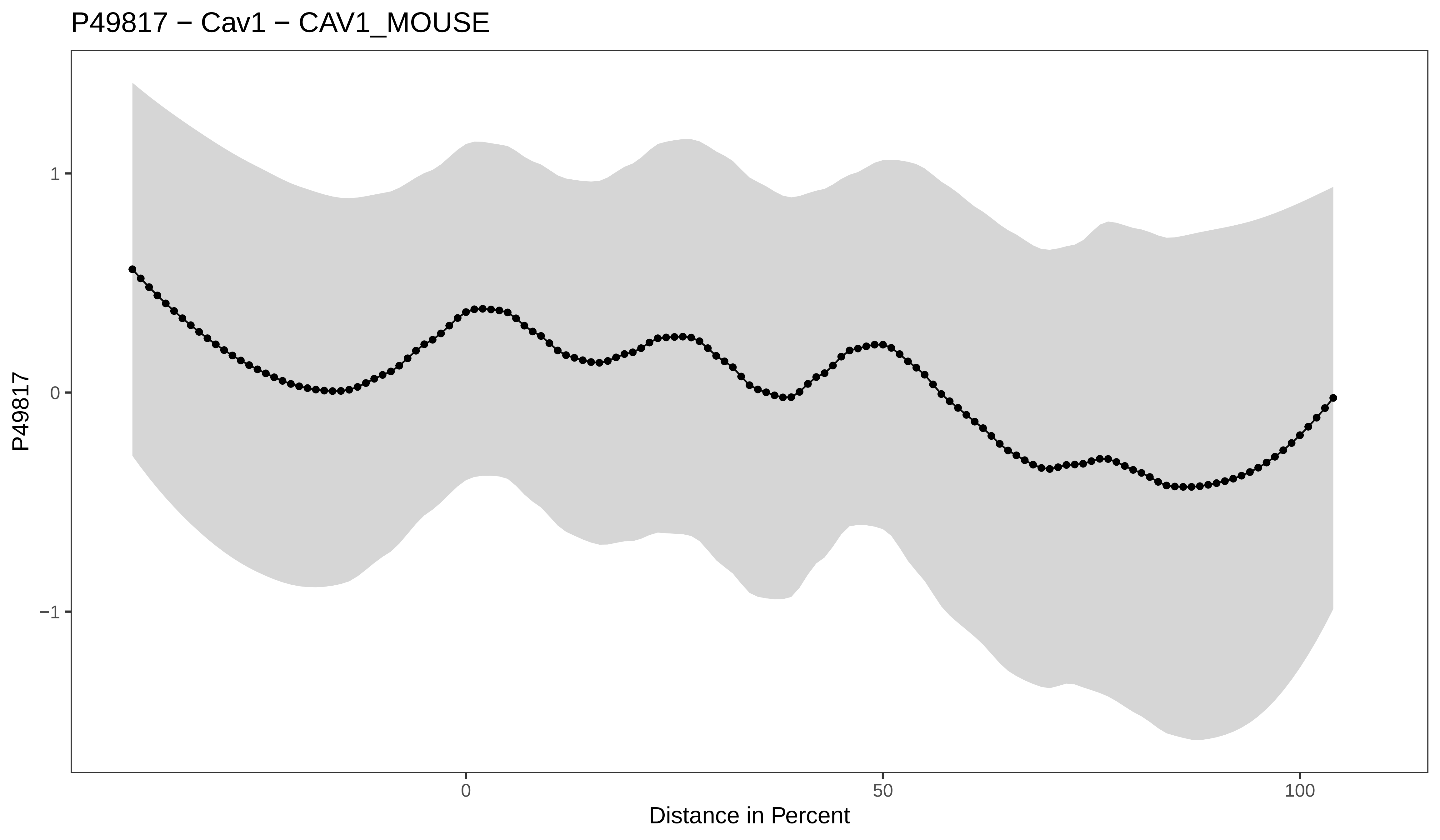

P49817 − Cav1 − CAV1_MOUSE
1
0
−1
P49817
0
50
100
Distance in Percent

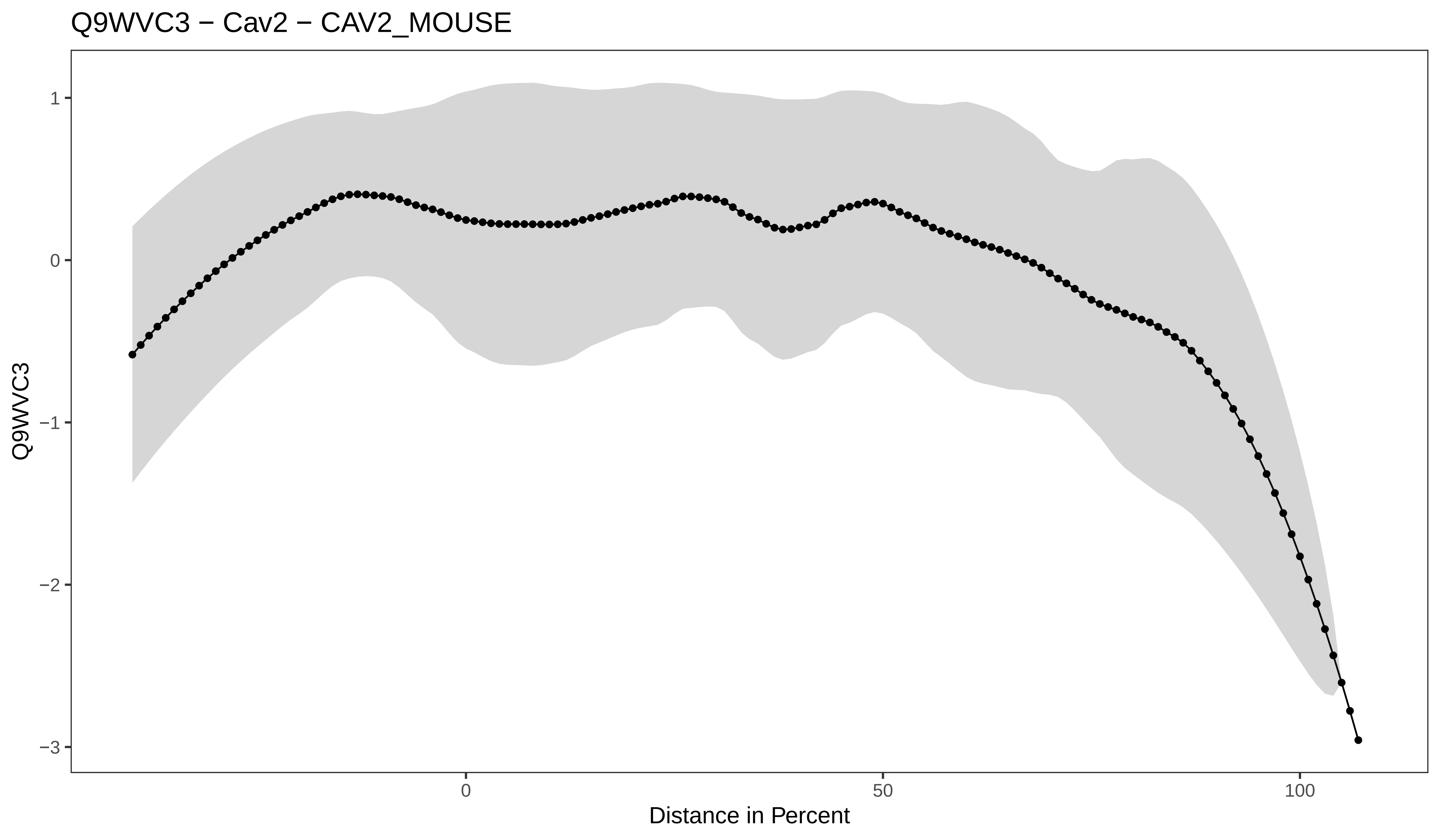

Q9WVC3 − Cav2 − CAV2_MOUSE
Q9WVC3
1
0
−1
−2
−3
0
50
100
Distance in Percent

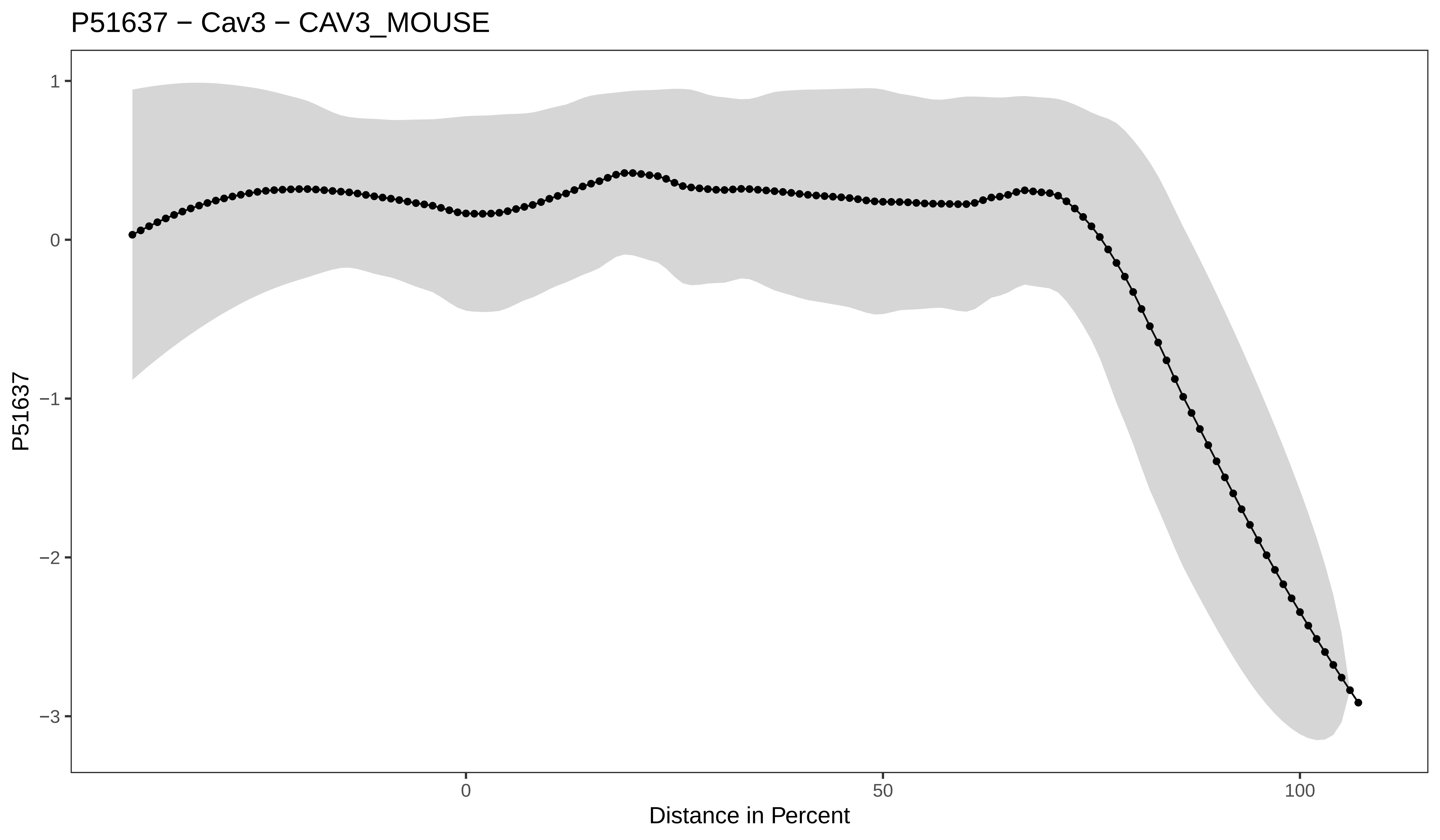

P51637 − Cav3 − CAV3_MOUSE
1
0
−1
−2
−3
P51637
0
50
100
Distance in Percent

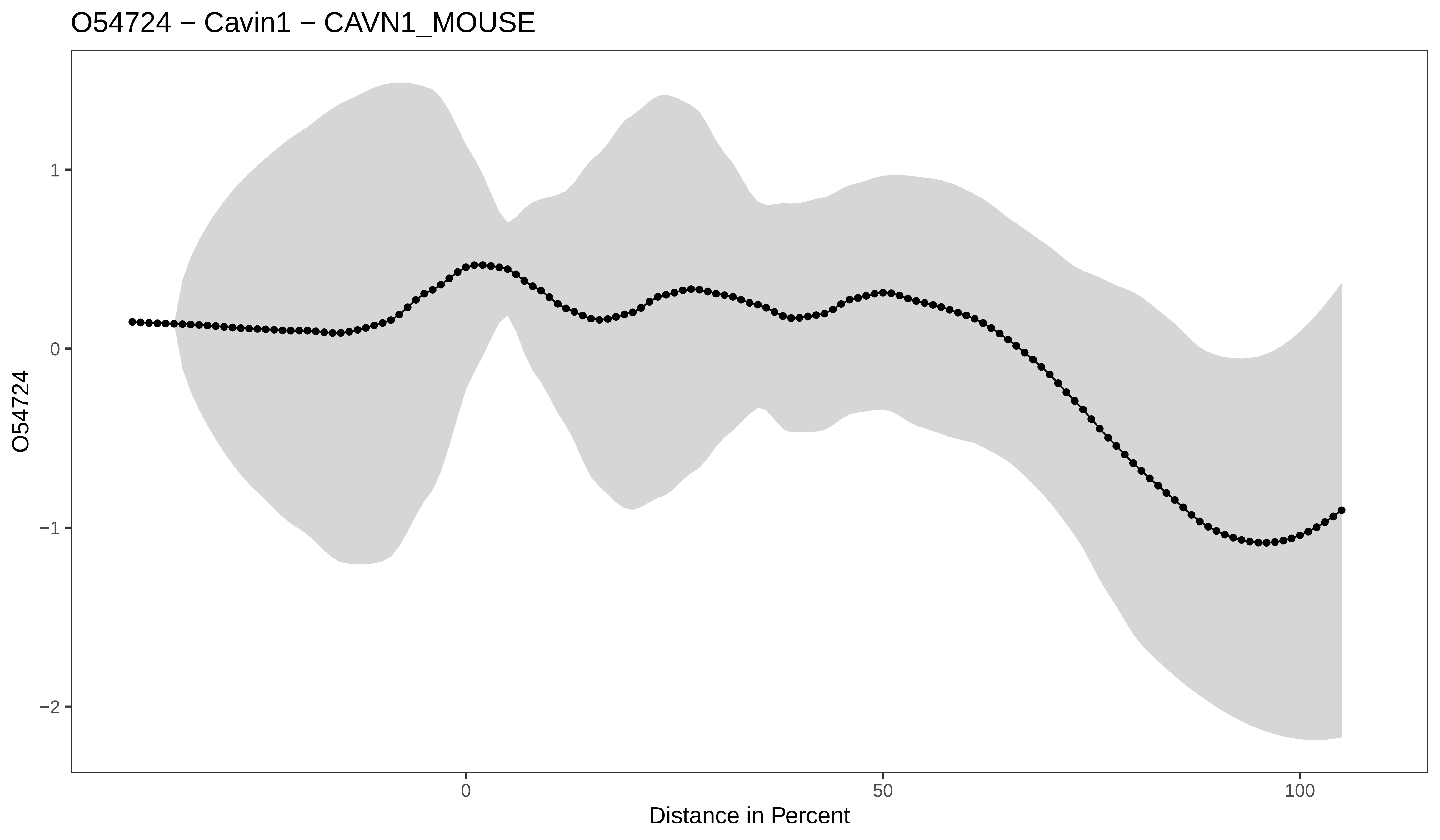
O54724 − Cavin1 − CAVN1_MOUSE
1
0
−1
−2
O54724
0
50
100
Distance in Percent

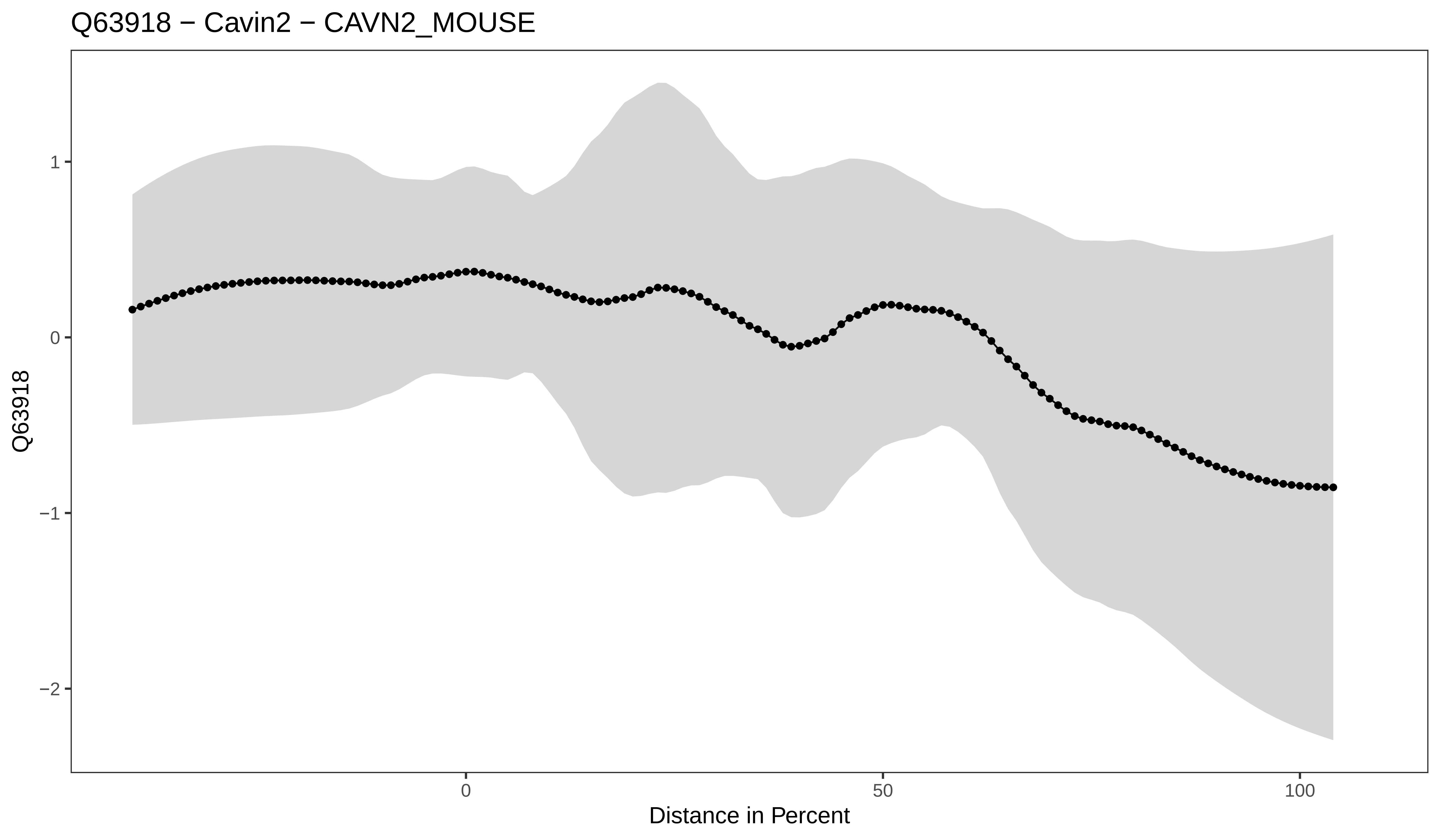
Q63918 − Cavin2 − CAVN2_MOUSE
Q63918
Distance in Percent
1
0
−1
−2
0
50
100

Q91VJ2 − Cavin3 − CAVN3_MOUSE

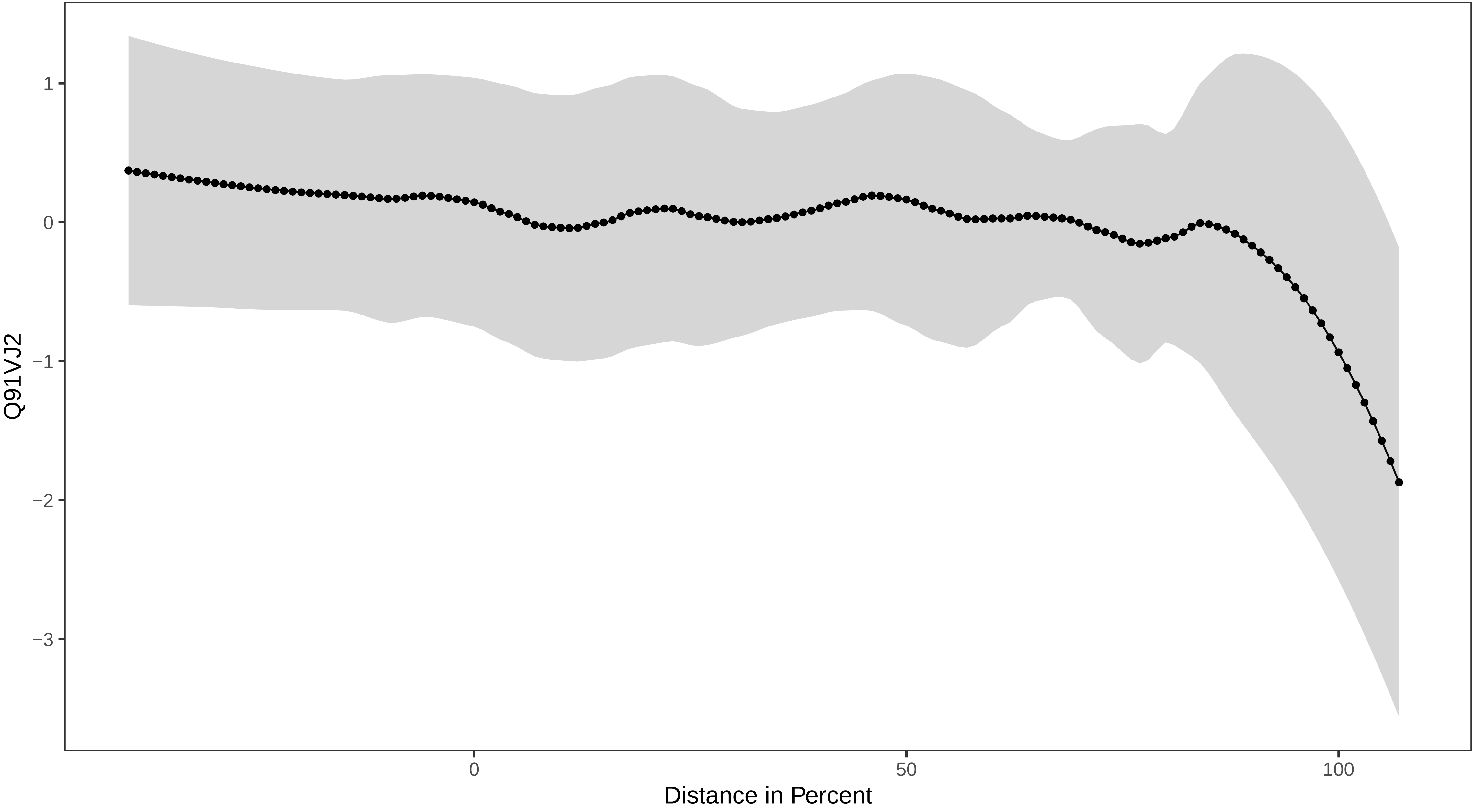

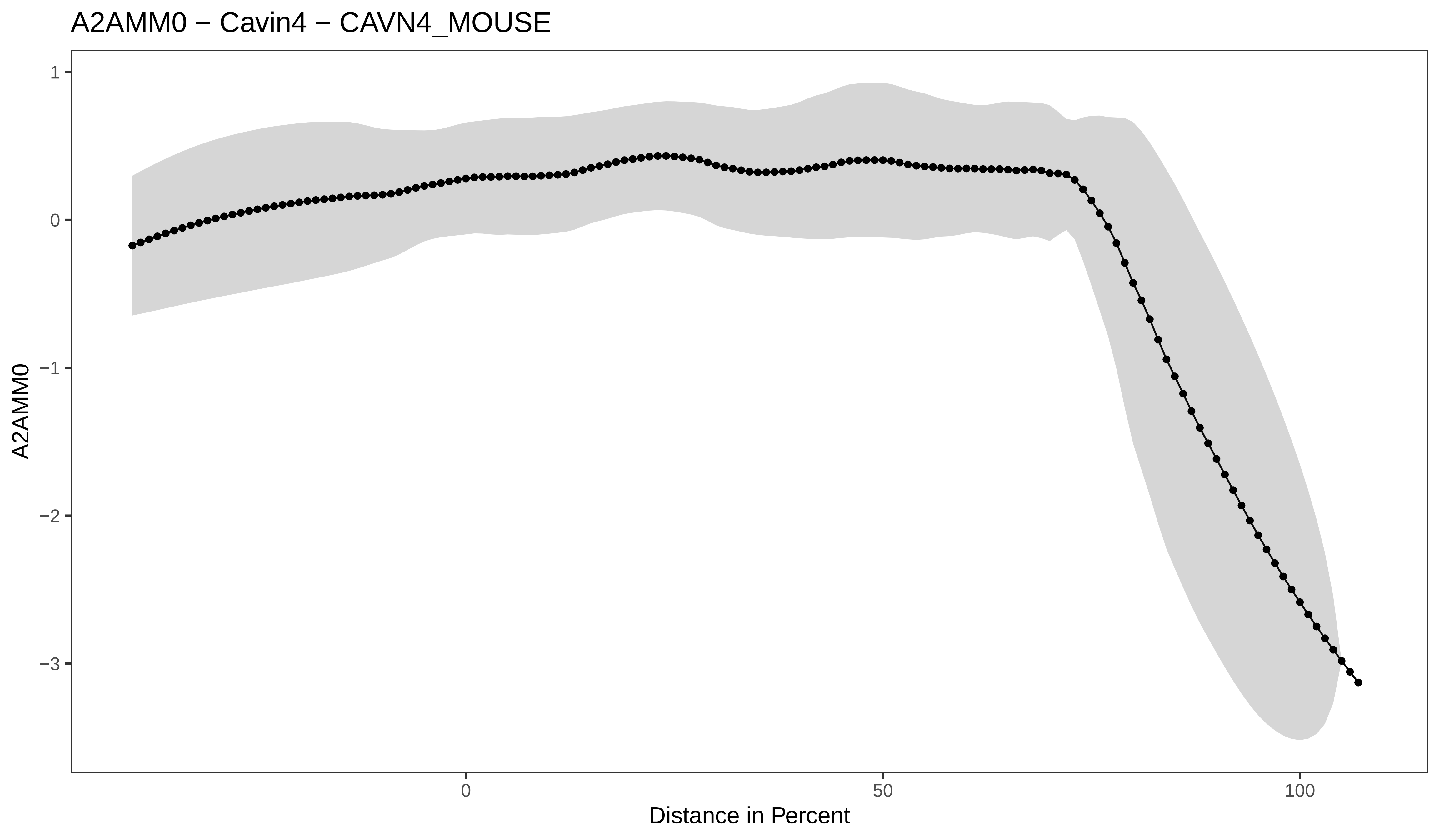

A2AMM0 − Cavin4 − CAVN4_MOUSE
A2AMM0
Distance in Percent
1
0
−1
−2
−3
0
50
100

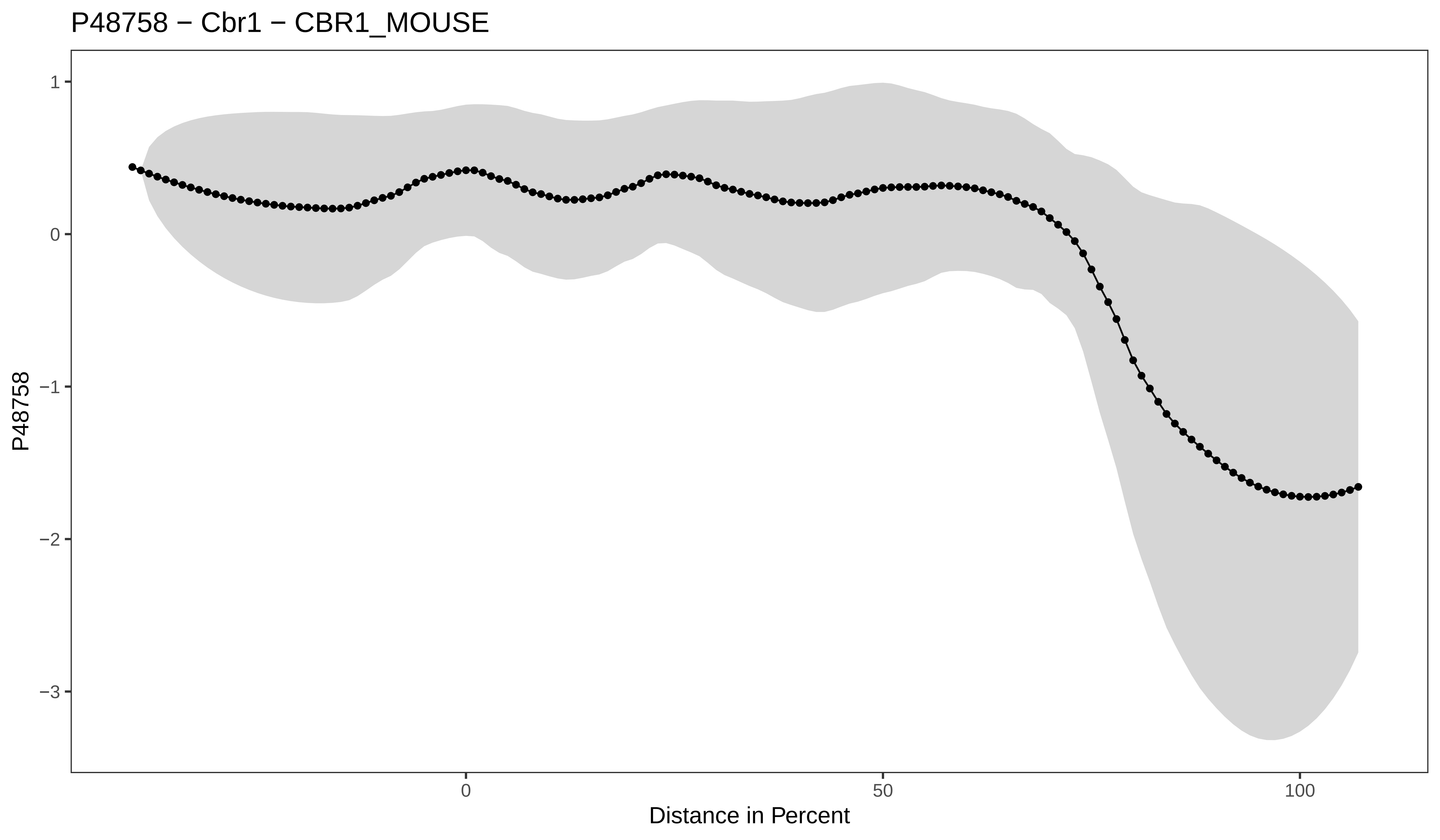
P48758 − Cbr1 − CBR1_MOUSE
1
0
−1
−2
−3
P48758
0
50
100
Distance in Percent

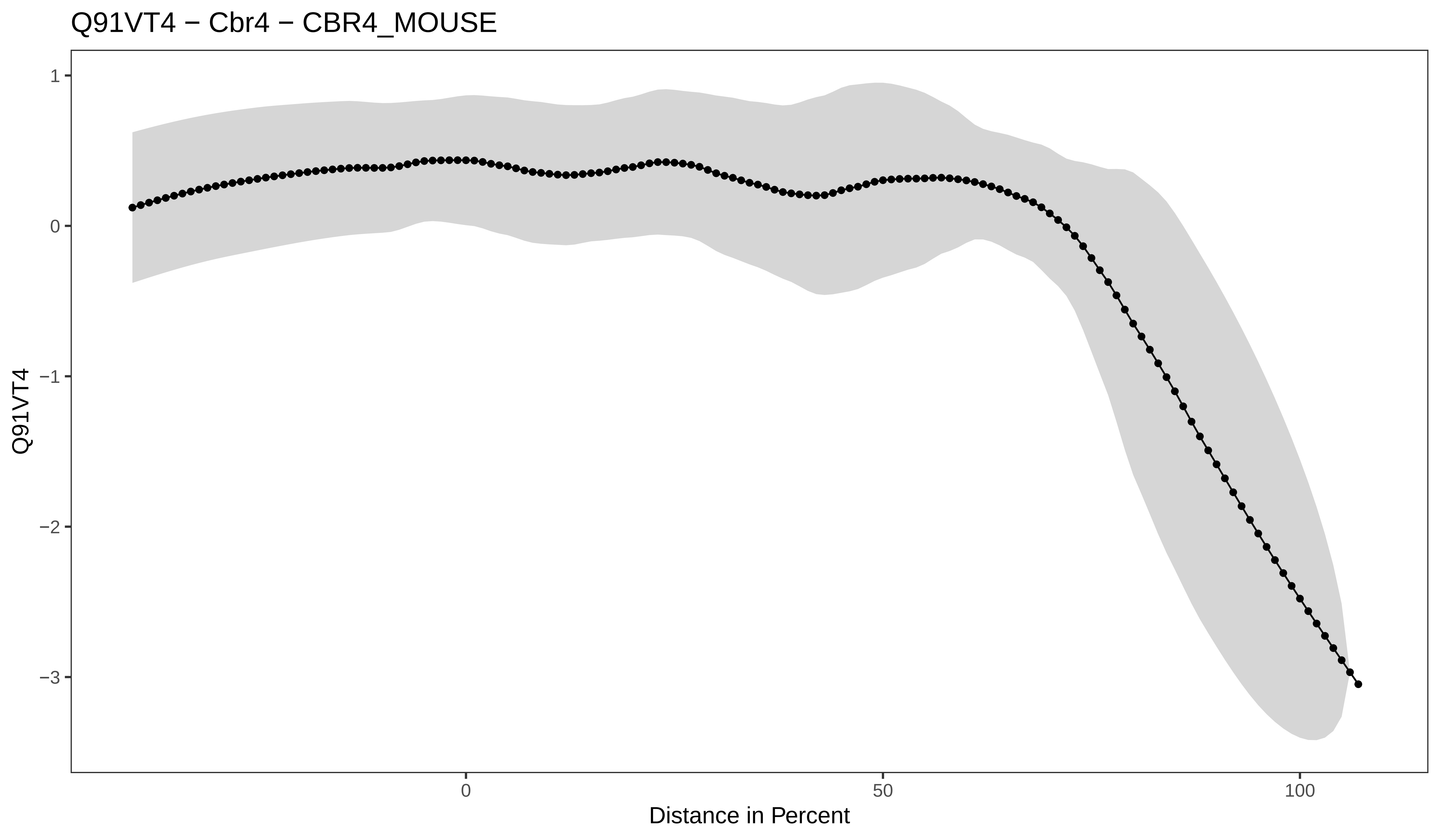

Q91VT4 − Cbr4 − CBR4_MOUSE
1
0
−1
−2
−3
Q91VT4
0
50
100
Distance in Percent

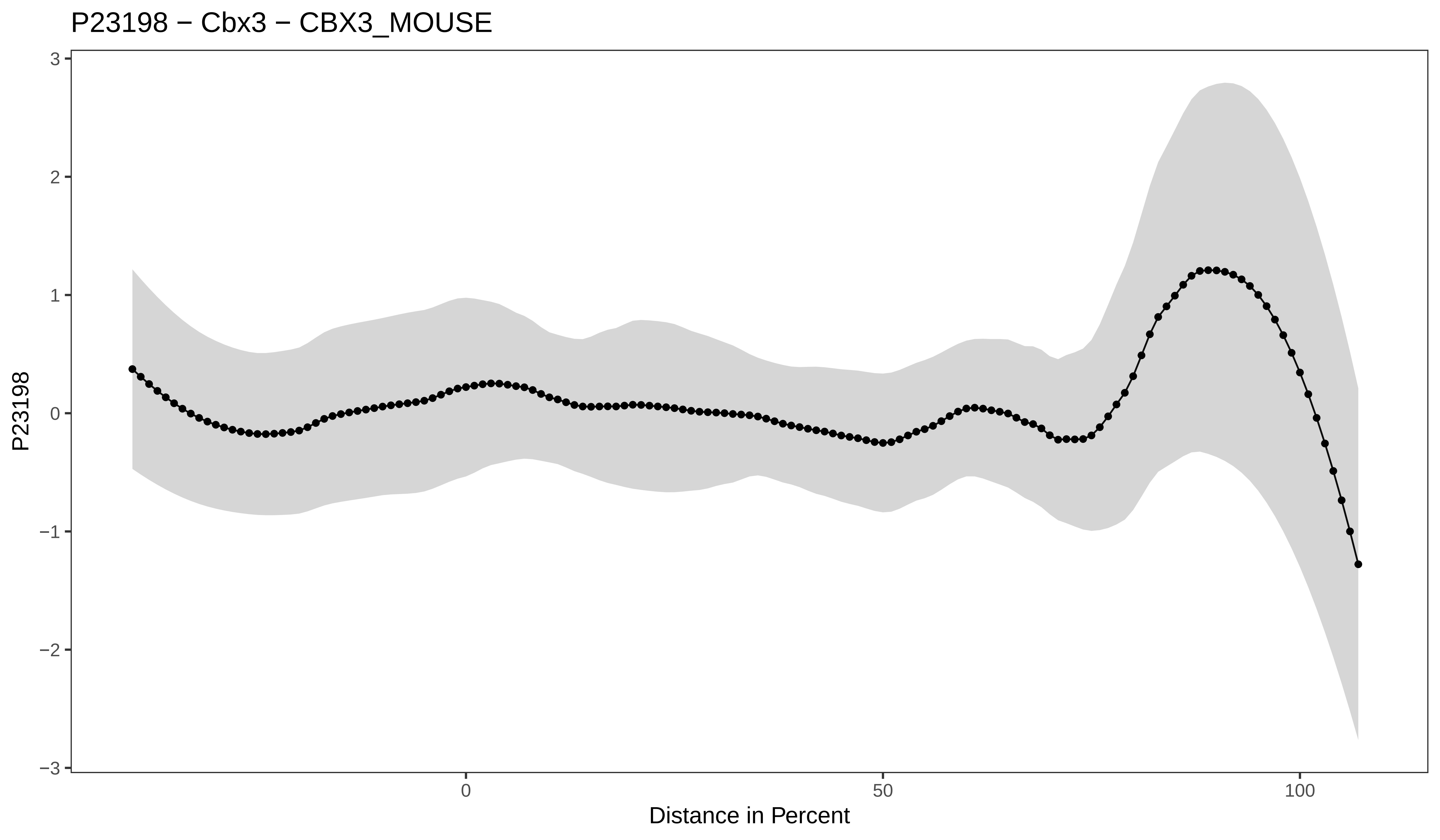

P23198 − Cbx3 − CBX3_MOUSE
3
2
1
0
−1
−2
−3
P23198
0
50
100
Distance in Percent

Q8CH18 − Ccar1 − CCAR1_MOUSE

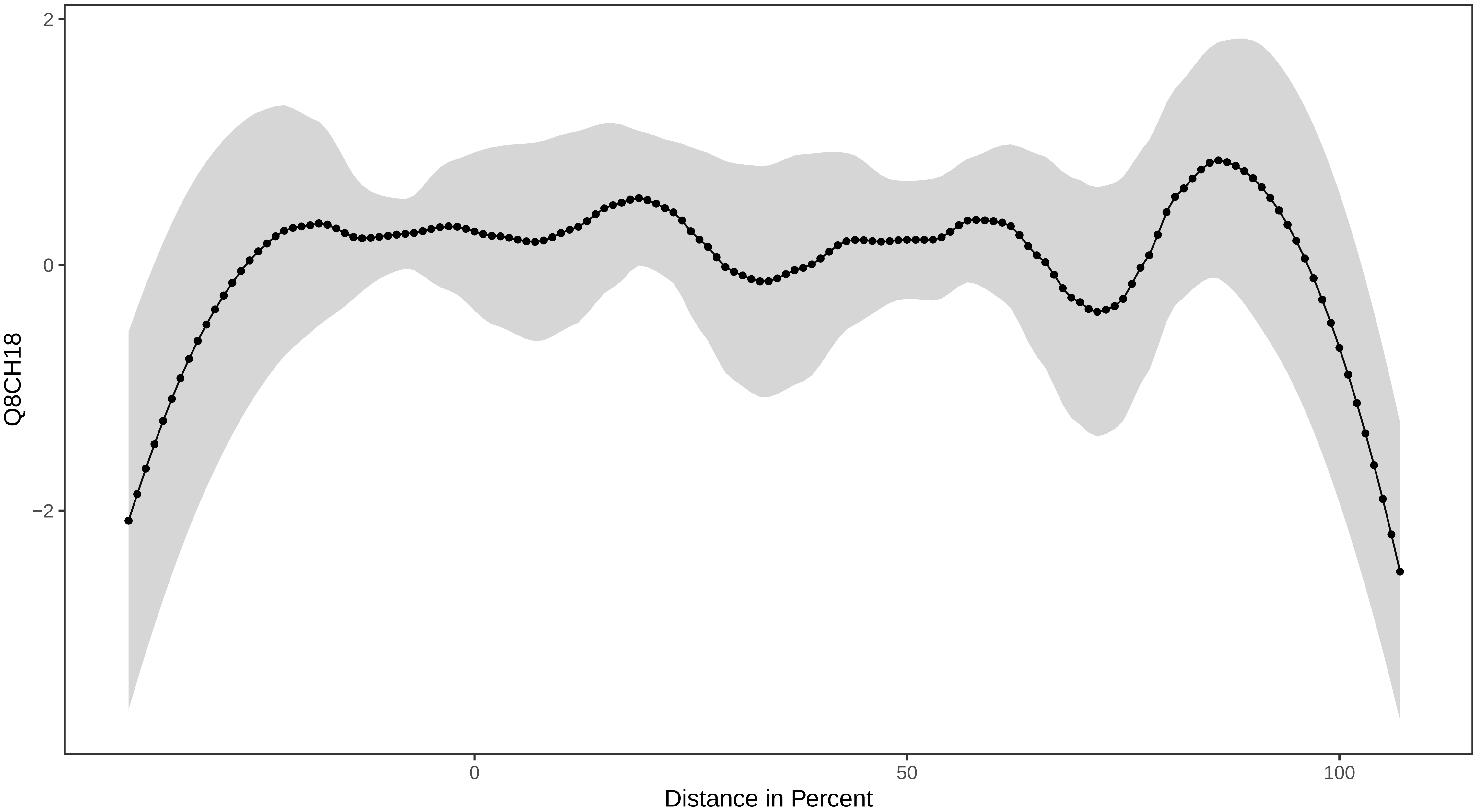

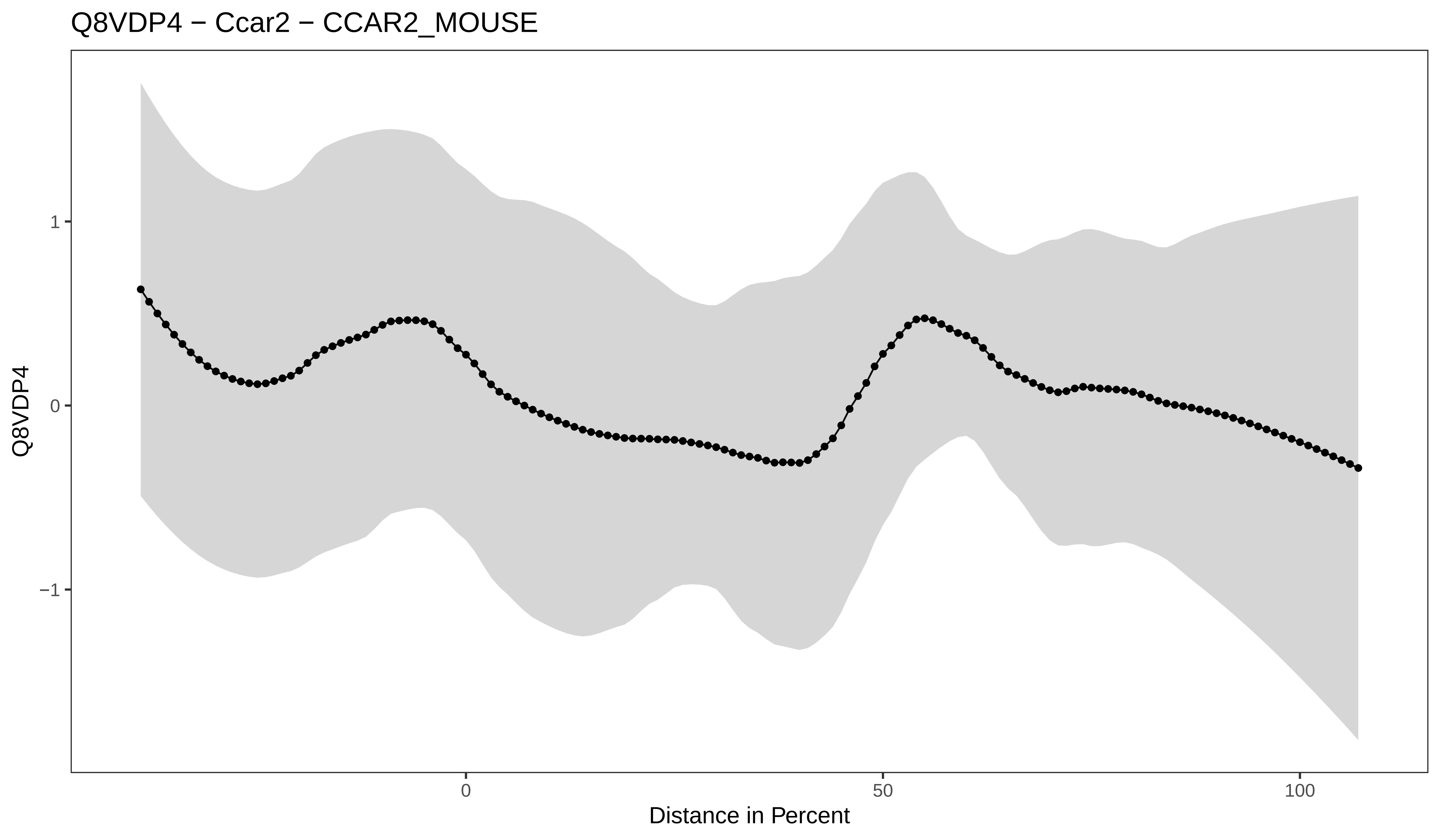

Q8VDP4 − Ccar2 − CCAR2_MOUSE
Q8VDP4
1
0
−1
0
50
100
Distance in Percent

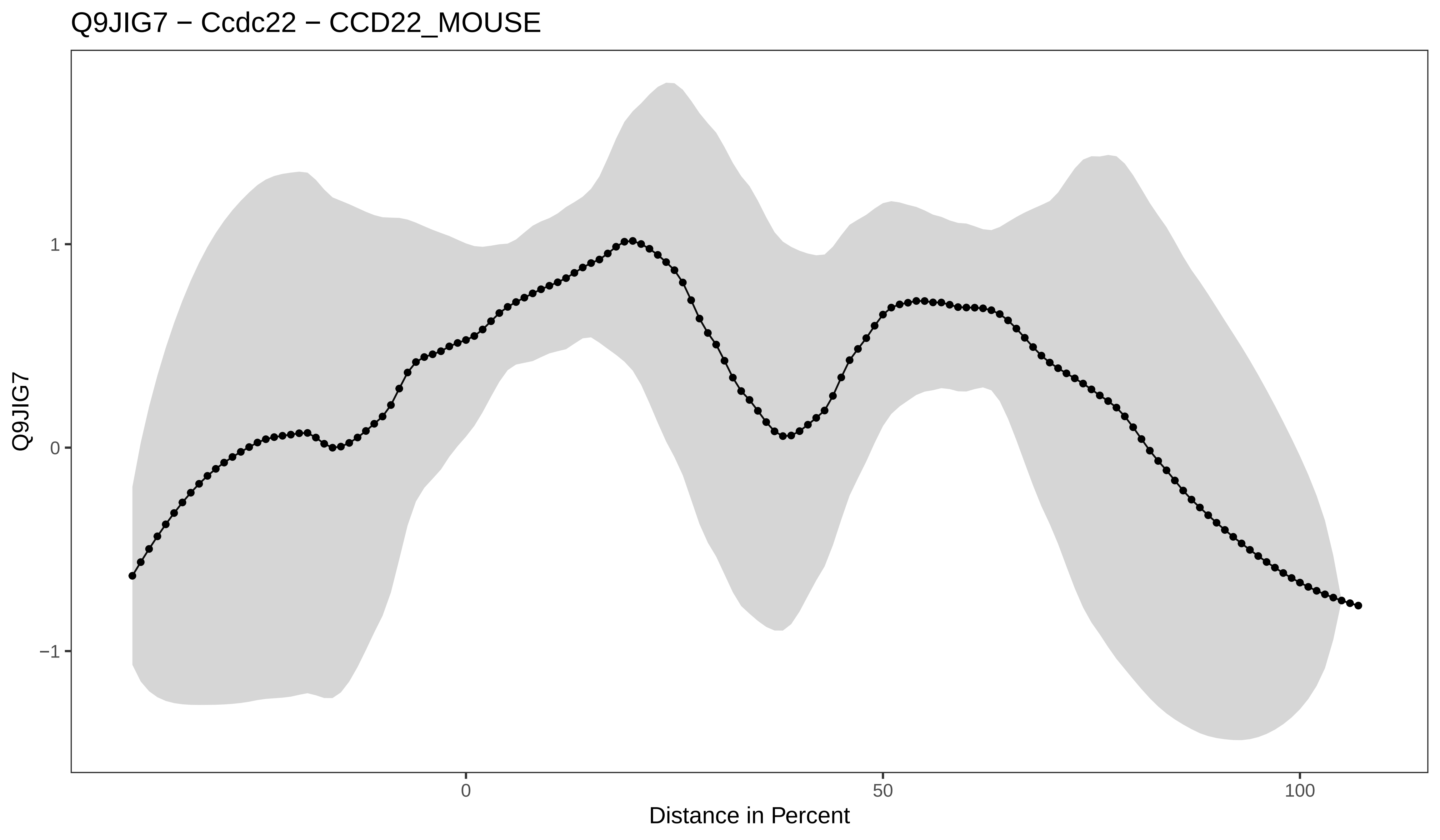

Q9JIG7 − Ccdc22 − CCD22_MOUSE
Q9JIG7
1
0
−1
0
50
100
Distance in Percent

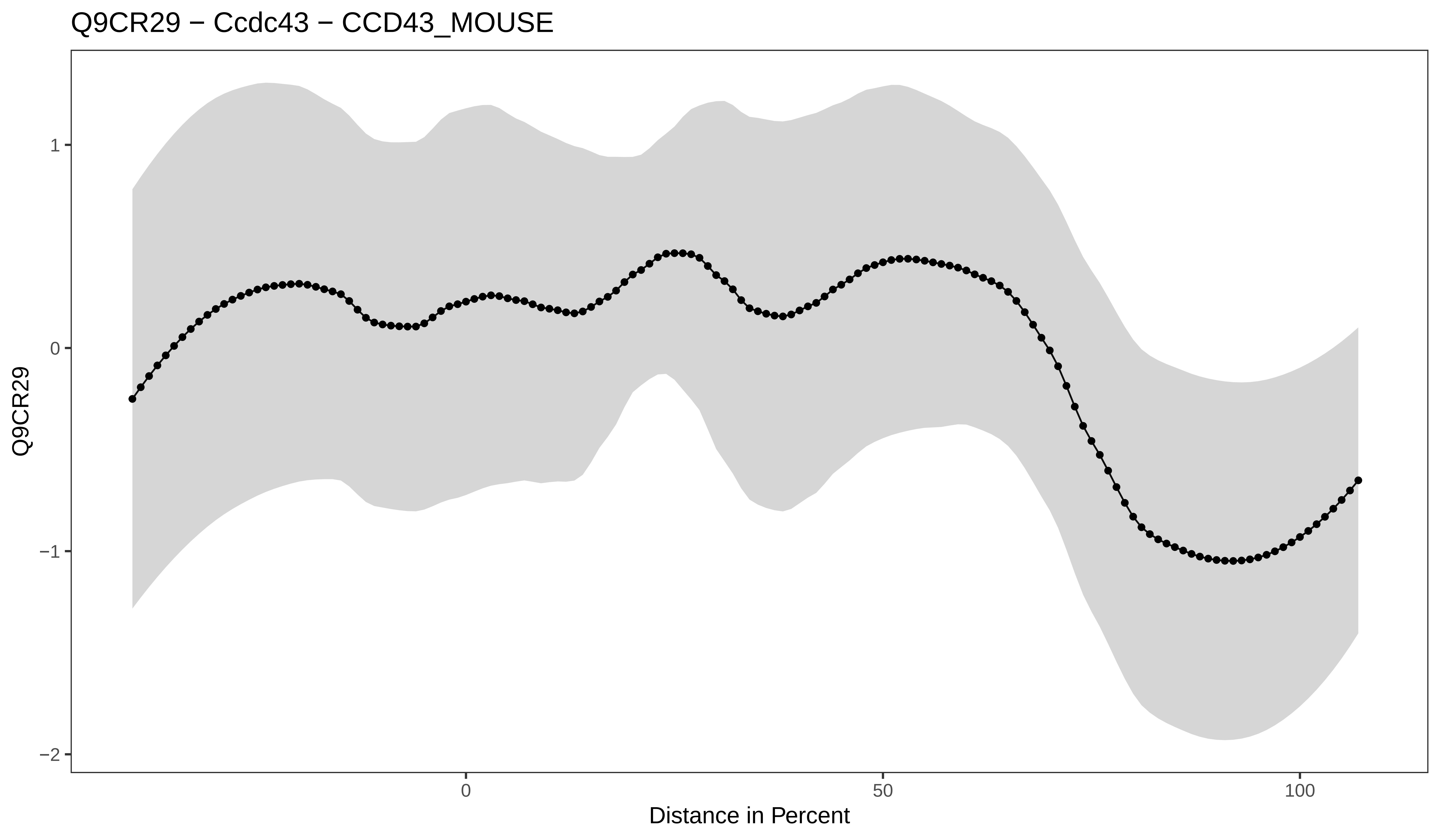

Q9CR29 − Ccdc43 − CCD43_MOUSE
Q9CR29
1
0
−1
−2
0
50
100
Distance in Percent

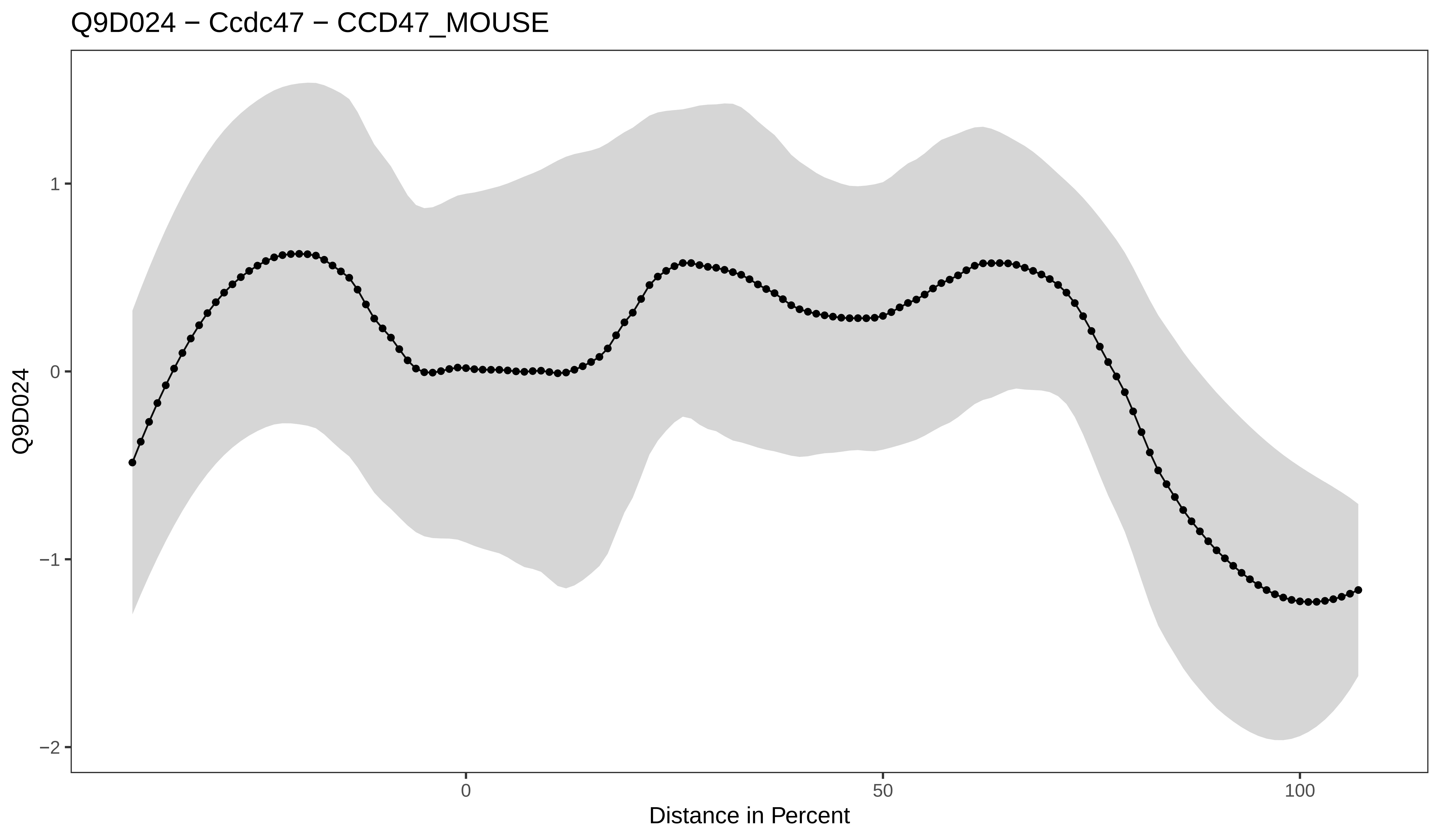
Q9D024 − Ccdc47 − CCD47_MOUSE
Q9D024
1
0
−1
−2
0
50
100
Distance in Percent

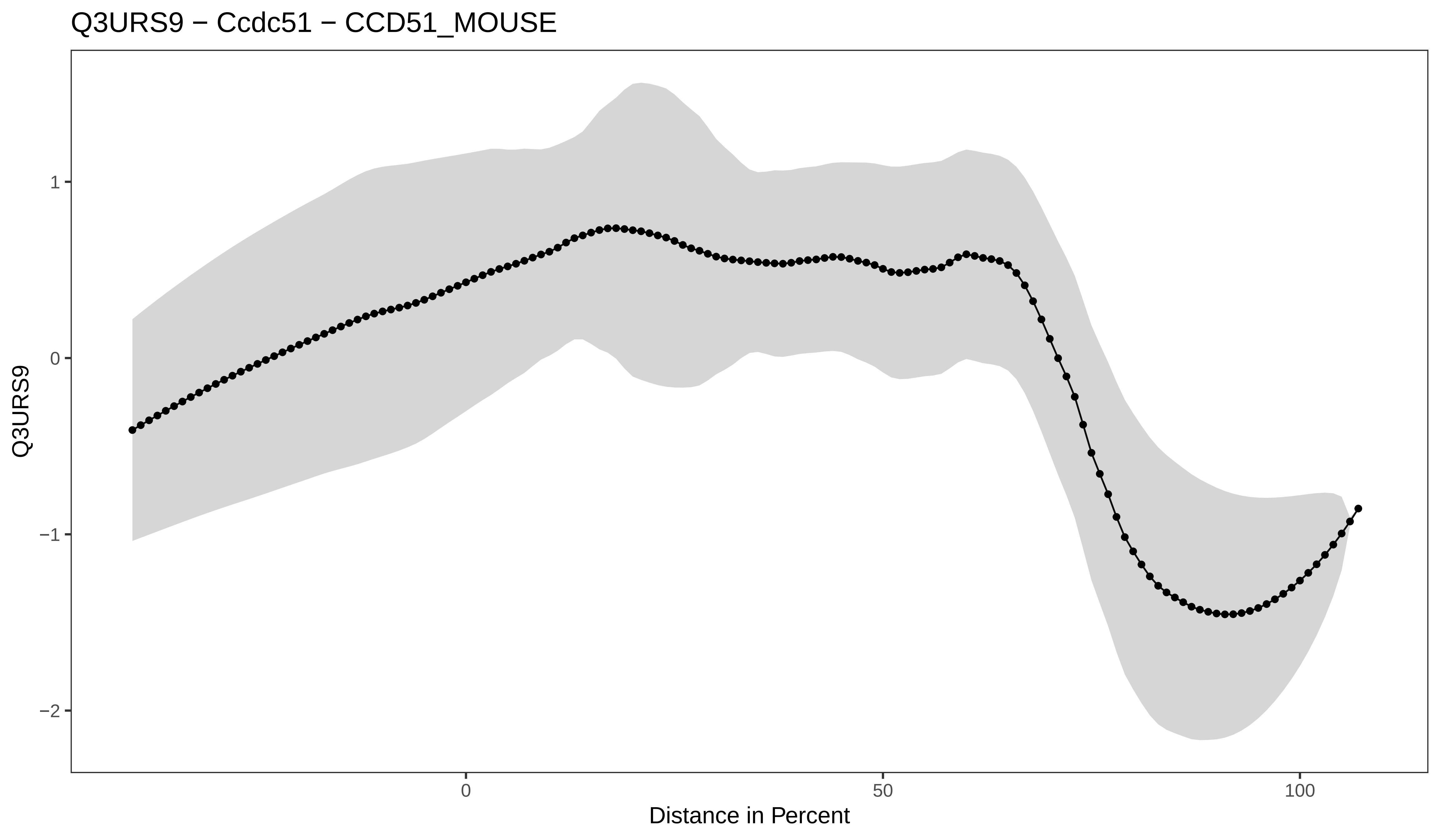

Q3URS9 − Ccdc51 − CCD51_MOUSE
1
0
−1
−2
0
50
100
Distance in Percent
Q3URS9

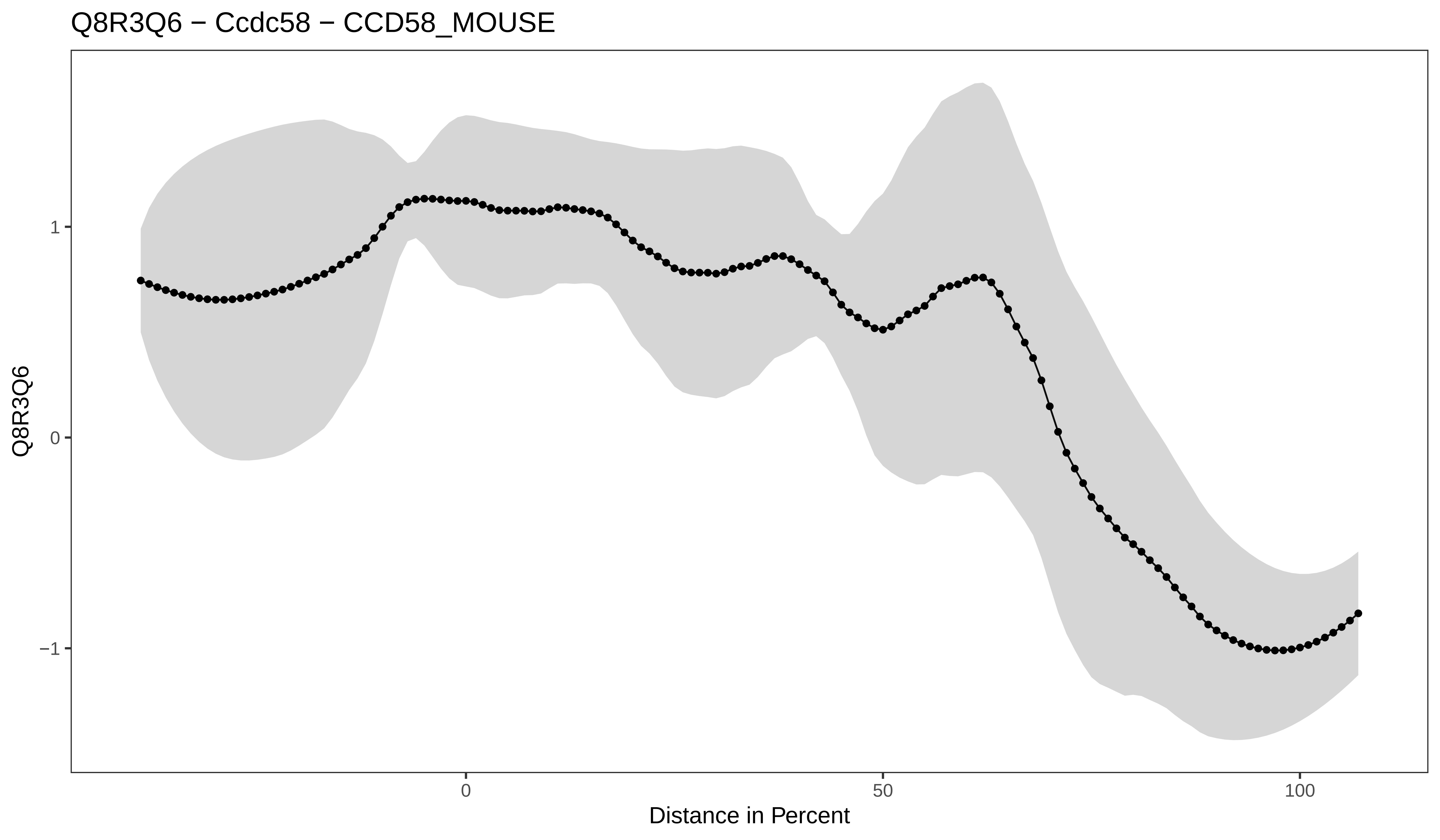
Q8R3Q6 − Ccdc58 − CCD58_MOUSE
Q8R3Q6
1
0
−1
0
50
100
Distance in Percent

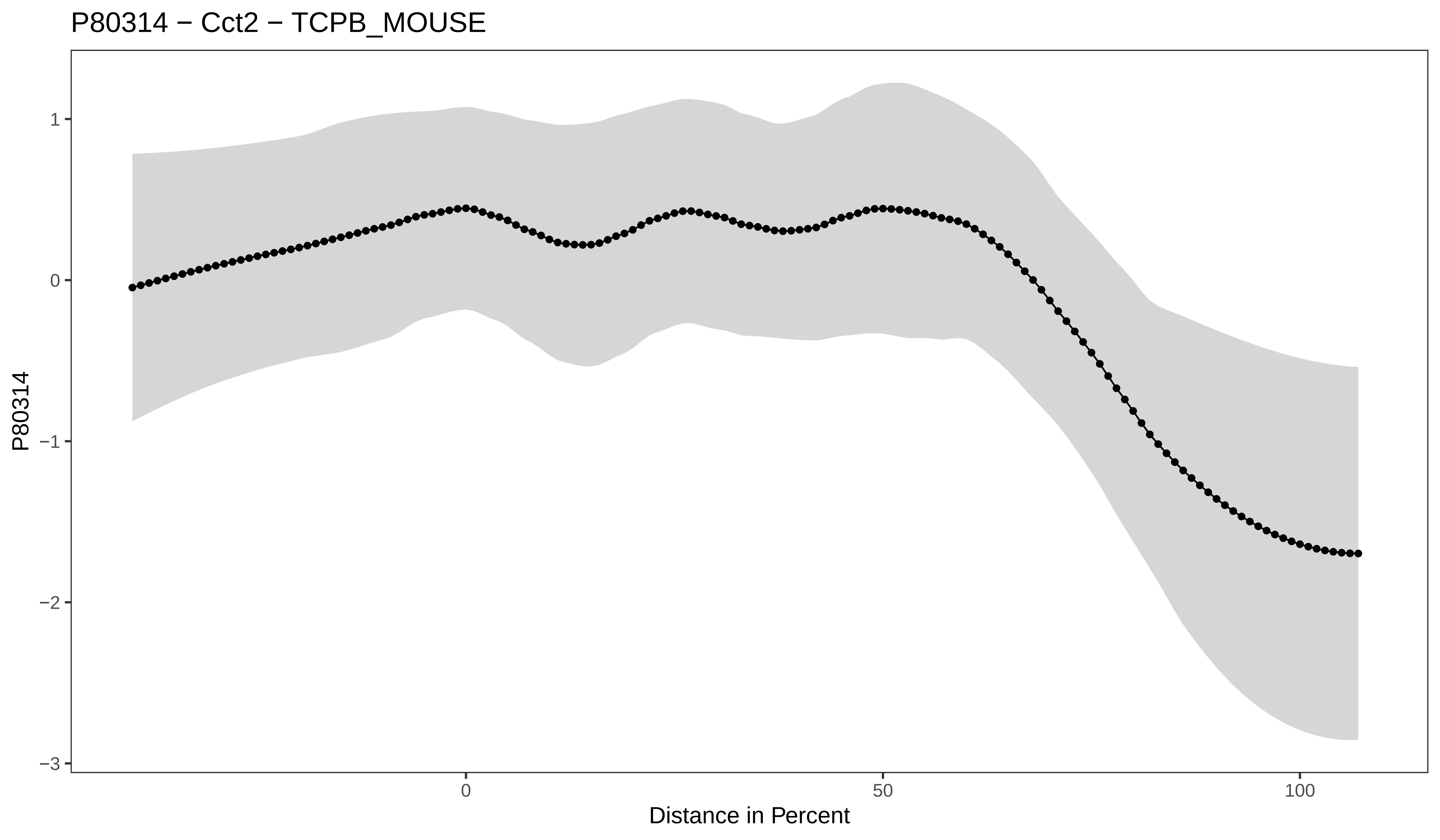
P80314 − Cct2 − TCPB_MOUSE
P80314
1
0
−1
−2
−3
0
50
100
Distance in Percent

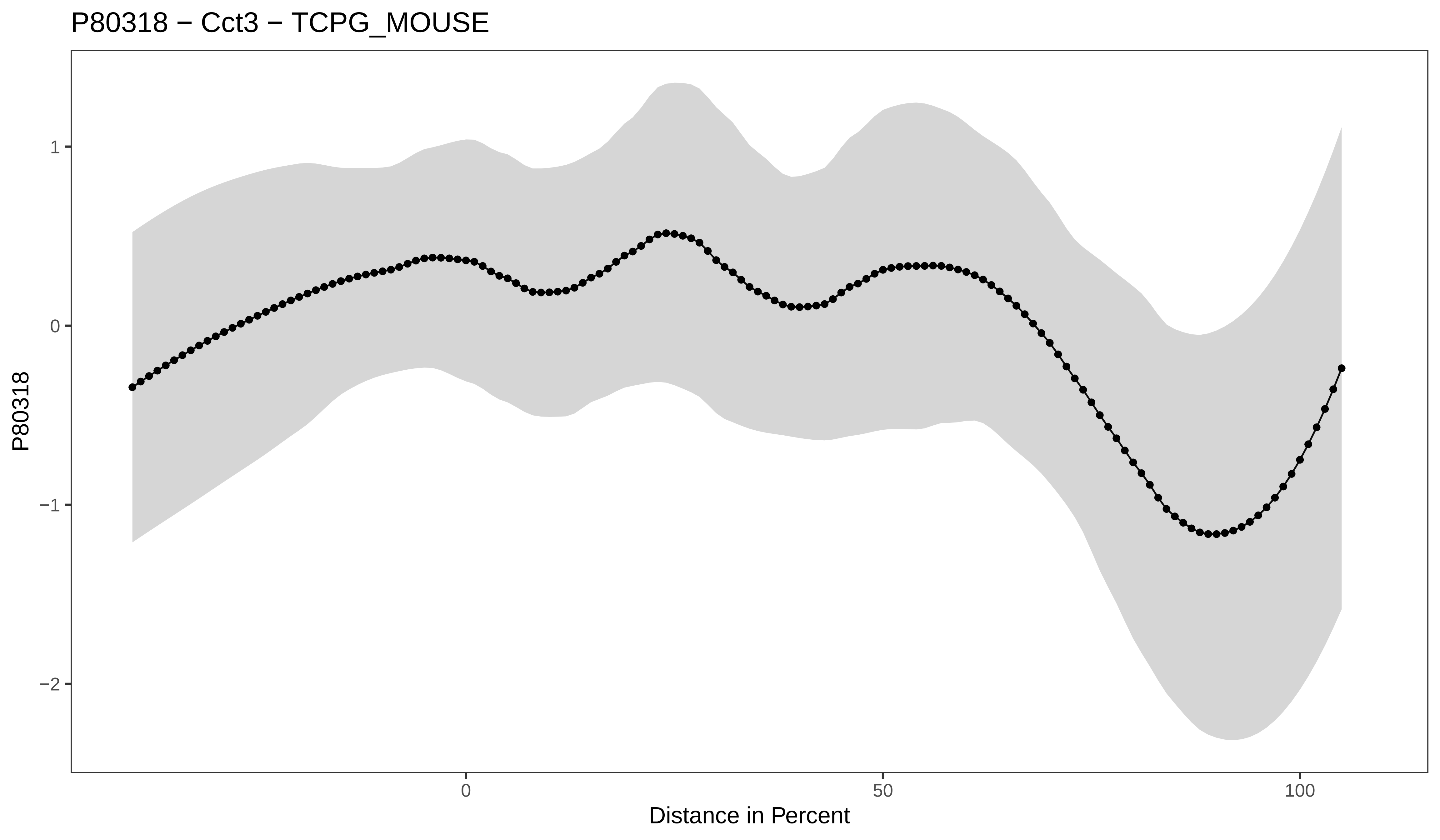

P80318 − Cct3 − TCPG_MOUSE
1
0
−1
−2
P80318
0
50
100
Distance in Percent

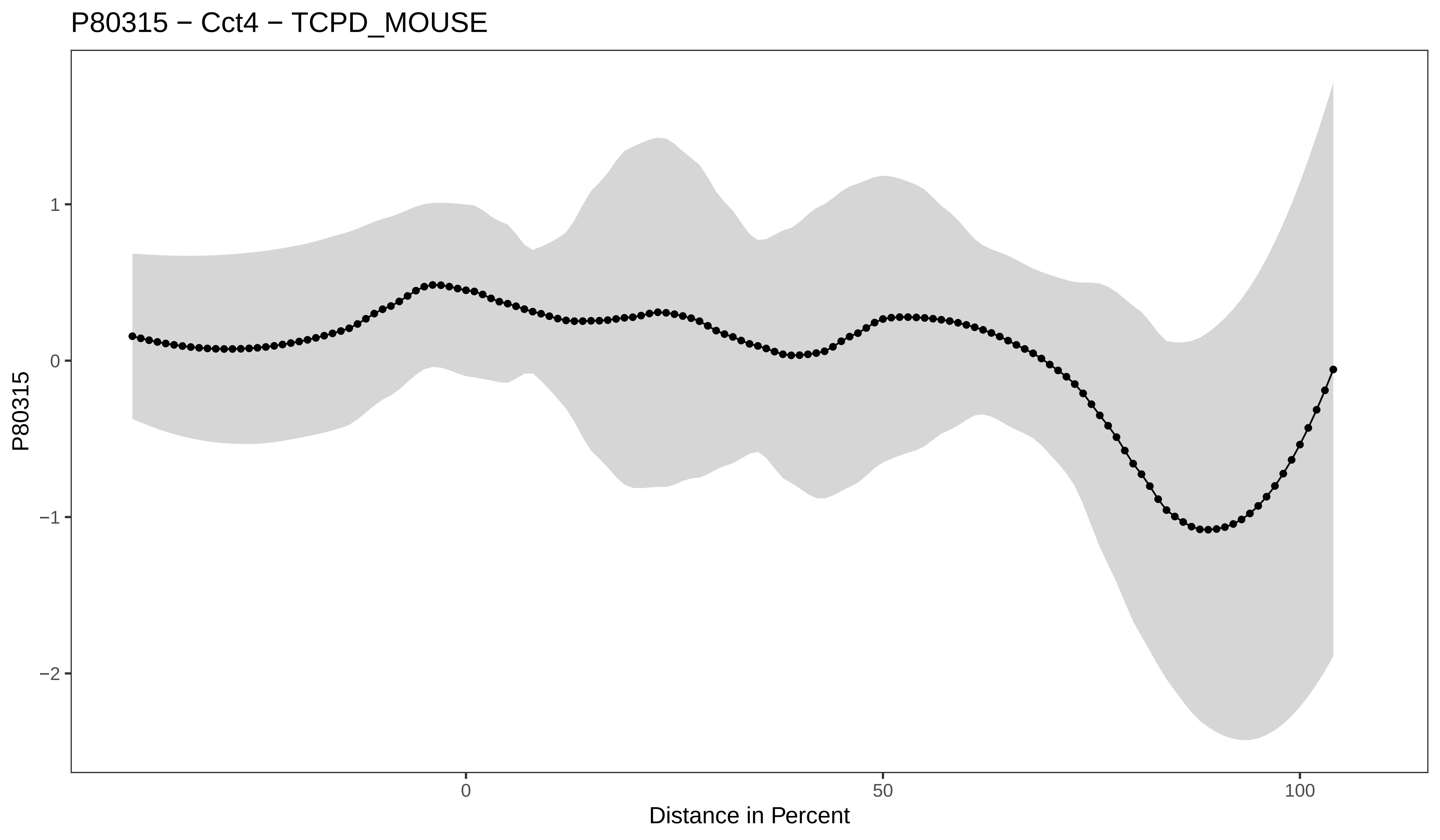

P80315 − Cct4 − TCPD_MOUSE
P80315
1
0
−1
−2
0
50
100
Distance in Percent

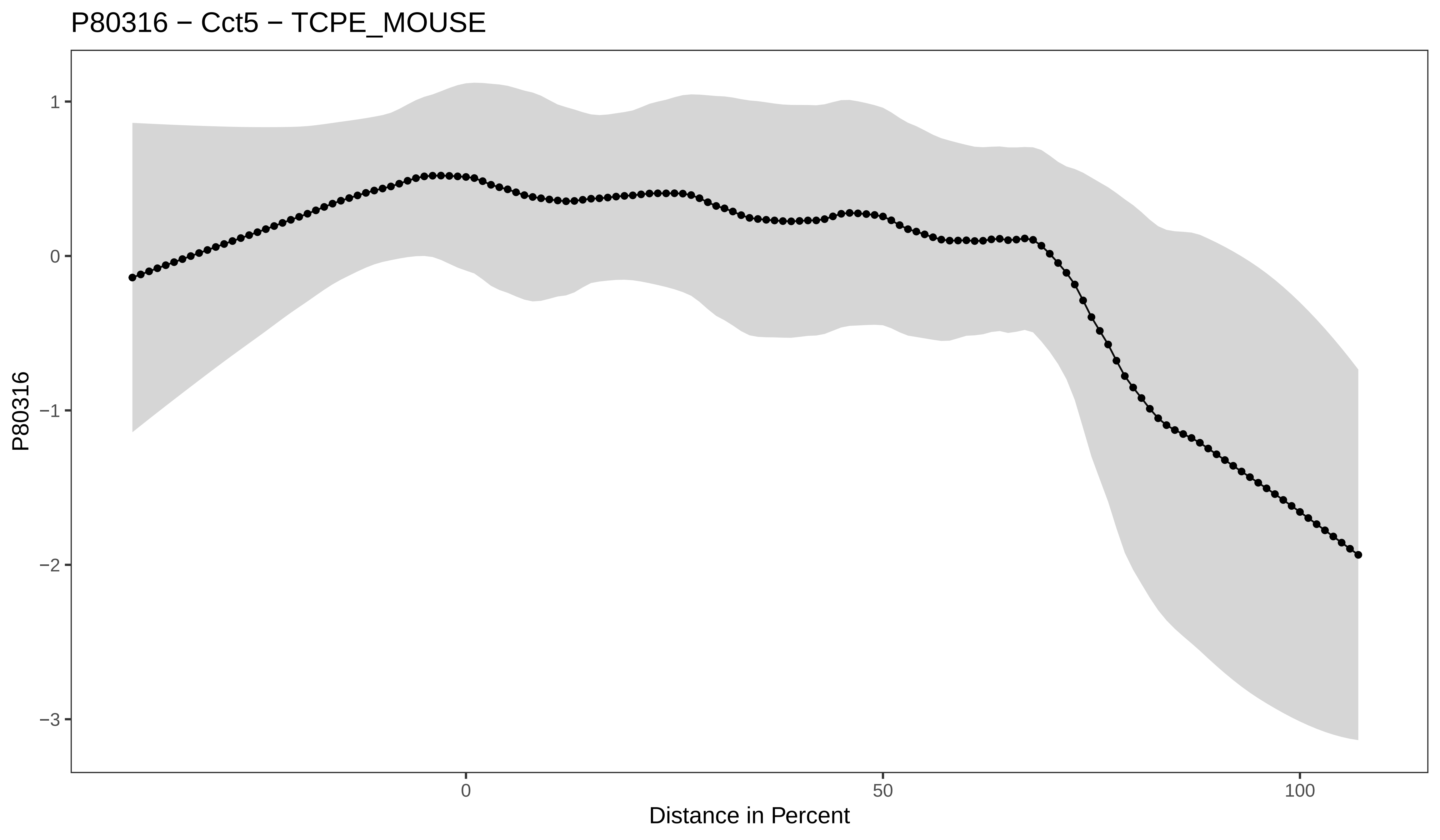

P80316 − Cct5 − TCPE_MOUSE
P80316
1
0
−1
−2
−3
0
50
100
Distance in Percent

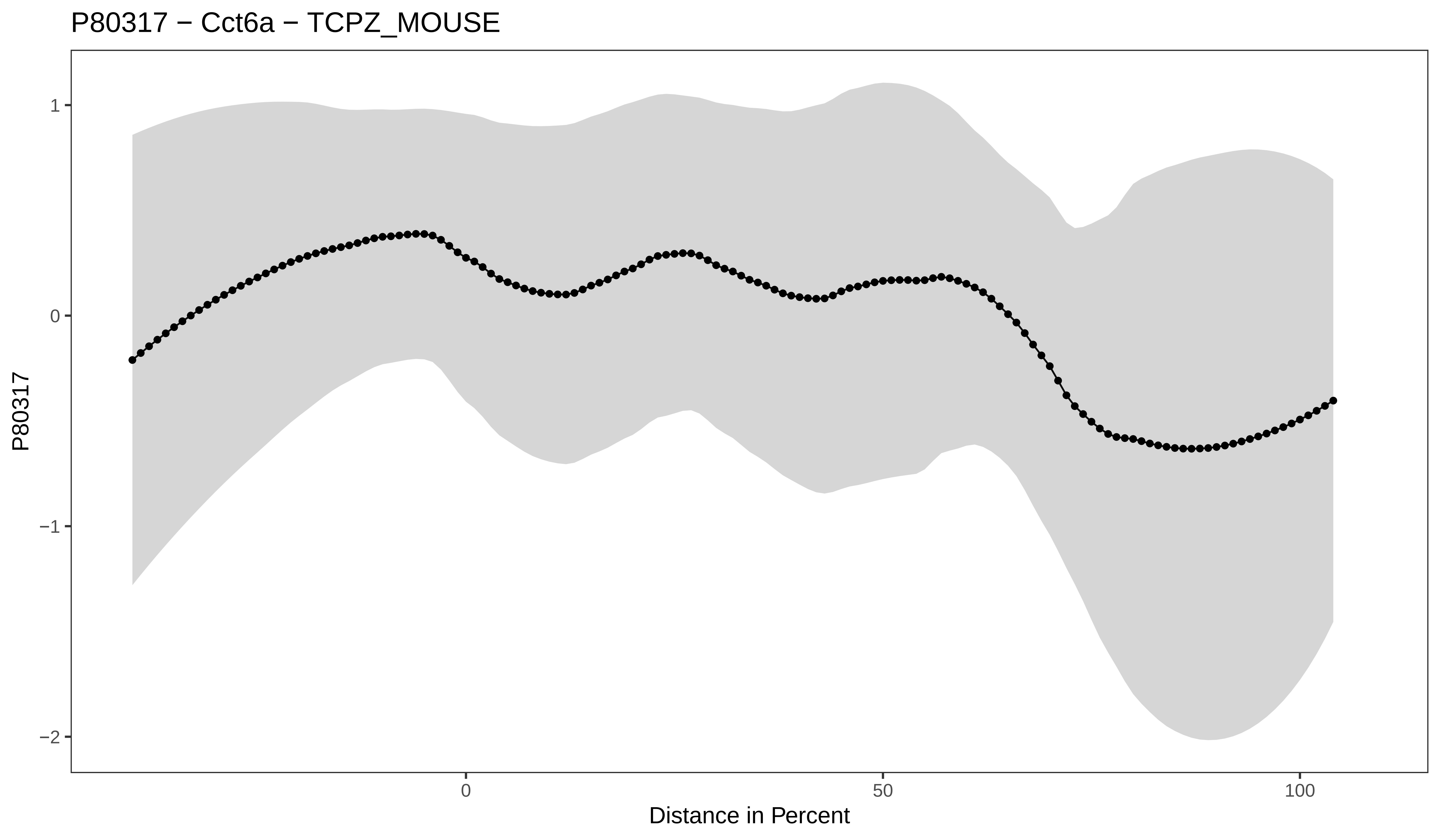
P80317 − Cct6a − TCPZ_MOUSE
1
0
−1
−2
P80317
0
50
100
Distance in Percent

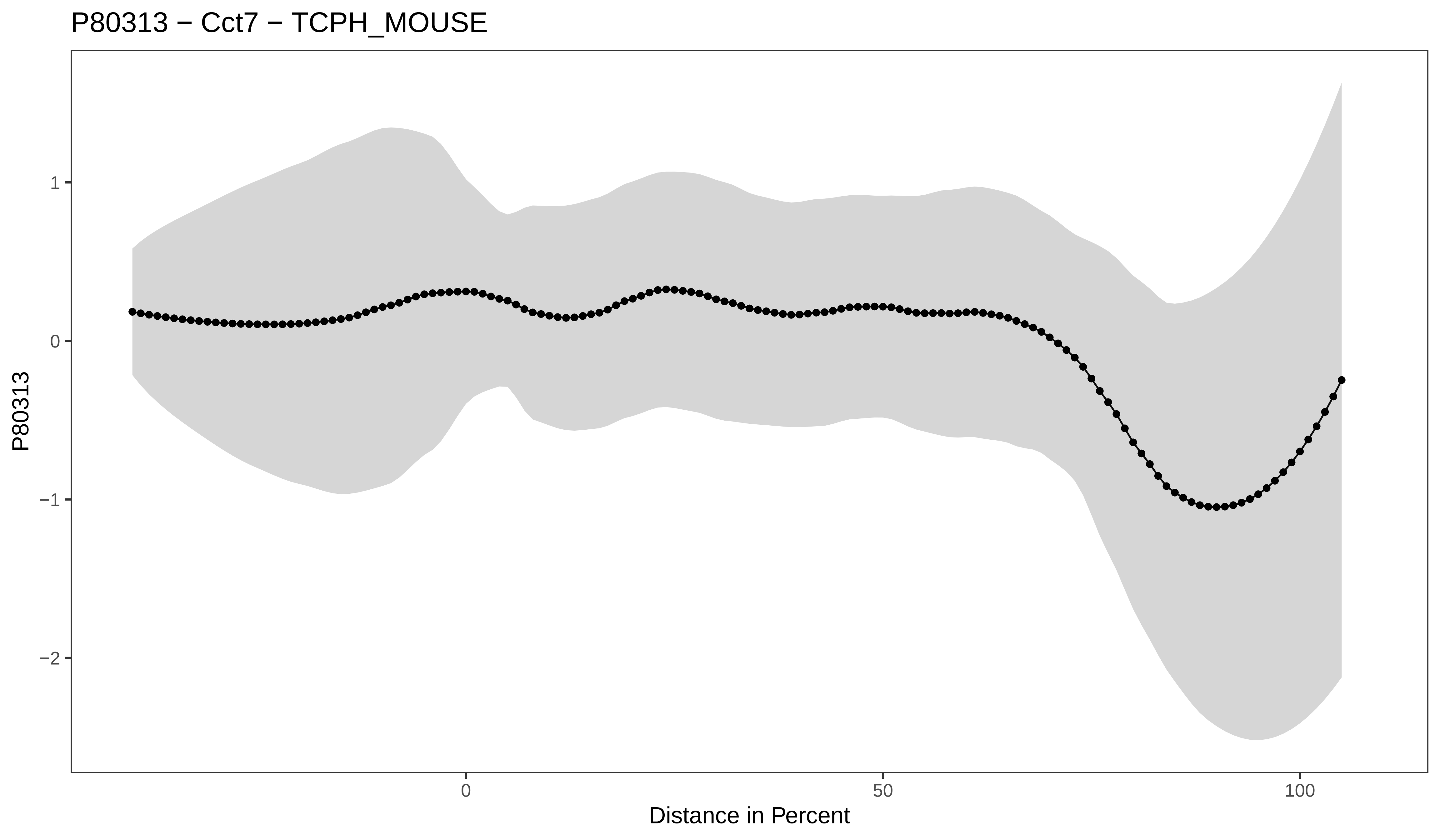

P80313 − Cct7 − TCPH_MOUSE
1
0
−1
−2
P80313
0
50
100
Distance in Percent

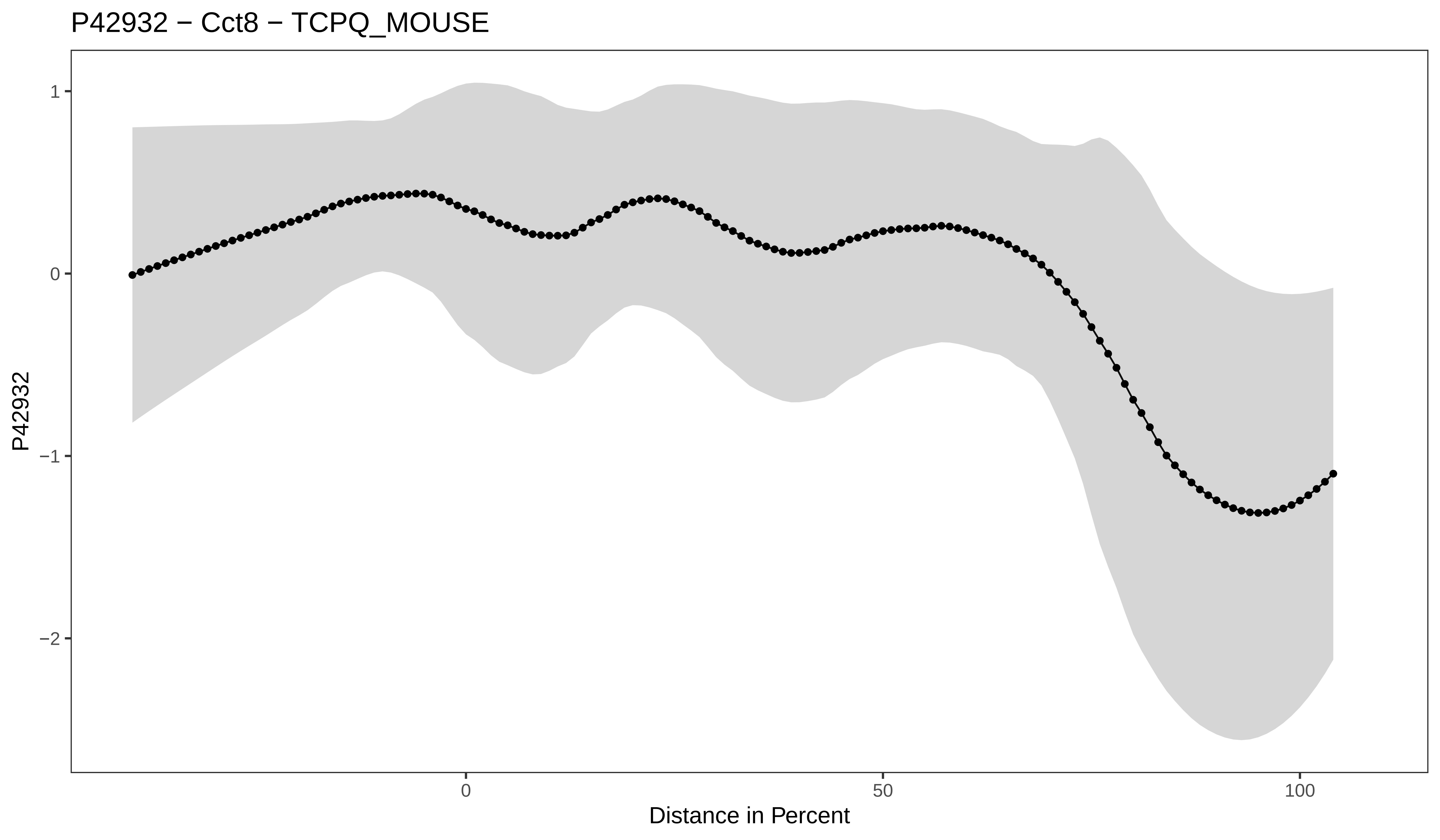

P42932 − Cct8 − TCPQ_MOUSE
1
0
−1
−2
P42932
0
50
100
Distance in Percent

Q8R422 − Cd109 − CD109_MOUSE

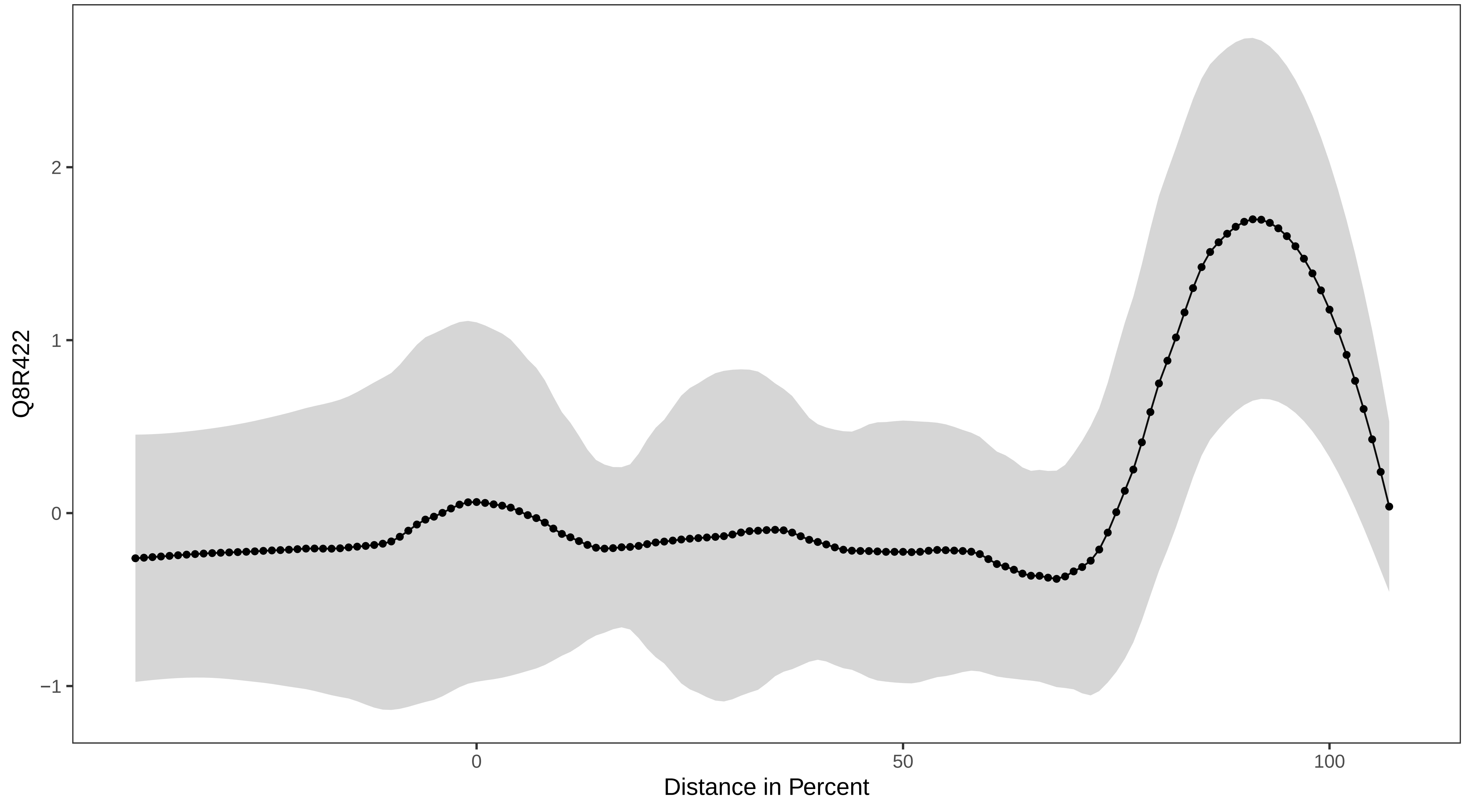

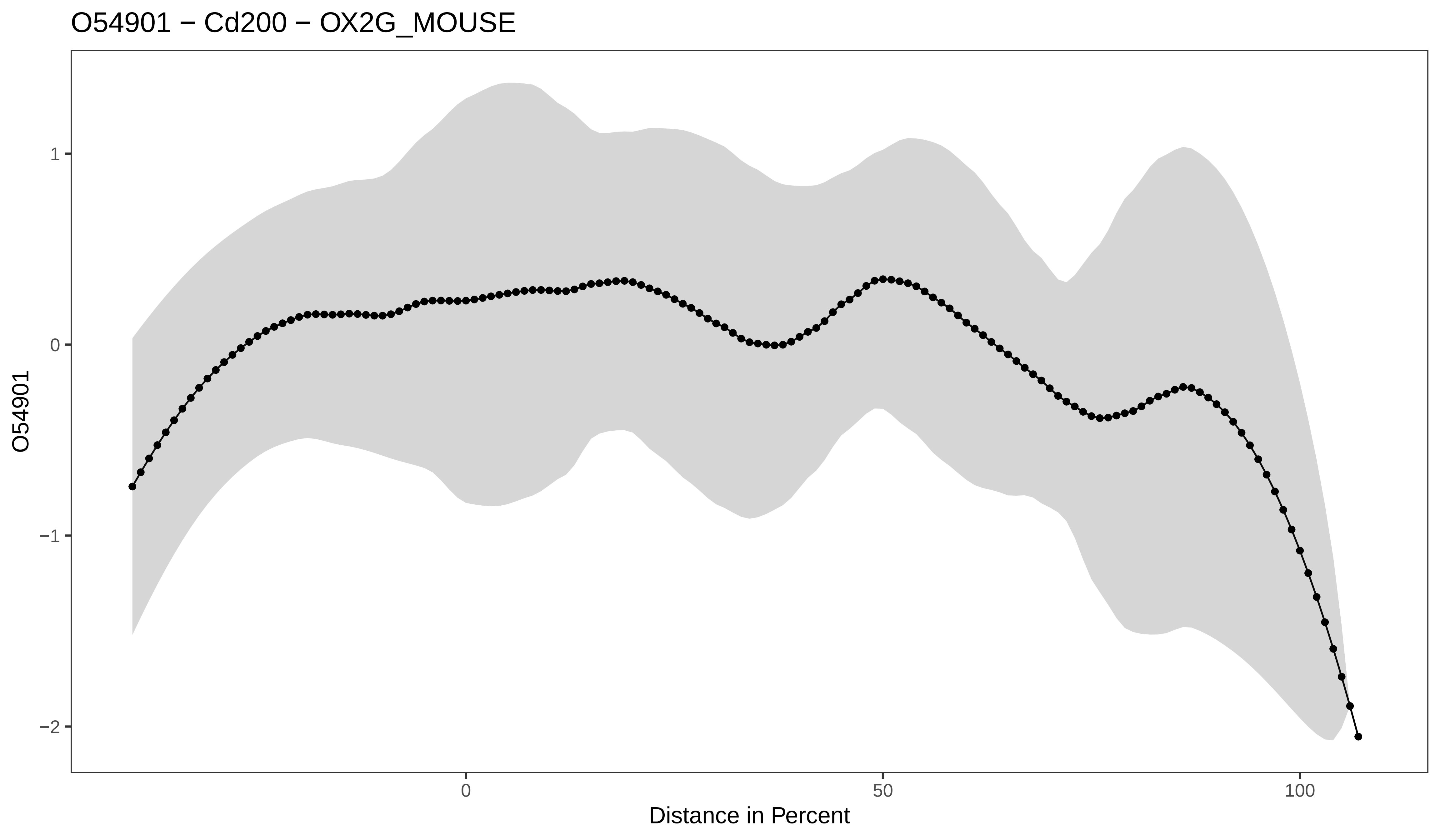

O54901 − Cd200 − OX2G_MOUSE
1
0
−1
−2
O54901
0
50
100
Distance in Percent

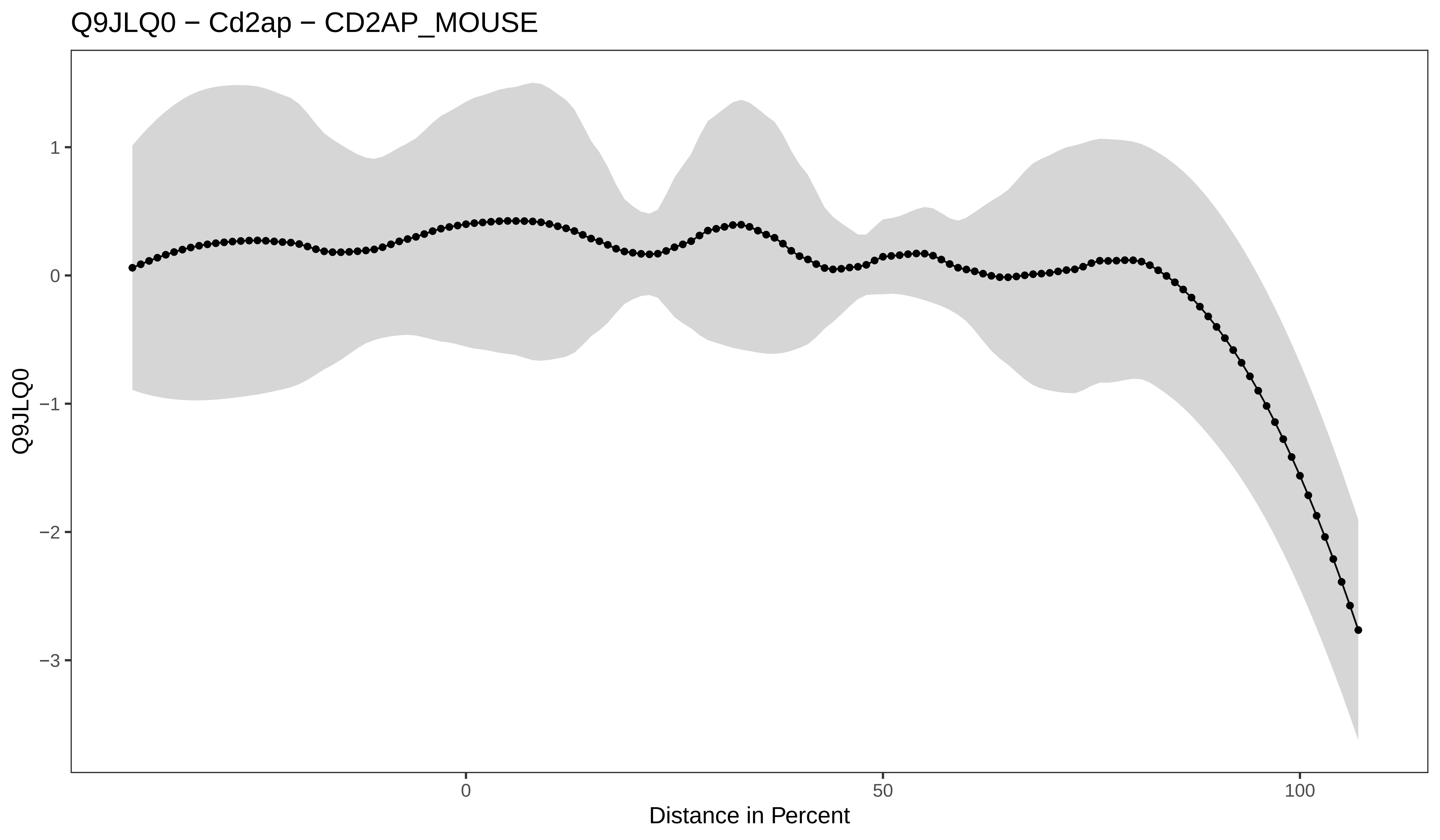
Q9JLQ0 − Cd2ap − CD2AP_MOUSE
Q9JLQ0
Distance in Percent
1
0
−1
−2
−3
0
50
100

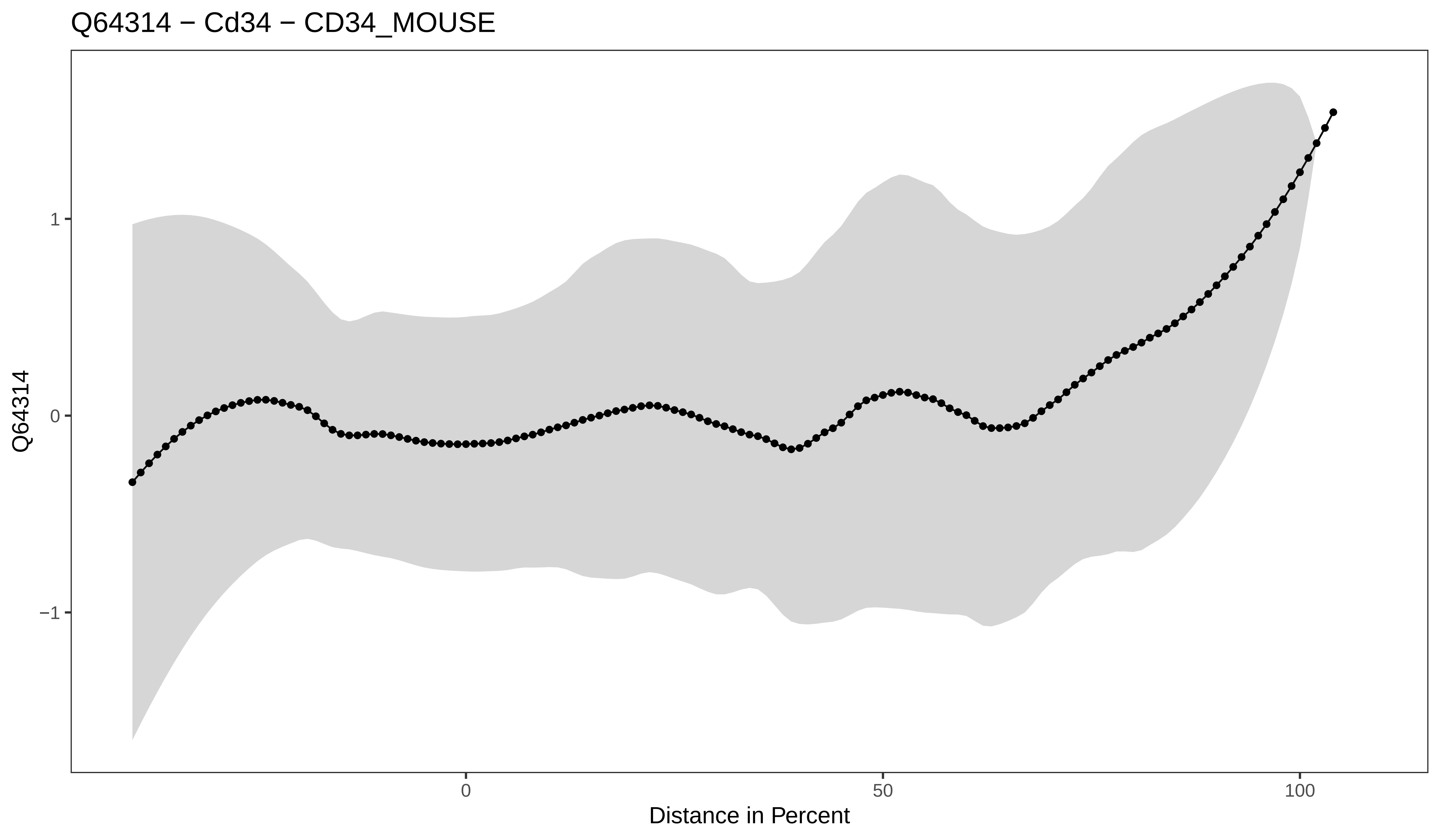
Q64314 − Cd34 − CD34_MOUSE
Q64314
1
0
−1
0
50
100
Distance in Percent

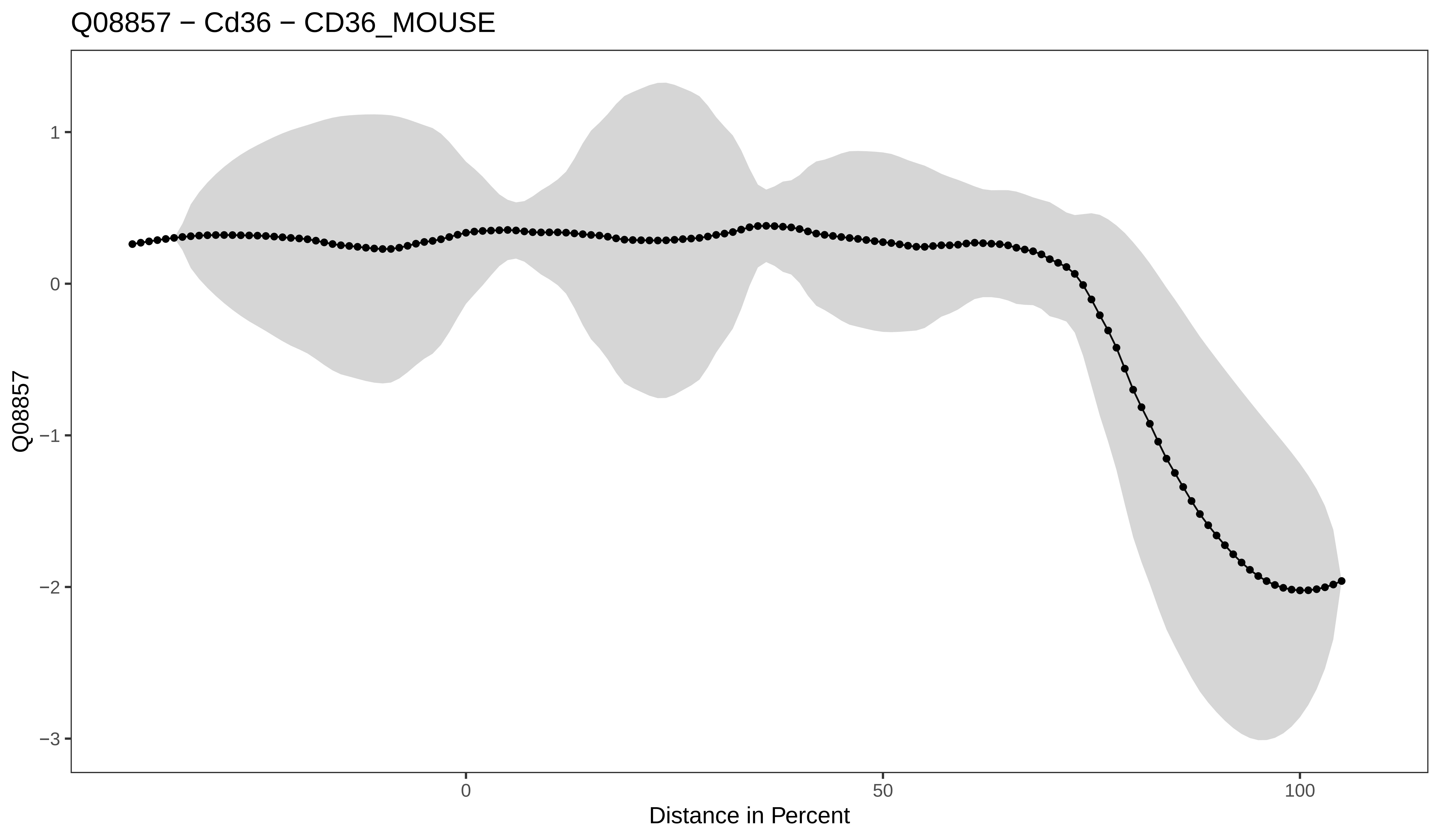

Q08857 − Cd36 − CD36_MOUSE
1
0
−1
−2
−3
Q08857
0
50
100
Distance in Percent

P15379 − Cd44 − CD44_MOUSE

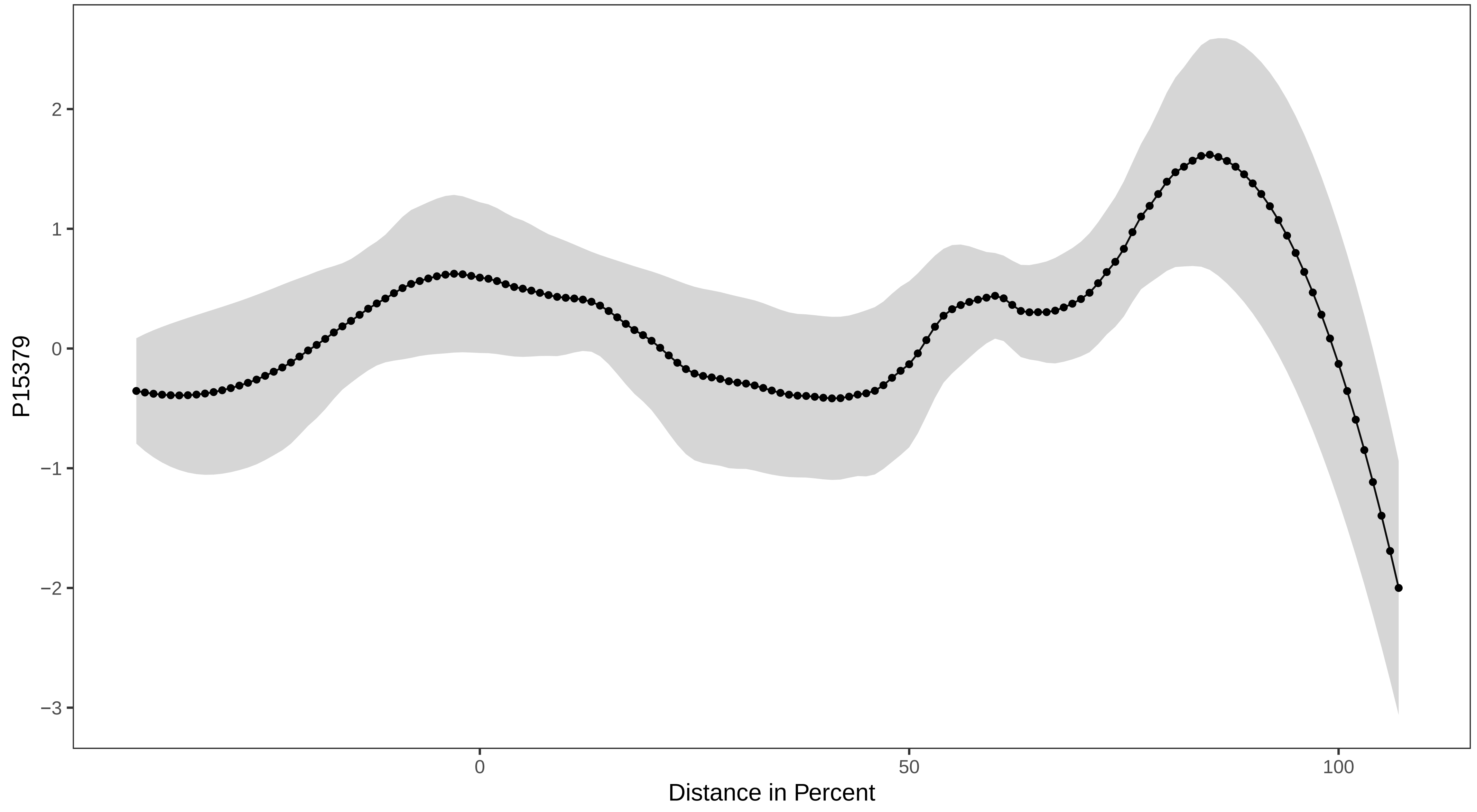

Q61735 − Cd47 − CD47_MOUSE

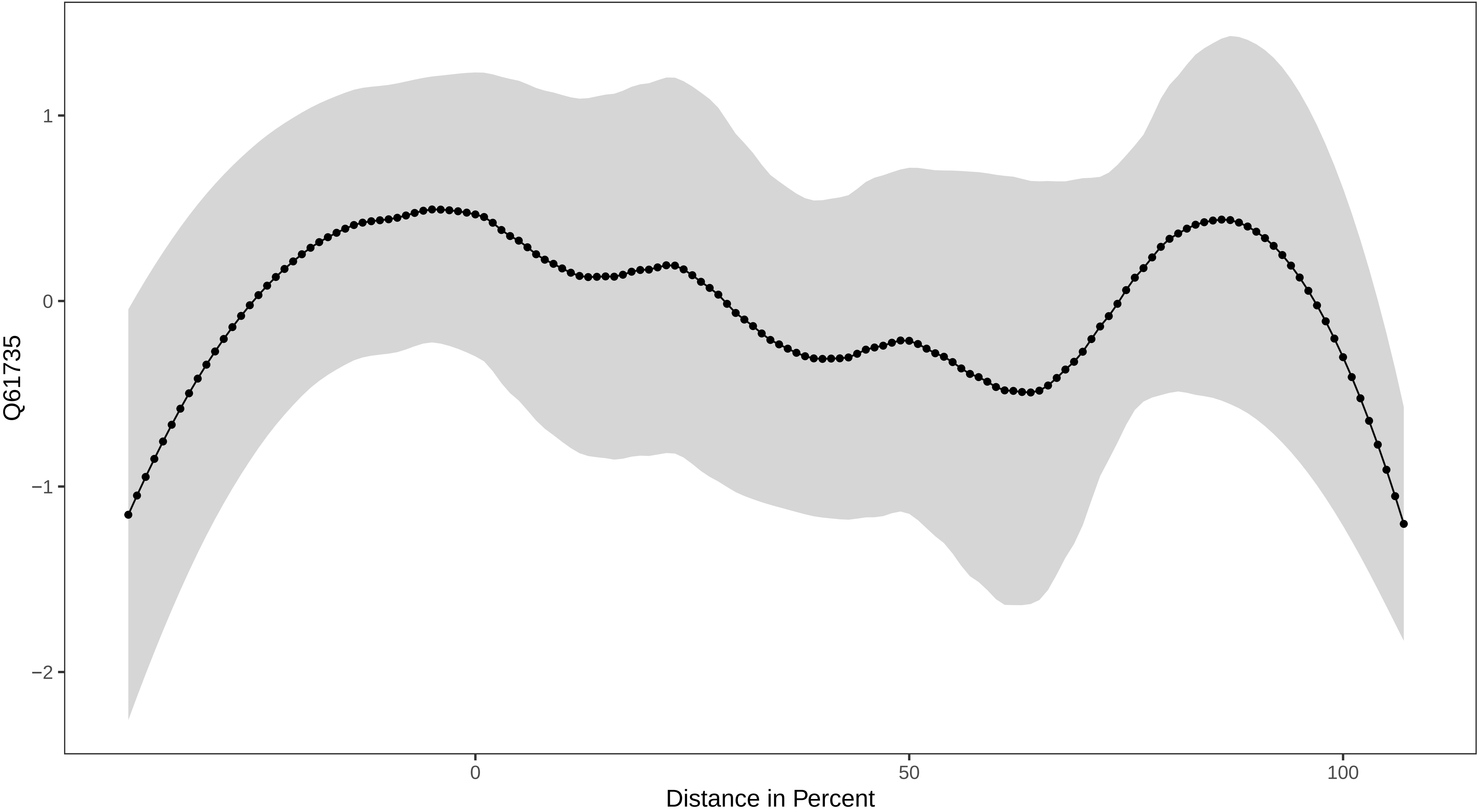

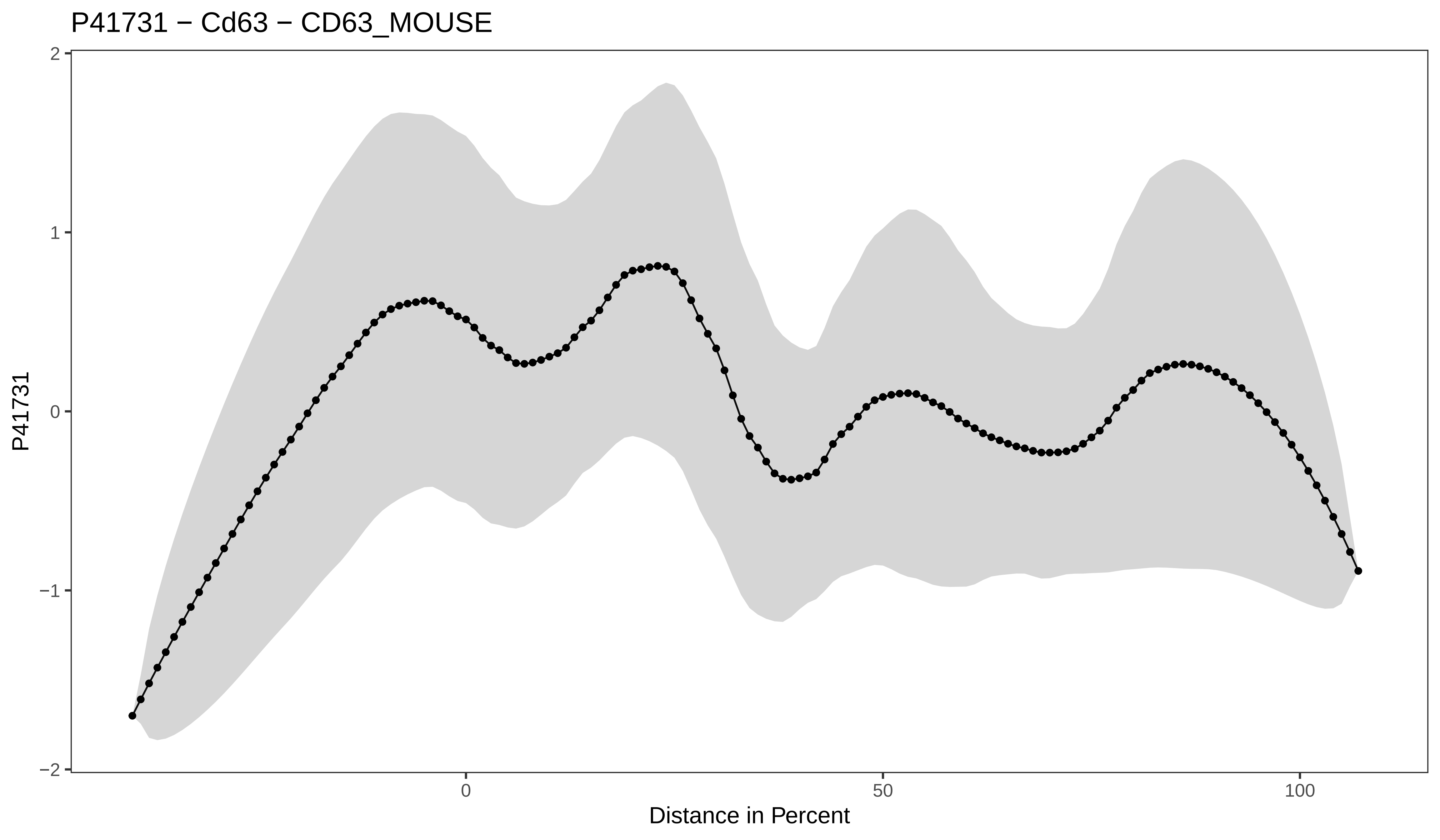

P41731 − Cd63 − CD63_MOUSE
2
1
0
−1
−2
P41731
0
50
100
Distance in Percent

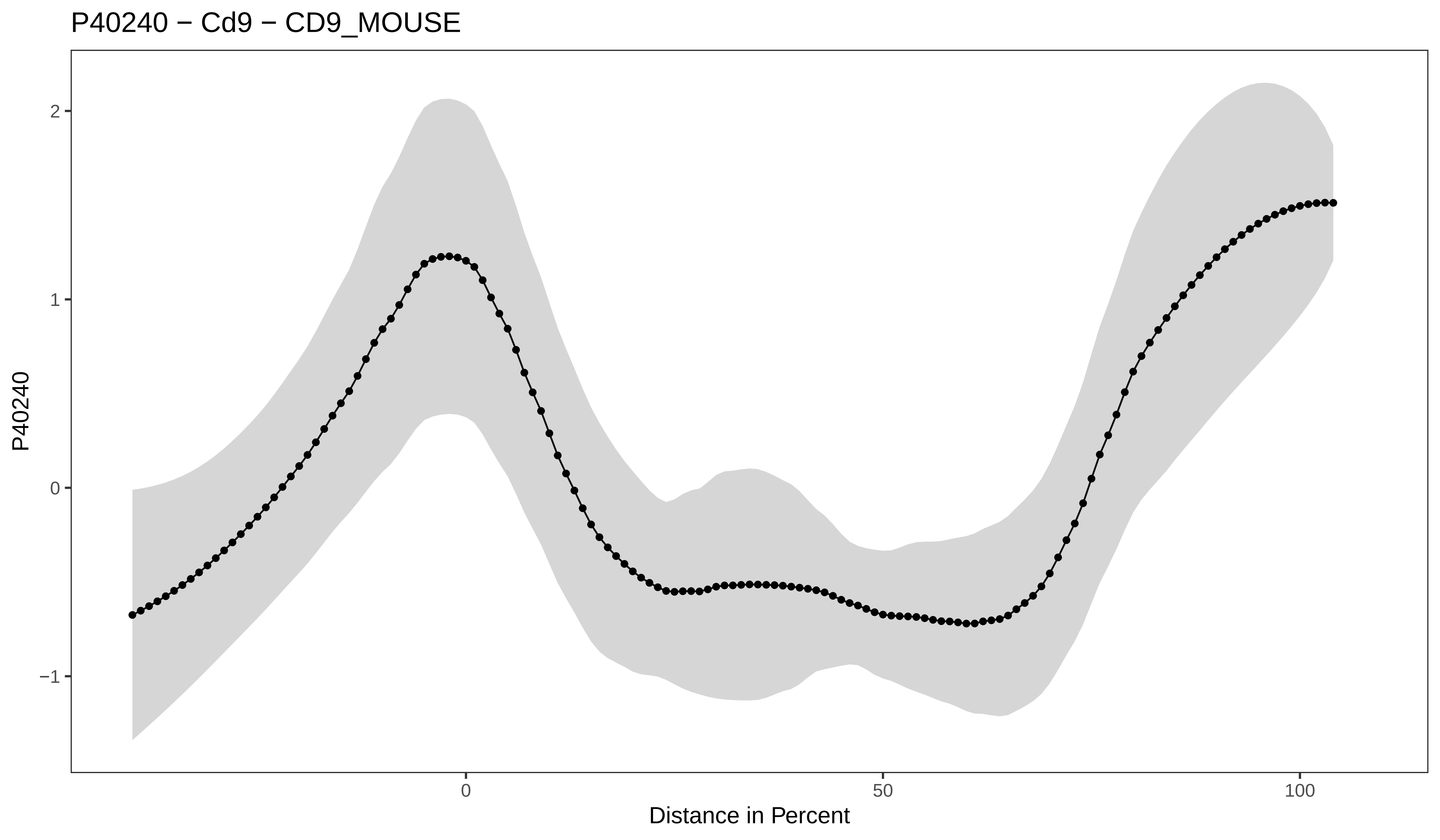
P40240 − Cd9 − CD9_MOUSE
P40240
2
1
0
−1
0
50
100
Distance in Percent

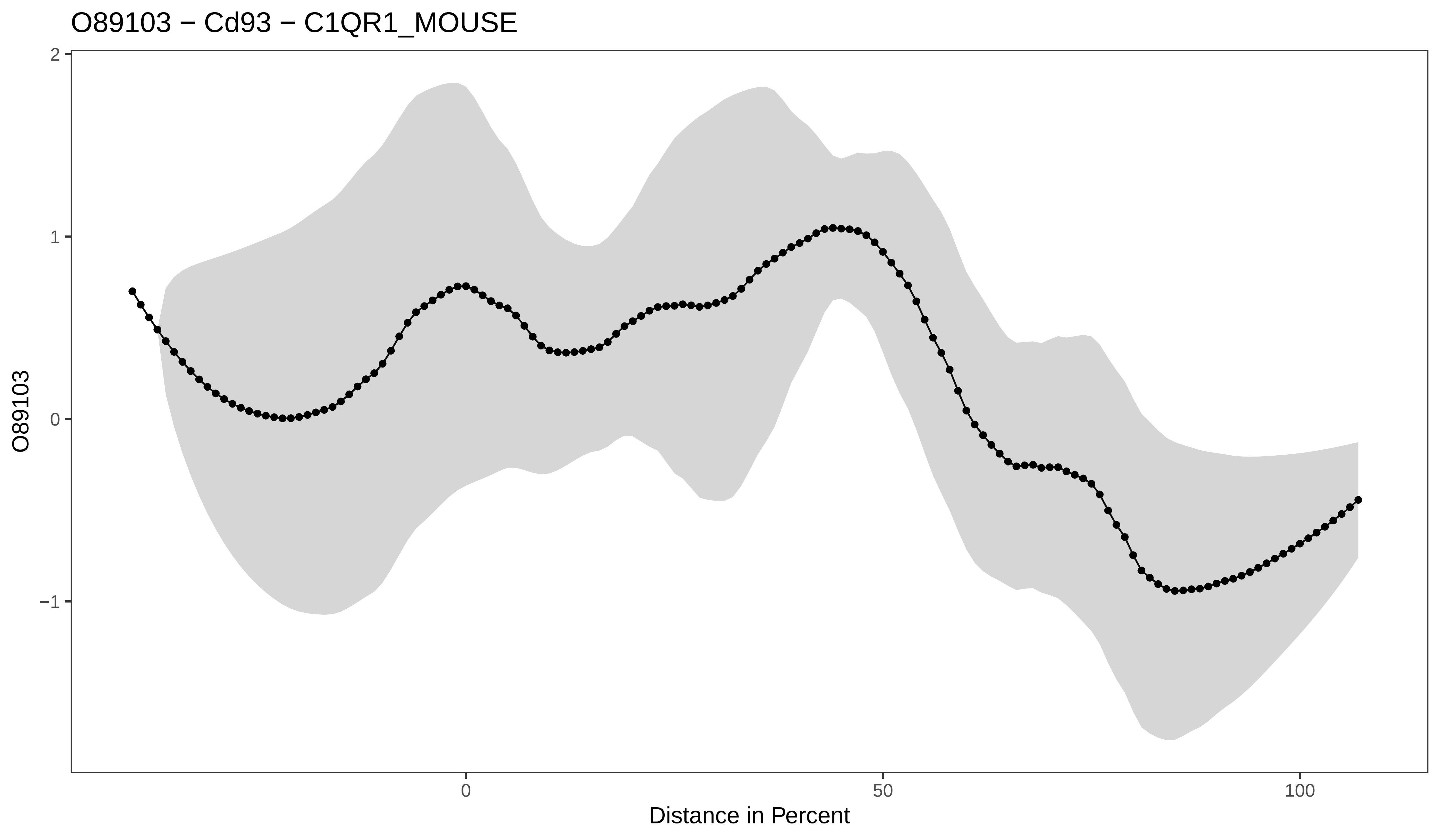

O89103 − Cd93 − C1QR1_MOUSE
2
1
0
−1
O89103
0
50
100
Distance in Percent

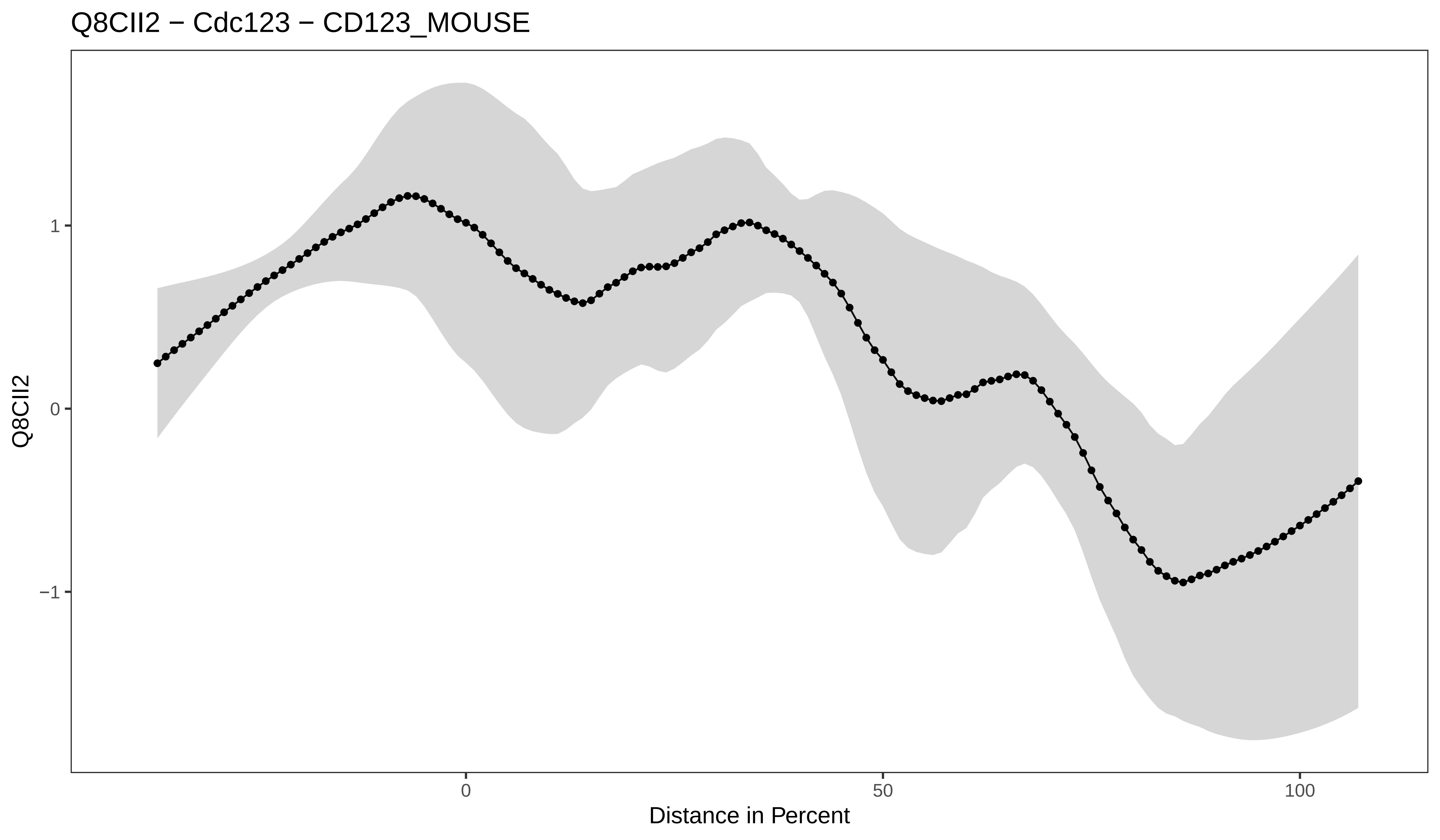
Q8CII2 − Cdc123 − CD123_MOUSE
Q8CII2
1
0
−1
0
50
100
Distance in Percent

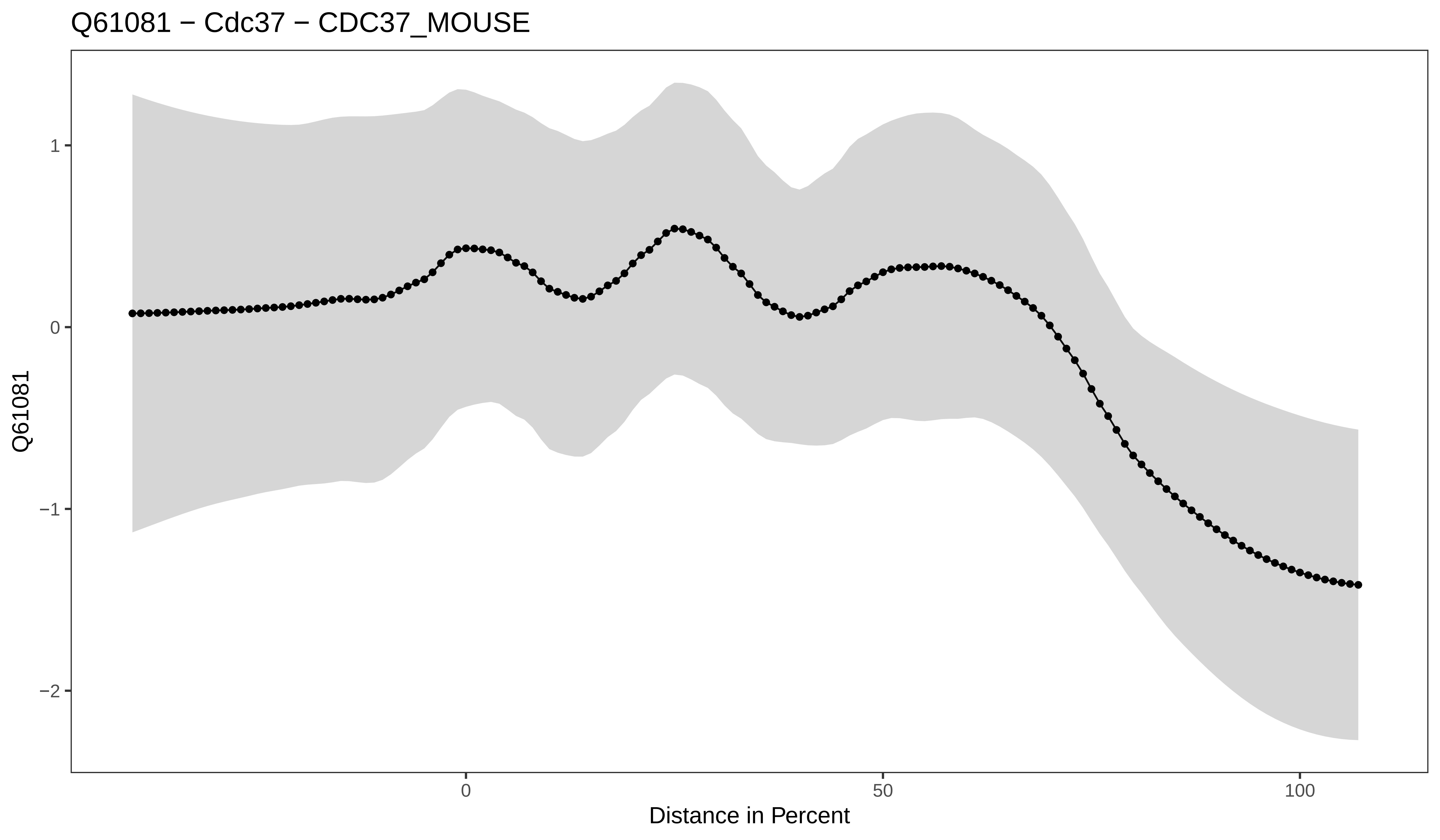

Q61081 − Cdc37 − CDC37_MOUSE
1
0
−1
−2
Q61081
0
50
100
Distance in Percent

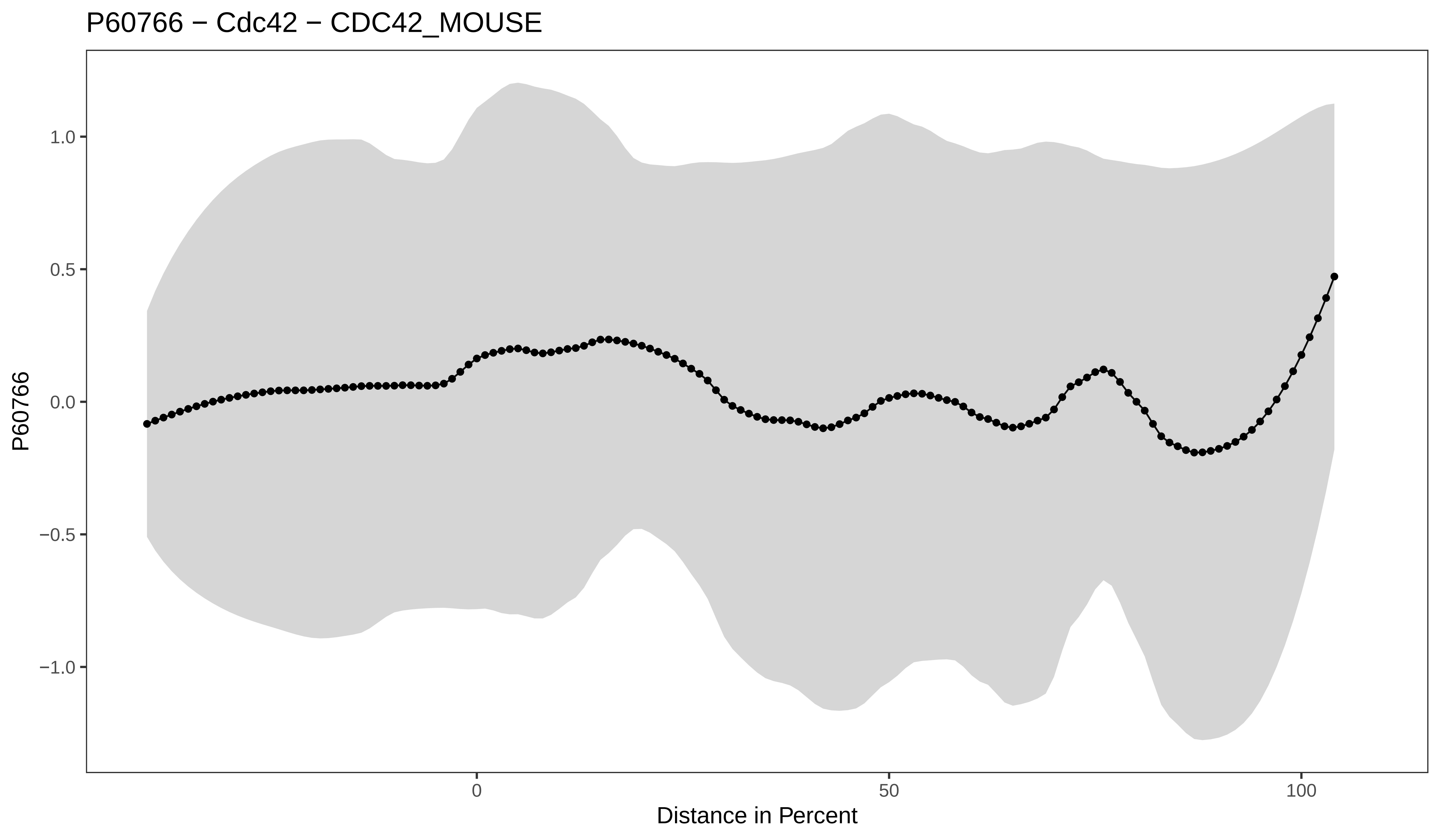
P60766 − Cdc42 − CDC42_MOUSE
P60766
1.0
0.5
0.0
−0.5
−1.0
0
50
100
Distance in Percent

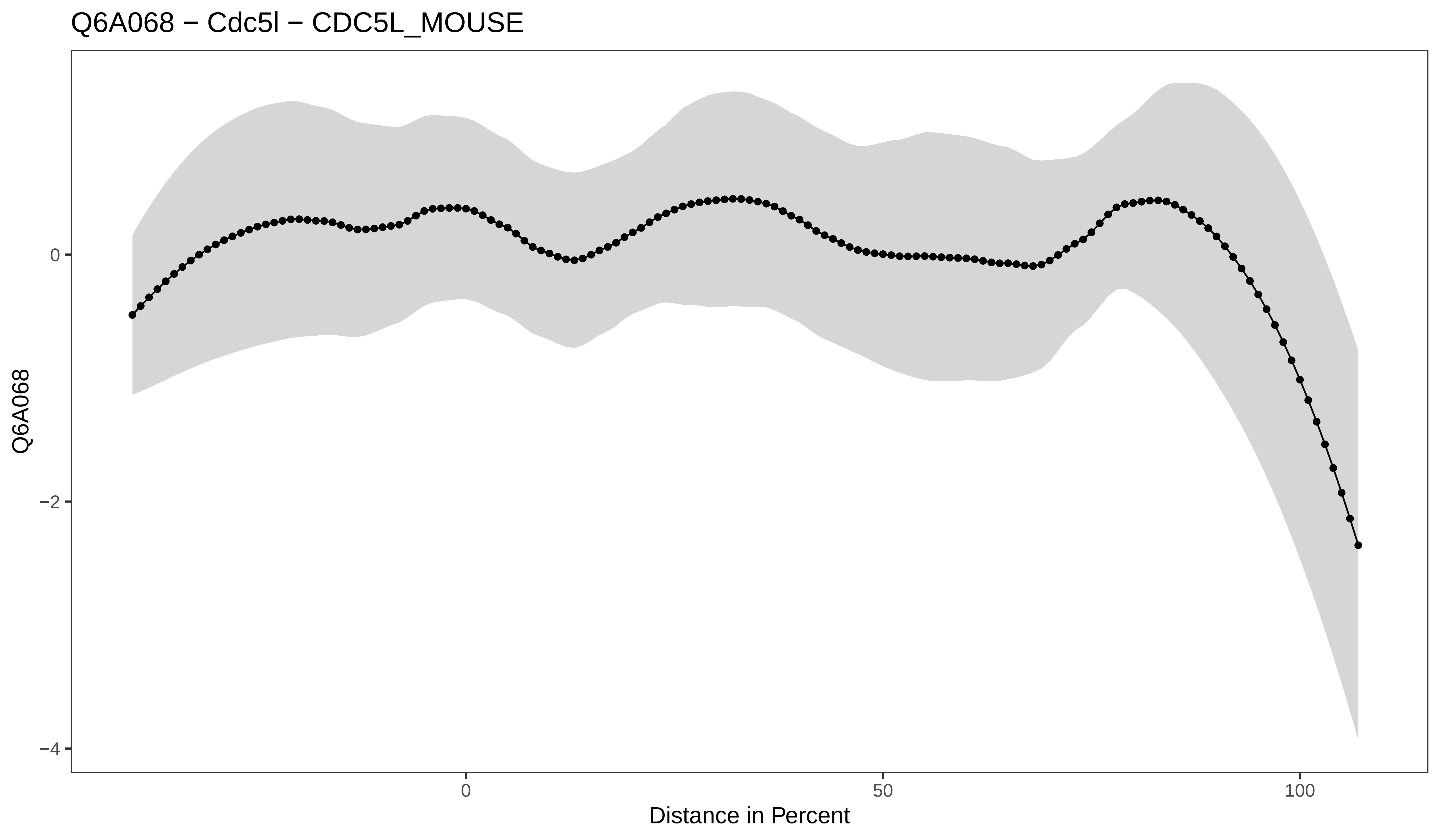

Q6A068 − Cdc5l − CDC5L_MOUSE
Q6A068
0
−2
−4
0
50
100
Distance in Percent

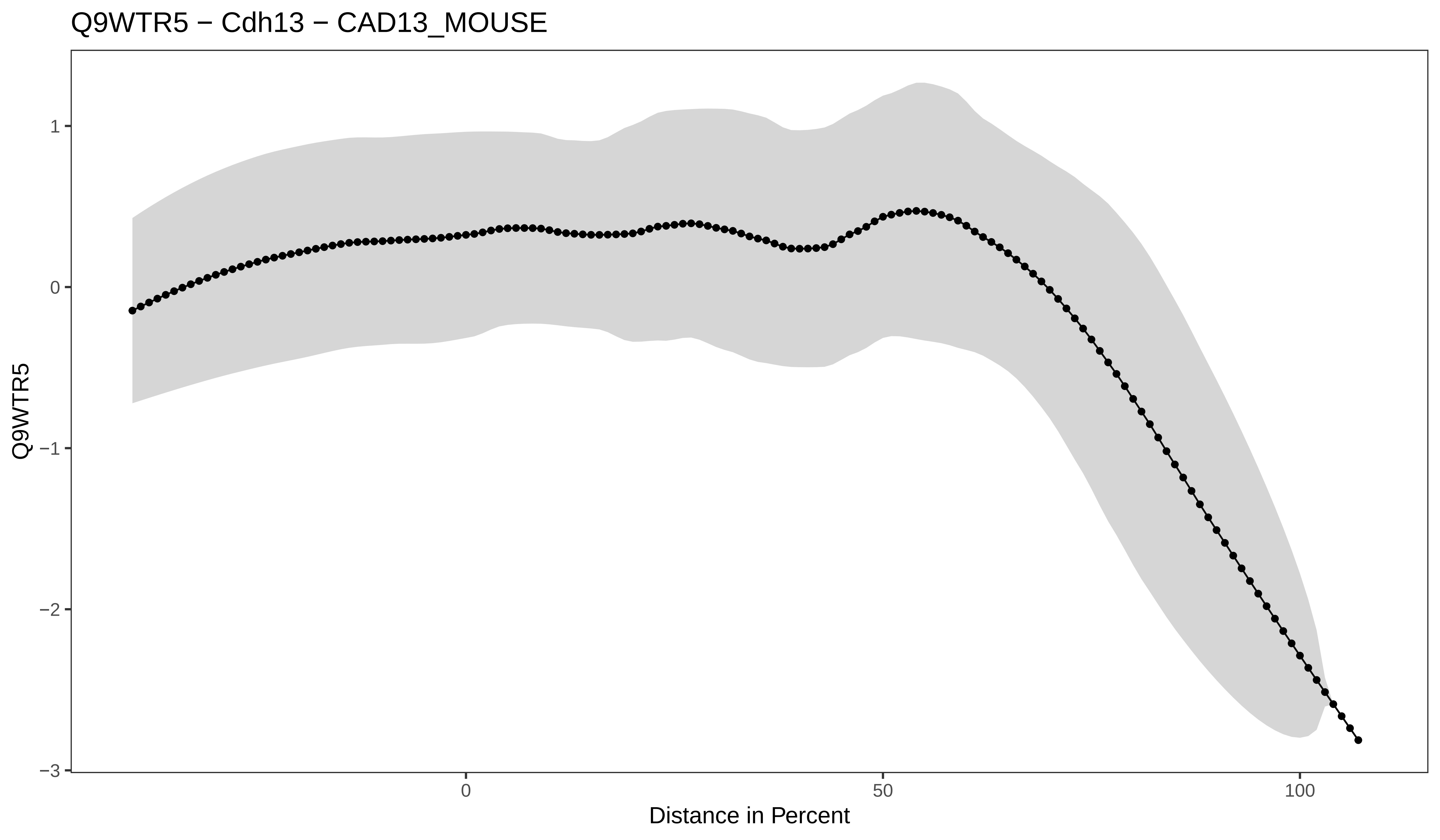

Q9WTR5 − Cdh13 − CAD13_MOUSE
Q9WTR5
1
0
−1
−2
−3
0
50
100
Distance in Percent

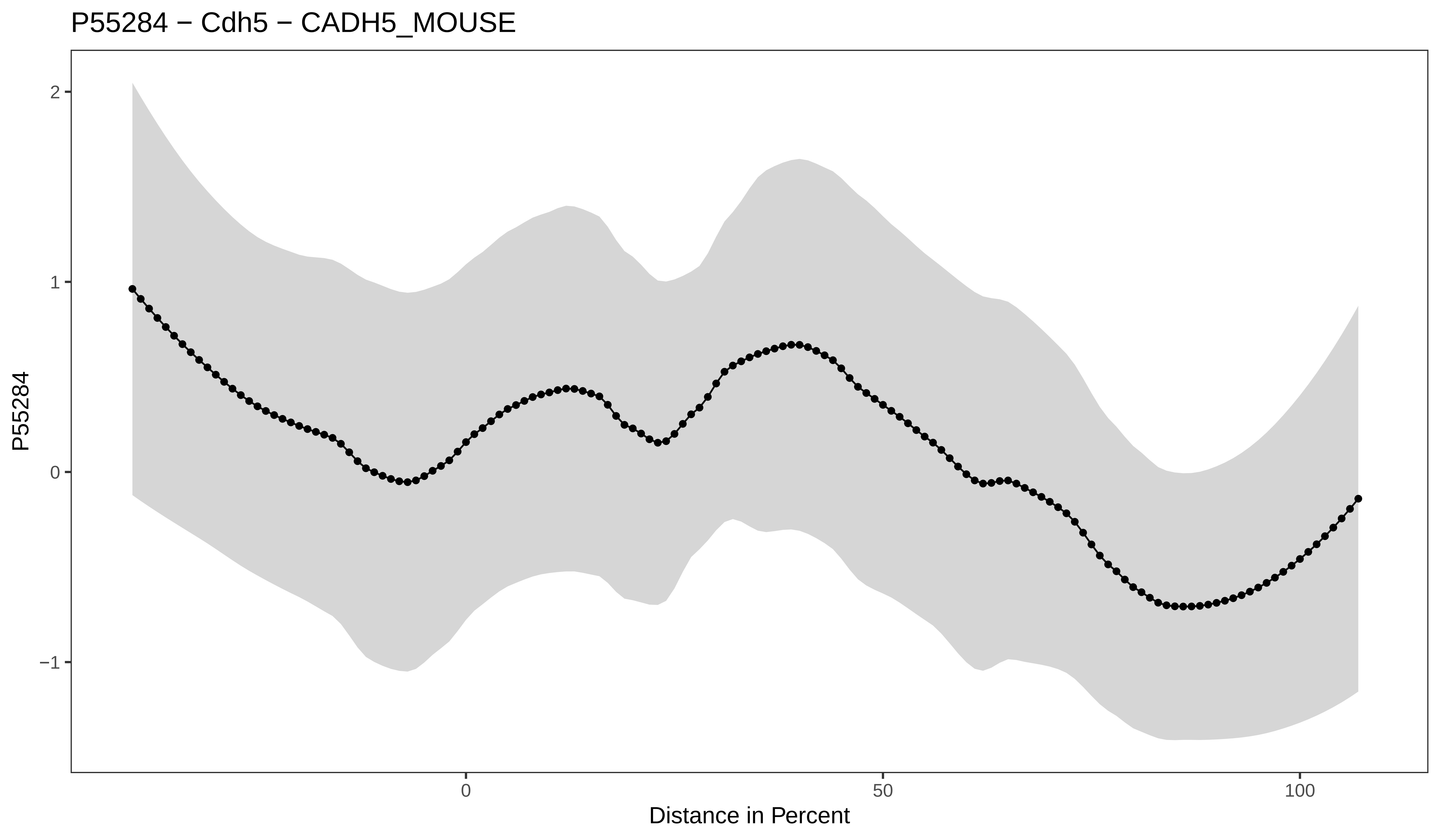

P55284 − Cdh5 − CADH5_MOUSE
P55284
2
1
0
−1
0
50
100
Distance in Percent

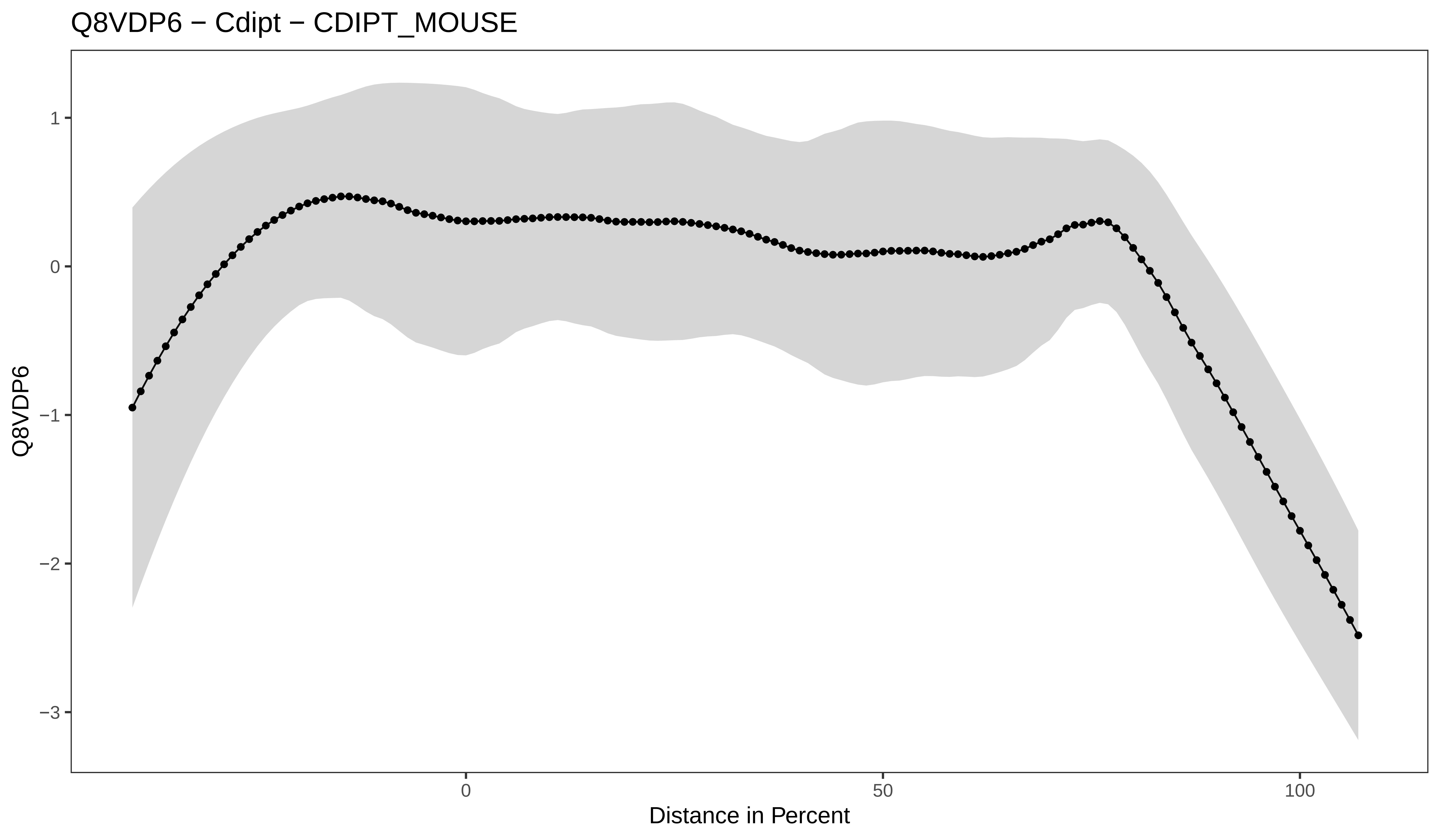

Q8VDP6 − Cdipt − CDIPT_MOUSE
1
0
−1
−2
−3
Q8VDP6
0
50
100
Distance in Percent

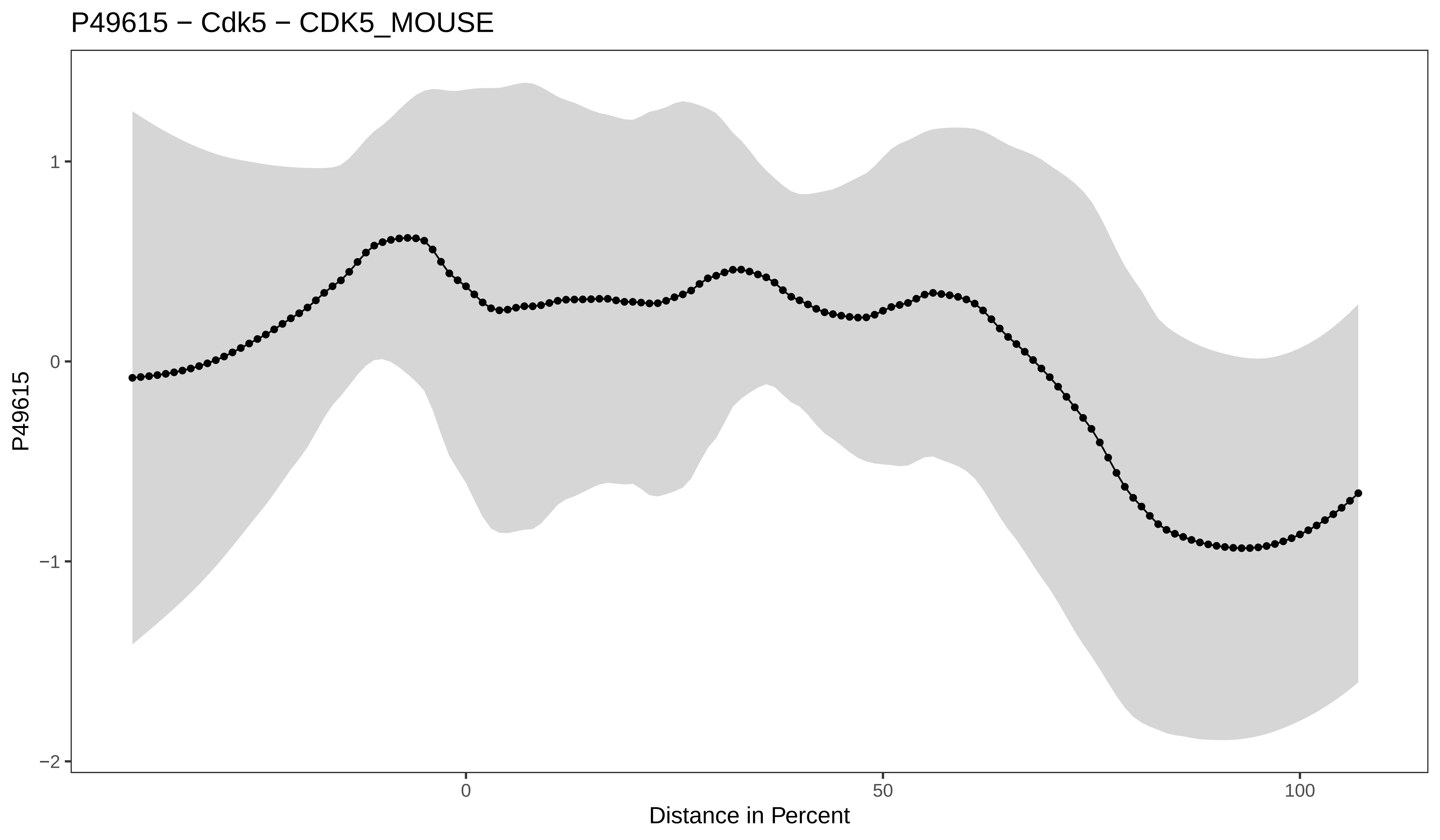

P49615 − Cdk5 − CDK5_MOUSE
P49615
1
0
−1
−2
0
50
100
Distance in Percent

Q4VAA2 − Cdv3 − CDV3_MOUSE

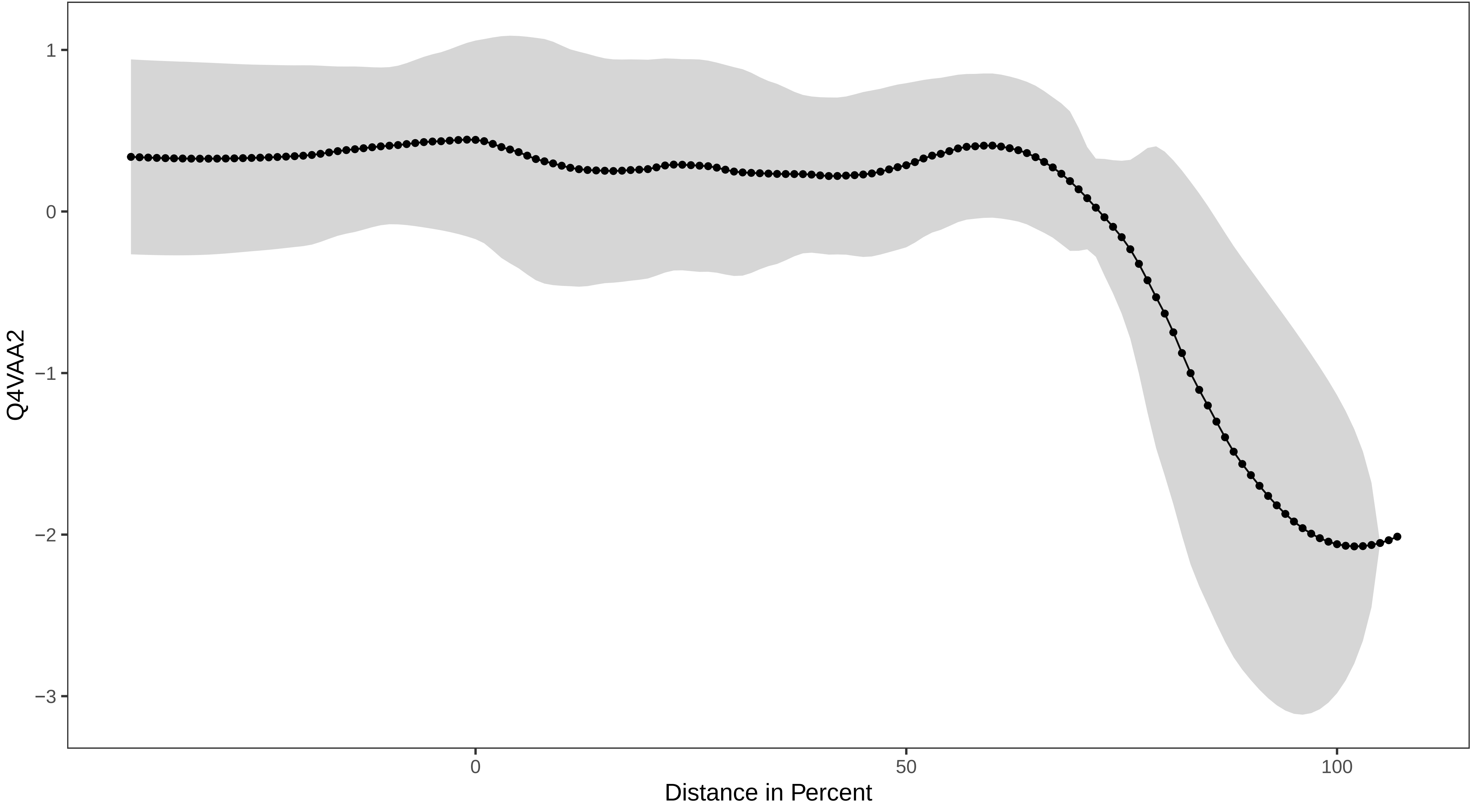

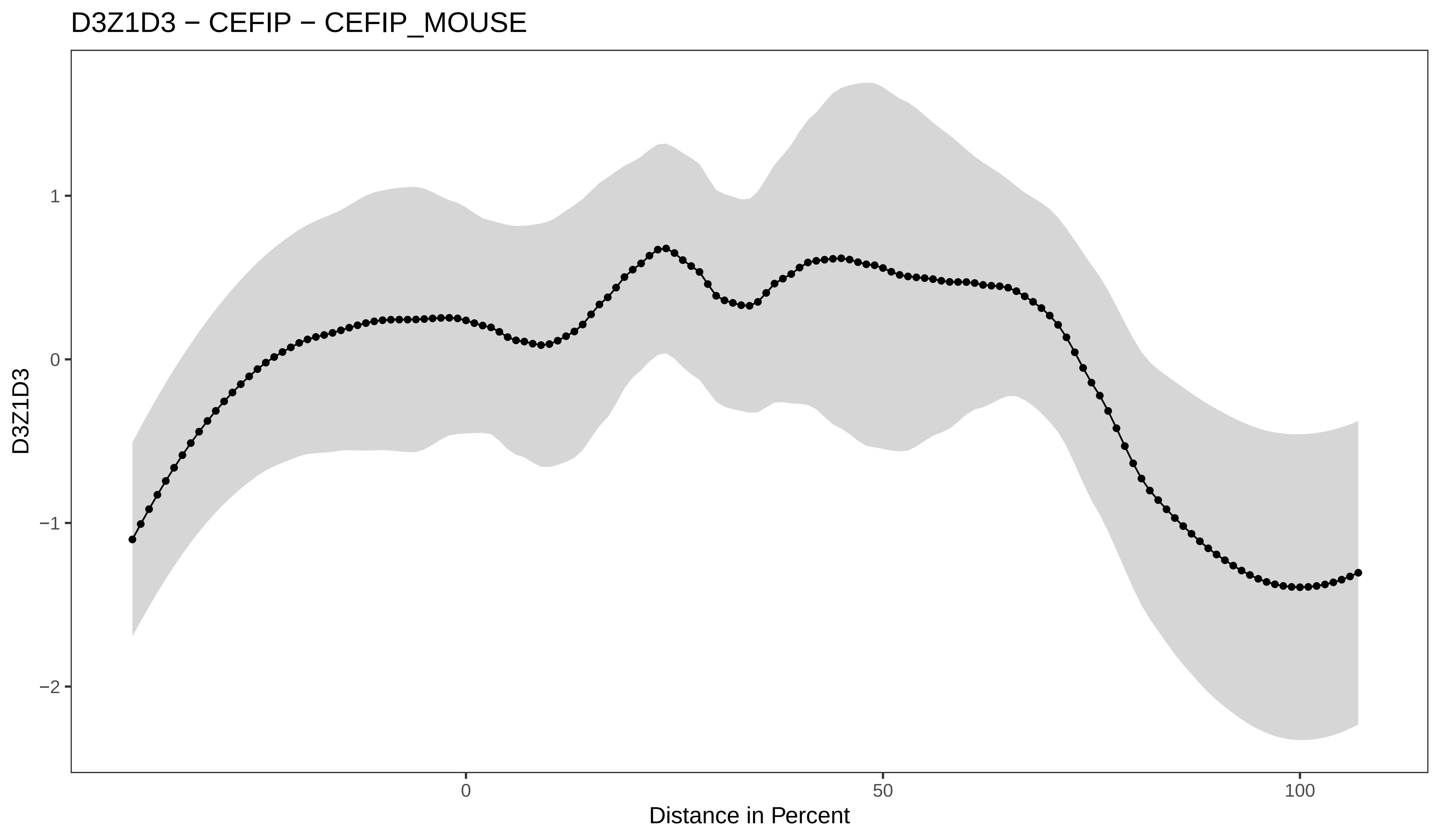

D3Z1D3 − CEFIP − CEFIP_MOUSE
D3Z1D3
1
0
−1
−2
0
50
100
Distance in Percent

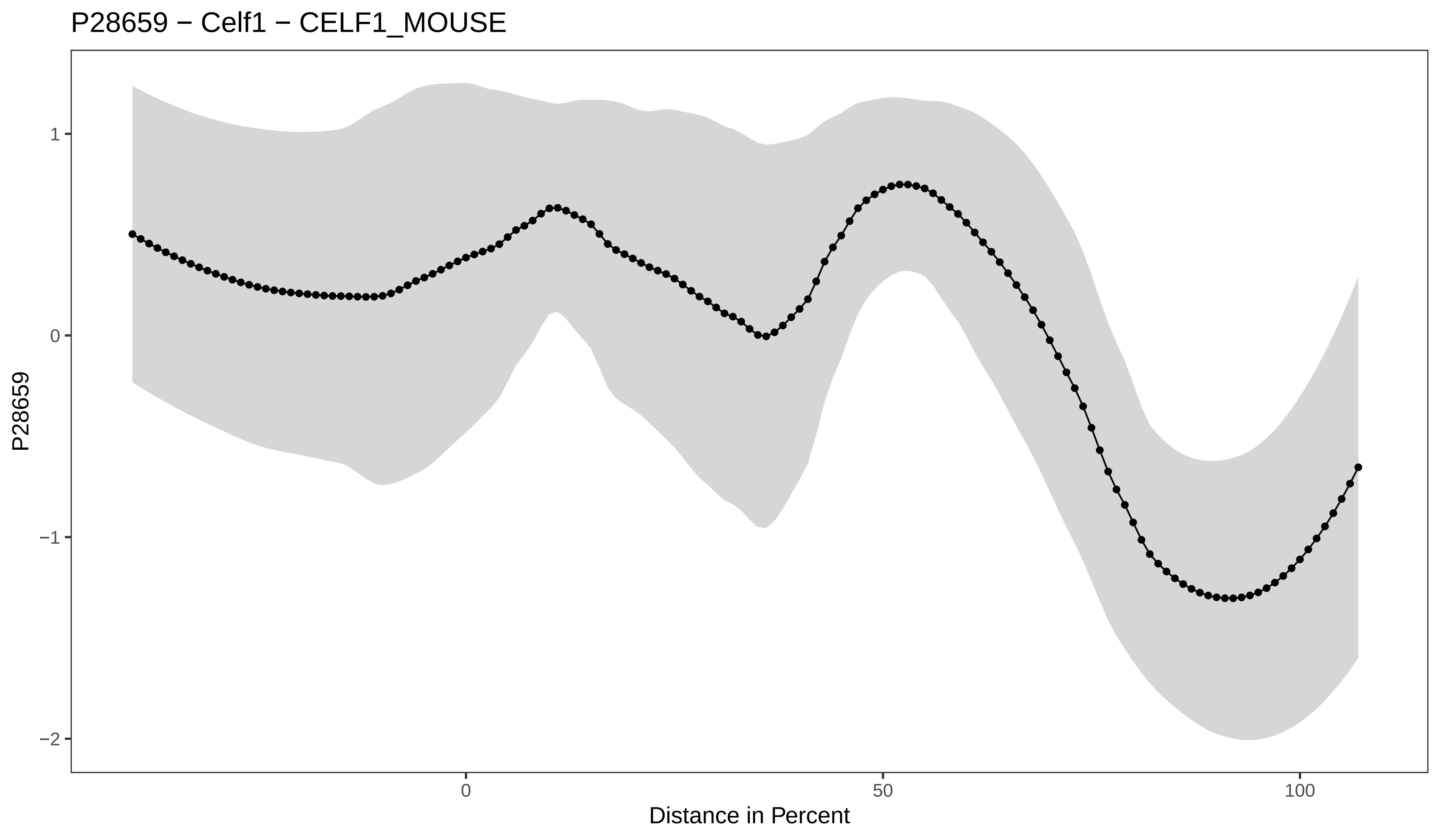
P28659 − Celf1 − CELF1_MOUSE
P28659
1
0
−1
−2
0
50
100
Distance in Percent

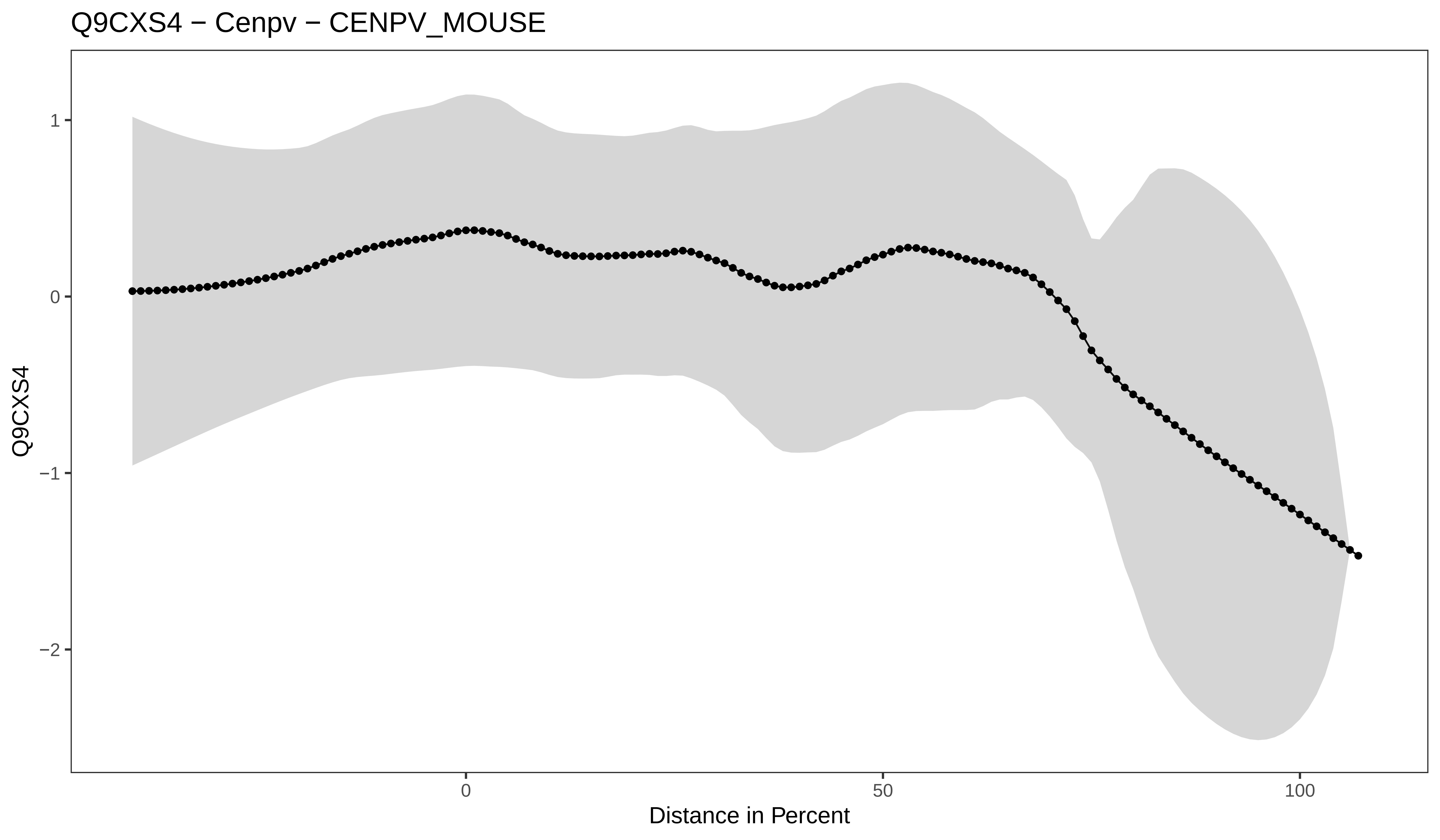

Q9CXS4 − Cenpv − CENPV_MOUSE
Q9CXS4
1
0
−1
−2
0
50
100
Distance in Percent

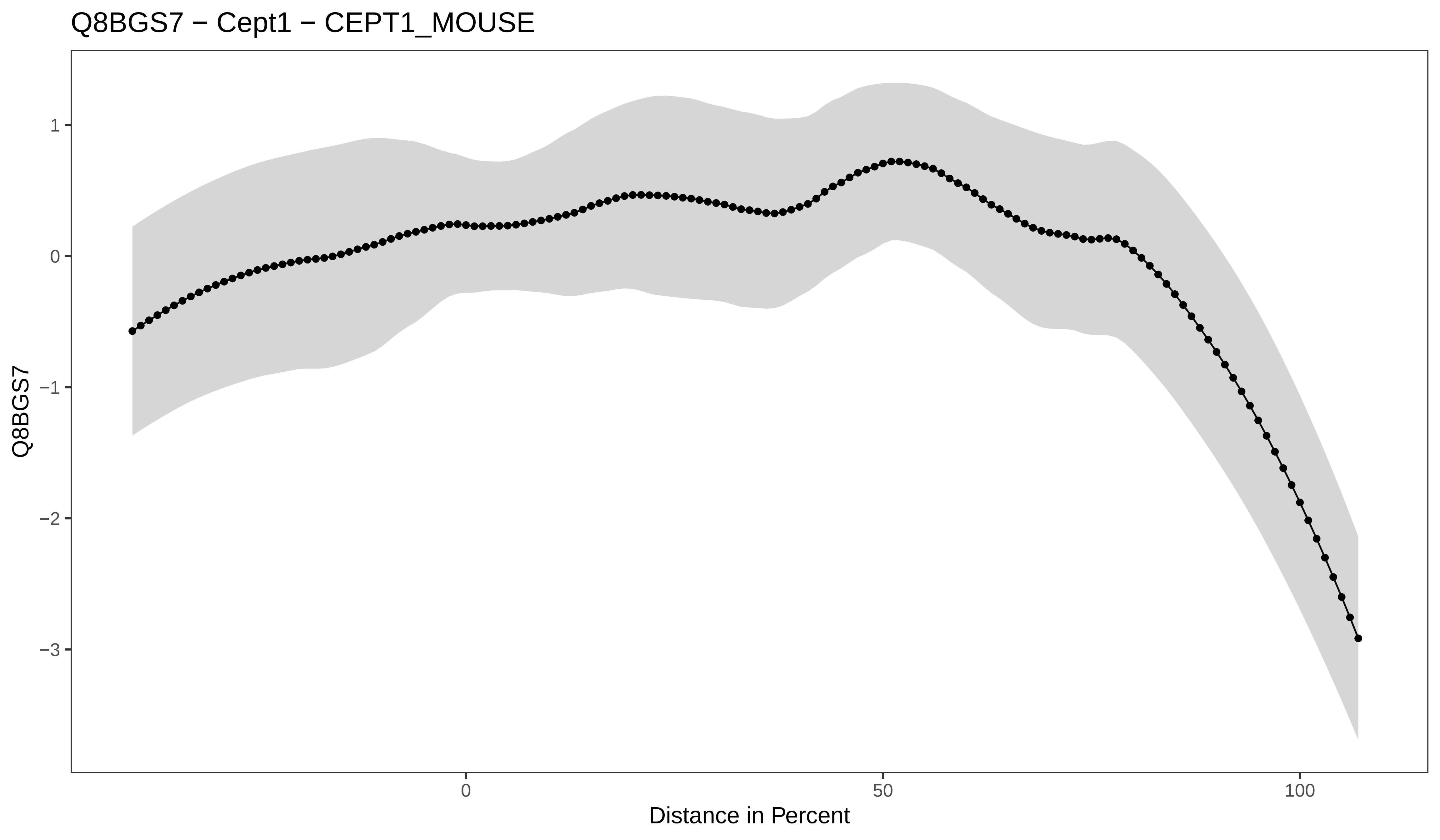

Q8BGS7 − Cept1 − CEPT1_MOUSE
Q8BGS7
Distance in Percent
1
0
−1
−2
−3
0
50
100

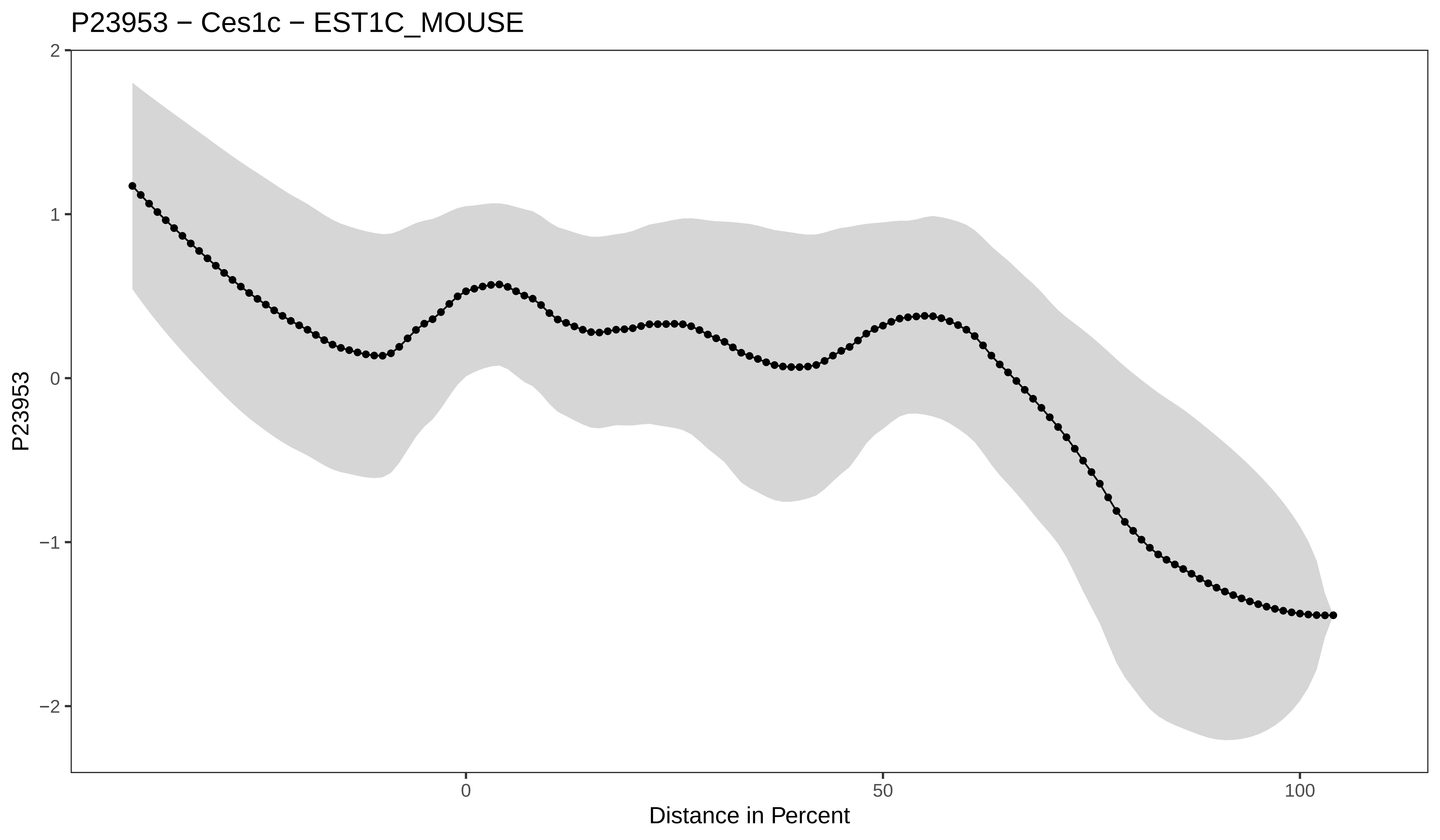

P23953 − Ces1c − EST1C_MOUSE
2
1
0
−1
−2
P23953
0
50
100
Distance in Percent

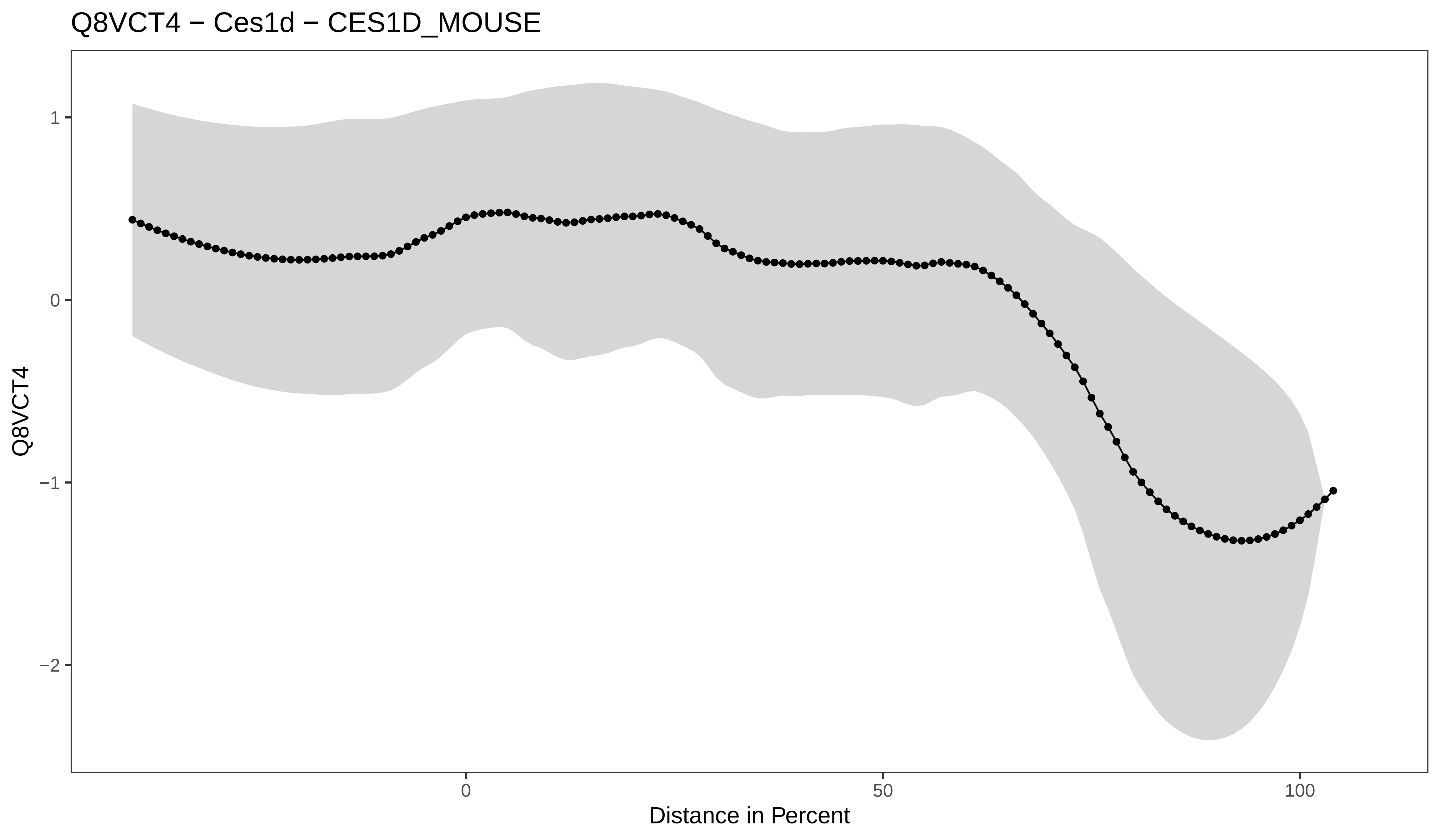
Q8VCT4 − Ces1d − CES1D_MOUSE
Q8VCT4
1
0
−1
−2
0
50
100
Distance in Percent

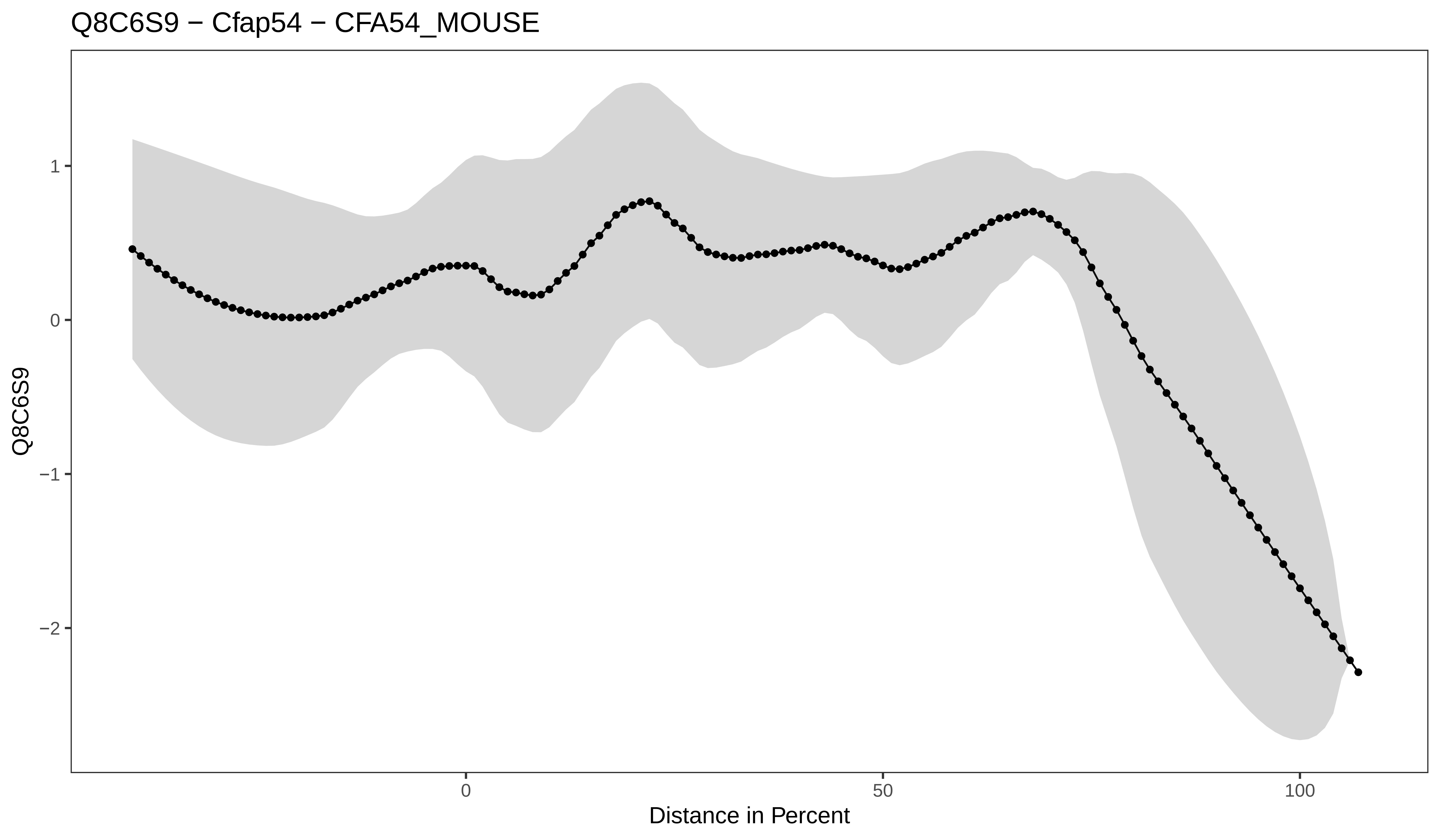

Q8C6S9 − Cfap54 − CFA54_MOUSE
Q8C6S9
1
0
−1
−2
0
50
100
Distance in Percent

P04186 − Cfb − CFAB_MOUSE

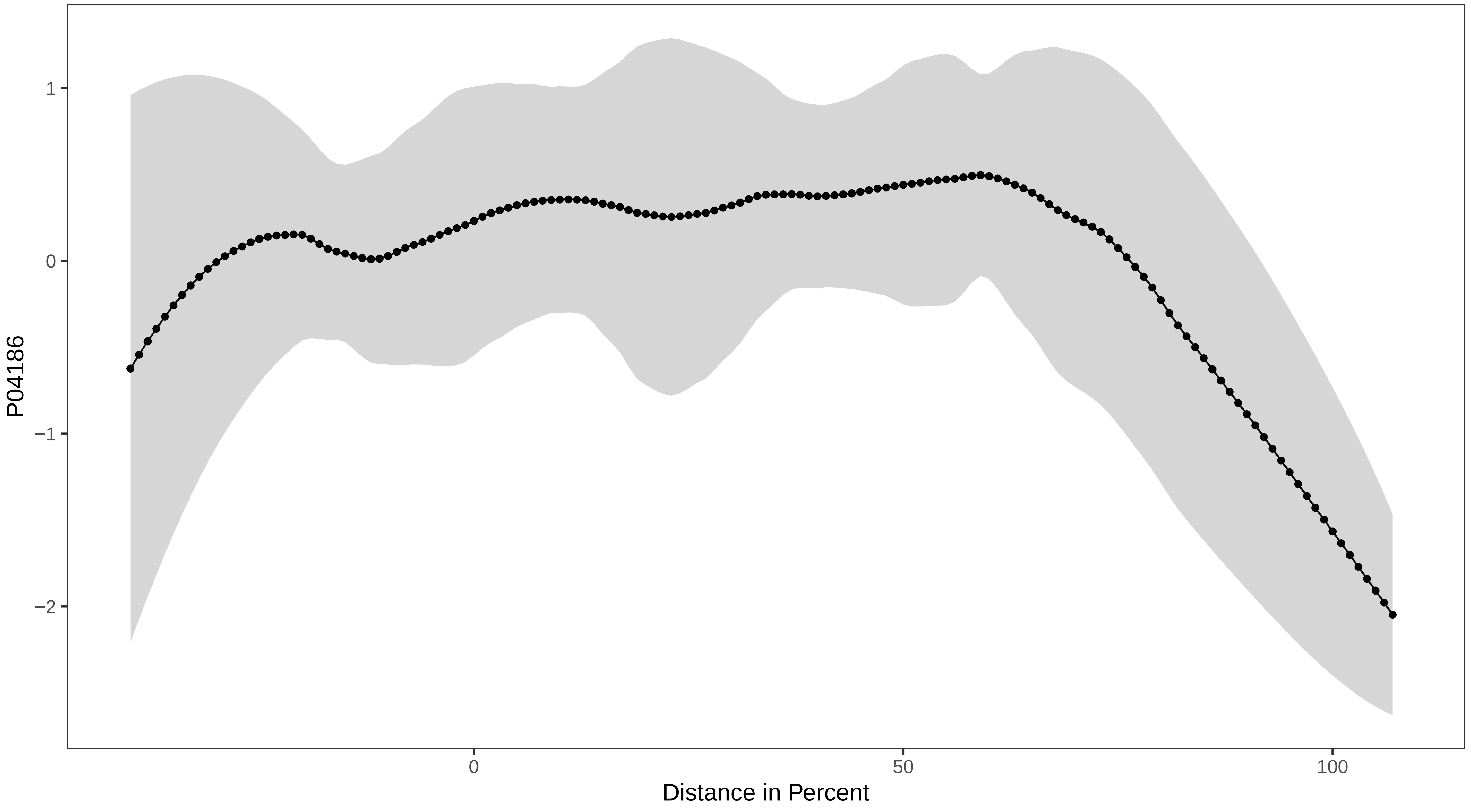

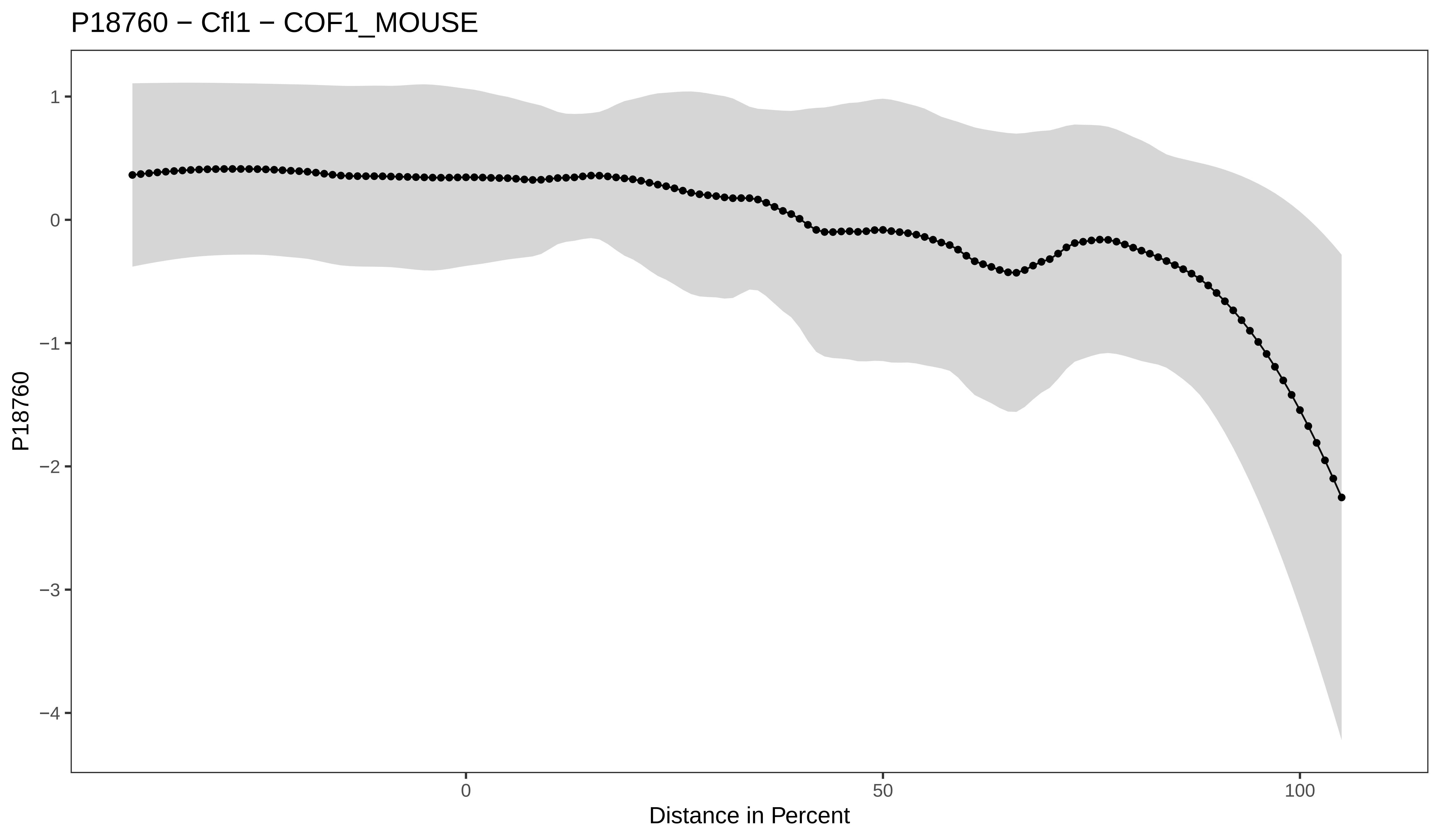
P18760 − Cfl1 − COF1_MOUSE
1
0
−1
−2
−3
−4
P18760
0
50
100
Distance in Percent

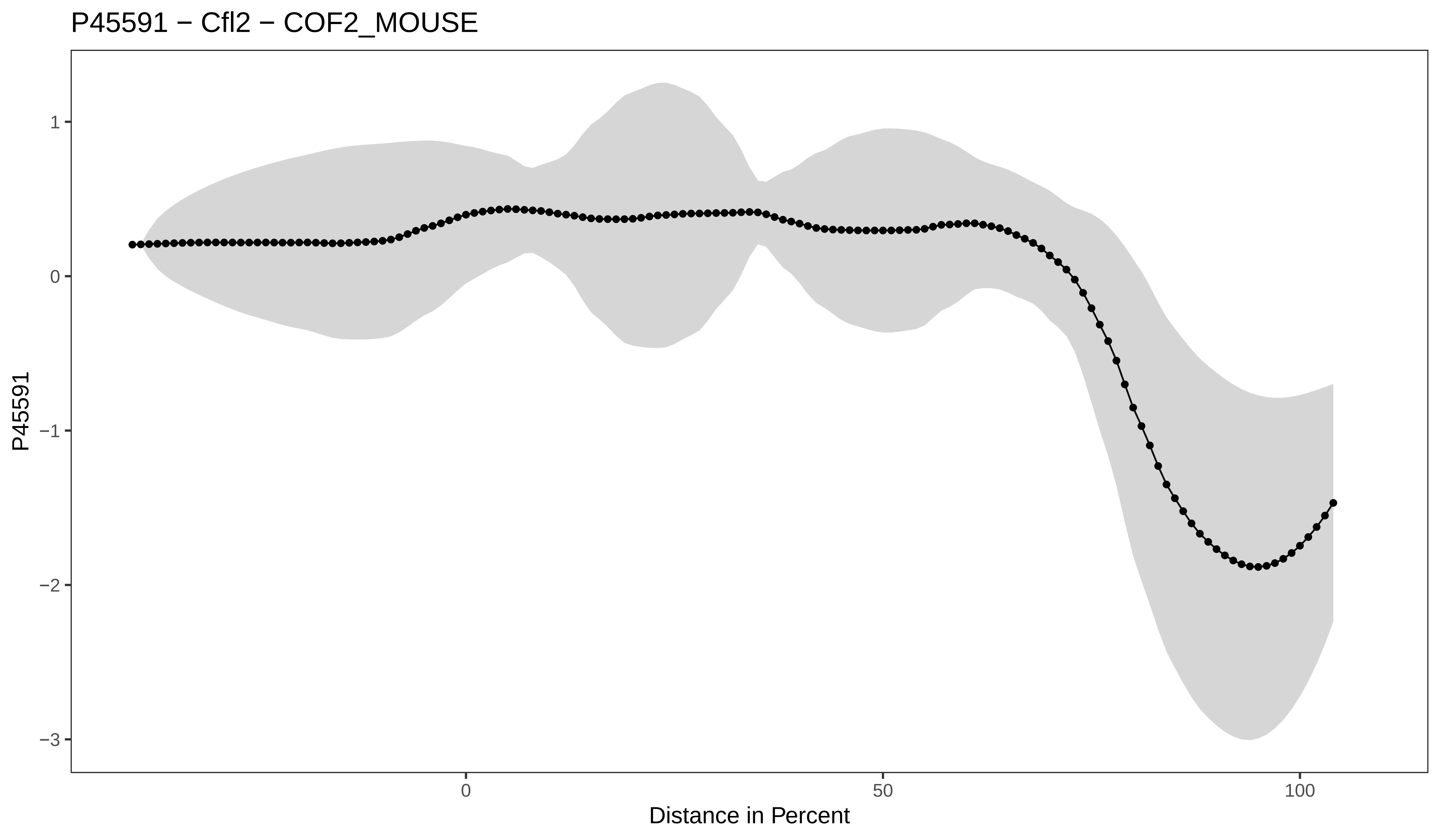

P45591 − Cfl2 − COF2_MOUSE
P45591
1
0
−1
−2
−3
0
50
100
Distance in Percent

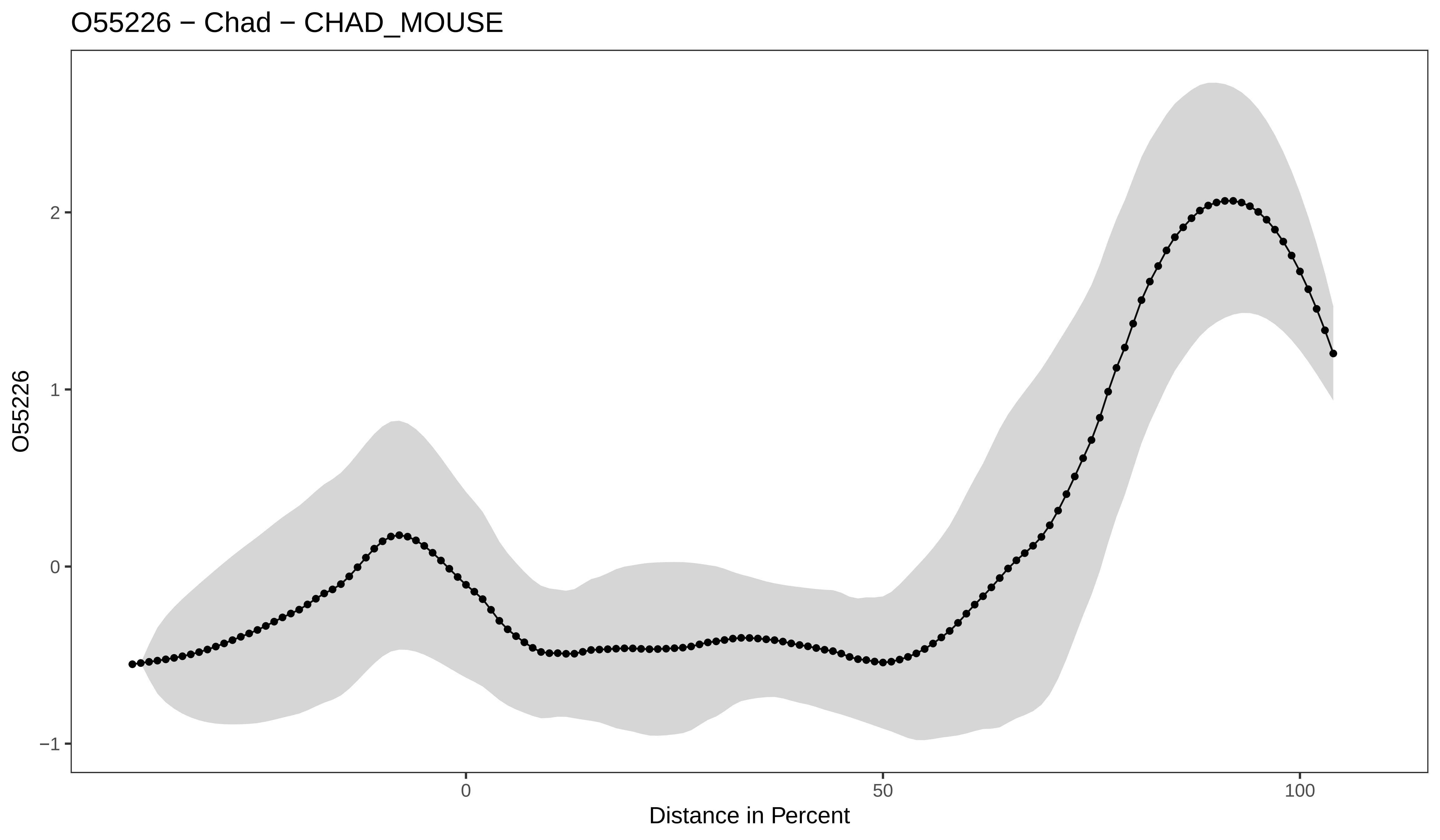

O55226 − Chad − CHAD_MOUSE
O55226
2
1
0
−1
0
50
100
Distance in Percent

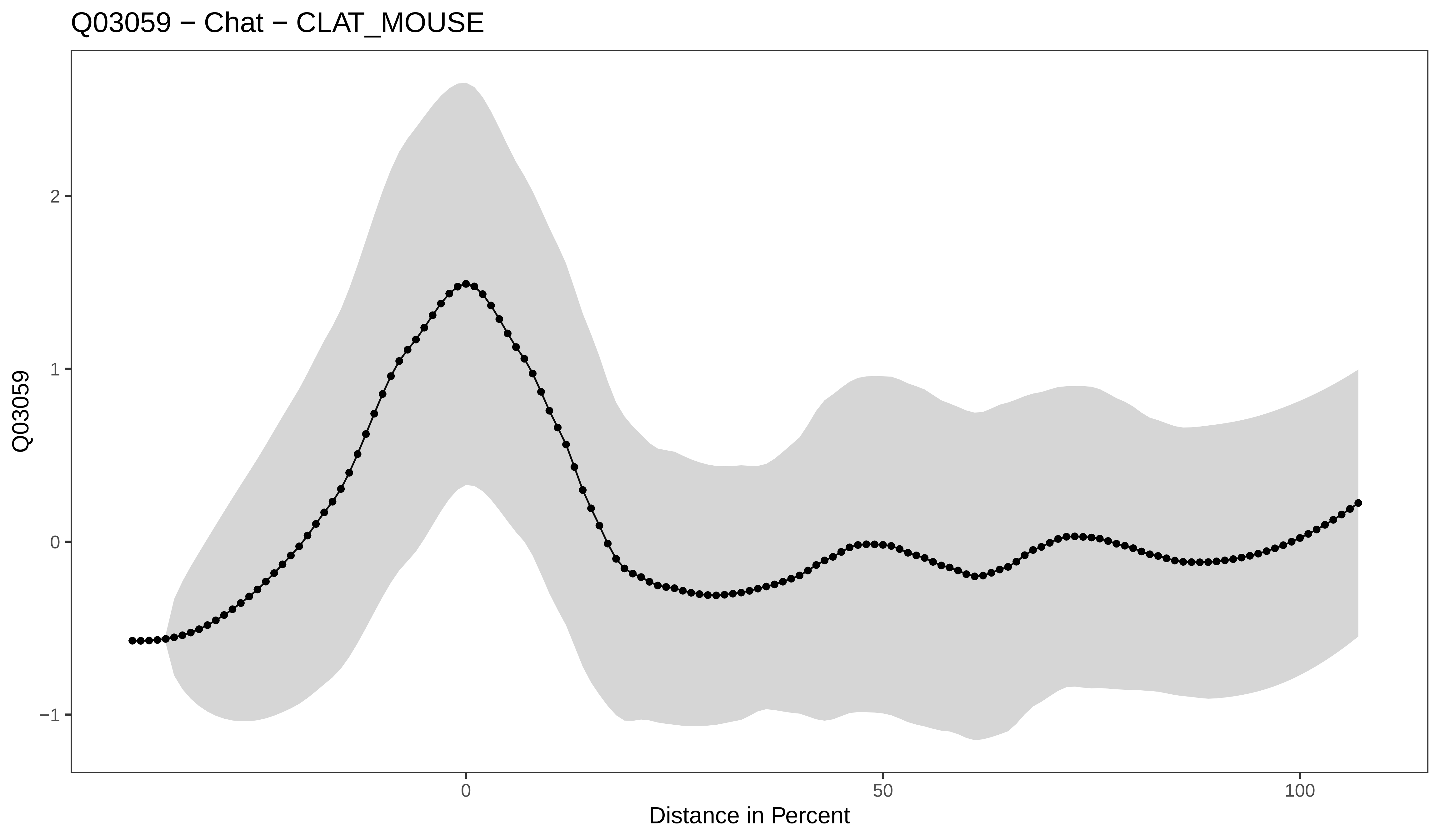
Q03059 − Chat − CLAT_MOUSE
Q03059
2
1
0
−1
0
50
100
Distance in Percent

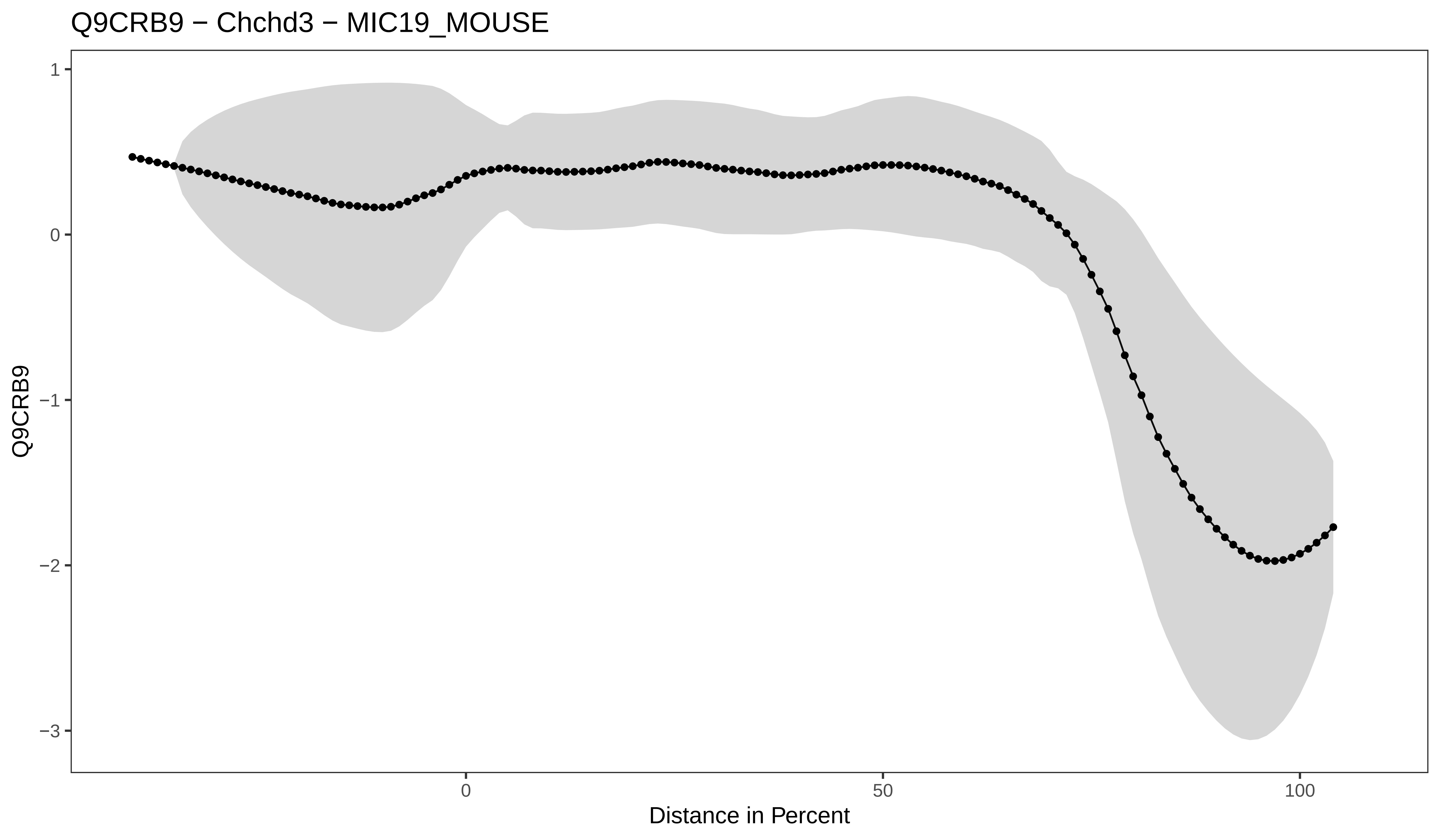
Q9CRB9 − Chchd3 − MIC19_MOUSE
1
0
−1
−2
−3
Q9CRB9
0
50
100
Distance in Percent

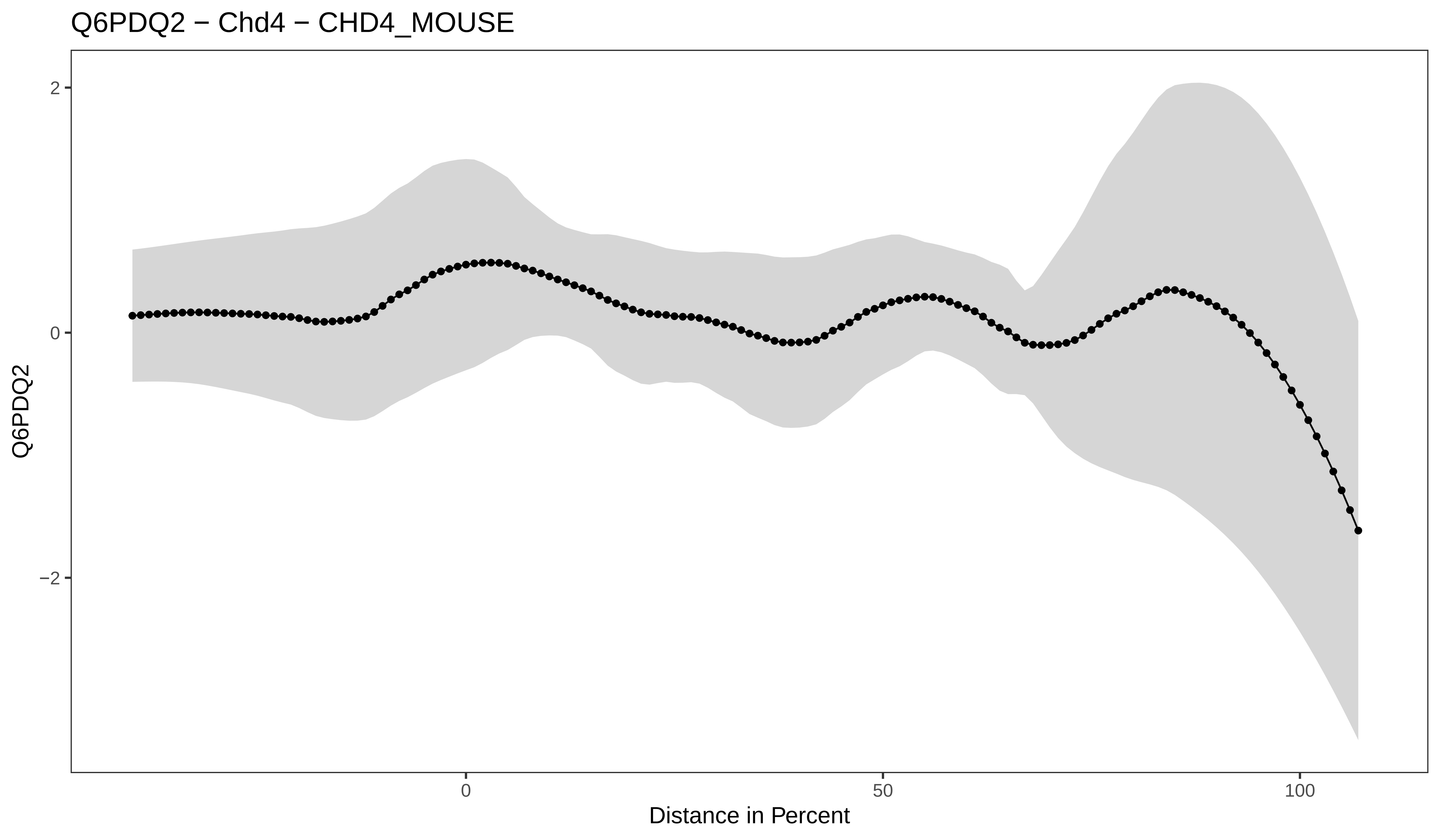

Q6PDQ2 − Chd4 − CHD4_MOUSE
2
0
−2
Q6PDQ2
0
50
100
Distance in Percent

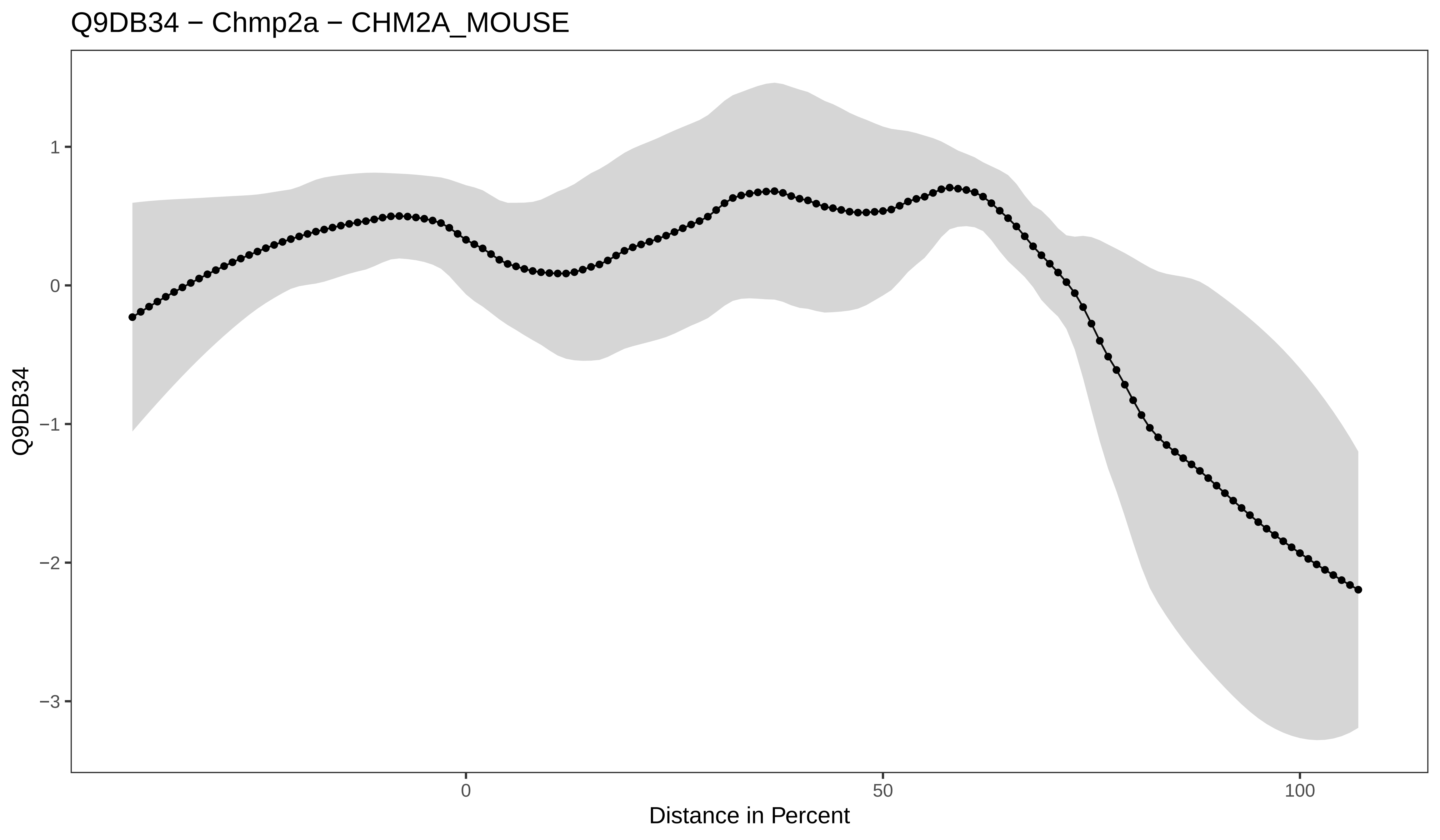
Q9DB34 − Chmp2a − CHM2A_MOUSE
Q9DB34
1
0
−1
−2
−3
0
50
100
Distance in Percent

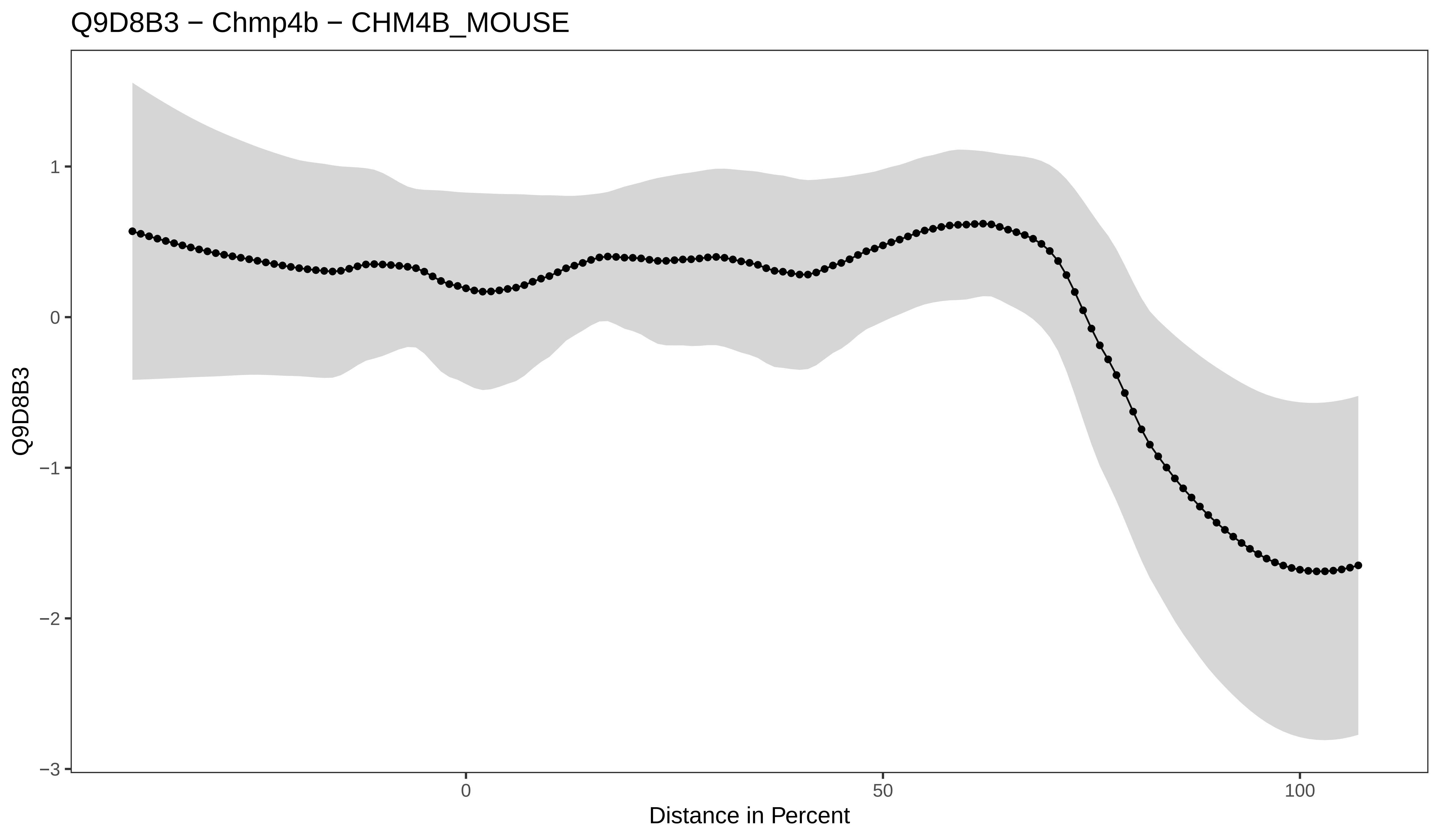

Q9D8B3 − Chmp4b − CHM4B_MOUSE
Q9D8B3
1
0
−1
−2
−3
0
50
100
Distance in Percent

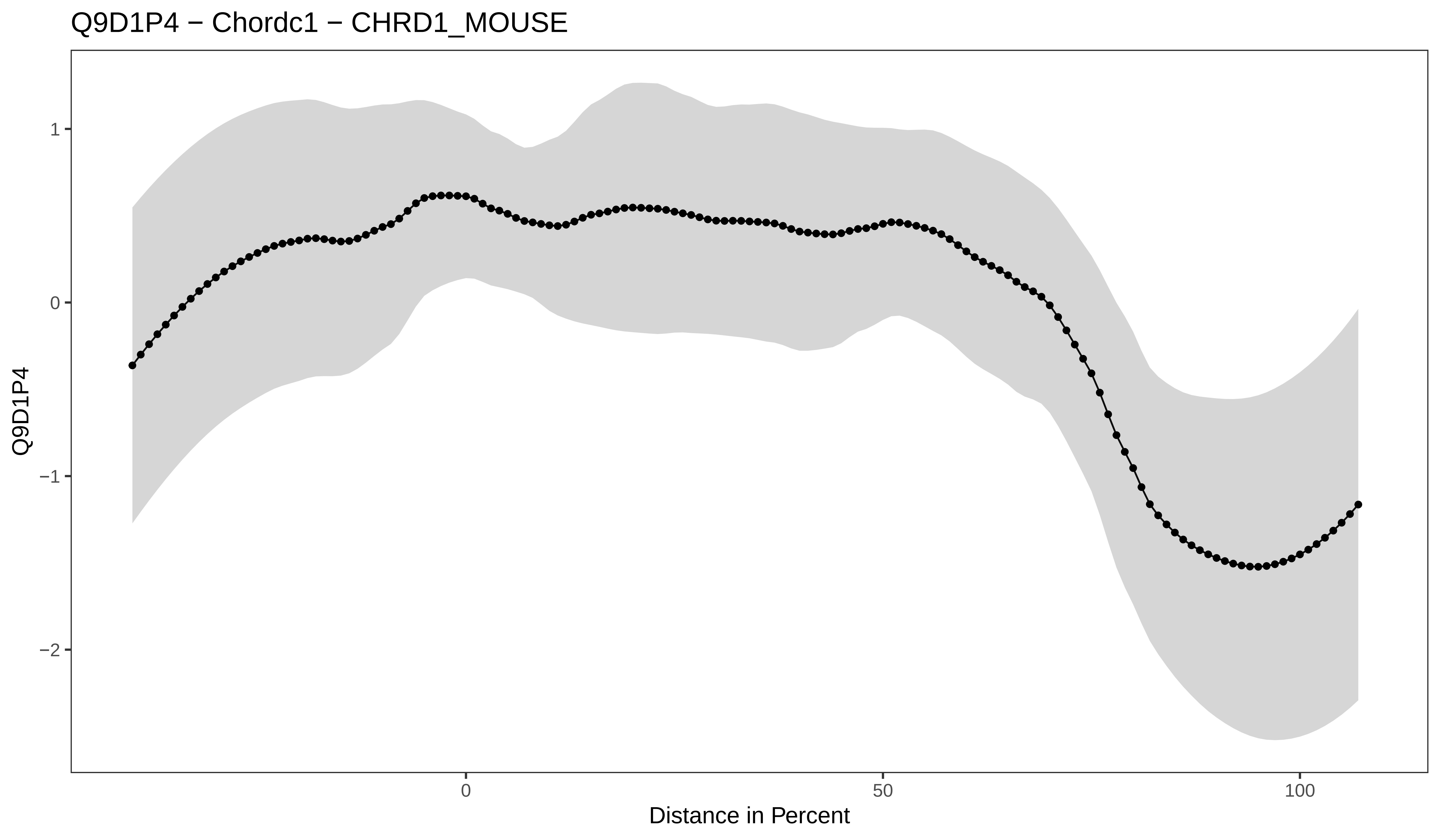
Q9D1P4 − Chordc1 − CHRD1_MOUSE
1
0
−1
−2
Q9D1P4
0
50
100
Distance in Percent

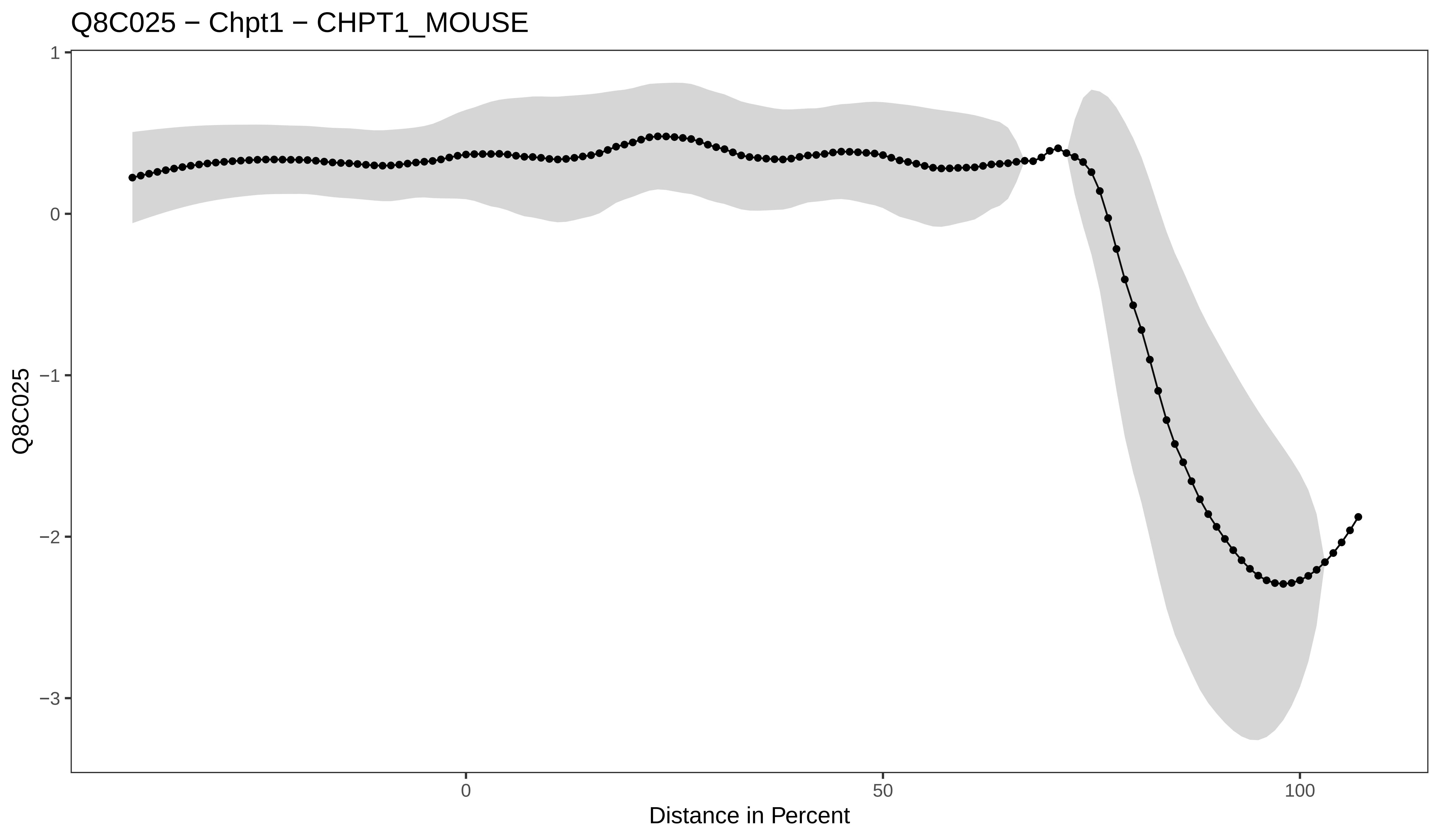

Q8C025 − Chpt1 − CHPT1_MOUSE
1
0
−1
−2
−3
Q8C025
0
50
100
Distance in Percent

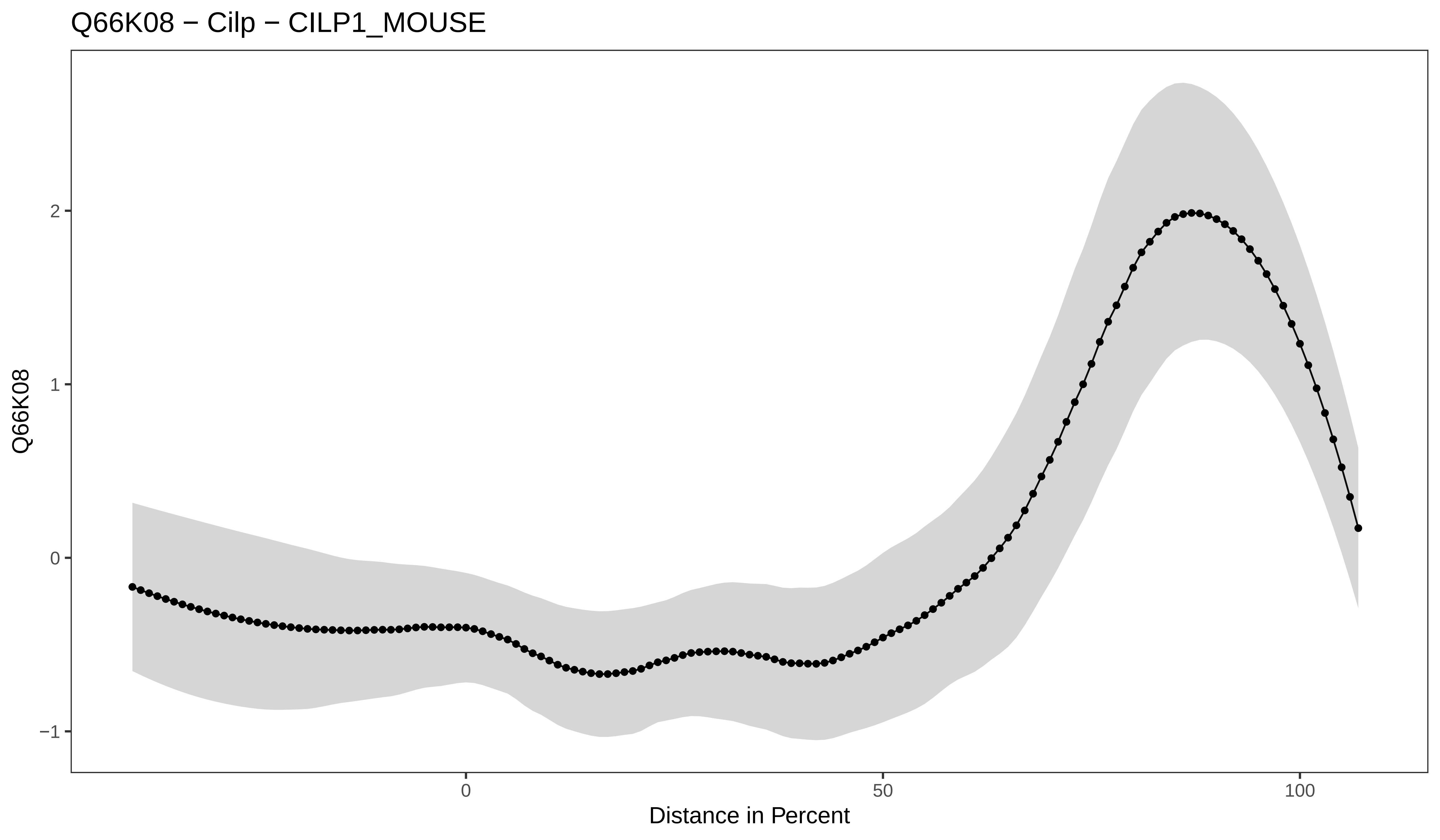
Q66K08 − Cilp − CILP1_MOUSE
Q66K08
2
1
0
−1
0
50
100
Distance in Percent

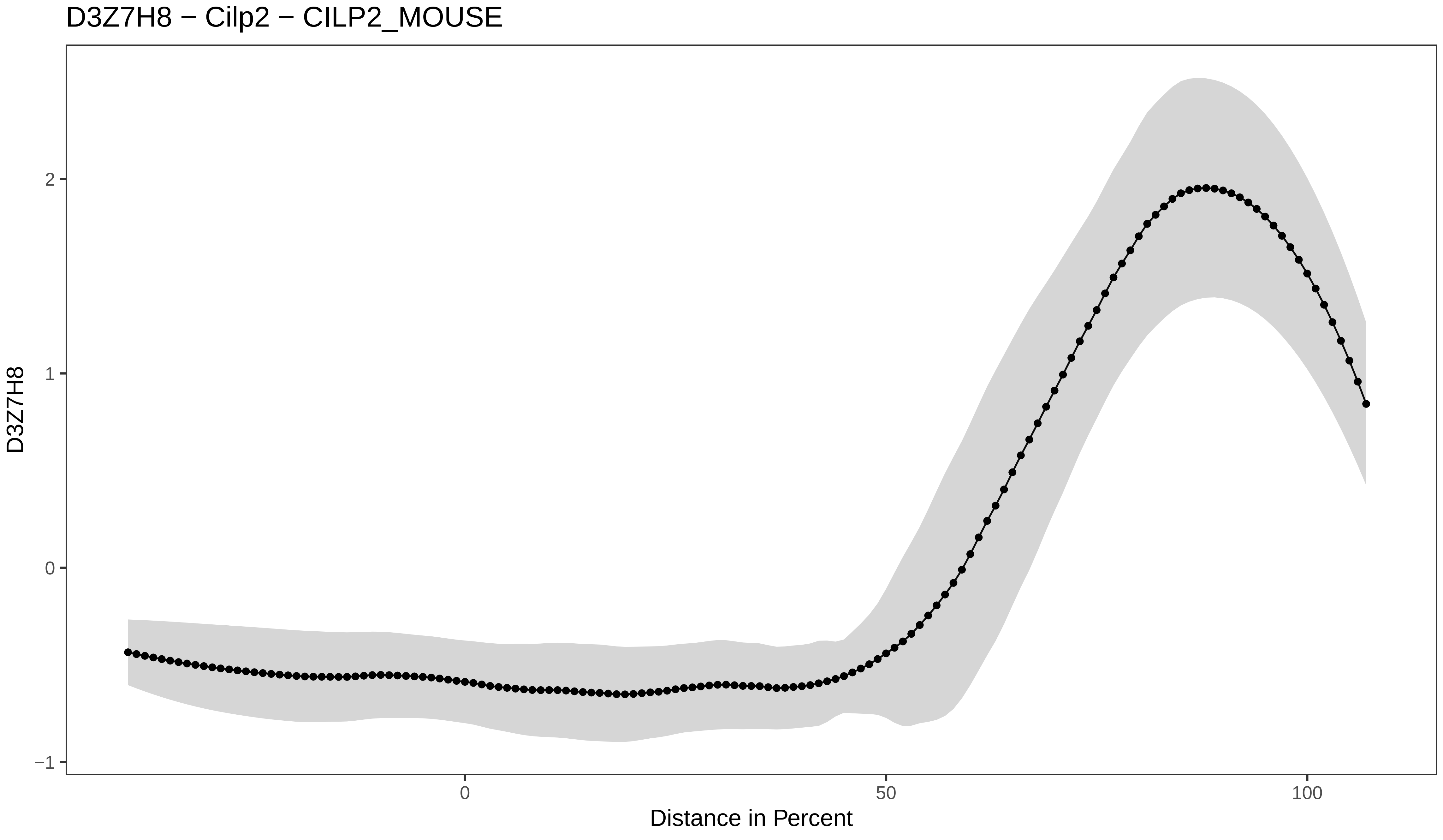
D3Z7H8 − Cilp2 − CILP2_MOUSE
D3Z7H8
Distance in Percent
2
1
0
−1
0
50
100

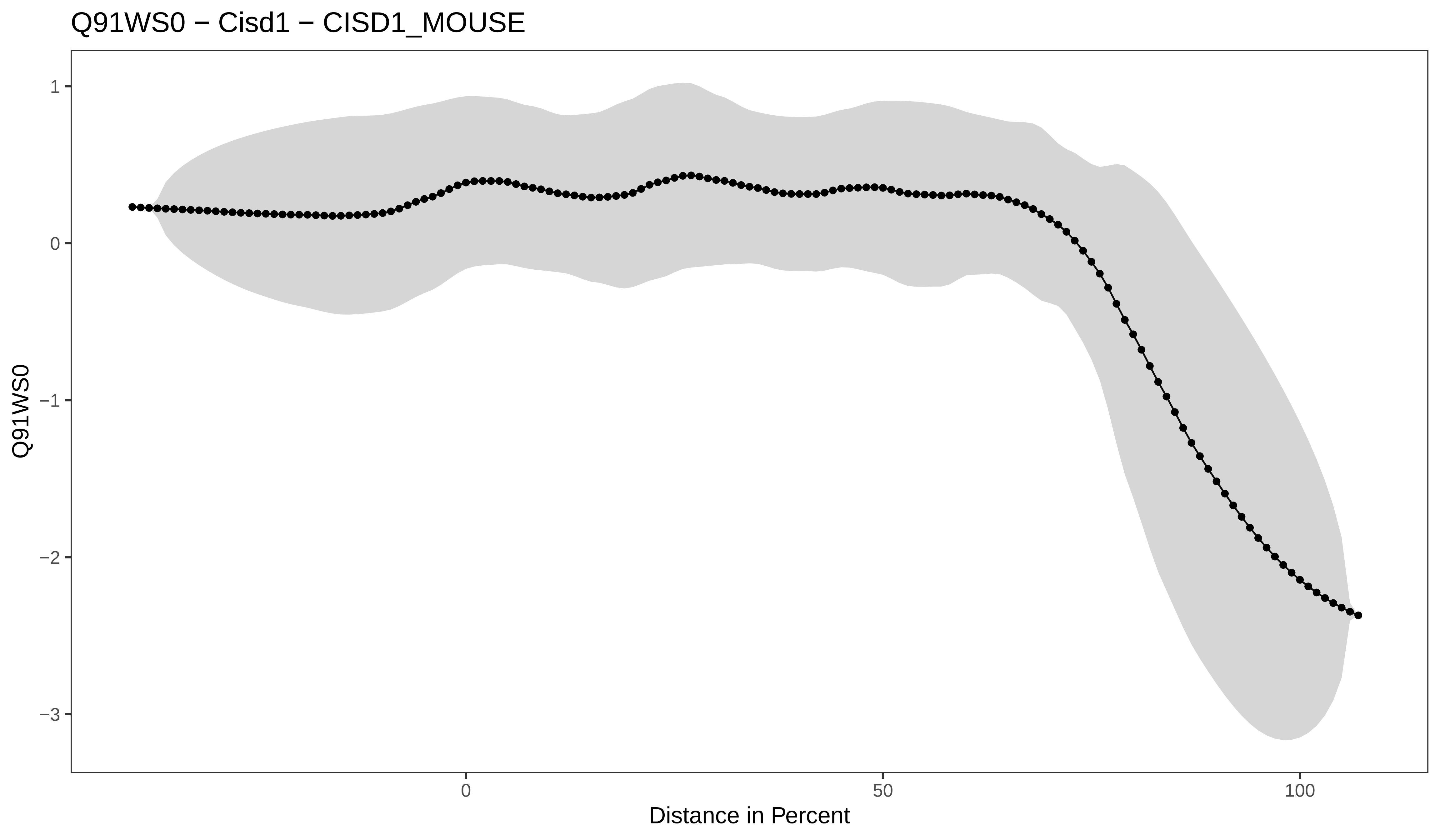
Q91WS0 − Cisd1 − CISD1_MOUSE
1
0
−1
−2
−3
Q91WS0
0
50
100
Distance in Percent

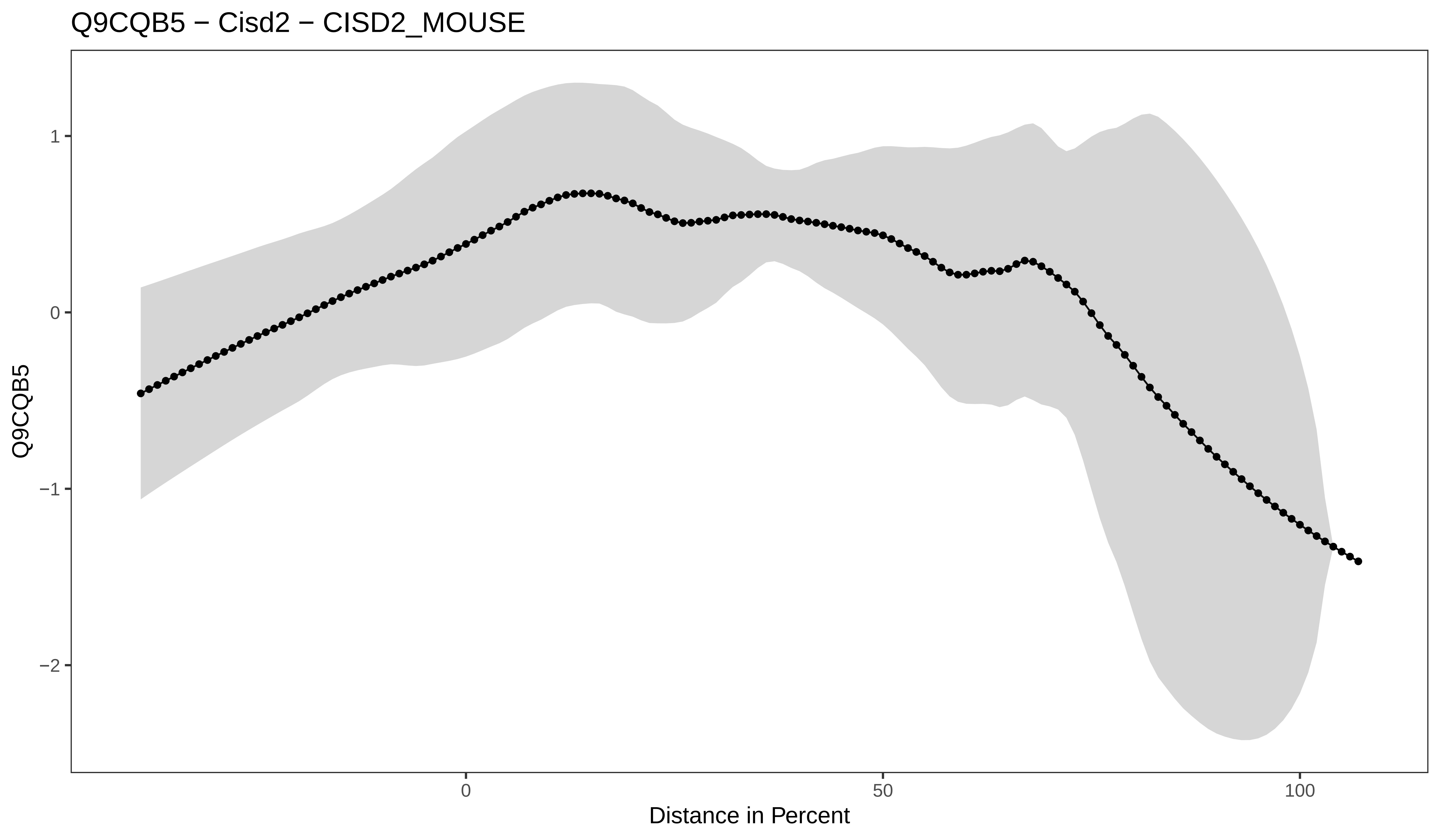

Q9CQB5 − Cisd2 − CISD2_MOUSE
1
0
−1
−2
Q9CQB5
0
50
100
Distance in Percent

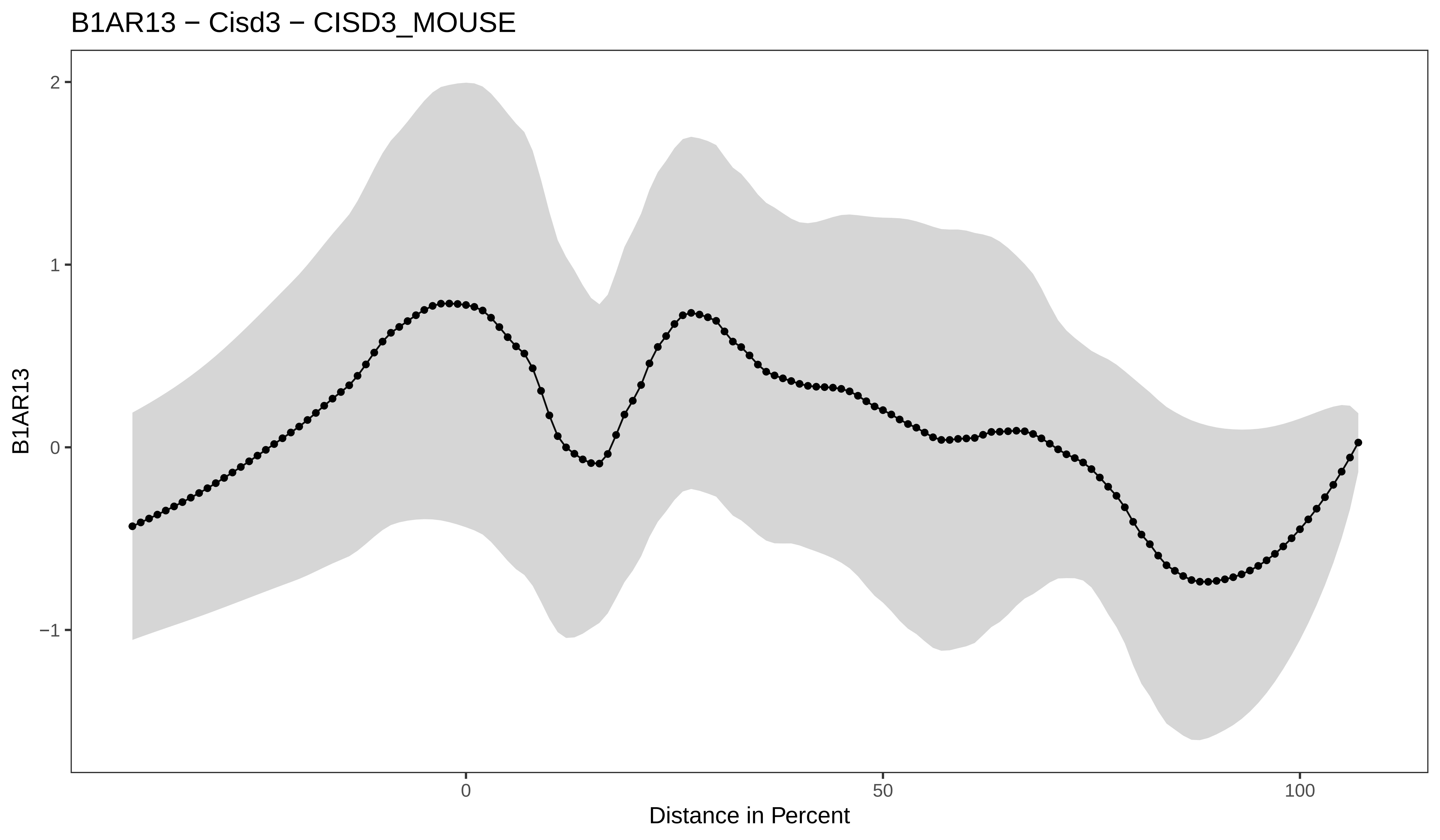
B1AR13 − Cisd3 − CISD3_MOUSE
B1AR13
2
1
0
−1
0
50
100
Distance in Percent

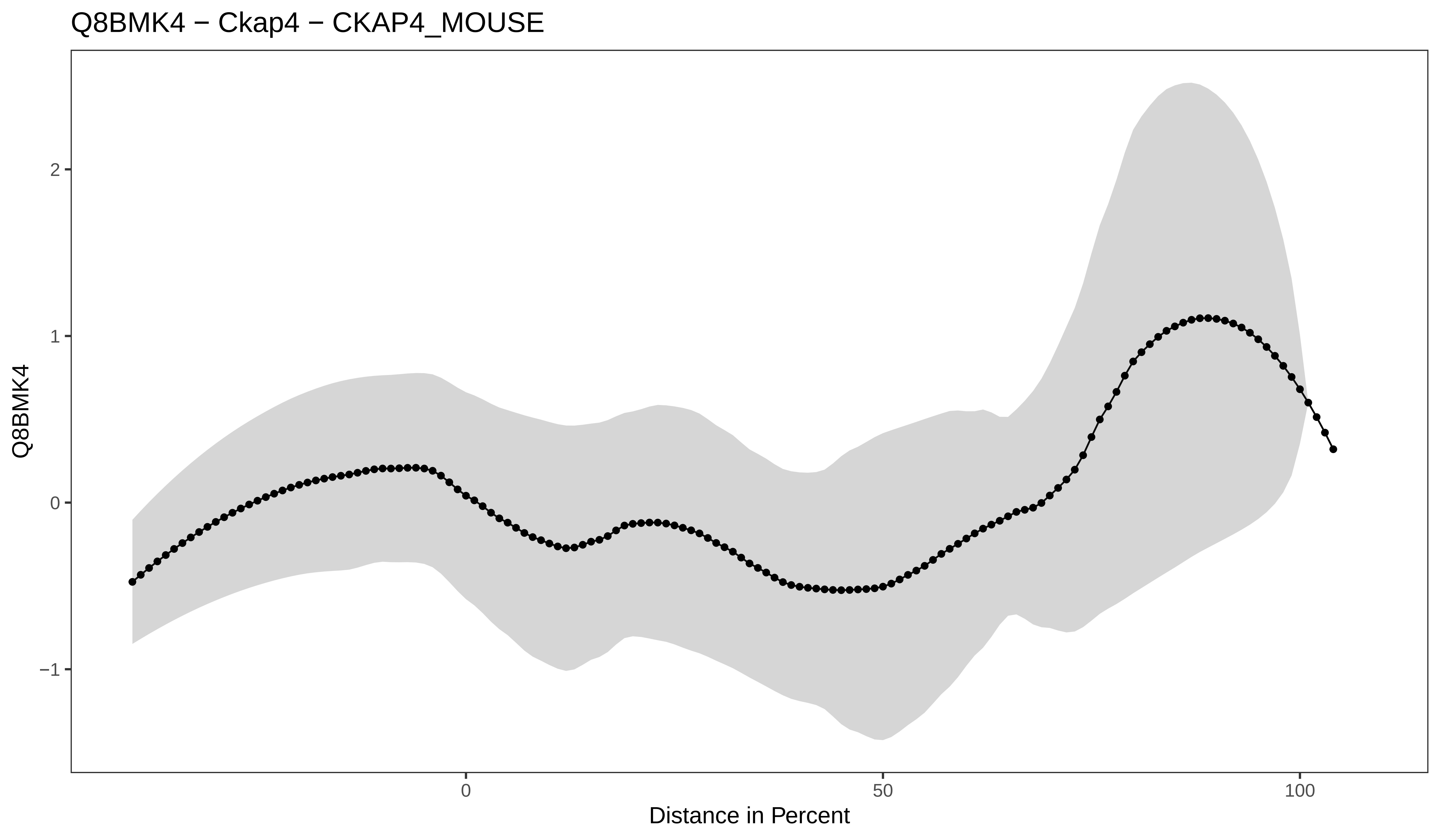

Q8BMK4 − Ckap4 − CKAP4_MOUSE
Q8BMK4
2
1
0
−1
0
50
100
Distance in Percent

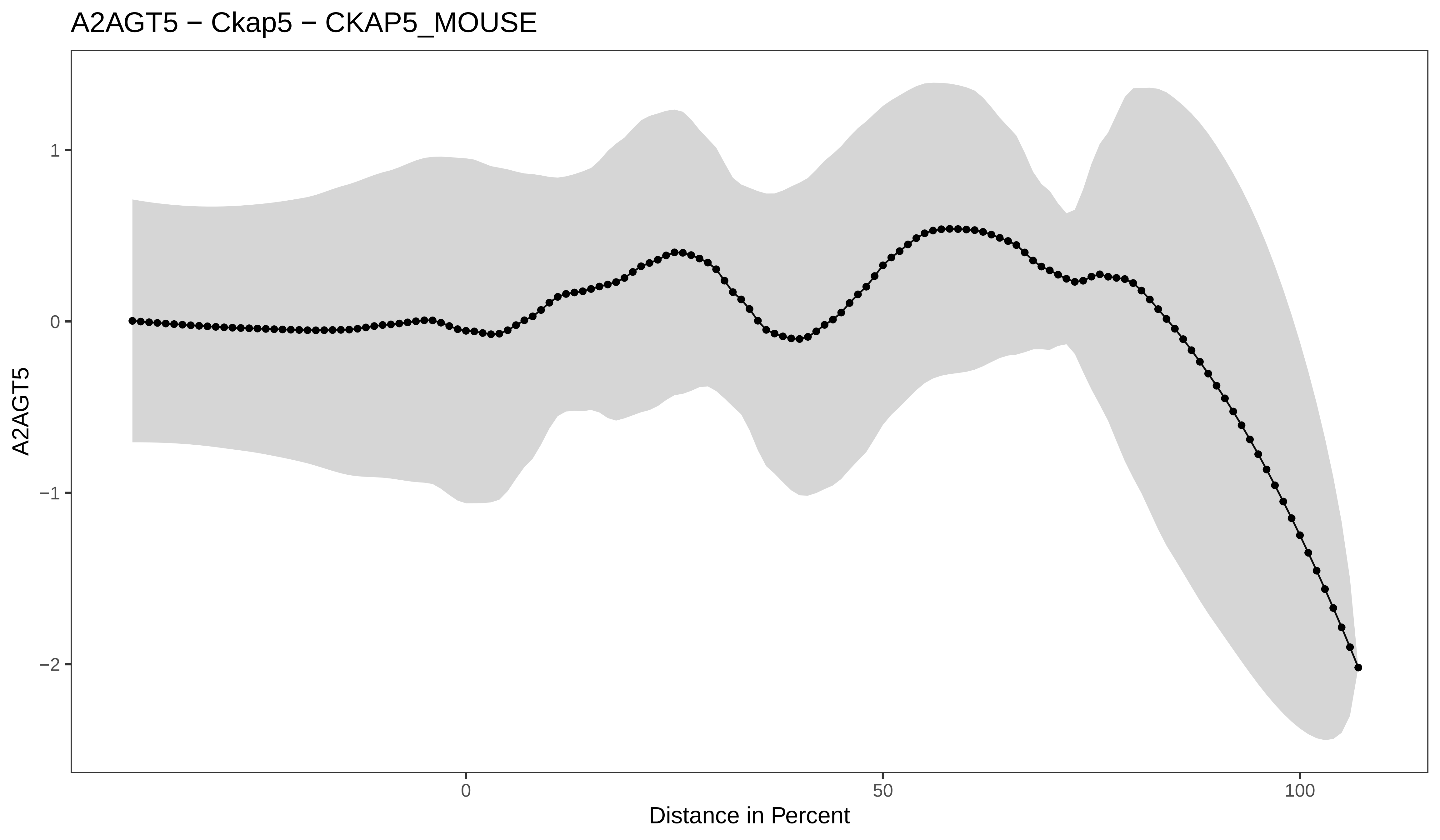
A2AGT5 − Ckap5 − CKAP5_MOUSE
A2AGT5
1
0
−1
−2
0
50
100
Distance in Percent

Q04447 − Ckb − KCRB_MOUSE

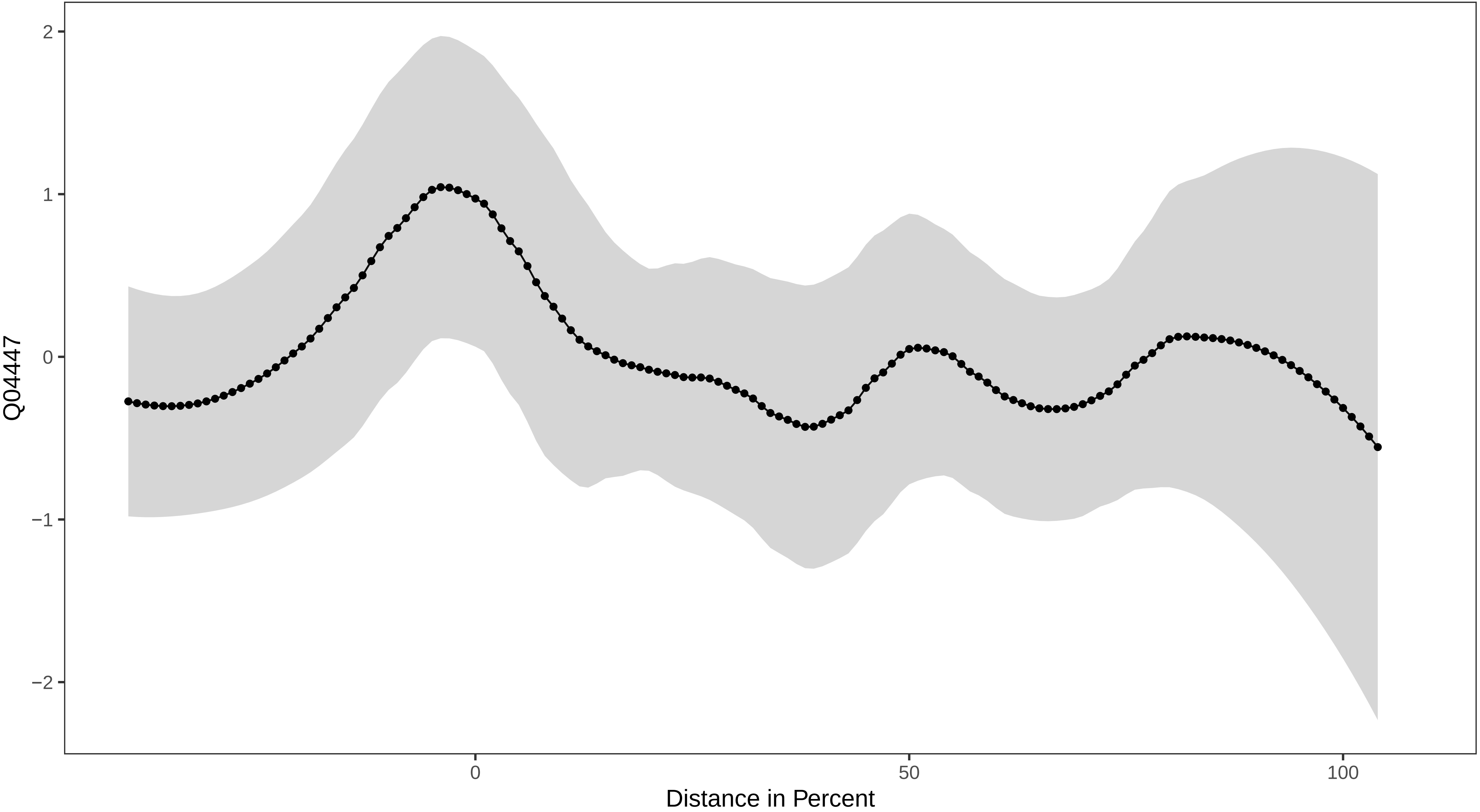

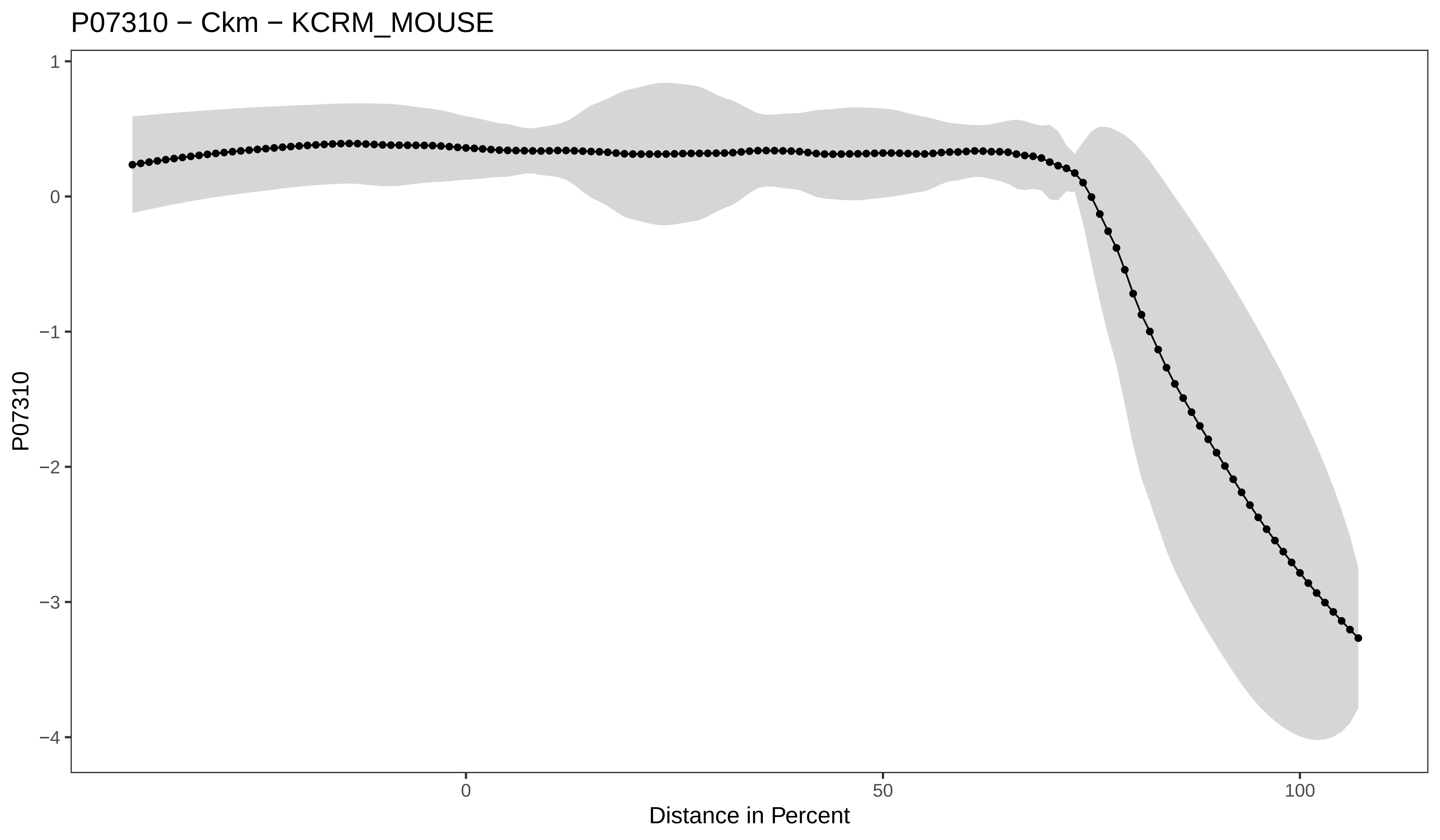

P07310 − Ckm − KCRM_MOUSE
P07310
Distance in Percent
1
0
−1
−2
−3
−4
0
50
100

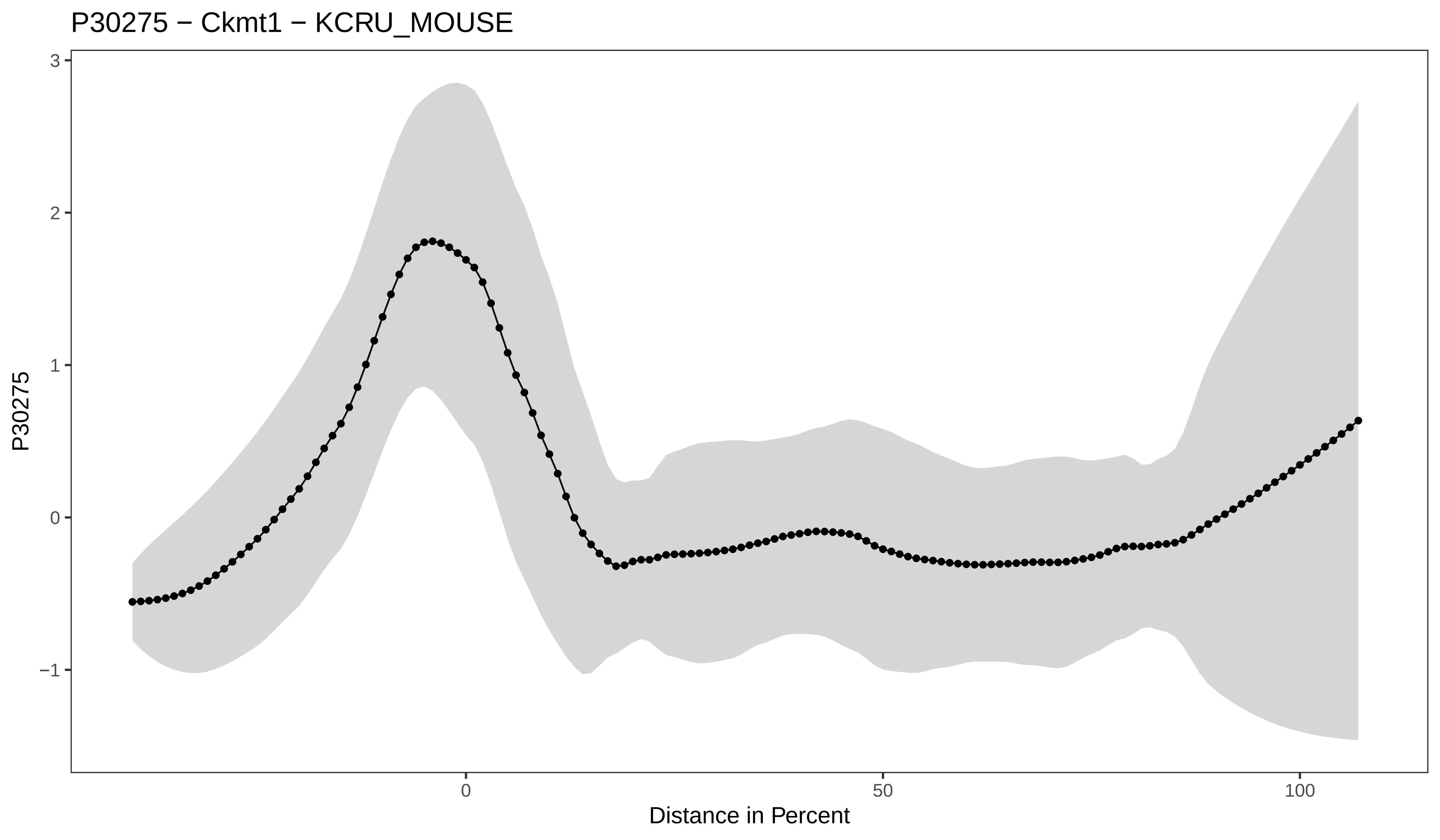
P30275 − Ckmt1 − KCRU_MOUSE
3
2
1
0
−1
P30275
0
50
100
Distance in Percent

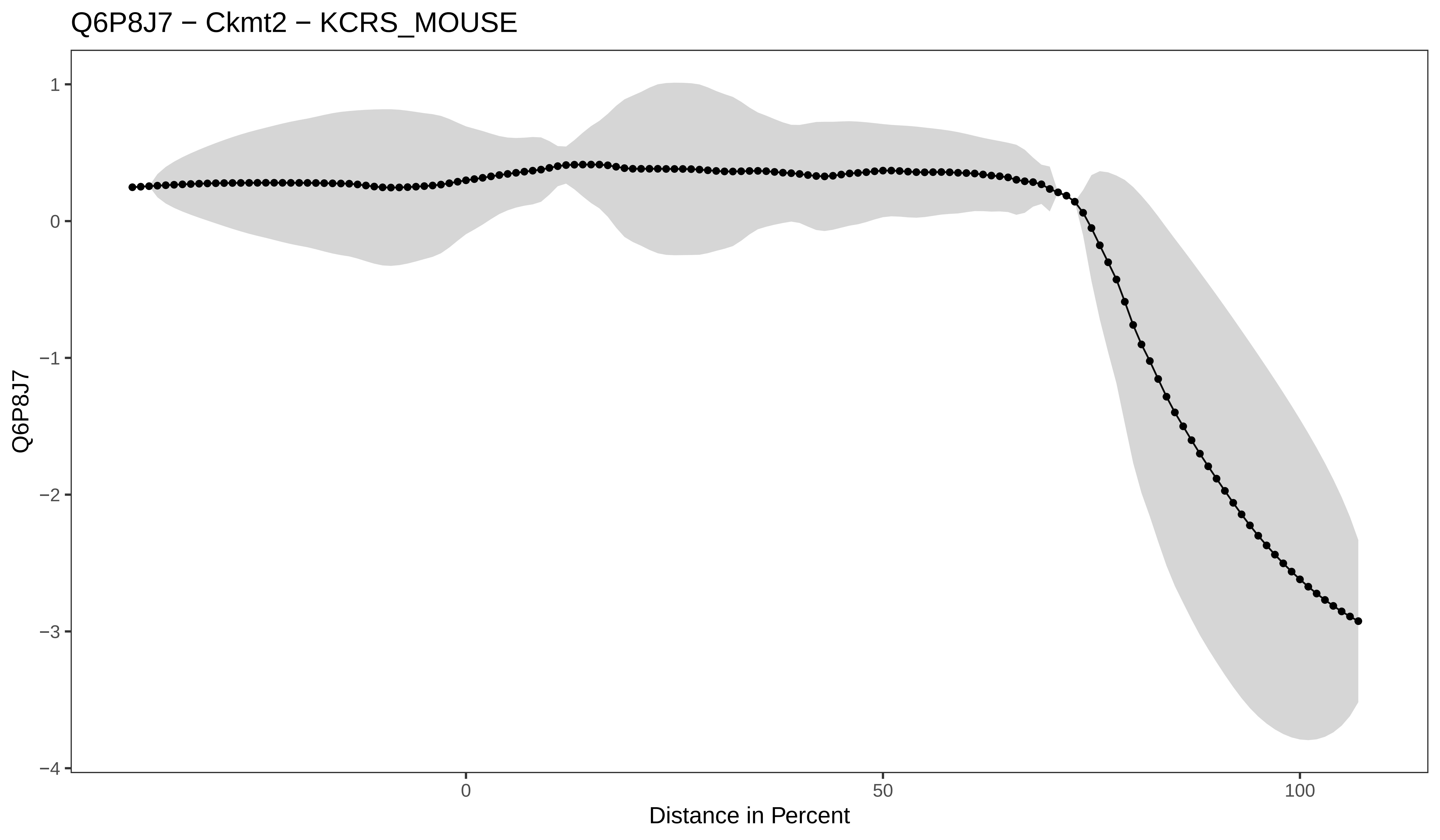

Q6P8J7 − Ckmt2 − KCRS_MOUSE
Q6P8J7
1
0
−1
−2
−3
−4
0
50
100
Distance in Percent

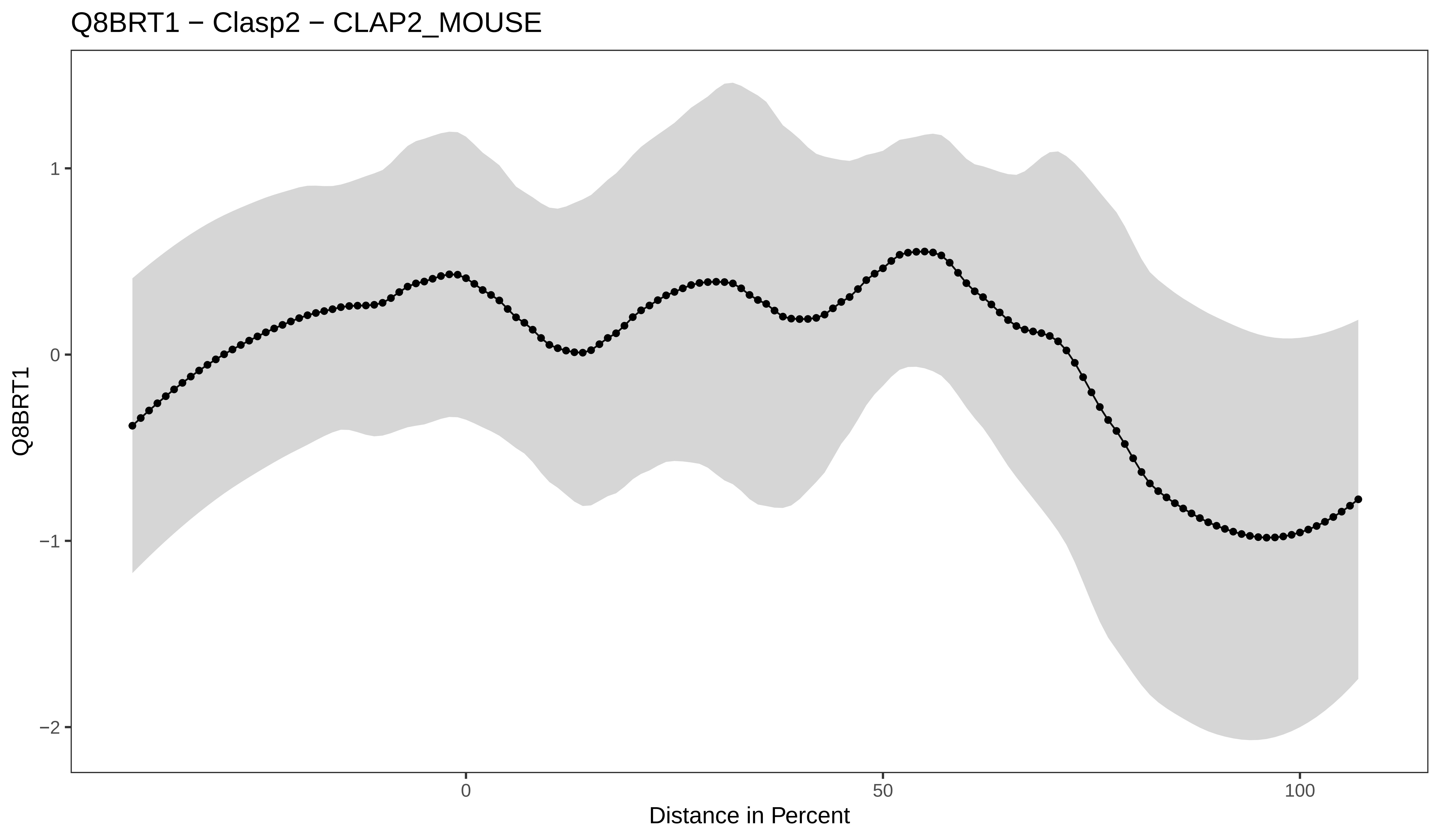
Q8BRT1 − Clasp2 − CLAP2_MOUSE
Q8BRT1
1
0
−1
−2
0
50
100
Distance in Percent

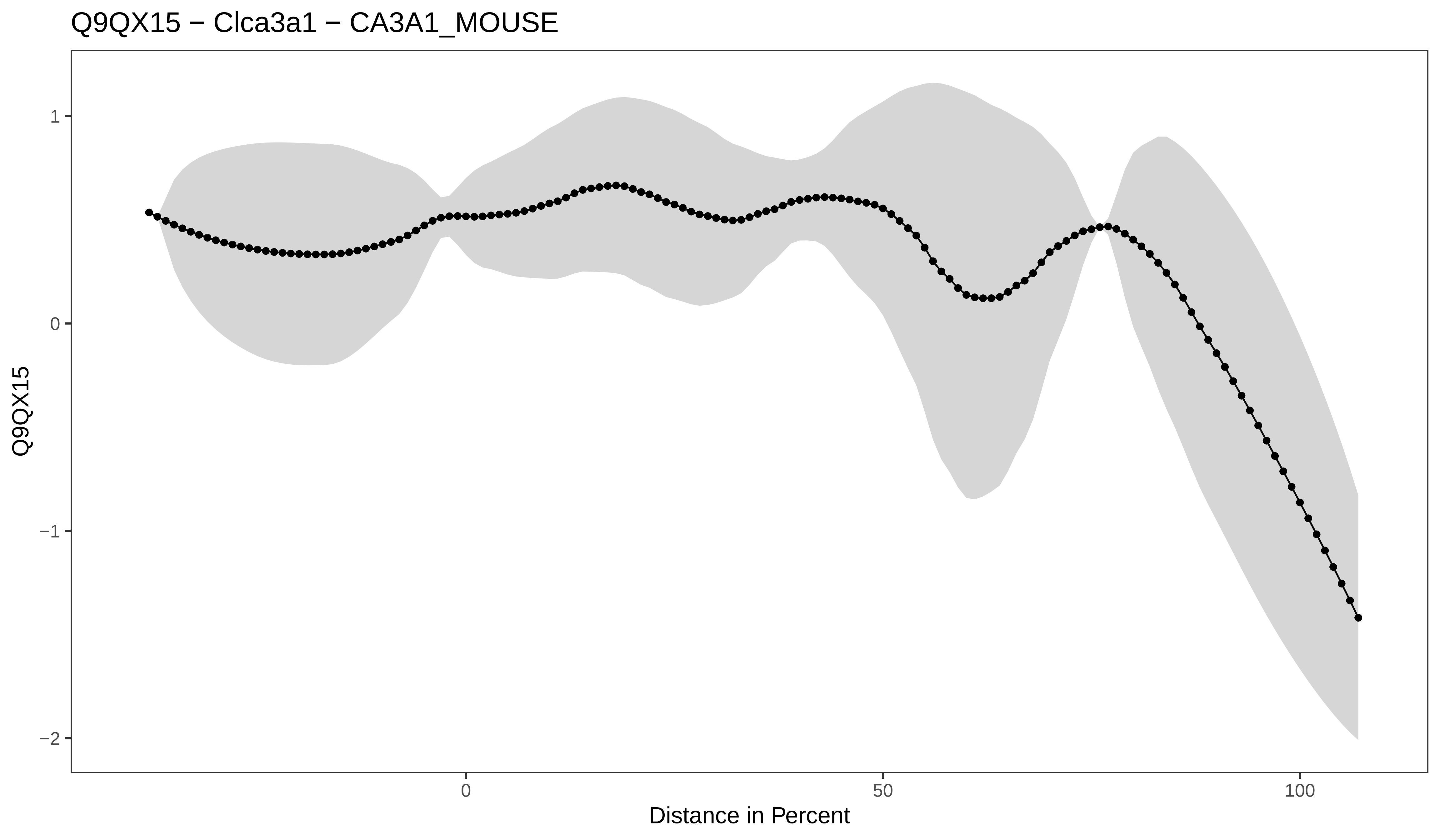

Q9QX15 − Clca3a1 − CA3A1_MOUSE
1
0
−1
−2
Q9QX15
0
50
100
Distance in Percent

Q9Z1Q5 − Clic1 − CLIC1_MOUSE

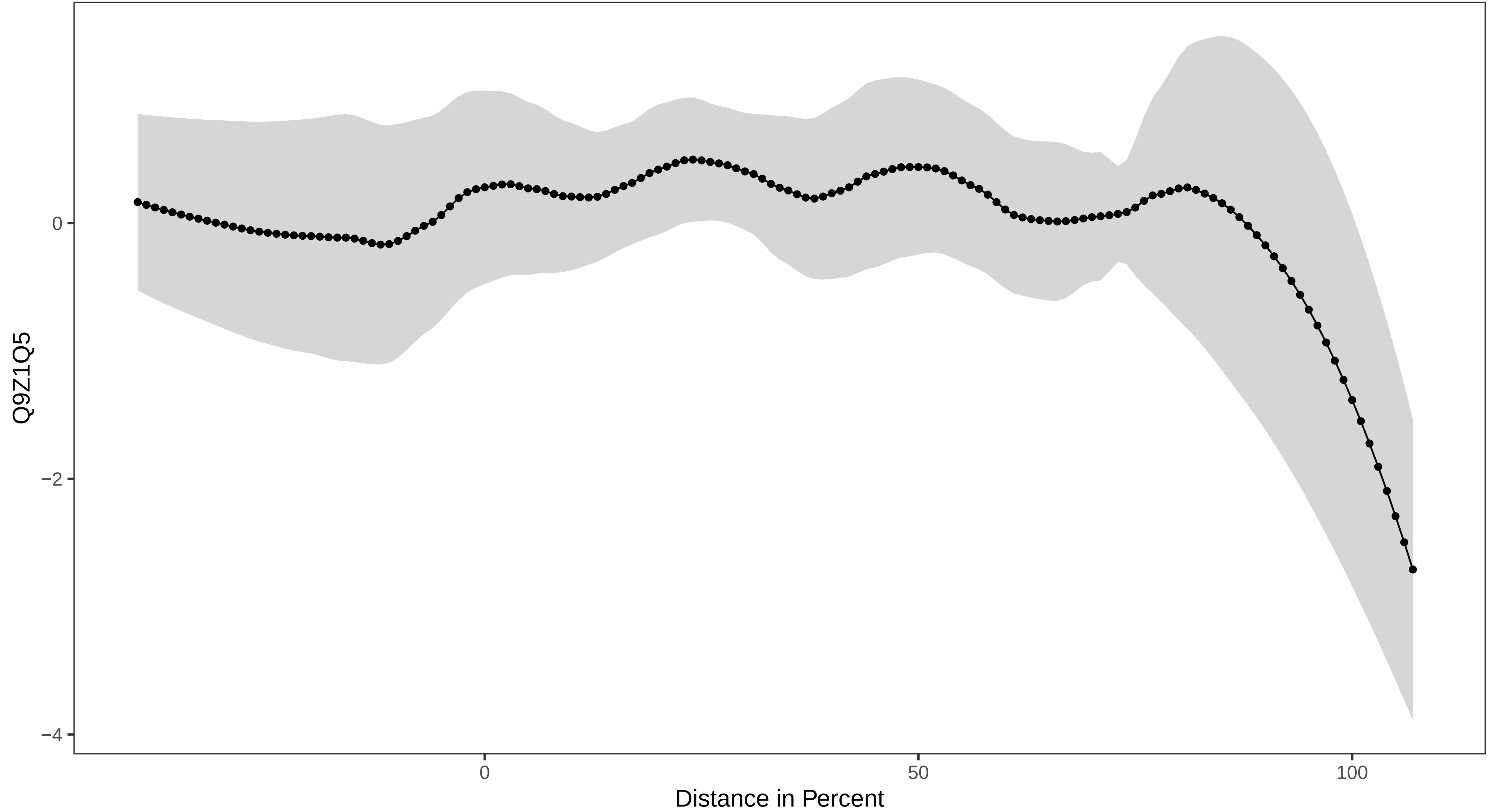

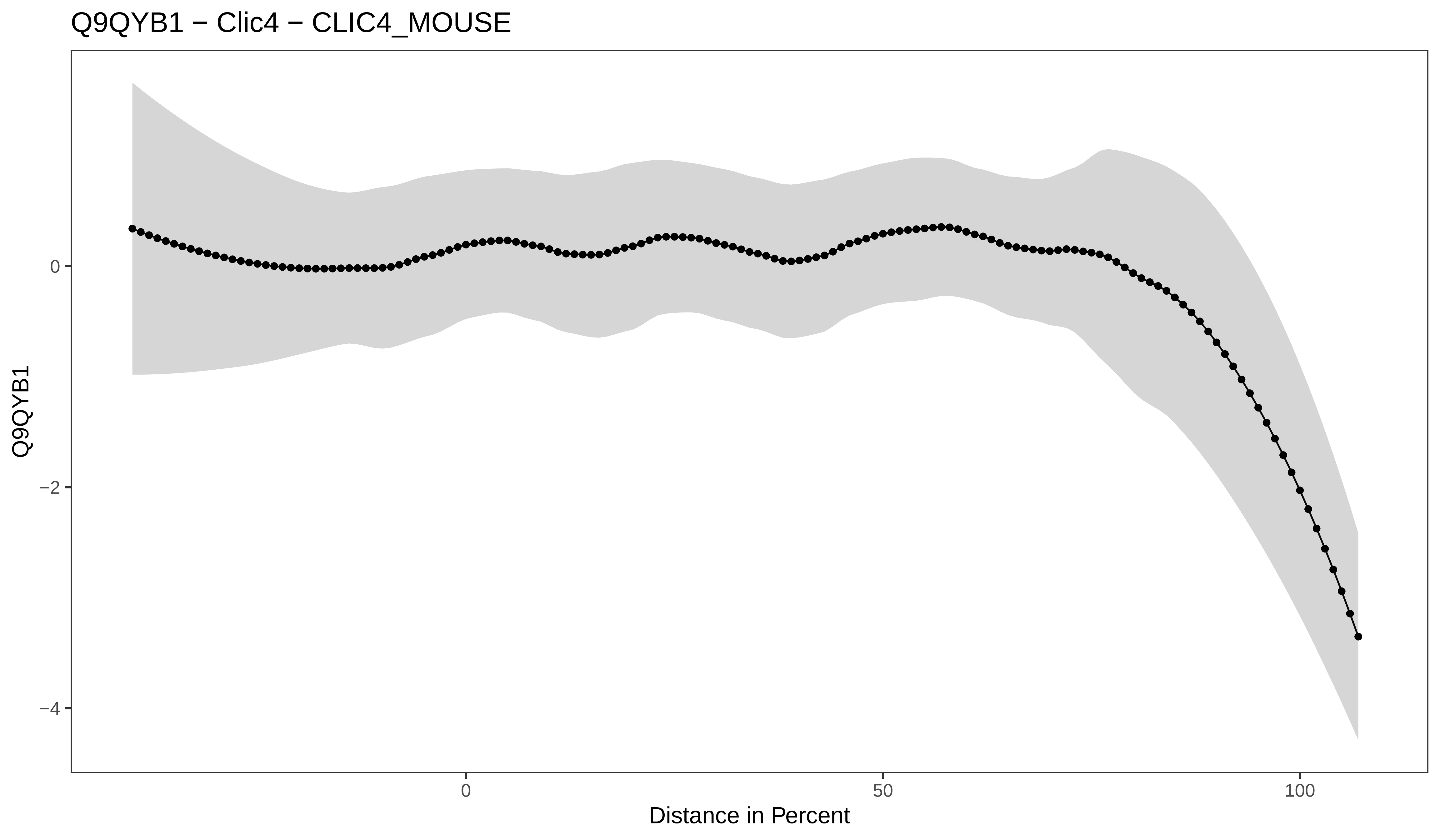

Q9QYB1 − Clic4 − CLIC4_MOUSE
Q9QYB1
0
−2
−4
0
50
100
Distance in Percent

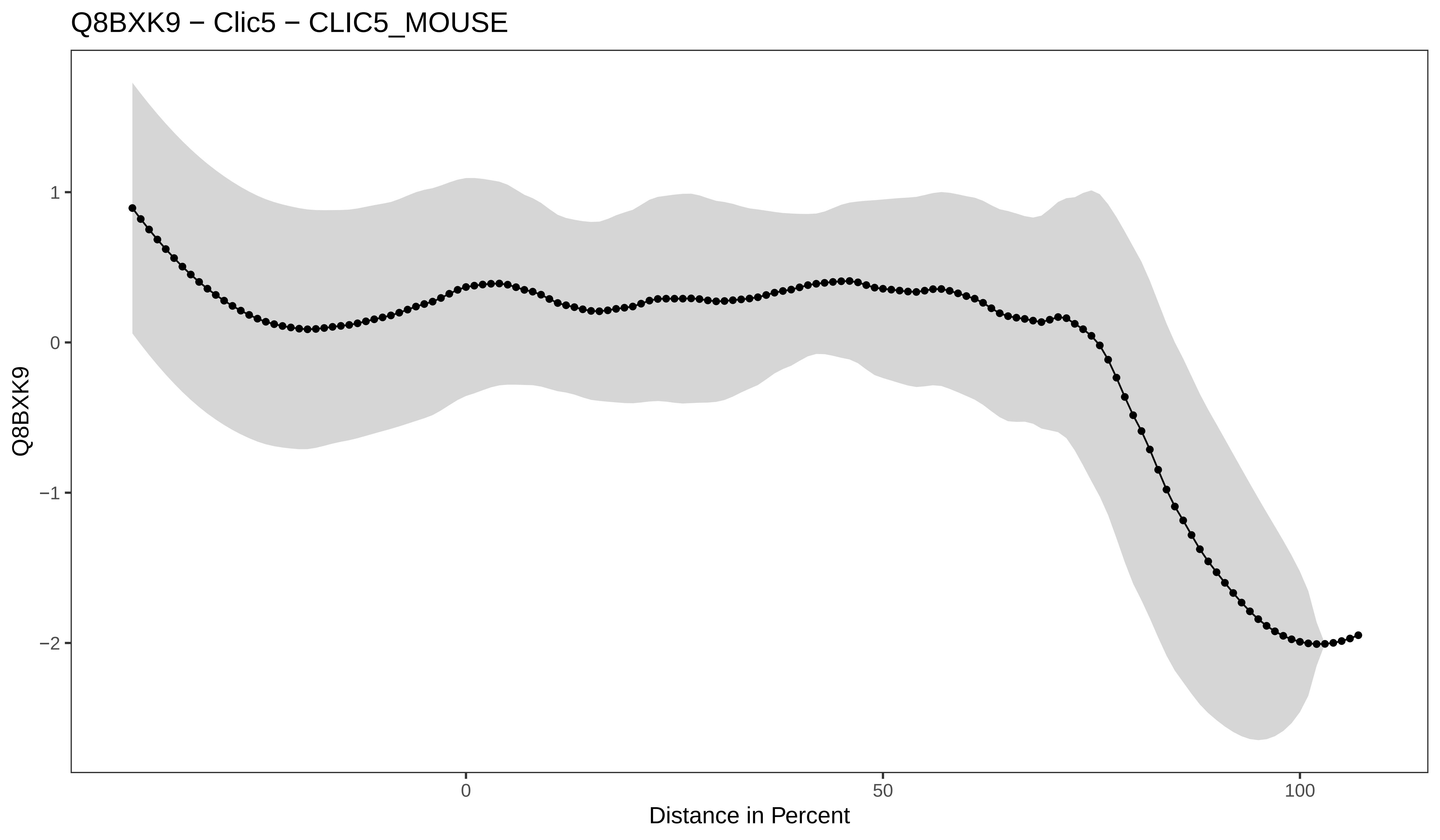

Q8BXK9 − Clic5 − CLIC5_MOUSE
1
0
−1
−2
Q8BXK9
0
50
100
Distance in Percent

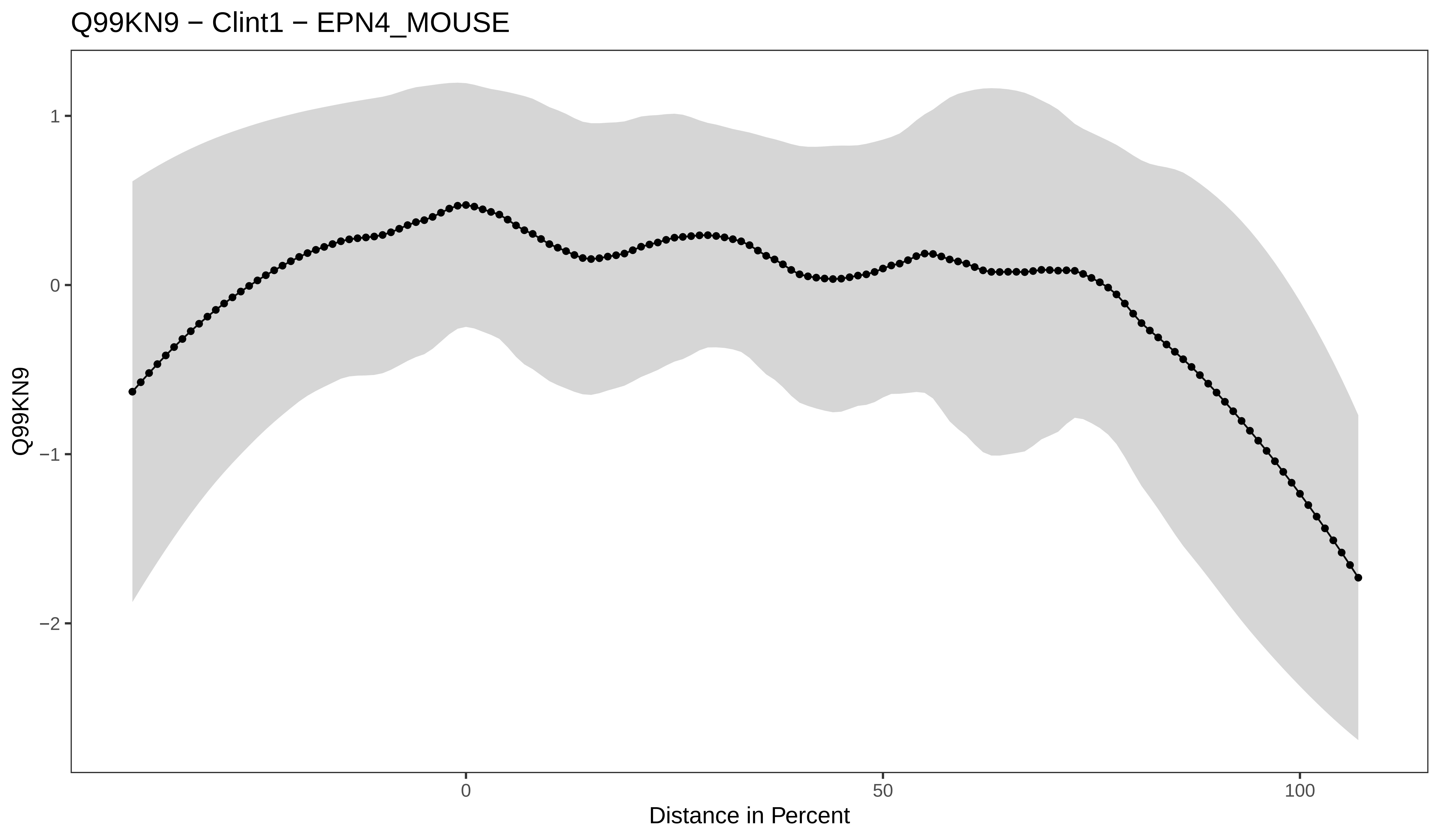

Q99KN9 − Clint1 − EPN4_MOUSE
1
0
−1
−2
Q99KN9
0
50
100
Distance in Percent

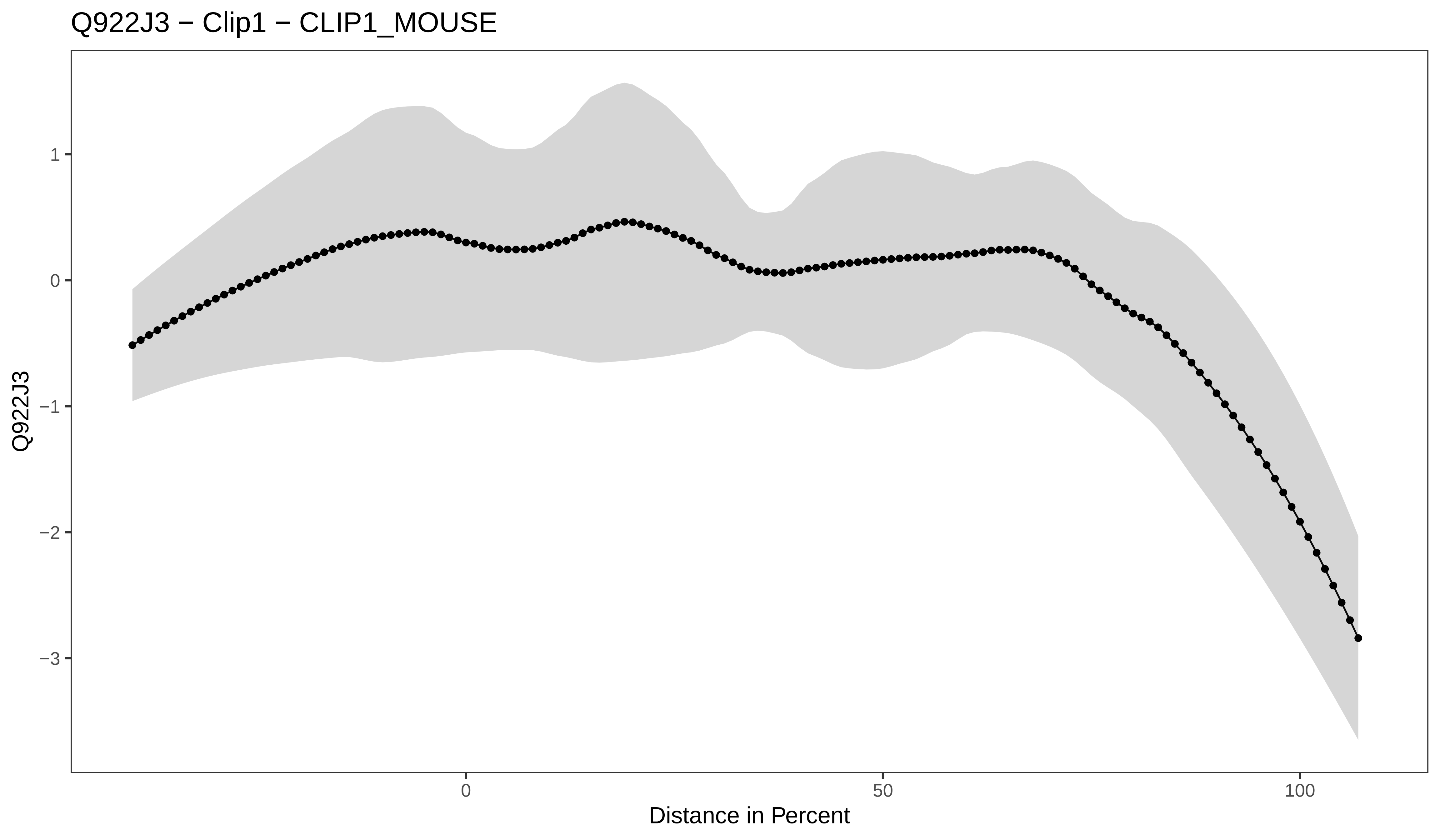

Q922J3 − Clip1 − CLIP1_MOUSE
Q922J3
1
0
−1
−2
−3
0
50
100
Distance in Percent

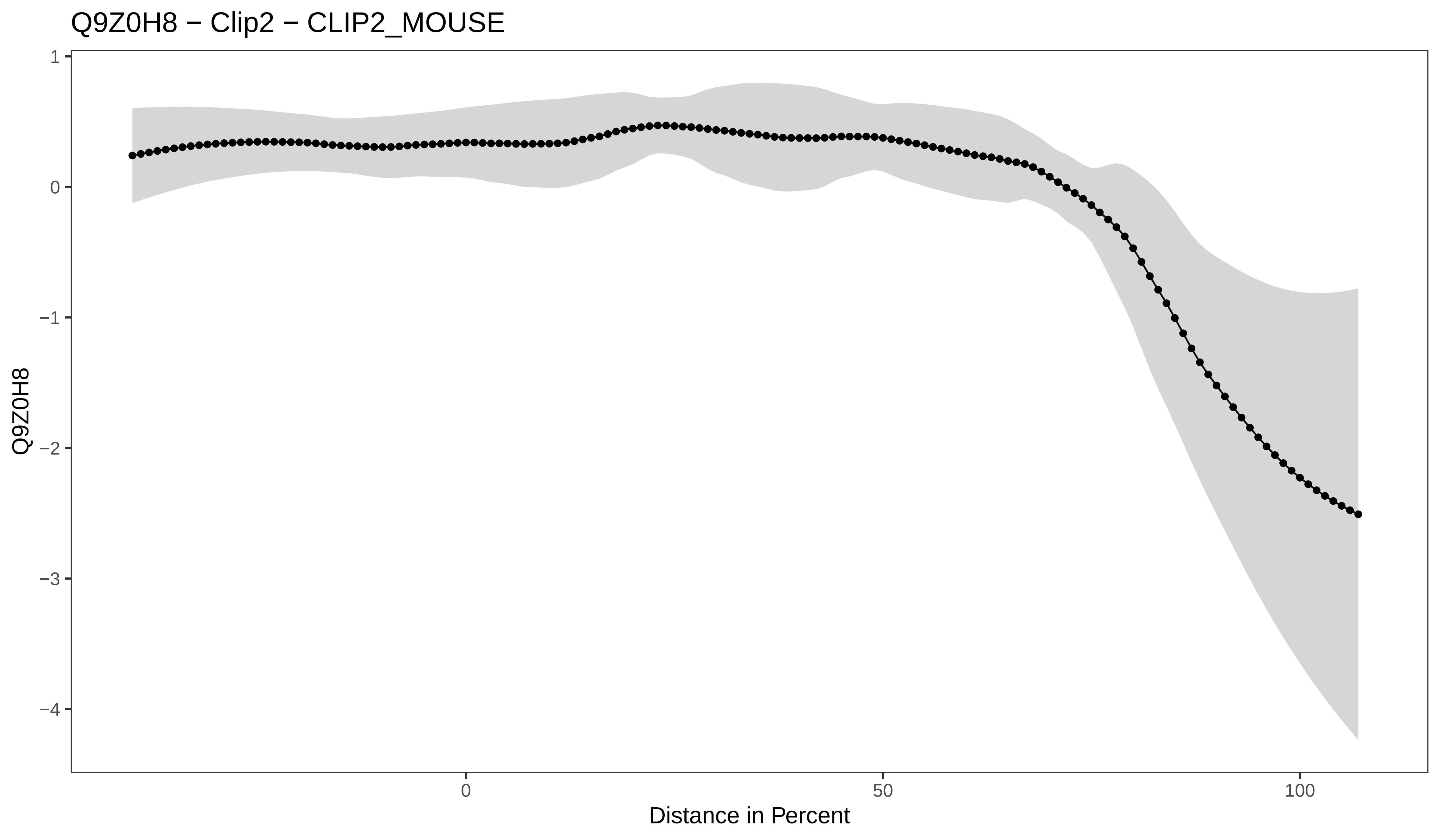

Q9Z0H8 − Clip2 − CLIP2_MOUSE
Q9Z0H8
1
0
−1
−2
−3
−4
0
50
100
Distance in Percent

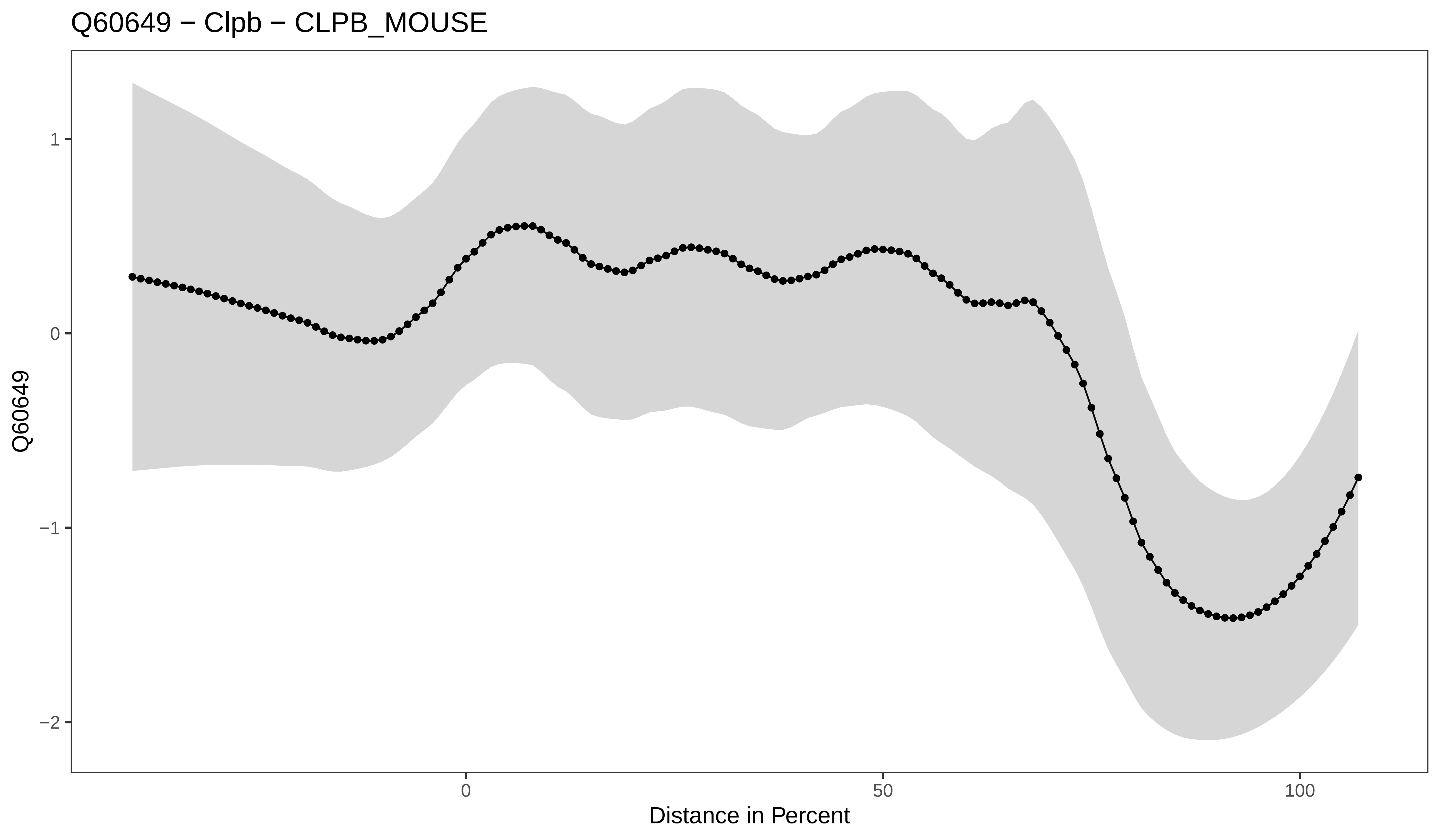

Q60649 − Clpb − CLPB_MOUSE
Q60649
1
0
−1
−2
0
50
100
Distance in Percent

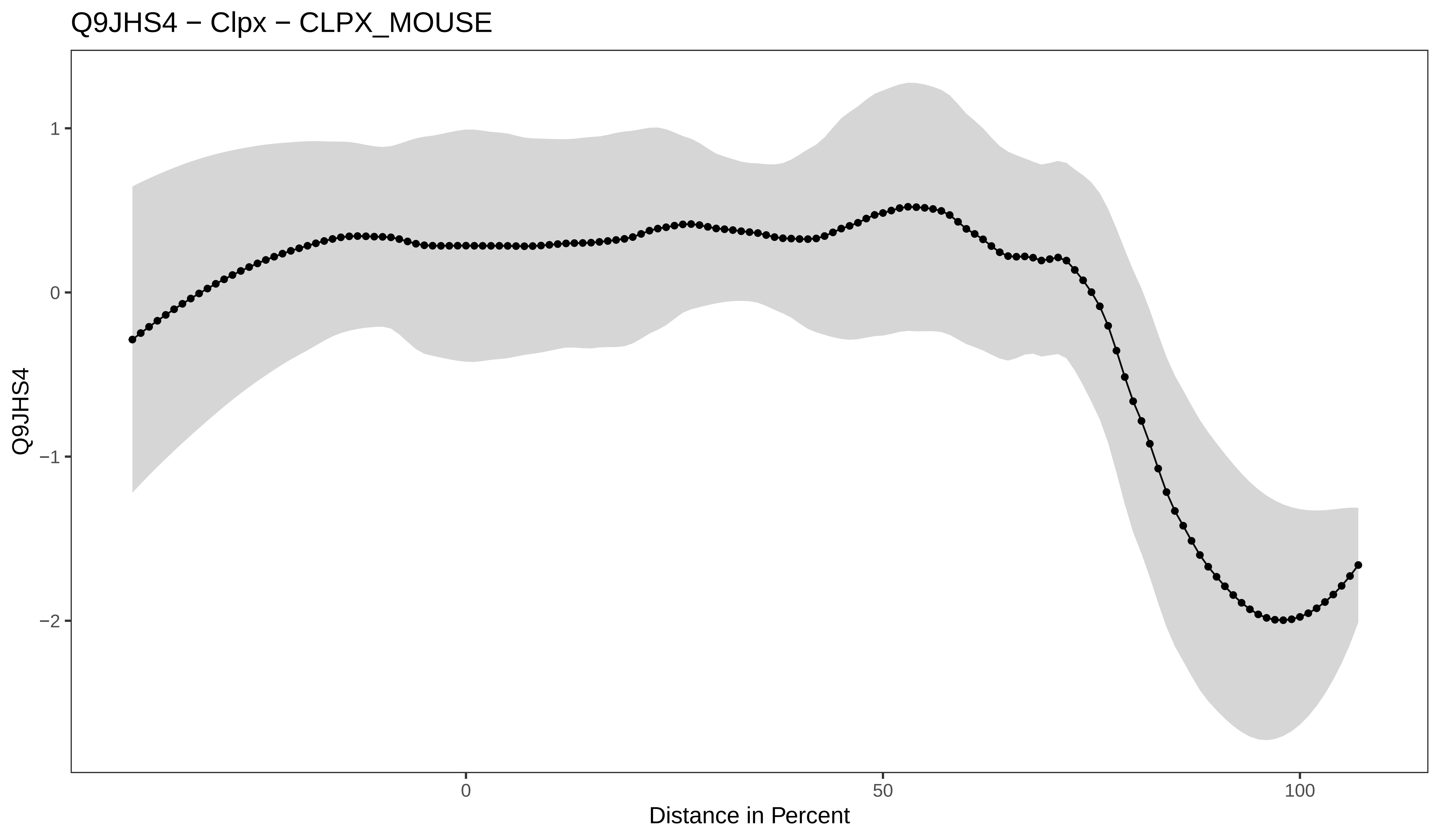

Q9JHS4 − Clpx − CLPX_MOUSE
Q9JHS4
Distance in Percent
1
0
−1
−2
0
50
100

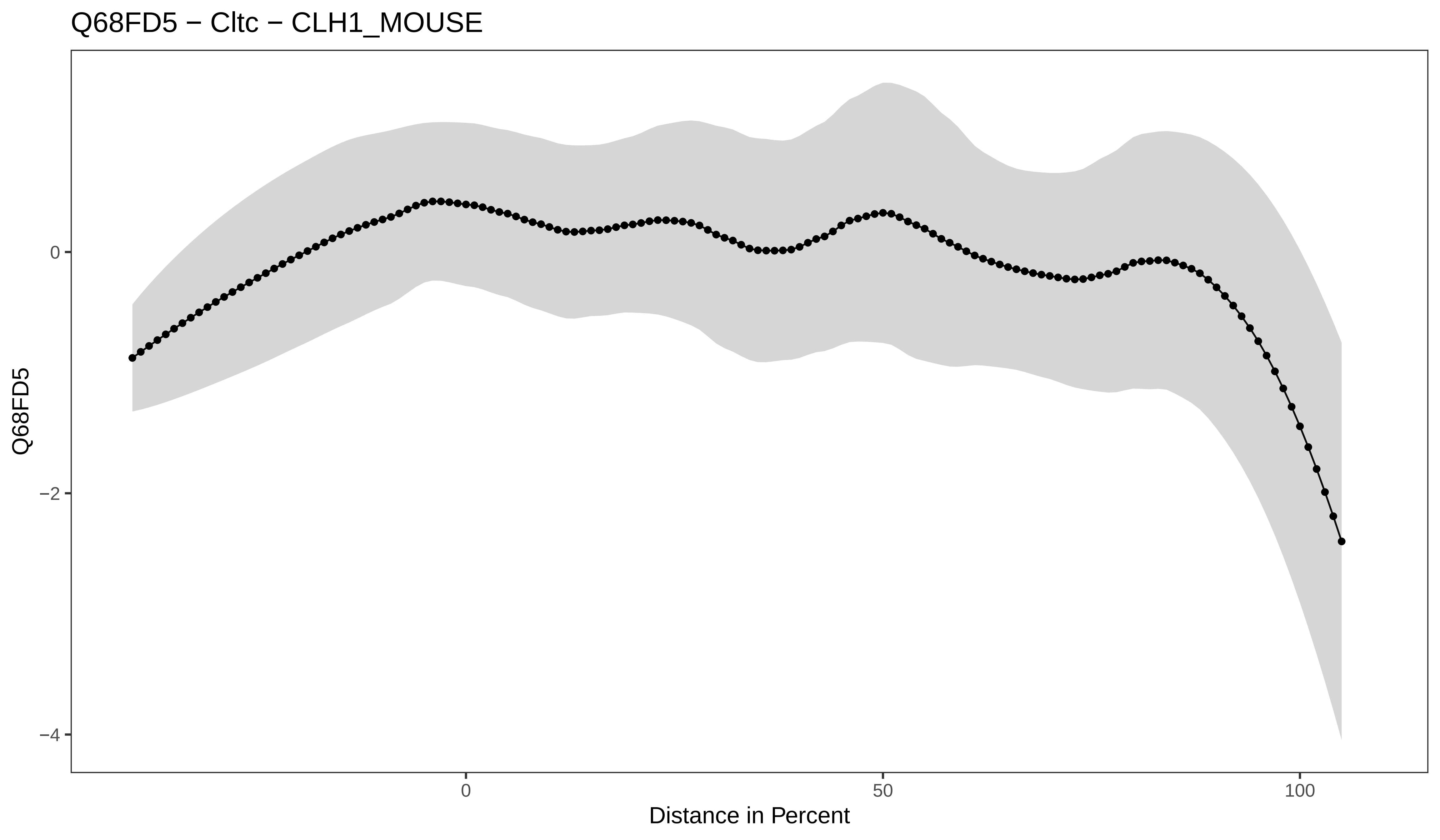
Q68FD5 − Cltc − CLH1_MOUSE
Q68FD5
0
−2
−4
0
50
100
Distance in Percent

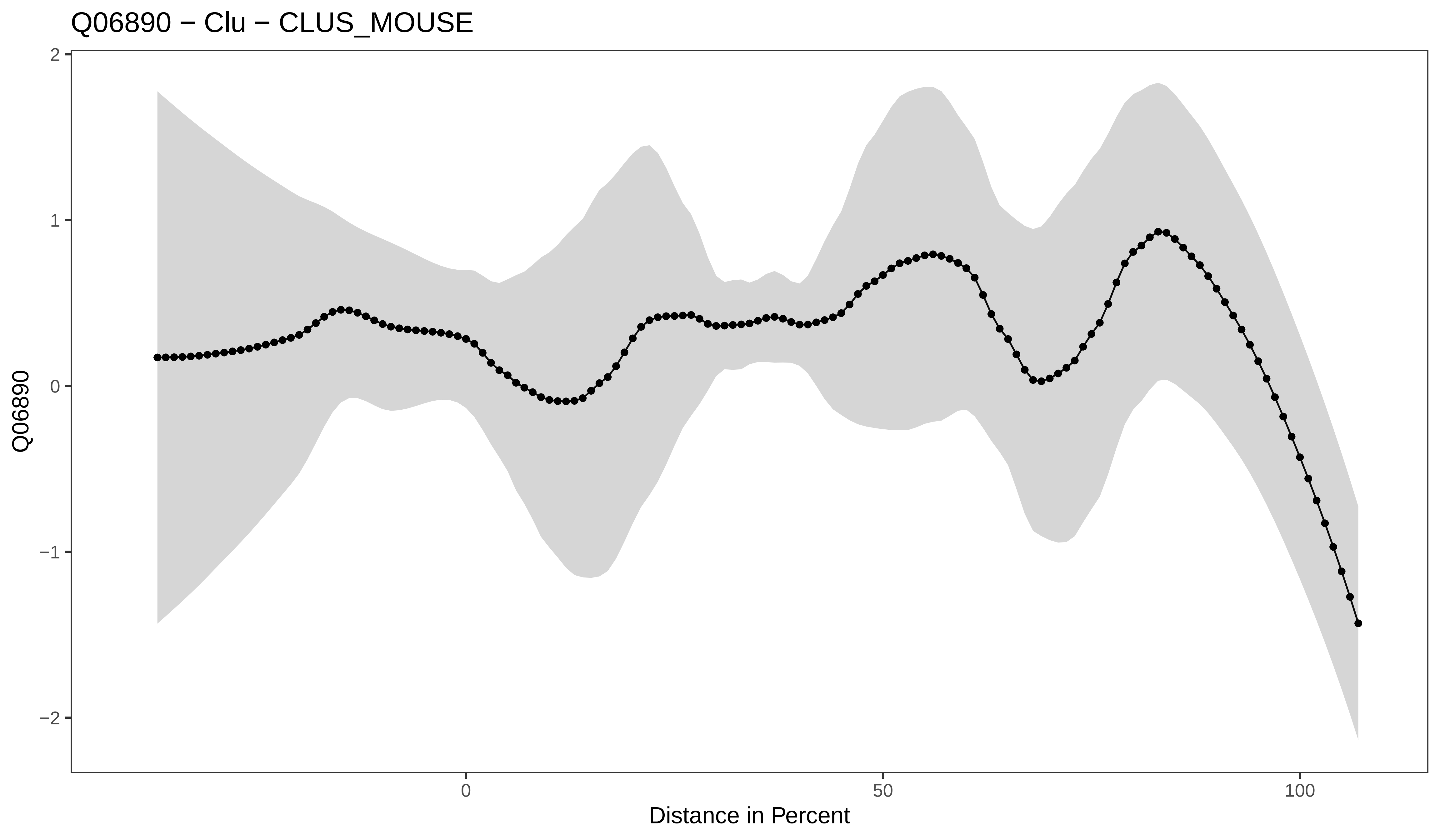

Q06890 − Clu − CLUS_MOUSE
2
1
0
−1
−2
Q06890
0
50
100
Distance in Percent

Q5SW19 − Cluh − CLU_MOUSE

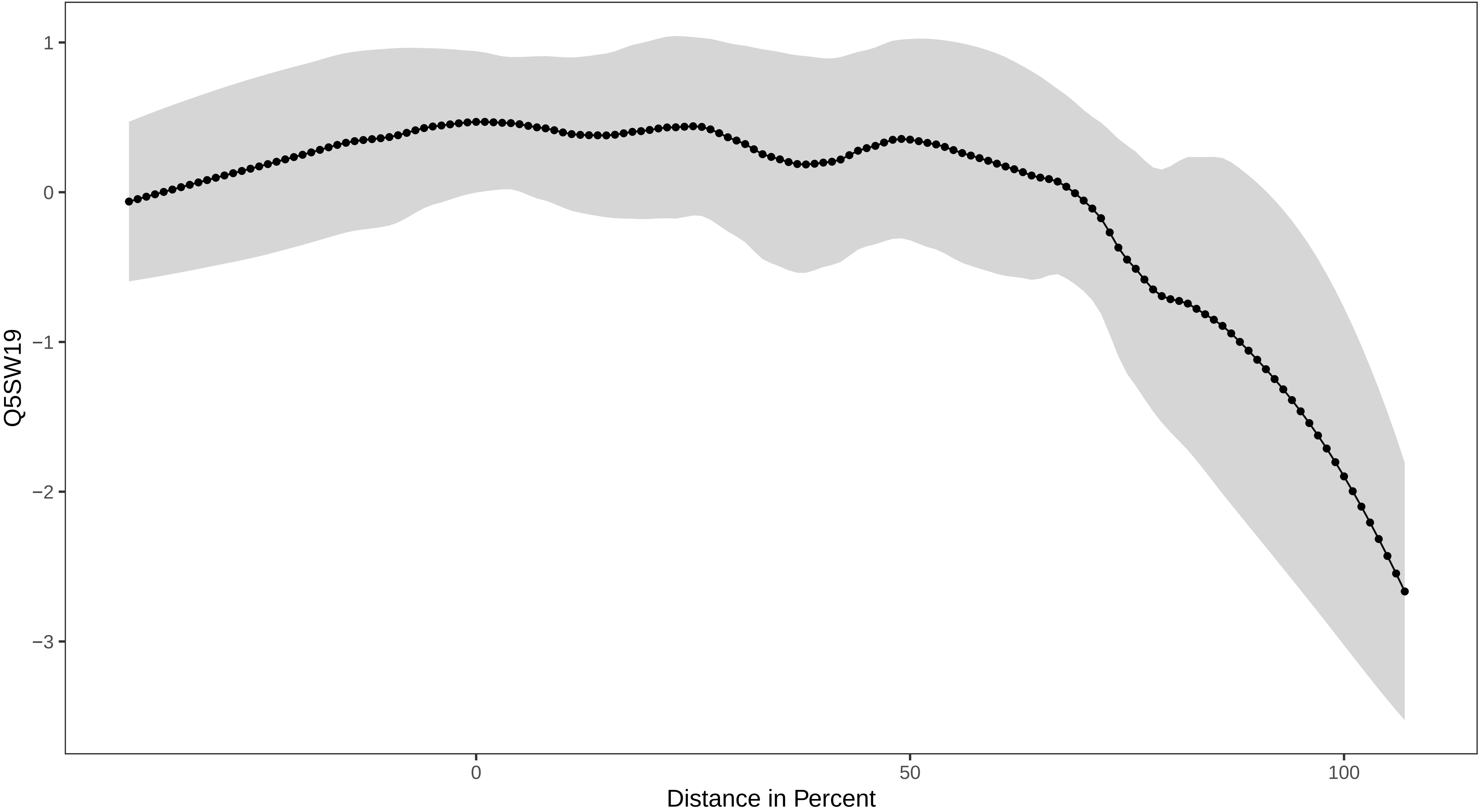

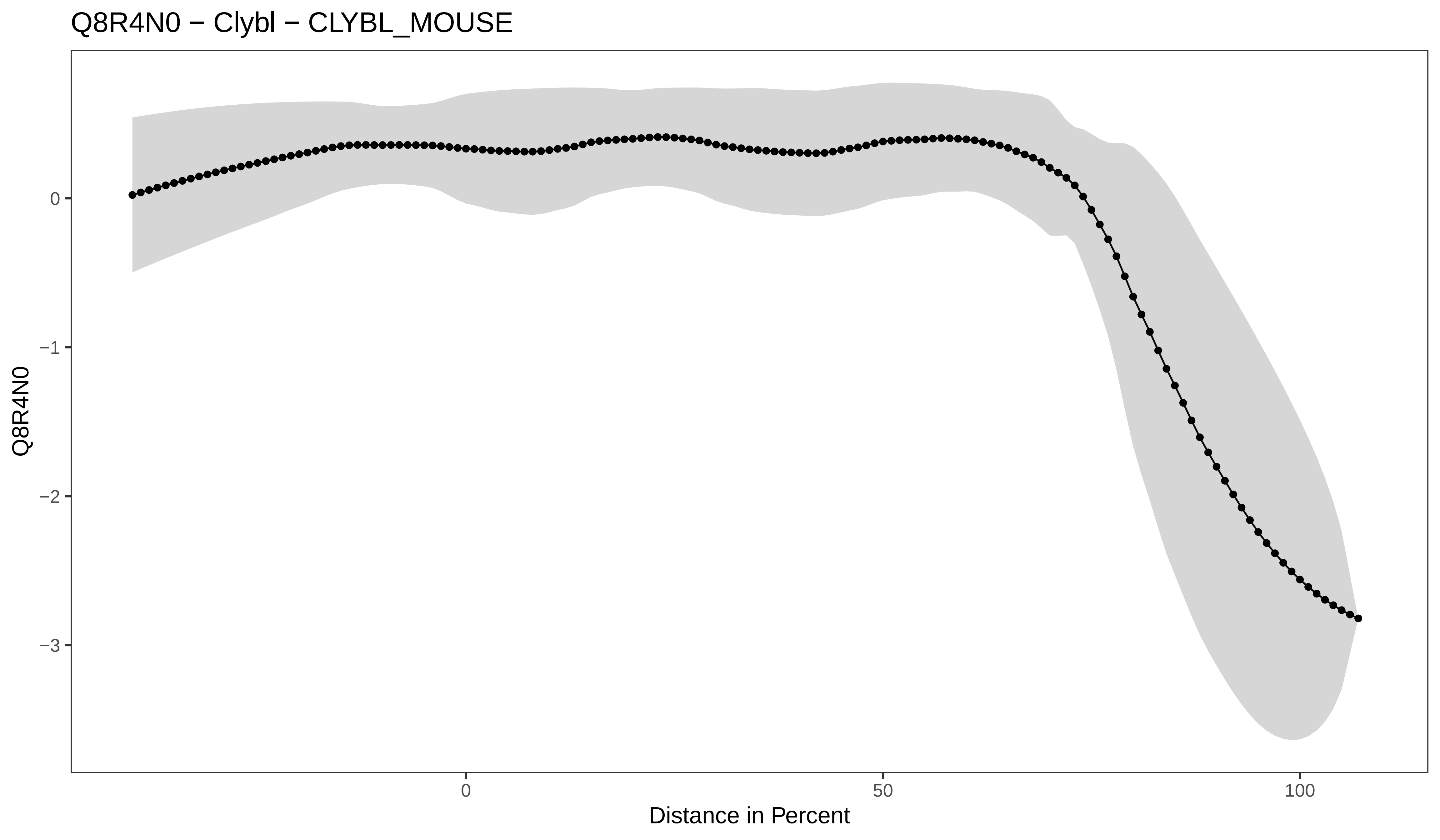

Q8R4N0 − Clybl − CLYBL_MOUSE
Q8R4N0
0
−1
−2
−3
0
50
100
Distance in Percent

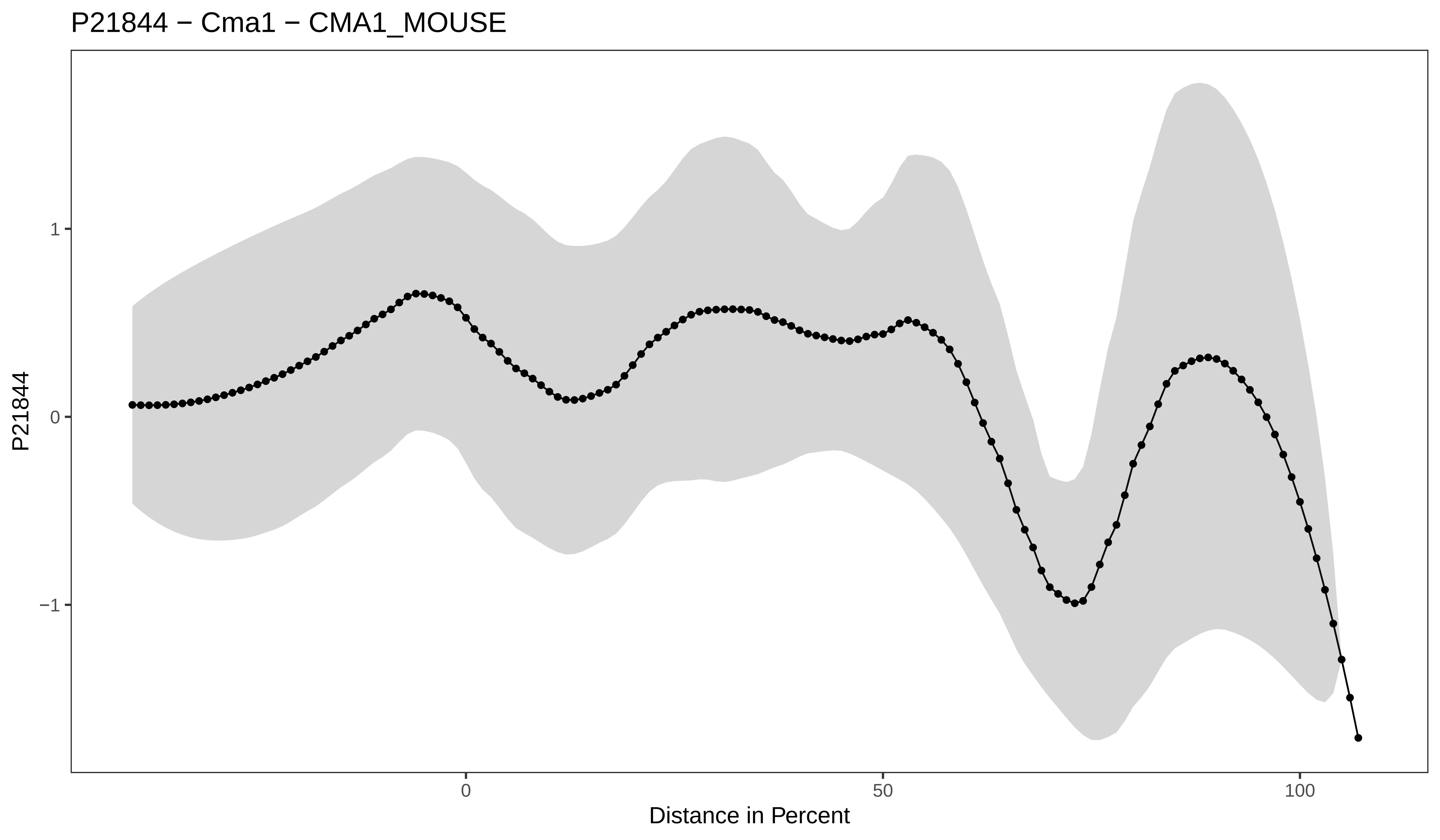
P21844 − Cma1 − CMA1_MOUSE
P21844
1
0
−1
0
50
100
Distance in Percent

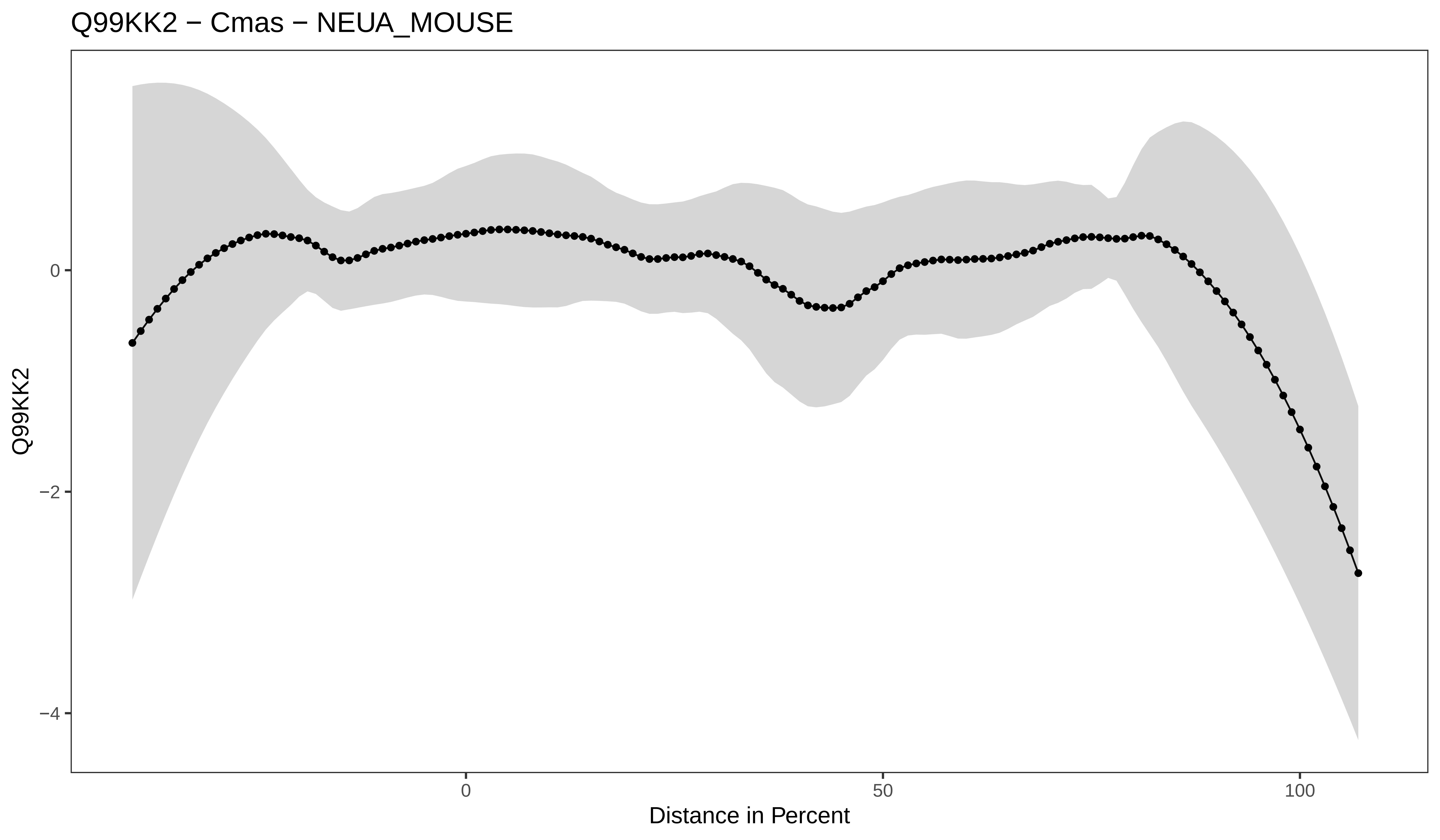
Q99KK2 − Cmas − NEUA_MOUSE
Q99KK2
0
−2
−4
0
50
100
Distance in Percent

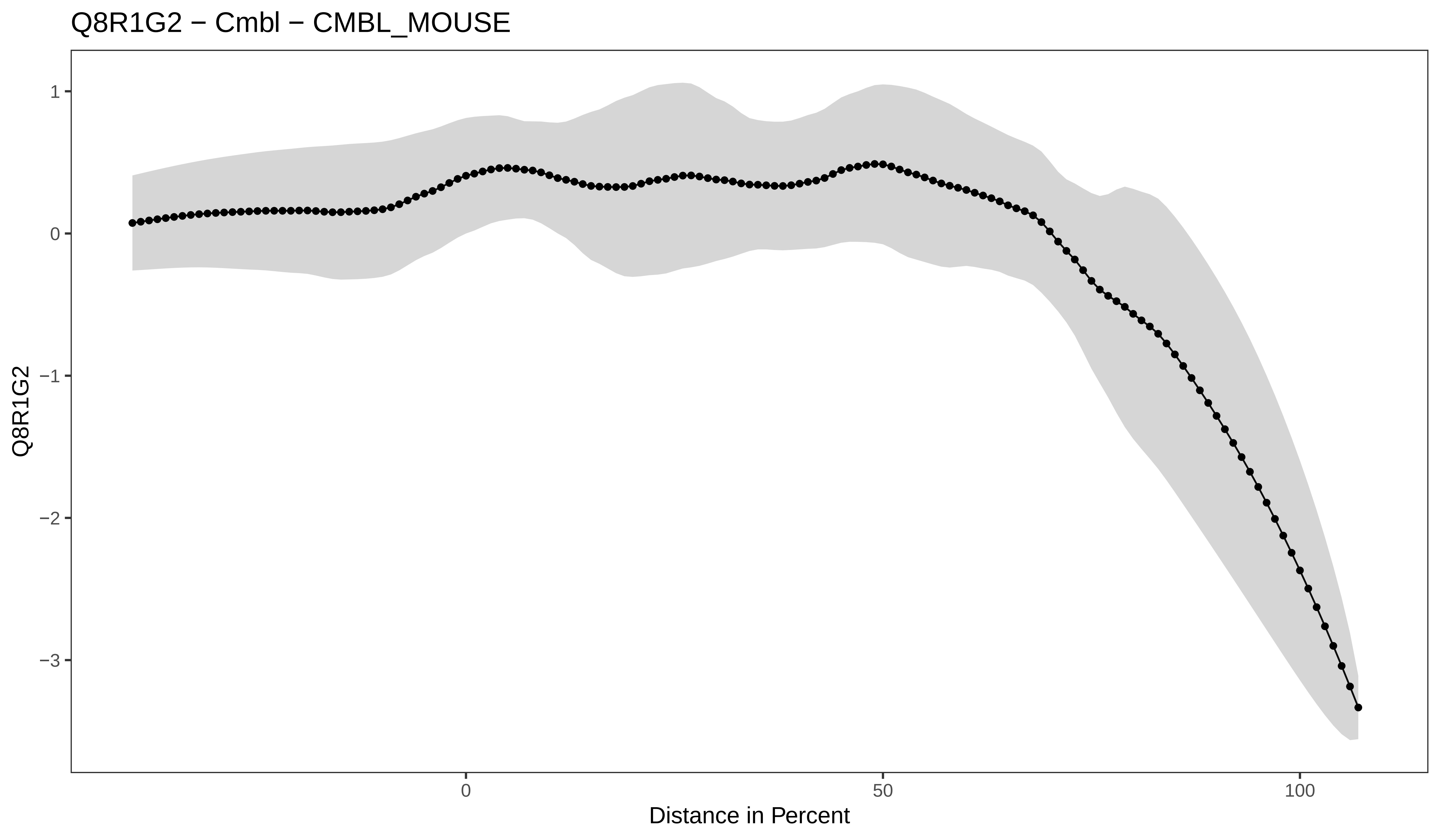

Q8R1G2 − Cmbl − CMBL_MOUSE
Q8R1G2
1
0
−1
−2
−3
0
50
100
Distance in Percent

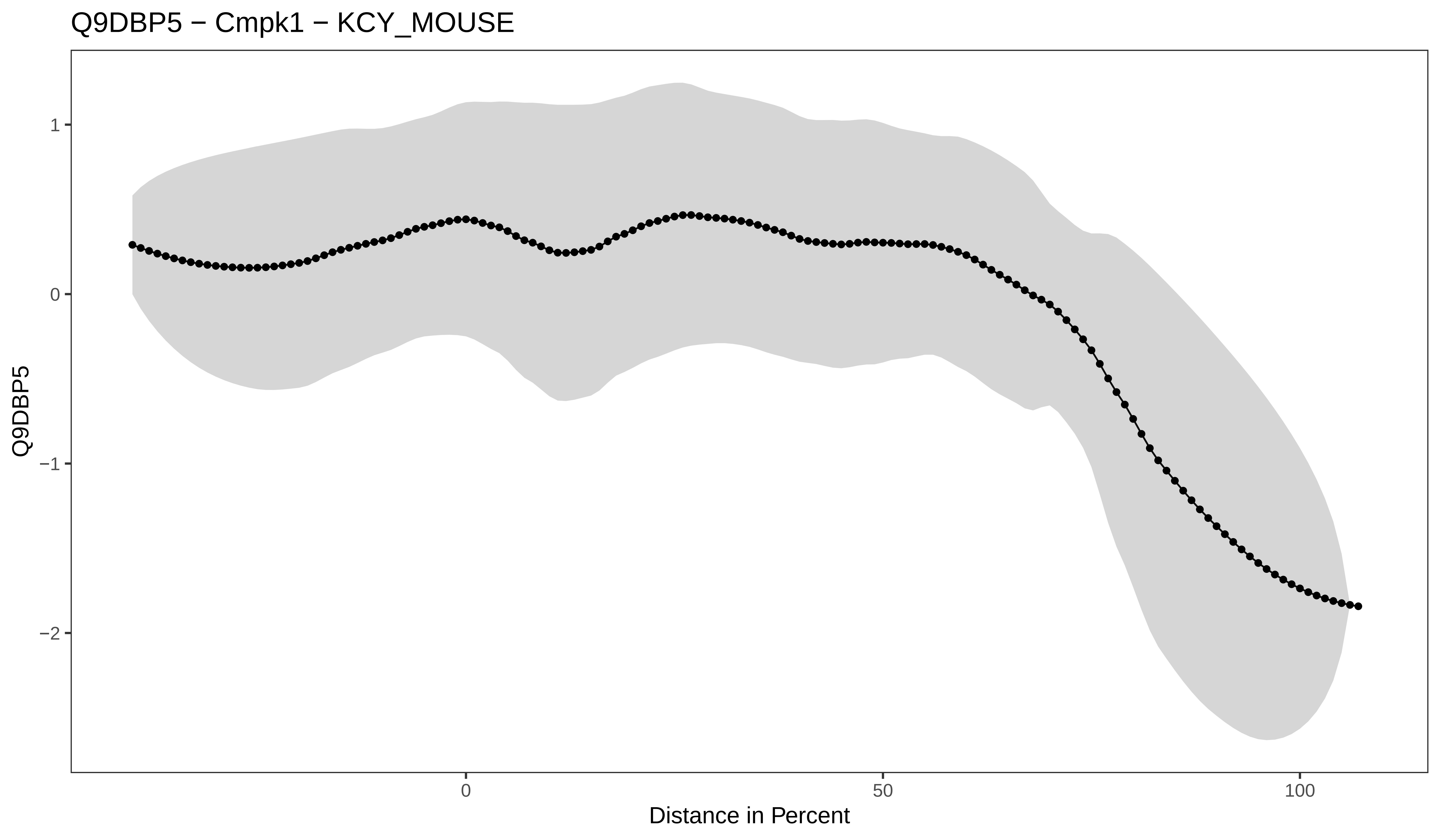

Q9DBP5 − Cmpk1 − KCY_MOUSE
Q9DBP5
1
0
−1
−2
0
50
100
Distance in Percent

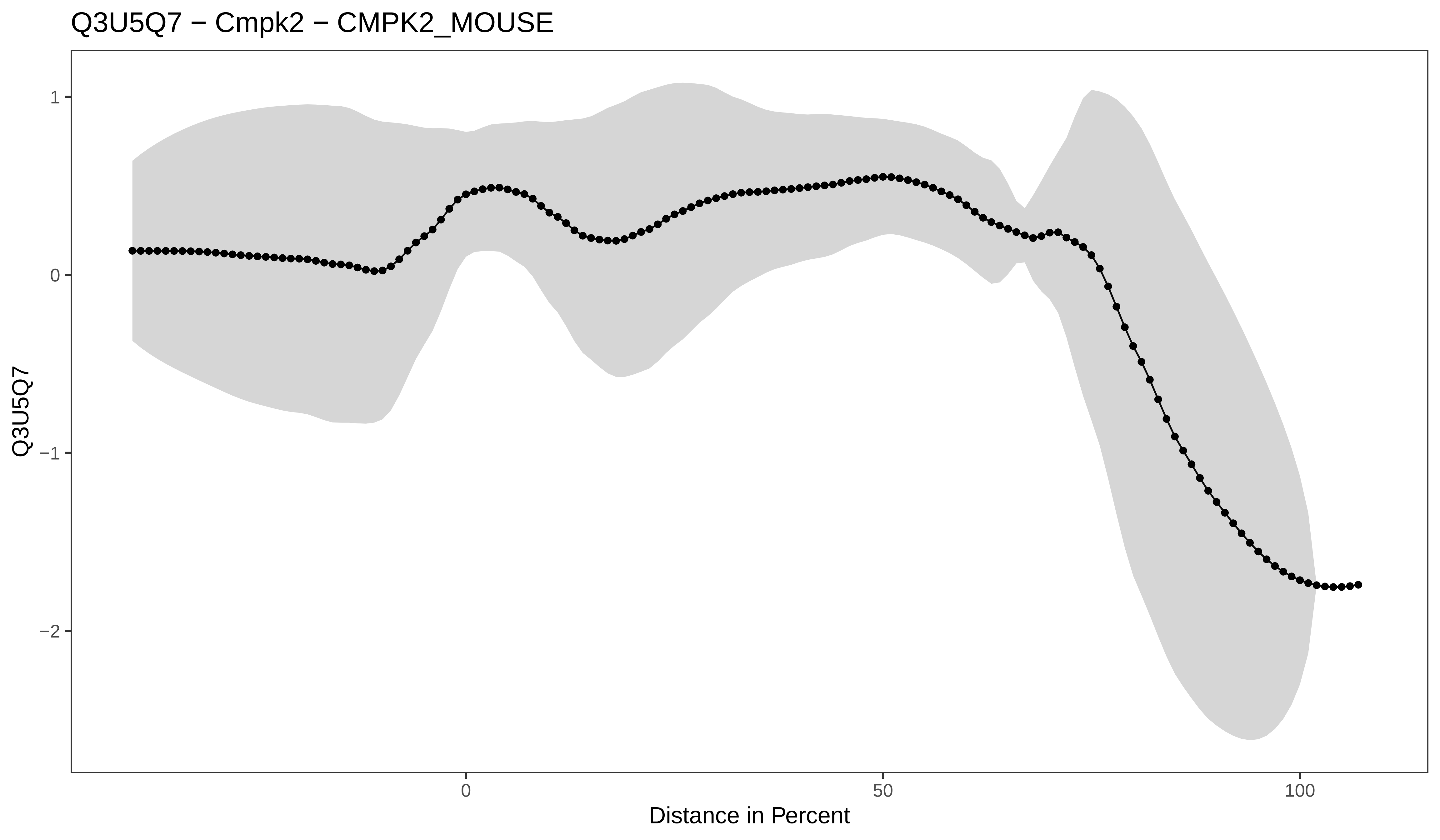

Q3U5Q7 − Cmpk2 − CMPK2_MOUSE
Q3U5Q7
1
0
−1
−2
0
50
100
Distance in Percent

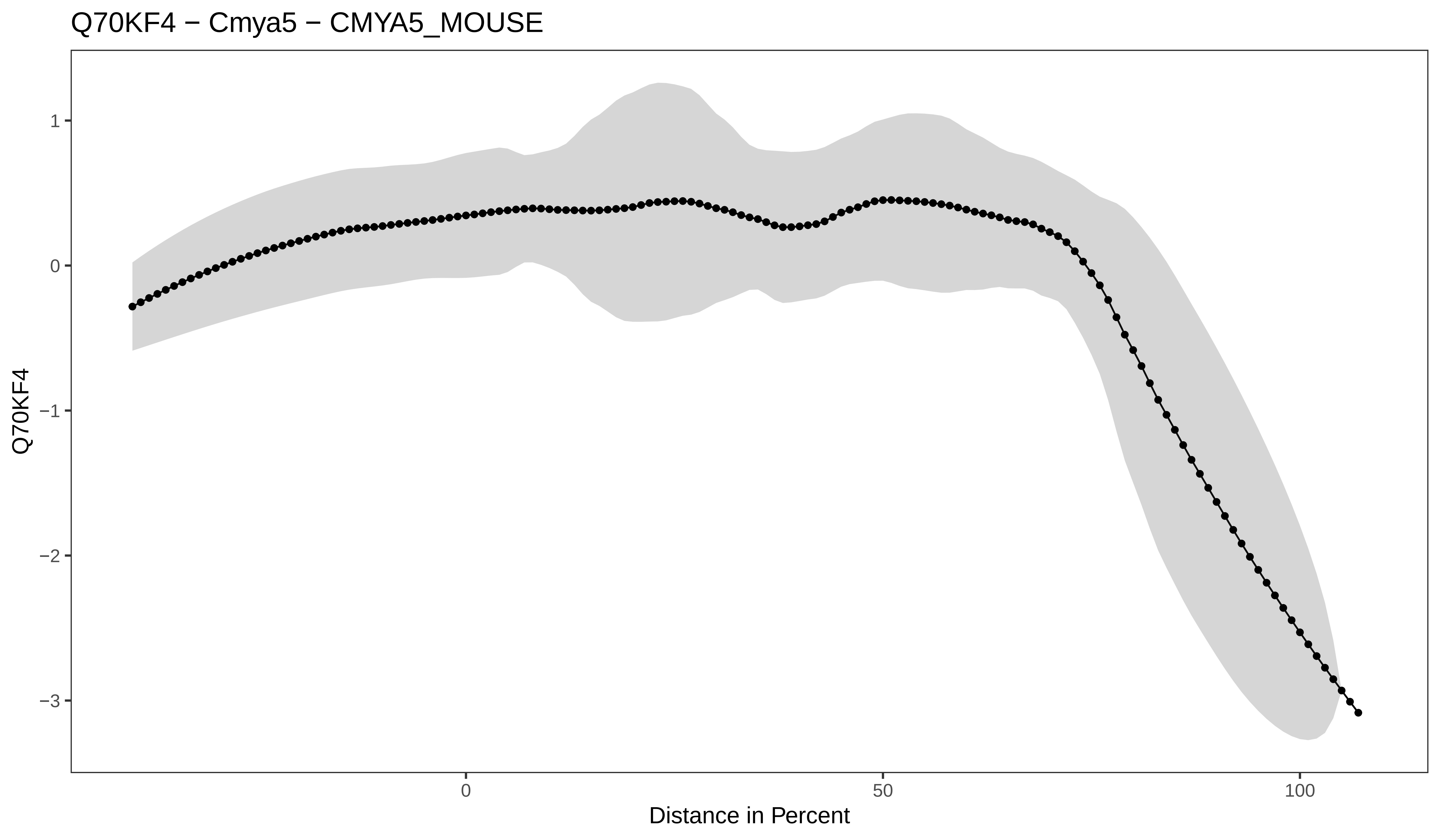
Q70KF4 − Cmya5 − CMYA5_MOUSE
Q70KF4
1
0
−1
−2
−3
0
50
100
Distance in Percent

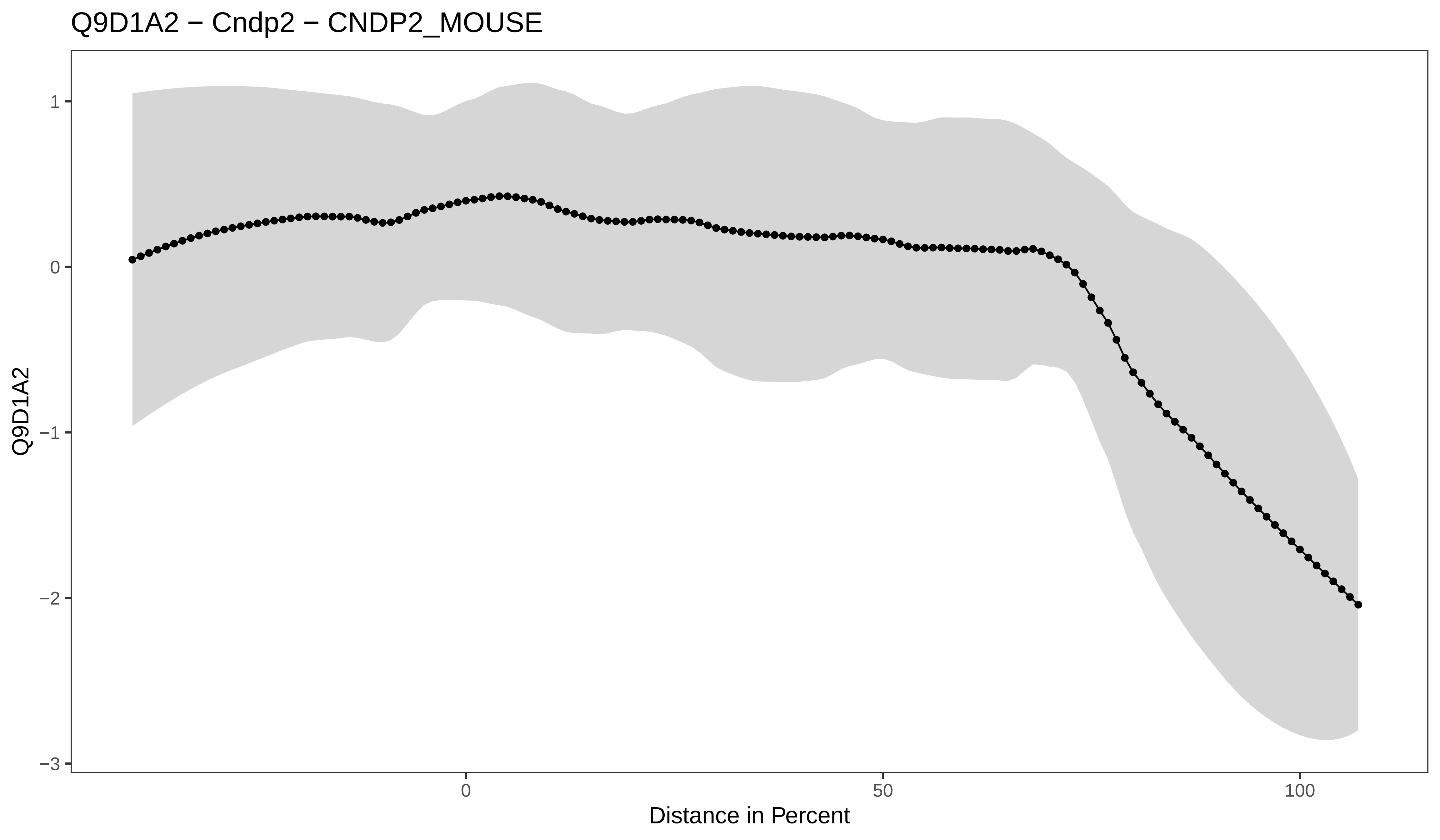
Q9D1A2 − Cndp2 − CNDP2_MOUSE
Q9D1A2
1
0
−1
−2
−3
0
50
100
Distance in Percent

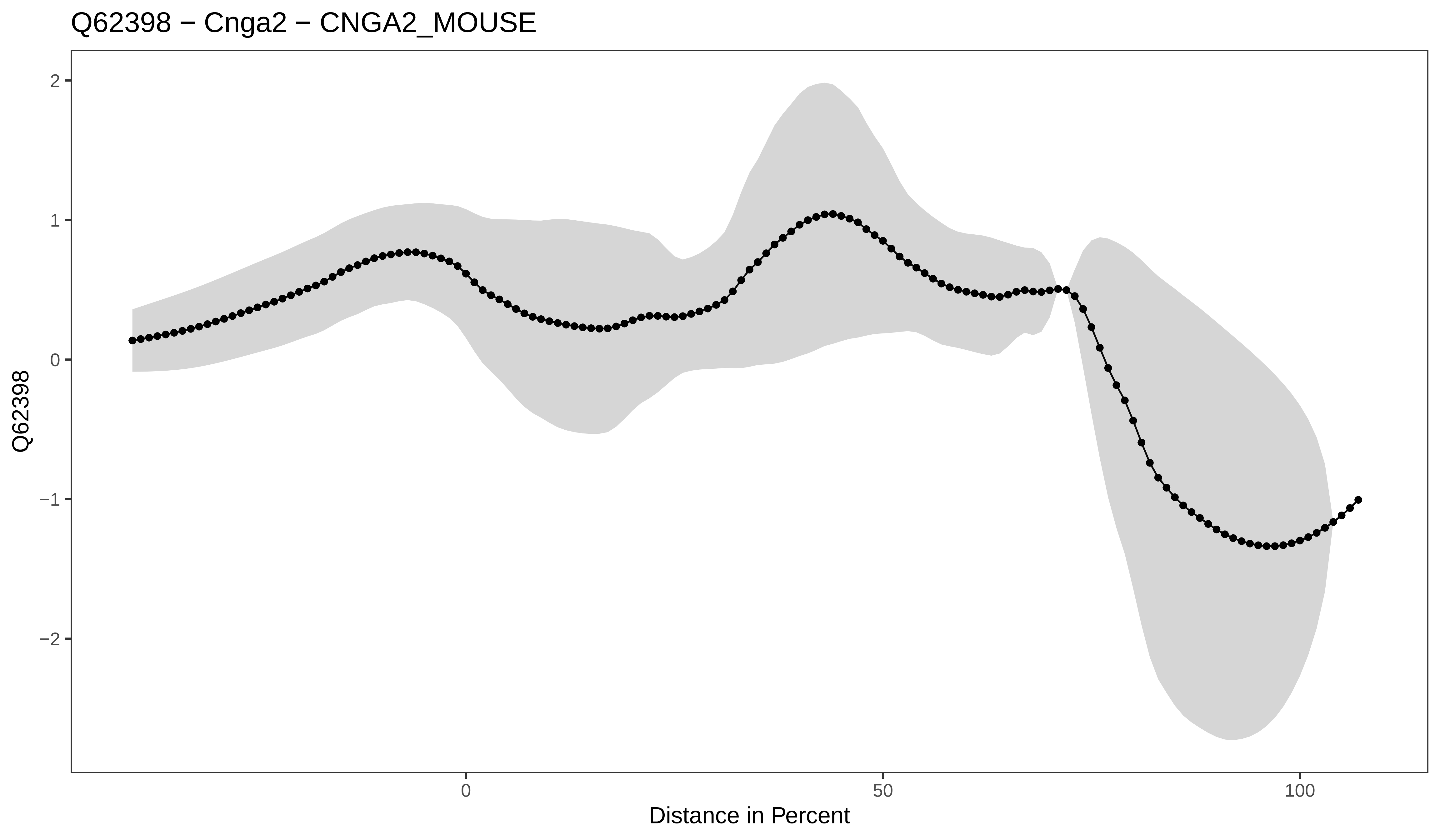

Q62398 − Cnga2 − CNGA2_MOUSE
Q62398
Distance in Percent
2
1
0
−1
−2
0
50
100

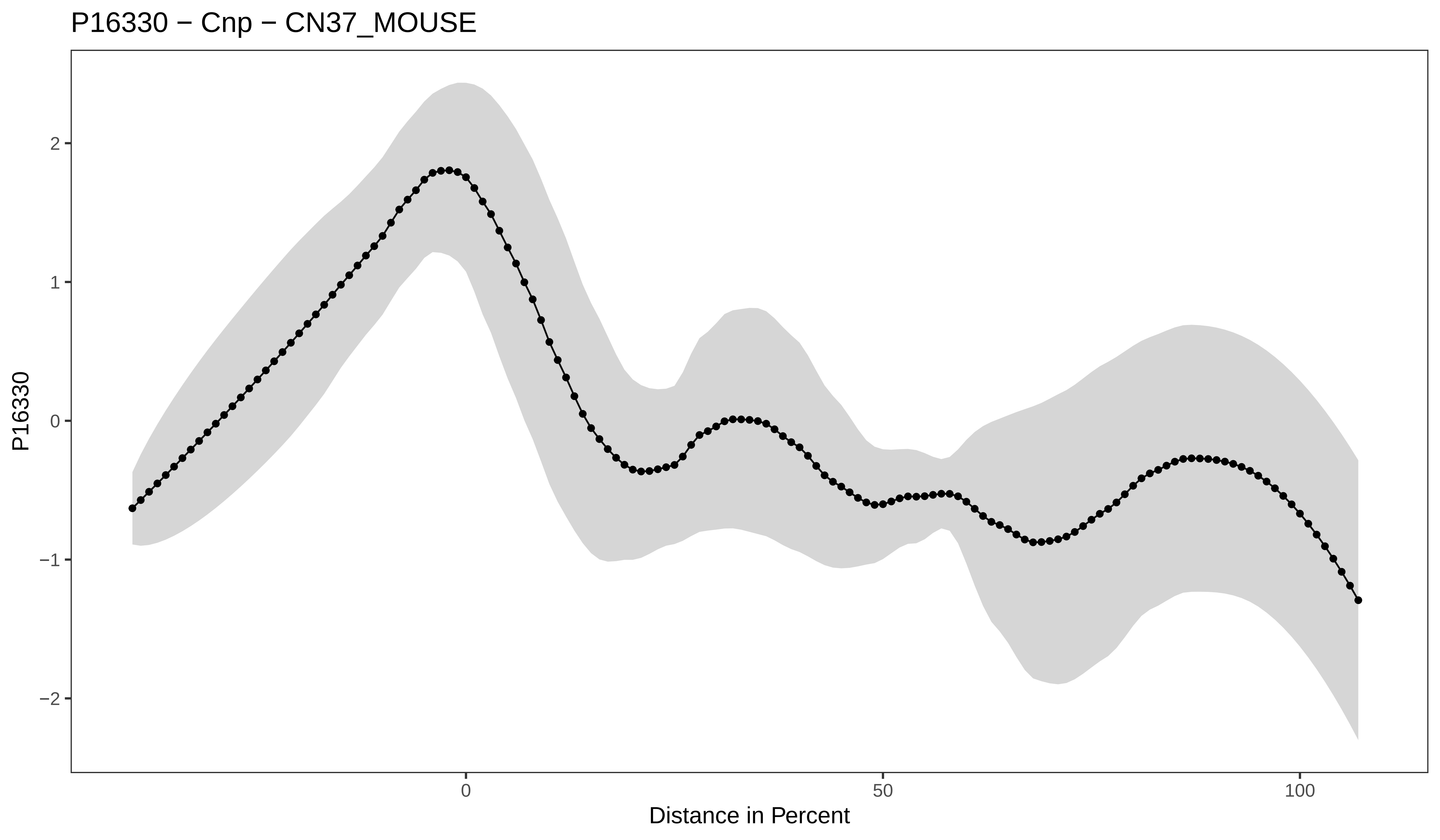

P16330 − Cnp − CN37_MOUSE
2
1
0
−1
−2
P16330
0
50
100
Distance in Percent

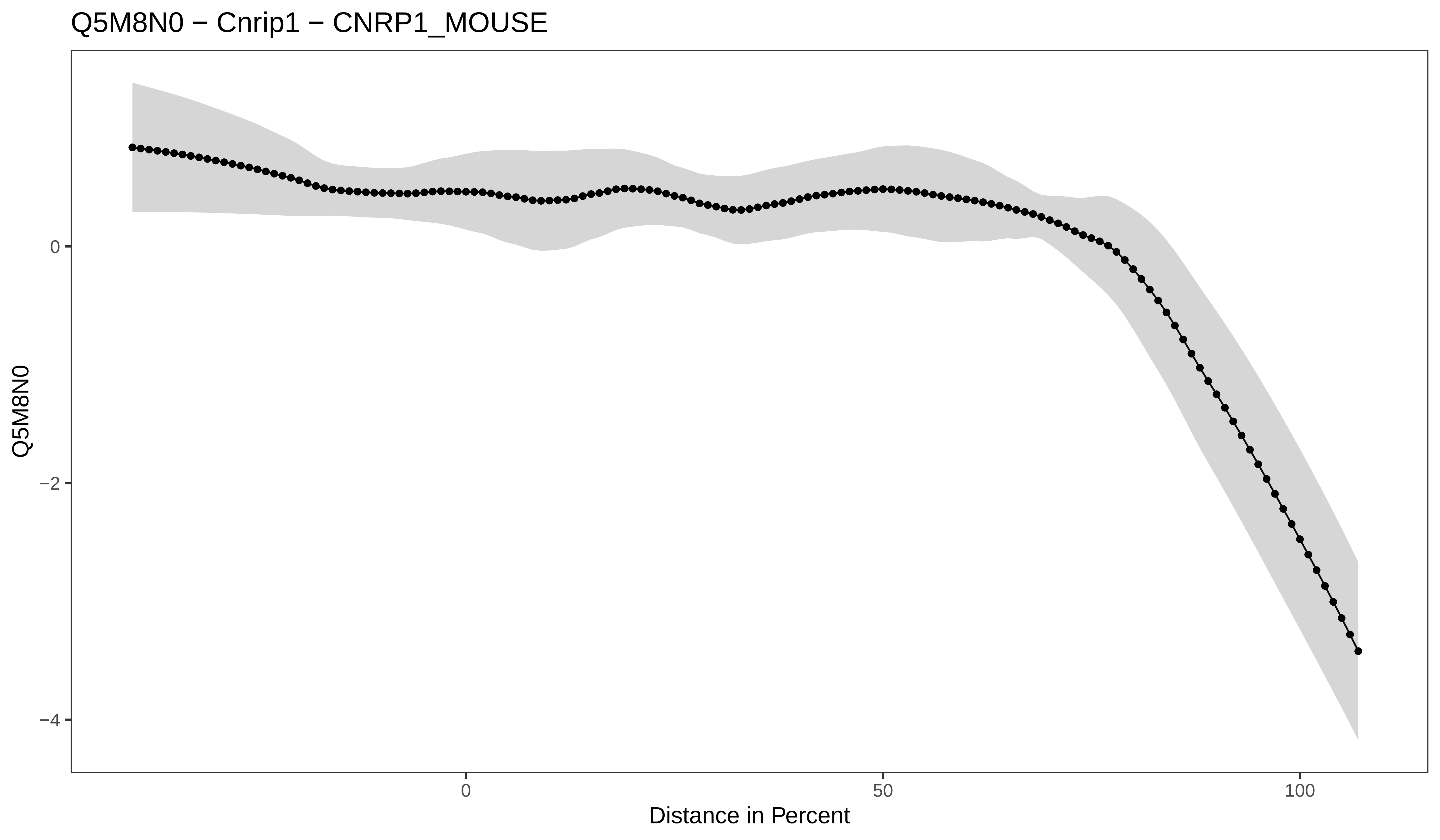

Q5M8N0 − Cnrip1 − CNRP1_MOUSE
Q5M8N0
0
−2
−4
0
50
100
Distance in Percent

Q9DBL7 − Coasy − COASY_MOUSE

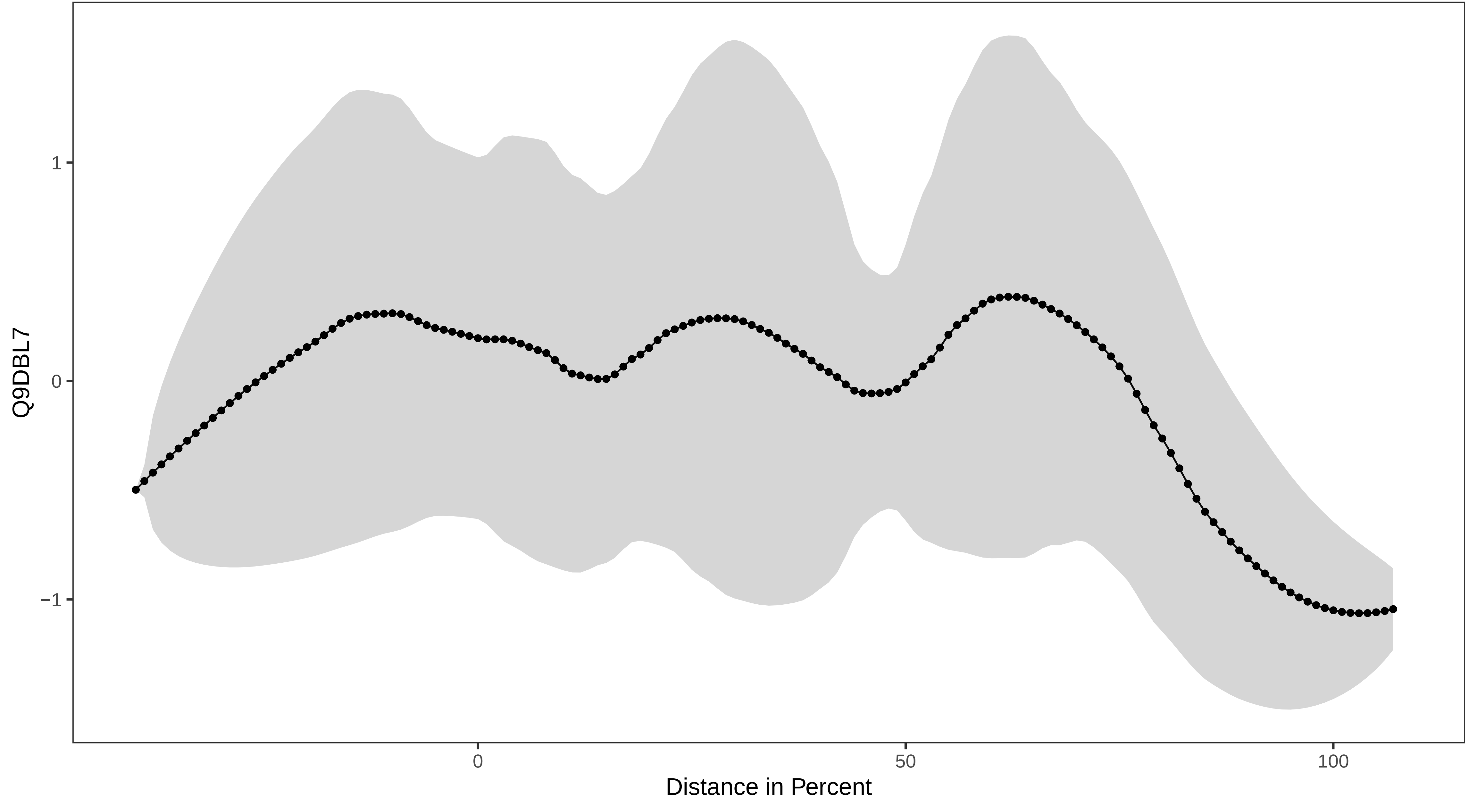

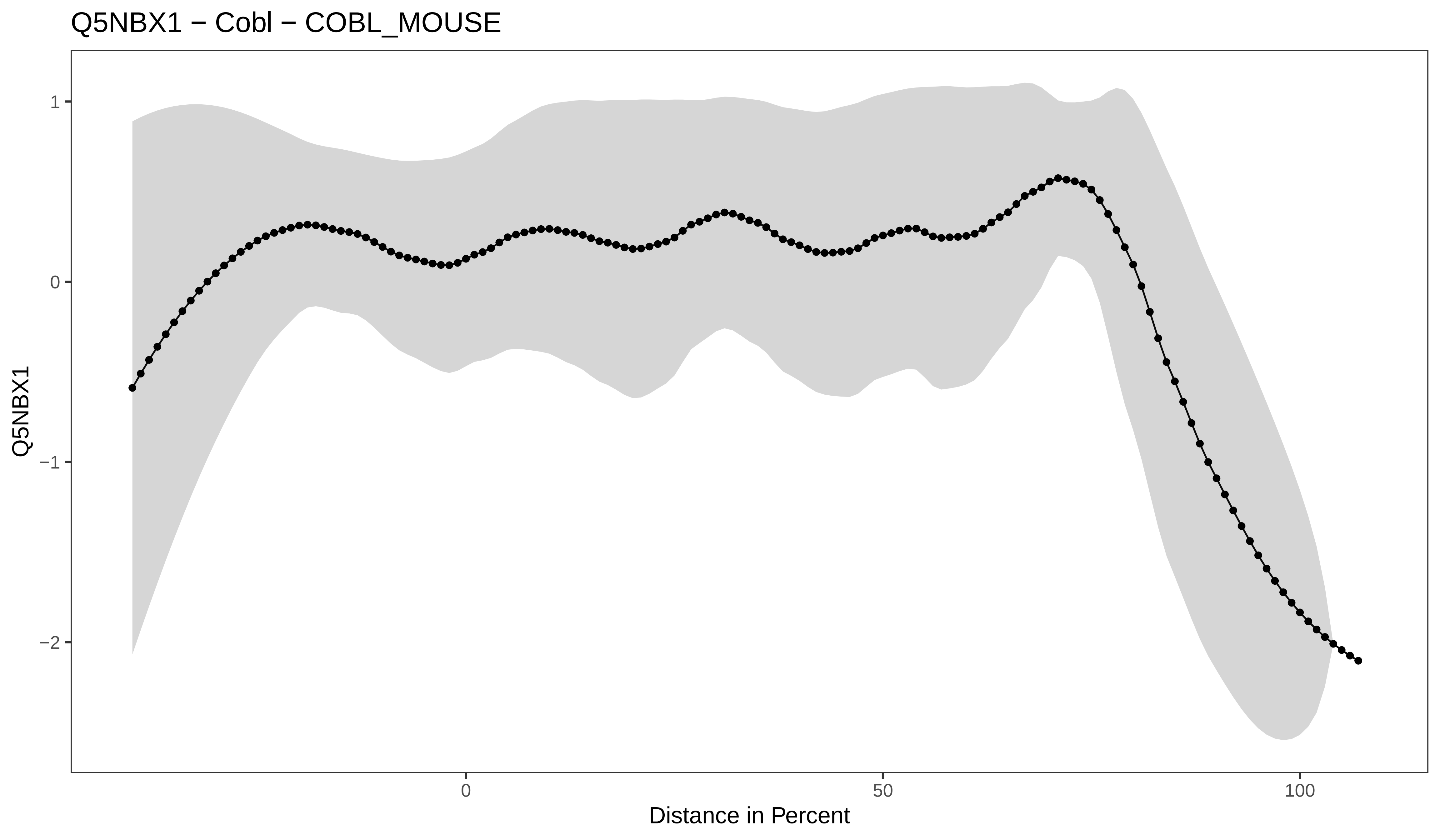
Q5NBX1 − Cobl − COBL_MOUSE
1
0
−1
−2
Q5NBX1
0
50
100
Distance in Percent

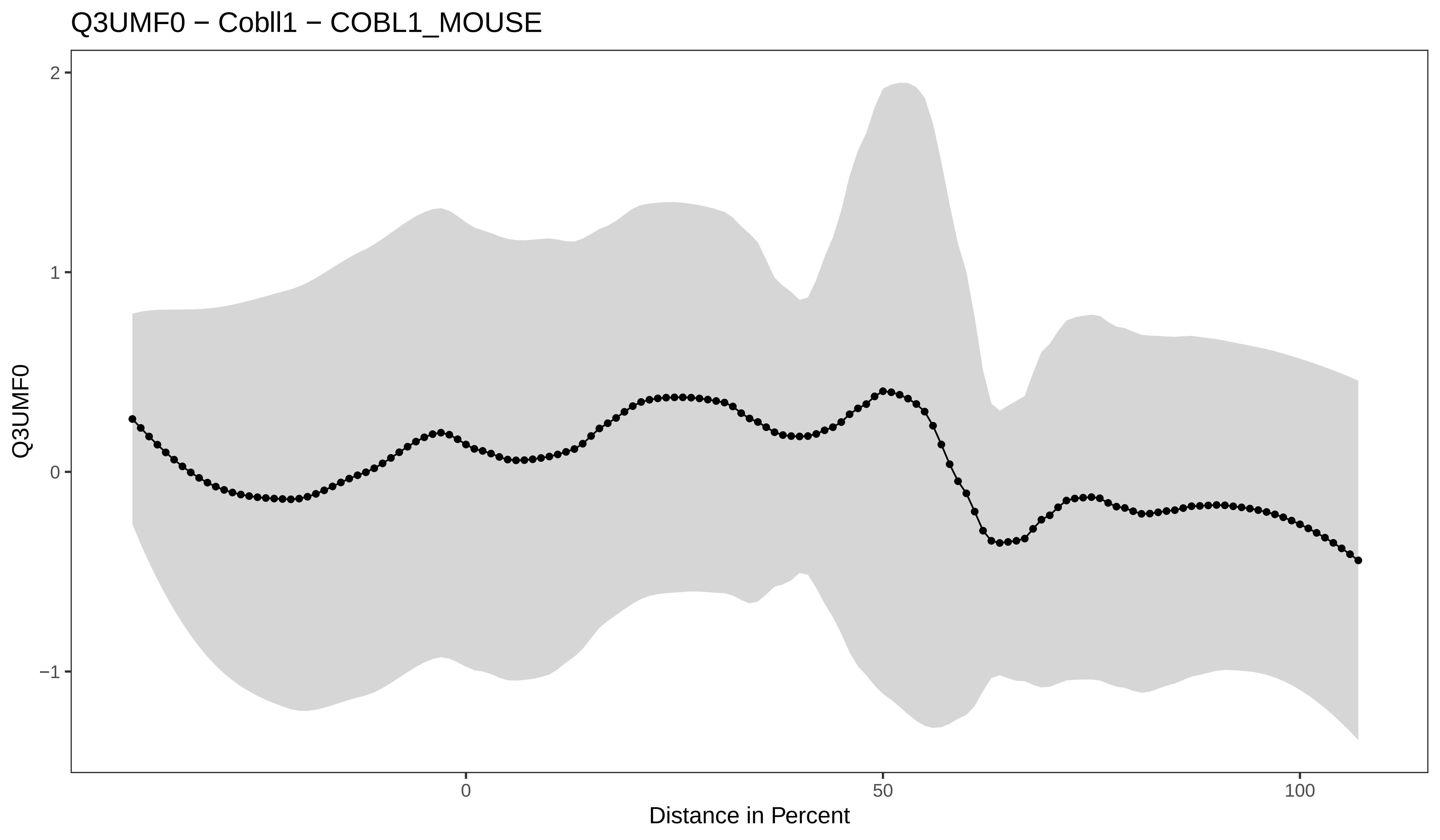
Q3UMF0 − Cobll1 − COBL1_MOUSE
Q3UMF0
2
1
0
−1
0
50
100
Distance in Percent

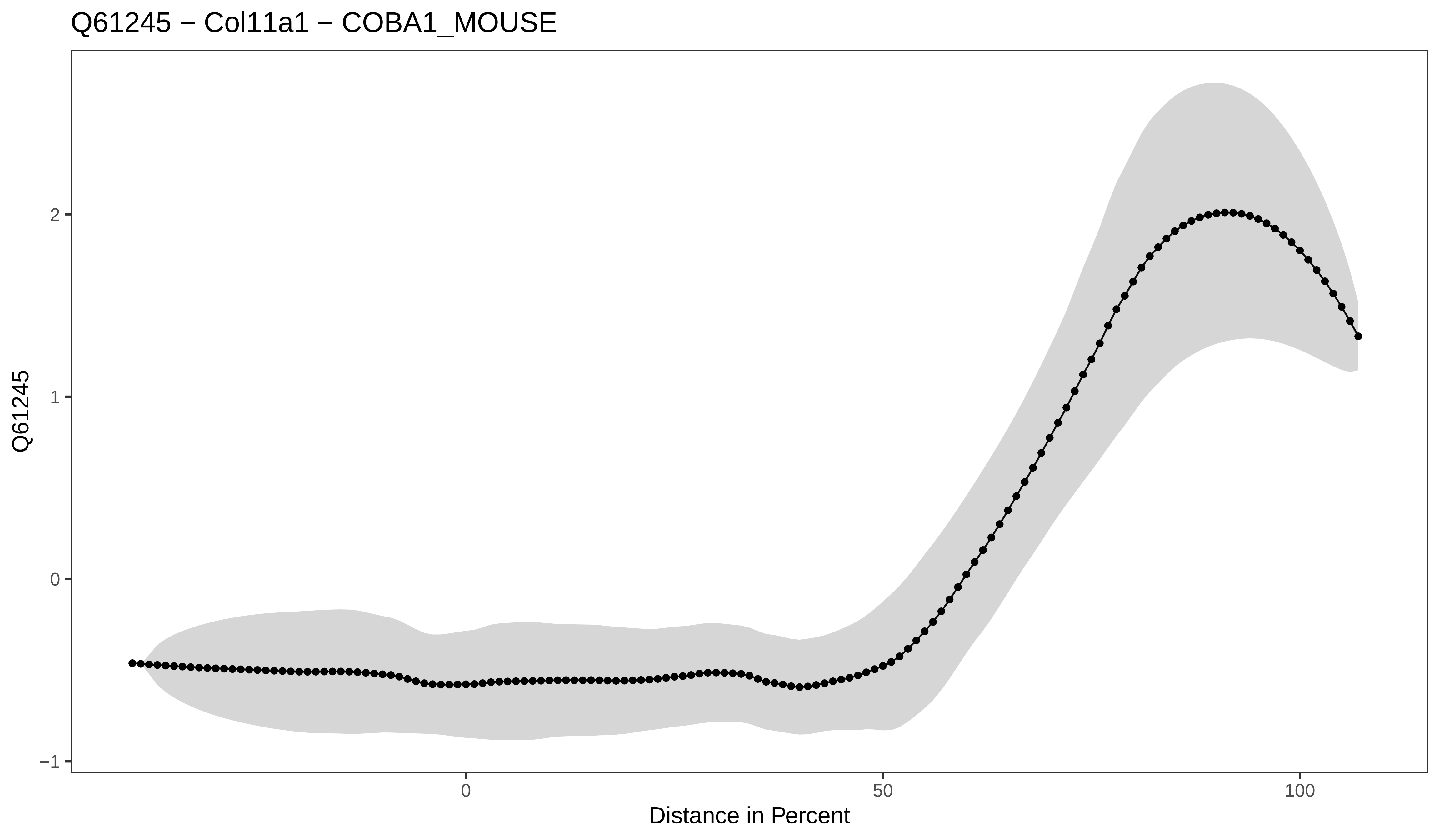

Q61245 − Col11a1 − COBA1_MOUSE
Q61245
2
1
0
−1
0
50
100
Distance in Percent

Q64739 − Col11a2 − COBA2_MOUSE

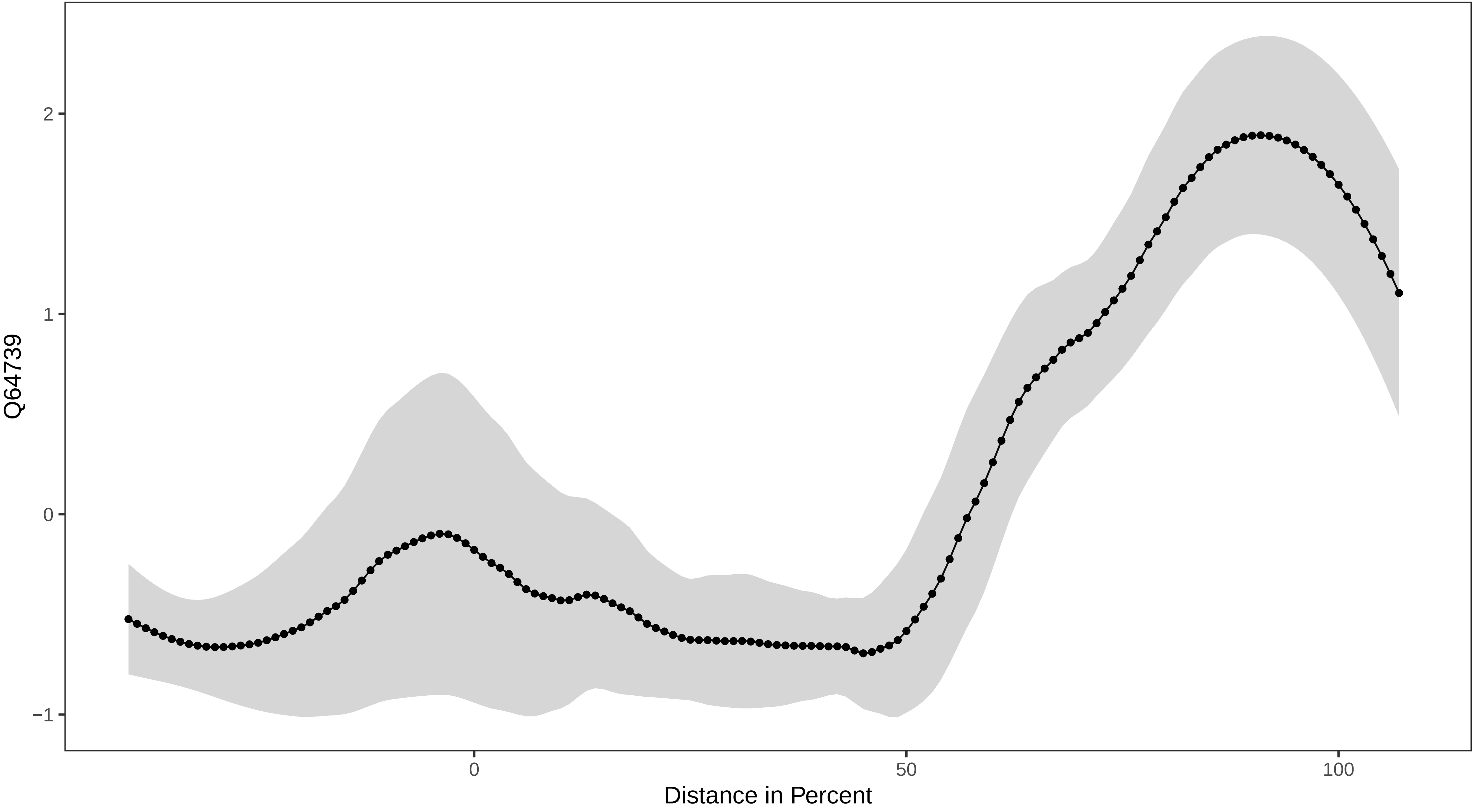

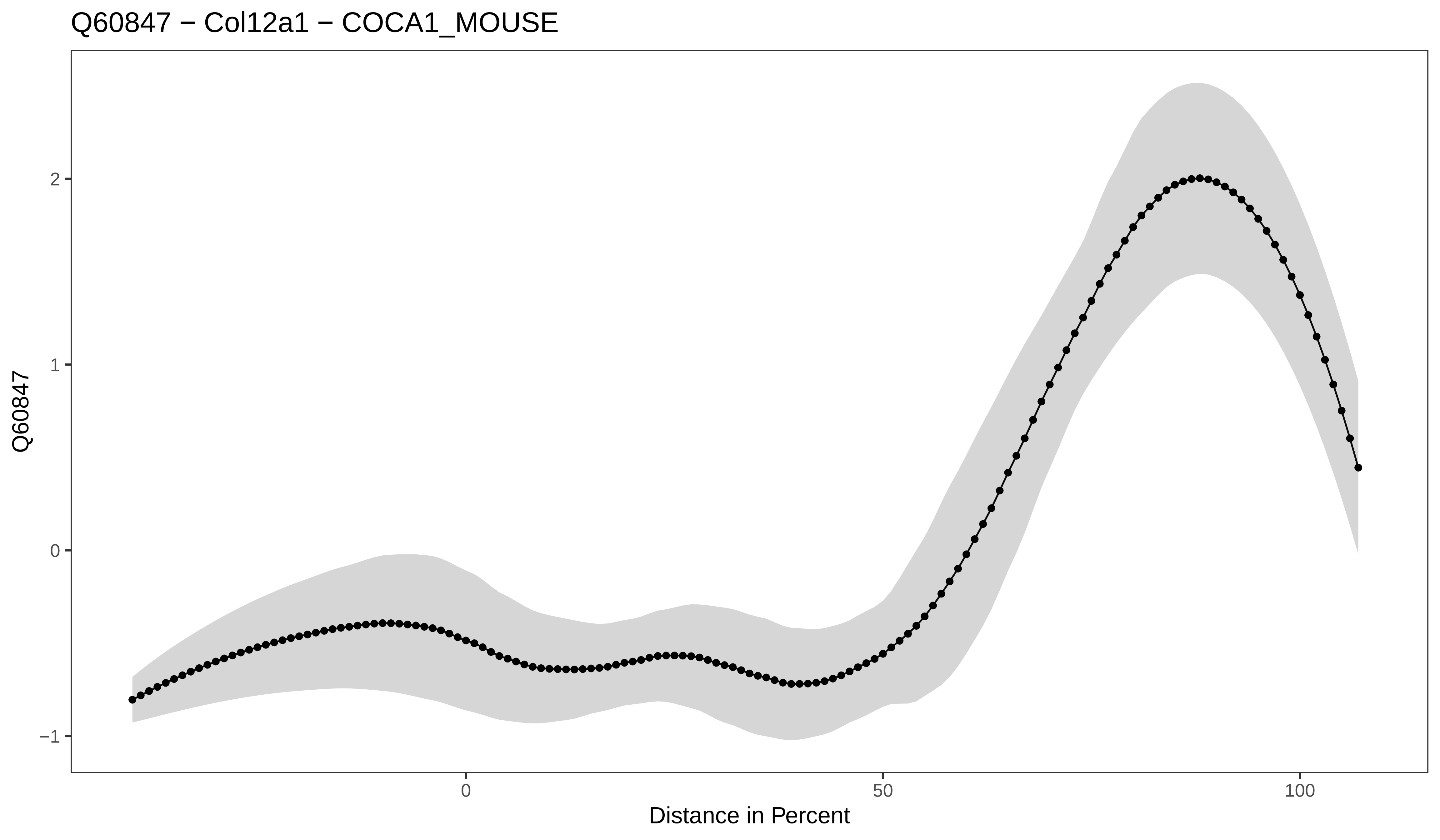

Q60847 − Col12a1 − COCA1_MOUSE
Q60847
2
1
0
−1
0
50
100
Distance in Percent

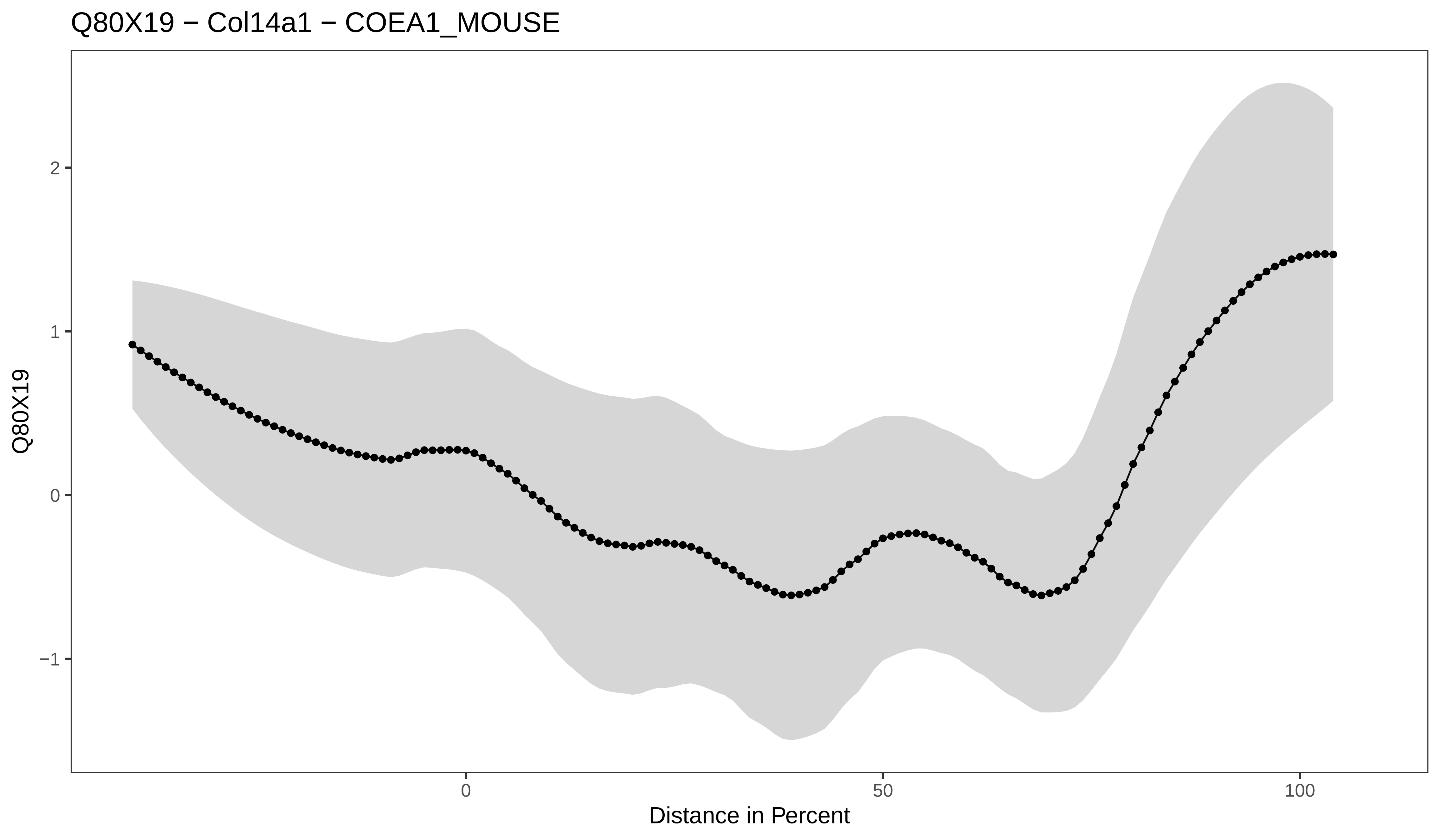
Q80X19 − Col14a1 − COEA1_MOUSE
Q80X19
2
1
0
−1
0
50
100
Distance in Percent

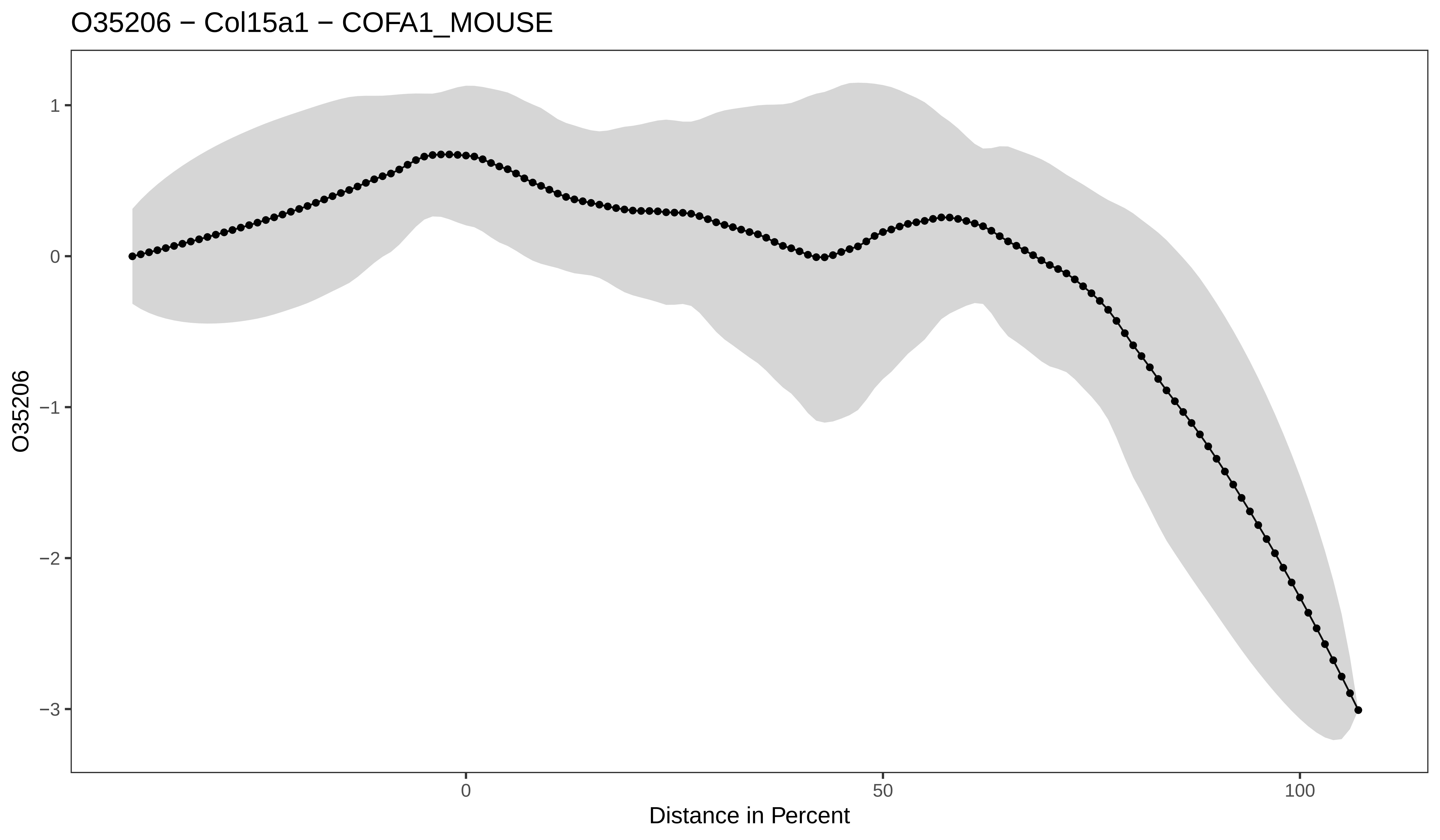
O35206 − Col15a1 − COFA1_MOUSE
O35206
1
0
−1
−2
−3
0
50
100
Distance in Percent

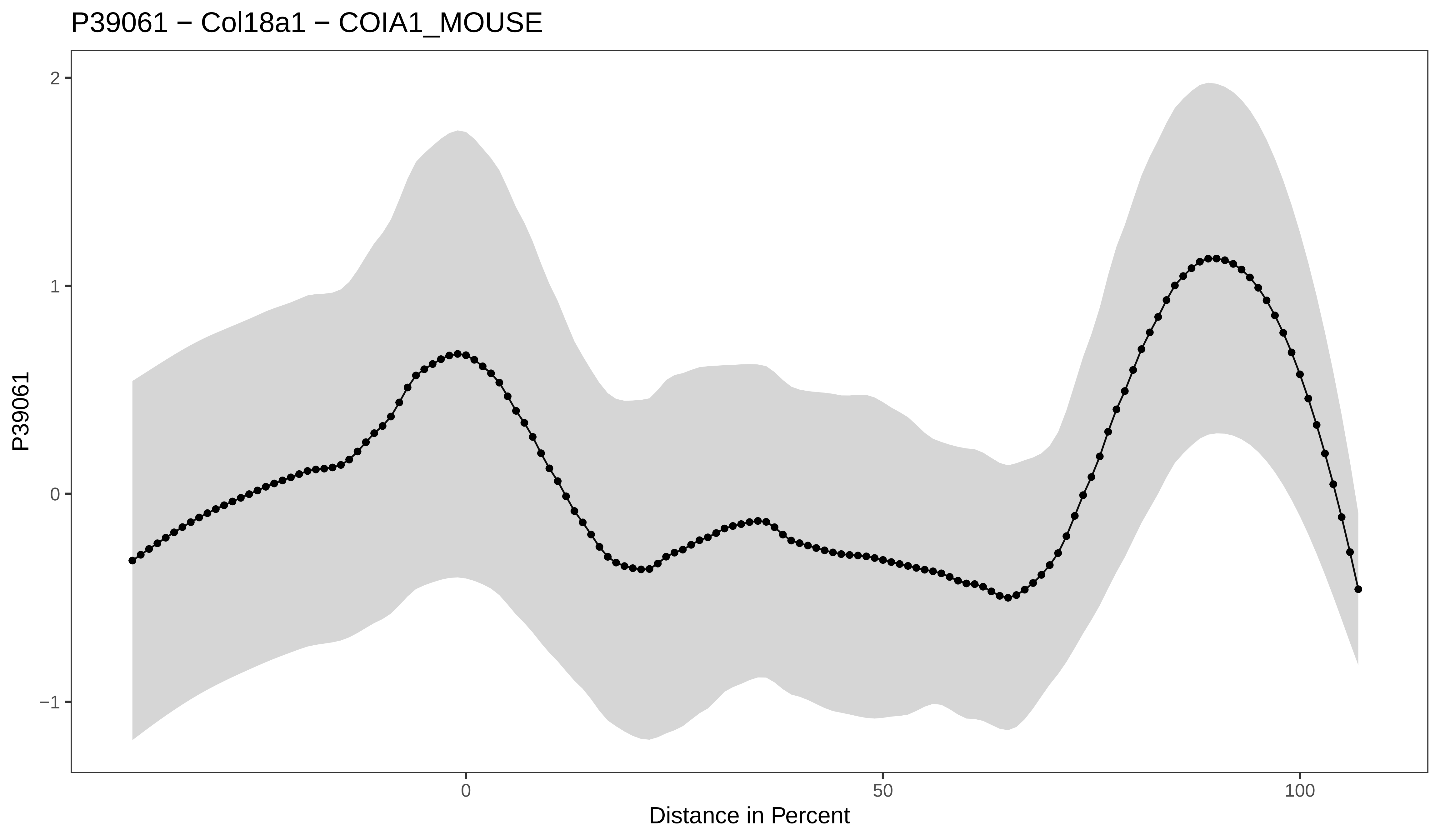

P39061 − Col18a1 − COIA1_MOUSE
P39061
2
1
0
−1
0
50
100
Distance in Percent

P11087 − Col1a1 − CO1A1_MOUSE

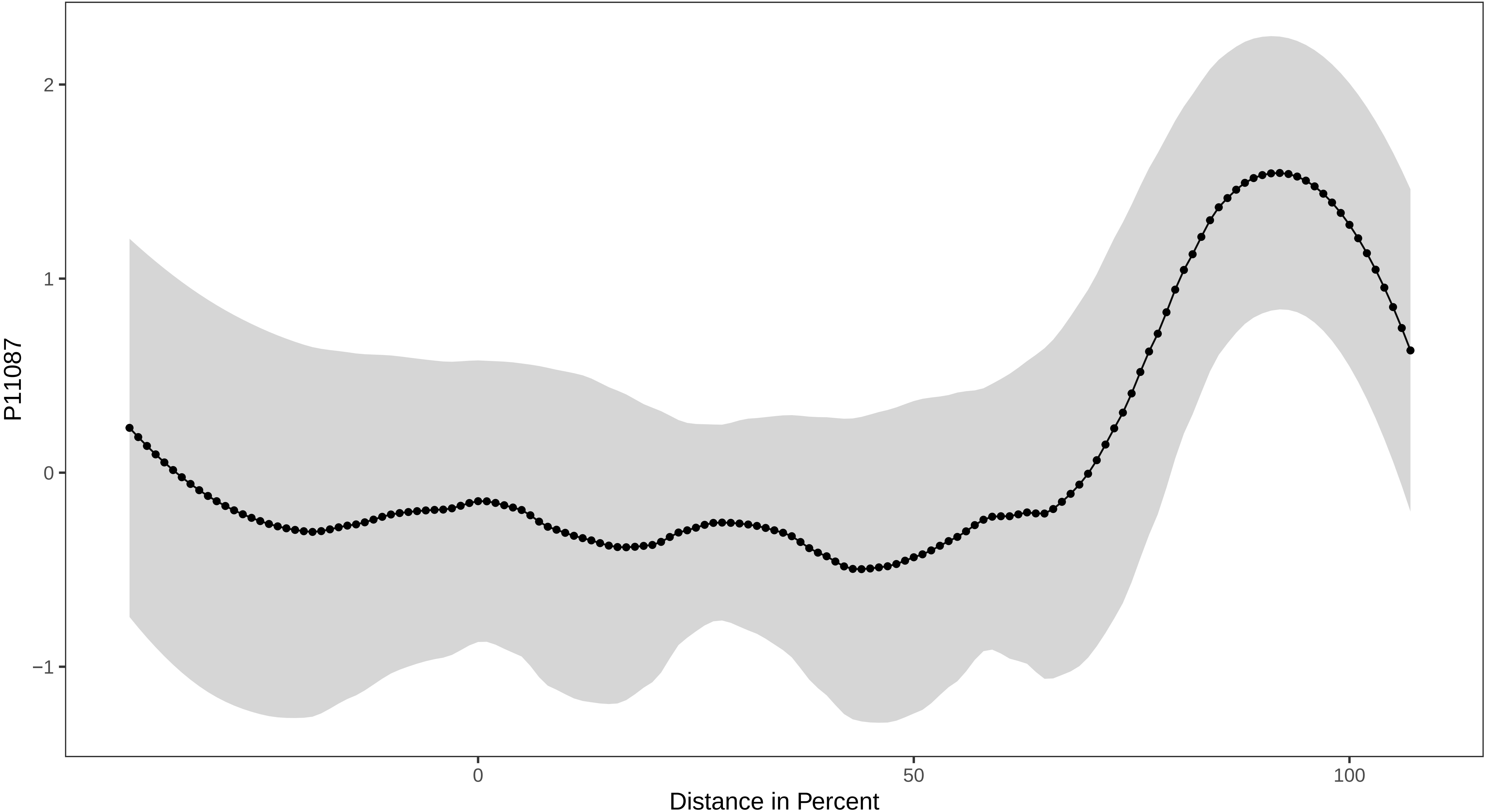

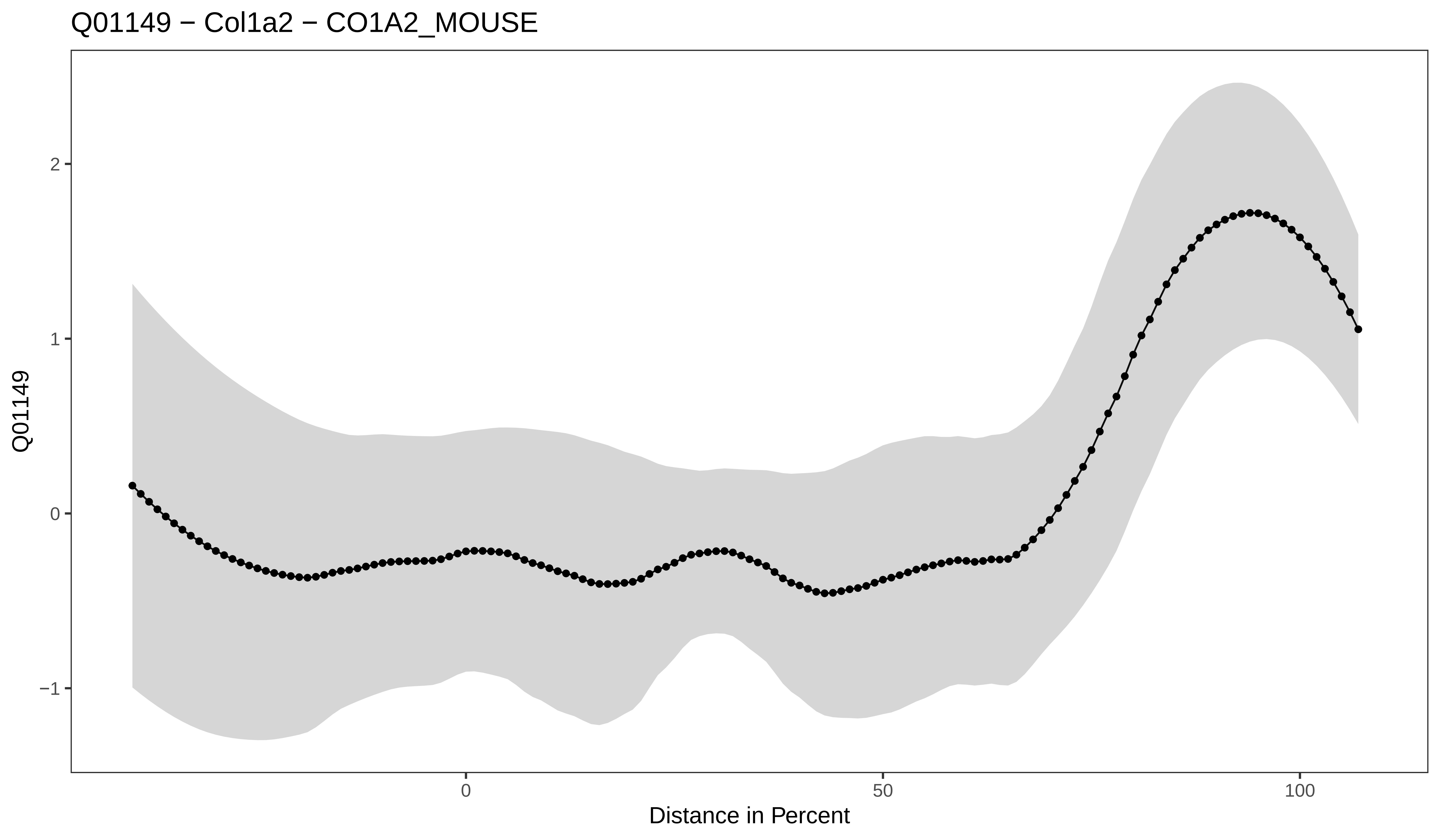
Q01149 − Col1a2 − CO1A2_MOUSE
Q01149
2
1
0
−1
0
50
100
Distance in Percent

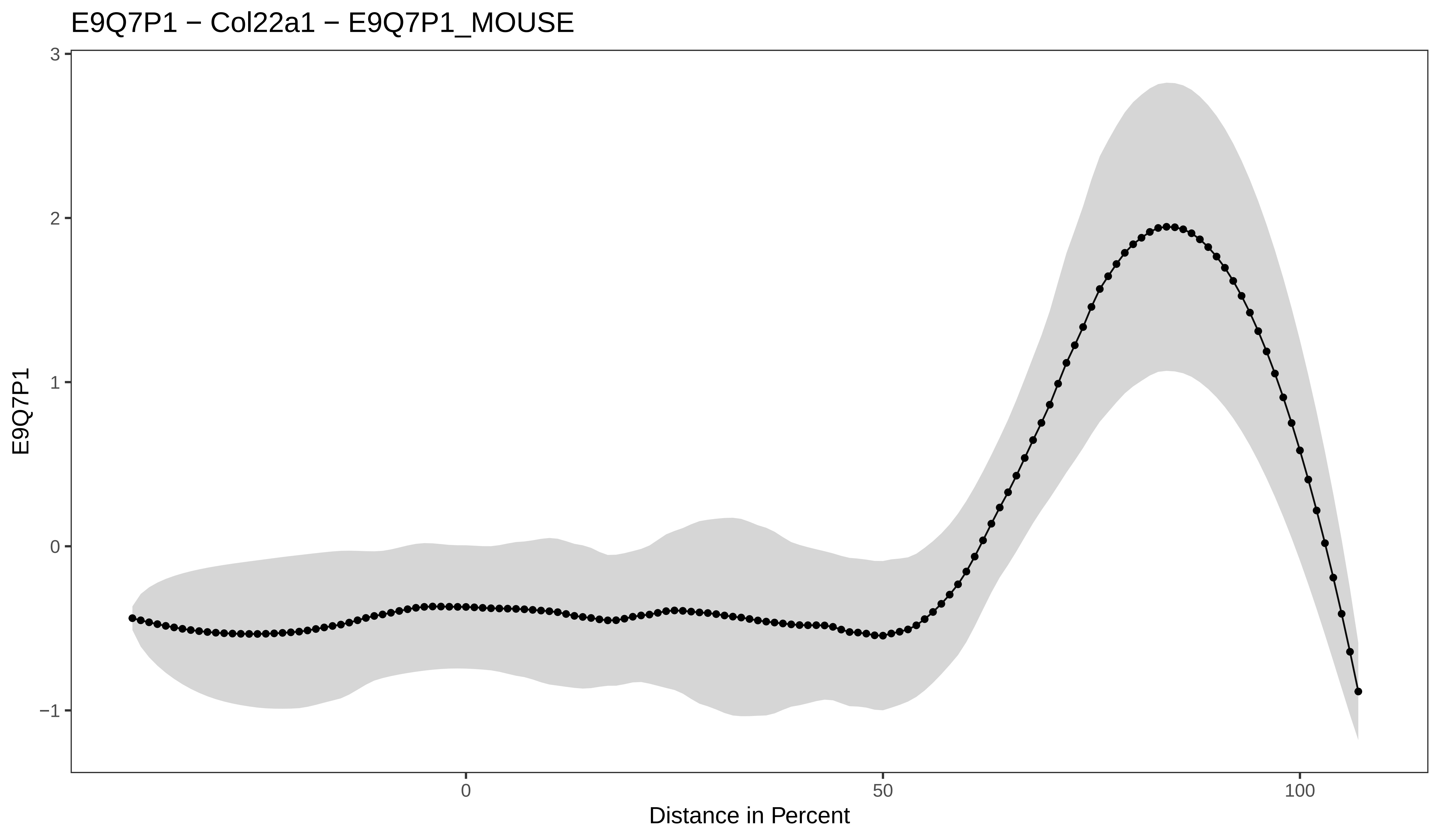

E9Q7P1 − Col22a1 − E9Q7P1_MOUSE
3
2
1
0
−1
E9Q7P1
0
50
100
Distance in Percent

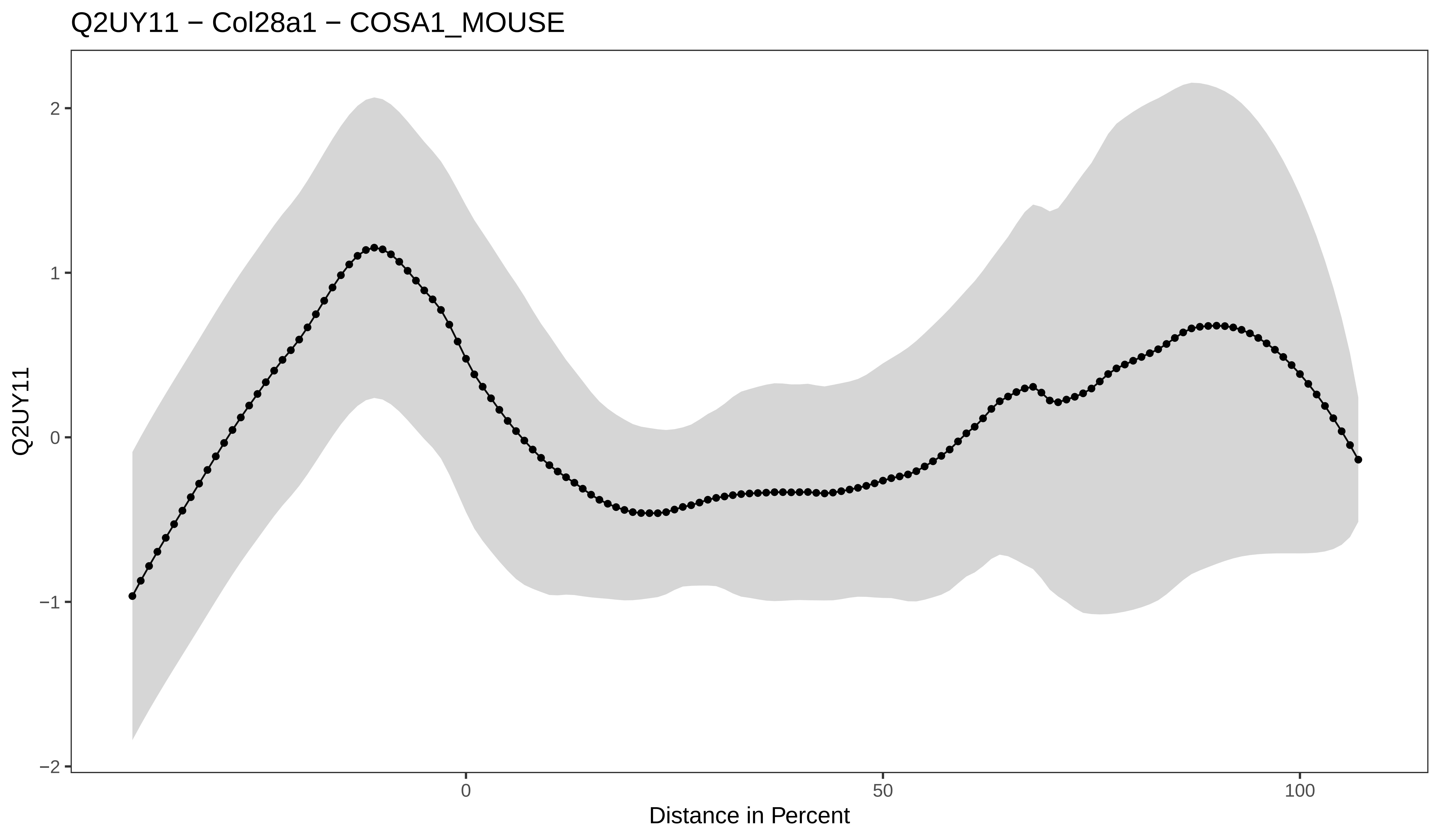

Q2UY11 − Col28a1 − COSA1_MOUSE
Q2UY11
2
1
0
−1
−2
0
50
100
Distance in Percent

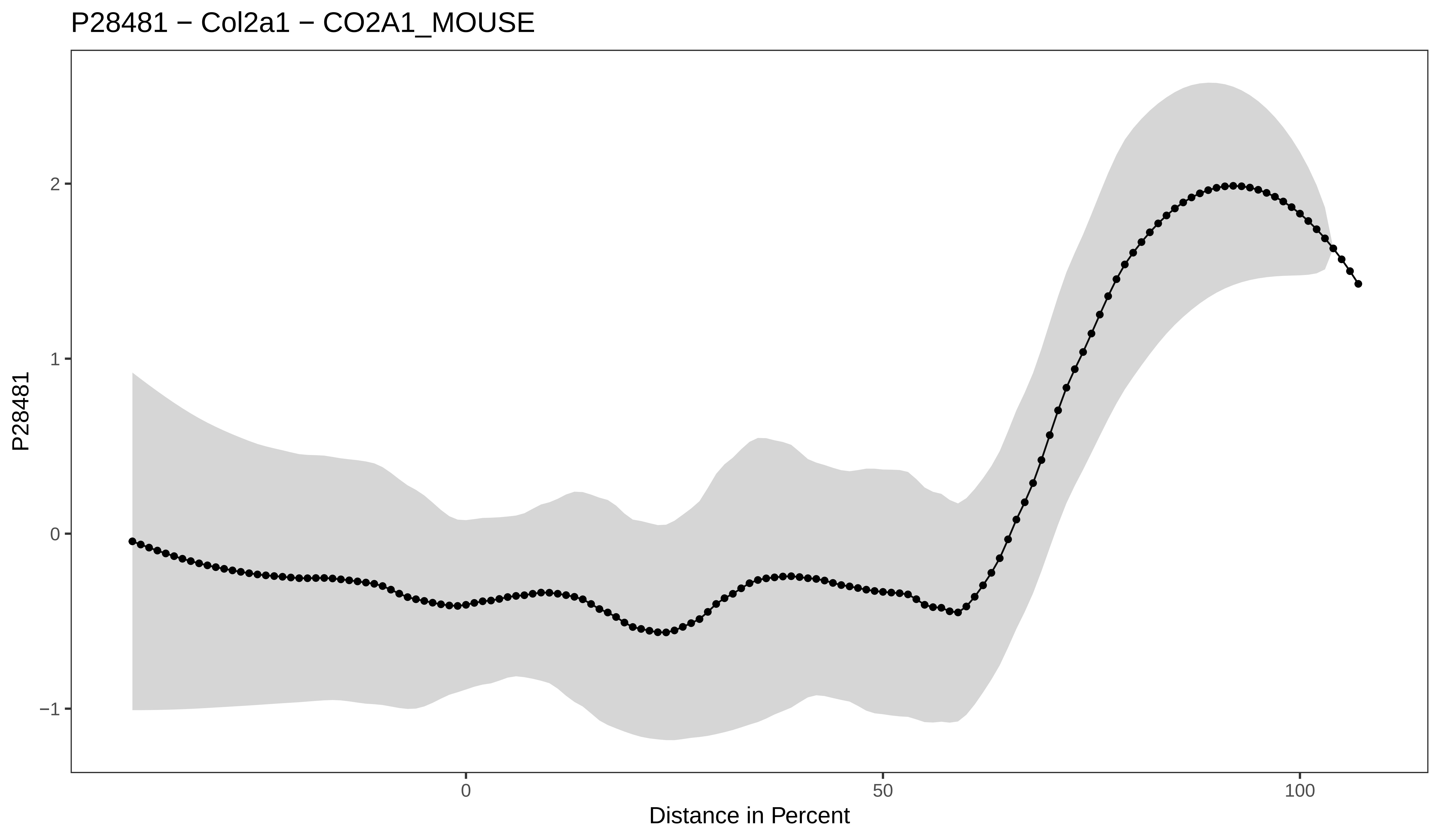

P28481 − Col2a1 − CO2A1_MOUSE
P28481
2
1
0
−1
0
50
100
Distance in Percent

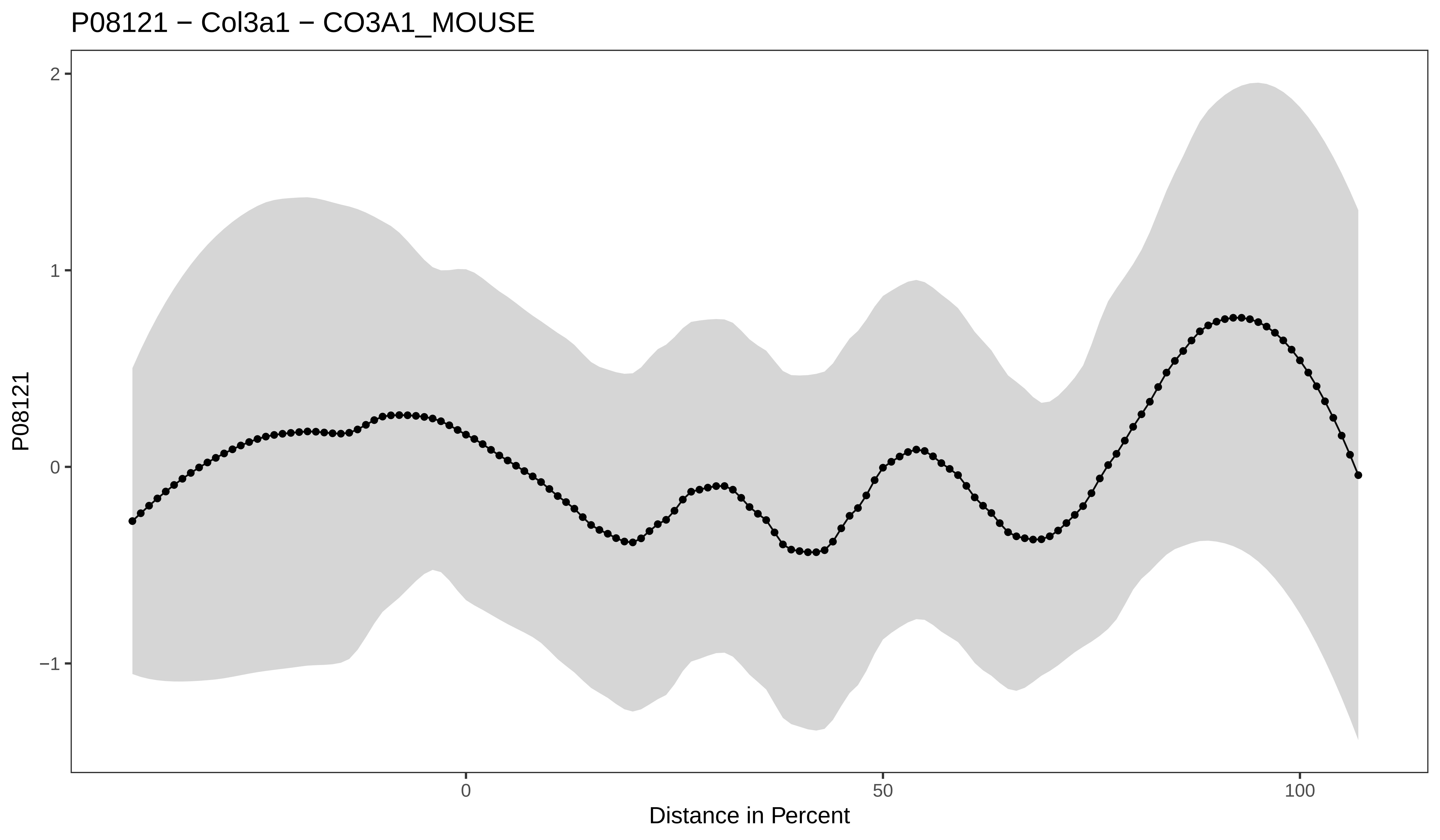
P08121 − Col3a1 − CO3A1_MOUSE
P08121
Distance in Percent
2
1
0
−1
0
50
100

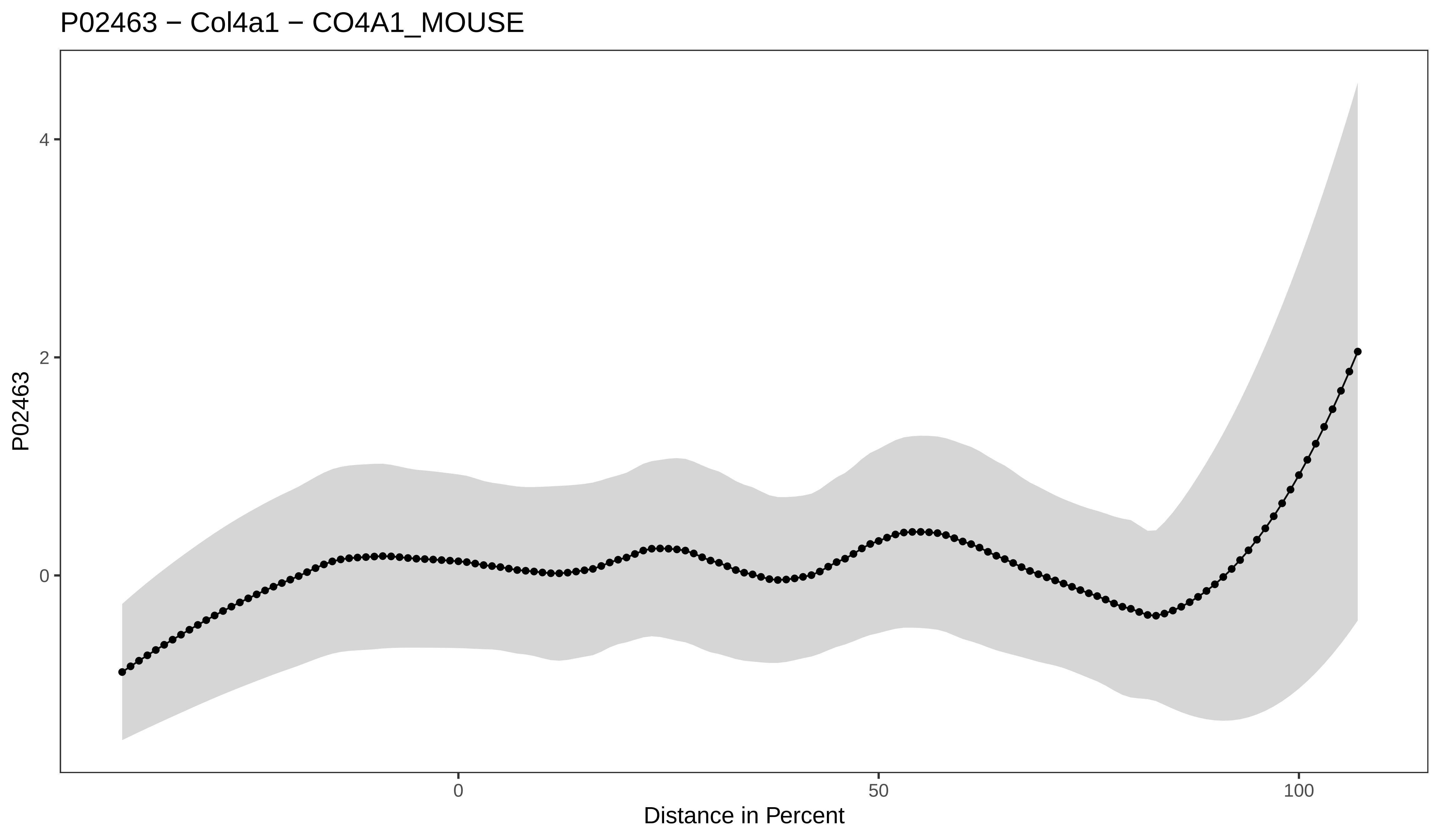

P02463 − Col4a1 − CO4A1_MOUSE
P02463
4
2
0
0
50
100
Distance in Percent

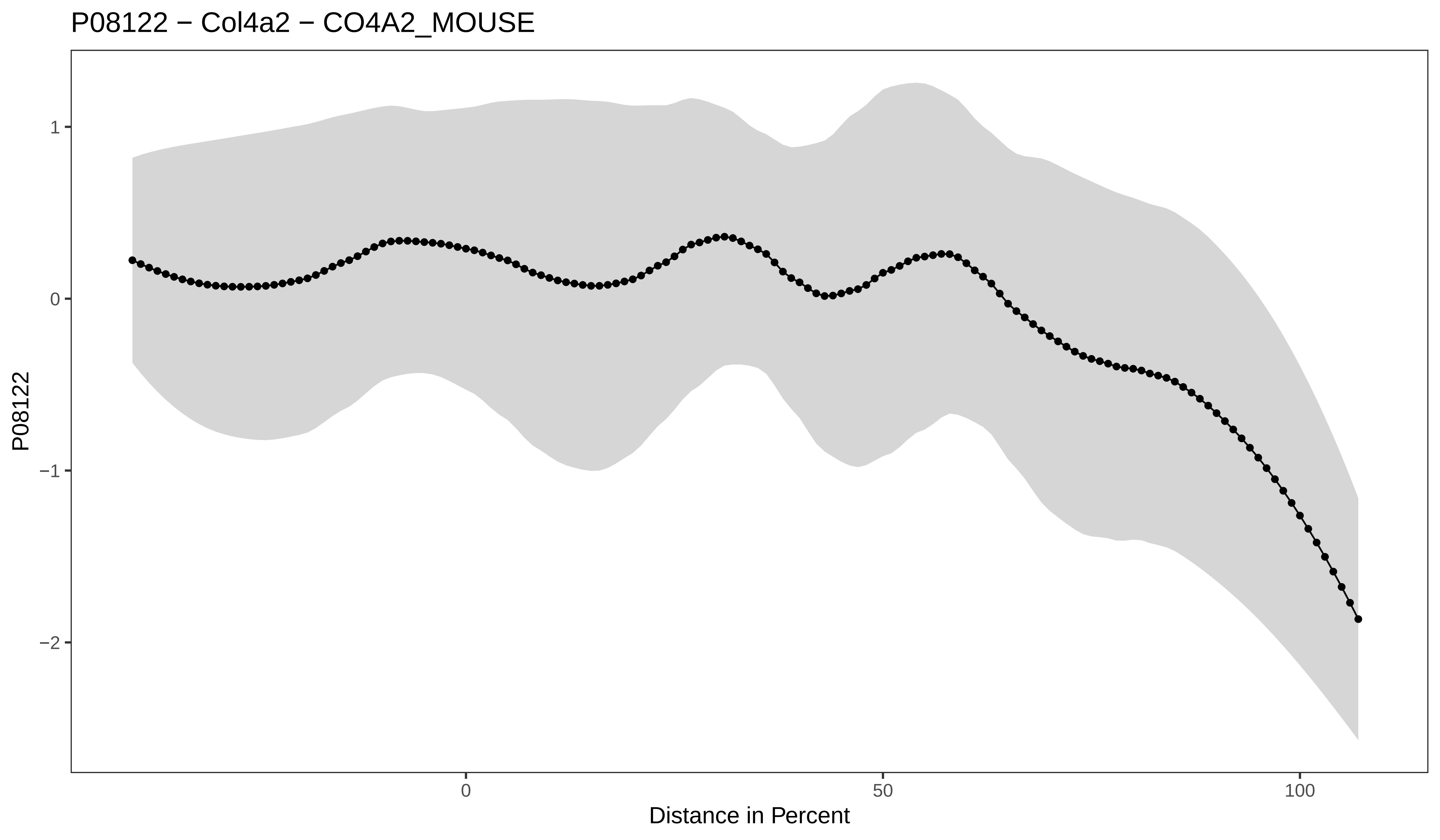

P08122 − Col4a2 − CO4A2_MOUSE
P08122
1
0
−1
−2
0
50
100
Distance in Percent

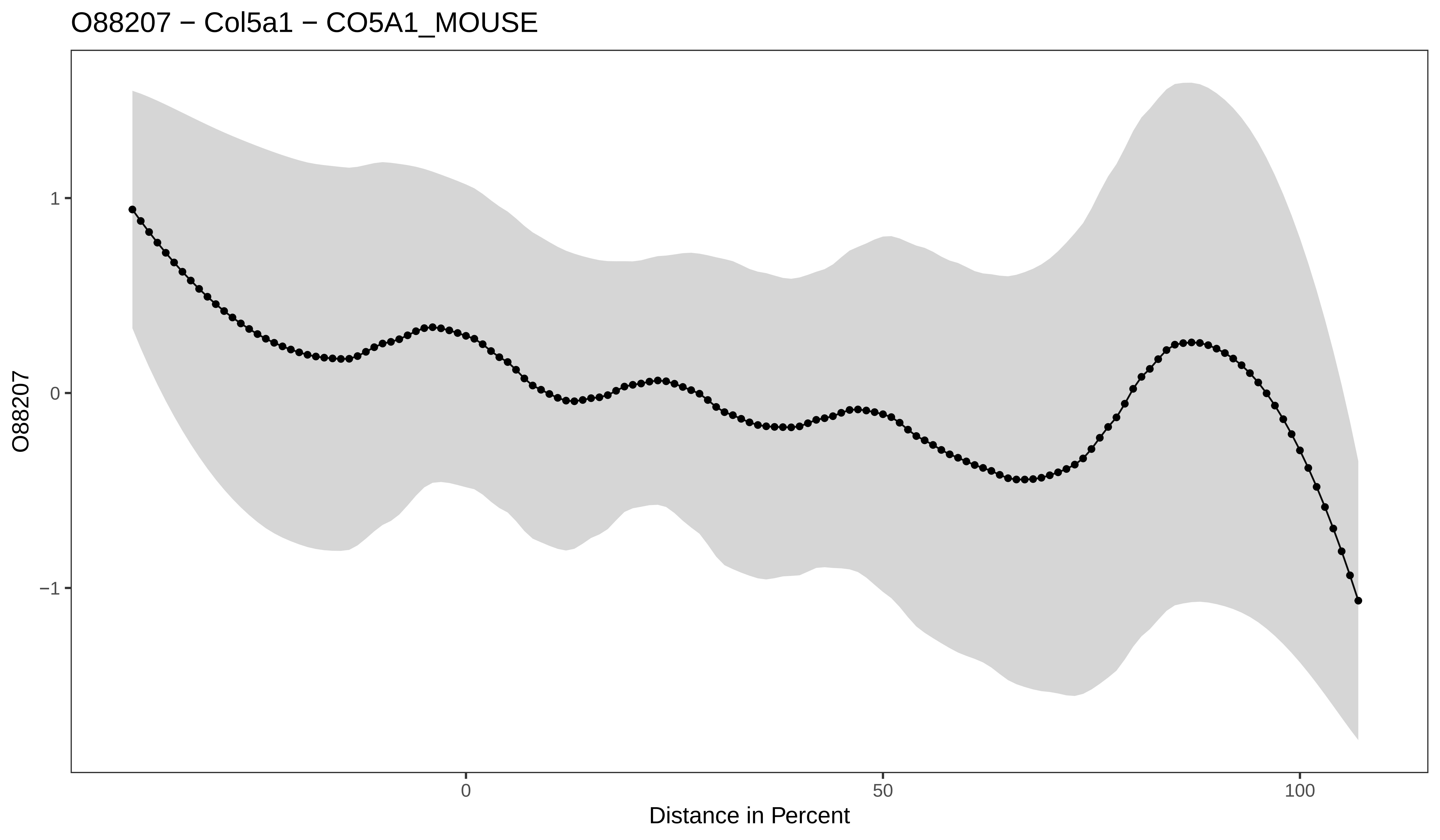

O88207 − Col5a1 − CO5A1_MOUSE
1
0
−1
O88207
0
50
100
Distance in Percent

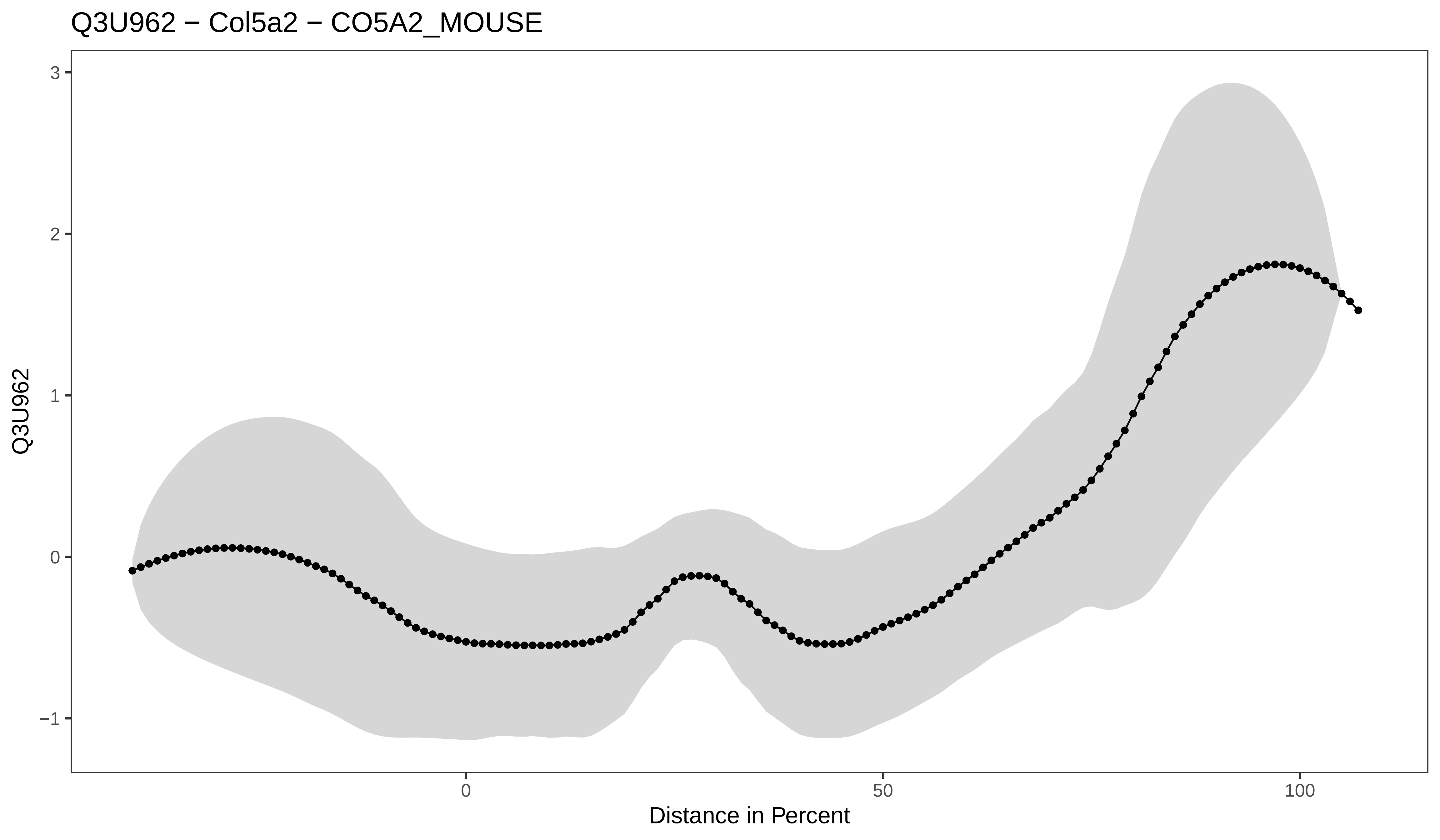
Q3U962 − Col5a2 − CO5A2_MOUSE
Q3U962
Distance in Percent
3
2
1
0
−1
0
50
100

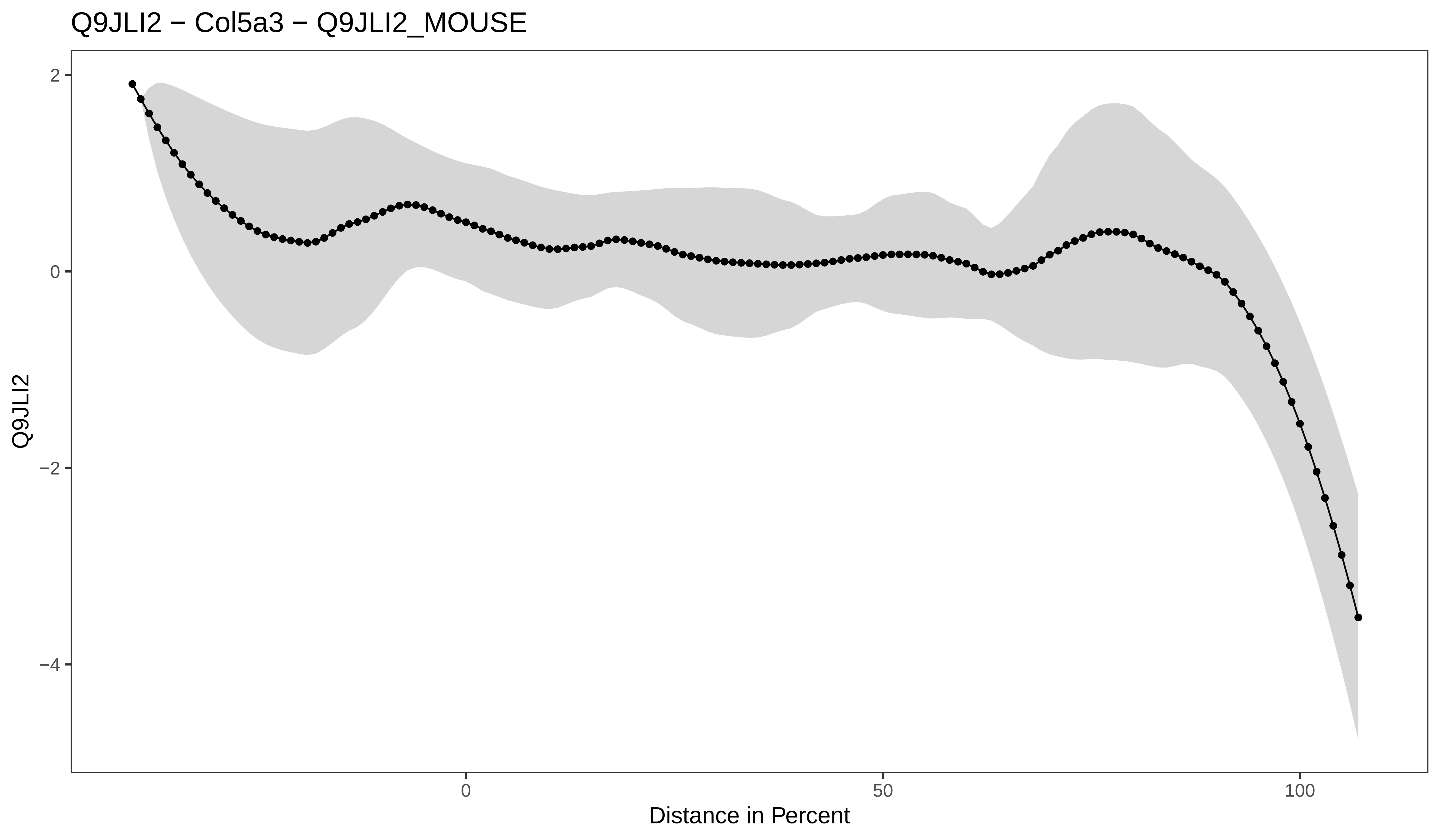

Q9JLI2 − Col5a3 − Q9JLI2_MOUSE
2
0
−2
−4
Q9JLI2
0
50
100
Distance in Percent

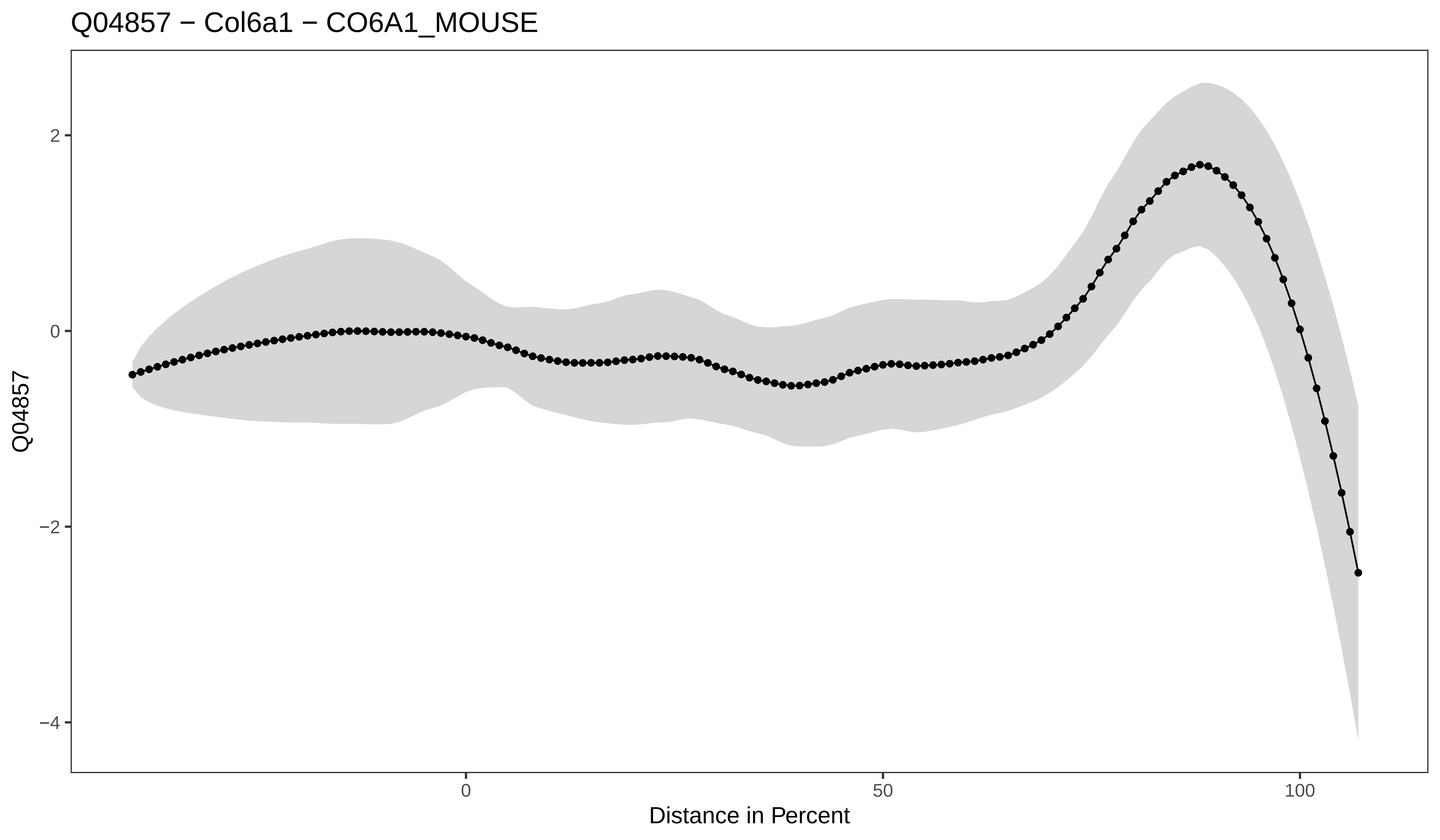
Q04857 − Col6a1 − CO6A1_MOUSE
Q04857
Distance in Percent
2
0
−2
−4
0
50
100

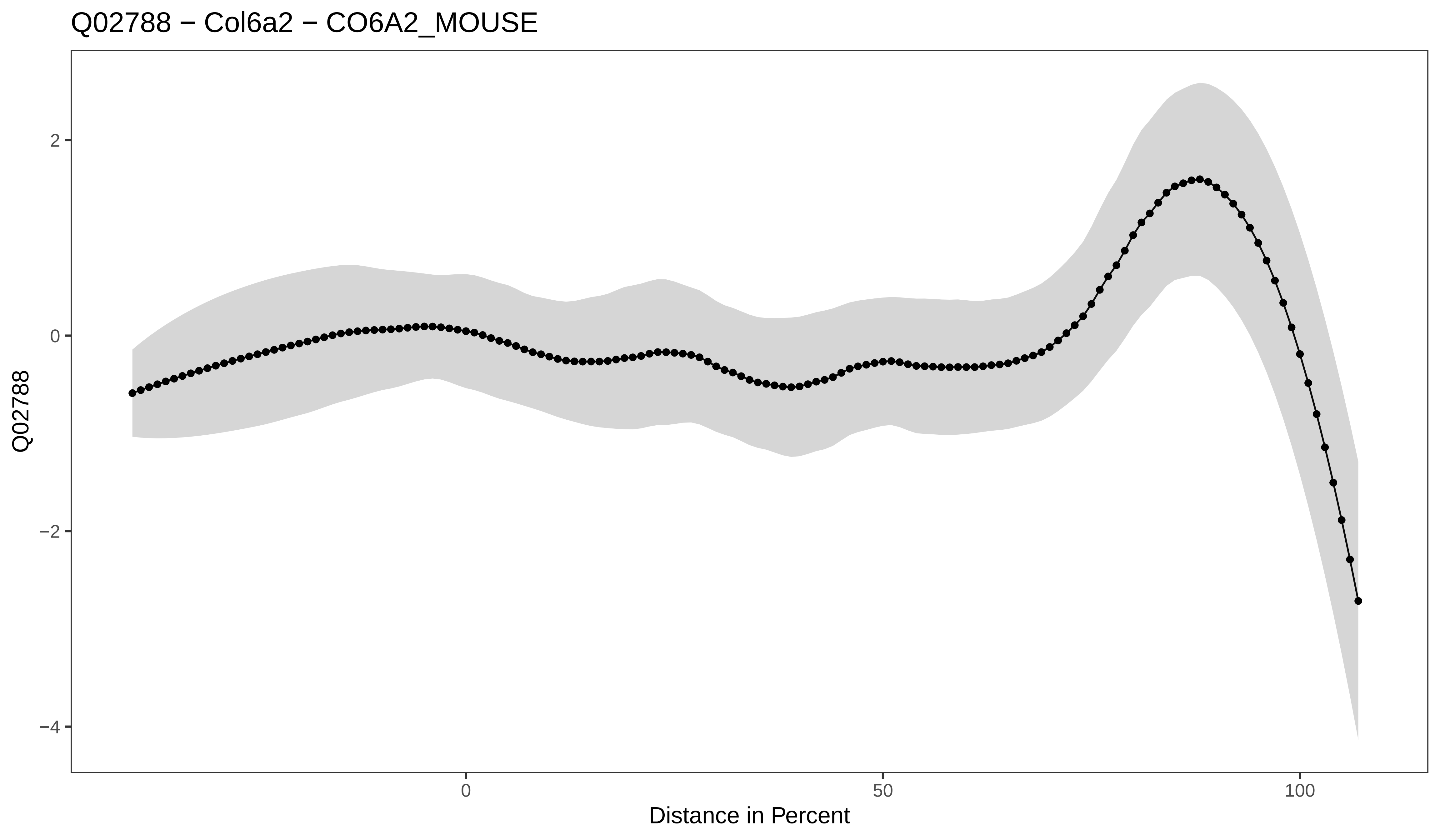

Q02788 − Col6a2 − CO6A2_MOUSE
Q02788
2
0
−2
−4
0
50
100
Distance in Percent

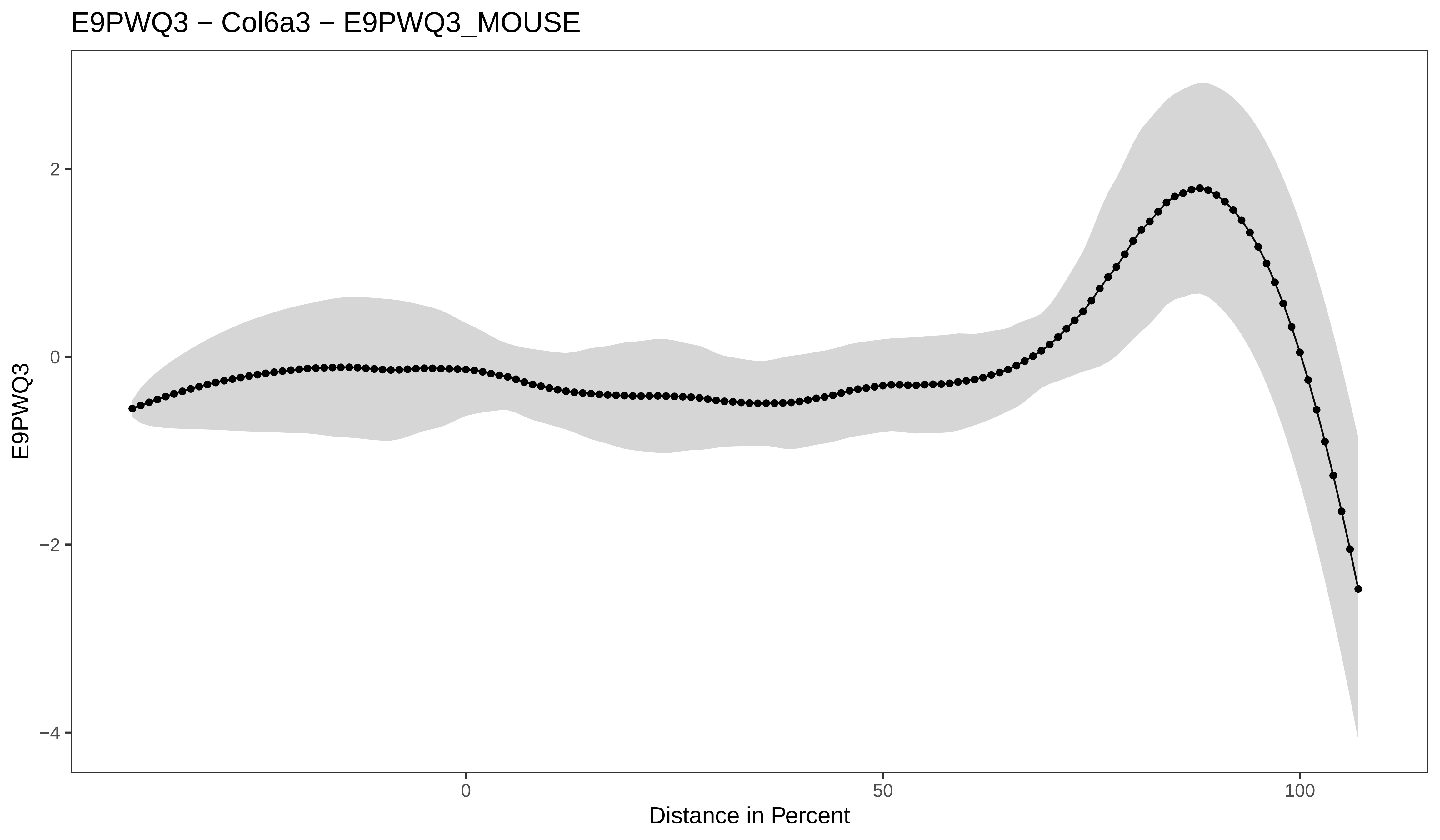
E9PWQ3 − Col6a3 − E9PWQ3_MOUSE
E9PWQ3
2
0
−2
−4
0
50
100
Distance in Percent

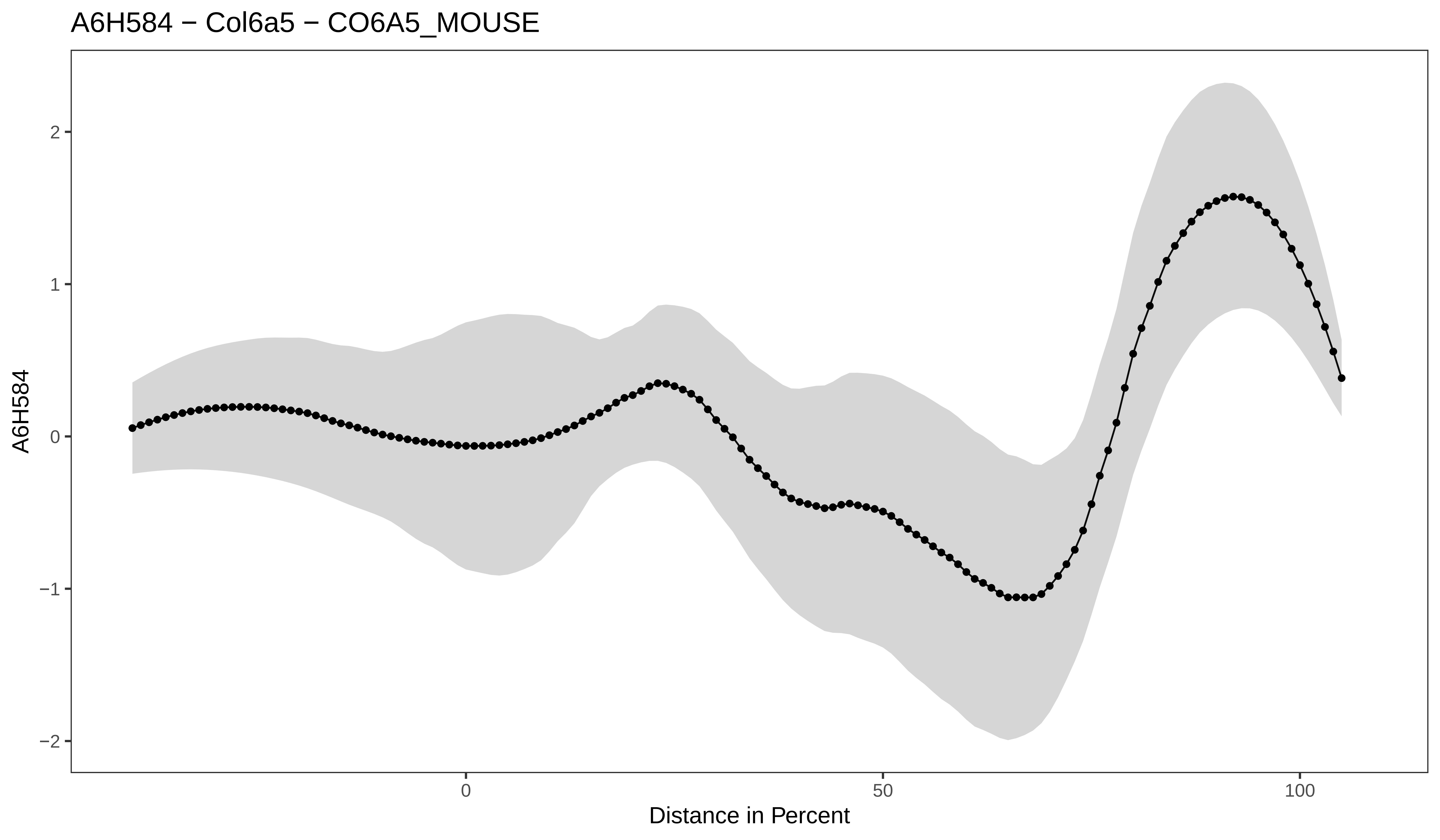

A6H584 − Col6a5 − CO6A5_MOUSE
A6H584
2
1
0
−1
−2
0
50
100
Distance in Percent

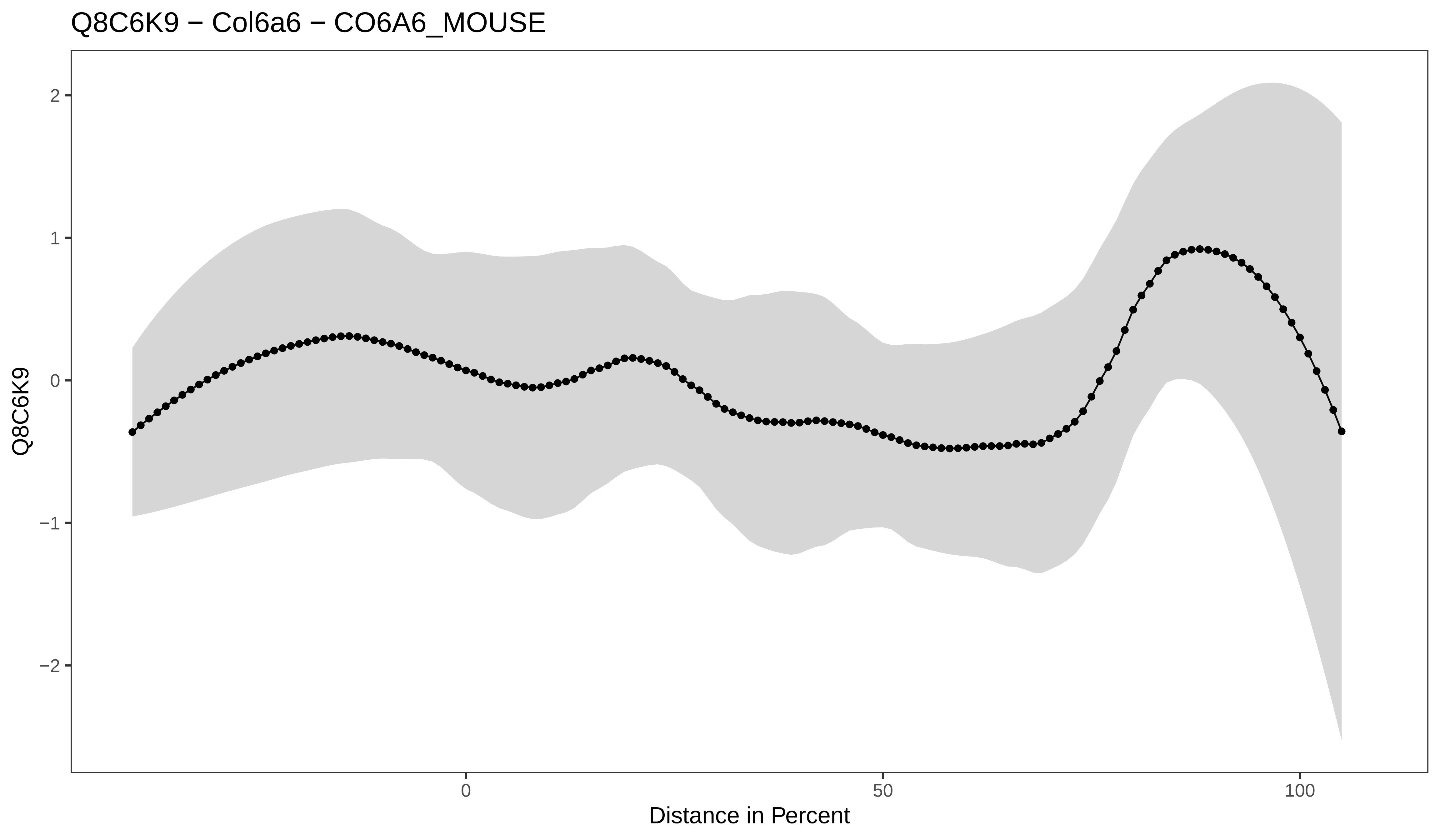

Q8C6K9 − Col6a6 − CO6A6_MOUSE
Q8C6K9
2
1
0
−1
−2
0
50
100
Distance in Percent

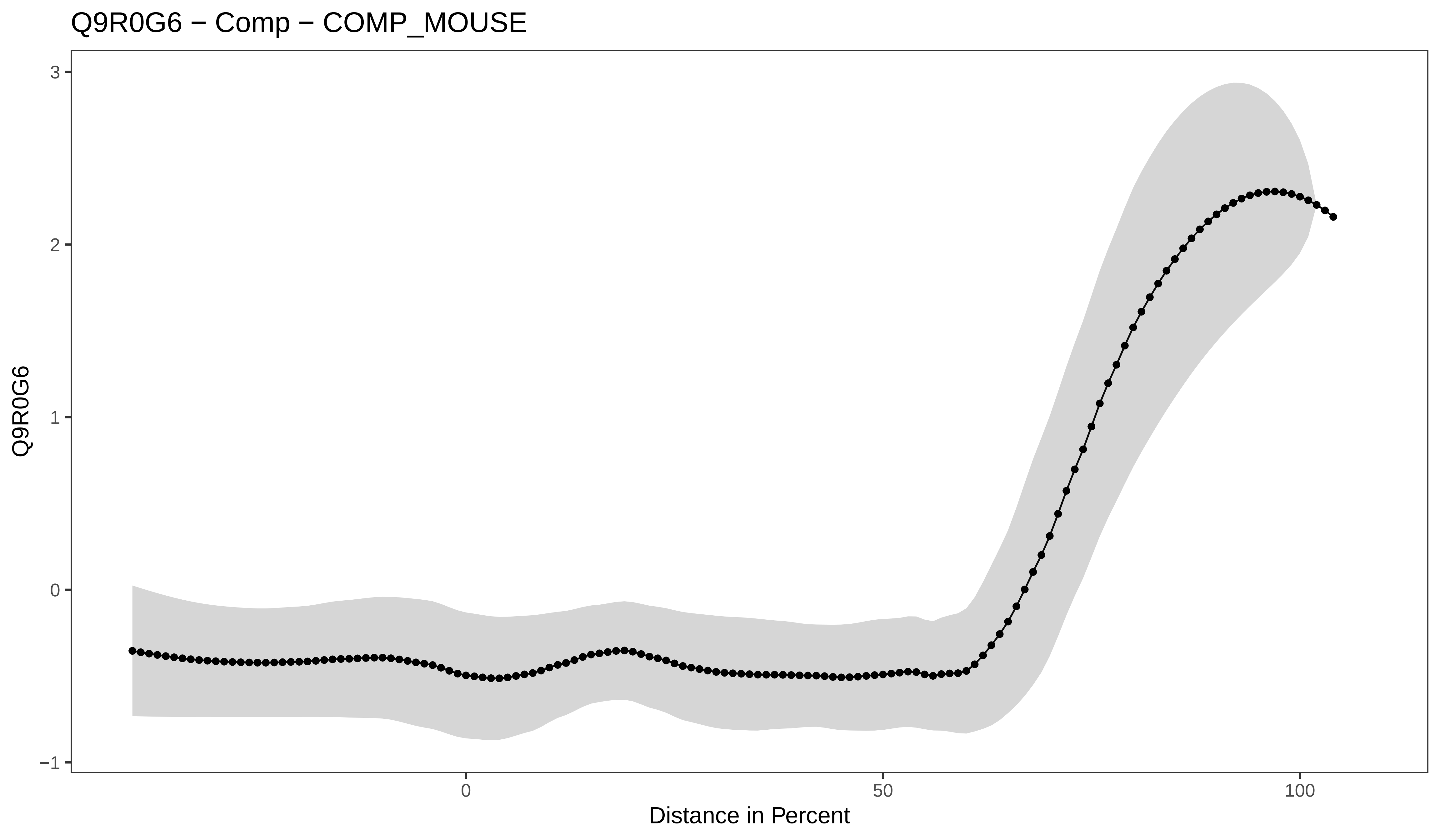
Q9R0G6 − Comp − COMP_MOUSE
Q9R0G6
3
2
1
0
−1
0
50
100
Distance in Percent

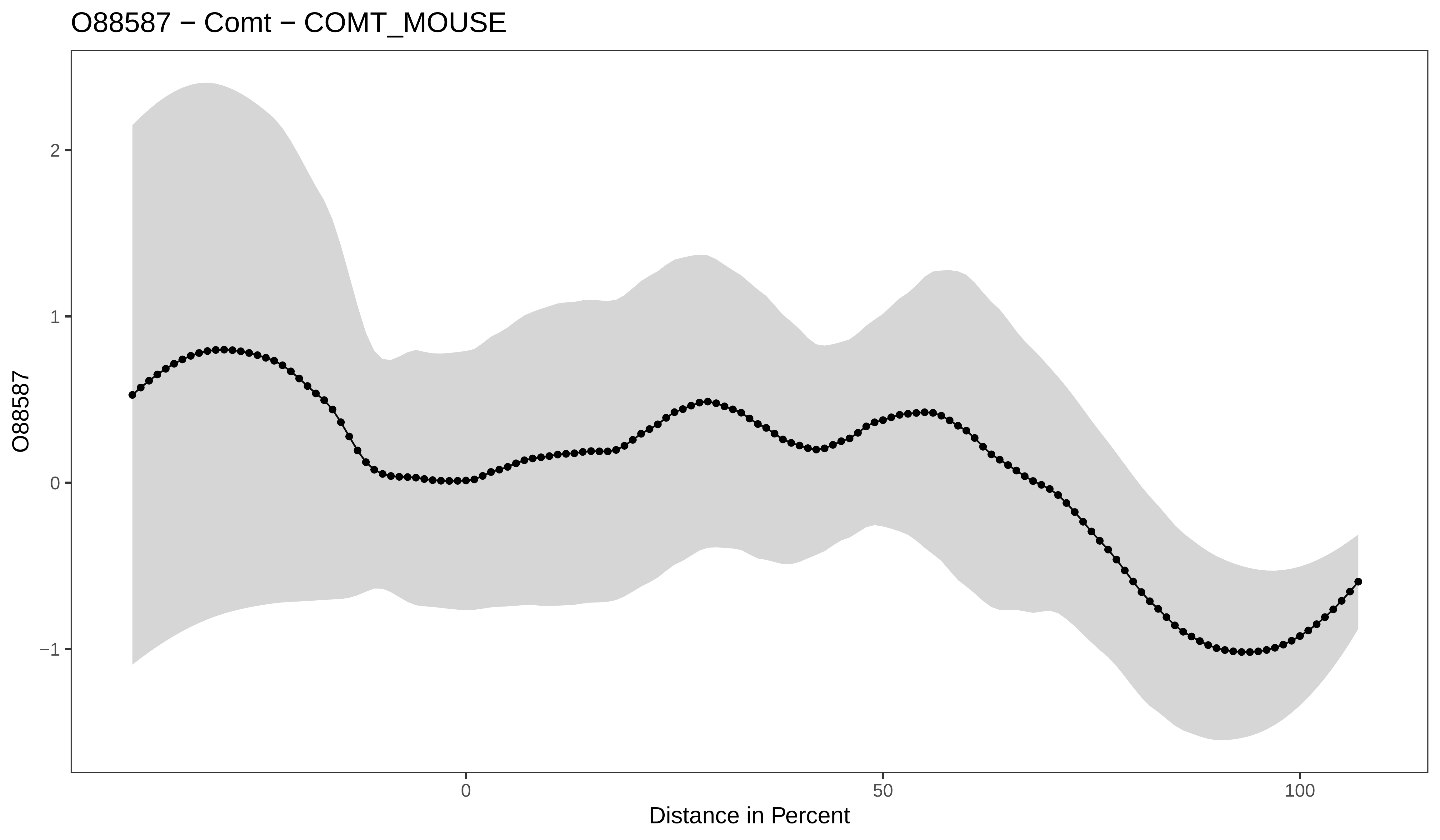
O88587 − Comt − COMT_MOUSE
O88587
2
1
0
−1
0
50
100
Distance in Percent

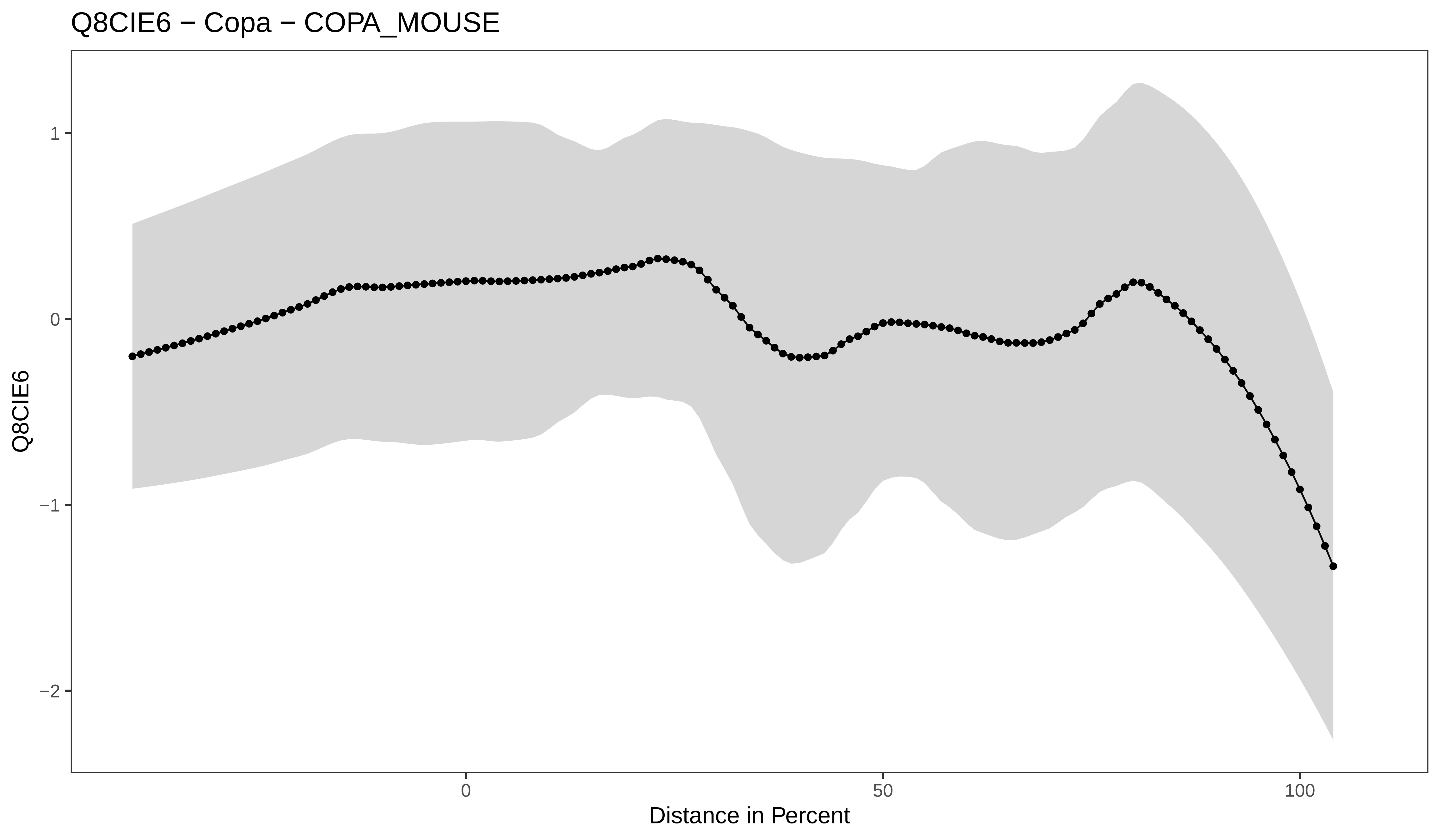

Q8CIE6 − Copa − COPA_MOUSE
Q8CIE6
1
0
−1
−2
0
50
100
Distance in Percent

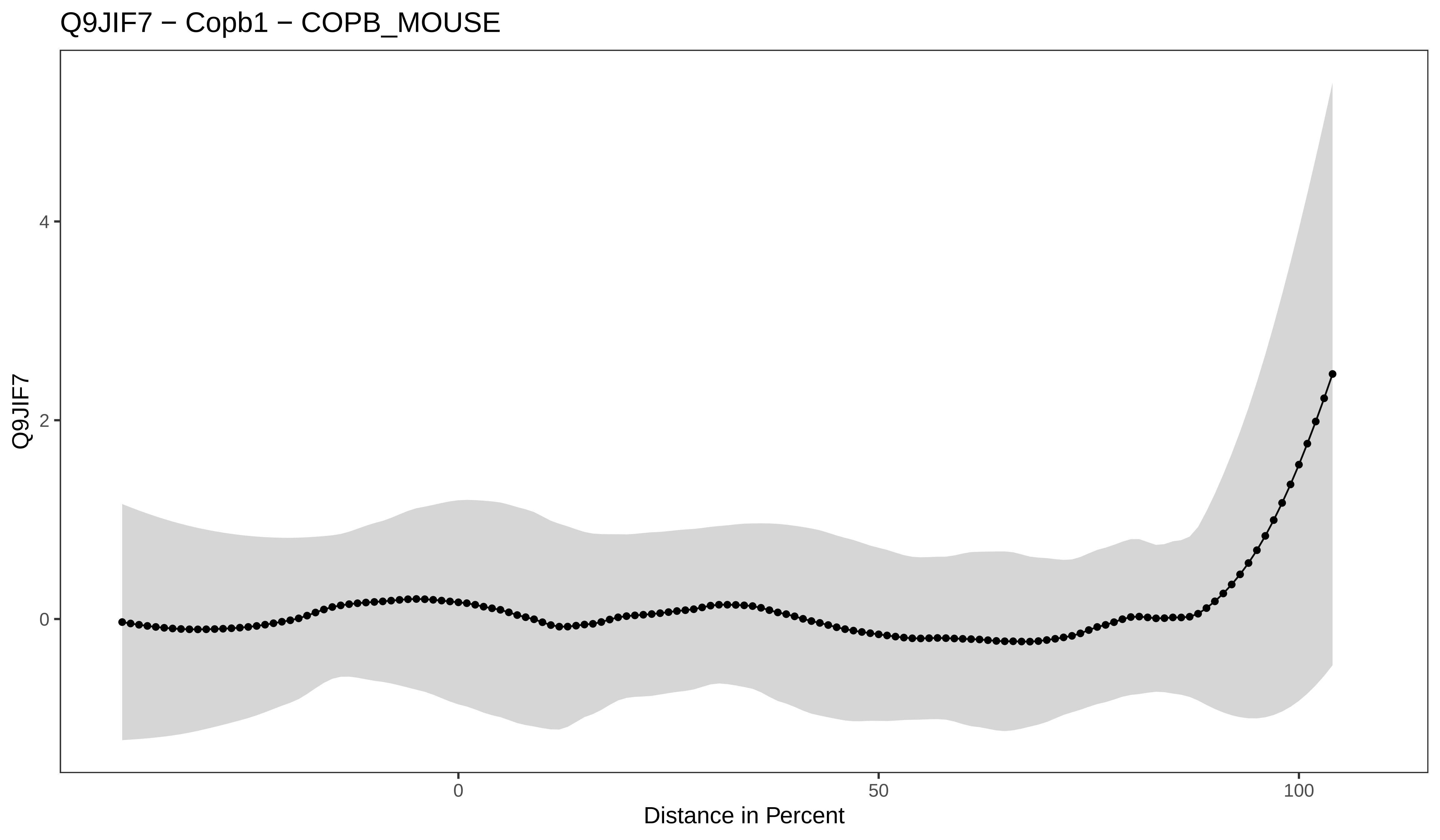
Q9JIF7 − Copb1 − COPB_MOUSE
Q9JIF7
4
2
0
0
50
100
Distance in Percent

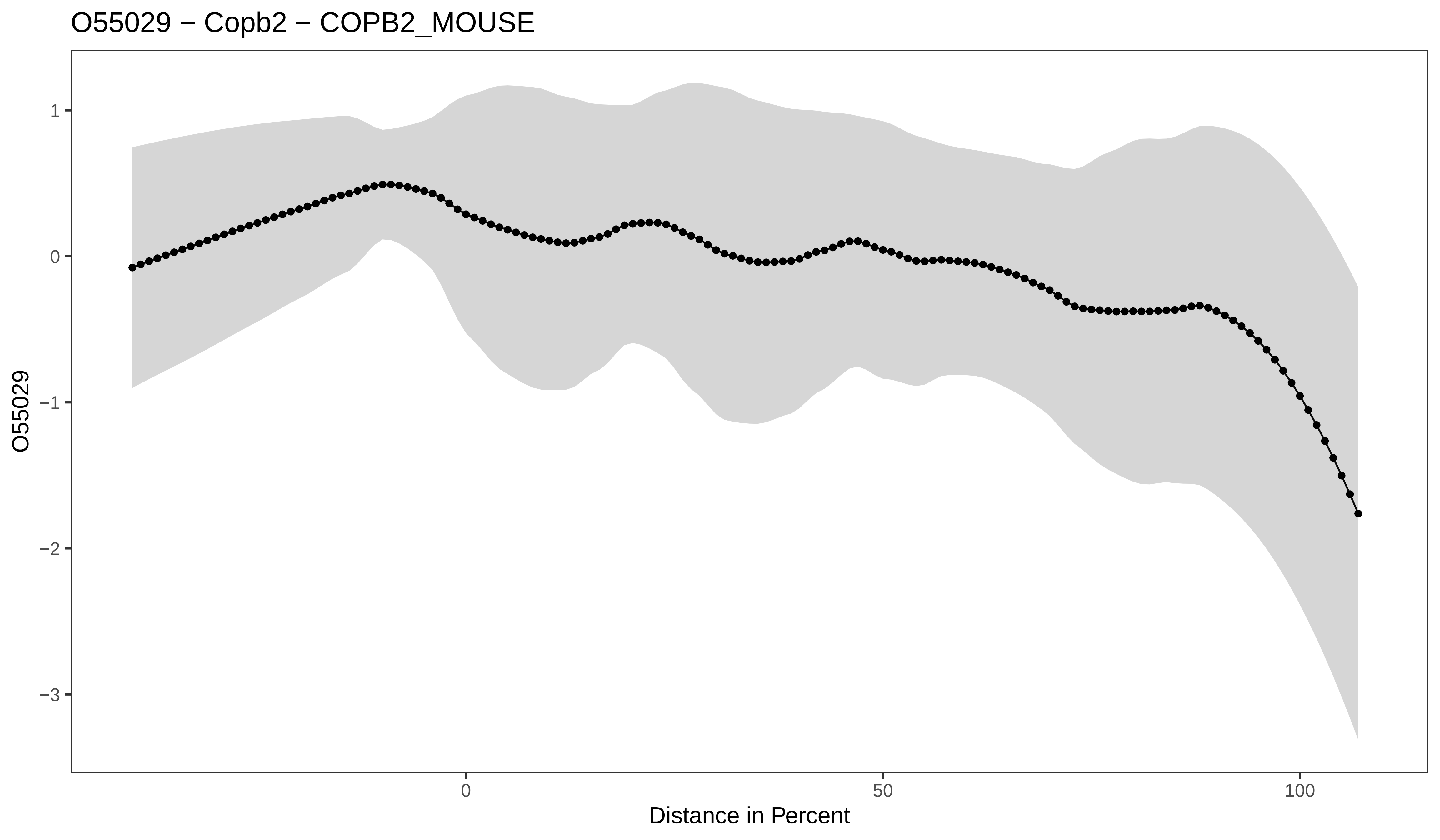

O55029 − Copb2 − COPB2_MOUSE
1
0
−1
−2
−3
O55029
0
50
100
Distance in Percent

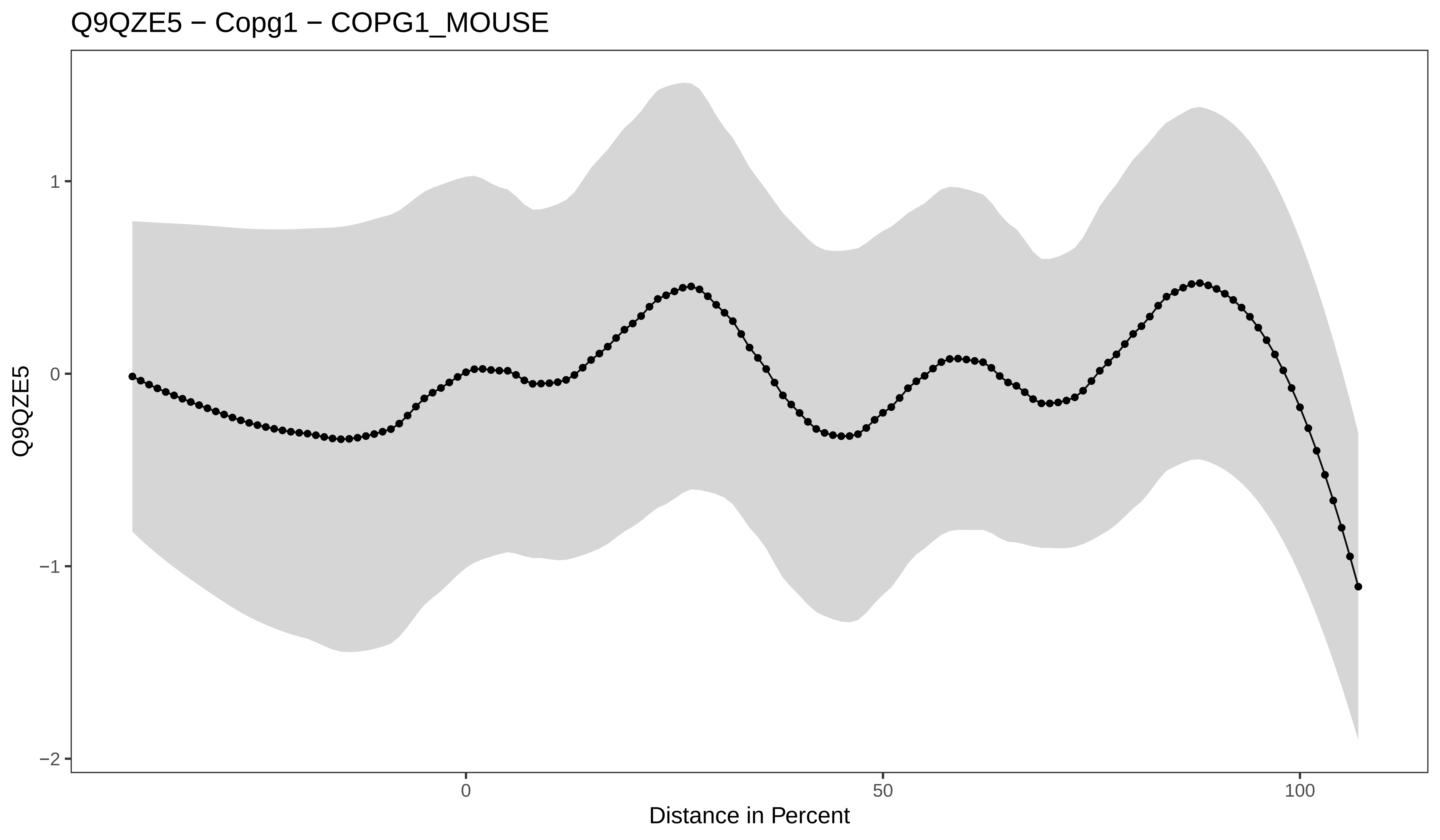
Q9QZE5 − Copg1 − COPG1_MOUSE
Q9QZE5
1
0
−1
−2
0
50
100
Distance in Percent

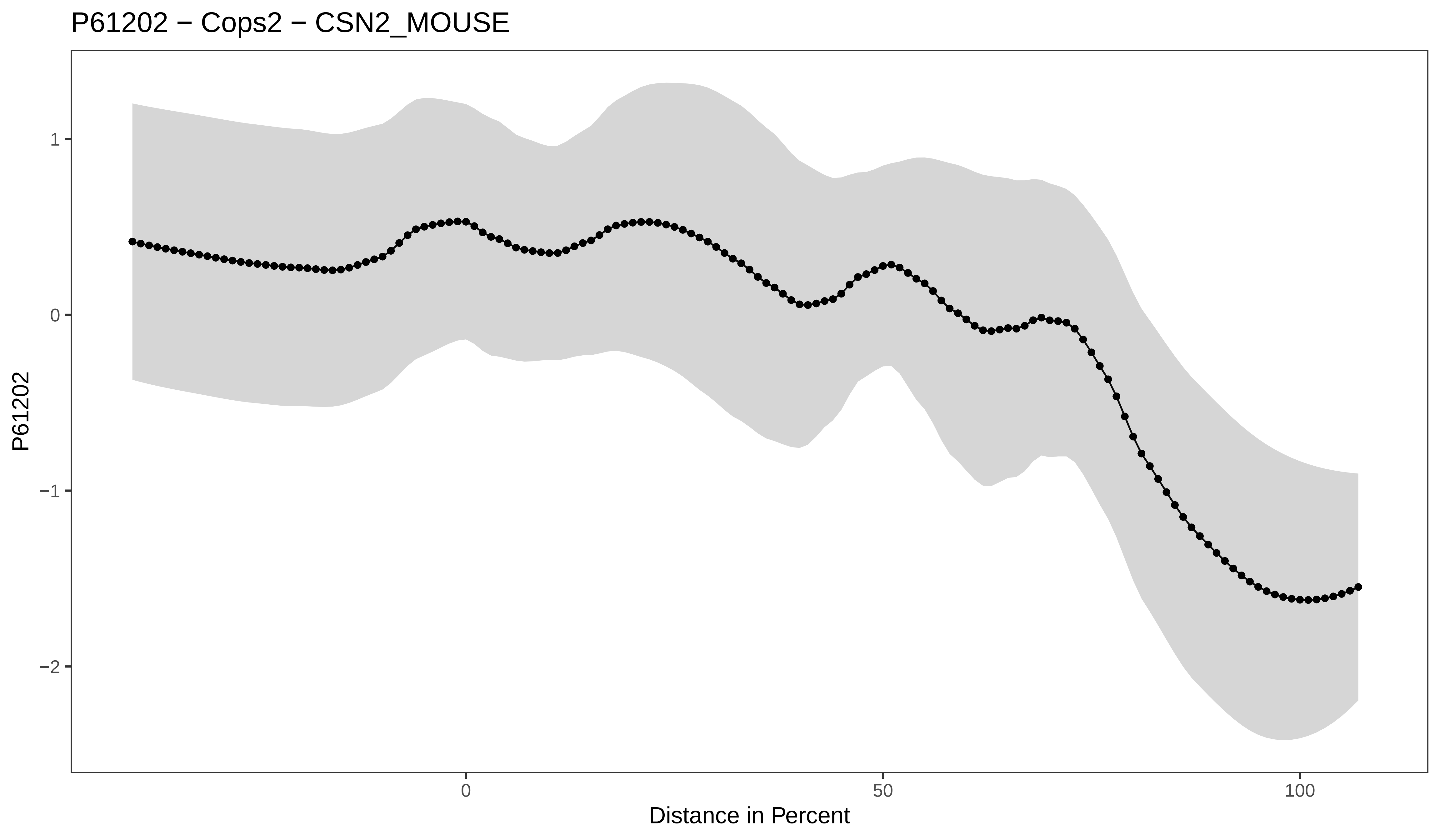

P61202 − Cops2 − CSN2_MOUSE
P61202
1
0
−1
−2
0
50
100
Distance in Percent

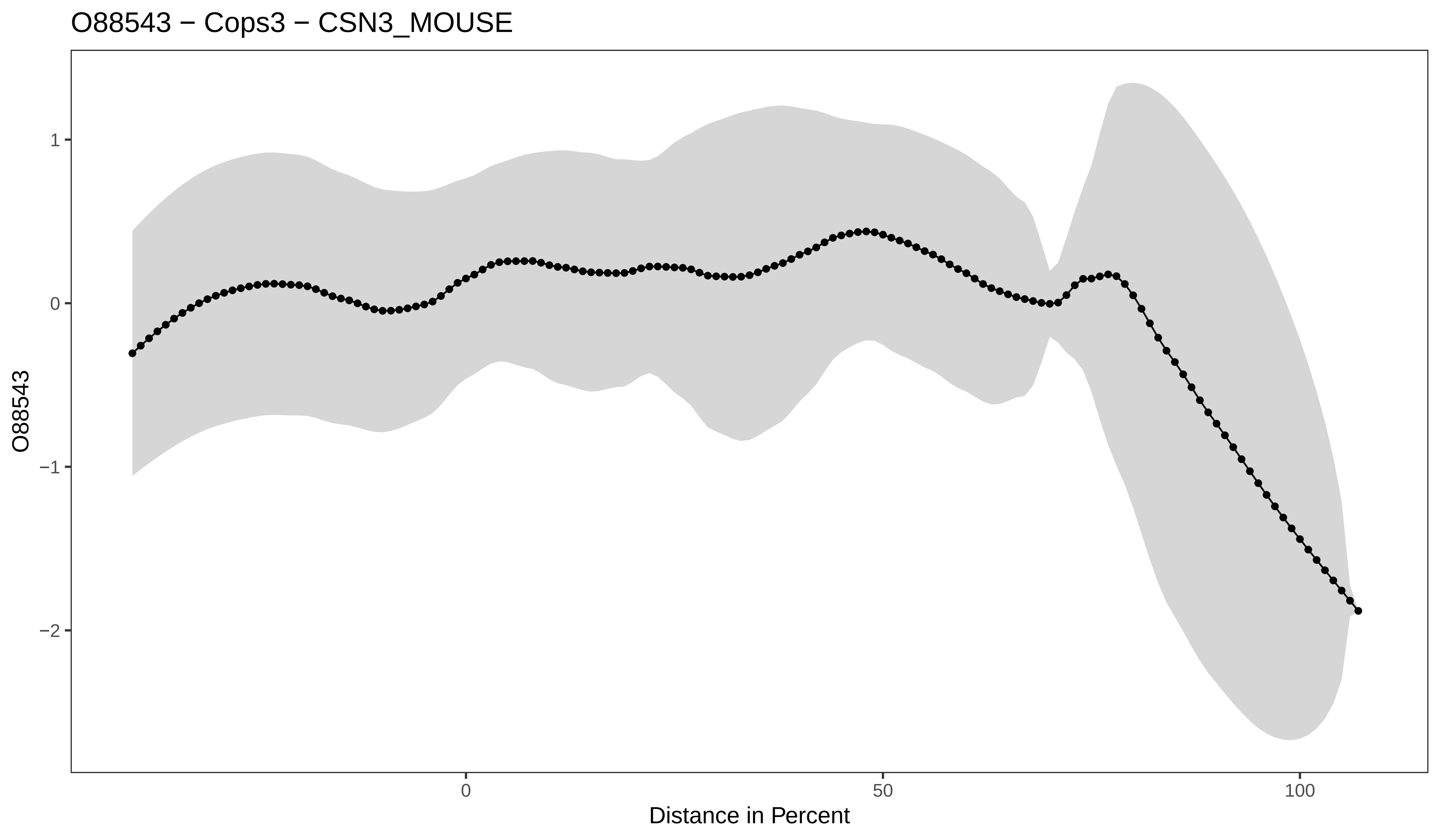

O88543 − Cops3 − CSN3_MOUSE
1
0
−1
−2
O88543
0
50
100
Distance in Percent

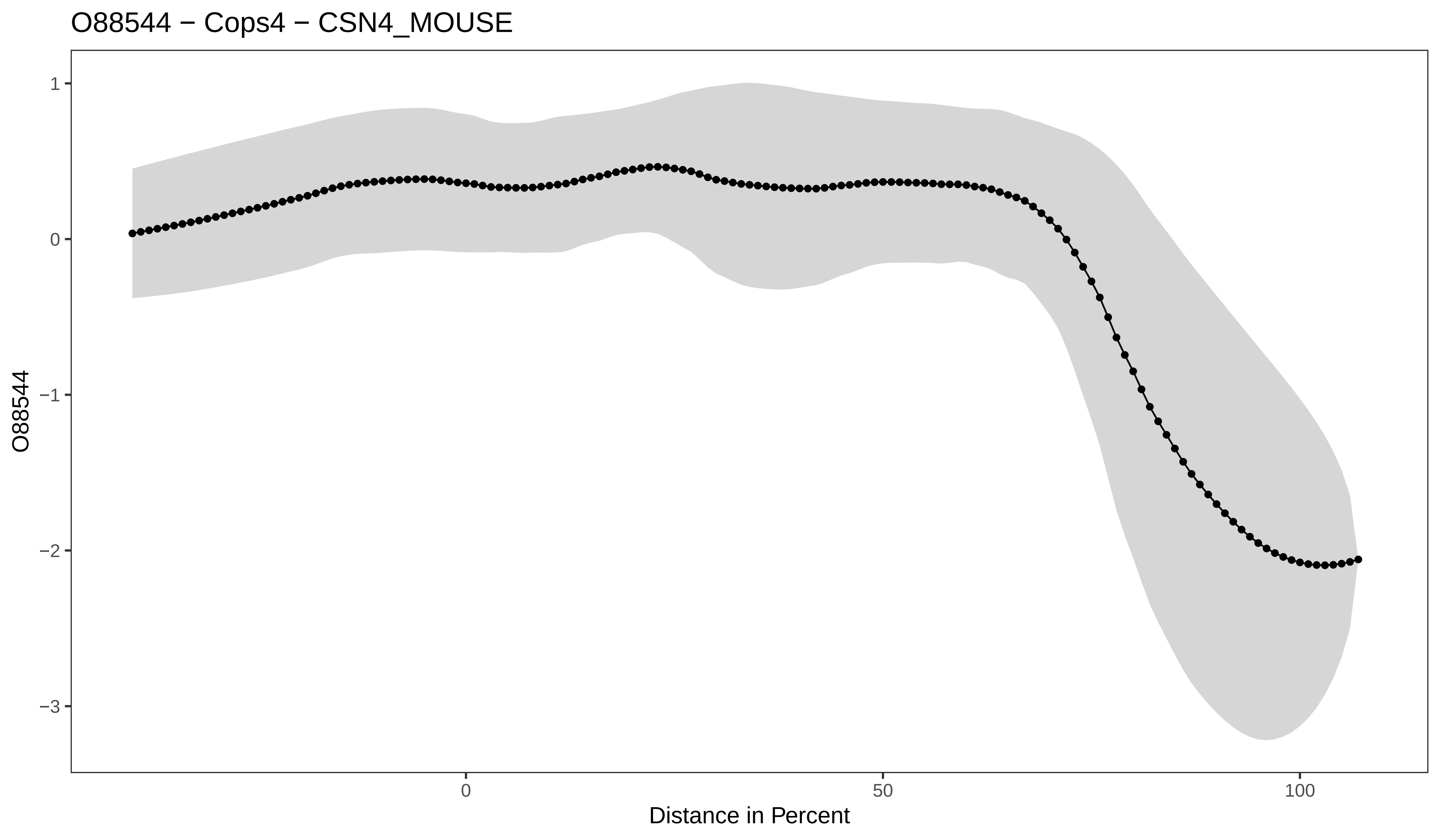

O88544 − Cops4 − CSN4_MOUSE
O88544
1
0
−1
−2
−3
0
50
100
Distance in Percent

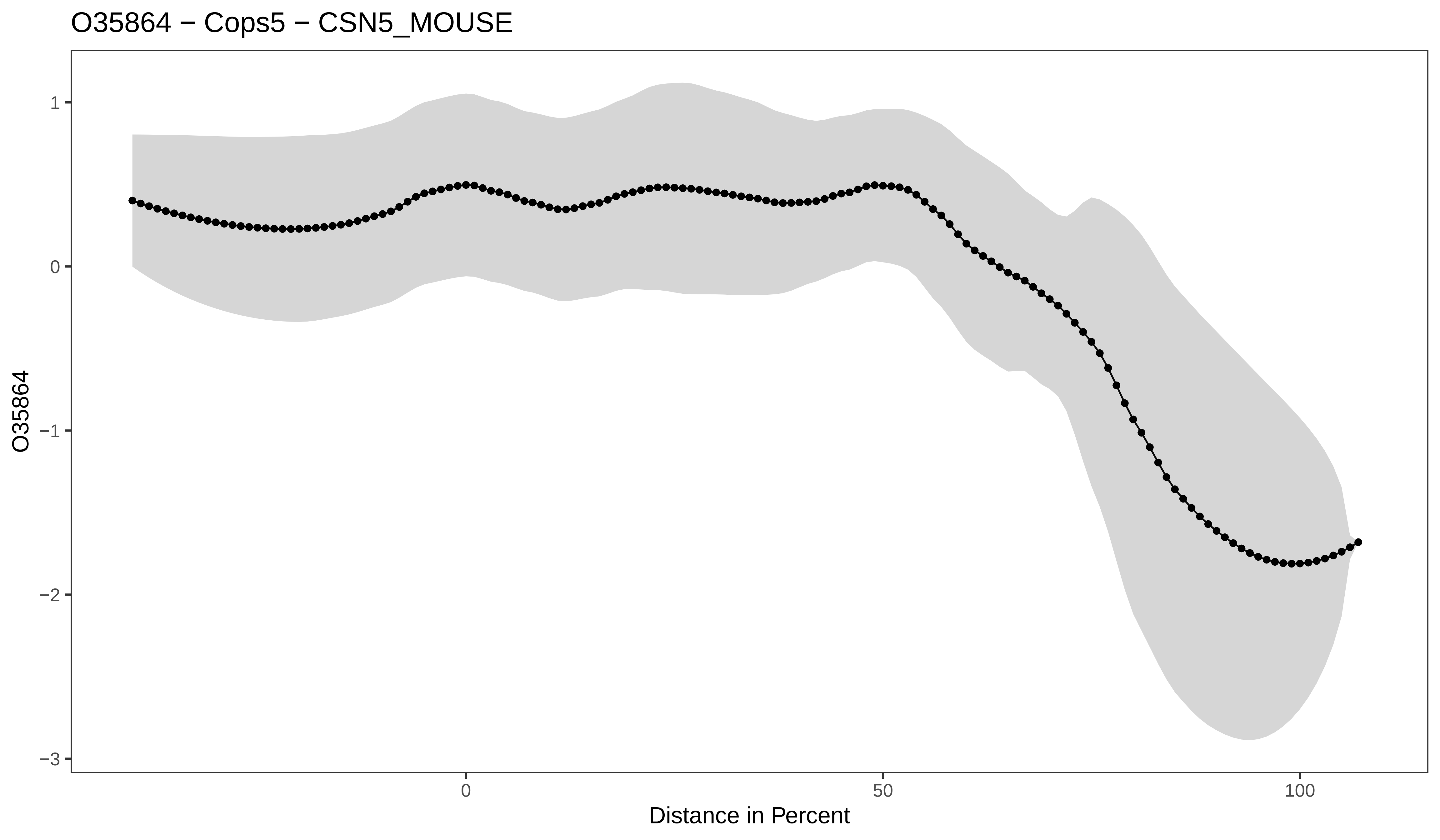
O35864 − Cops5 − CSN5_MOUSE
O35864
1
0
−1
−2
−3
0
50
100
Distance in Percent

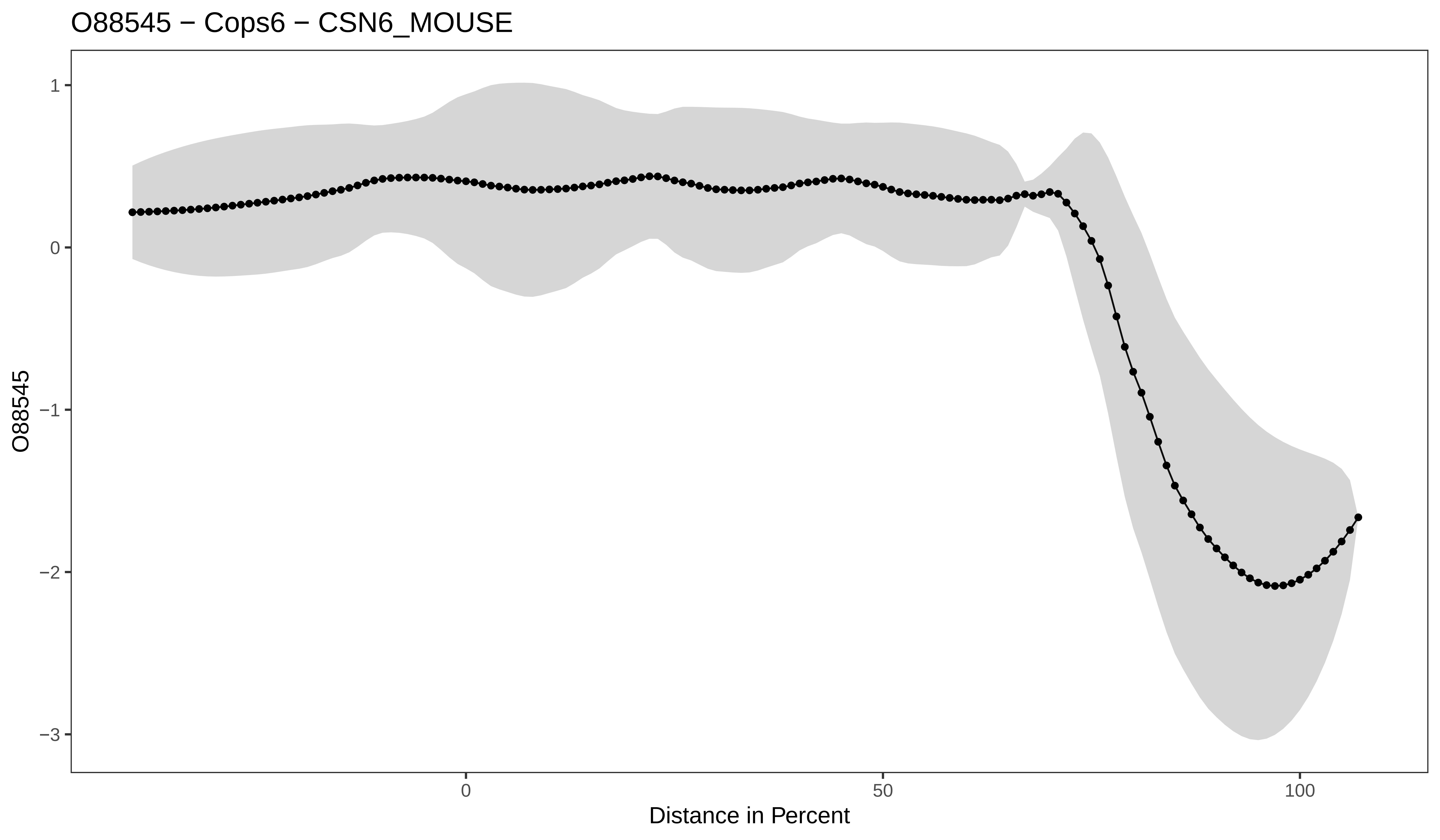

O88545 − Cops6 − CSN6_MOUSE
O88545
1
0
−1
−2
−3
0
50
100
Distance in Percent

Q9CZ04 − Cops7a − CSN7A_MOUSE

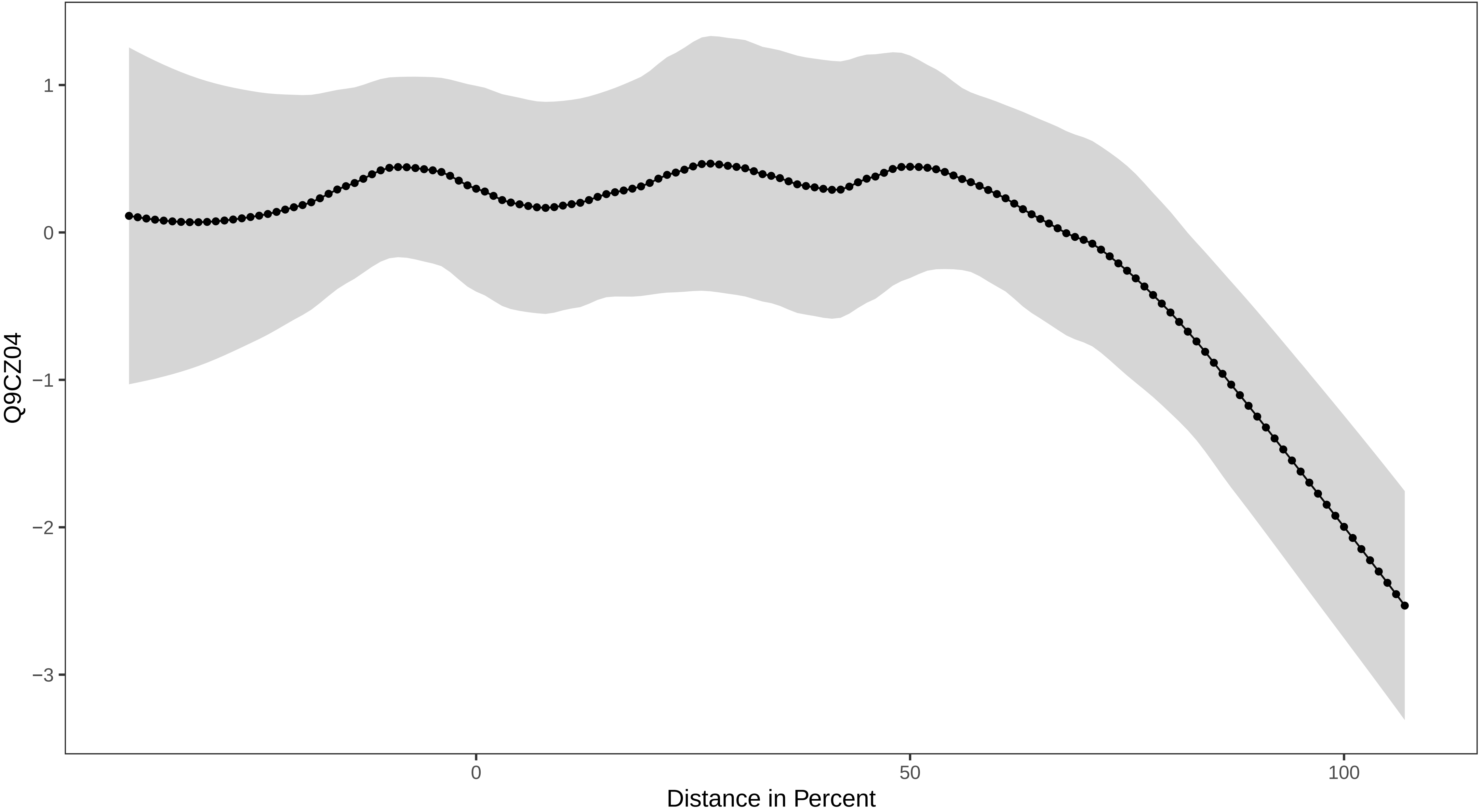

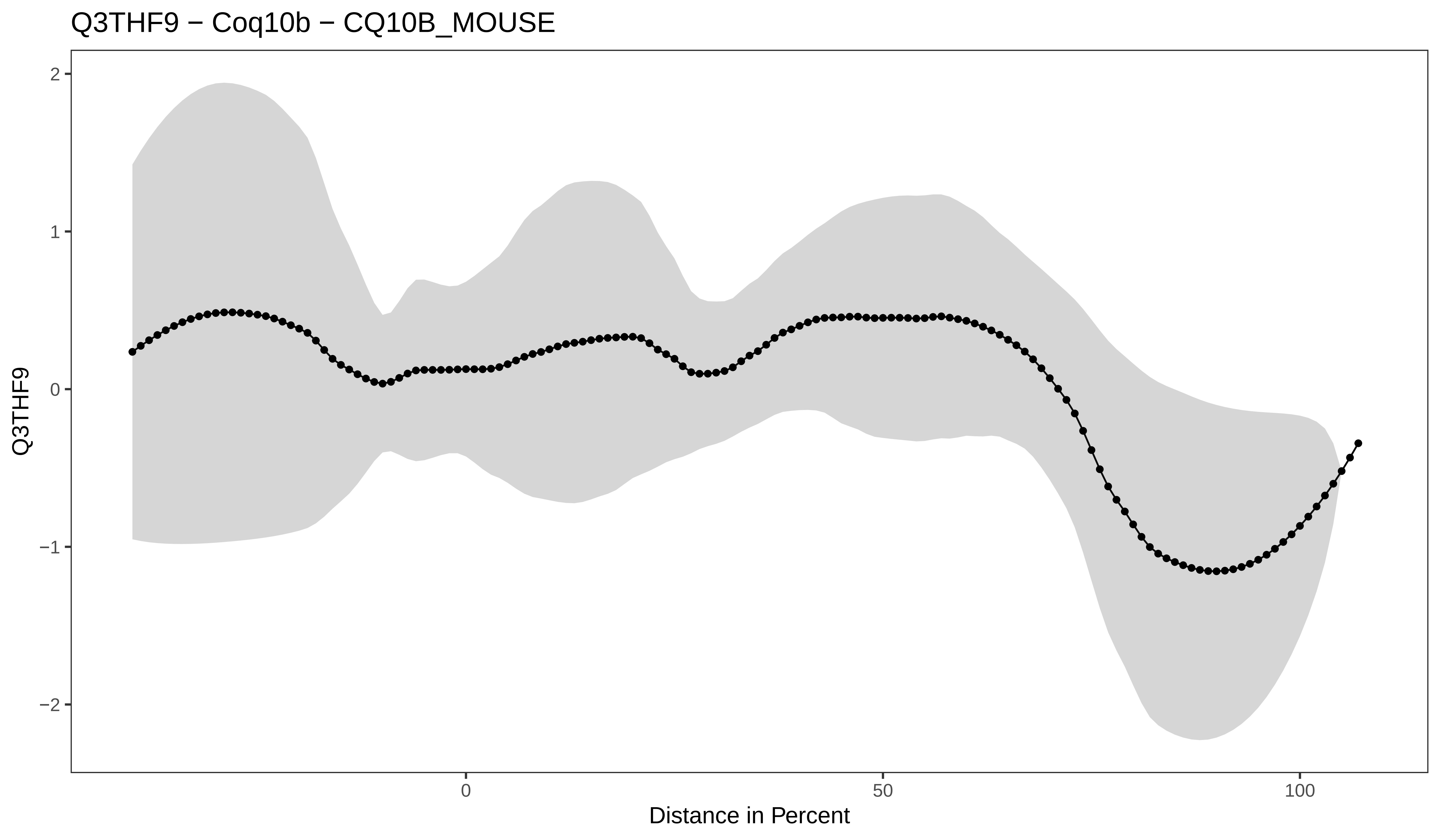

Q3THF9 − Coq10b − CQ10B_MOUSE
Q3THF9
2
1
0
−1
−2
0
50
100
Distance in Percent

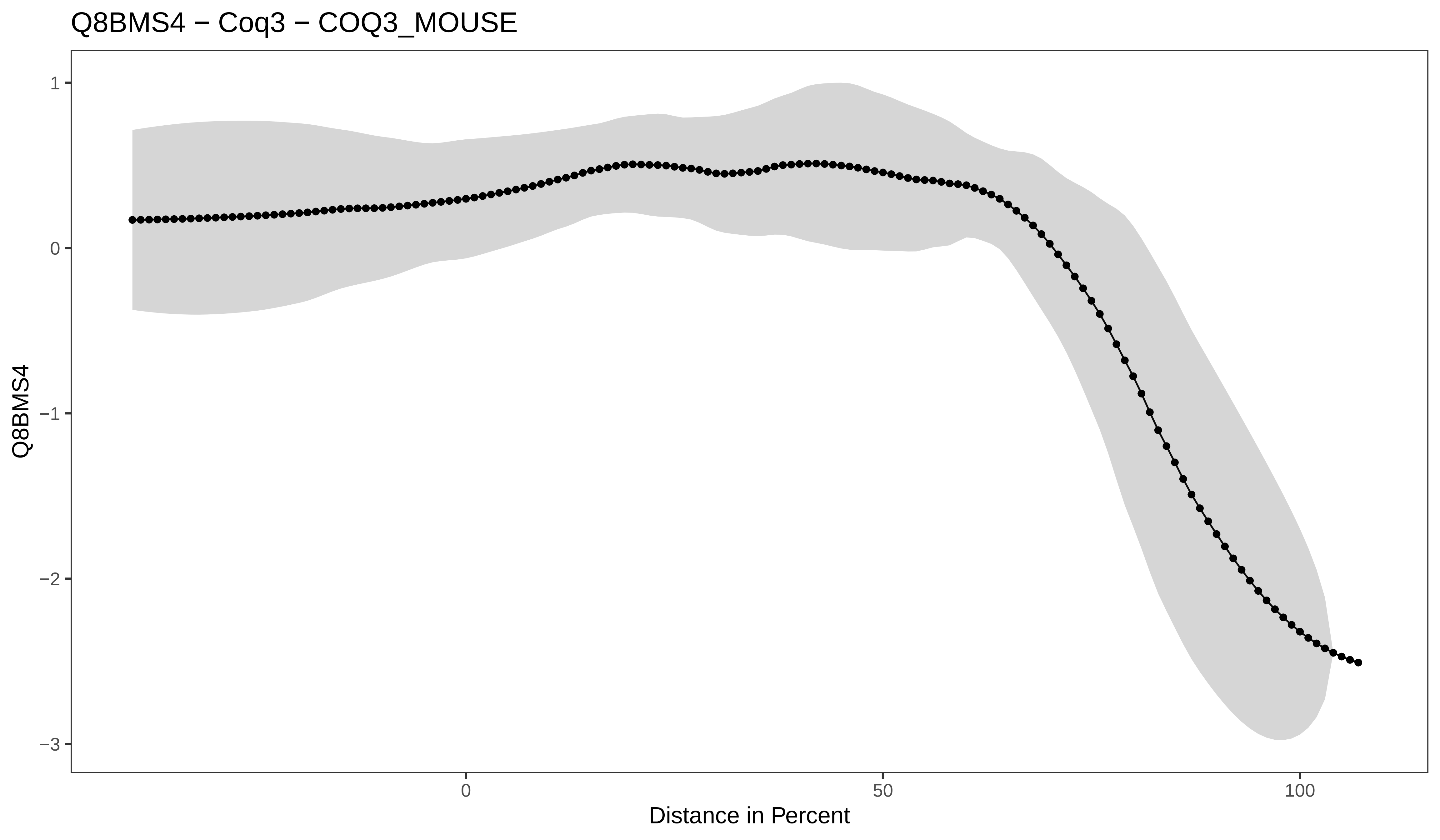
Q8BMS4 − Coq3 − COQ3_MOUSE
Q8BMS4
1
0
−1
−2
−3
0
50
100
Distance in Percent

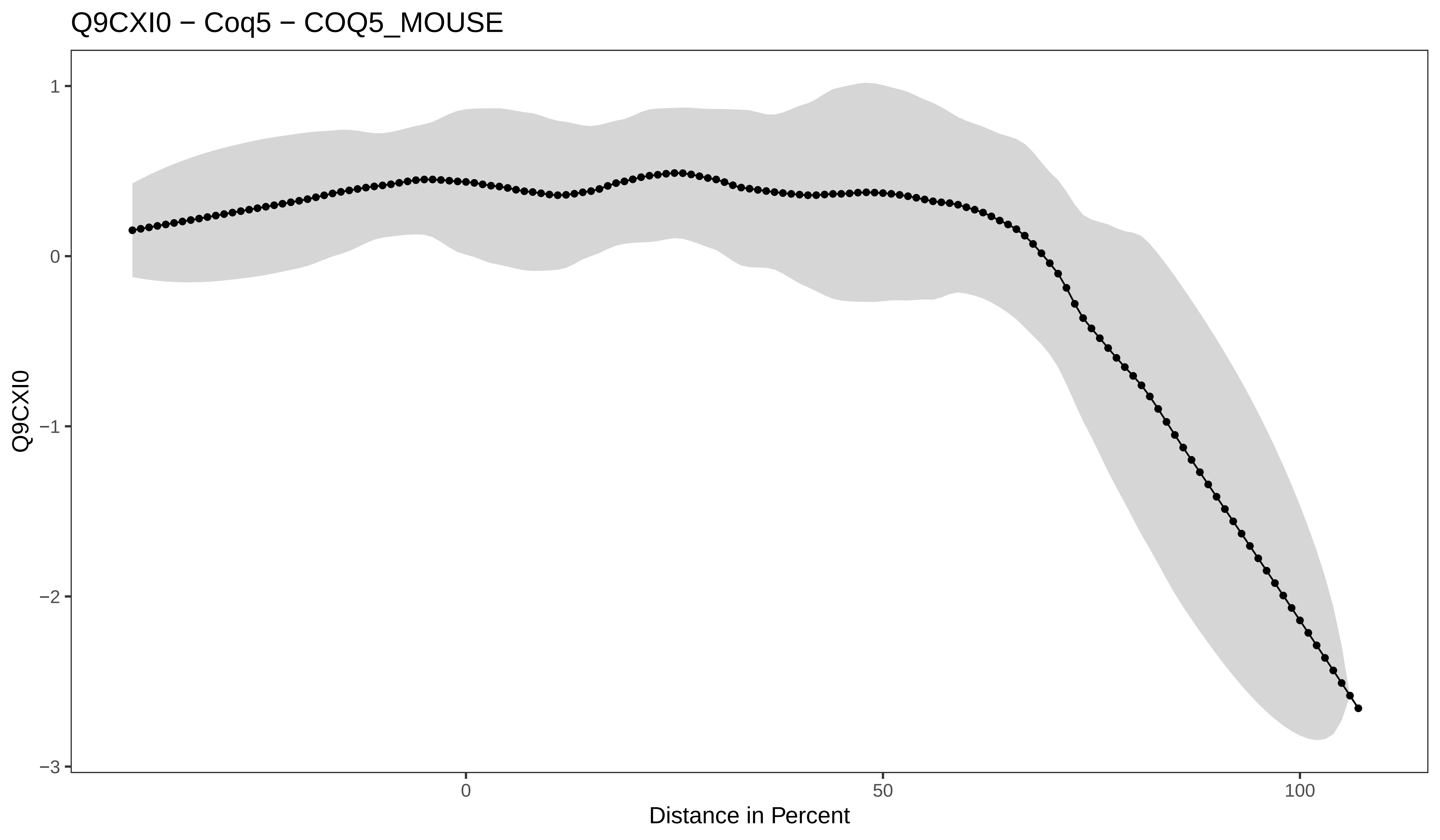

Q9CXI0 − Coq5 − COQ5_MOUSE
Q9CXI0
1
0
−1
−2
−3
0
50
100
Distance in Percent

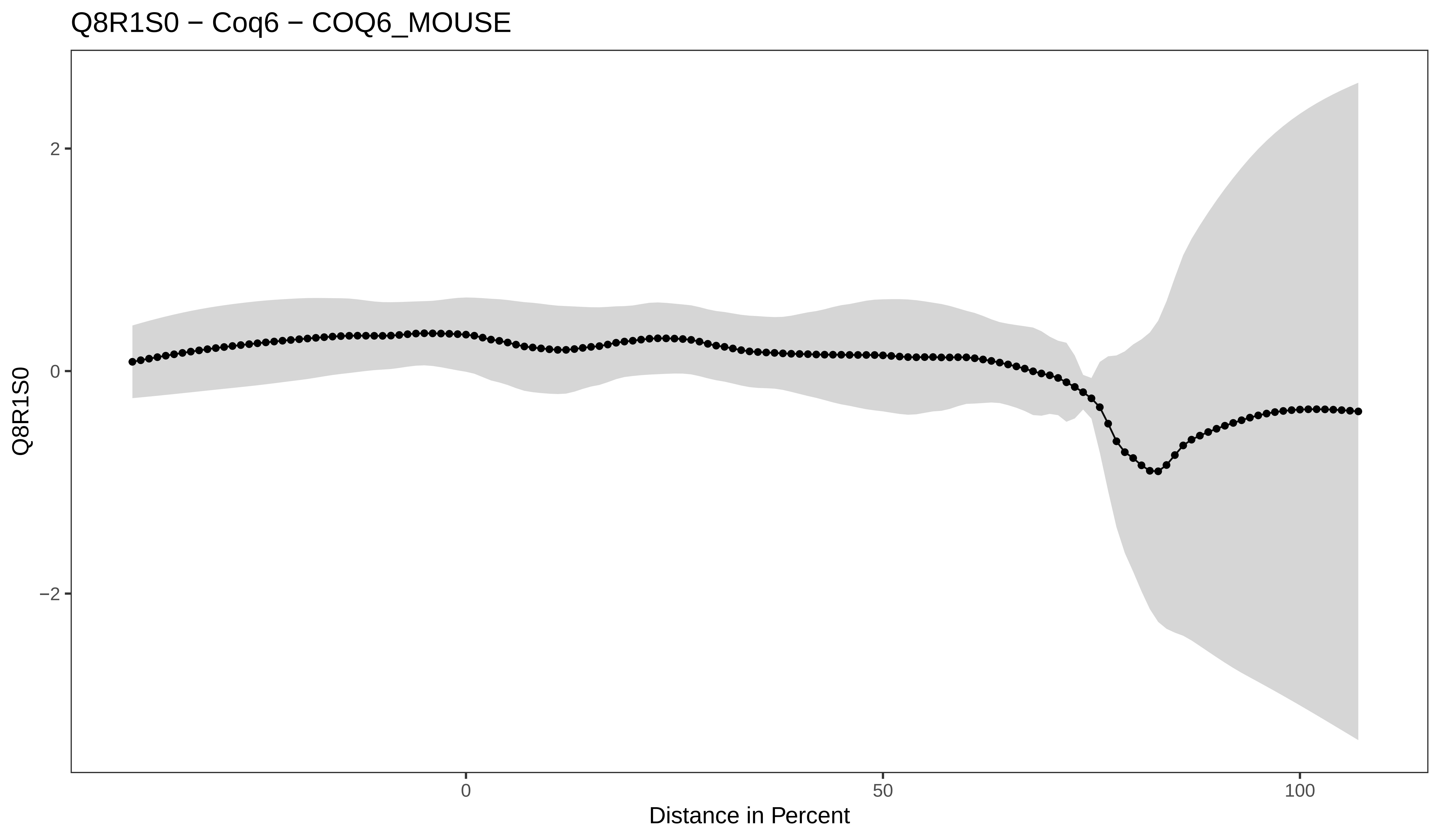
Q8R1S0 − Coq6 − COQ6_MOUSE
Q8R1S0
2
0
−2
0
50
100
Distance in Percent

P97478 − Coq7 − COQ7_MOUSE

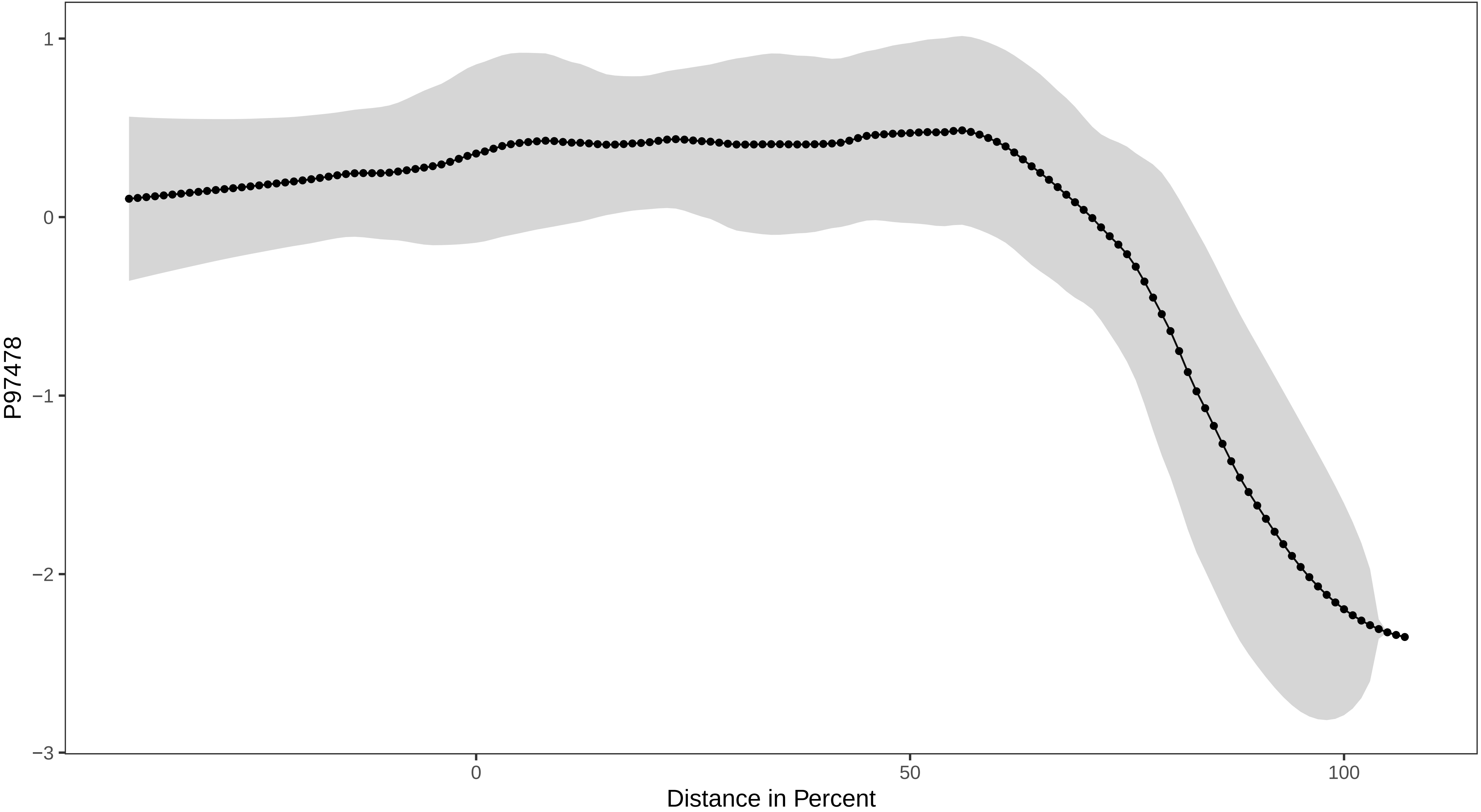

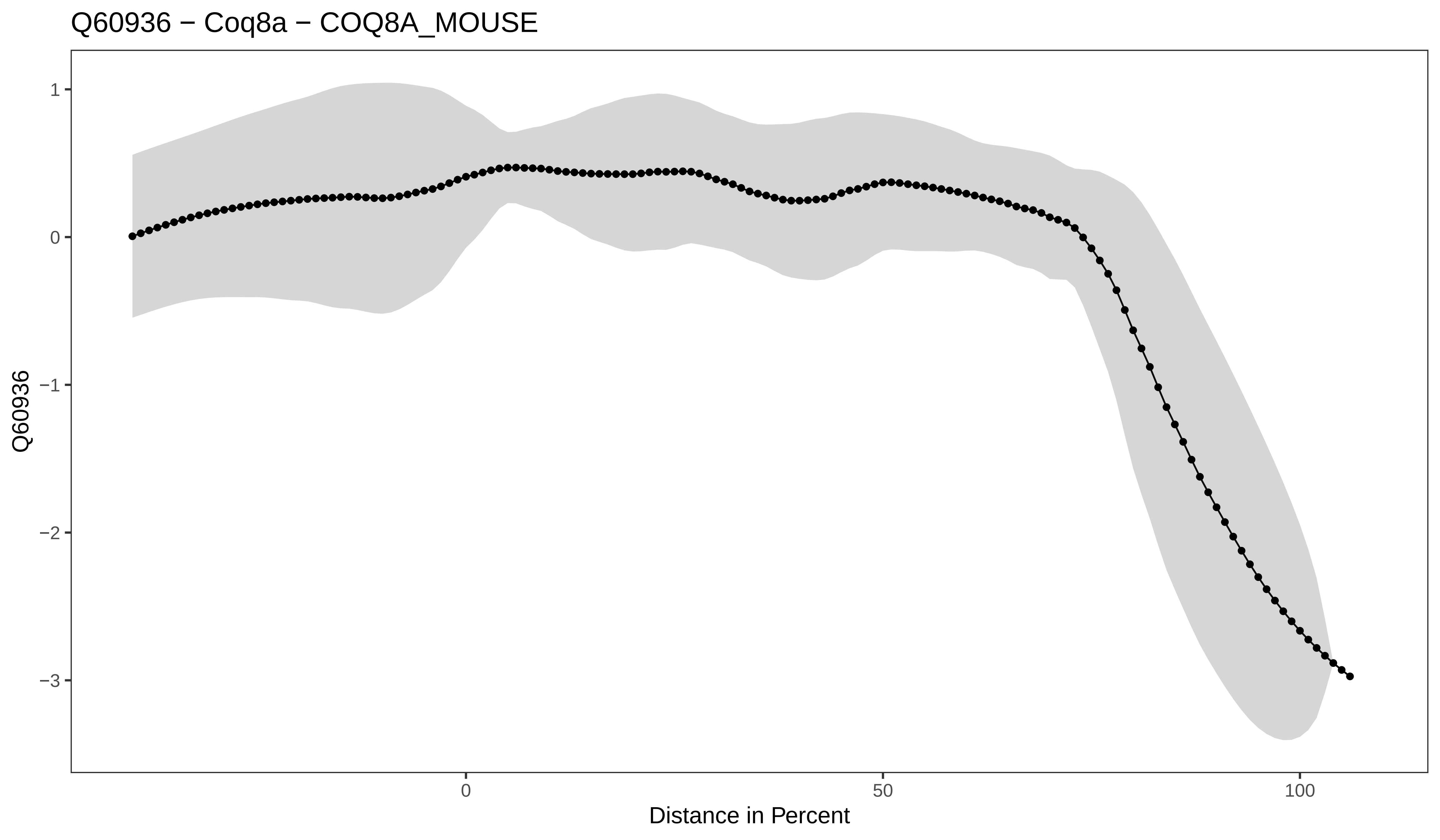

Q60936 − Coq8a − COQ8A_MOUSE
1
0
−1
−2
−3
Q60936
0
50
100
Distance in Percent

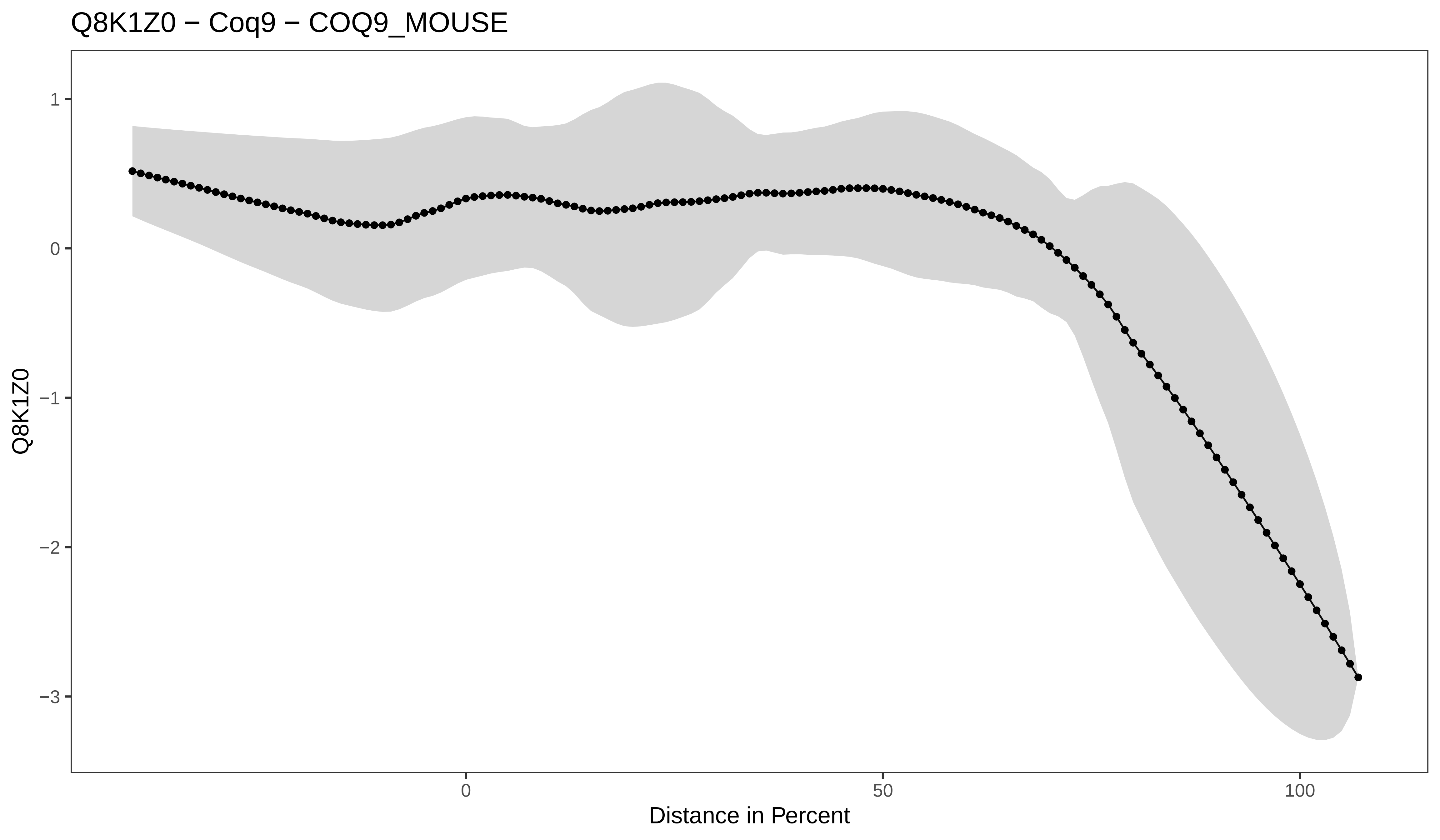

Q8K1Z0 − Coq9 − COQ9_MOUSE
1
0
−1
−2
−3
Q8K1Z0
0
50
100
Distance in Percent

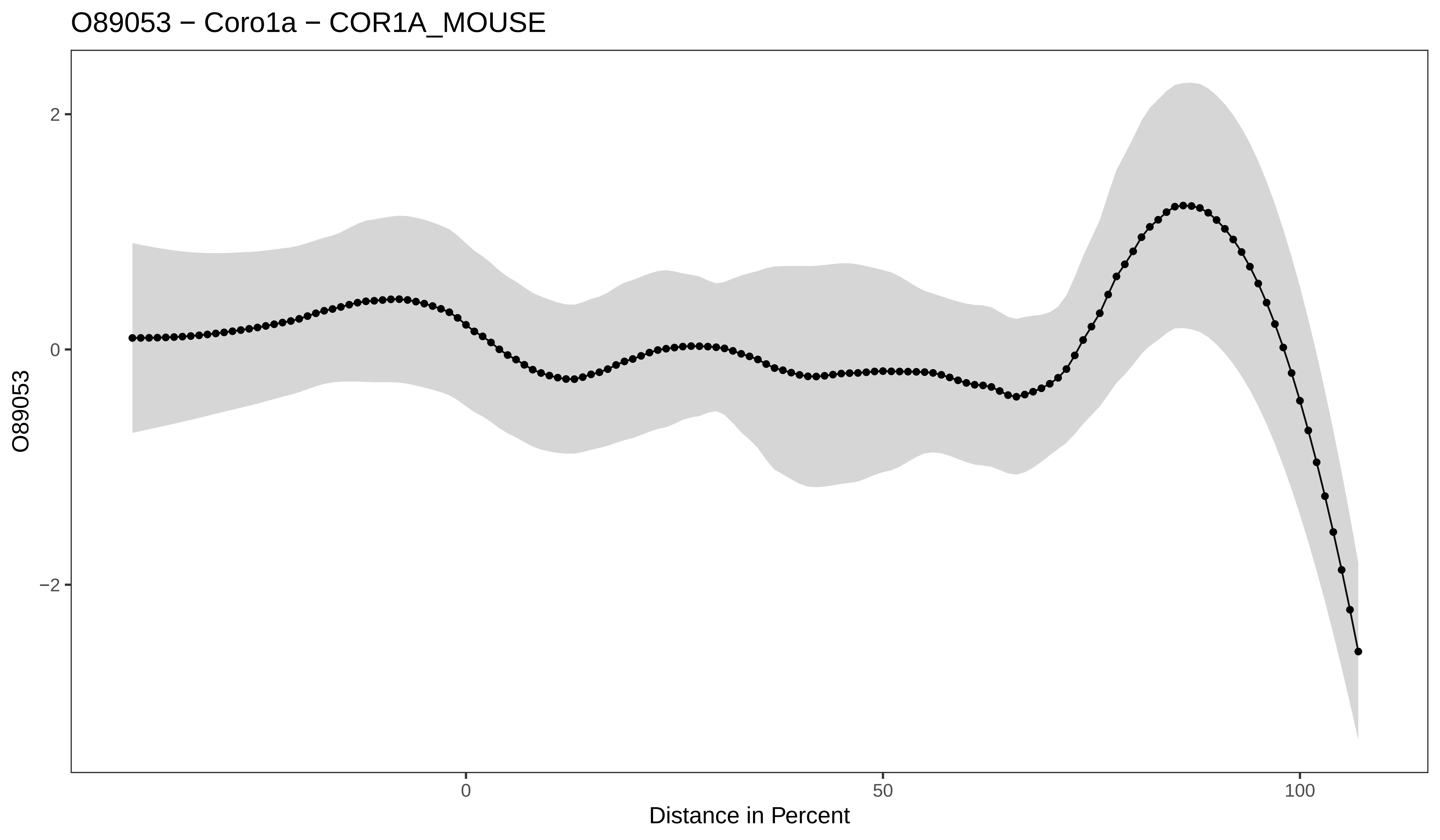
O89053 − Coro1a − COR1A_MOUSE
O89053
2
0
−2
0
50
100
Distance in Percent

Q9WUM3 − Coro1b − COR1B_MOUSE

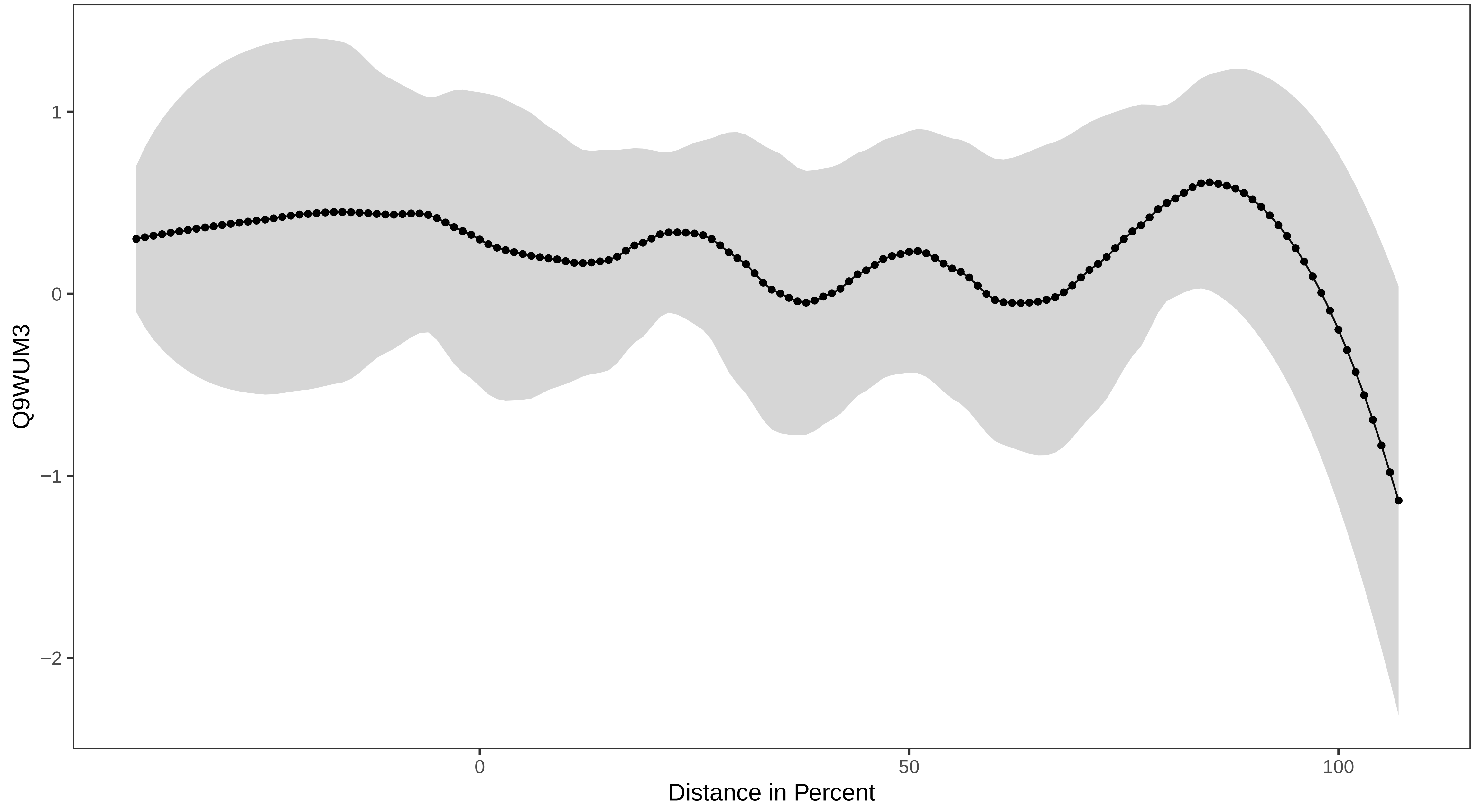

Q9WUM4 − Coro1c − COR1C_MOUSE

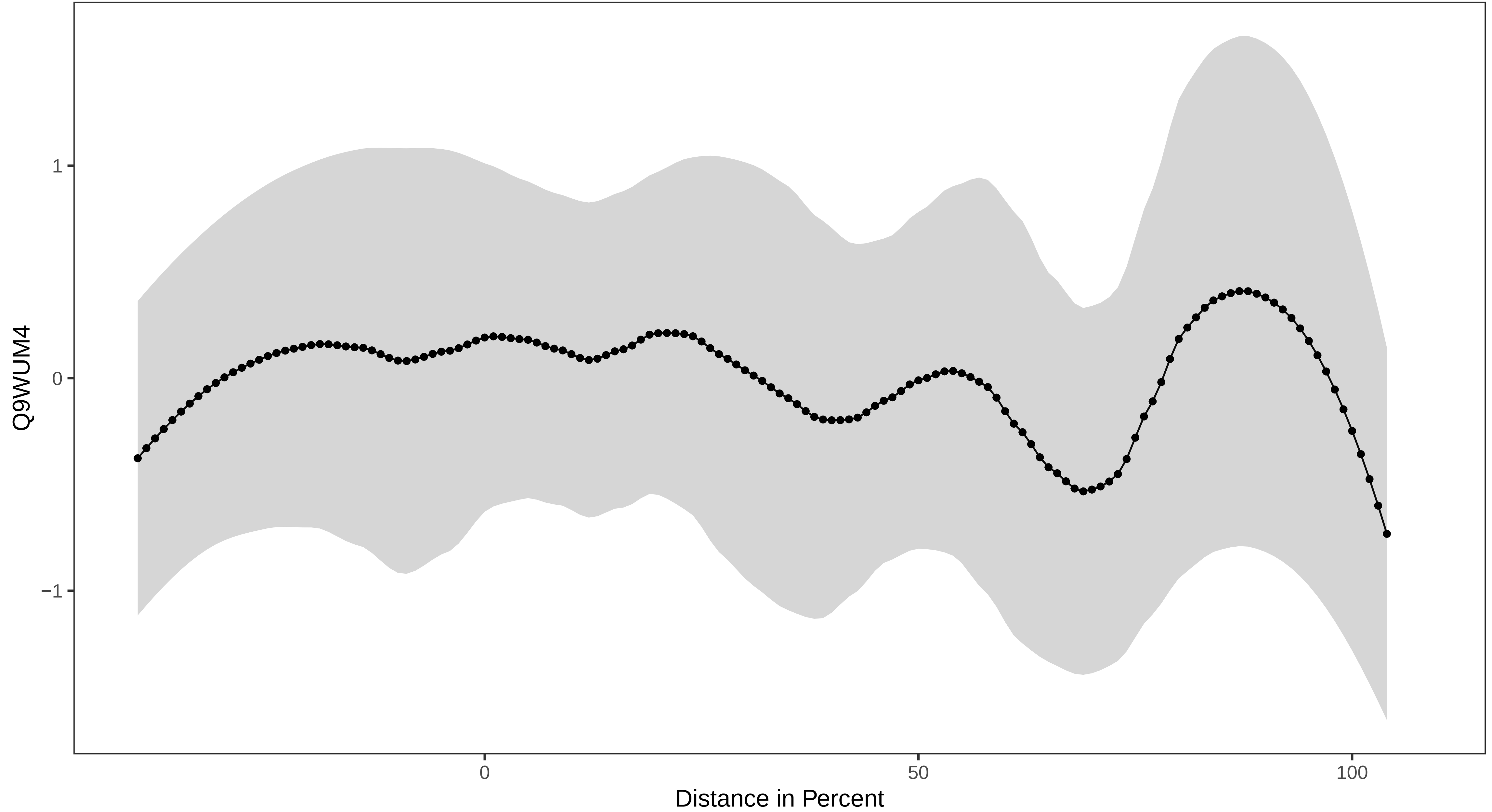

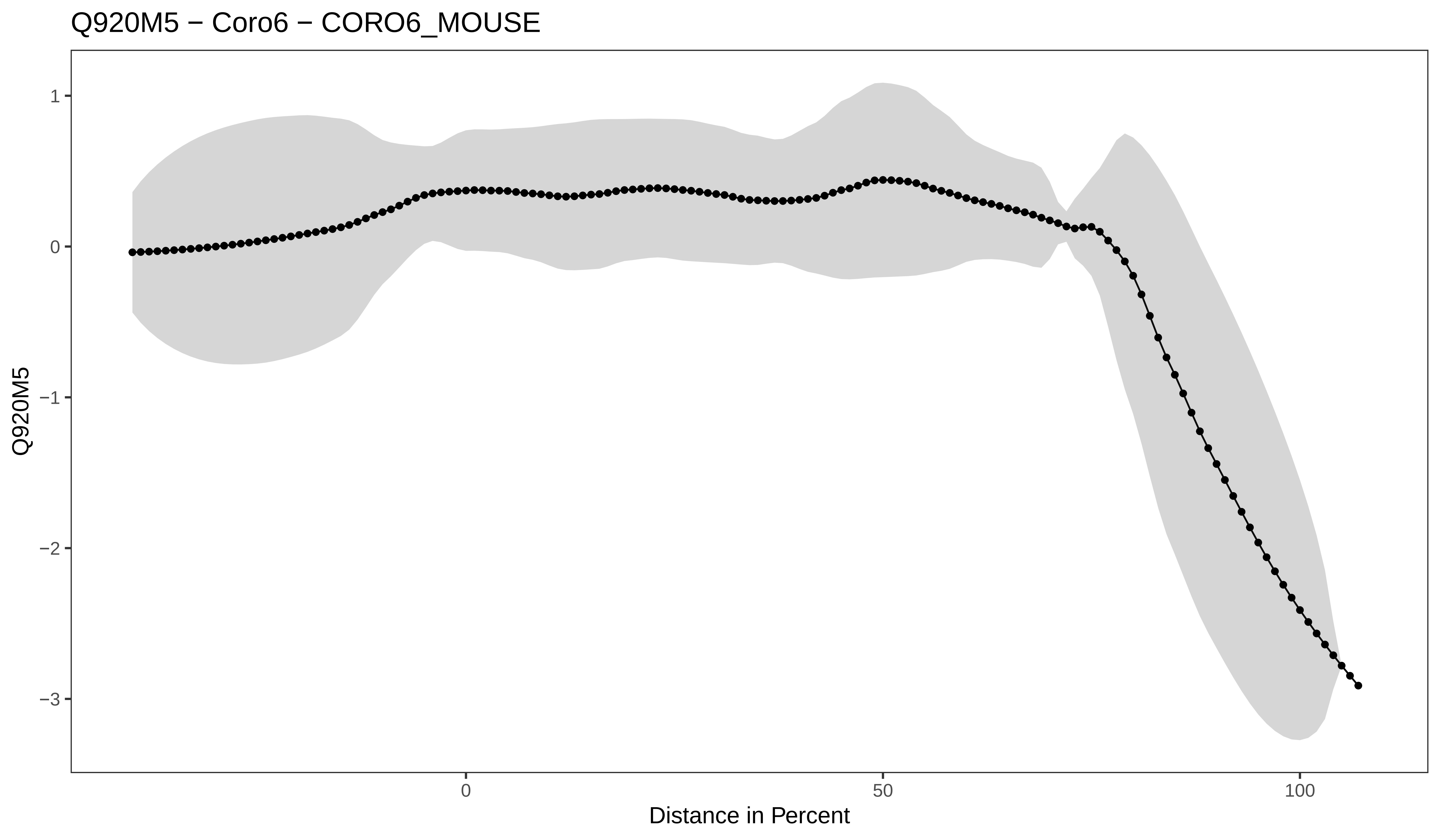

Q920M5 − Coro6 − CORO6_MOUSE
1
0
−1
−2
−3
Q920M5
0
50
100
Distance in Percent

Q9CQI6 − Cotl1 − COTL1_MOUSE

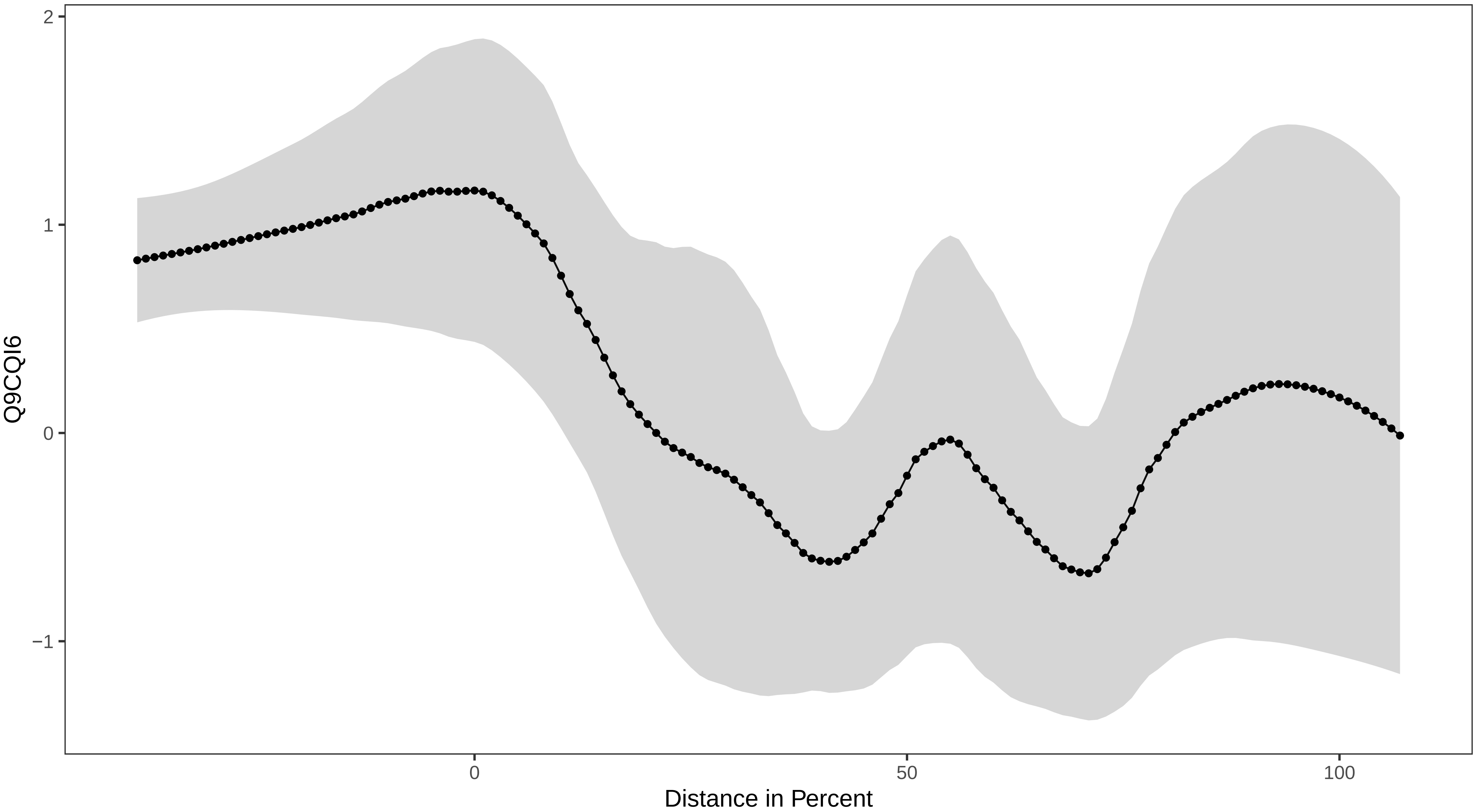

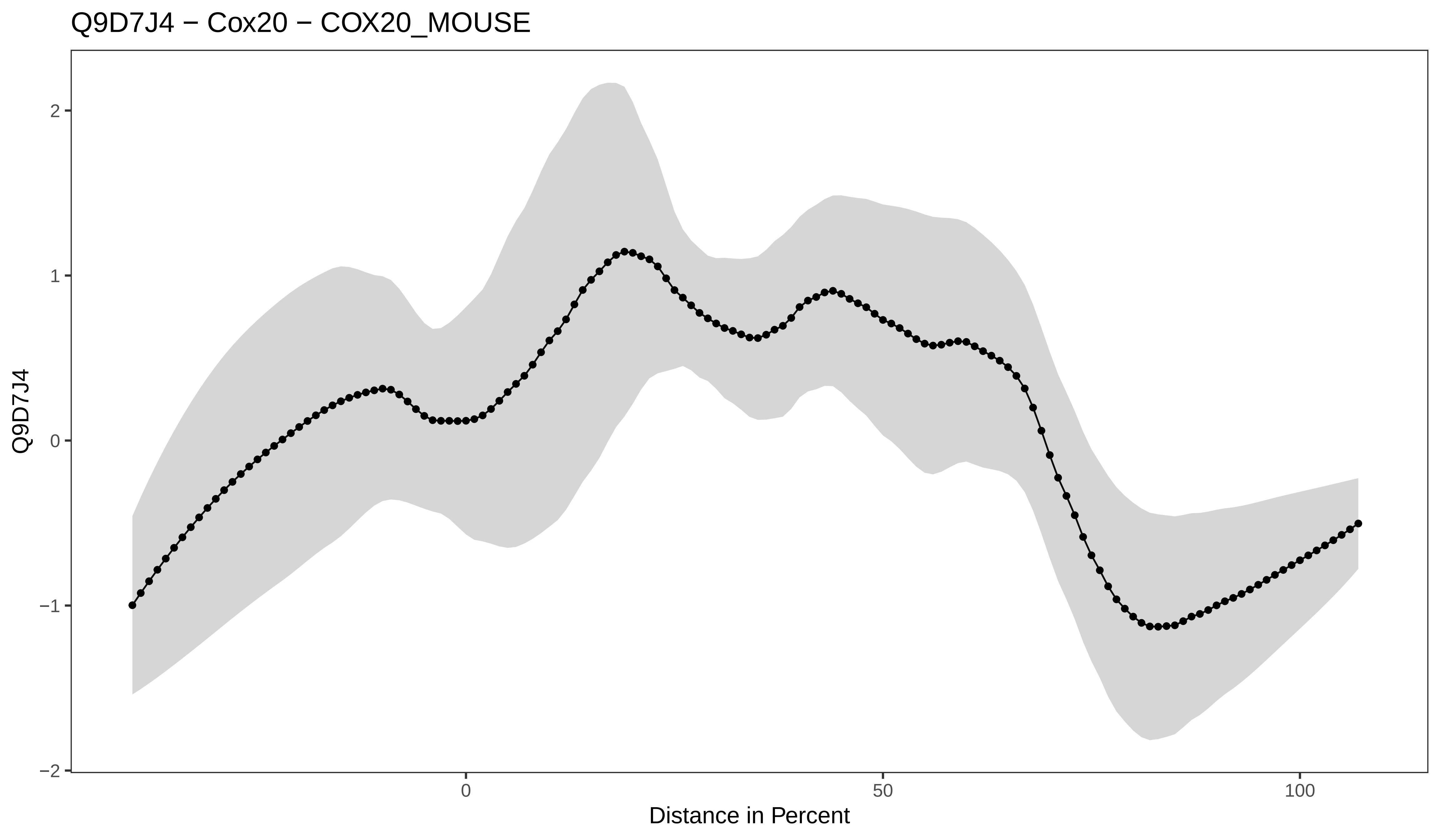

Q9D7J4 − Cox20 − COX20_MOUSE
Q9D7J4
Distance in Percent
2
1
0
−1
−2
0
50
100

P19783 − Cox4i1 − COX41_MOUSE

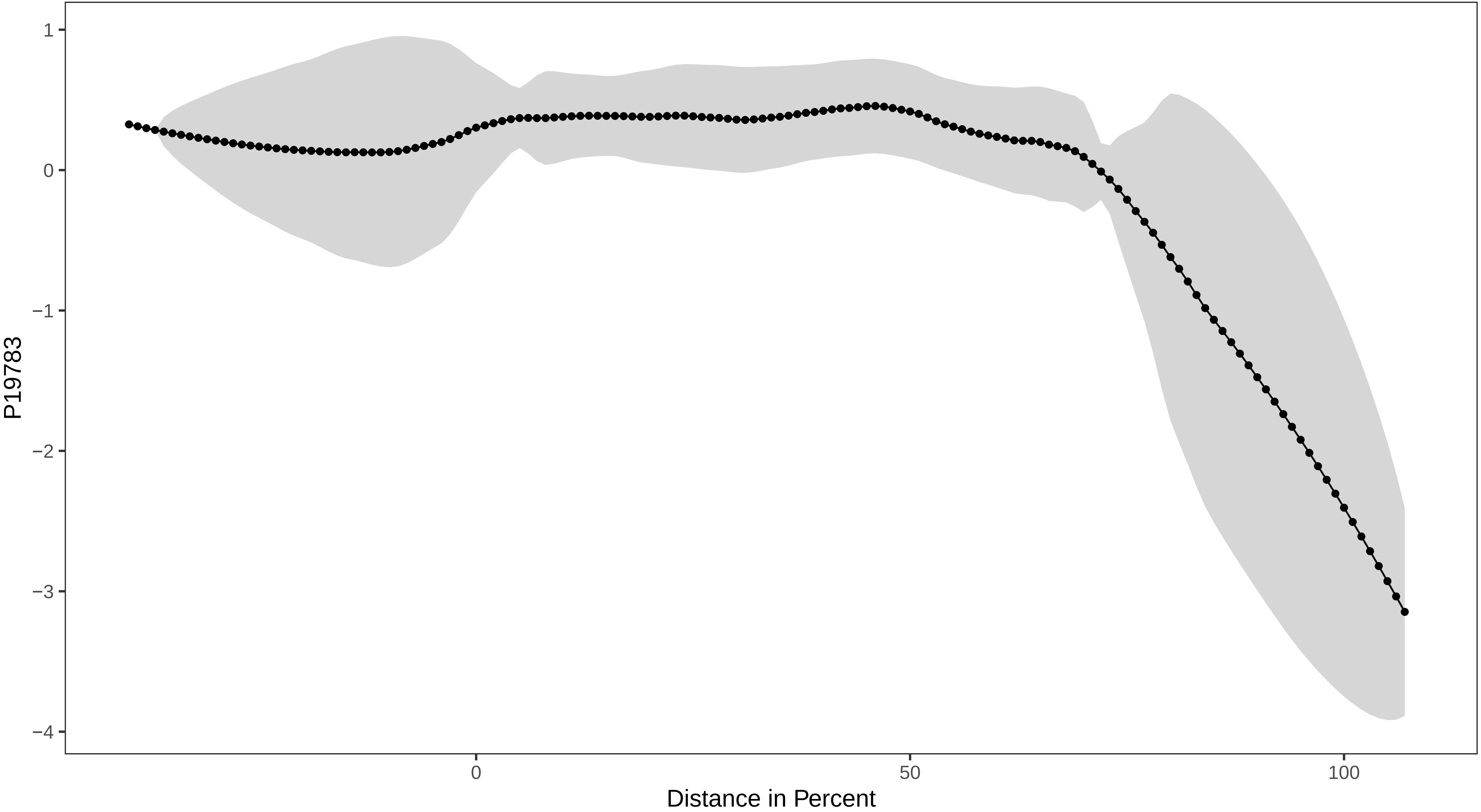

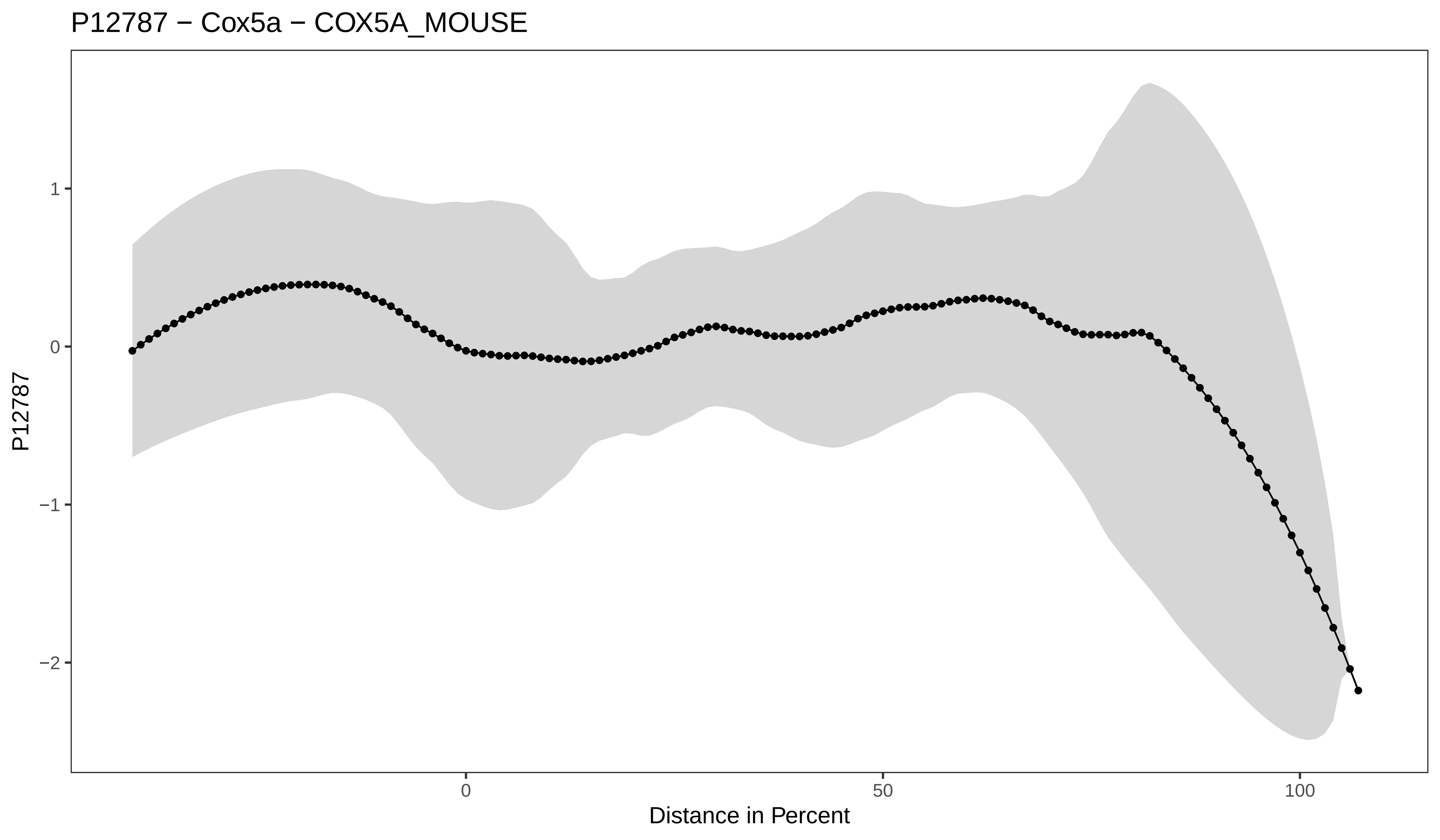
P12787 − Cox5a − COX5A_MOUSE
P12787
1
0
−1
−2
0
50
100
Distance in Percent

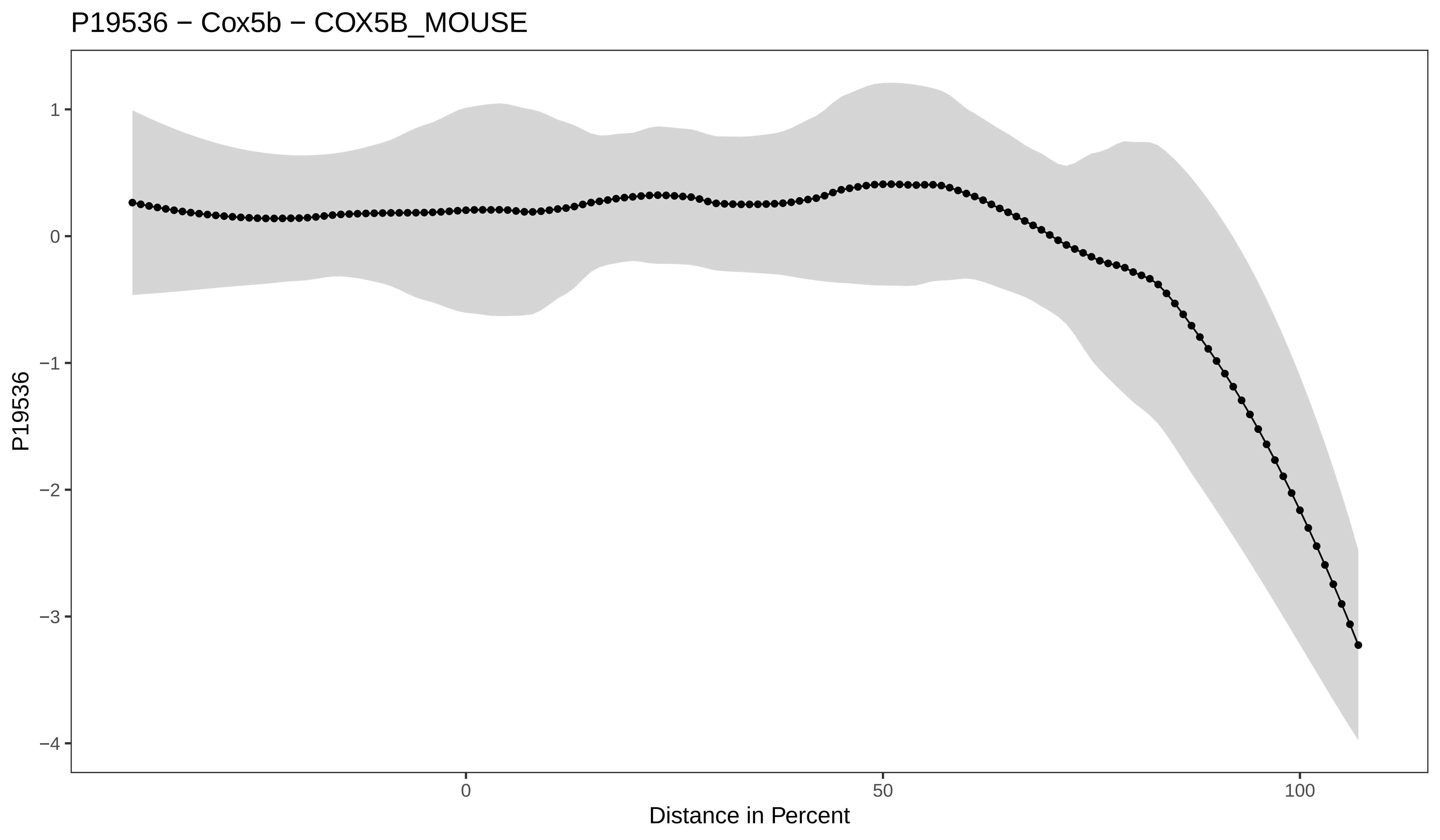
P19536 − Cox5b − COX5B_MOUSE
1
0
−1
−2
−3
−4
P19536
0
50
100
Distance in Percent

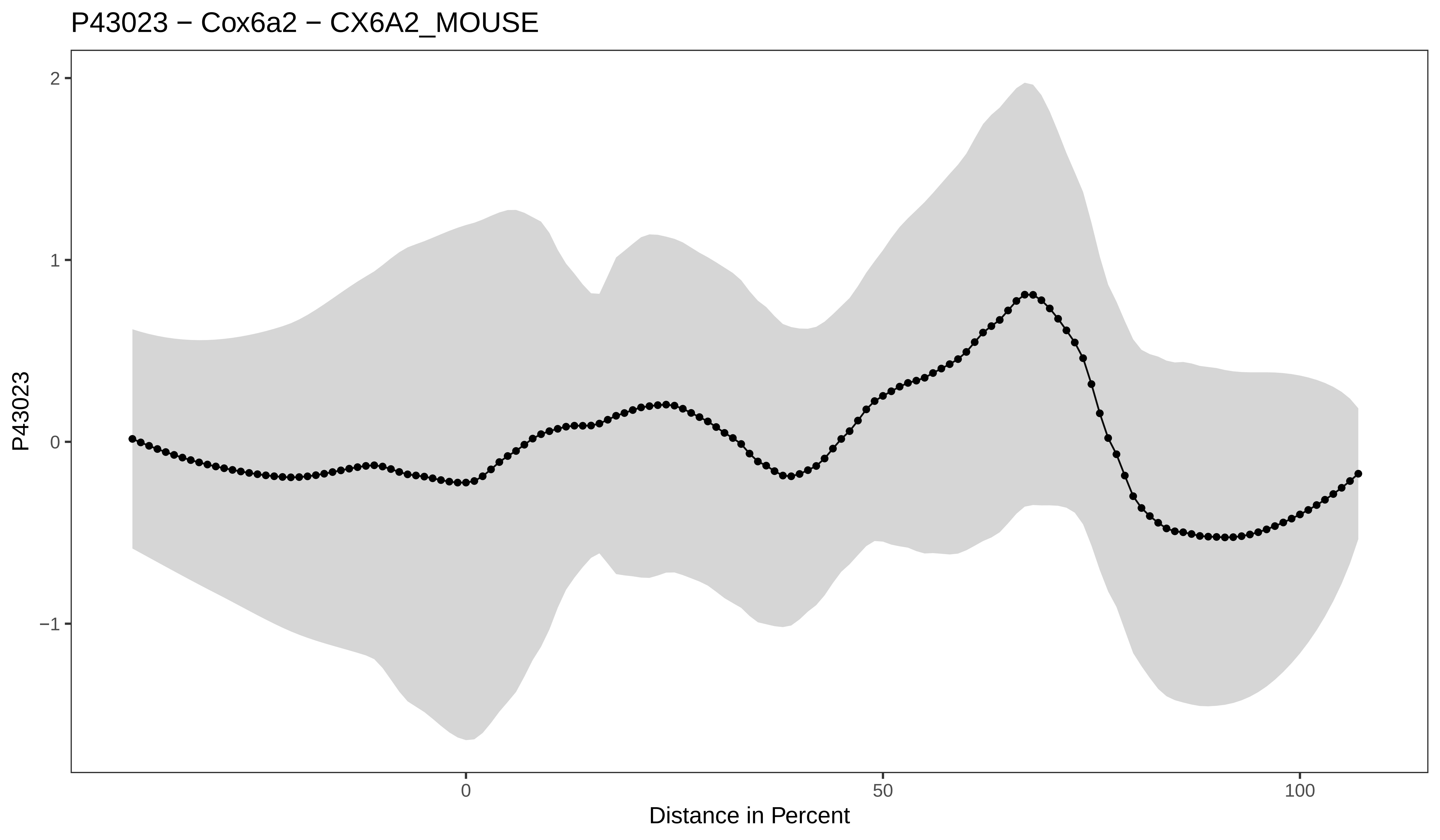
P43023 − Cox6a2 − CX6A2_MOUSE
P43023
2
1
0
−1
0
50
100
Distance in Percent

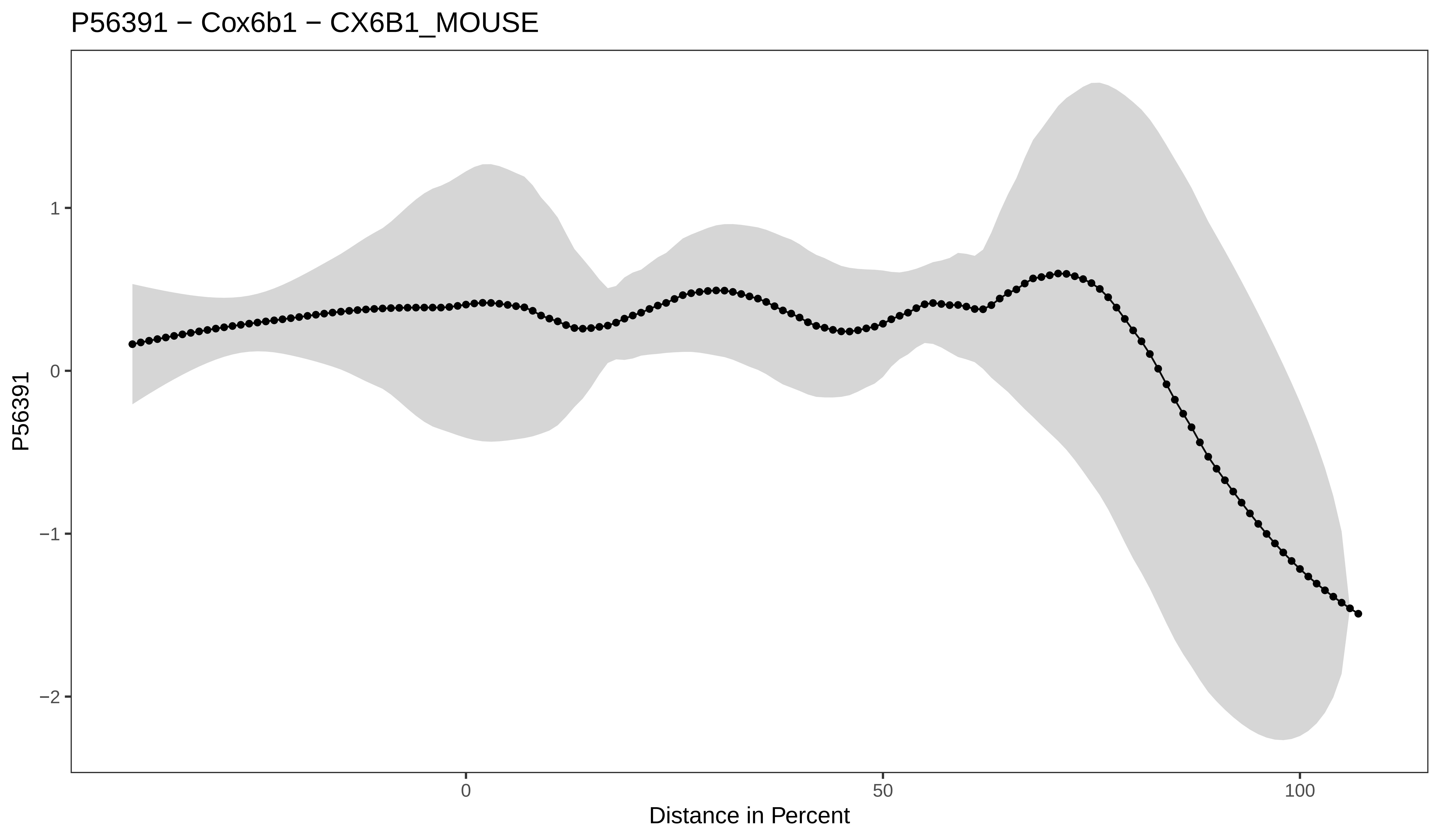

P56391 − Cox6b1 − CX6B1_MOUSE
P56391
1
0
−1
−2
0
50
100
Distance in Percent

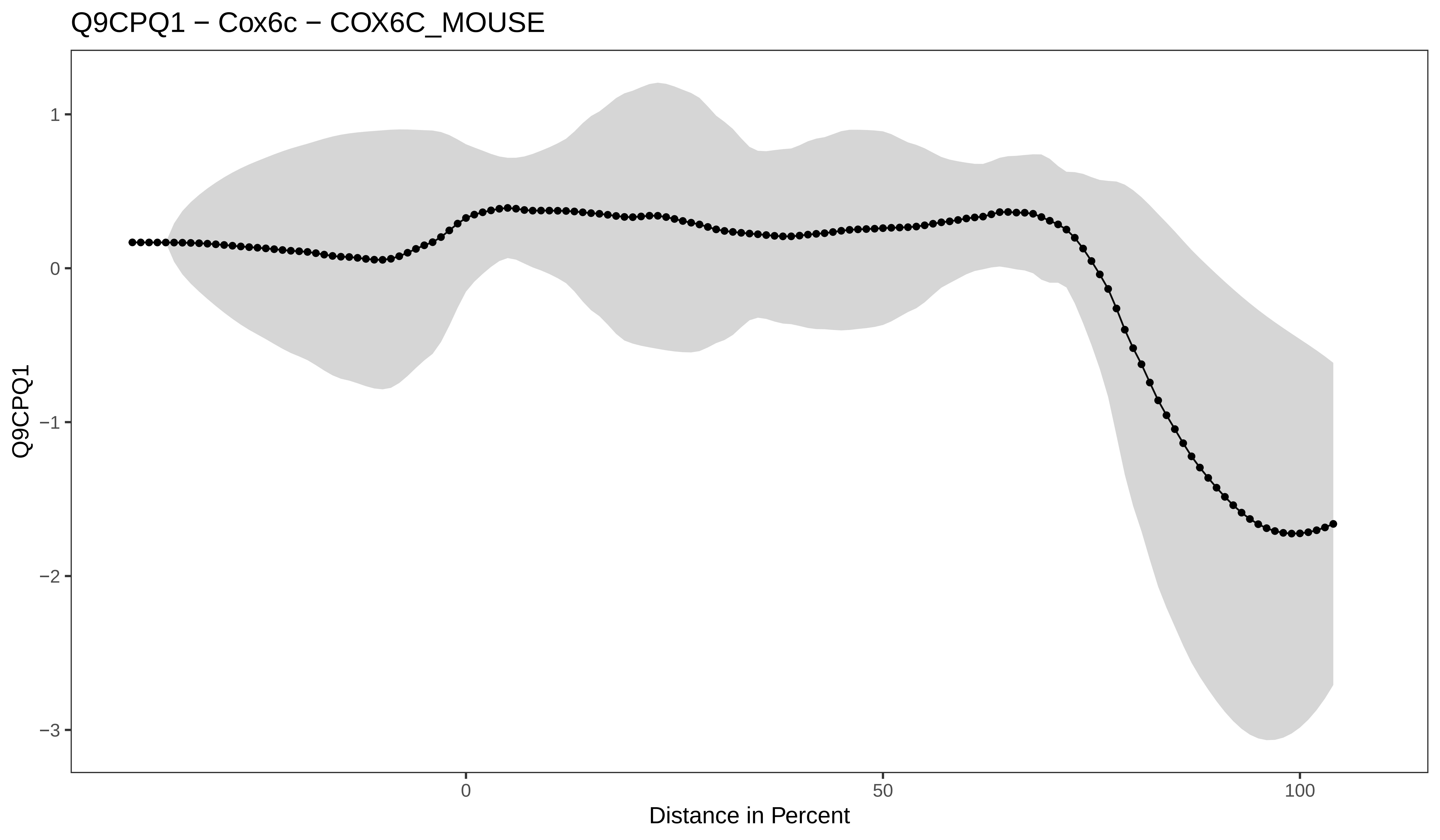
Q9CPQ1 − Cox6c − COX6C_MOUSE
1
0
−1
−2
−3
Q9CPQ1
0
50
100
Distance in Percent

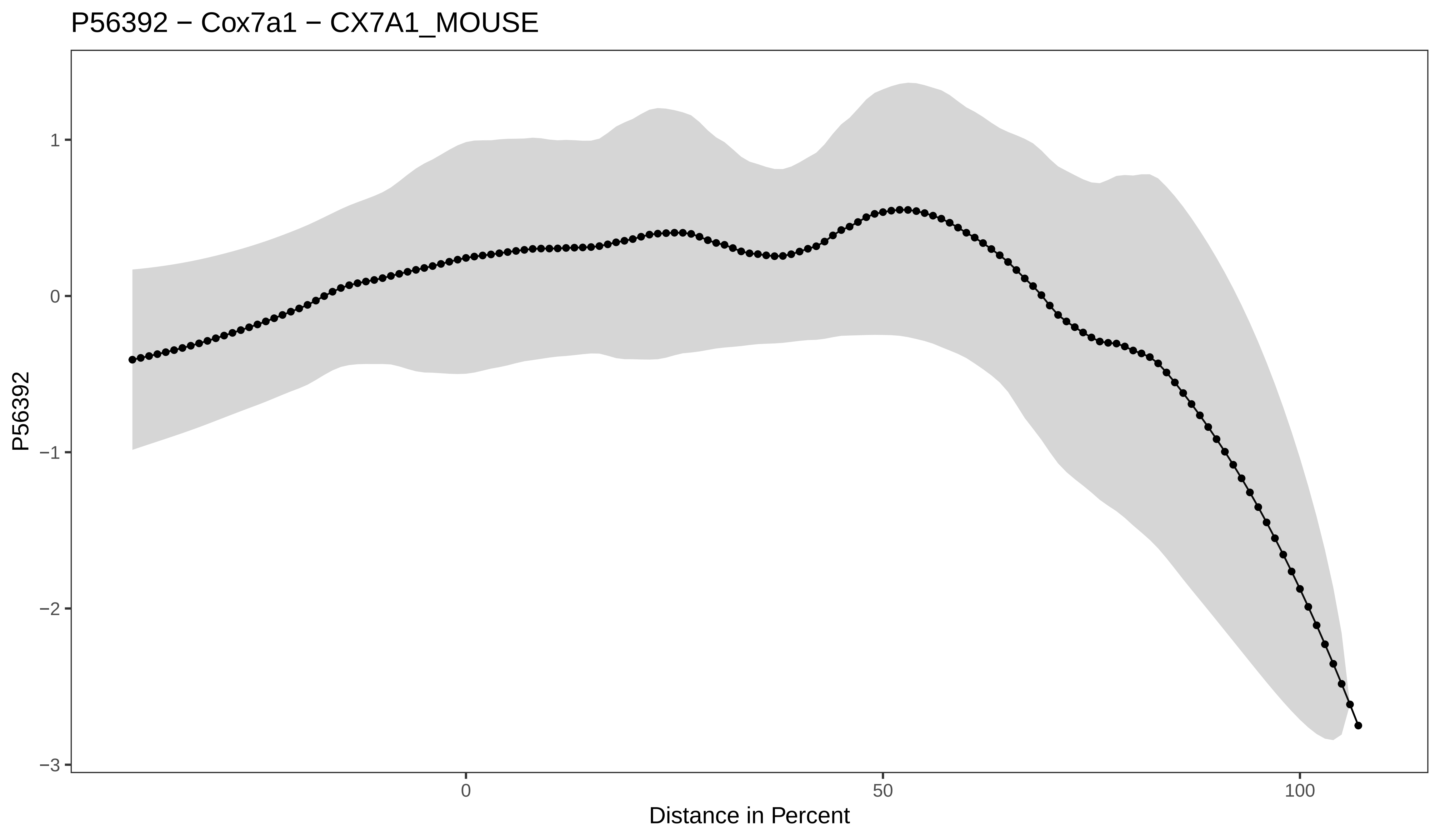
P56392 − Cox7a1 − CX7A1_MOUSE
1
0
−1
−2
−3
P56392
0
50
100
Distance in Percent

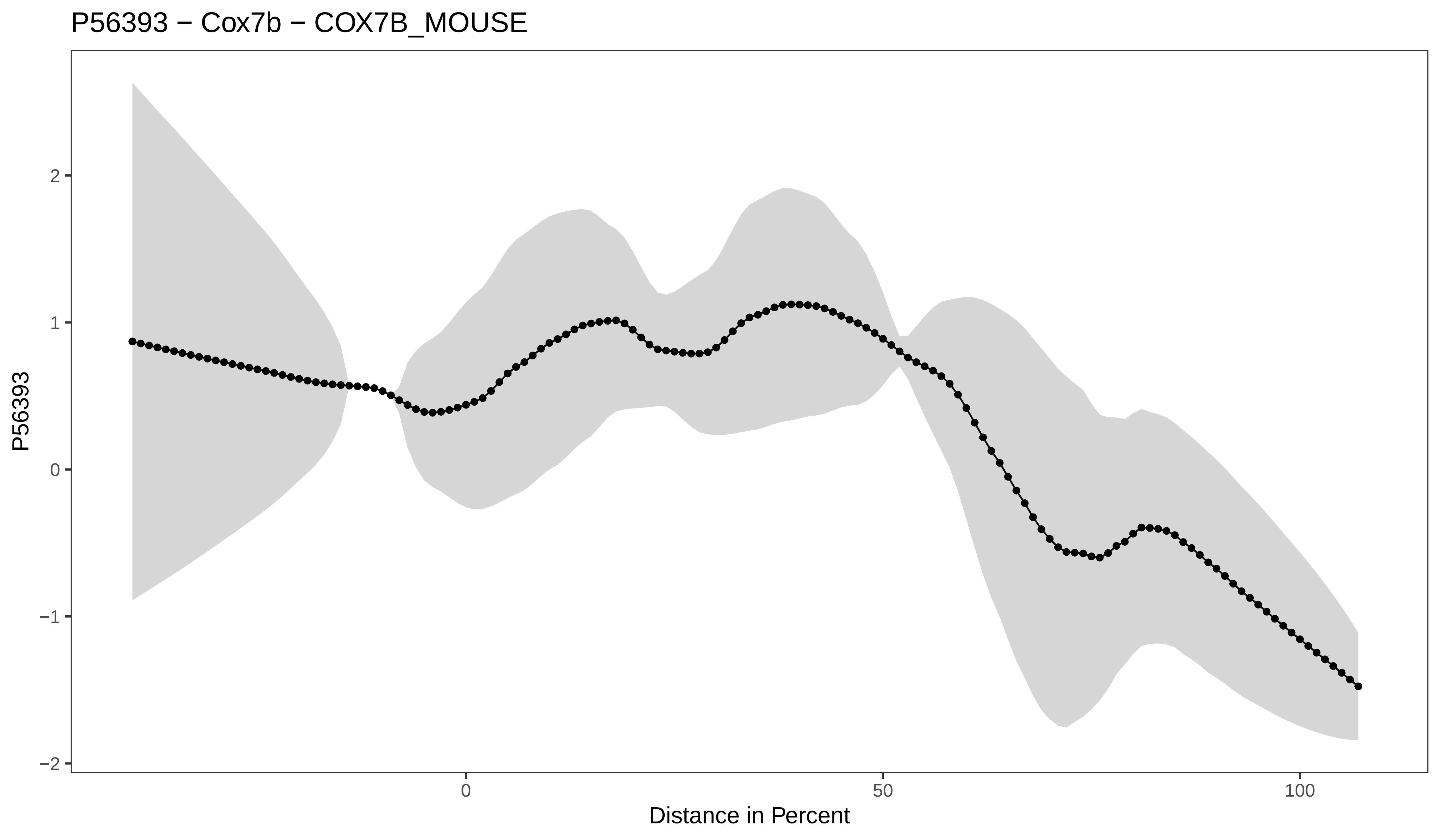
P56393 − Cox7b − COX7B_MOUSE
2
1
0
−1
−2
P56393
0
50
100
Distance in Percent

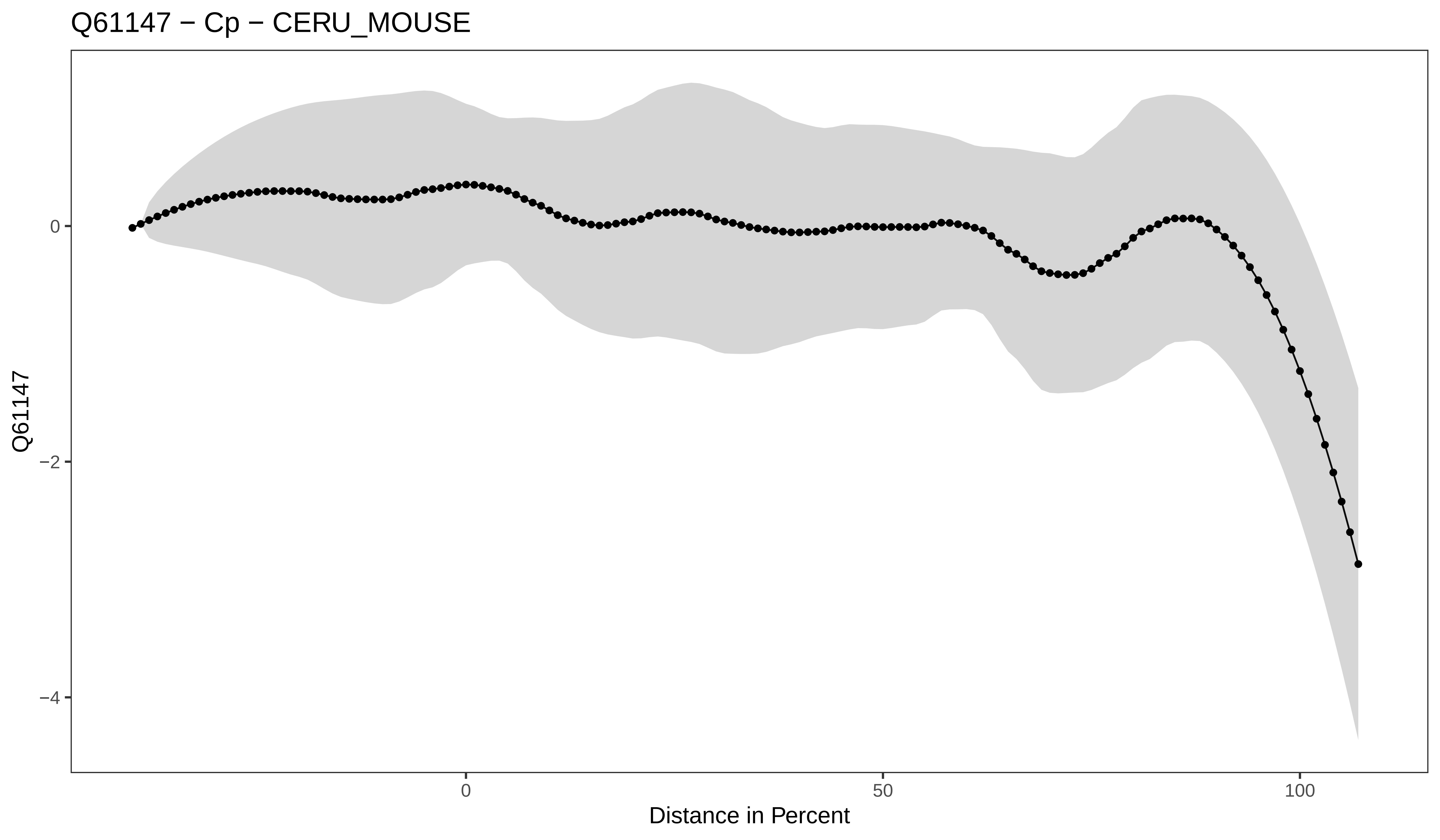

Q61147 − Cp − CERU_MOUSE
Q61147
0
−2
−4
0
50
100
Distance in Percent

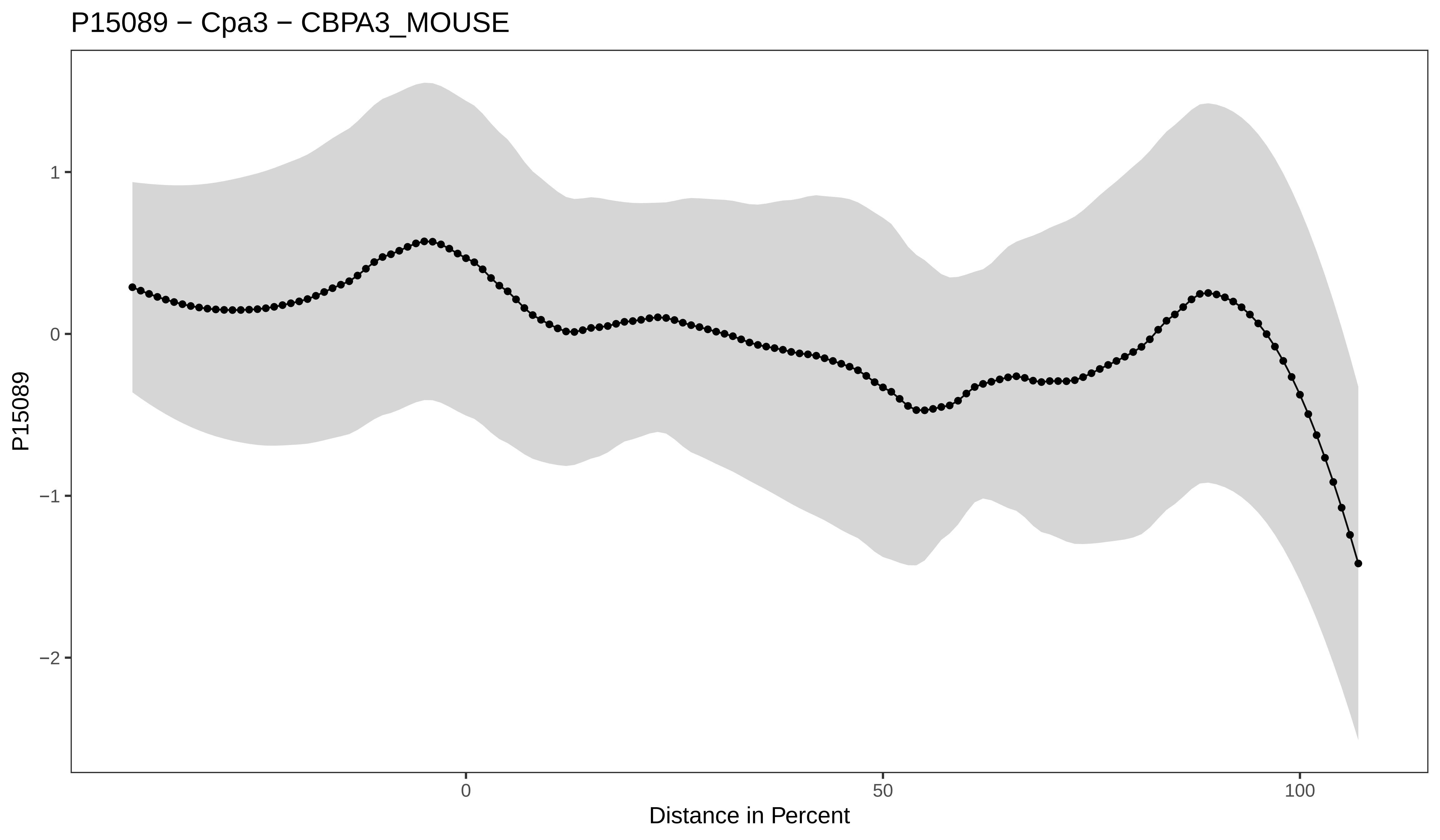

P15089 − Cpa3 − CBPA3_MOUSE
P15089
1
0
−1
−2
0
50
100
Distance in Percent

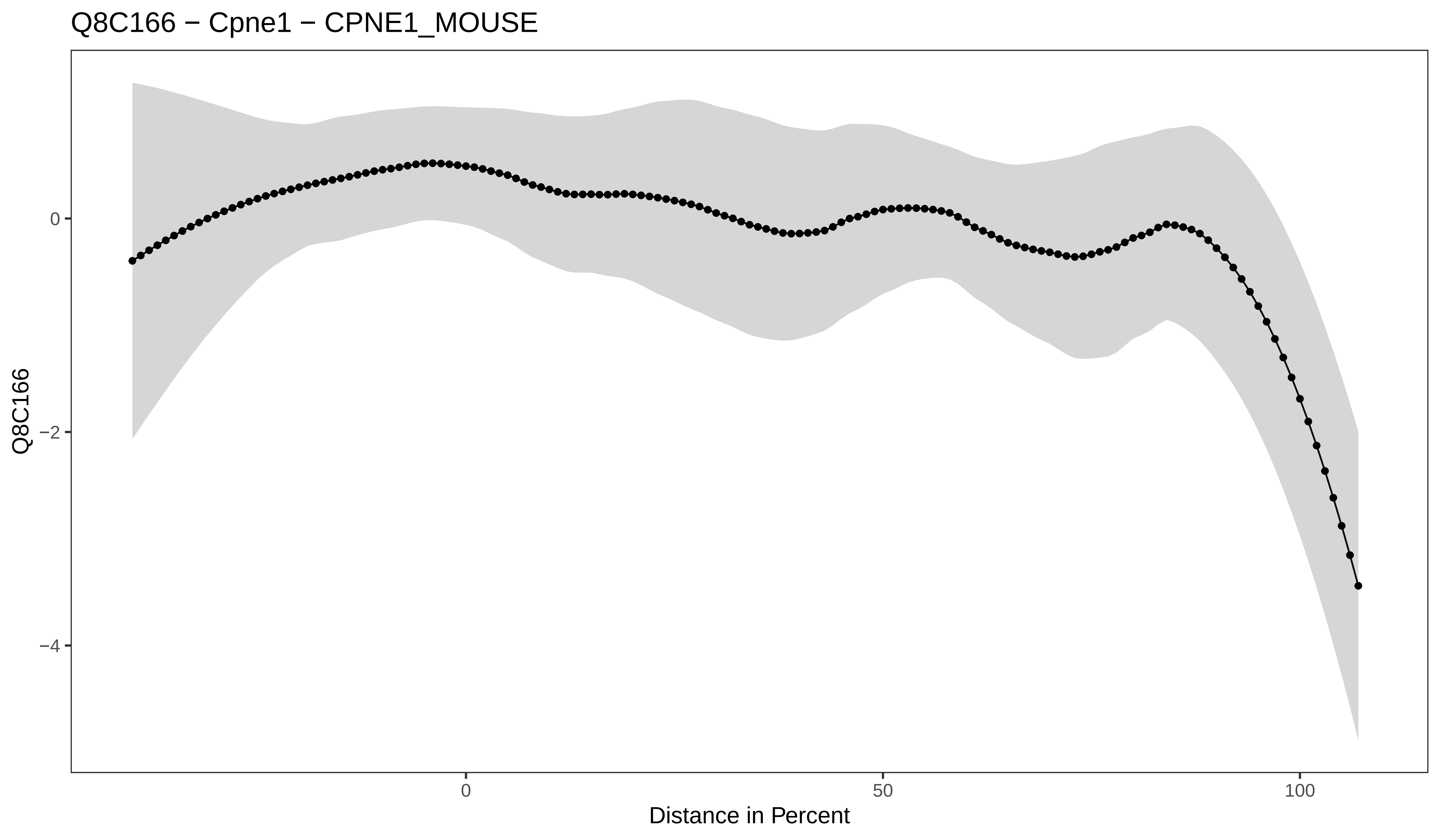

Q8C166 − Cpne1 − CPNE1_MOUSE
0
−2
−4
Q8C166
0
50
100
Distance in Percent

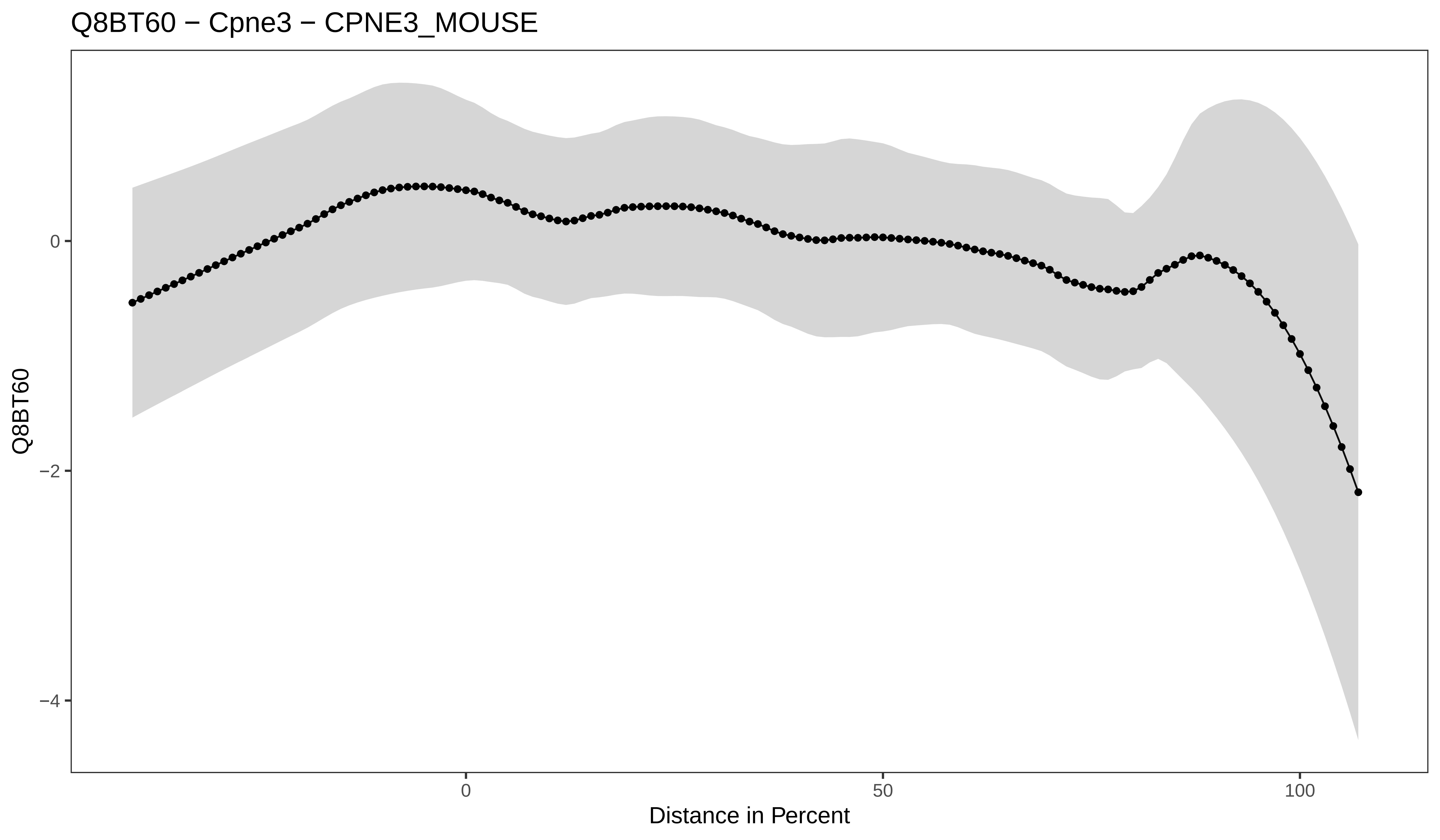

Q8BT60 − Cpne3 − CPNE3_MOUSE
Q8BT60
0
−2
−4
0
50
100
Distance in Percent

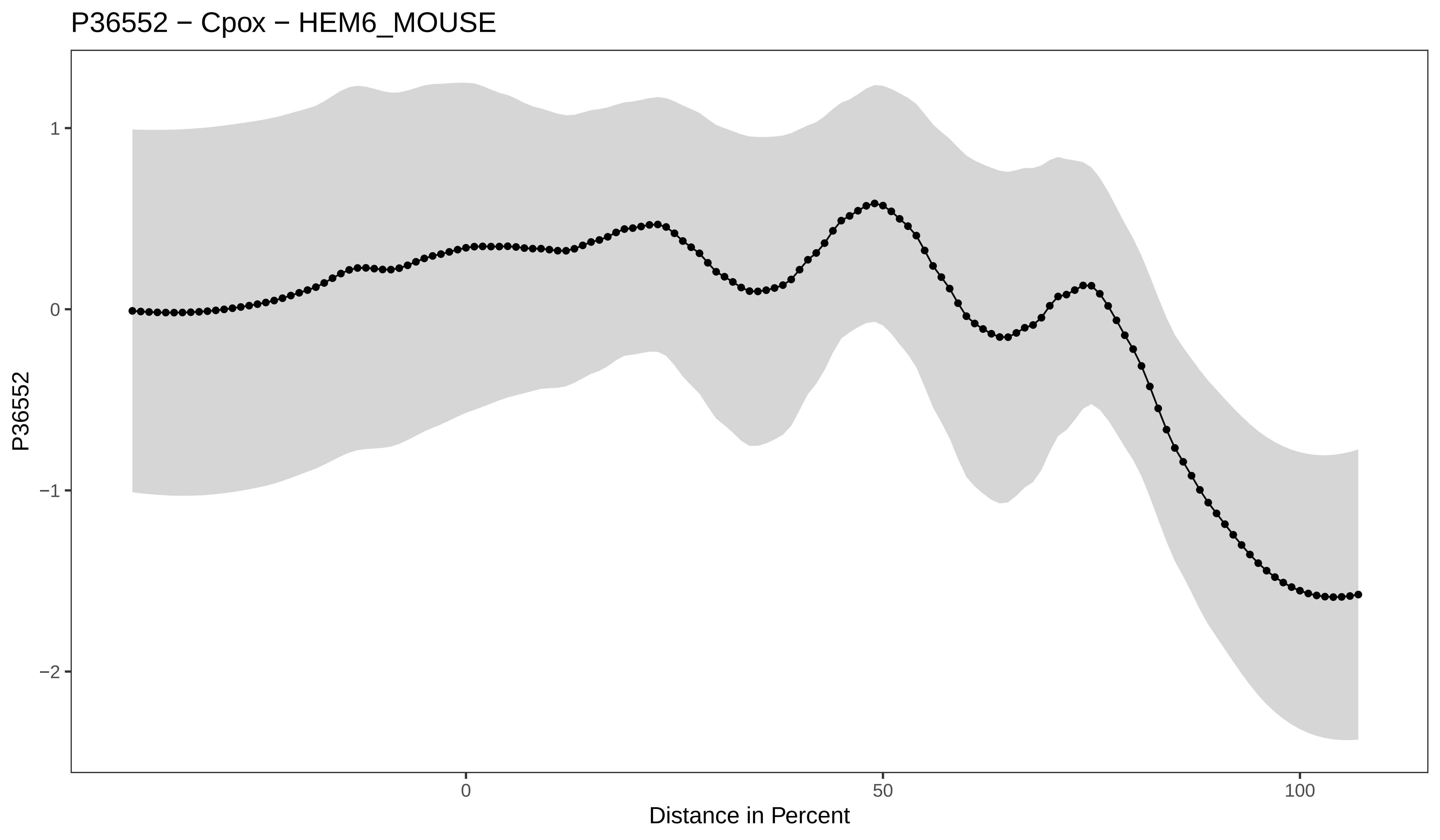
P36552 − Cpox − HEM6_MOUSE
1
0
−1
−2
P36552
0
50
100
Distance in Percent

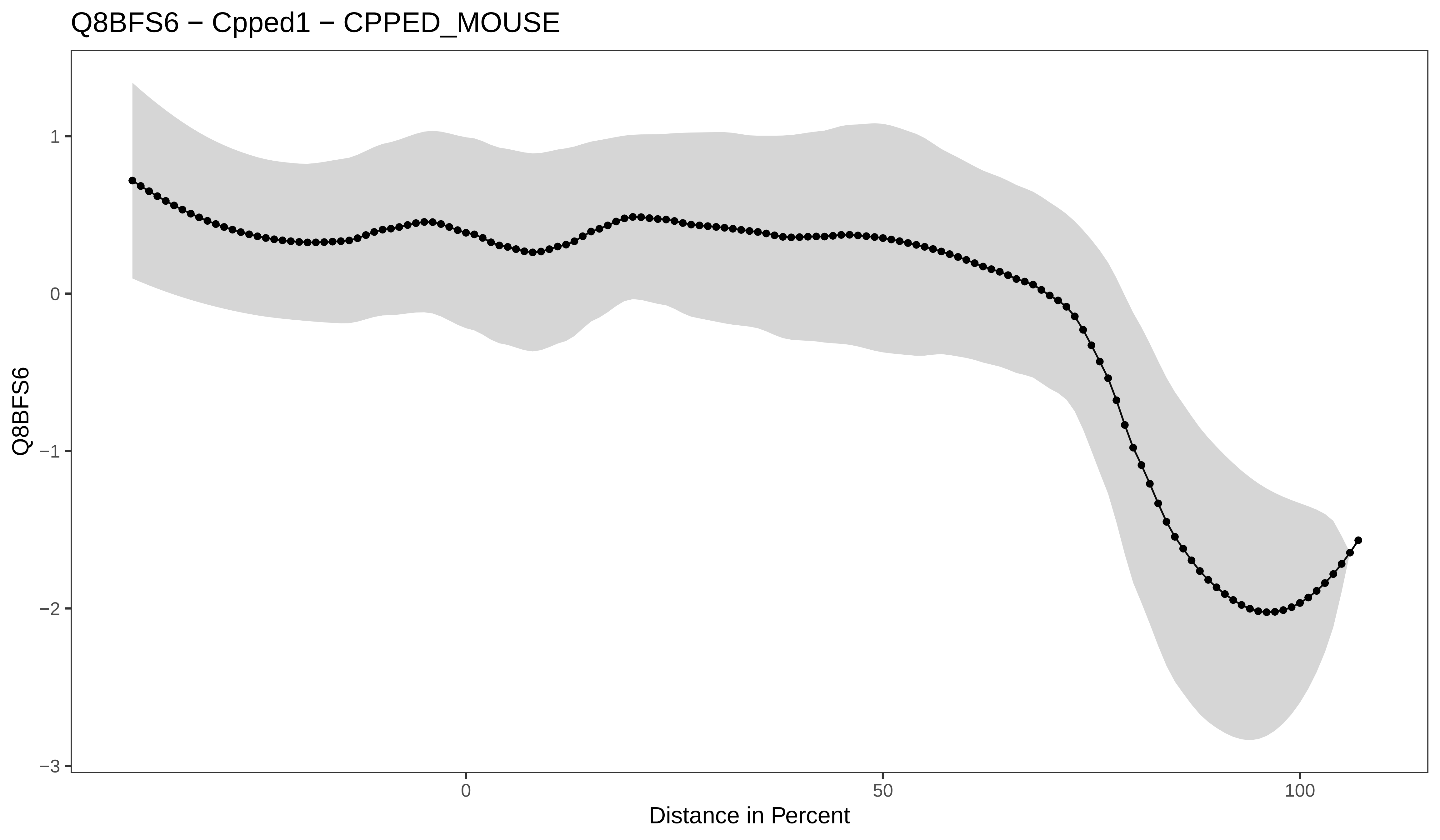

Q8BFS6 − Cpped1 − CPPED_MOUSE
Q8BFS6
1
0
−1
−2
−3
0
50
100
Distance in Percent

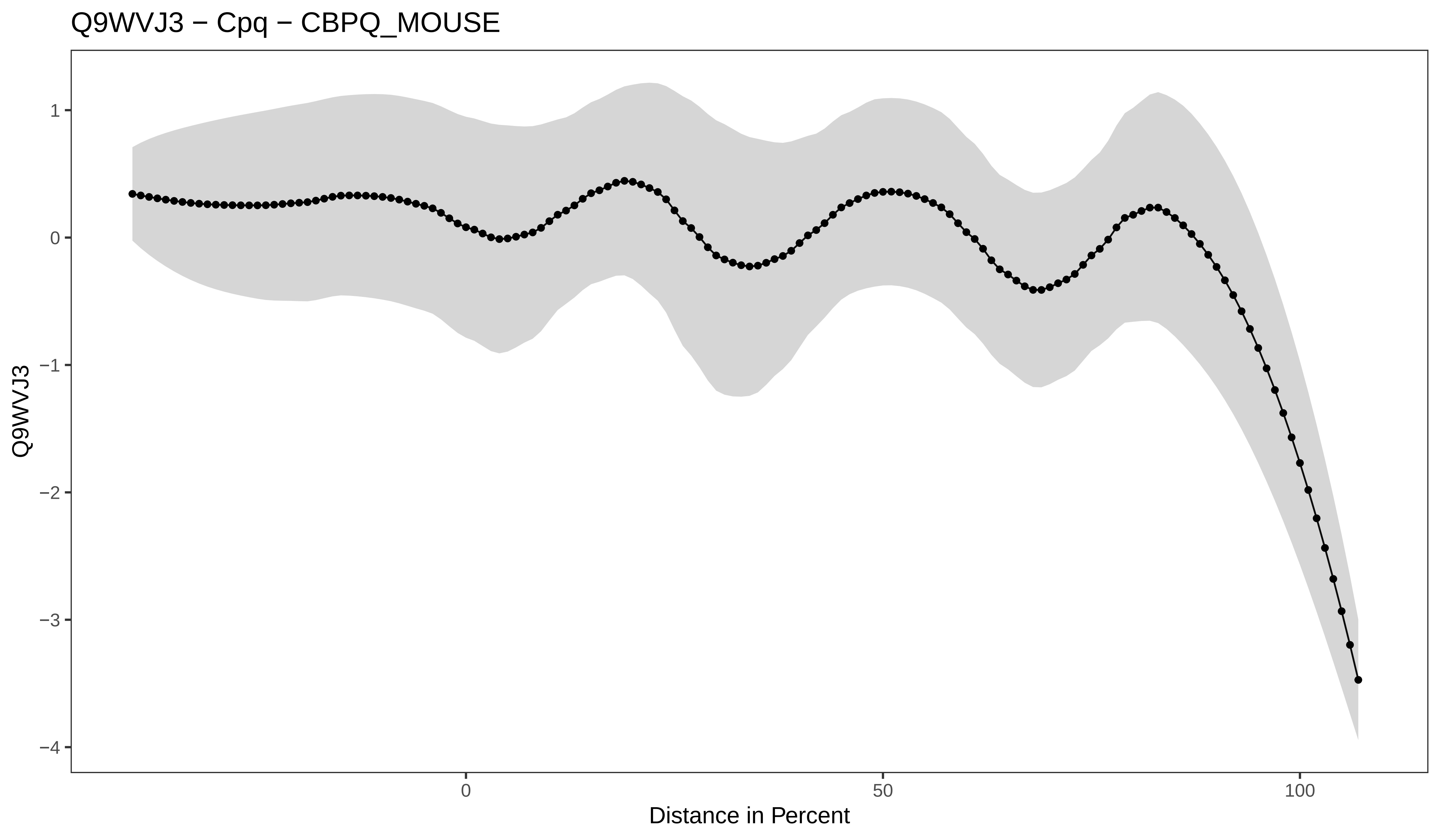

Q9WVJ3 − Cpq − CBPQ_MOUSE
1
0
−1
−2
−3
−4
Q9WVJ3
0
50
100
Distance in Percent

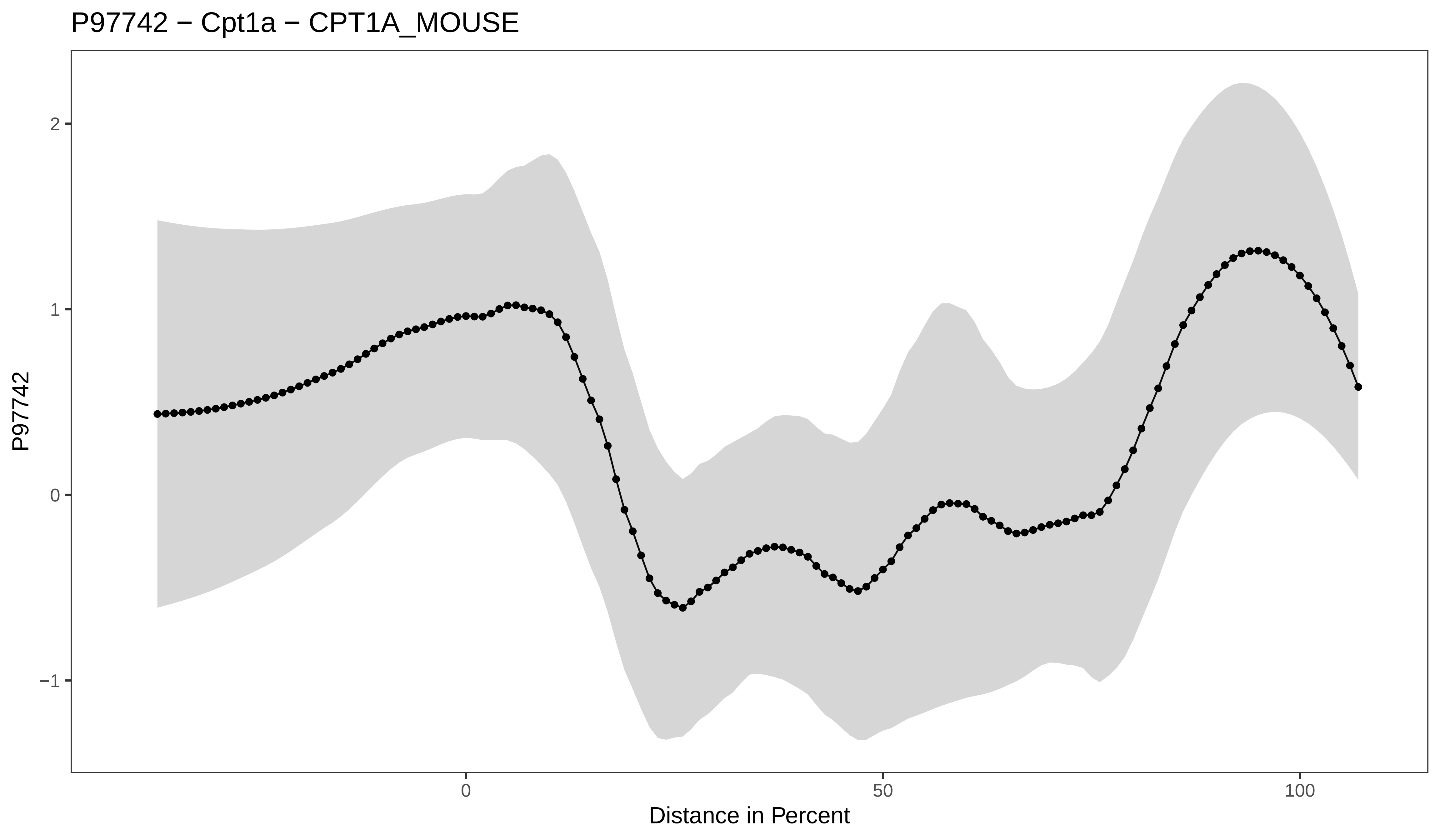

P97742 − Cpt1a − CPT1A_MOUSE
P97742
2
1
0
−1
0
50
100
Distance in Percent

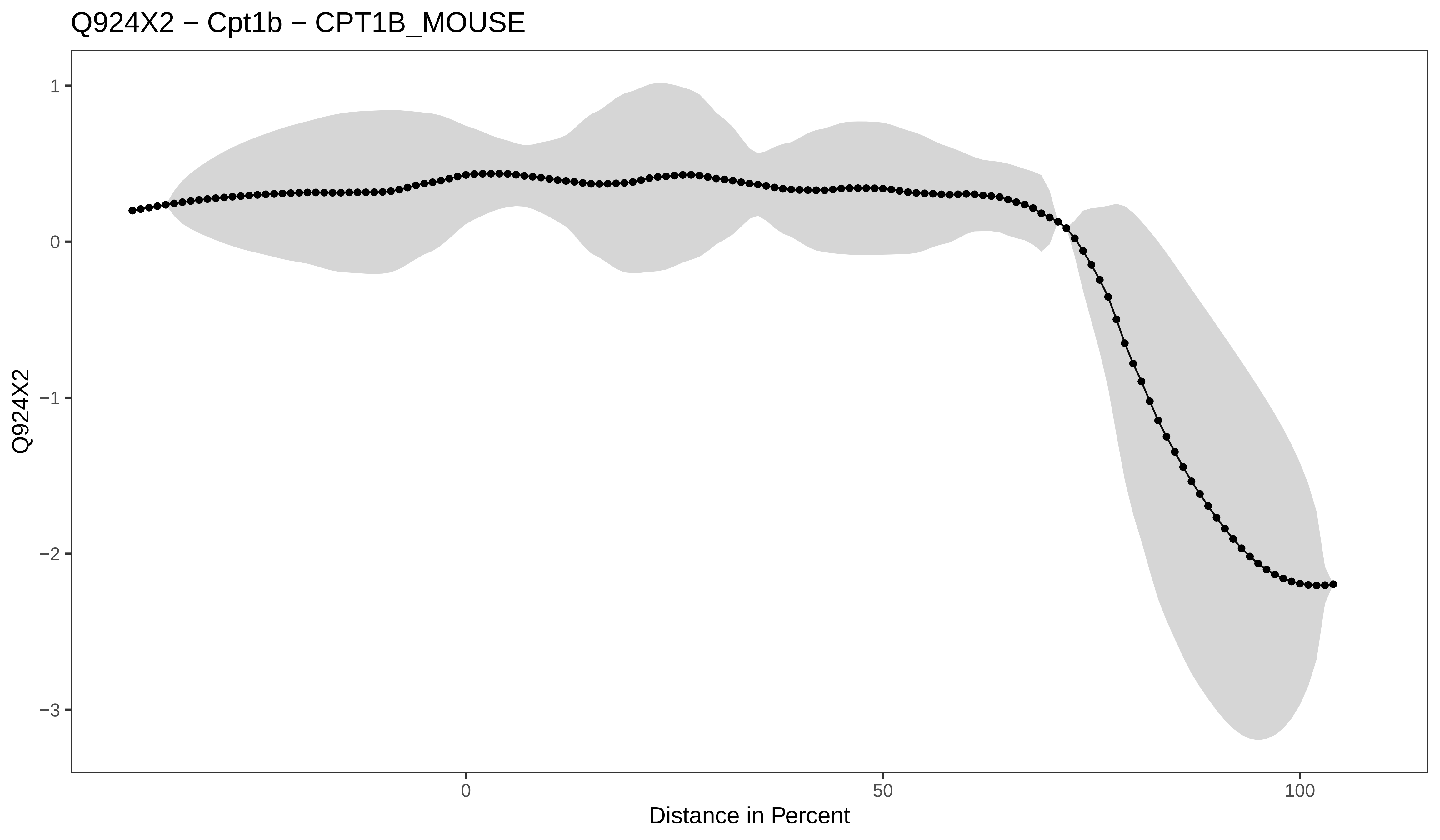

Q924X2 − Cpt1b − CPT1B_MOUSE
1
0
−1
−2
−3
Q924X2
0
50
100
Distance in Percent

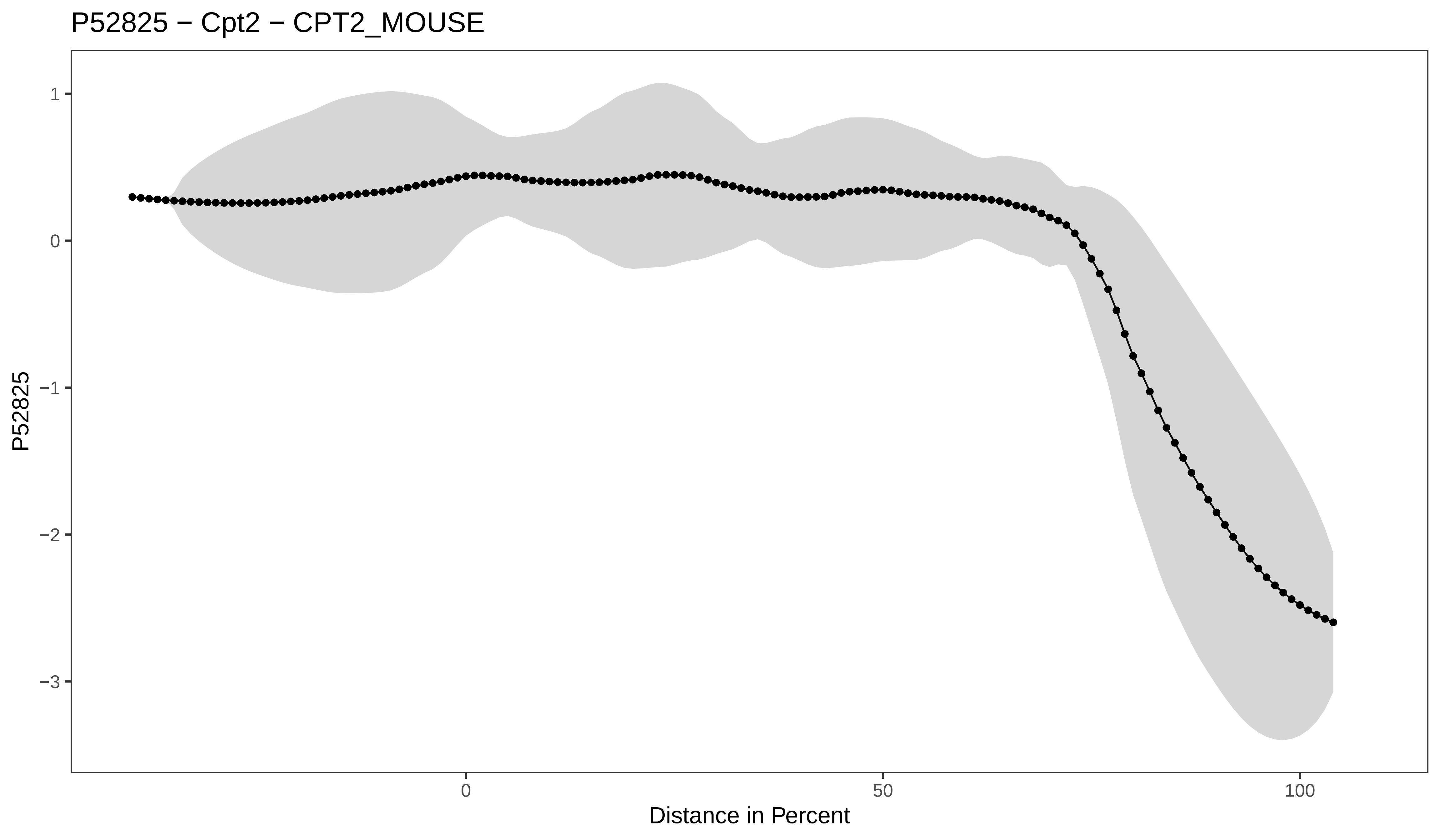

P52825 − Cpt2 − CPT2_MOUSE
P52825
1
0
−1
−2
−3
0
50
100
Distance in Percent

Q9D2L5 − Cpxm2 − CPXM2_MOUSE

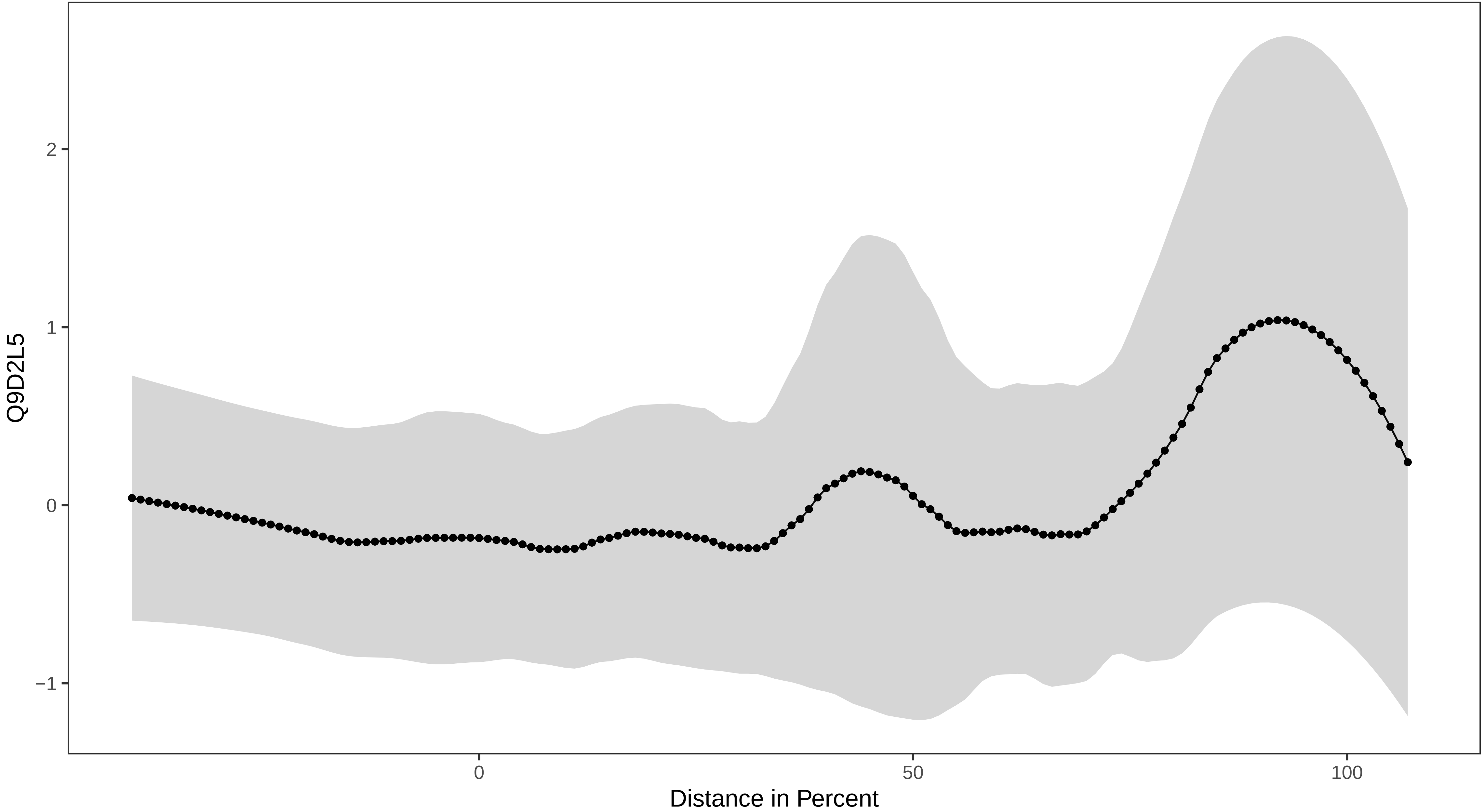

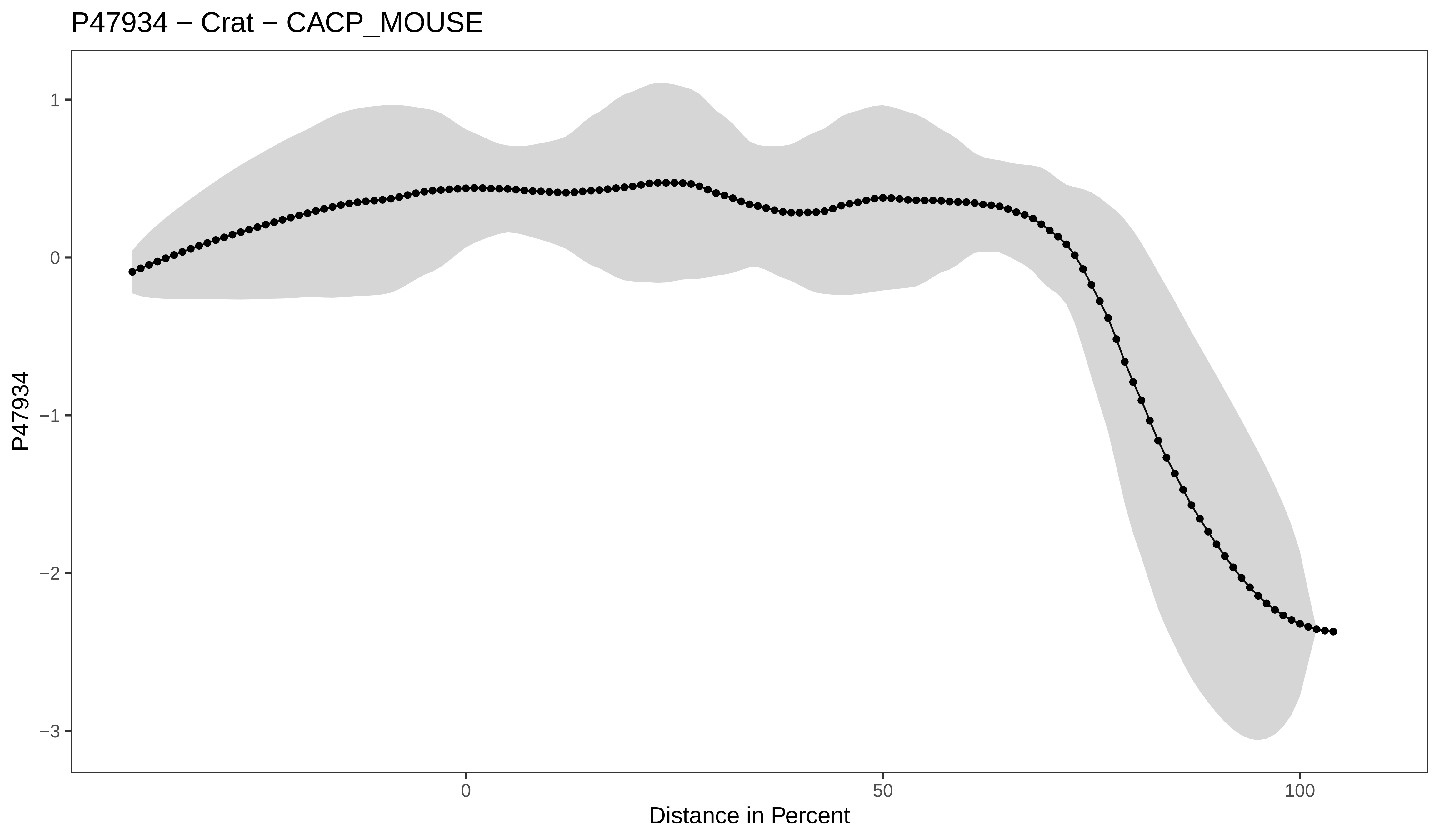

P47934 − Crat − CACP_MOUSE
P47934
1
0
−1
−2
−3
0
50
100
Distance in Percent

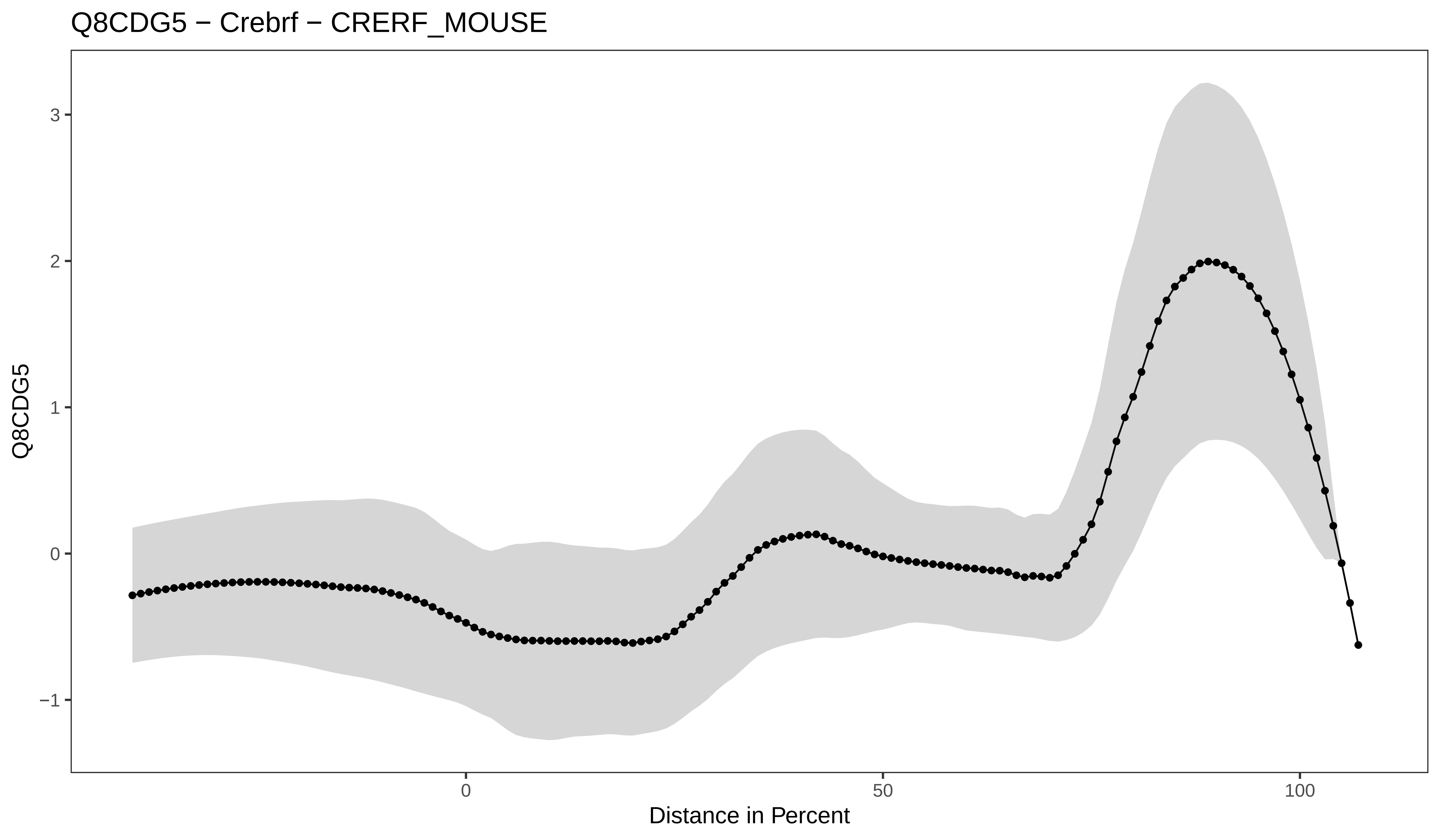

Q8CDG5 − Crebrf − CRERF_MOUSE
Q8CDG5
3
2
1
0
−1
0
50
100
Distance in Percent

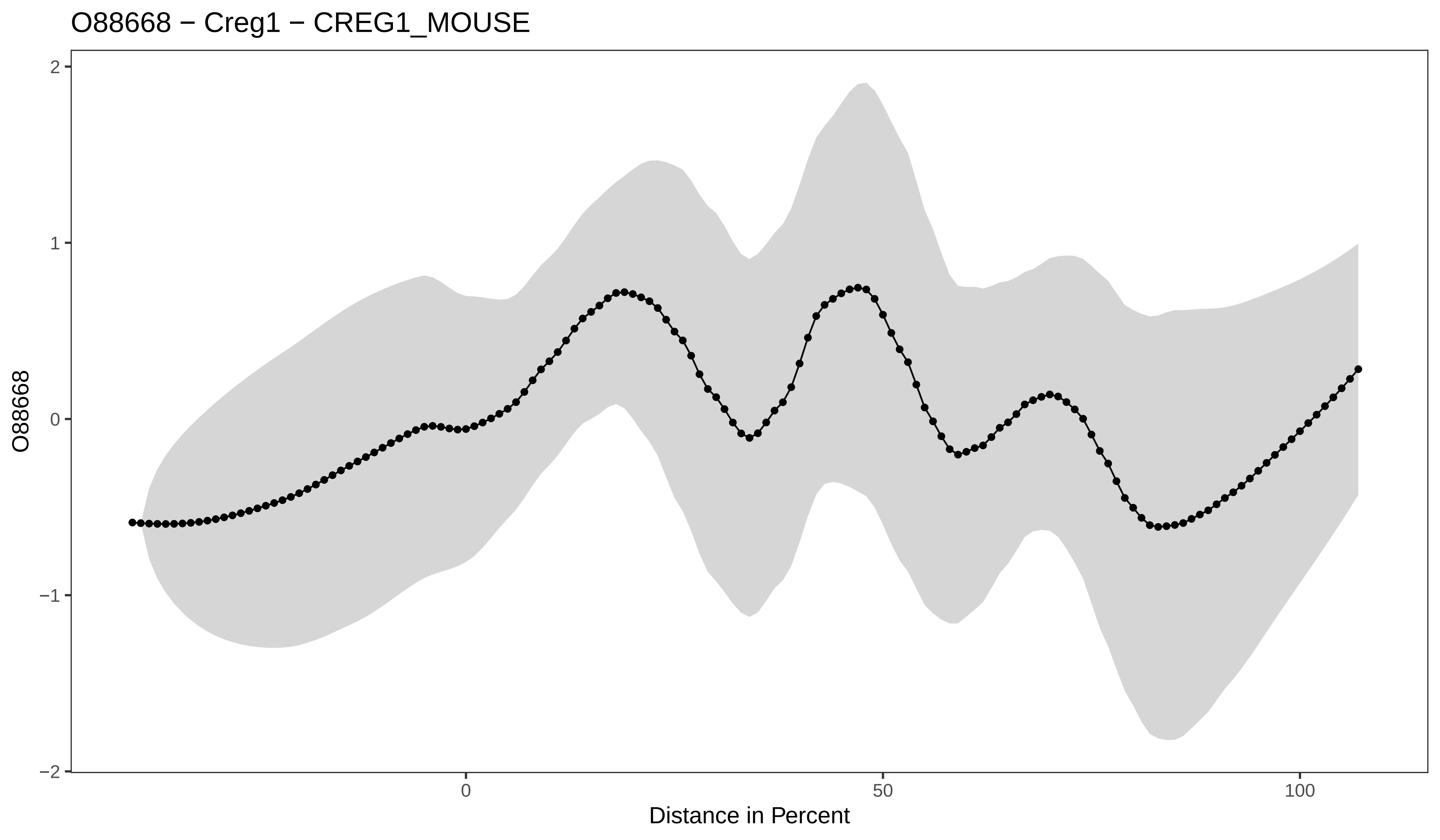
O88668 − Creg1 − CREG1_MOUSE
O88668
2
1
0
−1
−2
0
50
100
Distance in Percent

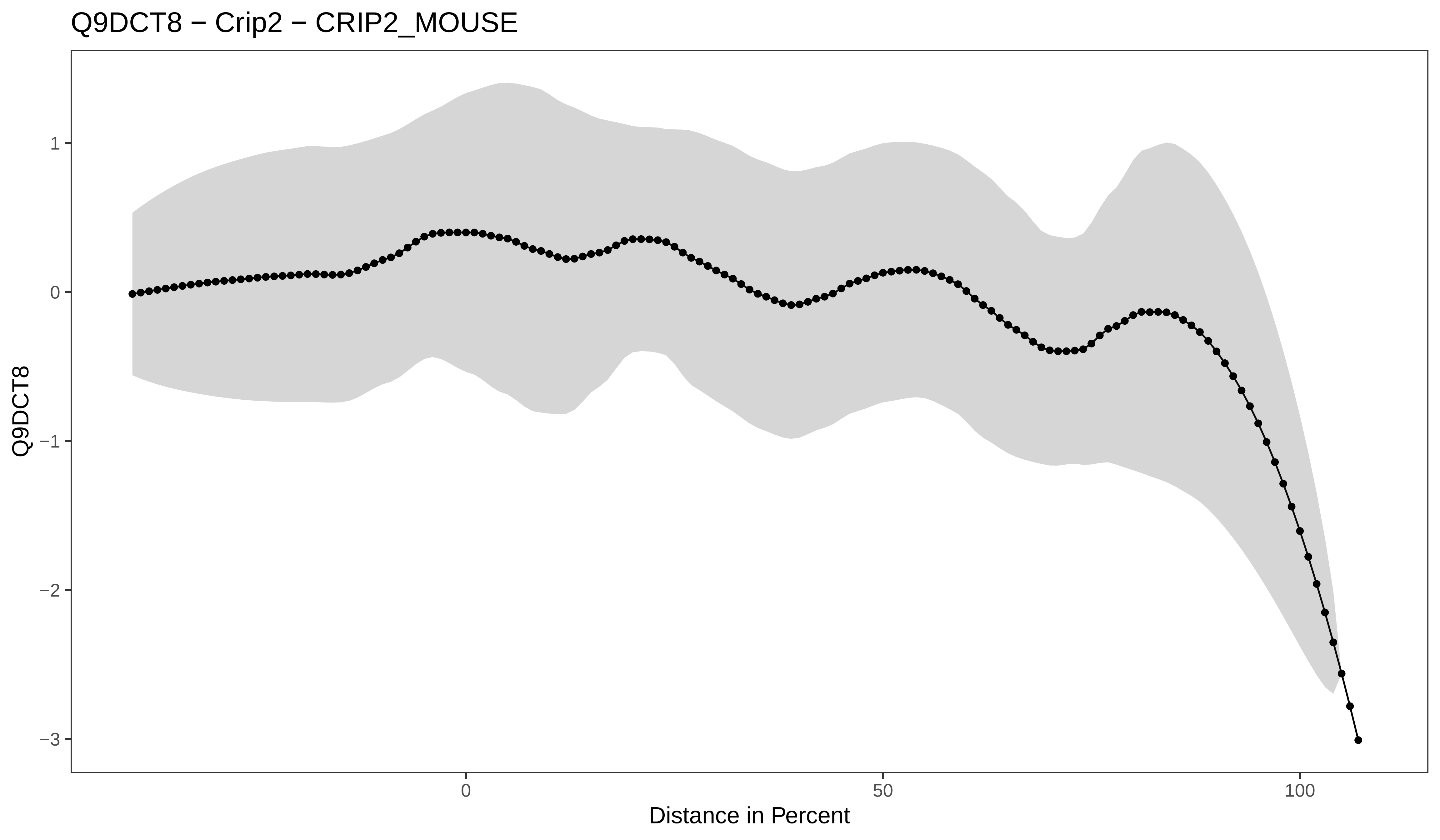
Q9DCT8 − Crip2 − CRIP2_MOUSE
Q9DCT8
Distance in Percent
1
0
−1
−2
−3
0
50
100

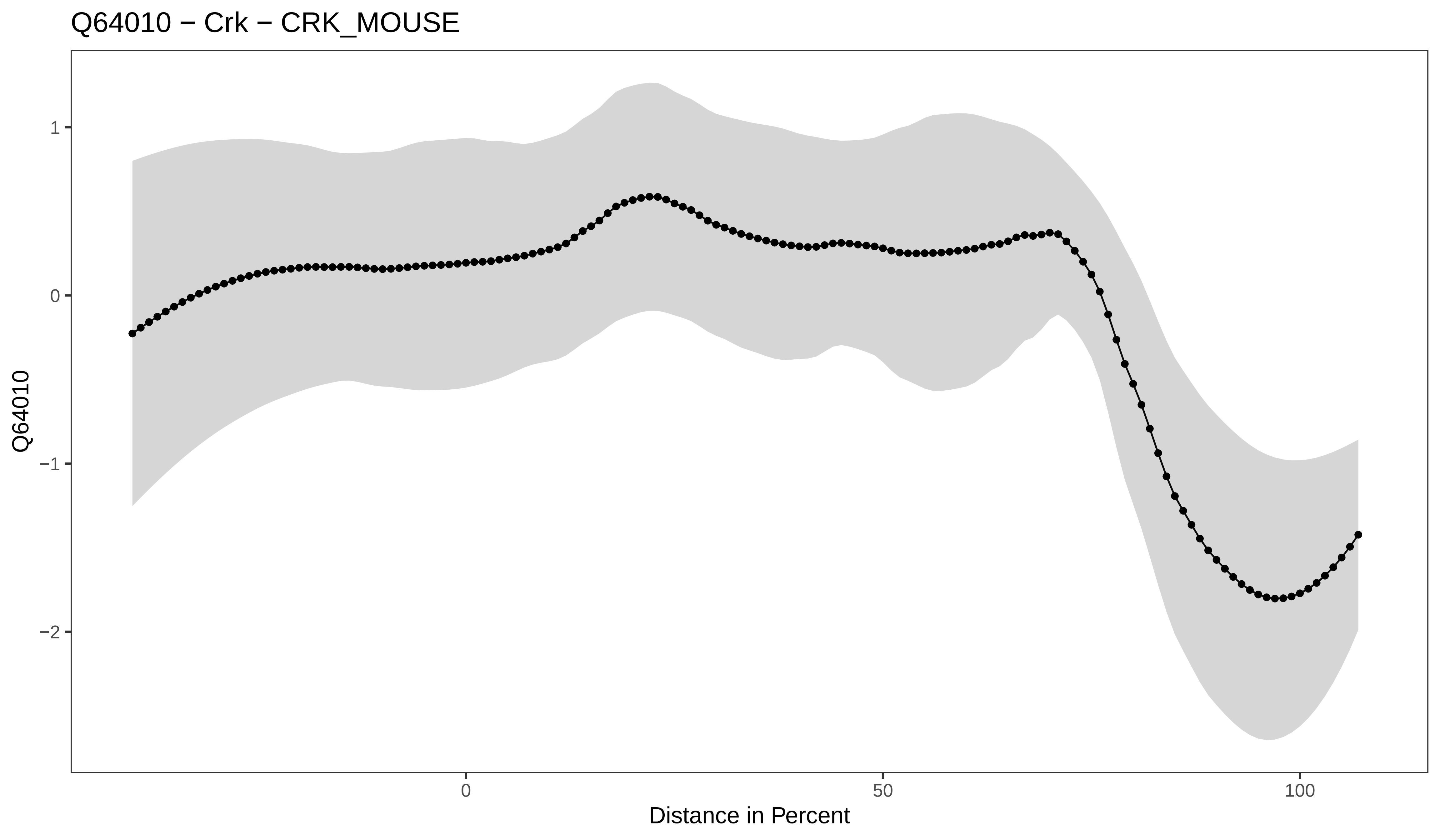

Q64010 − Crk − CRK_MOUSE
1
0
−1
−2
Q64010
0
50
100
Distance in Percent

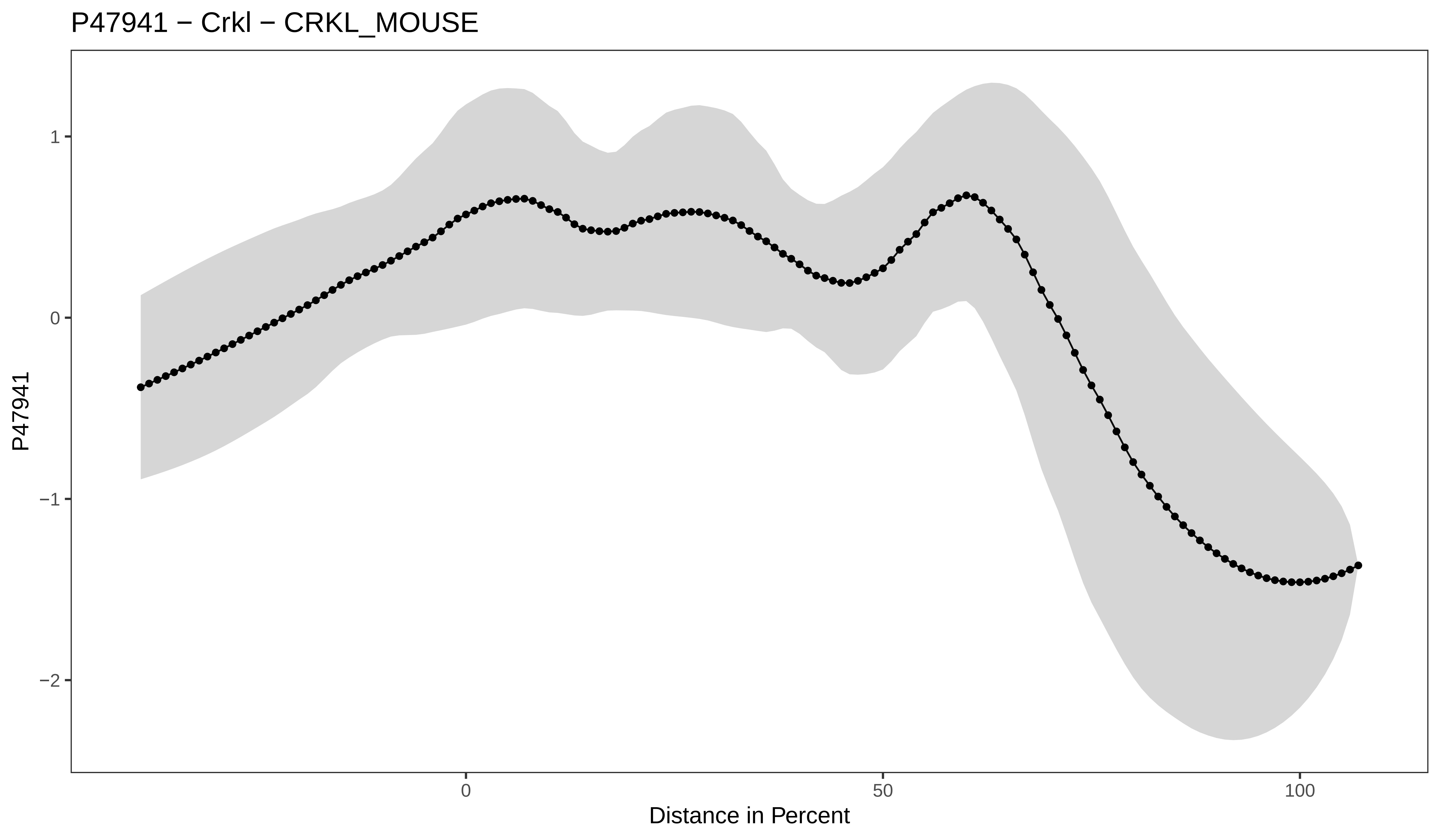

P47941 − Crkl − CRKL_MOUSE
1
0
−1
−2
P47941
0
50
100
Distance in Percent

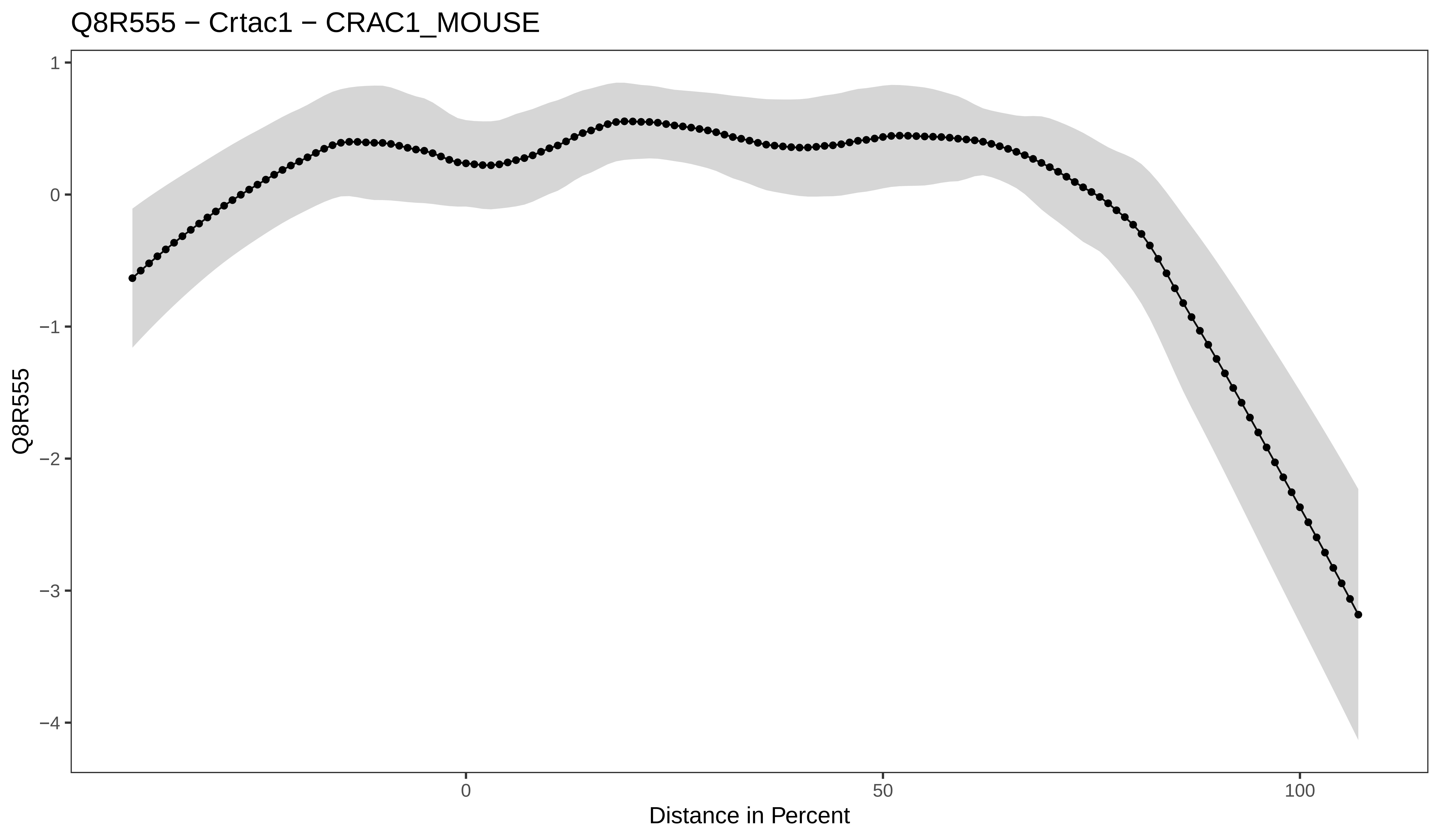
Q8R555 − Crtac1 − CRAC1_MOUSE
Q8R555
Distance in Percent
1
0
−1
−2
−3
−4
0
50
100

P23927 − Cryab − CRYAB_MOUSE

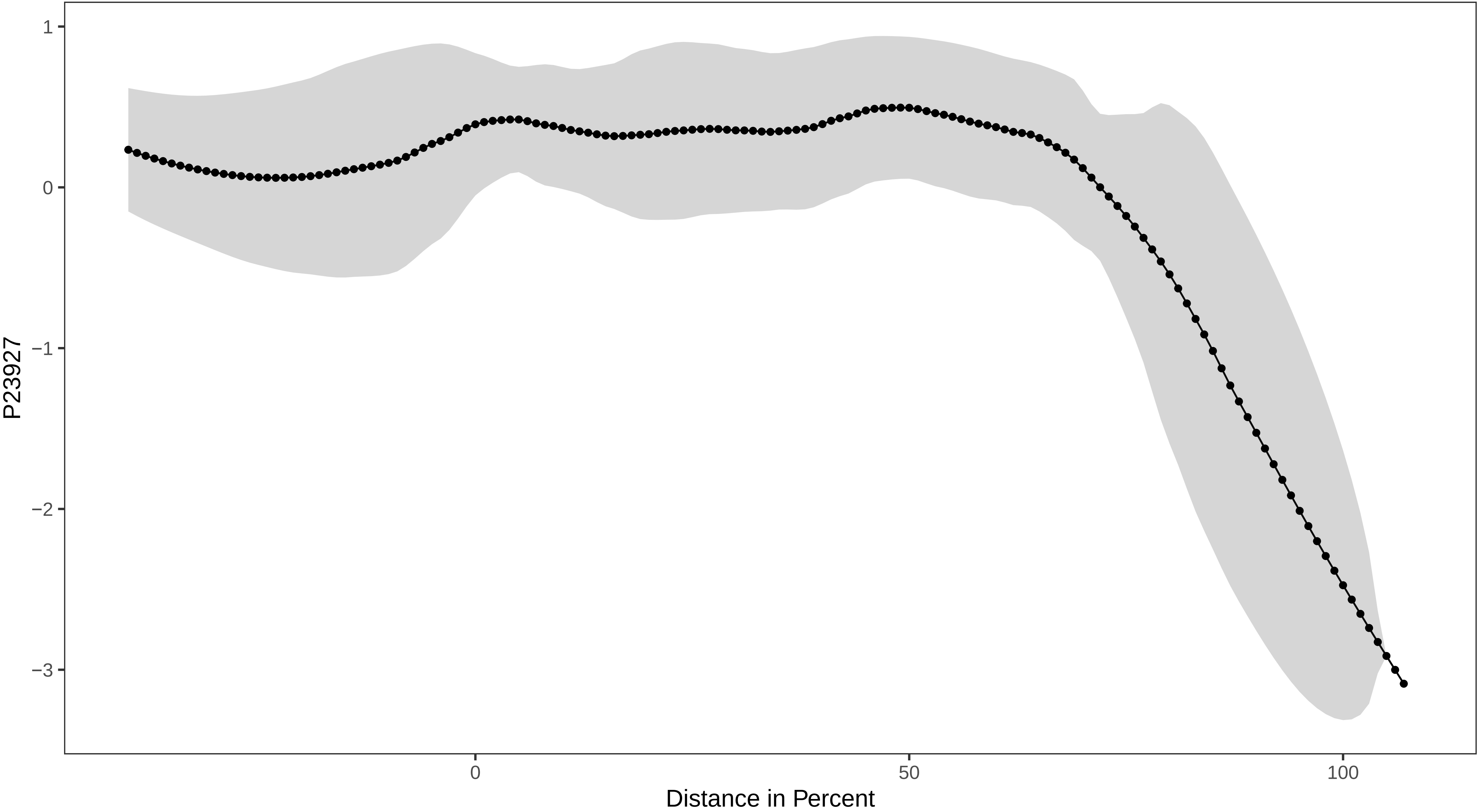

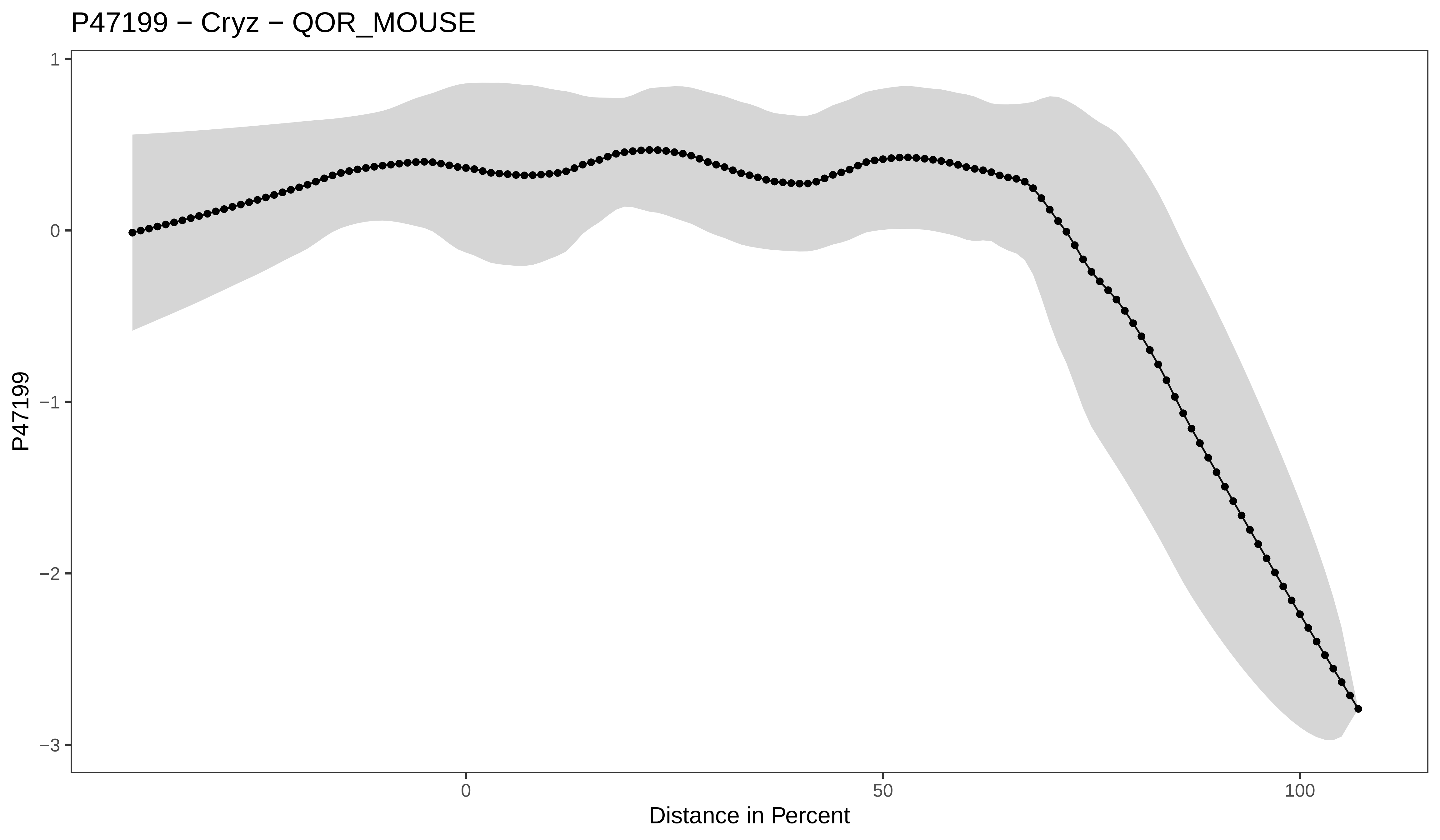

P47199 − Cryz − QOR_MOUSE
1
0
−1
−2
−3
P47199
0
50
100
Distance in Percent

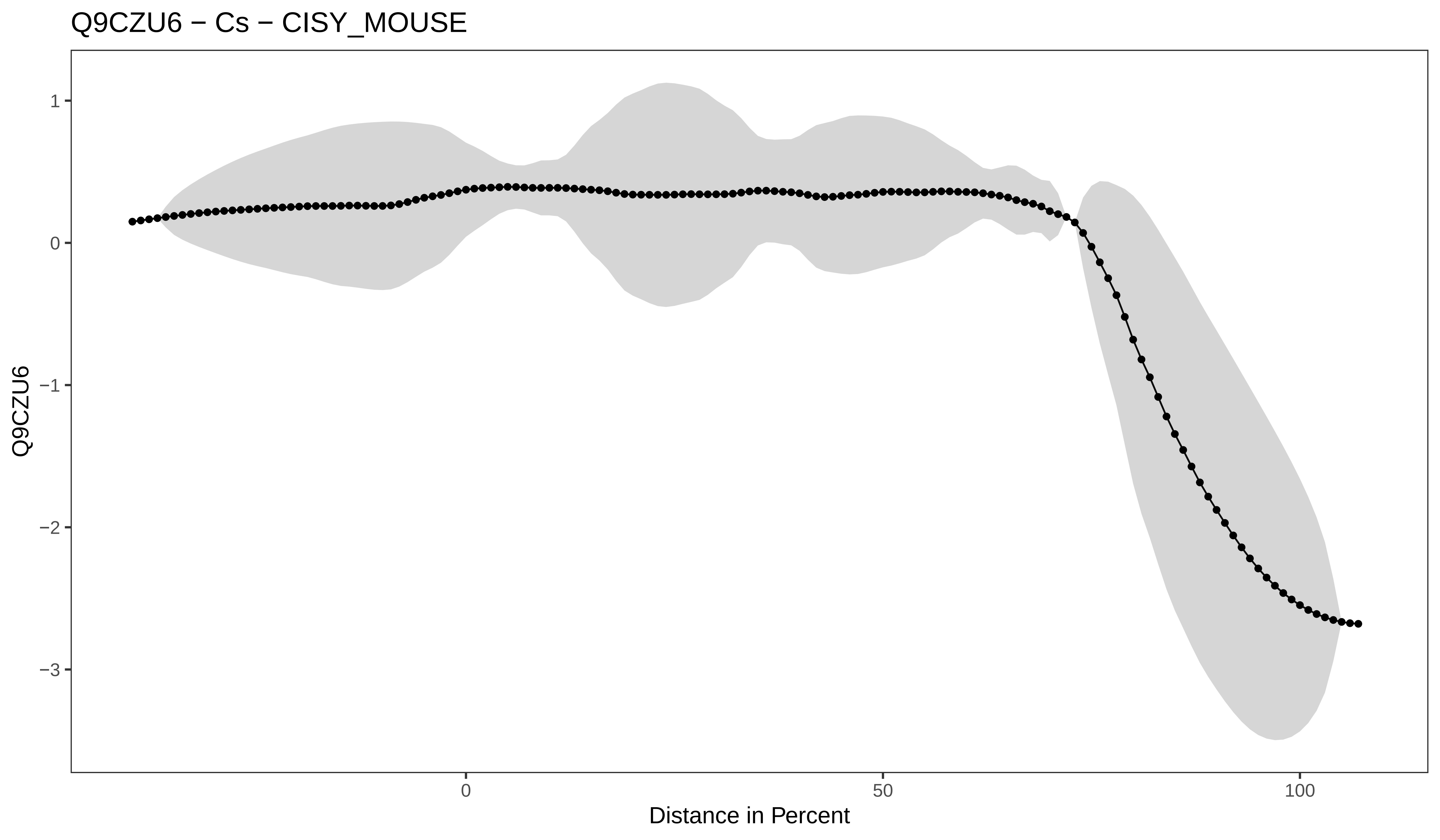

Q9CZU6 − Cs − CISY_MOUSE
Q9CZU6
1
0
−1
−2
−3
0
50
100
Distance in Percent

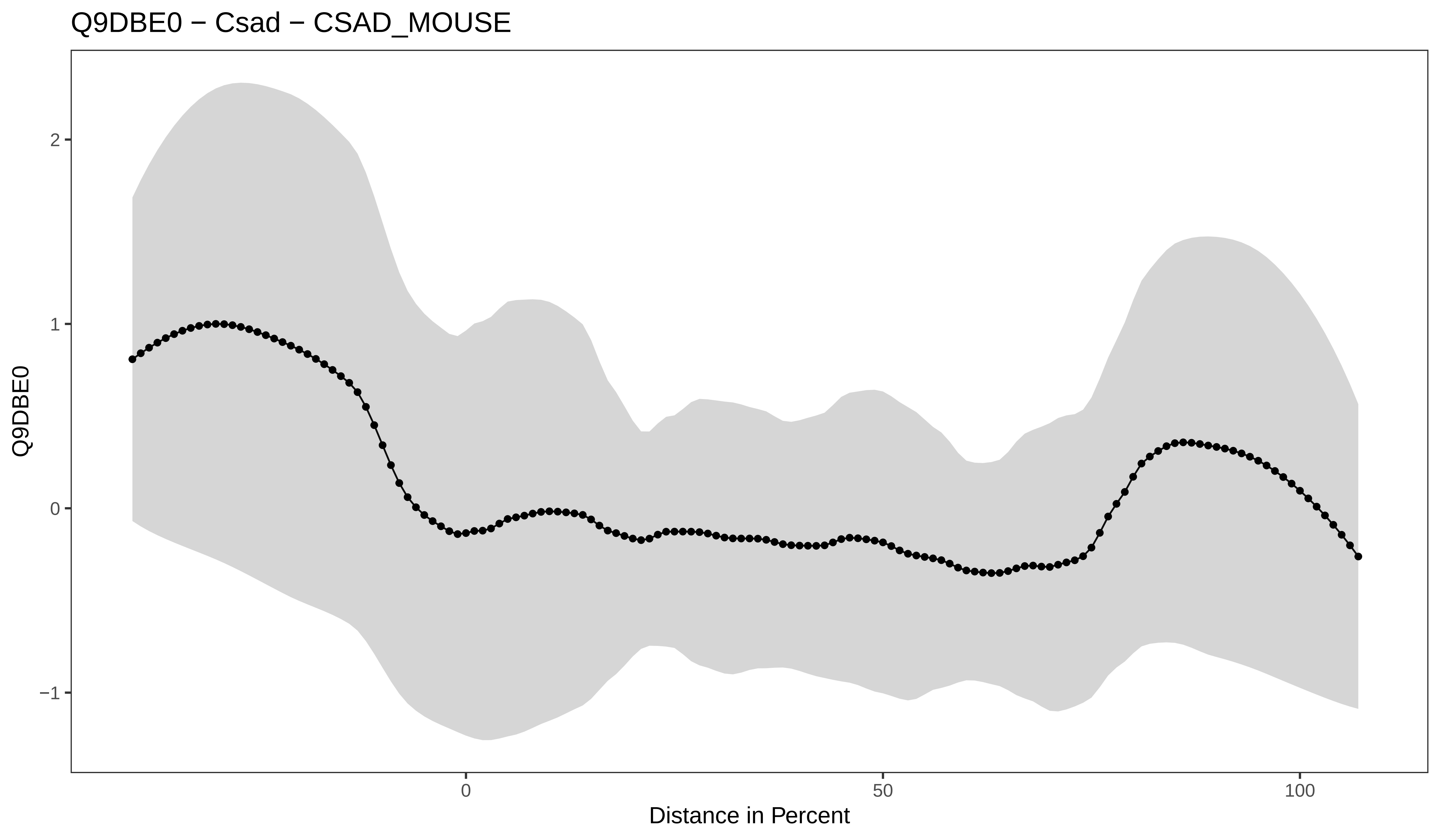

Q9DBE0 − Csad − CSAD_MOUSE
Q9DBE0
2
1
0
−1
0
50
100
Distance in Percent

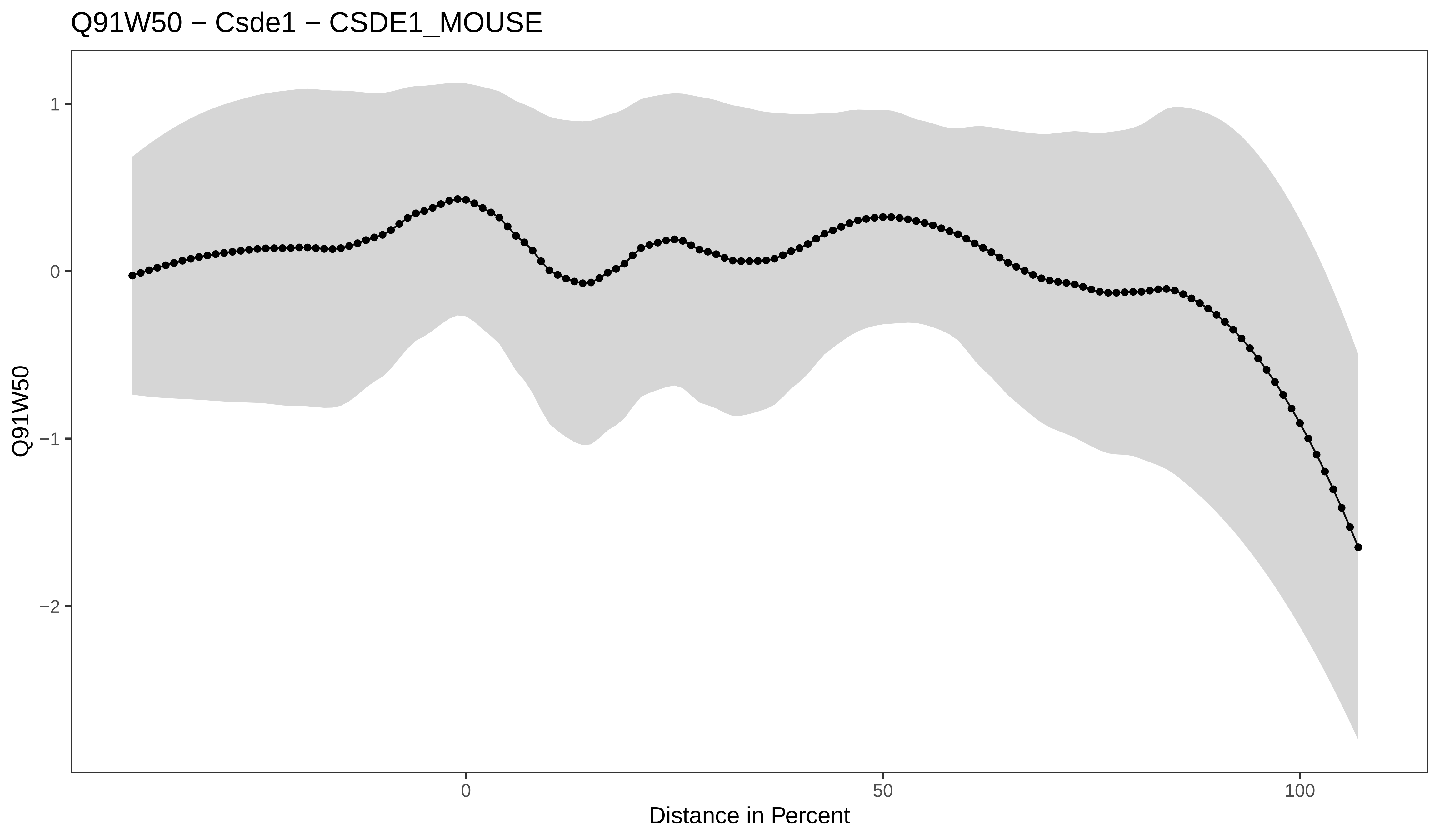
Q91W50 − Csde1 − CSDE1_MOUSE
Q91W50
1
0
−1
−2
0
50
100
Distance in Percent

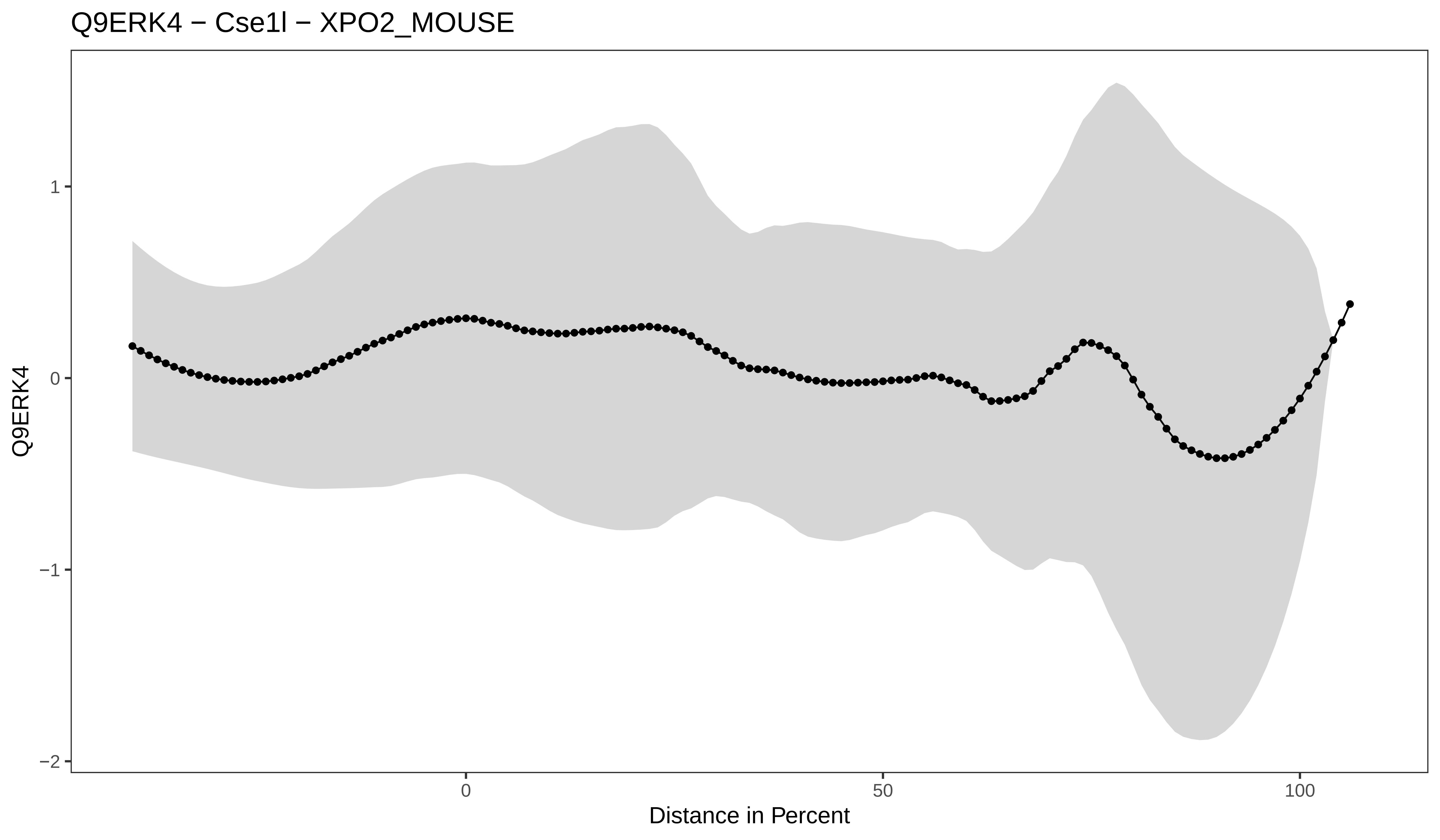
Q9ERK4 − Cse1l − XPO2_MOUSE
Q9ERK4
1
0
−1
−2
0
50
100
Distance in Percent

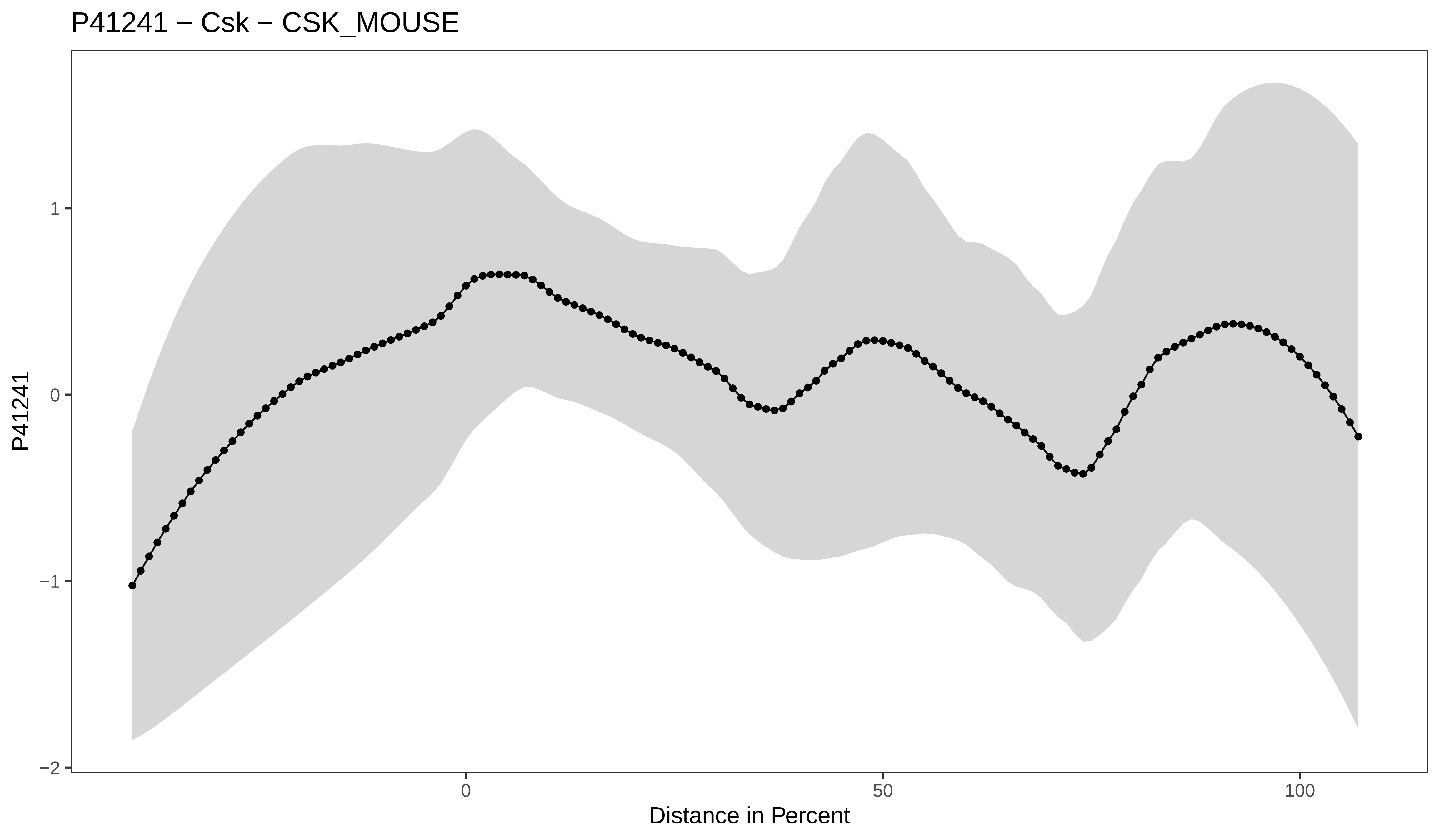
P41241 − Csk − CSK_MOUSE
P41241
1
0
−1
−2
0
50
100
Distance in Percent

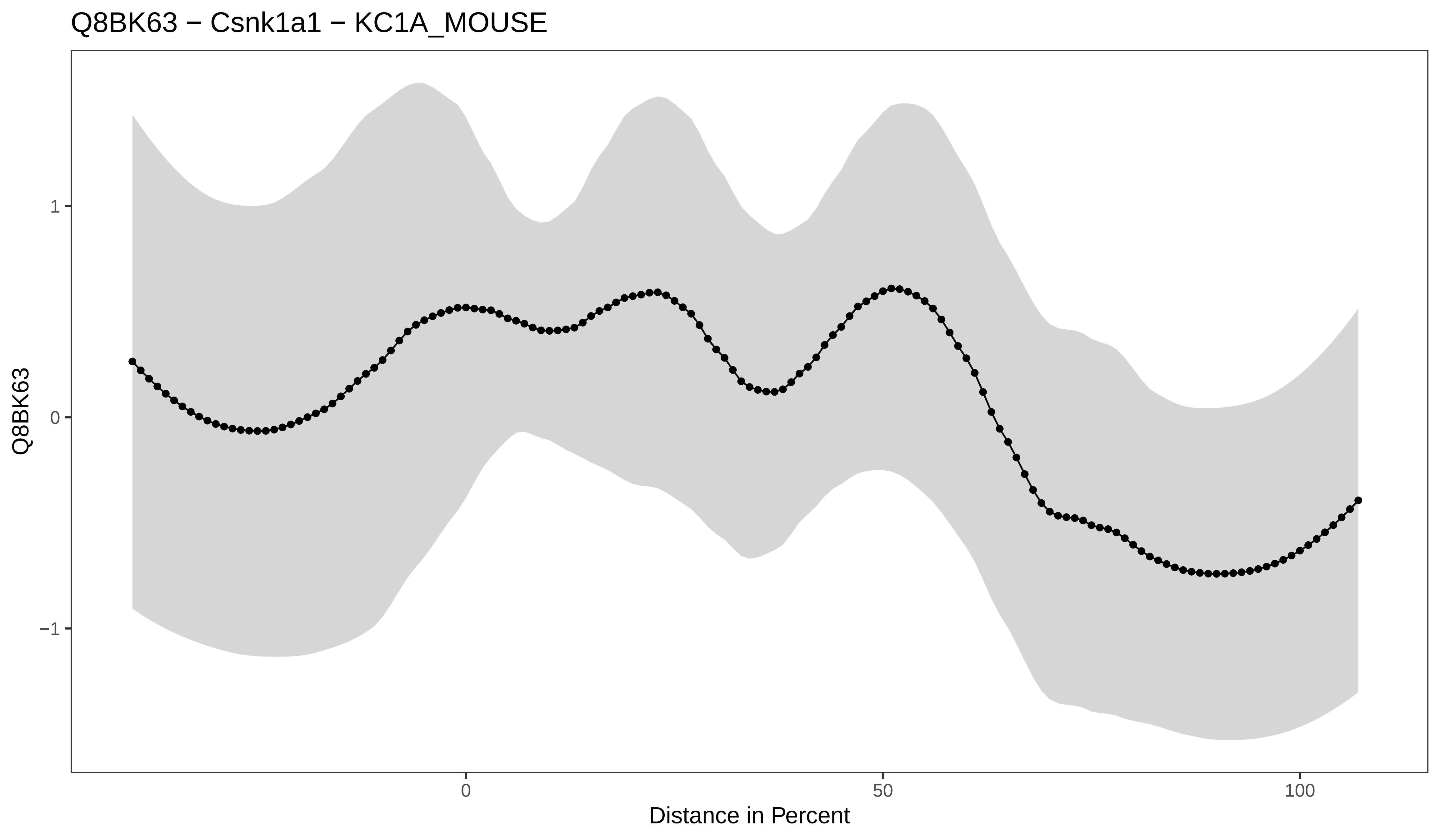

Q8BK63 − Csnk1a1 − KC1A_MOUSE
Q8BK63
1
0
−1
0
50
100
Distance in Percent

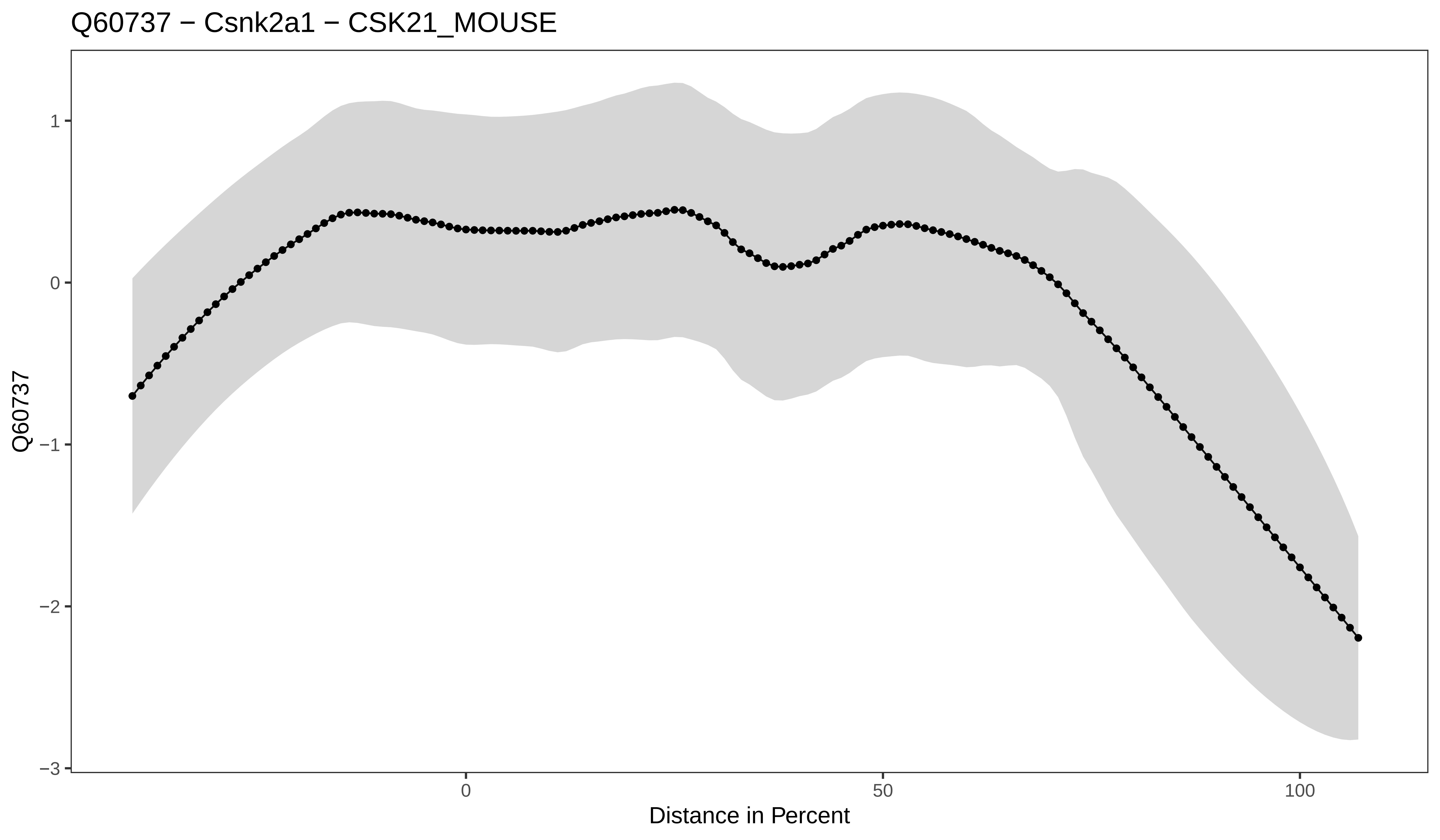
Q60737 − Csnk2a1 − CSK21_MOUSE
Q60737
1
0
−1
−2
−3
0
50
100
Distance in Percent

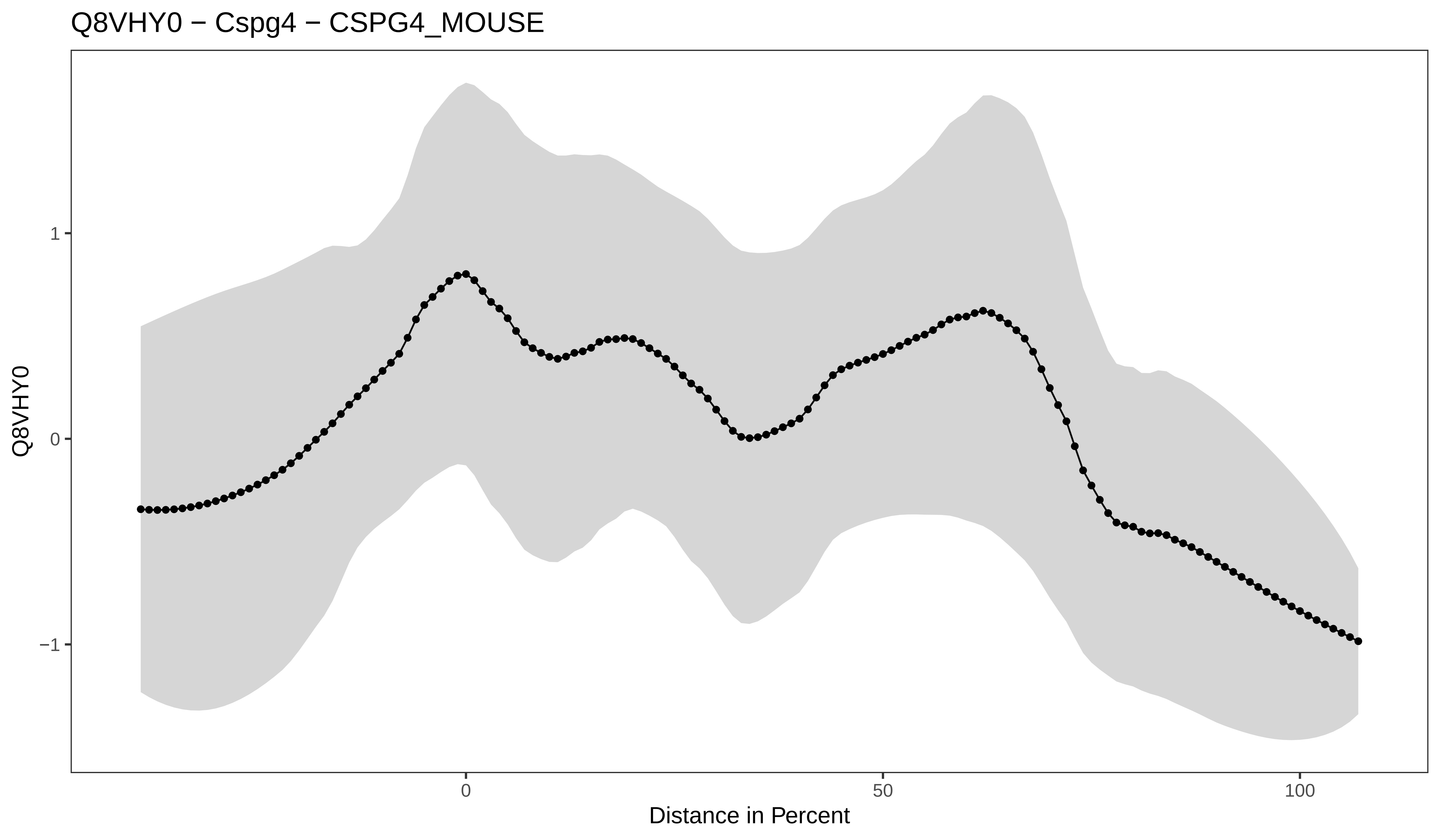
Q8VHY0 − Cspg4 − CSPG4_MOUSE
Q8VHY0
1
0
−1
0
50
100
Distance in Percent

P97315 − Csrp1 − CSRP1_MOUSE

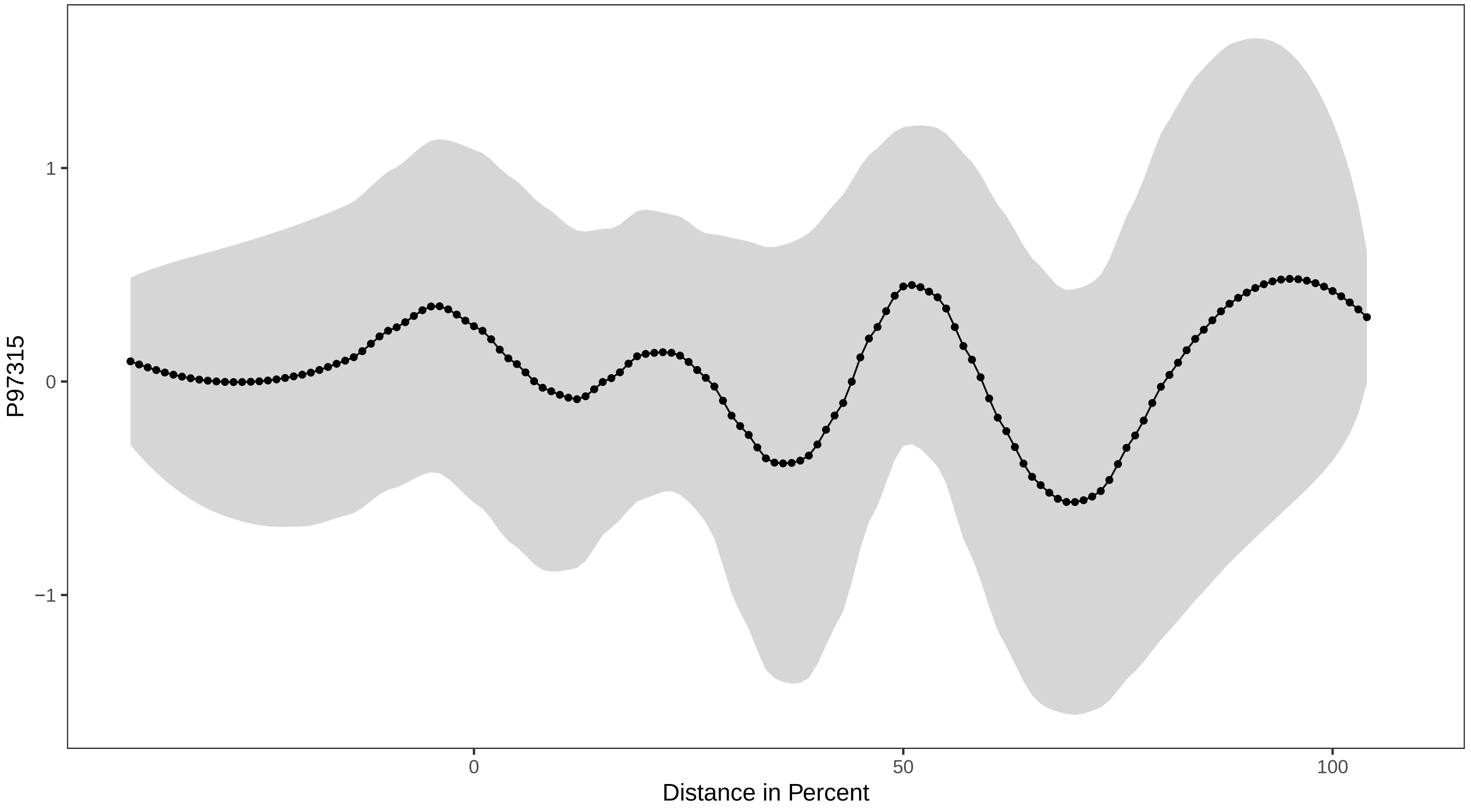

P50462 − Csrp3 − CSRP3_MOUSE

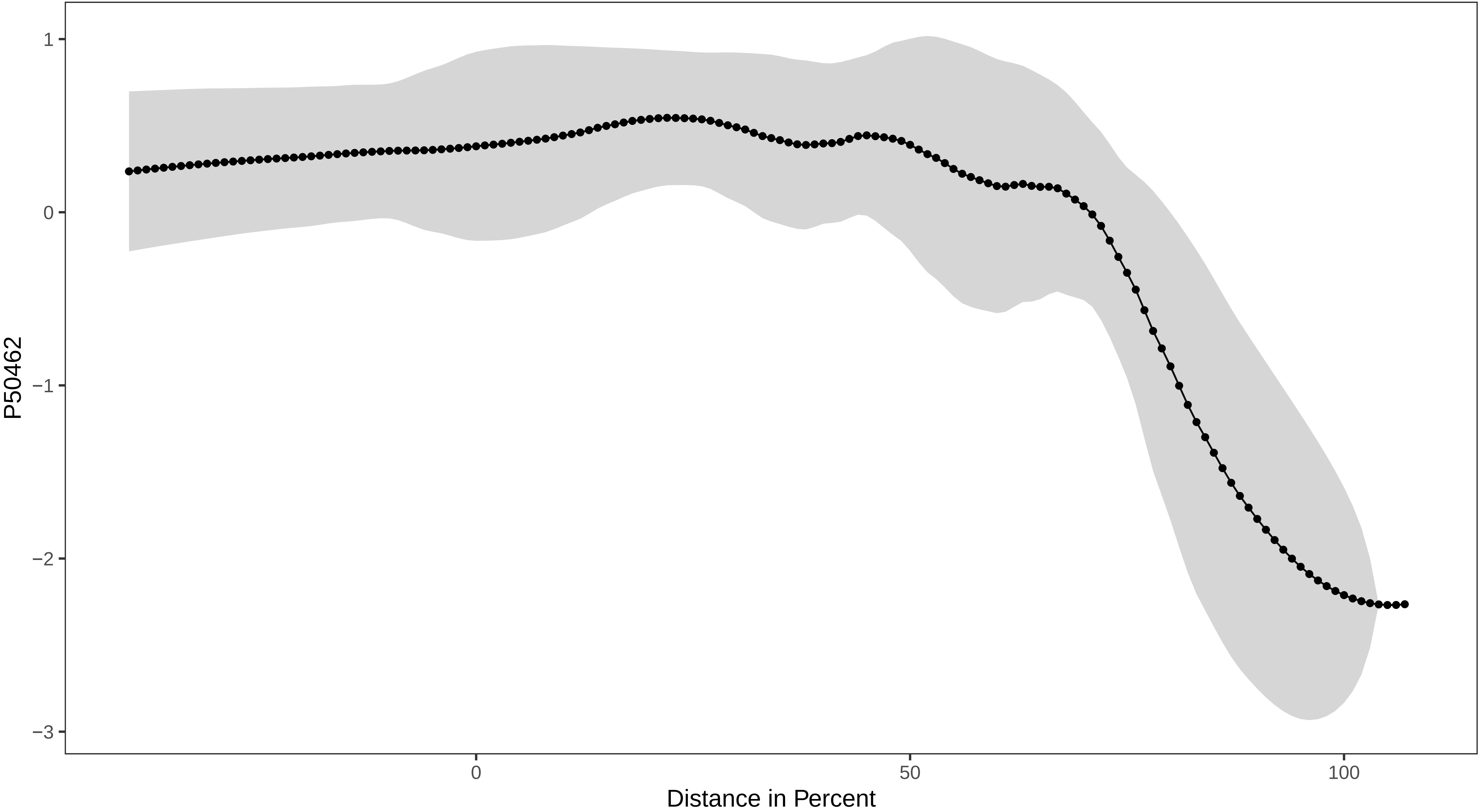

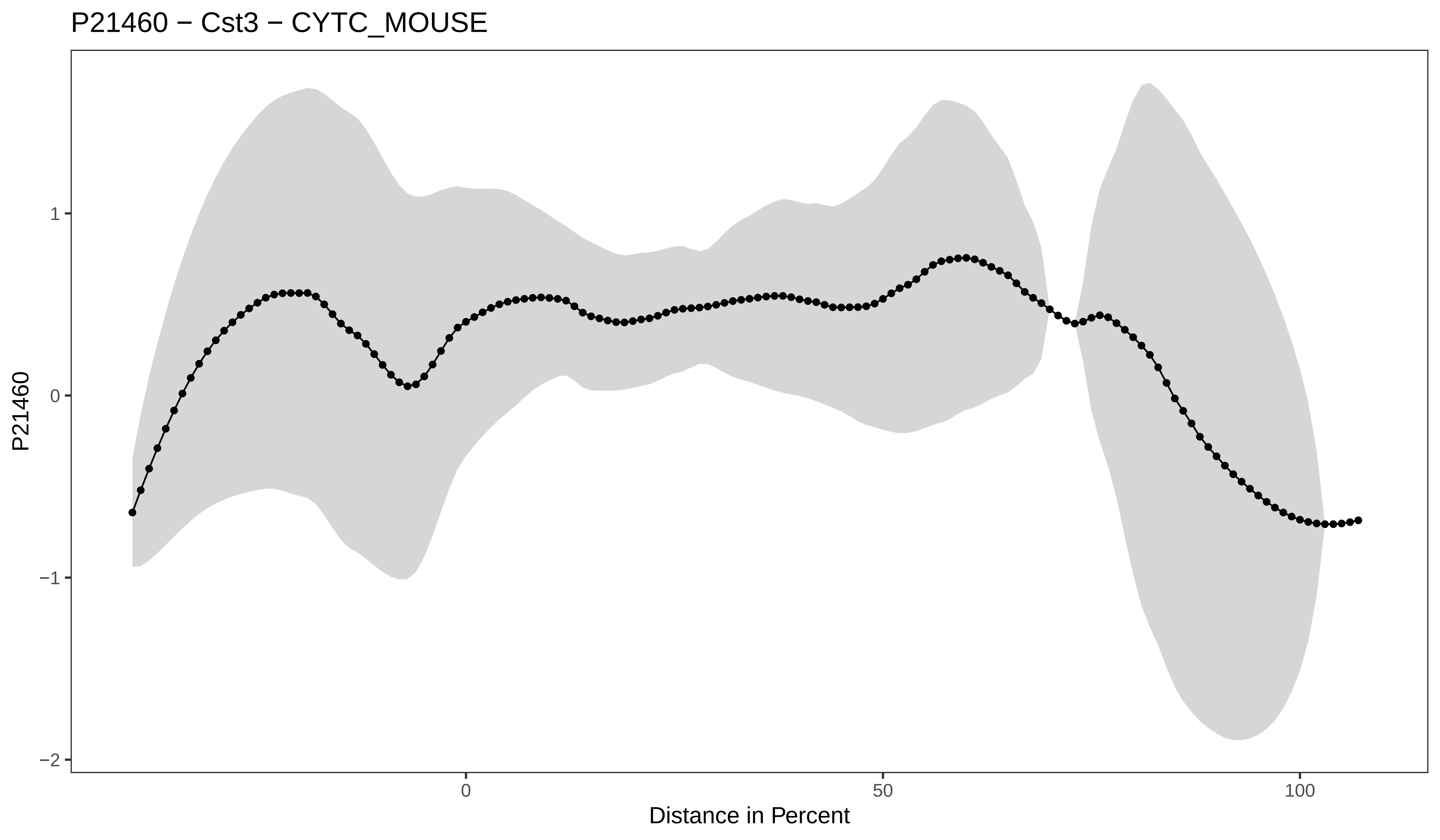

P21460 − Cst3 − CYTC_MOUSE
P21460
1
0
−1
−2
0
50
100
Distance in Percent

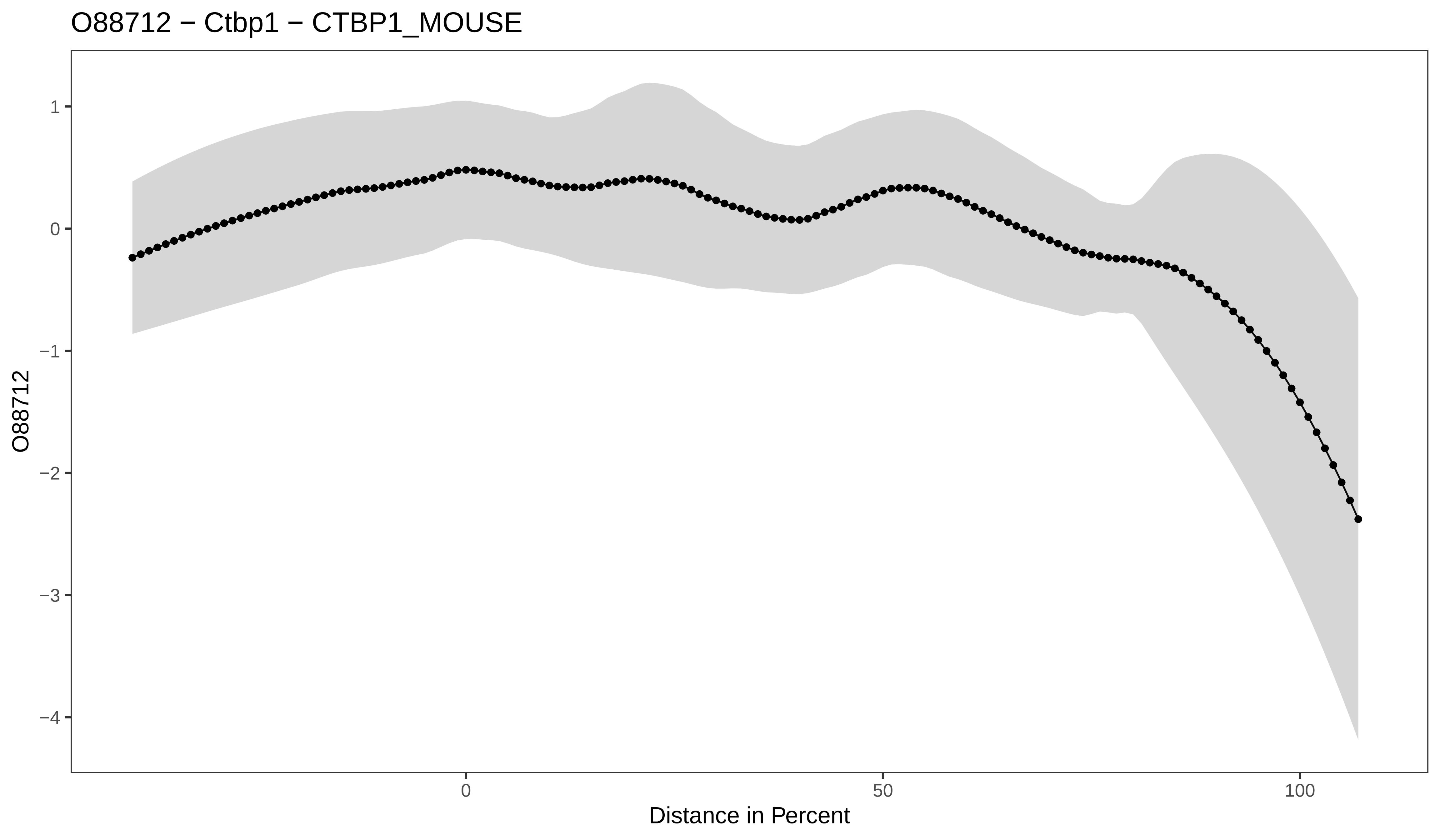

O88712 − Ctbp1 − CTBP1_MOUSE
1
0
−1
−2
−3
−4
O88712
0
50
100
Distance in Percent

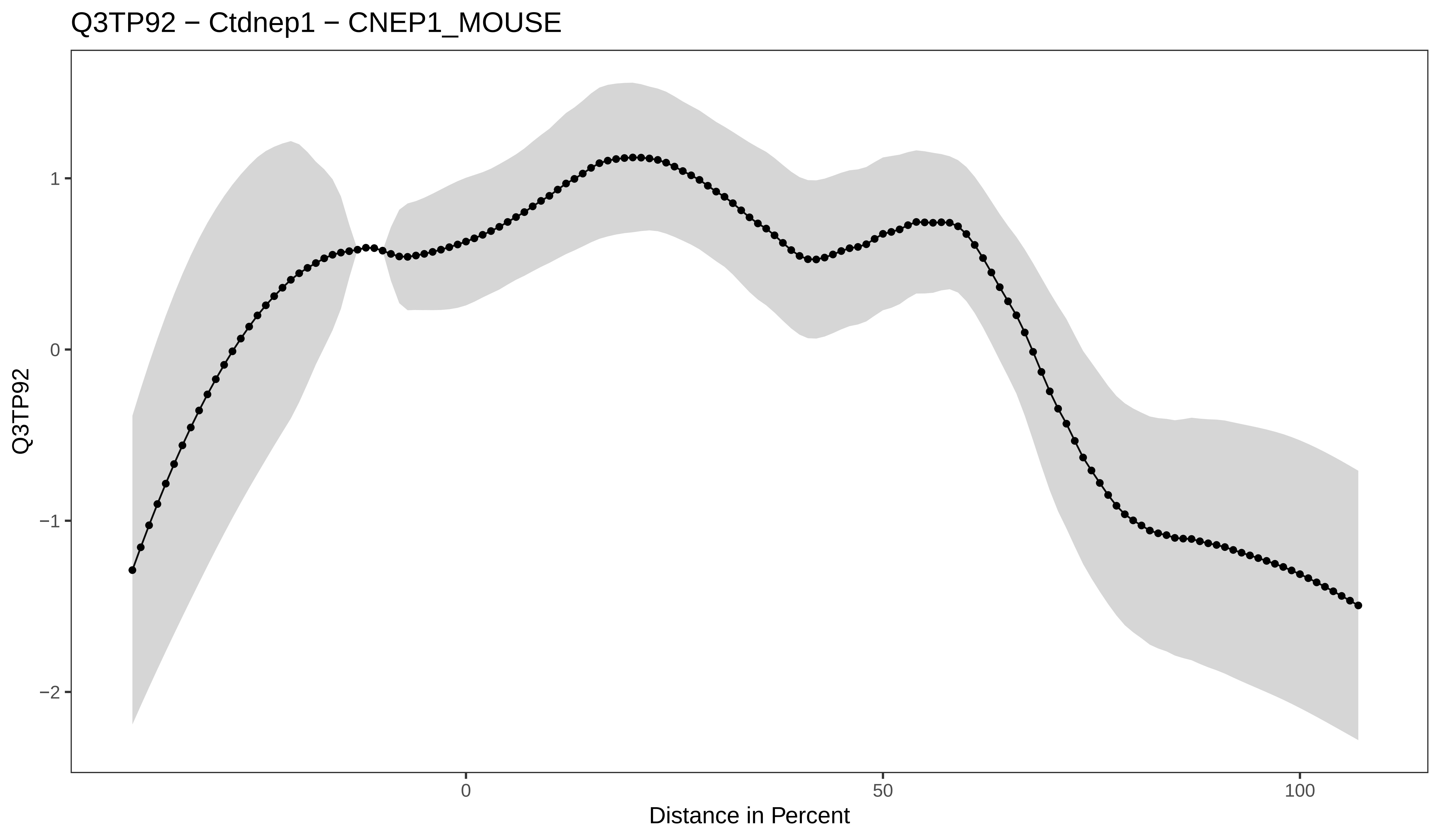

Q3TP92 − Ctdnep1 − CNEP1_MOUSE
Q3TP92
1
0
−1
−2
0
50
100
Distance in Percent

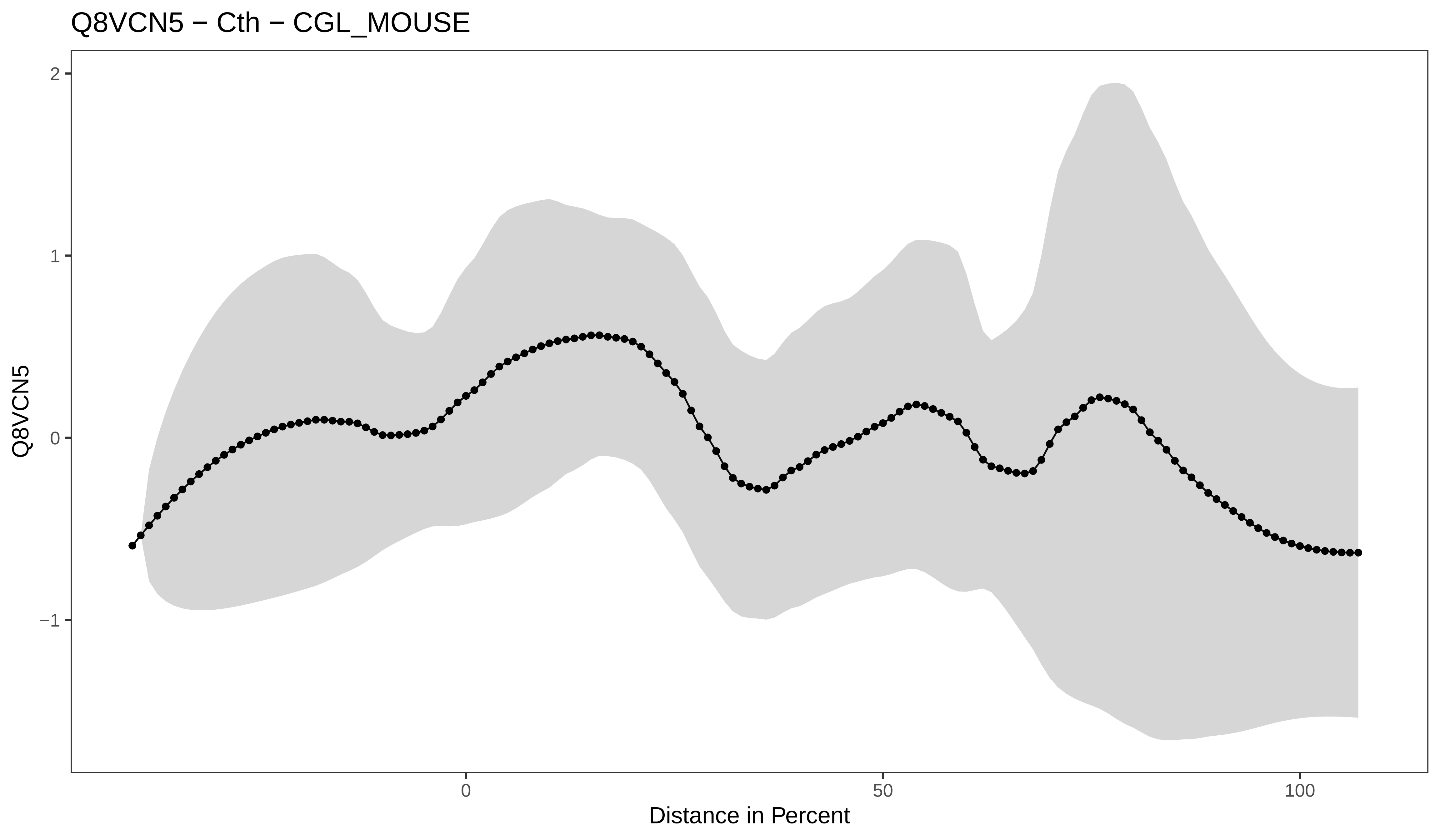

Q8VCN5 − Cth − CGL_MOUSE
Q8VCN5
2
1
0
−1
0
50
100
Distance in Percent

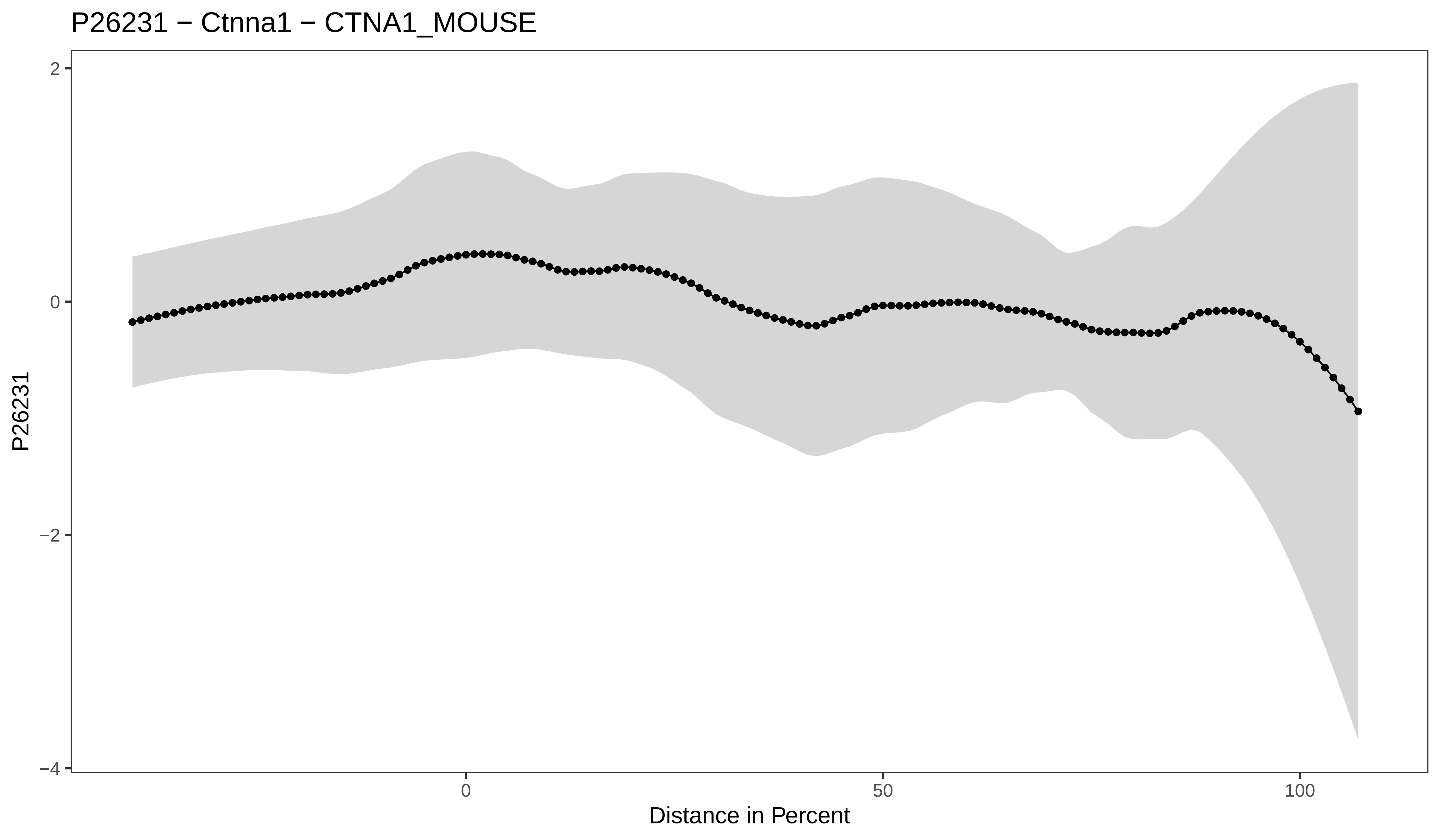

P26231 − Ctnna1 − CTNA1_MOUSE
2
0
−2
−4
P26231
0
50
100
Distance in Percent

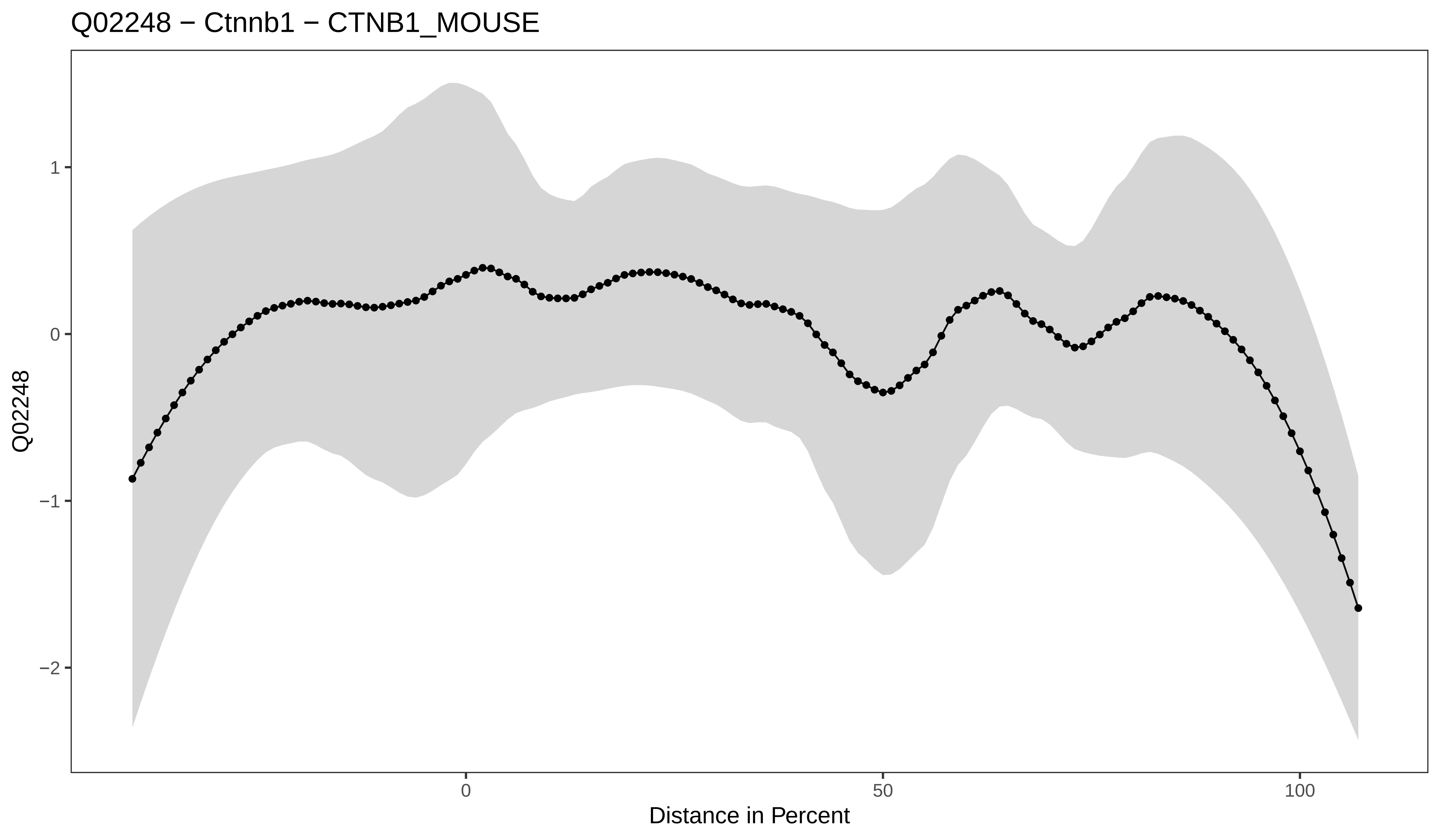
Q02248 − Ctnnb1 − CTNB1_MOUSE
Q02248
1
0
−1
−2
0
50
100
Distance in Percent

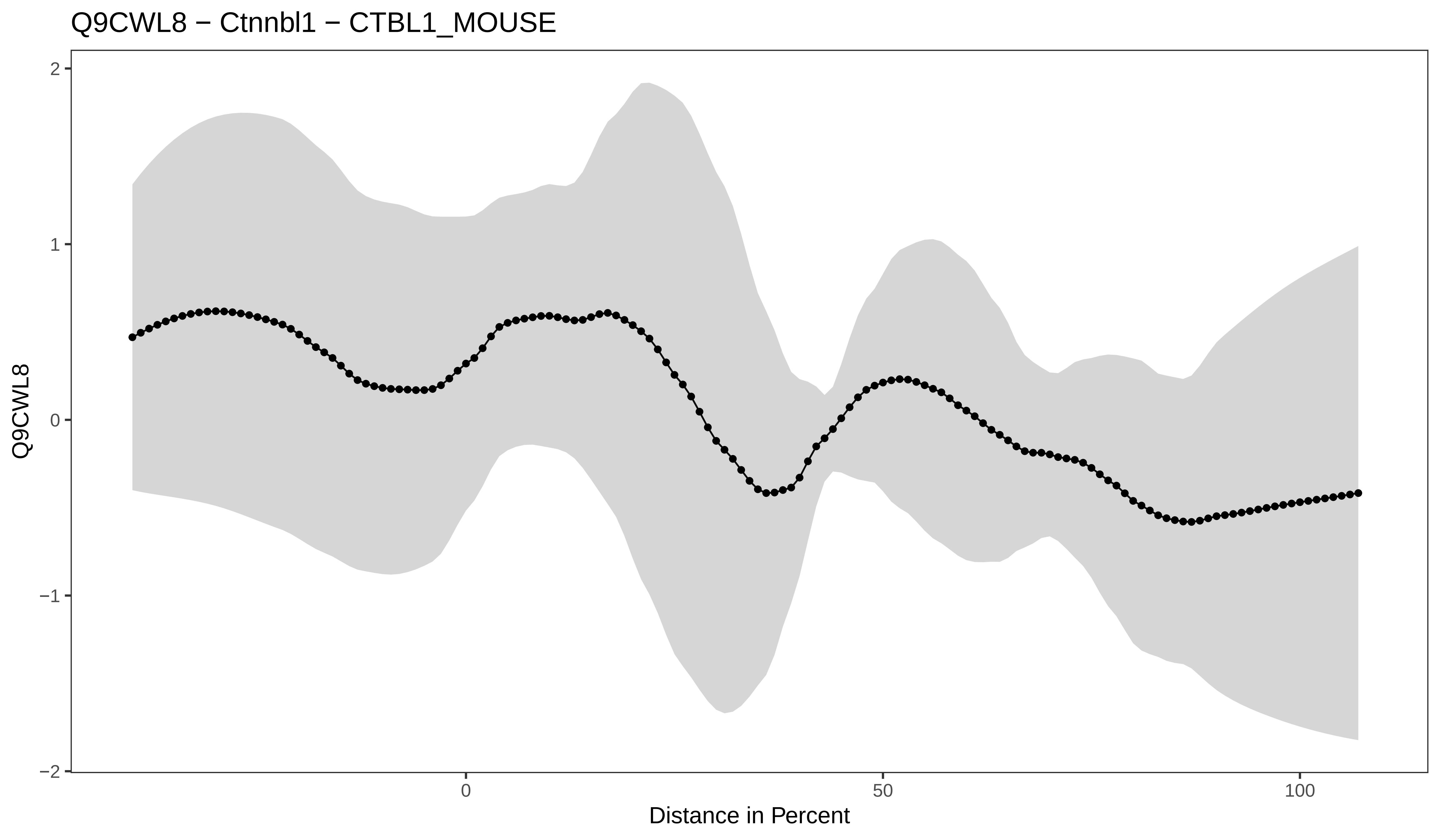
Q9CWL8 − Ctnnbl1 − CTBL1_MOUSE
Q9CWL8
Distance in Percent
2
1
0
−1
−2
0
50
100

P30999 − Ctnnd1 − CTND1_MOUSE

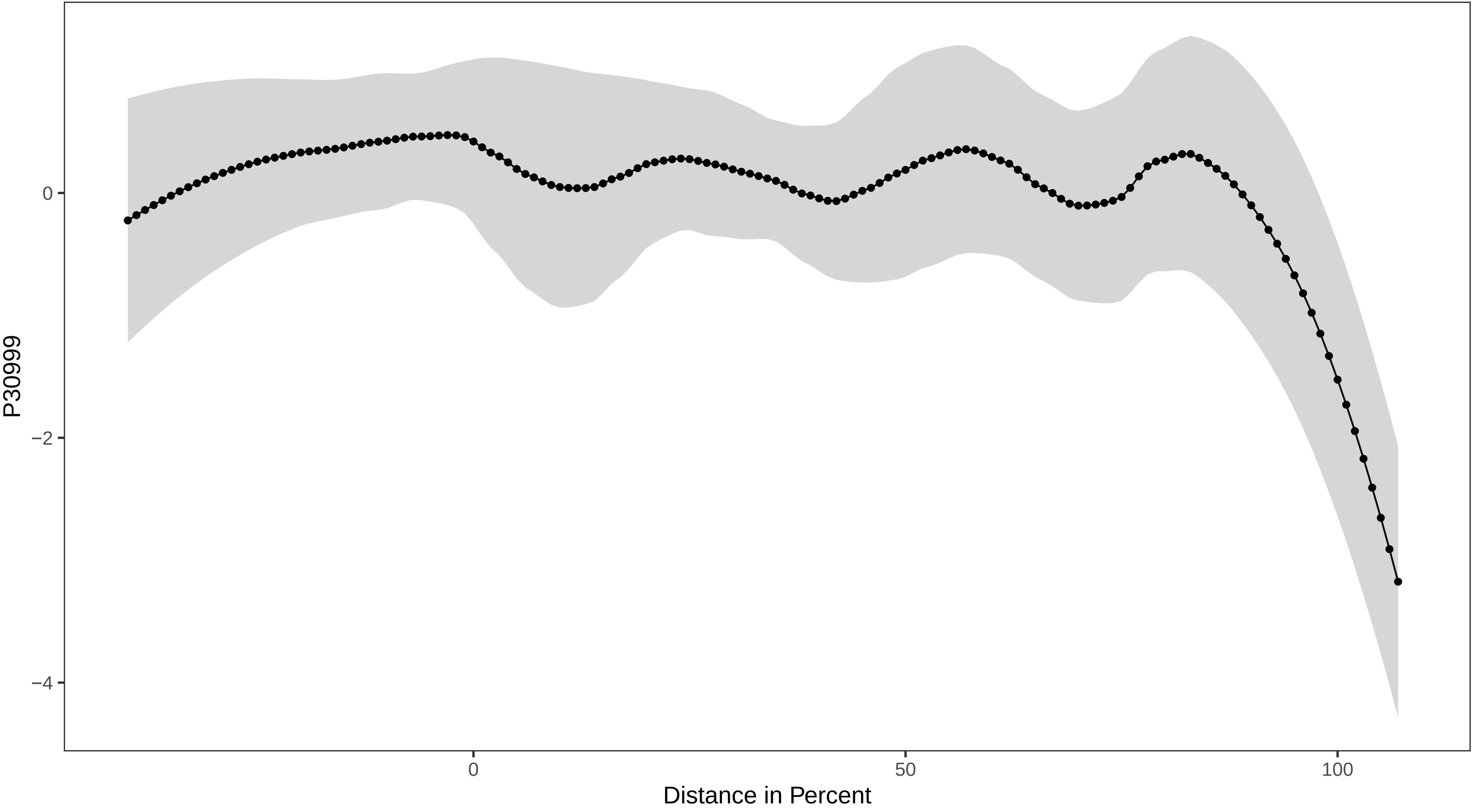

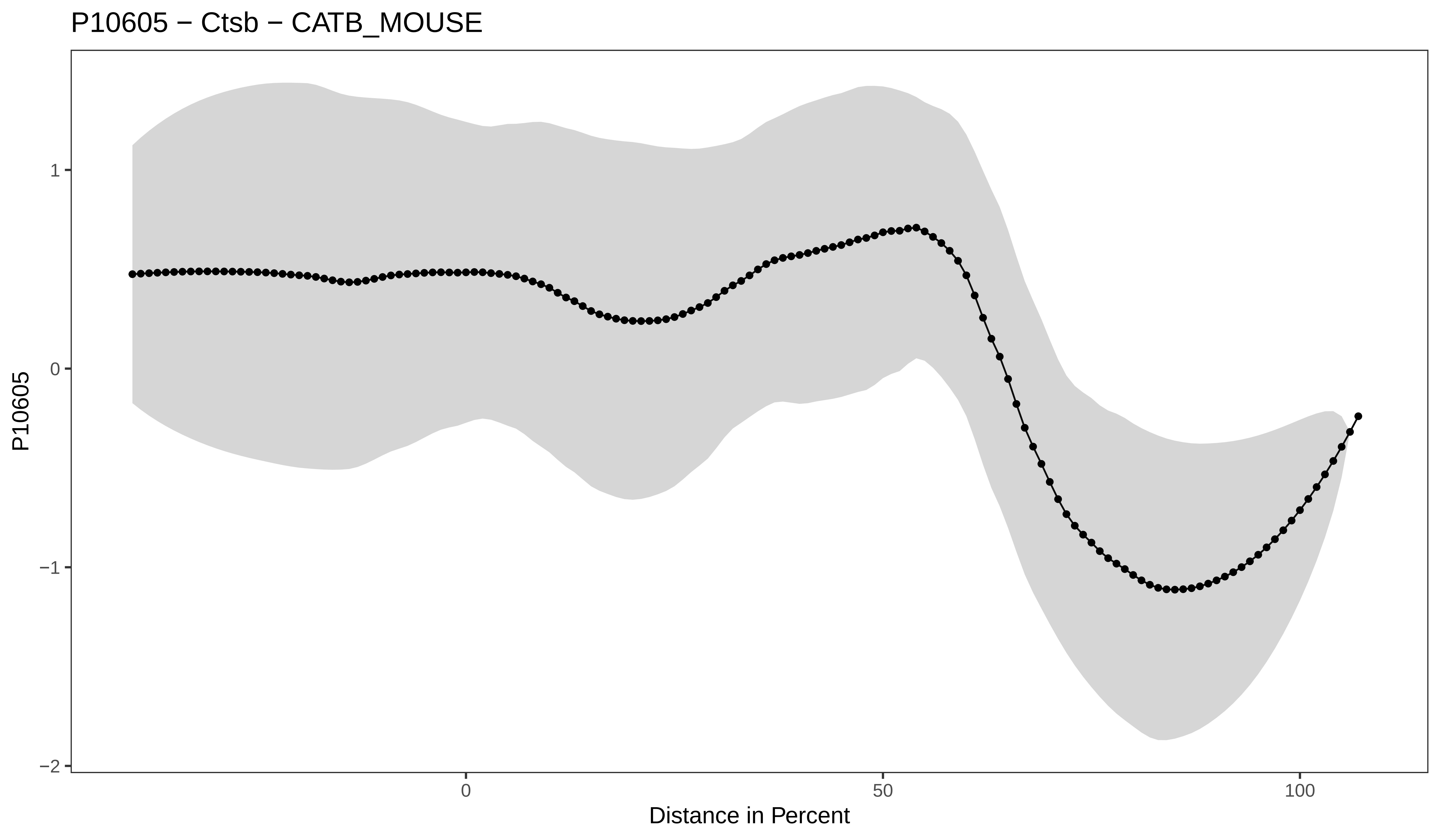

P10605 − Ctsb − CATB_MOUSE
P10605
1
0
−1
−2
0
50
100
Distance in Percent

P18242 − Ctsd − CATD_MOUSE

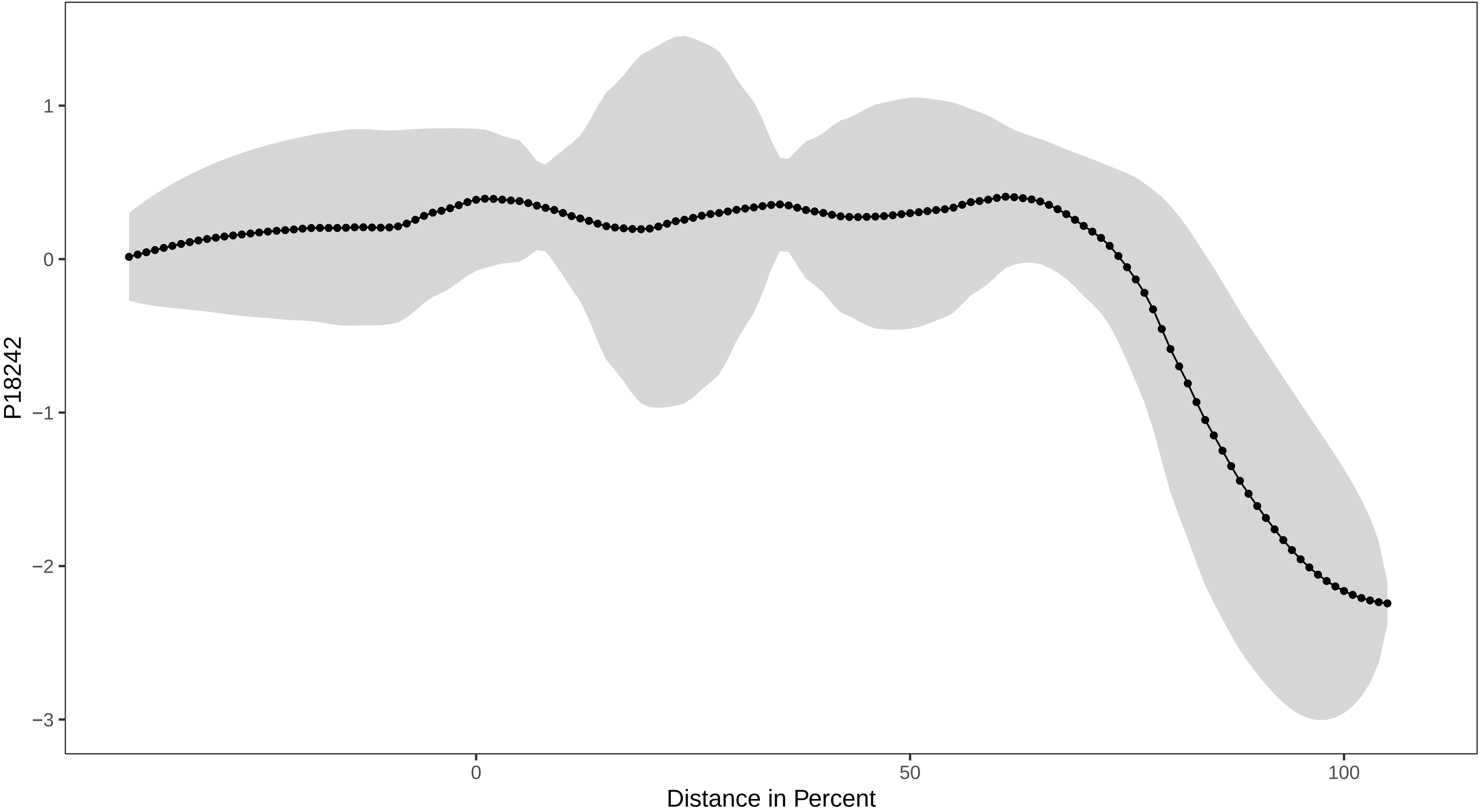

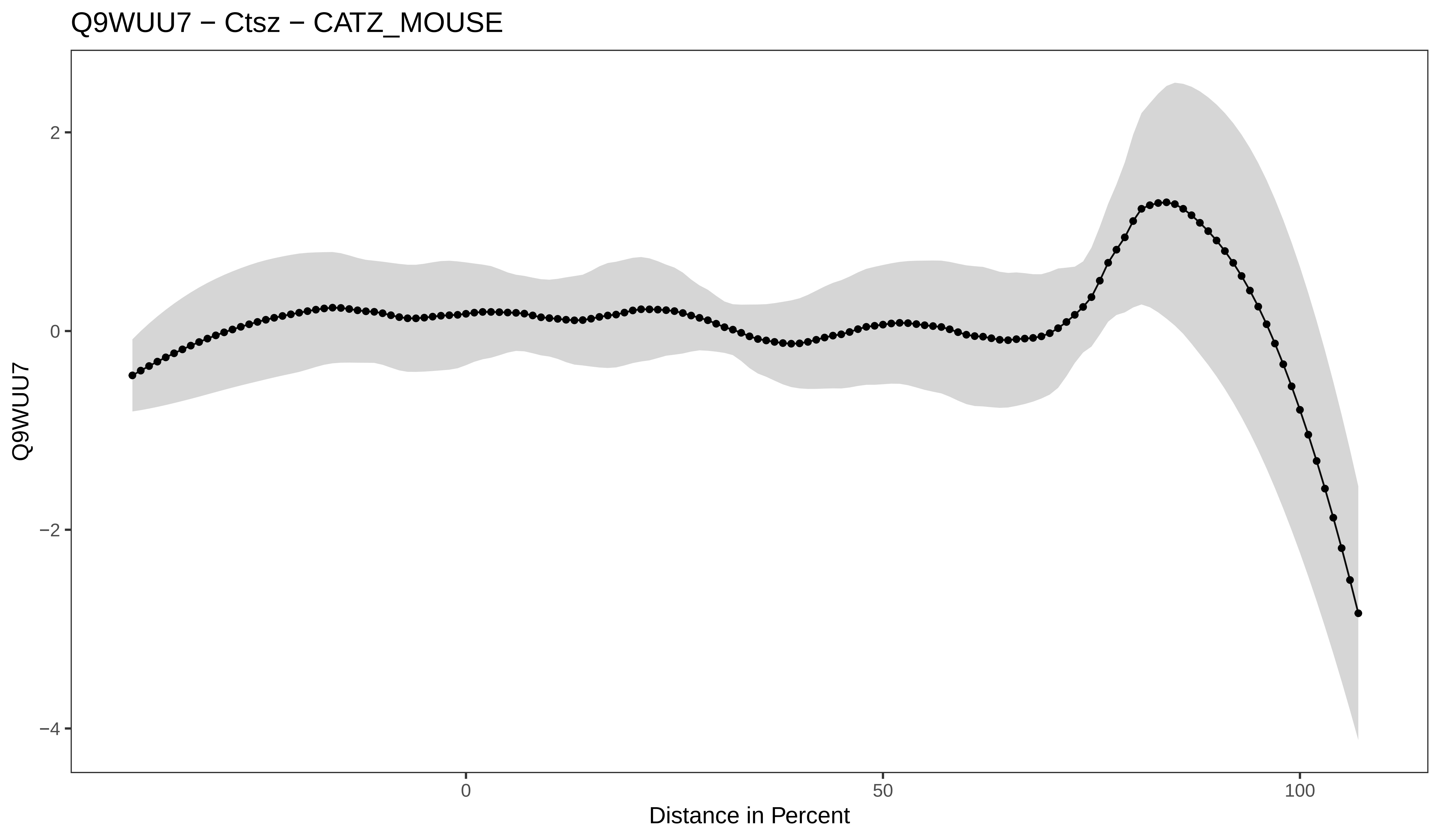
Q9WUU7 − Ctsz − CATZ_MOUSE
Q9WUU7
2
0
−2
−4
0
50
100
Distance in Percent

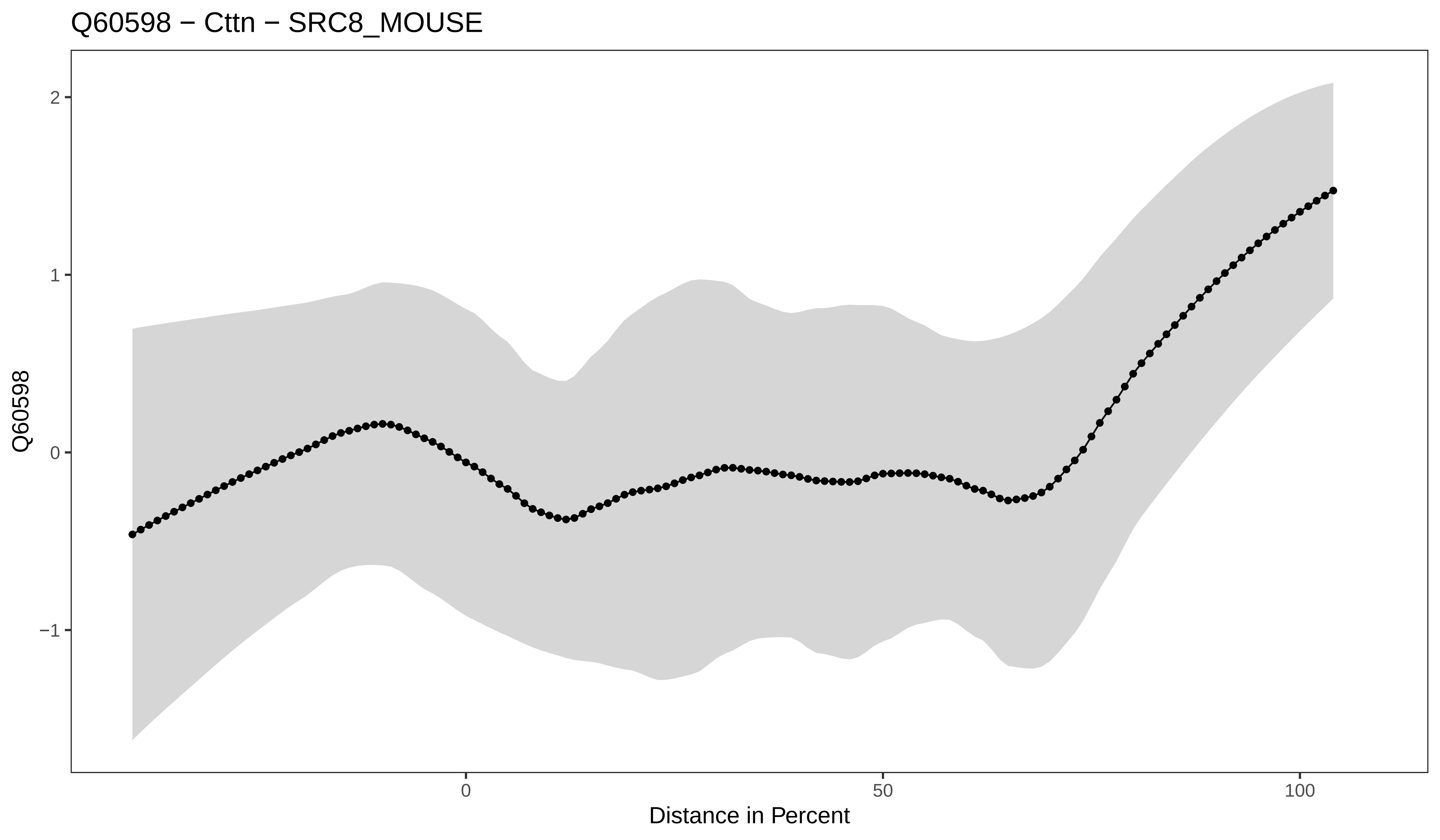

Q60598 − Cttn − SRC8_MOUSE
Q60598
2
1
0
−1
0
50
100
Distance in Percent

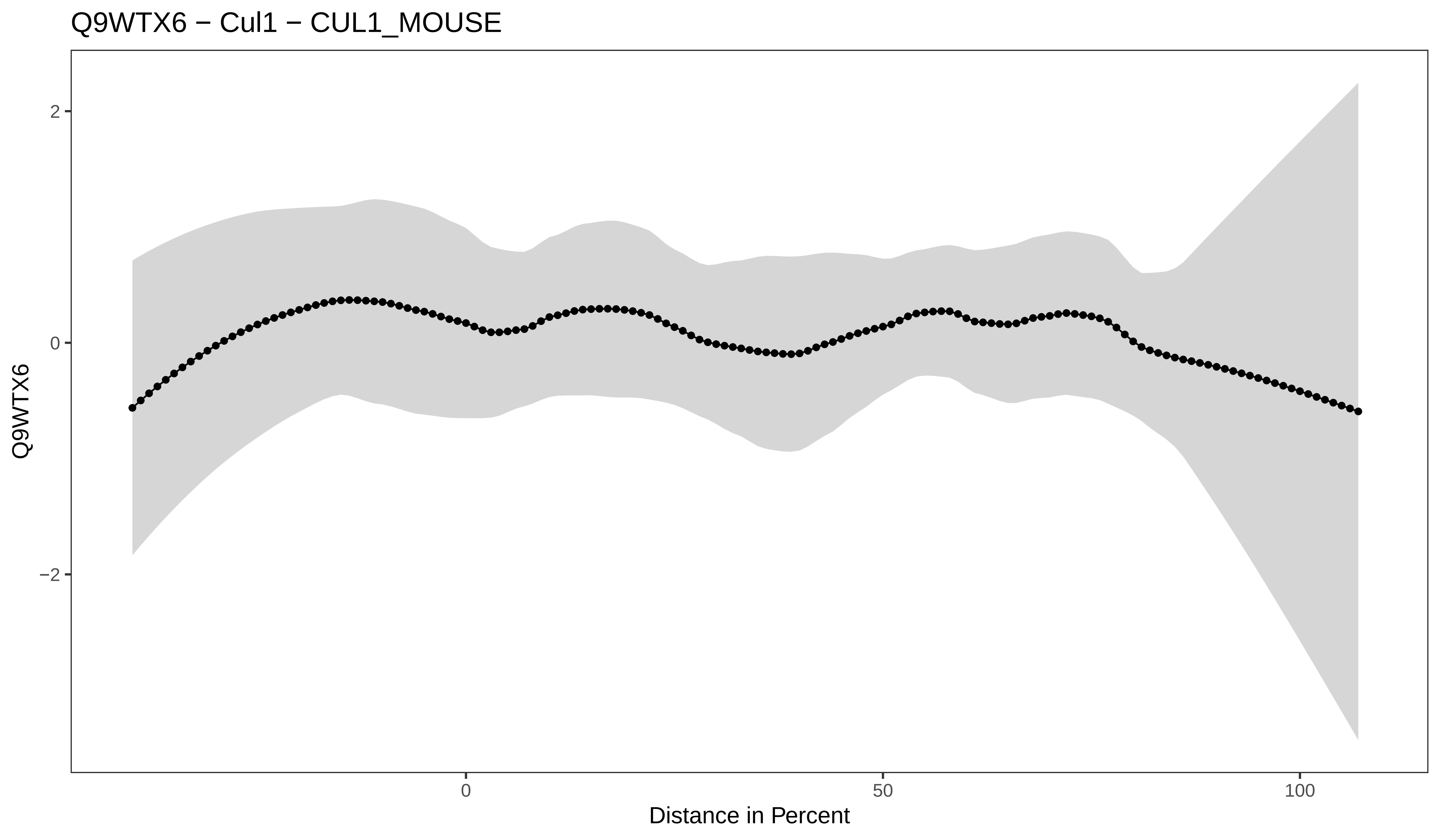

Q9WTX6 − Cul1 − CUL1_MOUSE
2
0
−2
0
50
100
Distance in Percent
Q9WTX6

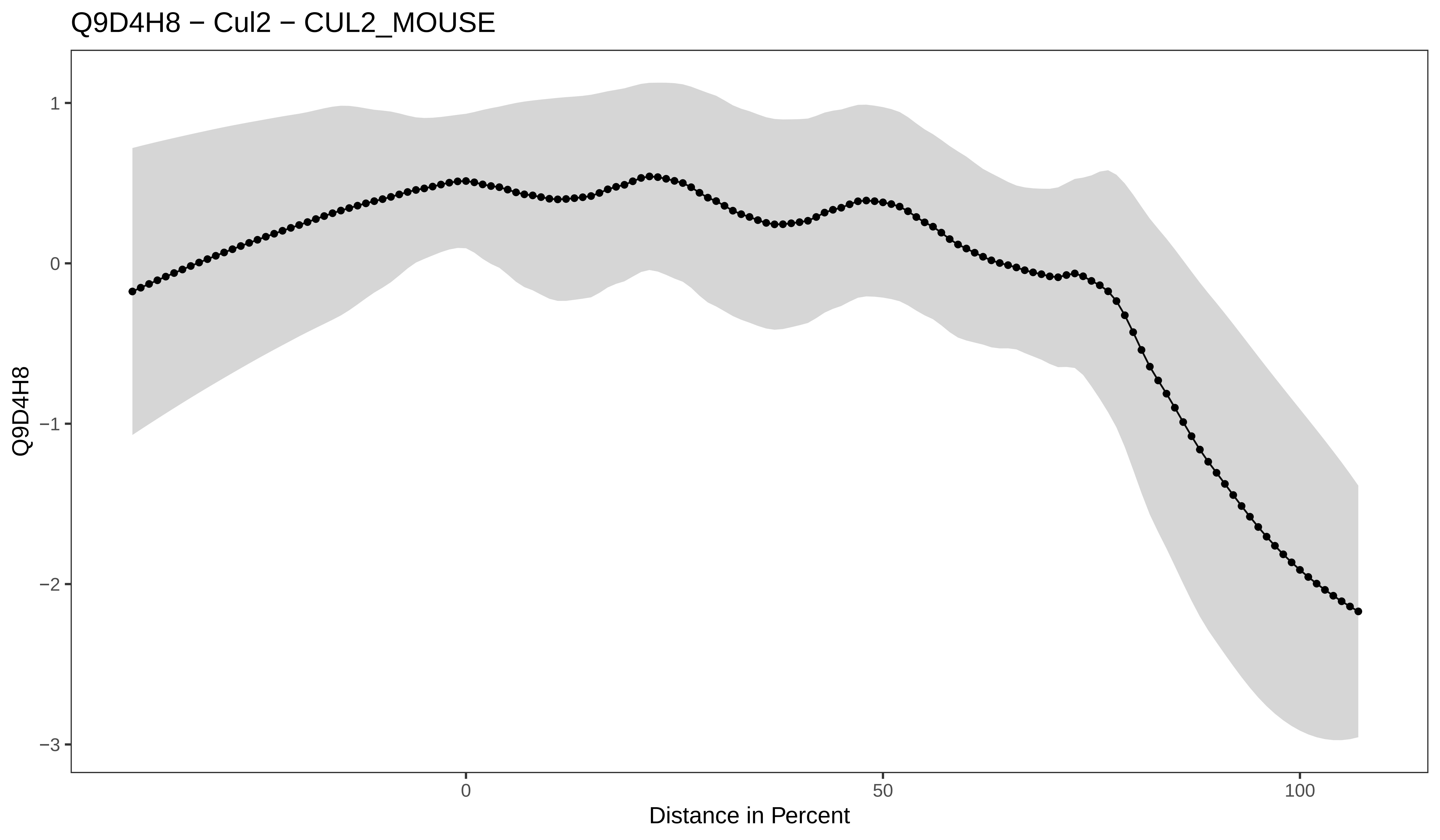

Q9D4H8 − Cul2 − CUL2_MOUSE
Q9D4H8
1
0
−1
−2
−3
0
50
100
Distance in Percent

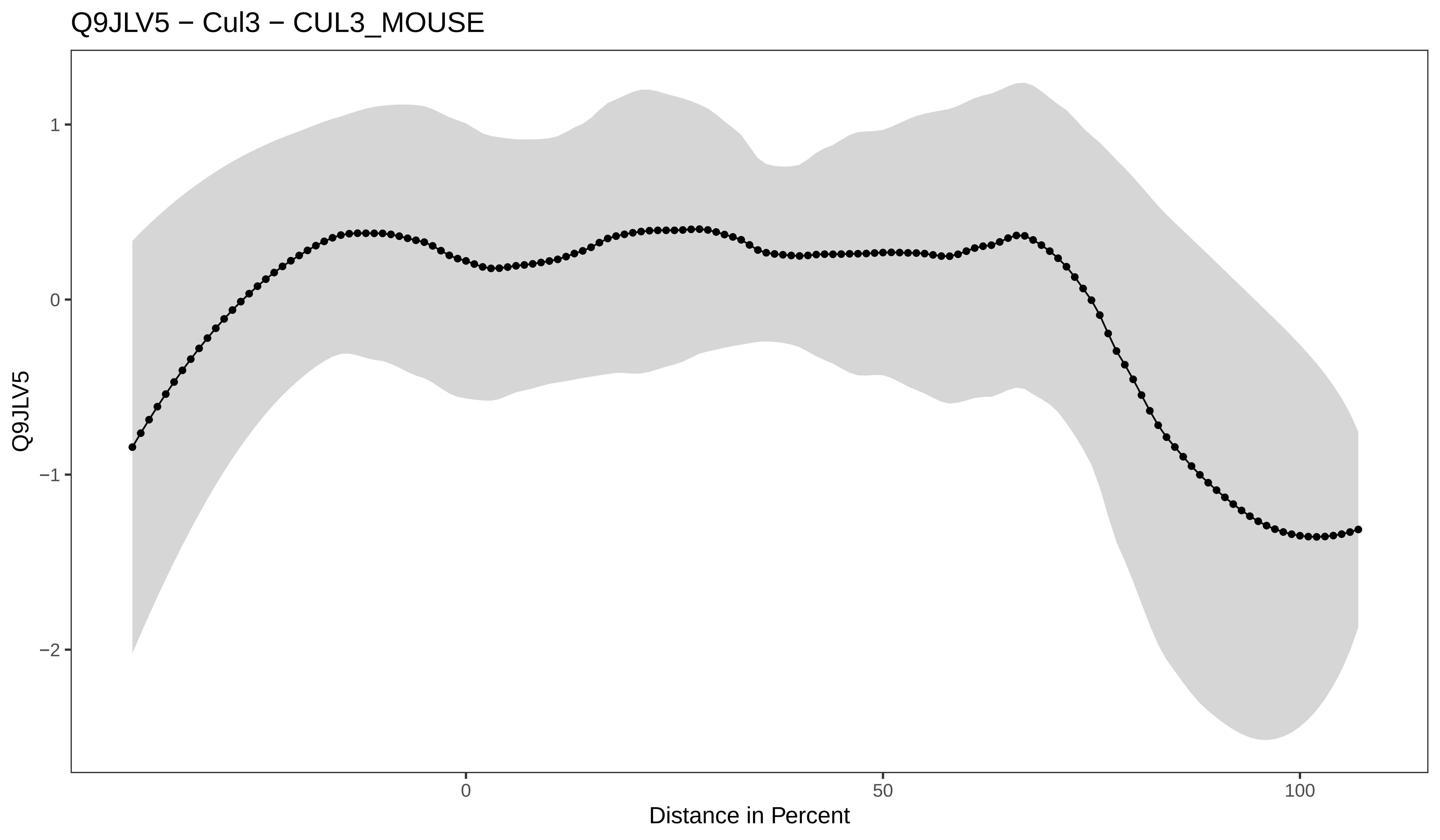
Q9JLV5 − Cul3 − CUL3_MOUSE
Q9JLV5
1
0
−1
−2
0
50
100
Distance in Percent

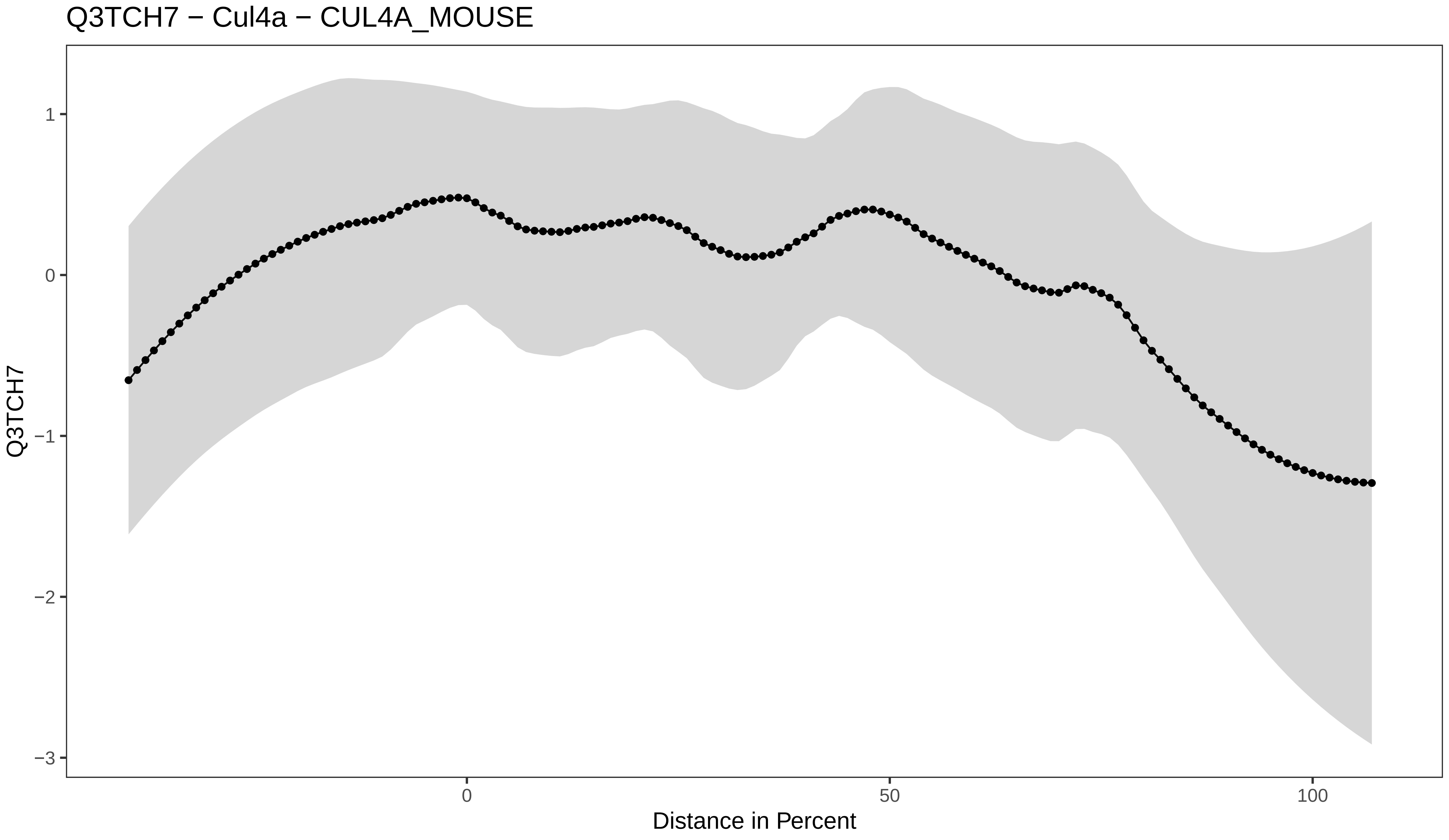

Q3TCH7 − Cul4a − CUL4A_MOUSE
Q3TCH7
1
0
−1
−2
−3
0
50
100
Distance in Percent

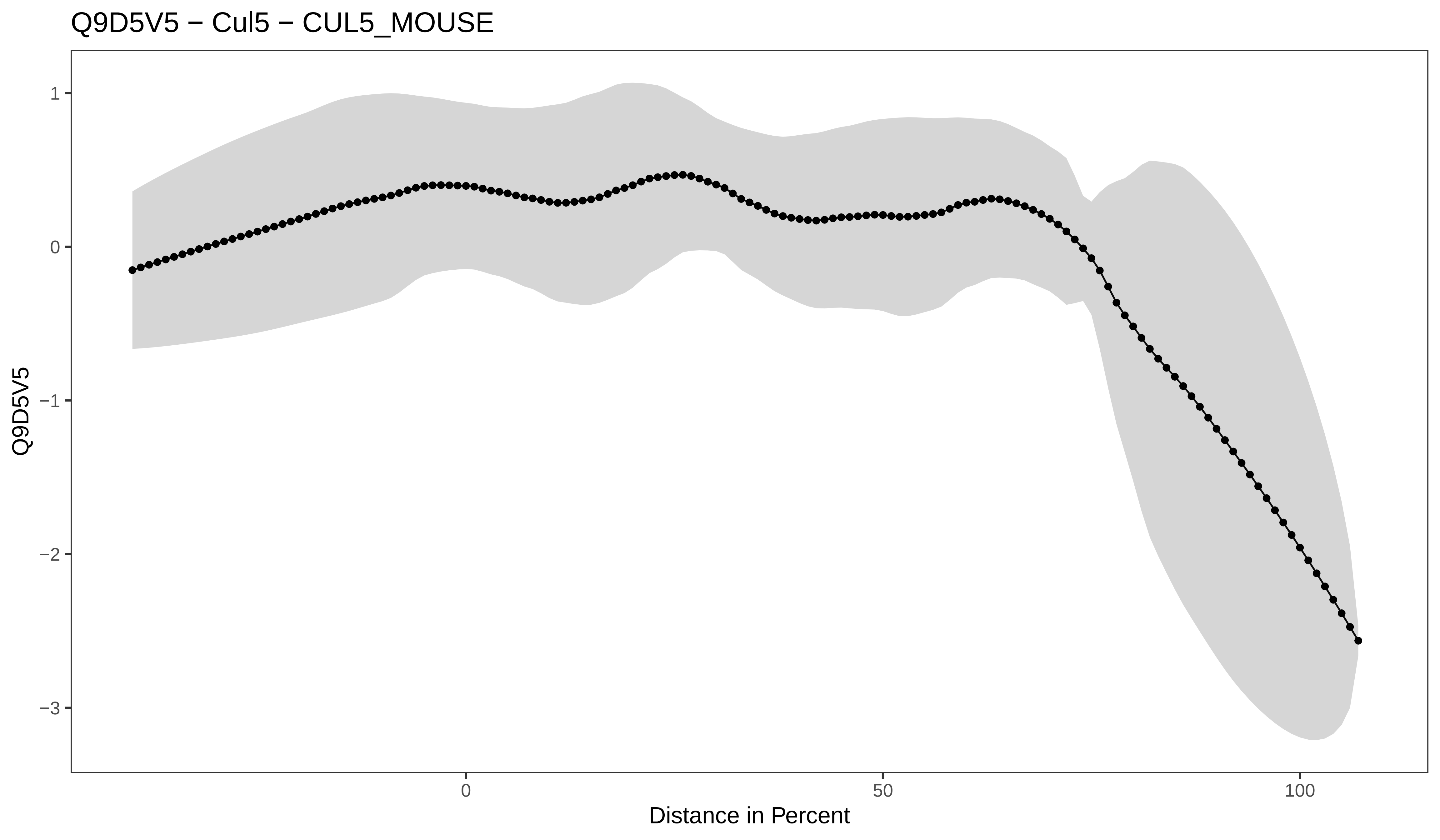
Q9D5V5 − Cul5 − CUL5_MOUSE
Q9D5V5
1
0
−1
−2
−3
0
50
100
Distance in Percent

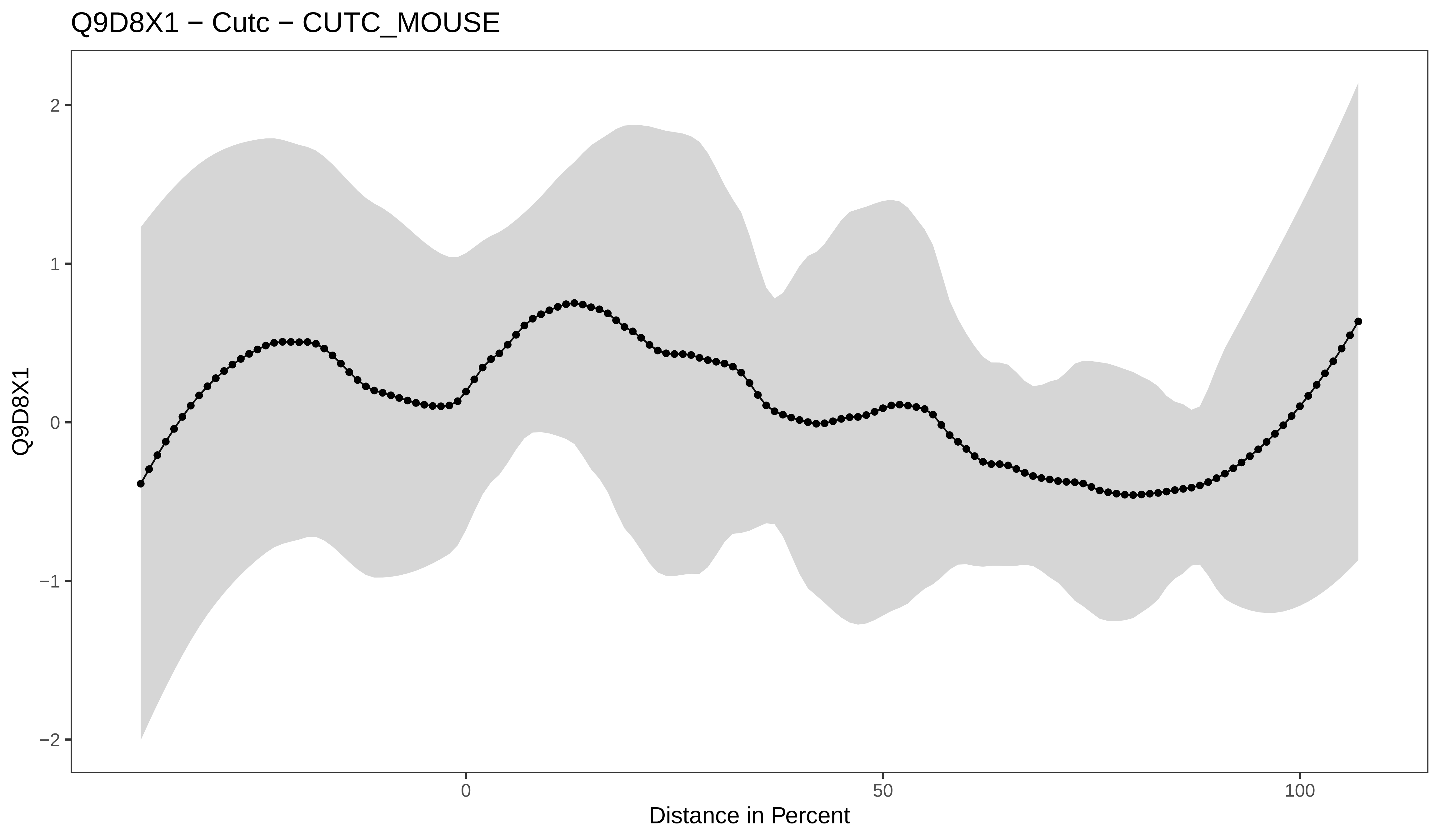
Q9D8X1 − Cutc − CUTC_MOUSE
Q9D8X1
Distance in Percent
2
1
0
−1
−2
0
50
100

Q8C5N3 − Cwc22 − CWC22_MOUSE

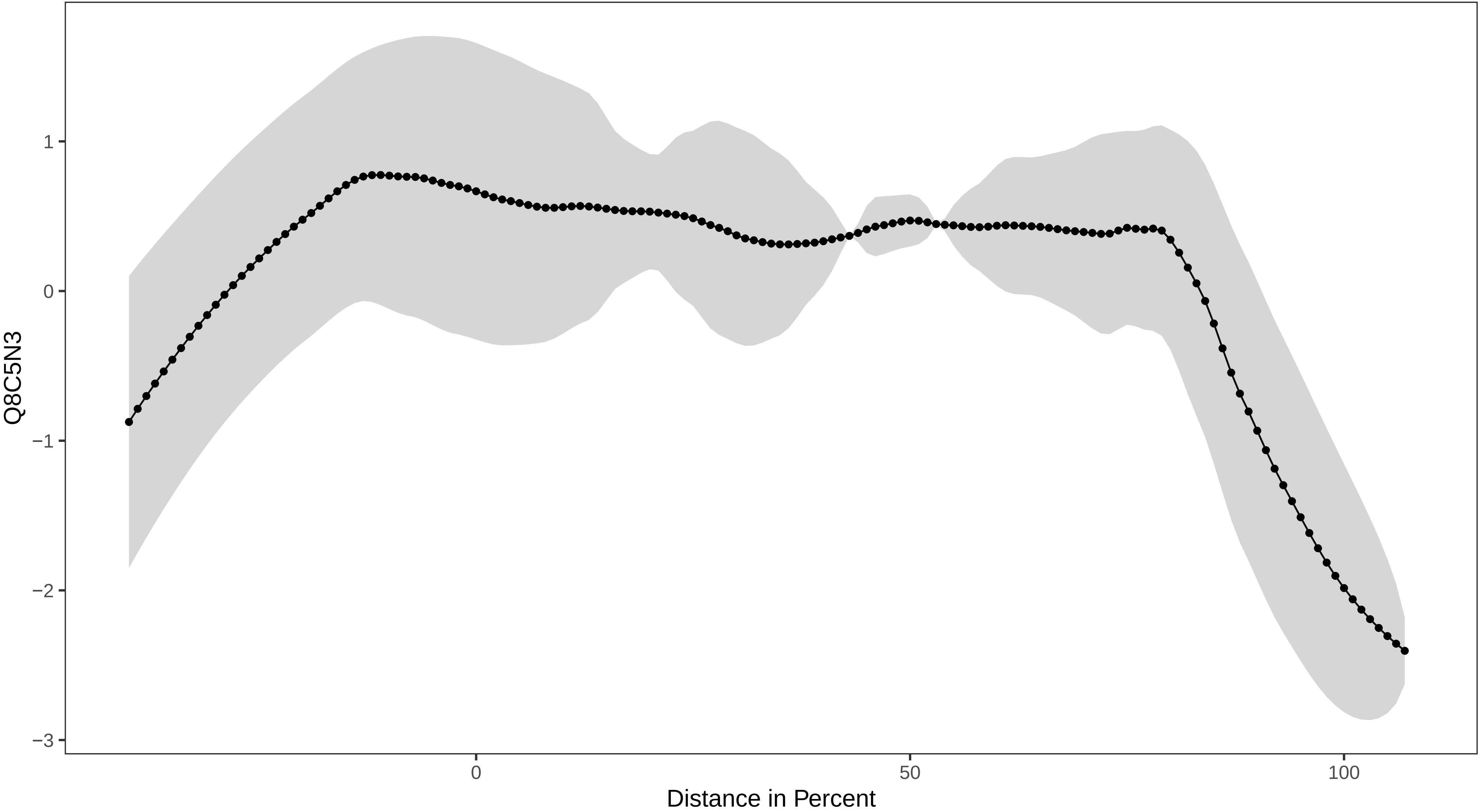

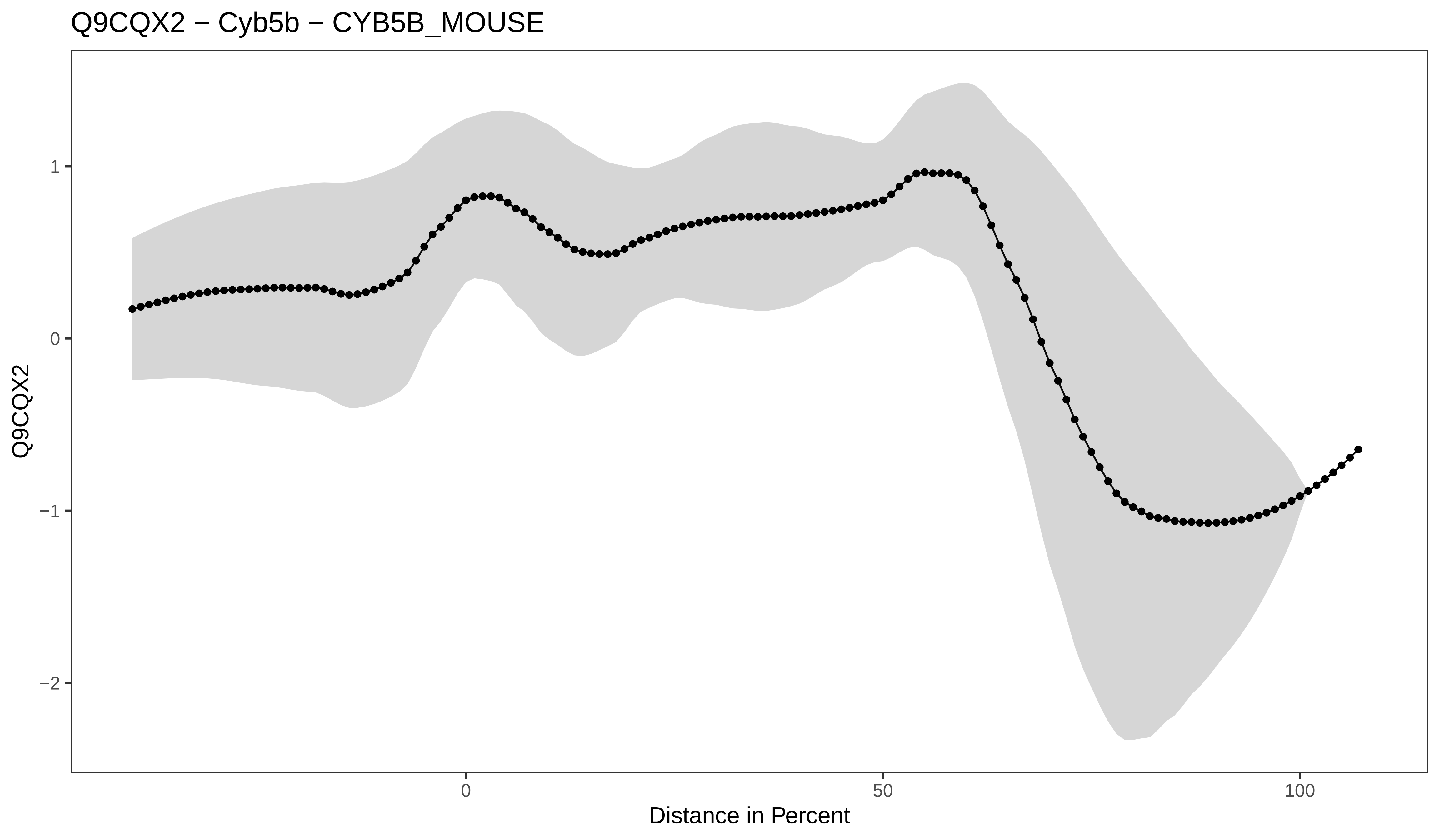

Q9CQX2 − Cyb5b − CYB5B_MOUSE
Q9CQX2
Distance in Percent
1
0
−1
−2
0
50
100

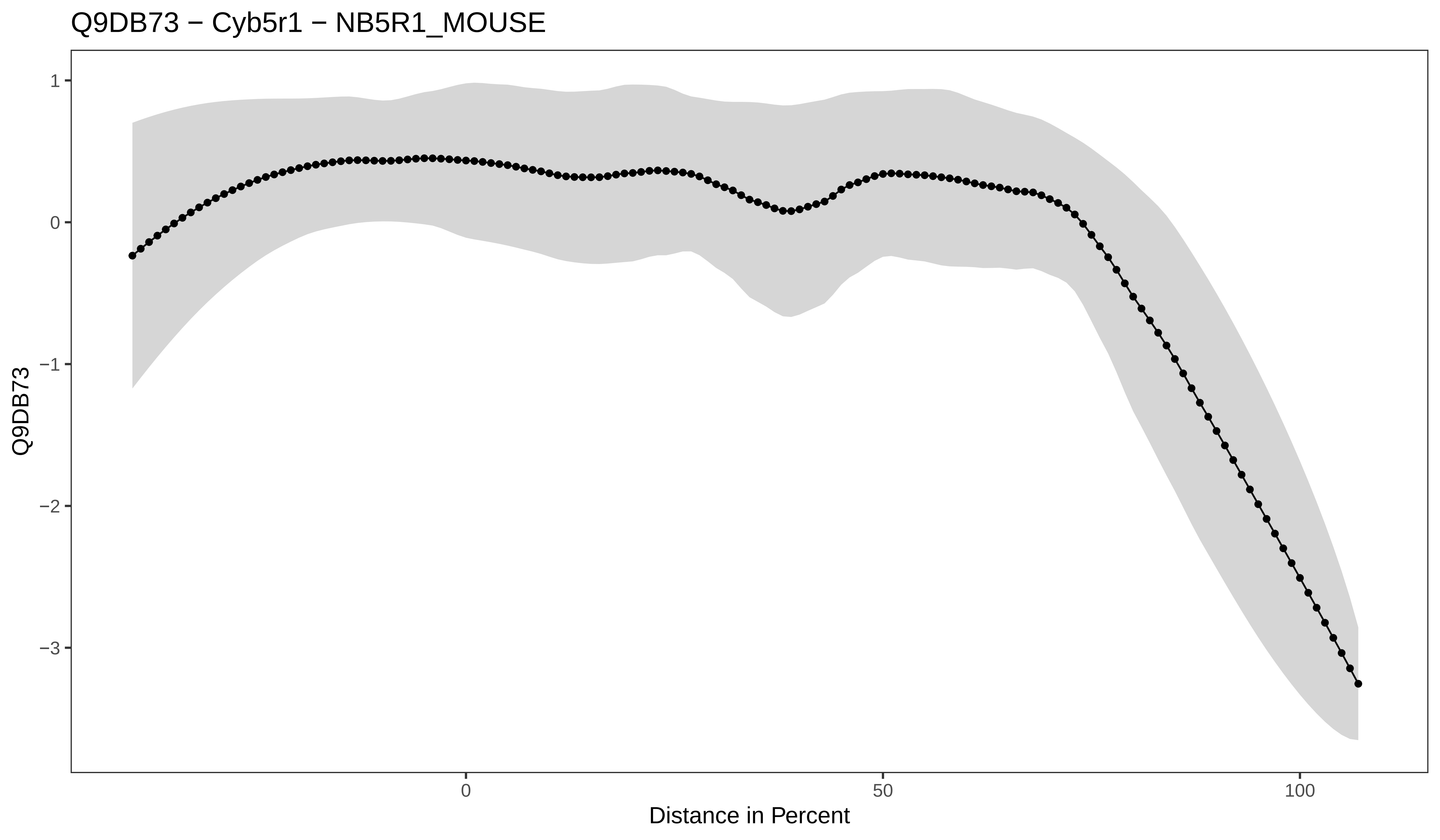

Q9DB73 − Cyb5r1 − NB5R1_MOUSE
1
0
−1
−2
−3
Q9DB73
0
50
100
Distance in Percent

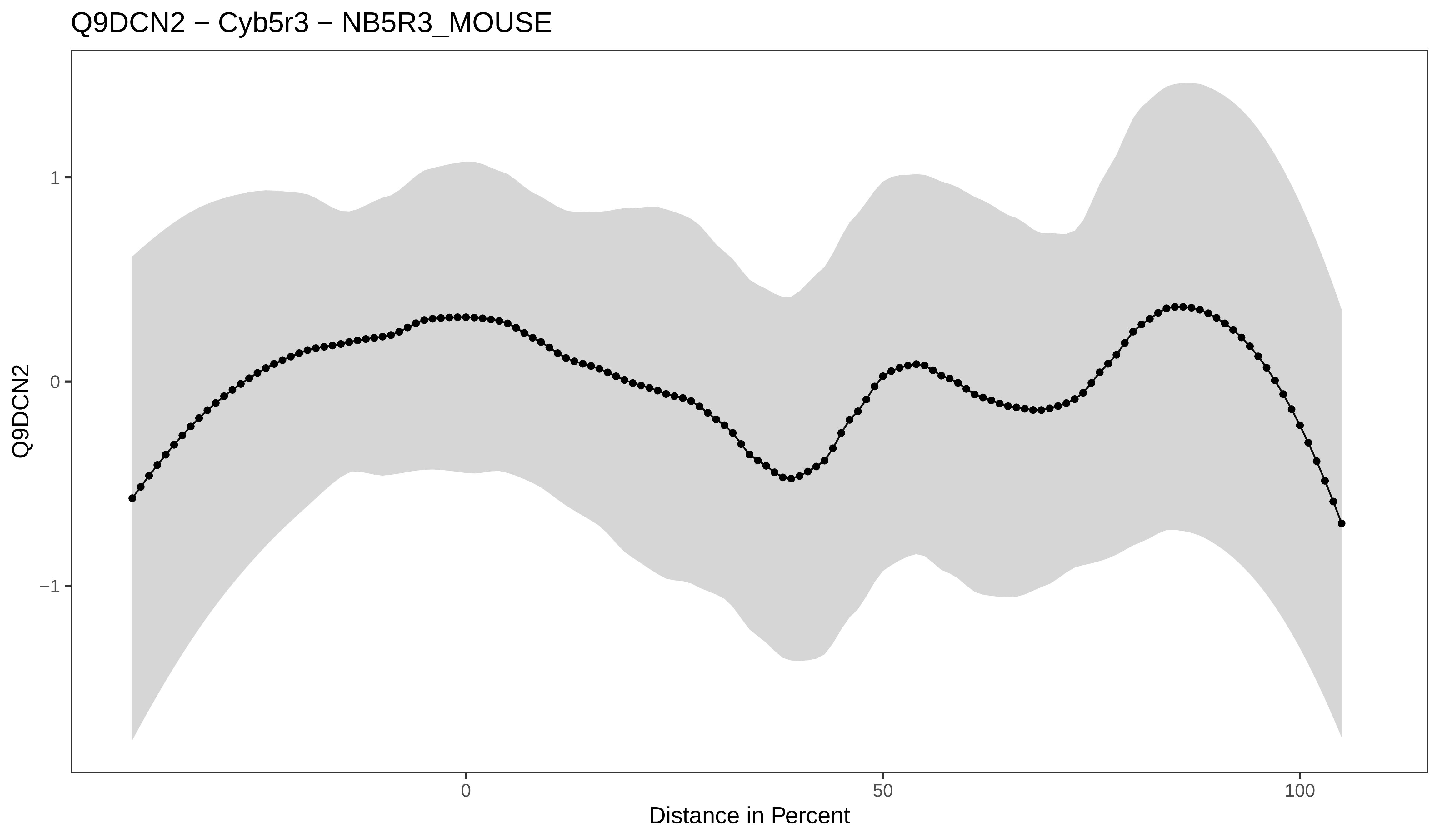
Q9DCN2 − Cyb5r3 − NB5R3_MOUSE
Q9DCN2
1
0
−1
0
50
100
Distance in Percent

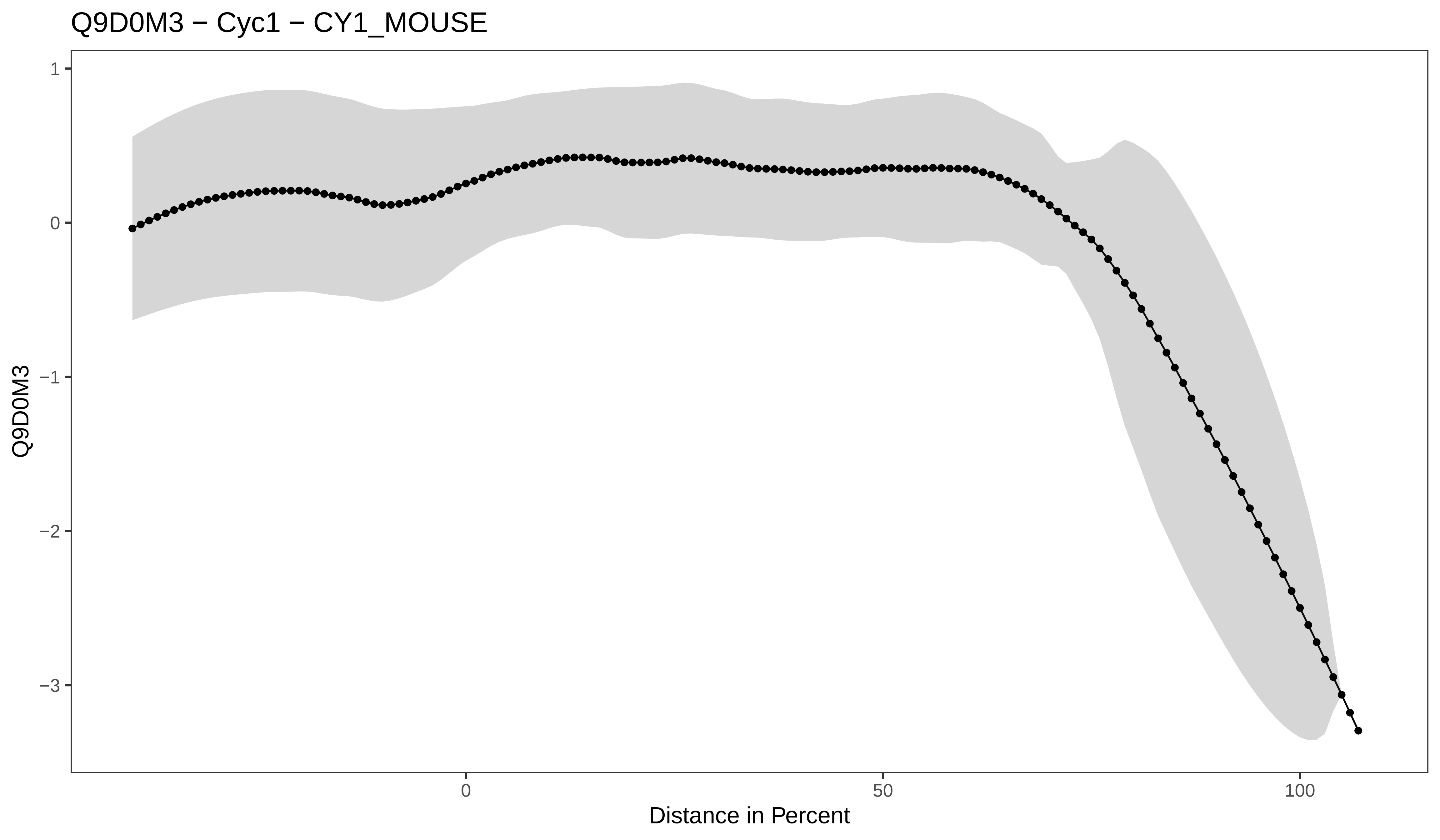

Q9D0M3 − Cyc1 − CY1_MOUSE
1
0
−1
−2
−3
Q9D0M3
0
50
100
Distance in Percent

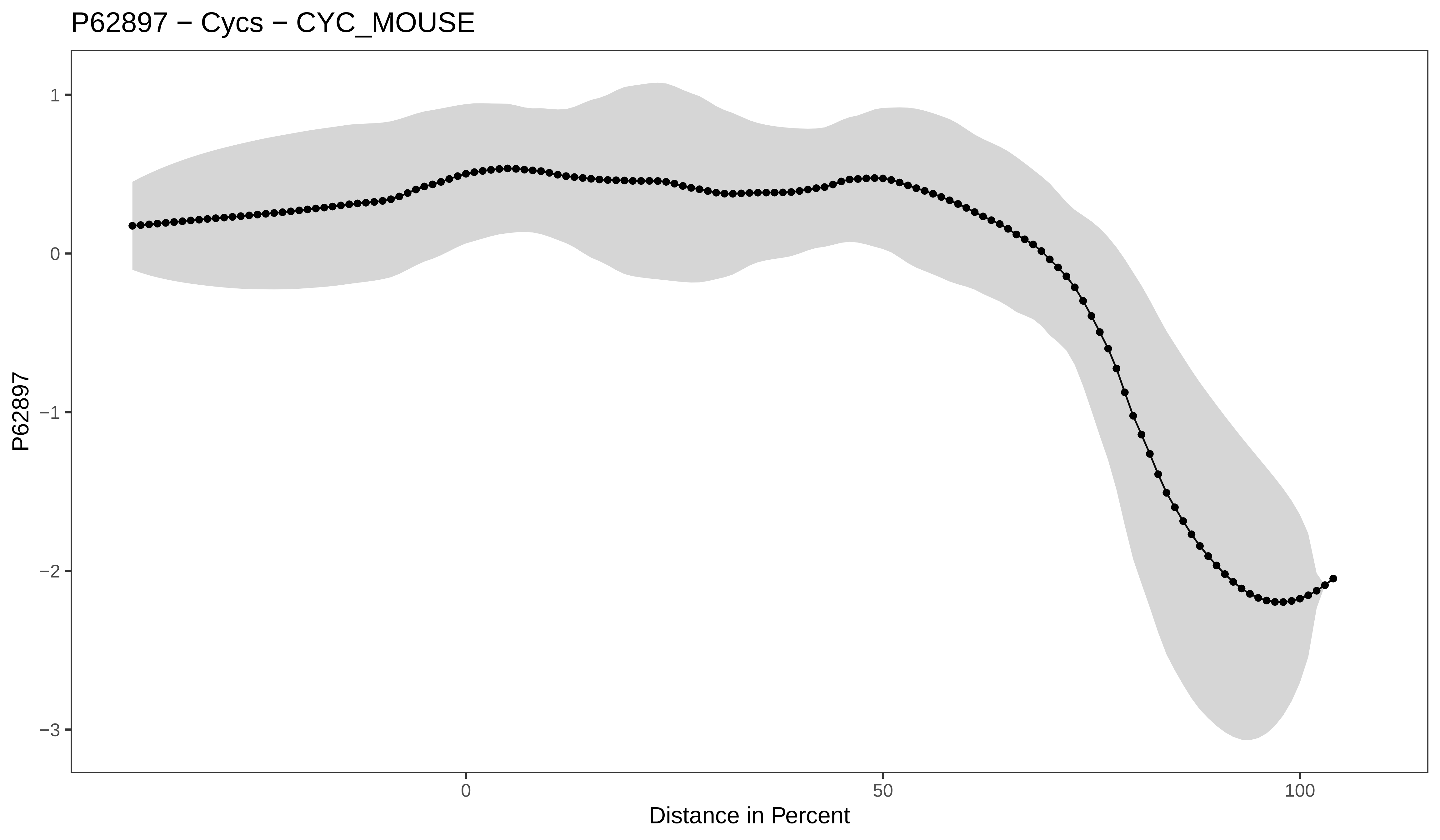

P62897 − Cycs − CYC_MOUSE
P62897
1
0
−1
−2
−3
0
50
100
Distance in Percent

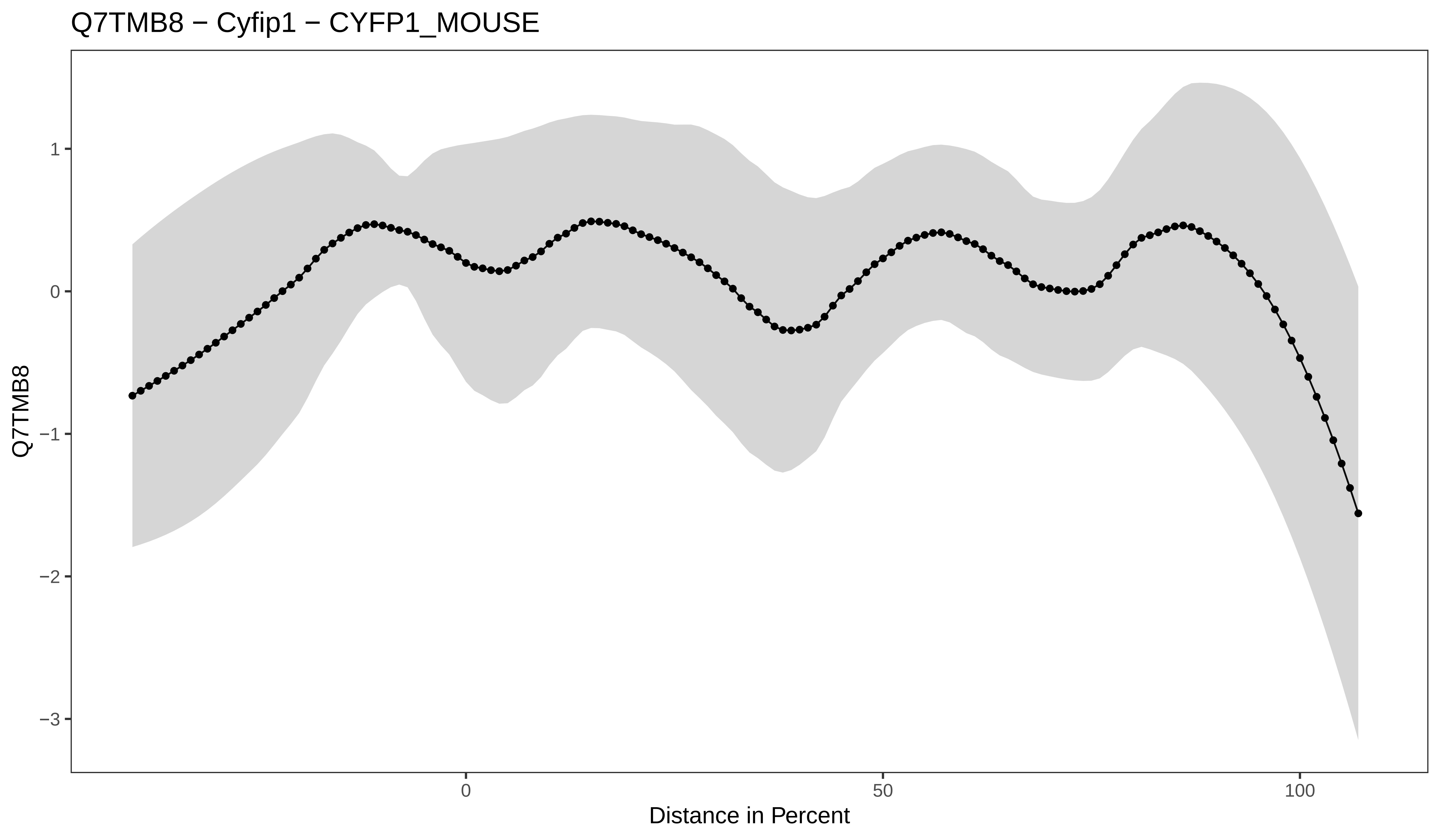

Q7TMB8 − Cyfip1 − CYFP1_MOUSE
Q7TMB8
1
0
−1
−2
−3
0
50
100
Distance in Percent

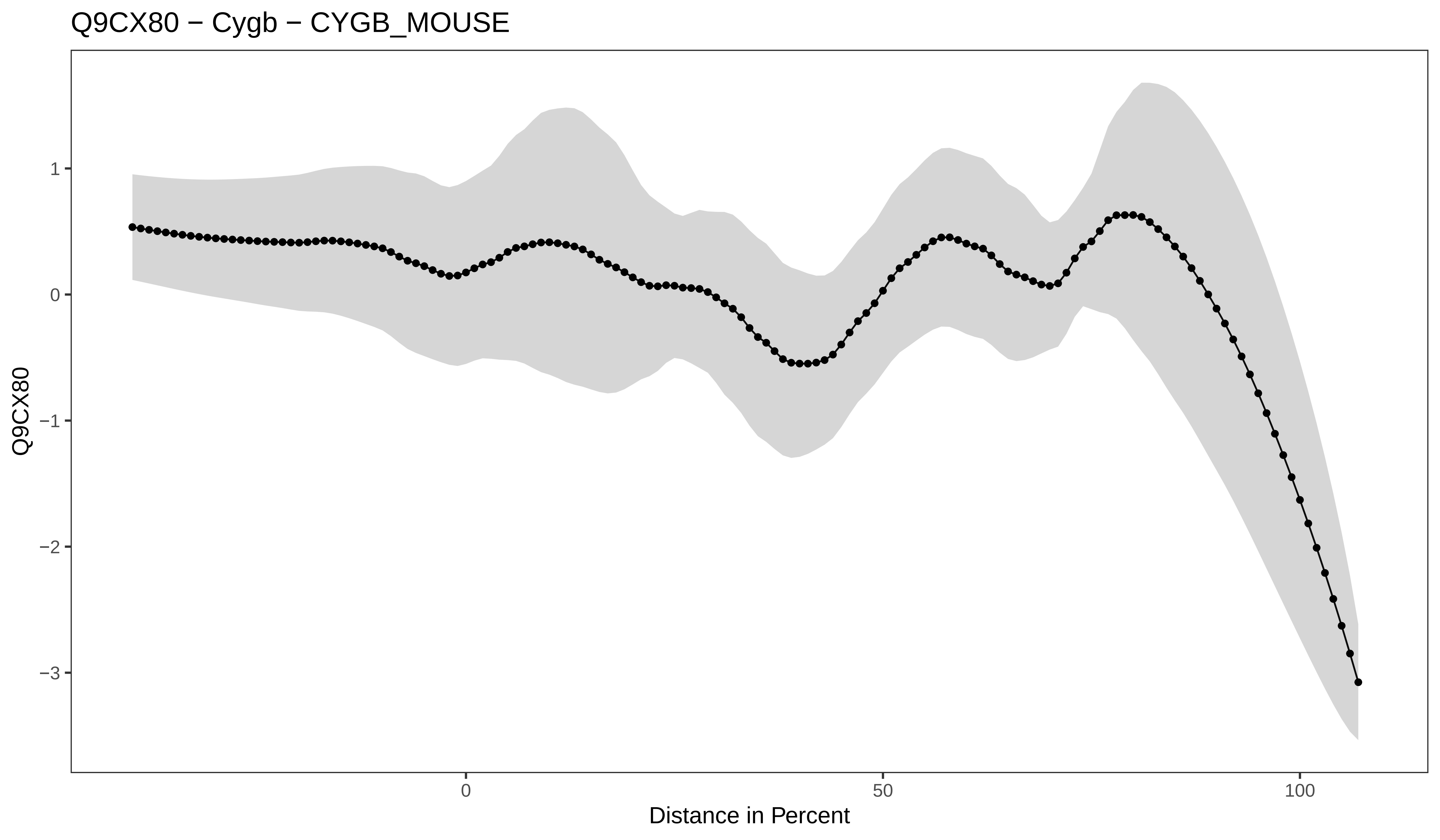

Q9CX80 − Cygb − CYGB_MOUSE
1
0
−1
−2
−3
Q9CX80
0
50
100
Distance in Percent

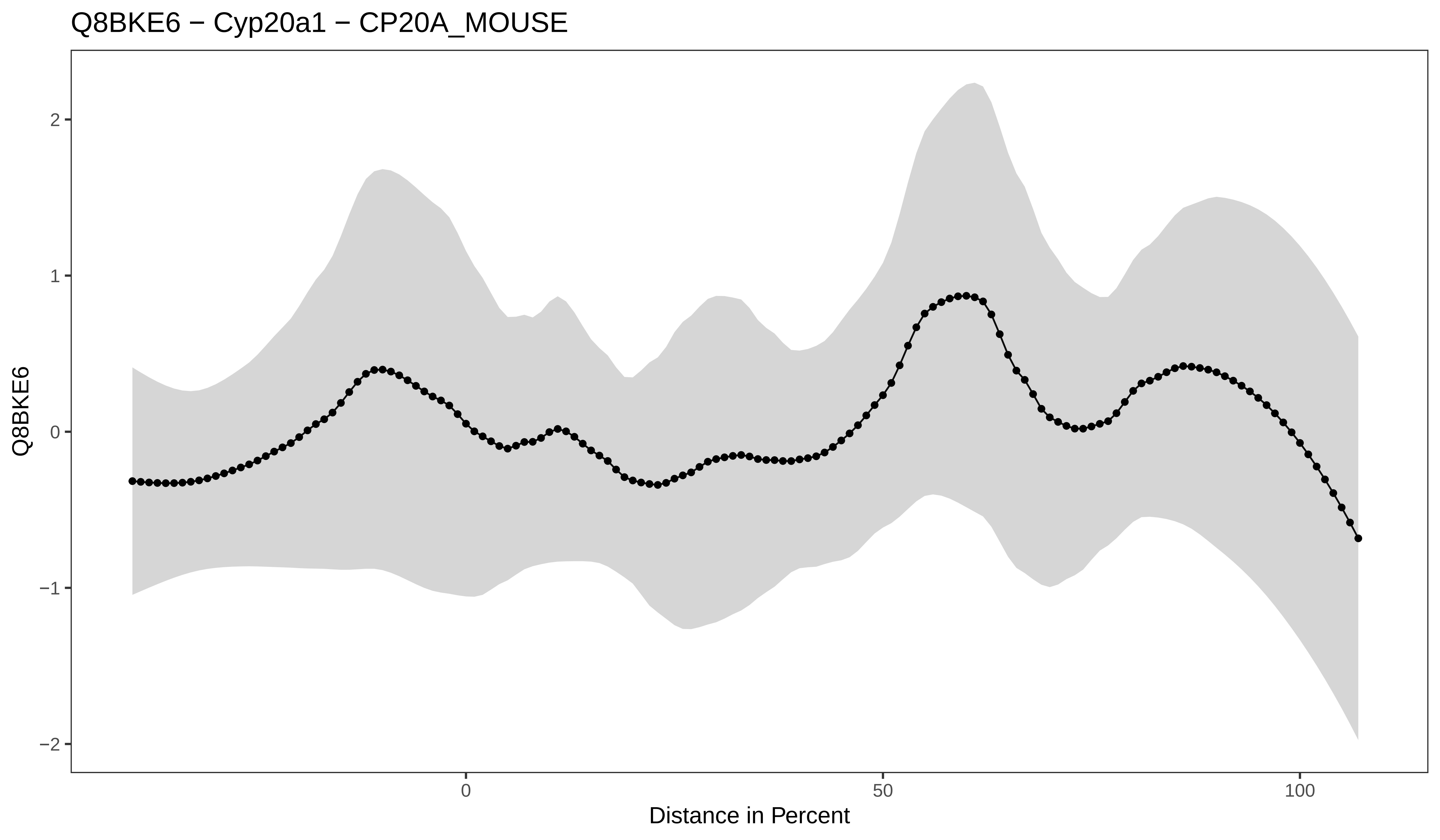

Q8BKE6 − Cyp20a1 − CP20A_MOUSE
Q8BKE6
Distance in Percent
2
1
0
−1
−2
0
50
100

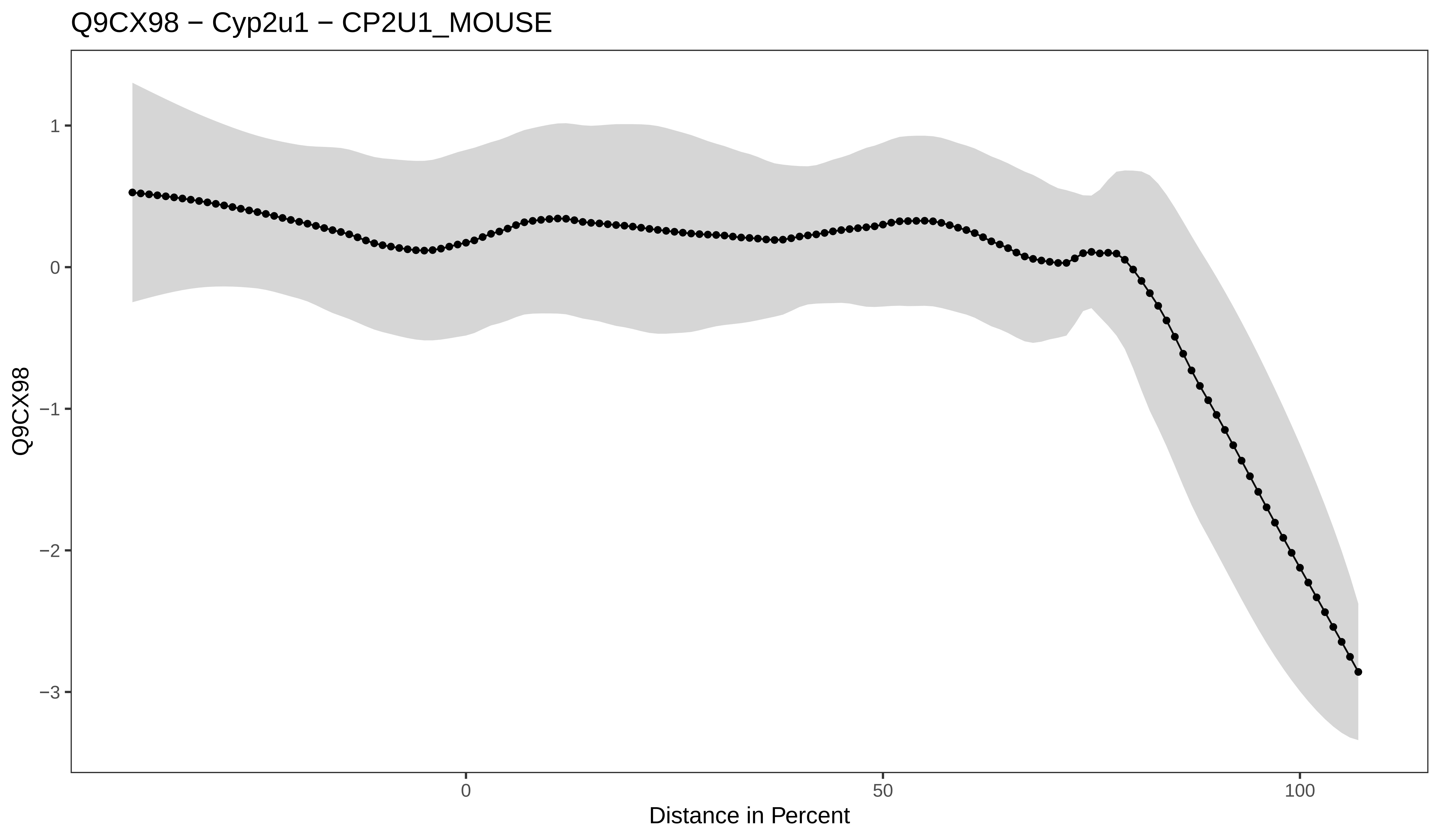

Q9CX98 − Cyp2u1 − CP2U1_MOUSE
Q9CX98
1
0
−1
−2
−3
0
50
100
Distance in Percent

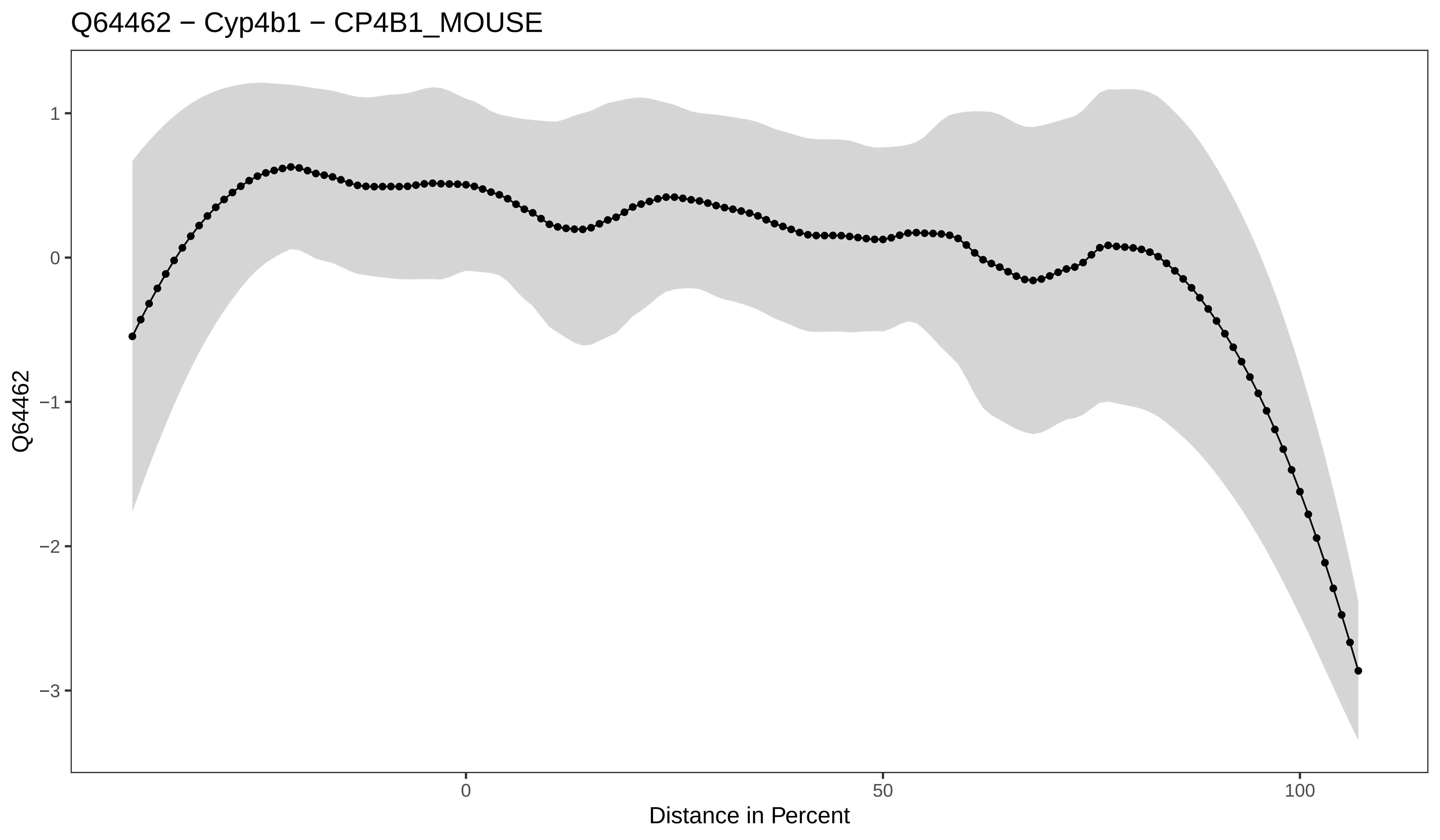

Q64462 − Cyp4b1 − CP4B1_MOUSE
1
0
−1
−2
−3
Q64462
0
50
100
Distance in Percent

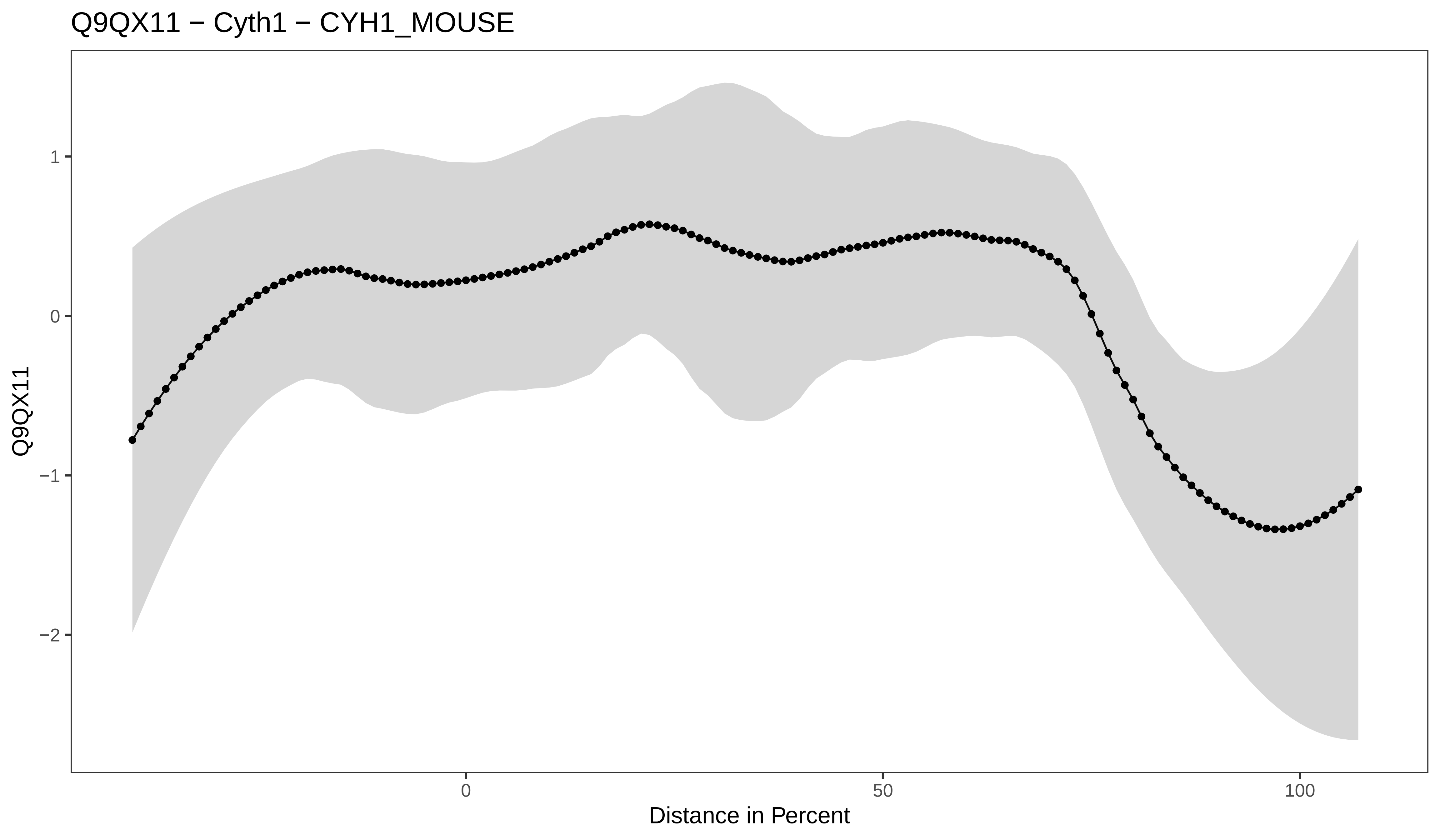
Q9QX11 − Cyth1 − CYH1_MOUSE
Q9QX11
1
0
−1
−2
0
50
100
Distance in Percent

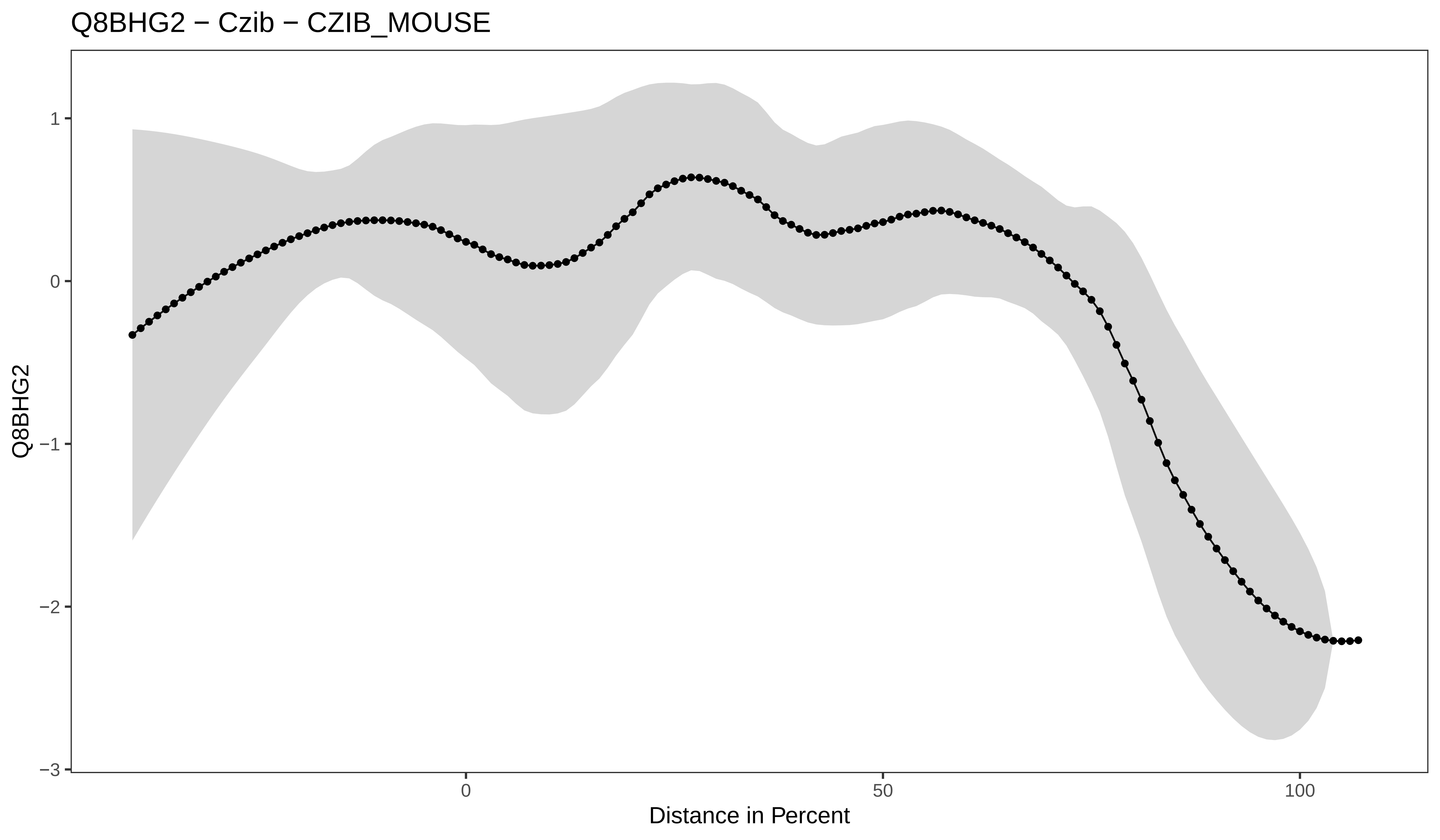

Q8BHG2 − Czib − CZIB_MOUSE
Q8BHG2
1
0
−1
−2
−3
0
50
100
Distance in Percent

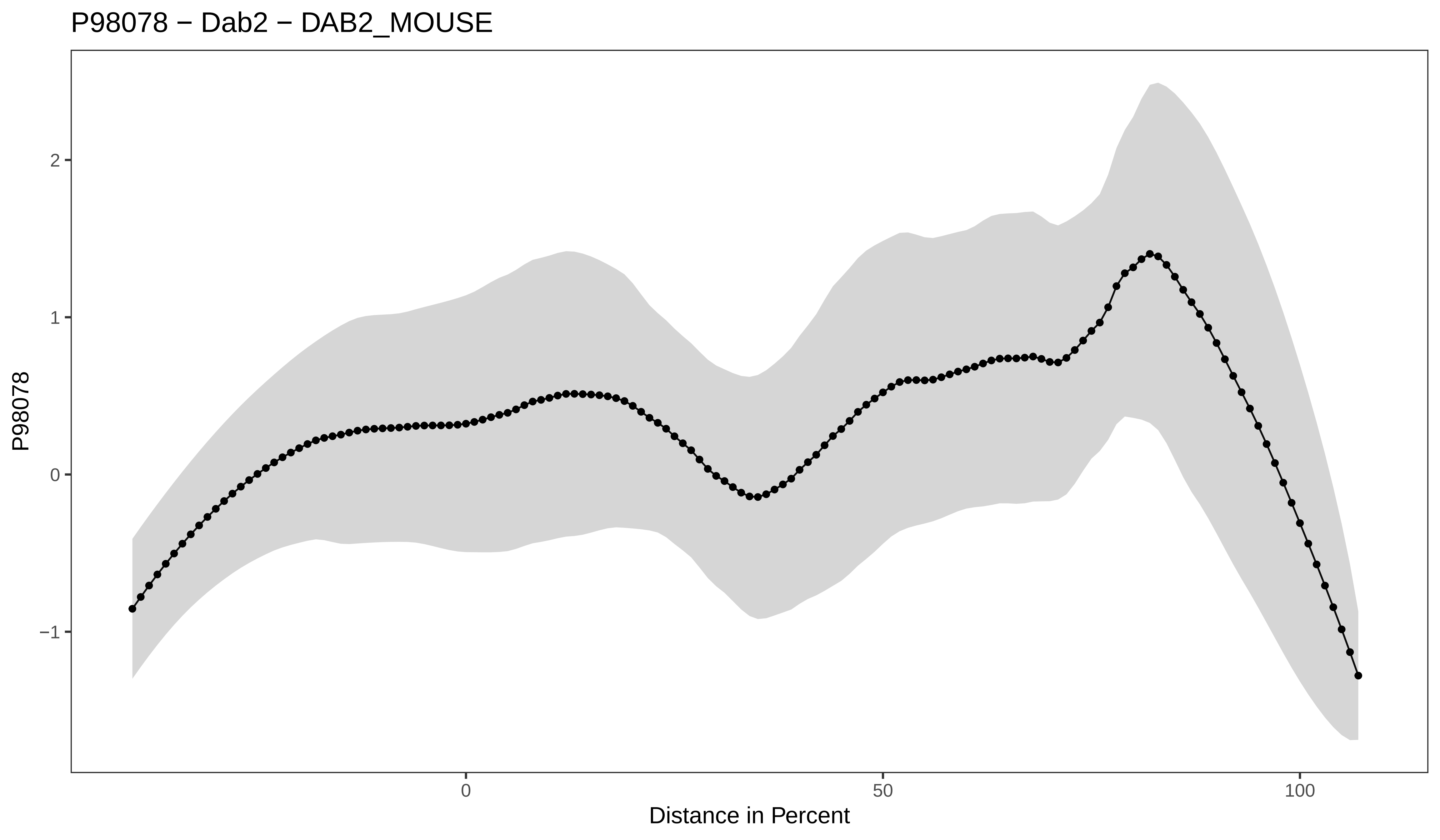

P98078 − Dab2 − DAB2_MOUSE
2
1
0
−1
P98078
0
50
100
Distance in Percent

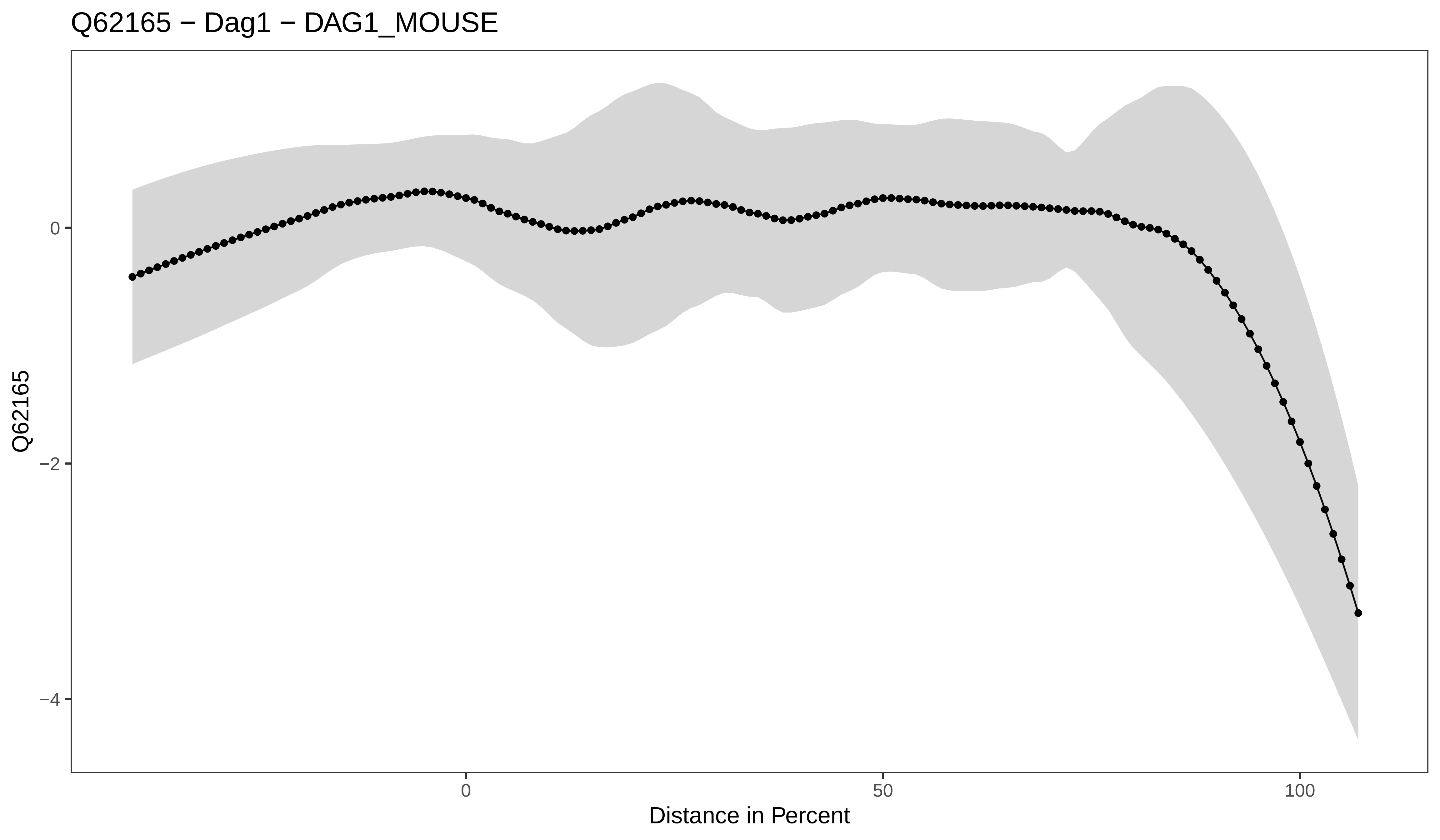
Q62165 − Dag1 − DAG1_MOUSE
0
−2
−4
Q62165
0
50
100
Distance in Percent

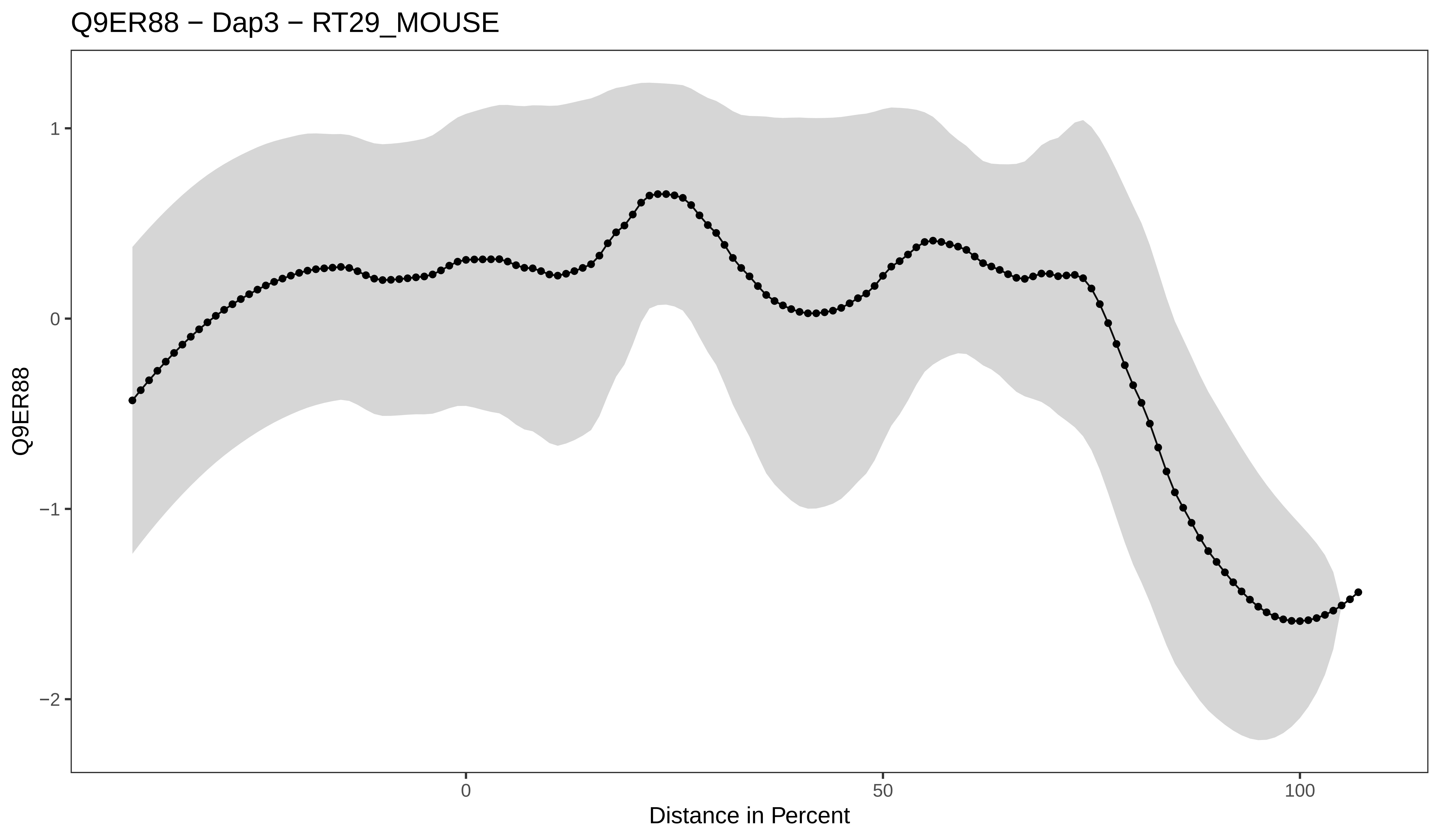

Q9ER88 − Dap3 − RT29_MOUSE
Q9ER88
1
0
−1
−2
0
50
100
Distance in Percent

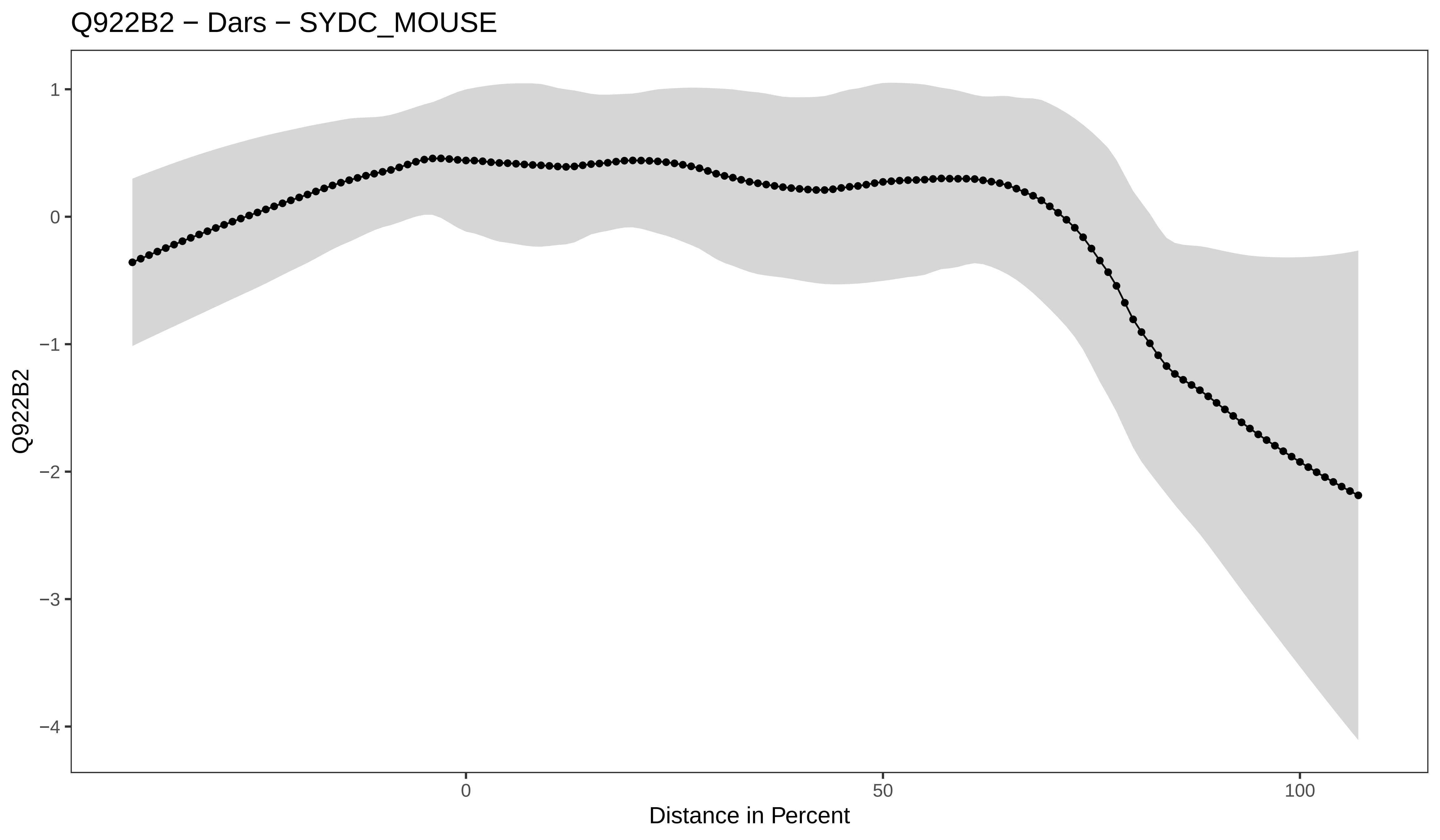
Q922B2 − Dars − SYDC_MOUSE
Q922B2
Distance in Percent
1
0
−1
−2
−3
−4
0
50
100

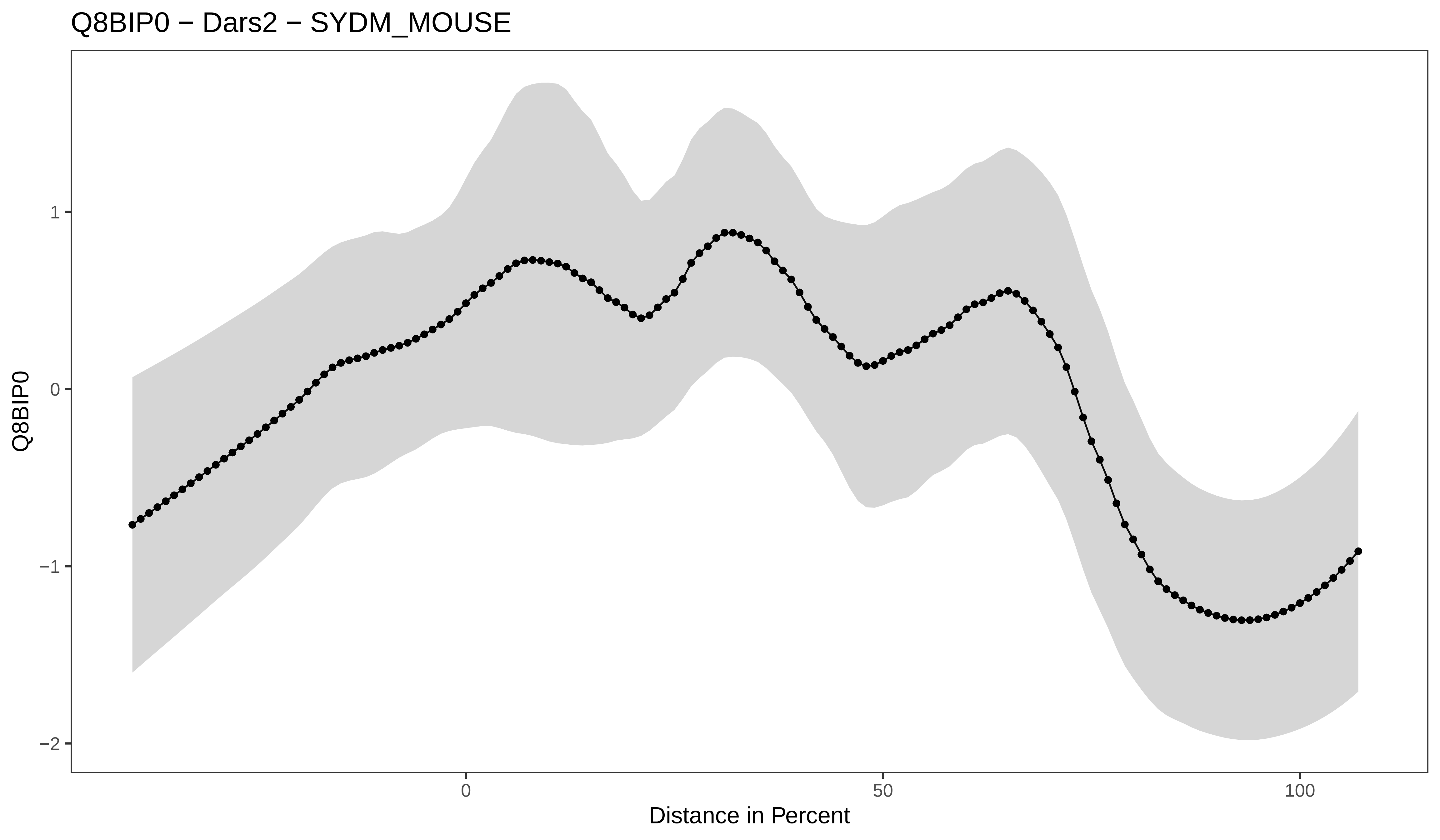
Q8BIP0 − Dars2 − SYDM_MOUSE
Q8BIP0
1
0
−1
−2
0
50
100
Distance in Percent

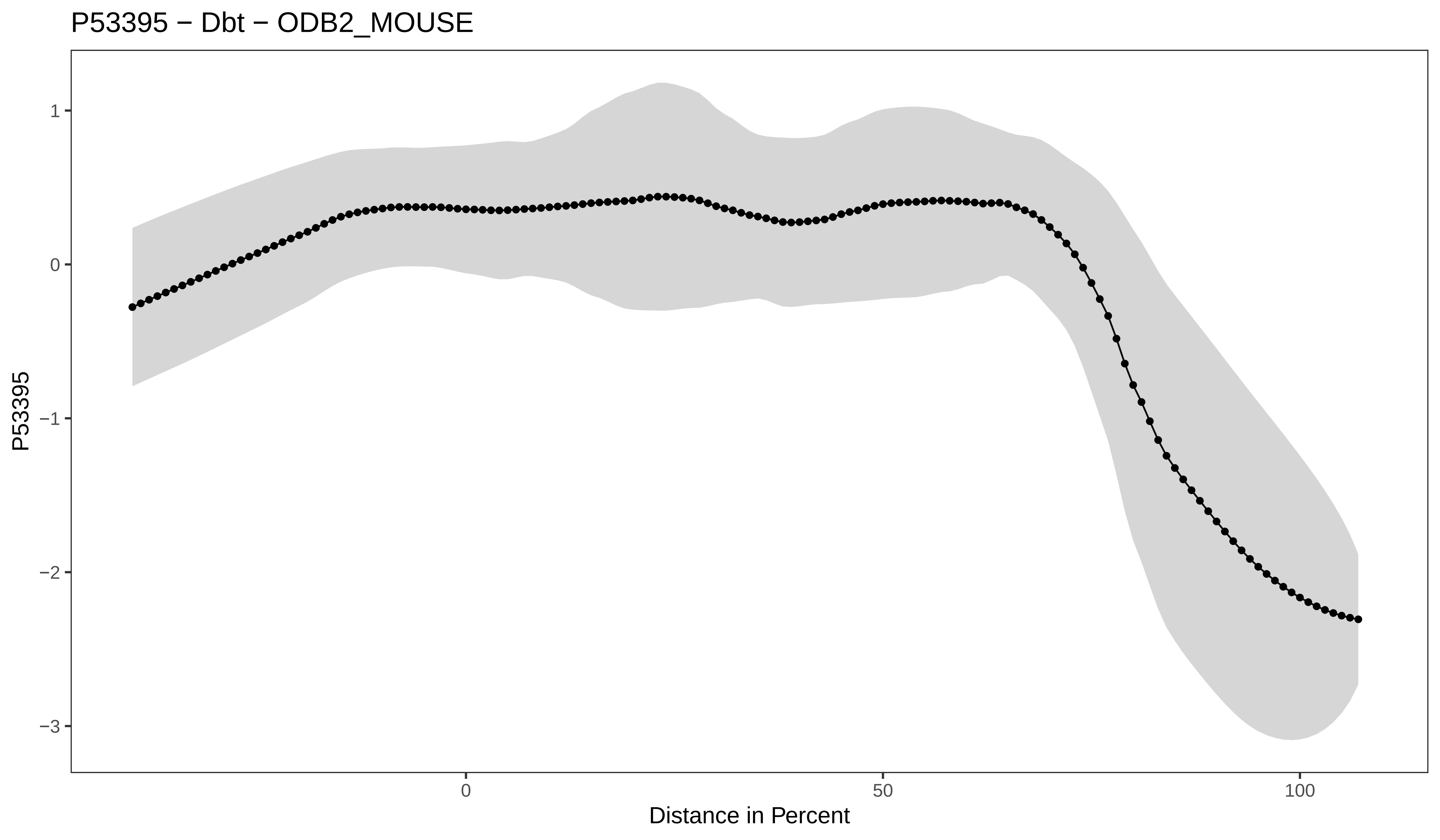

P53395 − Dbt − ODB2_MOUSE
P53395
1
0
−1
−2
−3
0
50
100
Distance in Percent

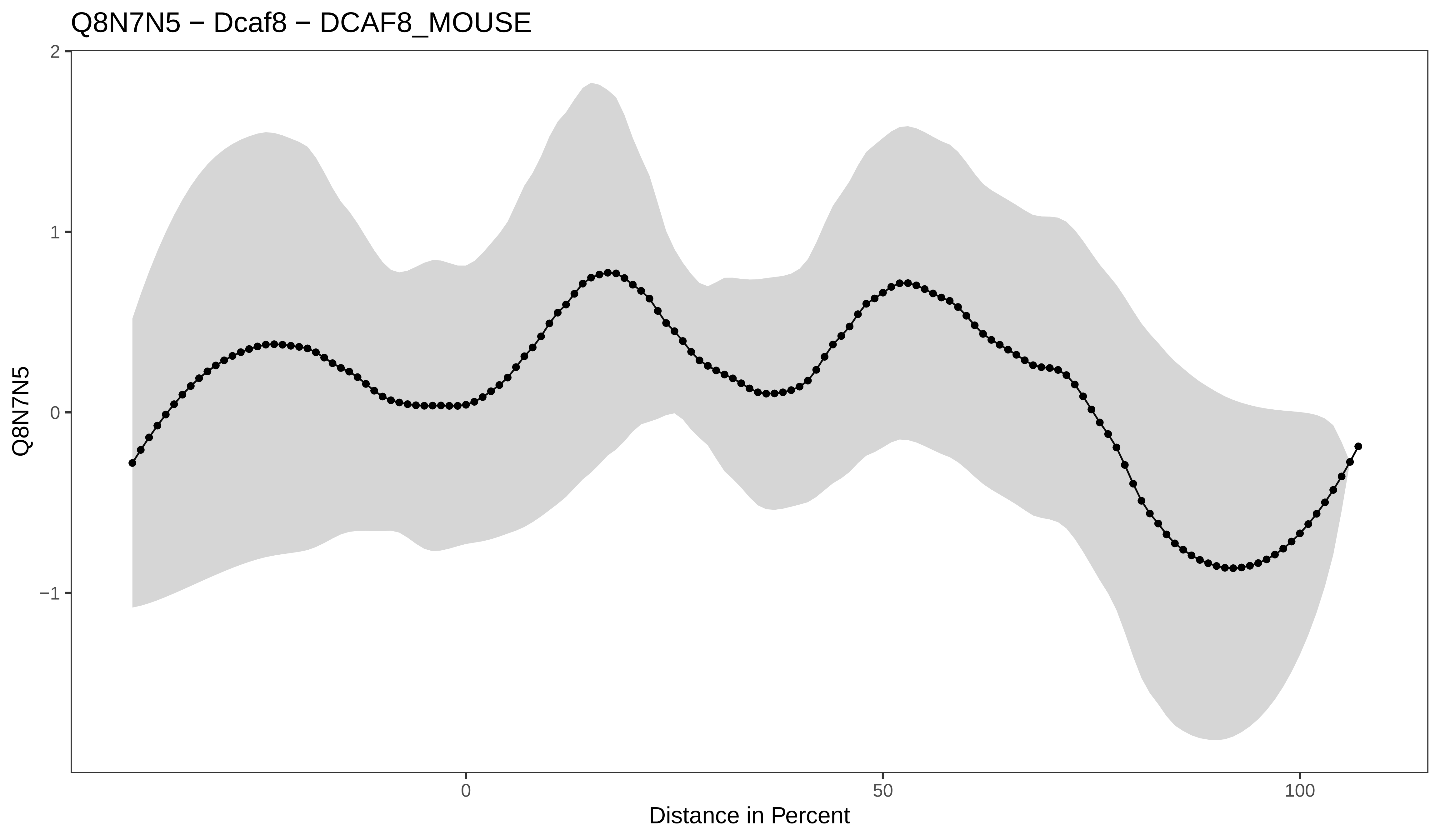
Q8N7N5 − Dcaf8 − DCAF8_MOUSE
2
1
0
−1
Q8N7N5
0
50
100
Distance in Percent

Q8BHC4 − Dcakd − DCAKD_MOUSE

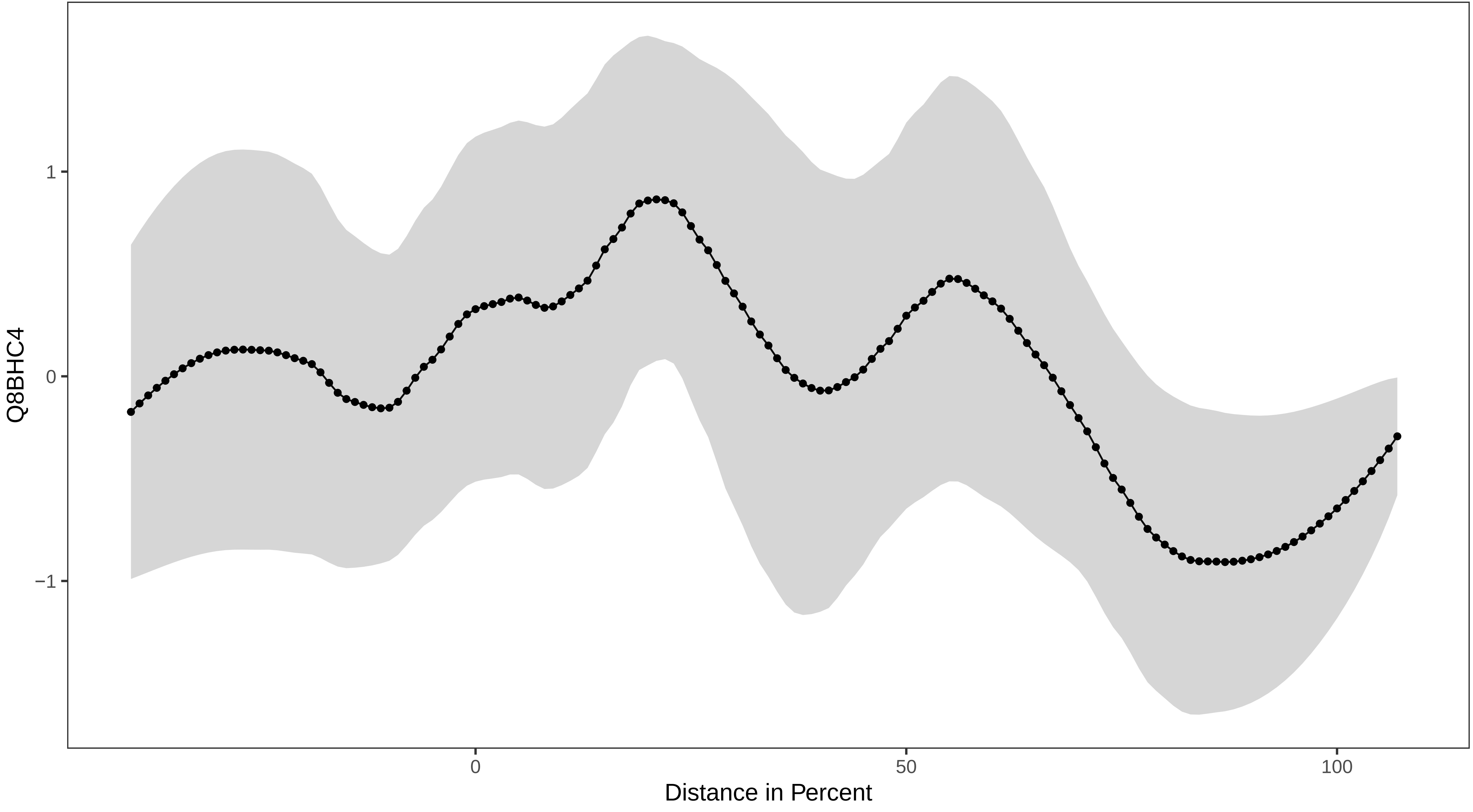

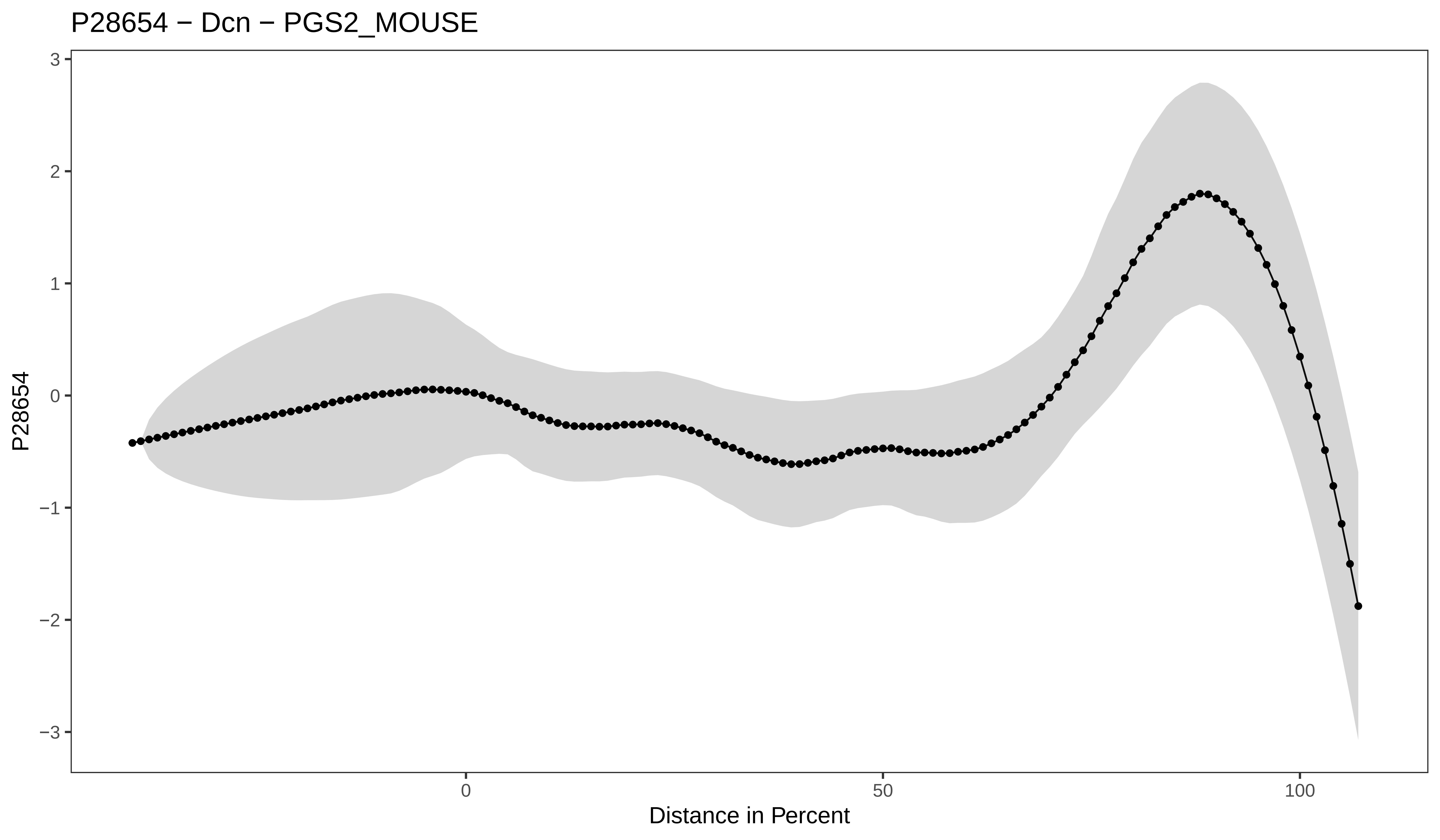
P28654 − Dcn − PGS2_MOUSE
3
2
1
0
−1
−2
−3
P28654
0
50
100
Distance in Percent

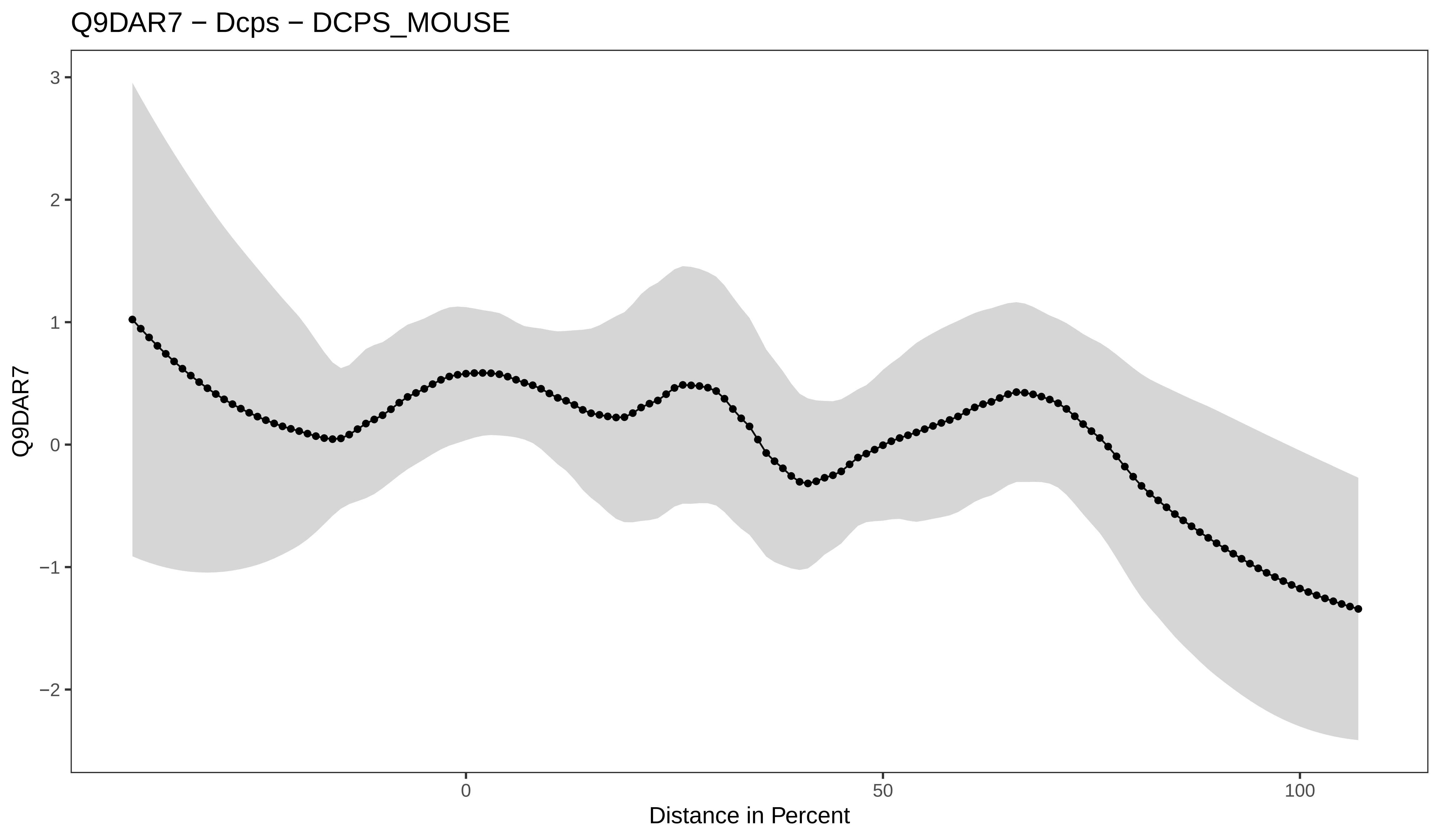

Q9DAR7 − Dcps − DCPS_MOUSE
Q9DAR7
3
2
1
0
−1
−2
0
50
100
Distance in Percent

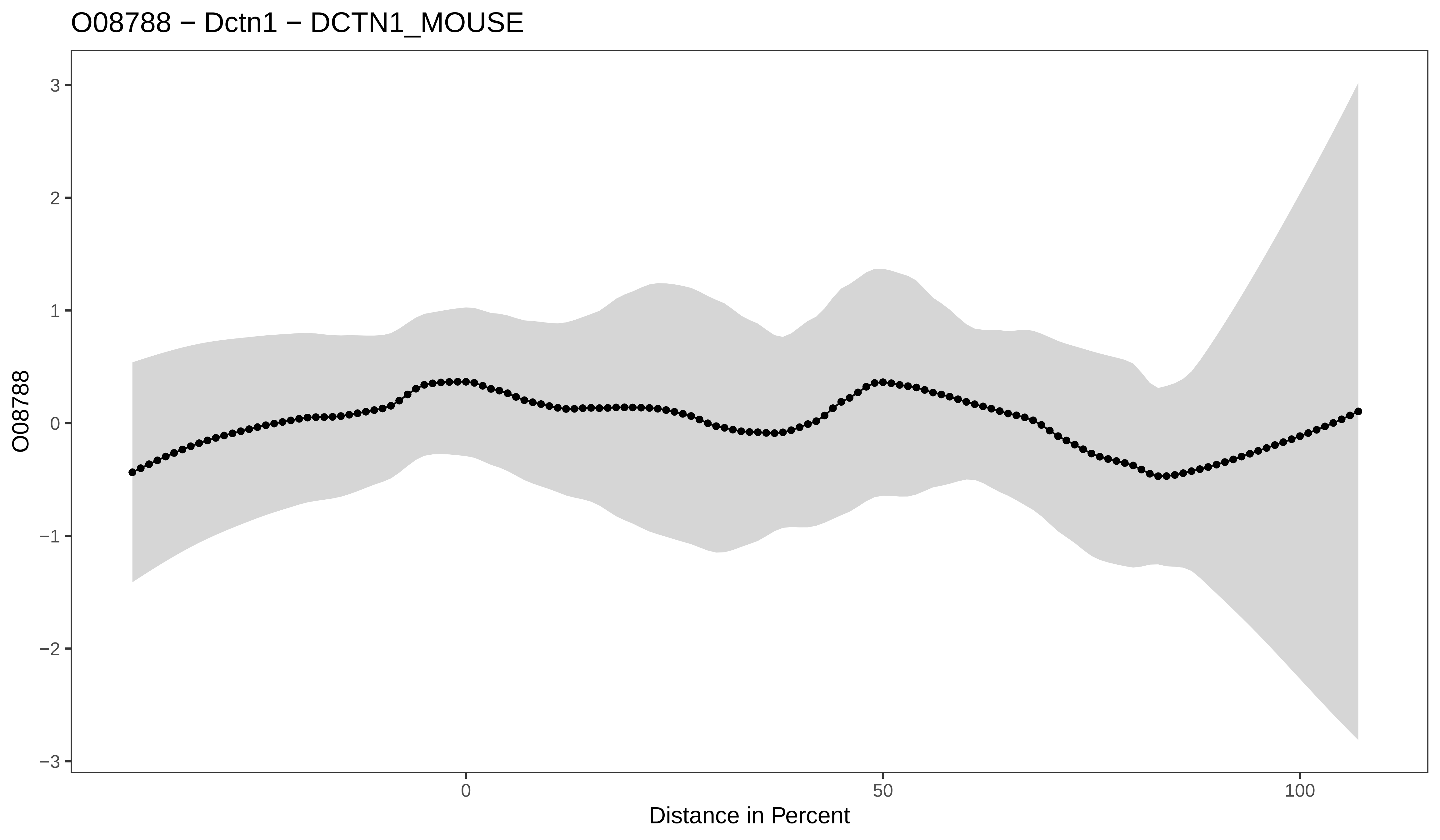
O08788 − Dctn1 − DCTN1_MOUSE
3
2
1
0
−1
−2
−3
O08788
0
50
100
Distance in Percent

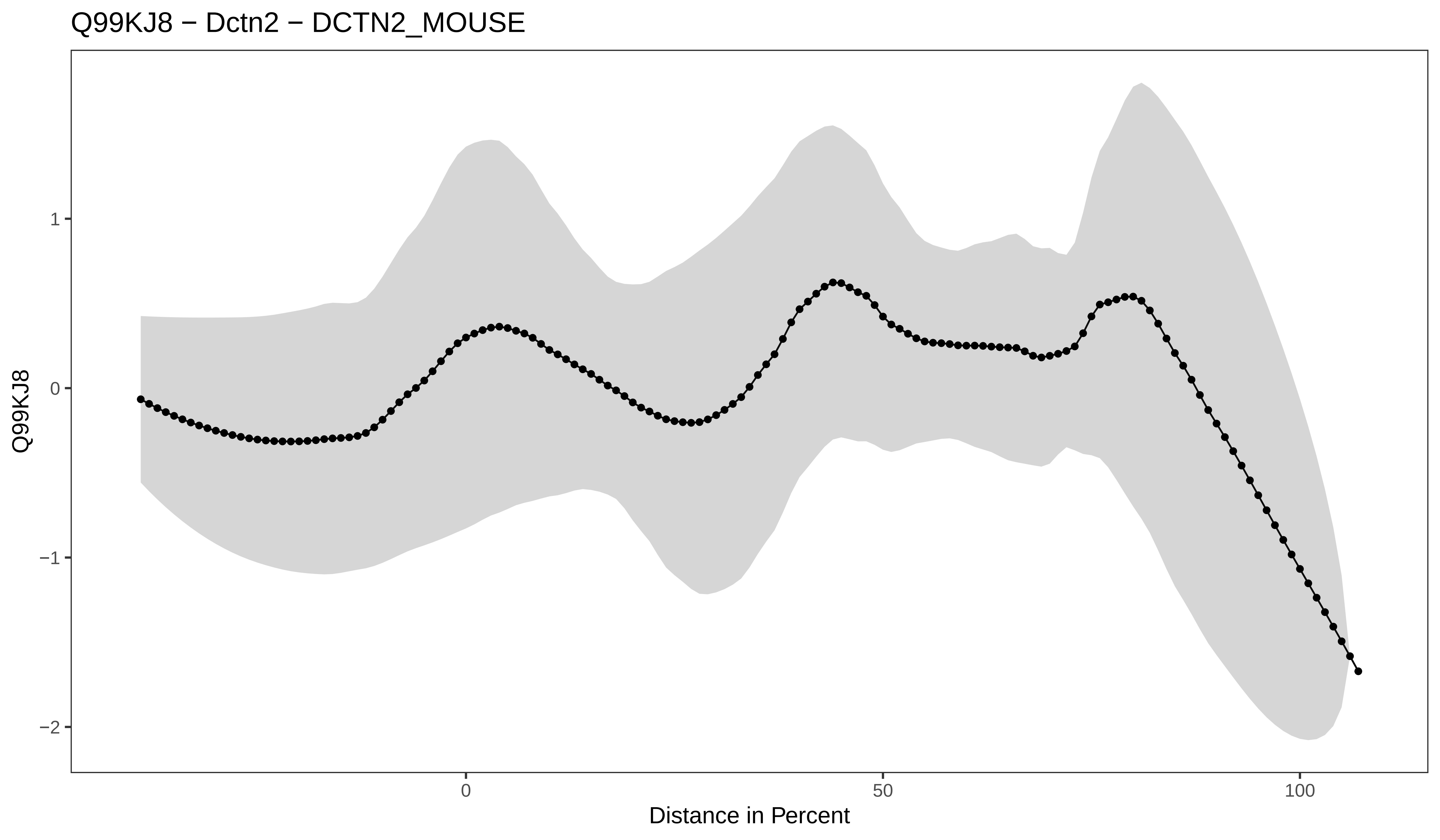

Q99KJ8 − Dctn2 − DCTN2_MOUSE
Q99KJ8
1
0
−1
−2
0
50
100
Distance in Percent

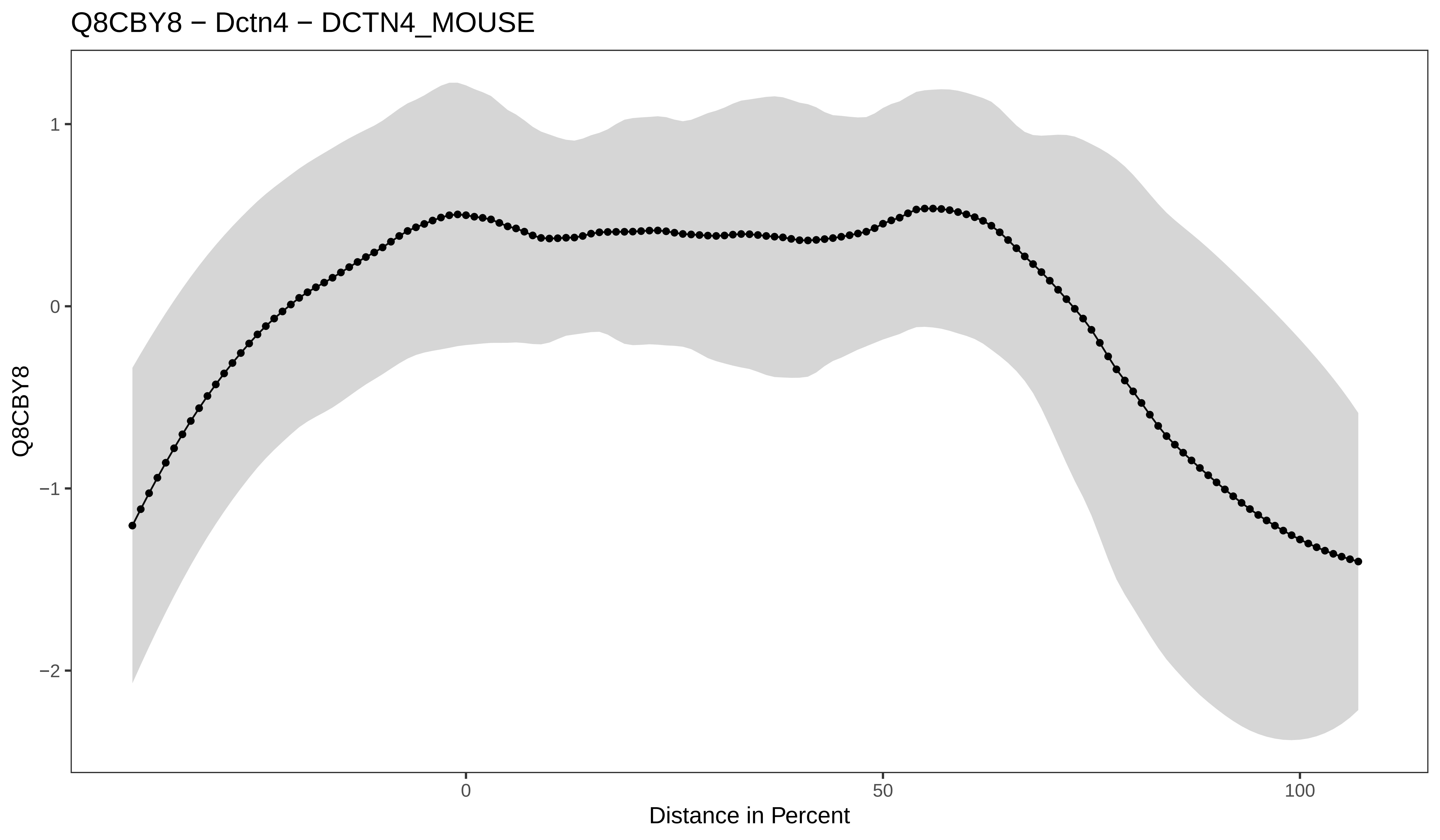

Q8CBY8 − Dctn4 − DCTN4_MOUSE
1
0
−1
−2
Q8CBY8
0
50
100
Distance in Percent

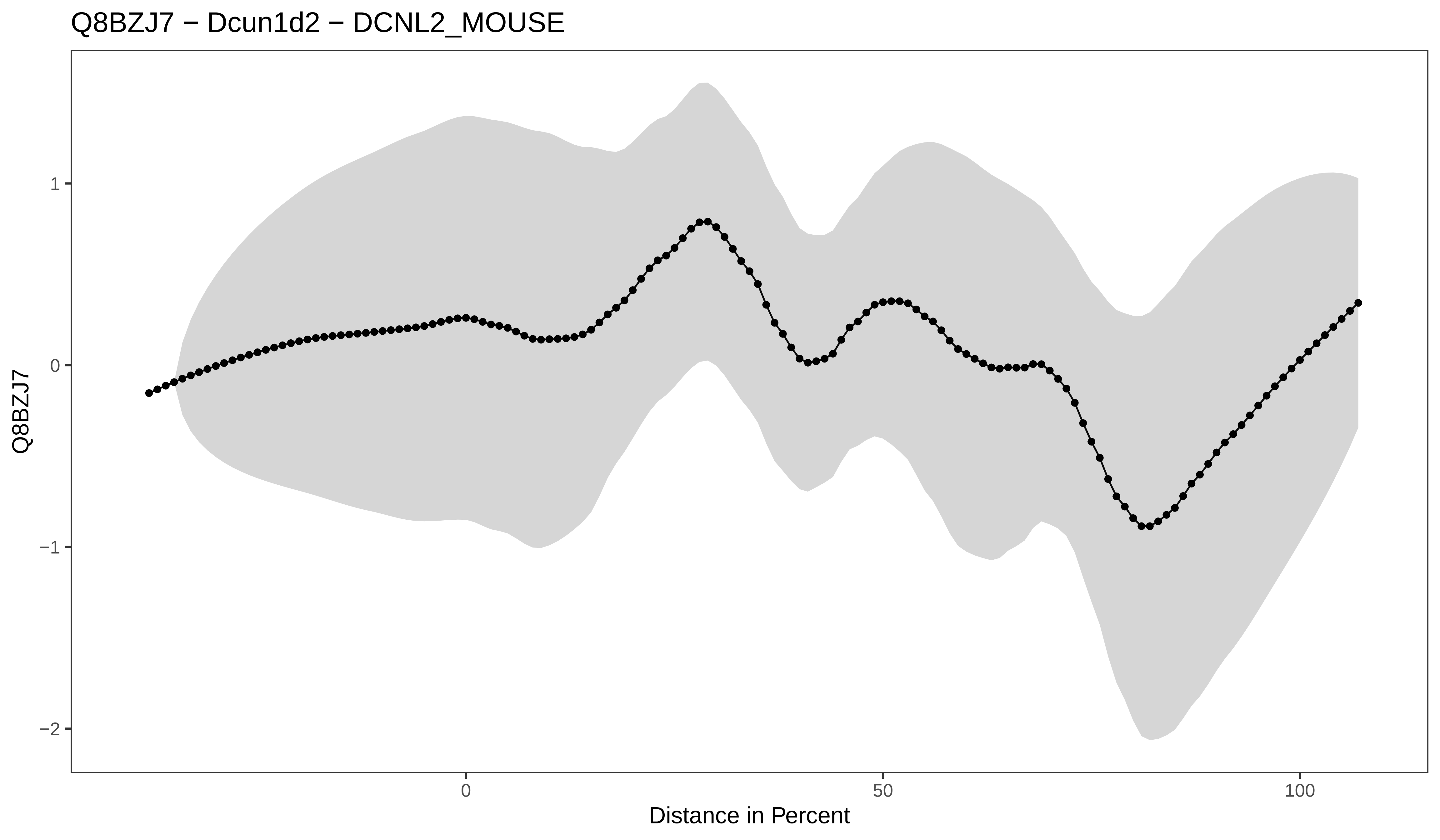
Q8BZJ7 − Dcun1d2 − DCNL2_MOUSE
Q8BZJ7
1
0
−1
−2
0
50
100
Distance in Percent

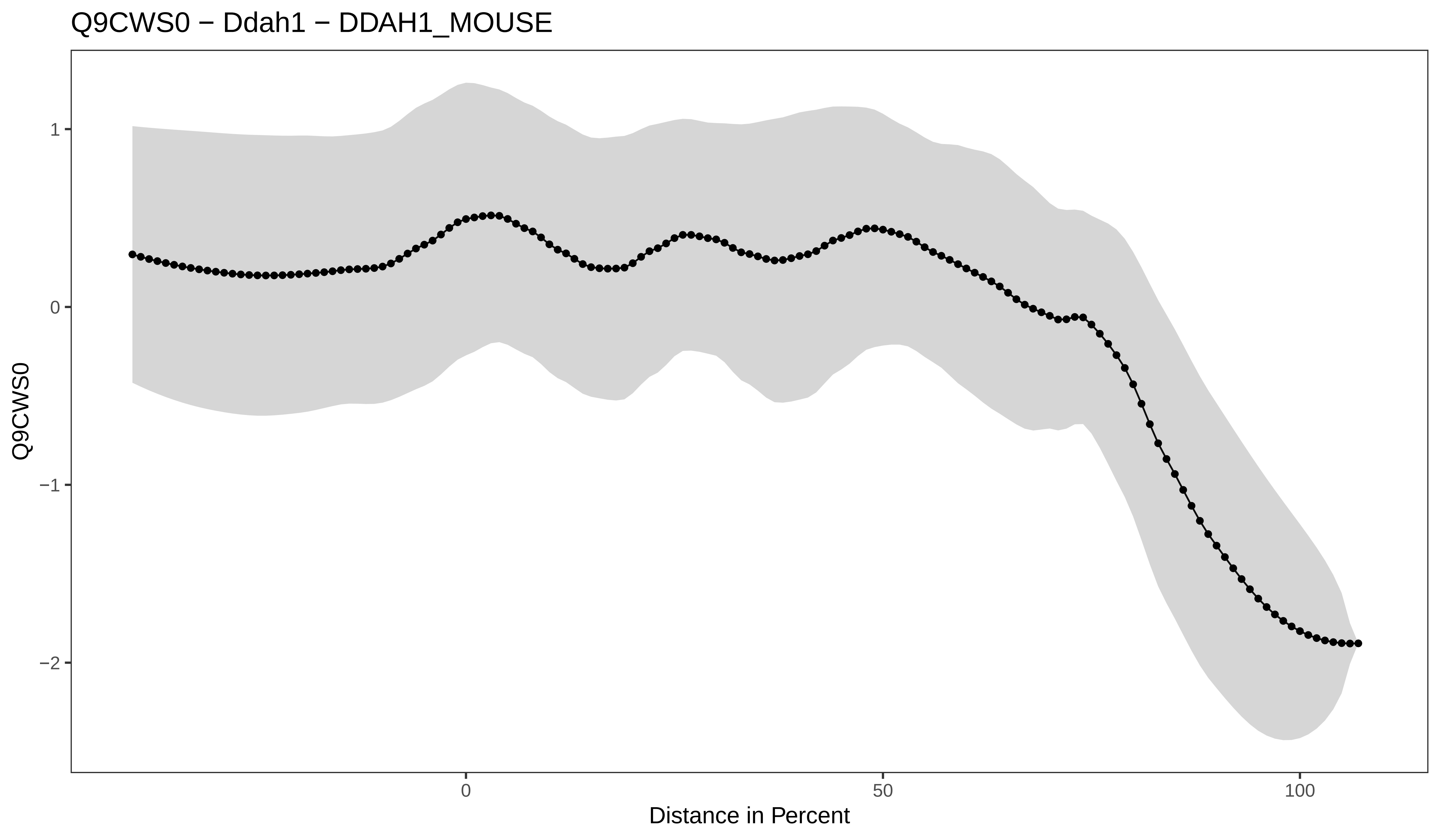

Q9CWS0 − Ddah1 − DDAH1_MOUSE
1
0
−1
−2
Q9CWS0
0
50
100
Distance in Percent

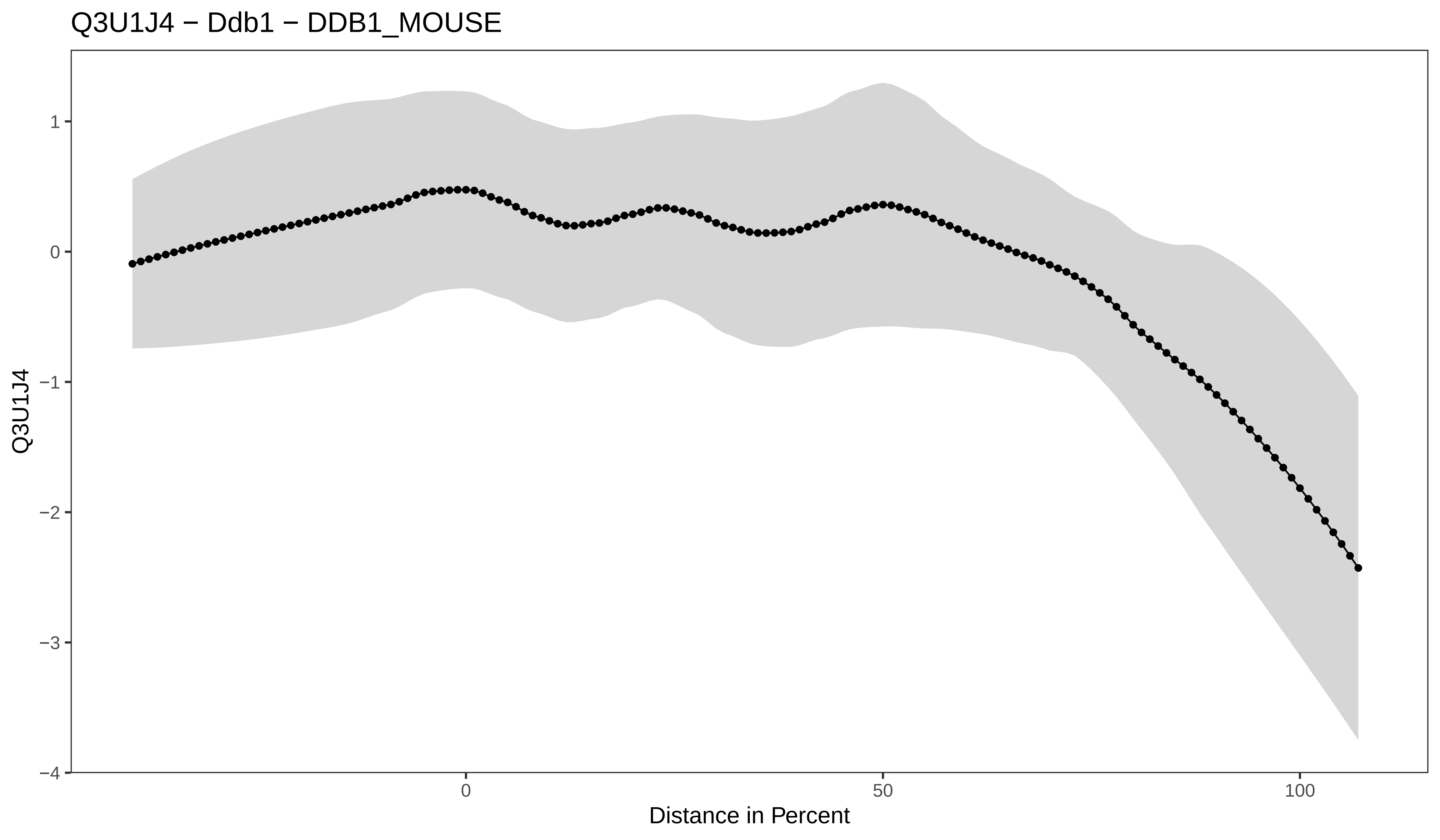

Q3U1J4 − Ddb1 − DDB1_MOUSE
Q3U1J4
1
0
−1
−2
−3
−4
0
50
100
Distance in Percent

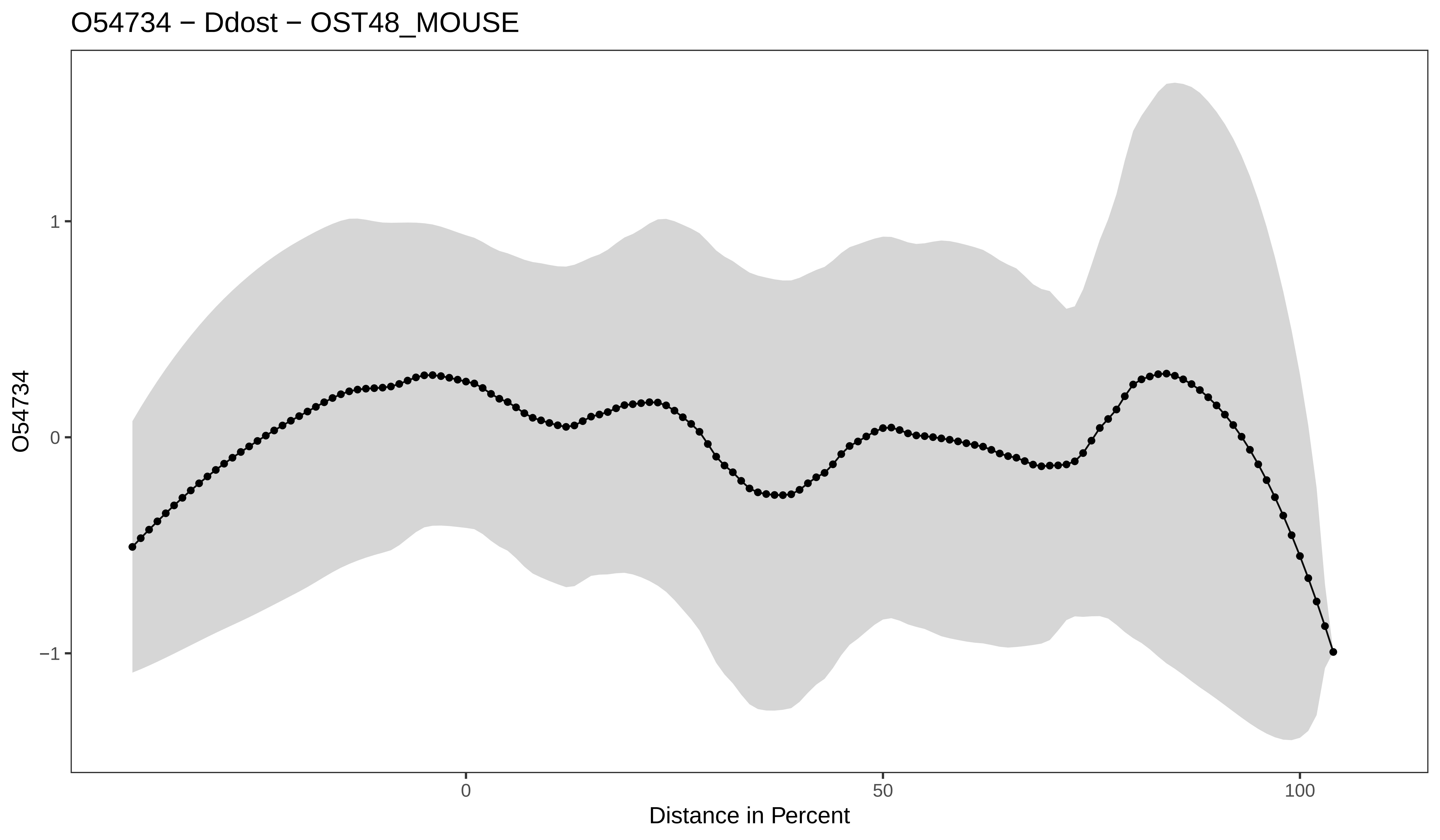

O54734 − Ddost − OST48_MOUSE
O54734
1
0
−1
0
50
100
Distance in Percent

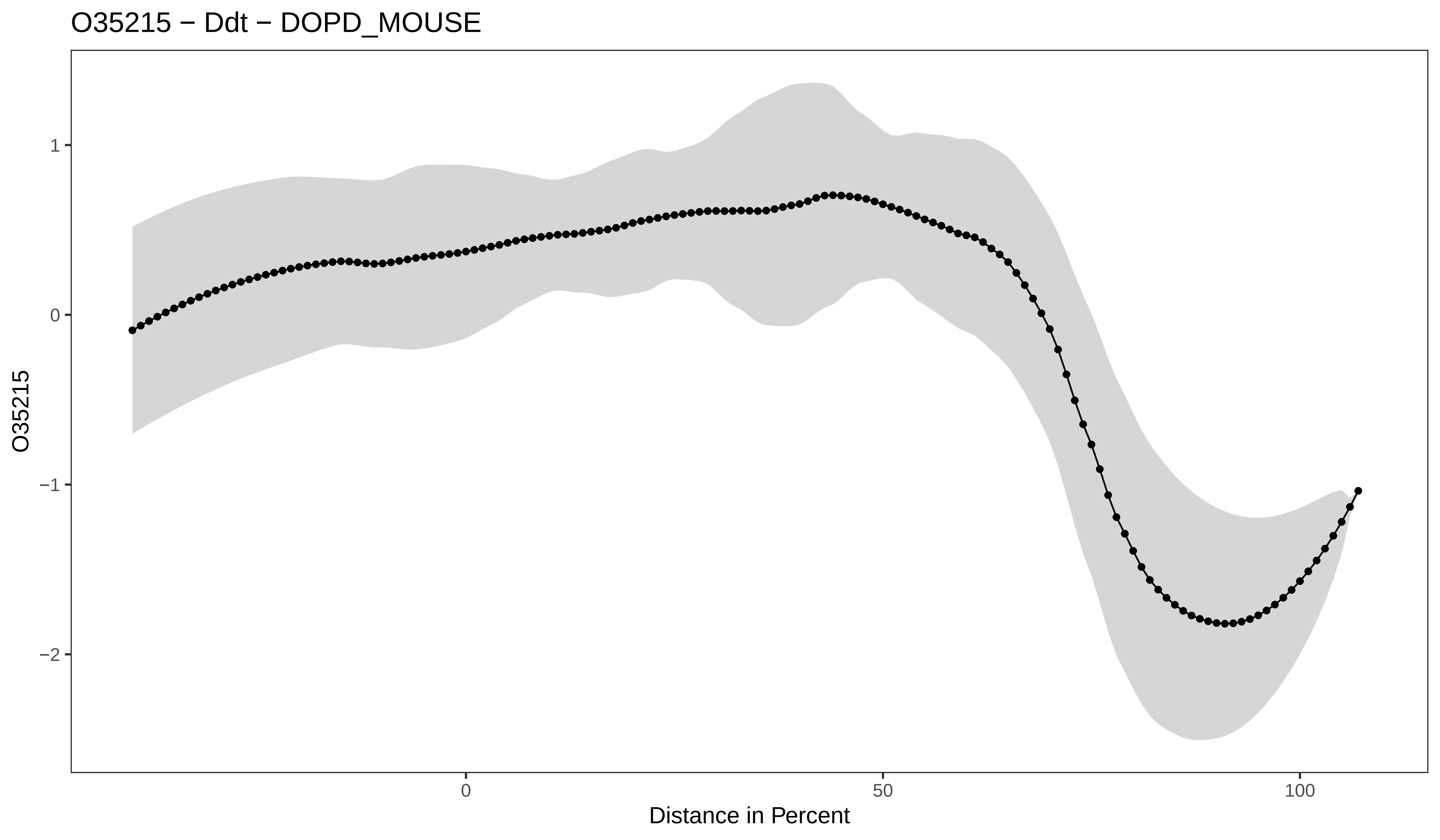
O35215 − Ddt − DOPD_MOUSE
O35215
1
0
−1
−2
0
50
100
Distance in Percent

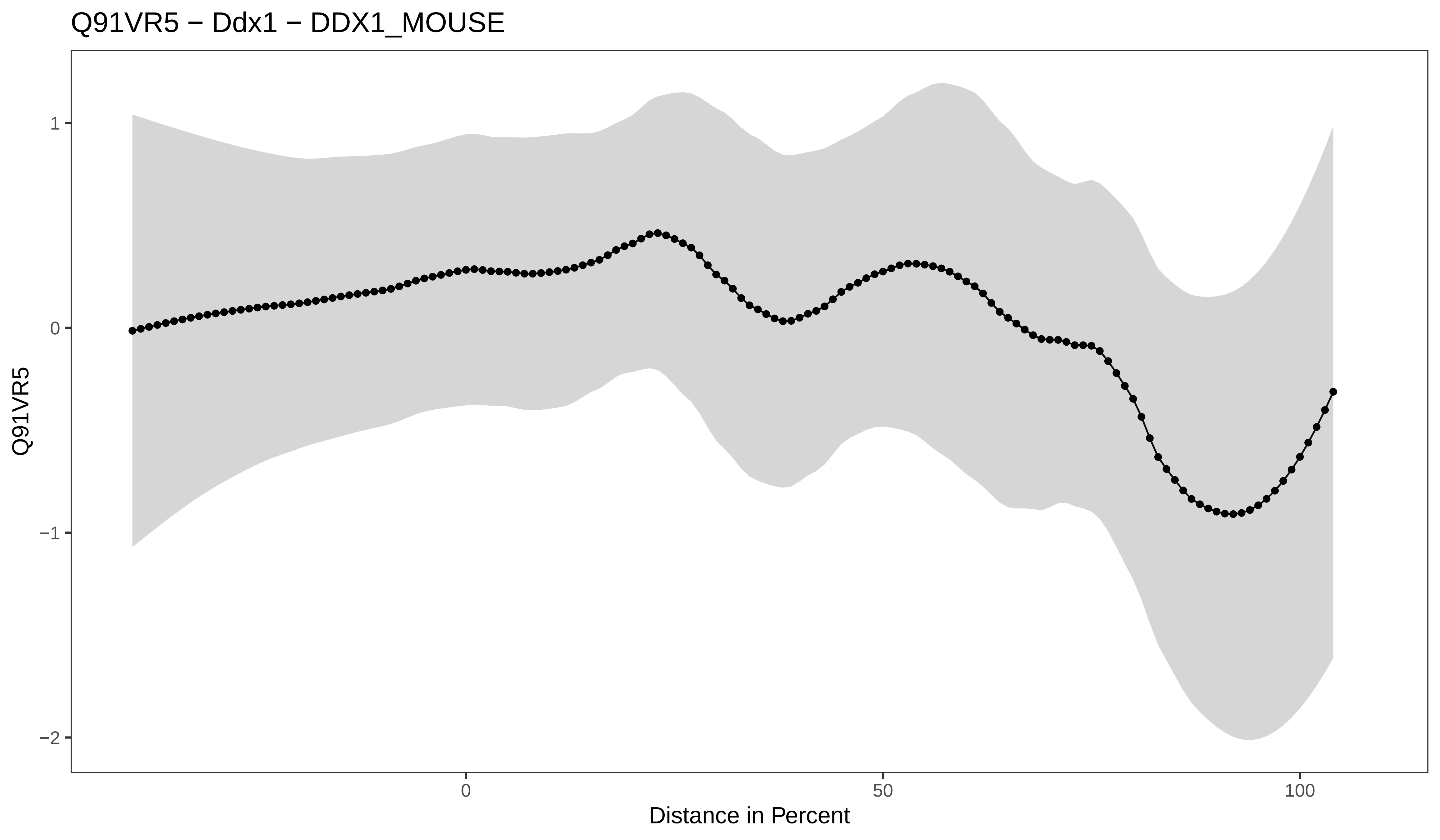

Q91VR5 − Ddx1 − DDX1_MOUSE
Q91VR5
1
0
−1
−2
0
50
100
Distance in Percent

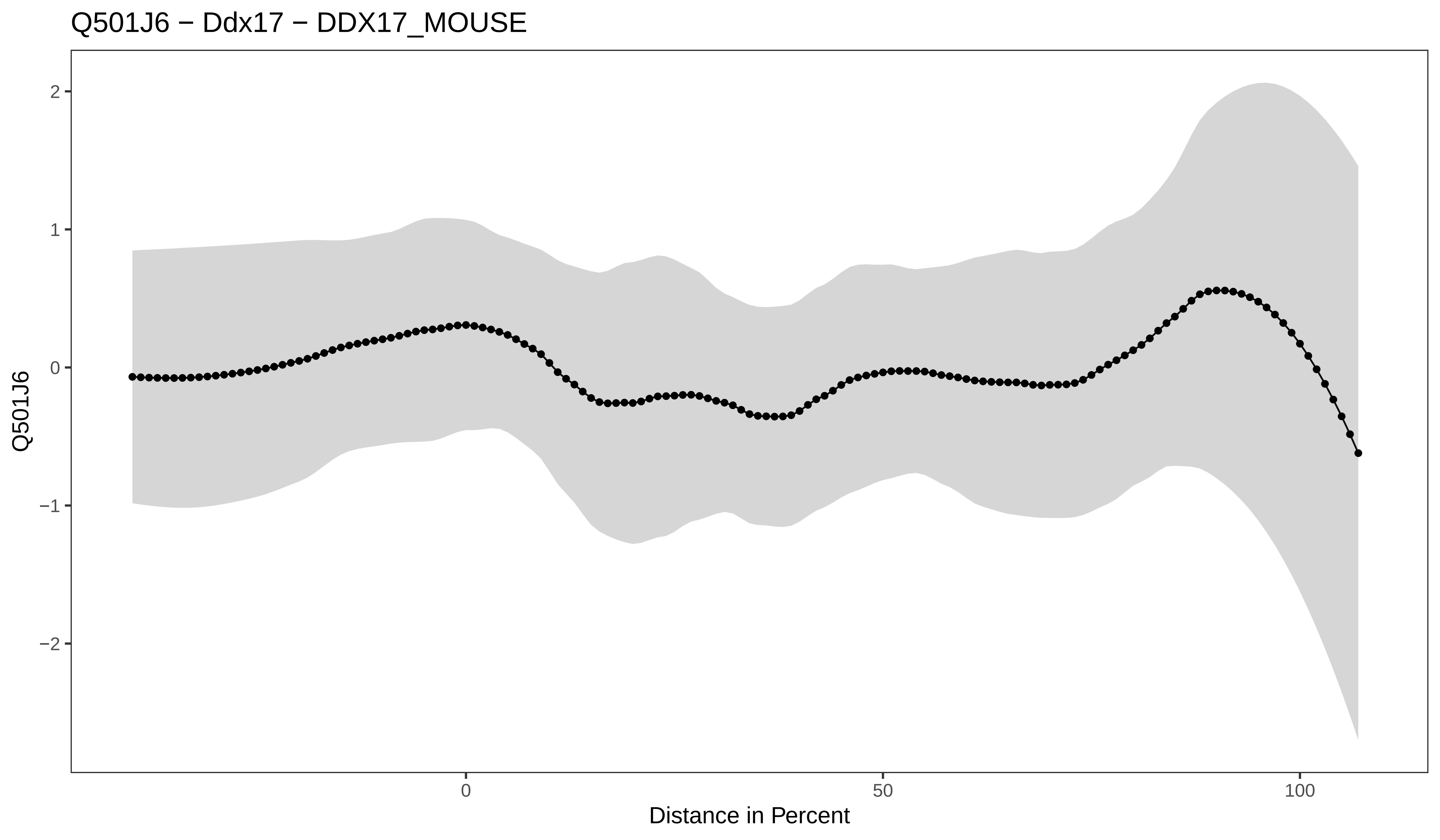

Q501J6 − Ddx17 − DDX17_MOUSE
2
1
0
−1
−2
Q501J6
0
50
100
Distance in Percent

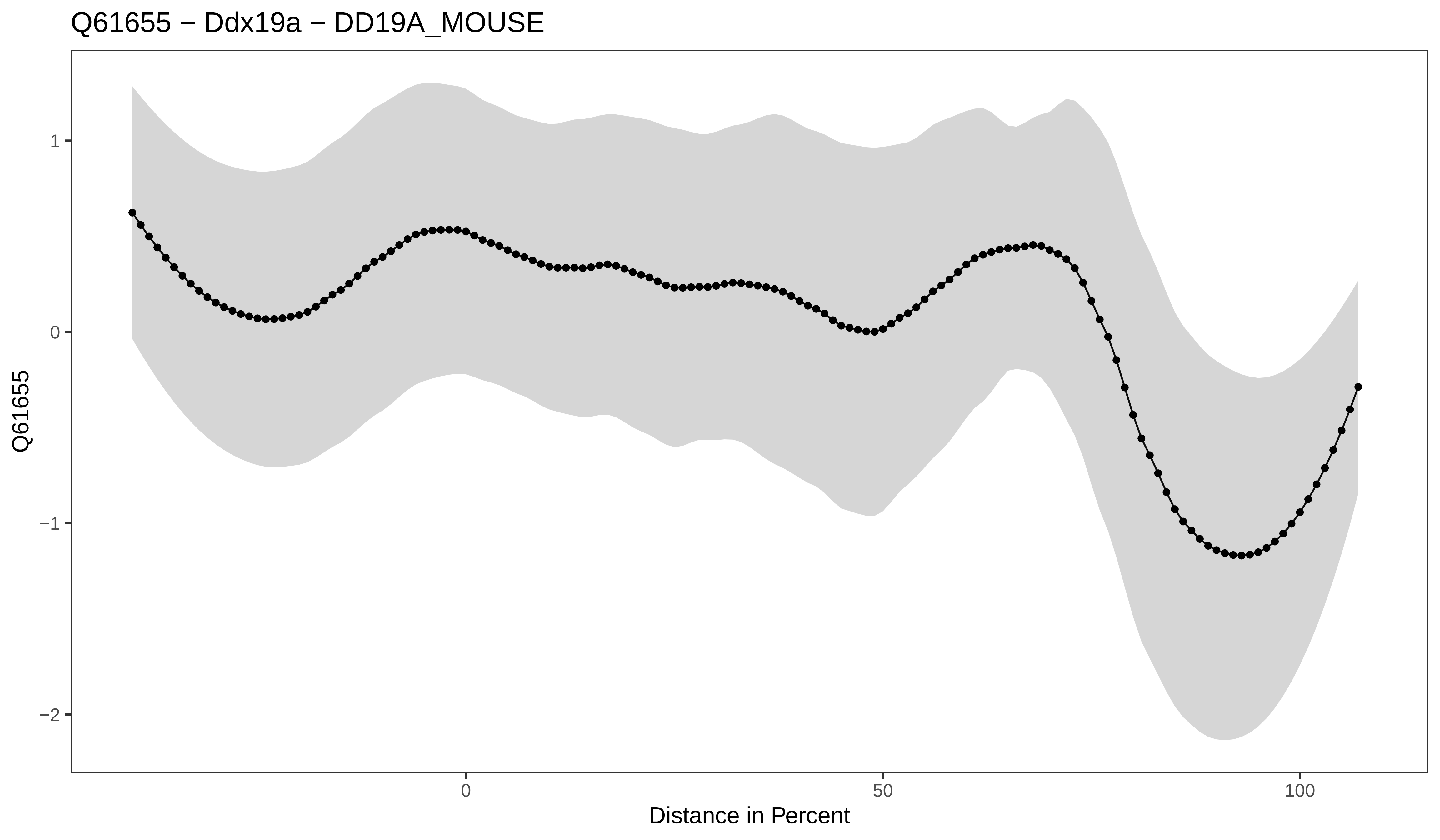
Q61655 − Ddx19a − DD19A_MOUSE
1
0
−1
−2
Q61655
0
50
100
Distance in Percent

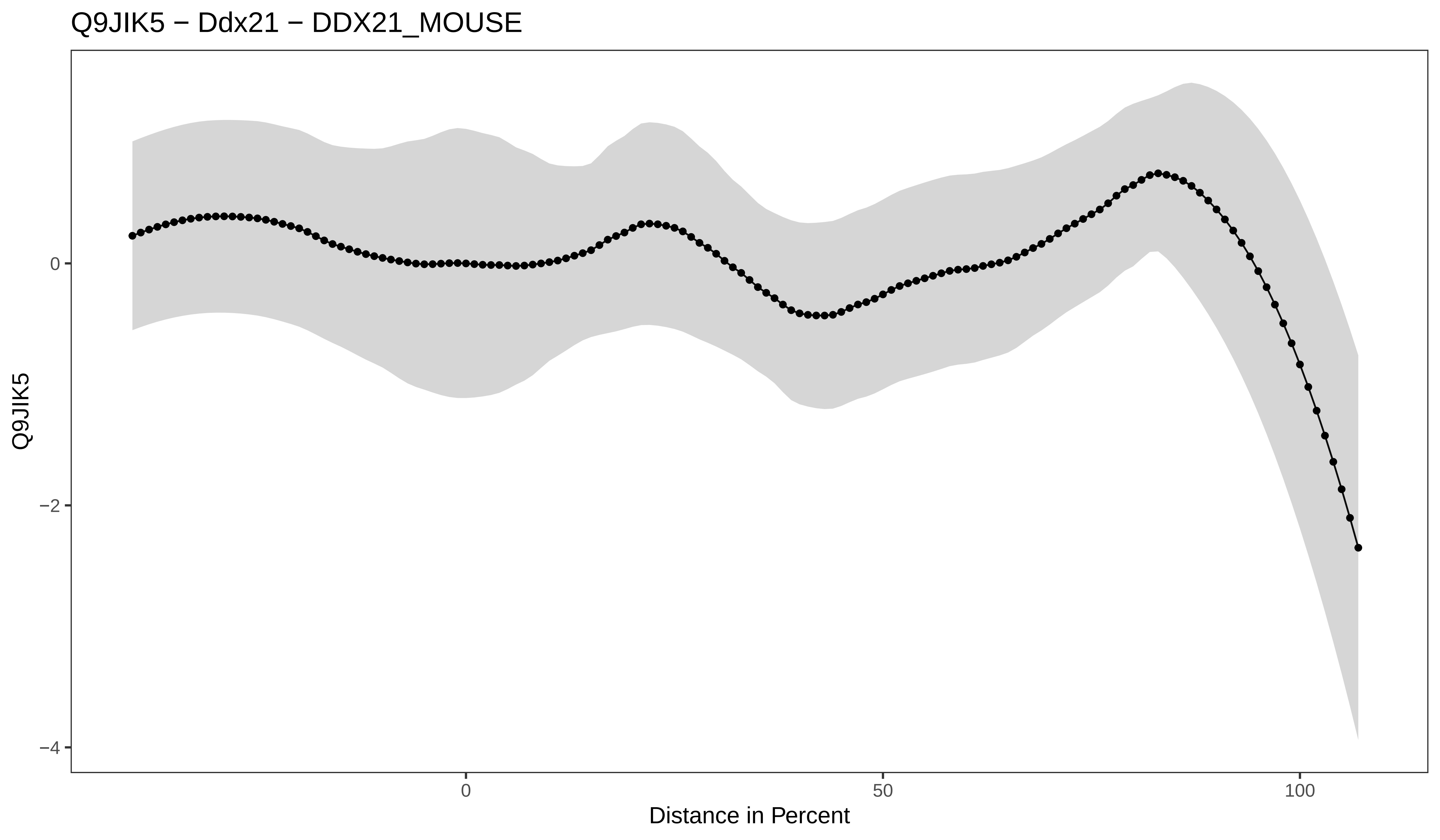
Q9JIK5 − Ddx21 − DDX21_MOUSE
Q9JIK5
0
−2
−4
0
50
100
Distance in Percent

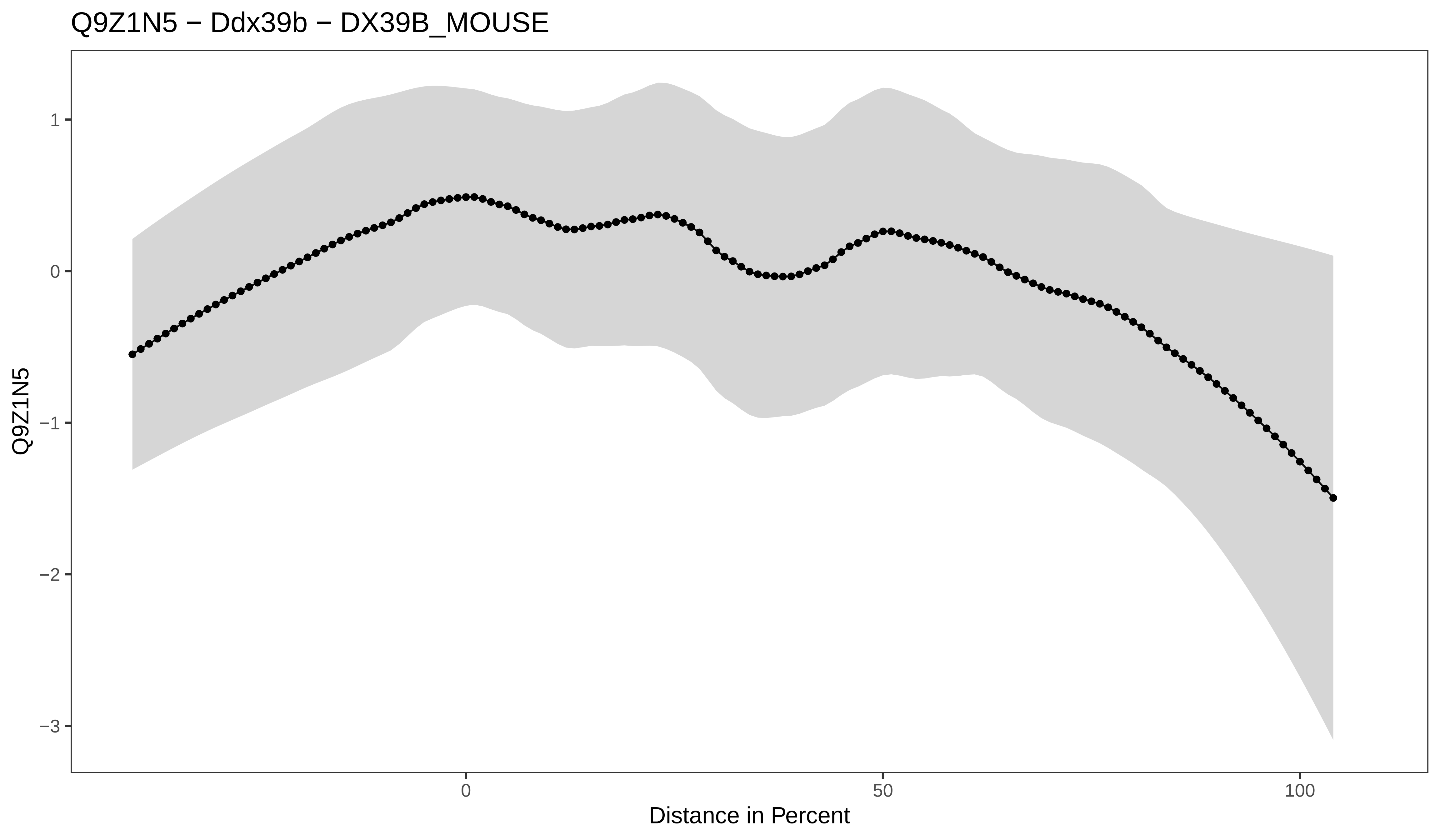
Q9Z1N5 − Ddx39b − DX39B_MOUSE
1
0
−1
−2
−3
Q9Z1N5
0
50
100
Distance in Percent

Q62167 − Ddx3x − DDX3X_MOUSE

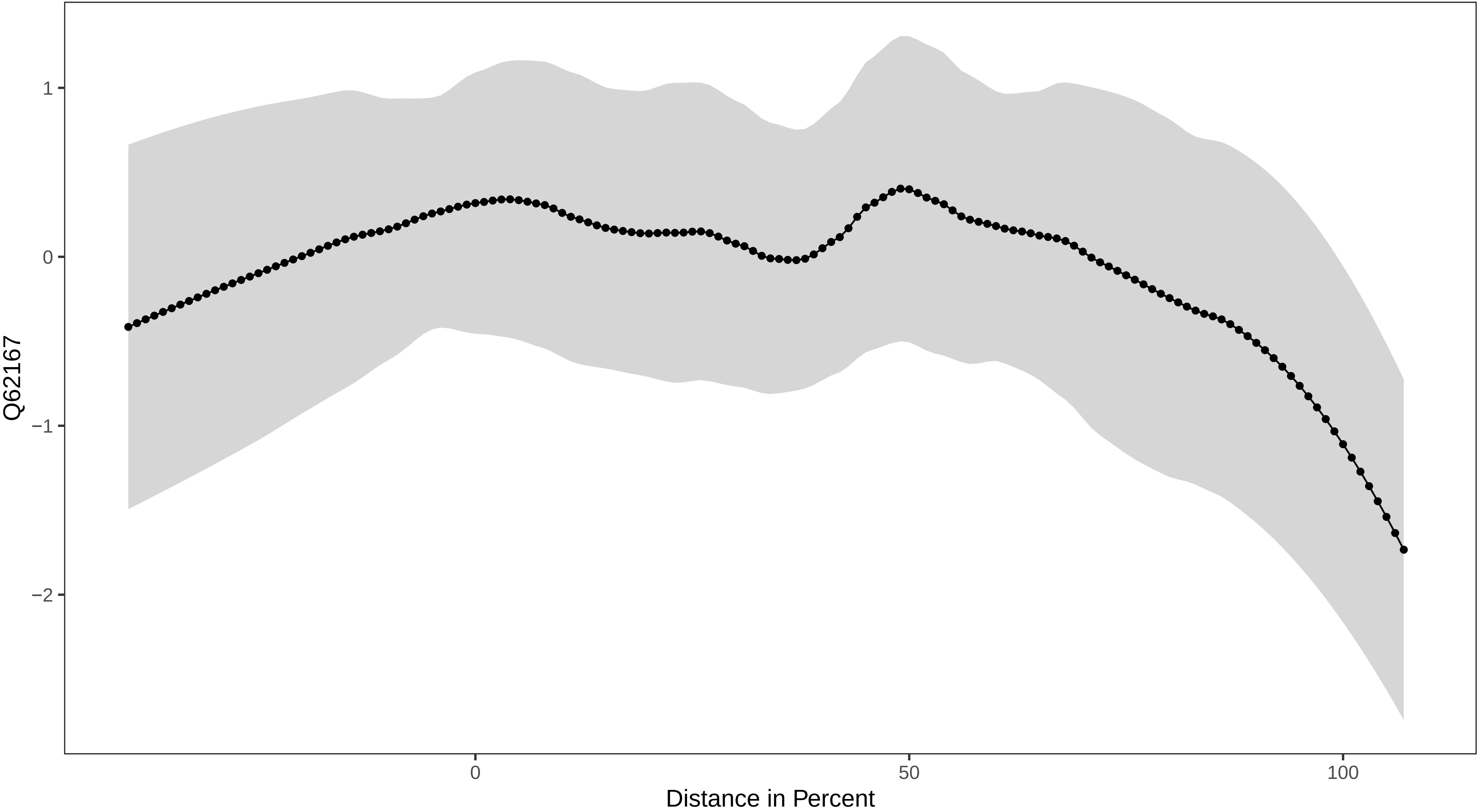

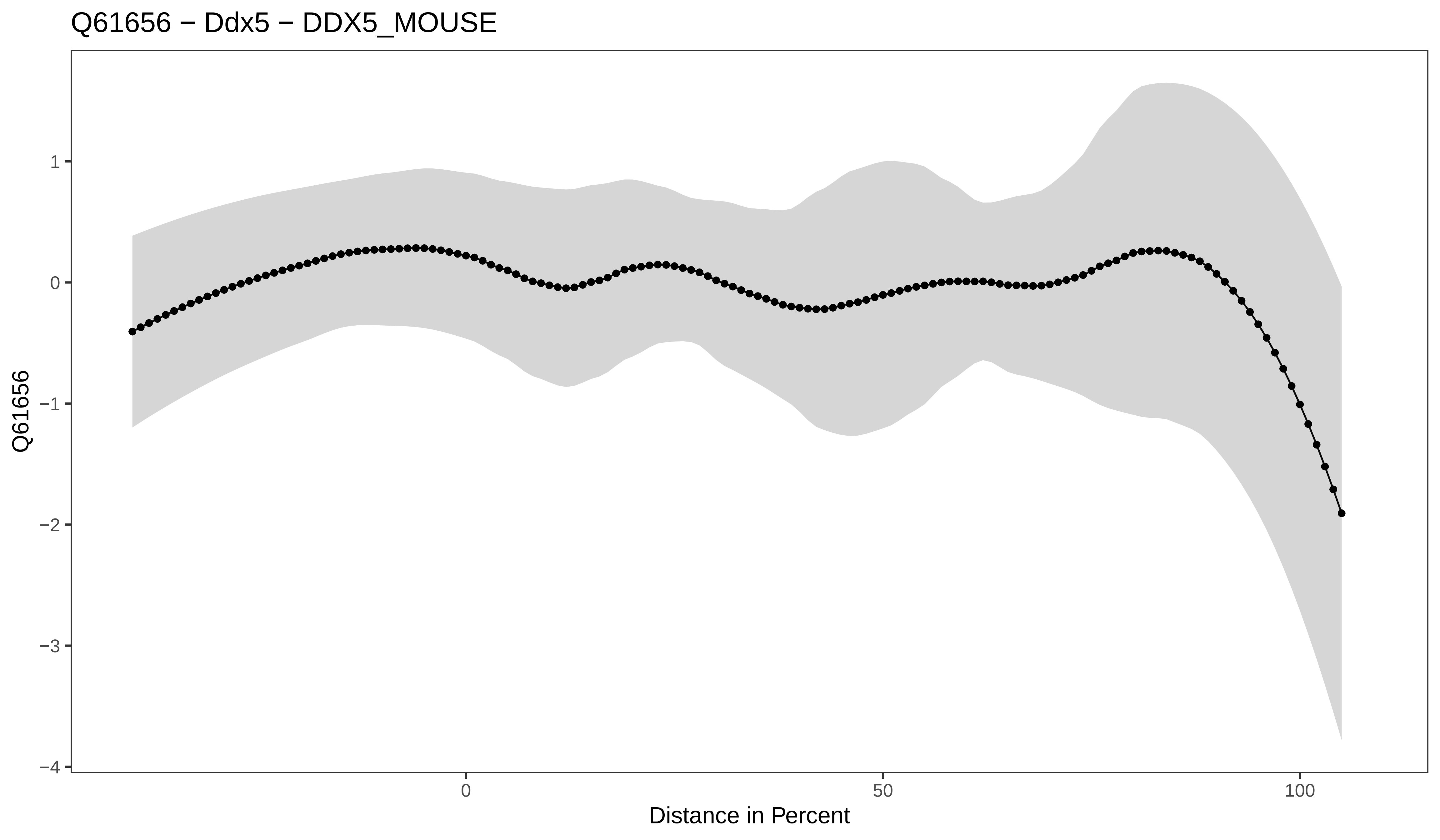
Q61656 − Ddx5 − DDX5_MOUSE
Q61656
1
0
−1
−2
−3
−4
0
50
100
Distance in Percent

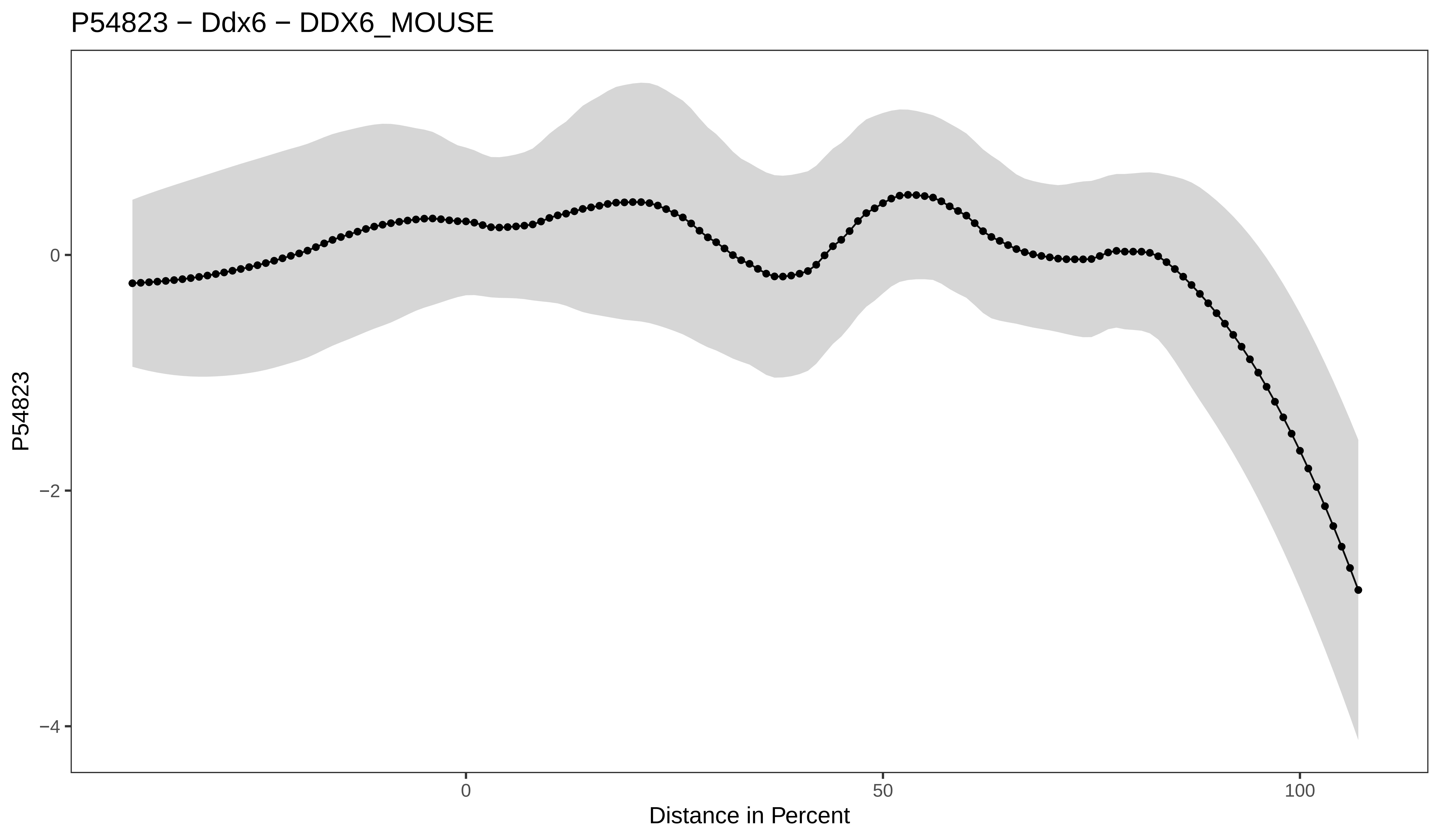
P54823 − Ddx6 − DDX6_MOUSE
0
−2
−4
P54823
0
50
100
Distance in Percent

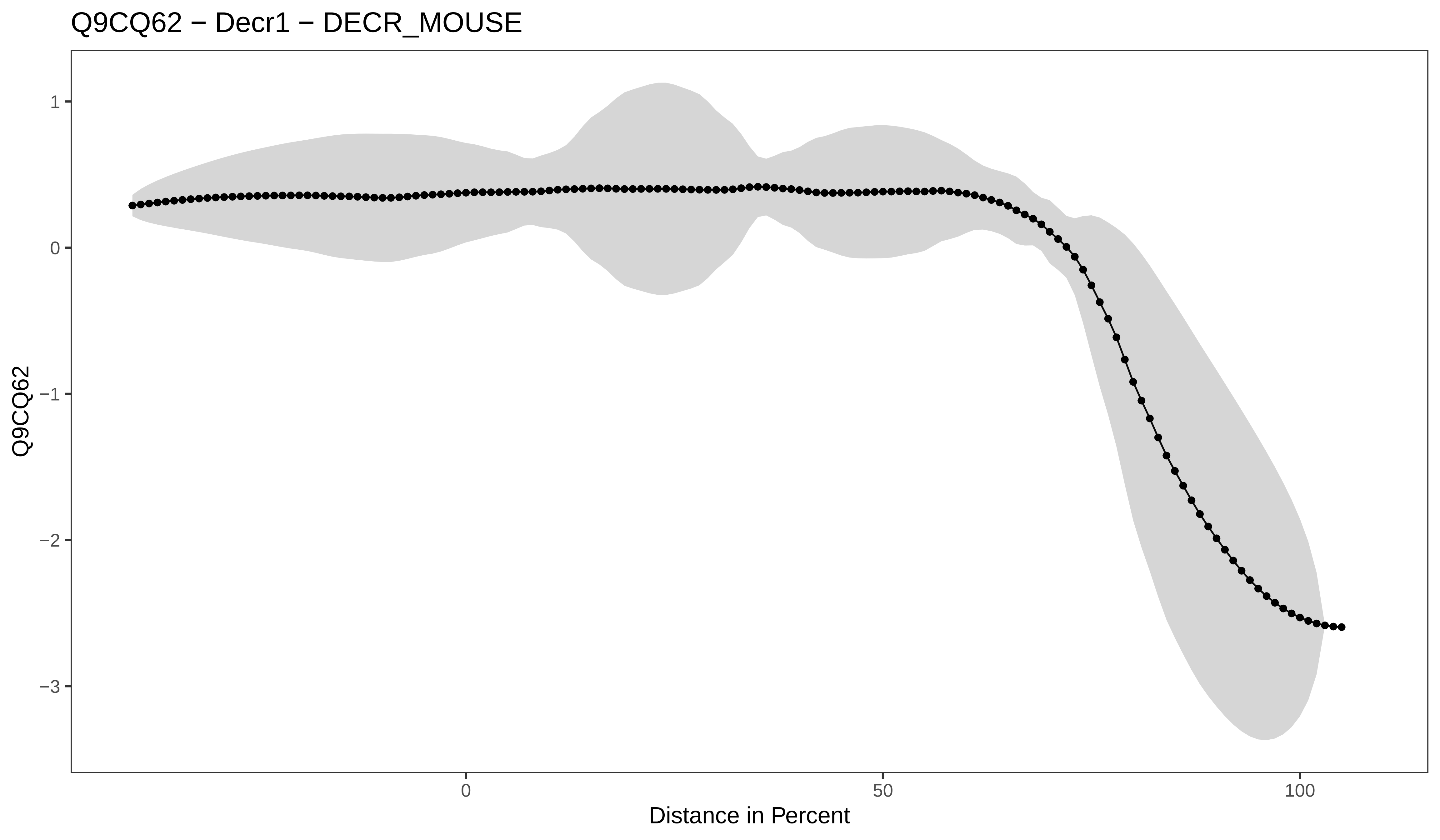

Q9CQ62 − Decr1 − DECR_MOUSE
Q9CQ62
Distance in Percent
1
0
−1
−2
−3
0
50
100

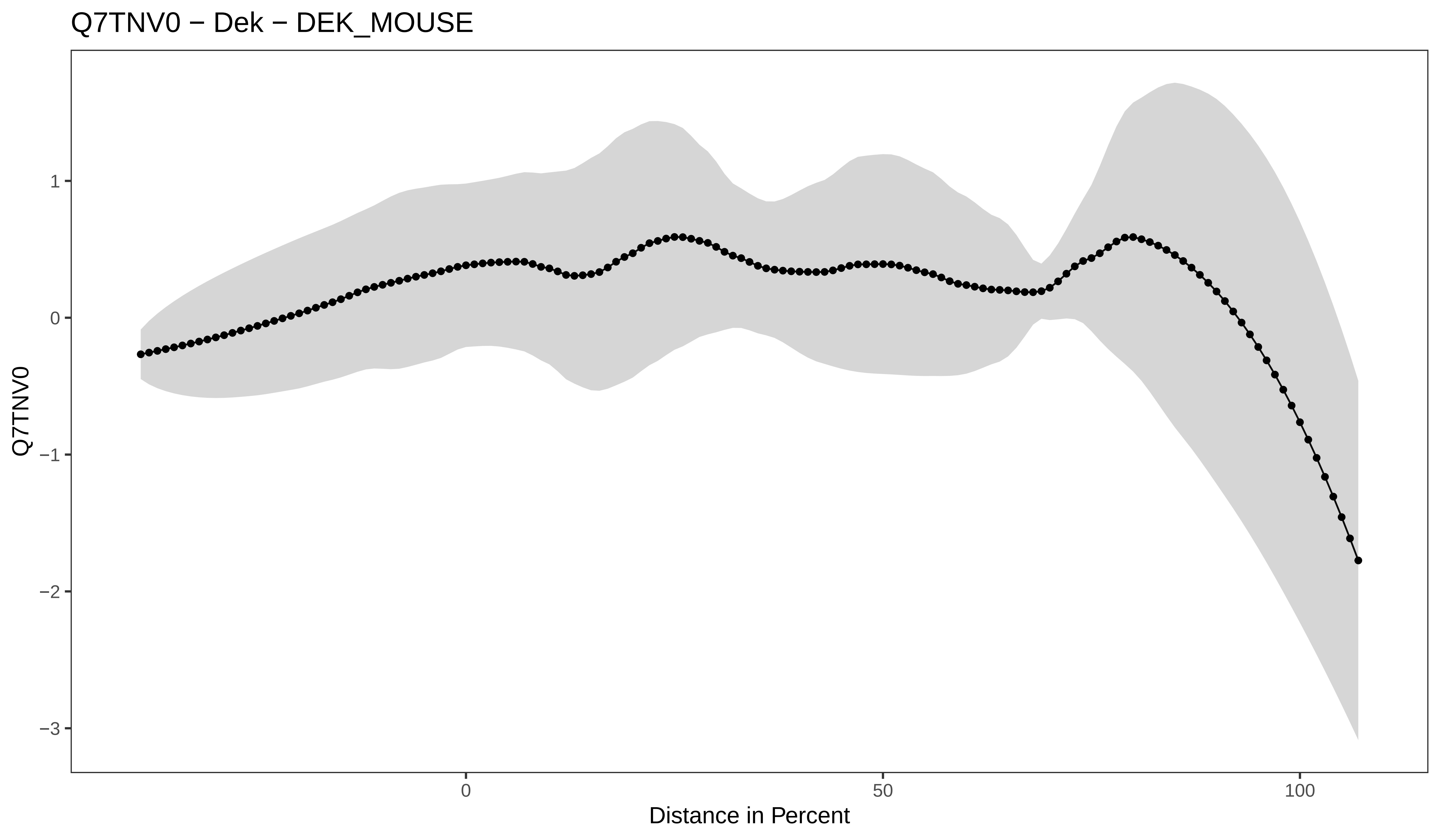
Q7TNV0 − Dek − DEK_MOUSE
Q7TNV0
1
0
−1
−2
−3
0
50
100
Distance in Percent

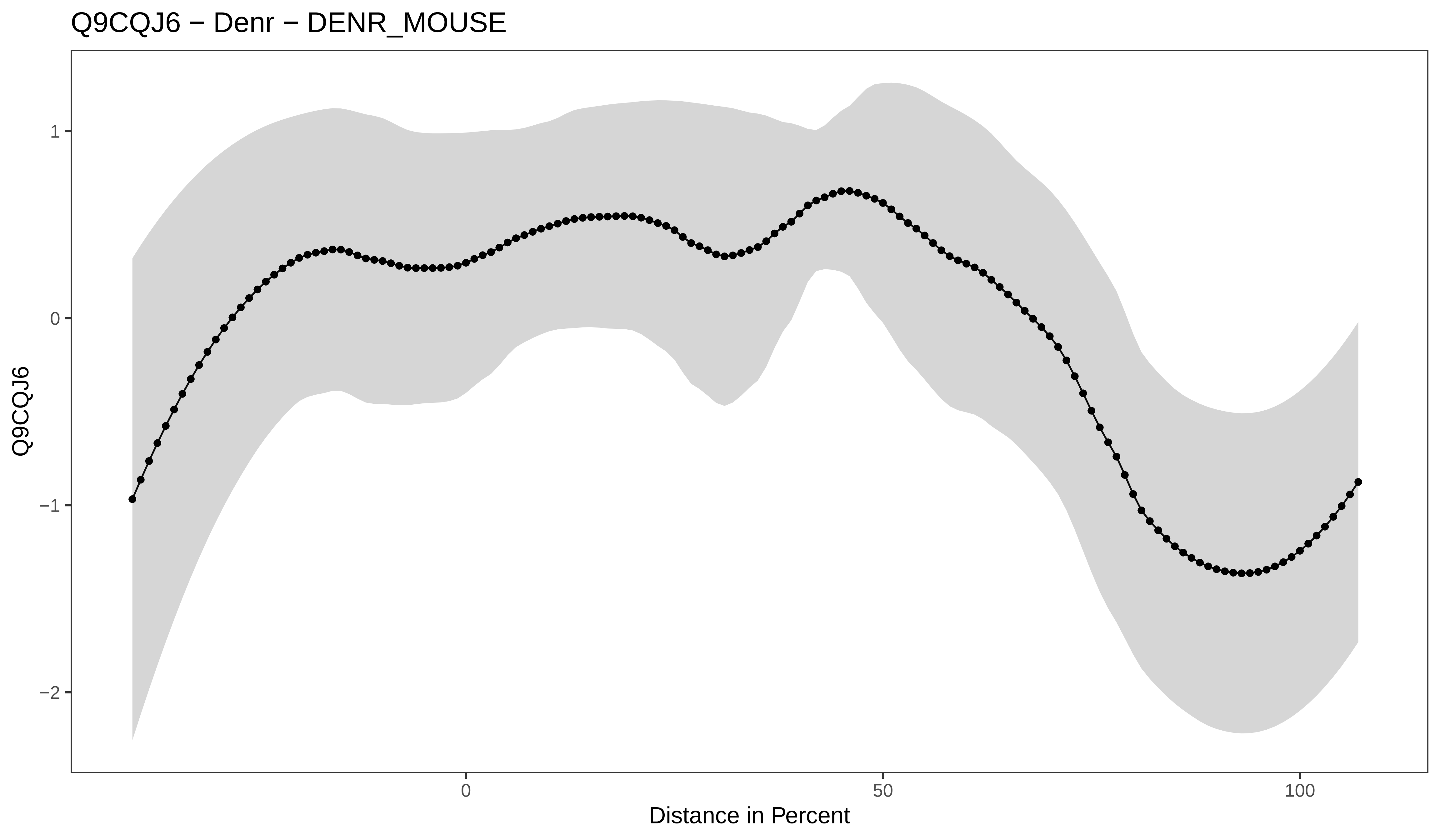
Q9CQJ6 − Denr − DENR_MOUSE
1
0
−1
−2
Q9CQJ6
0
50
100
Distance in Percent

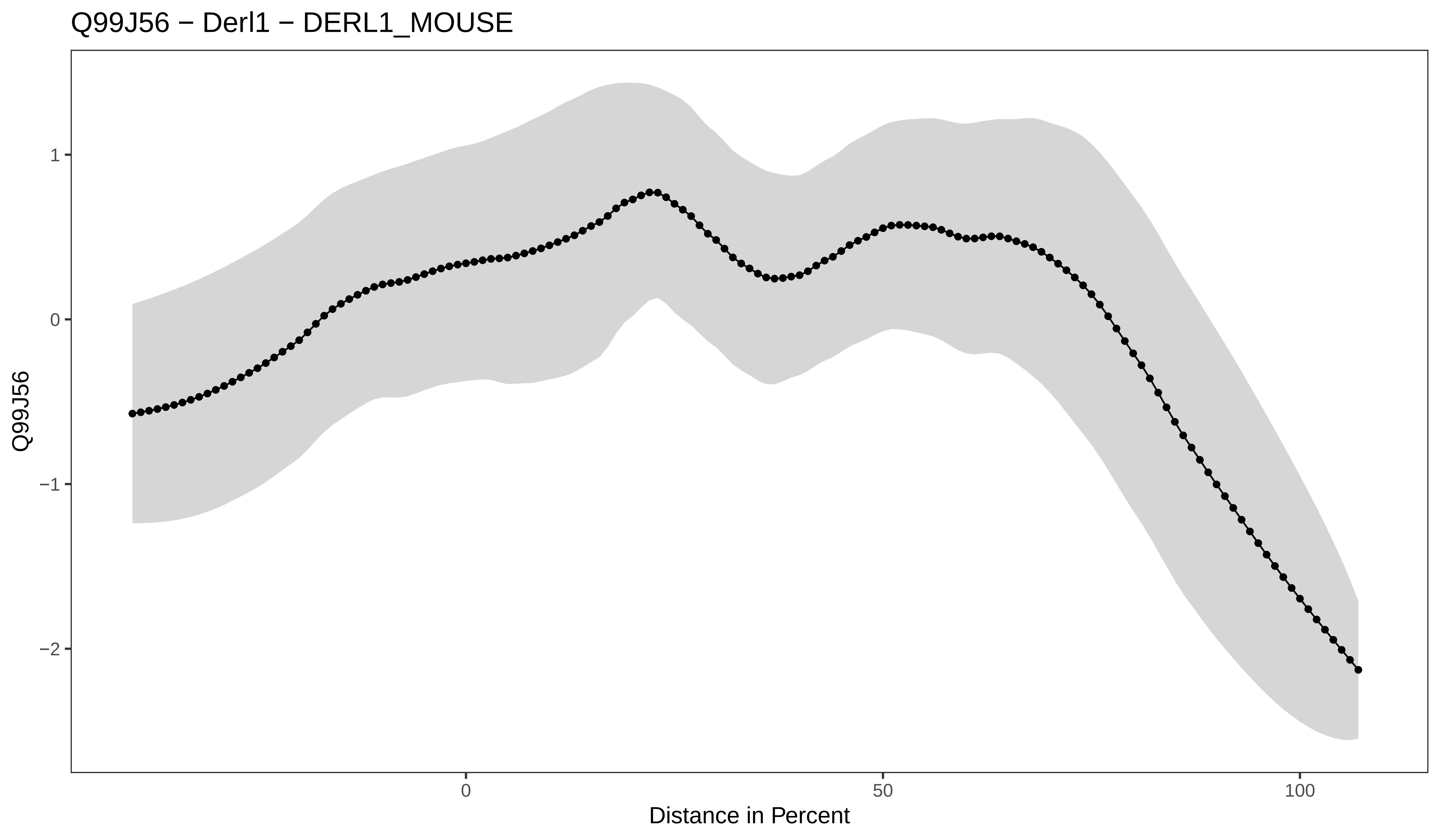
Q99J56 − Derl1 − DERL1_MOUSE
Q99J56
1
0
−1
−2
0
50
100
Distance in Percent

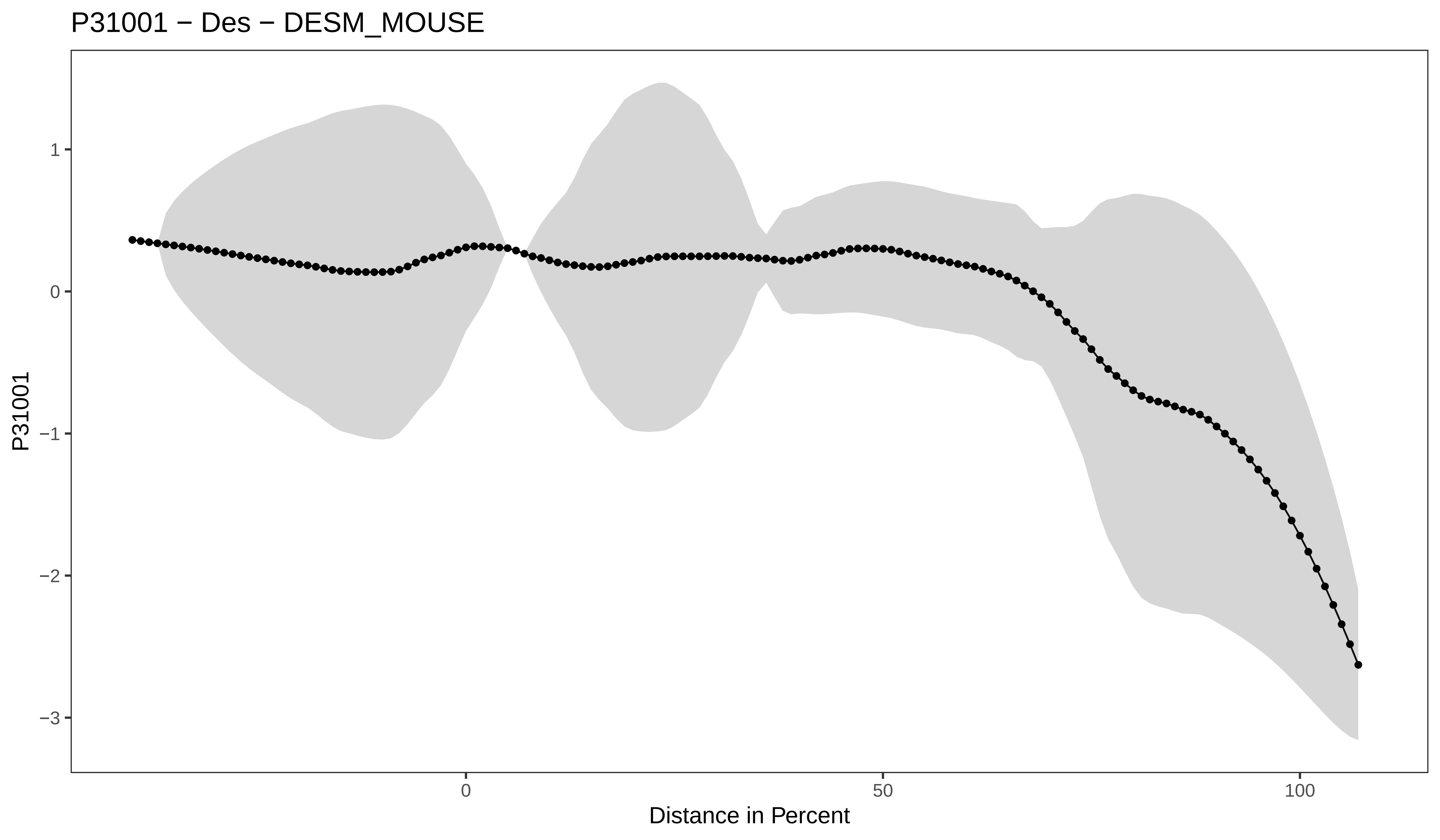

P31001 − Des − DESM_MOUSE
P31001
Distance in Percent
1
0
−1
−2
−3
0
50
100

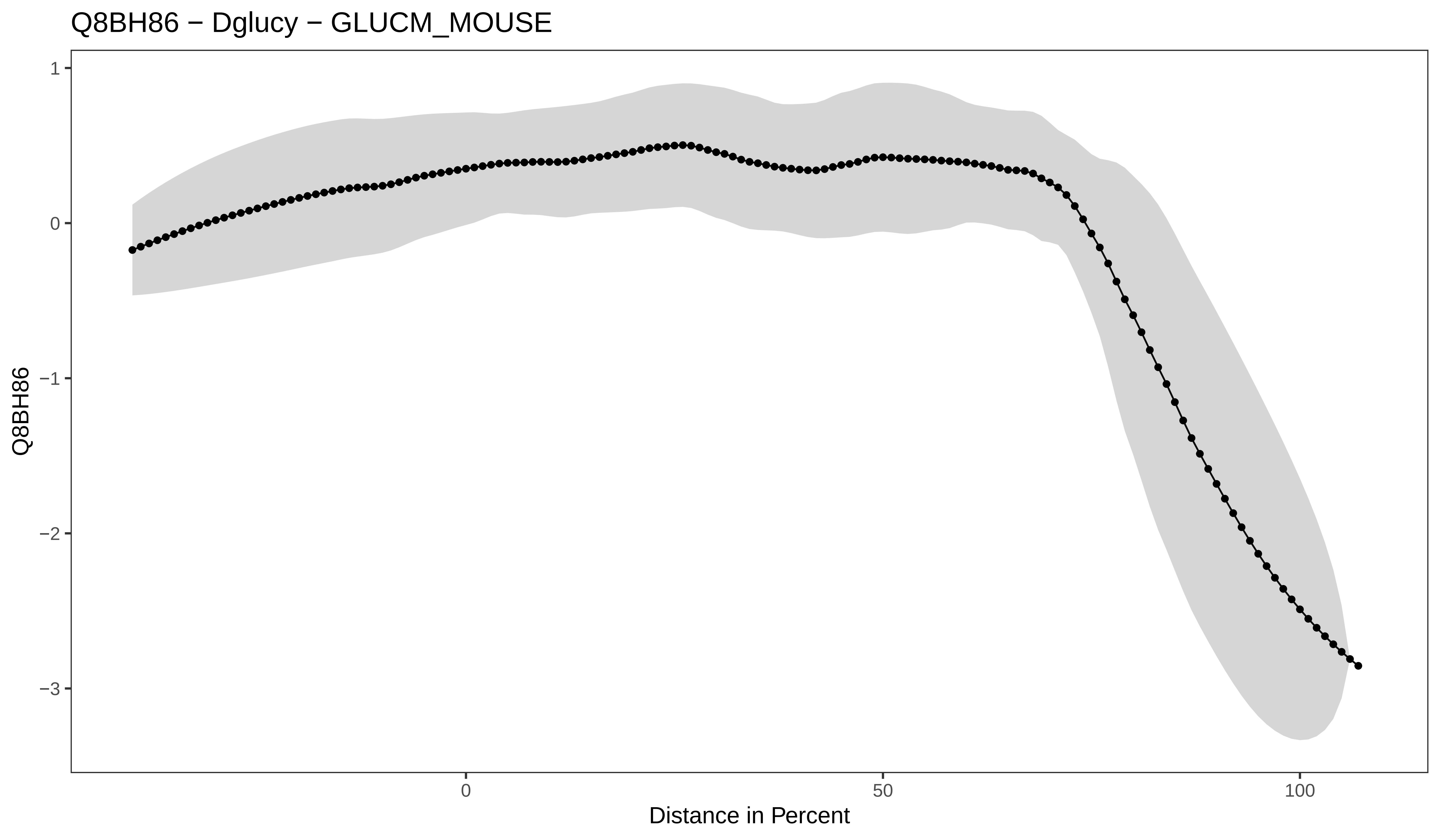

Q8BH86 − Dglucy − GLUCM_MOUSE
1
0
−1
−2
−3
Q8BH86
0
50
100
Distance in Percent

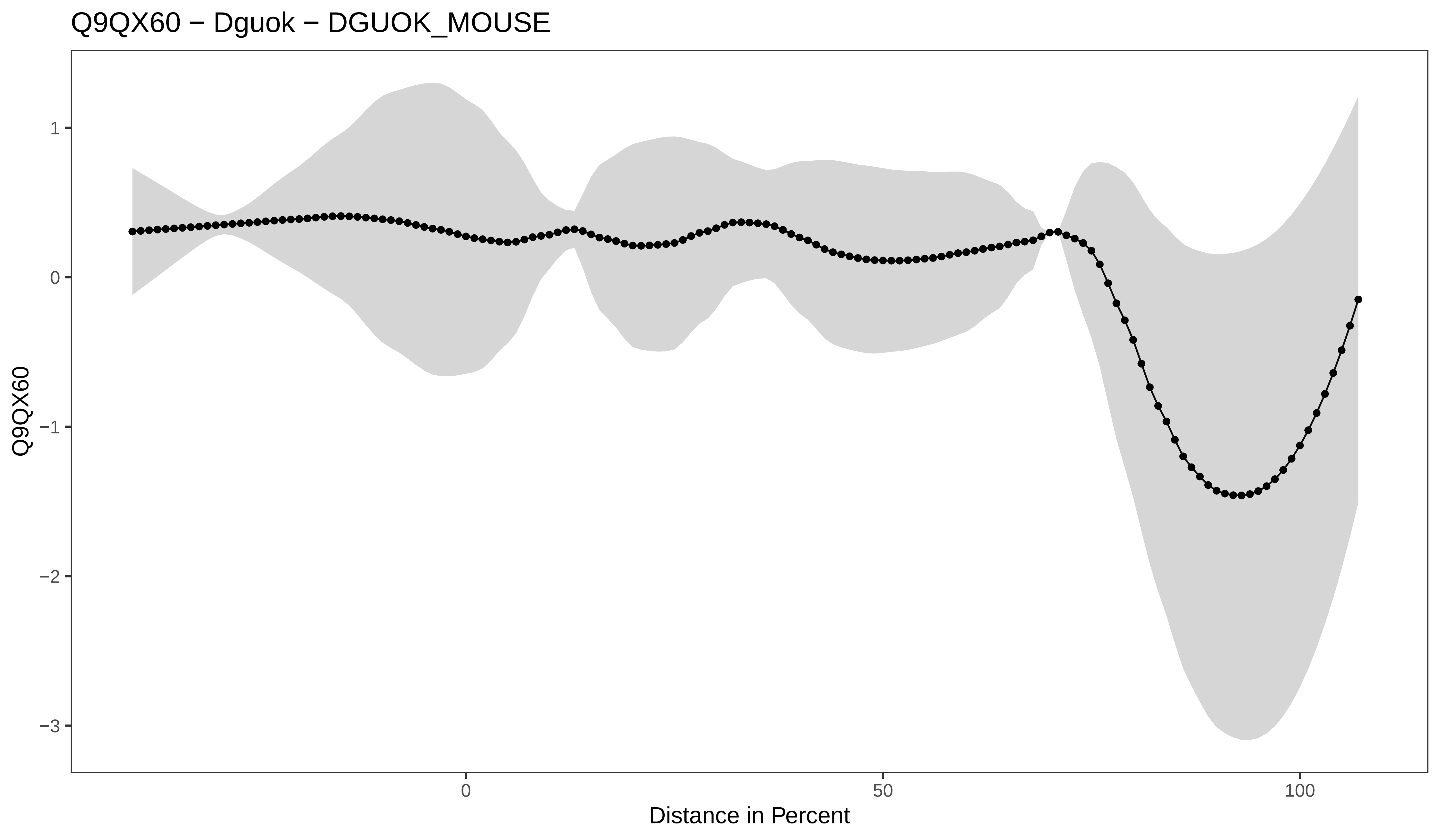
Q9QX60 − Dguok − DGUOK_MOUSE
1
0
−1
−2
−3
Q9QX60
0
50
100
Distance in Percent

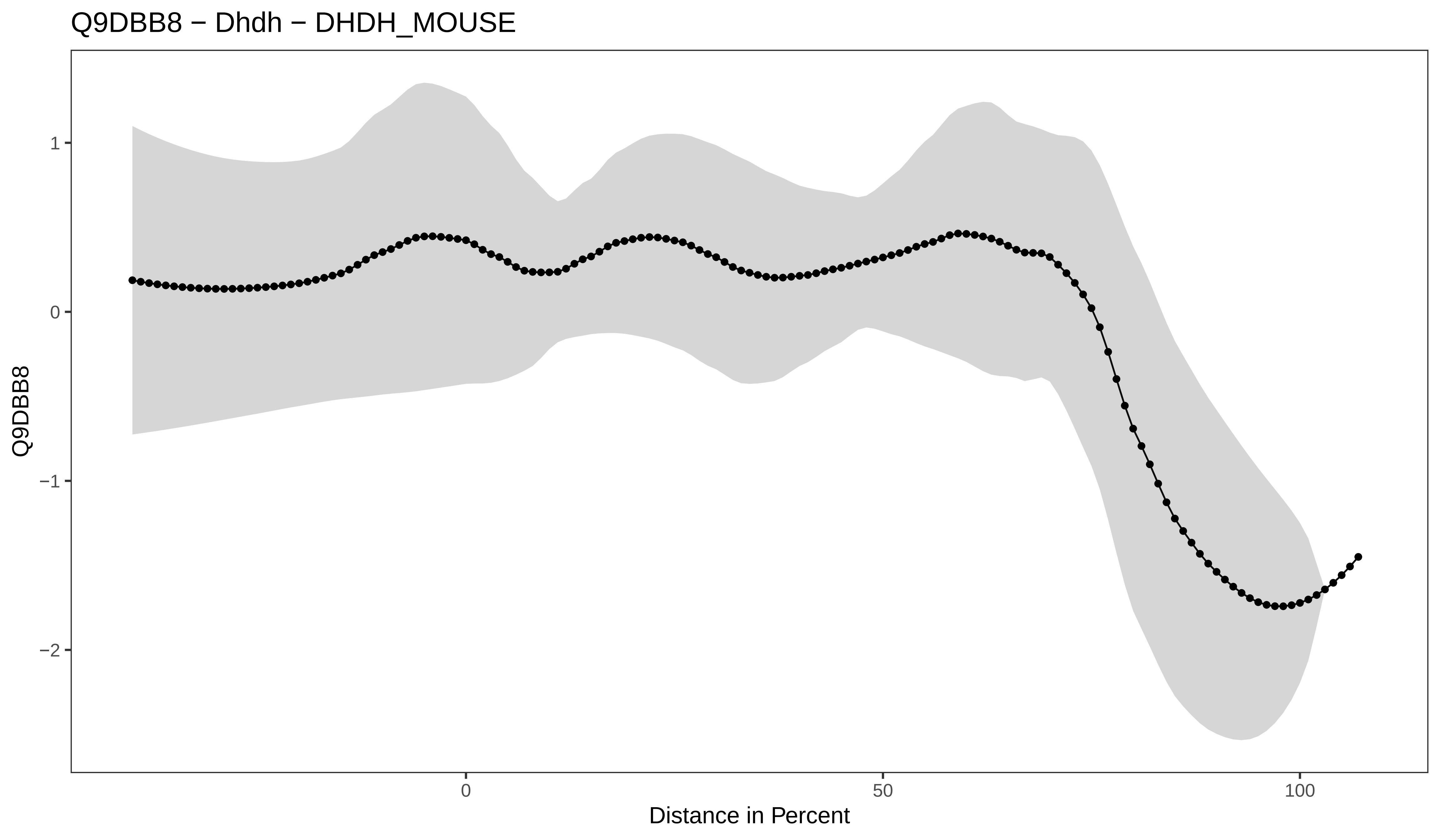
Q9DBB8 − Dhdh − DHDH_MOUSE
Q9DBB8
1
0
−1
−2
0
50
100
Distance in Percent

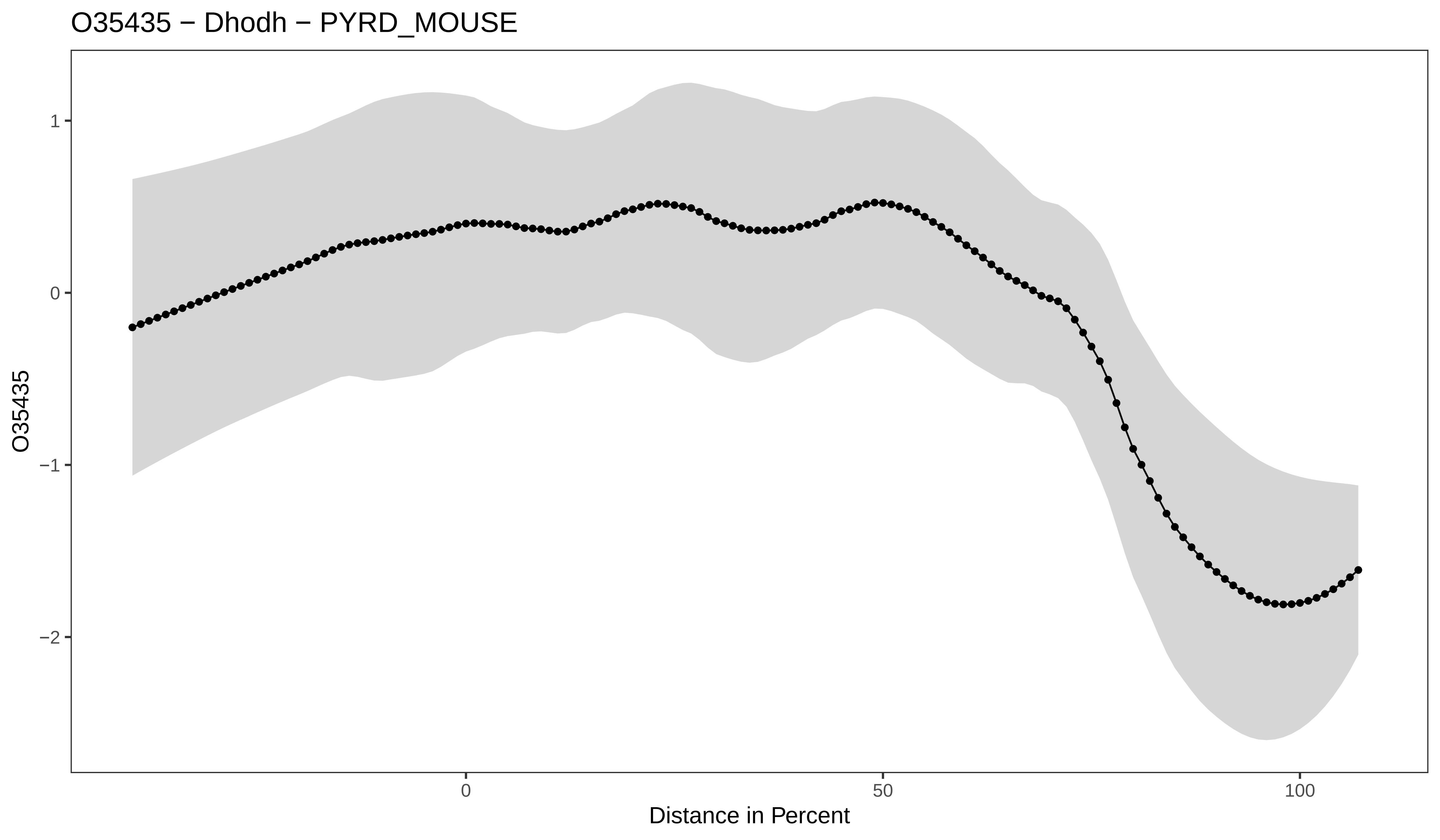
O35435 − Dhodh − PYRD_MOUSE
O35435
Distance in Percent
1
0
−1
−2
0
50
100

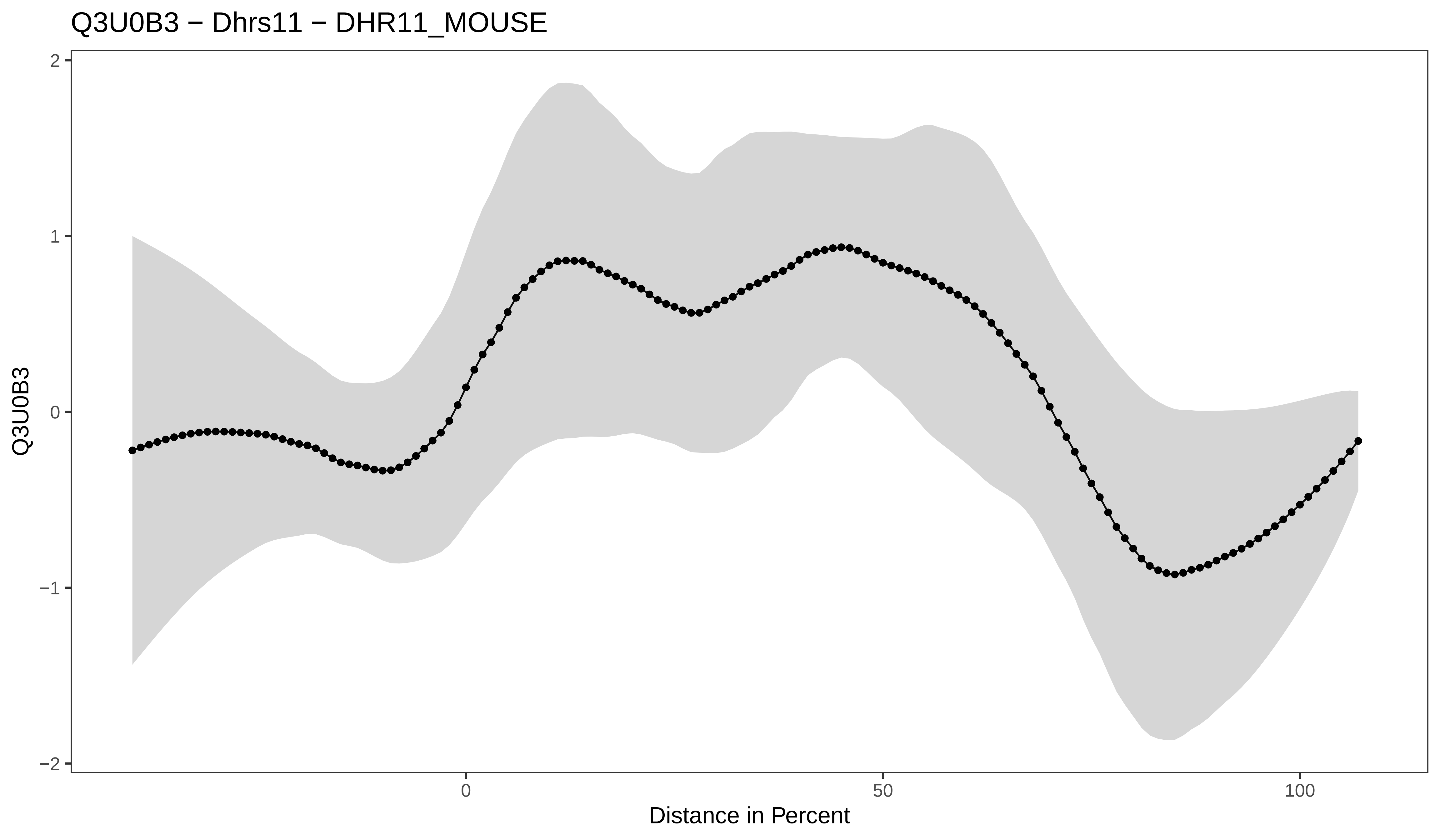

Q3U0B3 − Dhrs11 − DHR11_MOUSE
Q3U0B3
2
1
0
−1
−2
0
50
100
Distance in Percent

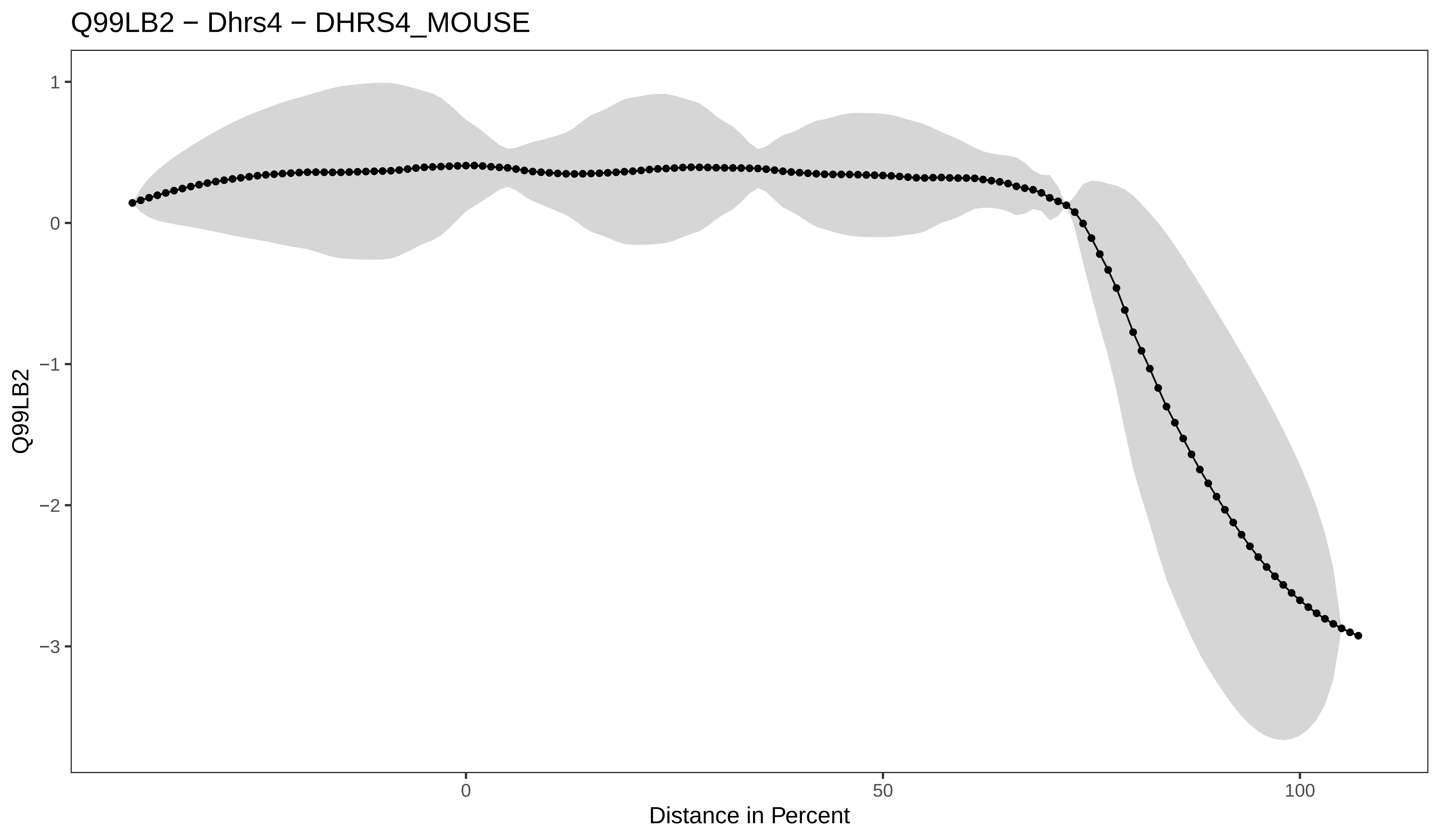
Q99LB2 − Dhrs4 − DHRS4_MOUSE
Q99LB2
1
0
−1
−2
−3
0
50
100
Distance in Percent

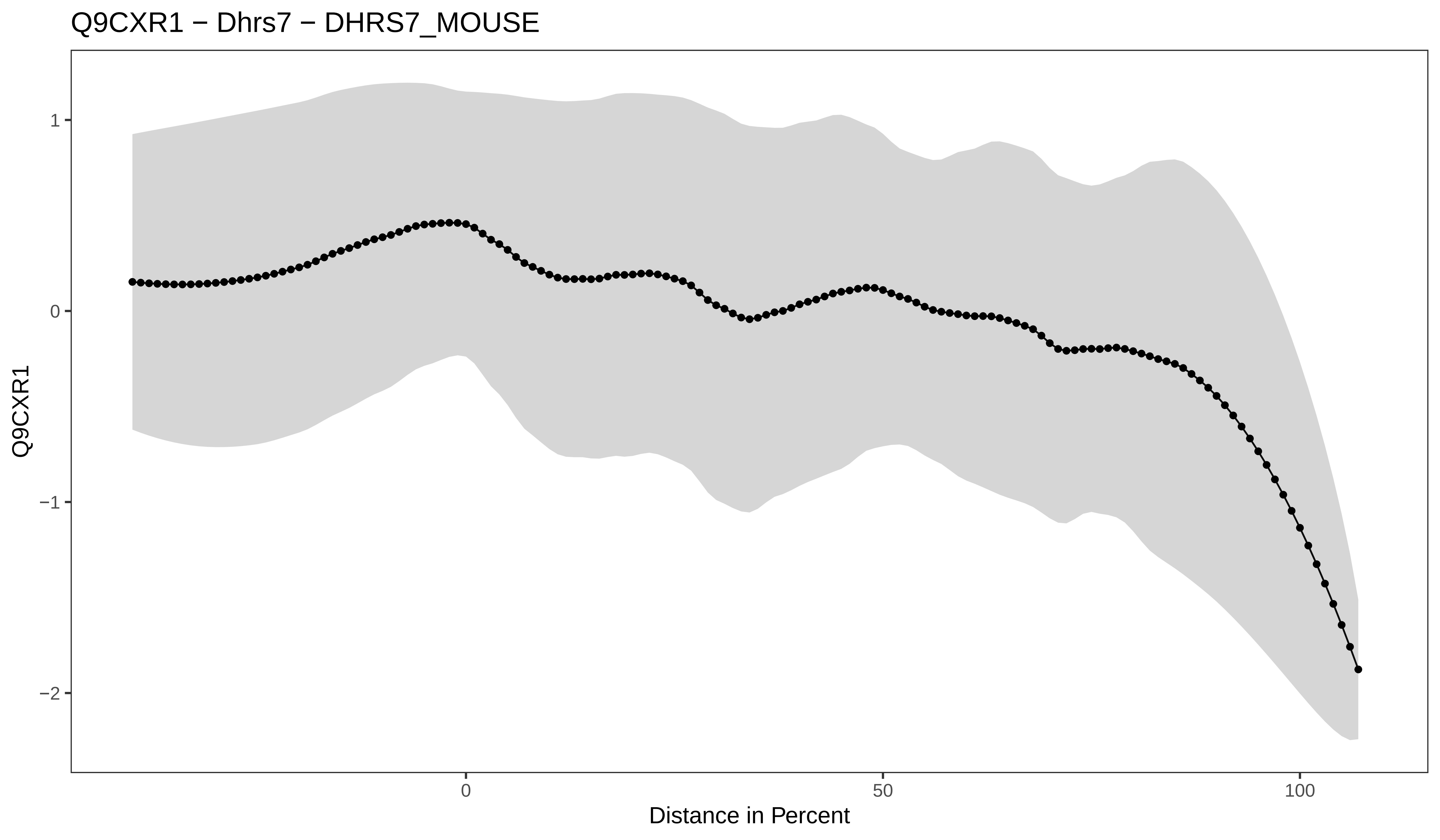

Q9CXR1 − Dhrs7 − DHRS7_MOUSE
Q9CXR1
1
0
−1
−2
0
50
100
Distance in Percent

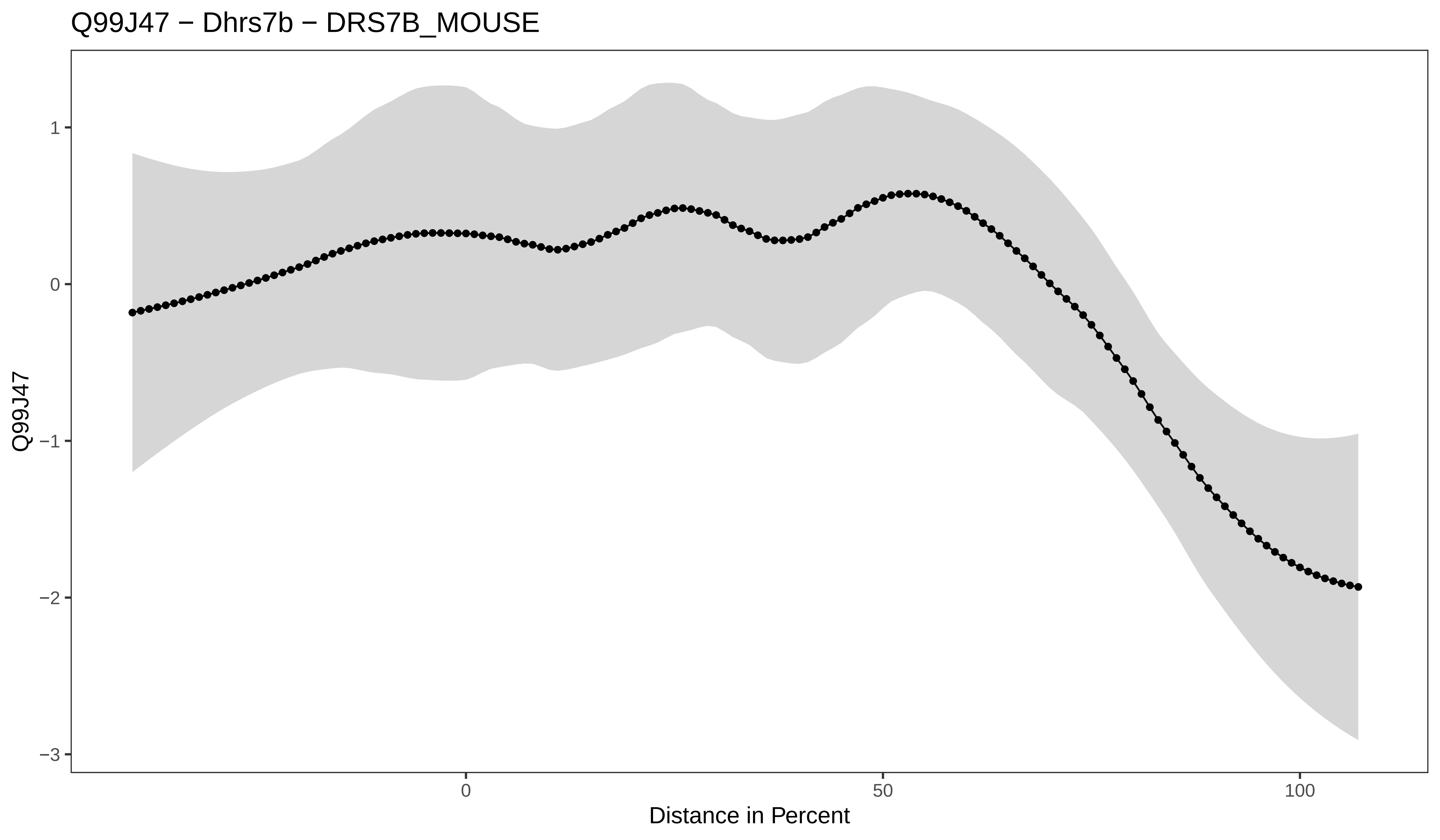

Q99J47 − Dhrs7b − DRS7B_MOUSE
1
0
−1
−2
−3
Q99J47
0
50
100
Distance in Percent

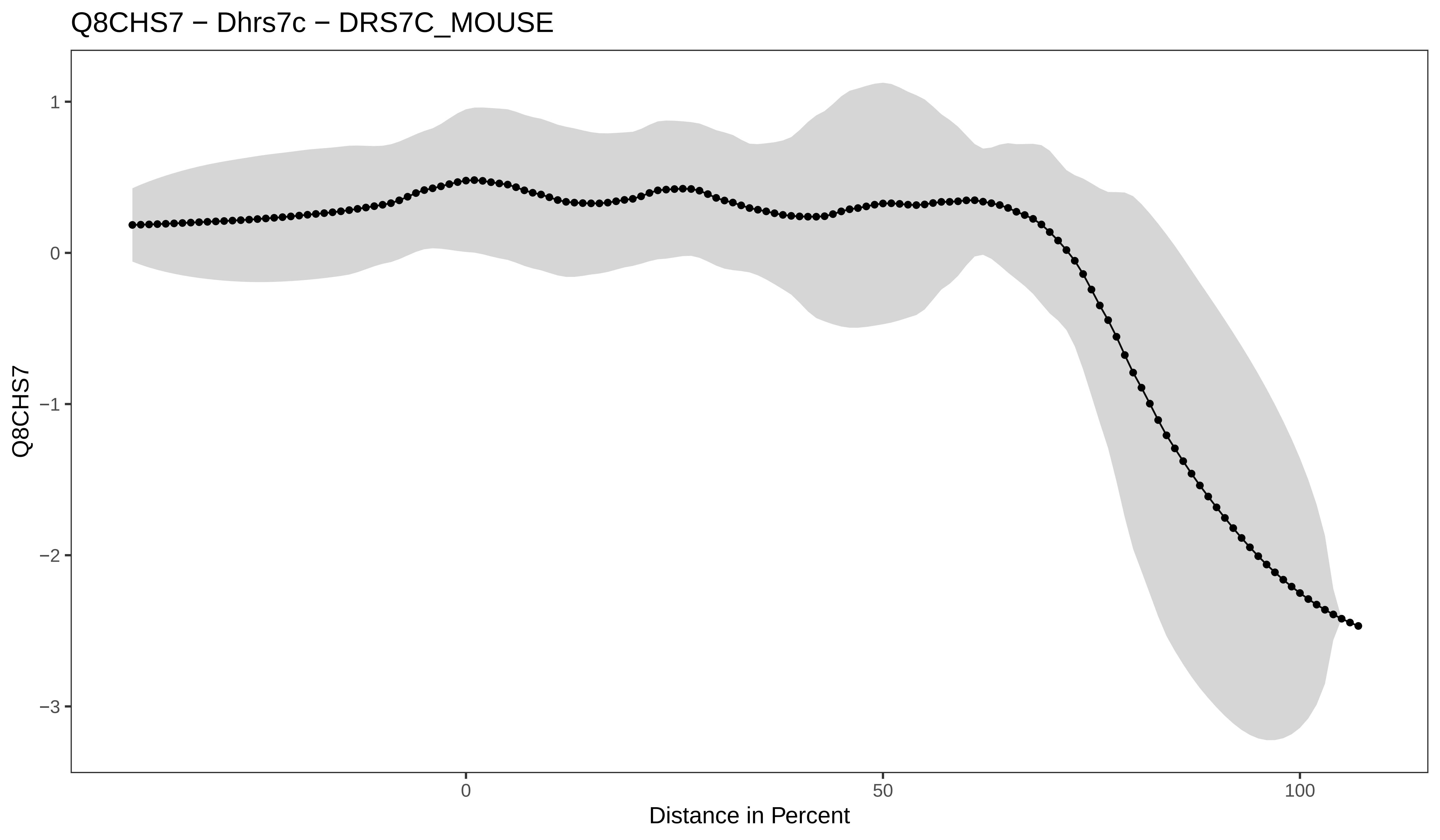

Q8CHS7 − Dhrs7c − DRS7C_MOUSE
Q8CHS7
1
0
−1
−2
−3
0
50
100
Distance in Percent

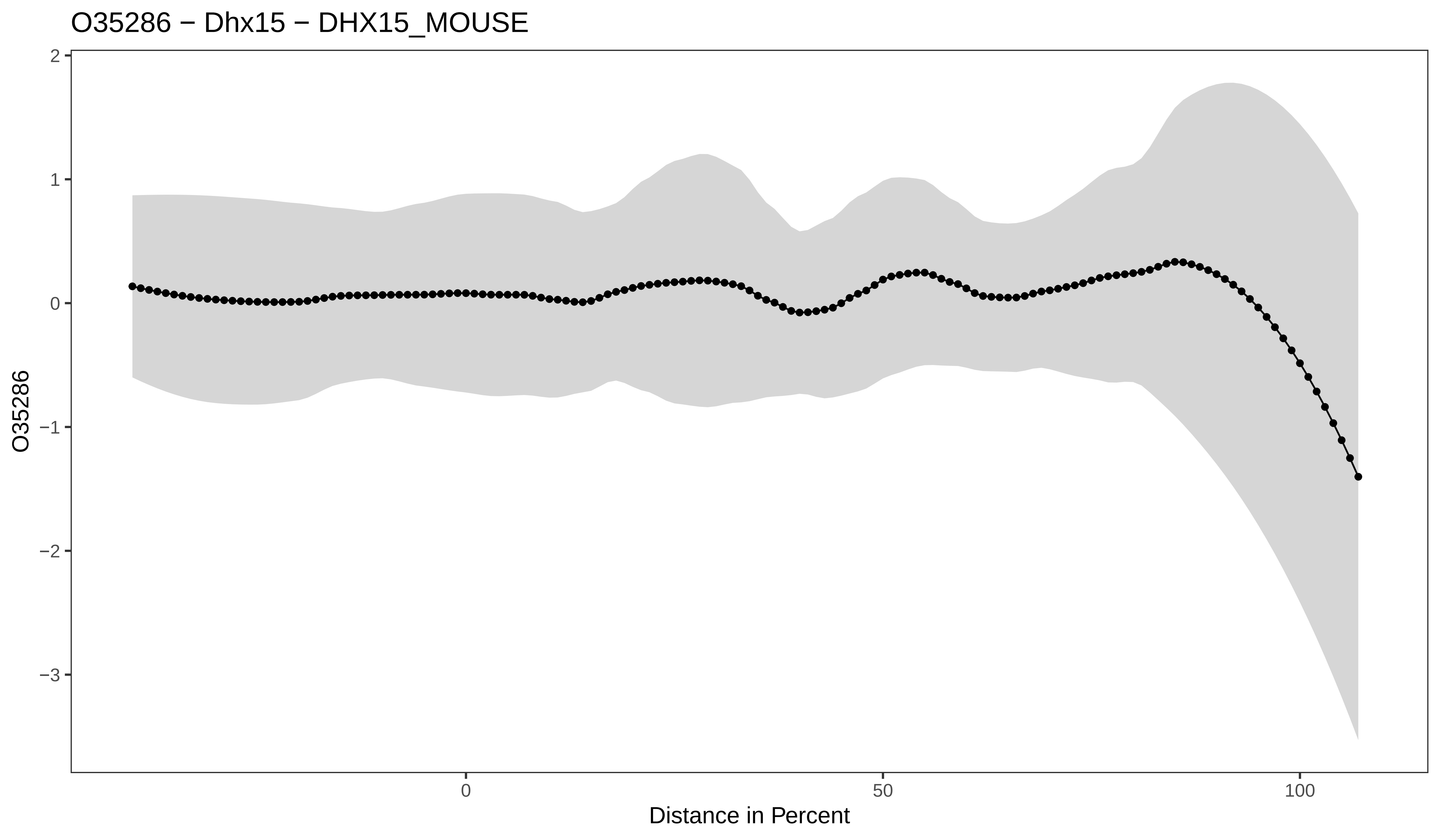

O35286 − Dhx15 − DHX15_MOUSE
O35286
2
1
0
−1
−2
−3
0
50
100
Distance in Percent

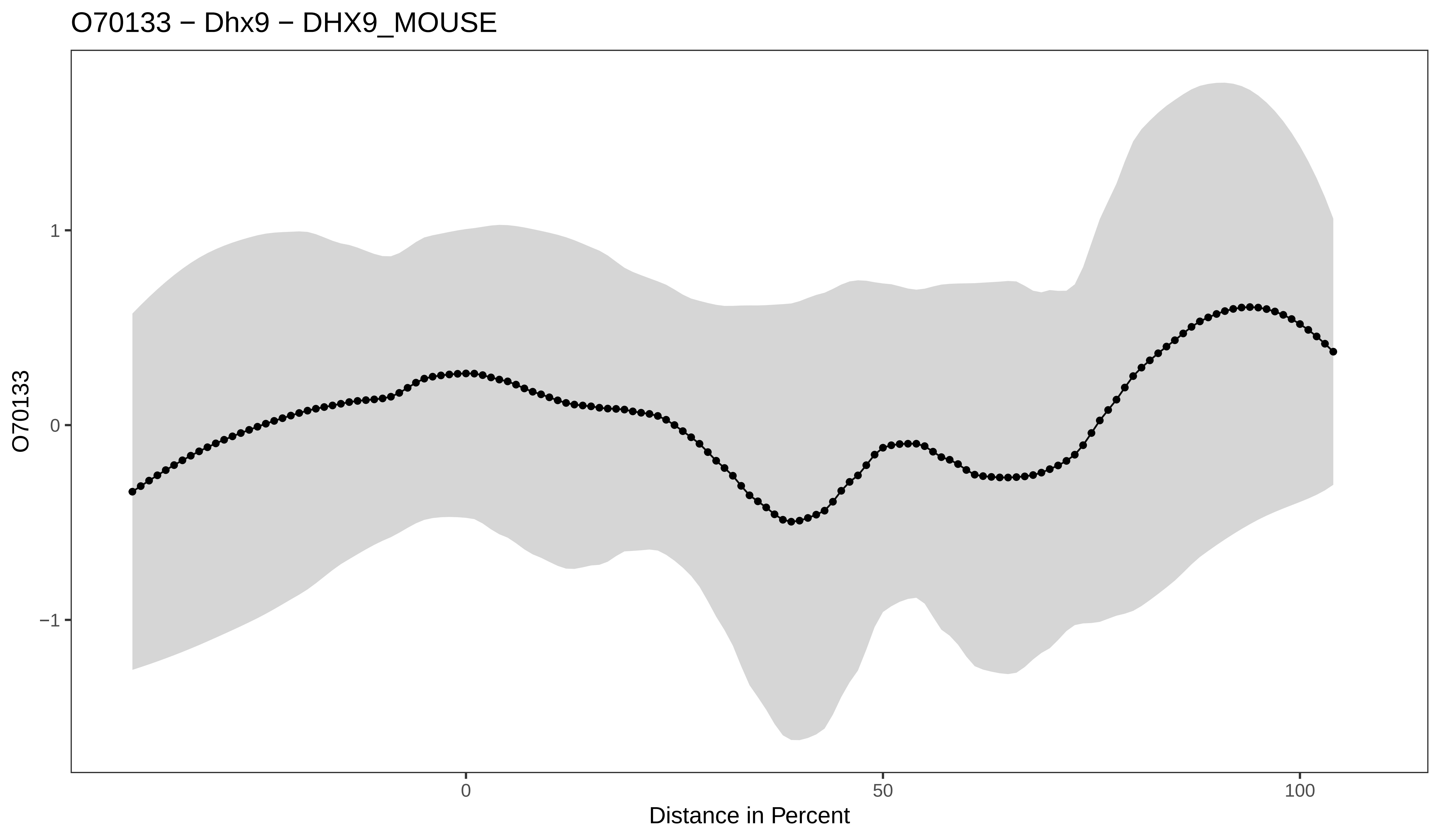
O70133 − Dhx9 − DHX9_MOUSE
1
0
−1
O70133
0
50
100
Distance in Percent

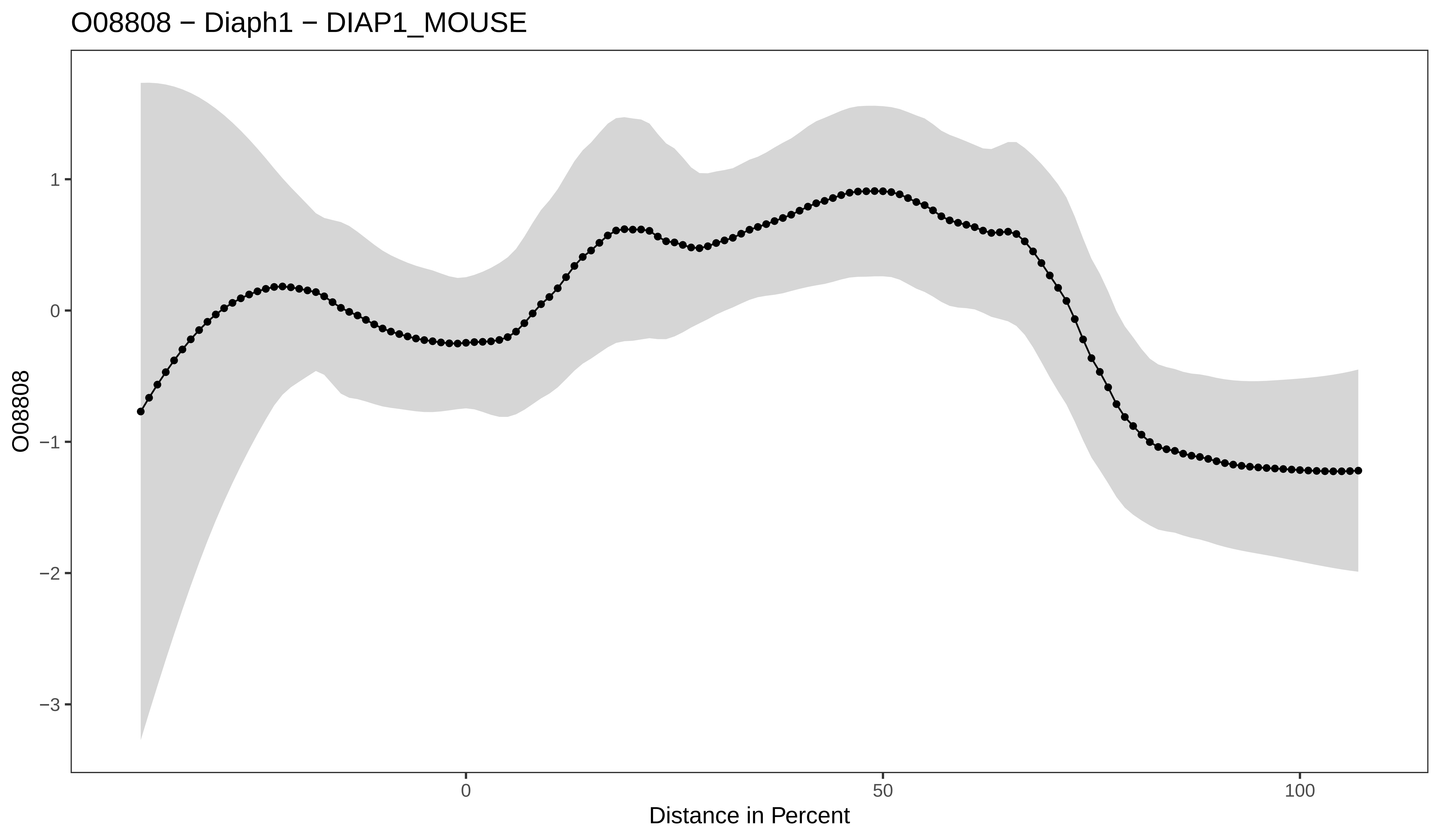
O08808 − Diaph1 − DIAP1_MOUSE
O08808
1
0
−1
−2
−3
0
50
100
Distance in Percent

Q8BWT5 − Dip2a − DIP2A_MOUSE

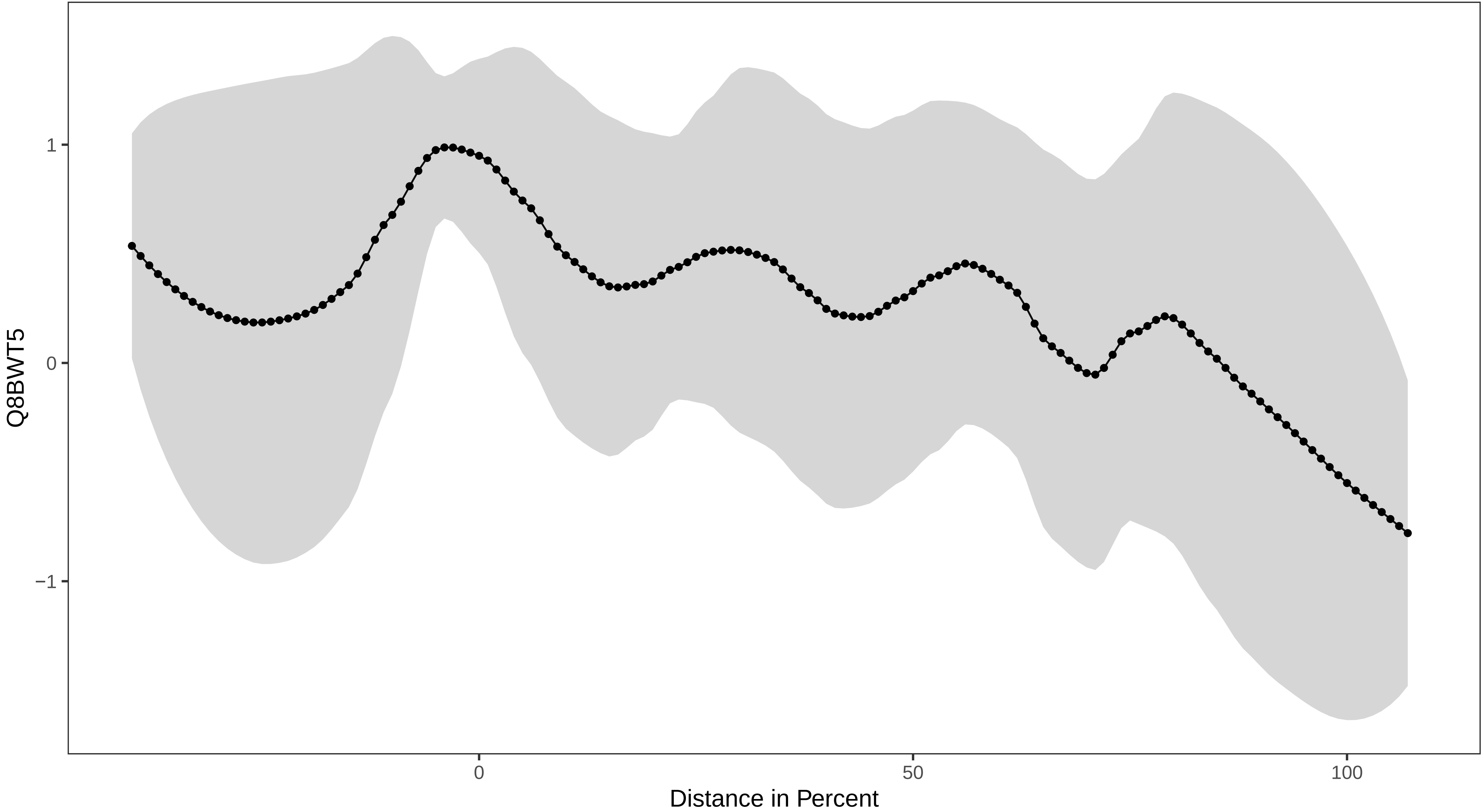

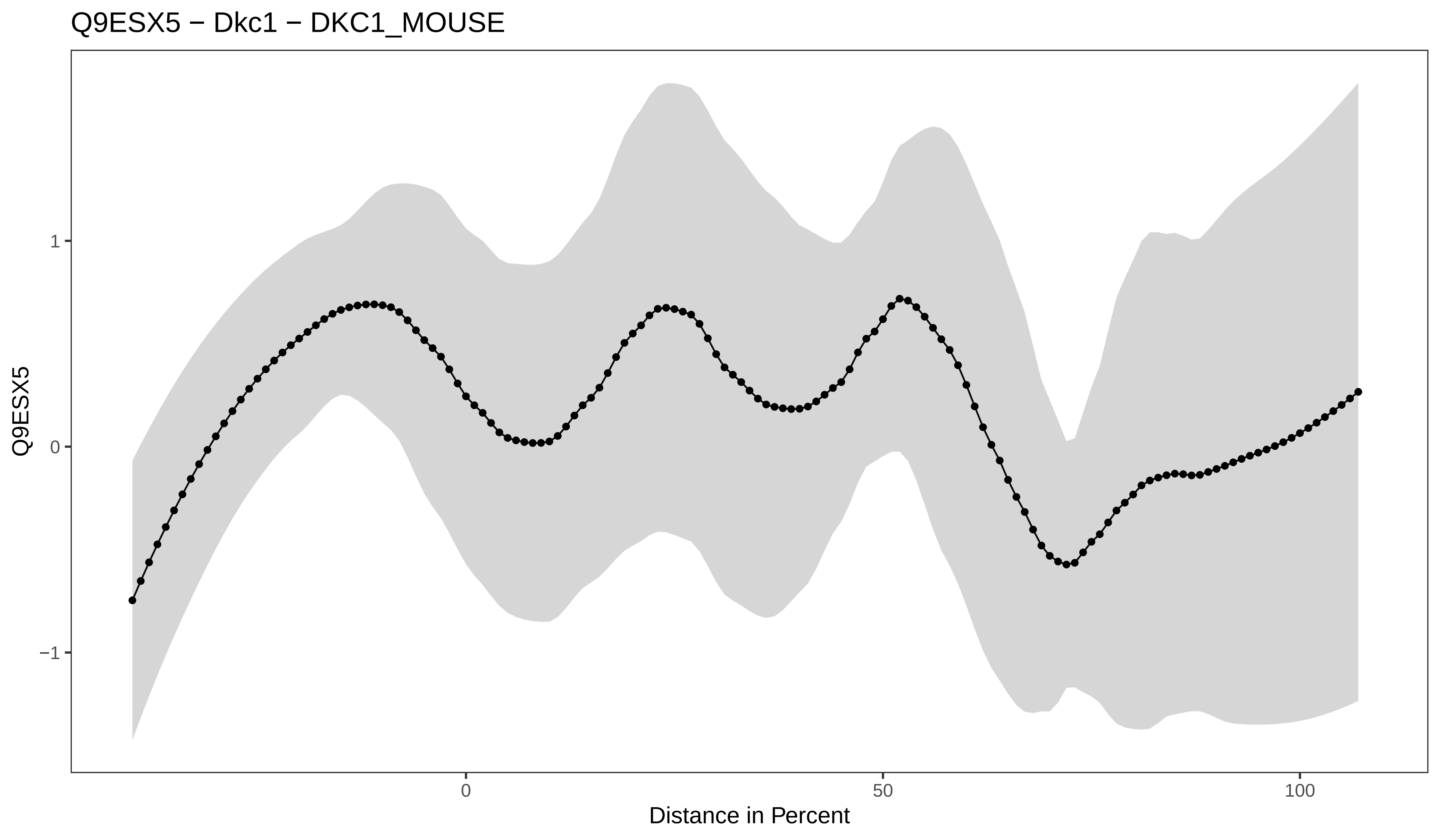
Q9ESX5 − Dkc1 − DKC1_MOUSE
Q9ESX5
1
0
−1
0
50
100
Distance in Percent

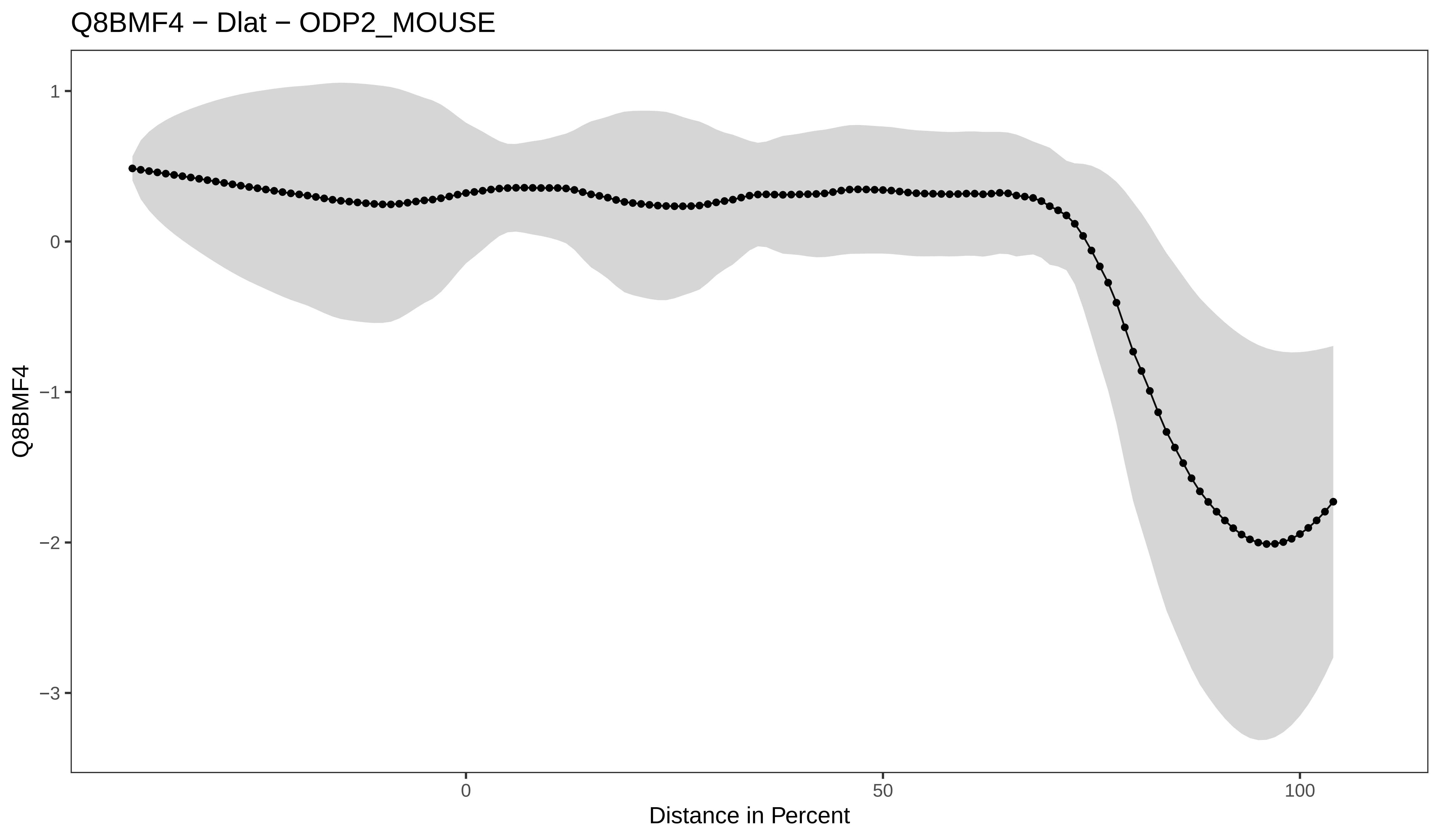

Q8BMF4 − Dlat − ODP2_MOUSE
Q8BMF4
1
0
−1
−2
−3
0
50
100
Distance in Percent

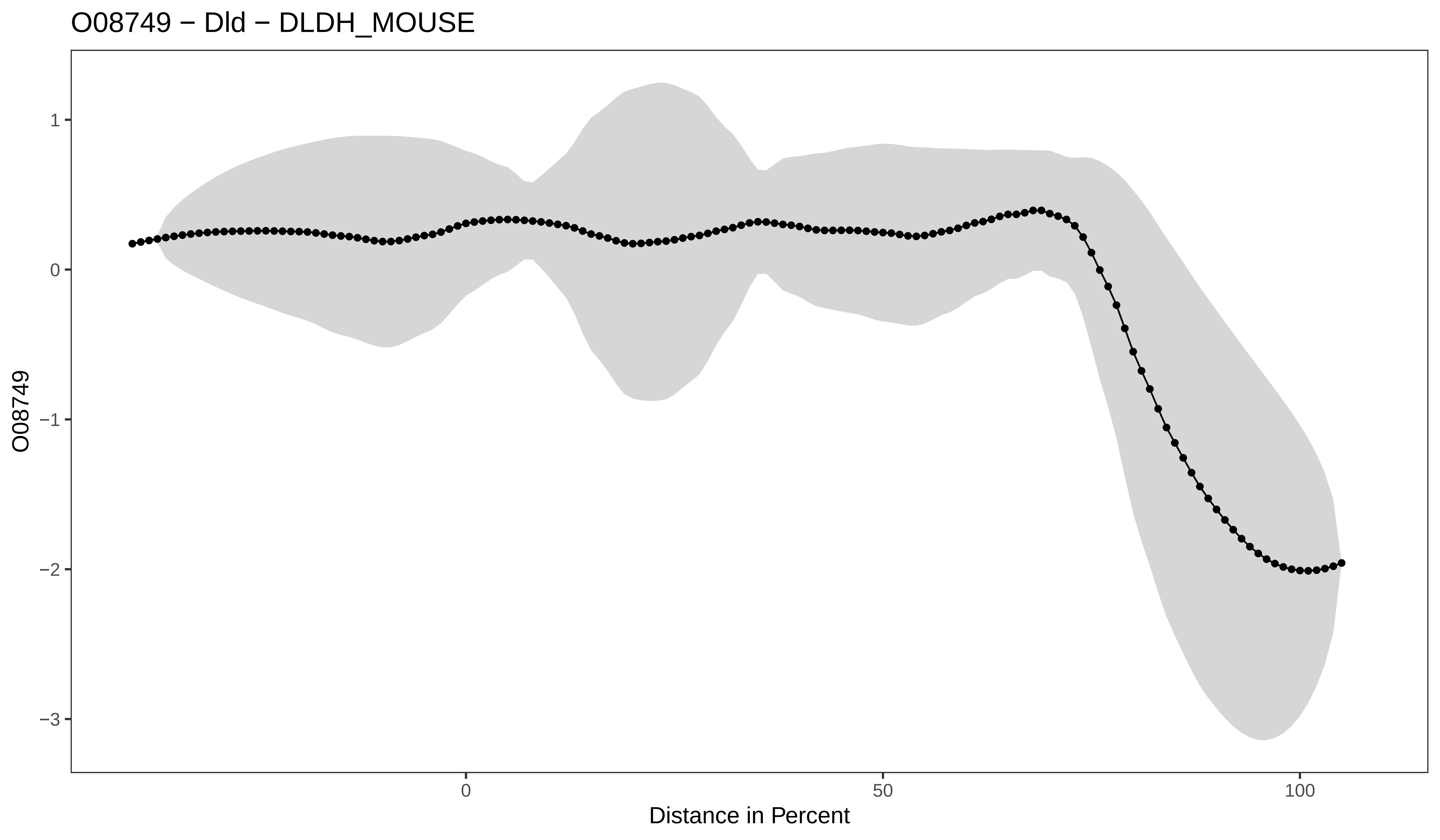
O08749 − Dld − DLDH_MOUSE
1
0
−1
−2
−3
O08749
0
50
100
Distance in Percent

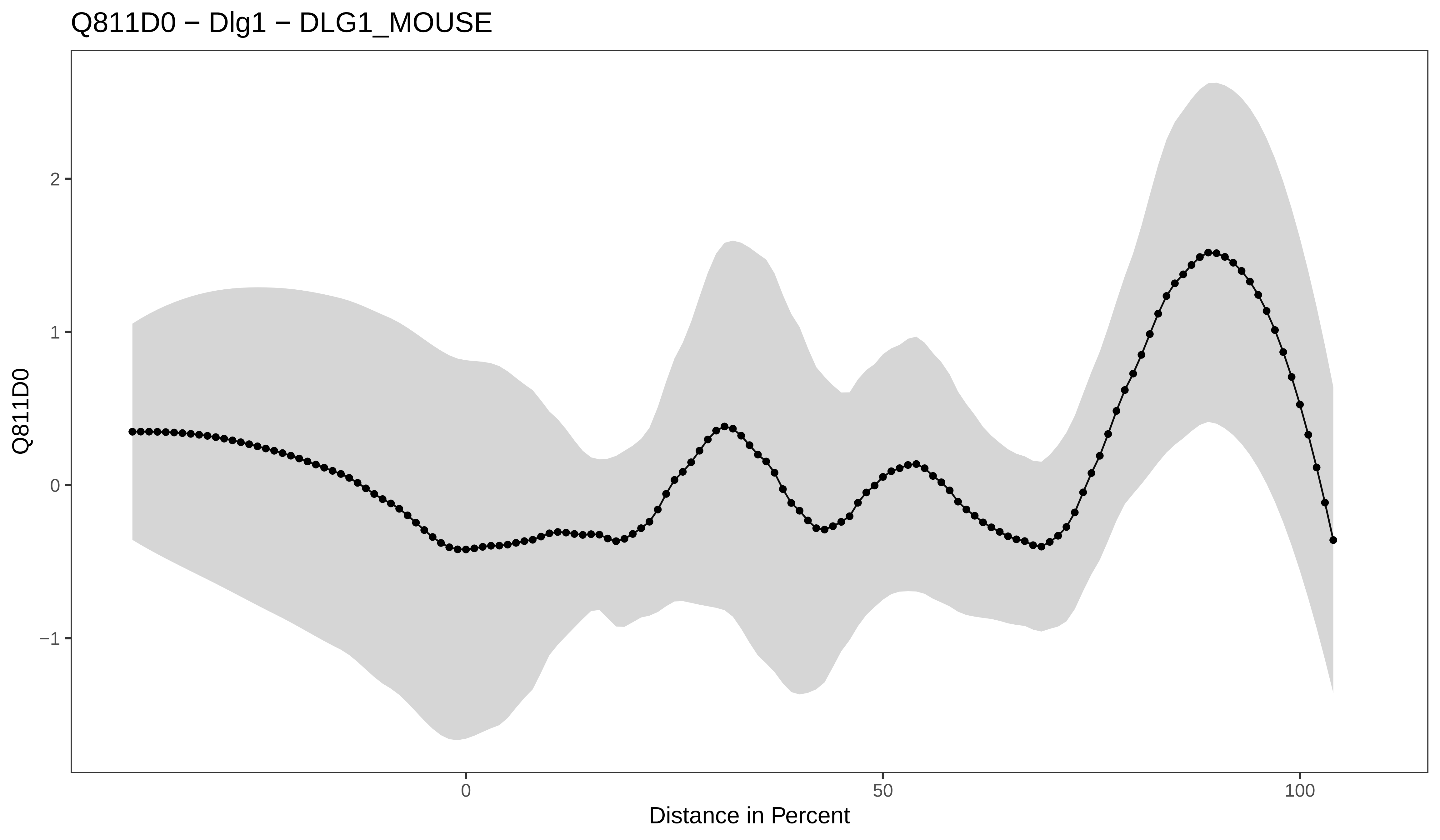

Q811D0 − Dlg1 − DLG1_MOUSE
Q811D0
2
1
0
−1
0
50
100
Distance in Percent

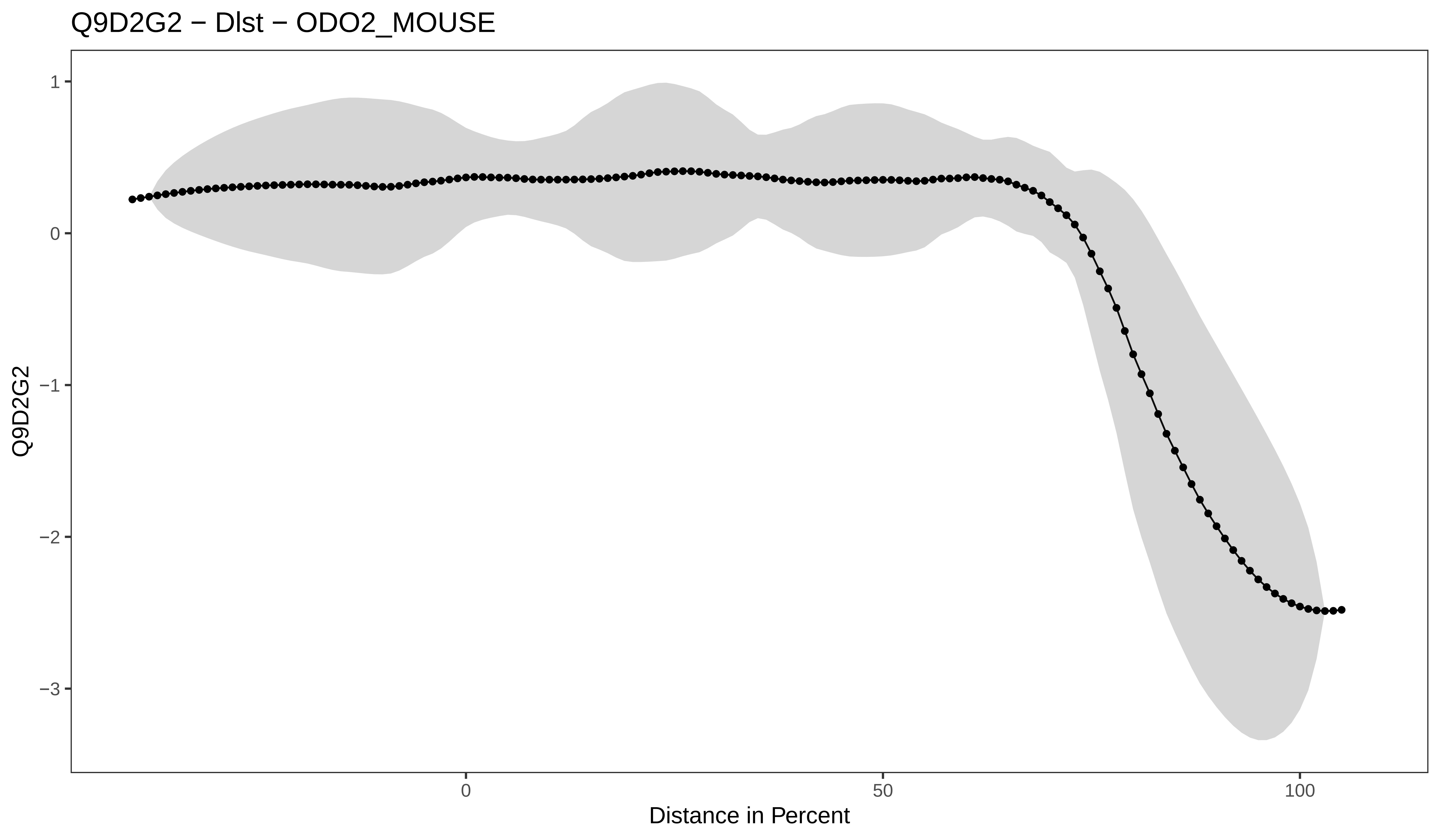

Q9D2G2 − Dlst − ODO2_MOUSE
1
0
−1
−2
−3
Q9D2G2
0
50
100
Distance in Percent

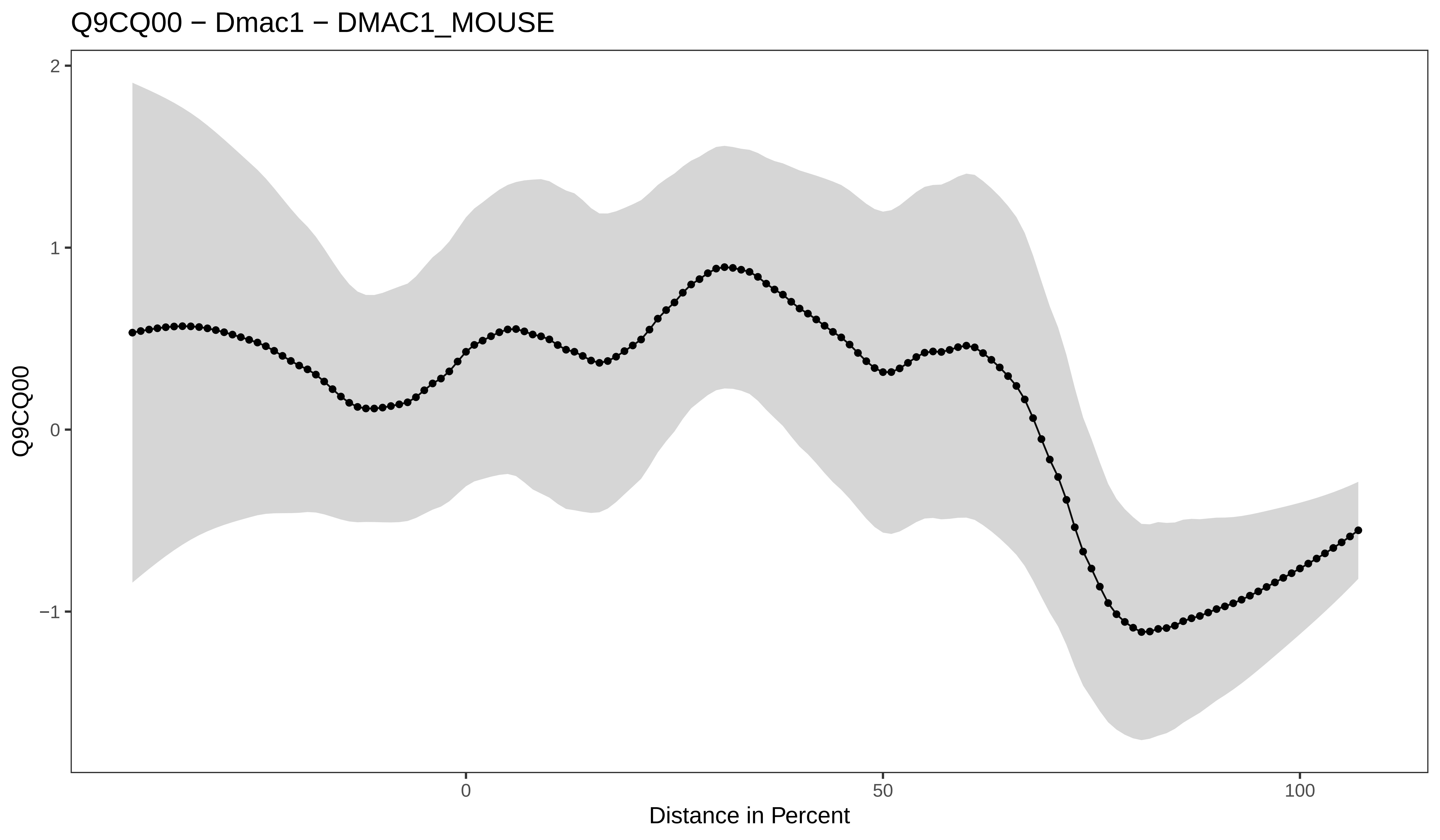

Q9CQ00 − Dmac1 − DMAC1_MOUSE
Q9CQ00
Distance in Percent
2
1
0
−1
0
50
100

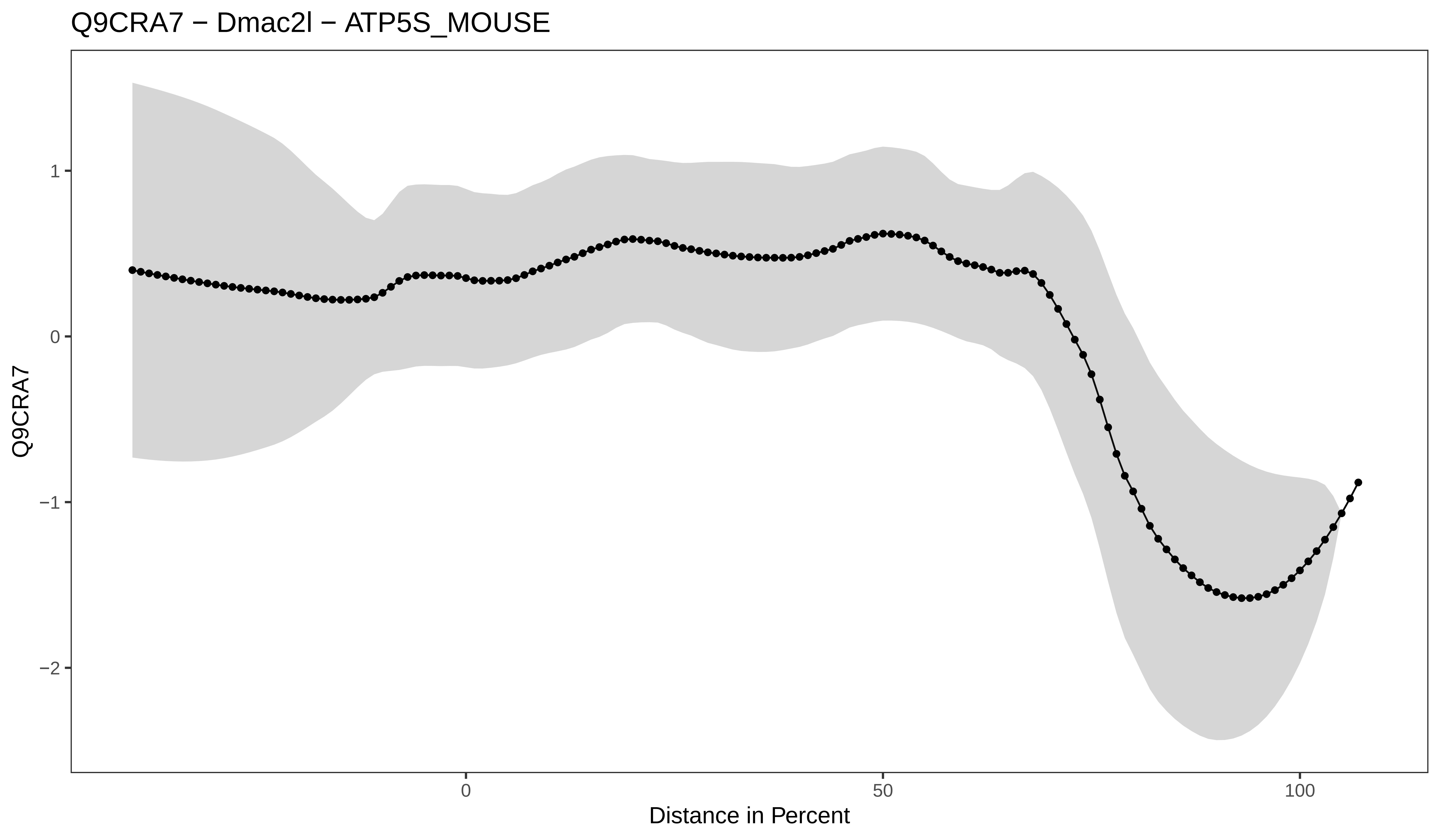
Q9CRA7 − Dmac2l − ATP5S_MOUSE
Q9CRA7
Distance in Percent
1
0
−1
−2
0
50
100

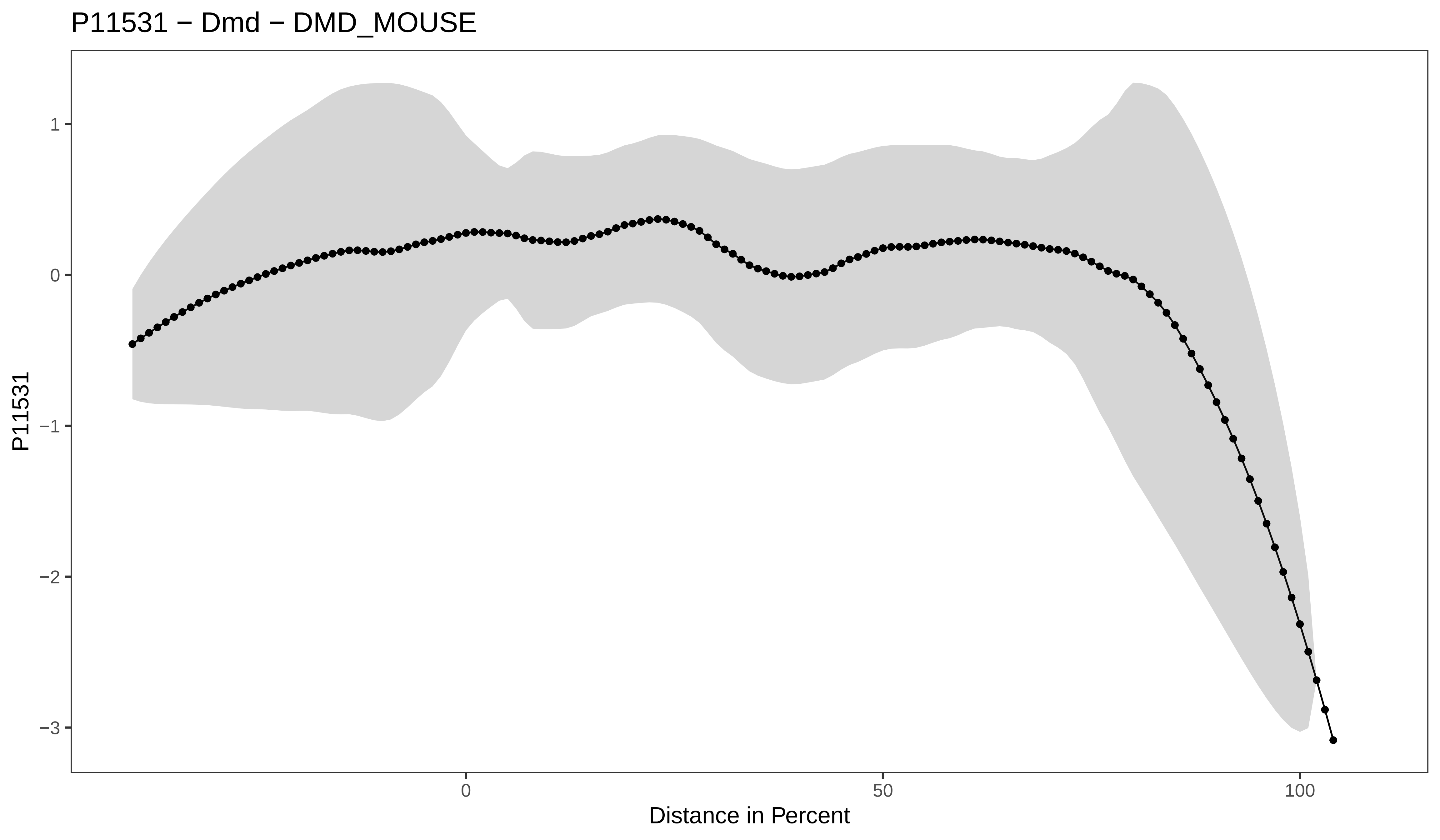
P11531 − Dmd − DMD_MOUSE
P11531
1
0
−1
−2
−3
0
50
100
Distance in Percent

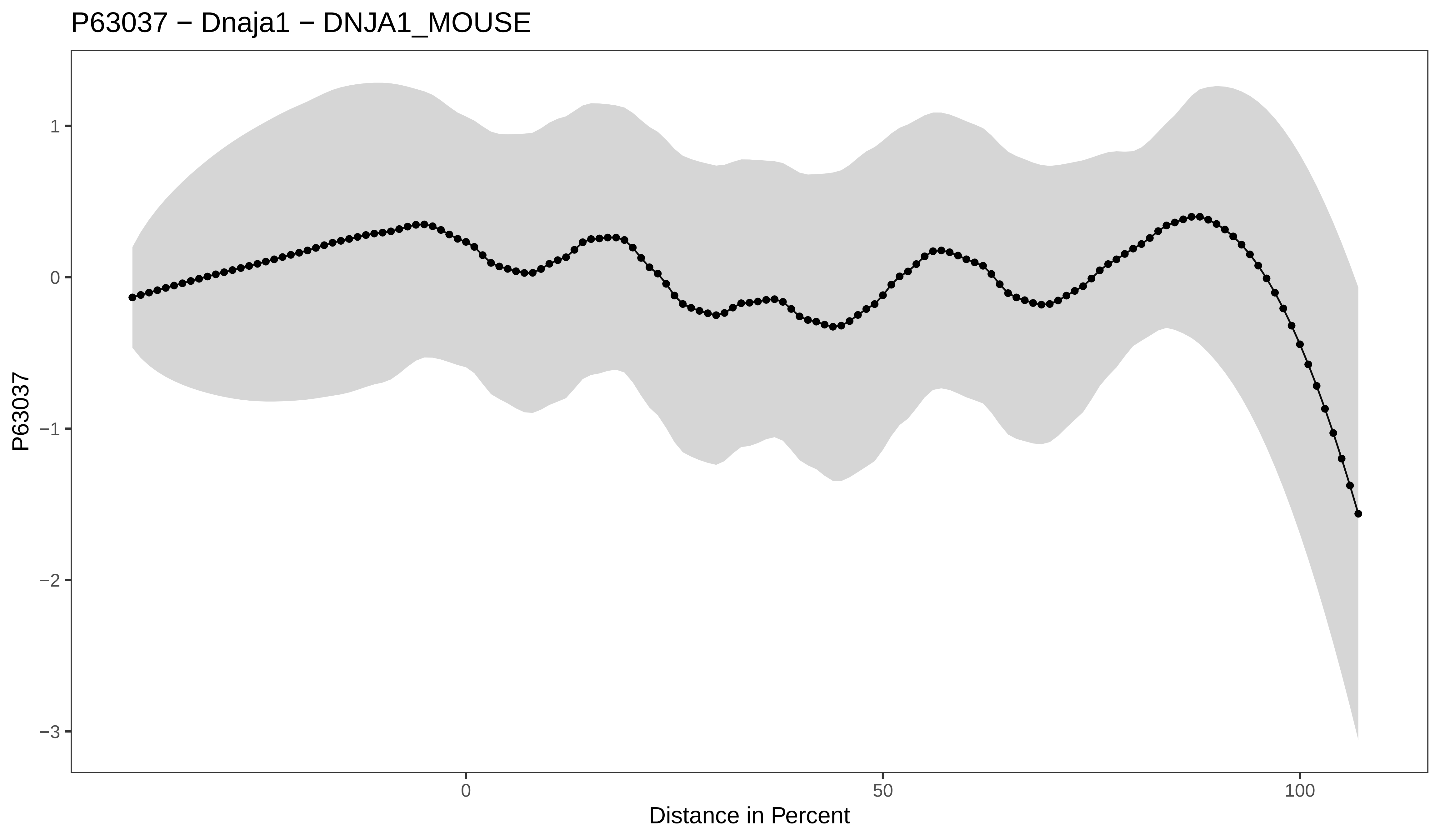
P63037 − Dnaja1 − DNJA1_MOUSE
P63037
1
0
−1
−2
−3
0
50
100
Distance in Percent

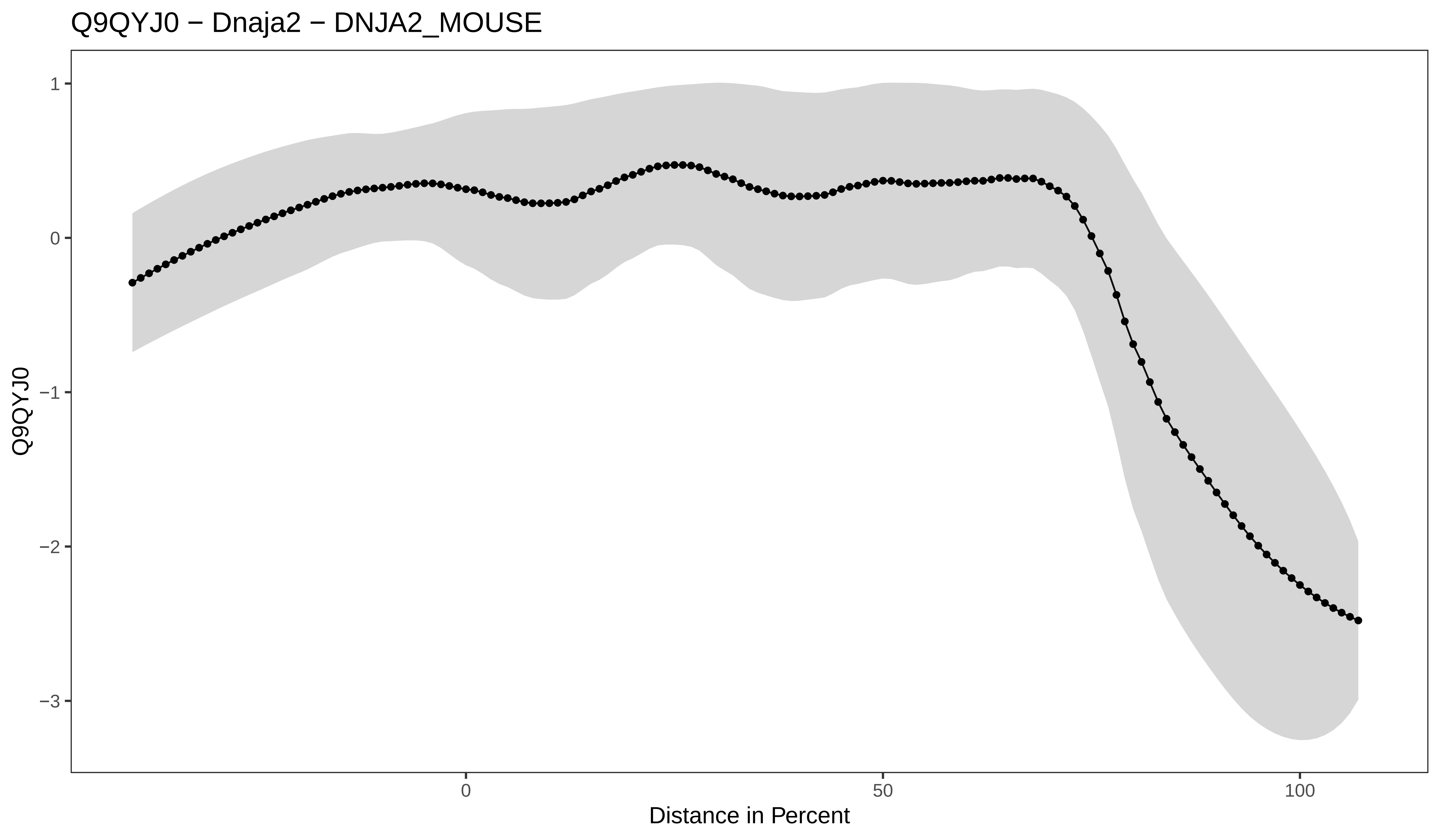

Q9QYJ0 − Dnaja2 − DNJA2_MOUSE
1
0
−1
−2
−3
Q9QYJ0
0
50
100
Distance in Percent

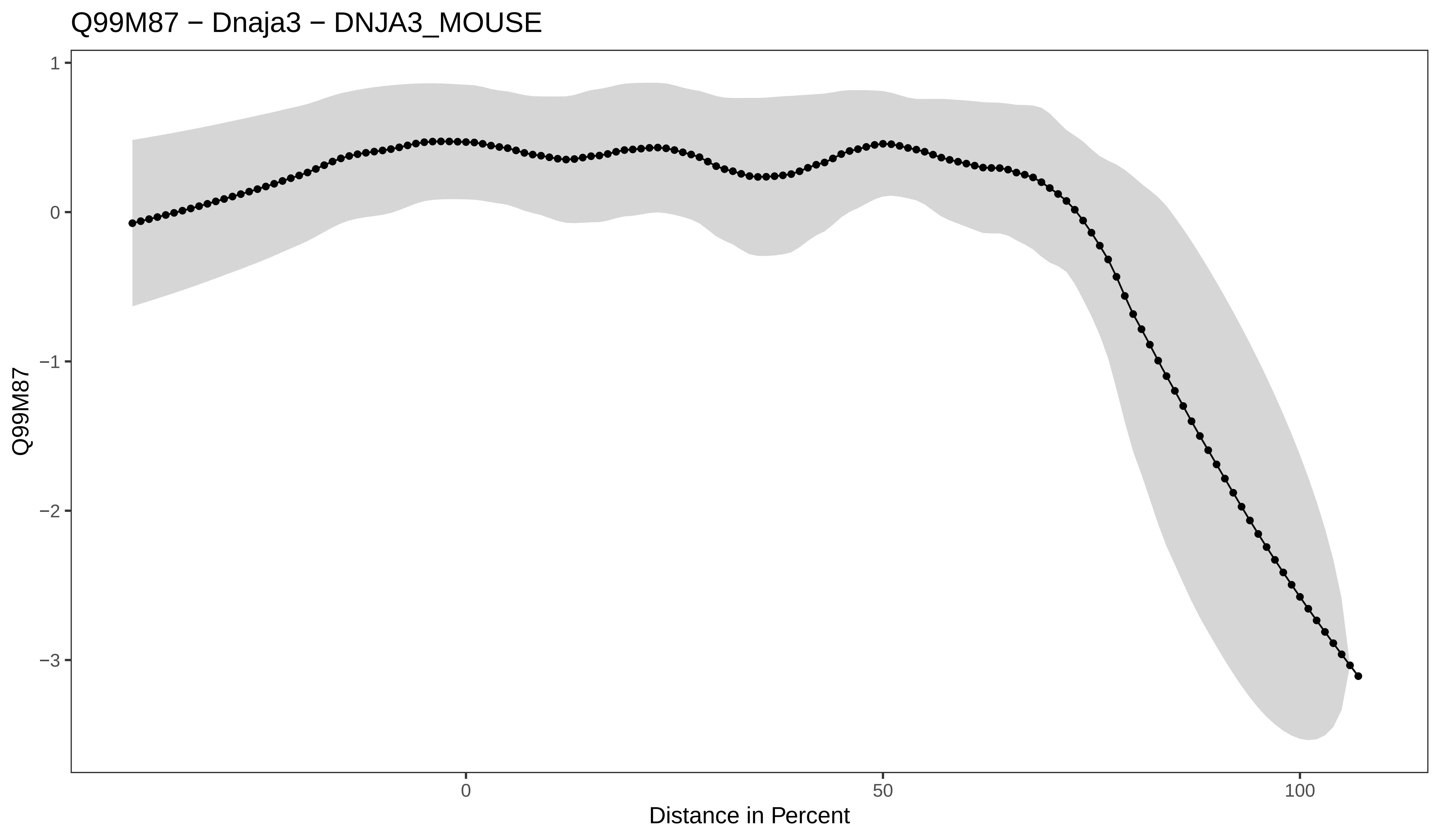

Q99M87 − Dnaja3 − DNJA3_MOUSE
1
0
−1
−2
−3
Q99M87
0
50
100
Distance in Percent

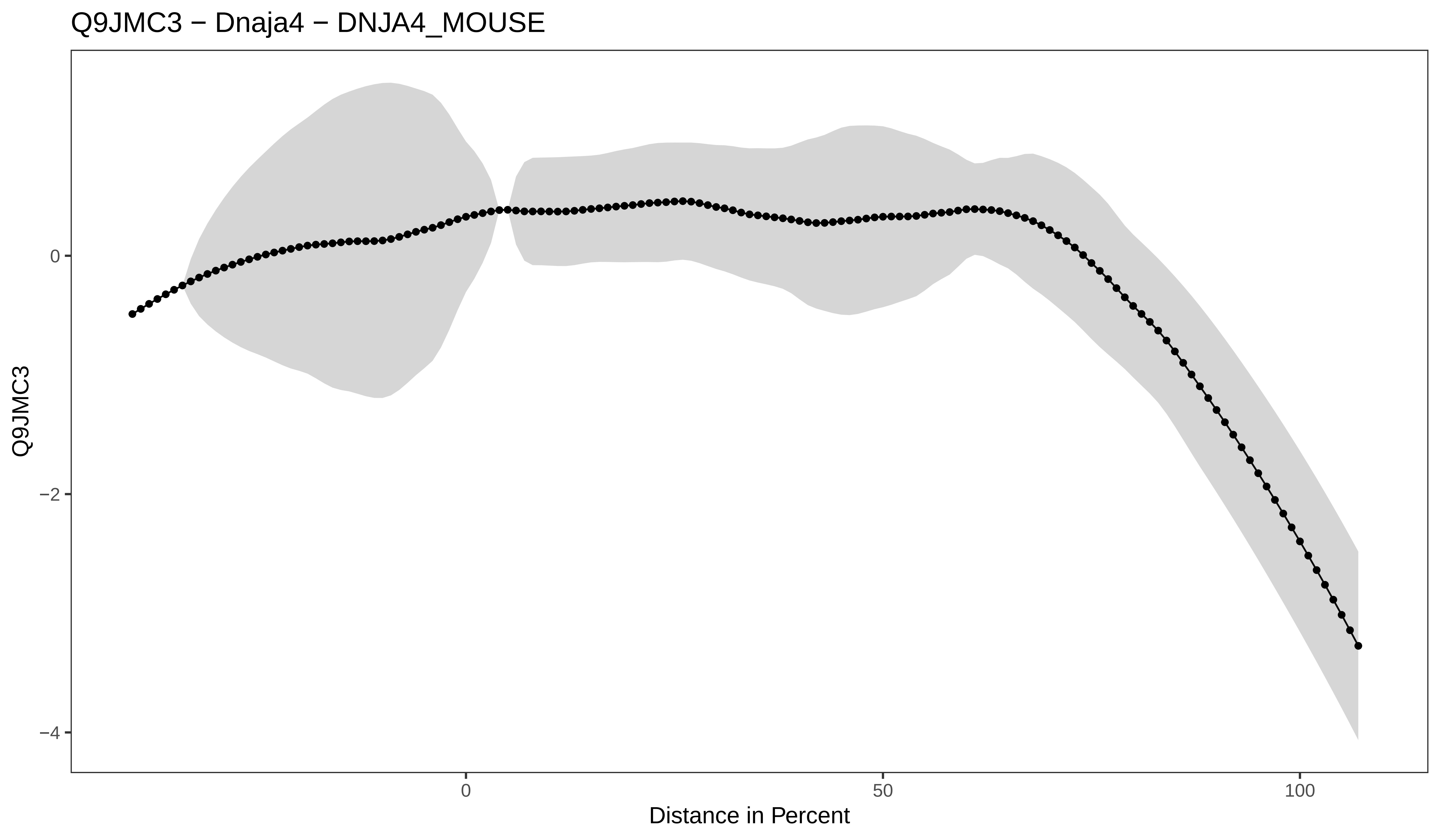
Q9JMC3 − Dnaja4 − DNJA4_MOUSE
Q9JMC3
0
−2
−4
0
50
100
Distance in Percent

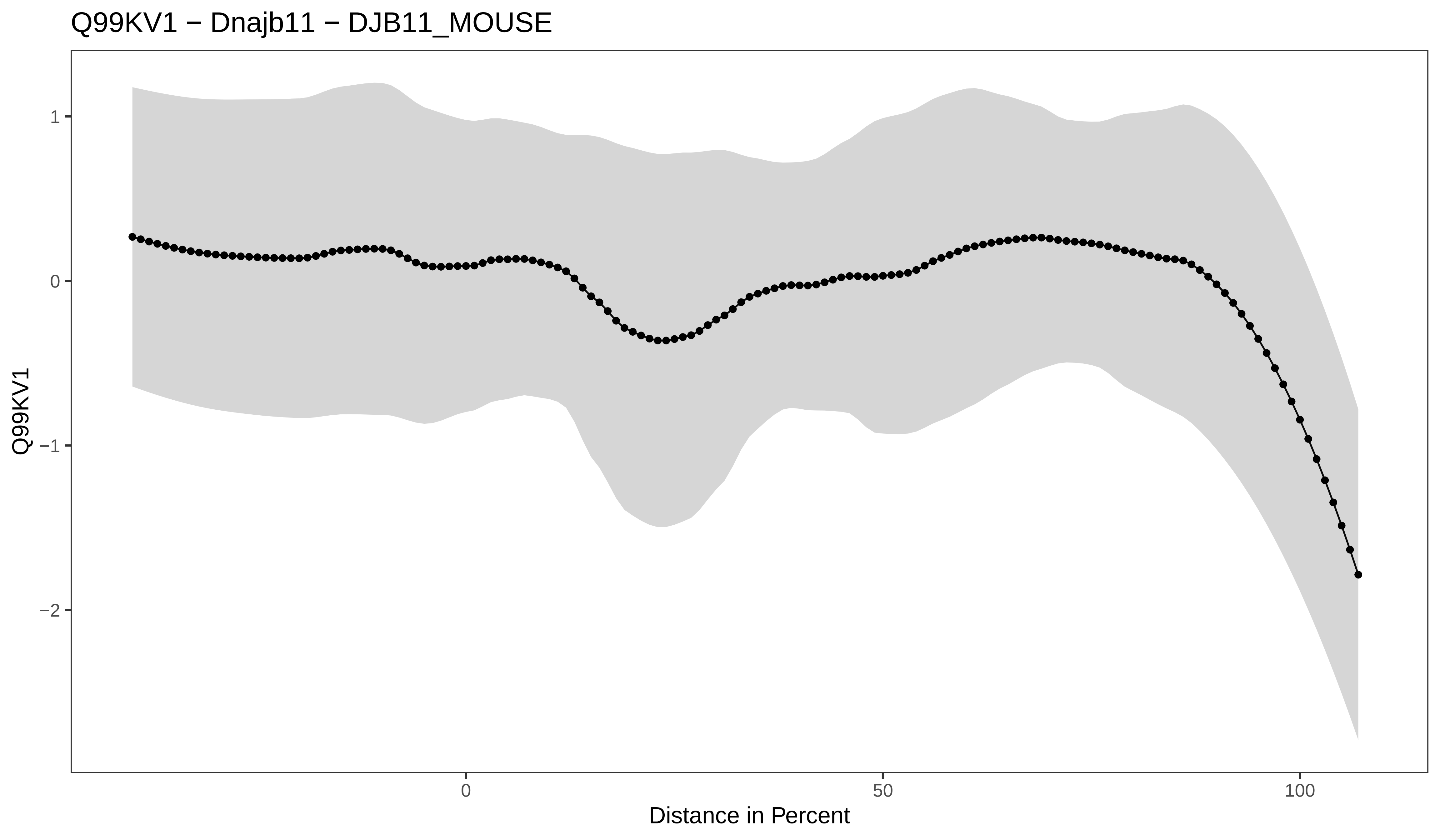
Q99KV1 − Dnajb11 − DJB11_MOUSE
1
0
−1
−2
Q99KV1
0
50
100
Distance in Percent

Q9QYI4 − Dnajb12 − DJB12_MOUSE

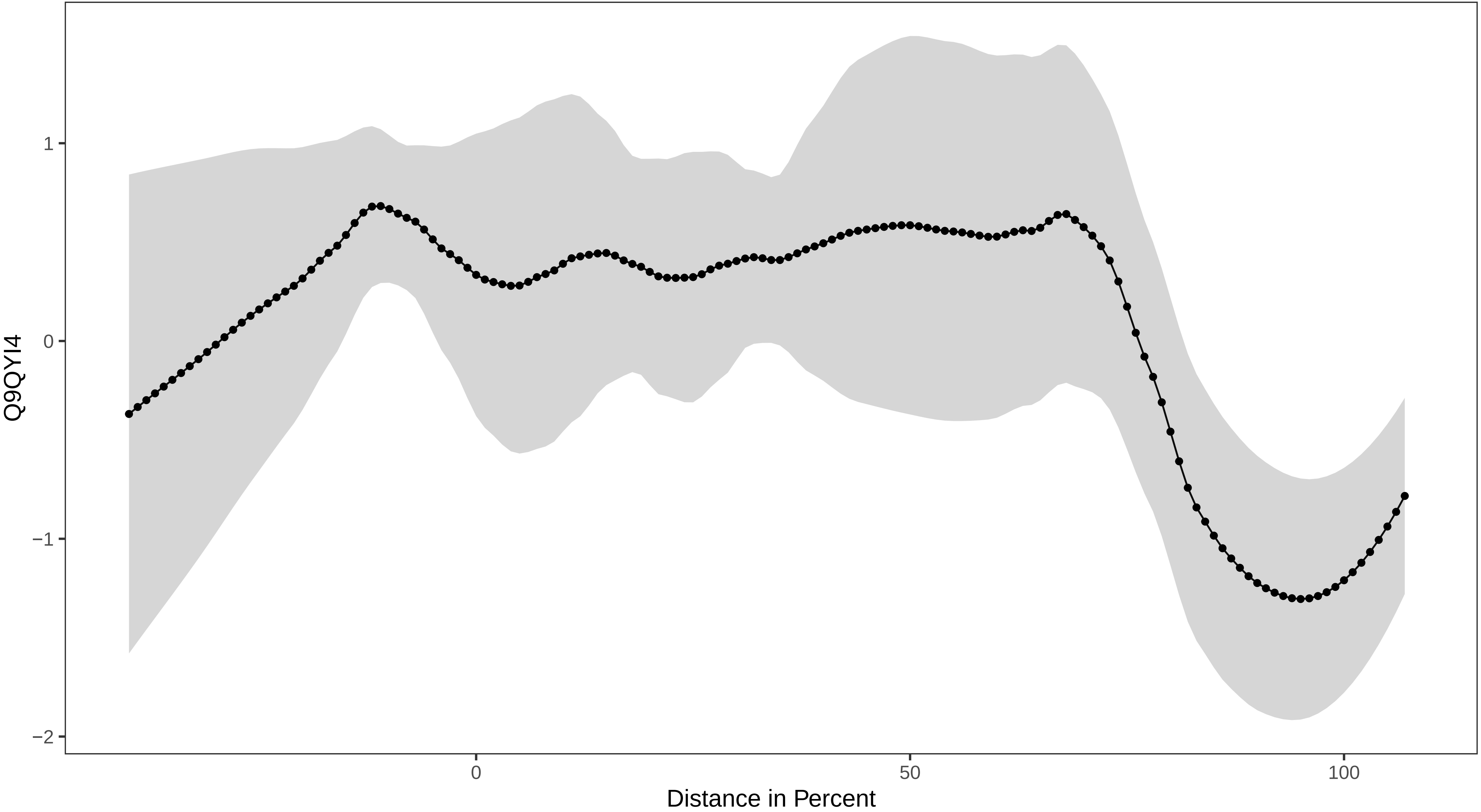

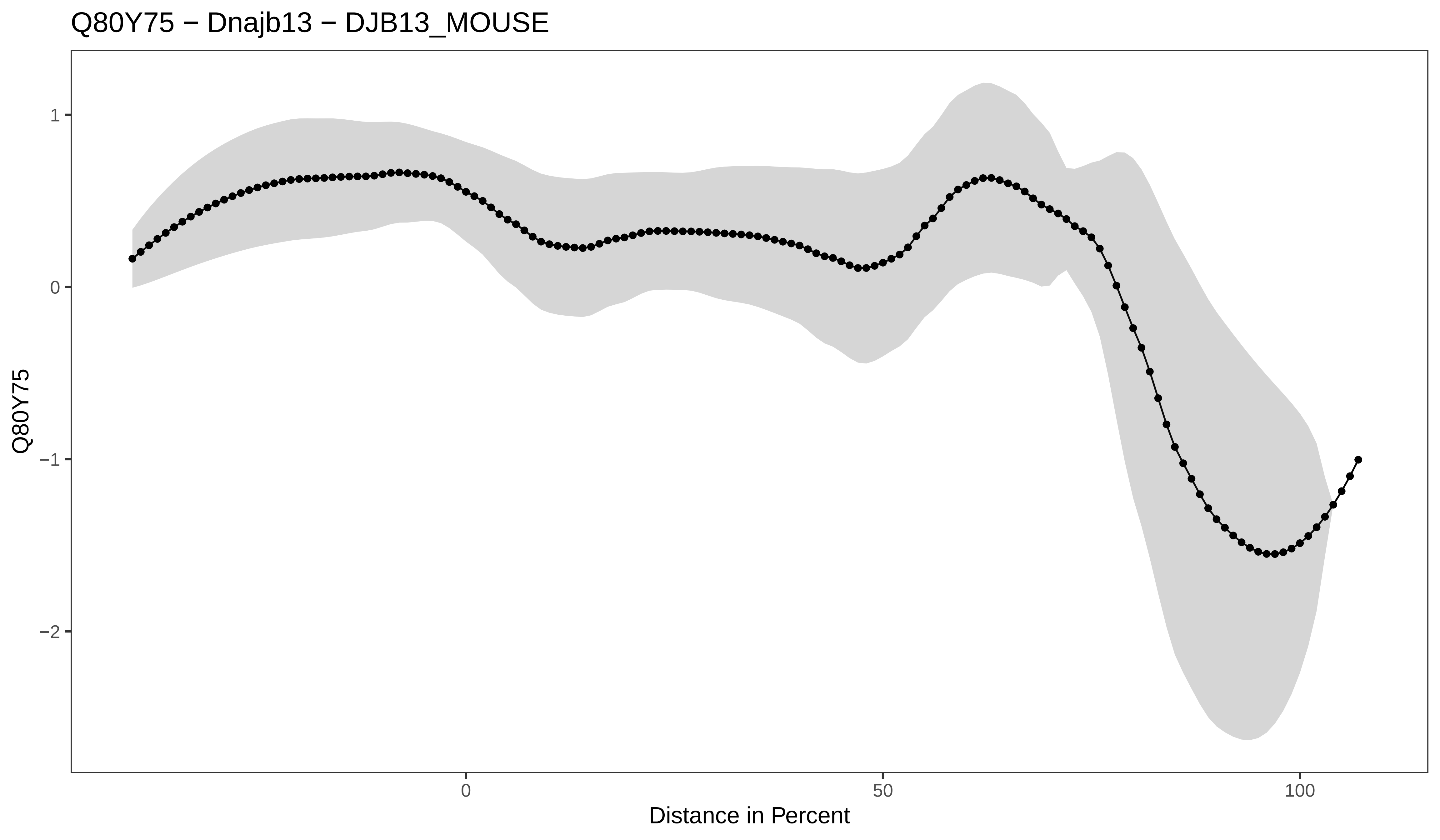

Q80Y75 − Dnajb13 − DJB13_MOUSE
1
0
−1
−2
Q80Y75
0
50
100
Distance in Percent

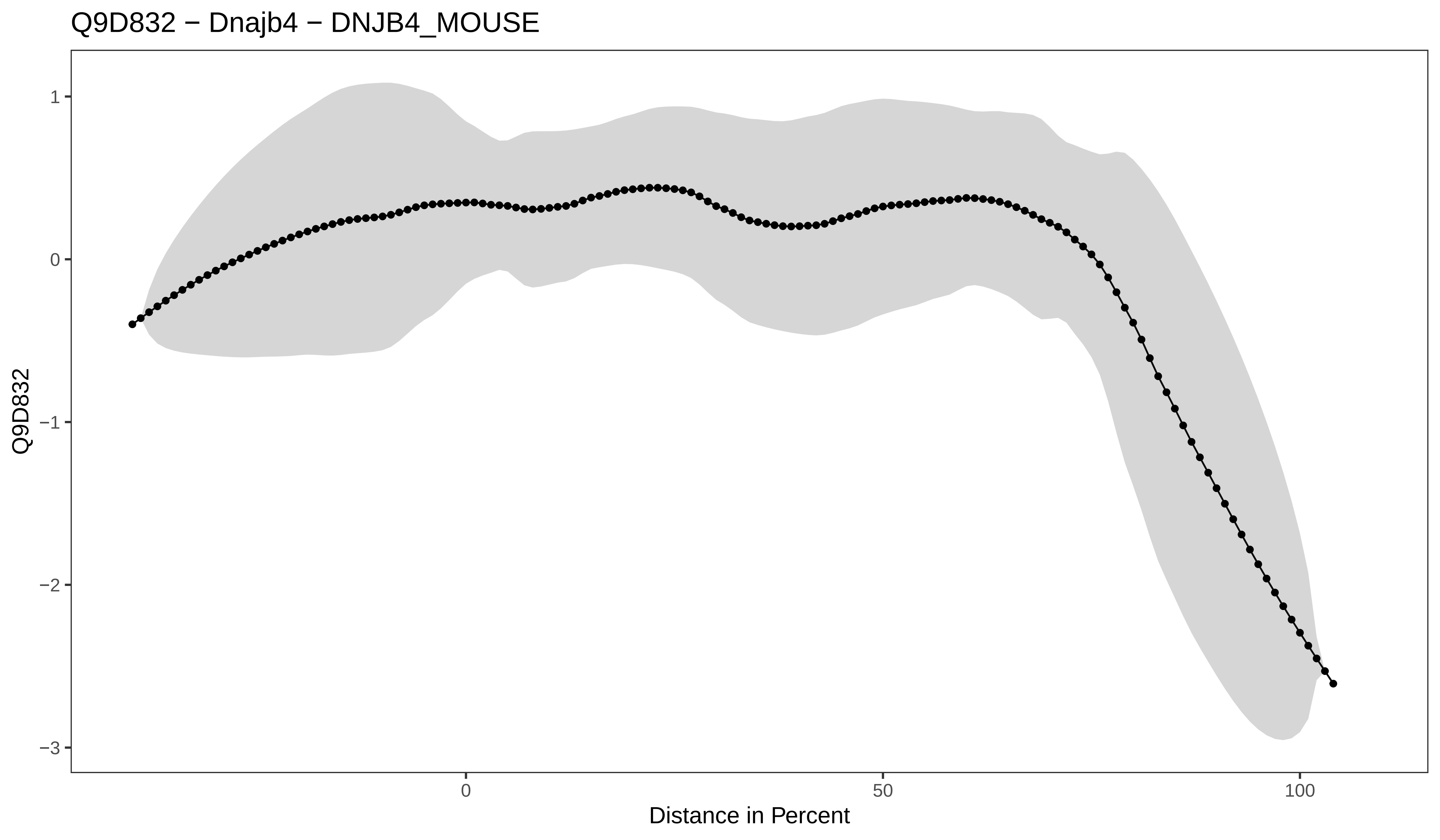

Q9D832 − Dnajb4 − DNJB4_MOUSE
1
0
−1
−2
−3
Q9D832
0
50
100
Distance in Percent

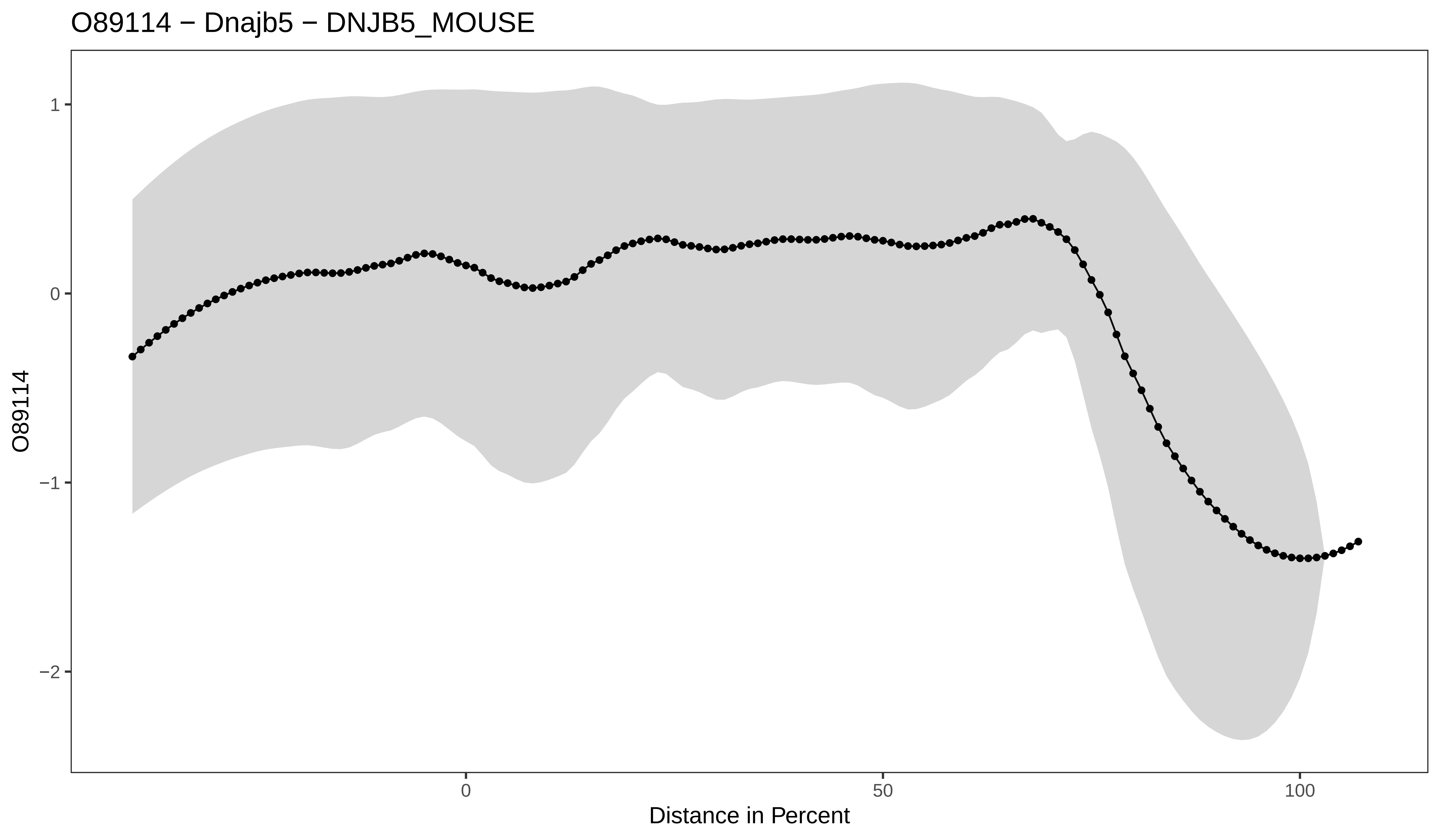

O89114 − Dnajb5 − DNJB5_MOUSE
1
0
−1
−2
O89114
0
50
100
Distance in Percent

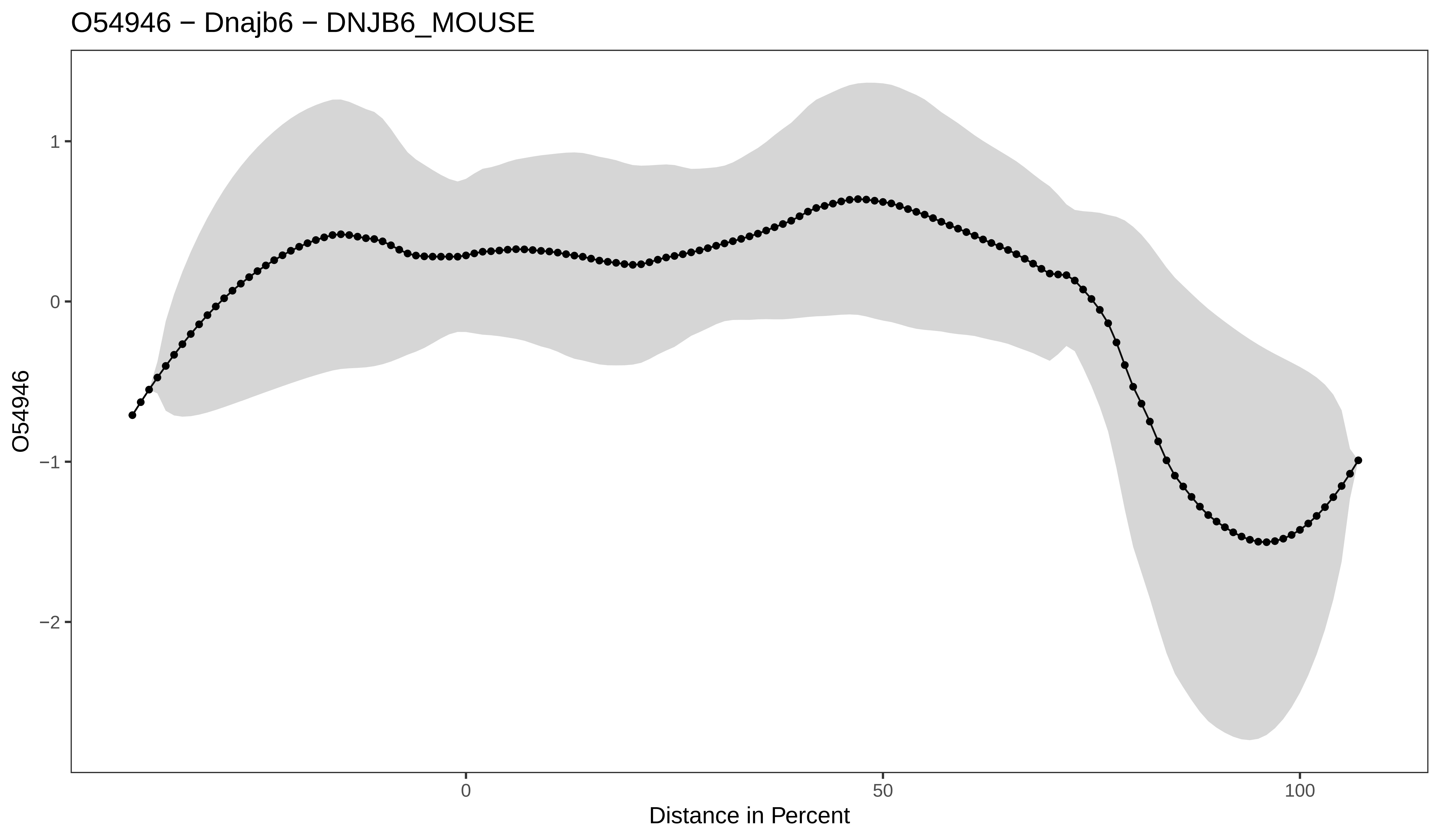

O54946 − Dnajb6 − DNJB6_MOUSE
1
0
−1
−2
O54946
0
50
100
Distance in Percent

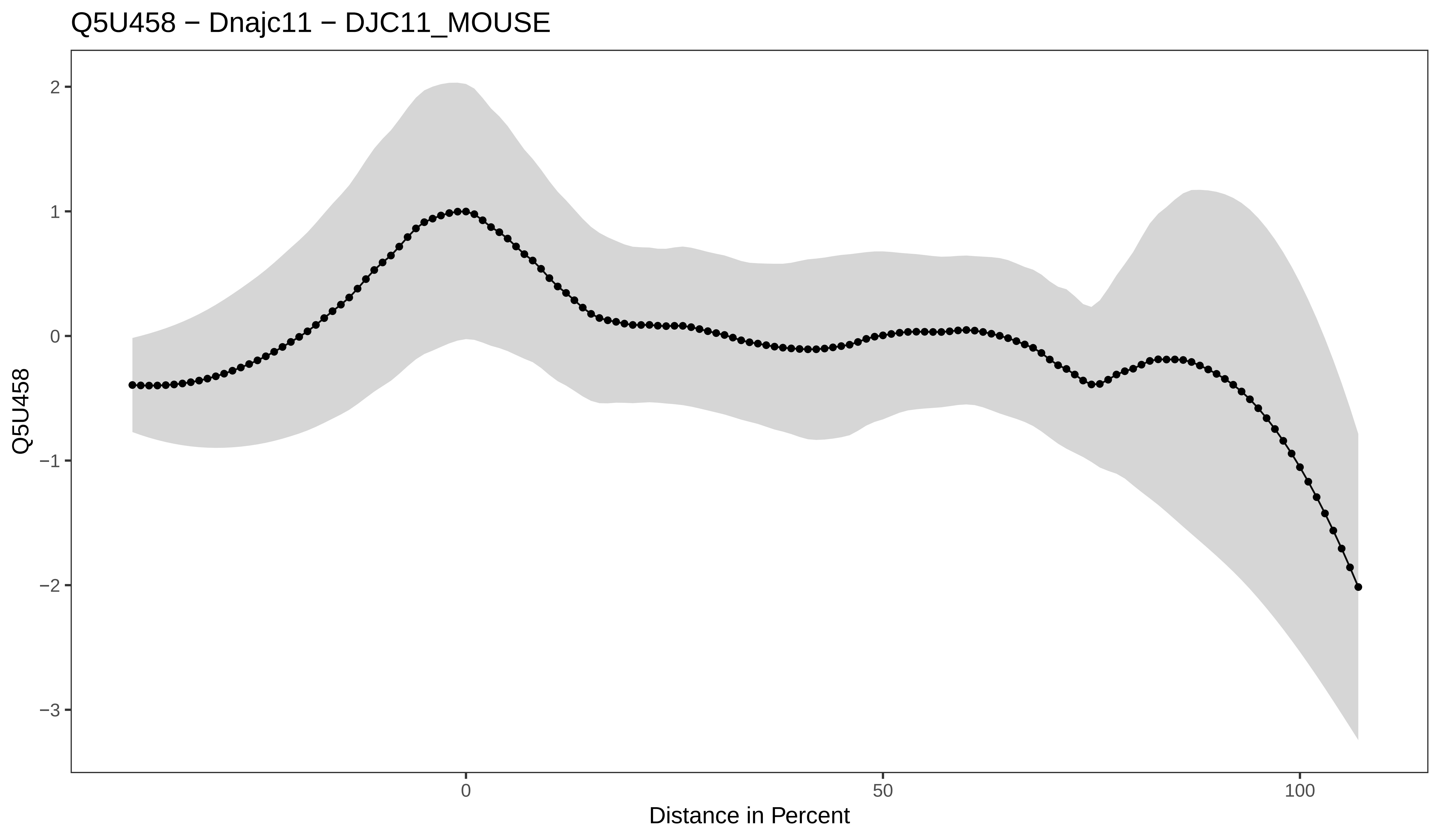
Q5U458 − Dnajc11 − DJC11_MOUSE
2
1
0
−1
−2
−3
Q5U458
0
50
100
Distance in Percent

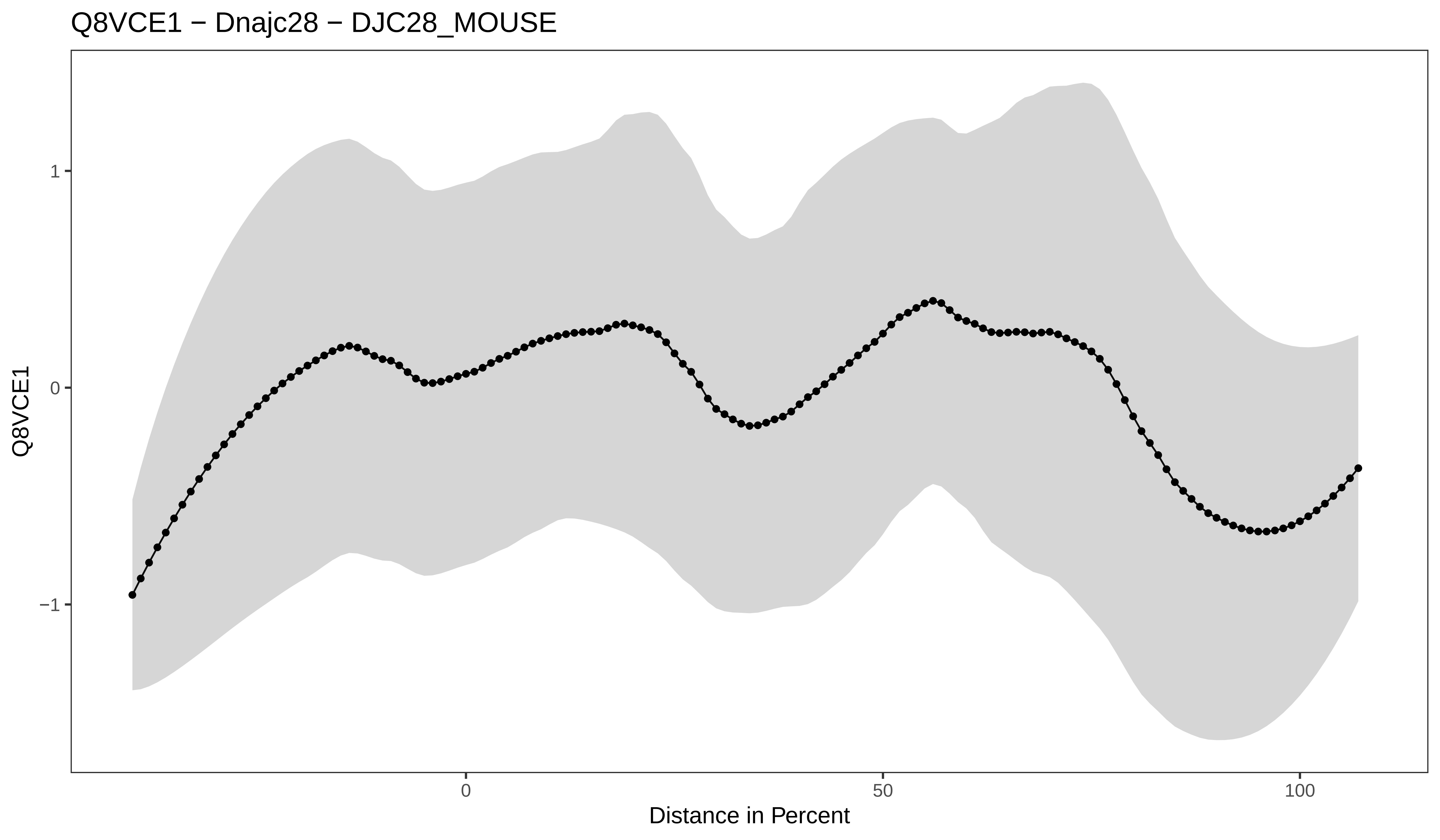
Q8VCE1 − Dnajc28 − DJC28_MOUSE
1
0
−1
Q8VCE1
0
50
100
Distance in Percent

Q6NZB0 − Dnajc8 − DNJC8_MOUSE

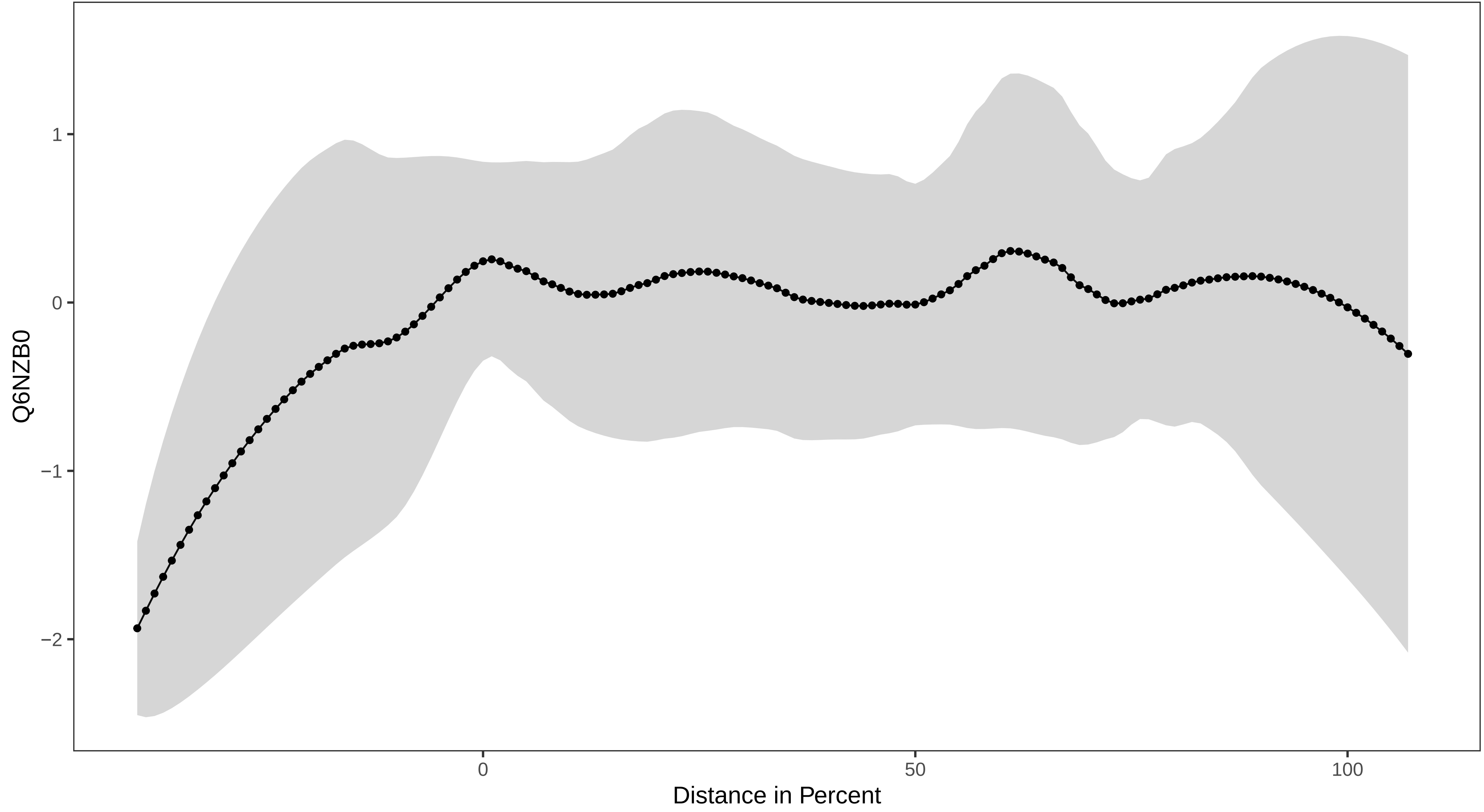

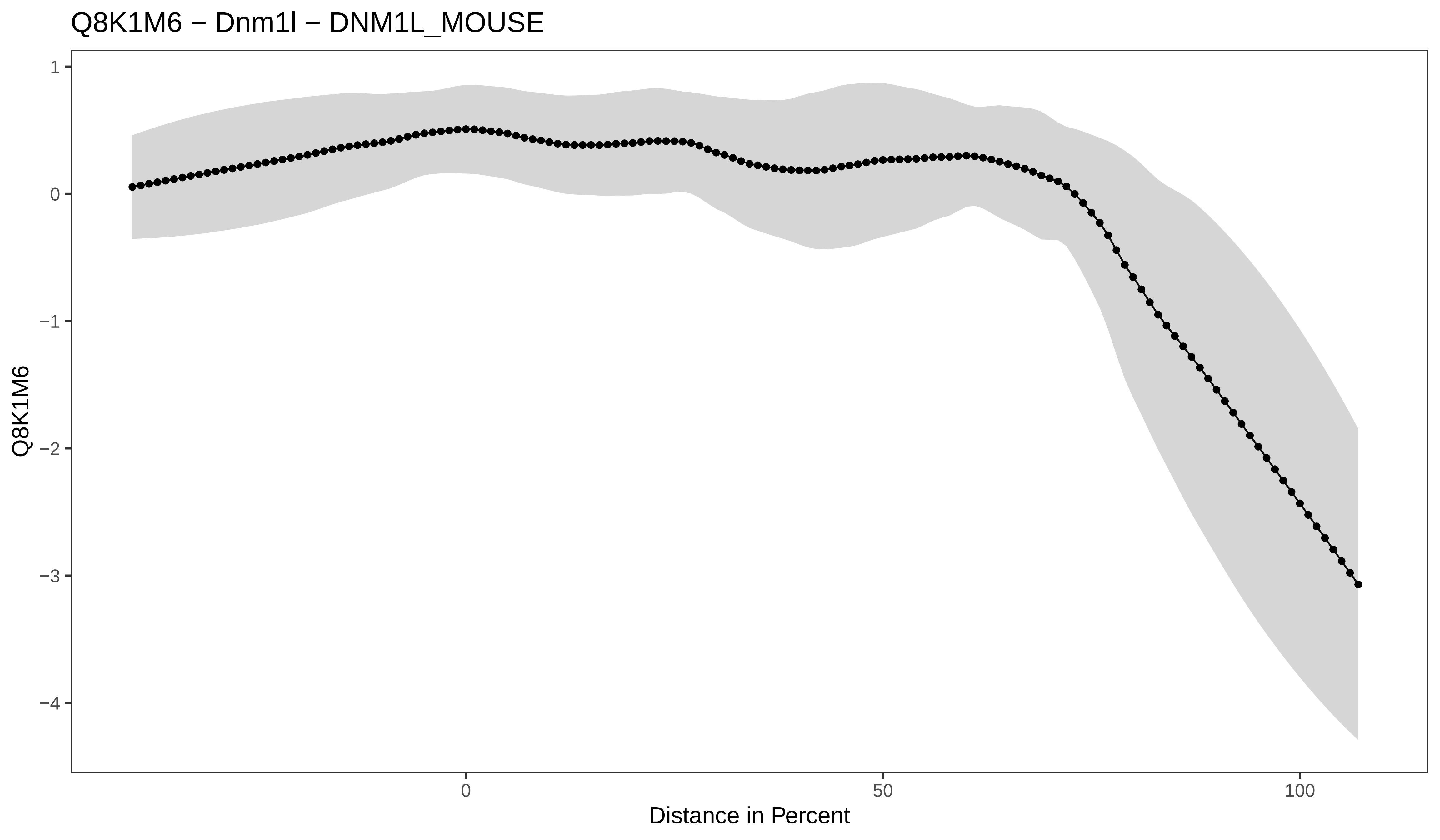

Q8K1M6 − Dnm1l − DNM1L_MOUSE
1
0
−1
−2
−3
−4
Q8K1M6
0
50
100
Distance in Percent

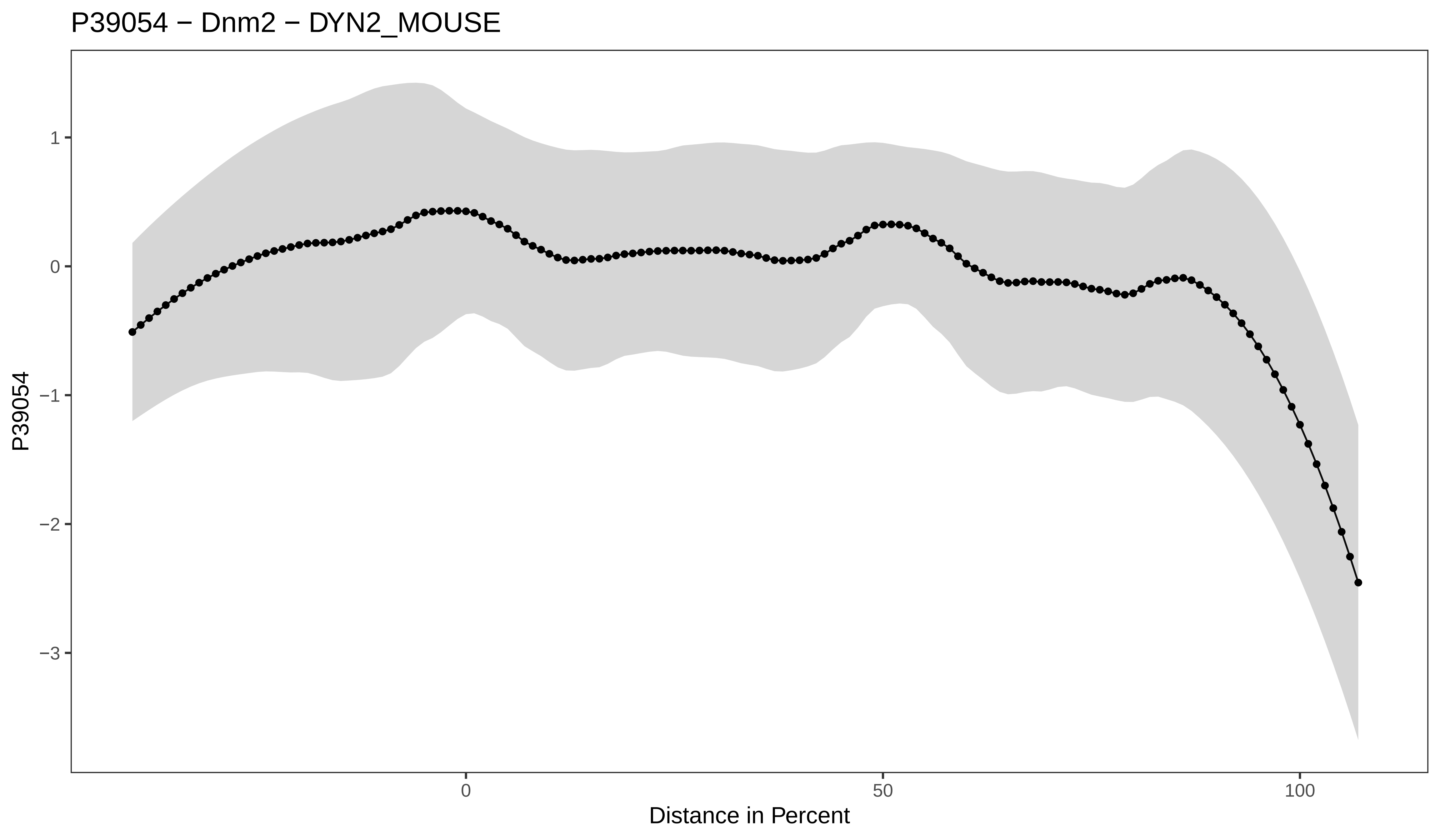
P39054 − Dnm2 − DYN2_MOUSE
1
0
−1
−2
−3
P39054
0
50
100
Distance in Percent

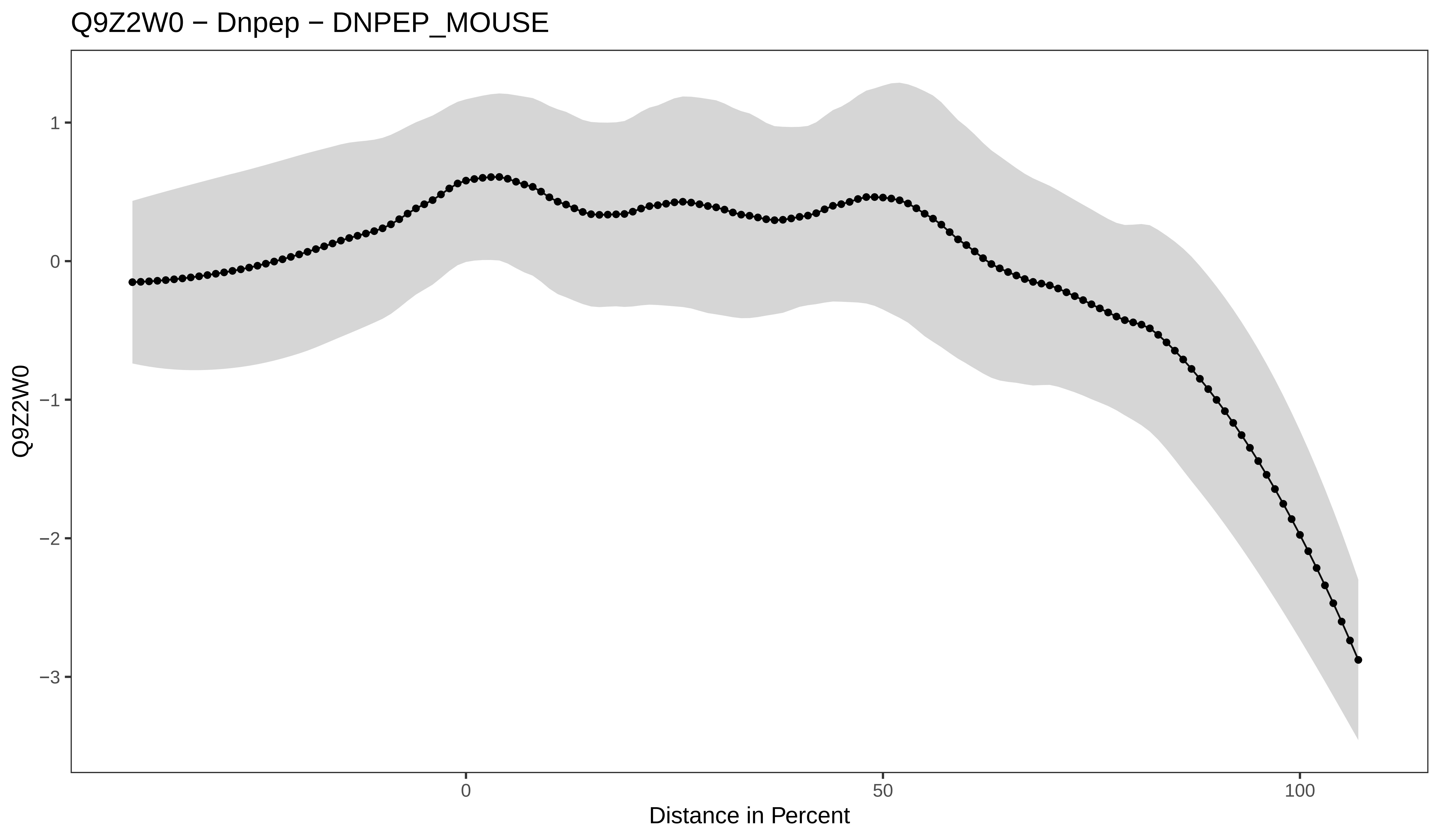
Q9Z2W0 − Dnpep − DNPEP_MOUSE
Q9Z2W0
1
0
−1
−2
−3
0
50
100
Distance in Percent

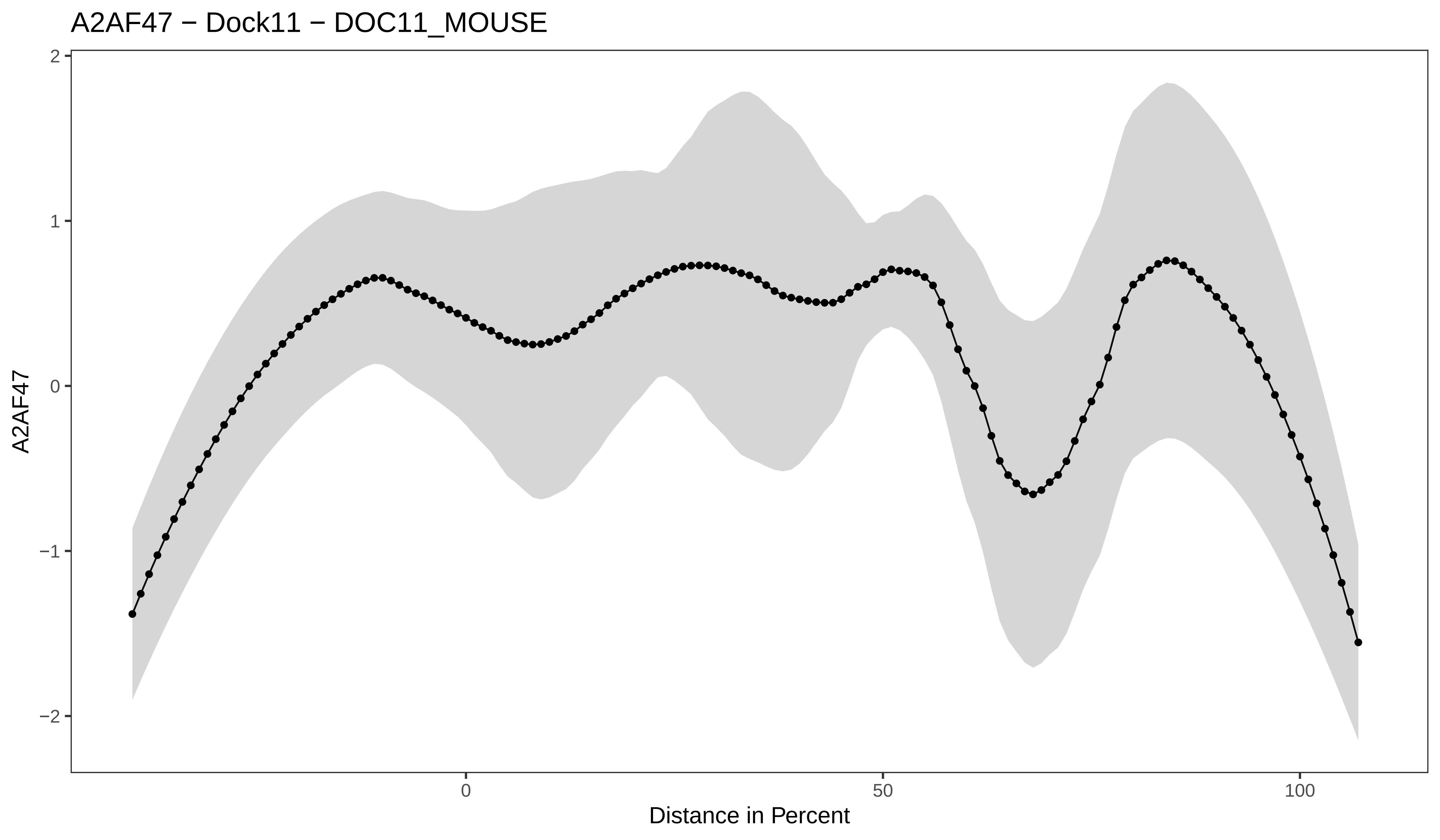

A2AF47 − Dock11 − DOC11_MOUSE
A2AF47
Distance in Percent
2
1
0
−1
−2
0
50
100

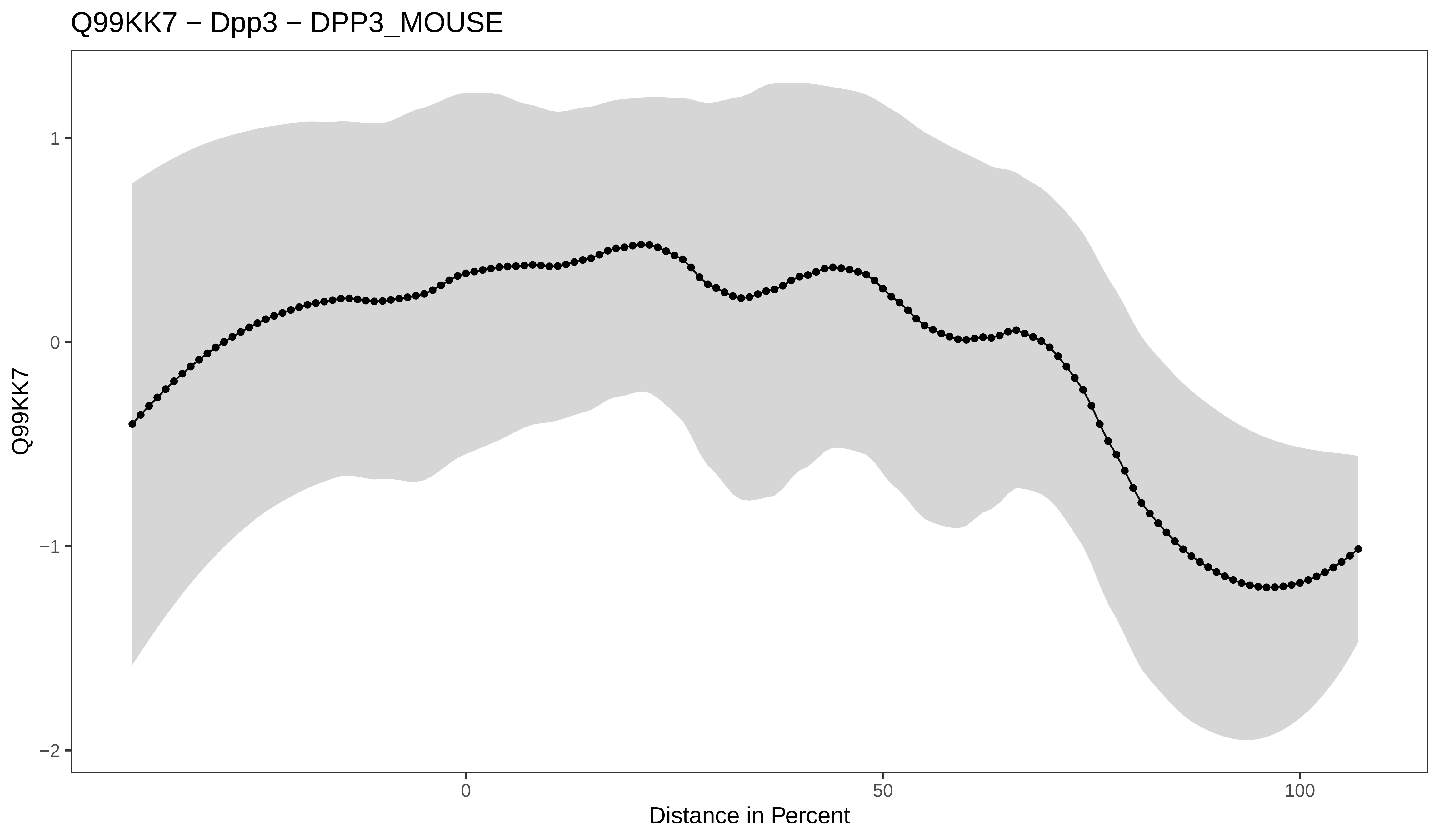

Q99KK7 − Dpp3 − DPP3_MOUSE
Q99KK7
1
0
−1
−2
0
50
100
Distance in Percent

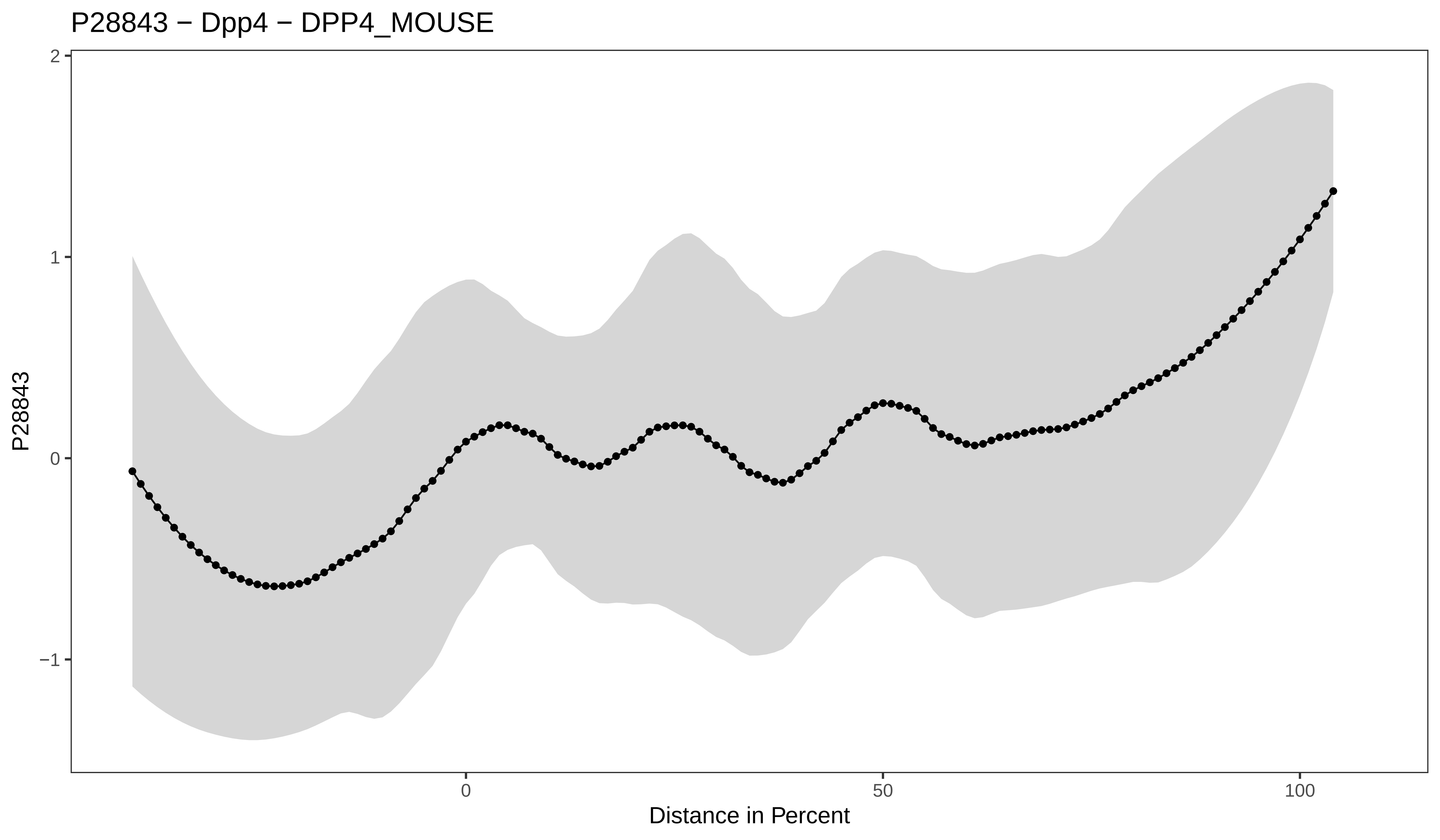

P28843 − Dpp4 − DPP4_MOUSE
2
1
0
−1
P28843
0
50
100
Distance in Percent

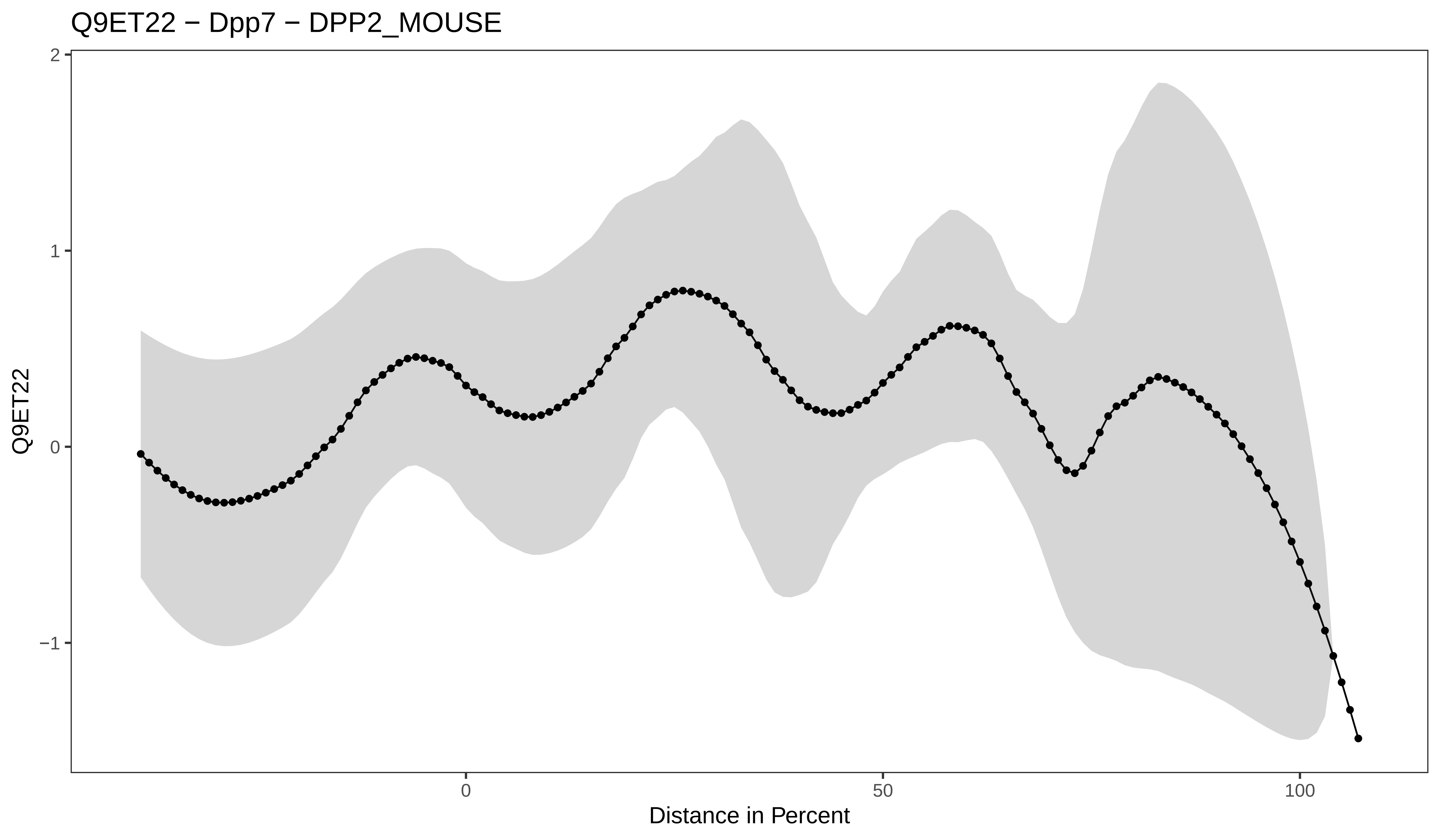

Q9ET22 − Dpp7 − DPP2_MOUSE
2
1
0
−1
Q9ET22
0
50
100
Distance in Percent

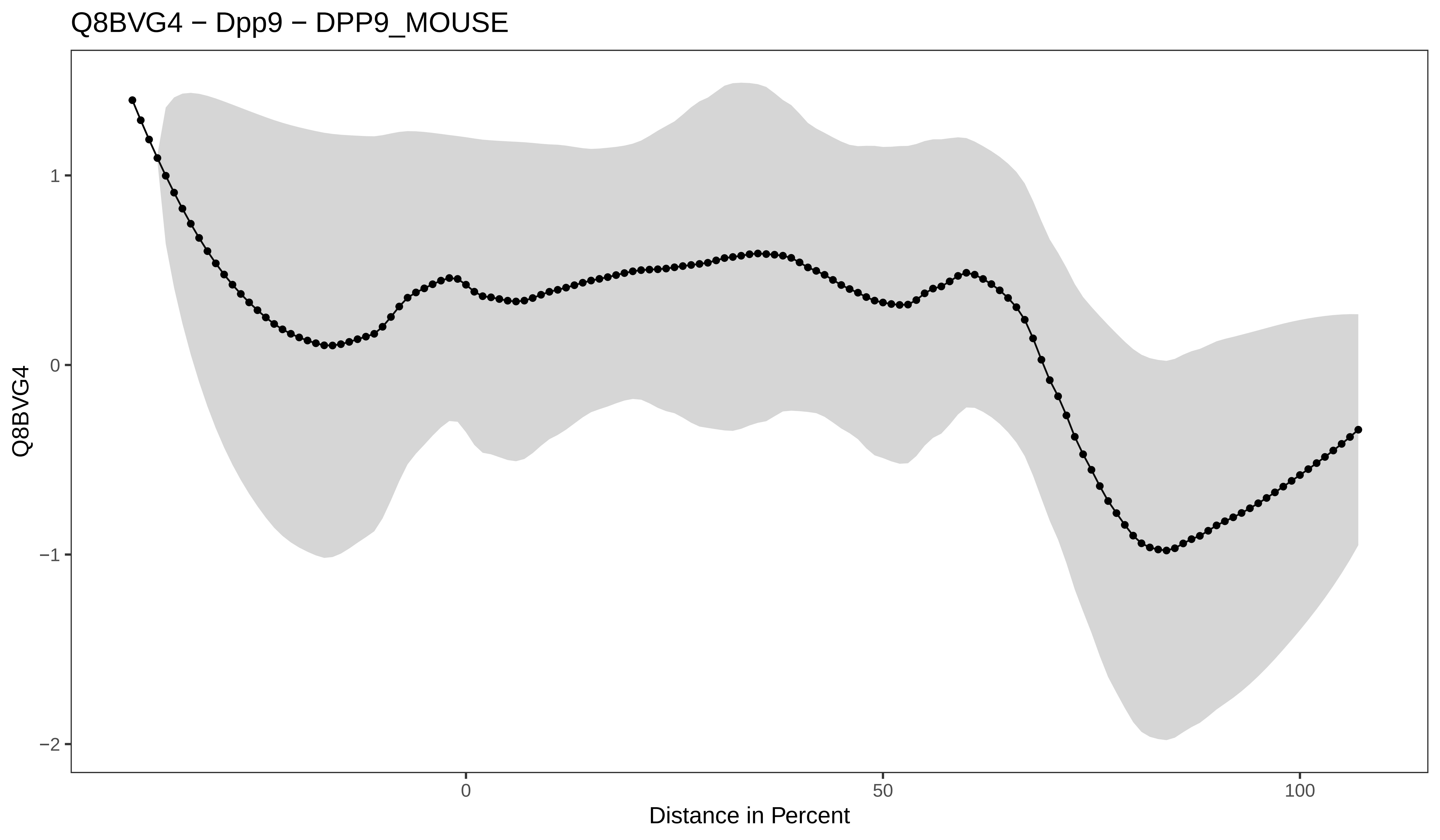

Q8BVG4 − Dpp9 − DPP9_MOUSE
Q8BVG4
1
0
−1
−2
0
50
100
Distance in Percent

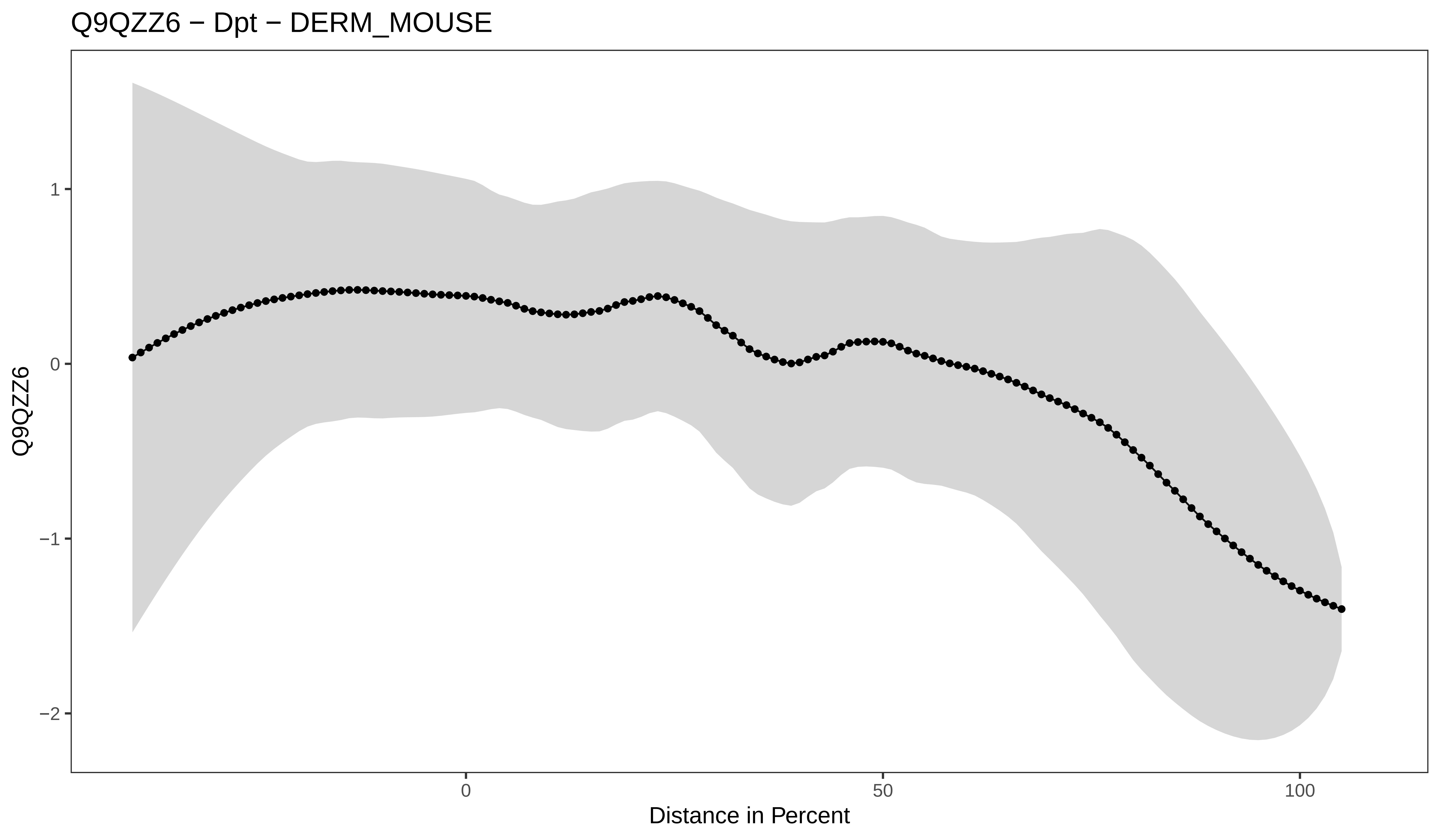

Q9QZZ6 − Dpt − DERM_MOUSE
Q9QZZ6
1
0
−1
−2
0
50
100
Distance in Percent

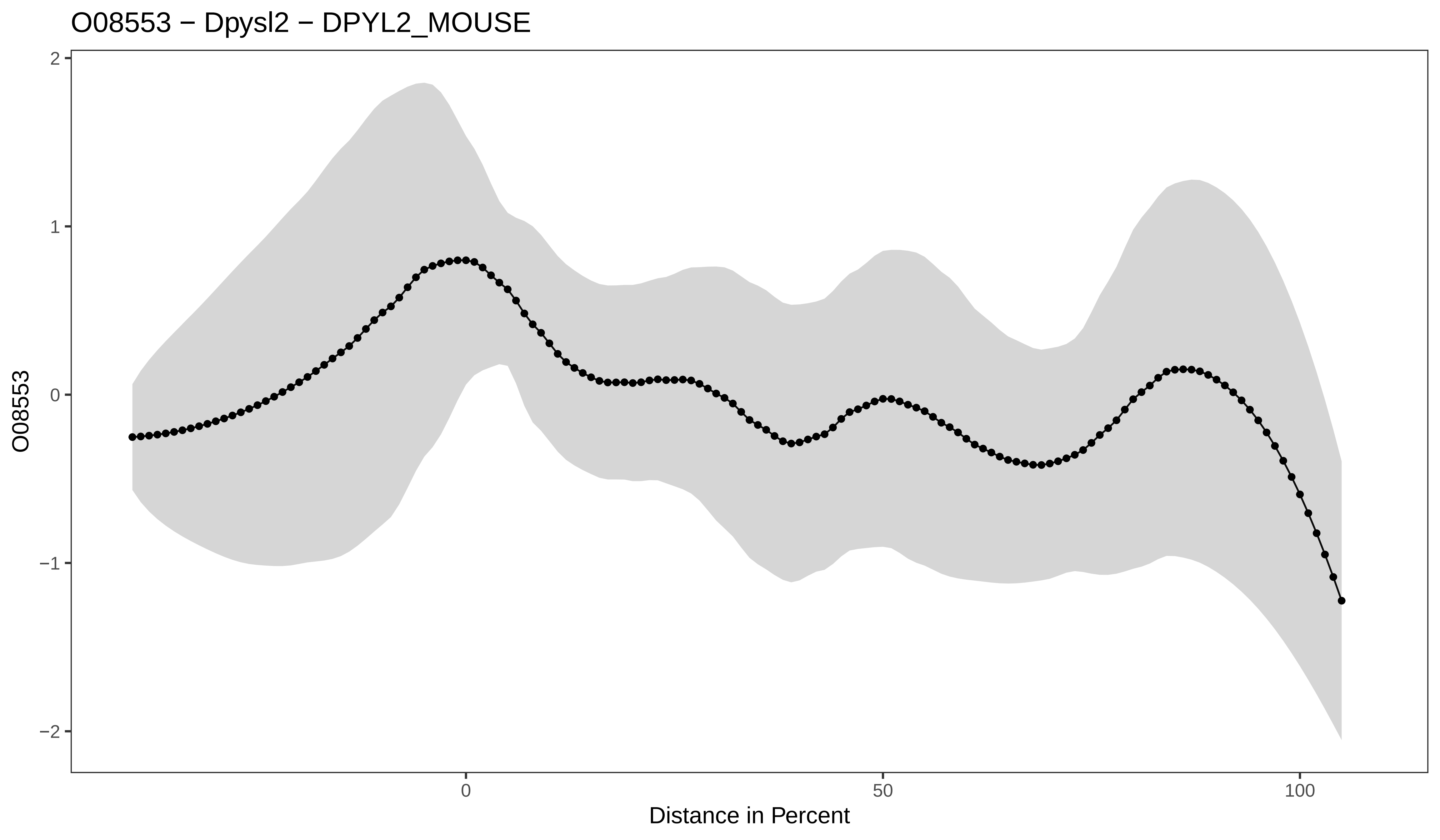
O08553 − Dpysl2 − DPYL2_MOUSE
O08553
2
1
0
−1
−2
0
50
100
Distance in Percent

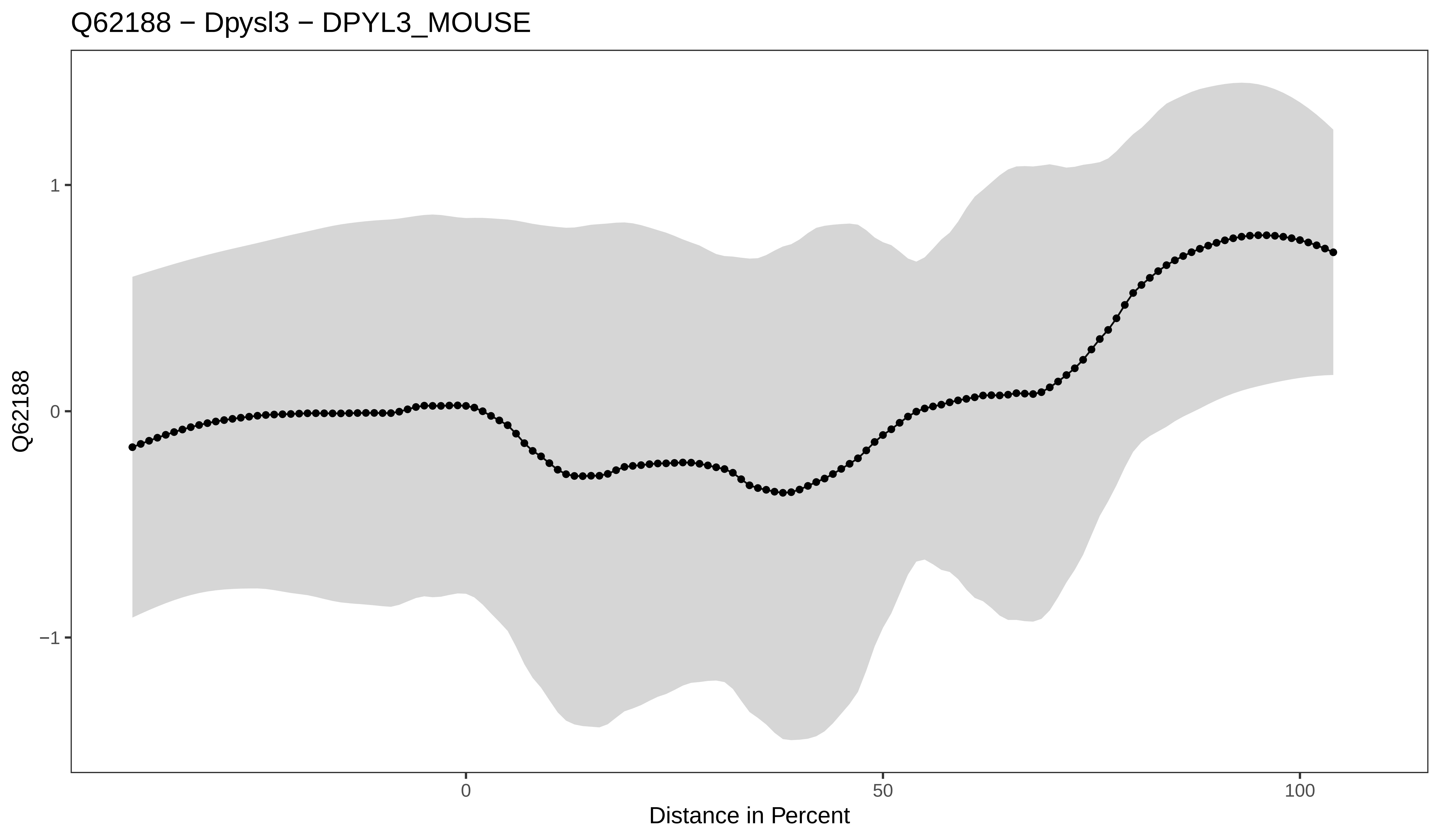

Q62188 − Dpysl3 − DPYL3_MOUSE
Q62188
1
0
−1
0
50
100
Distance in Percent

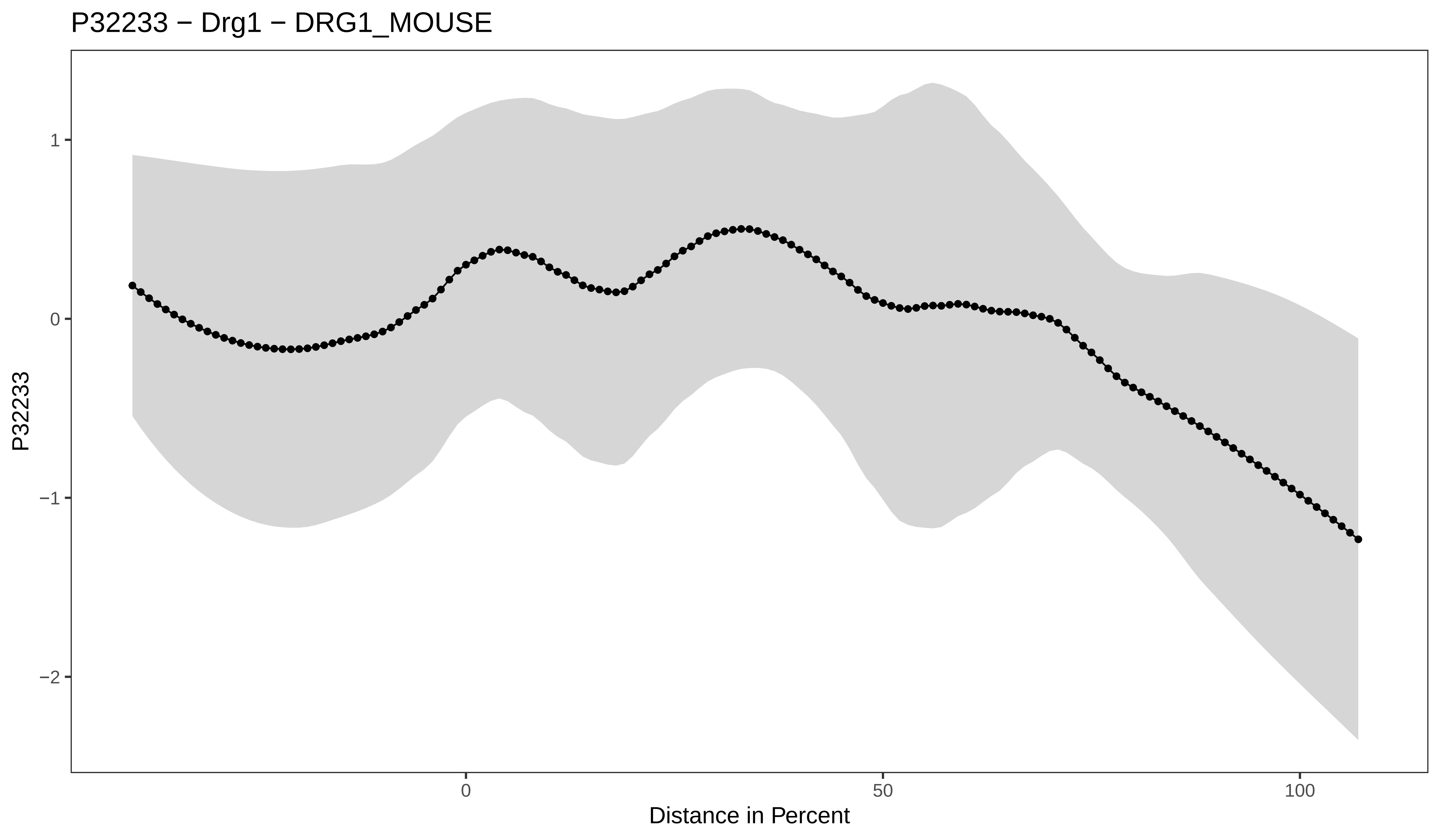
P32233 − Drg1 − DRG1_MOUSE
P32233
1
0
−1
−2
0
50
100
Distance in Percent

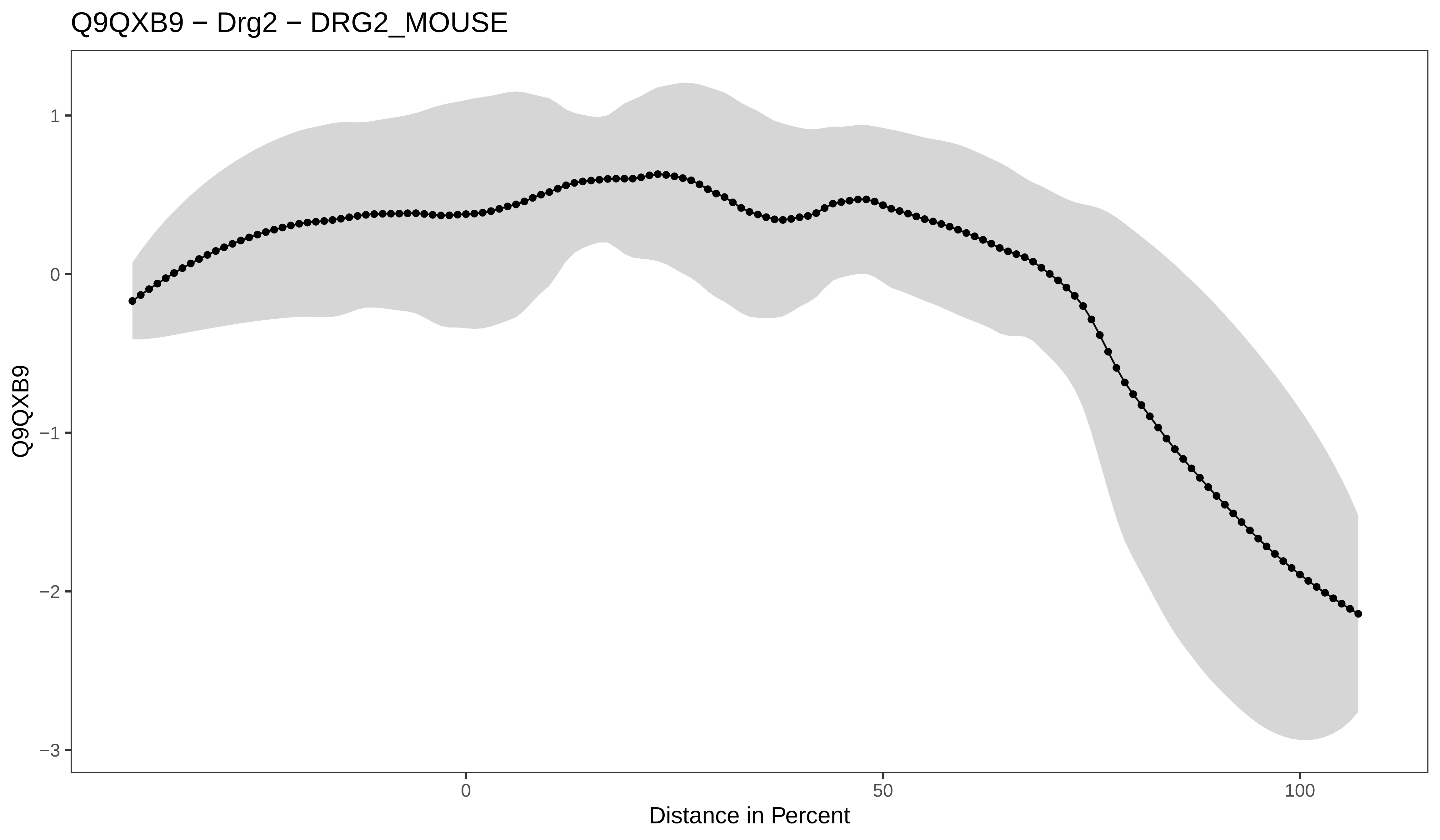

Q9QXB9 − Drg2 − DRG2_MOUSE
1
0
−1
−2
−3
Q9QXB9
0
50
100
Distance in Percent

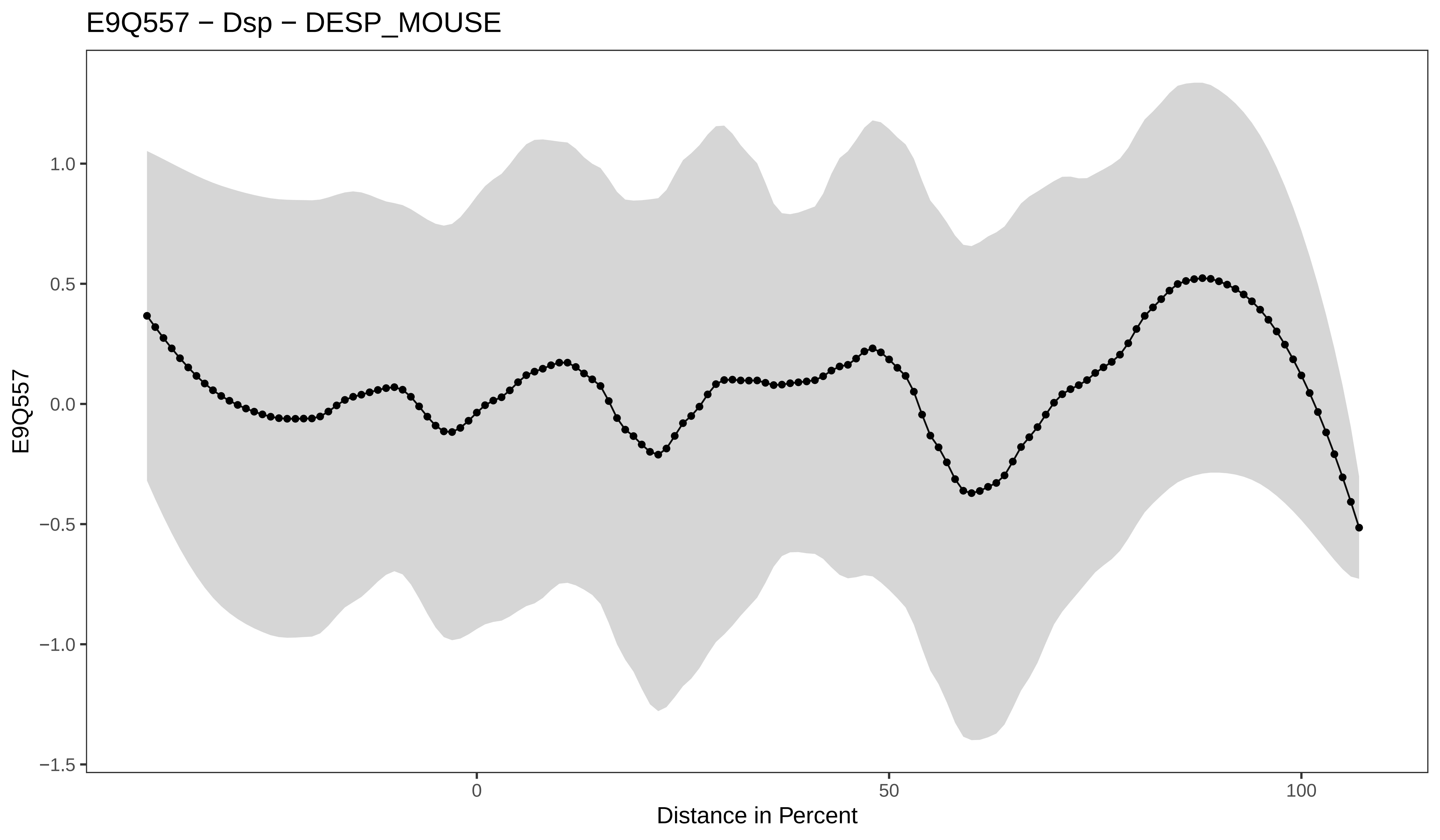
E9Q557 − Dsp − DESP_MOUSE
E9Q557
1.0
0.5
0.0
−0.5
−1.0
−1.5
0
50
100
Distance in Percent

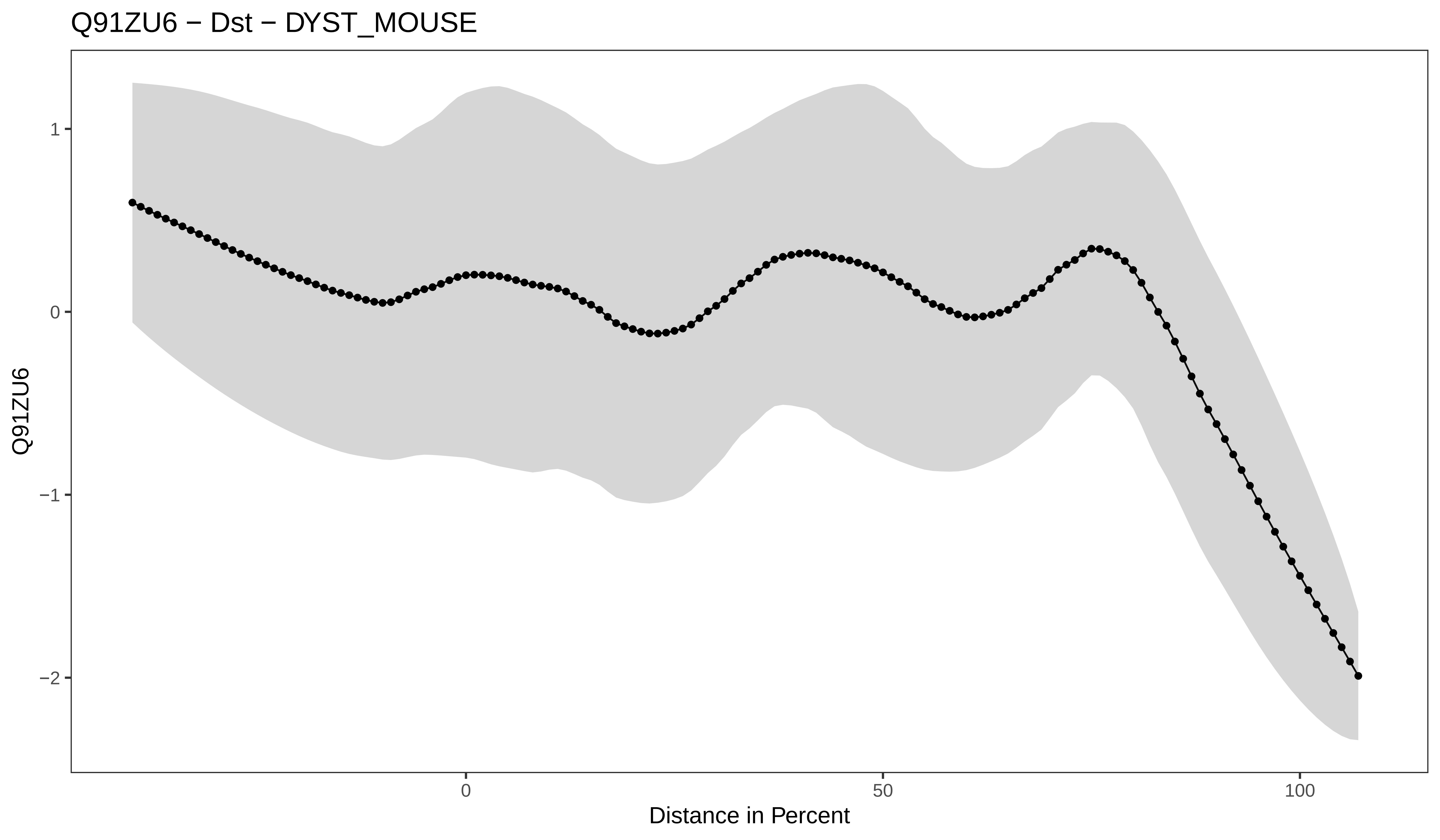

Q91ZU6 − Dst − DYST_MOUSE
1
0
−1
−2
Q91ZU6
0
50
100
Distance in Percent

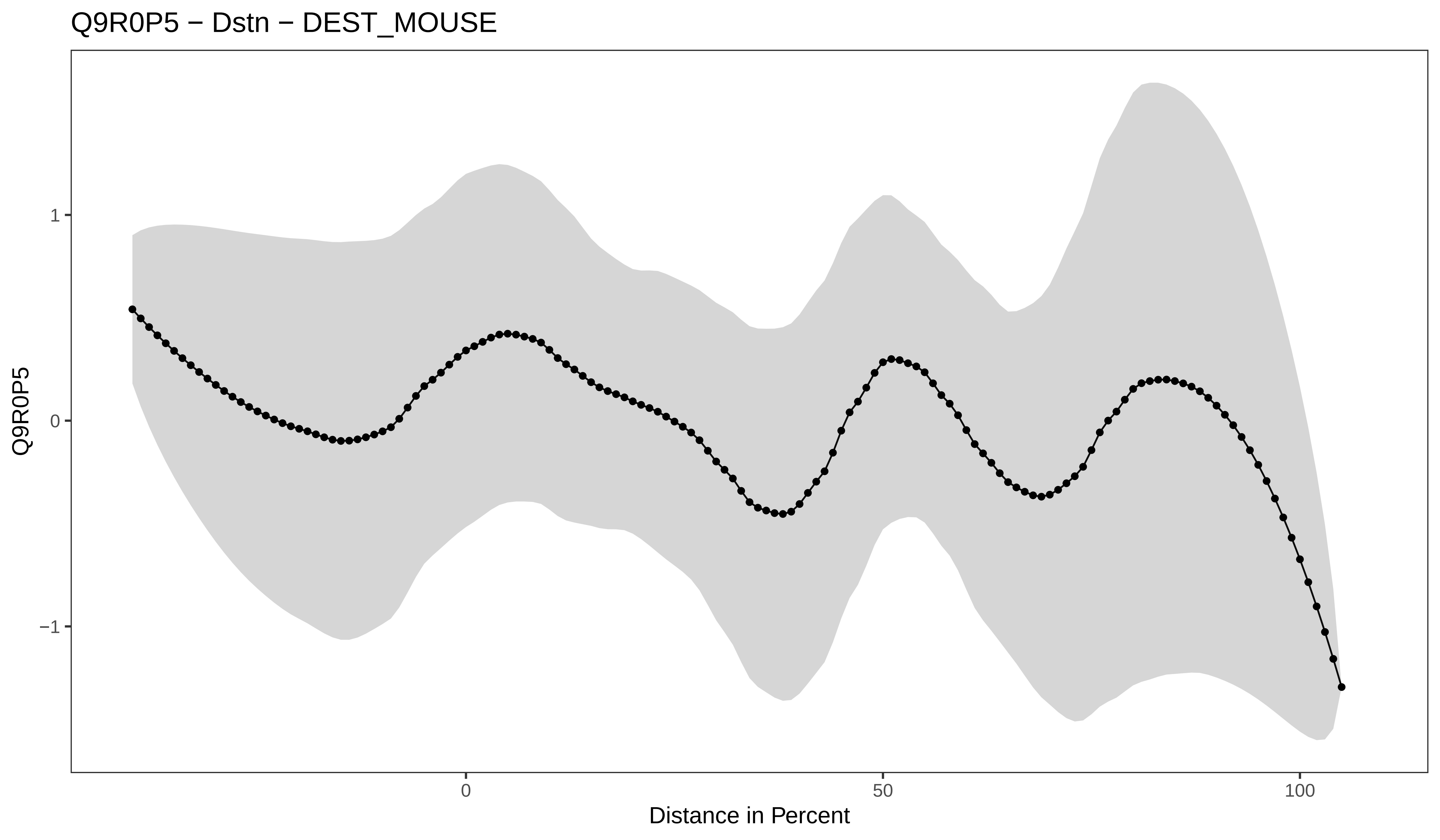
Q9R0P5 − Dstn − DEST_MOUSE
Q9R0P5
1
0
−1
0
50
100
Distance in Percent

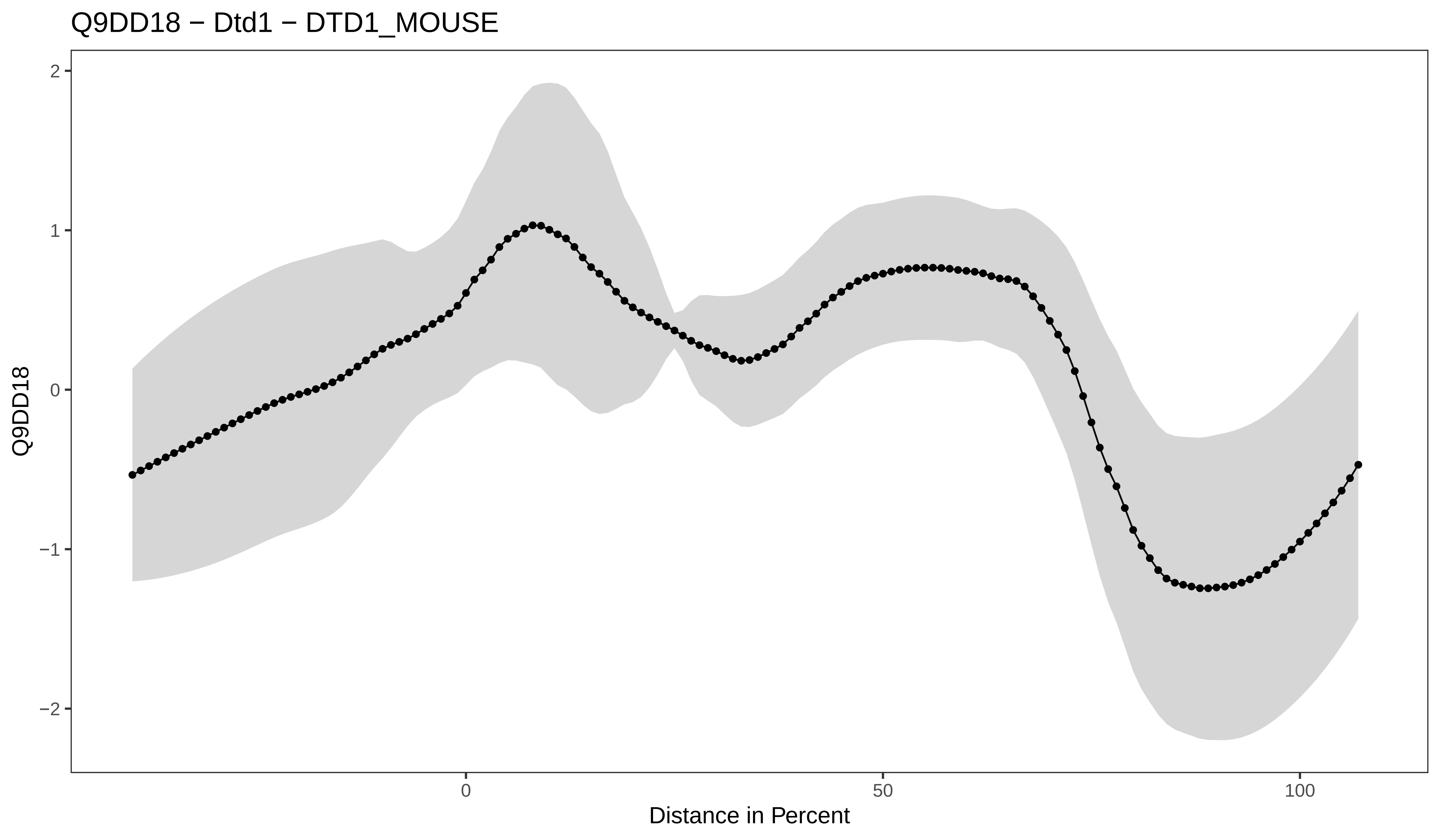

Q9DD18 − Dtd1 − DTD1_MOUSE
Q9DD18
2
1
0
−1
−2
0
50
100
Distance in Percent

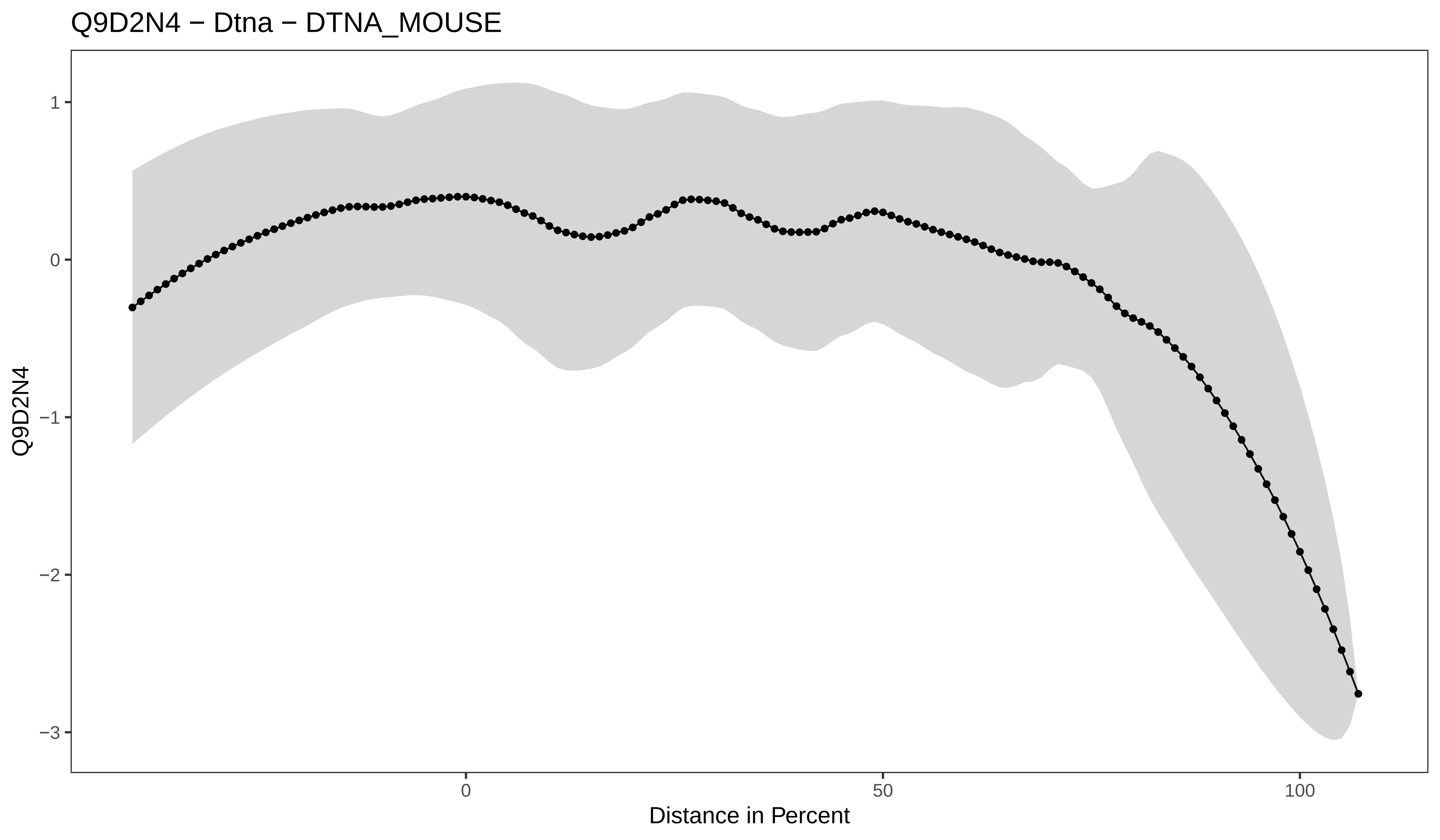

Q9D2N4 − Dtna − DTNA_MOUSE
Q9D2N4
1
0
−1
−2
−3
0
50
100
Distance in Percent

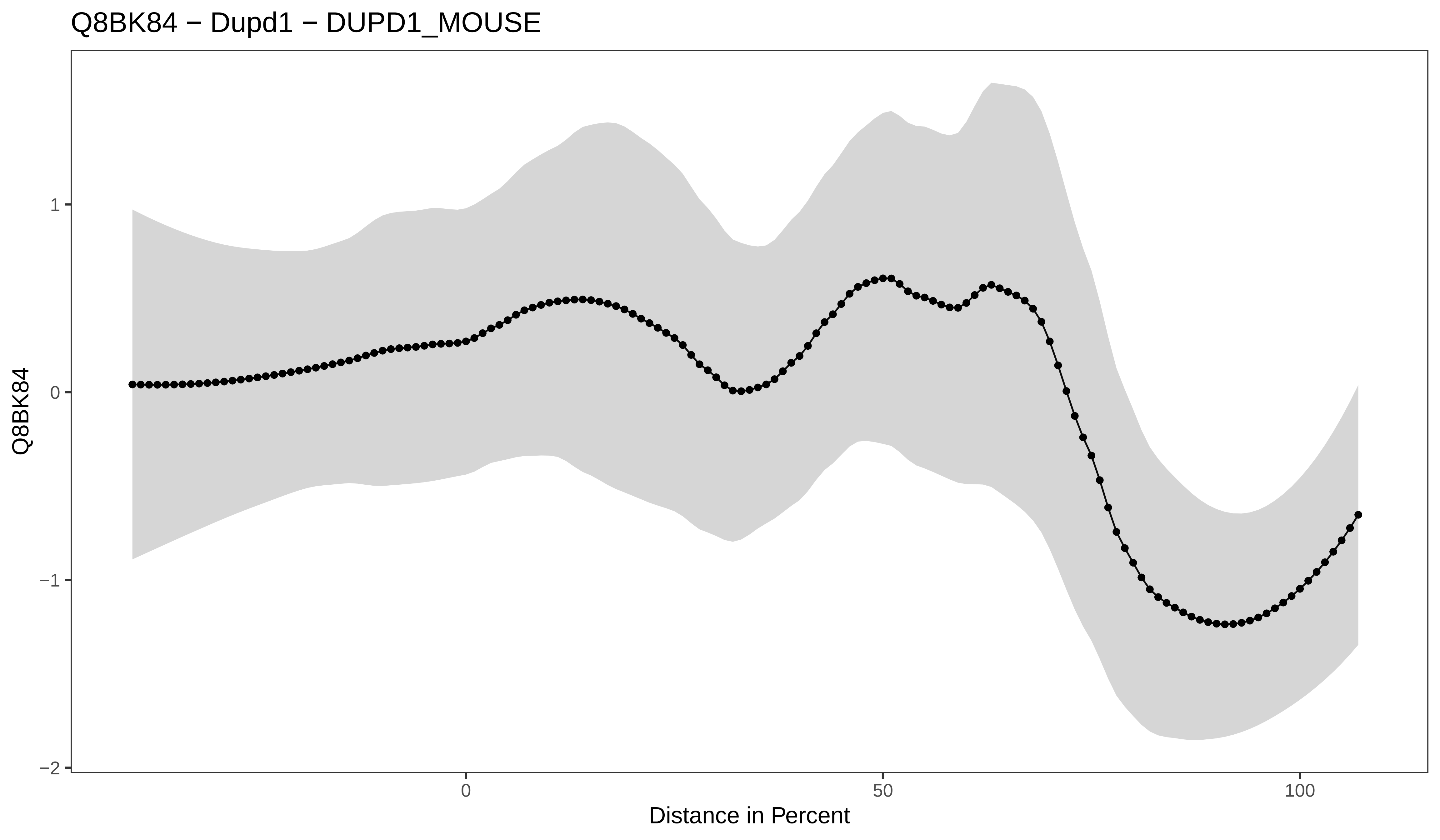

Q8BK84 − Dupd1 − DUPD1_MOUSE
Q8BK84
1
0
−1
−2
0
50
100
Distance in Percent

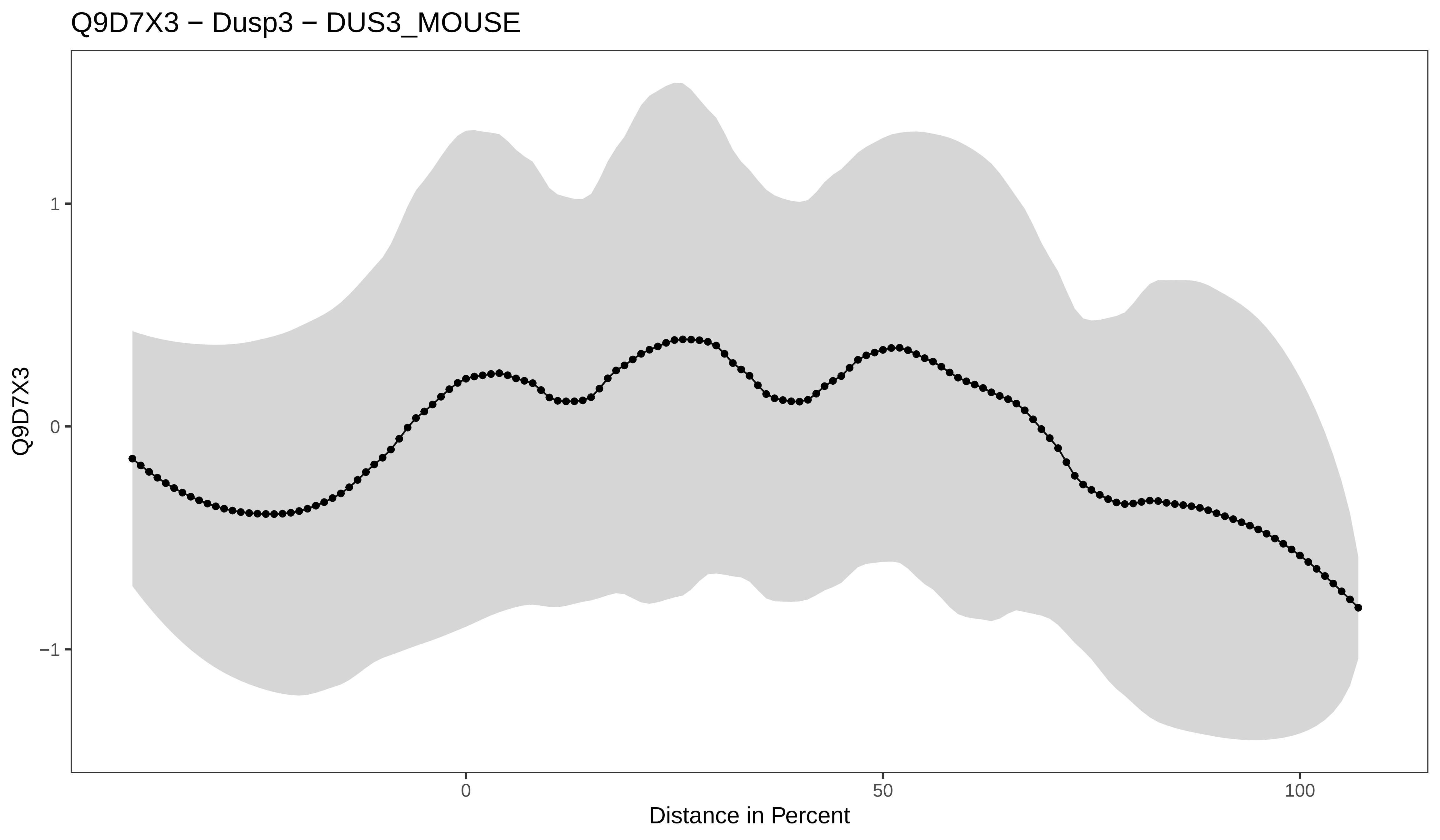
Q9D7X3 − Dusp3 − DUS3_MOUSE
Q9D7X3
1
0
−1
0
50
100
Distance in Percent

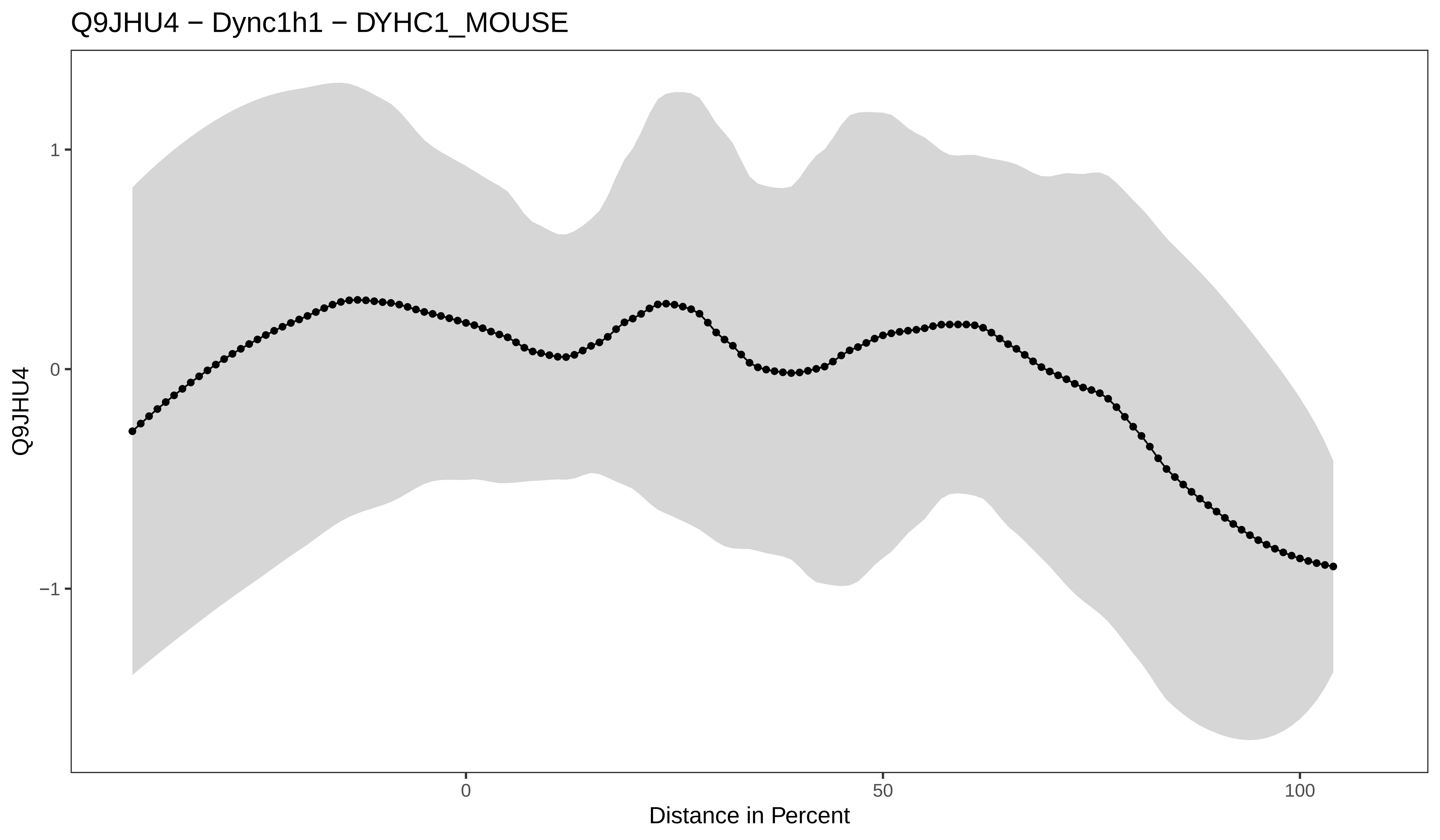

Q9JHU4 − Dync1h1 − DYHC1_MOUSE
1
0
−1
Q9JHU4
0
50
100
Distance in Percent

O88487 − Dync1i2 − DC1I2_MOUSE

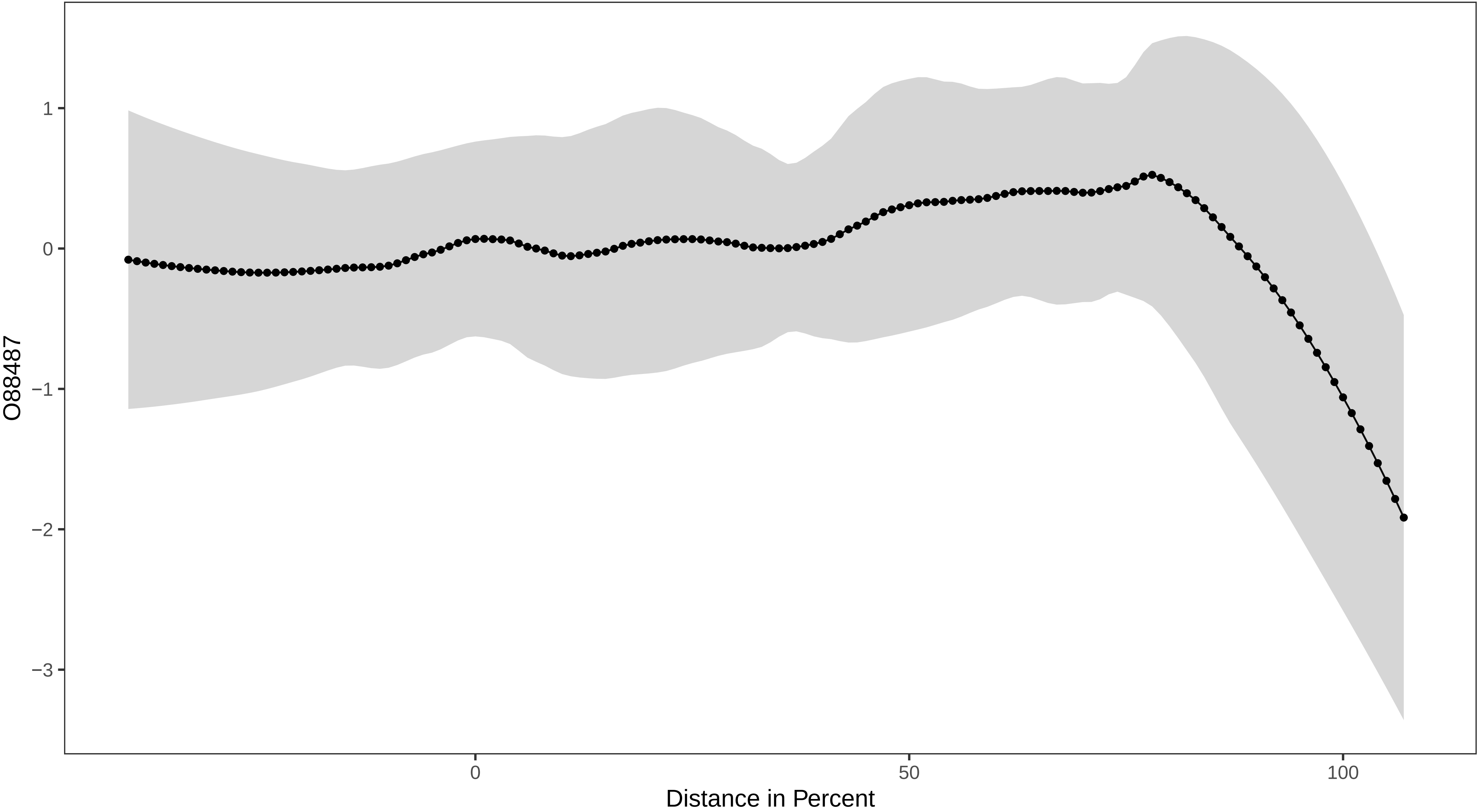

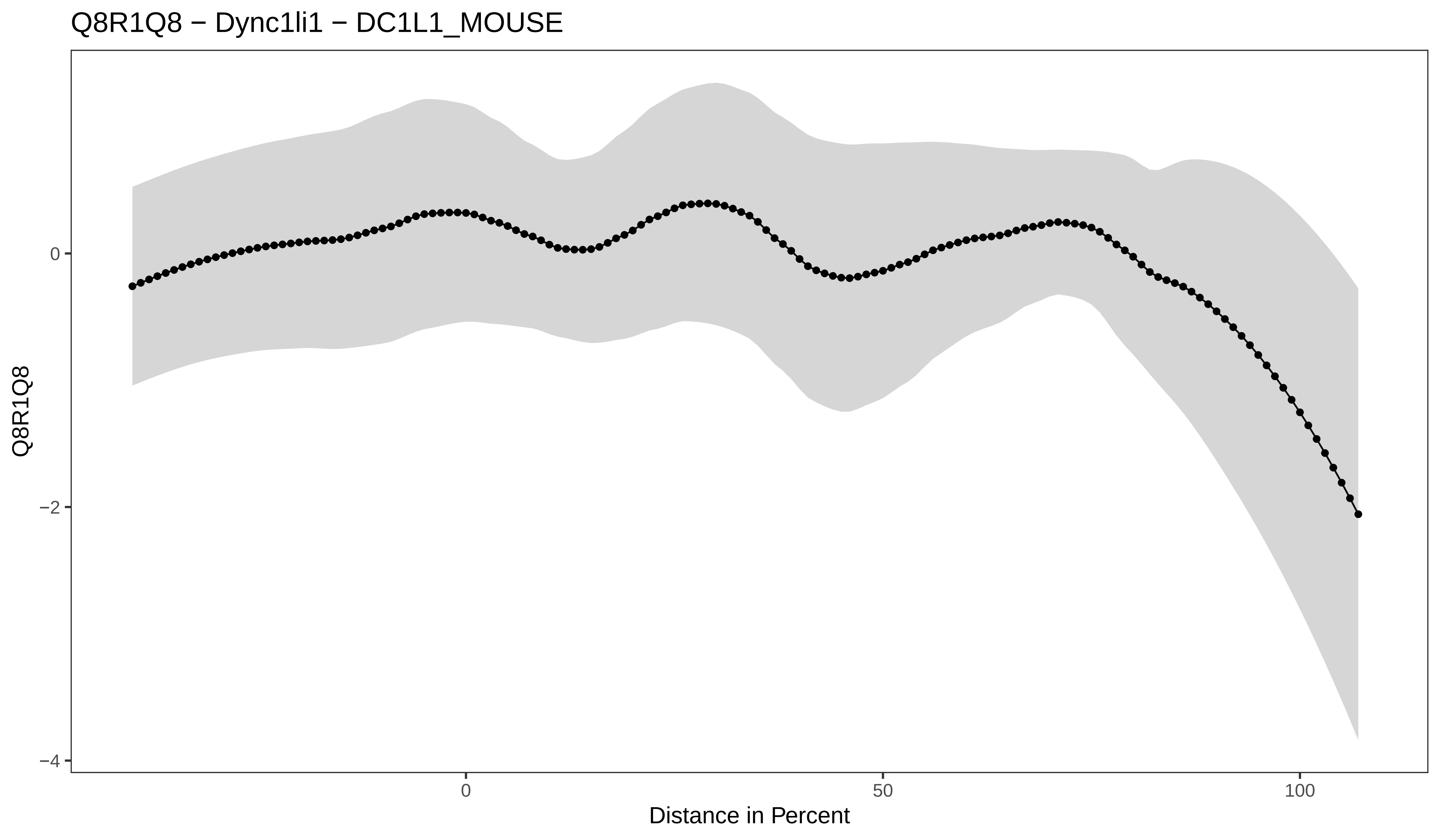
Q8R1Q8 − Dync1li1 − DC1L1_MOUSE
Q8R1Q8
0
−2
−4
0
50
100
Distance in Percent

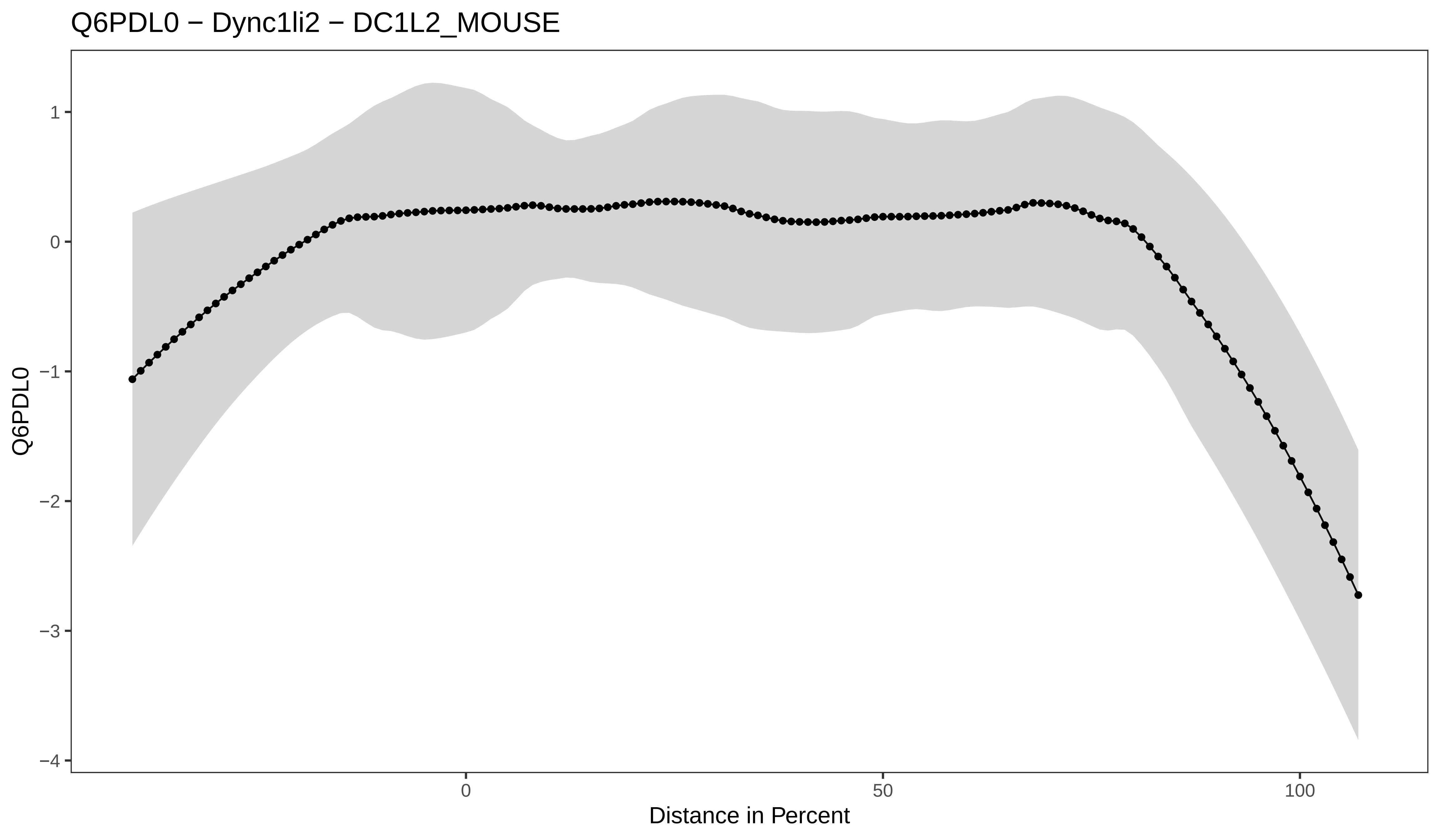

Q6PDL0 − Dync1li2 − DC1L2_MOUSE
Q6PDL0
1
0
−1
−2
−3
−4
0
50
100
Distance in Percent

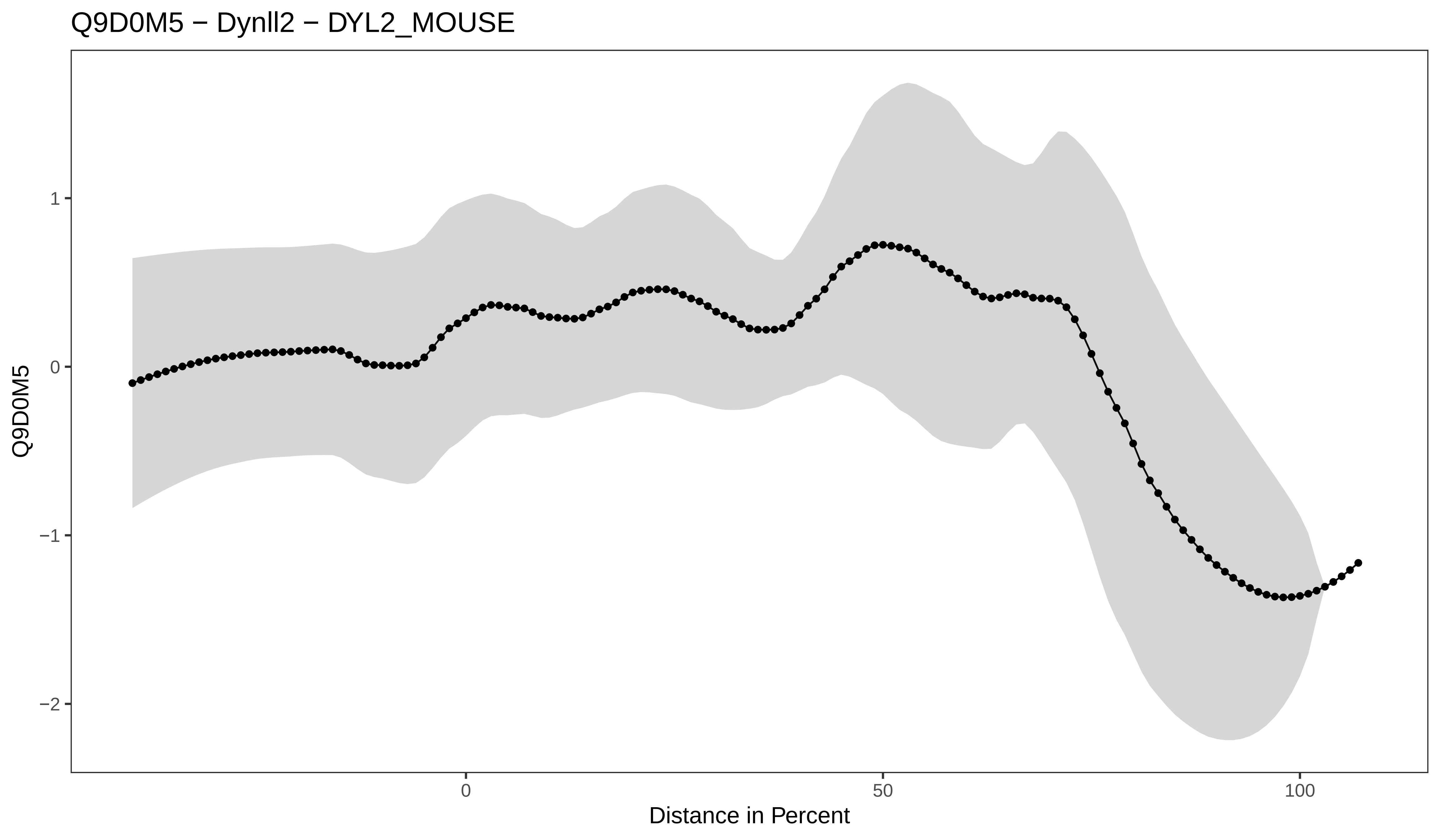

Q9D0M5 − Dynll2 − DYL2_MOUSE
Q9D0M5
1
0
−1
−2
0
50
100
Distance in Percent

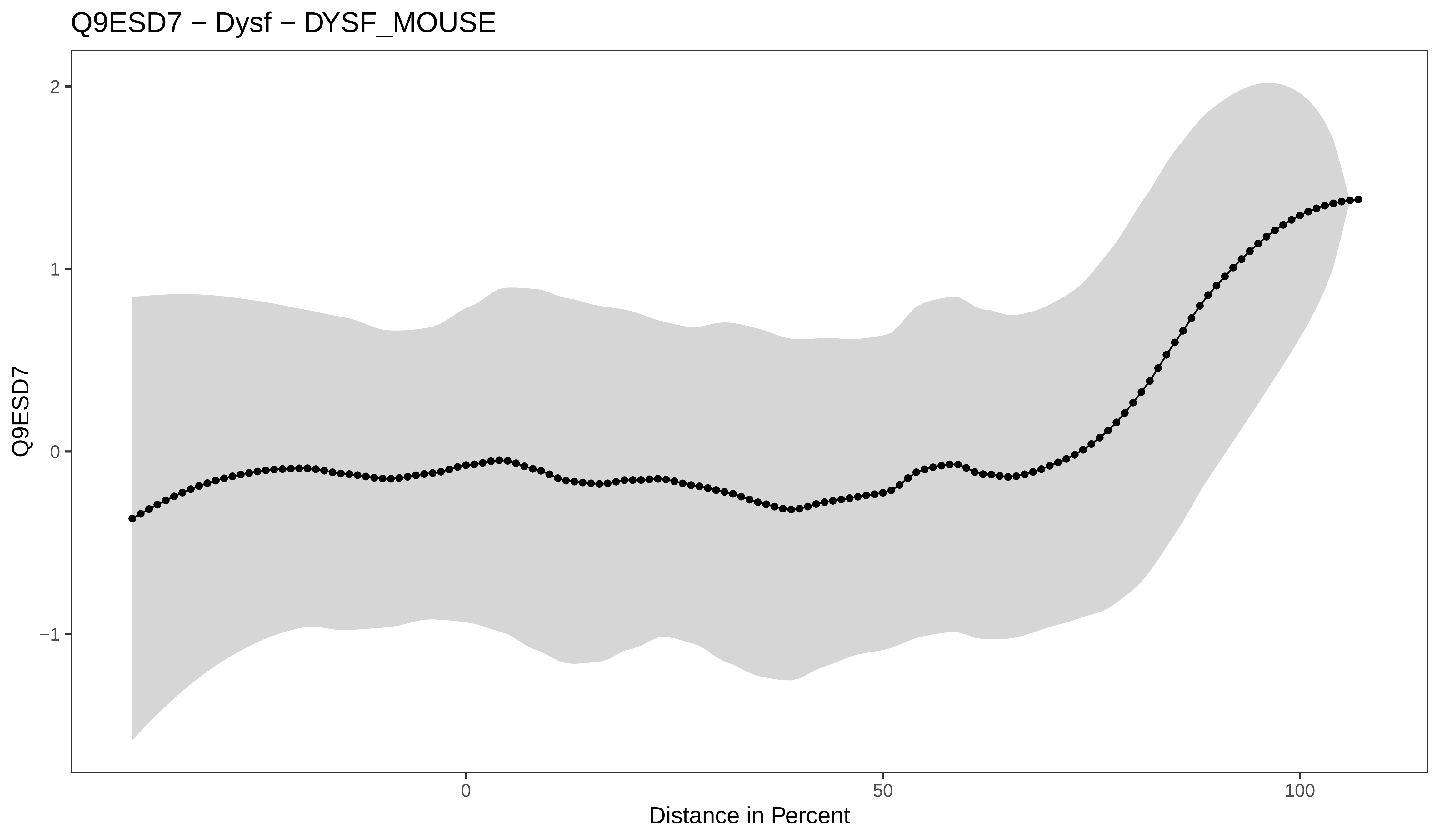

Q9ESD7 − Dysf − DYSF_MOUSE
Q9ESD7
2
1
0
−1
0
50
100
Distance in Percent

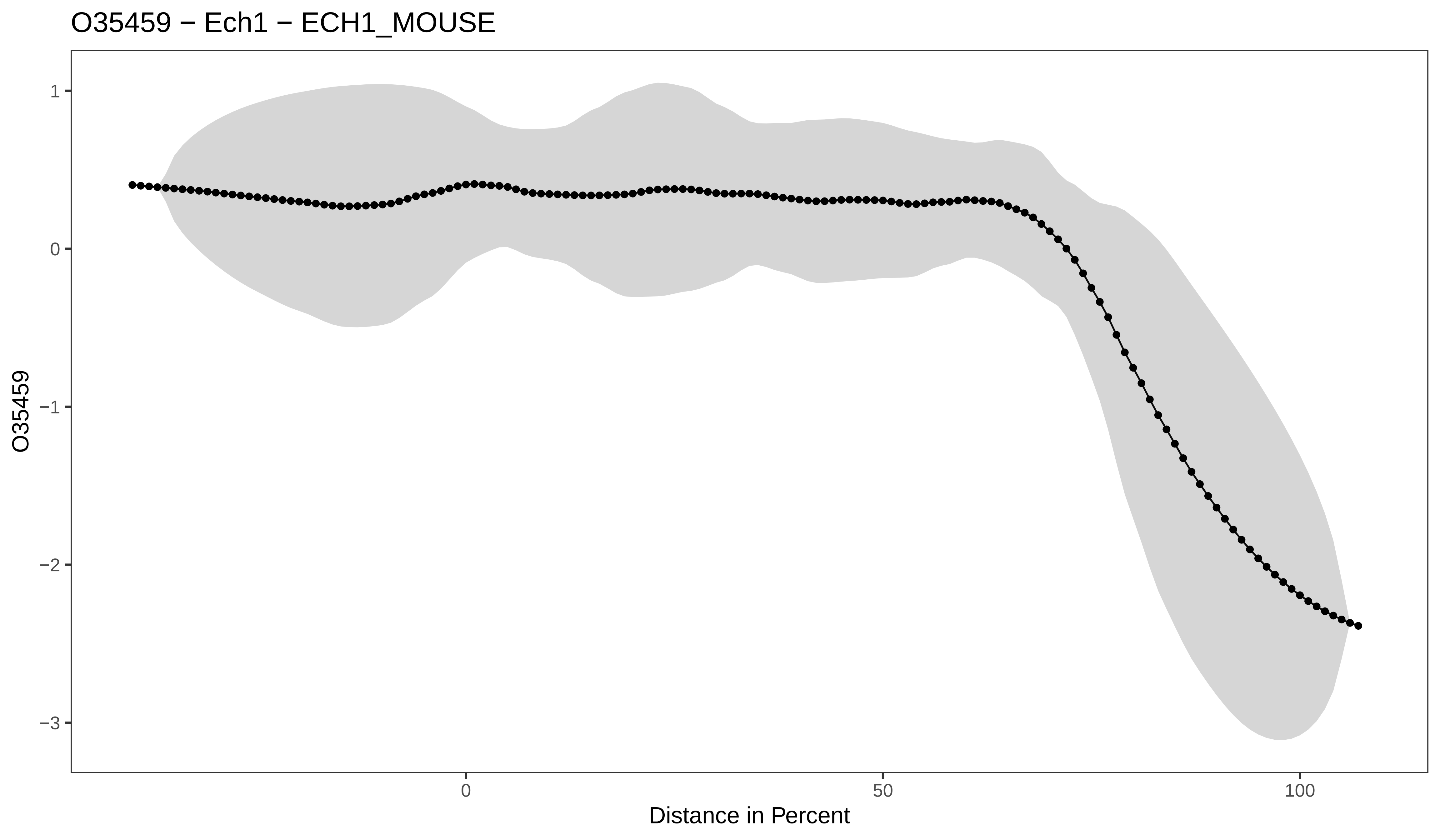

O35459 − Ech1 − ECH1_MOUSE
1
0
−1
−2
−3
O35459
0
50
100
Distance in Percent

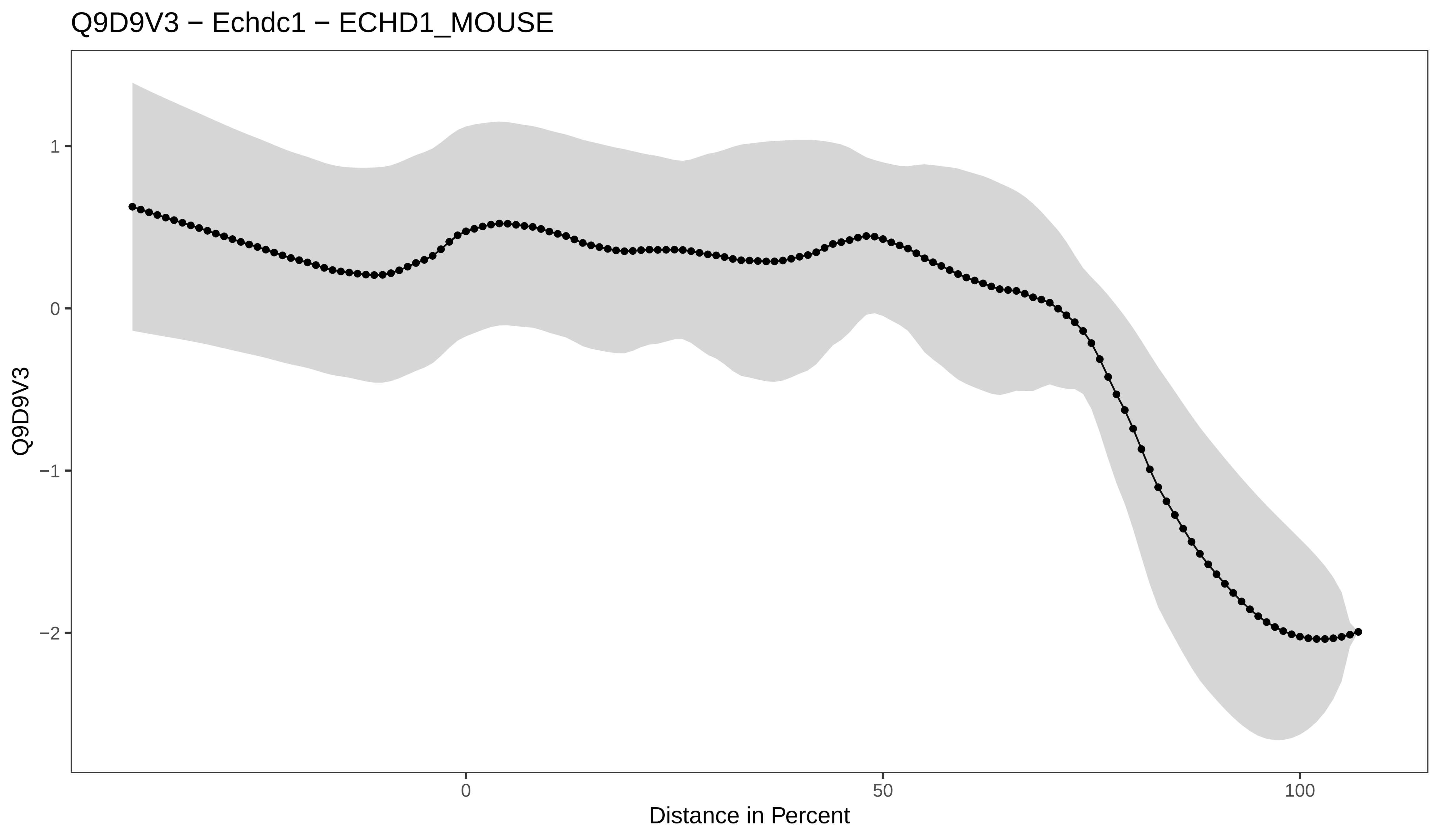

Q9D9V3 − Echdc1 − ECHD1_MOUSE
1
0
−1
−2
Q9D9V3
0
50
100
Distance in Percent

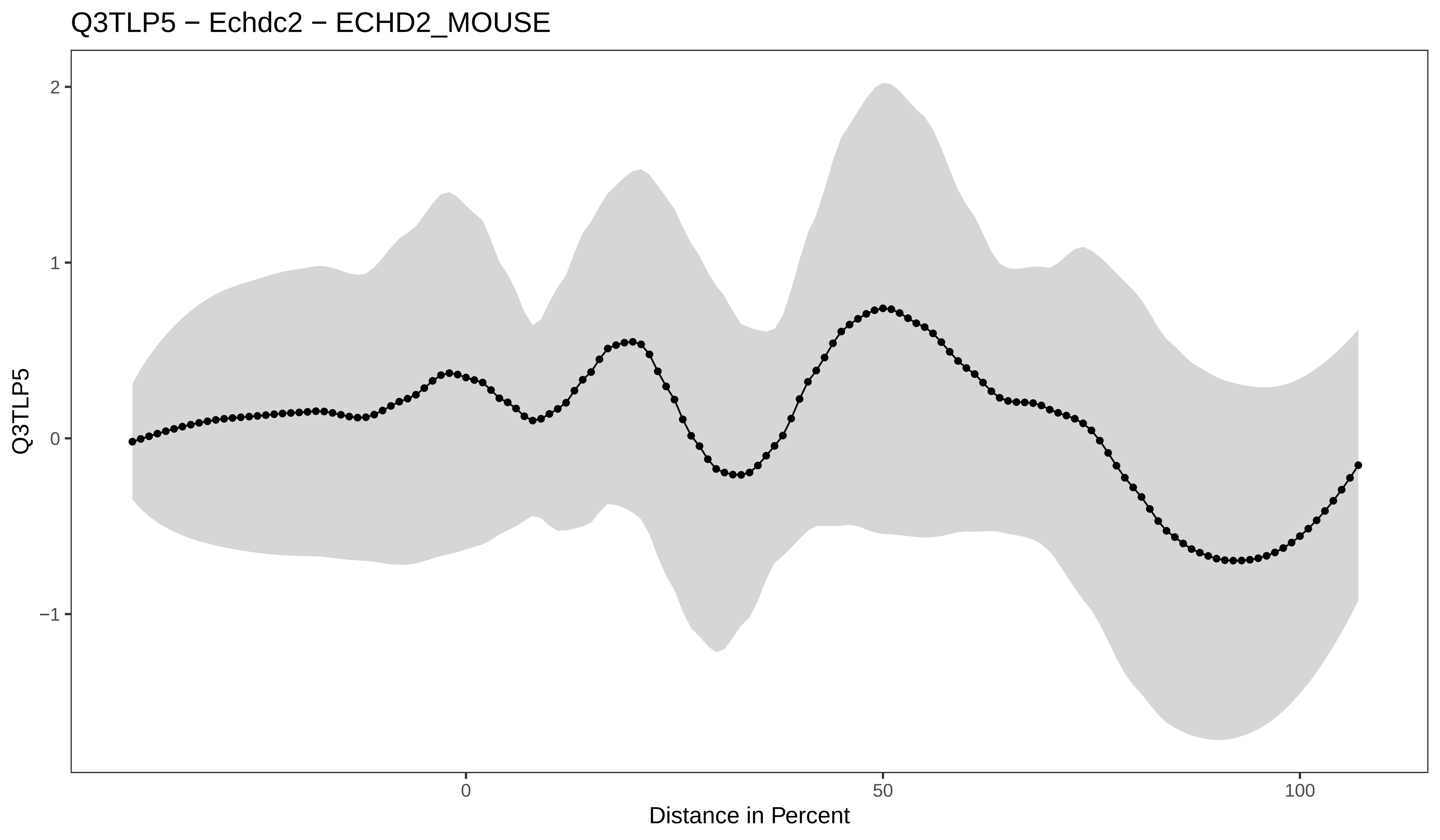
Q3TLP5 − Echdc2 − ECHD2_MOUSE
Q3TLP5
2
1
0
−1
0
50
100
Distance in Percent

Q9D7J9 − Echdc3 − ECHD3_MOUSE

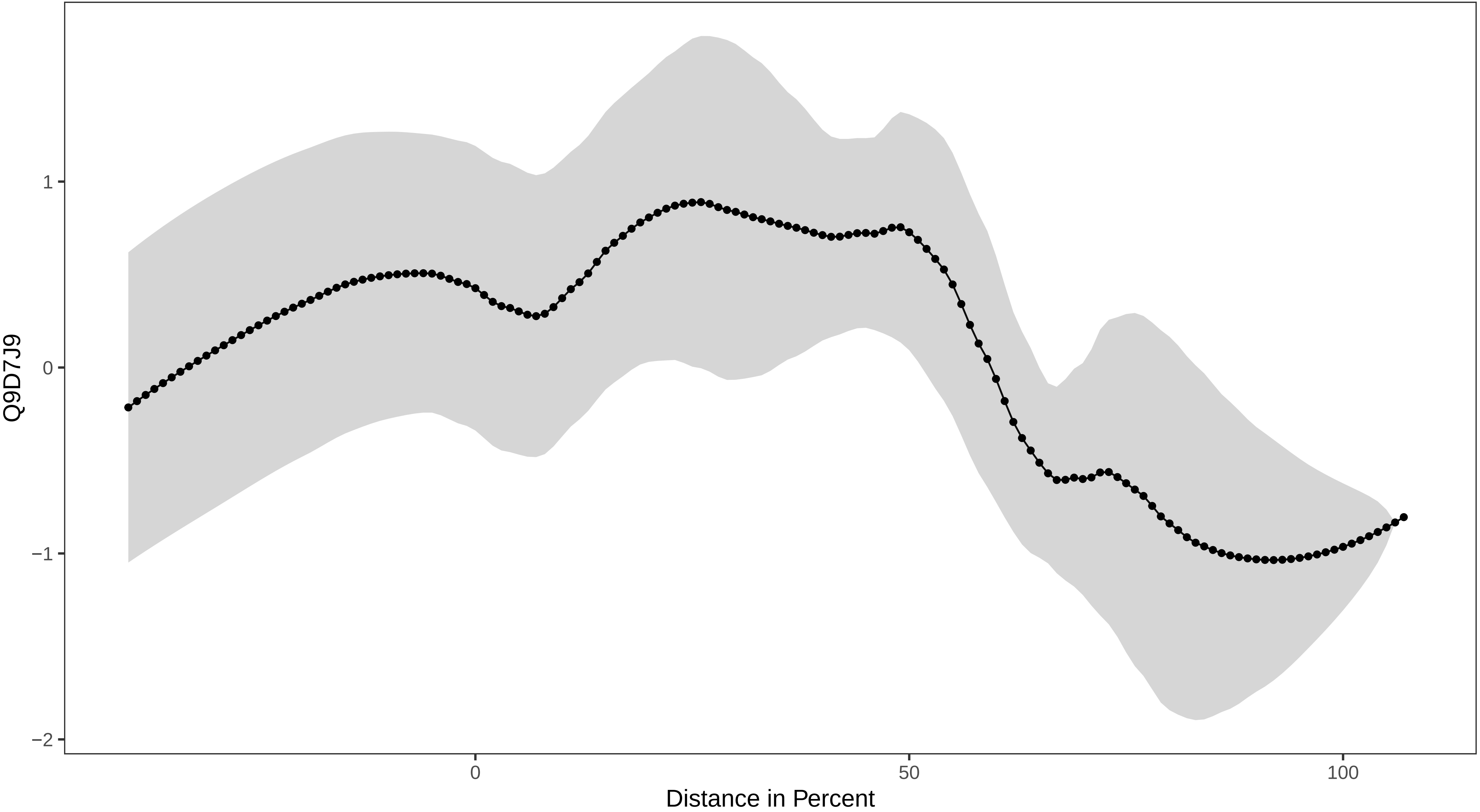

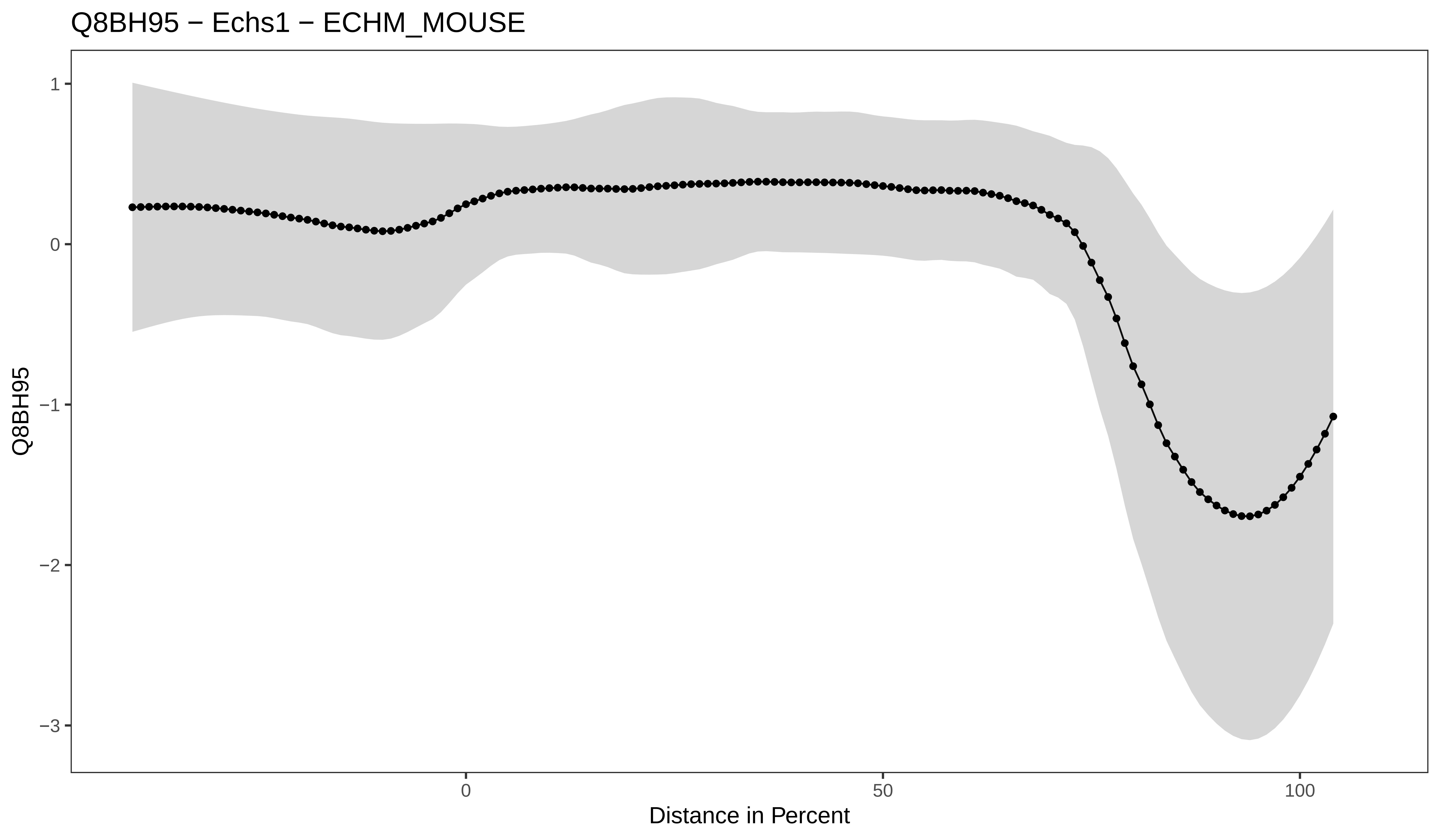

Q8BH95 − Echs1 − ECHM_MOUSE
1
0
−1
−2
−3
Q8BH95
0
50
100
Distance in Percent

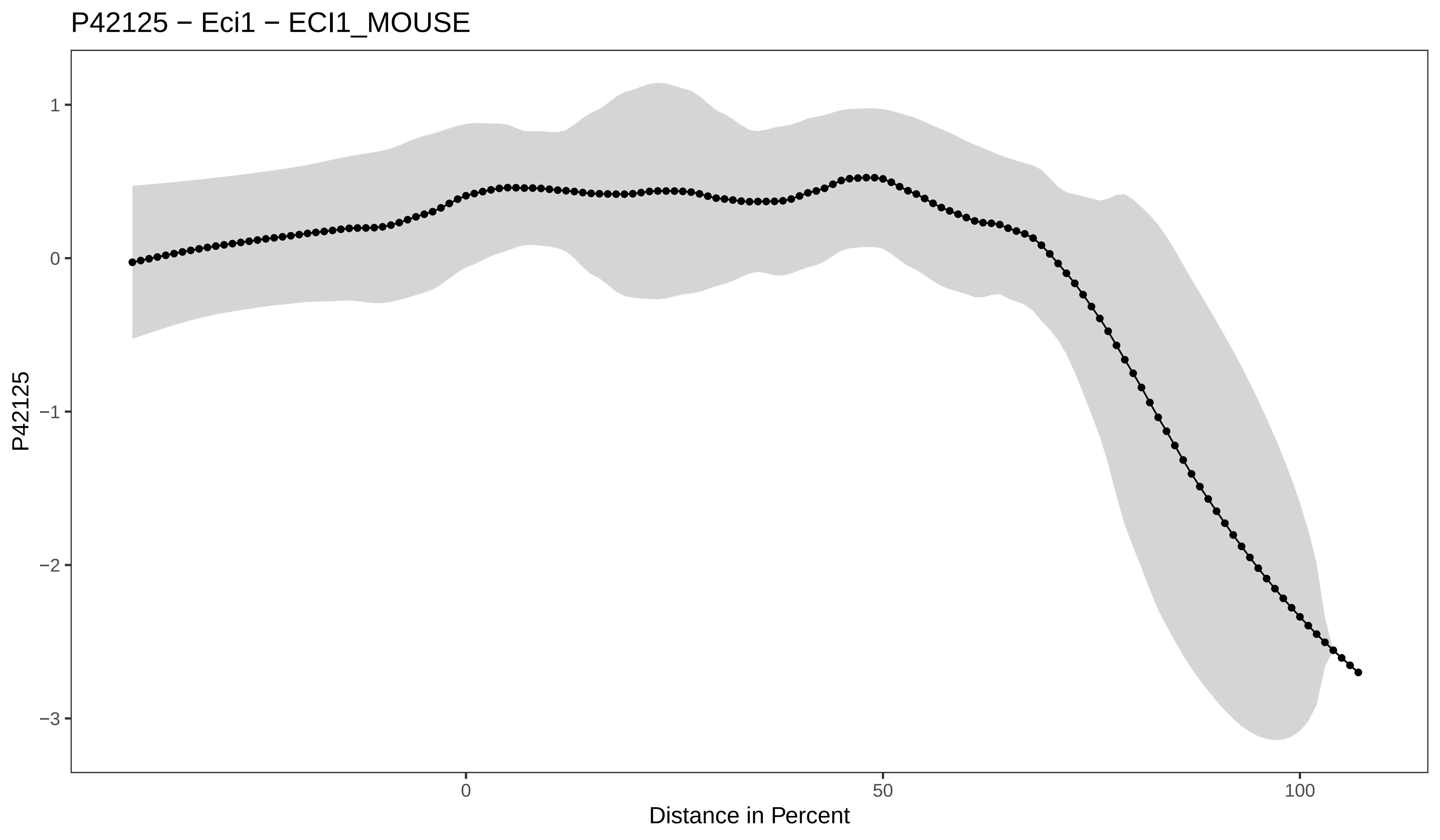
P42125 − Eci1 − ECI1_MOUSE
P42125
1
0
−1
−2
−3
0
50
100
Distance in Percent

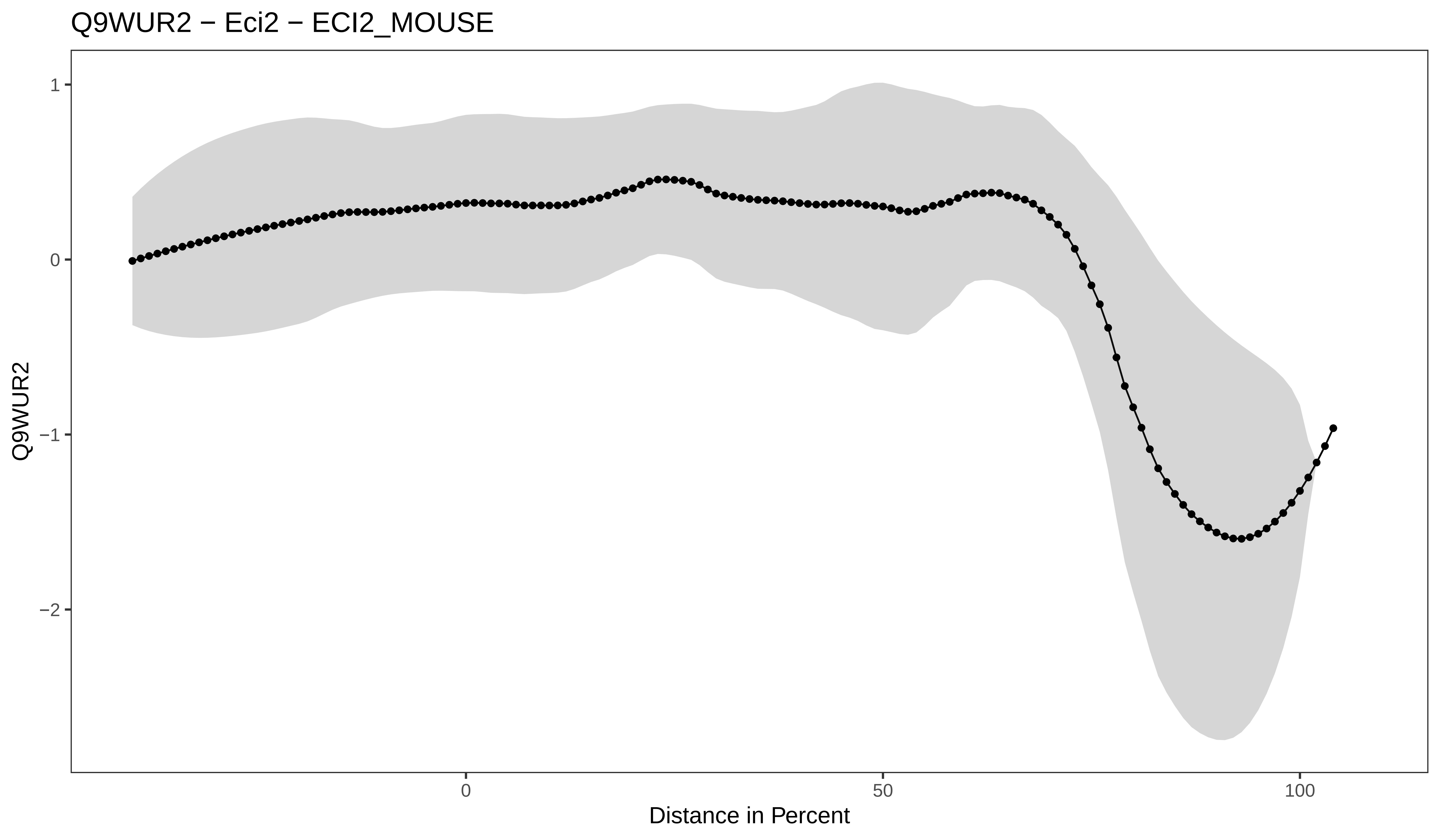

Q9WUR2 − Eci2 − ECI2_MOUSE
1
0
−1
−2
Q9WUR2
0
50
100
Distance in Percent

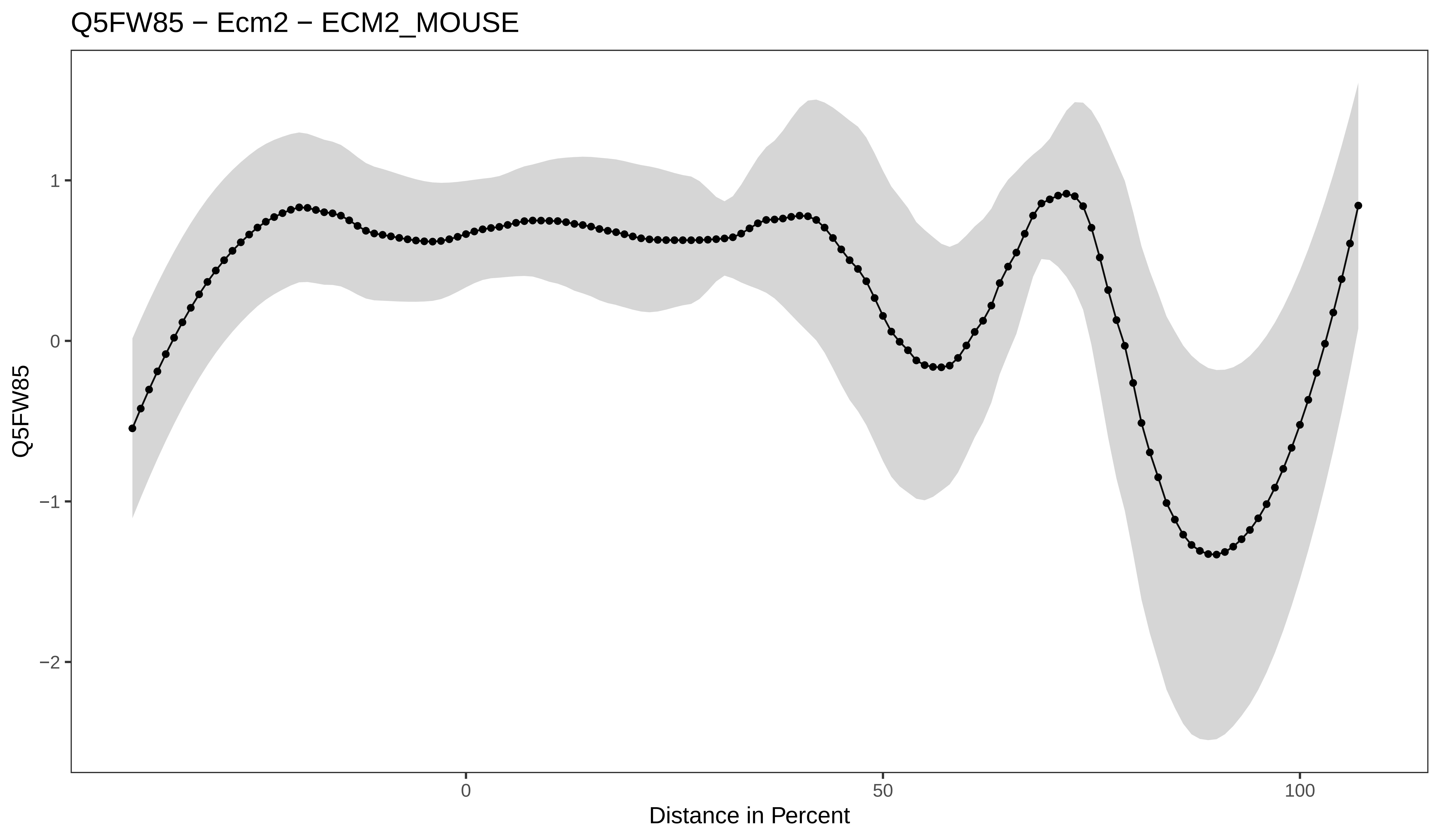

Q5FW85 − Ecm2 − ECM2_MOUSE
Q5FW85
1
0
−1
−2
0
50
100
Distance in Percent

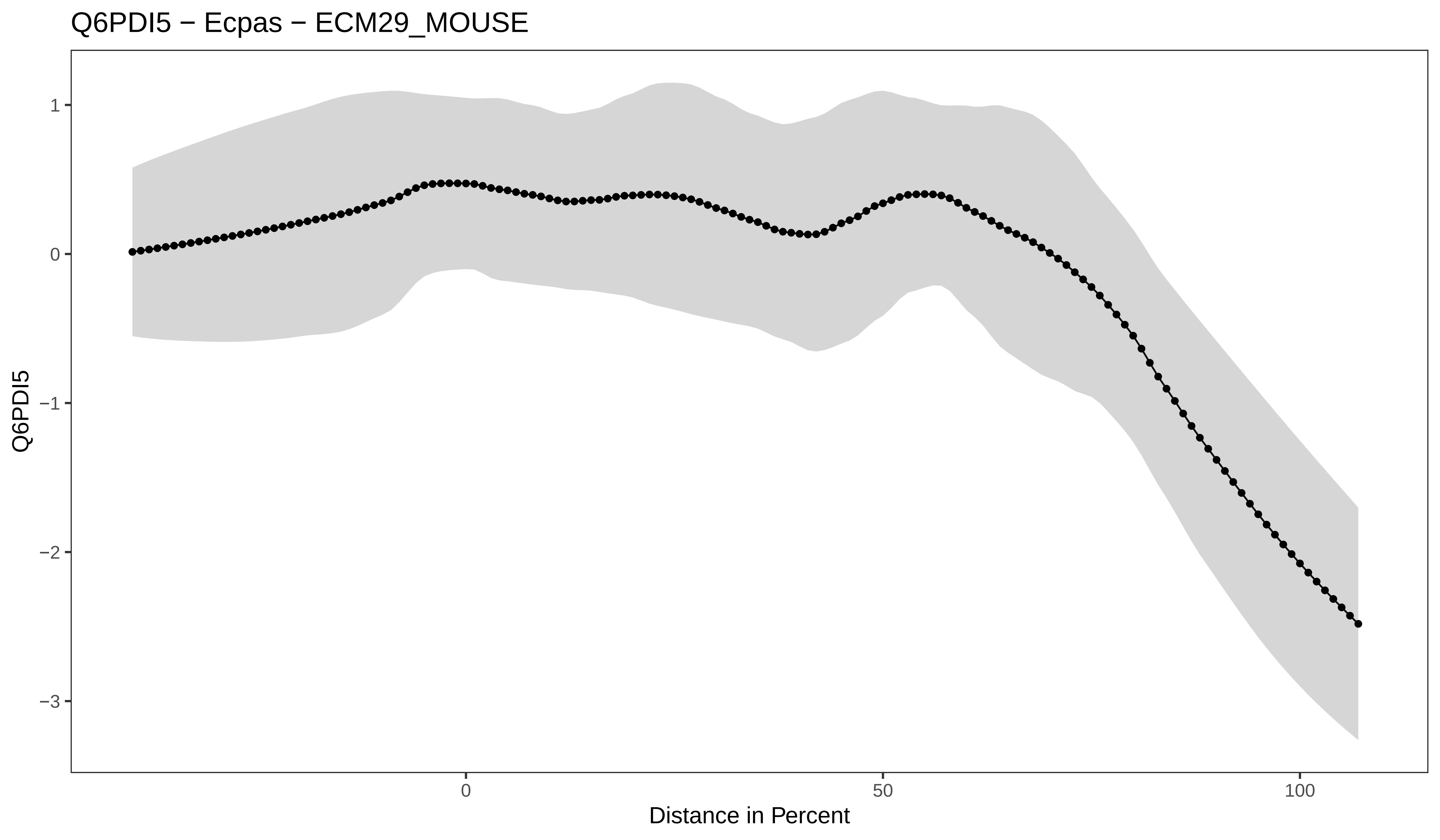

Q6PDI5 − Ecpas − ECM29_MOUSE
1
0
−1
−2
−3
Q6PDI5
0
50
100
Distance in Percent

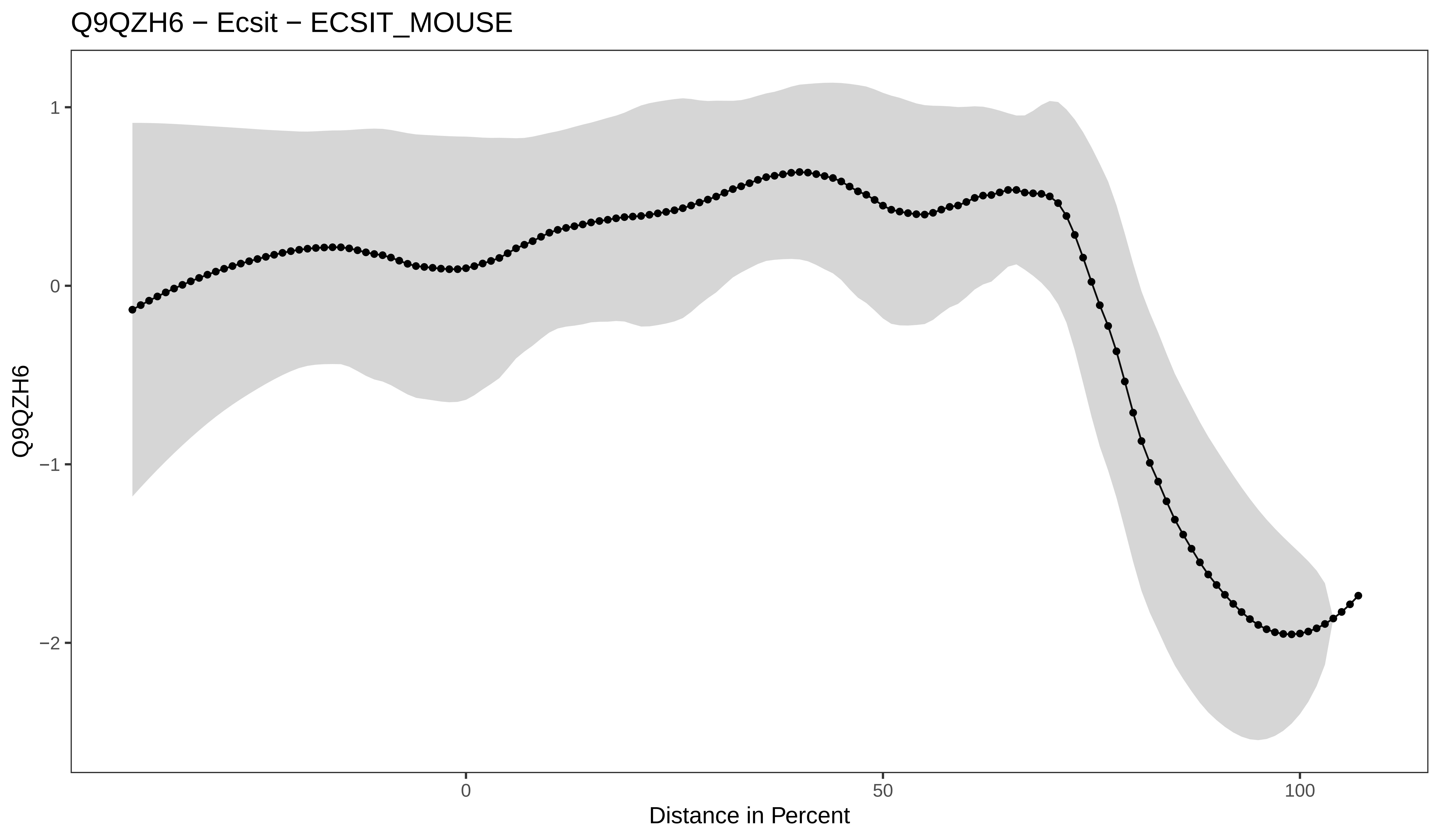
Q9QZH6 − Ecsit − ECSIT_MOUSE
1
0
−1
−2
Q9QZH6
0
50
100
Distance in Percent

Q3UJB9 − Edc4 − EDC4_MOUSE

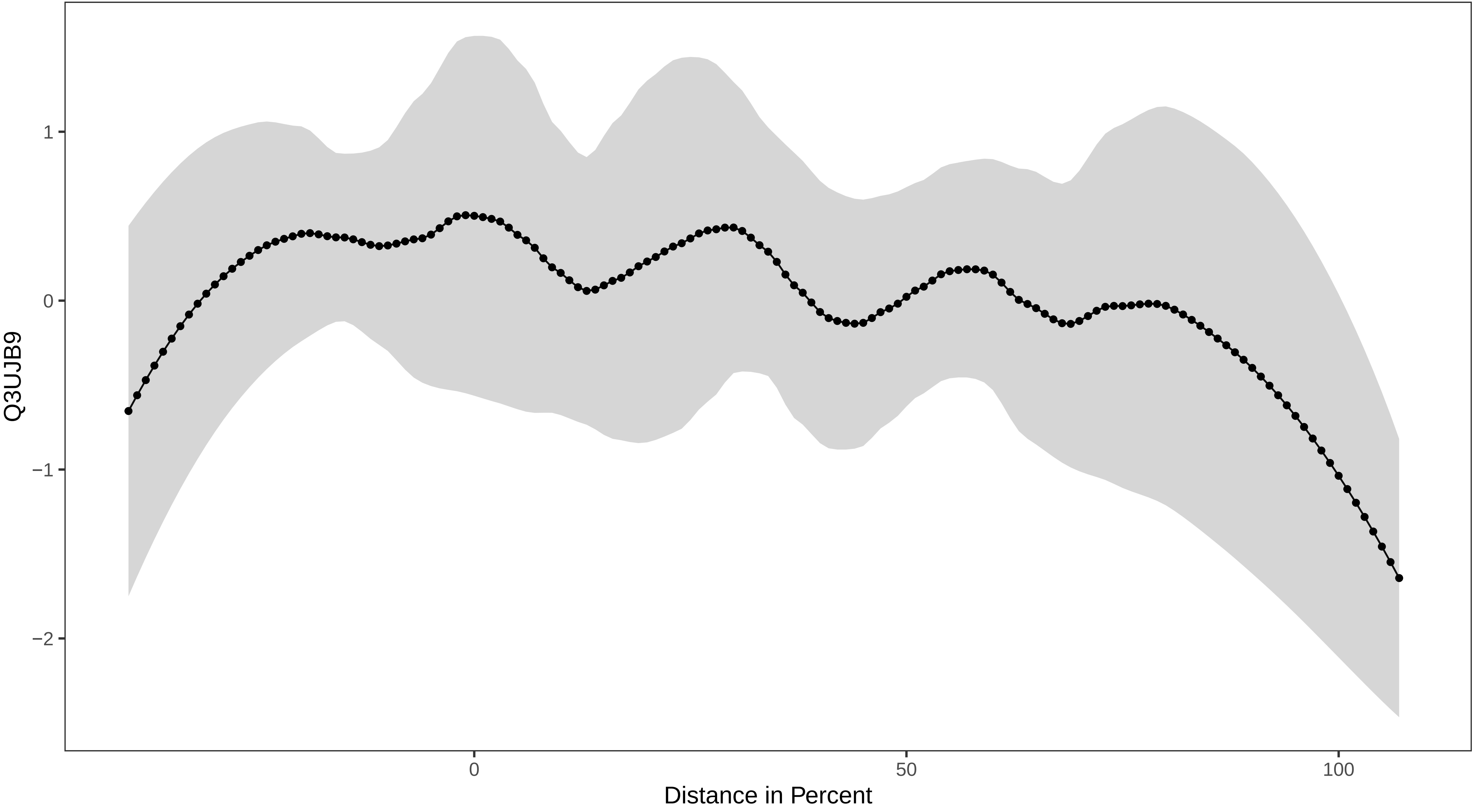

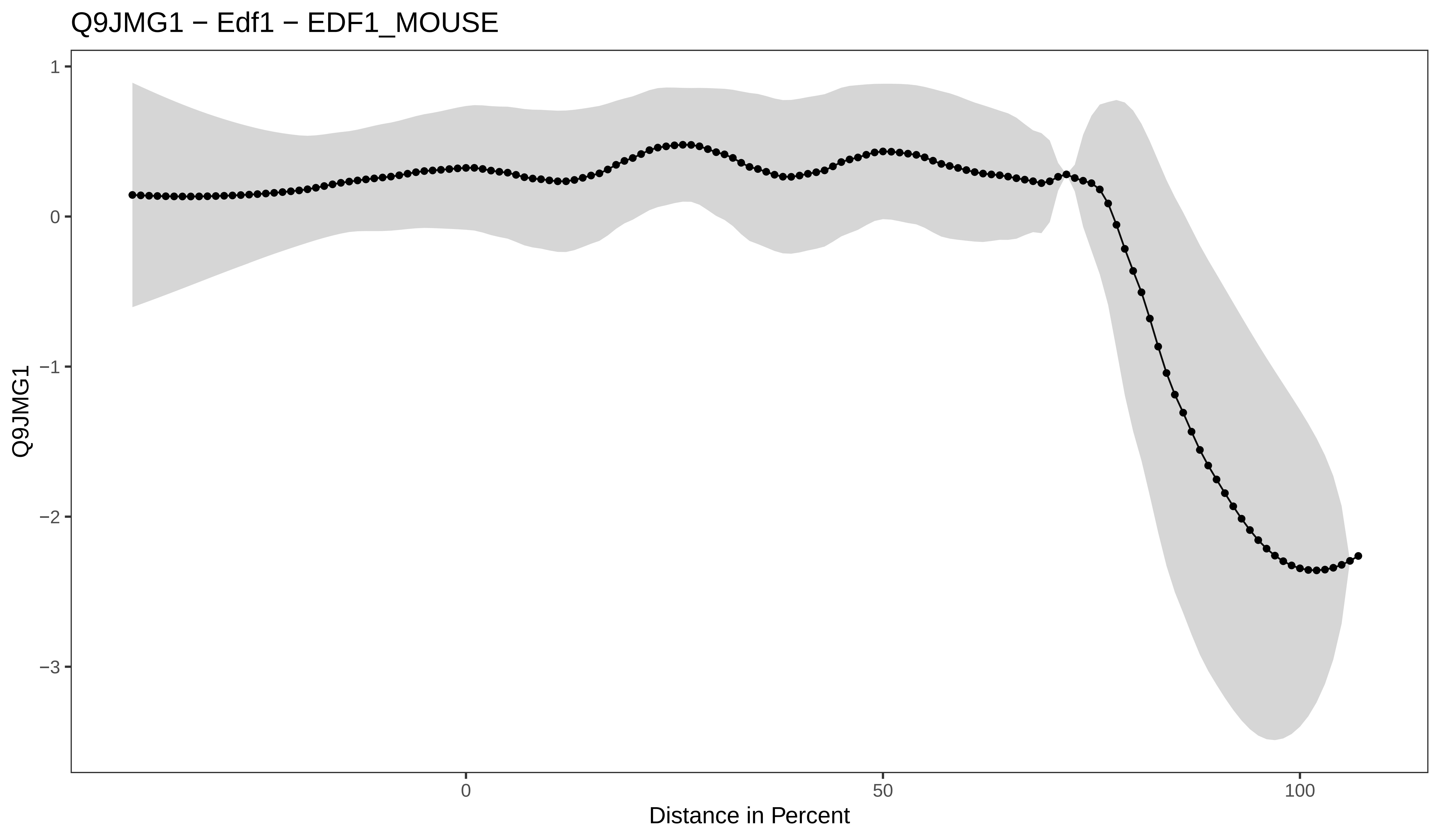
Q9JMG1 − Edf1 − EDF1_MOUSE
1
0
−1
−2
−3
Q9JMG1
0
50
100
Distance in Percent

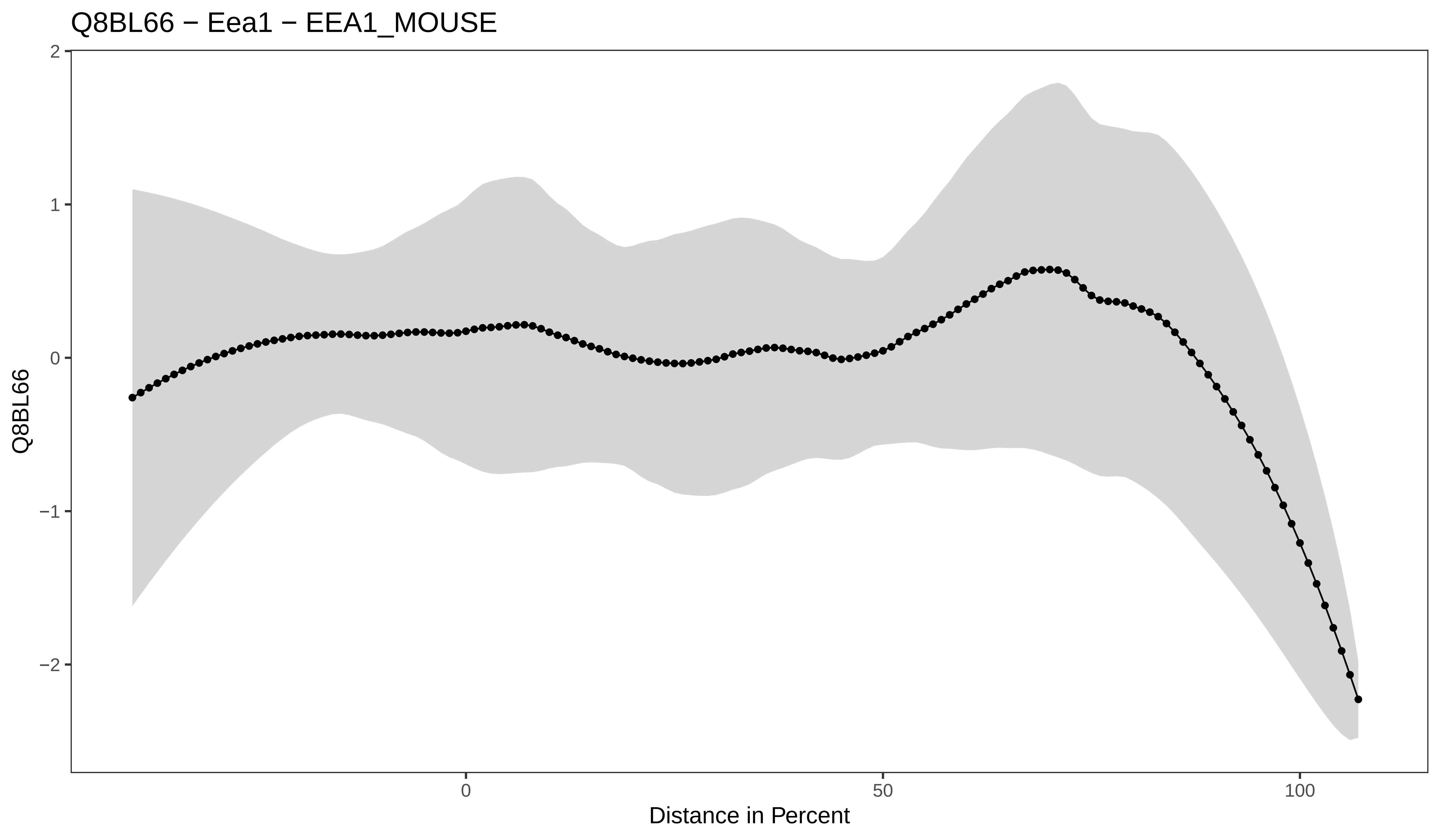

Q8BL66 − Eea1 − EEA1_MOUSE
2
1
0
−1
−2
Q8BL66
0
50
100
Distance in Percent

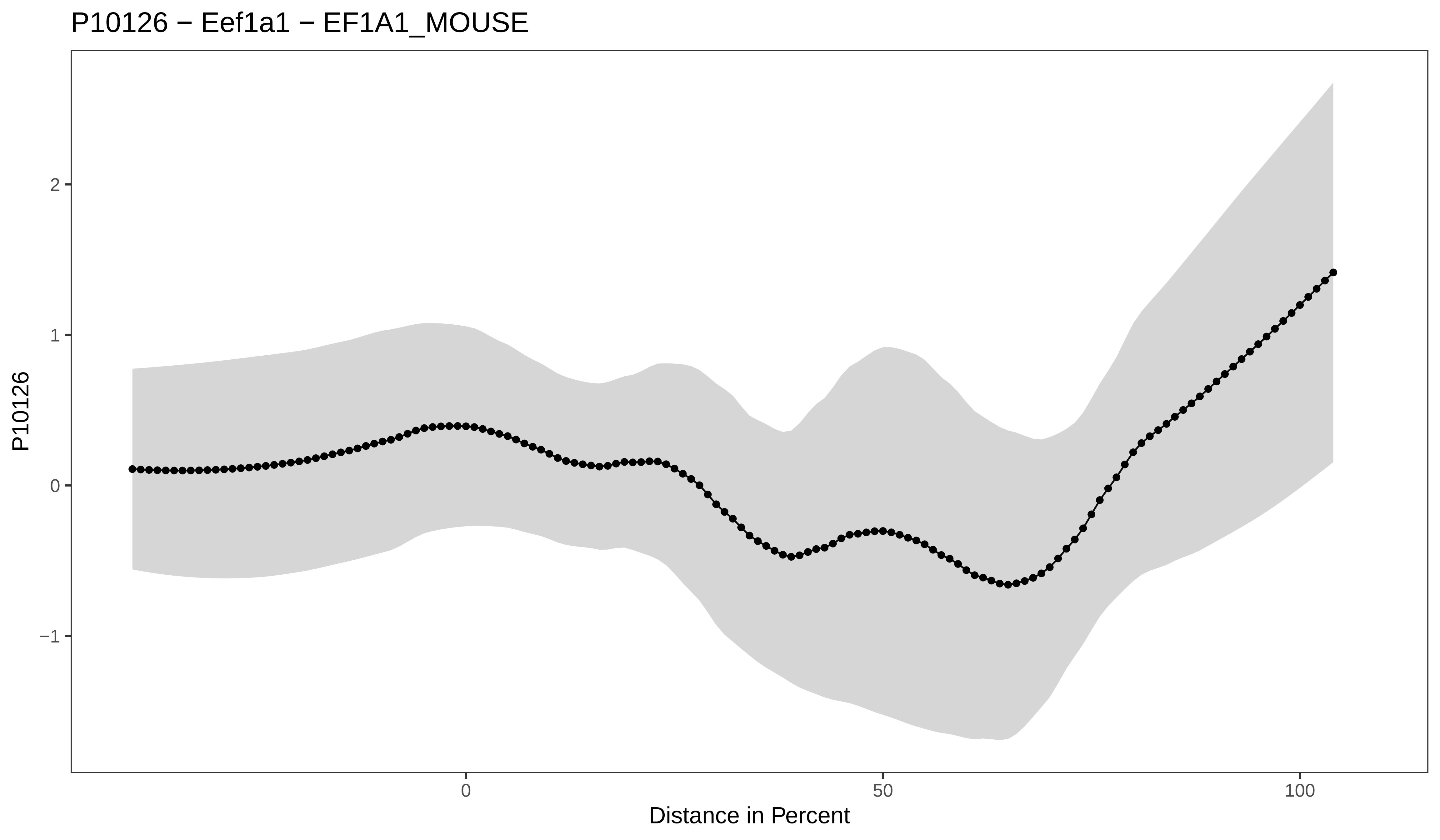

P10126 − Eef1a1 − EF1A1_MOUSE
P10126
2
1
0
−1
0
50
100
Distance in Percent

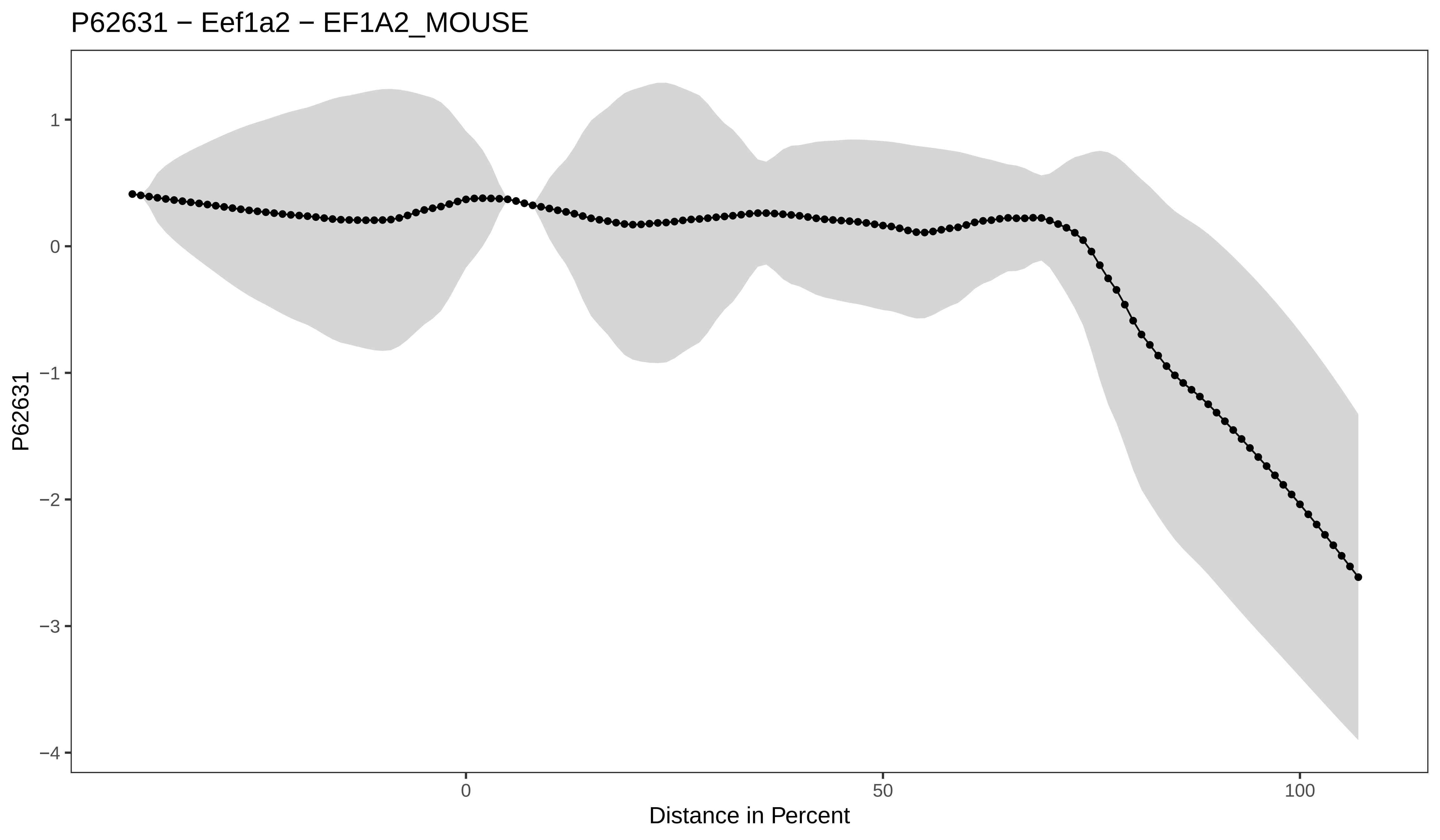
P62631 − Eef1a2 − EF1A2_MOUSE
P62631
1
0
−1
−2
−3
−4
0
50
100
Distance in Percent

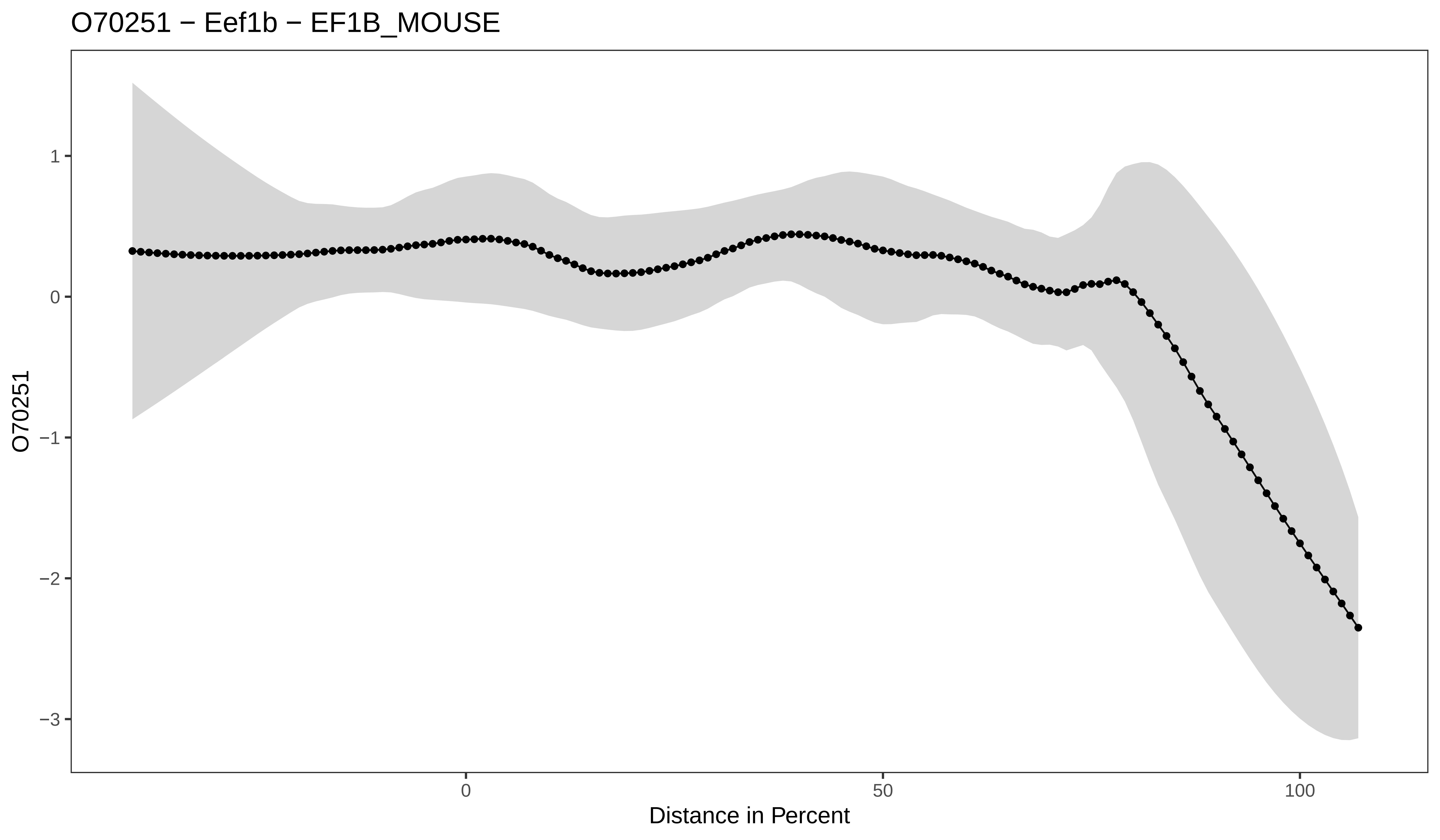

O70251 − Eef1b − EF1B_MOUSE
1
0
−1
−2
−3
O70251
0
50
100
Distance in Percent

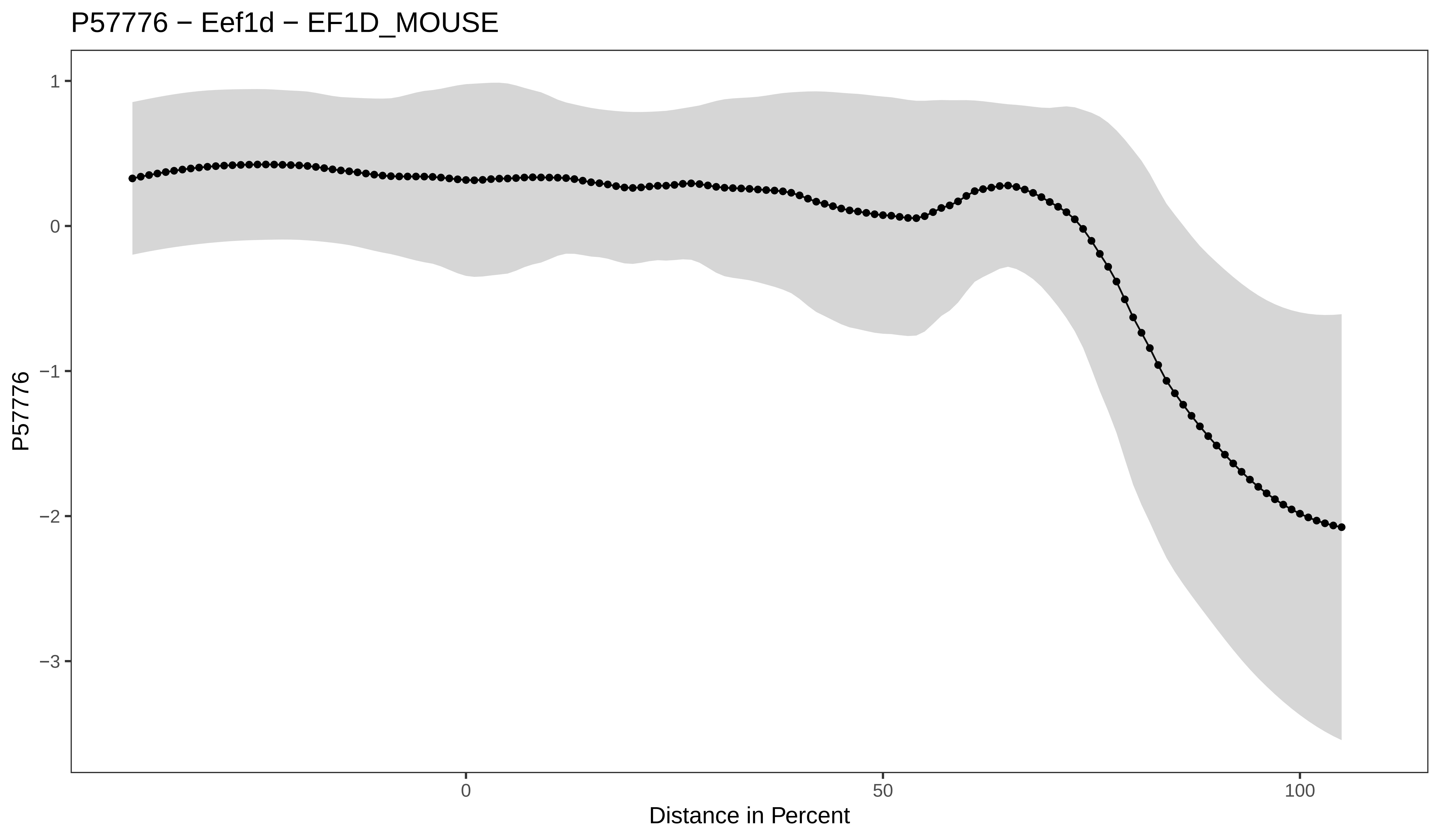

P57776 − Eef1d − EF1D_MOUSE
1
0
−1
−2
−3
P57776
0
50
100
Distance in Percent

Q9D8N0 − Eef1g − EF1G_MOUSE

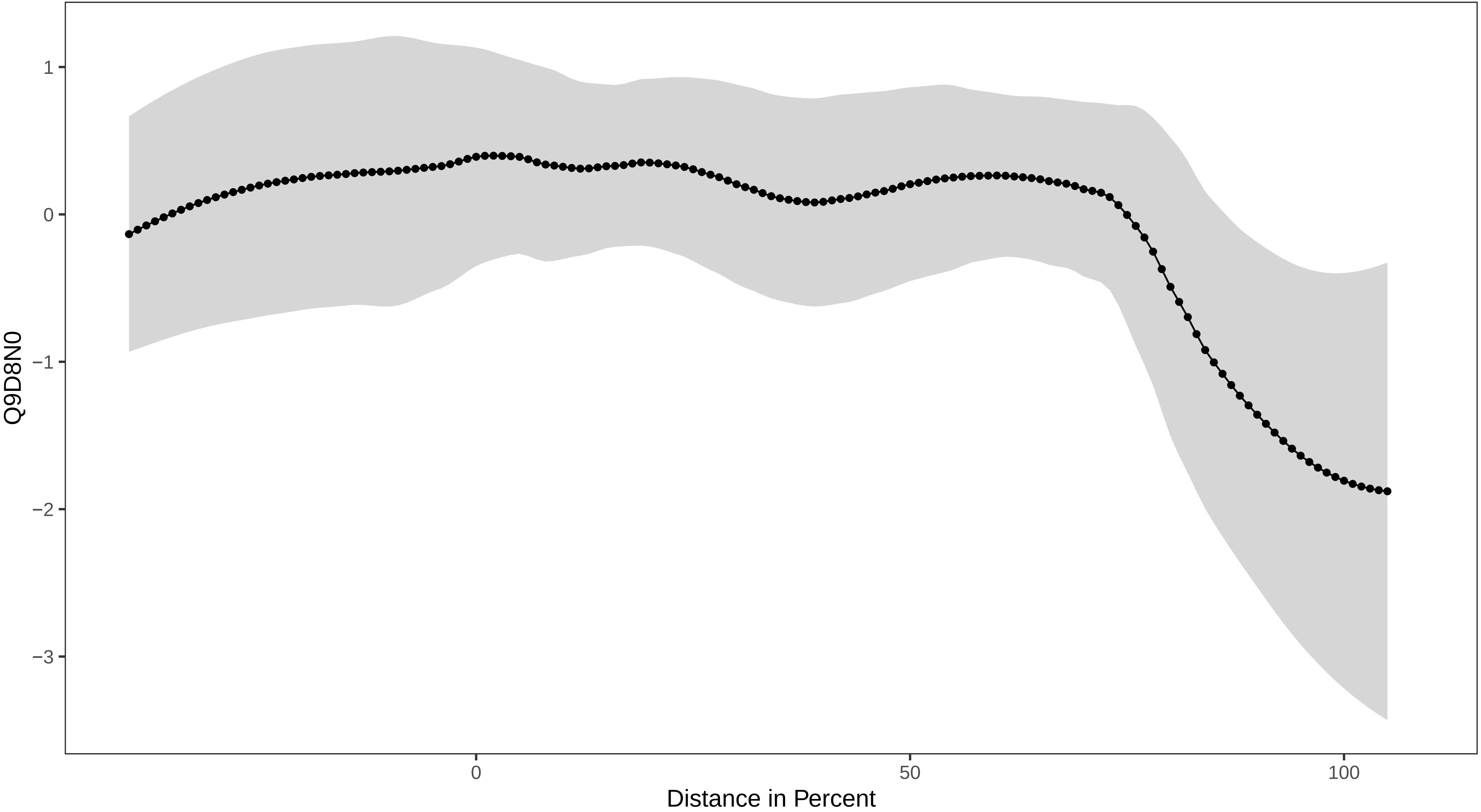

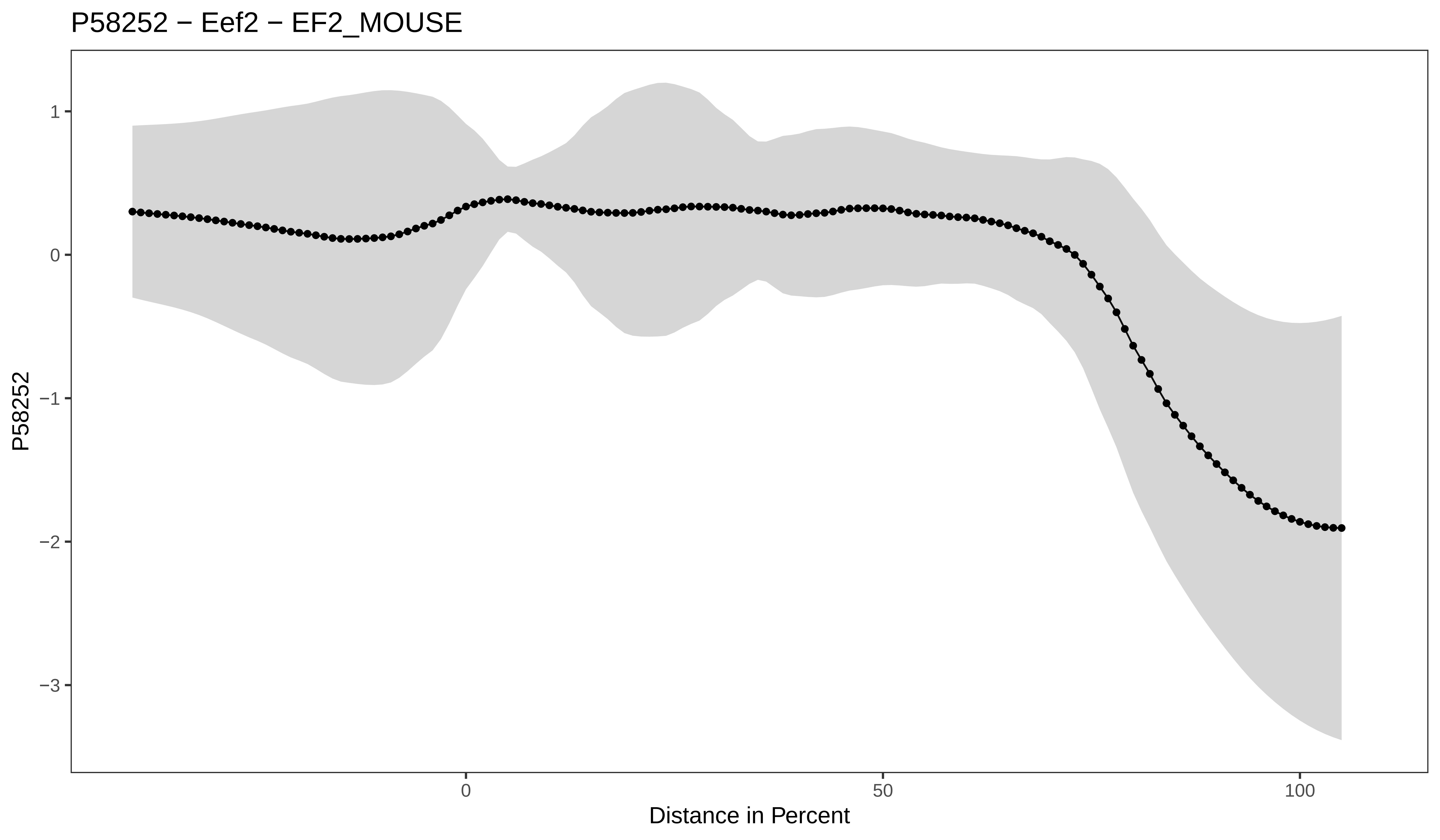

P58252 − Eef2 − EF2_MOUSE
1
0
−1
−2
−3
P58252
0
50
100
Distance in Percent

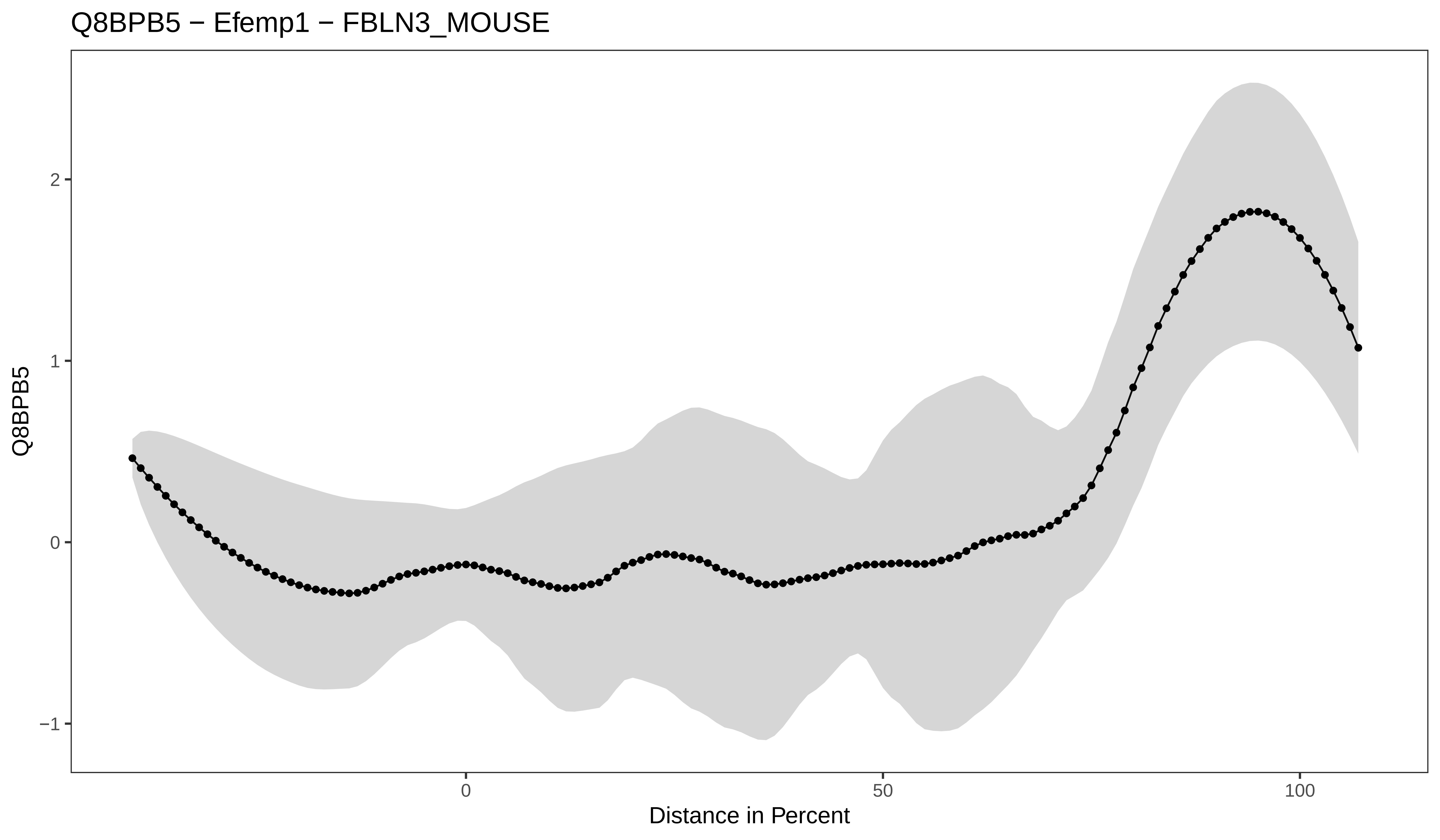

Q8BPB5 − Efemp1 − FBLN3_MOUSE
Q8BPB5
2
1
0
−1
0
50
100
Distance in Percent

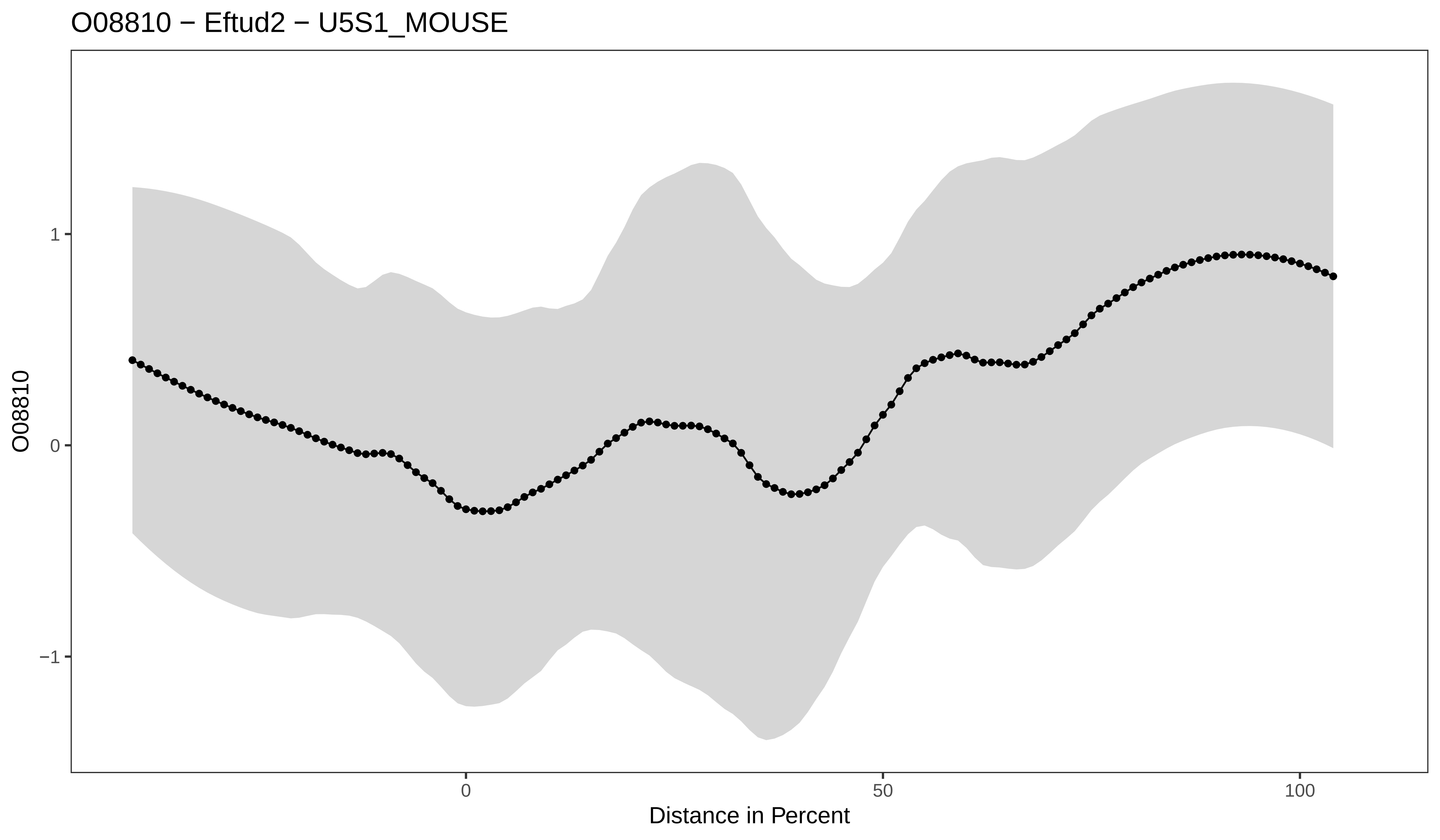

O08810 − Eftud2 − U5S1_MOUSE
O08810
1
0
−1
0
50
100
Distance in Percent

Q99MS7 − Ehbp1l1 − EH1L1_MOUSE

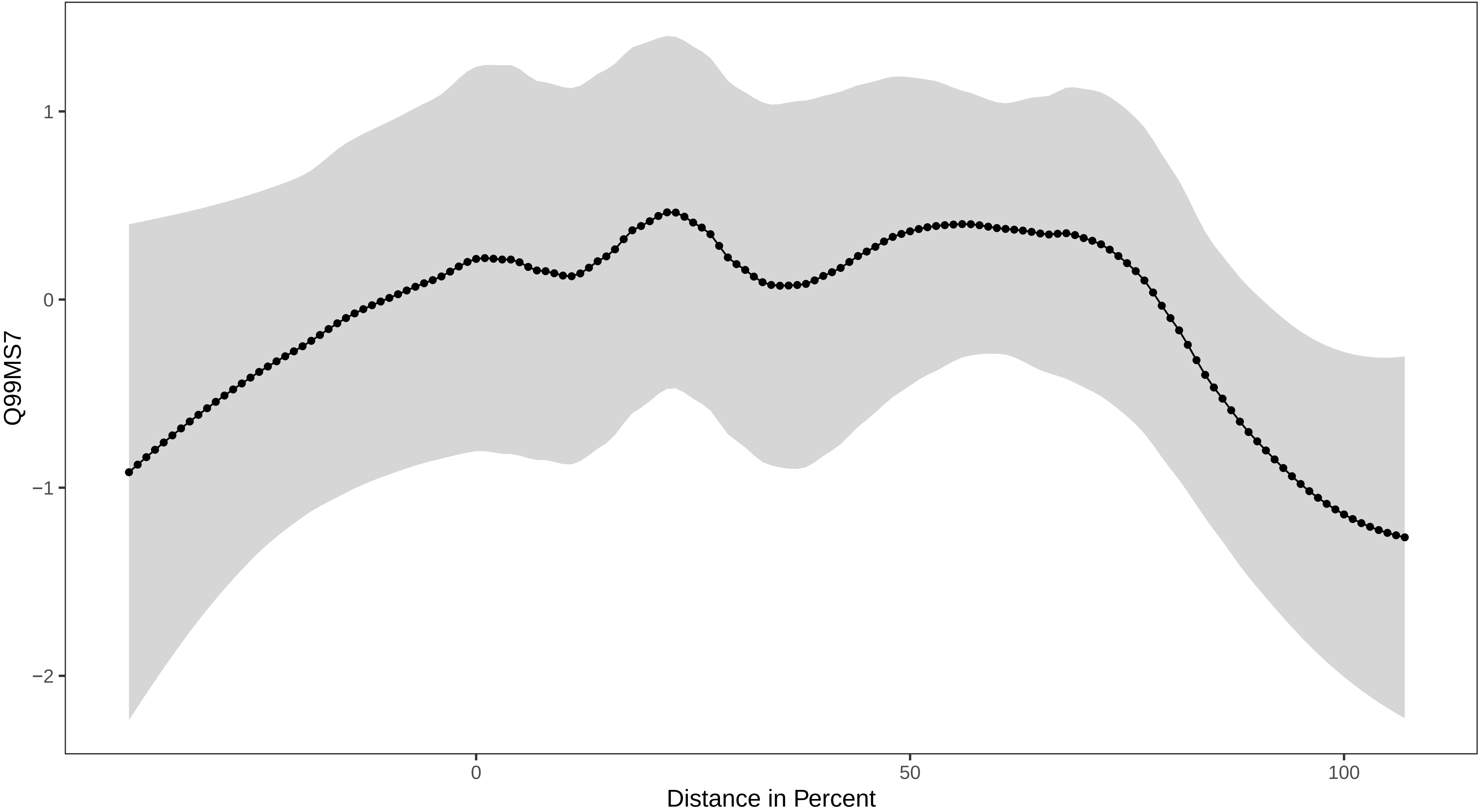

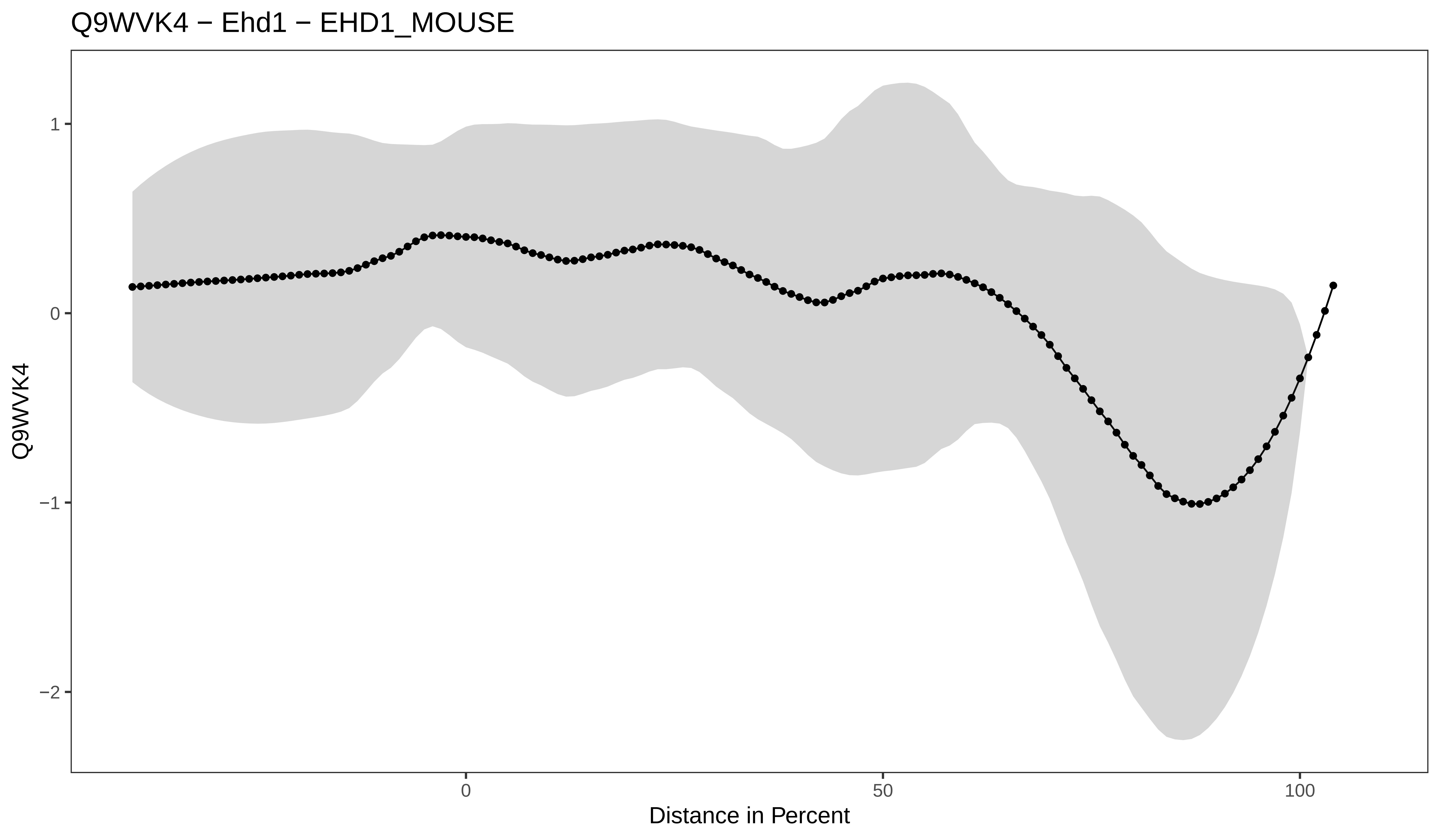
Q9WVK4 − Ehd1 − EHD1_MOUSE
1
0
−1
−2
Q9WVK4
0
50
100
Distance in Percent

Q8BH64 − Ehd2 − EHD2_MOUSE

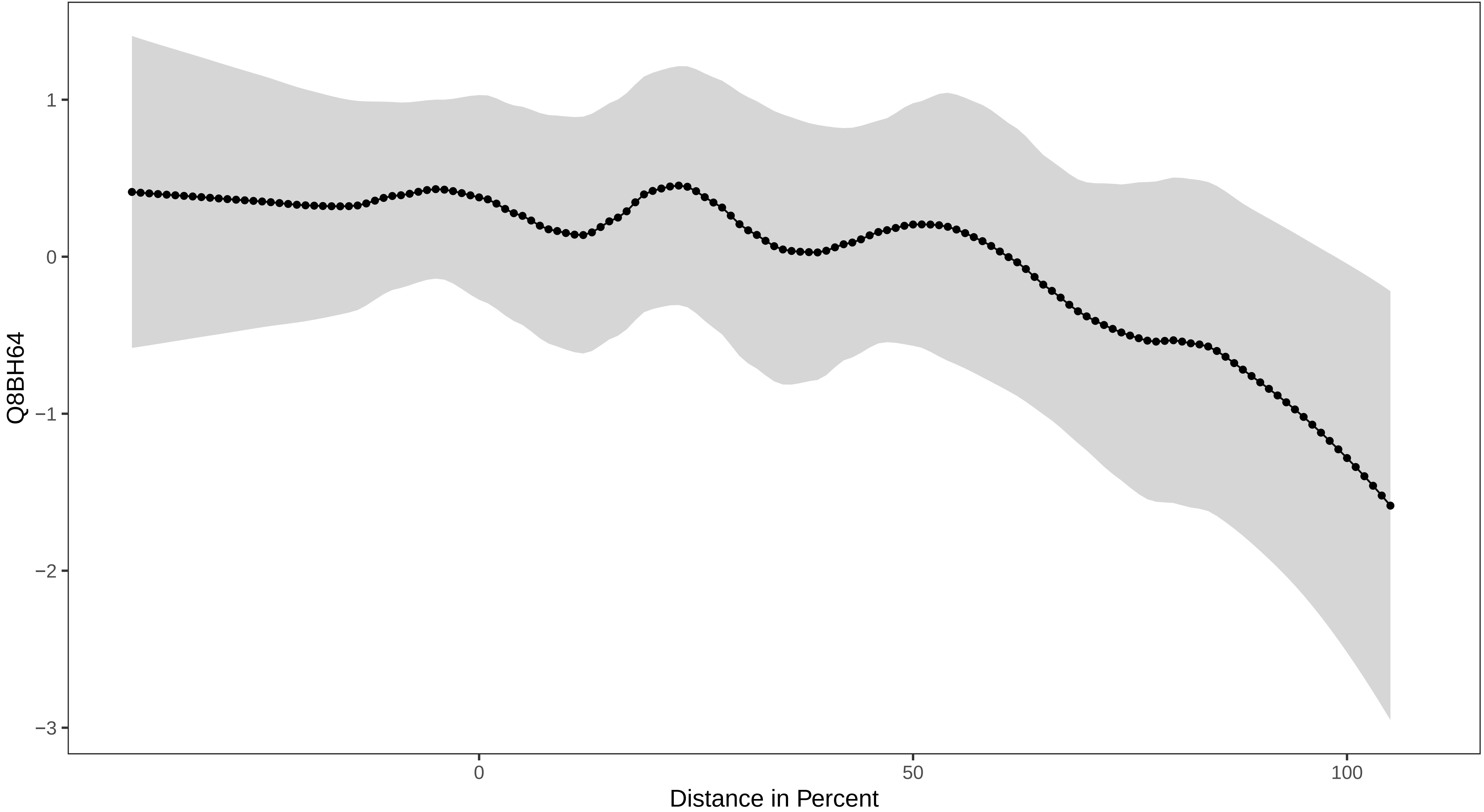

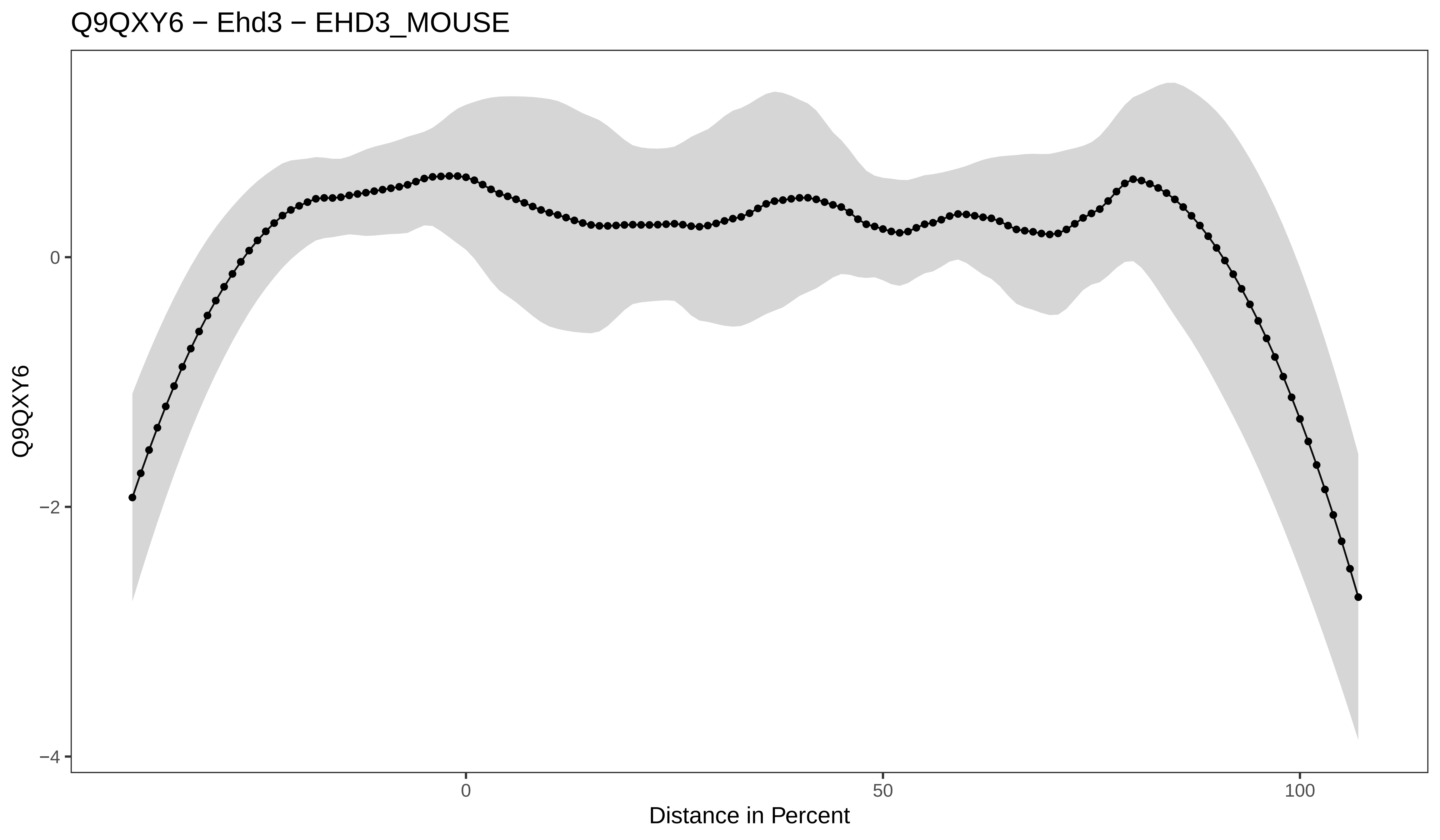
Q9QXY6 − Ehd3 − EHD3_MOUSE
0
−2
−4
Q9QXY6
0
50
100
Distance in Percent

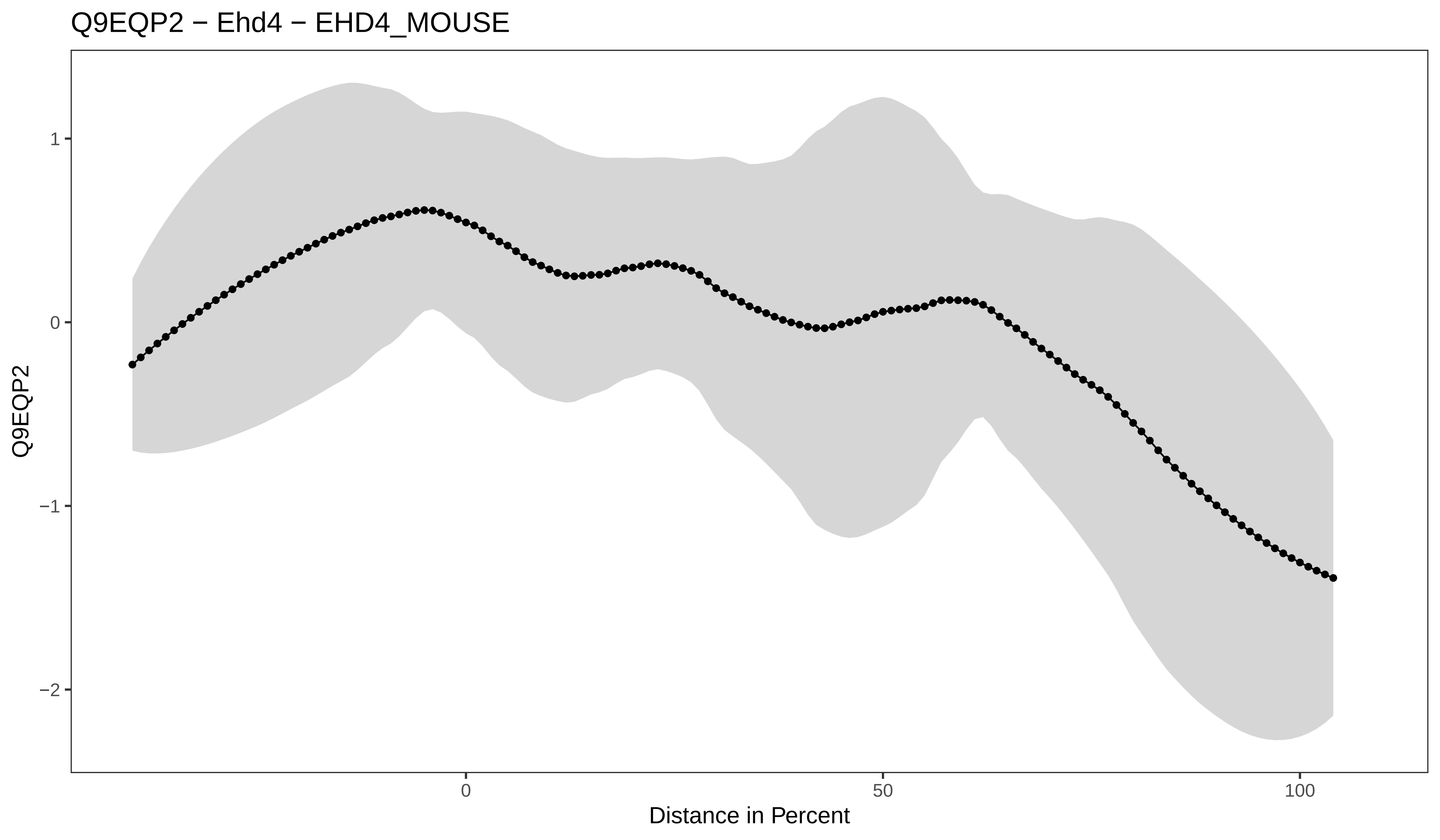
Q9EQP2 − Ehd4 − EHD4_MOUSE
Q9EQP2
1
0
−1
−2
0
50
100
Distance in Percent

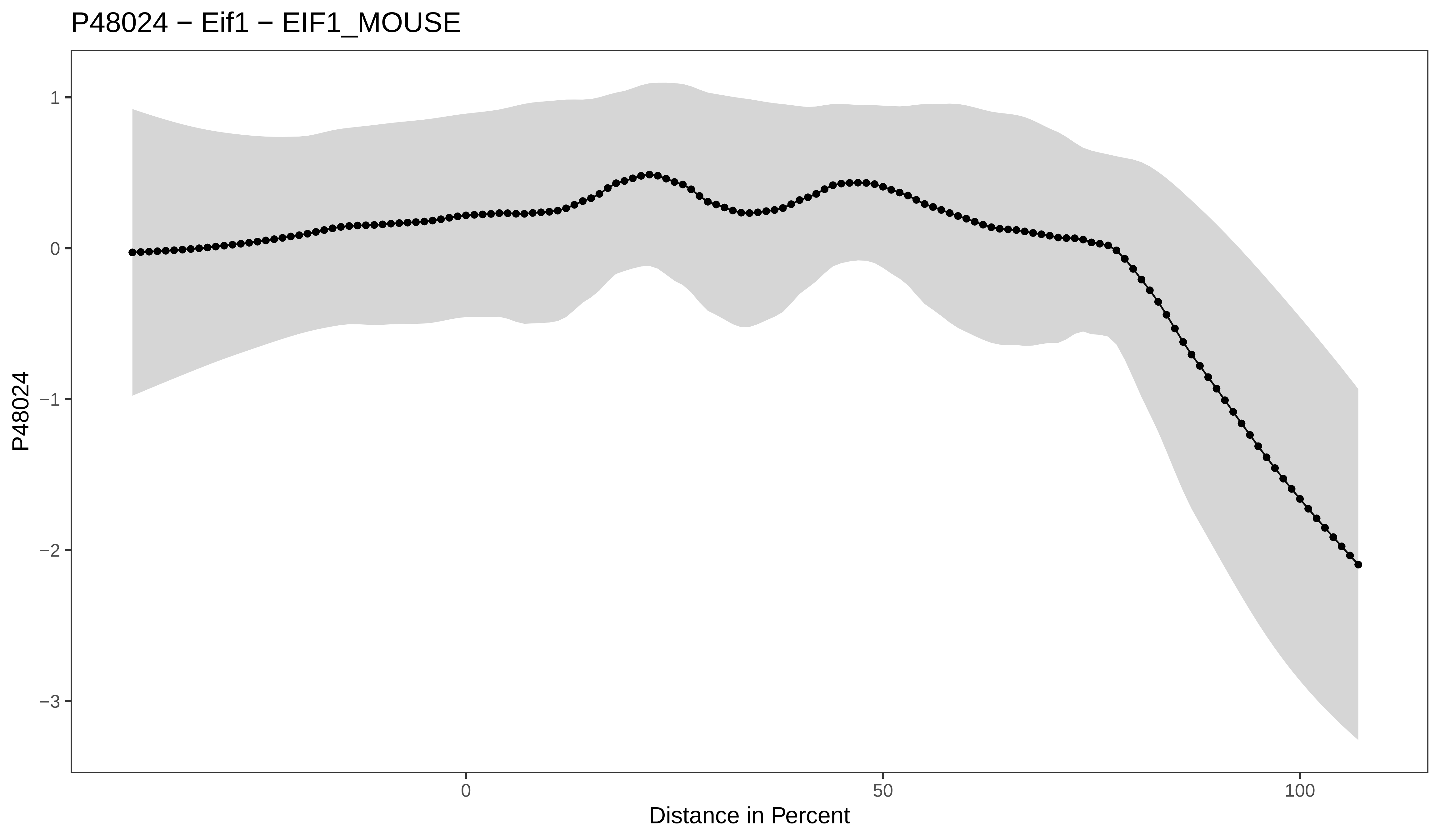
P48024 − Eif1 − EIF1_MOUSE
P48024
1
0
−1
−2
−3
0
50
100
Distance in Percent

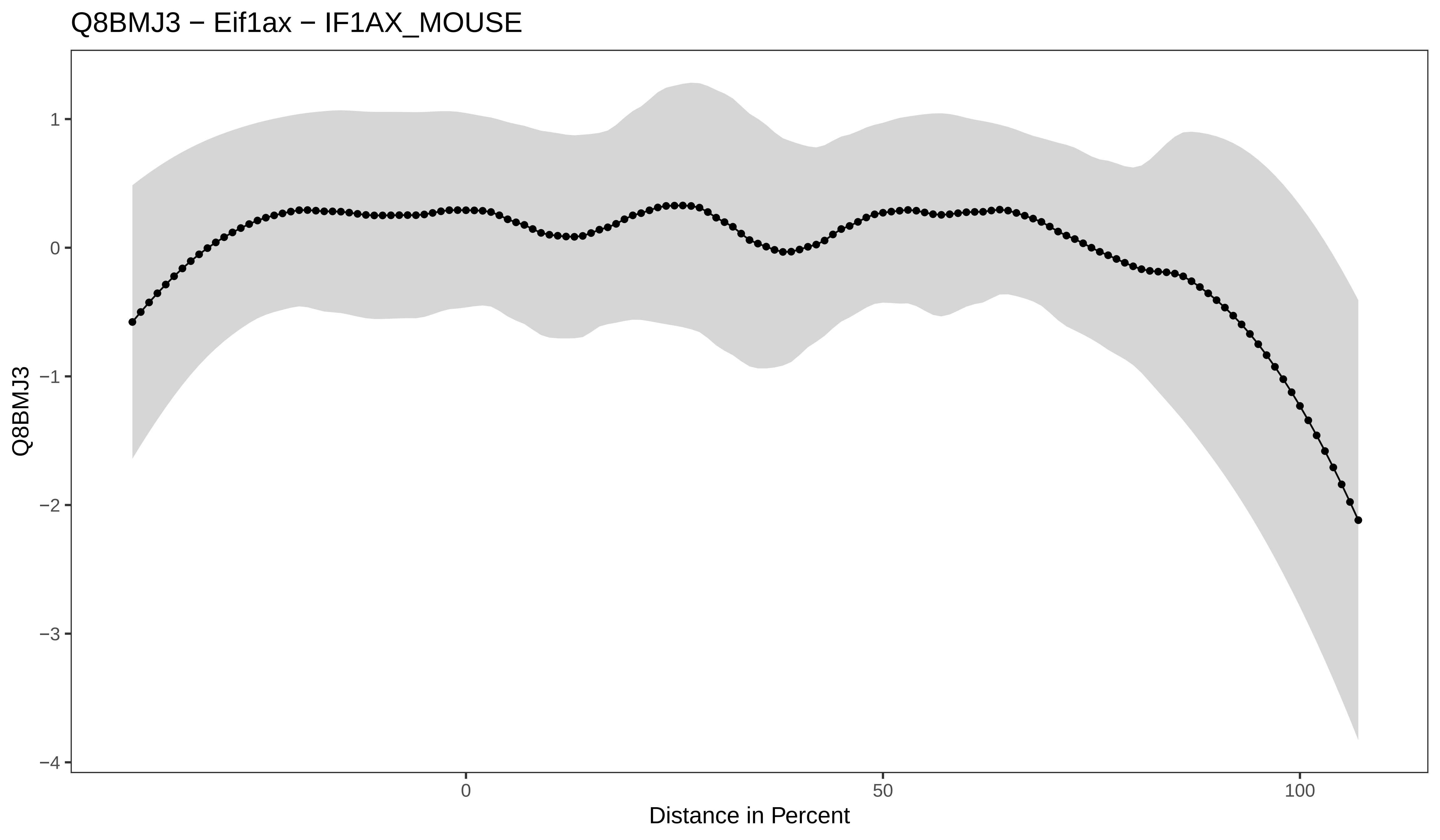

Q8BMJ3 − Eif1ax − IF1AX_MOUSE
Q8BMJ3
1
0
−1
−2
−3
−4
0
50
100
Distance in Percent

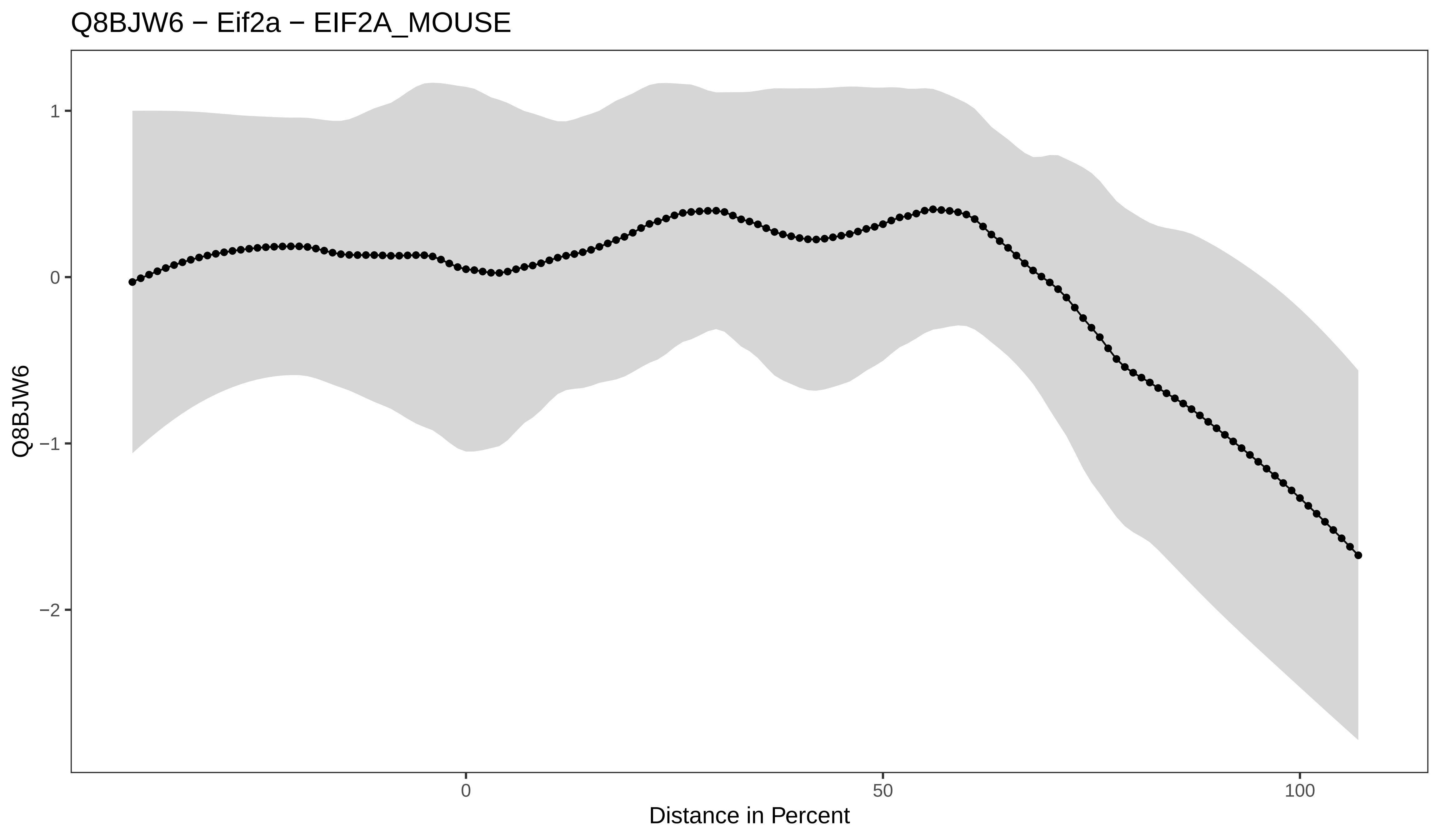

Q8BJW6 − Eif2a − EIF2A_MOUSE
Q8BJW6
1
0
−1
−2
0
50
100
Distance in Percent

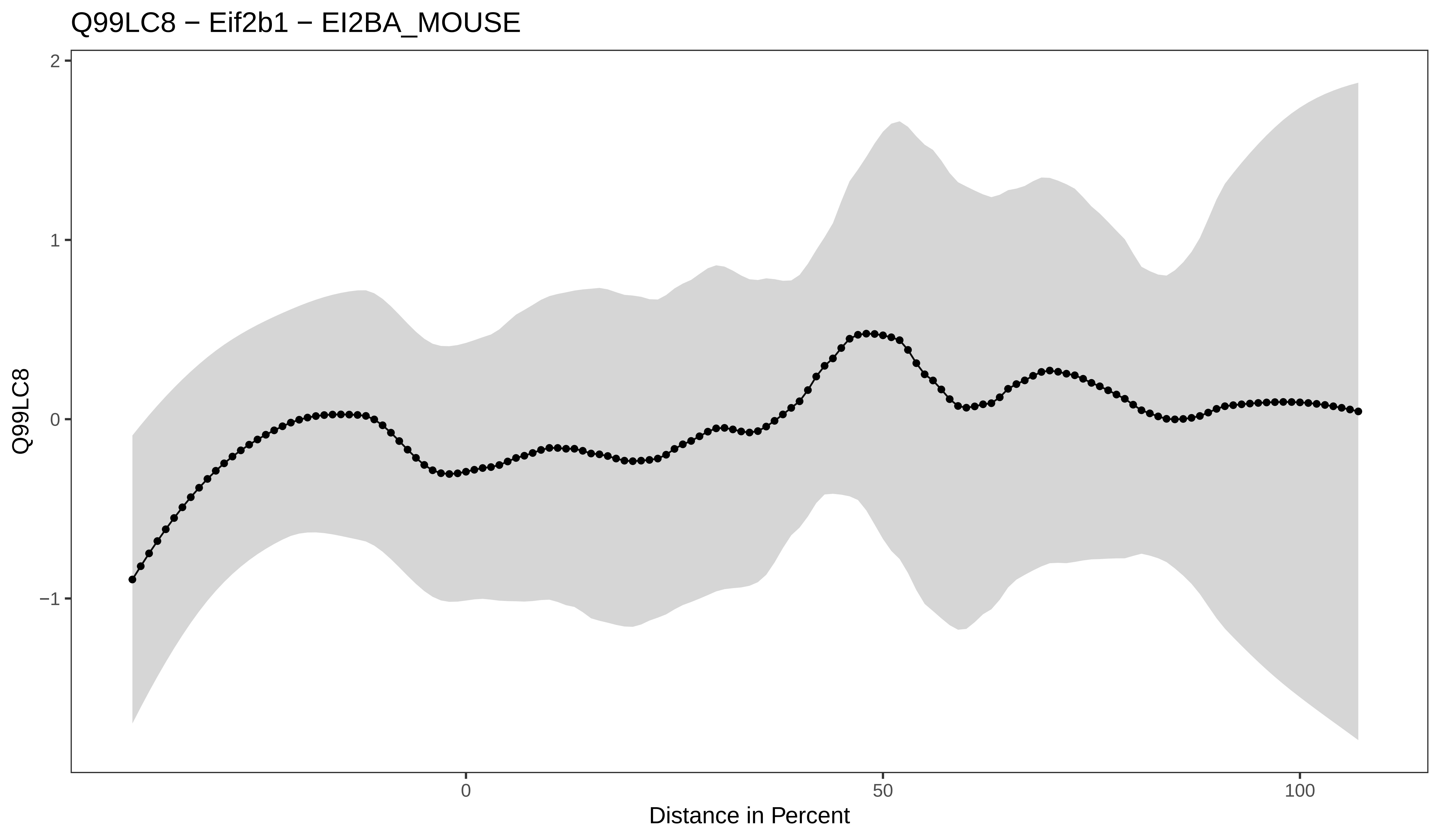

Q99LC8 − Eif2b1 − EI2BA_MOUSE
Q99LC8
2
1
0
−1
0
50
100
Distance in Percent

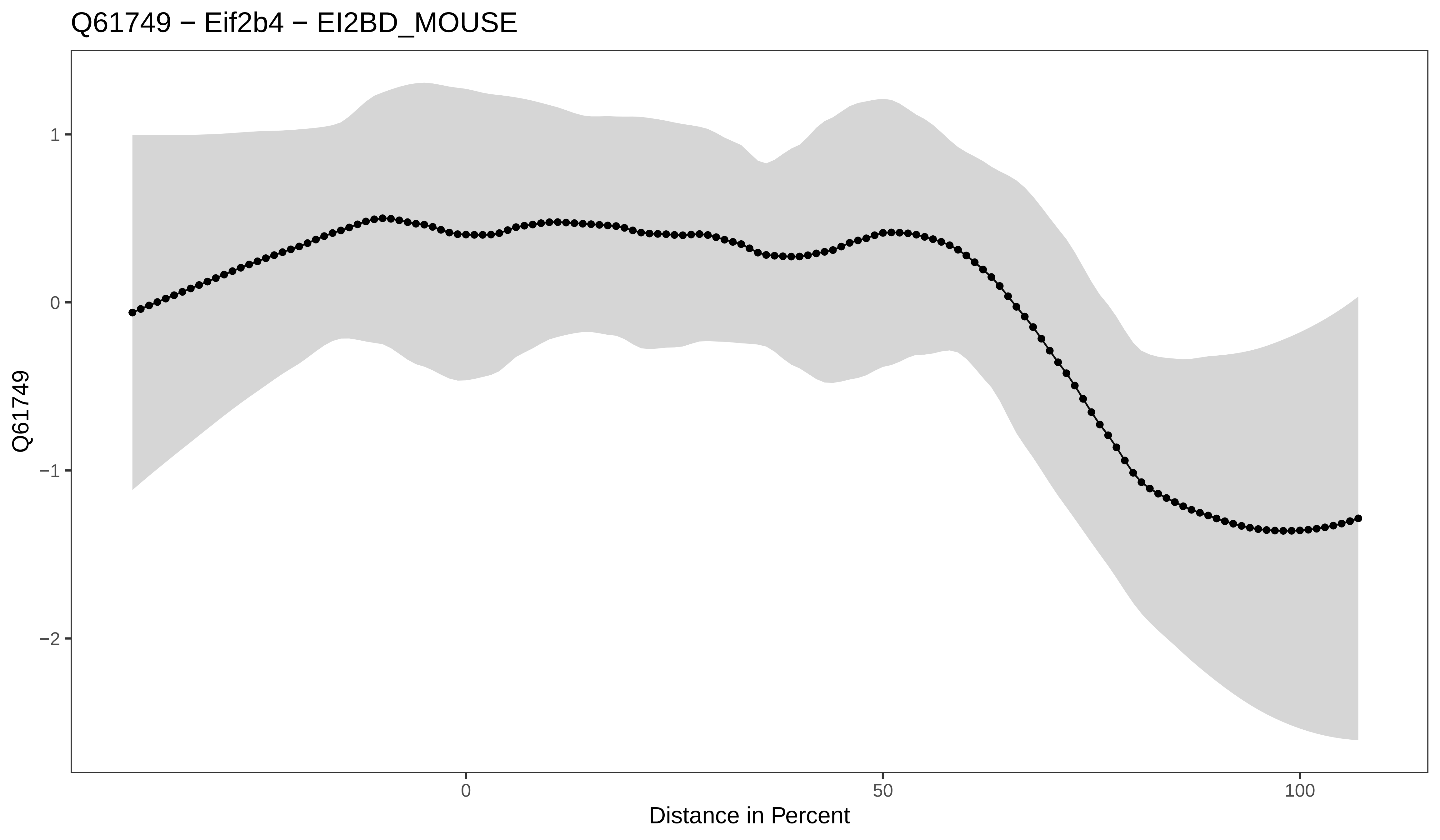
Q61749 − Eif2b4 − EI2BD_MOUSE
Q61749
1
0
−1
−2
0
50
100
Distance in Percent

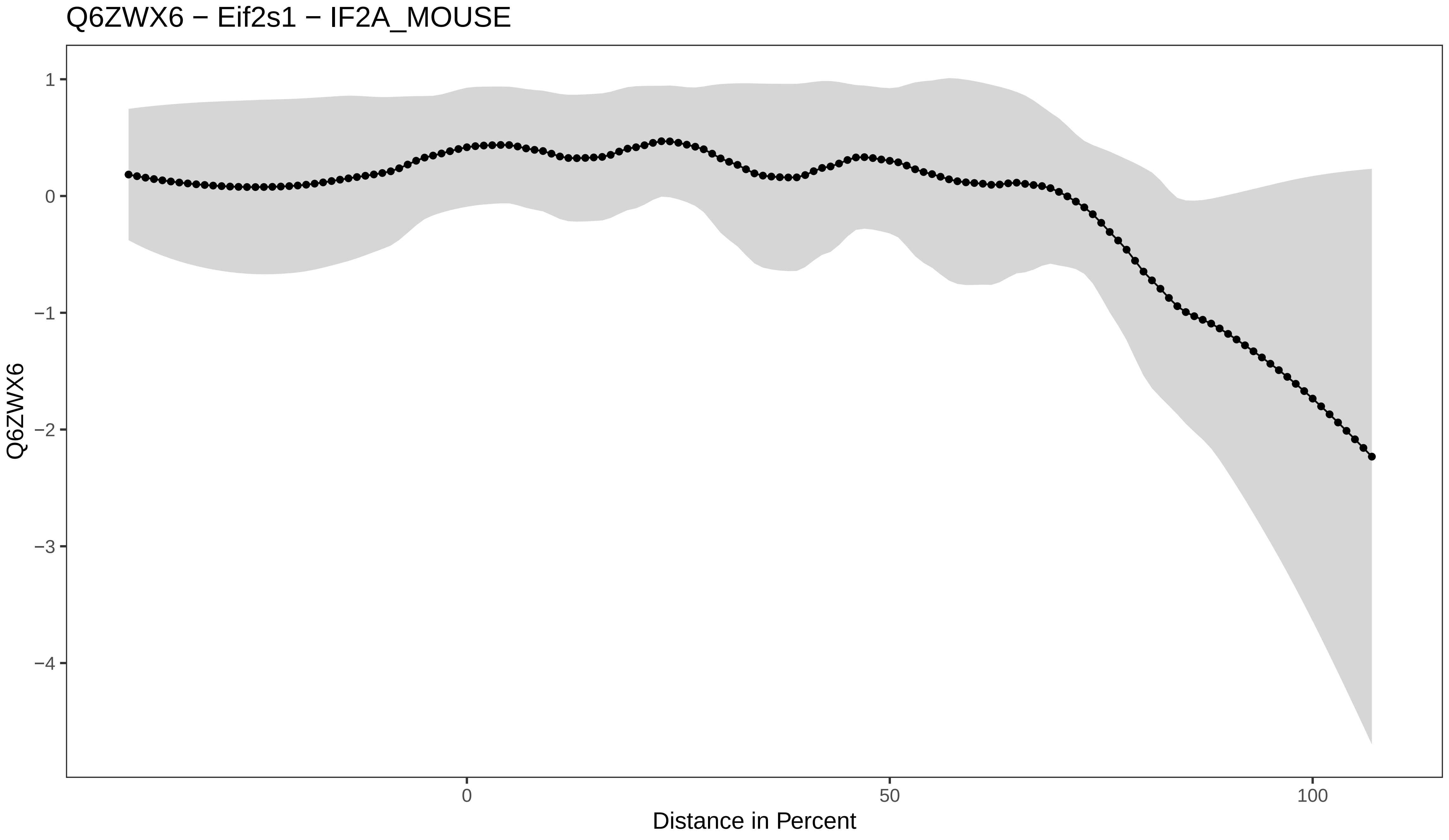

Q6ZWX6 − Eif2s1 − IF2A_MOUSE
1
0
−1
−2
−3
−4
Q6ZWX6
0
50
100
Distance in Percent

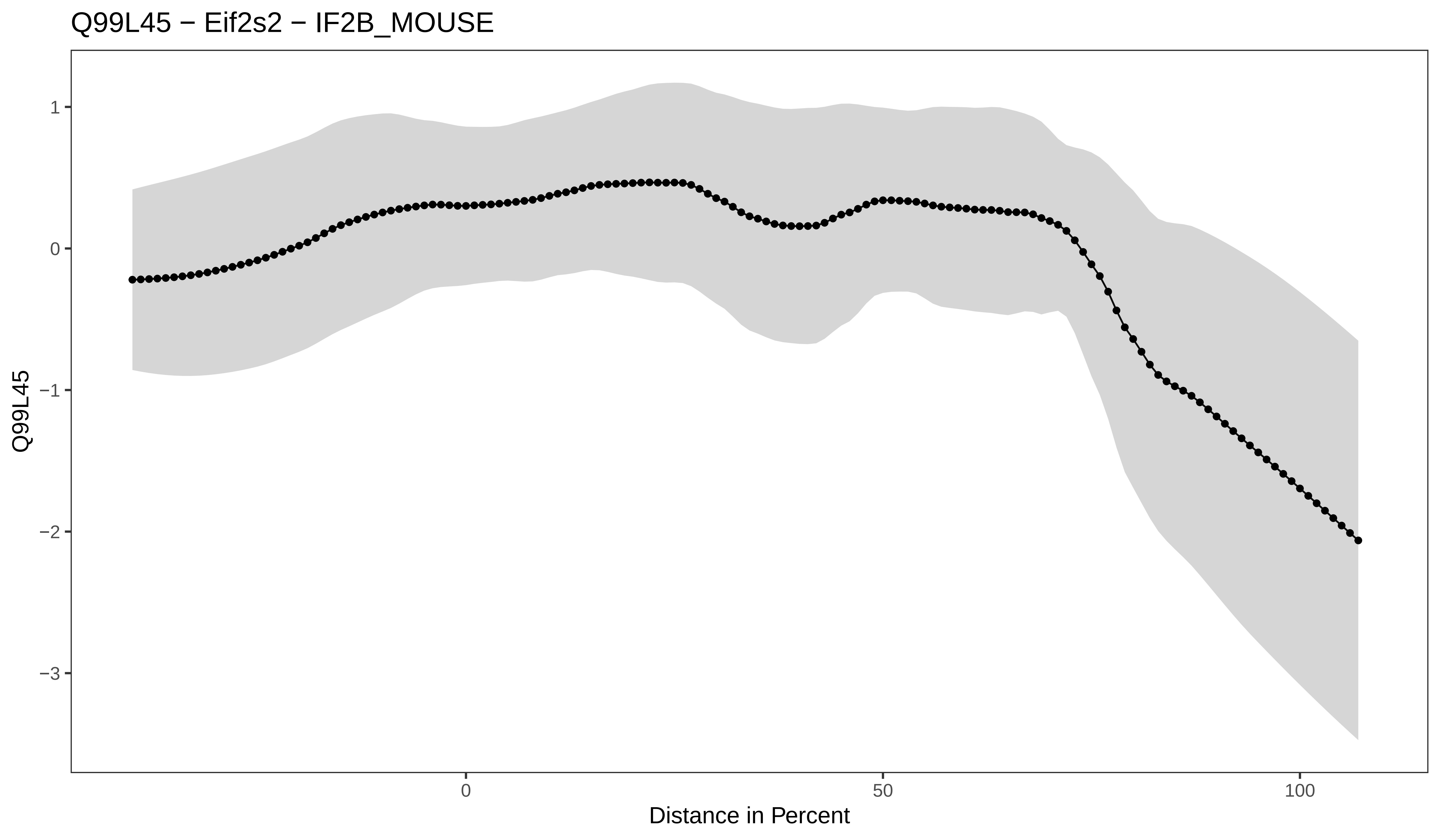
Q99L45 − Eif2s2 − IF2B_MOUSE
1
0
−1
−2
−3
Q99L45
0
50
100
Distance in Percent

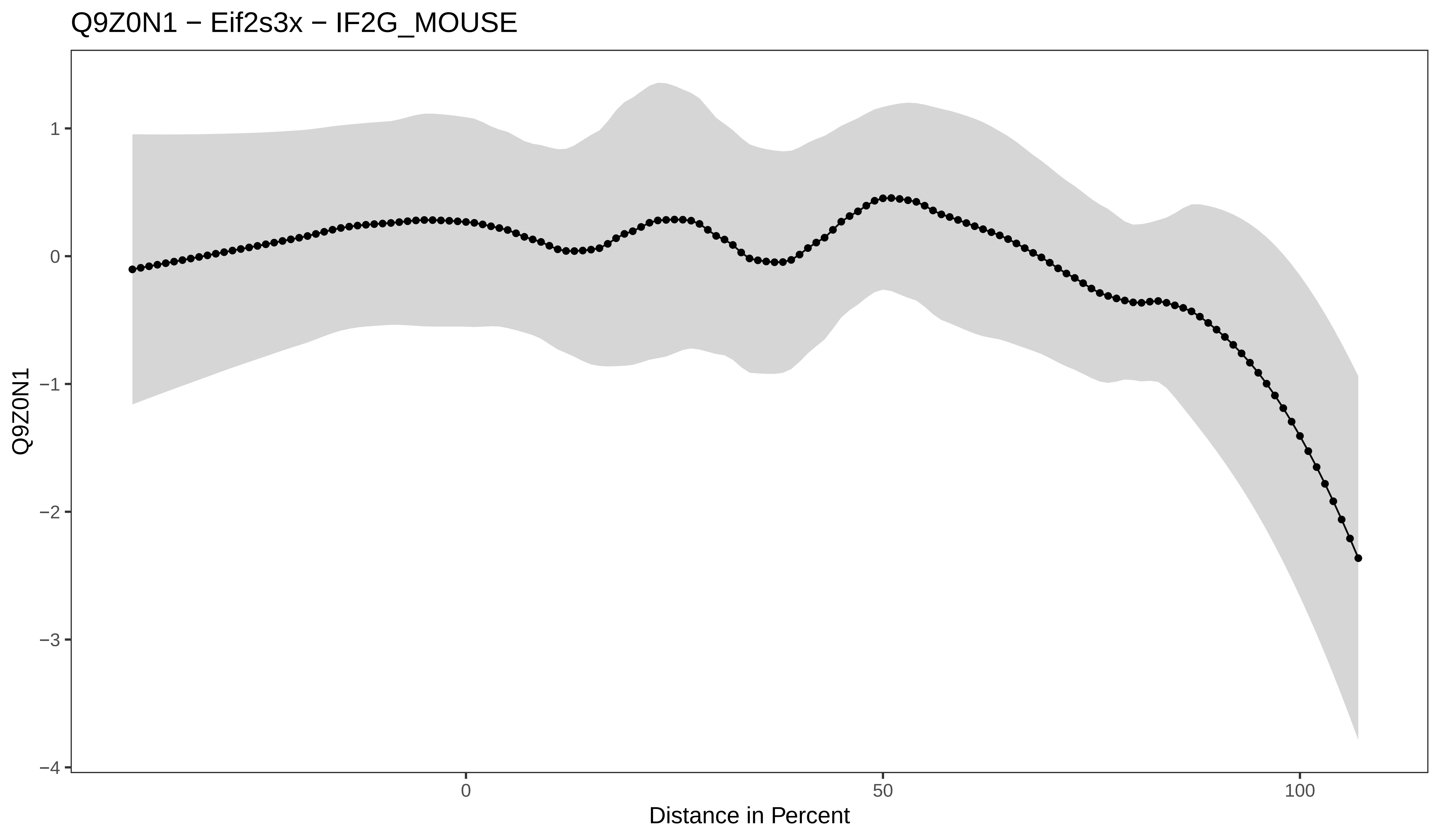

Q9Z0N1 − Eif2s3x − IF2G_MOUSE
Q9Z0N1
1
0
−1
−2
−3
−4
0
50
100
Distance in Percent

P23116 − Eif3a − EIF3A_MOUSE

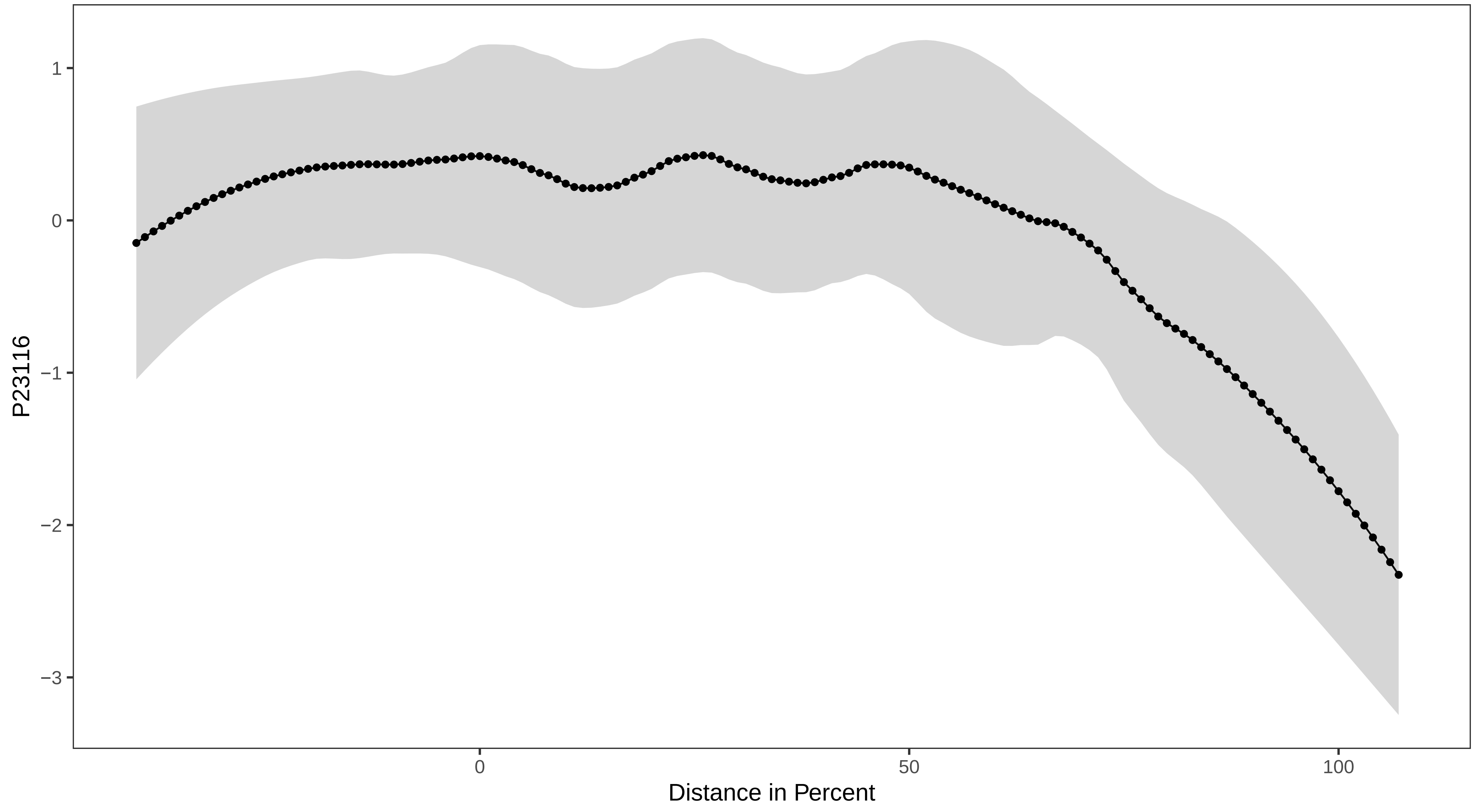

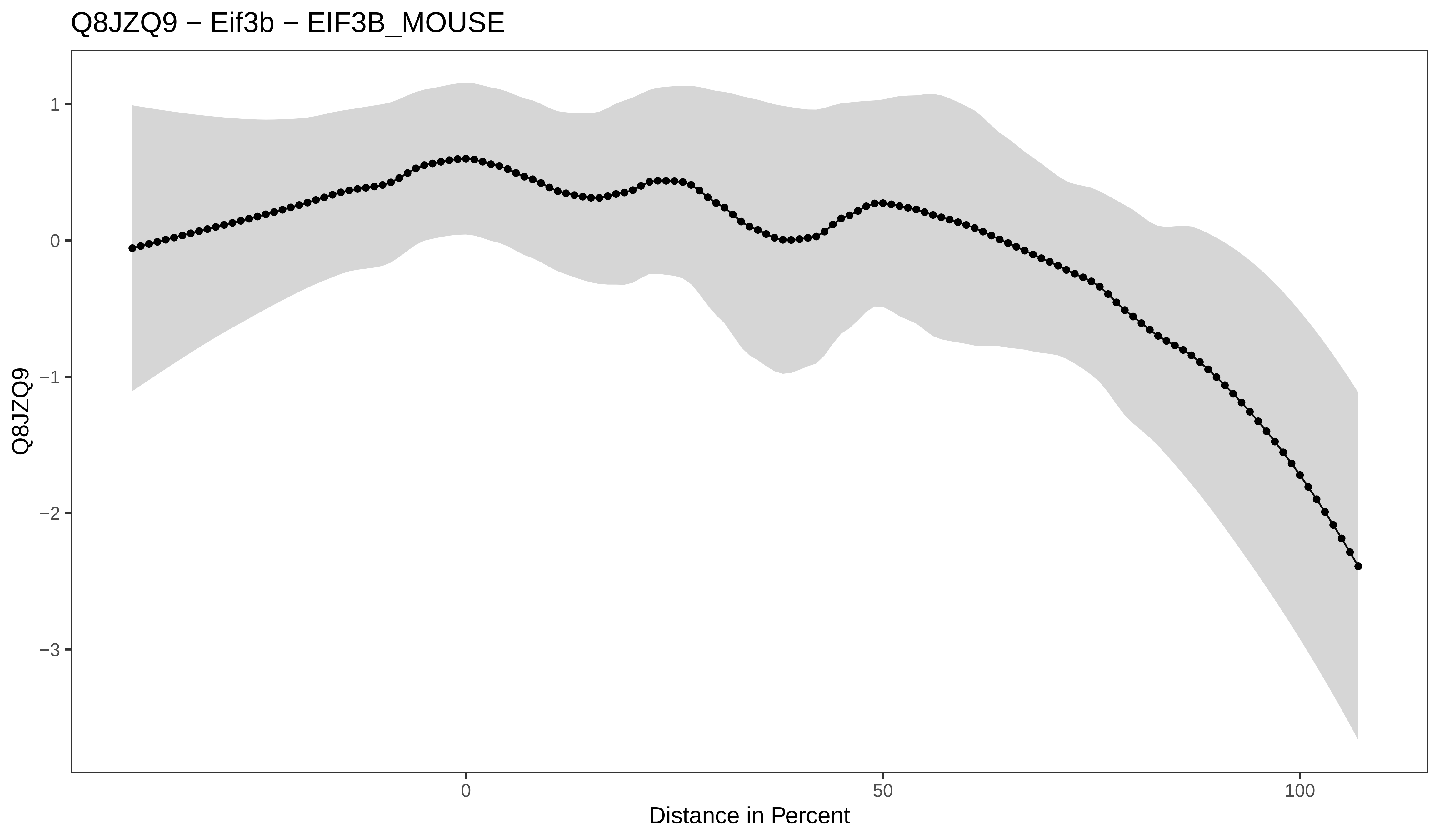
Q8JZQ9 − Eif3b − EIF3B_MOUSE
Q8JZQ9
Distance in Percent
1
0
−1
−2
−3
0
50
100

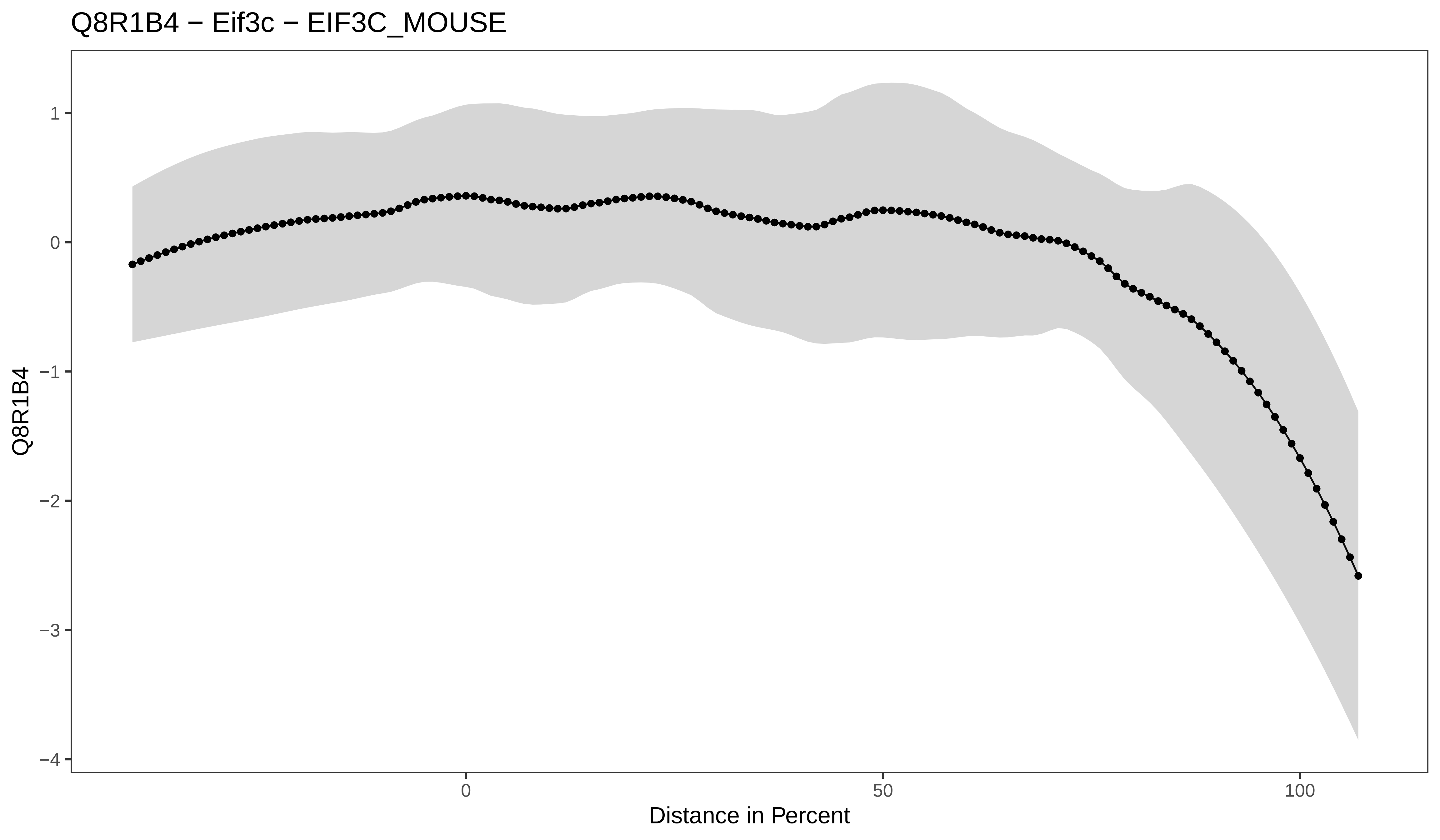
Q8R1B4 − Eif3c − EIF3C_MOUSE
Q8R1B4
Distance in Percent
1
0
−1
−2
−3
−4
0
50
100

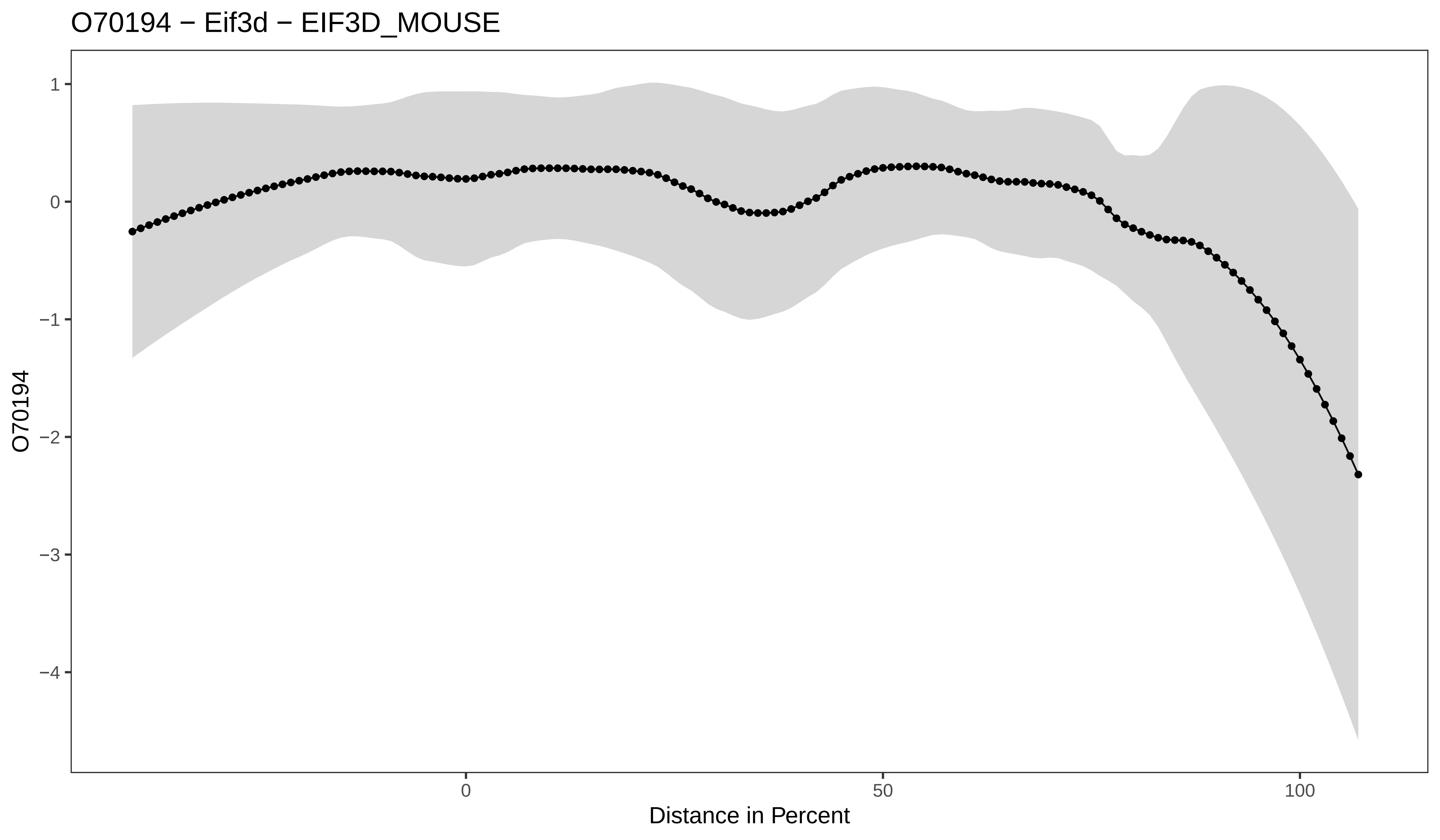
O70194 − Eif3d − EIF3D_MOUSE
O70194
Distance in Percent
1
0
−1
−2
−3
−4
0
50
100

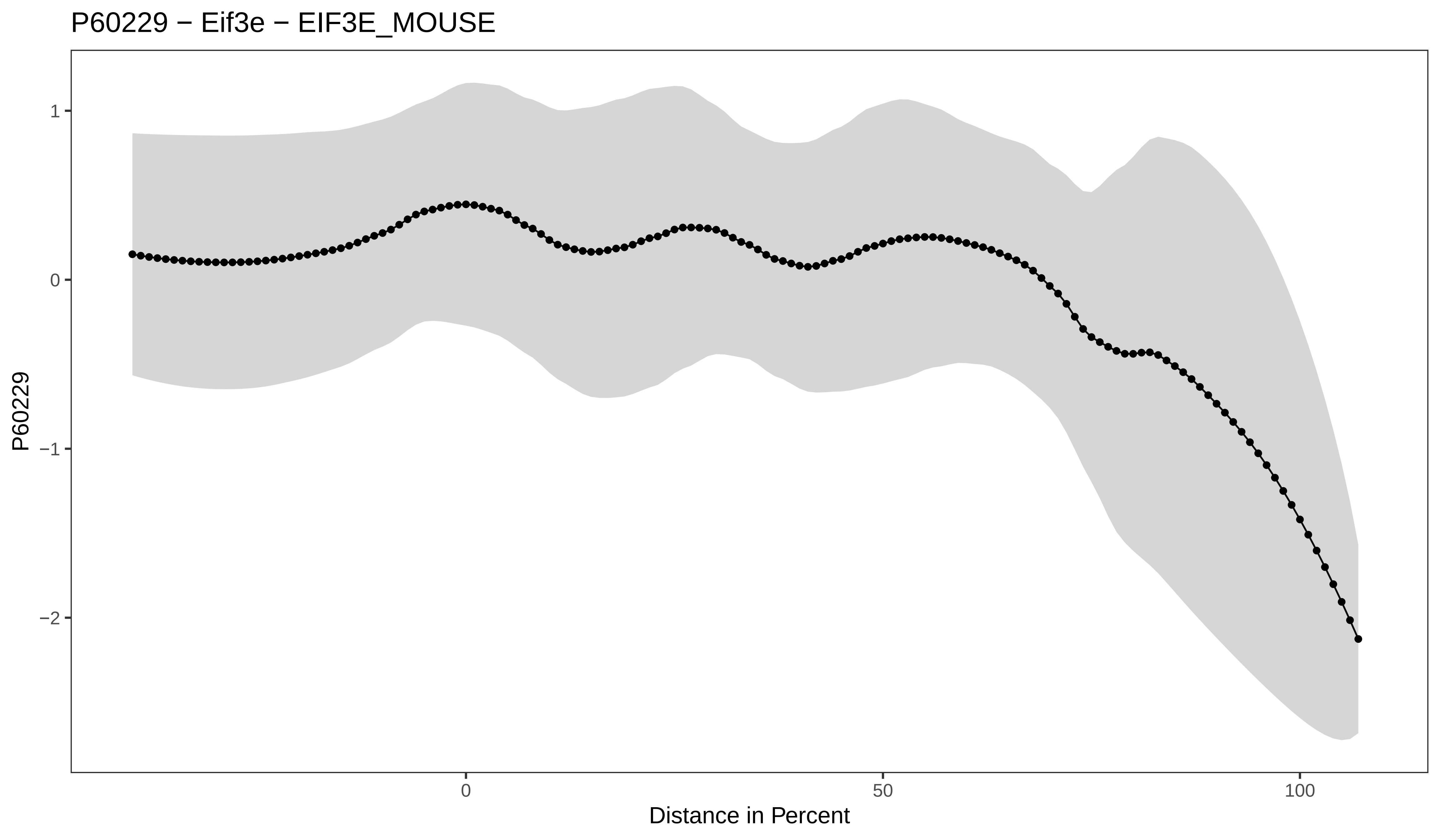

P60229 − Eif3e − EIF3E_MOUSE
P60229
1
0
−1
−2
0
50
100
Distance in Percent

Q9DCH4 − Eif3f − EIF3F_MOUSE

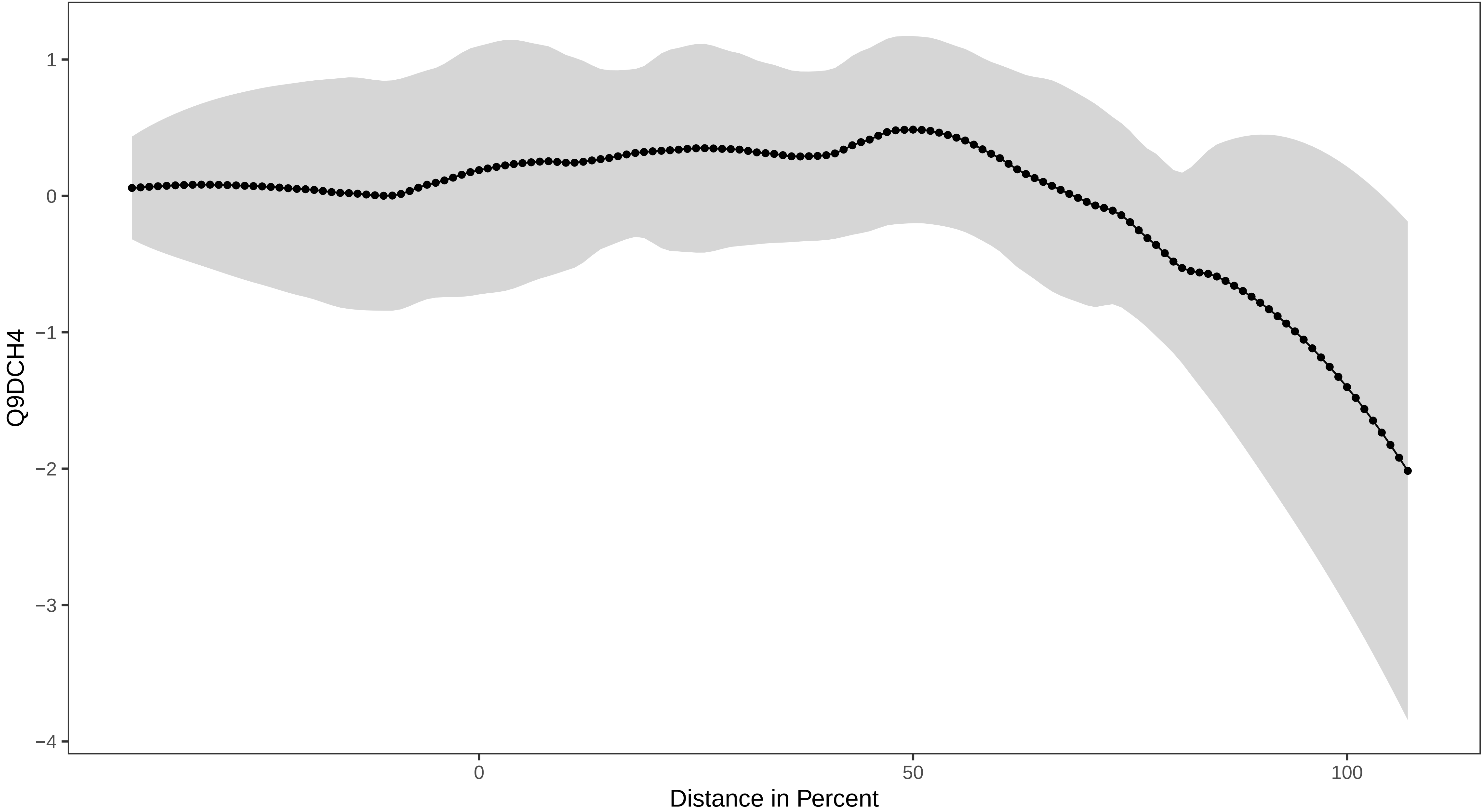

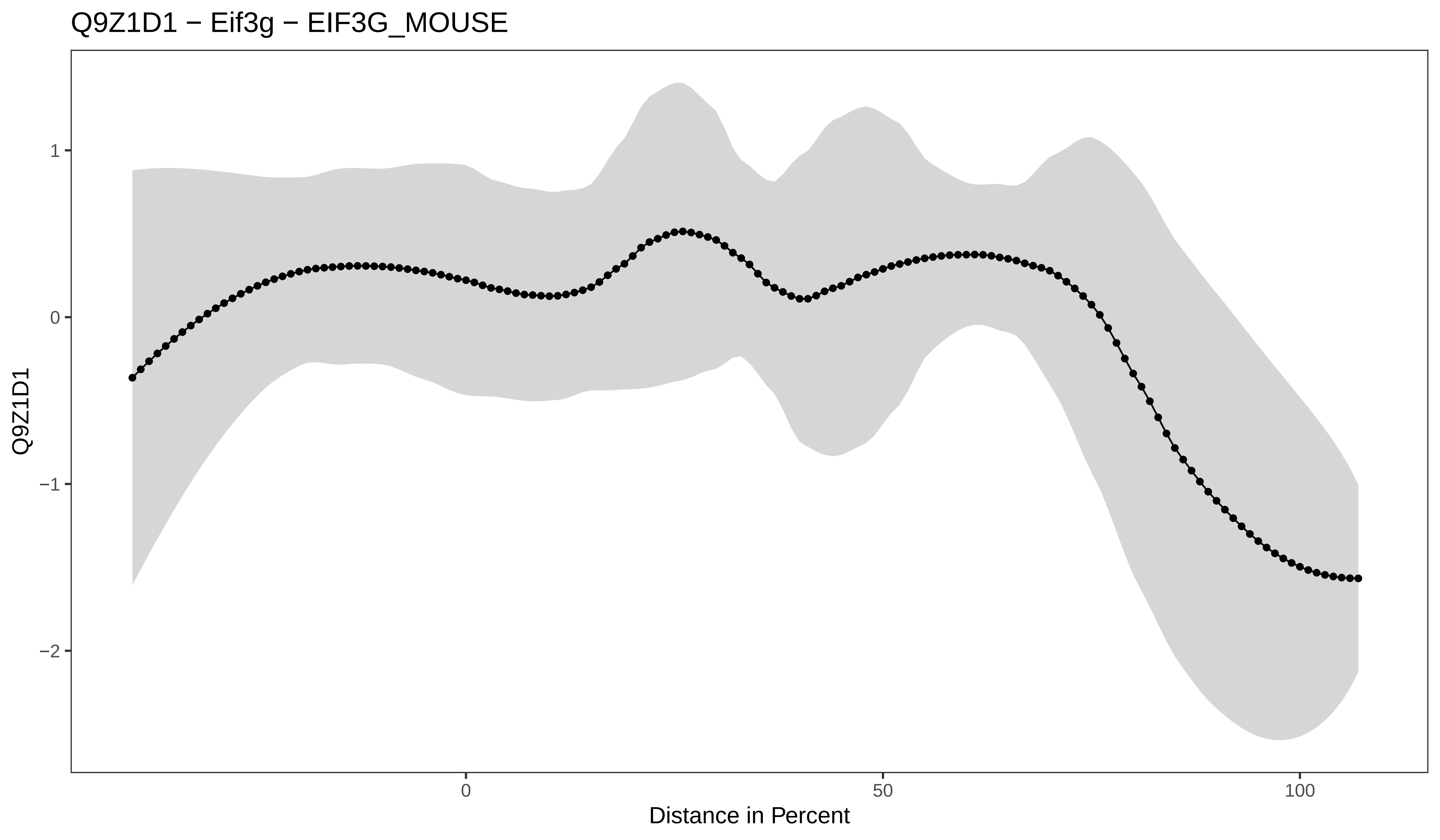

Q9Z1D1 − Eif3g − EIF3G_MOUSE
1
0
−1
−2
0
50
100
Q9Z1D1
Distance in Percent

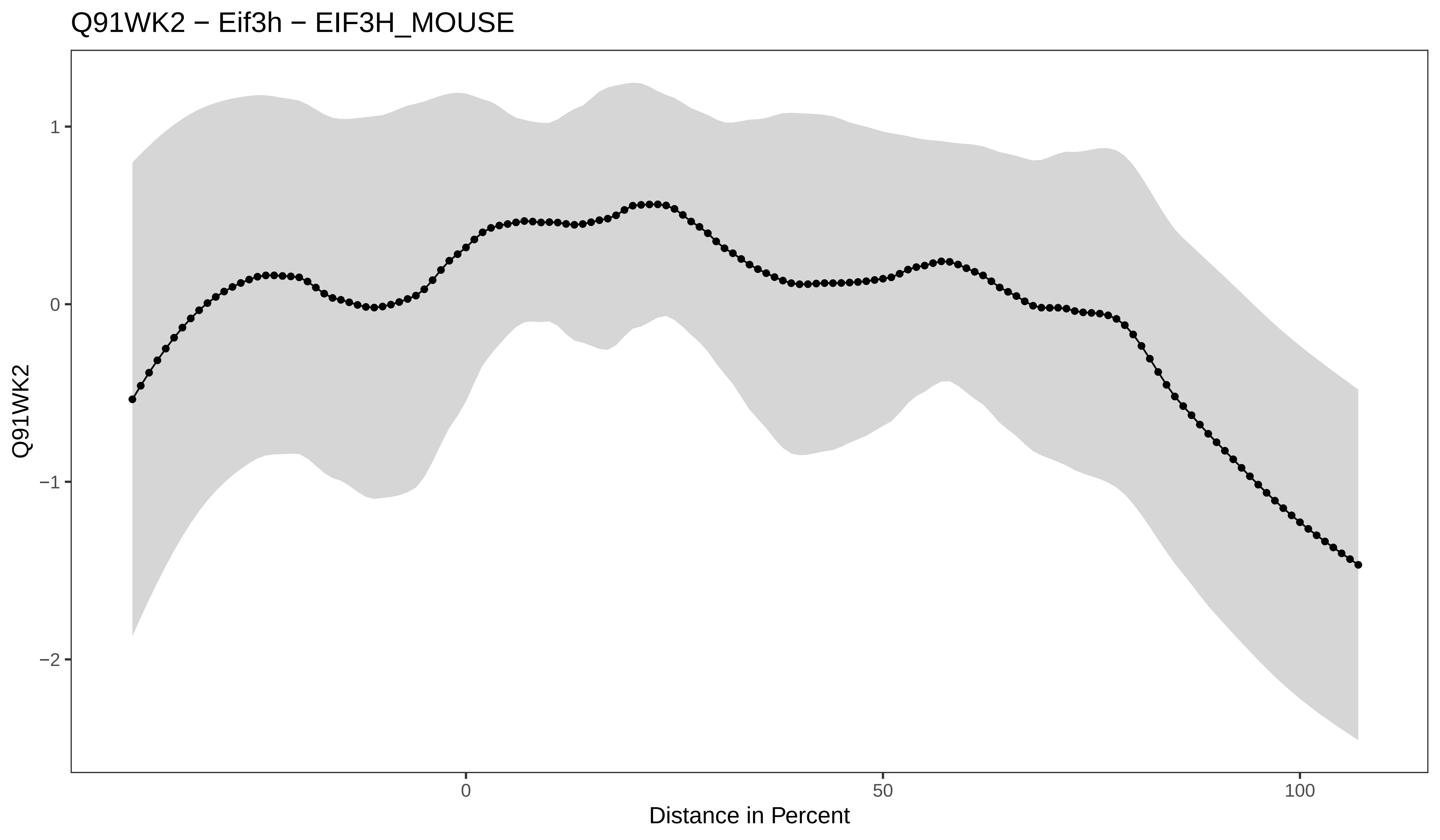

Q91WK2 − Eif3h − EIF3H_MOUSE
1
0
−1
−2
Q91WK2
0
50
100
Distance in Percent

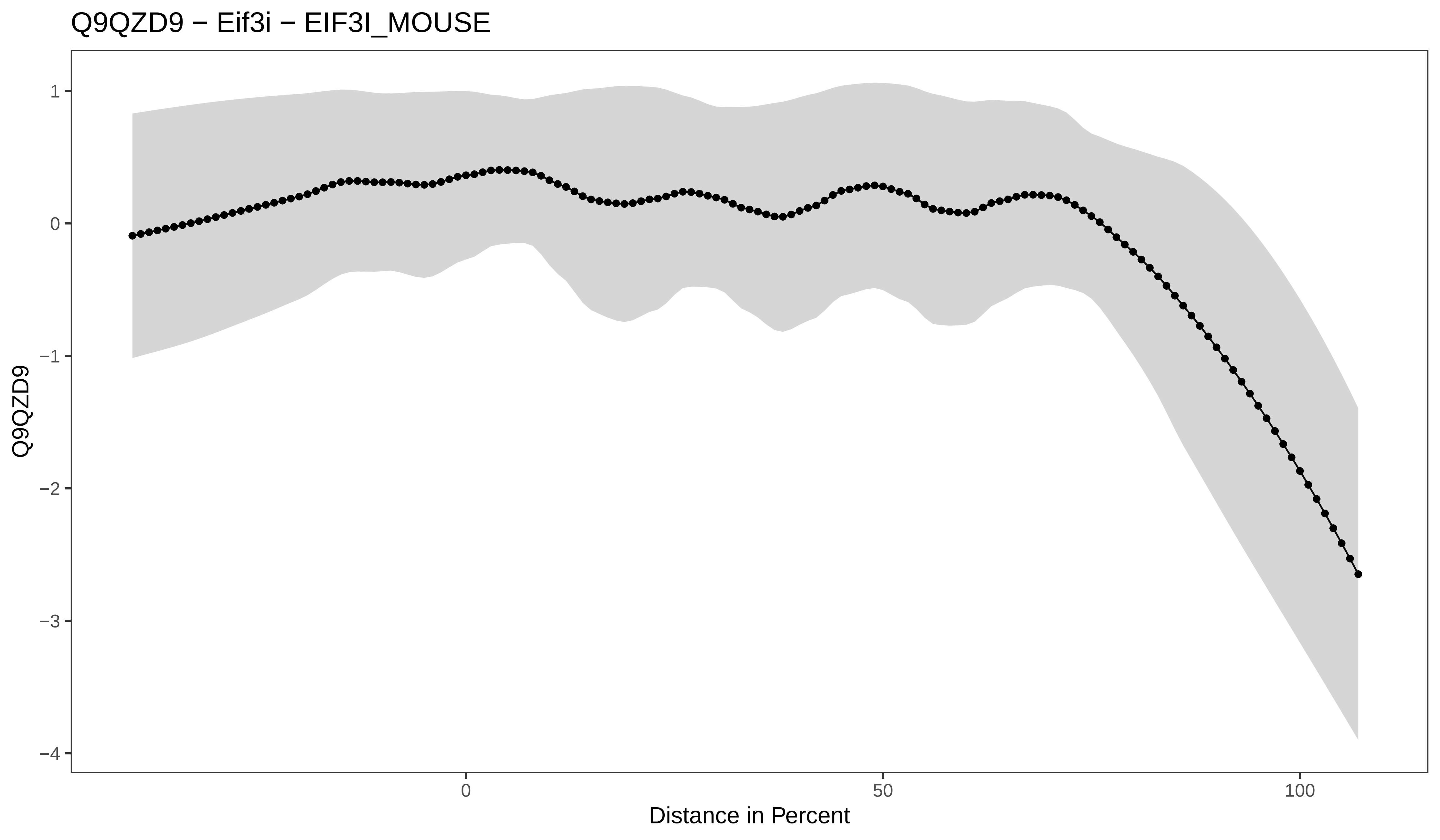

Q9QZD9 − Eif3i − EIF3I_MOUSE
1
0
−1
−2
−3
−4
Q9QZD9
0
50
100
Distance in Percent

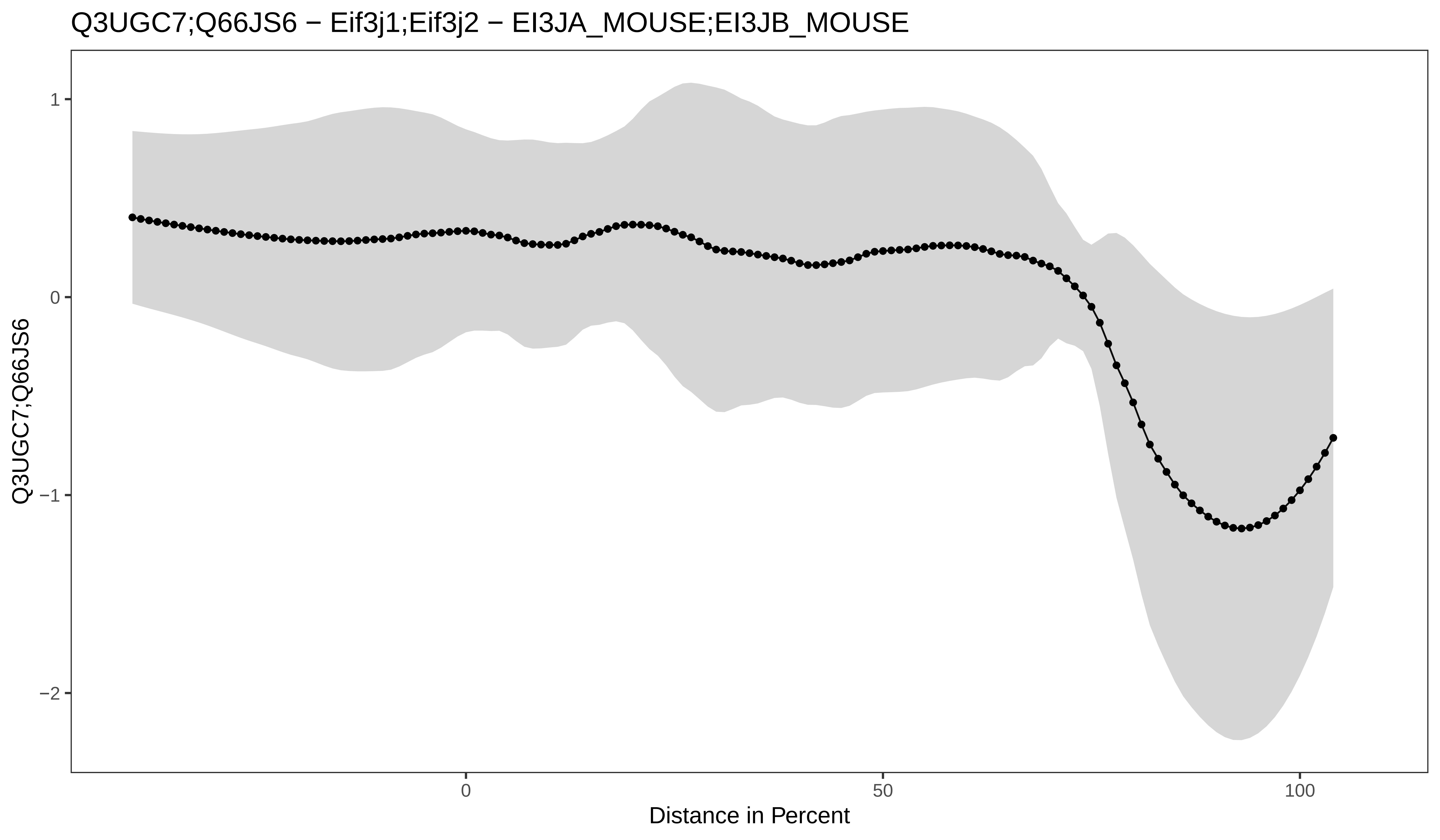
Q3UGC7;Q66JS6 − Eif3j1;Eif3j2 − EI3JA_MOUSE;EI3JB_MOUSE
1
0
−1
−2
Q3UGC7;Q66JS6
0
50
100
Distance in Percent

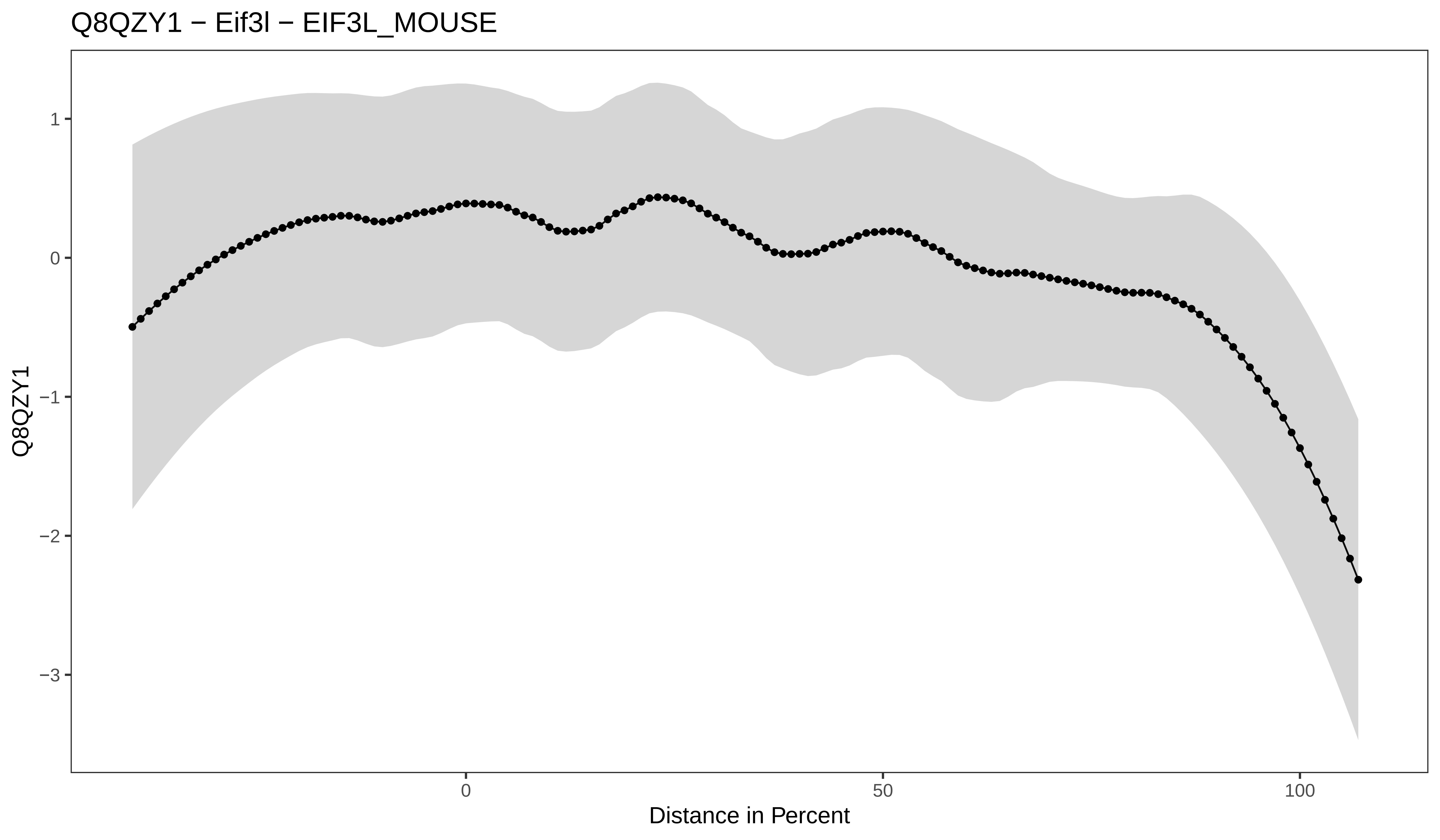

Q8QZY1 − Eif3l − EIF3L_MOUSE
1
0
−1
−2
−3
Q8QZY1
0
50
100
Distance in Percent

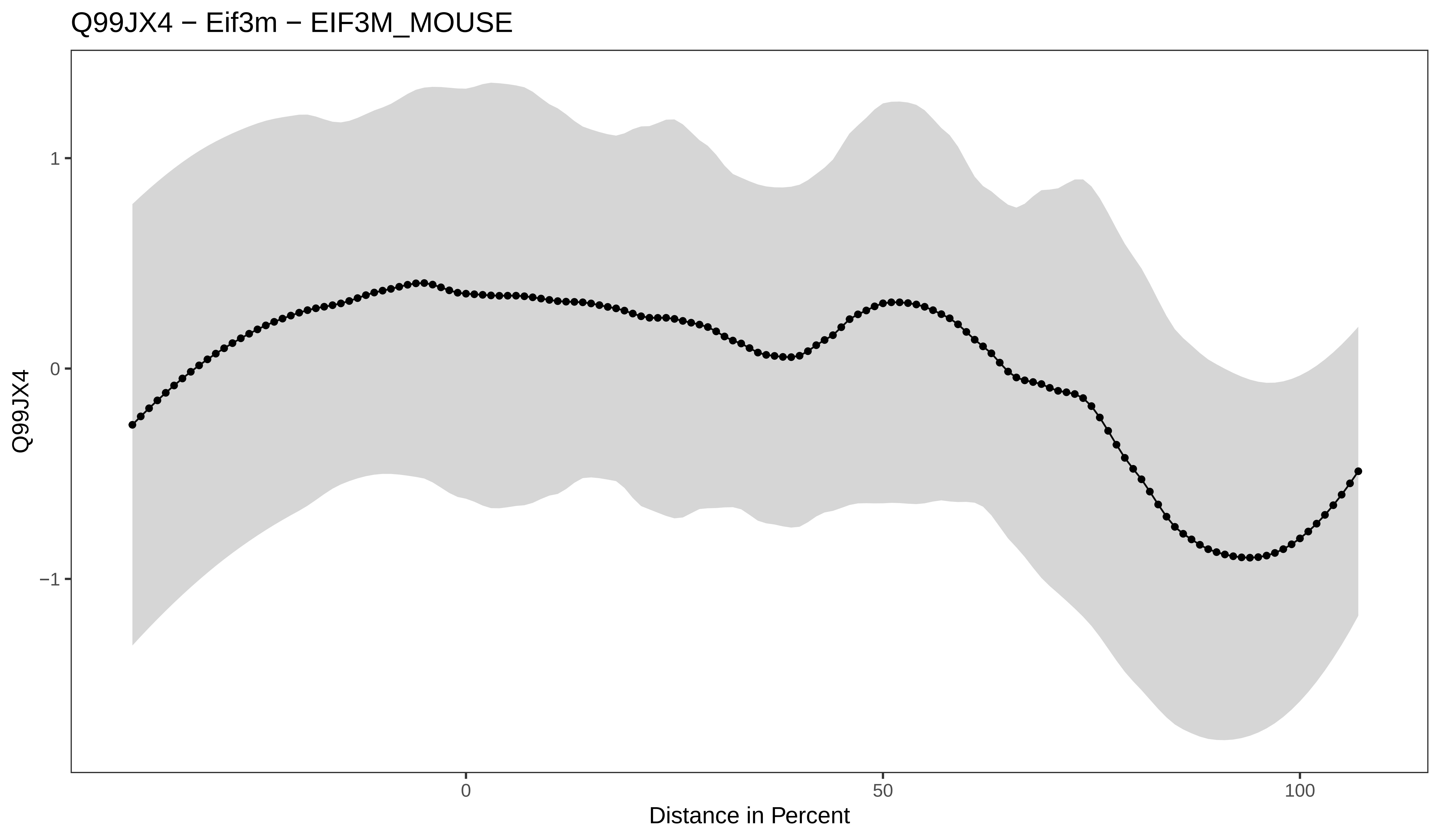

Q99JX4 − Eif3m − EIF3M_MOUSE
Q99JX4
1
0
−1
0
50
100
Distance in Percent

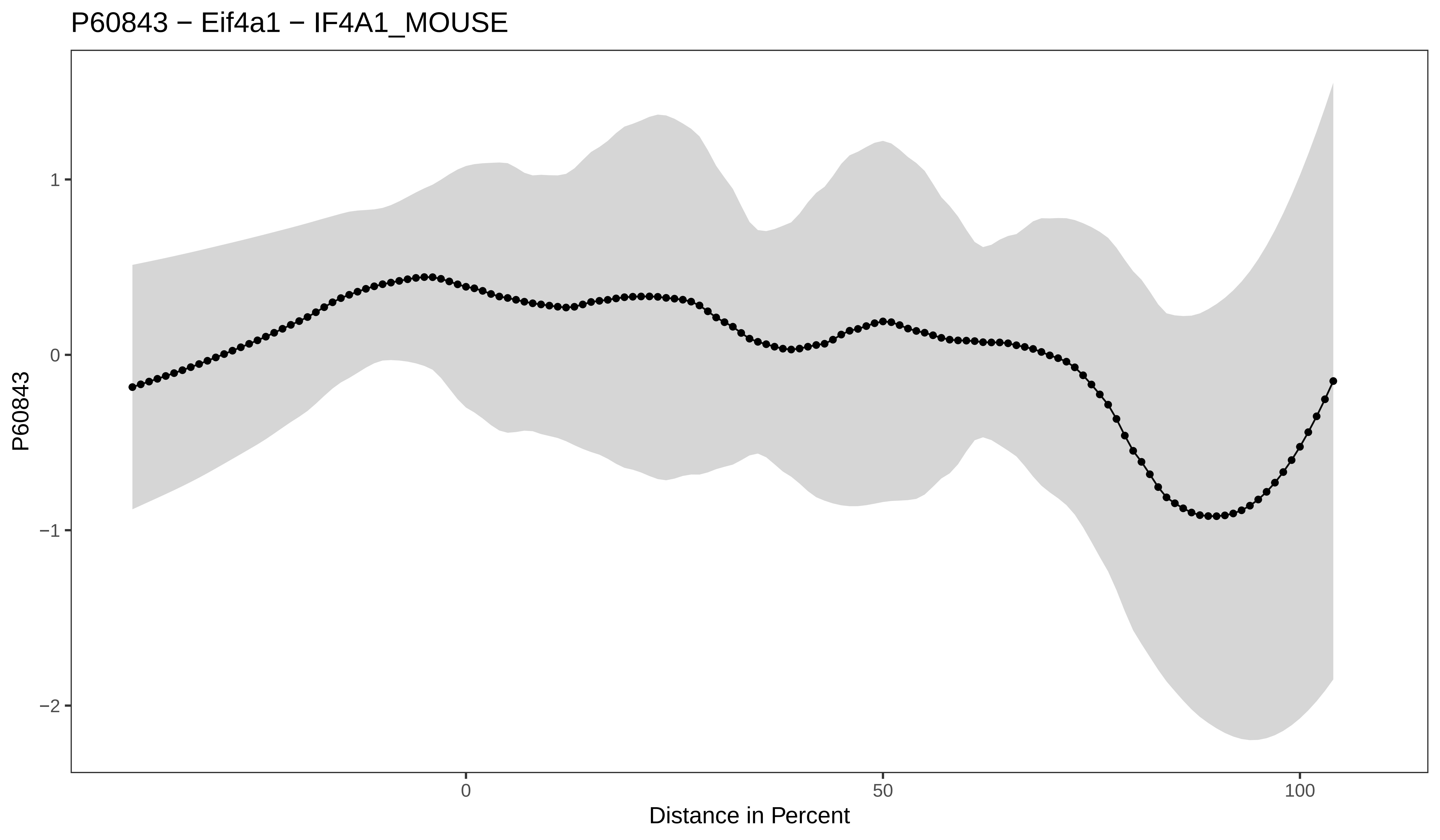
P60843 − Eif4a1 − IF4A1_MOUSE
1
0
−1
−2
P60843
0
50
100
Distance in Percent

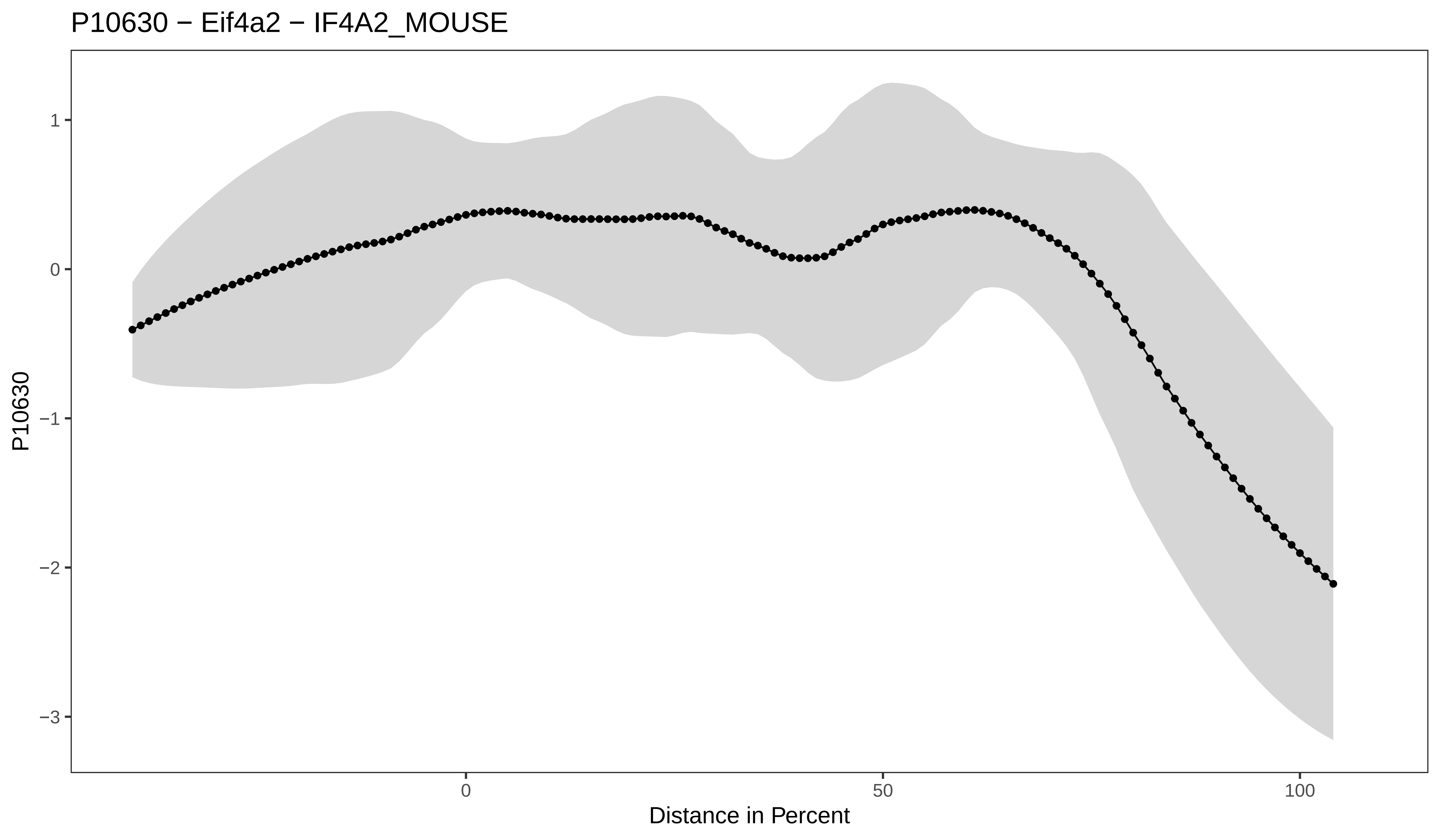

P10630 − Eif4a2 − IF4A2_MOUSE
P10630
Distance in Percent
1
0
−1
−2
−3
0
50
100

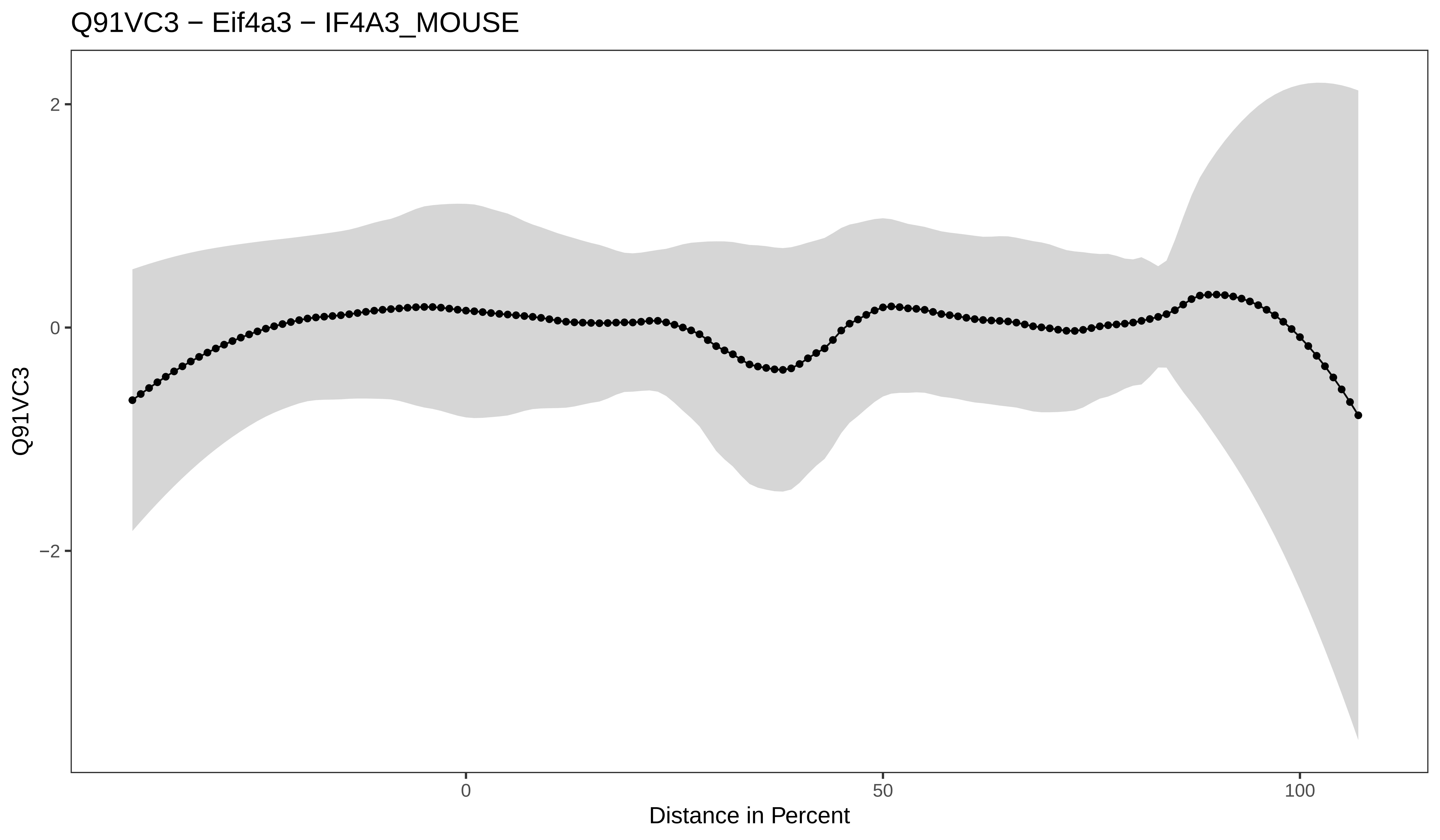

Q91VC3 − Eif4a3 − IF4A3_MOUSE
2
0
−2
Q91VC3
0
50
100
Distance in Percent

Q8BGD9 − Eif4b − IF4B_MOUSE

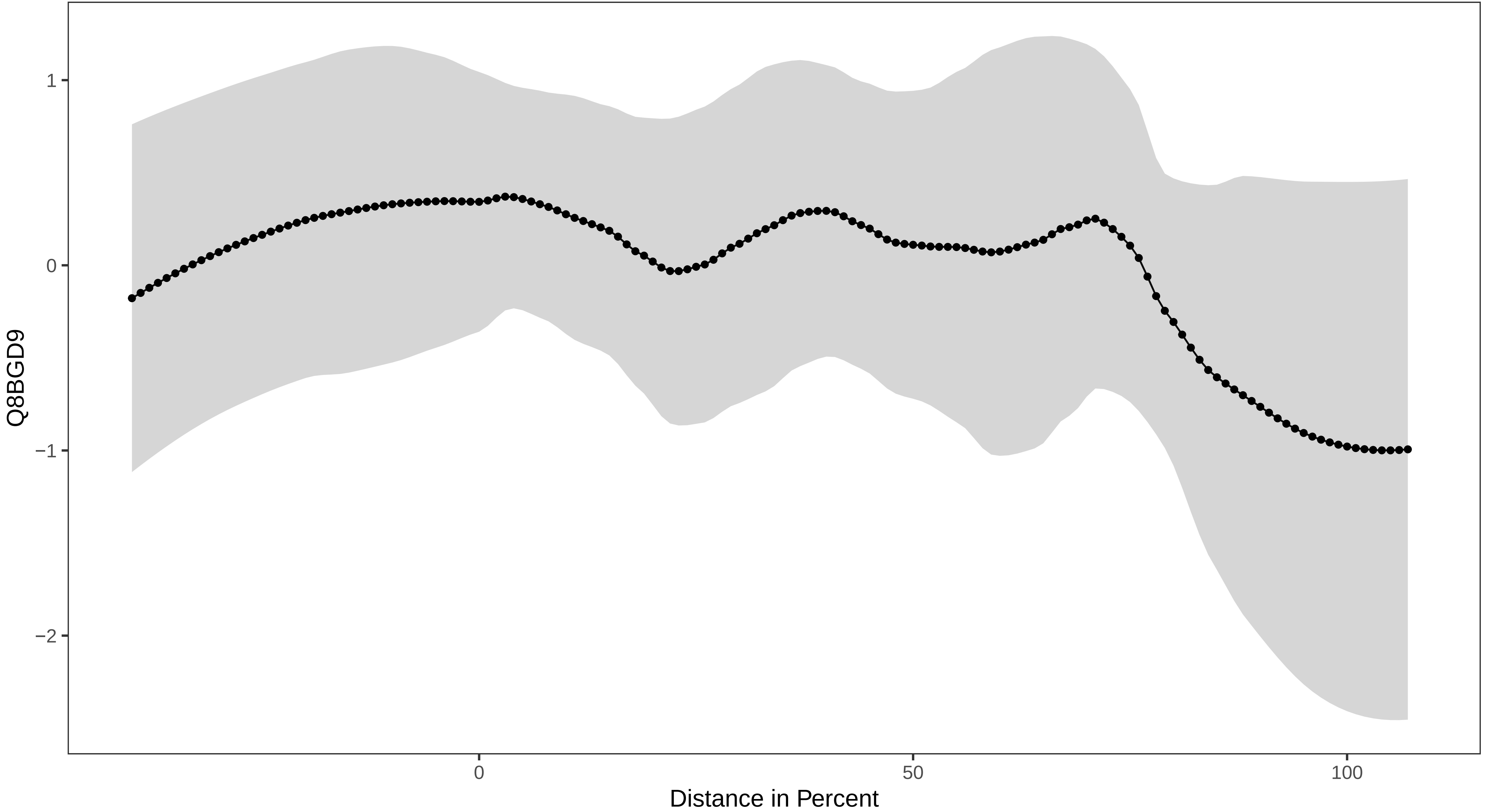

P63073 − Eif4e − IF4E_MOUSE

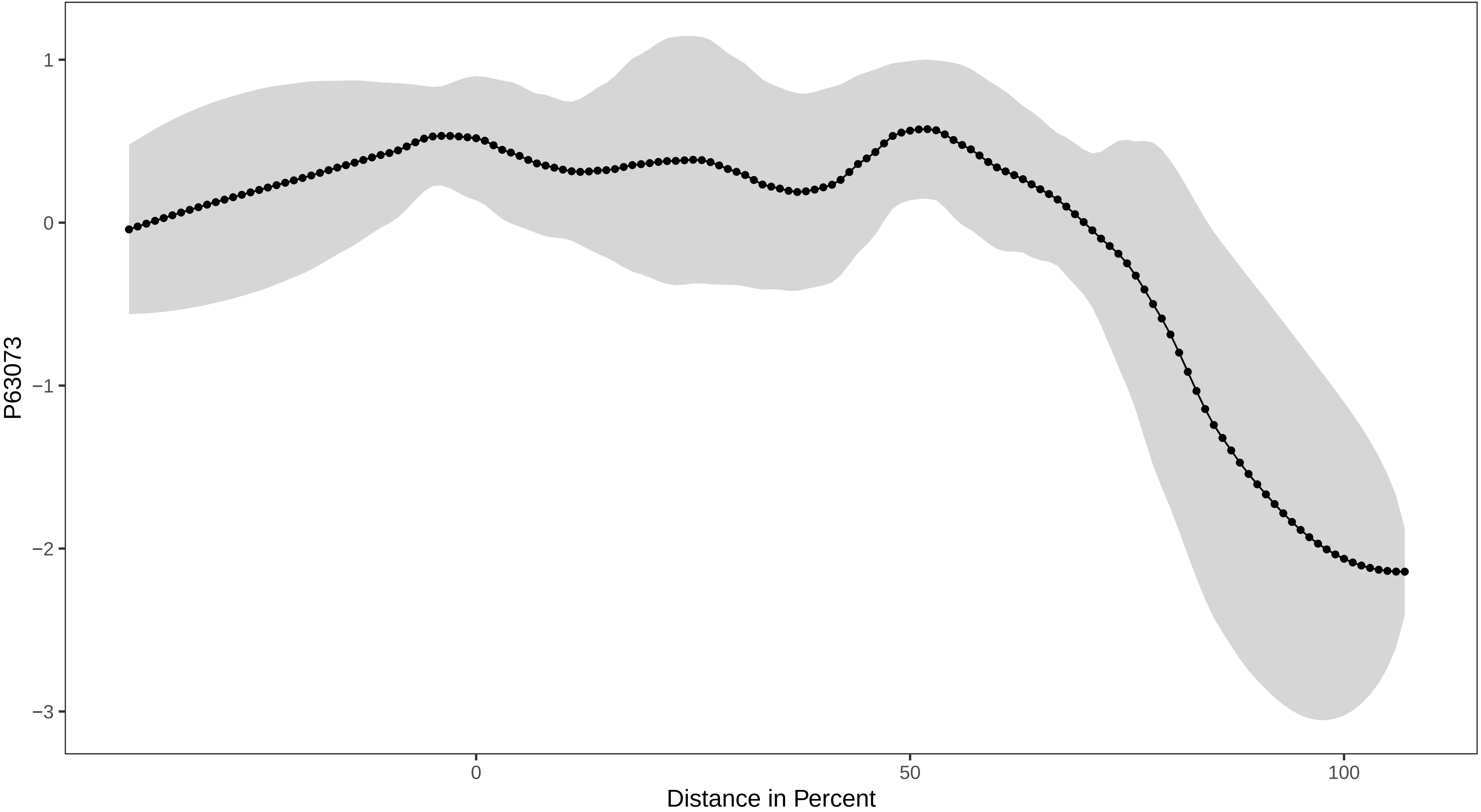

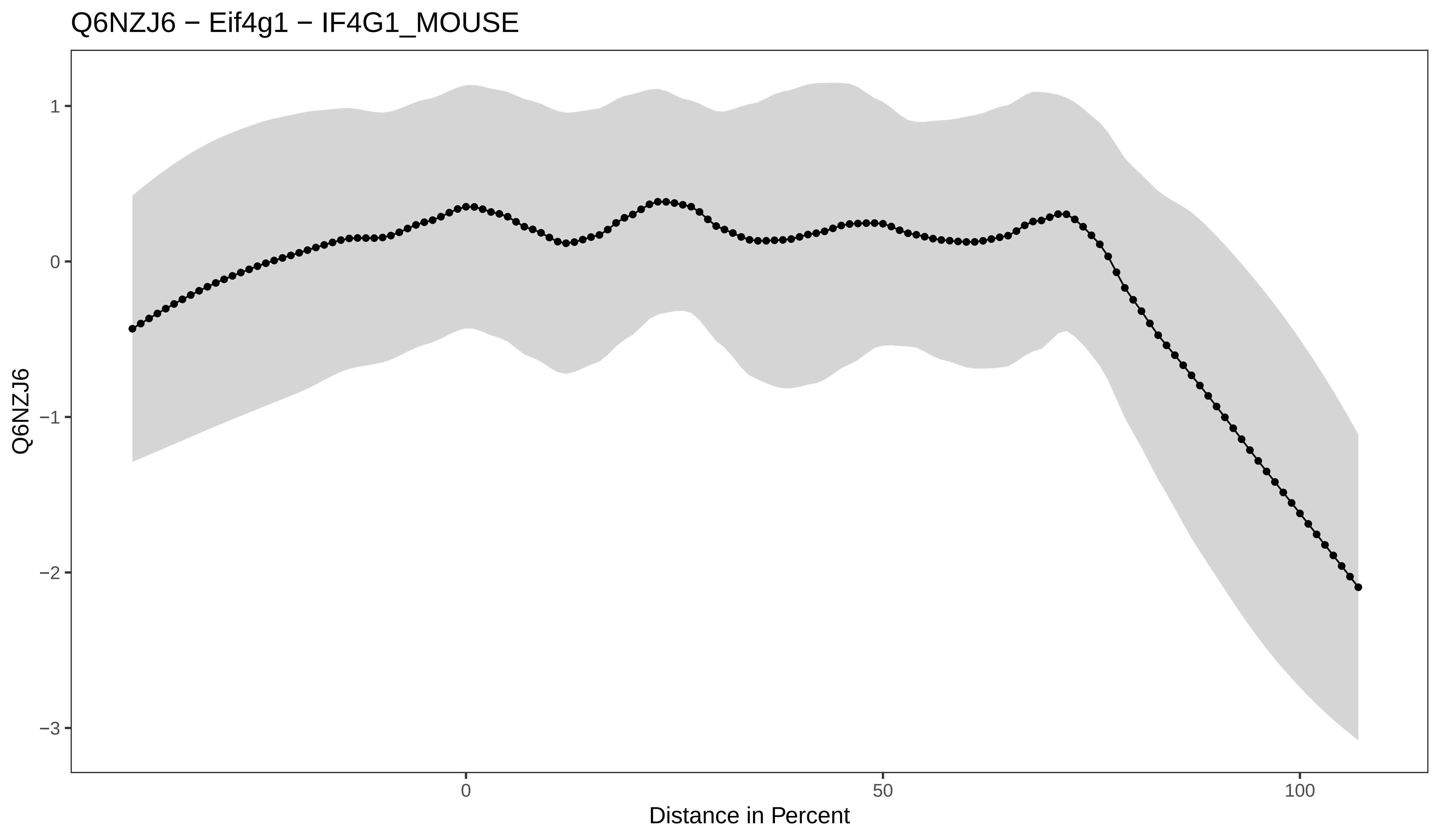
Q6NZJ6 − Eif4g1 − IF4G1_MOUSE
Q6NZJ6
1
0
−1
−2
−3
0
50
100
Distance in Percent

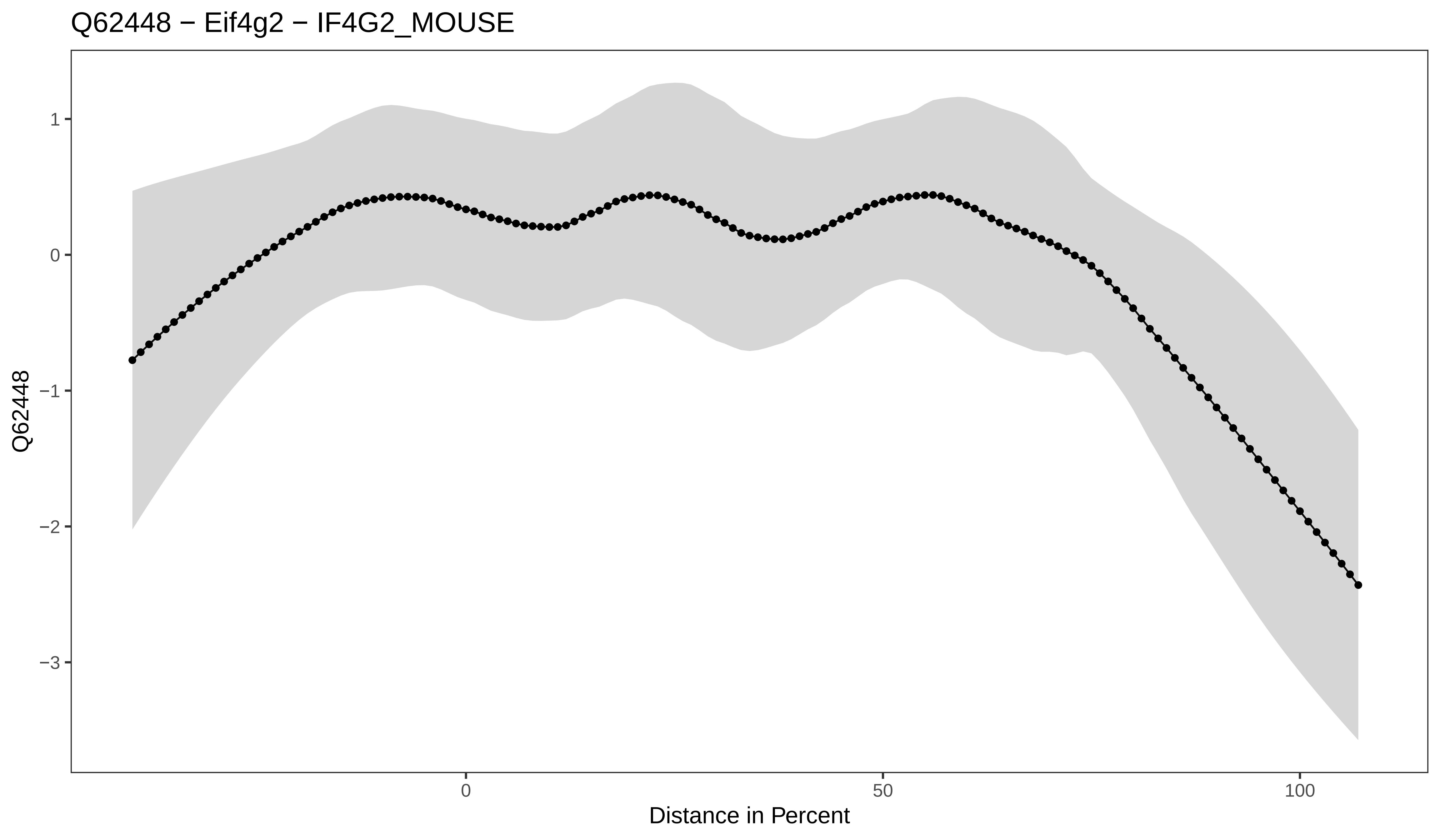
Q62448 − Eif4g2 − IF4G2_MOUSE
Q62448
1
0
−1
−2
−3
0
50
100
Distance in Percent

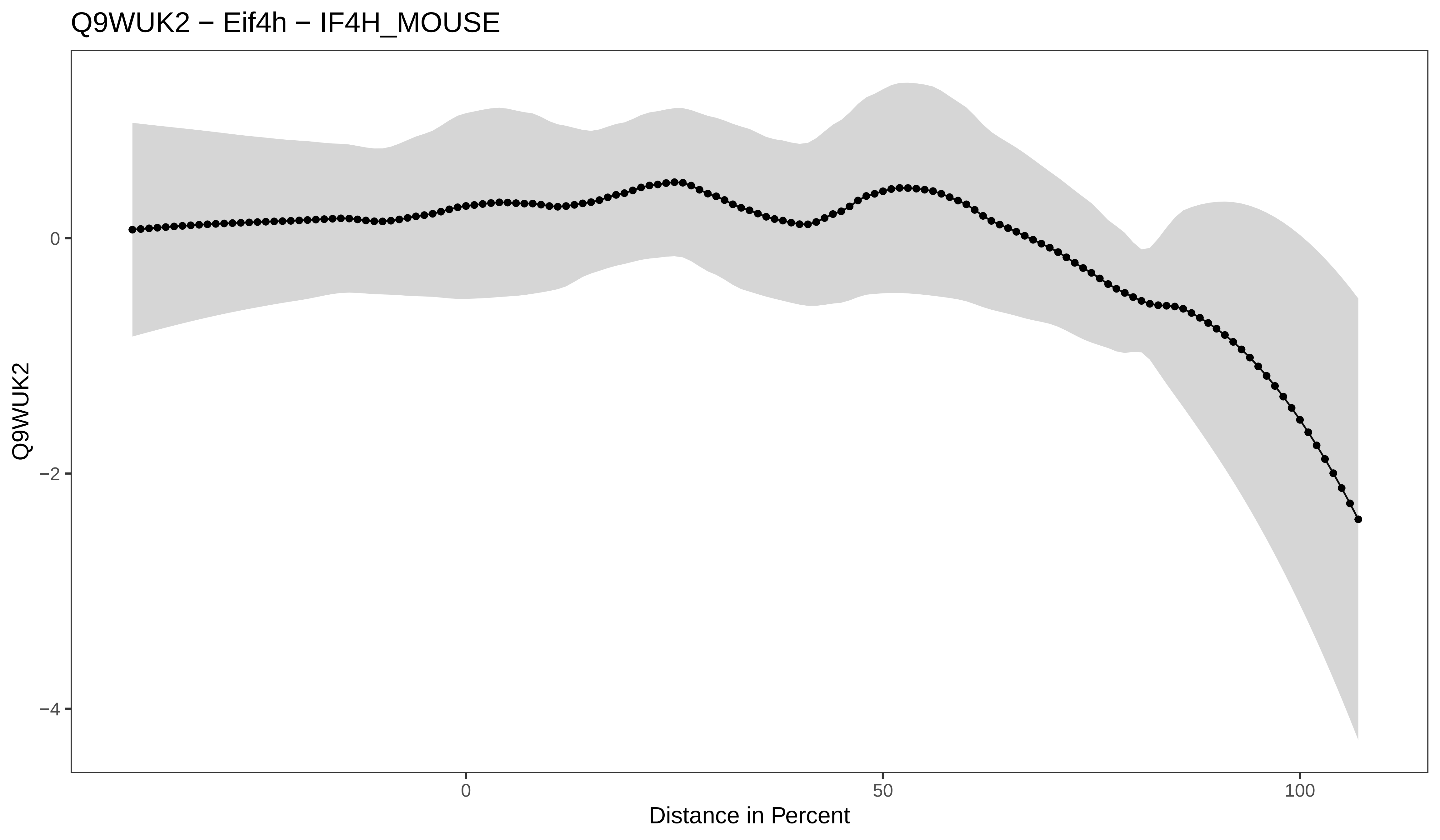

Q9WUK2 − Eif4h − IF4H_MOUSE
Q9WUK2
0
−2
−4
0
50
100
Distance in Percent

P59325 − Eif5 − IF5_MOUSE

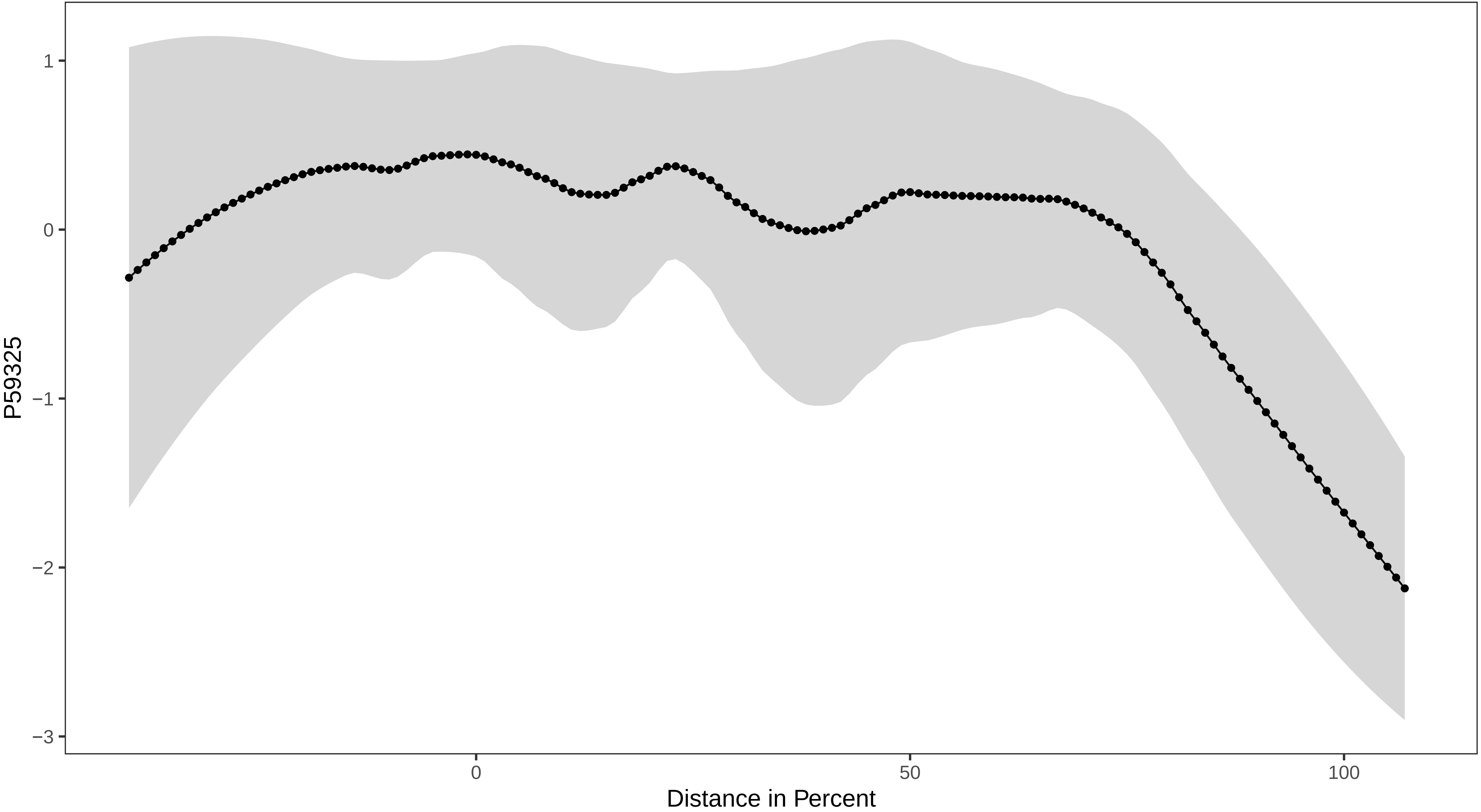

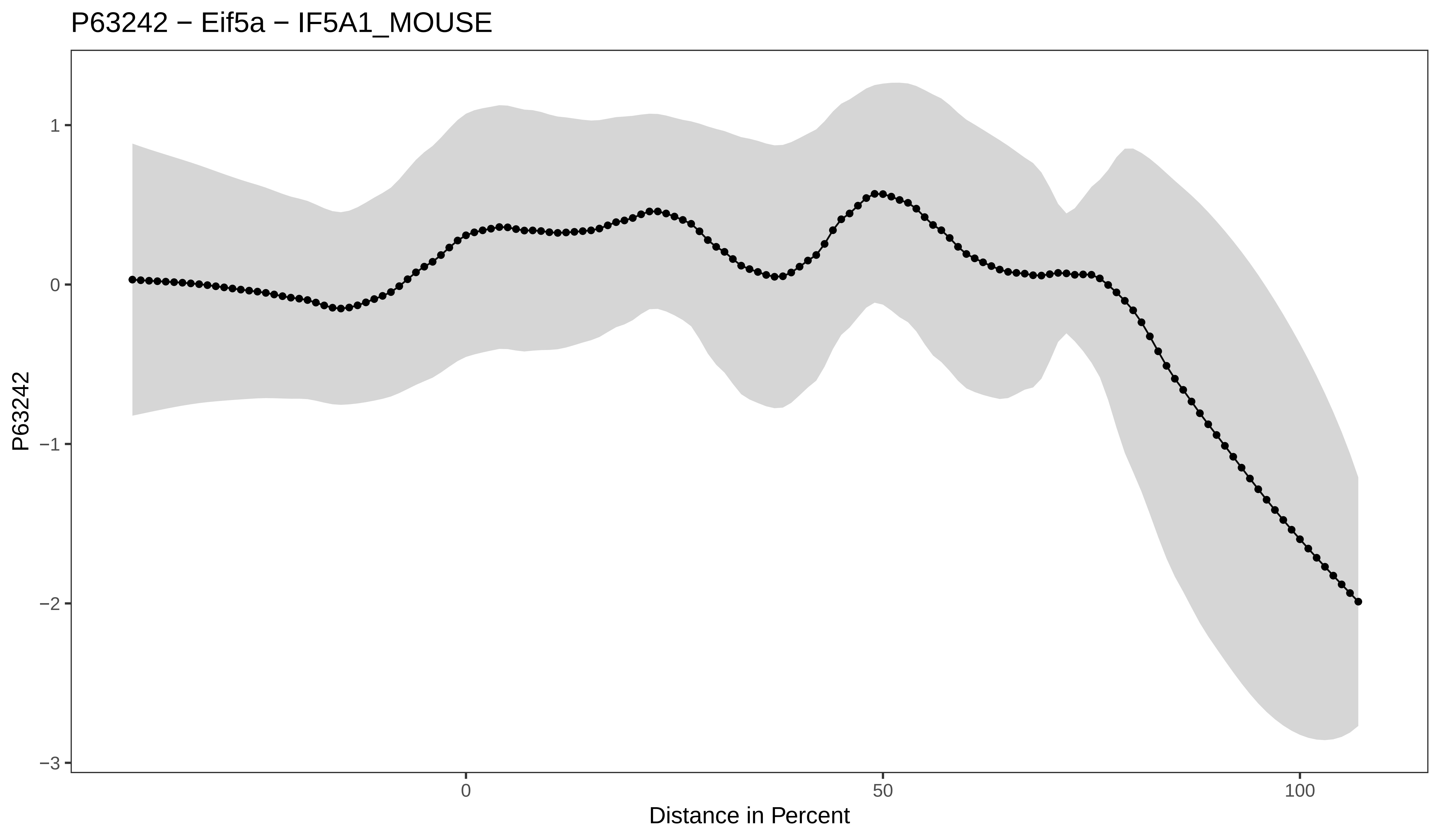
P63242 − Eif5a − IF5A1_MOUSE
P63242
1
0
−1
−2
−3
0
50
100
Distance in Percent

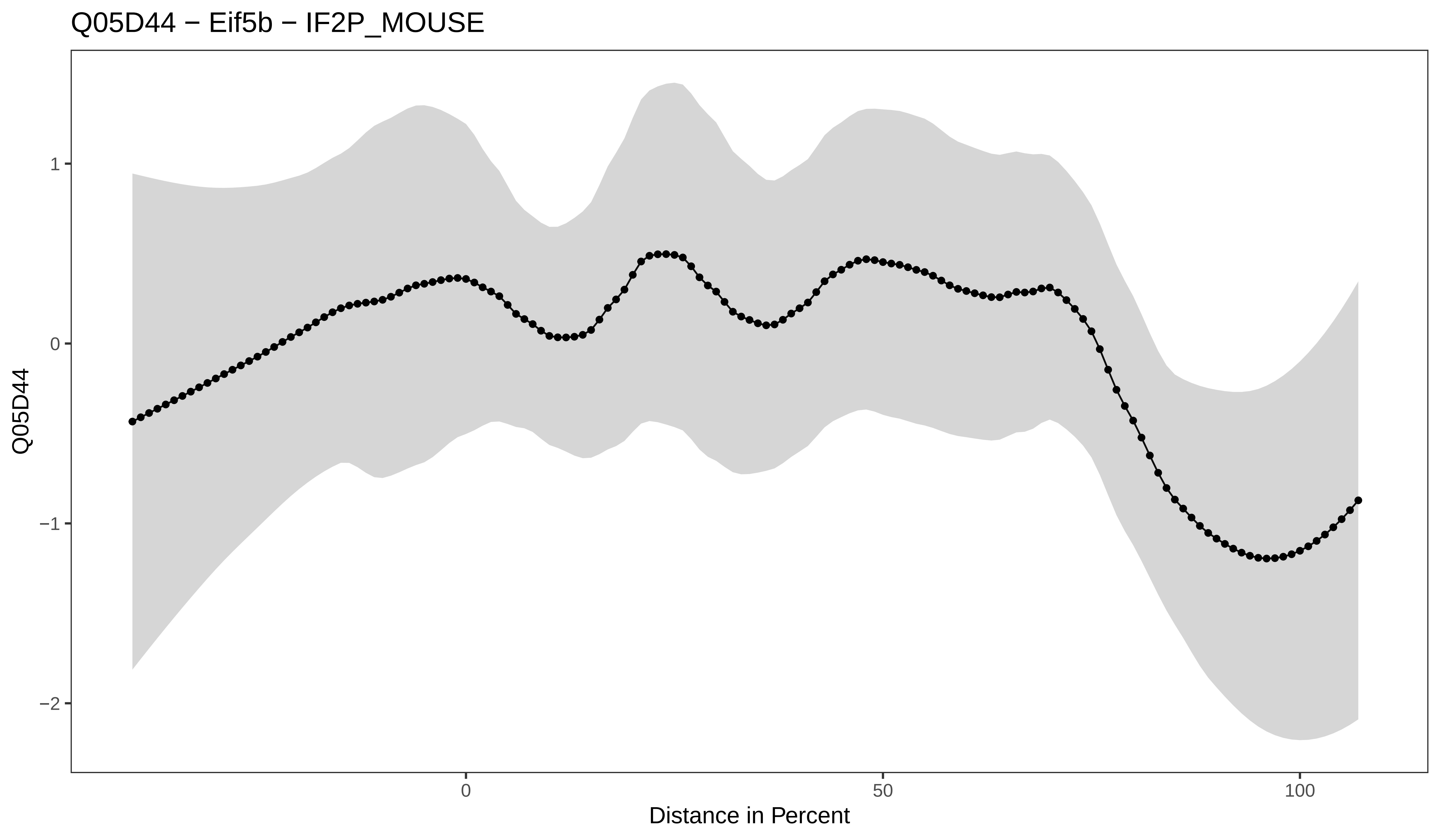
Q05D44 − Eif5b − IF2P_MOUSE
Q05D44
1
0
−1
−2
0
50
100
Distance in Percent

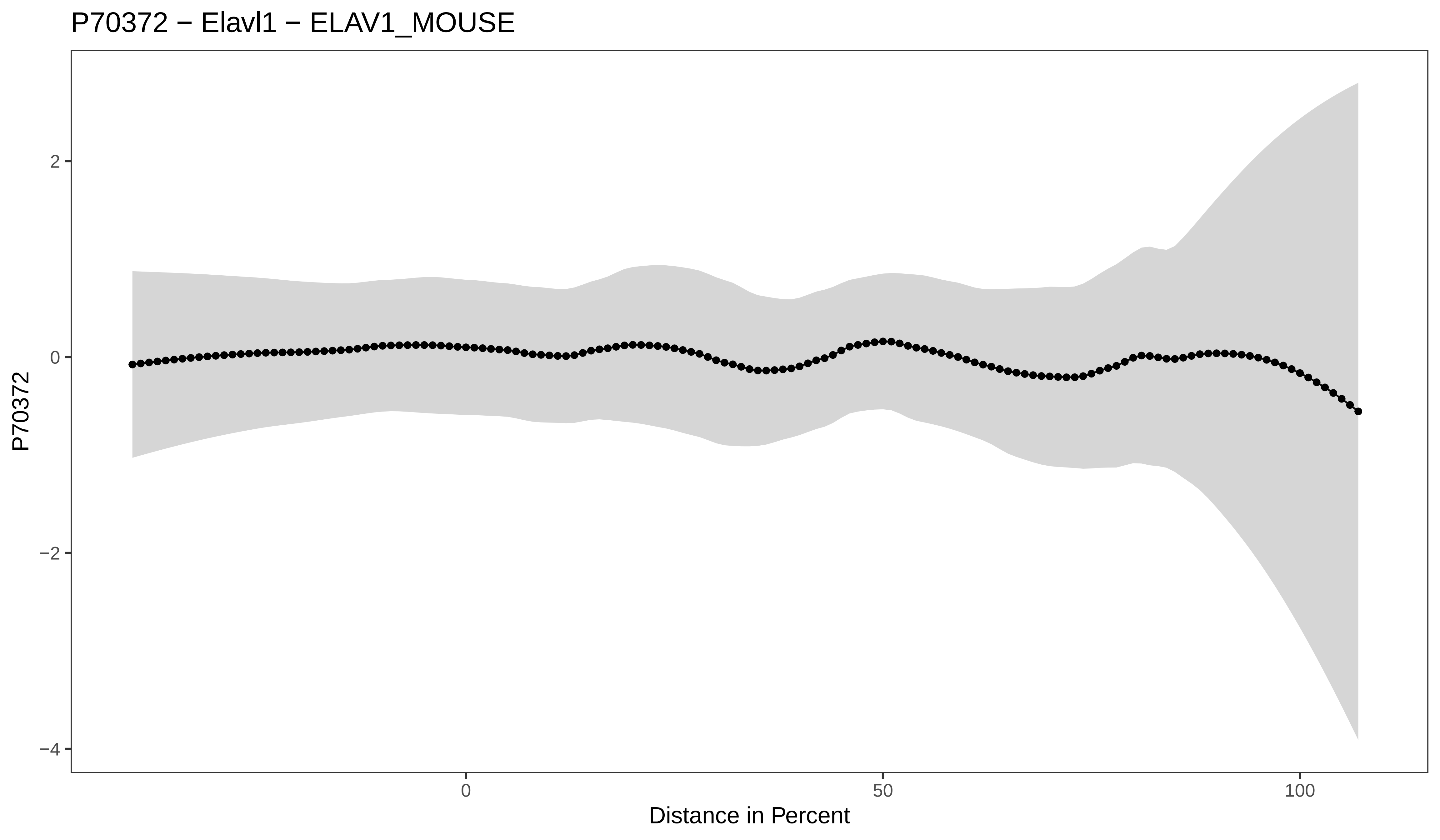
P70372 − Elavl1 − ELAV1_MOUSE
P70372
2
0
−2
−4
0
50
100
Distance in Percent

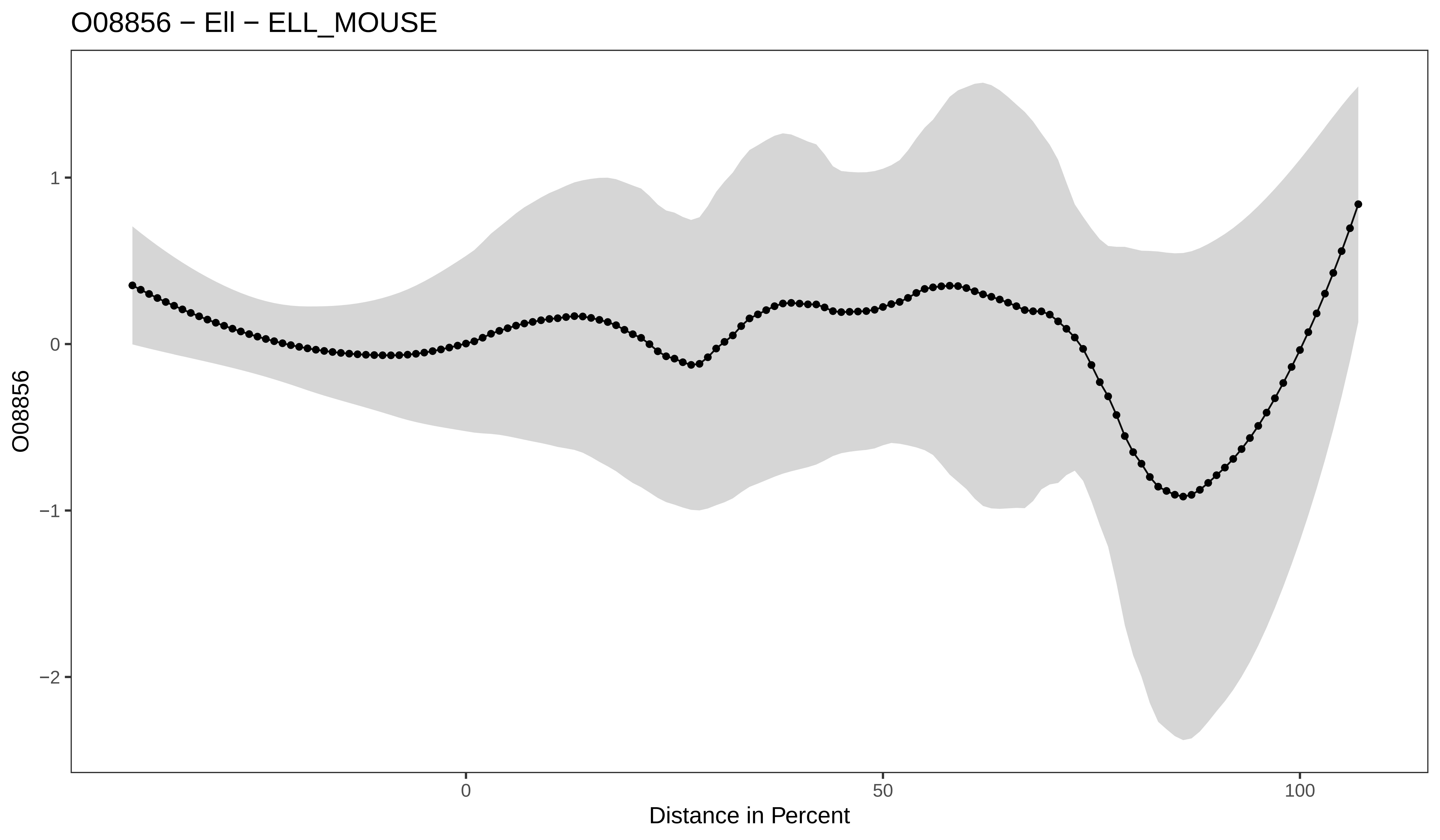
O08856 − Ell − ELL_MOUSE
1
0
−1
−2
O08856
0
50
100
Distance in Percent

P54320 − Eln − ELN_MOUSE

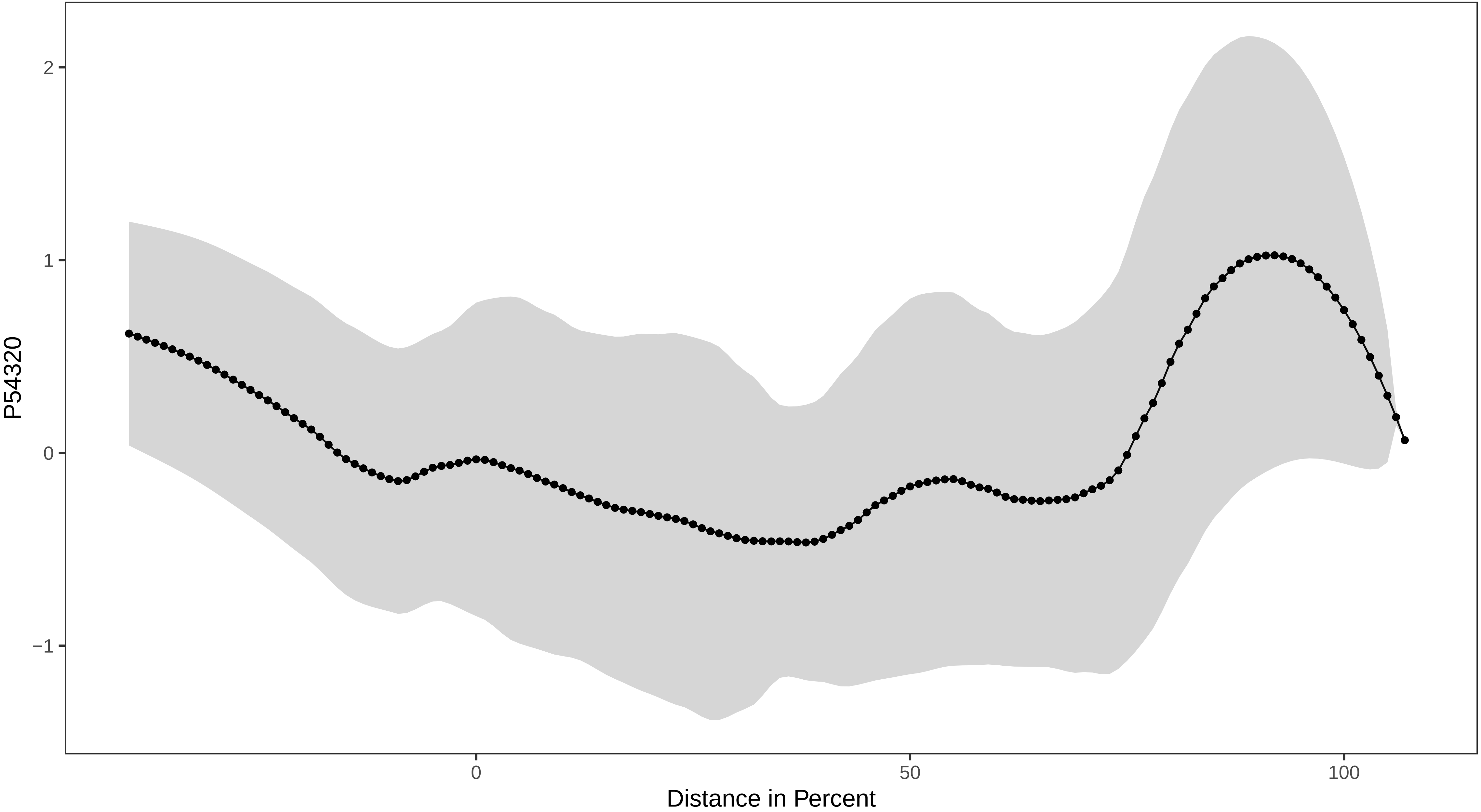

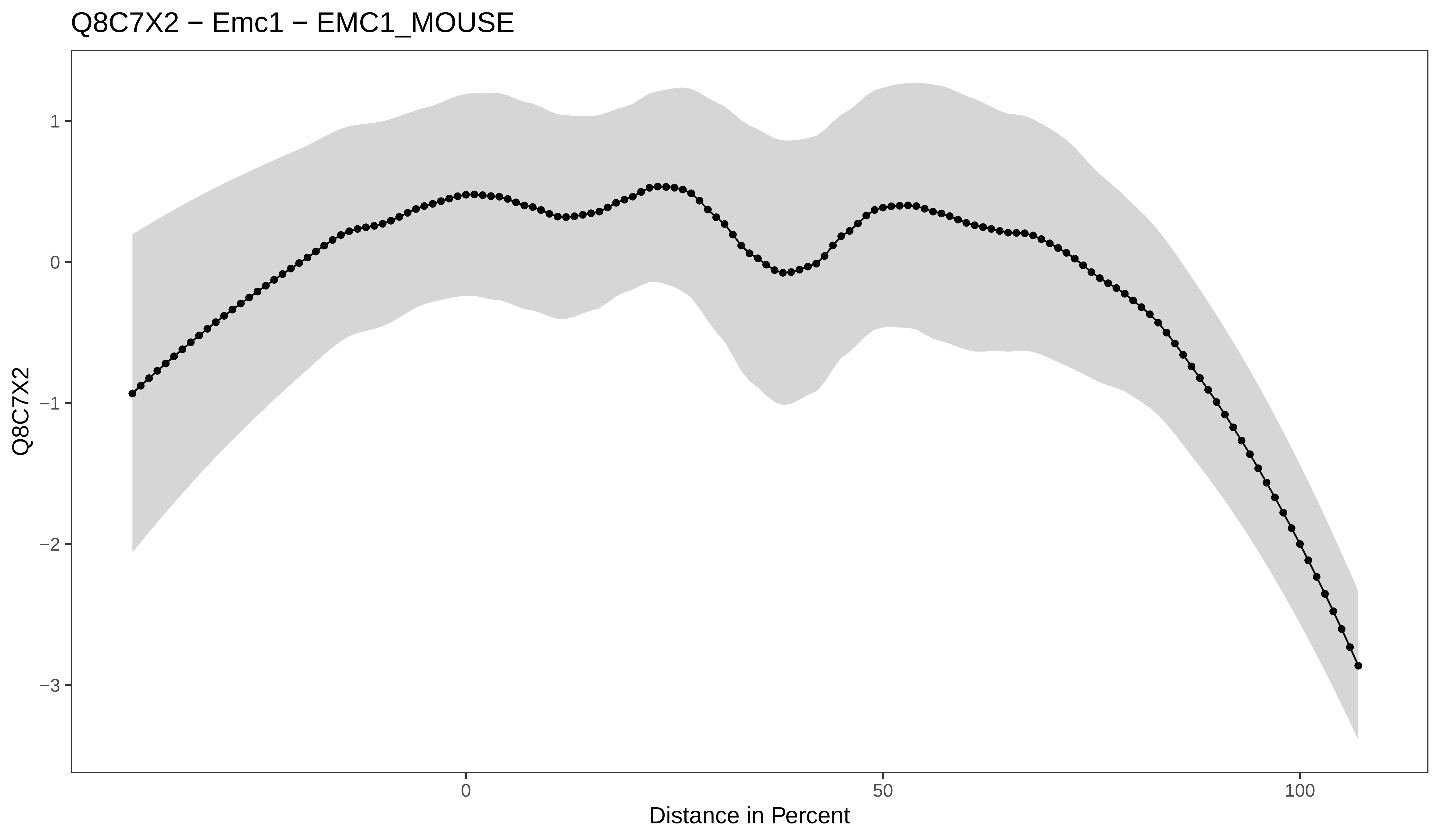

Q8C7X2 − Emc1 − EMC1_MOUSE
Q8C7X2
Distance in Percent
1
0
−1
−2
−3
0
50
100

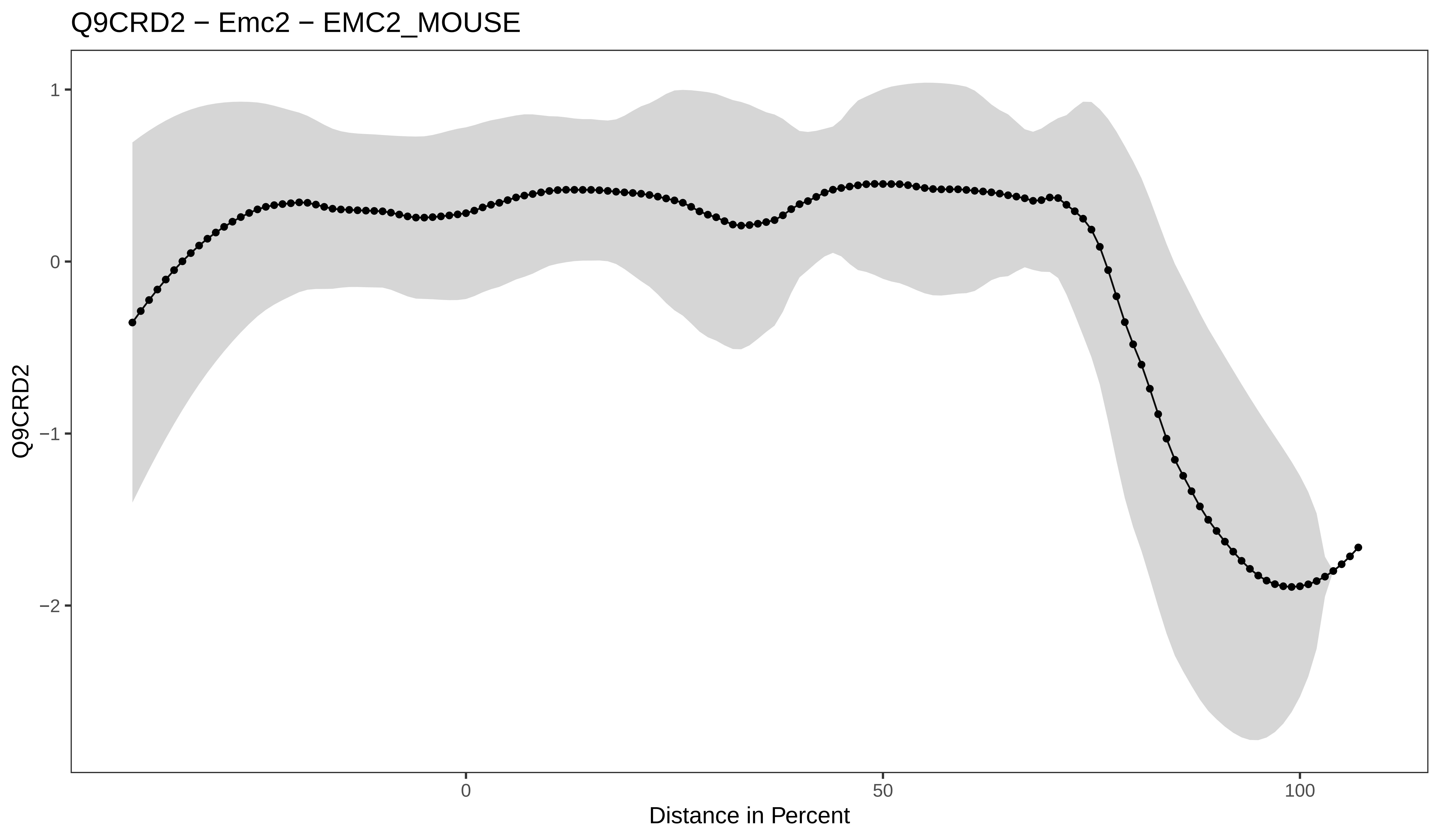
Q9CRD2 − Emc2 − EMC2_MOUSE
1
0
−1
−2
Q9CRD2
0
50
100
Distance in Percent

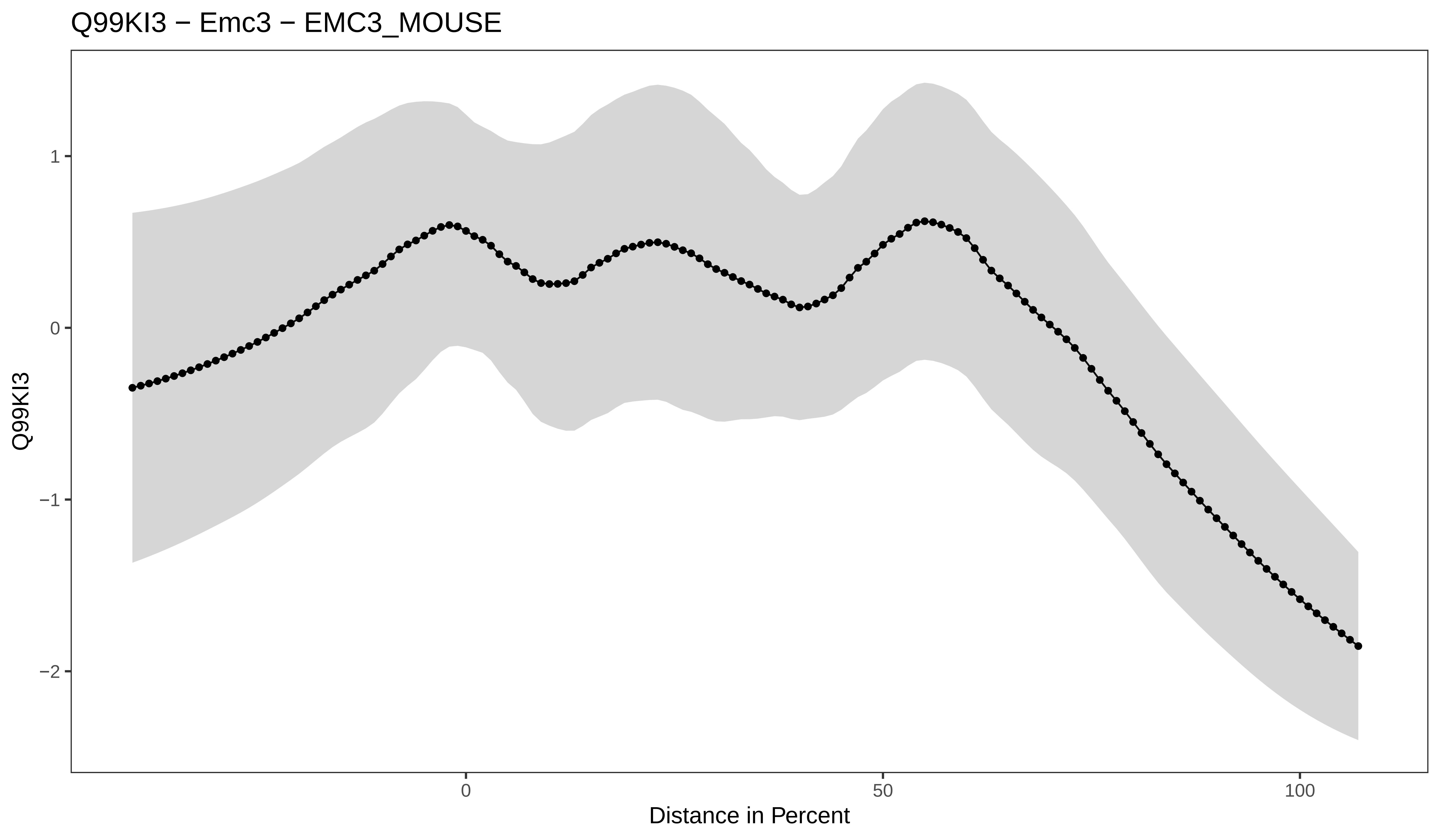
Q99KI3 − Emc3 − EMC3_MOUSE
1
0
−1
−2
Q99KI3
0
50
100
Distance in Percent

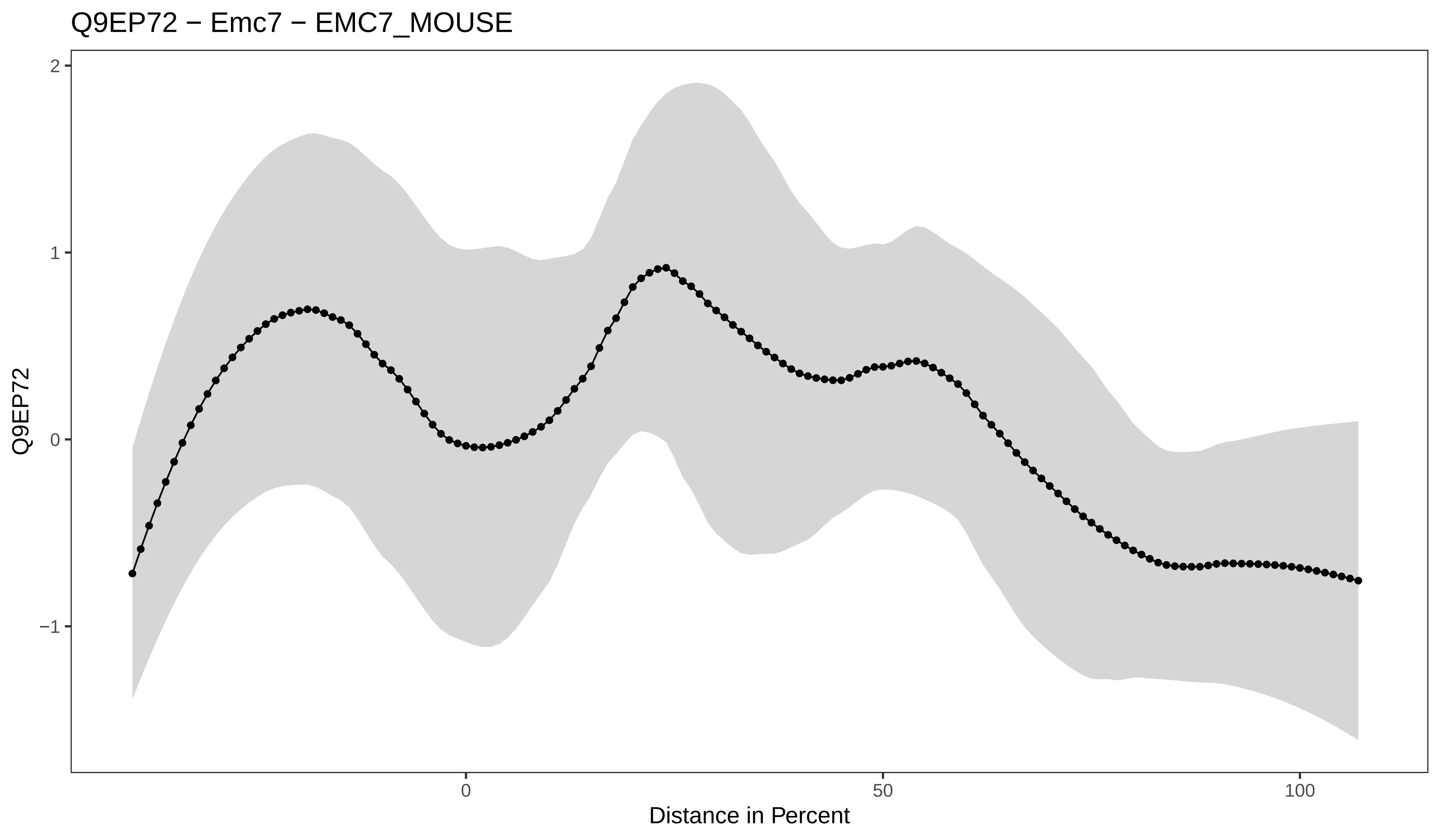
Q9EP72 − Emc7 − EMC7_MOUSE
2
1
0
−1
Q9EP72
0
50
100
Distance in Percent

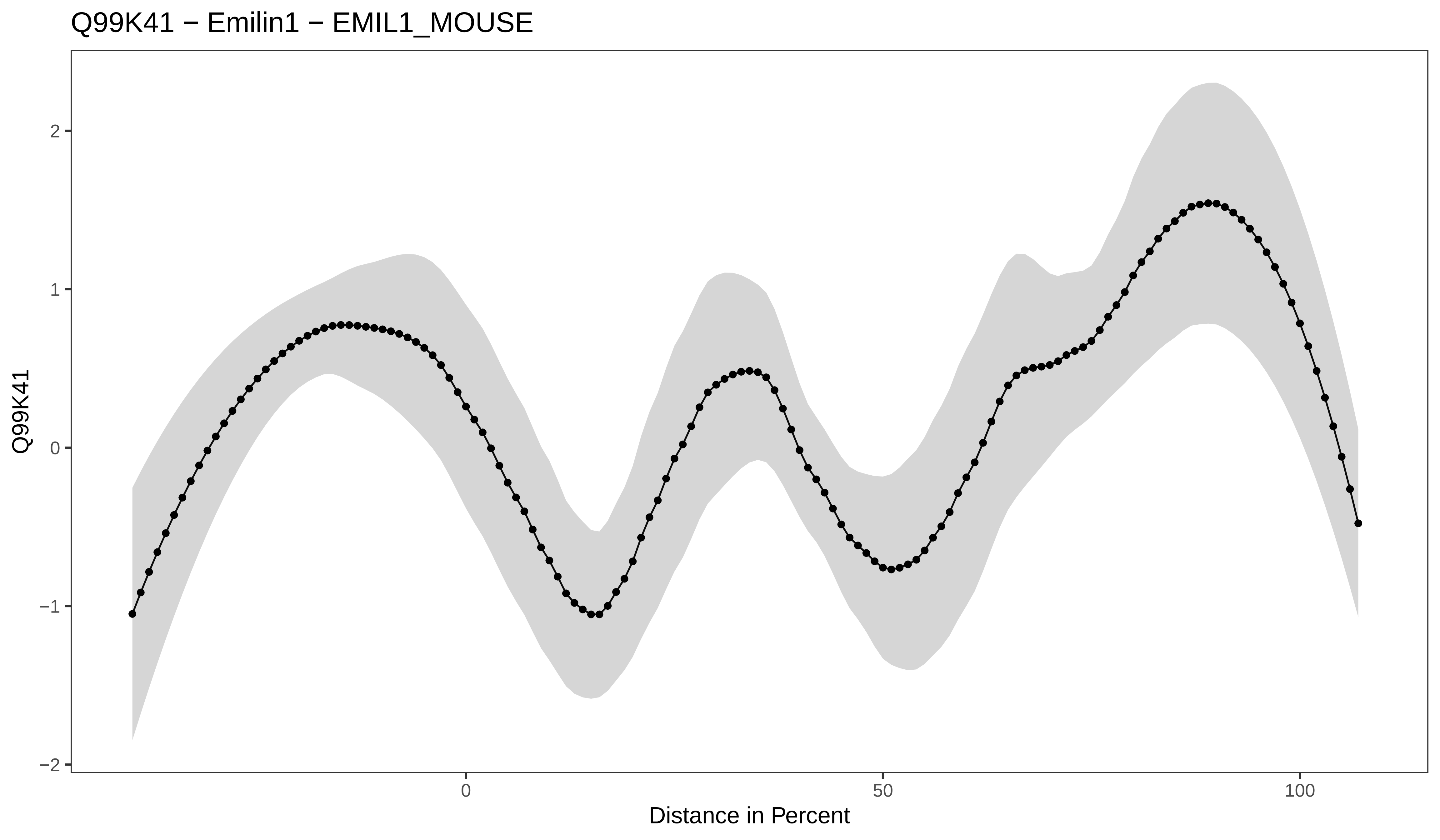

Q99K41 − Emilin1 − EMIL1_MOUSE
Q99K41
2
1
0
−1
−2
0
50
100
Distance in Percent

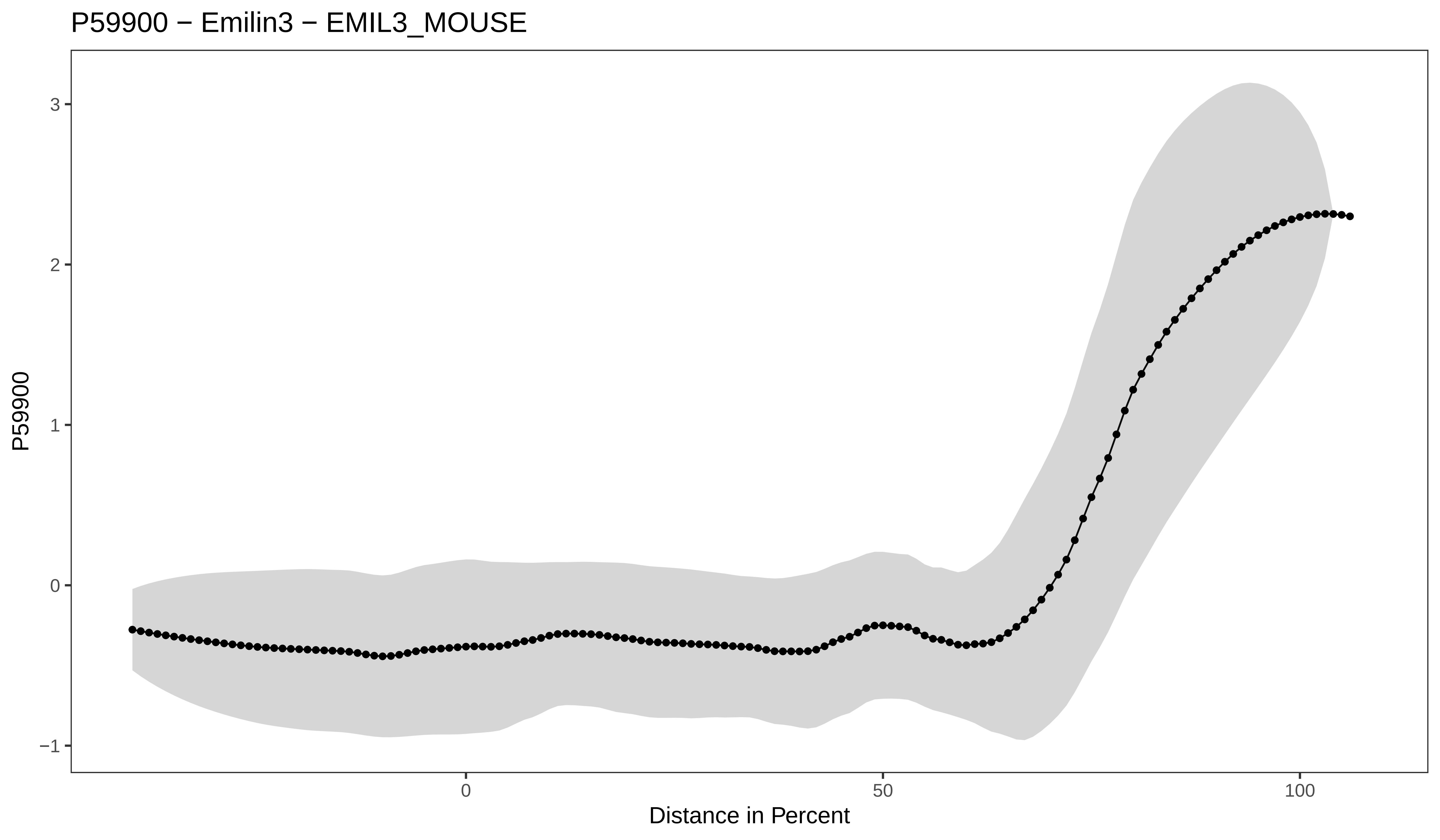
P59900 − Emilin3 − EMIL3_MOUSE
P59900
3
2
1
0
−1
0
50
100
Distance in Percent

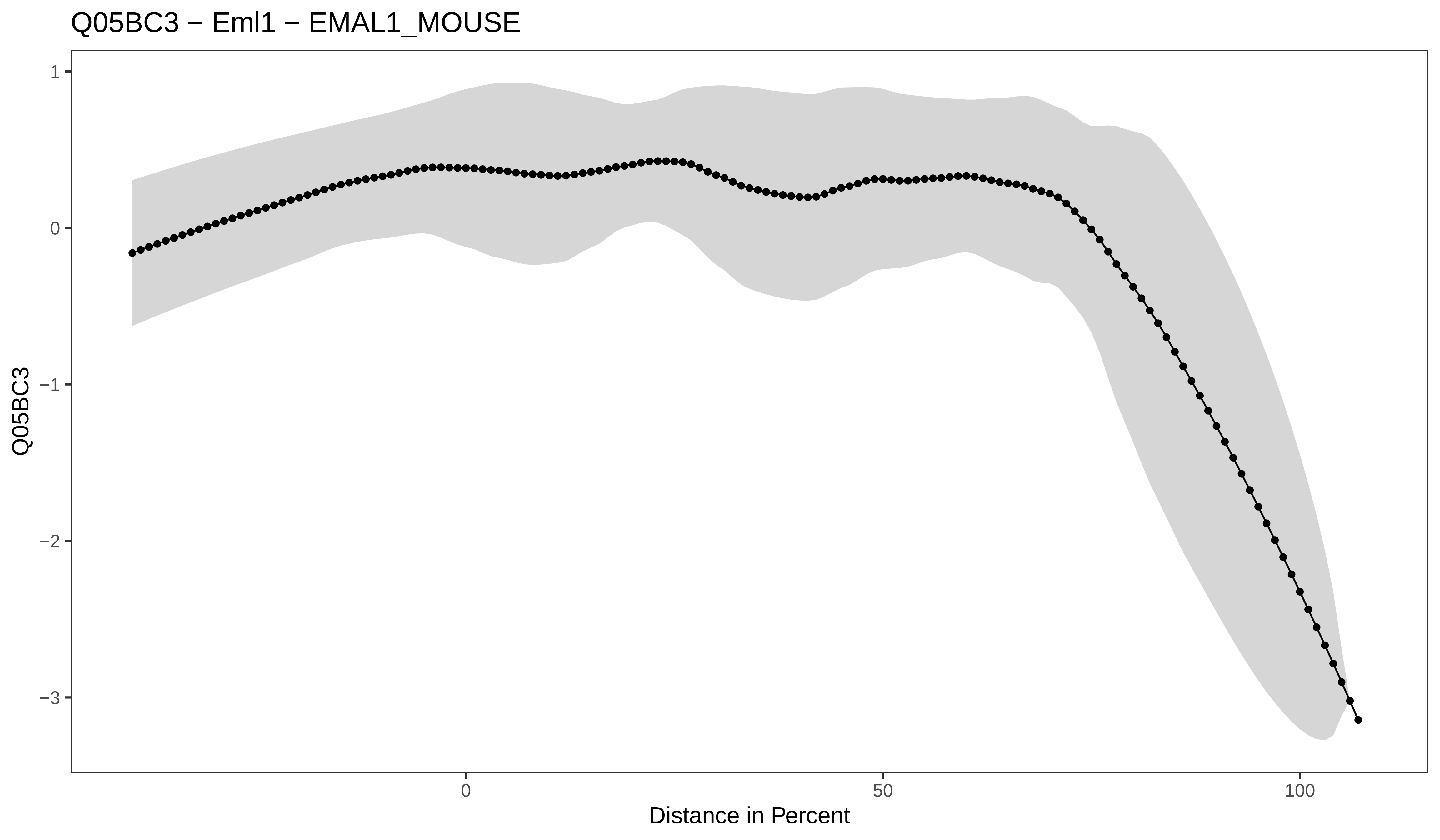
Q05BC3 − Eml1 − EMAL1_MOUSE
1
0
−1
−2
−3
Q05BC3
0
50
100
Distance in Percent

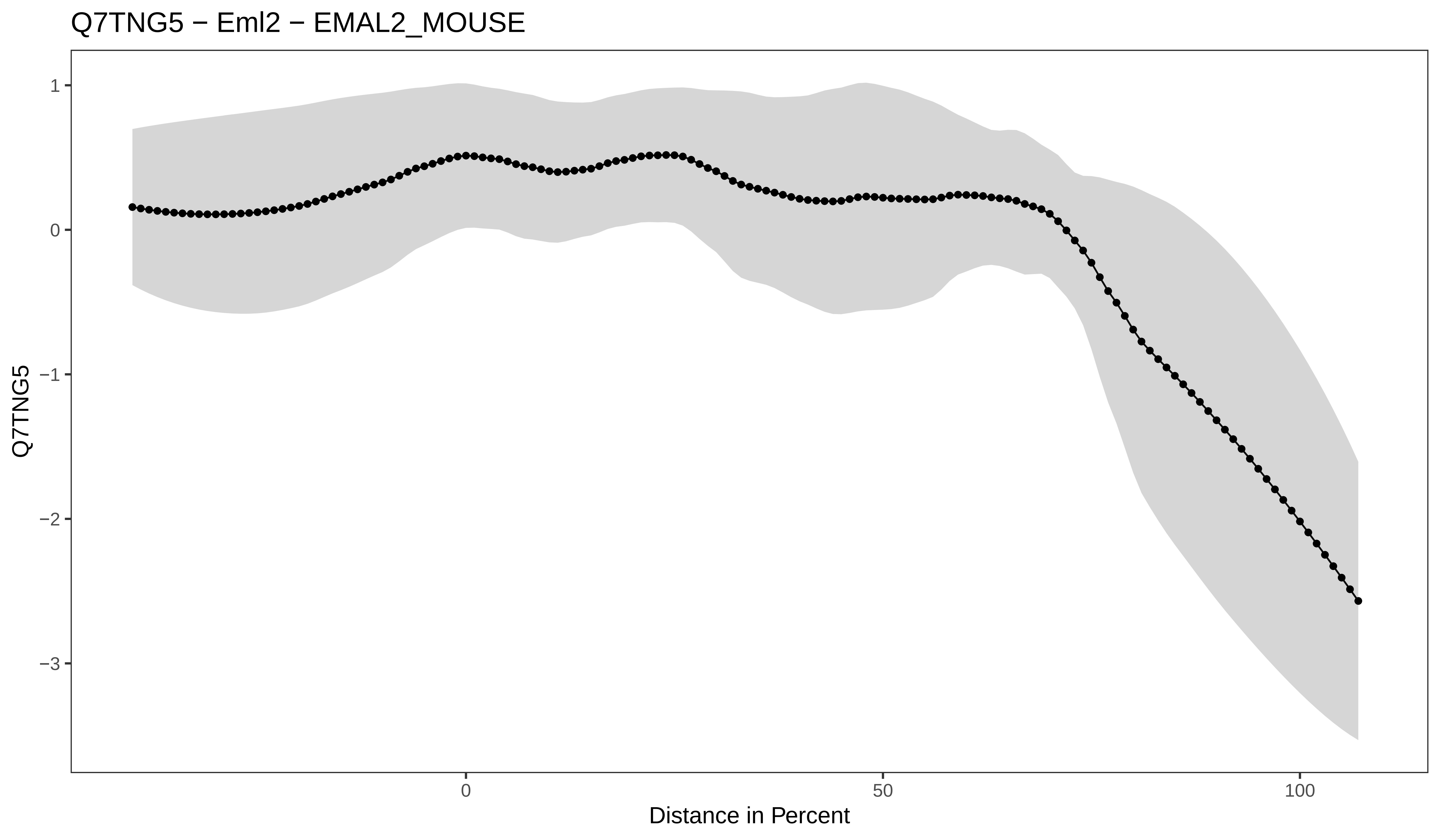
Q7TNG5 − Eml2 − EMAL2_MOUSE
1
0
−1
−2
−3
Q7TNG5
0
50
100
Distance in Percent

Q8C522 − Endod1 − ENDD1_MOUSE

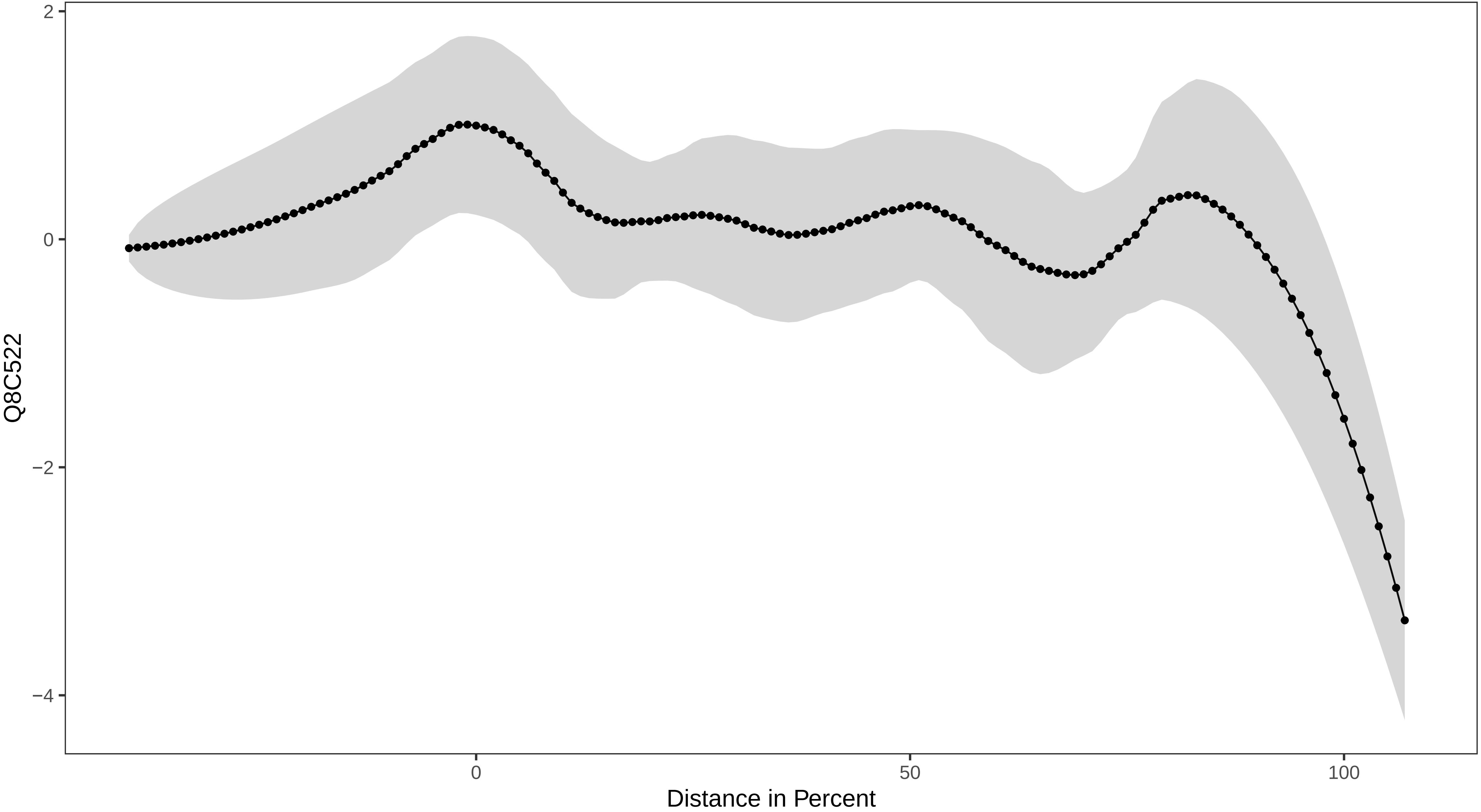

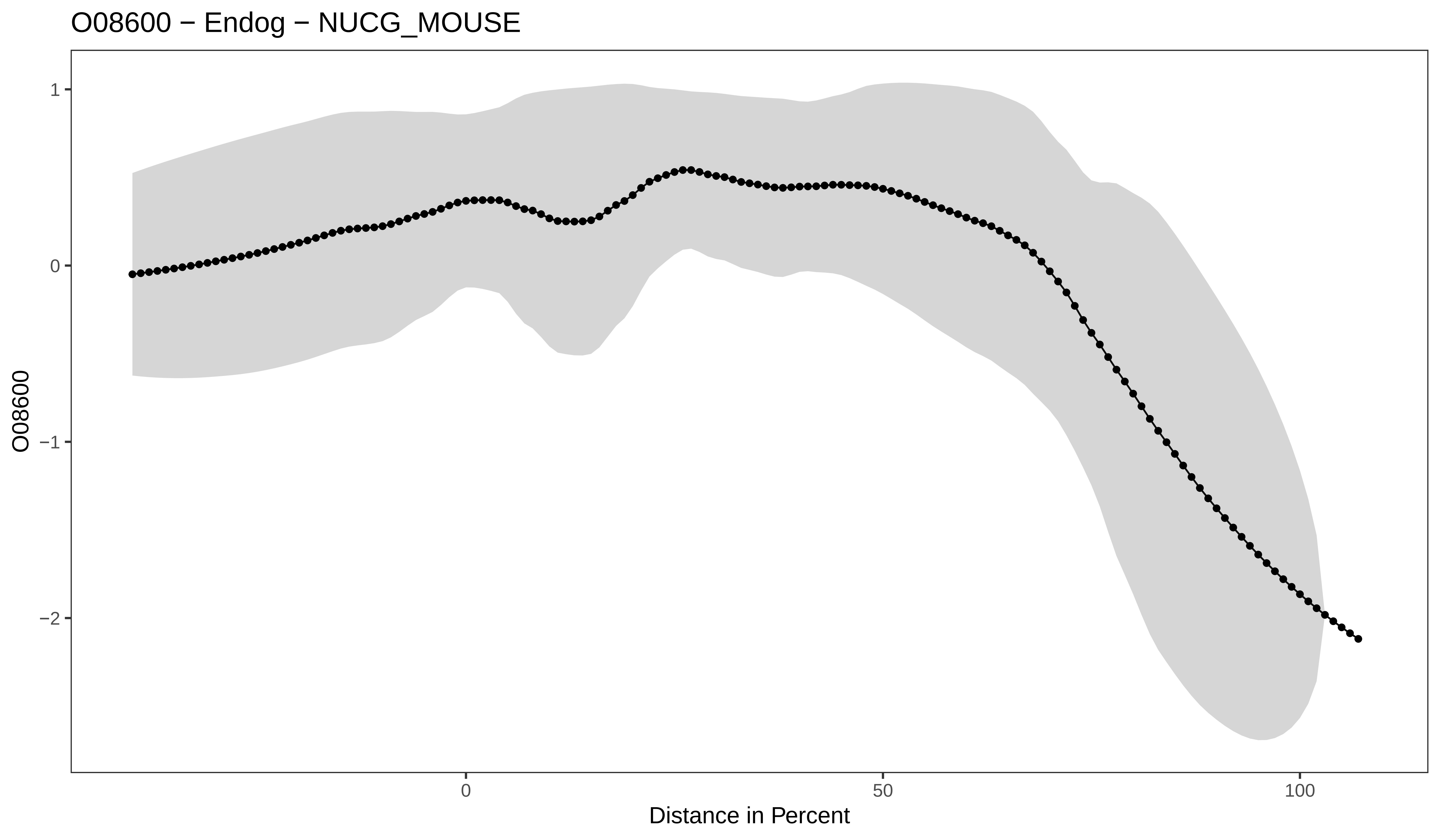

O08600 − Endog − NUCG_MOUSE
O08600
1
0
−1
−2
0
50
100
Distance in Percent

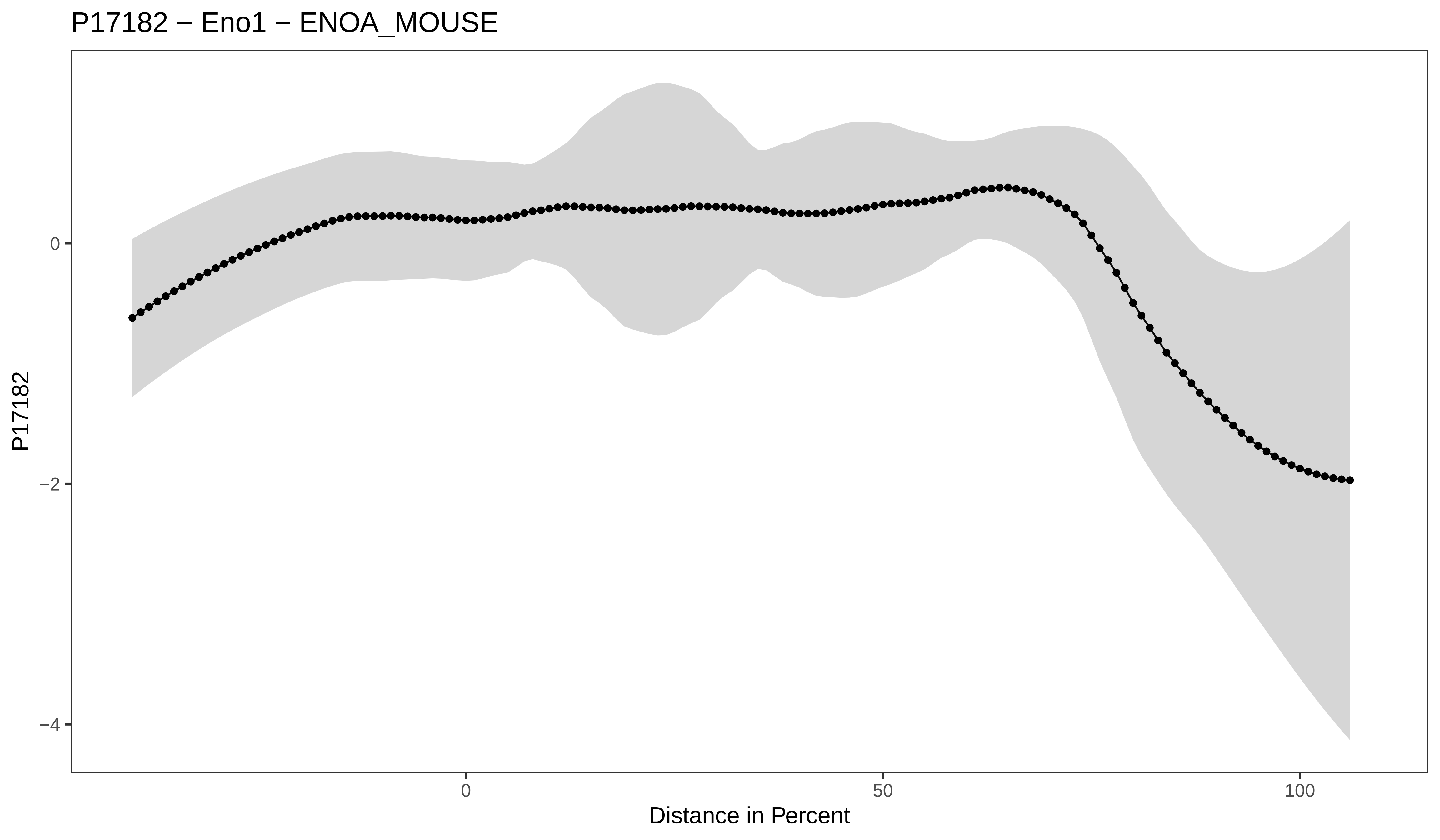

P17182 − Eno1 − ENOA_MOUSE
P17182
0
−2
−4
0
50
100
Distance in Percent

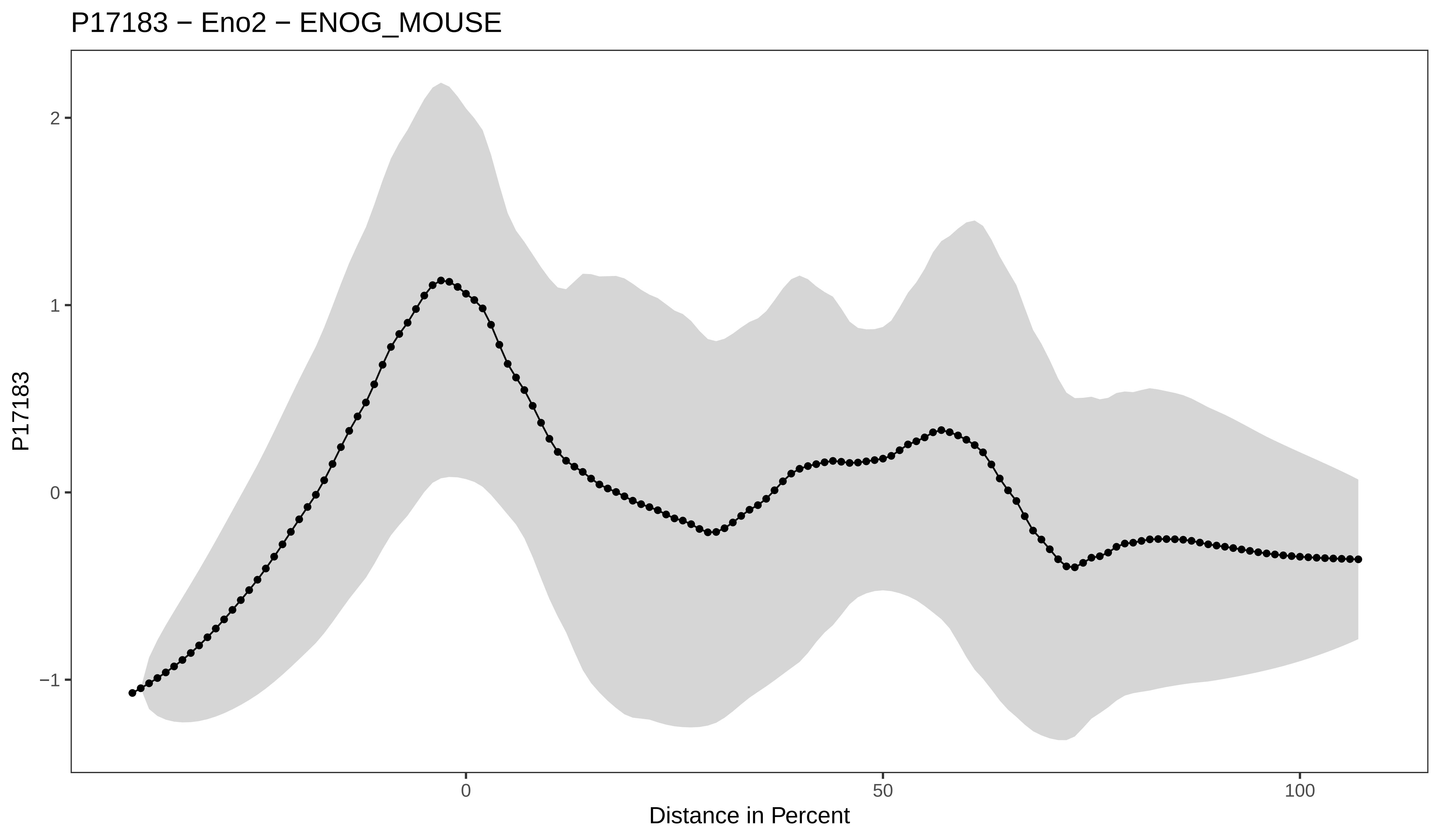

P17183 − Eno2 − ENOG_MOUSE
P17183
2
1
0
−1
0
50
100
Distance in Percent

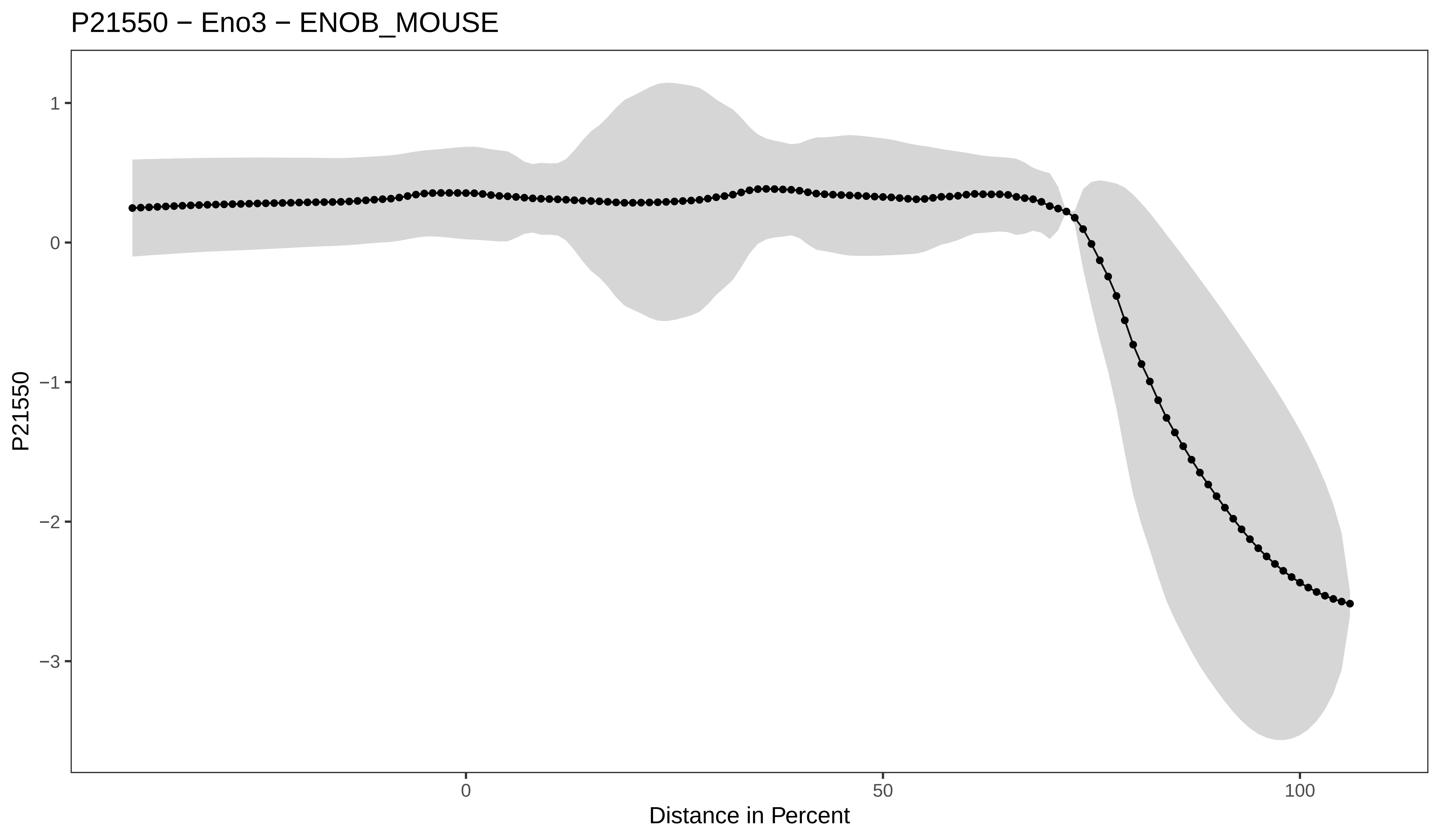

P21550 − Eno3 − ENOB_MOUSE
1
0
−1
−2
−3
P21550
0
50
100
Distance in Percent

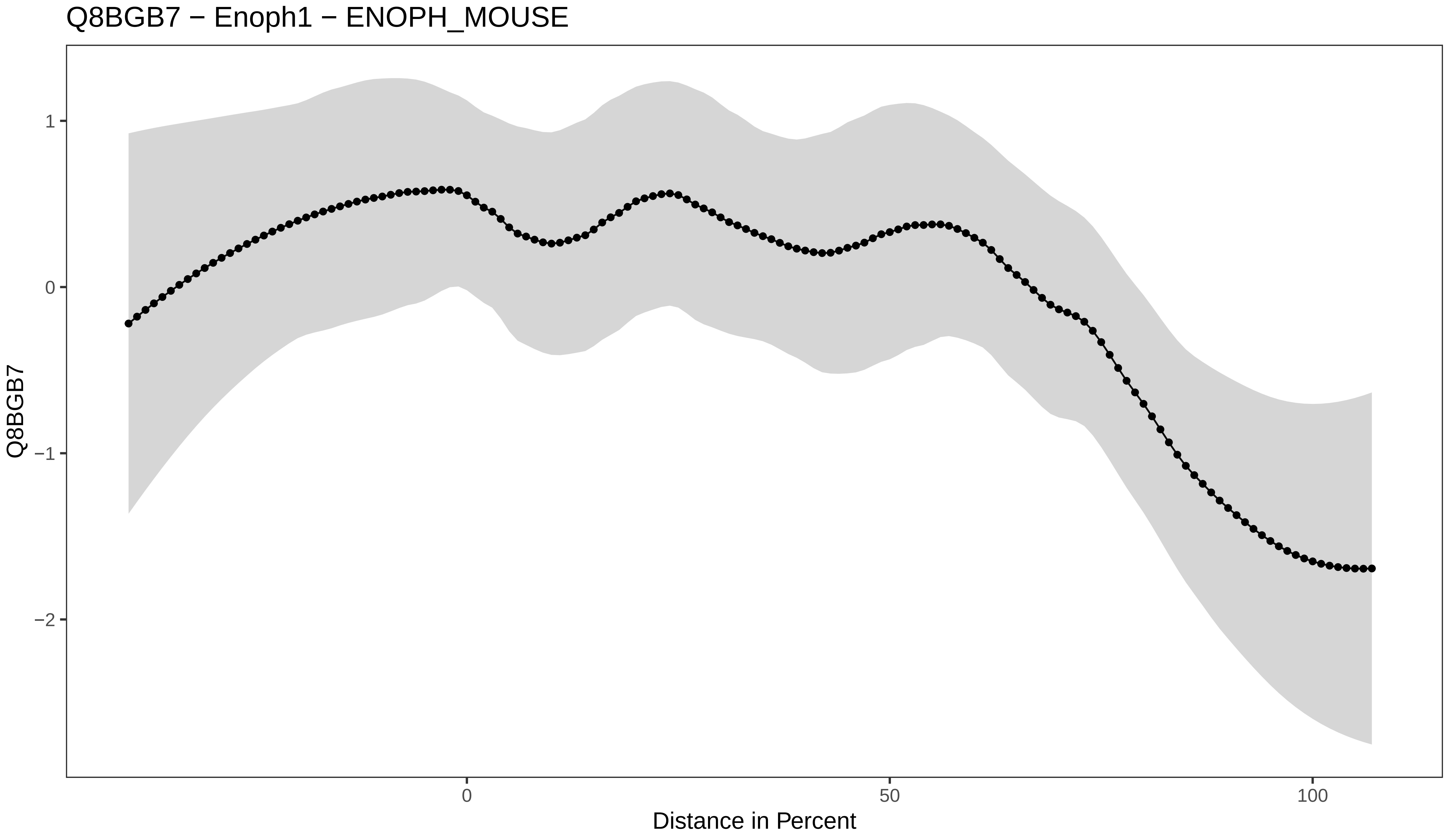

Q8BGB7 − Enoph1 − ENOPH_MOUSE
Q8BGB7
1
0
−1
−2
0
50
100
Distance in Percent

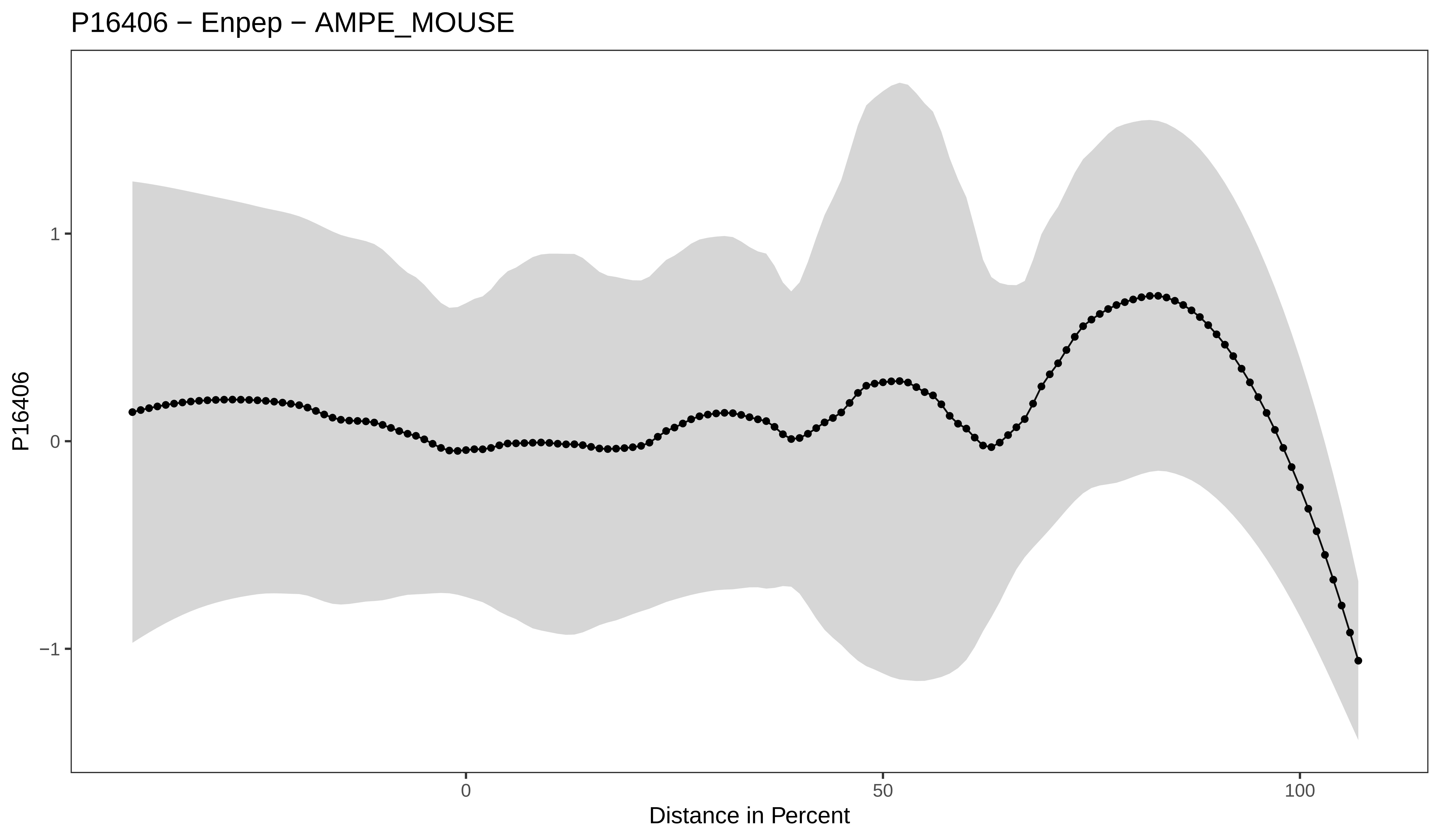
P16406 − Enpep − AMPE_MOUSE
P16406
1
0
−1
0
50
100
Distance in Percent

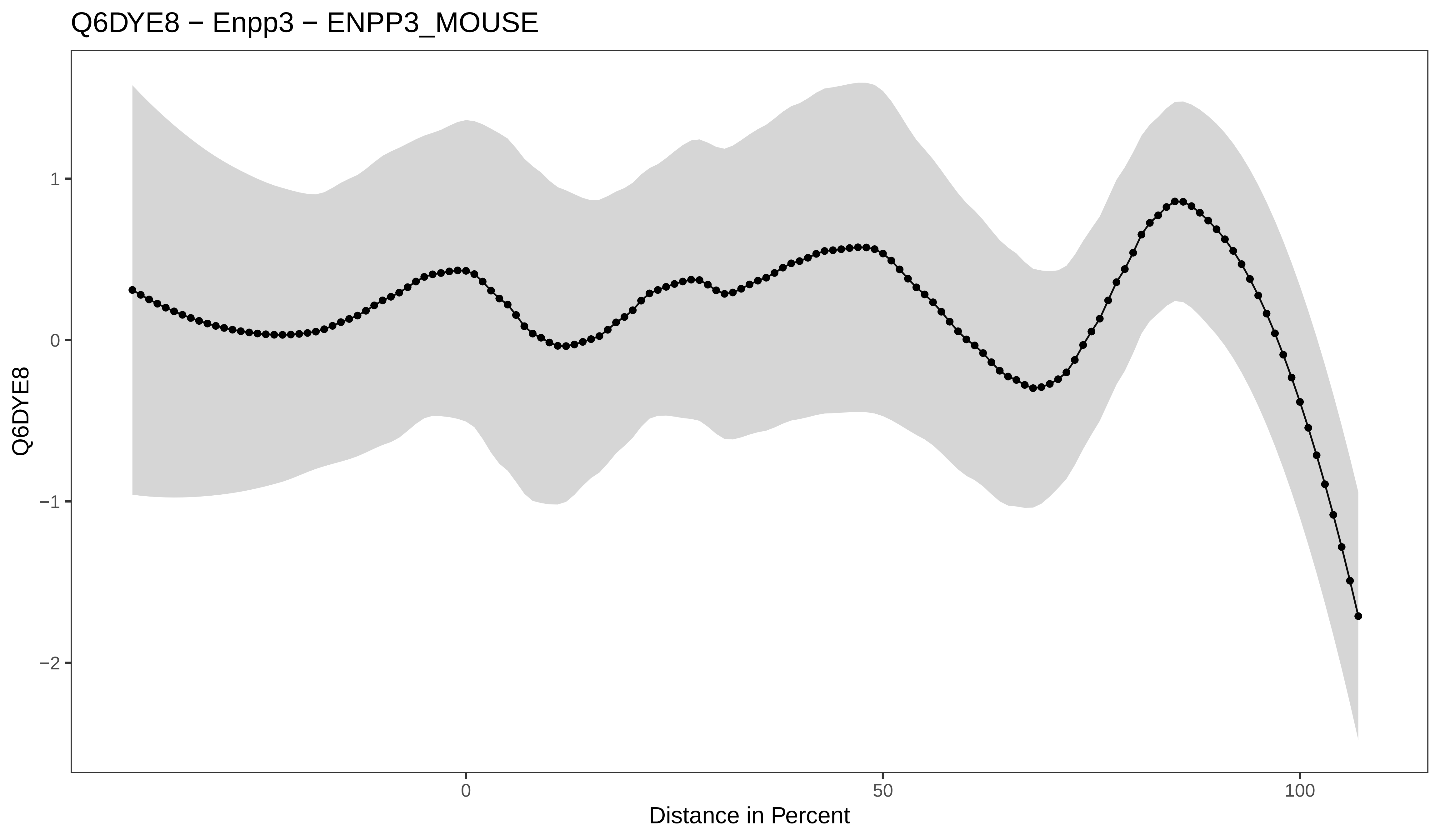
Q6DYE8 − Enpp3 − ENPP3_MOUSE
Q6DYE8
Distance in Percent
1
0
−1
−2
0
50
100

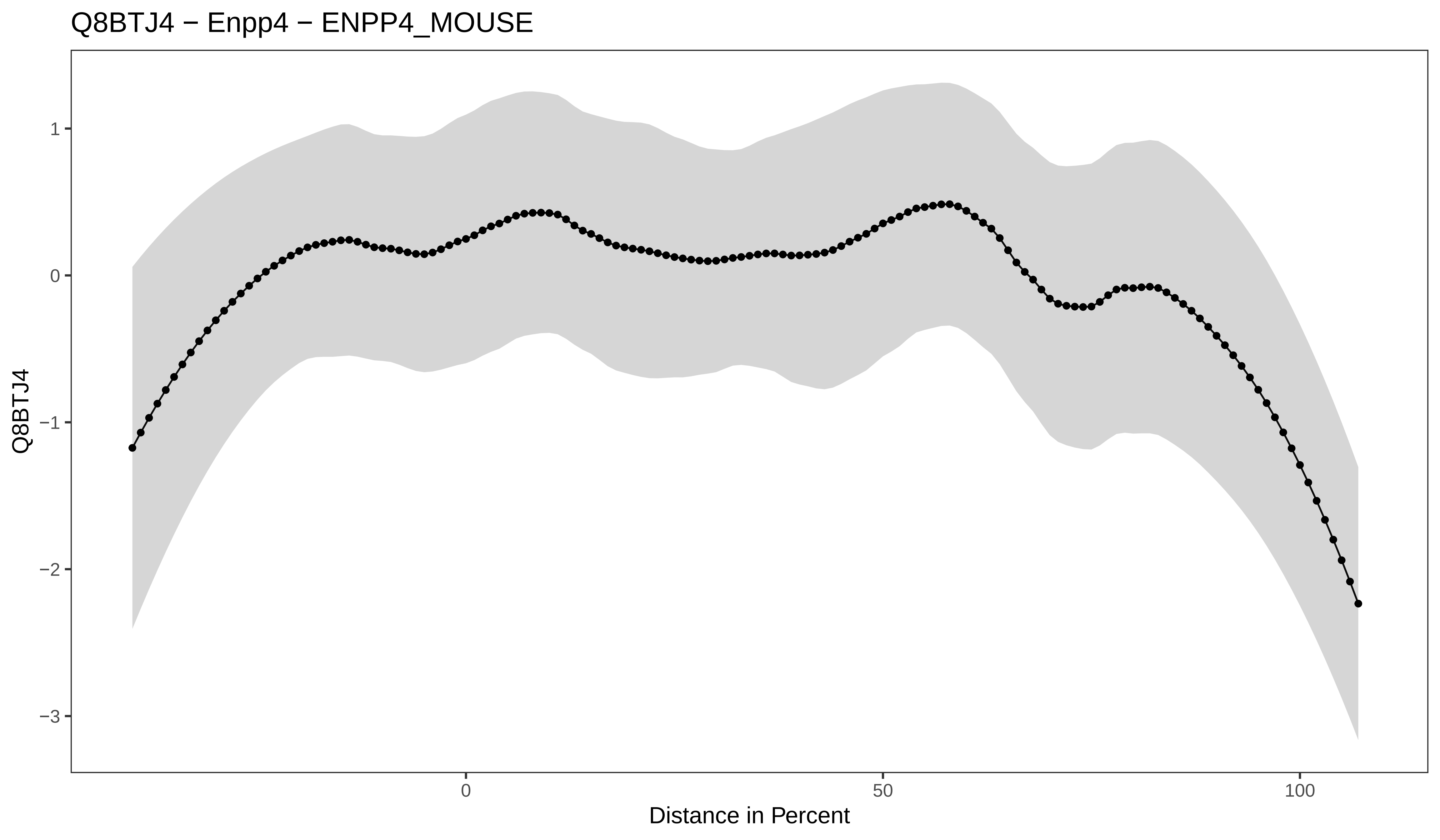

Q8BTJ4 − Enpp4 − ENPP4_MOUSE
1
0
−1
−2
−3
Q8BTJ4
0
50
100
Distance in Percent

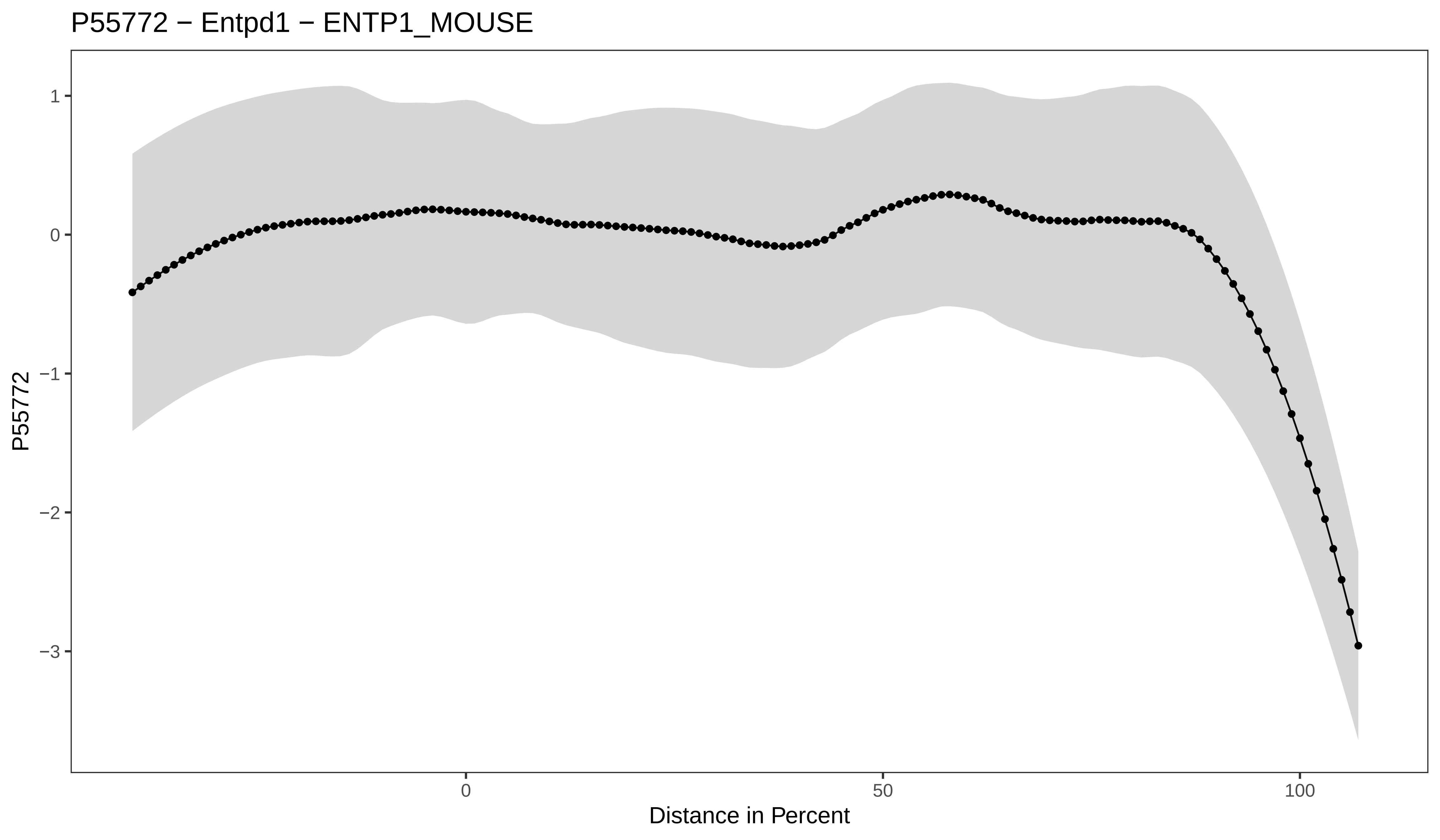

P55772 − Entpd1 − ENTP1_MOUSE
P55772
1
0
−1
−2
−3
0
50
100
Distance in Percent

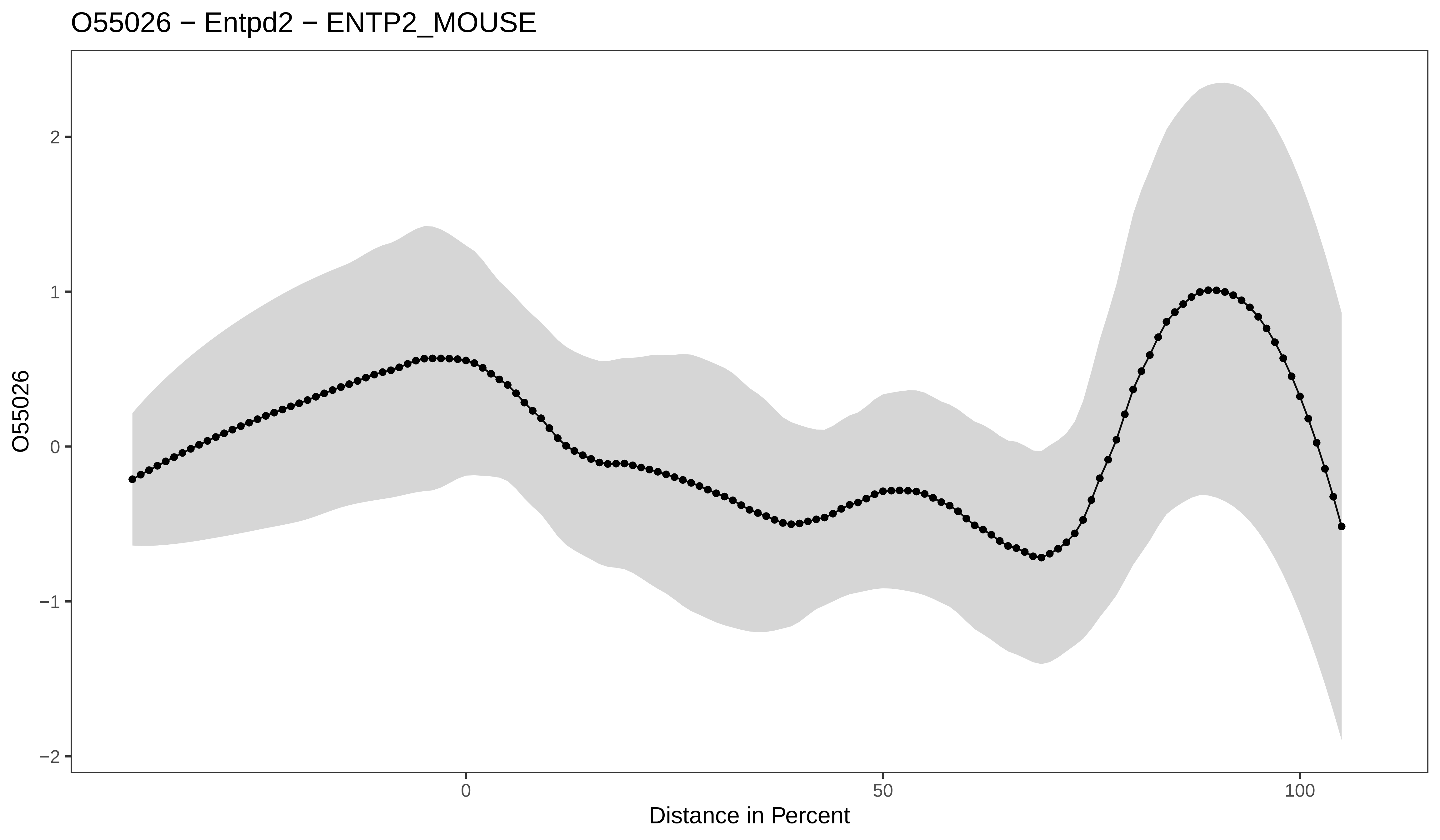
O55026 − Entpd2 − ENTP2_MOUSE
O55026
2
1
0
−1
−2
0
50
100
Distance in Percent

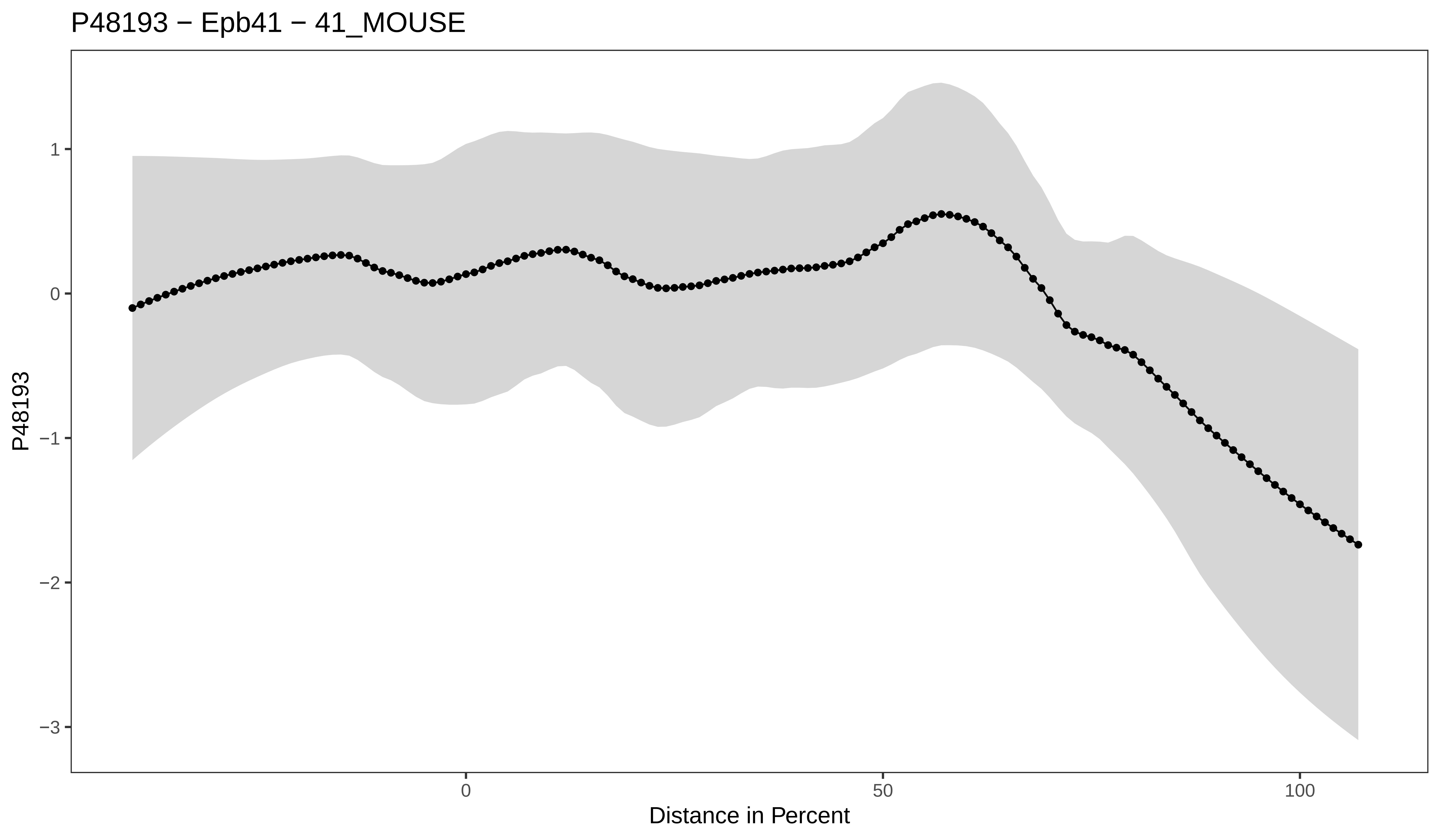

P48193 − Epb41 − 41_MOUSE
P48193
1
0
−1
−2
−3
0
50
100
Distance in Percent

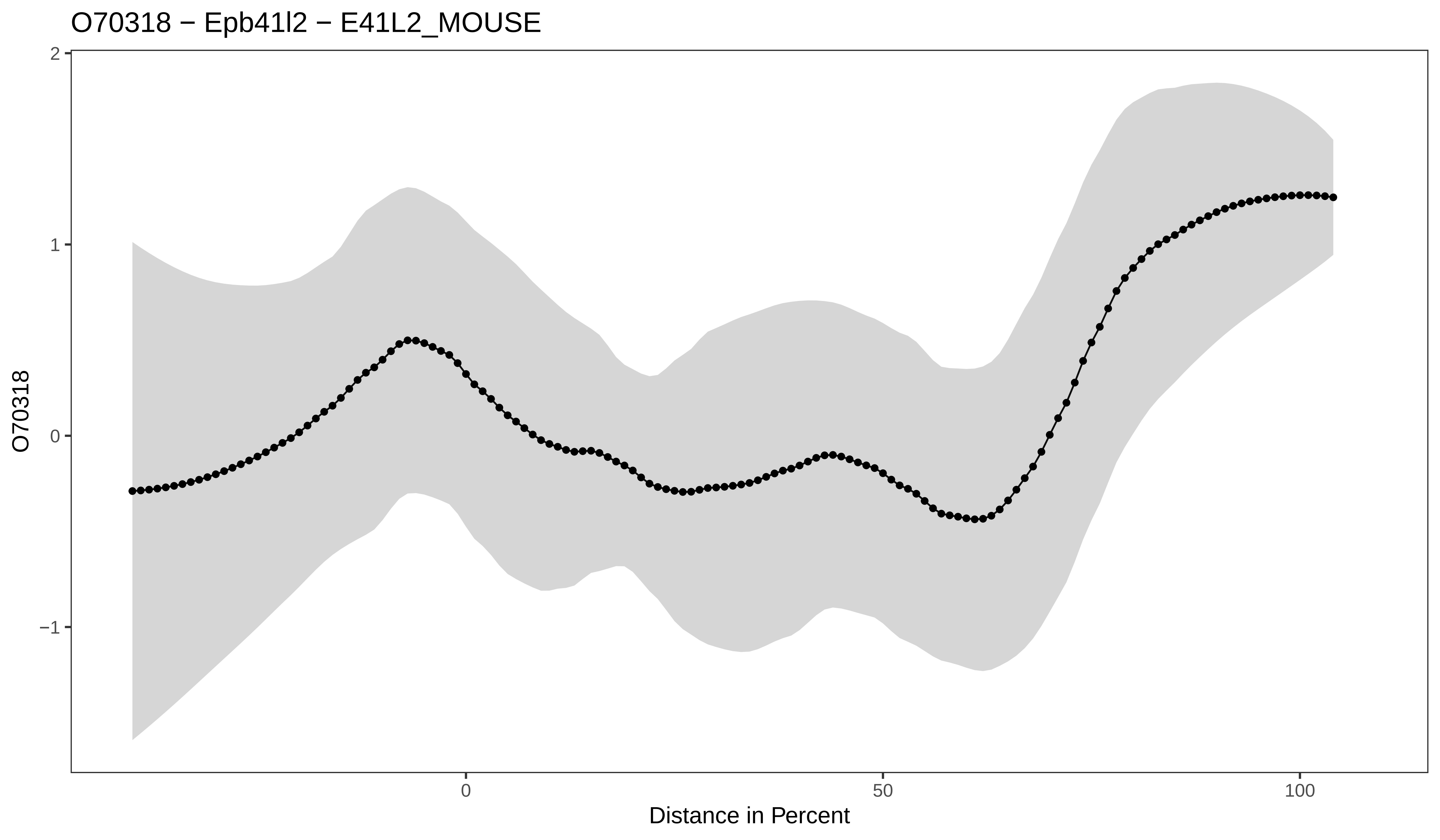

O70318 − Epb41l2 − E41L2_MOUSE
2
1
0
−1
O70318
0
50
100
Distance in Percent

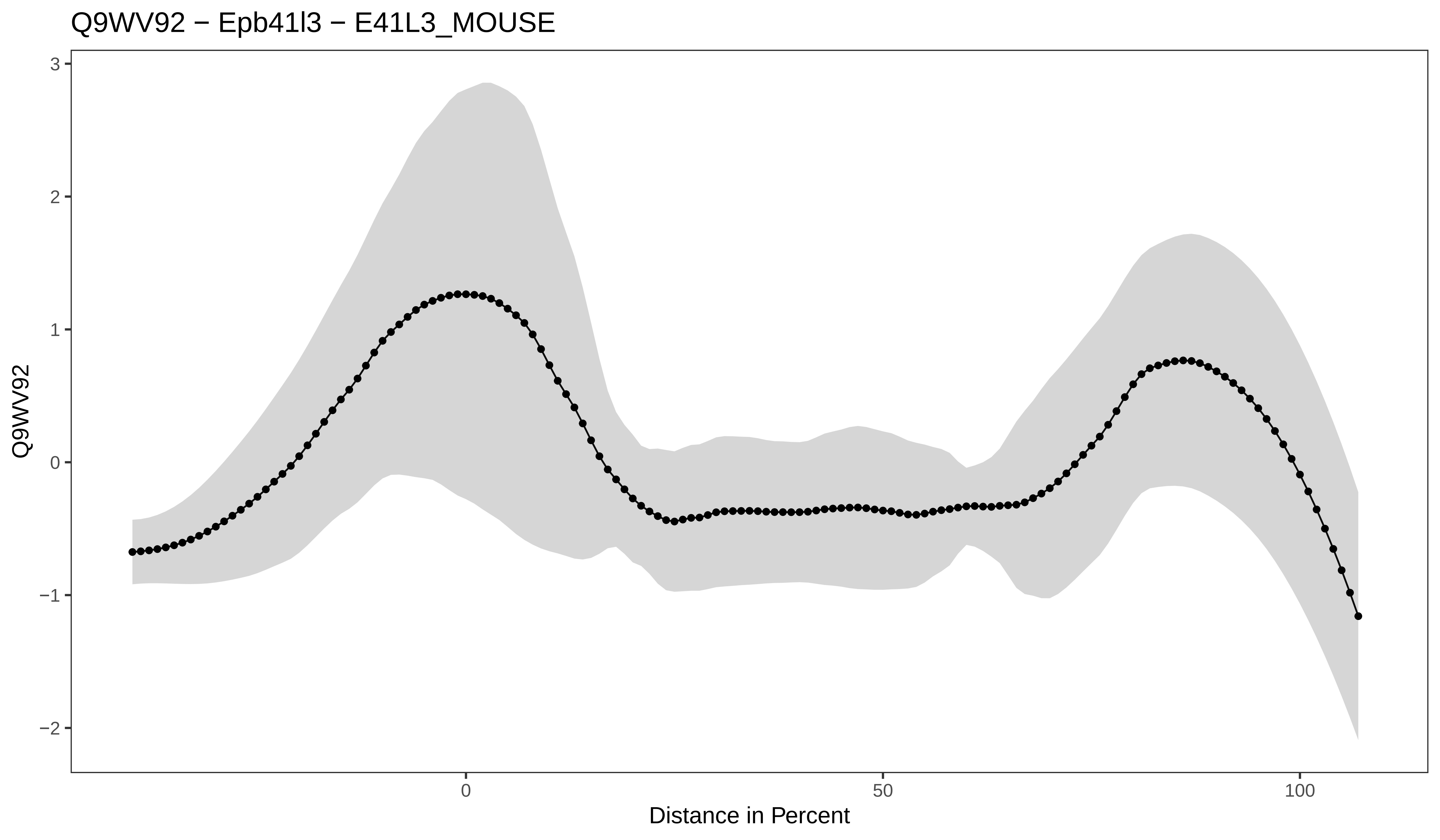

Q9WV92 − Epb41l3 − E41L3_MOUSE
Q9WV92
3
2
1
0
−1
−2
0
50
100
Distance in Percent

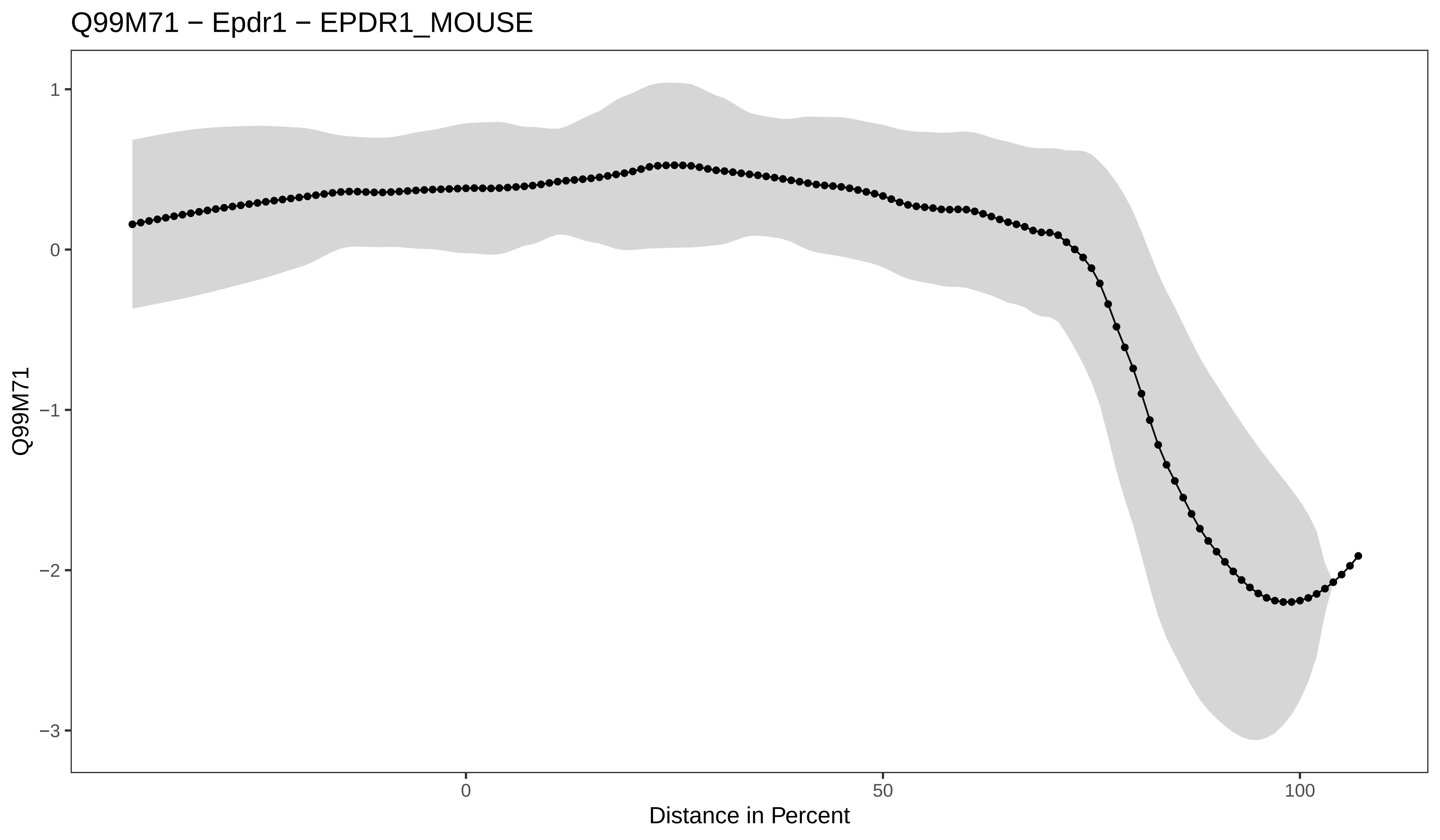

Q99M71 − Epdr1 − EPDR1_MOUSE
1
0
−1
−2
−3
Q99M71
0
50
100
Distance in Percent

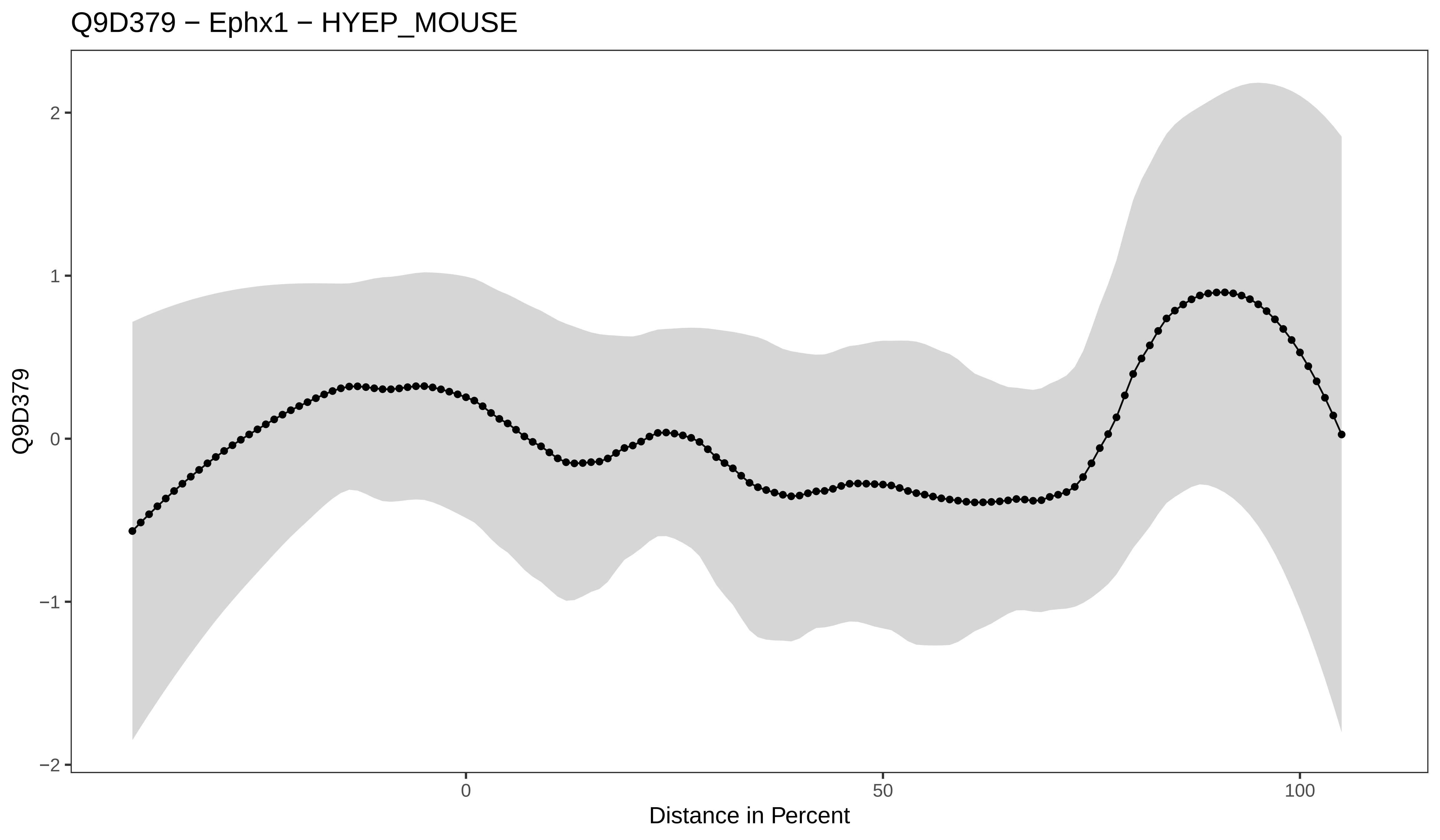
Q9D379 − Ephx1 − HYEP_MOUSE
Q9D379
Distance in Percent
2
1
0
−1
−2
0
50
100

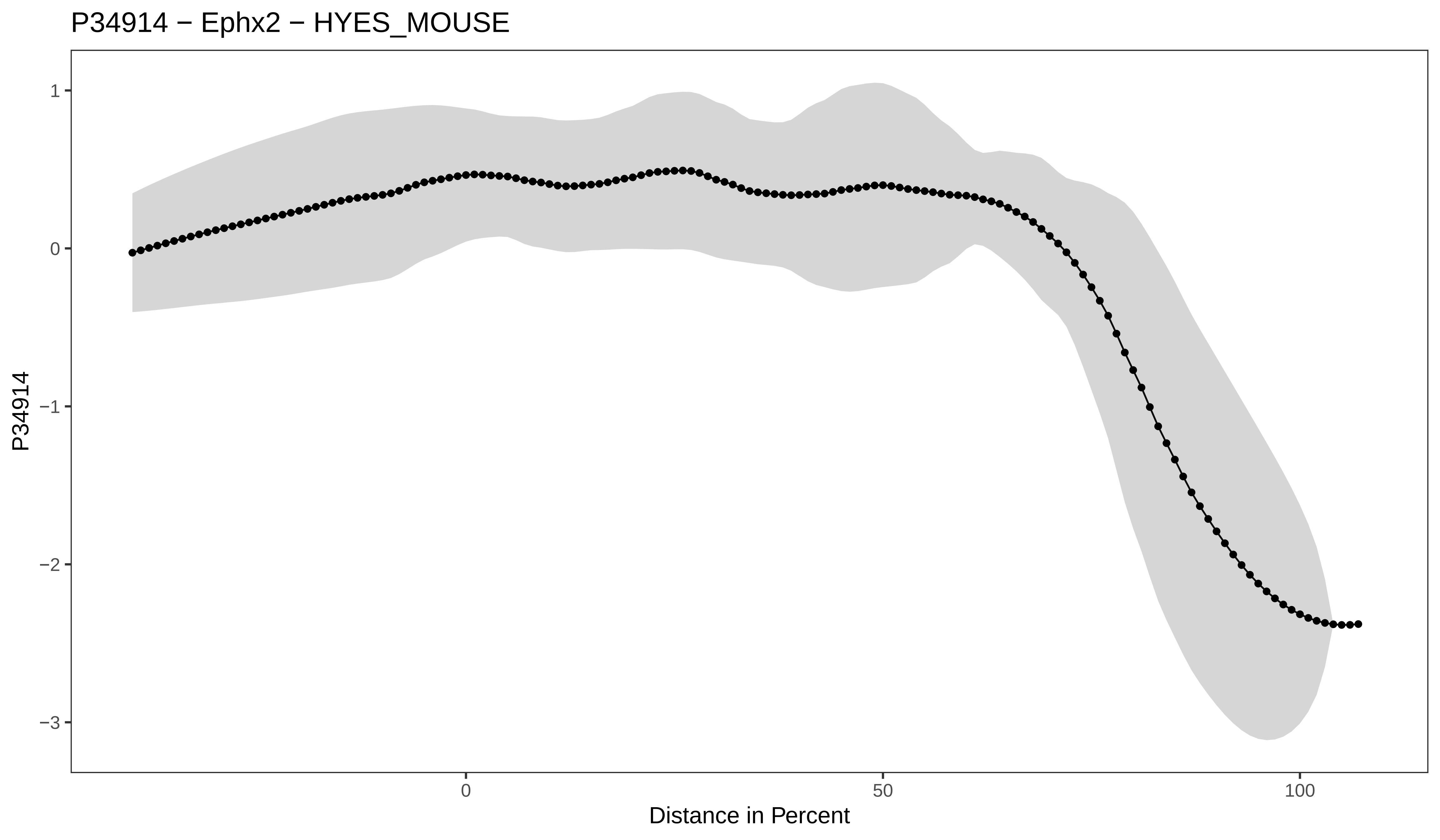

P34914 − Ephx2 − HYES_MOUSE
P34914
1
0
−1
−2
−3
0
50
100
Distance in Percent

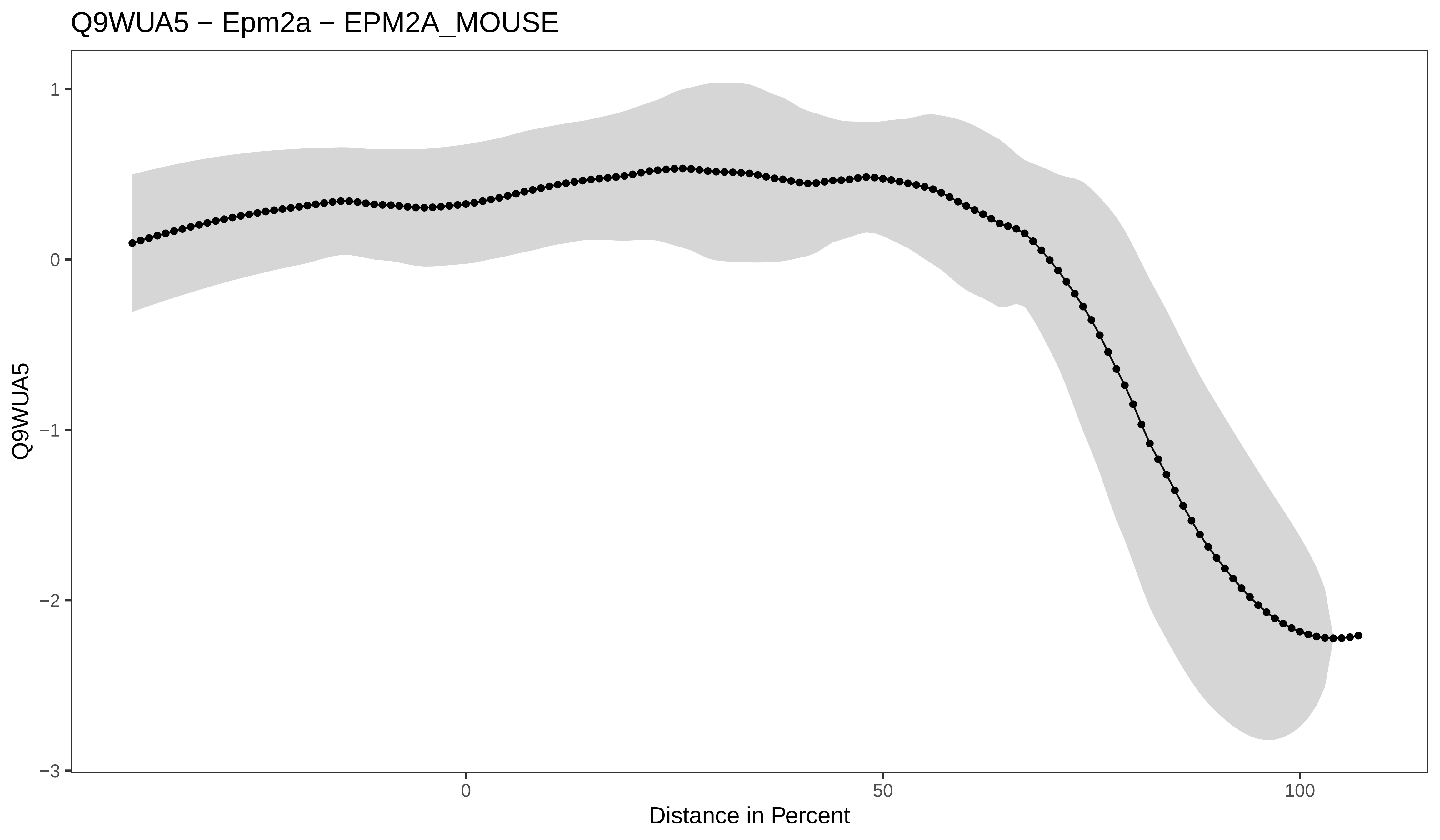
Q9WUA5 − Epm2a − EPM2A_MOUSE
Q9WUA5
1
0
−1
−2
−3
0
50
100
Distance in Percent

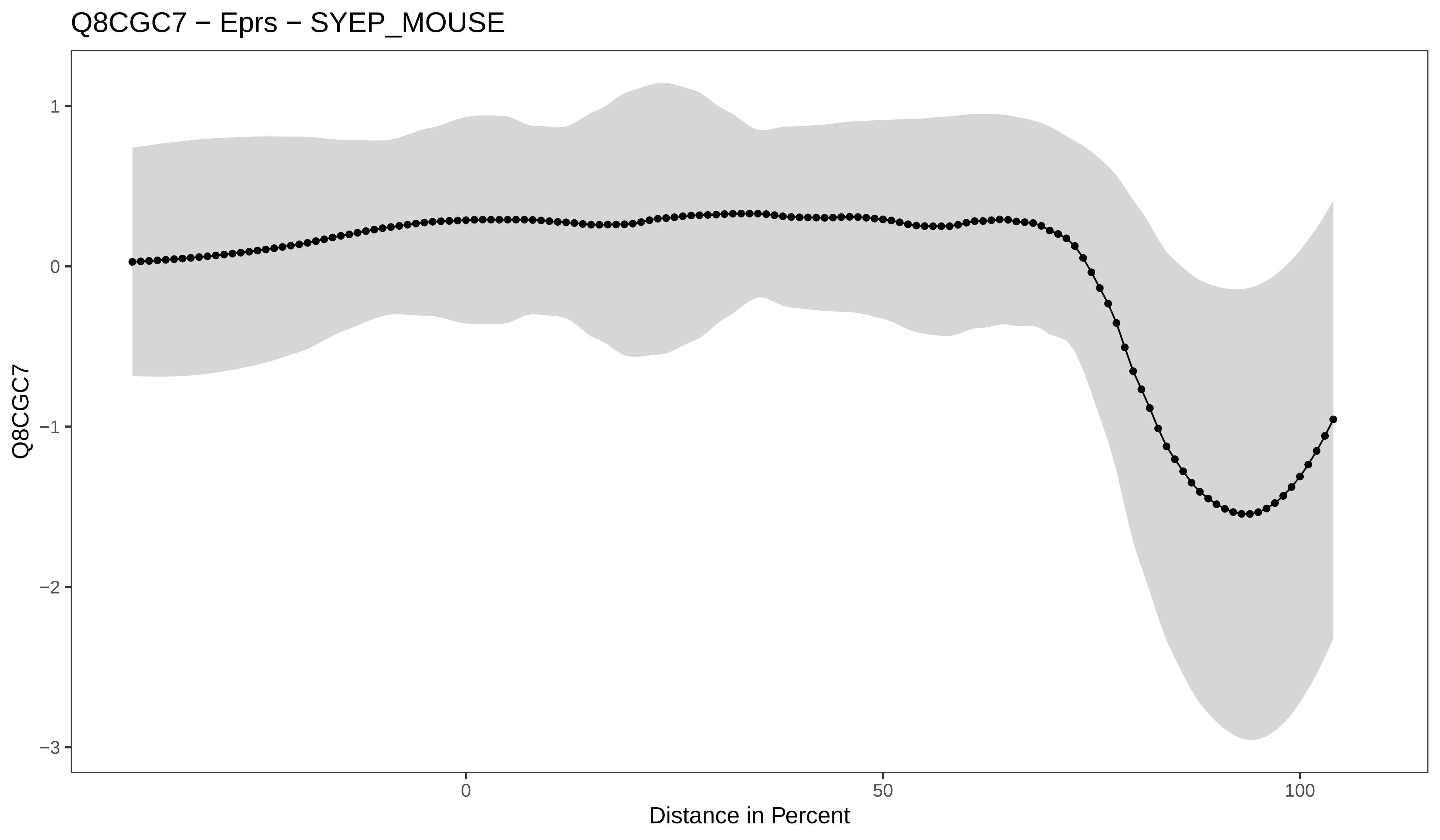
Q8CGC7 − Eprs − SYEP_MOUSE
Q8CGC7
1
0
−1
−2
−3
0
50
100
Distance in Percent

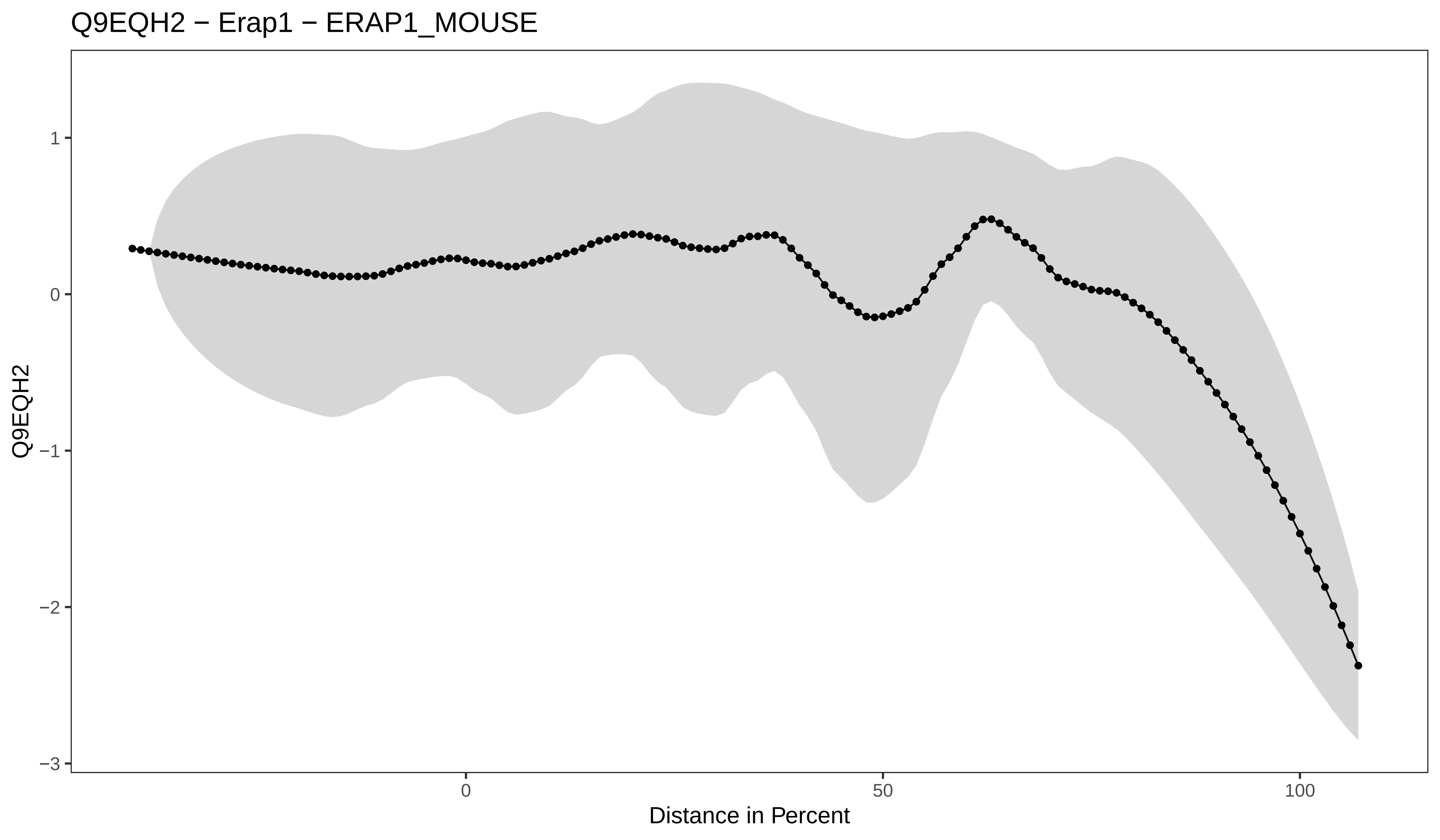

Q9EQH2 − Erap1 − ERAP1_MOUSE
Q9EQH2
Distance in Percent
1
0
−1
−2
−3
0
50
100

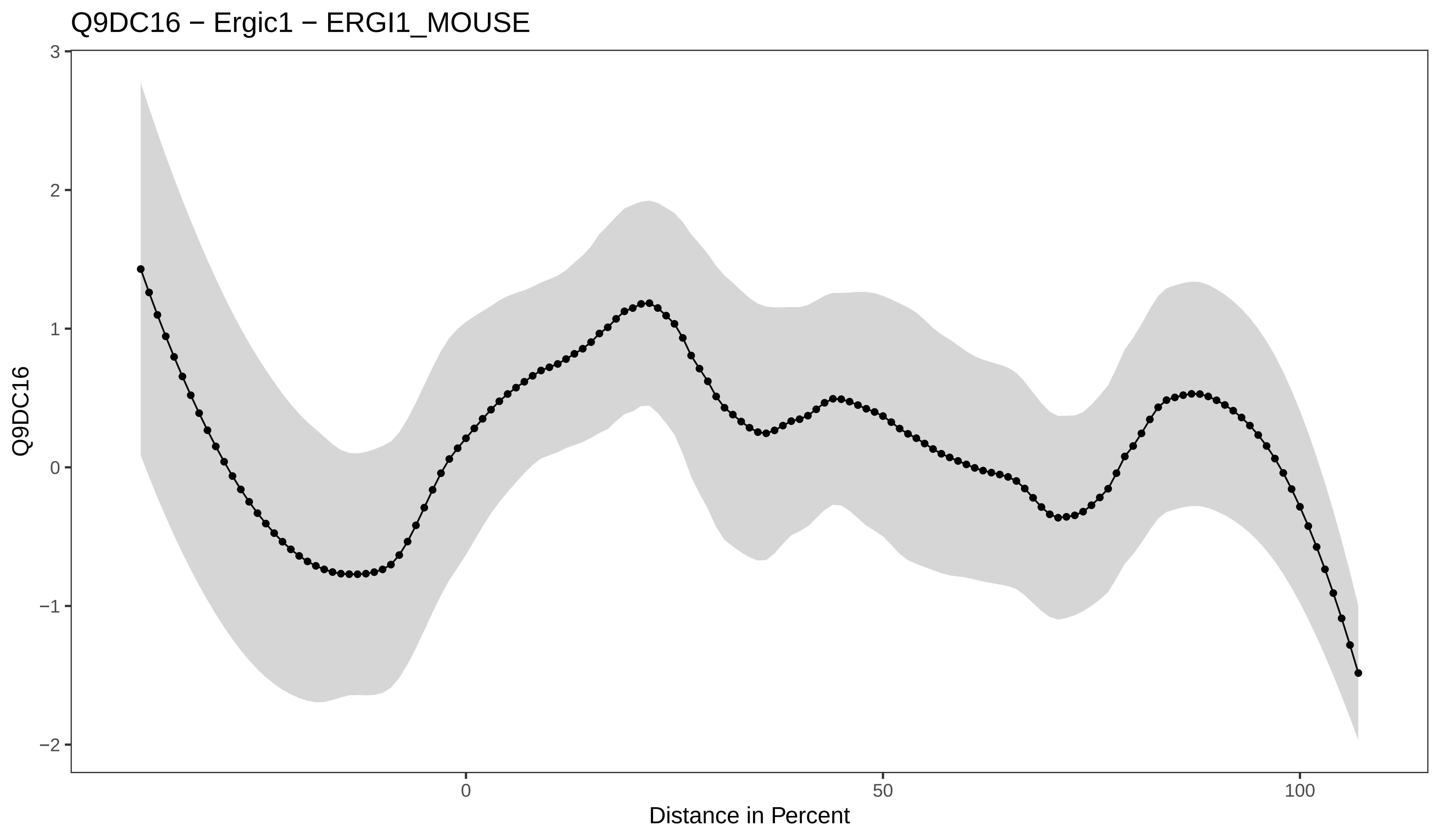
Q9DC16 − Ergic1 − ERGI1_MOUSE
3
2
1
0
−1
−2
Q9DC16
0
50
100
Distance in Percent

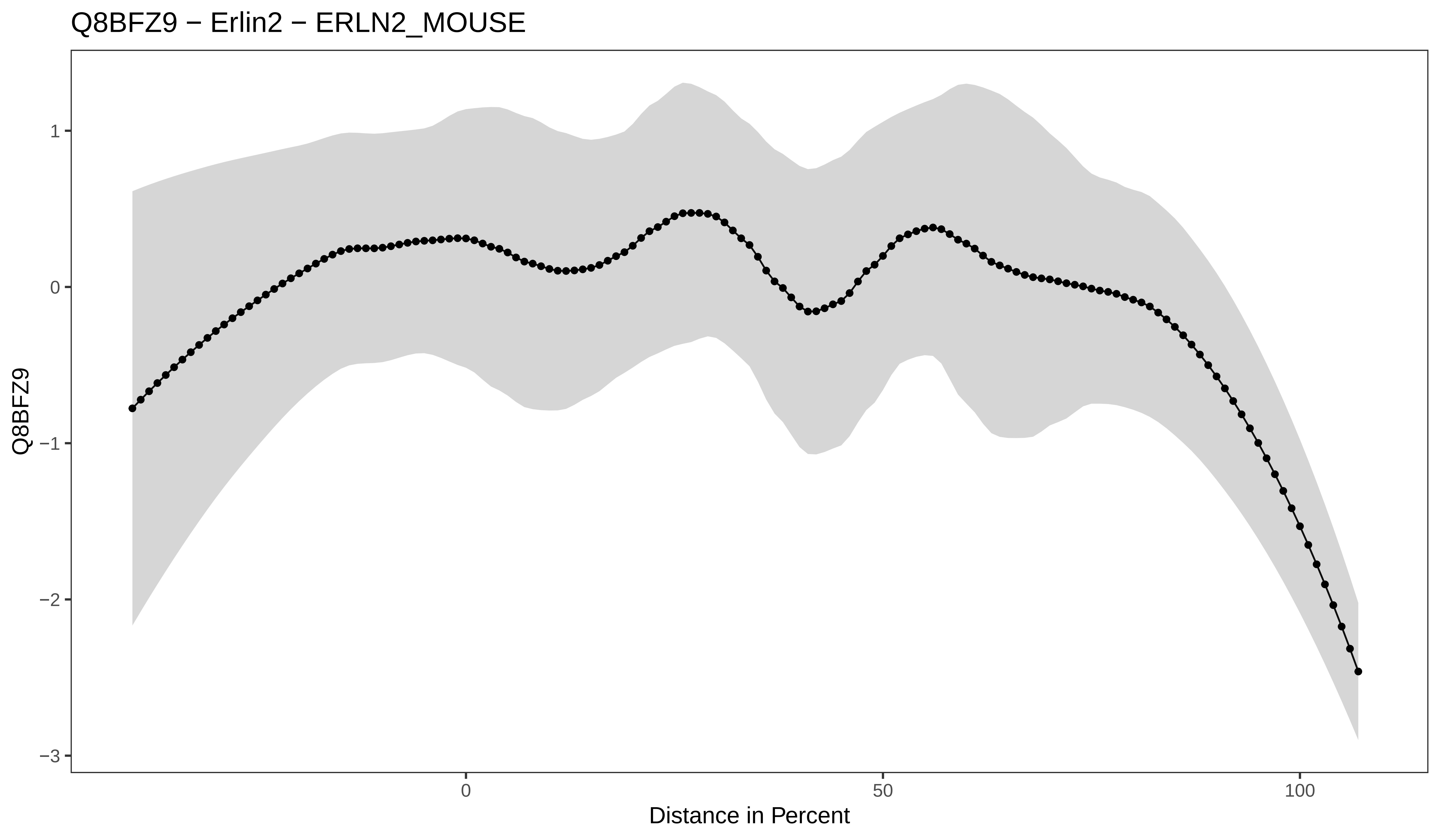
Q8BFZ9 − Erlin2 − ERLN2_MOUSE
Q8BFZ9
1
0
−1
−2
−3
0
50
100
Distance in Percent

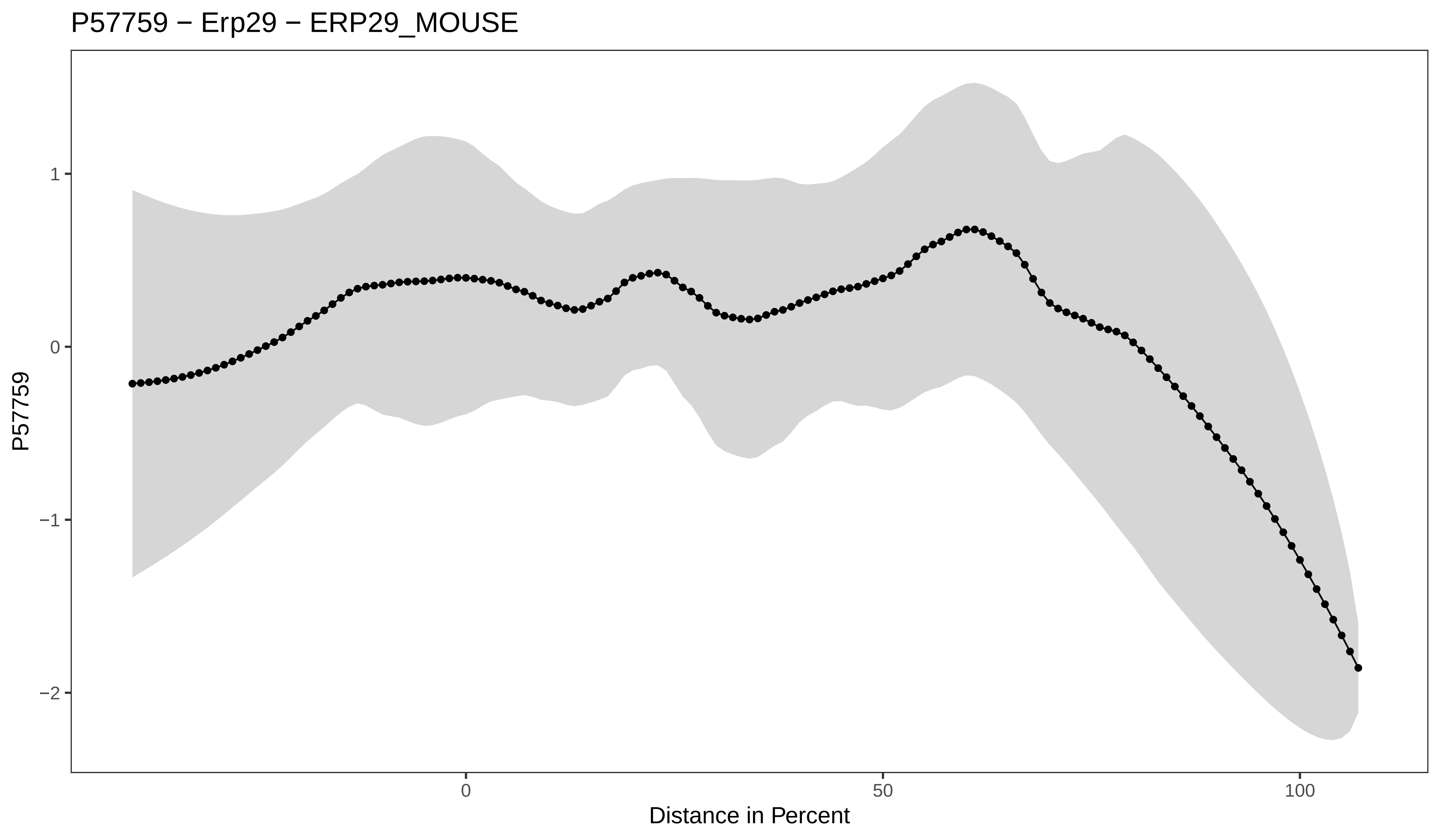
P57759 − Erp29 − ERP29_MOUSE
P57759
1
0
−1
−2
0
50
100
Distance in Percent

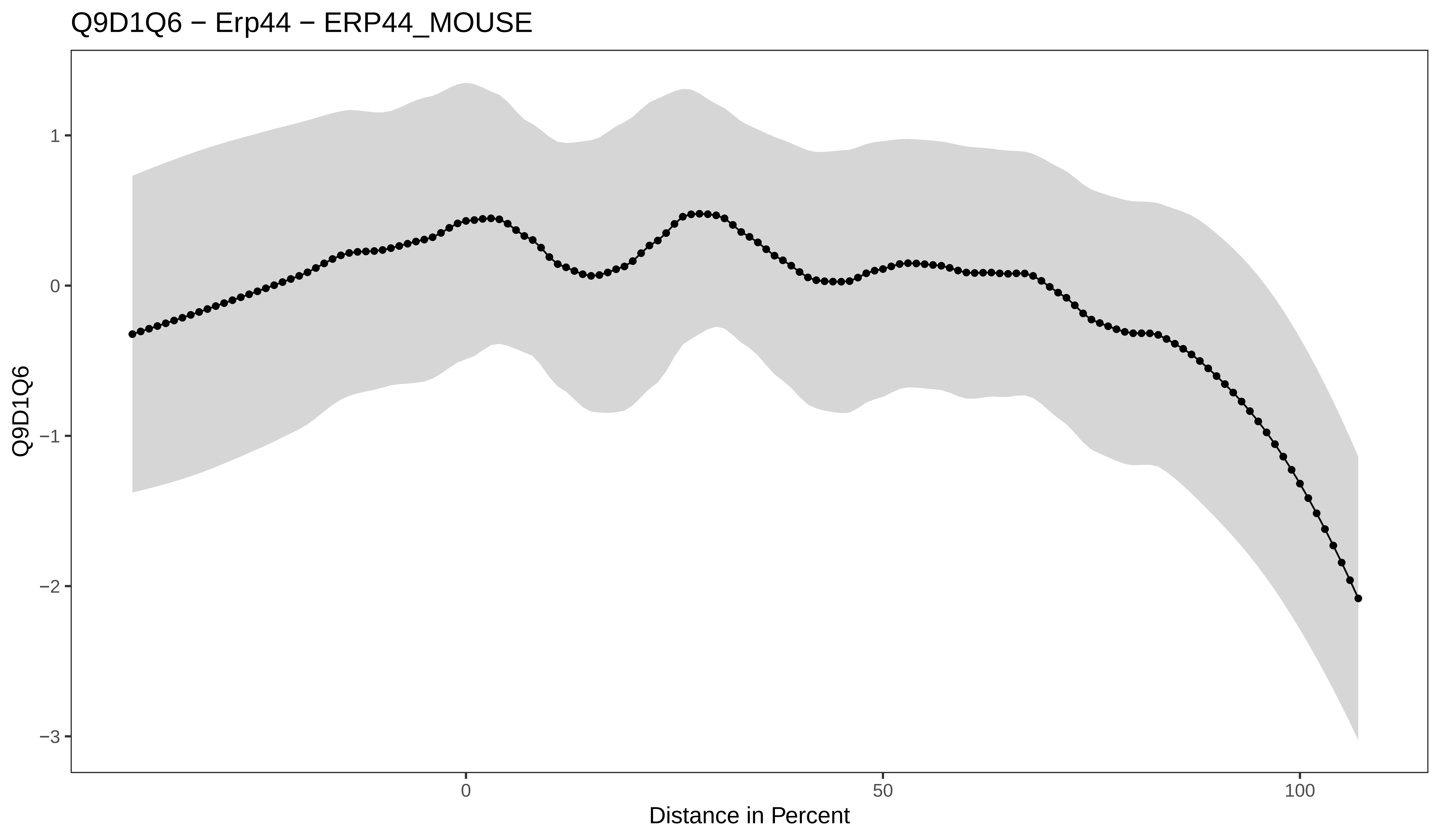
Q9D1Q6 − Erp44 − ERP44_MOUSE
1
0
−1
−2
−3
Q9D1Q6
0
50
100
Distance in Percent

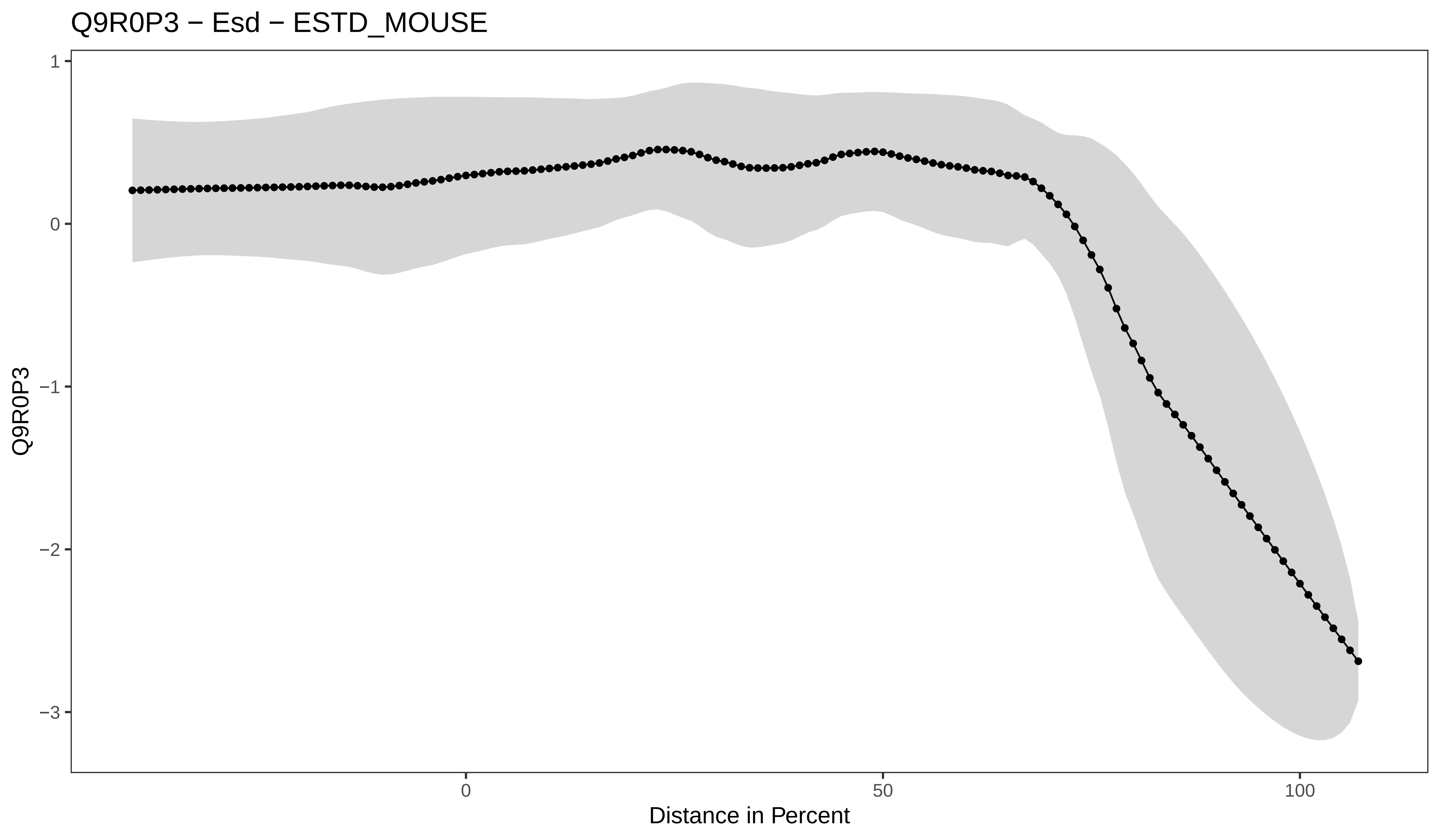
Q9R0P3 − Esd − ESTD_MOUSE
Q9R0P3
Distance in Percent
1
0
−1
−2
−3
0
50
100

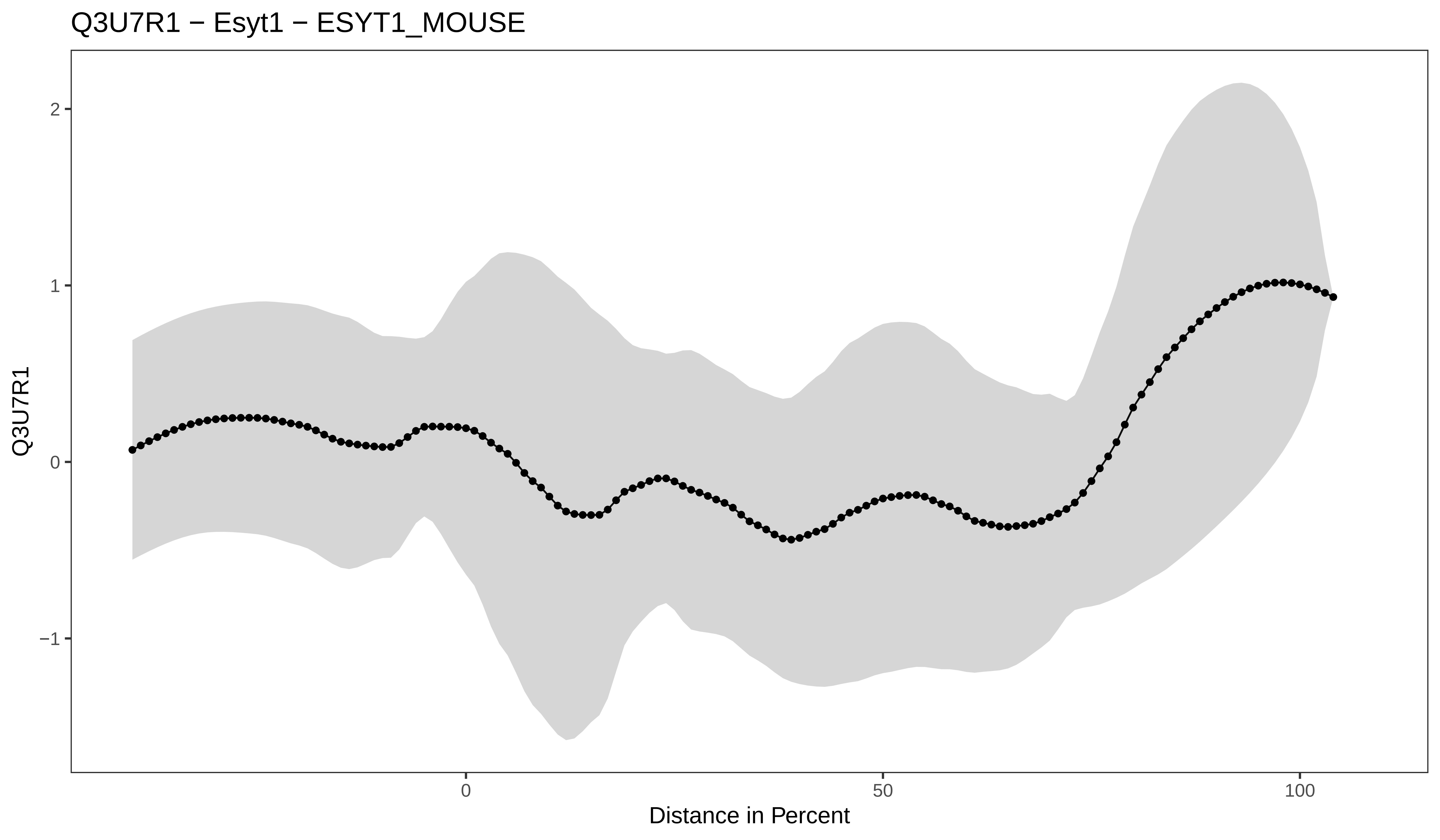

Q3U7R1 − Esyt1 − ESYT1_MOUSE
2
1
0
−1
Q3U7R1
0
50
100
Distance in Percent

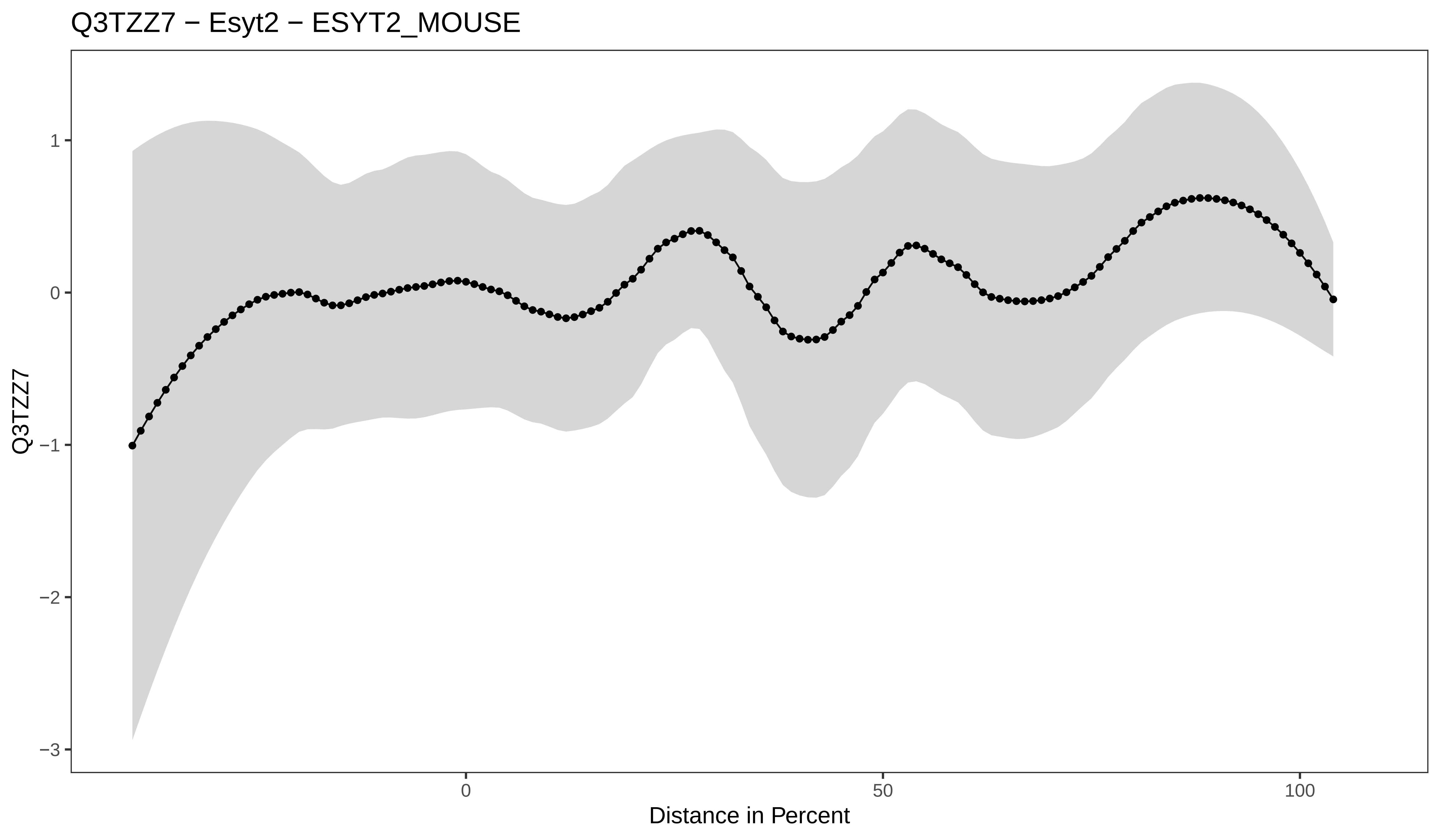

Q3TZZ7 − Esyt2 − ESYT2_MOUSE
1
0
−1
−2
−3
Q3TZZ7
0
50
100
Distance in Percent

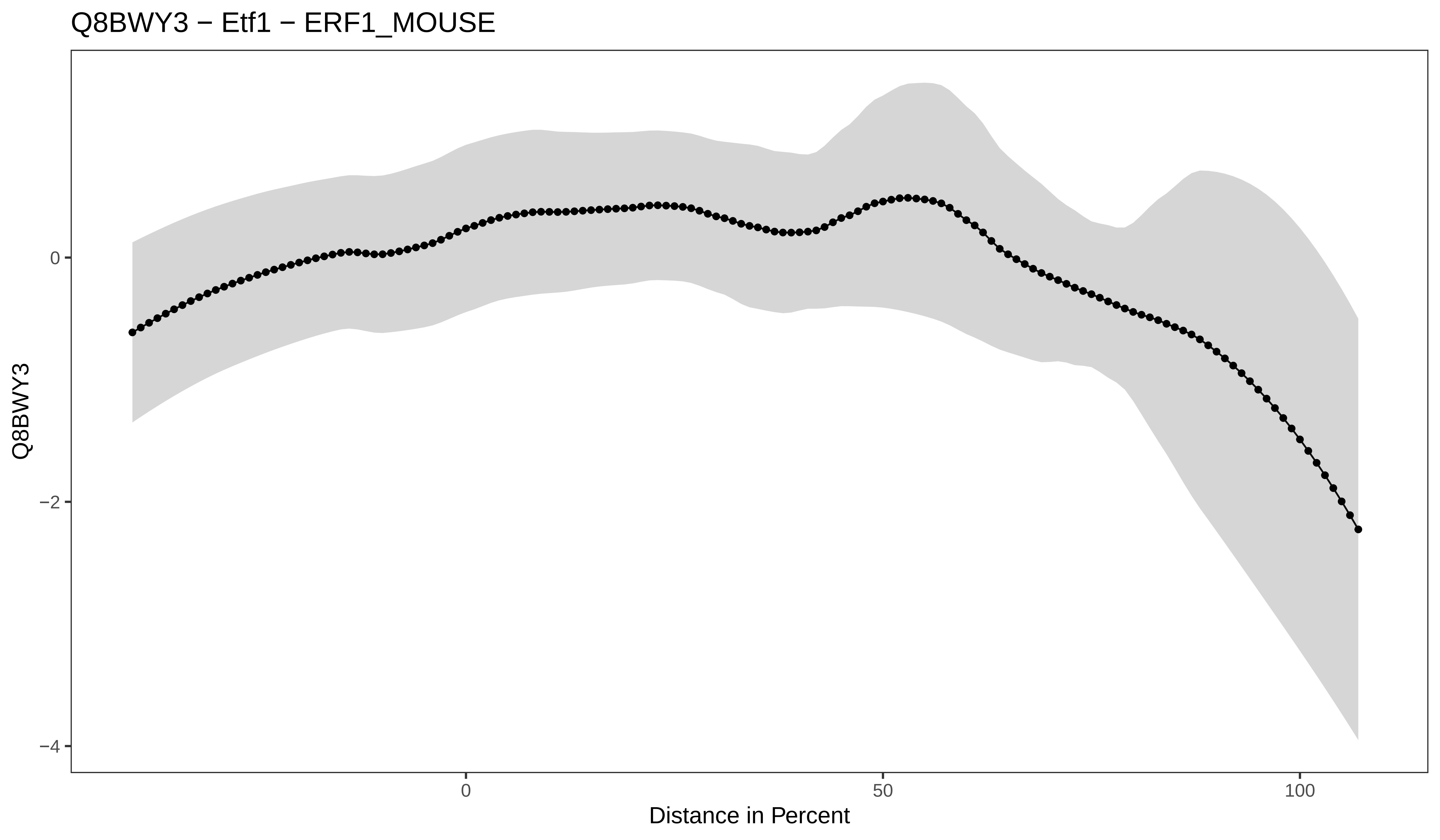
Q8BWY3 − Etf1 − ERF1_MOUSE
Q8BWY3
0
−2
−4
0
50
100
Distance in Percent

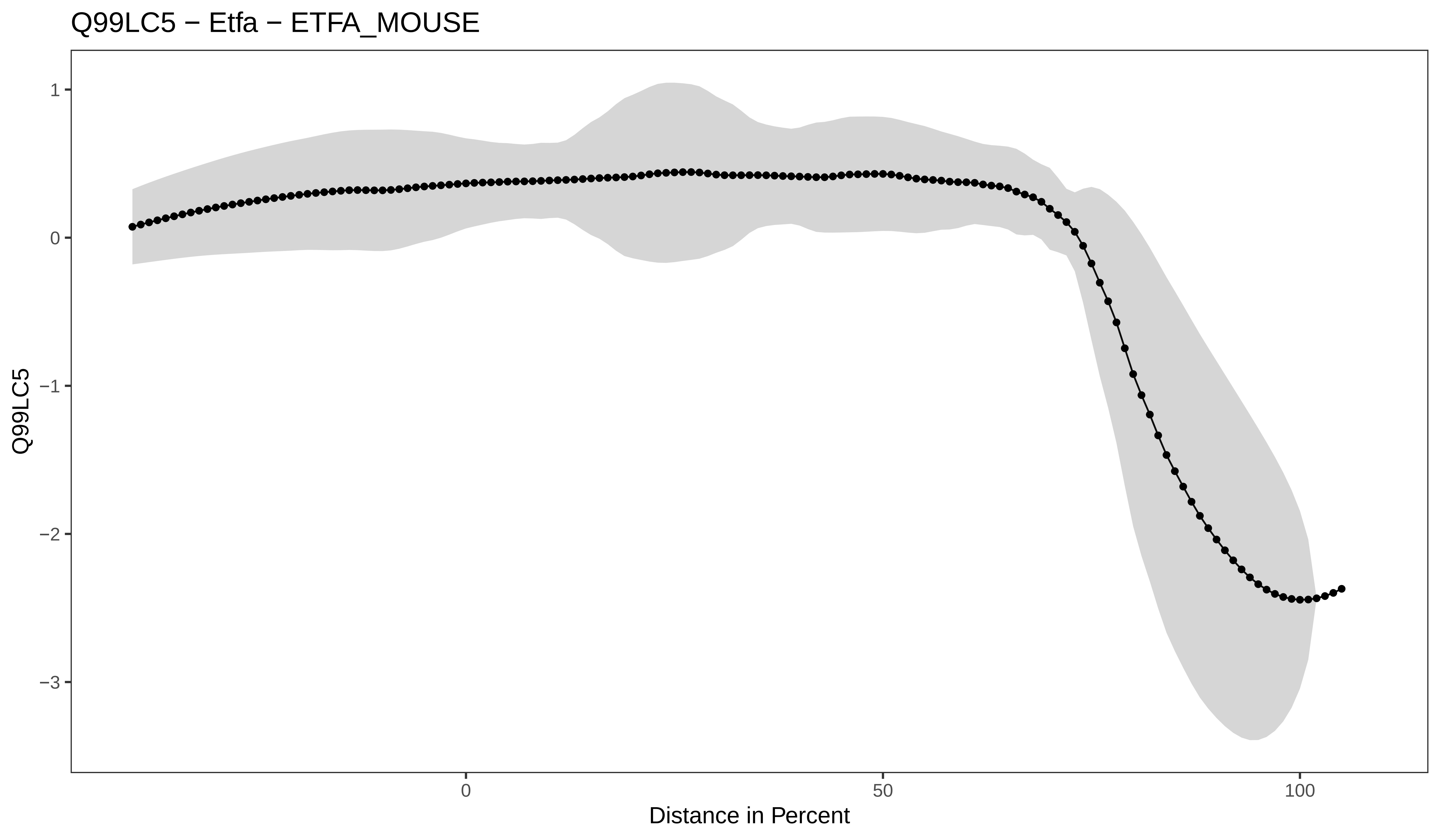

Q99LC5 − Etfa − ETFA_MOUSE
1
0
−1
−2
−3
Q99LC5
0
50
100
Distance in Percent

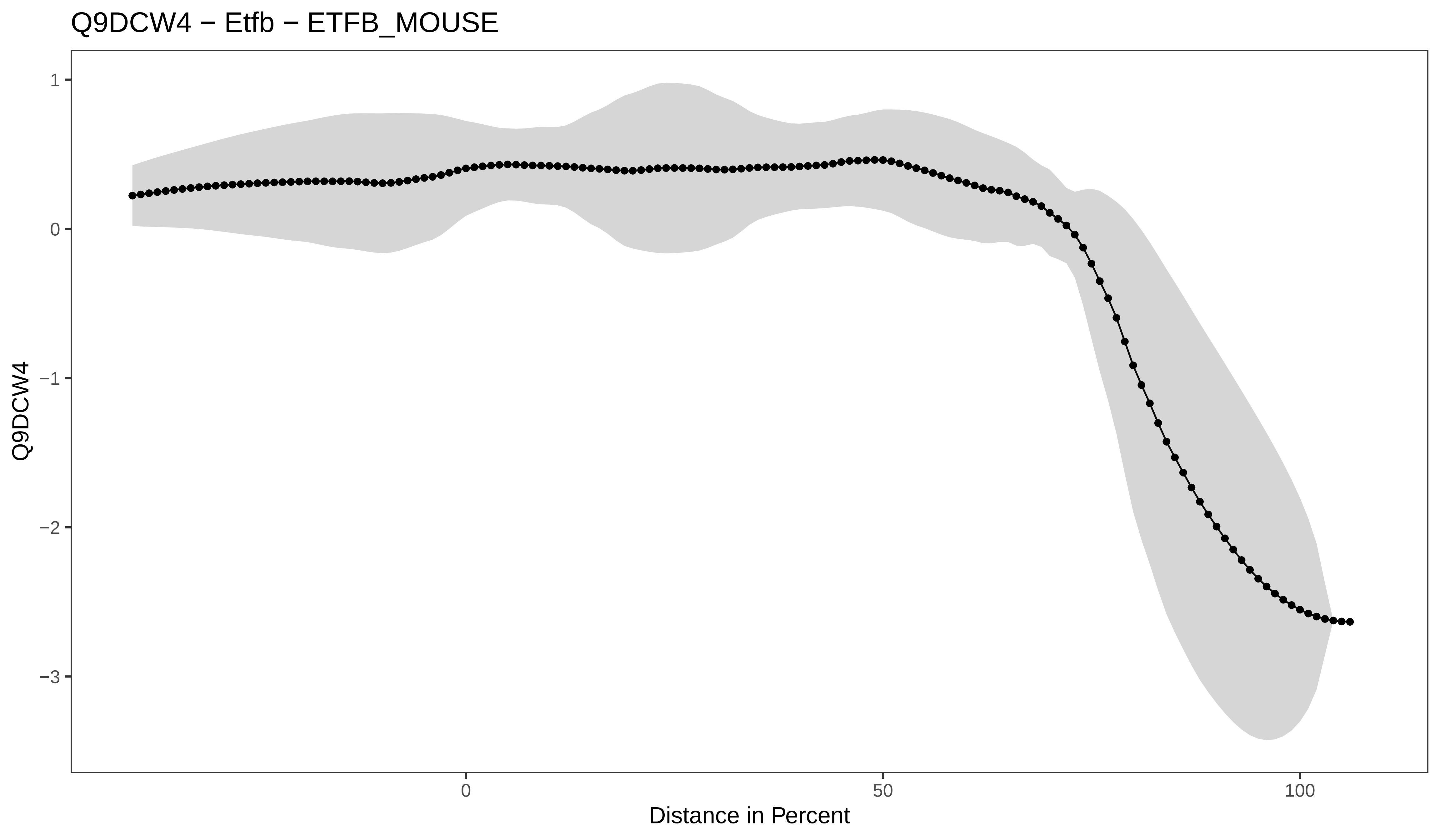

Q9DCW4 − Etfb − ETFB_MOUSE
Q9DCW4
Distance in Percent
1
0
−1
−2
−3
0
50
100

Q921G7 − Etfdh − ETFD_MOUSE

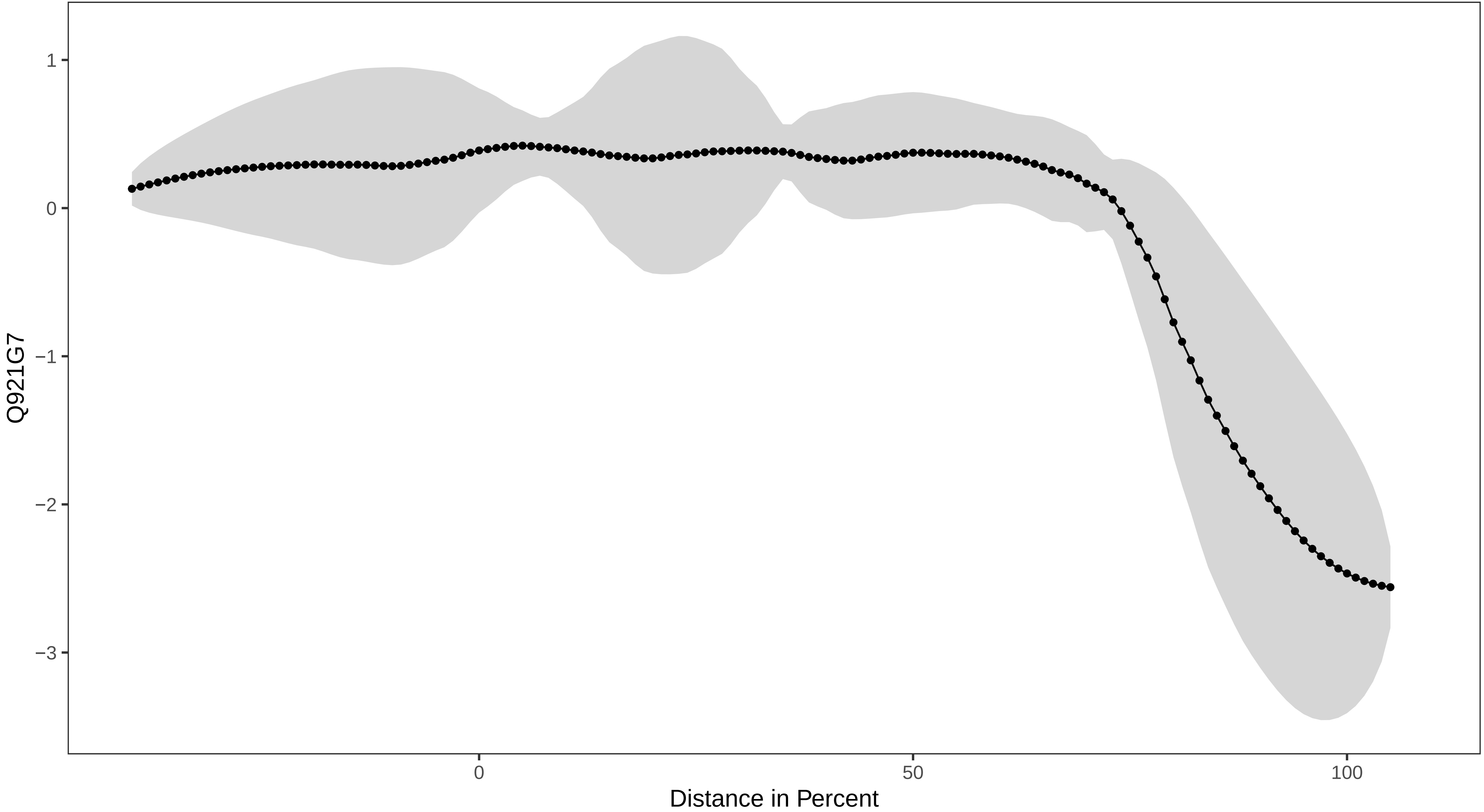

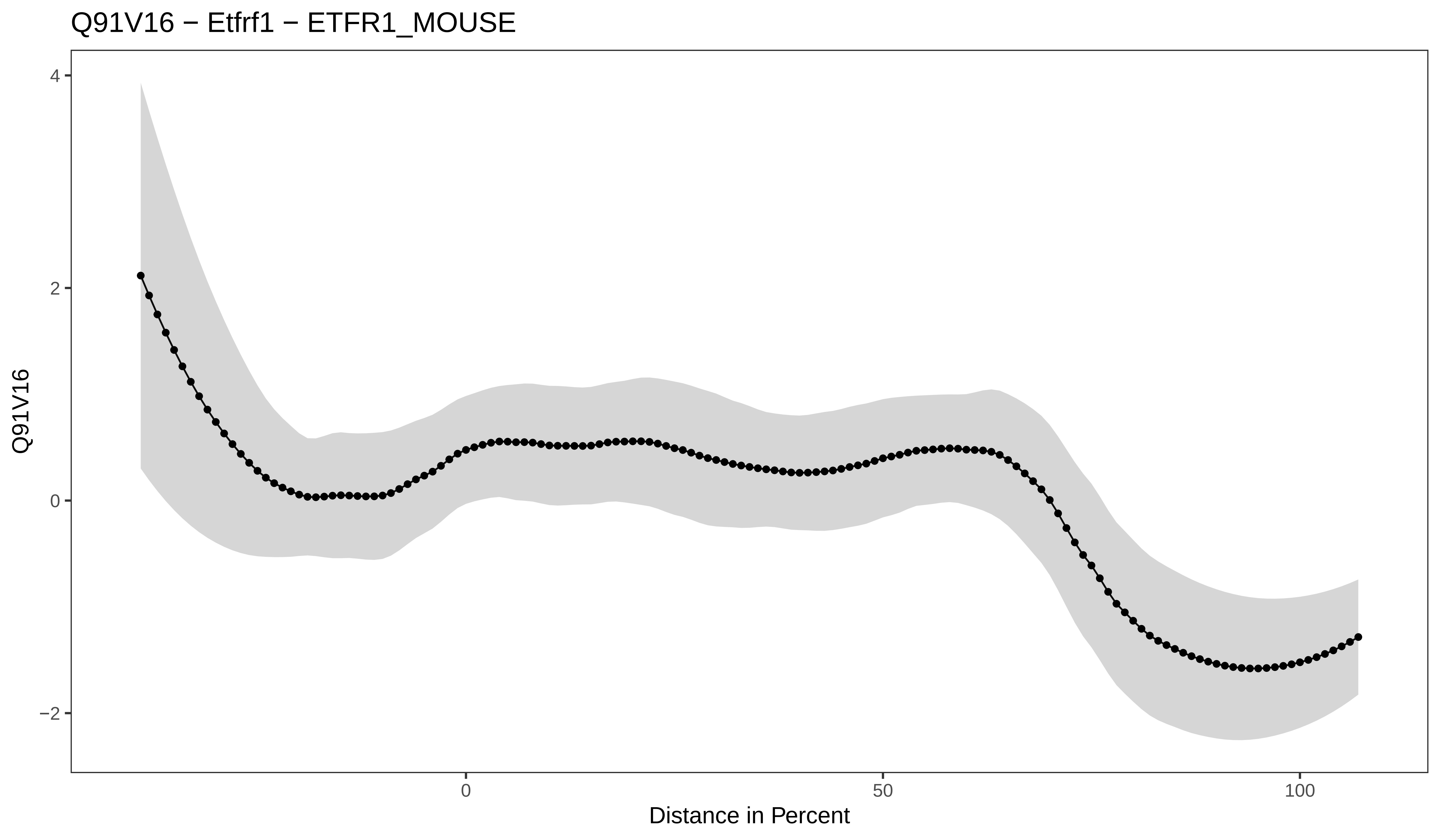

Q91V16 − Etfrf1 − ETFR1_MOUSE
Q91V16
4
2
0
−2
0
50
100
Distance in Percent

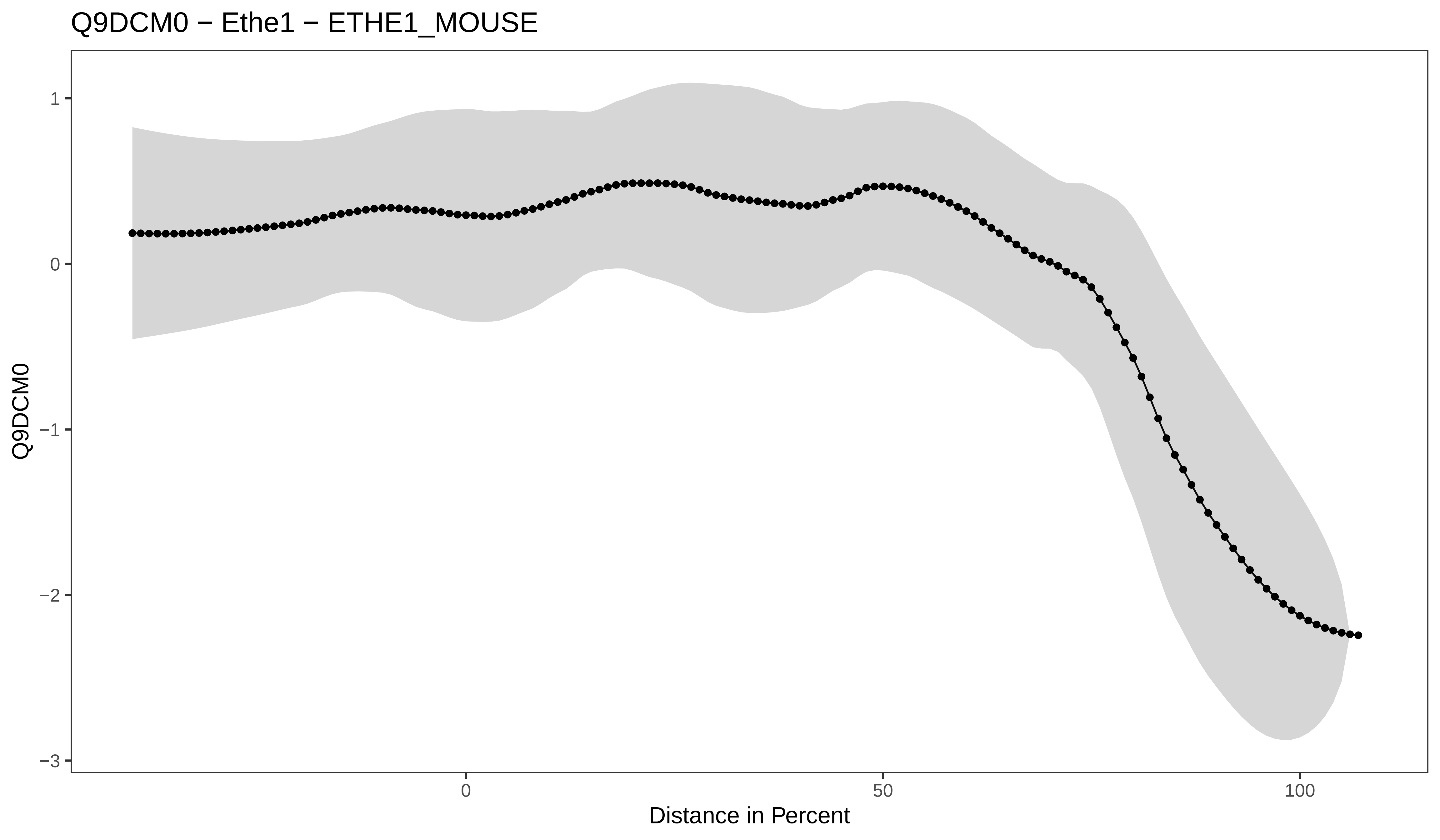

Q9DCM0 − Ethe1 − ETHE1_MOUSE
Q9DCM0
Distance in Percent
1
0
−1
−2
−3
0
50
100

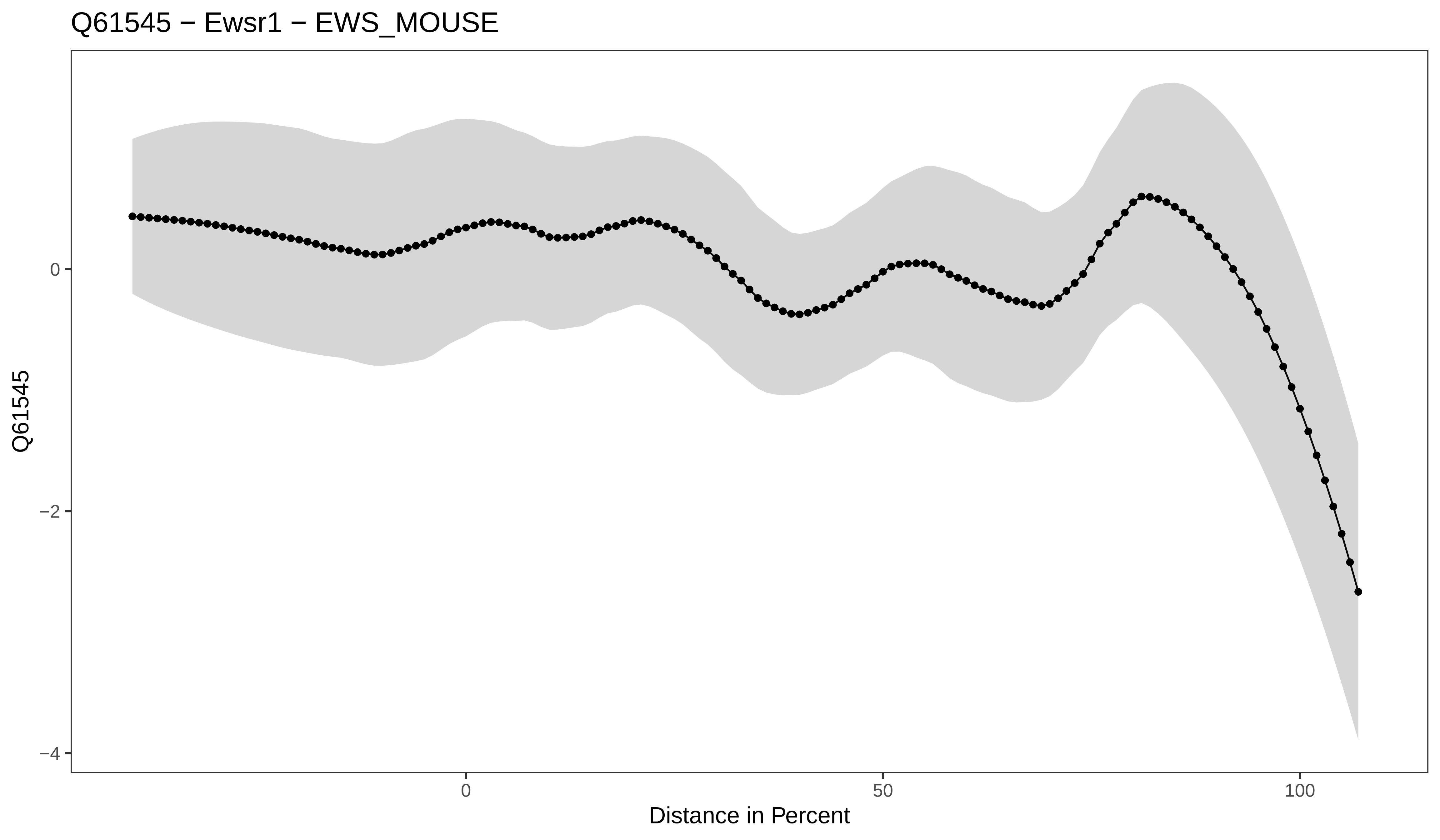

Q61545 − Ewsr1 − EWS_MOUSE
Q61545
0
−2
−4
0
50
100
Distance in Percent

O35382 − Exoc4 − EXOC4_MOUSE

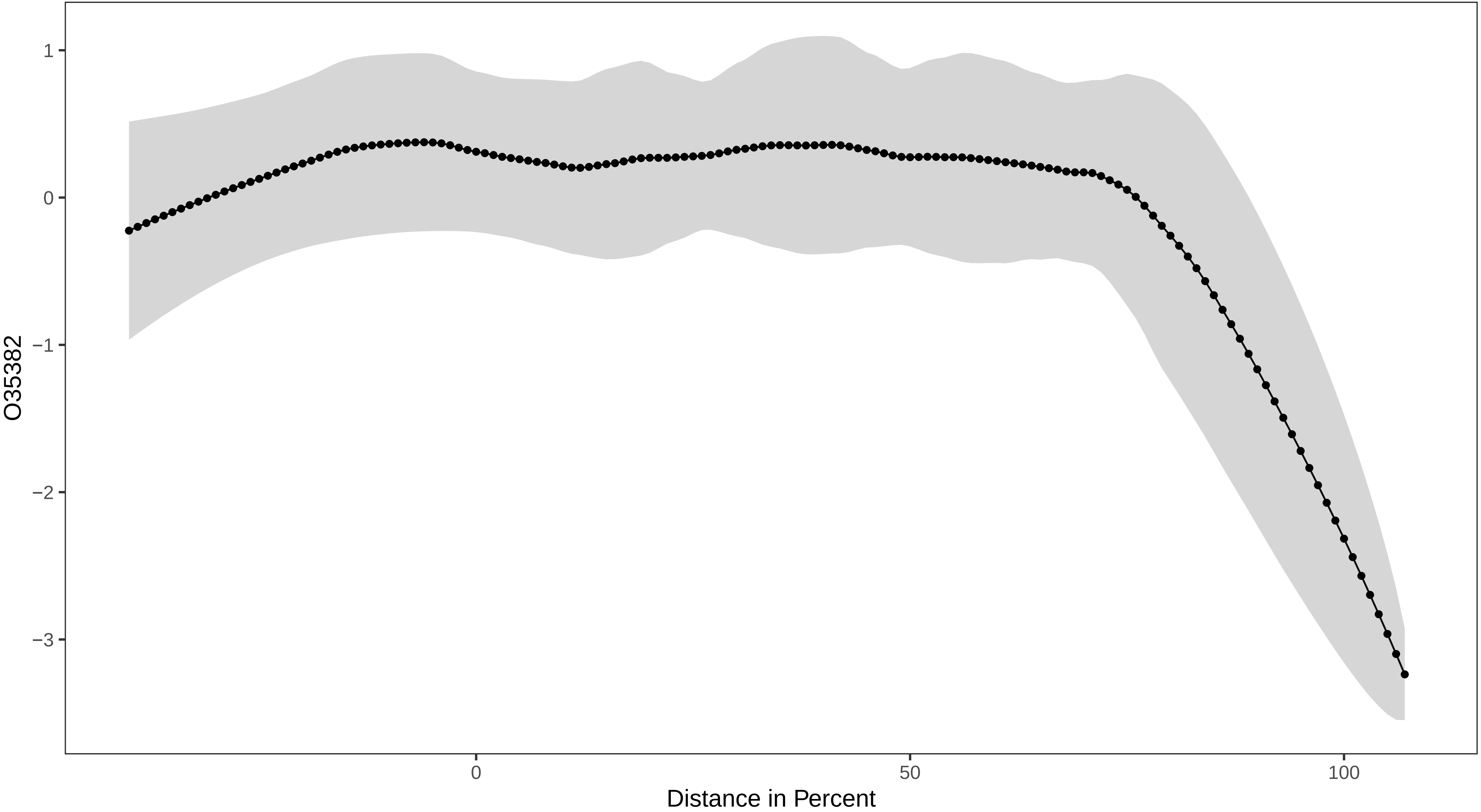

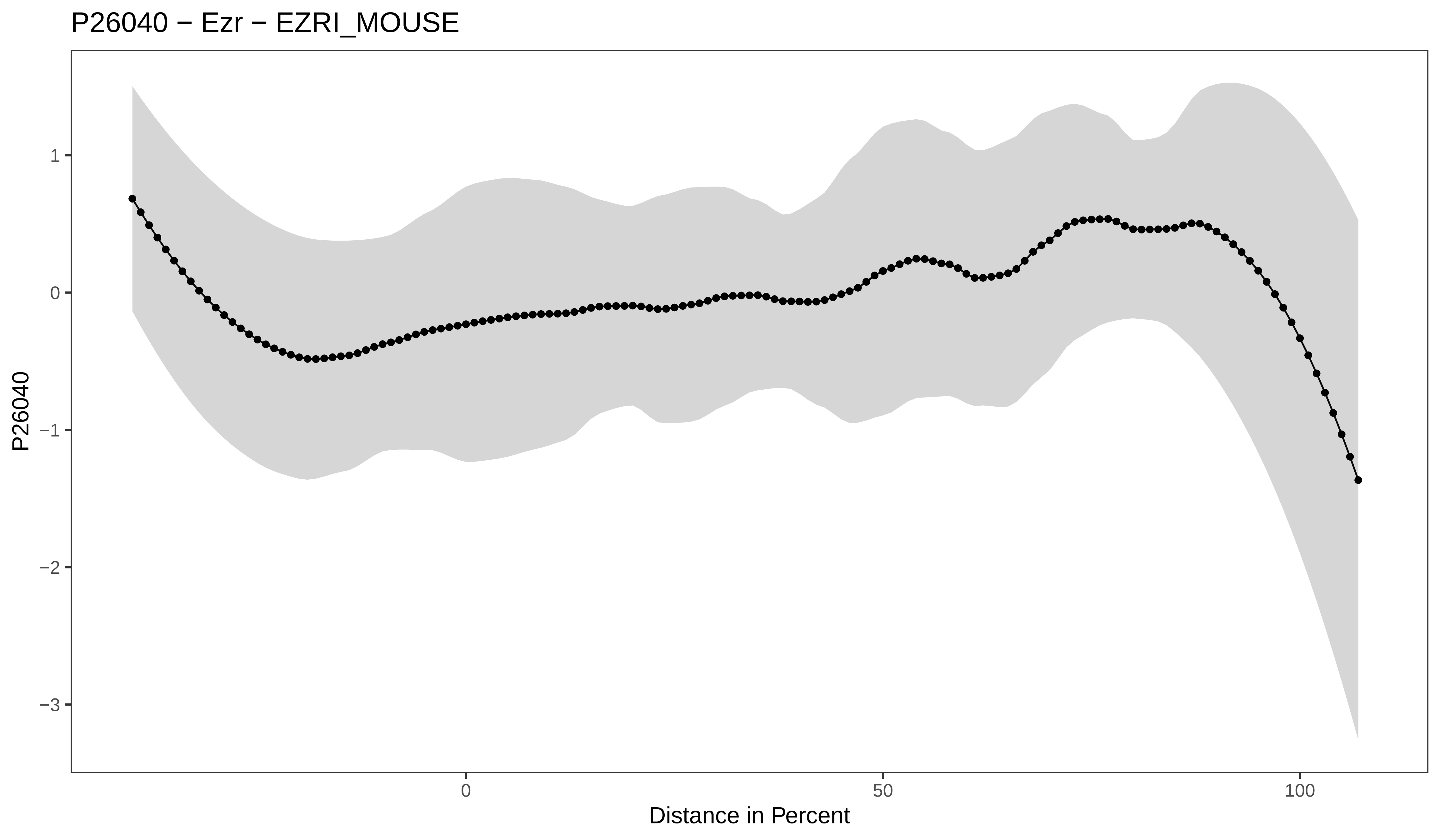
P26040 − Ezr − EZRI_MOUSE
P26040
Distance in Percent
1
0
−1
−2
−3
0
50
100

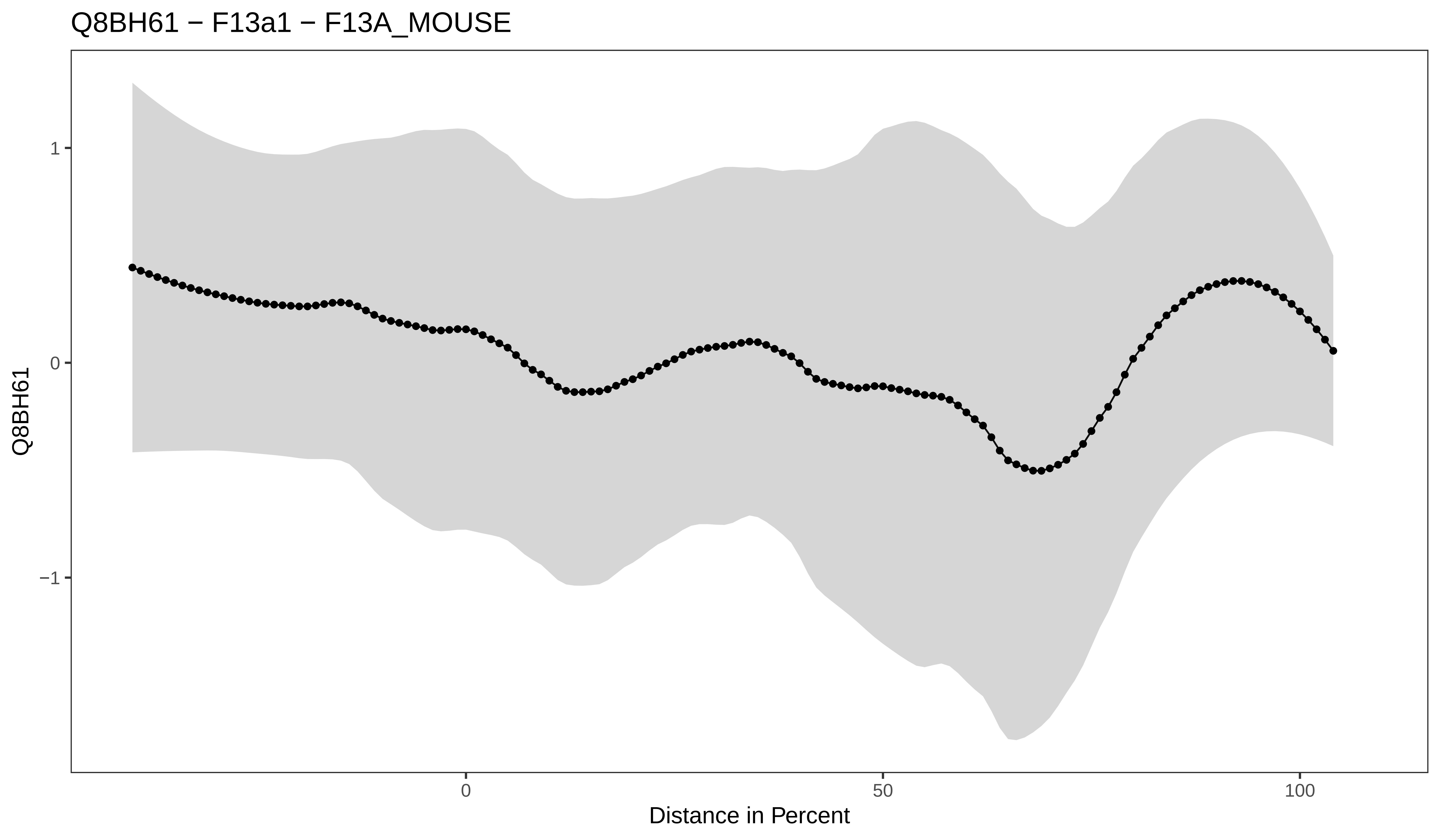

Q8BH61 − F13a1 − F13A_MOUSE
1
0
−1
Q8BH61
0
50
100
Distance in Percent

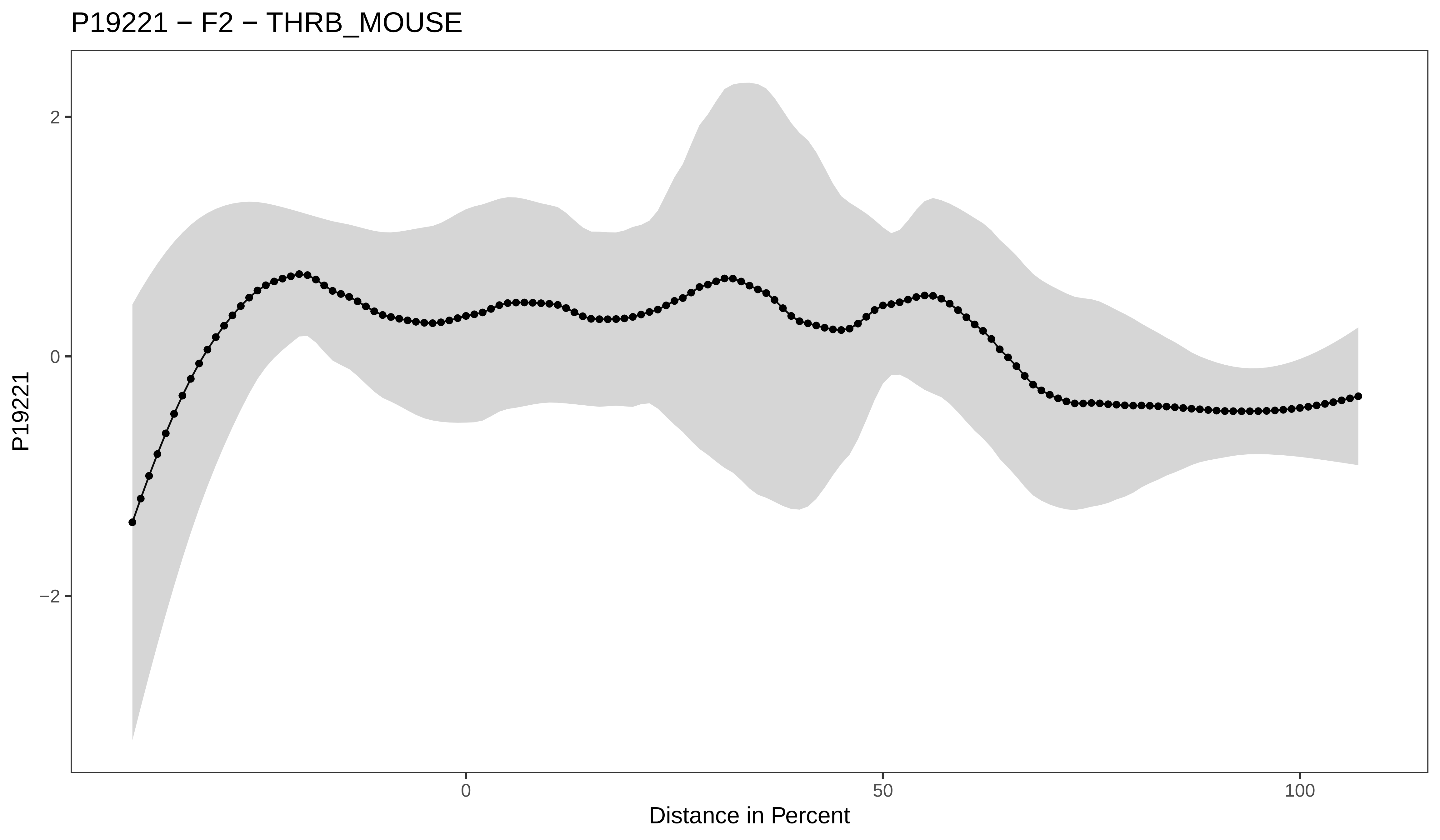
P19221 − F2 − THRB_MOUSE
2
0
−2
P19221
0
50
100
Distance in Percent

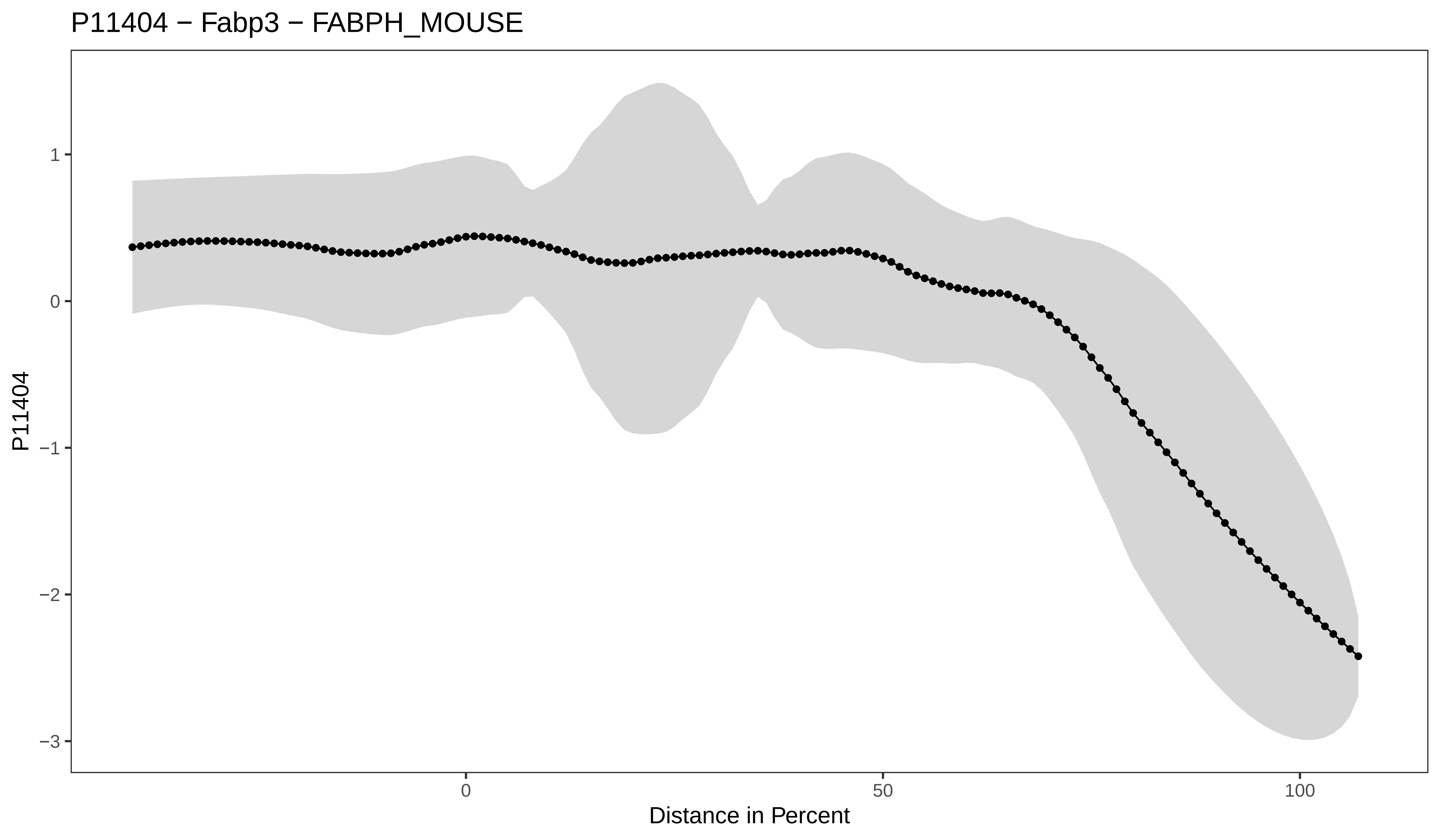

P11404 − Fabp3 − FABPH_MOUSE
1
0
−1
−2
−3
P11404
0
50
100
Distance in Percent

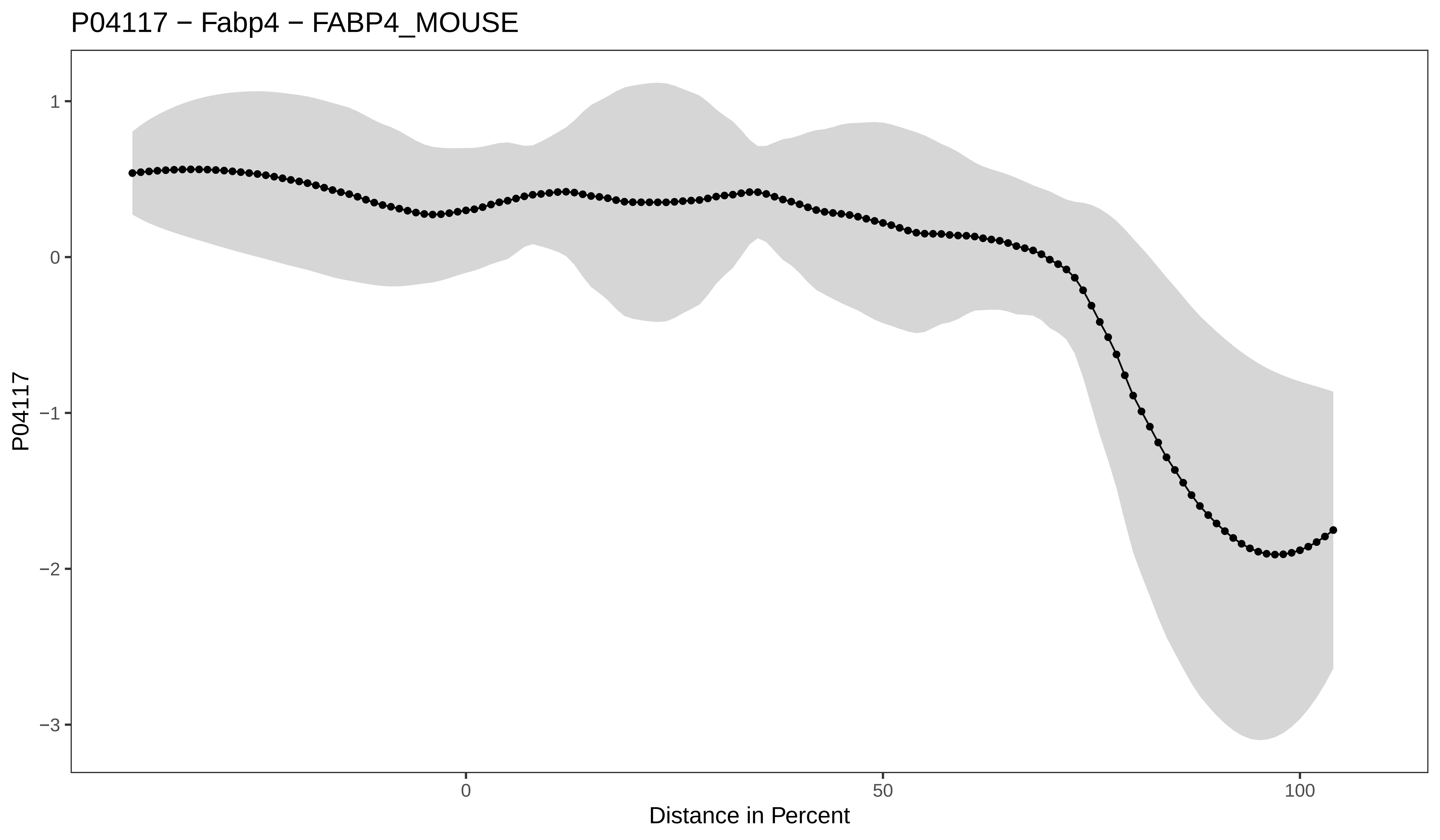
P04117 − Fabp4 − FABP4_MOUSE
P04117
1
0
−1
−2
−3
0
50
100
Distance in Percent

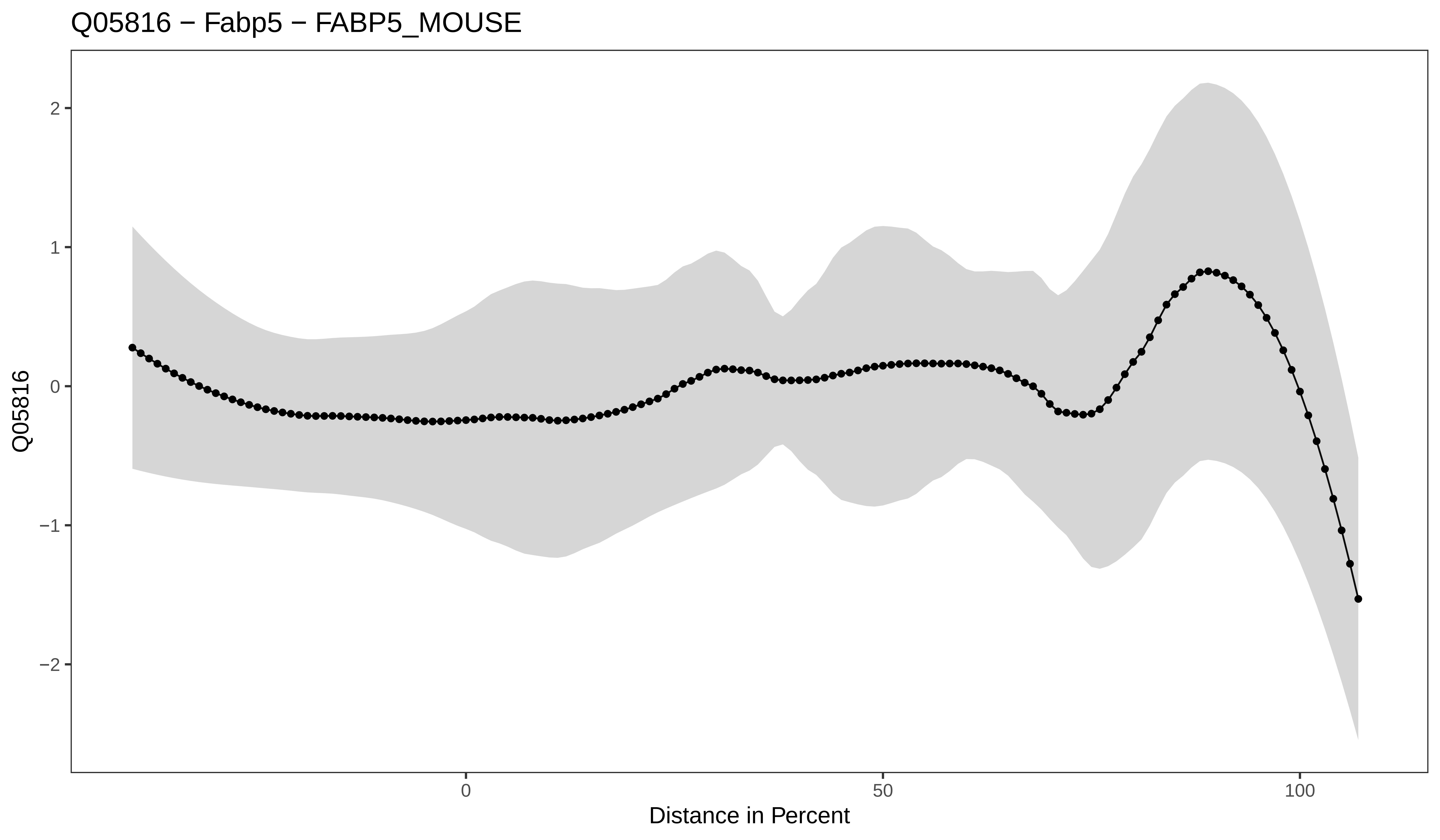
Q05816 − Fabp5 − FABP5_MOUSE
Q05816
2
1
0
−1
−2
0
50
100
Distance in Percent

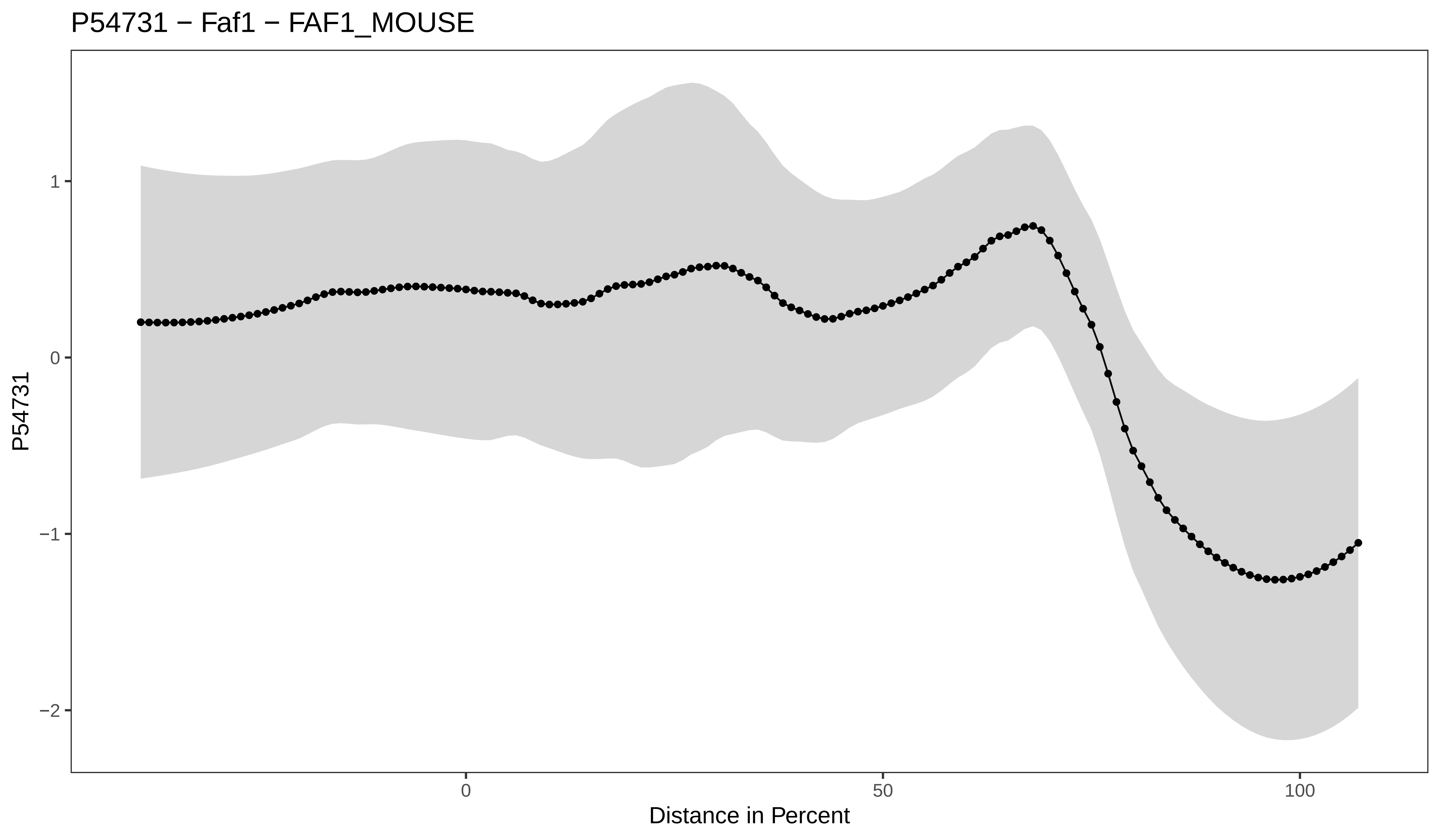

P54731 − Faf1 − FAF1_MOUSE
1
0
−1
−2
P54731
0
50
100
Distance in Percent

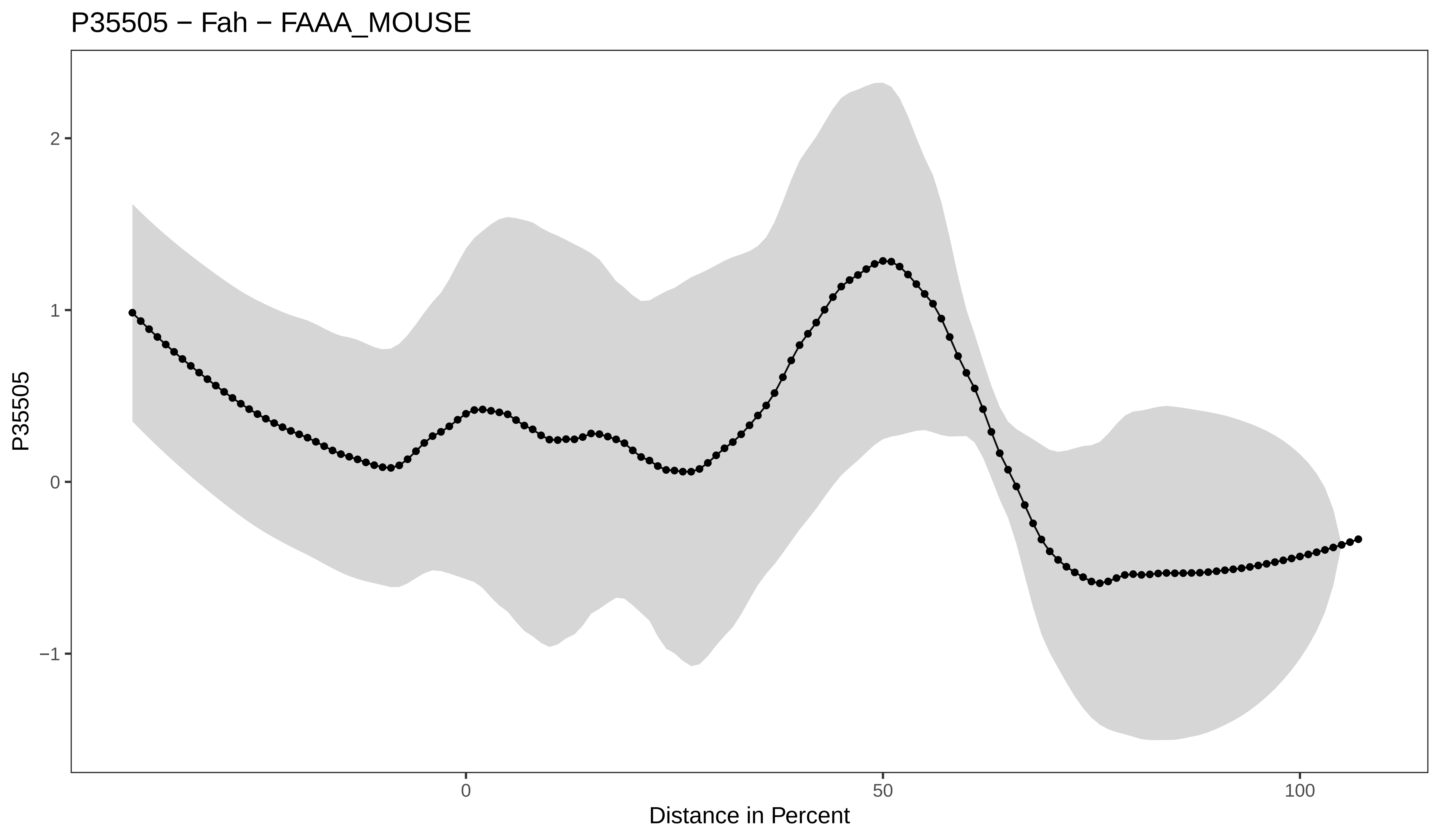
P35505 − Fah − FAAA_MOUSE
P35505
2
1
0
−1
0
50
100
Distance in Percent

Q8R0F8 − Fahd1 − FAHD1_MOUSE

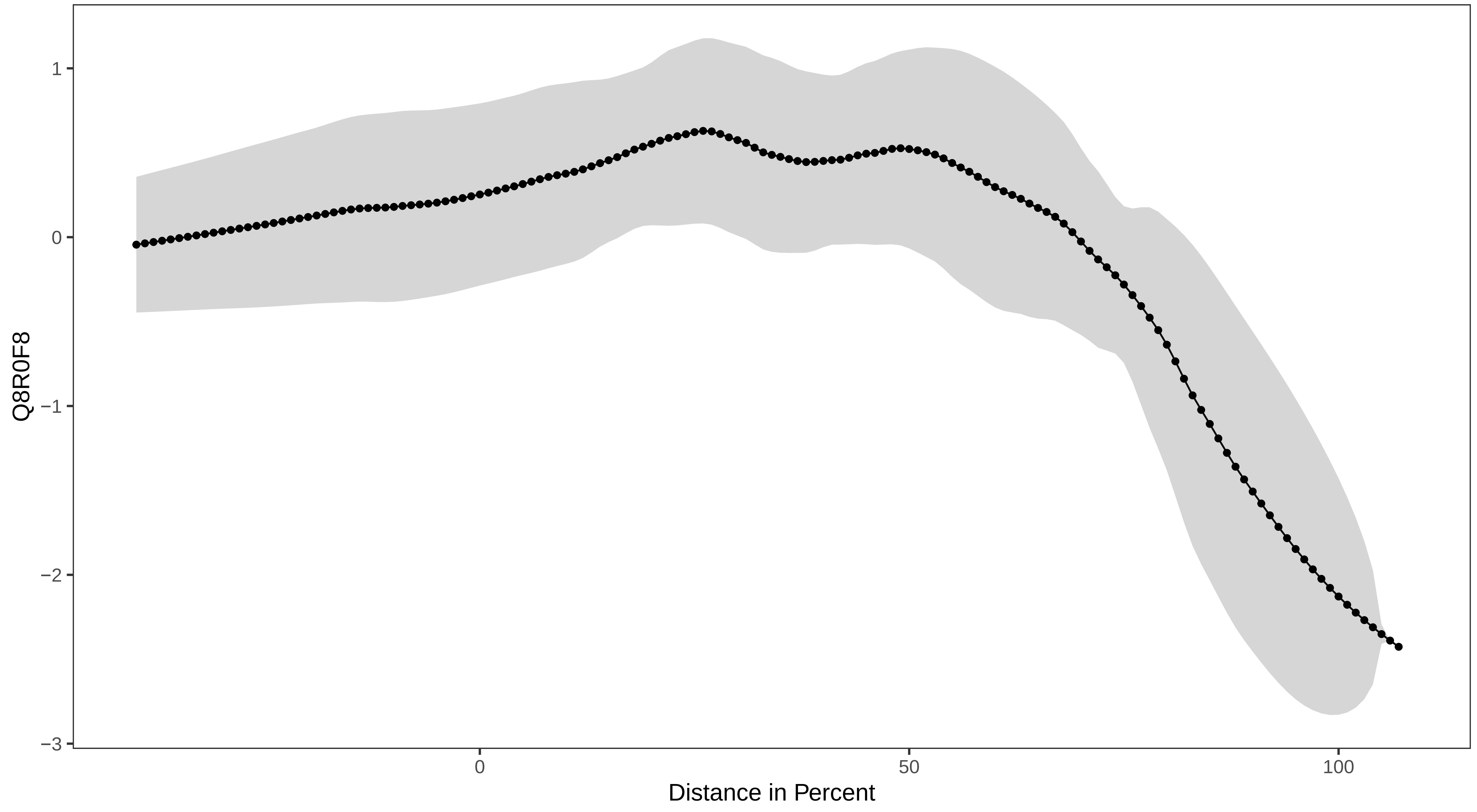

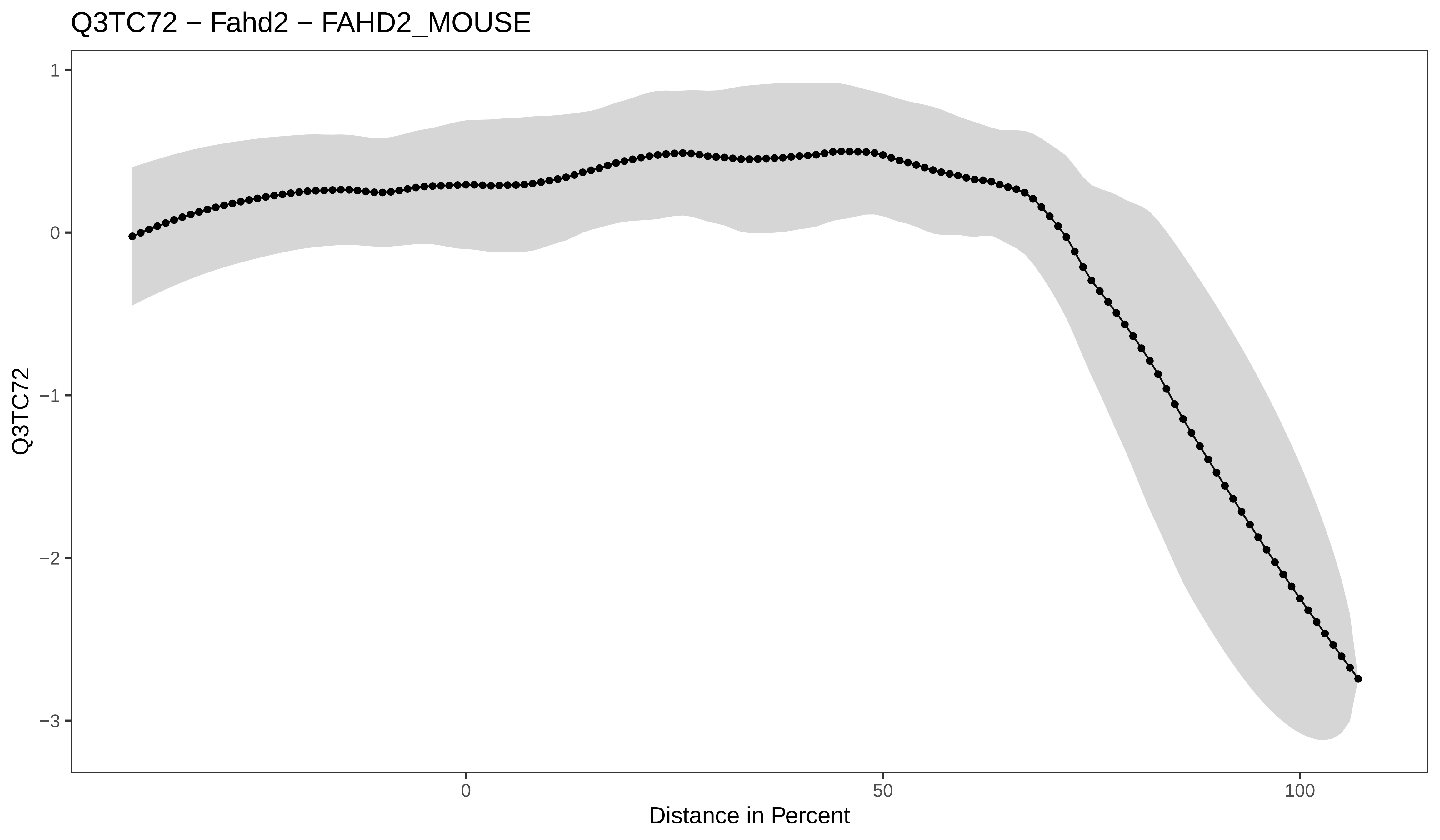

Q3TC72 − Fahd2 − FAHD2_MOUSE
1
0
−1
−2
−3
Q3TC72
0
50
100
Distance in Percent

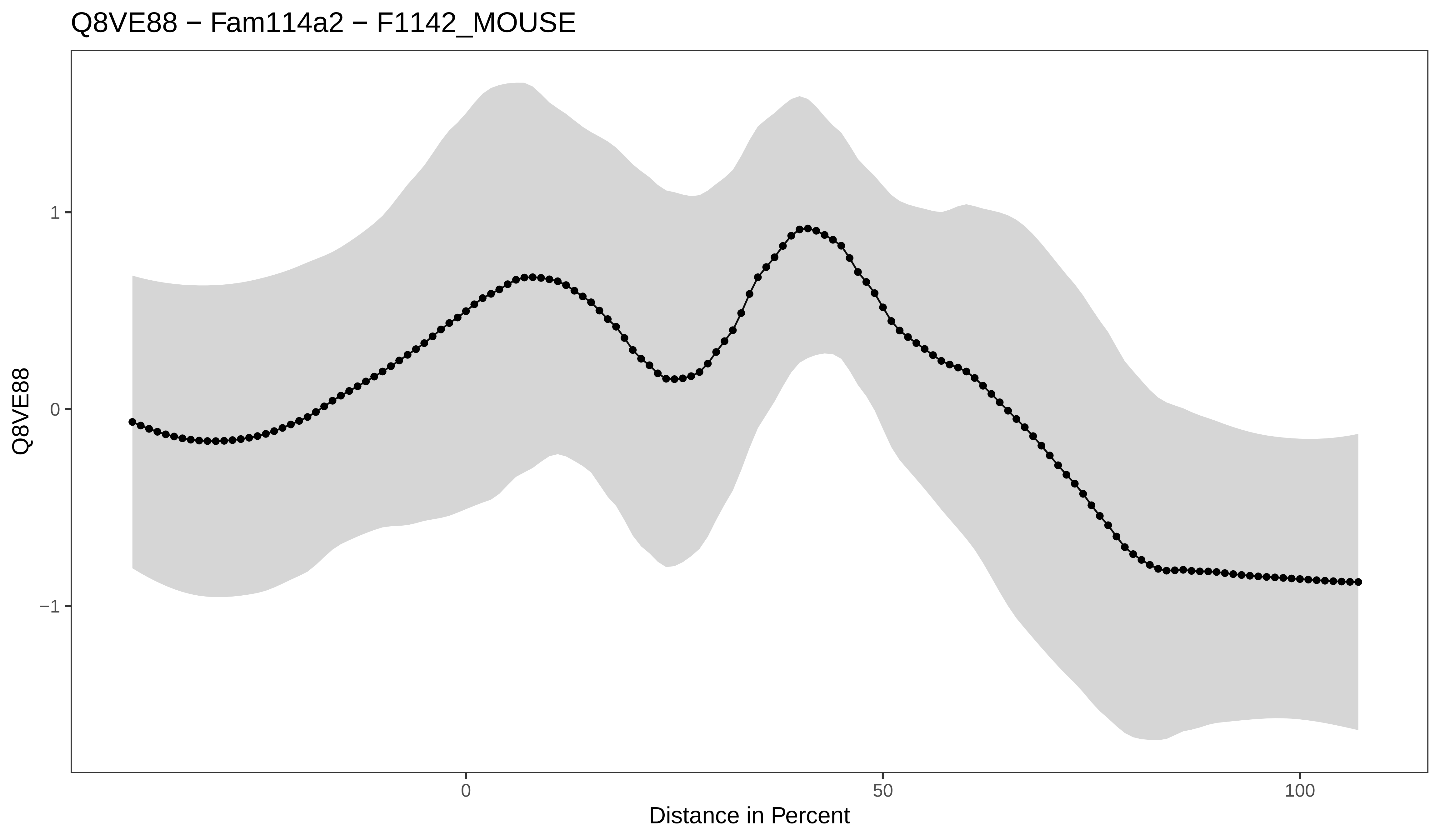

Q8VE88 − Fam114a2 − F1142_MOUSE
Q8VE88
1
0
−1
0
50
100
Distance in Percent

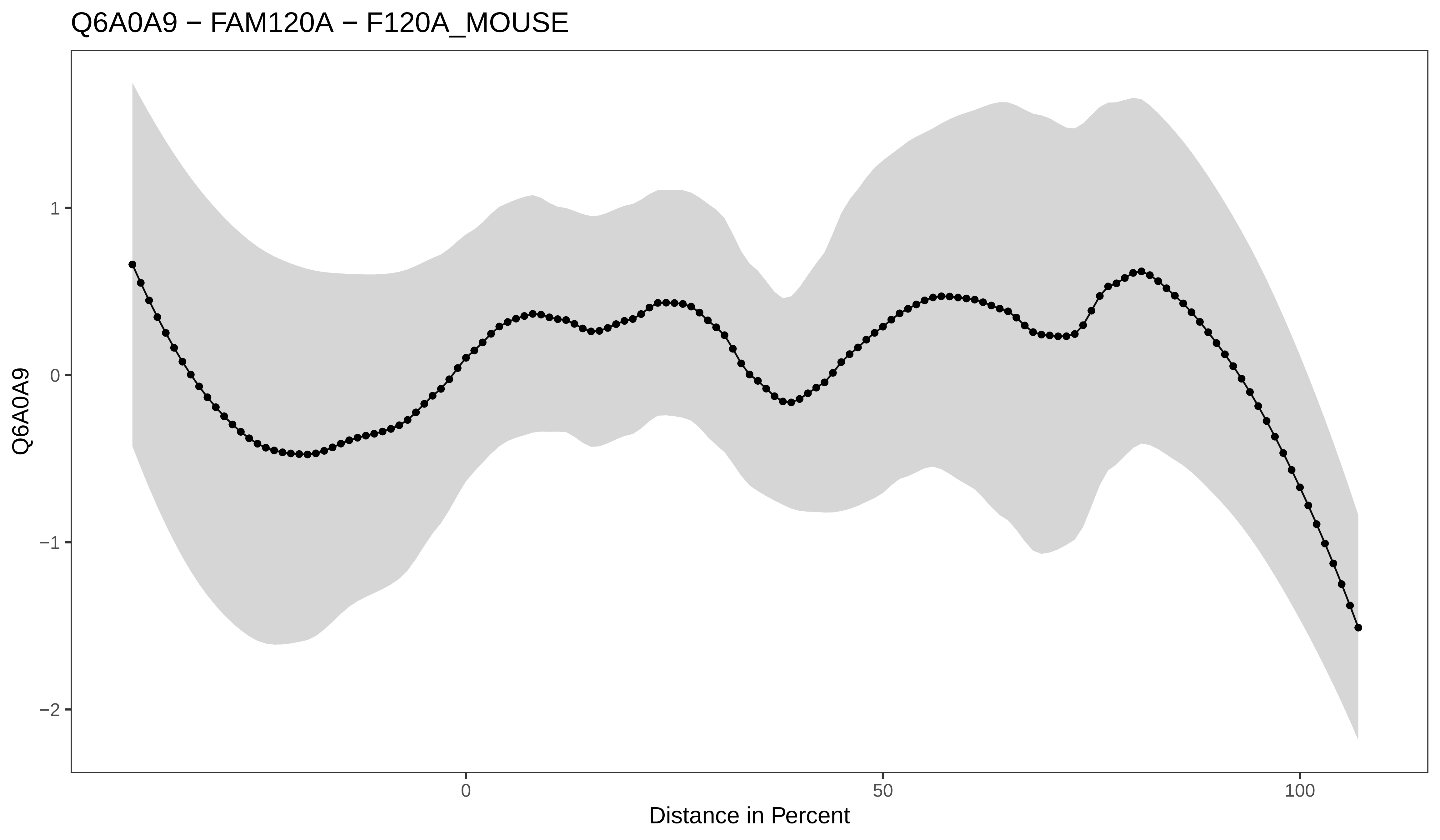

Q6A0A9 − FAM120A − F120A_MOUSE
Q6A0A9
1
0
−1
−2
0
50
100
Distance in Percent

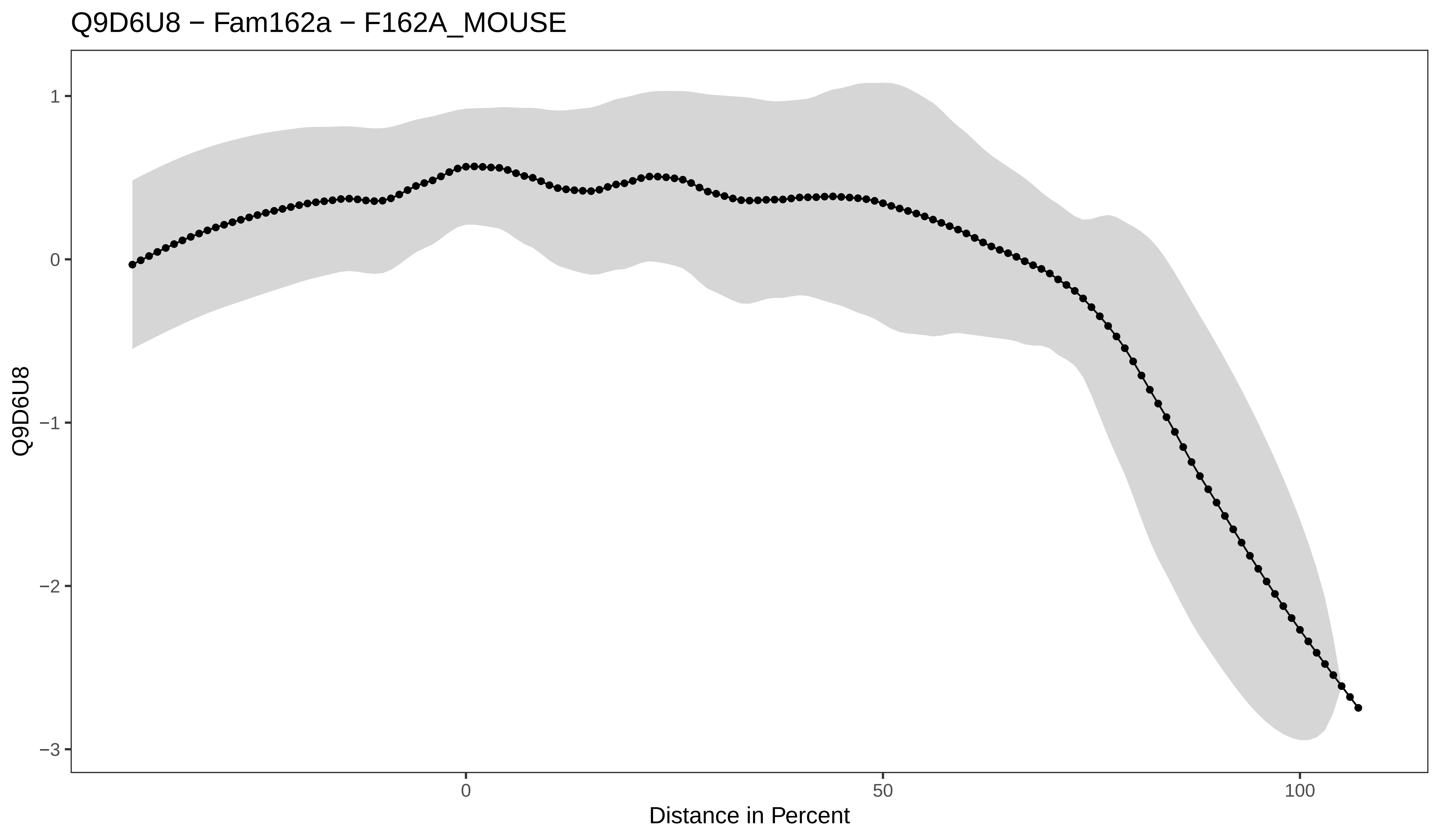
Q9D6U8 − Fam162a − F162A_MOUSE
1
0
−1
−2
−3
Q9D6U8
0
50
100
Distance in Percent

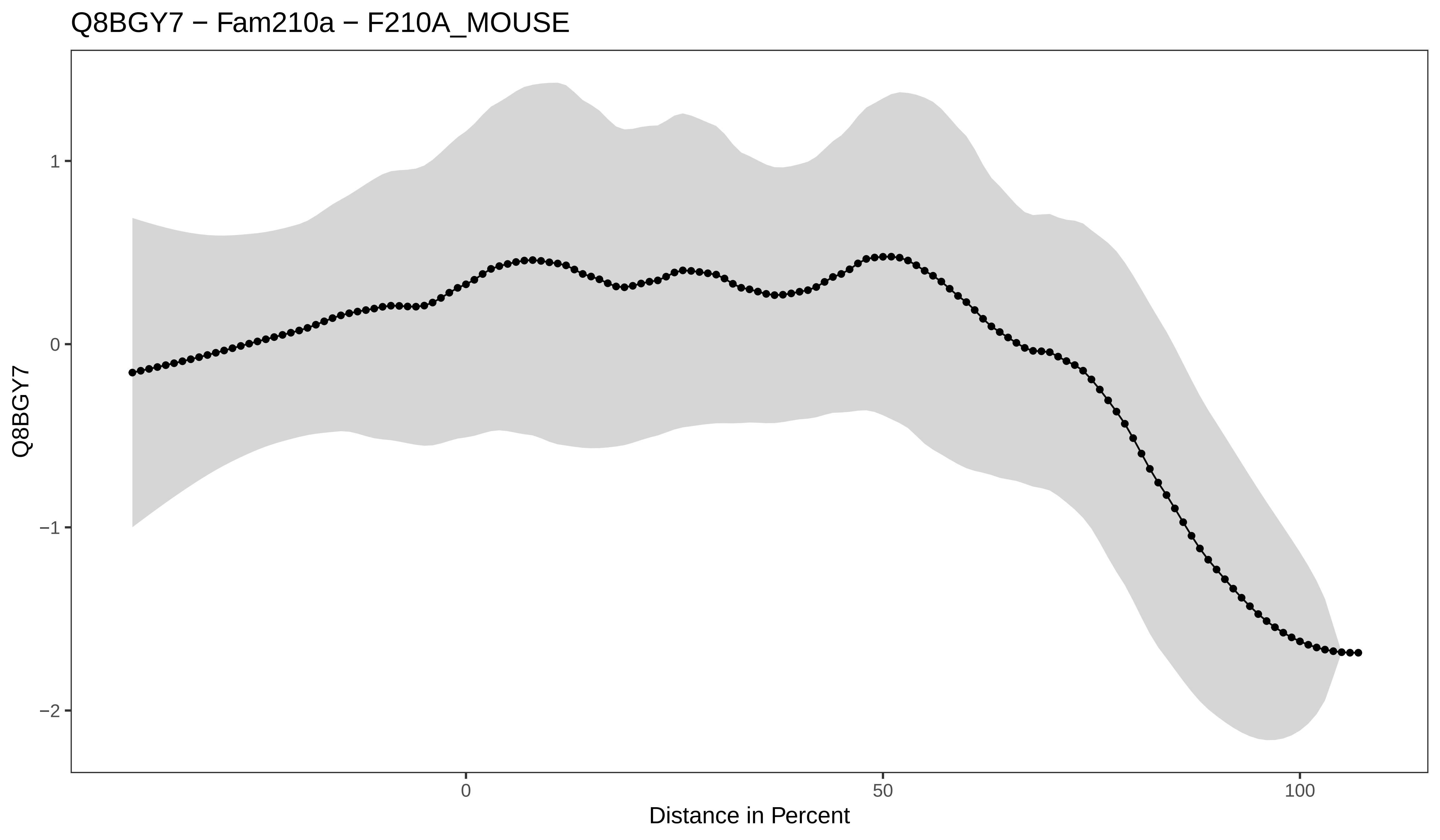
Q8BGY7 − Fam210a − F210A_MOUSE
Q8BGY7
1
0
−1
−2
0
50
100
Distance in Percent

Q8C0Z1 − Fam234a − F234A_MOUSE

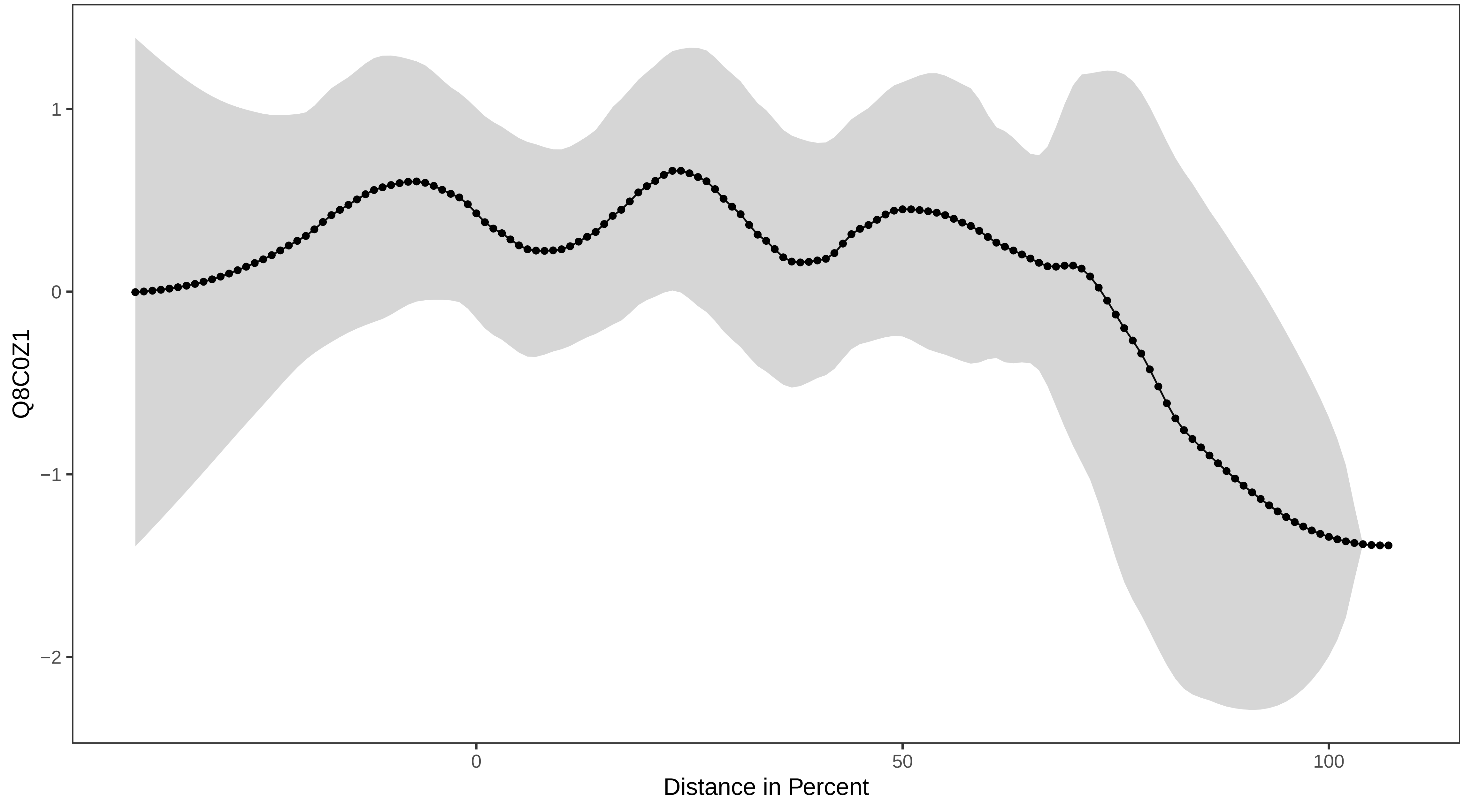

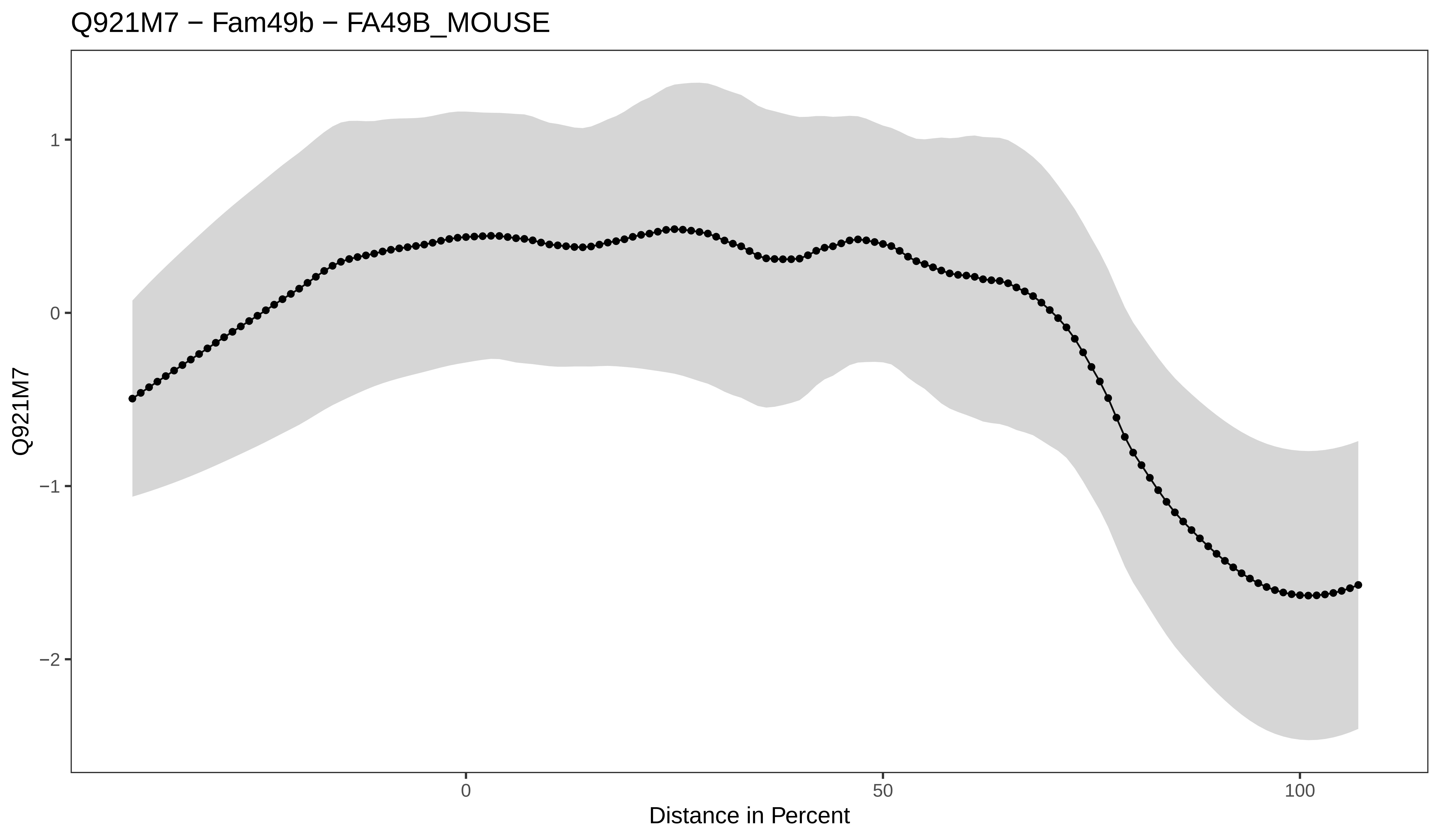

Q921M7 − Fam49b − FA49B_MOUSE
1
0
−1
−2
Q921M7
0
50
100
Distance in Percent

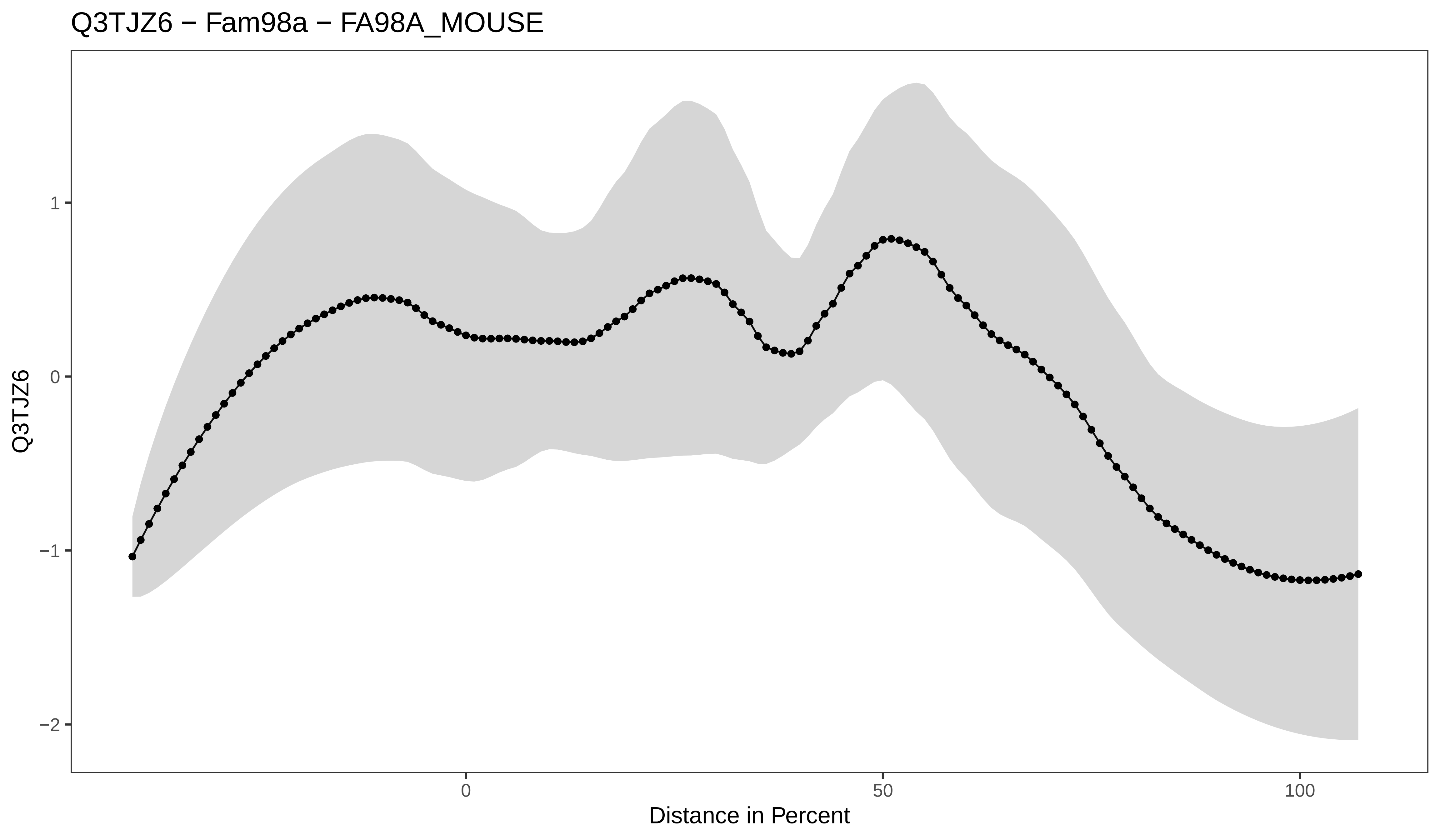
Q3TJZ6 − Fam98a − FA98A_MOUSE
1
0
−1
−2
Q3TJZ6
0
50
100
Distance in Percent

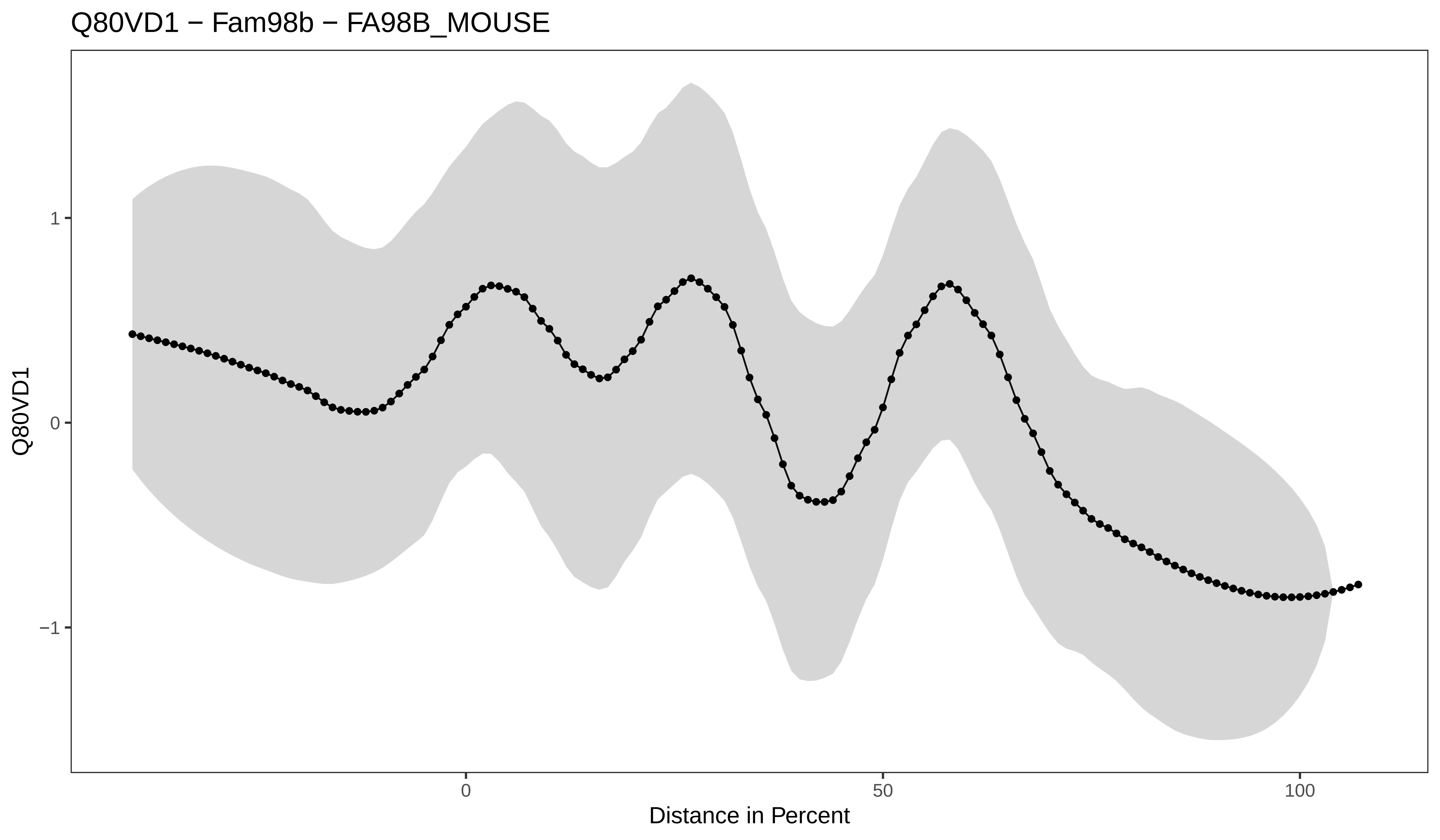

Q80VD1 − Fam98b − FA98B_MOUSE
Q80VD1
1
0
−1
0
50
100
Distance in Percent

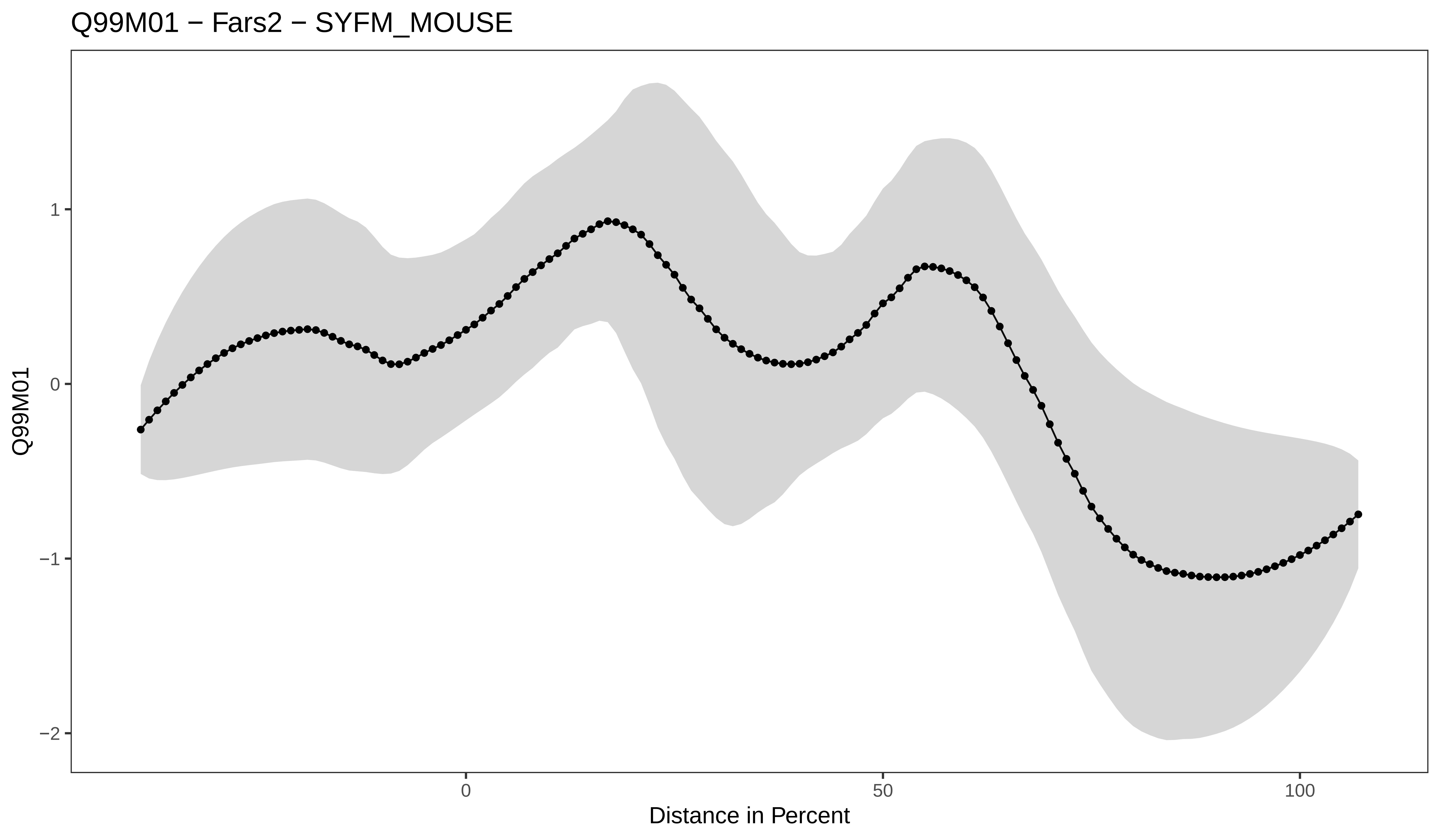
Q99M01 − Fars2 − SYFM_MOUSE
Q99M01
1
0
−1
−2
0
50
100
Distance in Percent

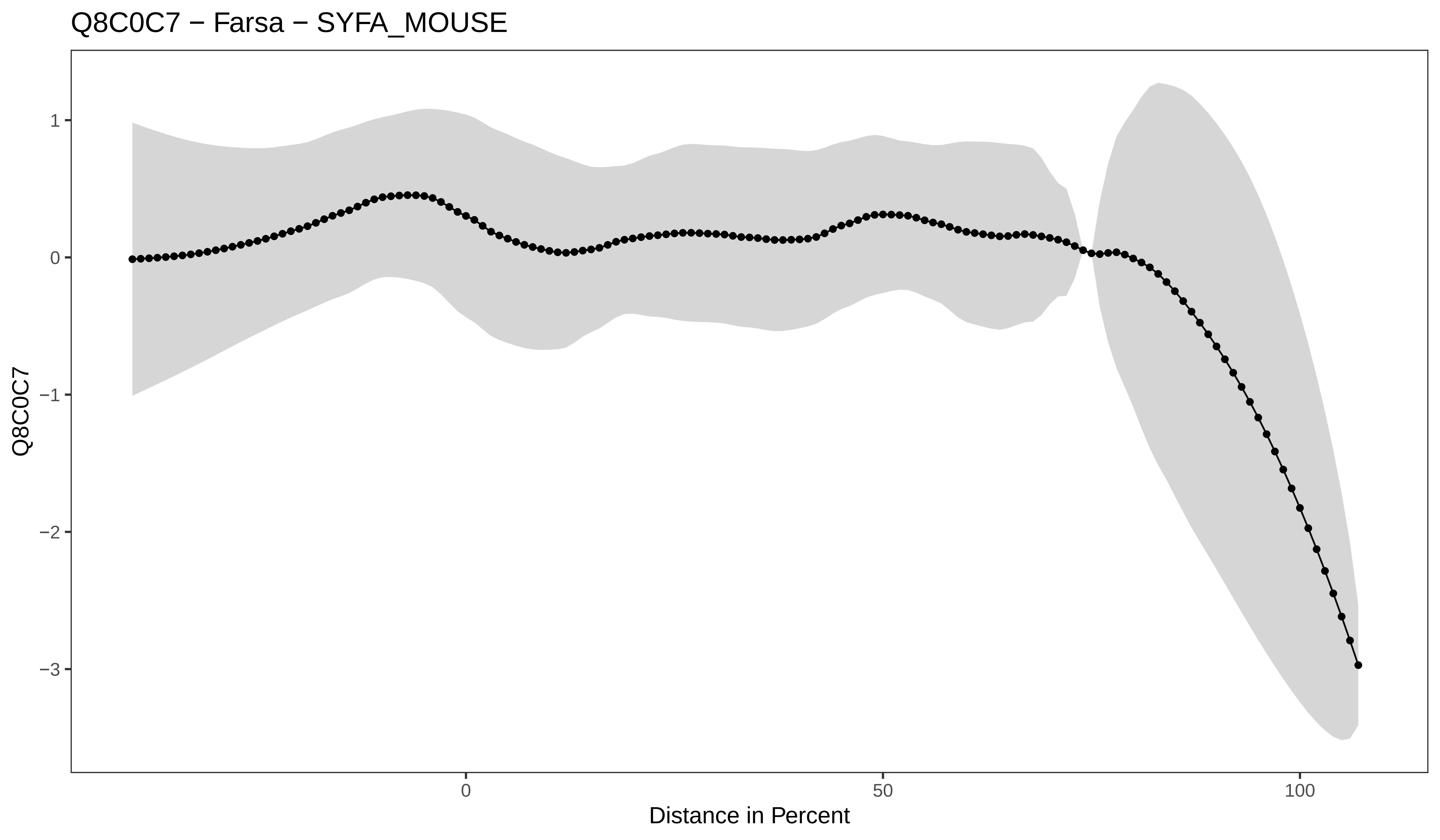
Q8C0C7 − Farsa − SYFA_MOUSE
Q8C0C7
1
0
−1
−2
−3
0
50
100
Distance in Percent

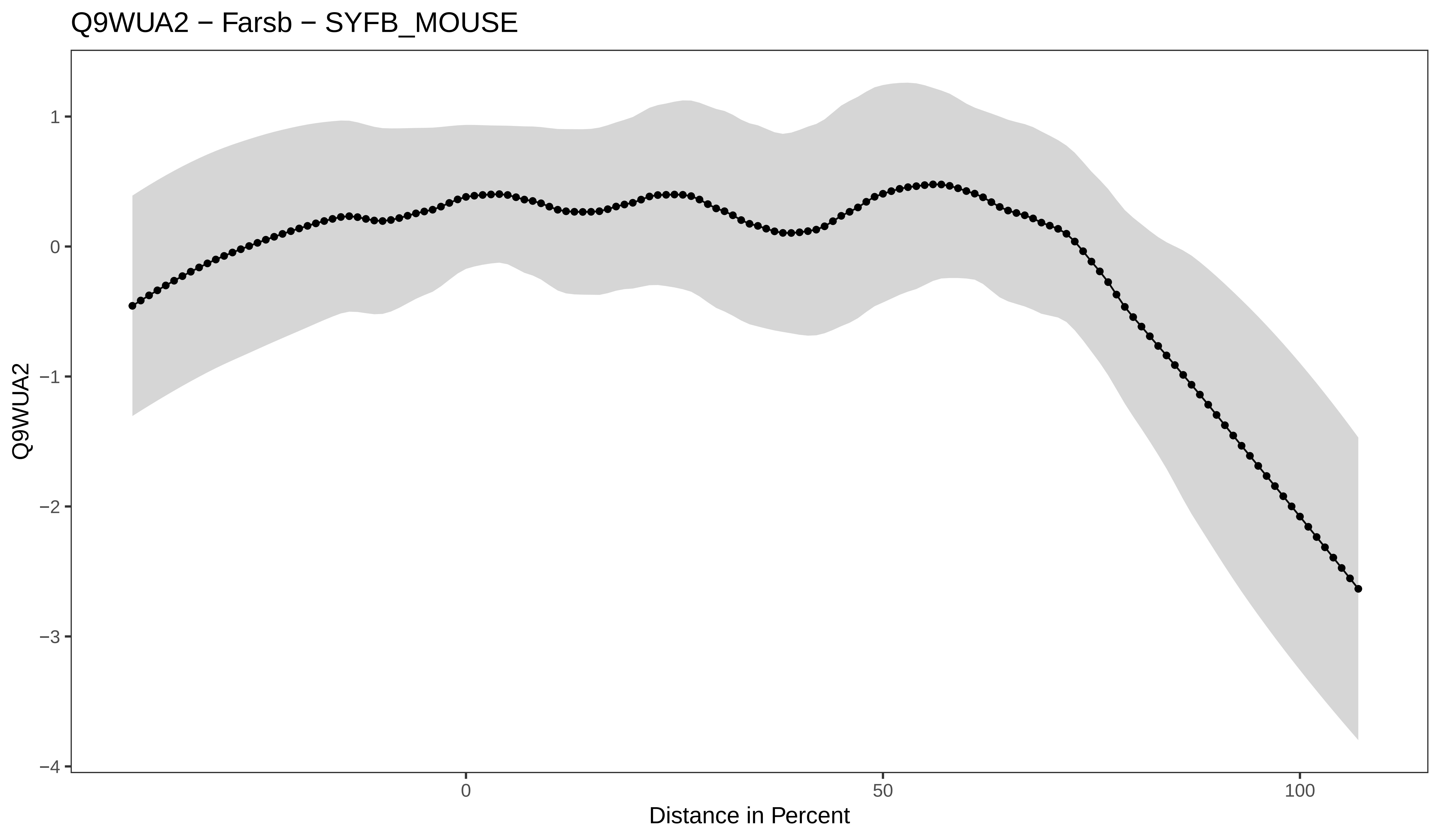

Q9WUA2 − Farsb − SYFB_MOUSE
Q9WUA2
Distance in Percent
1
0
−1
−2
−3
−4
0
50
100

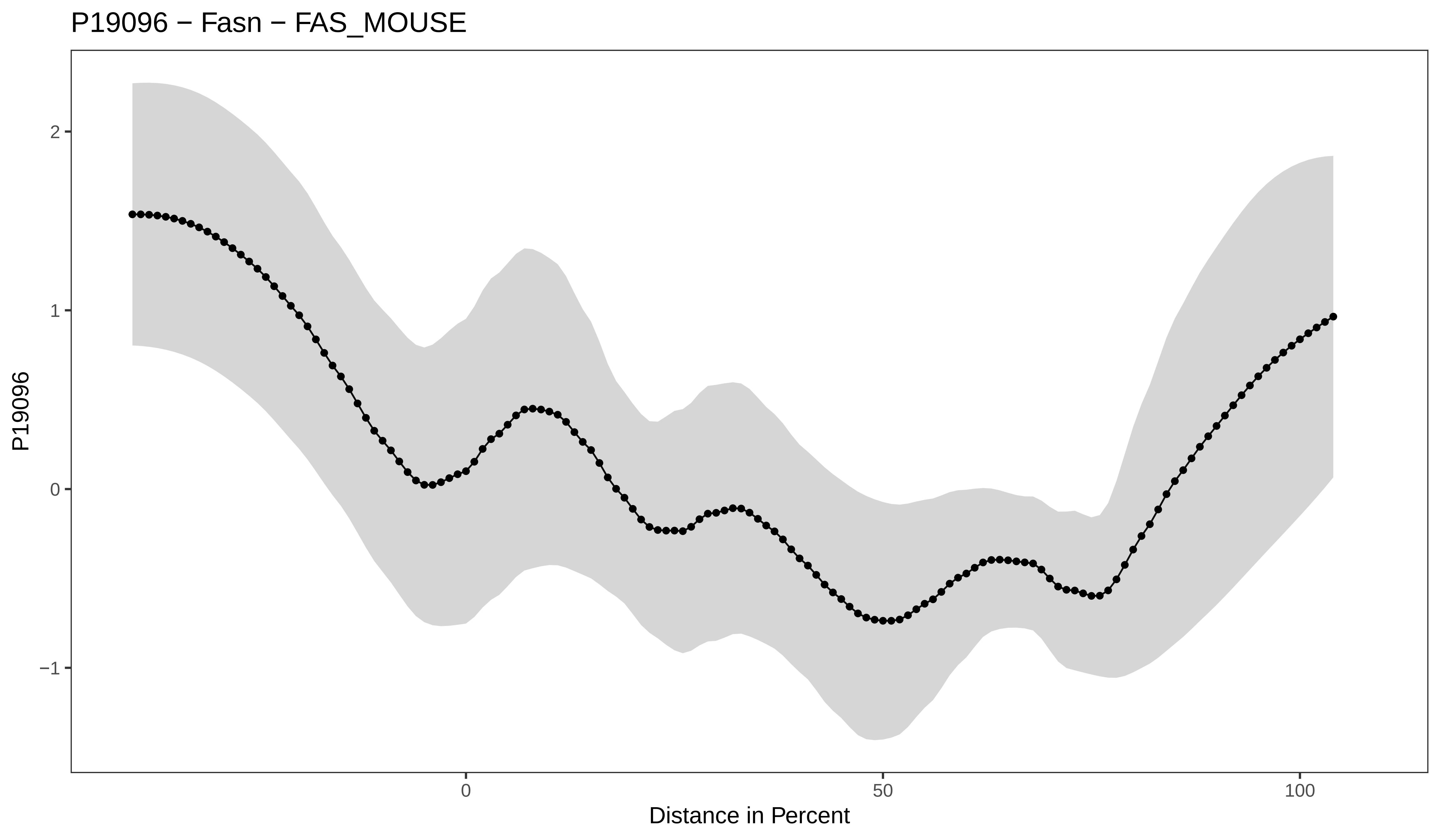

P19096 − Fasn − FAS_MOUSE
2
1
0
−1
P19096
0
50
100
Distance in Percent

Q922E6 − Fastkd2 − FAKD2_MOUSE

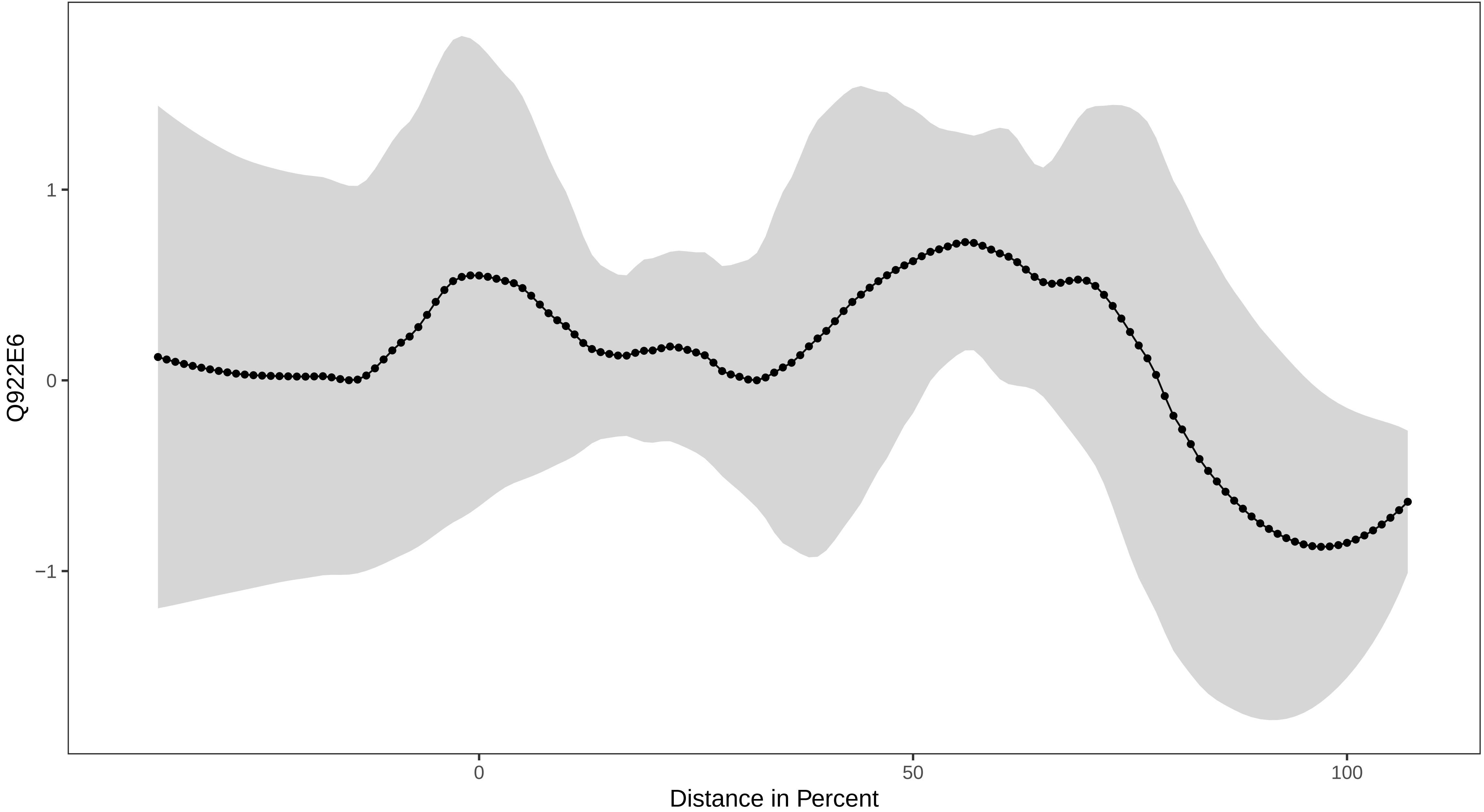

P62862 − Fau − RS30_MOUSE

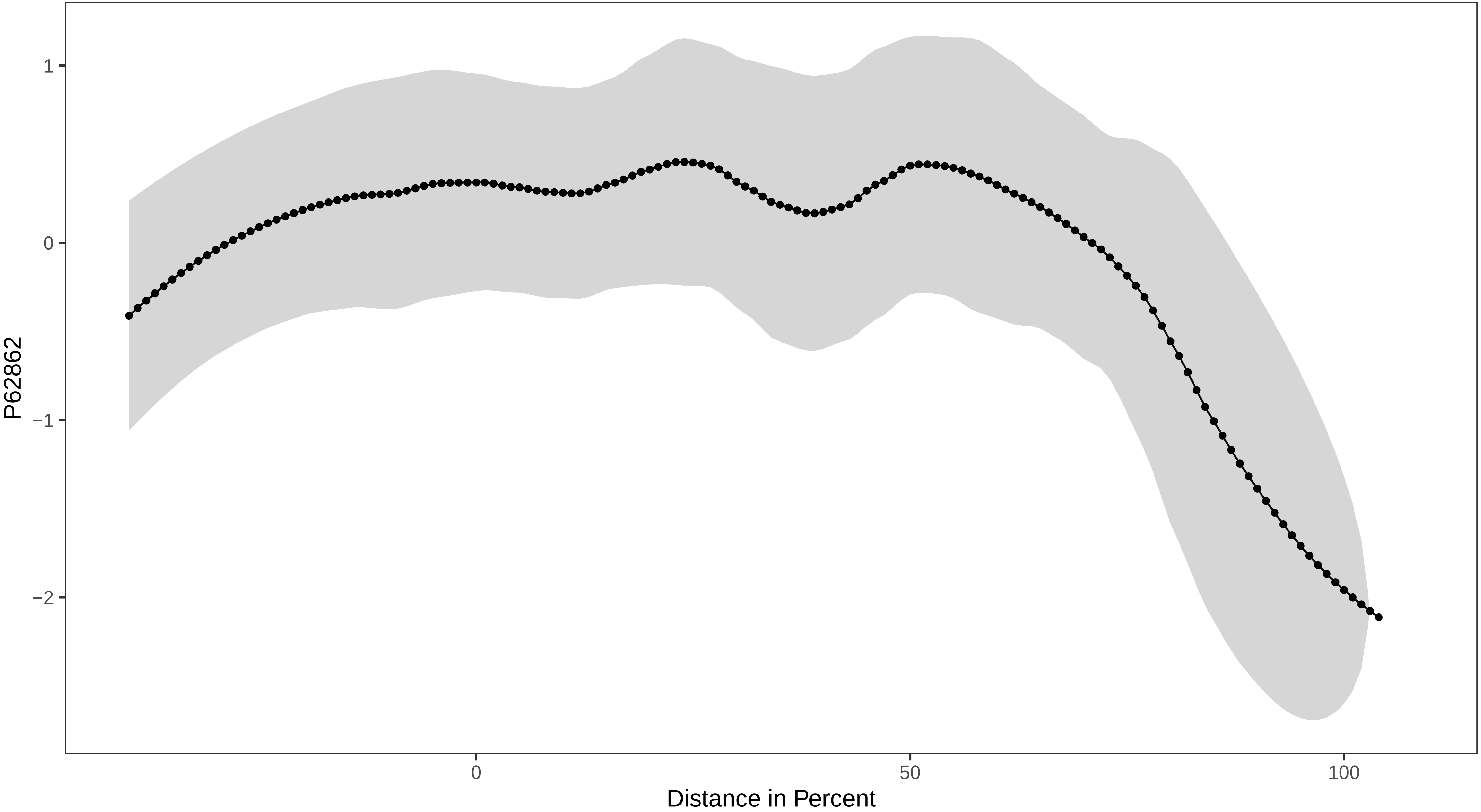

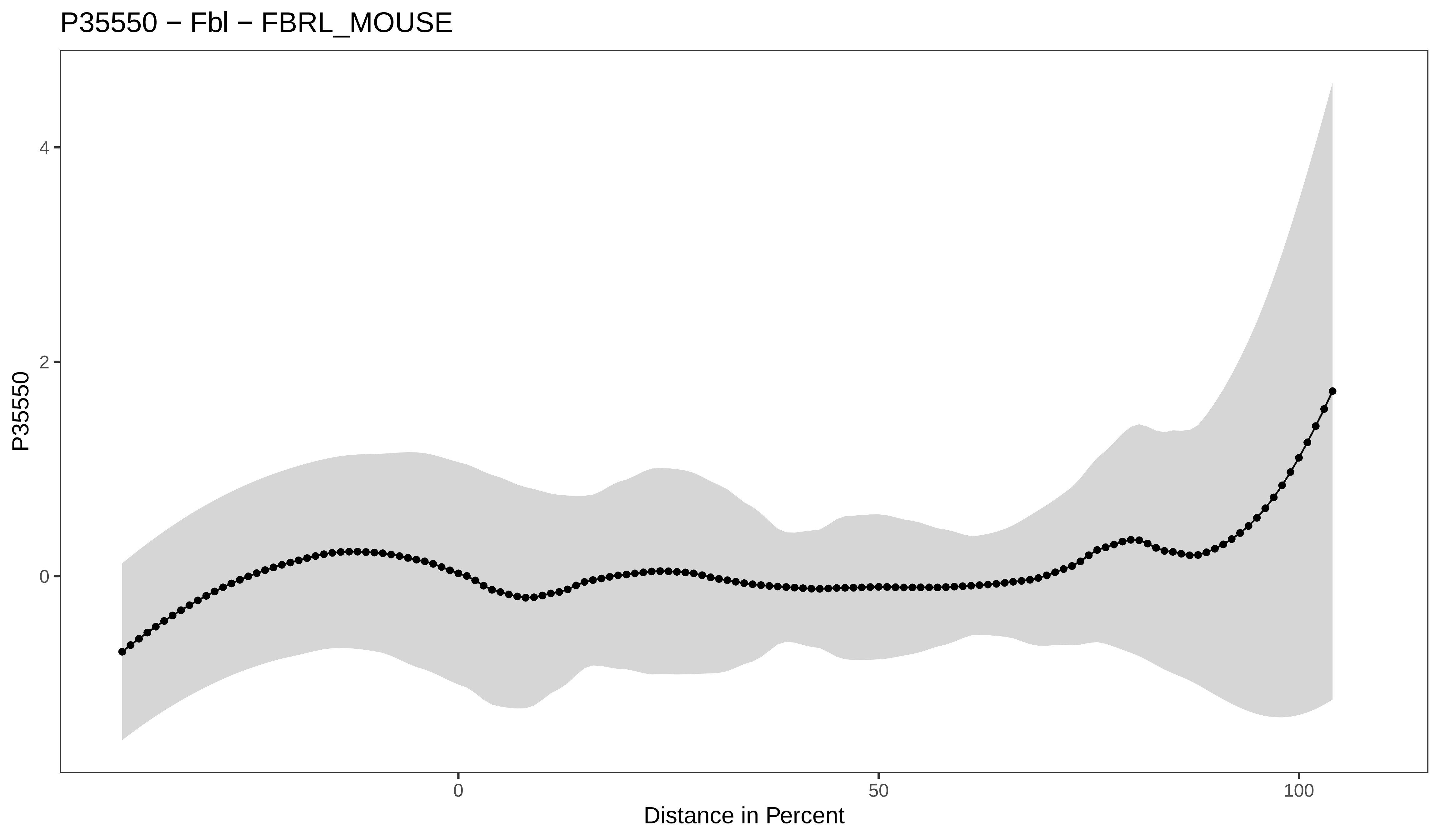

P35550 − Fbl − FBRL_MOUSE
P35550
4
2
0
0
50
100
Distance in Percent

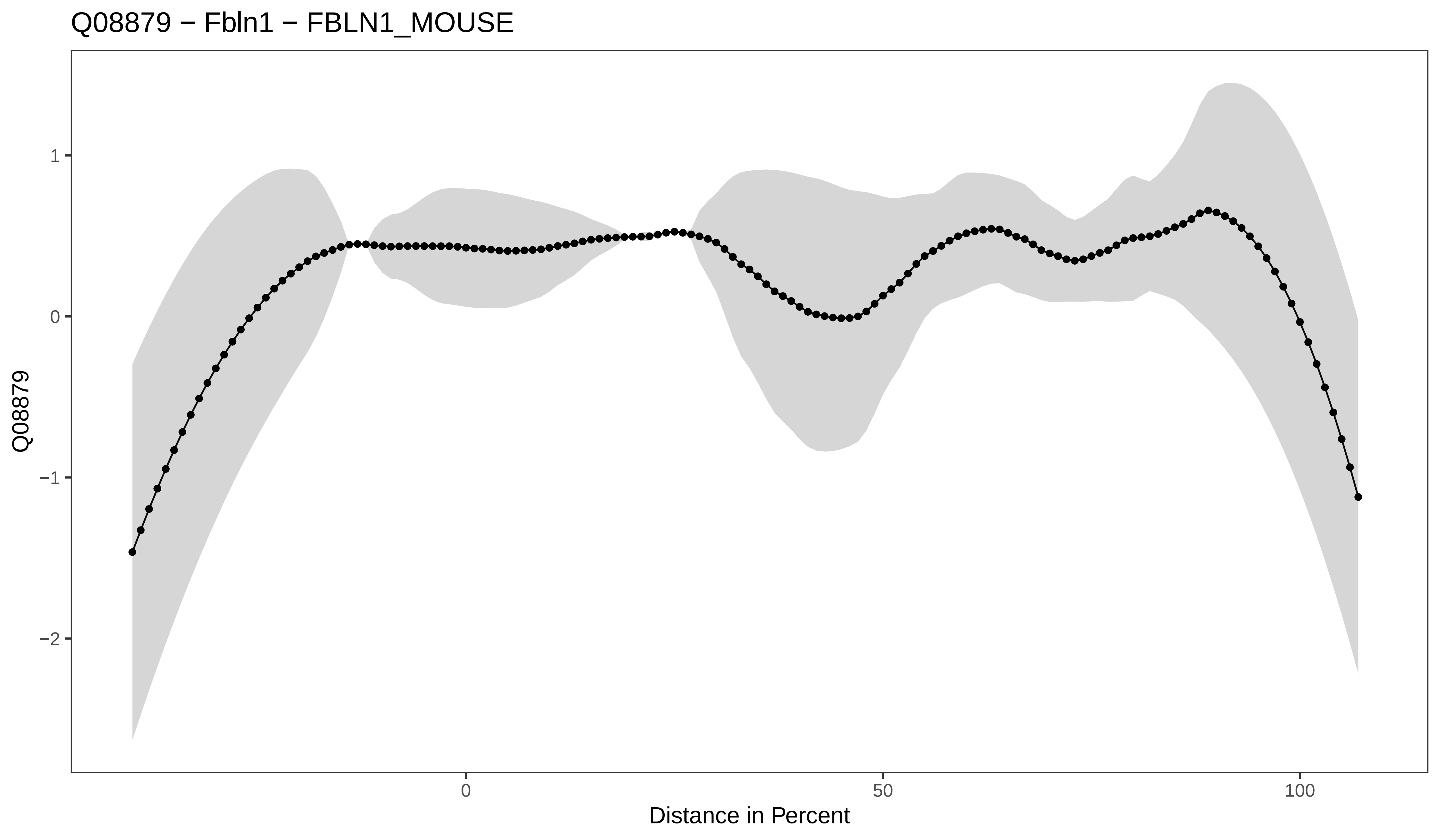

Q08879 − Fbln1 − FBLN1_MOUSE
1
0
−1
−2
Q08879
0
50
100
Distance in Percent

P37889 − Fbln2 − FBLN2_MOUSE

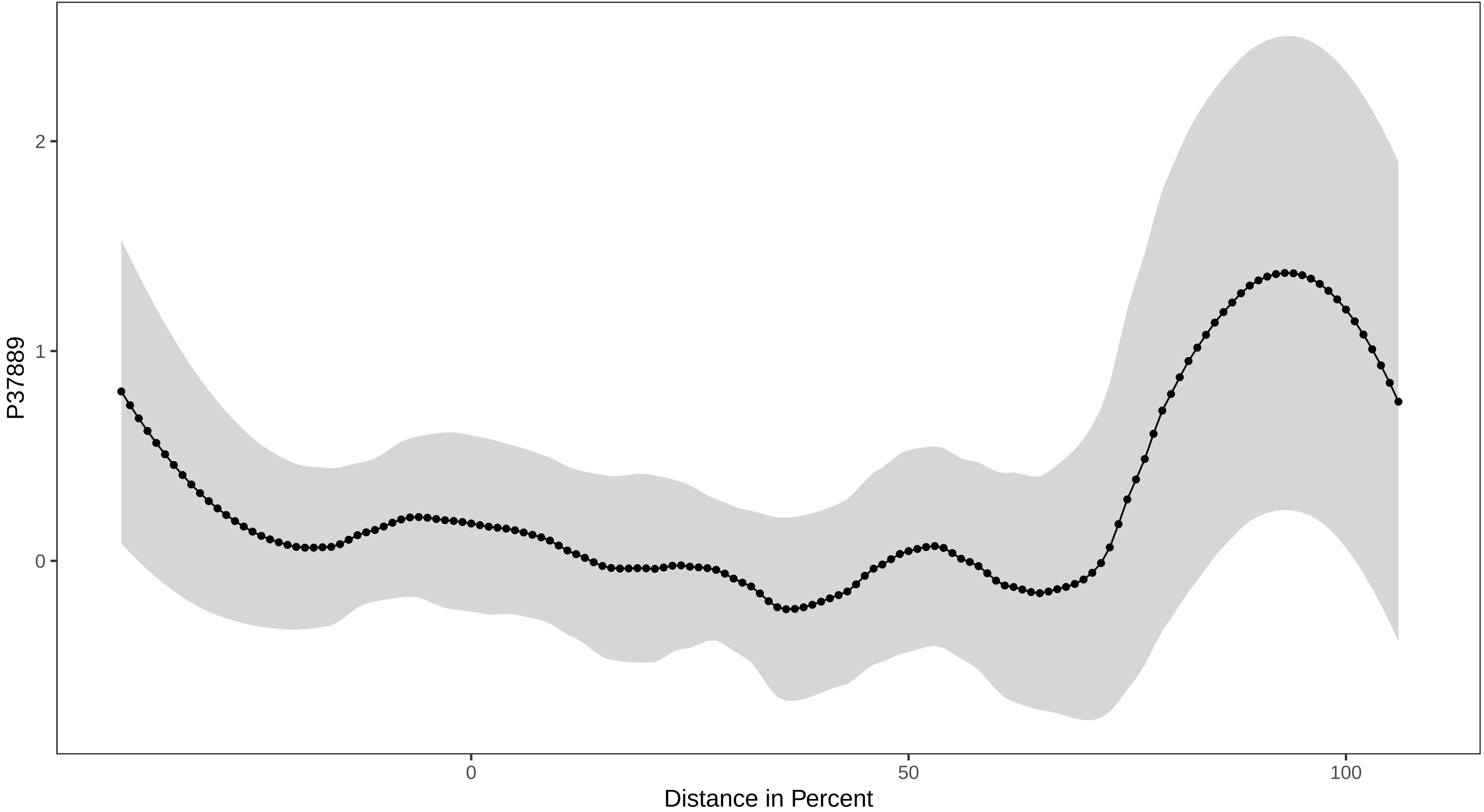

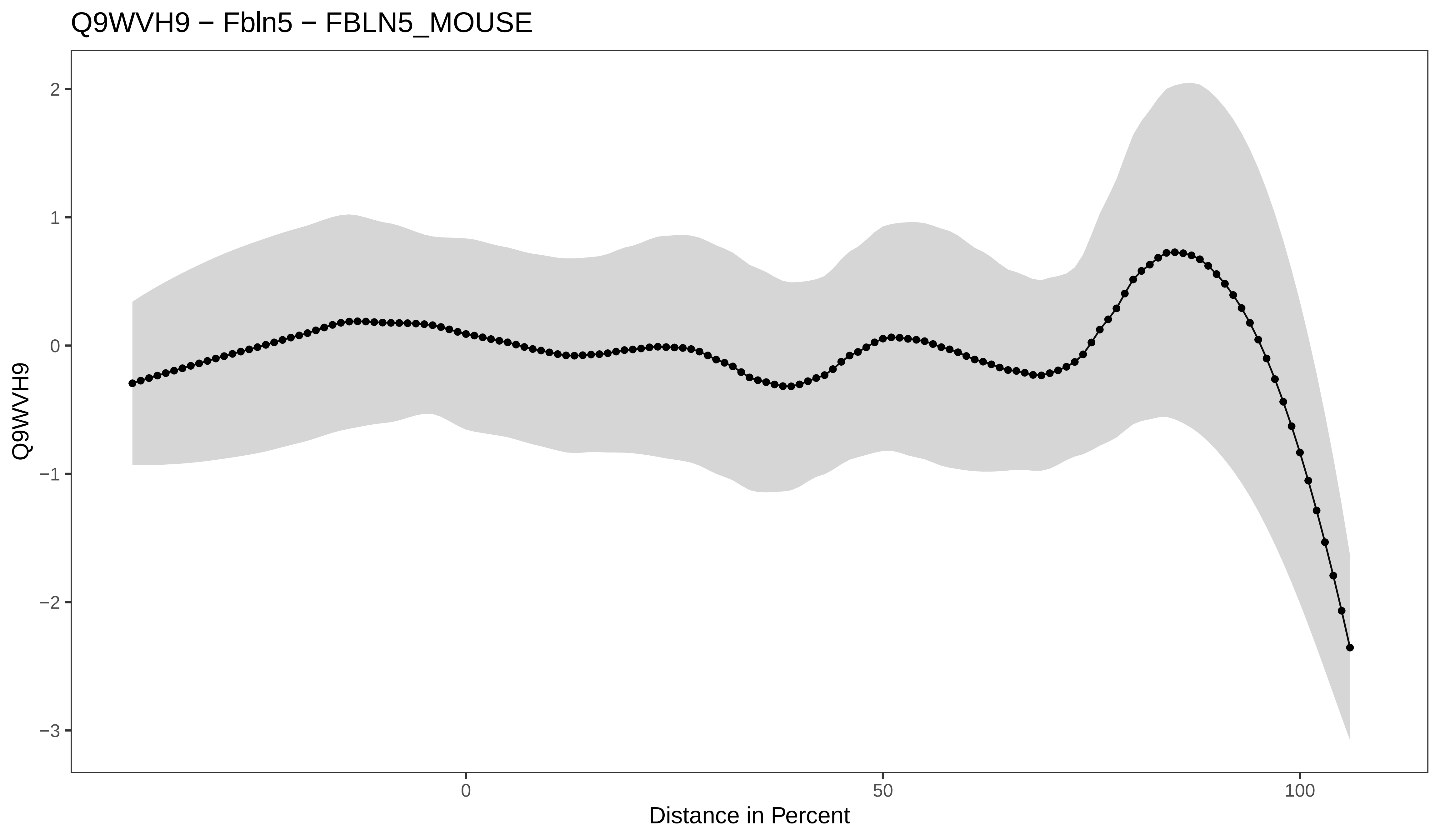

Q9WVH9 − Fbln5 − FBLN5_MOUSE
2
1
0
−1
−2
−3
Q9WVH9
0
50
100
Distance in Percent

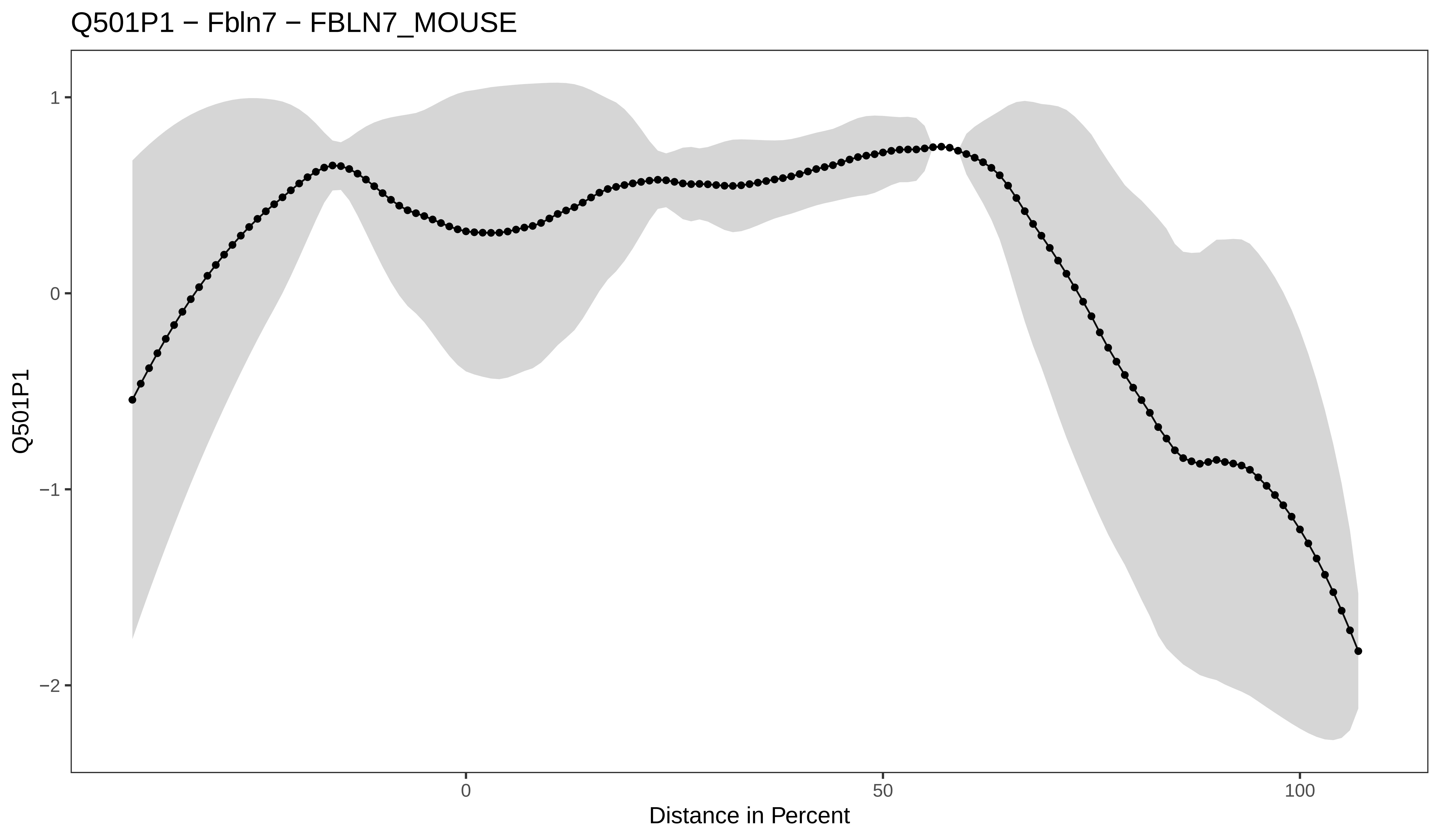

Q501P1 − Fbln7 − FBLN7_MOUSE
1
0
−1
−2
Q501P1
0
50
100
Distance in Percent

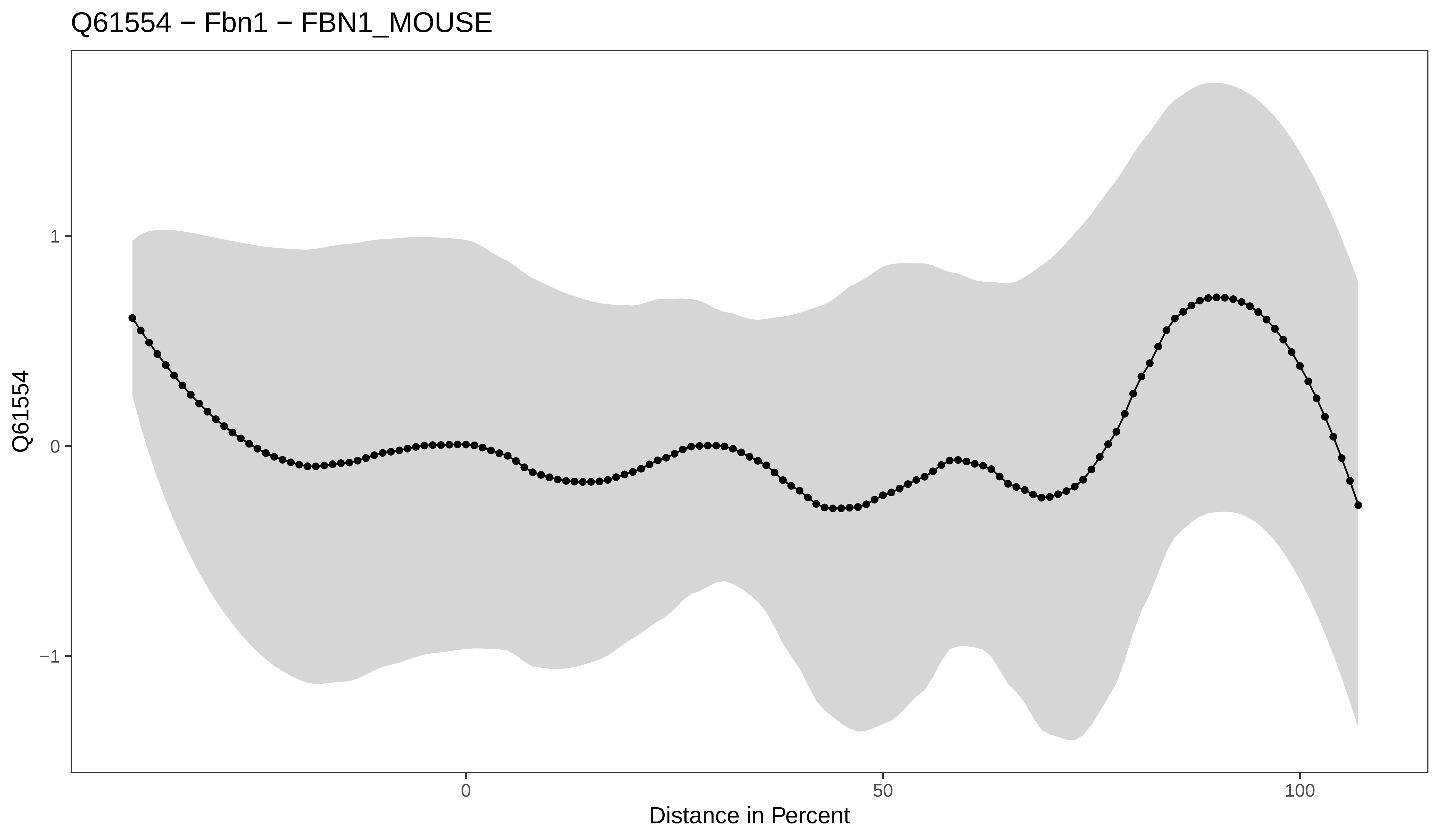

Q61554 − Fbn1 − FBN1_MOUSE
Q61554
1
0
−1
0
50
100
Distance in Percent

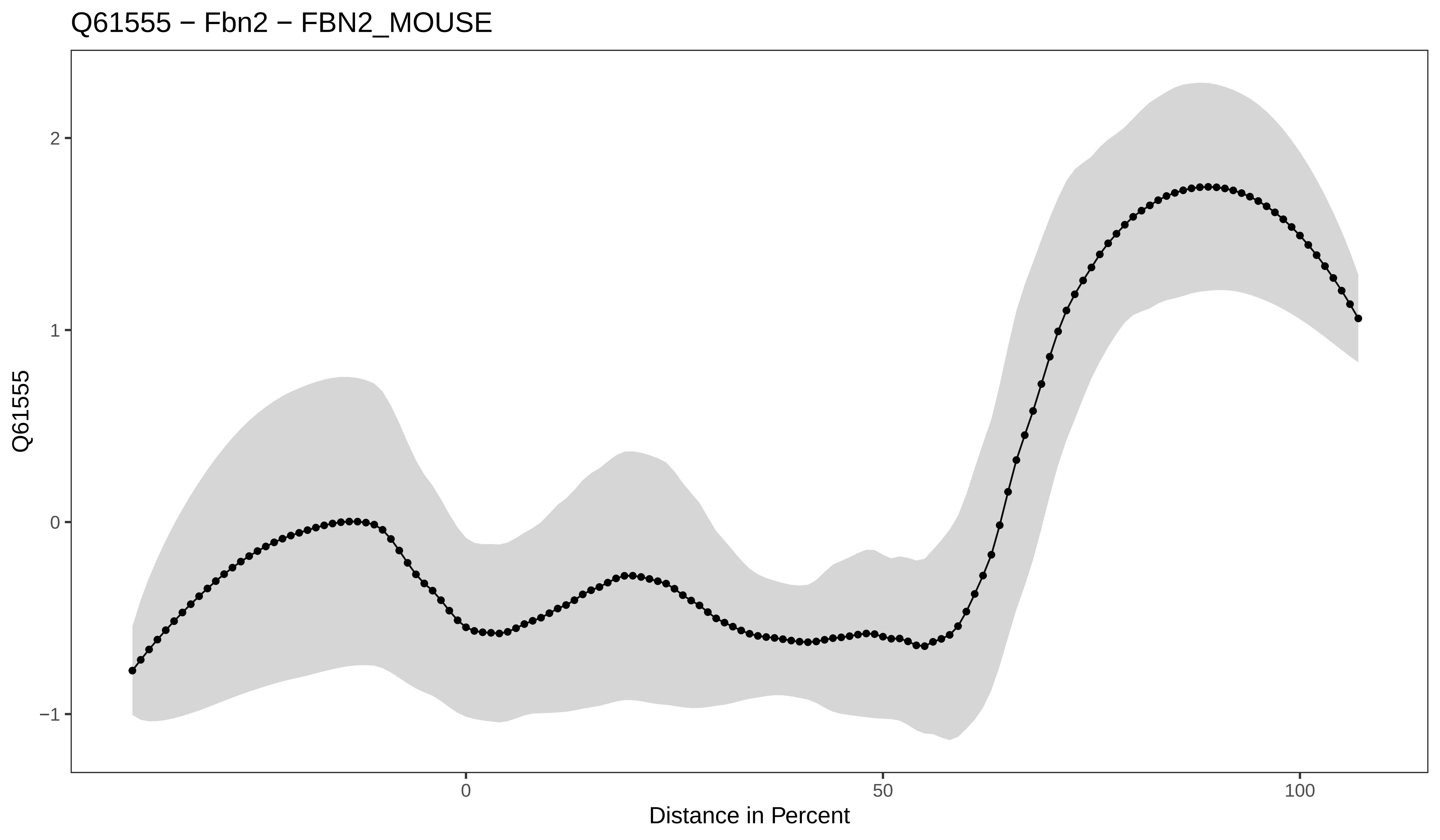
Q61555 − Fbn2 − FBN2_MOUSE
Q61555
Distance in Percent
2
1
0
−1
0
50
100

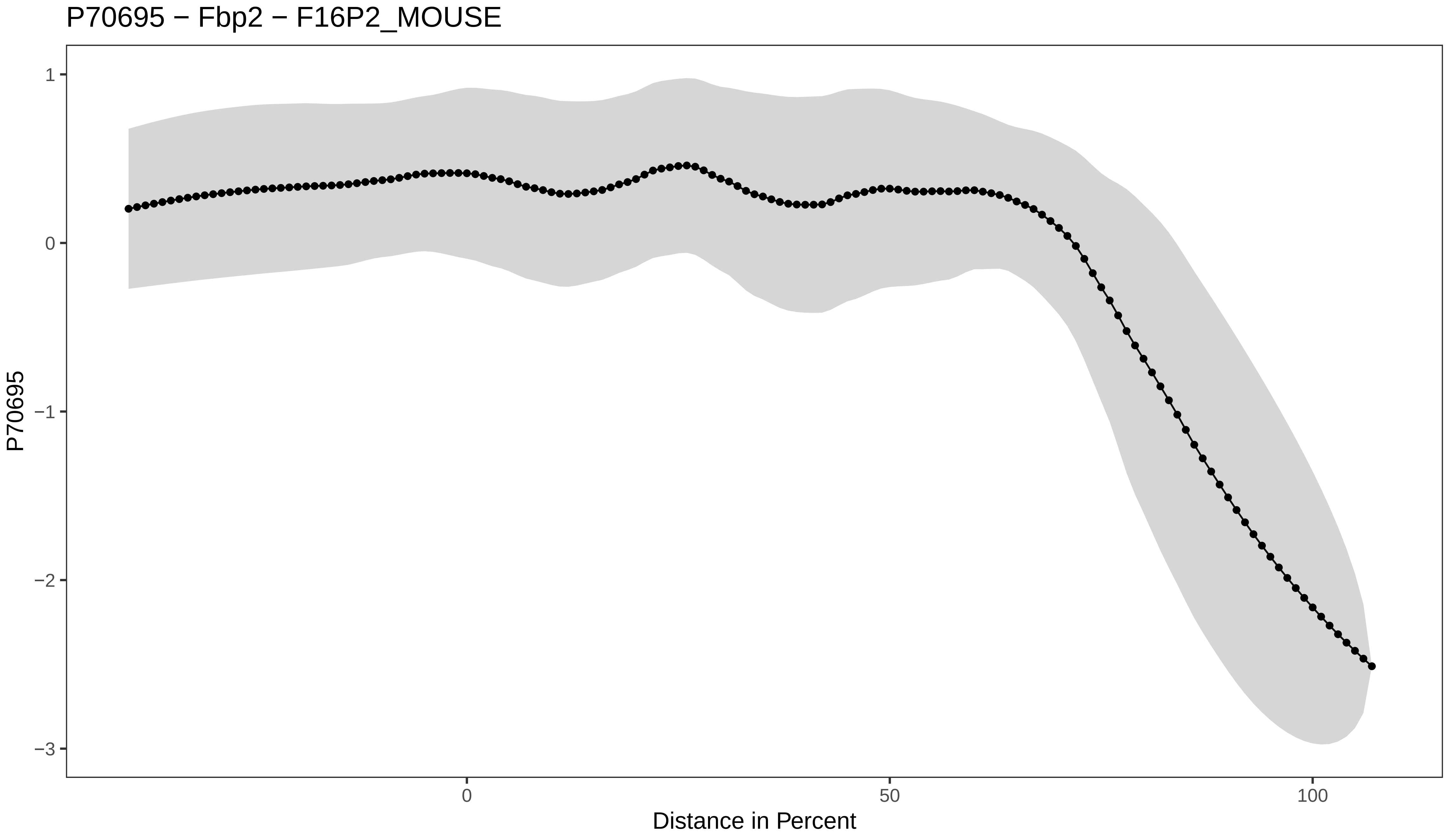

P70695 − Fbp2 − F16P2_MOUSE
1
0
−1
−2
−3
P70695
0
50
100
Distance in Percent

P62932 − Fbxo40 − FBX40_MOUSE

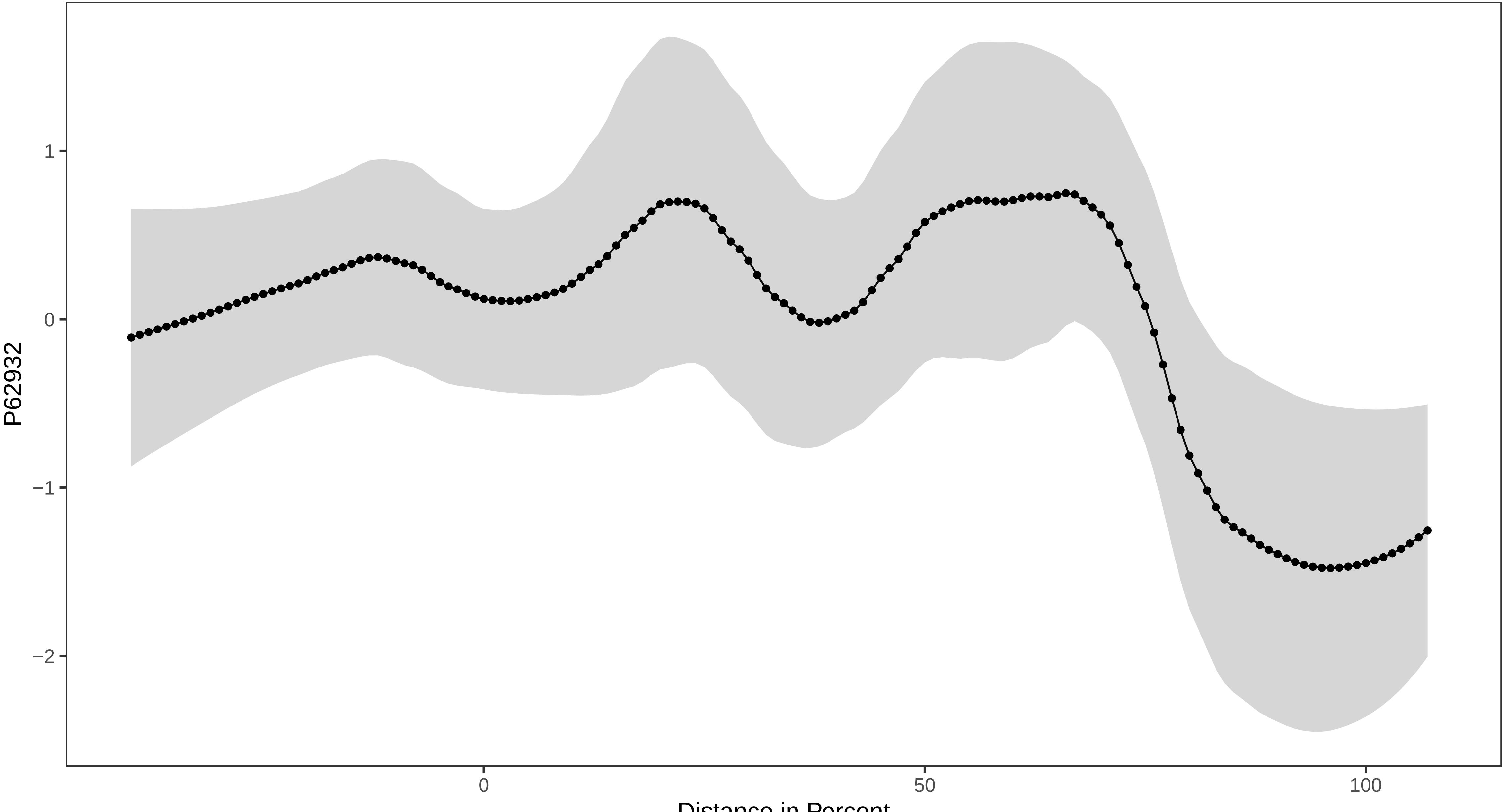

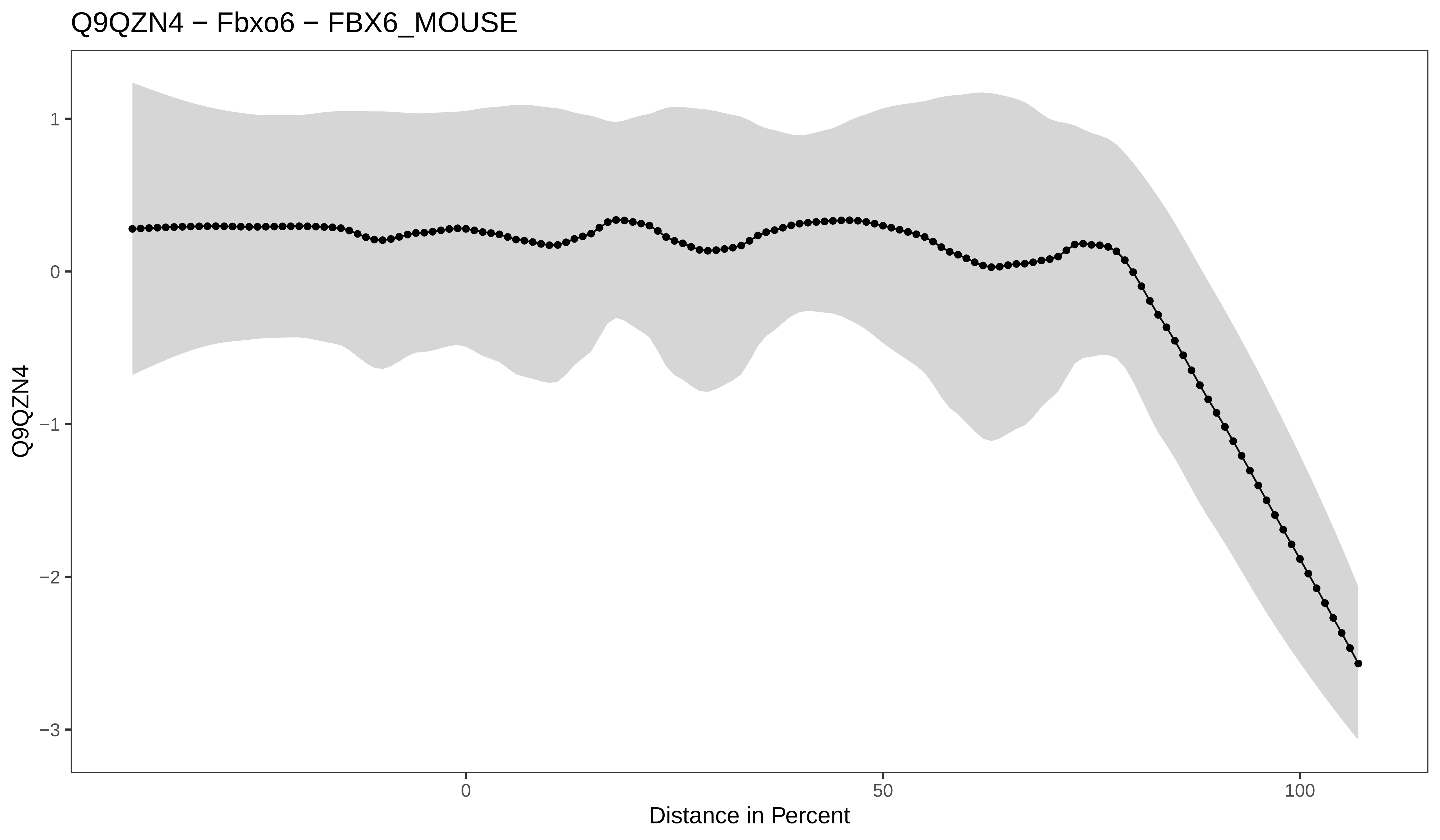

Q9QZN4 − Fbxo6 − FBX6_MOUSE
Q9QZN4
1
0
−1
−2
−3
0
50
100
Distance in Percent

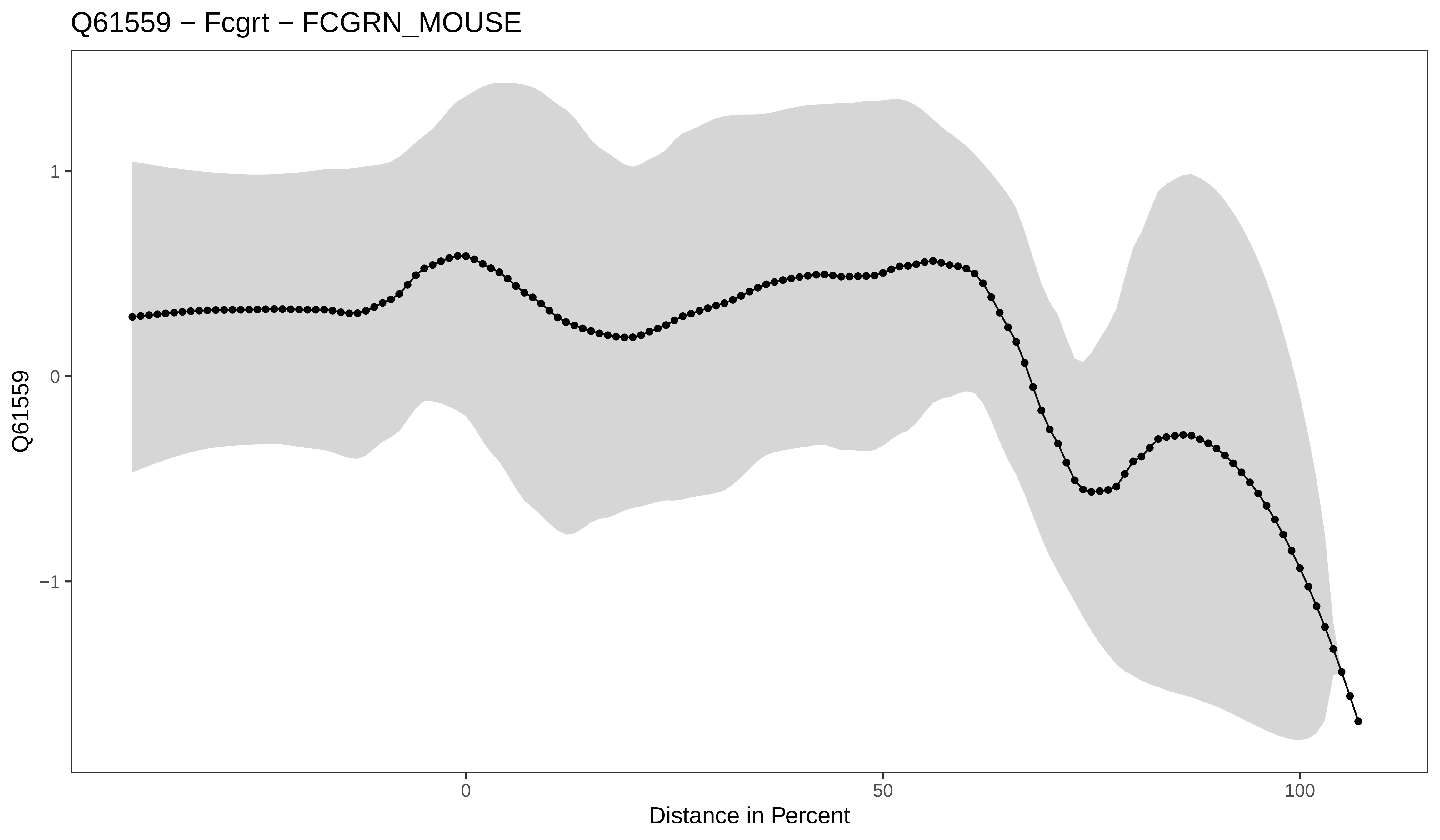

Q61559 − Fcgrt − FCGRN_MOUSE
1
0
−1
Q61559
0
50
100
Distance in Percent

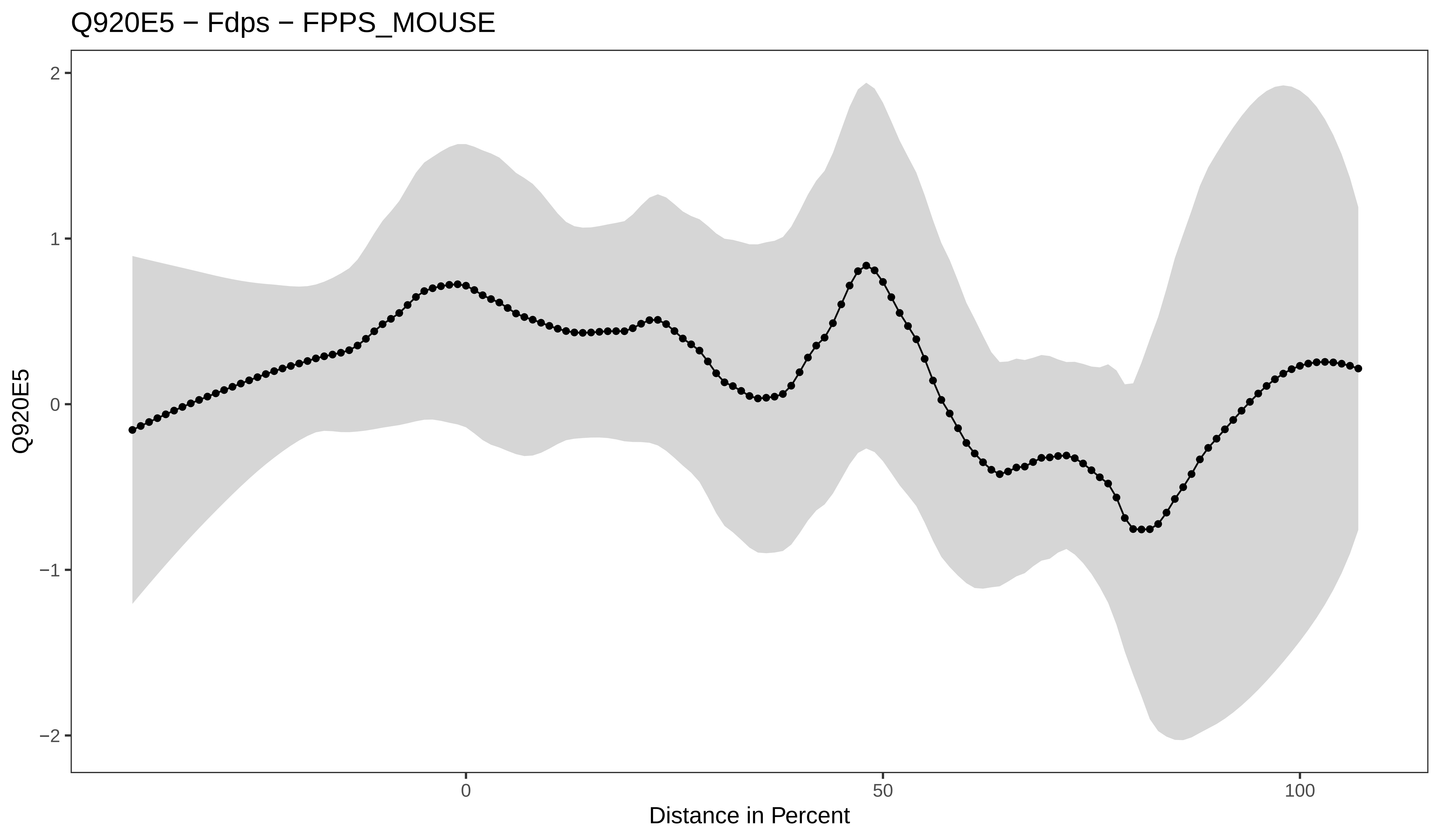

Q920E5 − Fdps − FPPS_MOUSE
2
1
0
−1
−2
Q920E5
0
50
100
Distance in Percent

Q61578 − Fdxr − ADRO_MOUSE

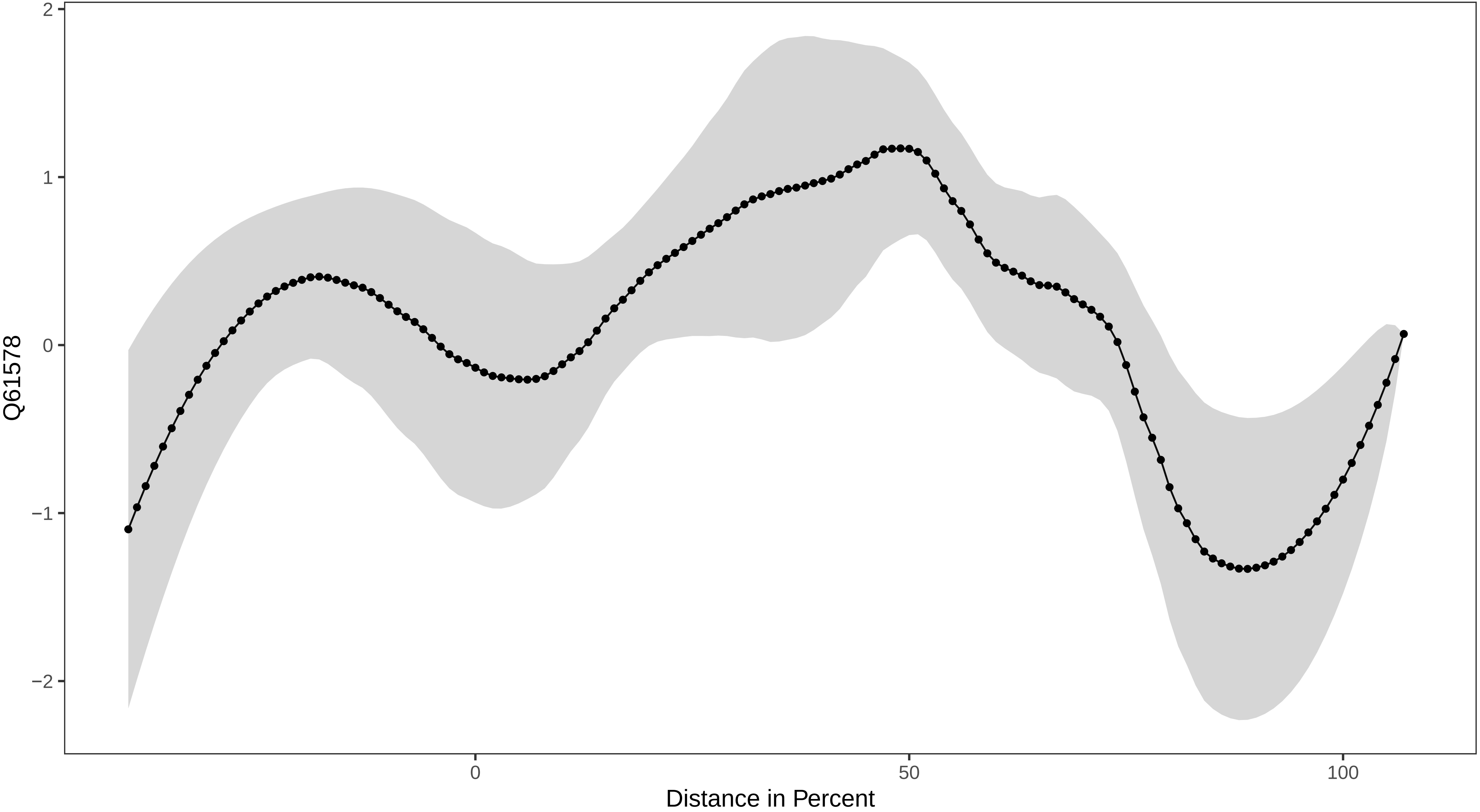

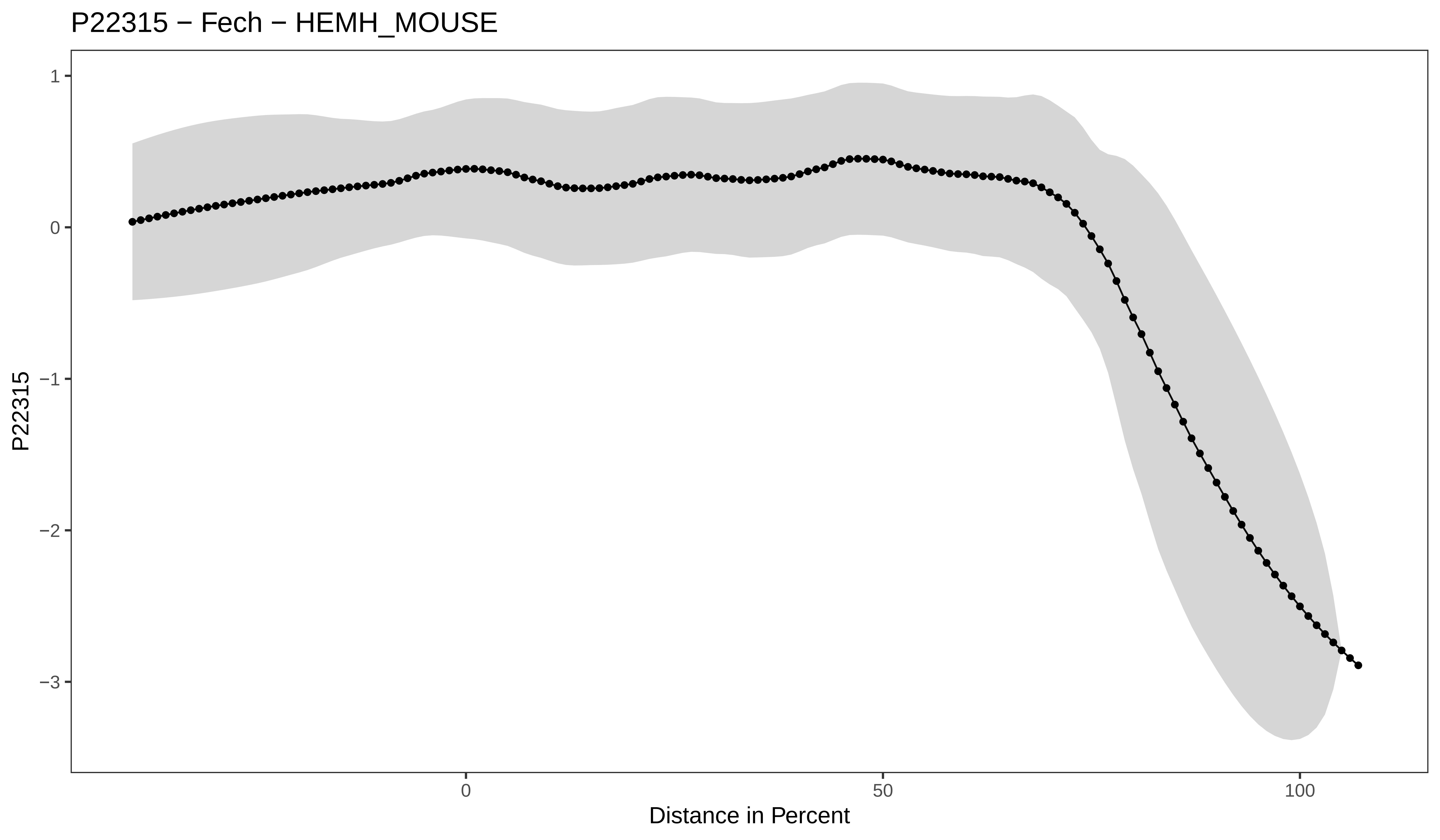
P22315 − Fech − HEMH_MOUSE
1
0
−1
−2
−3
P22315
0
50
100
Distance in Percent

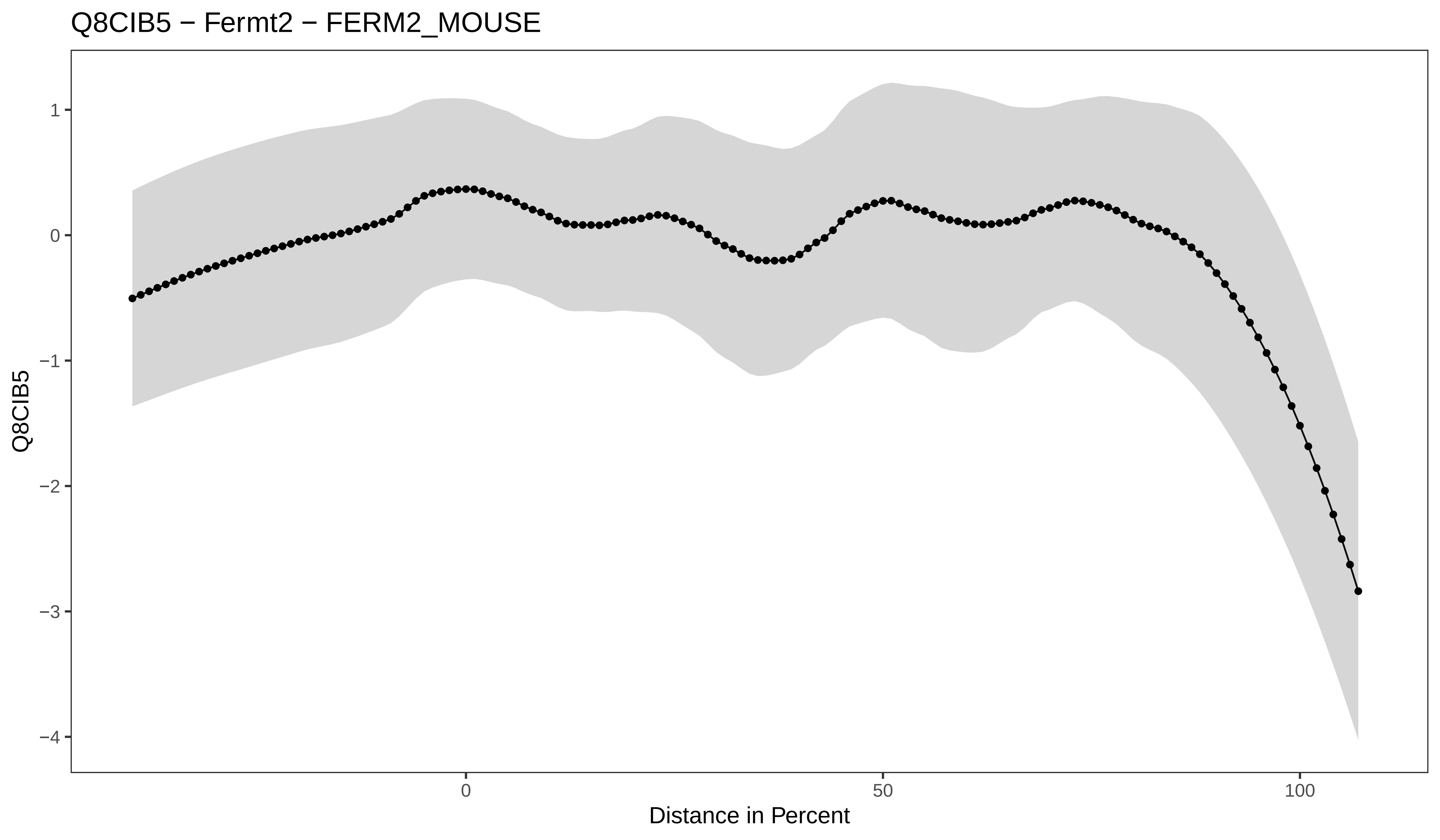
Q8CIB5 − Fermt2 − FERM2_MOUSE
Q8CIB5
Distance in Percent
1
0
−1
−2
−3
−4
0
50
100

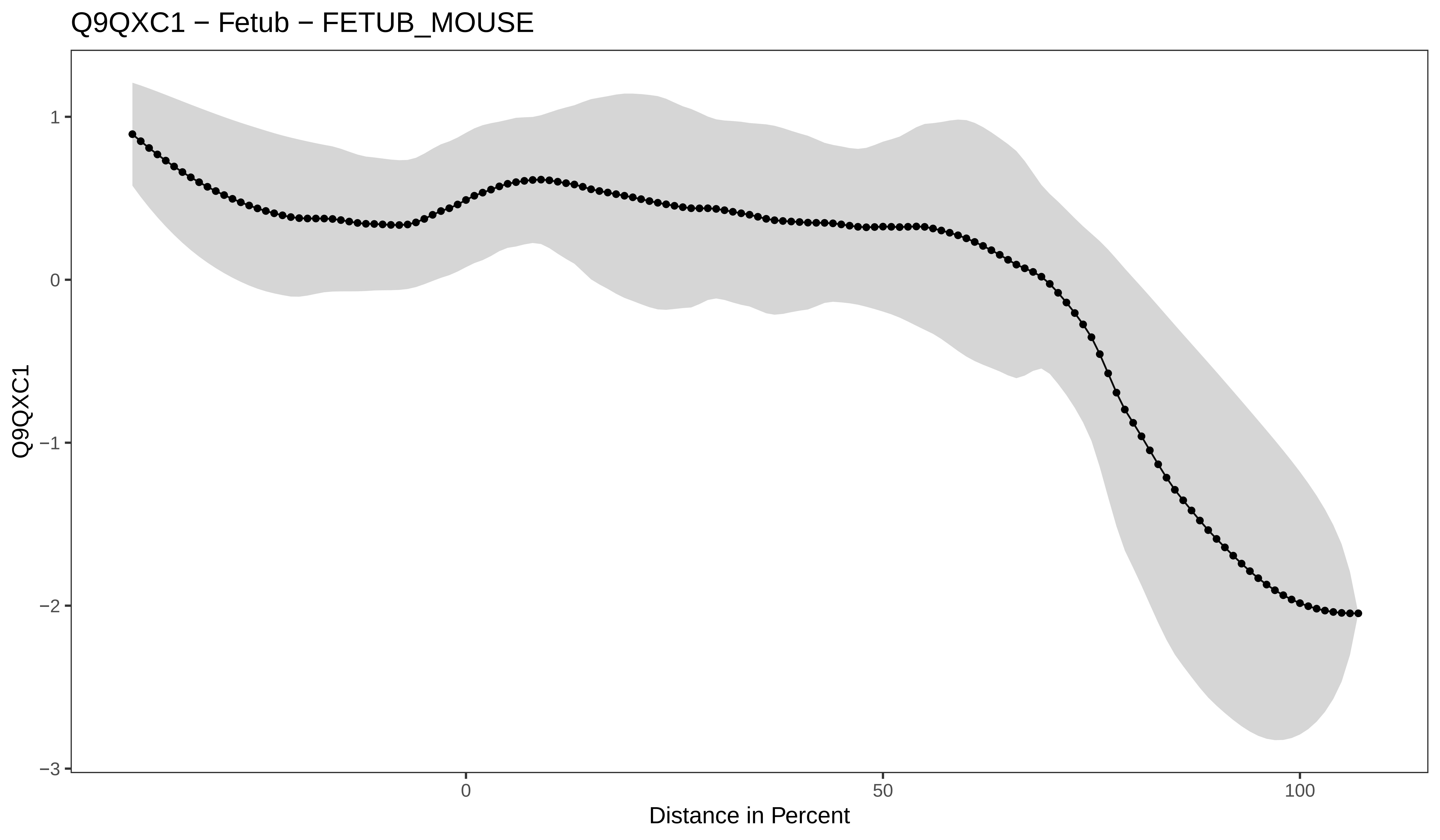

Q9QXC1 − Fetub − FETUB_MOUSE
1
0
−1
−2
−3
Q9QXC1
0
50
100
Distance in Percent

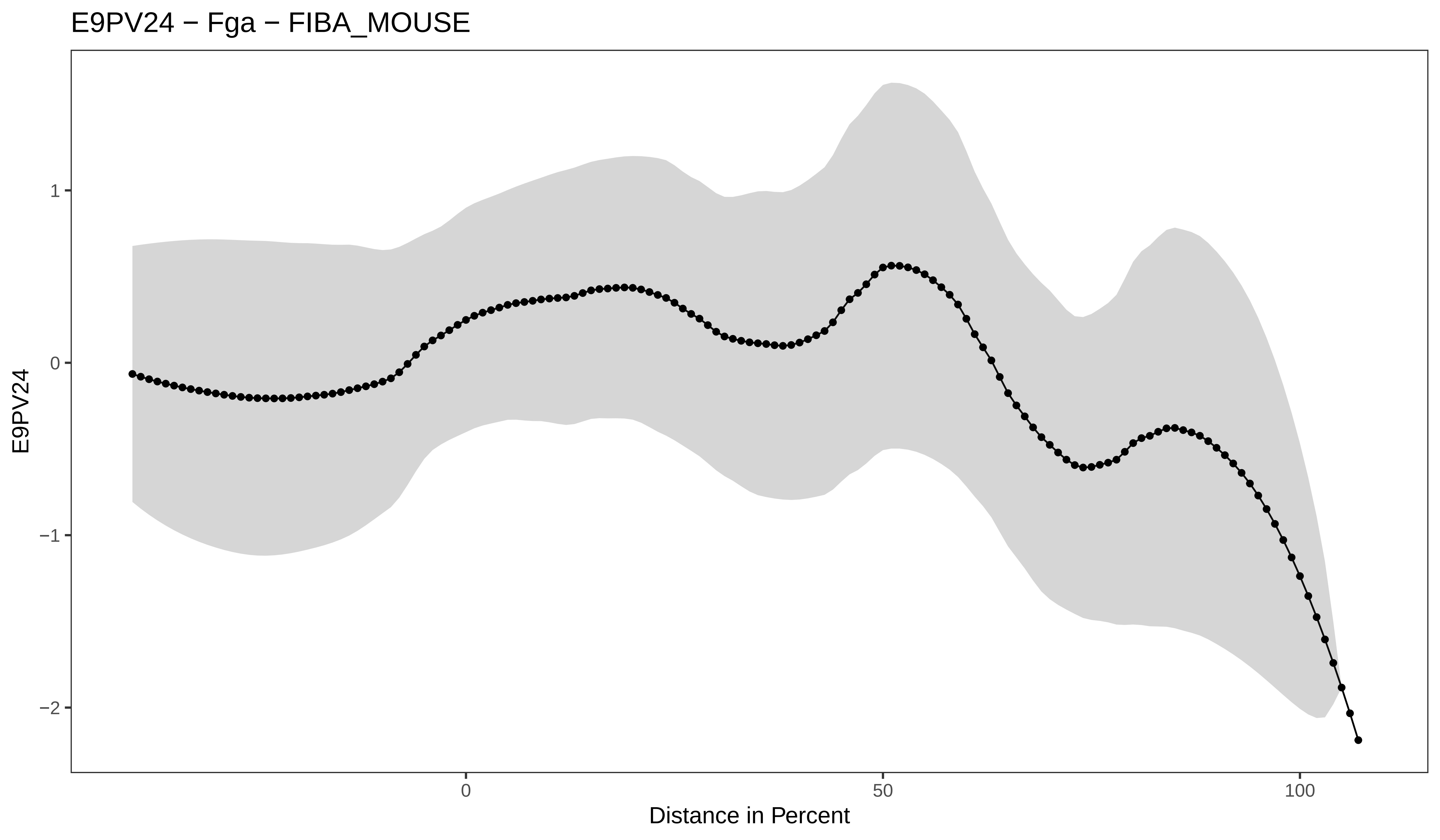
E9PV24 − Fga − FIBA_MOUSE
E9PV24
1
0
−1
−2
0
50
100
Distance in Percent

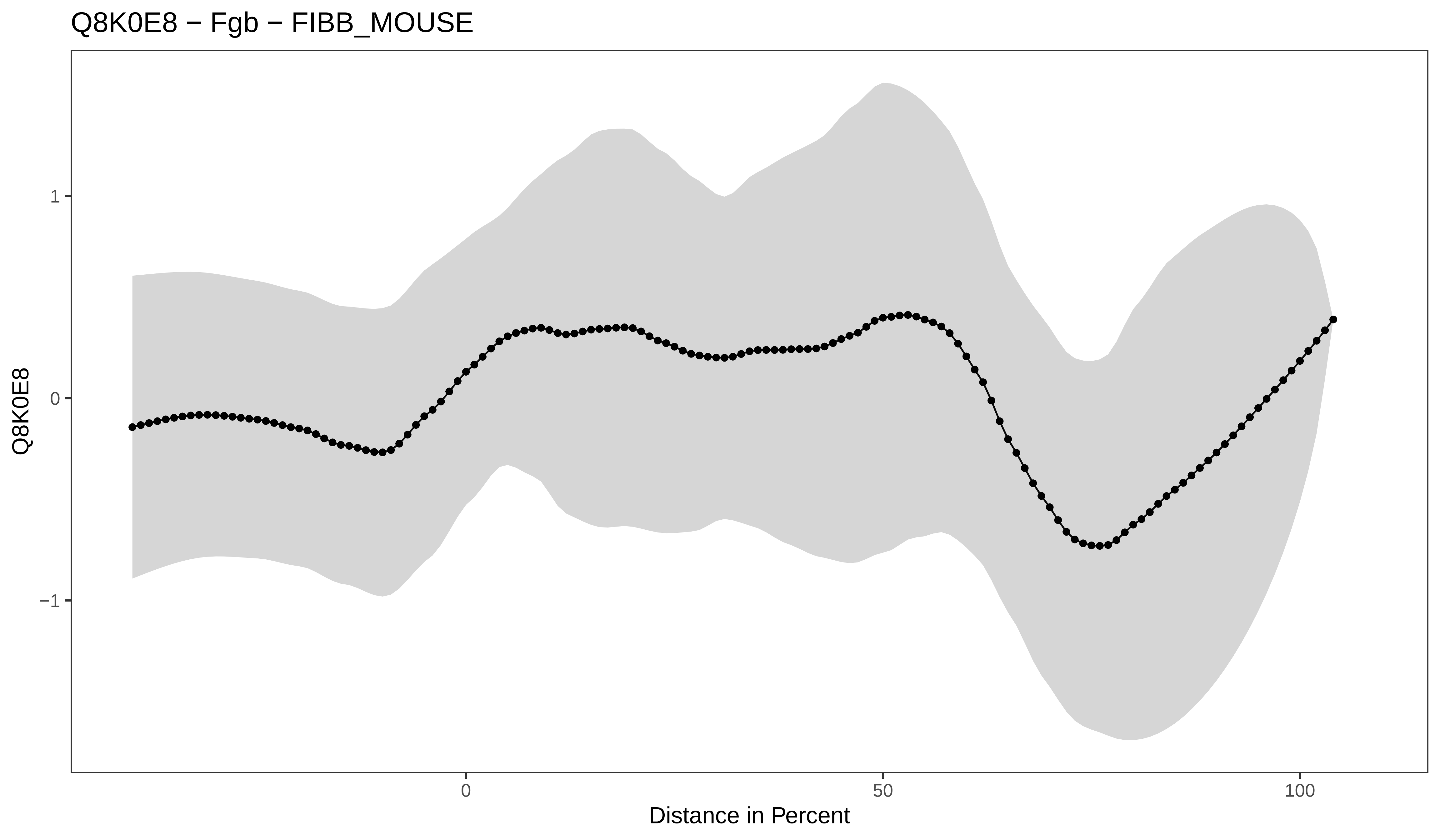
Q8K0E8 − Fgb − FIBB_MOUSE
Q8K0E8
1
0
−1
0
50
100
Distance in Percent

Q8VCM7 − Fgg − FIBG_MOUSE

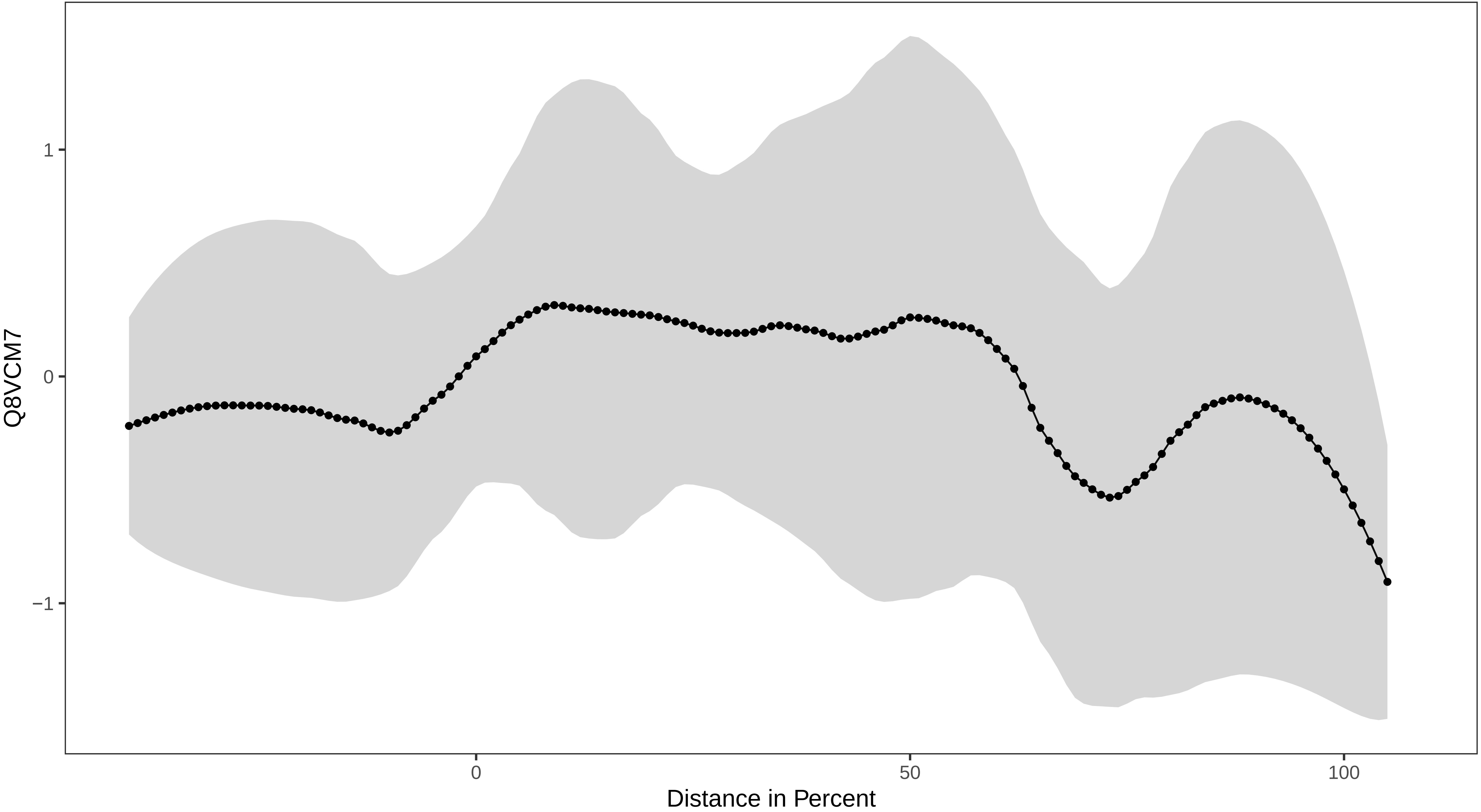

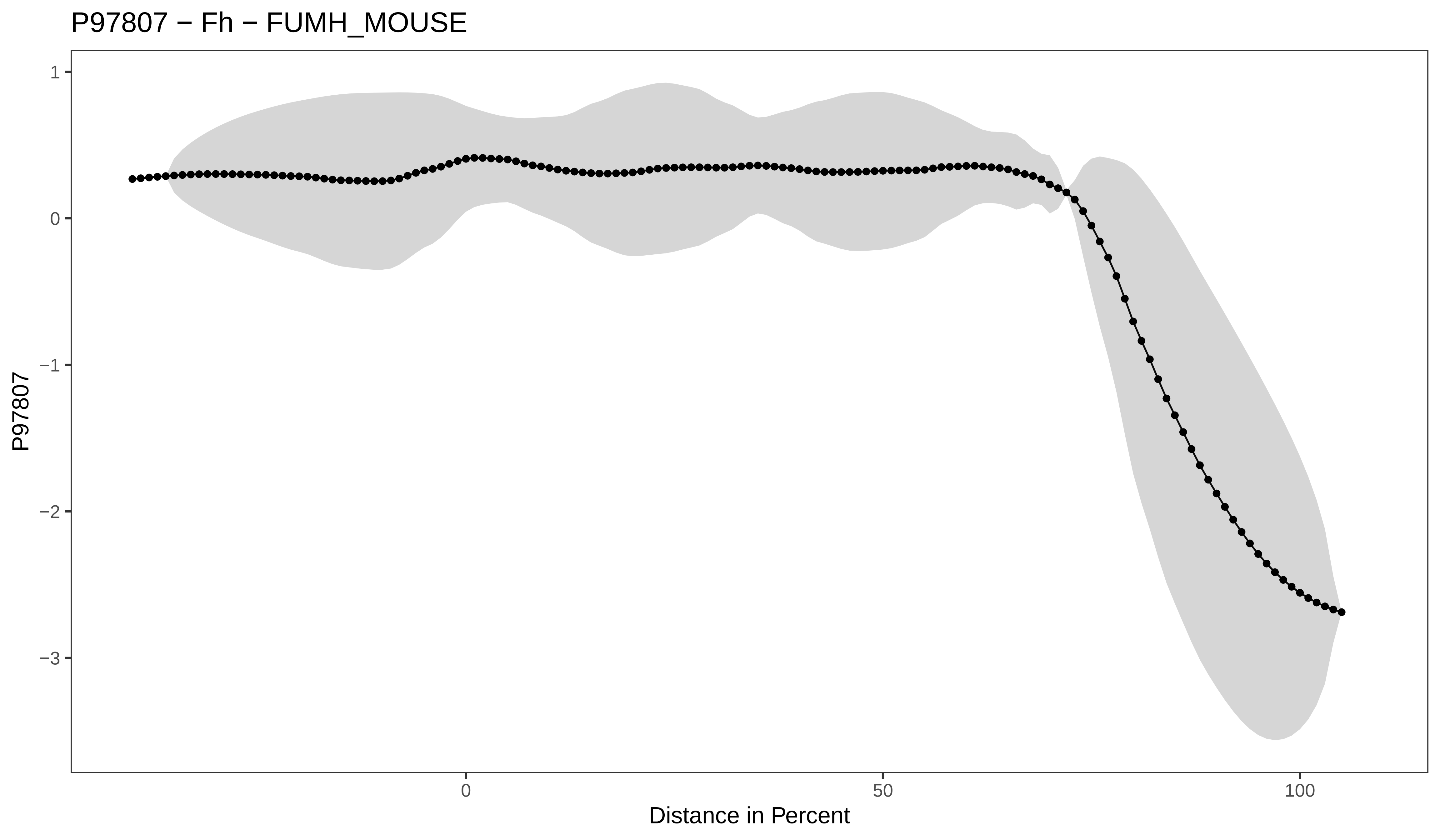

P97807 − Fh − FUMH_MOUSE
1
0
−1
−2
−3
P97807
0
50
100
Distance in Percent

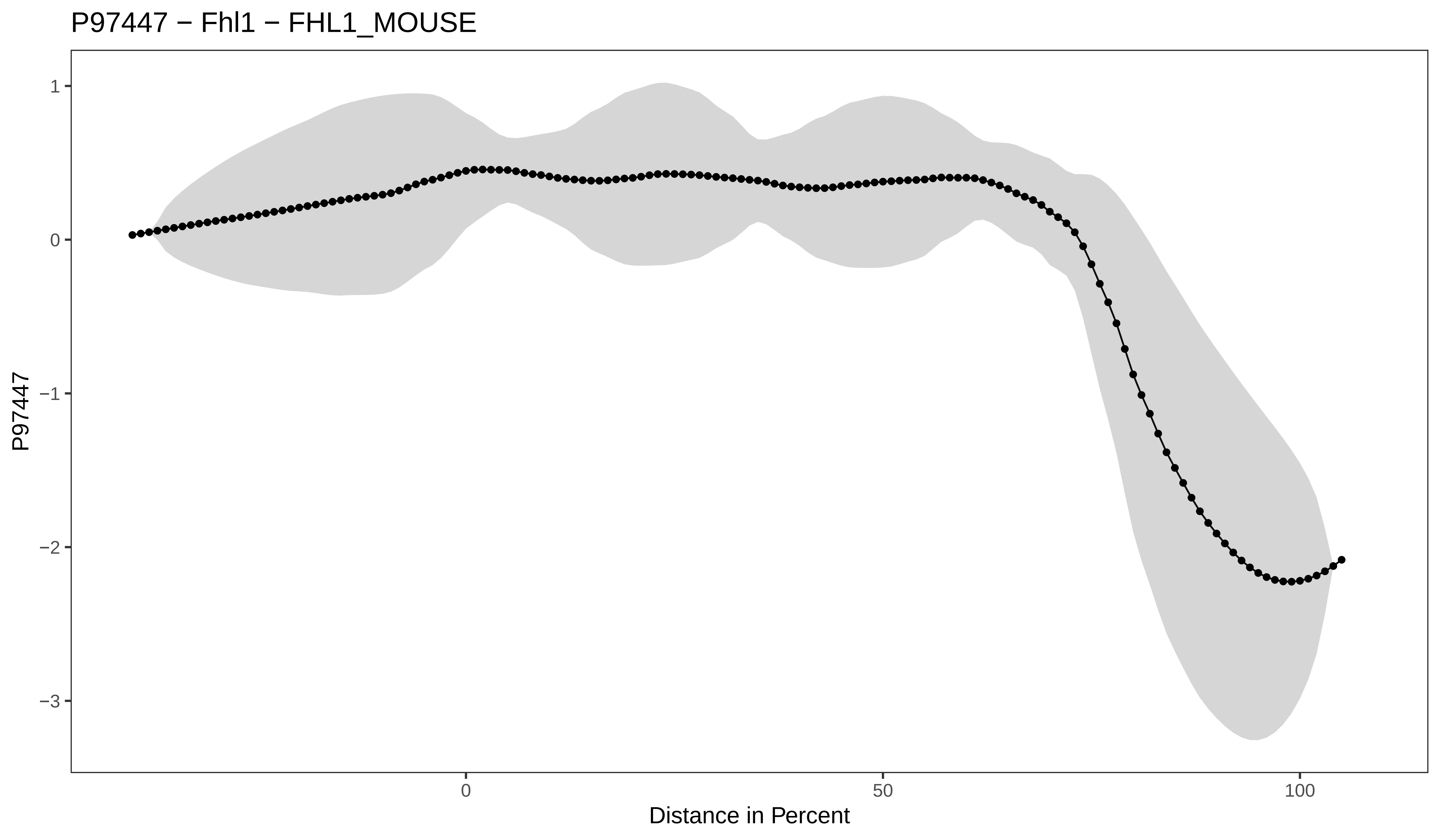

P97447 − Fhl1 − FHL1_MOUSE
P97447
1
0
−1
−2
−3
0
50
100
Distance in Percent

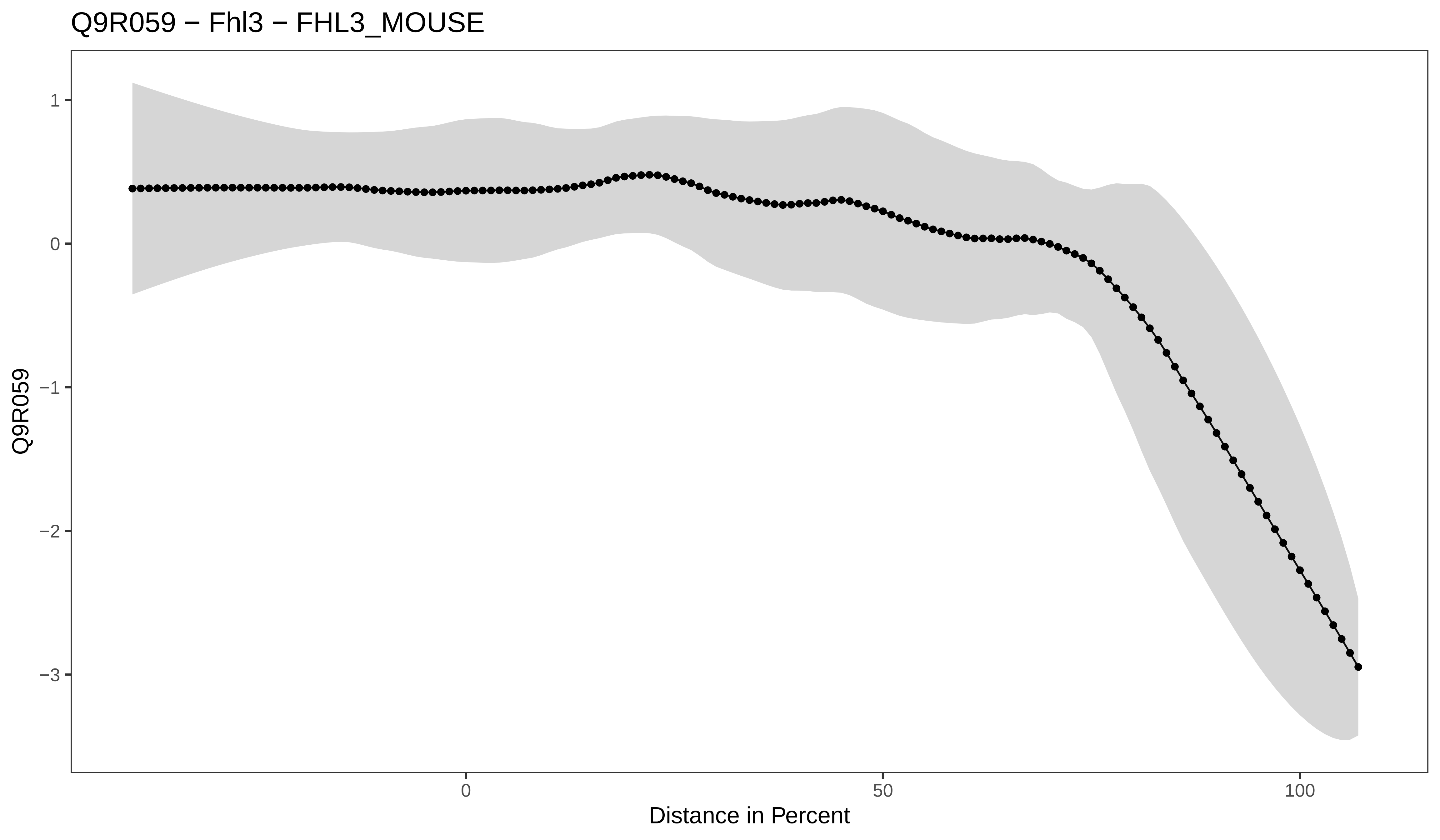

Q9R059 − Fhl3 − FHL3_MOUSE
Q9R059
1
0
−1
−2
−3
0
50
100
Distance in Percent

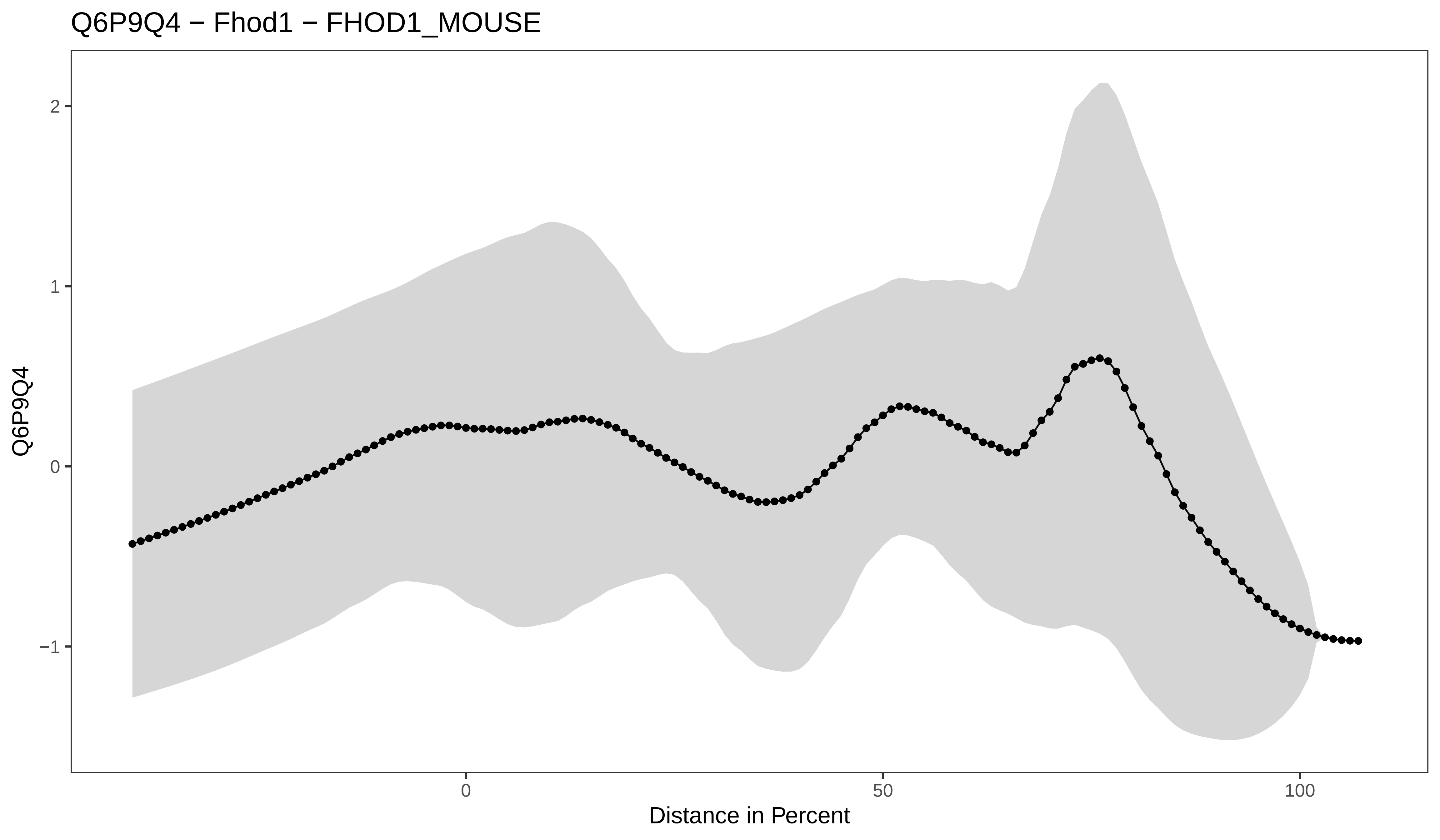

Q6P9Q4 − Fhod1 − FHOD1_MOUSE
Q6P9Q4
2
1
0
−1
0
50
100
Distance in Percent

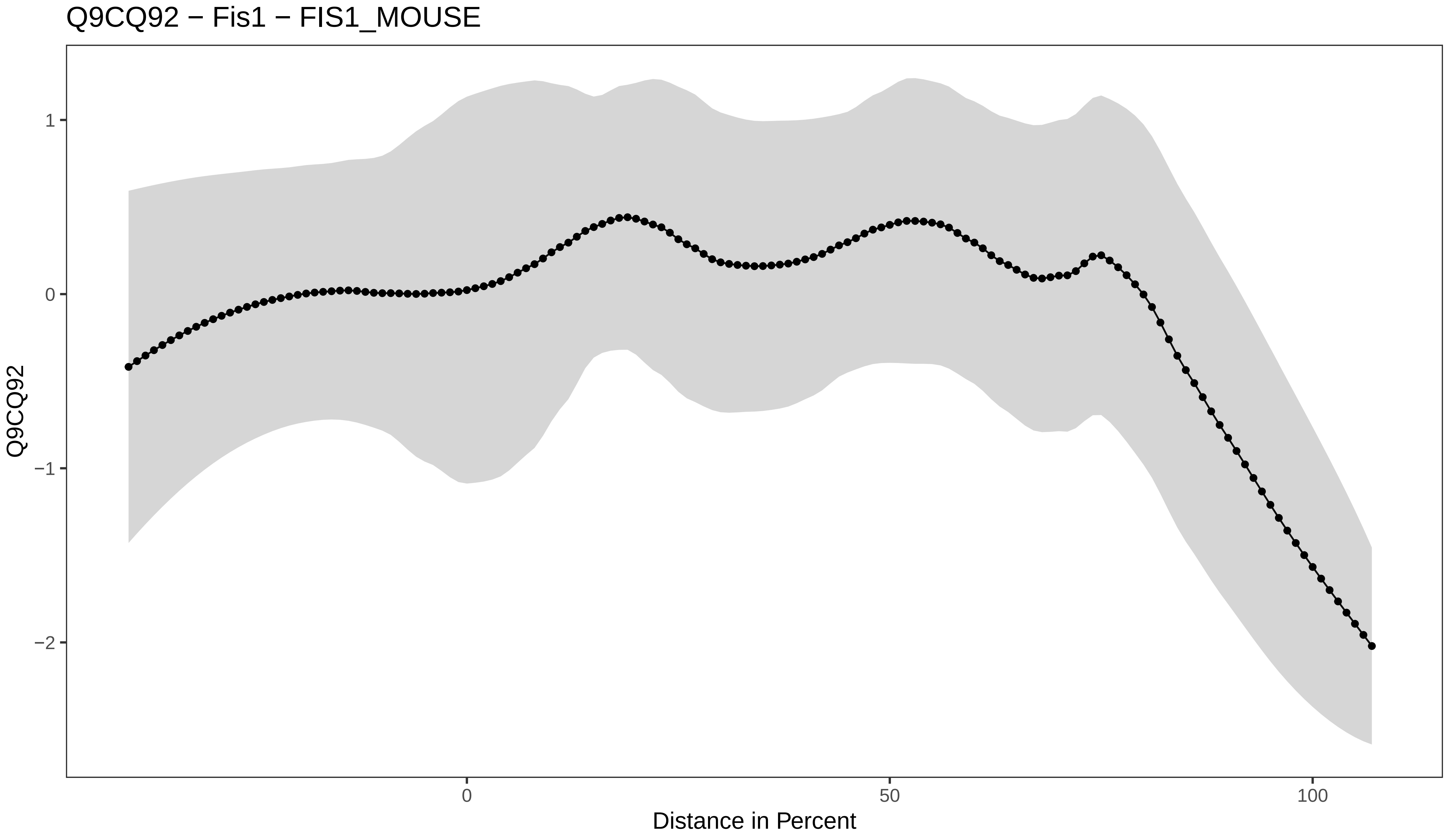
Q9CQ92 − Fis1 − FIS1_MOUSE
Q9CQ92
1
0
−1
−2
0
50
100
Distance in Percent

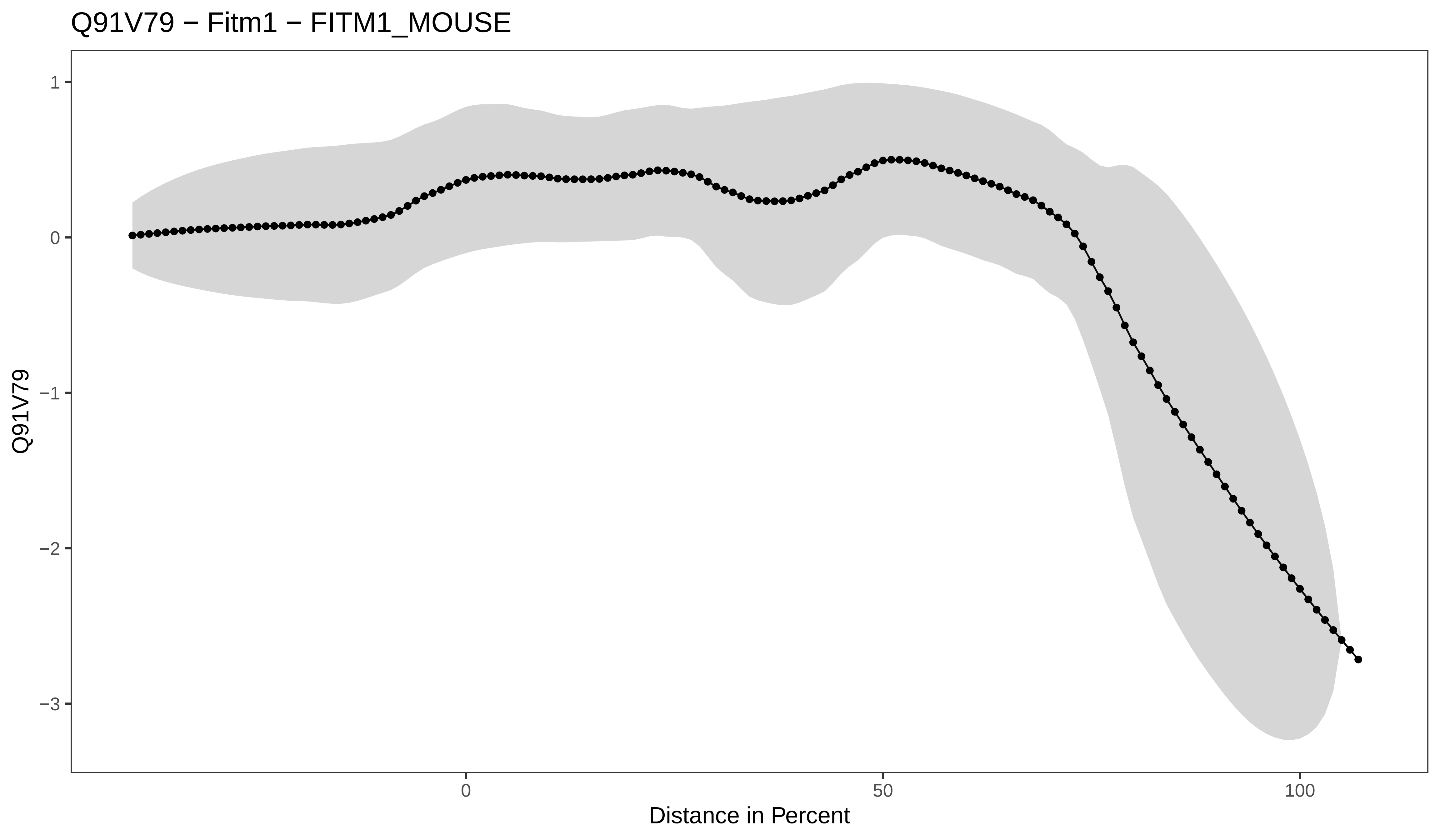
Q91V79 − Fitm1 − FITM1_MOUSE
1
0
−1
−2
−3
Q91V79
0
50
100
Distance in Percent

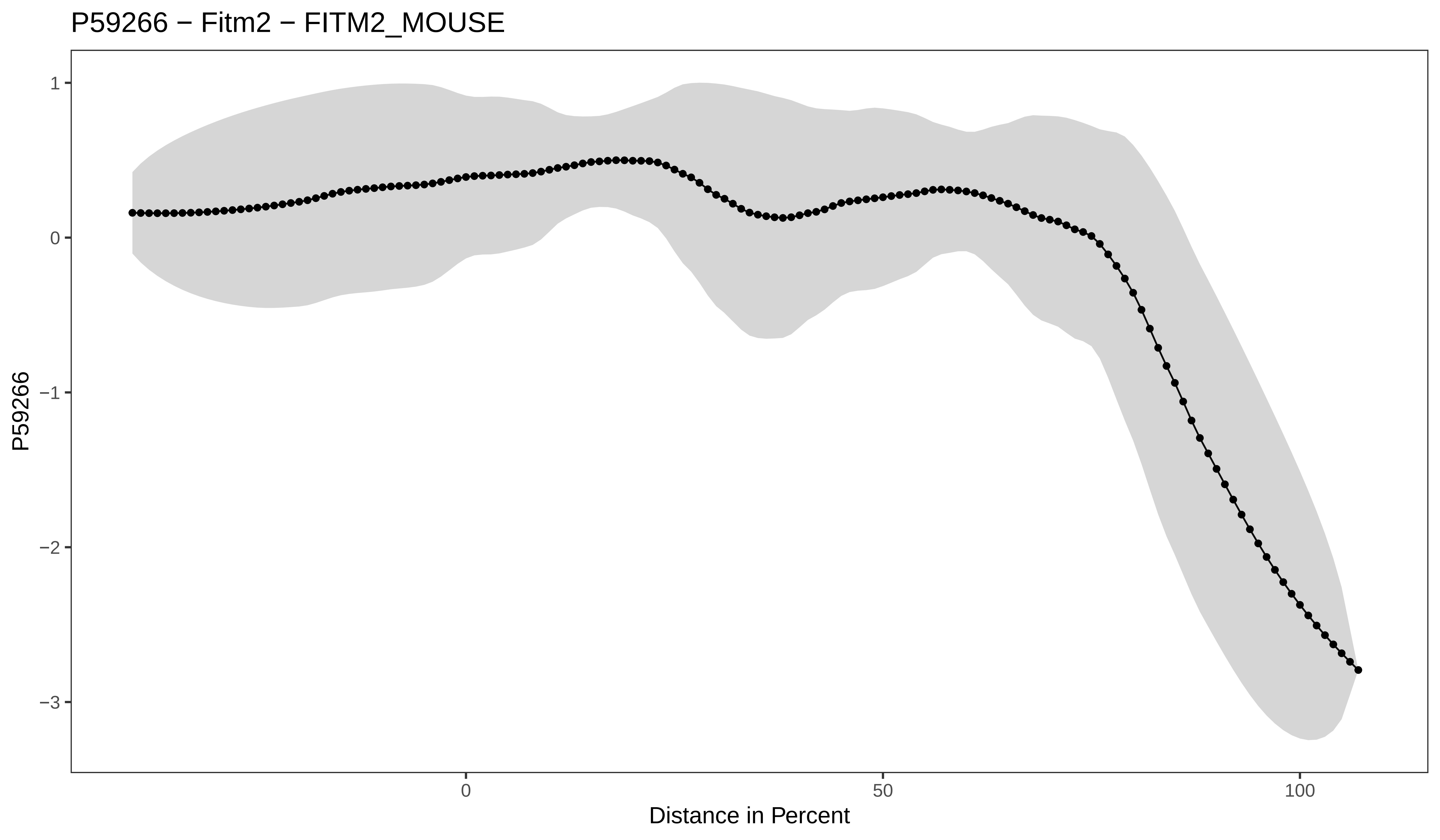
P59266 − Fitm2 − FITM2_MOUSE
P59266
1
0
−1
−2
−3
0
50
100
Distance in Percent

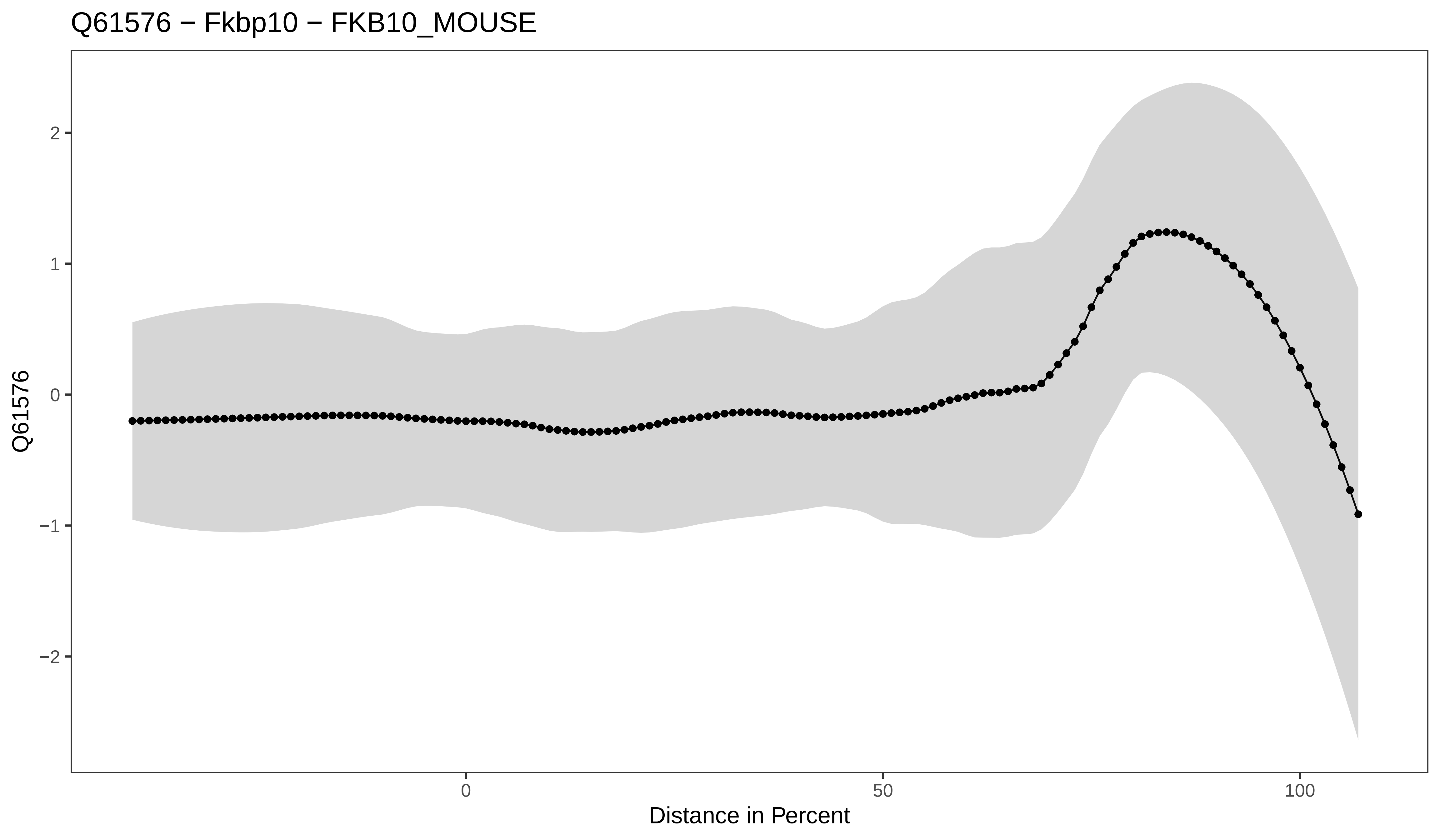

Q61576 − Fkbp10 − FKB10_MOUSE
2
1
0
−1
−2
Q61576
0
50
100
Distance in Percent

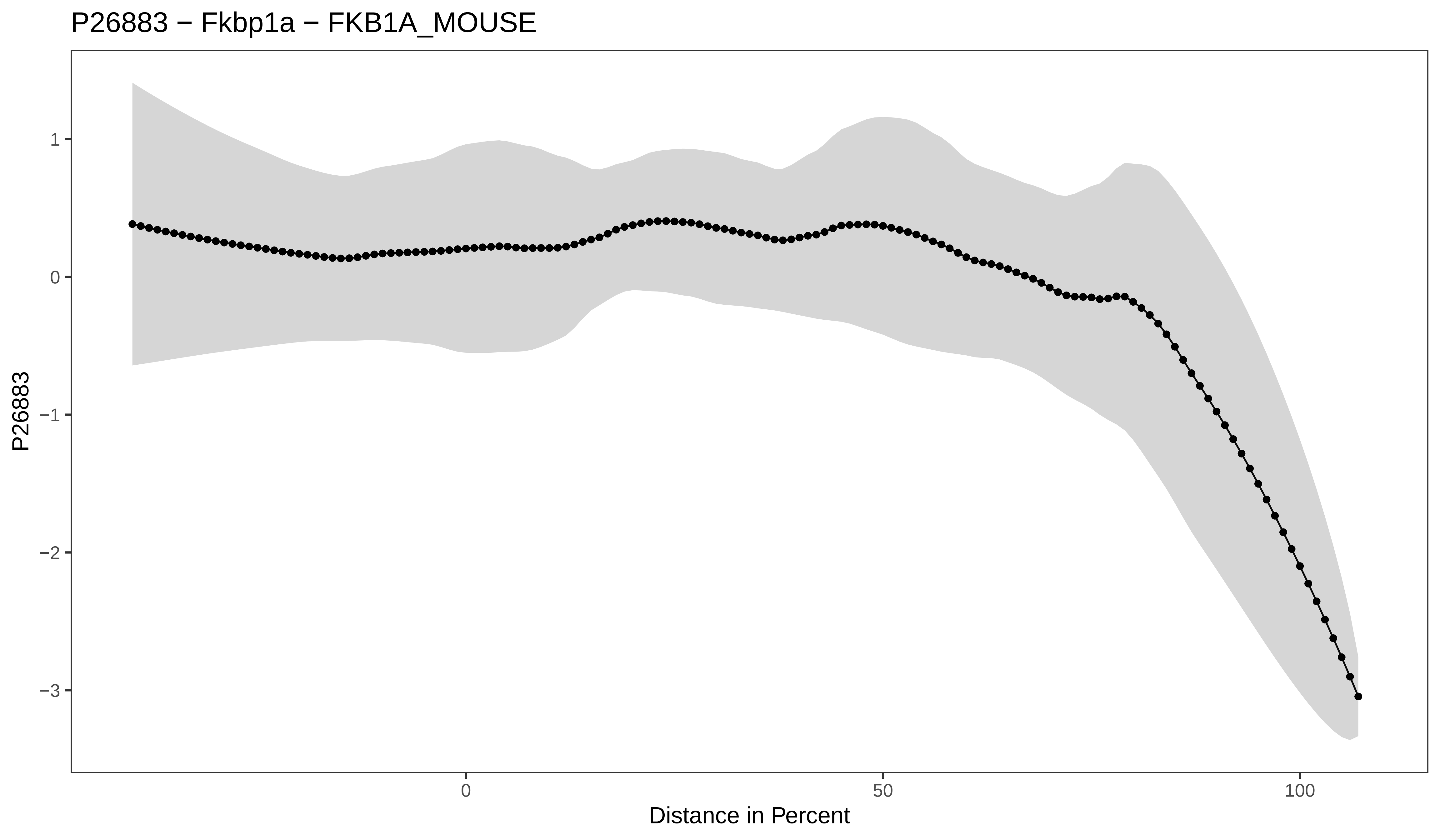
P26883 − Fkbp1a − FKB1A_MOUSE
P26883
1
0
−1
−2
−3
0
50
100
Distance in Percent

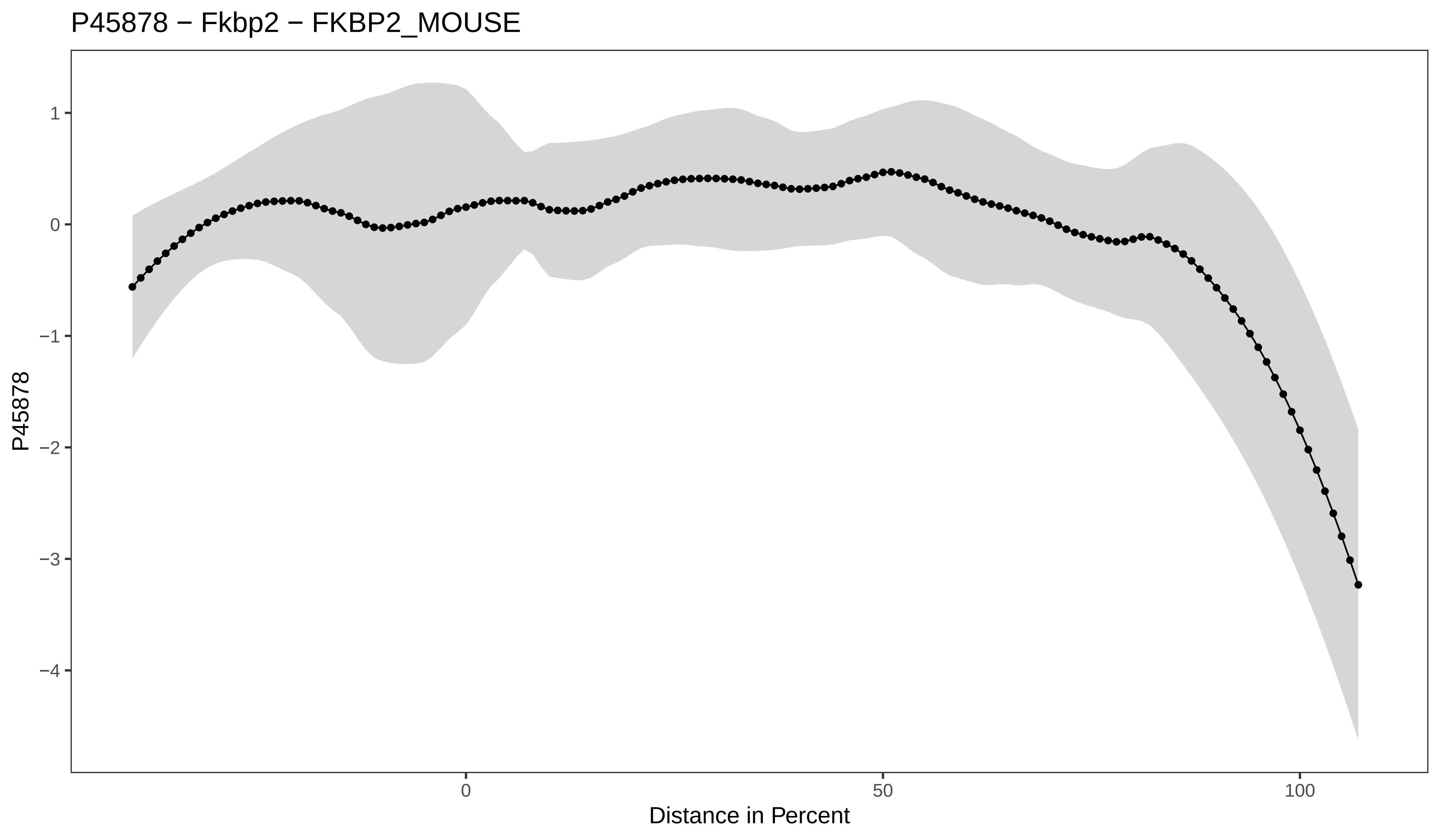

P45878 − Fkbp2 − FKBP2_MOUSE
1
0
−1
−2
−3
−4
P45878
0
50
100
Distance in Percent

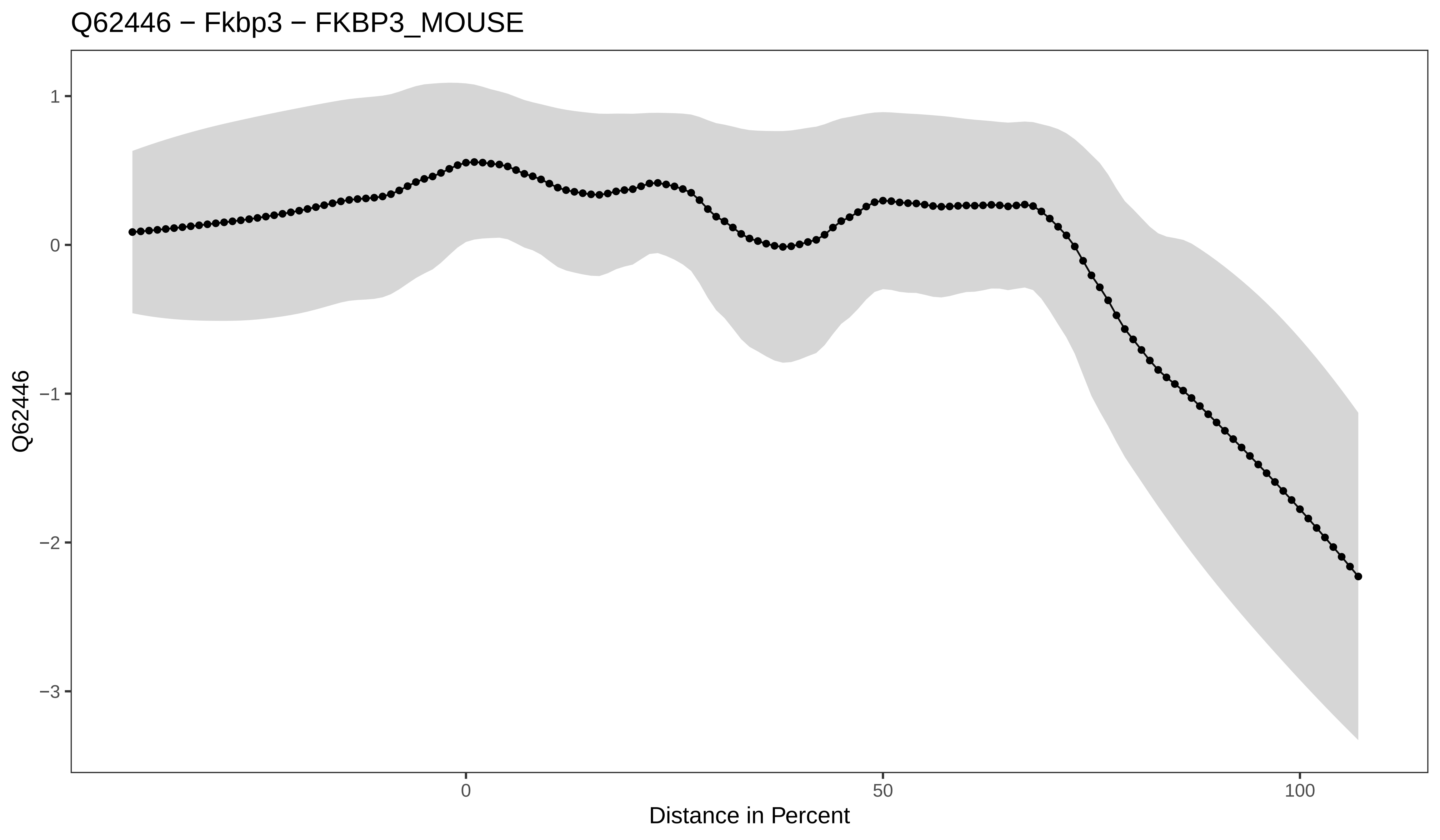

Q62446 − Fkbp3 − FKBP3_MOUSE
1
0
−1
−2
−3
Q62446
0
50
100
Distance in Percent

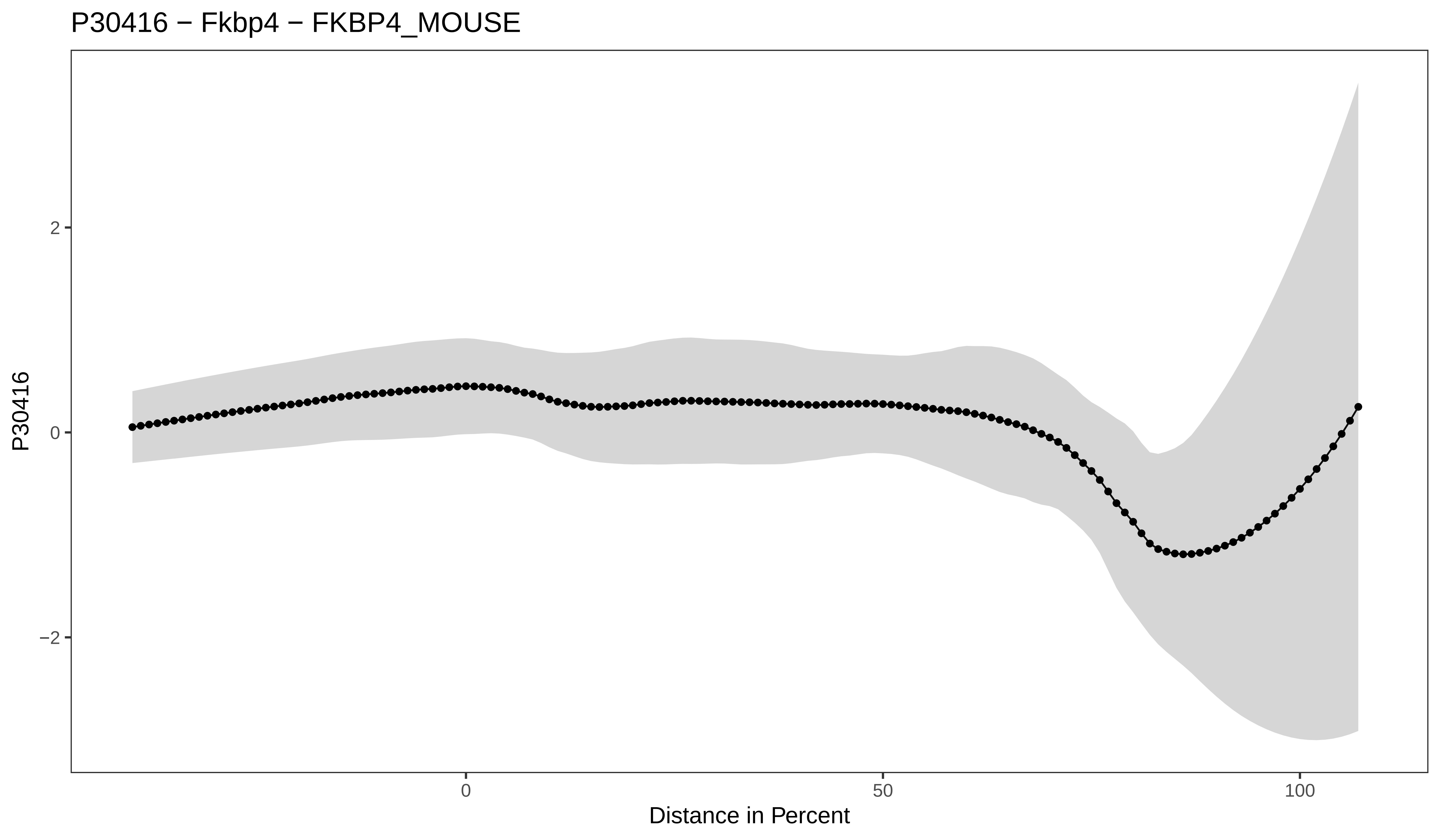
P30416 − Fkbp4 − FKBP4_MOUSE
P30416
2
0
−2
0
50
100
Distance in Percent

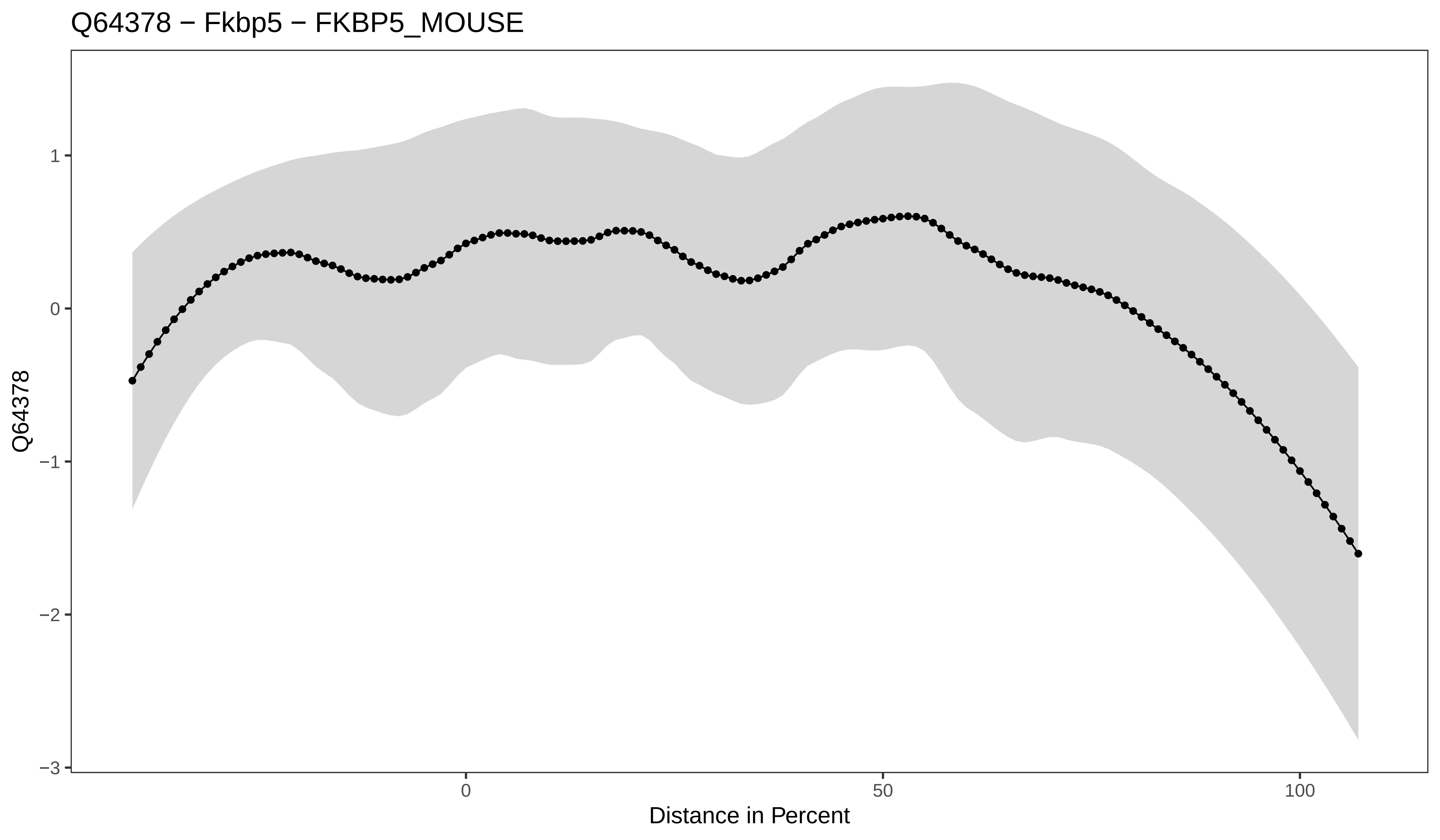
Q64378 − Fkbp5 − FKBP5_MOUSE
Q64378
1
0
−1
−2
−3
0
50
100
Distance in Percent

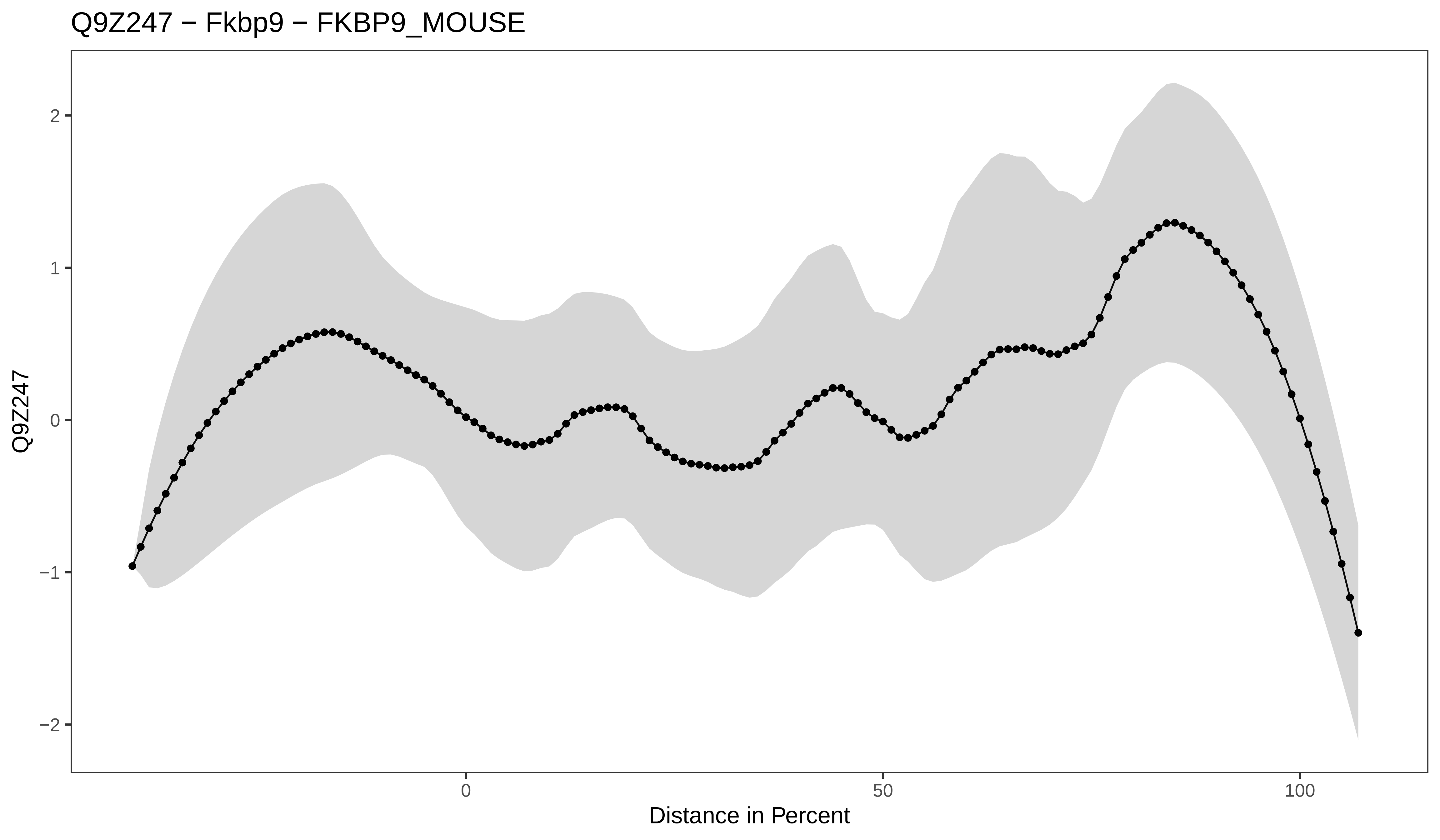
Q9Z247 − Fkbp9 − FKBP9_MOUSE
Q9Z247
2
1
0
−1
−2
0
50
100
Distance in Percent

Q8R123 − Flad1 − FAD1_MOUSE

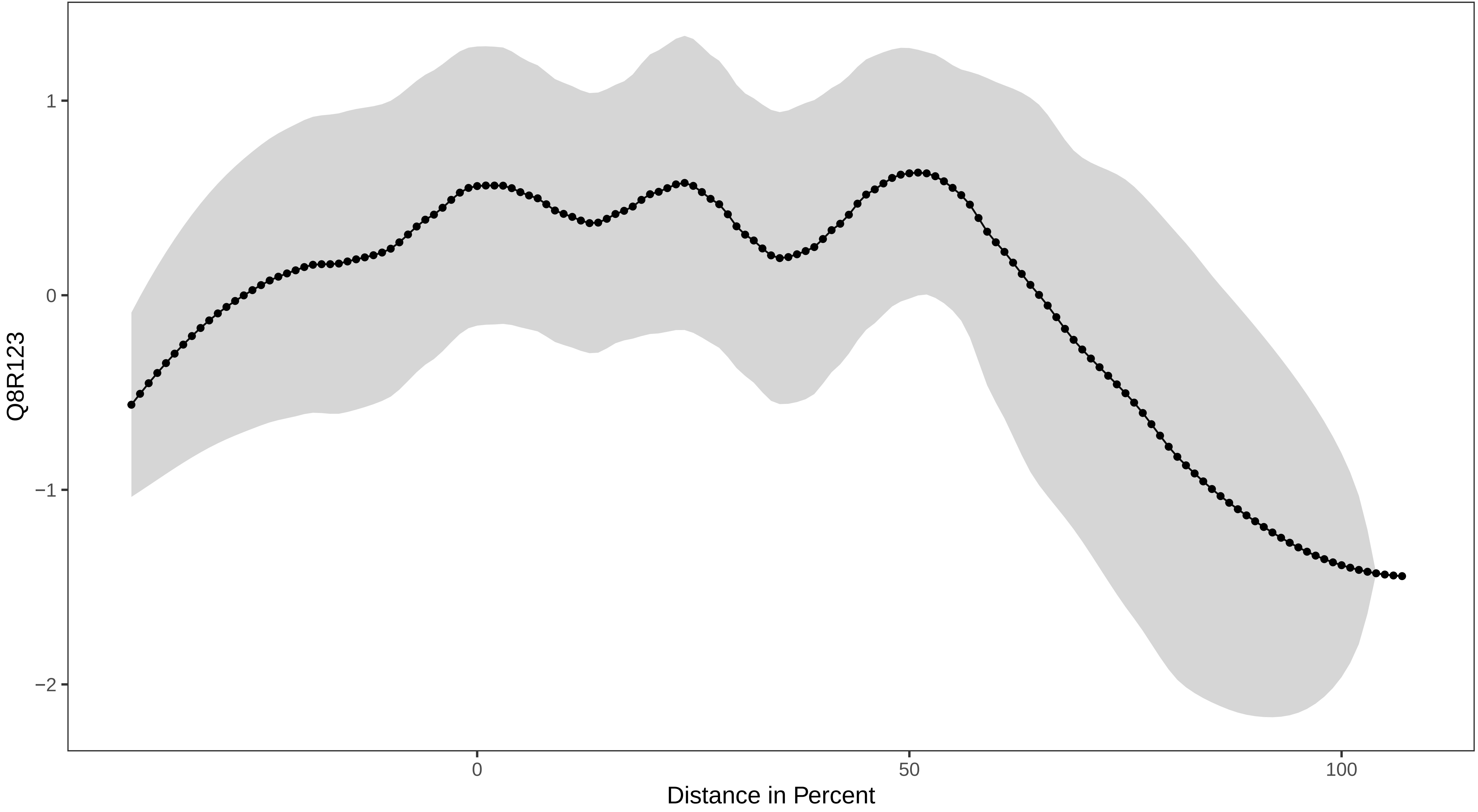

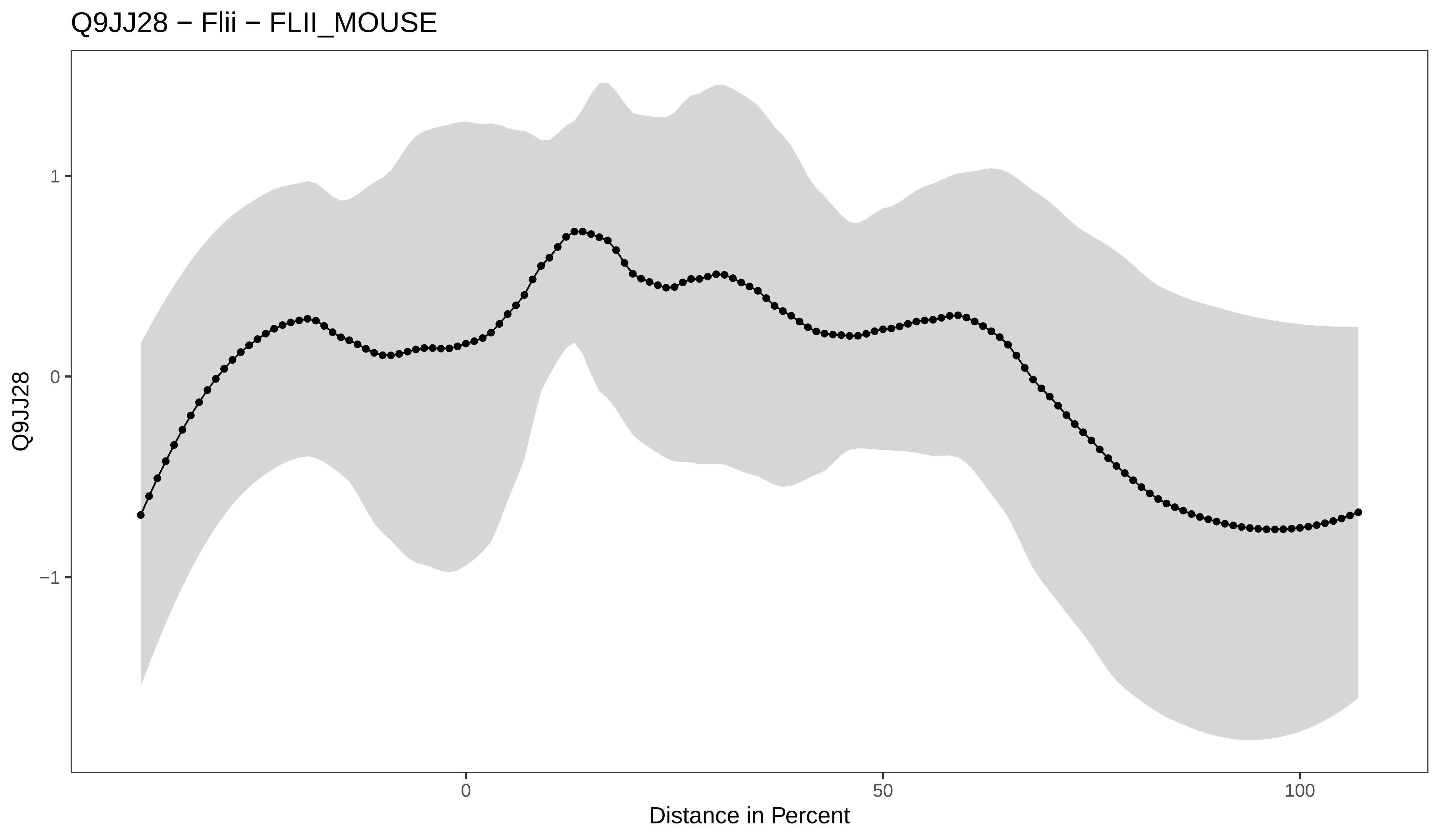

Q9JJ28 − Flii − FLII_MOUSE
Q9JJ28
1
0
−1
0
50
100
Distance in Percent

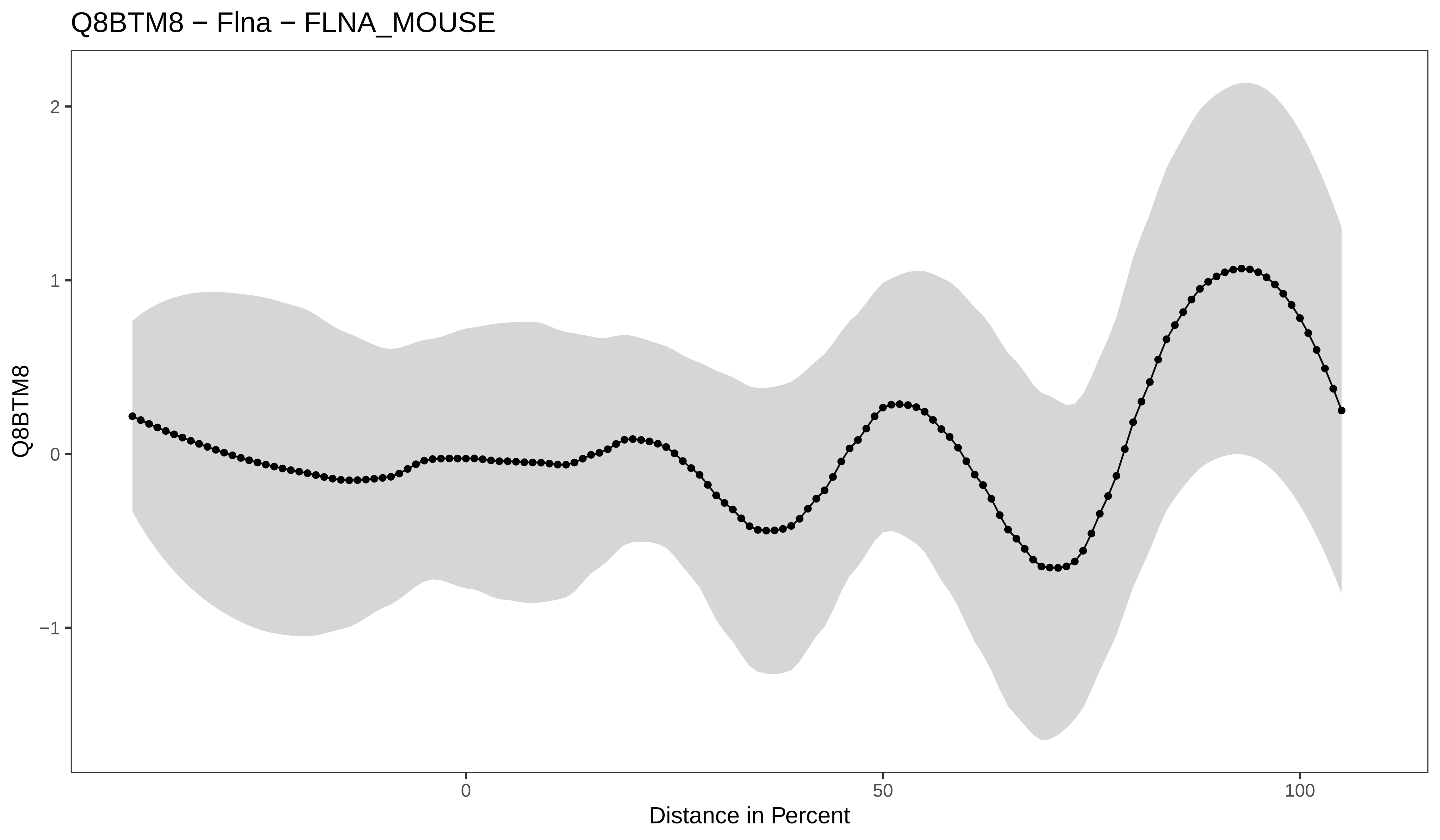

Q8BTM8 − Flna − FLNA_MOUSE
Q8BTM8
2
1
0
−1
0
50
100
Distance in Percent

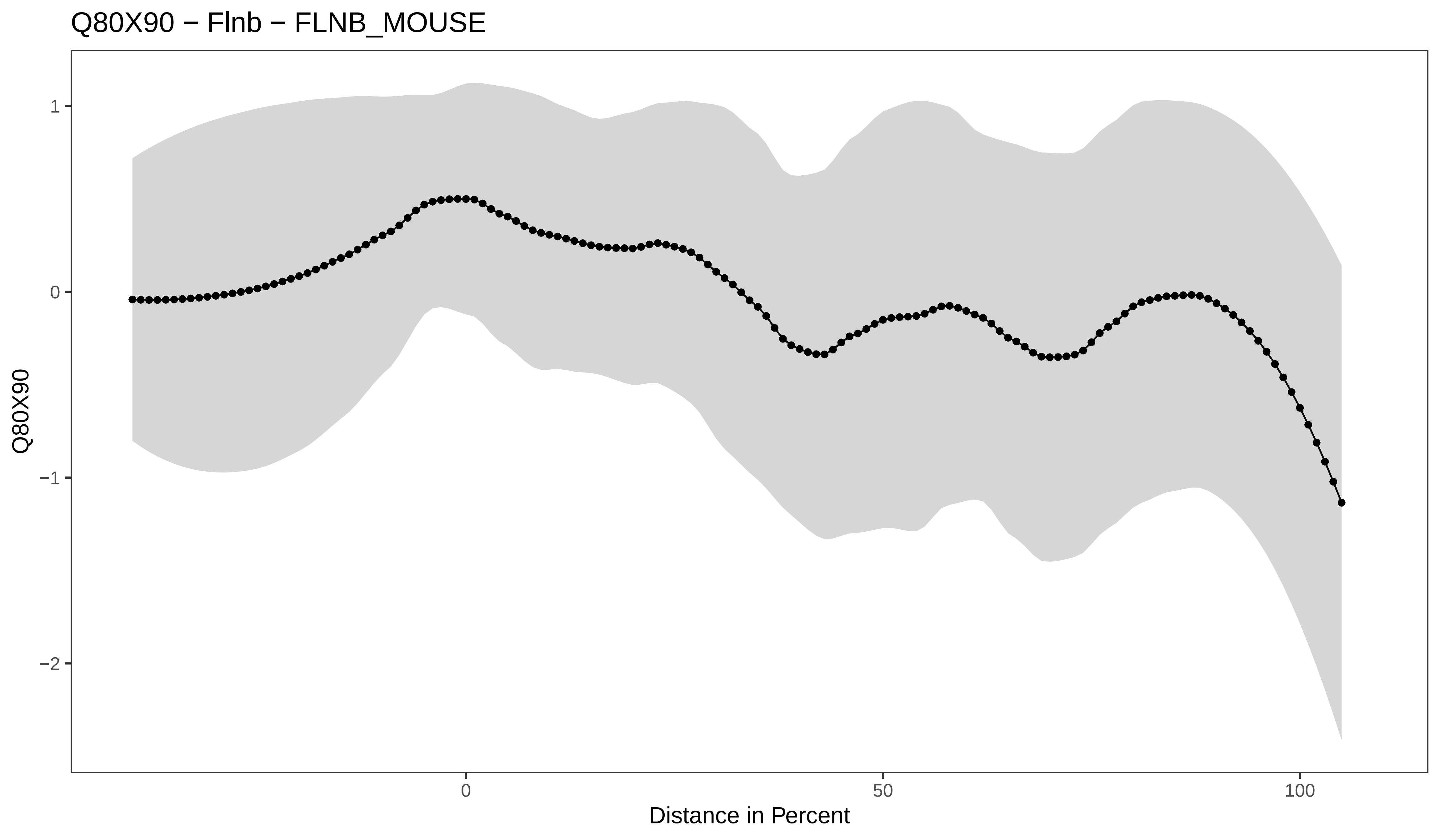

Q80X90 − Flnb − FLNB_MOUSE
Q80X90
1
0
−1
−2
0
50
100
Distance in Percent

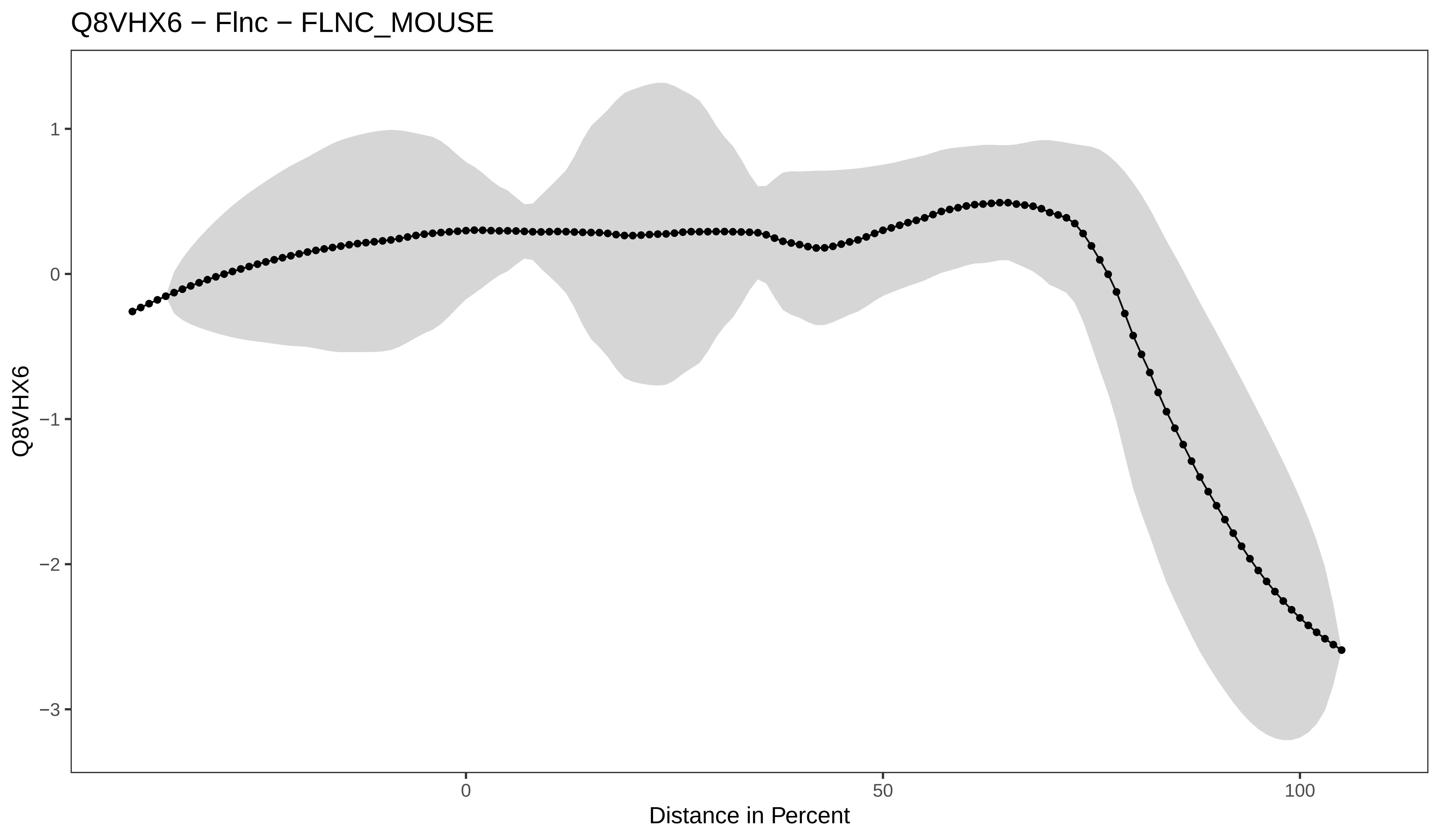
Q8VHX6 − Flnc − FLNC_MOUSE
Q8VHX6
1
0
−1
−2
−3
0
50
100
Distance in Percent

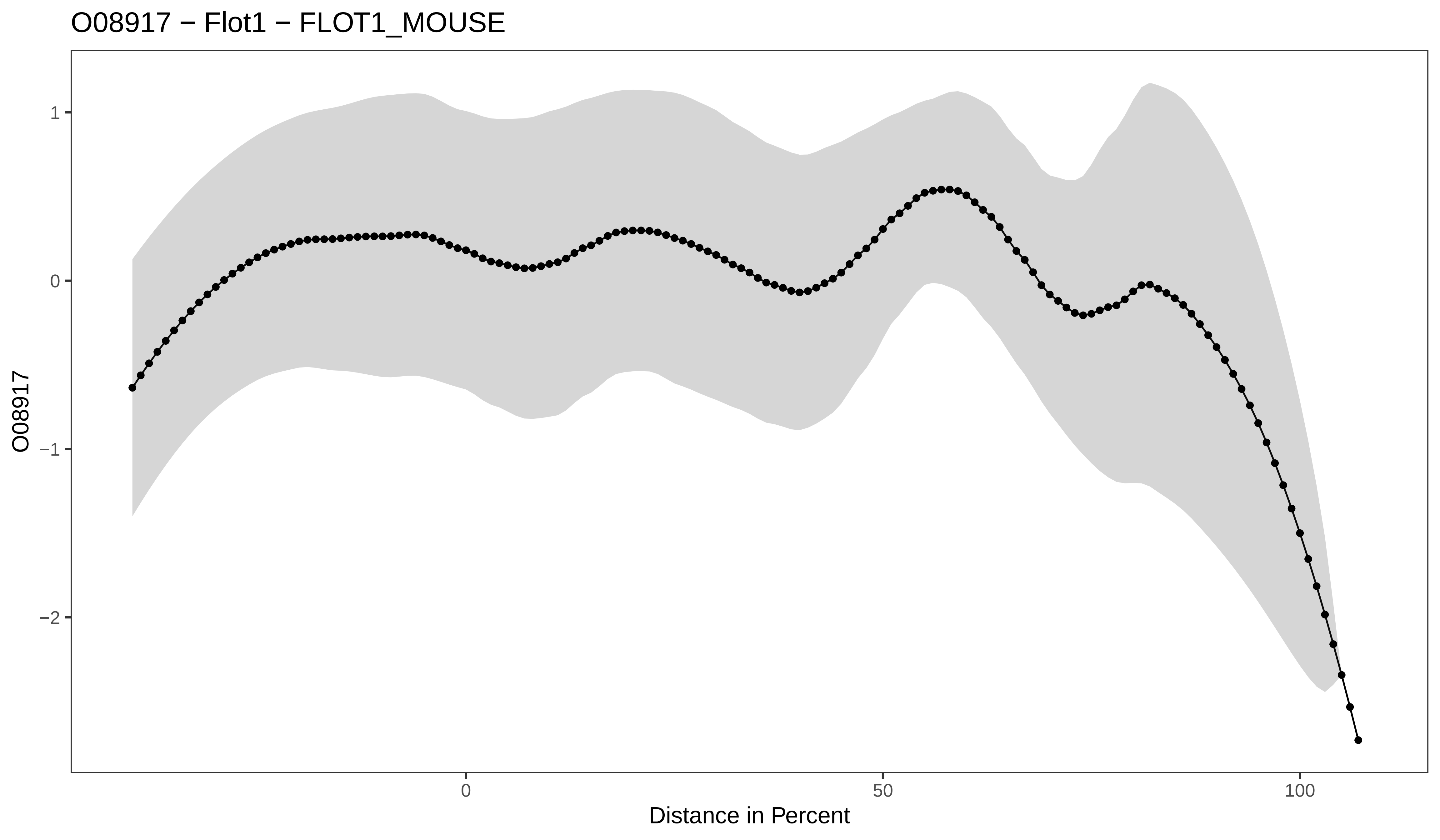

O08917 − Flot1 − FLOT1_MOUSE
1
0
−1
−2
O08917
0
50
100
Distance in Percent

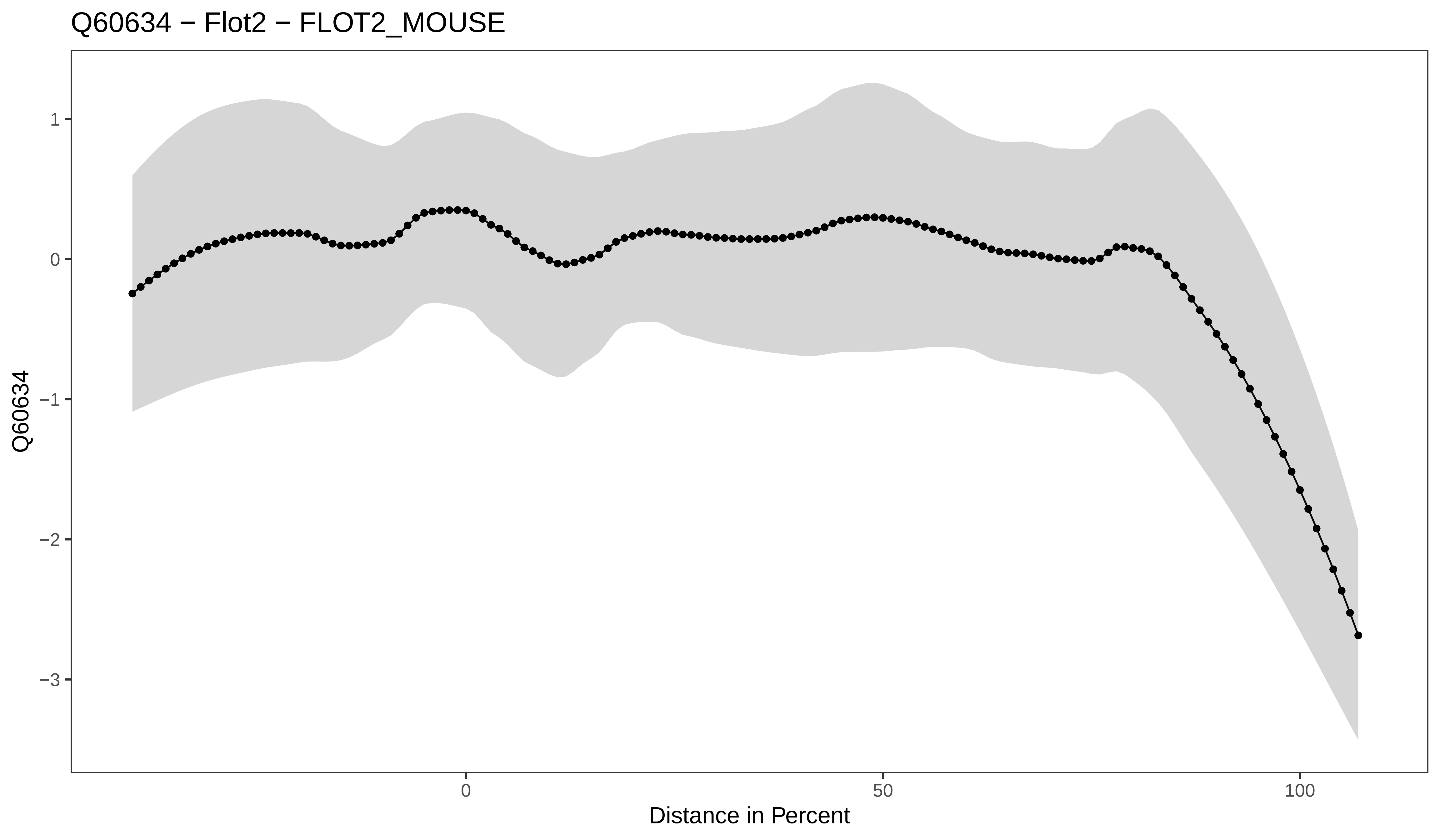
Q60634 − Flot2 − FLOT2_MOUSE
Q60634
Distance in Percent
1
0
−1
−2
−3
0
50
100

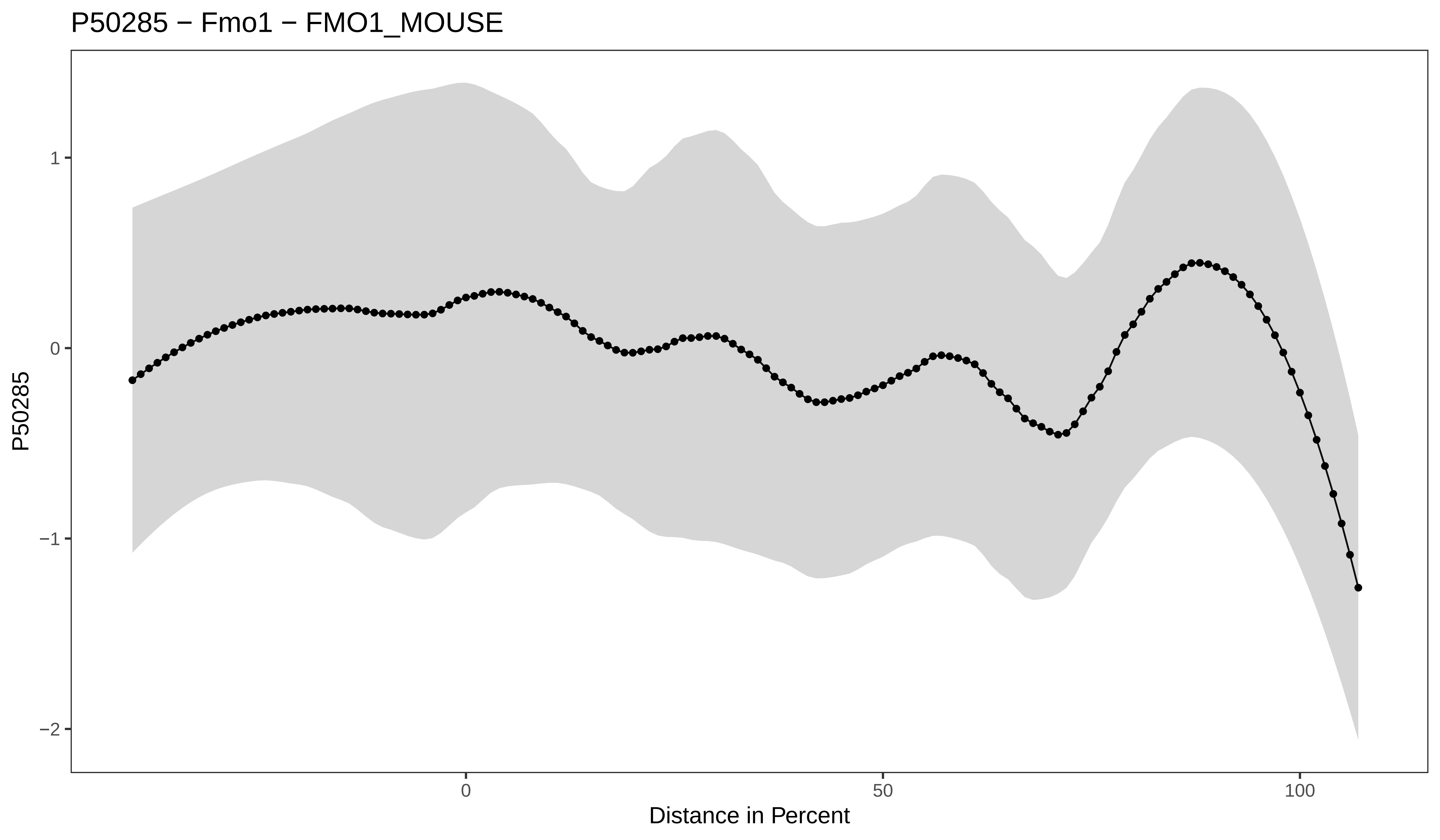

P50285 − Fmo1 − FMO1_MOUSE
P50285
Distance in Percent
1
0
−1
−2
0
50
100

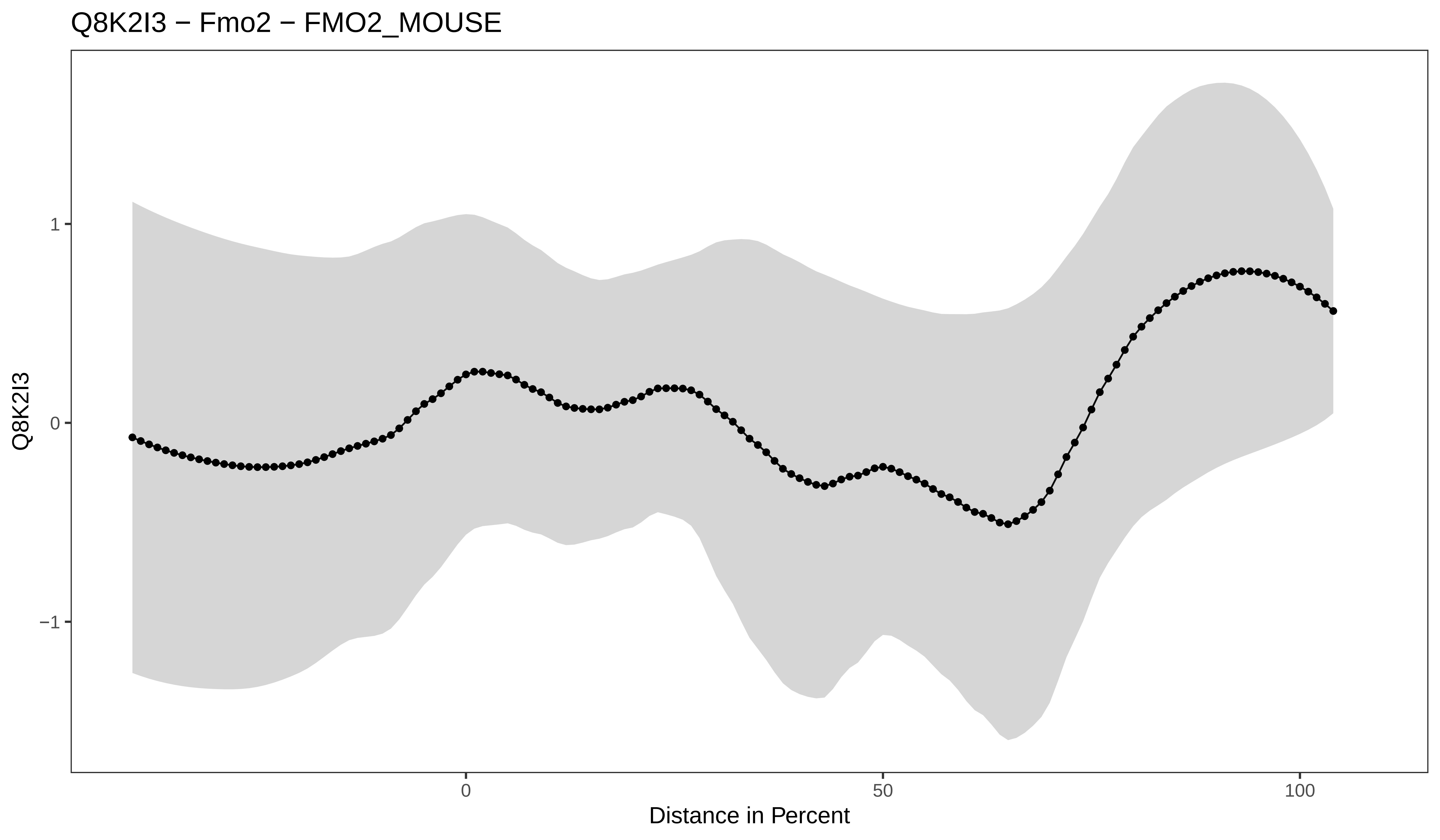
Q8K2I3 − Fmo2 − FMO2_MOUSE
Q8K2I3
1
0
−1
0
50
100
Distance in Percent

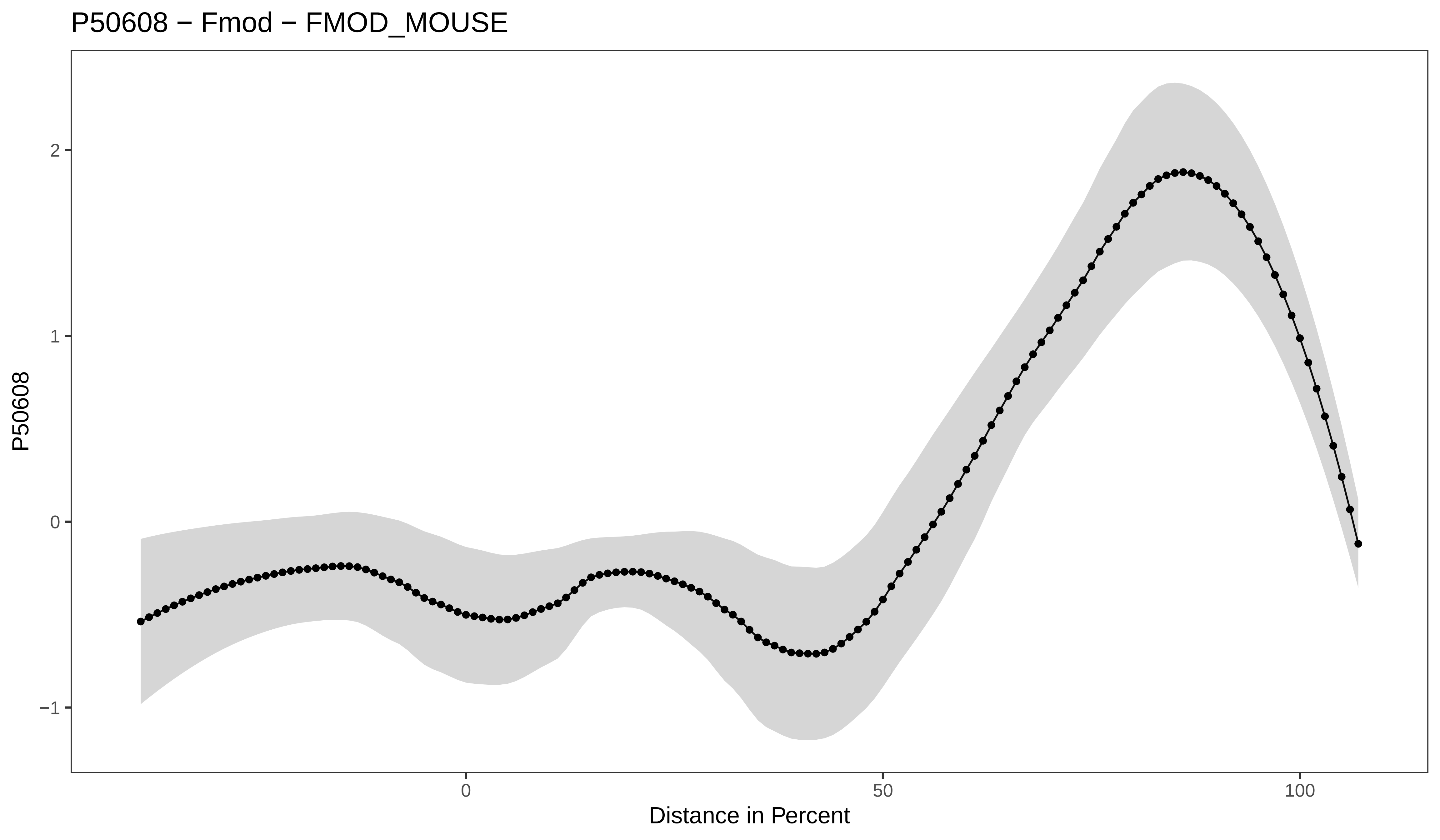

P50608 − Fmod − FMOD_MOUSE
P50608
Distance in Percent
2
1
0
−1
0
50
100

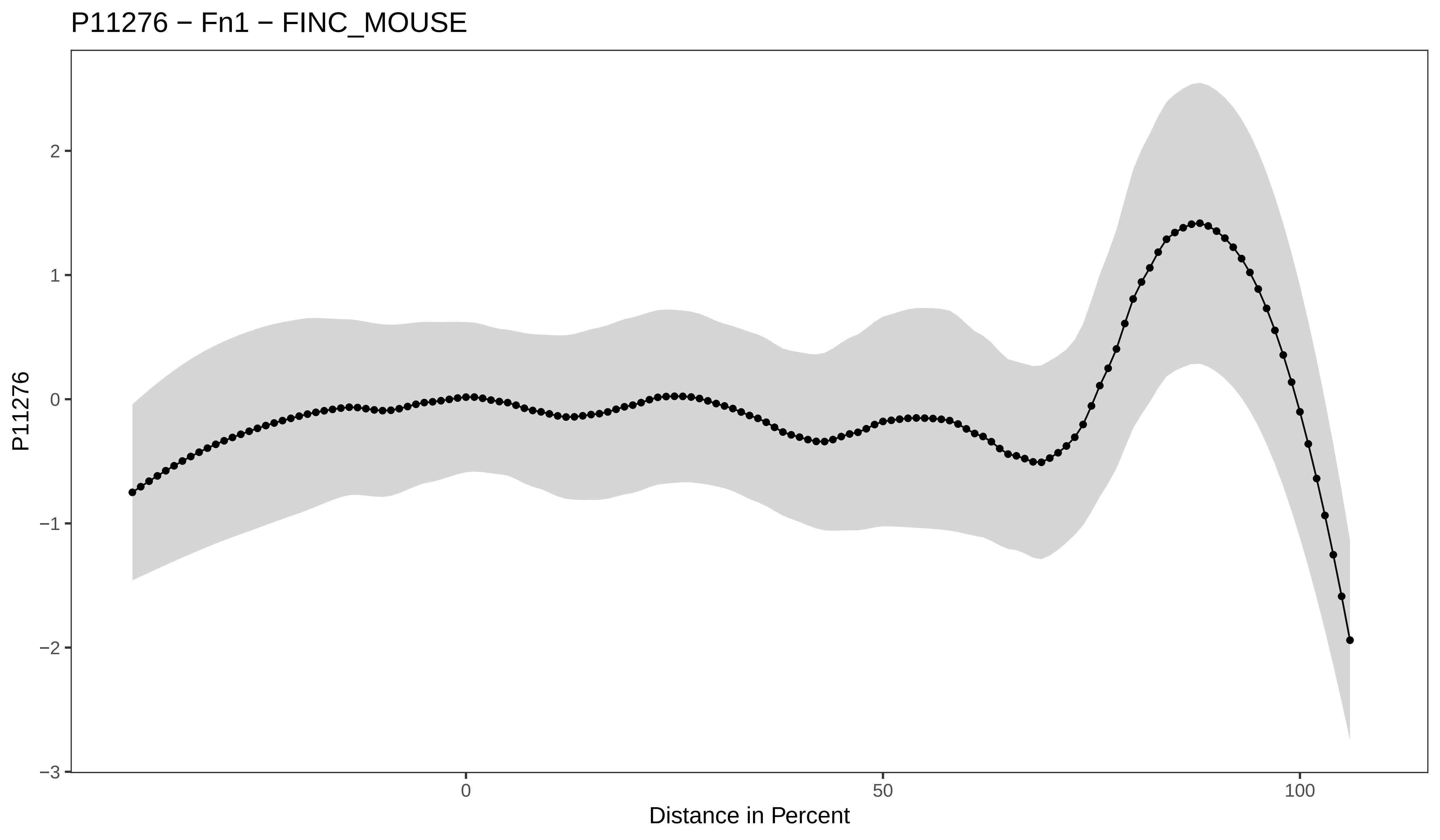
P11276 − Fn1 − FINC_MOUSE
P11276
Distance in Percent
2
1
0
−1
−2
−3
0
50
100

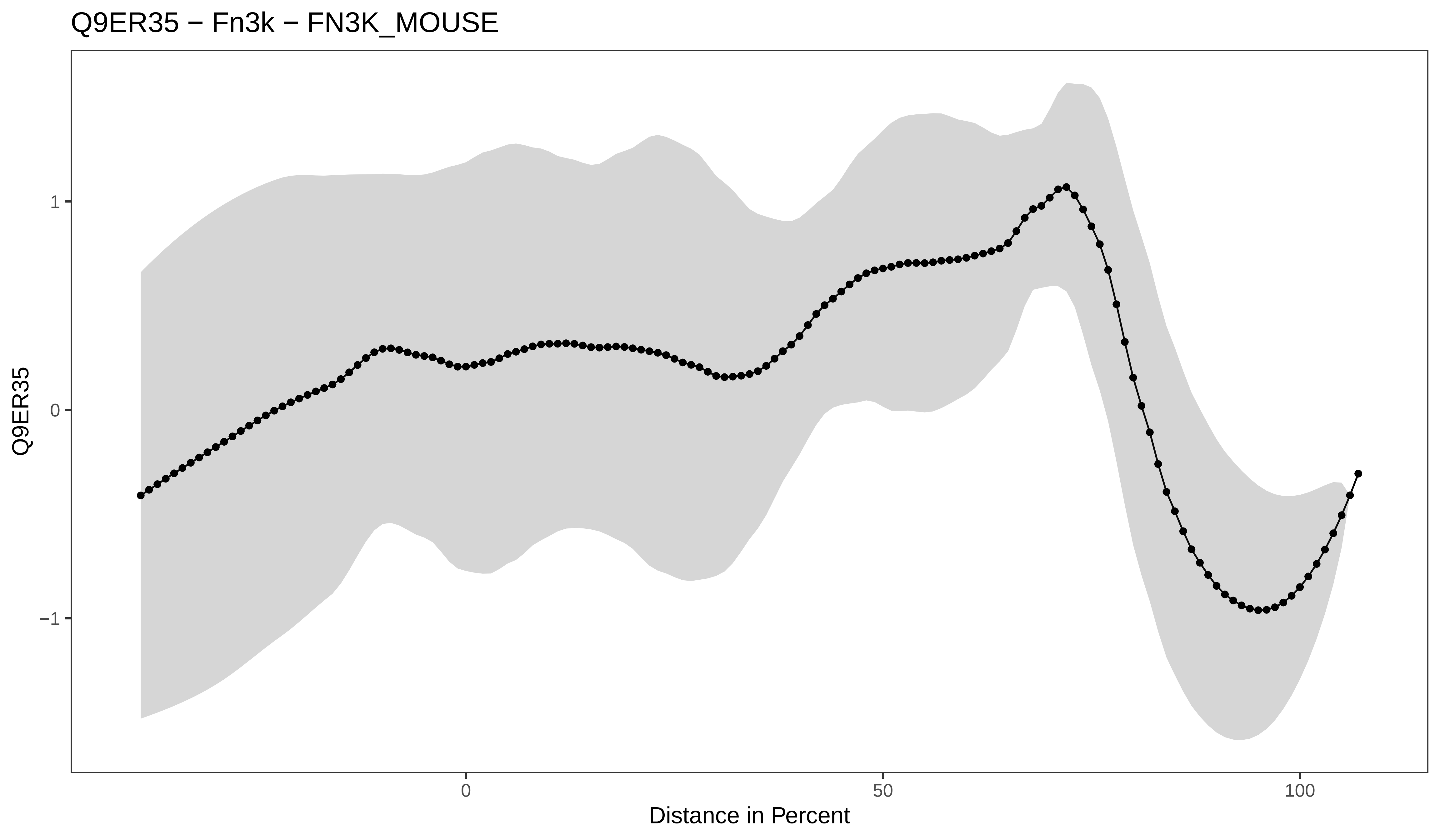

Q9ER35 − Fn3k − FN3K_MOUSE
Q9ER35
1
0
−1
0
50
100
Distance in Percent

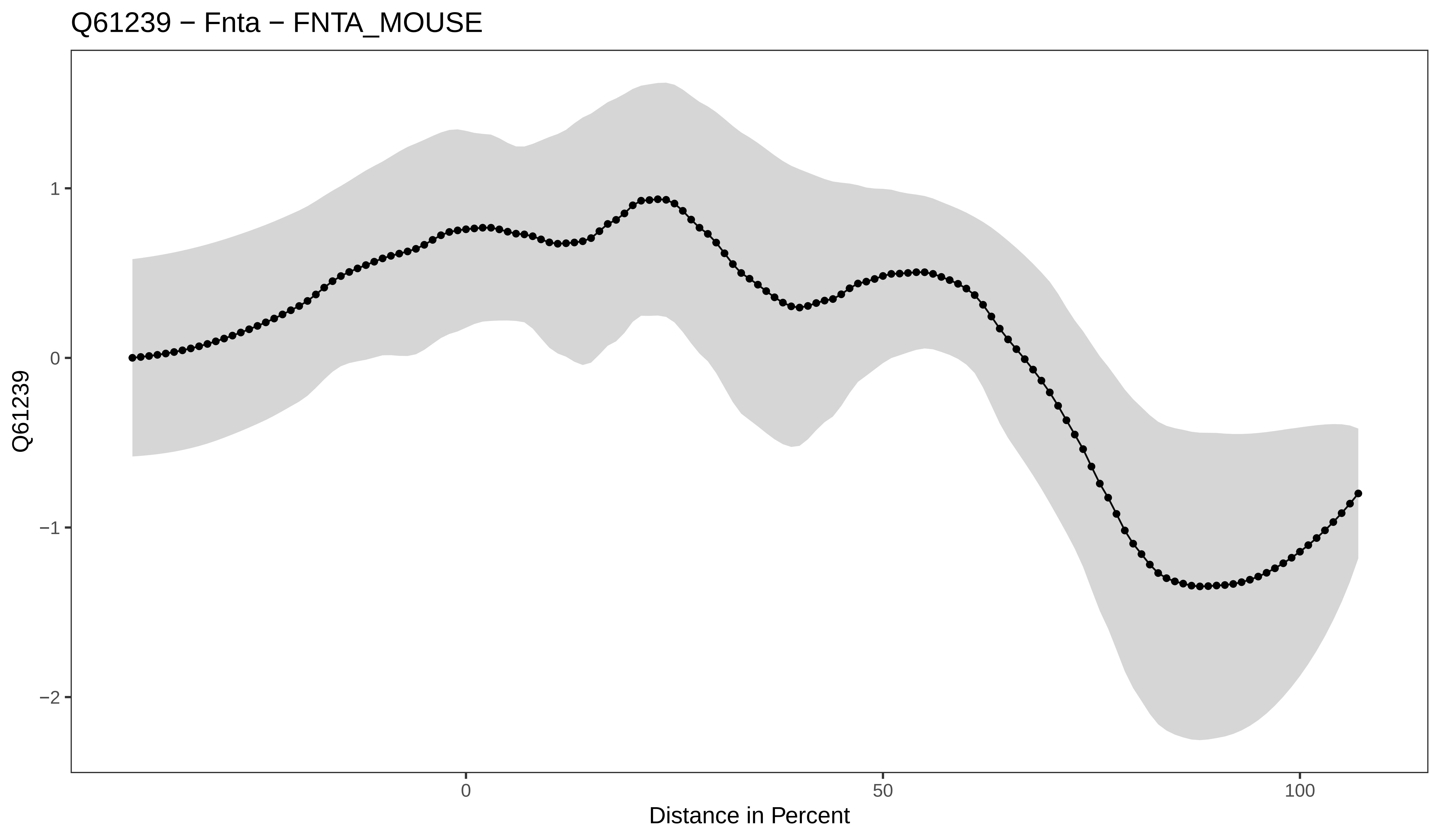
Q61239 − Fnta − FNTA_MOUSE
1
0
−1
−2
Q61239
0
50
100
Distance in Percent

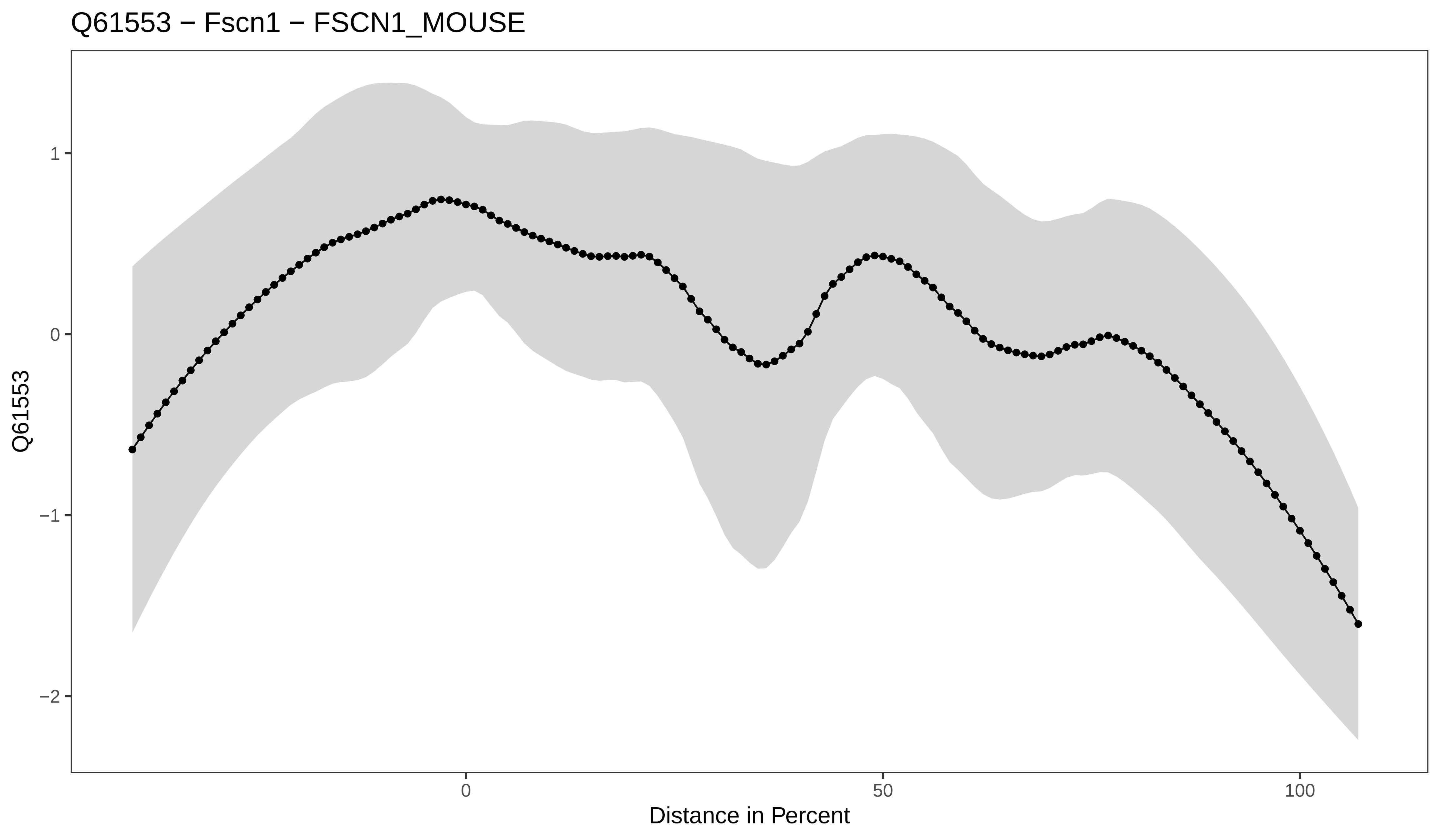
Q61553 − Fscn1 − FSCN1_MOUSE
Q61553
1
0
−1
−2
0
50
100
Distance in Percent

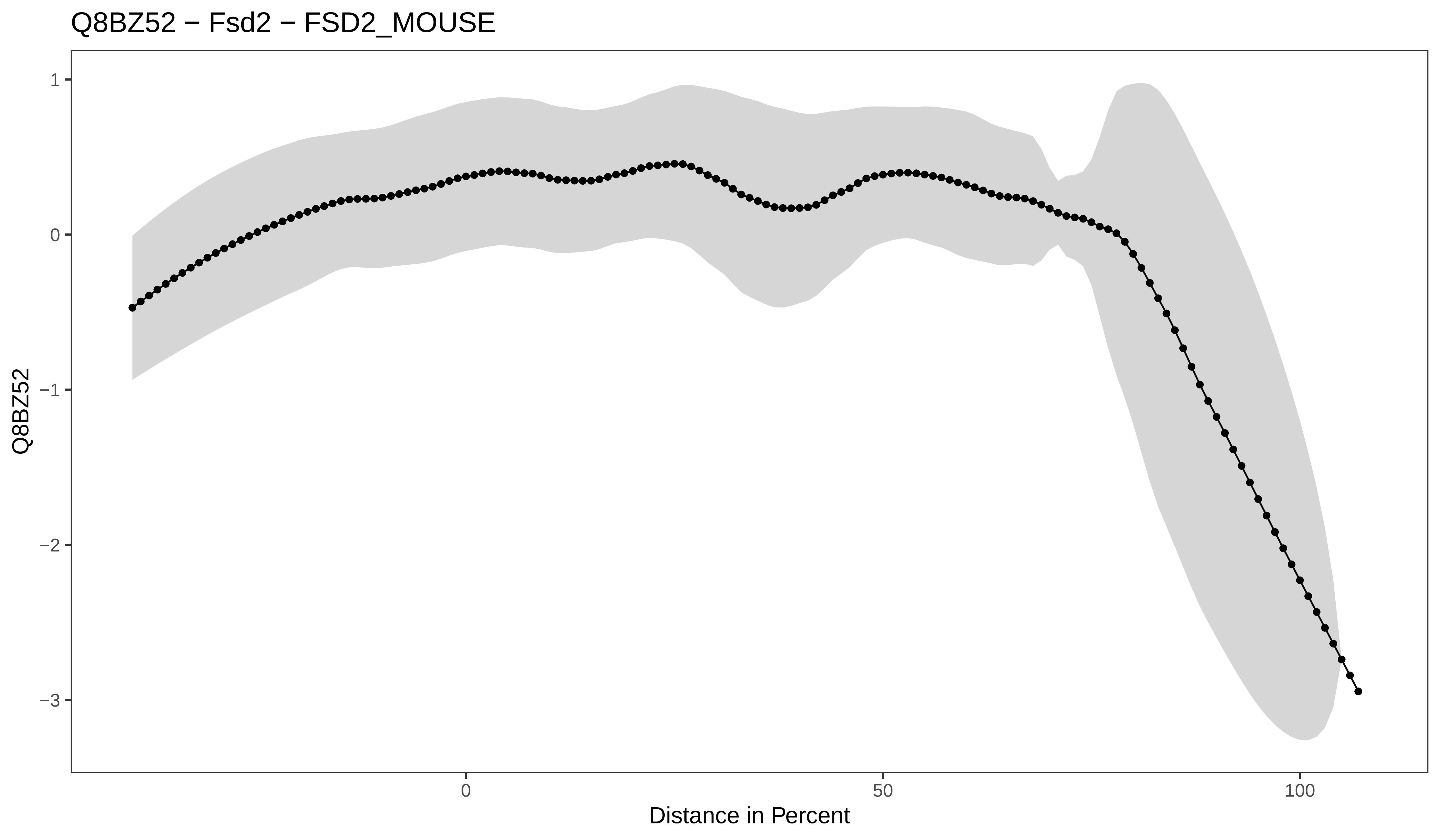

Q8BZ52 − Fsd2 − FSD2_MOUSE
Q8BZ52
Distance in Percent
1
0
−1
−2
−3
0
50
100

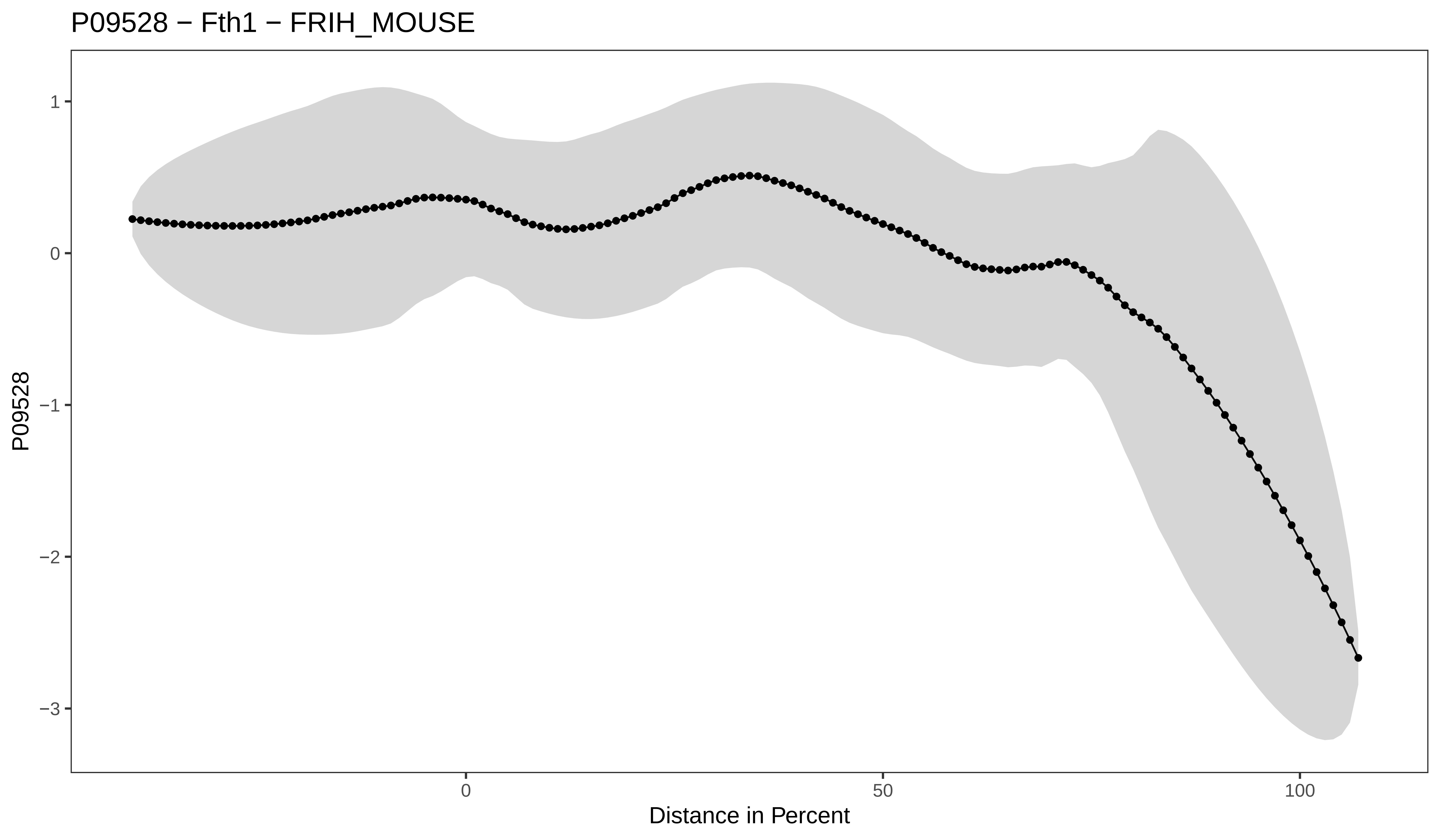

P09528 − Fth1 − FRIH_MOUSE
P09528
1
0
−1
−2
−3
0
50
100
Distance in Percent

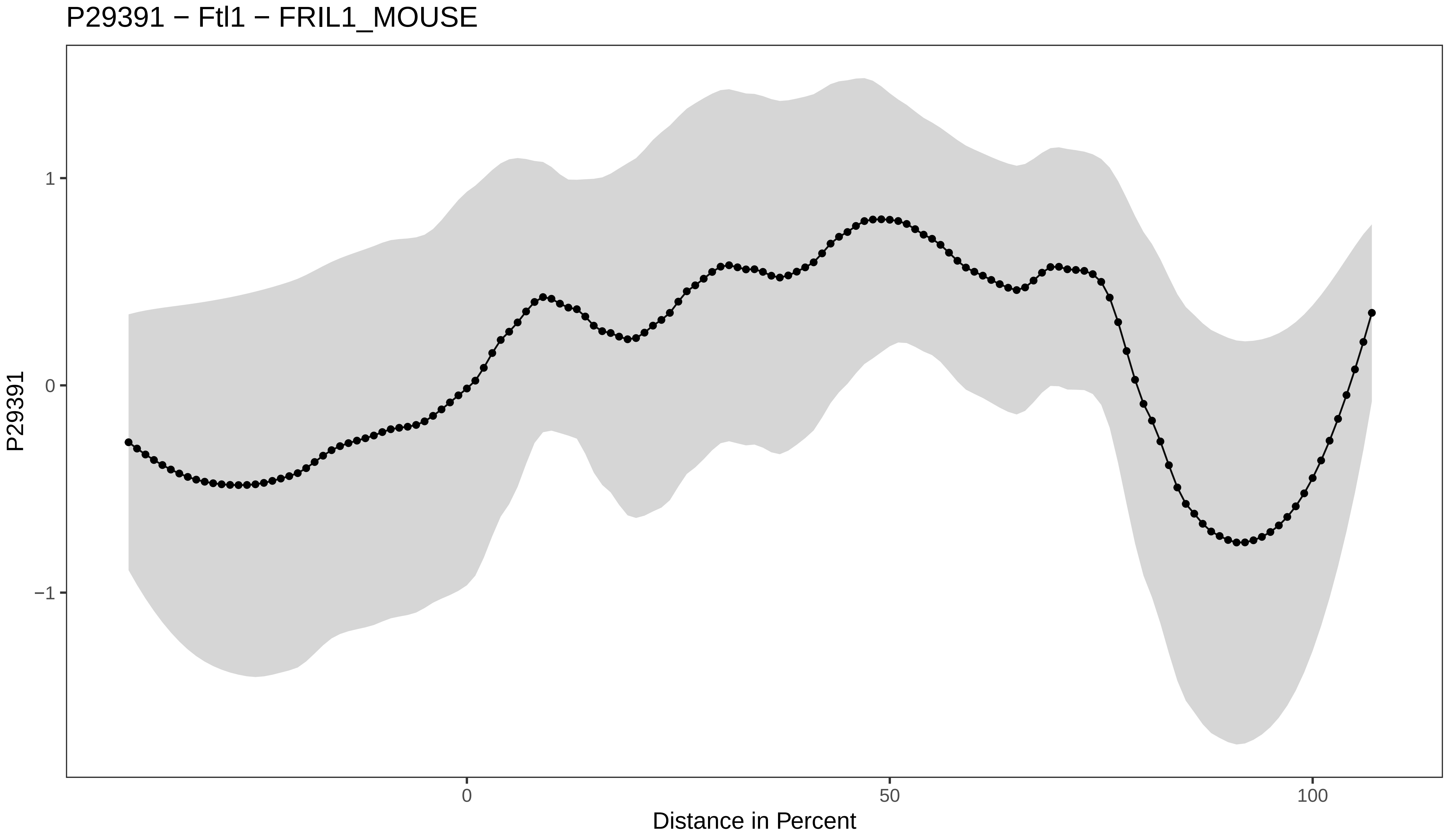
P29391 − Ftl1 − FRIL1_MOUSE
P29391
1
0
−1
0
50
100
Distance in Percent

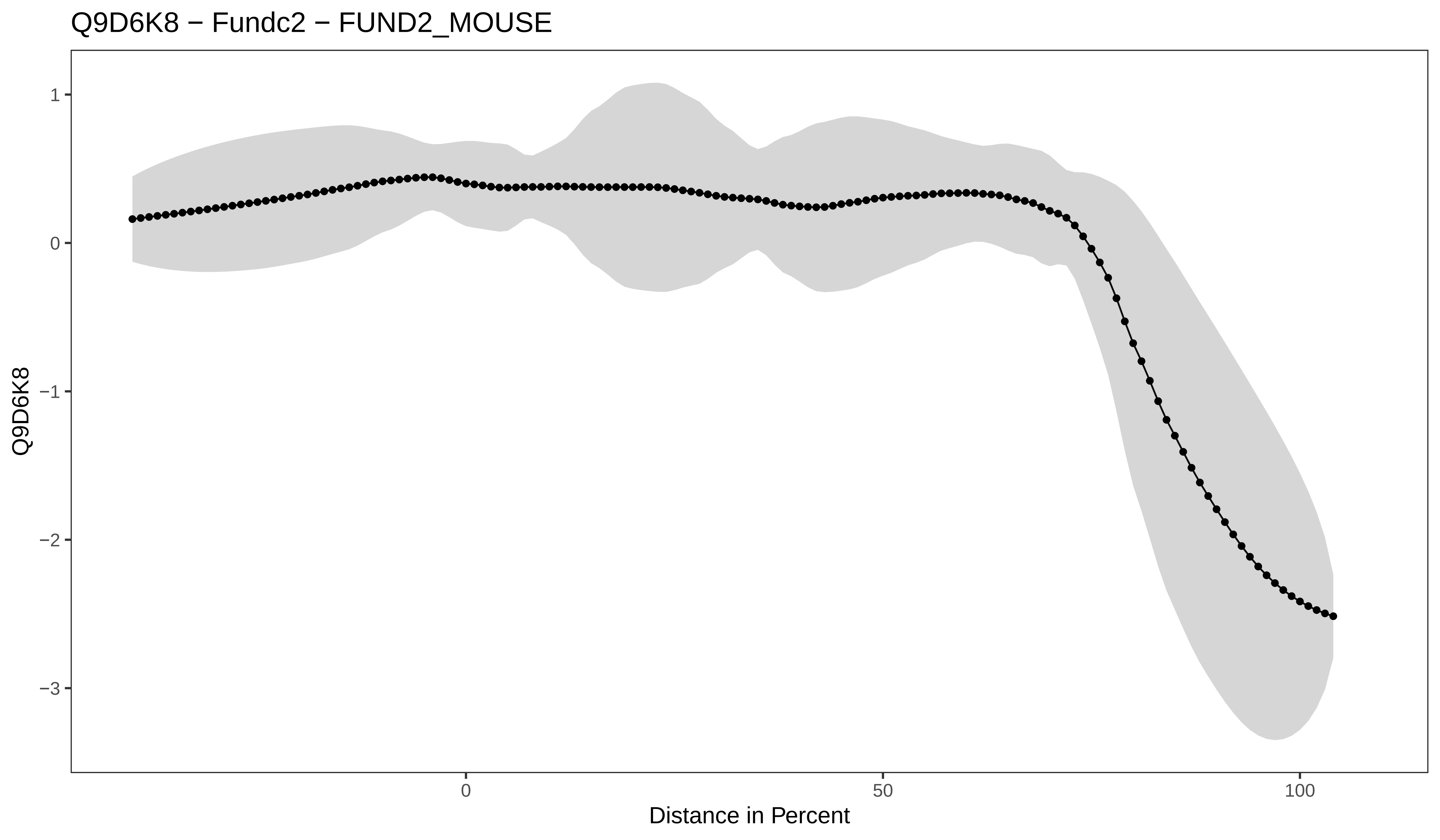
Q9D6K8 − Fundc2 − FUND2_MOUSE
1
0
−1
−2
−3
Q9D6K8
0
50
100
Distance in Percent

P56959 − Fus − FUS_MOUSE

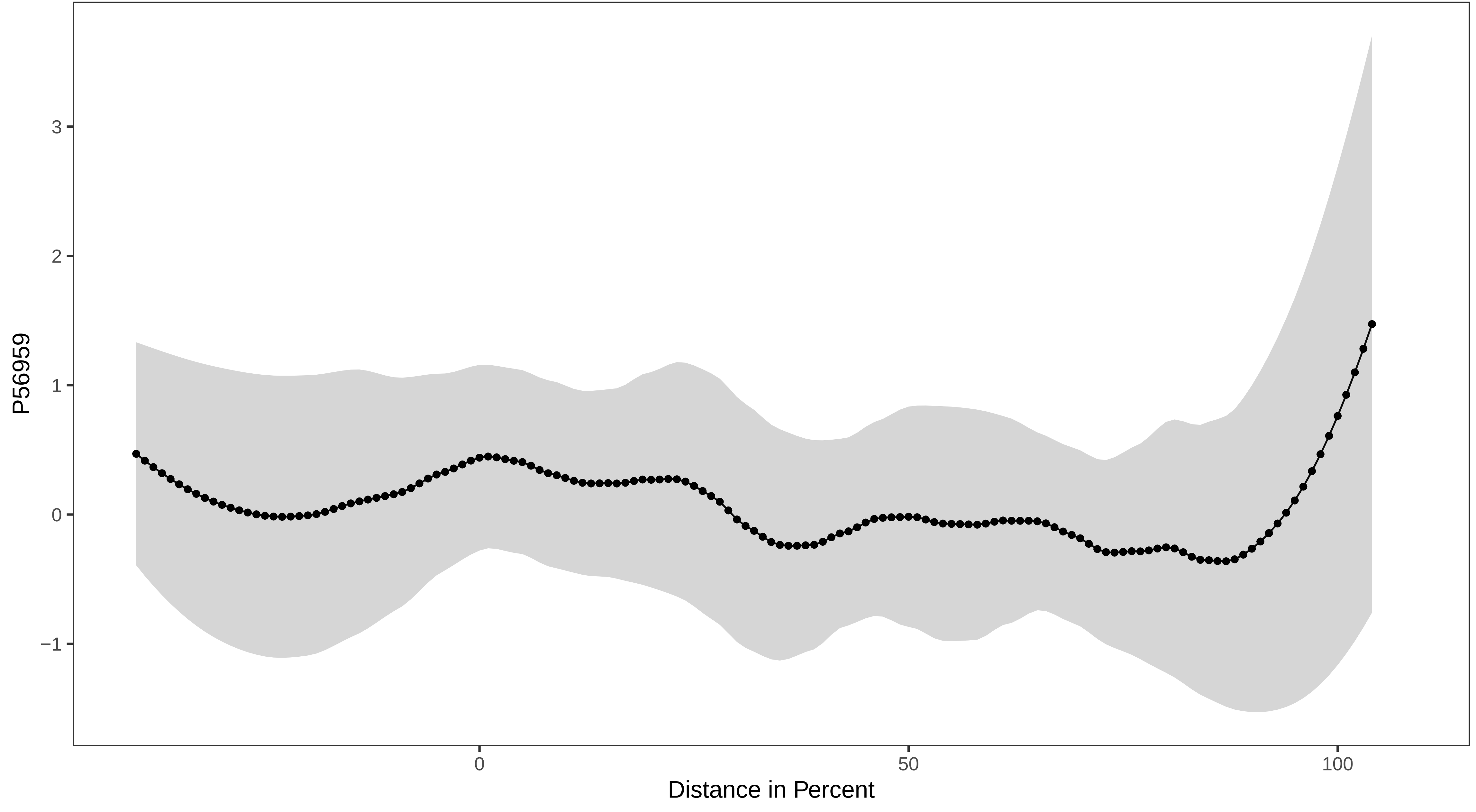

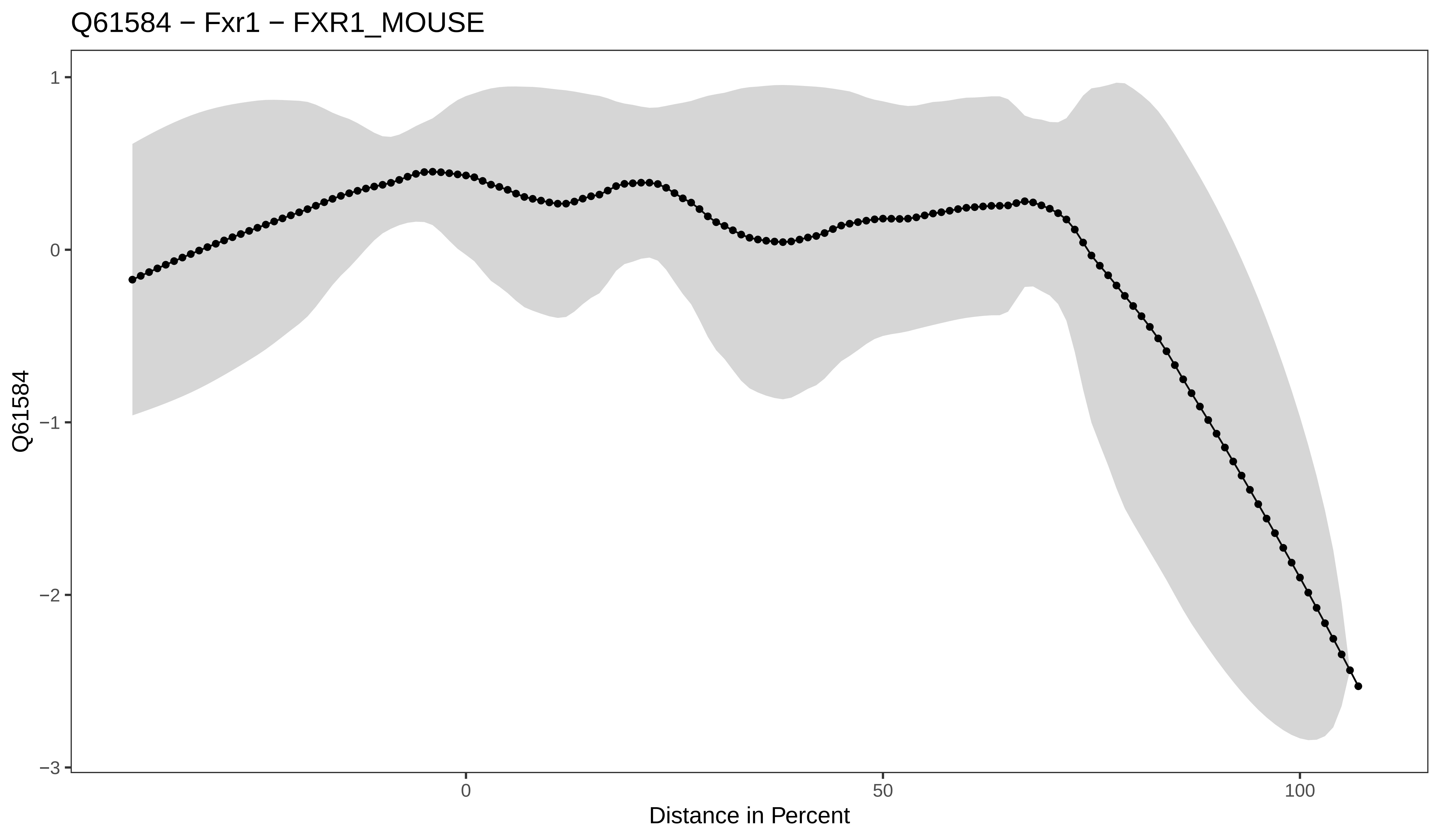
Q61584 − Fxr1 − FXR1_MOUSE
Q61584
Distance in Percent
1
0
−1
−2
−3
0
50
100

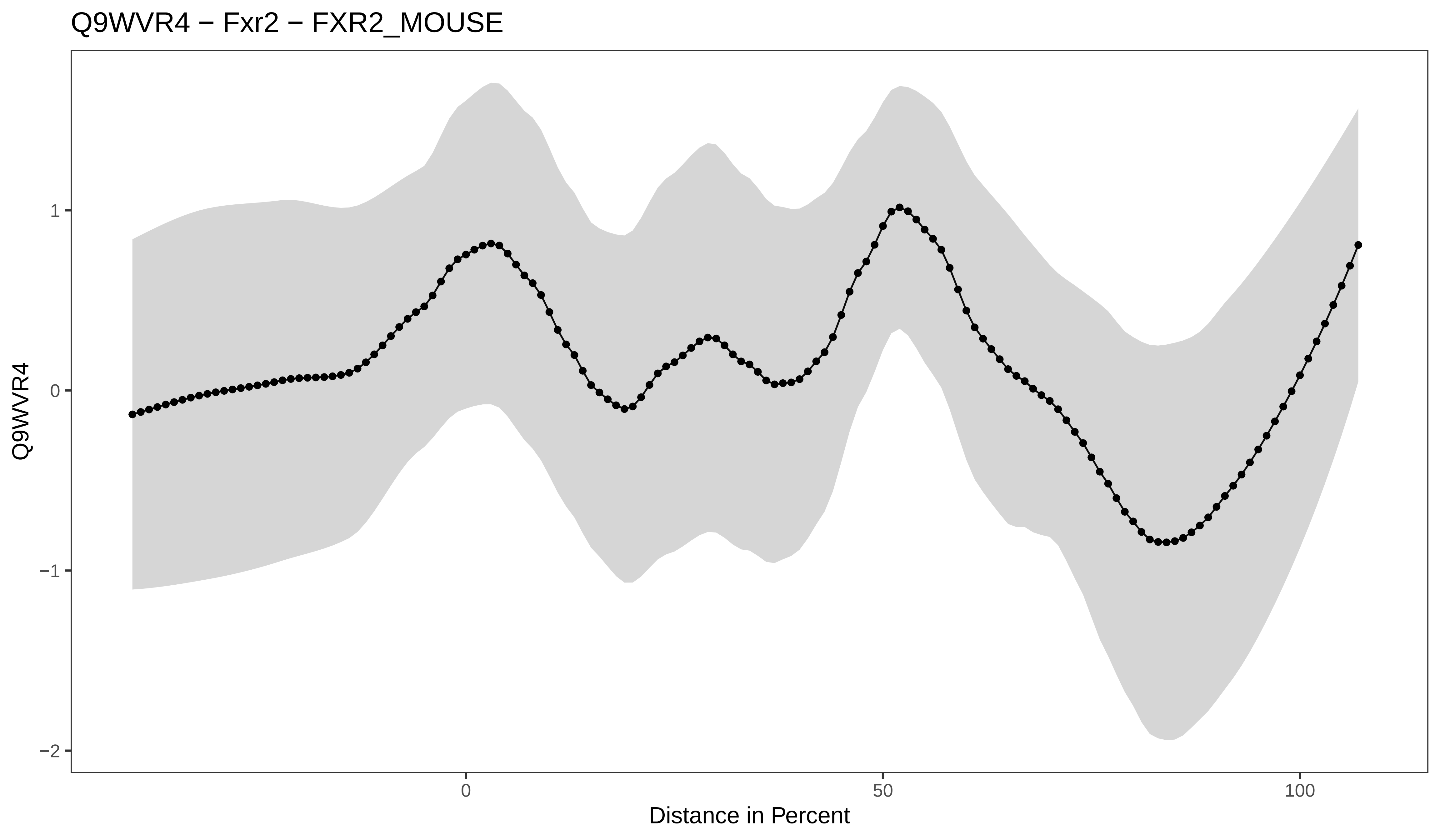

Q9WVR4 − Fxr2 − FXR2_MOUSE
Q9WVR4
1
0
−1
−2
0
50
100
Distance in Percent

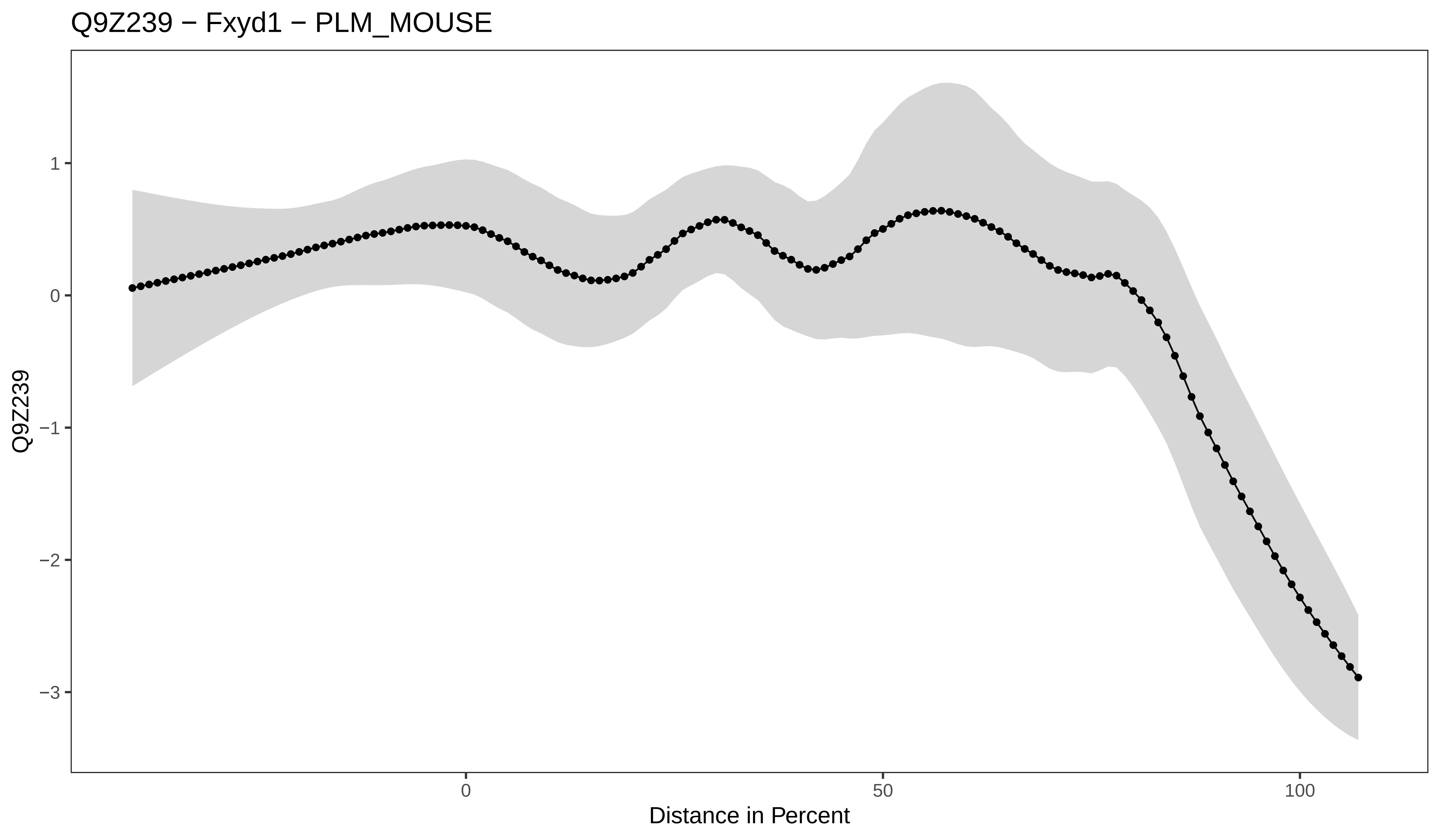

Q9Z239 − Fxyd1 − PLM_MOUSE
1
0
−1
−2
−3
Q9Z239
0
50
100
Distance in Percent

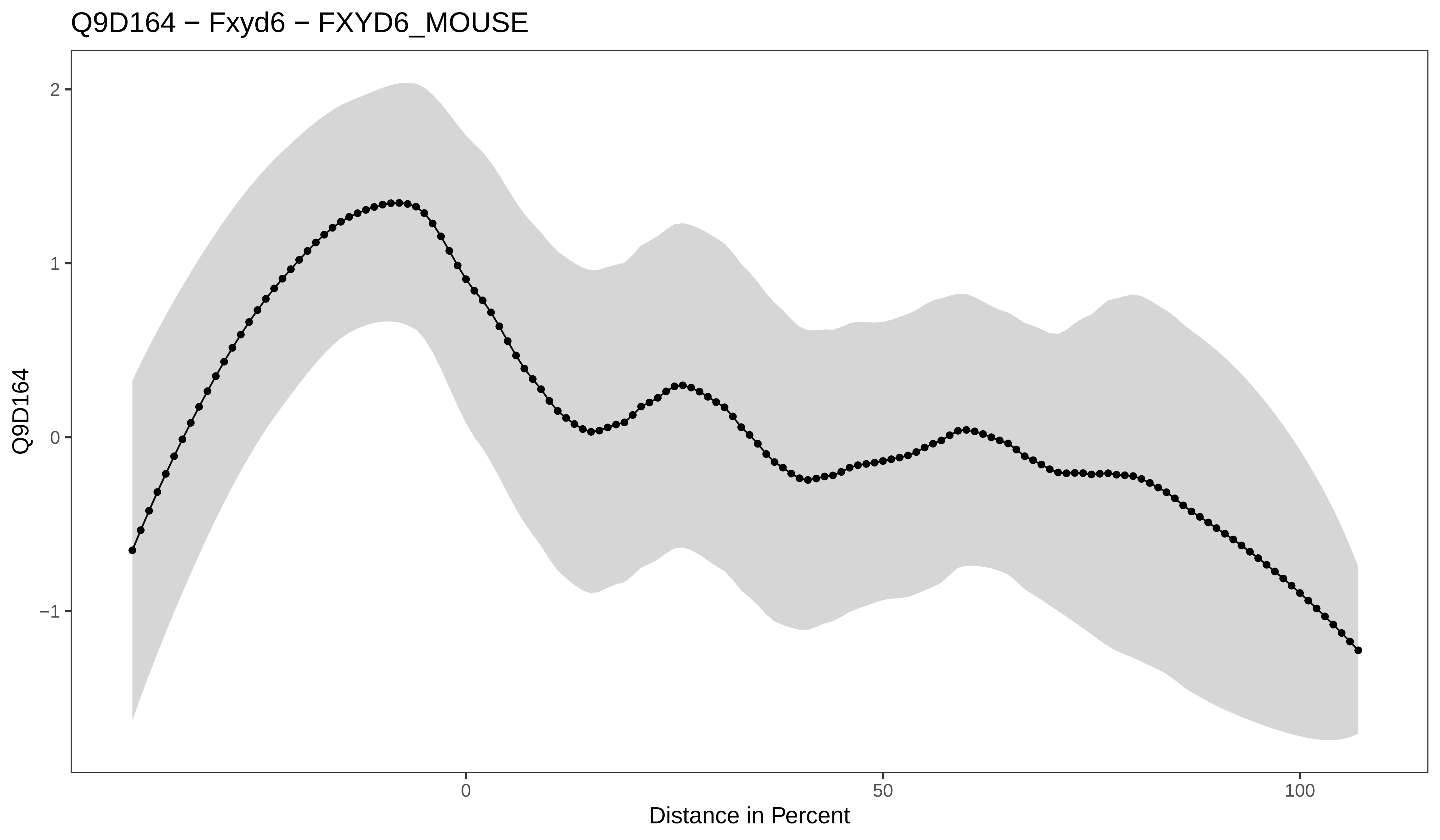
Q9D164 − Fxyd6 − FXYD6_MOUSE
Q9D164
2
1
0
−1
0
50
100
Distance in Percent

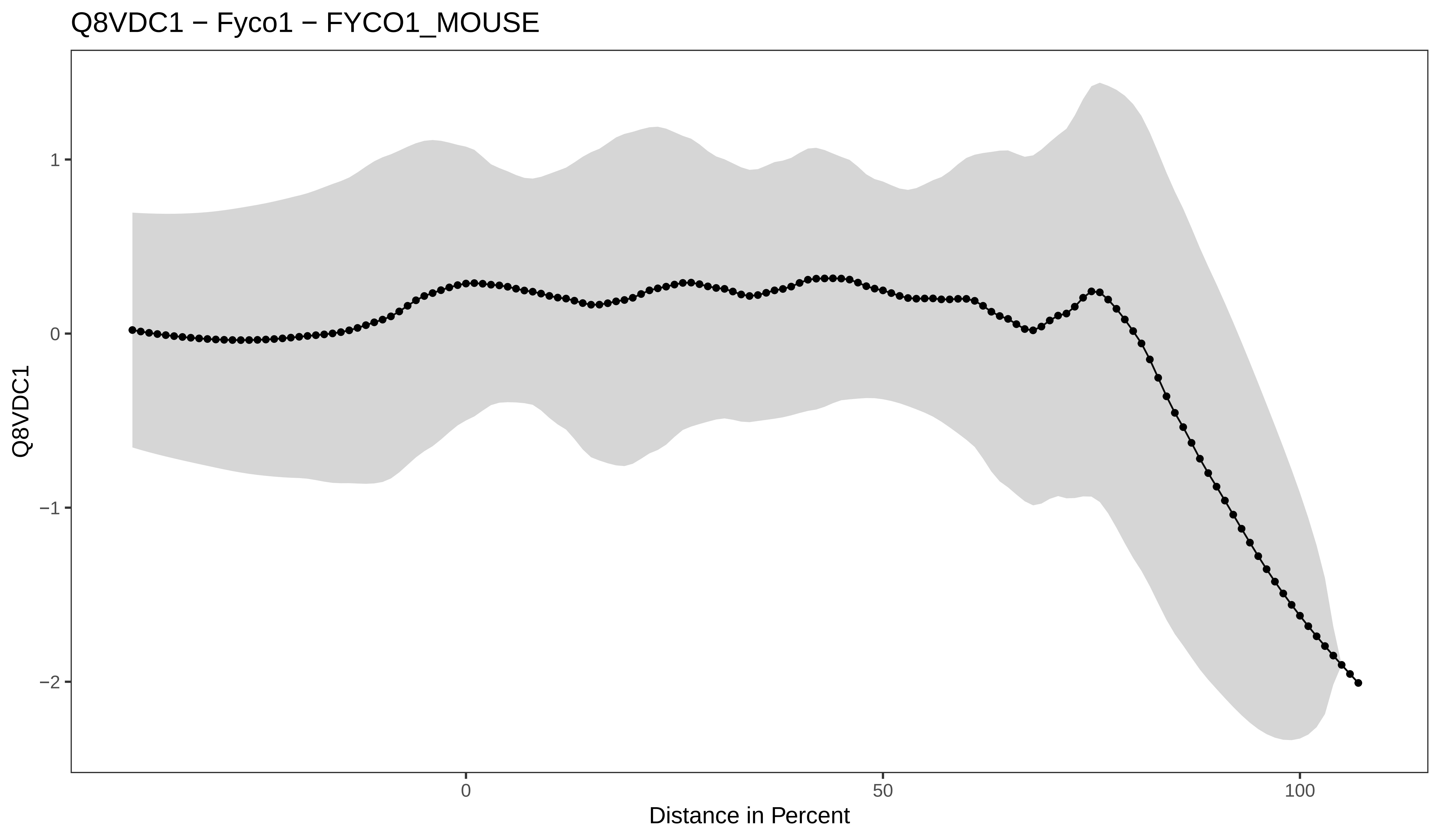
Q8VDC1 − Fyco1 − FYCO1_MOUSE
Q8VDC1
1
0
−1
−2
0
50
100
Distance in Percent

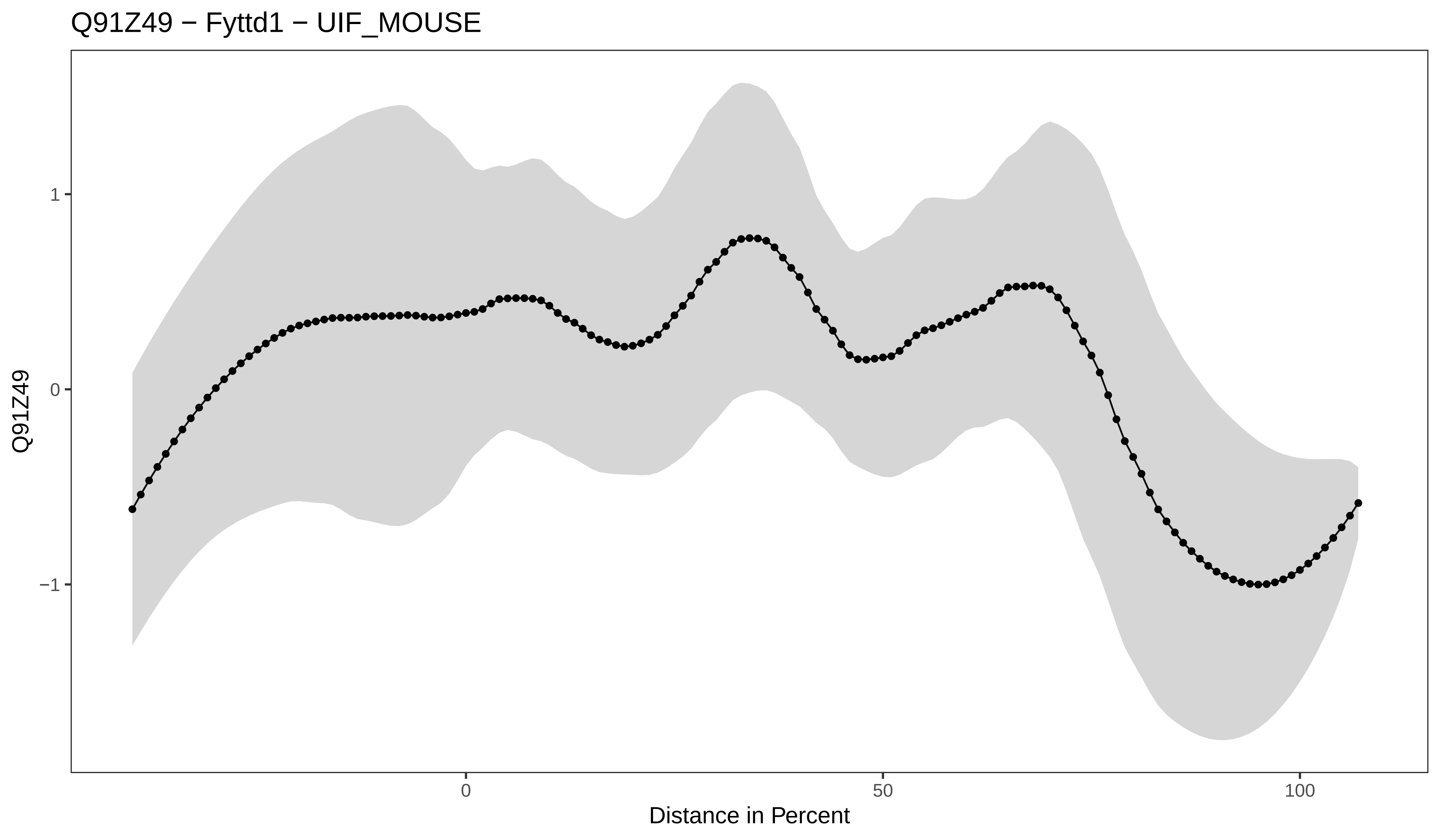

Q91Z49 − Fyttd1 − UIF_MOUSE
Q91Z49
1
0
−1
0
50
100
Distance in Percent

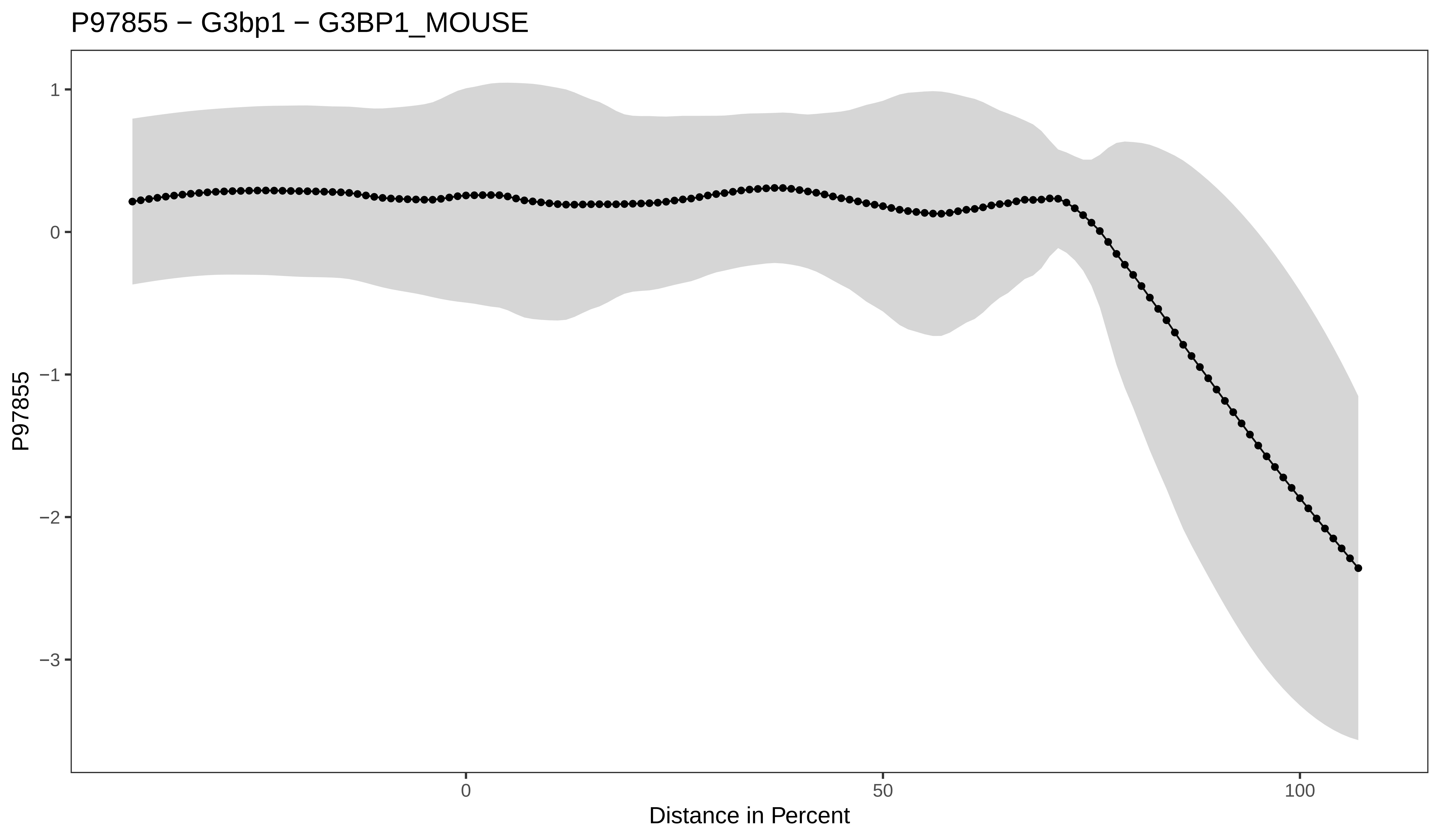

P97855 − G3bp1 − G3BP1_MOUSE
1
0
−1
−2
−3
P97855
0
50
100
Distance in Percent

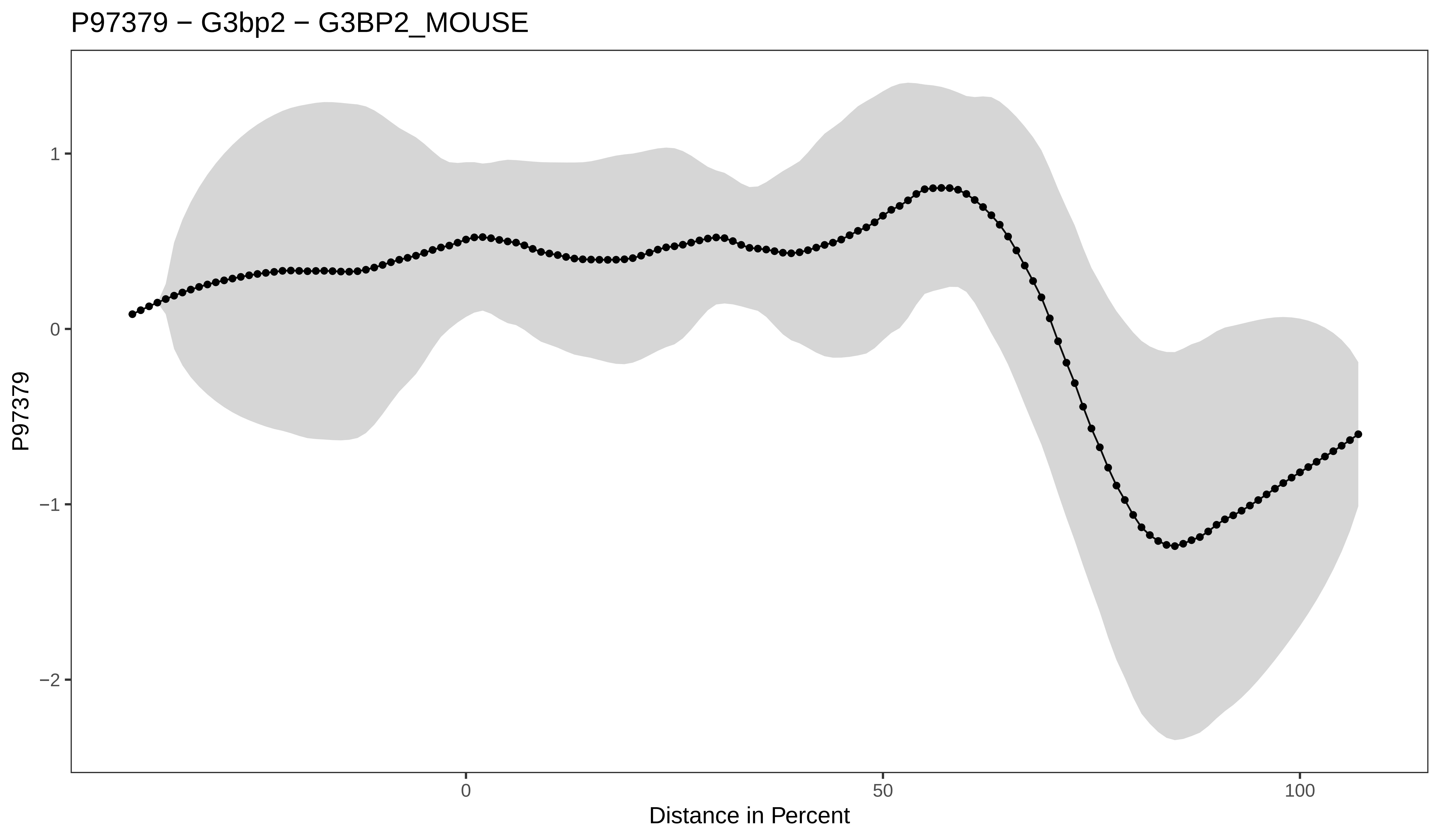

P97379 − G3bp2 − G3BP2_MOUSE
P97379
1
0
−1
−2
0
50
100
Distance in Percent

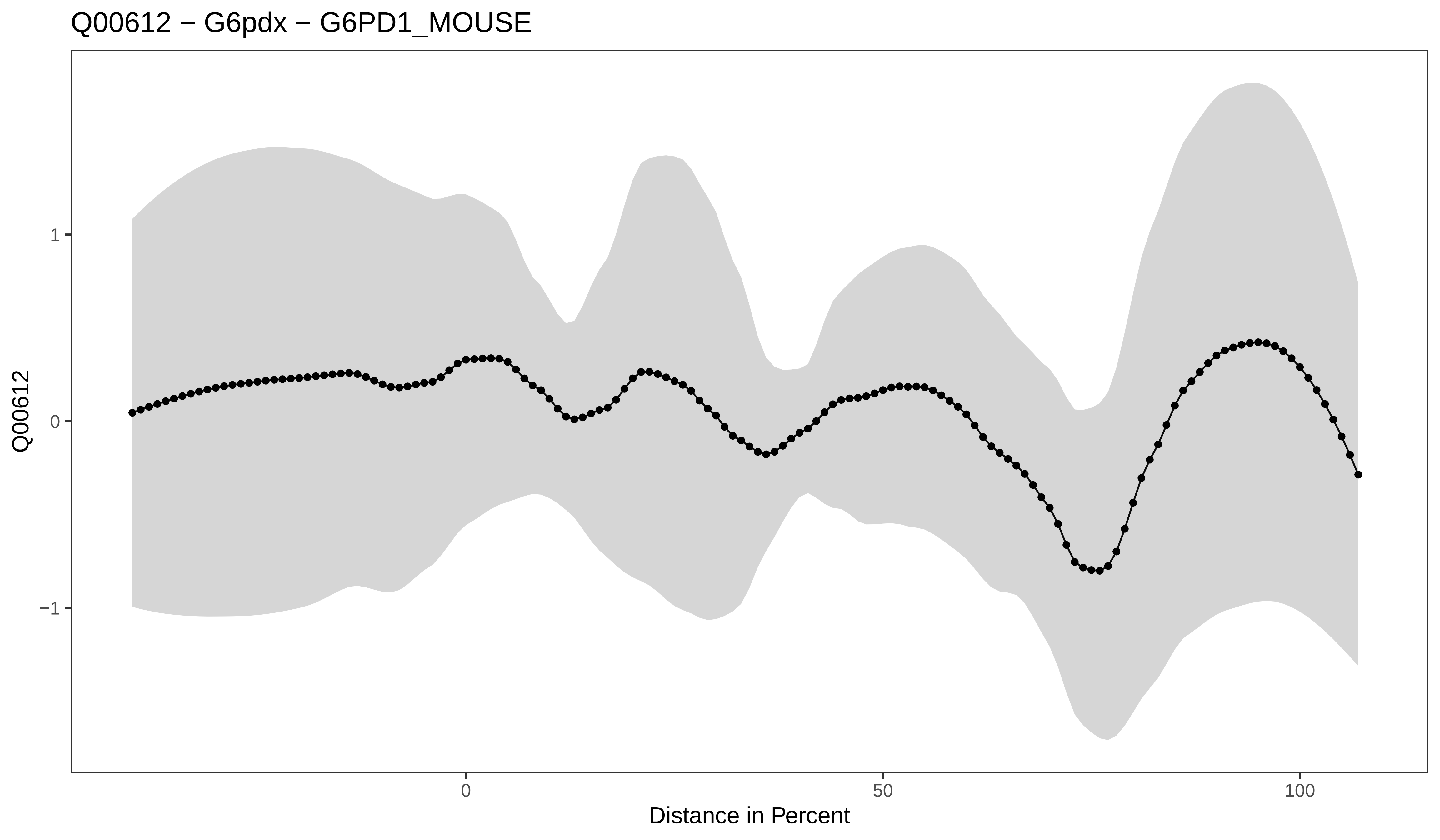
Q00612 − G6pdx − G6PD1_MOUSE
1
0
−1
Q00612
0
50
100
Distance in Percent

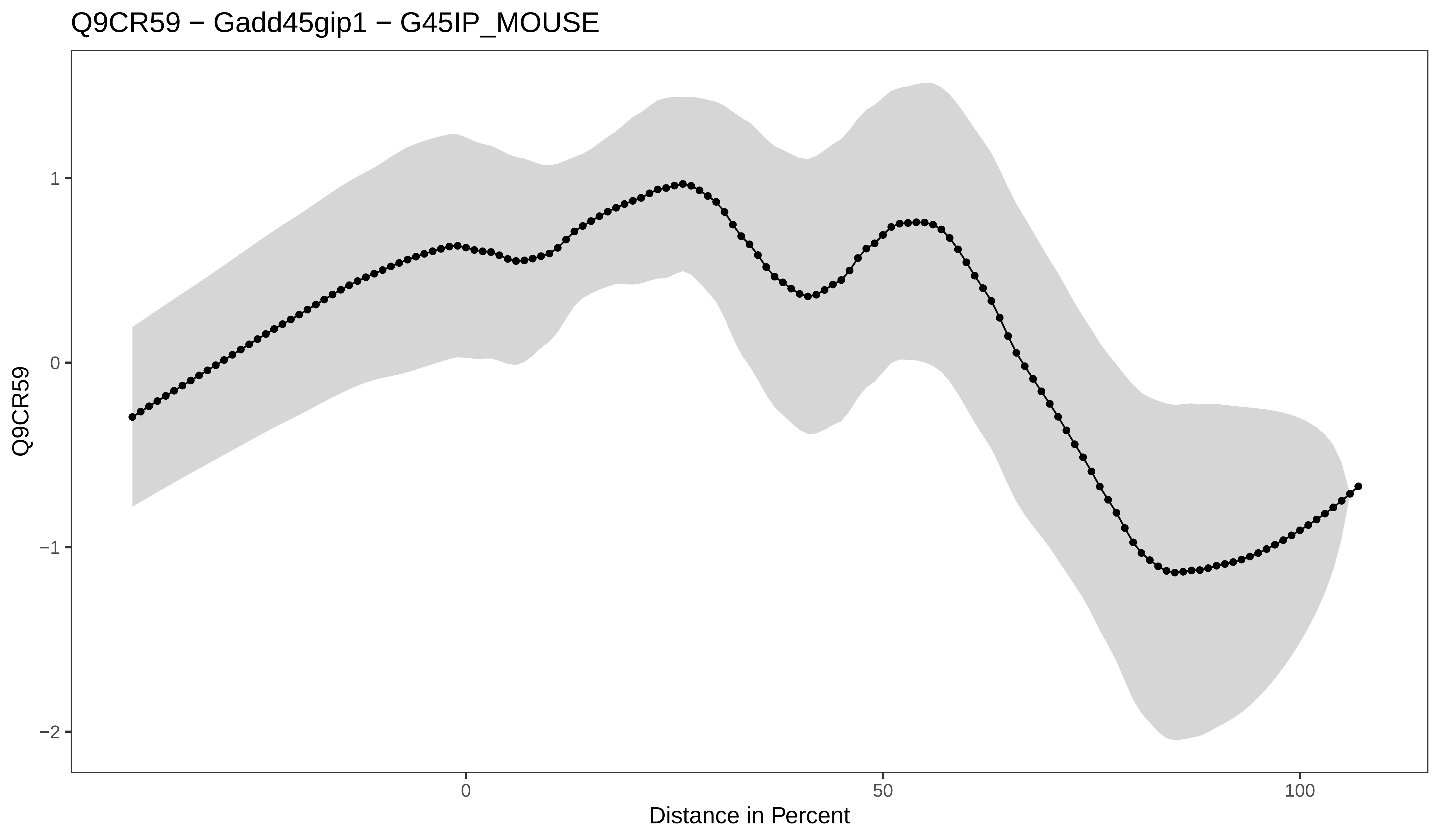
Q9CR59 − Gadd45gip1 − G45IP_MOUSE
Q9CR59
1
0
−1
−2
0
50
100
Distance in Percent

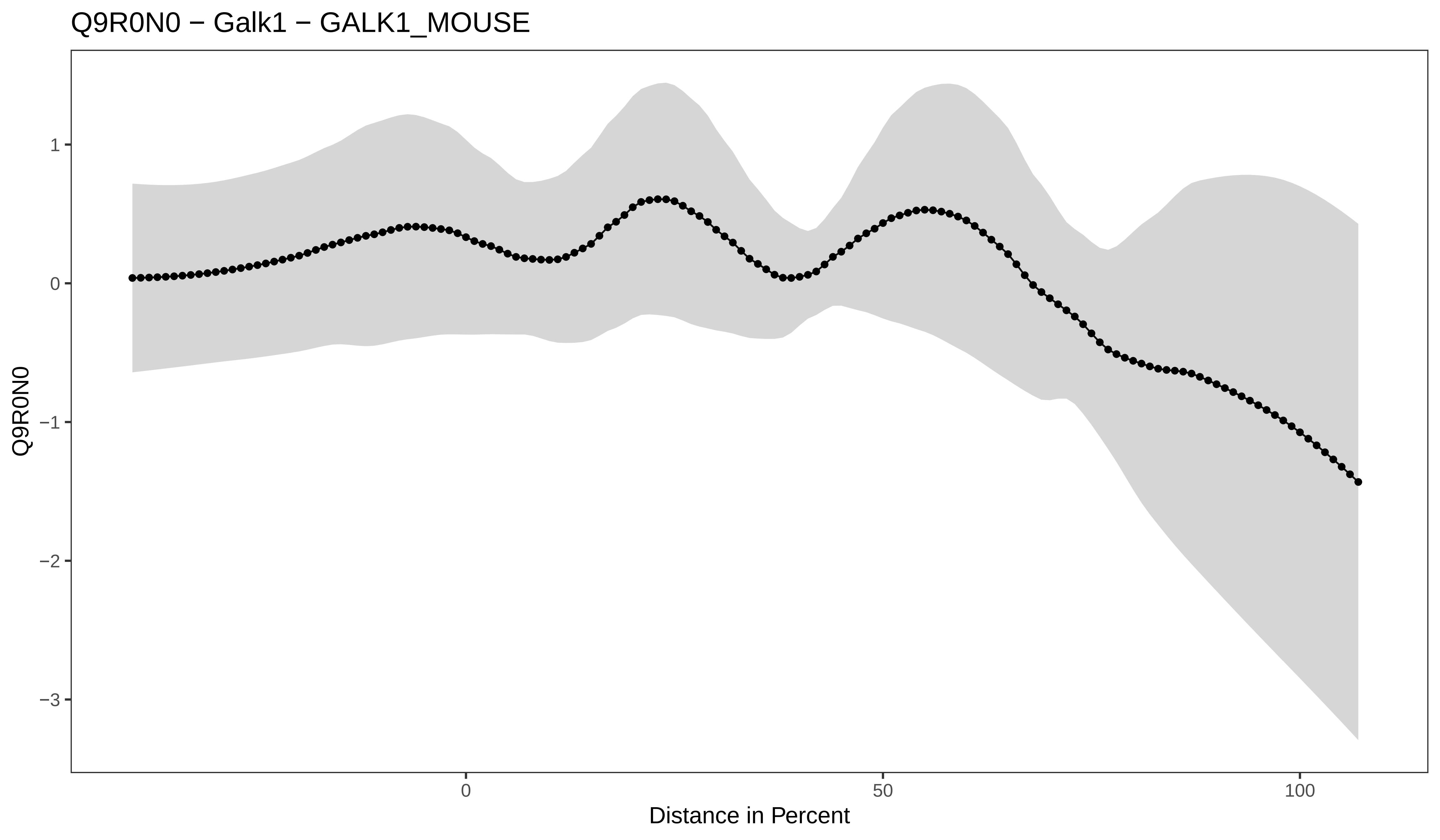
Q9R0N0 − Galk1 − GALK1_MOUSE
1
0
−1
−2
−3
Q9R0N0
0
50
100
Distance in Percent

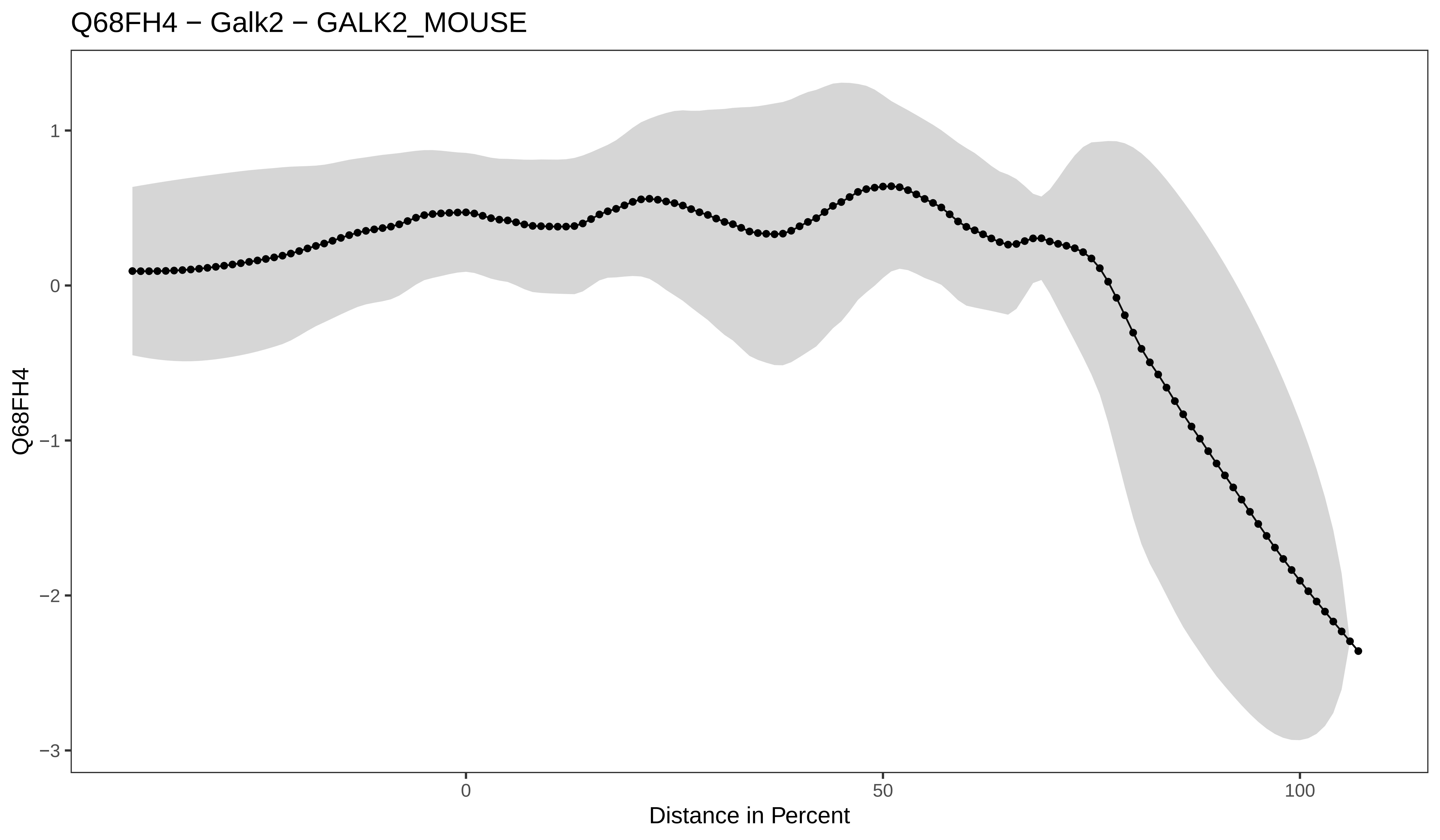

Q68FH4 − Galk2 − GALK2_MOUSE
Q68FH4
1
0
−1
−2
−3
0
50
100
Distance in Percent

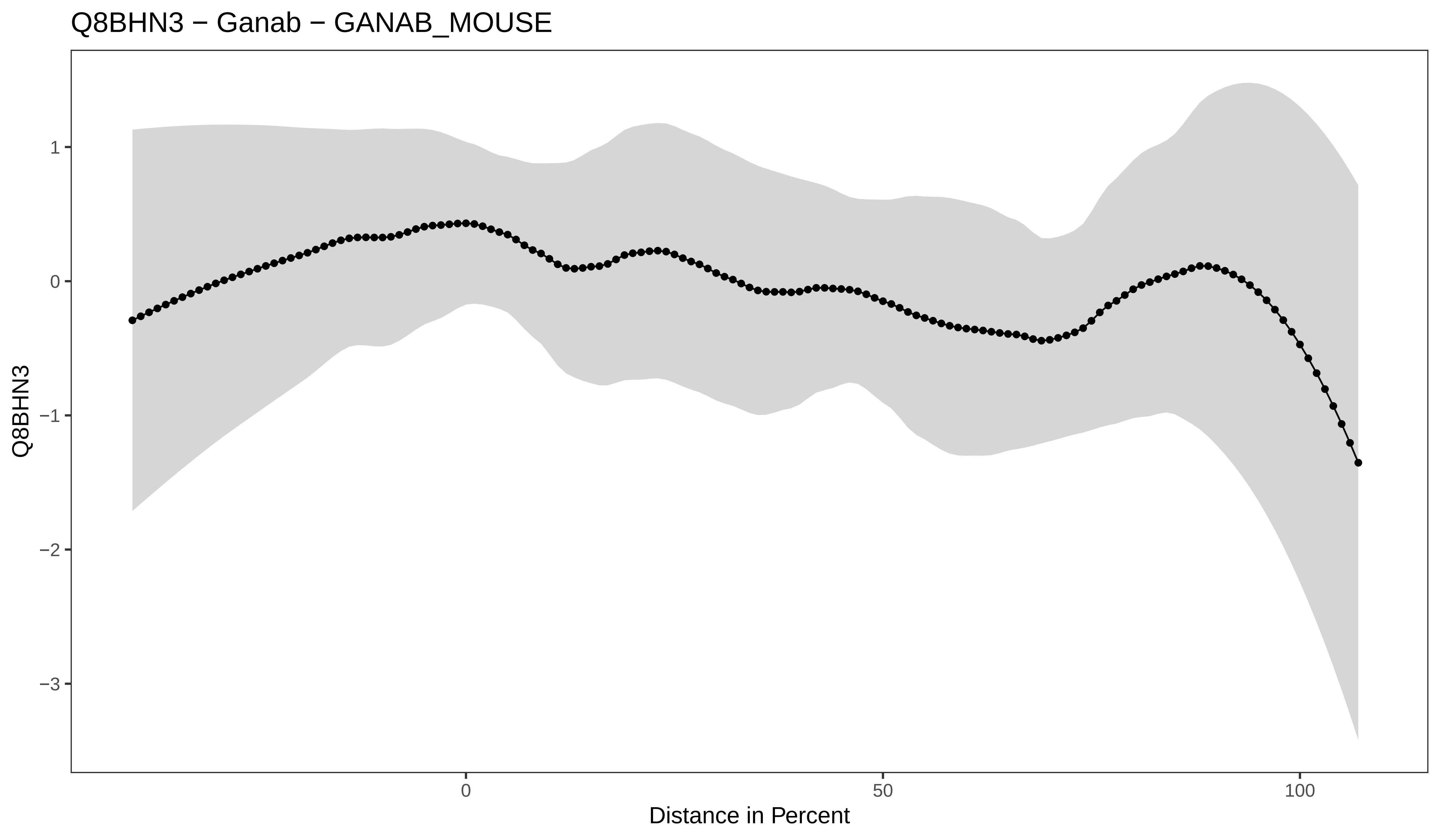
Q8BHN3 − Ganab − GANAB_MOUSE
Q8BHN3
1
0
−1
−2
−3
0
50
100
Distance in Percent

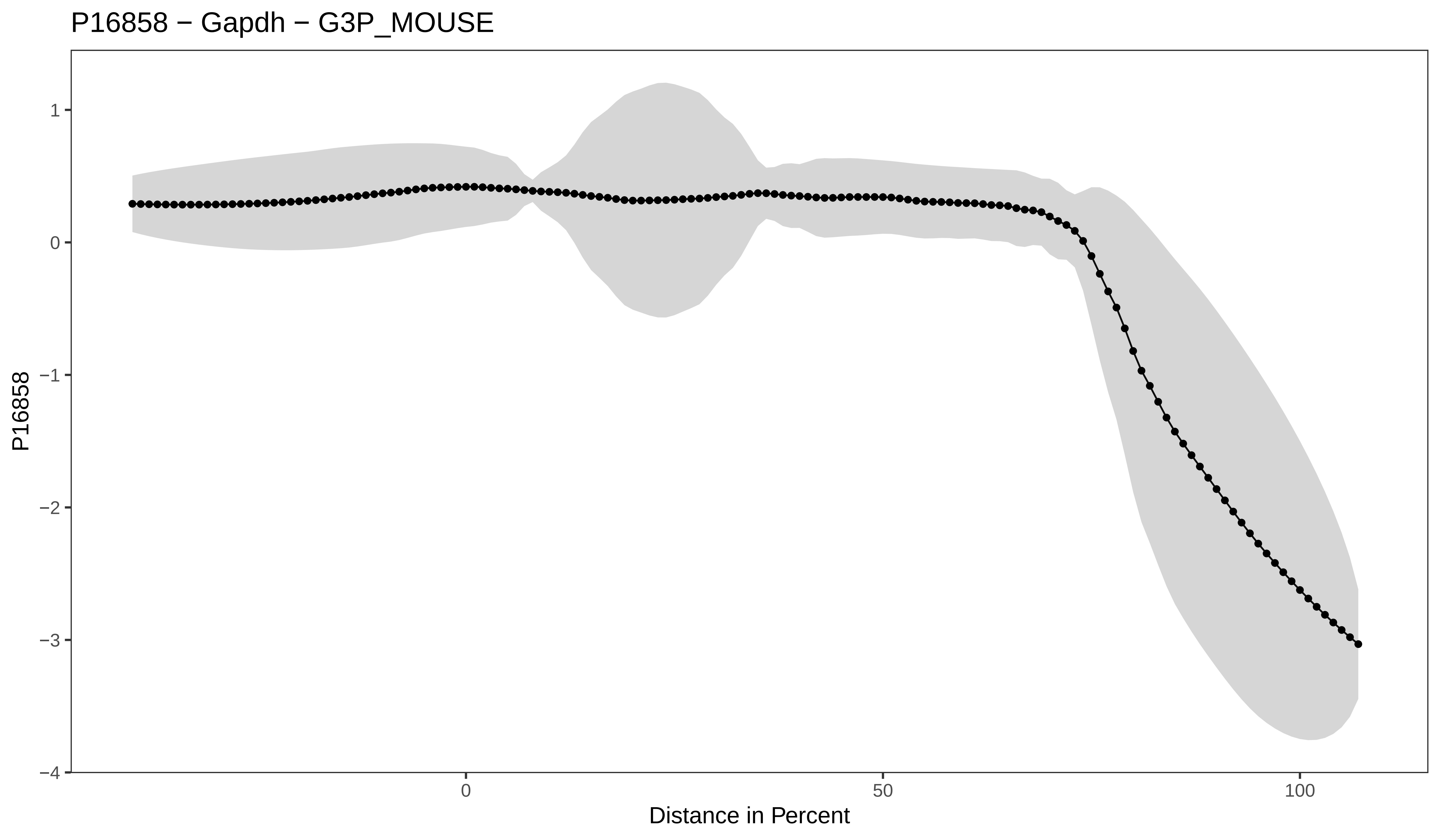

P16858 − Gapdh − G3P_MOUSE
1
0
−1
−2
−3
−4
P16858
0
50
100
Distance in Percent

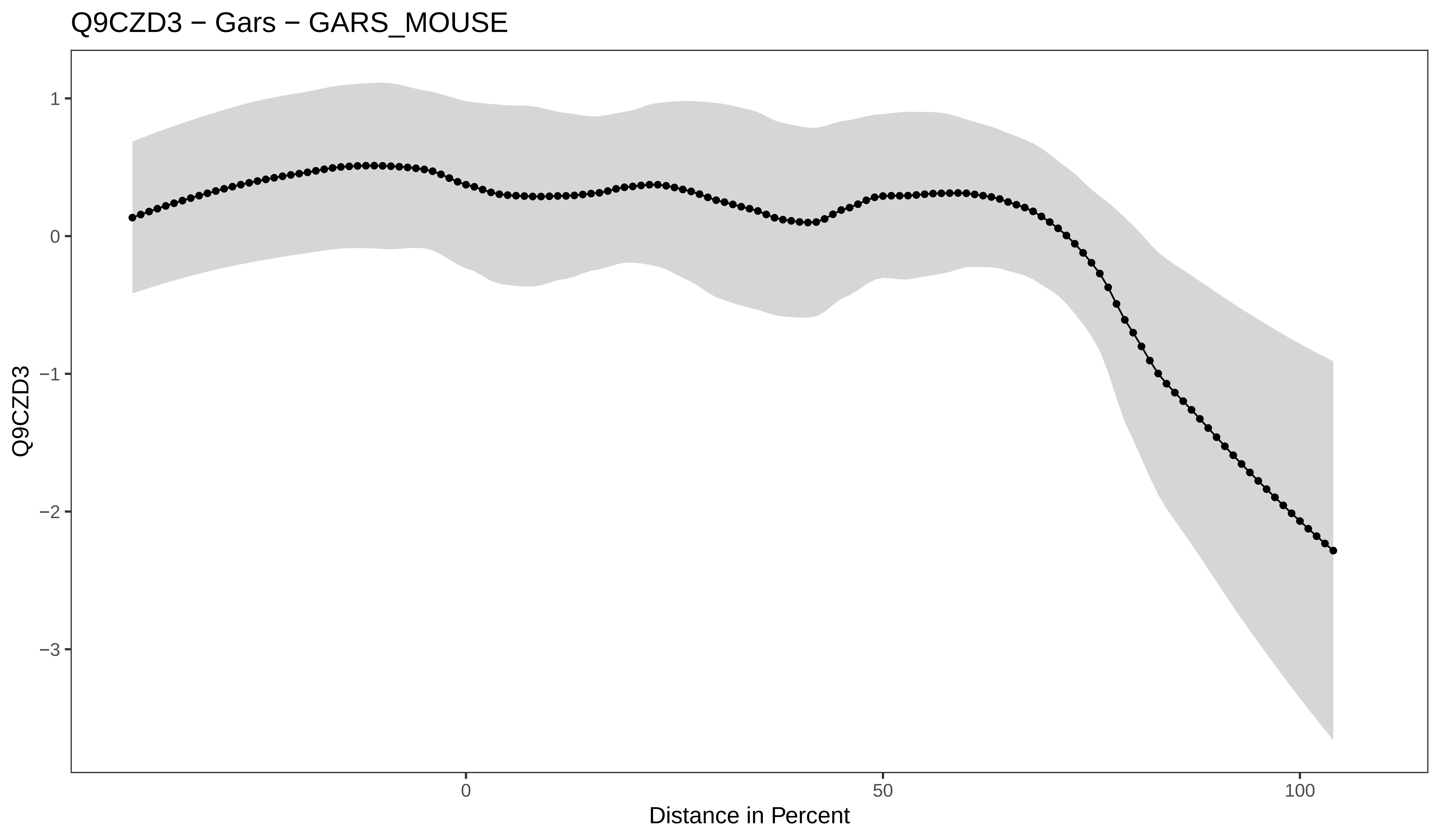

Q9CZD3 − Gars − GARS_MOUSE
Q9CZD3
Distance in Percent
1
0
−1
−2
−3
0
50
100

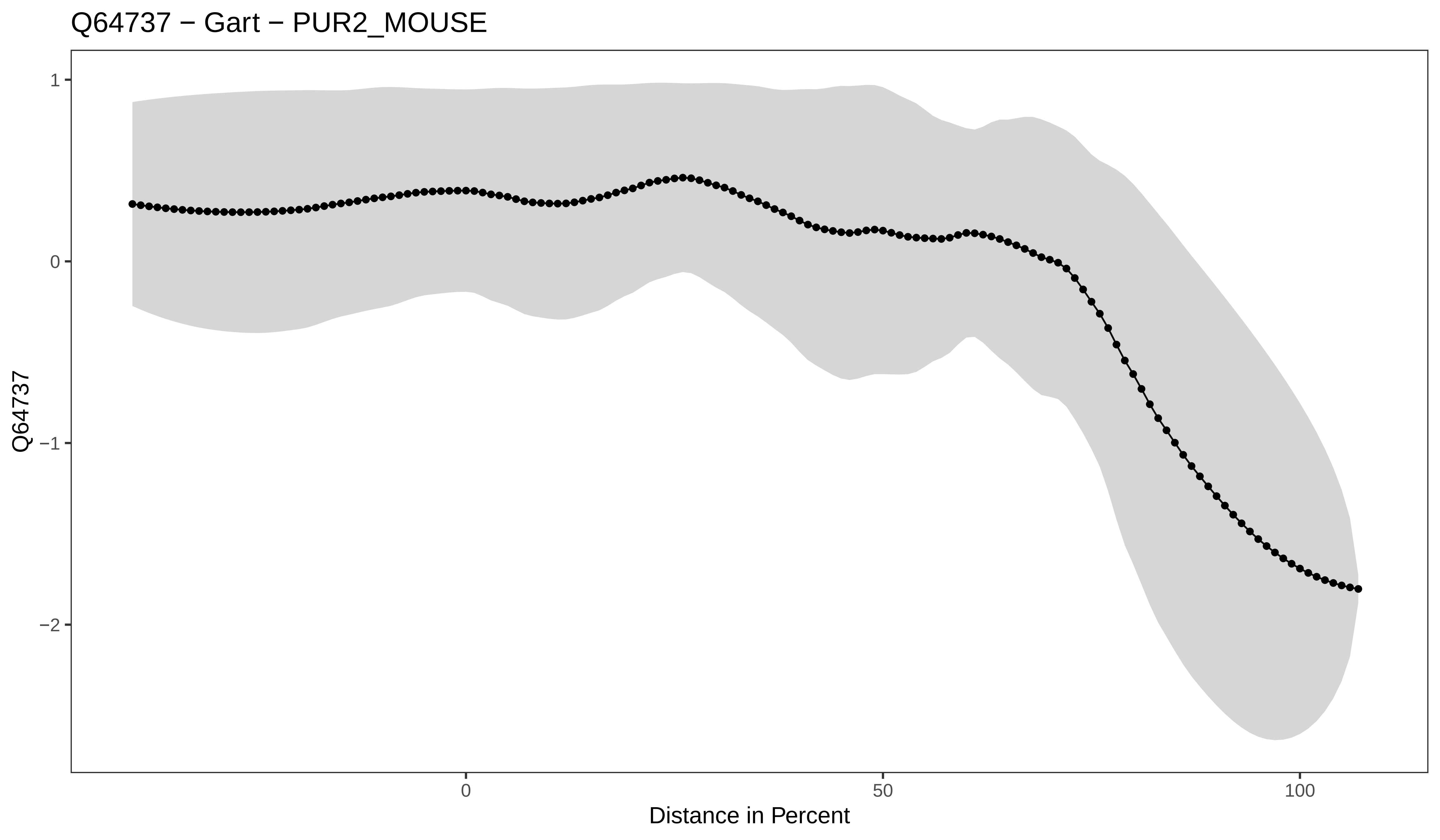
Q64737 − Gart − PUR2_MOUSE
1
0
−1
−2
Q64737
0
50
100
Distance in Percent

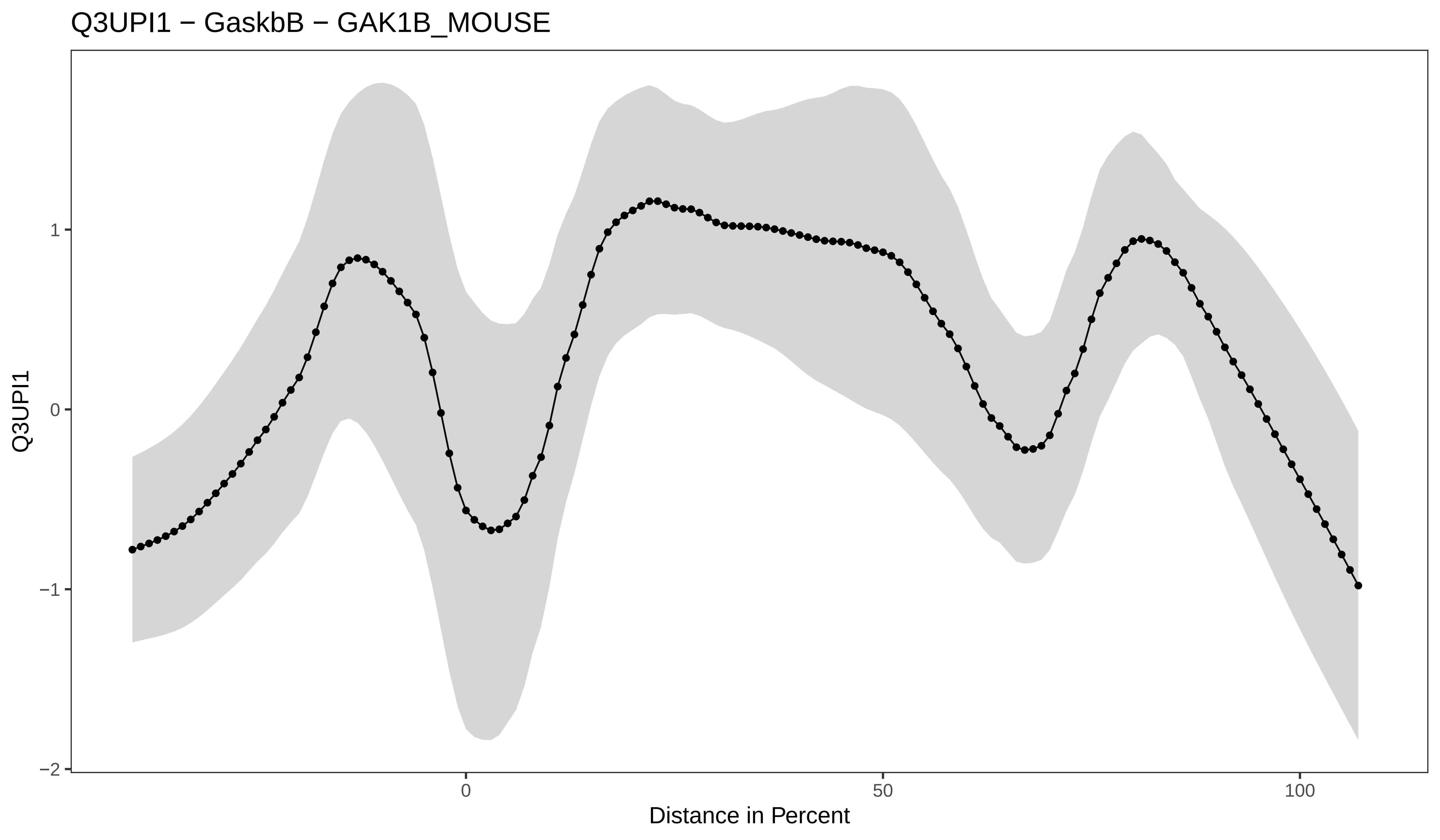

Q3UPI1 − GaskbB − GAK1B_MOUSE
Q3UPI1
1
0
−1
−2
0
50
100
Distance in Percent

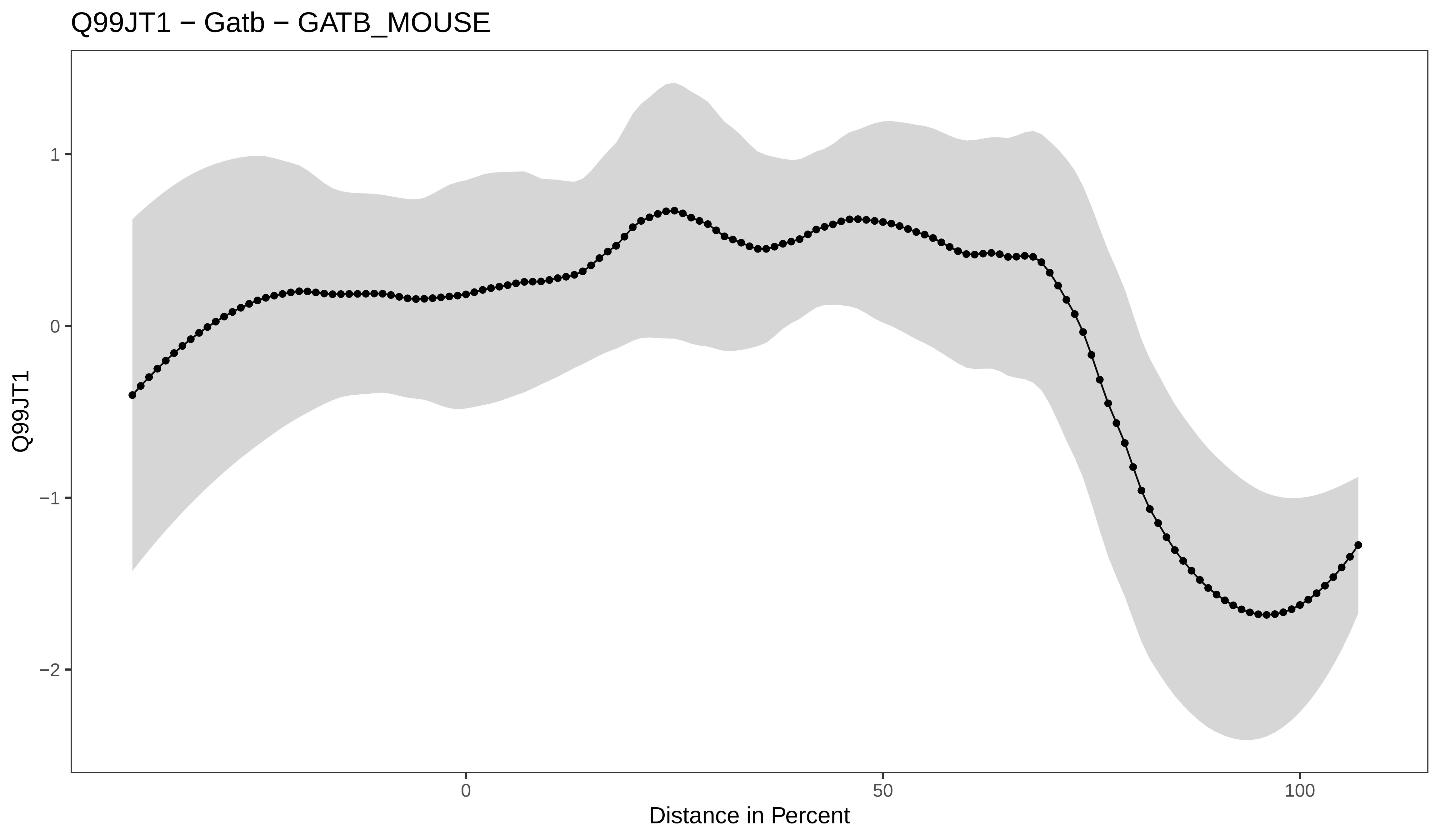
Q99JT1 − Gatb − GATB_MOUSE
Q99JT1
1
0
−1
−2
0
50
100
Distance in Percent

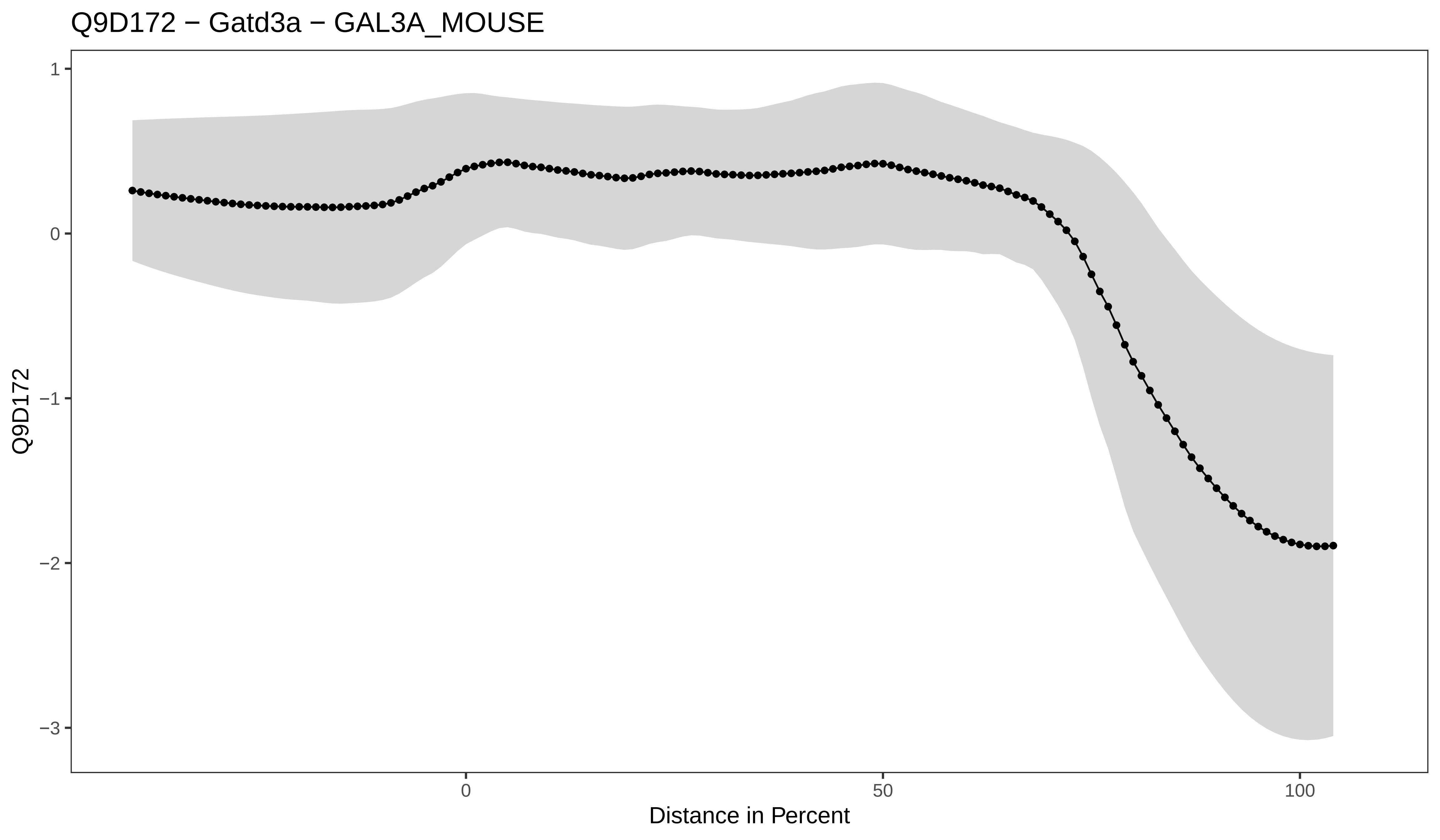

Q9D172 − Gatd3a − GAL3A_MOUSE
Q9D172
Distance in Percent
1
0
−1
−2
−3
0
50
100

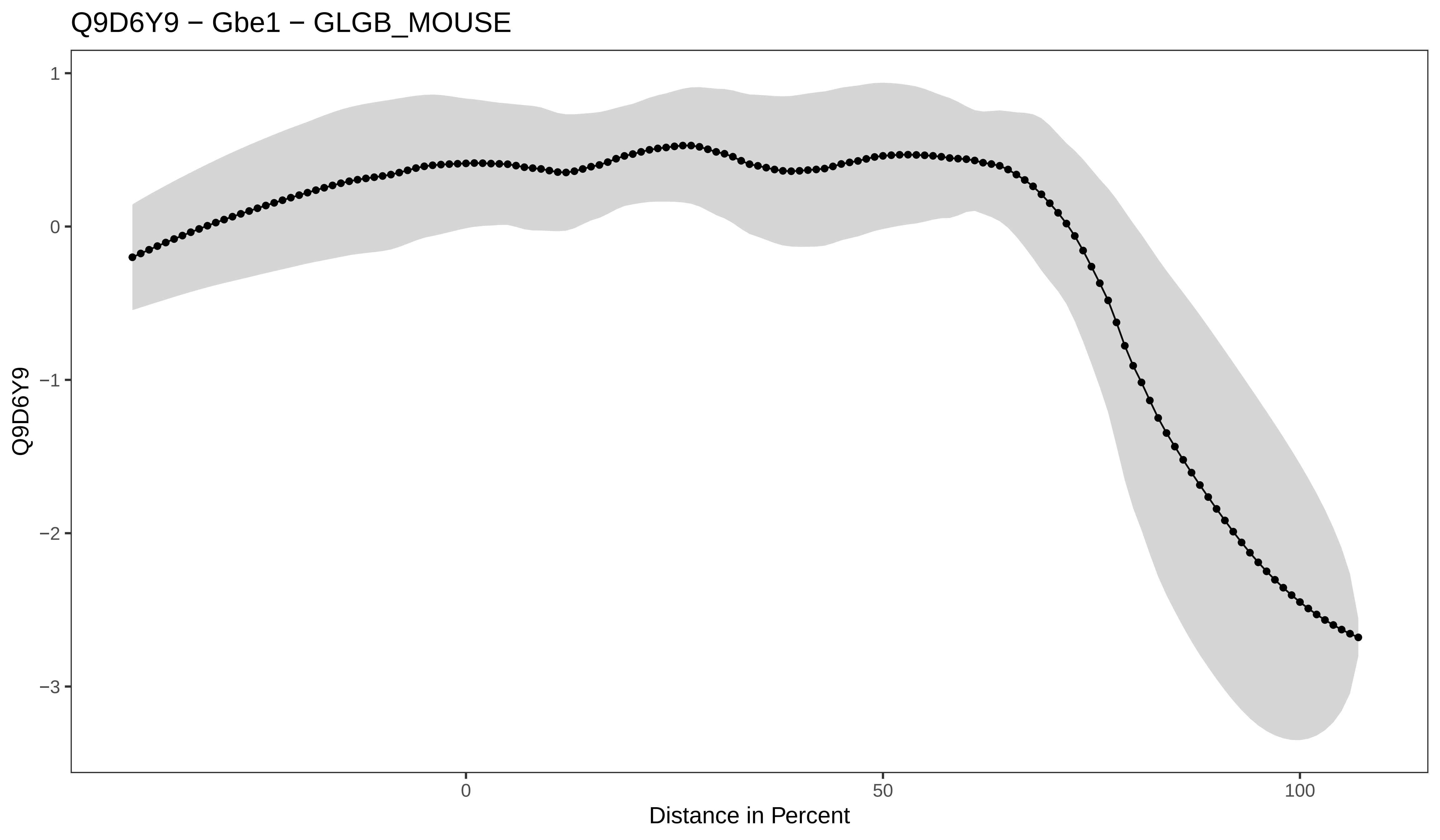

Q9D6Y9 − Gbe1 − GLGB_MOUSE
1
0
−1
−2
−3
Q9D6Y9
0
50
100
Distance in Percent

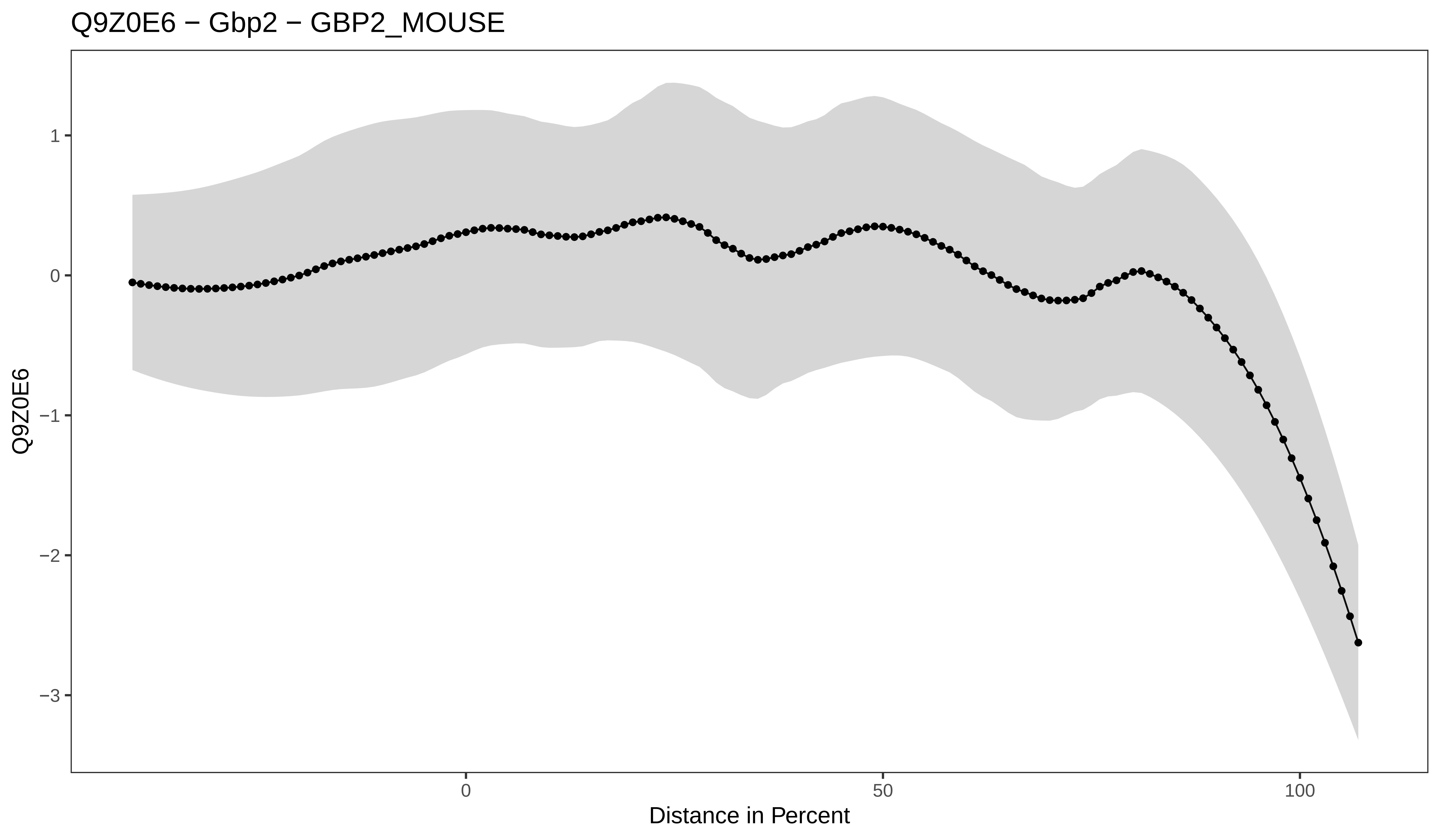
Q9Z0E6 − Gbp2 − GBP2_MOUSE
Q9Z0E6
1
0
−1
−2
−3
0
50
100
Distance in Percent

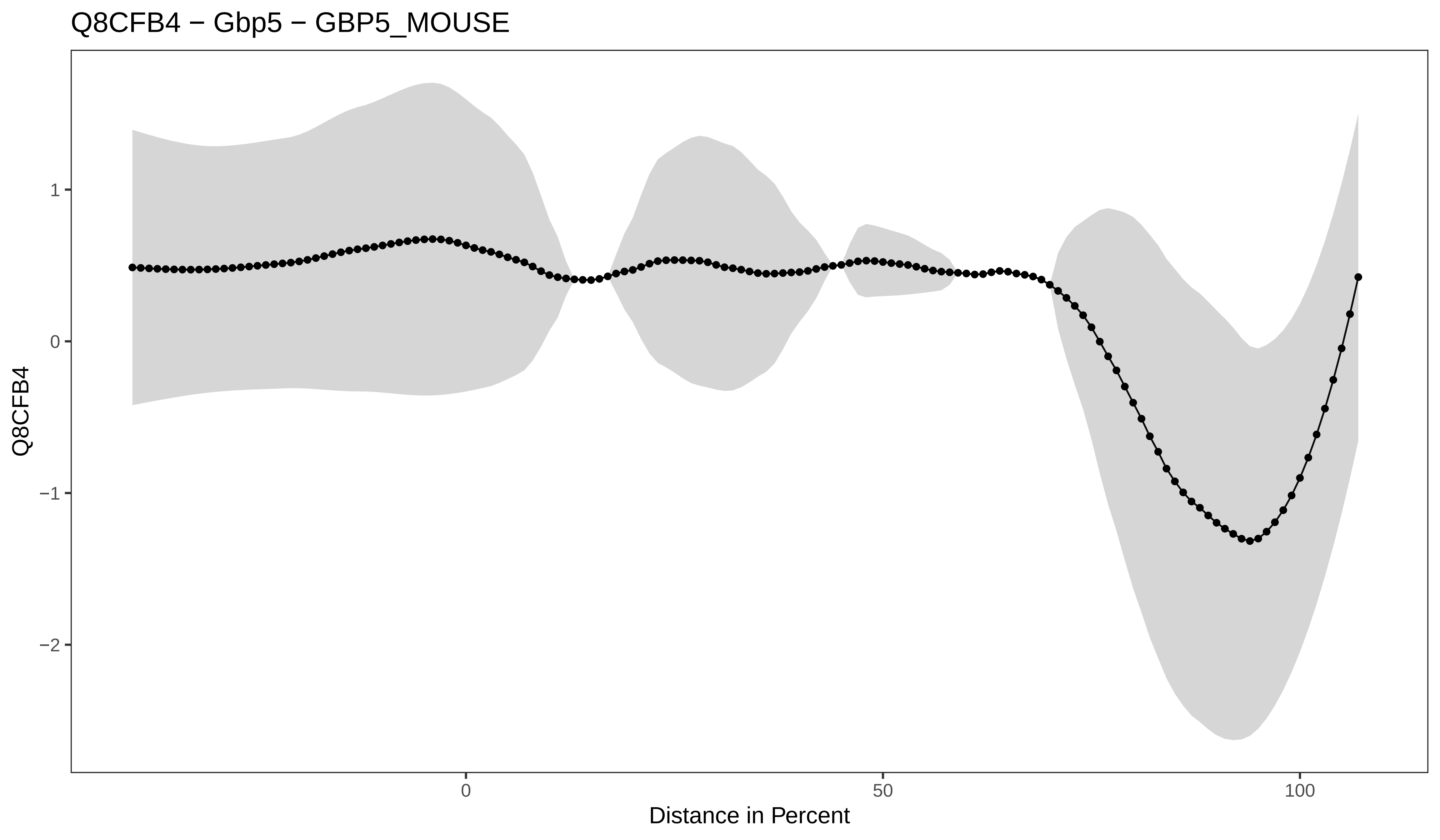

Q8CFB4 − Gbp5 − GBP5_MOUSE
1
0
−1
−2
Q8CFB4
0
50
100
Distance in Percent

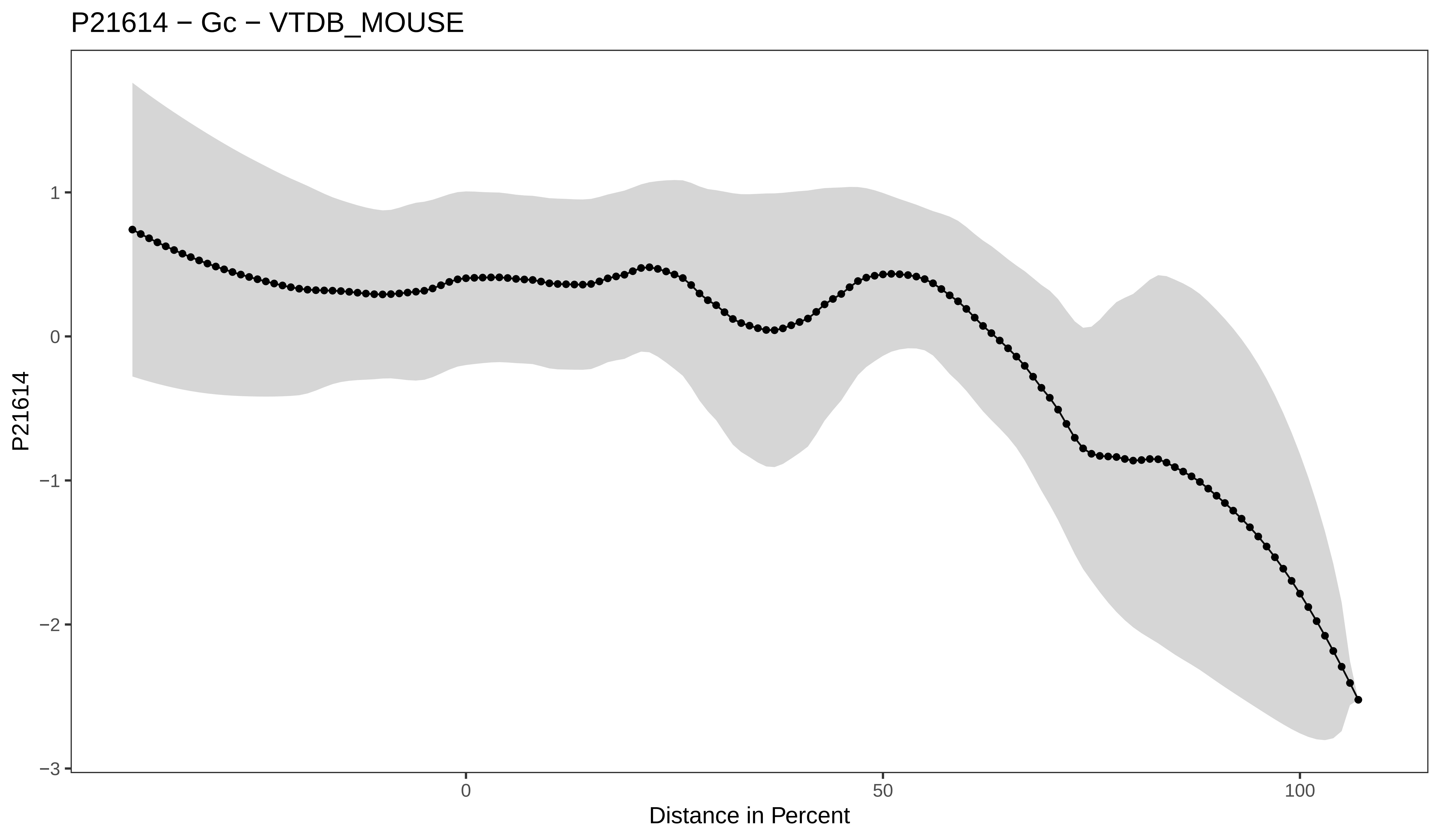
P21614 − Gc − VTDB_MOUSE
P21614
1
0
−1
−2
−3
0
50
100
Distance in Percent

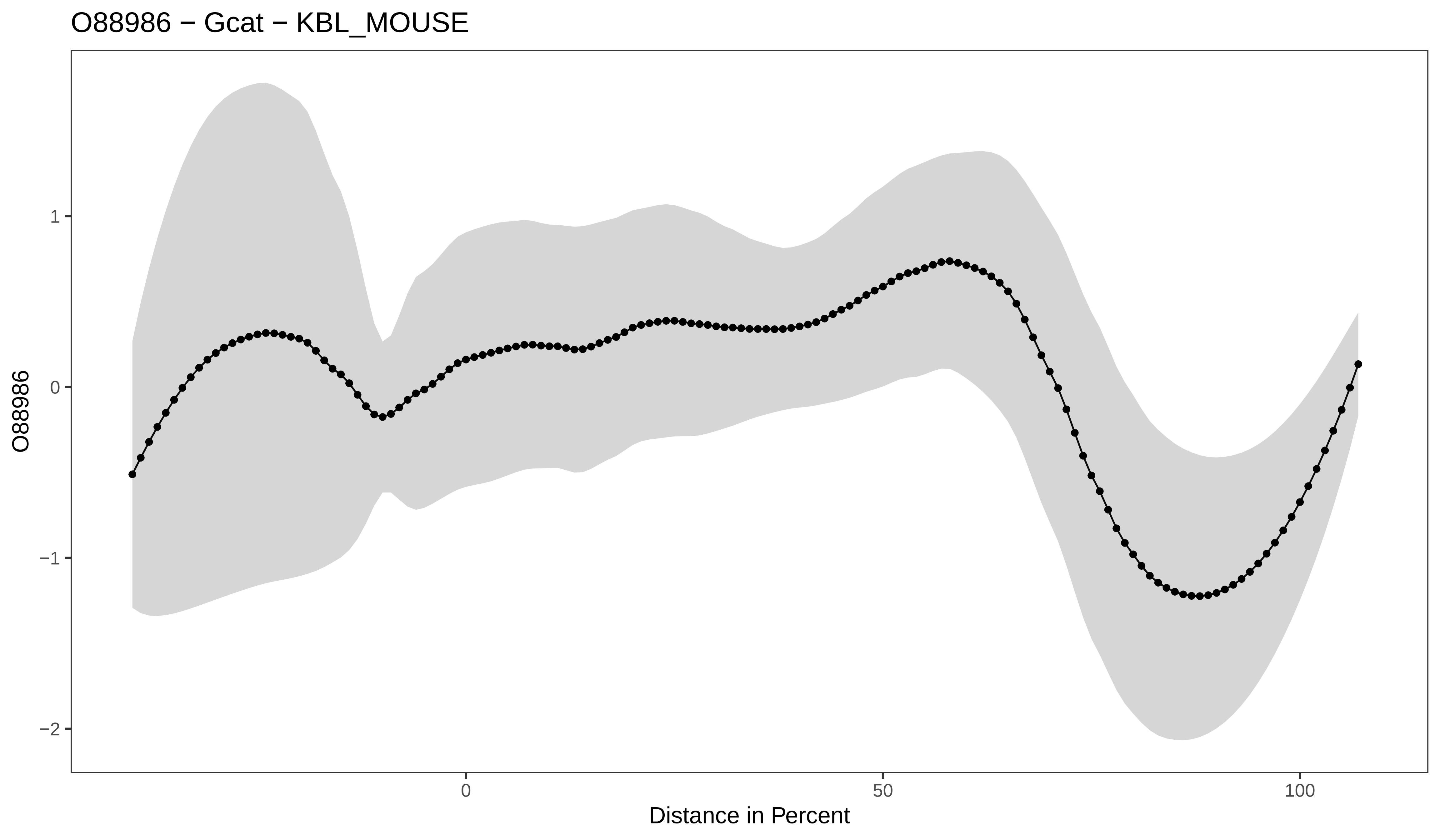
O88986 − Gcat − KBL_MOUSE
O88986
1
0
−1
−2
0
50
100
Distance in Percent

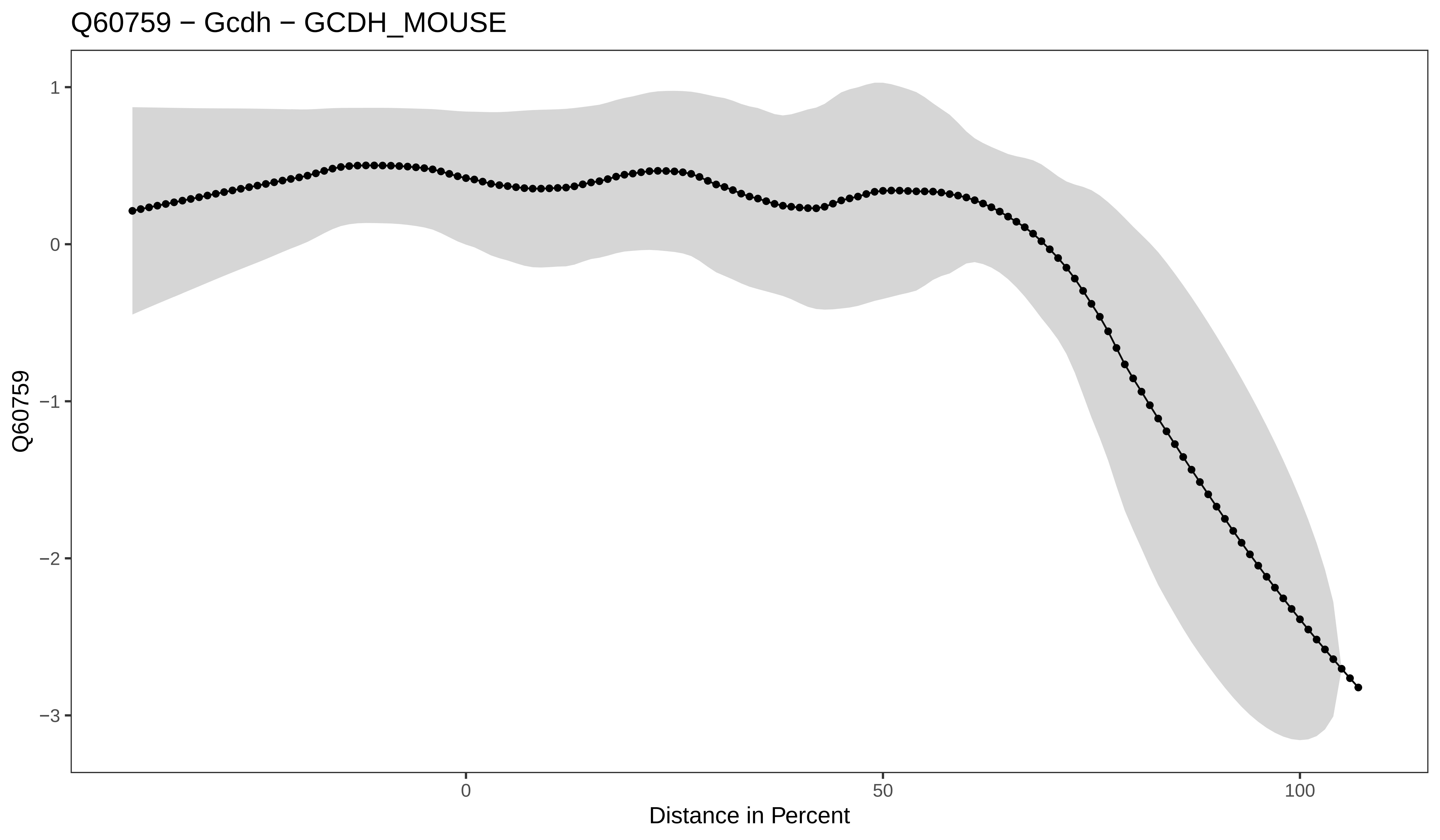

Q60759 − Gcdh − GCDH_MOUSE
1
0
−1
−2
−3
Q60759
0
50
100
Distance in Percent

P97494 − Gclc − GSH1_MOUSE

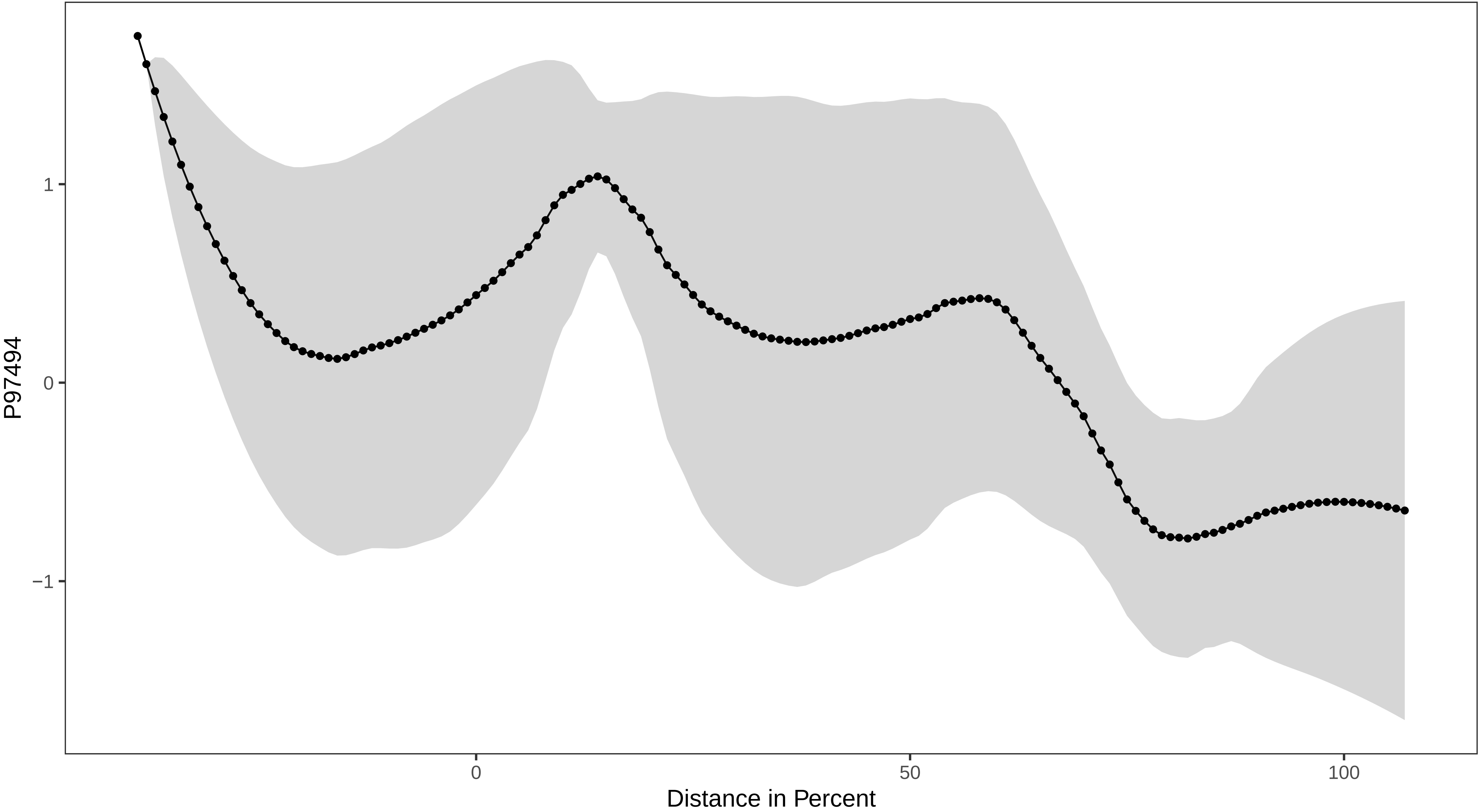

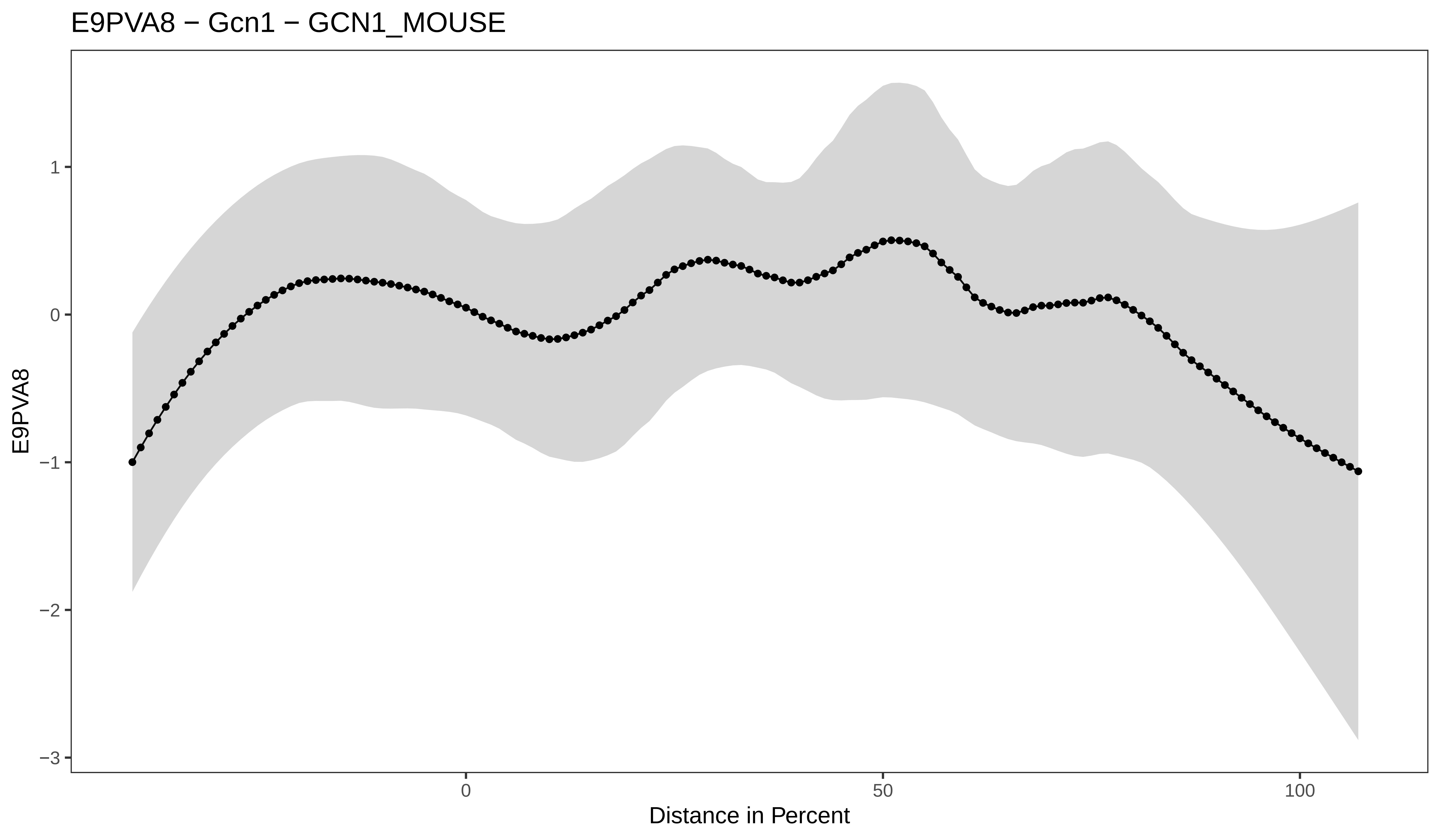
E9PVA8 − Gcn1 − GCN1_MOUSE
E9PVA8
1
0
−1
−2
−3
0
50
100
Distance in Percent

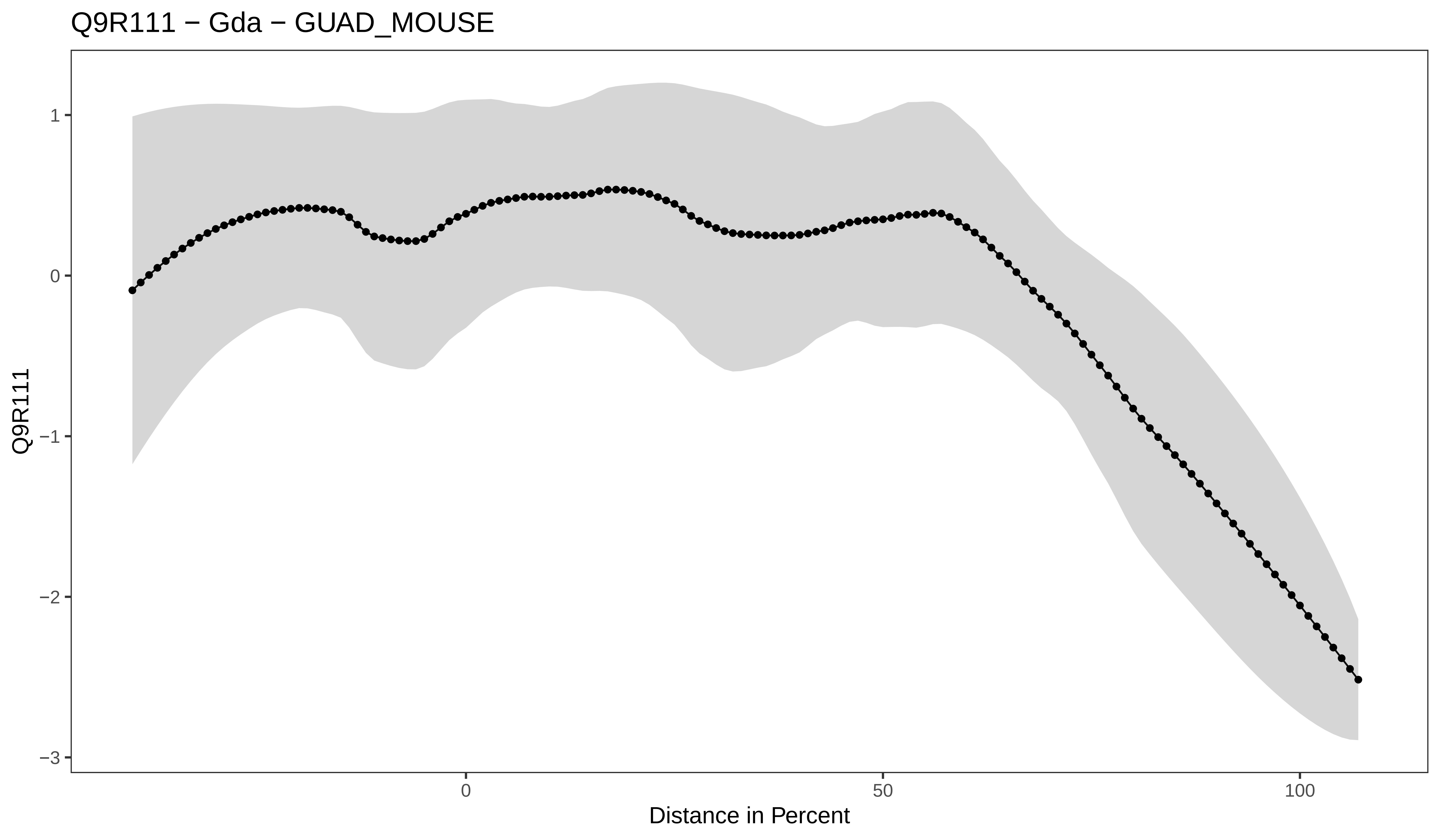

Q9R111 − Gda − GUAD_MOUSE
1
0
−1
−2
−3
Q9R111
0
50
100
Distance in Percent

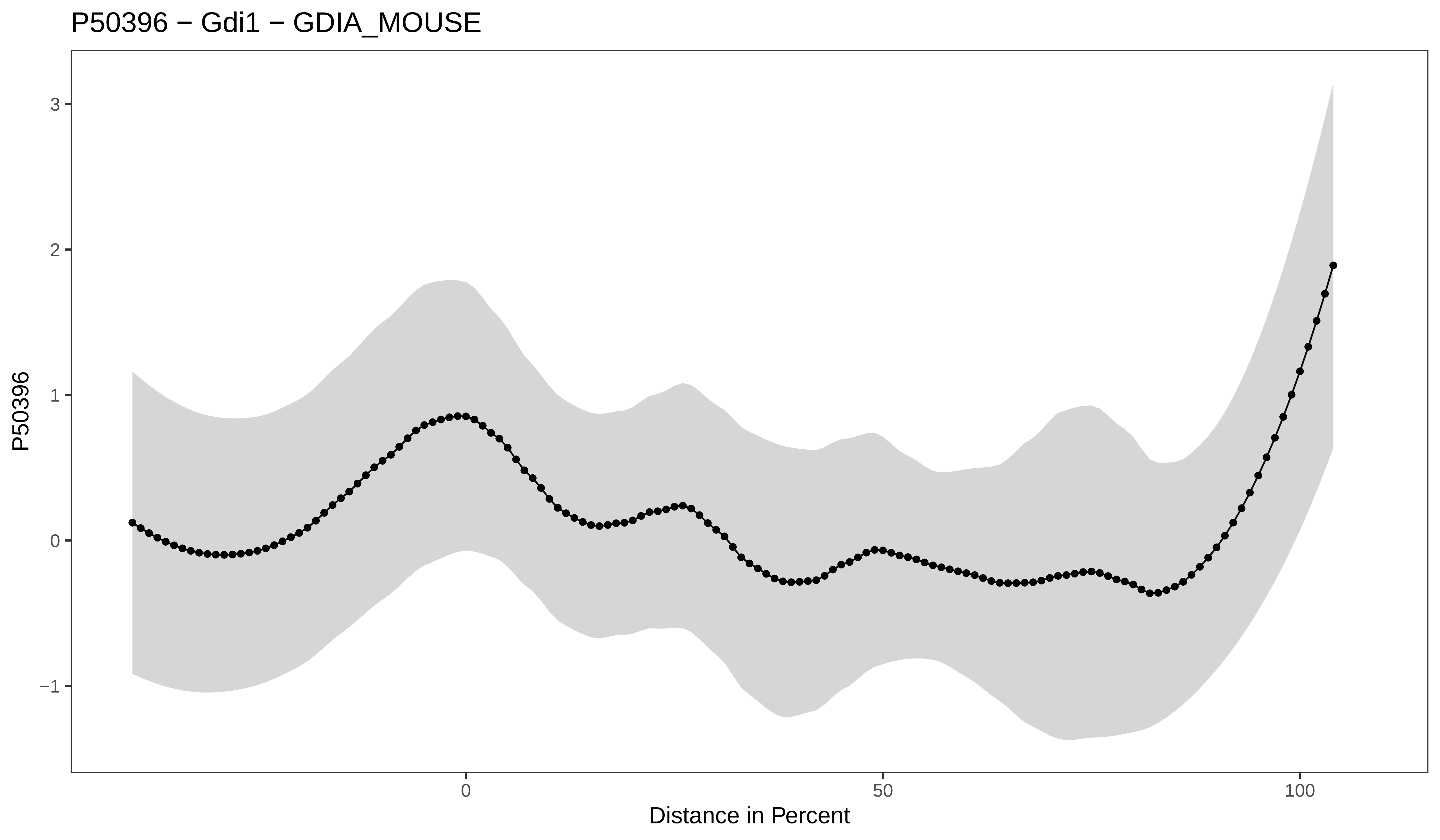
P50396 − Gdi1 − GDIA_MOUSE
3
2
1
0
−1
P50396
0
50
100
Distance in Percent

Q61598 − Gdi2 − GDIB_MOUSE

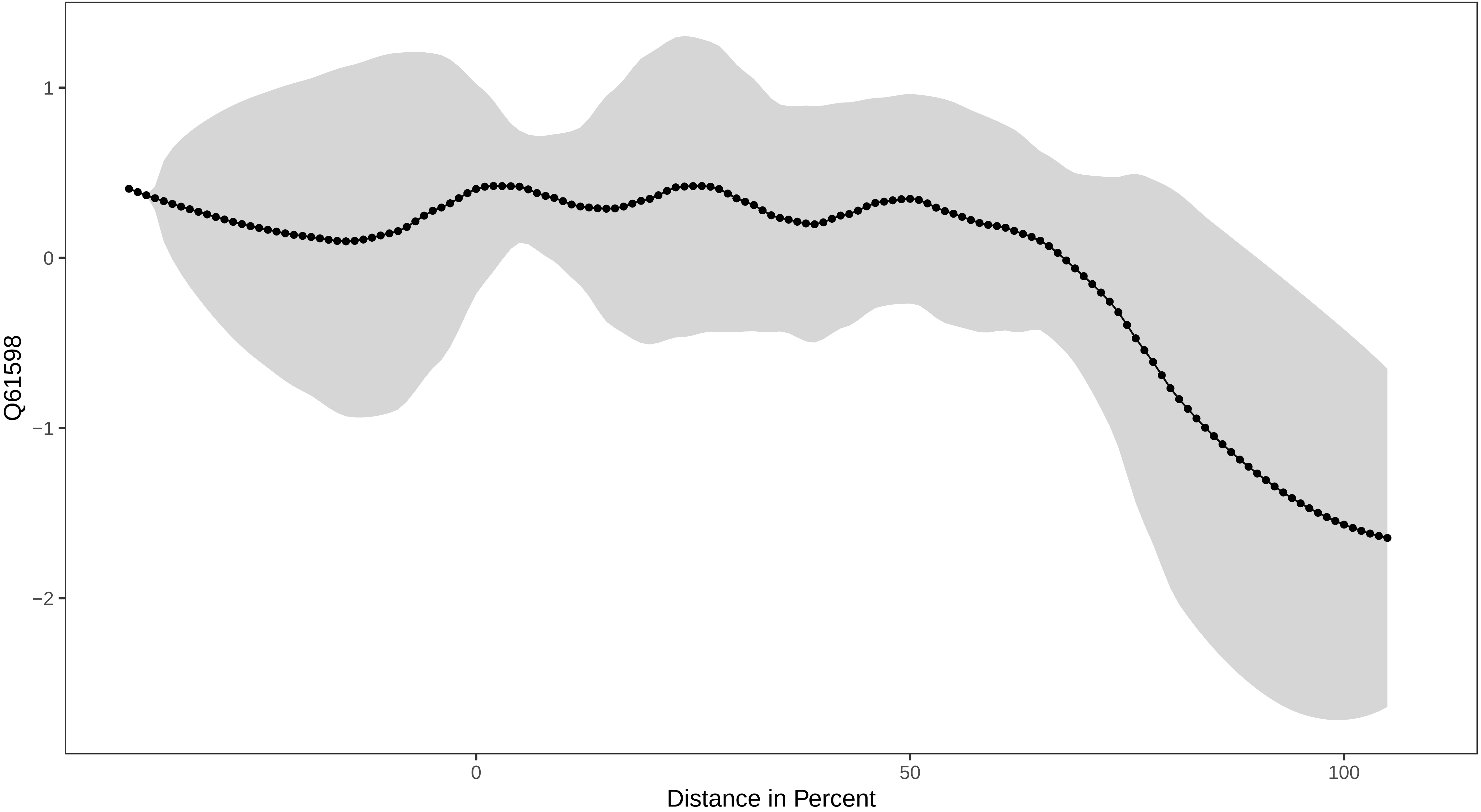

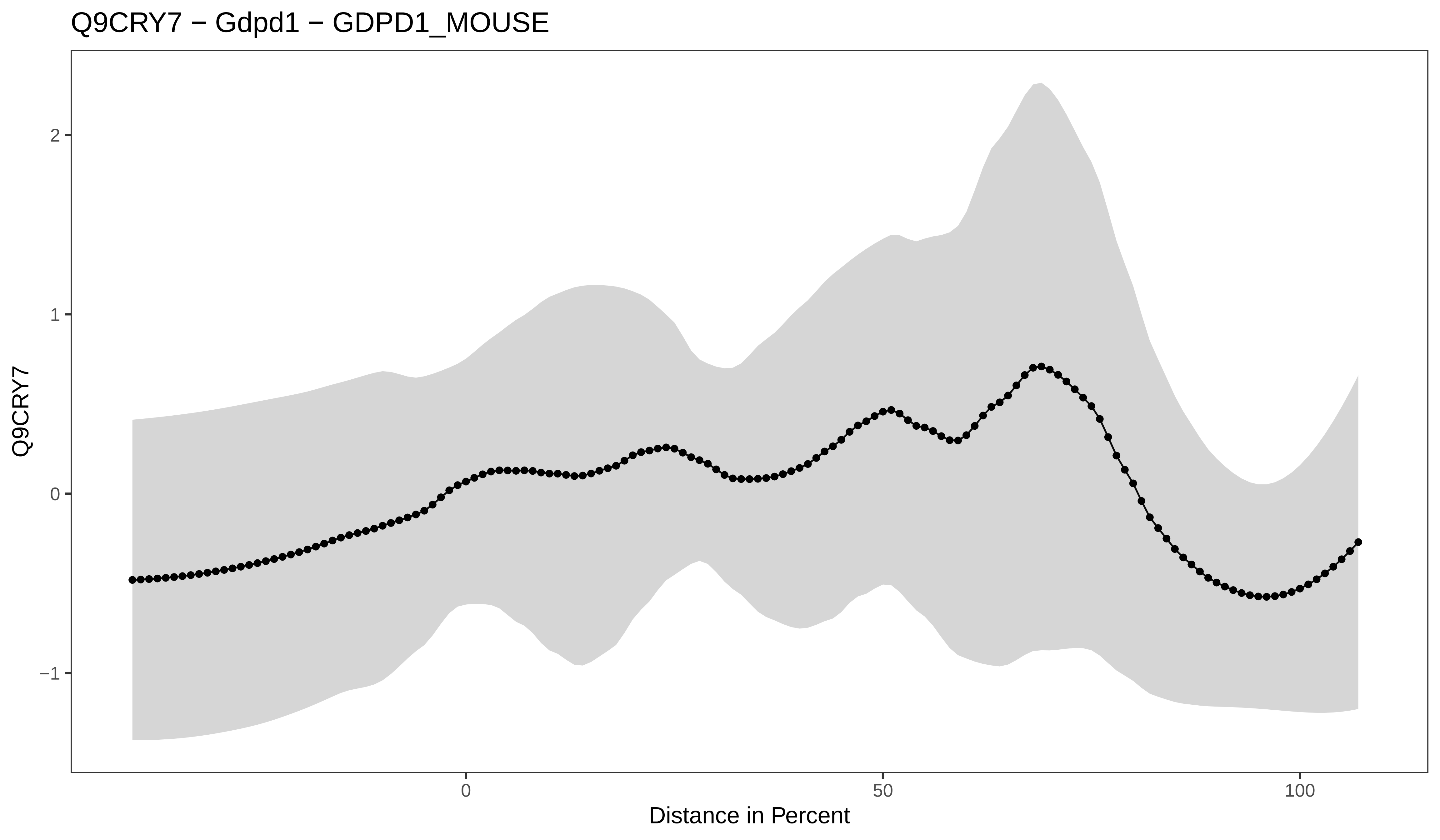
Q9CRY7 − Gdpd1 − GDPD1_MOUSE
Q9CRY7
2
1
0
−1
0
50
100
Distance in Percent

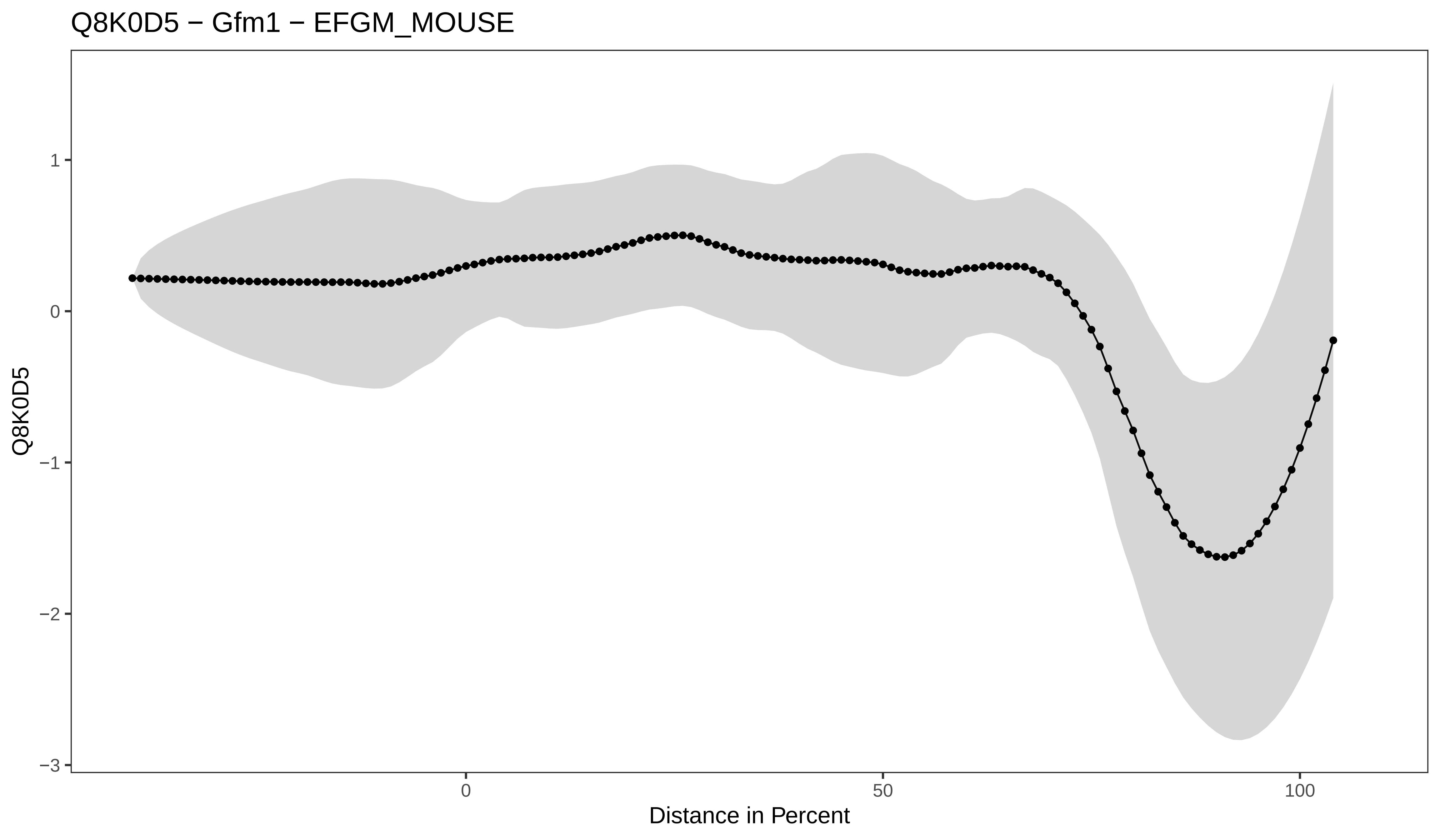

Q8K0D5 − Gfm1 − EFGM_MOUSE
1
0
−1
−2
−3
Q8K0D5
0
50
100
Distance in Percent

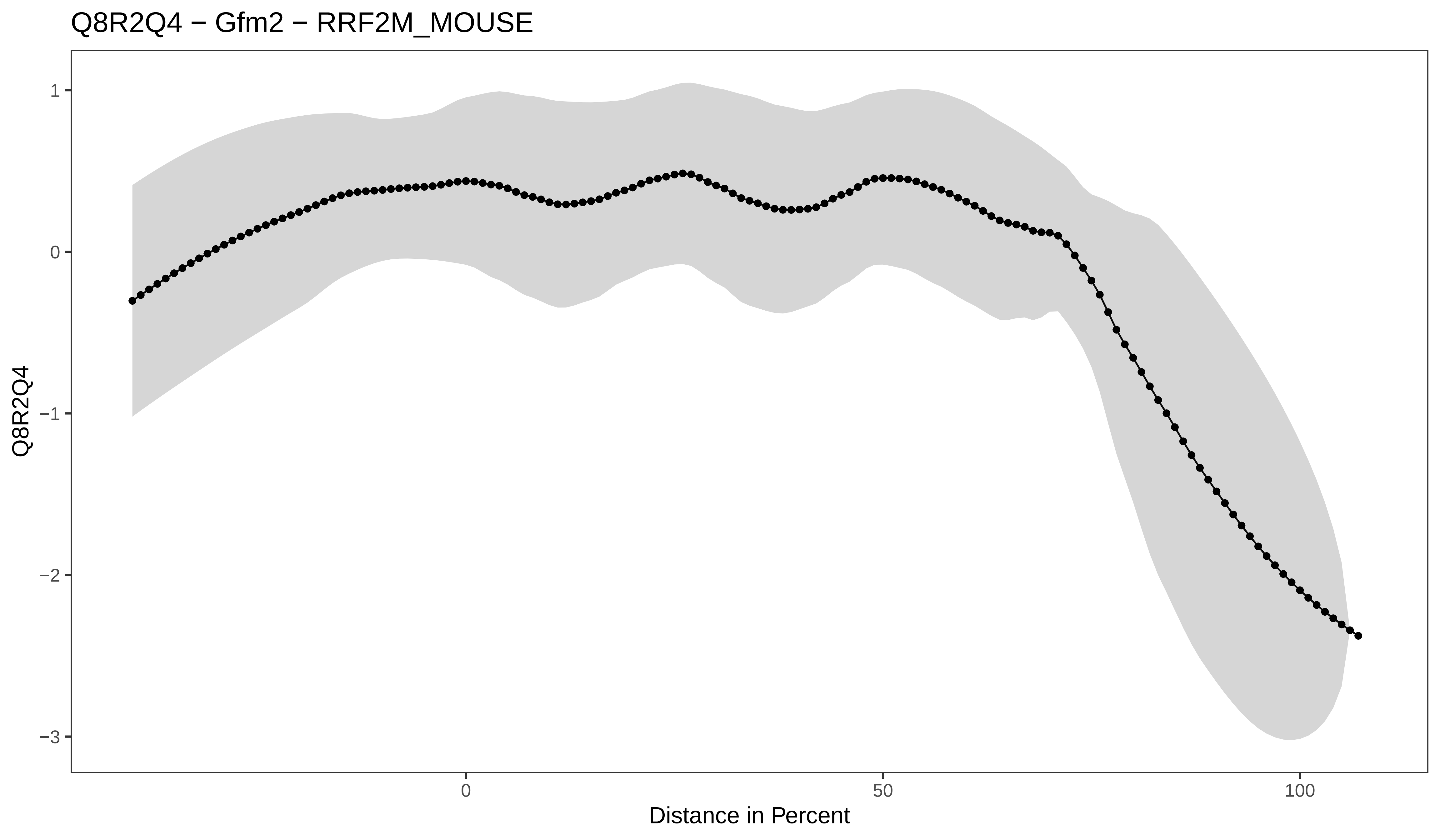

Q8R2Q4 − Gfm2 − RRF2M_MOUSE
1
0
−1
−2
−3
Q8R2Q4
0
50
100
Distance in Percent

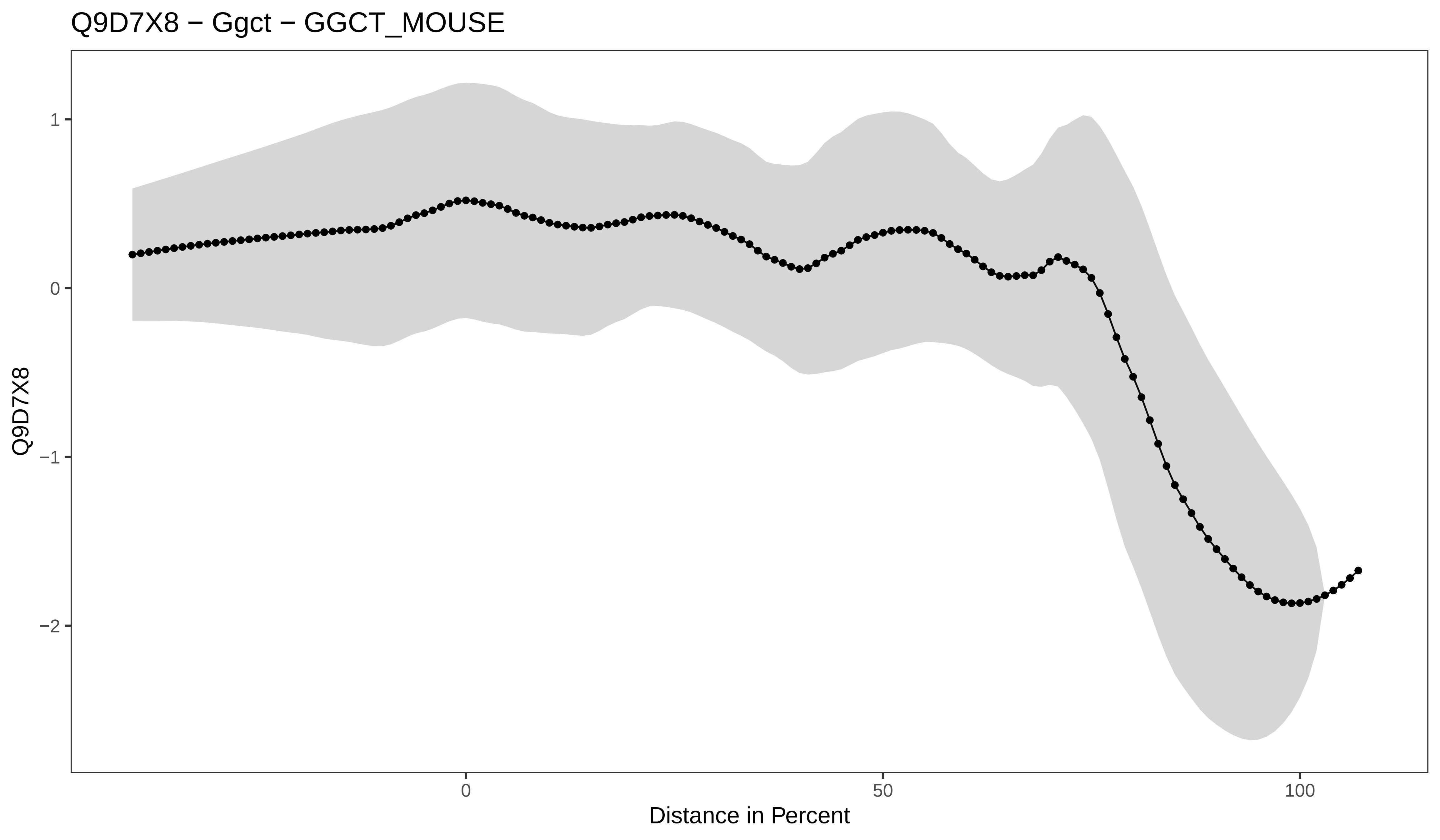

Q9D7X8 − Ggct − GGCT_MOUSE
1
0
−1
−2
Q9D7X8
0
50
100
Distance in Percent

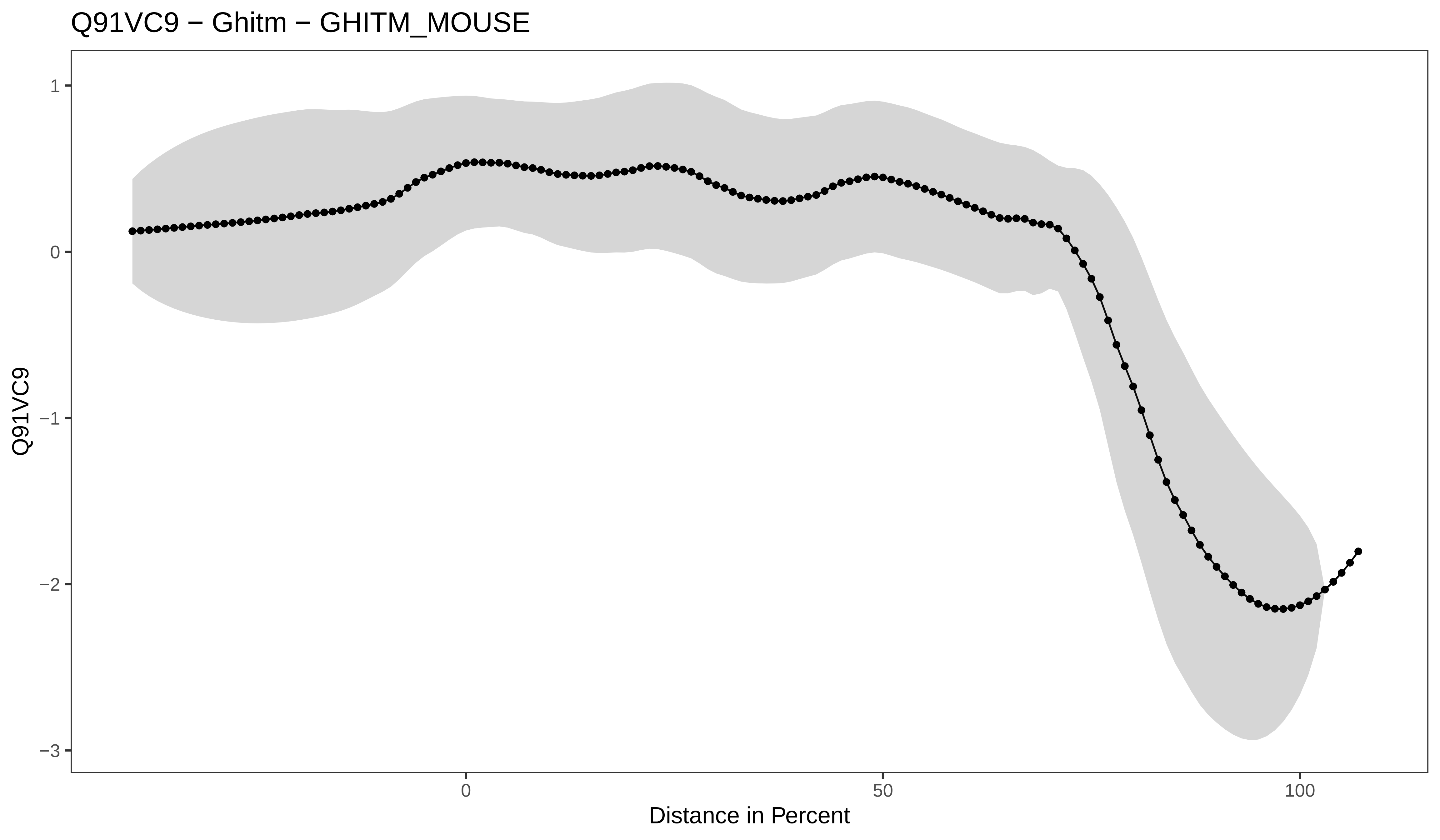

Q91VC9 − Ghitm − GHITM_MOUSE
1
0
−1
−2
−3
Q91VC9
0
50
100
Distance in Percent

Q99JY3 − Gimap4 − GIMA4_MOUSE

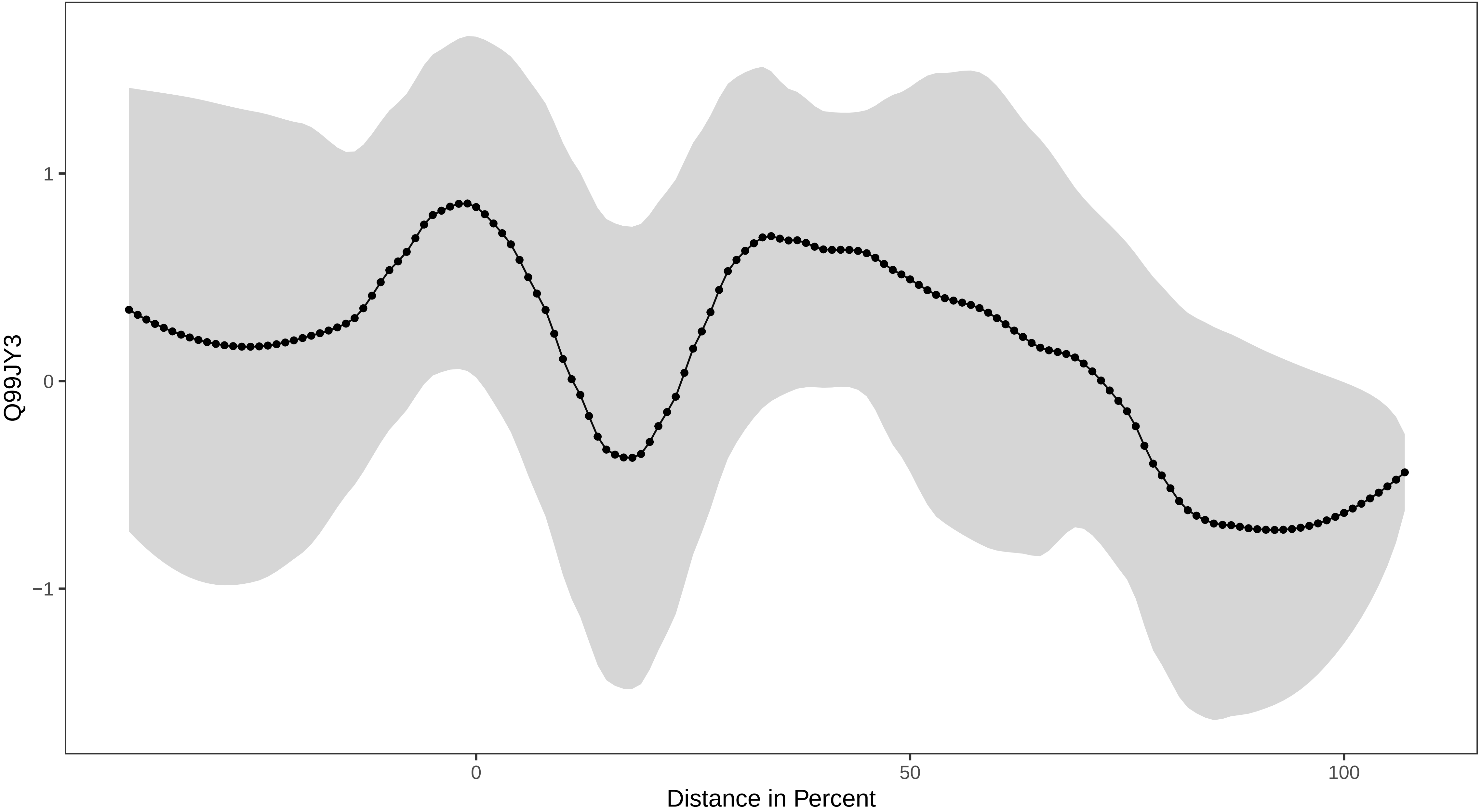

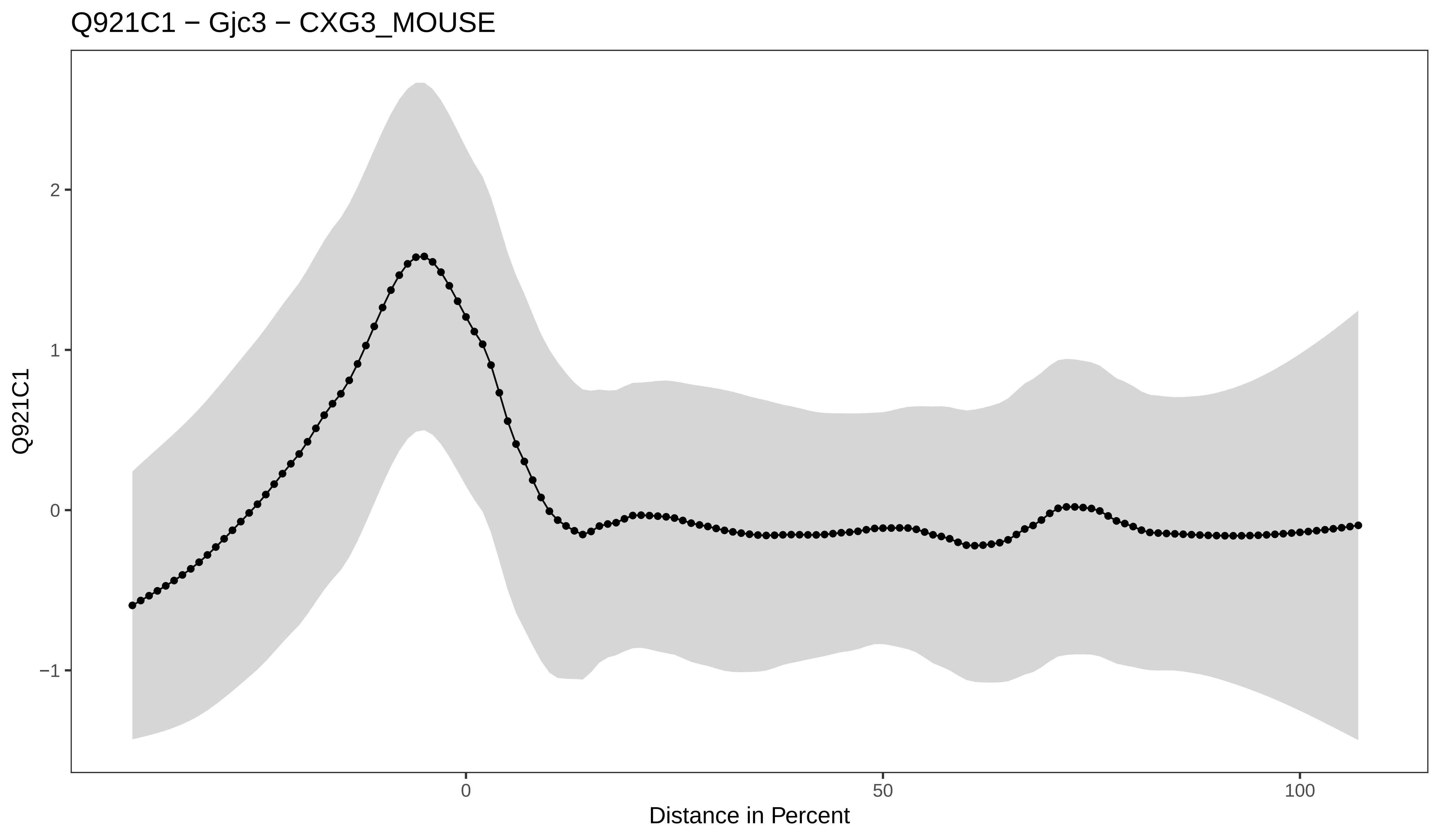

Q921C1 − Gjc3 − CXG3_MOUSE
Q921C1
2
1
0
−1
0
50
100
Distance in Percent

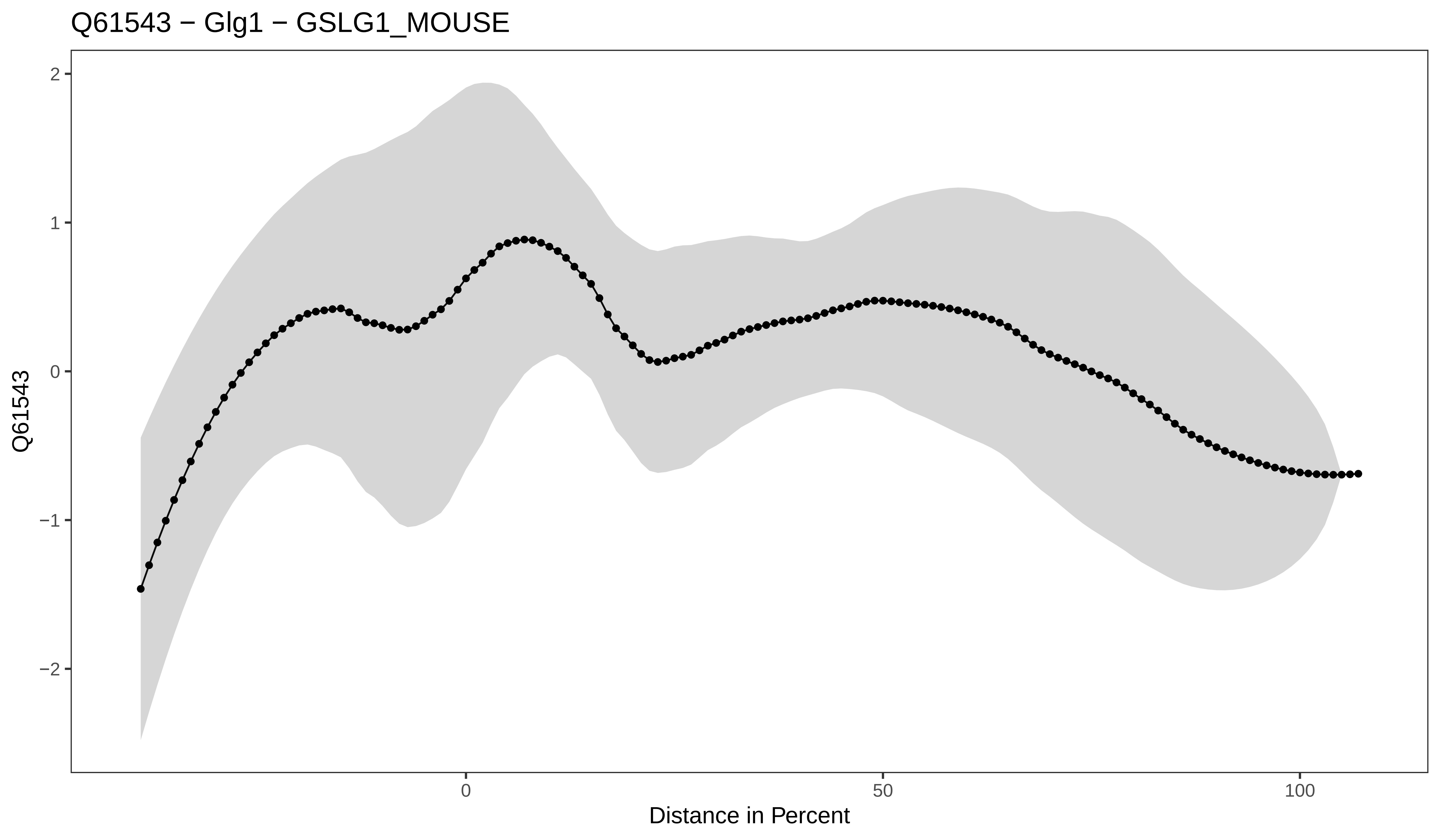
Q61543 − Glg1 − GSLG1_MOUSE
Q61543
Distance in Percent
2
1
0
−1
−2
0
50
100

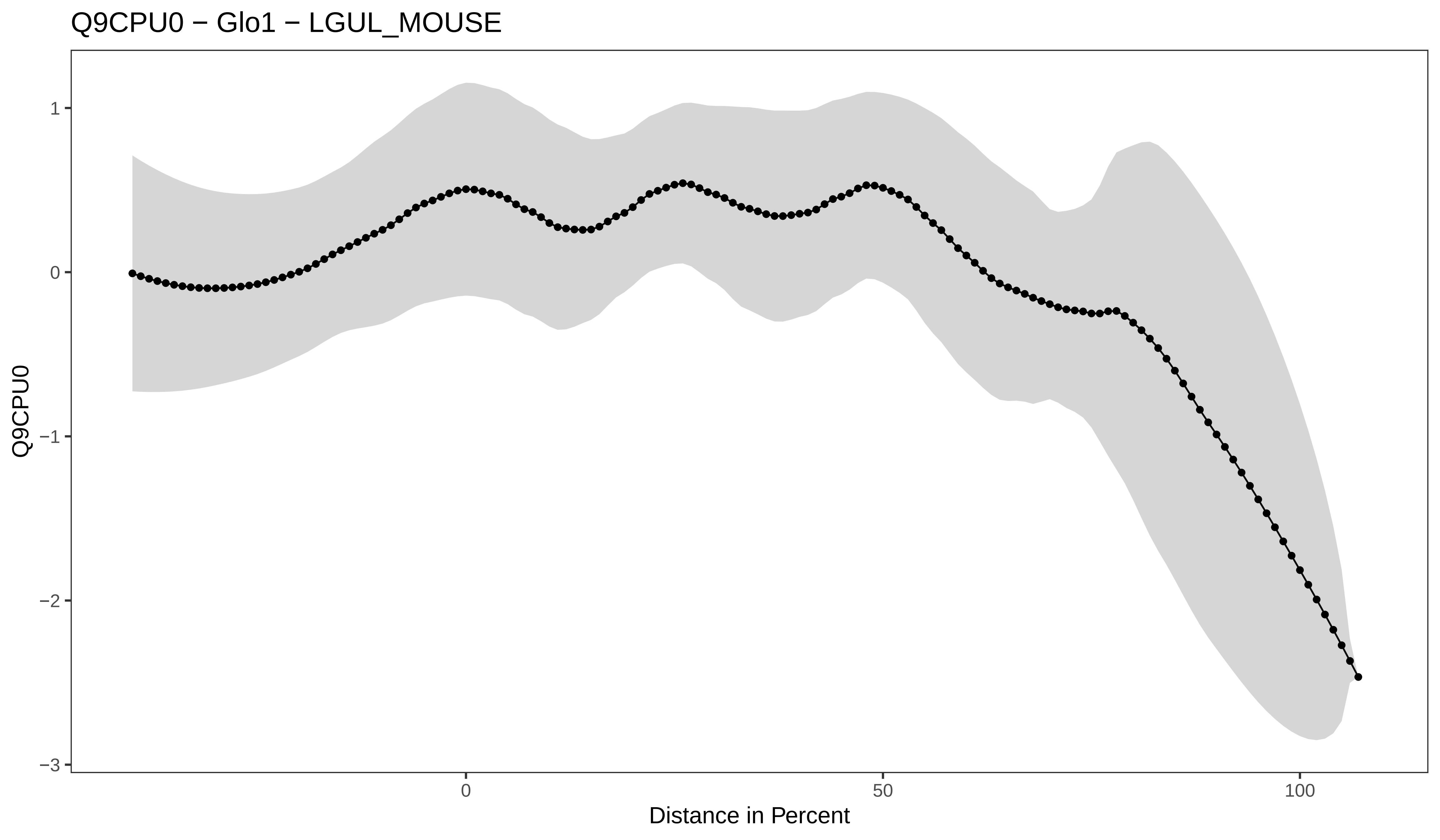
Q9CPU0 − Glo1 − LGUL_MOUSE
Q9CPU0
Distance in Percent
1
0
−1
−2
−3
0
50
100

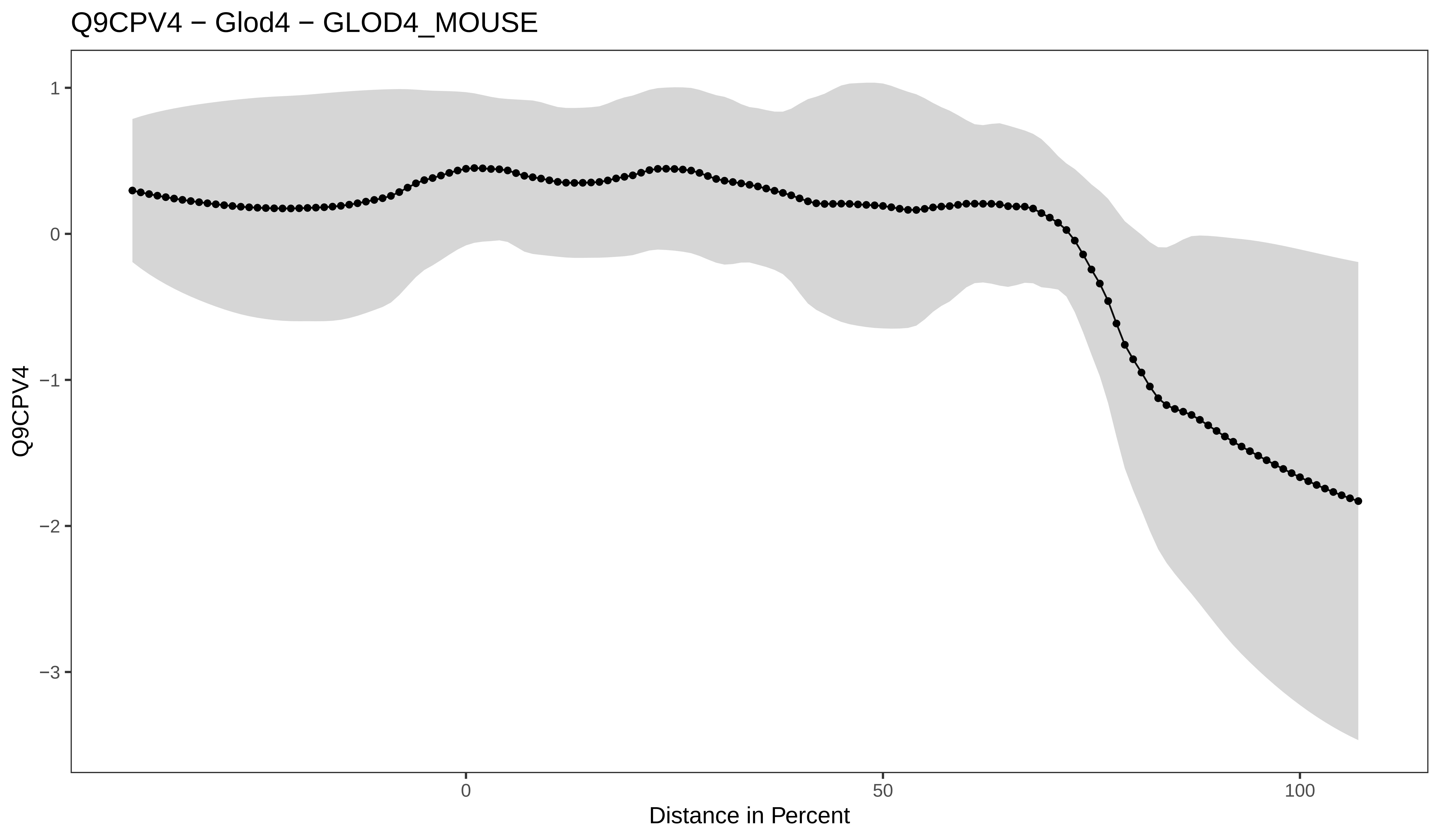

Q9CPV4 − Glod4 − GLOD4_MOUSE
1
0
−1
−2
−3
Q9CPV4
0
50
100
Distance in Percent

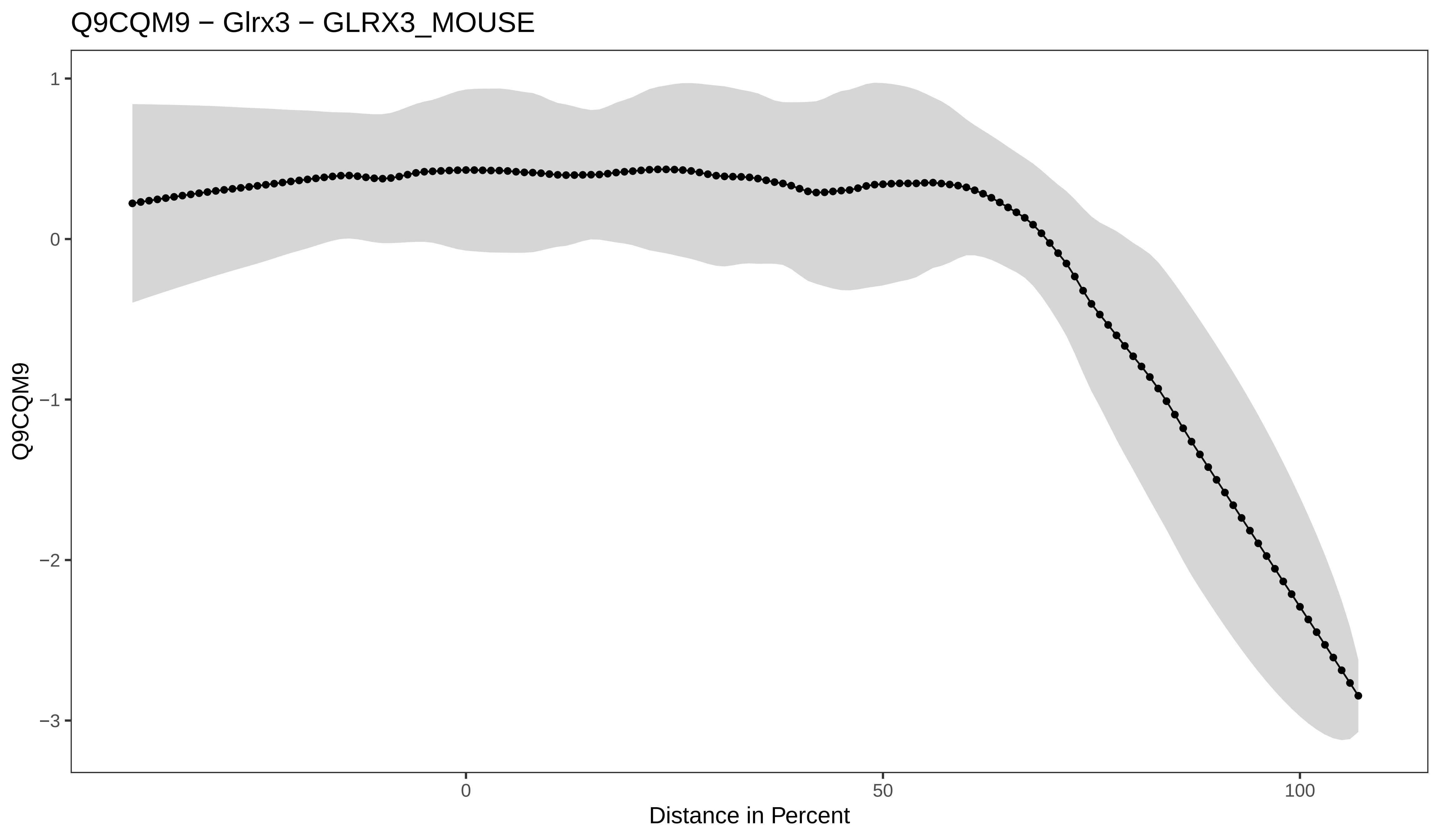
Q9CQM9 − Glrx3 − GLRX3_MOUSE
1
0
−1
−2
−3
Q9CQM9
0
50
100
Distance in Percent

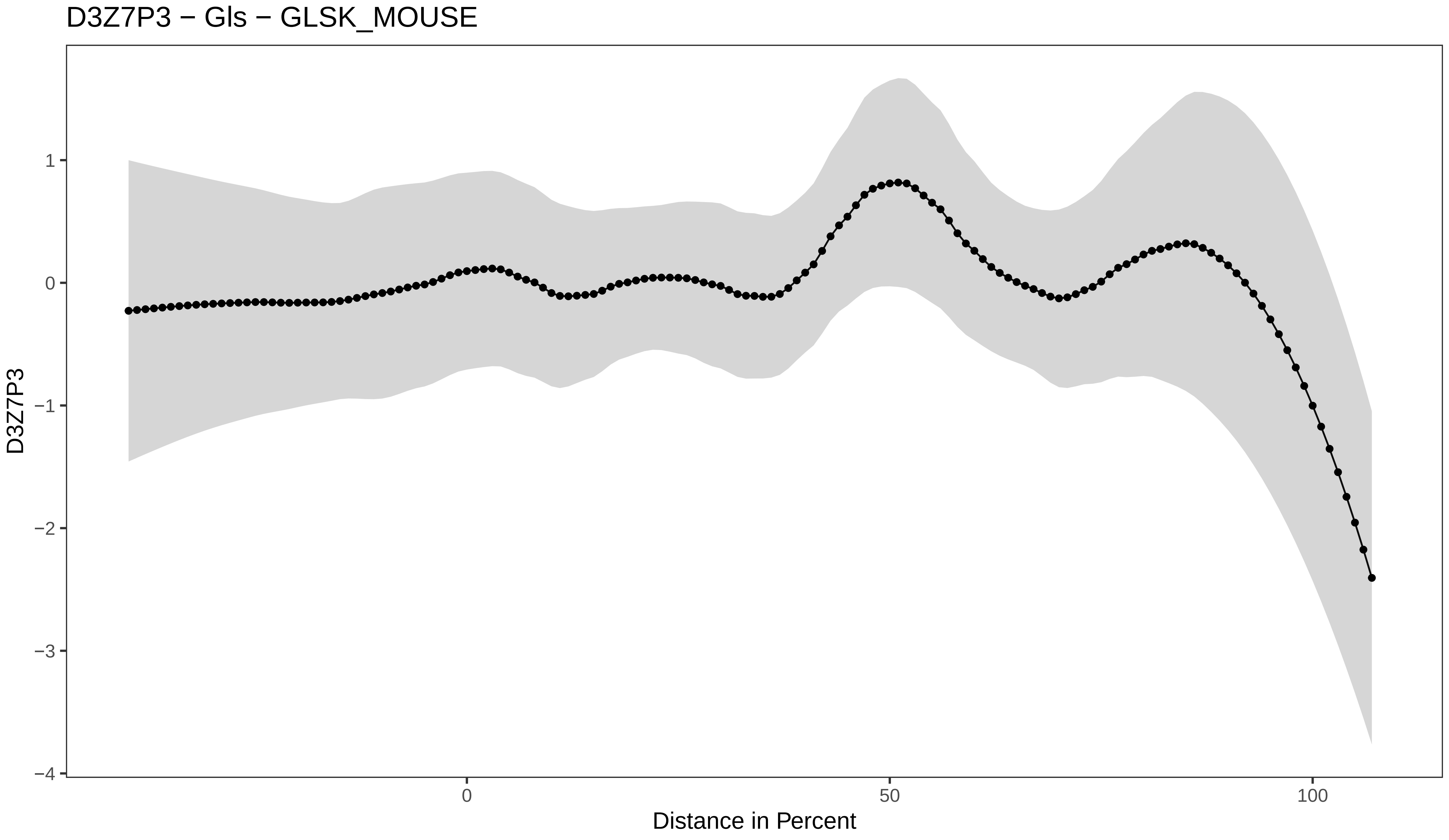

D3Z7P3 − Gls − GLSK_MOUSE
1
0
−1
−2
−3
−4
D3Z7P3
0
50
100
Distance in Percent

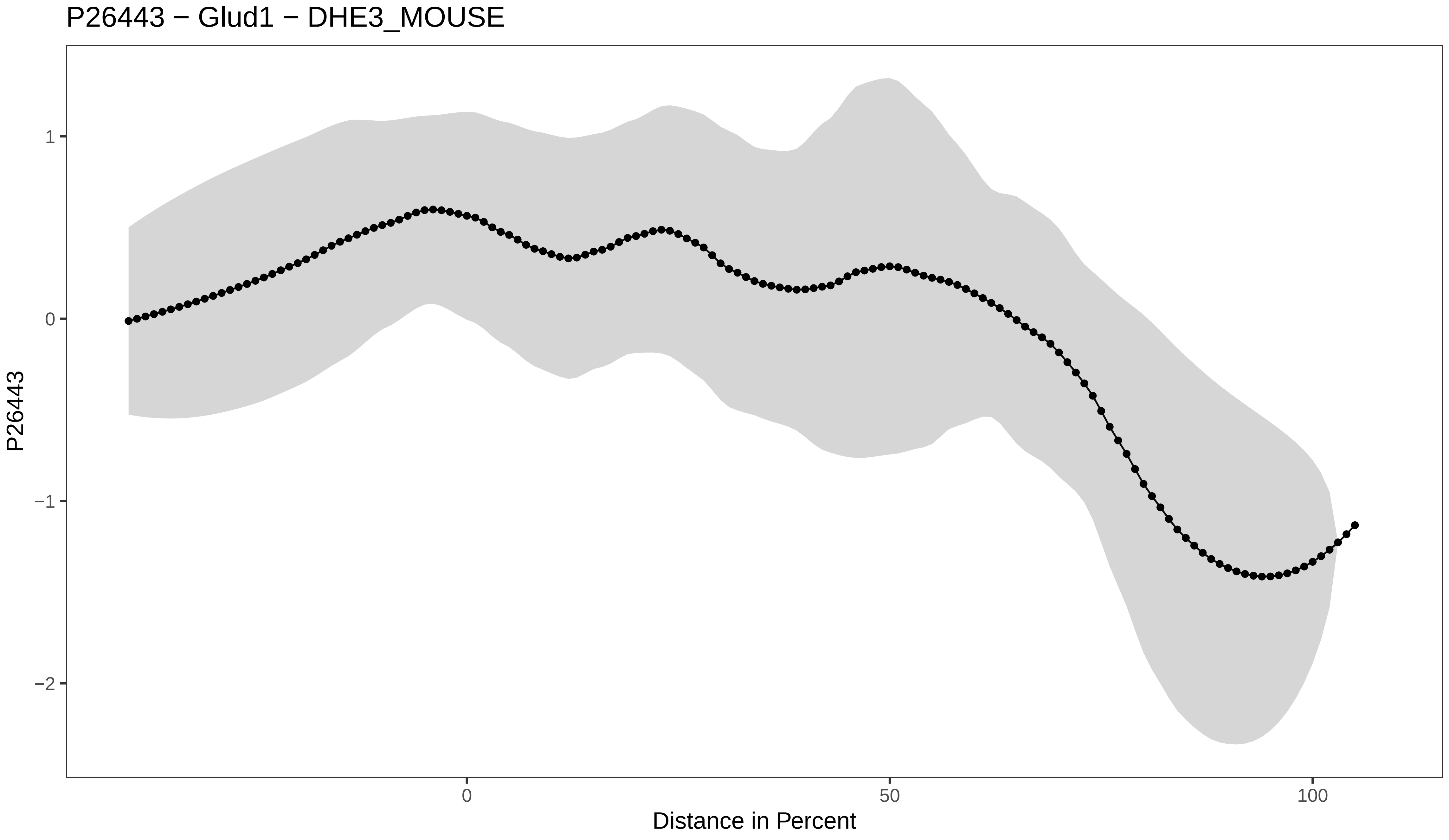

P26443 − Glud1 − DHE3_MOUSE
P26443
1
0
−1
−2
0
50
100
Distance in Percent

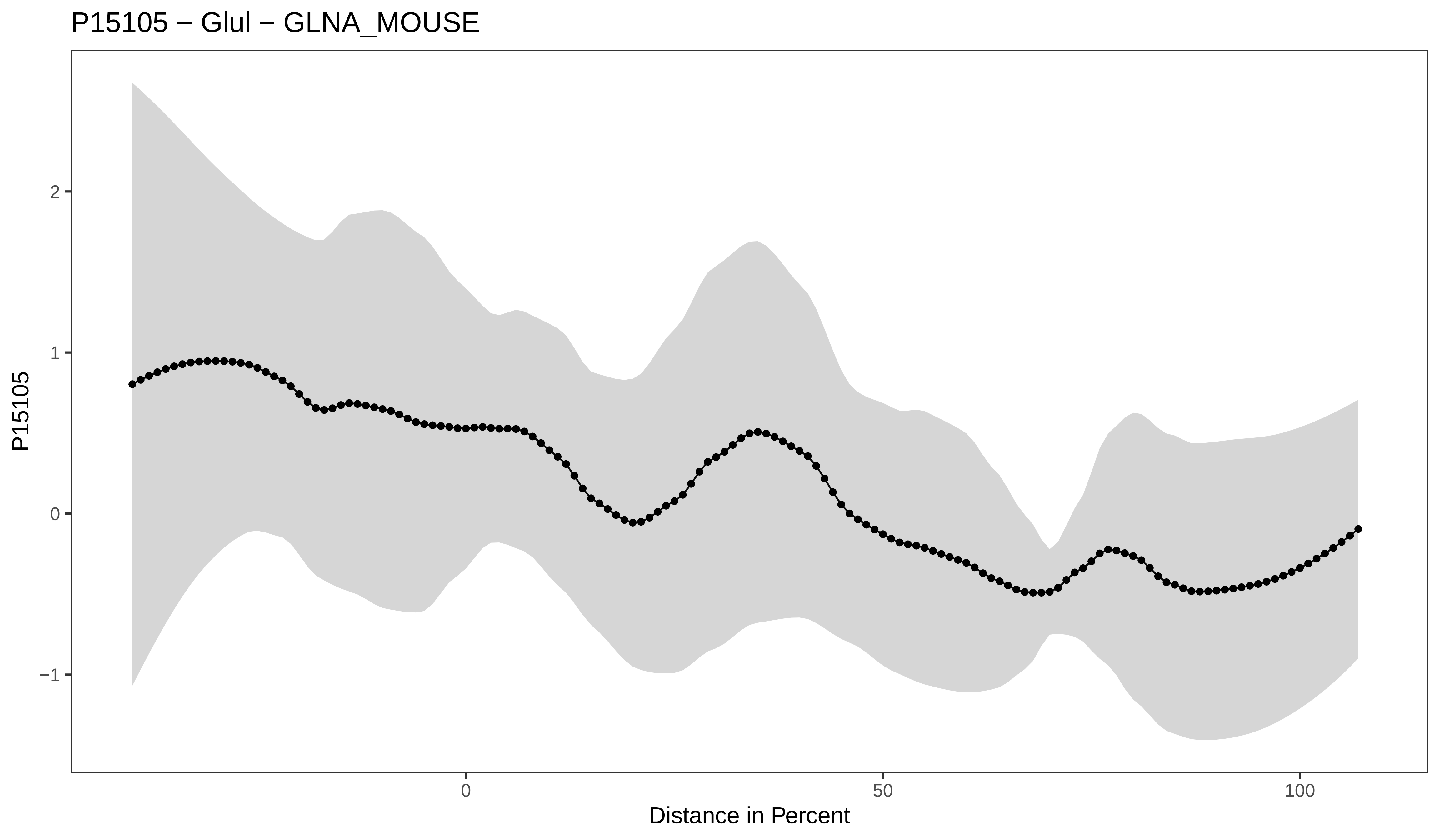
P15105 − Glul − GLNA_MOUSE
2
1
0
−1
P15105
0
50
100
Distance in Percent

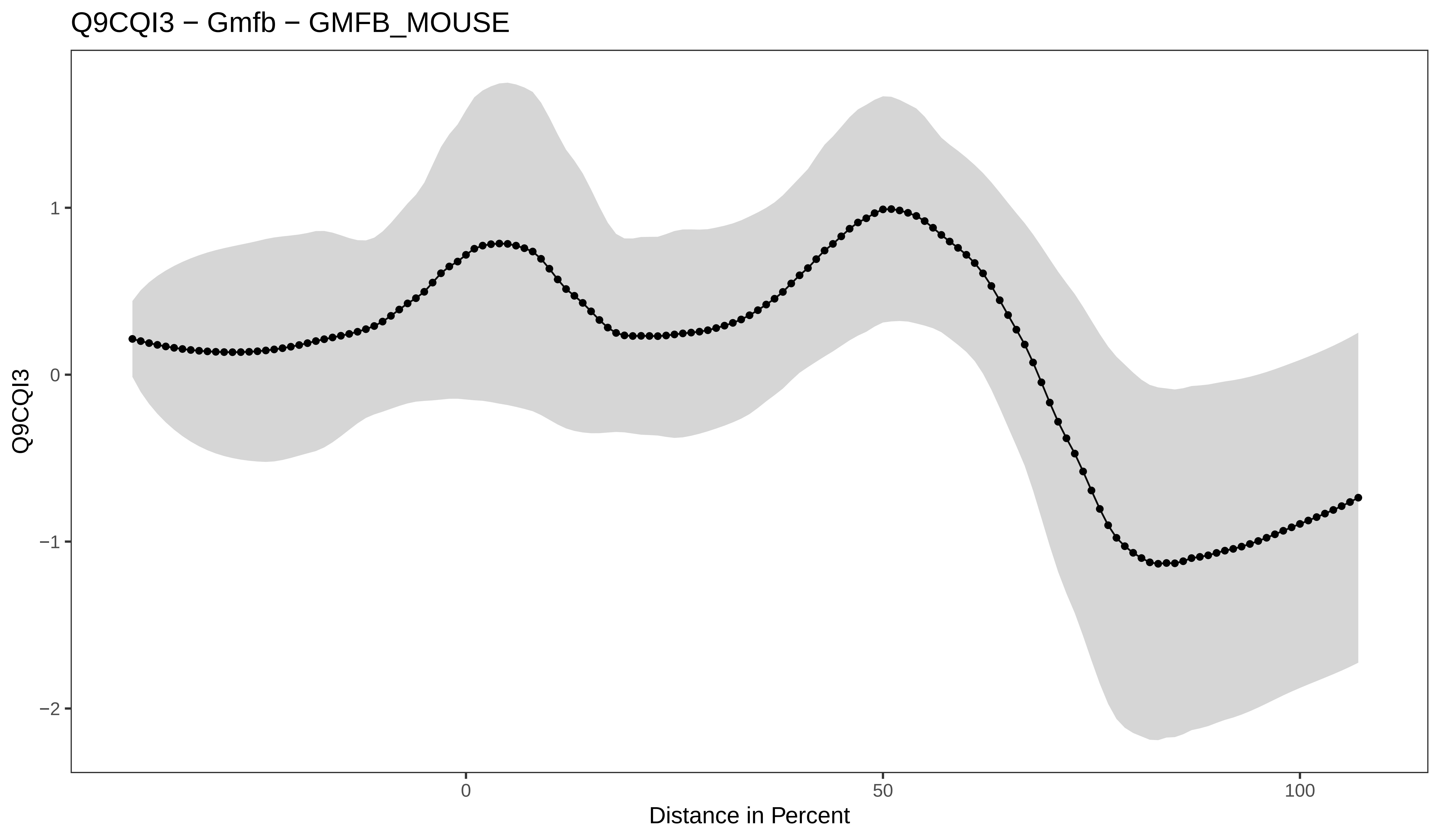
Q9CQI3 − Gmfb − GMFB_MOUSE
Q9CQI3
1
0
−1
−2
0
50
100
Distance in Percent

Q922H4 − Gmppa − GMPPA_MOUSE

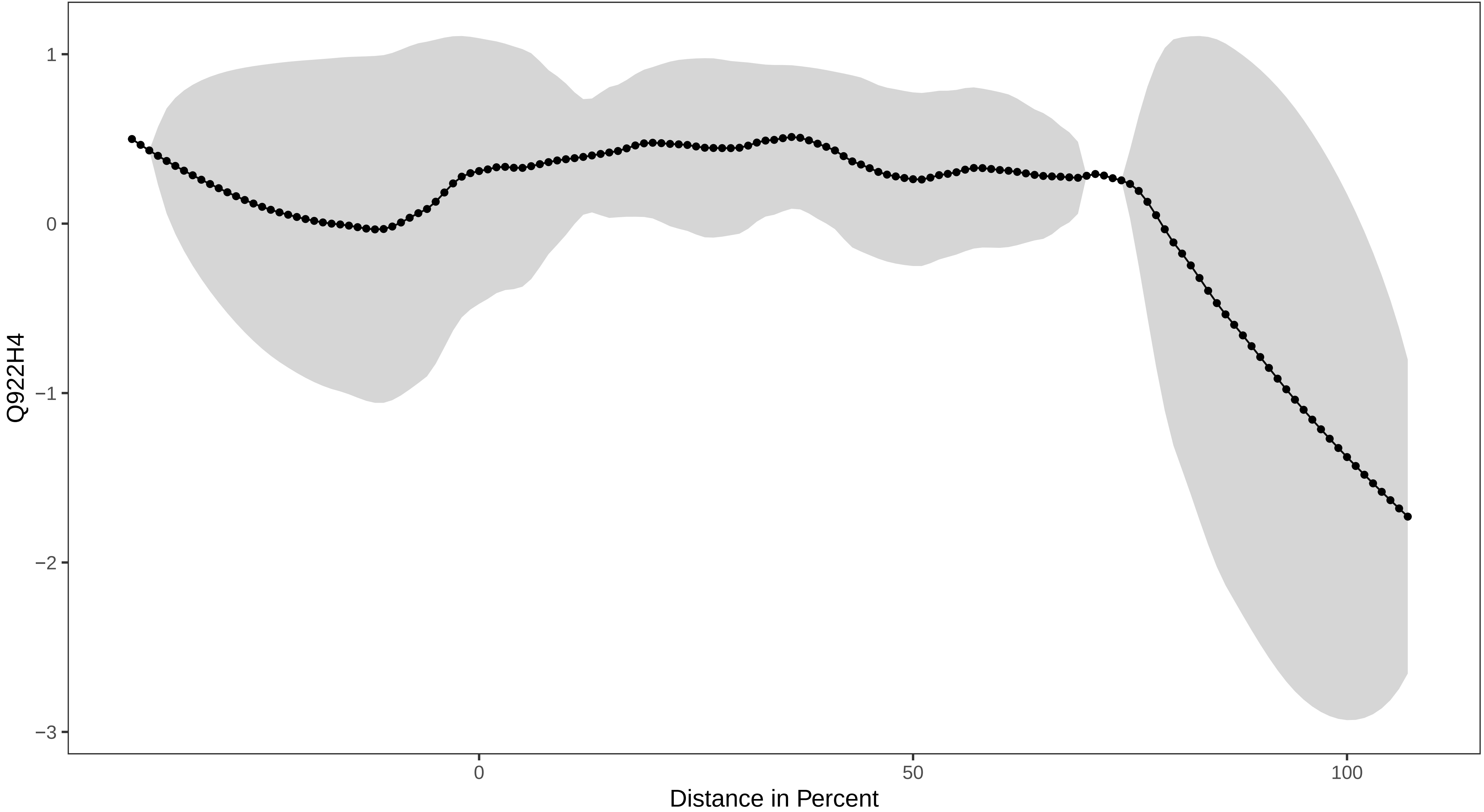

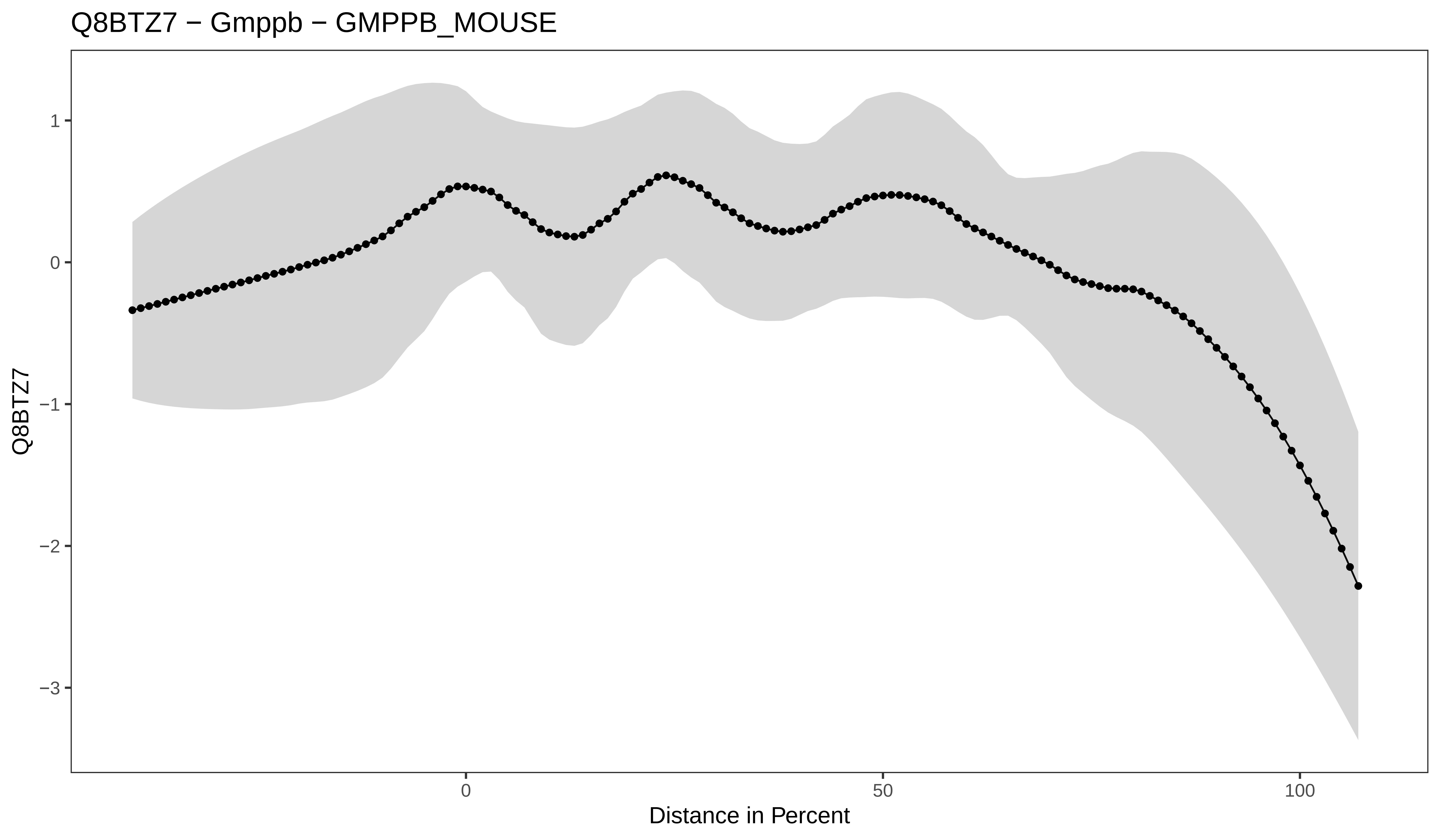

Q8BTZ7 − Gmppb − GMPPB_MOUSE
1
0
−1
−2
−3
Q8BTZ7
0
50
100
Distance in Percent

Q9DCZ1 − Gmpr − GMPR1_MOUSE

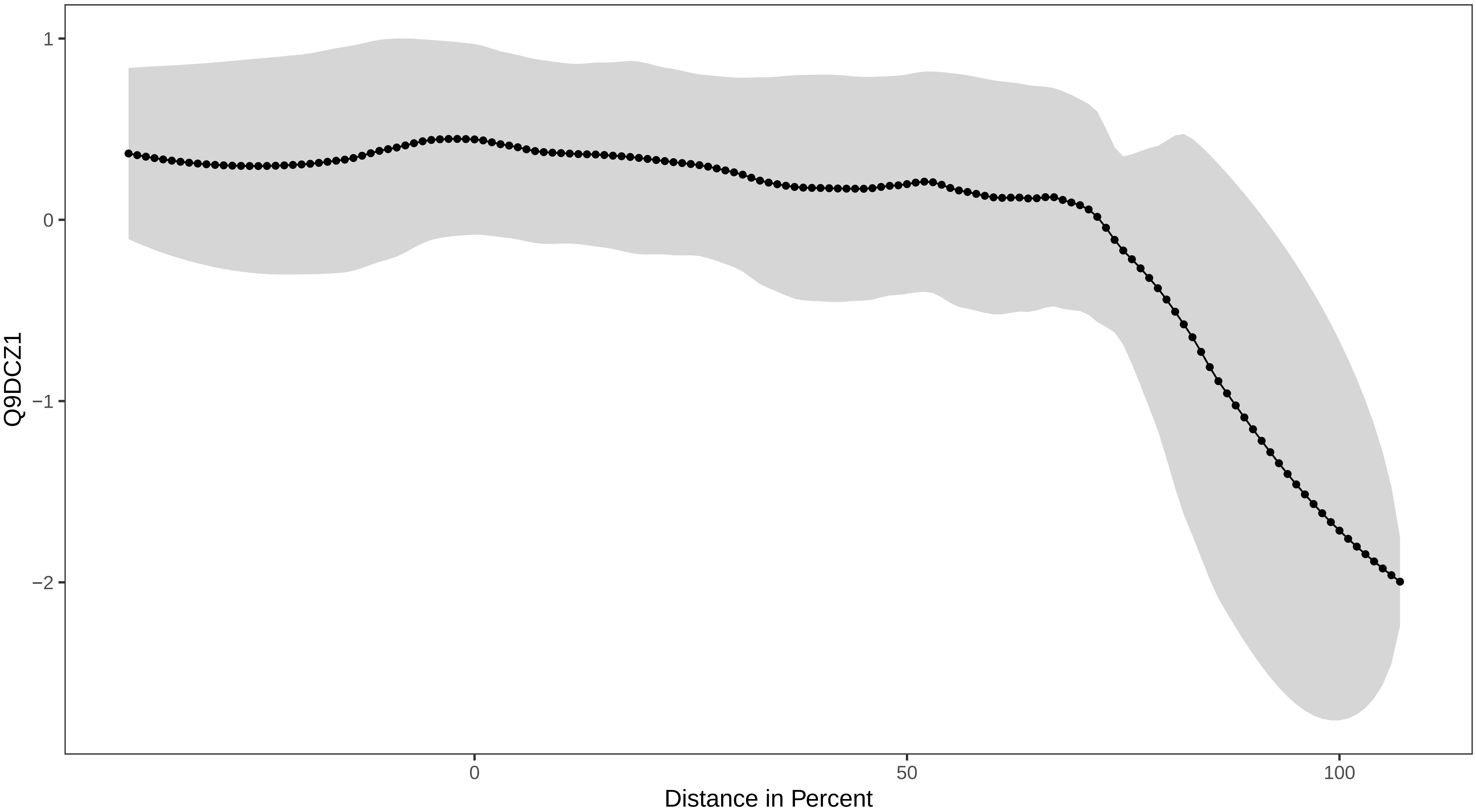

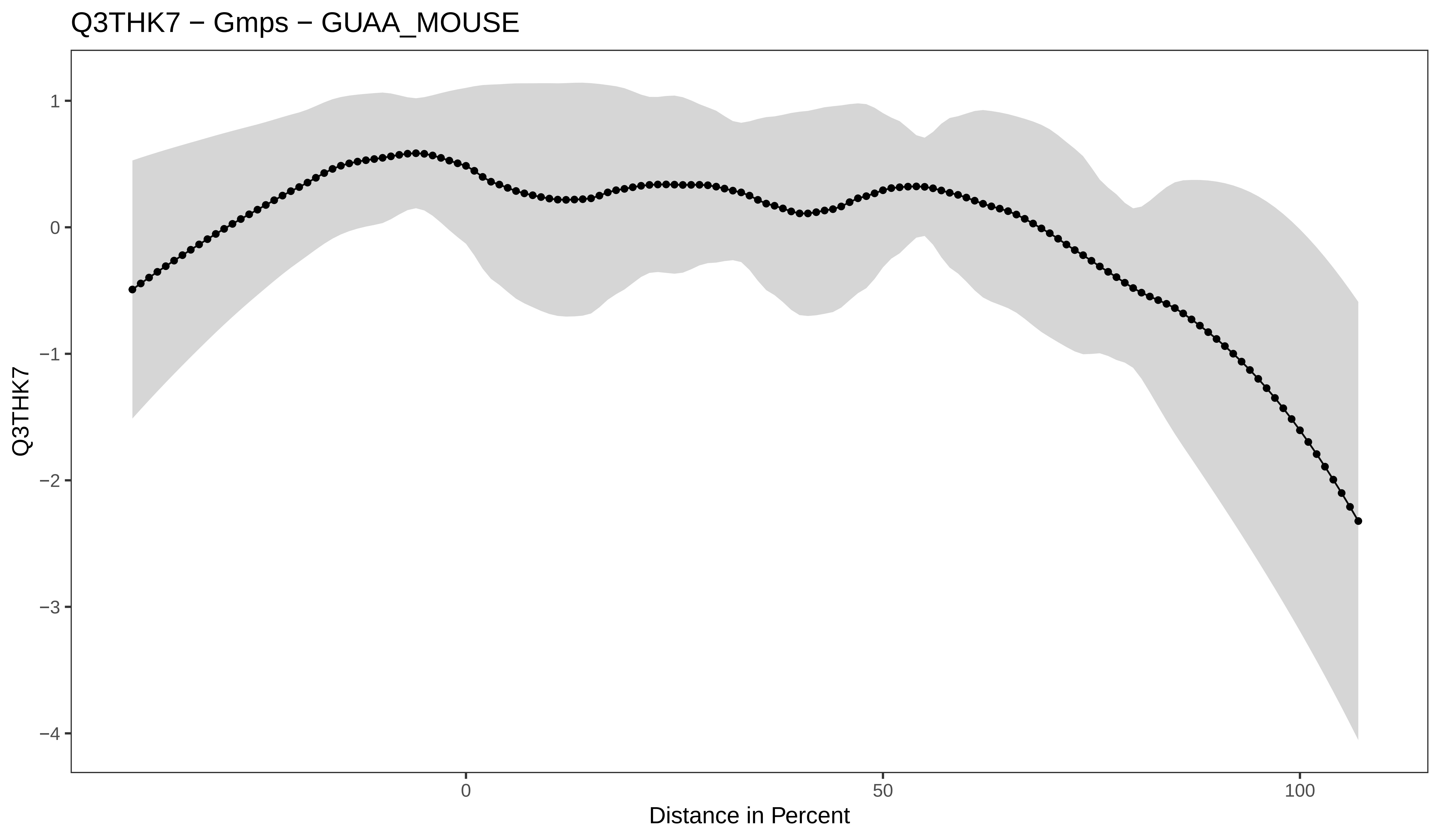

Q3THK7 − Gmps − GUAA_MOUSE
Q3THK7
Distance in Percent
1
0
−1
−2
−3
−4
0
50
100

P21278 − Gna11 − GNA11_MOUSE

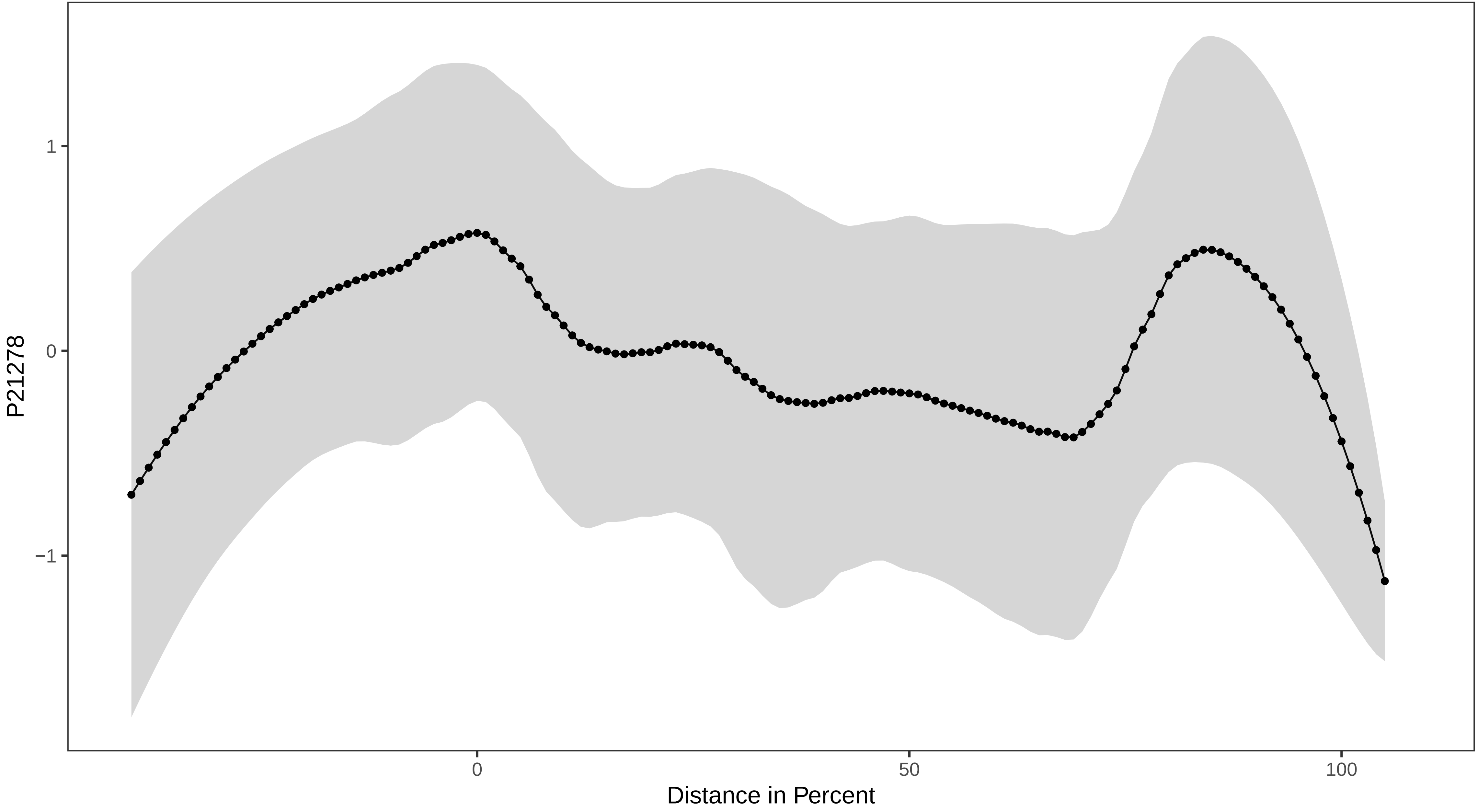

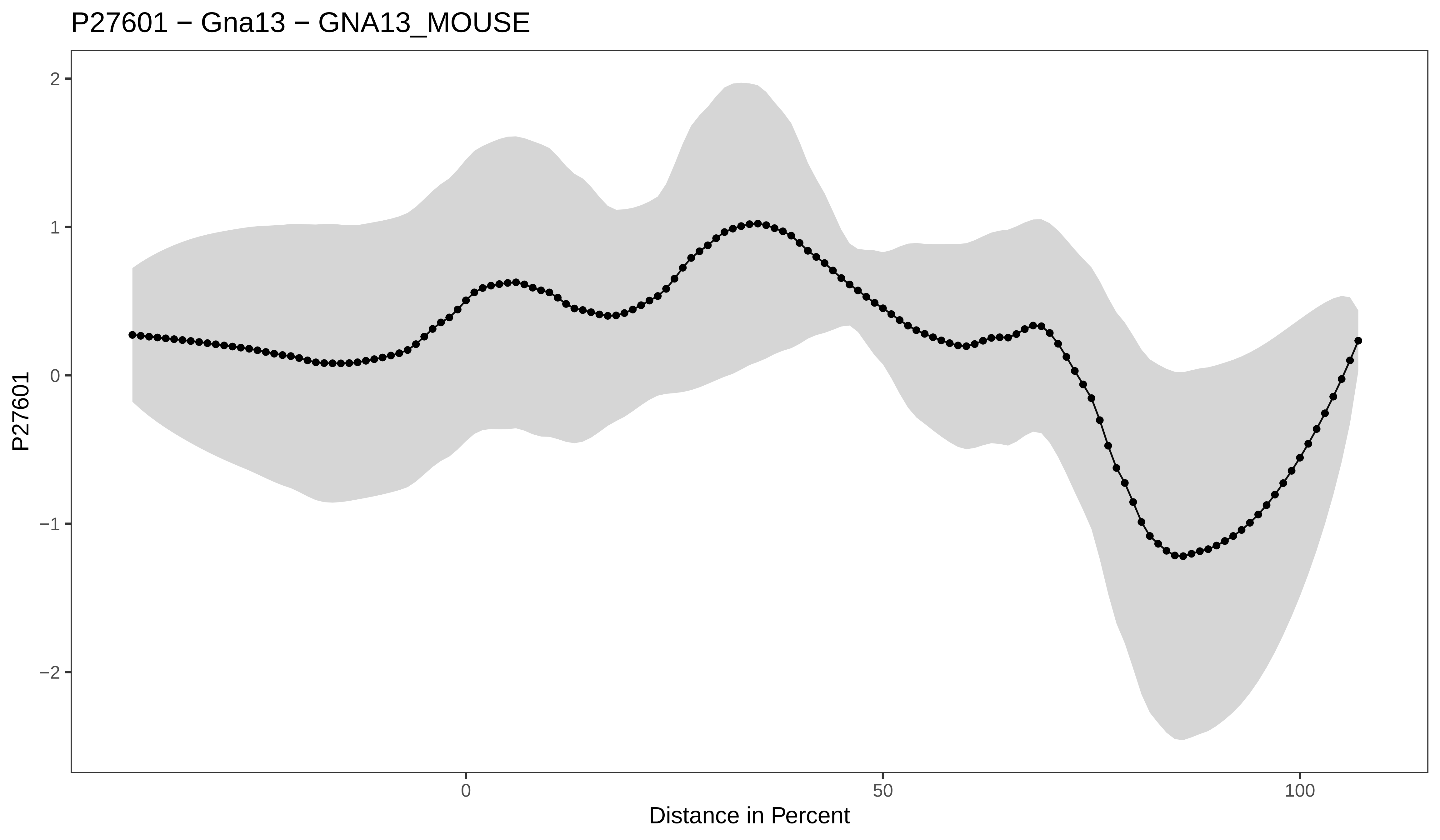

P27601 − Gna13 − GNA13_MOUSE
2
1
0
−1
−2
P27601
0
50
100
Distance in Percent

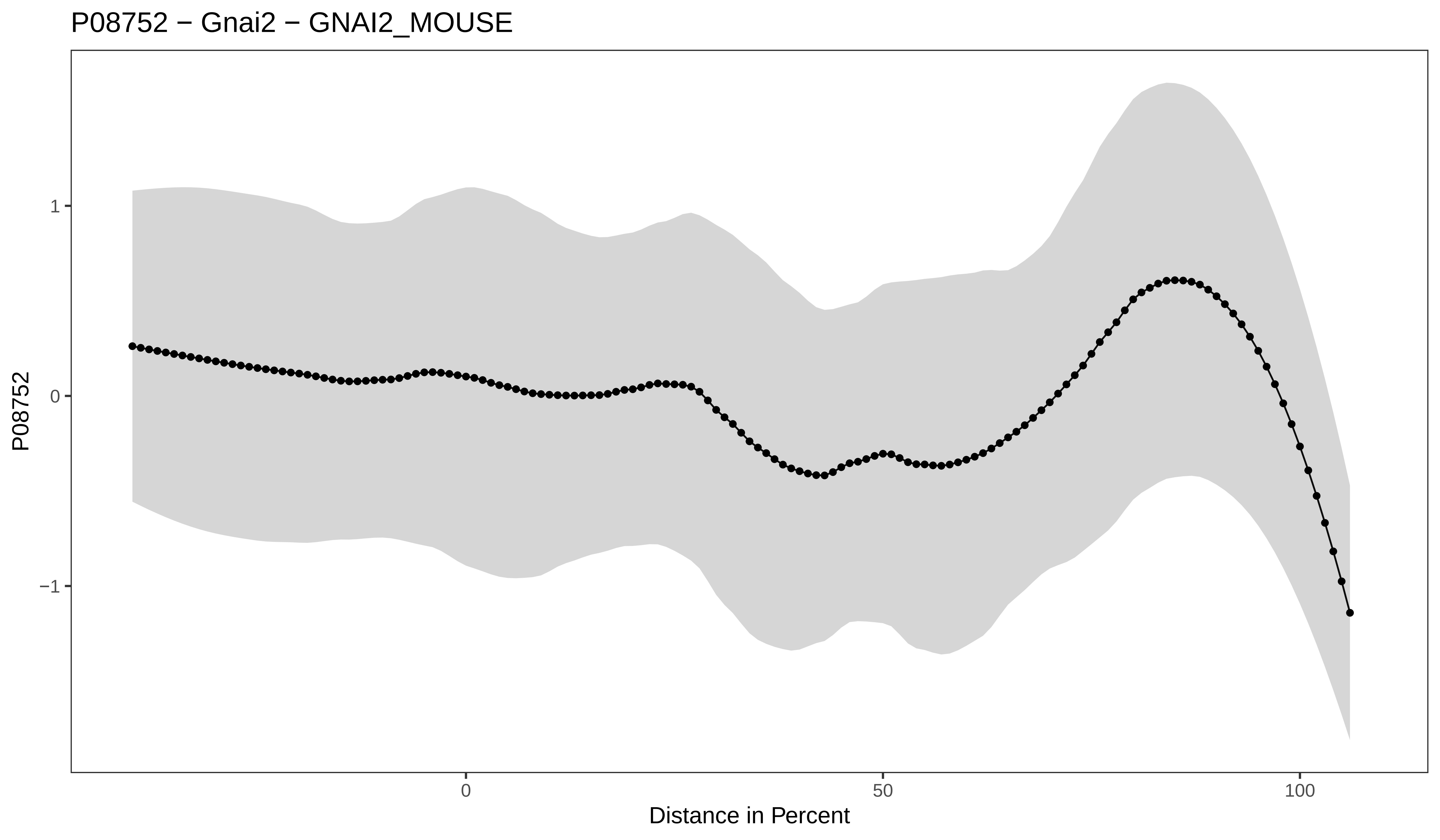

P08752 − Gnai2 − GNAI2_MOUSE
1
0
−1
P08752
0
50
100
Distance in Percent

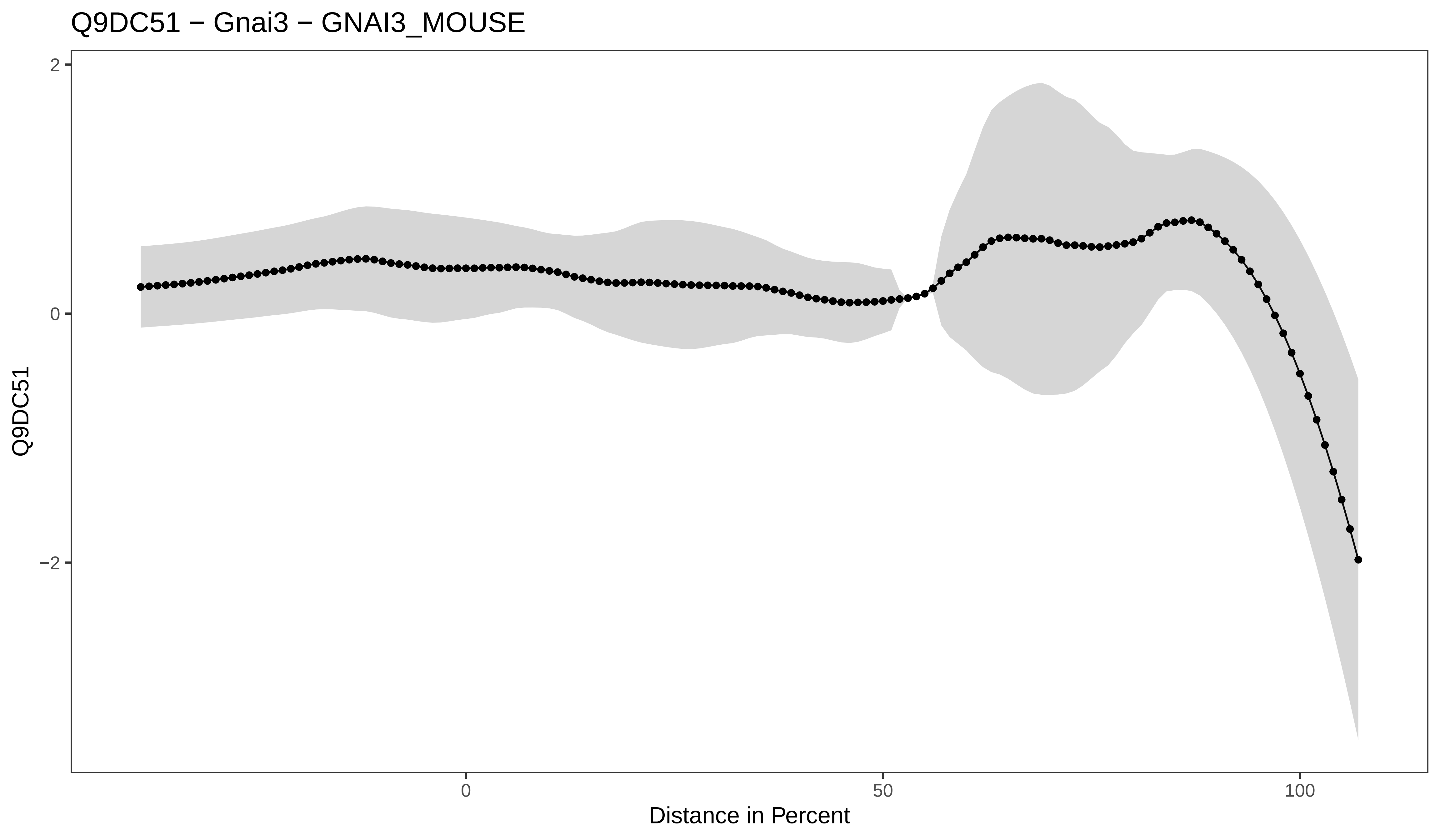
Q9DC51 − Gnai3 − GNAI3_MOUSE
Q9DC51
Distance in Percent
2
0
−2
0
50
100

P21279 − Gnaq − GNAQ_MOUSE

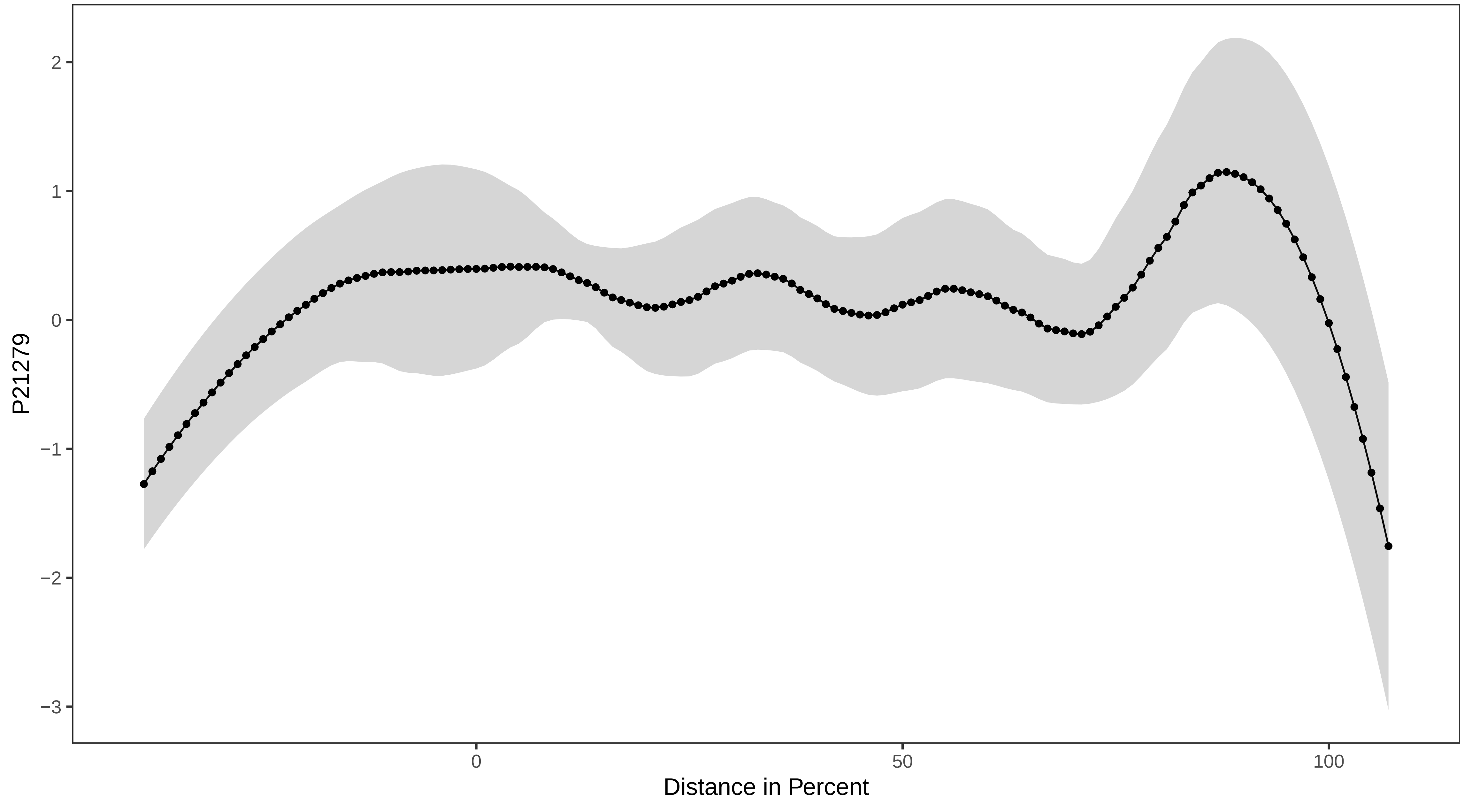

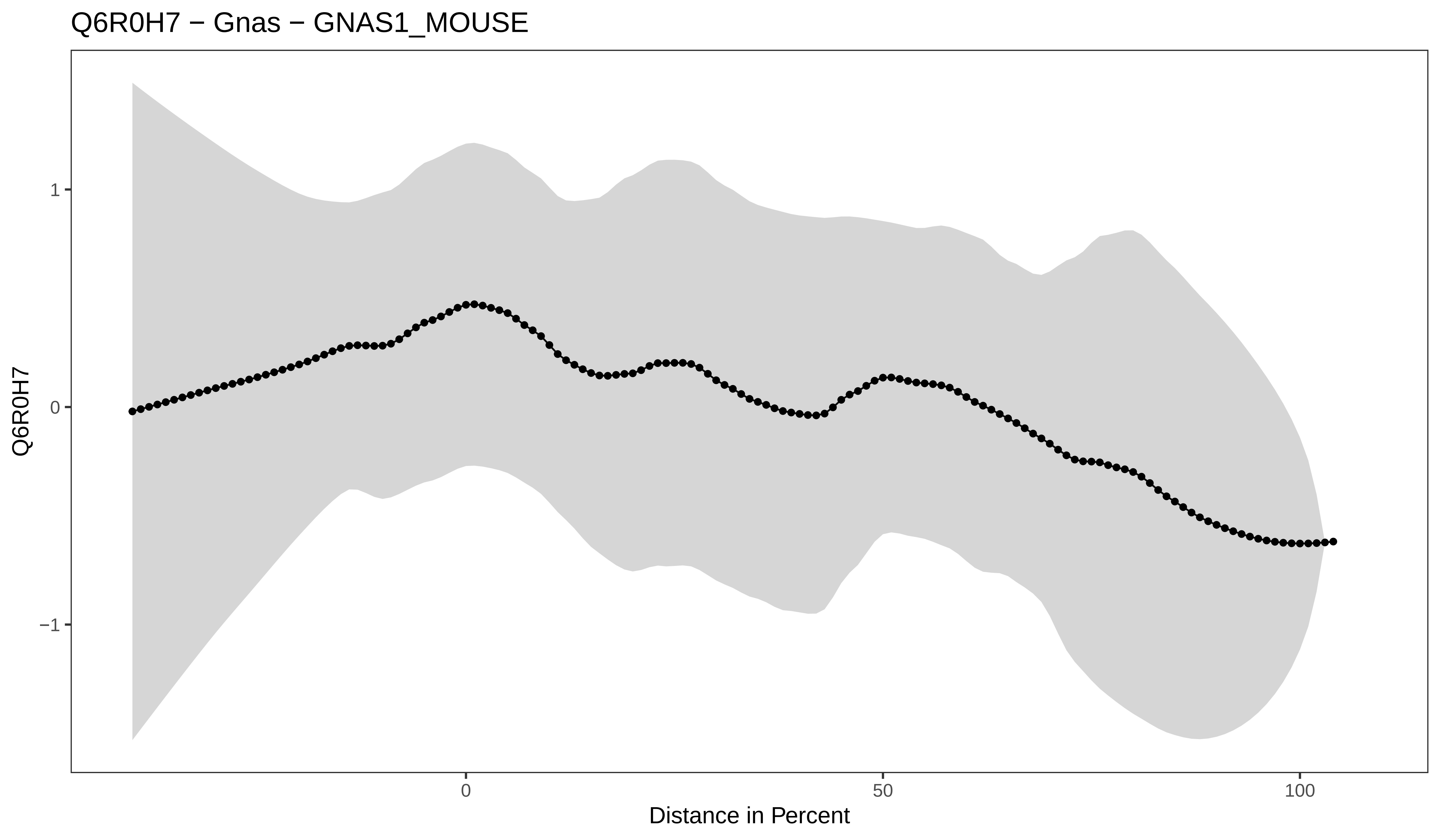
Q6R0H7 − Gnas − GNAS1_MOUSE
Q6R0H7
1
0
−1
0
50
100
Distance in Percent

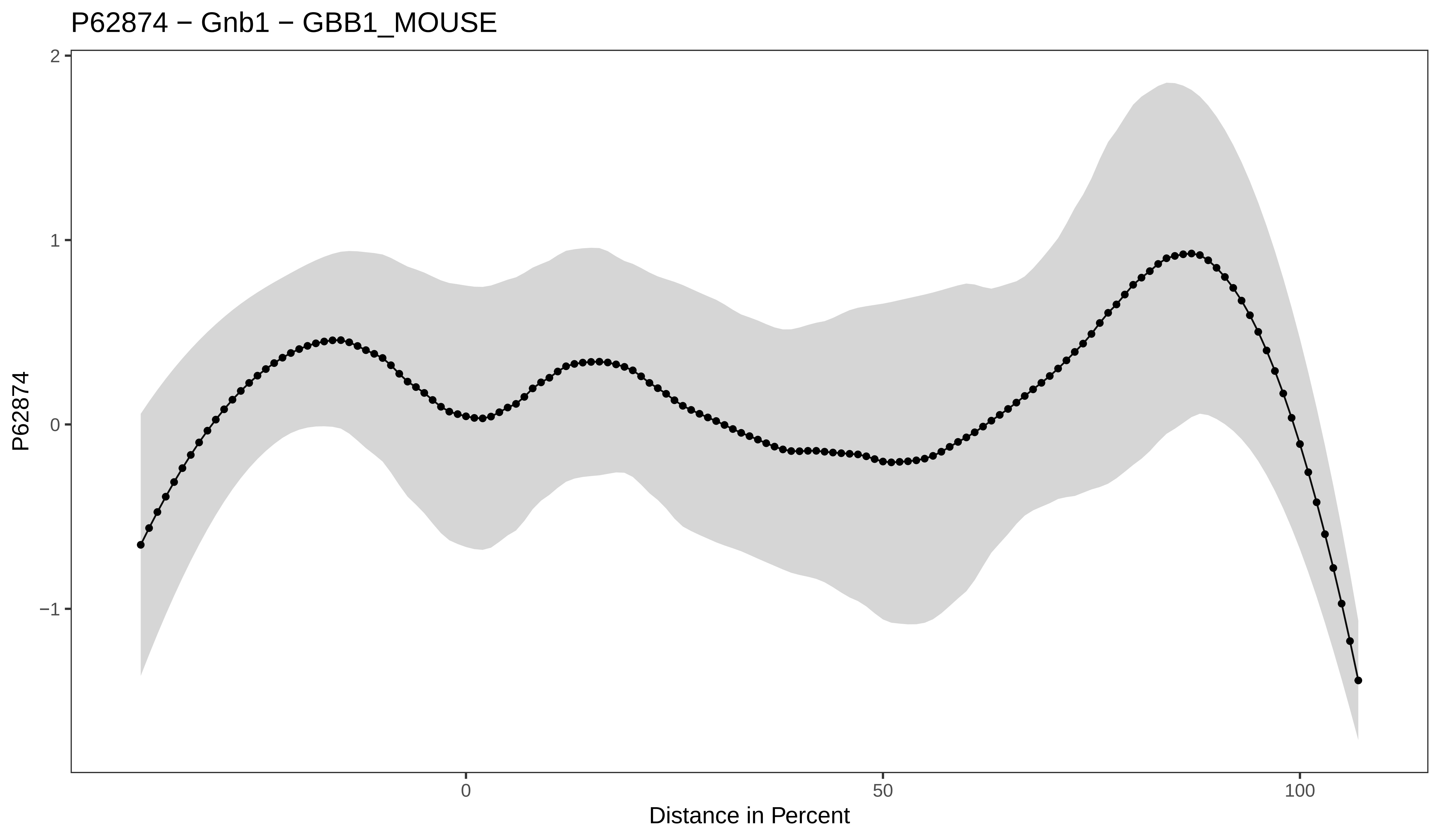
P62874 − Gnb1 − GBB1_MOUSE
2
1
0
−1
P62874
0
50
100
Distance in Percent

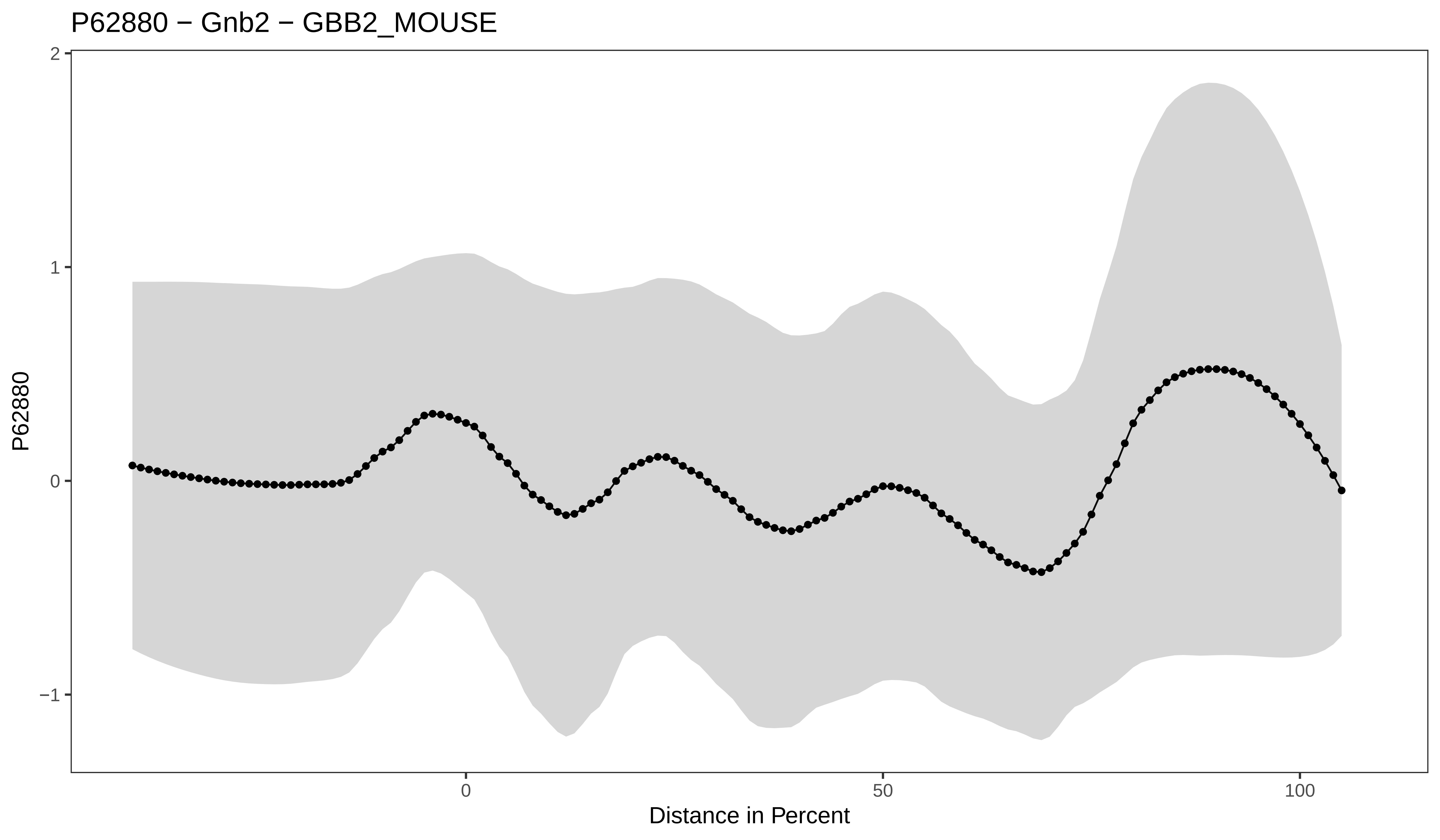

P62880 − Gnb2 − GBB2_MOUSE
2
1
0
−1
P62880
0
50
100
Distance in Percent

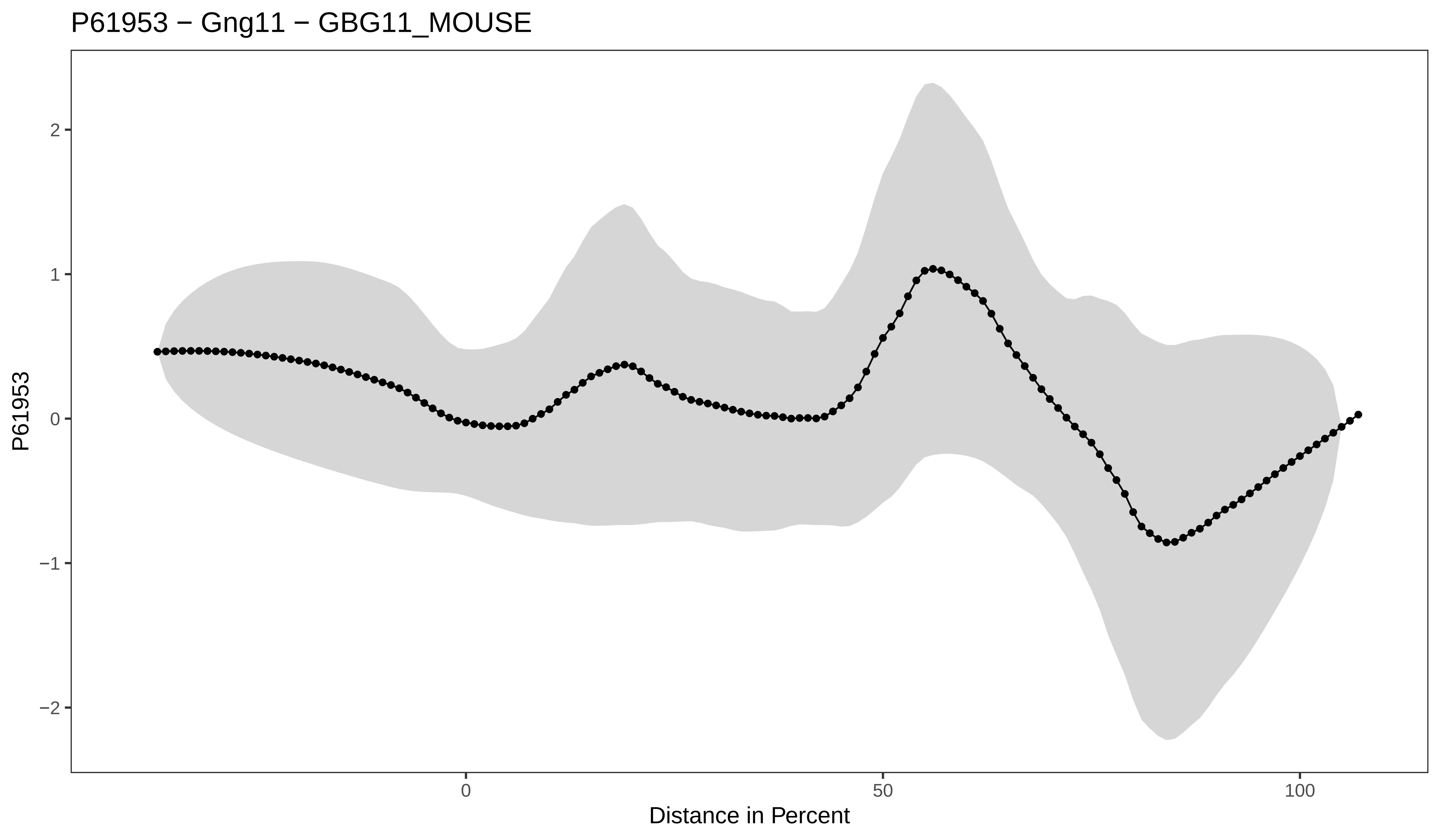

P61953 − Gng11 − GBG11_MOUSE
P61953
2
1
0
−1
−2
0
50
100
Distance in Percent

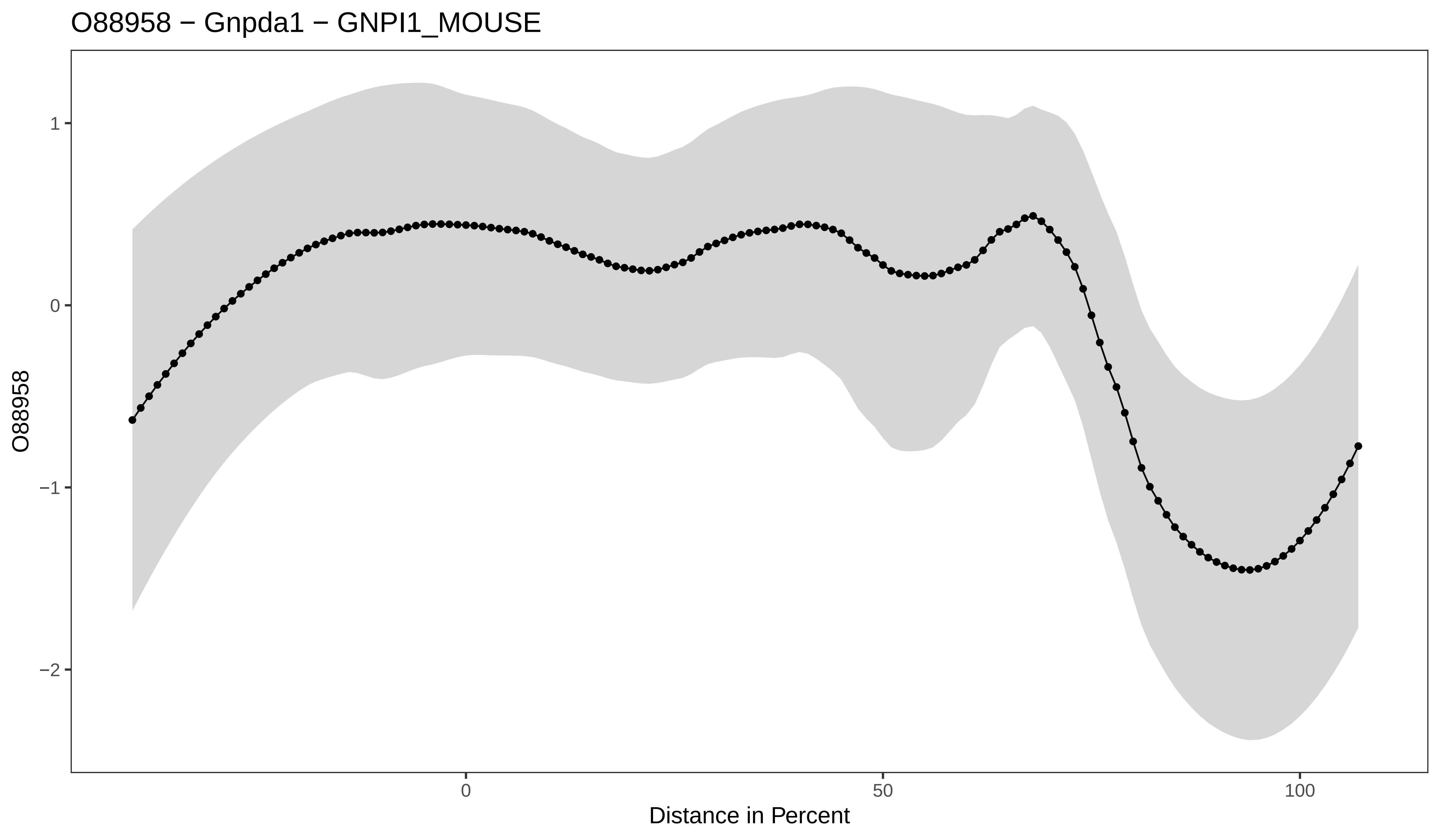
O88958 − Gnpda1 − GNPI1_MOUSE
1
0
−1
−2
O88958
0
50
100
Distance in Percent

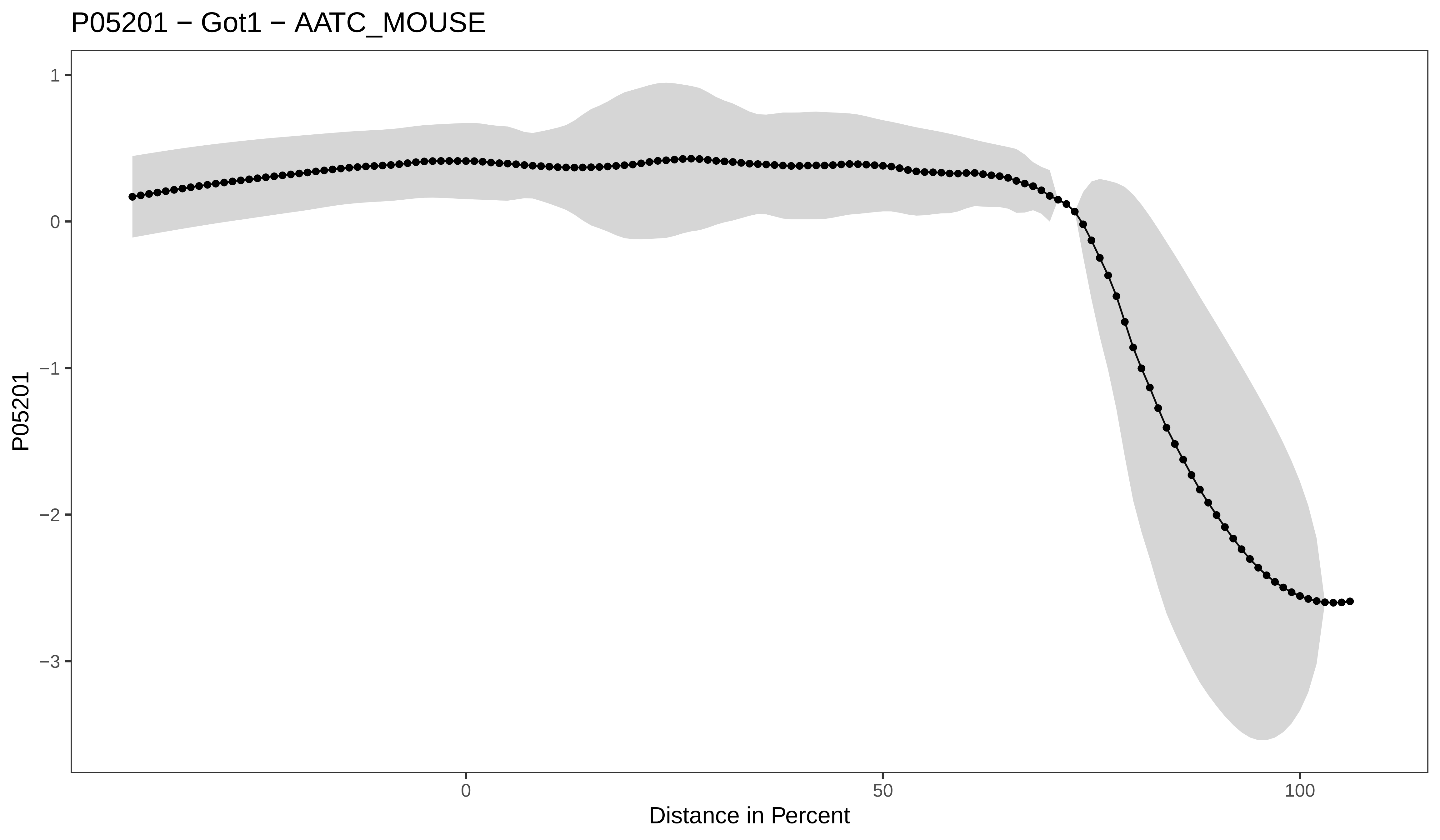

P05201 − Got1 − AATC_MOUSE
P05201
1
0
−1
−2
−3
0
50
100
Distance in Percent

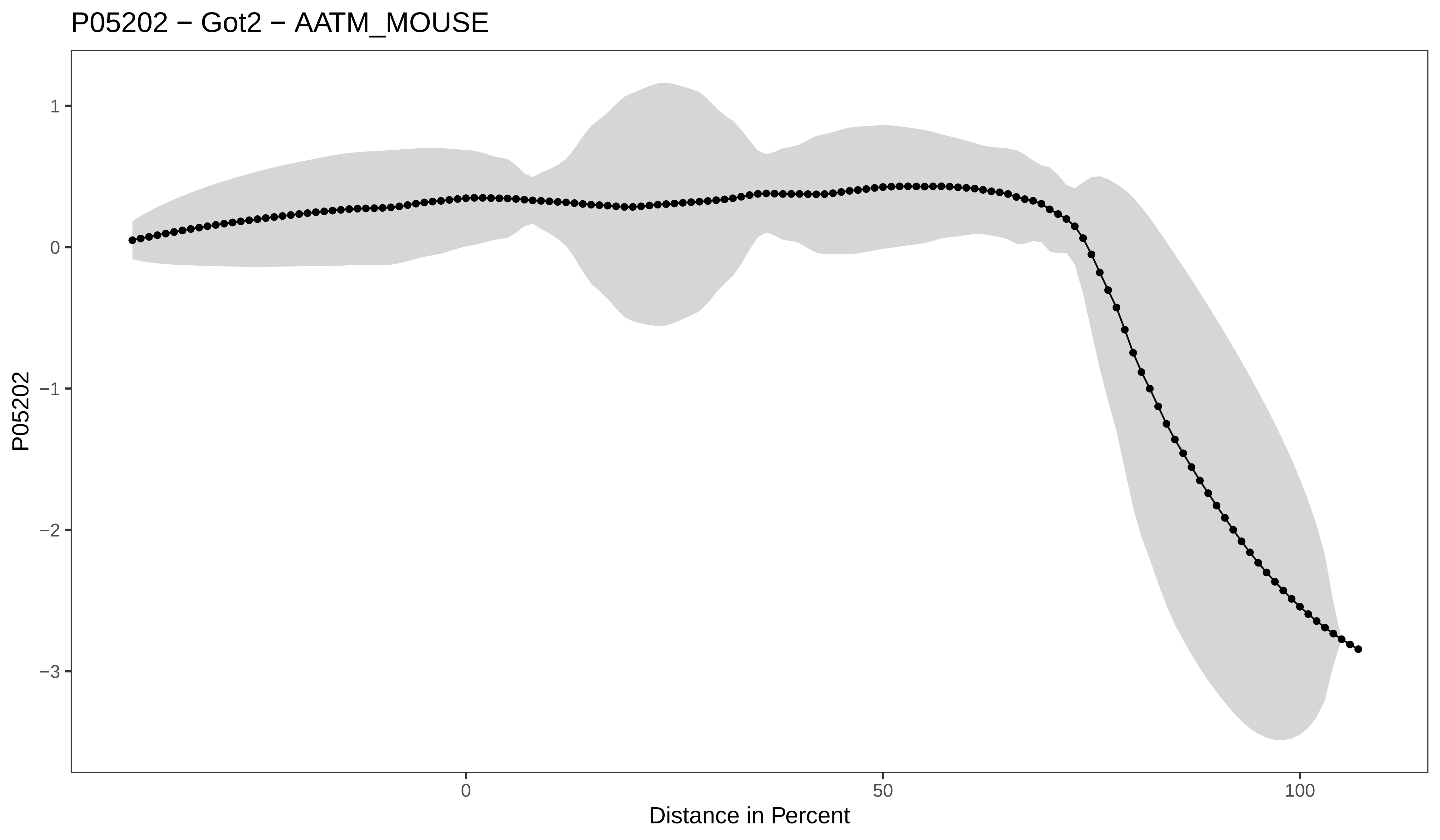

P05202 − Got2 − AATM_MOUSE
P05202
1
0
−1
−2
−3
0
50
100
Distance in Percent

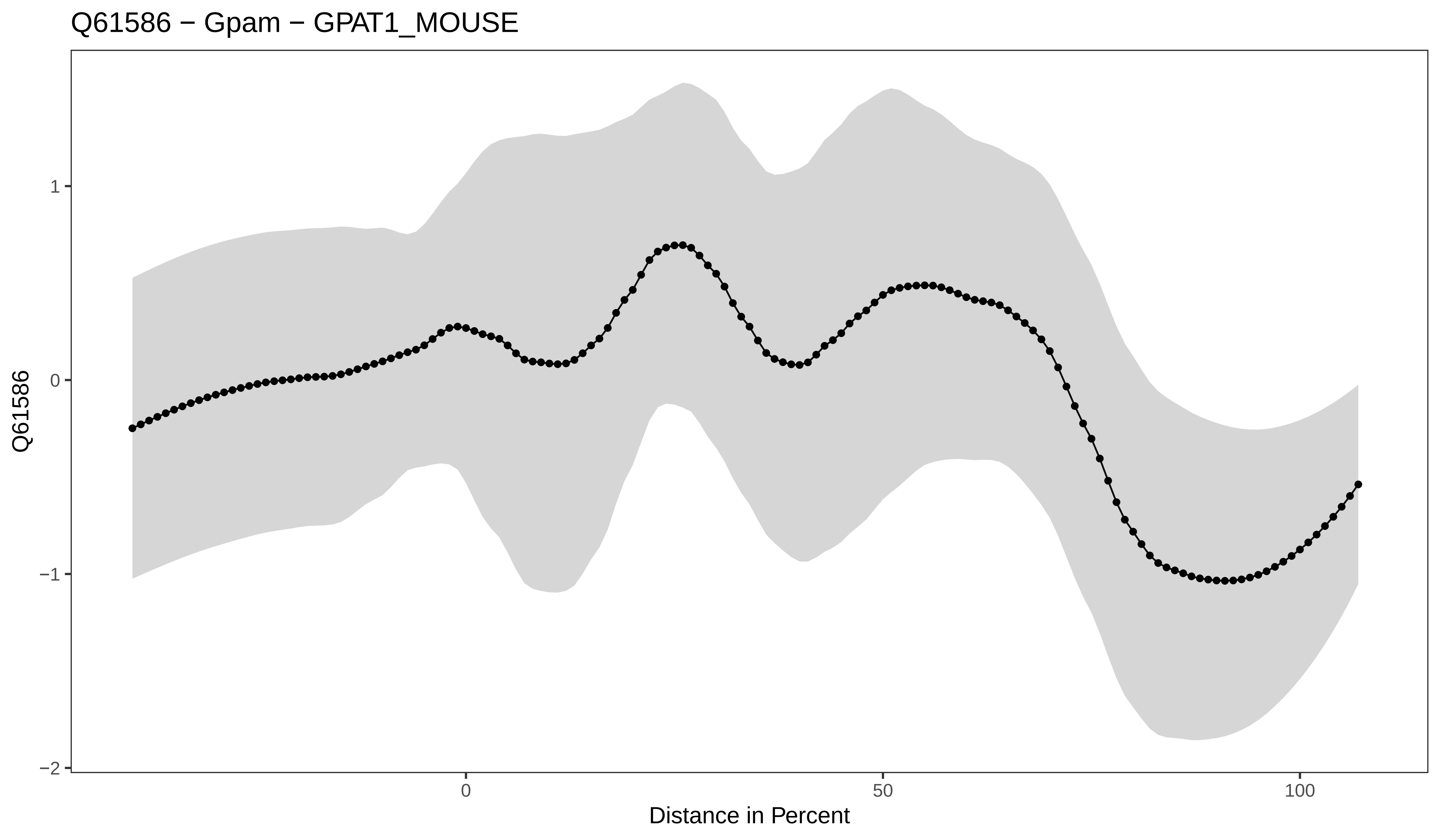

Q61586 − Gpam − GPAT1_MOUSE
Q61586
1
0
−1
−2
0
50
100
Distance in Percent

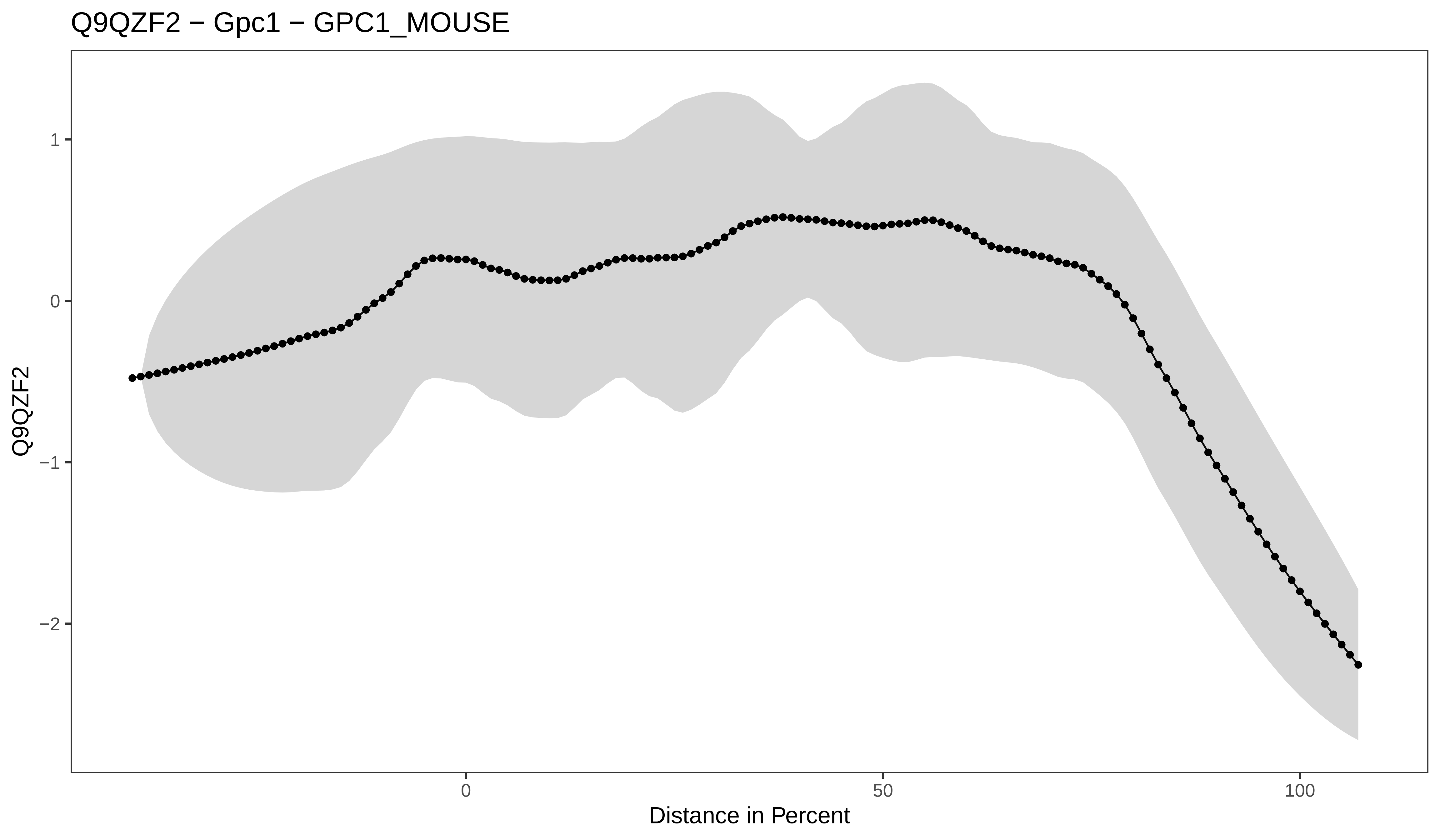

Q9QZF2 − Gpc1 − GPC1_MOUSE
1
0
−1
−2
Q9QZF2
0
50
100
Distance in Percent

Q8C0L9 − Gpcpd1 − GPCP1_MOUSE

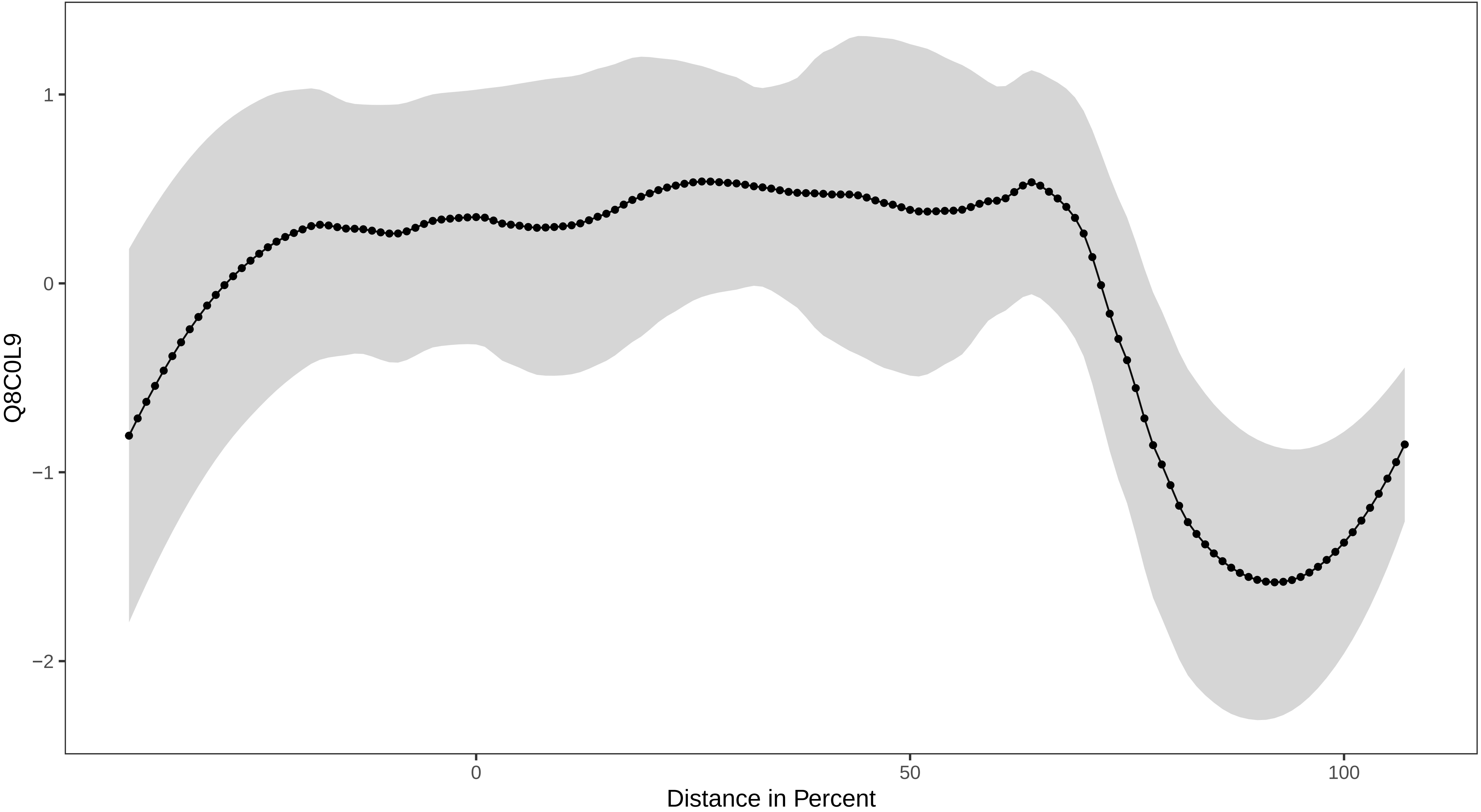

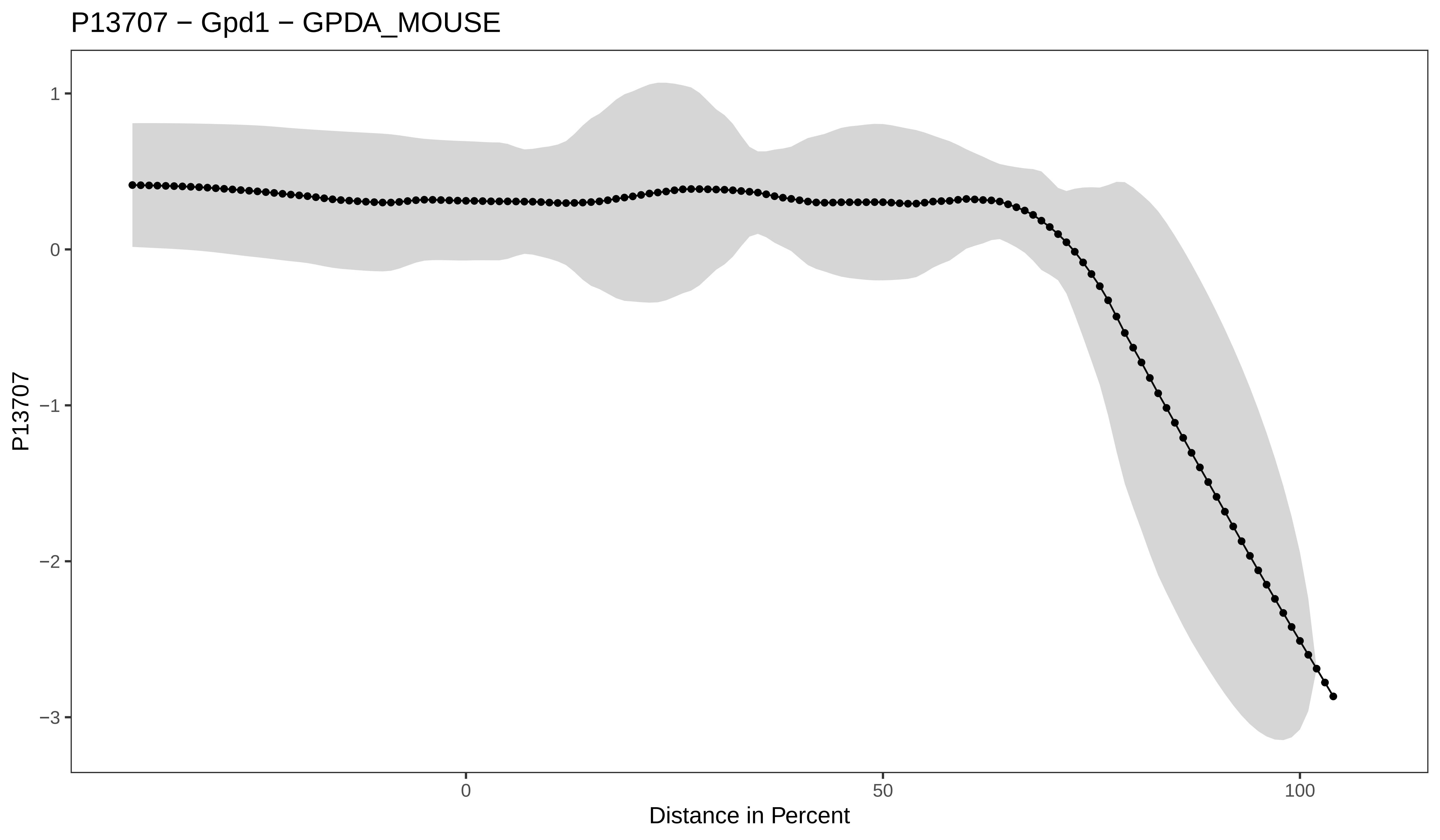

P13707 − Gpd1 − GPDA_MOUSE
1
0
−1
−2
−3
P13707
0
50
100
Distance in Percent

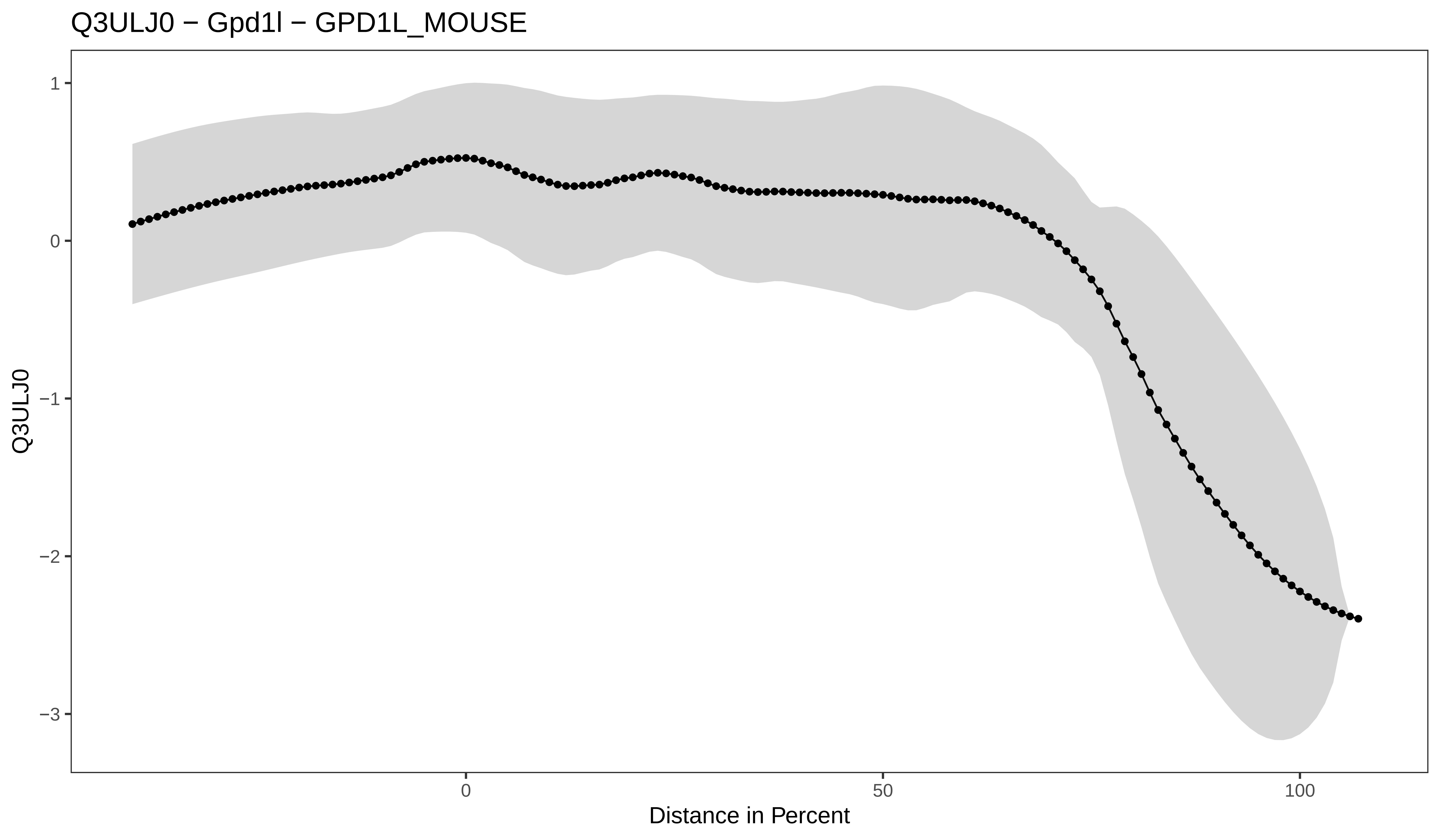
Q3ULJ0 − Gpd1l − GPD1L_MOUSE
Q3ULJ0
Distance in Percent
1
0
−1
−2
−3
0
50
100

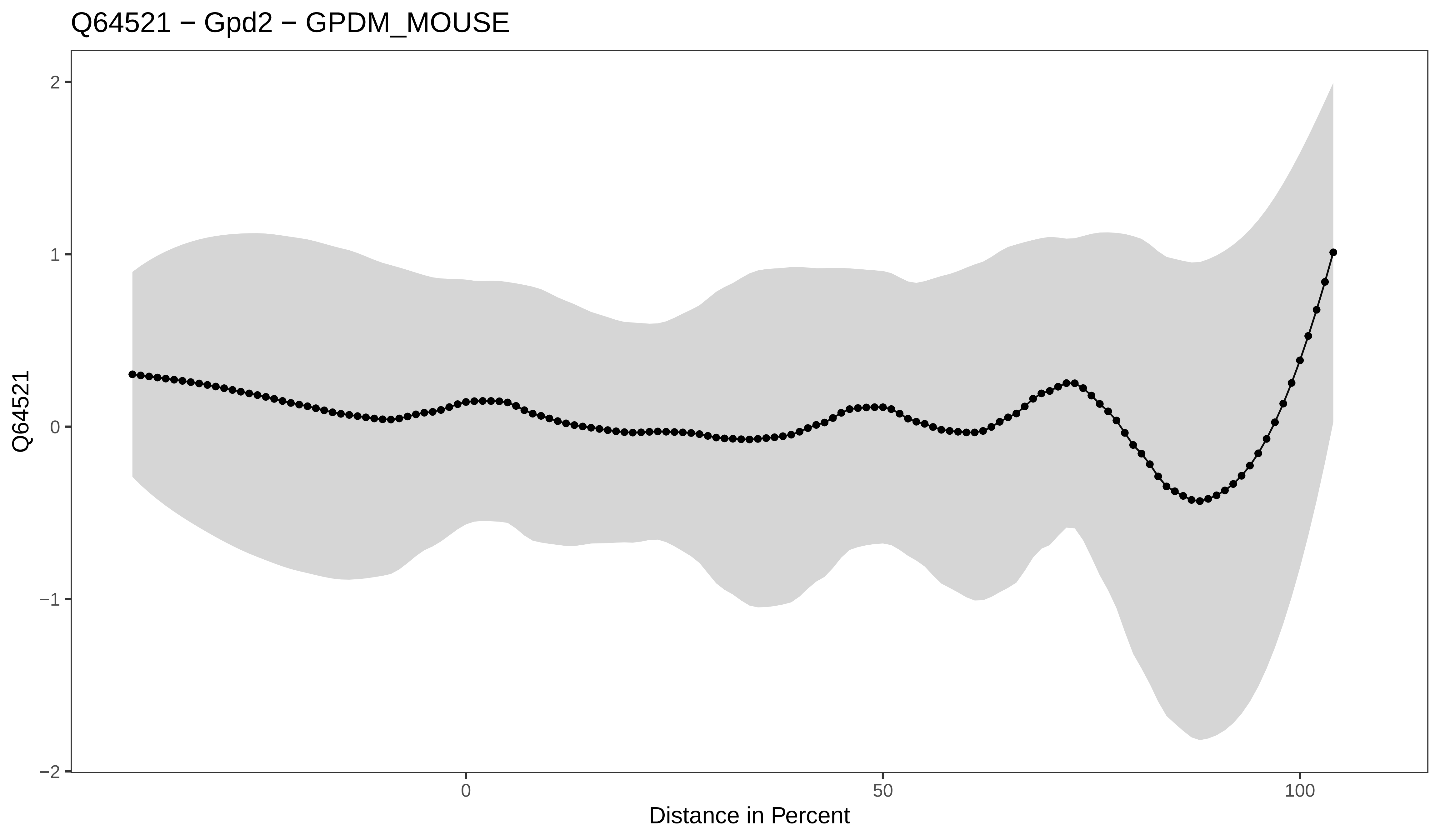
Q64521 − Gpd2 − GPDM_MOUSE
Q64521
2
1
0
−1
−2
0
50
100
Distance in Percent

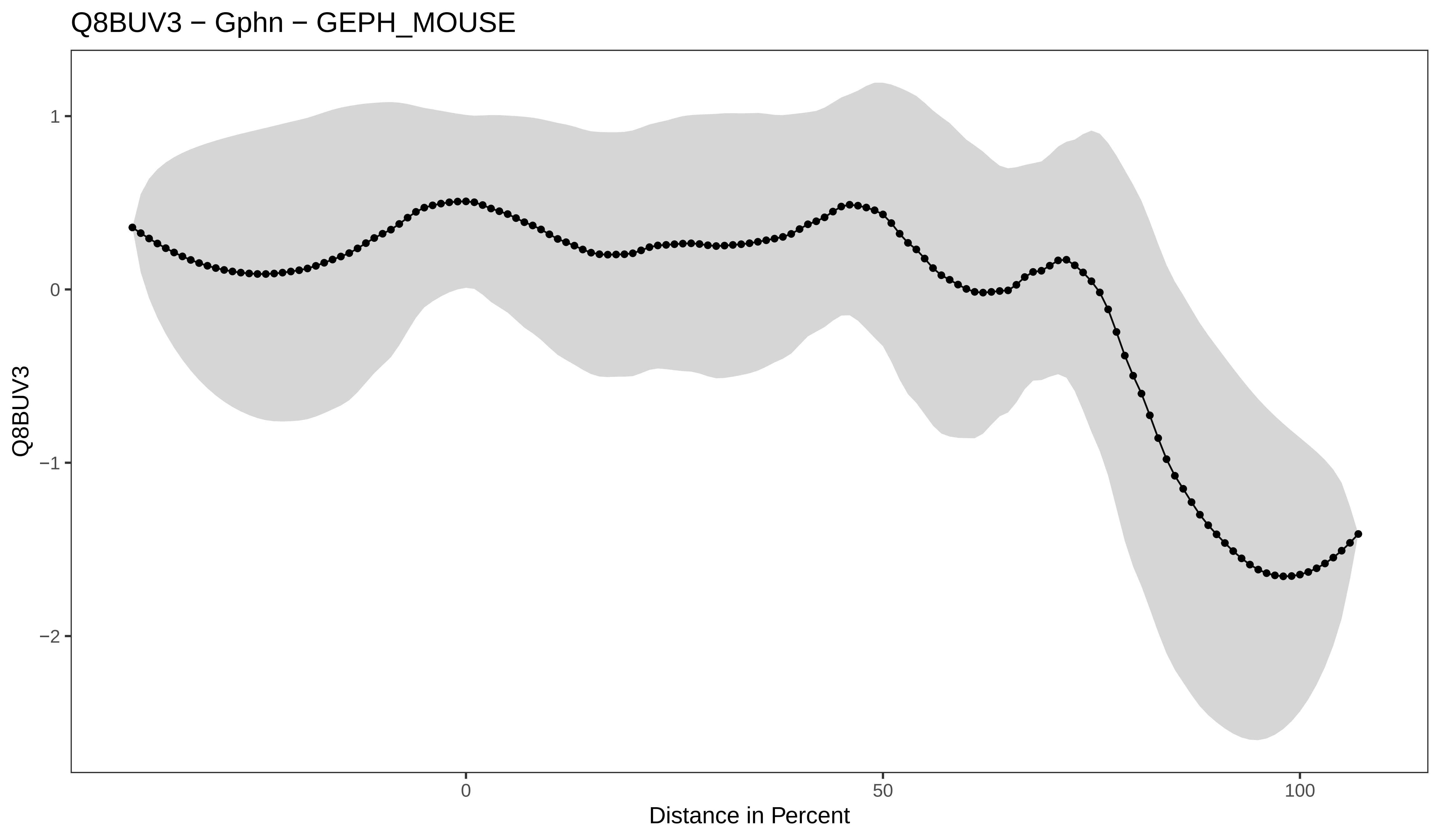

Q8BUV3 − Gphn − GEPH_MOUSE
Q8BUV3
1
0
−1
−2
0
50
100
Distance in Percent

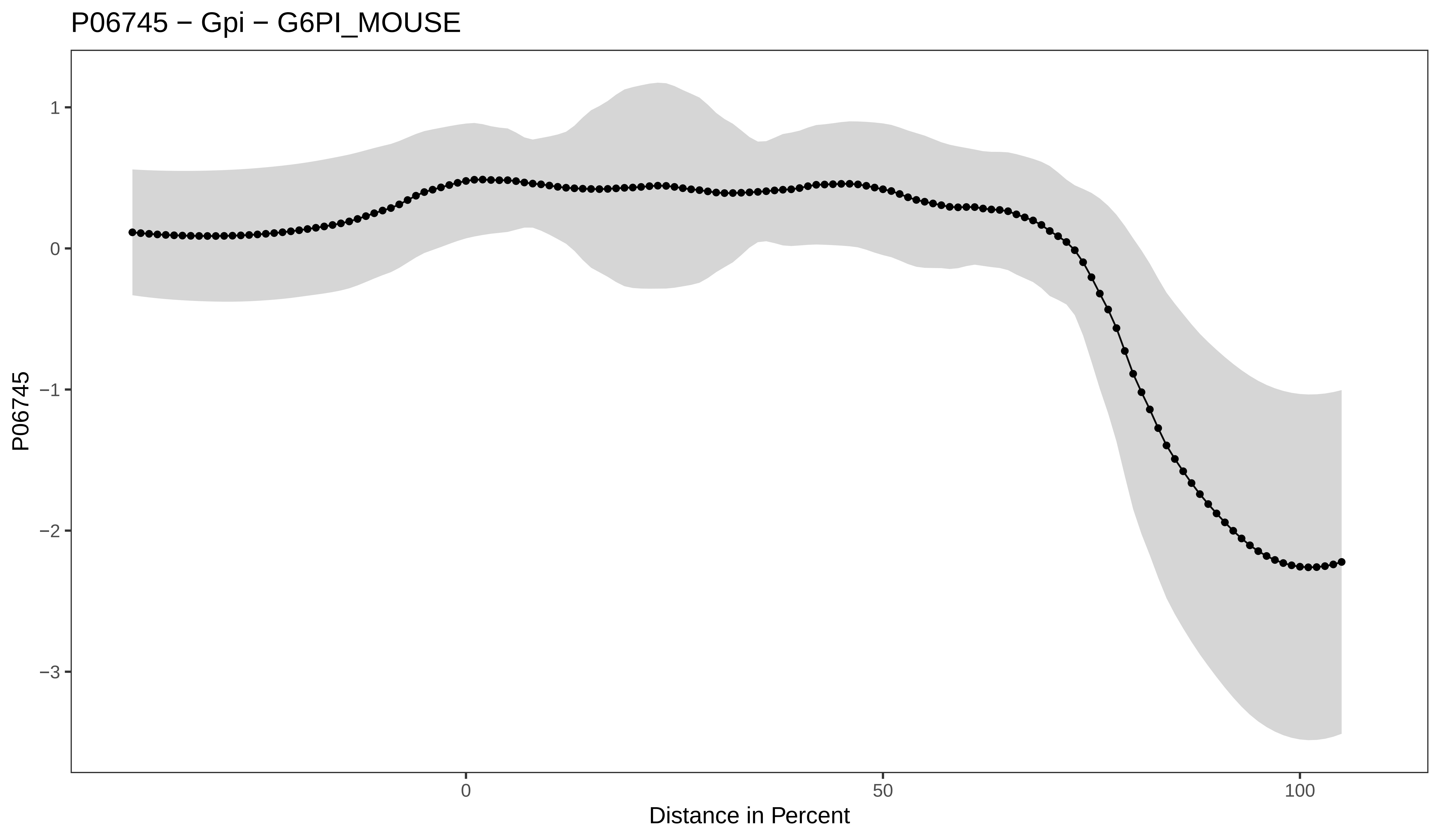

P06745 − Gpi − G6PI_MOUSE
P06745
1
0
−1
−2
−3
0
50
100
Distance in Percent

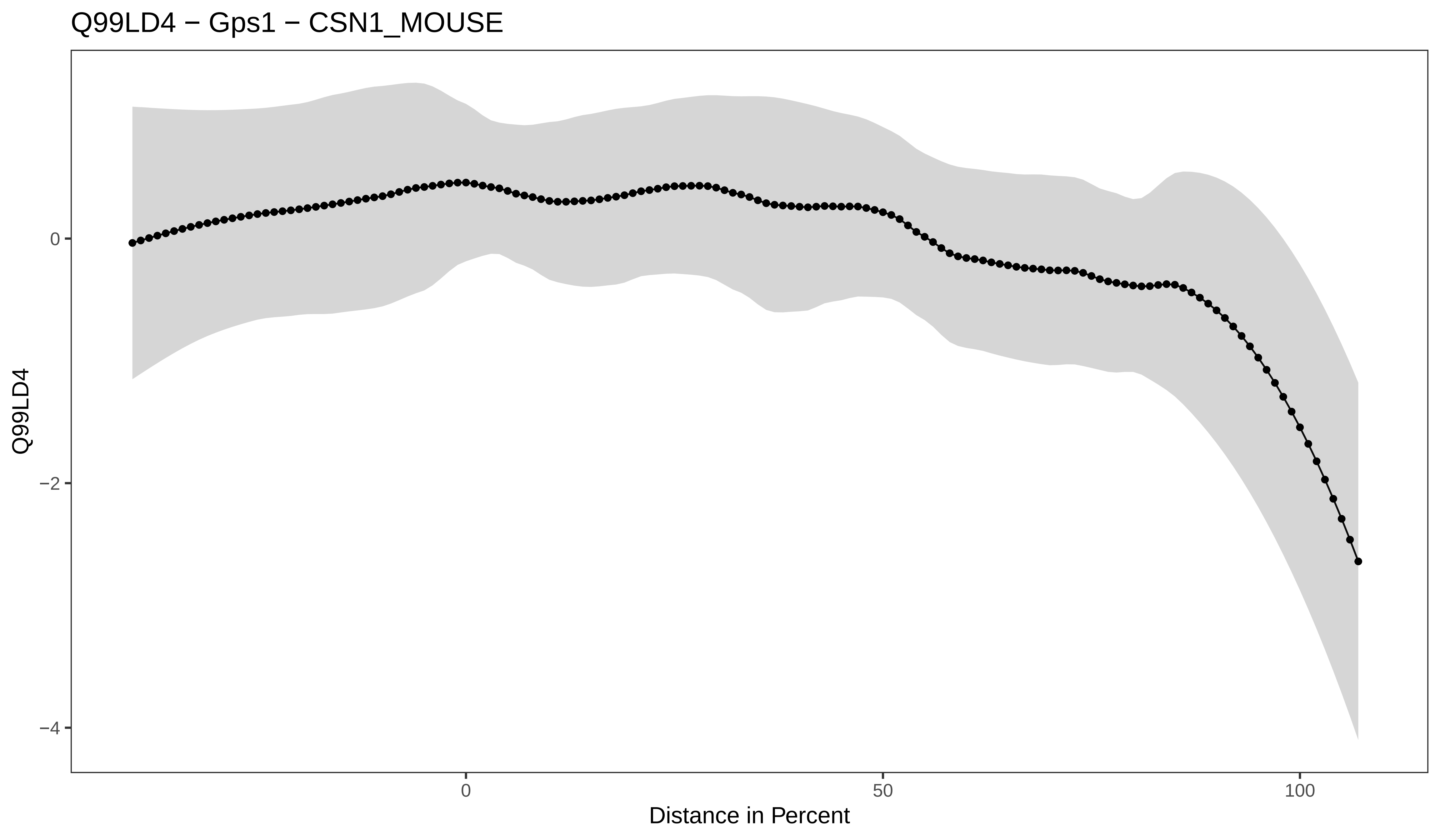

Q99LD4 − Gps1 − CSN1_MOUSE
Q99LD4
0
−2
−4
0
50
100
Distance in Percent

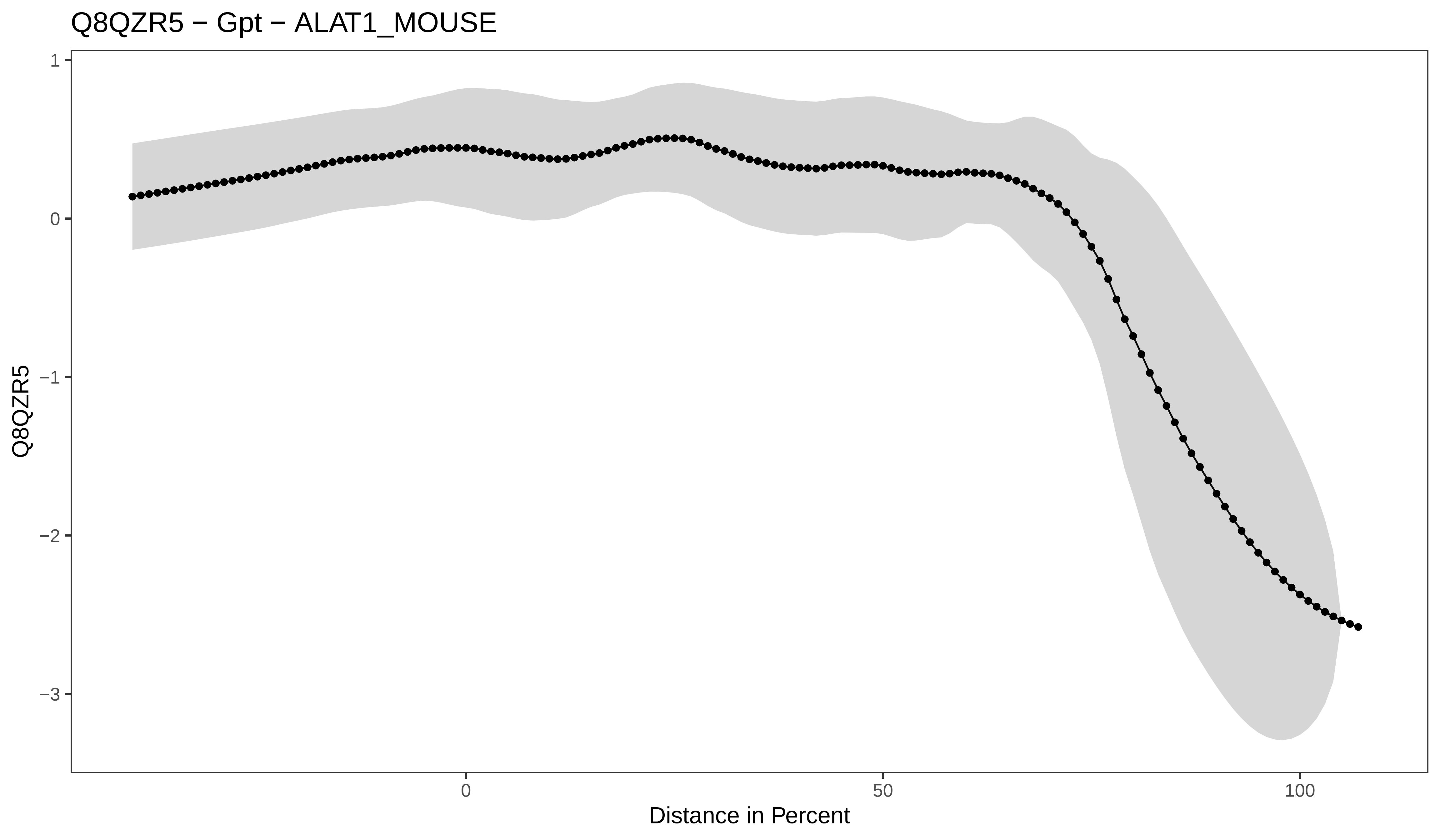

Q8QZR5 − Gpt − ALAT1_MOUSE
1
0
−1
−2
−3
Q8QZR5
0
50
100
Distance in Percent

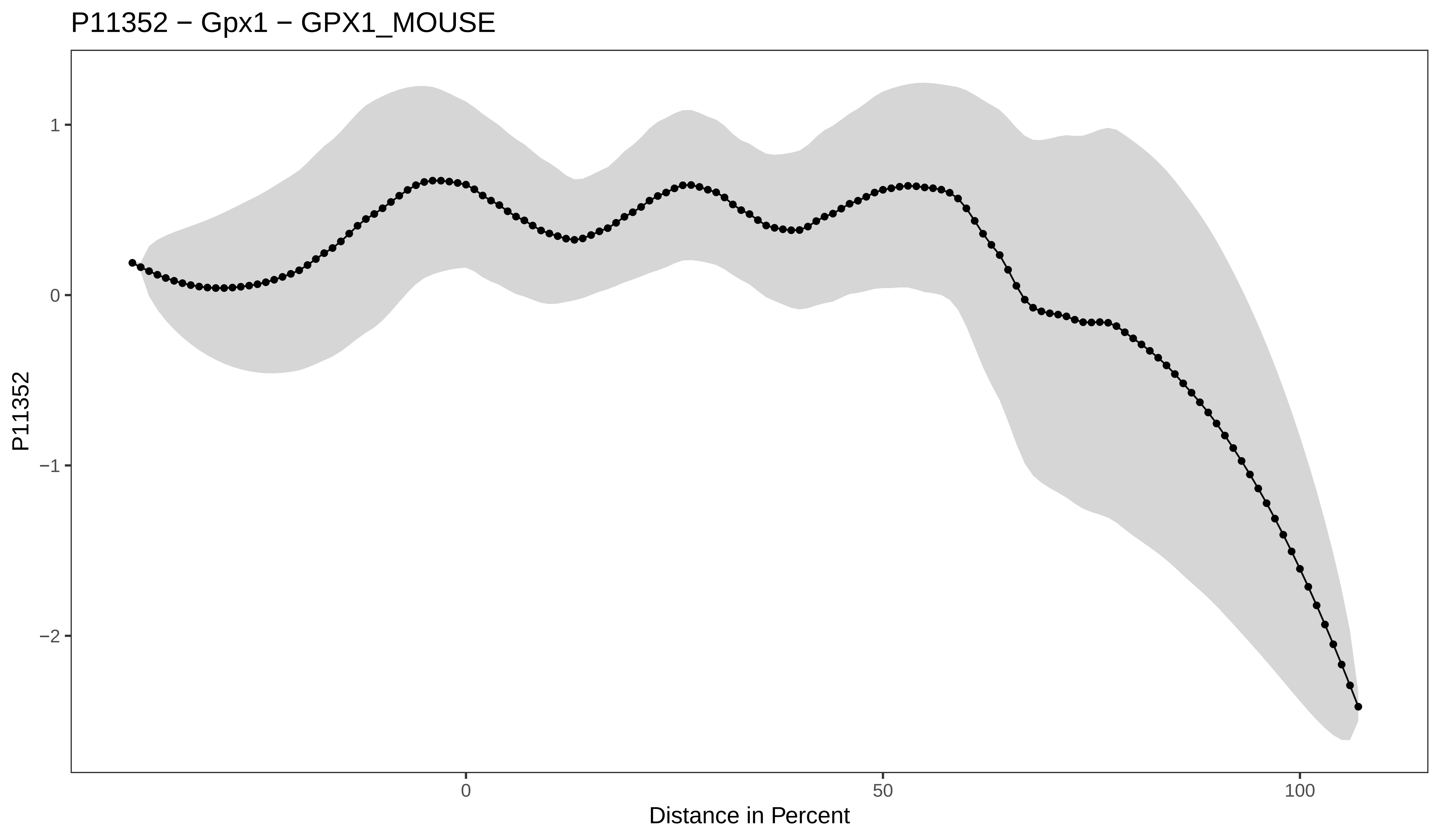
P11352 − Gpx1 − GPX1_MOUSE
1
0
−1
−2
P11352
0
50
100
Distance in Percent

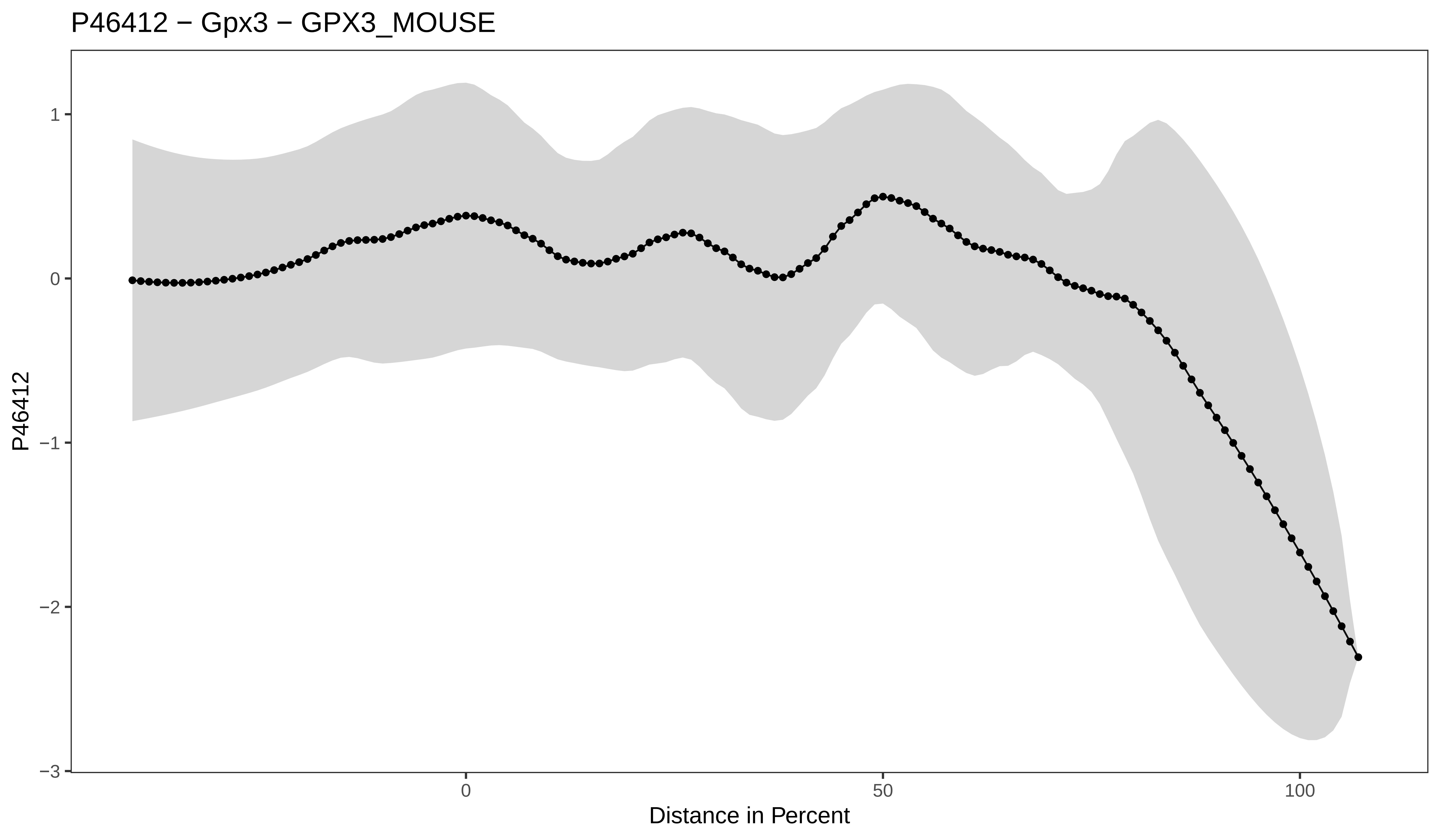

P46412 − Gpx3 − GPX3_MOUSE
1
0
−1
−2
−3
P46412
0
50
100
Distance in Percent

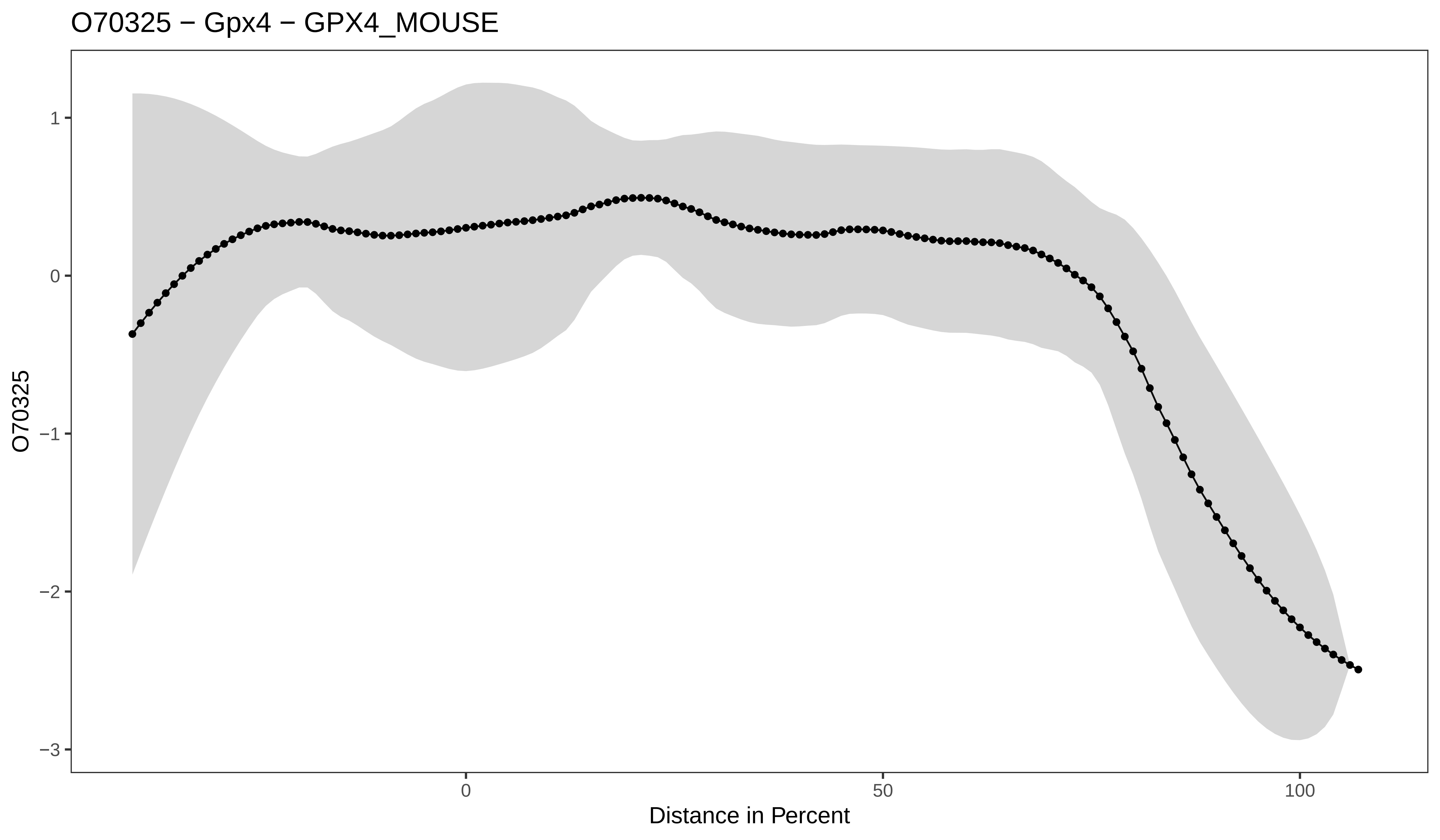

O70325 − Gpx4 − GPX4_MOUSE
1
0
−1
−2
−3
O70325
0
50
100
Distance in Percent

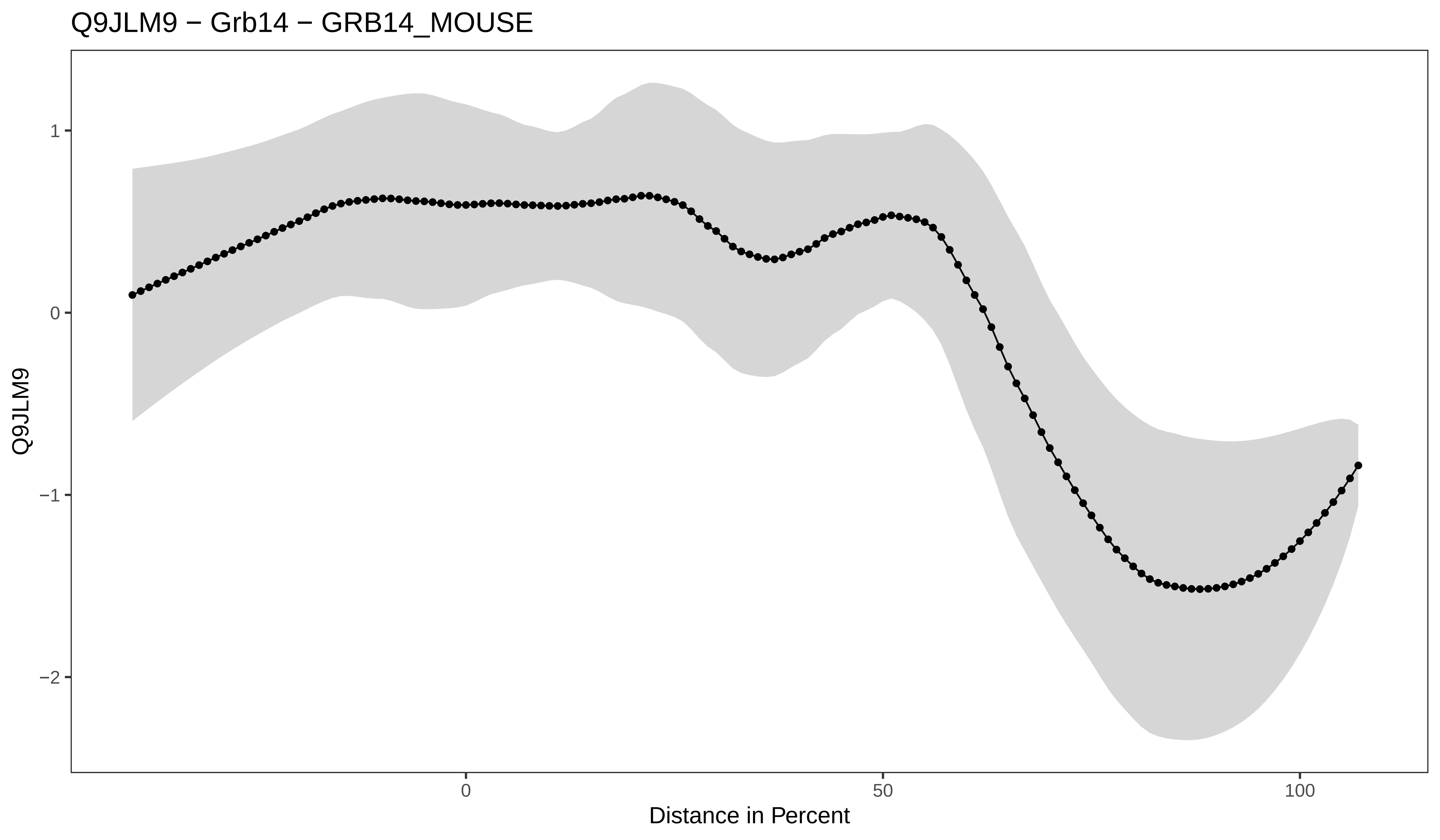
Q9JLM9 − Grb14 − GRB14_MOUSE
Q9JLM9
1
0
−1
−2
0
50
100
Distance in Percent

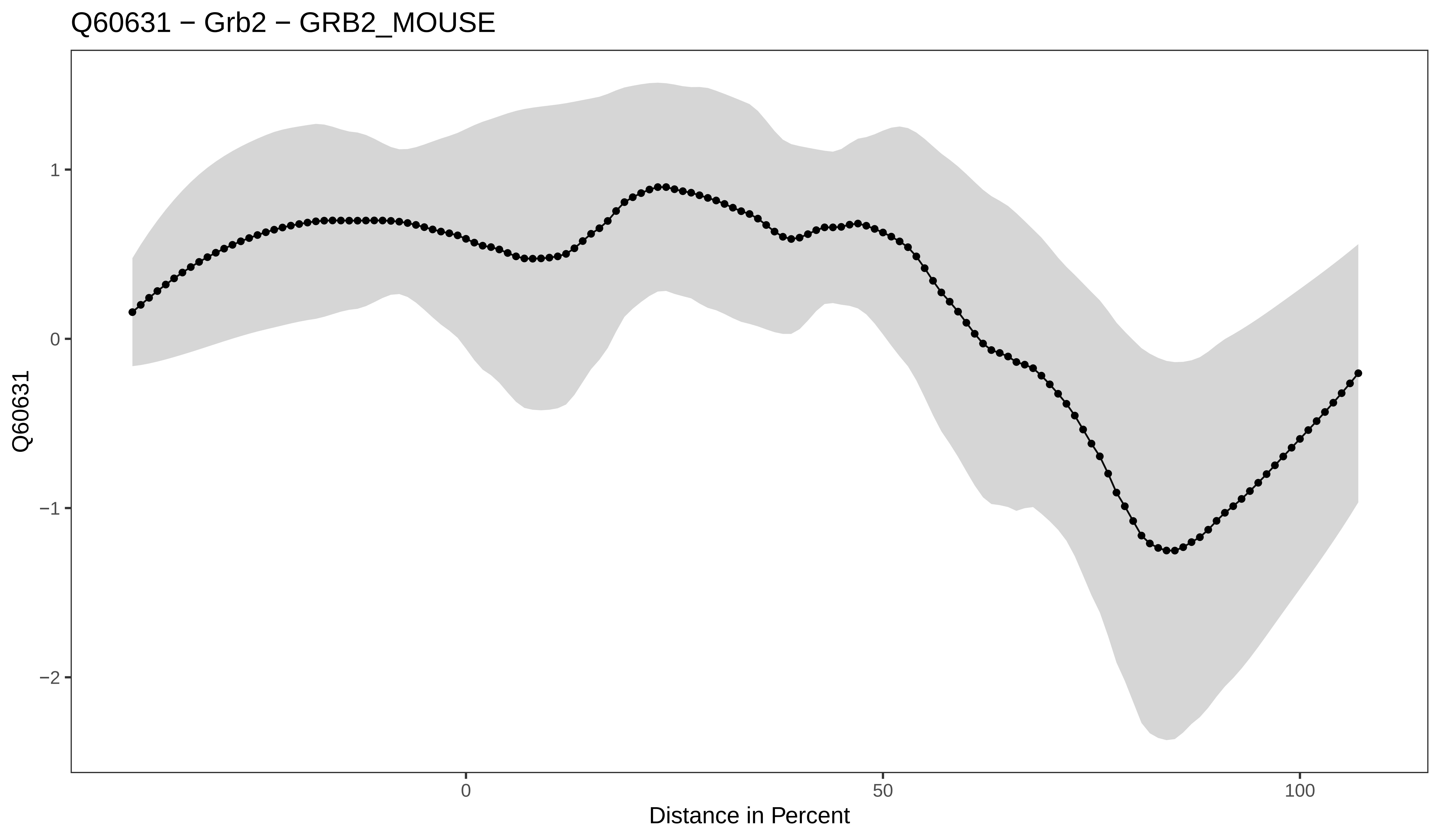

Q60631 − Grb2 − GRB2_MOUSE
1
0
−1
−2
Q60631
0
50
100
Distance in Percent

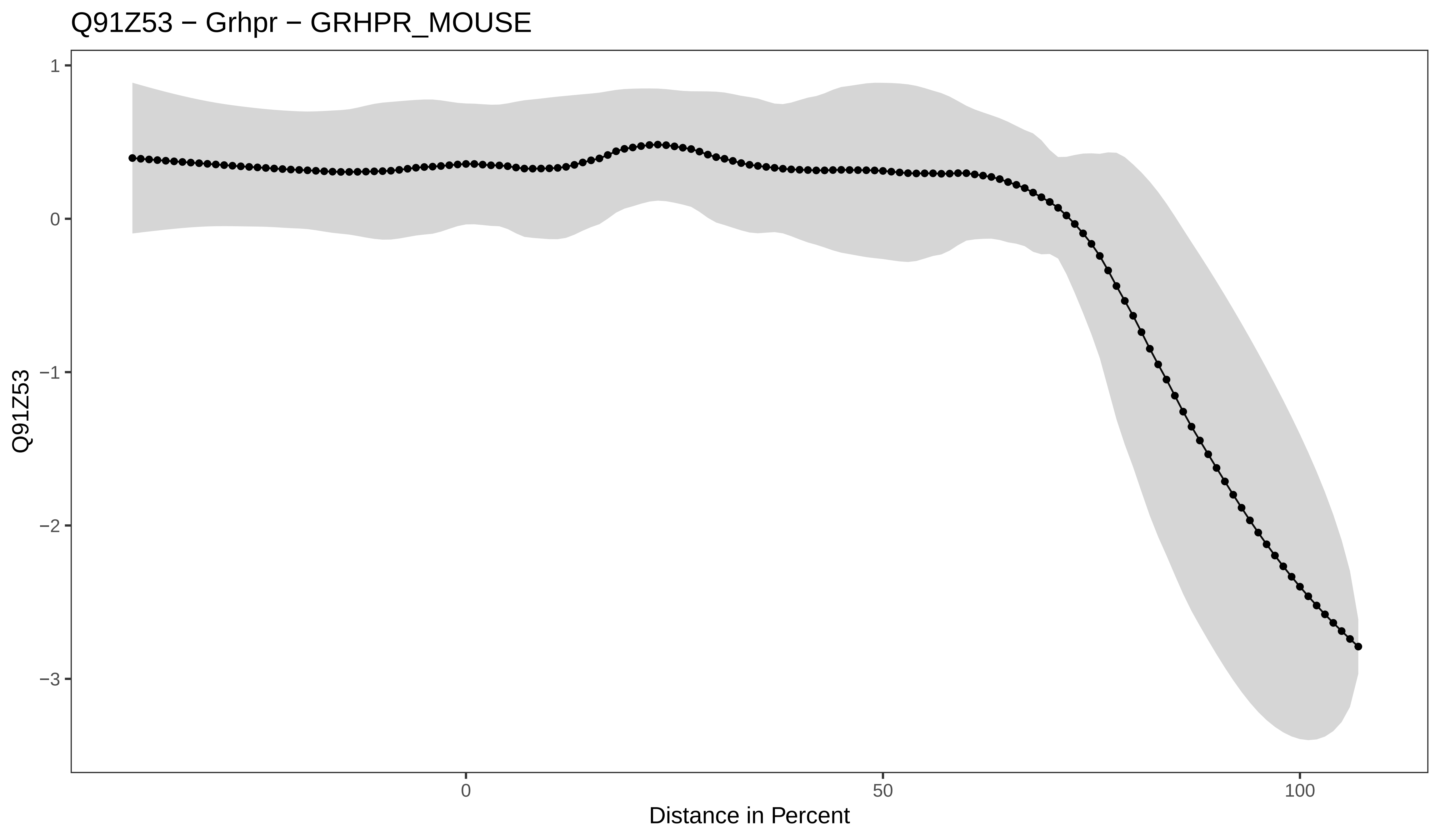
Q91Z53 − Grhpr − GRHPR_MOUSE
Q91Z53
Distance in Percent
1
0
−1
−2
−3
0
50
100

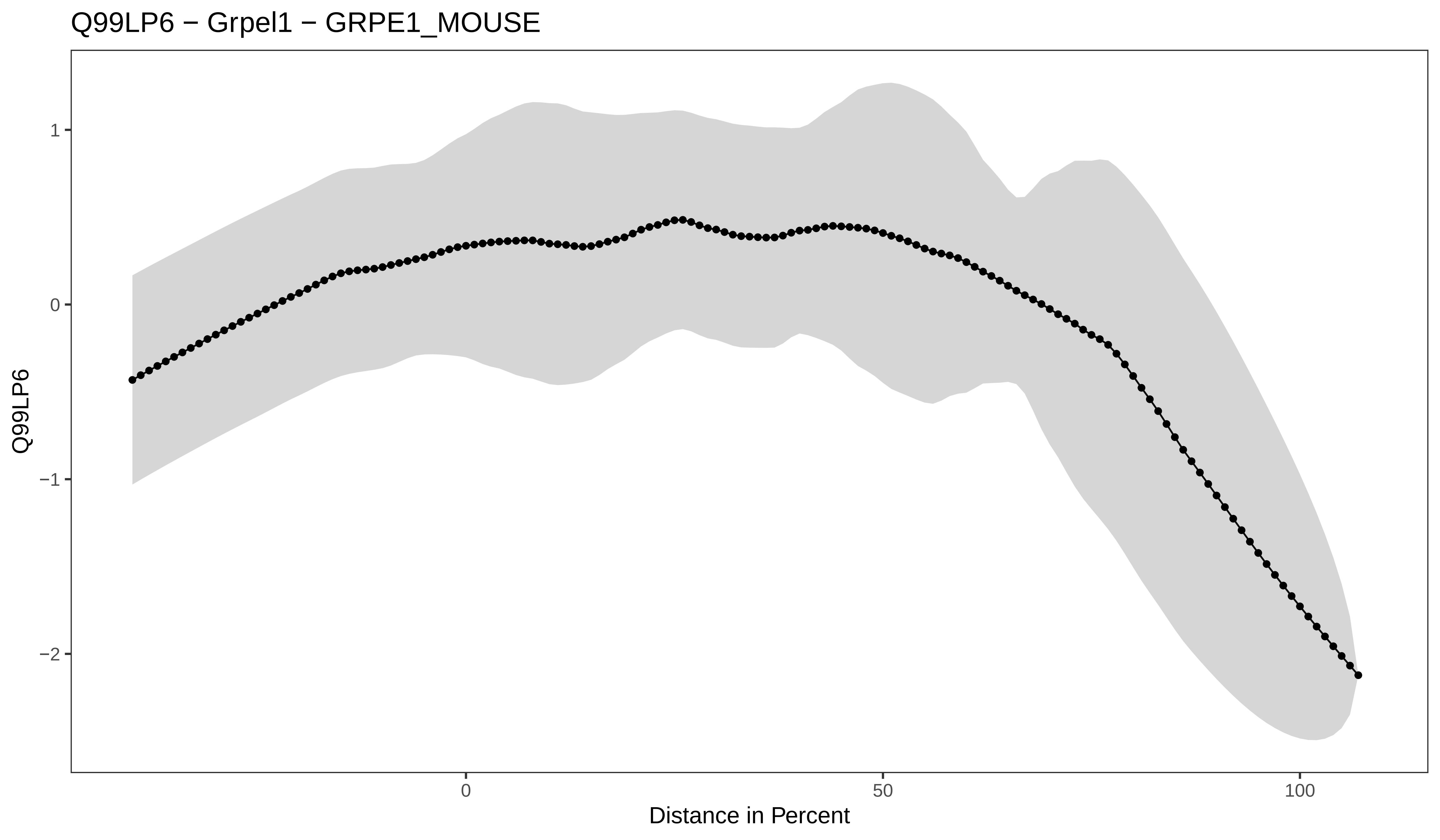

Q99LP6 − Grpel1 − GRPE1_MOUSE
1
0
−1
−2
Q99LP6
0
50
100
Distance in Percent

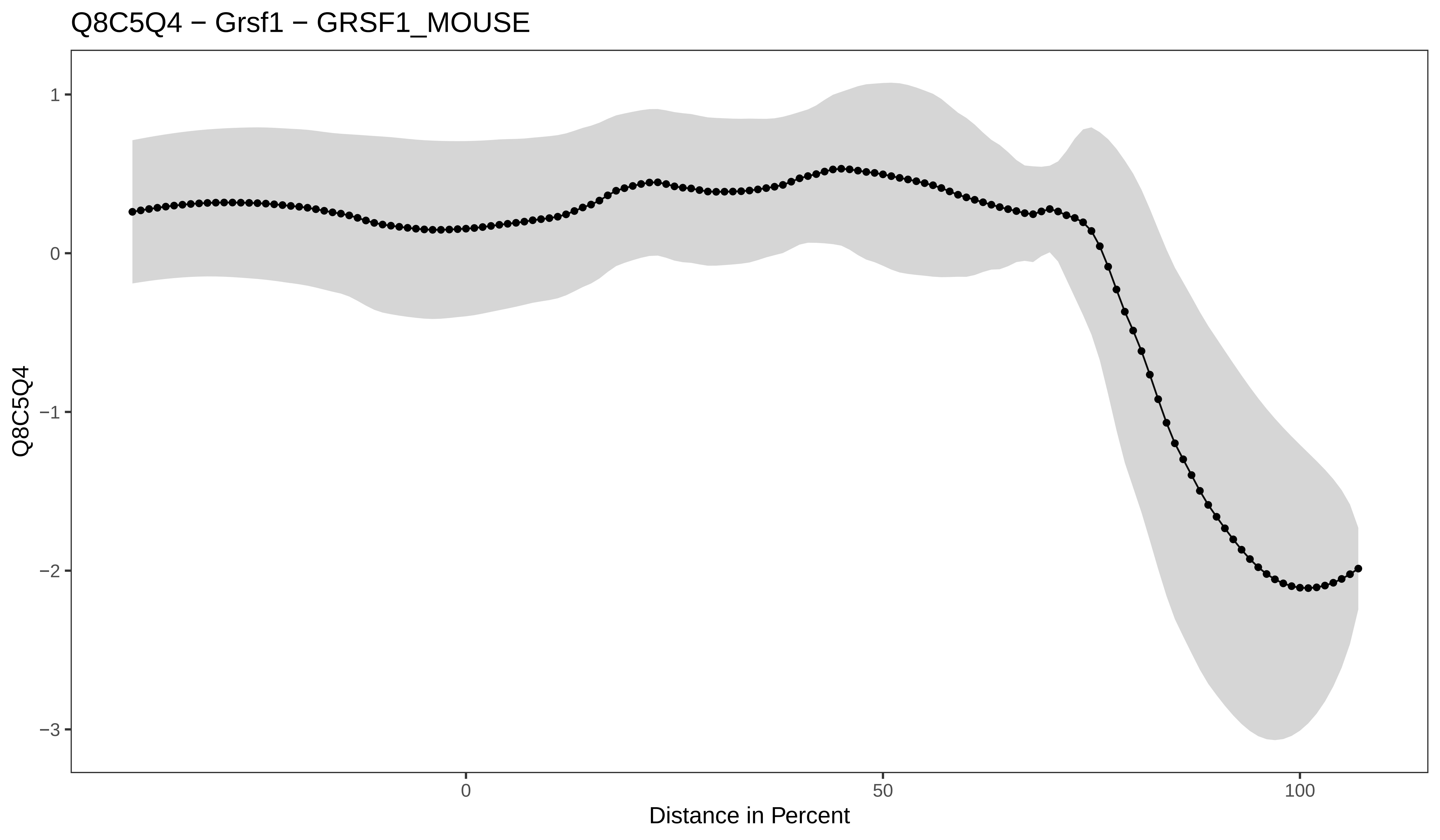

Q8C5Q4 − Grsf1 − GRSF1_MOUSE
Q8C5Q4
1
0
−1
−2
−3
0
50
100
Distance in Percent

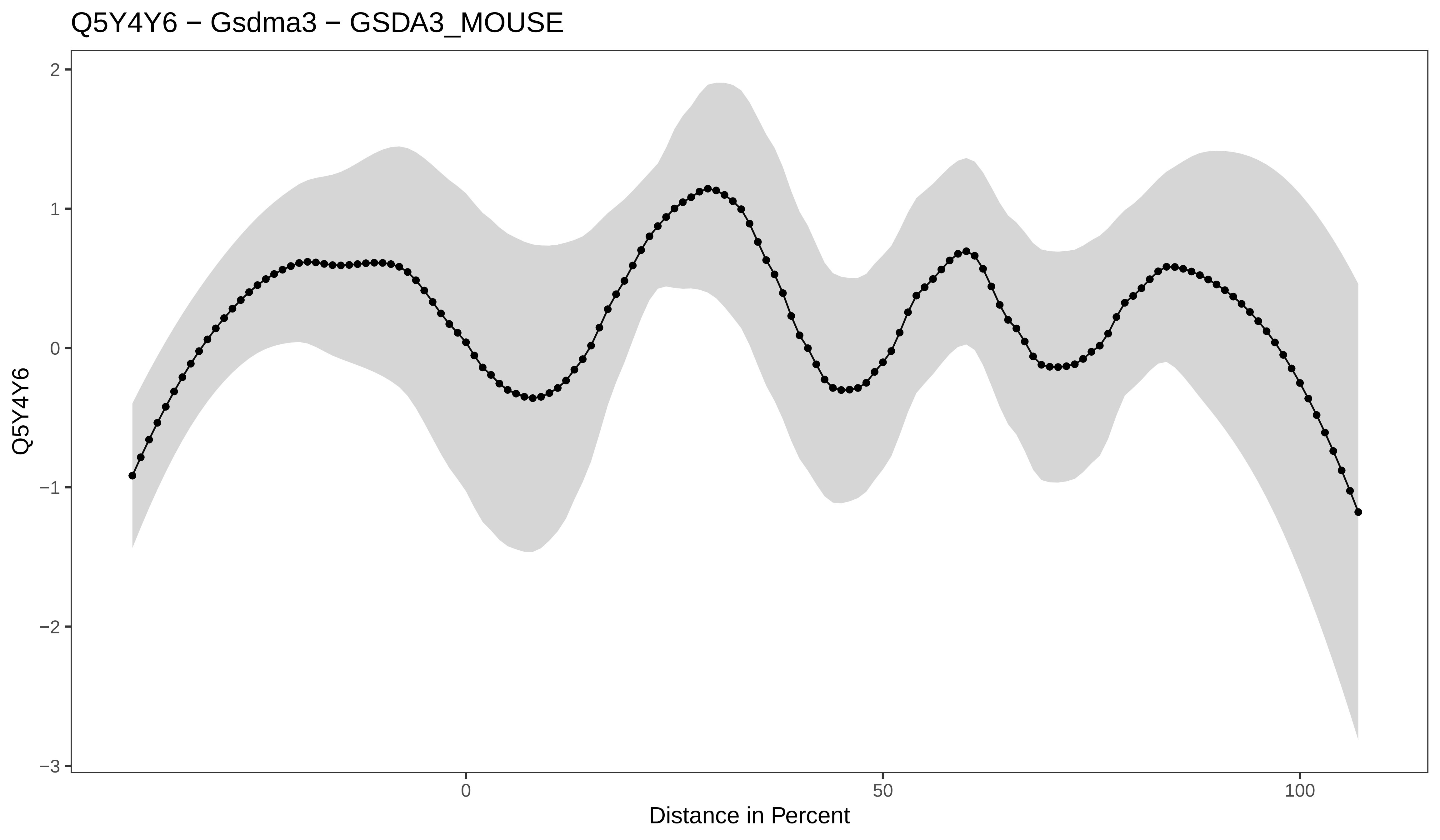

Q5Y4Y6 − Gsdma3 − GSDA3_MOUSE
Q5Y4Y6
2
1
0
−1
−2
−3
0
50
100
Distance in Percent

Q2NL51 − Gsk3a − GSK3A_MOUSE

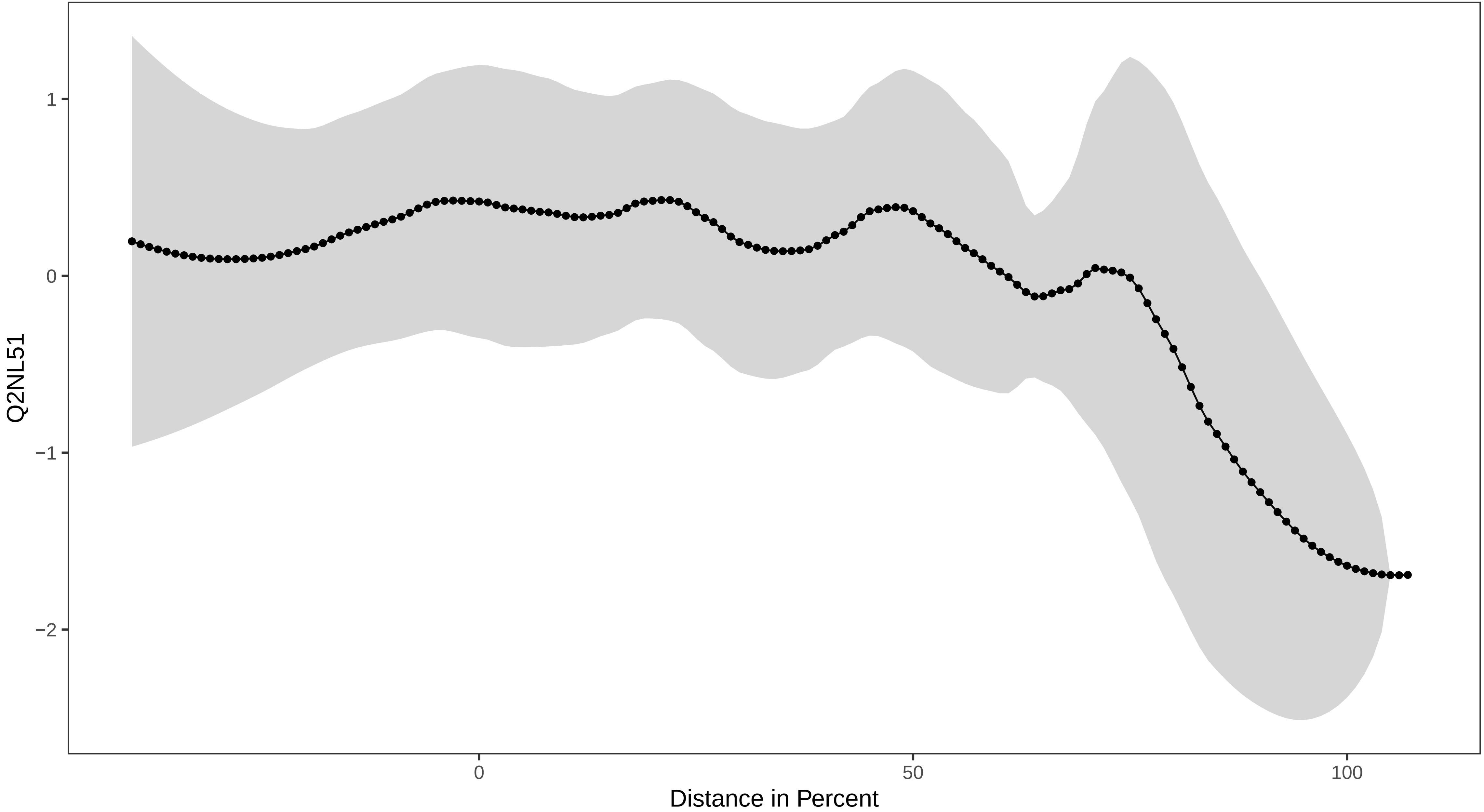

Q9WV60 − Gsk3b − GSK3B_MOUSE

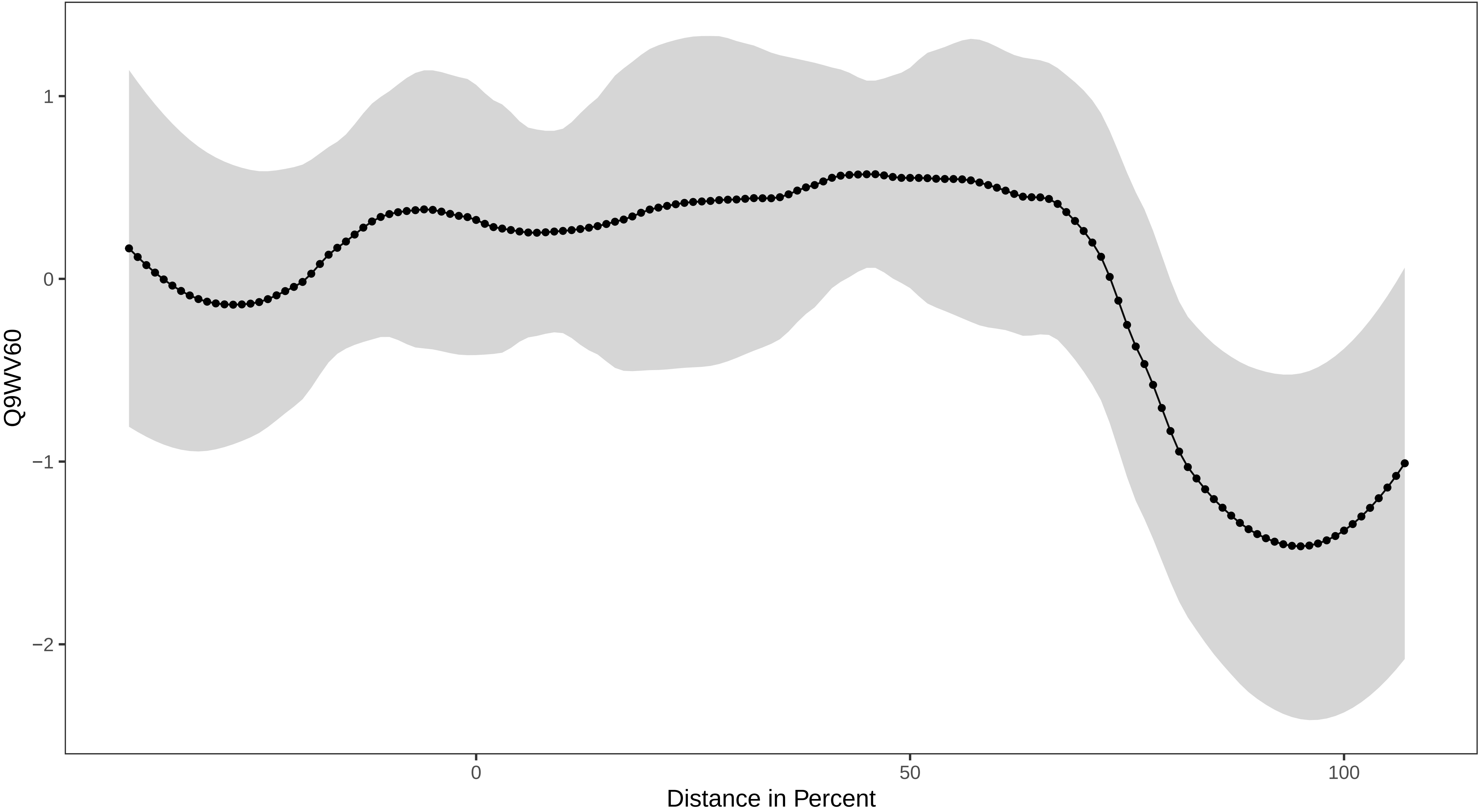

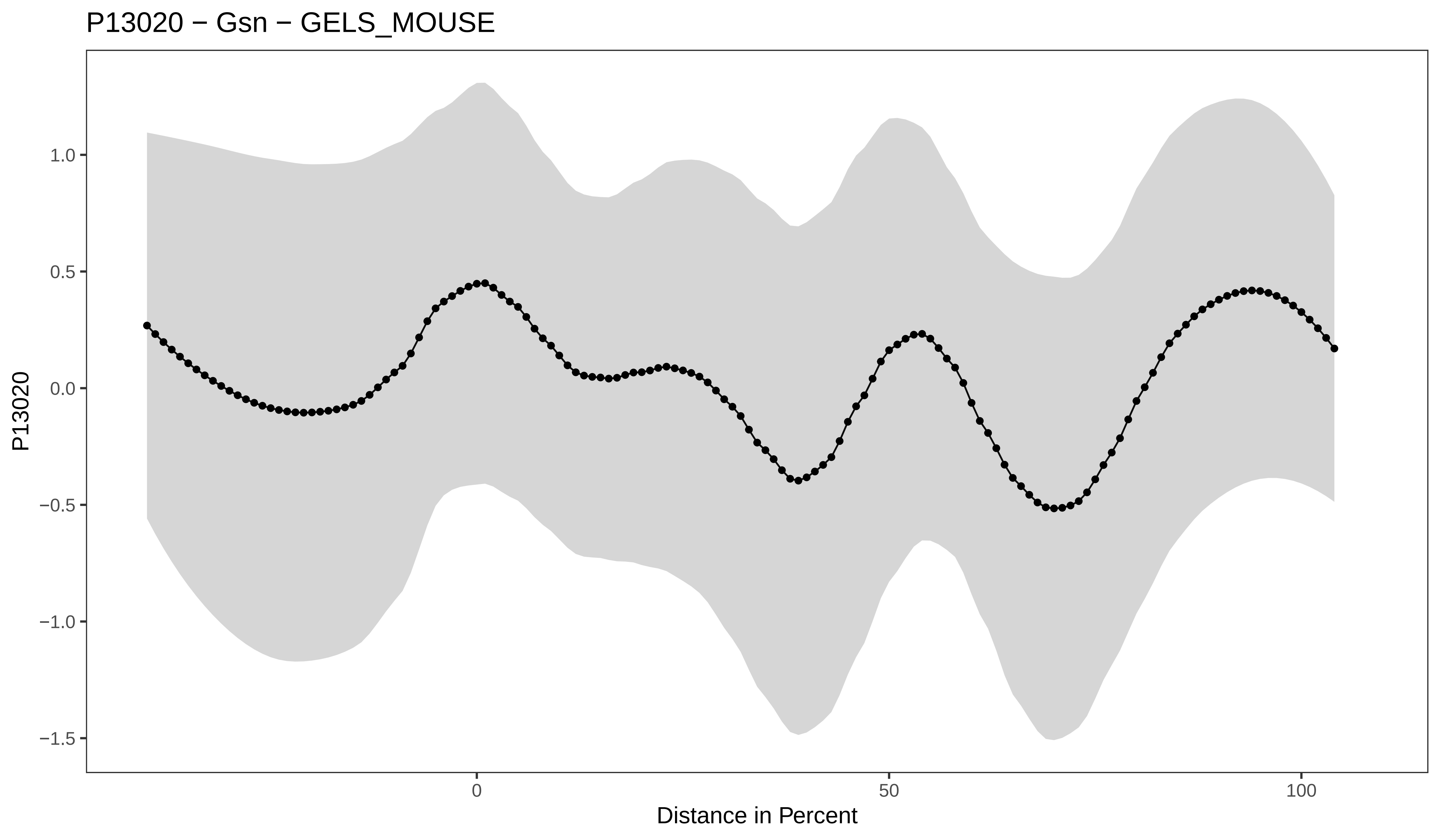
P13020 − Gsn − GELS_MOUSE
P13020
1.0
0.5
0.0
−0.5
−1.0
−1.5
0
50
100
Distance in Percent

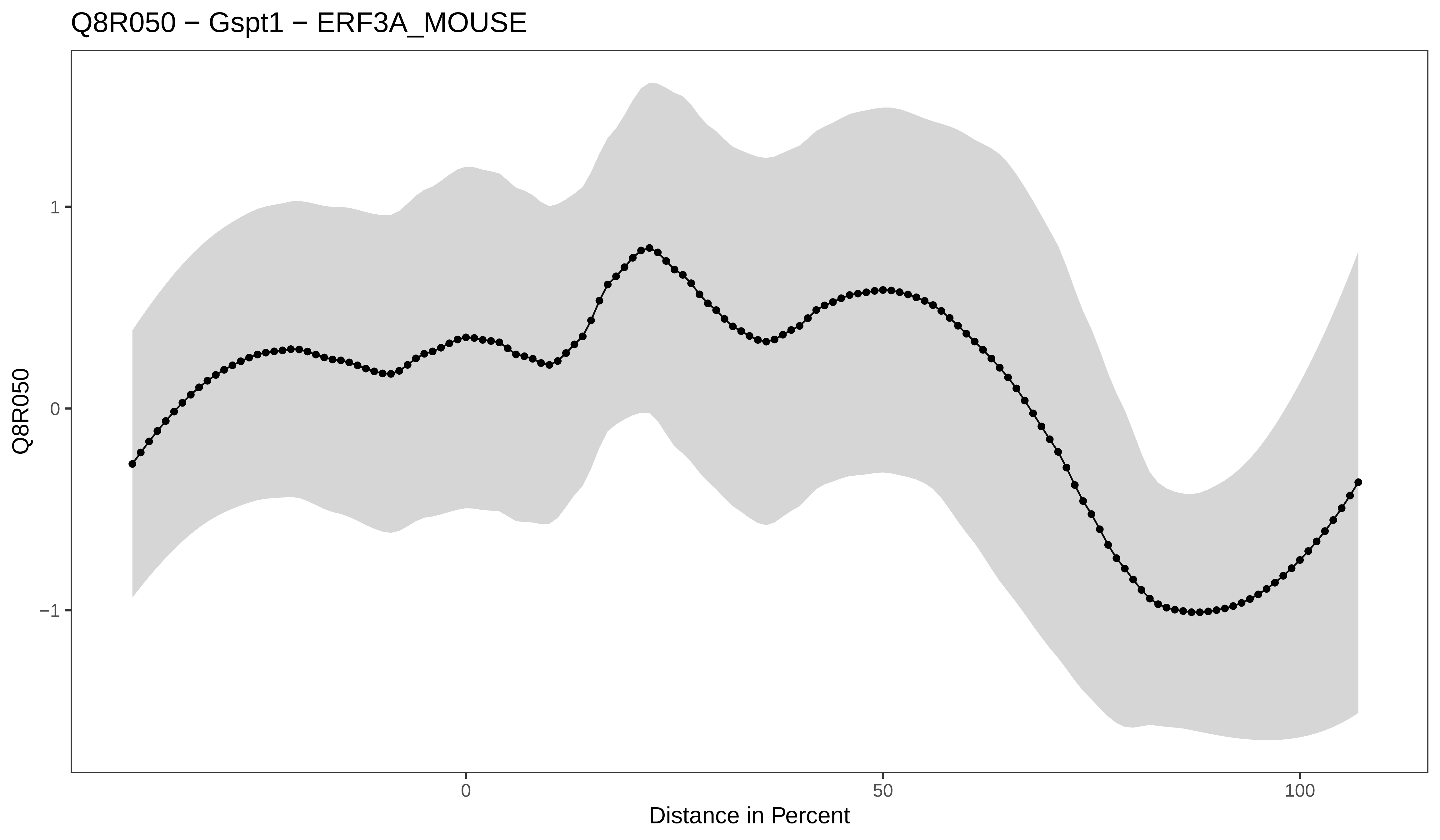

Q8R050 − Gspt1 − ERF3A_MOUSE
Q8R050
1
0
−1
0
50
100
Distance in Percent

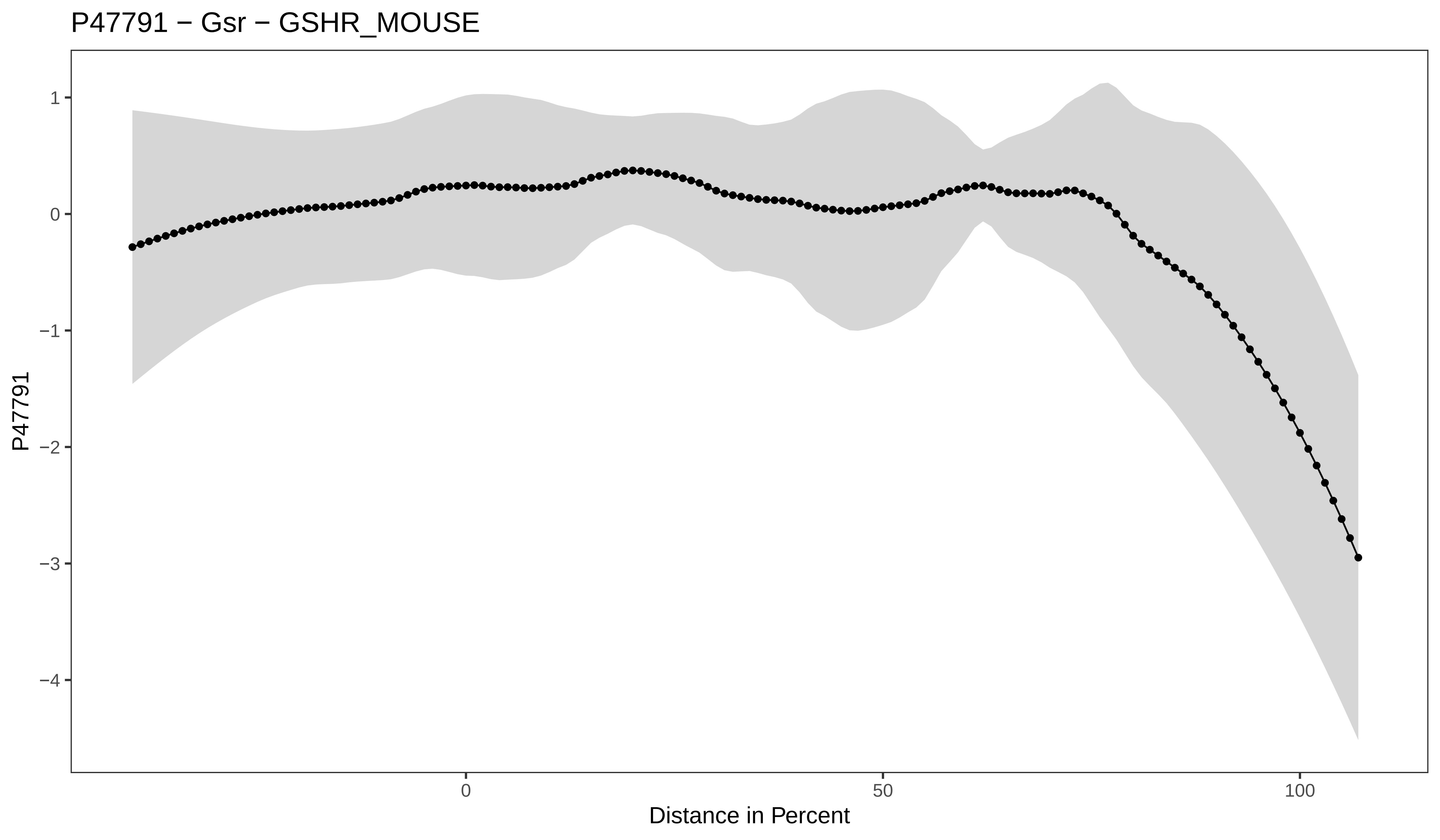

P47791 − Gsr − GSHR_MOUSE
1
0
−1
−2
−3
−4
P47791
0
50
100
Distance in Percent

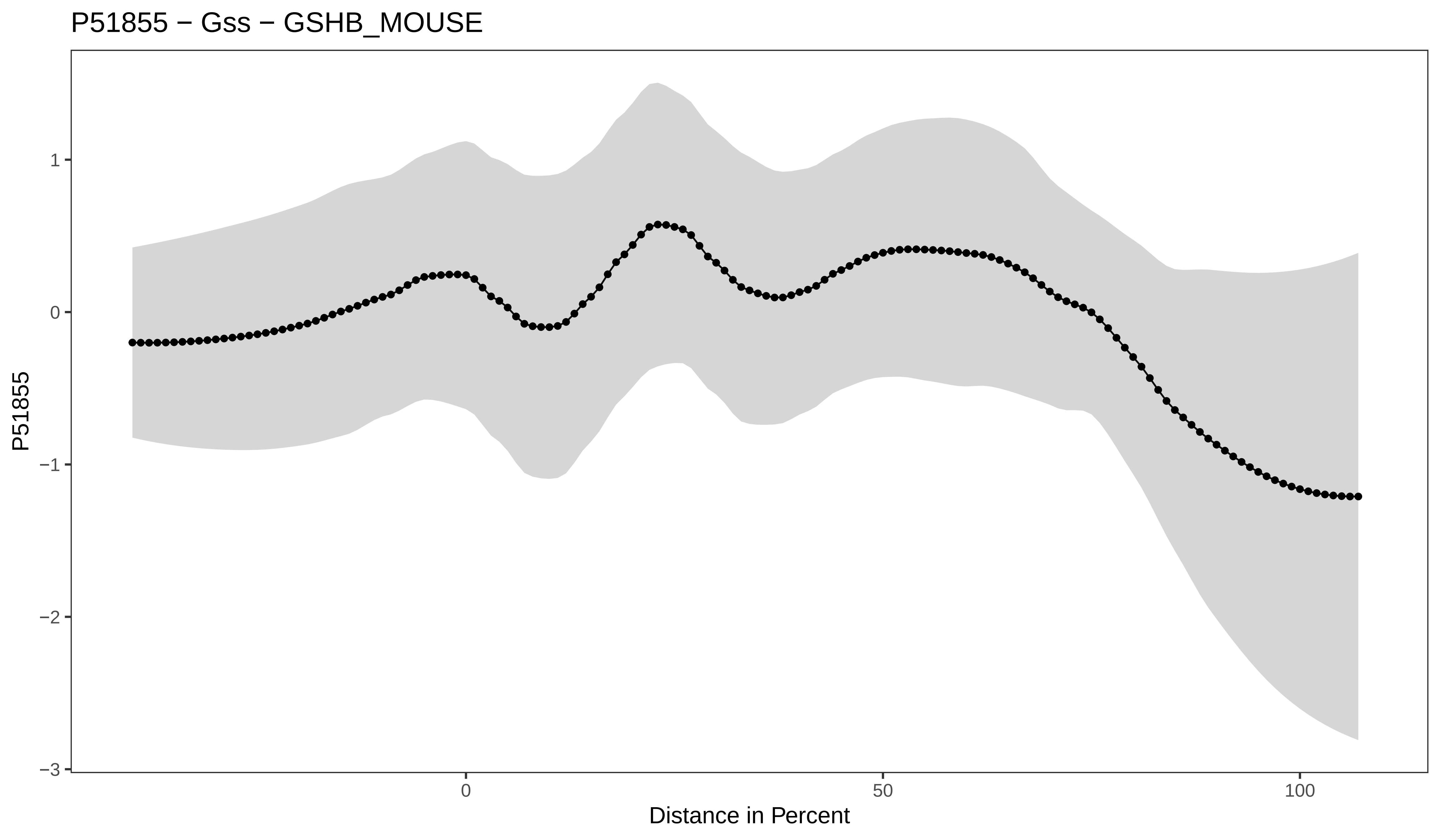
P51855 − Gss − GSHB_MOUSE
P51855
1
0
−1
−2
−3
0
50
100
Distance in Percent

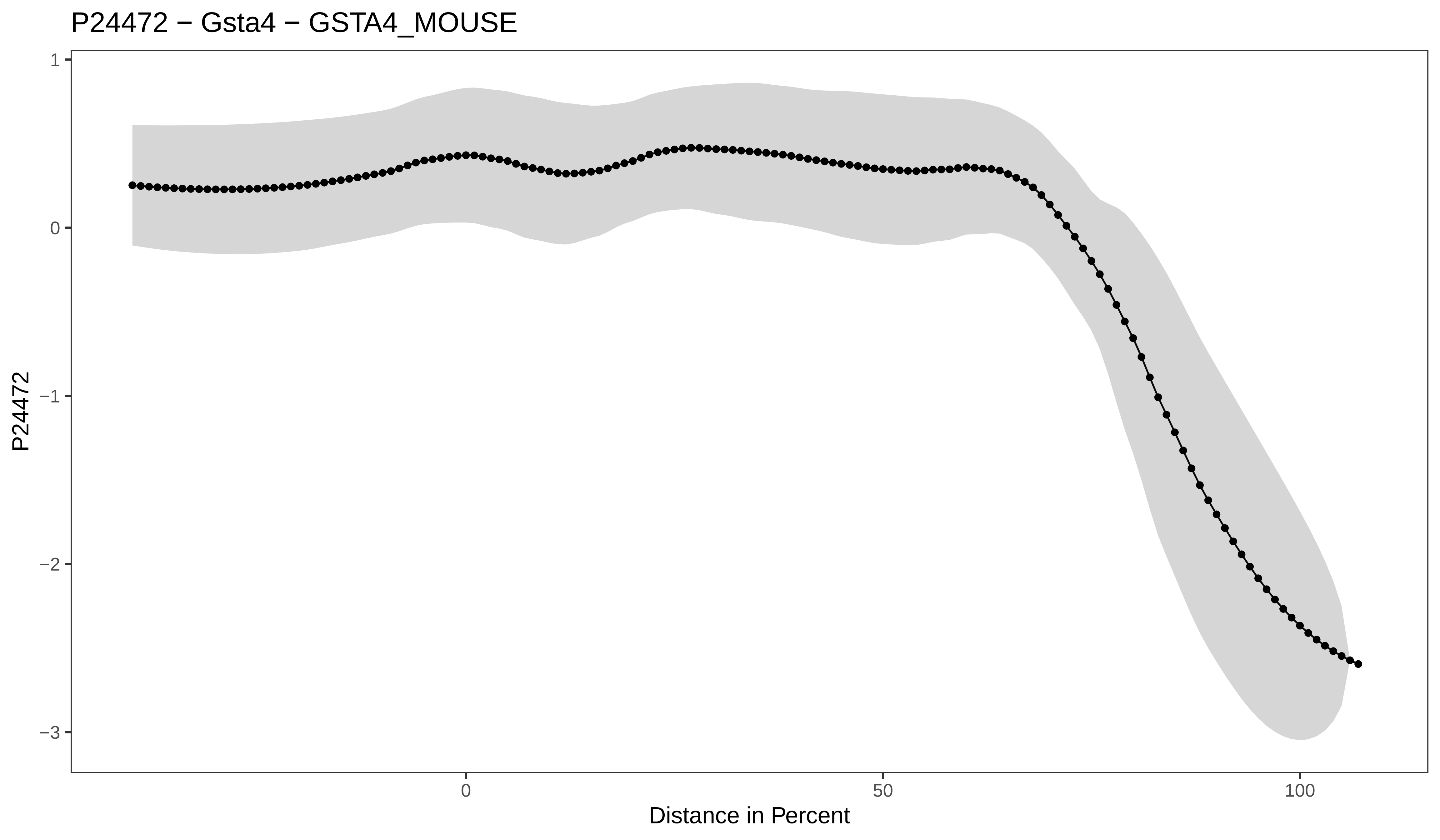
P24472 − Gsta4 − GSTA4_MOUSE
P24472
Distance in Percent
1
0
−1
−2
−3
0
50
100

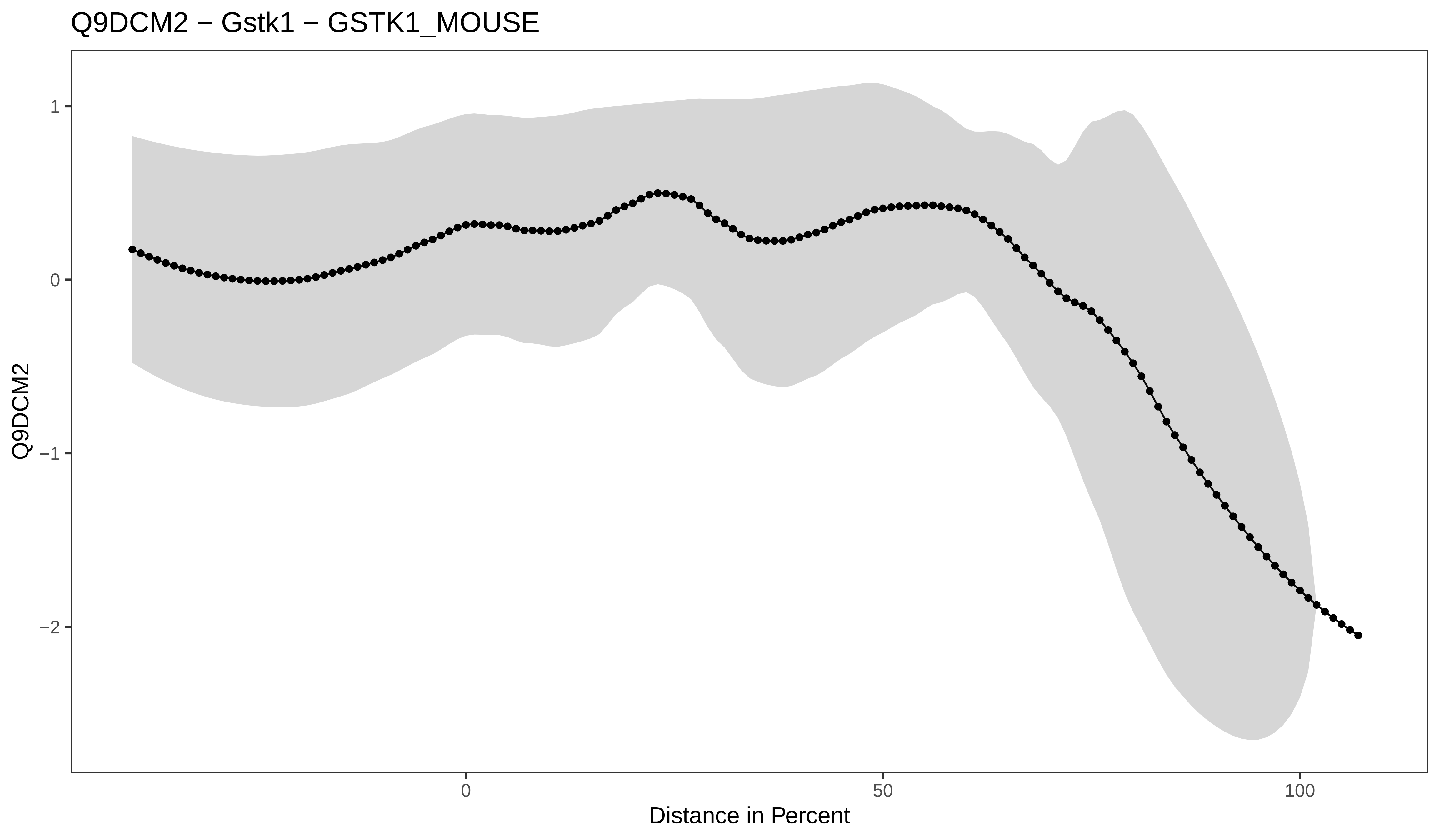

Q9DCM2 − Gstk1 − GSTK1_MOUSE
Q9DCM2
Distance in Percent
1
0
−1
−2
0
50
100

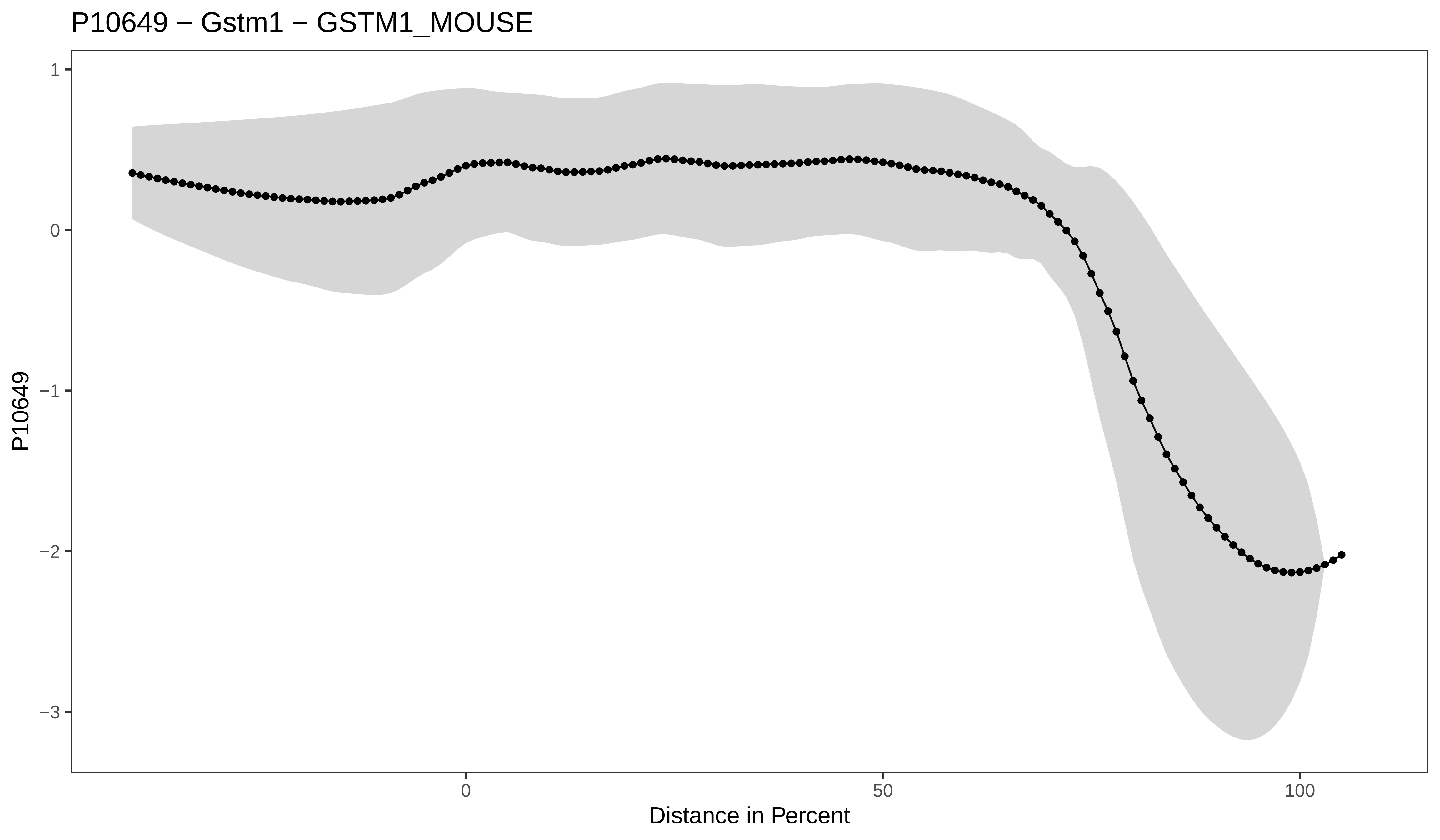

P10649 − Gstm1 − GSTM1_MOUSE
1
0
−1
−2
−3
P10649
0
50
100
Distance in Percent

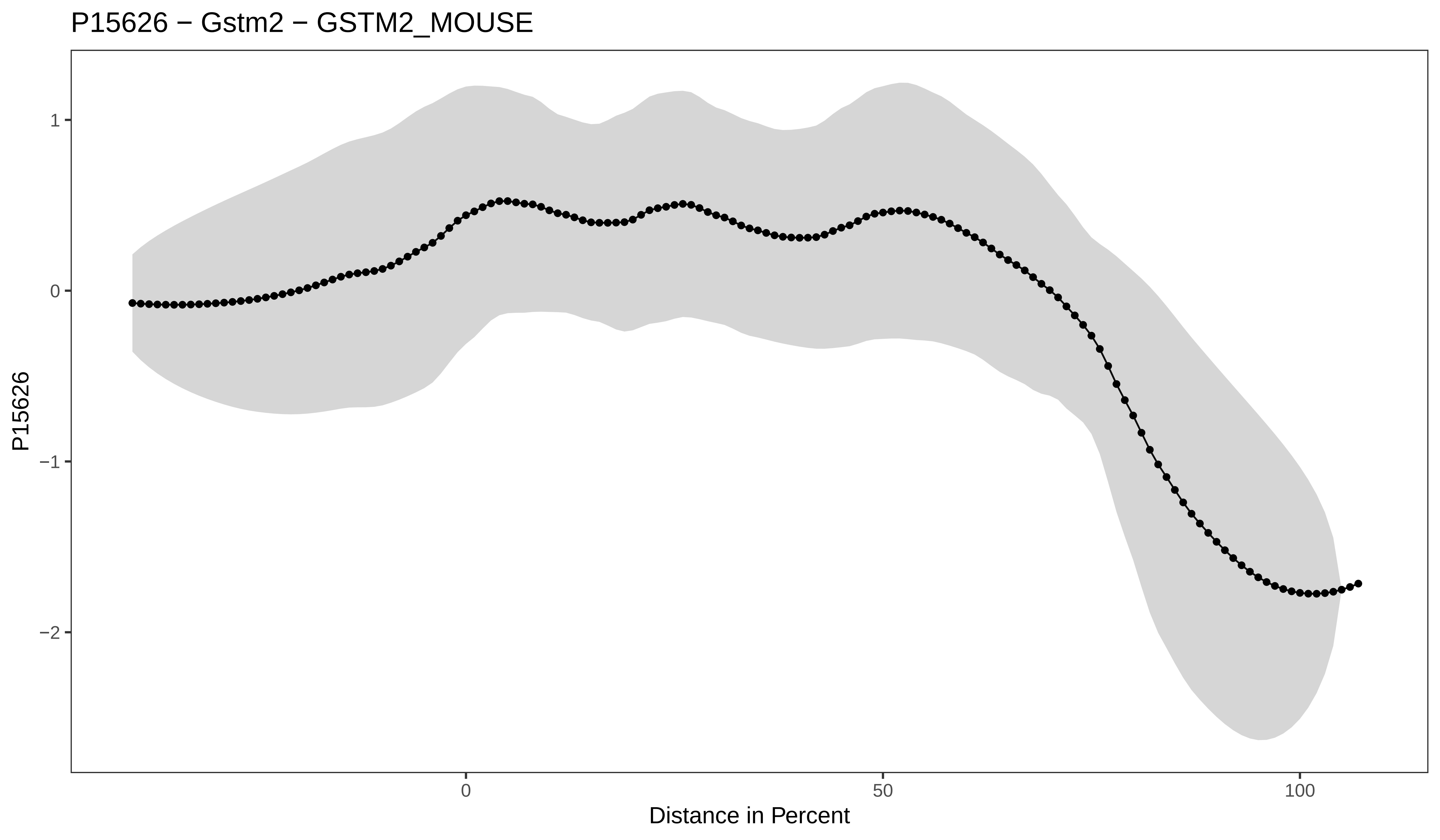
P15626 − Gstm2 − GSTM2_MOUSE
P15626
1
0
−1
−2
0
50
100
Distance in Percent

P19639 − Gstm3 − GSTM3_MOUSE

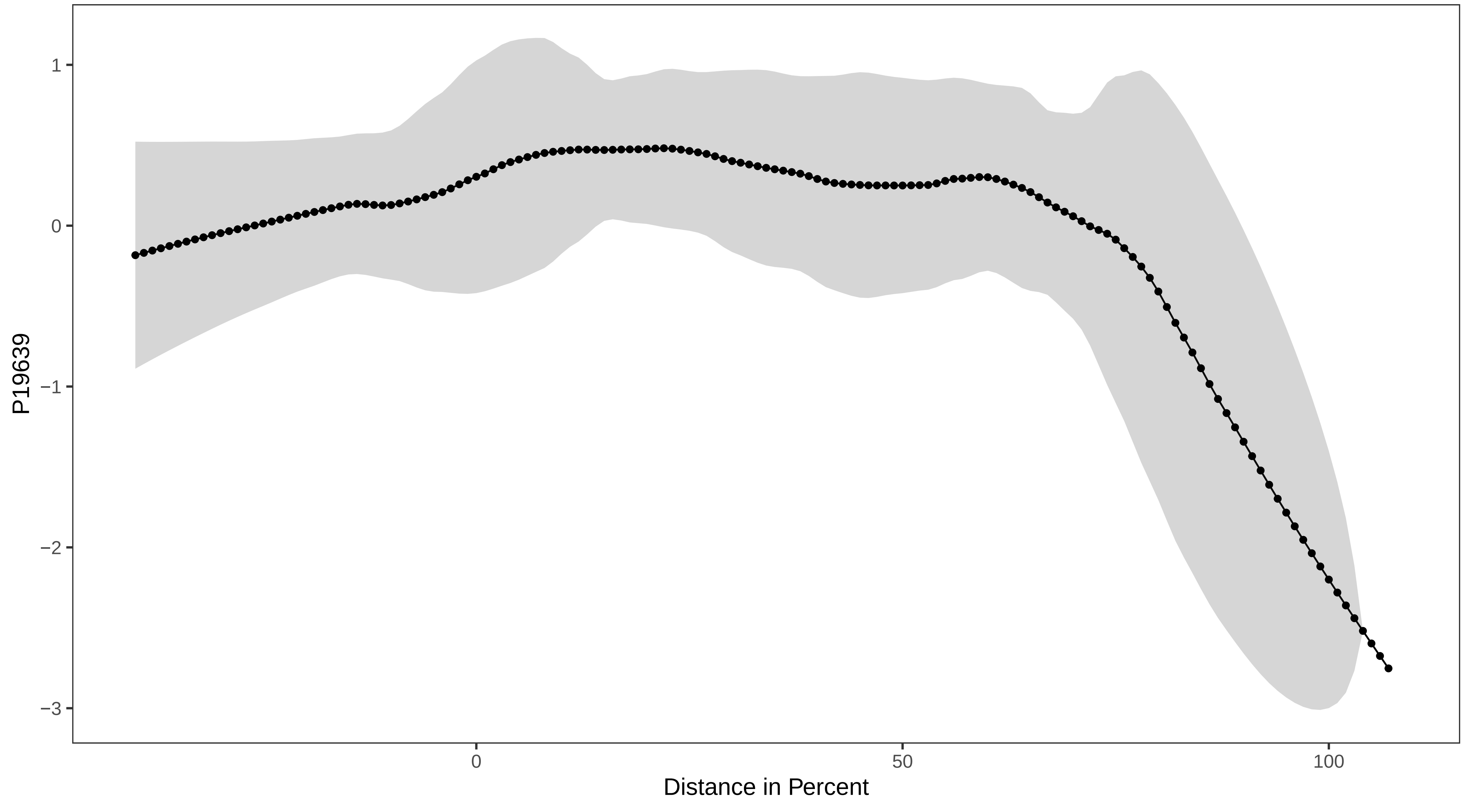

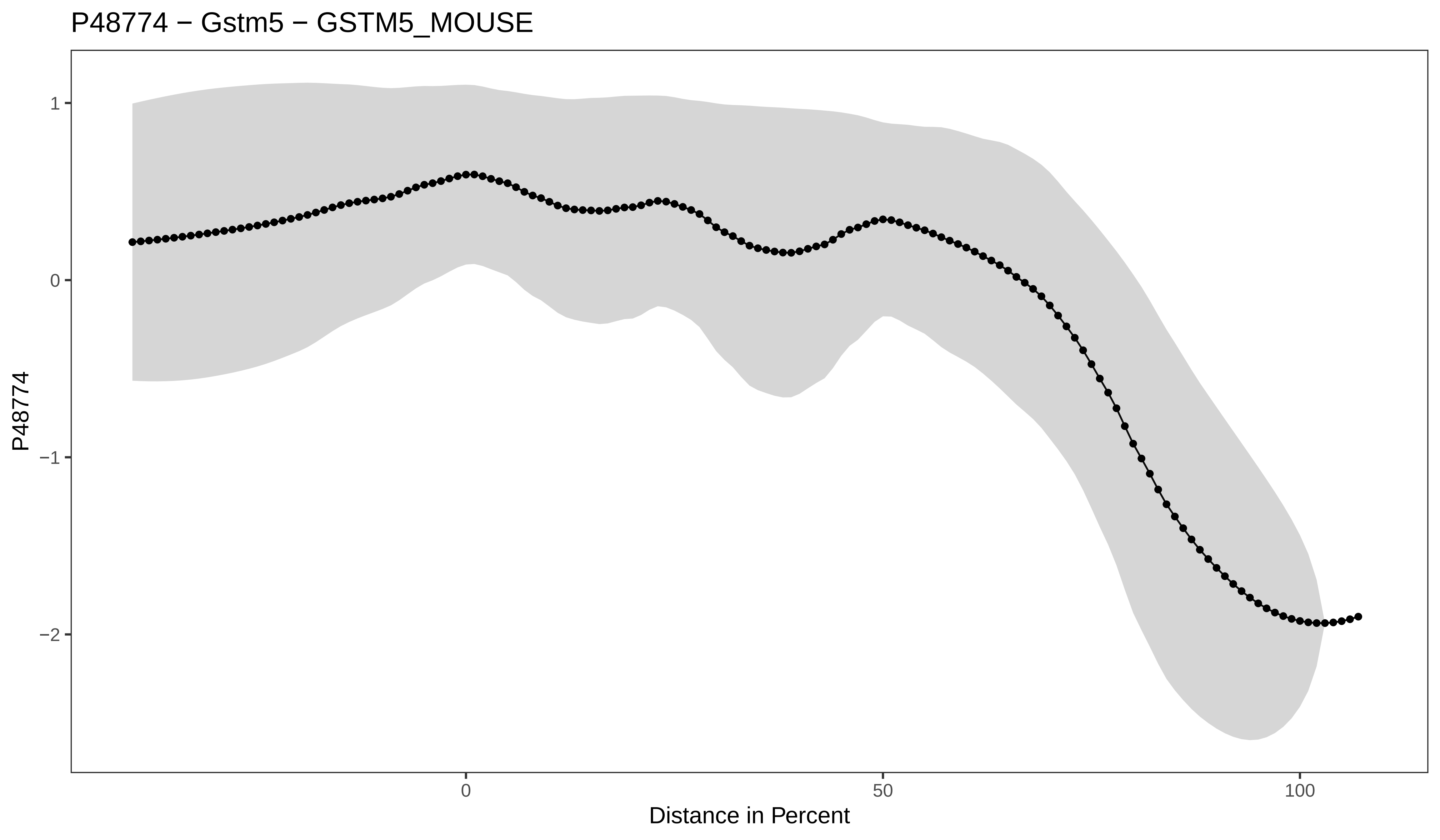

P48774 − Gstm5 − GSTM5_MOUSE
1
0
−1
−2
P48774
0
50
100
Distance in Percent

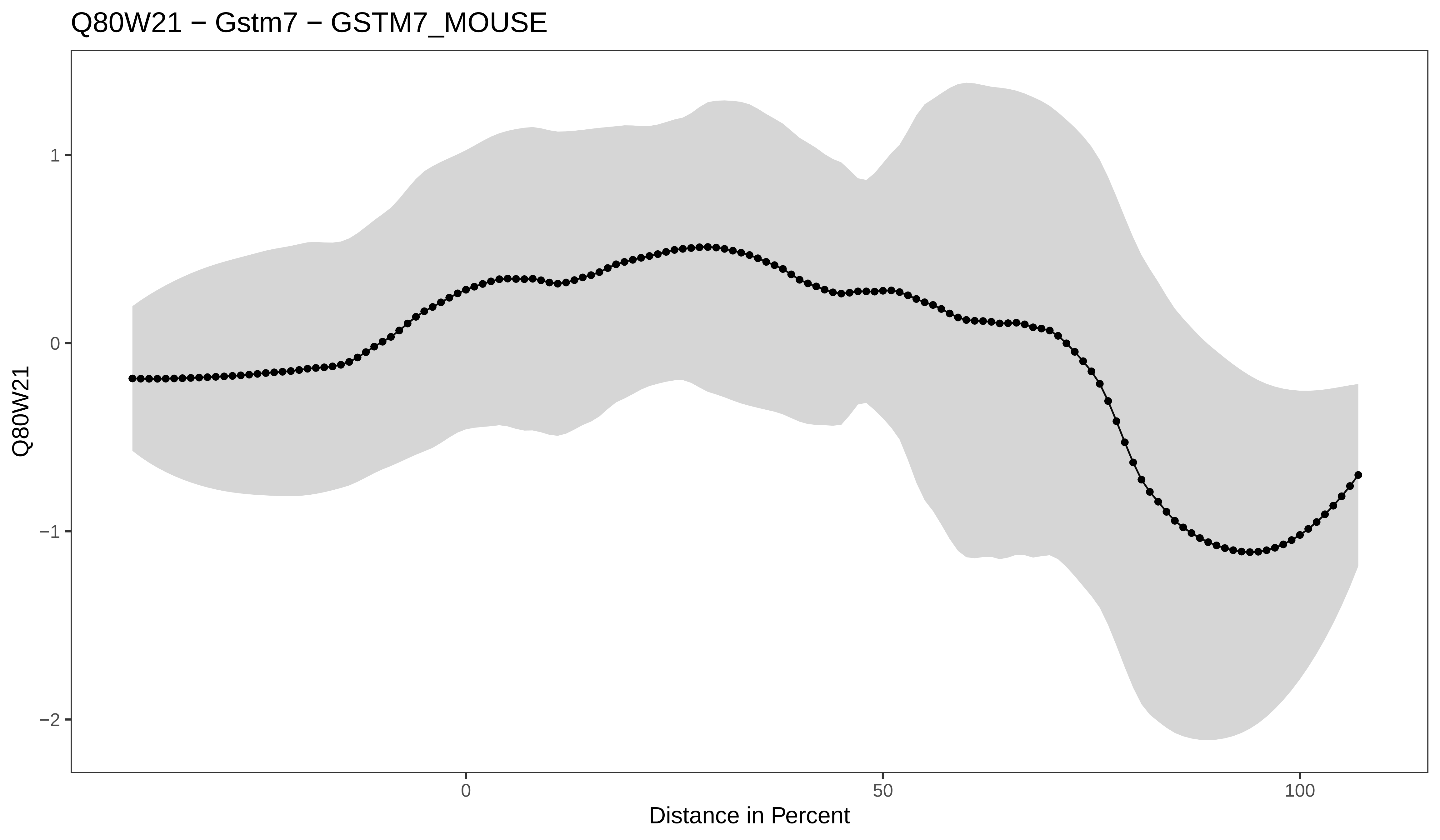

Q80W21 − Gstm7 − GSTM7_MOUSE
1
0
−1
−2
Q80W21
0
50
100
Distance in Percent

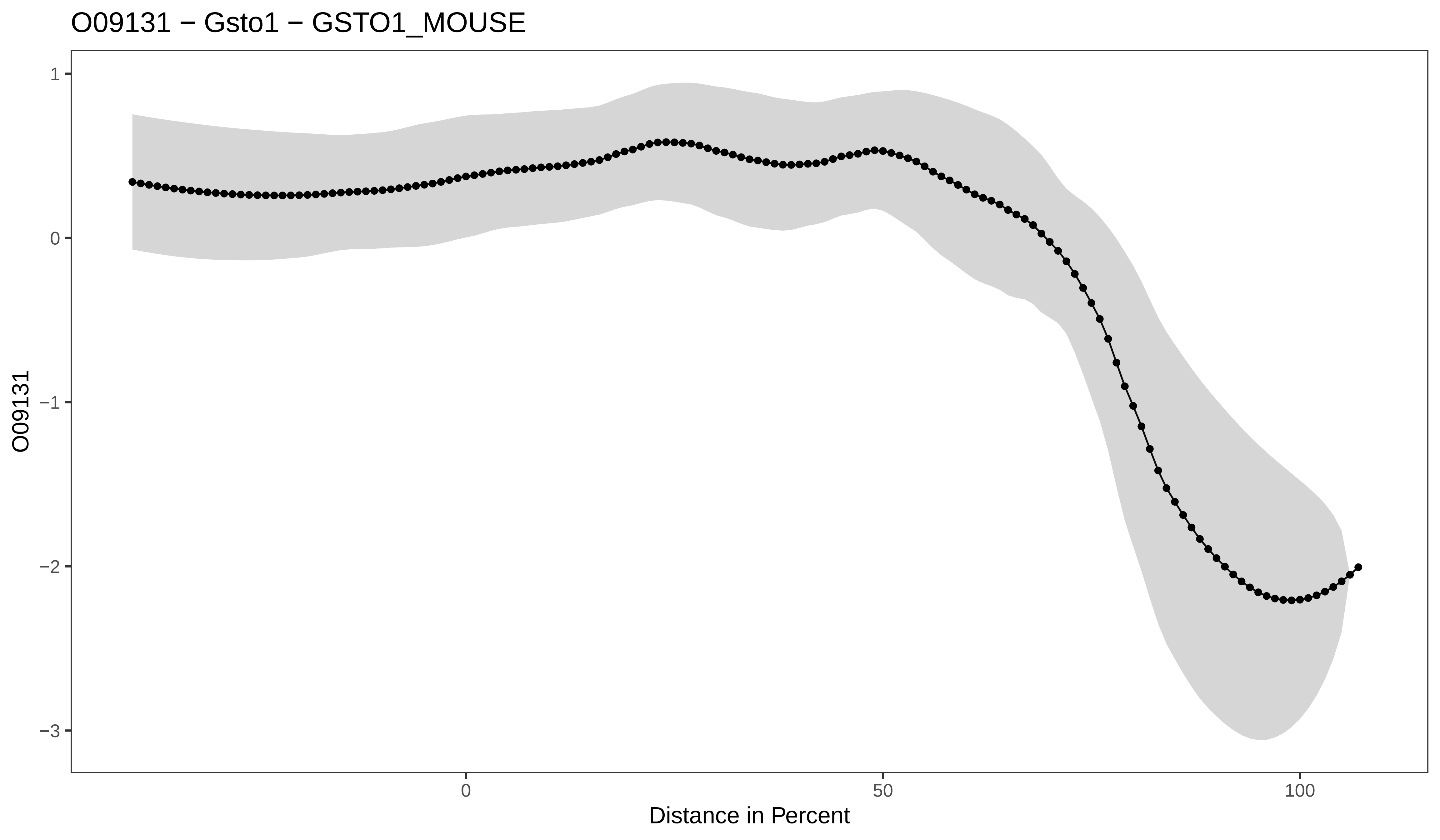

O09131 − Gsto1 − GSTO1_MOUSE
O09131
1
0
−1
−2
−3
0
50
100
Distance in Percent

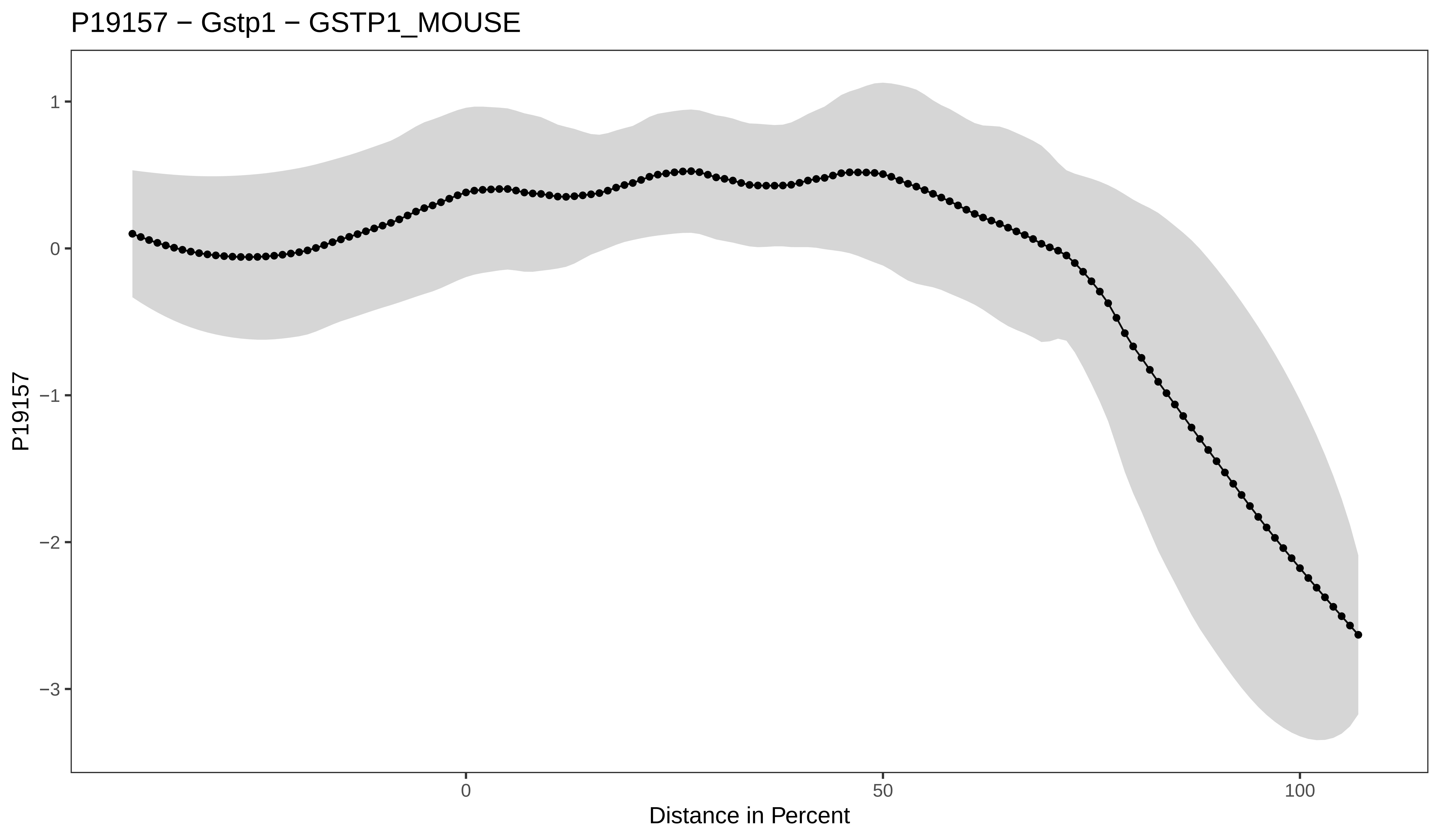
P19157 − Gstp1 − GSTP1_MOUSE
P19157
1
0
−1
−2
−3
0
50
100
Distance in Percent

Q61133 − Gstt2 − GSTT2_MOUSE

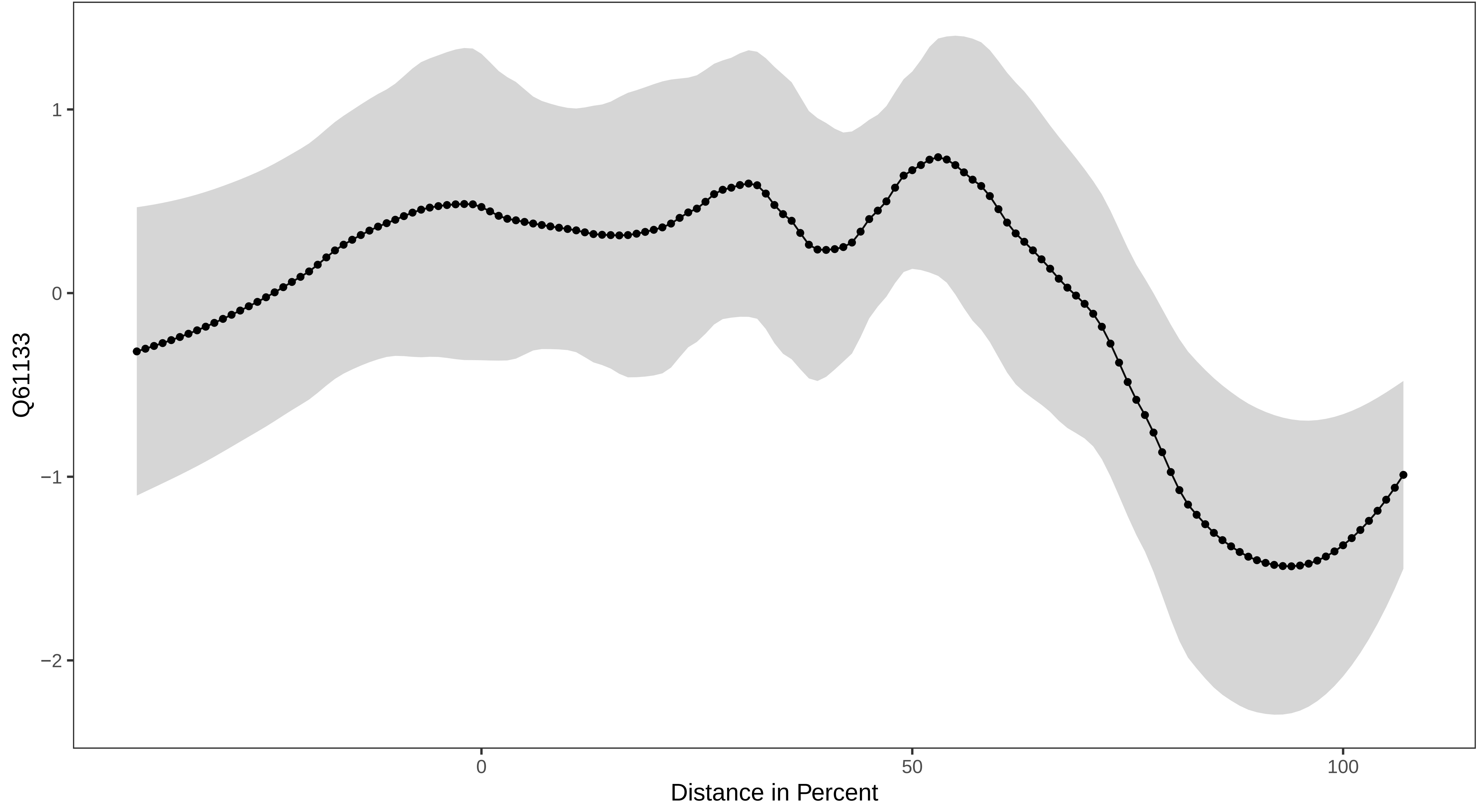

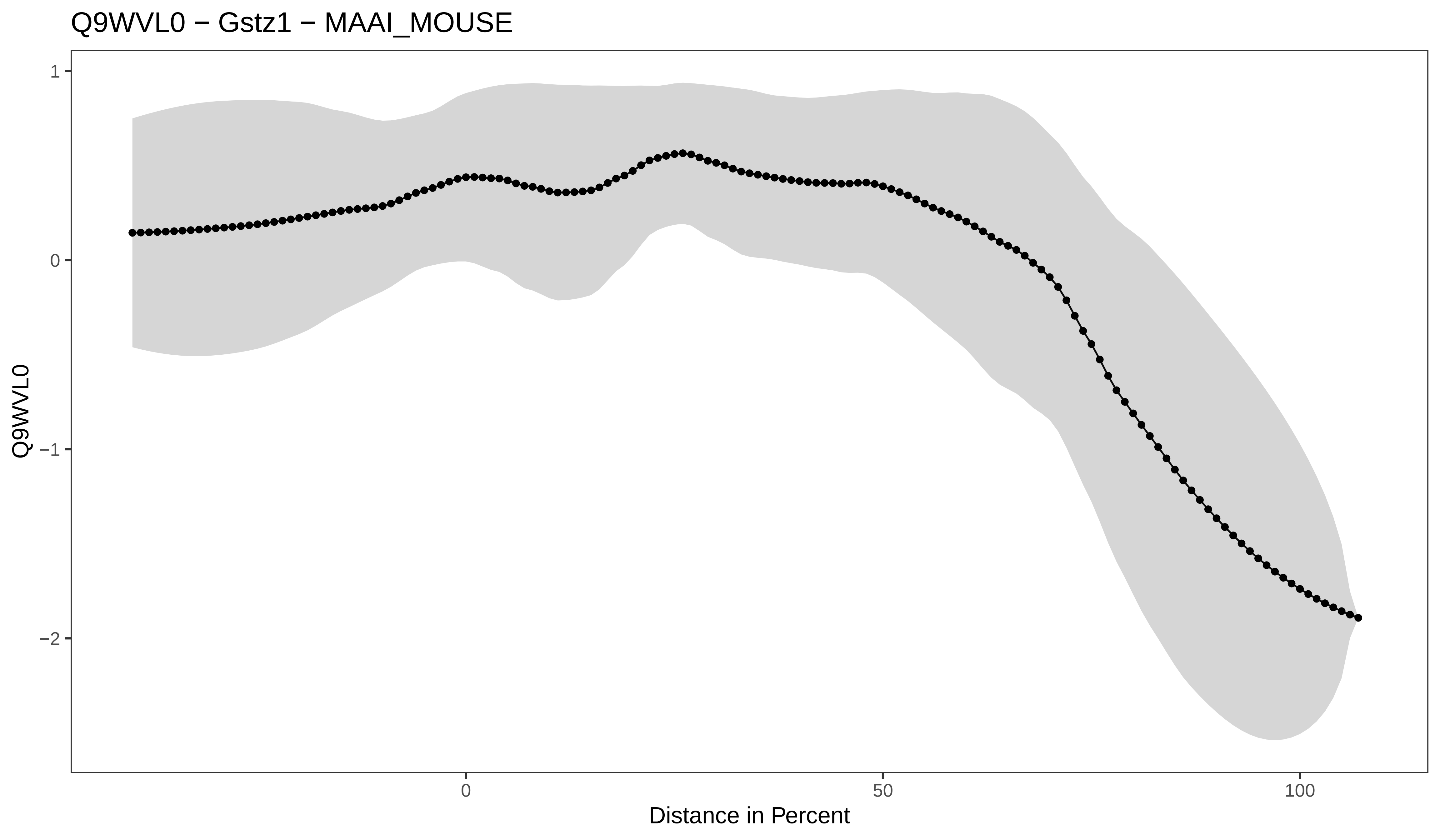

Q9WVL0 − Gstz1 − MAAI_MOUSE
Q9WVL0
1
0
−1
−2
0
50
100
Distance in Percent

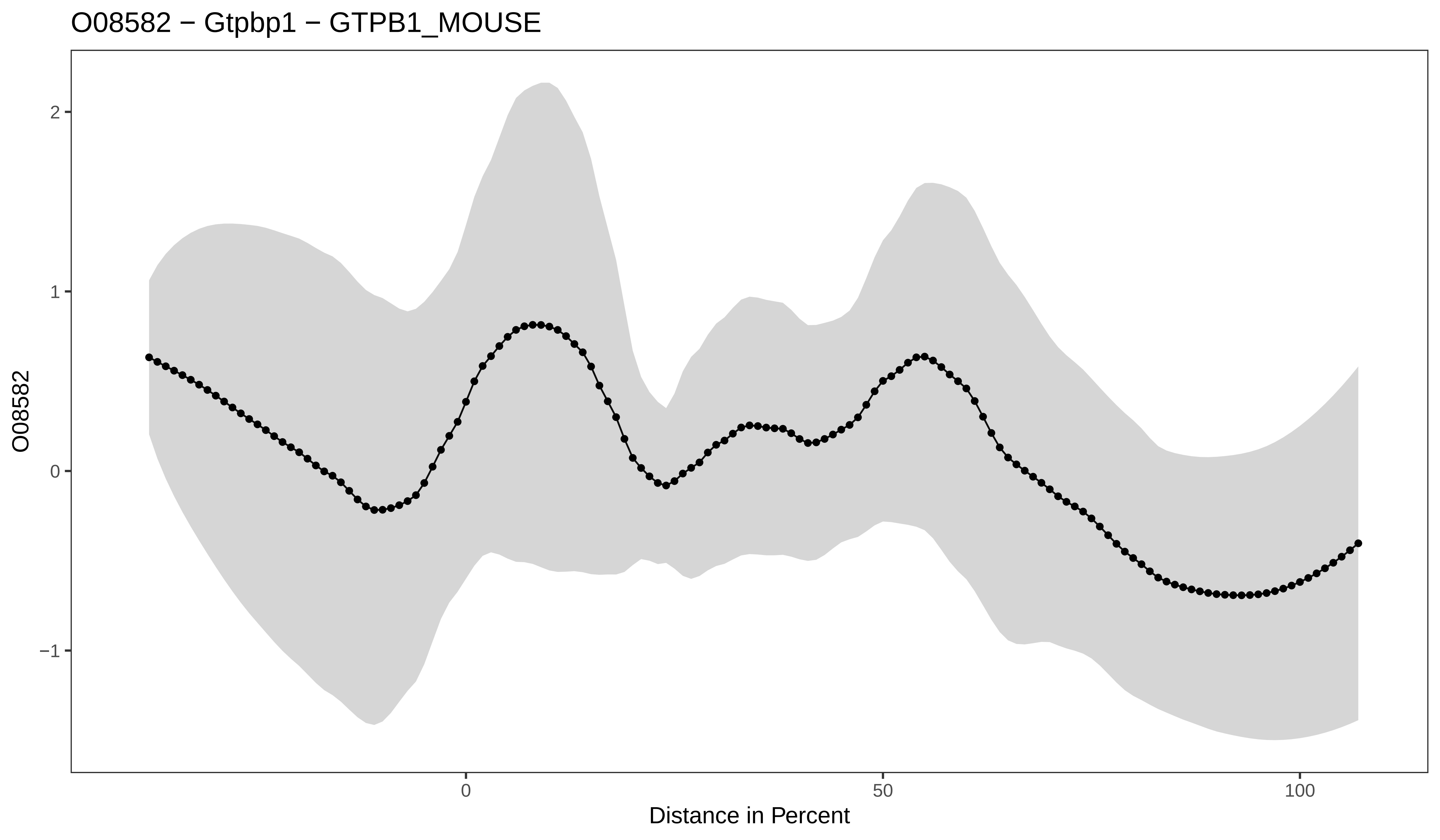
O08582 − Gtpbp1 − GTPB1_MOUSE
2
1
0
−1
O08582
0
50
100
Distance in Percent

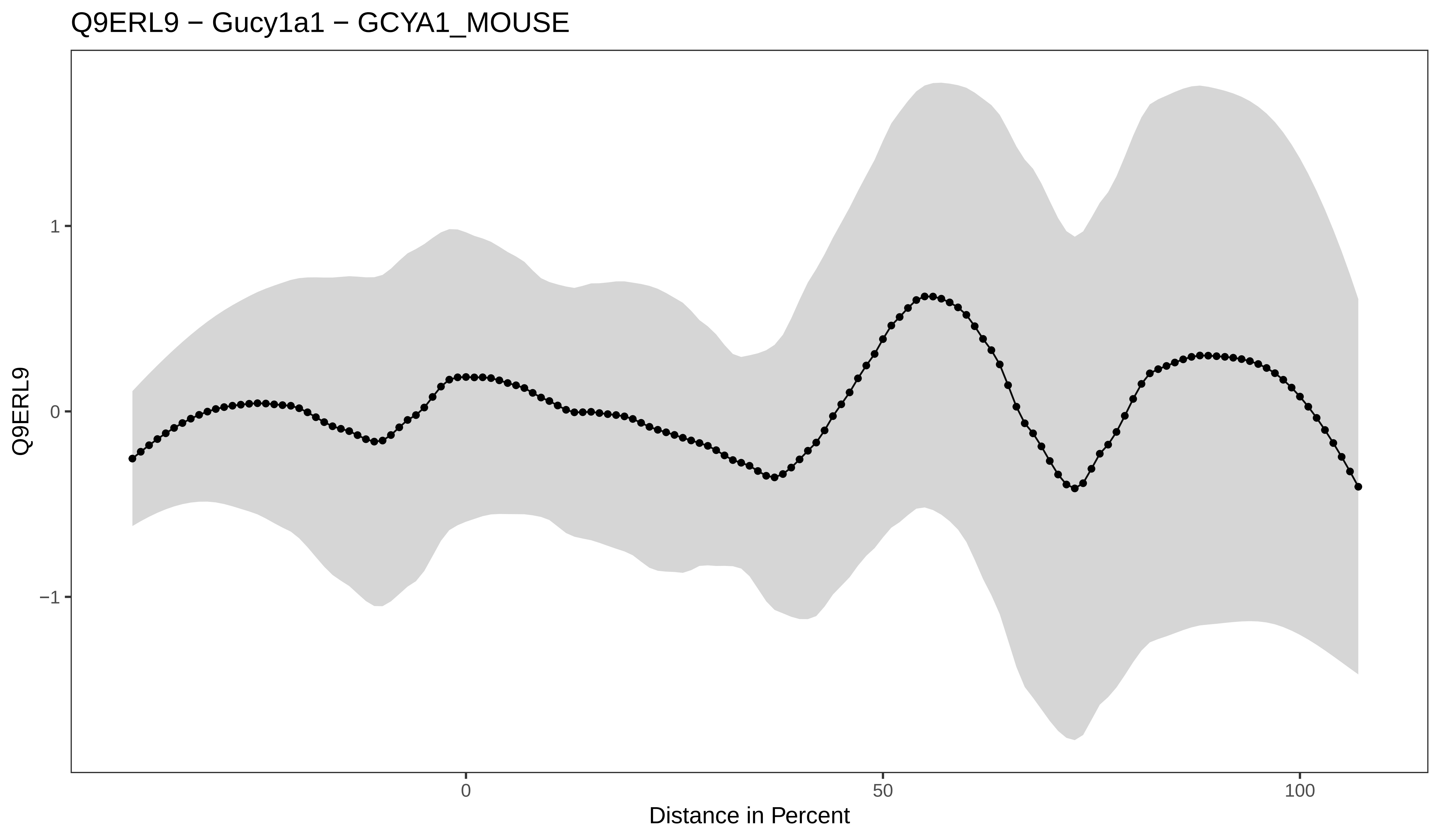

Q9ERL9 − Gucy1a1 − GCYA1_MOUSE
1
0
−1
Q9ERL9
0
50
100
Distance in Percent

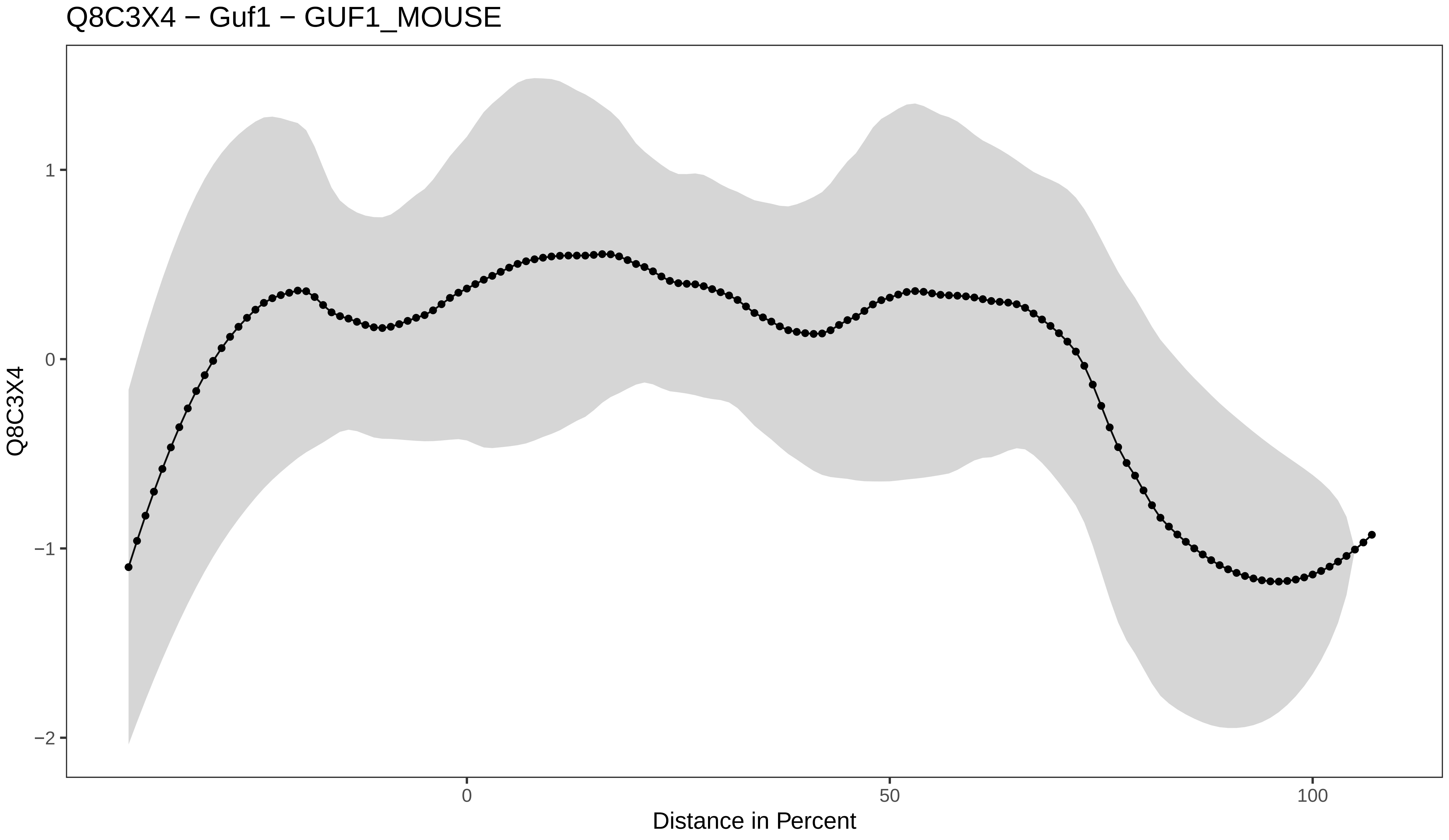

Q8C3X4 − Guf1 − GUF1_MOUSE
Q8C3X4
1
0
−1
−2
0
50
100
Distance in Percent

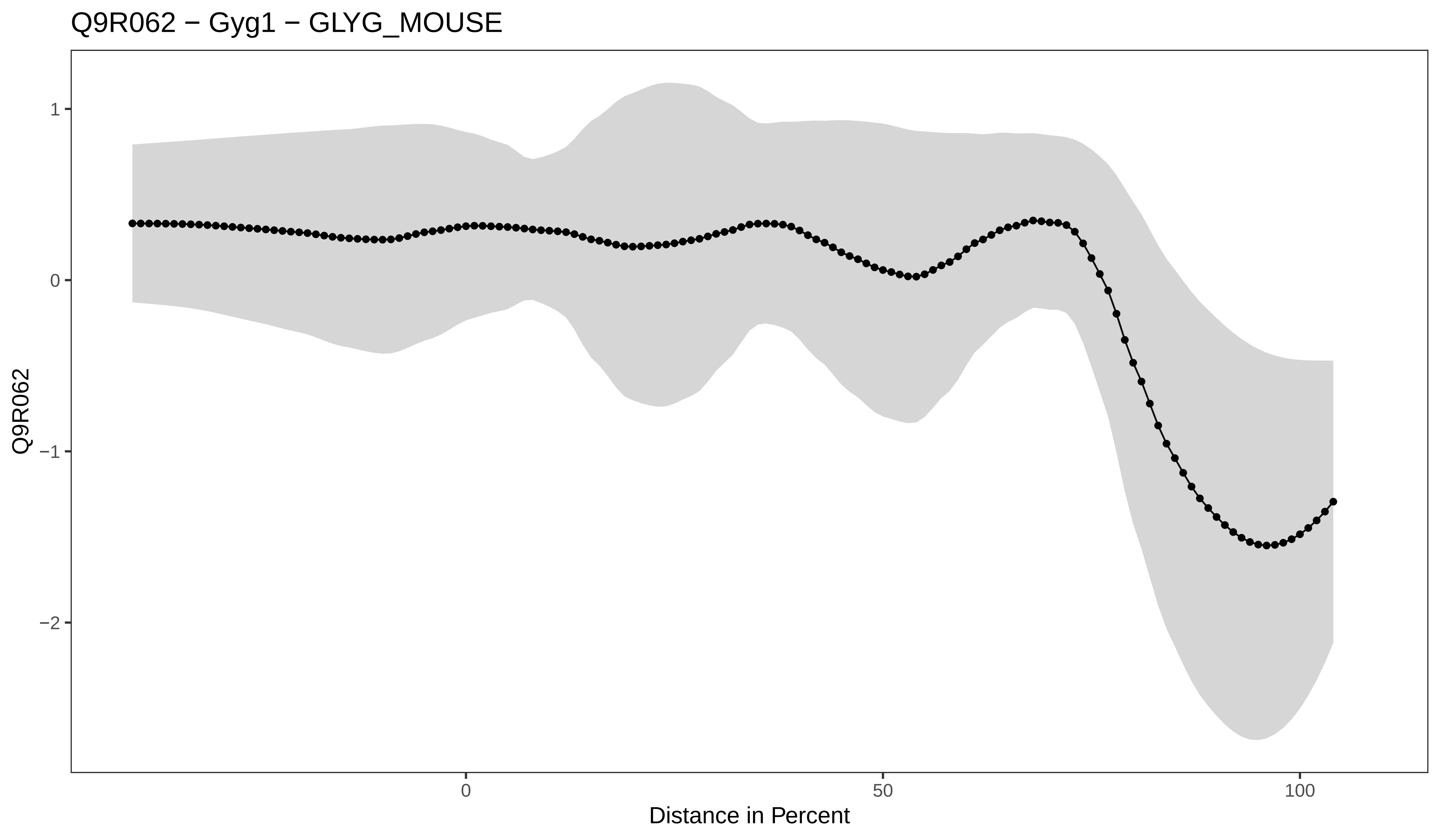
Q9R062 − Gyg1 − GLYG_MOUSE
1
0
−1
−2
Q9R062
0
50
100
Distance in Percent

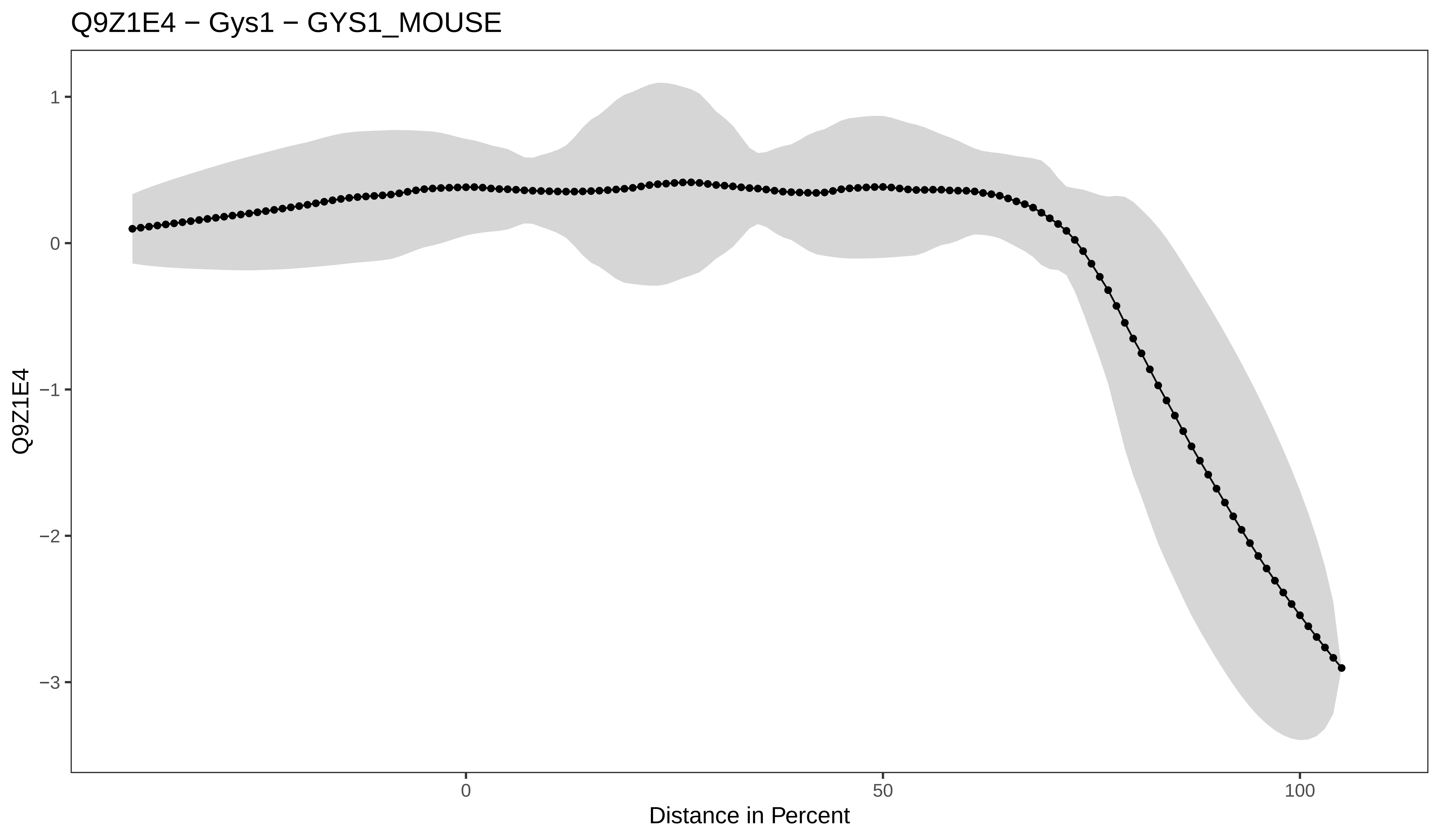

Q9Z1E4 − Gys1 − GYS1_MOUSE
1
0
−1
−2
−3
Q9Z1E4
0
50
100
Distance in Percent

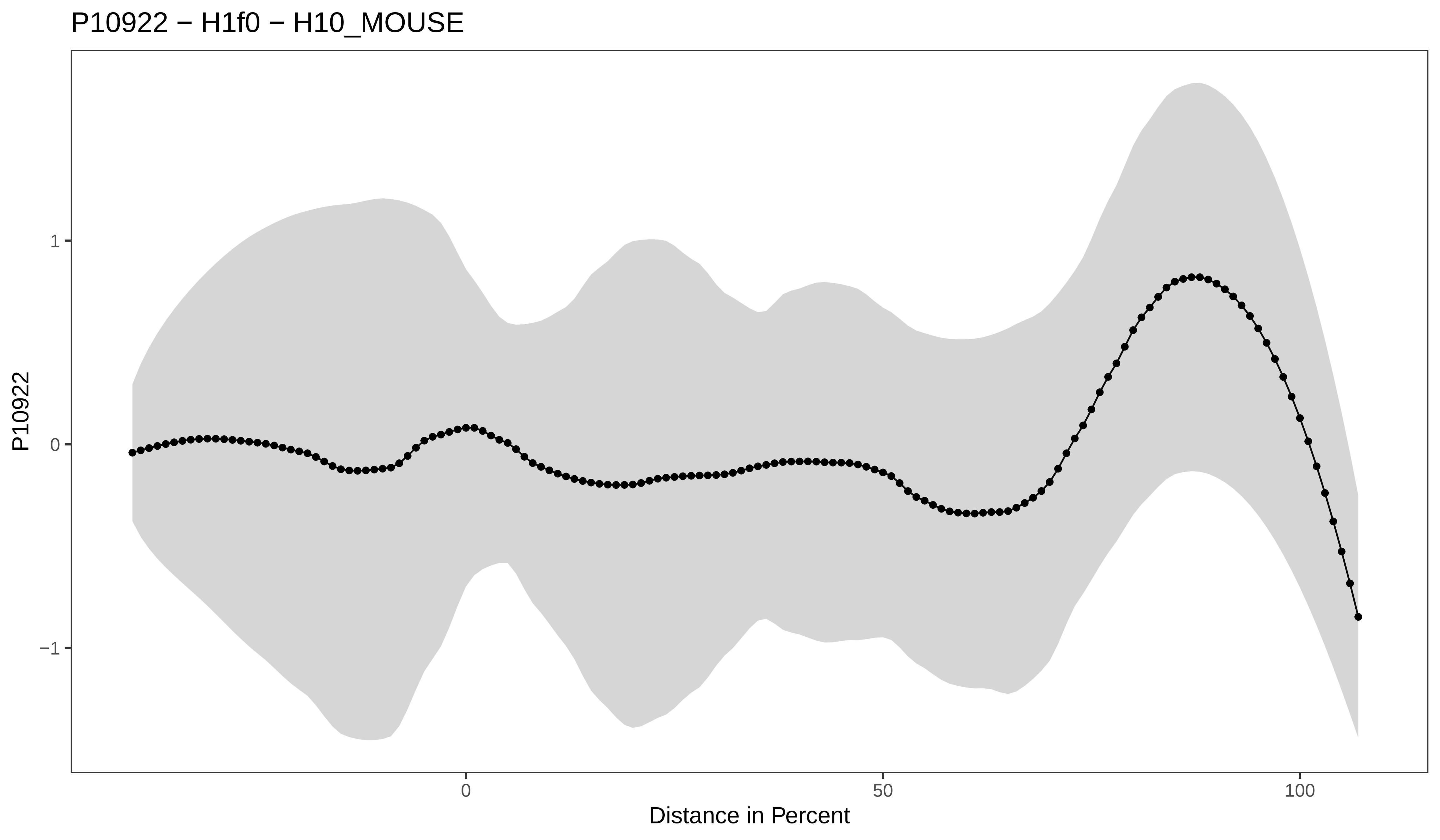

P10922 − H1f0 − H10_MOUSE
P10922
1
0
−1
0
50
100
Distance in Percent

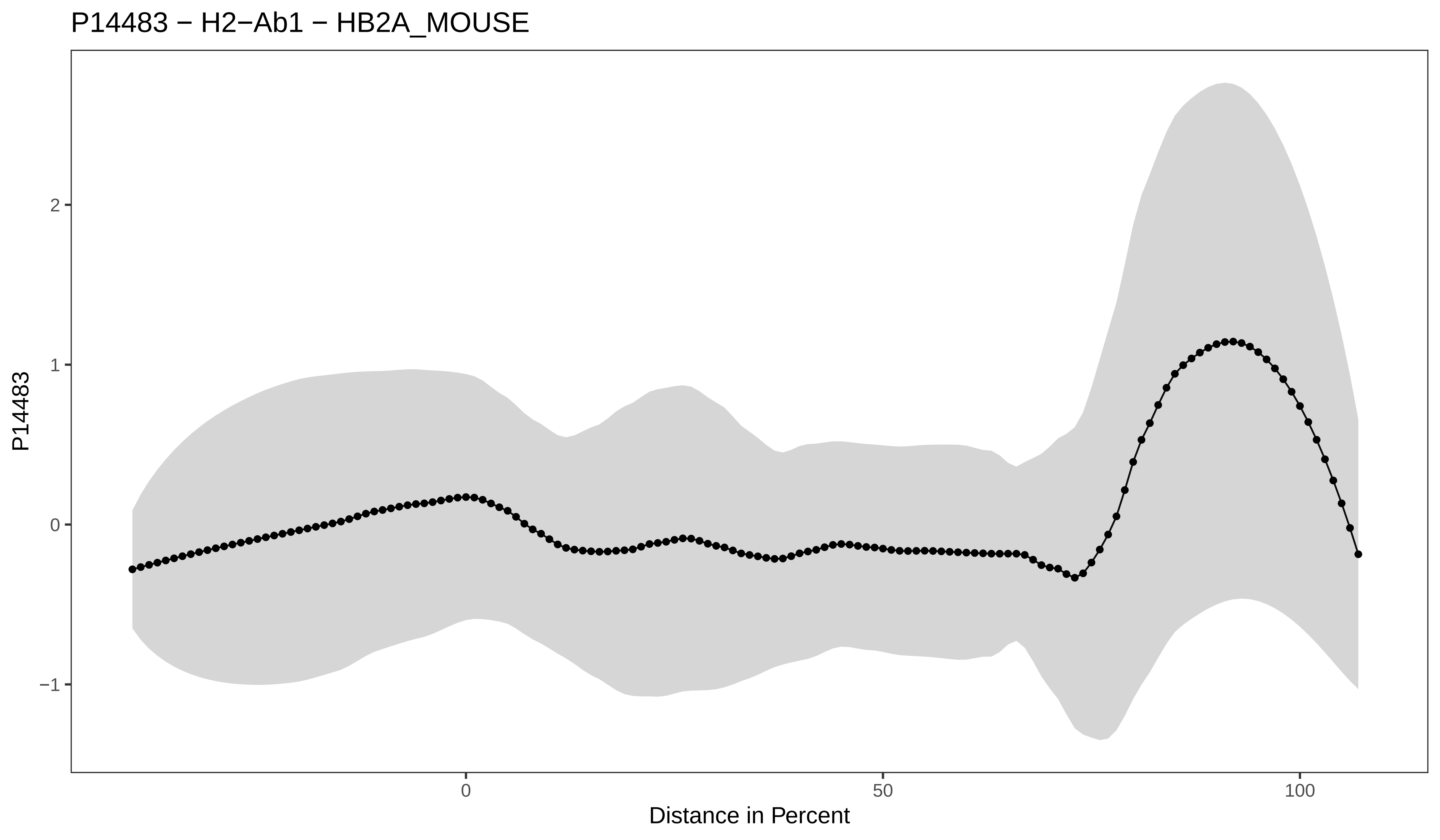

P14483 − H2−Ab1 − HB2A_MOUSE
P14483
Distance in Percent
2
1
0
−1
0
50
100

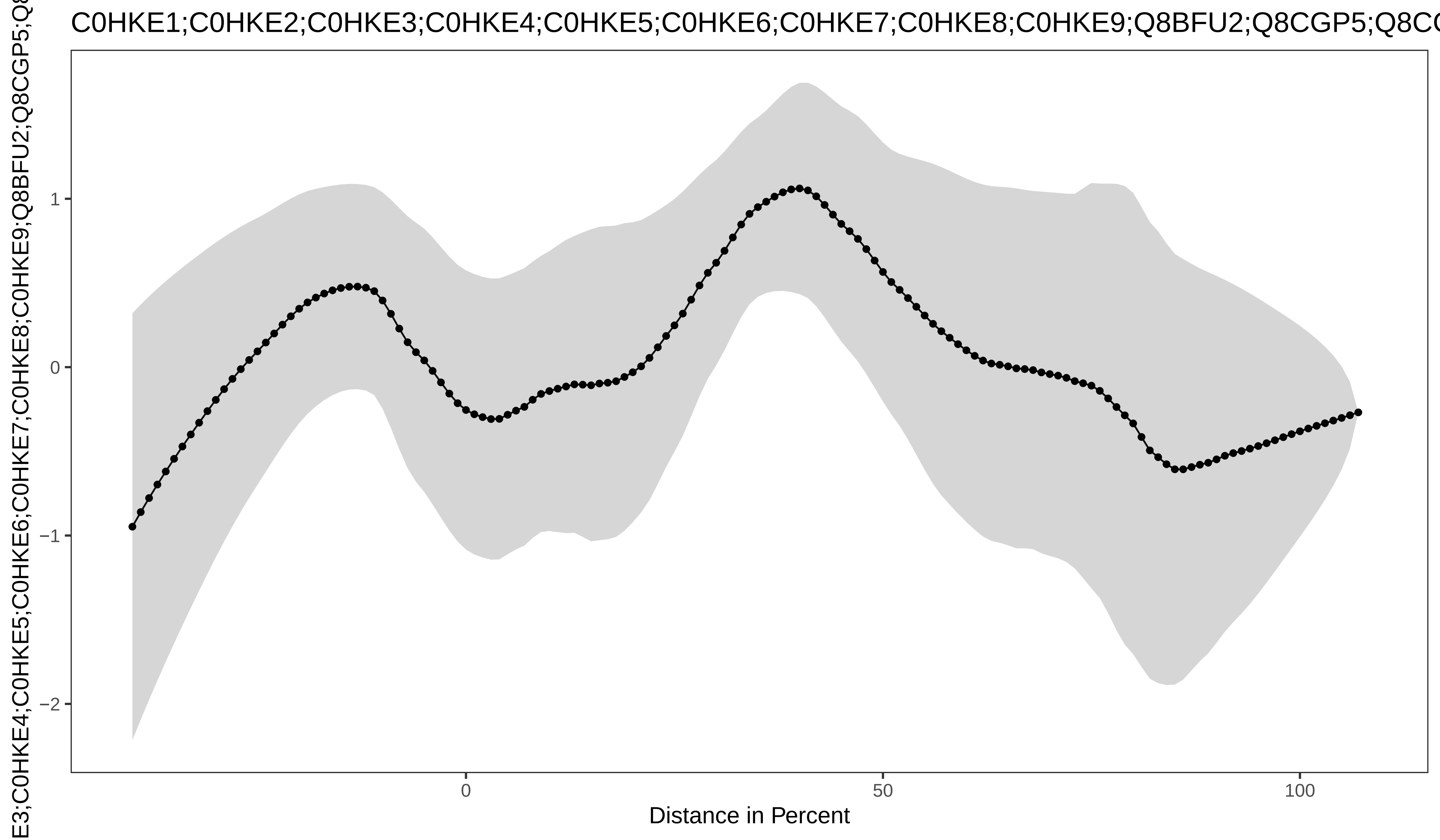
C0HKE1;C0HKE2;C0HKE3;C0HKE4;C0HKE5;C0HKE6;C0HKE7;C0HKE8;C0HKE9;Q8BFU2;Q8CGP5;Q8C
E3;C0HKE4;C0HKE5;C0HKE6;C0HKE7;C0HKE8;C0HKE9;Q8BFU2;Q8CGP5;Q8
1
0
−1
−2
0
50
100
Distance in Percent

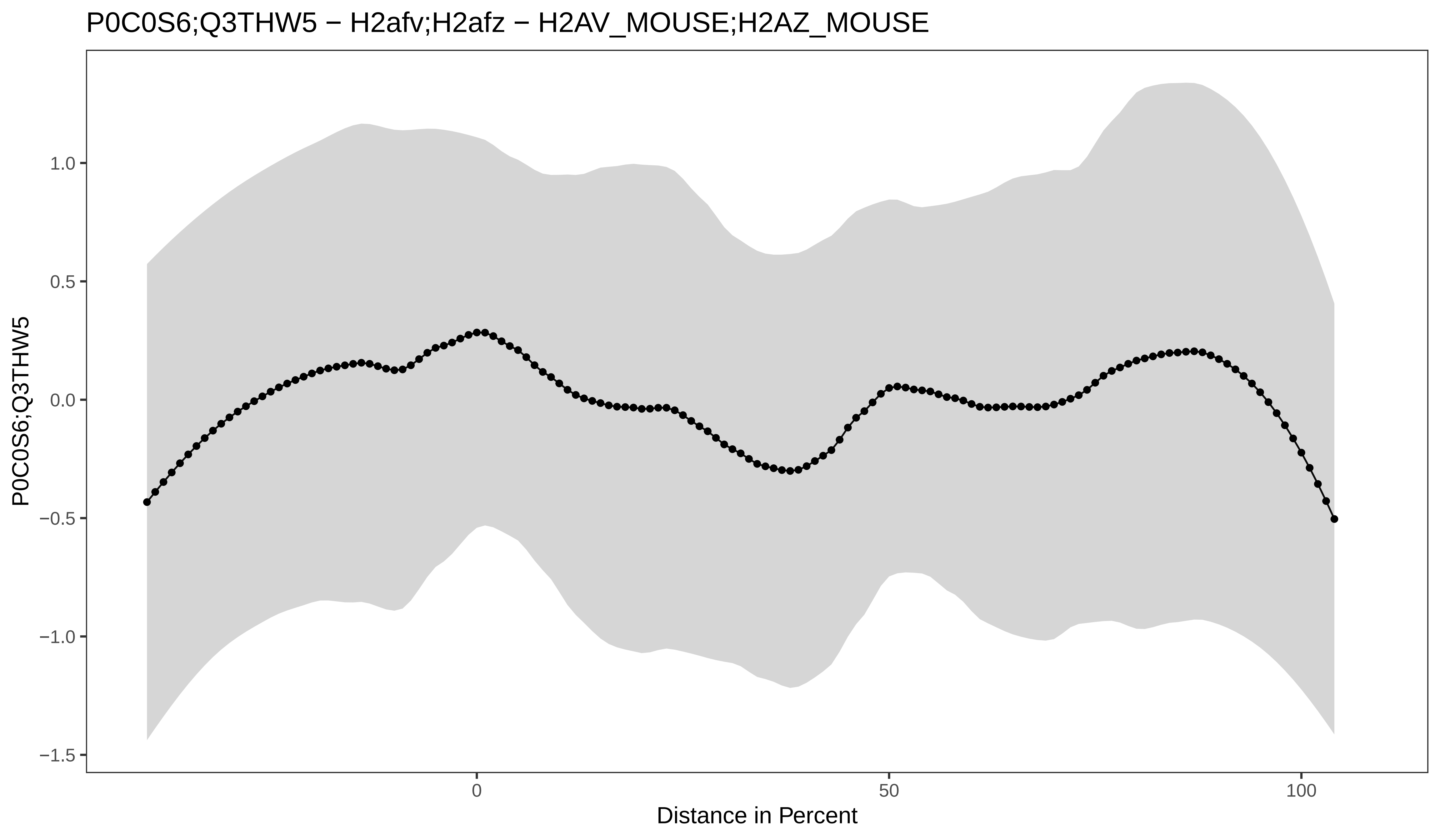
P0C0S6;Q3THW5 − H2afv;H2afz − H2AV_MOUSE;H2AZ_MOUSE
P0C0S6;Q3THW5
1.0
0.5
0.0
−0.5
−1.0
−1.5
0
50
100
Distance in Percent

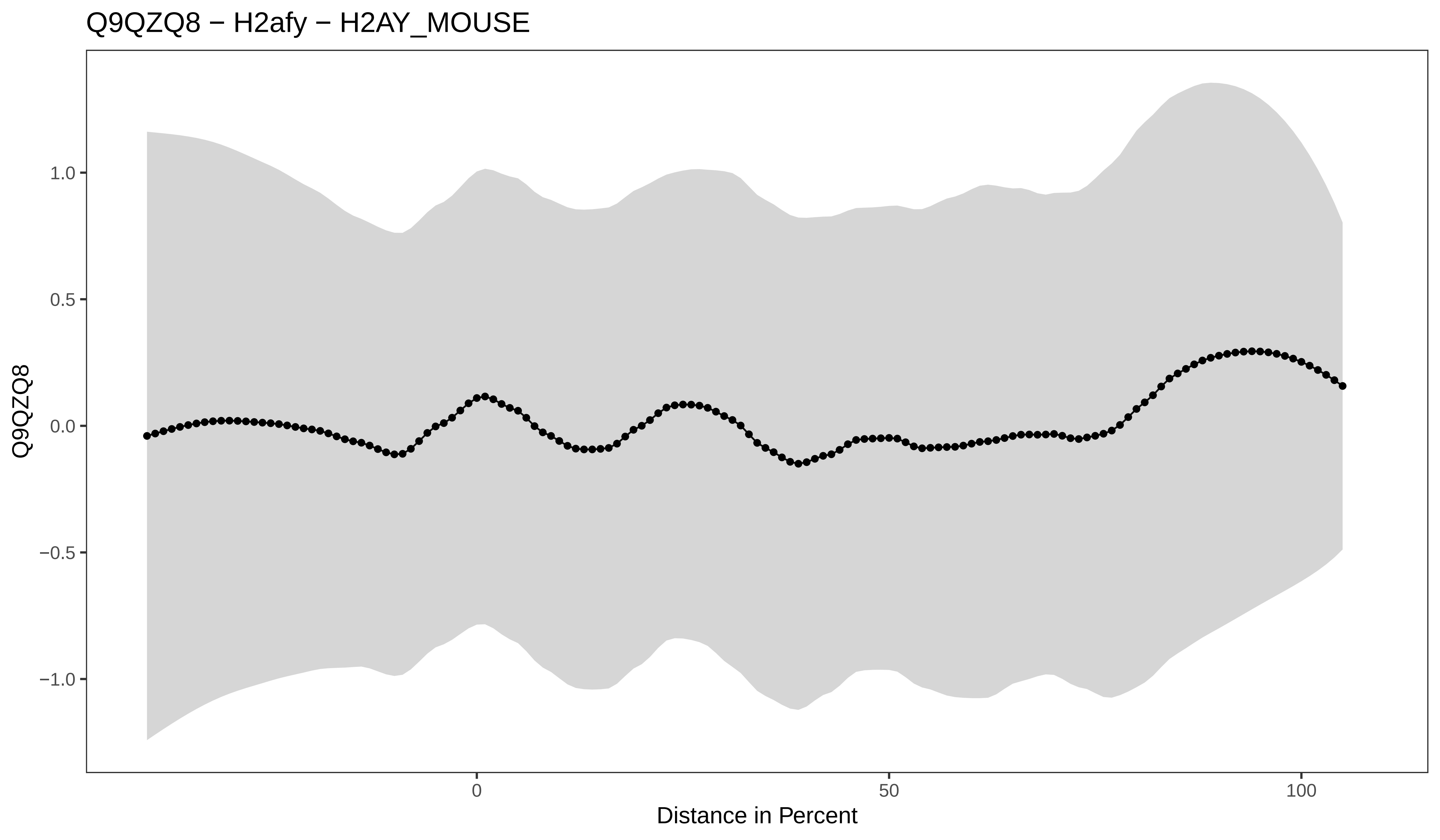
Q9QZQ8 − H2afy − H2AY_MOUSE
Q9QZQ8
1.0
0.5
0.0
−0.5
−1.0
0
50
100
Distance in Percent

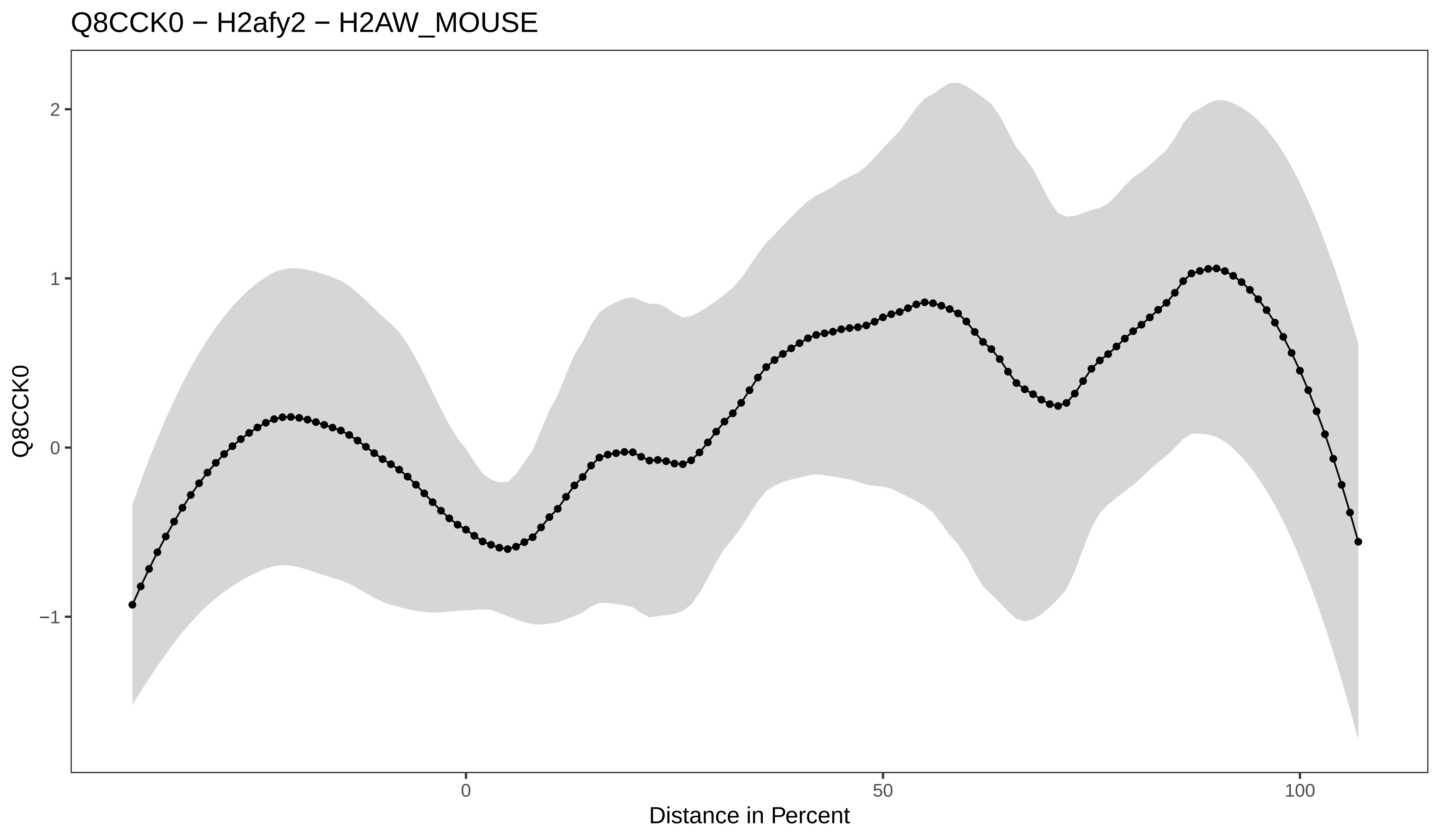
Q8CCK0 − H2afy2 − H2AW_MOUSE
Q8CCK0
2
1
0
−1
0
50
100
Distance in Percent

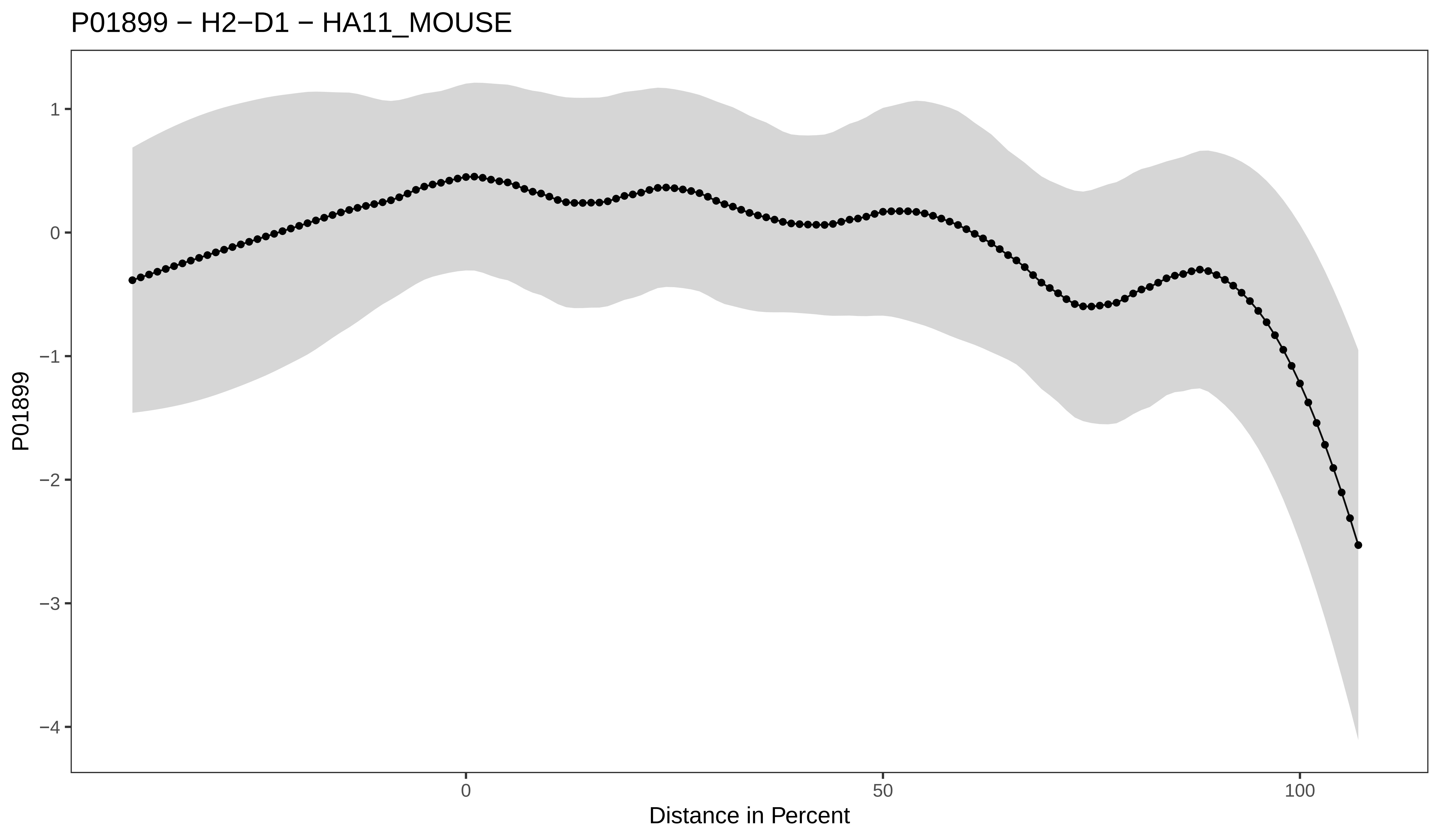
P01899 − H2−D1 − HA11_MOUSE
P01899
1
0
−1
−2
−3
−4
0
50
100
Distance in Percent

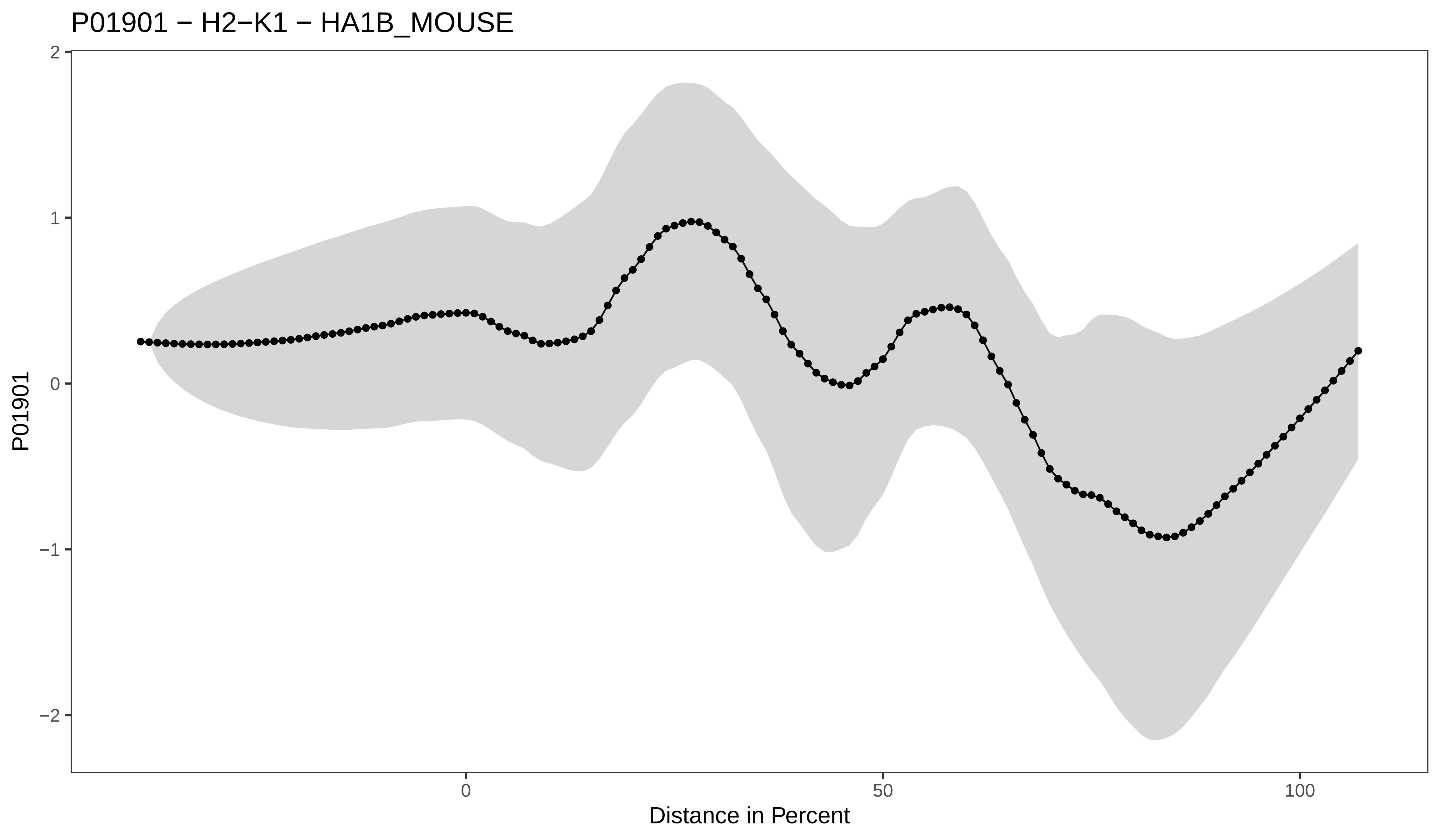
P01901 − H2−K1 − HA1B_MOUSE
2
1
0
−1
−2
P01901
0
50
100
Distance in Percent

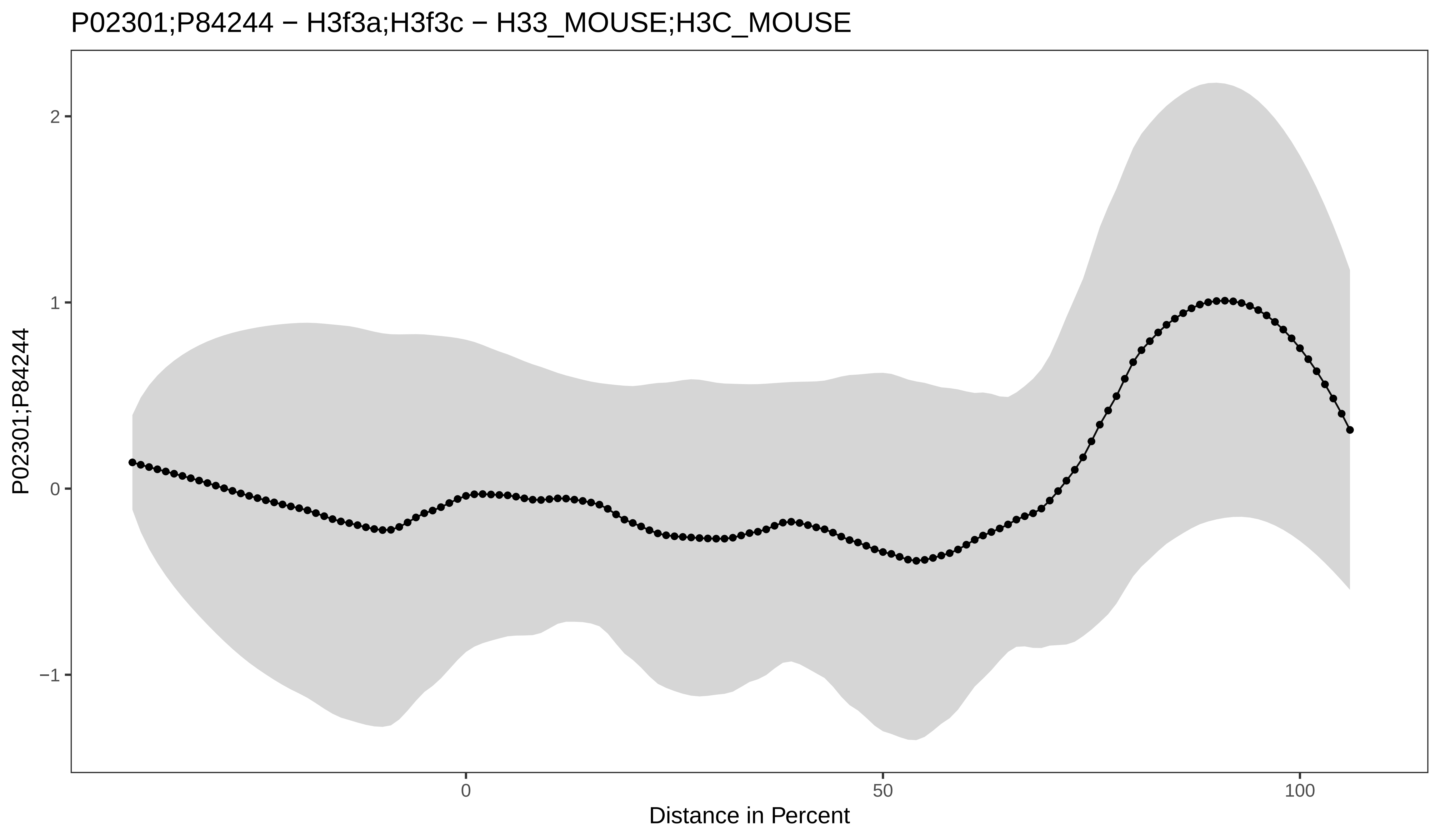

P02301;P84244 − H3f3a;H3f3c − H33_MOUSE;H3C_MOUSE
P02301;P84244
2
1
0
−1
0
50
100
Distance in Percent

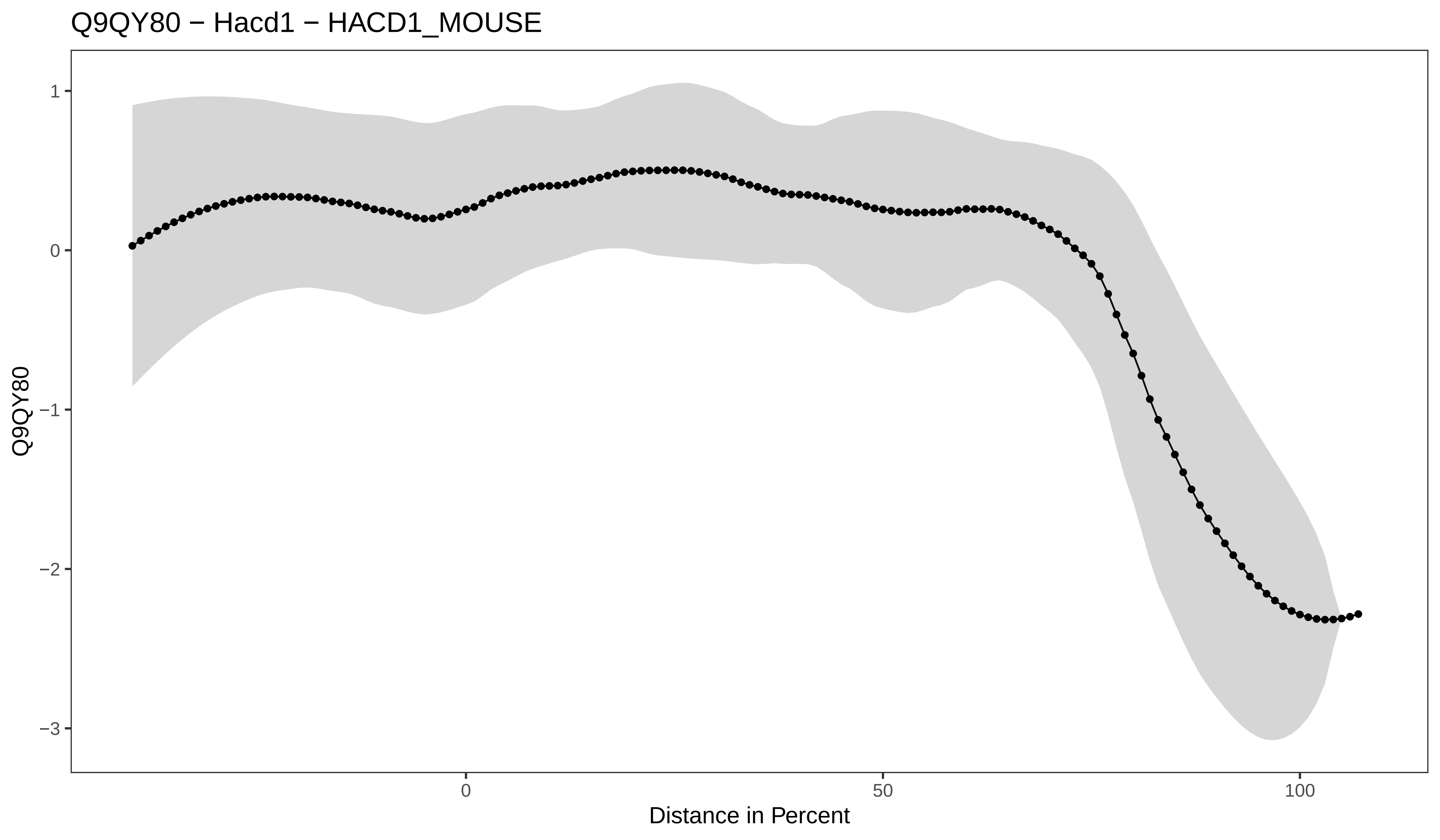
Q9QY80 − Hacd1 − HACD1_MOUSE
1
0
−1
−2
−3
Q9QY80
0
50
100
Distance in Percent

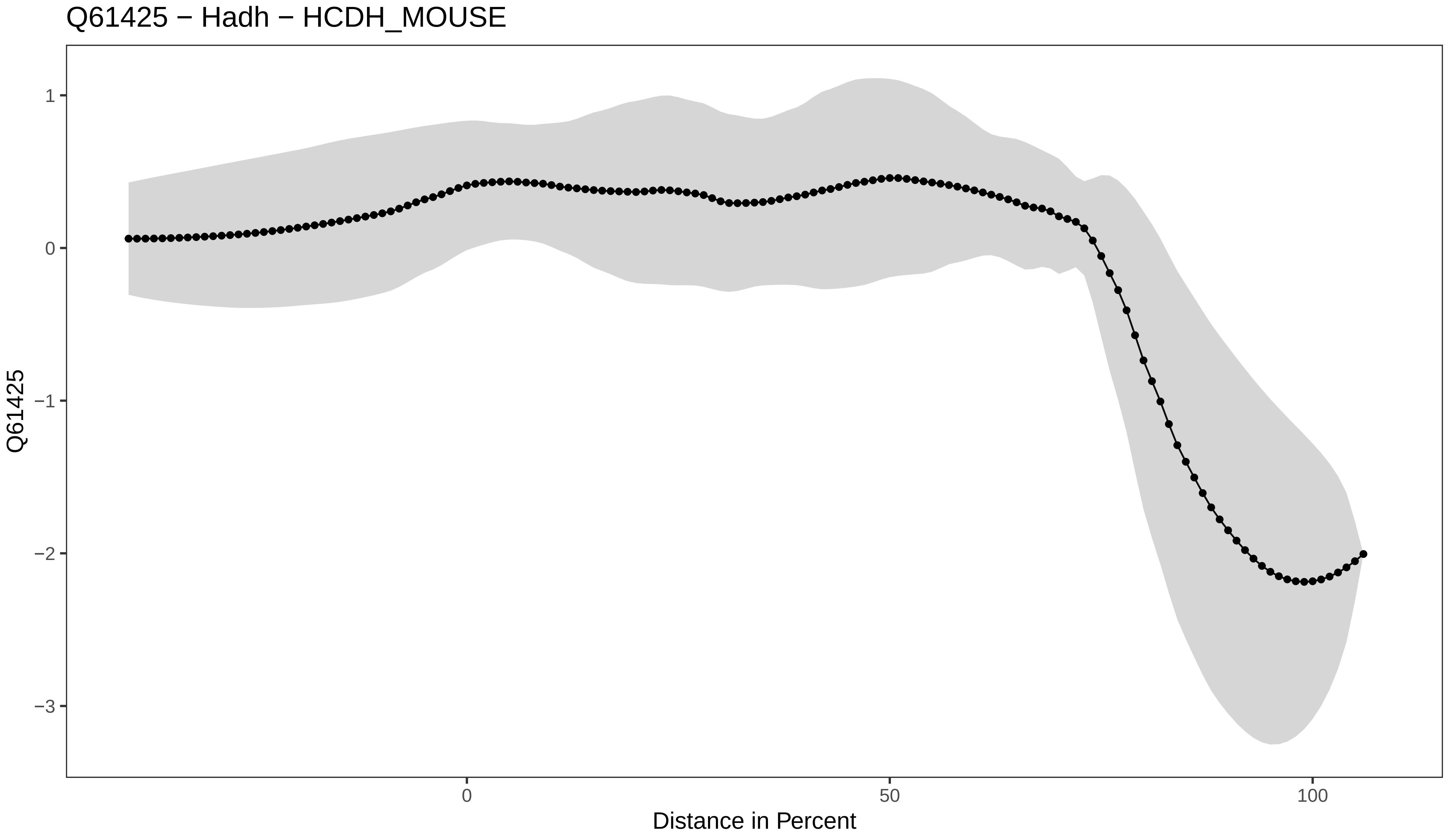

Q61425 − Hadh − HCDH_MOUSE
Q61425
1
0
−1
−2
−3
0
50
100
Distance in Percent

Q8BMS1 − Hadha − ECHA_MOUSE

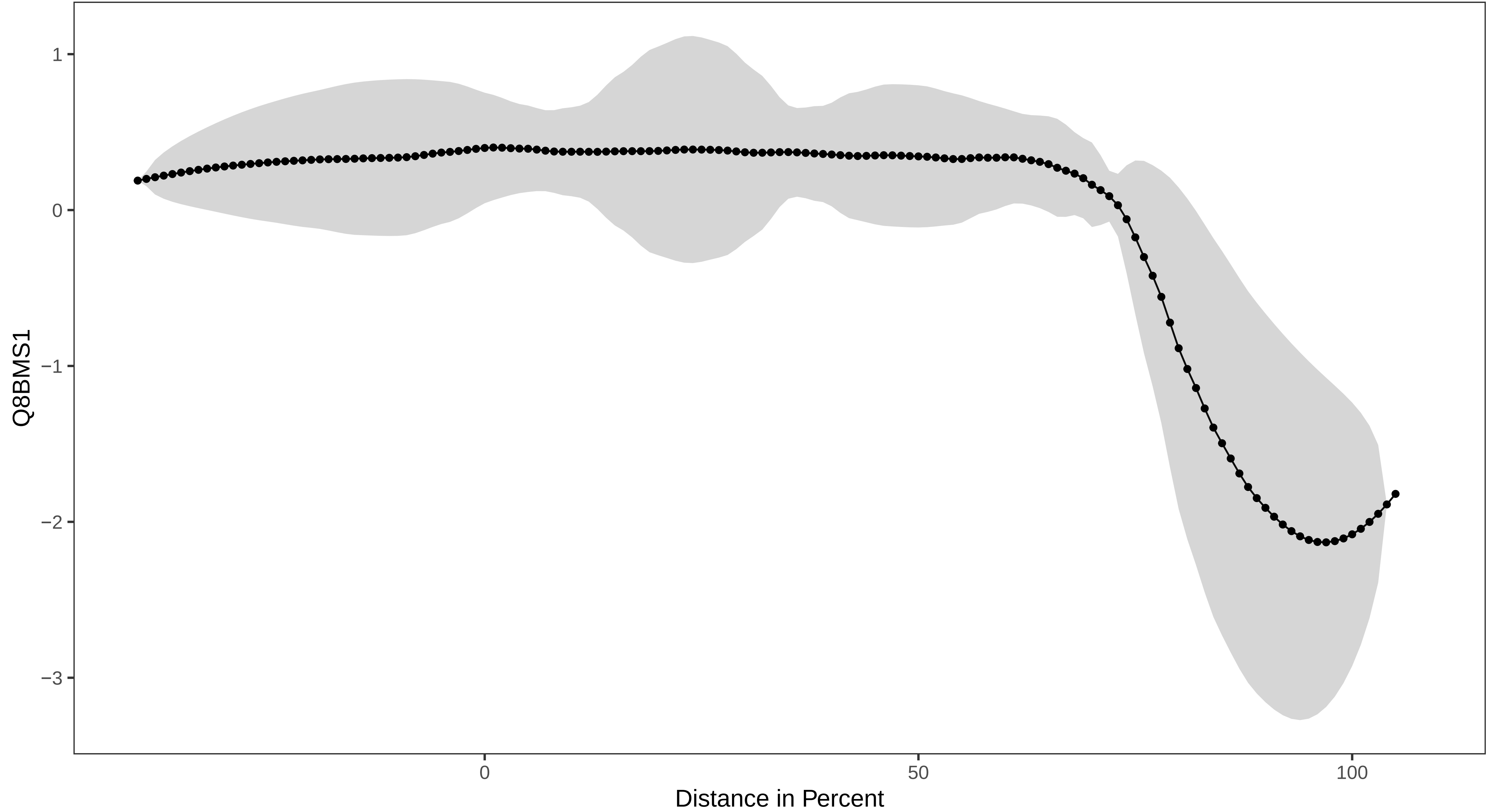

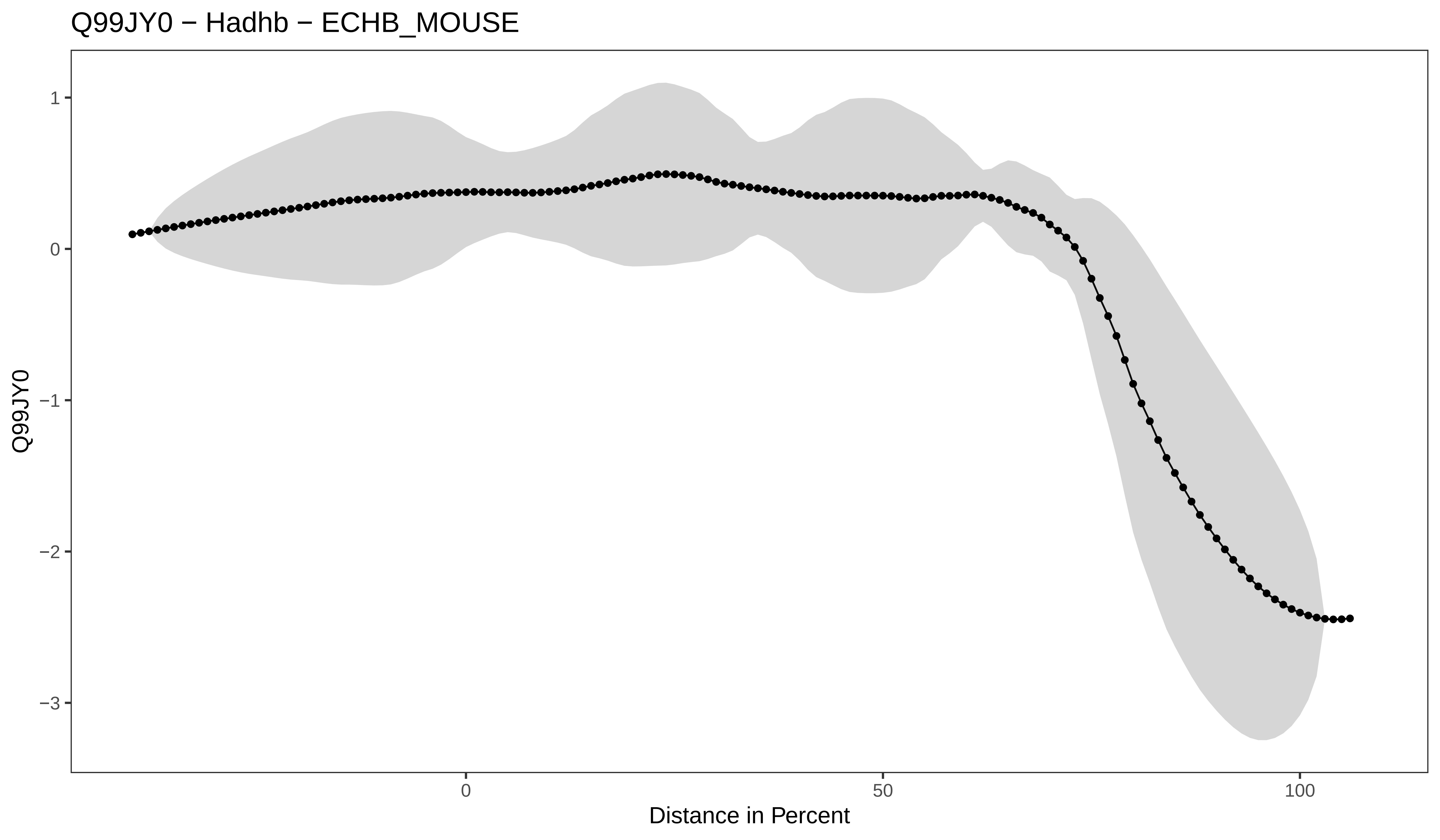

Q99JY0 − Hadhb − ECHB_MOUSE
1
0
−1
−2
−3
Q99JY0
0
50
100
Distance in Percent

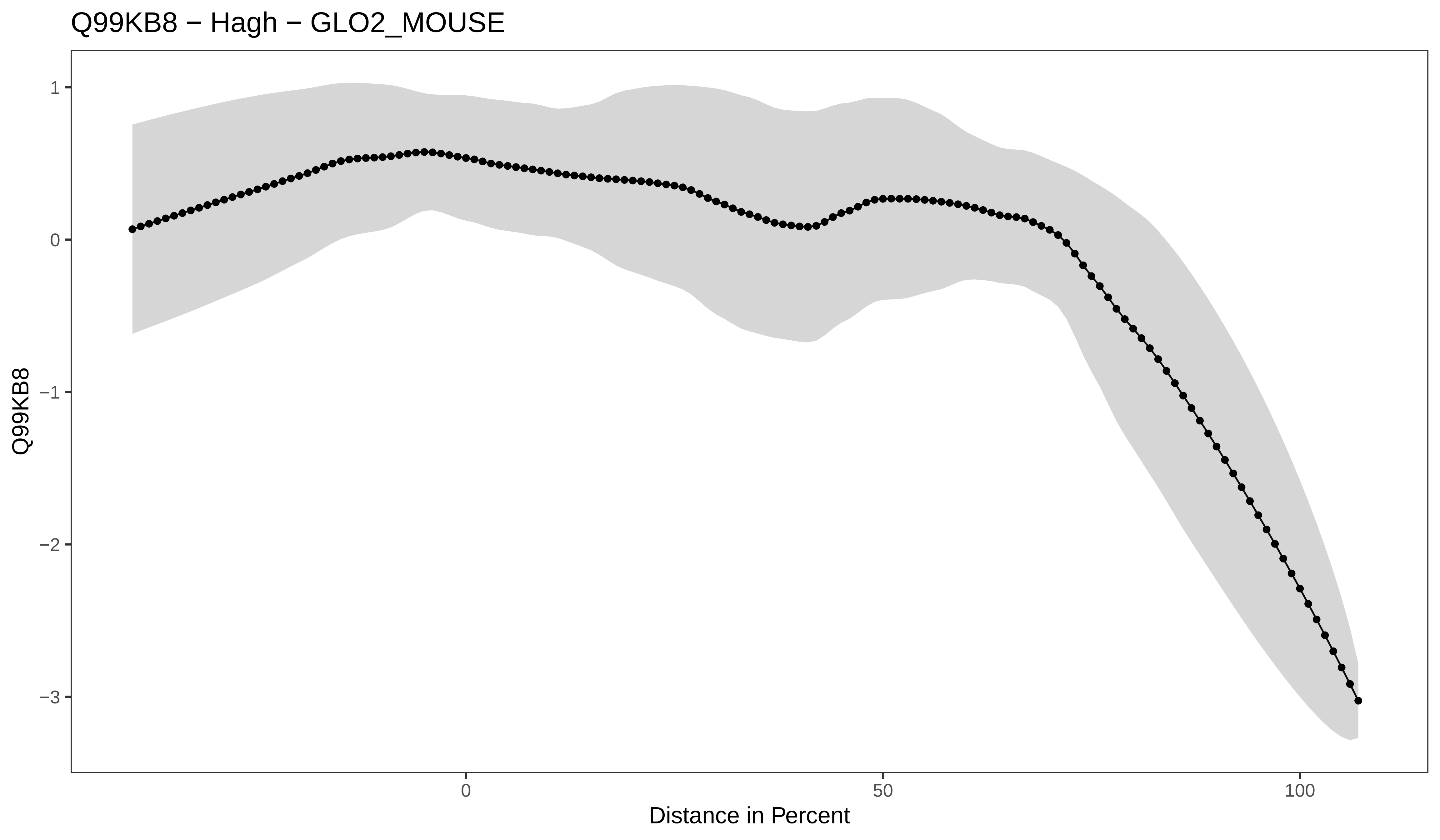

Q99KB8 − Hagh − GLO2_MOUSE
Q99KB8
Distance in Percent
1
0
−1
−2
−3
0
50
100

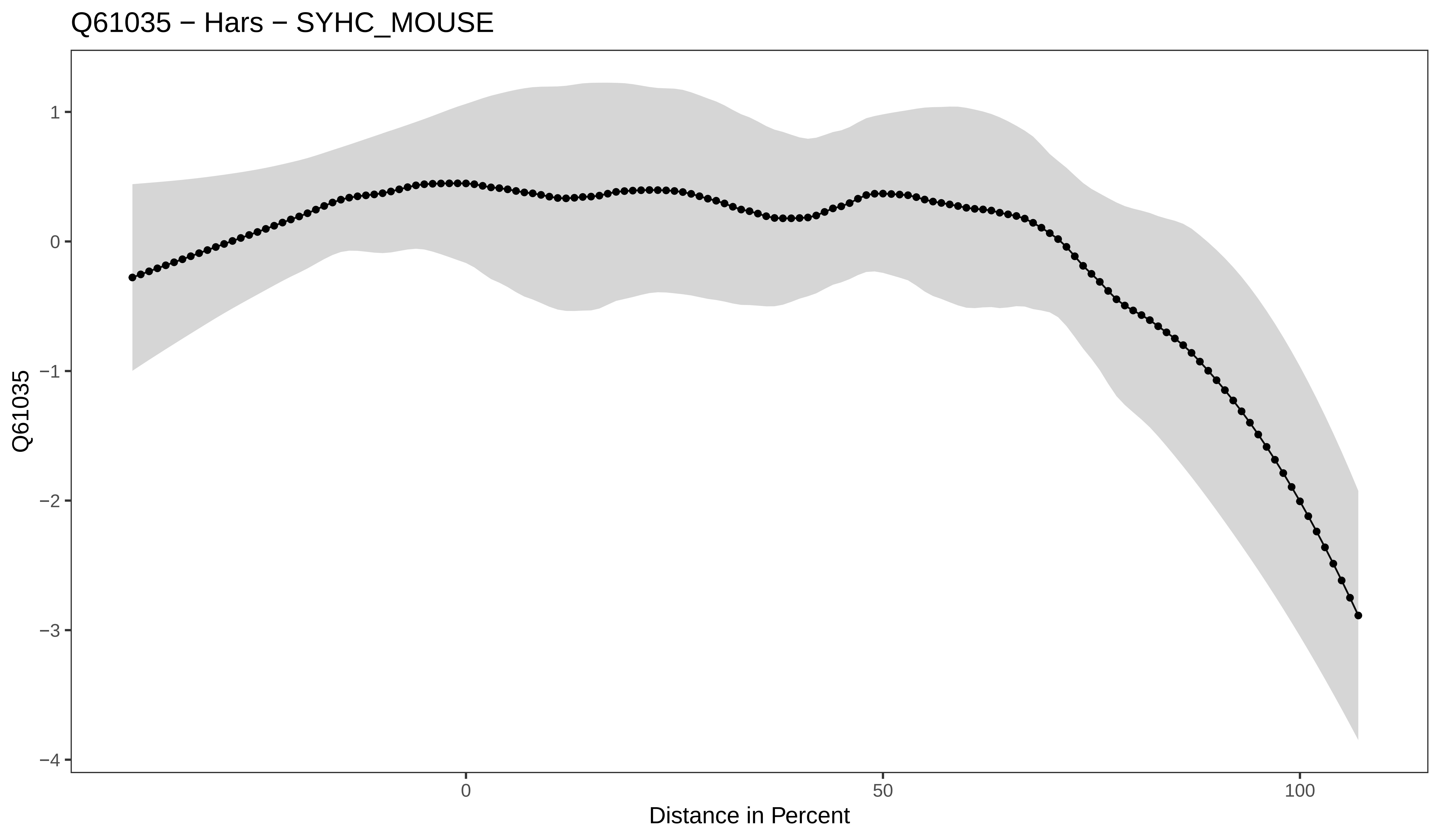

Q61035 − Hars − SYHC_MOUSE
Q61035
1
0
−1
−2
−3
−4
0
50
100
Distance in Percent

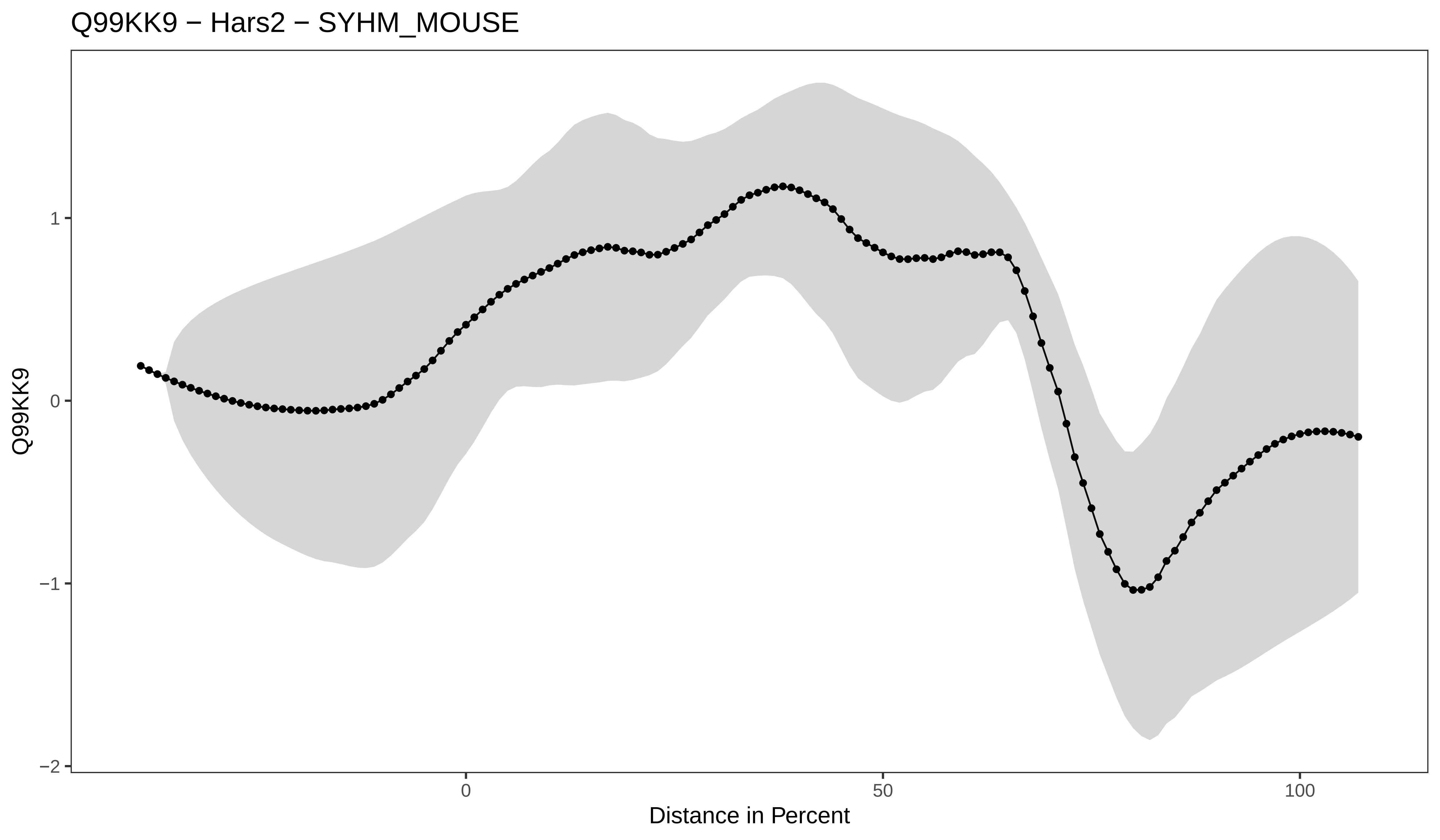

Q99KK9 − Hars2 − SYHM_MOUSE
Q99KK9
1
0
−1
−2
0
50
100
Distance in Percent

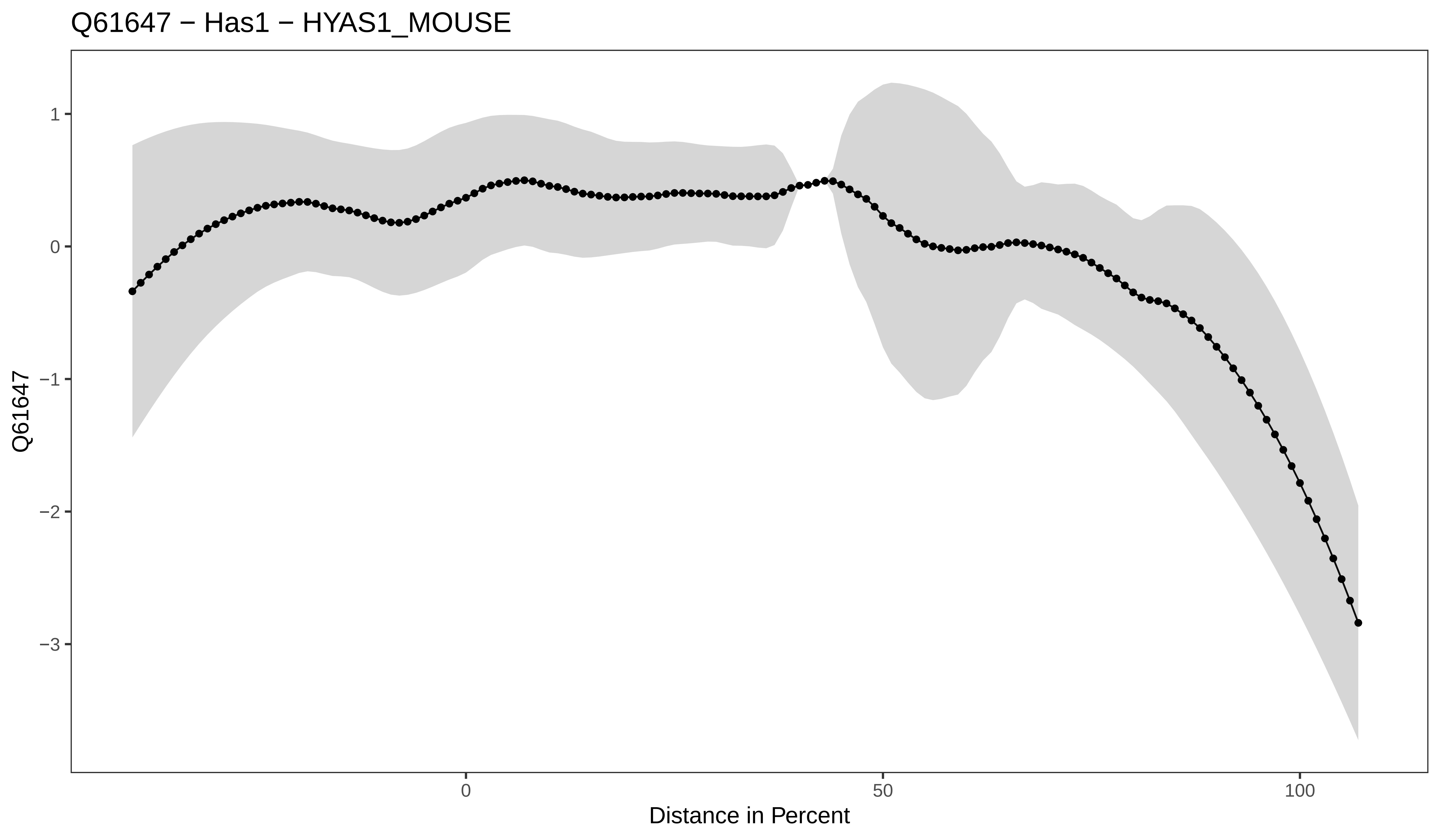
Q61647 − Has1 − HYAS1_MOUSE
1
0
−1
−2
−3
Q61647
0
50
100
Distance in Percent

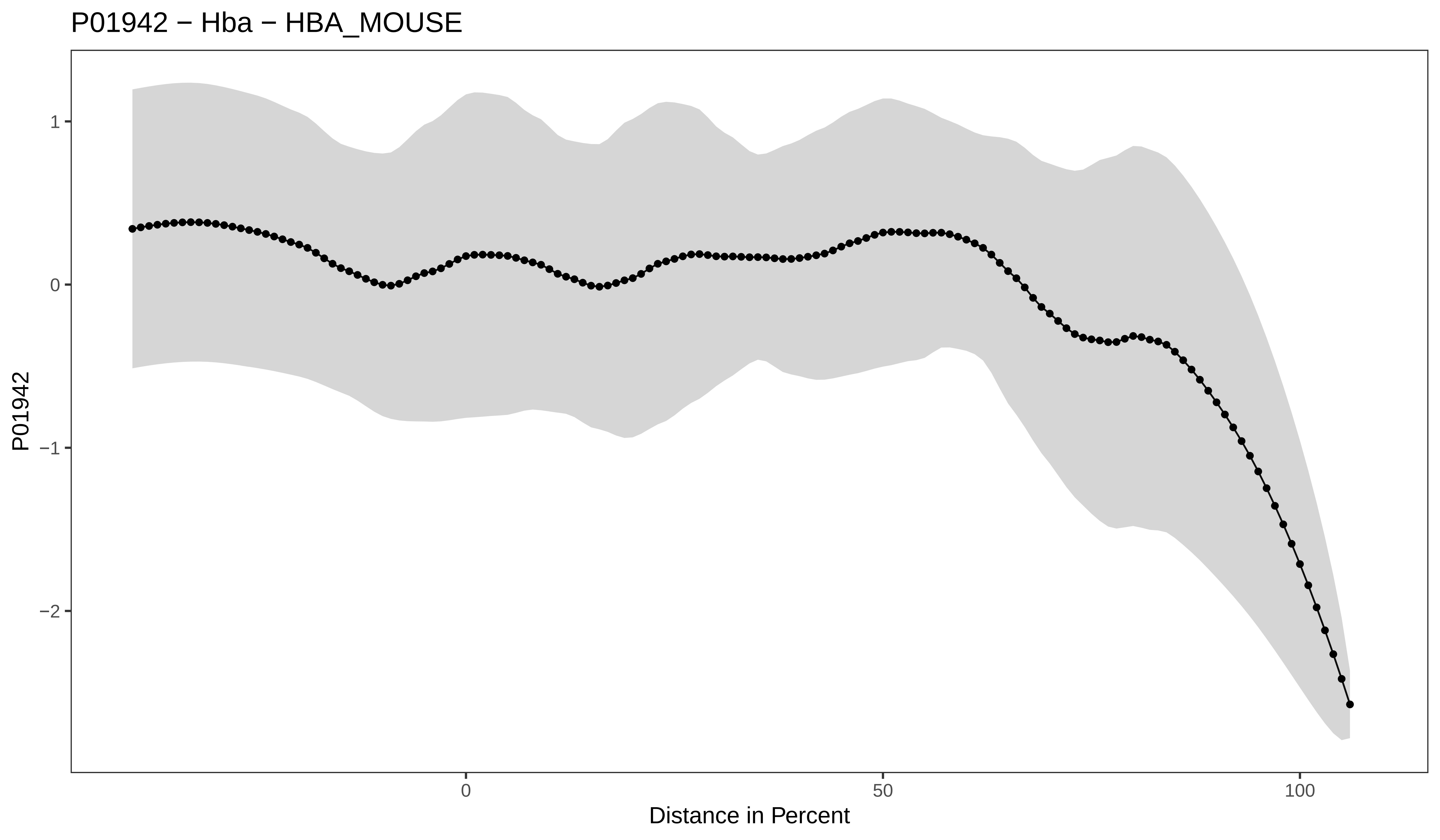

P01942 − Hba − HBA_MOUSE
P01942
1
0
−1
−2
0
50
100
Distance in Percent

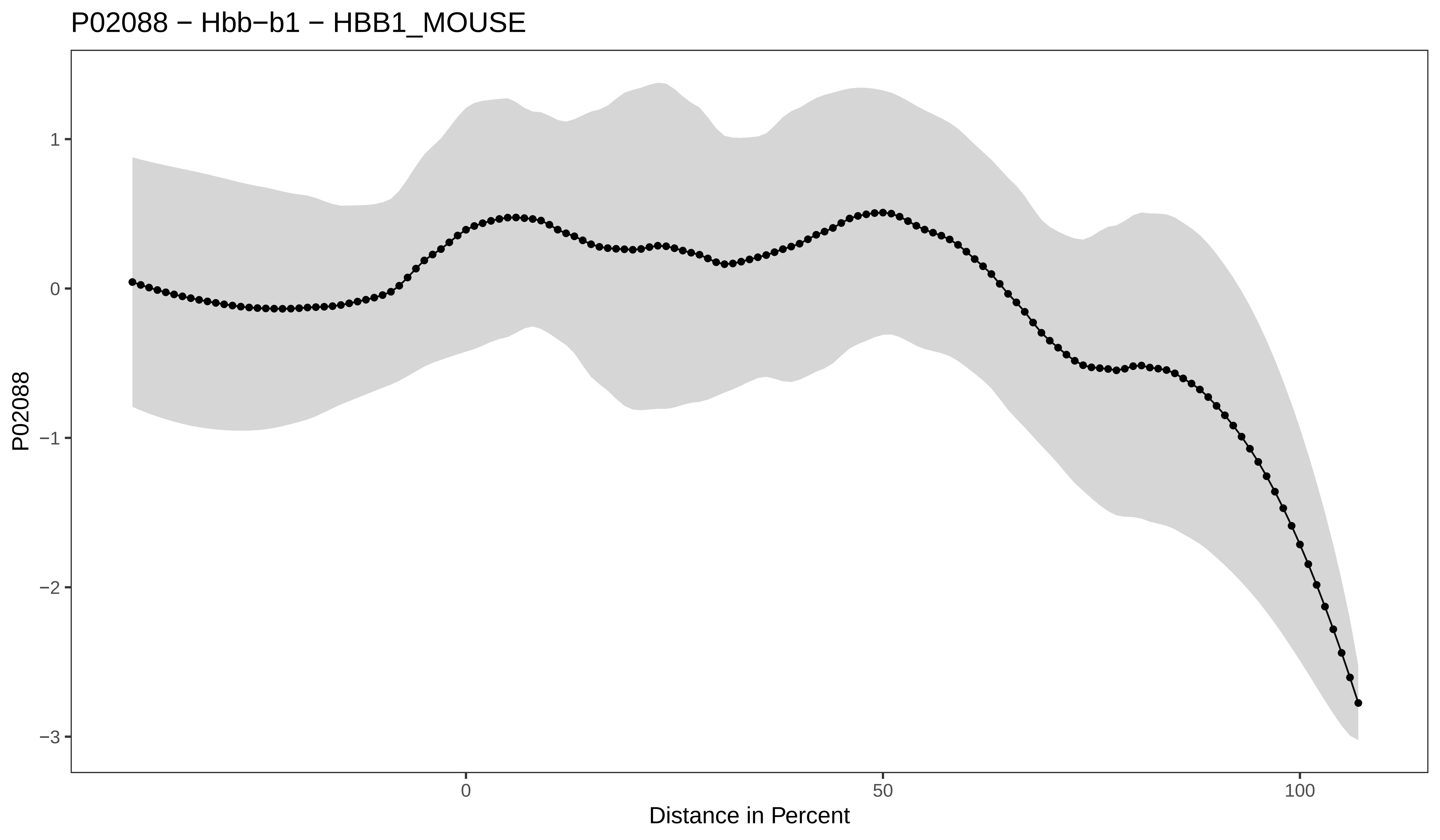

P02088 − Hbb−b1 − HBB1_MOUSE
P02088
1
0
−1
−2
−3
0
50
100
Distance in Percent

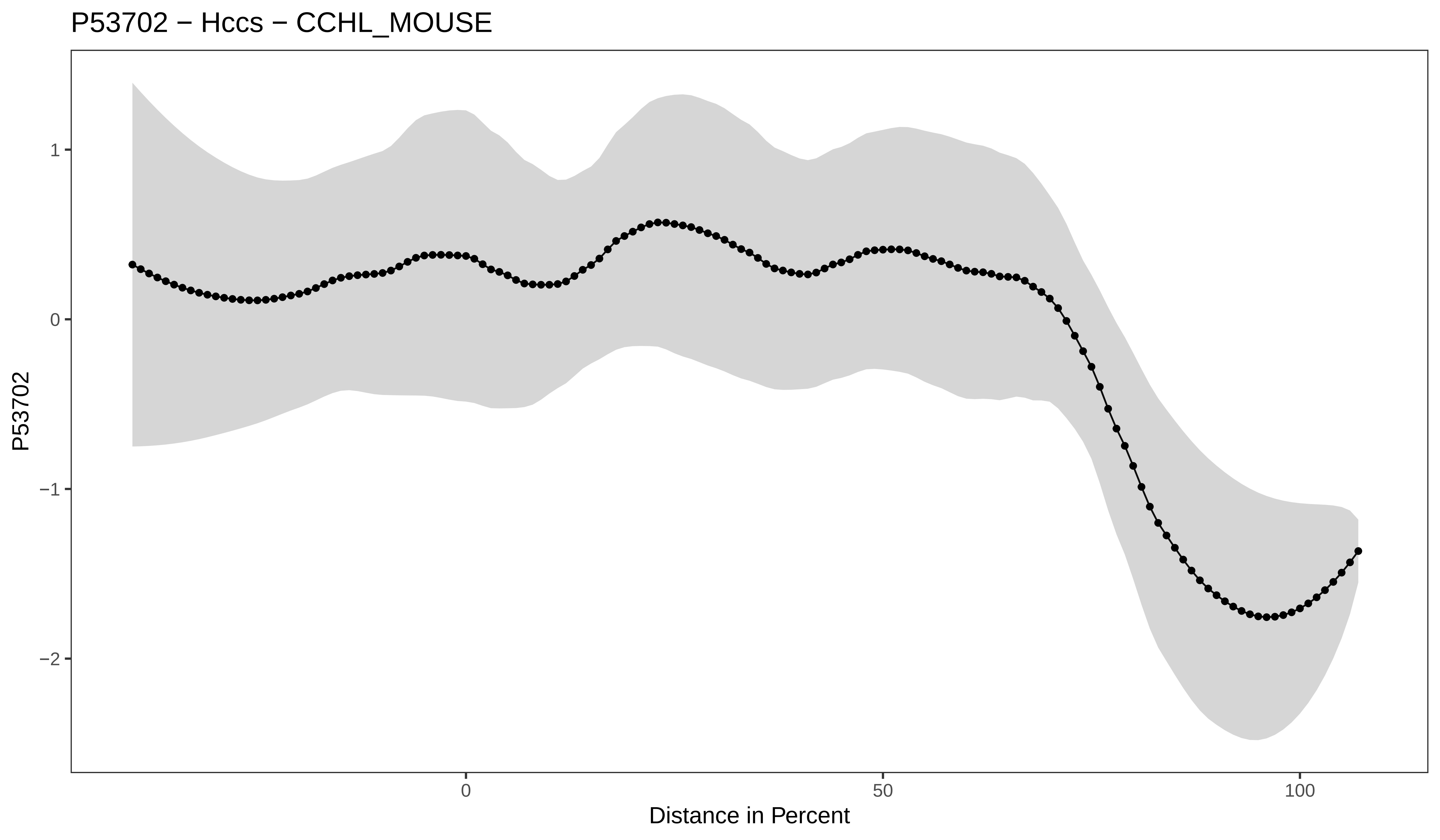

P53702 − Hccs − CCHL_MOUSE
1
0
−1
−2
P53702
0
50
100
Distance in Percent

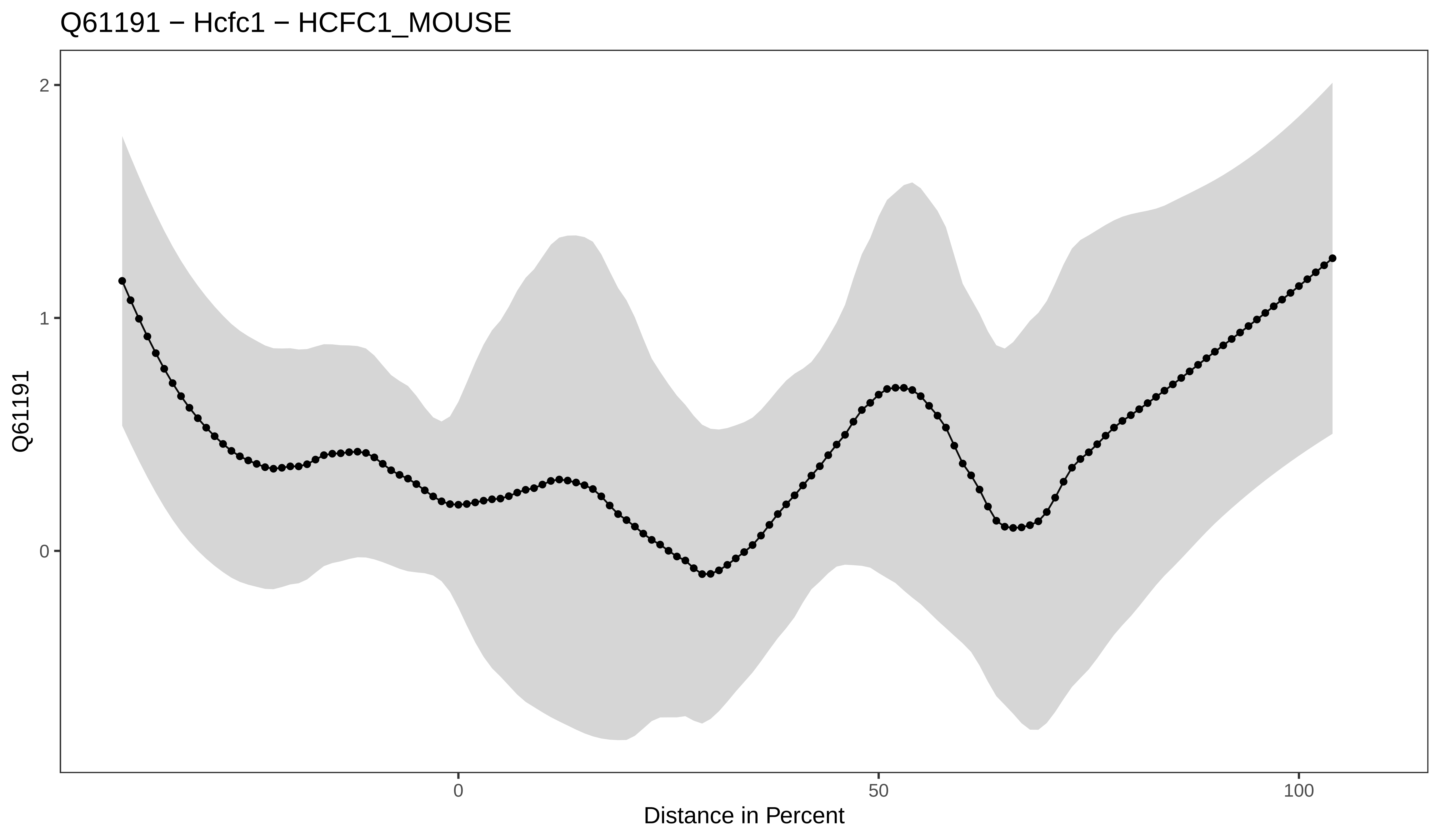
Q61191 − Hcfc1 − HCFC1_MOUSE
Q61191
2
1
0
0
50
100
Distance in Percent

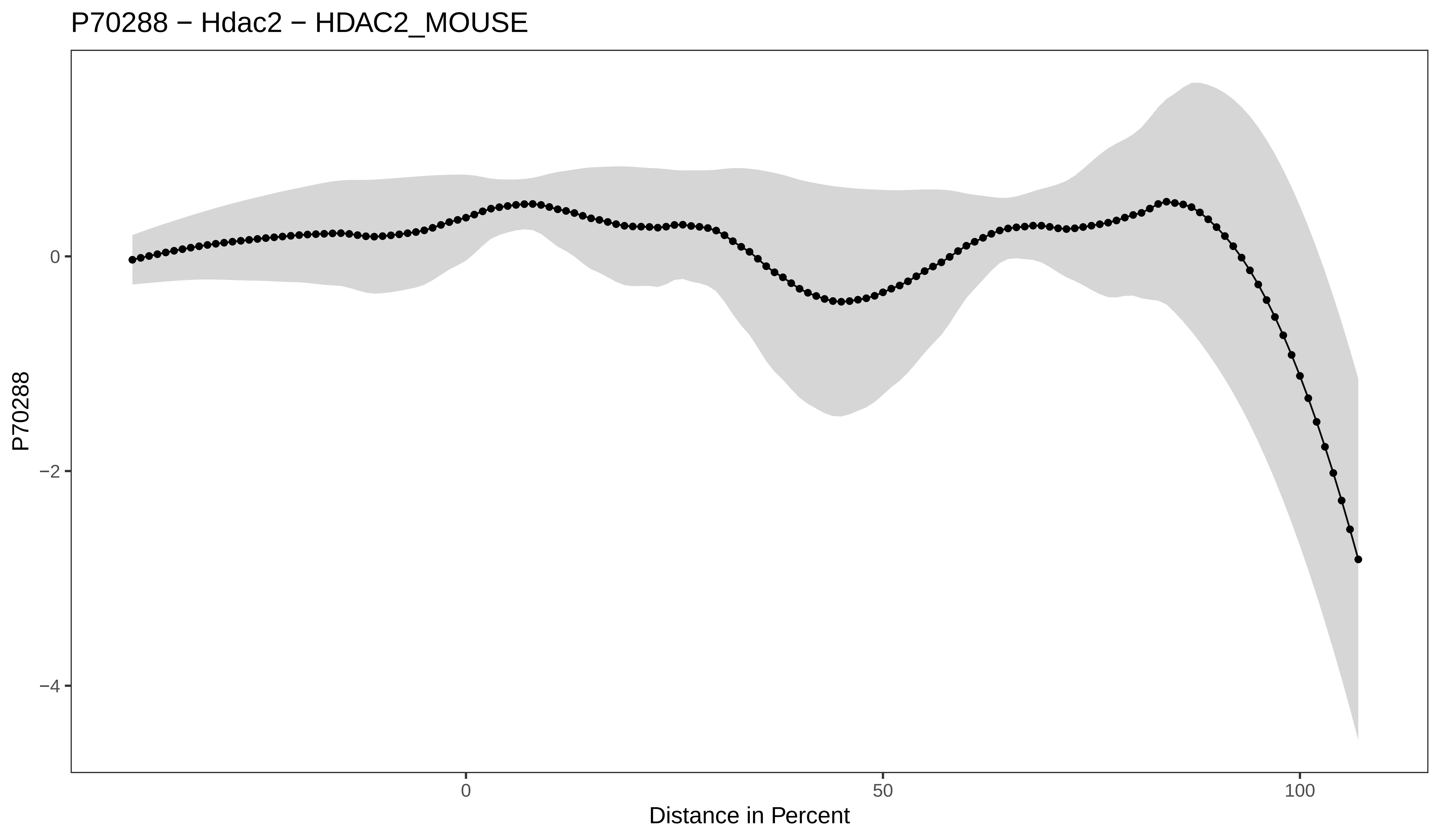
P70288 − Hdac2 − HDAC2_MOUSE
P70288
0
−2
−4
0
50
100
Distance in Percent

P51859 − Hdgf − HDGF_MOUSE

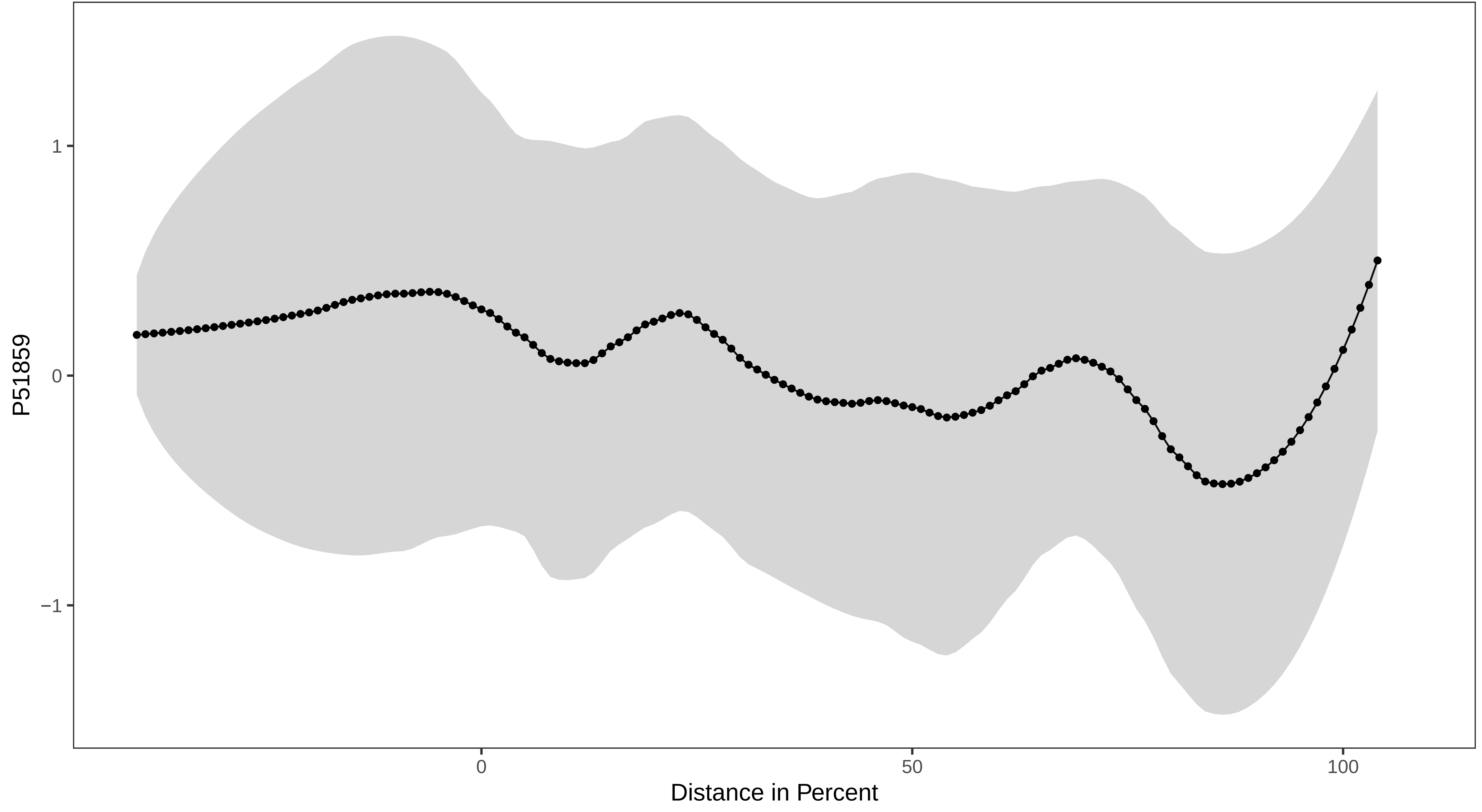

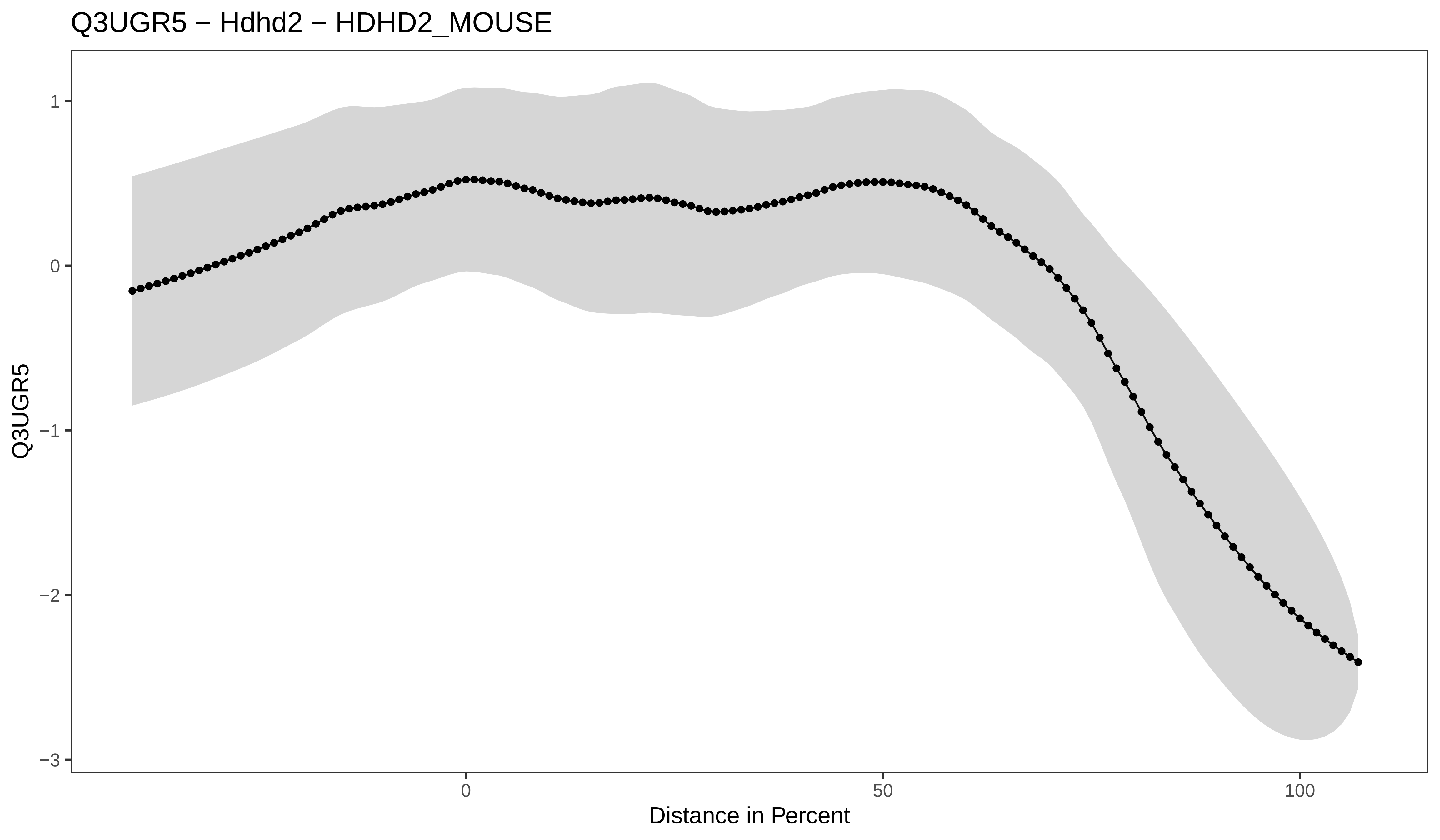

Q3UGR5 − Hdhd2 − HDHD2_MOUSE
1
0
−1
−2
−3
Q3UGR5
0
50
100
Distance in Percent

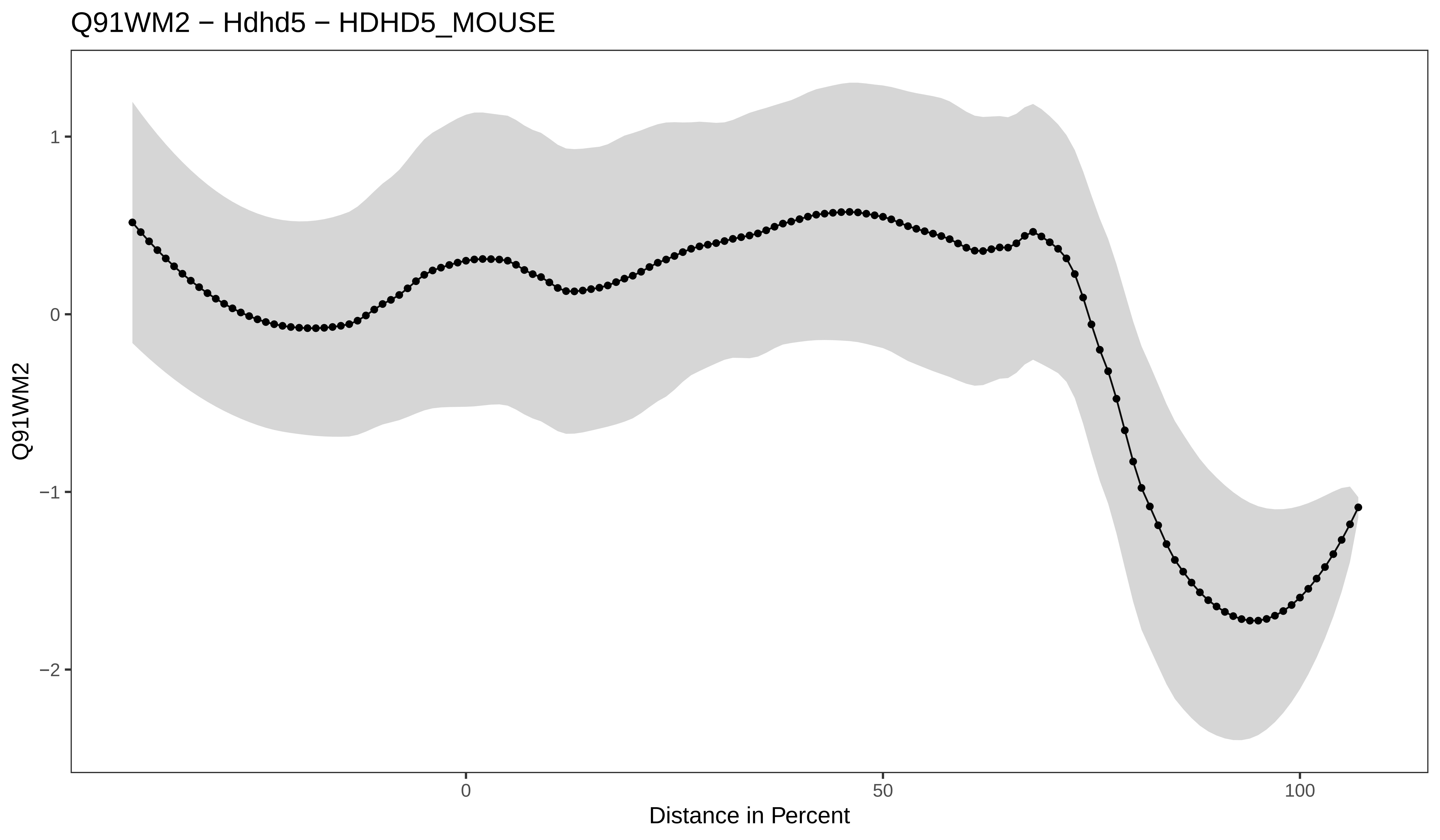
Q91WM2 − Hdhd5 − HDHD5_MOUSE
Q91WM2
Distance in Percent
1
0
−1
−2
0
50
100

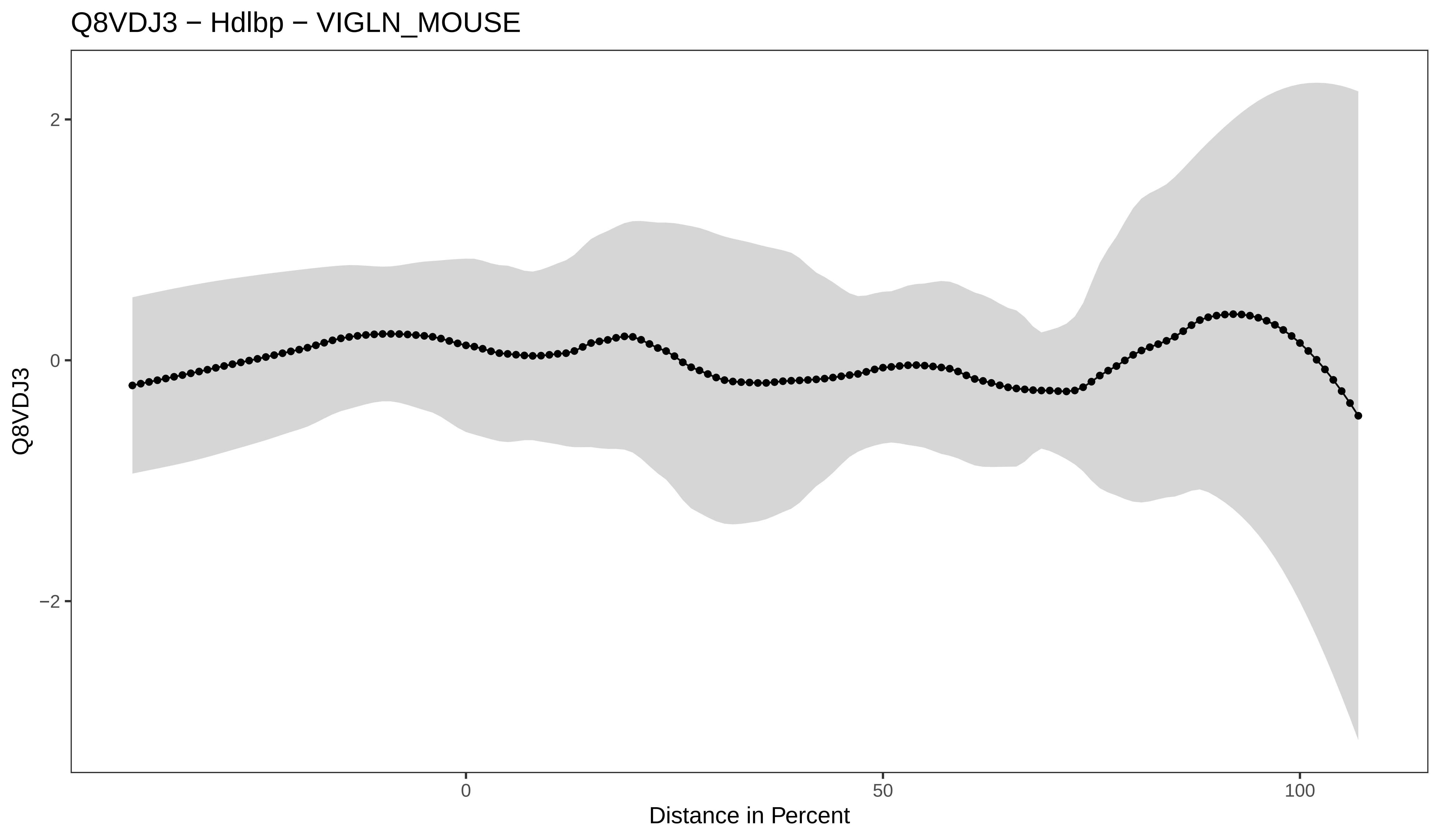
Q8VDJ3 − Hdlbp − VIGLN_MOUSE
Q8VDJ3
2
0
−2
0
50
100
Distance in Percent

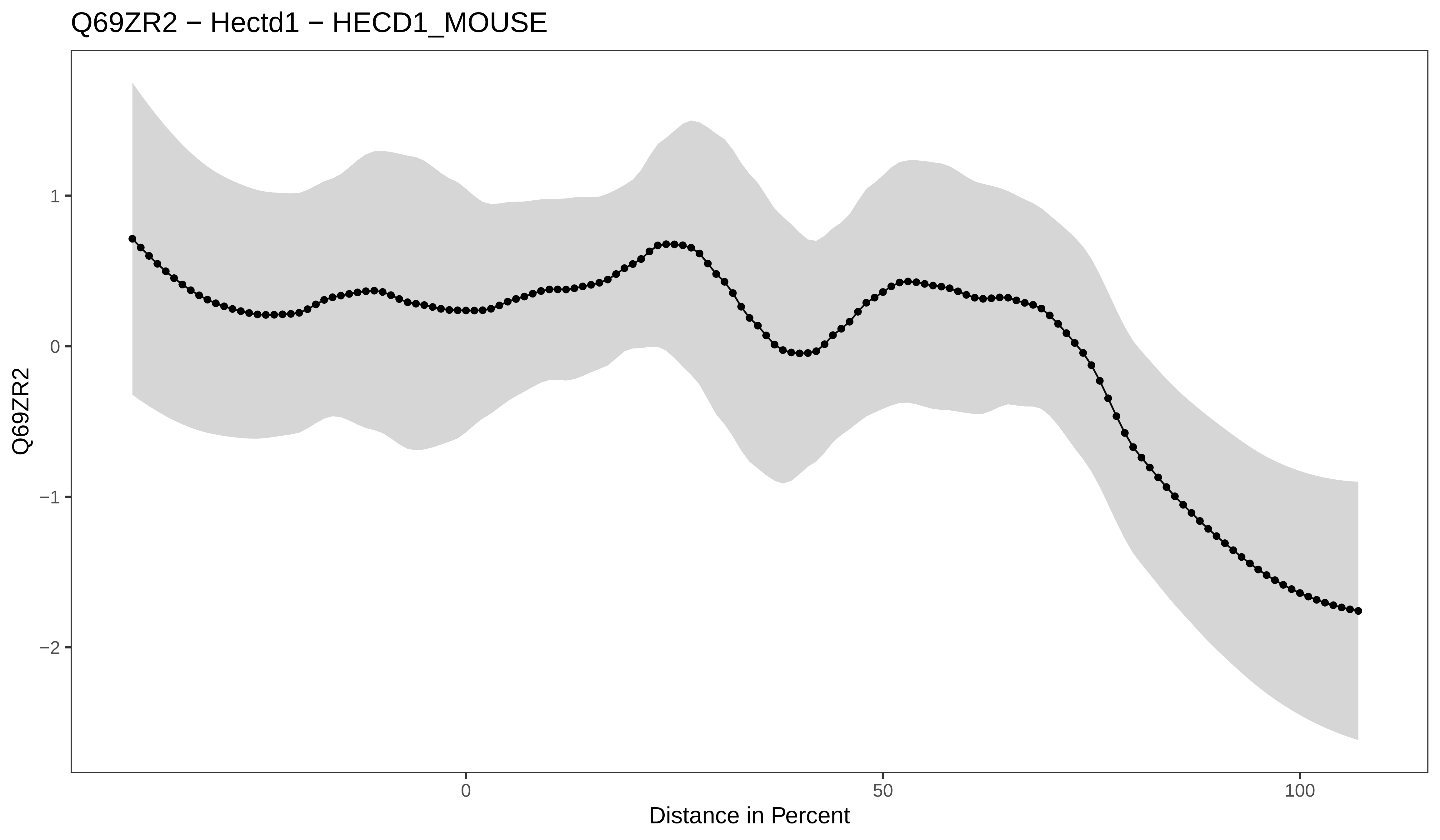
Q69ZR2 − Hectd1 − HECD1_MOUSE
Q69ZR2
Distance in Percent
1
0
−1
−2
0
50
100

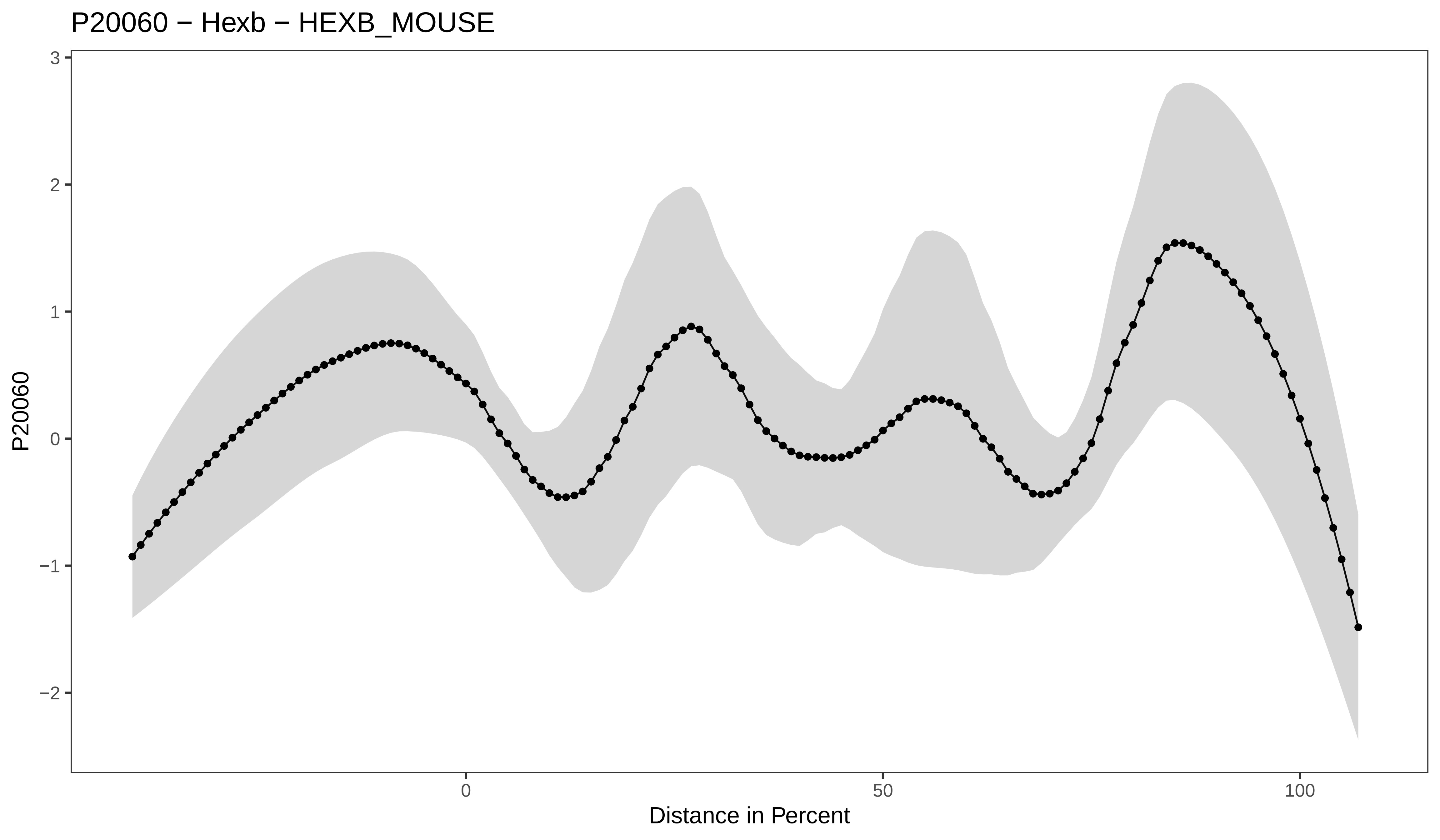

P20060 − Hexb − HEXB_MOUSE
3
2
1
0
−1
−2
P20060
0
50
100
Distance in Percent

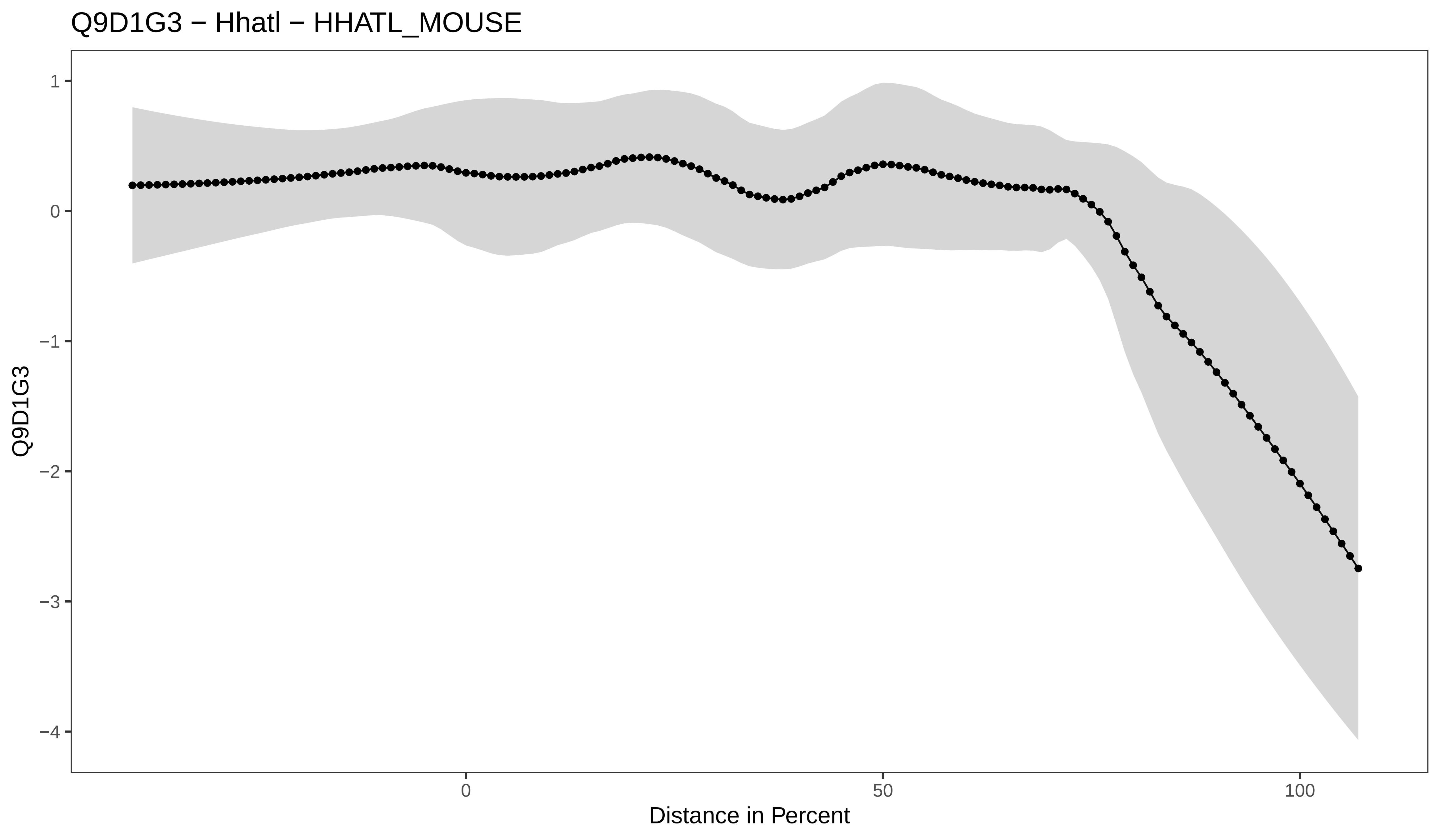
Q9D1G3 − Hhatl − HHATL_MOUSE
Q9D1G3
1
0
−1
−2
−3
−4
0
50
100
Distance in Percent

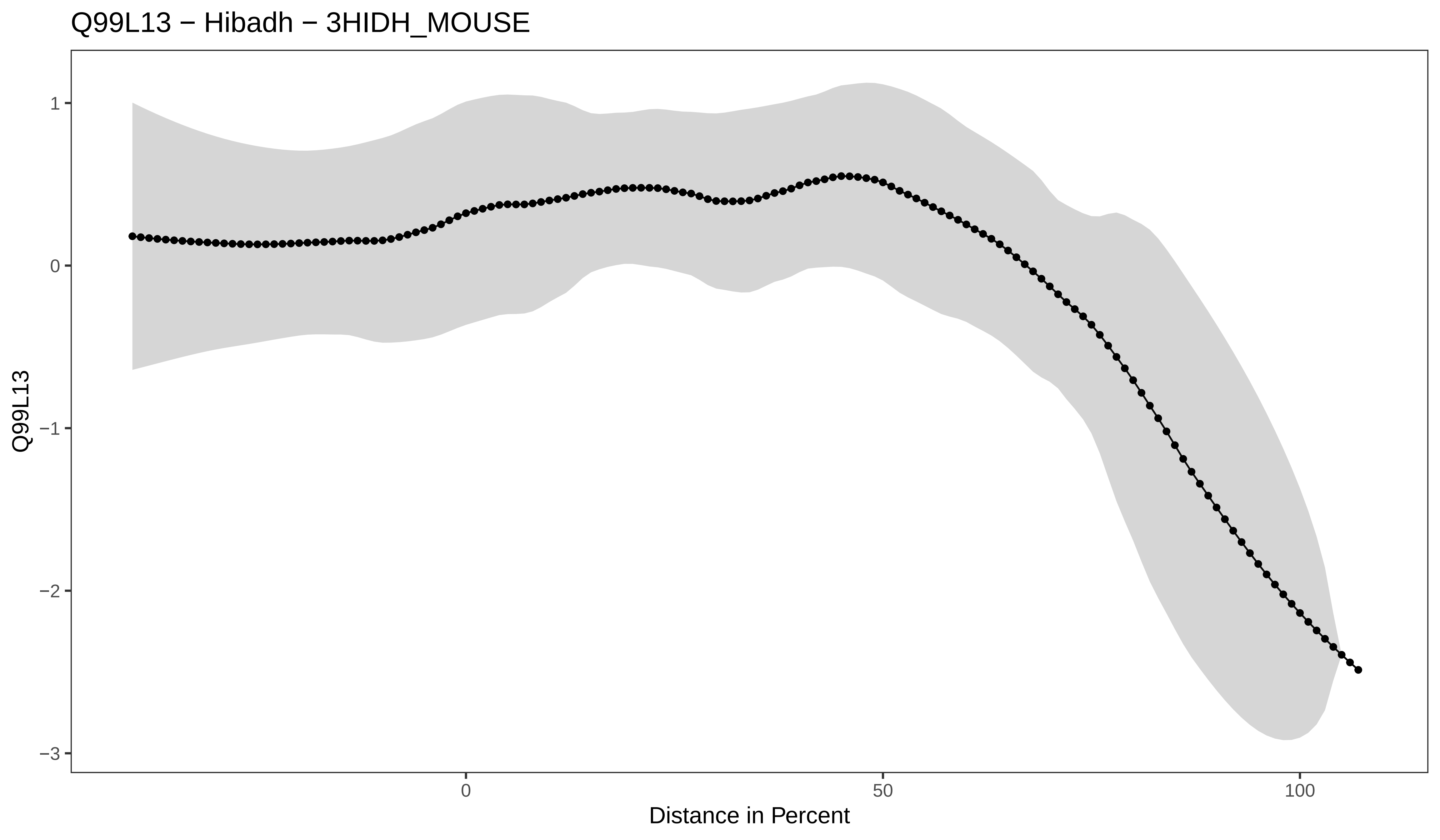

Q99L13 − Hibadh − 3HIDH_MOUSE
Q99L13
1
0
−1
−2
−3
0
50
100
Distance in Percent

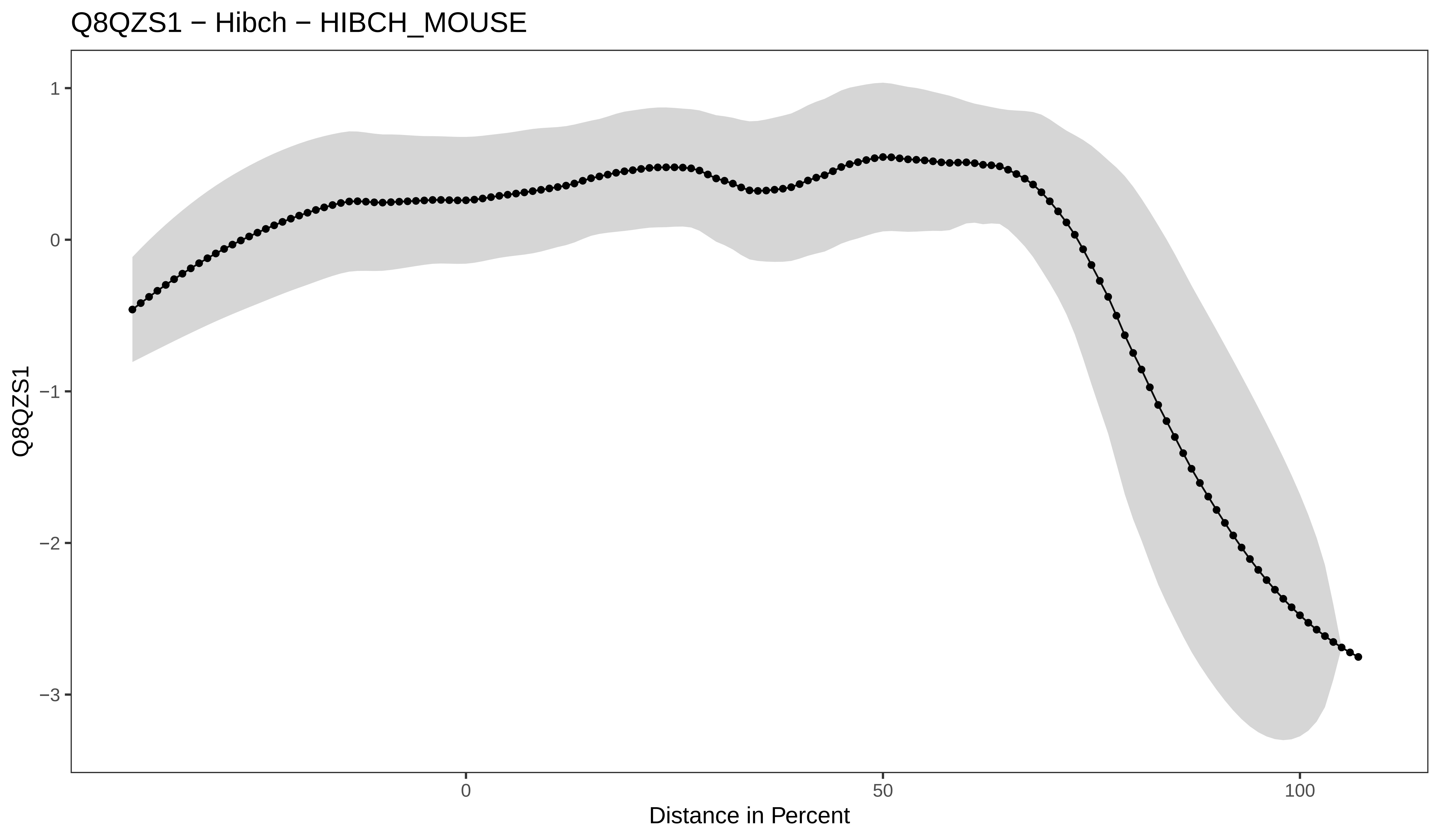

Q8QZS1 − Hibch − HIBCH_MOUSE
Q8QZS1
Distance in Percent
1
0
−1
−2
−3
0
50
100

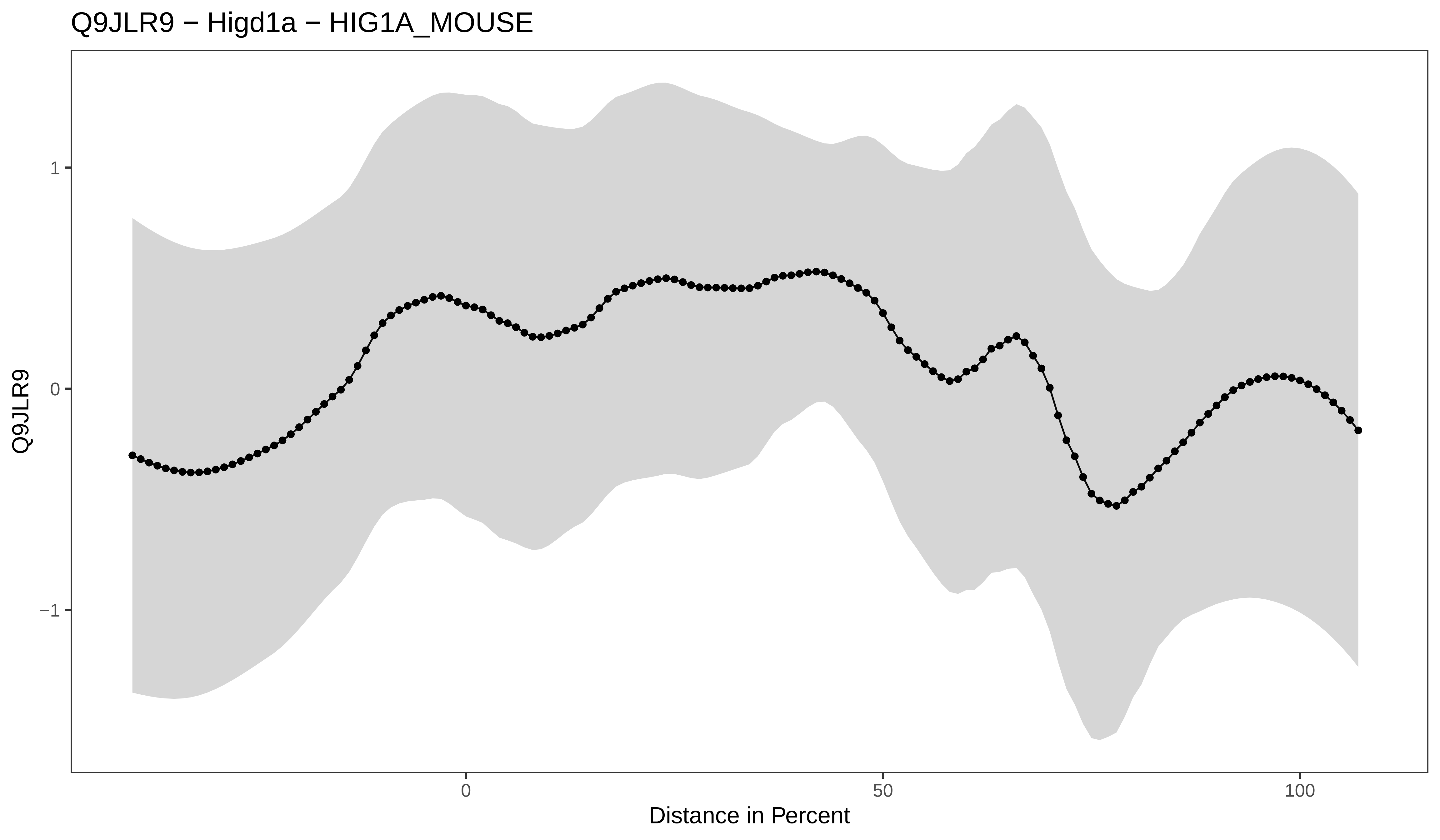

Q9JLR9 − Higd1a − HIG1A_MOUSE
1
0
−1
Q9JLR9
0
50
100
Distance in Percent

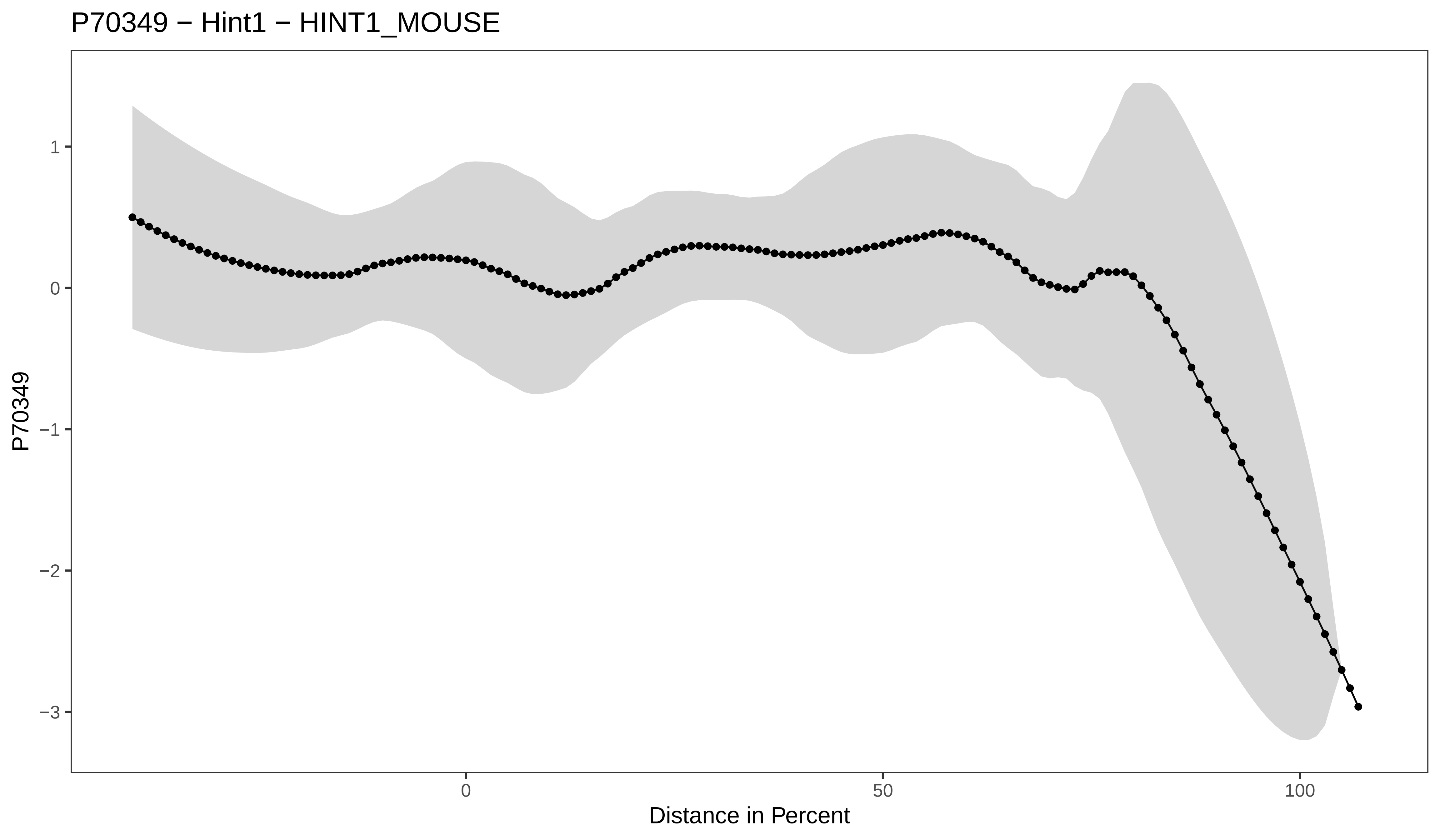

P70349 − Hint1 − HINT1_MOUSE
P70349
1
0
−1
−2
−3
0
50
100
Distance in Percent

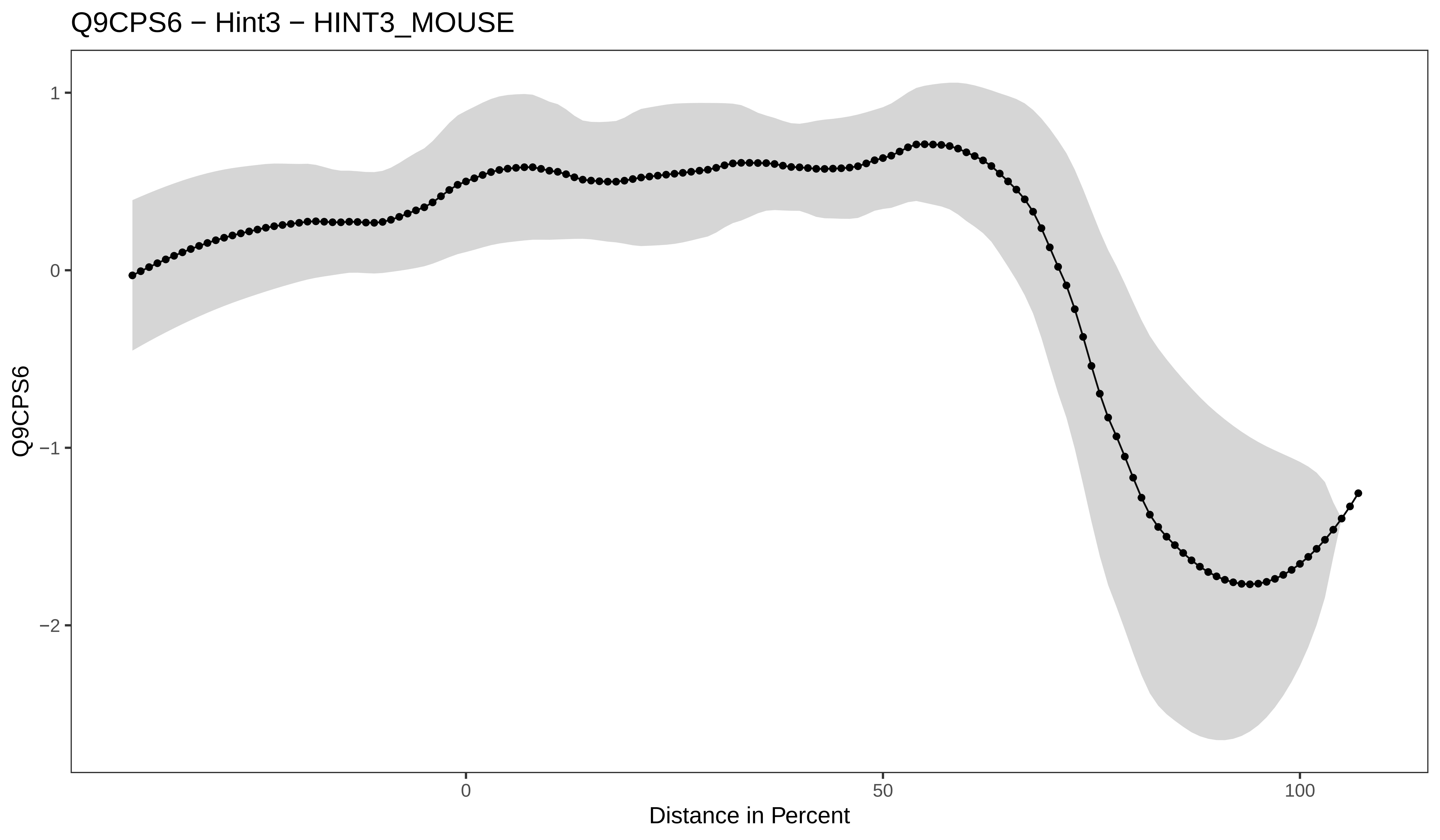
Q9CPS6 − Hint3 − HINT3_MOUSE
Q9CPS6
1
0
−1
−2
0
50
100
Distance in Percent

P43276 − Hist1h1b − H15_MOUSE

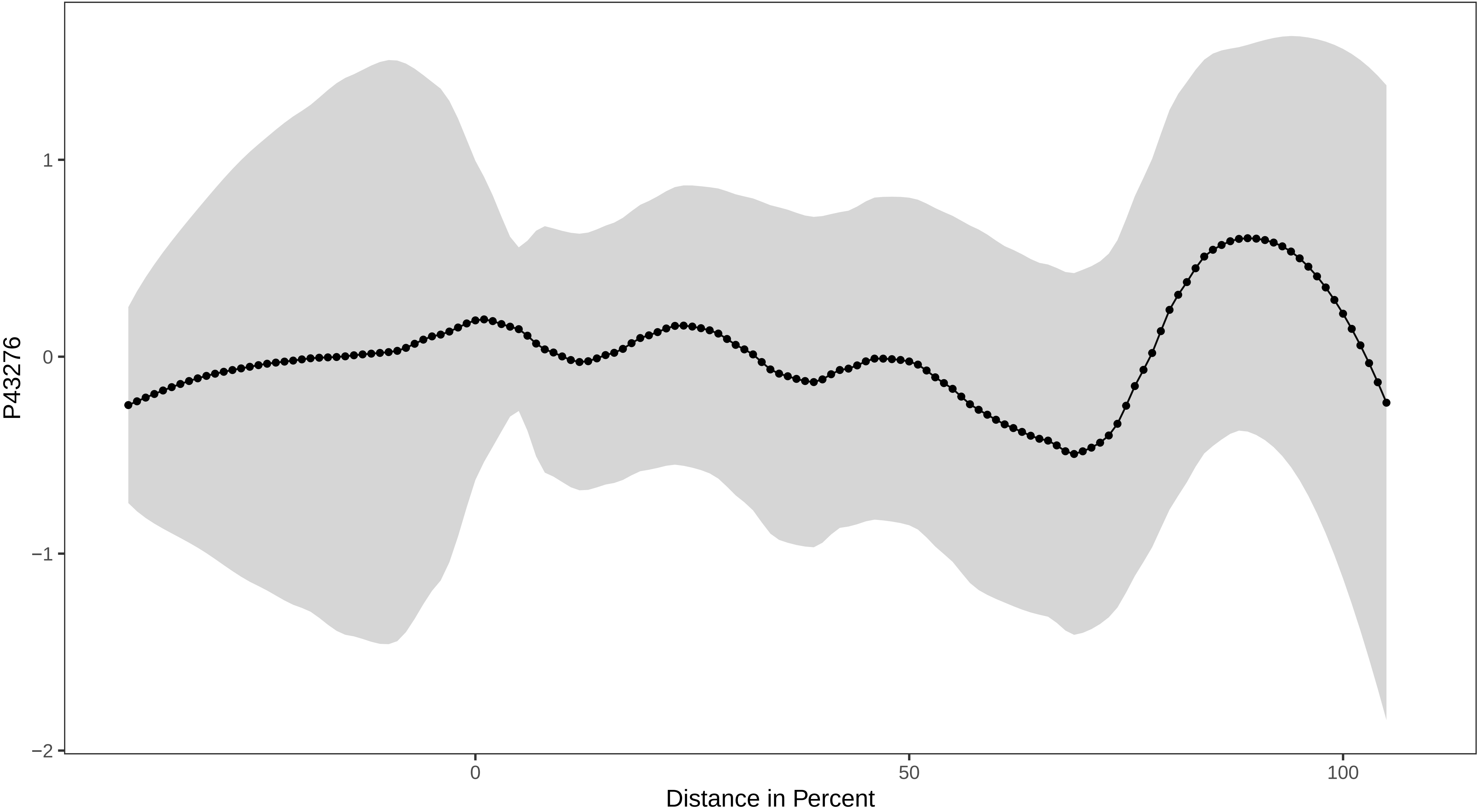

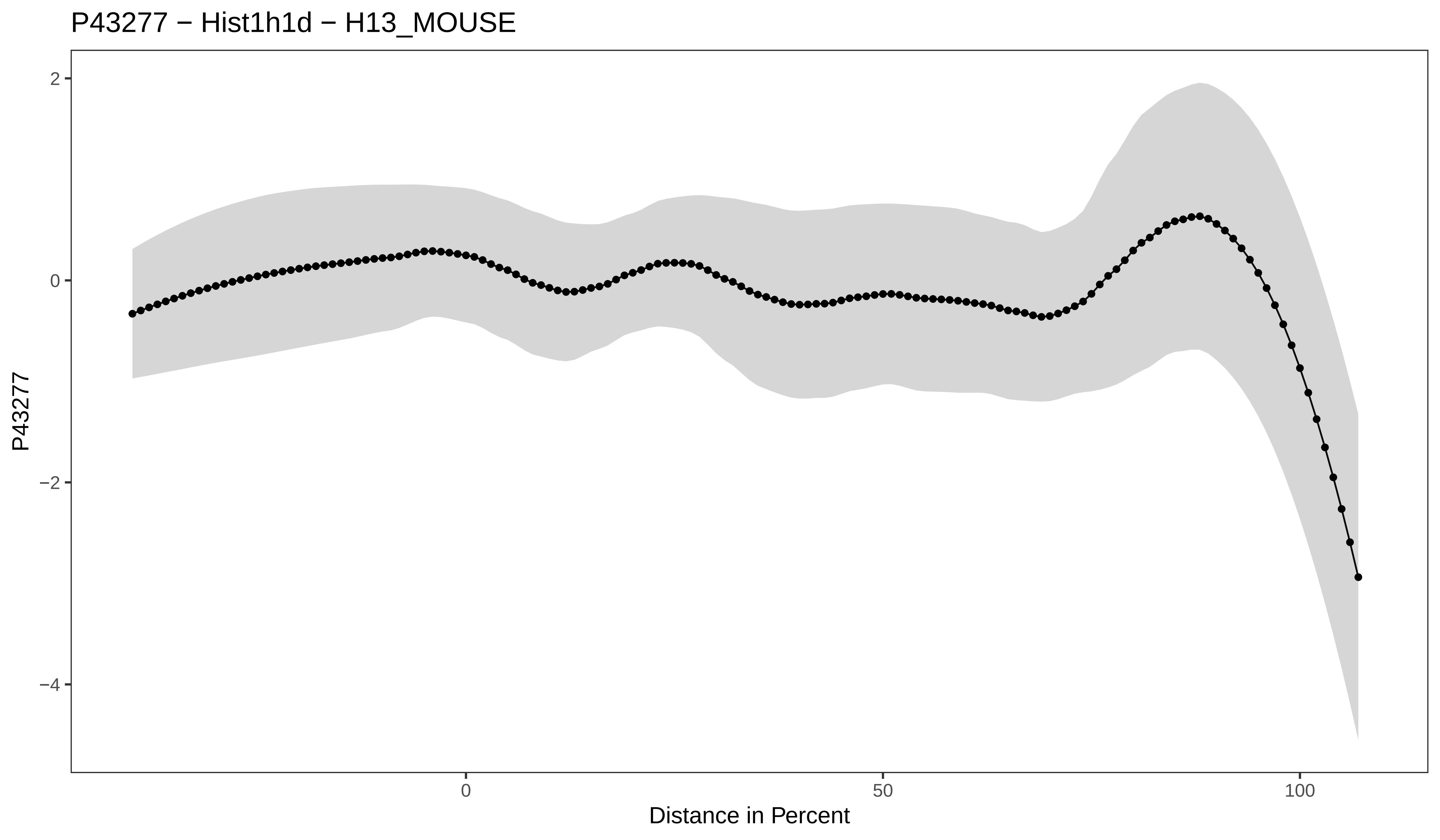
P43277 − Hist1h1d − H13_MOUSE
2
0
−2
−4
P43277
0
50
100
Distance in Percent

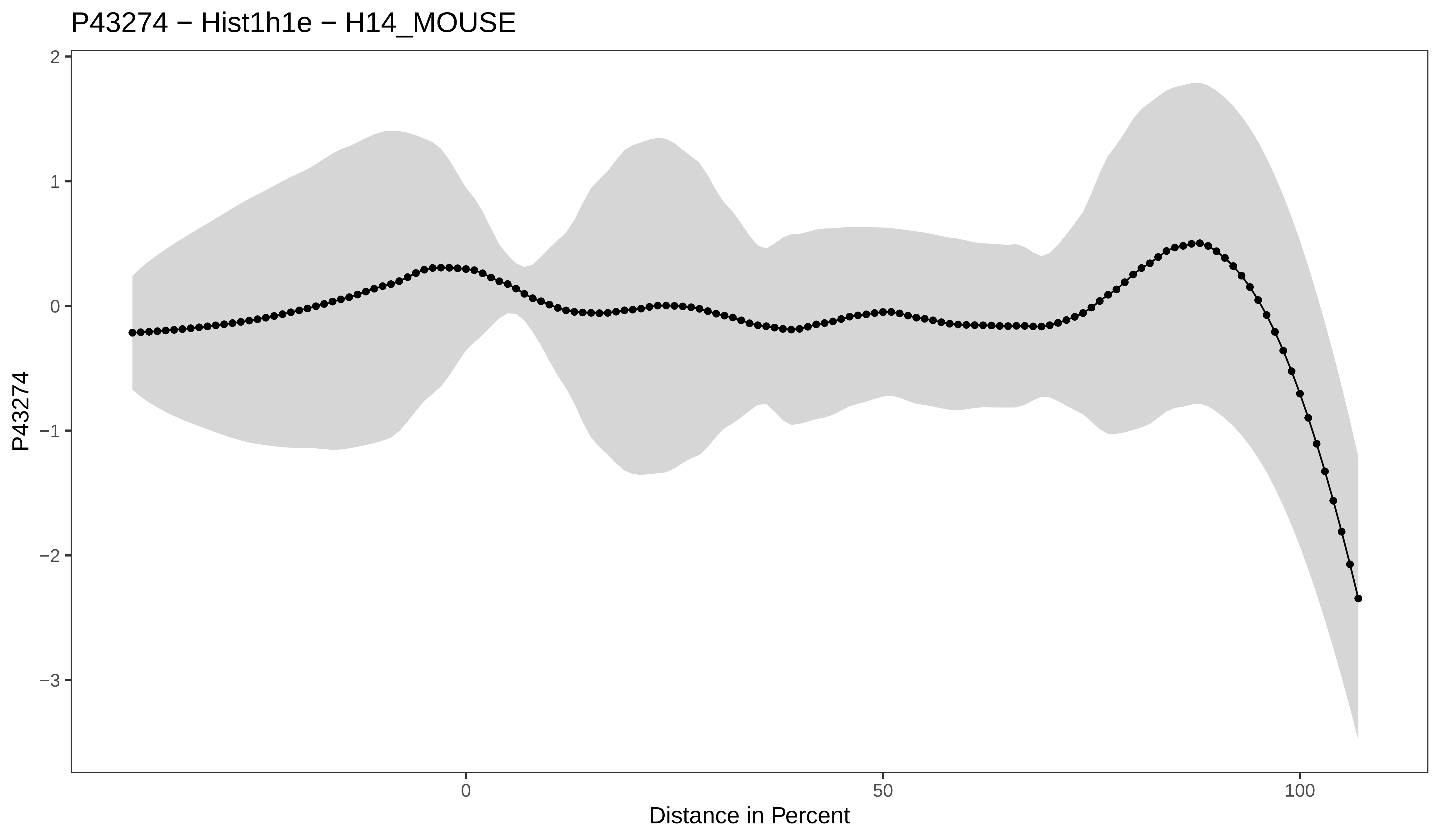

P43274 − Hist1h1e − H14_MOUSE
P43274
Distance in Percent
2
1
0
−1
−2
−3
0
50
100

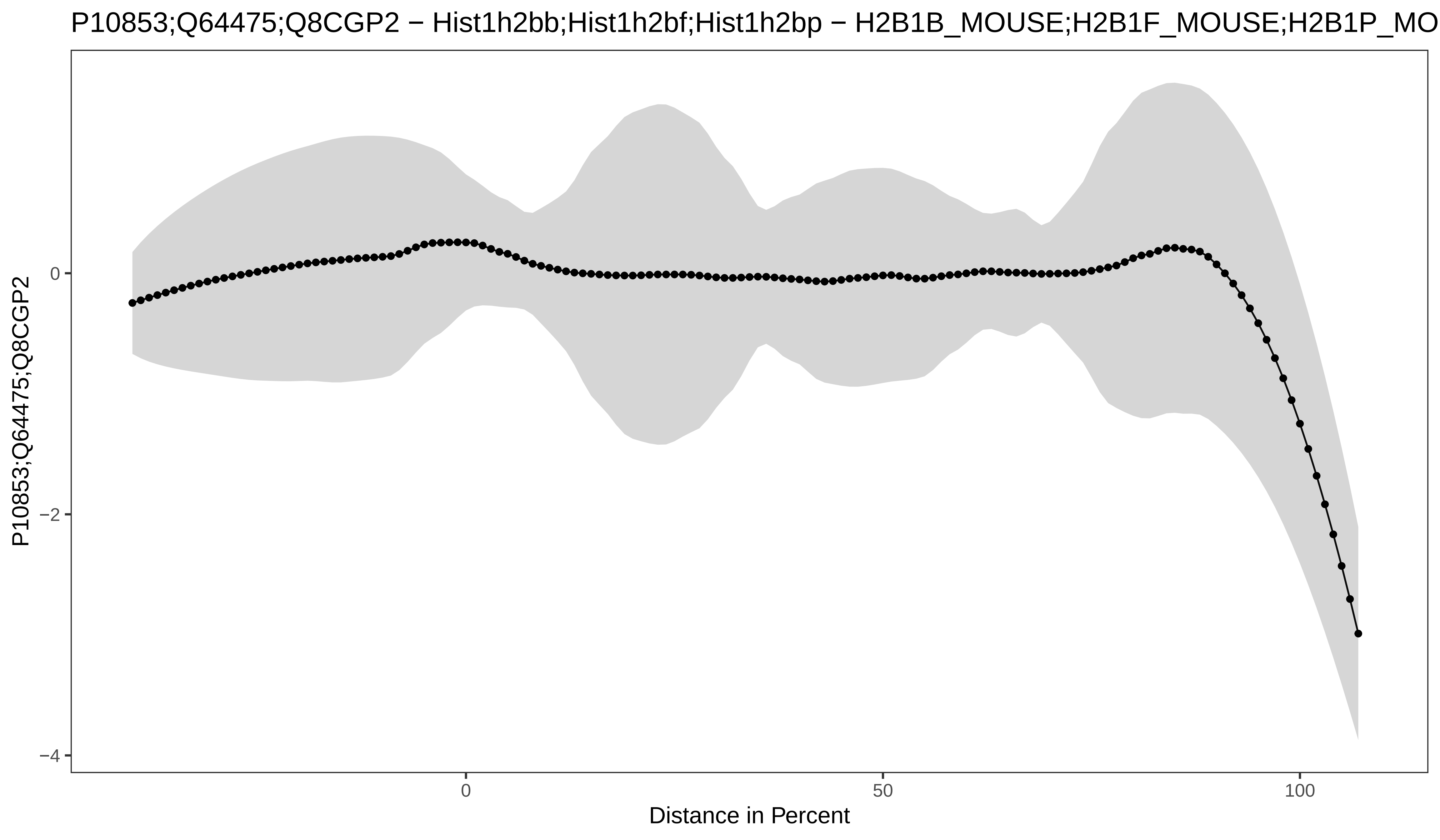

P10853;Q64475;Q8CGP2 − Hist1h2bb;Hist1h2bf;Hist1h2bp − H2B1B_MOUSE;H2B1F_MOUSE;H2B1P_MO
P10853;Q64475;Q8CGP2
0
−2
−4
0
50
100
Distance in Percent

P62806 − Hist1h4a − H4_MOUSE

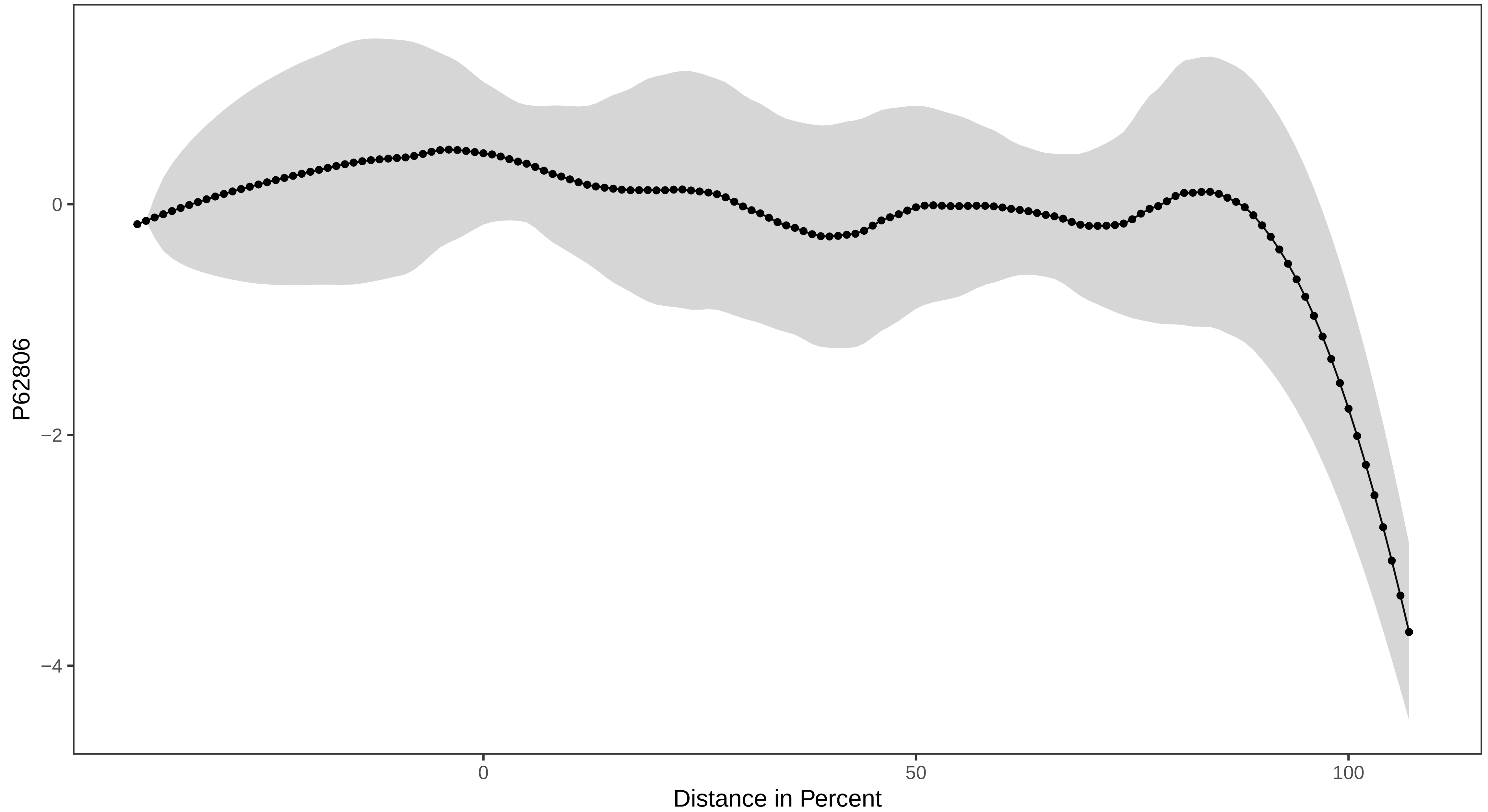

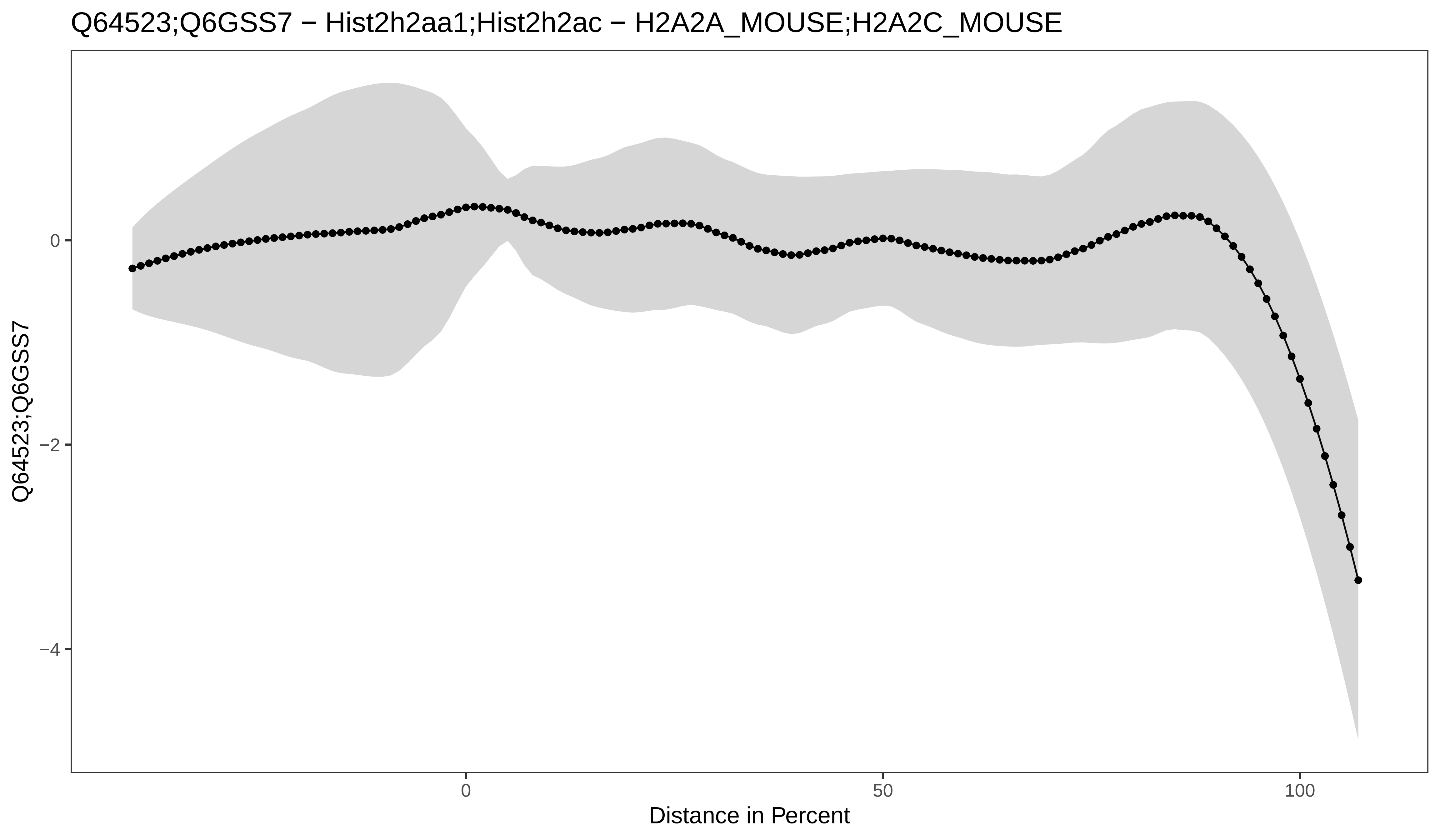
Q64523;Q6GSS7 − Hist2h2aa1;Hist2h2ac − H2A2A_MOUSE;H2A2C_MOUSE
Q64523;Q6GSS7
0
−2
−4
0
50
100
Distance in Percent

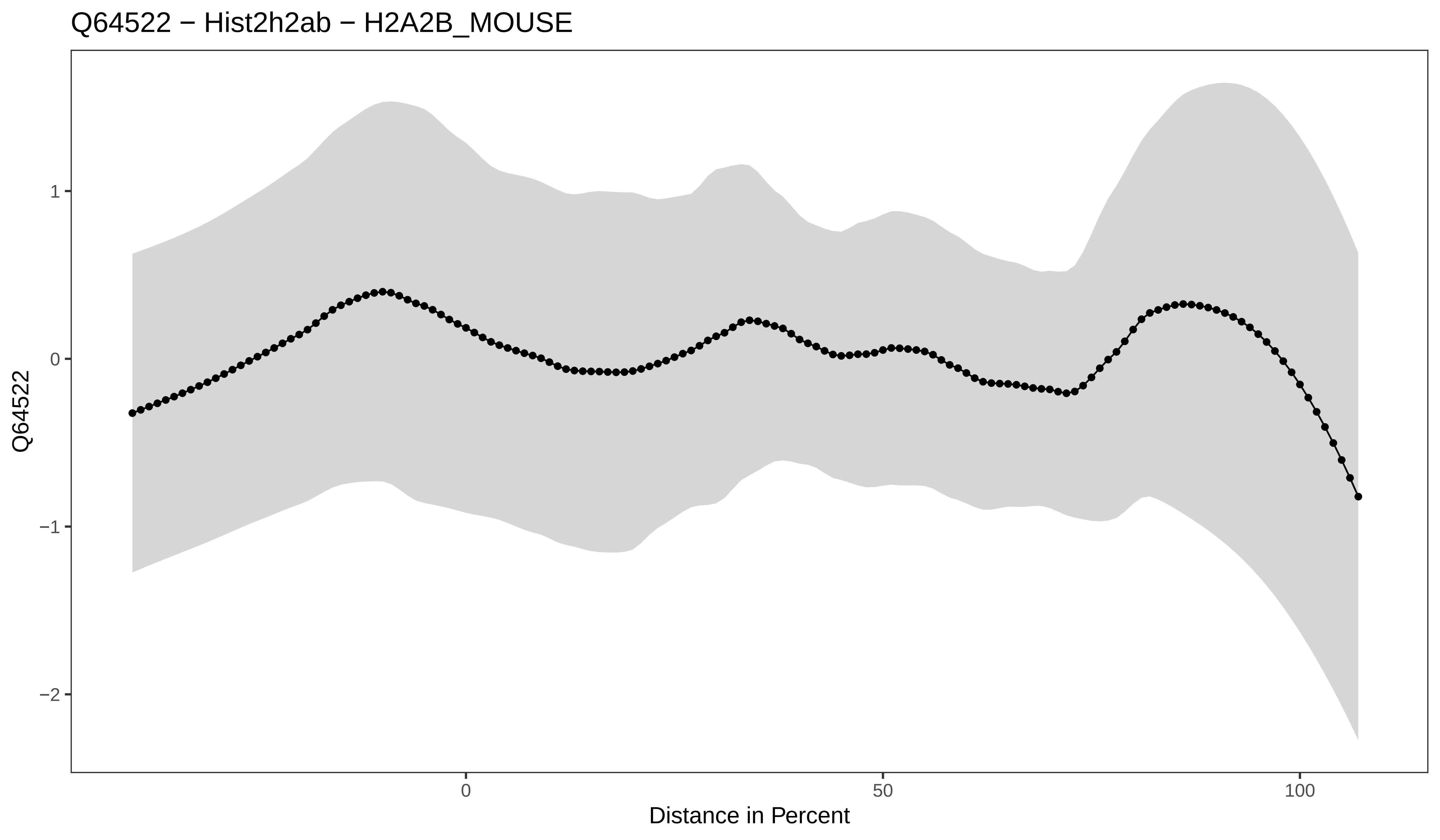

Q64522 − Hist2h2ab − H2A2B_MOUSE
1
0
−1
−2
Q64522
0
50
100
Distance in Percent

Q8CGP0;Q9D2U9 − Hist3h2ba;Hist3h2bb − H2B3A_MOUSE;H2B3B_MOUSE

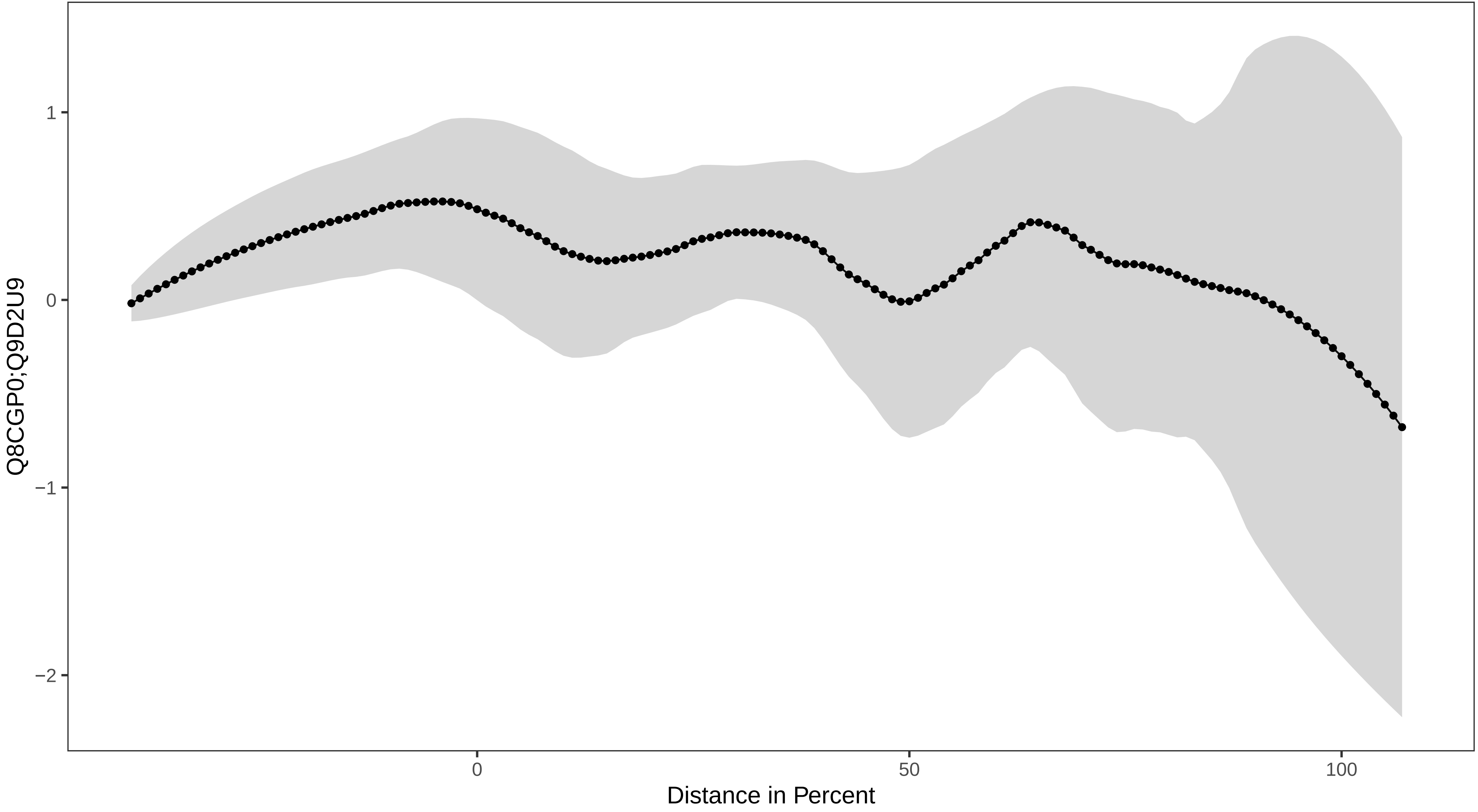

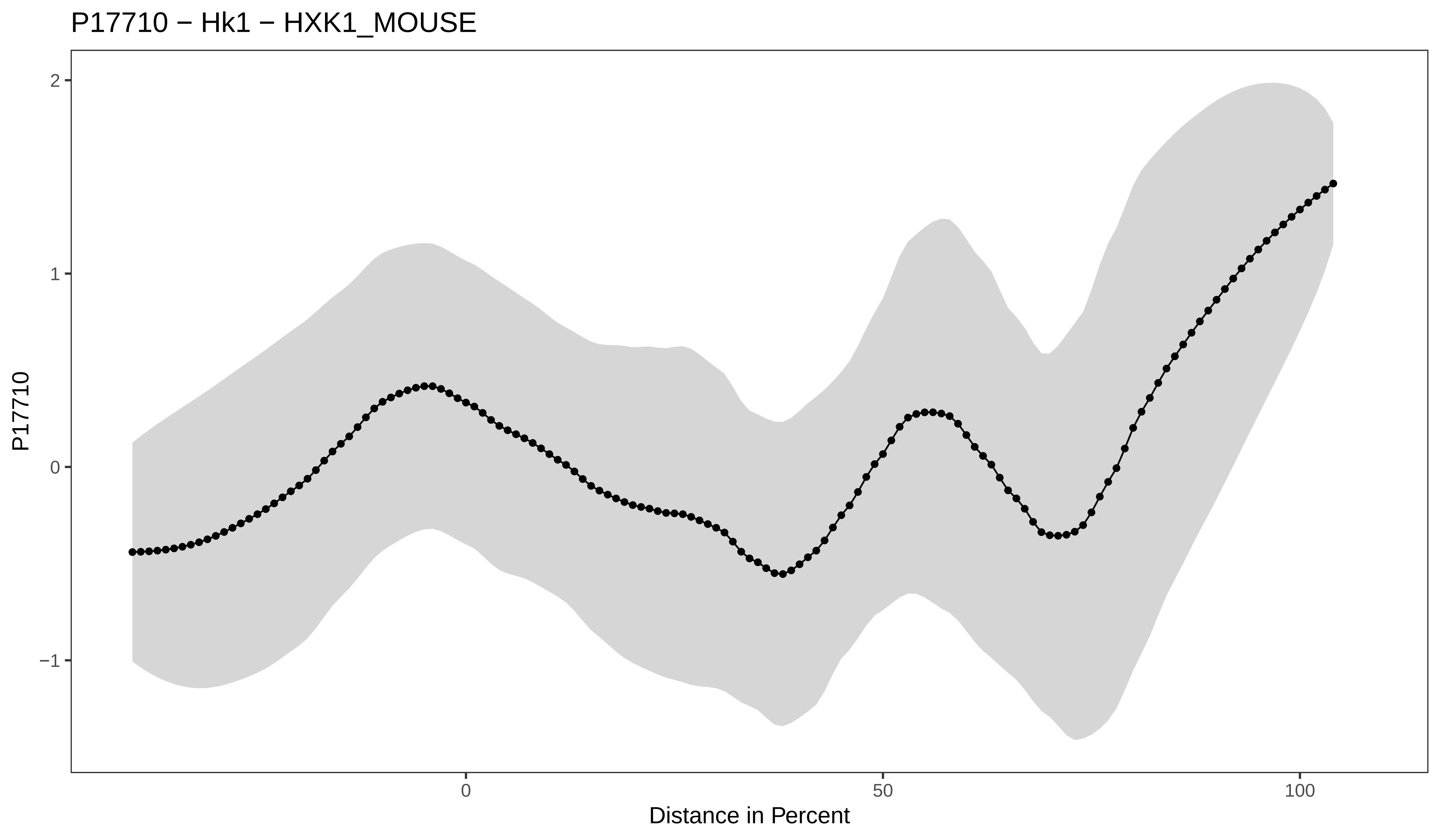
P17710 − Hk1 − HXK1_MOUSE
P17710
2
1
0
−1
0
50
100
Distance in Percent

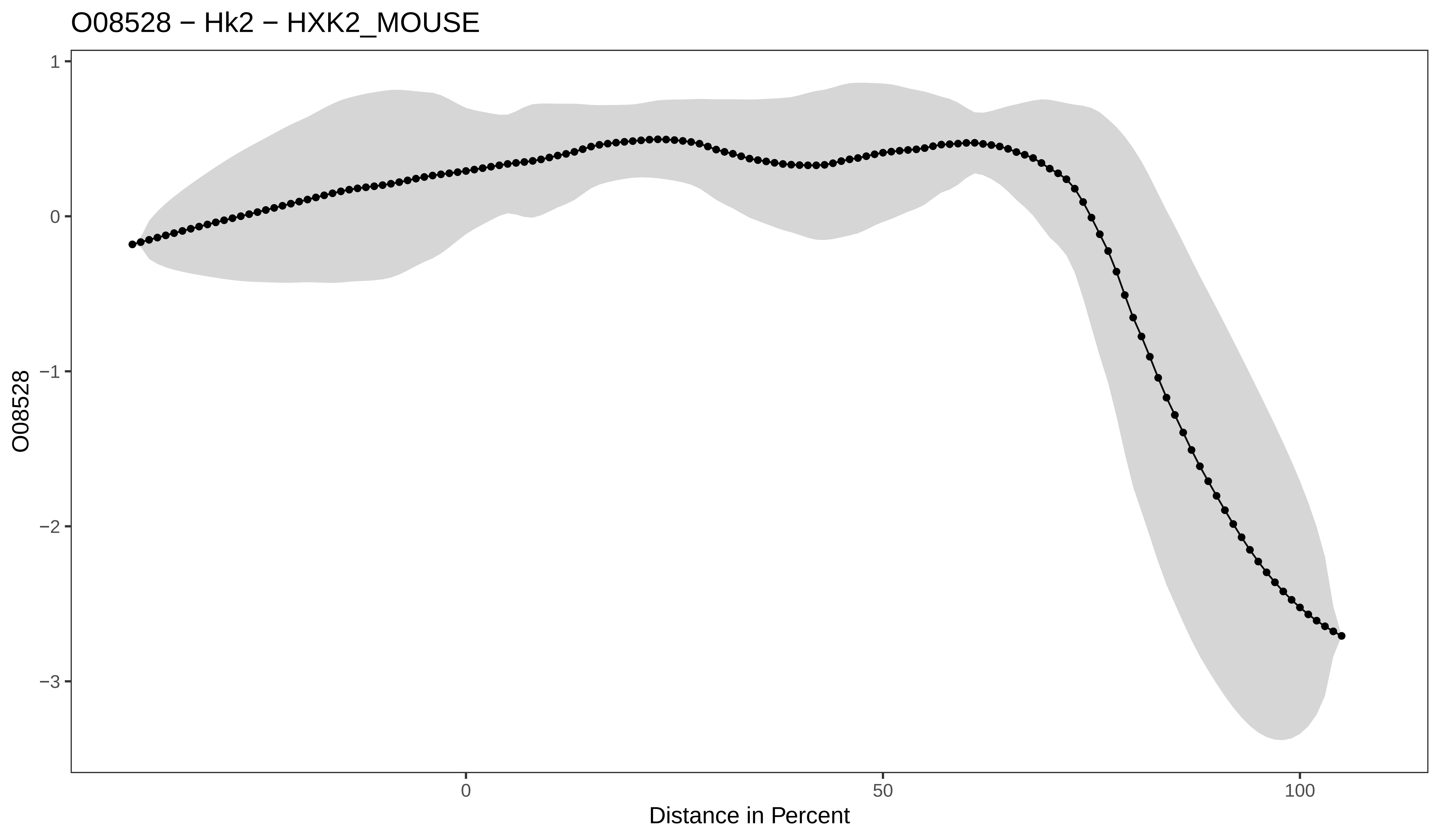

O08528 − Hk2 − HXK2_MOUSE
O08528
1
0
−1
−2
−3
0
50
100
Distance in Percent

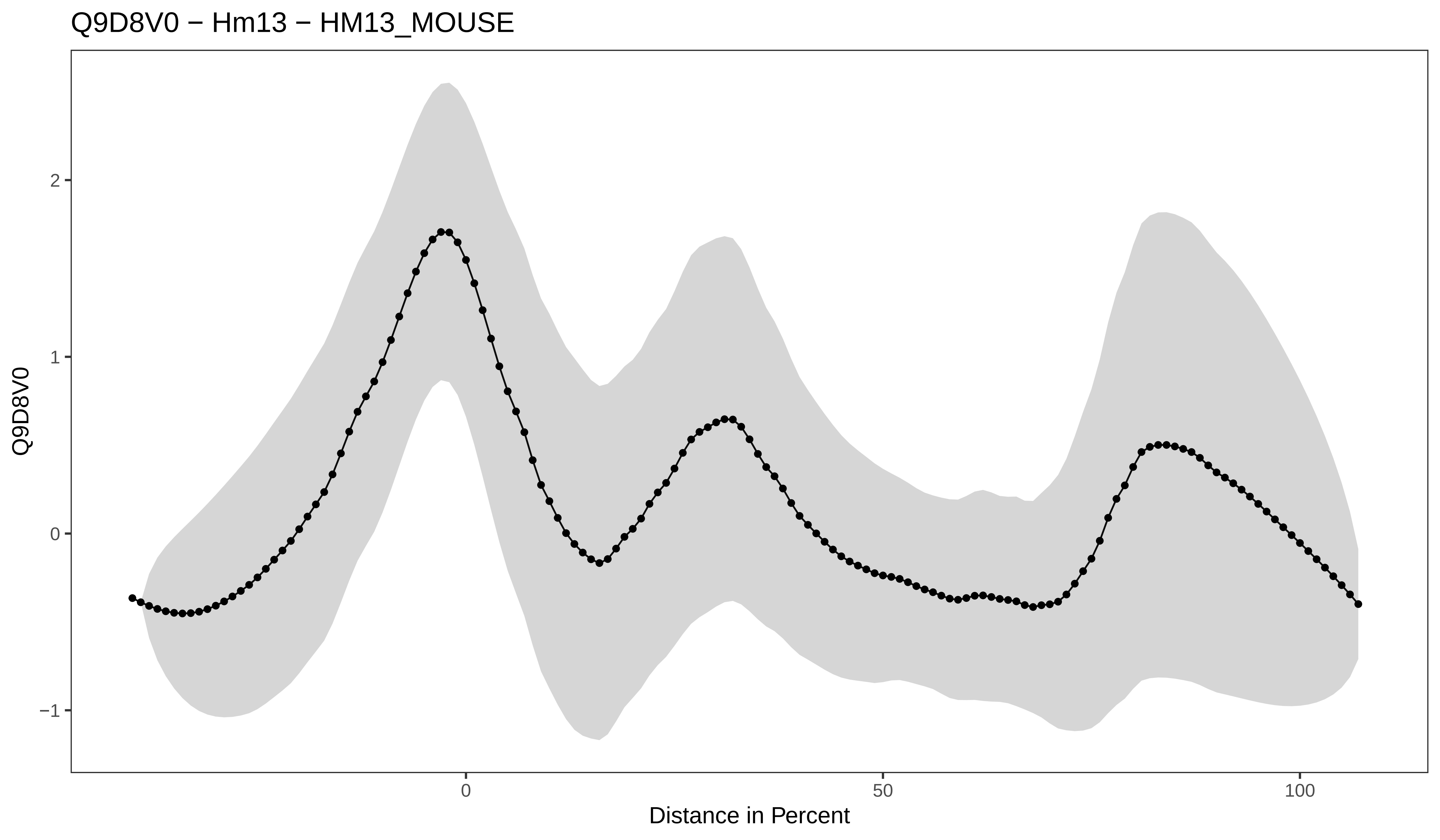

Q9D8V0 − Hm13 − HM13_MOUSE
Q9D8V0
2
1
0
−1
0
50
100
Distance in Percent

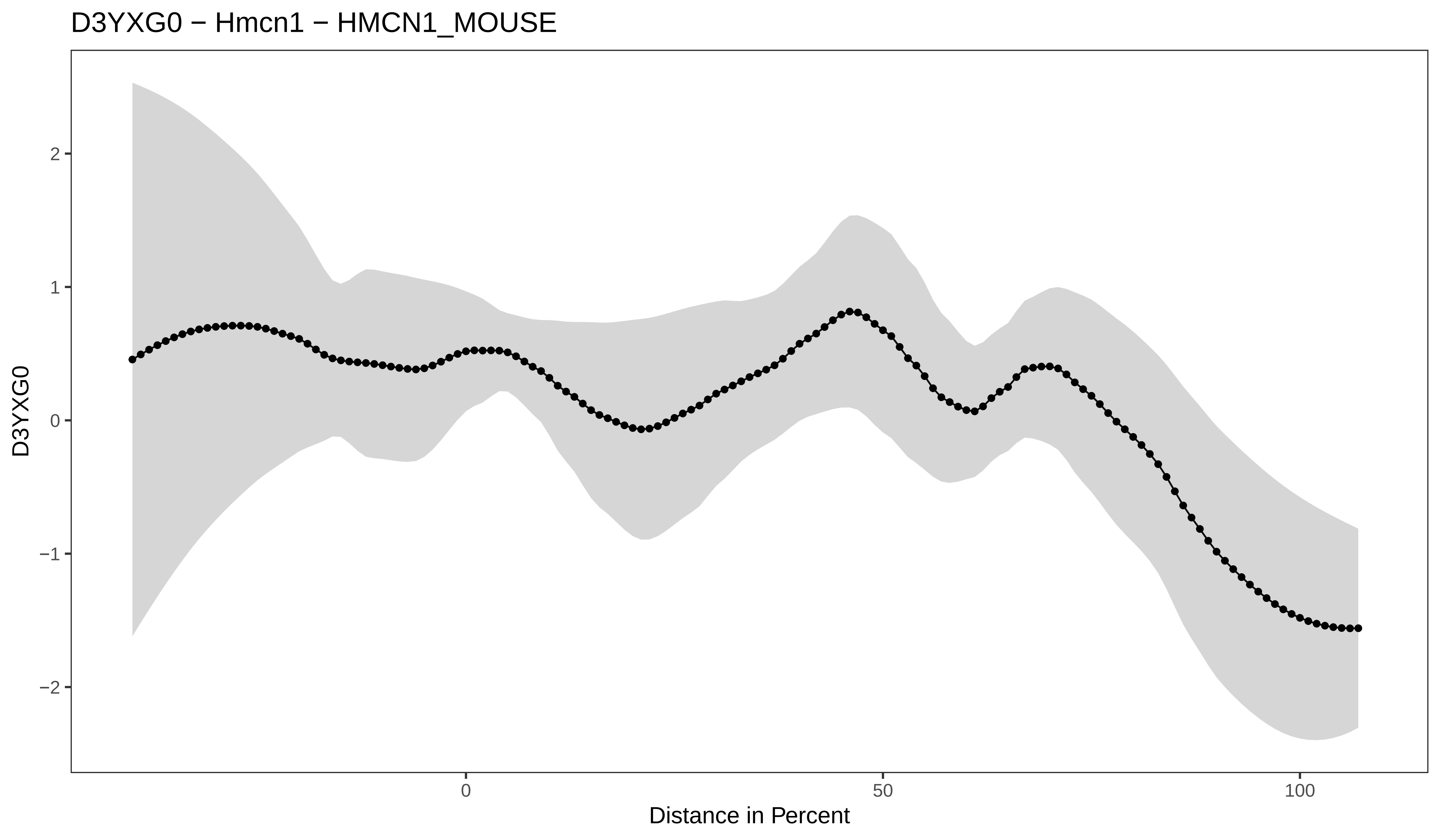

D3YXG0 − Hmcn1 − HMCN1_MOUSE
D3YXG0
2
1
0
−1
−2
0
50
100
Distance in Percent

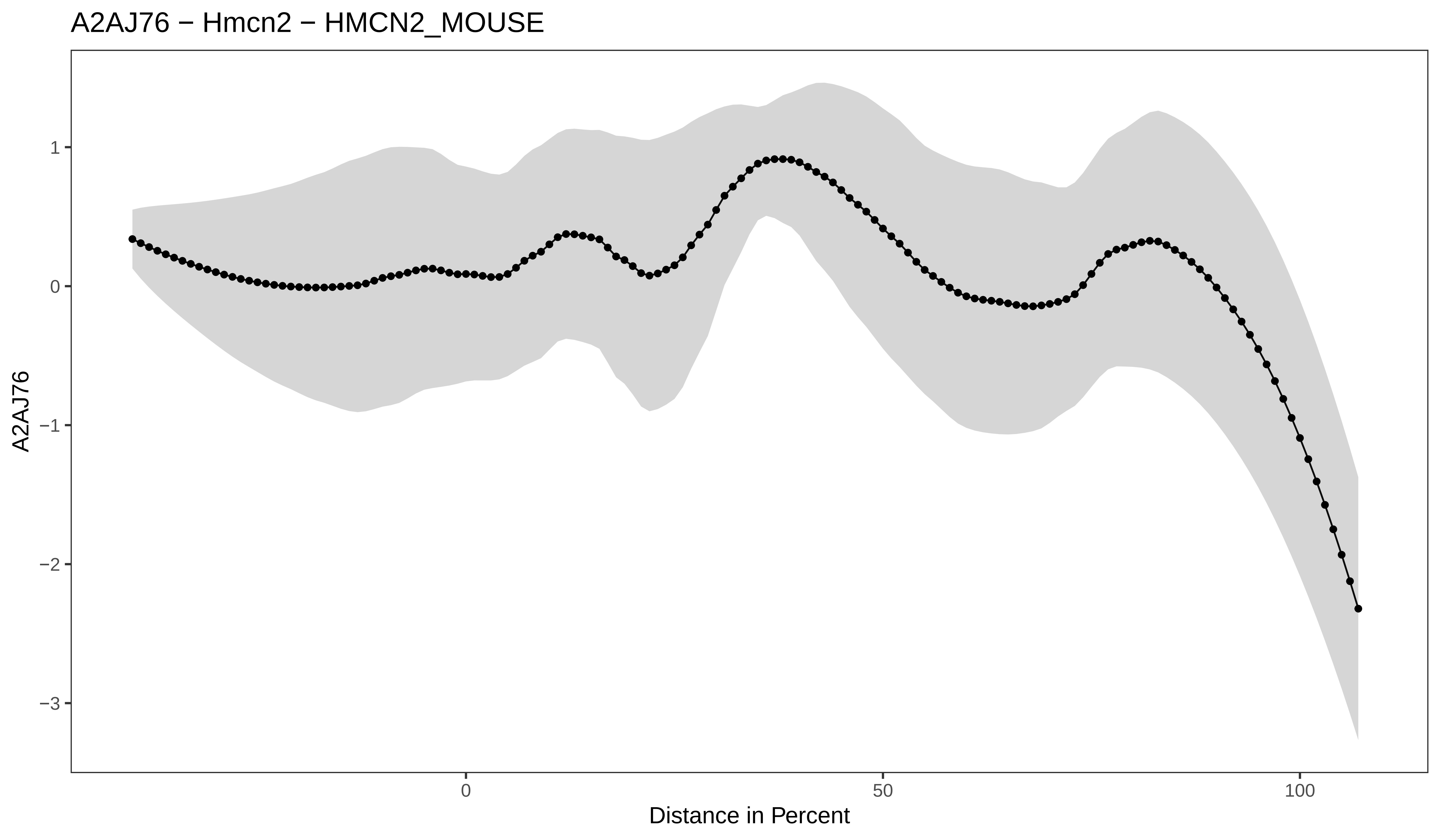
A2AJ76 − Hmcn2 − HMCN2_MOUSE
1
0
−1
−2
−3
A2AJ76
0
50
100
Distance in Percent

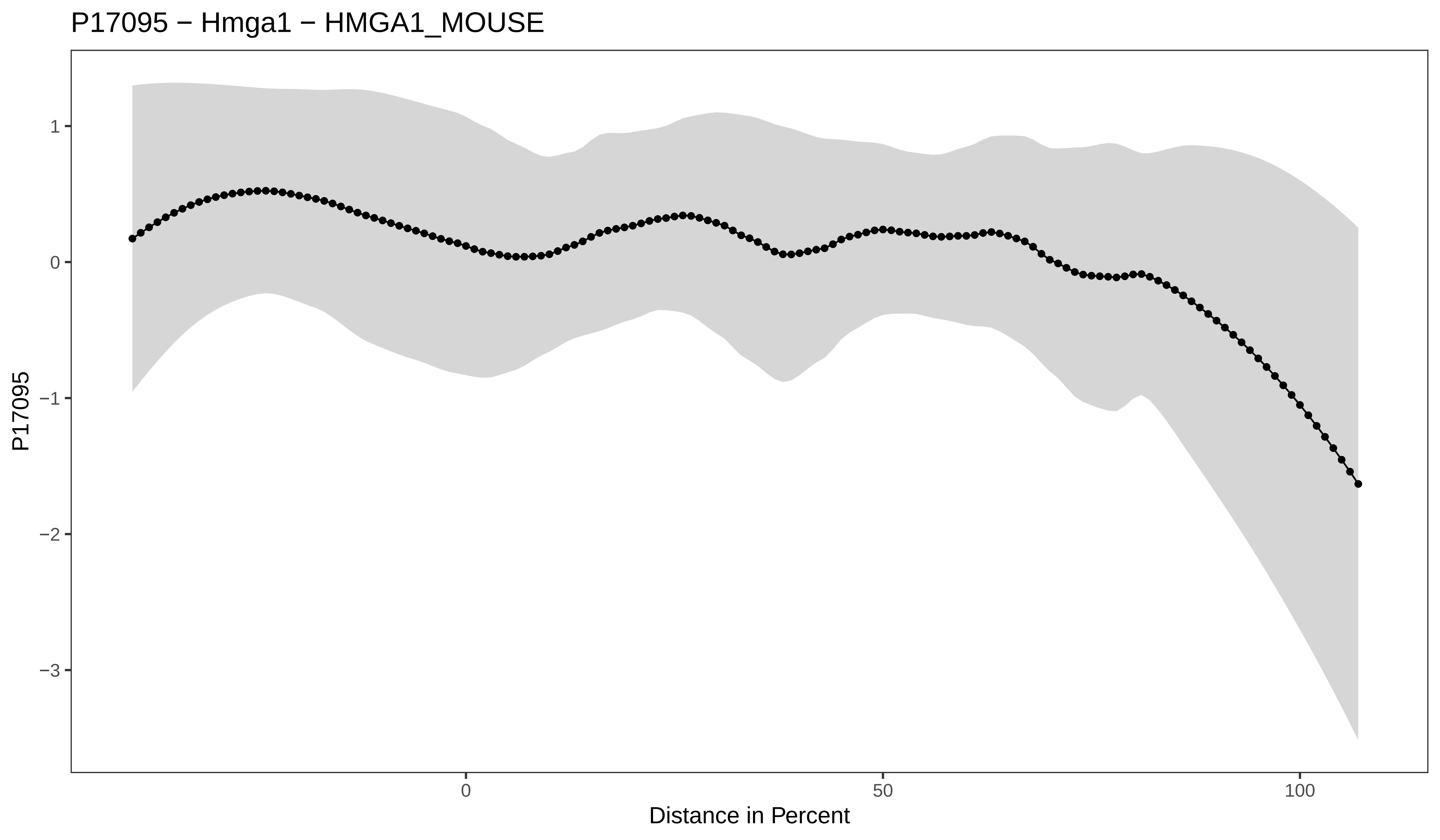
P17095 − Hmga1 − HMGA1_MOUSE
1
0
−1
−2
−3
P17095
0
50
100
Distance in Percent

P63158 − Hmgb1 − HMGB1_MOUSE

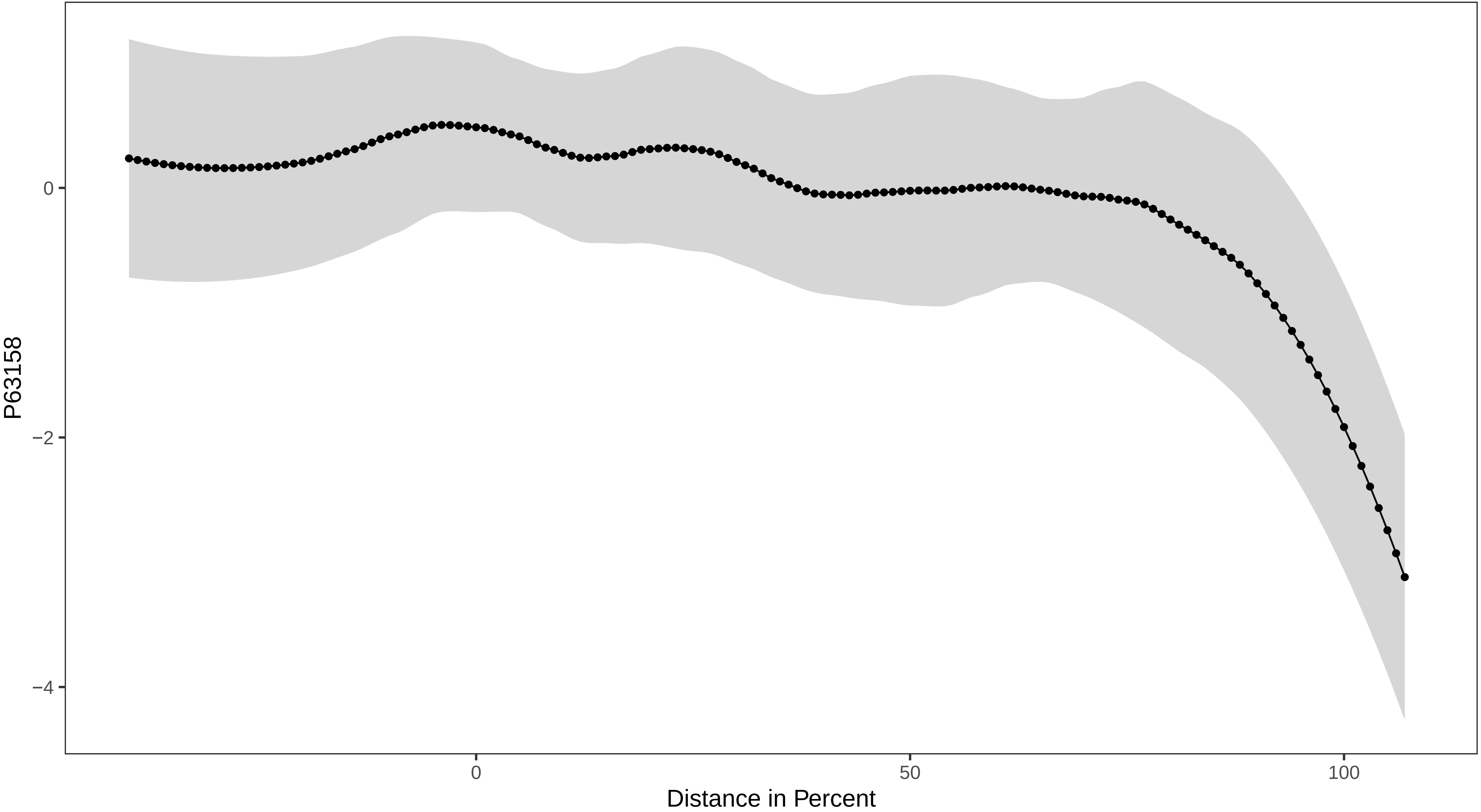

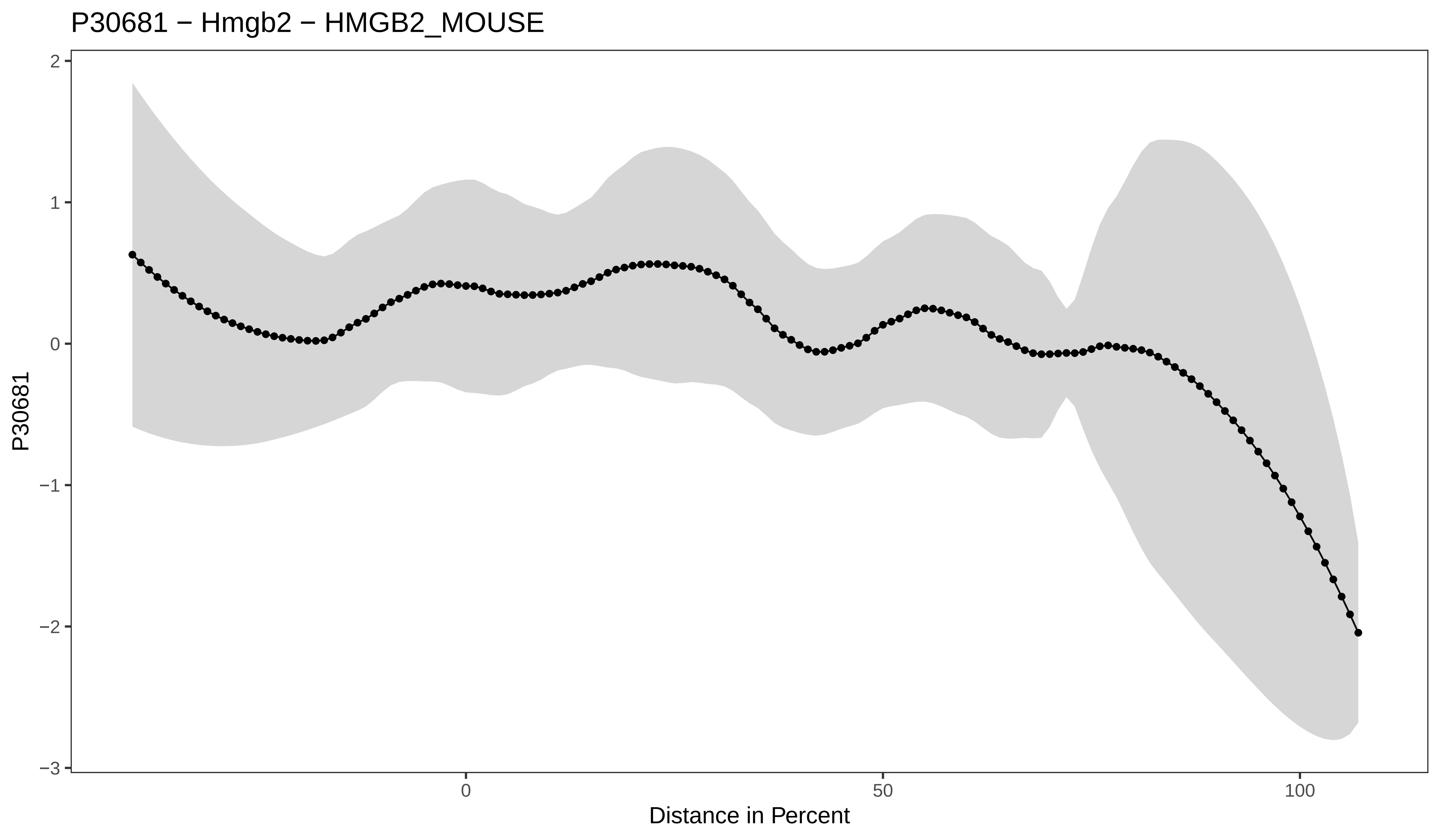

P30681 − Hmgb2 − HMGB2_MOUSE
P30681
Distance in Percent
2
1
0
−1
−2
−3
0
50
100

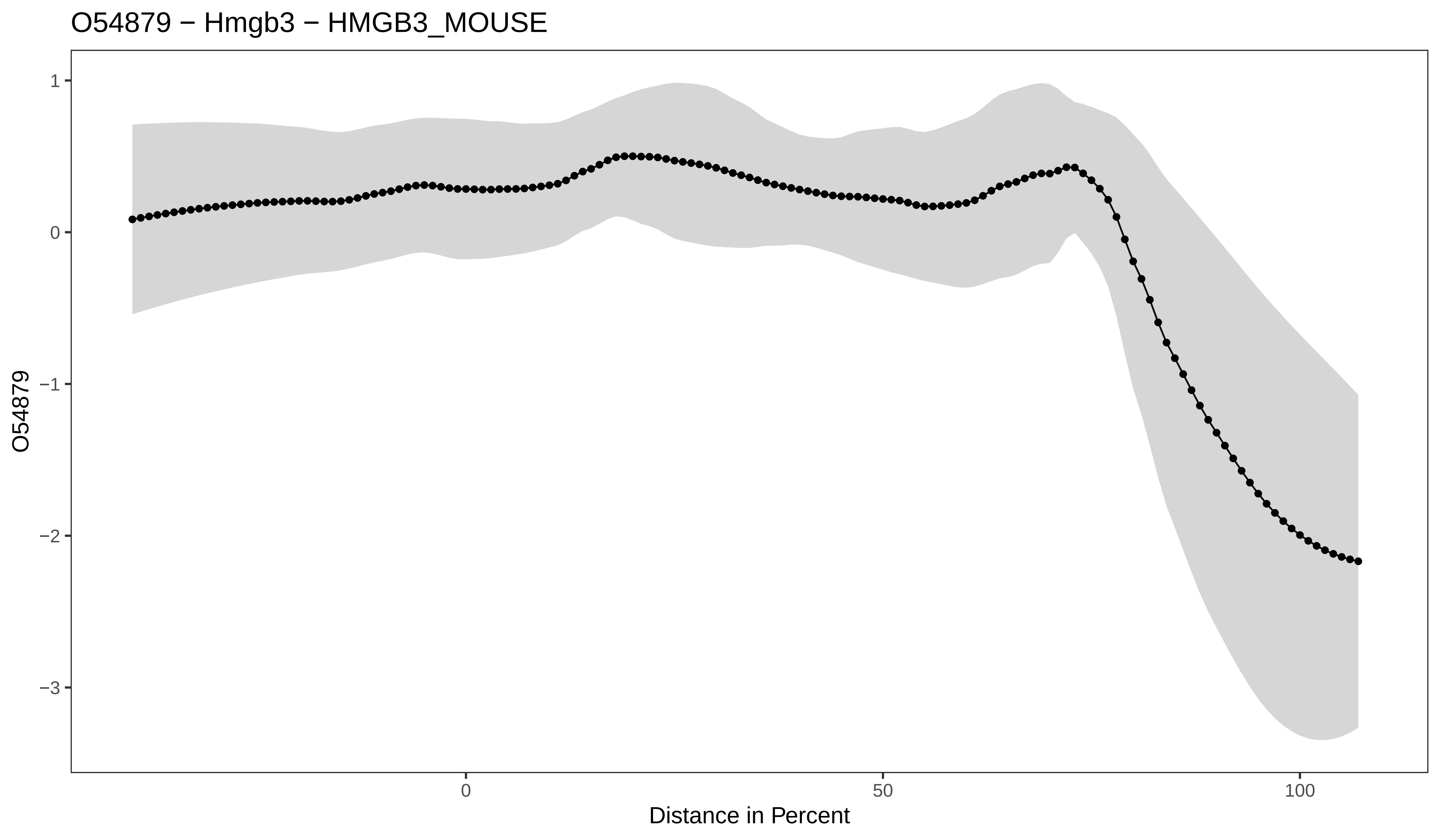

O54879 − Hmgb3 − HMGB3_MOUSE
1
0
−1
−2
−3
O54879
0
50
100
Distance in Percent

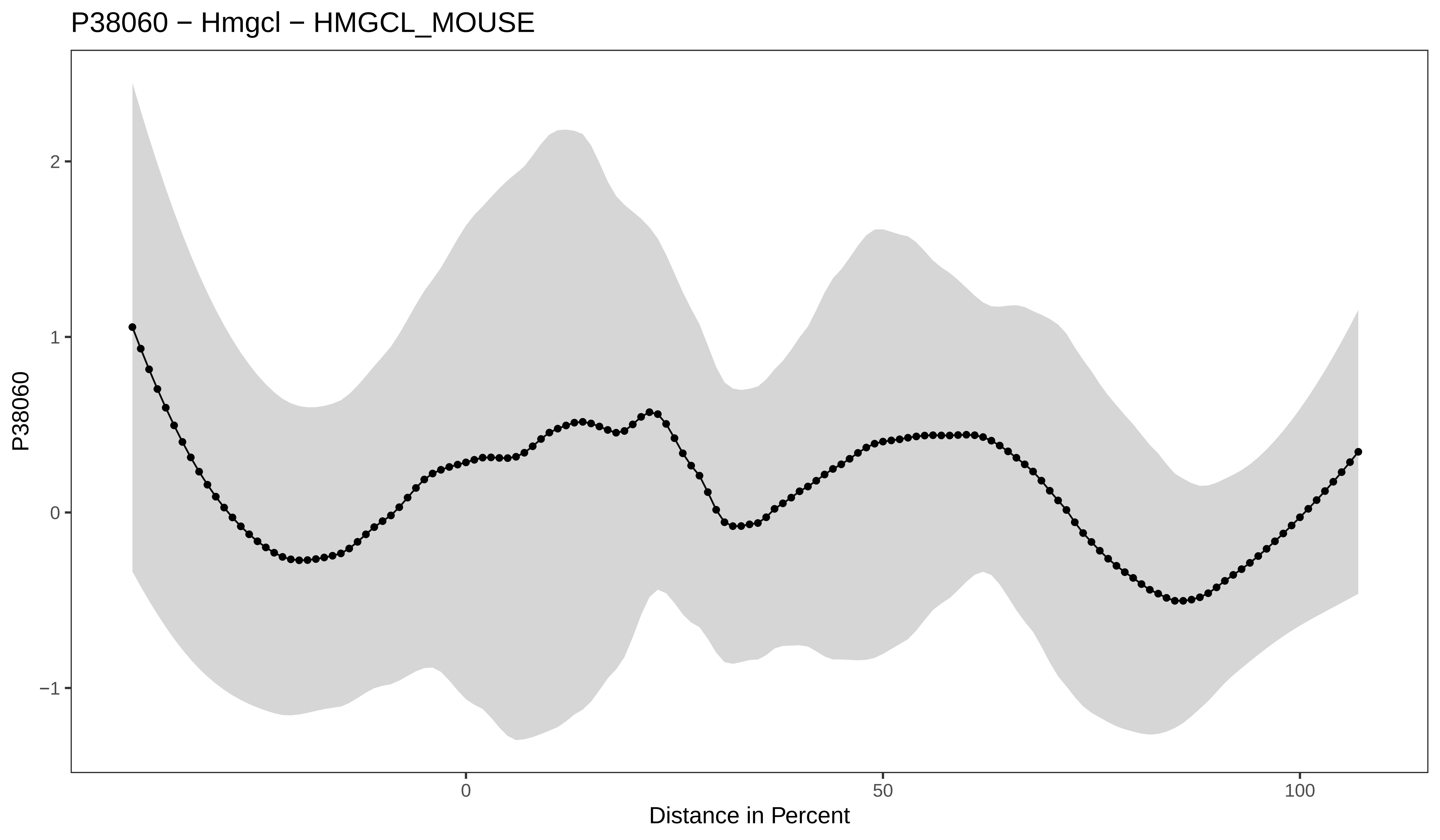

P38060 − Hmgcl − HMGCL_MOUSE
P38060
2
1
0
−1
0
50
100
Distance in Percent

P09602 − Hmgn2 − HMGN2_MOUSE

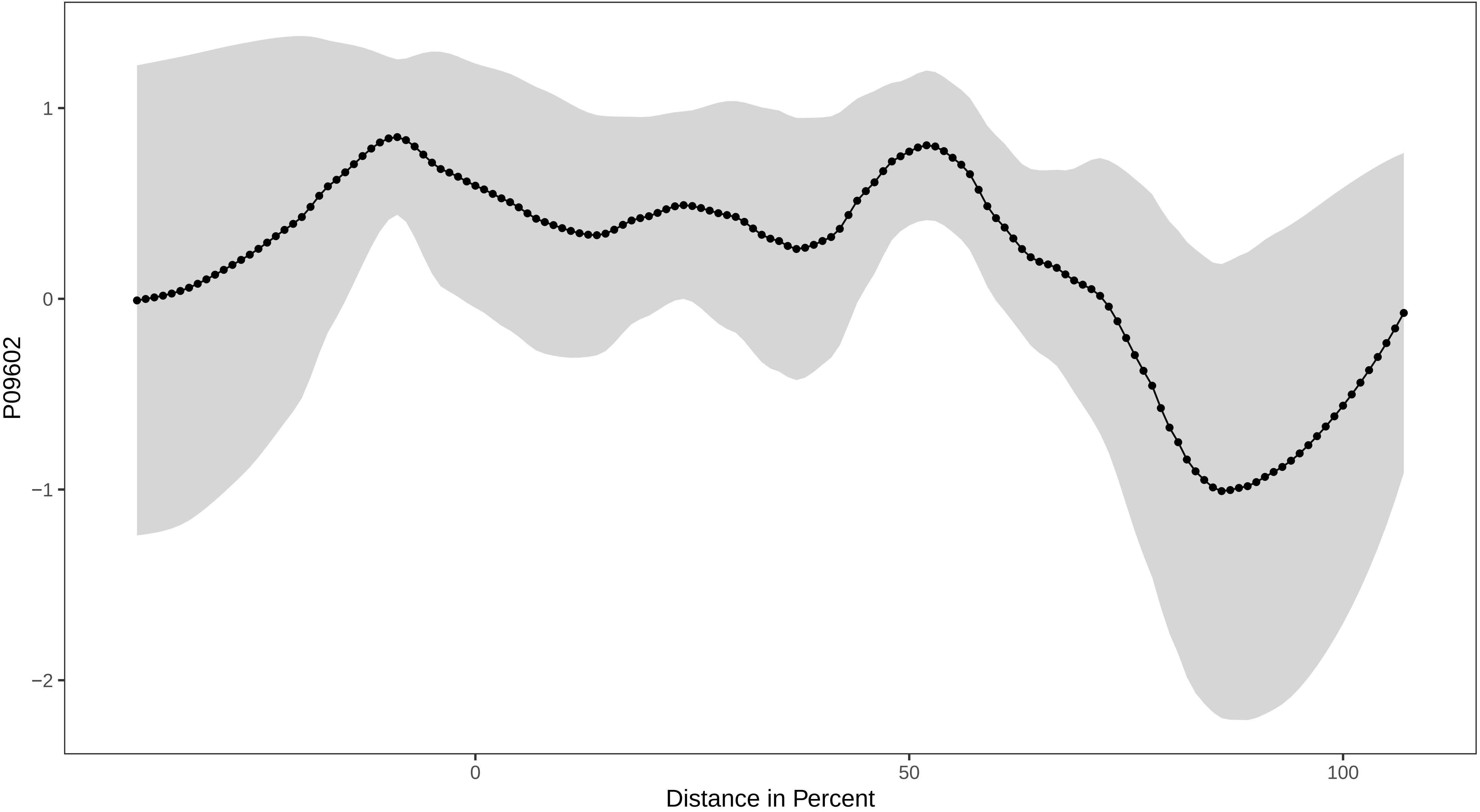

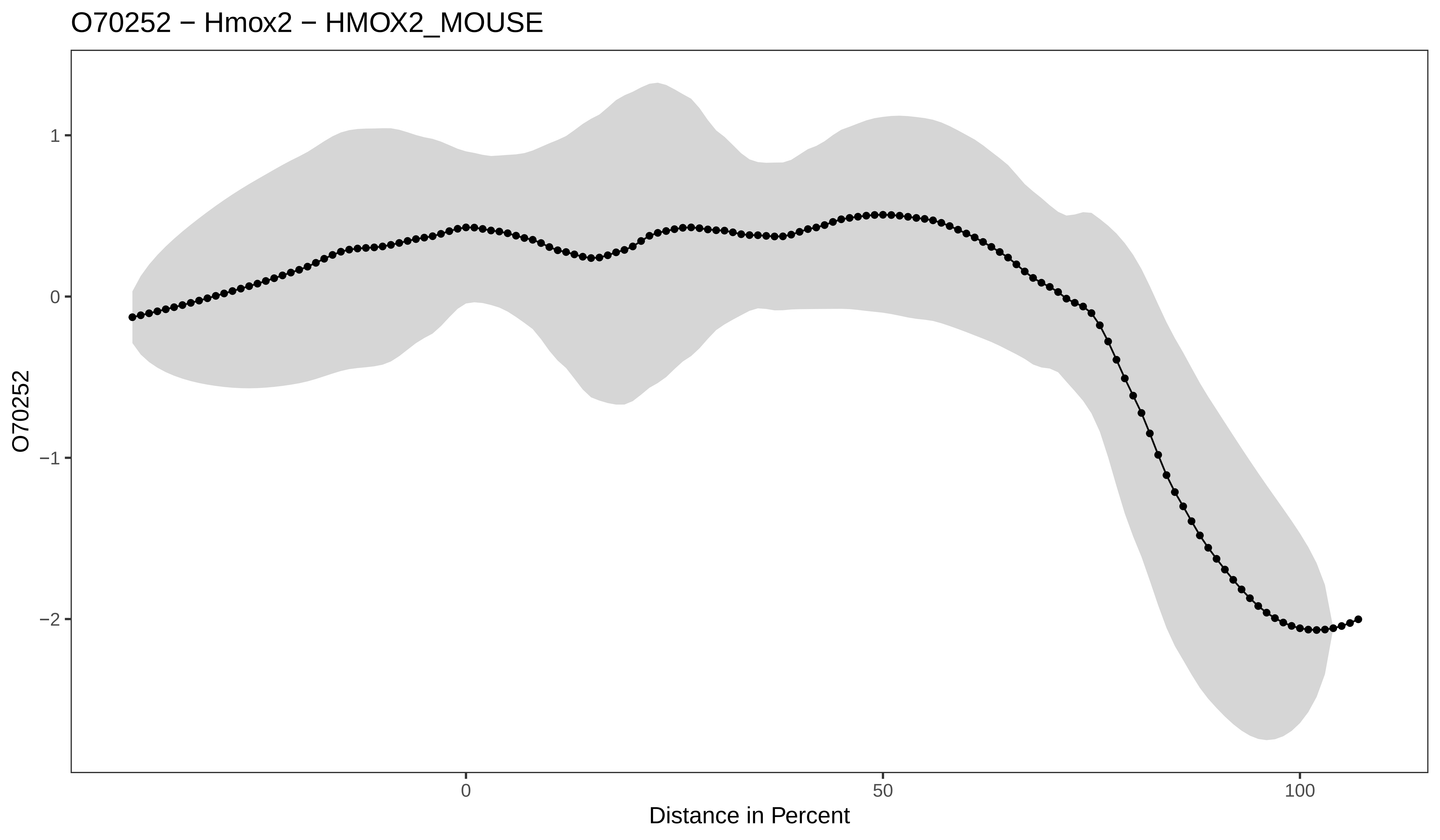

O70252 − Hmox2 − HMOX2_MOUSE
O70252
1
0
−1
−2
0
50
100
Distance in Percent

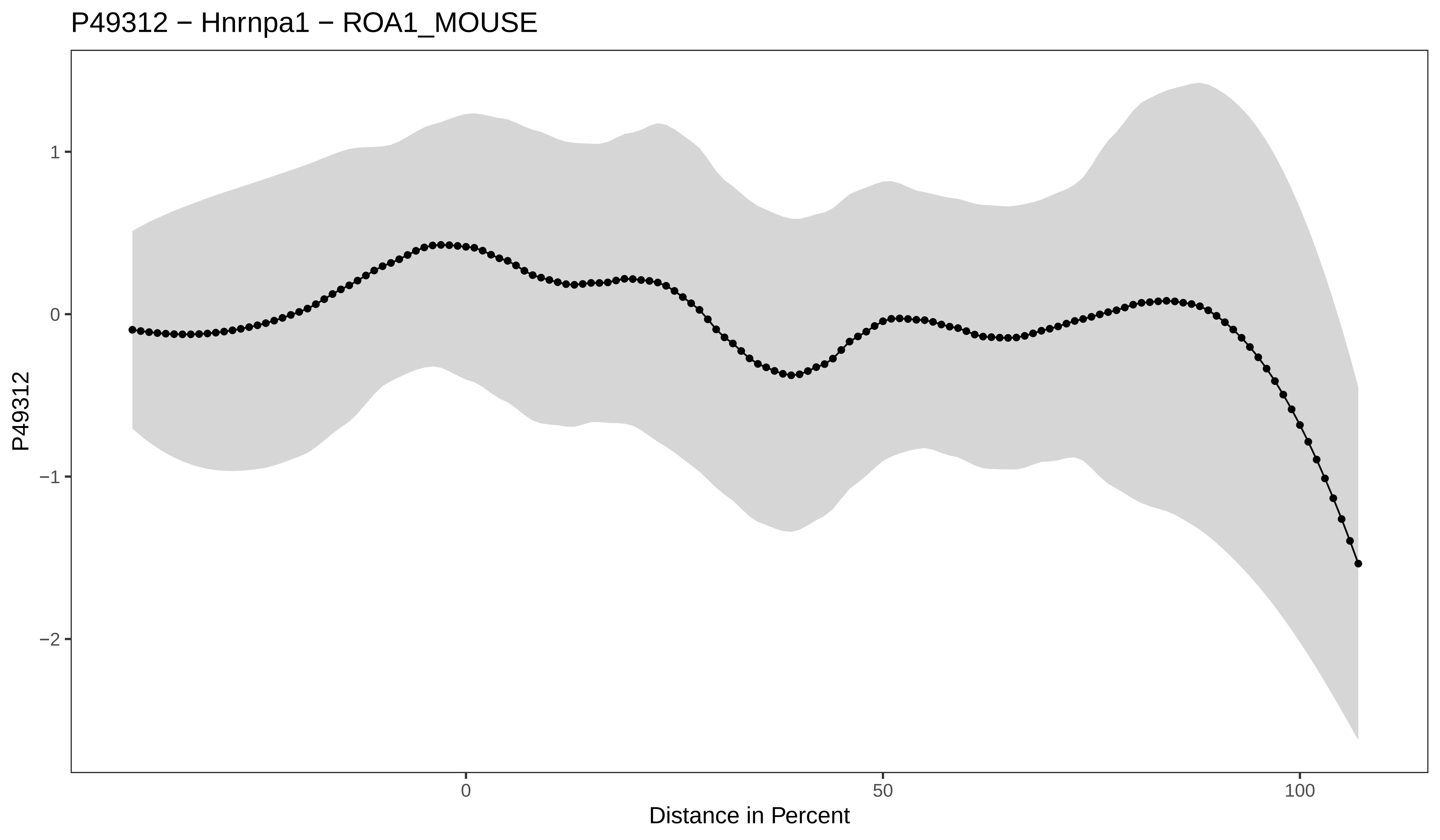
P49312 − Hnrnpa1 − ROA1_MOUSE
1
0
−1
−2
P49312
0
50
100
Distance in Percent

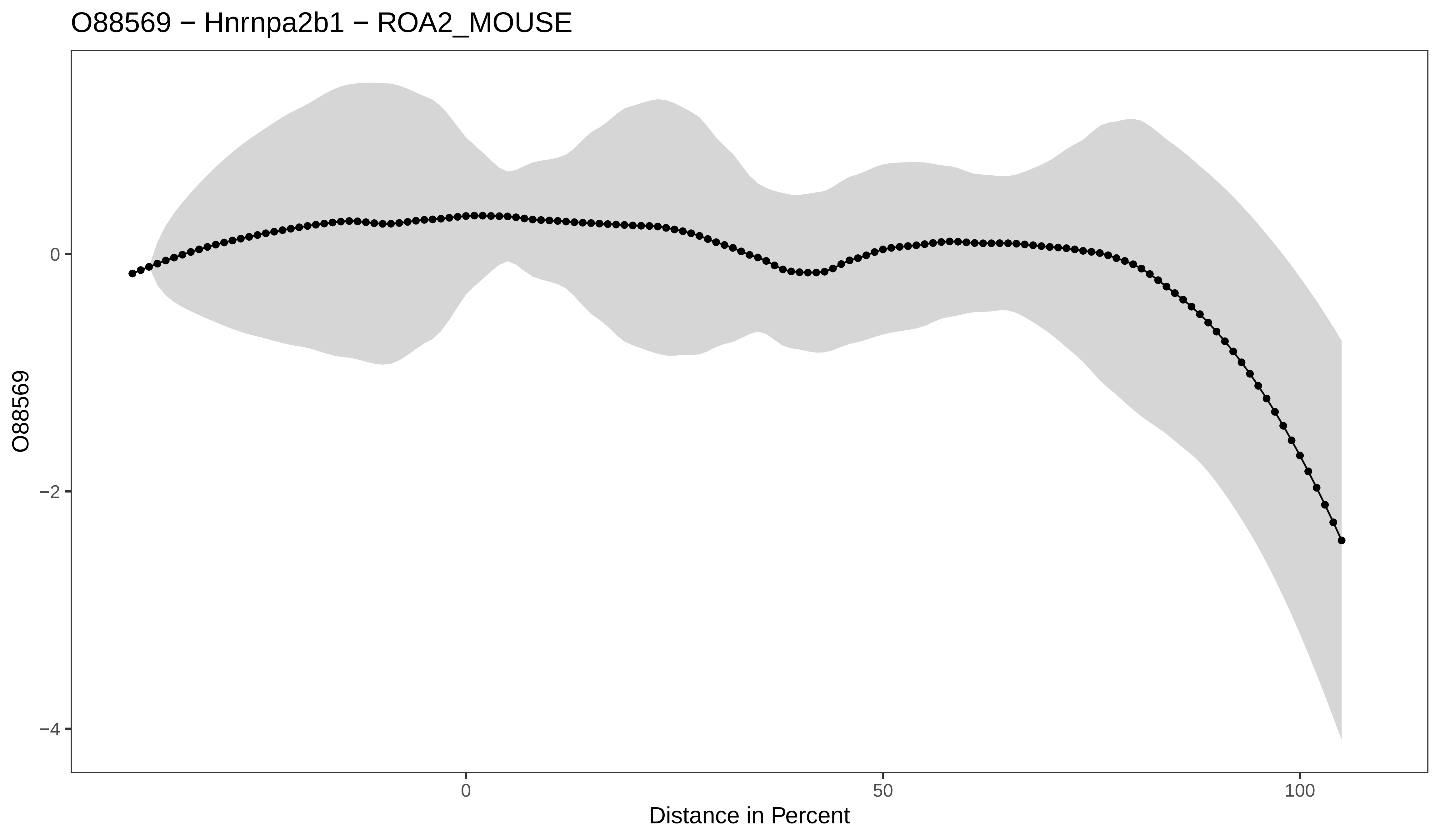
O88569 − Hnrnpa2b1 − ROA2_MOUSE
0
−2
−4
O88569
0
50
100
Distance in Percent

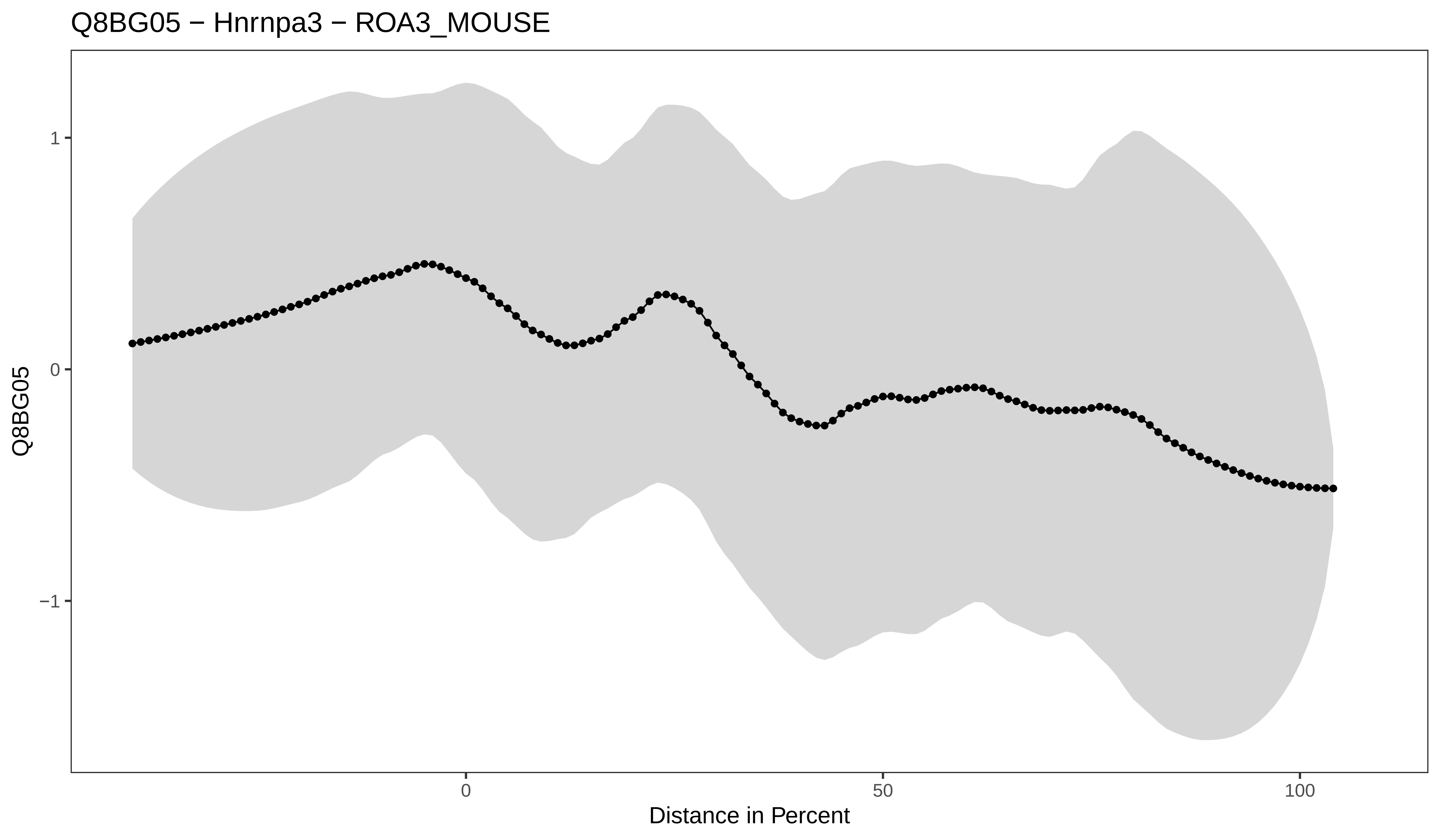

Q8BG05 − Hnrnpa3 − ROA3_MOUSE
1
0
−1
Q8BG05
0
50
100
Distance in Percent

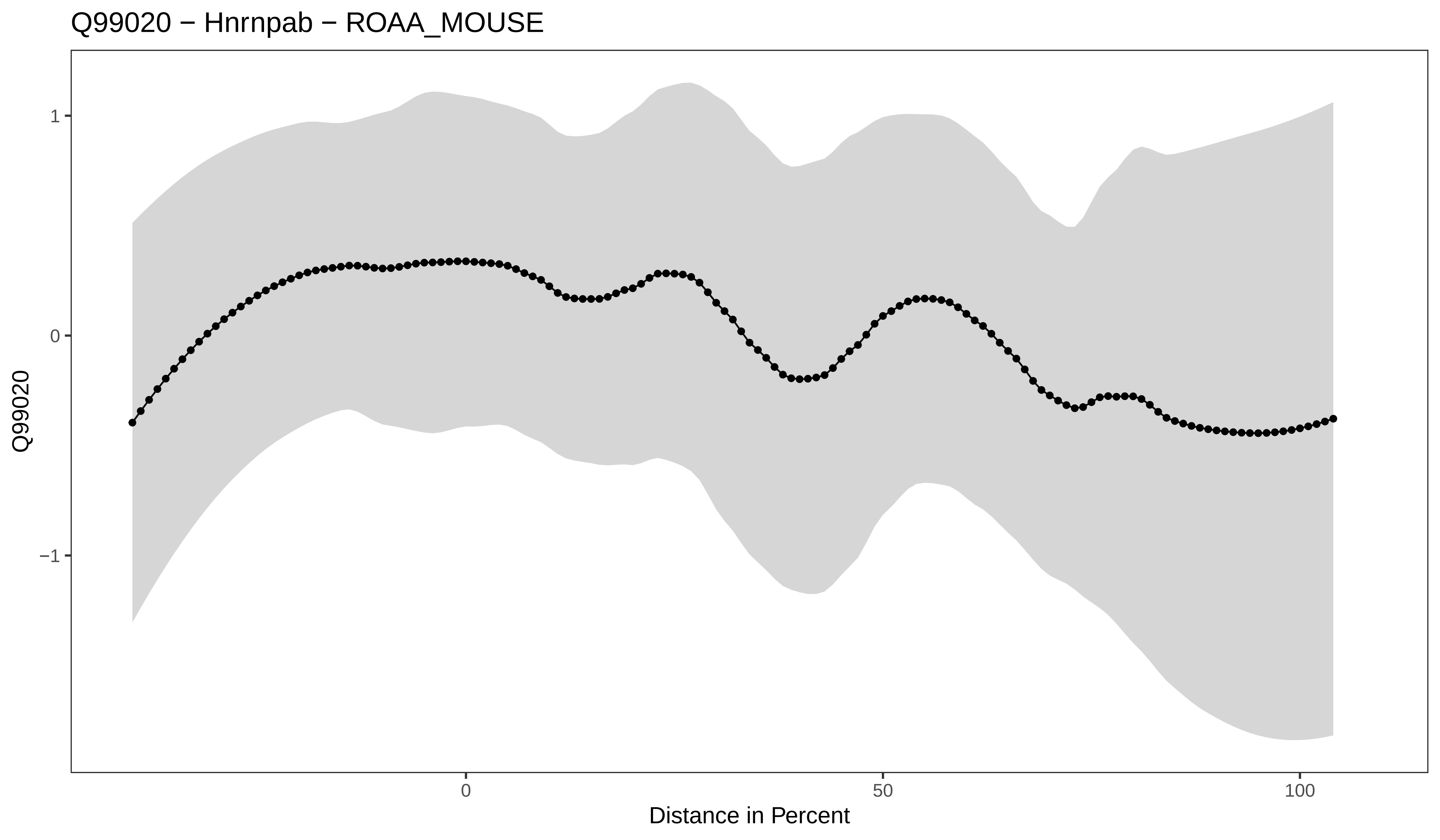

Q99020 − Hnrnpab − ROAA_MOUSE
1
0
−1
Q99020
0
50
100
Distance in Percent

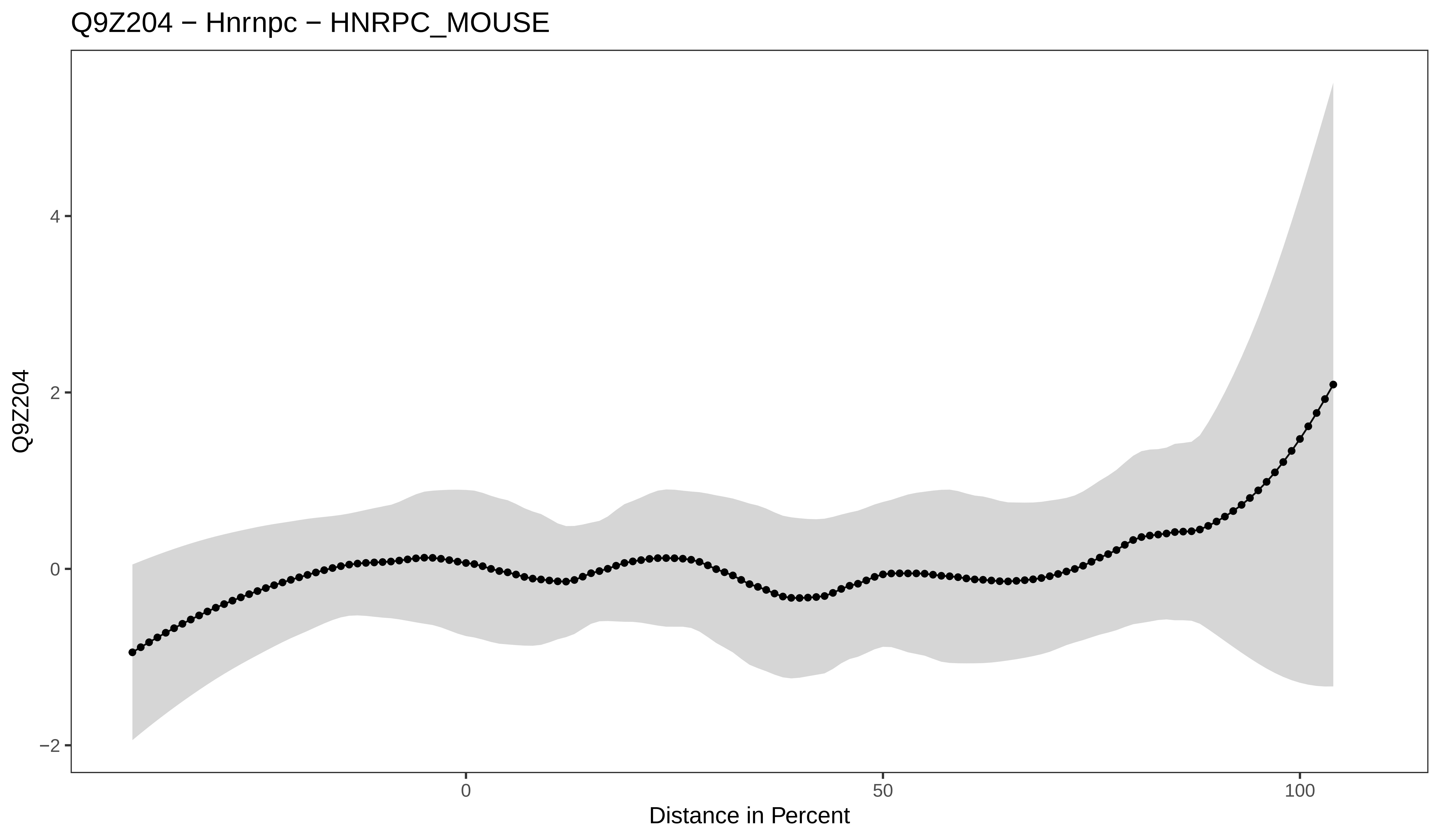

Q9Z204 − Hnrnpc − HNRPC_MOUSE
Q9Z204
4
2
0
−2
0
50
100
Distance in Percent

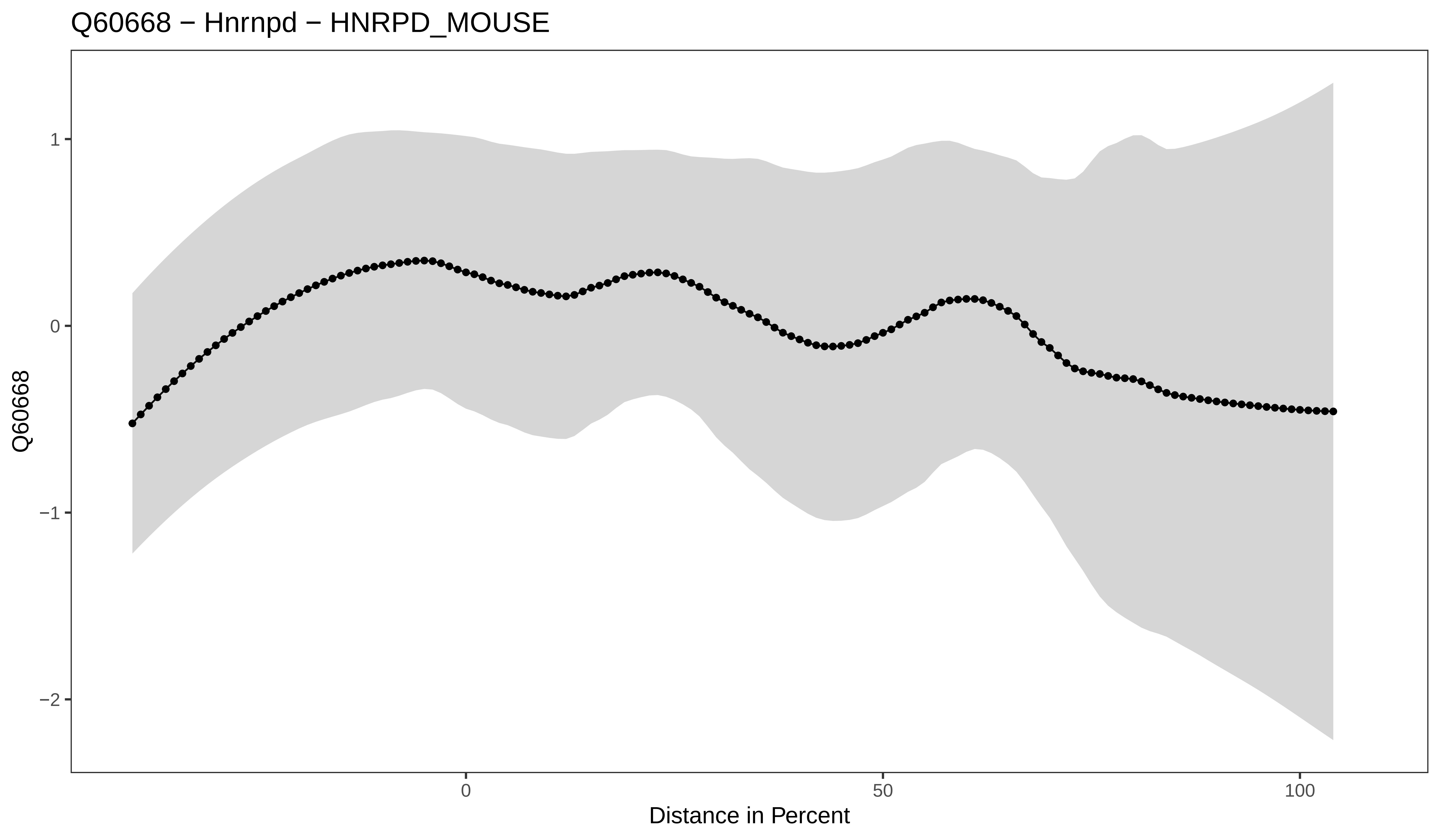
Q60668 − Hnrnpd − HNRPD_MOUSE
Q60668
1
0
−1
−2
0
50
100
Distance in Percent

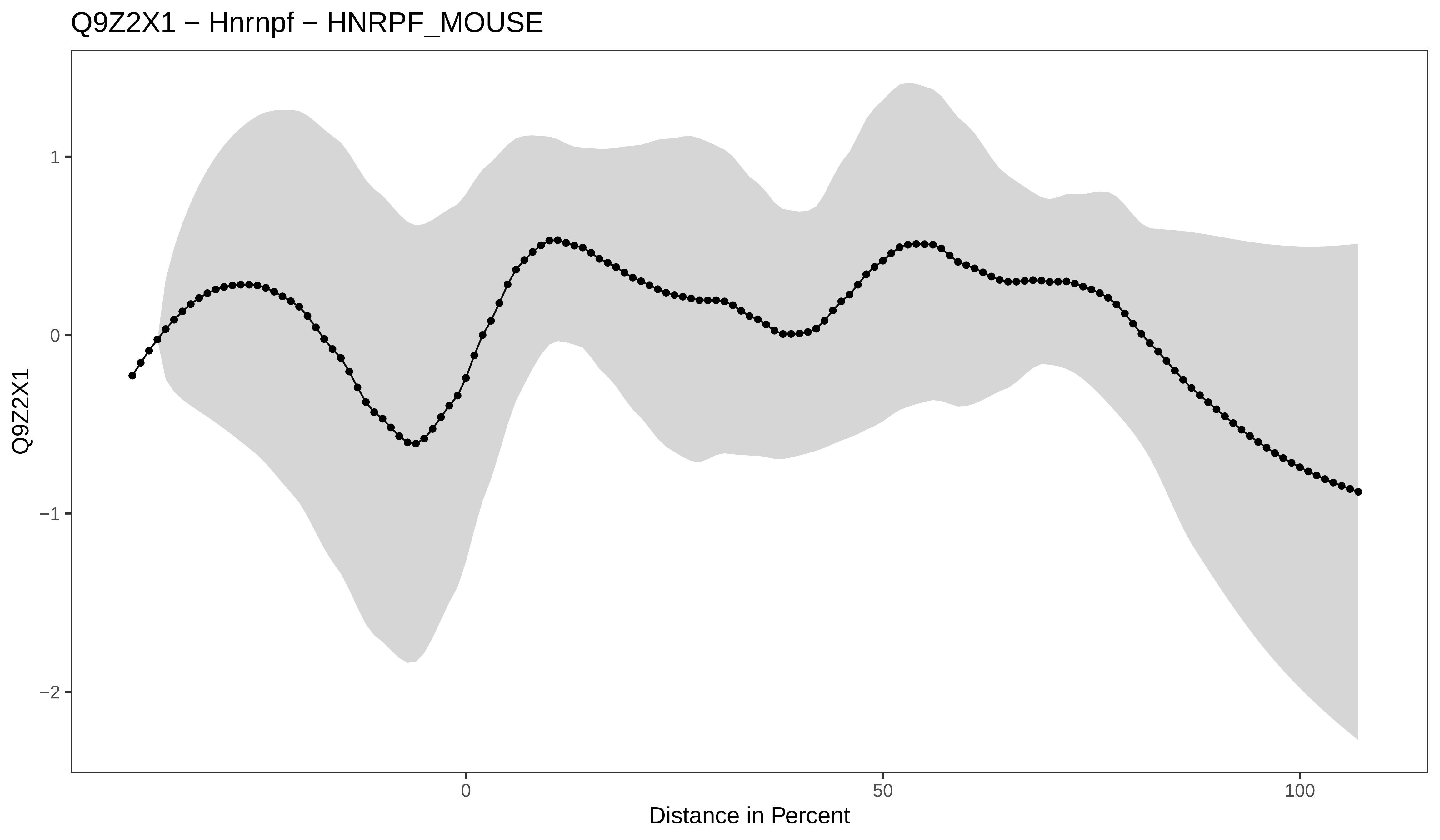
Q9Z2X1 − Hnrnpf − HNRPF_MOUSE
1
0
−1
−2
Q9Z2X1
0
50
100
Distance in Percent

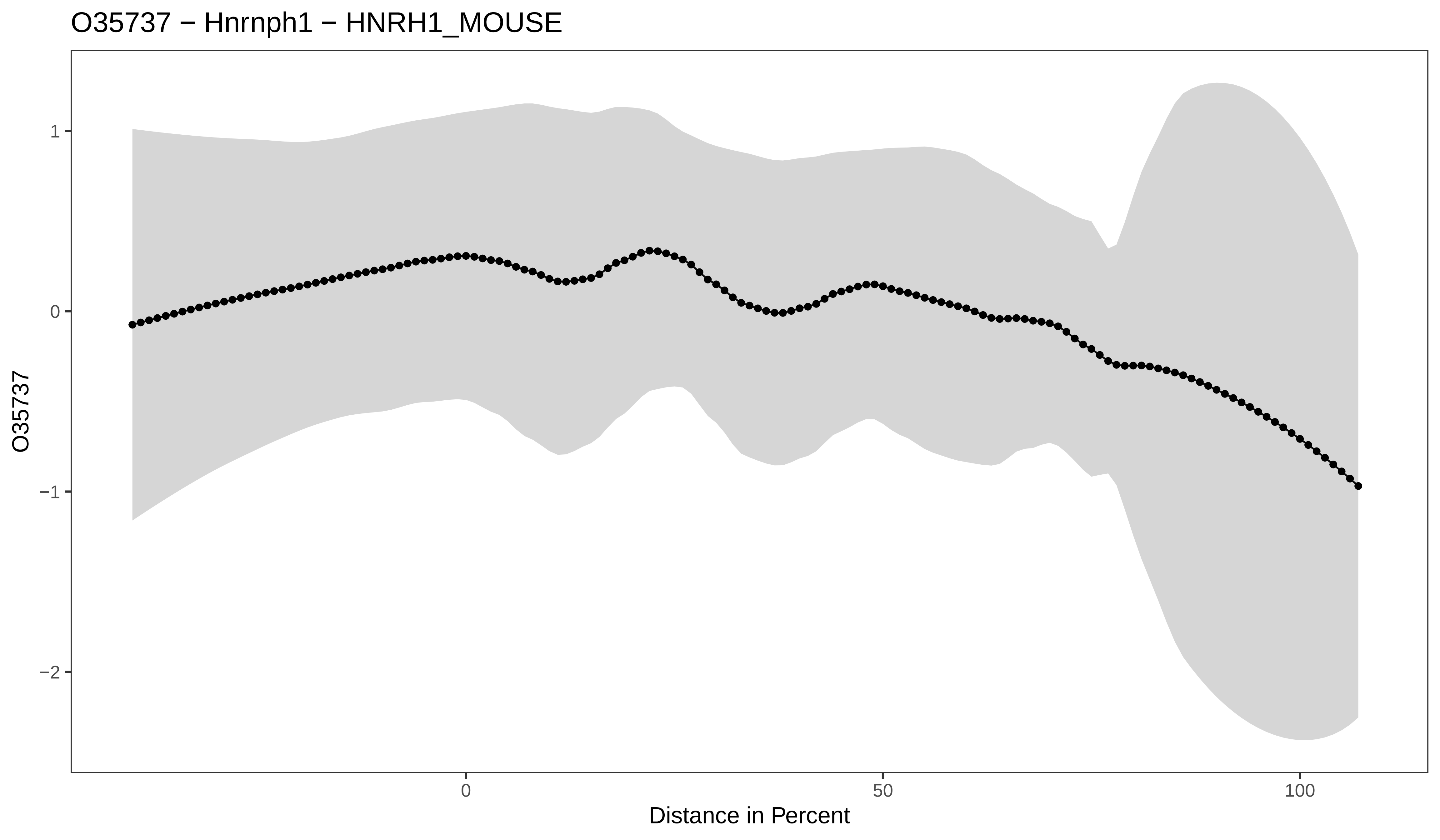
O35737 − Hnrnph1 − HNRH1_MOUSE
O35737
1
0
−1
−2
0
50
100
Distance in Percent

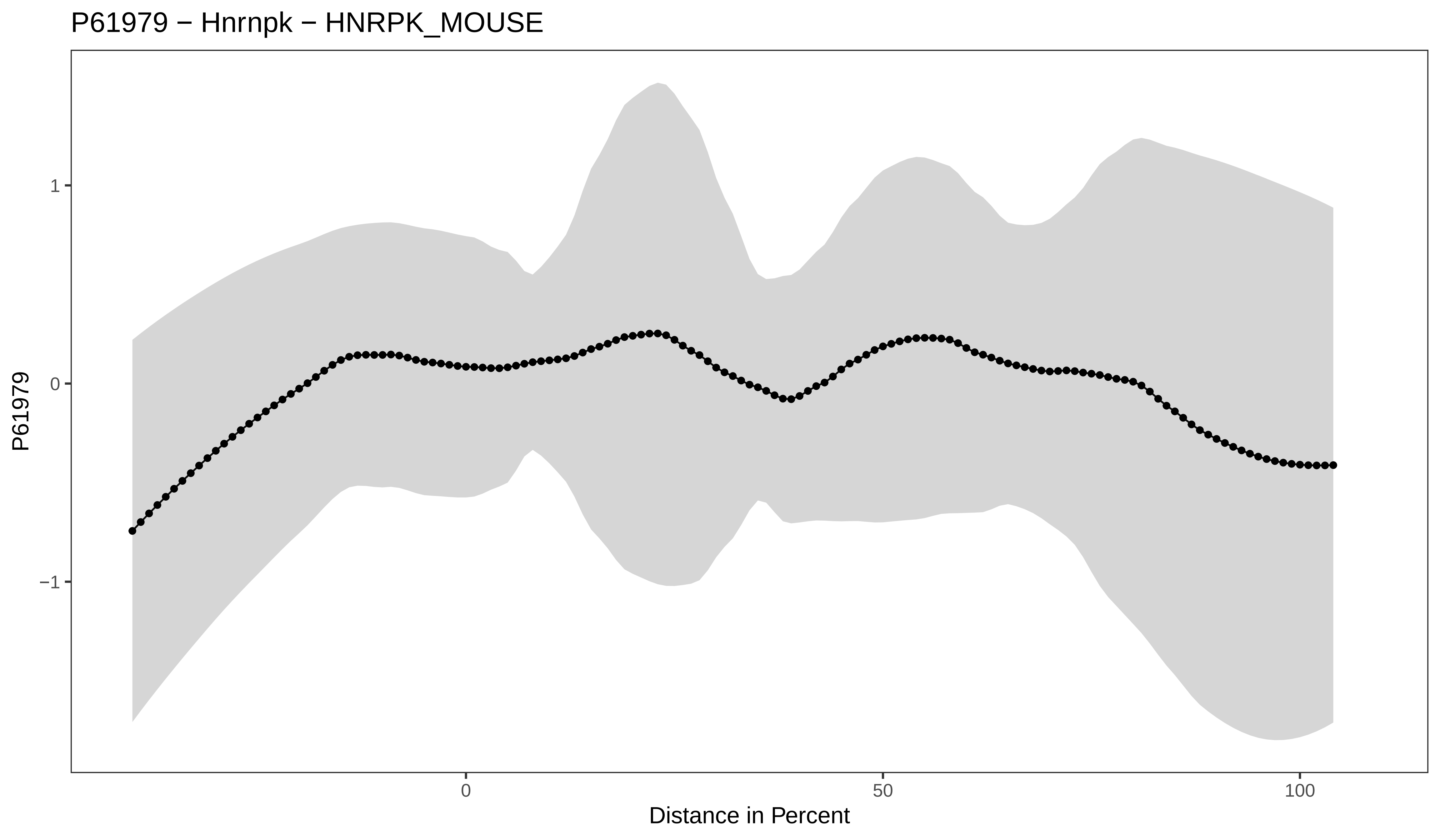
P61979 − Hnrnpk − HNRPK_MOUSE
1
0
−1
P61979
0
50
100
Distance in Percent

Q8R081 − Hnrnpl − HNRPL_MOUSE

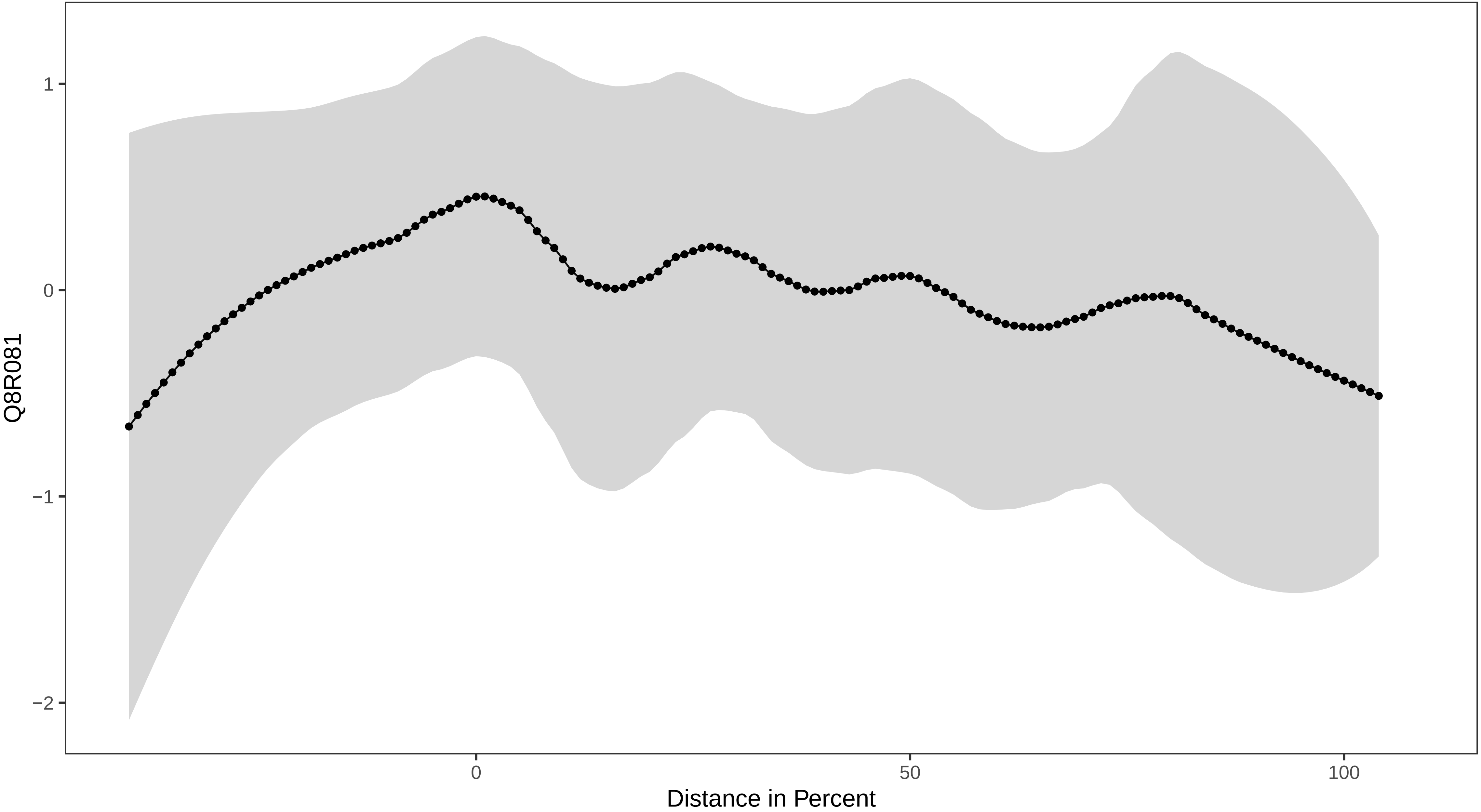

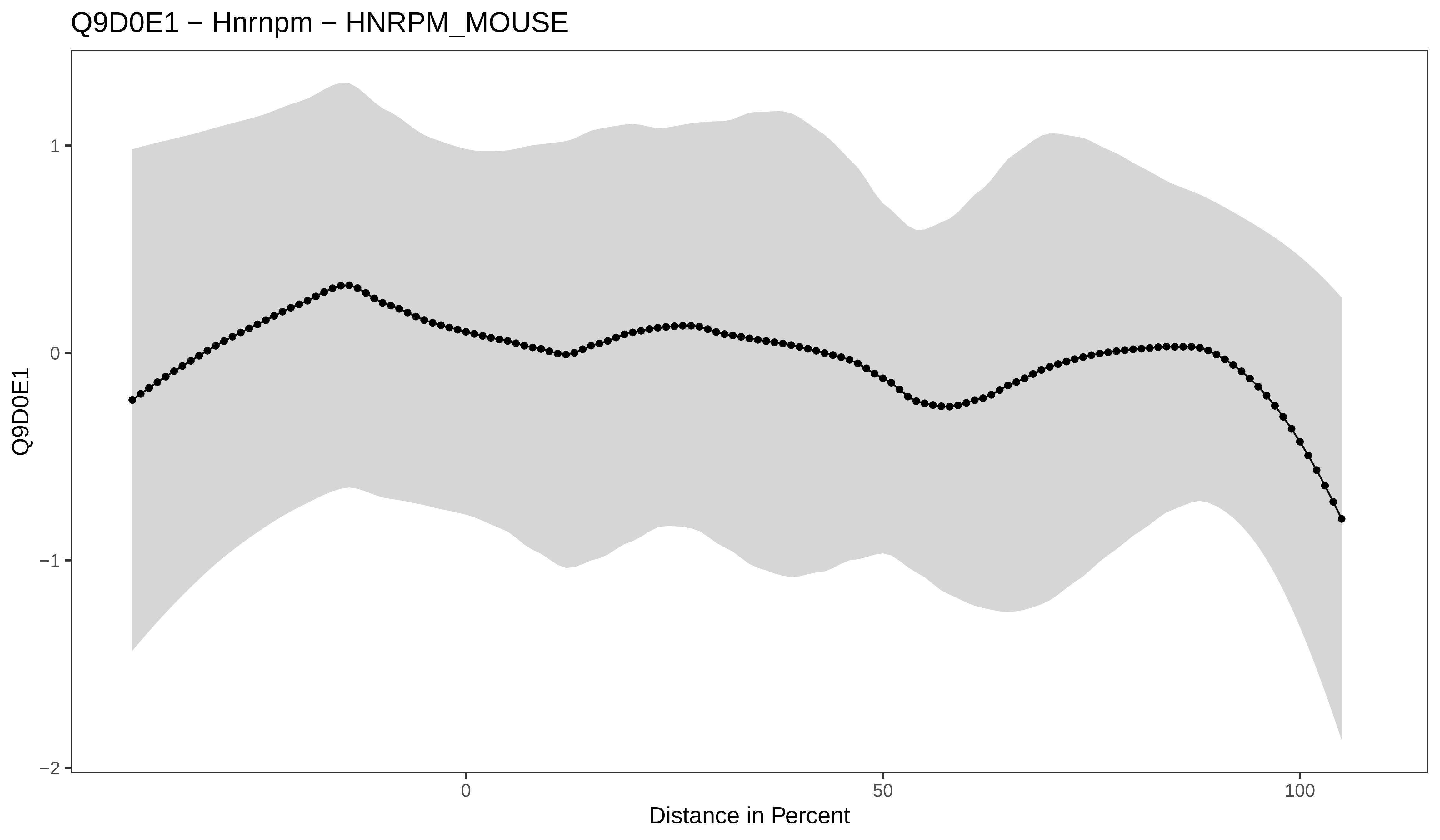

Q9D0E1 − Hnrnpm − HNRPM_MOUSE
Q9D0E1
1
0
−1
−2
0
50
100
Distance in Percent

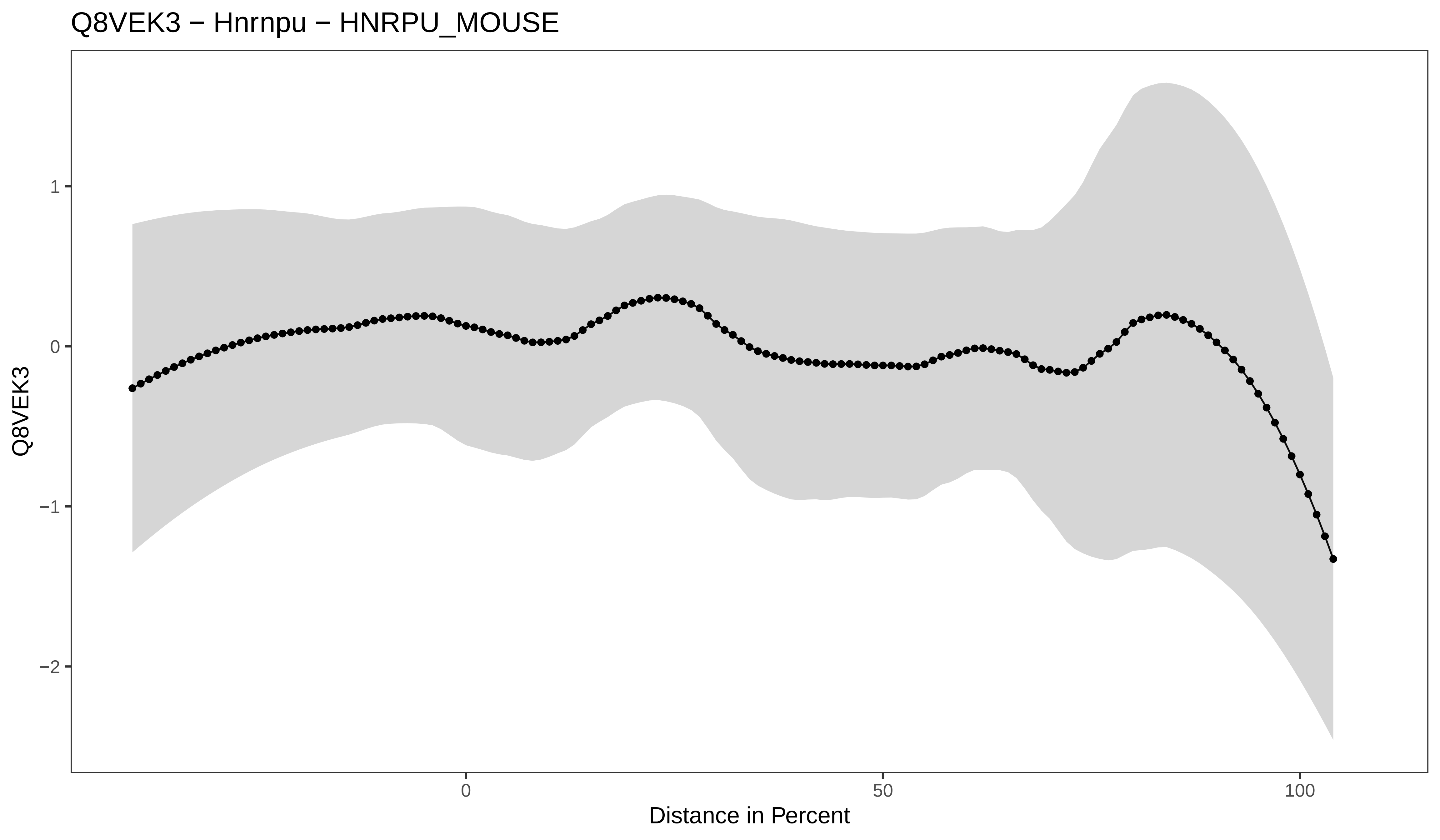

Q8VEK3 − Hnrnpu − HNRPU_MOUSE
1
0
−1
−2
Q8VEK3
0
50
100
Distance in Percent

Q8VDM6 − Hnrnpul1 − HNRL1_MOUSE

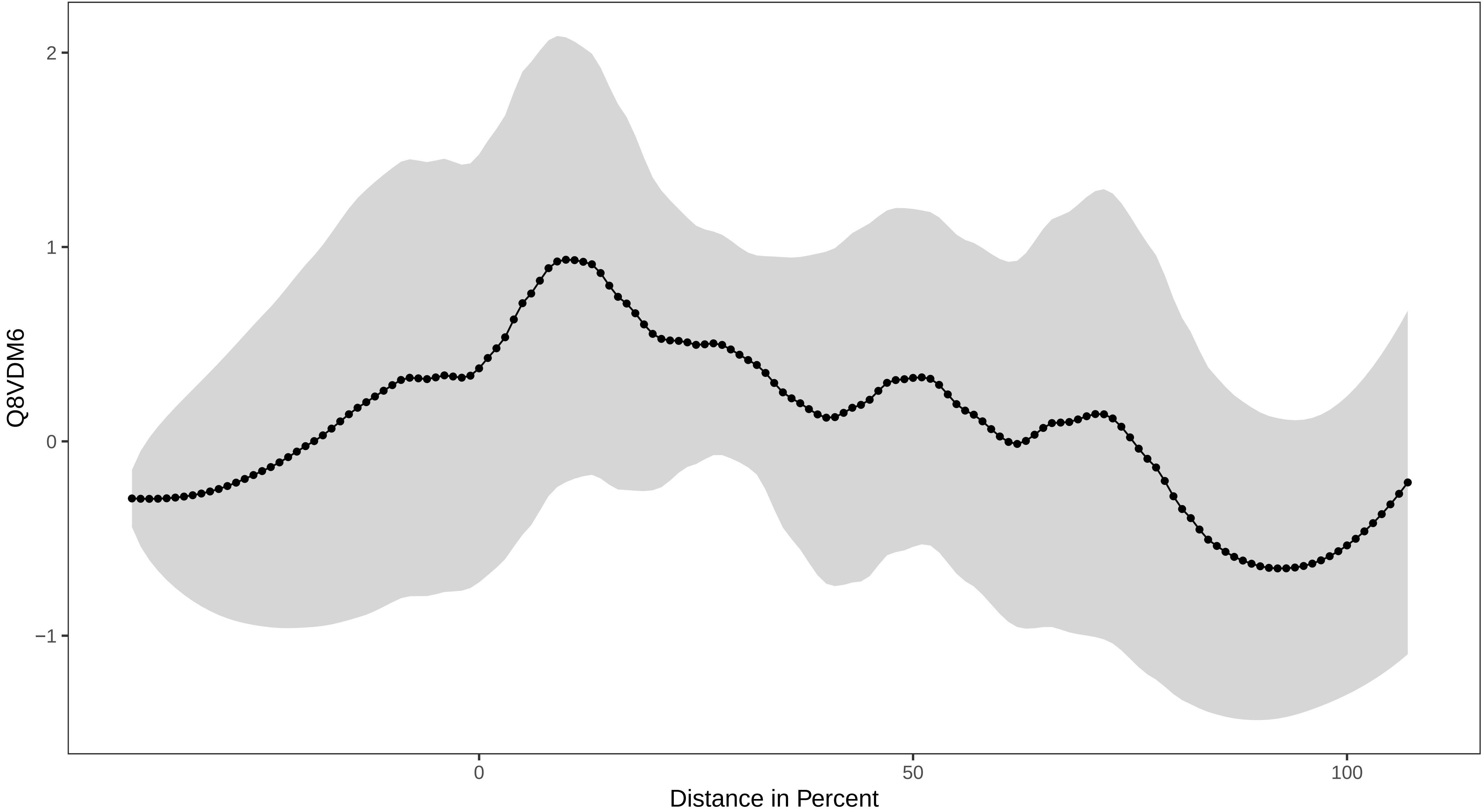

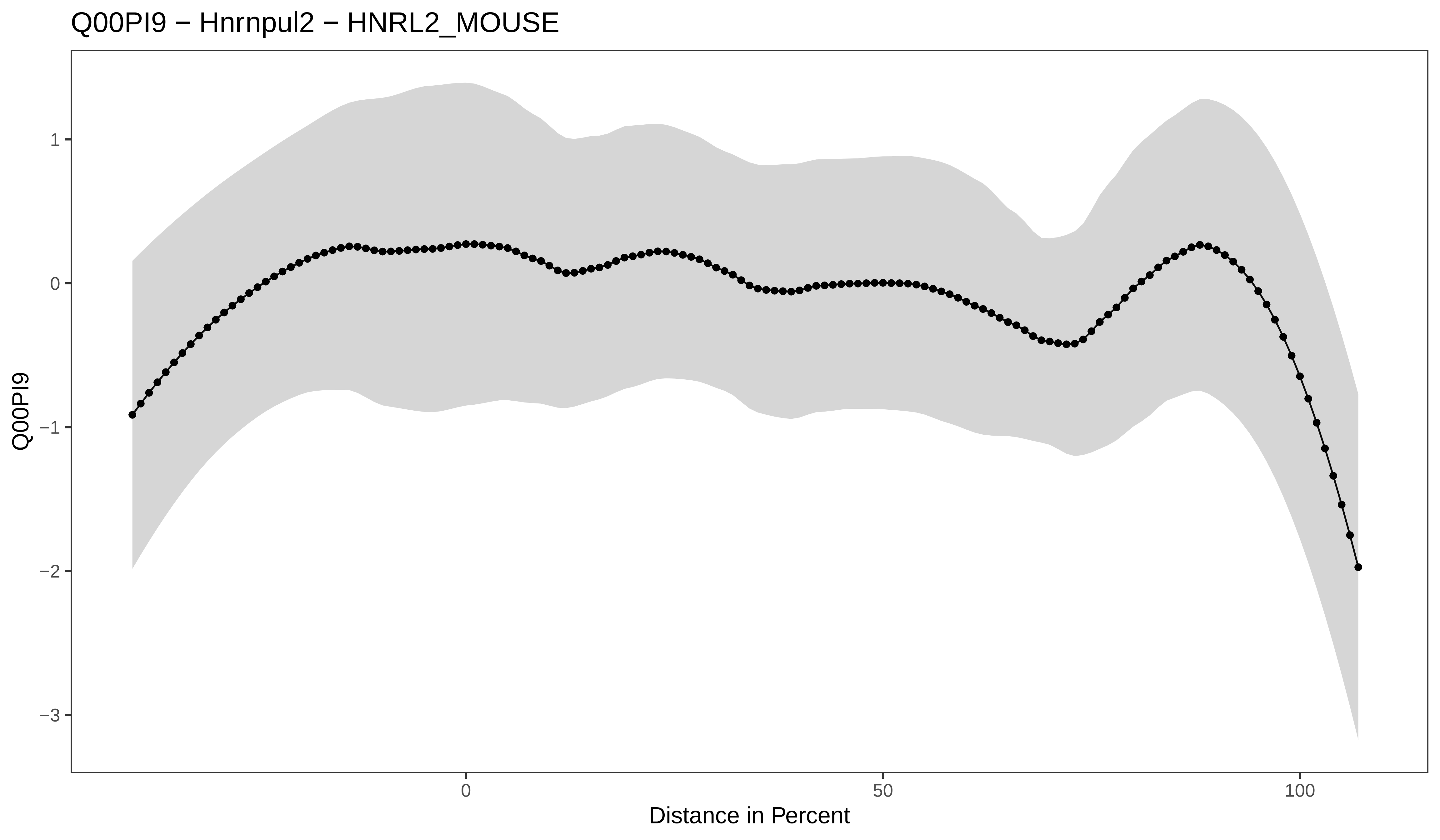
Q00PI9 − Hnrnpul2 − HNRL2_MOUSE
Q00PI9
1
0
−1
−2
−3
0
50
100
Distance in Percent

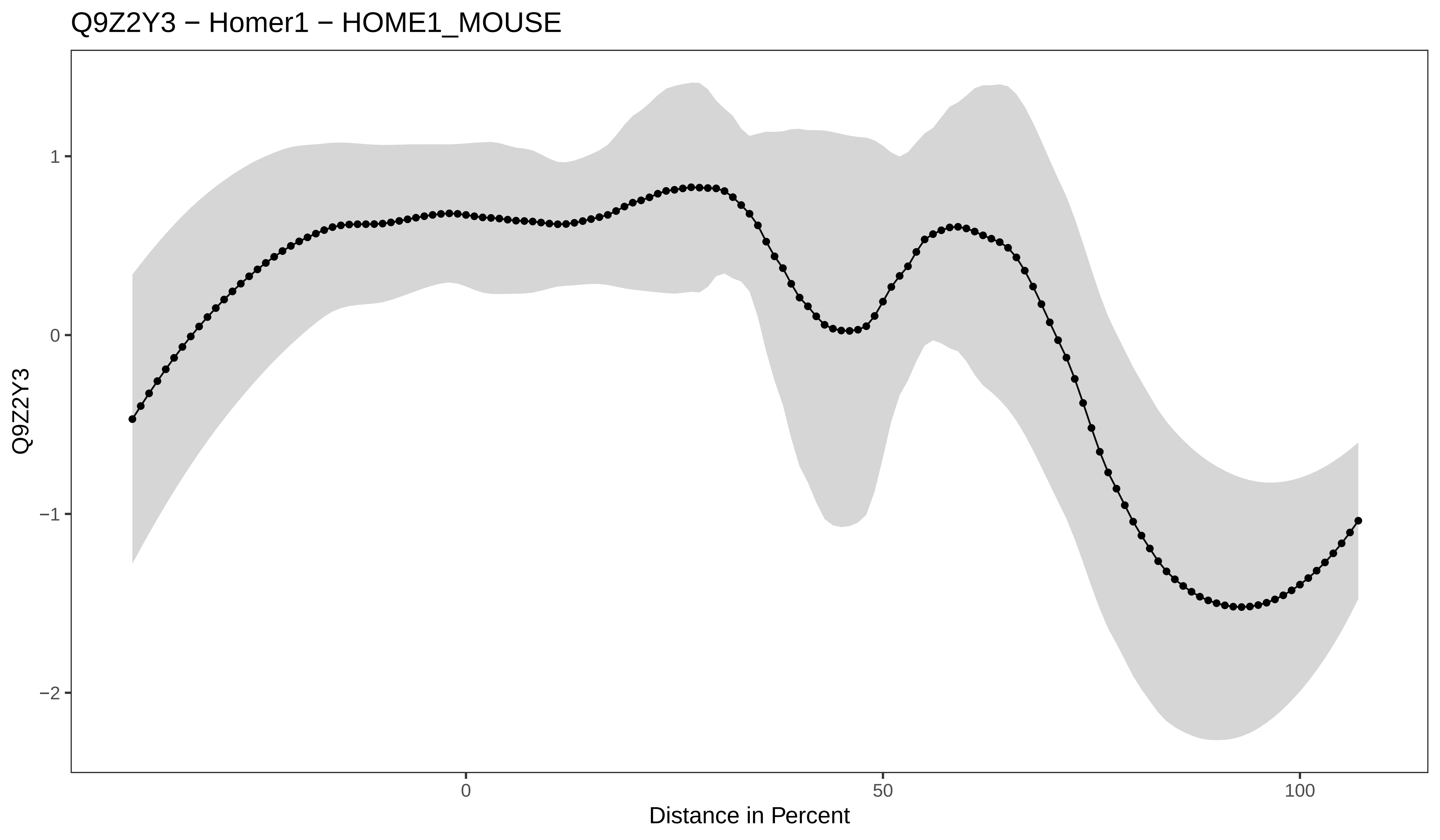
Q9Z2Y3 − Homer1 − HOME1_MOUSE
Q9Z2Y3
1
0
−1
−2
0
50
100
Distance in Percent

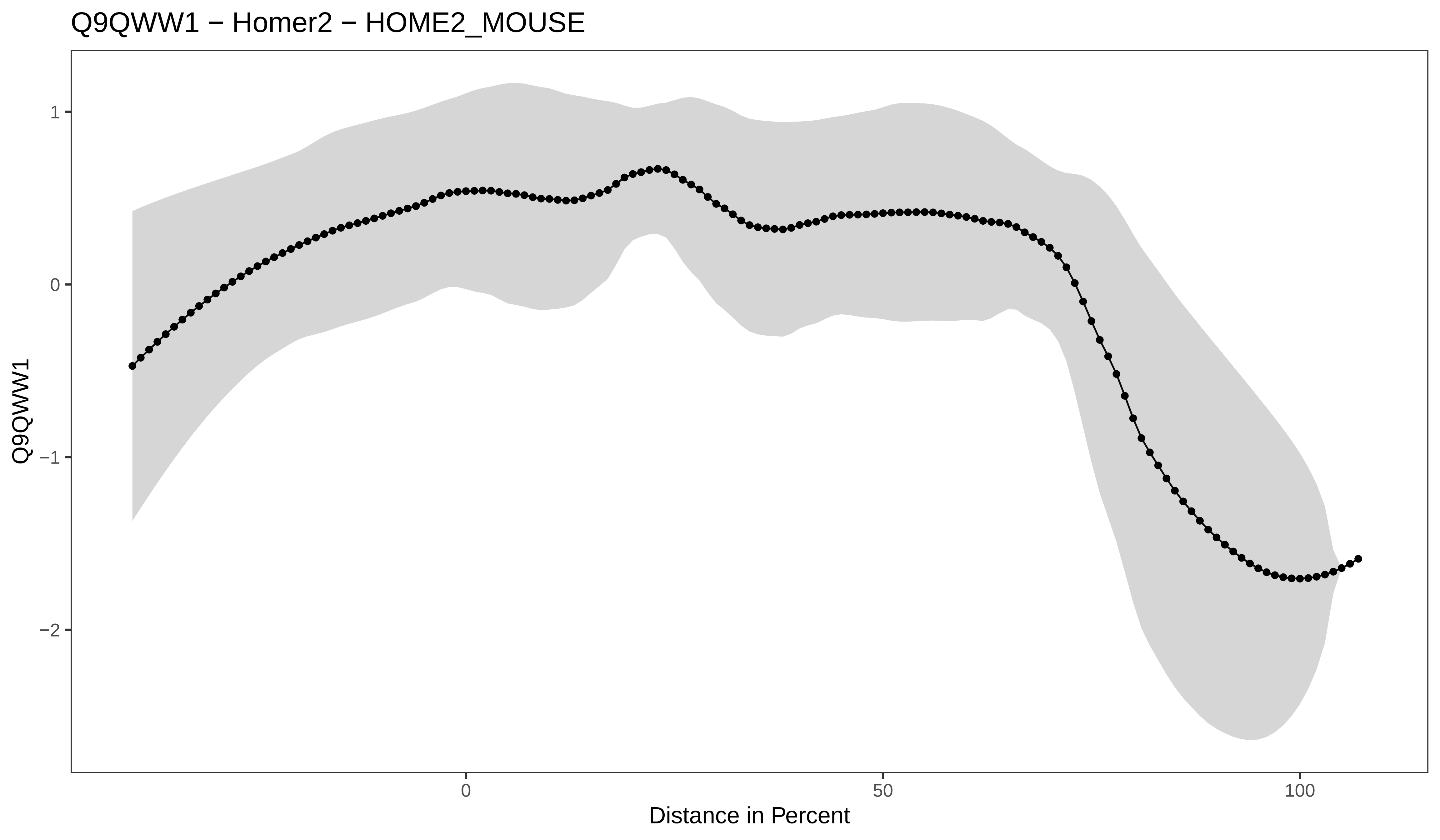

Q9QWW1 − Homer2 − HOME2_MOUSE
1
0
−1
−2
Q9QWW1
0
50
100
Distance in Percent

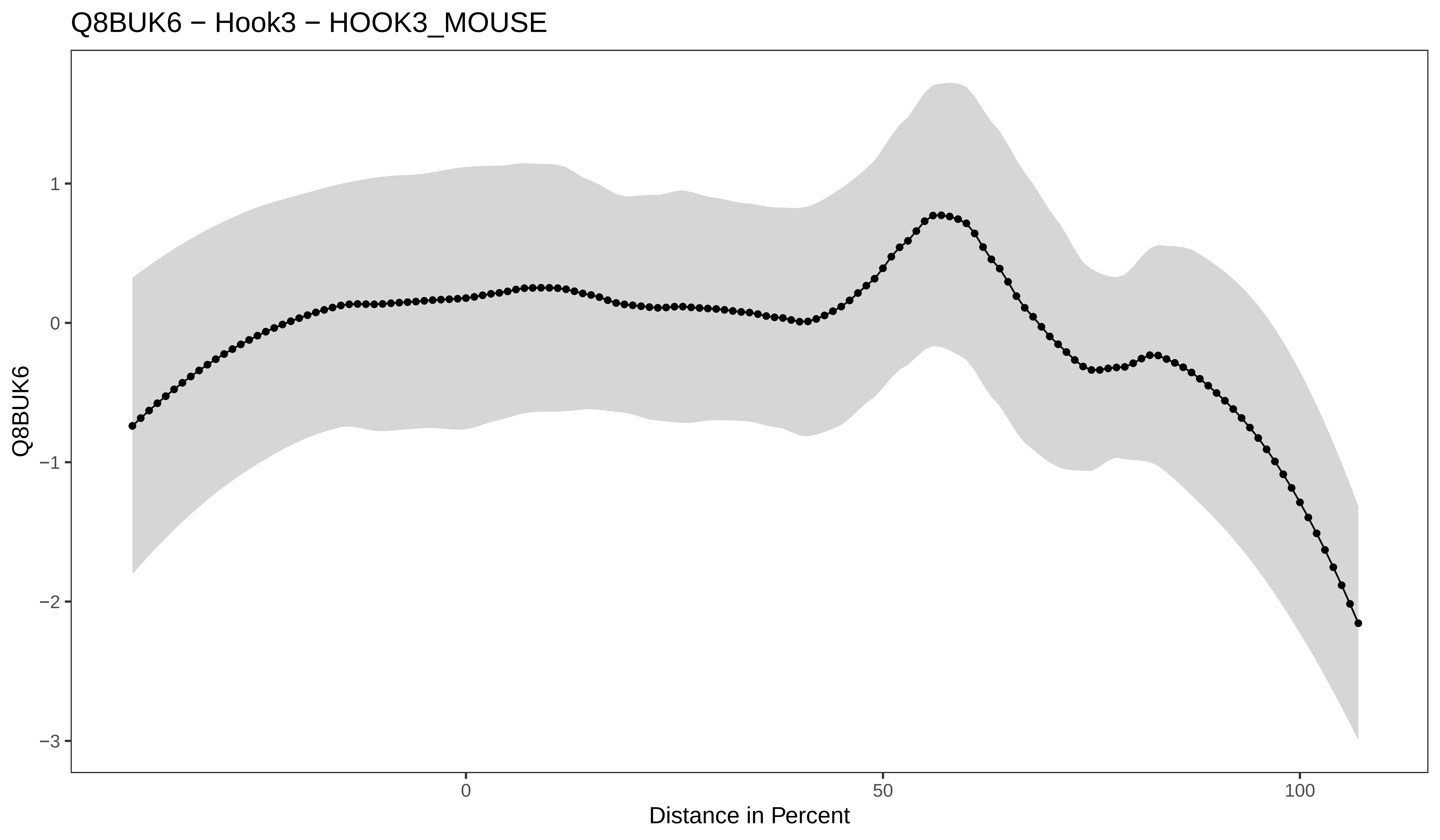
Q8BUK6 − Hook3 − HOOK3_MOUSE
Q8BUK6
1
0
−1
−2
−3
0
50
100
Distance in Percent

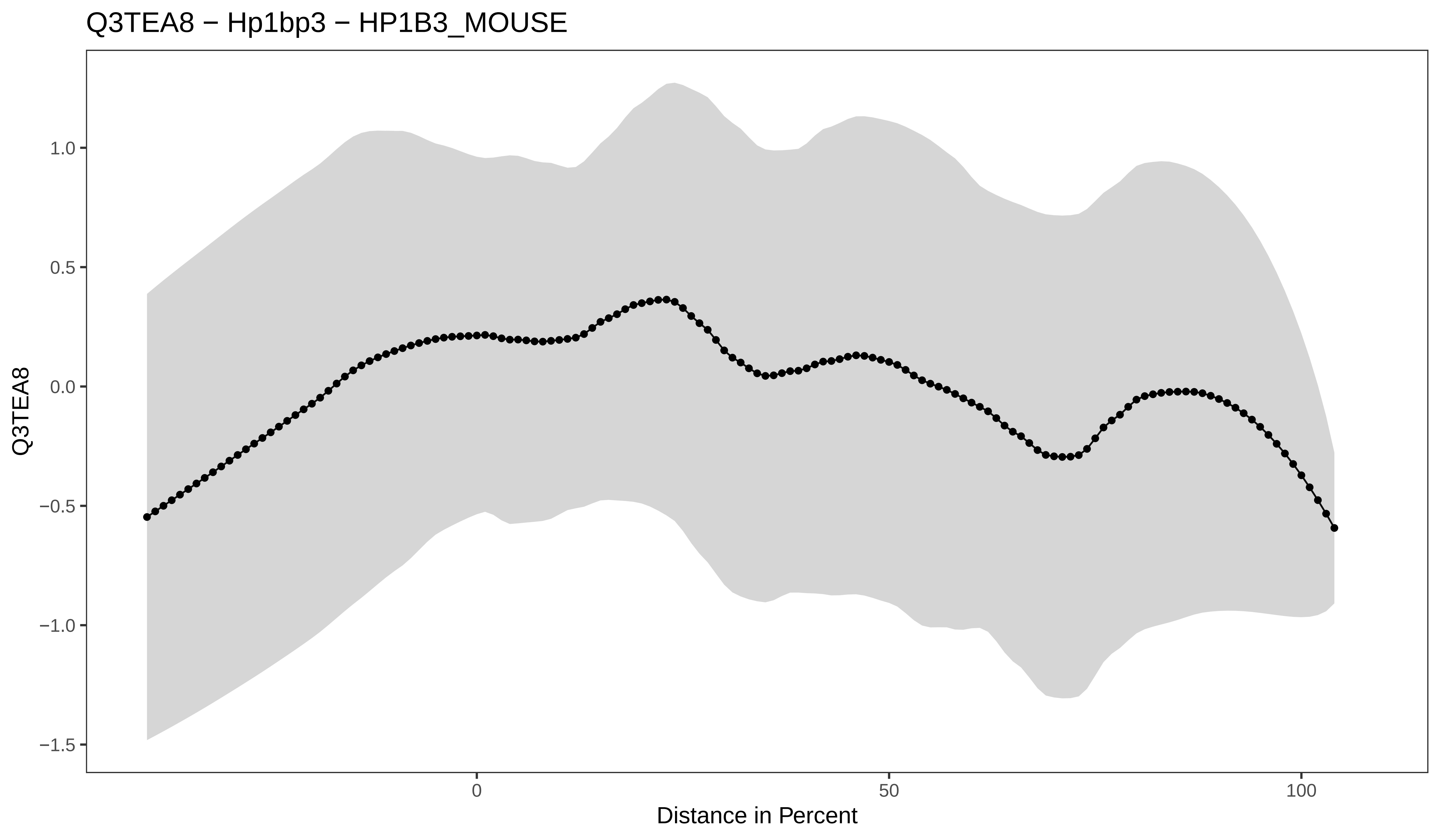

Q3TEA8 − Hp1bp3 − HP1B3_MOUSE
Q3TEA8
1.0
0.5
0.0
−0.5
−1.0
−1.5
0
50
100
Distance in Percent

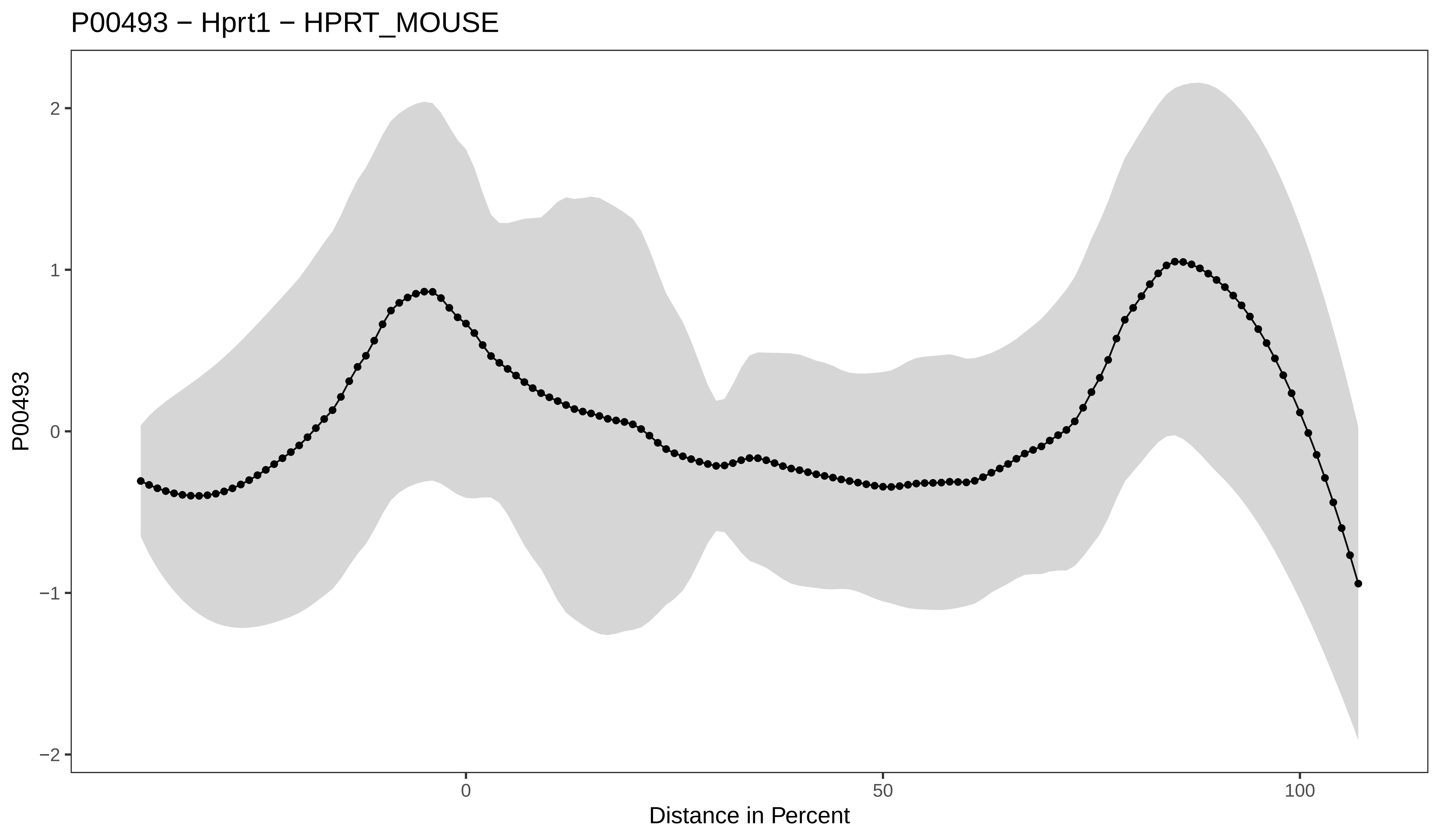
P00493 − Hprt1 − HPRT_MOUSE
P00493
2
1
0
−1
−2
0
50
100
Distance in Percent

Q91X72 − Hpx − HEMO_MOUSE

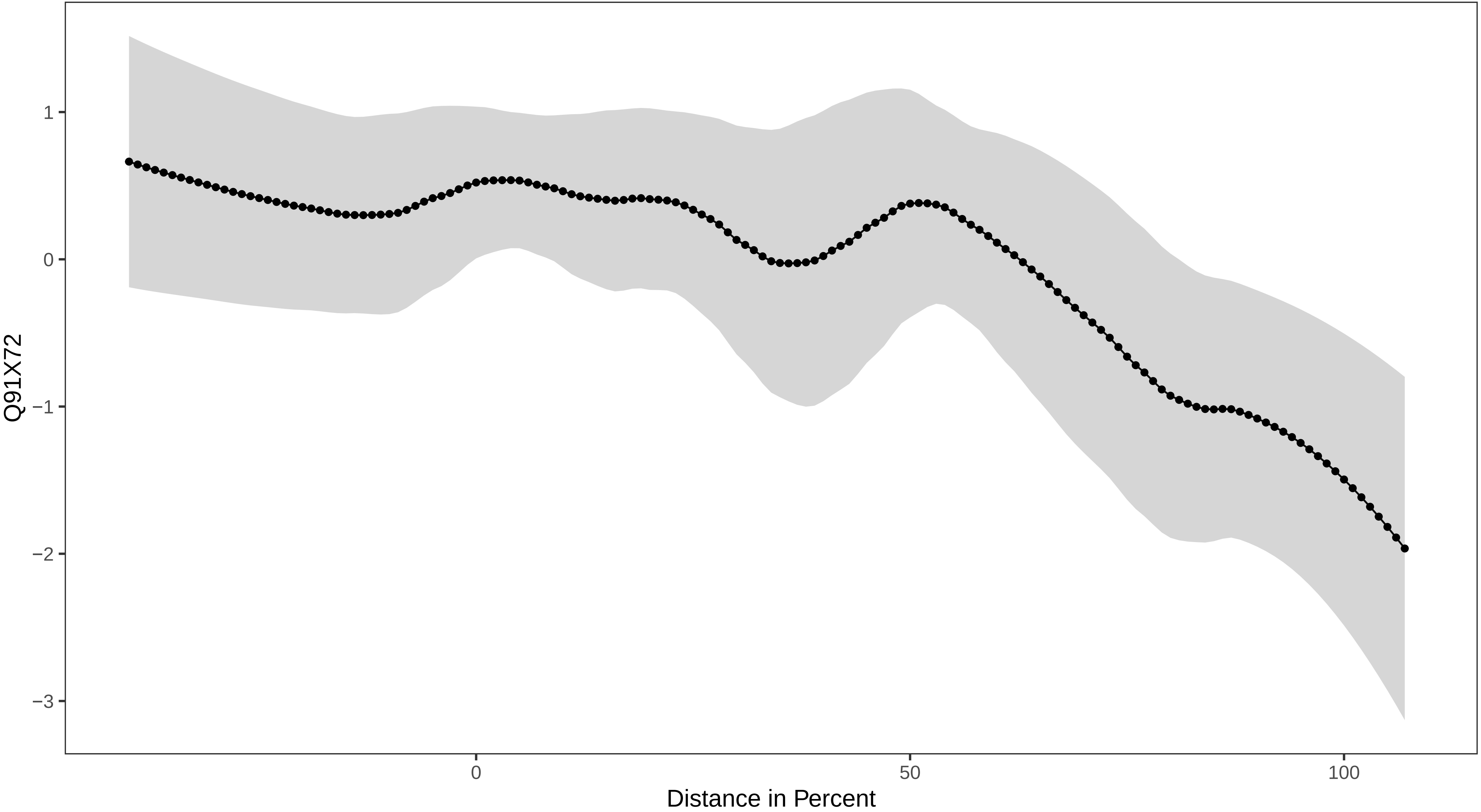

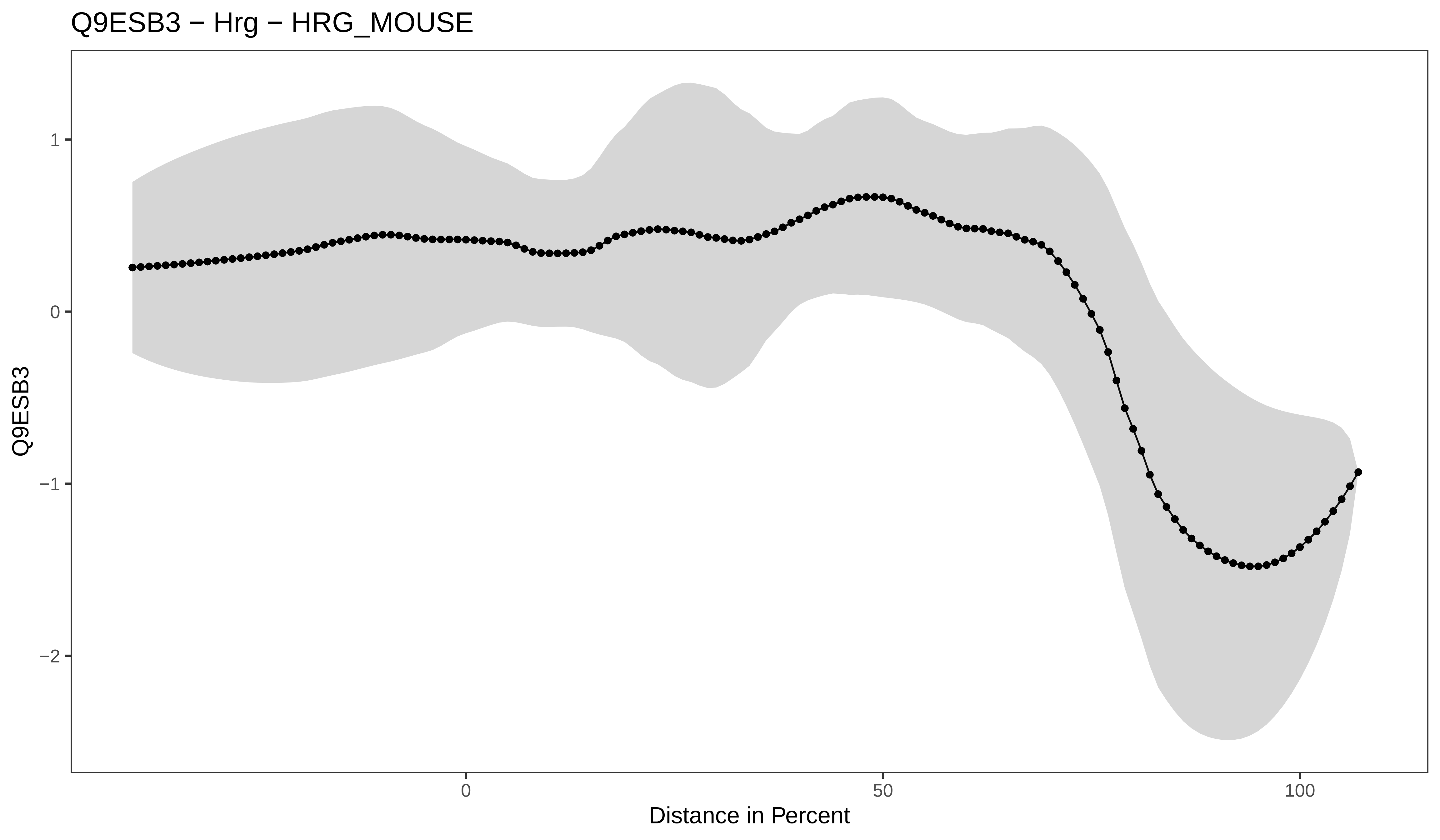

Q9ESB3 − Hrg − HRG_MOUSE
1
0
−1
−2
Q9ESB3
0
50
100
Distance in Percent

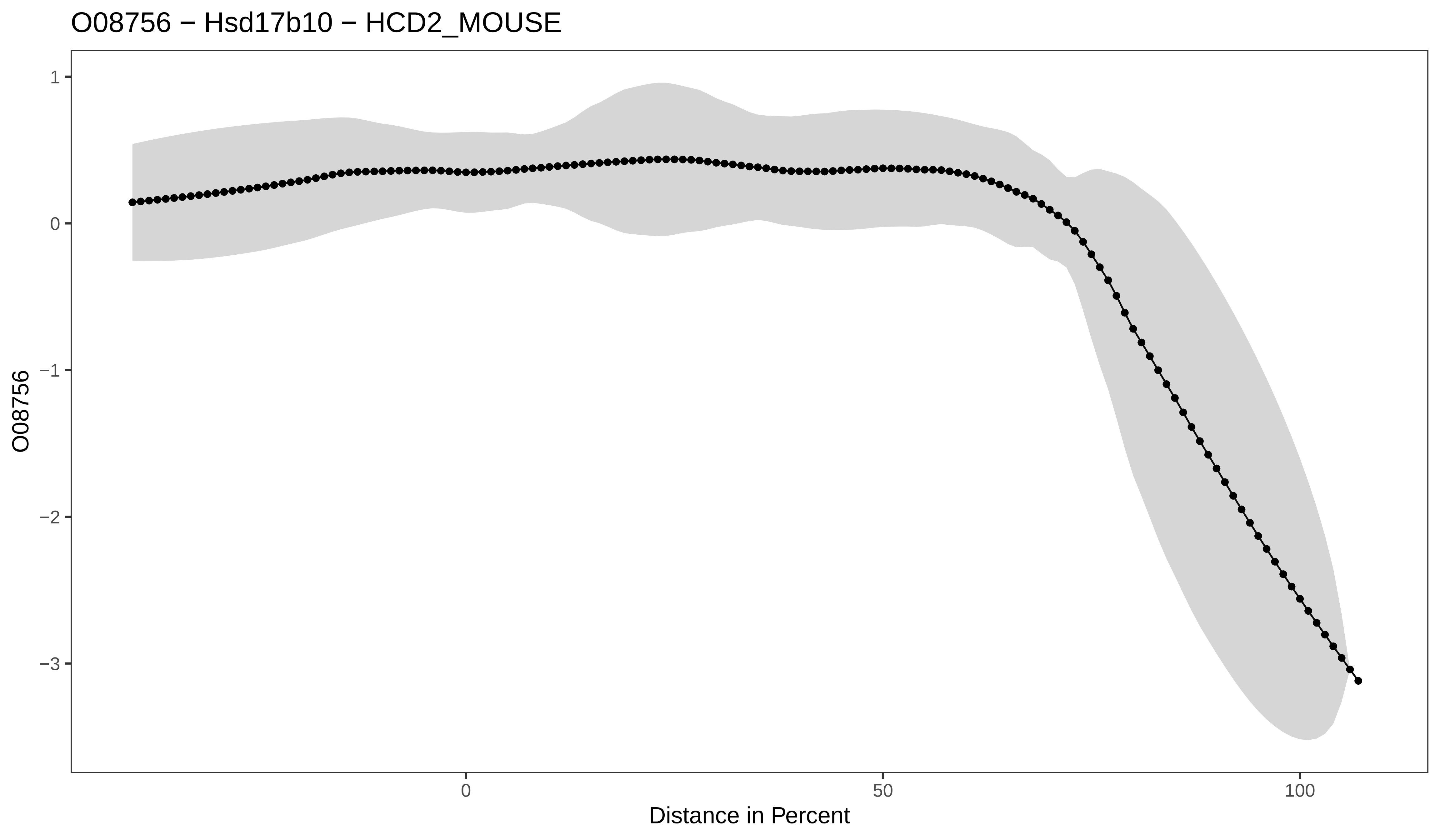

O08756 − Hsd17b10 − HCD2_MOUSE
1
0
−1
−2
−3
O08756
0
50
100
Distance in Percent

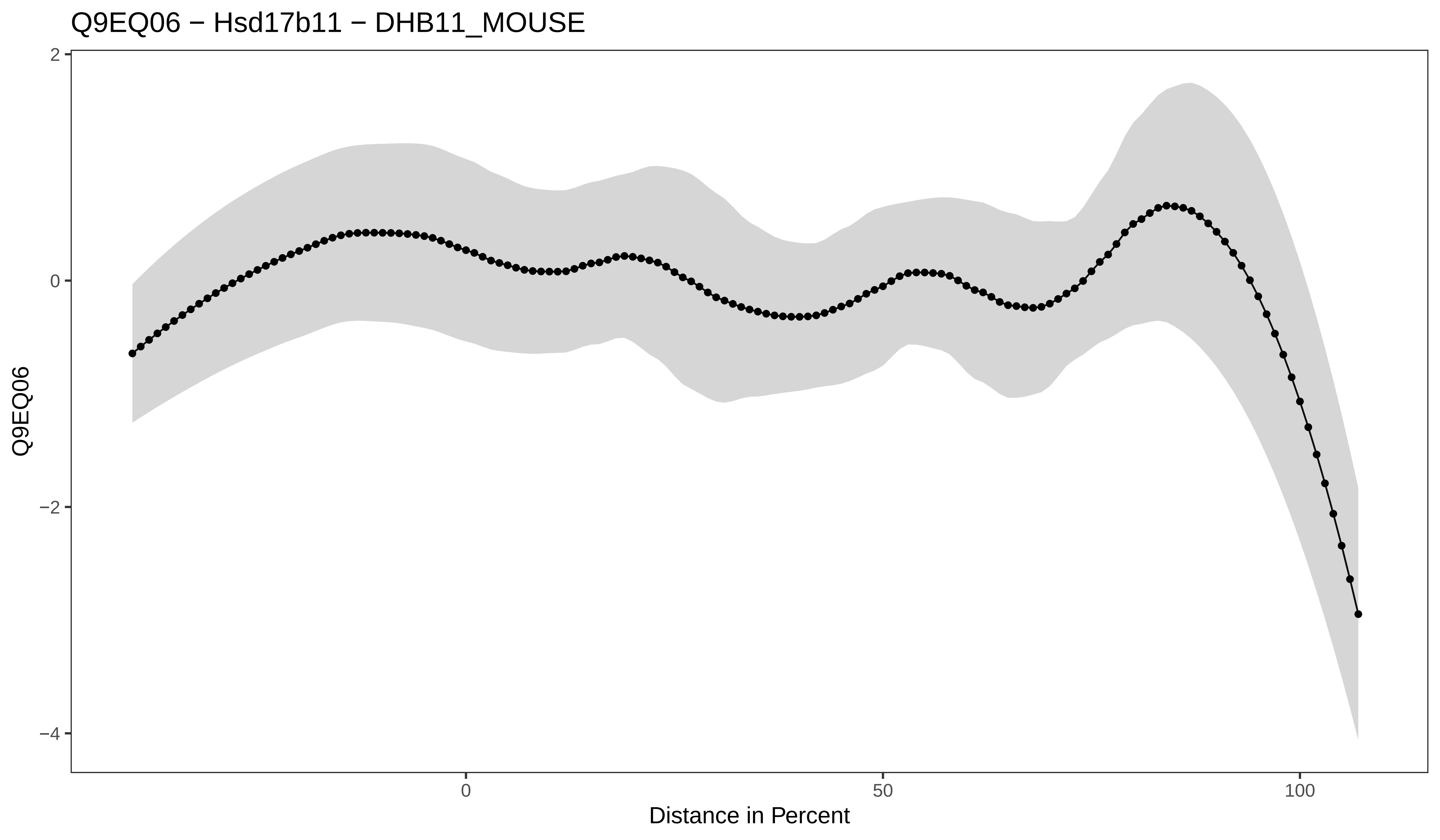

Q9EQ06 − Hsd17b11 − DHB11_MOUSE
2
0
−2
−4
Q9EQ06
0
50
100
Distance in Percent

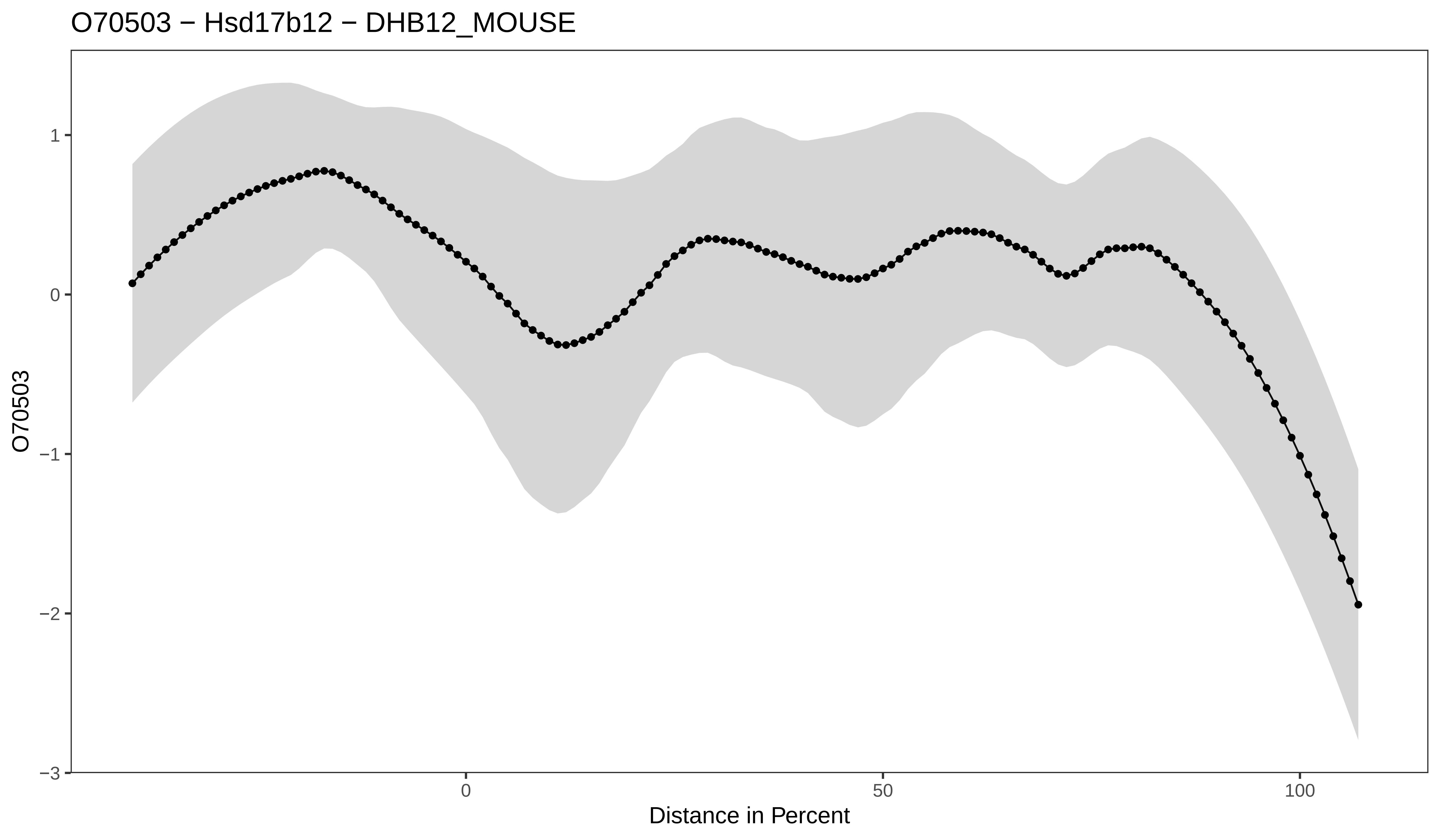
O70503 − Hsd17b12 − DHB12_MOUSE
O70503
Distance in Percent
1
0
−1
−2
−3
0
50
100

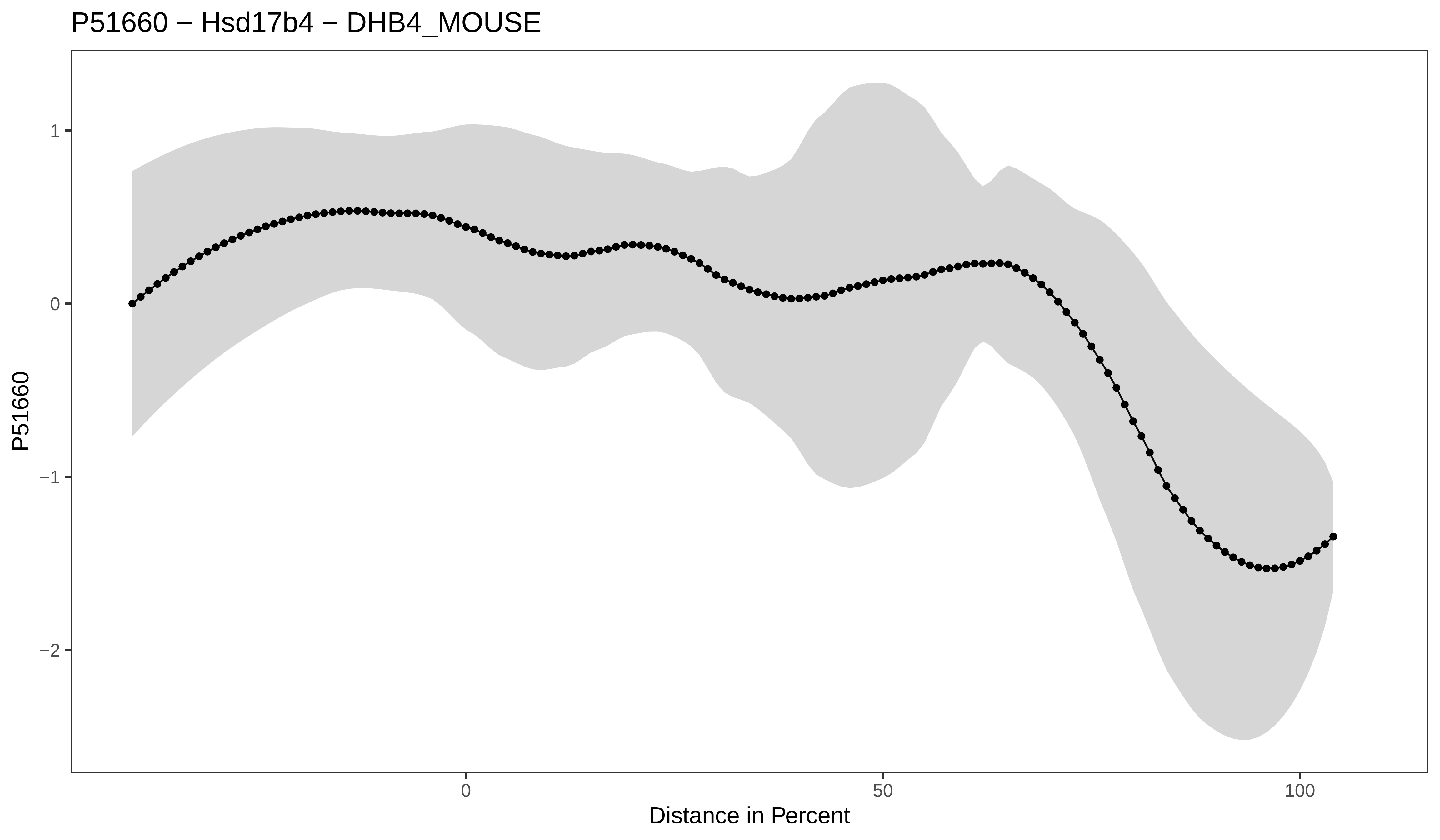

P51660 − Hsd17b4 − DHB4_MOUSE
1
0
−1
−2
P51660
0
50
100
Distance in Percent

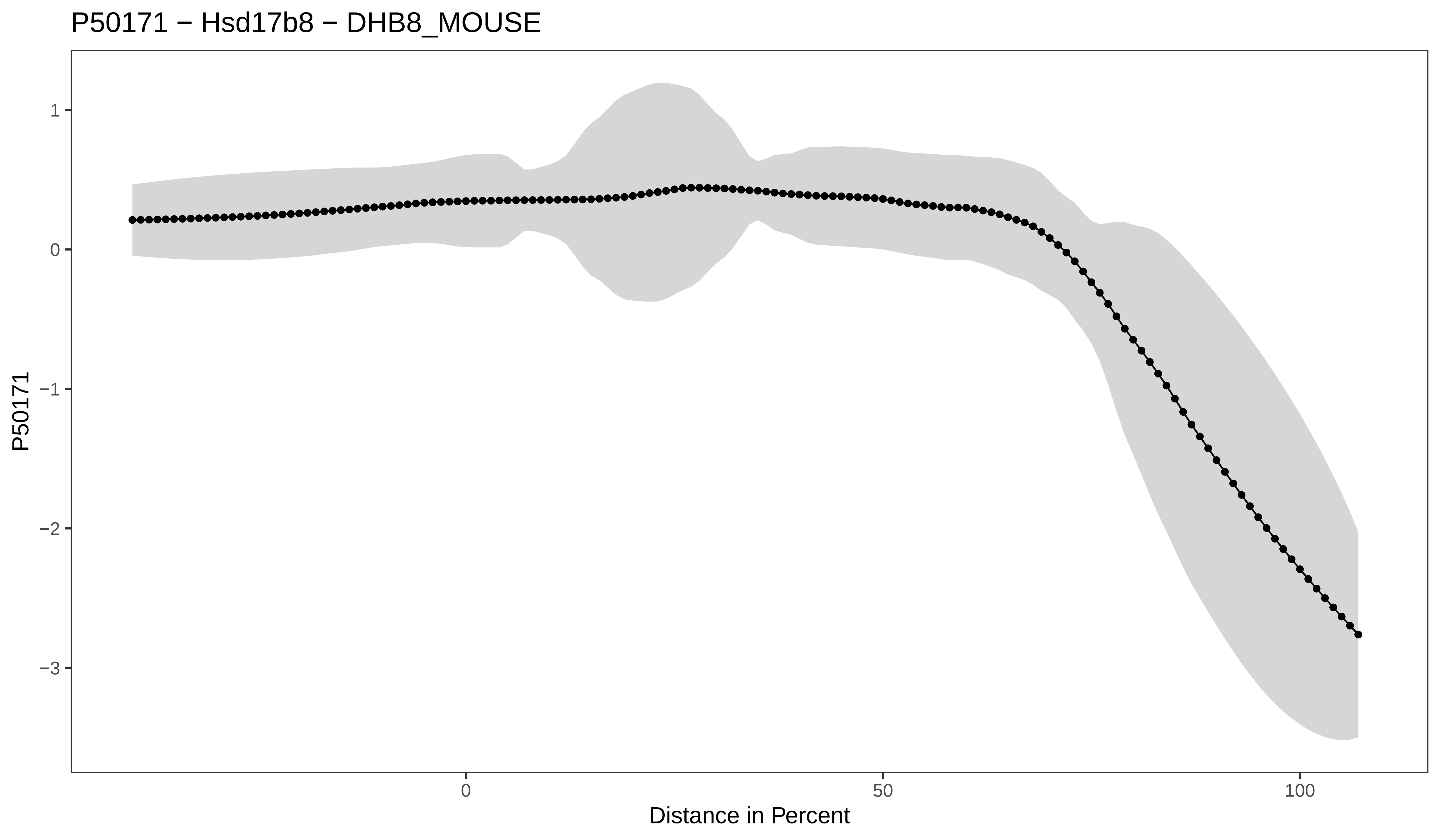
P50171 − Hsd17b8 − DHB8_MOUSE
P50171
1
0
−1
−2
−3
0
50
100
Distance in Percent

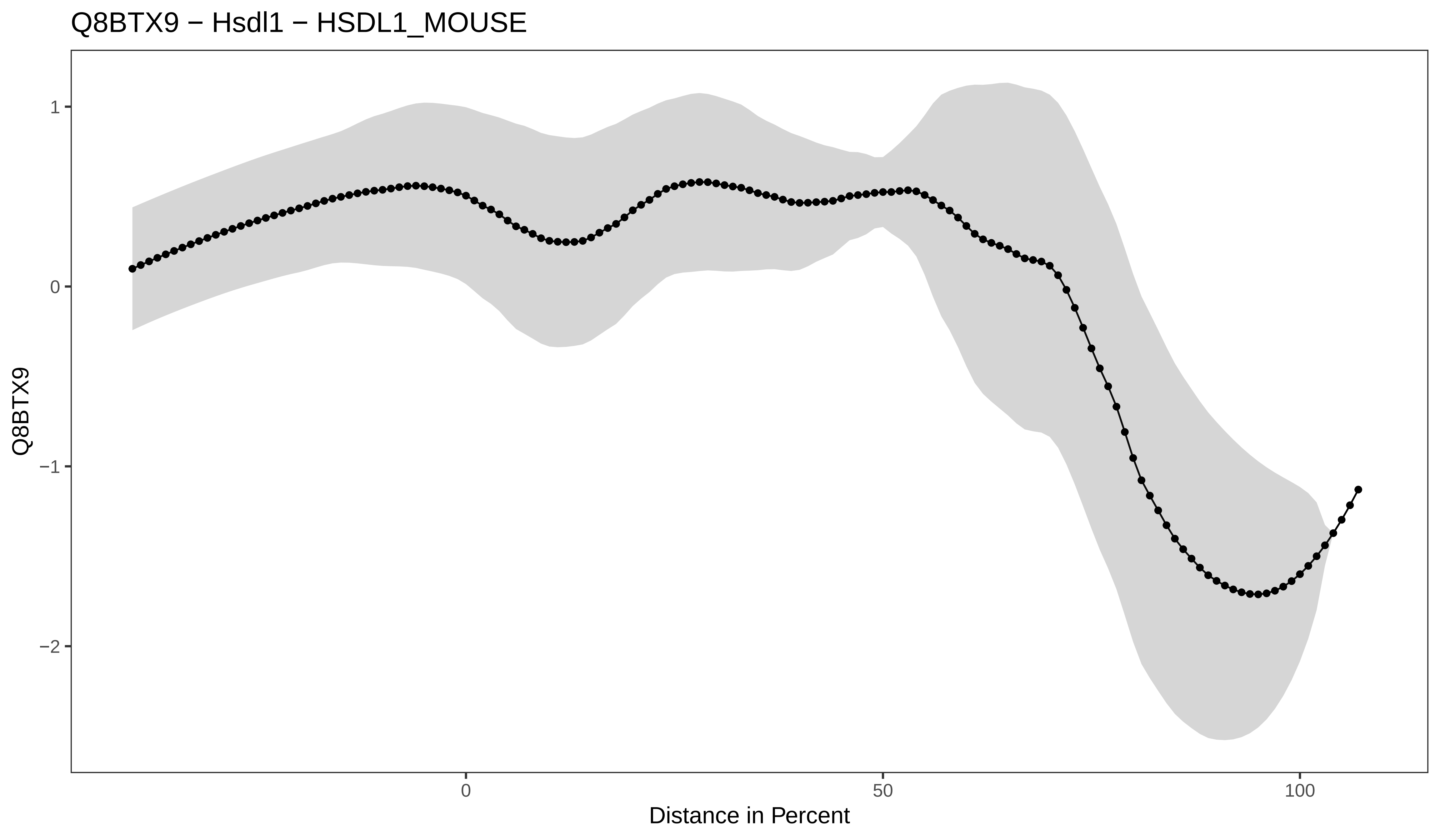
Q8BTX9 − Hsdl1 − HSDL1_MOUSE
Q8BTX9
1
0
−1
−2
0
50
100
Distance in Percent

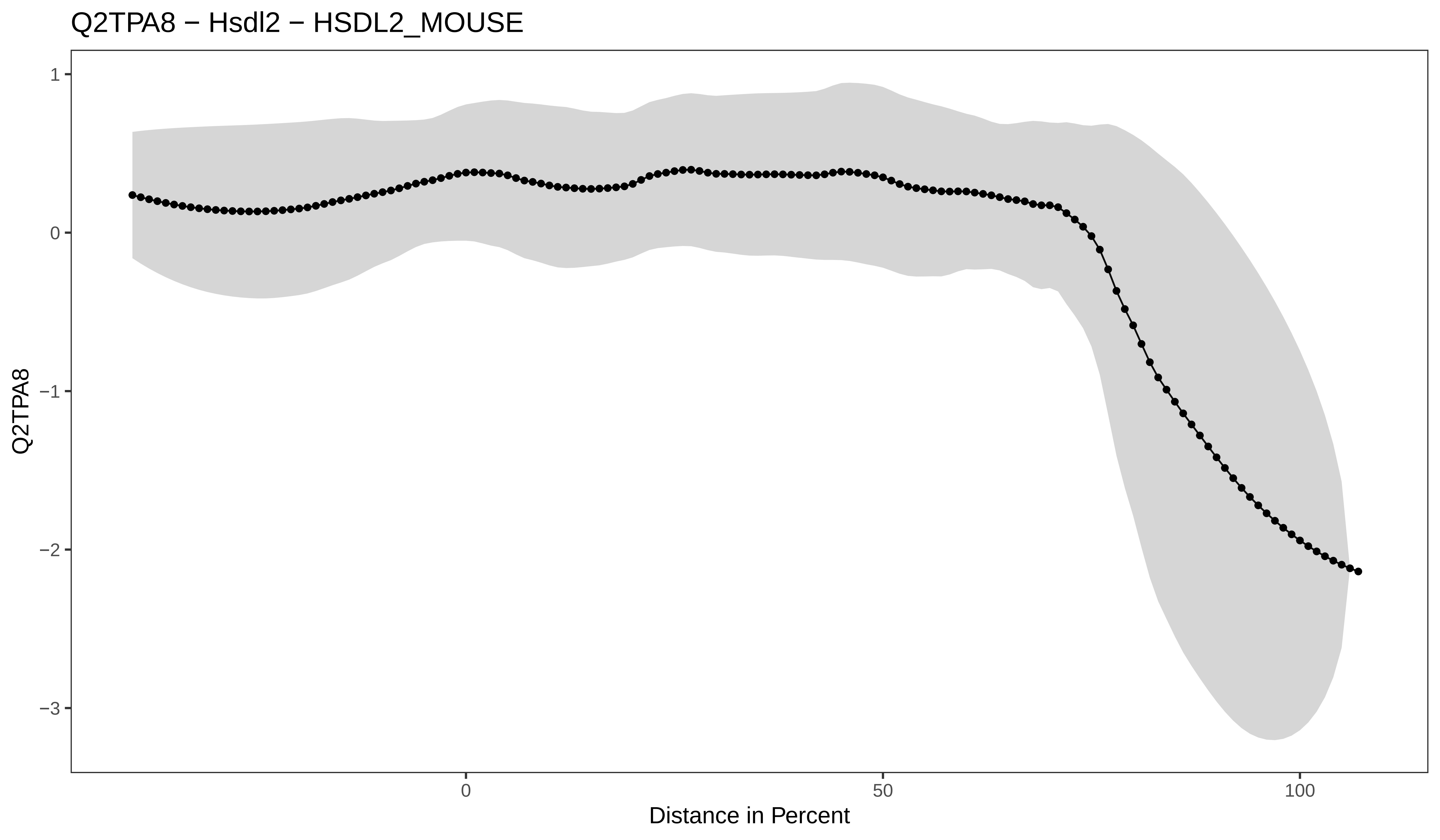
Q2TPA8 − Hsdl2 − HSDL2_MOUSE
Q2TPA8
1
0
−1
−2
−3
0
50
100
Distance in Percent

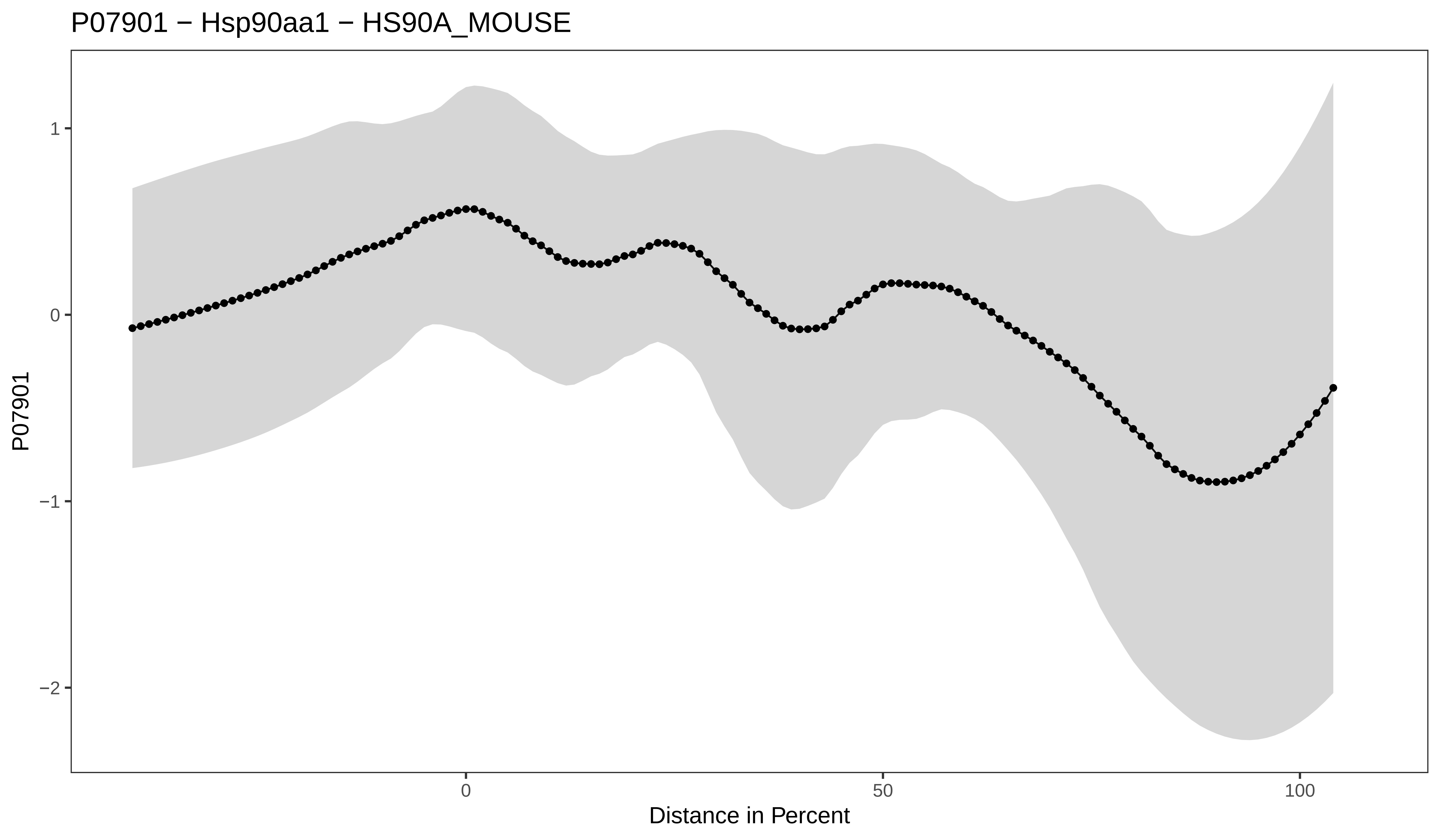
P07901 − Hsp90aa1 − HS90A_MOUSE
P07901
1
0
−1
−2
0
50
100
Distance in Percent

P11499 − Hsp90ab1 − HS90B_MOUSE

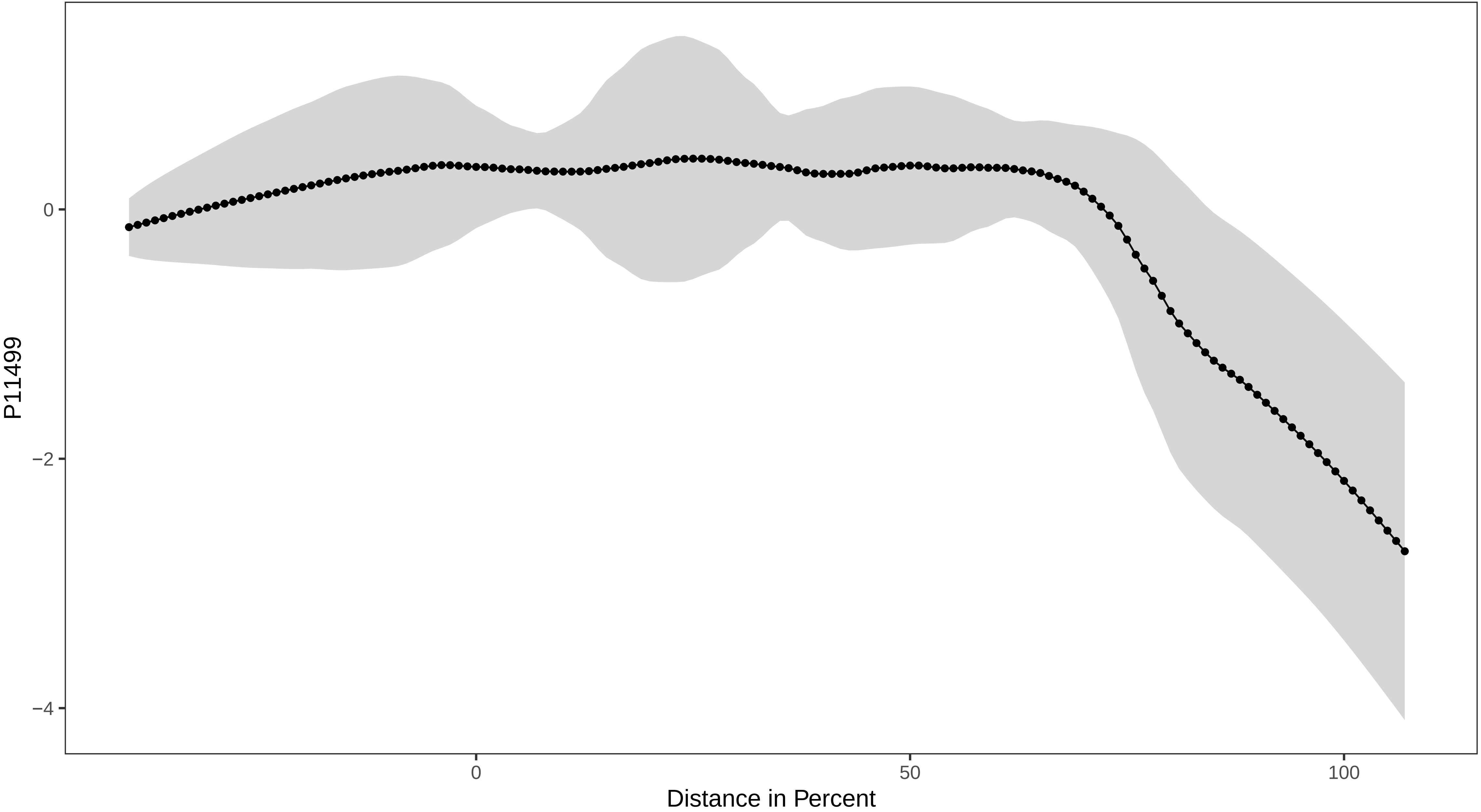

P08113 − Hsp90b1 − ENPL_MOUSE

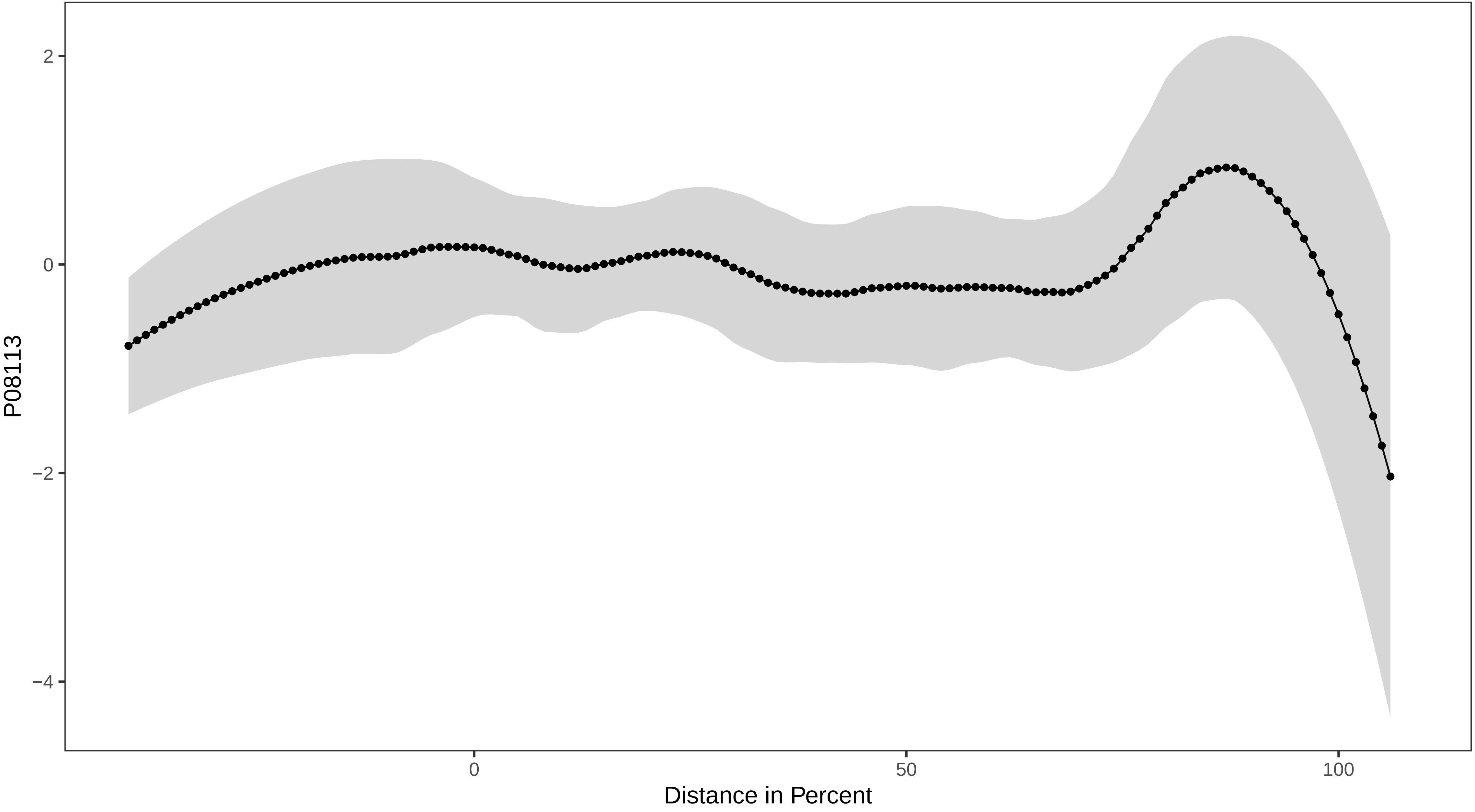

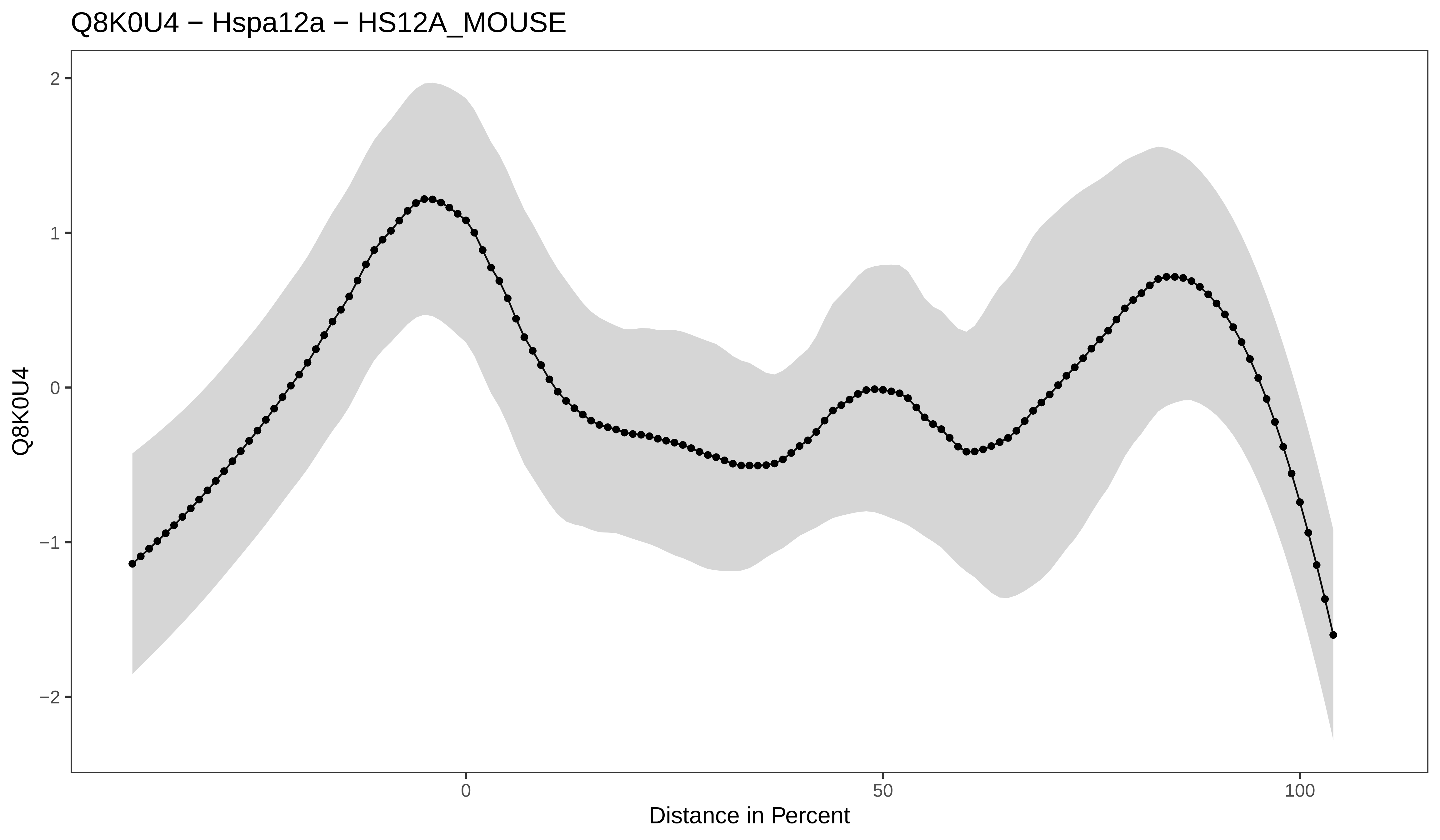
Q8K0U4 − Hspa12a − HS12A_MOUSE
Q8K0U4
2
1
0
−1
−2
0
50
100
Distance in Percent

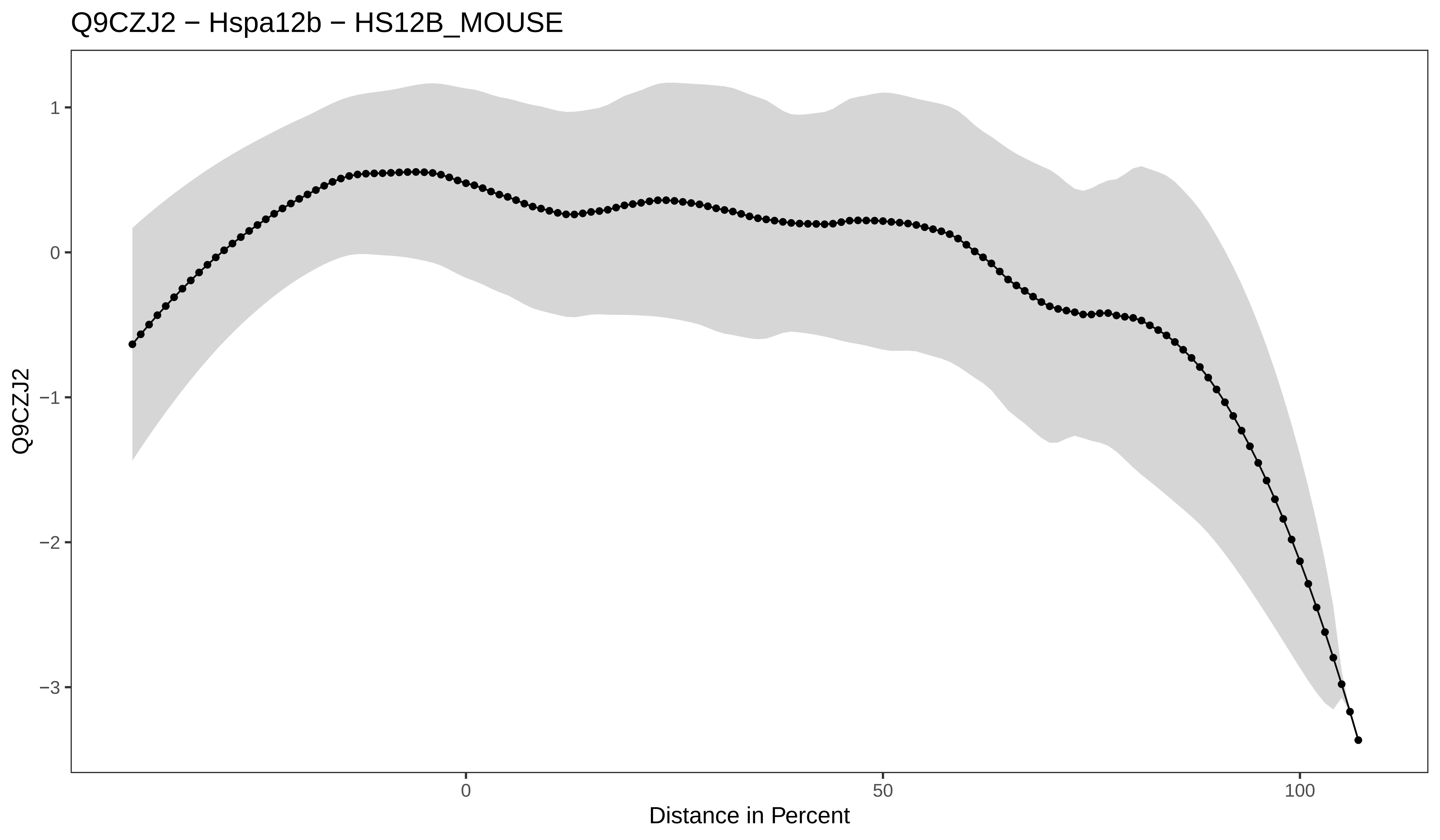
Q9CZJ2 − Hspa12b − HS12B_MOUSE
Q9CZJ2
Distance in Percent
1
0
−1
−2
−3
0
50
100

P17879;Q61696 − Hspa1a;Hspa1b − HS71A_MOUSE;HS71B_MOUSE

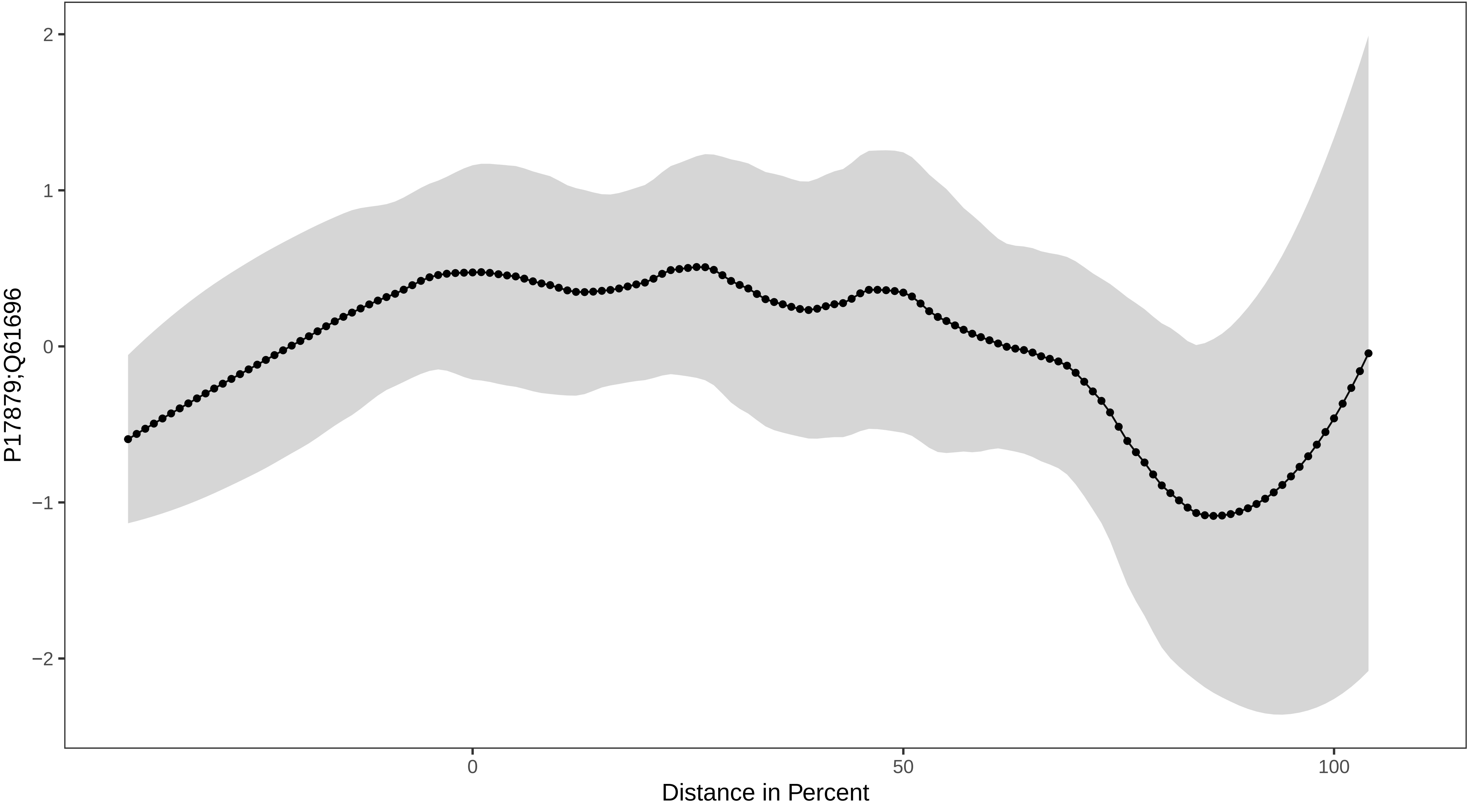

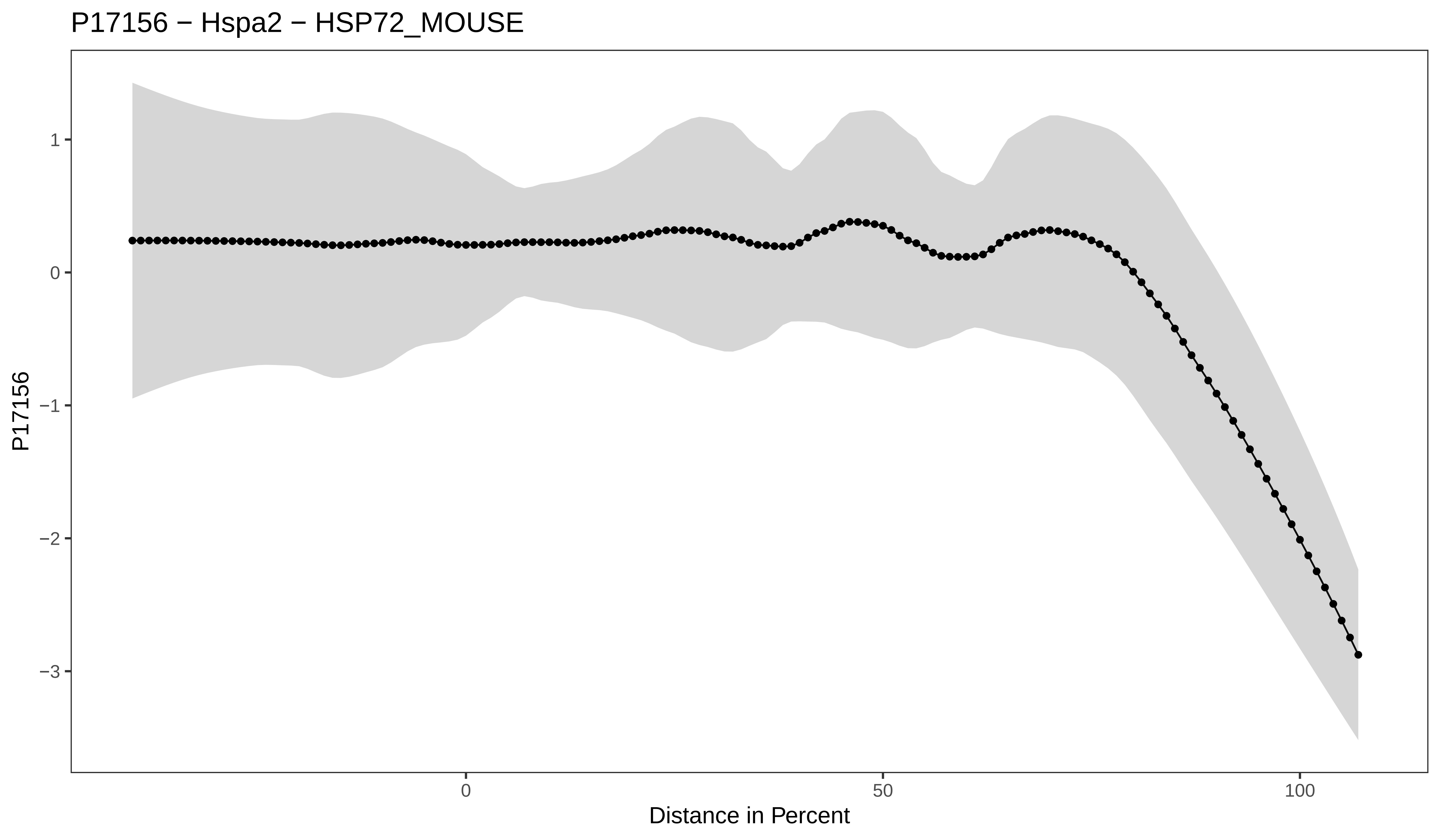

P17156 − Hspa2 − HSP72_MOUSE
P17156
1
0
−1
−2
−3
0
50
100
Distance in Percent

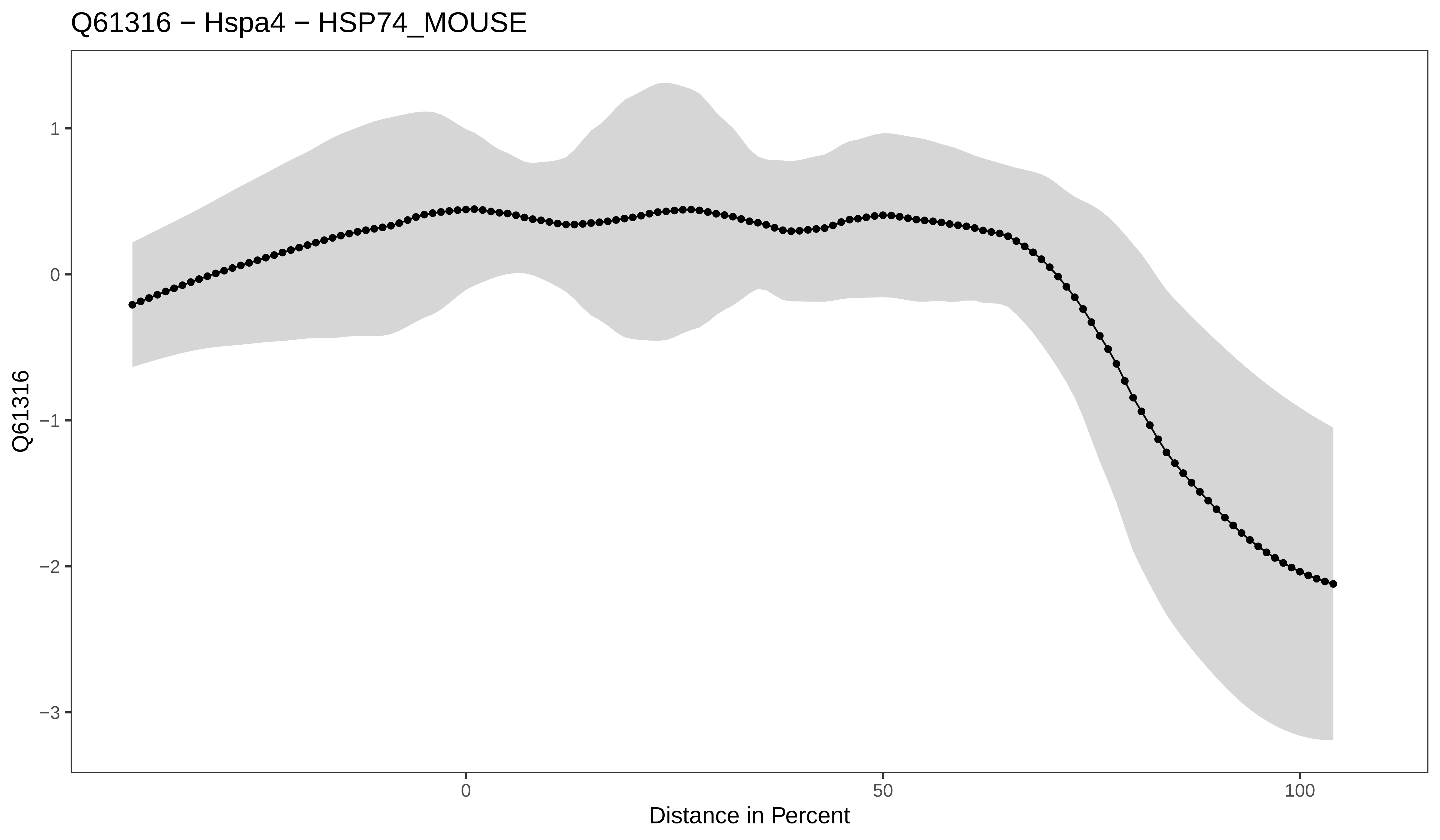
Q61316 − Hspa4 − HSP74_MOUSE
Q61316
1
0
−1
−2
−3
0
50
100
Distance in Percent

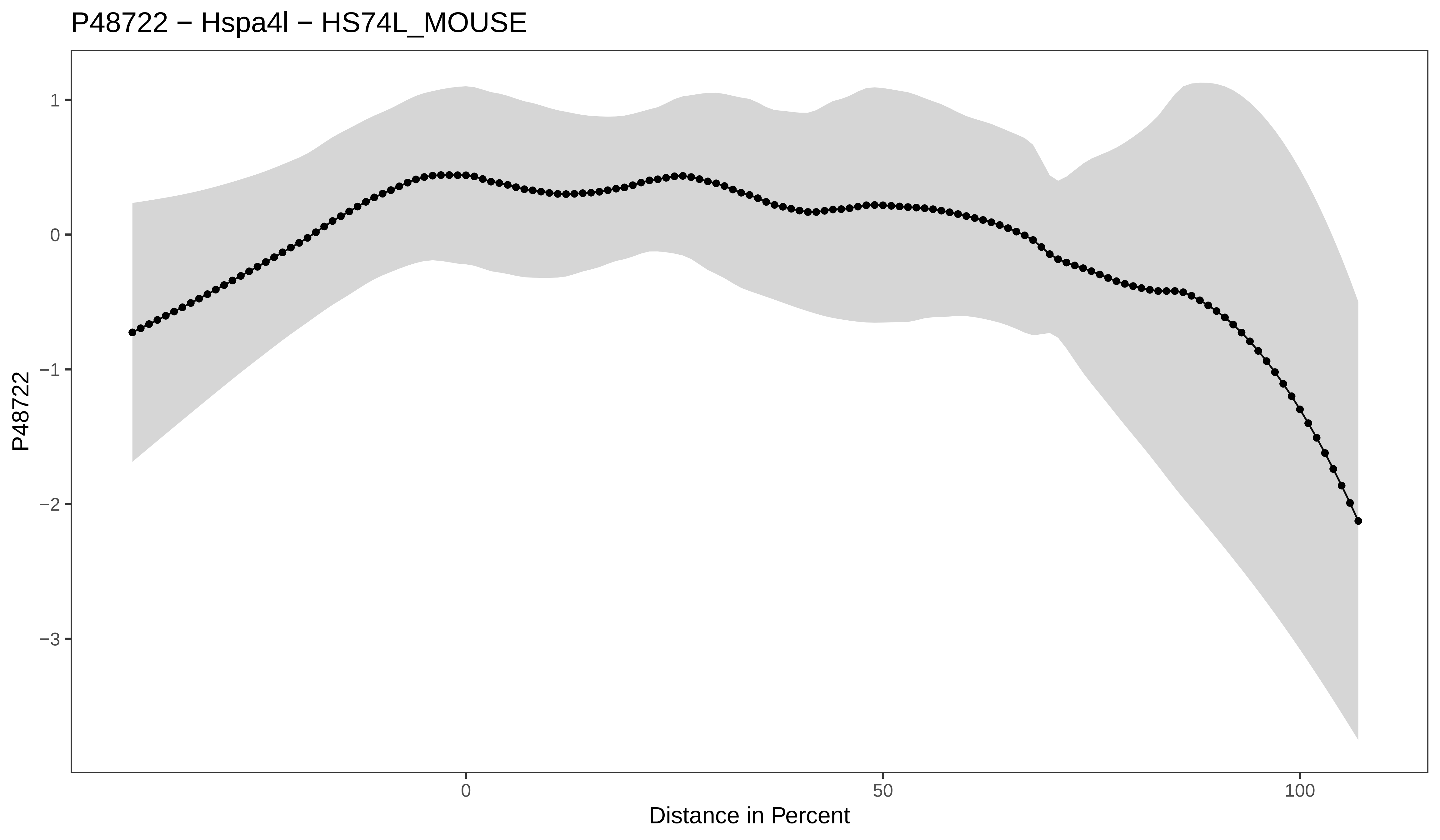
P48722 − Hspa4l − HS74L_MOUSE
1
0
−1
−2
−3
P48722
0
50
100
Distance in Percent

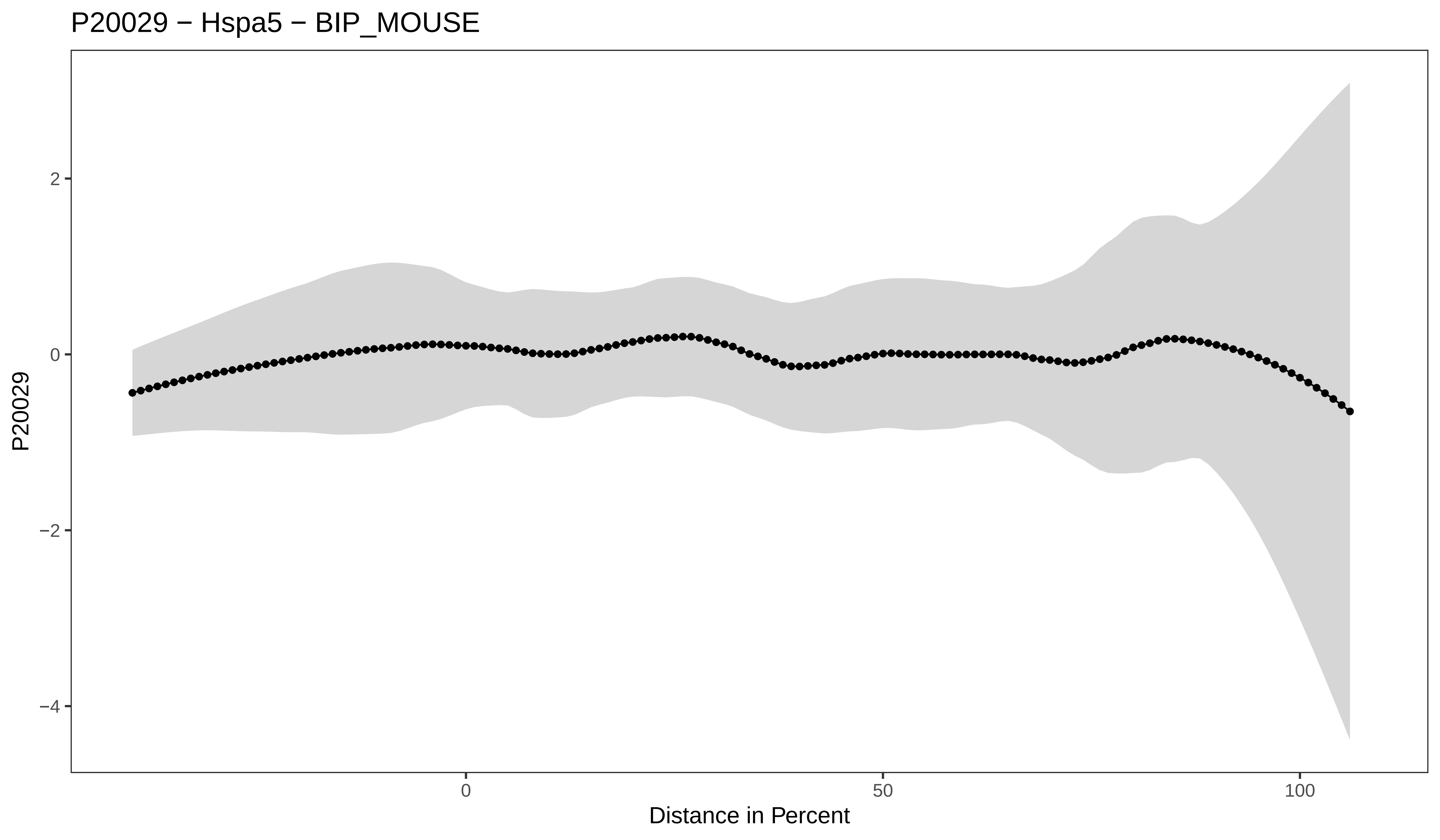

P20029 − Hspa5 − BIP_MOUSE
2
0
−2
−4
P20029
0
50
100
Distance in Percent

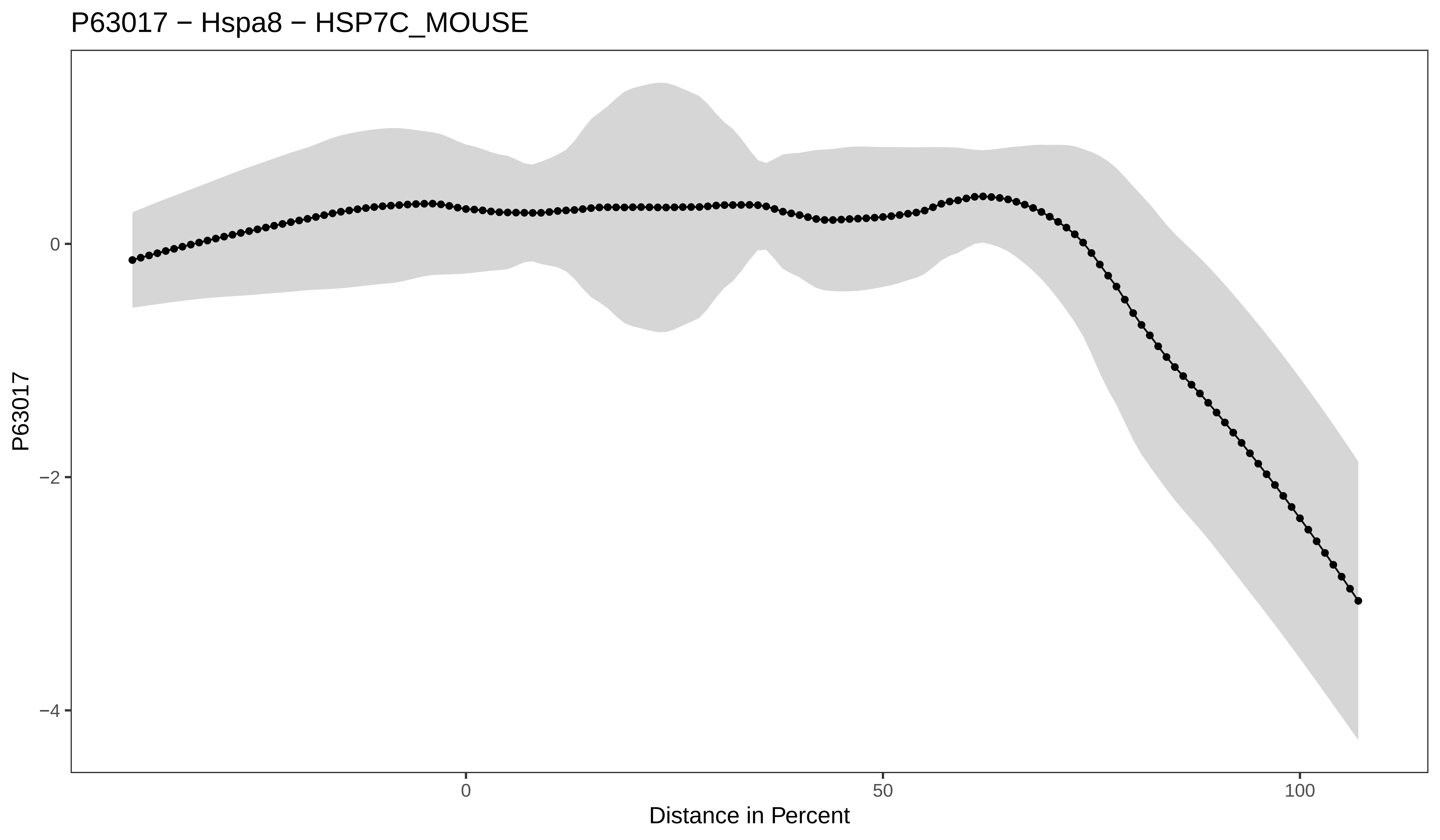

P63017 − Hspa8 − HSP7C_MOUSE
0
−2
−4
P63017
0
50
100
Distance in Percent

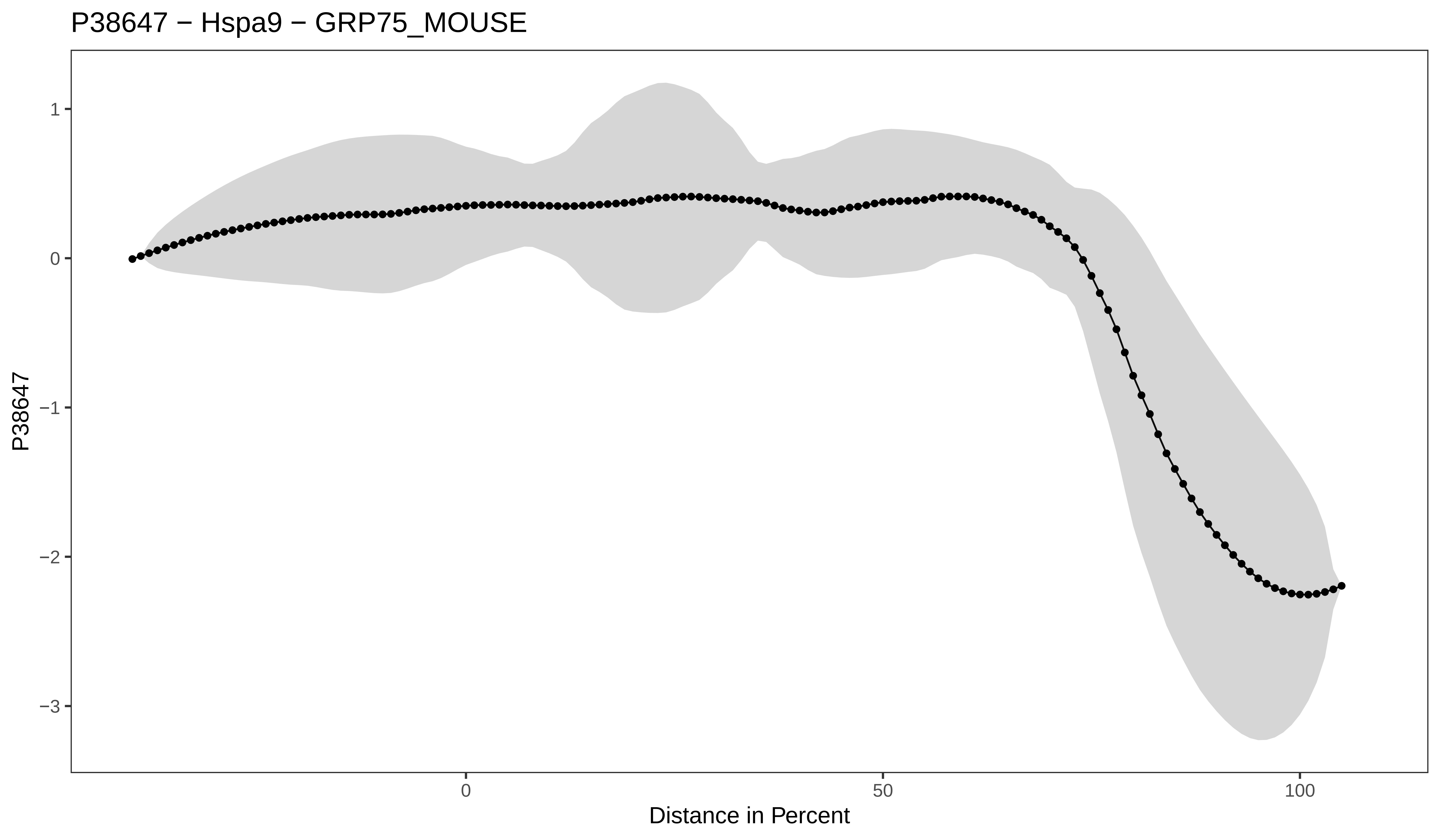

P38647 − Hspa9 − GRP75_MOUSE
1
0
−1
−2
−3
P38647
0
50
100
Distance in Percent

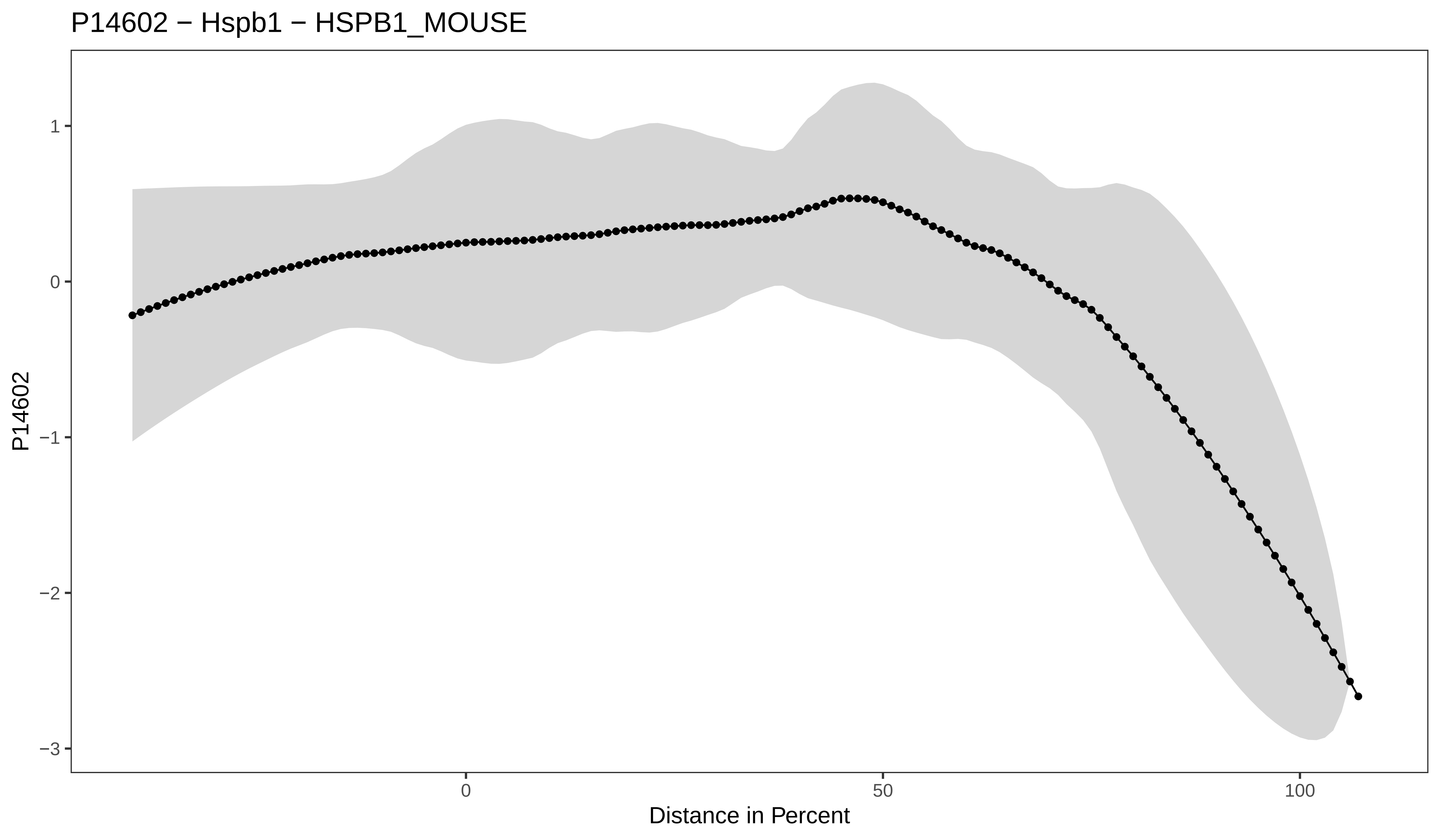

P14602 − Hspb1 − HSPB1_MOUSE
1
0
−1
−2
−3
P14602
0
50
100
Distance in Percent

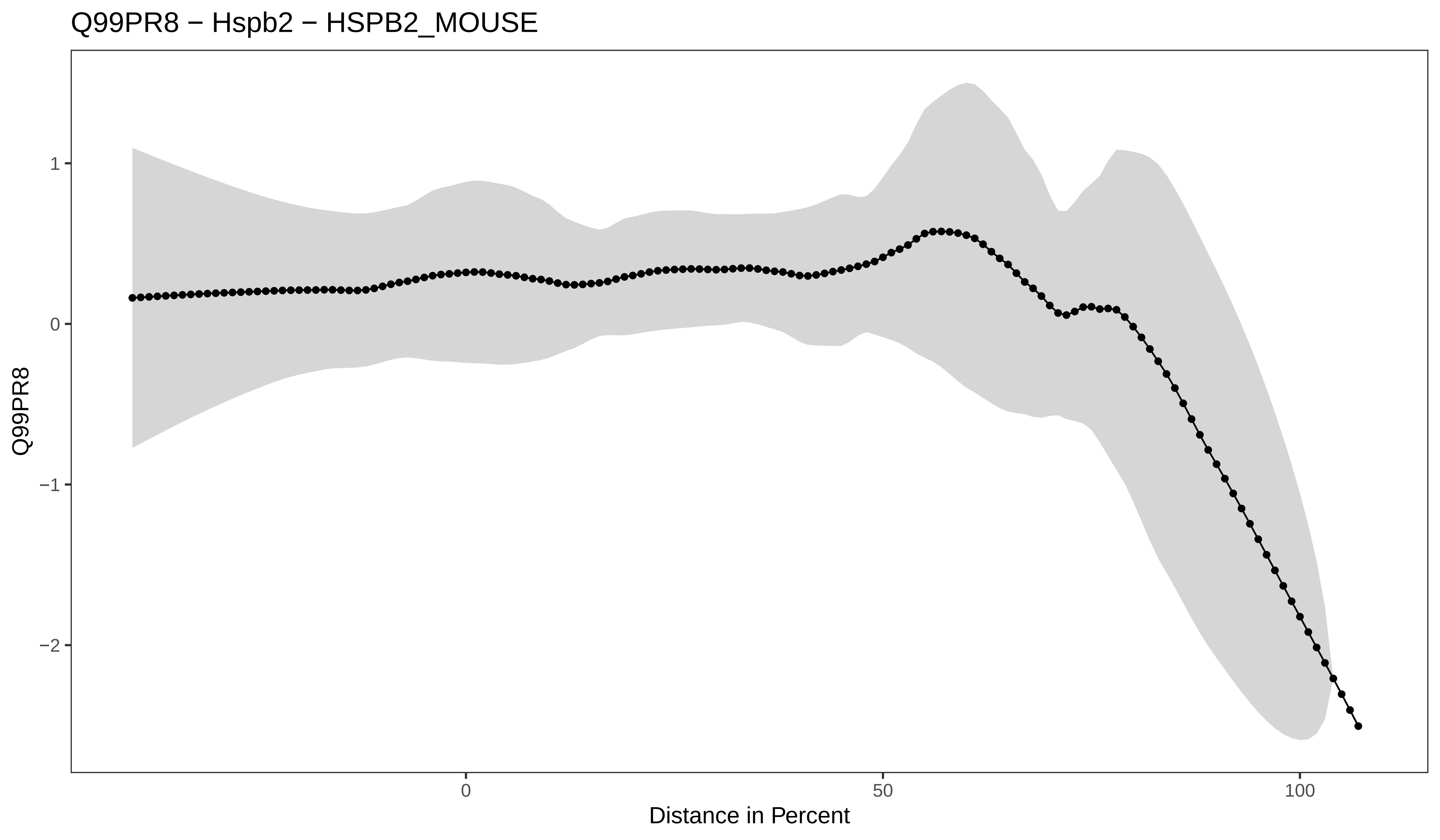

Q99PR8 − Hspb2 − HSPB2_MOUSE
Q99PR8
1
0
−1
−2
0
50
100
Distance in Percent

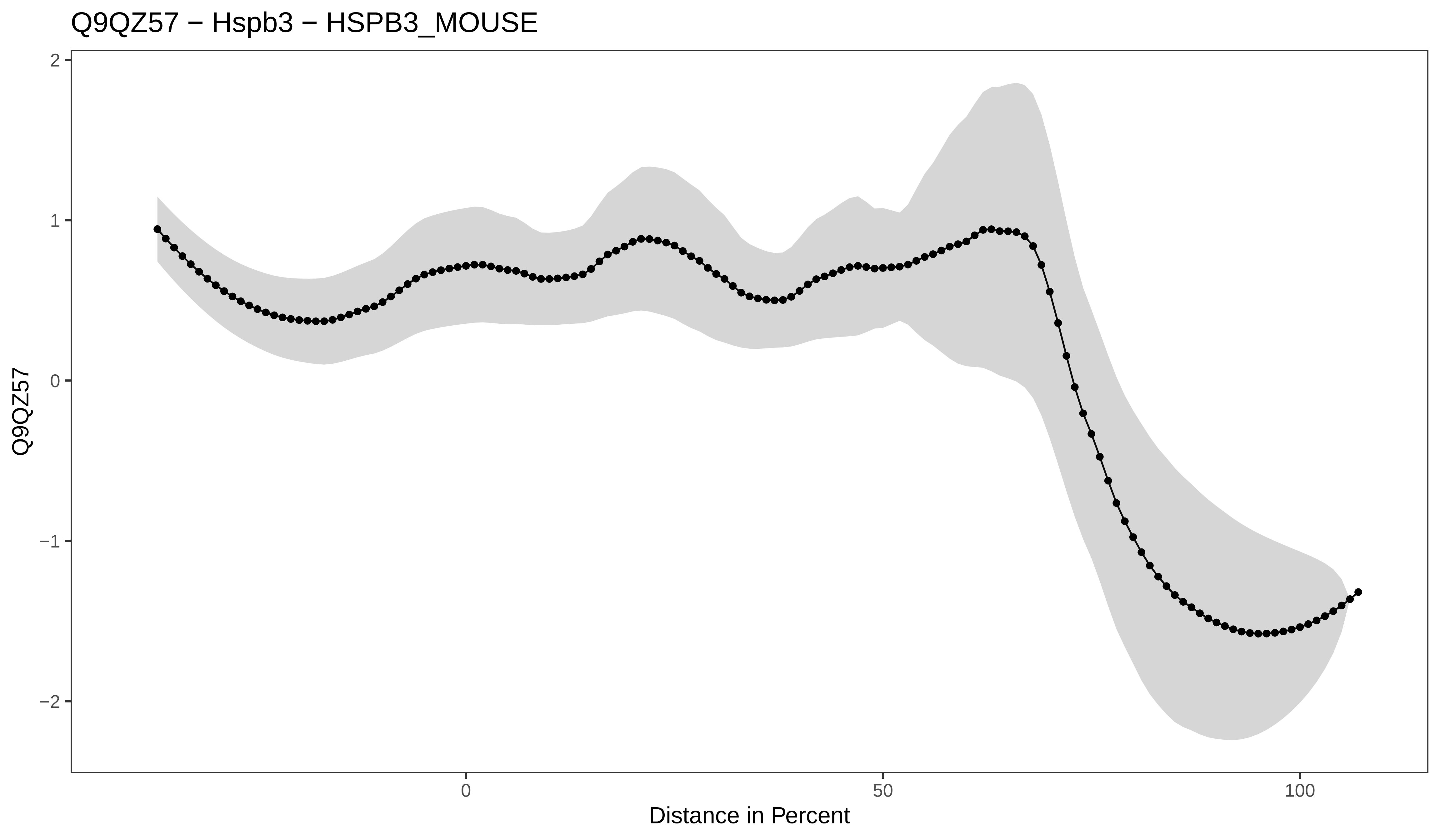

Q9QZ57 − Hspb3 − HSPB3_MOUSE
Q9QZ57
Distance in Percent
2
1
0
−1
−2
0
50
100

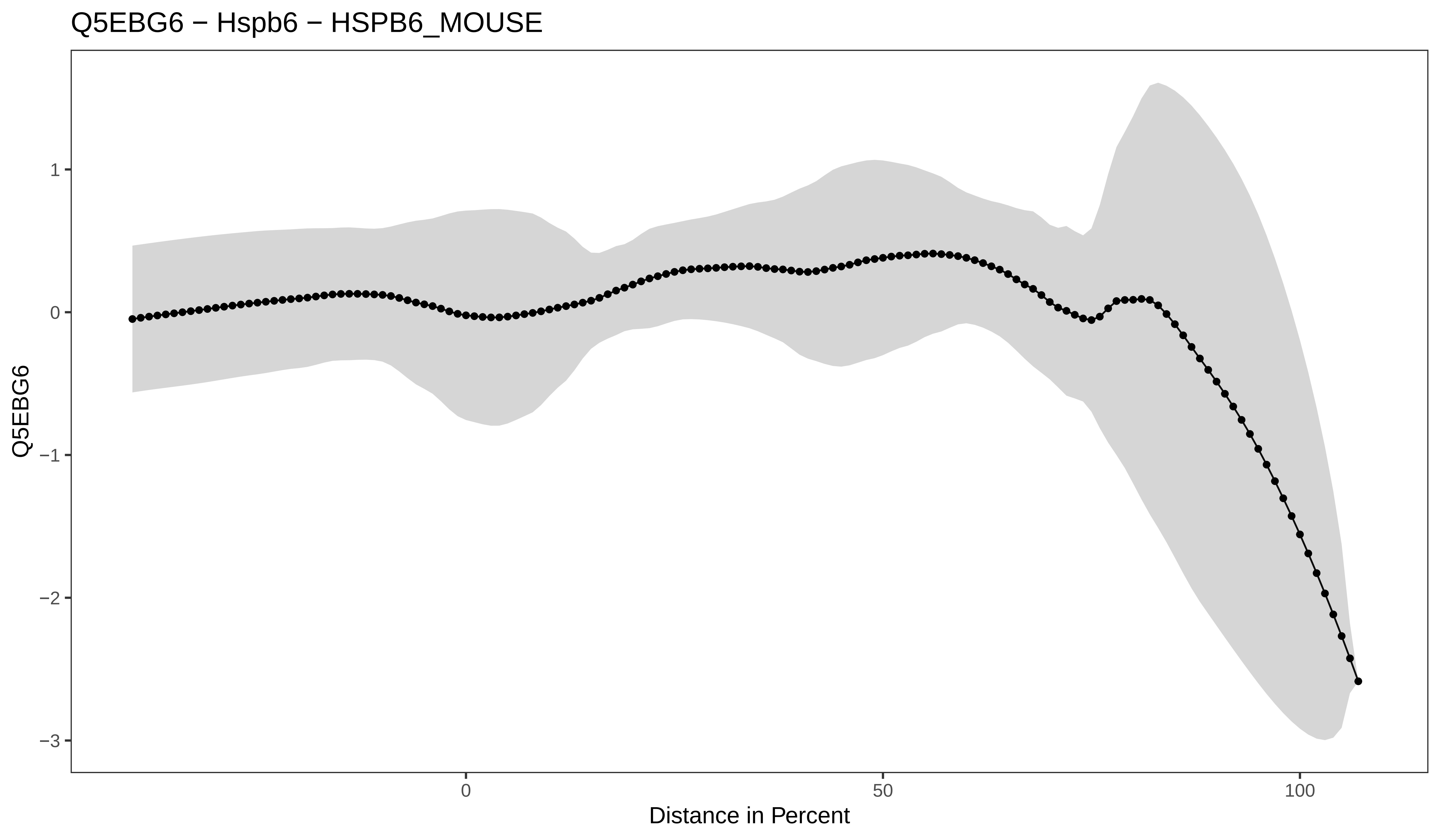

Q5EBG6 − Hspb6 − HSPB6_MOUSE
Q5EBG6
1
0
−1
−2
−3
0
50
100
Distance in Percent

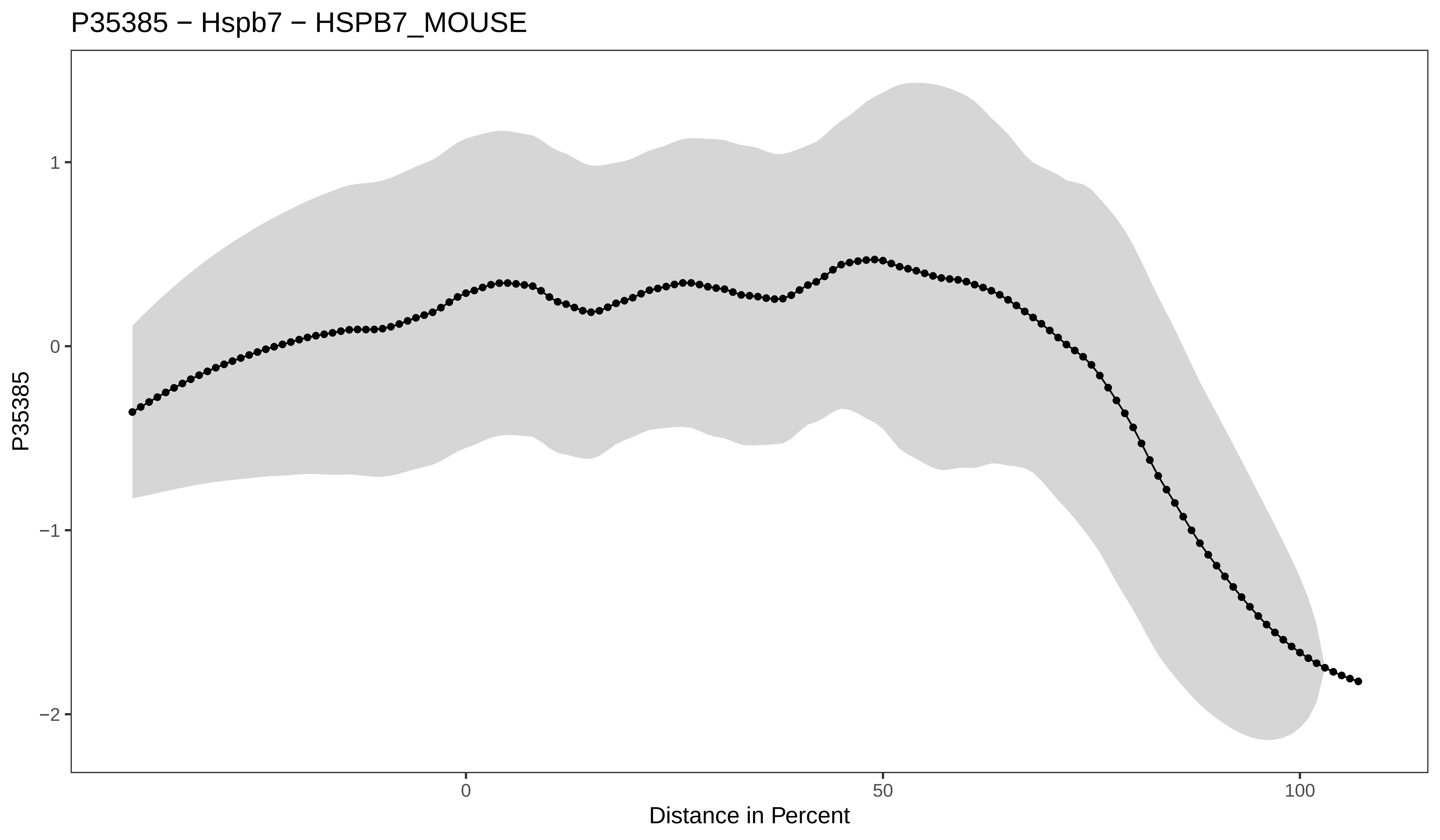
P35385 − Hspb7 − HSPB7_MOUSE
P35385
1
0
−1
−2
0
50
100
Distance in Percent

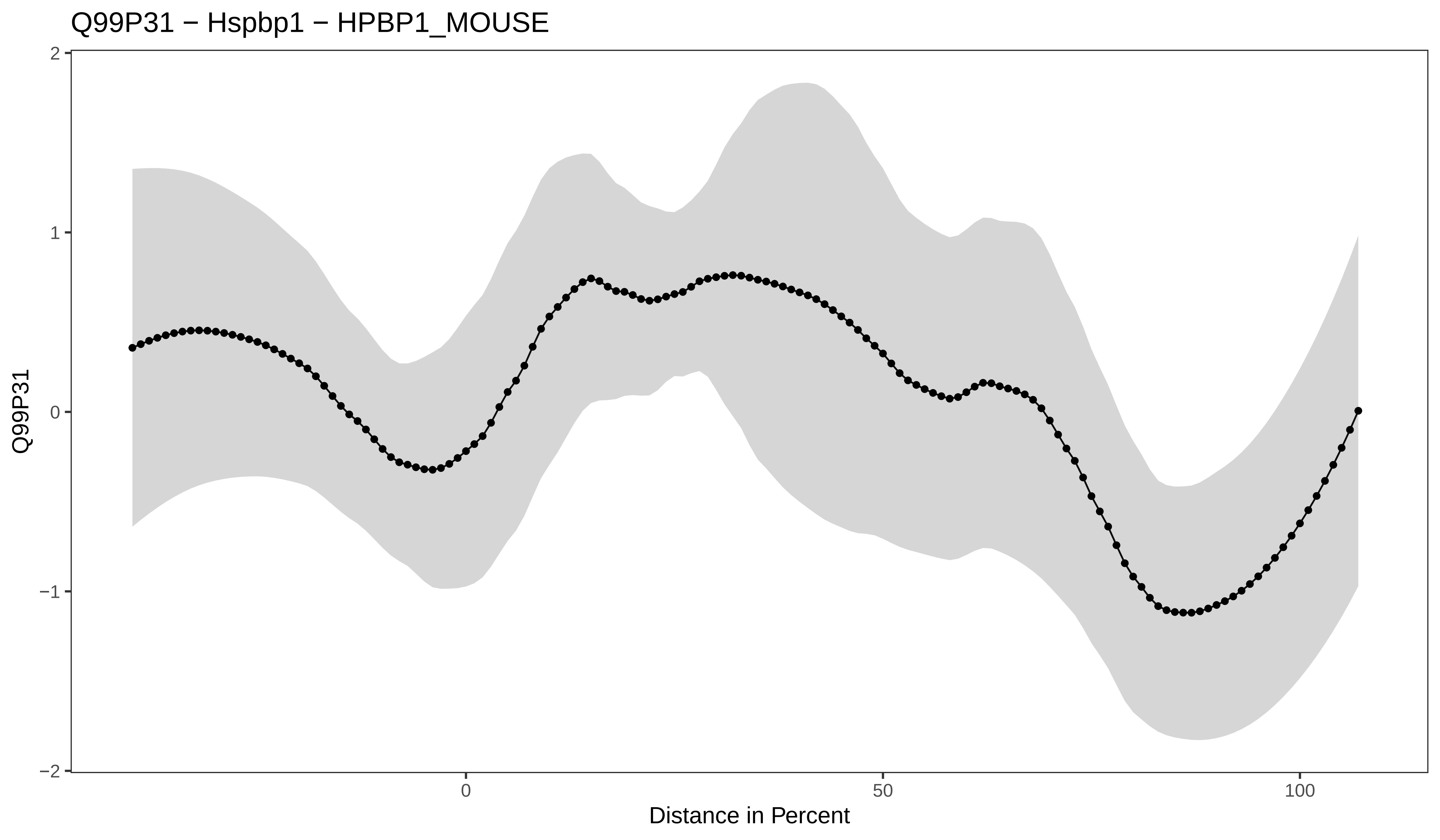

Q99P31 − Hspbp1 − HPBP1_MOUSE
Q99P31
Distance in Percent
2
1
0
−1
−2
0
50
100

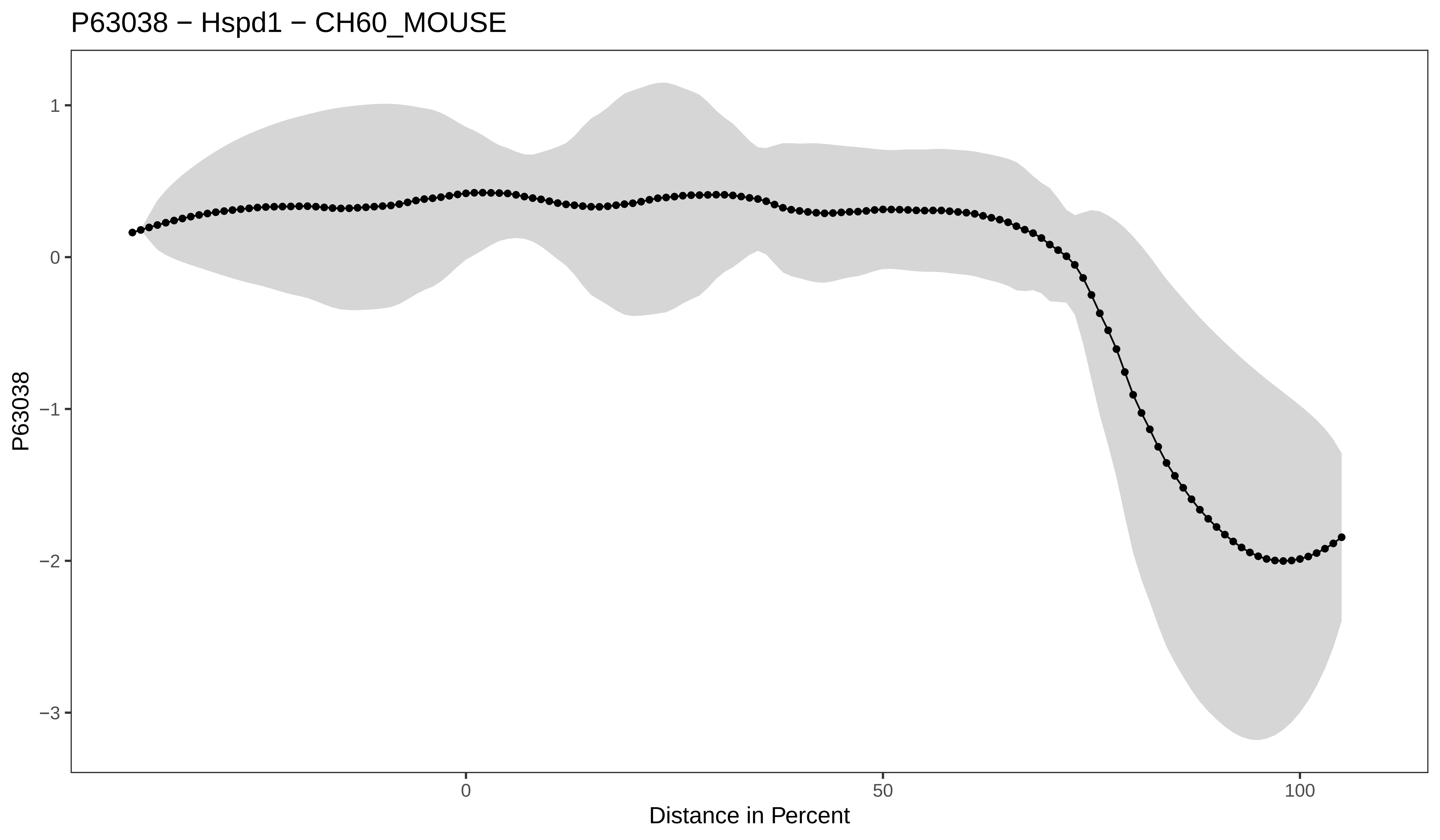
P63038 − Hspd1 − CH60_MOUSE
P63038
Distance in Percent
1
0
−1
−2
−3
0
50
100

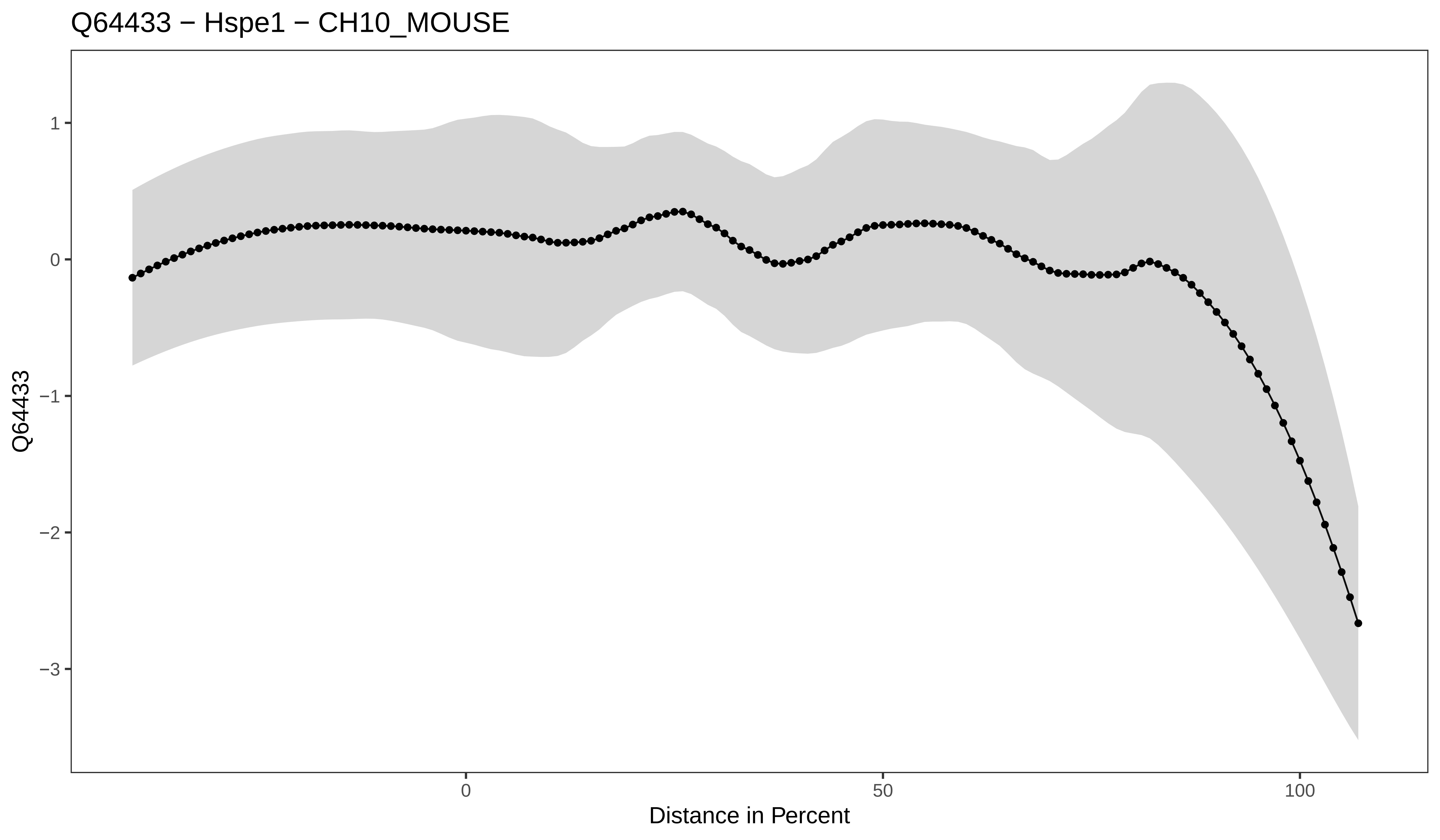
Q64433 − Hspe1 − CH10_MOUSE
Q64433
1
0
−1
−2
−3
0
50
100
Distance in Percent

Q05793 − Hspg2 − PGBM_MOUSE

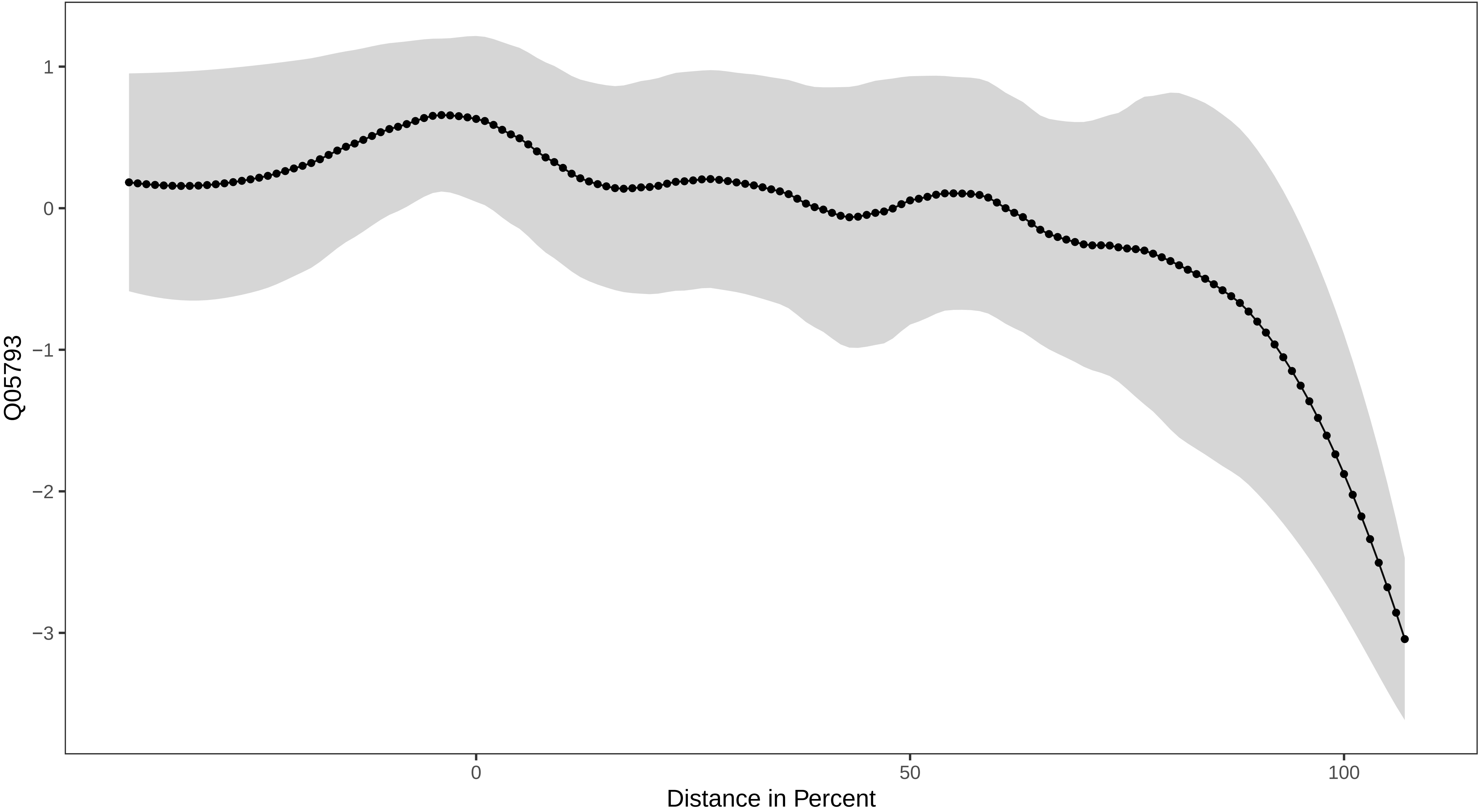

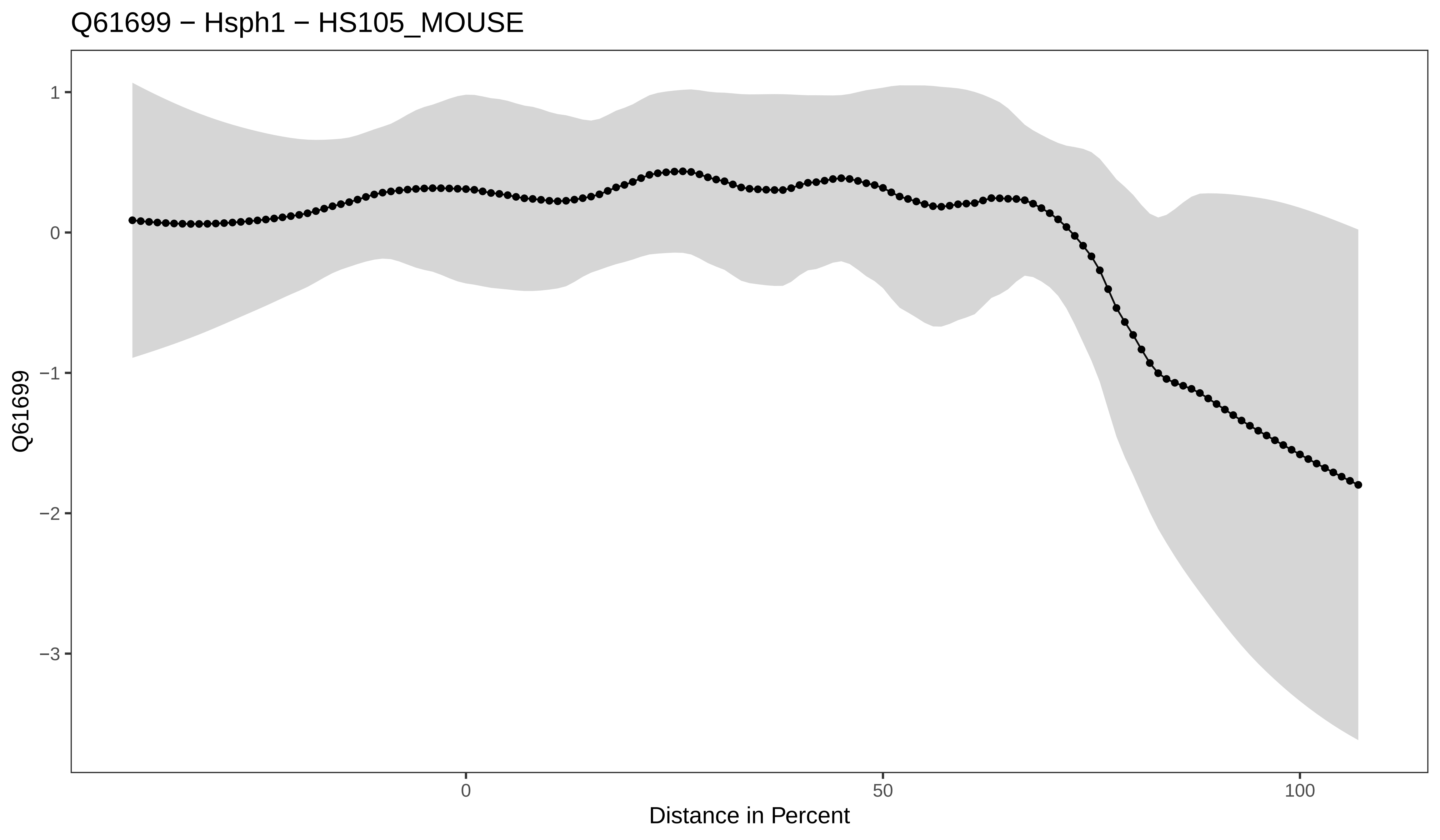
Q61699 − Hsph1 − HS105_MOUSE
Q61699
1
0
−1
−2
−3
0
50
100
Distance in Percent

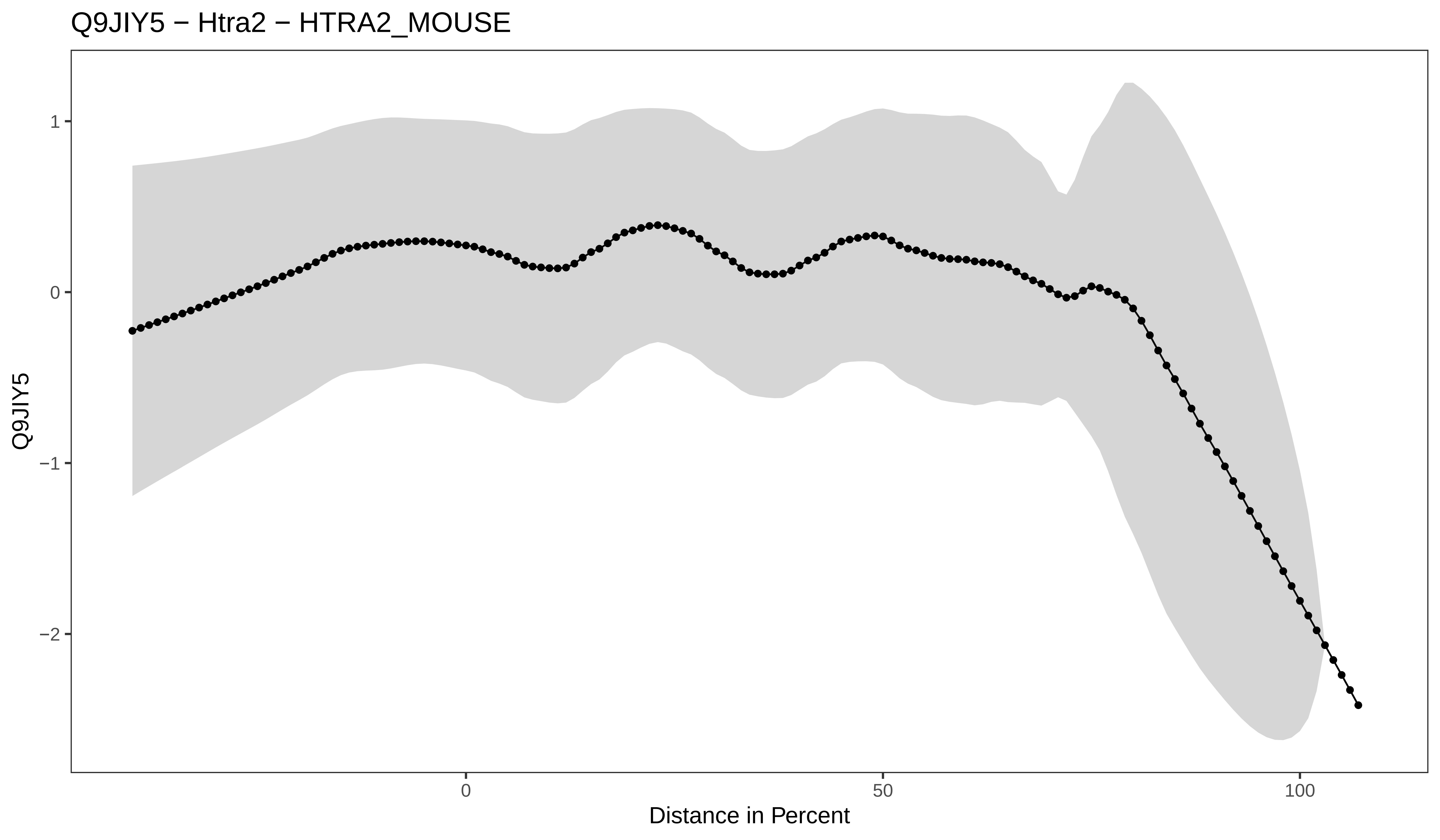
Q9JIY5 − Htra2 − HTRA2_MOUSE
1
0
−1
−2
Q9JIY5
0
50
100
Distance in Percent

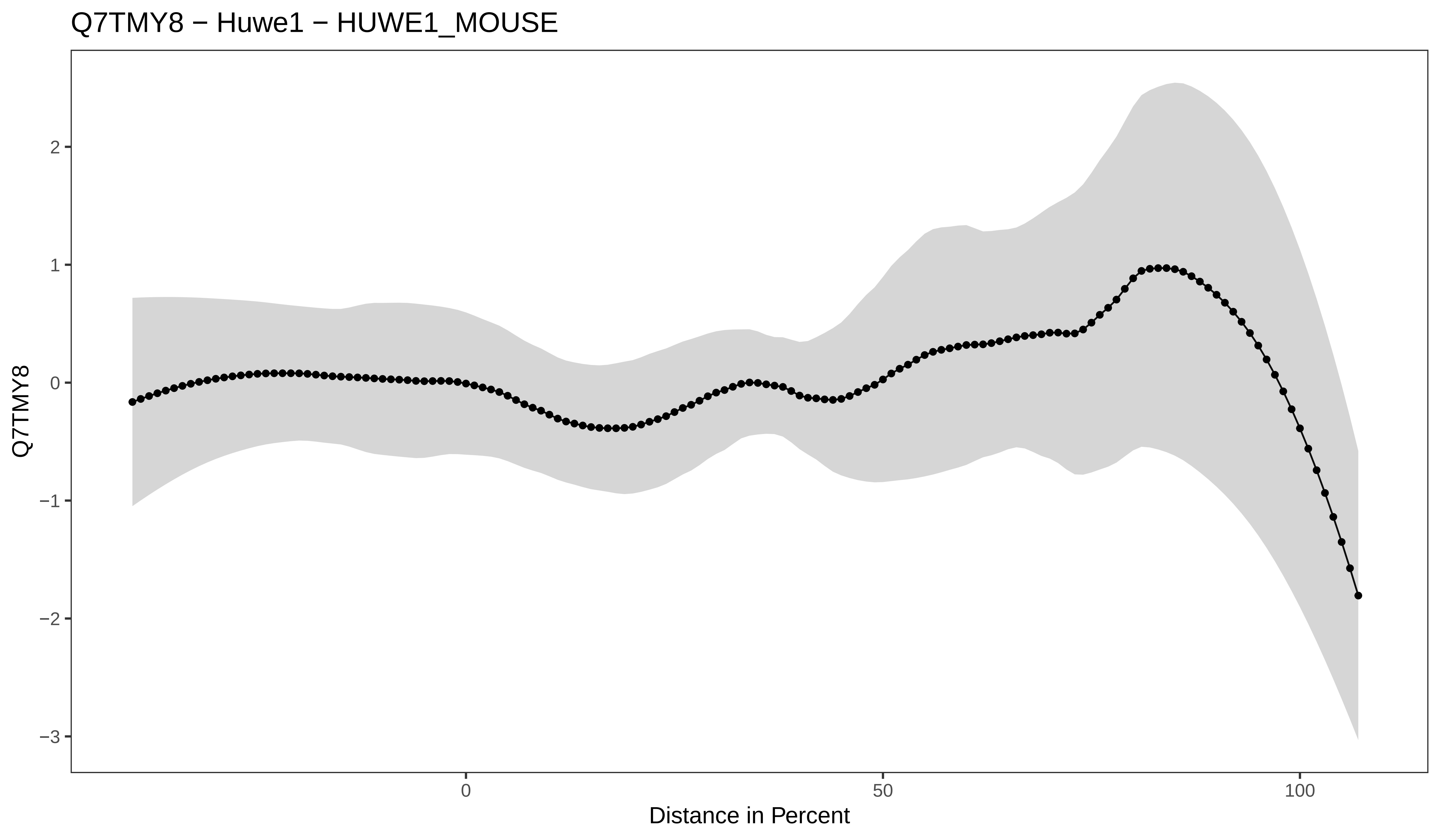

Q7TMY8 − Huwe1 − HUWE1_MOUSE
Q7TMY8
Distance in Percent
2
1
0
−1
−2
−3
0
50
100

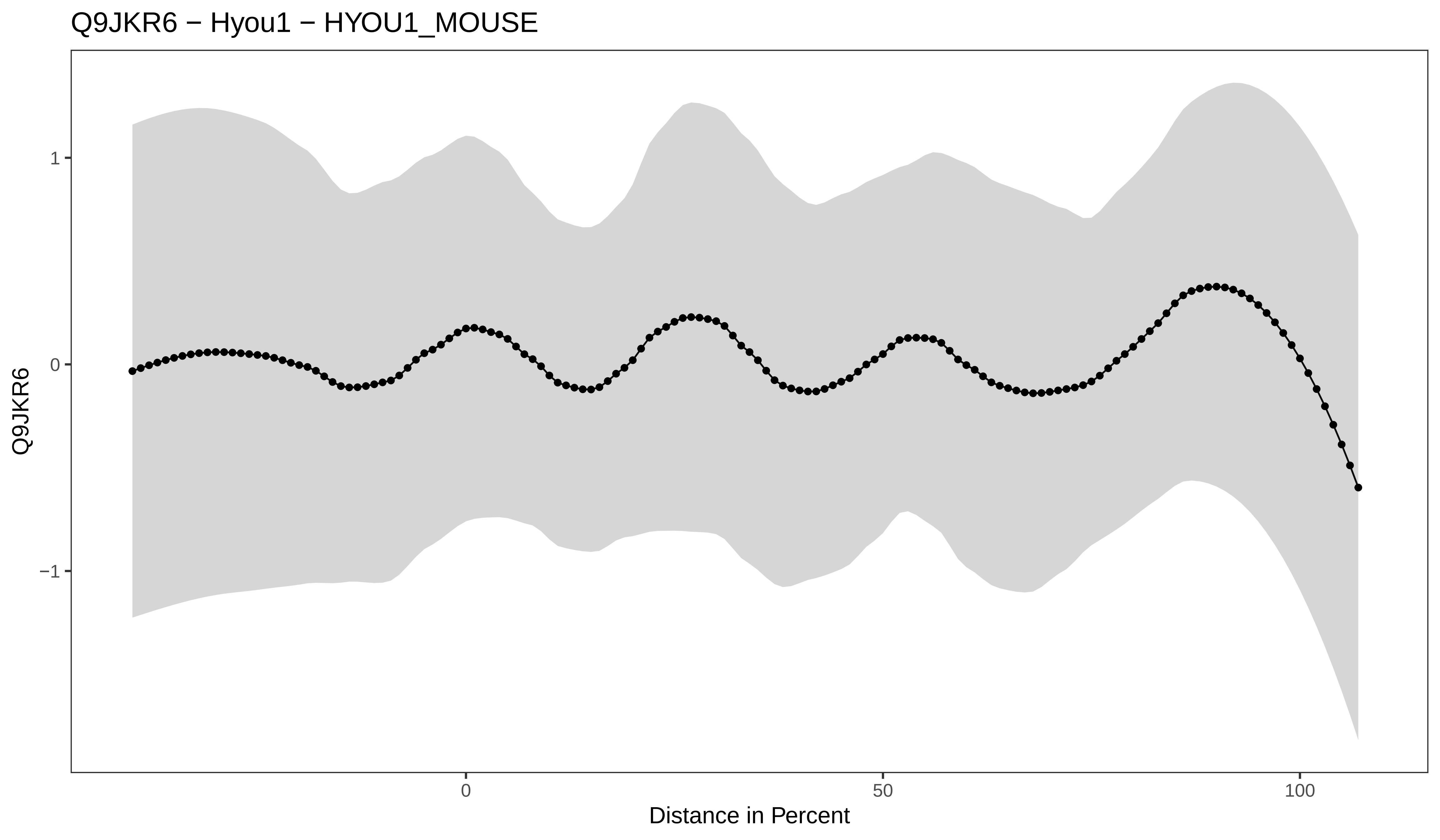
Q9JKR6 − Hyou1 − HYOU1_MOUSE
Q9JKR6
1
0
−1
0
50
100
Distance in Percent

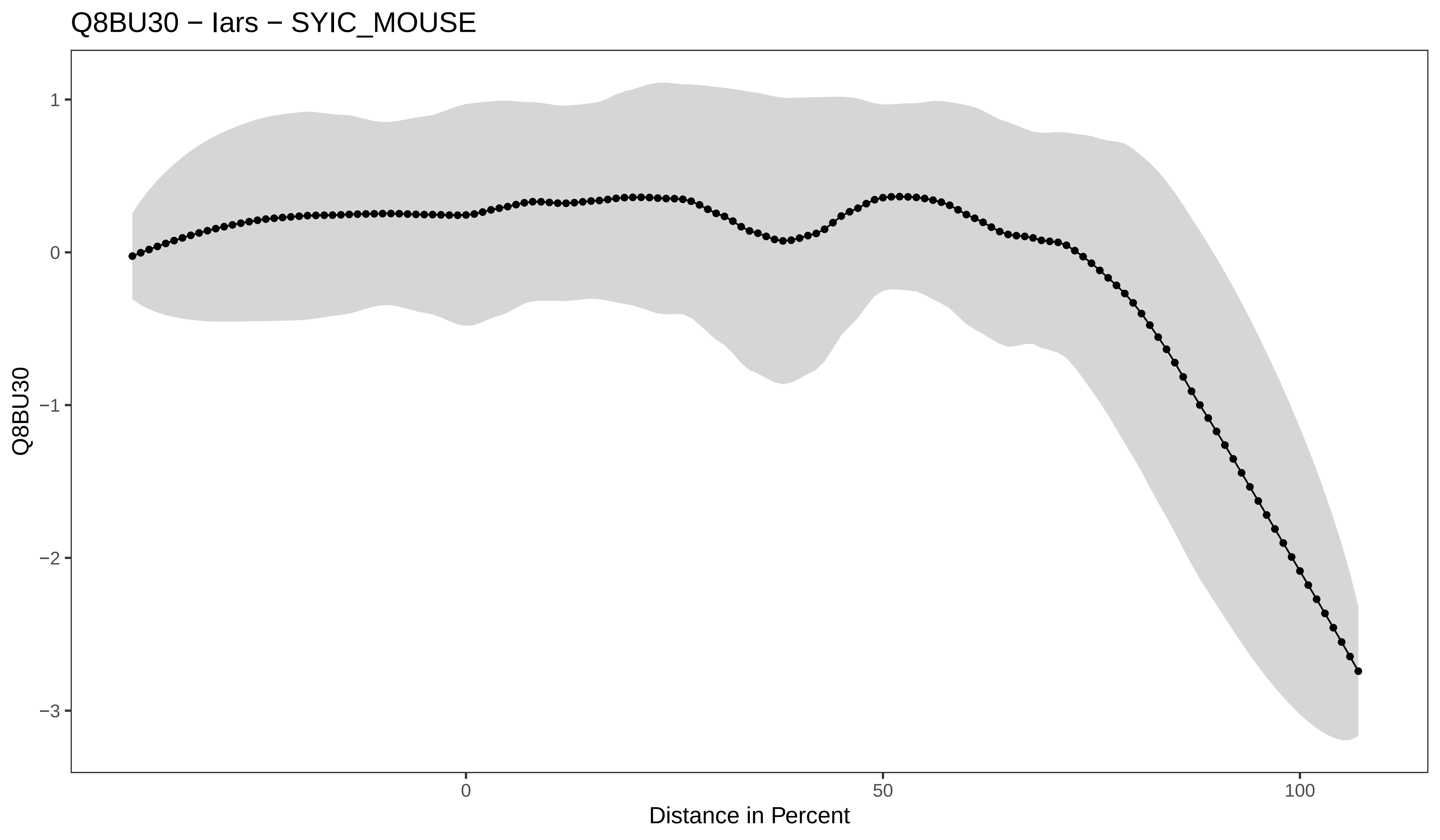

Q8BU30 − Iars − SYIC_MOUSE
Q8BU30
1
0
−1
−2
−3
0
50
100
Distance in Percent

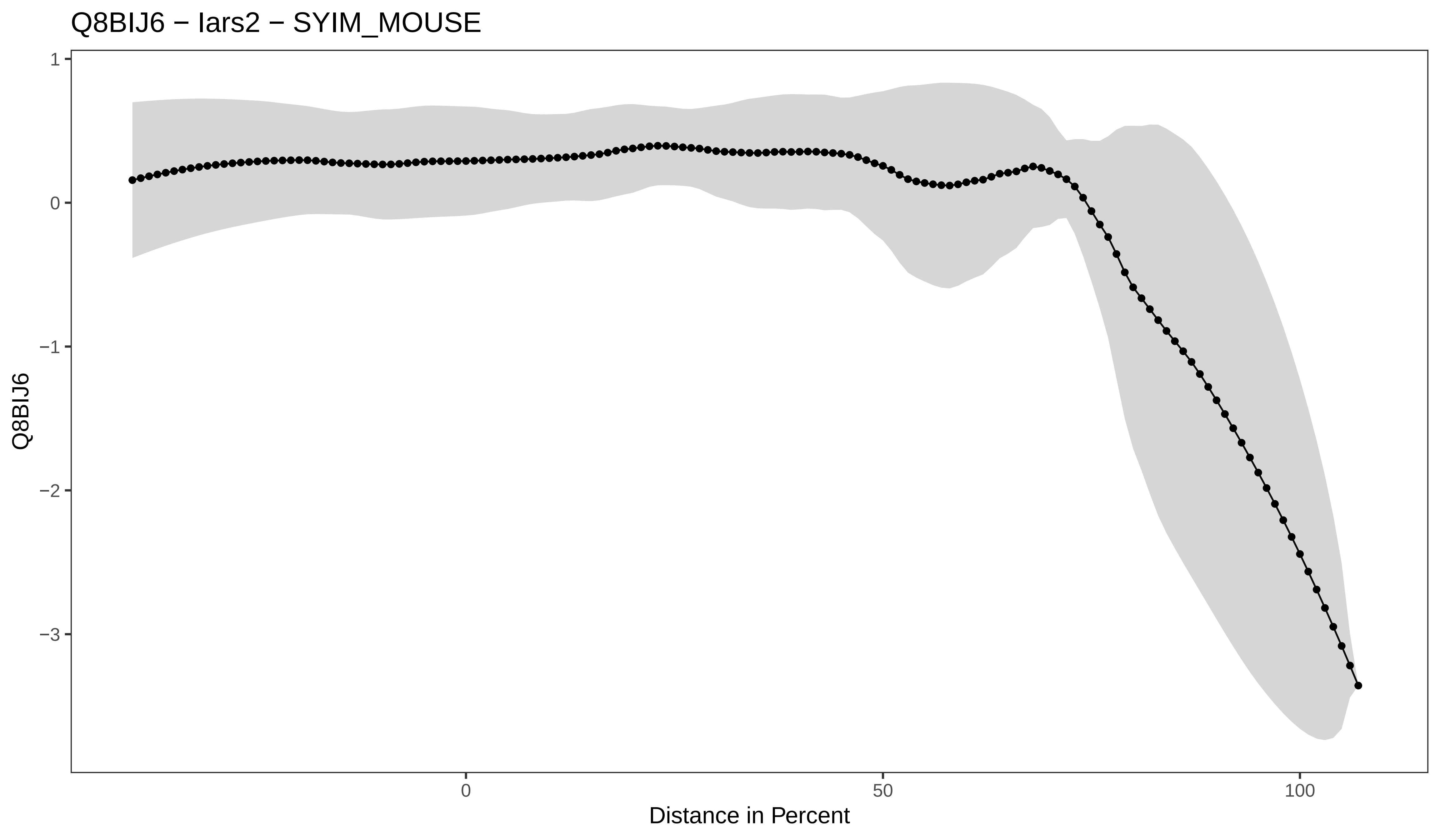

Q8BIJ6 − Iars2 − SYIM_MOUSE
1
0
−1
−2
−3
Q8BIJ6
0
50
100
Distance in Percent

Q8CAK1 − Iba57 − CAF17_MOUSE

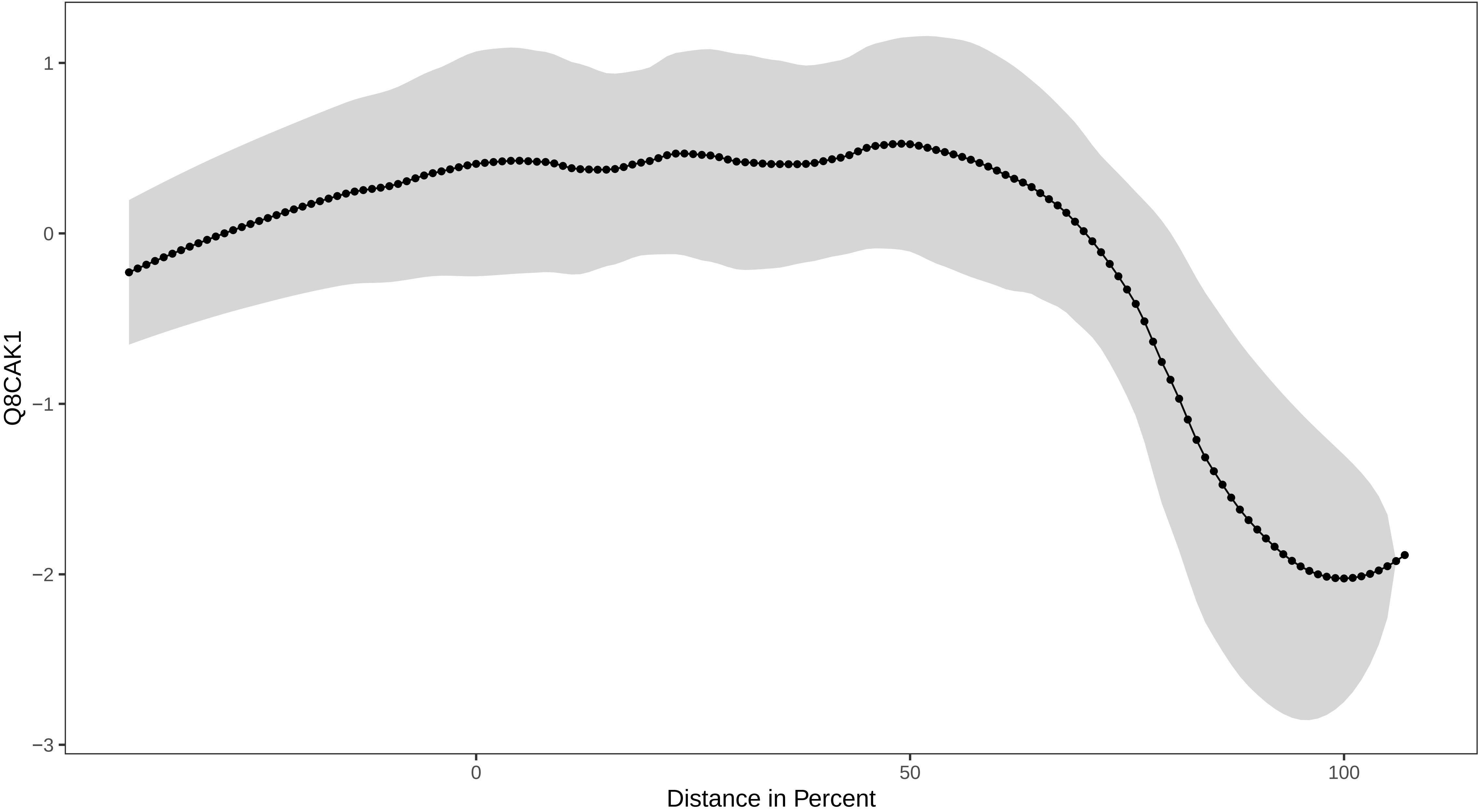

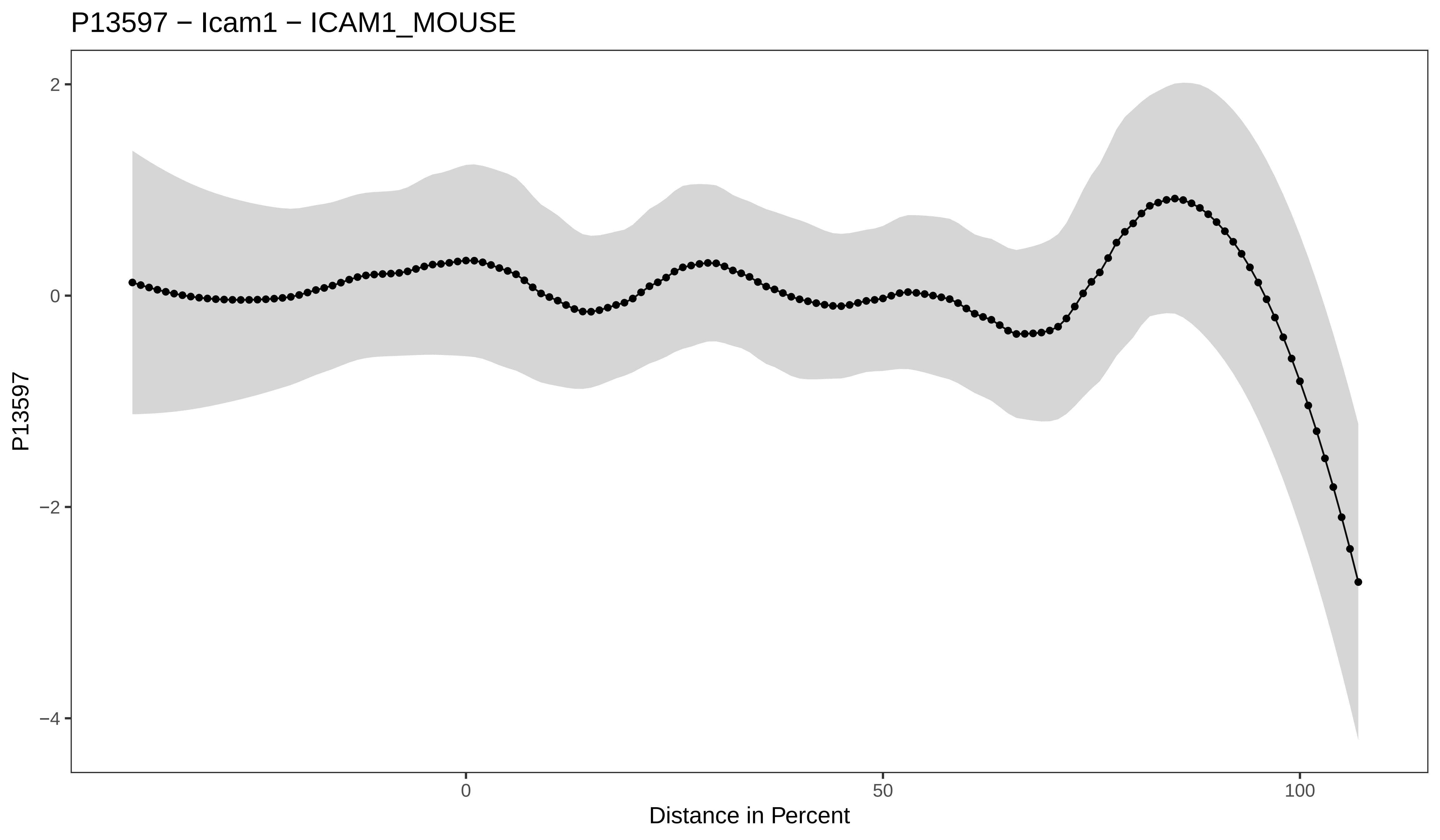
P13597 − Icam1 − ICAM1_MOUSE
P13597
2
0
−2
−4
0
50
100
Distance in Percent

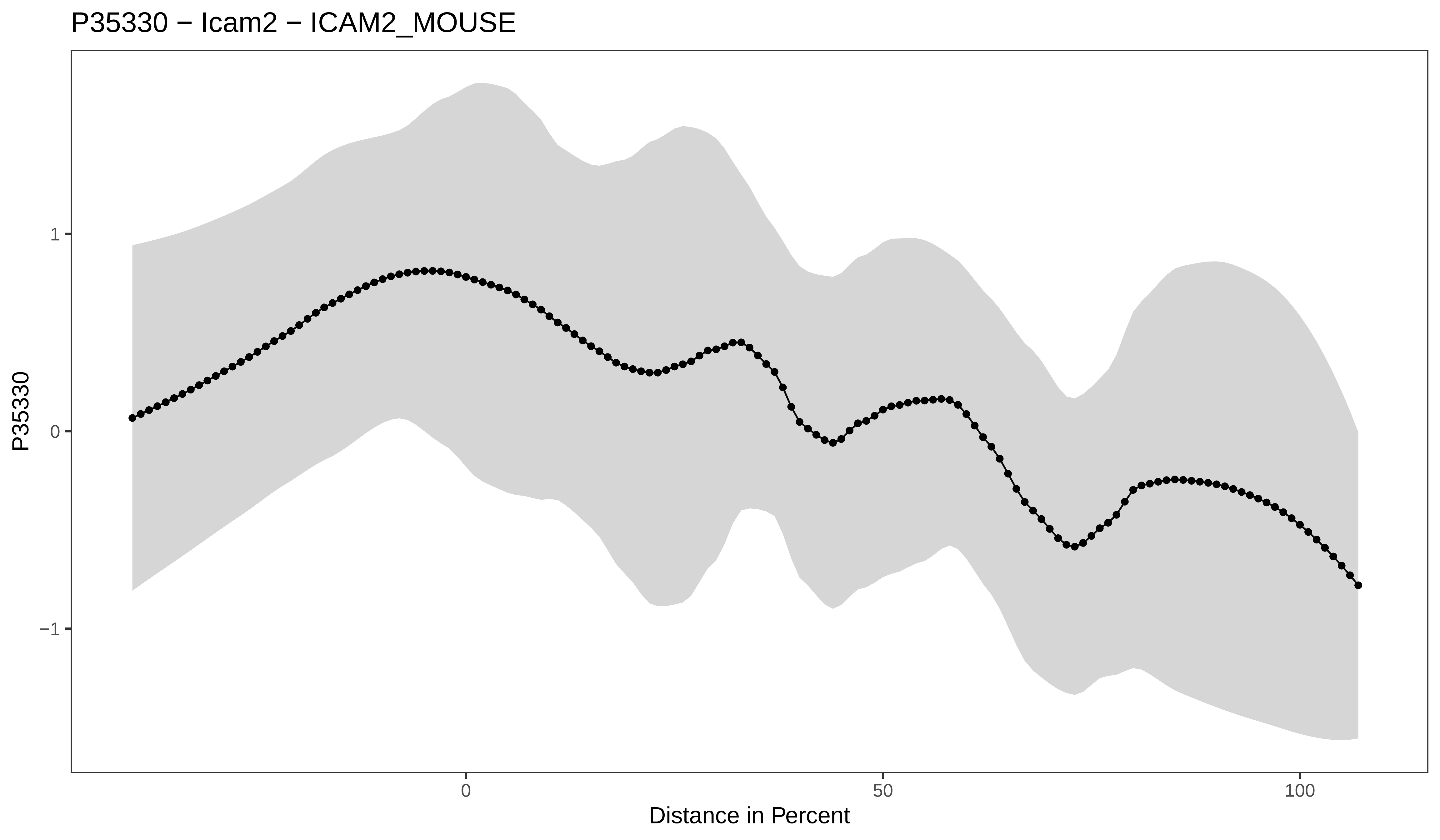

P35330 − Icam2 − ICAM2_MOUSE
P35330
1
0
−1
0
50
100
Distance in Percent

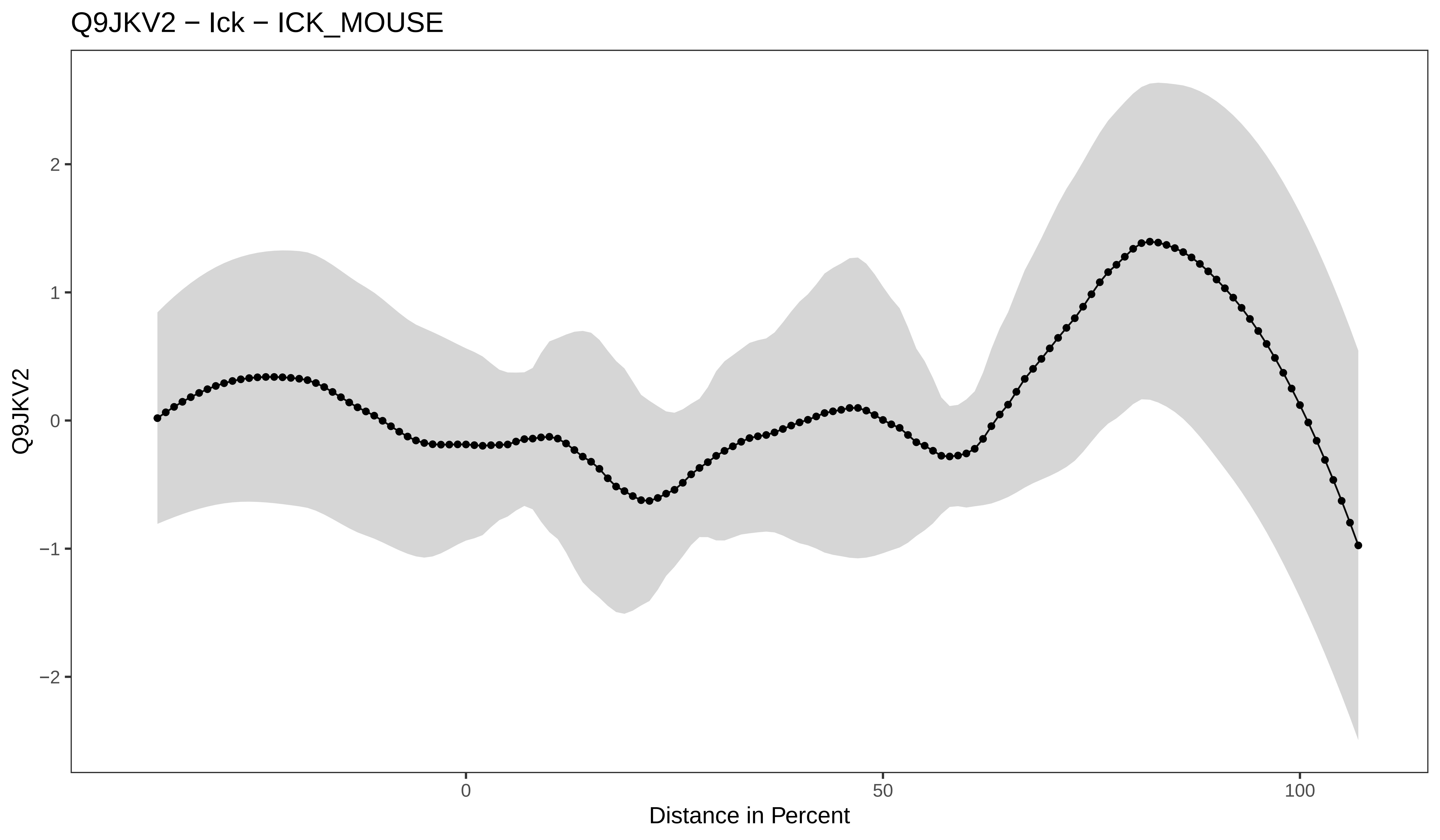

Q9JKV2 − lck − ICK_MOUSE
2
1
0
−1
−2
Q9JKV2
0
50
100
Distance in Percent

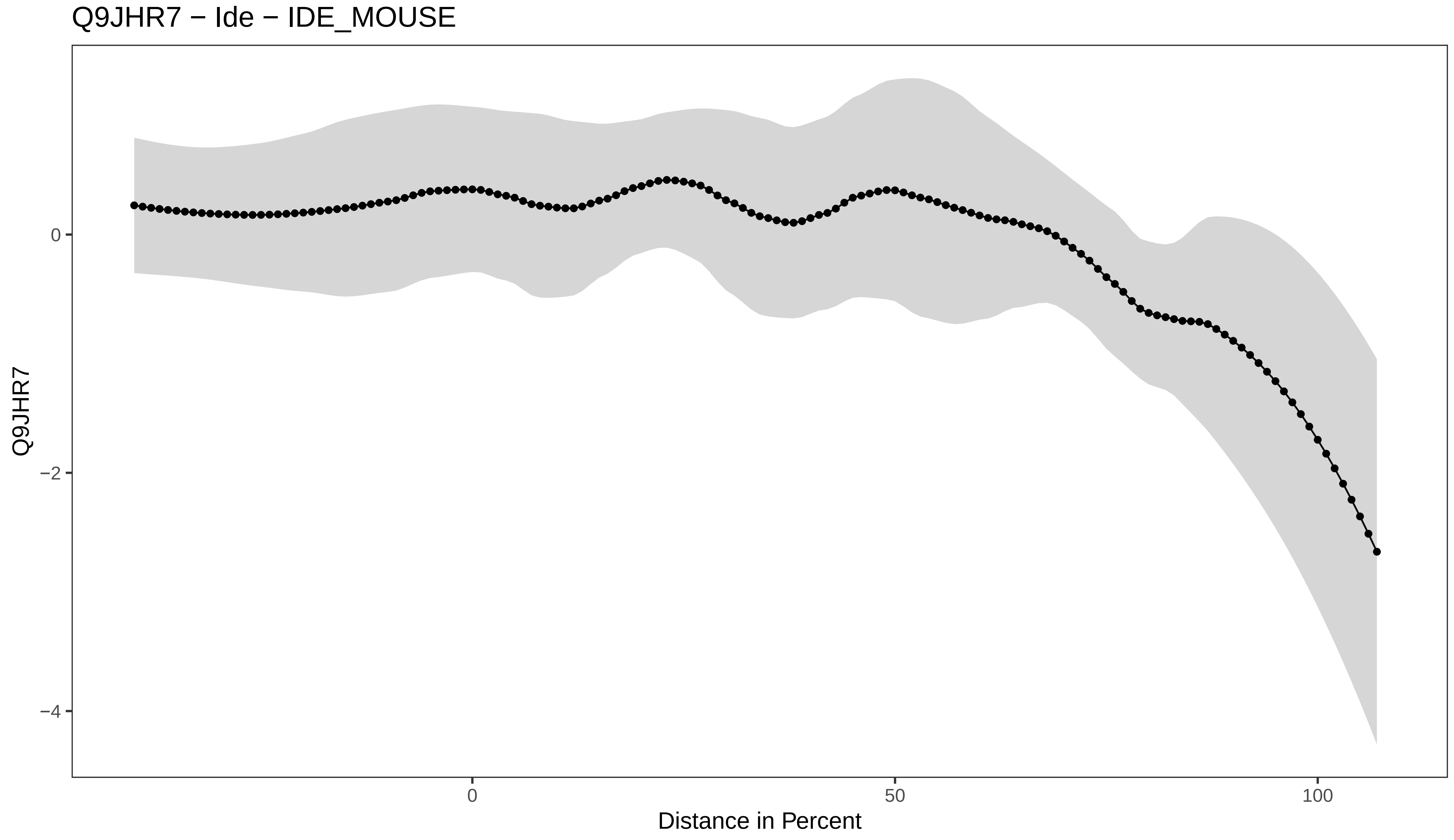
Q9JHR7 − Ide − IDE_MOUSE
Q9JHR7
0
−2
−4
0
50
100
Distance in Percent

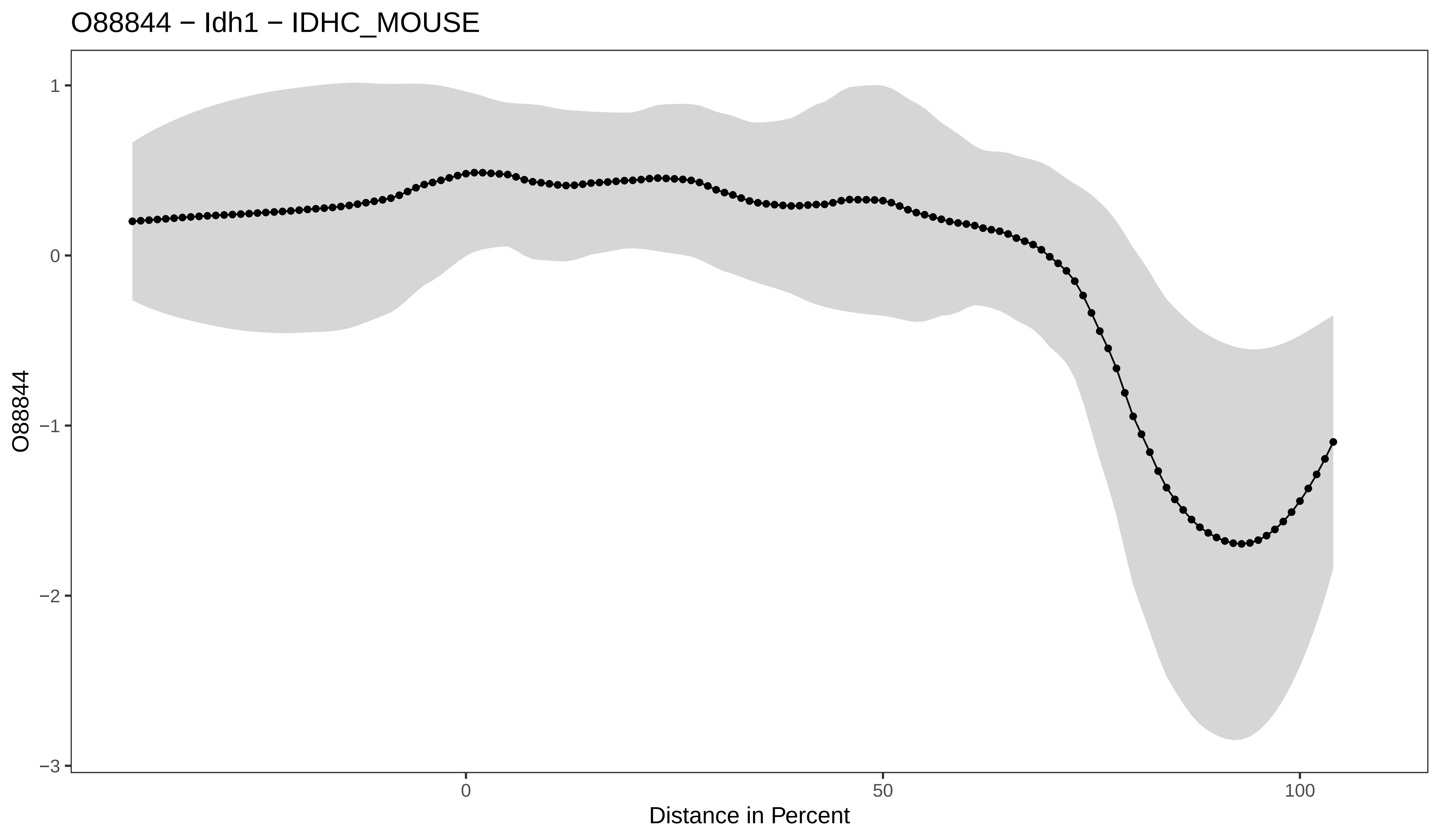
O88844 − Idh1 − IDHC_MOUSE
O88844
1
0
−1
−2
−3
0
50
100
Distance in Percent

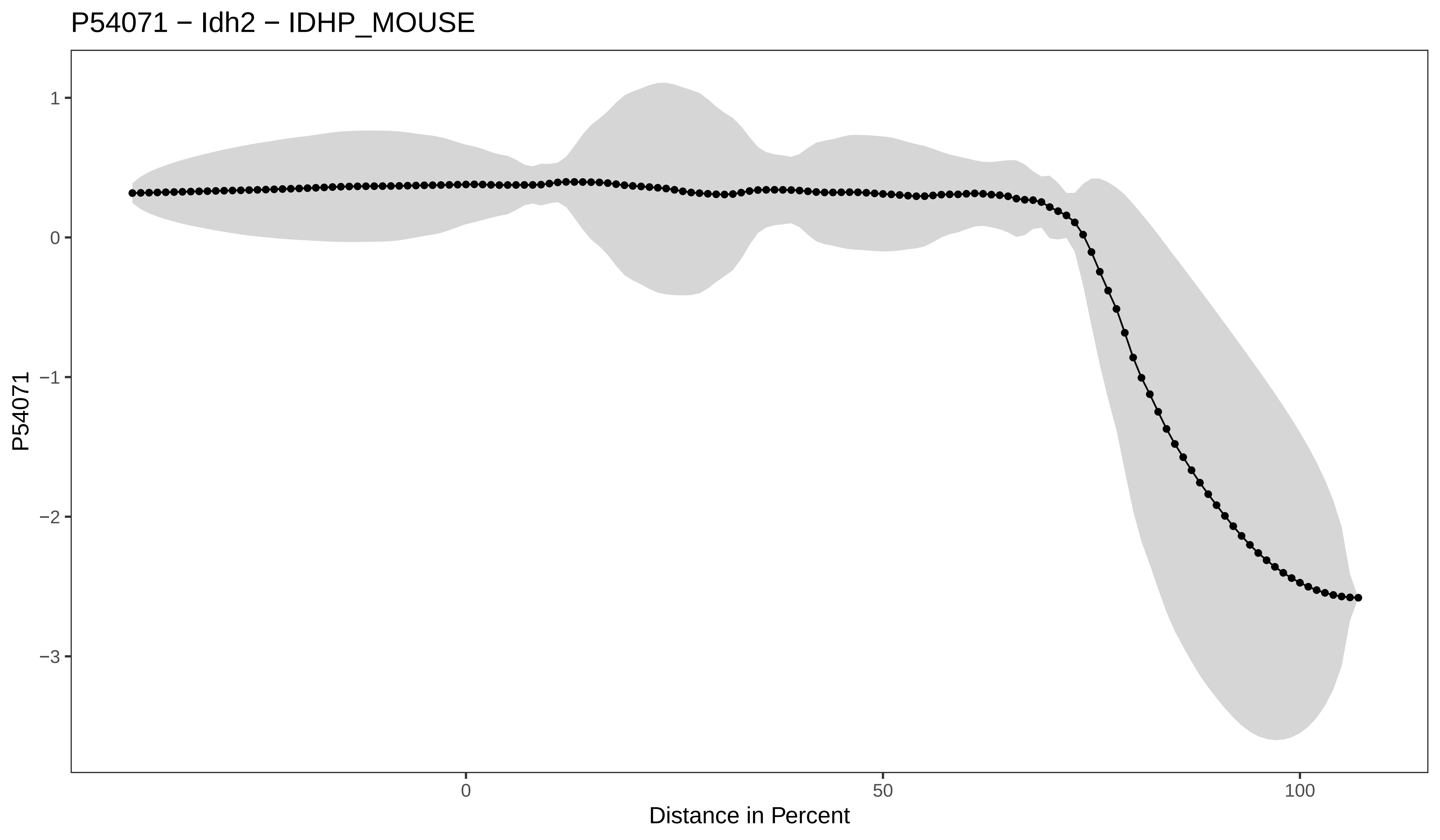
P54071 − Idh2 − IDHP_MOUSE
P54071
Distance in Percent
1
0
−1
−2
−3
0
50
100

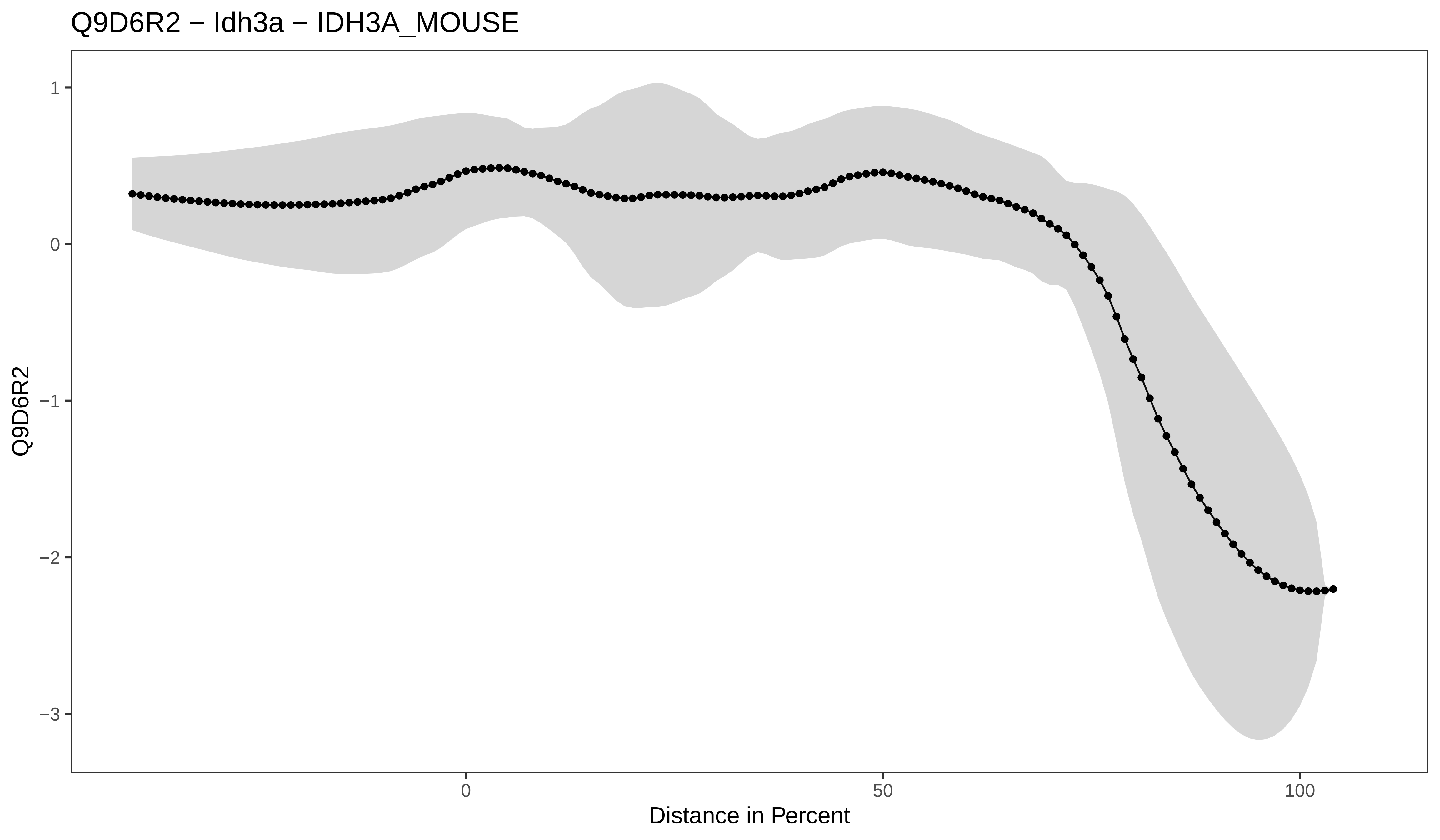
Q9D6R2 − Idh3a − IDH3A_MOUSE
Q9D6R2
Distance in Percent
1
0
−1
−2
−3
0
50
100

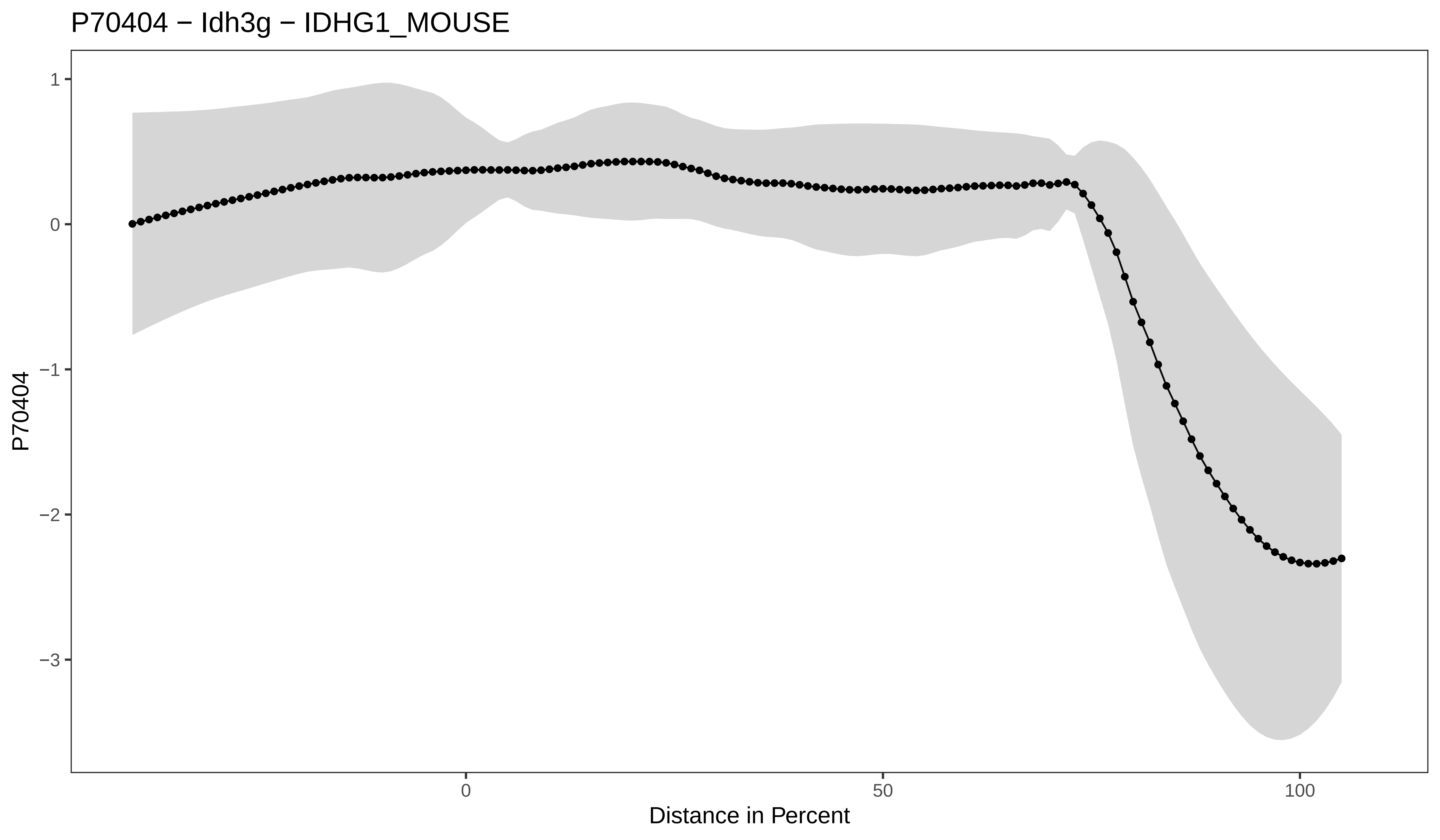

P70404 − Idh3g − IDHG1_MOUSE
P70404
Distance in Percent
1
0
−1
−2
−3
0
50
100

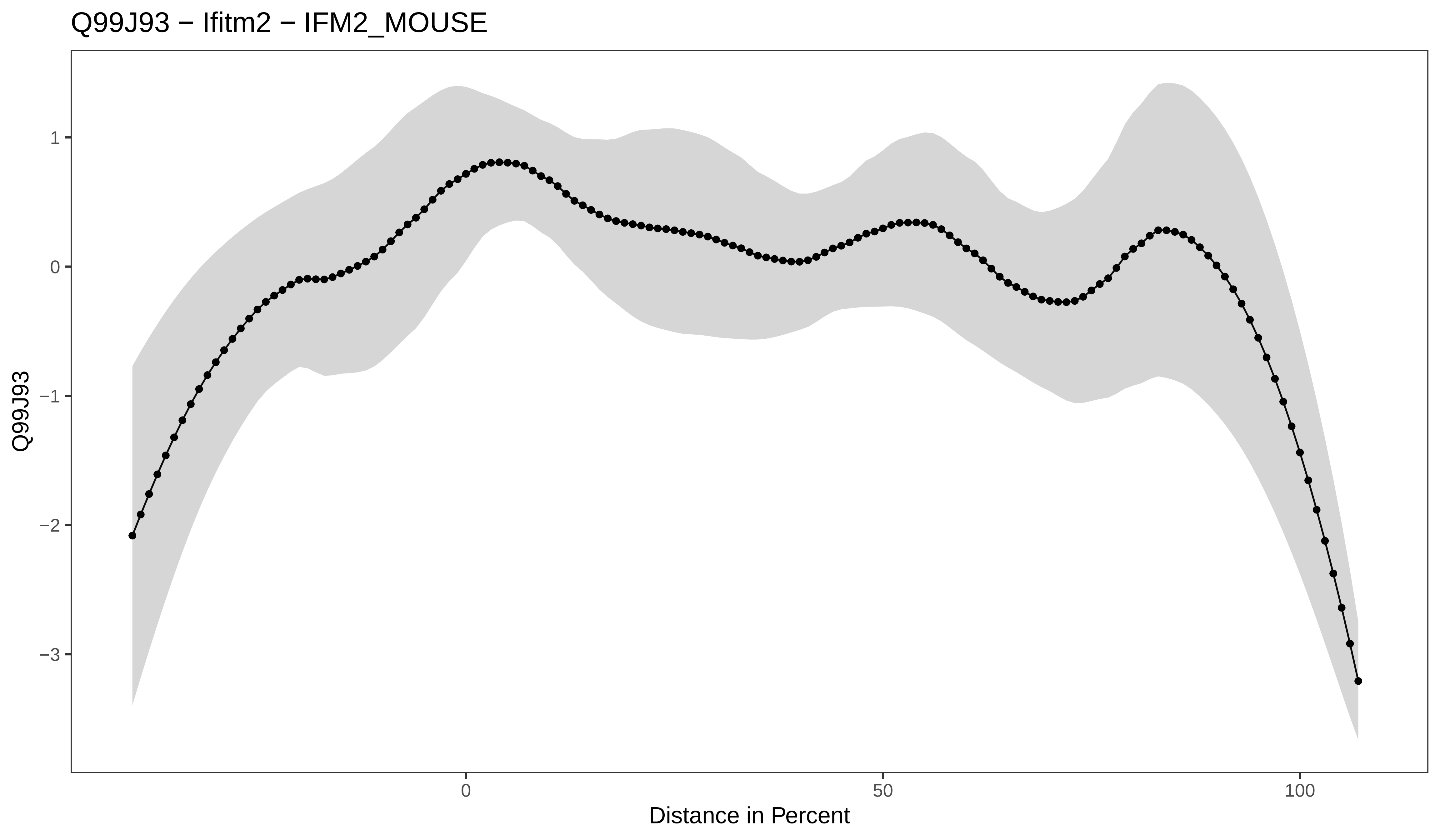
Q99J93 − Ifitm2 − IFM2_MOUSE
Q99J93
1
0
−1
−2
−3
0
50
100
Distance in Percent

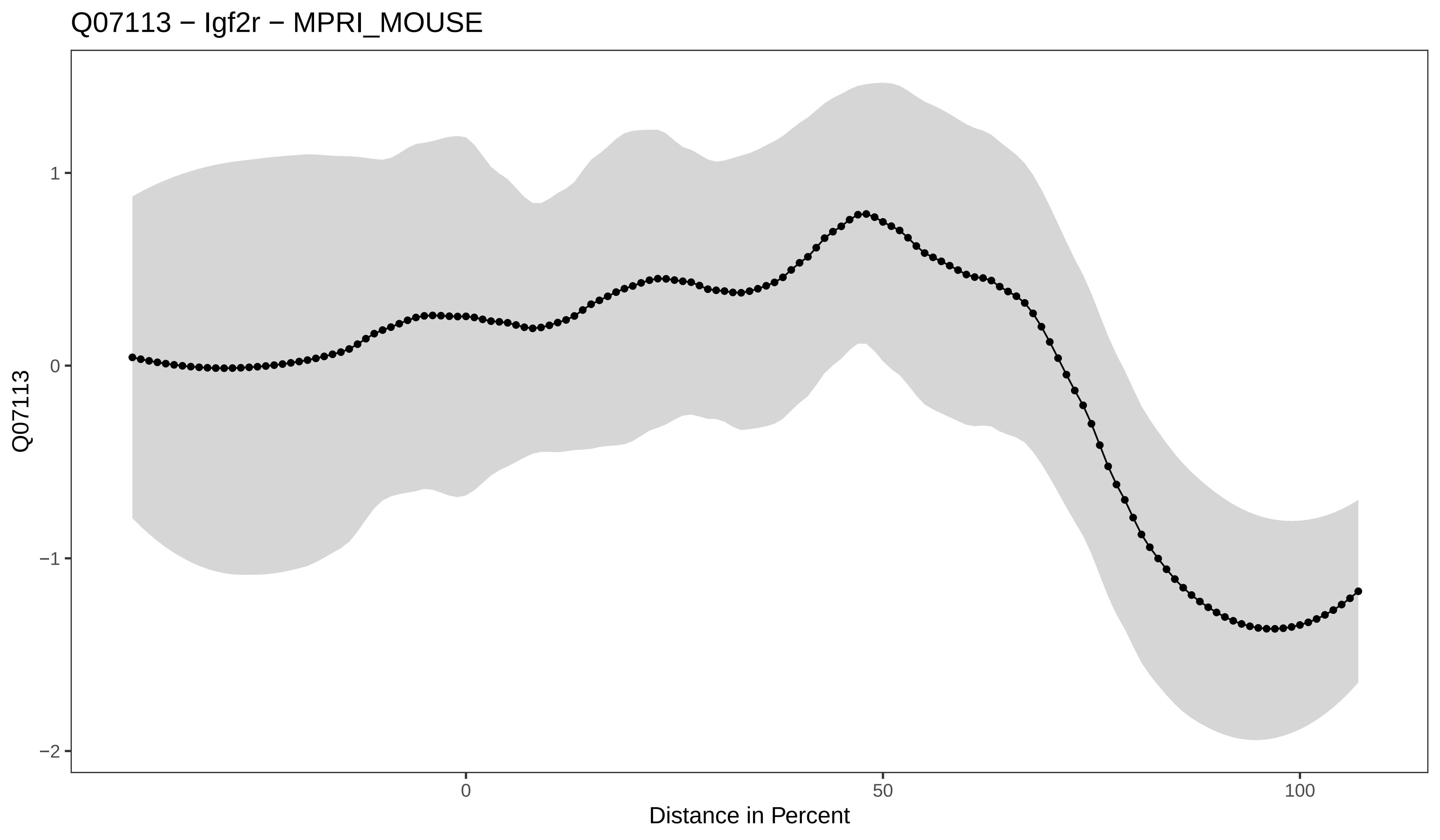

Q07113 − Igf2r − MPRI_MOUSE
1
0
−1
−2
Q07113
0
50
100
Distance in Percent

P01867 − Igh−3 − IGG2B_MOUSE

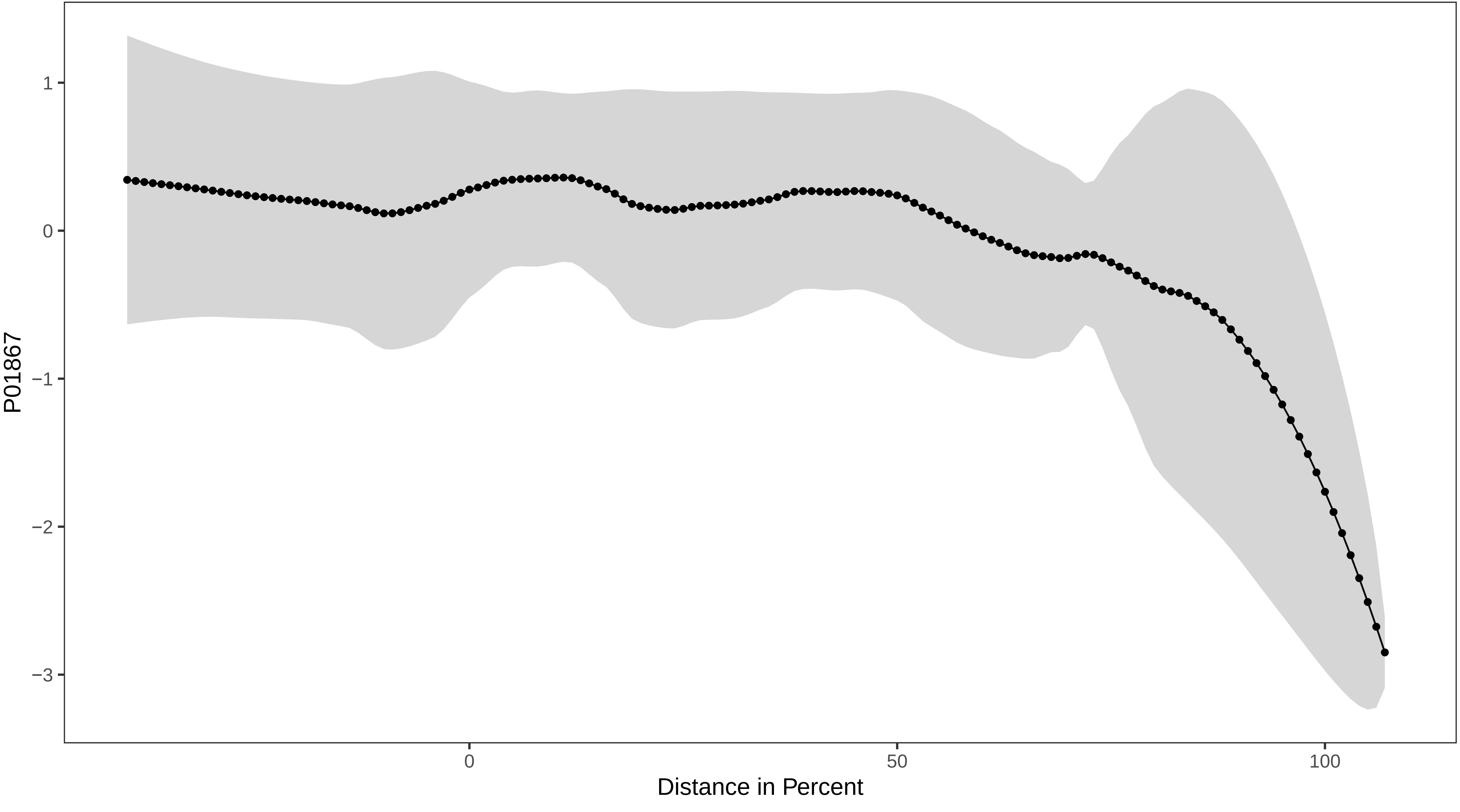

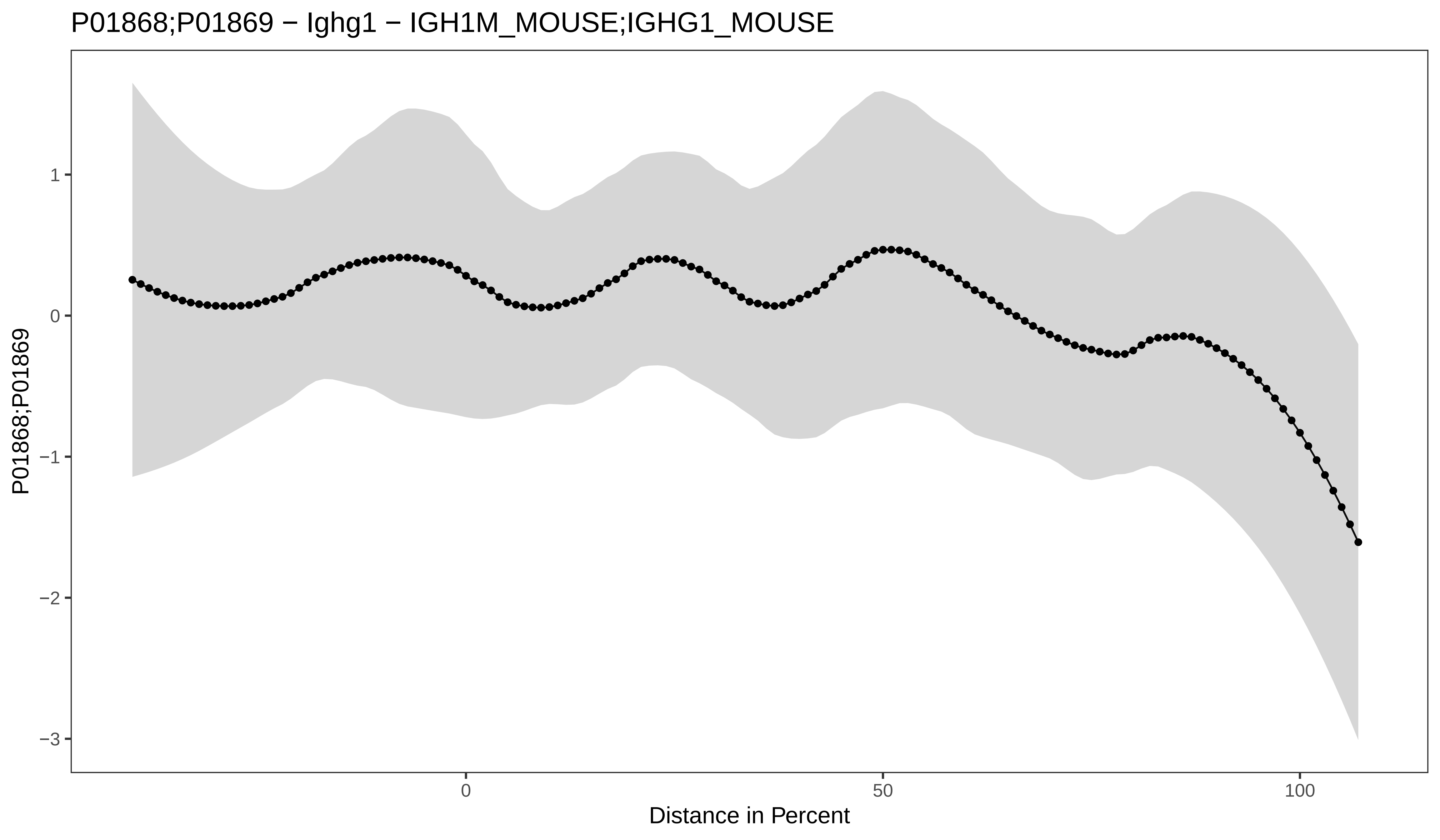
P01868;P01869 − Ighg1 − IGH1M_MOUSE;IGHG1_MOUSE
P01868;P01869
1
0
−1
−2
−3
0
50
100
Distance in Percent

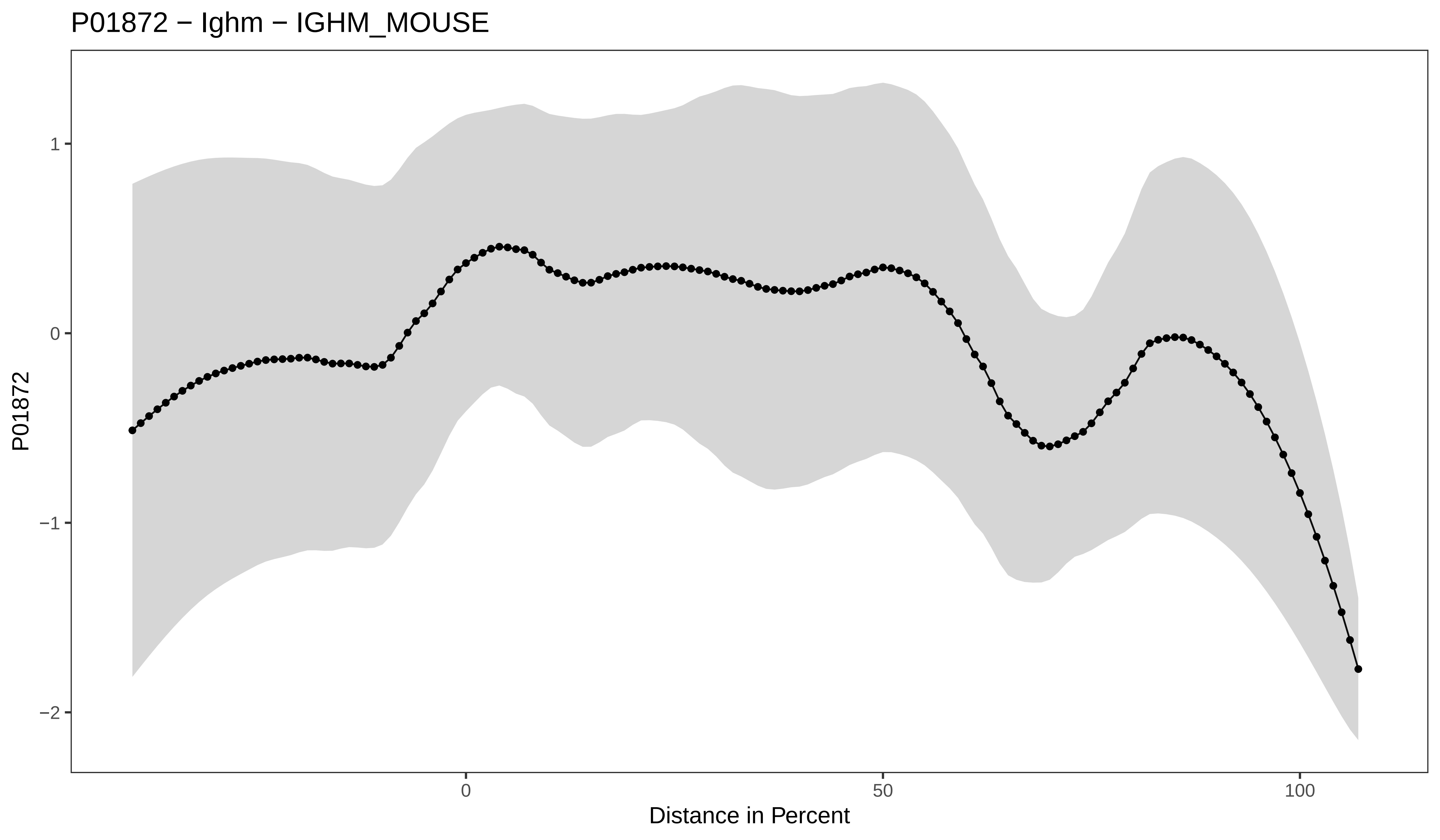
P01872 − Ighm − IGHM_MOUSE
P01872
1
0
−1
−2
0
50
100
Distance in Percent

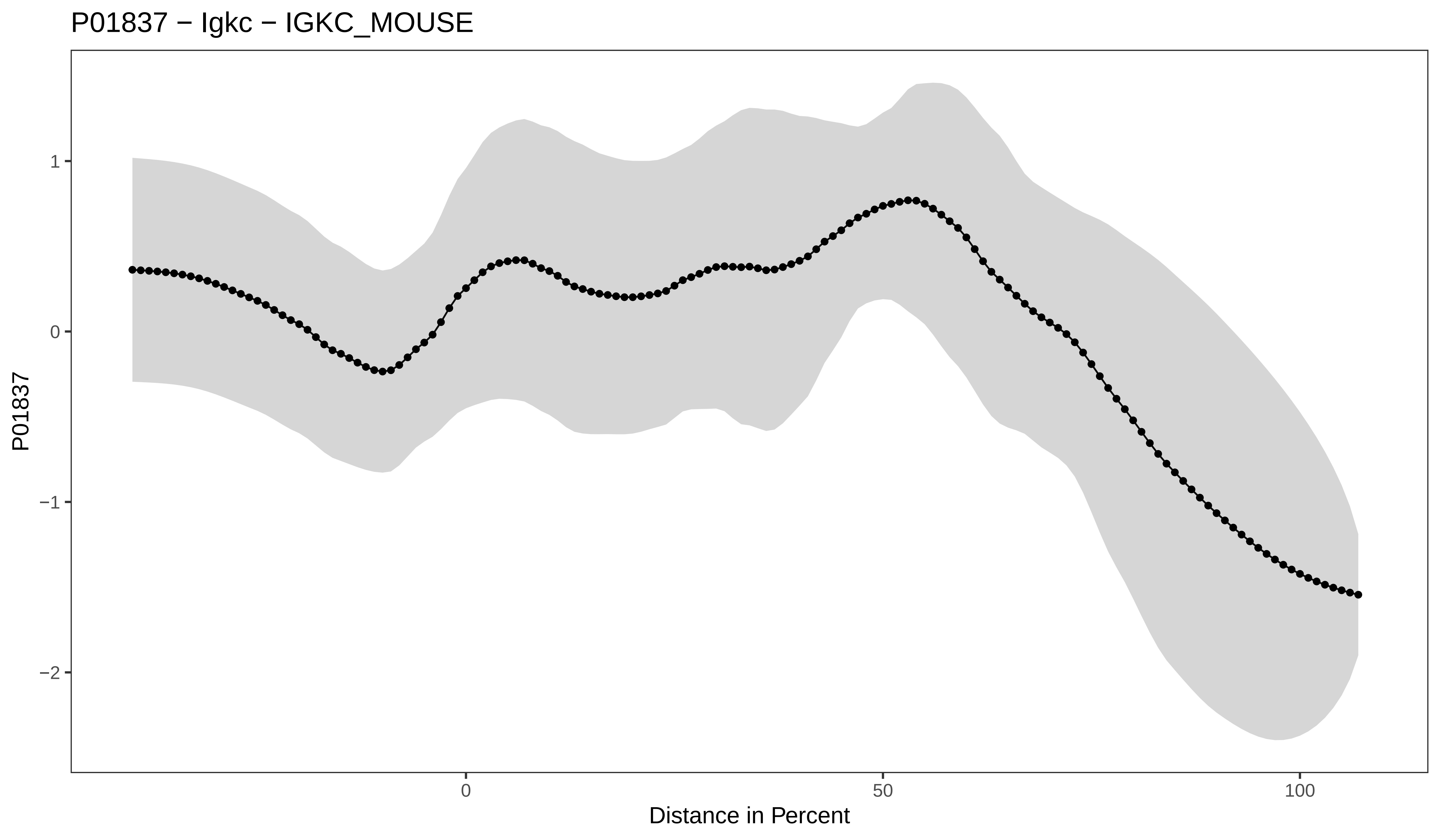
P01837 − Igkc − IGKC_MOUSE
1
0
−1
−2
P01837
0
50
100
Distance in Percent

Q9DBZ1 − Ikbip − IKIP_MOUSE

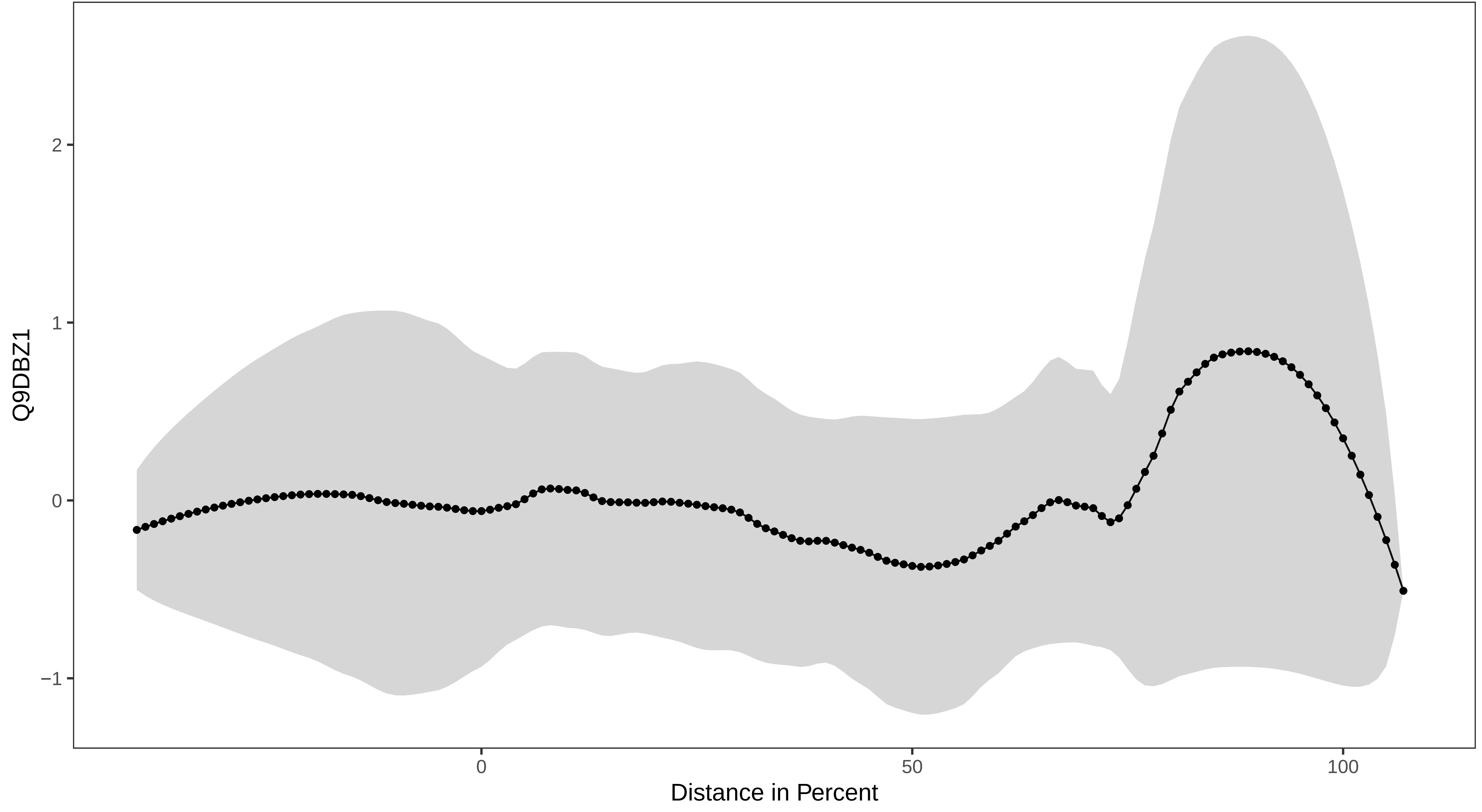

P13504 − Il1r1 − IL1R1_MOUSE

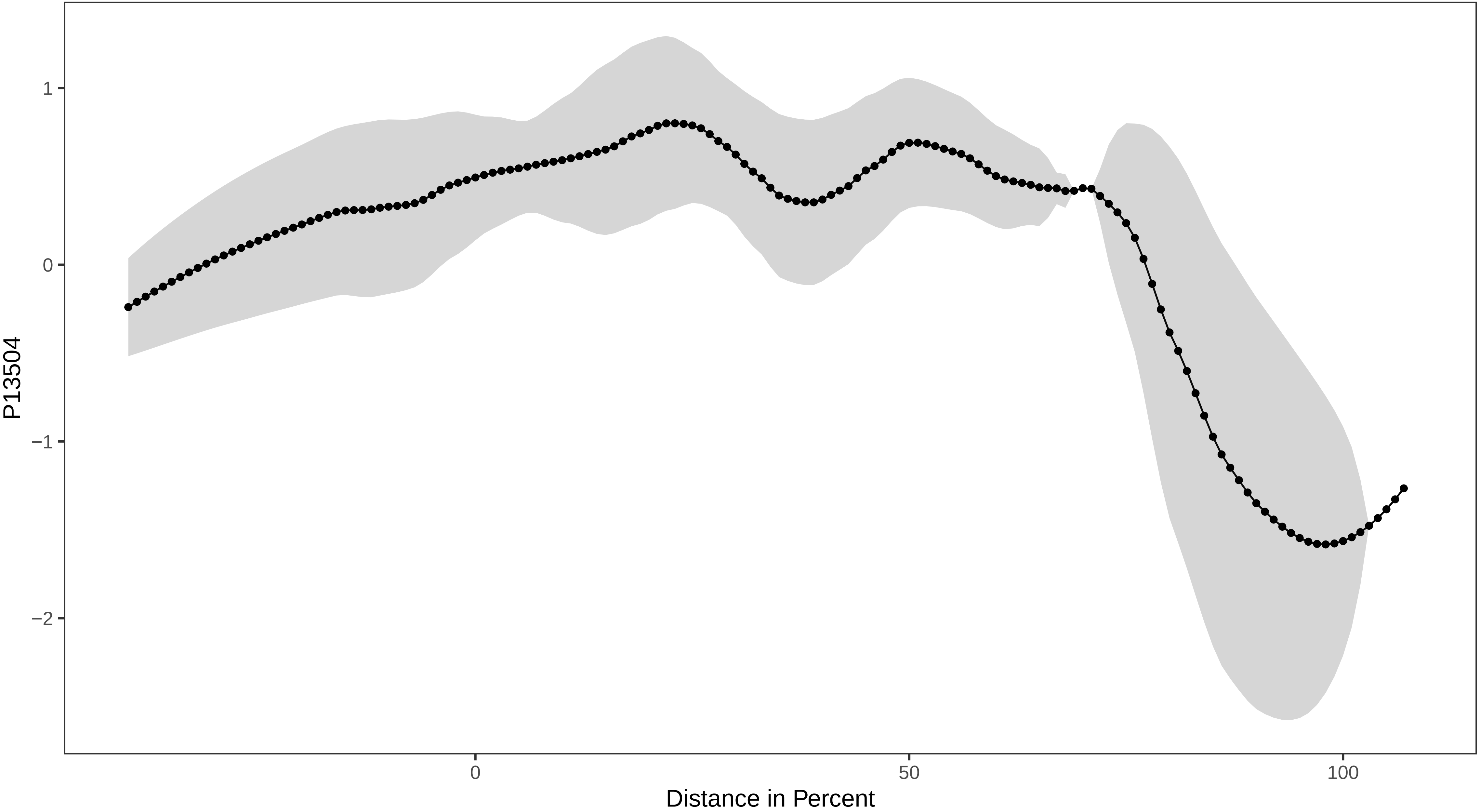

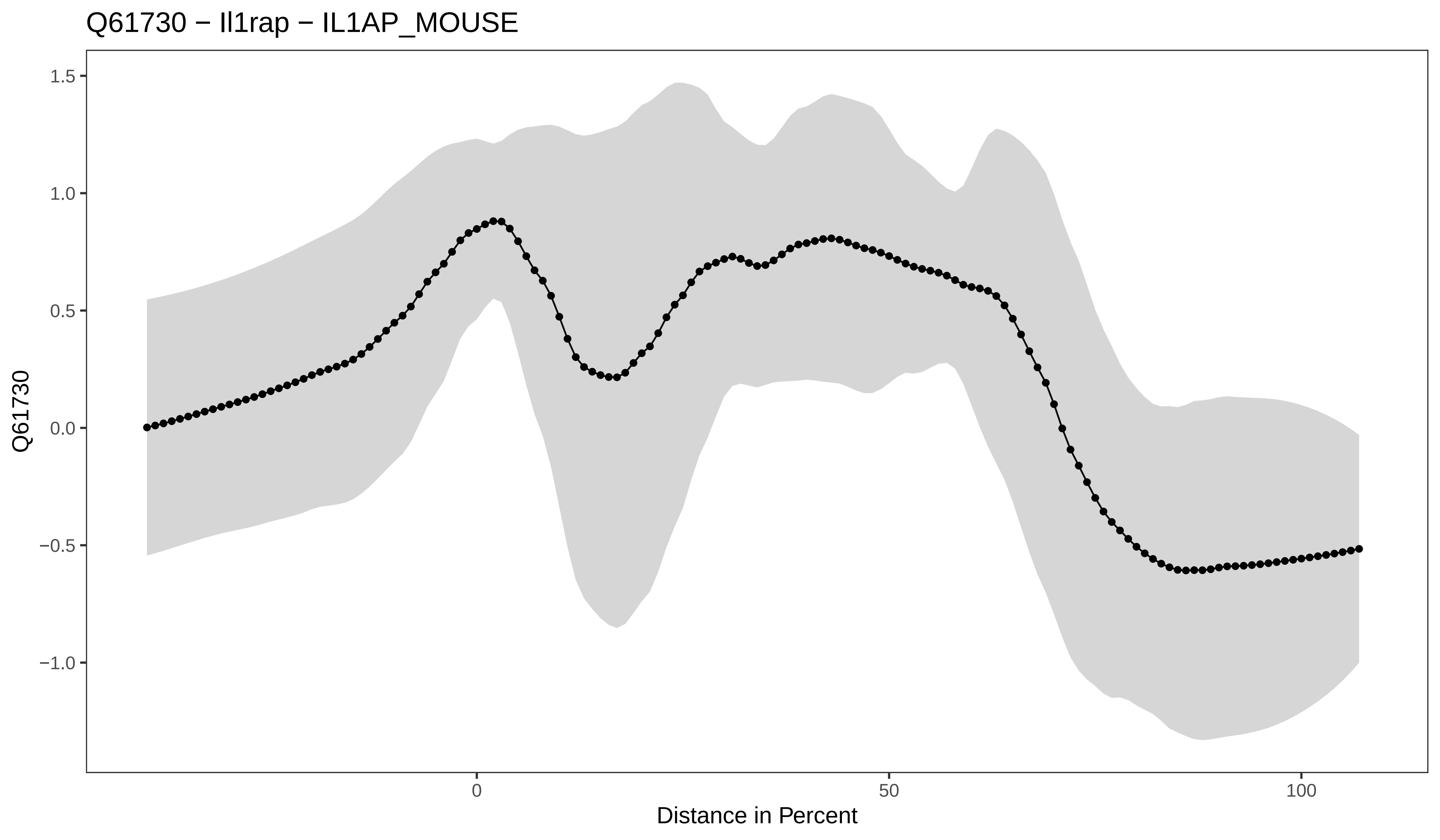

Q61730 − Il1rap − IL1AP_MOUSE
1.5
1.0
0.5
0.0
−0.5
−1.0
Q61730
0
50
100
Distance in Percent

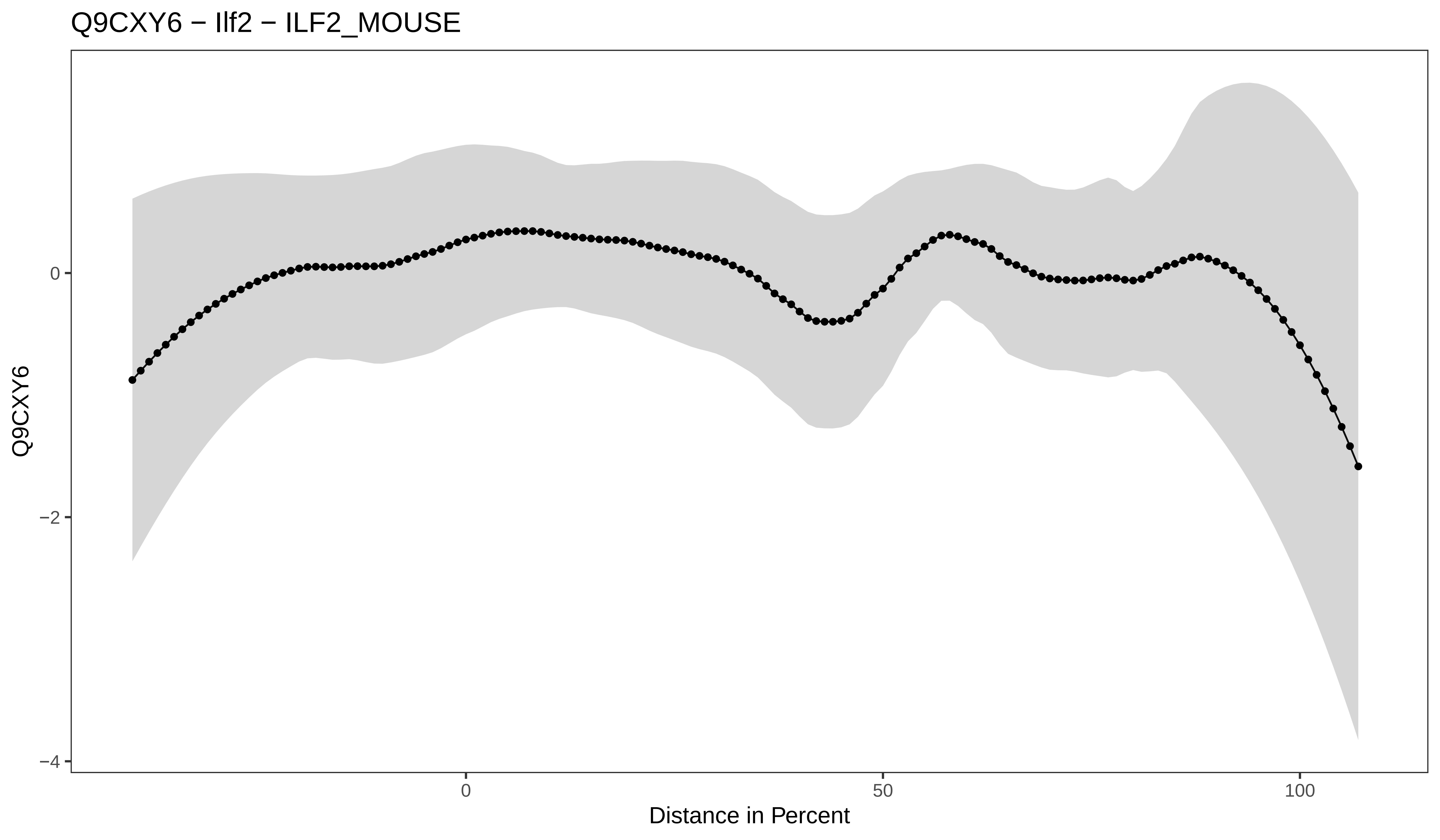

Q9CXY6 − Ilf2 − ILF2_MOUSE
0
−2
−4
Q9CXY6
0
50
100
Distance in Percent

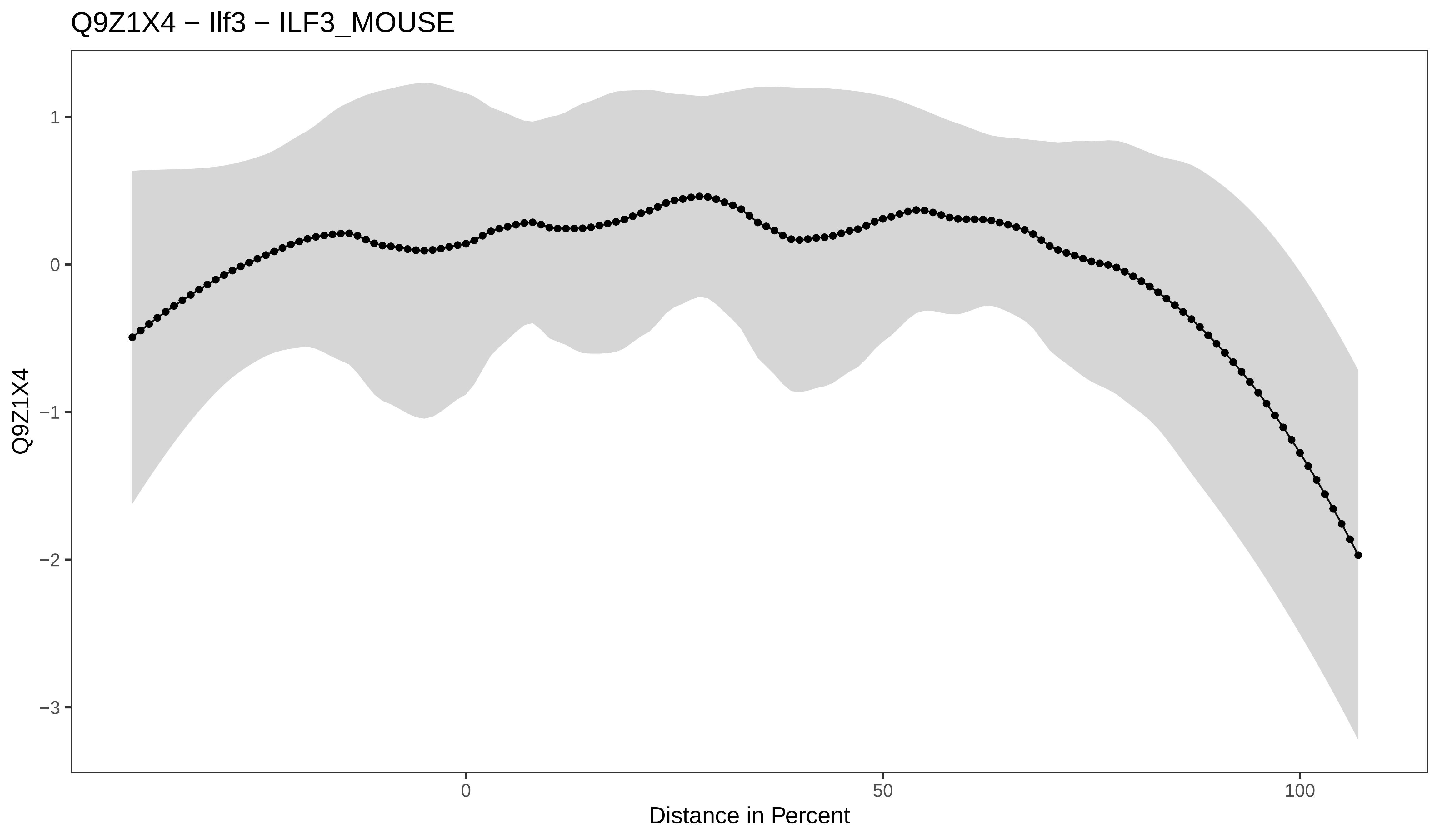

Q9Z1X4 − Ilf3 − ILF3_MOUSE
1
0
−1
−2
−3
Q9Z1X4
0
50
100
Distance in Percent

O55222 − Ilk − ILK_MOUSE

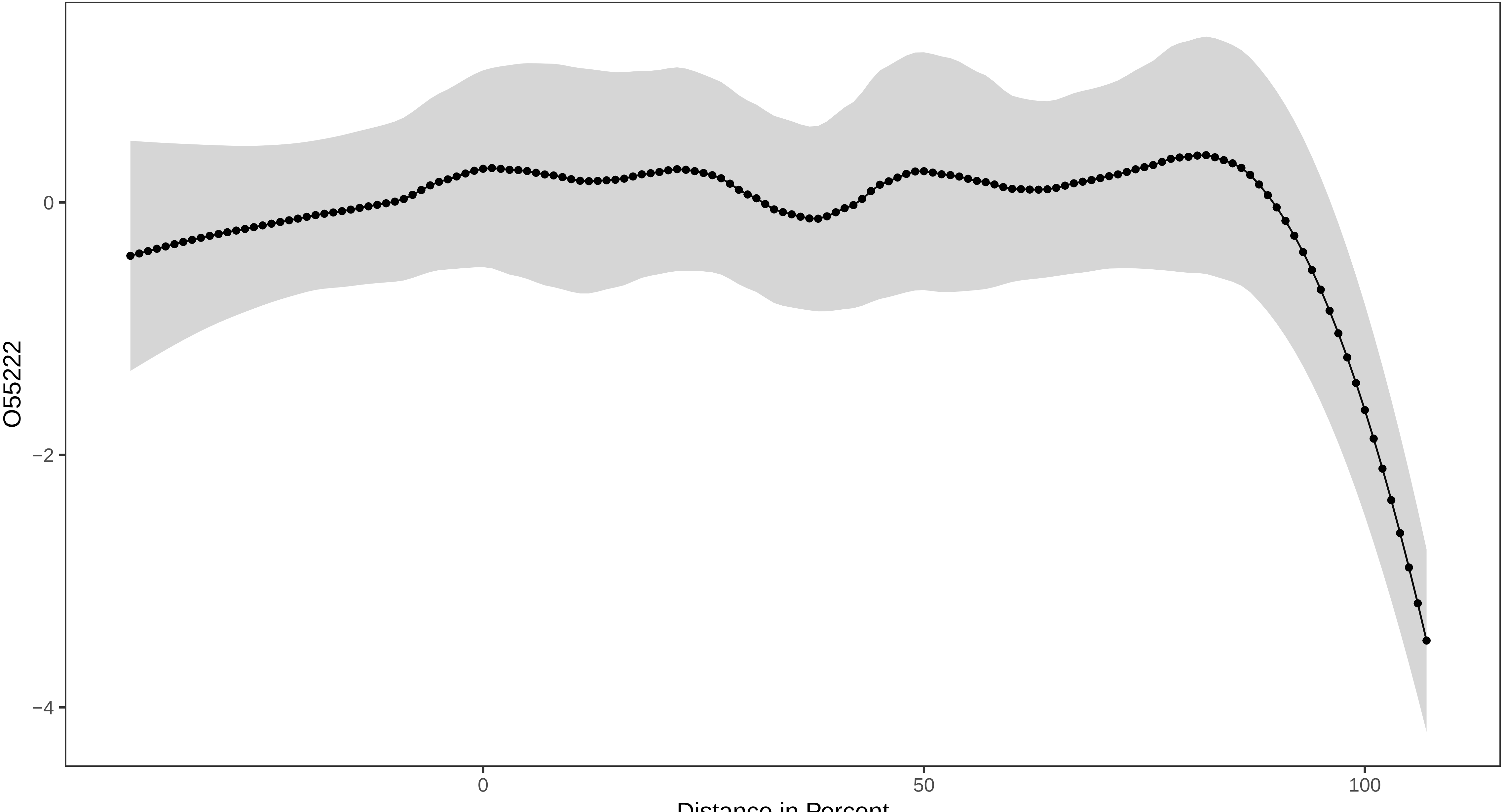

Q8CAQ8 − Immt − MIC60_MOUSE

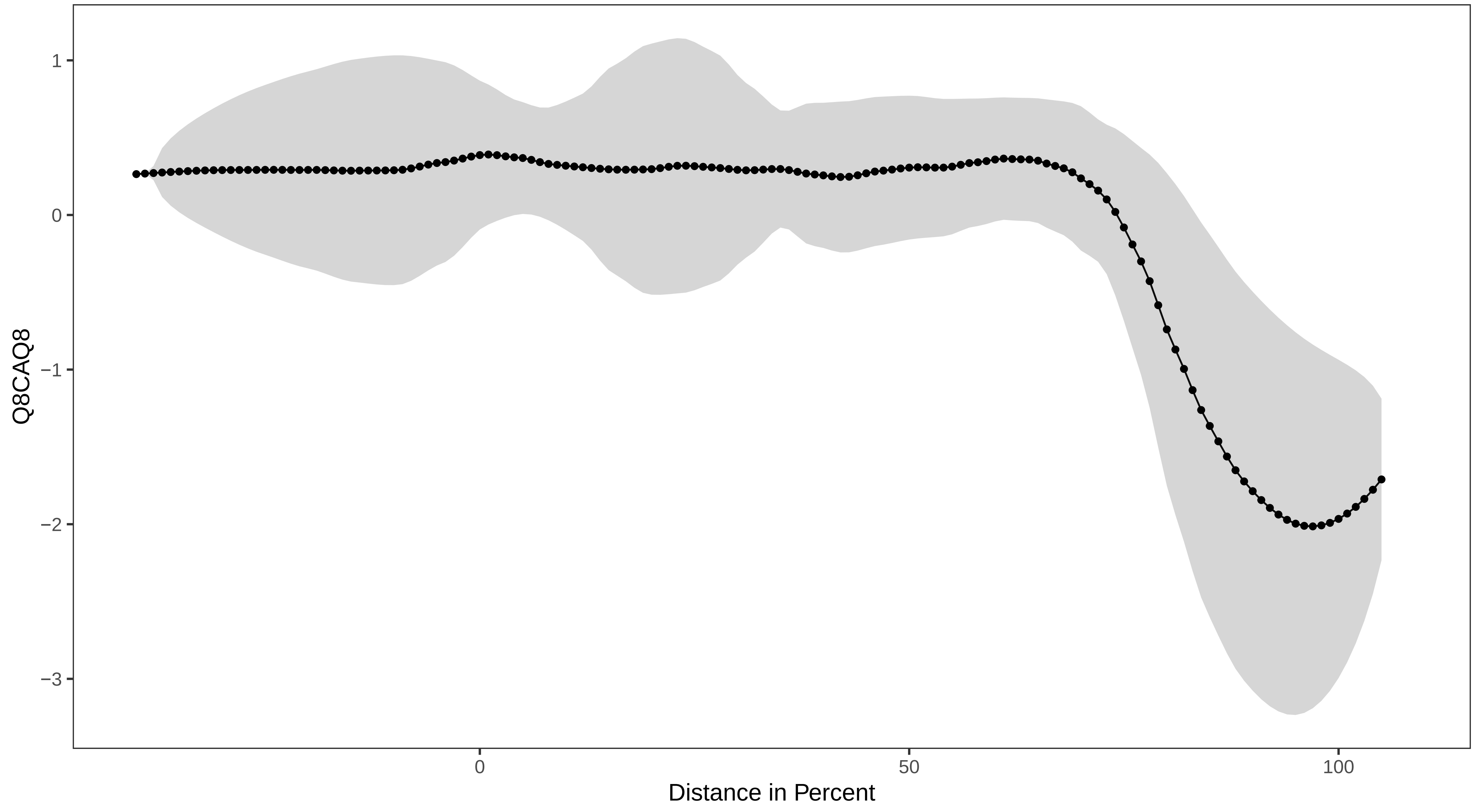

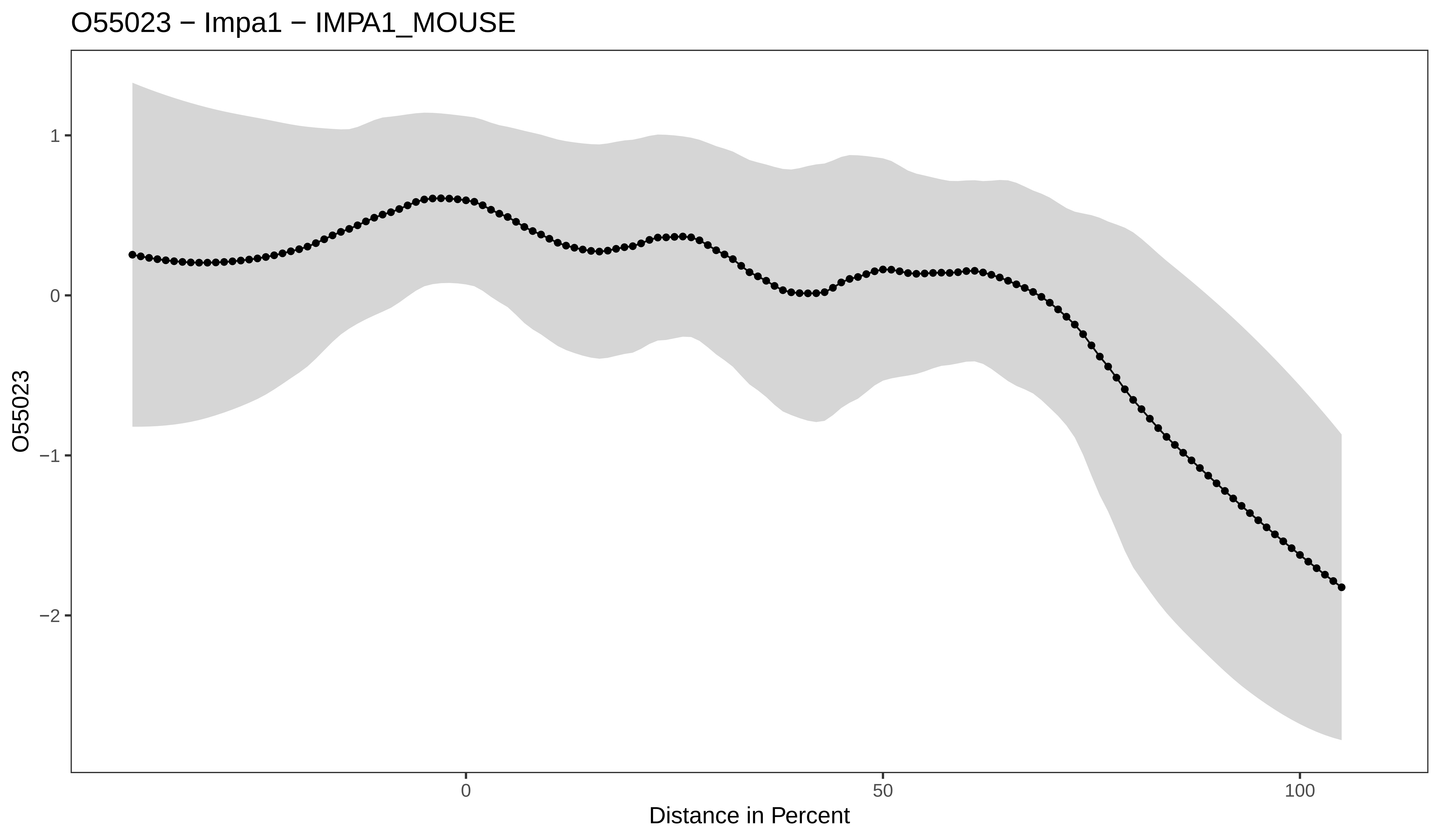
O55023 − Impa1 − IMPA1_MOUSE
1
0
−1
−2
O55023
0
50
100
Distance in Percent

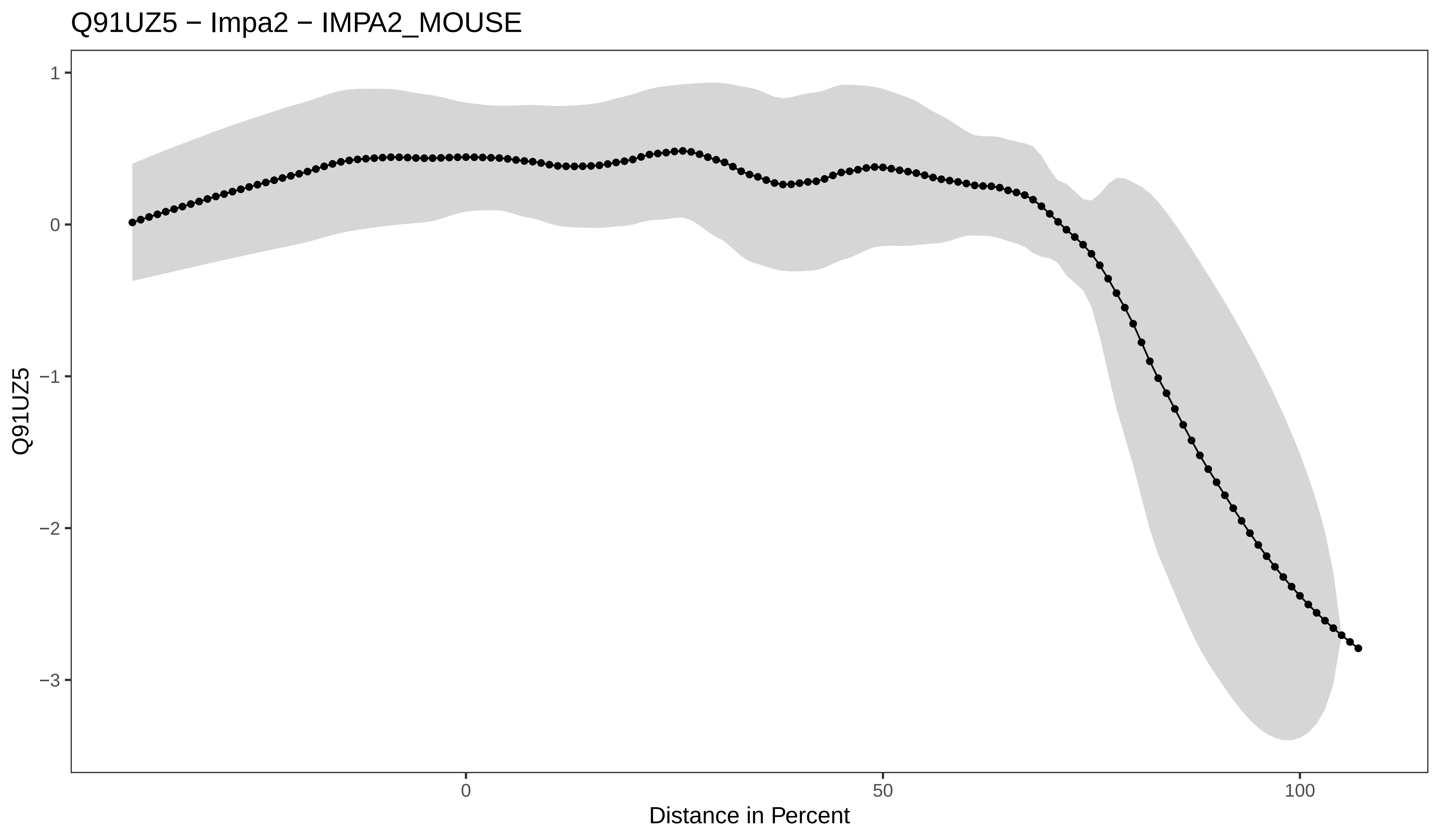

Q91UZ5 − Impa2 − IMPA2_MOUSE
1
0
−1
−2
−3
Q91UZ5
0
50
100
Distance in Percent

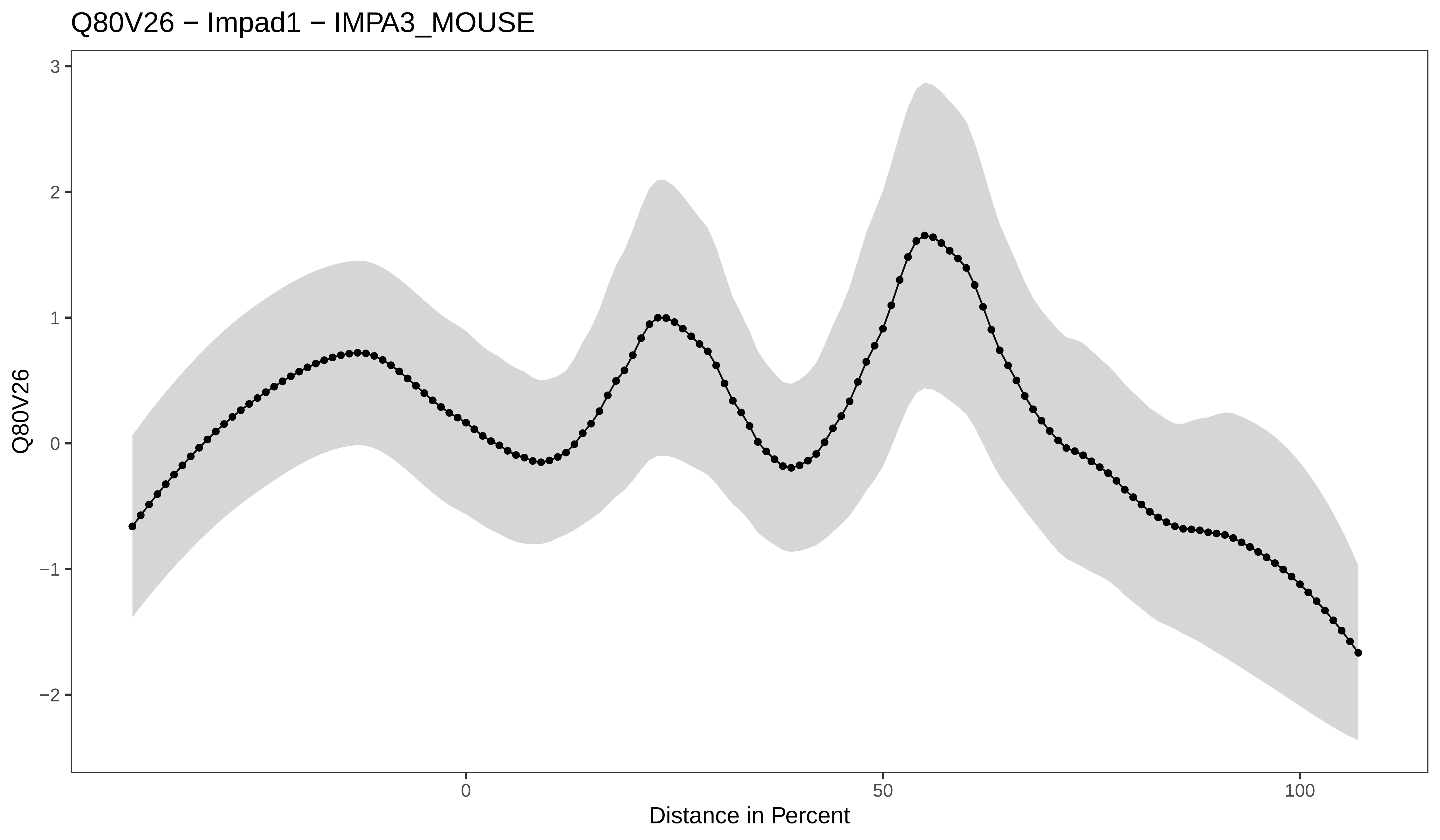
Q80V26 − Impad1 − IMPA3_MOUSE
3
2
1
0
−1
−2
Q80V26
0
50
100
Distance in Percent

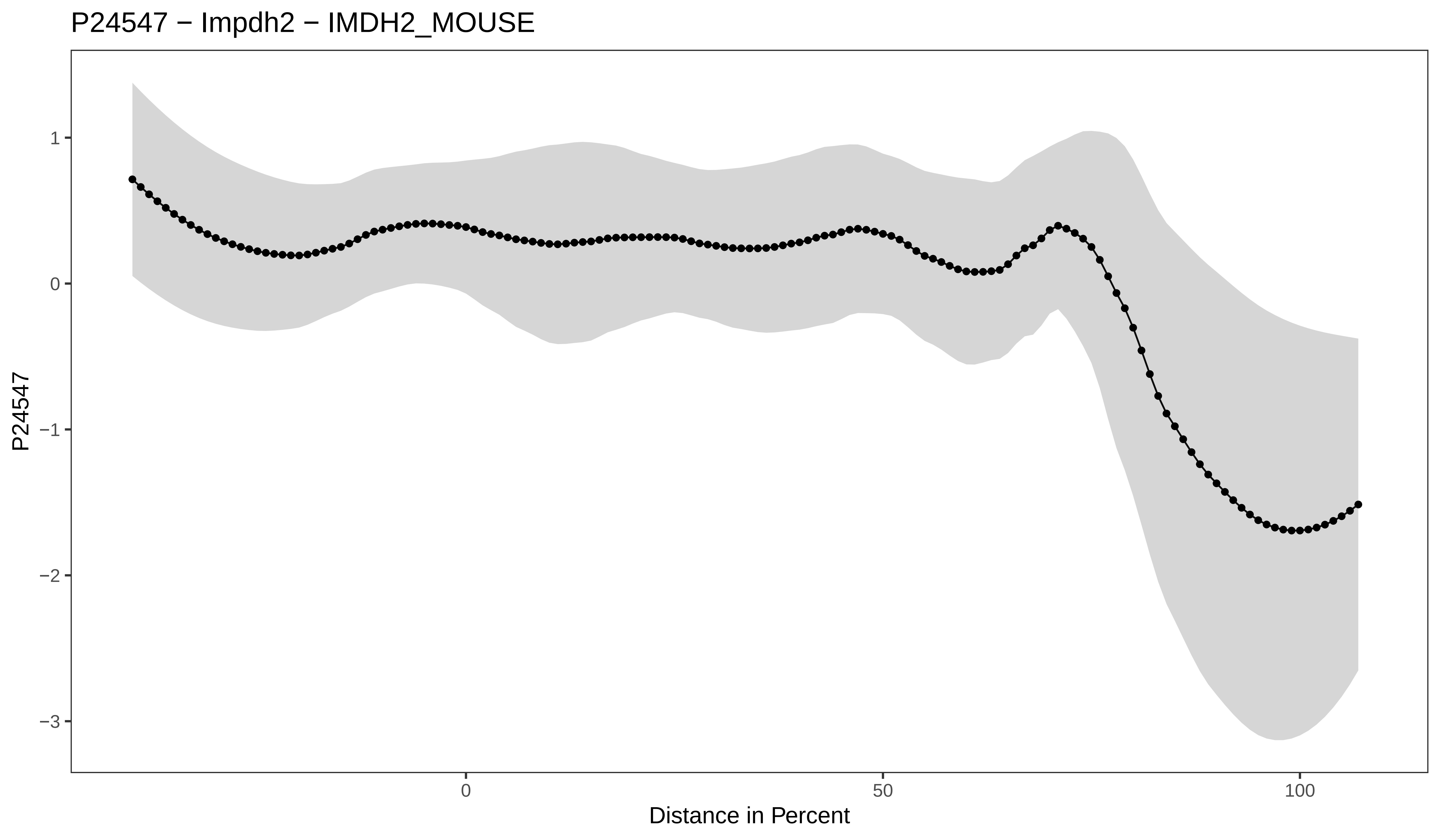
P24547 − Impdh2 − IMDH2_MOUSE
P24547
1
0
−1
−2
−3
0
50
100
Distance in Percent

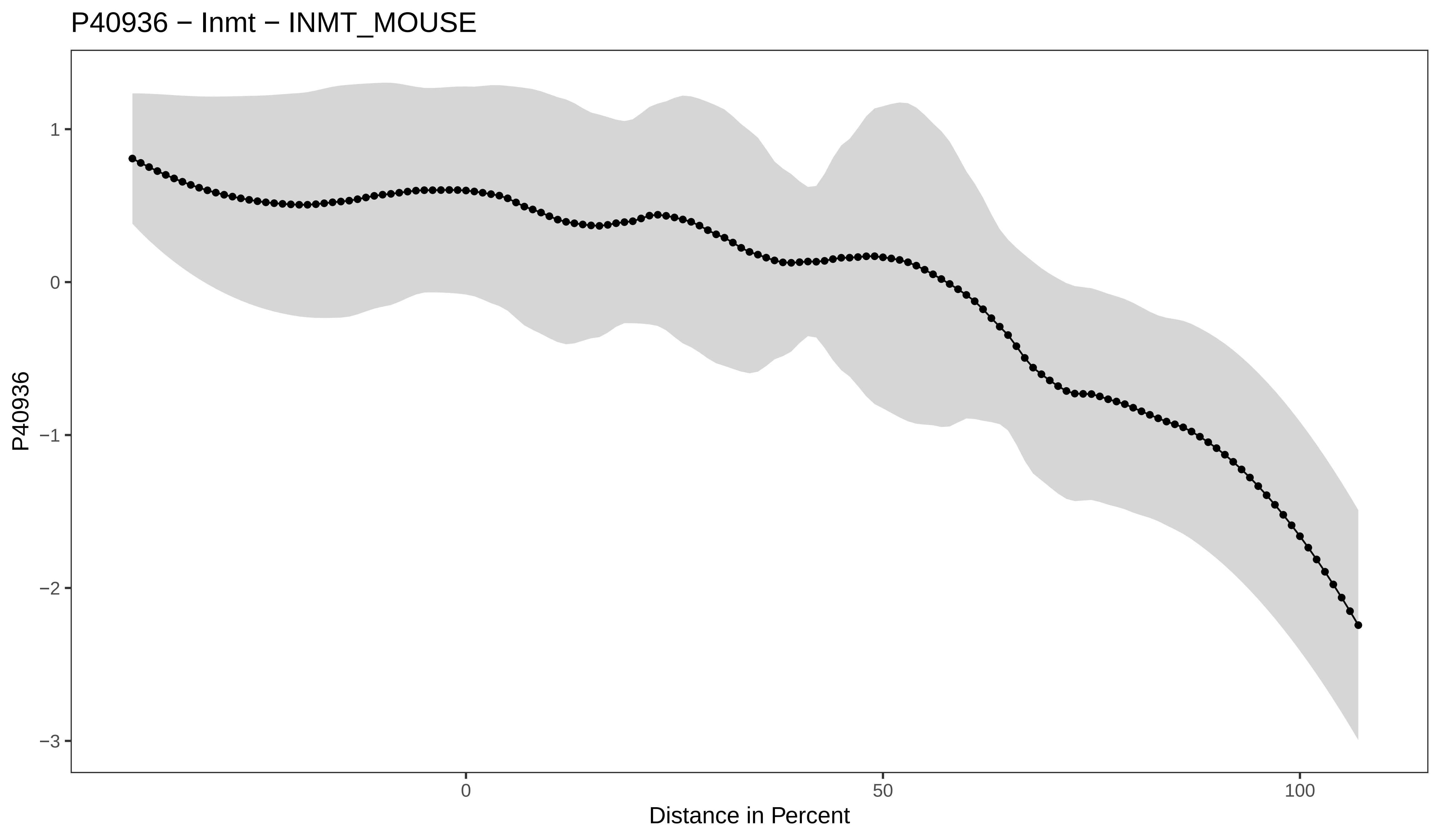

P40936 − Inmt − INMT_MOUSE
P40936
1
0
−1
−2
−3
0
50
100
Distance in Percent

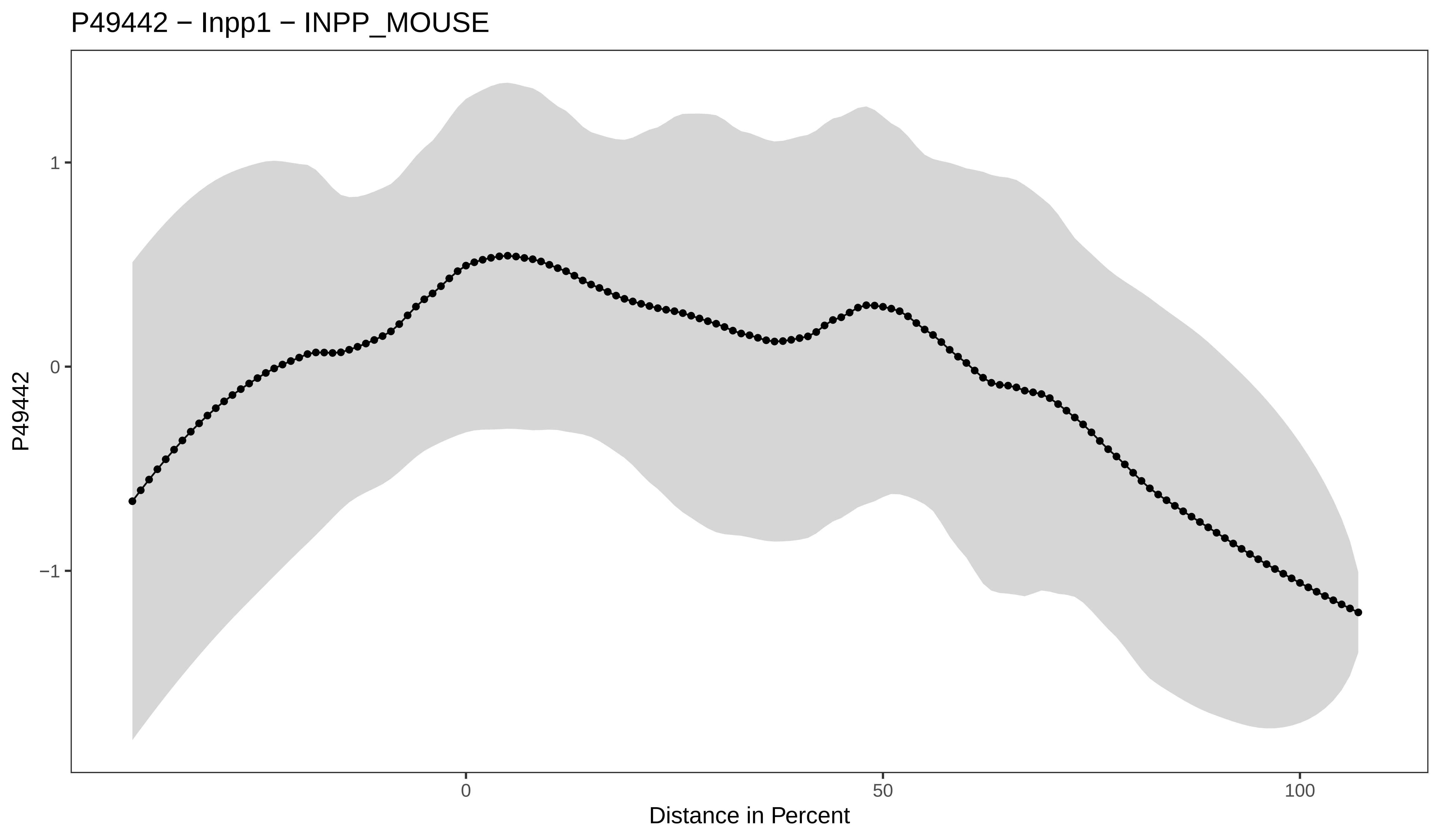
P49442 − Inpp1 − INPP_MOUSE
1
0
−1
P49442
0
50
100
Distance in Percent

Q8CDA1 − Inpp5f − SAC2_MOUSE

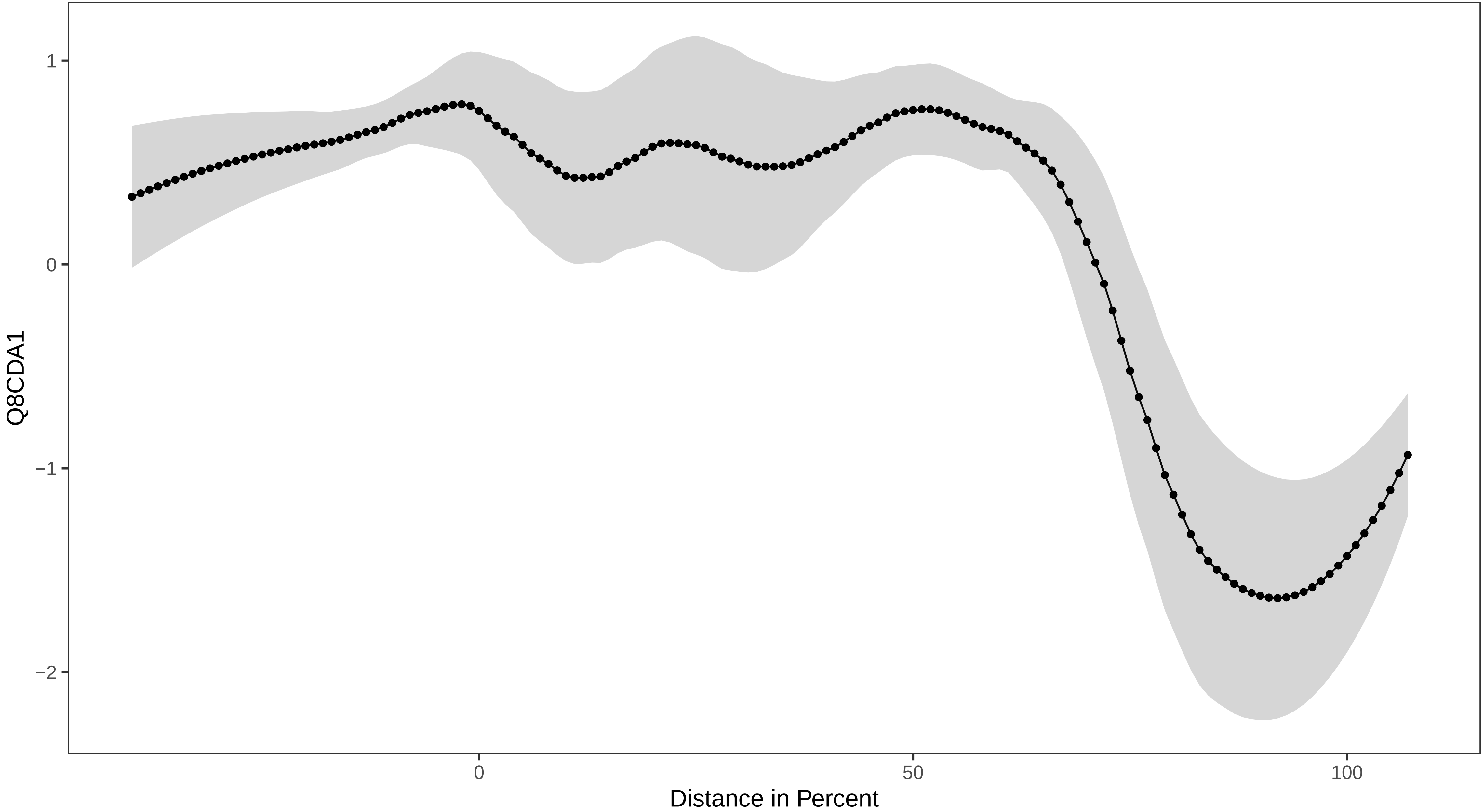

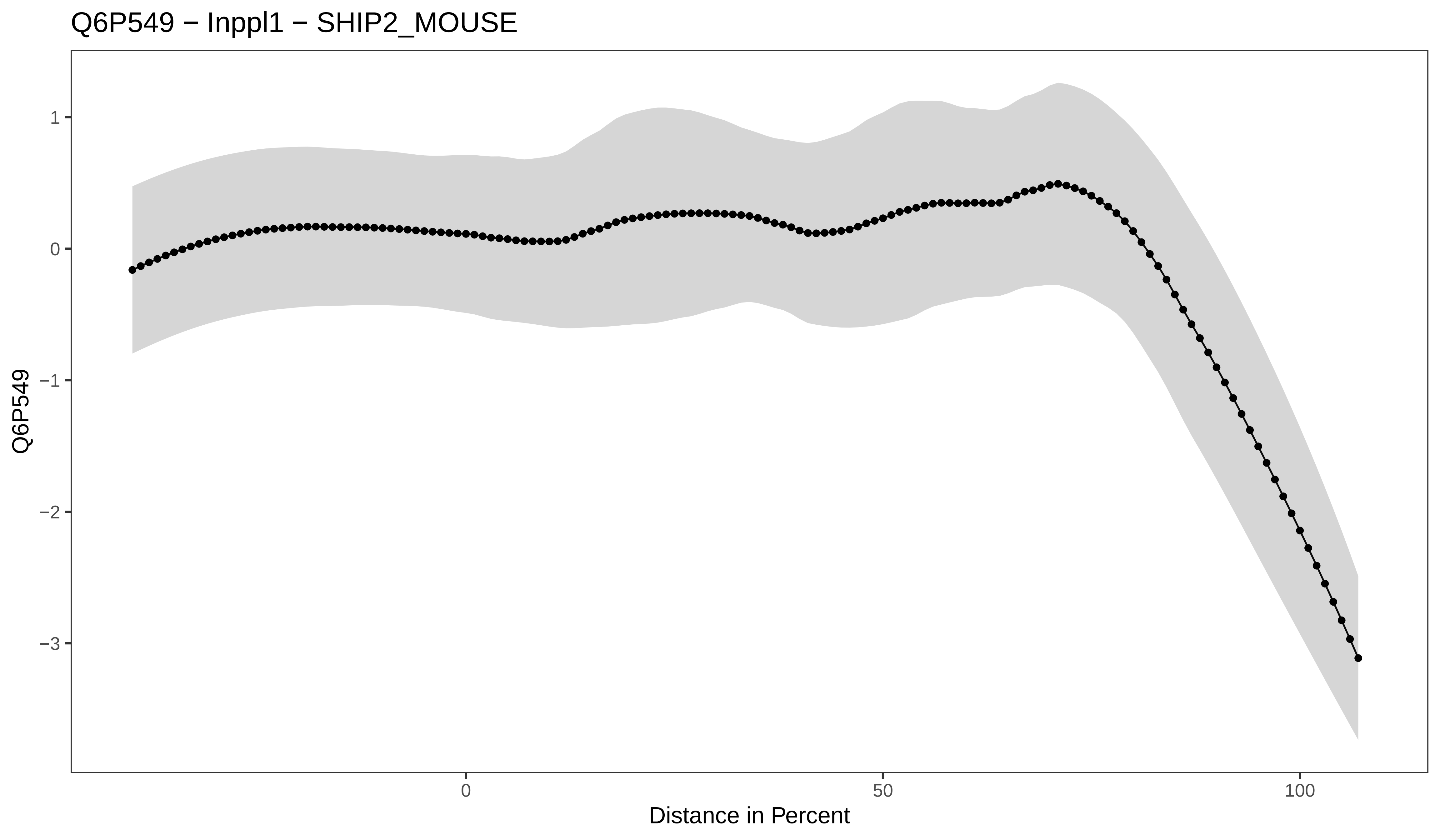
Q6P549 − Inppl1 − SHIP2_MOUSE
Q6P549
Distance in Percent
1
0
−1
−2
−3
0
50
100

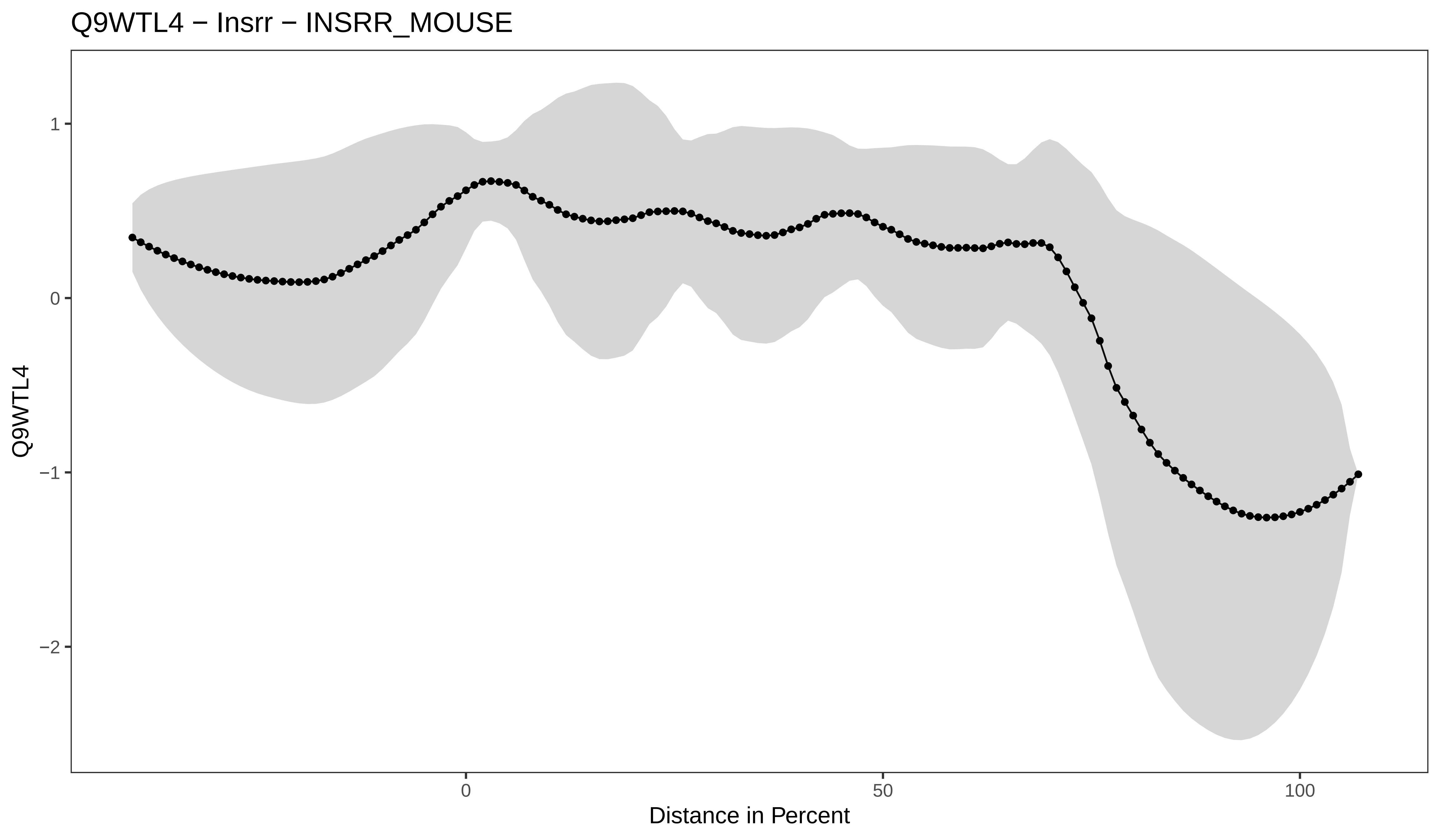

Q9WTL4 − Insrr − INSRR_MOUSE
1
0
−1
−2
Q9WTL4
0
50
100
Distance in Percent

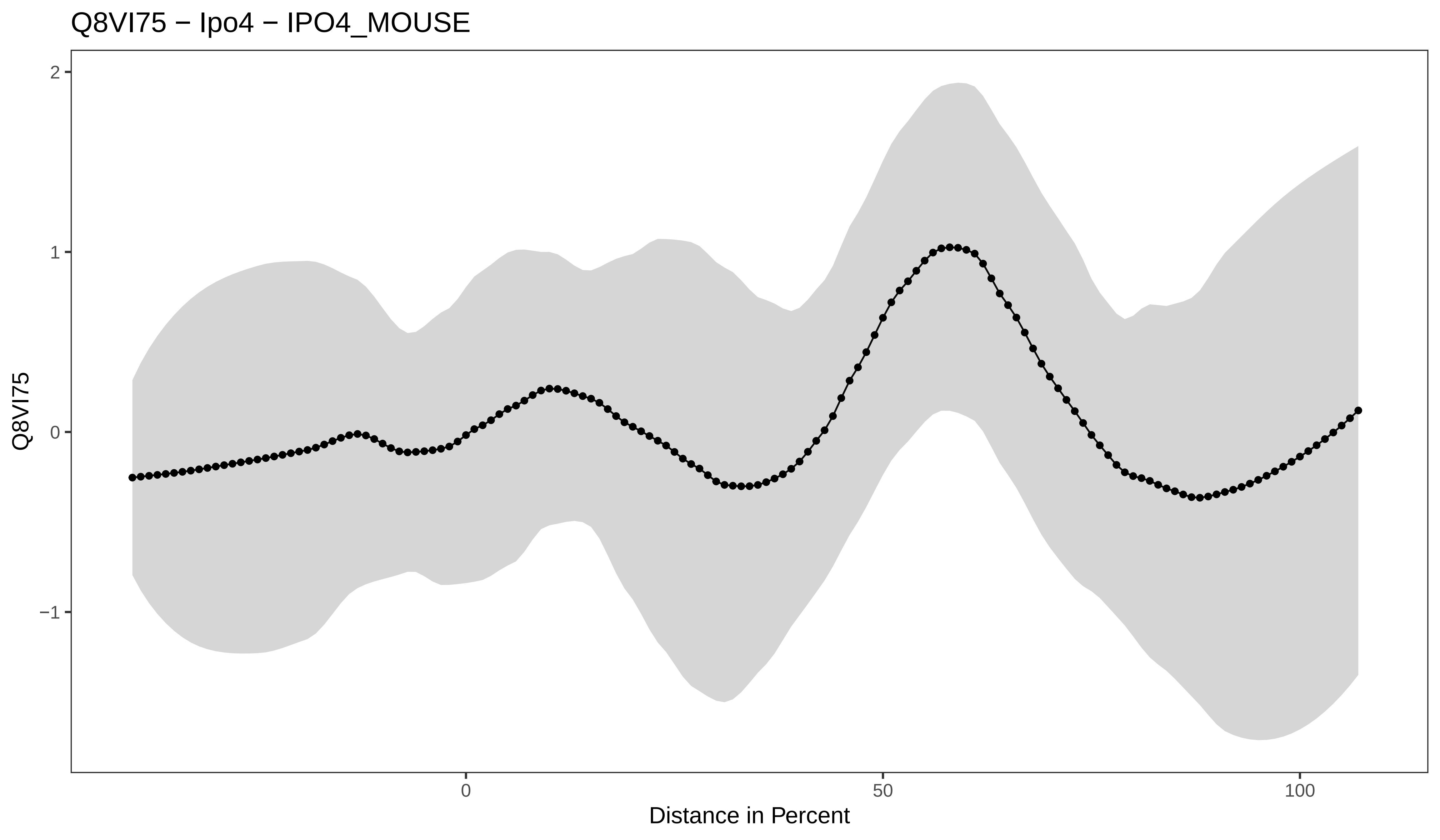

Q8VI75 − Ipo4 − IPO4_MOUSE
Q8VI75
2
1
0
−1
0
50
100
Distance in Percent

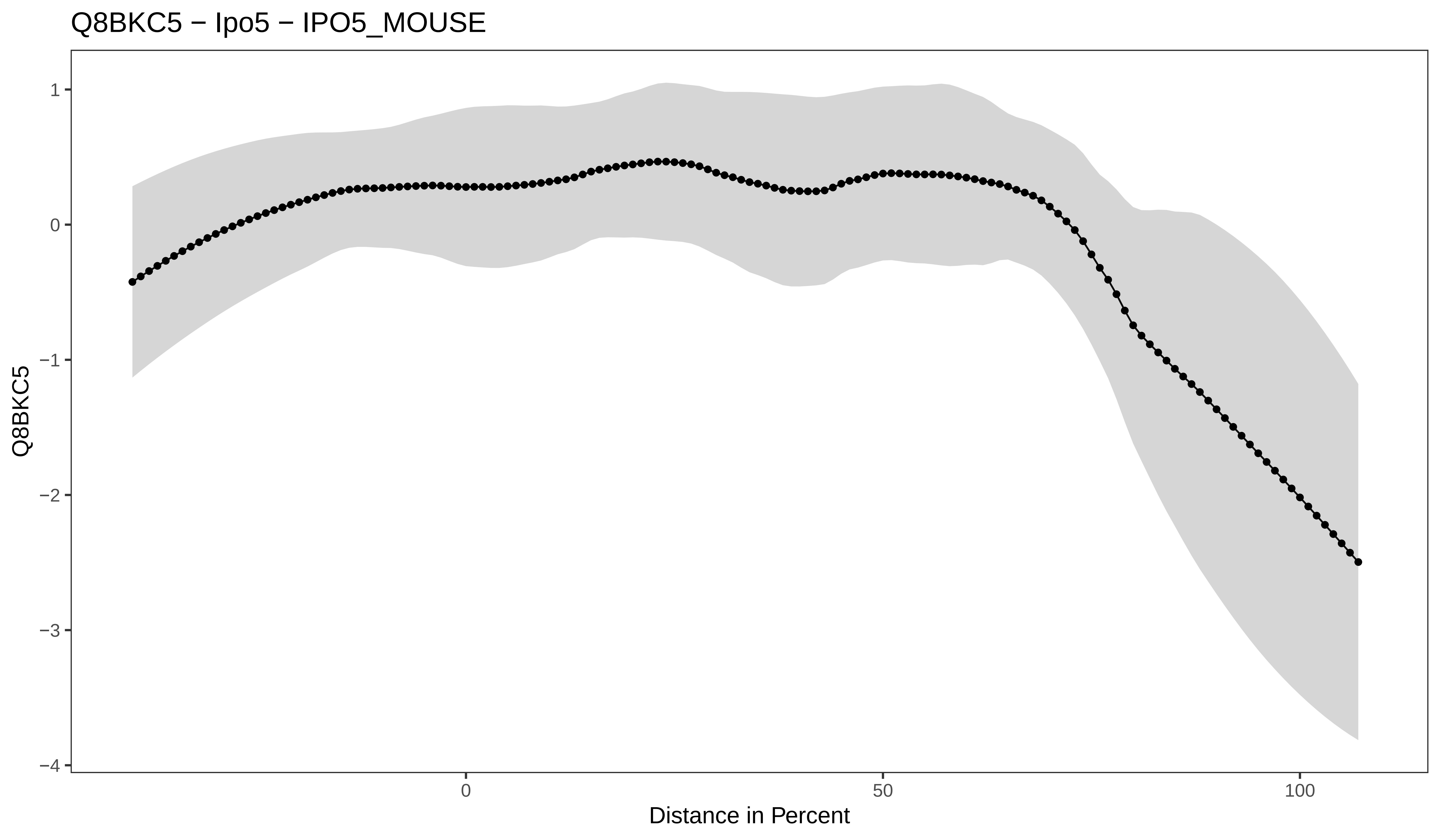

Q8BKC5 − Ipo5 − IPO5_MOUSE
Q8BKC5
Distance in Percent
1
0
−1
−2
−3
−4
0
50
100

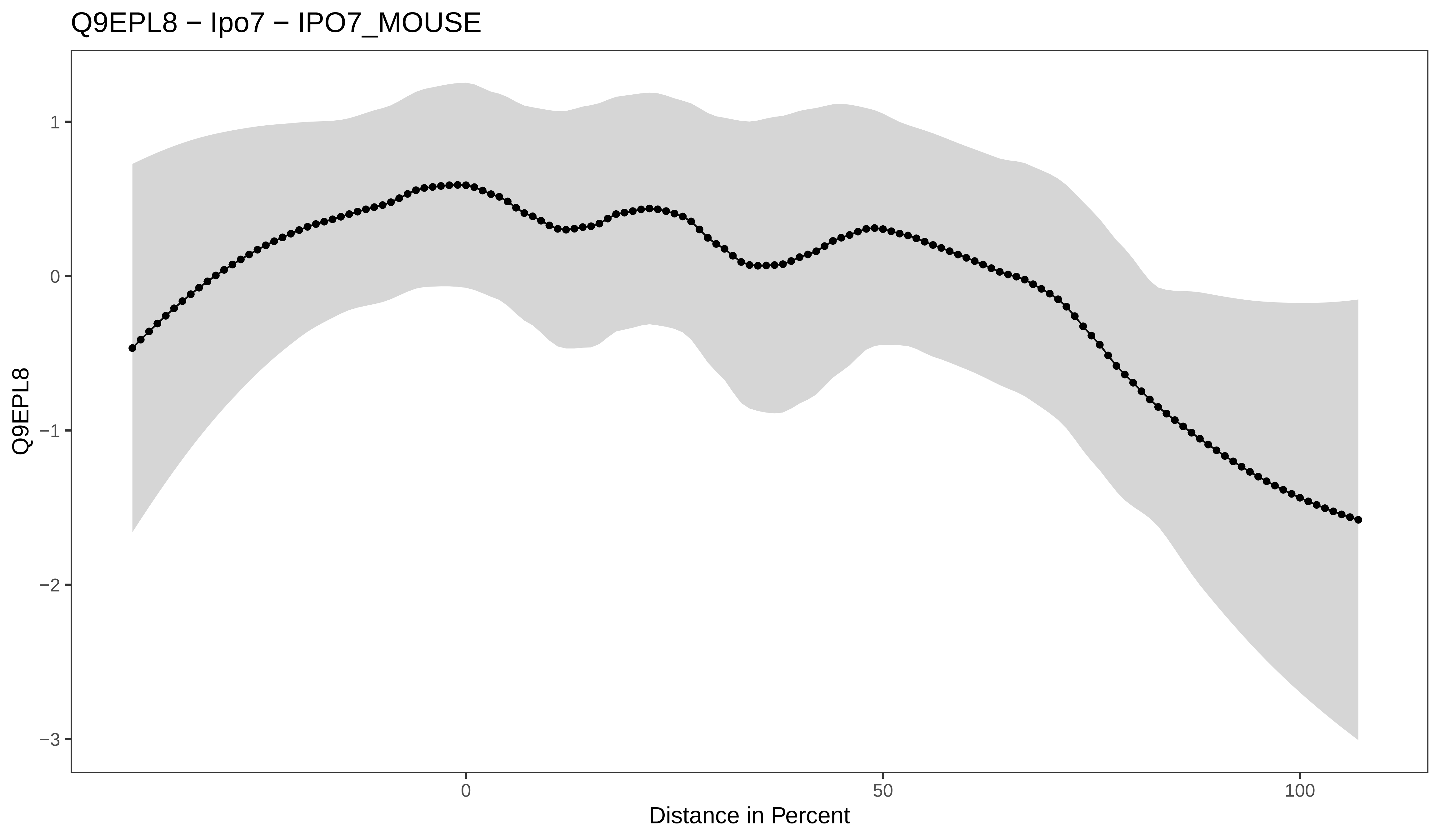
Q9EPL8 − Ipo7 − IPO7_MOUSE
Q9EPL8
Distance in Percent
1
0
−1
−2
−3
0
50
100

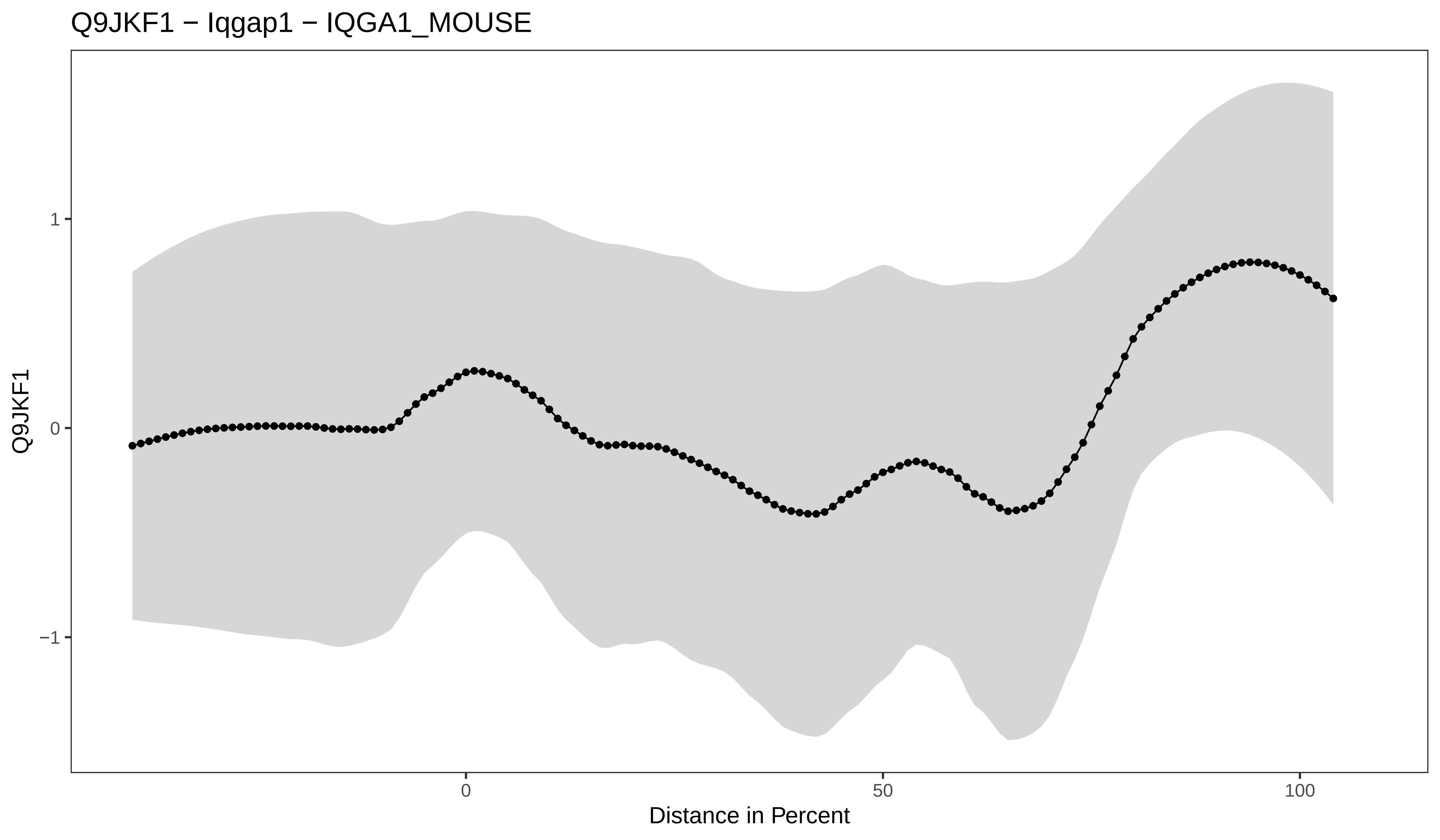

Q9JKF1 − Iqgap1 − IQGA1_MOUSE
Q9JKF1
1
0
−1
0
50
100
Distance in Percent

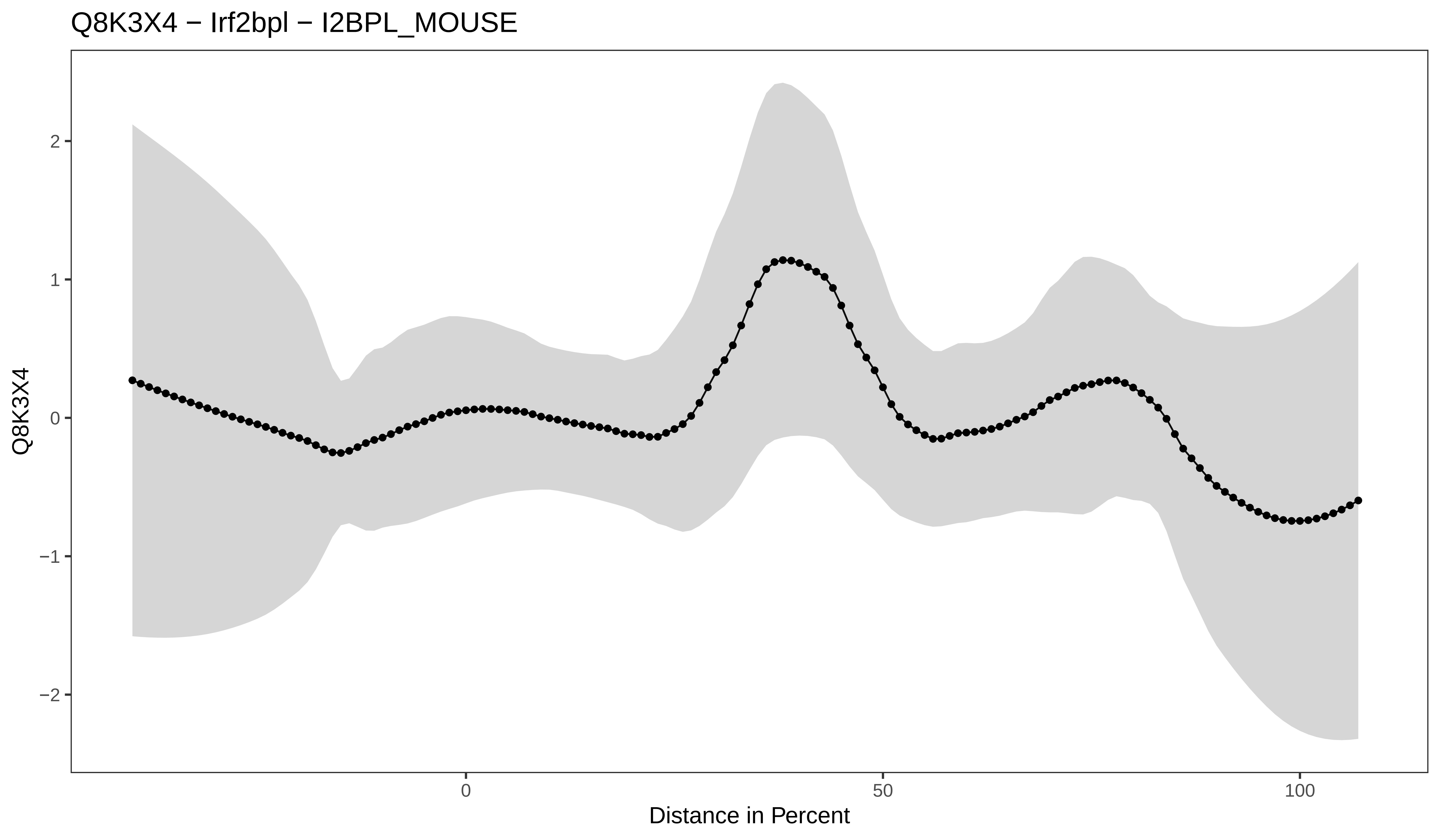
Q8K3X4 − Irf2bpl − I2BPL_MOUSE
Q8K3X4
2
1
0
−1
−2
0
50
100
Distance in Percent

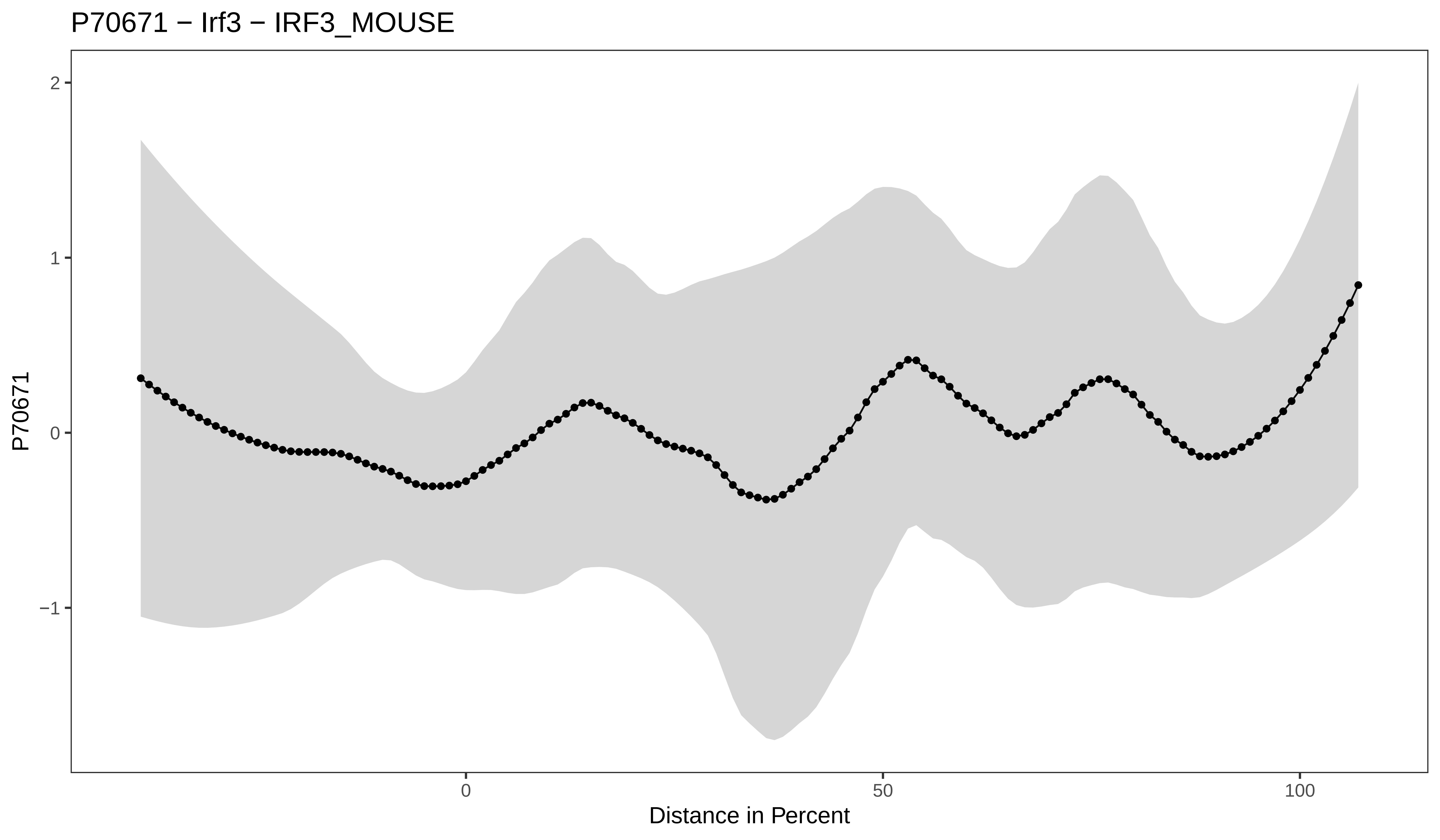

P70671 − Irf3 − IRF3_MOUSE
P70671
Distance in Percent
2
1
0
−1
0
50
100

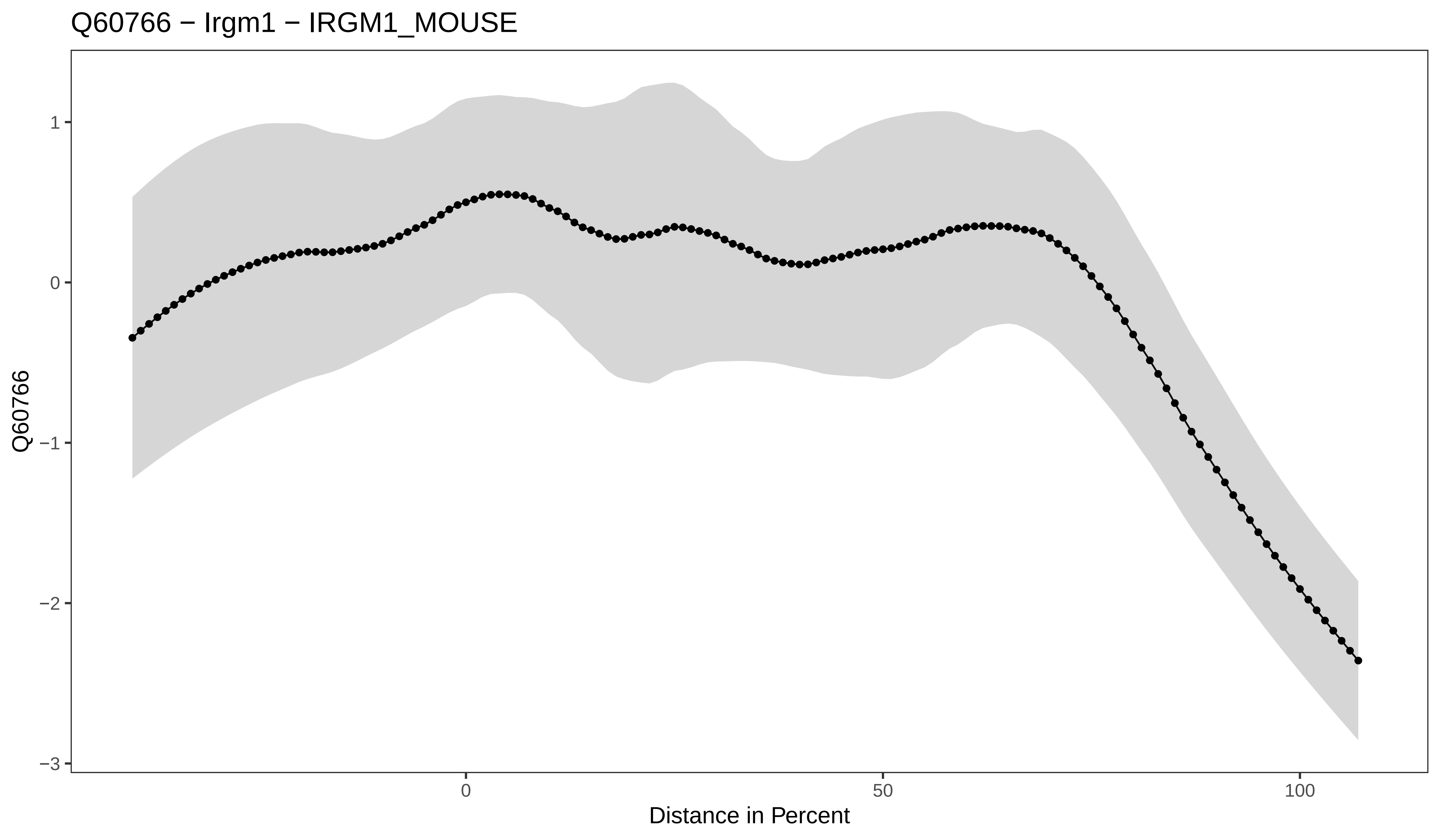

Q60766 − Irgm1 − IRGM1_MOUSE
1
0
−1
−2
−3
Q60766
0
50
100
Distance in Percent

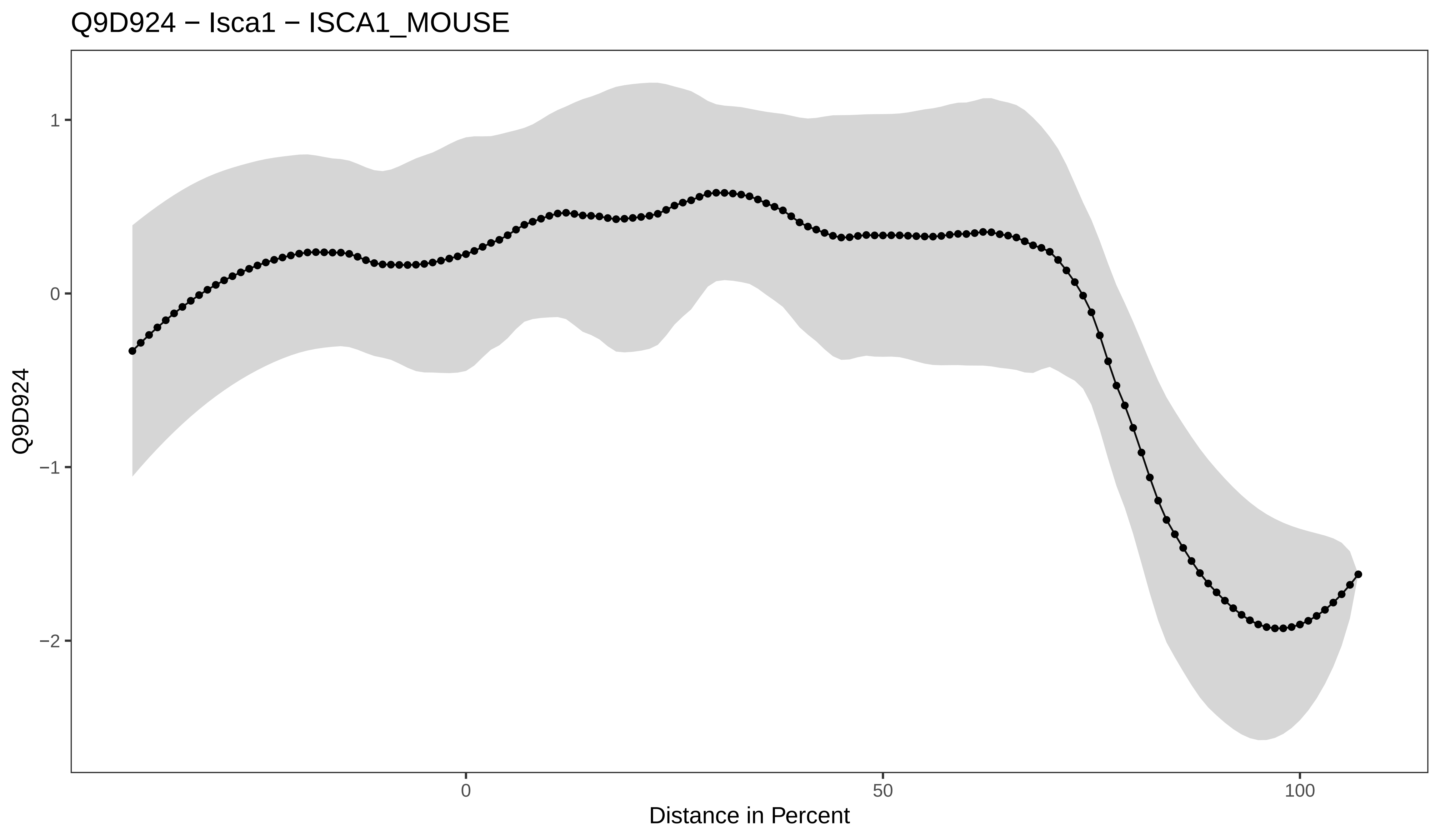
Q9D924 − Isca1 − ISCA1_MOUSE
1
0
−1
−2
Q9D924
0
50
100
Distance in Percent

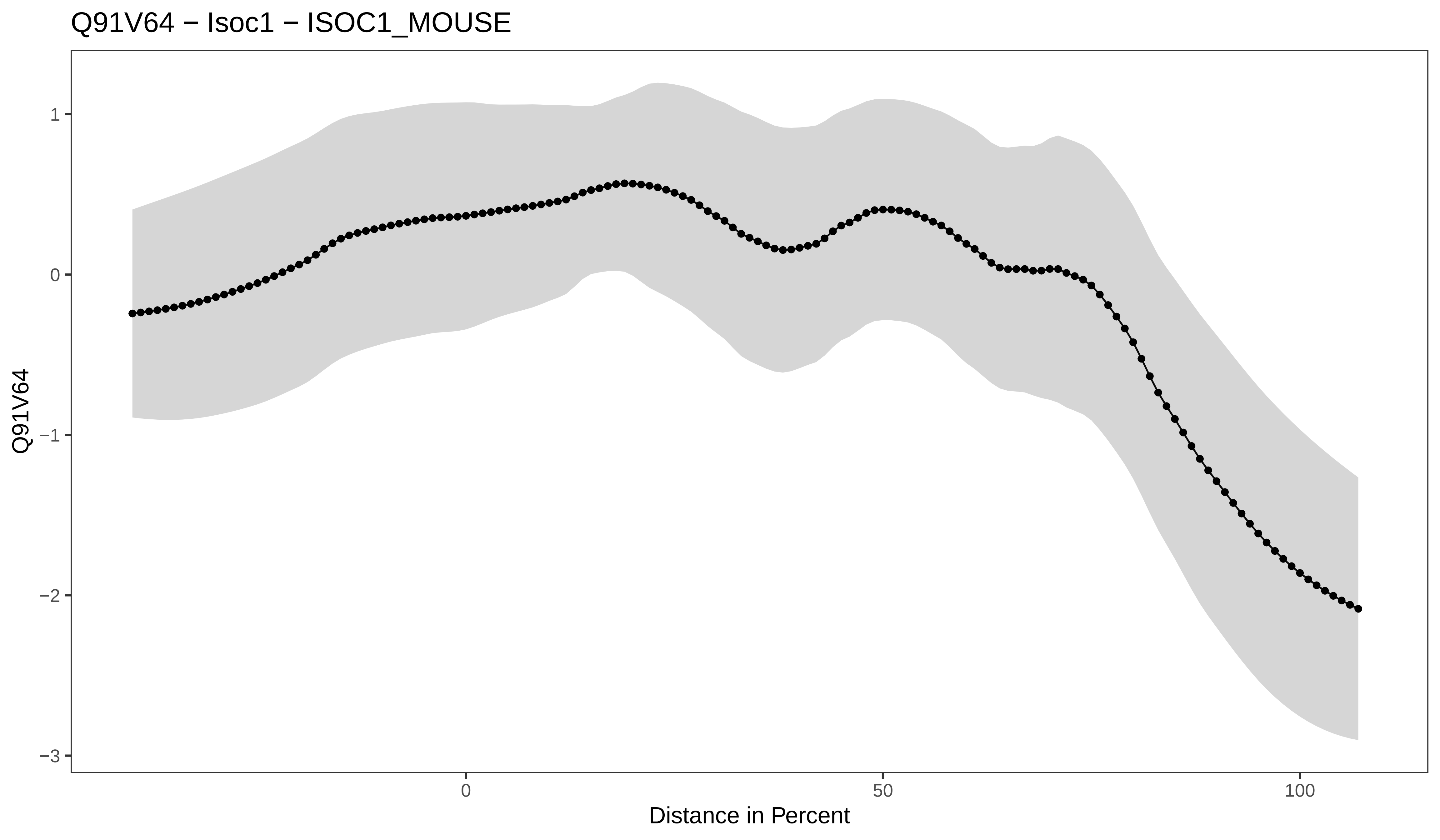
Q91V64 − Isoc1 − ISOC1_MOUSE
Q91V64
1
0
−1
−2
−3
0
50
100
Distance in Percent

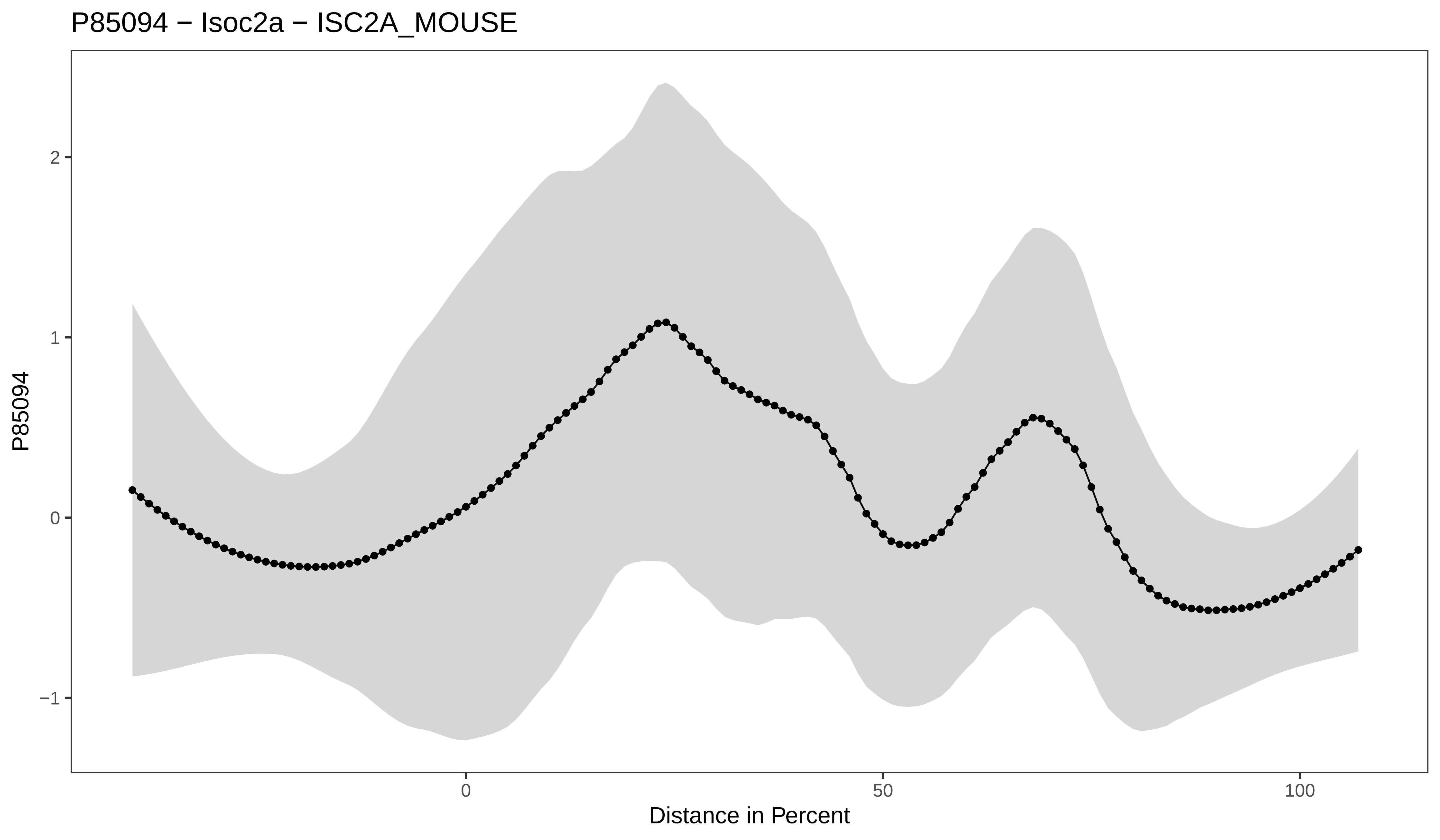
P85094 − Isoc2a − ISC2A_MOUSE
P85094
2
1
0
−1
0
50
100
Distance in Percent

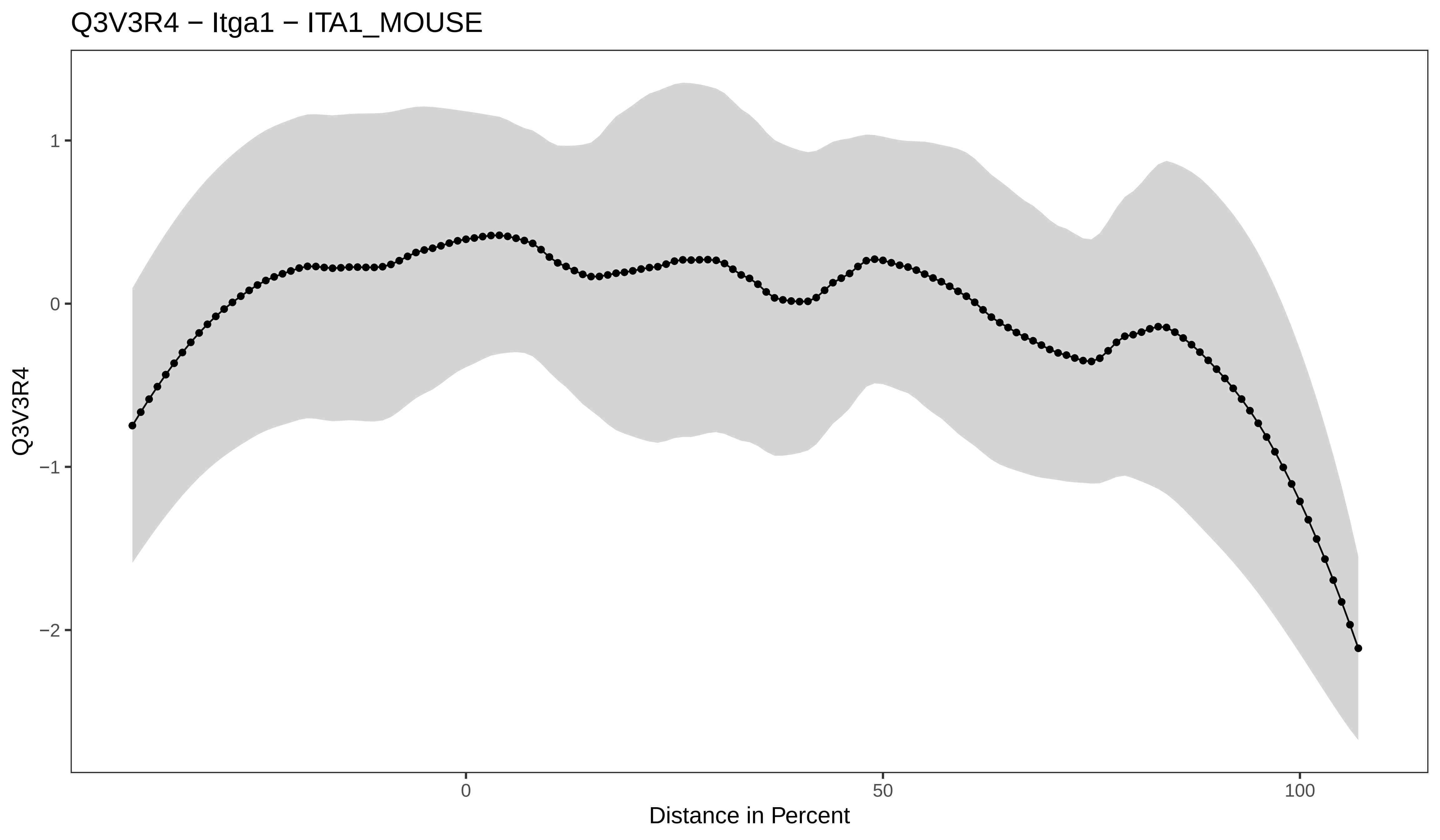

Q3V3R4 − Itga1 − ITA1_MOUSE
1
0
−1
−2
Q3V3R4
0
50
100
Distance in Percent

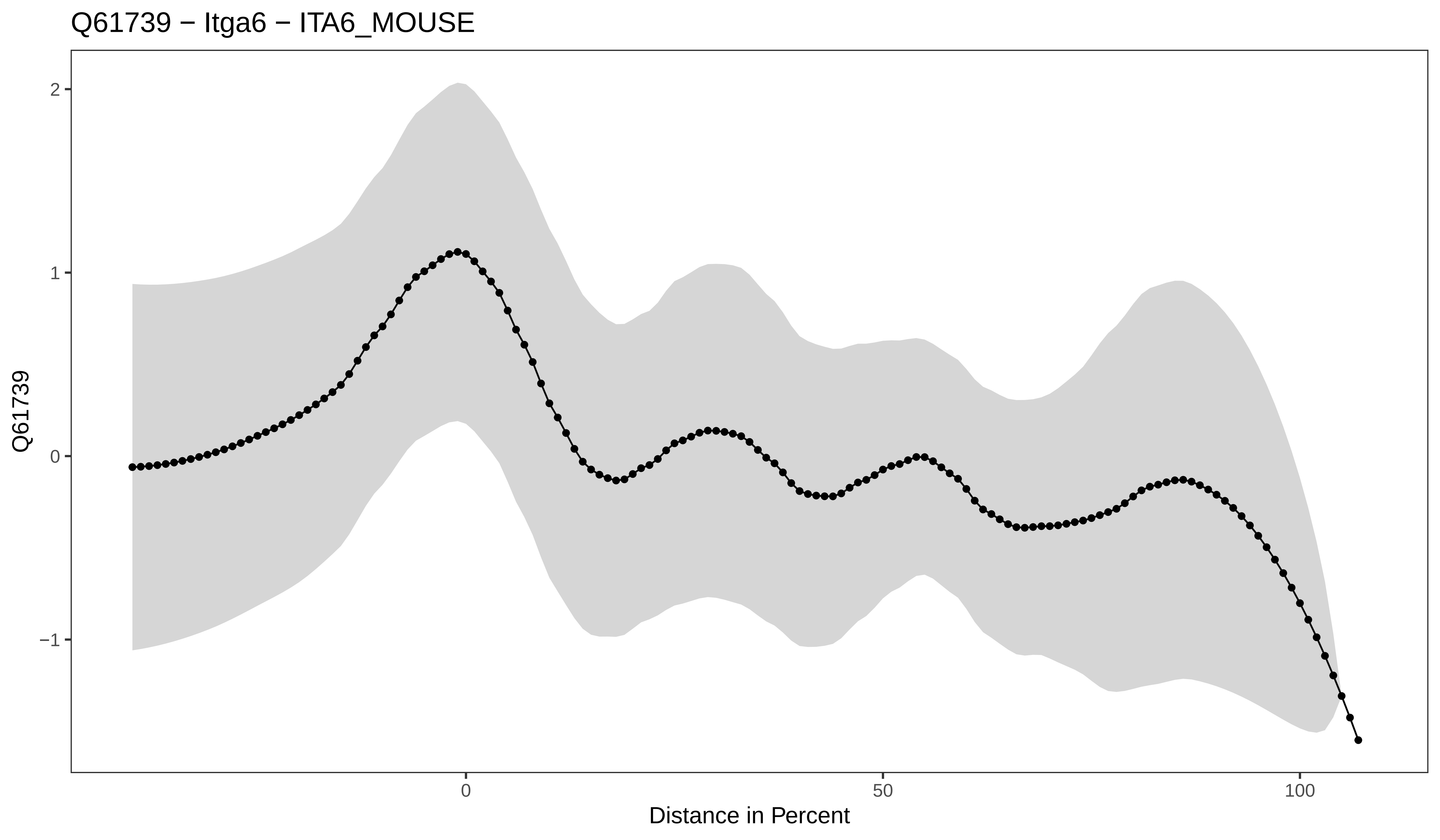

Q61739 − Itga6 − ITA6_MOUSE
Q61739
2
1
0
−1
0
50
100
Distance in Percent

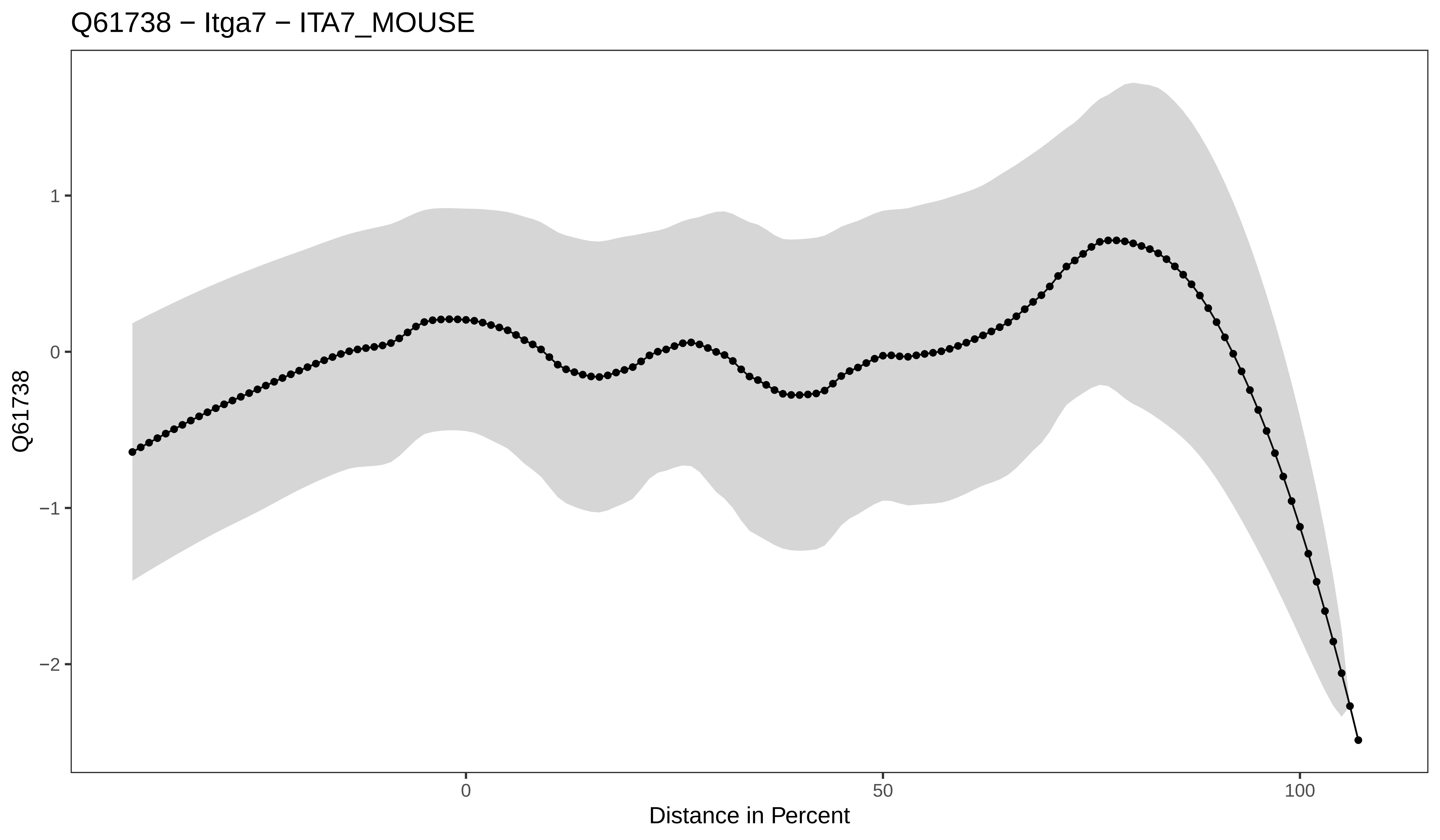
Q61738 − Itga7 − ITA7_MOUSE
1
0
−1
−2
Q61738
0
50
100
Distance in Percent

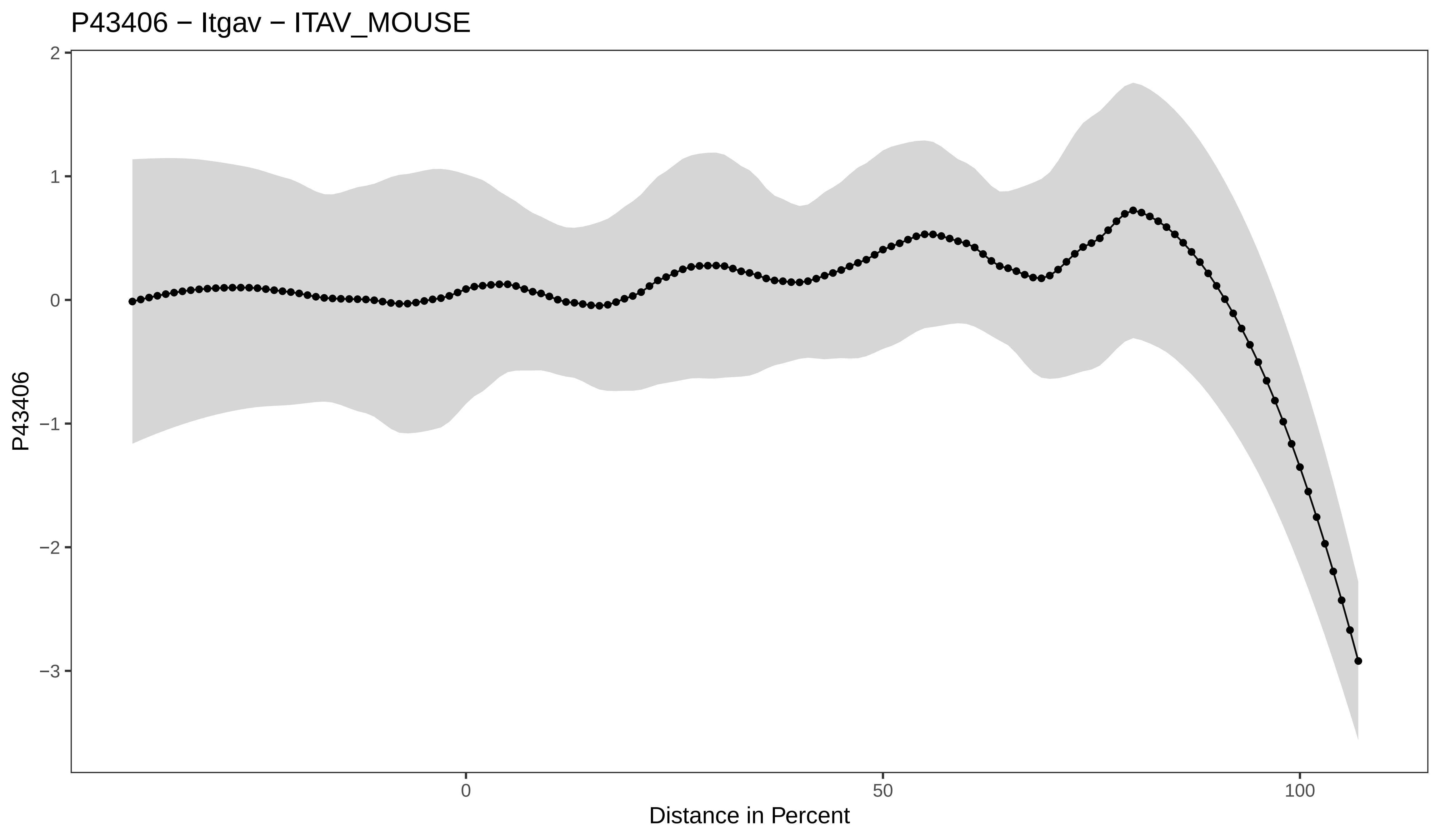
P43406 − Itgav − ITAV_MOUSE
P43406
Distance in Percent
2
1
0
−1
−2
−3
0
50
100

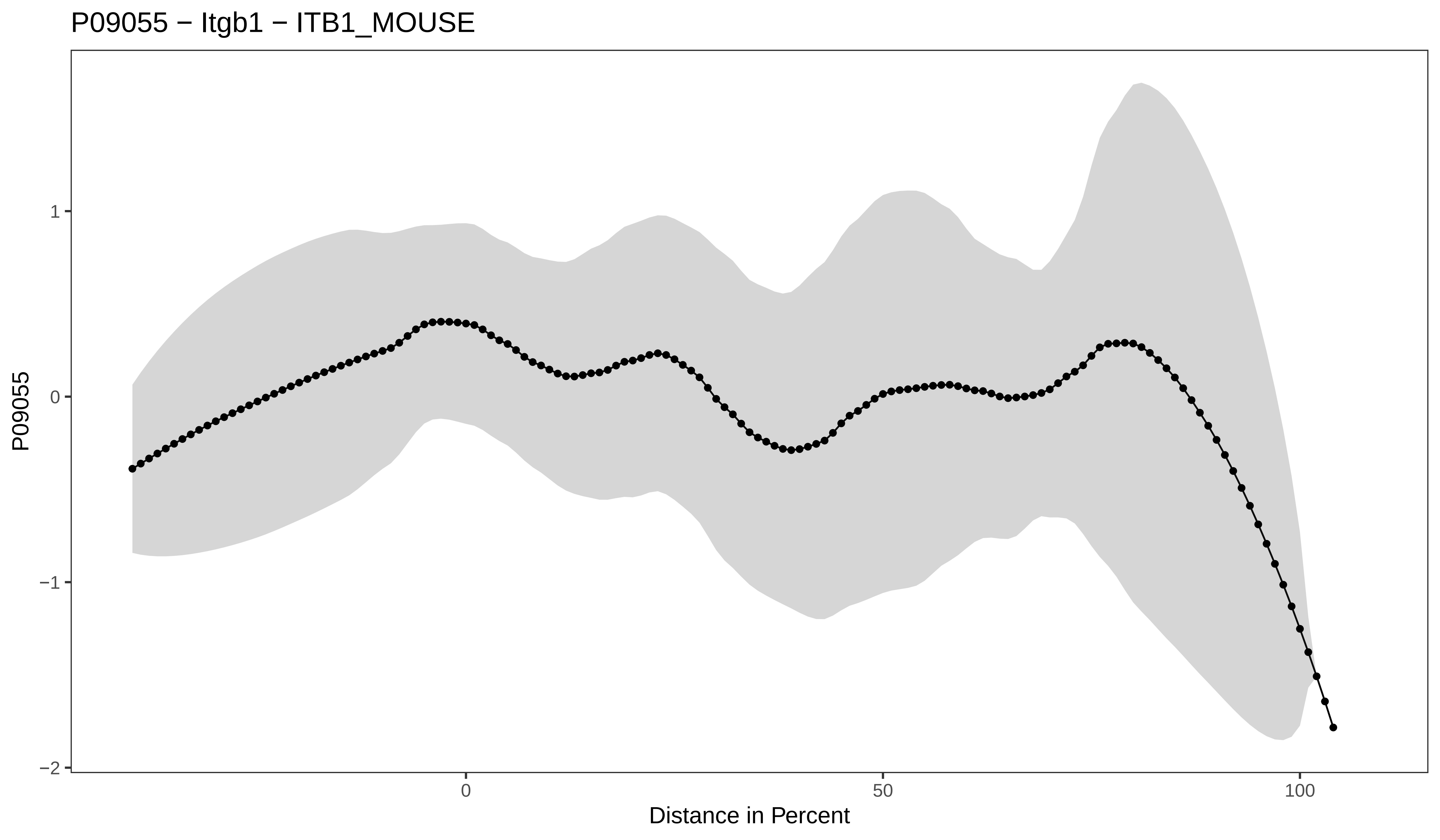

P09055 − Itgb1 − ITB1_MOUSE
1
0
−1
−2
P09055
0
50
100
Distance in Percent

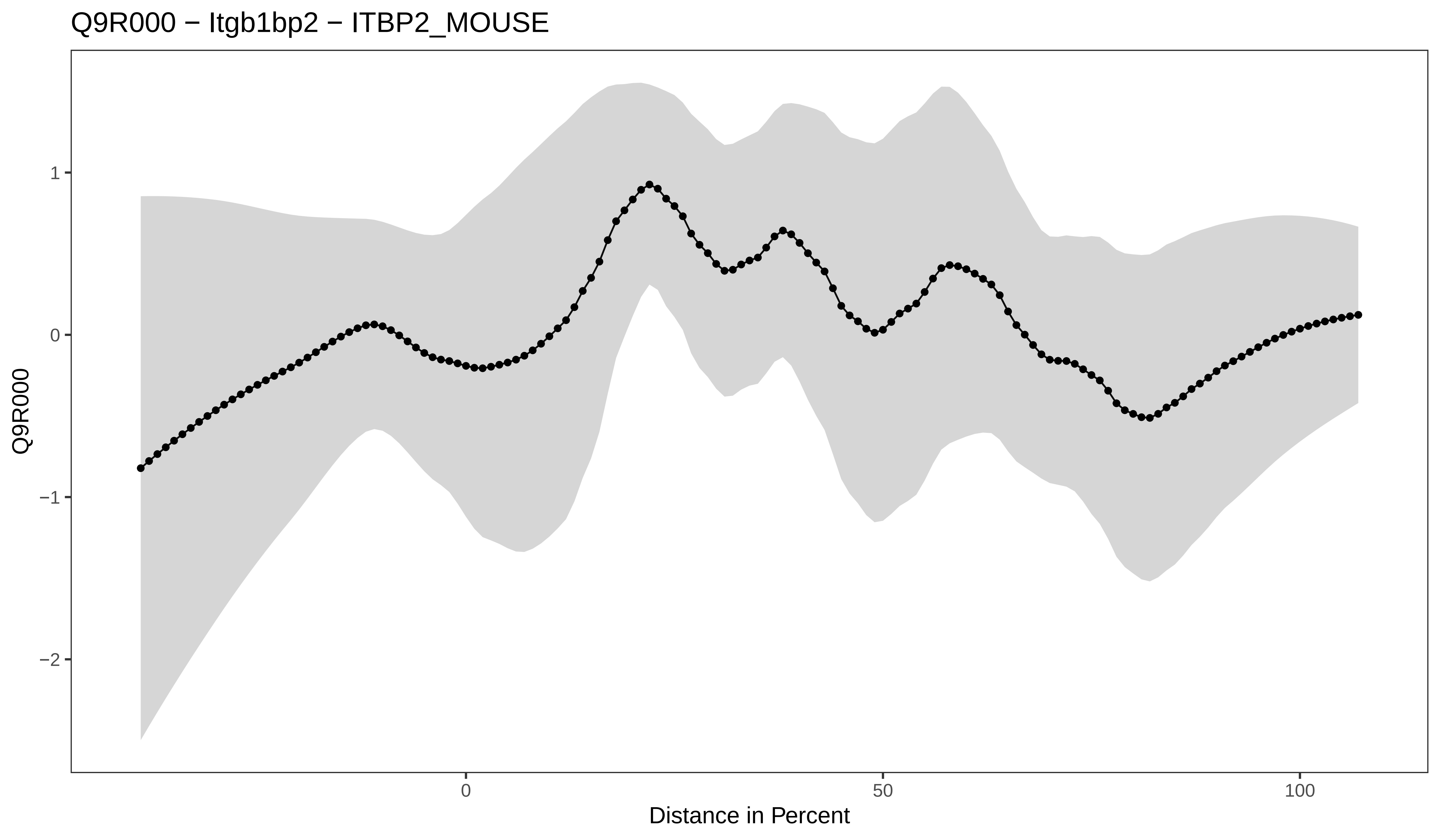

Q9R000 − Itgb1bp2 − ITBP2_MOUSE
Q9R000
1
0
−1
−2
0
50
100
Distance in Percent

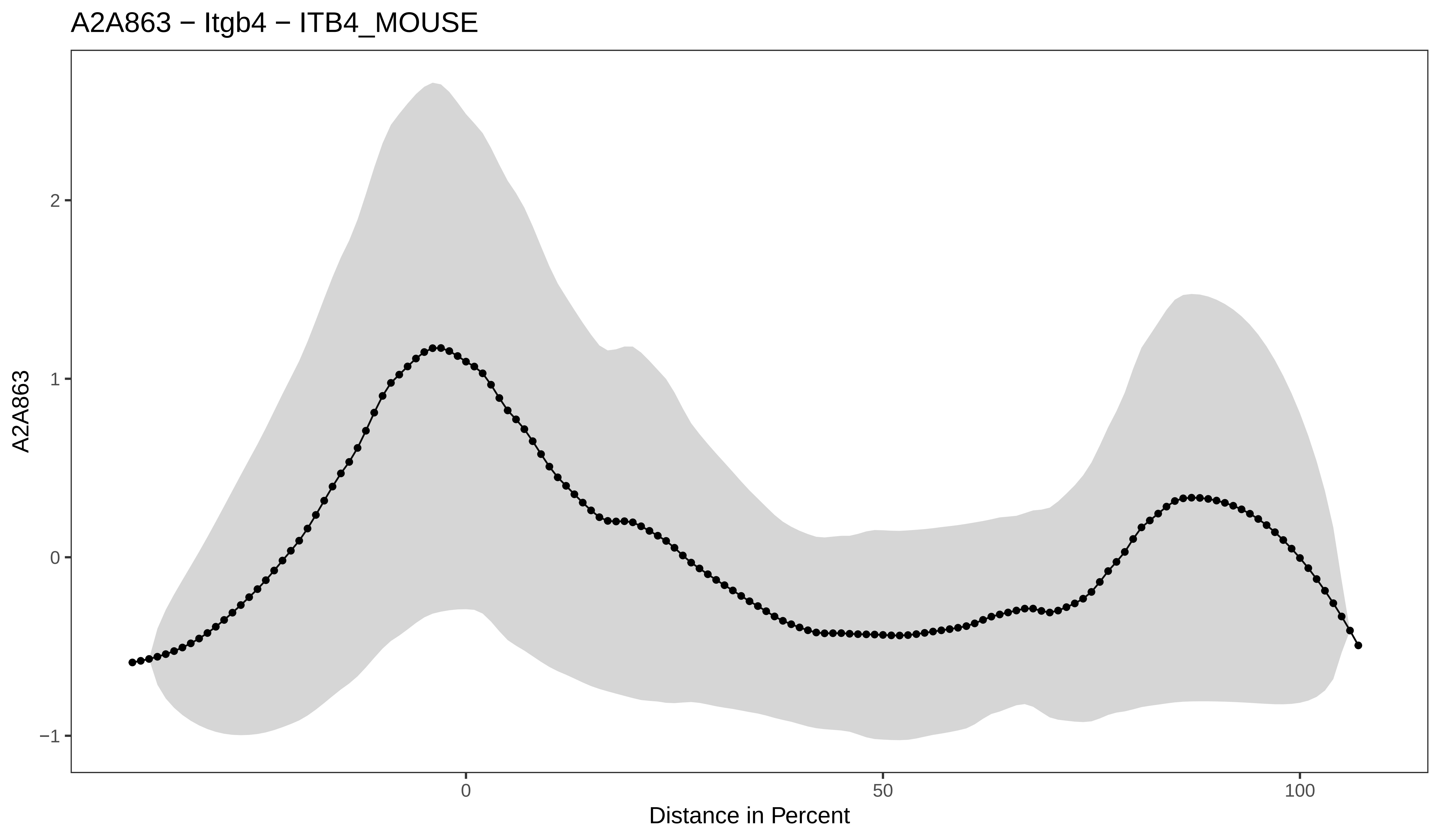
A2A863 − Itgb4 − ITB4_MOUSE
A2A863
2
1
0
−1
0
50
100
Distance in Percent

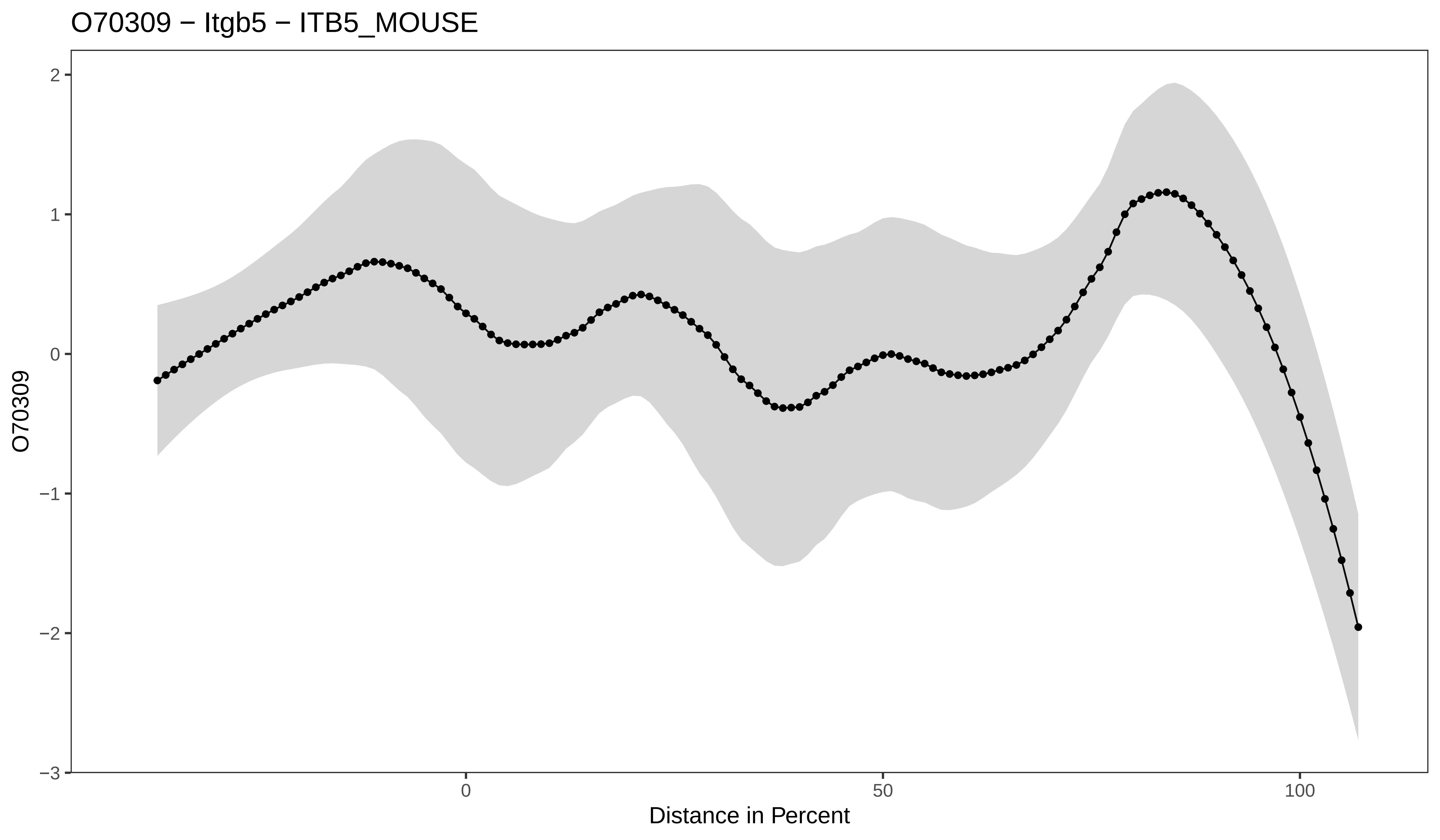

O70309 − Itgb5 − ITB5_MOUSE
O70309
2
1
0
−1
−2
−3
0
50
100
Distance in Percent

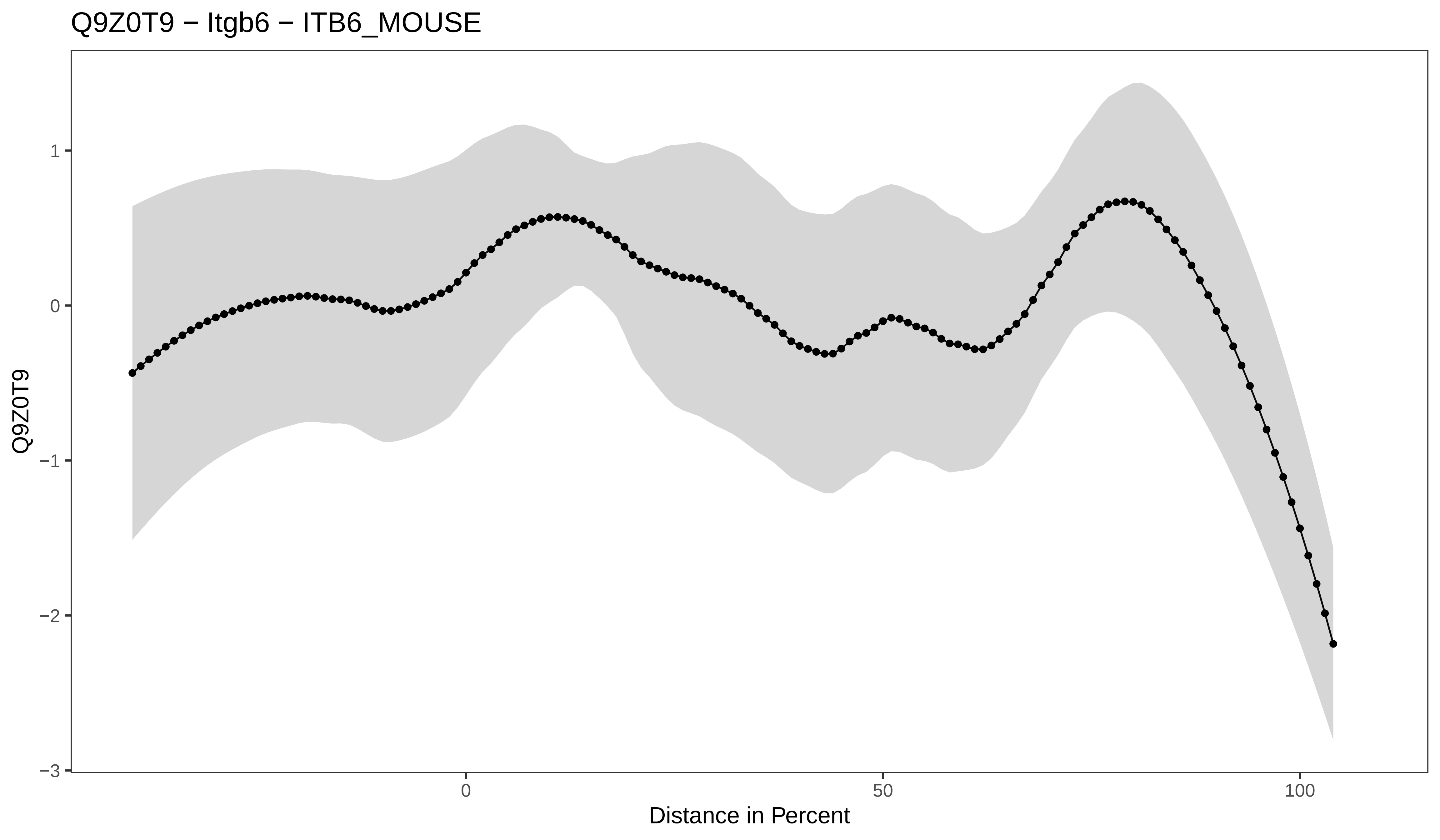
Q9Z0T9 − Itgb6 − ITB6_MOUSE
Q9Z0T9
1
0
−1
−2
−3
0
50
100
Distance in Percent

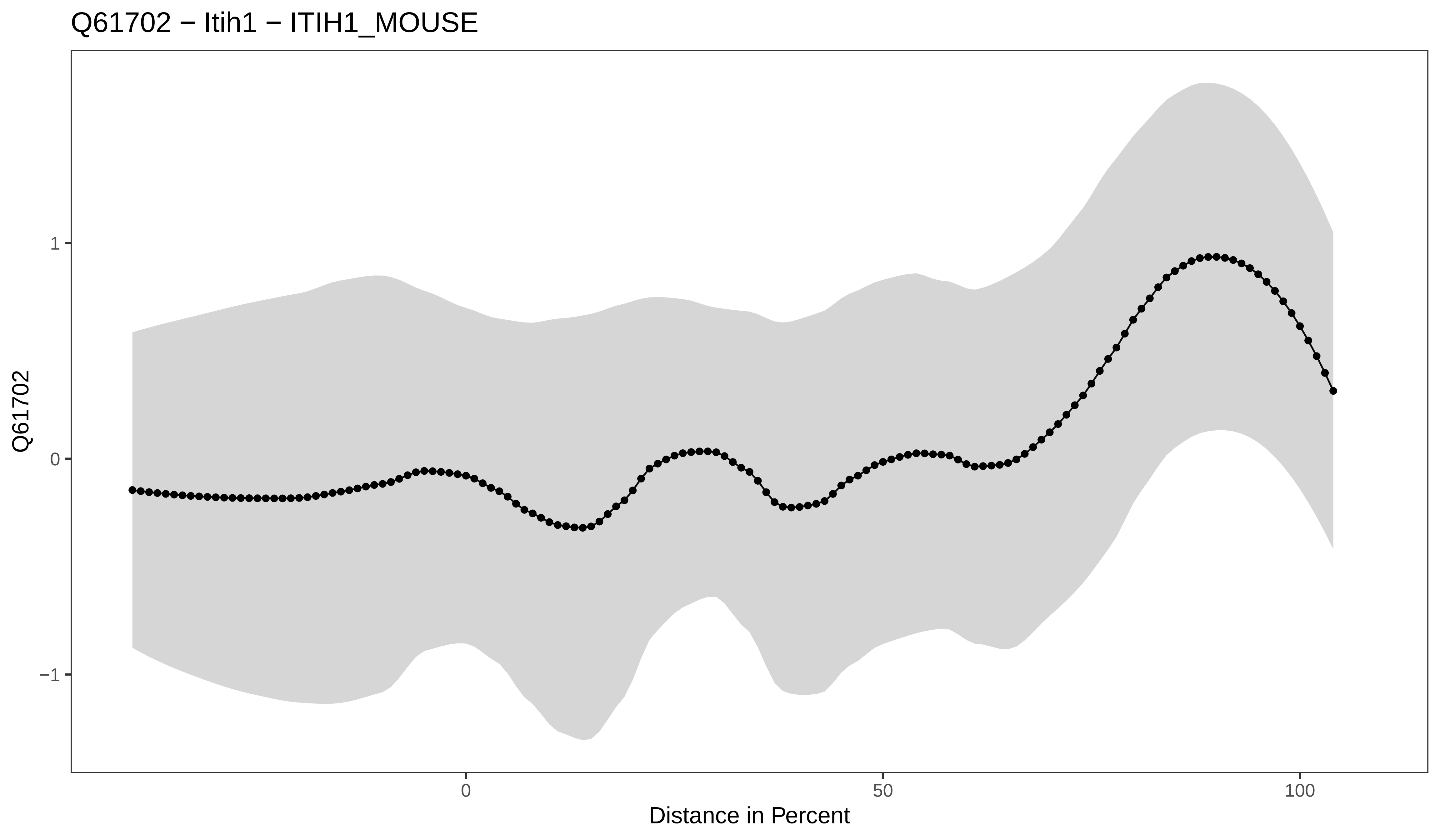

Q61702 − Itih1 − ITIH1_MOUSE
Q61702
1
0
−1
0
50
100
Distance in Percent

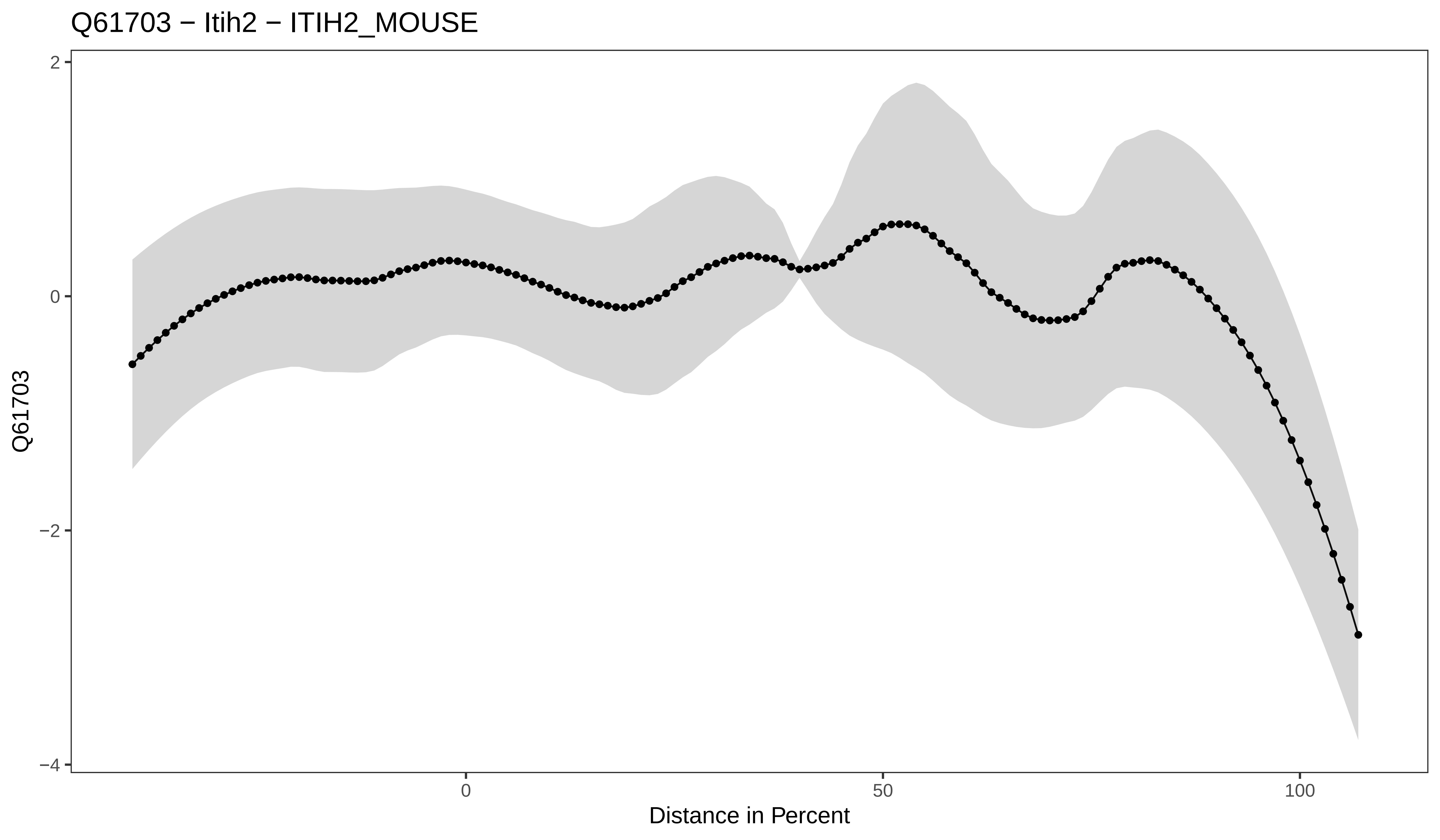

Q61703 − Itih2 − ITIH2_MOUSE
2
0
−2
−4
Q61703
0
50
100
Distance in Percent

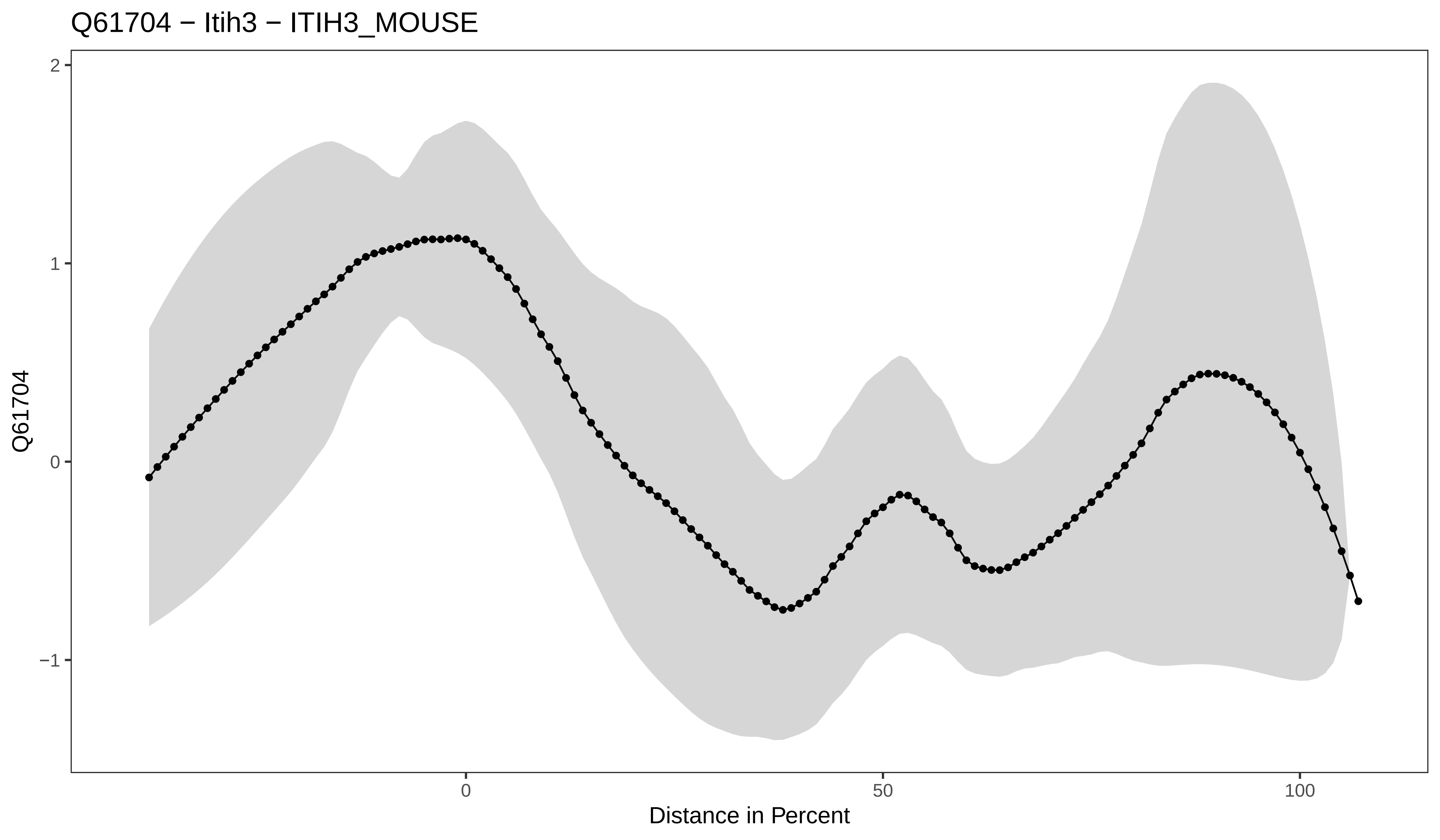

Q61704 − Itih3 − ITIH3_MOUSE
2
1
0
−1
Q61704
0
50
100
Distance in Percent

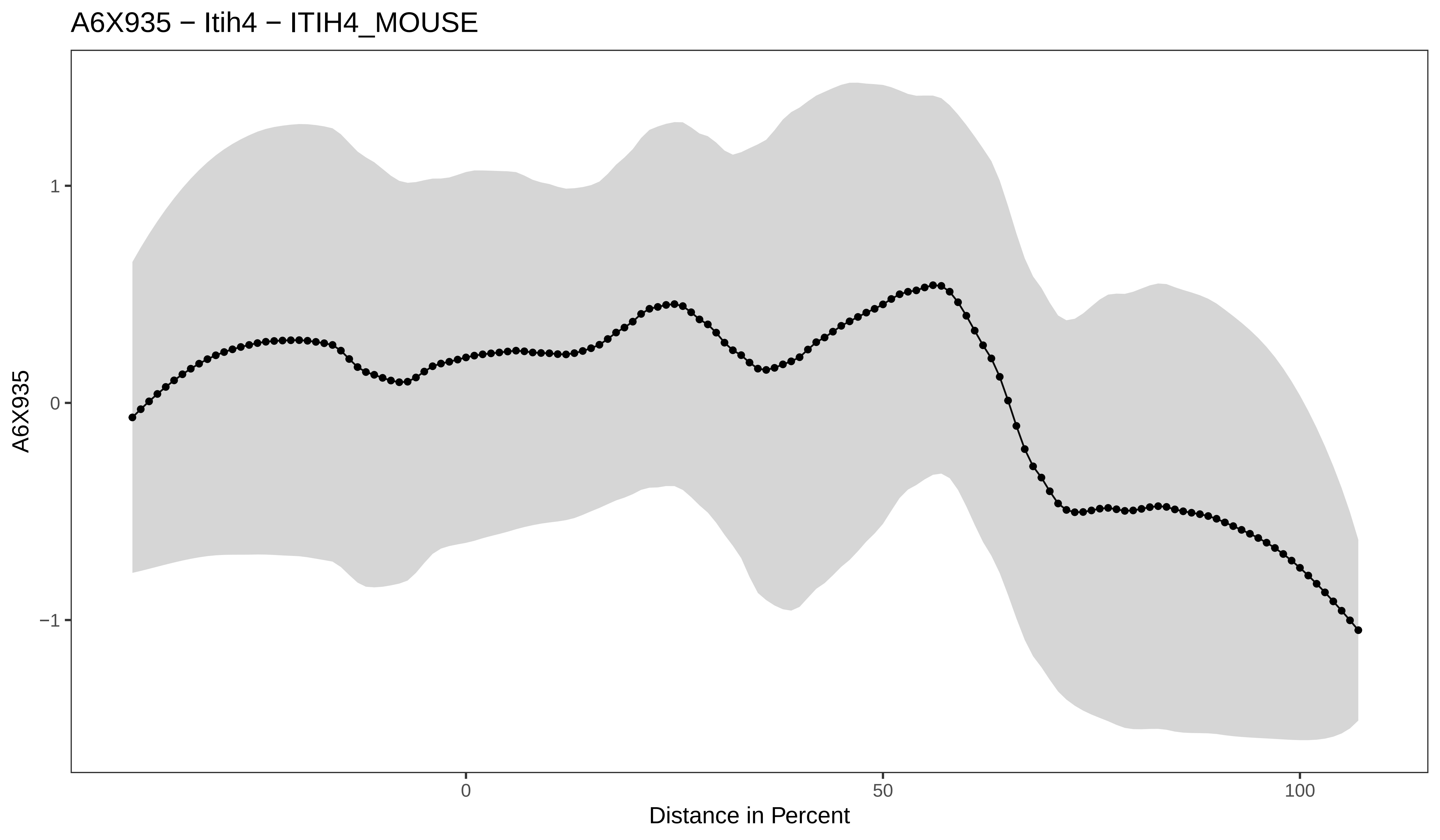

A6X935 − Itih4 − ITIH4_MOUSE
A6X935
1
0
−1
0
50
100
Distance in Percent

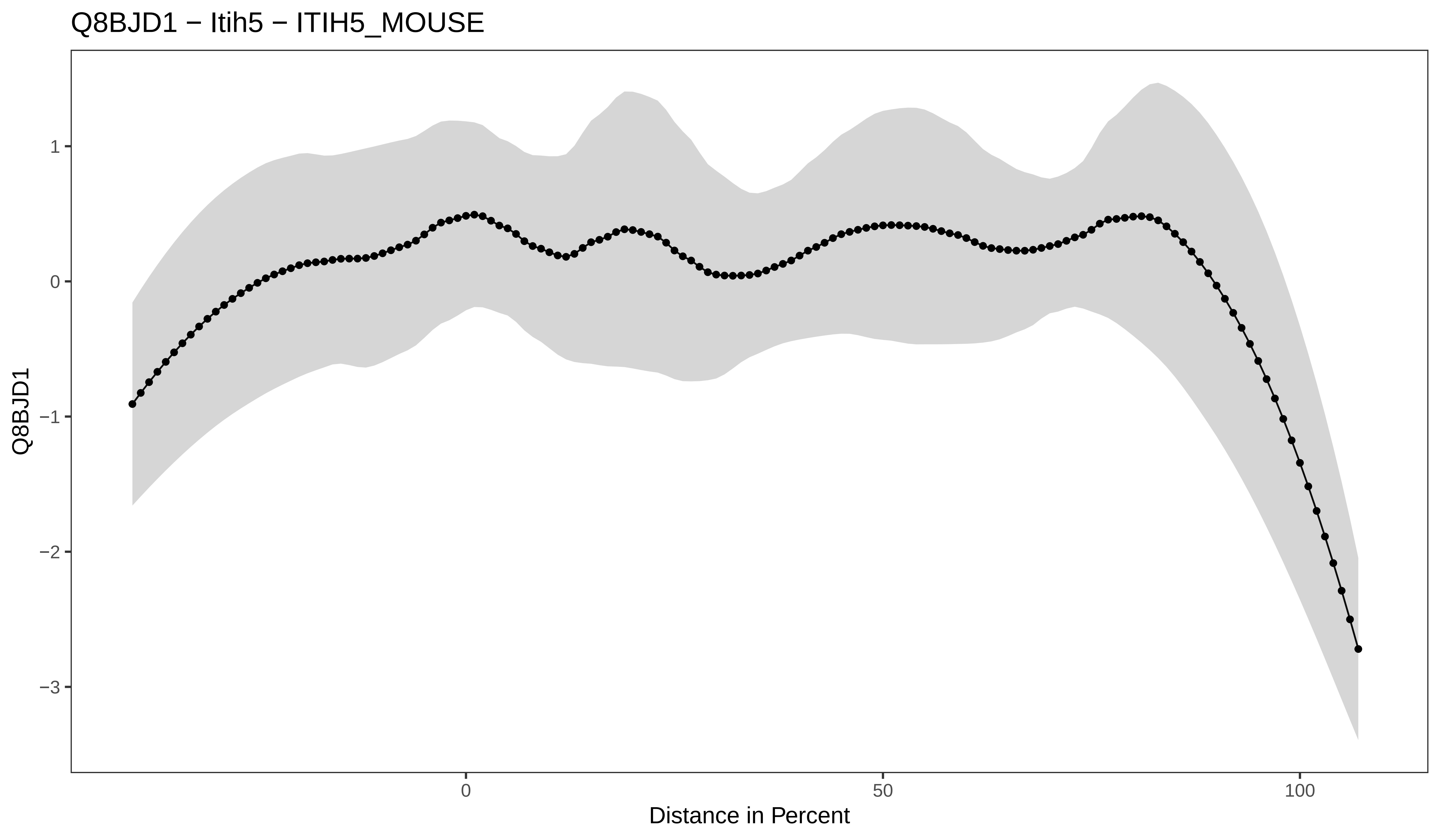

Q8BJD1 − Itih5 − ITIH5_MOUSE
Q8BJD1
Distance in Percent
1
0
−1
−2
−3
0
50
100

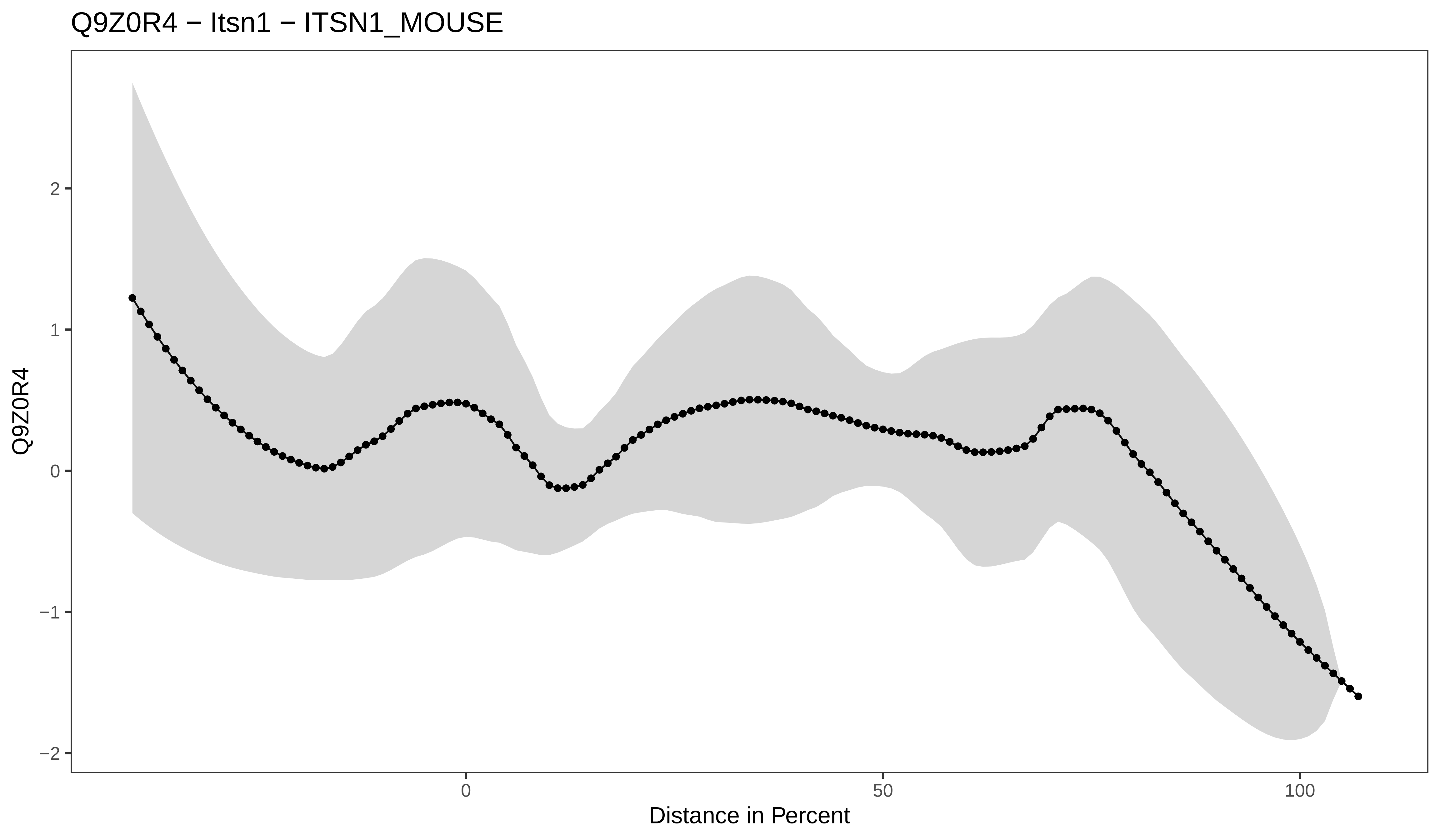

Q9Z0R4 − Itsn1 − ITSN1_MOUSE
Q9Z0R4
2
1
0
−1
−2
0
50
100
Distance in Percent

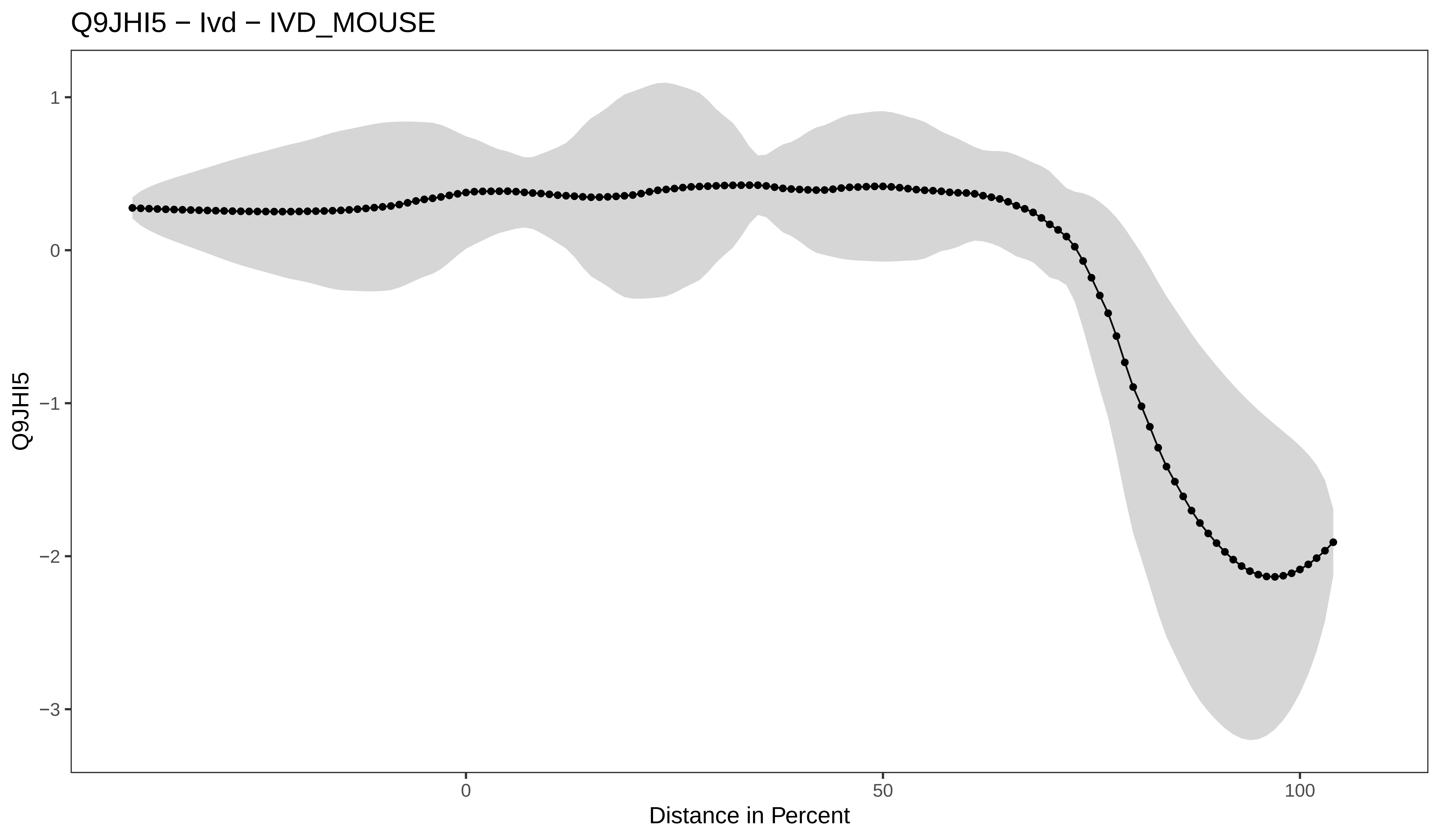

Q9JHI5 − Ivd − IVD_MOUSE
Q9JHI5
Distance in Percent
1
0
−1
−2
−3
0
50
100

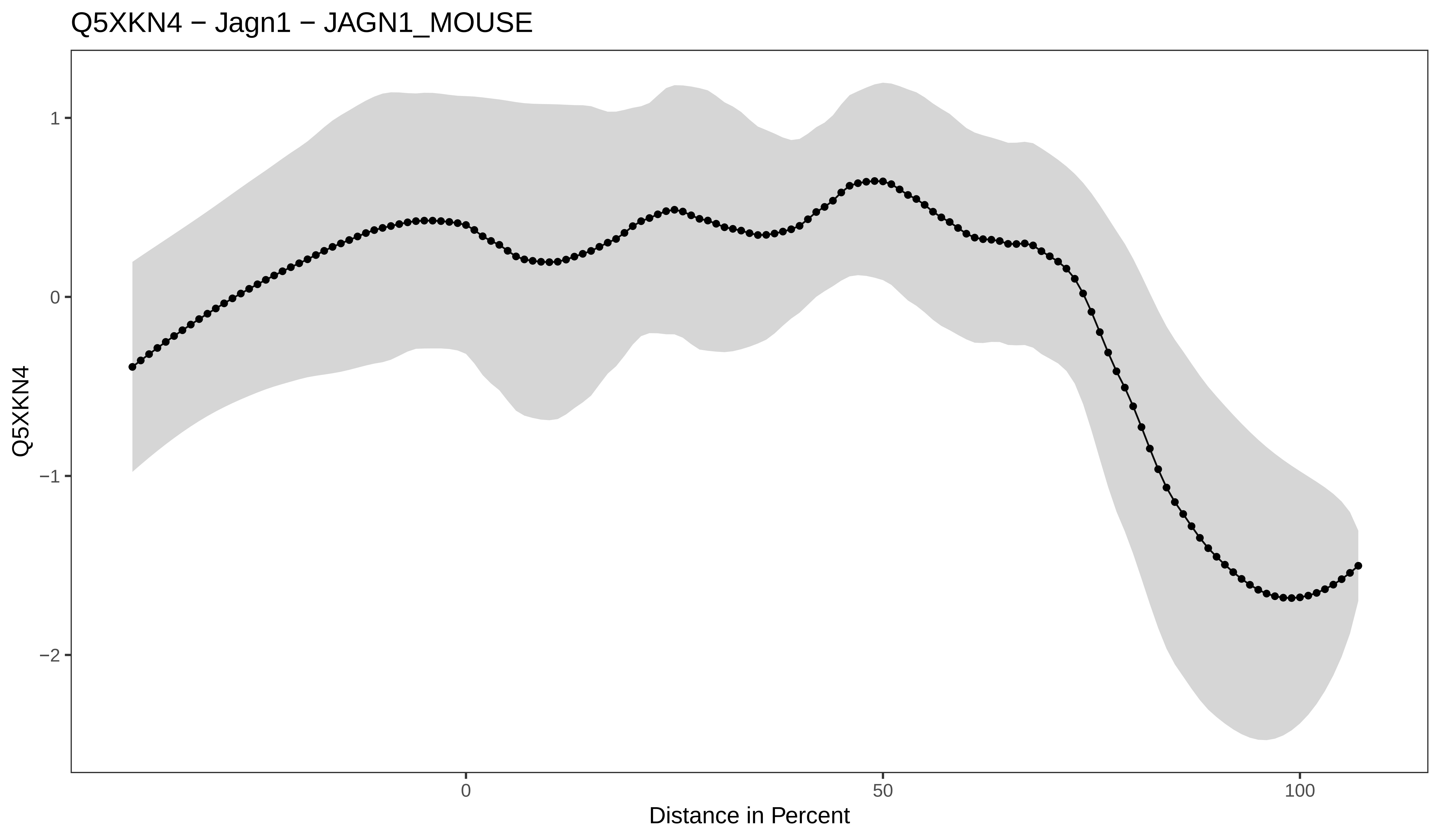
Q5XKN4 − Jagn1 − JAGN1_MOUSE
Q5XKN4
1
0
−1
−2
0
50
100
Distance in Percent

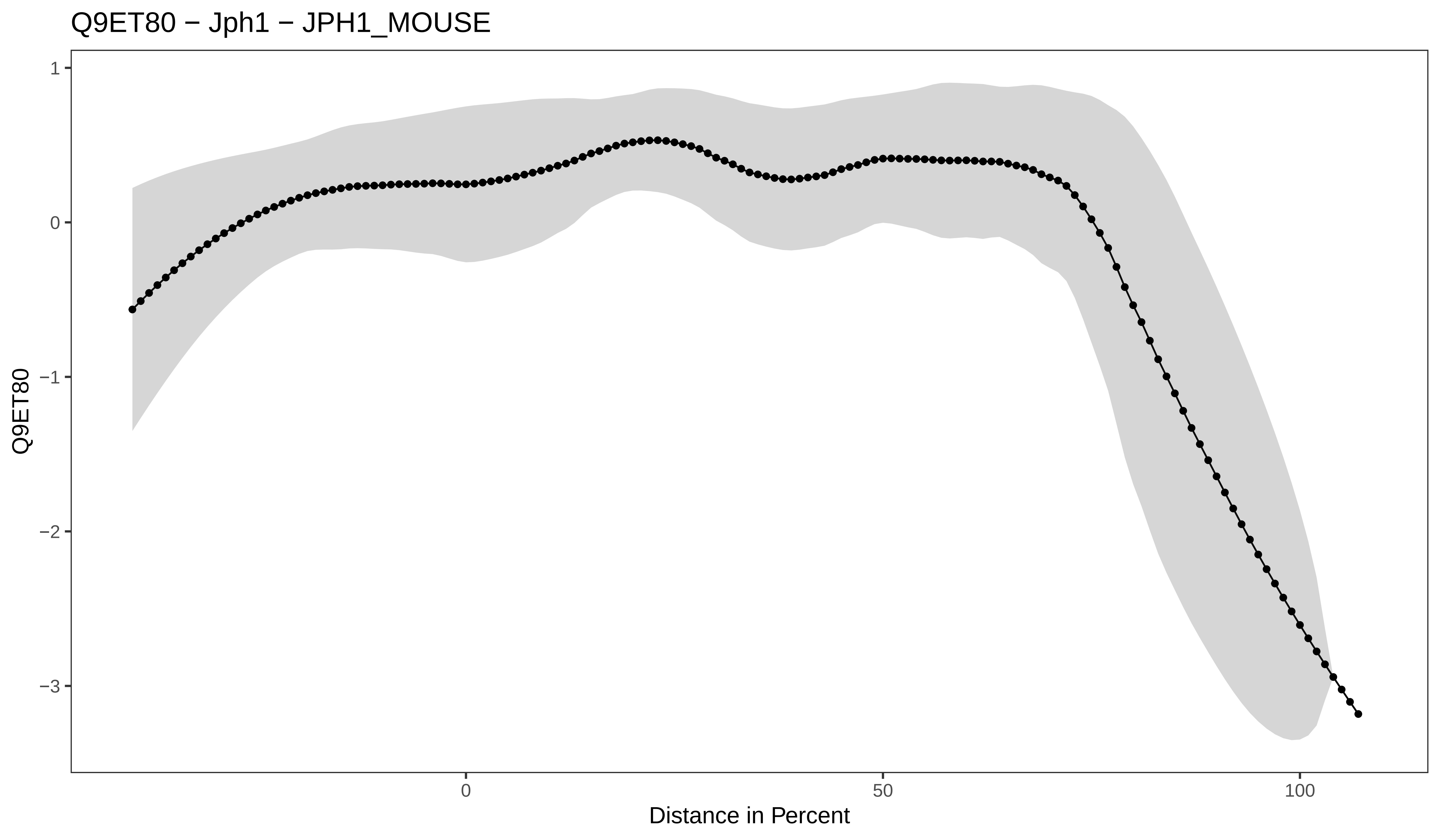

Q9ET80 − Jph1 − JPH1_MOUSE
Q9ET80
Distance in Percent
1
0
−1
−2
−3
0
50
100

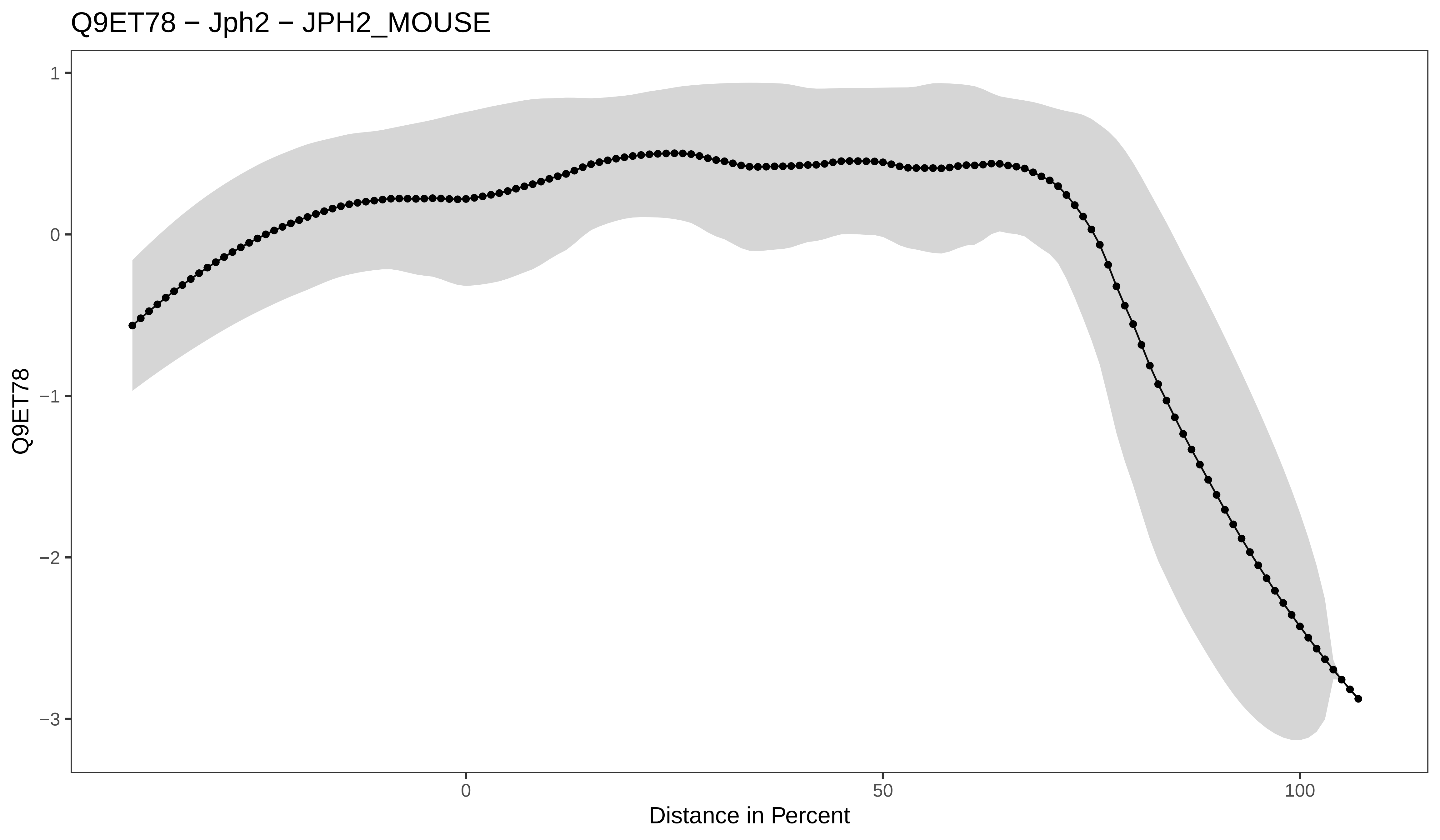
Q9ET78 − Jph2 − JPH2_MOUSE
1
0
−1
−2
−3
Q9ET78
0
50
100
Distance in Percent

Q3MI48 − Jsrp1 − JSPR1_MOUSE

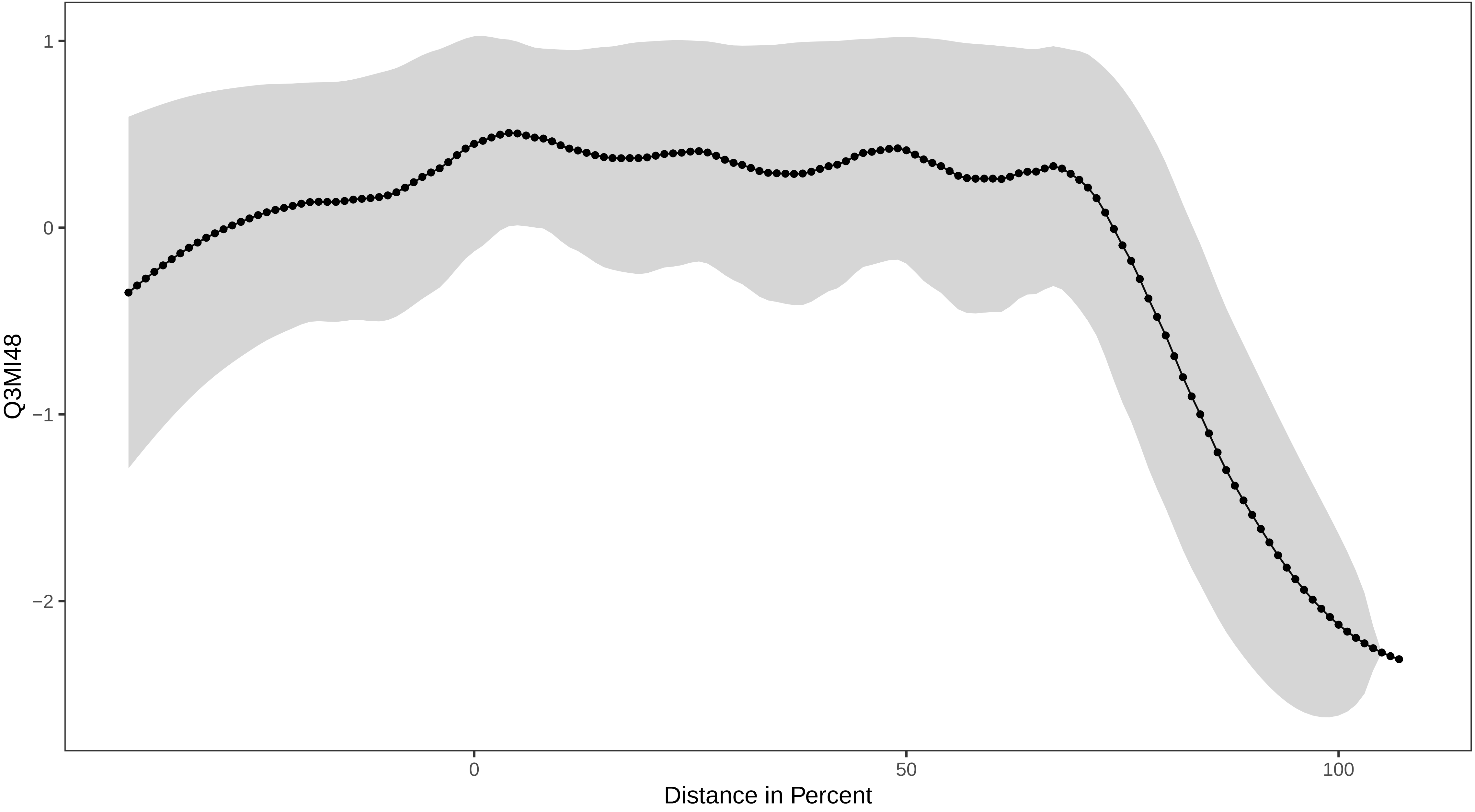

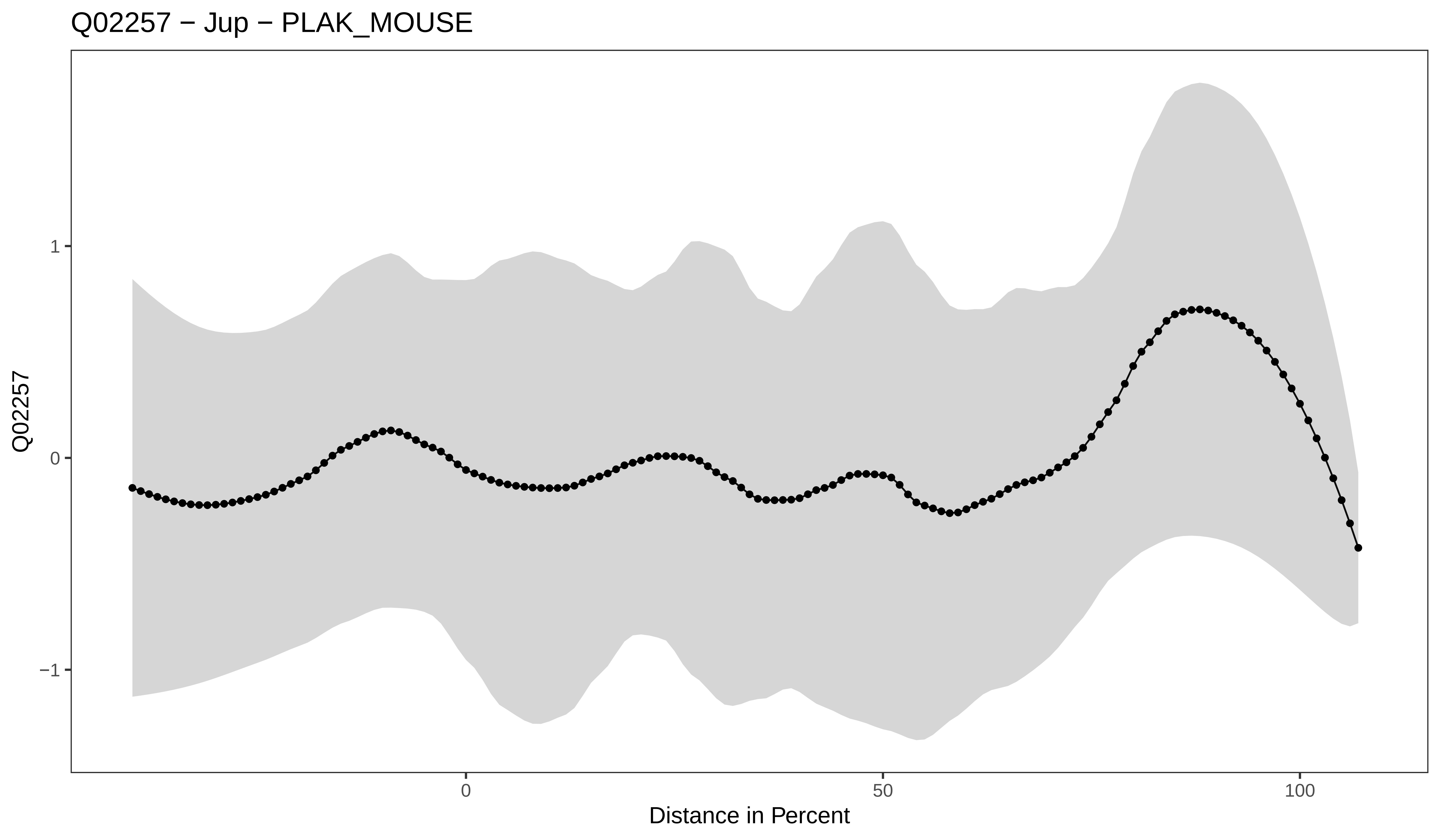

Q02257 − Jup − PLAK_MOUSE
Q02257
1
0
−1
0
50
100
Distance in Percent

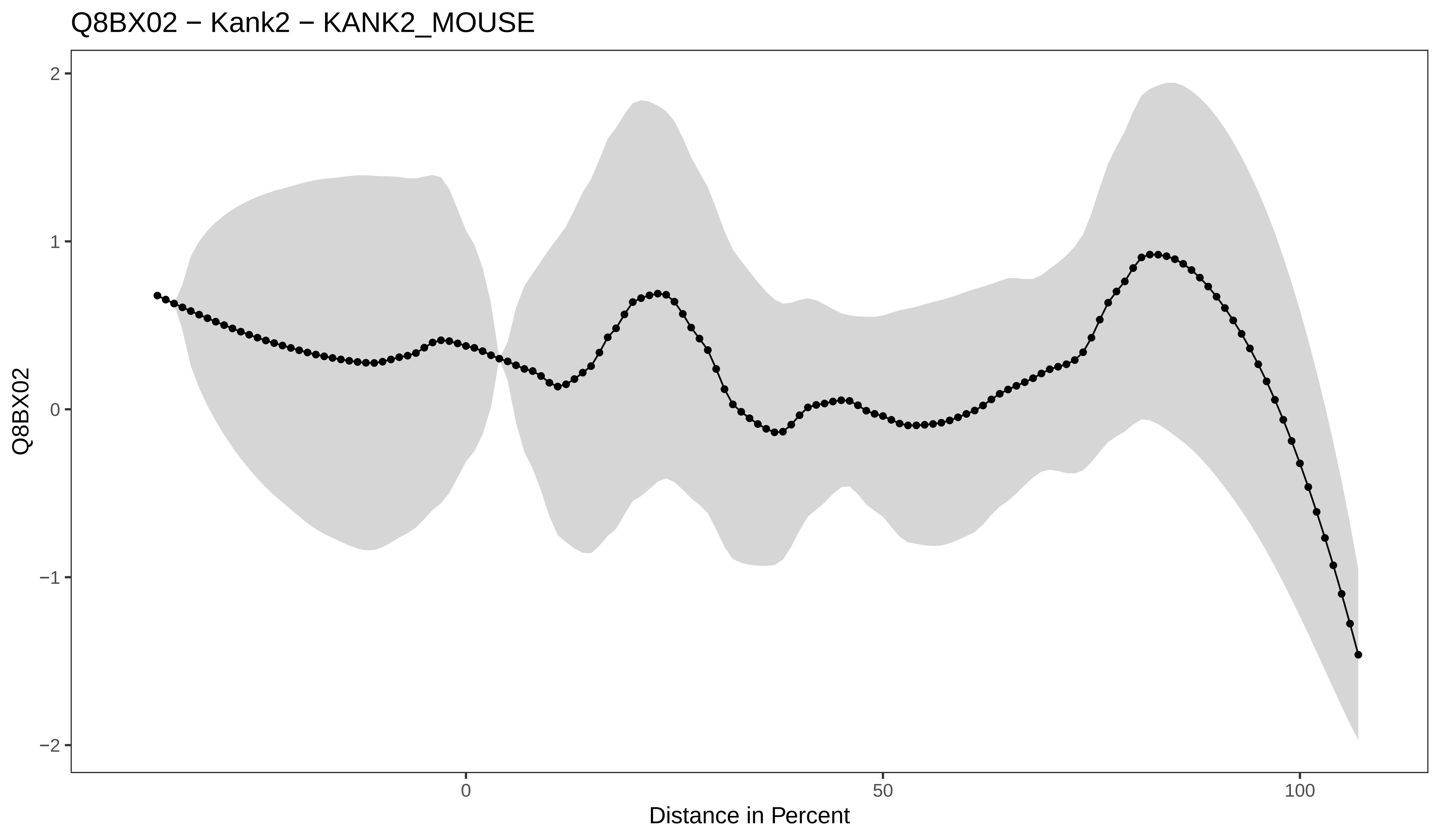

Q8BX02 − Kank2 − KANK2_MOUSE
Q8BX02
Distance in Percent
2
1
0
−1
−2
0
50
100

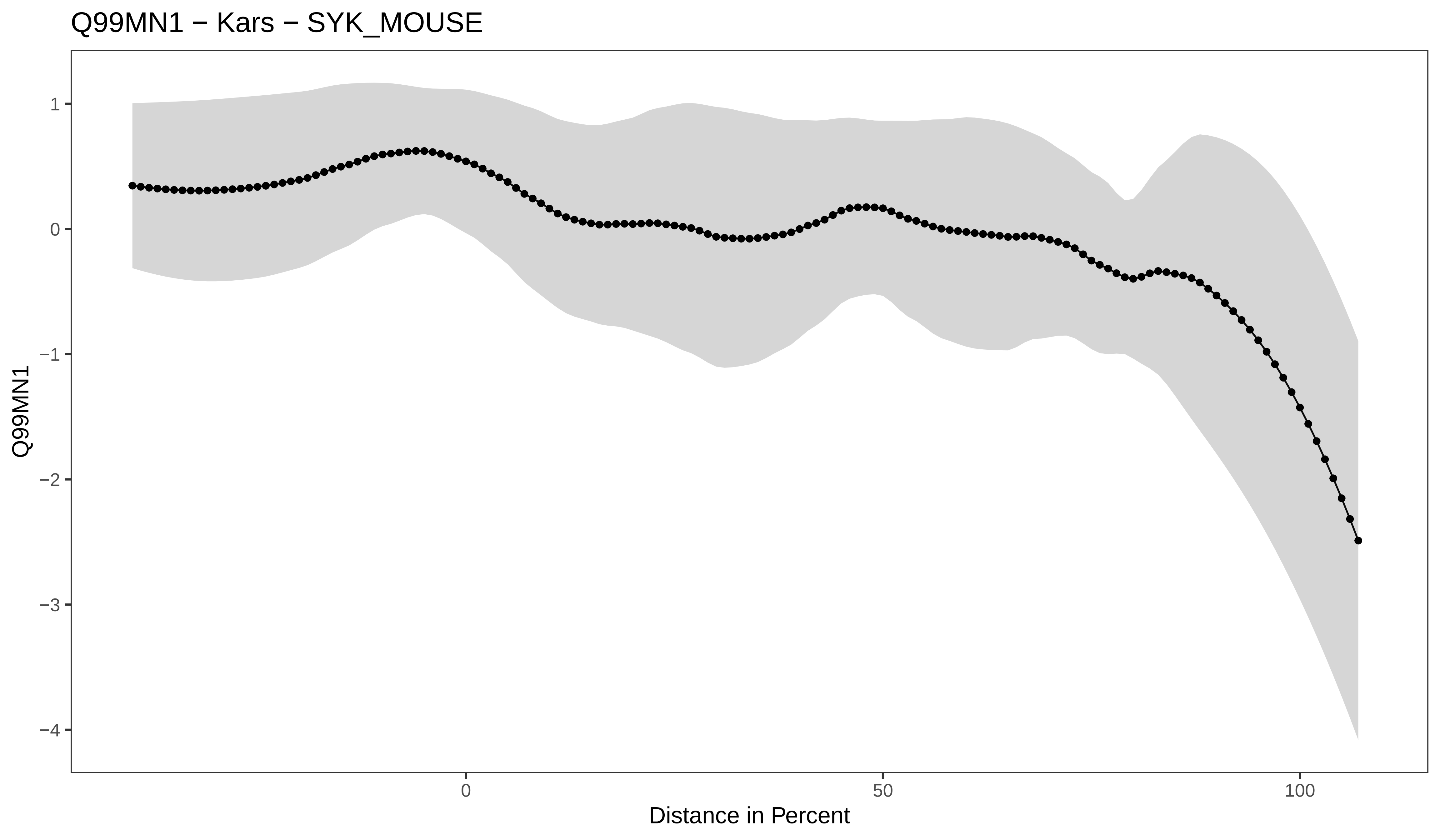

Q99MN1 − Kars − SYK_MOUSE
1
0
−1
−2
−3
−4
Q99MN1
0
50
100
Distance in Percent

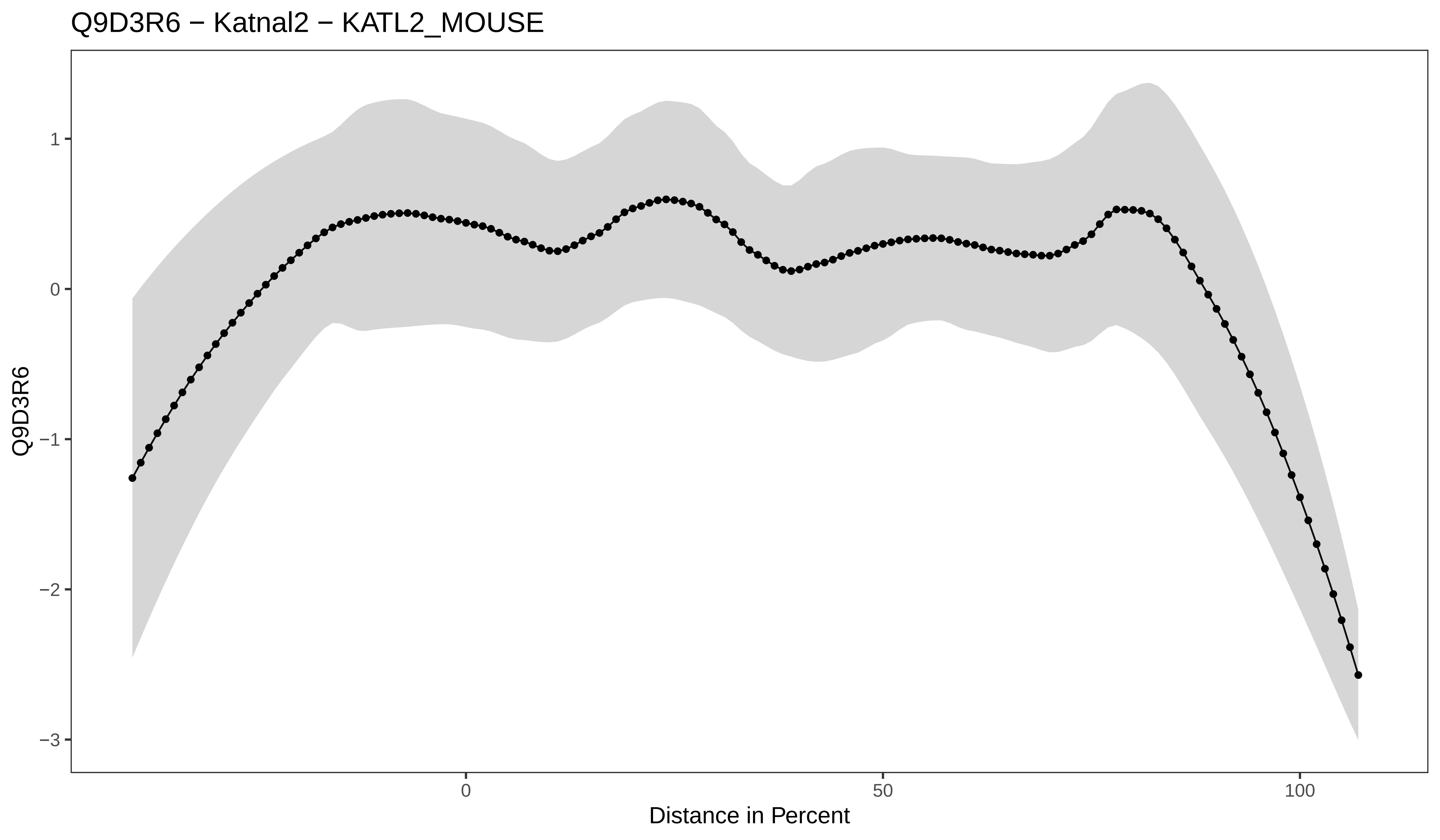
Q9D3R6 − Katnal2 − KATL2_MOUSE
Q9D3R6
Distance in Percent
1
0
−1
−2
−3
0
50
100

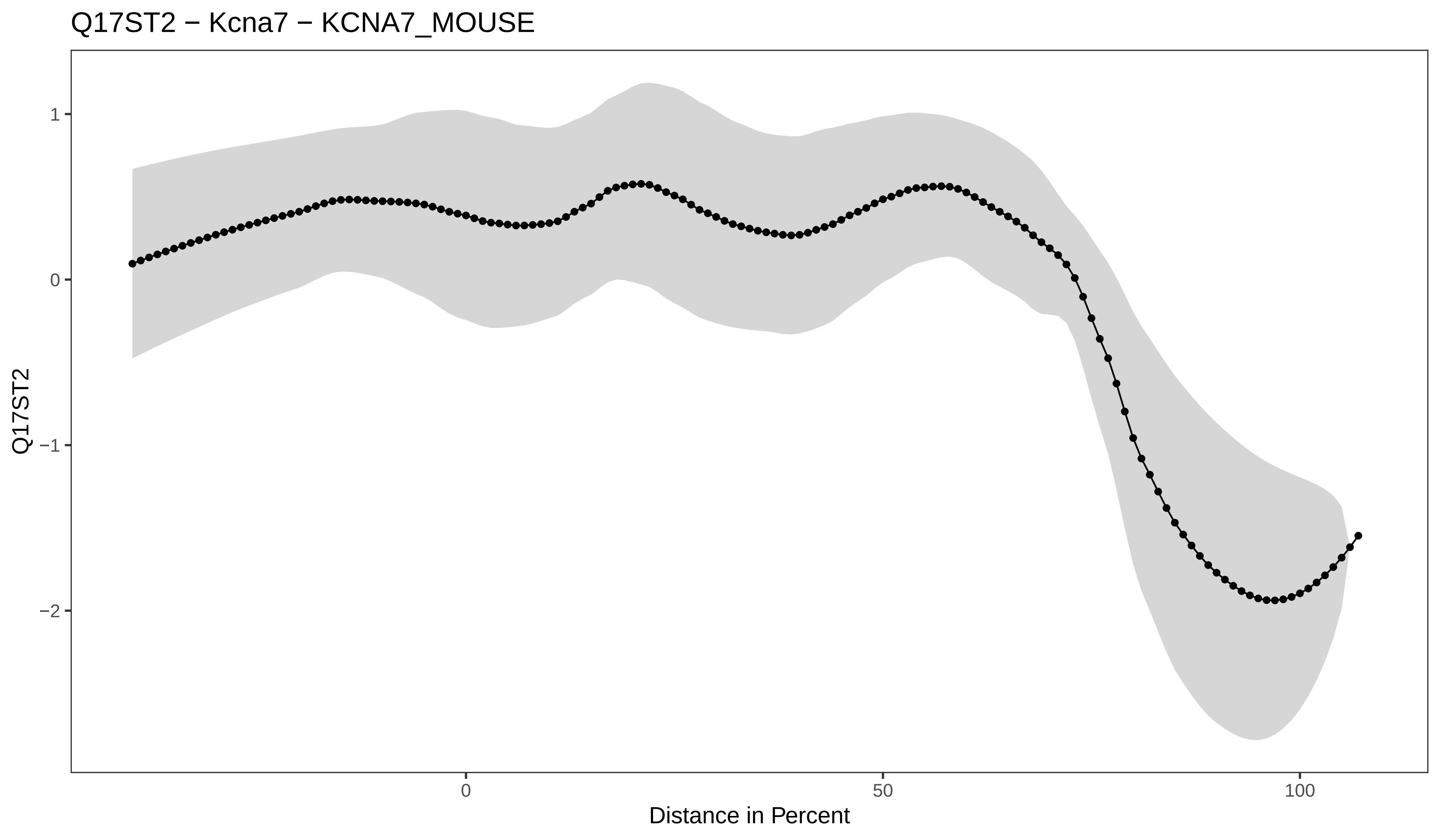

Q17ST2 − Kcna7 − KCNA7_MOUSE
Q17ST2
1
0
−1
−2
0
50
100
Distance in Percent

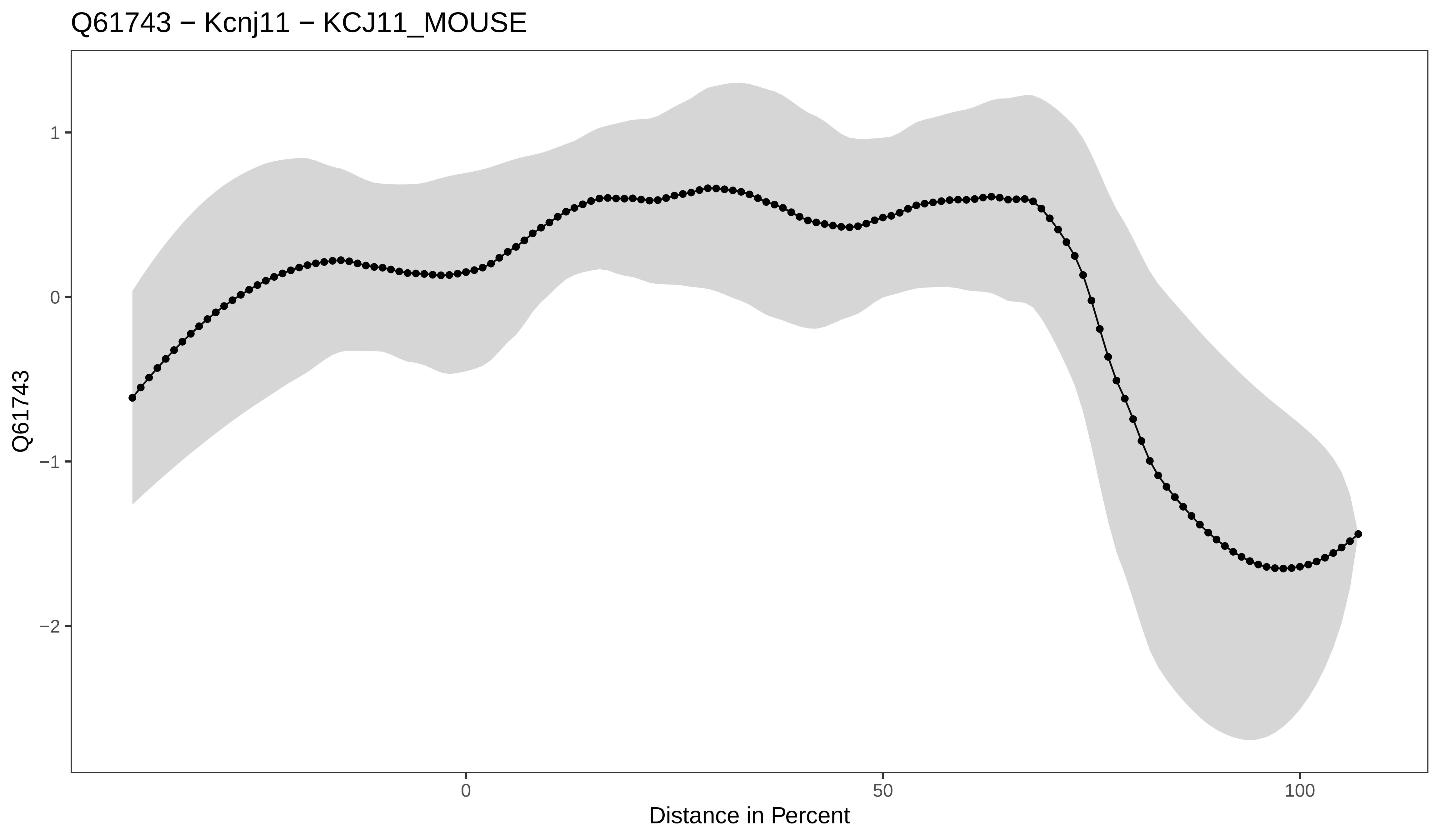

Q61743 − Kcnj11 − KCJ11_MOUSE
1
0
−1
−2
0
50
100
Q61743
Distance in Percent

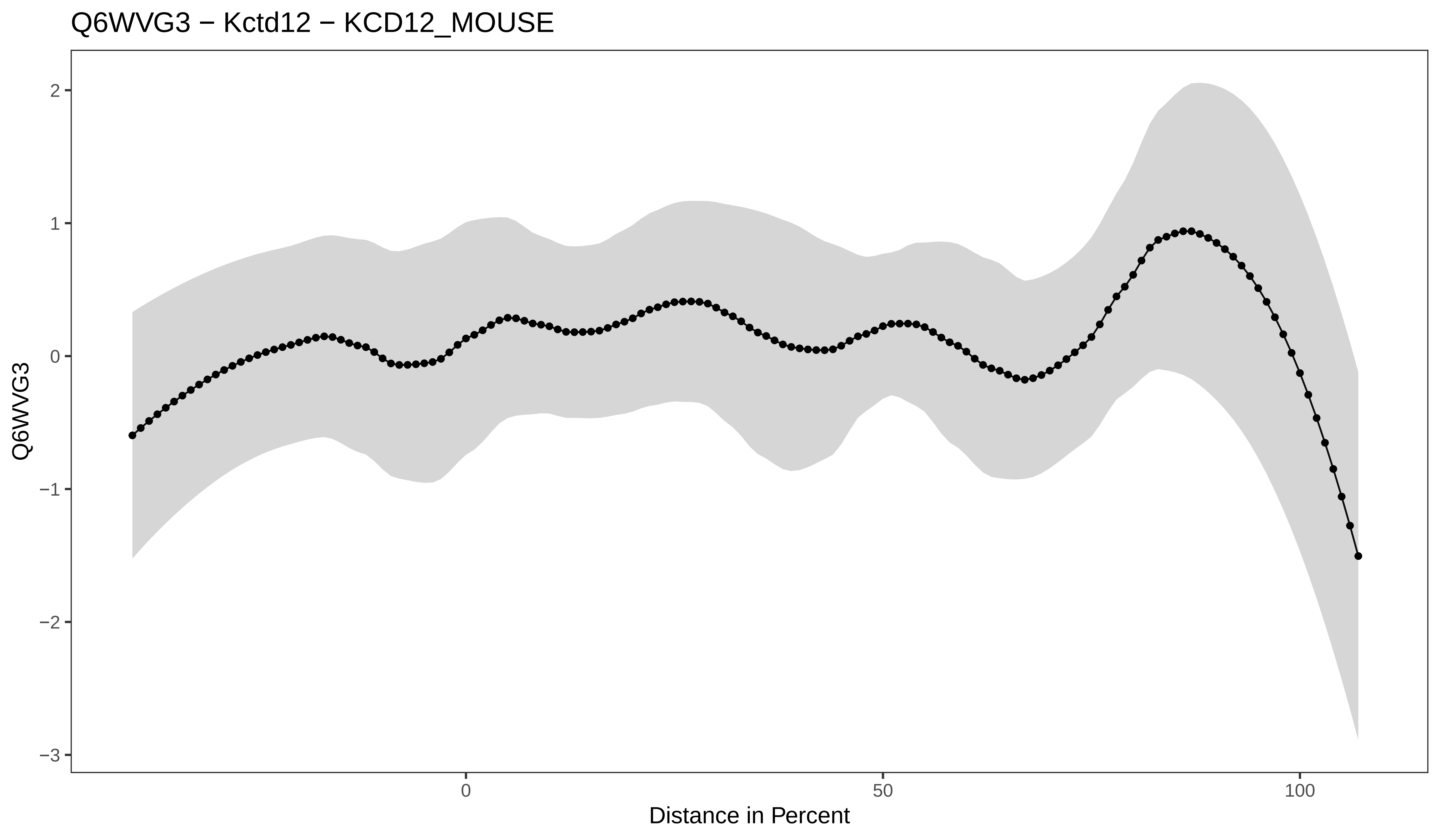
Q6WVG3 − Kctd12 − KCD12_MOUSE
Q6WVG3
2
1
0
-1
-2
-3
0
50
100
Distance in Percent

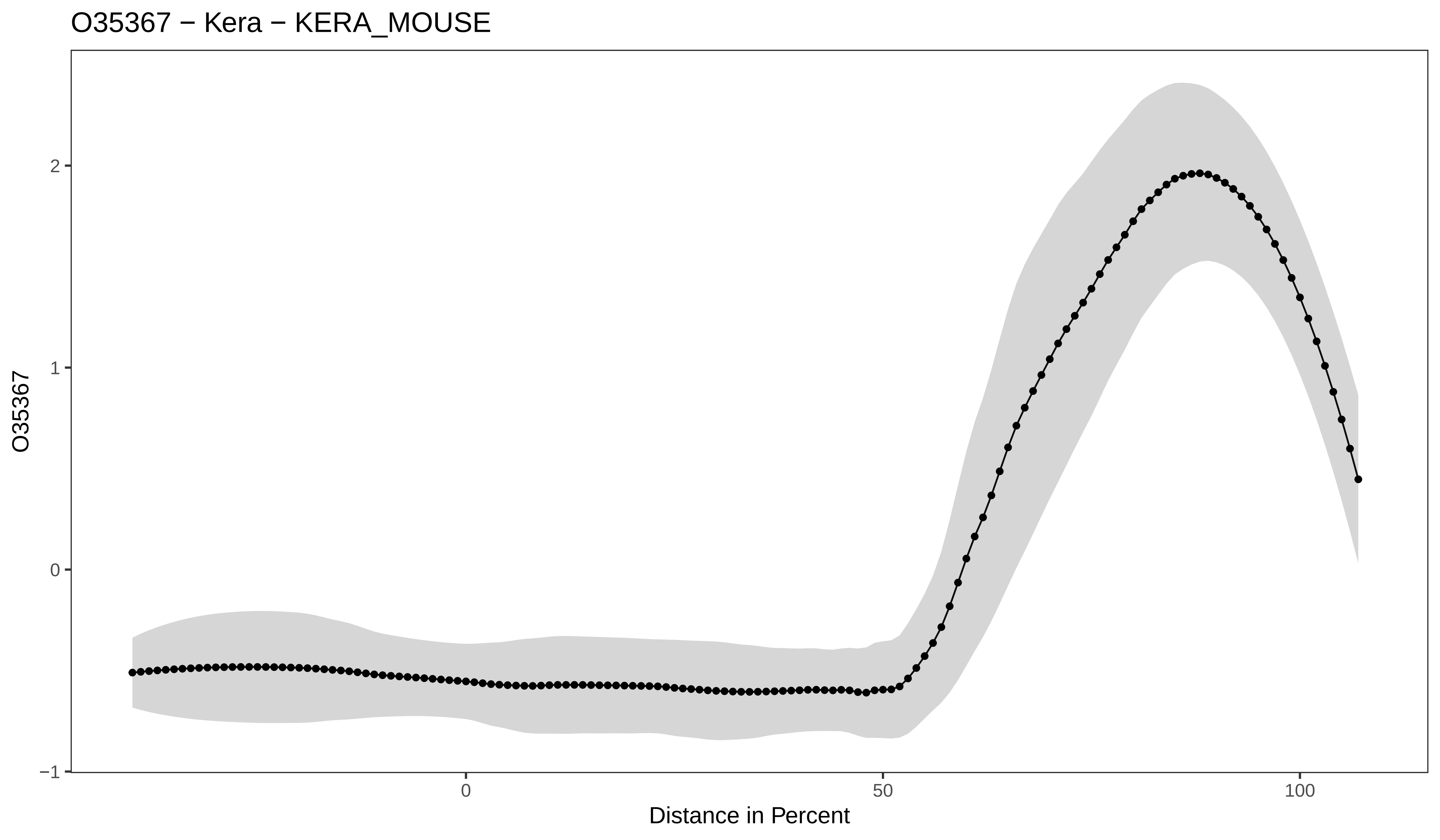
O35367 − Kera − KERA_MOUSE
O35367
2
1
0
−1
0
50
100
Distance in Percent

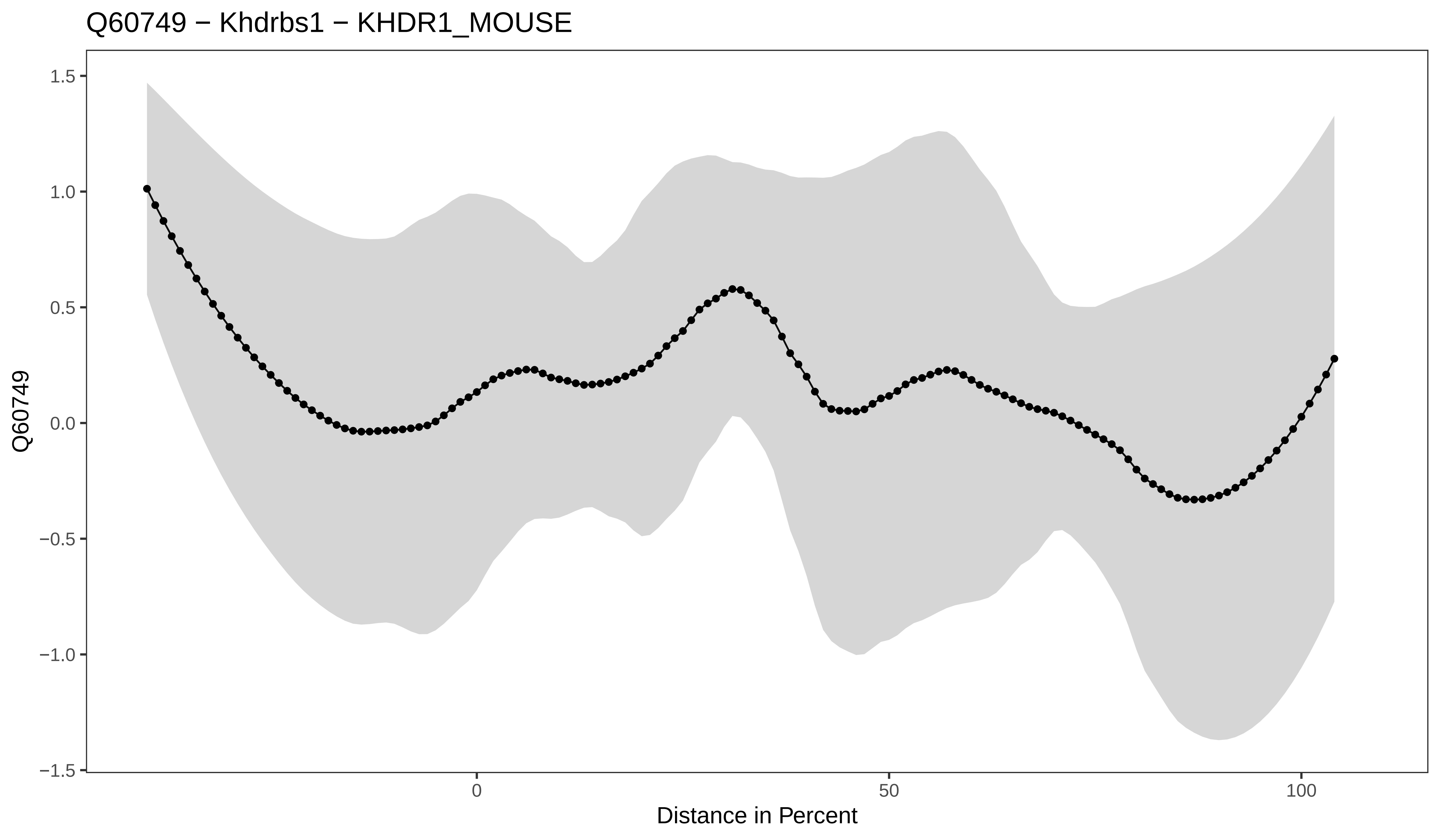

Q60749 − Khdrbs1 − KHDR1_MOUSE
Q60749
1.5
1.0
0.5
0.0
−0.5
−1.0
−1.5
0
50
100
Distance in Percent

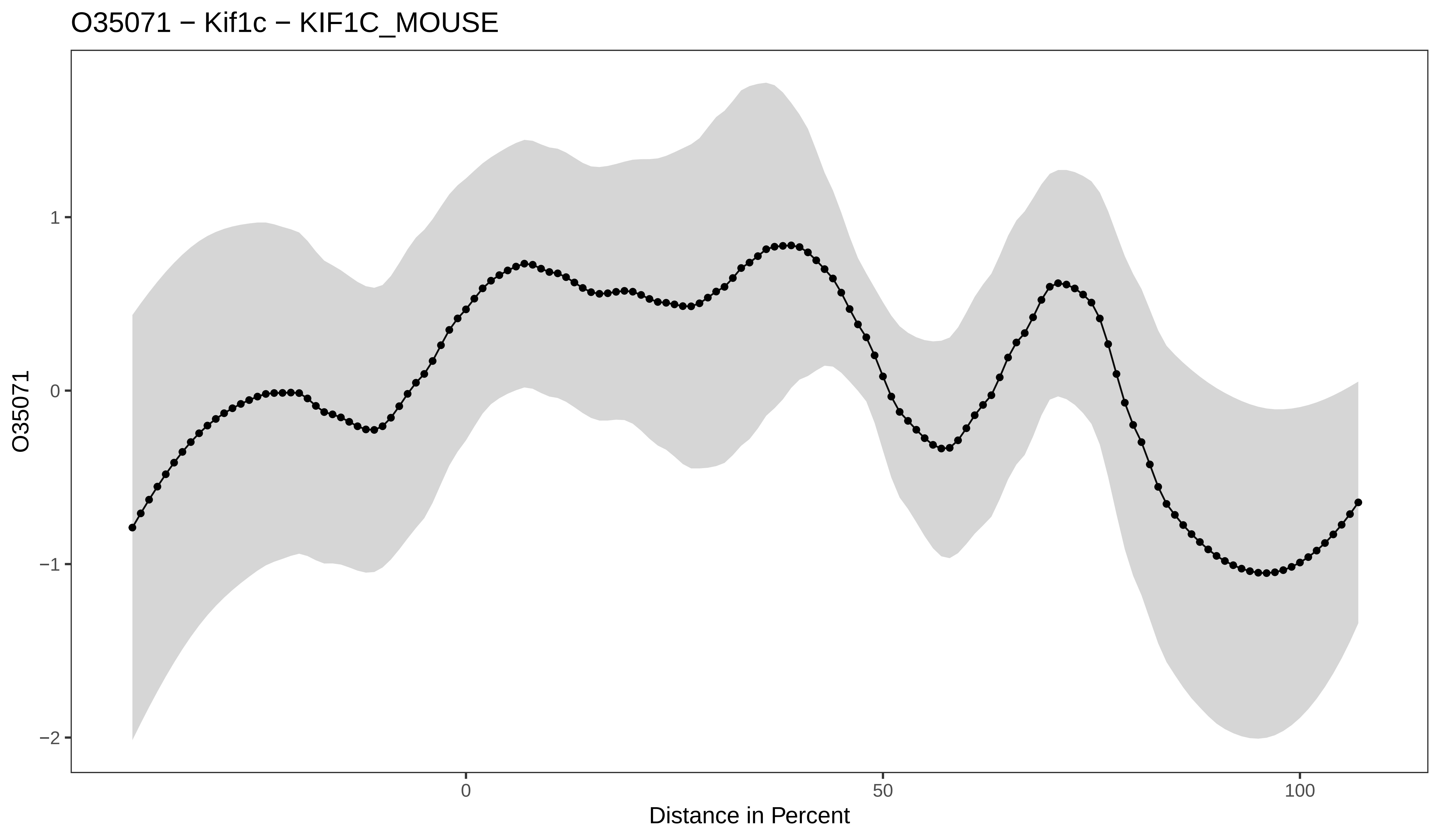

O35071 − Kif1c − KIF1C_MOUSE
O35071
1
0
−1
−2
0
50
100
Distance in Percent

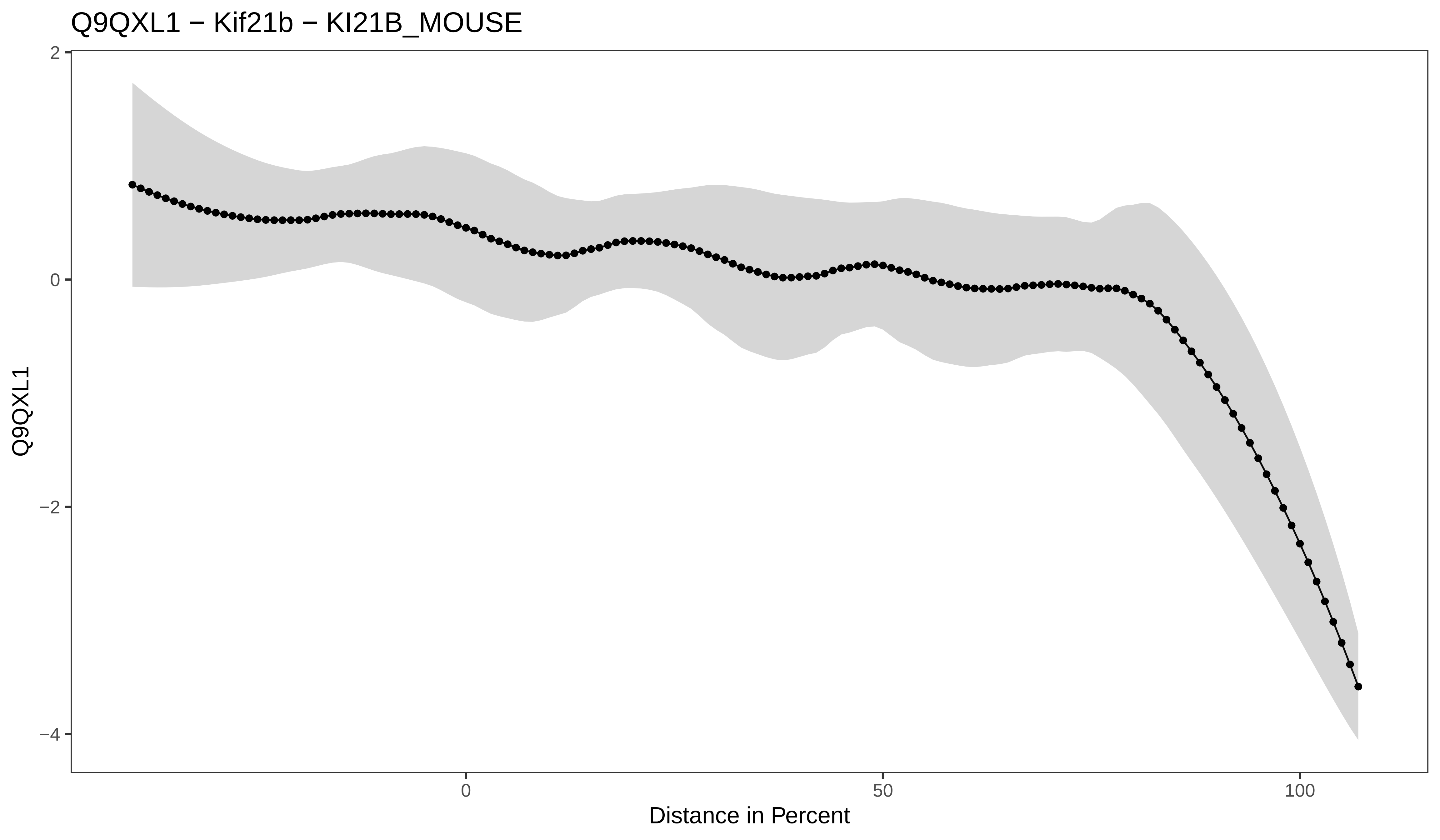

Q9QXL1 − Kif21b − KI21B_MOUSE
2
0
−2
−4
Q9QXL1
0
50
100
Distance in Percent

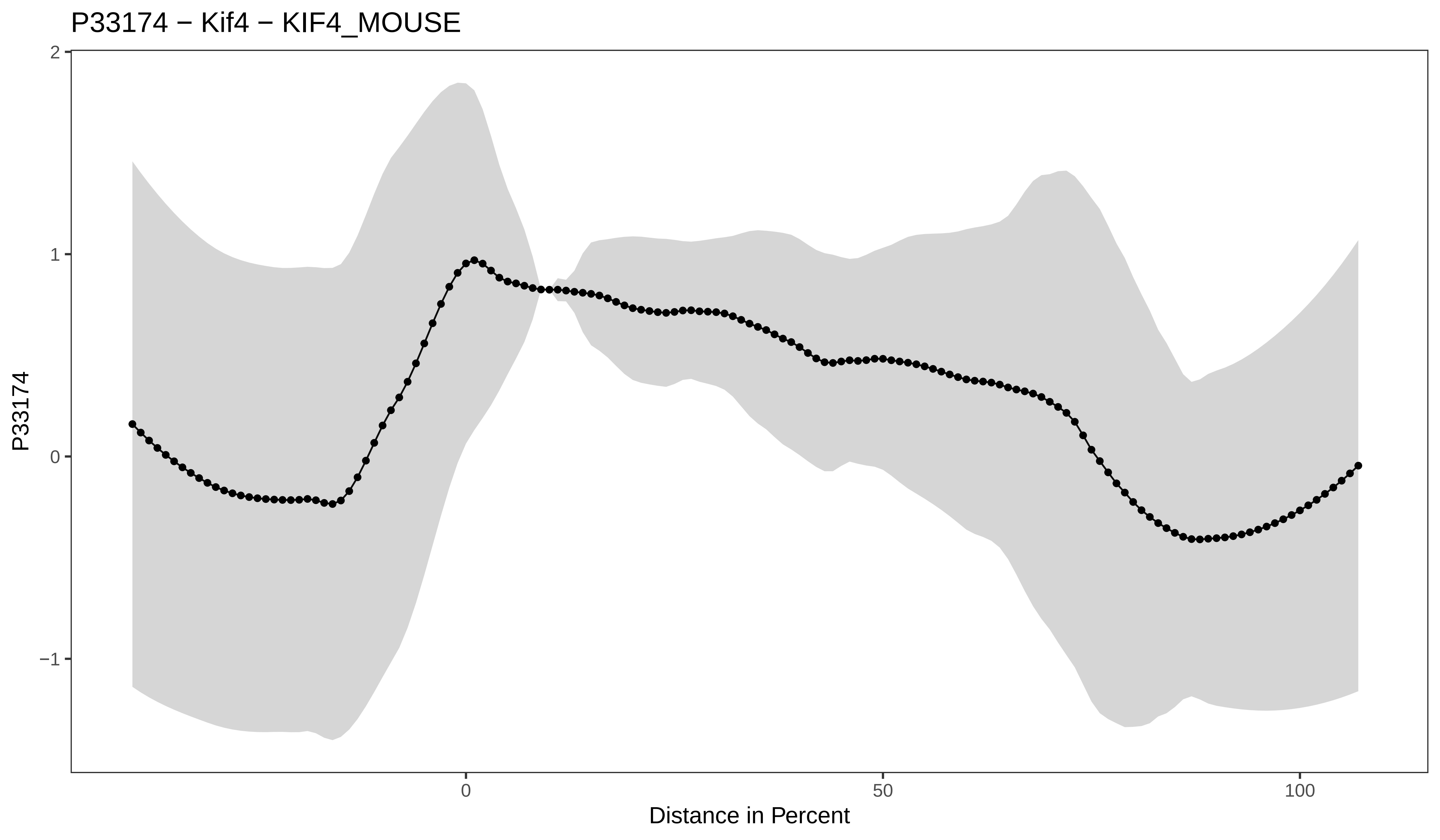

P33174 − Kif4 − KIF4_MOUSE
P33174
Distance in Percent
2
1
0
−1
0
50
100

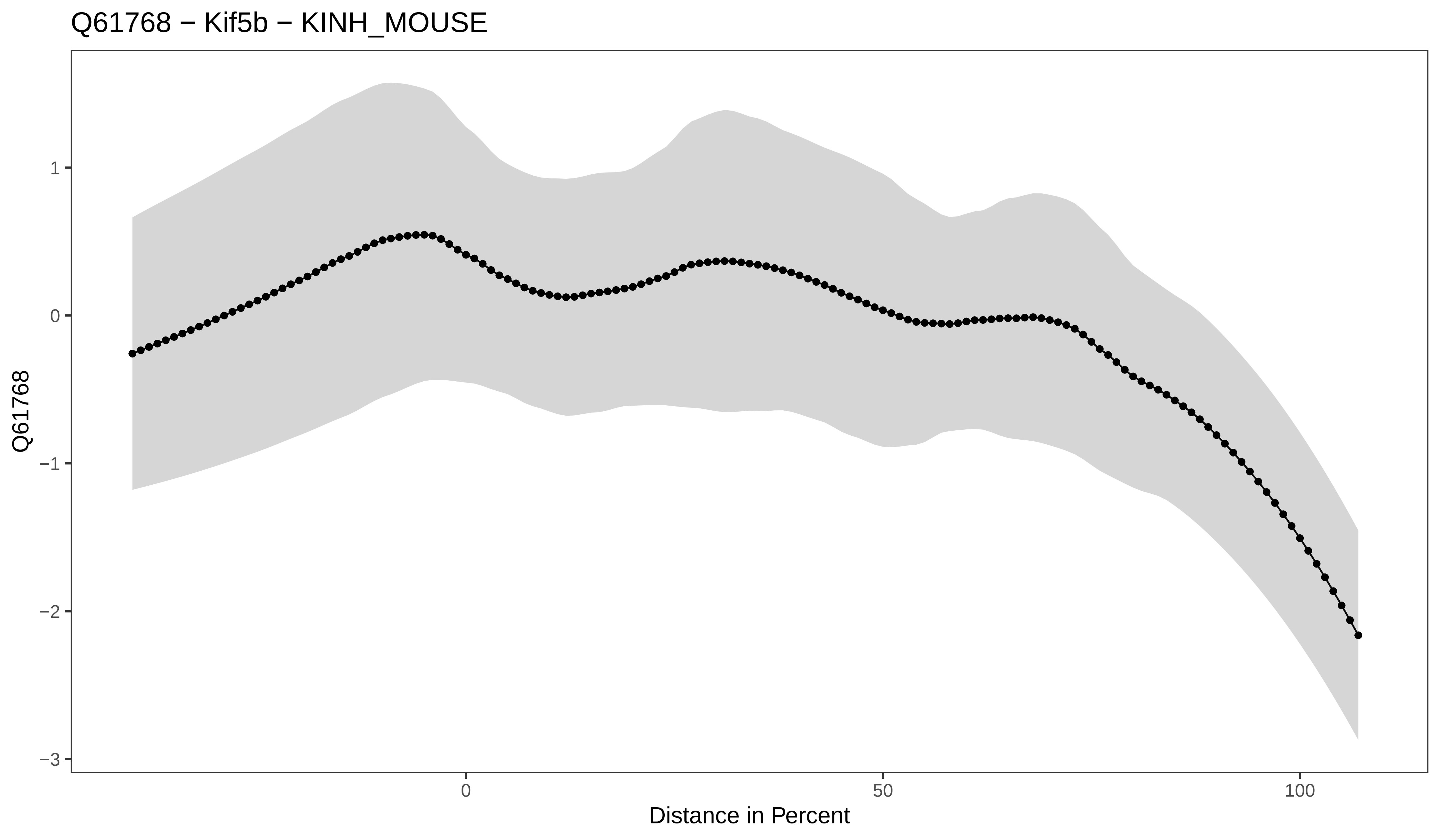
Q61768 − Kif5b − KINH_MOUSE
Q61768
Distance in Percent
1
0
−1
−2
−3
0
50
100

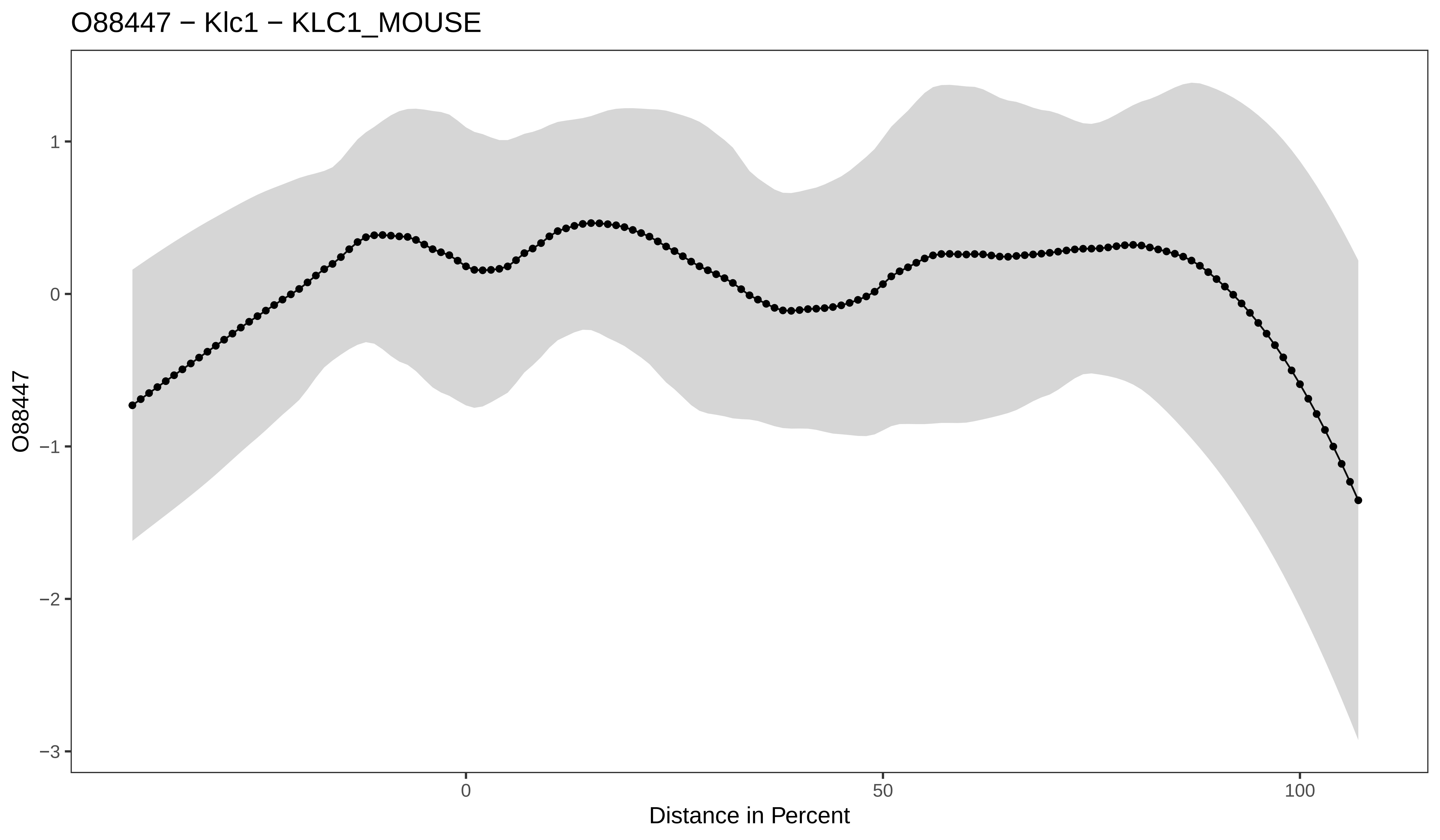
O88447 − Klc1 − KLC1_MOUSE
O88447
1
0
−1
−2
−3
0
50
100
Distance in Percent

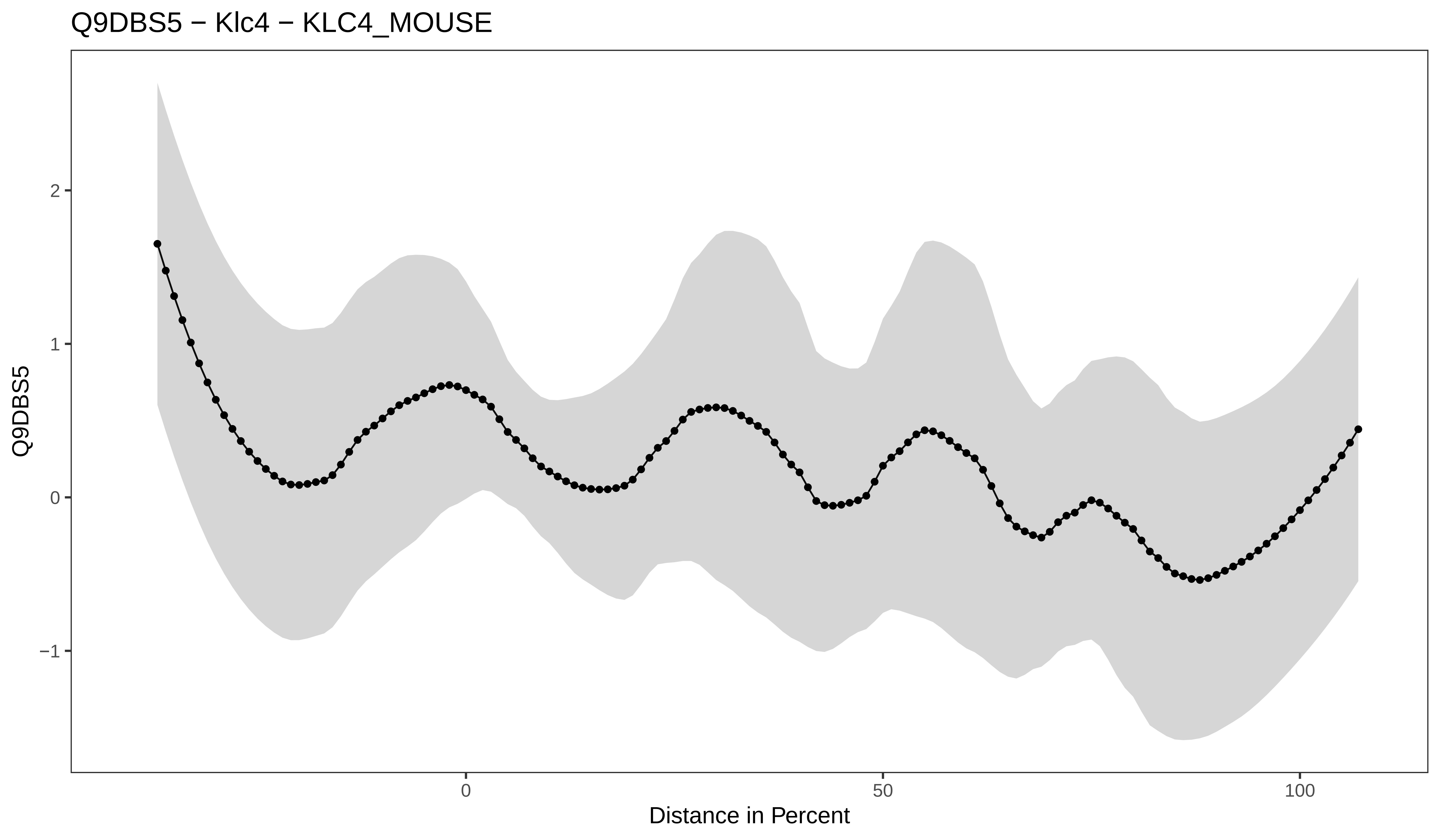

Q9DBS5 − Klc4 − KLC4_MOUSE
Q9DBS5
2
1
0
−1
0
50
100
Distance in Percent

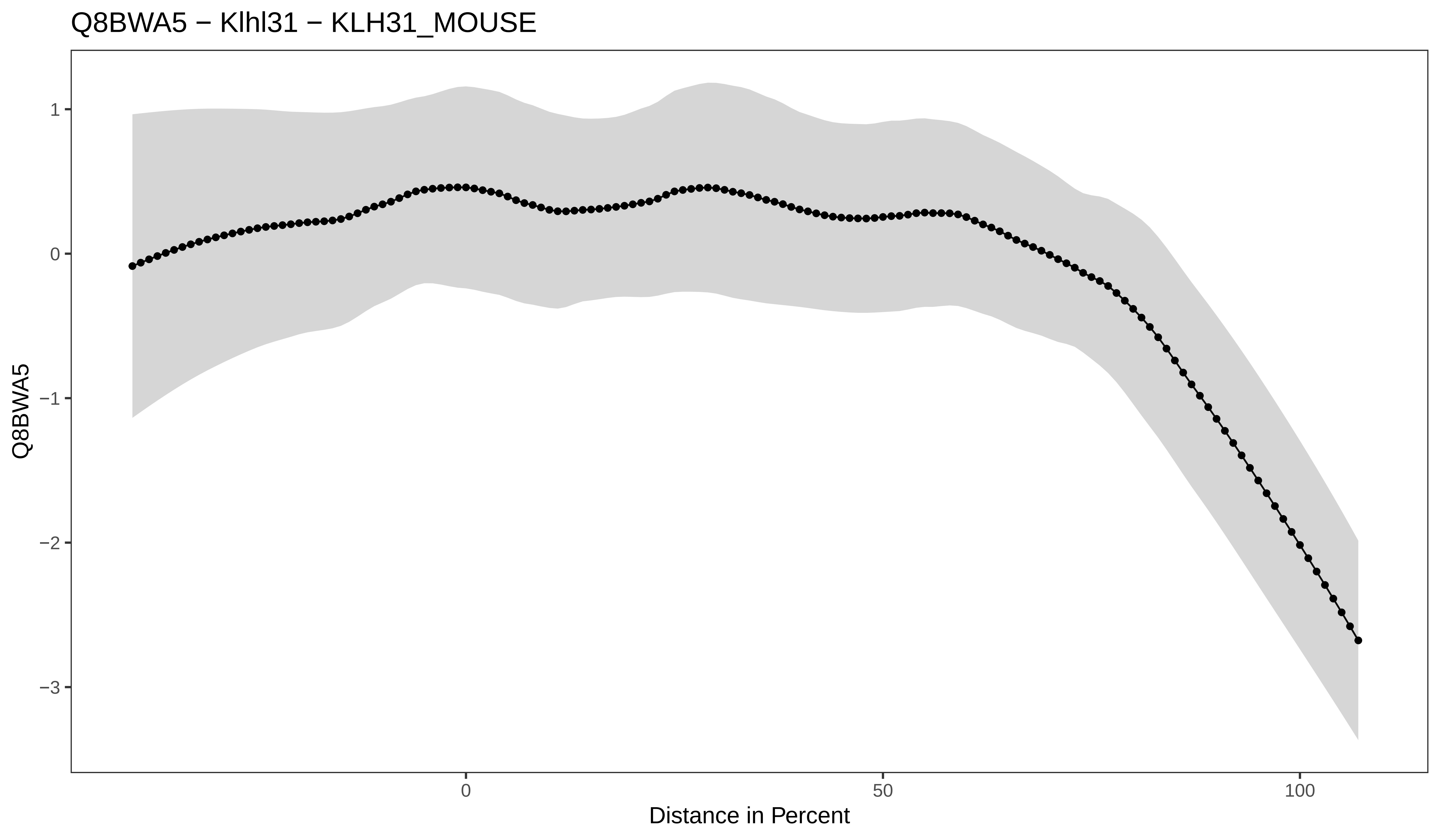

Q8BWA5 − Klhl31 − KLH31_MOUSE
1
0
−1
−2
−3
Q8BWA5
0
50
100
Distance in Percent

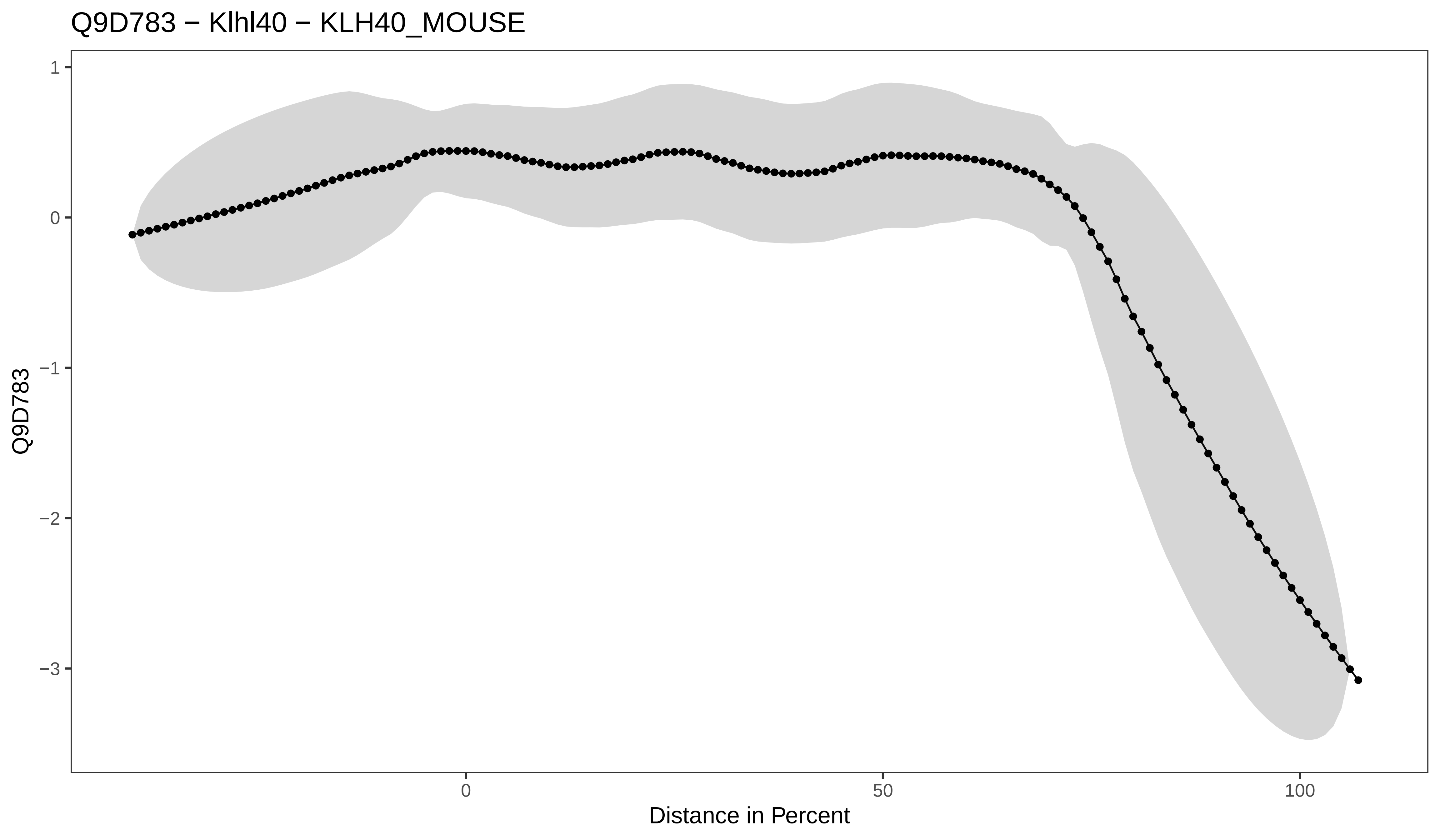
Q9D783 − Klhl40 − KLH40_MOUSE
1
0
−1
−2
−3
Q9D783
0
50
100
Distance in Percent

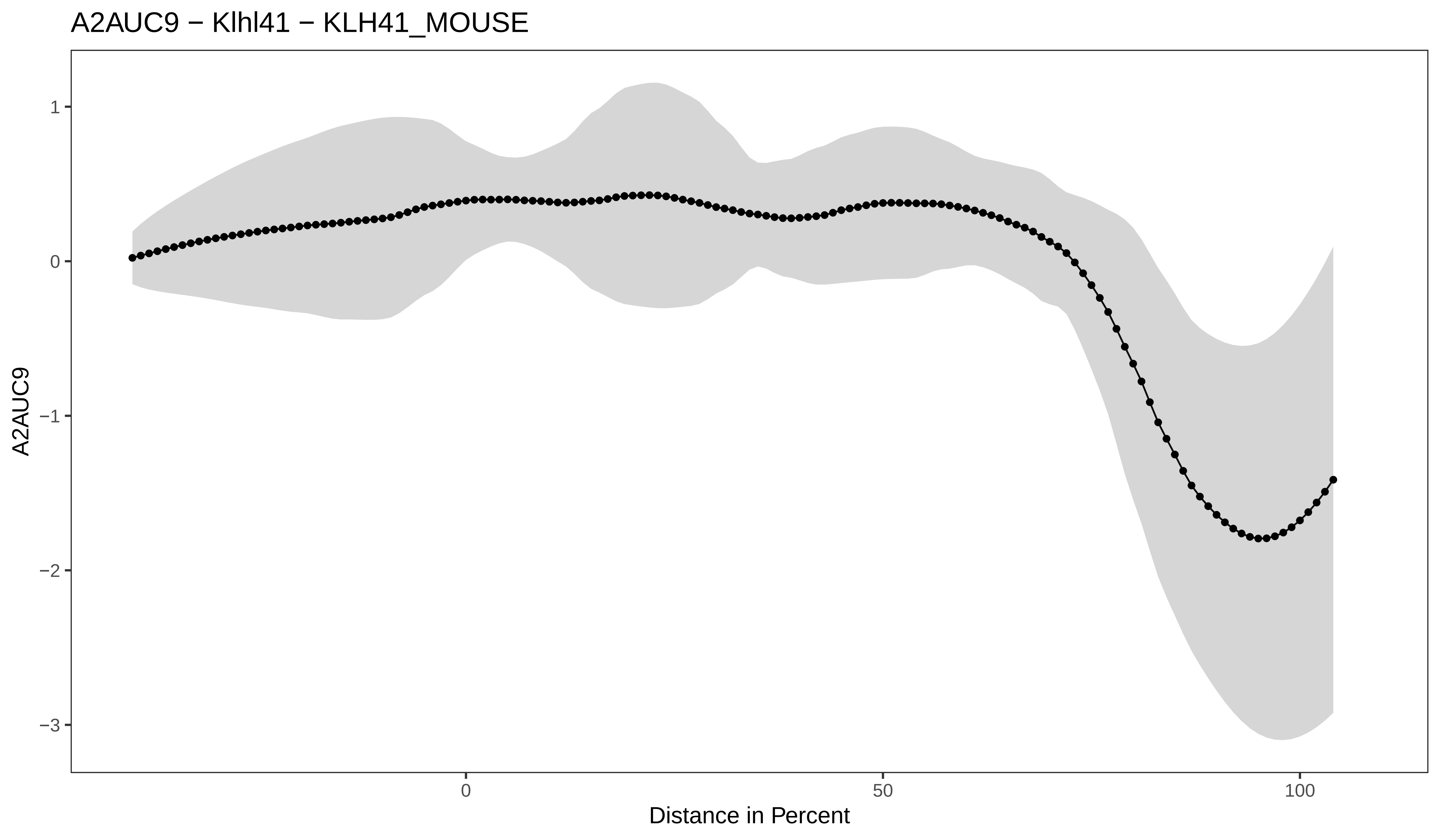

A2AUC9 − Klhl41 − KLH41_MOUSE
1
0
−1
−2
−3
A2AUC9
0
50
100
Distance in Percent

O08677 − Kng1 − KNG1_MOUSE

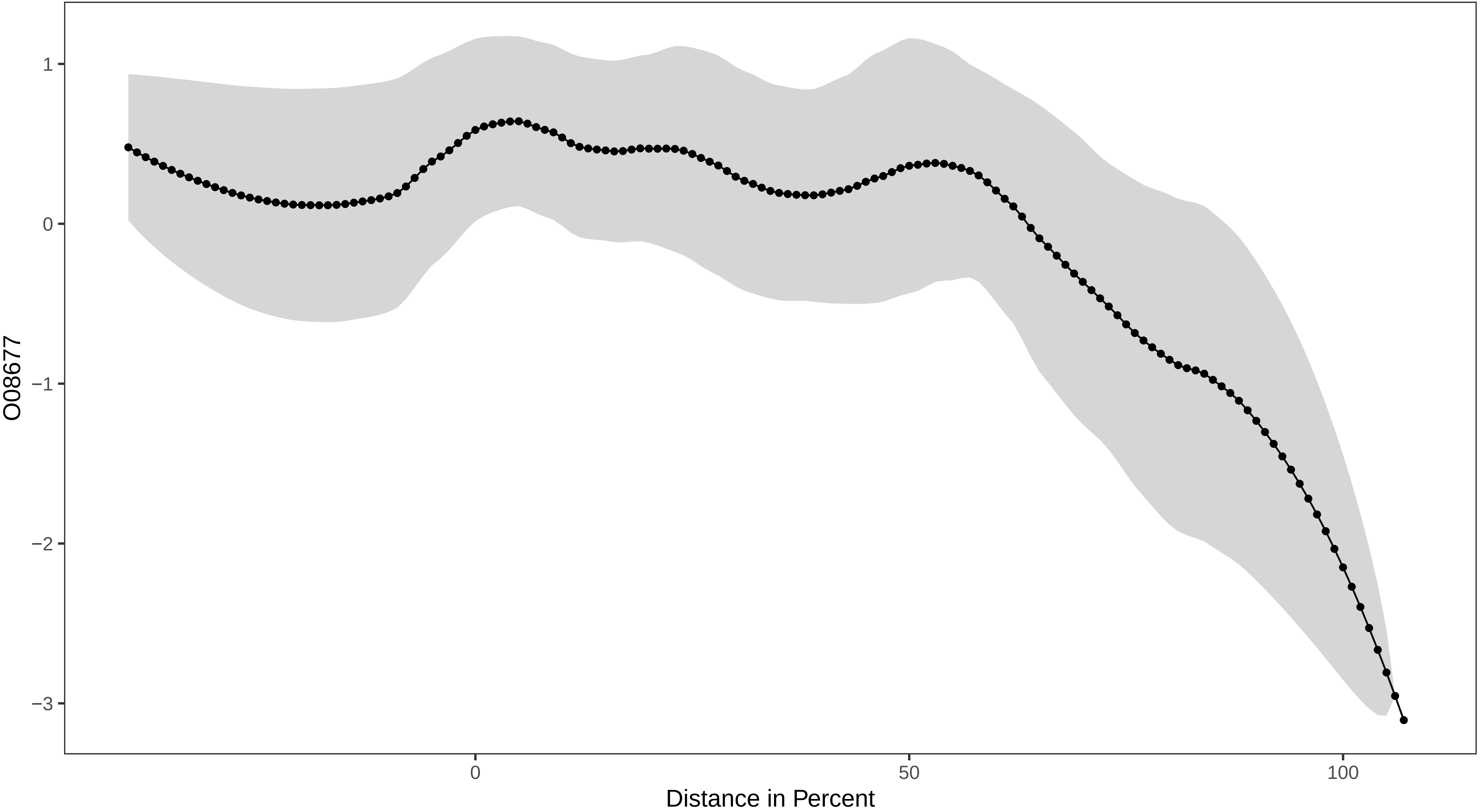

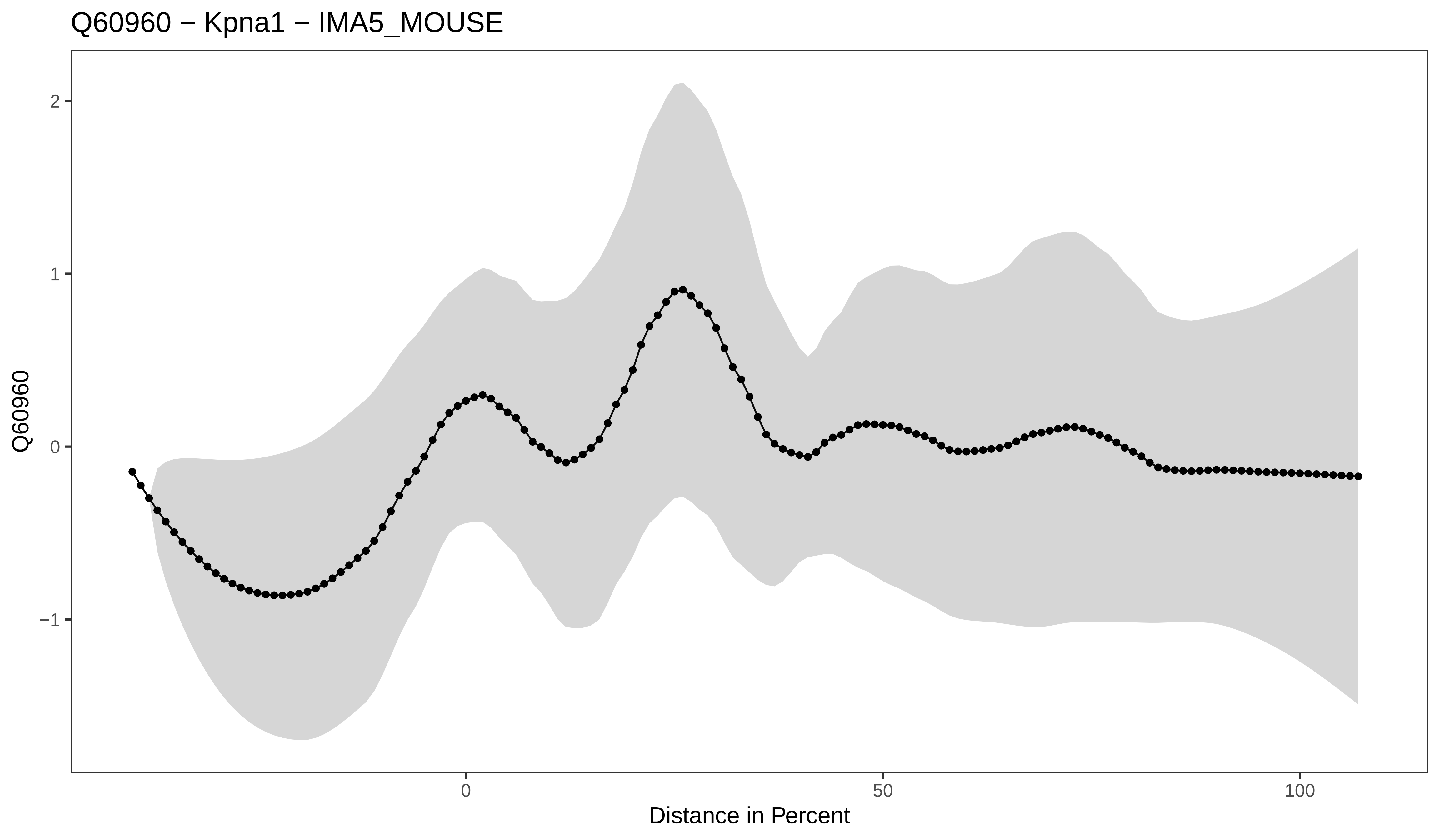

Q60960 − Kpna1 − IMA5_MOUSE
Q60960
2
1
0
−1
0
50
100
Distance in Percent

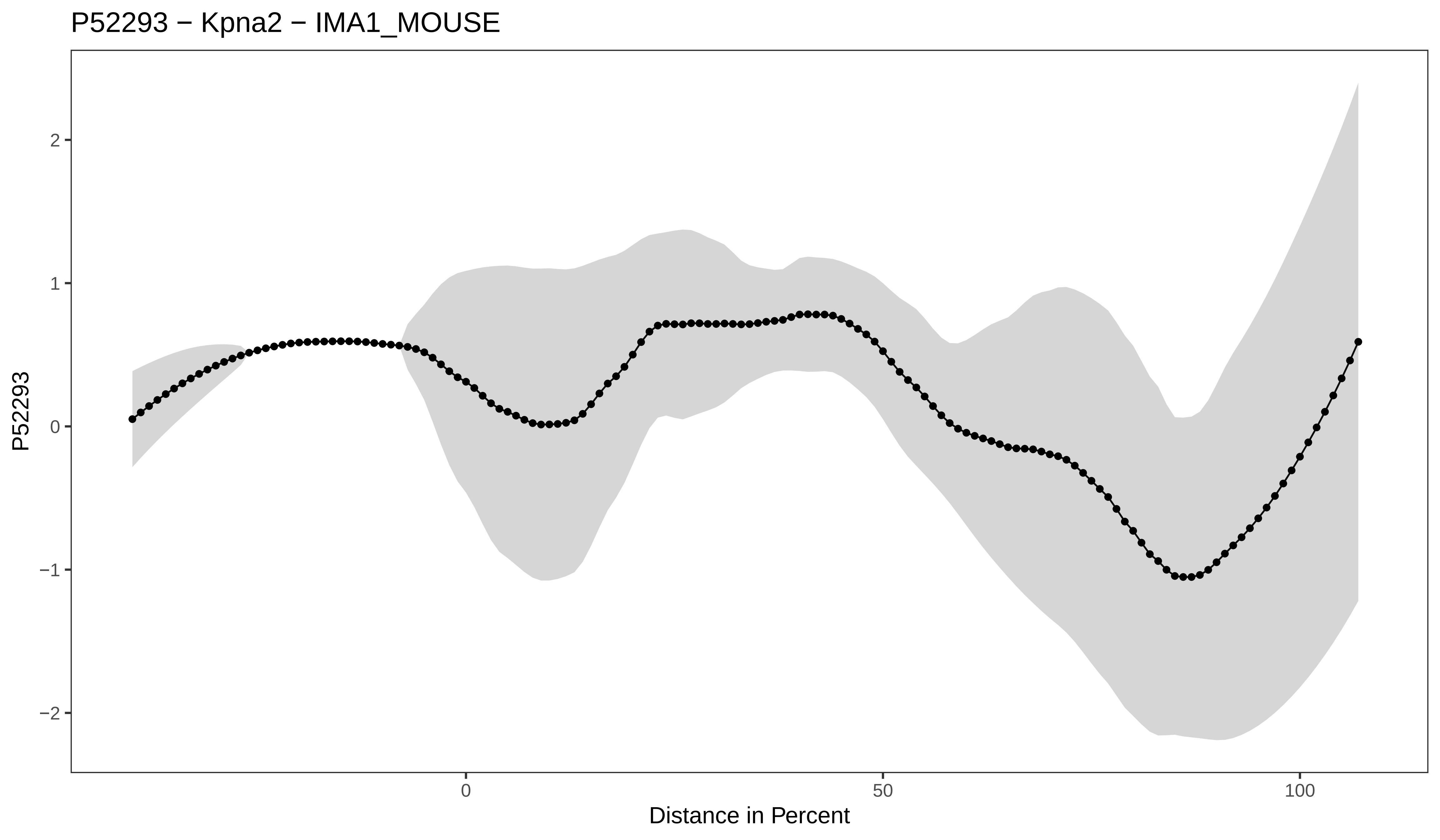
P52293 − Kpna2 − IMA1_MOUSE
P52293
2
1
0
−1
−2
0
50
100
Distance in Percent

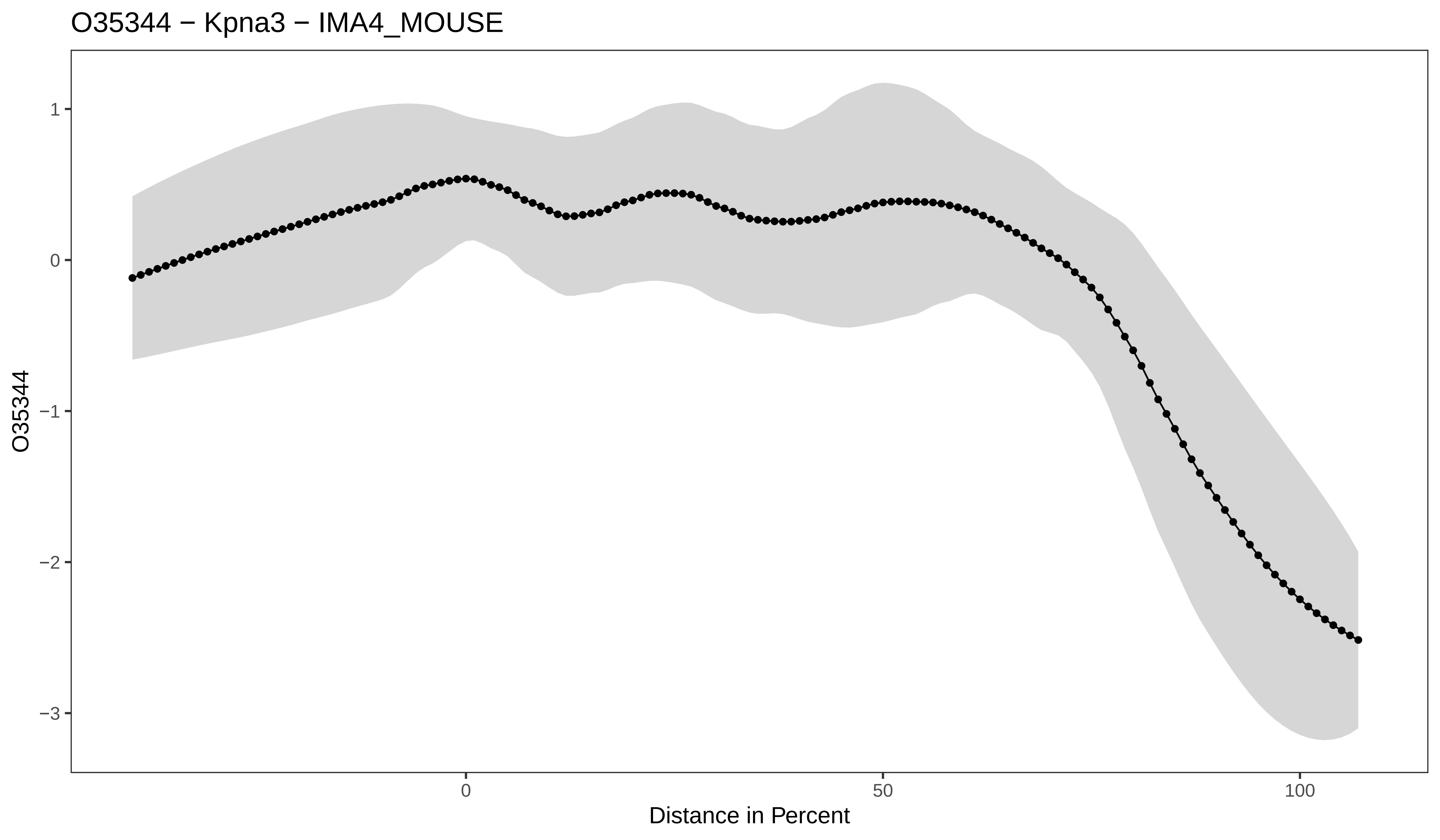

O35344 − Kpna3 − IMA4_MOUSE
1
0
−1
−2
−3
O35344
0
50
100
Distance in Percent

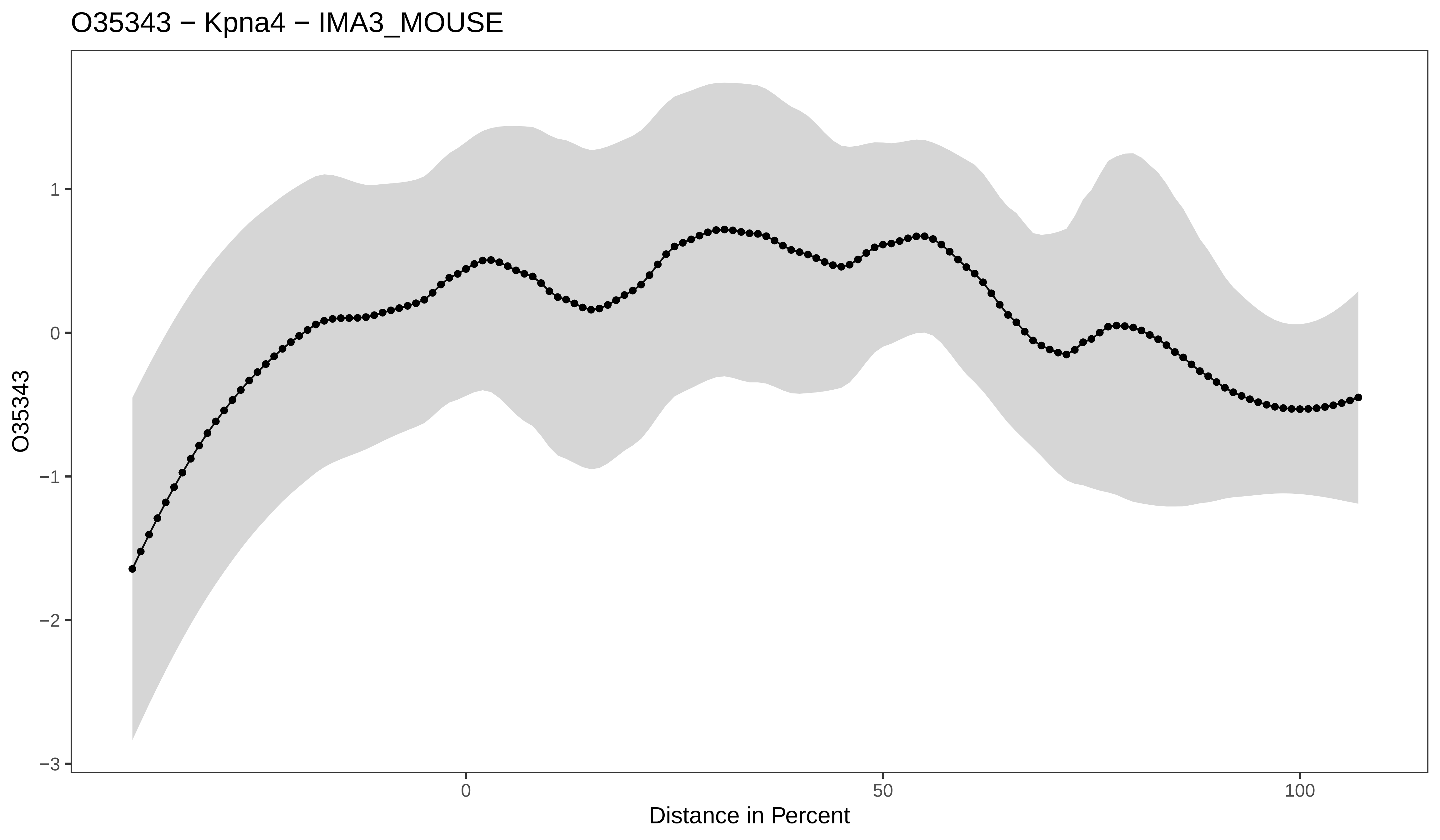
O35343 − Kpna4 − IMA3_MOUSE
O35343
1
0
−1
−2
−3
0
50
100
Distance in Percent

P70168 − Kpnb1 − IMB1_MOUSE

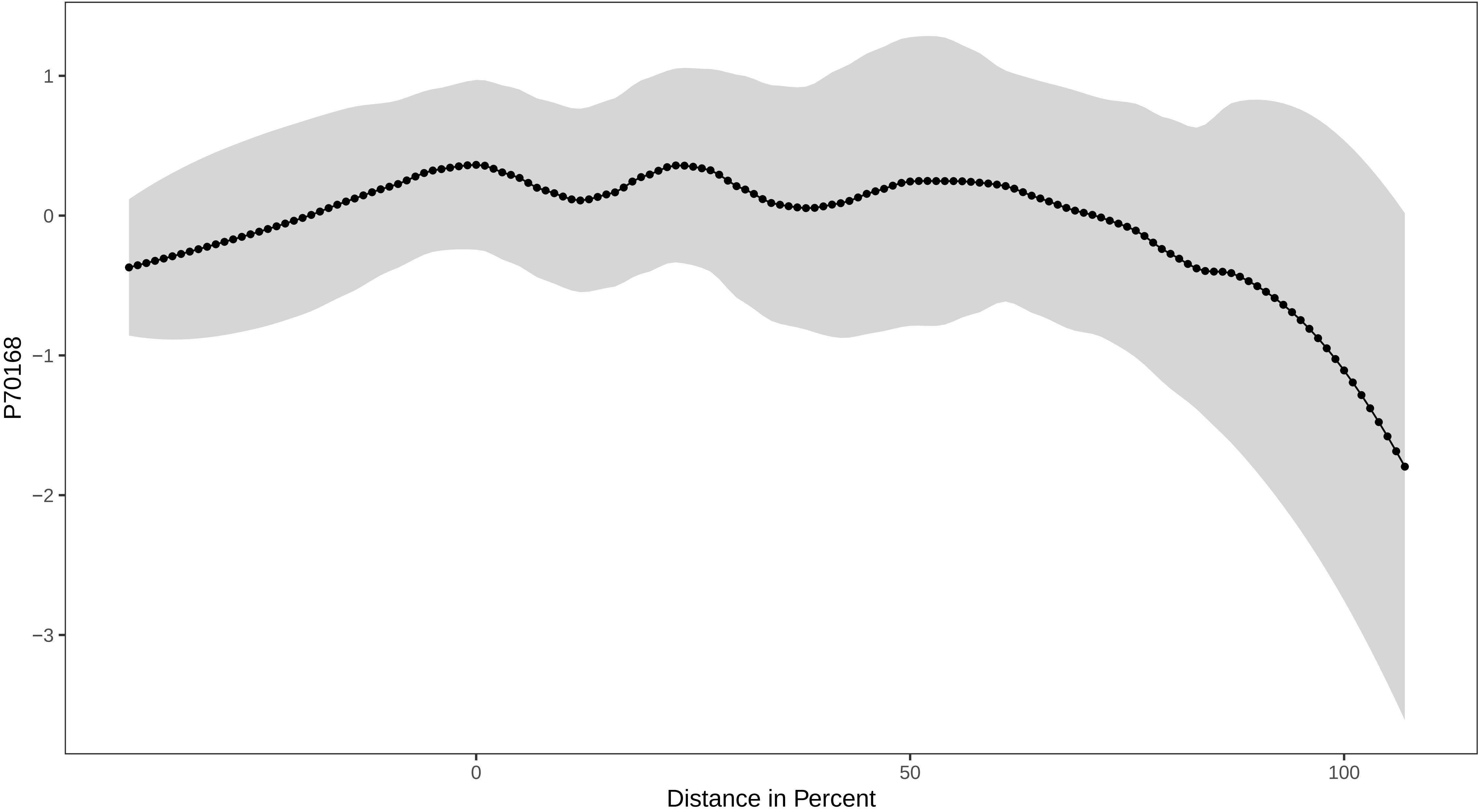

P04104 − Krt1 − K2C1_MOUSE

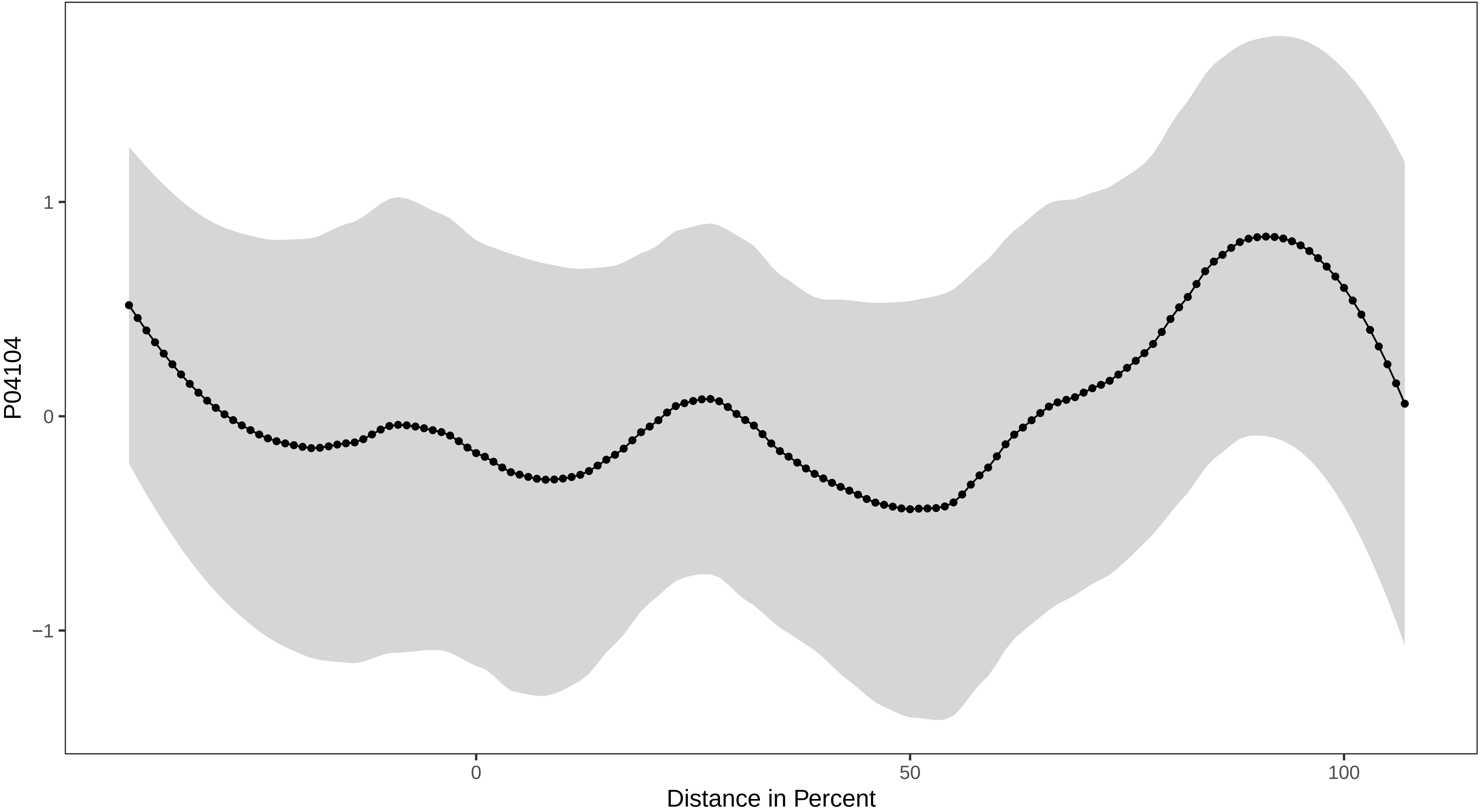

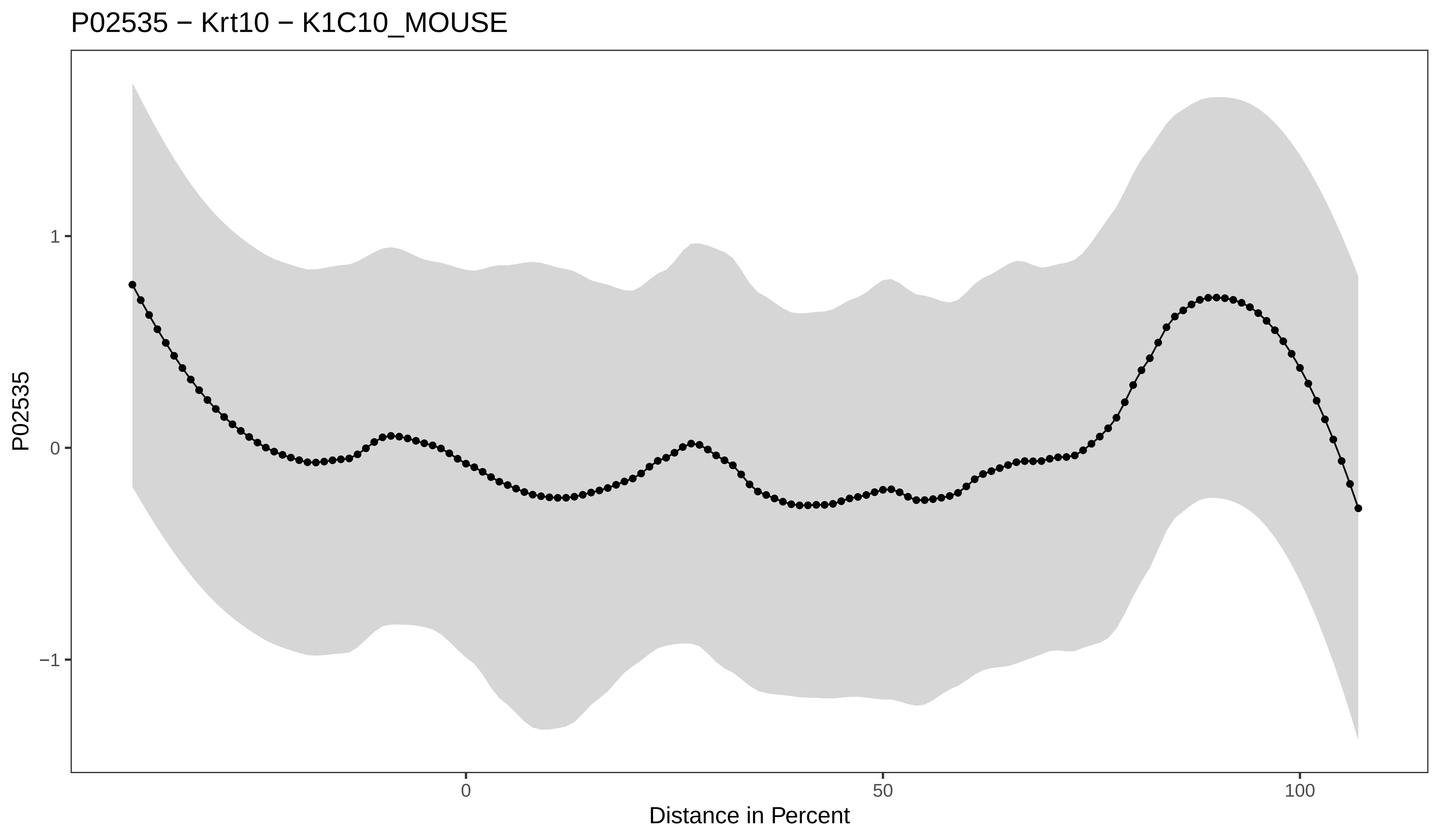

P02535 − Krt10 − K1C10_MOUSE
1
0
−1
P02535
0
50
100
Distance in Percent

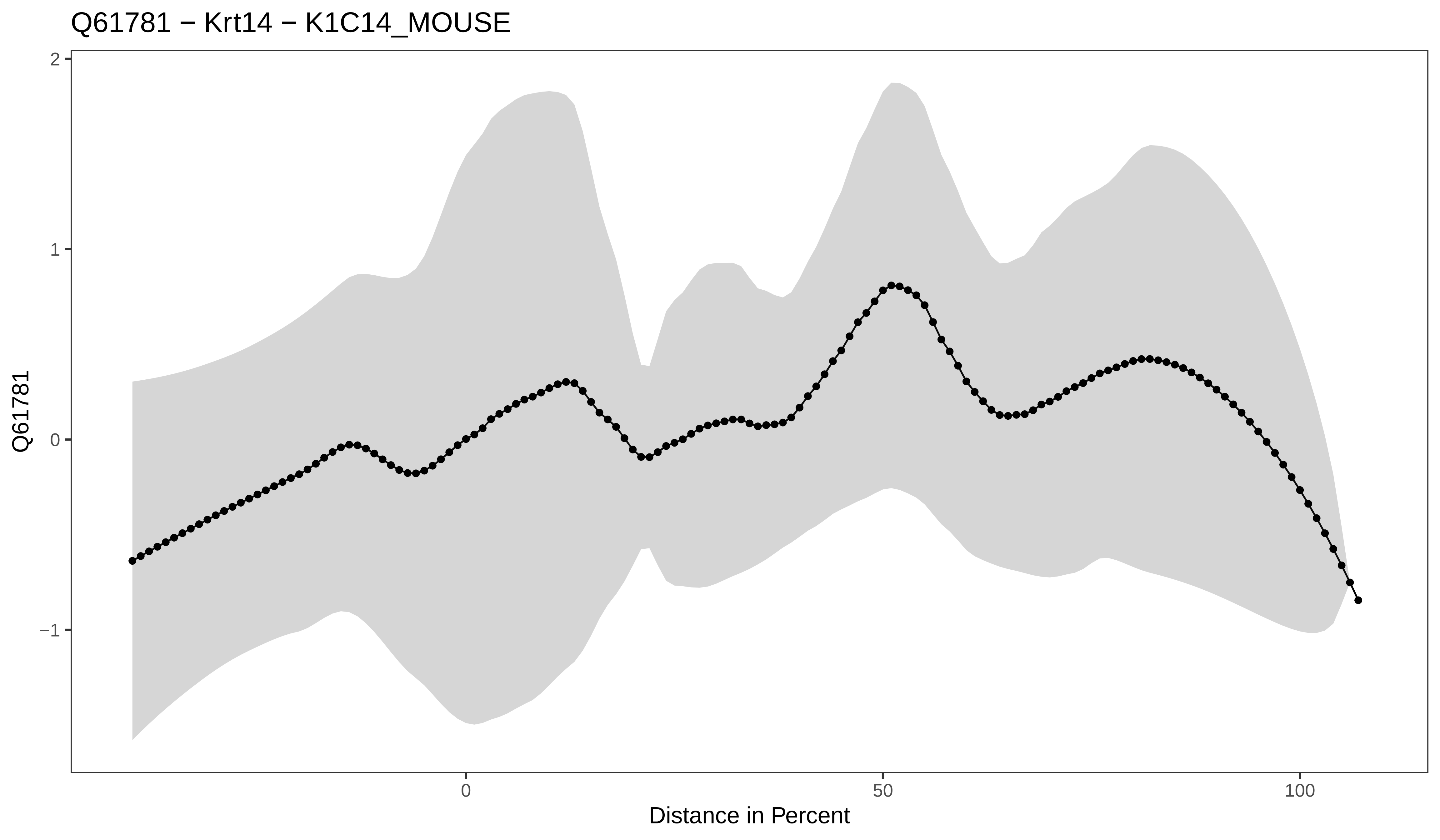

Q61781 − Krt14 − K1C14_MOUSE
Q61781
Distance in Percent
2
1
0
−1
0
50
100

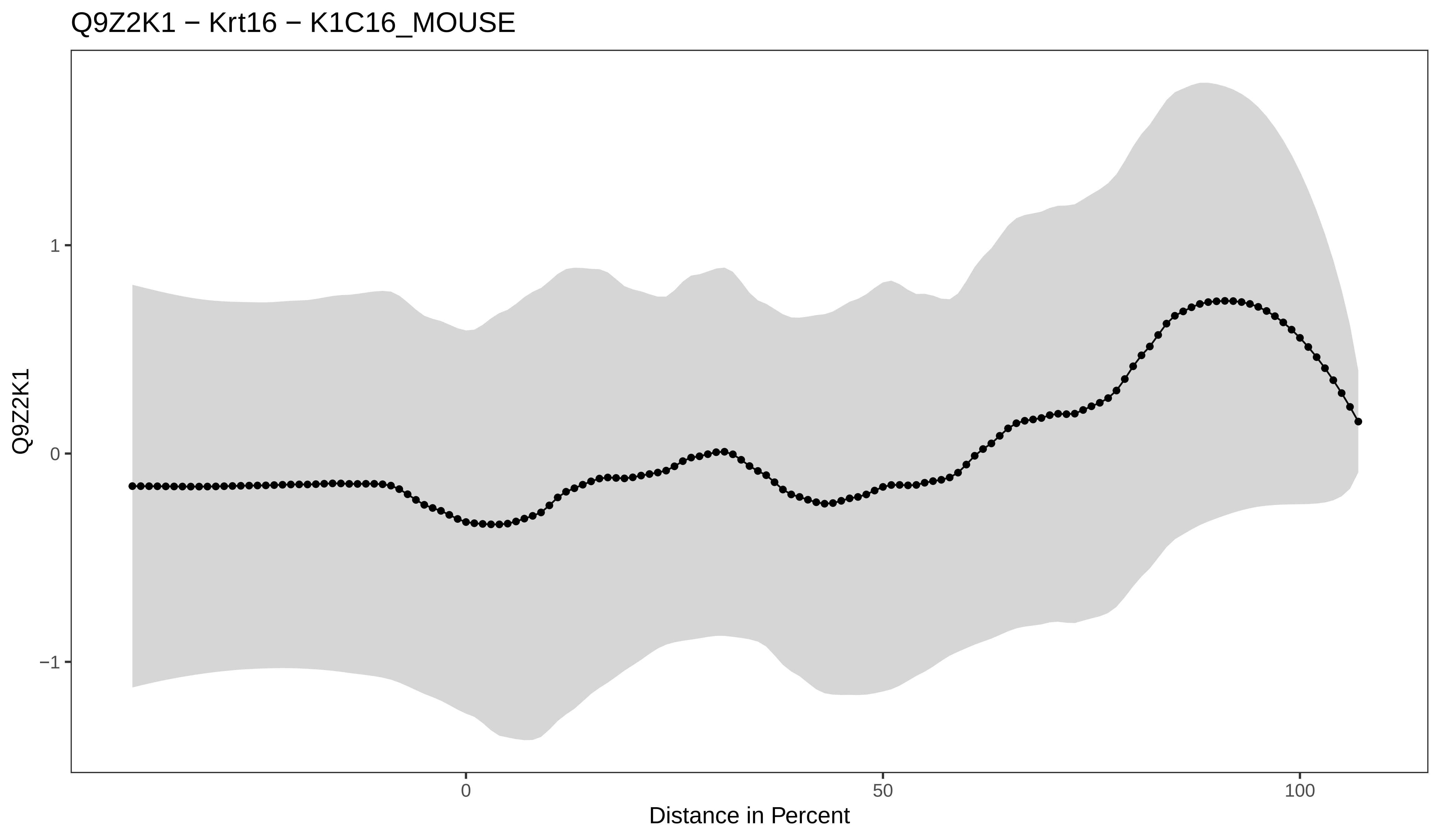

Q9Z2K1 − Krt16 − K1C16_MOUSE
Q9Z2K1
1
0
−1
0
50
100
Distance in Percent

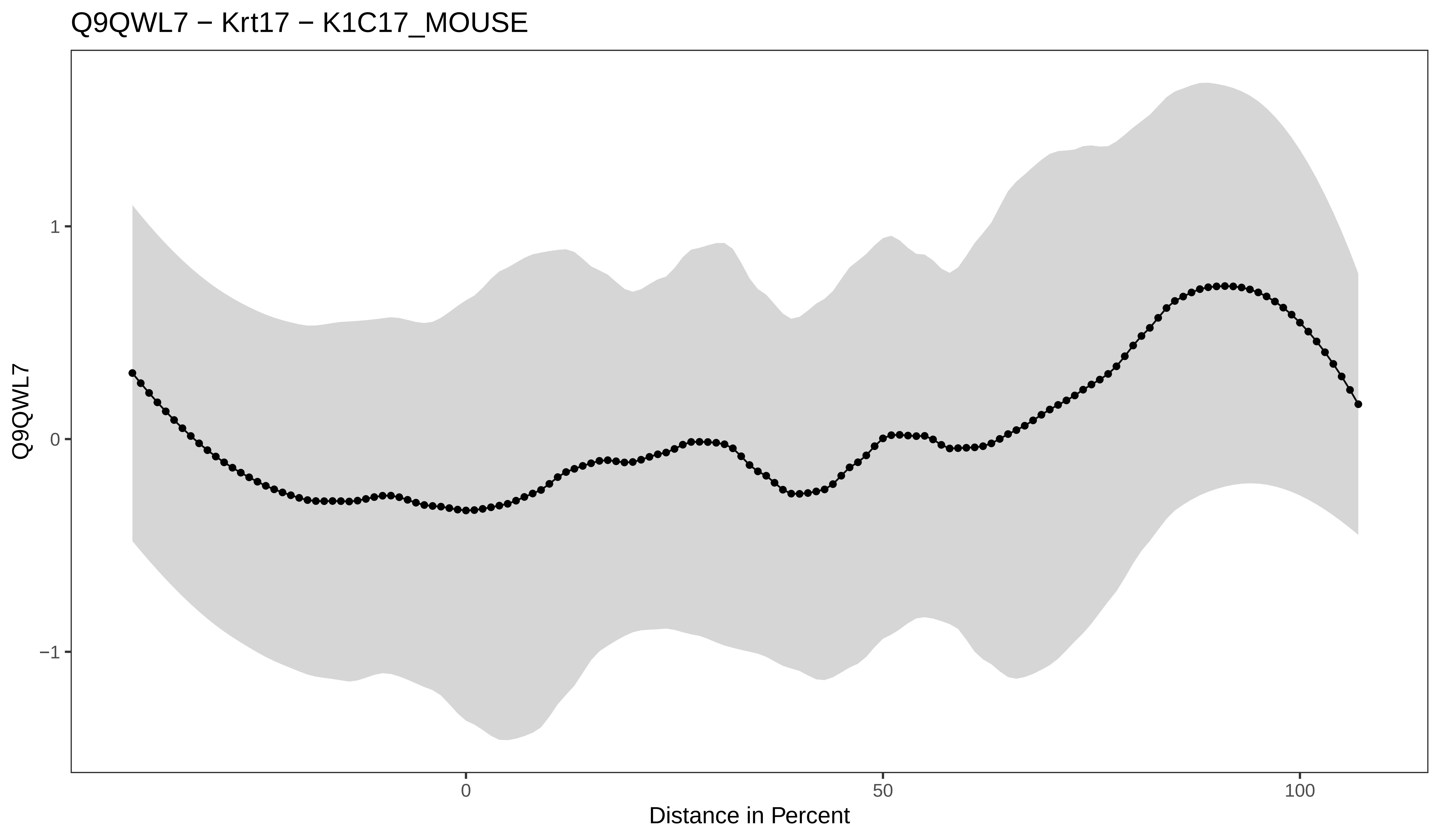

Q9QWL7 − Krt17 − K1C17_MOUSE
1
0
−1
Q9QWL7
0
50
100
Distance in Percent

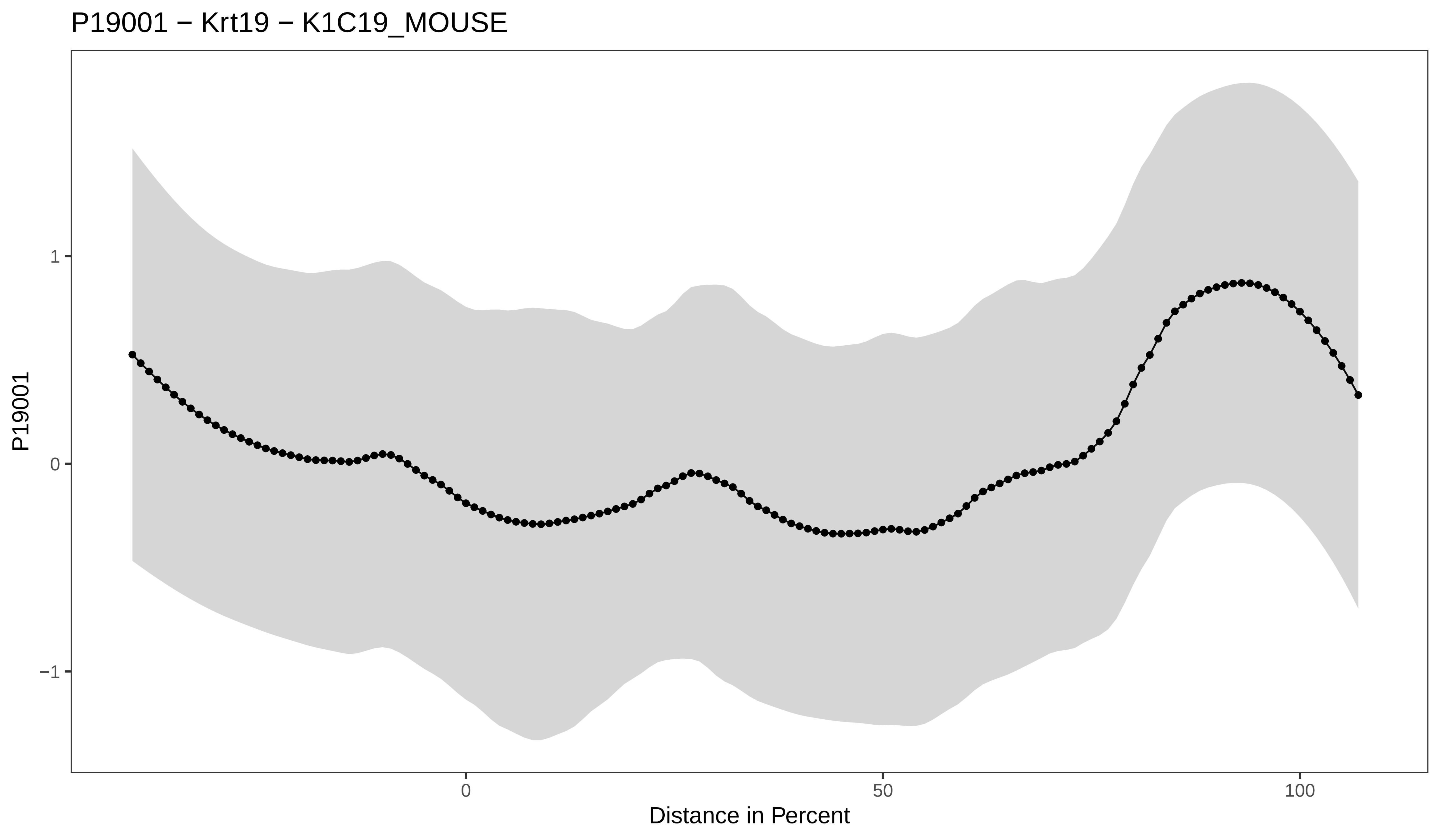
P19001 − Krt19 − K1C19_MOUSE
P19001
1
0
−1
0
50
100
Distance in Percent

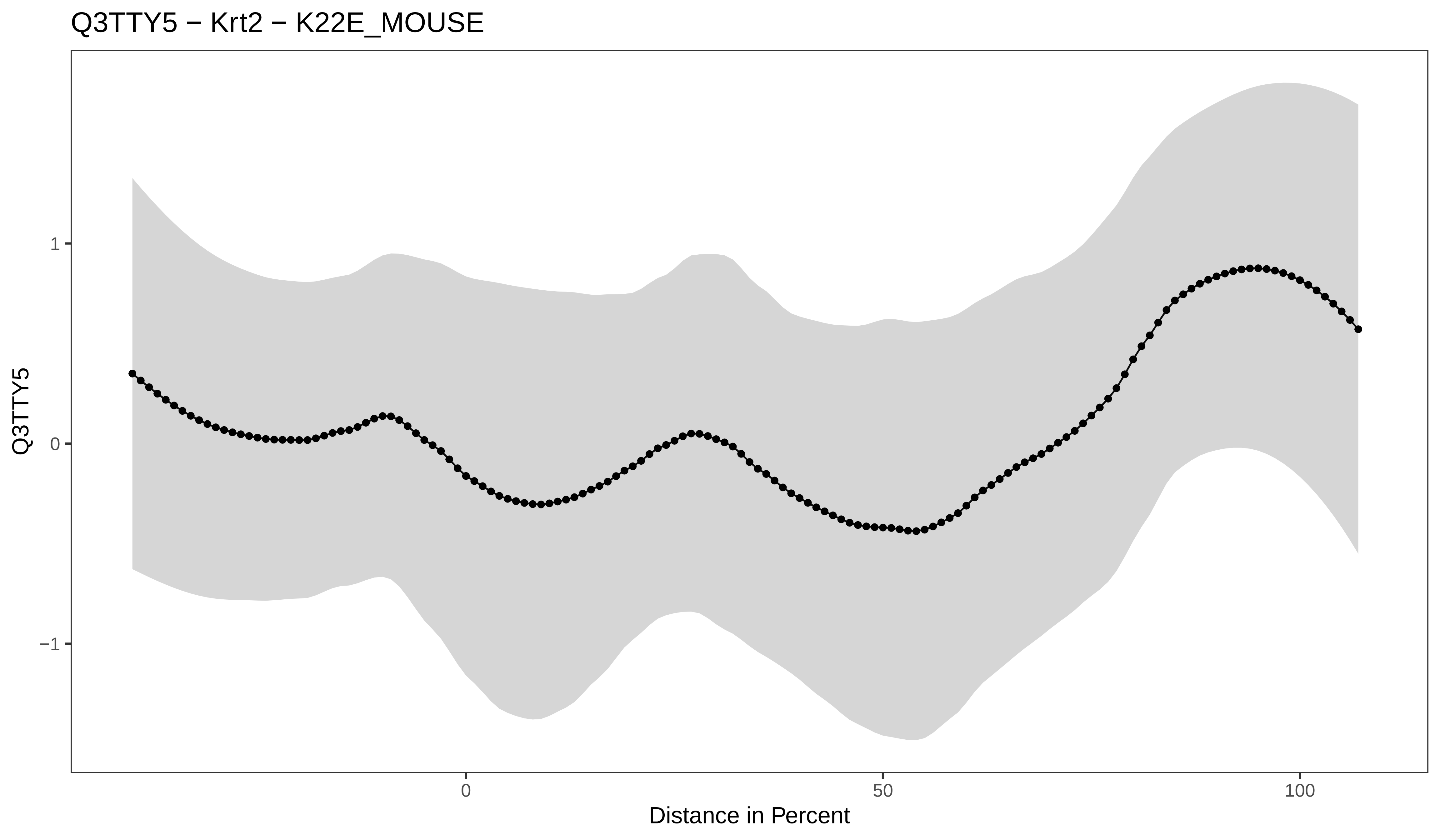

Q3TTY5 − Krt2 − K22E_MOUSE
Q3TTY5
1
0
−1
0
50
100
Distance in Percent

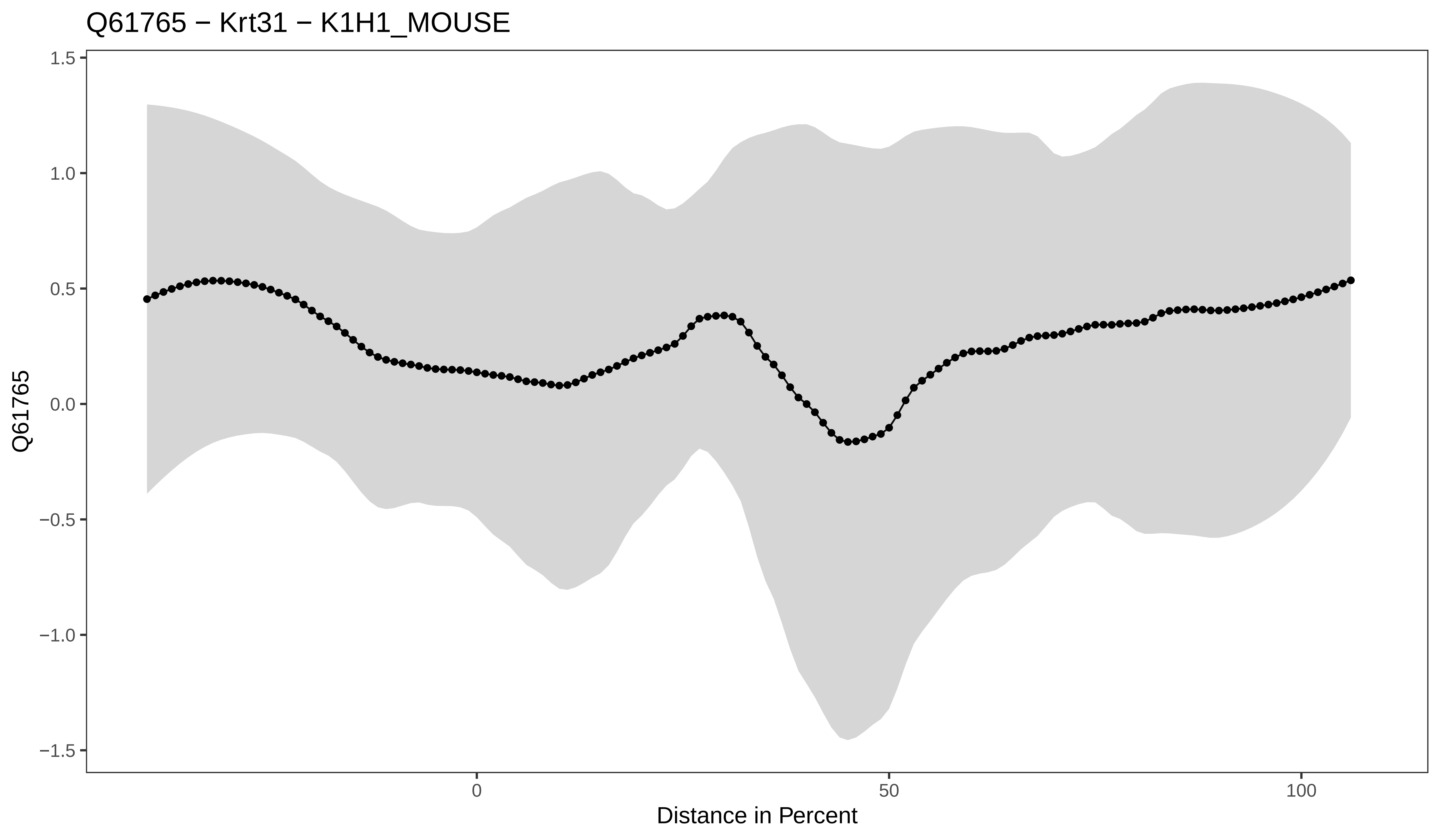
Q61765 − Krt31 − K1H1_MOUSE
1.5
1.0
0.5
0.0
−0.5
−1.0
−1.5
Q61765
0
50
100
Distance in Percent

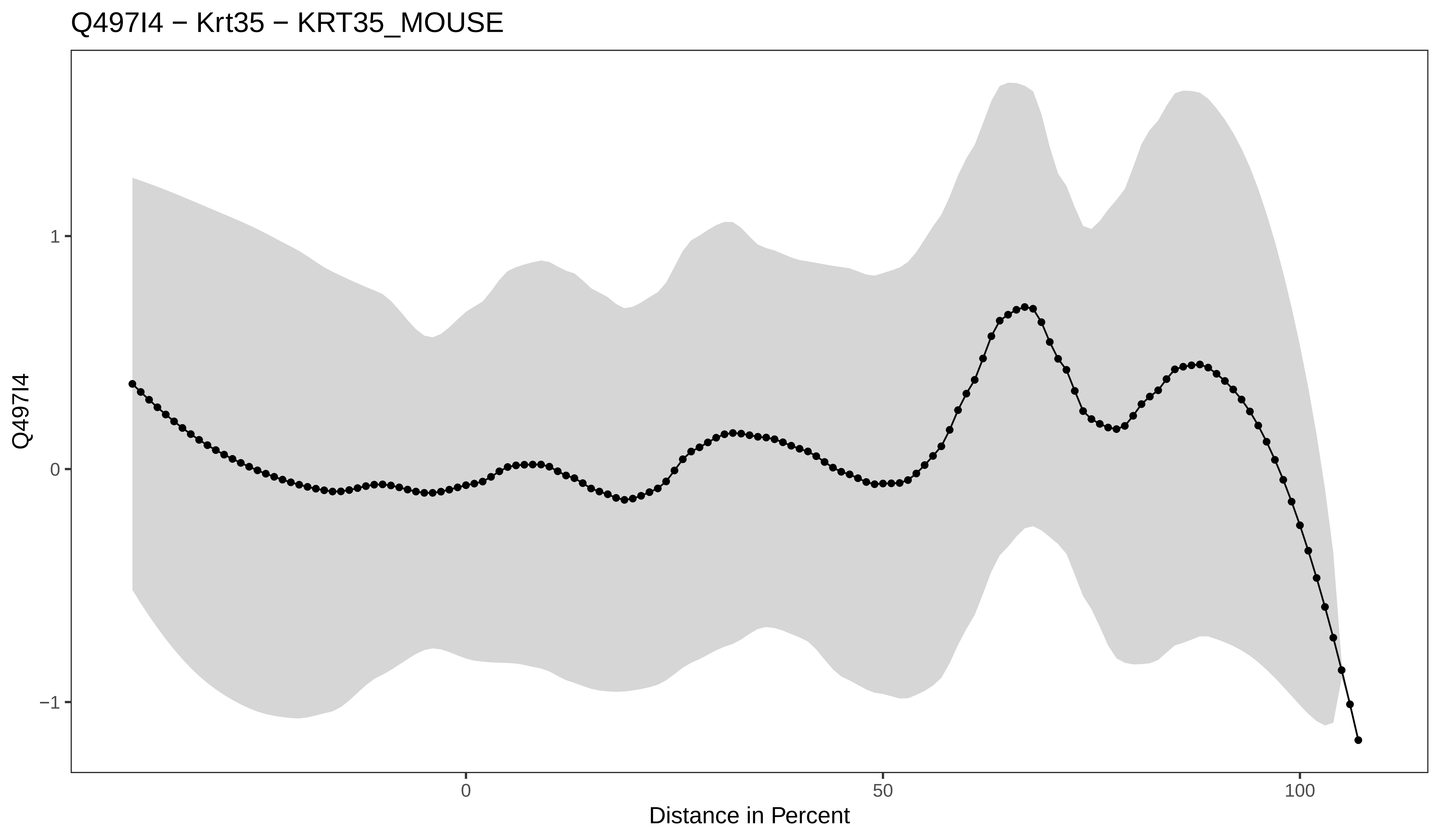

Q497I4 − Krt35 − KRT35_MOUSE
Q497I4
1
0
−1
0
50
100
Distance in Percent

B1AQ75 − Krt36 − KRT36_MOUSE

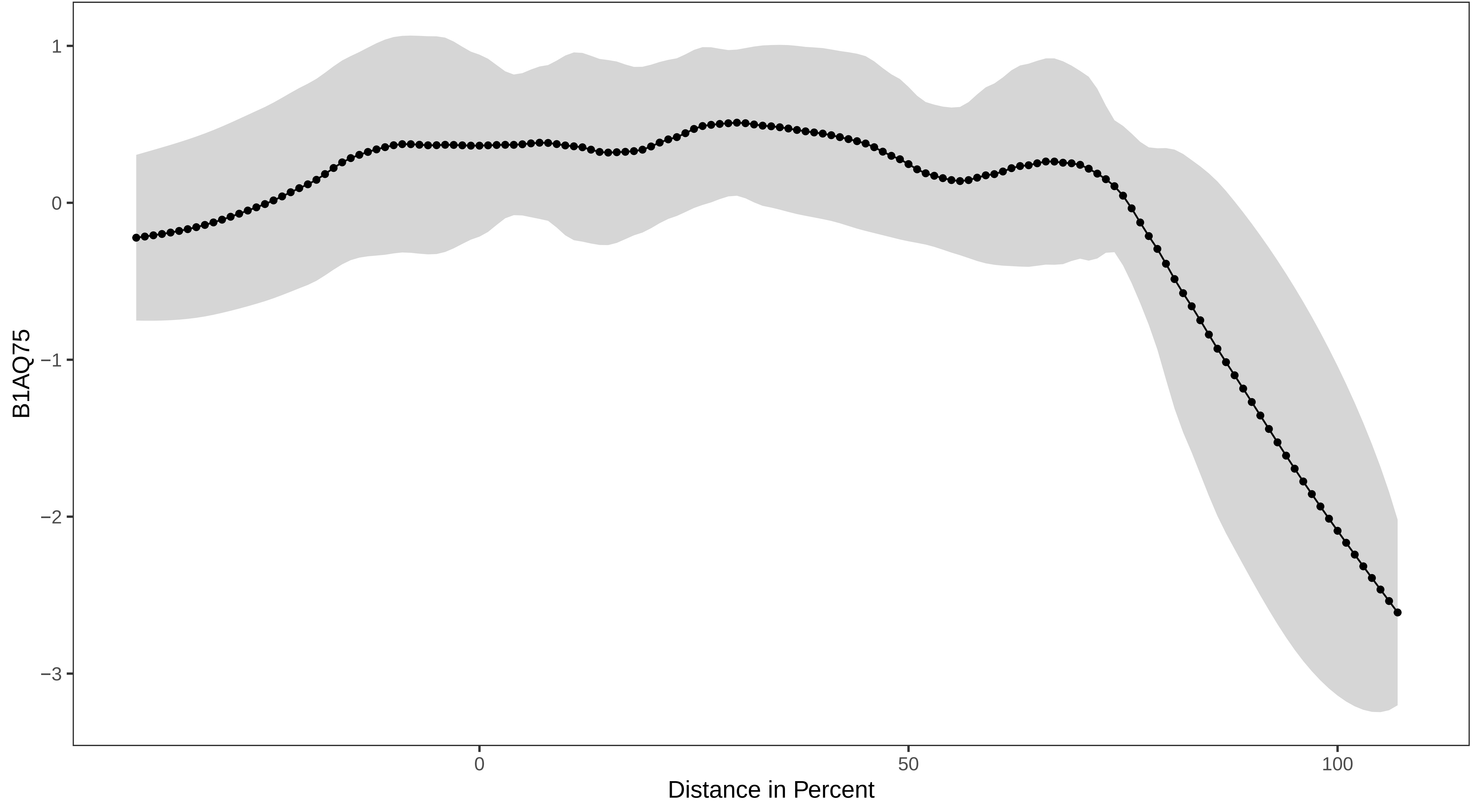

Q6IFX2 − Krt42 − K1C42_MOUSE

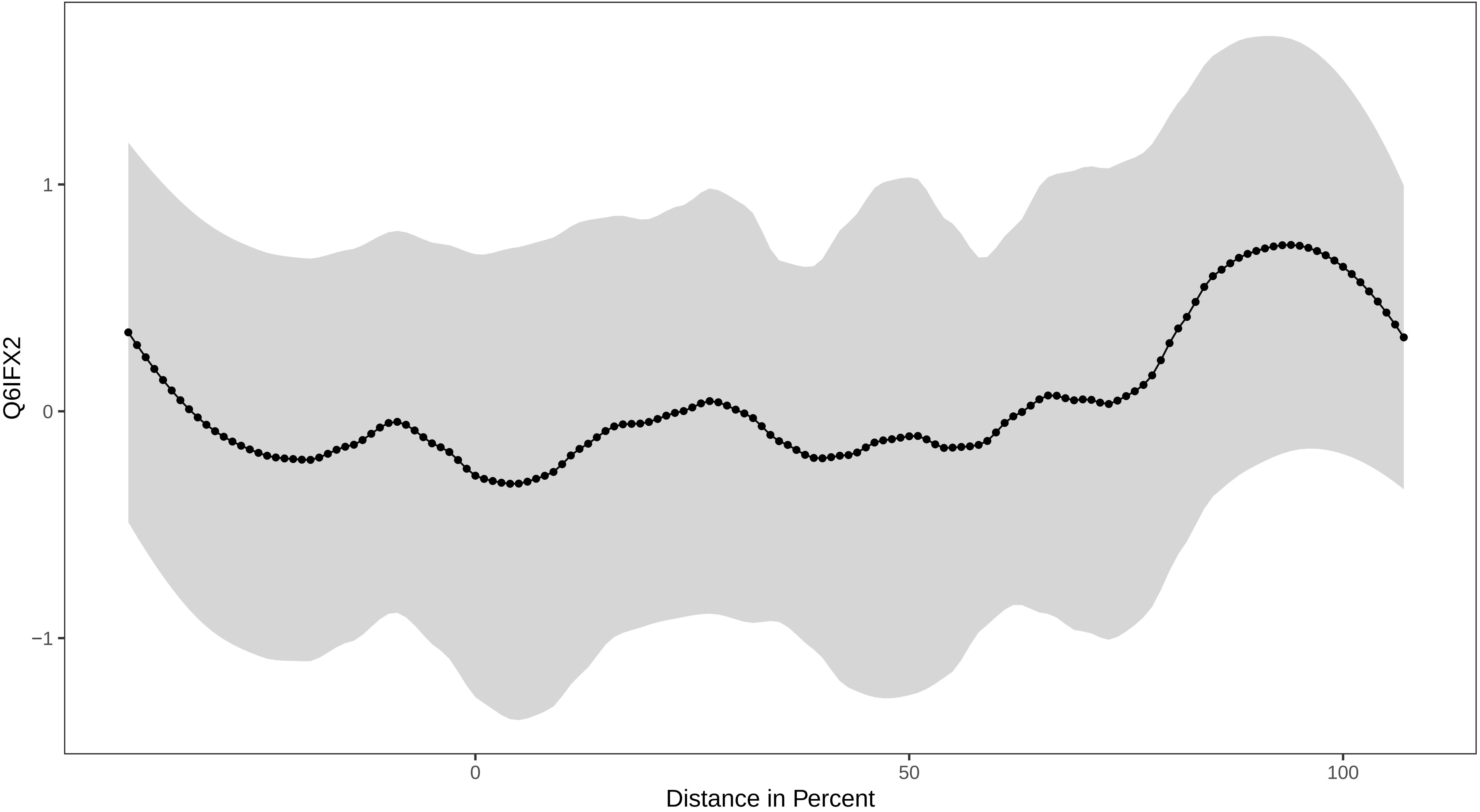

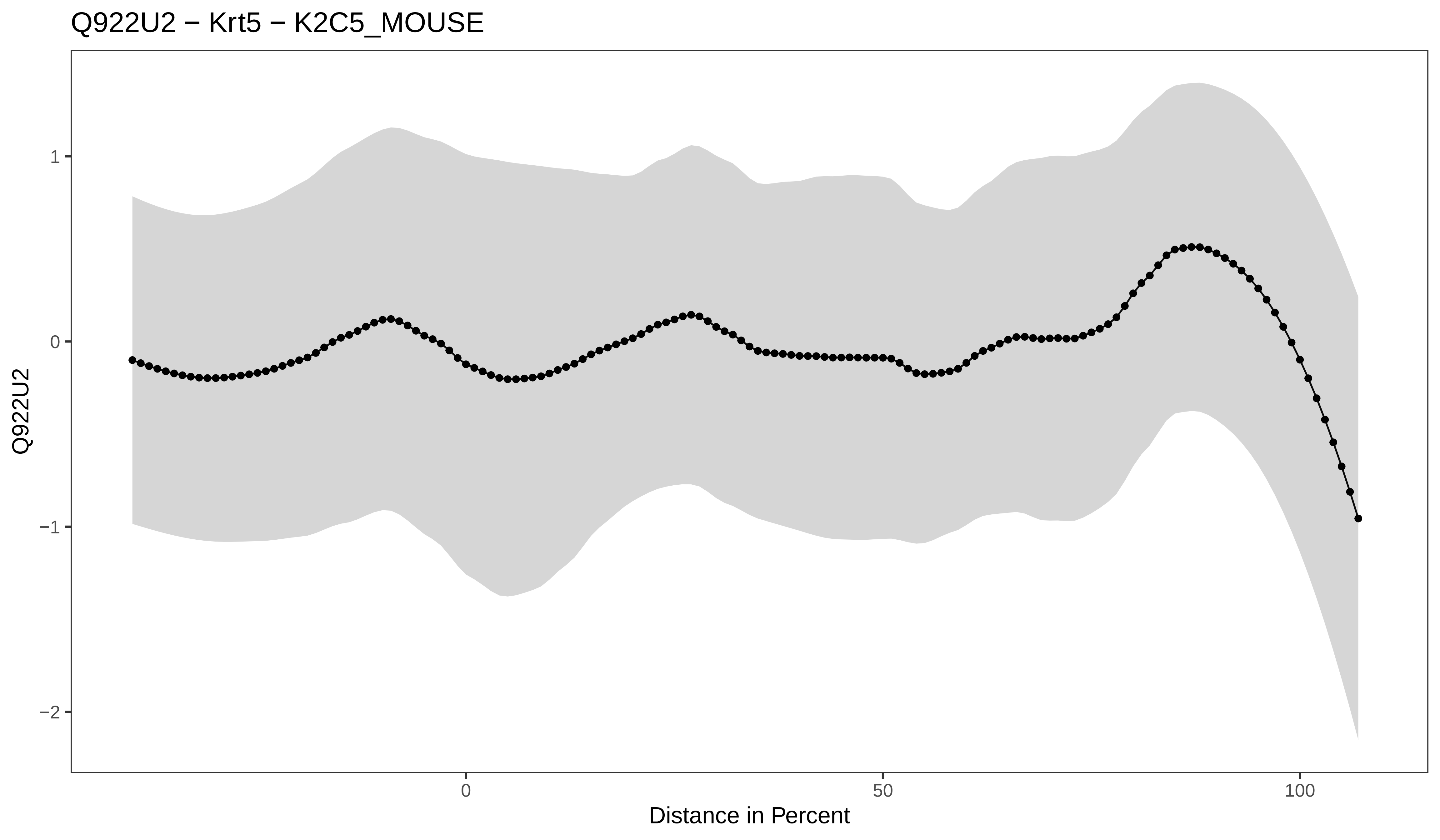

Q922U2 − Krt5 − K2C5_MOUSE
Q922U2
1
0
−1
−2
0
50
100
Distance in Percent

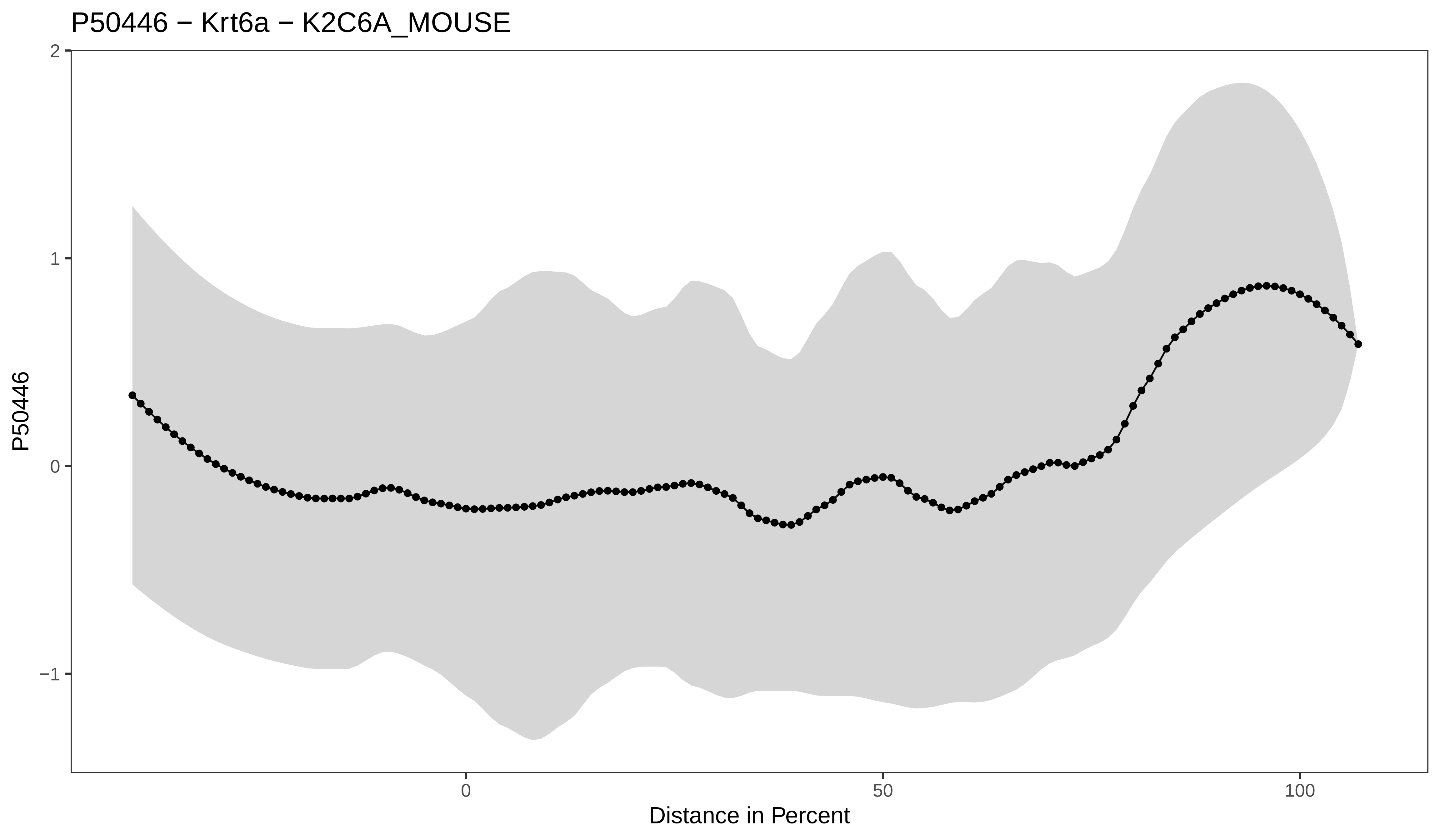

P50446 − Krt6a − K2C6A_MOUSE
P50446
2
1
0
−1
0
50
100
Distance in Percent

Q9DCV7 − Krt7 − K2C7_MOUSE

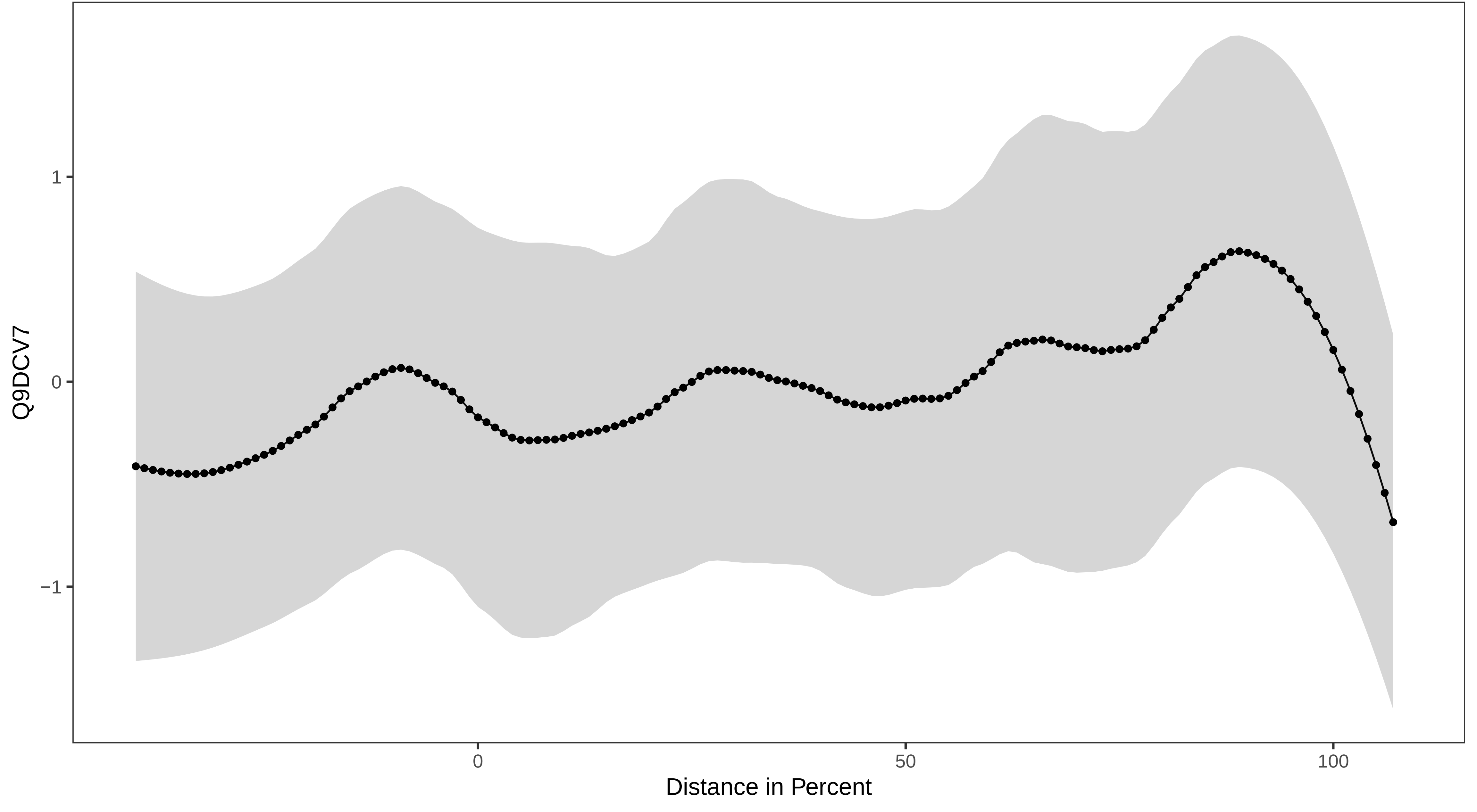

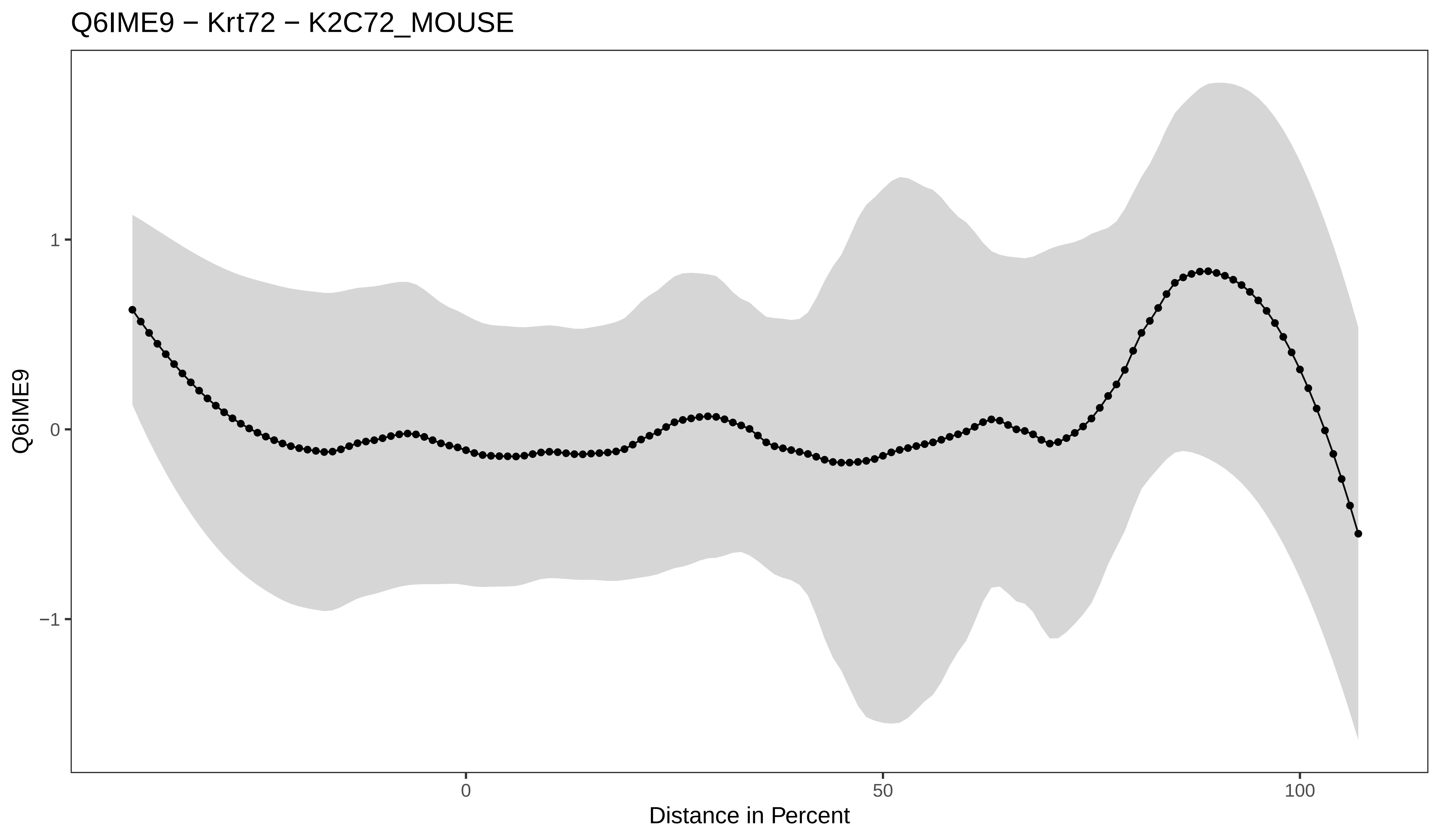
Q6IME9 − Krt72 − K2C72_MOUSE
Q6IME9
1
0
−1
0
50
100
Distance in Percent

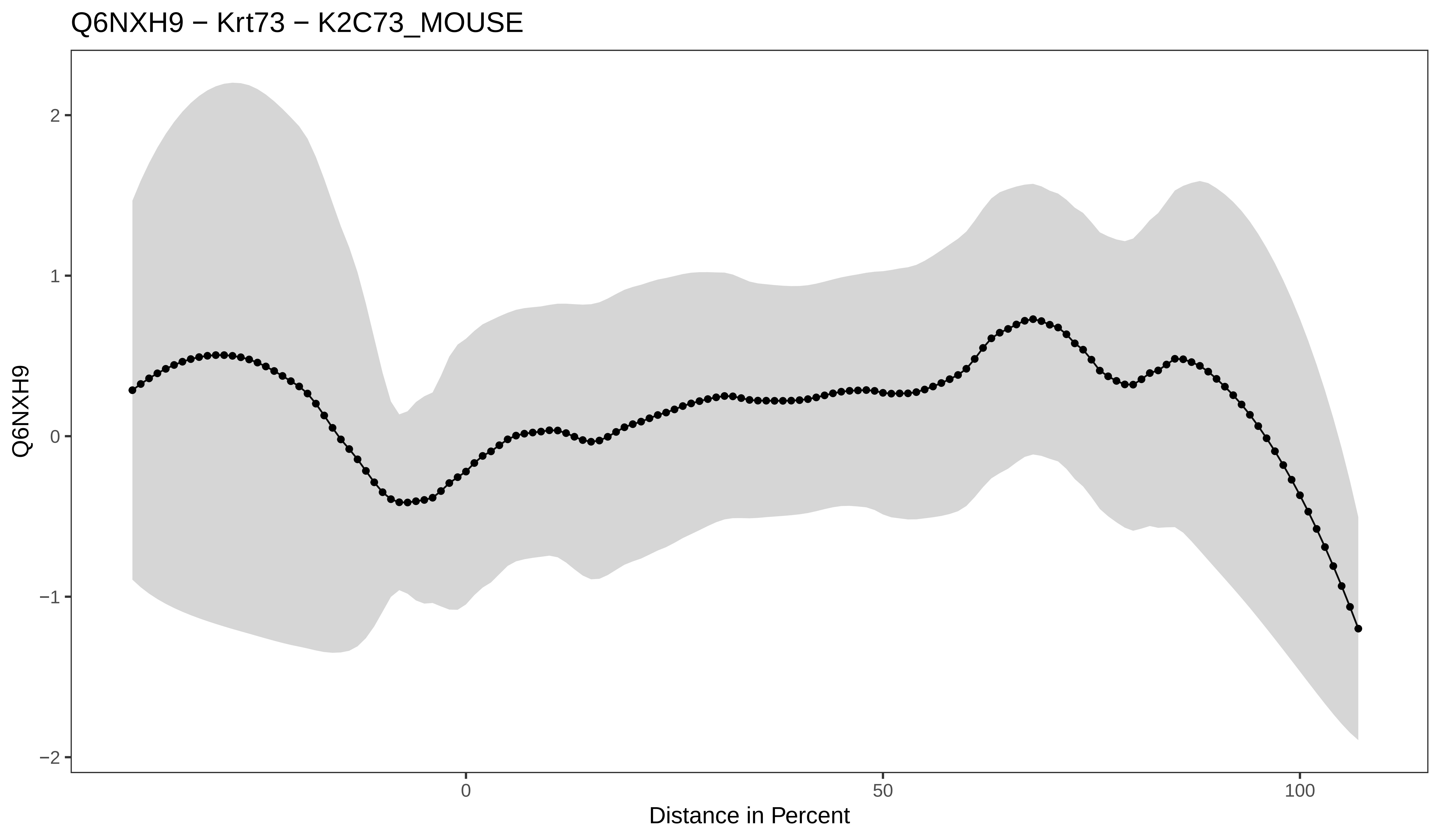

Q6NXH9 − Krt73 − K2C73_MOUSE
Q6NXH9
2
1
0
−1
−2
0
50
100
Distance in Percent

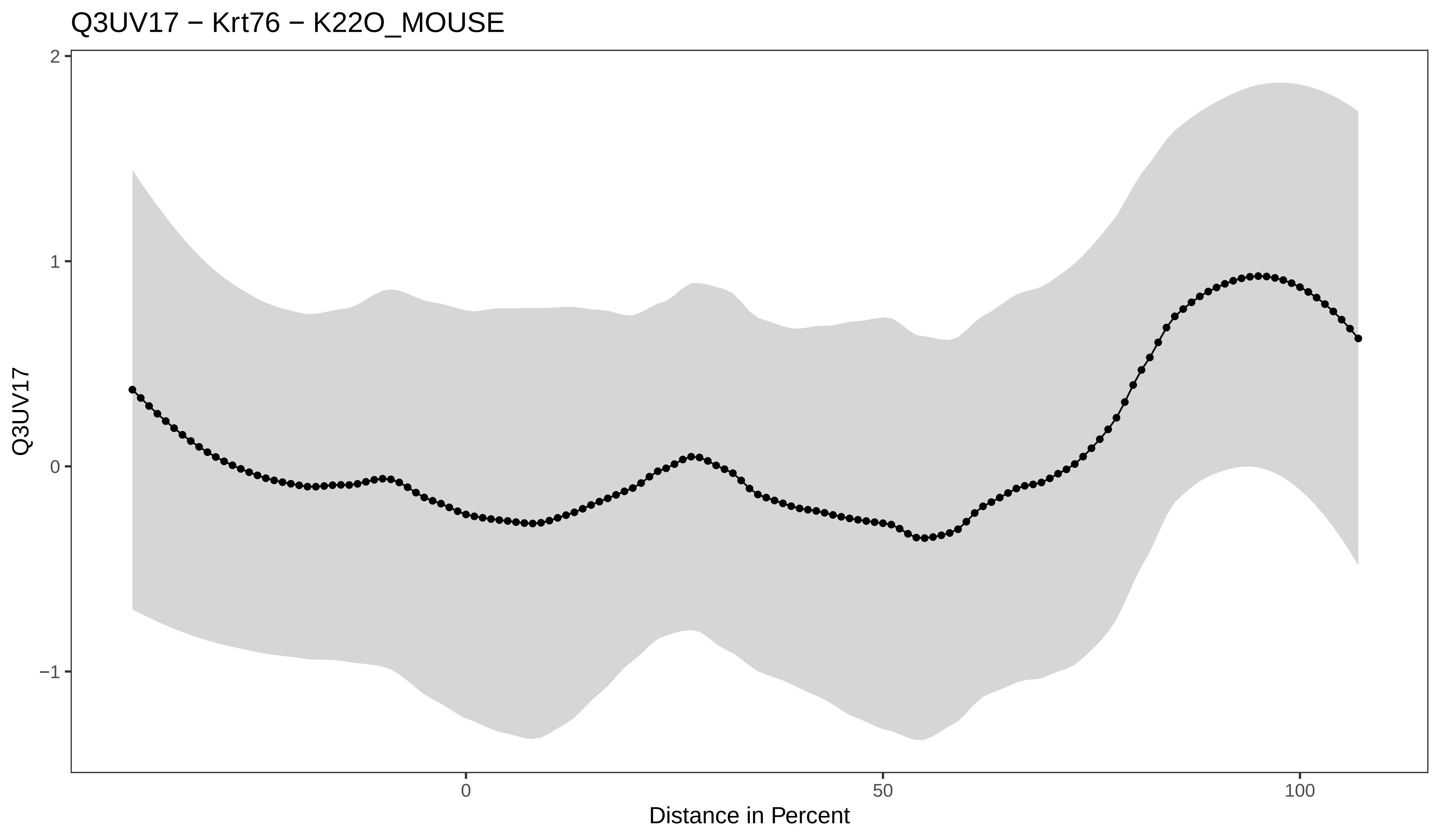

Q3UV17 − Krt76 − K22O_MOUSE
2
1
0
−1
Q3UV17
0
50
100
Distance in Percent

Q6IFZ6 − Krt77 − K2C1B_MOUSE

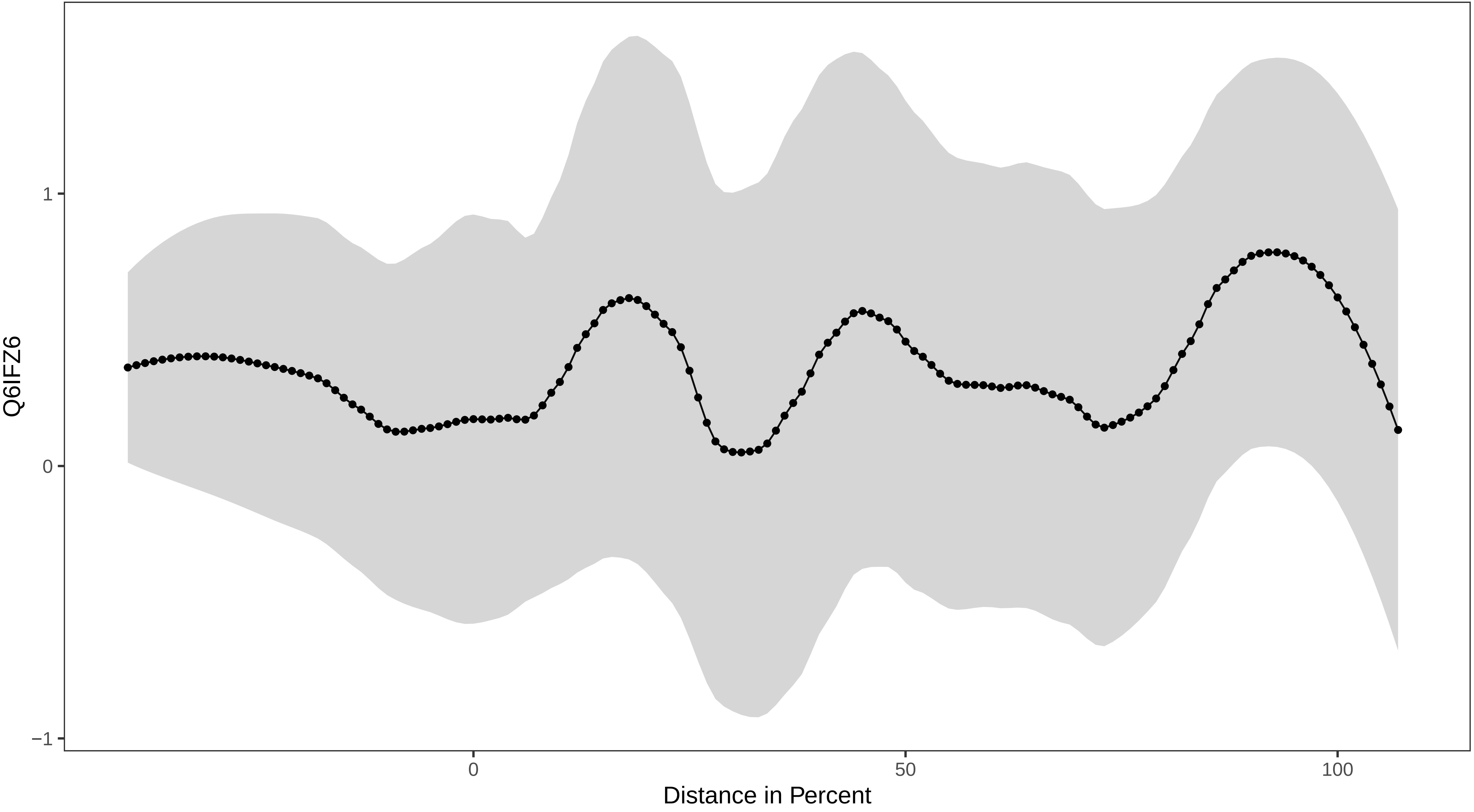

Q8VED5 − Krt79 − K2C79_MOUSE

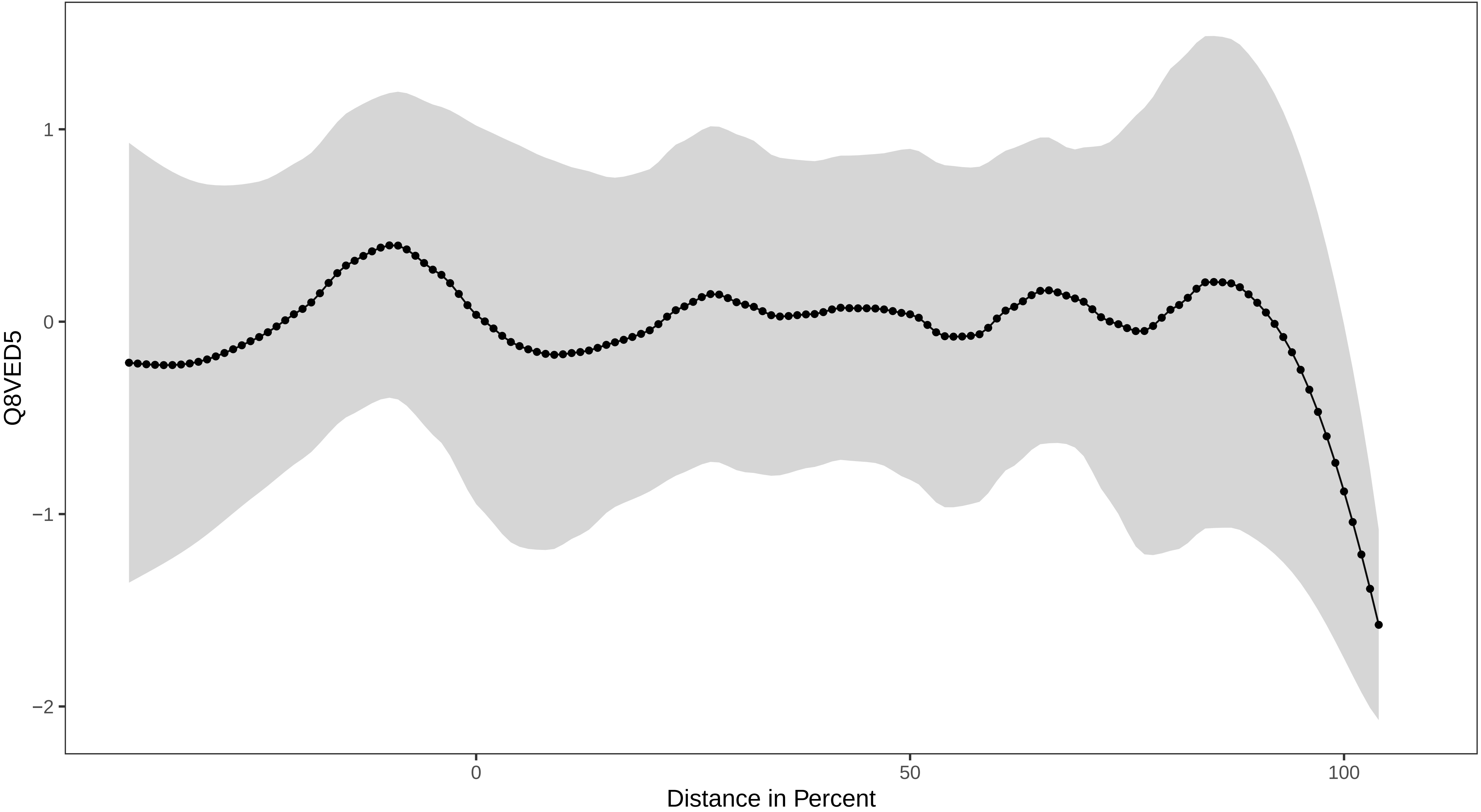

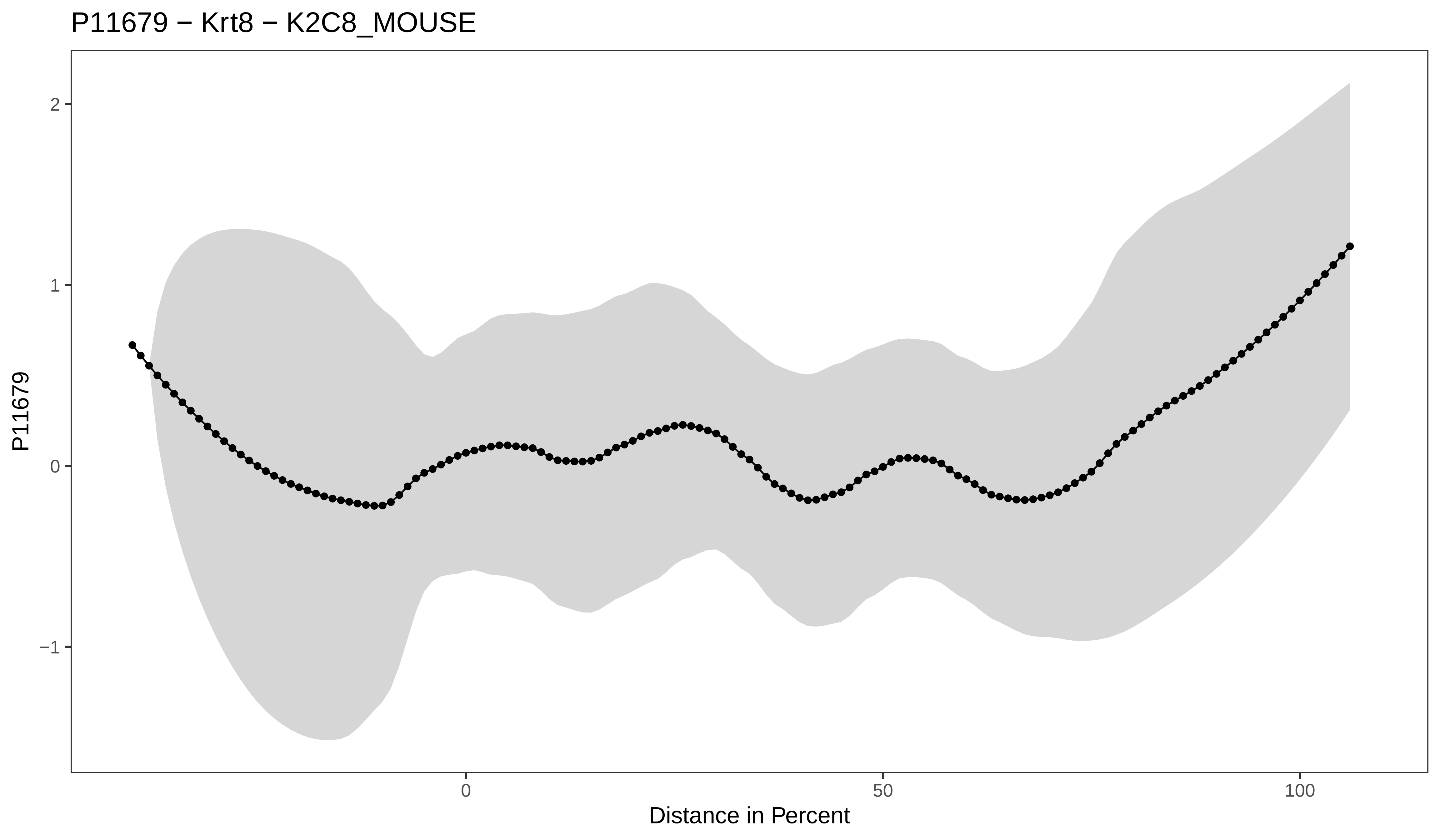

P11679 − Krt8 − K2C8_MOUSE
P11679
2
1
0
−1
0
50
100
Distance in Percent

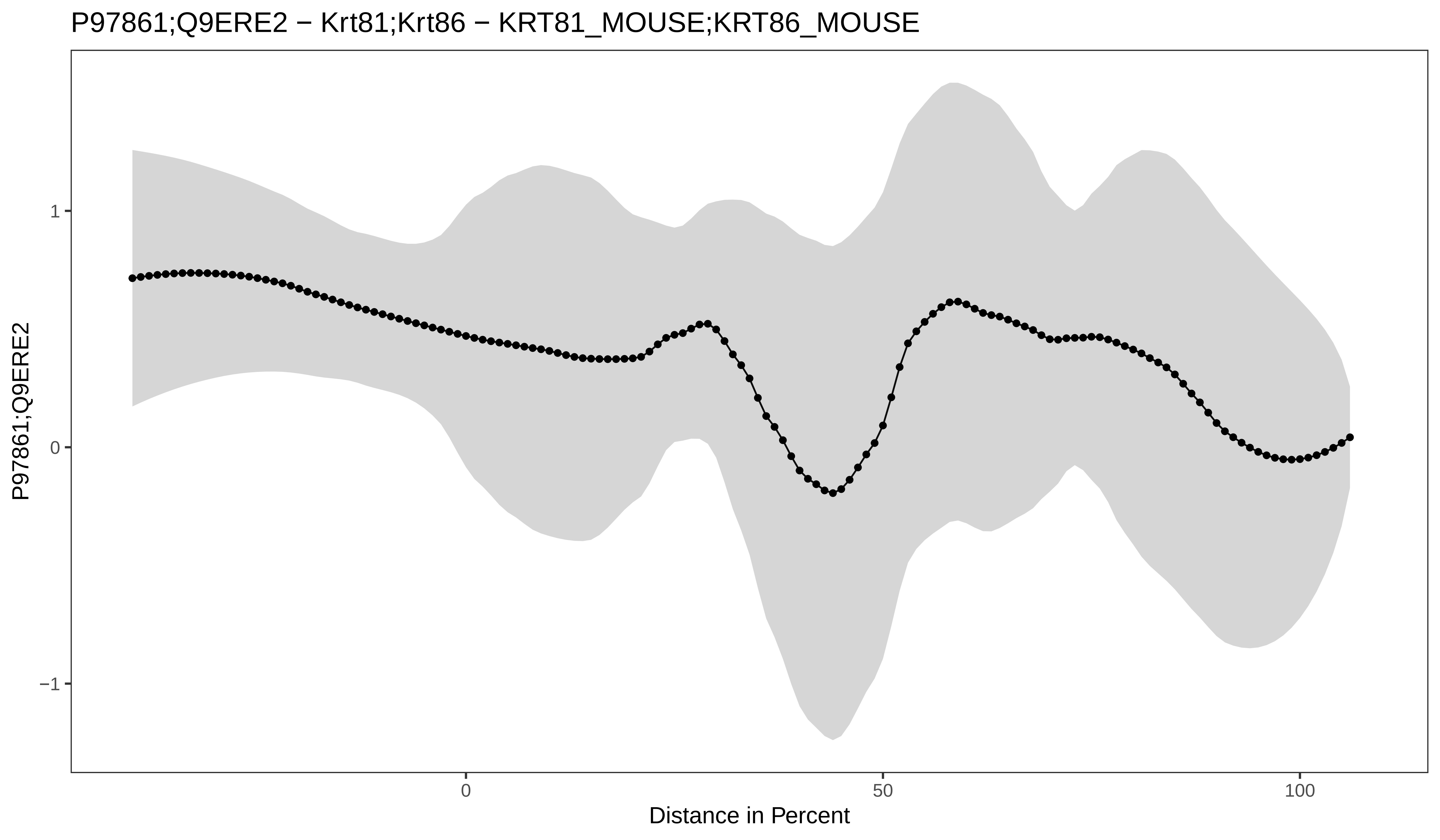
P97861;Q9ERE2 − Krt81;Krt86 − KRT81_MOUSE;KRT86_MOUSE
P97861;Q9ERE2
1
0
−1
0
50
100
Distance in Percent

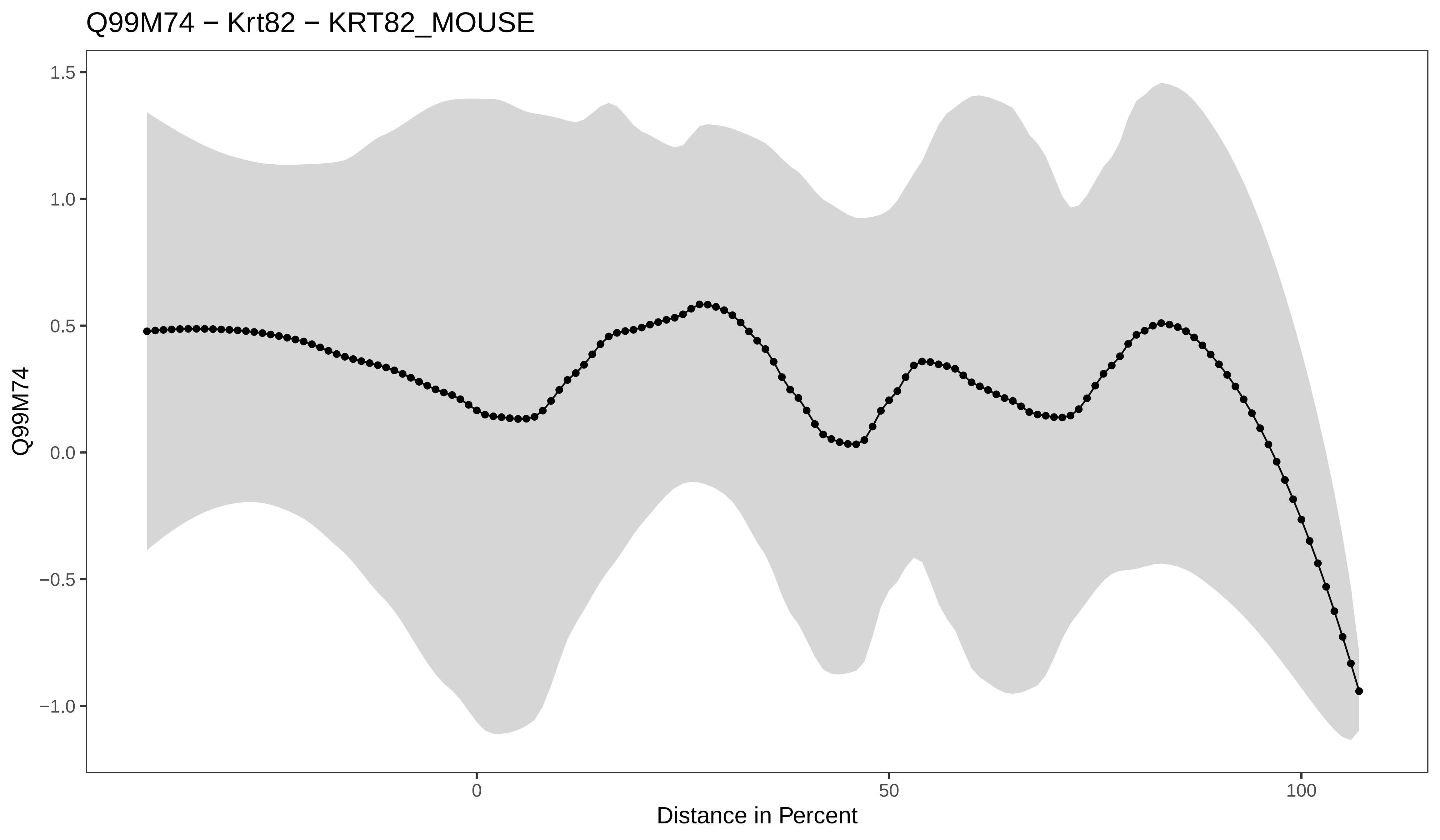
Q99M74 − Krt82 − KRT82_MOUSE
Q99M74
Distance in Percent
1.5
1.0
0.5
0.0
−0.5
−1.0
0
50
100

Q9Z2T6 − Krt85 − KRT85_MOUSE

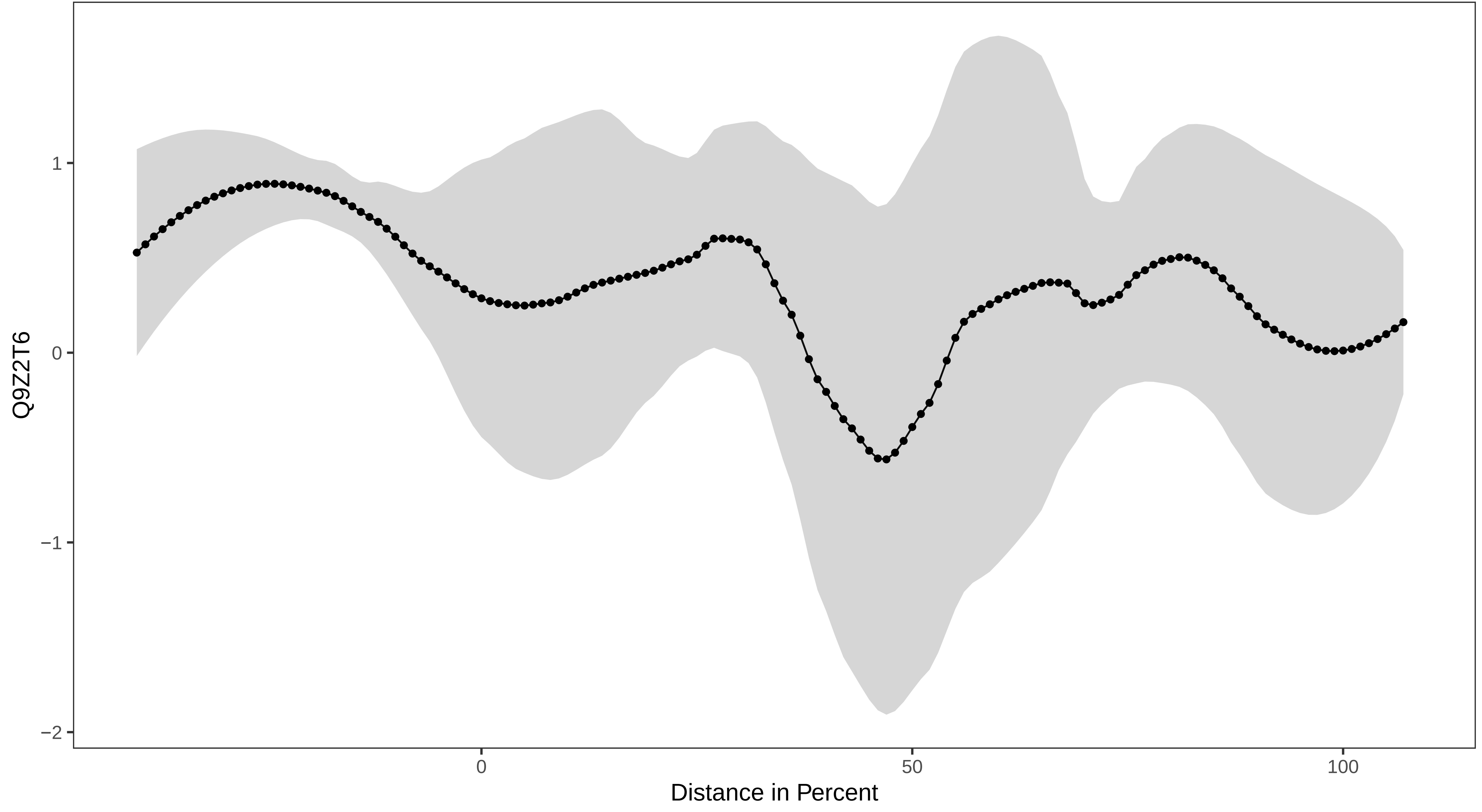

Q61595 − Ktn1 − KTN1_MOUSE

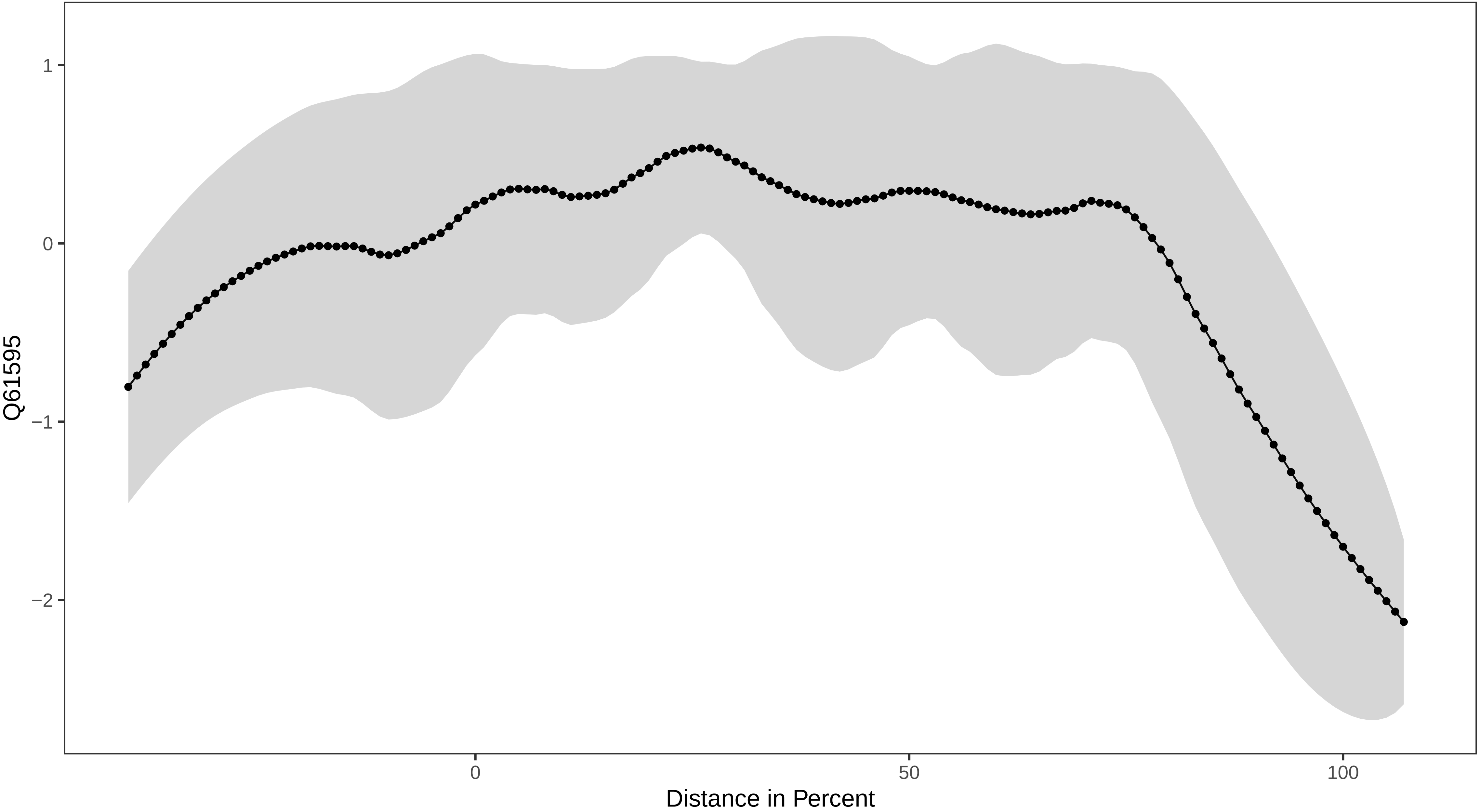

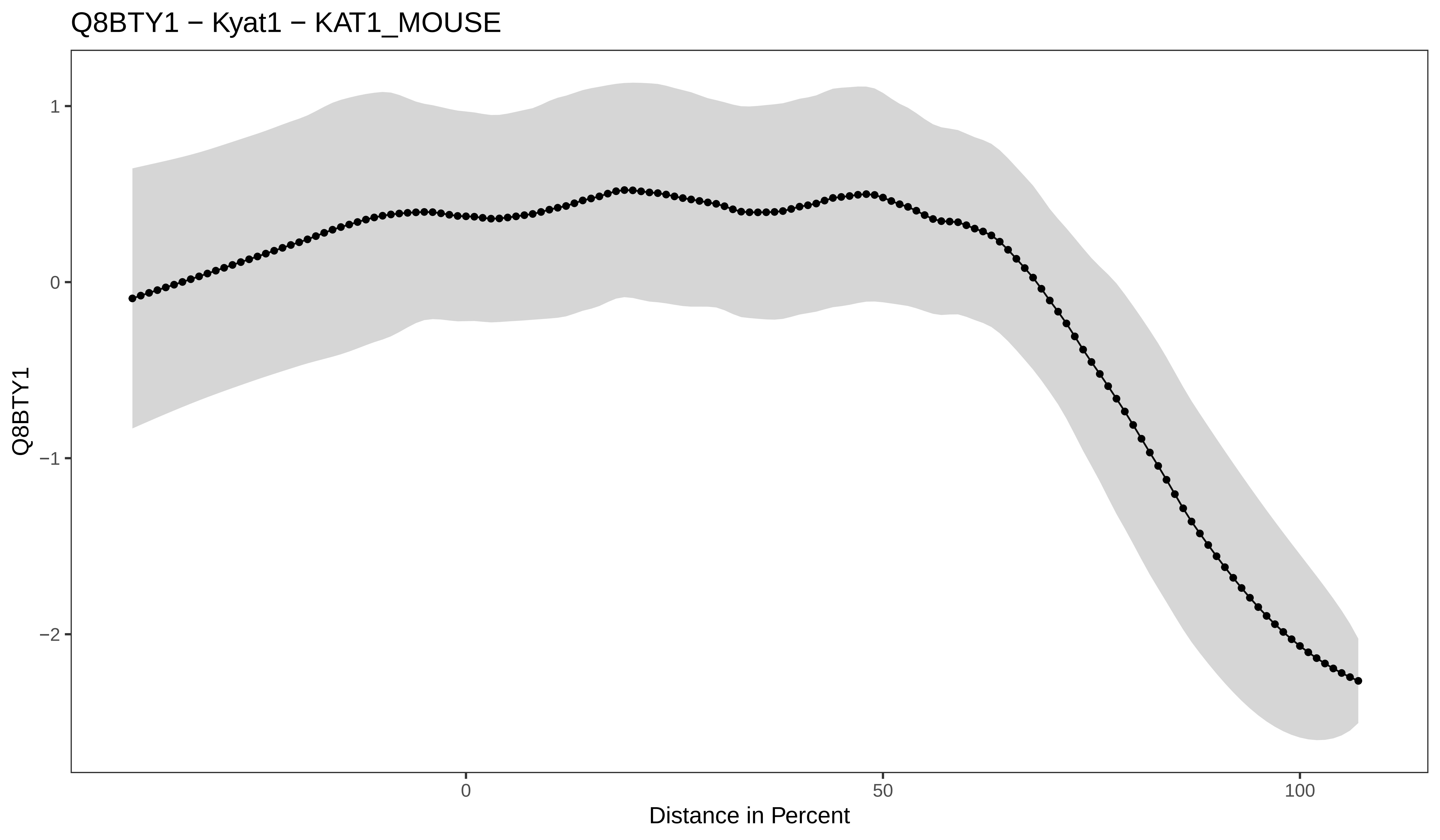

Q8BTY1 − Kyat1 − KAT1_MOUSE
1
0
−1
−2
Q8BTY1
0
50
100
Distance in Percent

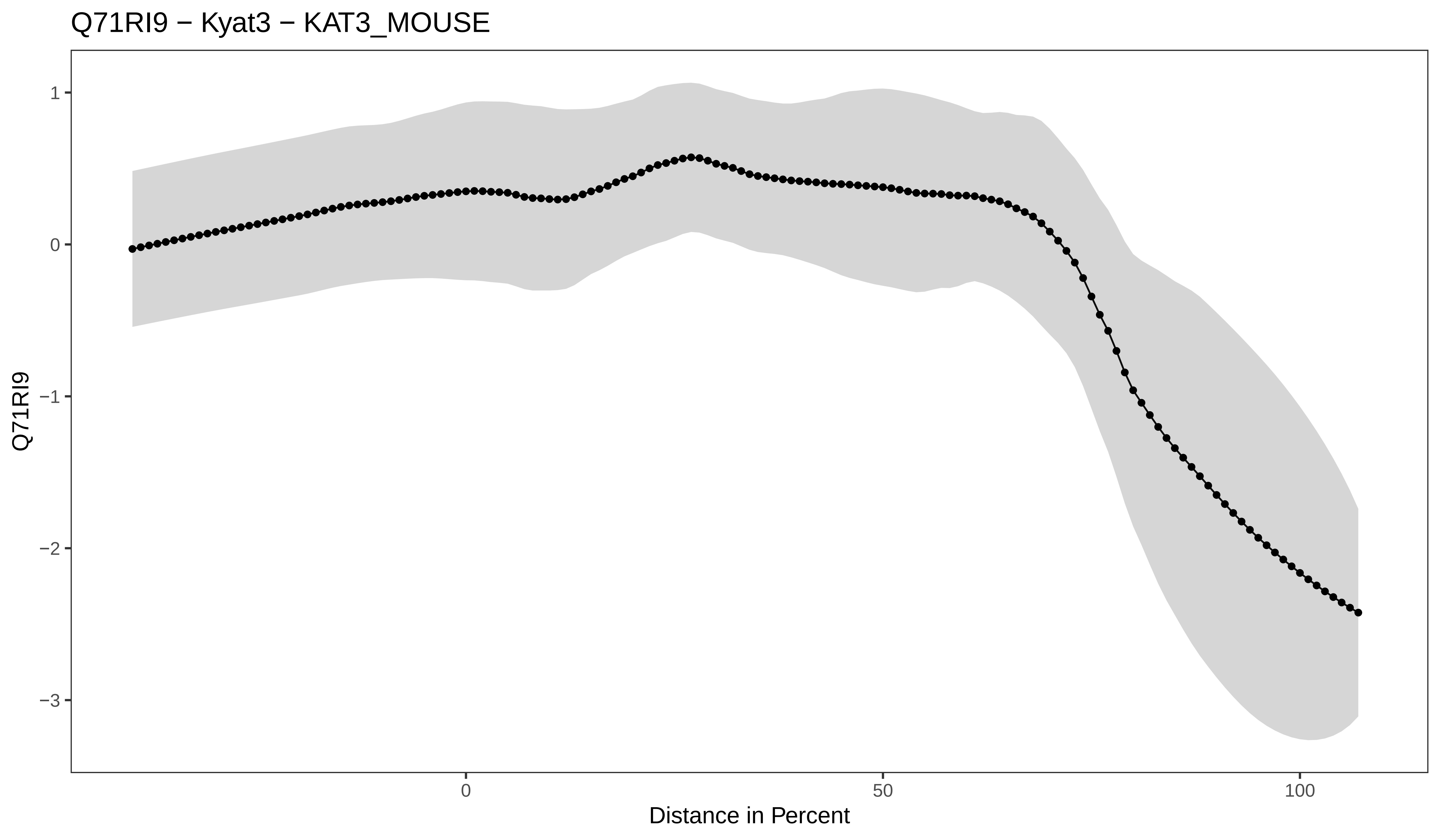

Q71RI9 − Kyat3 − KAT3_MOUSE
1
0
−1
−2
−3
Q71RI9
0
50
100
Distance in Percent

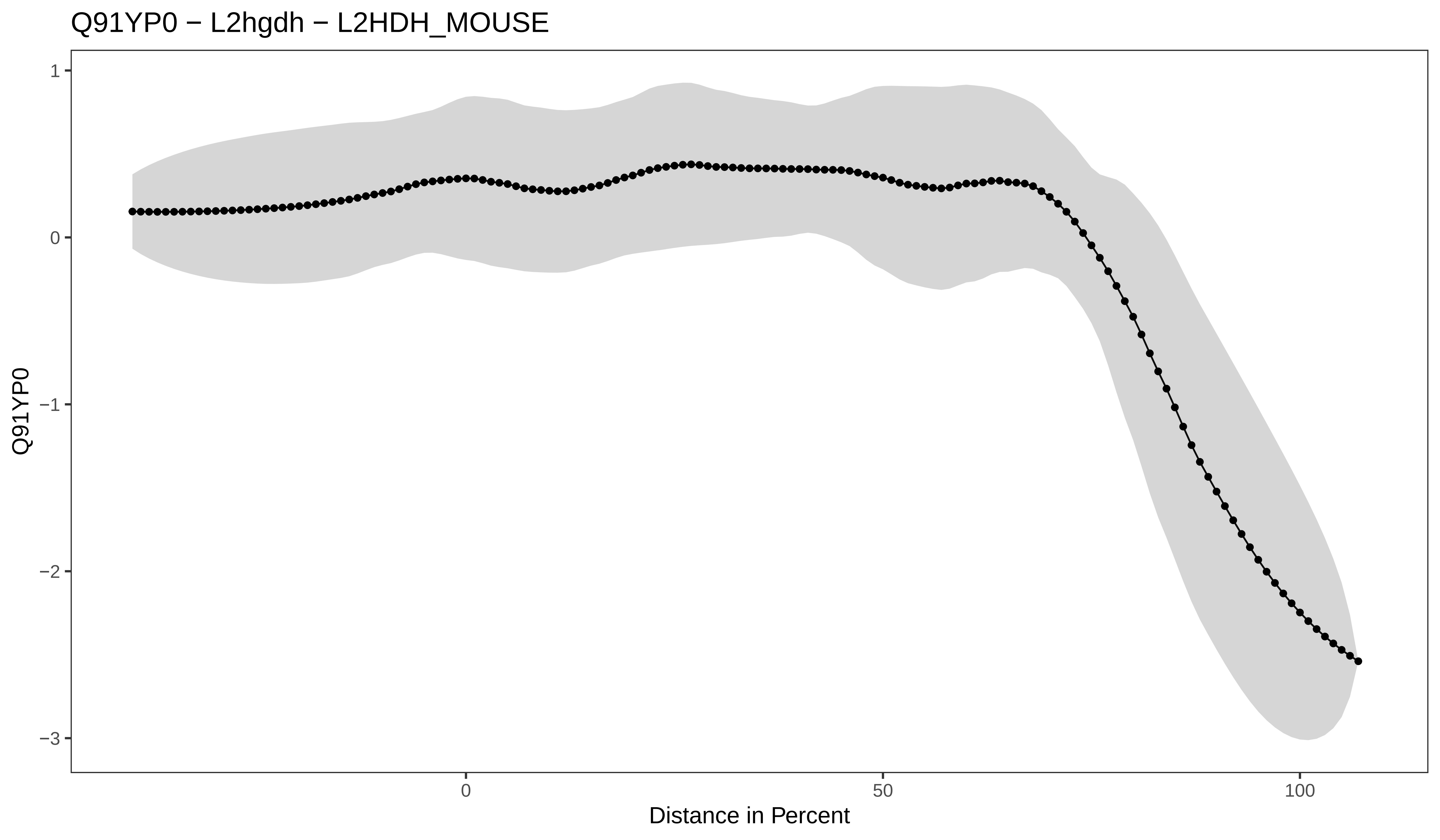
Q91YP0 − L2hgdh − L2HDH_MOUSE
Q91YP0
1
0
−1
−2
−3
0
50
100
Distance in Percent

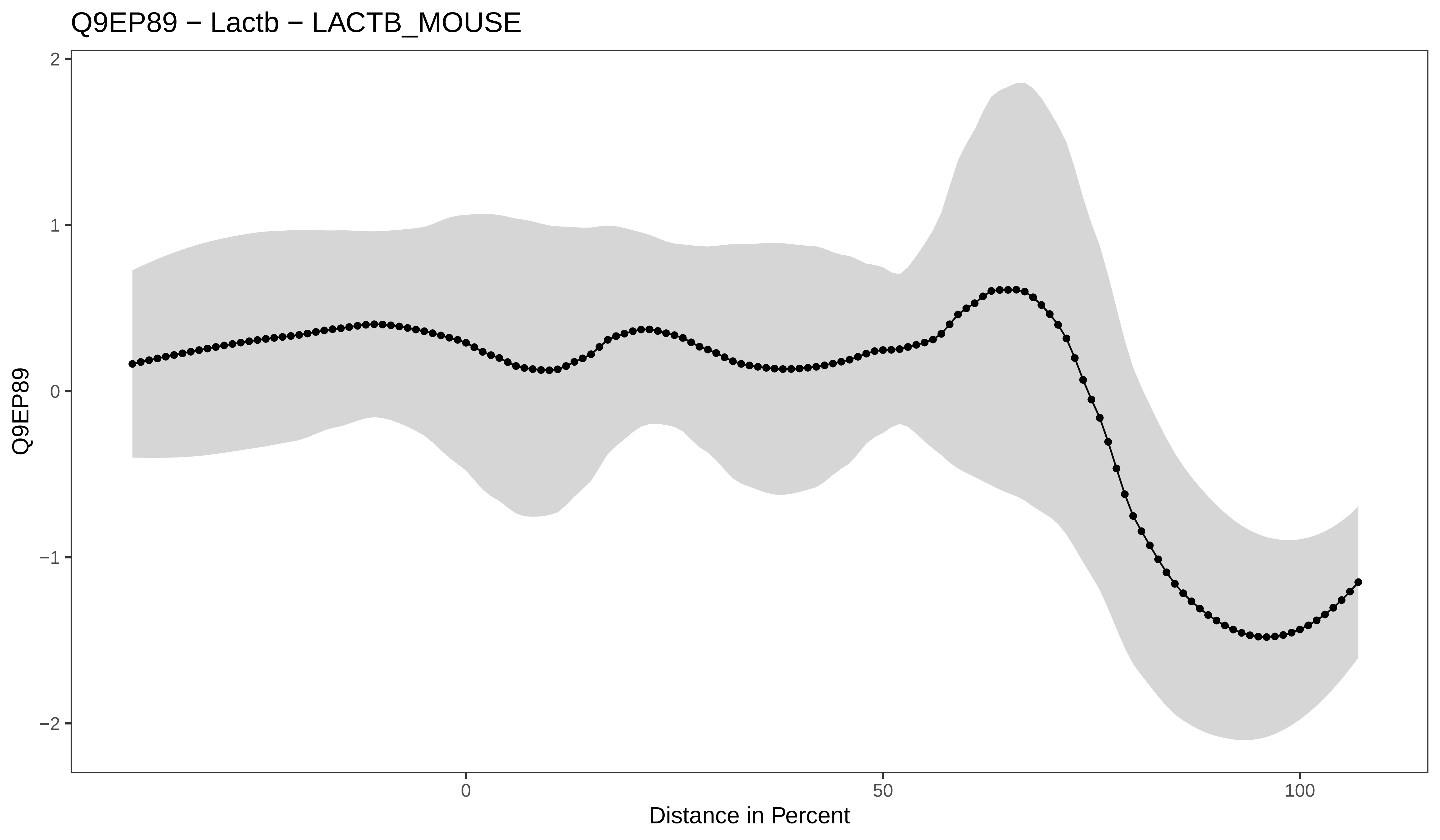

Q9EP89 − Lactb − LACTB_MOUSE
2
1
0
−1
−2
Q9EP89
0
50
100
Distance in Percent

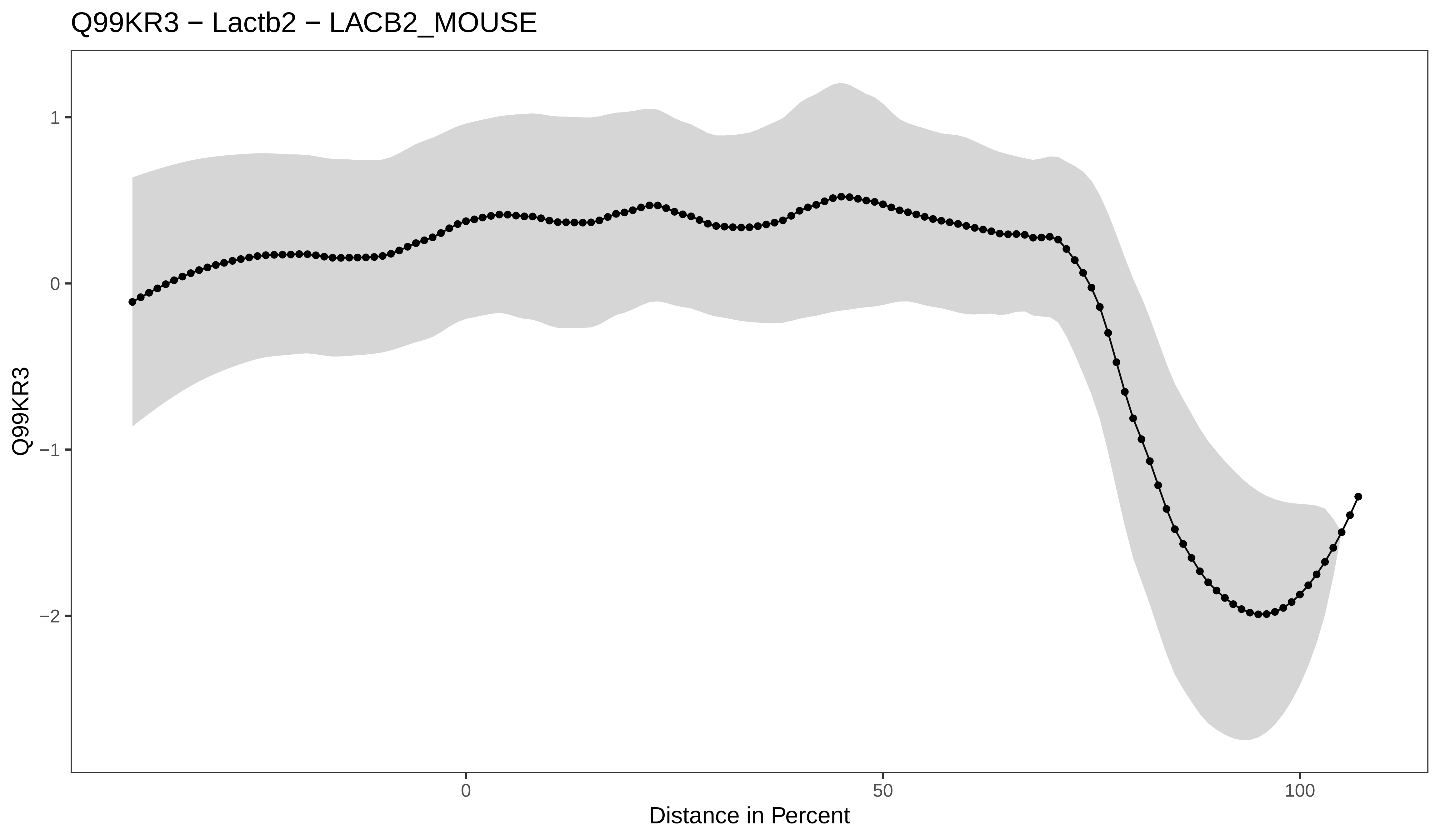
Q99KR3 − Lactb2 − LACB2_MOUSE
Q99KR3
1
0
−1
−2
0
50
100
Distance in Percent

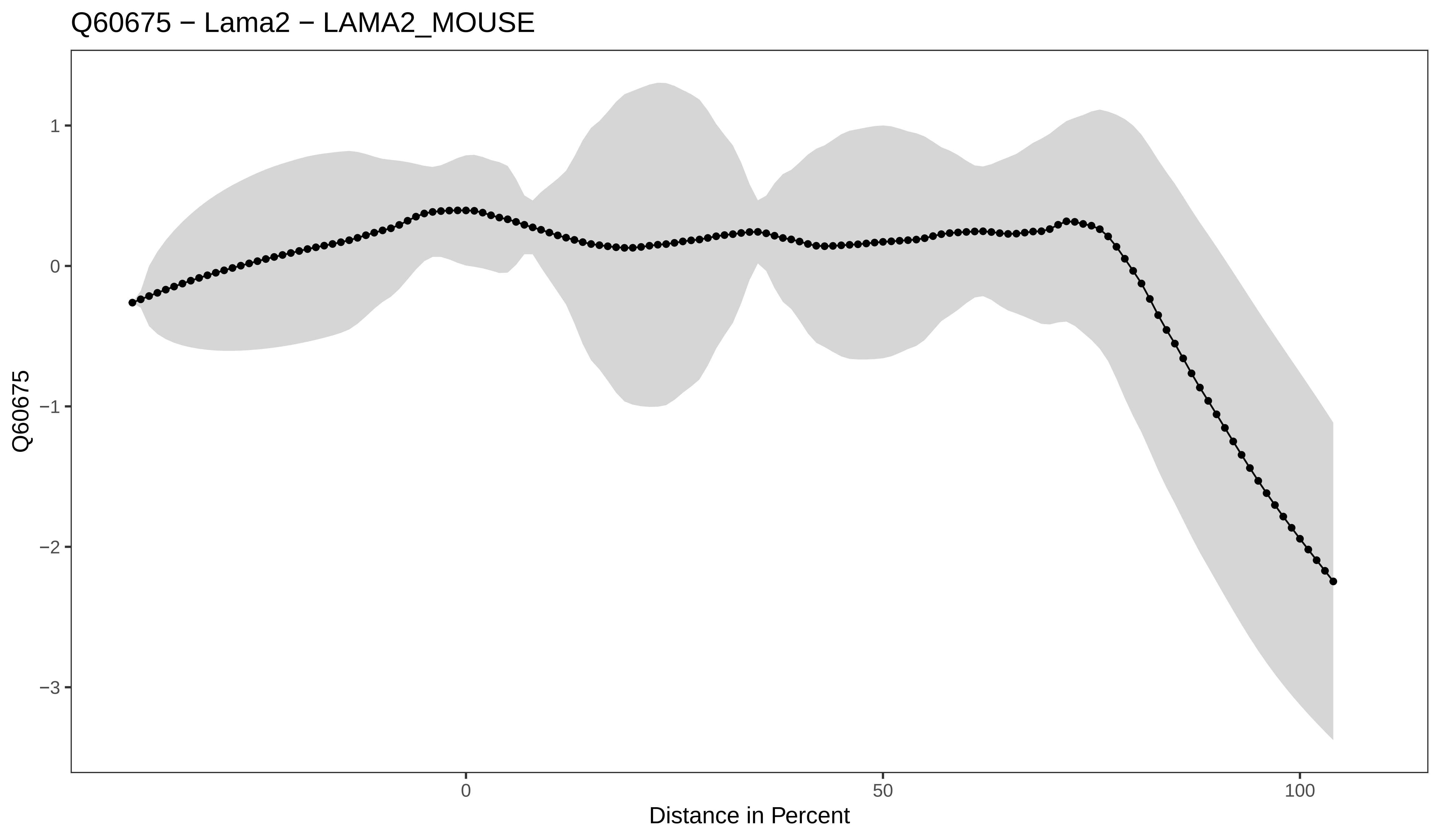
Q60675 − Lama2 − LAMA2_MOUSE
1
0
−1
−2
−3
Q60675
0
50
100
Distance in Percent

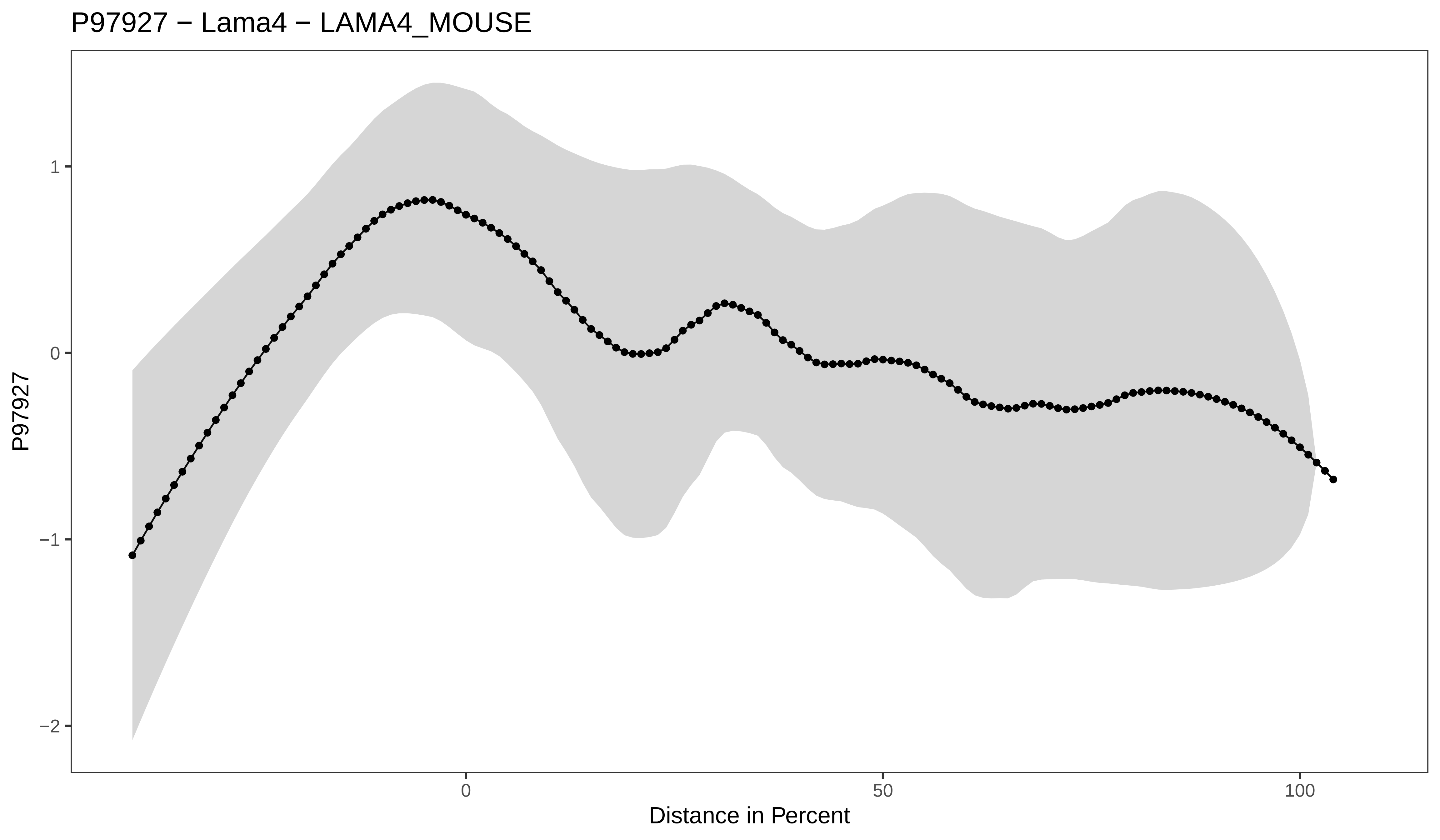
P97927 − Lama4 − LAMA4_MOUSE
P97927
1
0
−1
−2
0
50
100
Distance in Percent

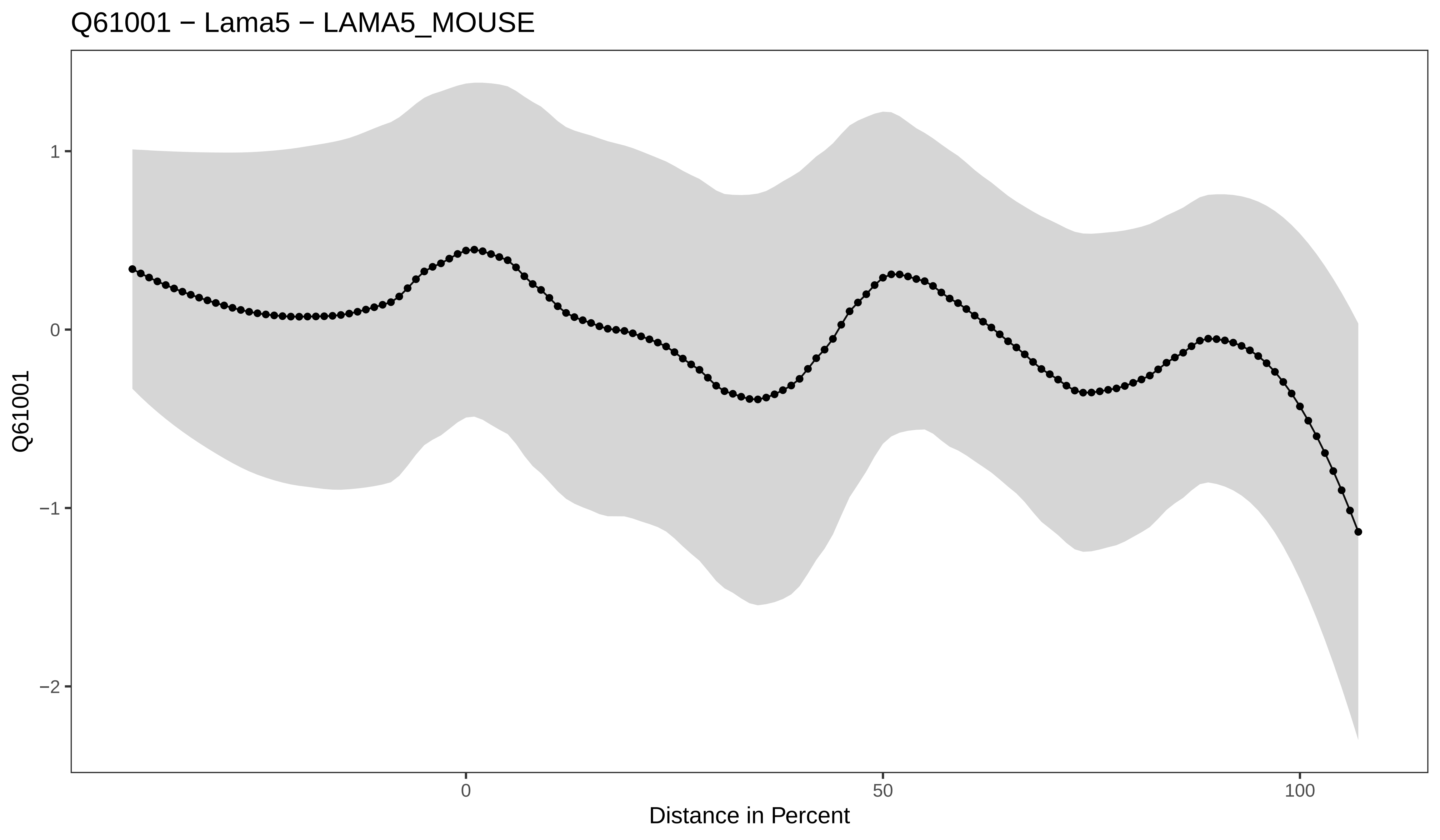
Q61001 − Lama5 − LAMA5_MOUSE
Q61001
1
0
−1
−2
0
50
100
Distance in Percent

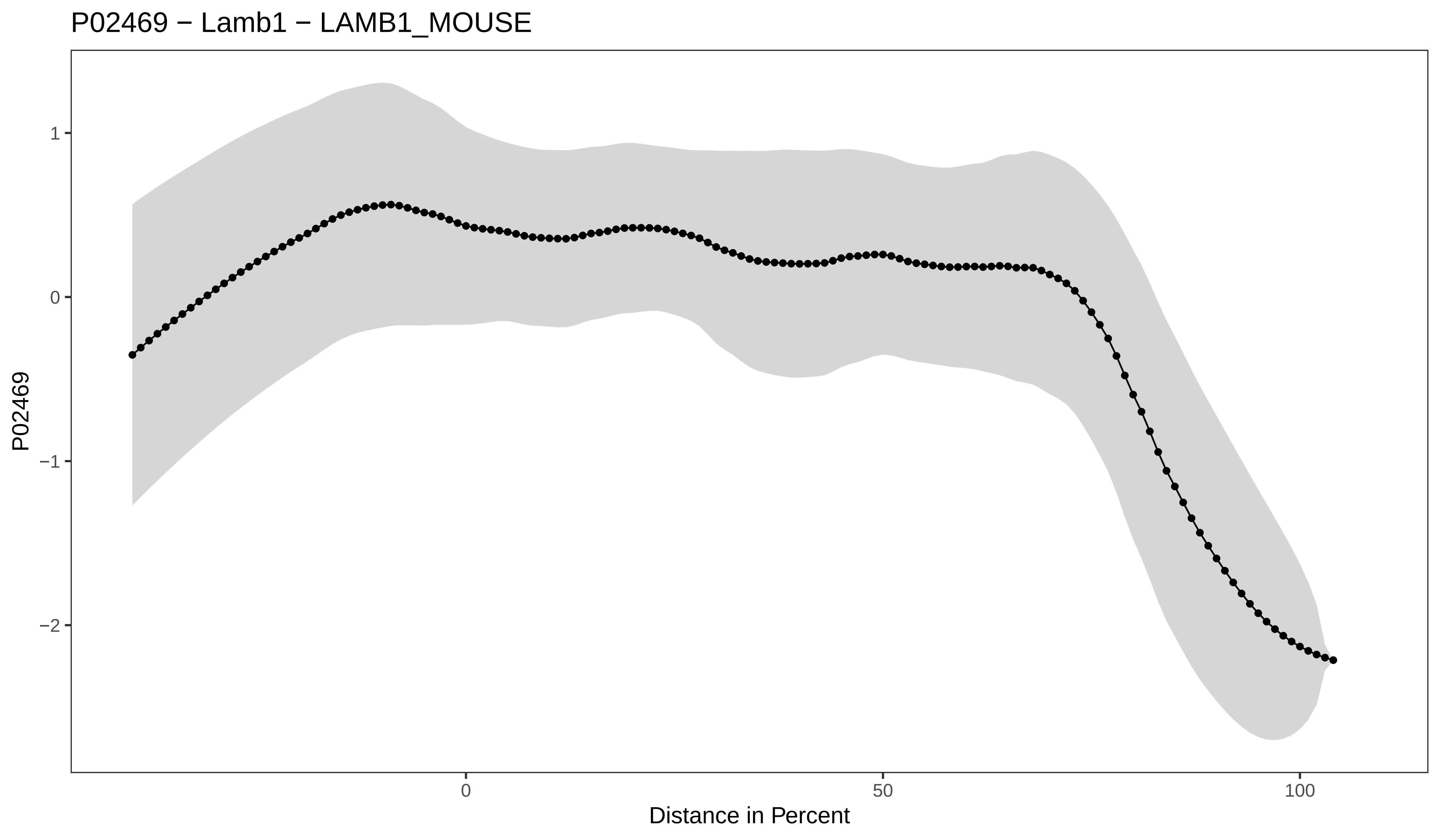

P02469 − Lamb1 − LAMB1_MOUSE
1
0
−1
−2
P02469
0
50
100
Distance in Percent

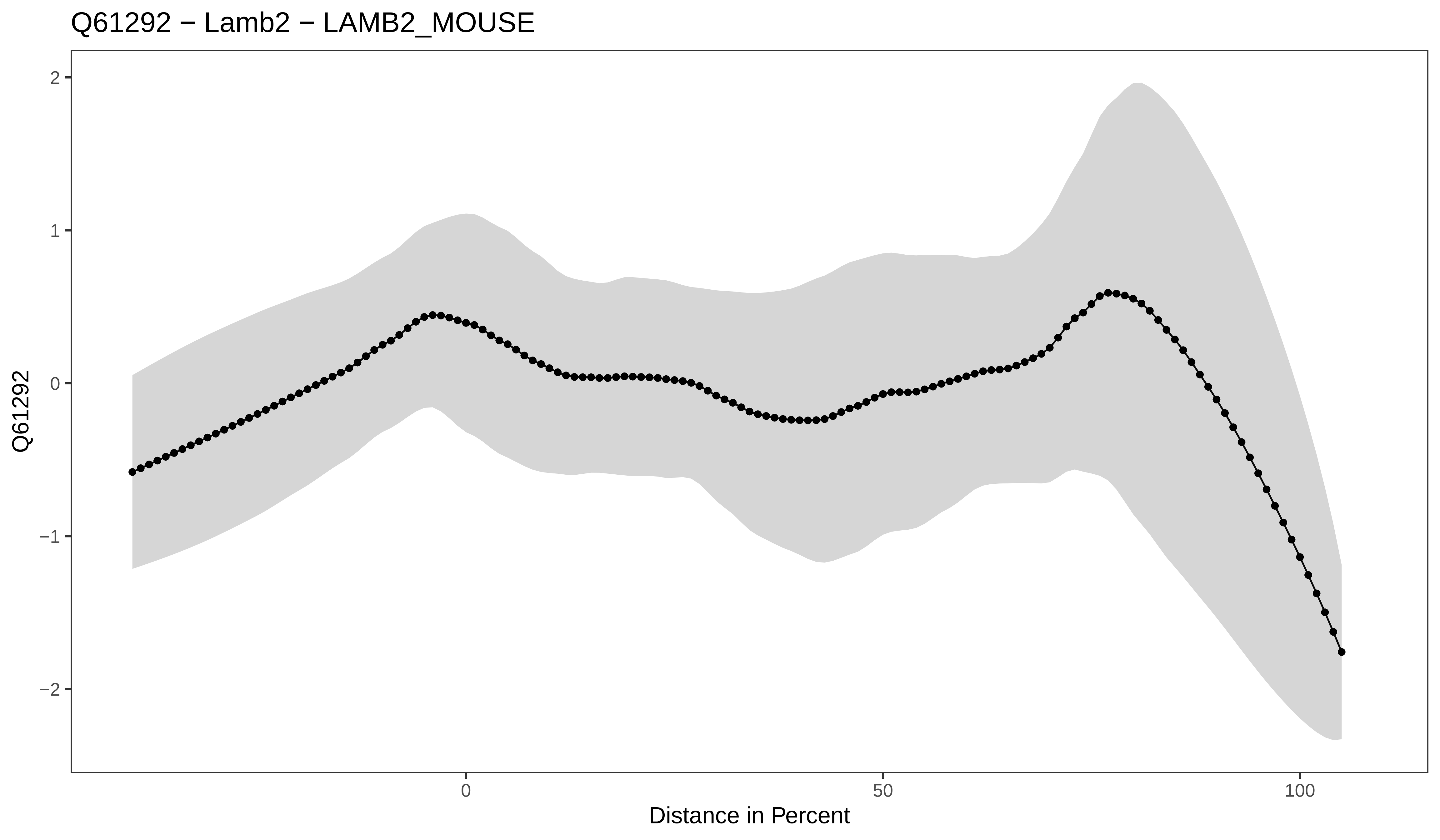
Q61292 − Lamb2 − LAMB2_MOUSE
Q61292
Distance in Percent
2
1
0
−1
−2
0
50
100

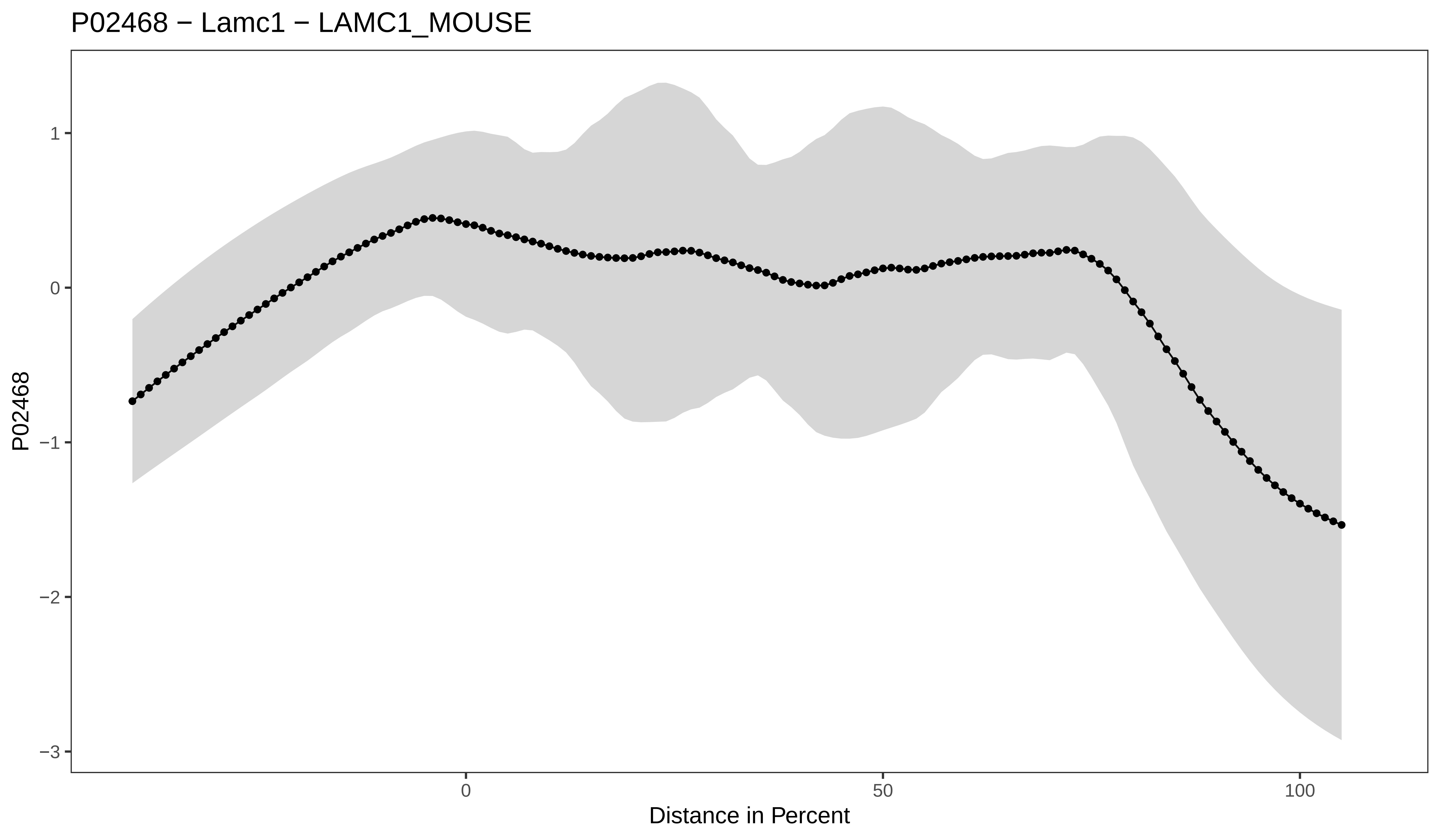

P02468 − Lamc1 − LAMC1_MOUSE
P02468
1
0
−1
−2
−3
0
50
100
Distance in Percent

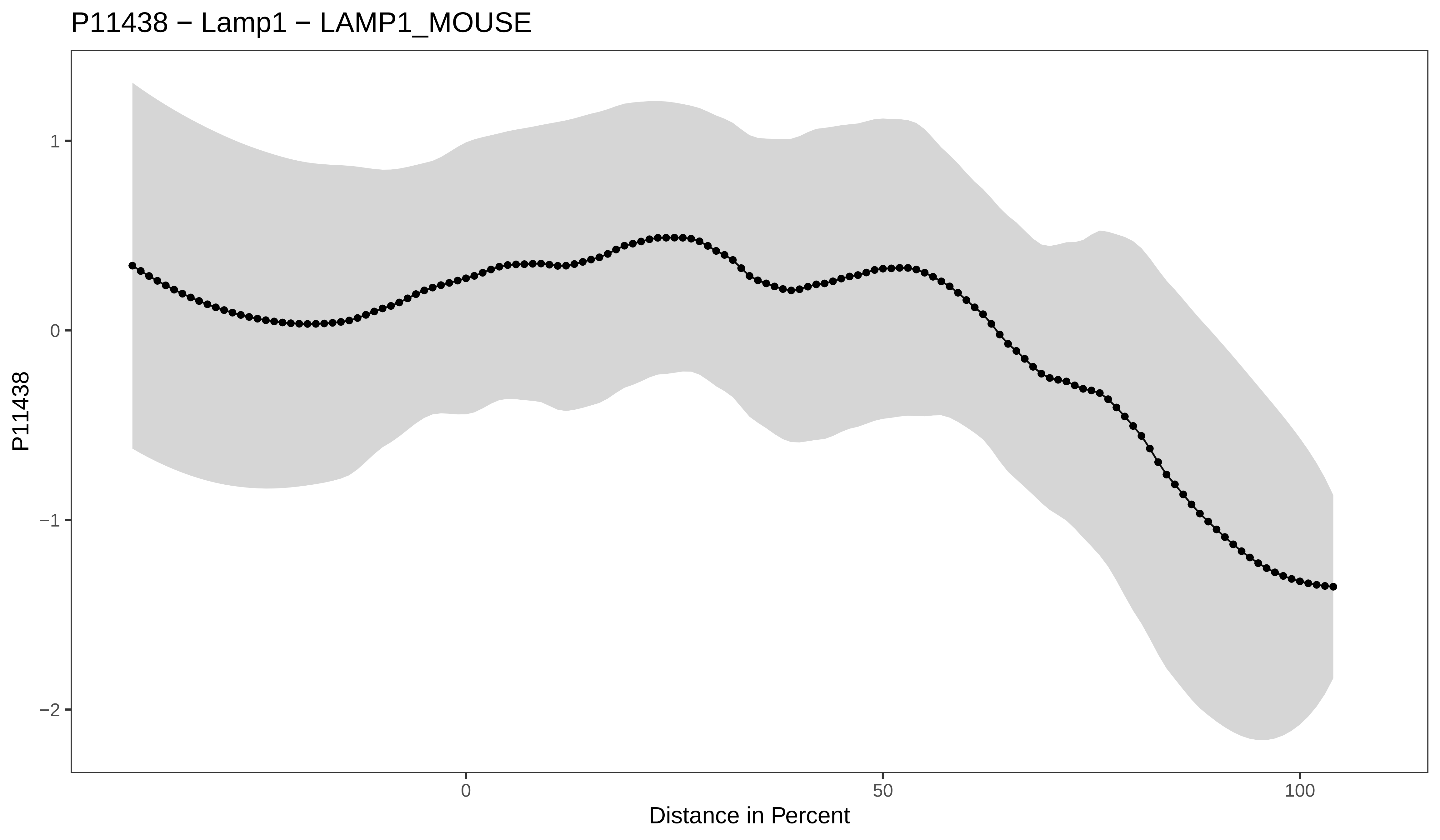

P11438 − Lamp1 − LAMP1_MOUSE
1
0
−1
−2
P11438
0
50
100
Distance in Percent

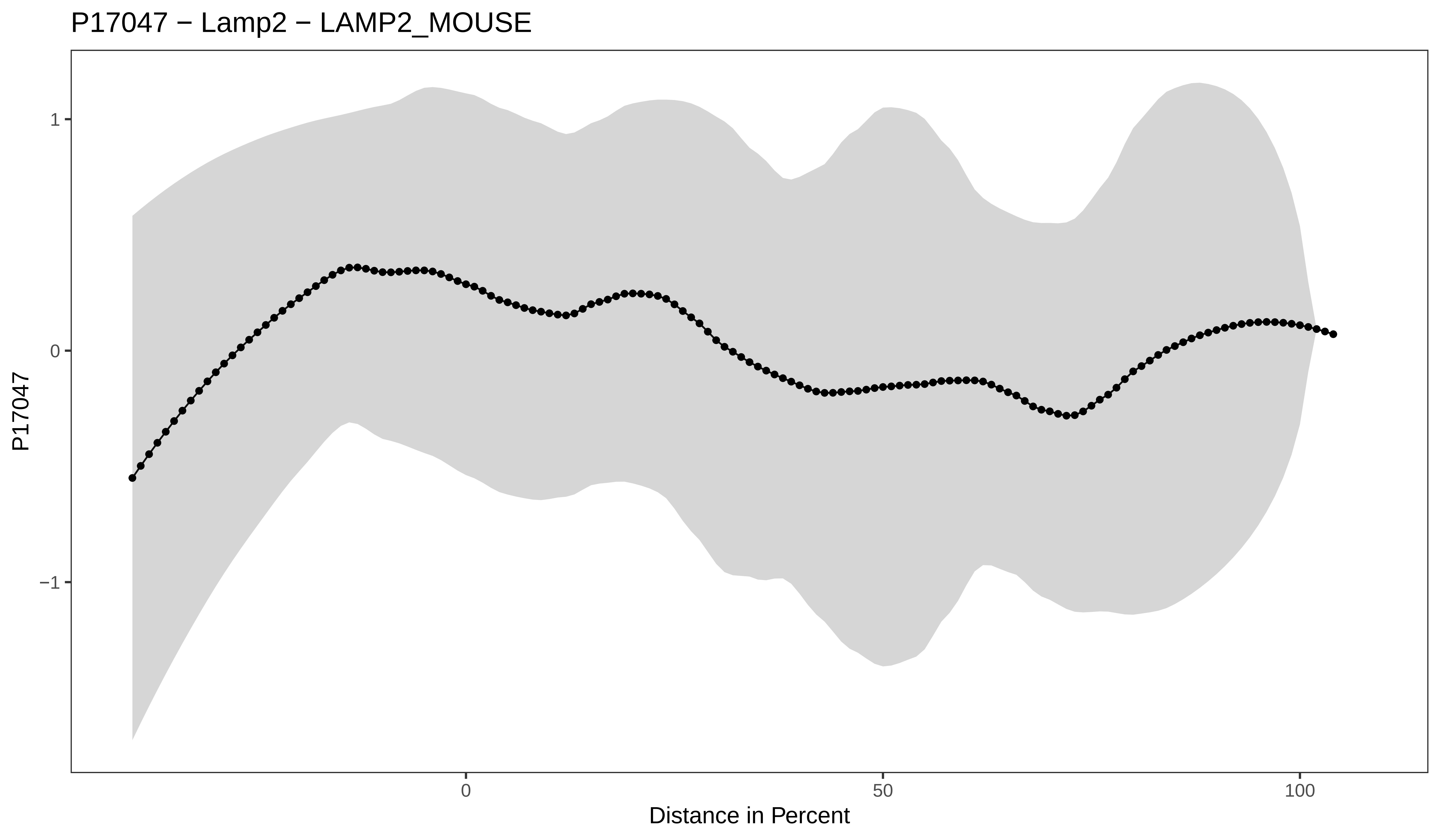

P17047 − Lamp2 − LAMP2_MOUSE
1
0
−1
P17047
0
50
100
Distance in Percent

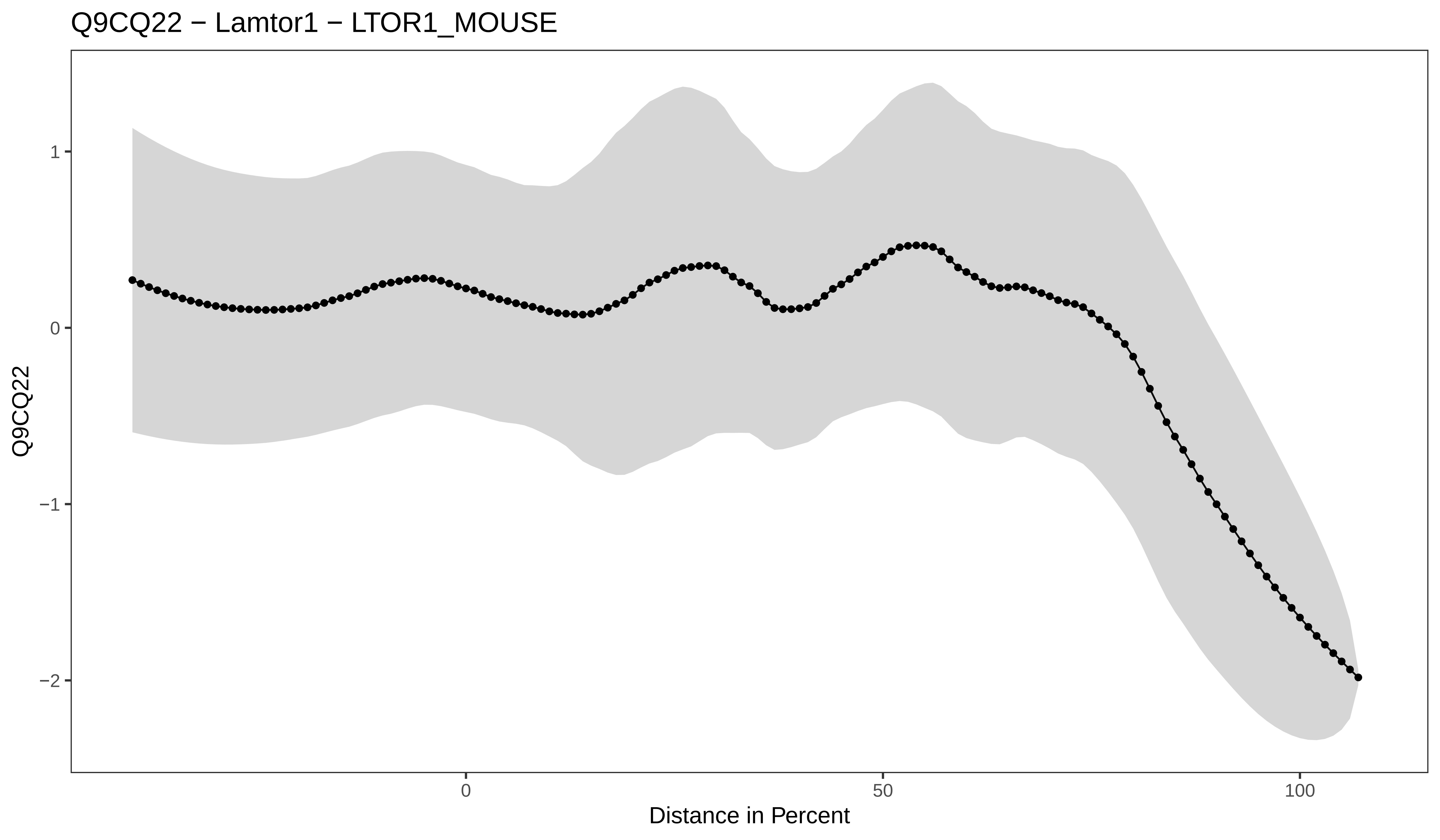

Q9CQ22 − Lamtor1 − LTOR1_MOUSE
Q9CQ22
1
0
−1
−2
0
50
100
Distance in Percent

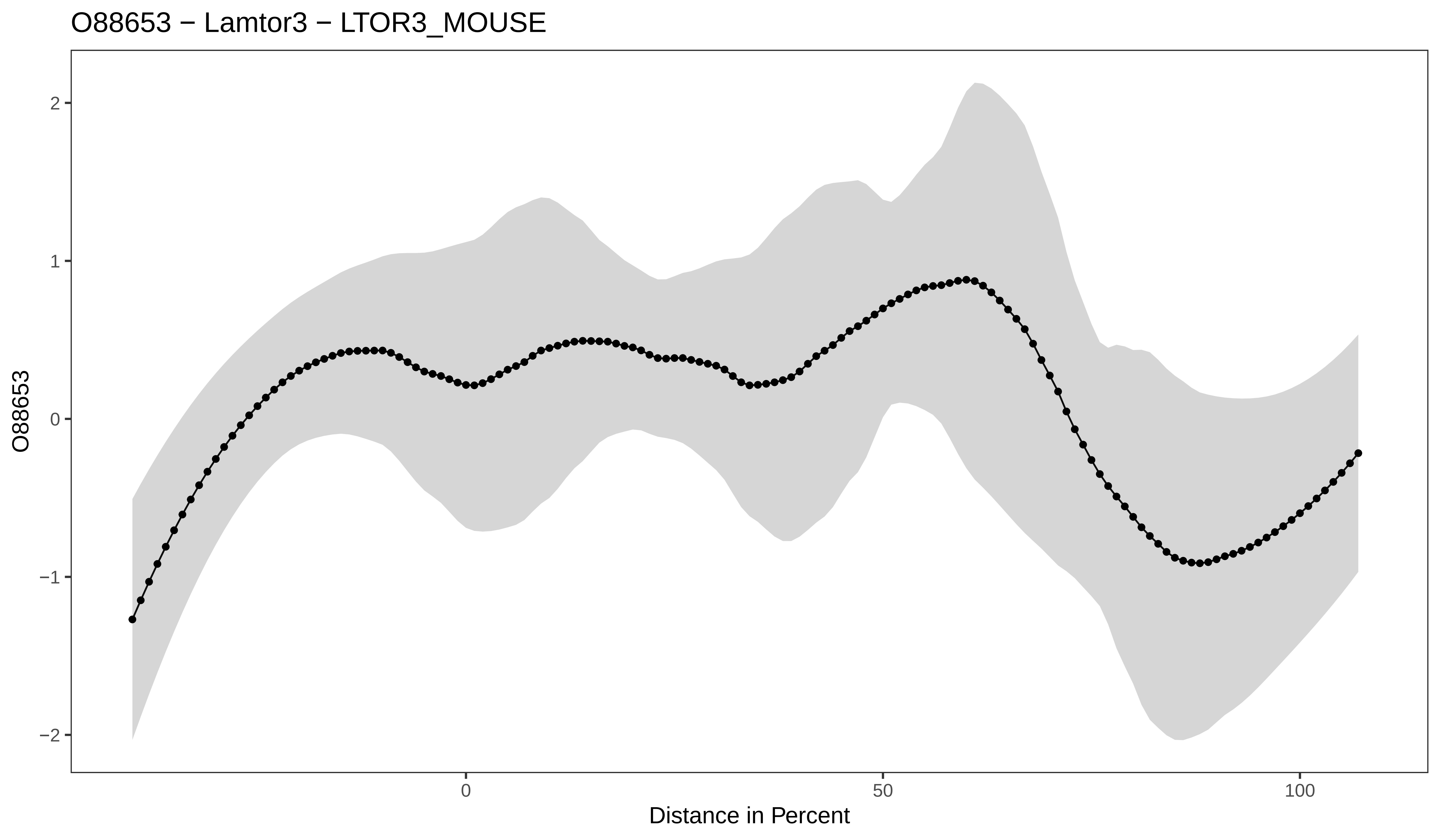

O88653 − Lamtor3 − LTOR3_MOUSE
O88653
Distance in Percent
2
1
0
−1
−2
0
50
100

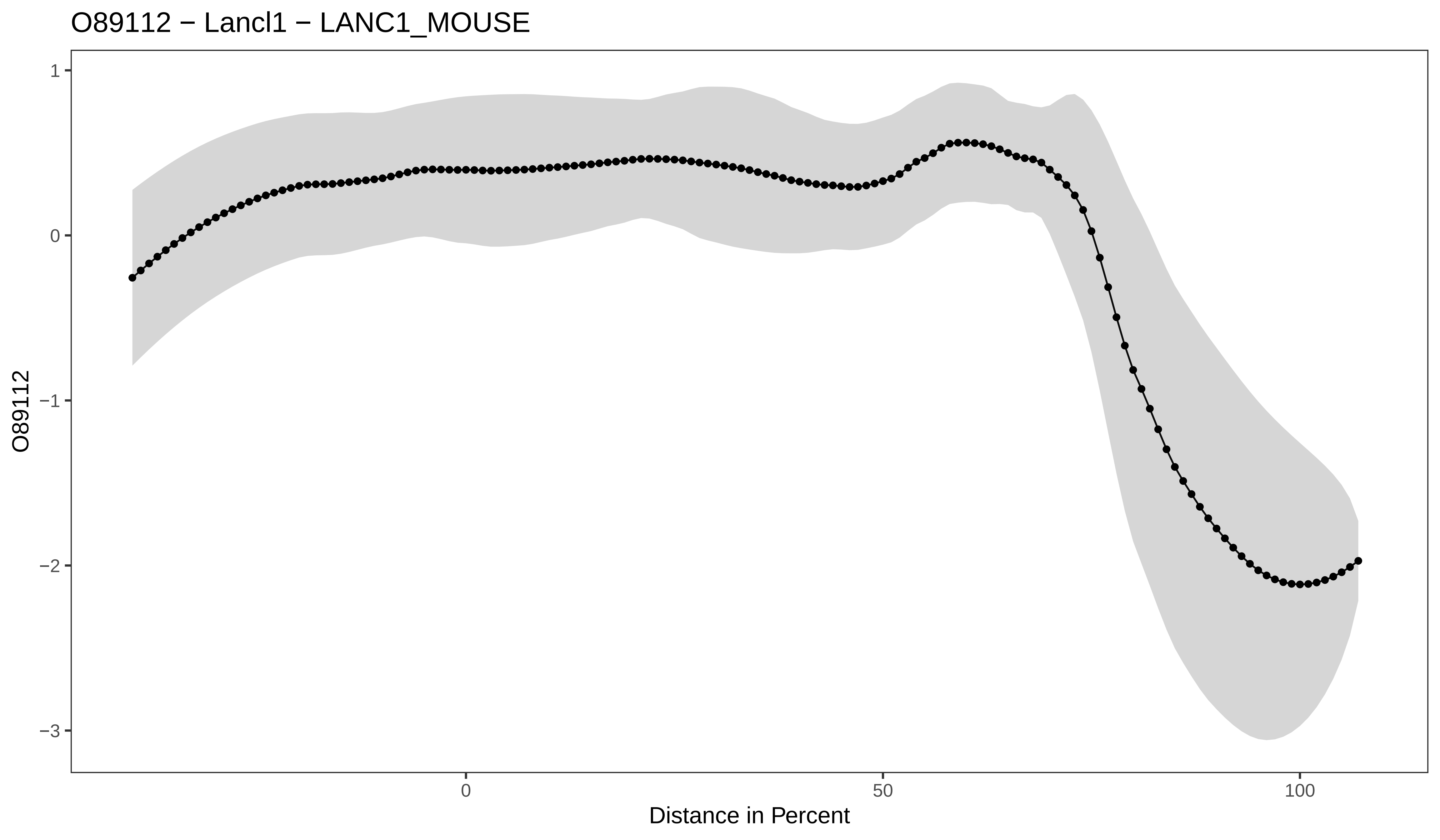

O89112 − Lancl1 − LANC1_MOUSE
1
0
−1
−2
−3
O89112
0
50
100
Distance in Percent

Q9JJK2 − Lancl2 − LANC2_MOUSE

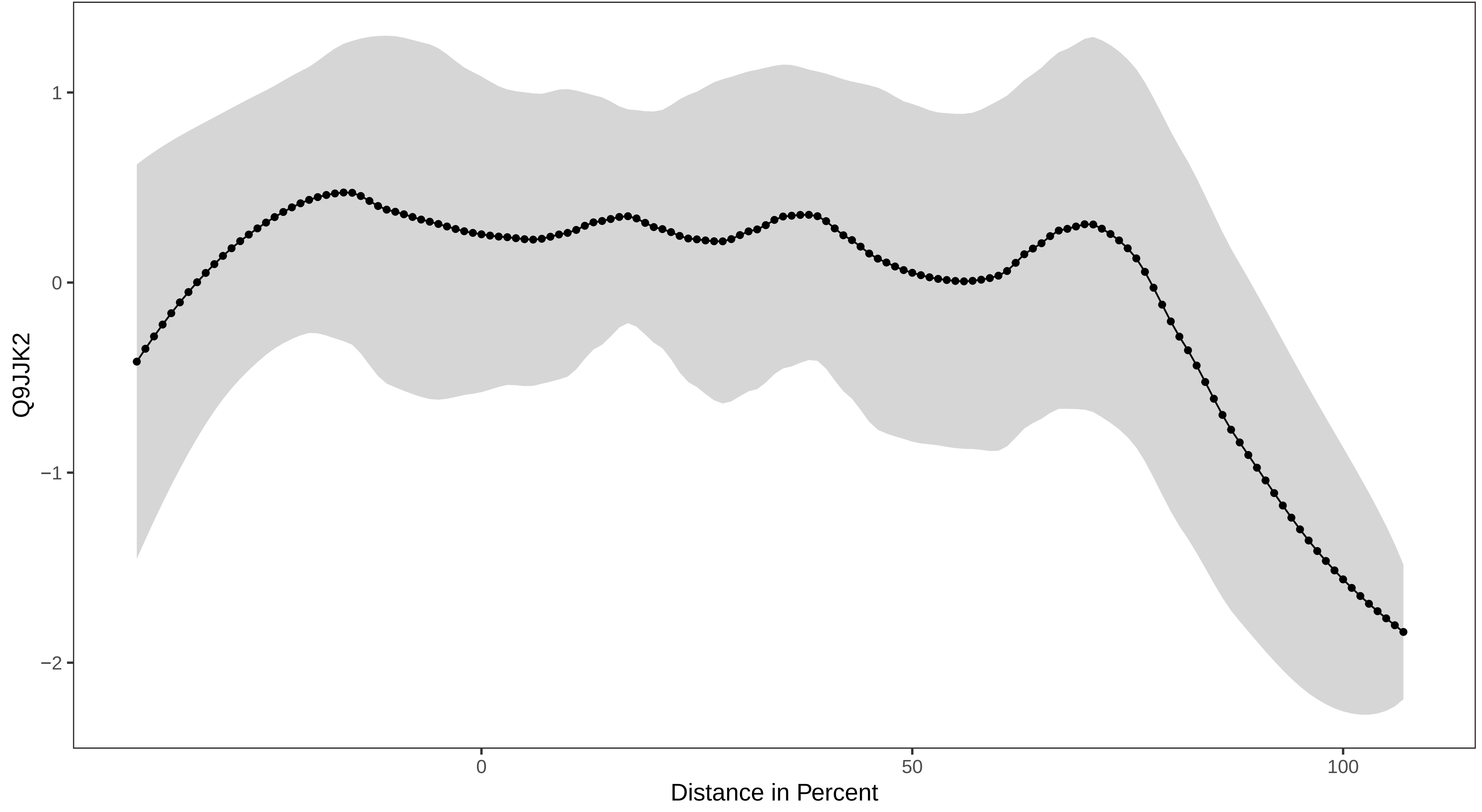

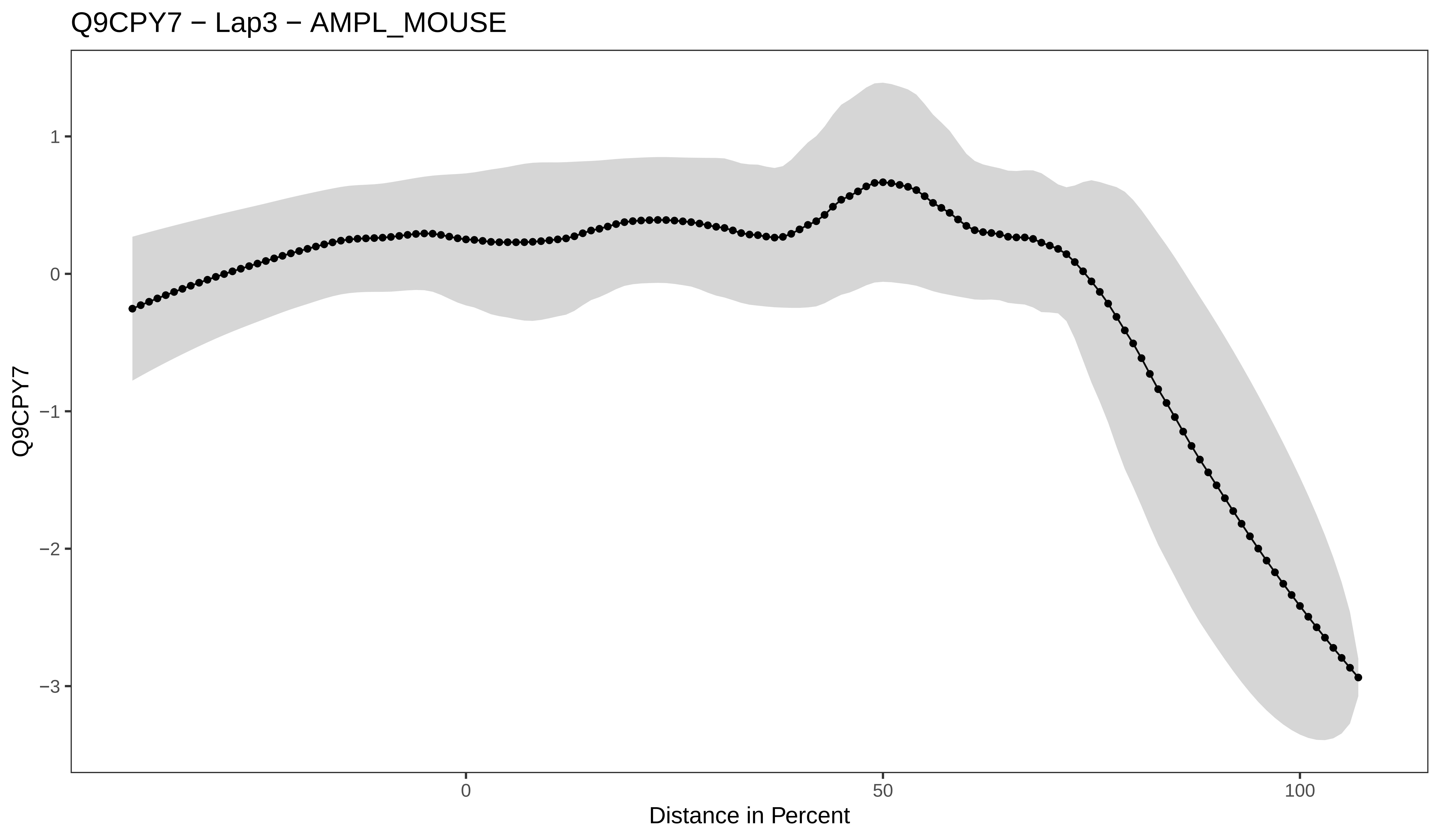

Q9CPY7 − Lap3 − AMPL_MOUSE
Q9CPY7
Distance in Percent
1
0
−1
−2
−3
0
50
100

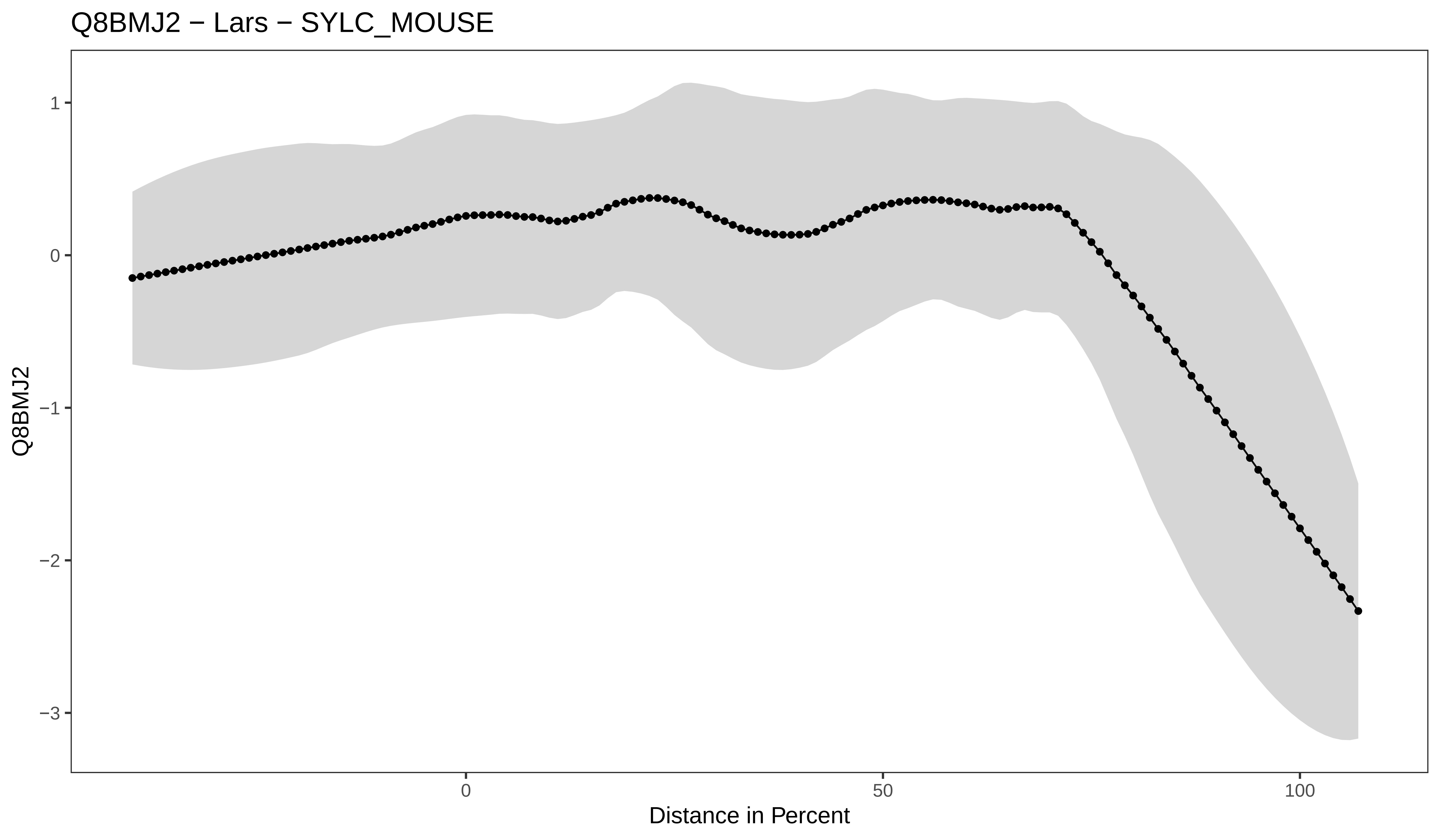

Q8BMJ2 − Lars − SYLC_MOUSE
Q8BMJ2
1
0
−1
−2
−3
0
50
100
Distance in Percent

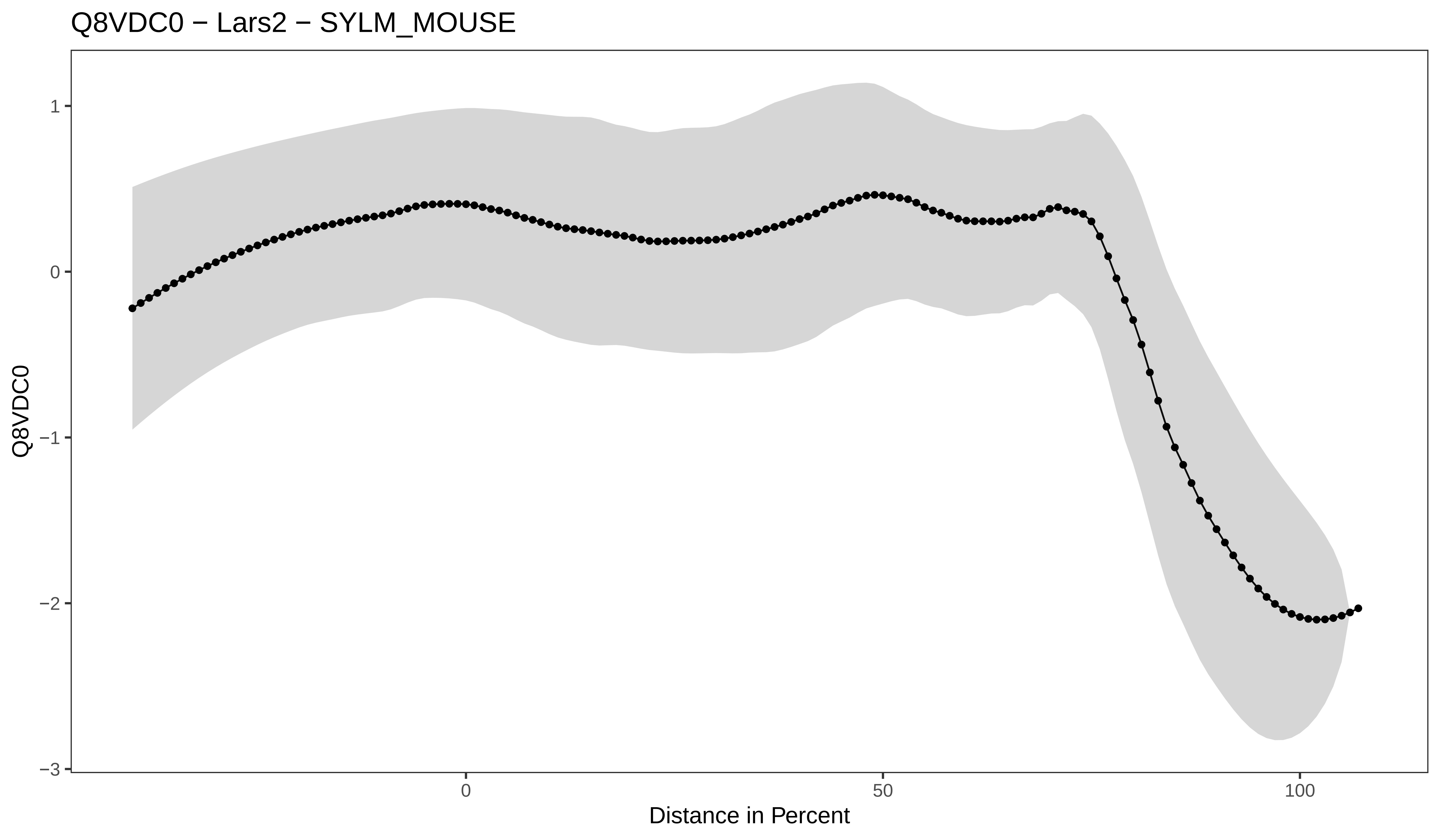
Q8VDC0 − Lars2 − SYLM_MOUSE
Q8VDC0
1
0
−1
−2
−3
0
50
100
Distance in Percent

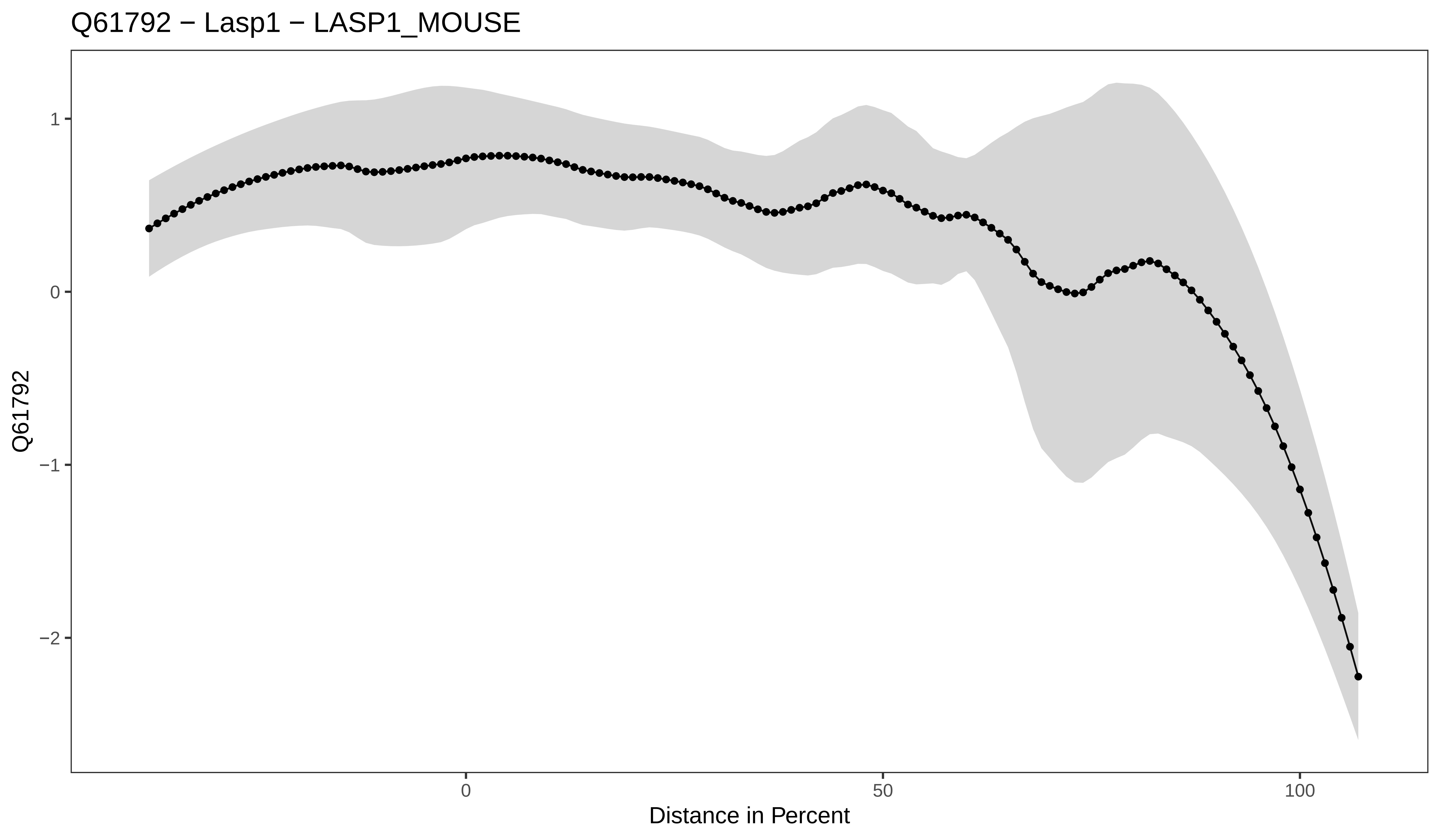
Q61792 − Lasp1 − LASP1_MOUSE
Q61792
1
0
−1
−2
0
50
100
Distance in Percent

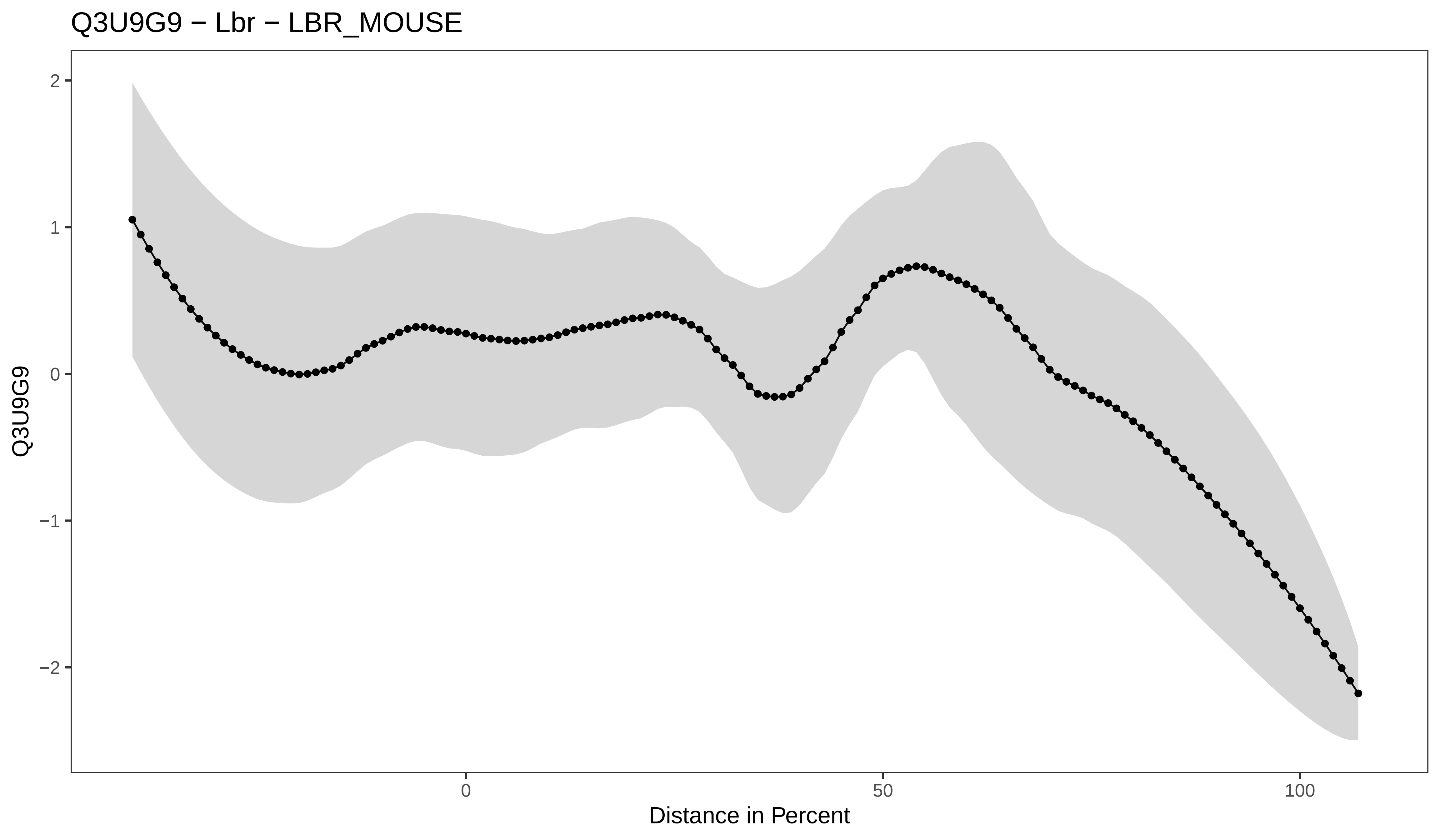

Q3U9G9 − Lbr − LBR_MOUSE
Q3U9G9
Distance in Percent
2
1
0
−1
−2
0
50
100

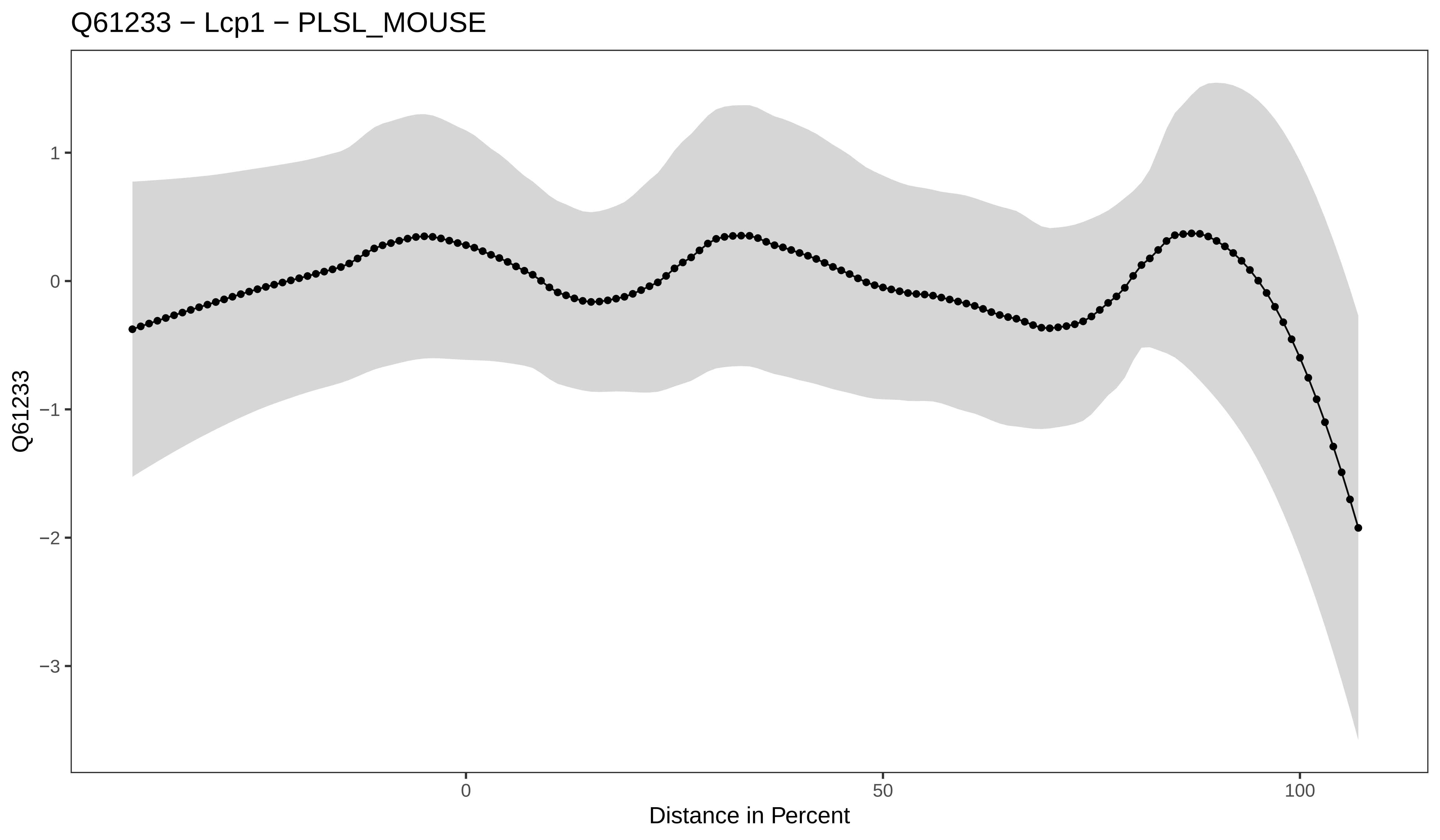

Q61233 − Lcp1 − PLSL_MOUSE
Q61233
1
0
−1
−2
−3
0
50
100
Distance in Percent

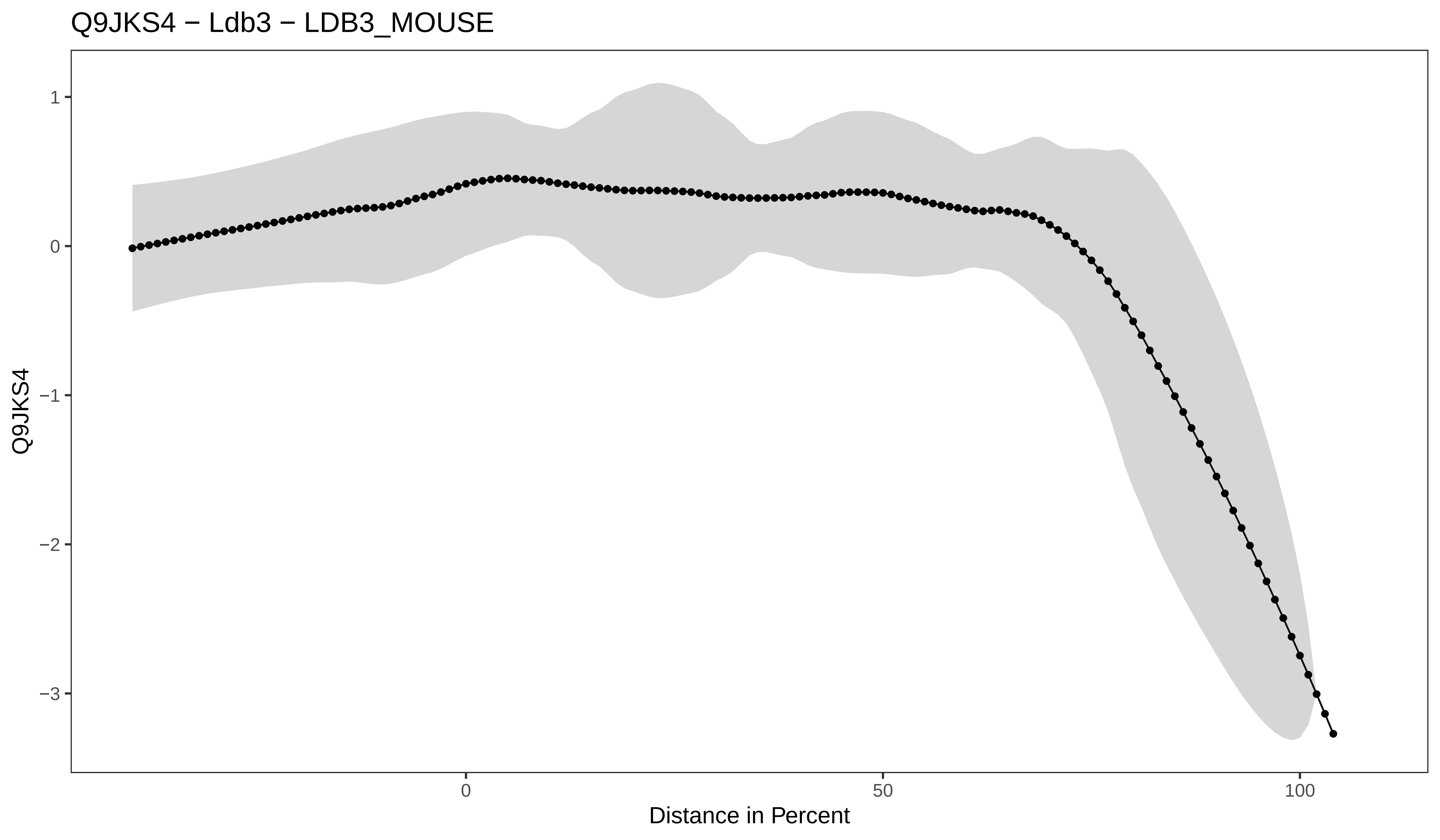

Q9JKS4 − Ldb3 − LDB3_MOUSE
1
0
−1
−2
−3
Q9JKS4
0
50
100
Distance in Percent

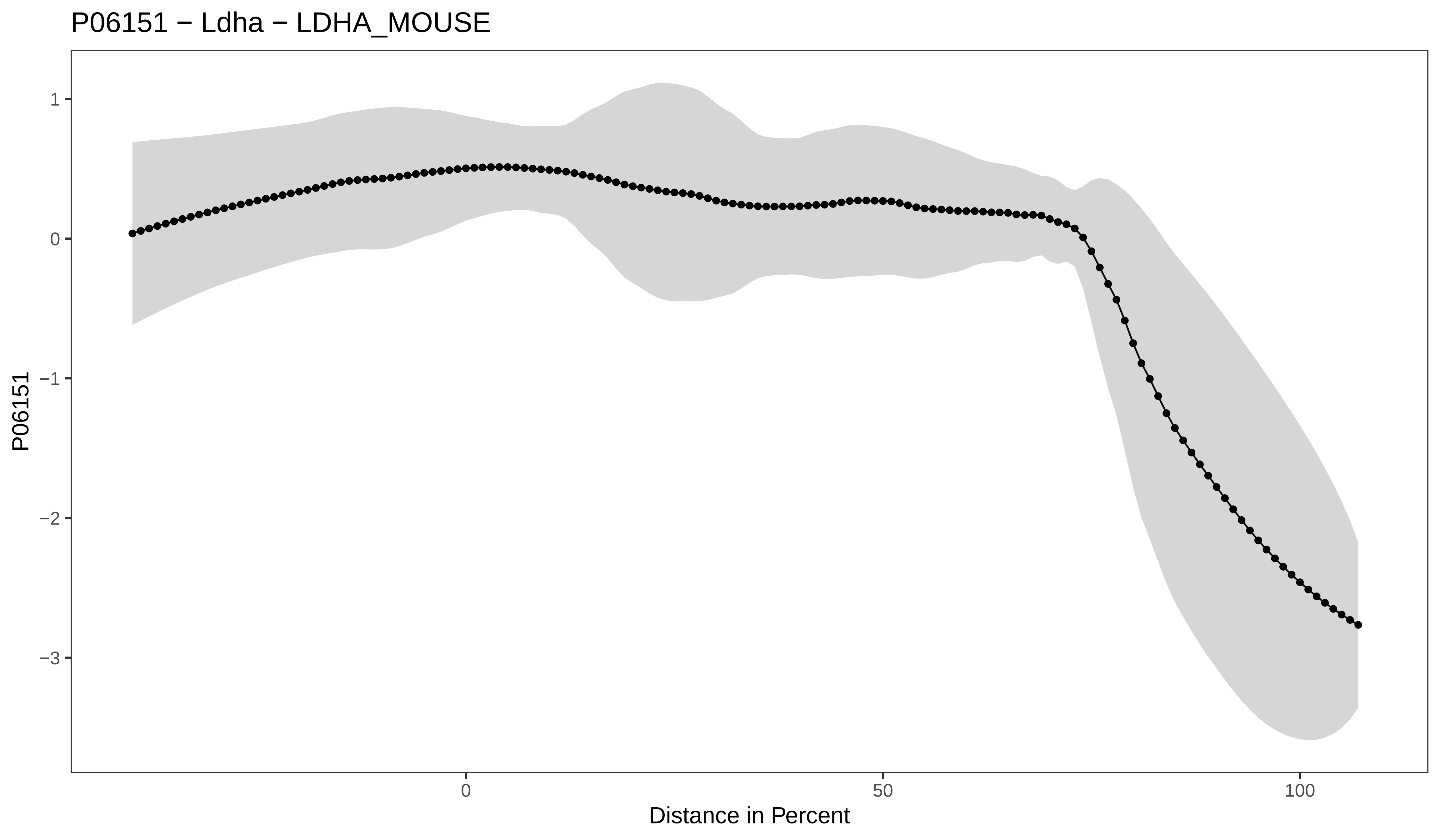

P06151 − Ldha − LDHA_MOUSE
P06151
Distance in Percent
1
0
−1
−2
−3
0
50
100

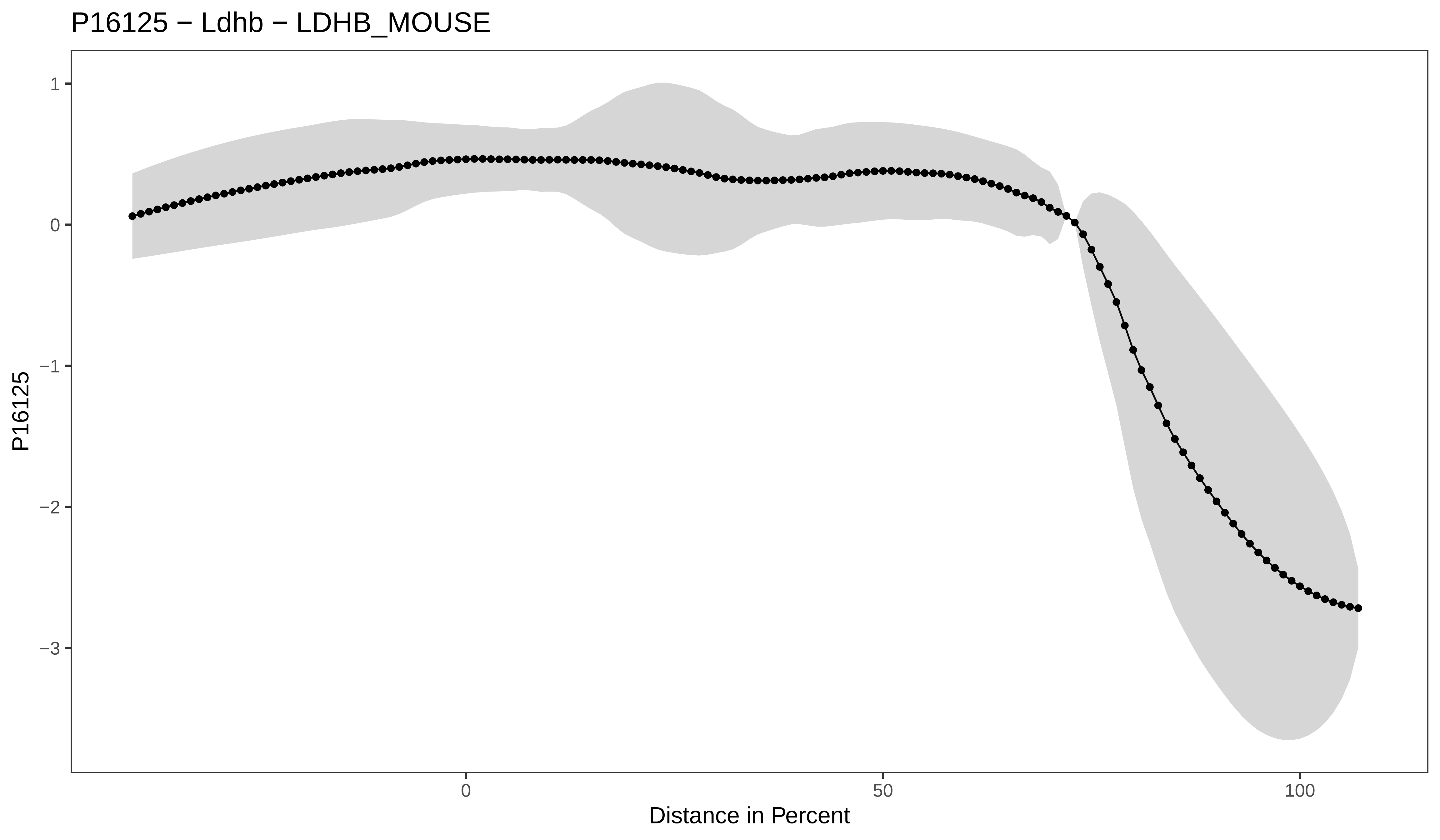
P16125 − Ldhb − LDHB_MOUSE
1
0
−1
−2
−3
P16125
0
50
100
Distance in Percent

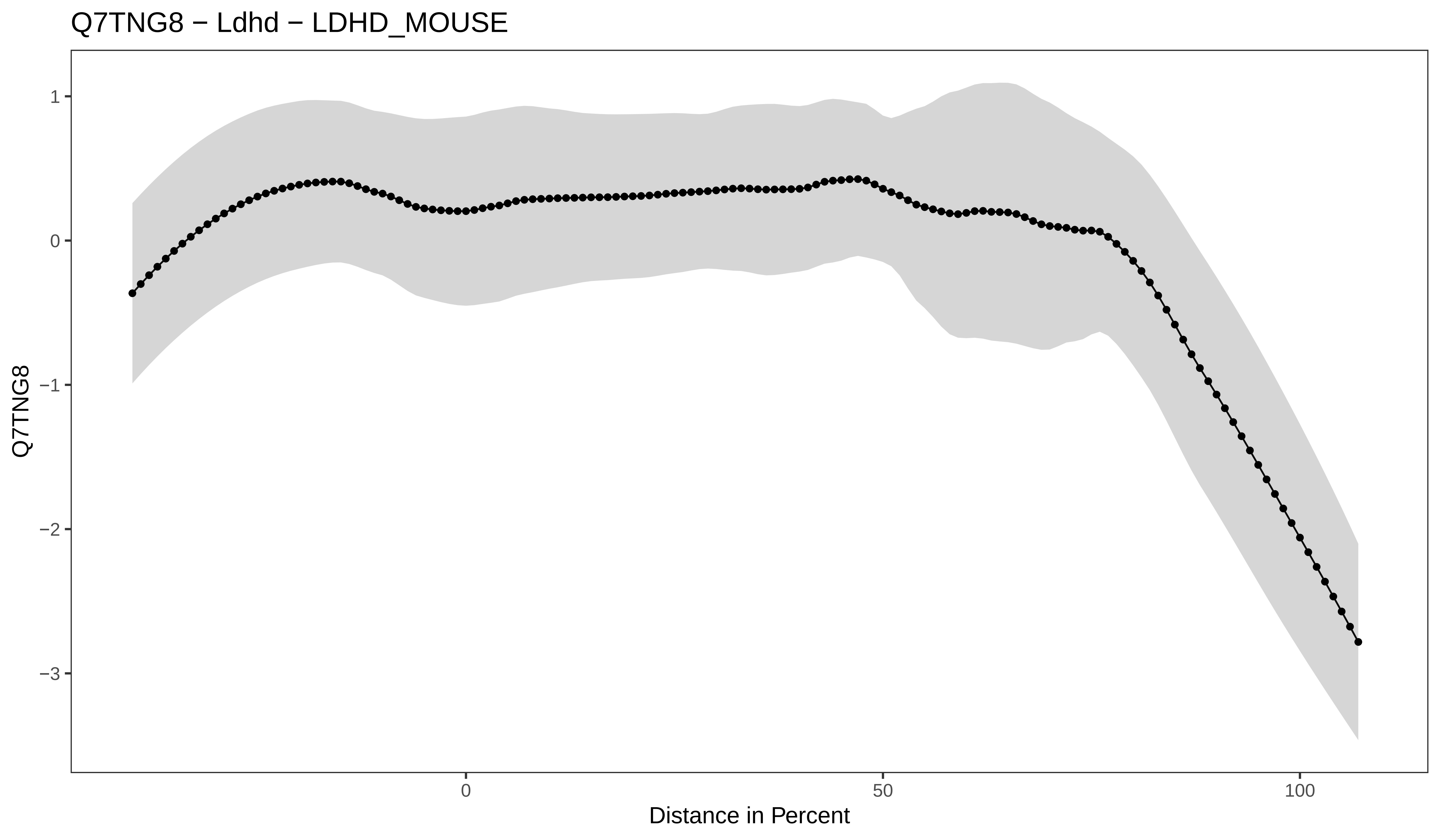
Q7TNG8 − Ldhd − LDHD_MOUSE
1
0
−1
−2
−3
Q7TNG8
0
50
100
Distance in Percent

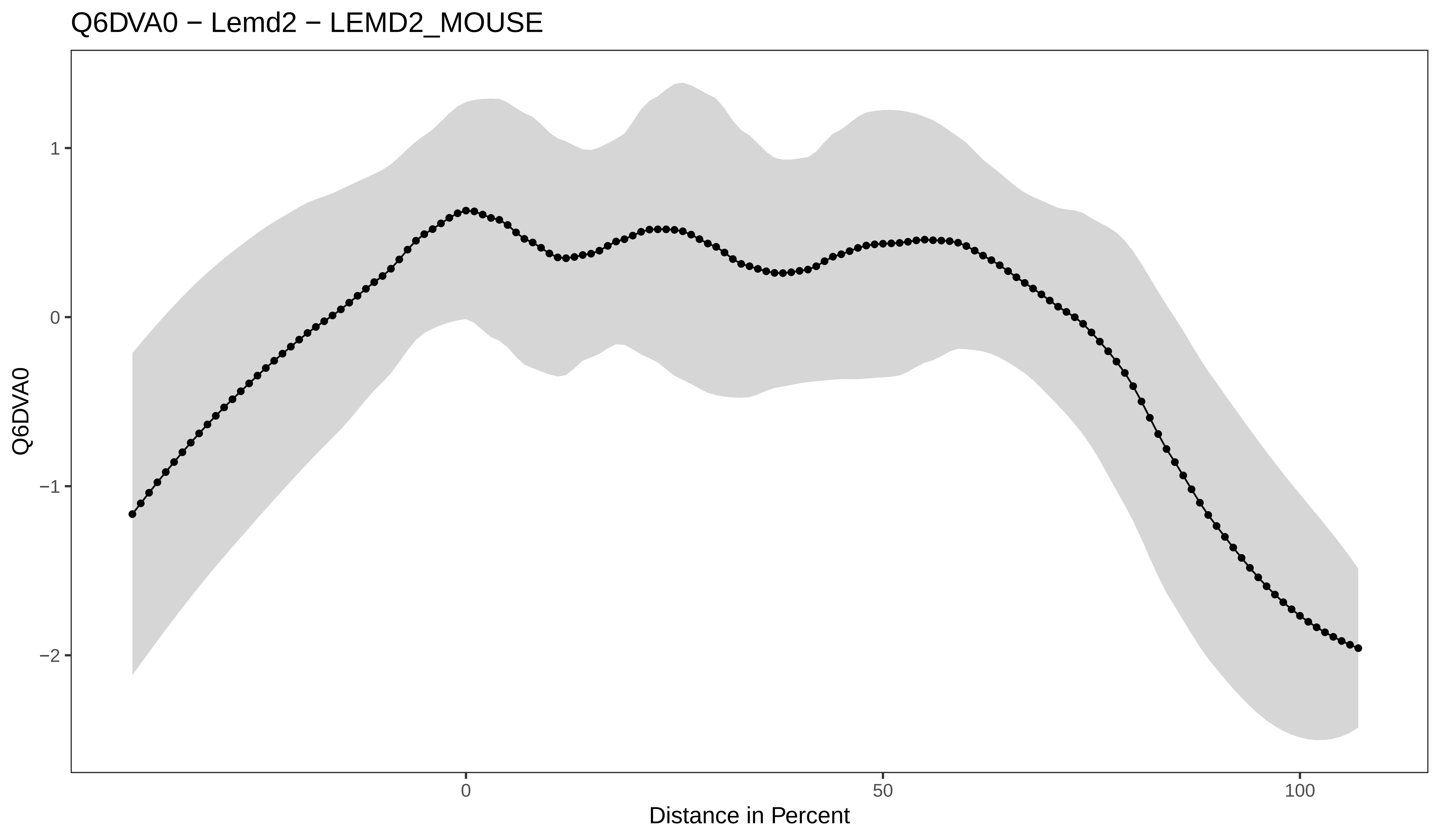

Q6DVA0 − Lemd2 − LEMD2_MOUSE
1
0
−1
−2
Q6DVA0
0
50
100
Distance in Percent

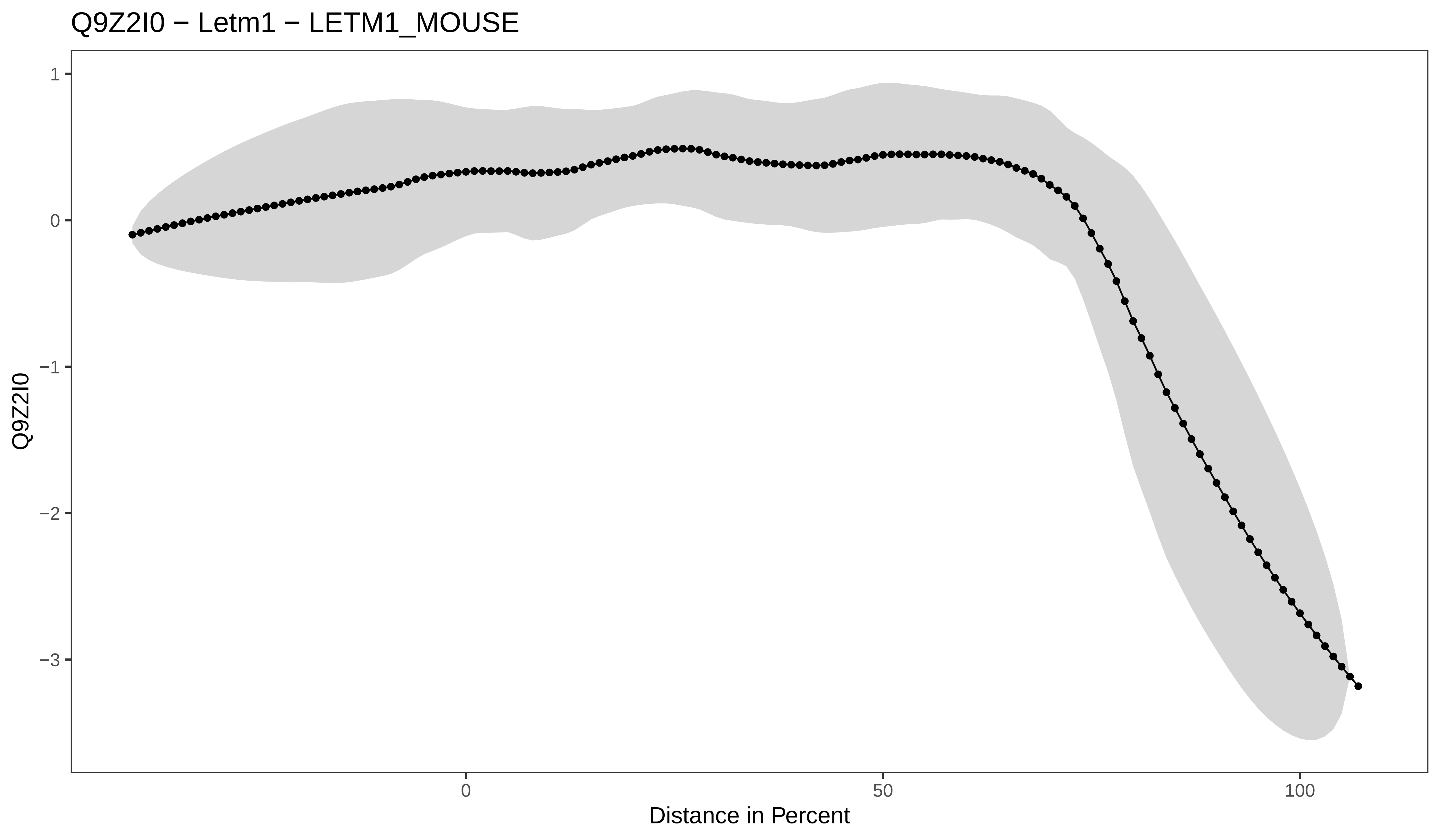
Q9Z2I0 − Letm1 − LETM1_MOUSE
1
0
−1
−2
−3
Q9Z2I0
0
50
100
Distance in Percent

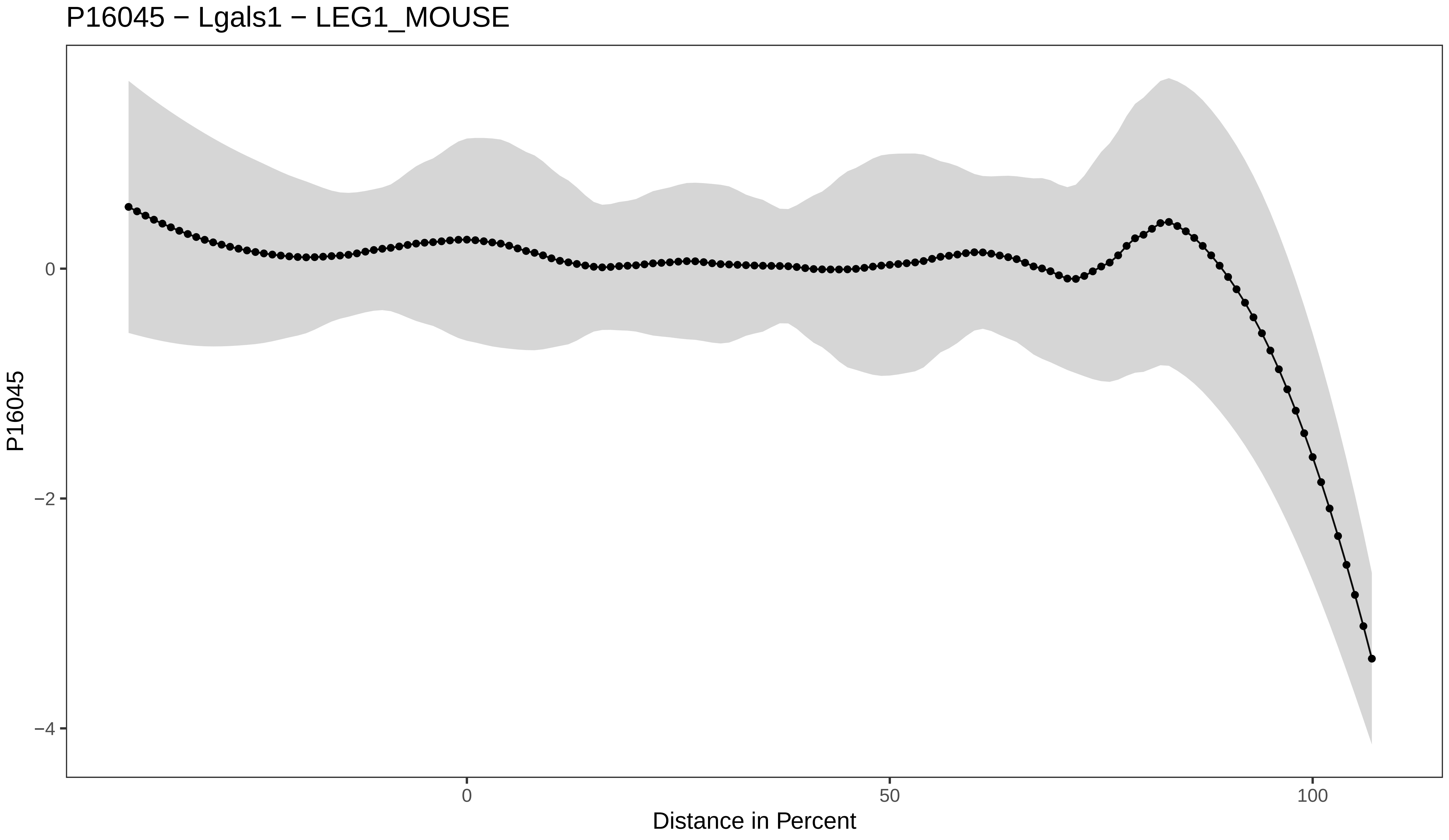

P16045 − Lgals1 − LEG1_MOUSE
P16045
0
−2
−4
0
50
100
Distance in Percent

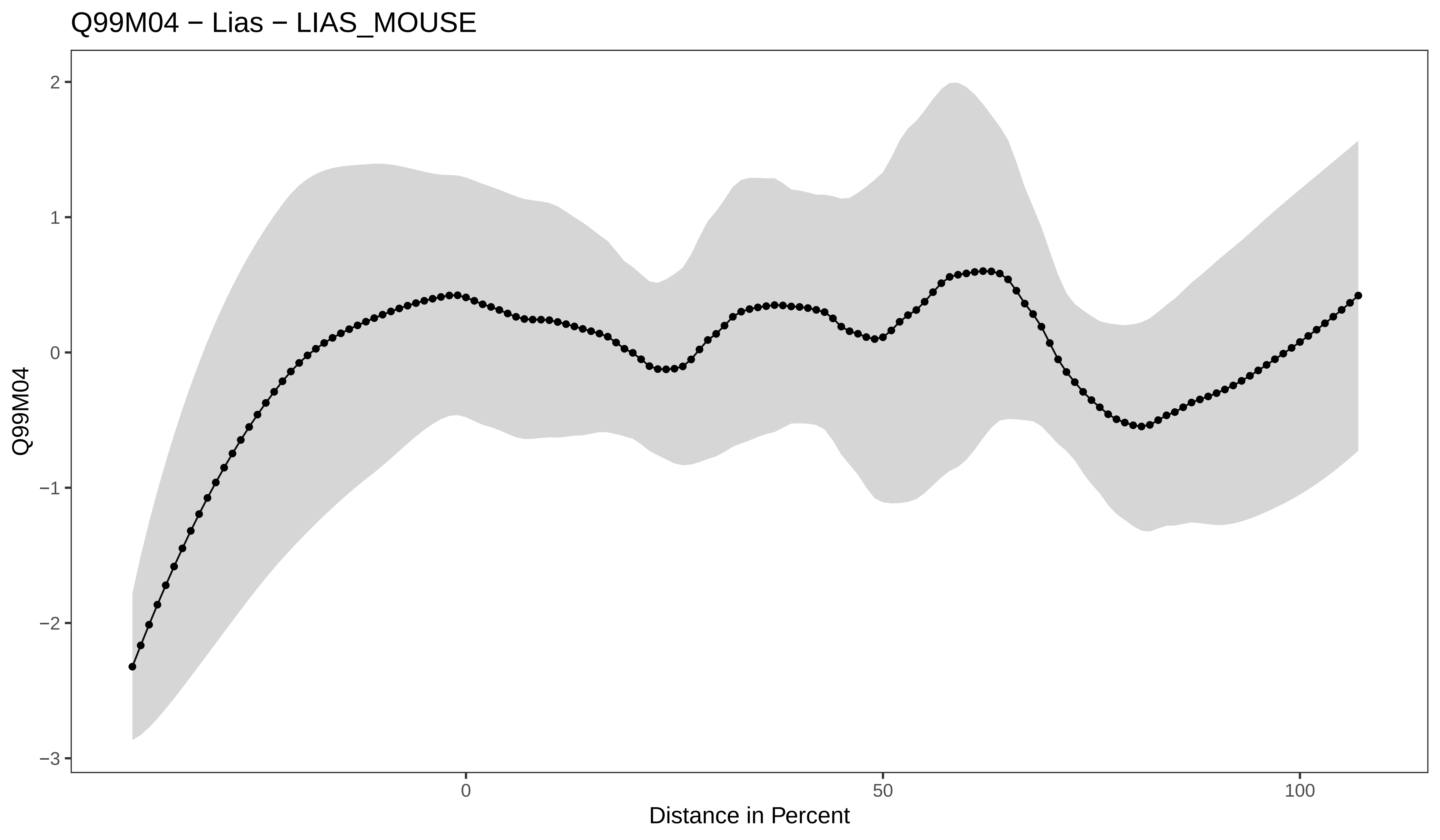

Q99M04 − Lias − LIAS_MOUSE
Q99M04
Distance in Percent
2
1
0
−1
−2
−3
0
50
100

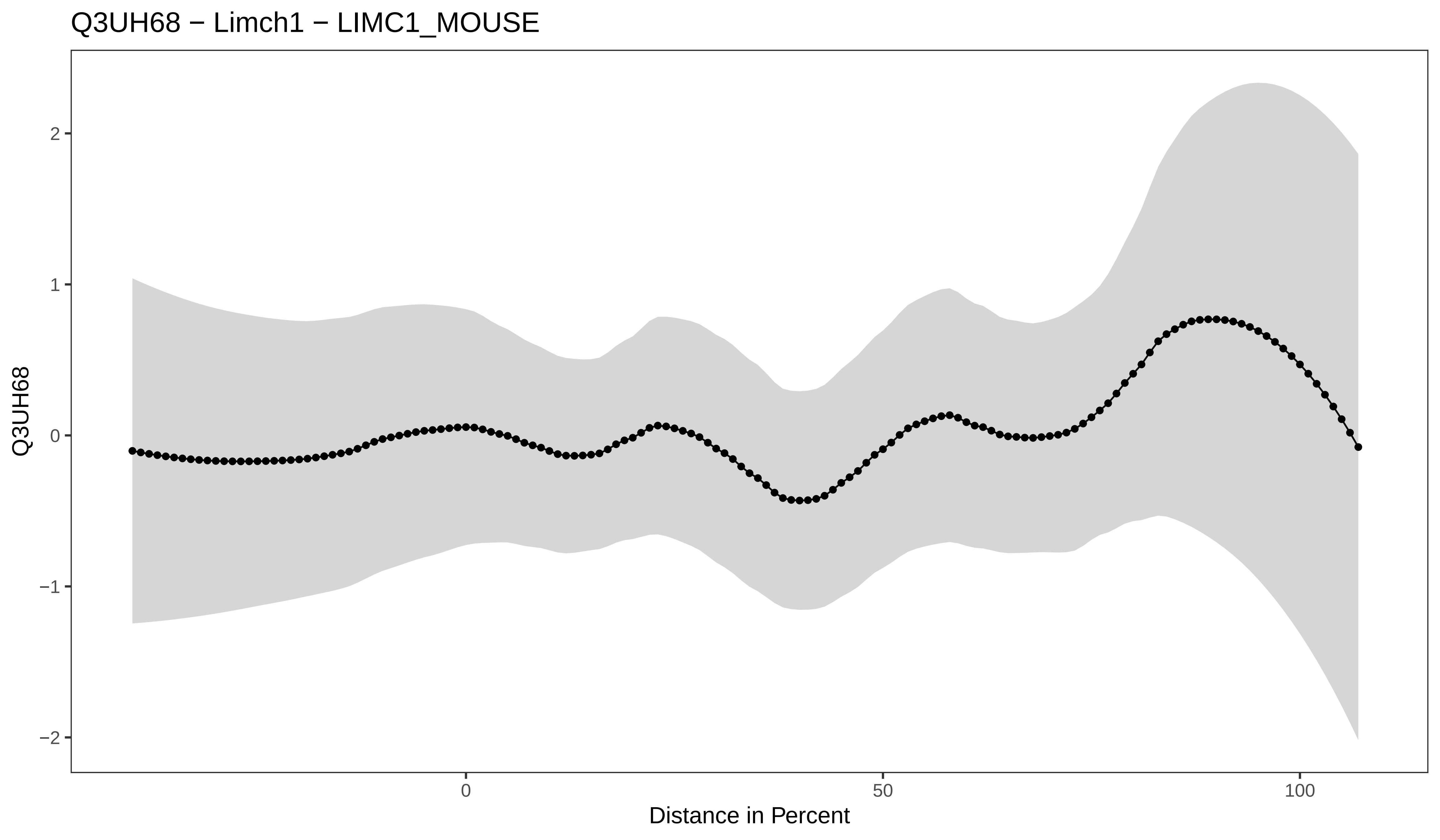

Q3UH68 − Limch1 − LIMC1_MOUSE
Q3UH68
2
1
0
−1
−2
0
50
100
Distance in Percent

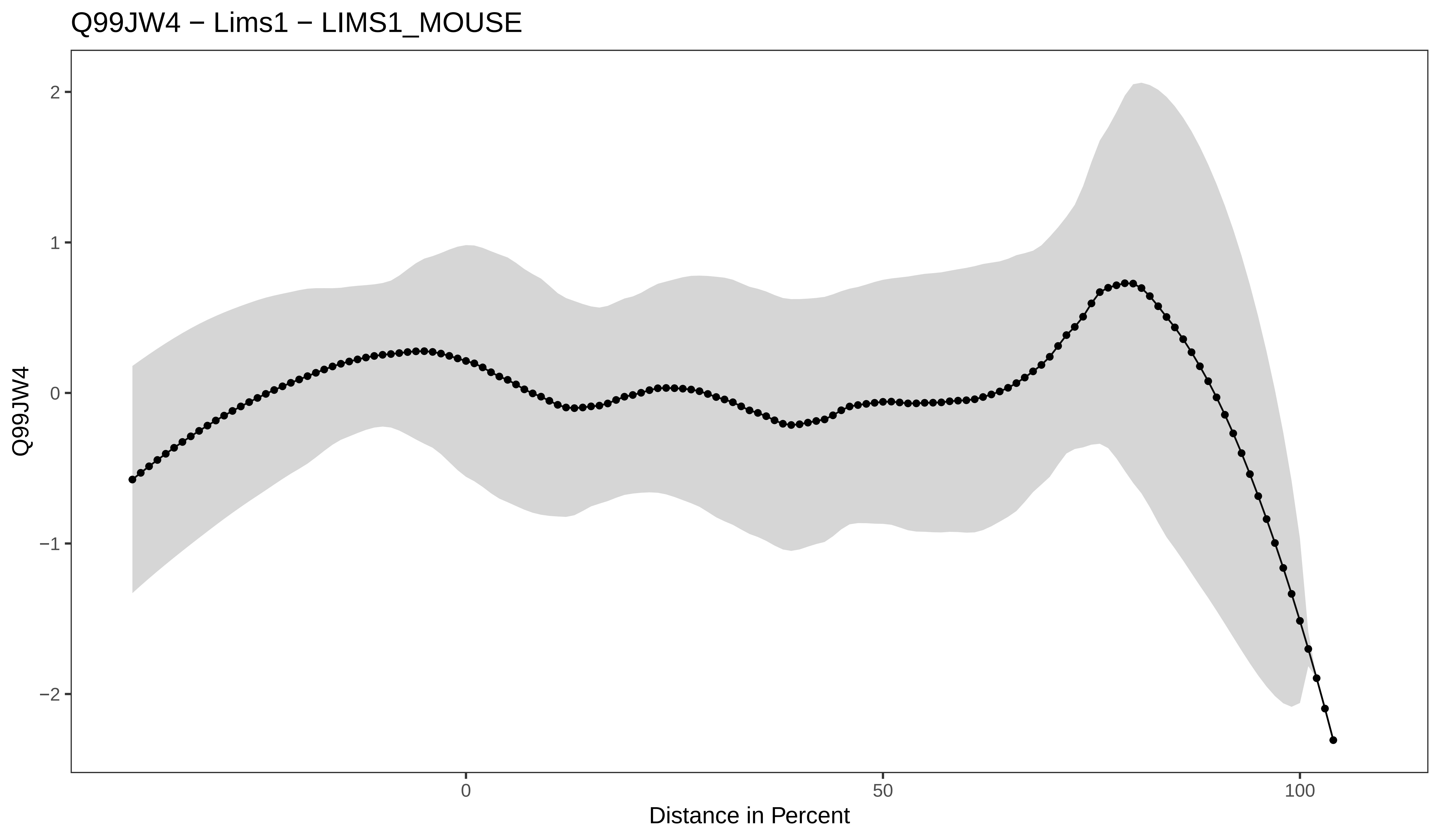

Q99JW4 − Lims1 − LIMS1_MOUSE
Q99JW4
2
1
0
−1
−2
0
50
100
Distance in Percent

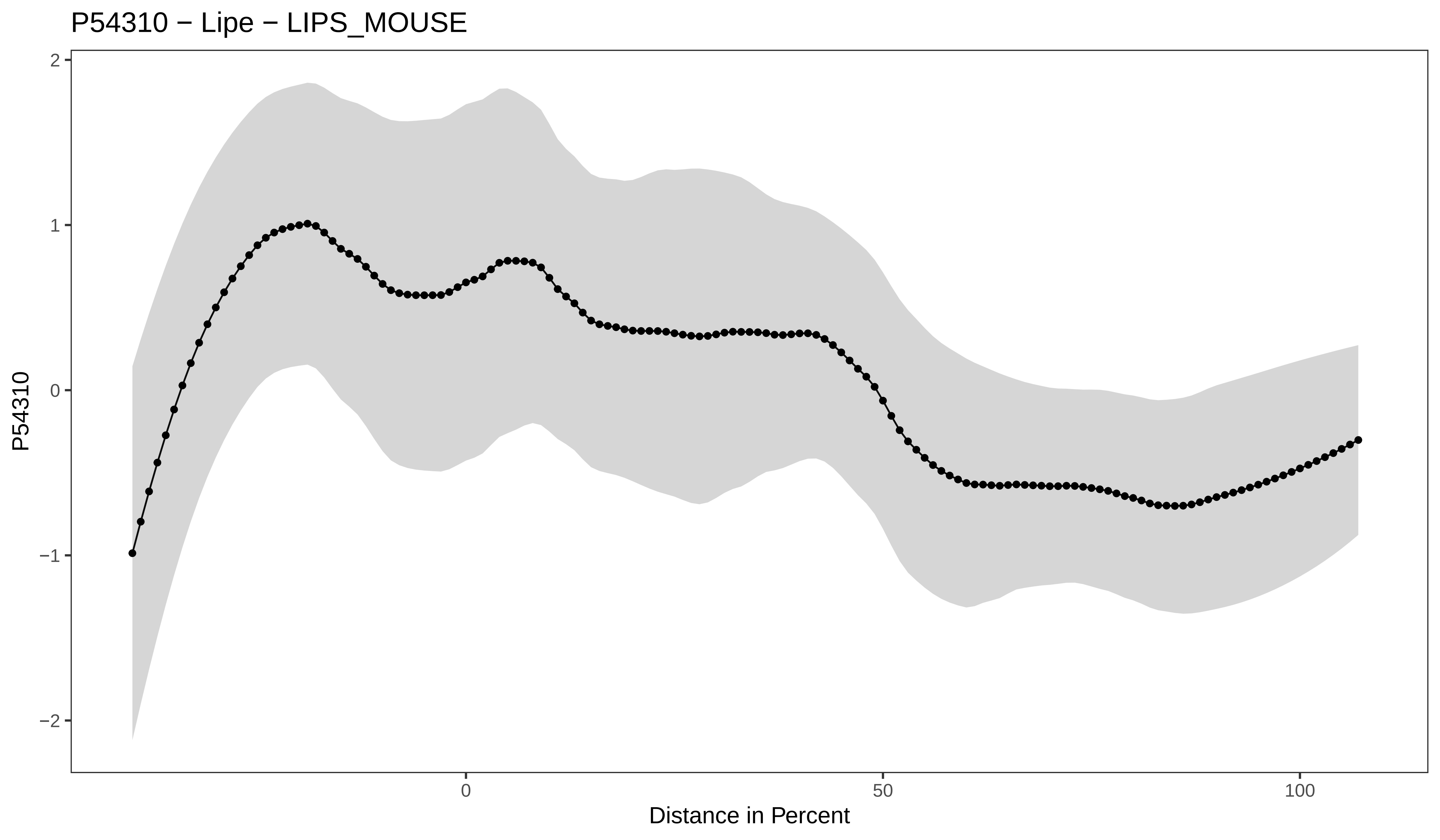
P54310 − Lipe − LIPS_MOUSE
2
1
0
−1
−2
P54310
0
50
100
Distance in Percent

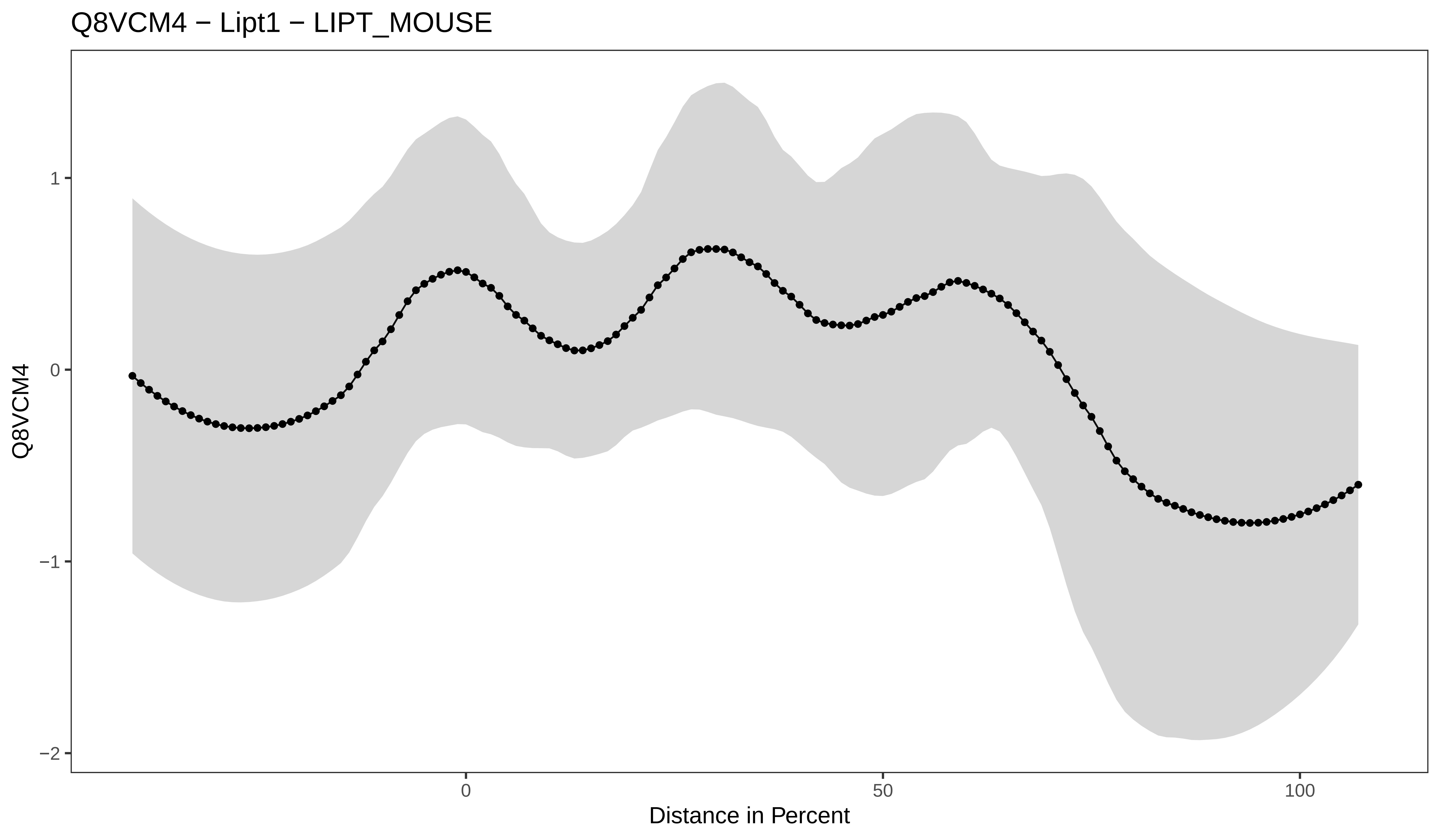
Q8VCM4 − Lipt1 − LIPT_MOUSE
Q8VCM4
Distance in Percent
1
0
−1
−2
0
50
100

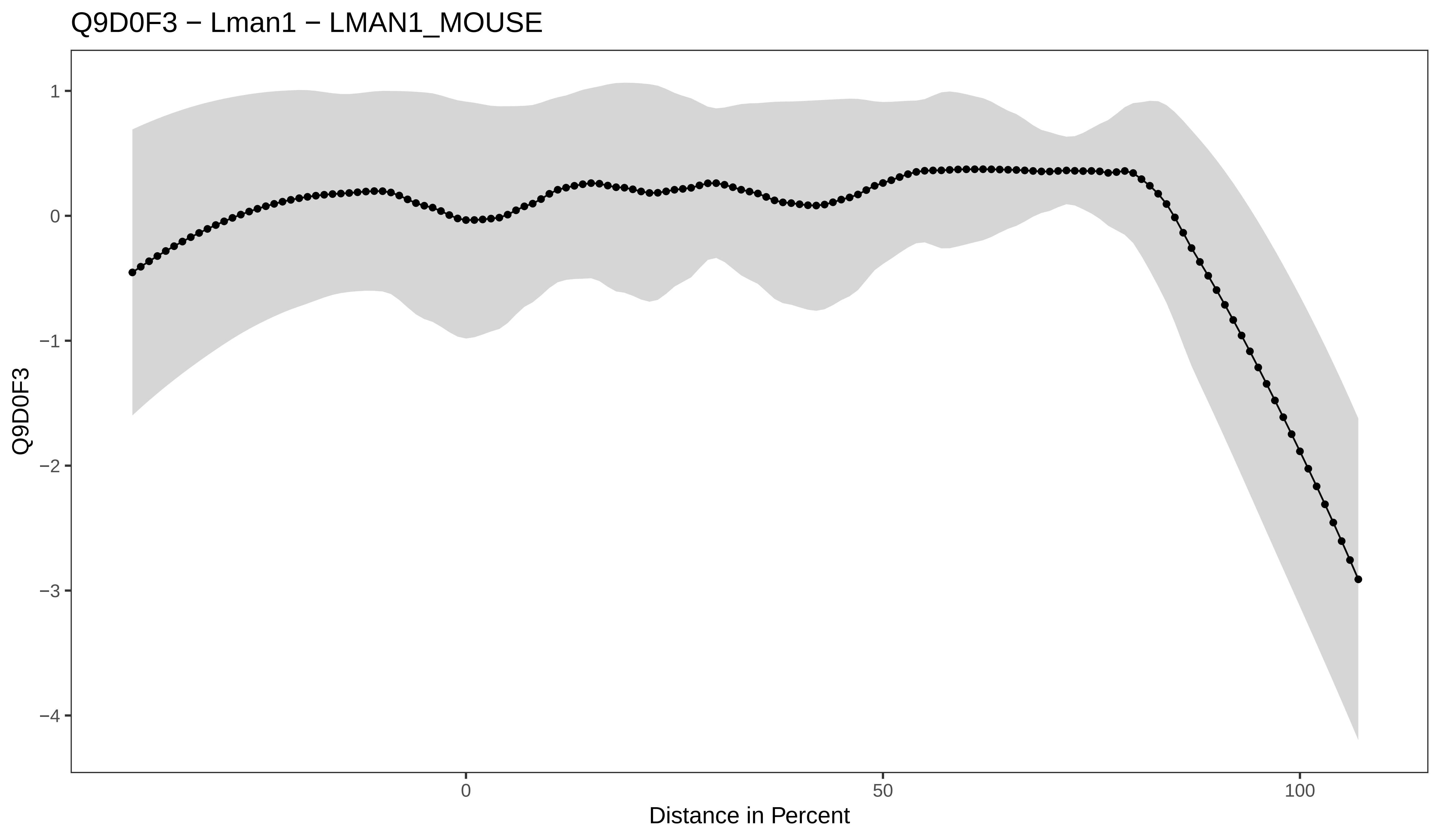
Q9D0F3 − Lman1 − LMAN1_MOUSE
1
0
−1
−2
−3
−4
Q9D0F3
0
50
100
Distance in Percent

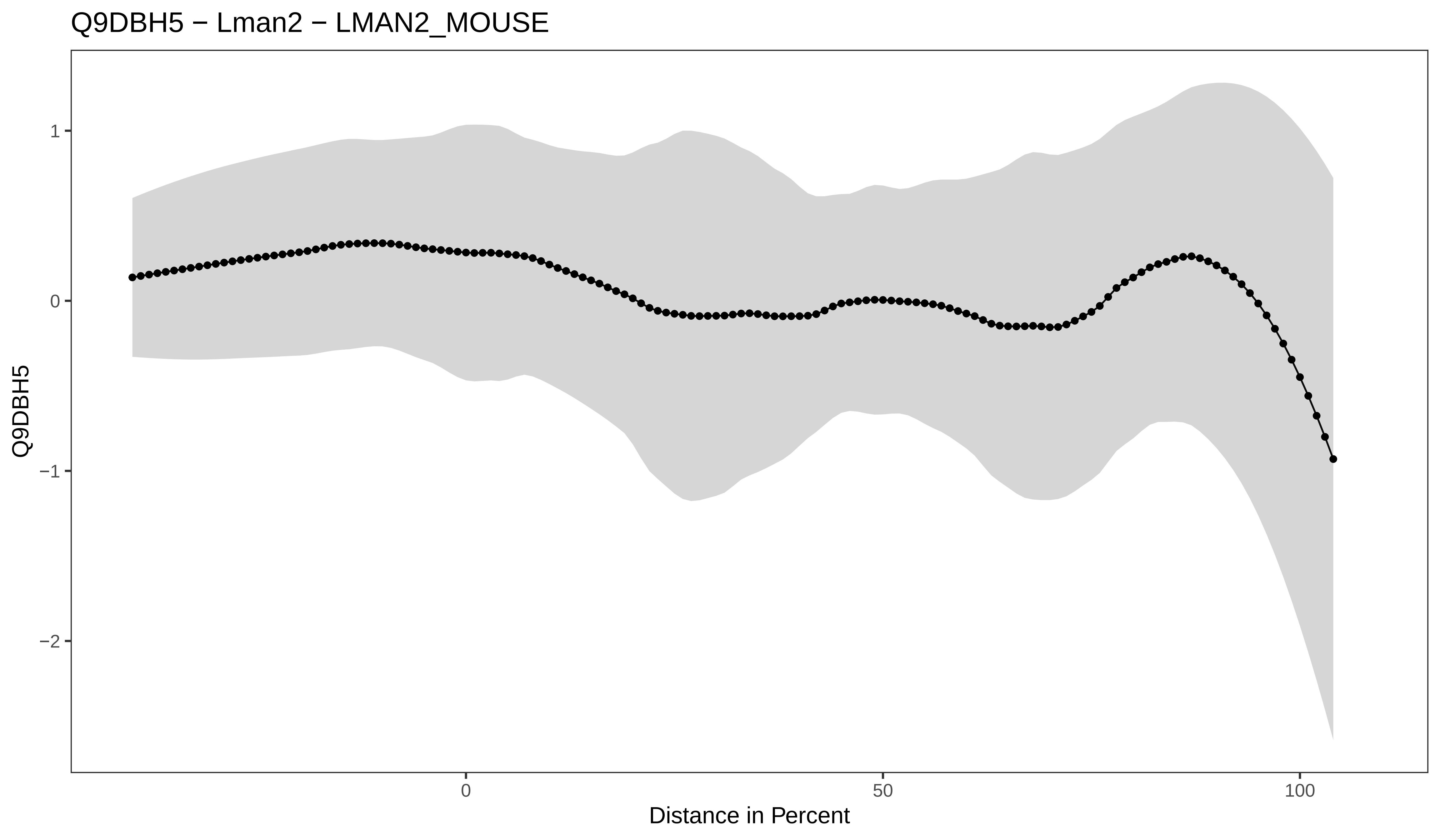
Q9DBH5 − Lman2 − LMAN2_MOUSE
1
0
−1
−2
Q9DBH5
0
50
100
Distance in Percent

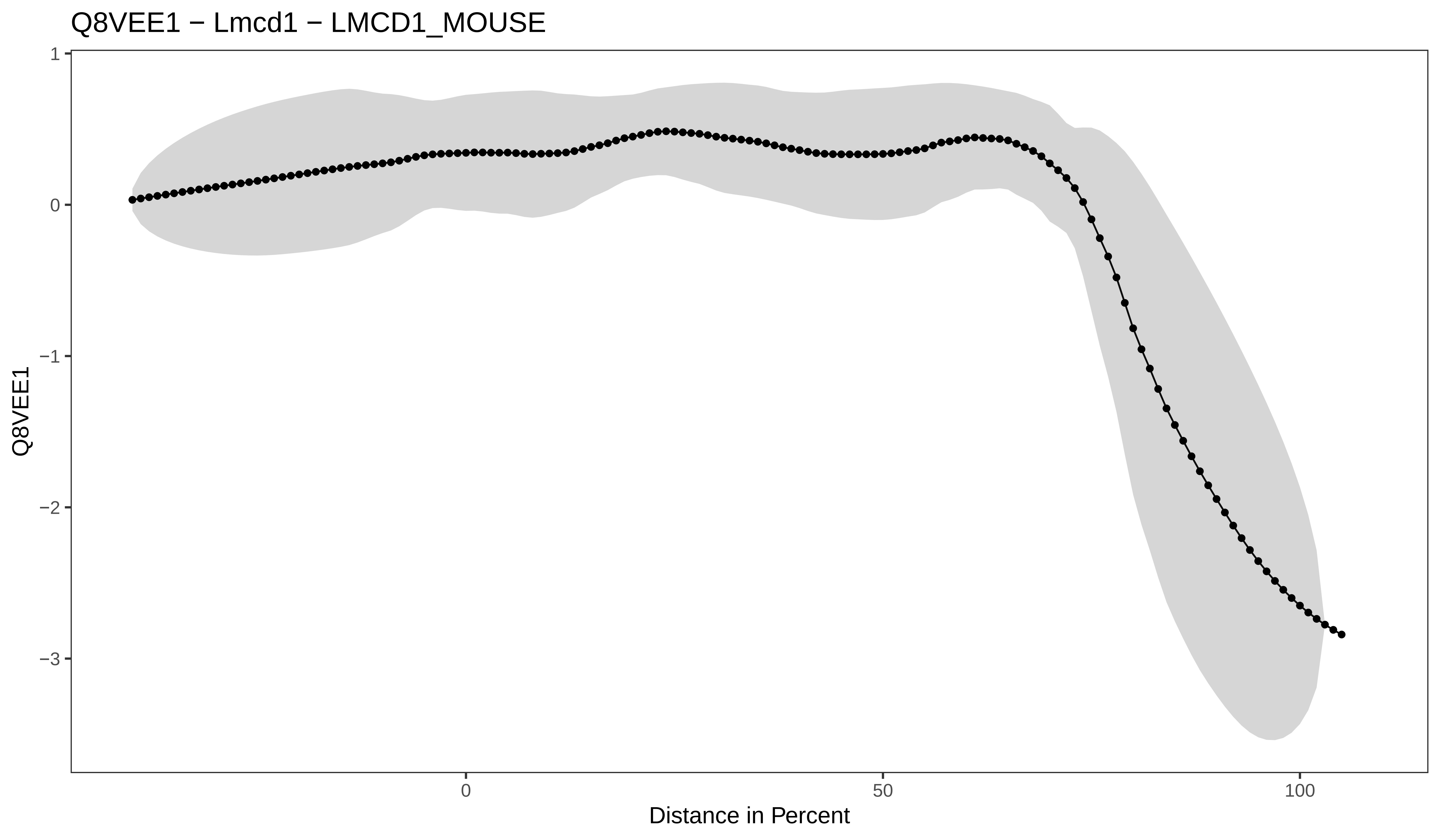

Q8VEE1 − Lmcd1 − LMCD1_MOUSE
1
0
−1
−2
−3
Q8VEE1
0
50
100
Distance in Percent

P48678 − Lmna − LMNA_MOUSE

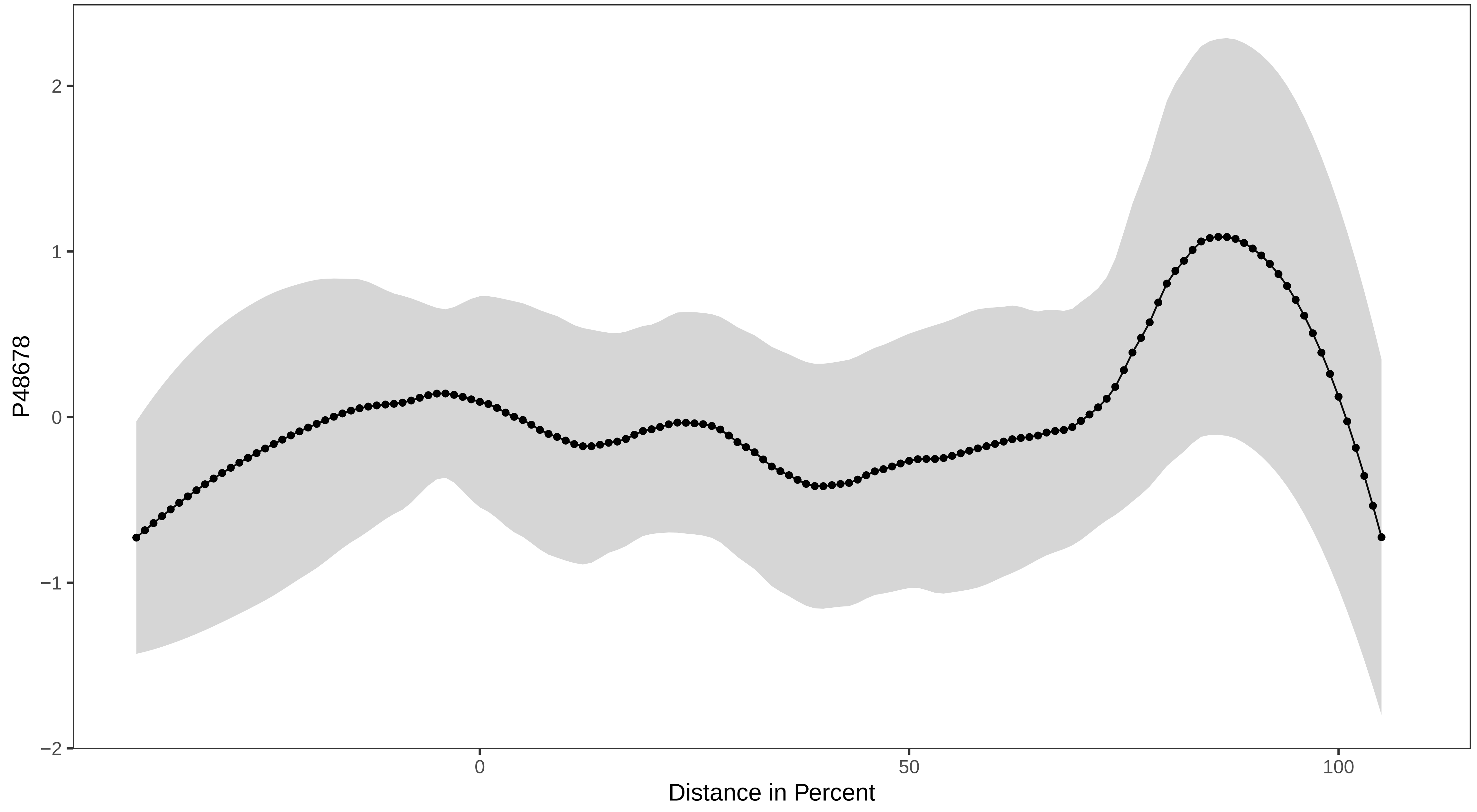

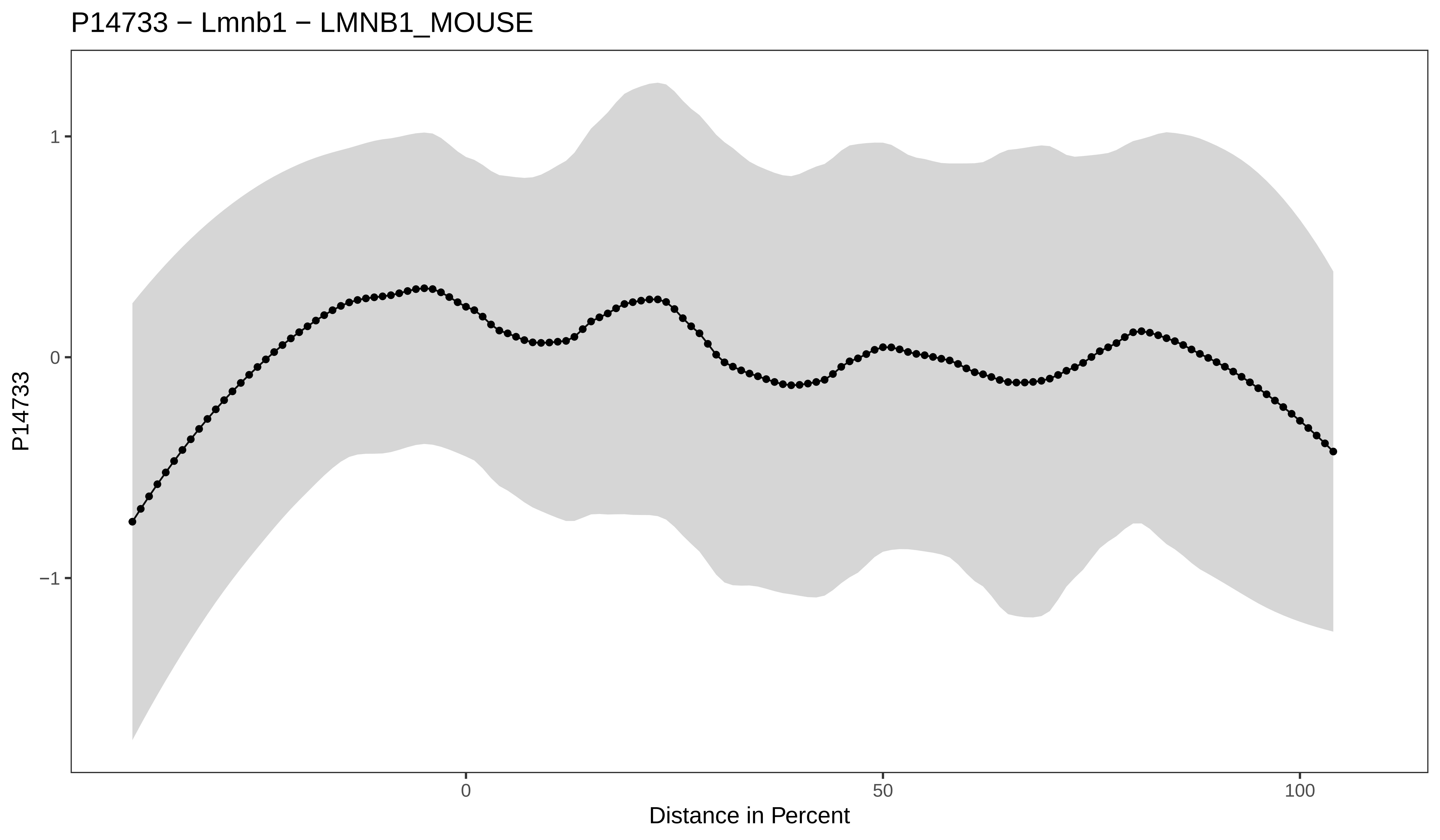

P14733 − Lmnb1 − LMNB1_MOUSE
1
0
−1
P14733
0
50
100
Distance in Percent

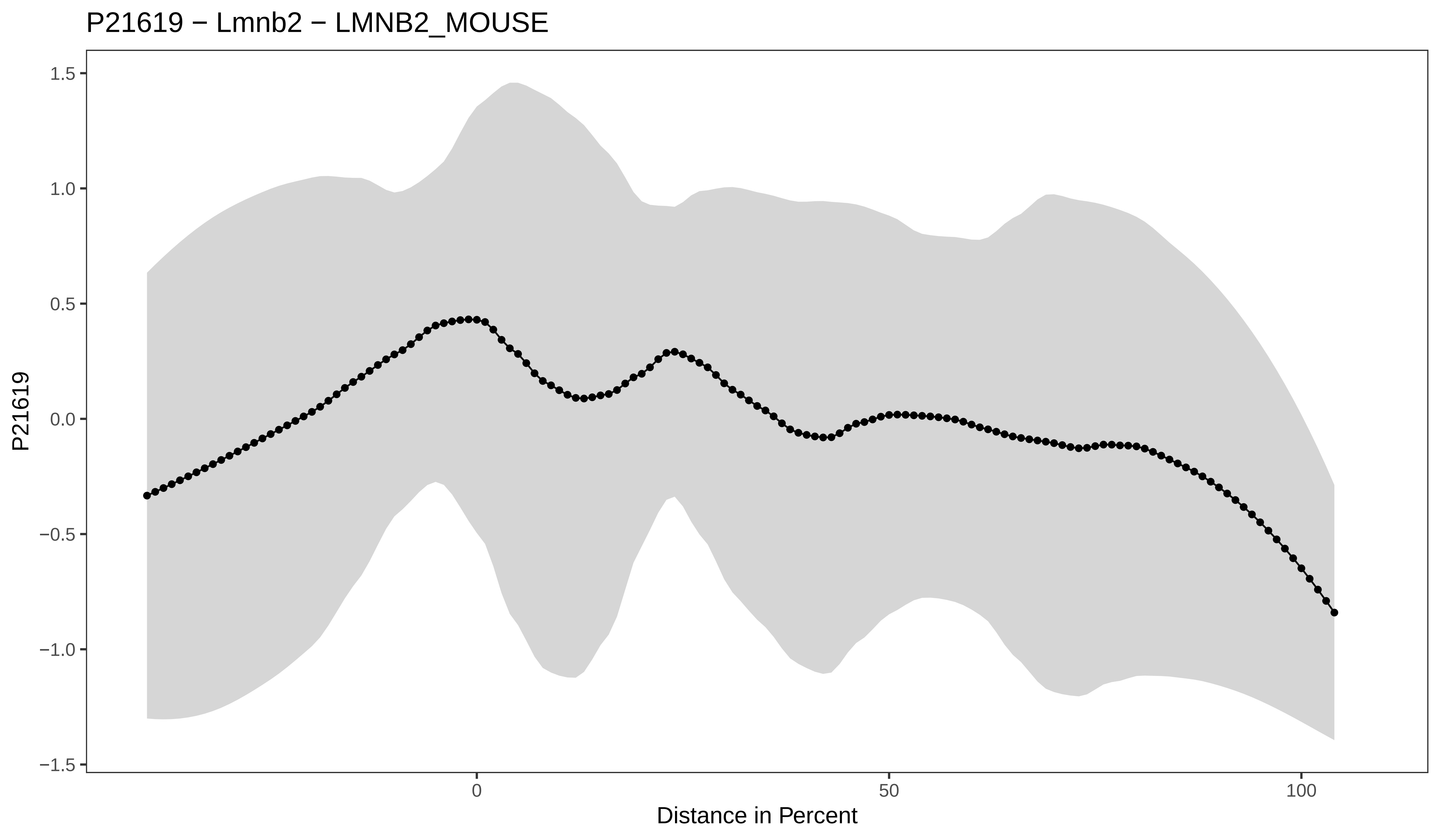

P21619 − Lmnb2 − LMNB2_MOUSE
1.5
1.0
0.5
0.0
−0.5
−1.0
−1.5
P21619
0
50
100
Distance in Percent

Q8BVA4 − Lmod1 − LMOD1_MOUSE

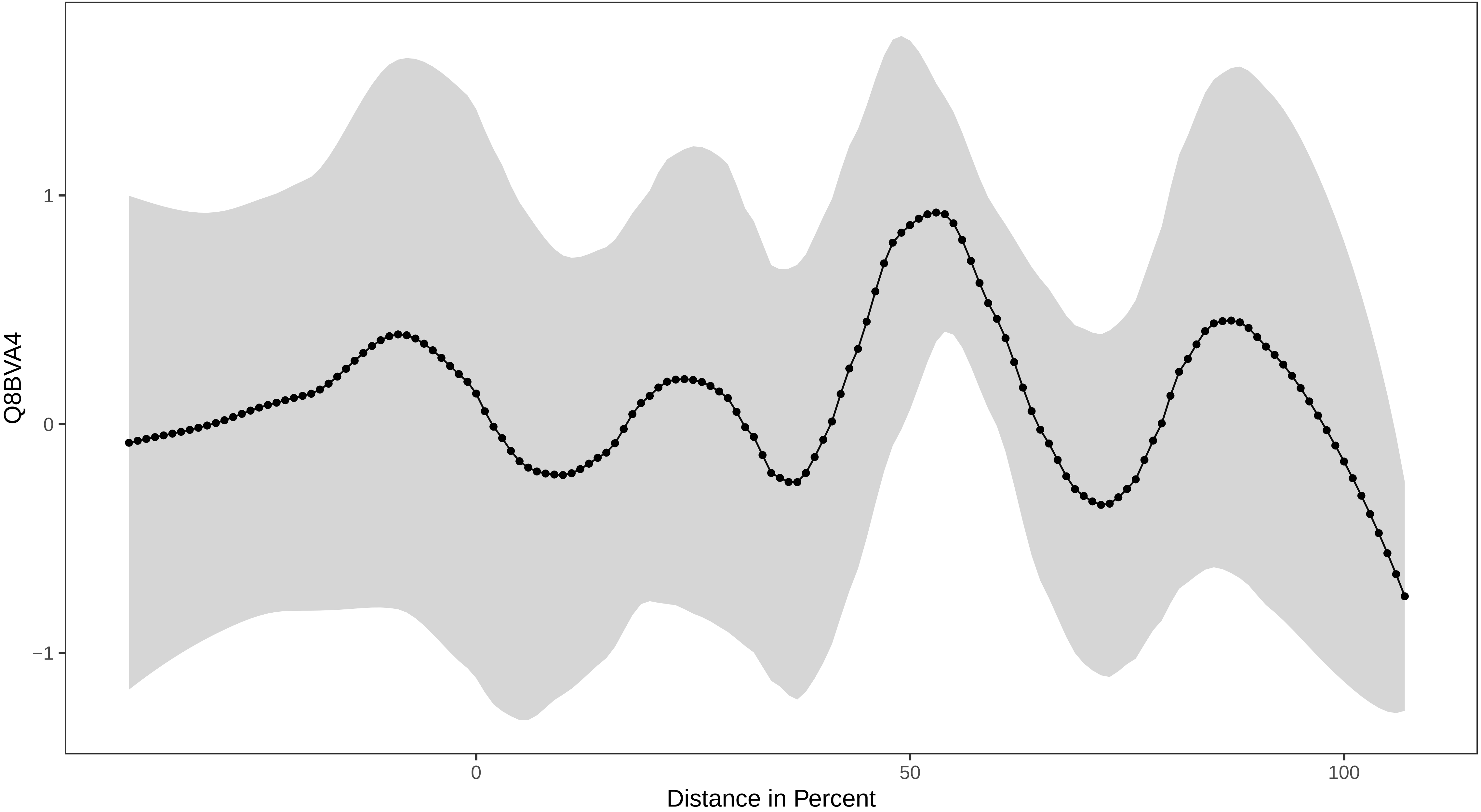

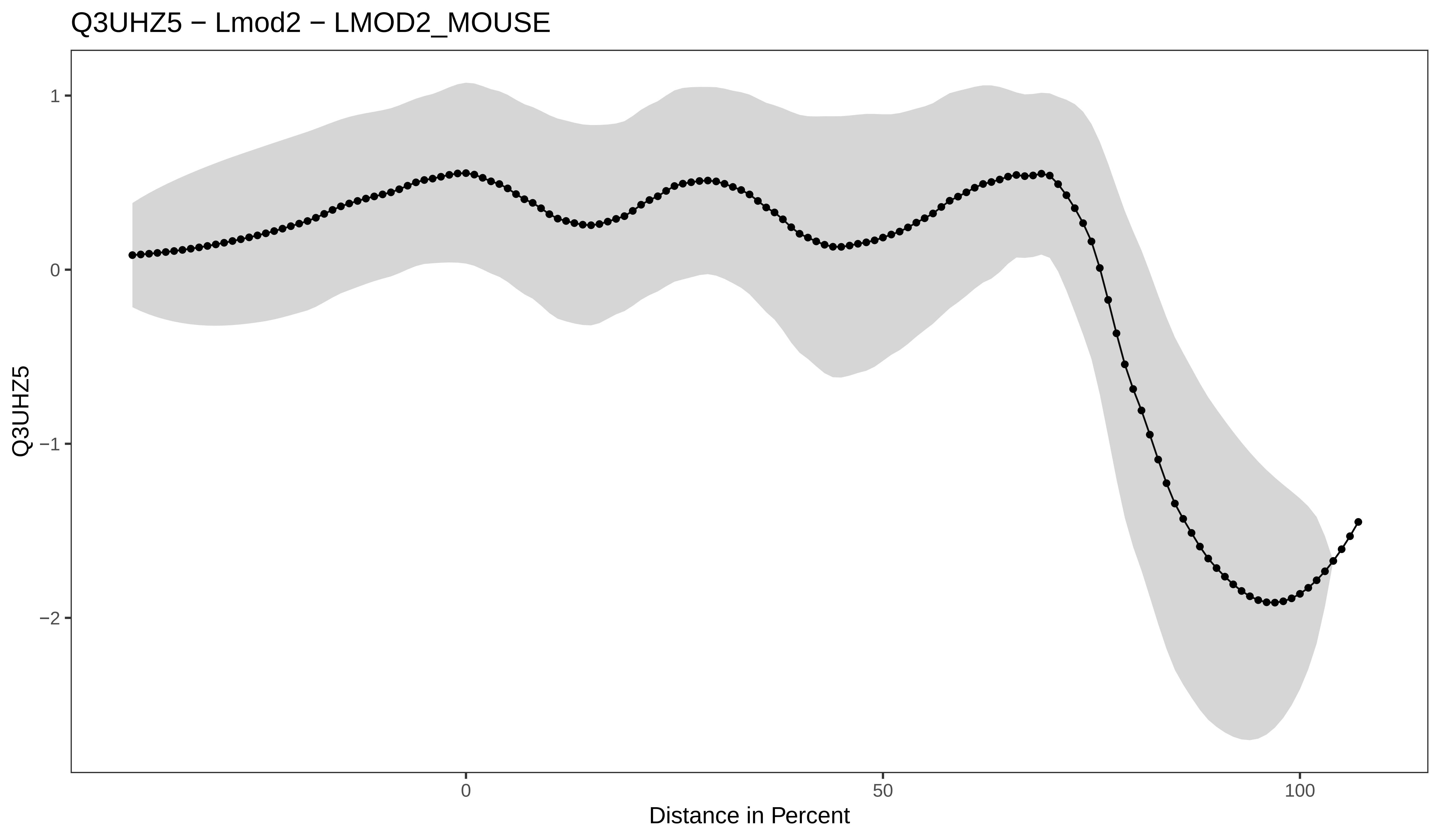

Q3UHZ5 − Lmod2 − LMOD2_MOUSE
1
0
−1
−2
Q3UHZ5
0
50
100
Distance in Percent

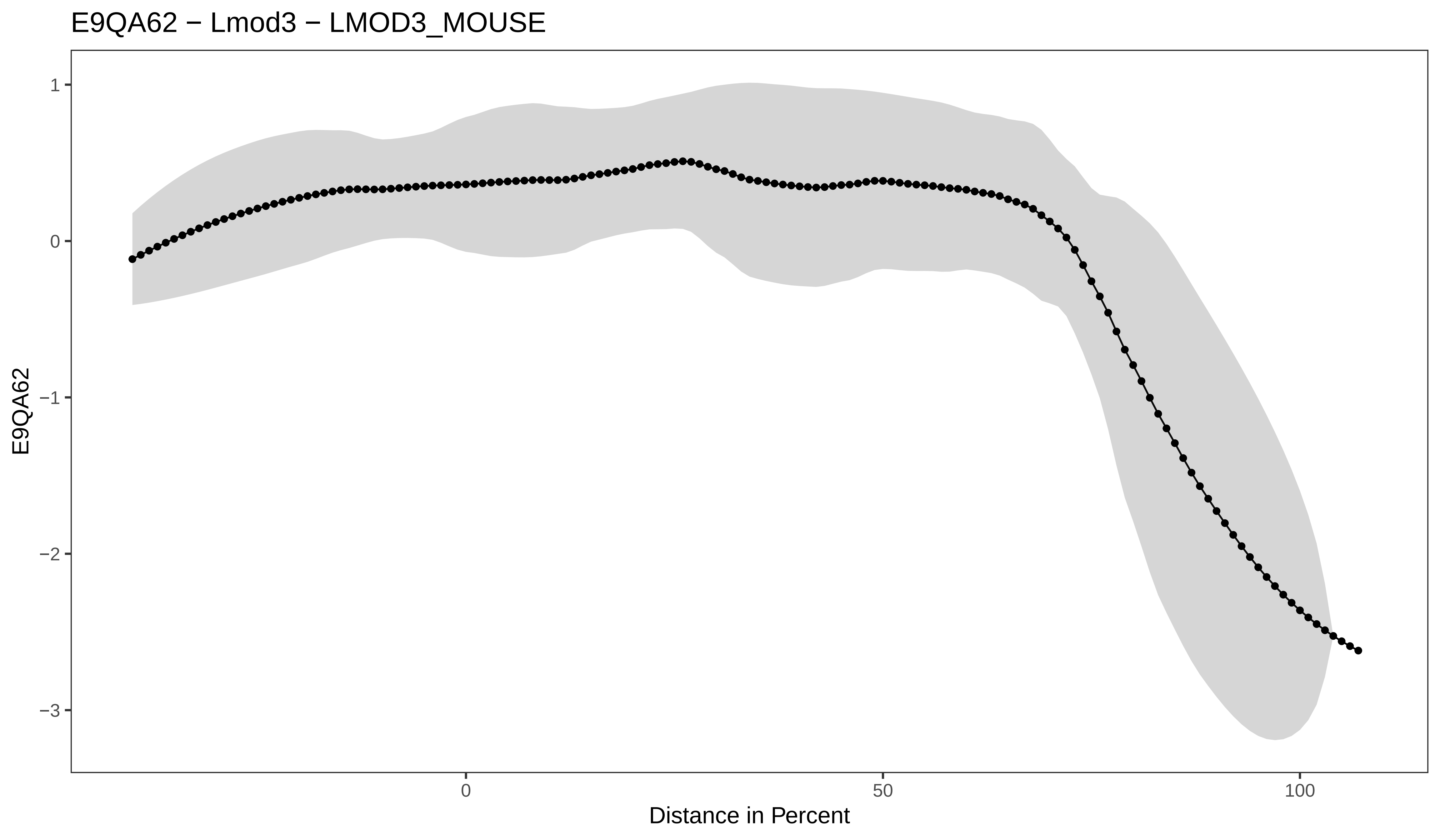

E9QA62 − Lmod3 − LMOD3_MOUSE
1
0
−1
−2
−3
E9QA62
0
50
100
Distance in Percent

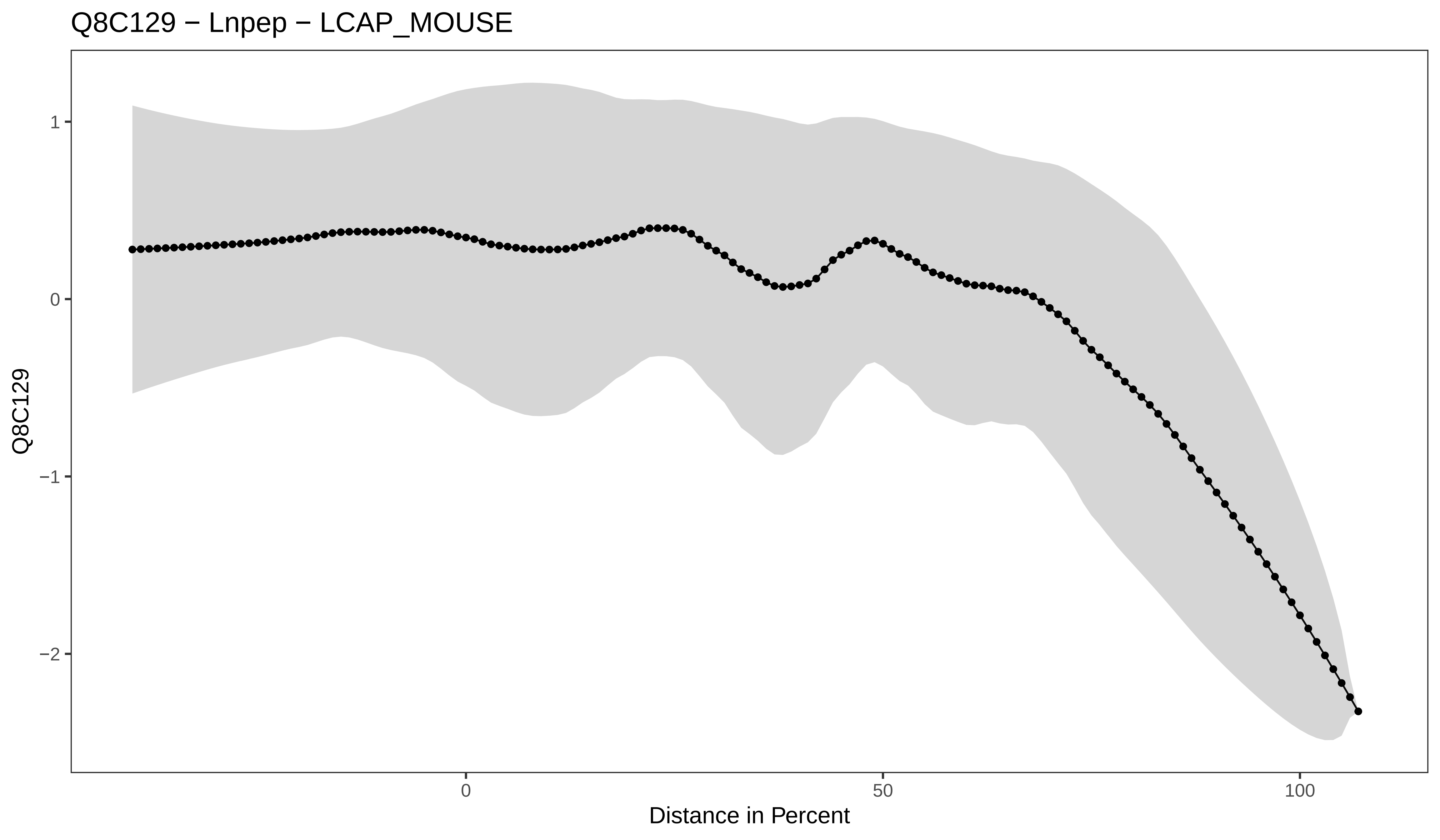

Q8C129 − Lnpep − LCAP_MOUSE
1
0
−1
−2
Q8C129
0
50
100
Distance in Percent

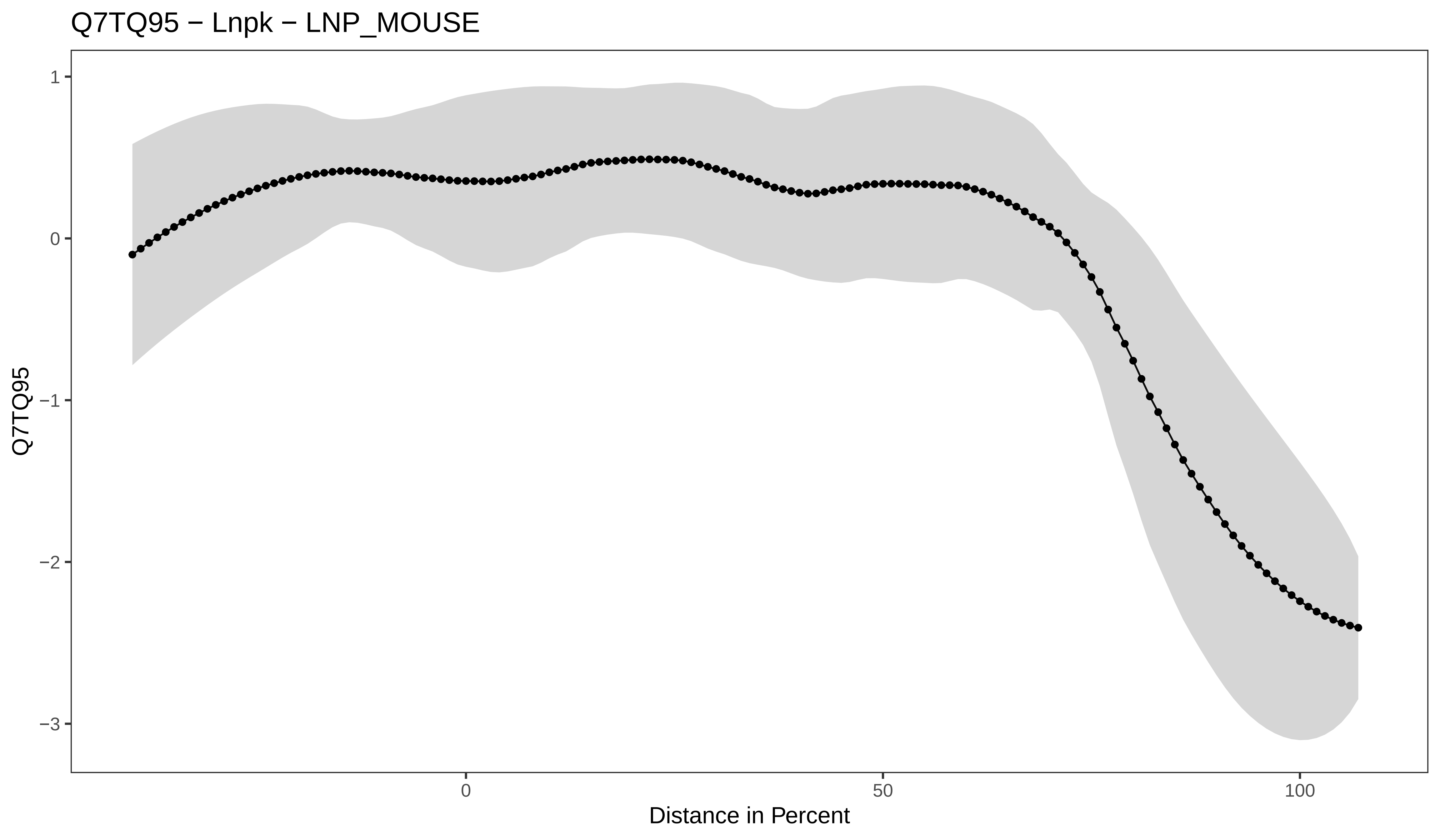

Q7TQ95 − Lnpk − LNP_MOUSE
Q7TQ95
1
0
−1
−2
−3
0
50
100
Distance in Percent

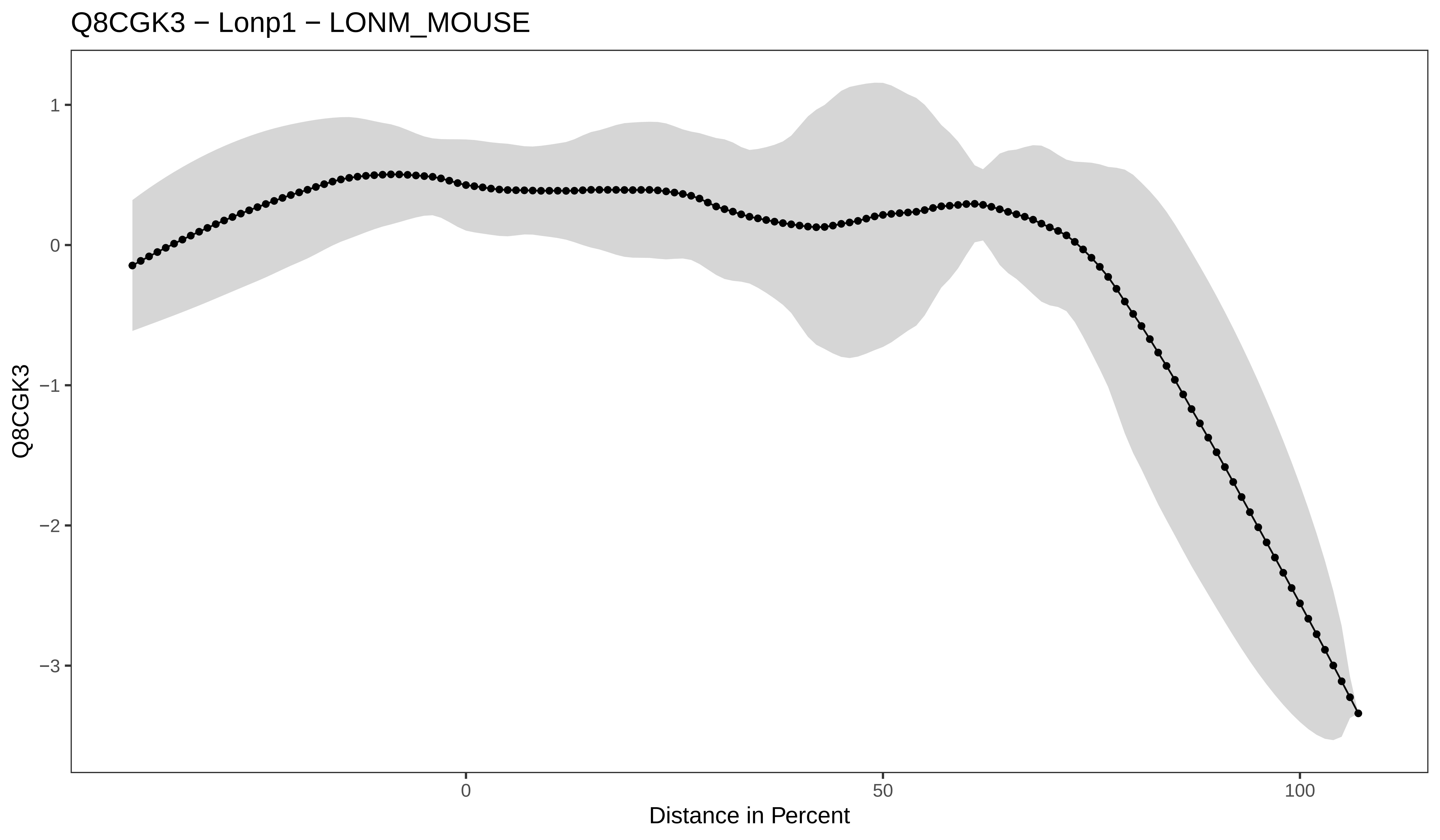

Q8CGK3 − Lonp1 − LONM_MOUSE
1
0
−1
−2
−3
Q8CGK3
0
50
100
Distance in Percent

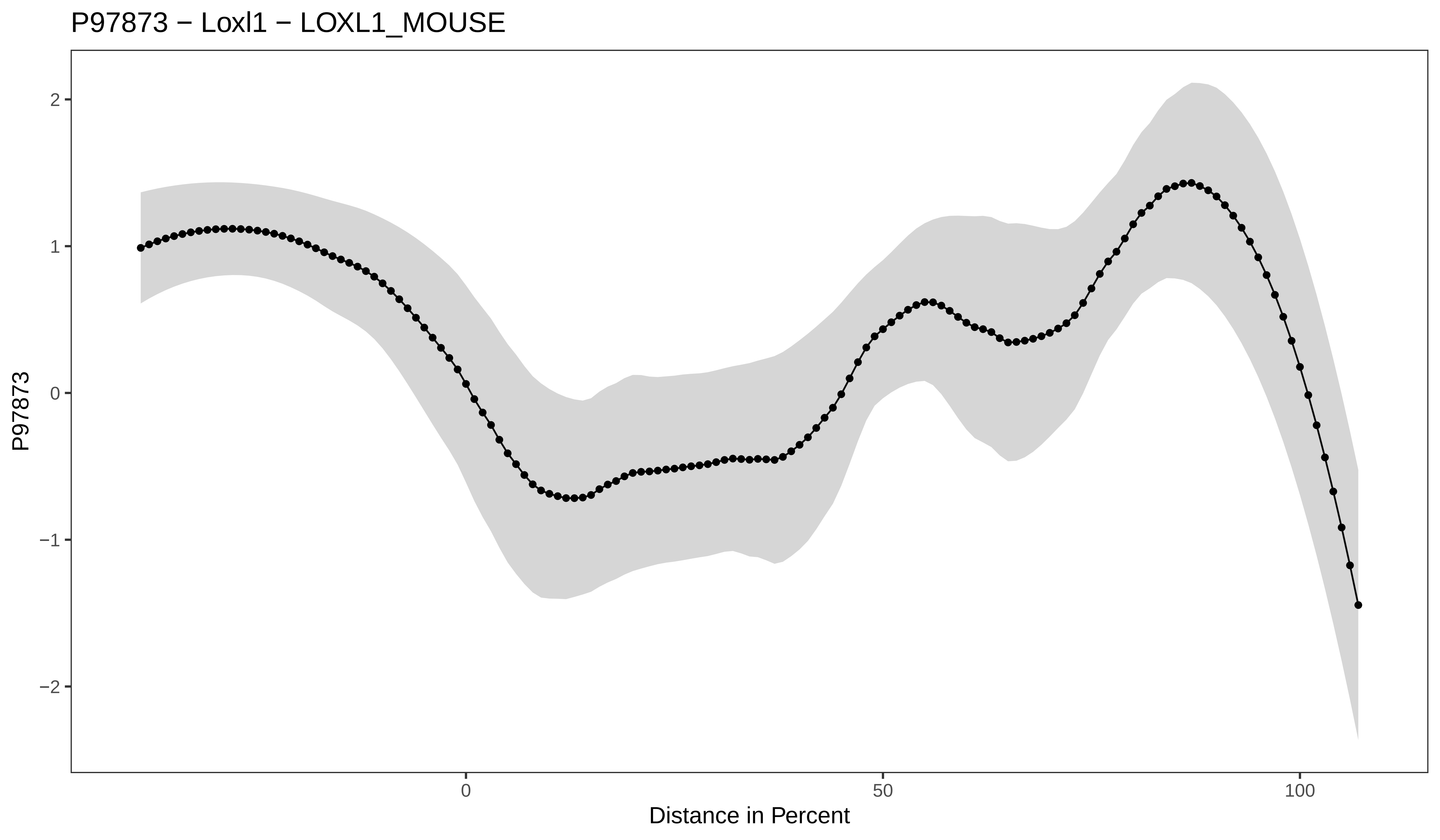
P97873 − Loxl1 − LOXL1_MOUSE
P97873
Distance in Percent
2
1
0
−1
−2
0
50
100

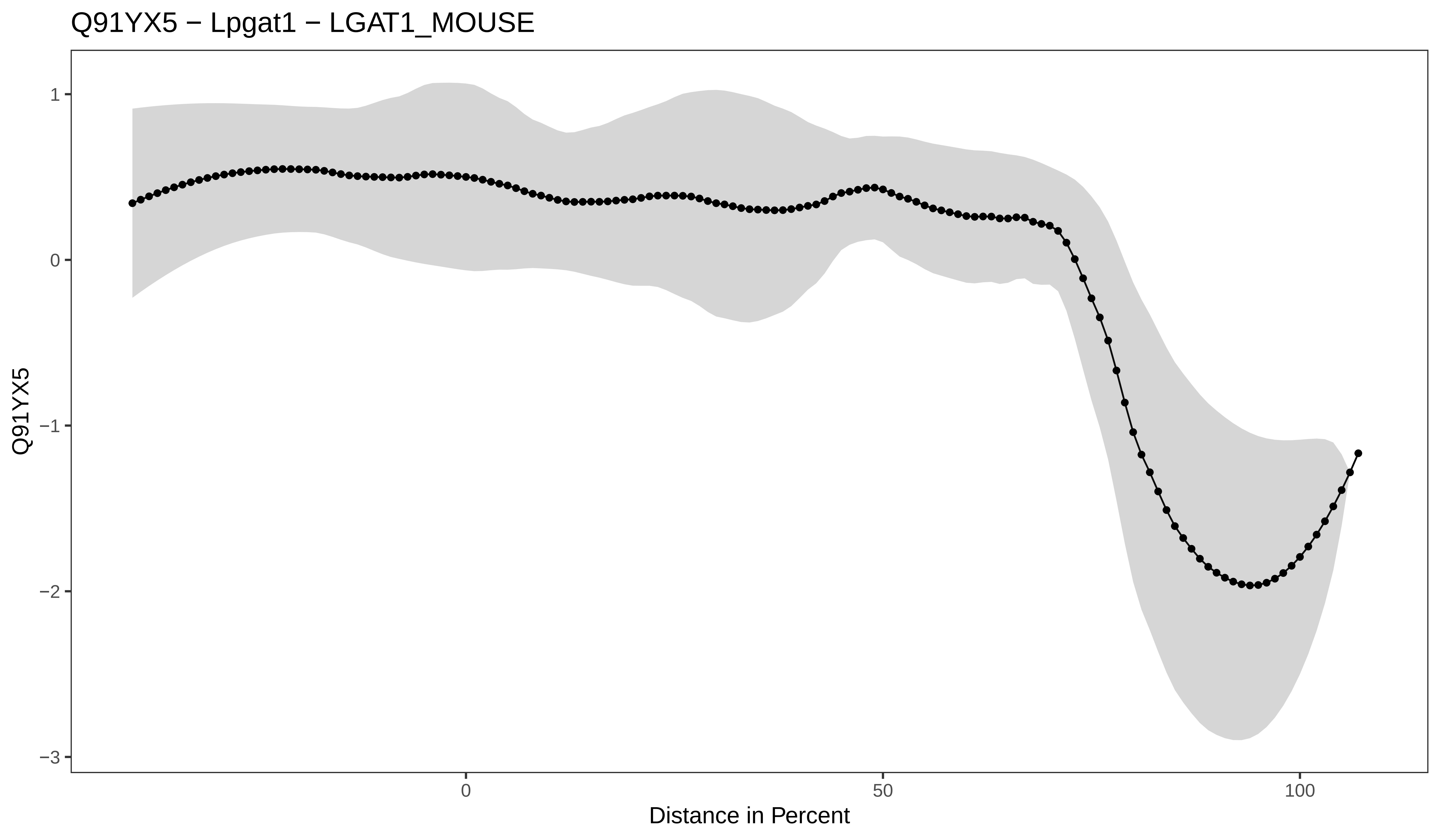

Q91YX5 − Lpgat1 − LGAT1_MOUSE
1
0
−1
−2
−3
Q91YX5
0
50
100
Distance in Percent

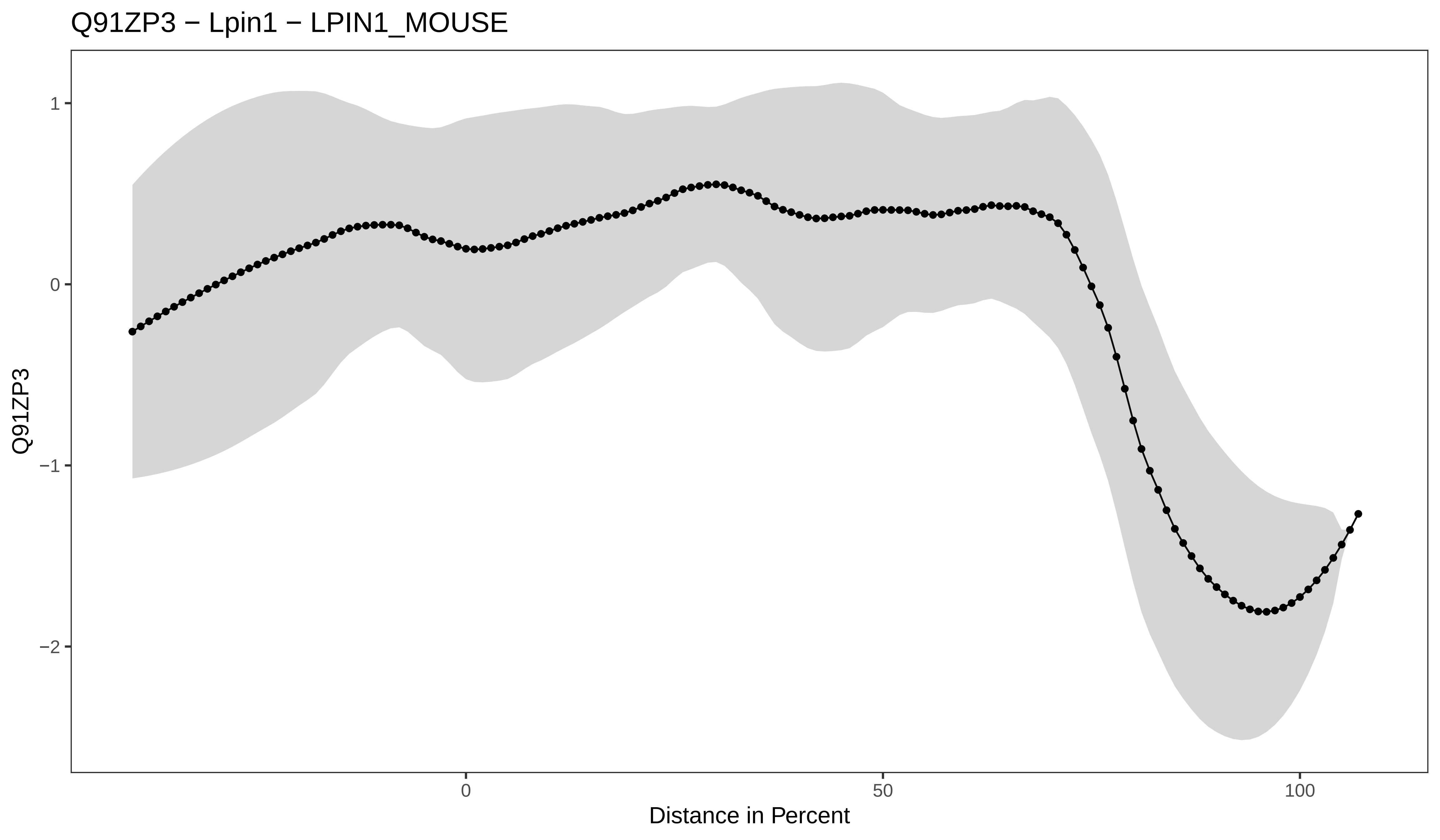

Q91ZP3 − Lpin1 − LPIN1_MOUSE
1
0
−1
−2
Q91ZP3
0
50
100
Distance in Percent

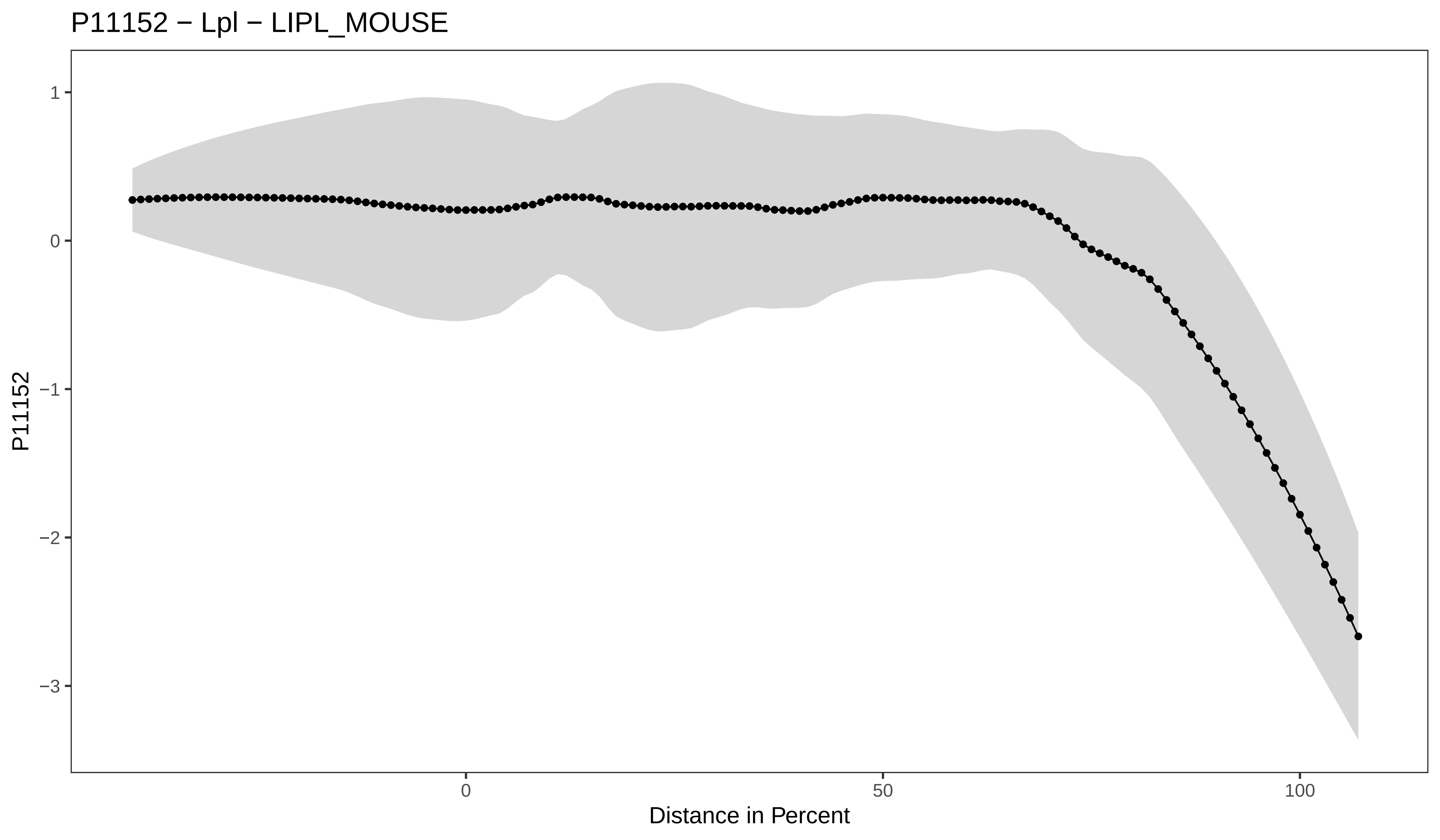
P11152 − Lpl − LIPL_MOUSE
P11152
1
0
−1
−2
−3
0
50
100
Distance in Percent

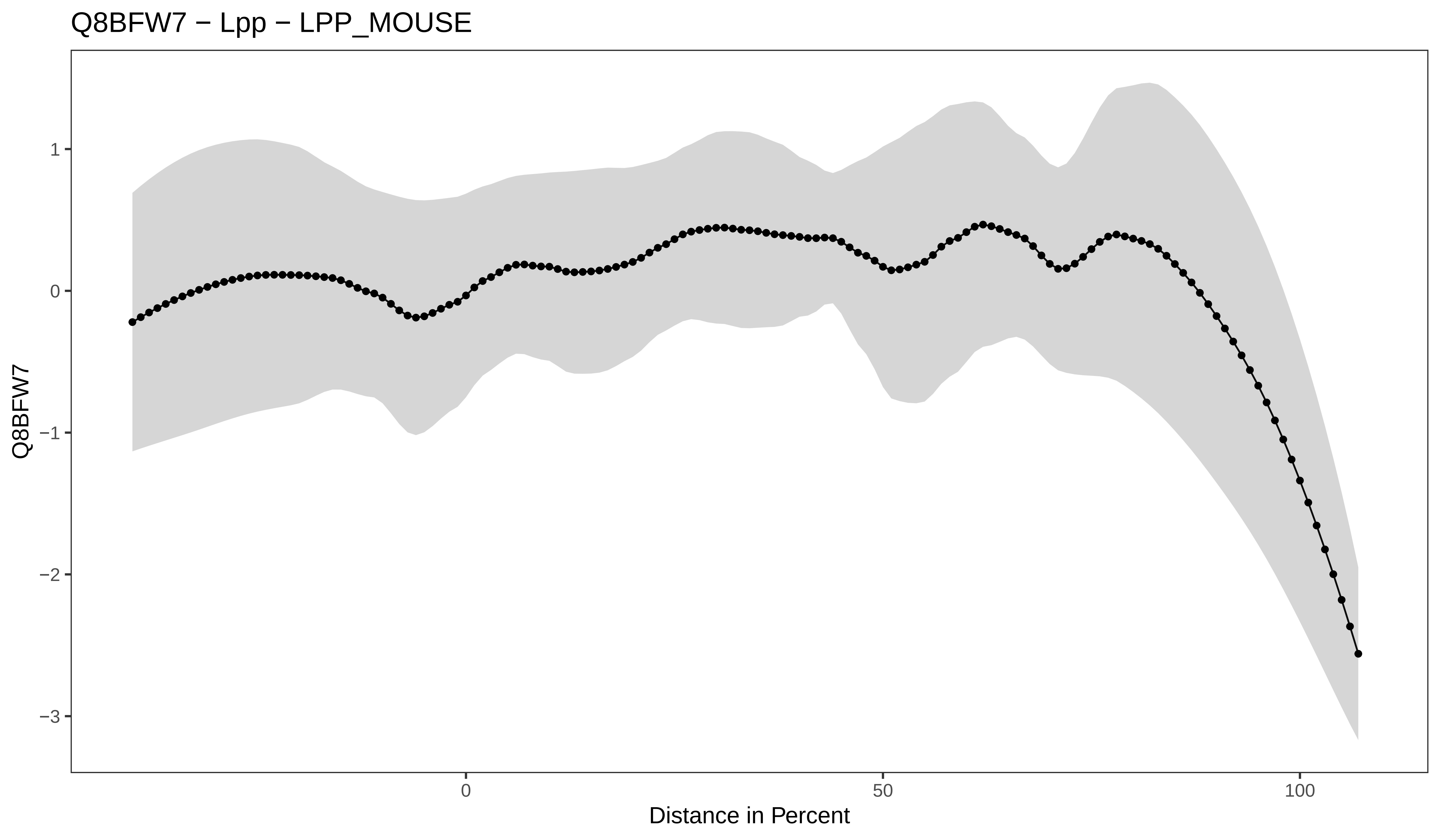

Q8BFW7 − Lpp − LPP_MOUSE
Q8BFW7
1
0
−1
−2
−3
0
50
100
Distance in Percent

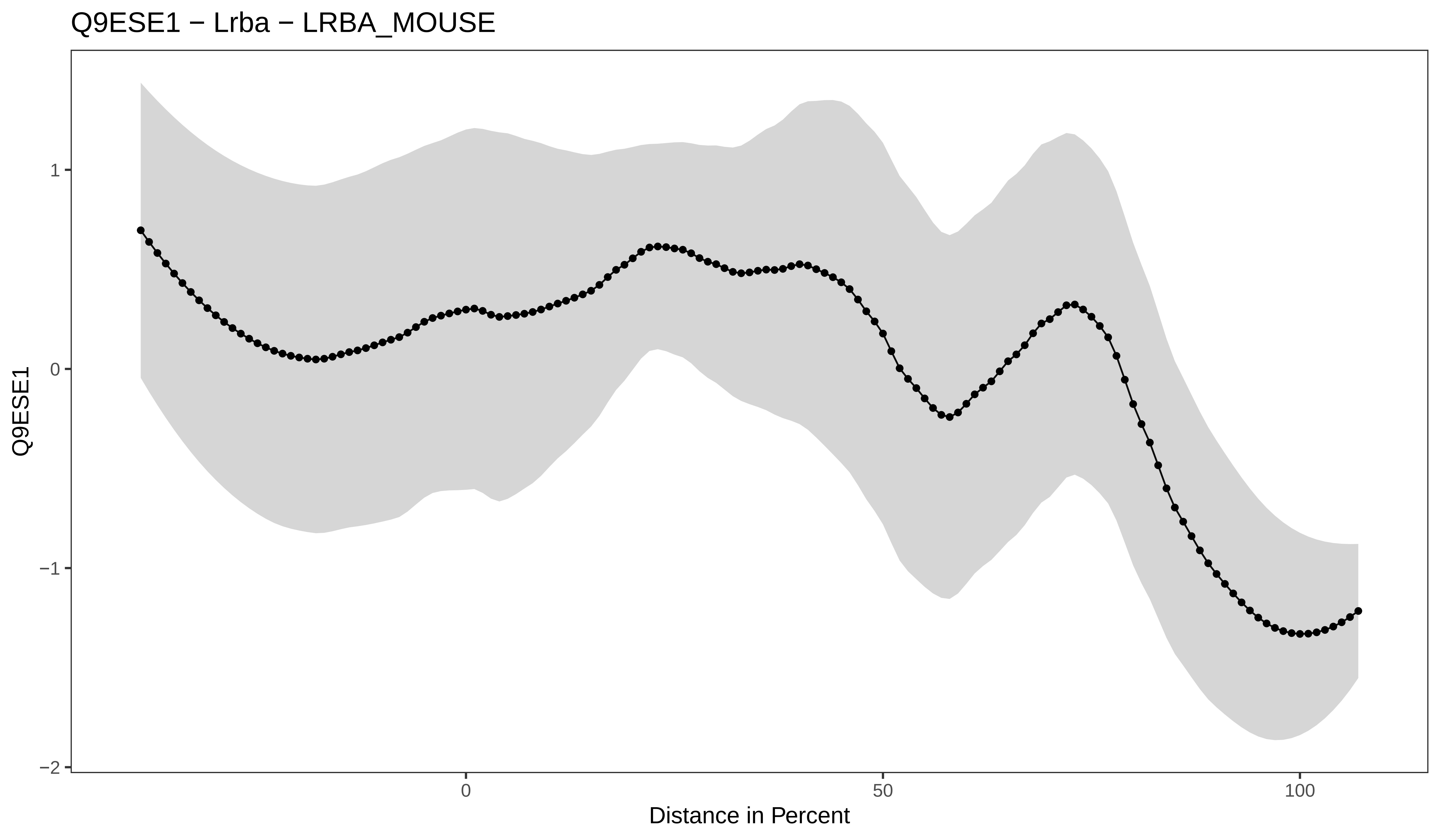

Q9ESE1 − Lrba − LRBA_MOUSE
Q9ESE1
1
0
−1
−2
0
50
100
Distance in Percent

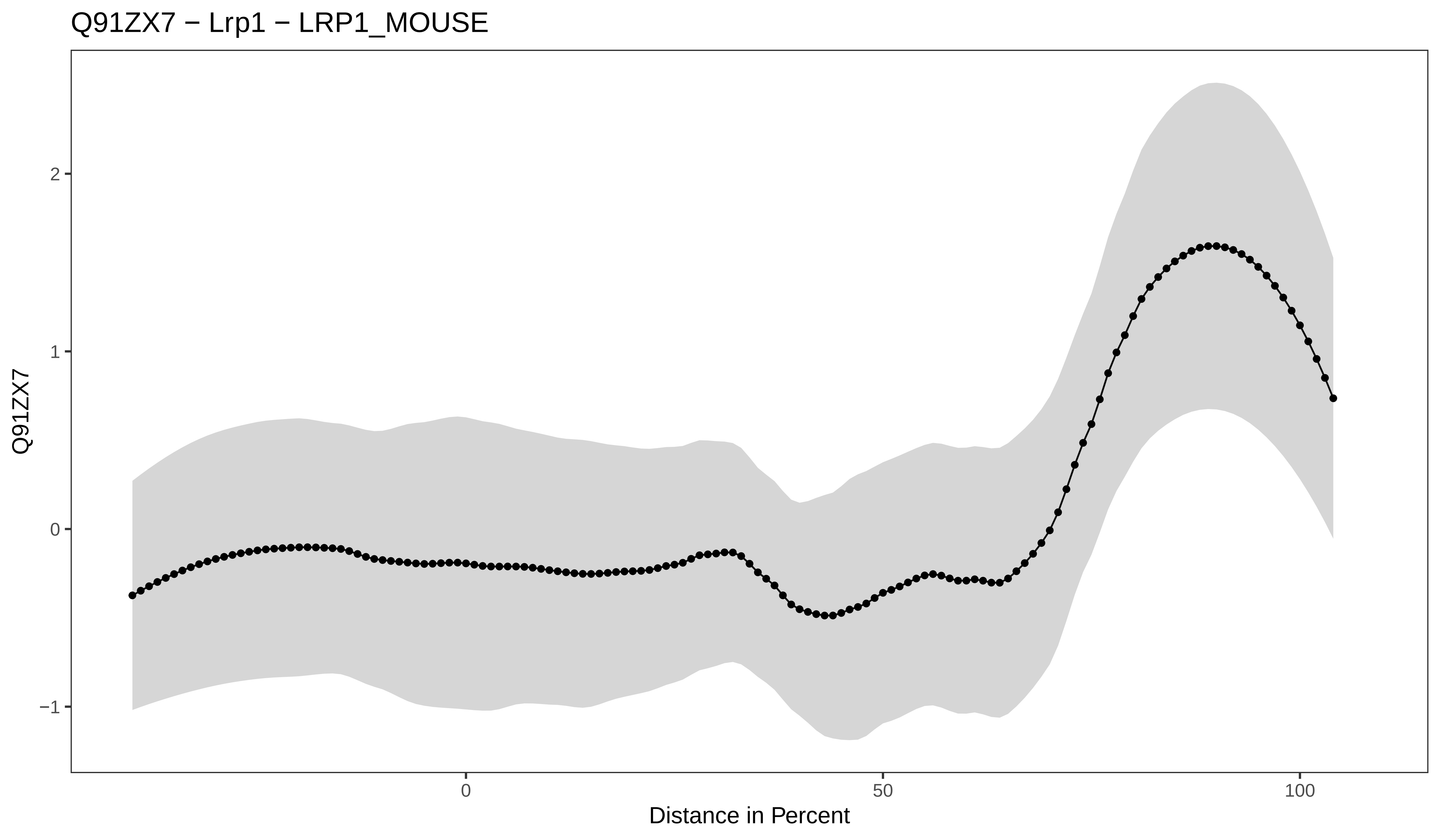

Q91ZX7 − Lrp1 − LRP1_MOUSE
Q91ZX7
2
1
0
−1
0
50
100
Distance in Percent

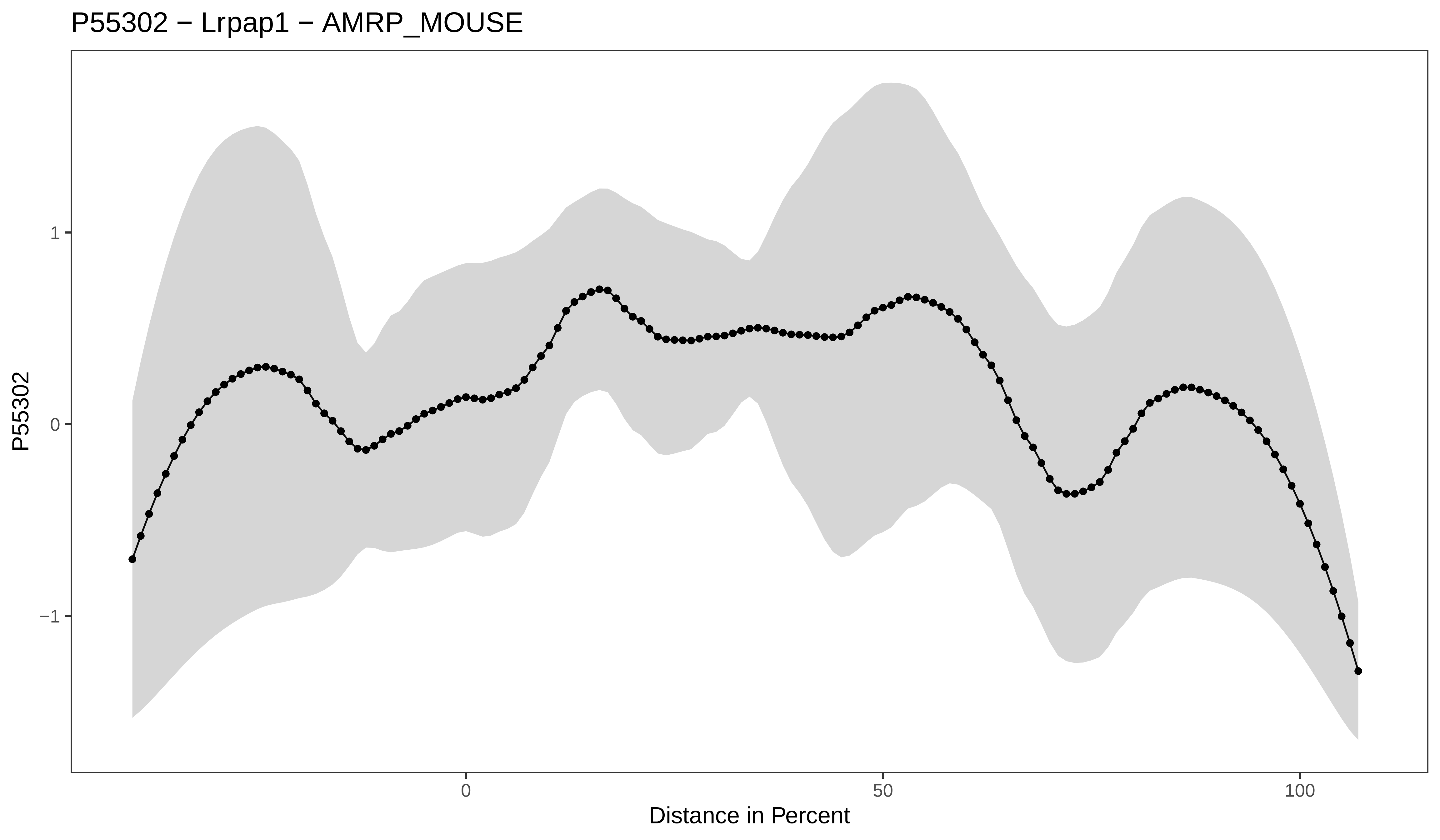
P55302 − Lrpap1 − AMRP_MOUSE
P55302
1
0
−1
0
50
100
Distance in Percent

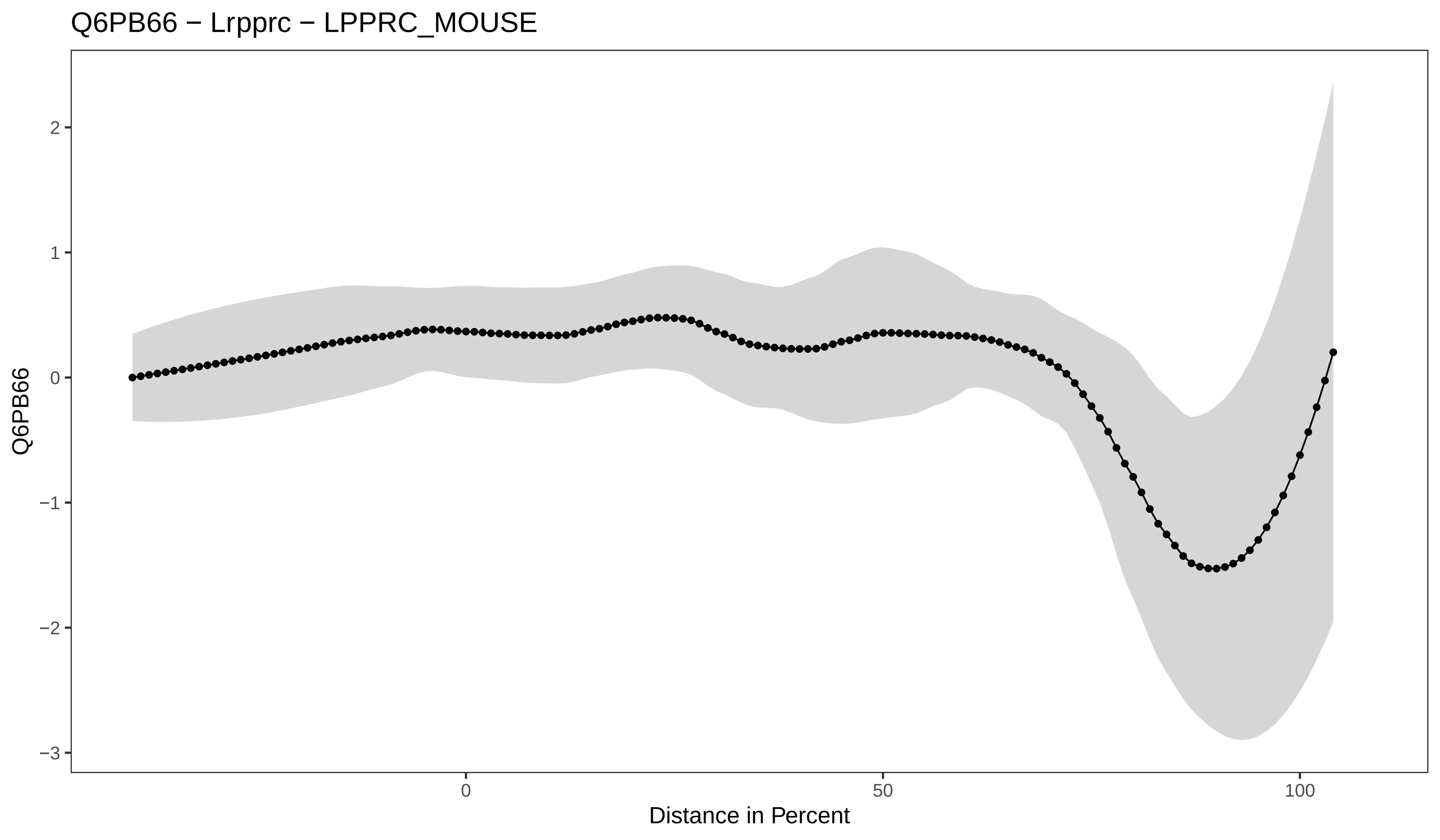
Q6PB66 − Lrpprc − LPPRC_MOUSE
2
1
0
−1
−2
−3
Q6PB66
0
50
100
Distance in Percent

Q8VDB8 − Lrrc2 − LRRC2_MOUSE

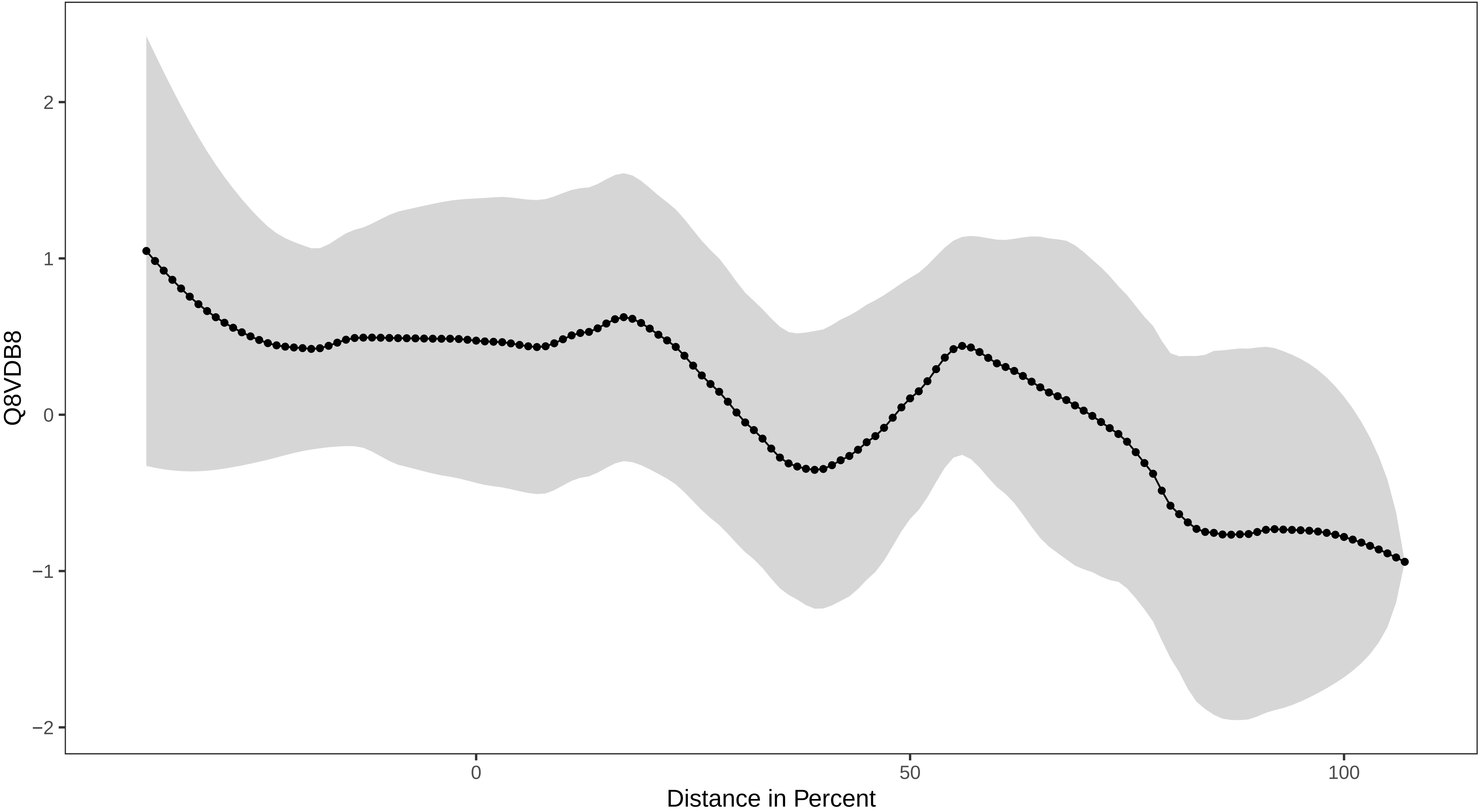

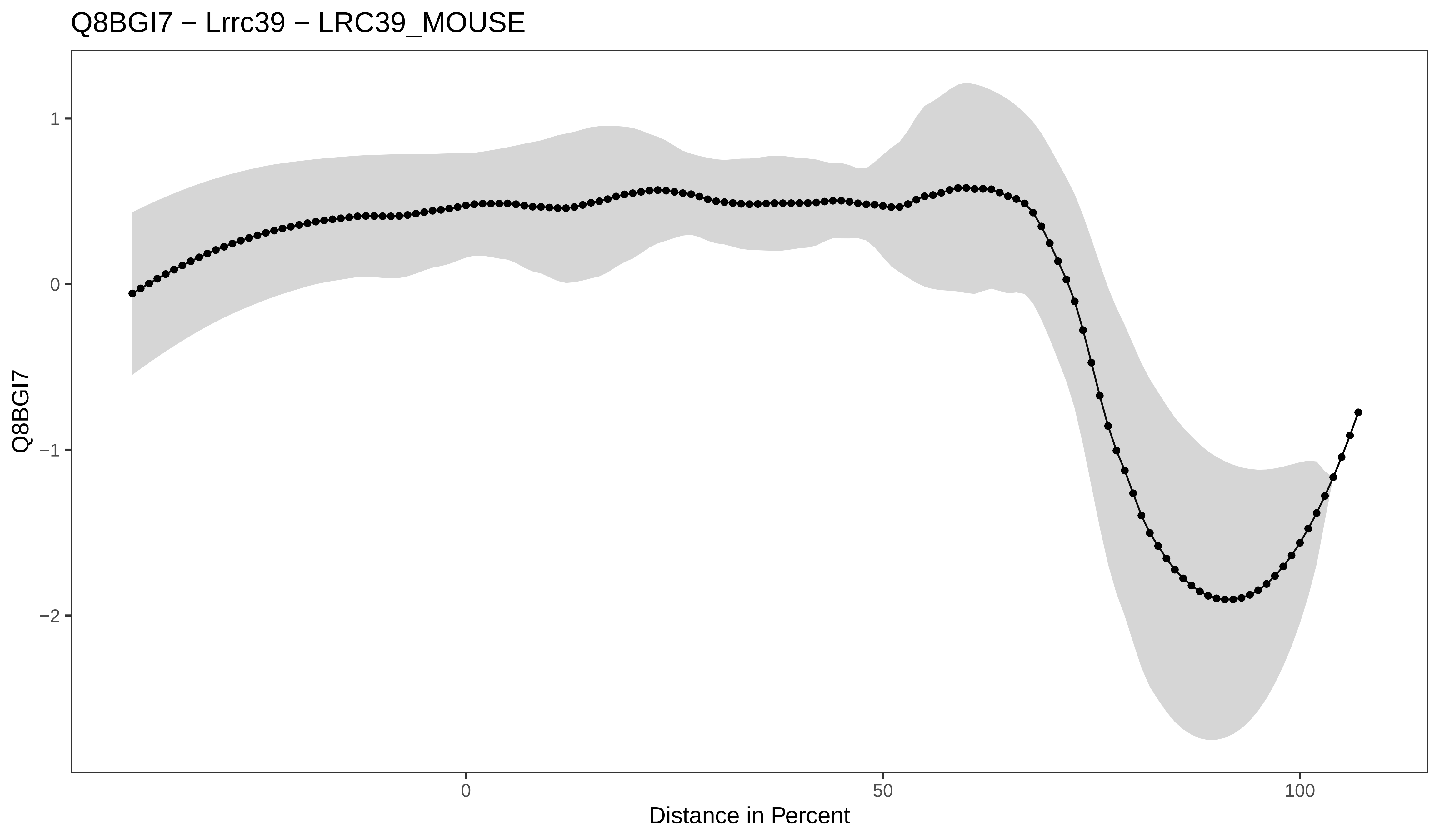

Q8BGI7 − Lrrc39 − LRC39_MOUSE
Q8BGI7
1
0
−1
−2
0
50
100
Distance in Percent

Q505F5 − Lrrc47 − LRC47_MOUSE

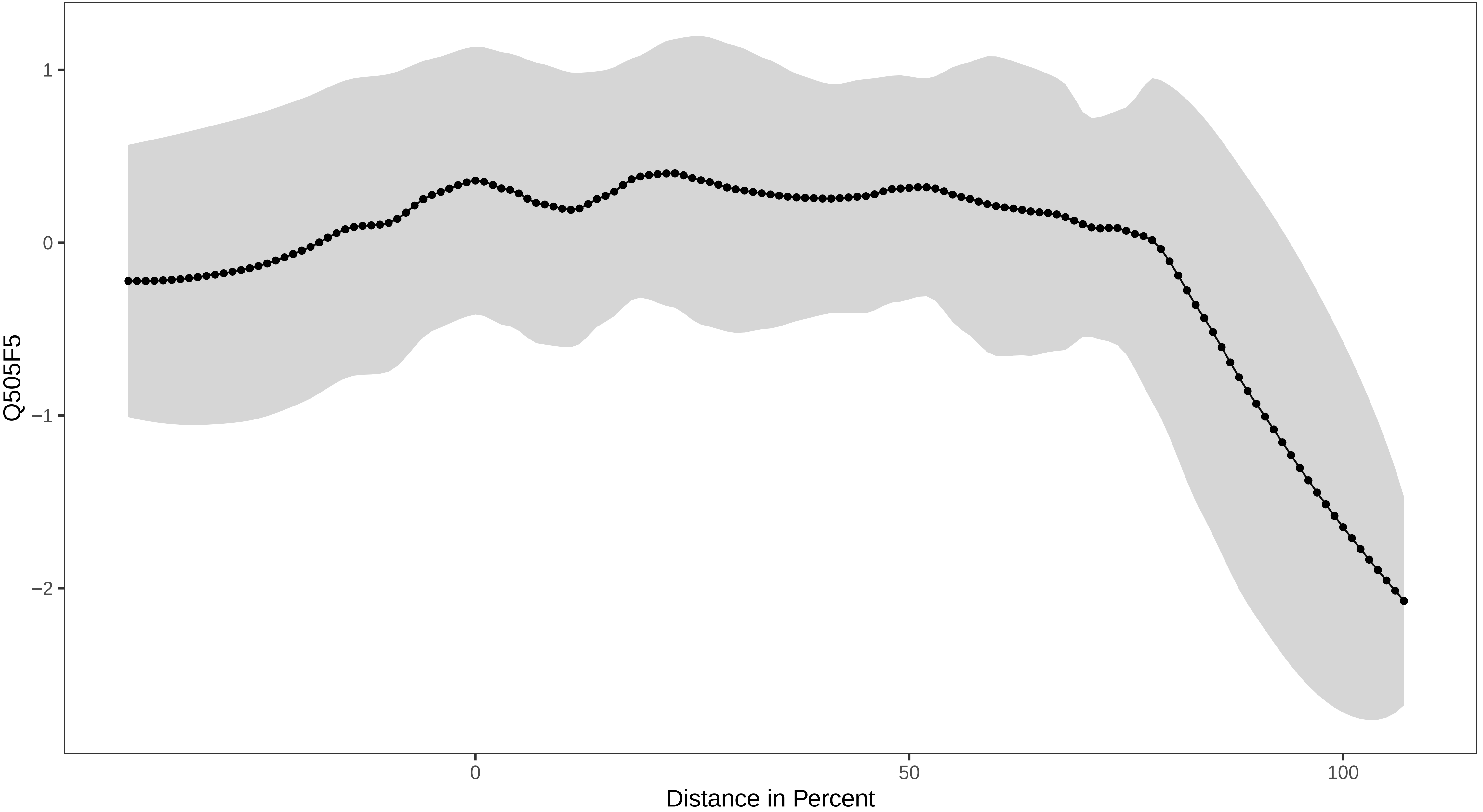

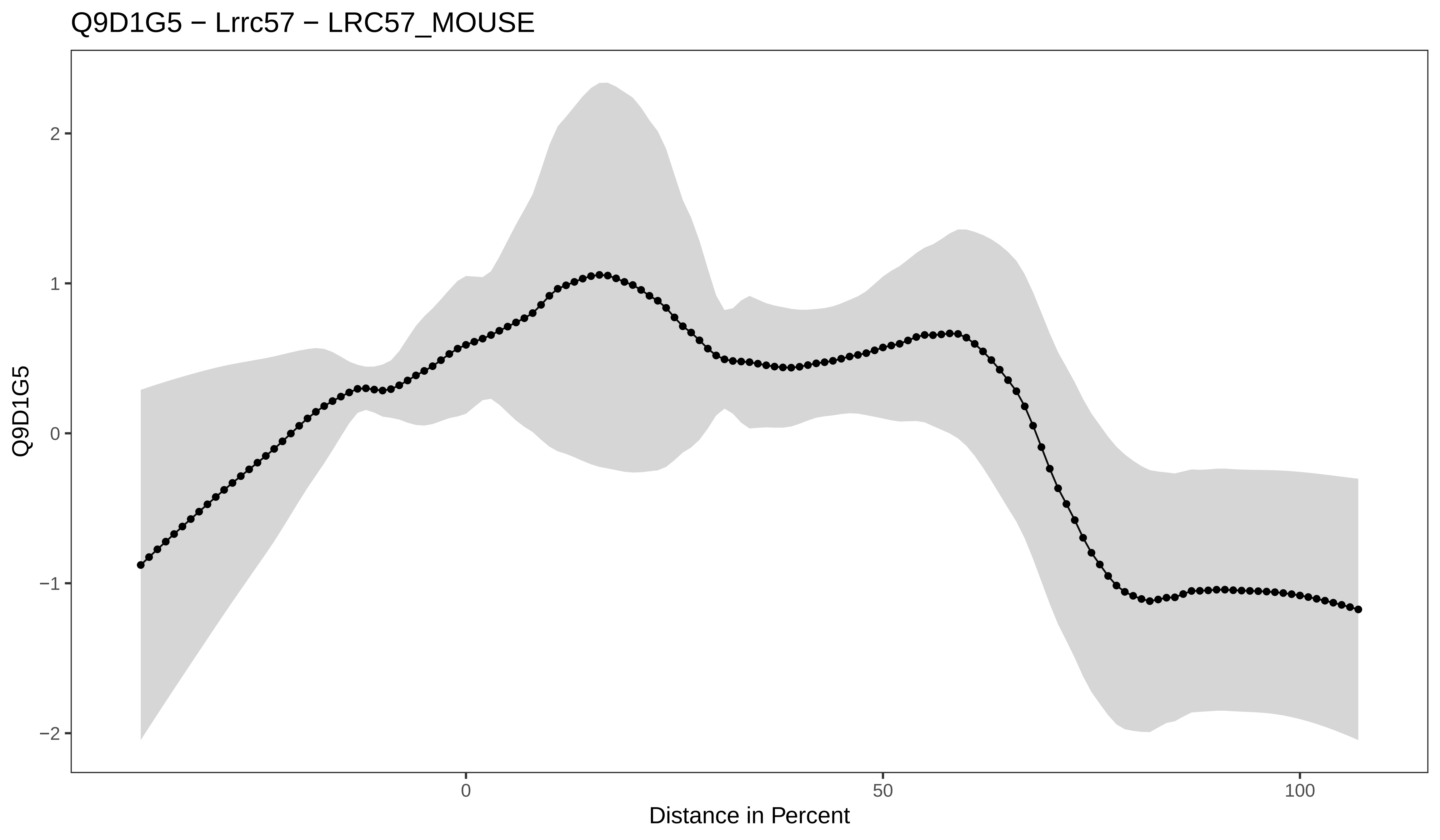

Q9D1G5 − Lrrc57 − LRC57_MOUSE
Q9D1G5
2
1
0
−1
−2
0
50
100
Distance in Percent

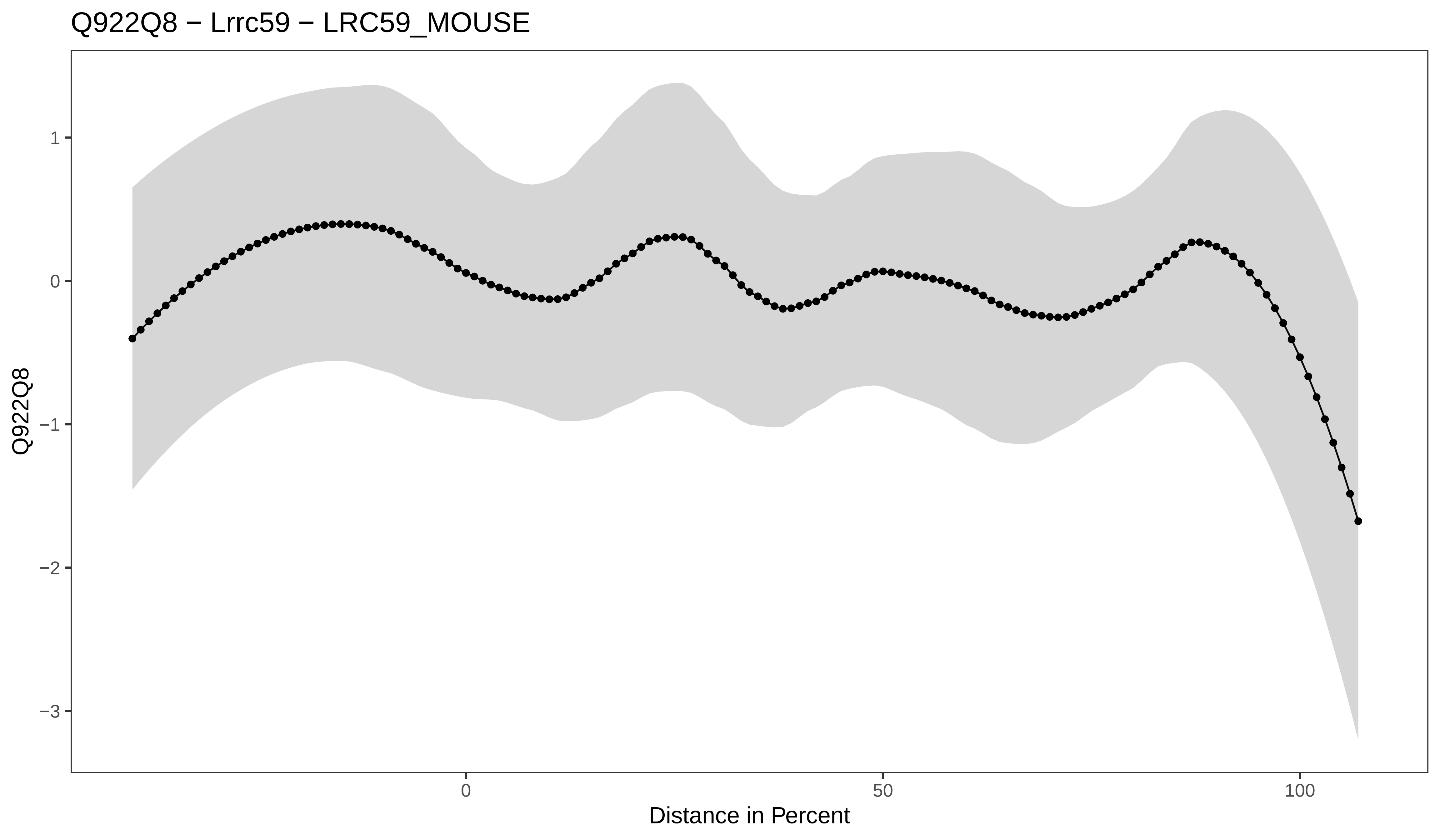

Q922Q8 − Lrrc59 − LRC59_MOUSE
Q922Q8
Distance in Percent
1
0
−1
−2
−3
0
50
100

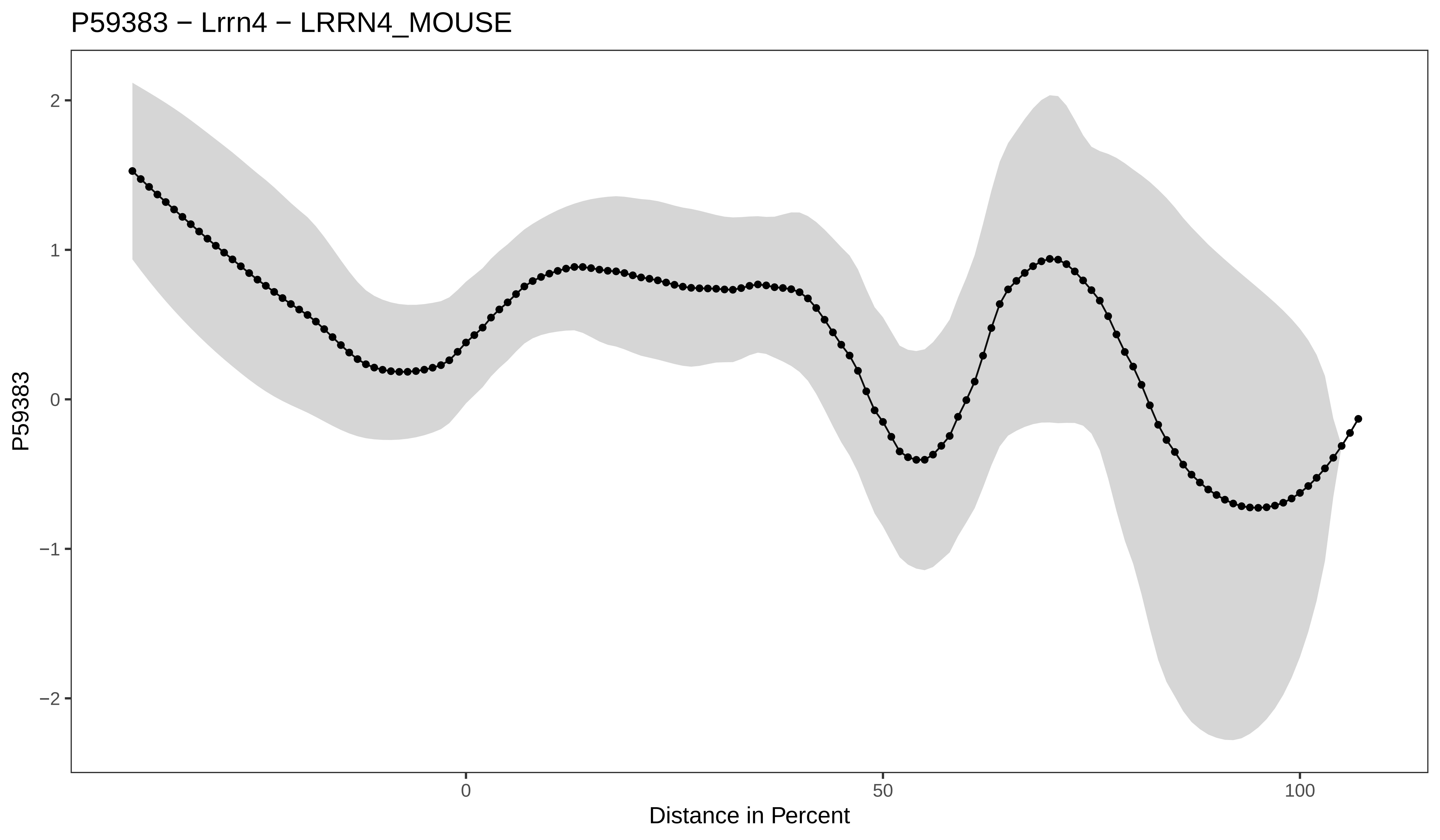
P59383 − Lrrn4 − LRRN4_MOUSE
P59383
2
1
0
−1
−2
0
50
100
Distance in Percent

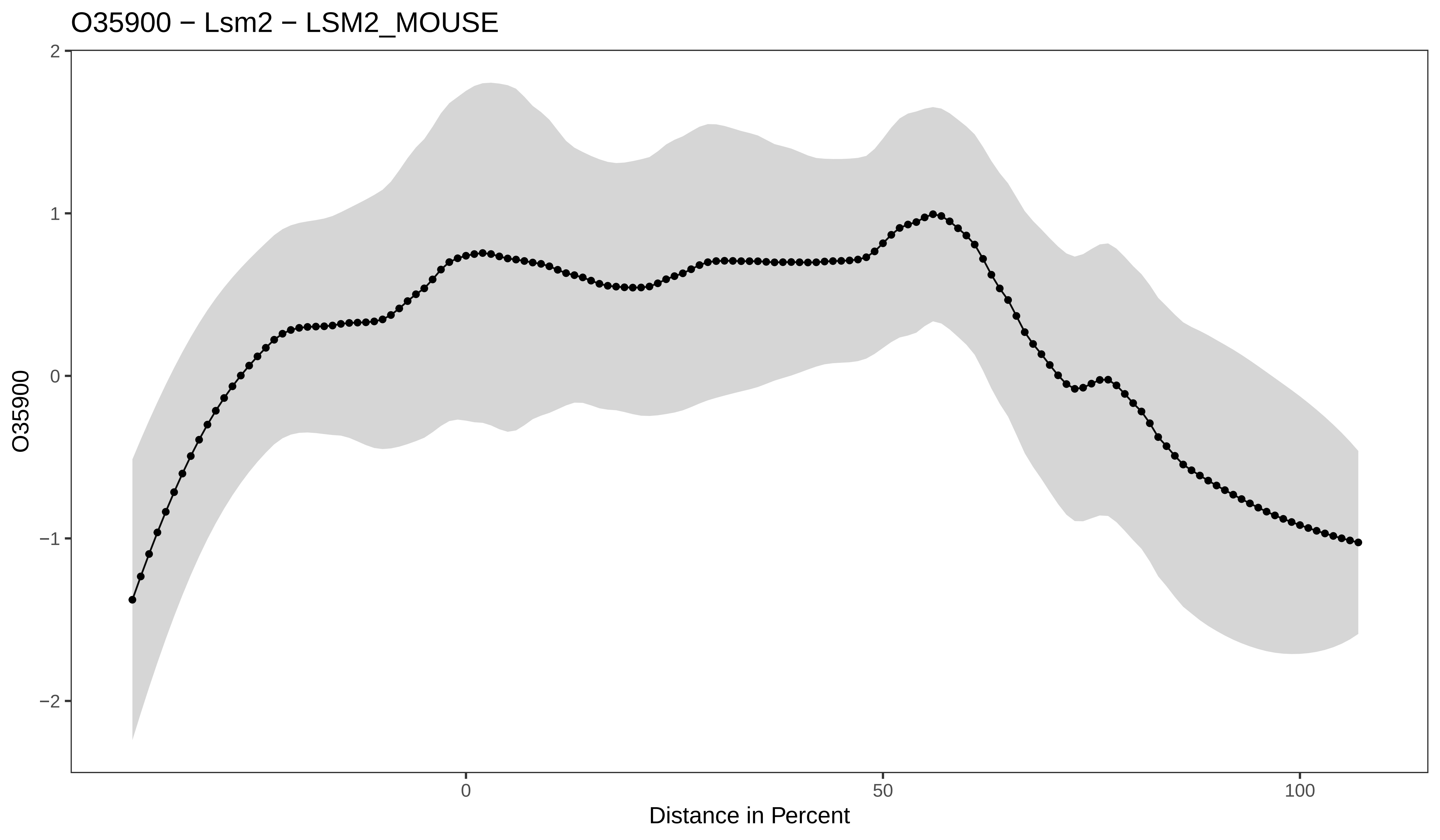
O35900 − Lsm2 − LSM2_MOUSE
O35900
2
1
0
−1
−2
0
50
100
Distance in Percent

P24527 − Lta4h − LKHA4_MOUSE

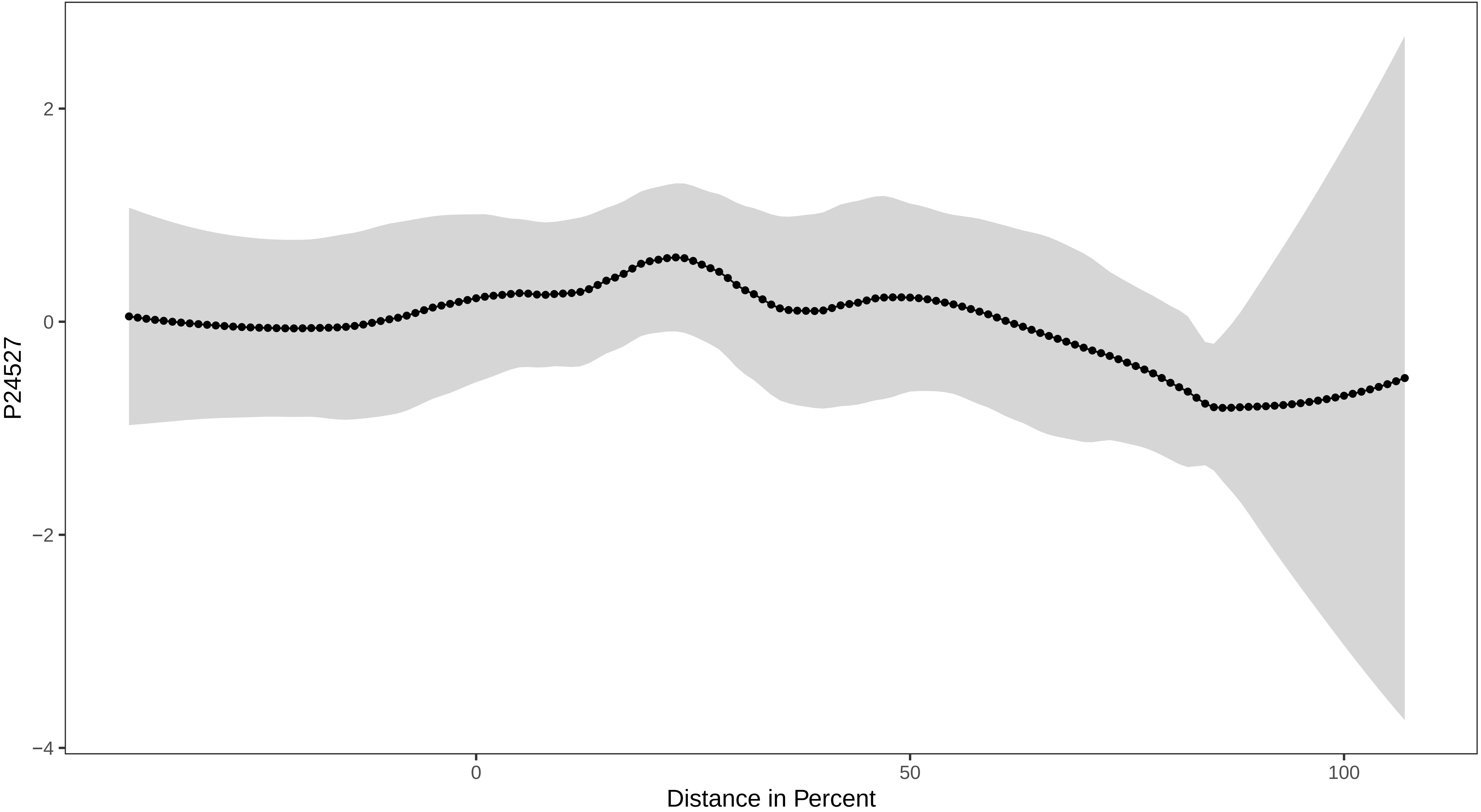

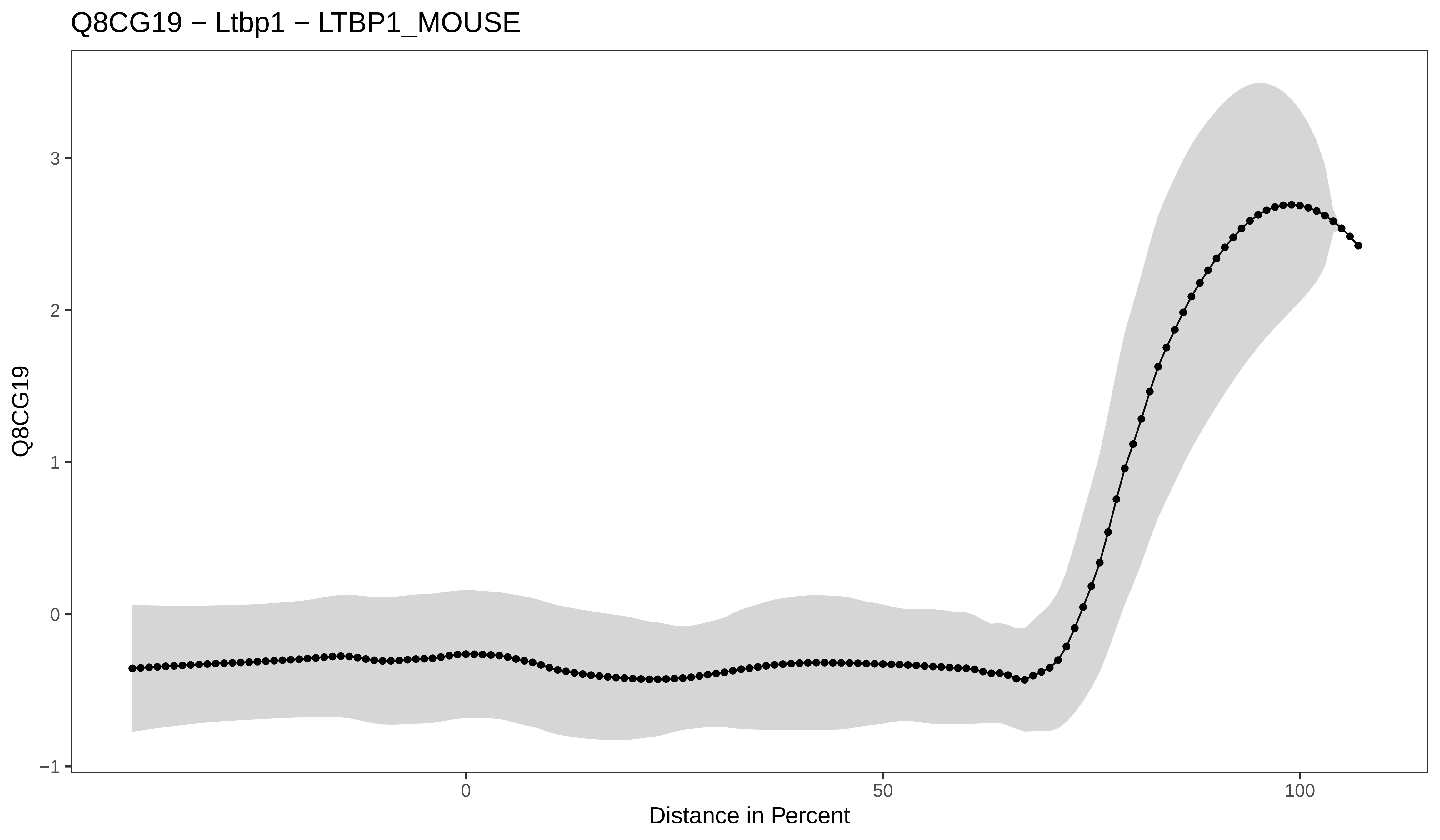

Q8CG19 − Ltbp1 − LTBP1_MOUSE
Q8CG19
3
2
1
0
−1
0
50
100
Distance in Percent

Q8K4G1 − Ltbp4 − LTBP4_MOUSE

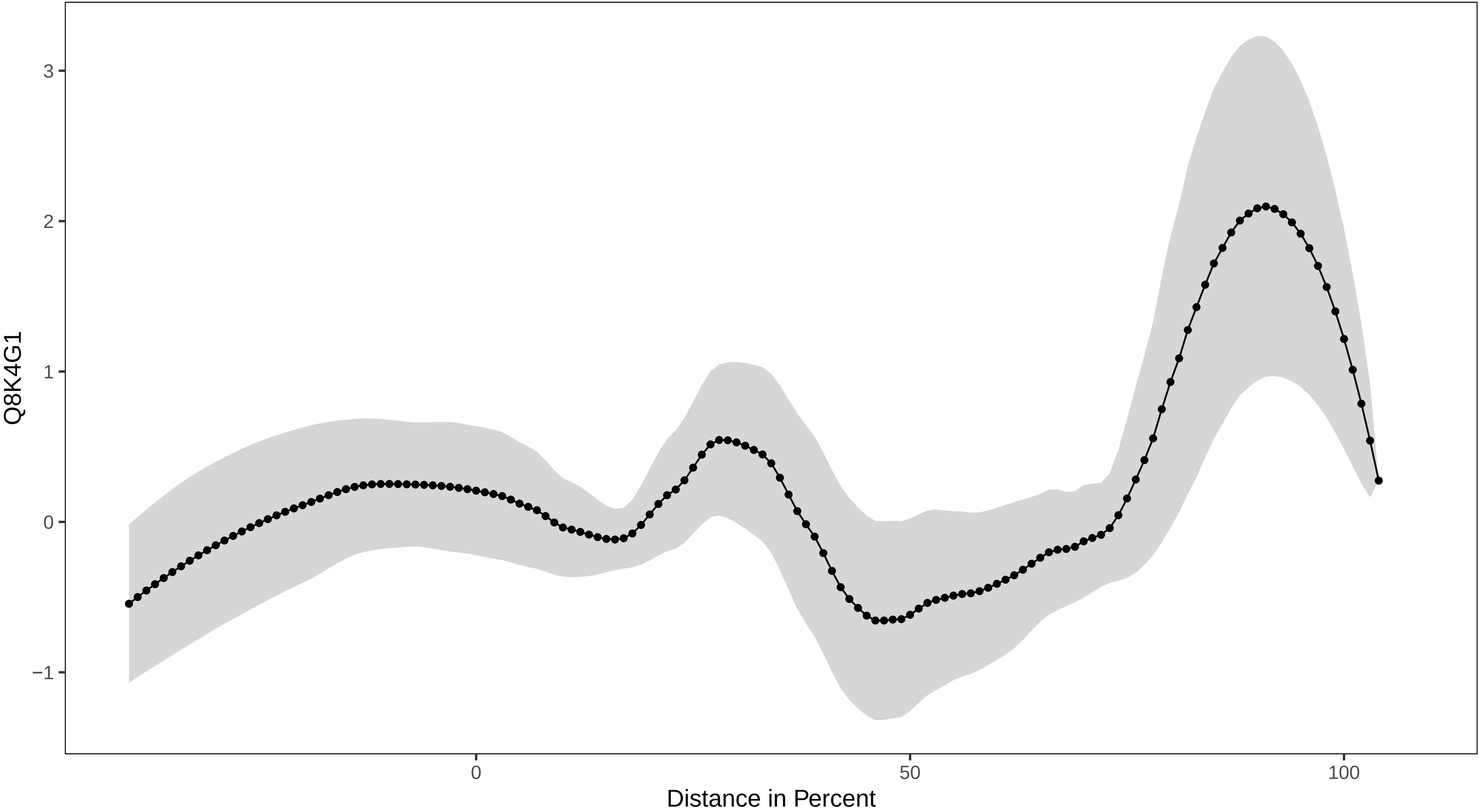

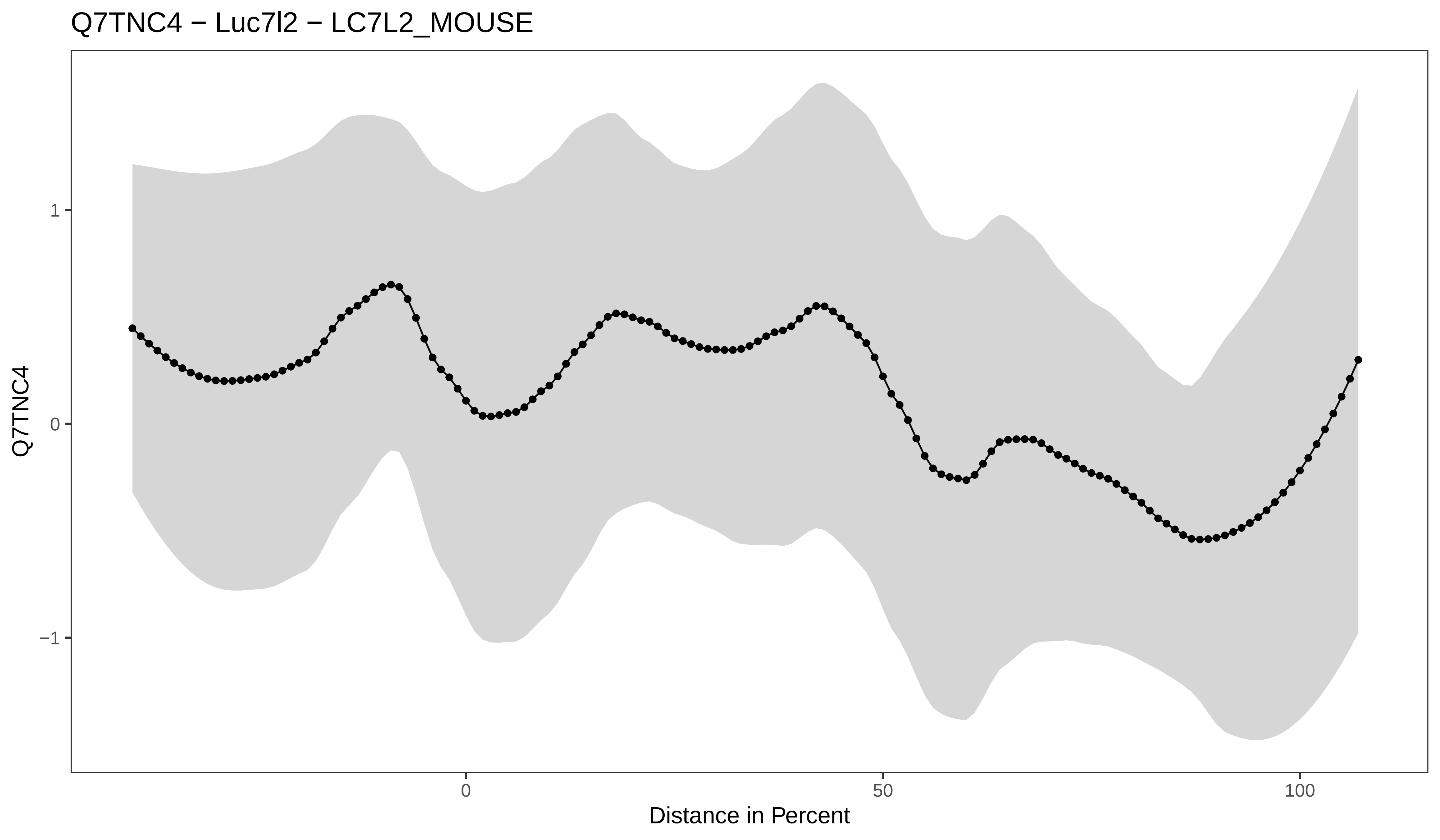

Q7TNC4 − Luc7l2 − LC7L2_MOUSE
Q7TNC4
1
0
−1
0
50
100
Distance in Percent

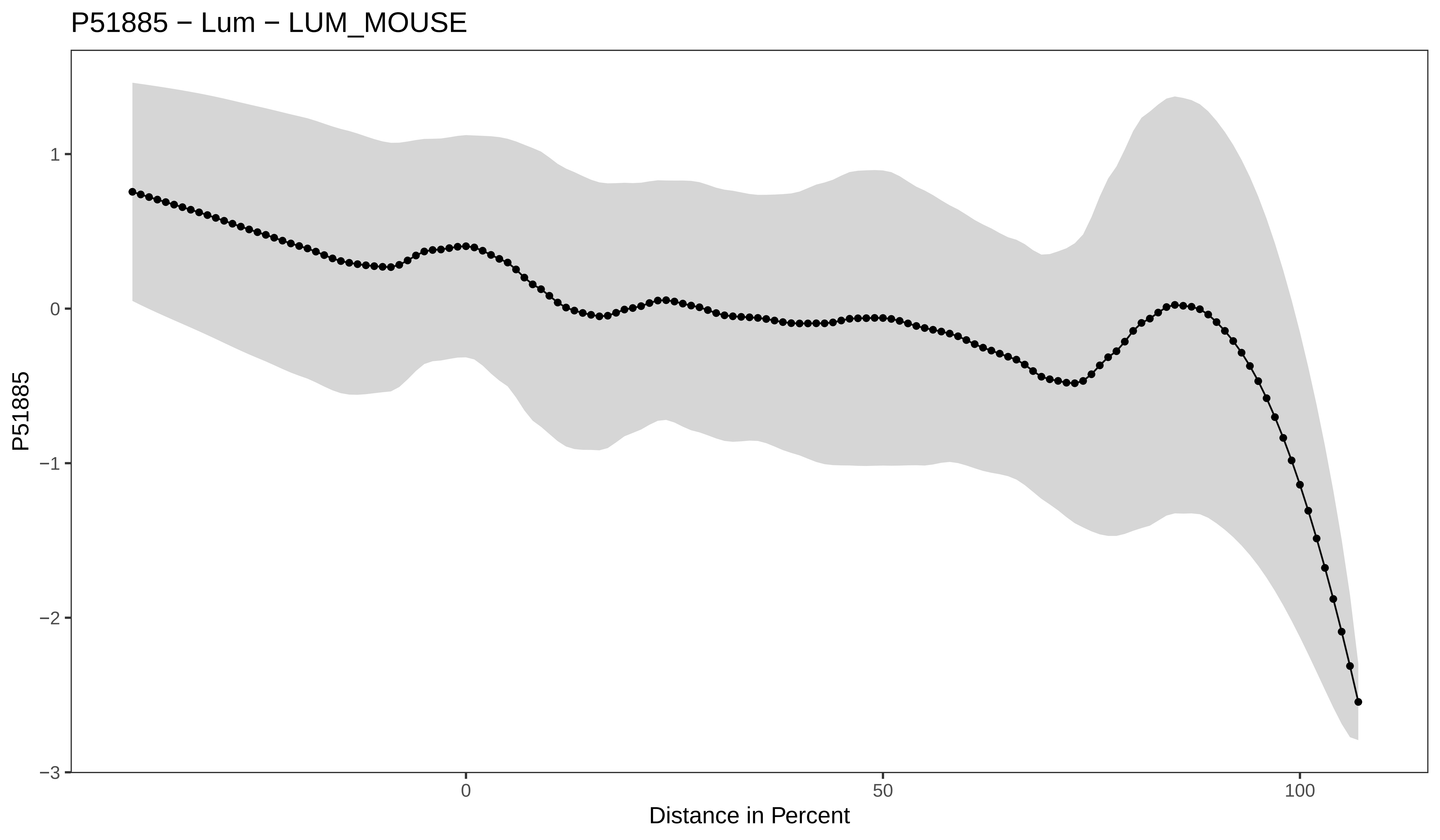

P51885 − Lum − LUM_MOUSE
P51885
1
0
−1
−2
−3
0
50
100
Distance in Percent

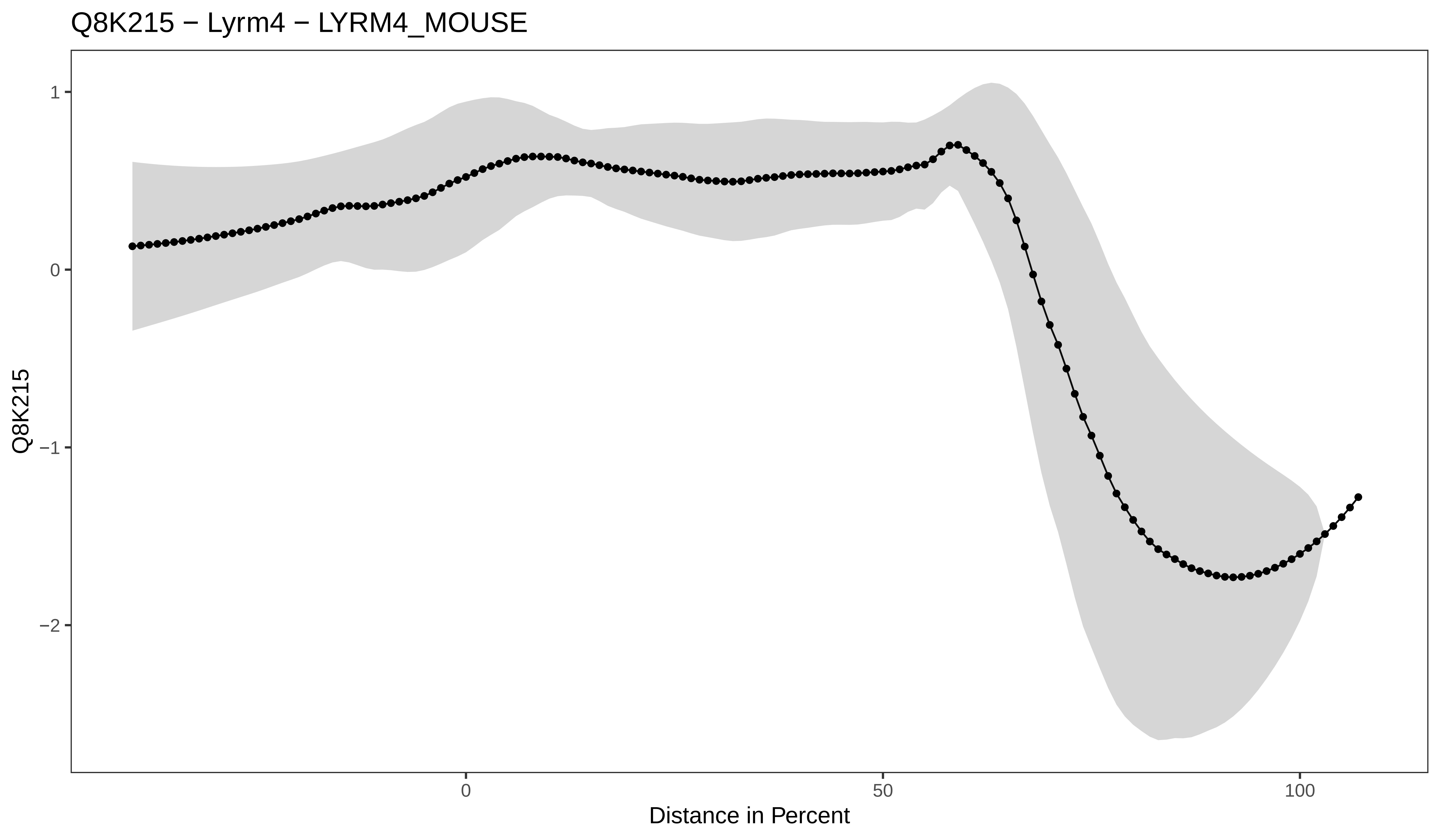
Q8K215 − Lyrm4 − LYRM4_MOUSE
Q8K215
Distance in Percent
1
0
−1
−2
0
50
100

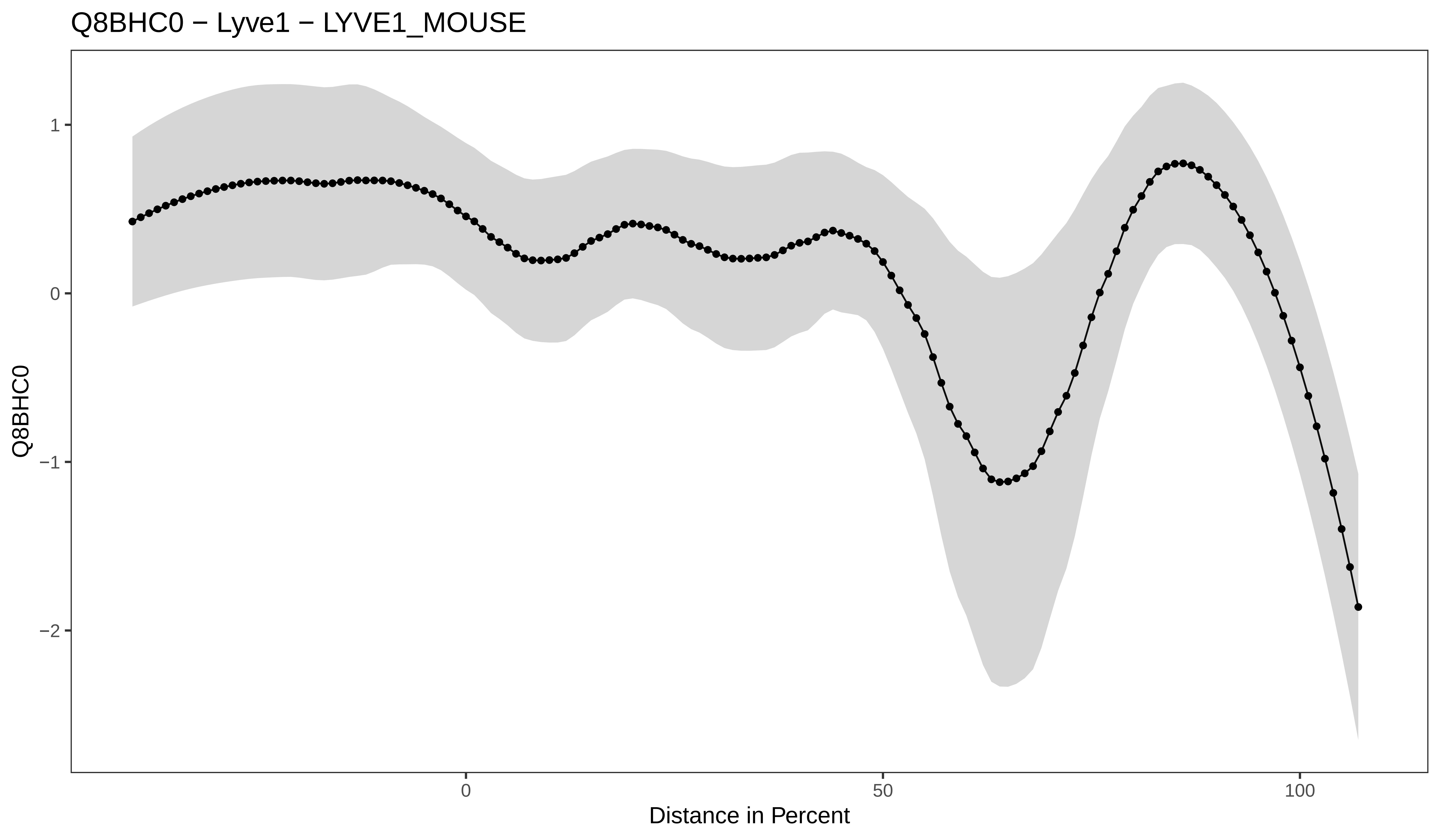
Q8BHC0 − Lyve1 − LYVE1_MOUSE
Q8BHC0
1
0
−1
−2
0
50
100
Distance in Percent

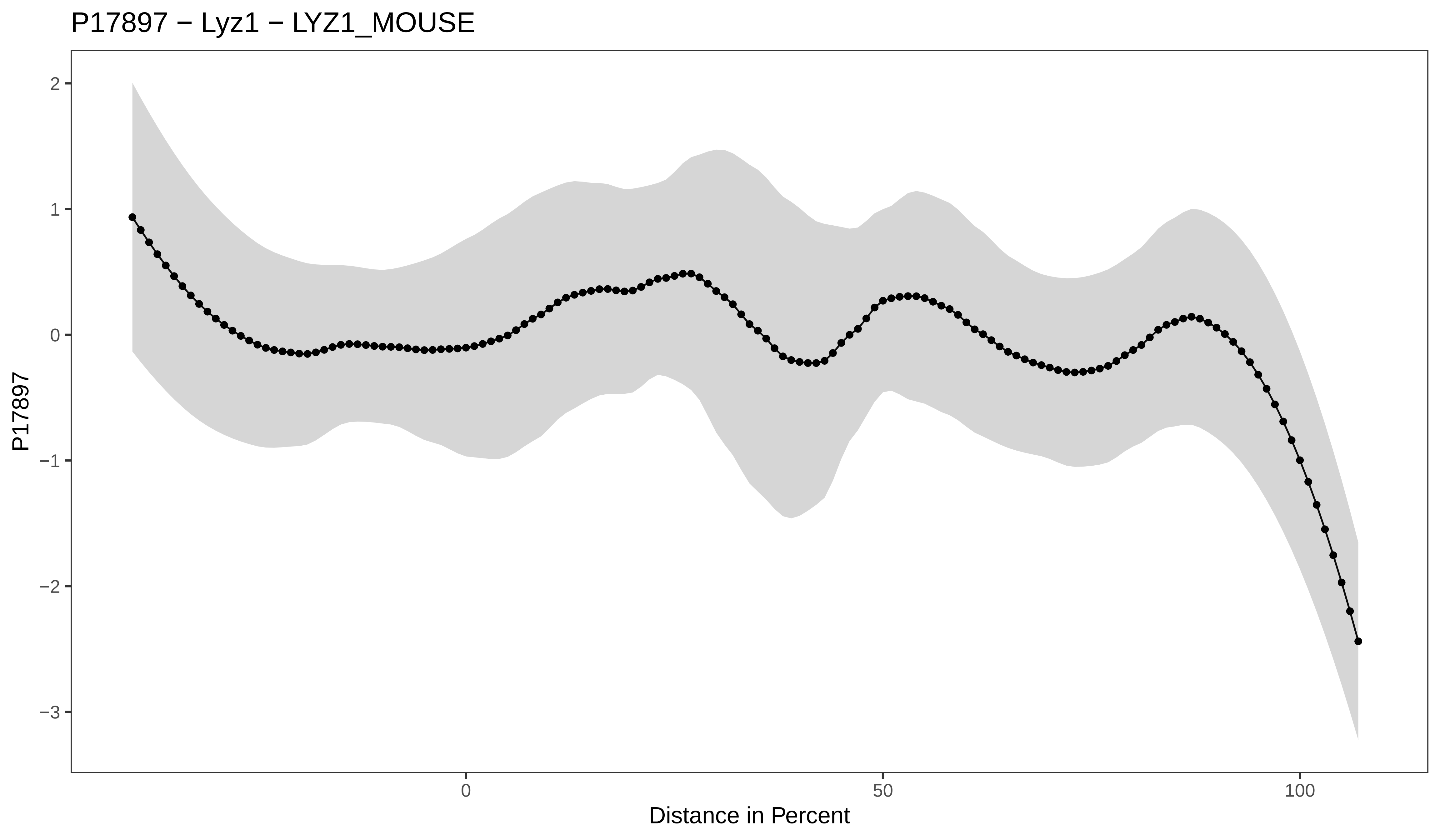
P17897 − Lyz1 − LYZ1_MOUSE
2
1
0
−1
−2
−3
P17897
0
50
100
Distance in Percent

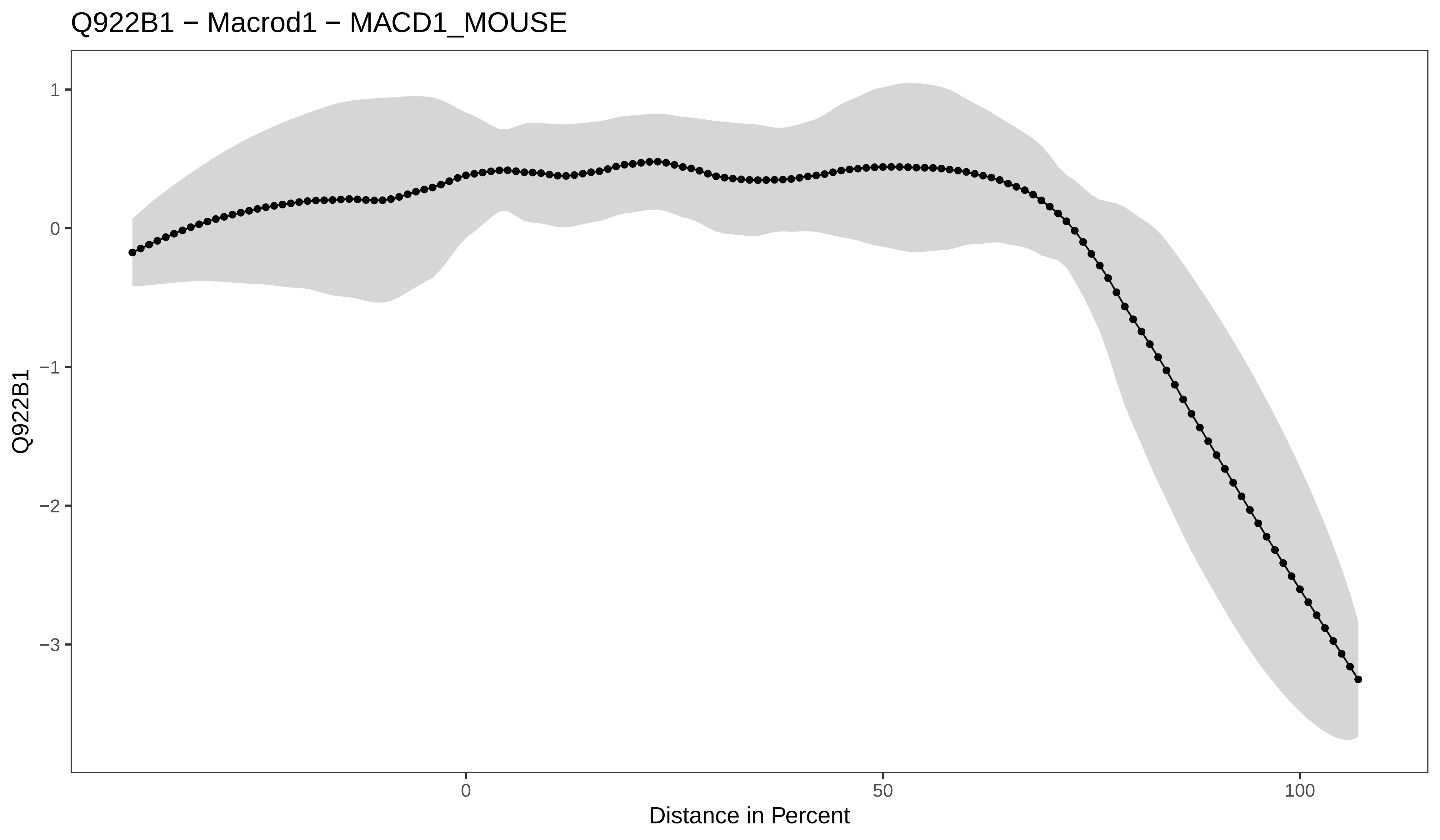

Q922B1 − Macrod1 − MACD1_MOUSE
1
0
−1
−2
−3
Q922B1
0
50
100
Distance in Percent

P20917 − Mag − MAG_MOUSE

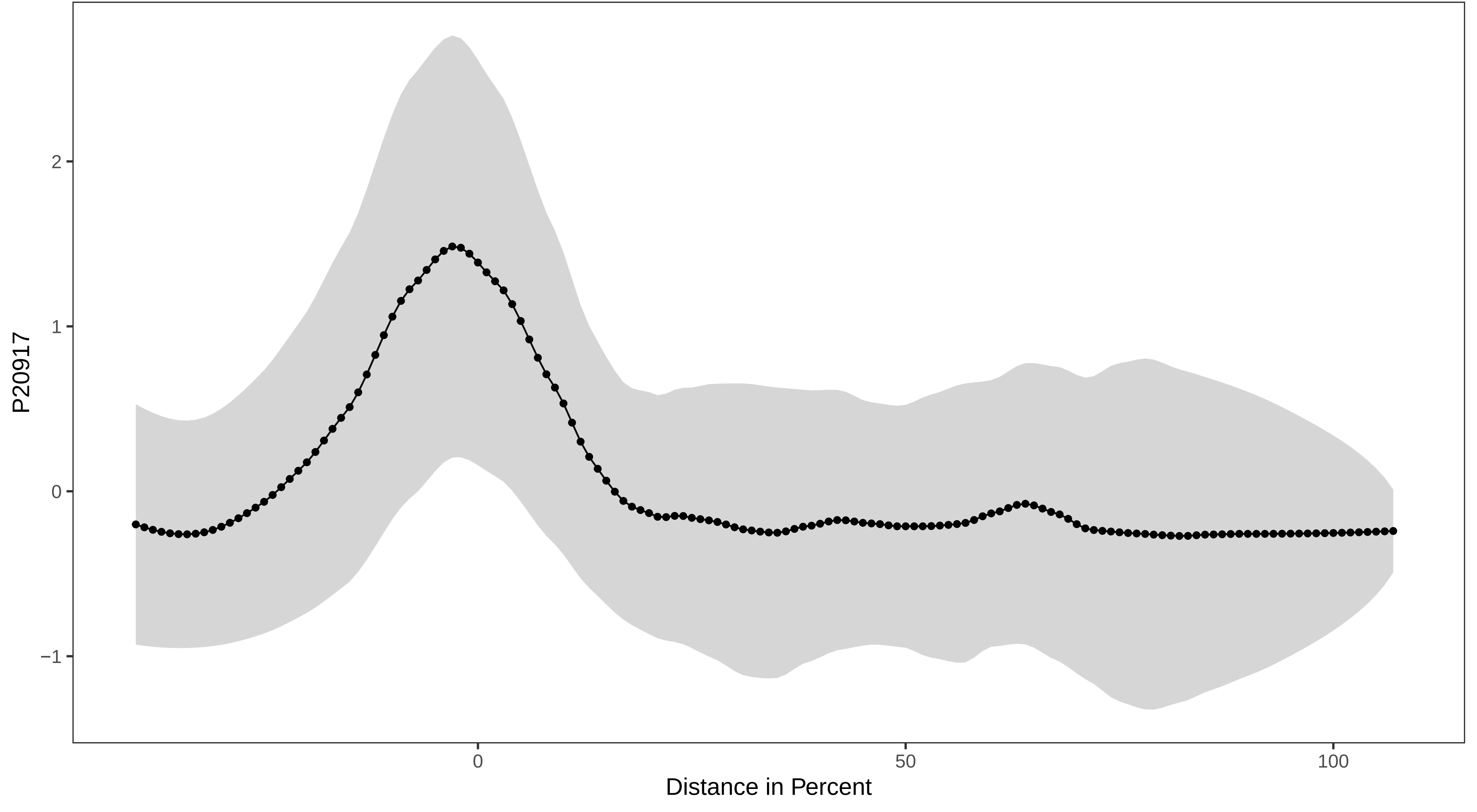

P61327;Q9CQL1 − Magoh;Magohb − MGN2_MOUSE;MGN_MOUSE

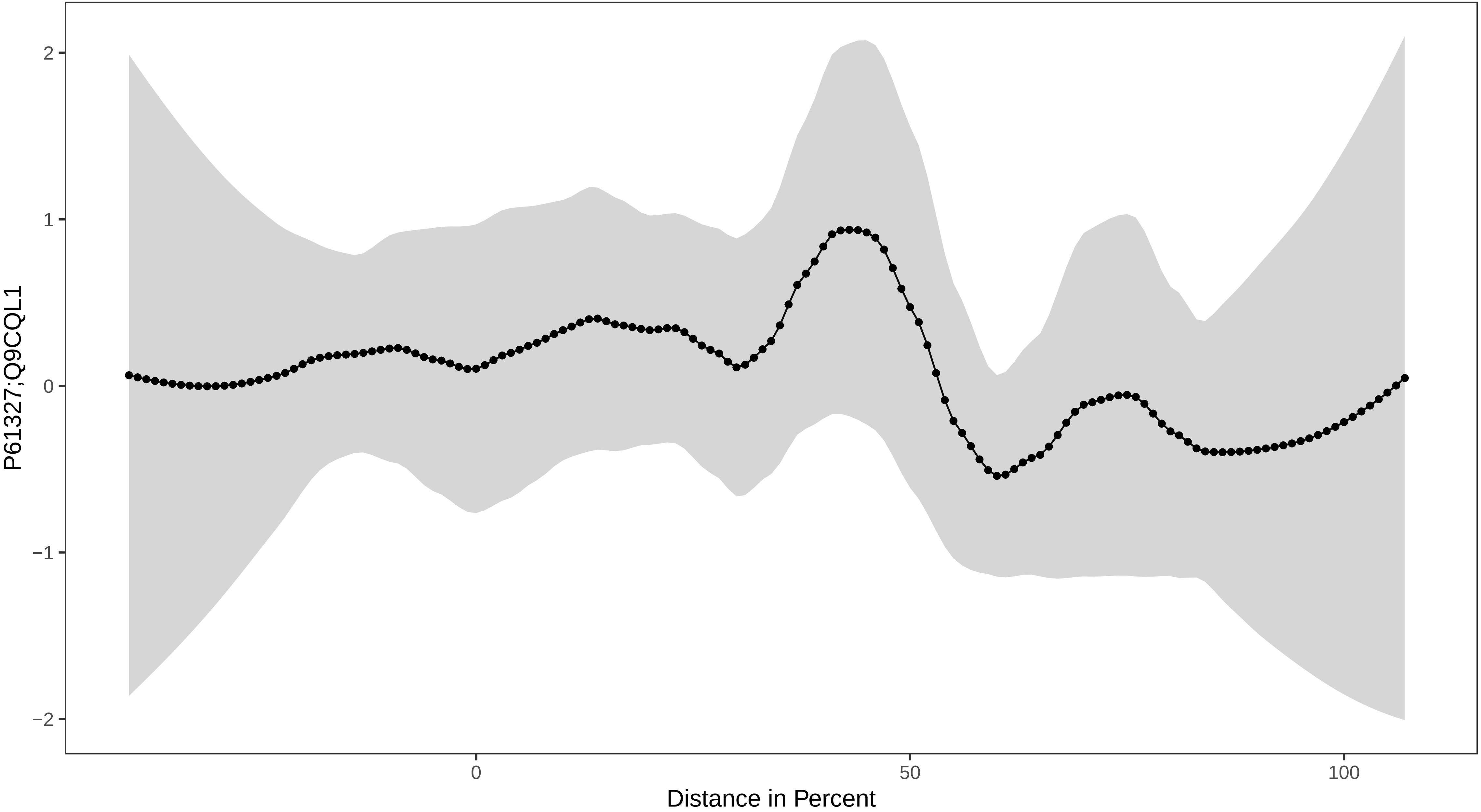

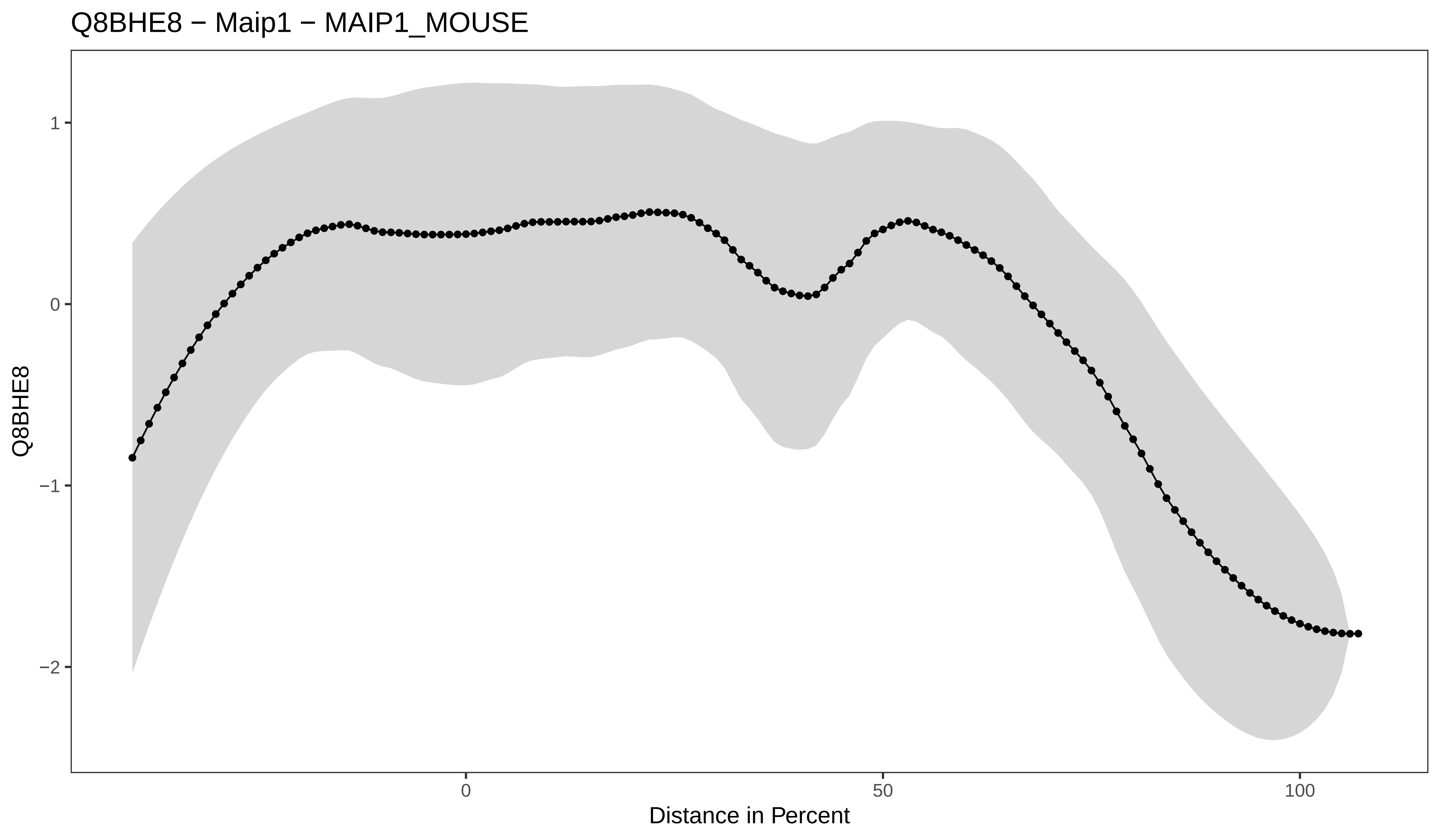

Q8BHE8 − Maip1 − MAIP1_MOUSE
1
0
−1
−2
Q8BHE8
0
50
100
Distance in Percent

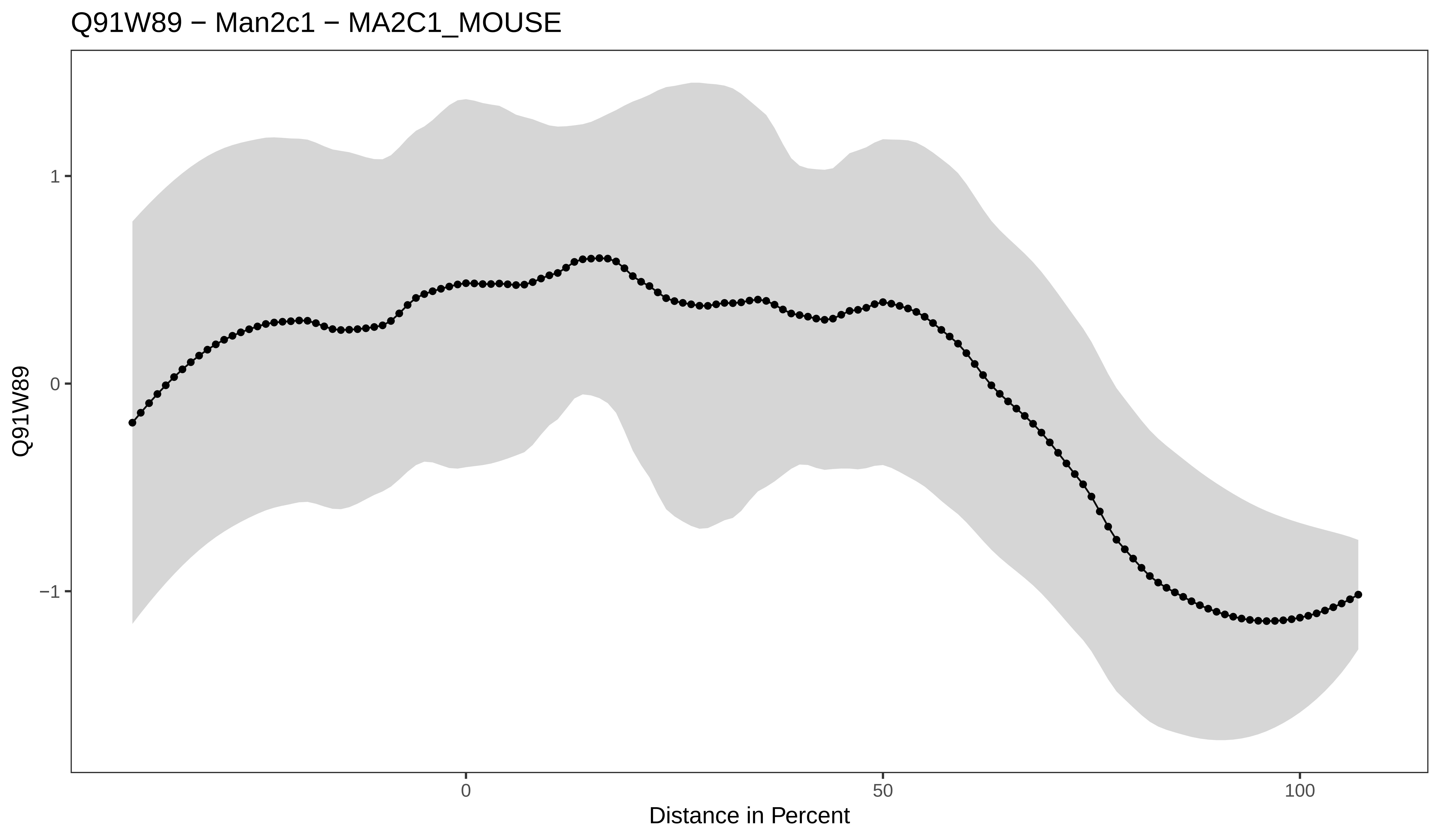

Q91W89 − Man2c1 − MA2C1_MOUSE
1
0
−1
Q91W89
0
50
100
Distance in Percent

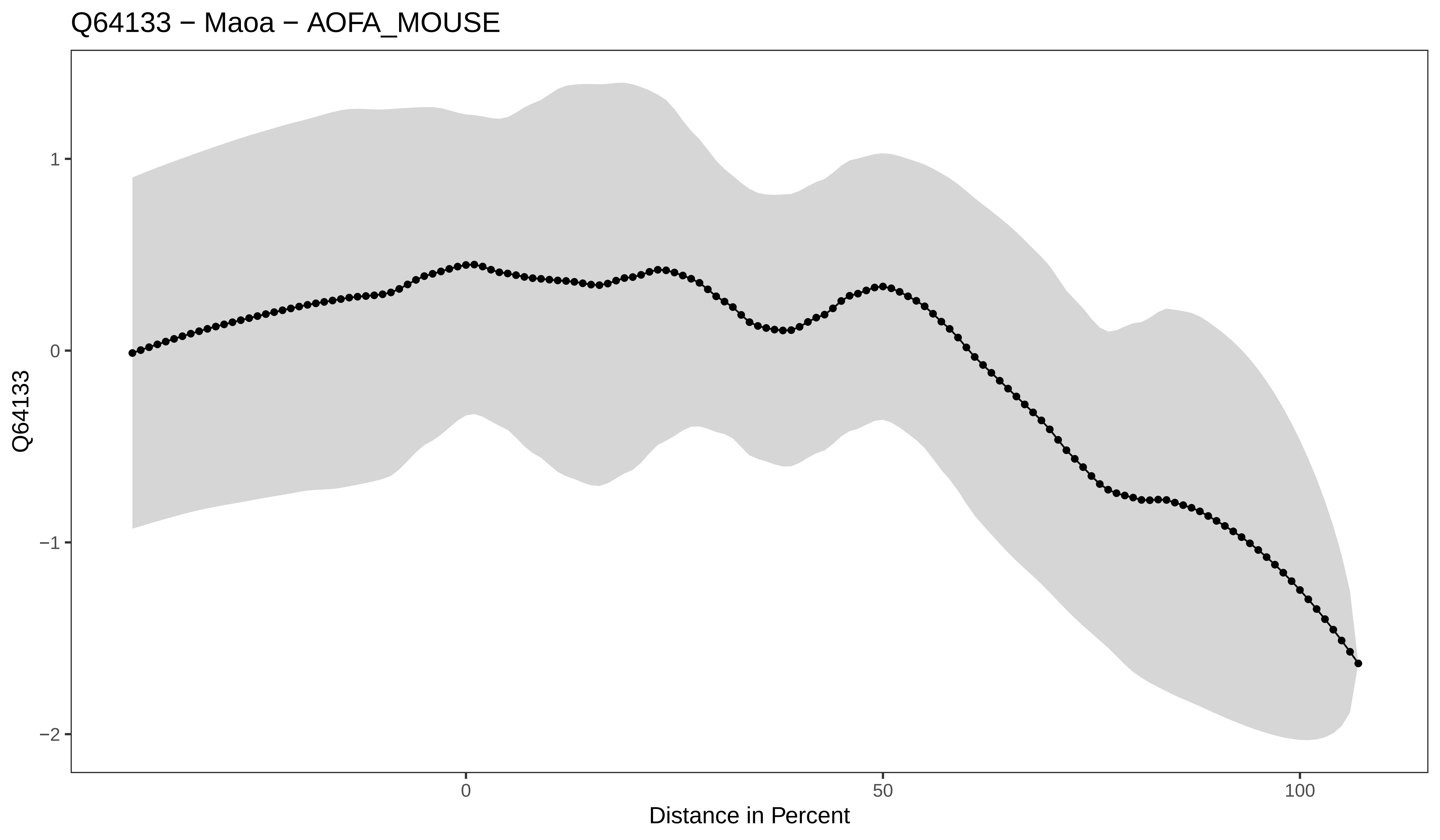

Q64133 − Maoa − AOFA_MOUSE
Q64133
1
0
−1
−2
0
50
100
Distance in Percent

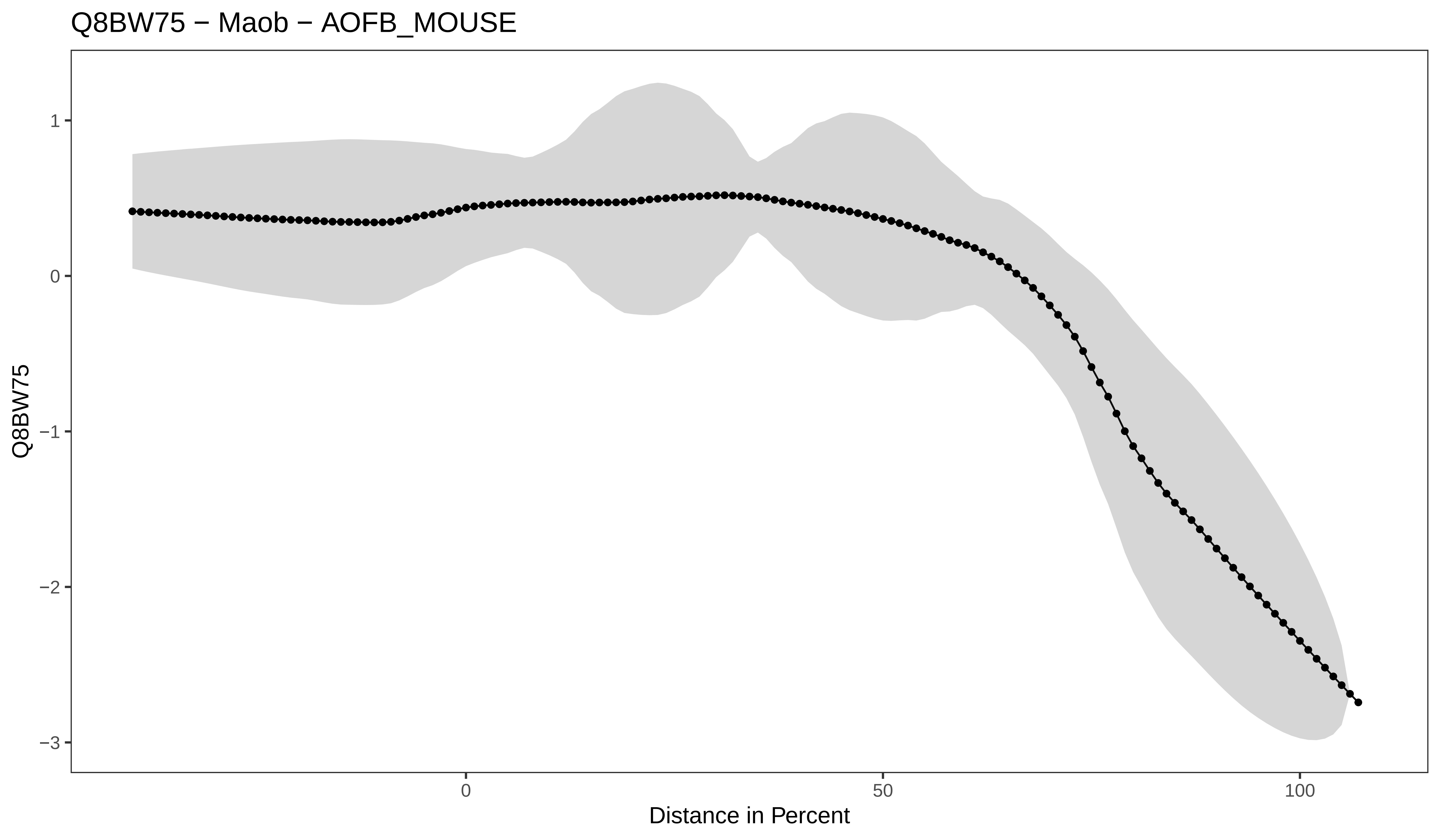
Q8BW75 − Maob − AOFB_MOUSE
Q8BW75
Distance in Percent
1
0
−1
−2
−3
0
50
100

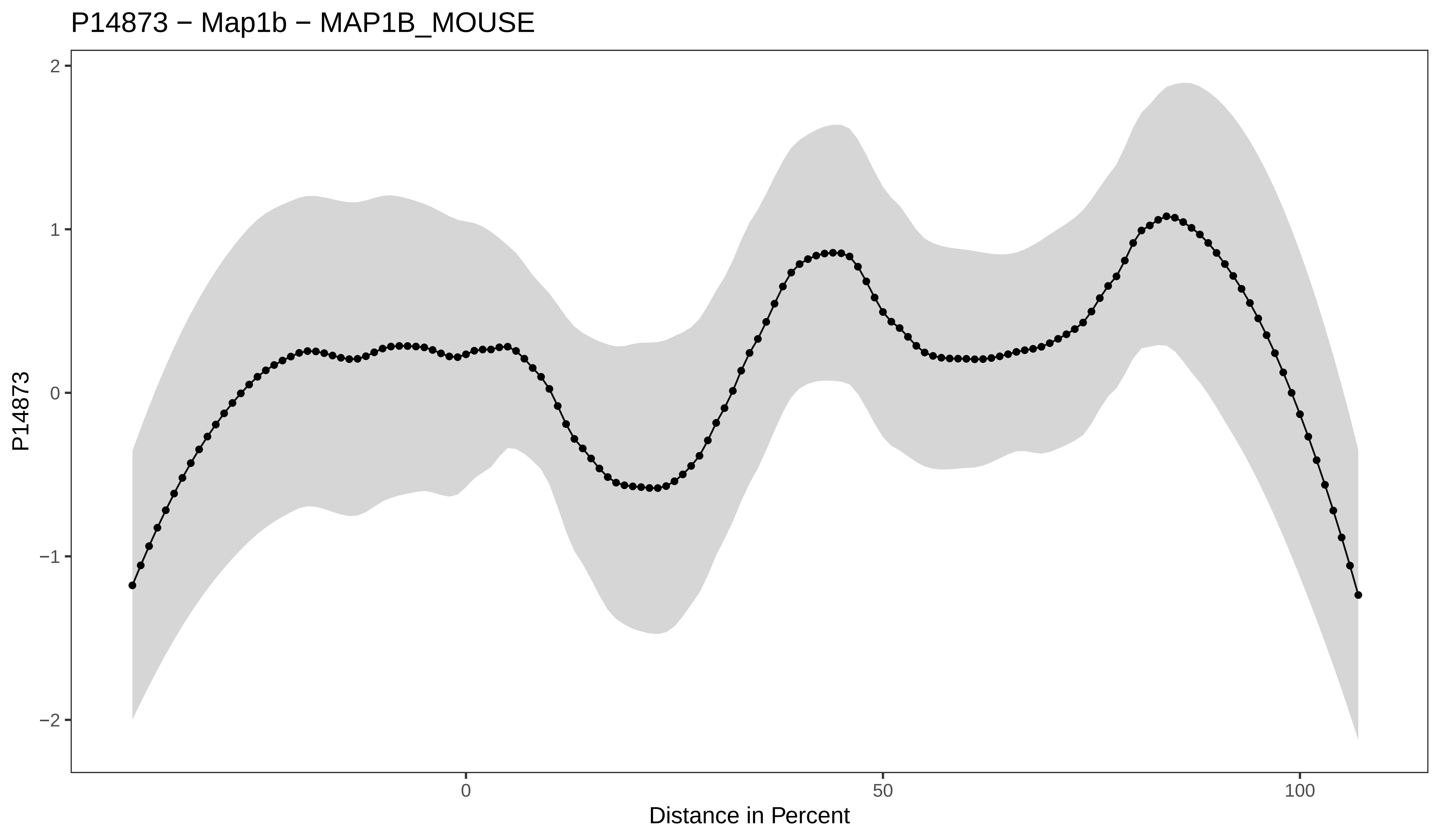

P14873 − Map1b − MAP1B_MOUSE
P14873
2
1
0
−1
−2
0
50
100
Distance in Percent

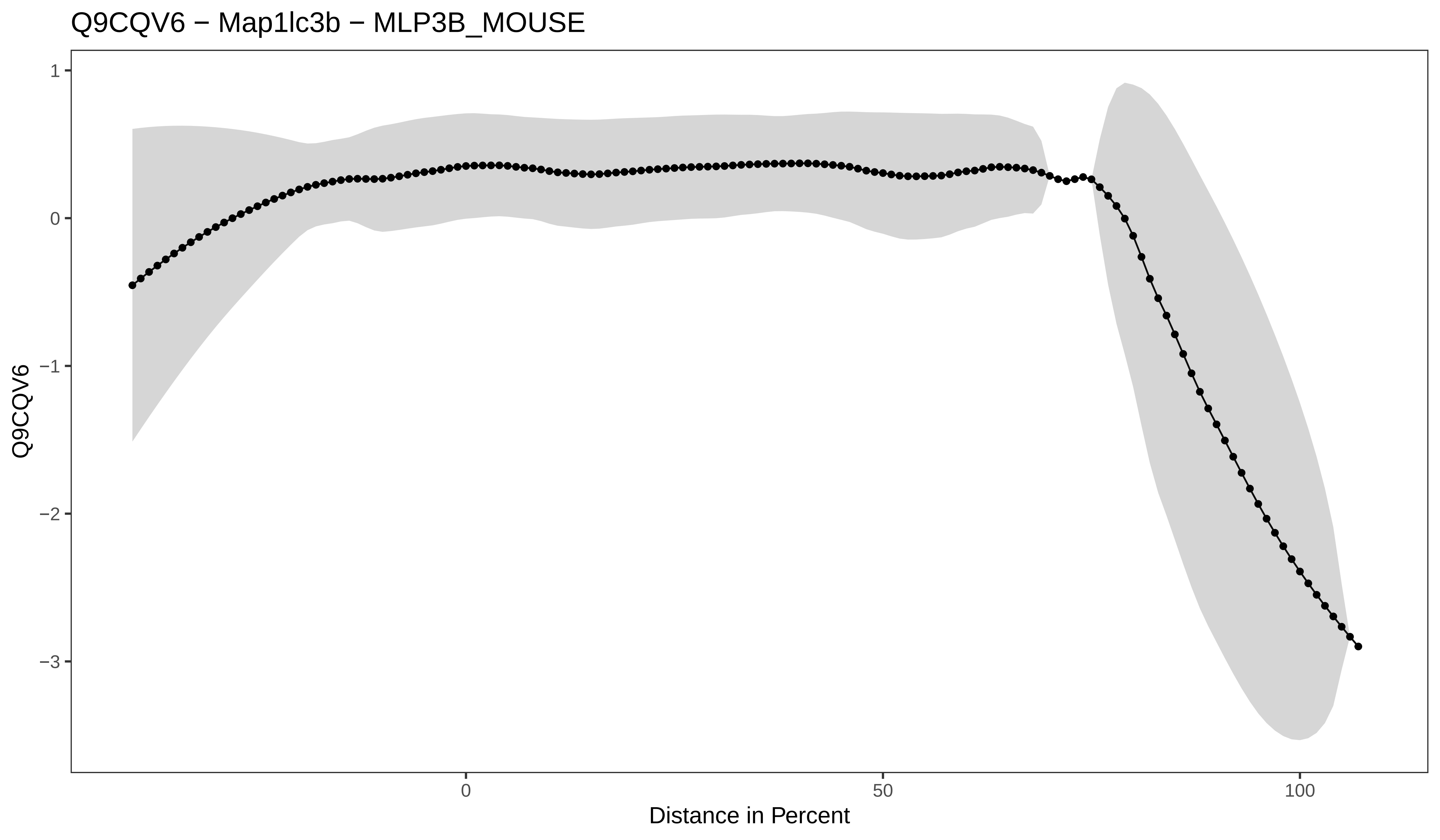
Q9CQV6 − Map1lc3b − MLP3B_MOUSE
Q9CQV6
Distance in Percent
1
0
−1
−2
−3
0
50
100

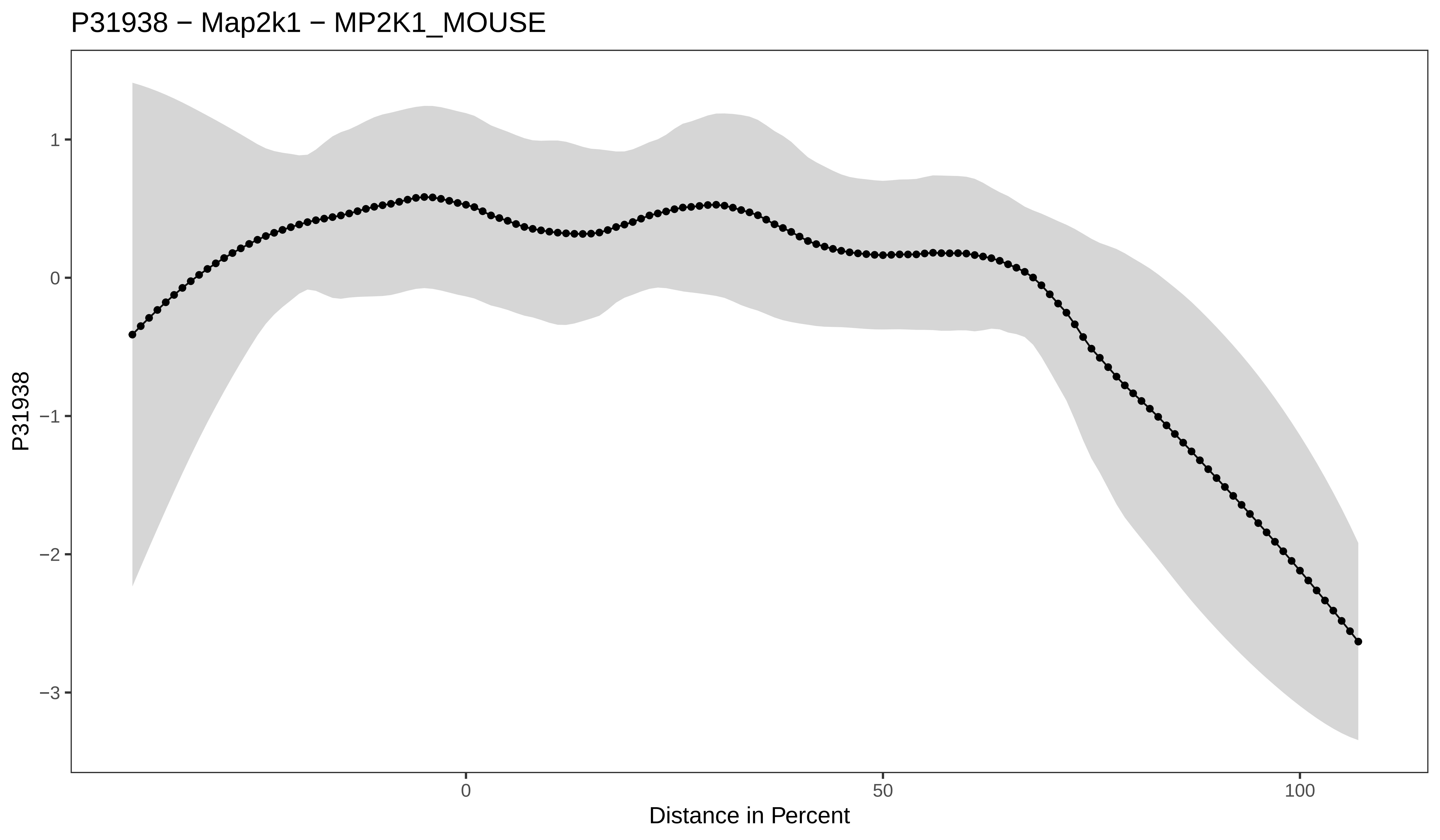

P31938 − Map2k1 − MP2K1_MOUSE
P31938
1
0
−1
−2
−3
0
50
100
Distance in Percent

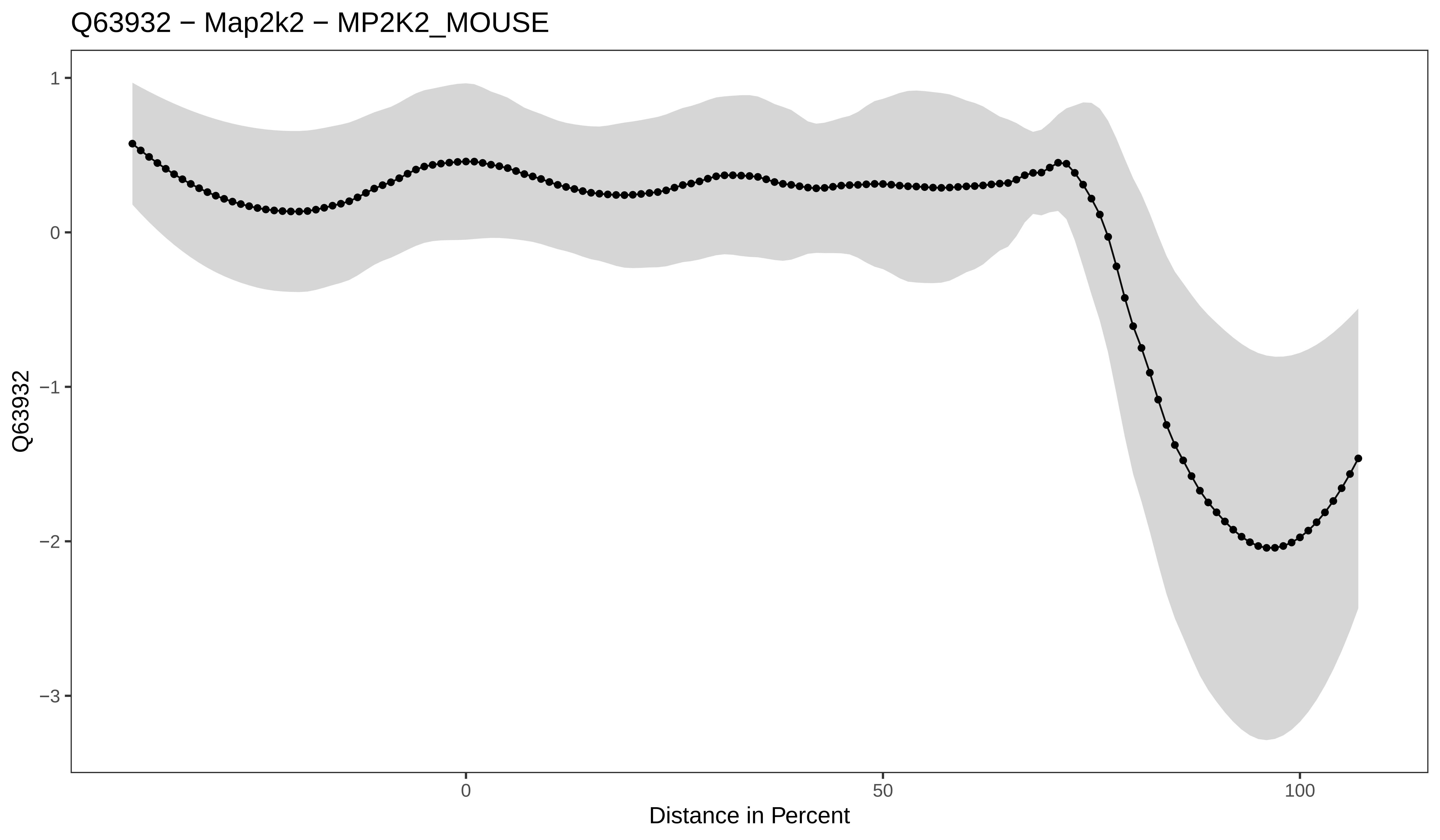

Q63932 − Map2k2 − MP2K2_MOUSE
1
0
−1
−2
−3
Q63932
0
50
100
Distance in Percent

O09110 − Map2k3 − MP2K3_MOUSE

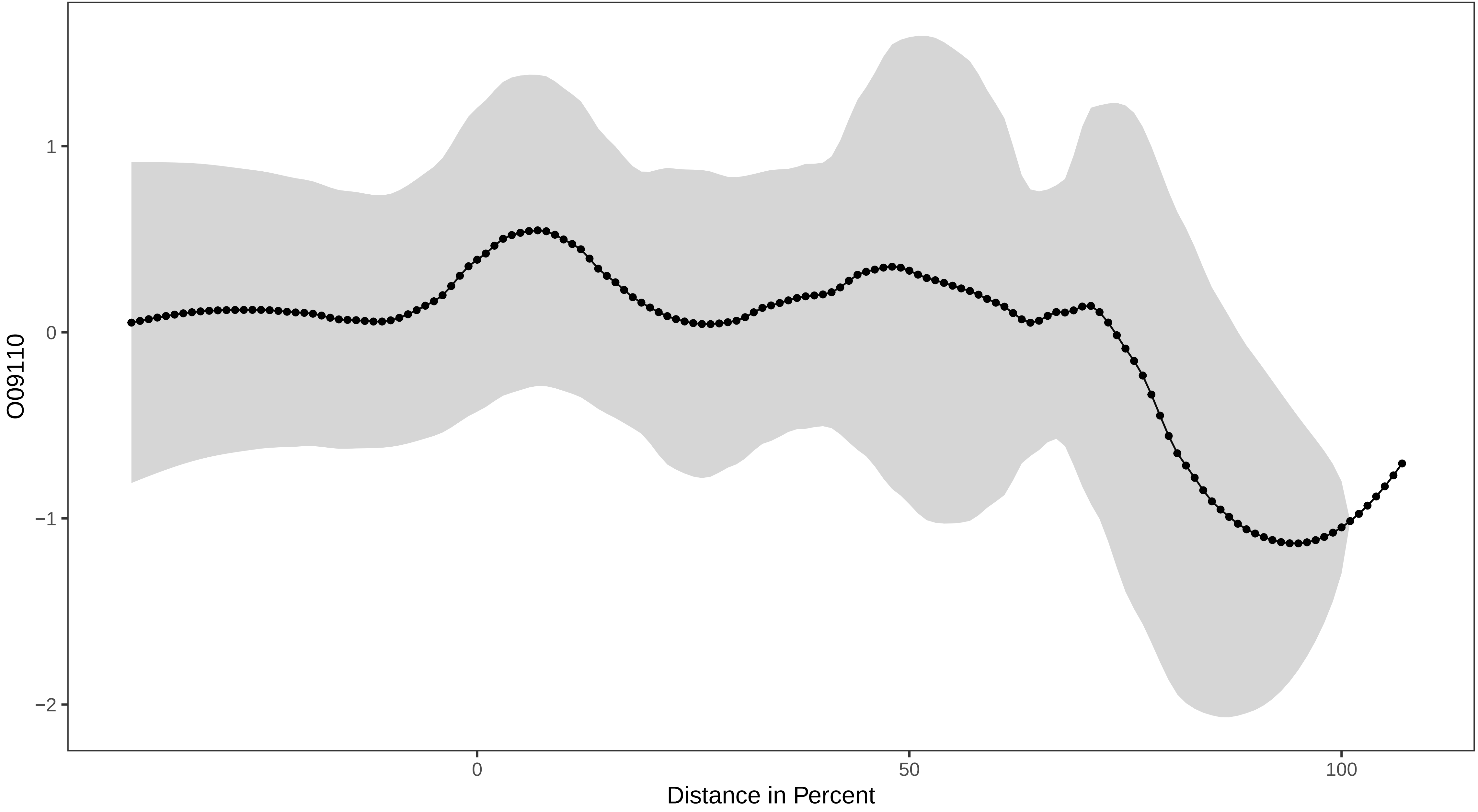

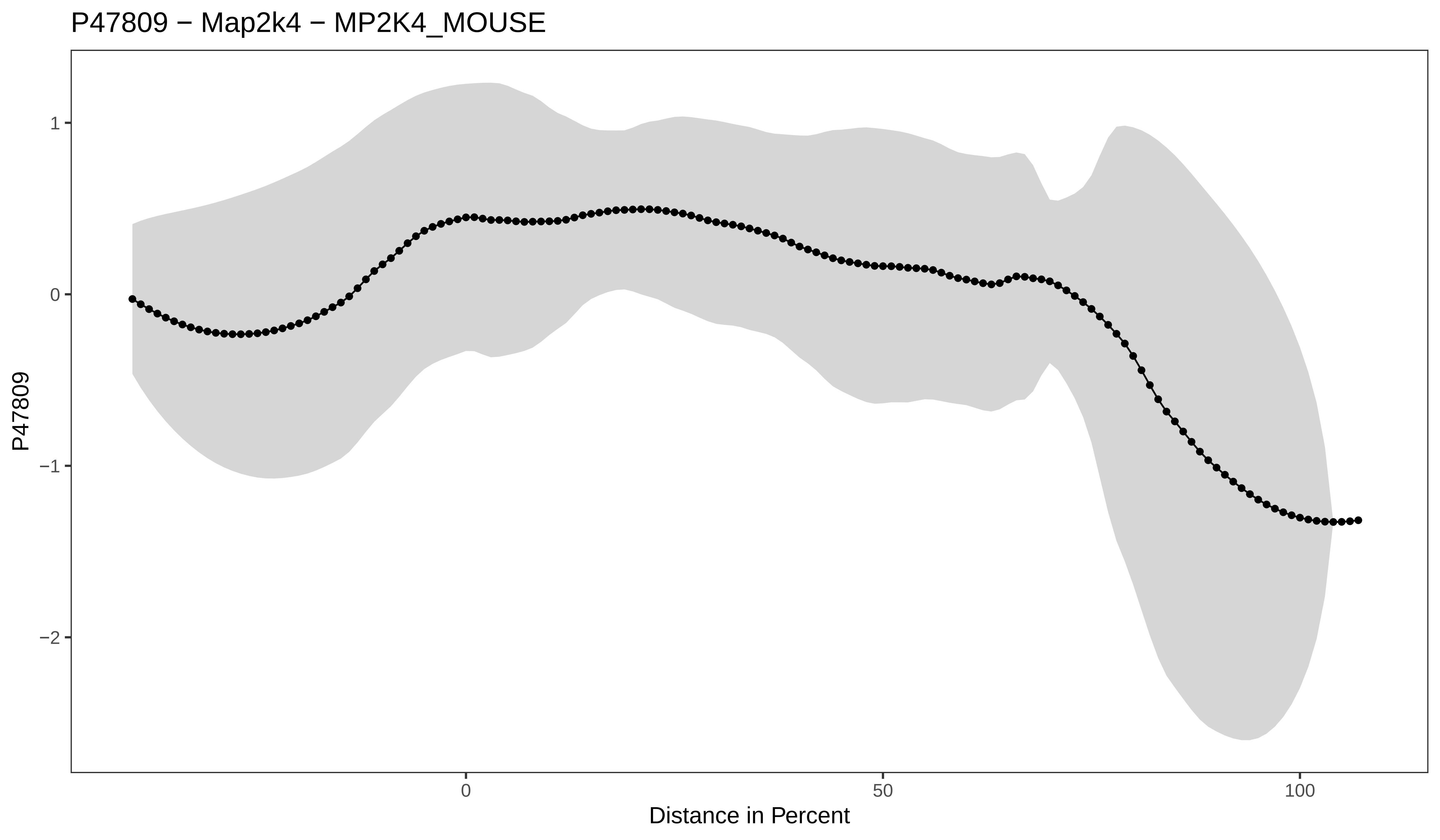

P47809 − Map2k4 − MP2K4_MOUSE
1
0
−1
−2
P47809
0
50
100
Distance in Percent

P70236 − Map2k6 − MP2K6_MOUSE

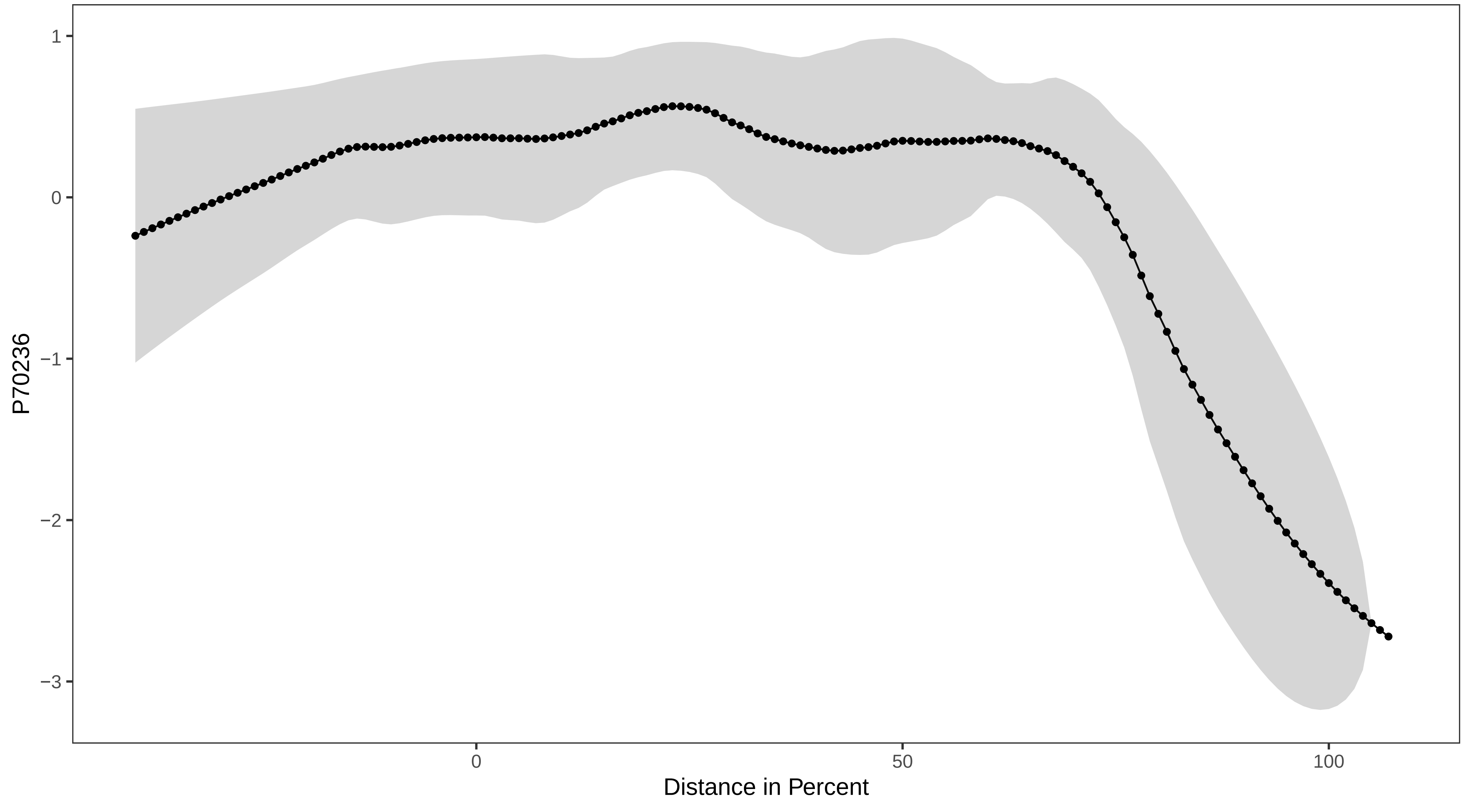

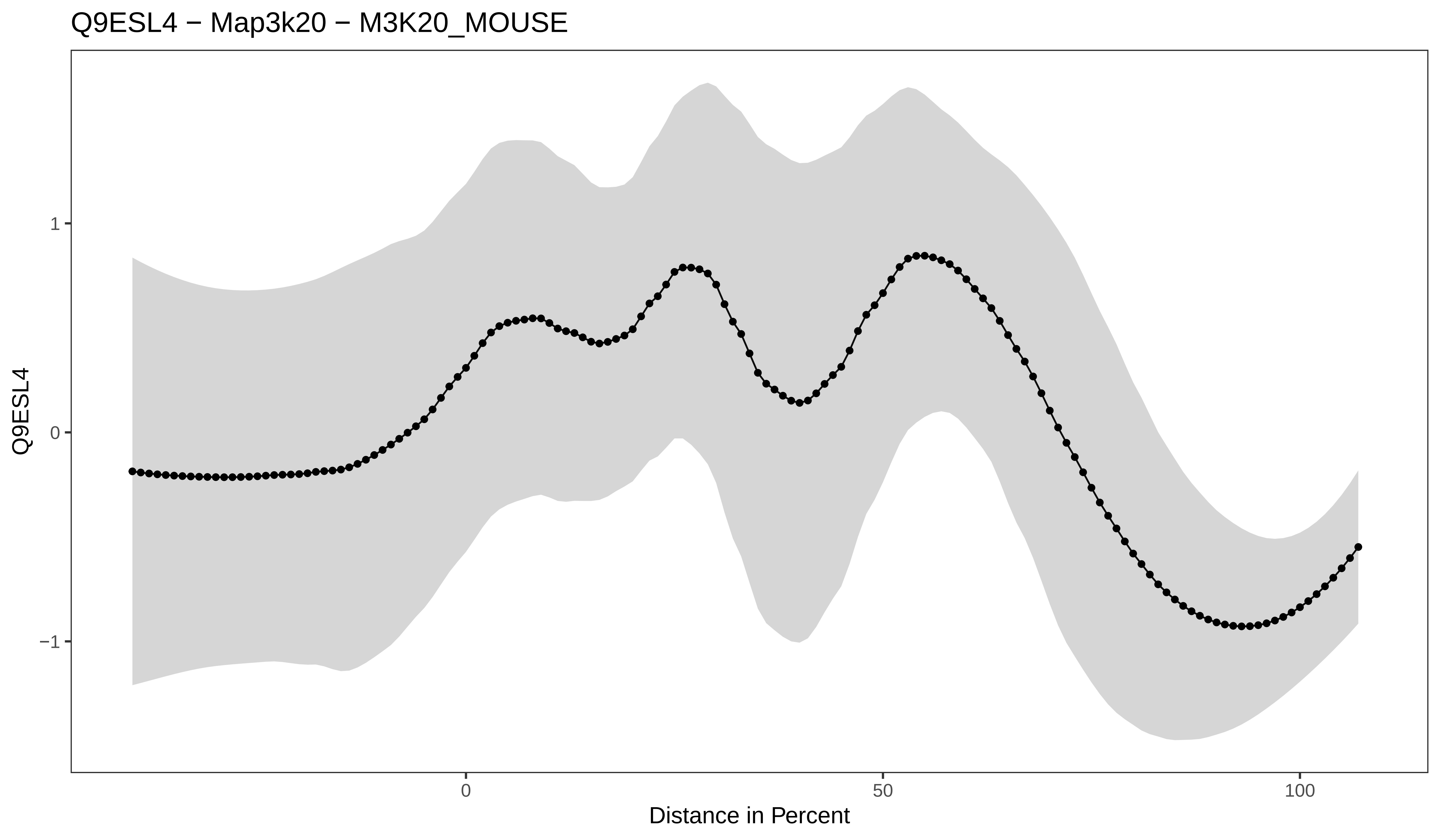
Q9ESL4 − Map3k20 − M3K20_MOUSE
Q9ESL4
1
0
−1
0
50
100
Distance in Percent

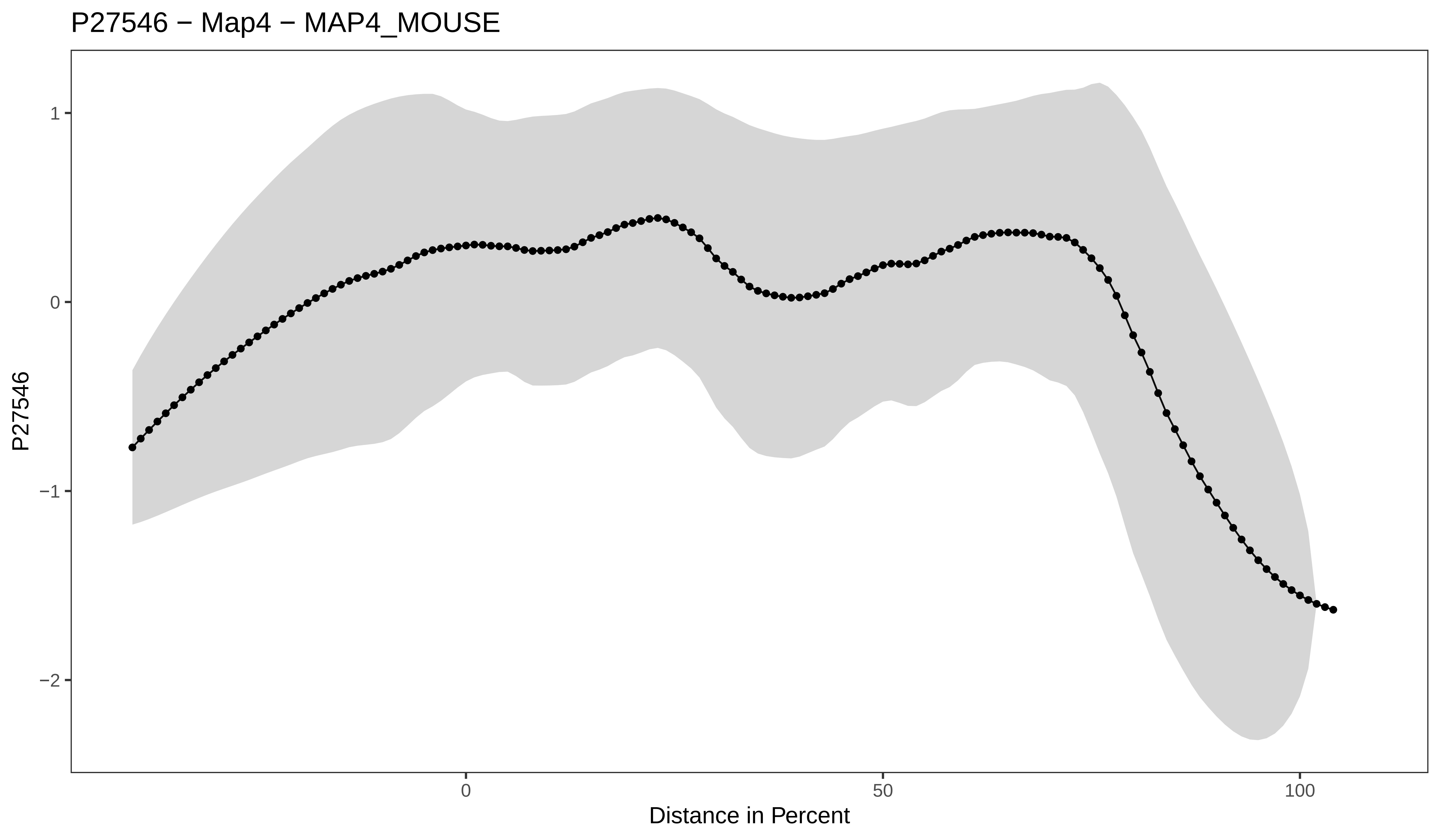

P27546 − Map4 − MAP4_MOUSE
1
0
−1
−2
P27546
0
50
100
Distance in Percent

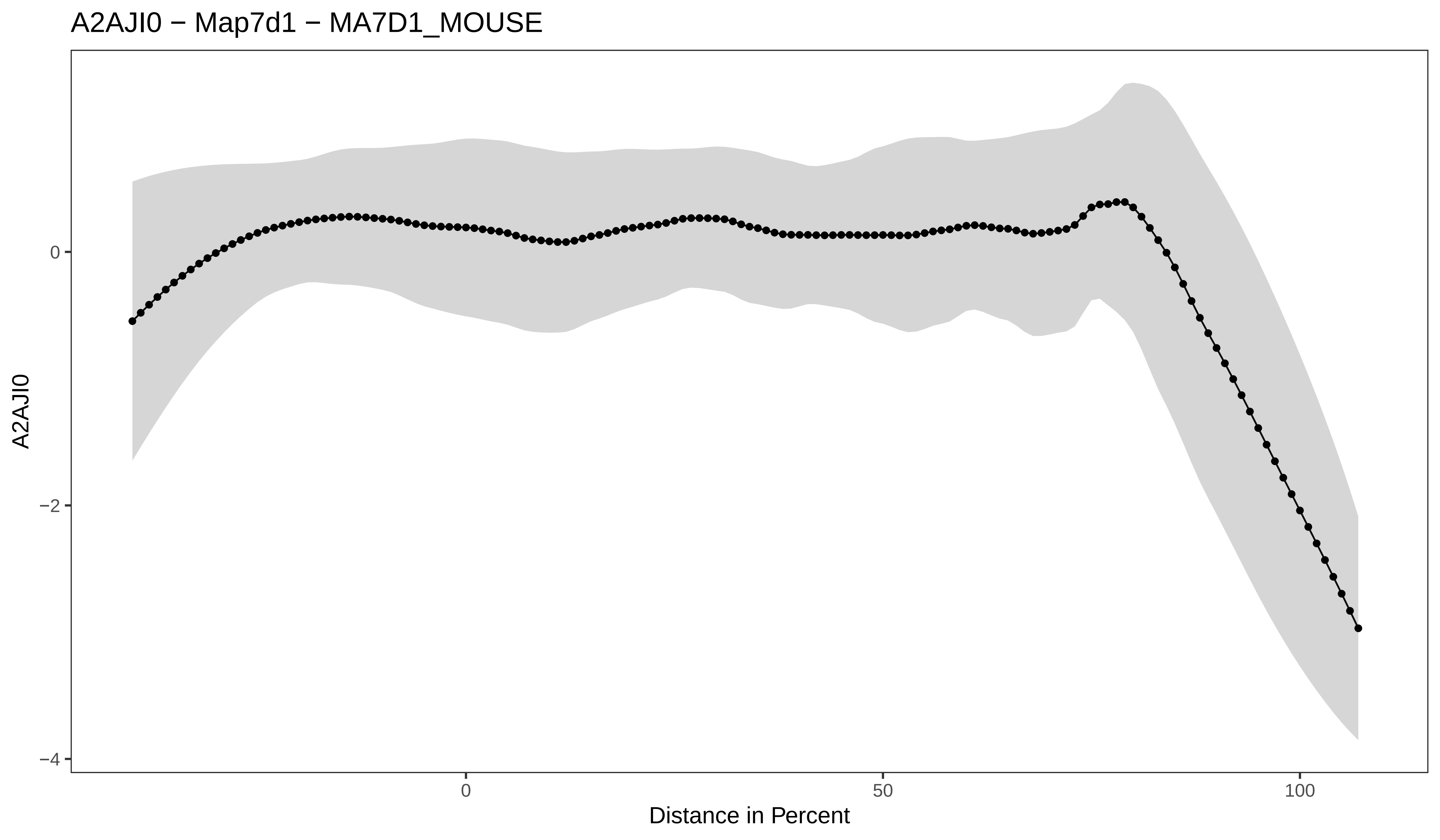
A2AJI0 − Map7d1 − MA7D1_MOUSE
A2AJI0
0
−2
−4
0
50
100
Distance in Percent

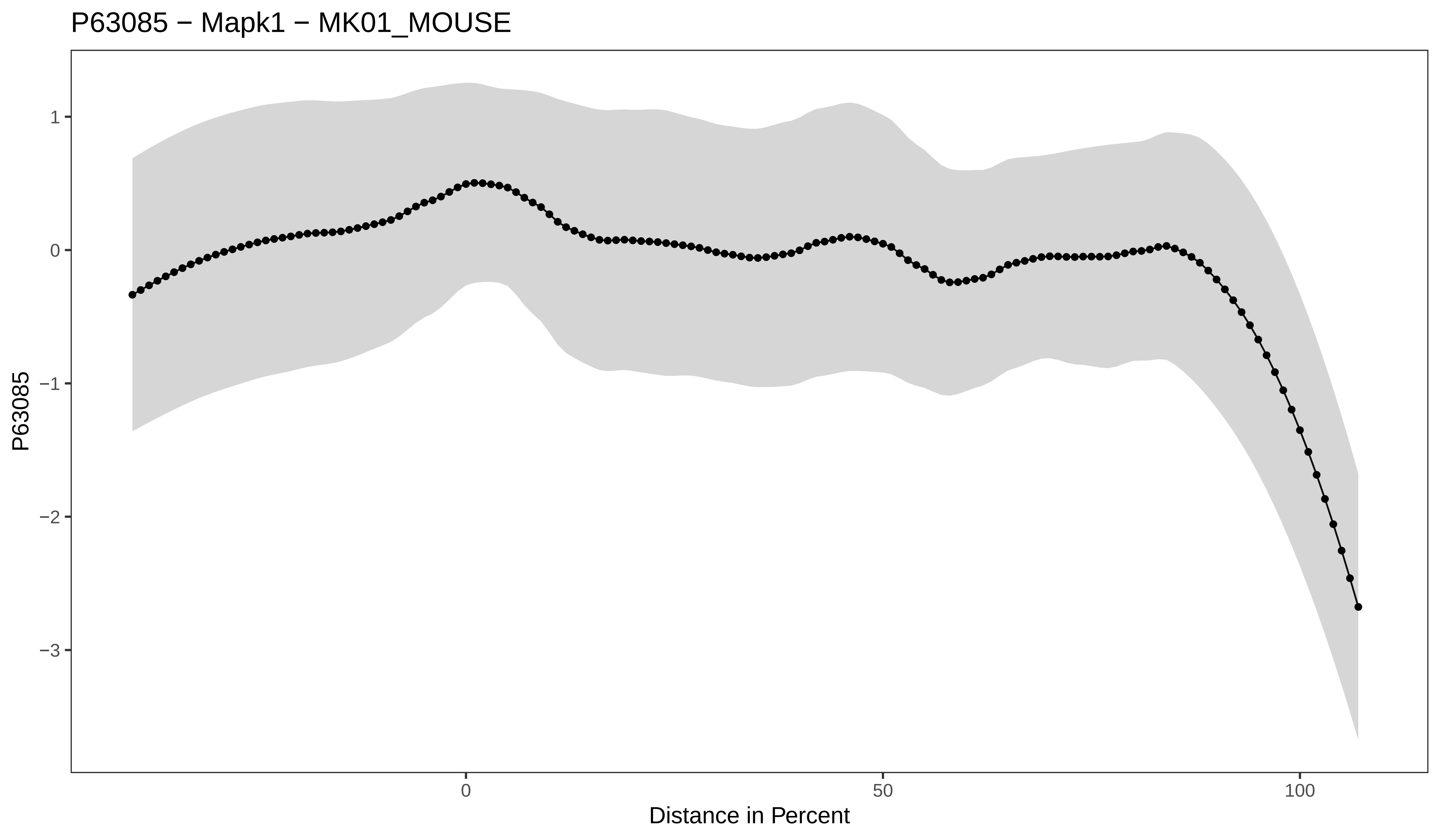
P63085 − Mapk1 − MK01_MOUSE
P63085
Distance in Percent
1
0
−1
−2
−3
0
50
100

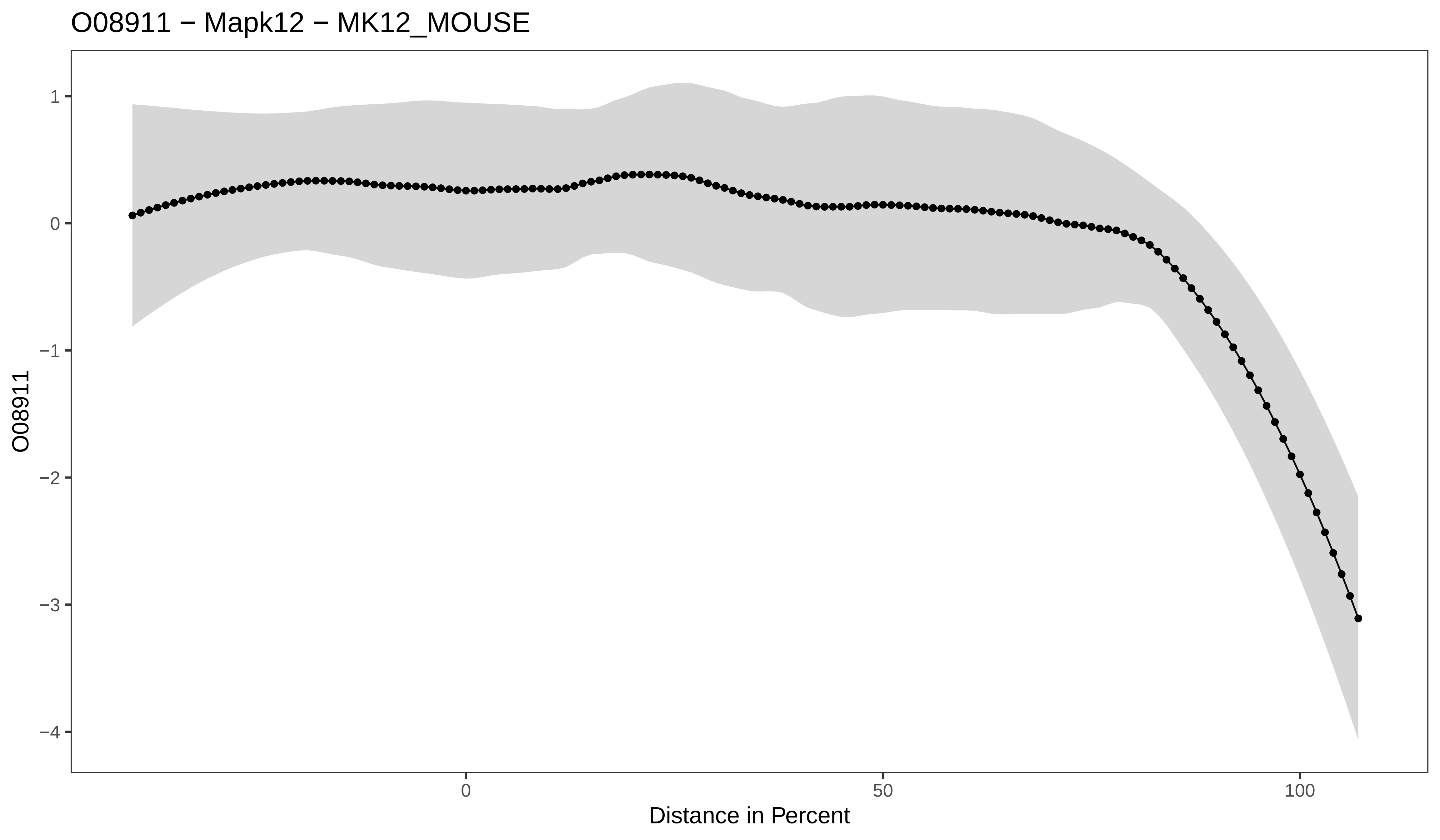

O08911 − Mapk12 − MK12_MOUSE
1
0
−1
−2
−3
−4
O08911
0
50
100
Distance in Percent

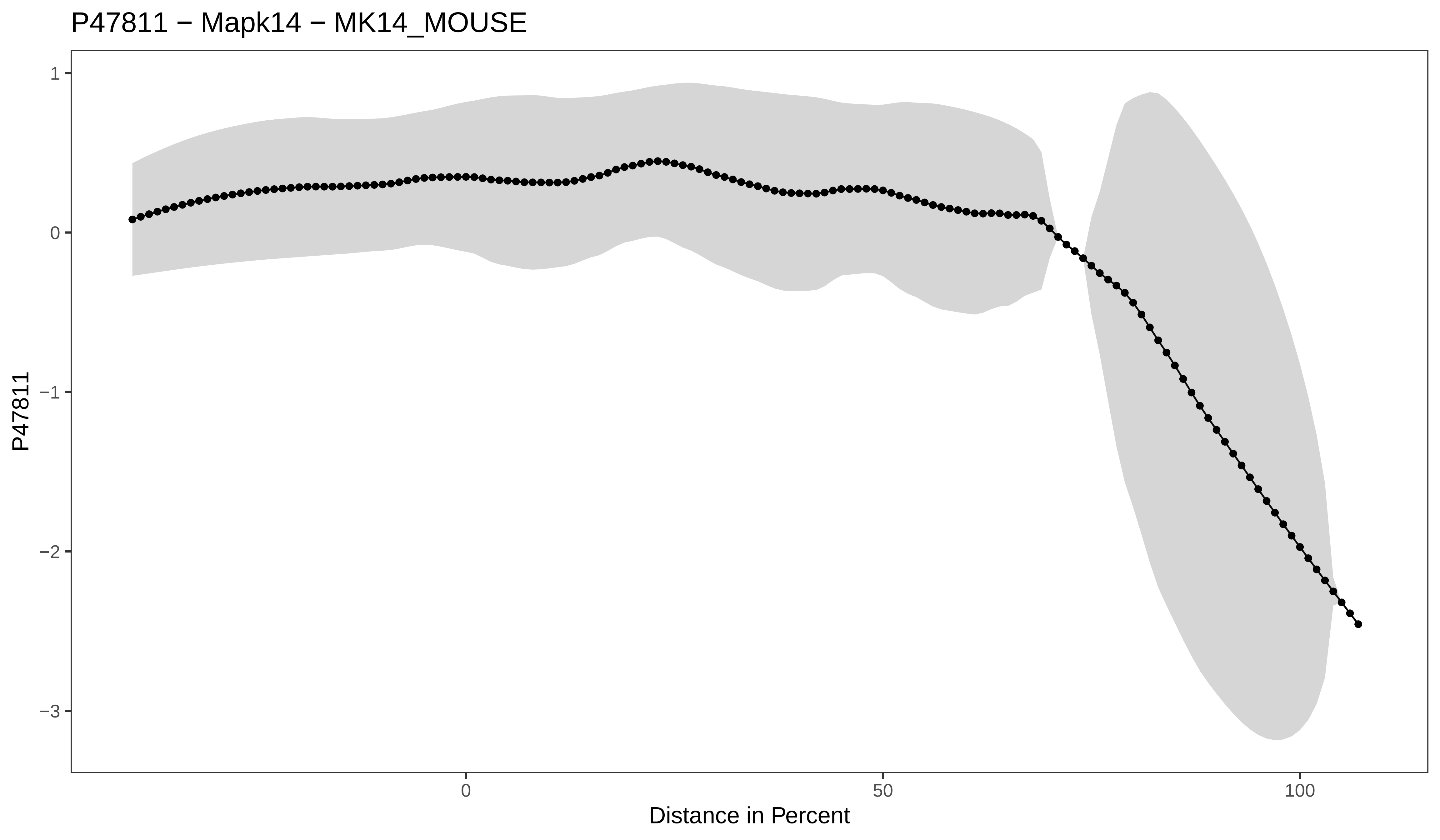

P47811 − Mapk14 − MK14_MOUSE
P47811
1
0
−1
−2
−3
0
50
100
Distance in Percent

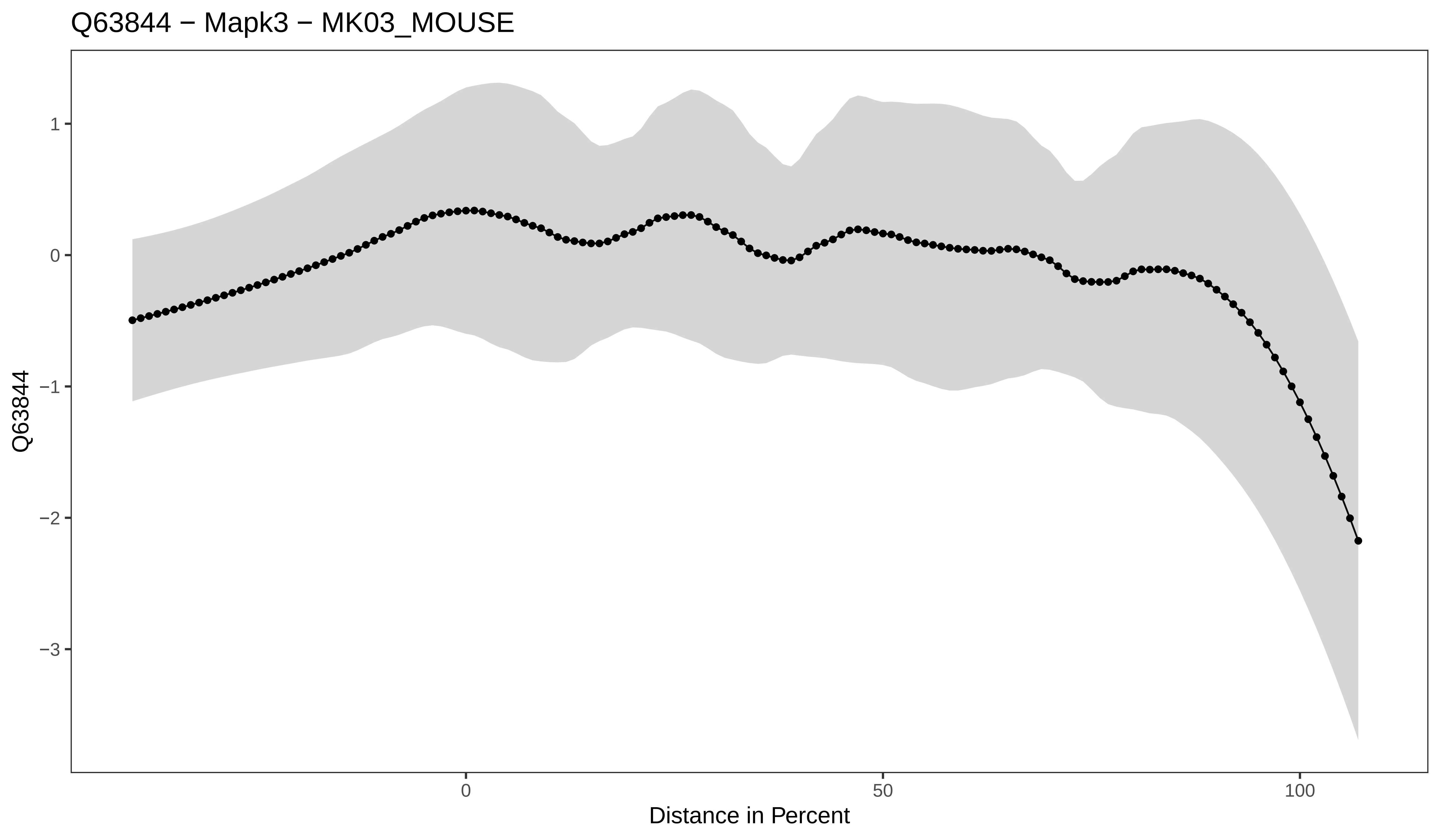
Q63844 − Mapk3 − MK03_MOUSE
Q63844
1
0
−1
−2
−3
0
50
100
Distance in Percent

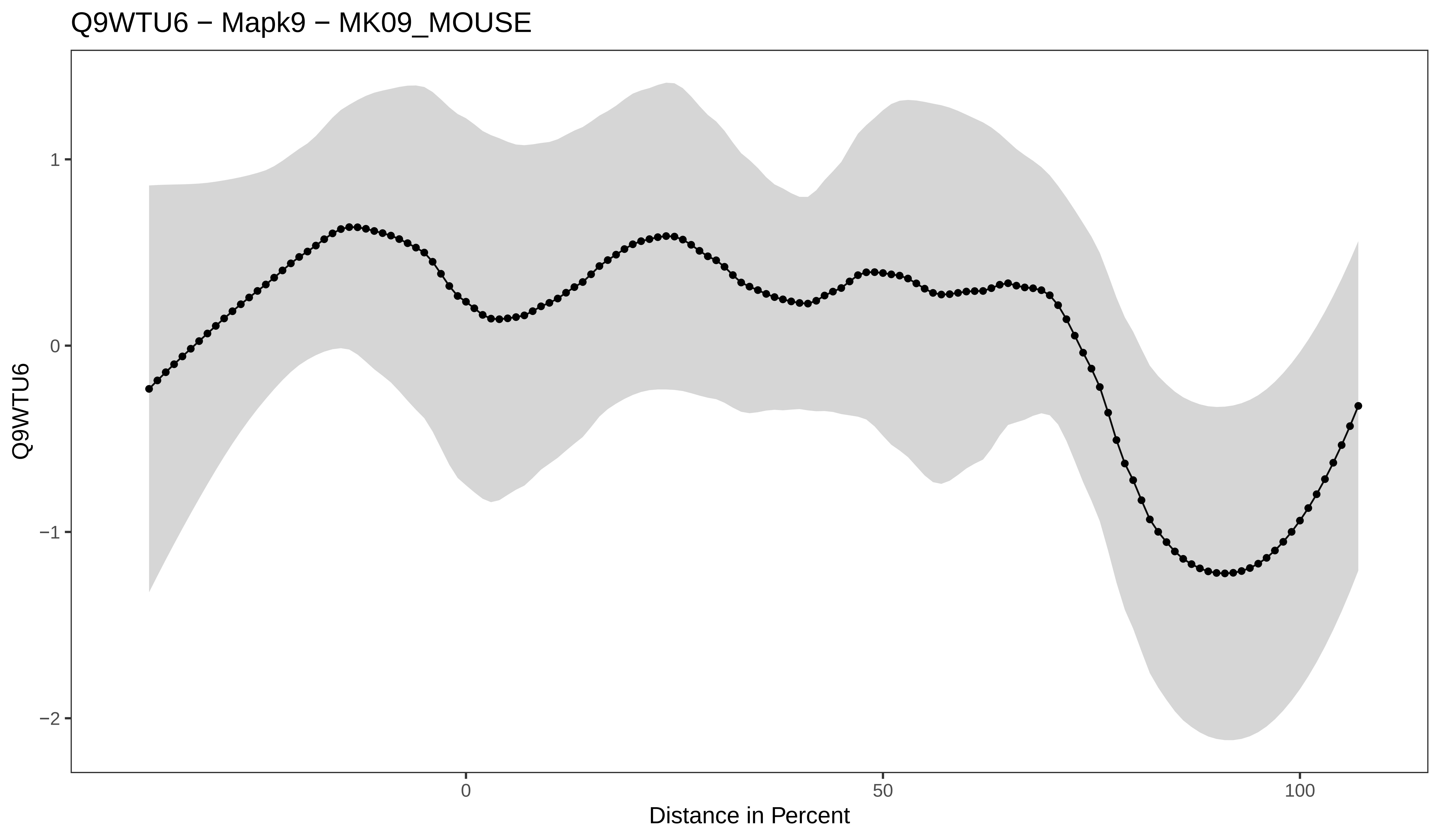

Q9WTU6 − Mapk9 − MK09_MOUSE
Q9WTU6
1
0
−1
−2
0
50
100
Distance in Percent

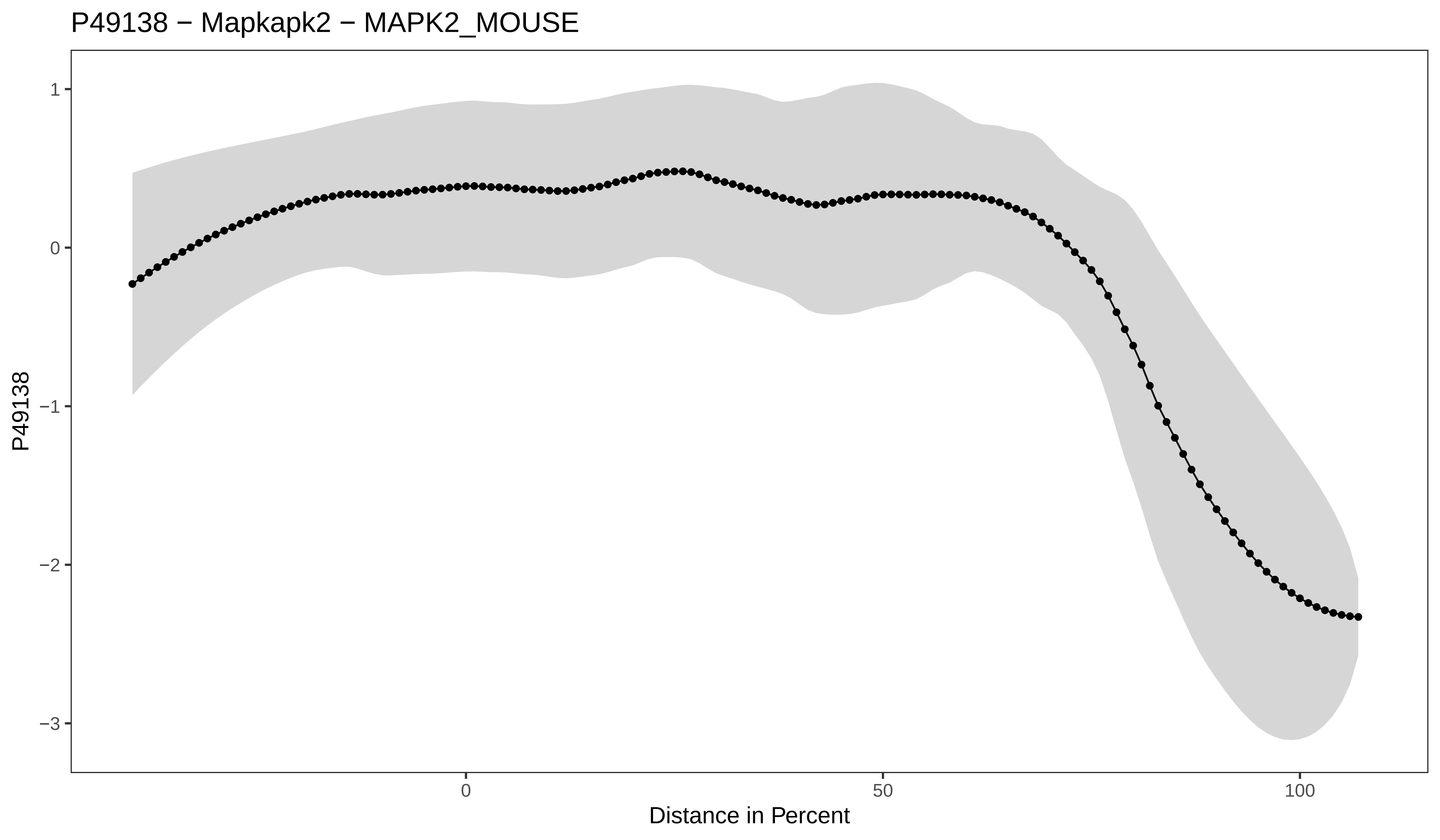
P49138 − Mapkapk2 − MAPK2_MOUSE
1
0
−1
−2
−3
P49138
0
50
100
Distance in Percent

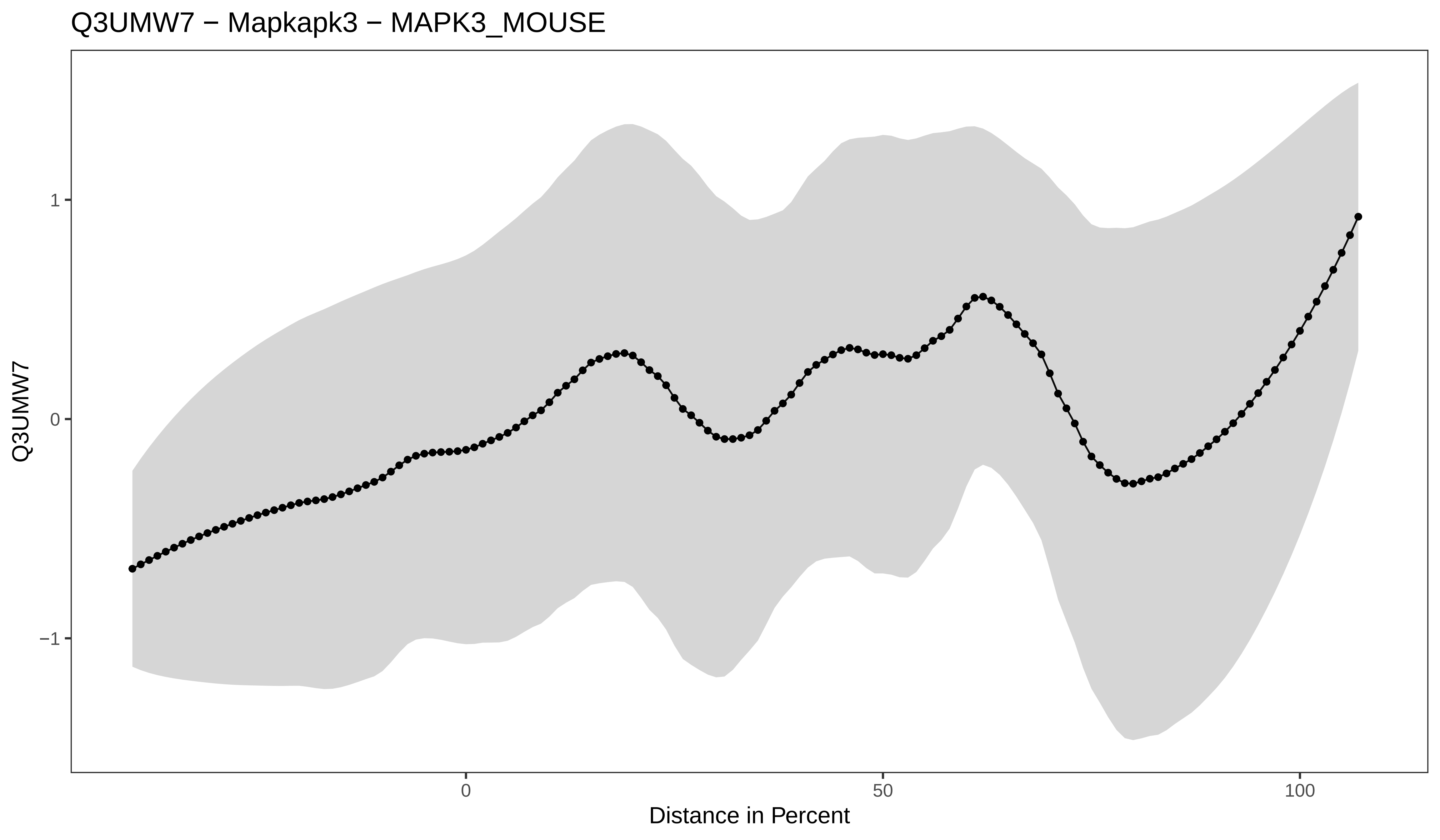
Q3UMW7 − Mapkapk3 − MAPK3_MOUSE
Q3UMW7
1
0
−1
0
50
100
Distance in Percent

Q61166 − Mapre1 − MARE1_MOUSE

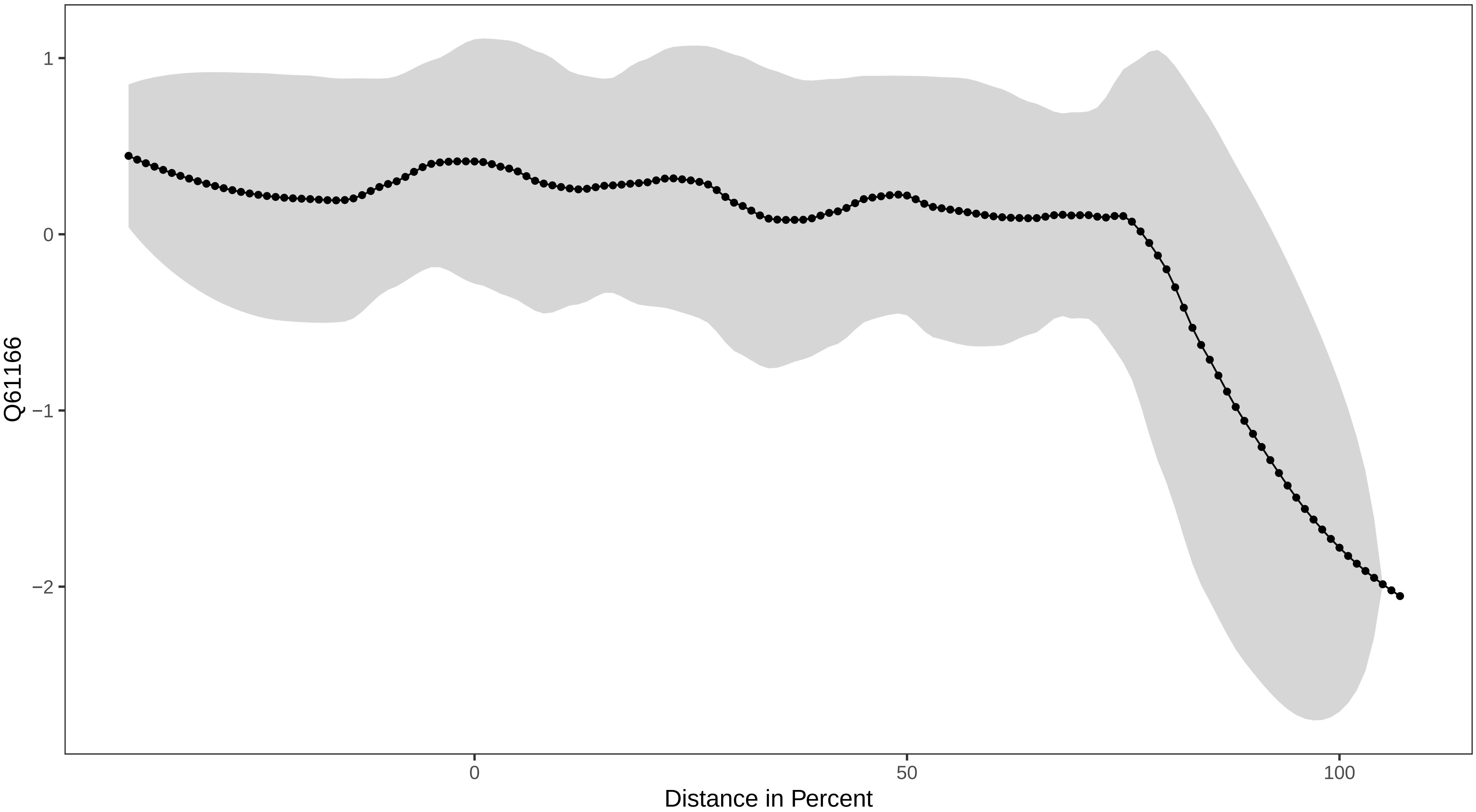

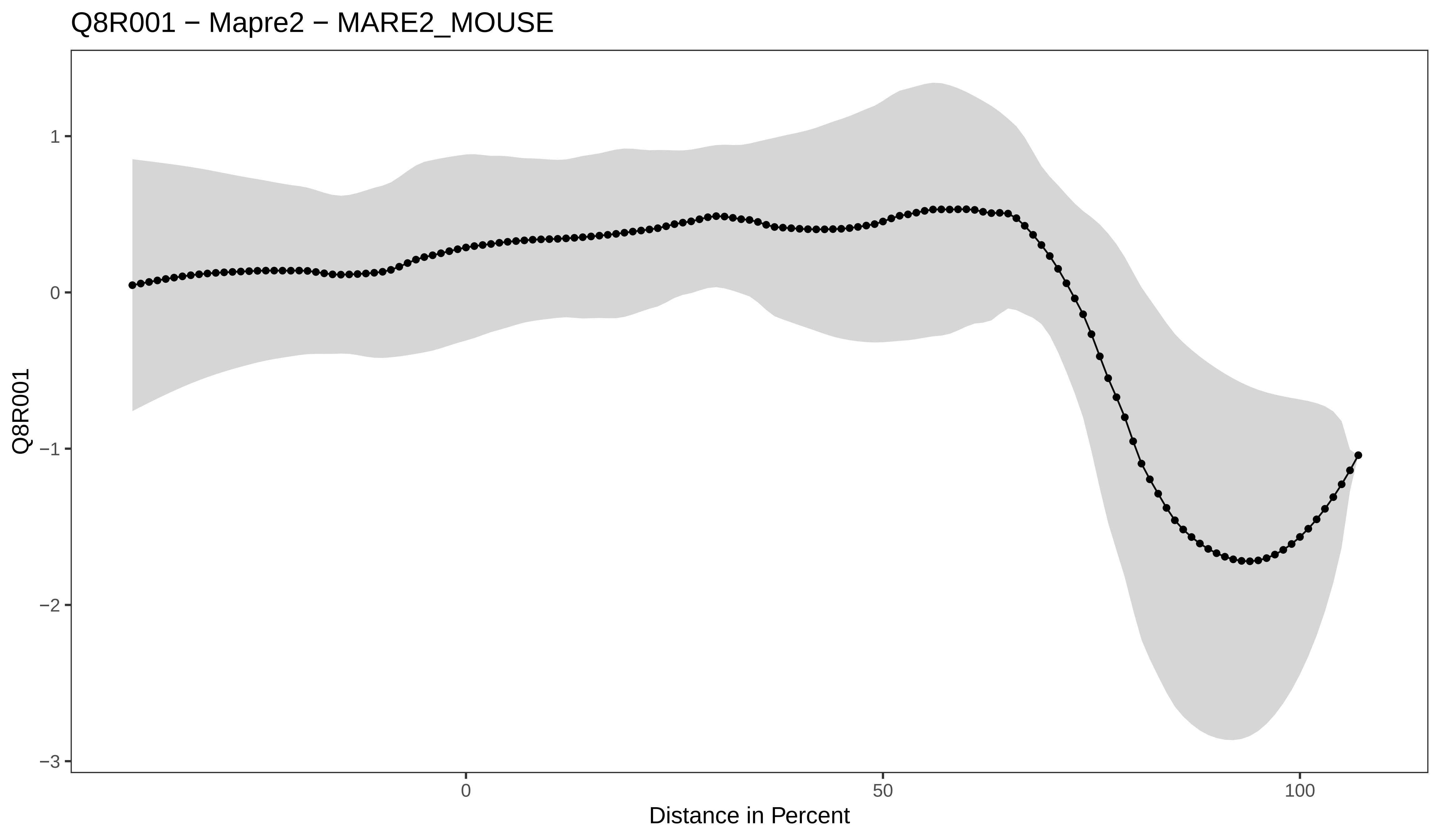

Q8R001 − Mapre2 − MARE2_MOUSE
1
0
−1
−2
−3
Q8R001
0
50
100
Distance in Percent

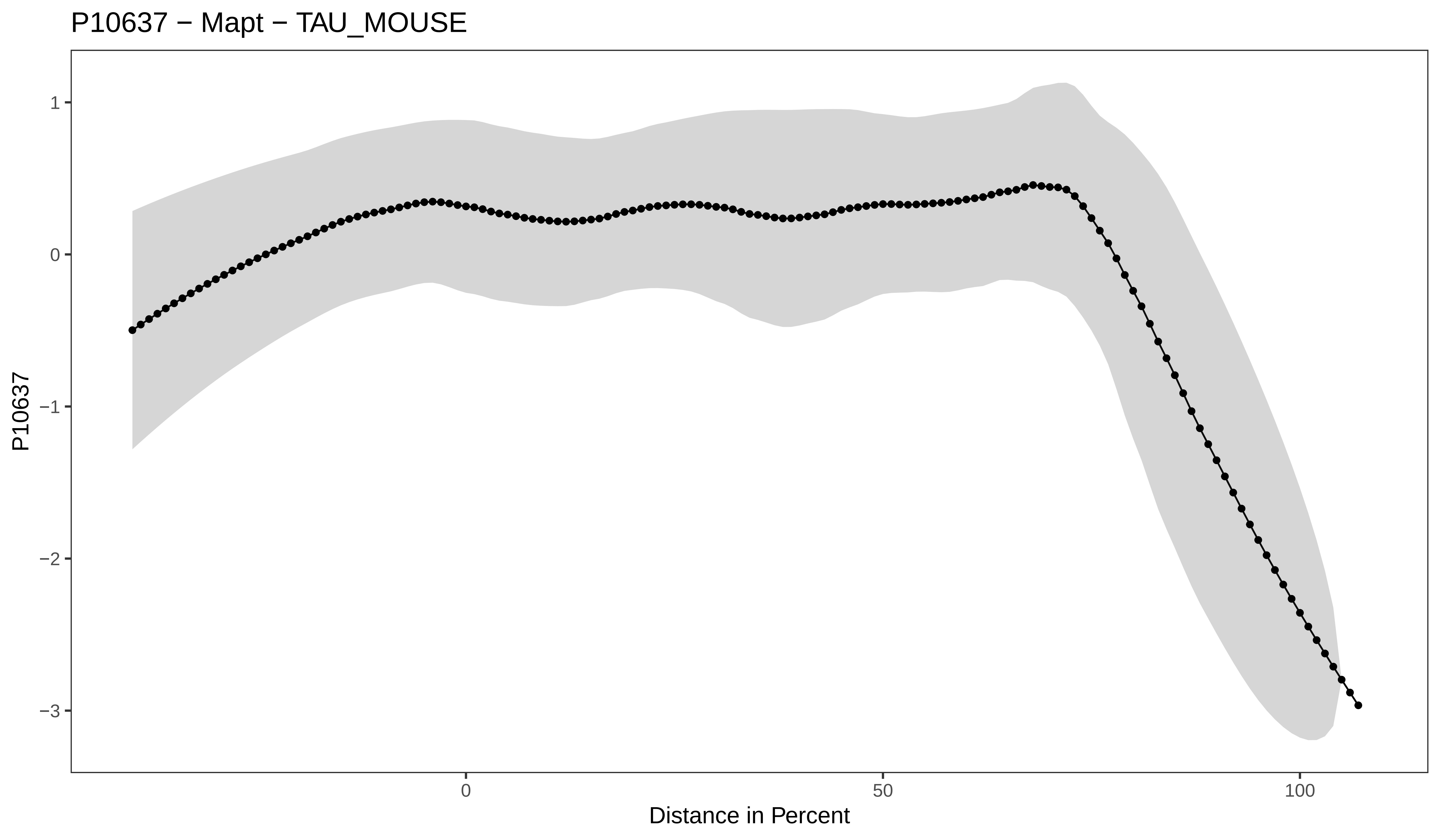
P10637 − Mapt − TAU_MOUSE
1
0
−1
−2
−3
P10637
0
50
100
Distance in Percent

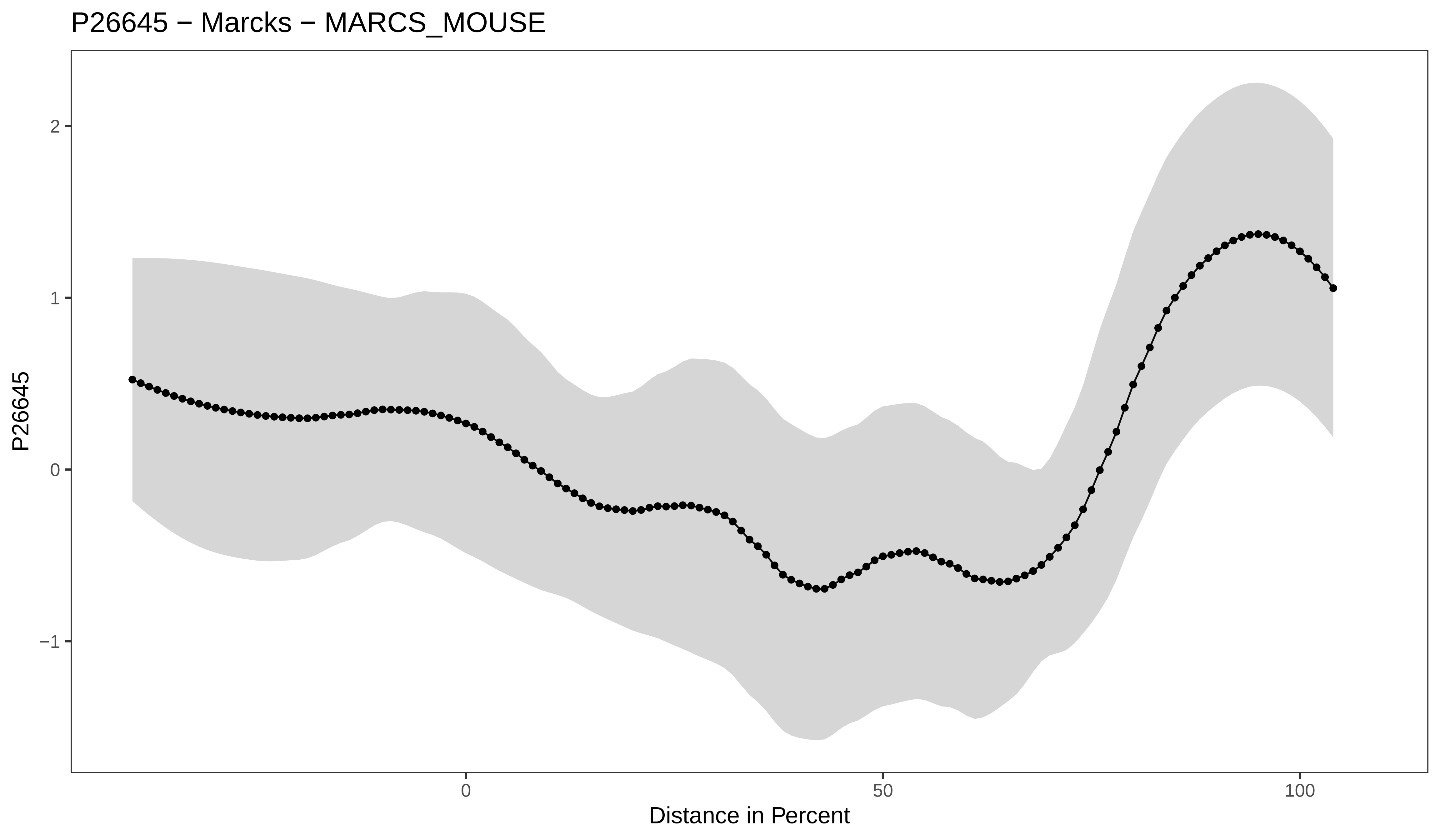
P26645 − Marcks − MARCS_MOUSE
P26645
2
1
0
−1
0
50
100
Distance in Percent

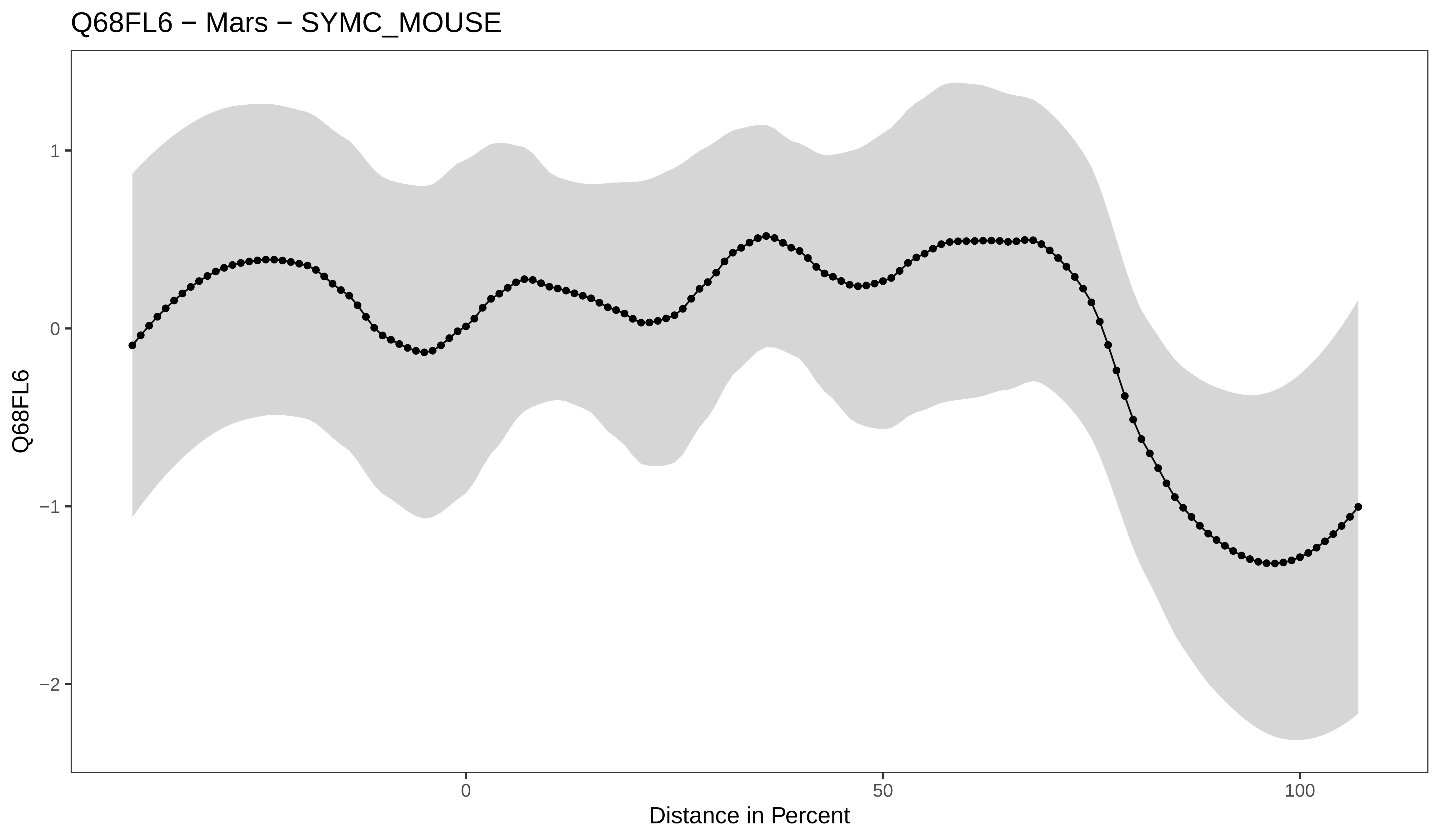
Q68FL6 − Mars − SYMC_MOUSE
Q68FL6
1
0
−1
−2
0
50
100
Distance in Percent

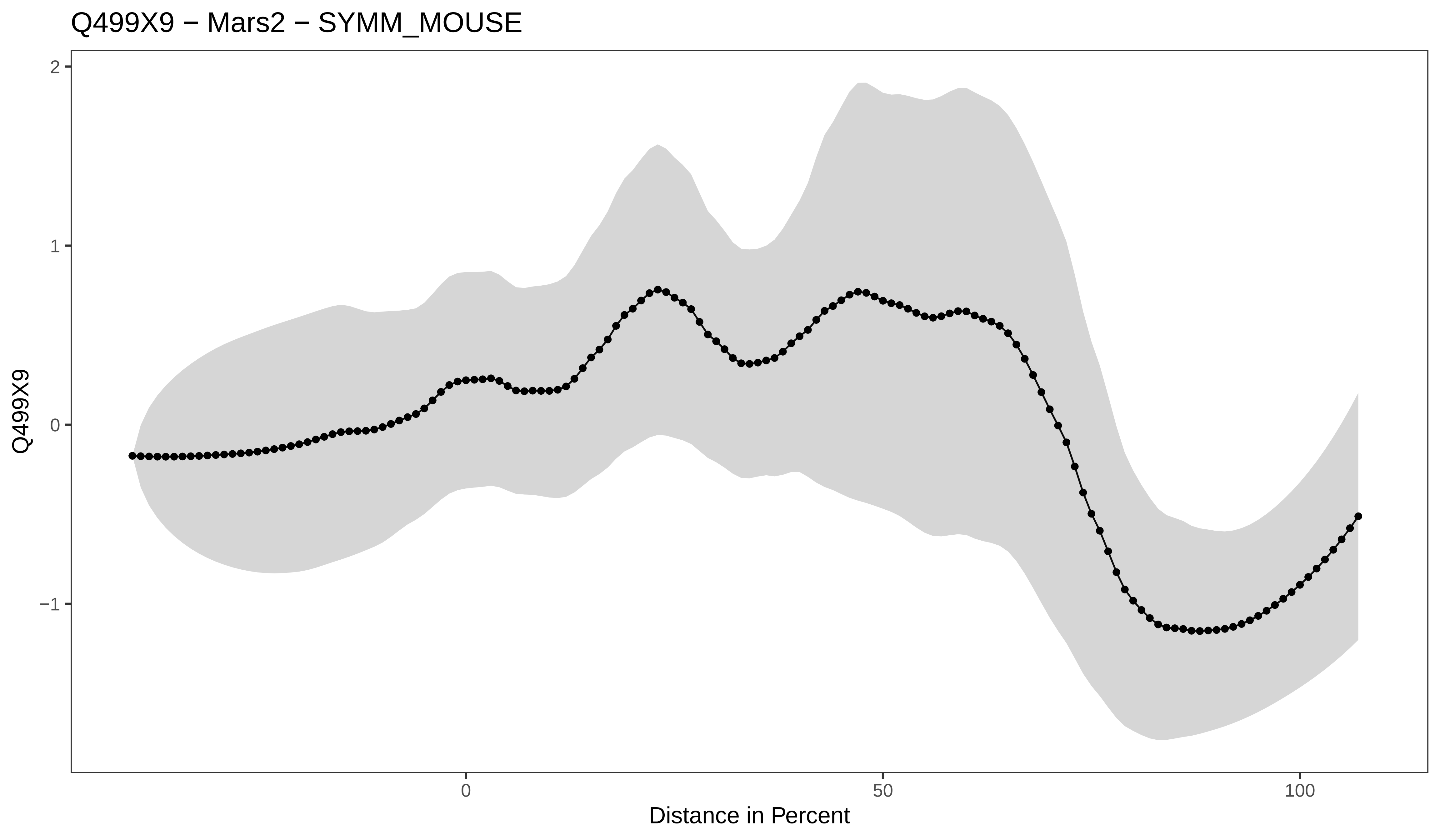

Q499X9 − Mars2 − SYMM_MOUSE
Q499X9
2
1
0
−1
0
50
100
Distance in Percent

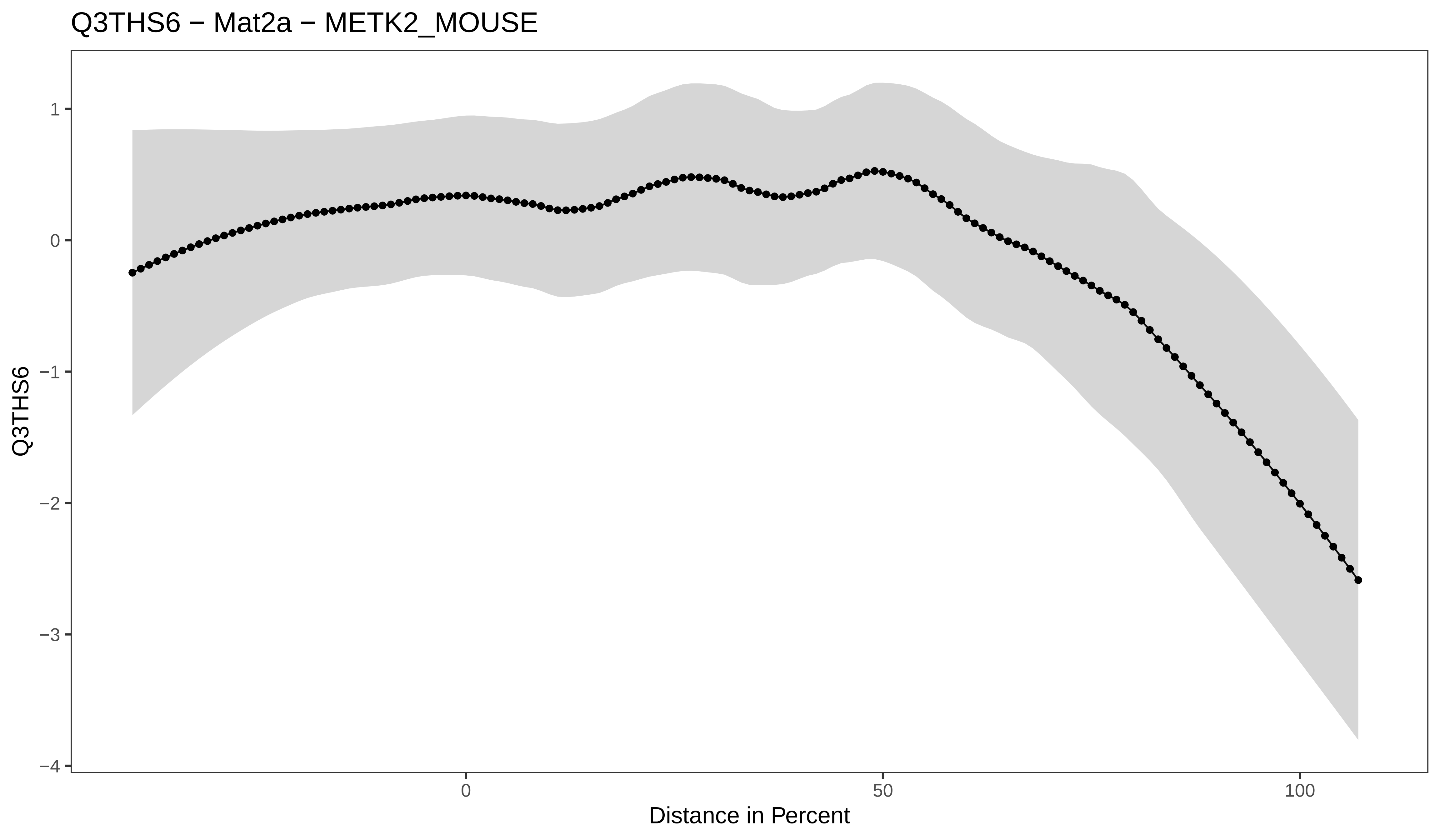
Q3THS6 − Mat2a − METK2_MOUSE
Q3THS6
Distance in Percent
1
0
−1
−2
−3
−4
0
50
100

Q99LB6 − Mat2b − MAT2B_MOUSE

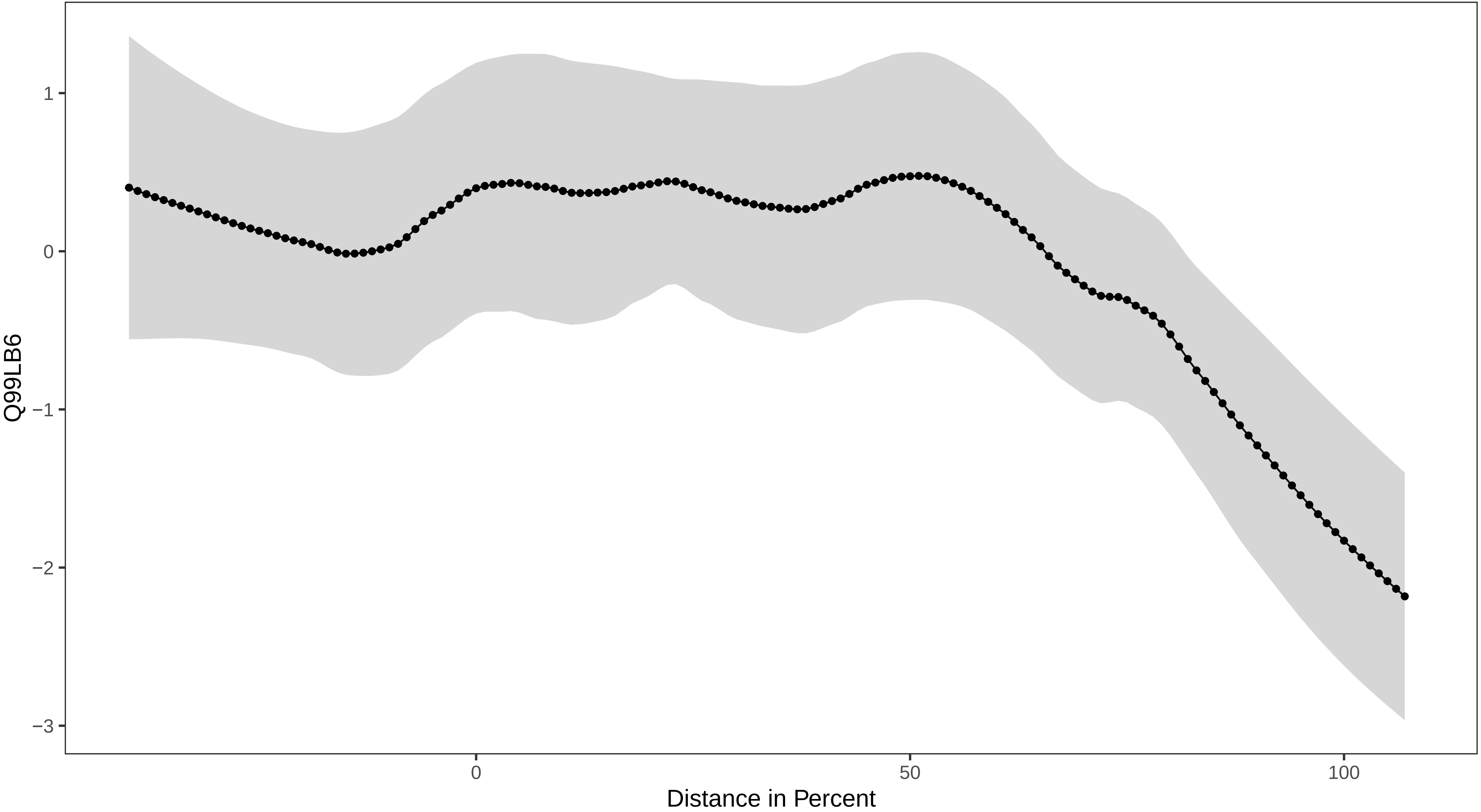

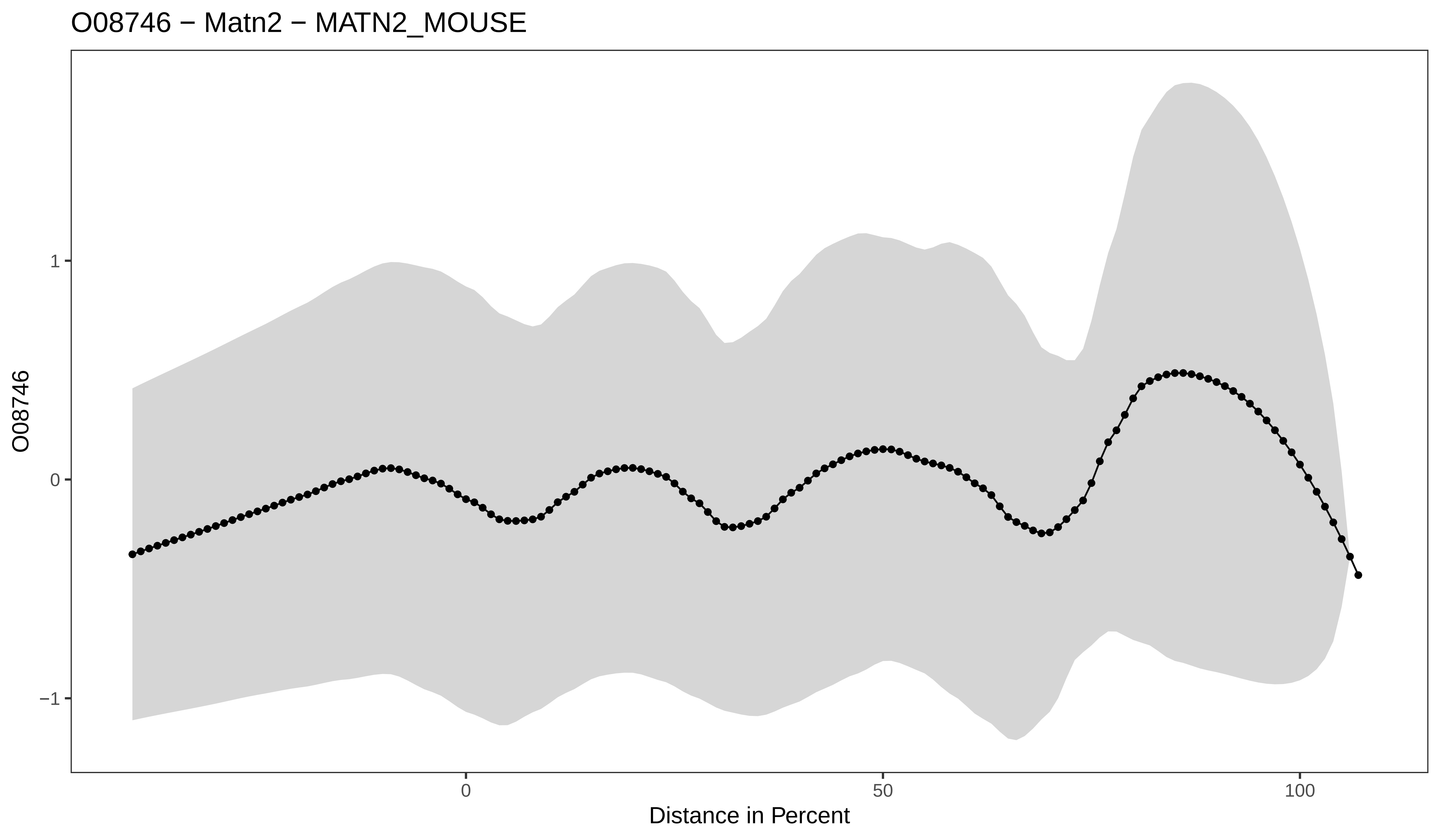

O08746 − Matn2 − MATN2_MOUSE
O08746
1
0
−1
0
50
100
Distance in Percent

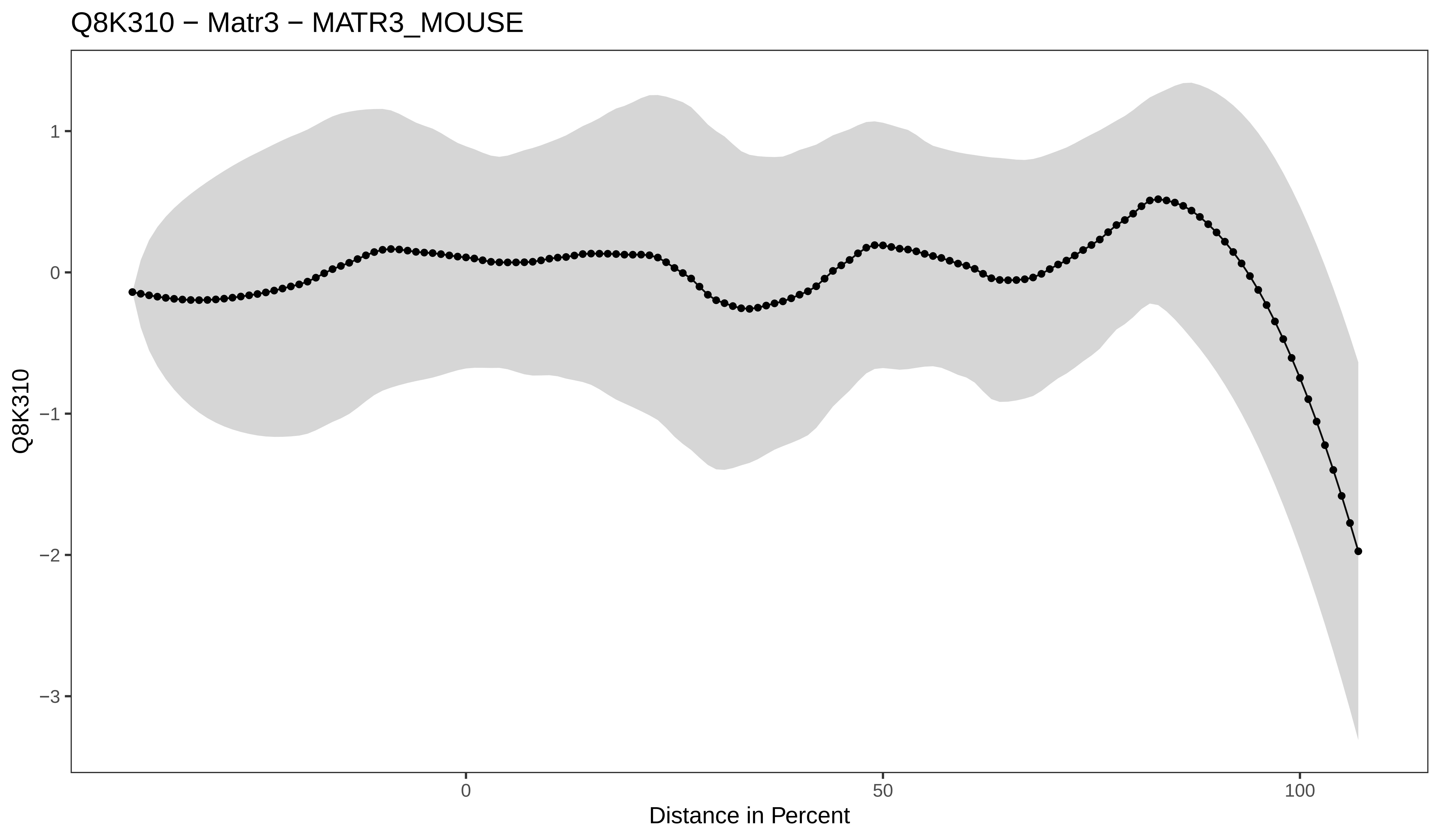

Q8K310 − Matr3 − MATR3_MOUSE
1
0
−1
−2
−3
Q8K310
0
50
100
Distance in Percent

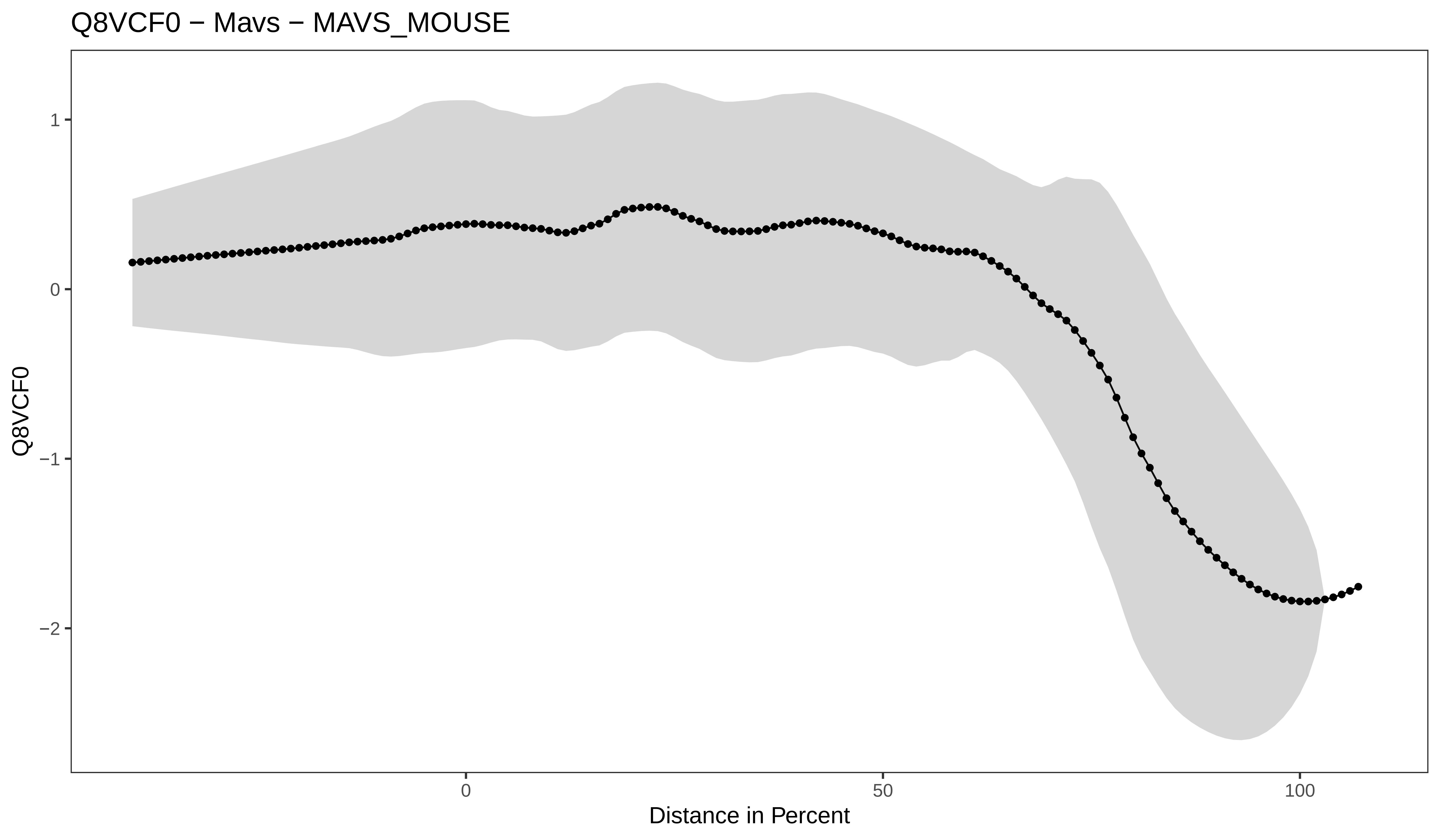
Q8VCF0 − Mavs − MAVS_MOUSE
Q8VCF0
1
0
−1
−2
0
50
100
Distance in Percent

P04247 − Mb − MYG_MOUSE

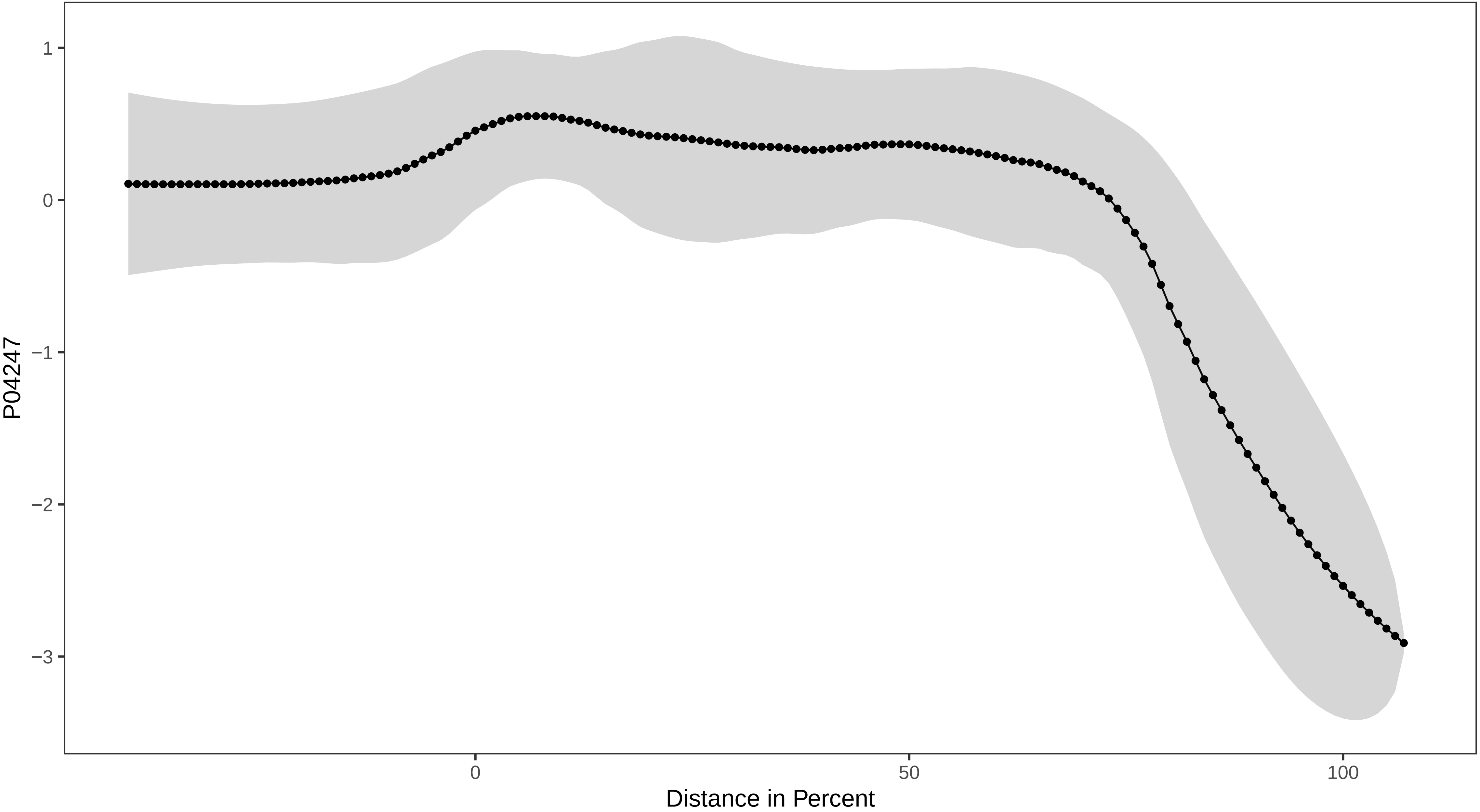

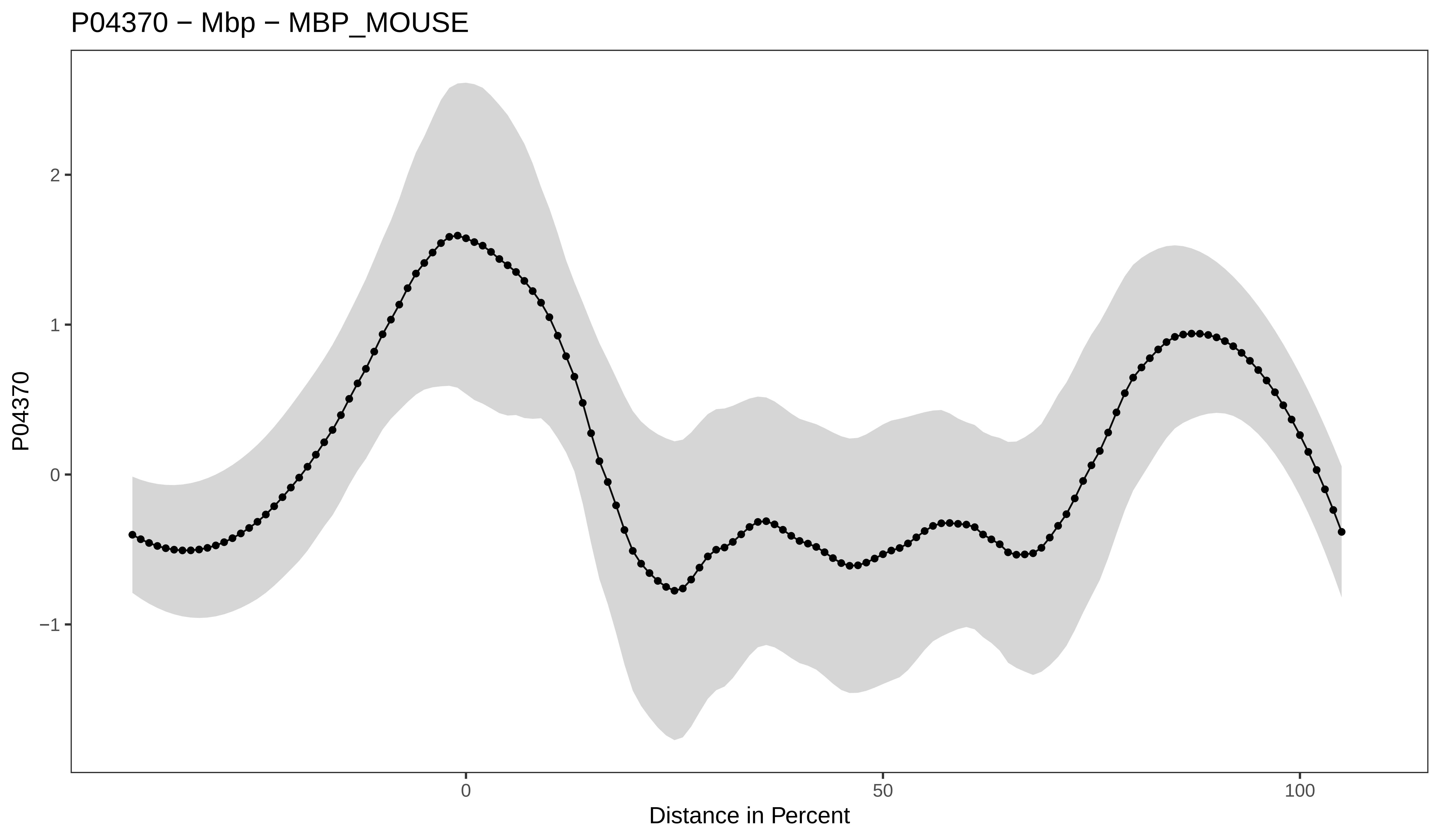

P04370 − Mbp − MBP_MOUSE
P04370
Distance in Percent
2
1
0
−1
0
50
100

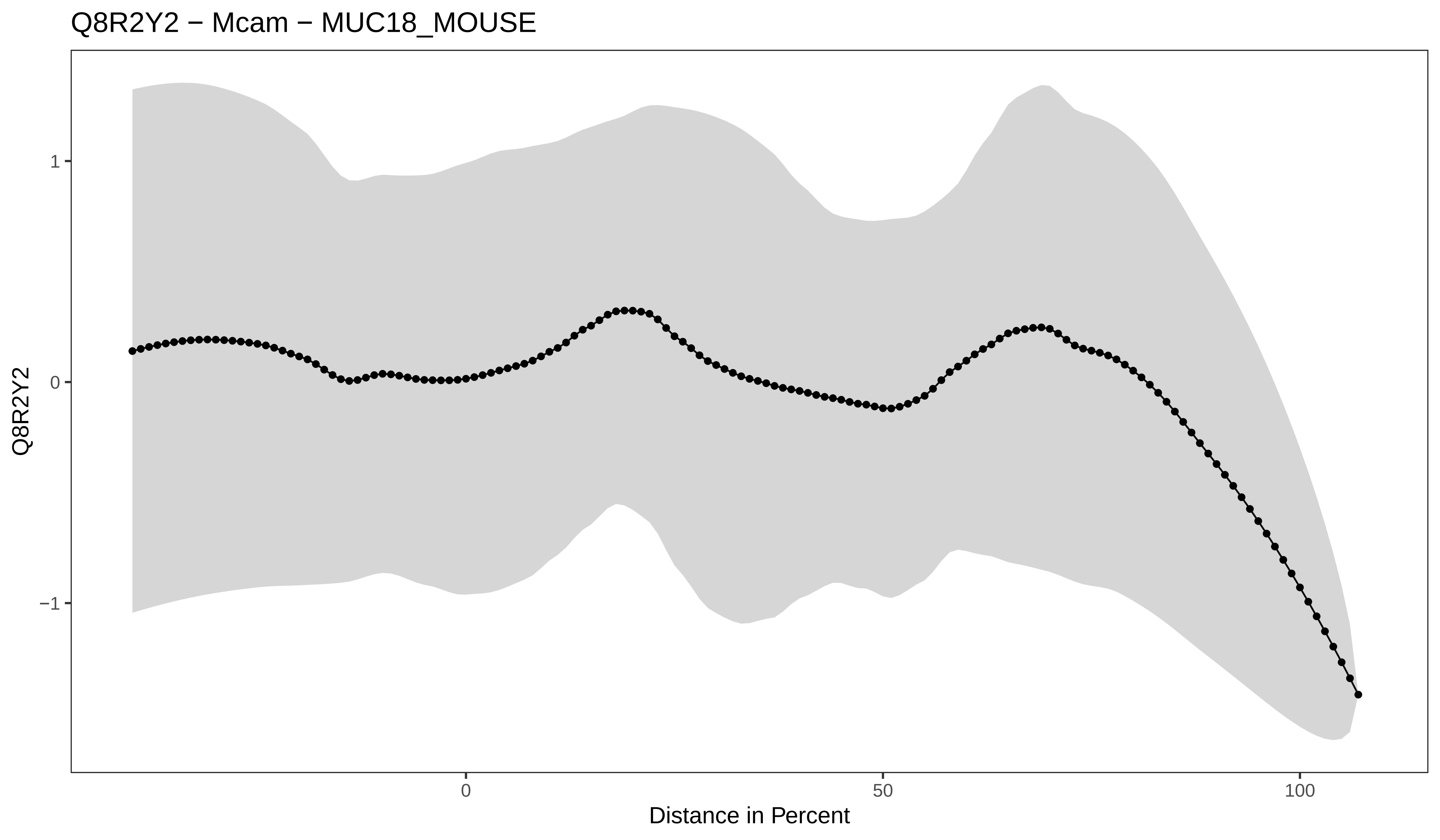

Q8R2Y2 − Mcam − MUC18_MOUSE
Q8R2Y2
1
0
−1
0
50
100
Distance in Percent

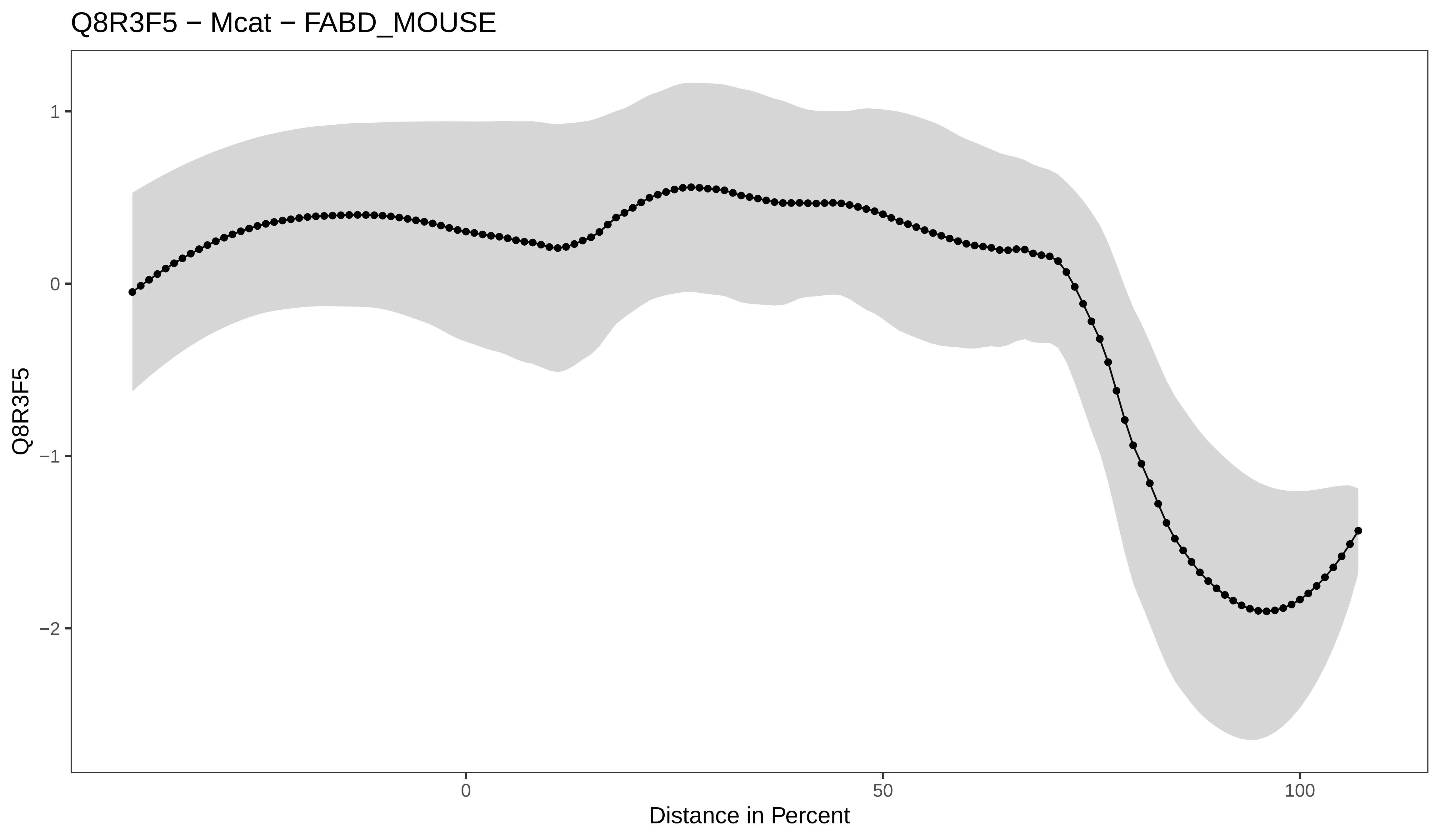

Q8R3F5 − Mcat − FABD_MOUSE
Q8R3F5
1
0
−1
−2
0
50
100
Distance in Percent

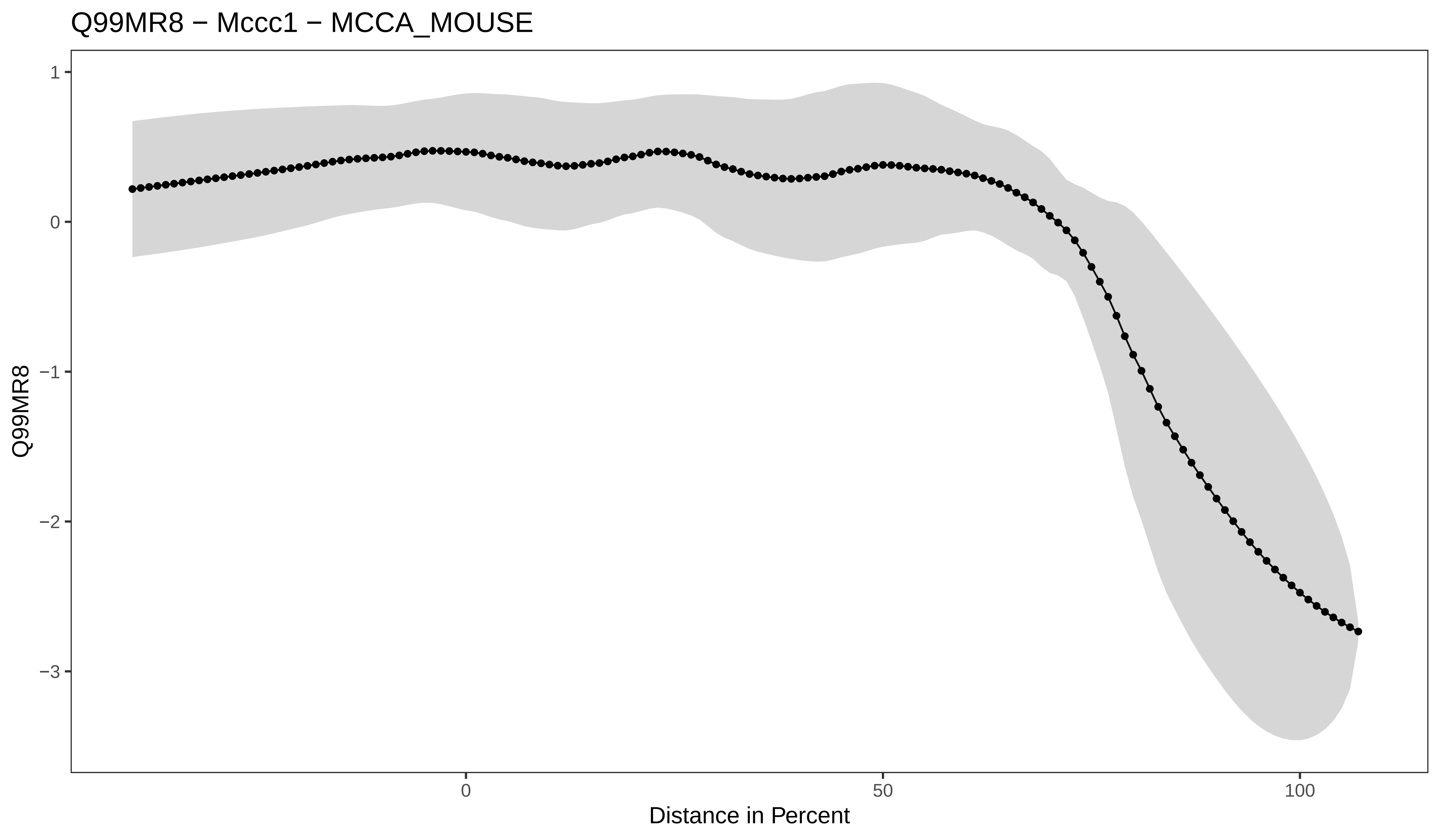
Q99MR8 − Mccc1 − MCCA_MOUSE
1
0
−1
−2
−3
Q99MR8
0
50
100
Distance in Percent

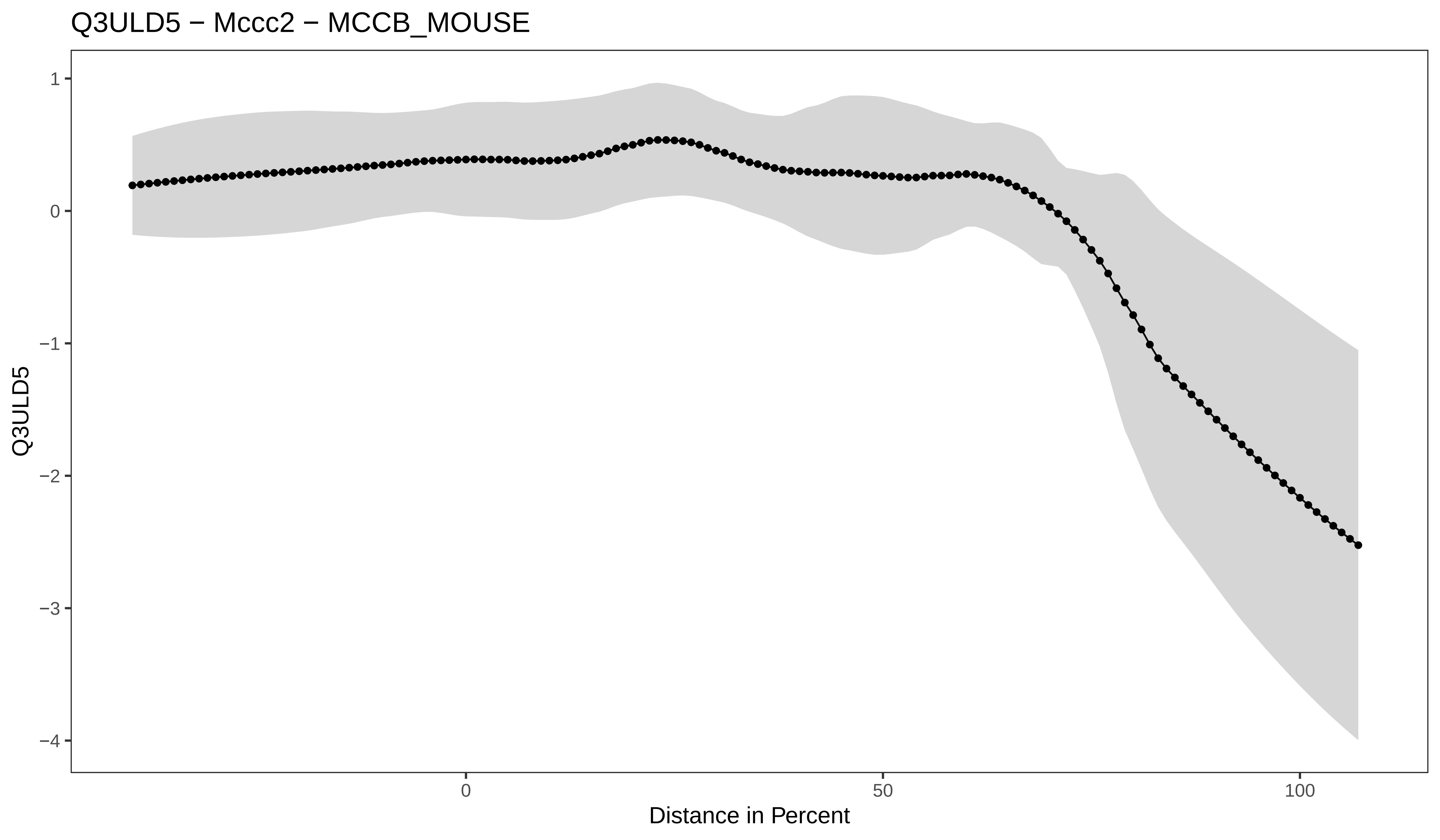

Q3ULD5 − Mccc2 − MCCB_MOUSE
Q3ULD5
1
0
−1
−2
−3
−4
0
50
100
Distance in Percent

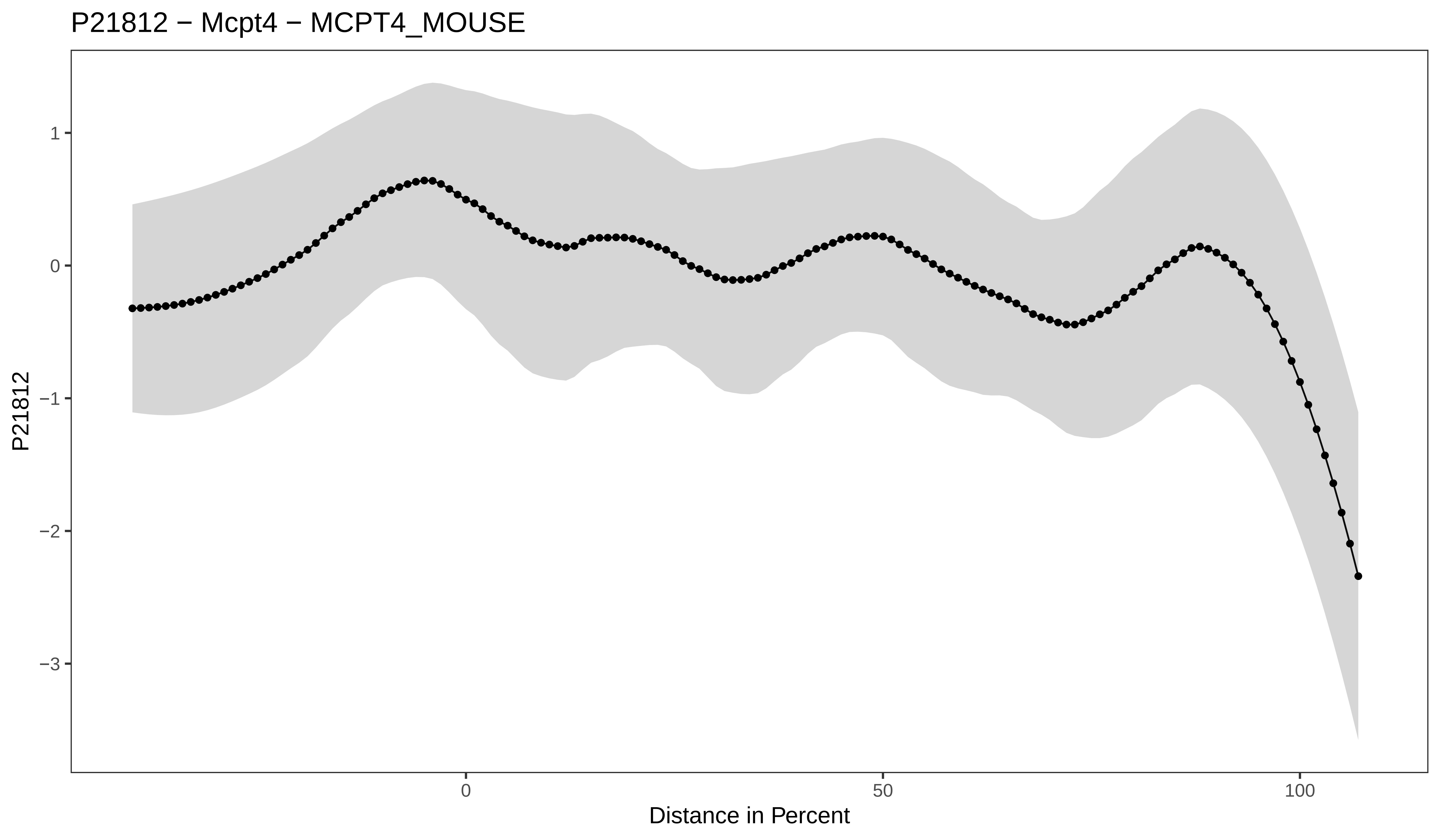
P21812 − Mcpt4 − MCPT4_MOUSE
P21812
Distance in Percent
1
0
−1
−2
−3
0
50
100

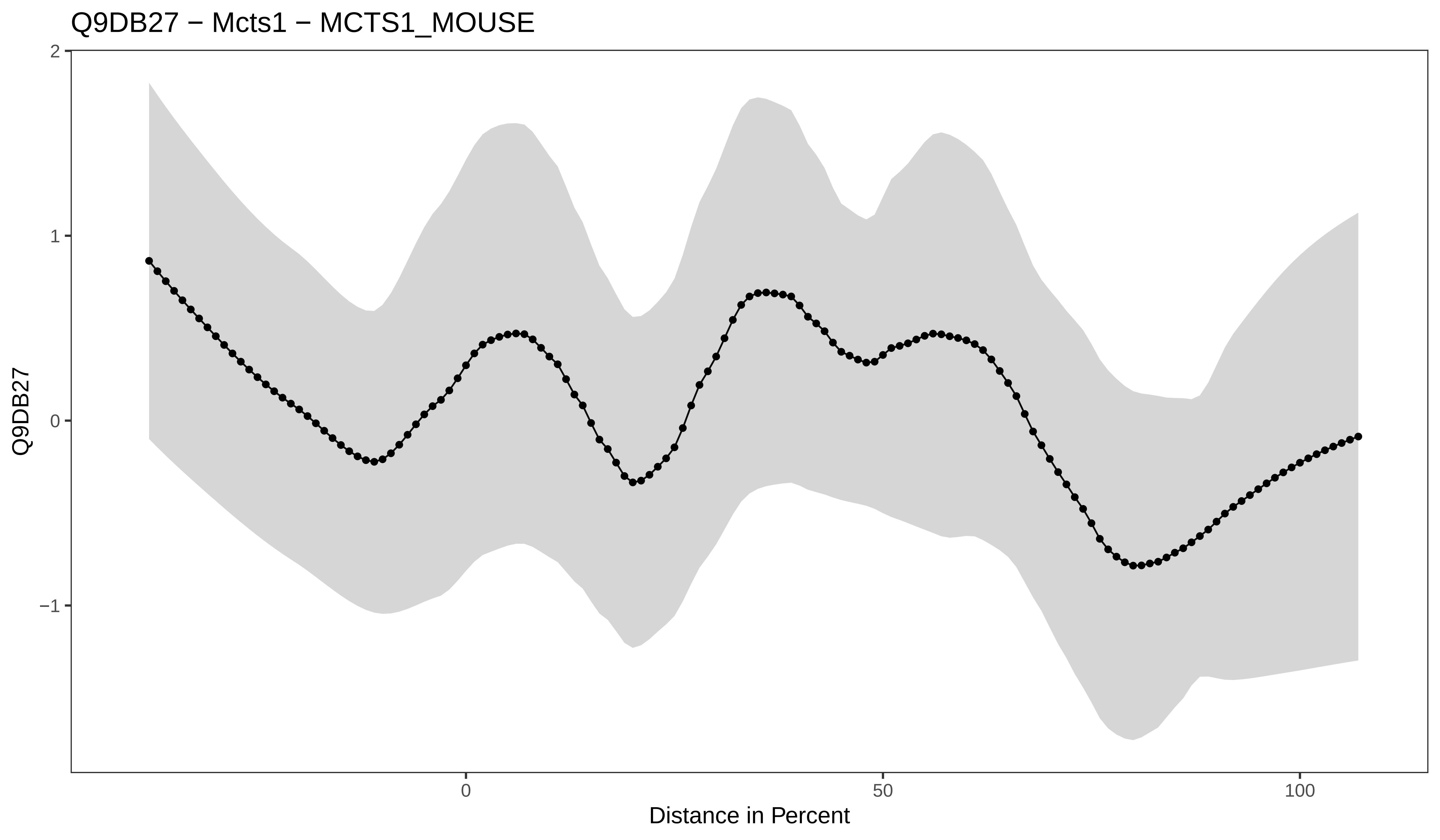

Q9DB27 − Mcts1 − MCTS1_MOUSE
2
1
0
−1
Q9DB27
0
50
100
Distance in Percent

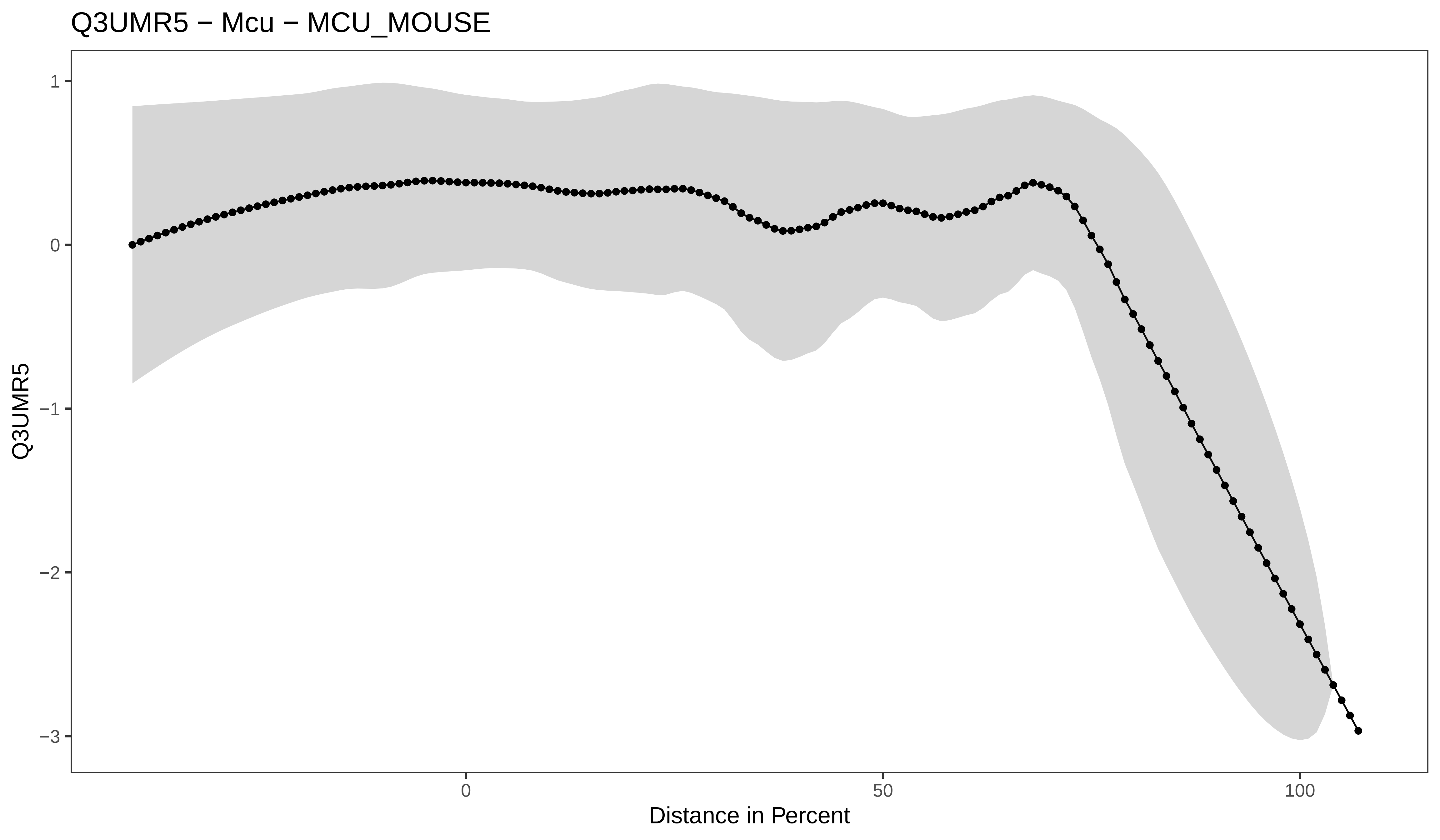

Q3UMR5 − Mcu − MCU_MOUSE
Q3UMR5
1
0
−1
−2
−3
0
50
100
Distance in Percent

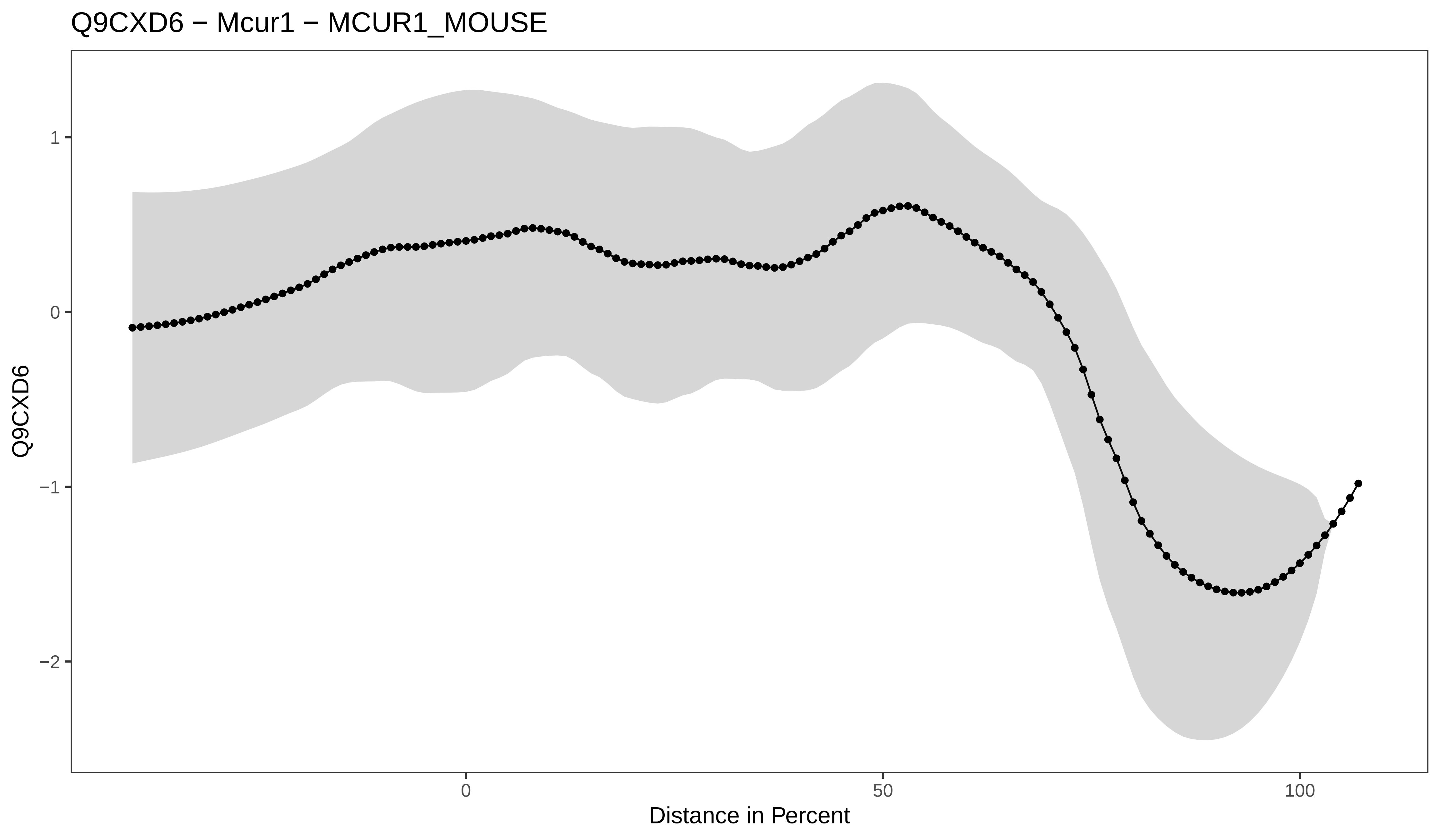

Q9CXD6 − Mcur1 − MCUR1_MOUSE
1
0
−1
−2
Q9CXD6
0
50
100
Distance in Percent

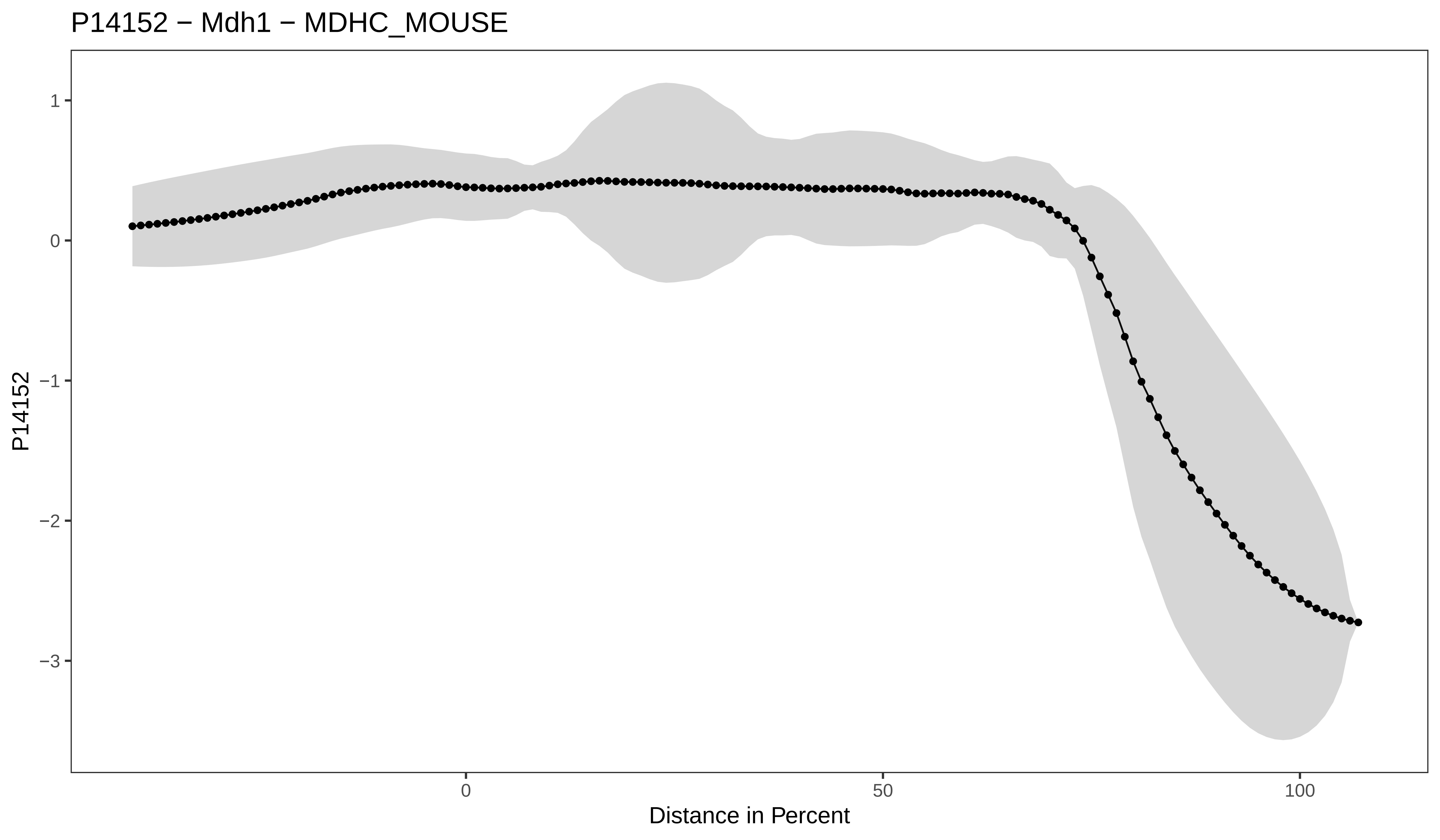

P14152 − Mdh1 − MDHC_MOUSE
P14152
1
0
−1
−2
−3
0
50
100
Distance in Percent

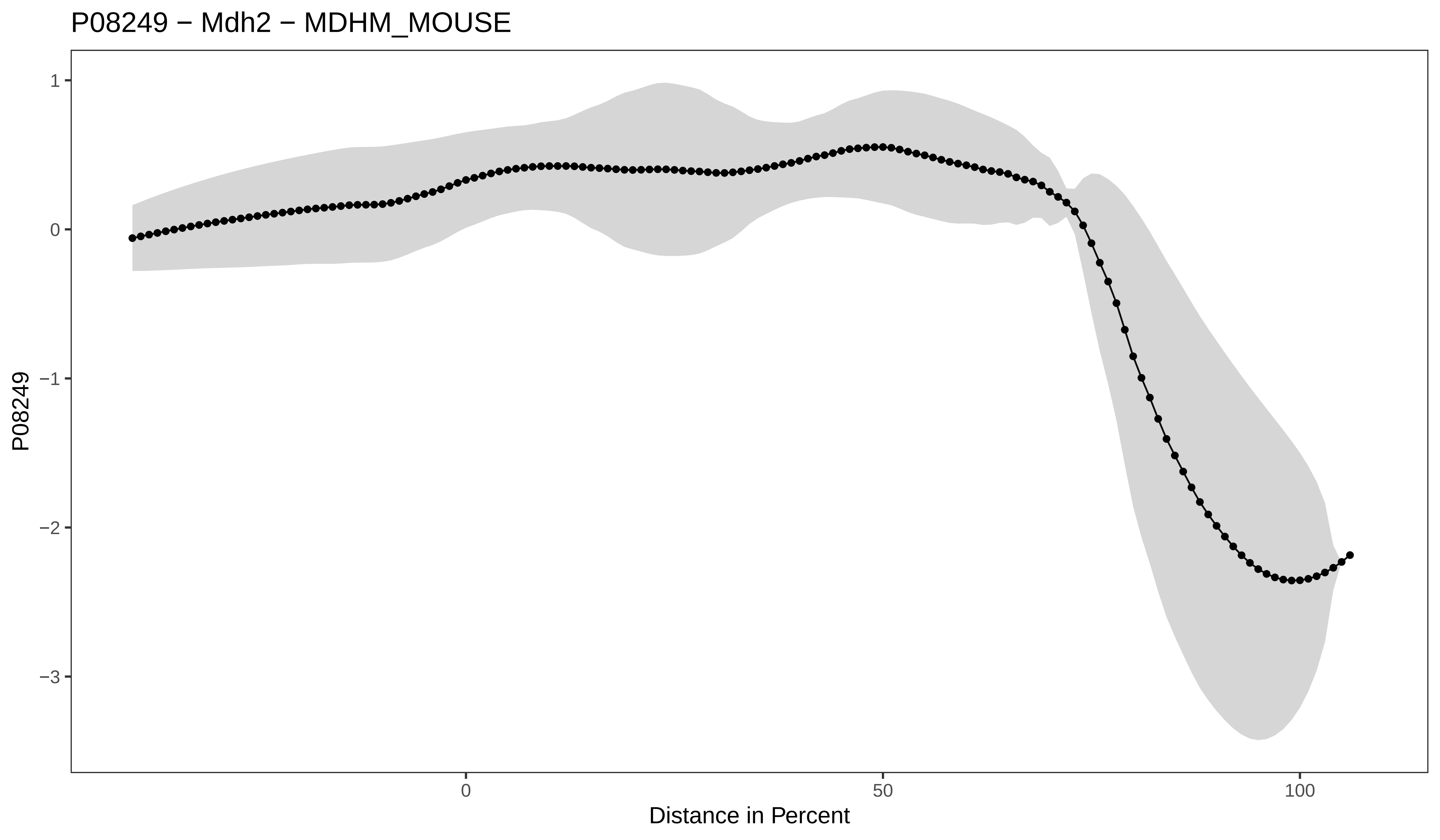
P08249 − Mdh2 − MDHM_MOUSE
1
0
−1
−2
−3
P08249
0
50
100
Distance in Percent

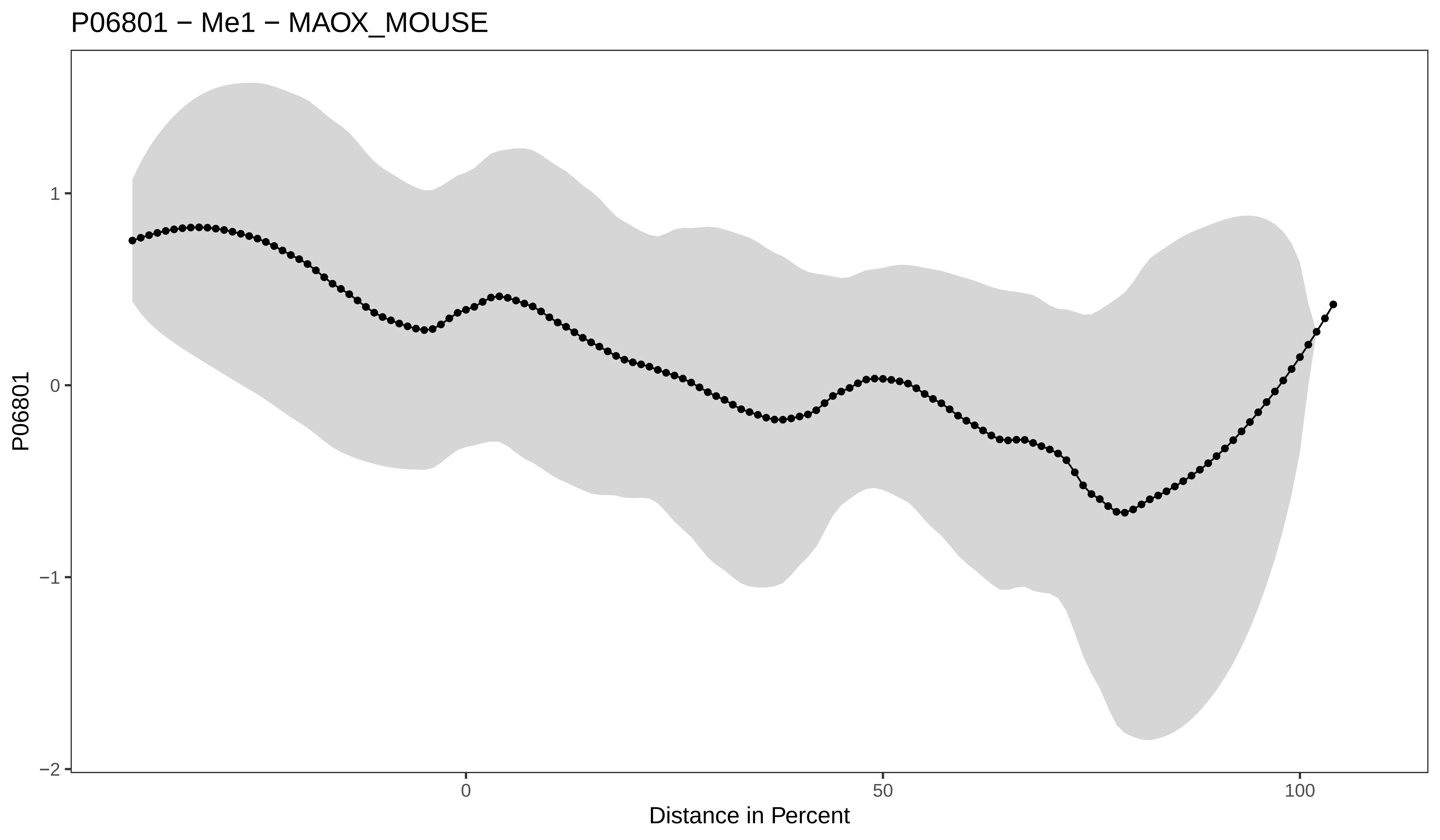

P06801 − Me1 − MAOX_MOUSE
P06801
Distance in Percent
1
0
−1
−2
0
50
100

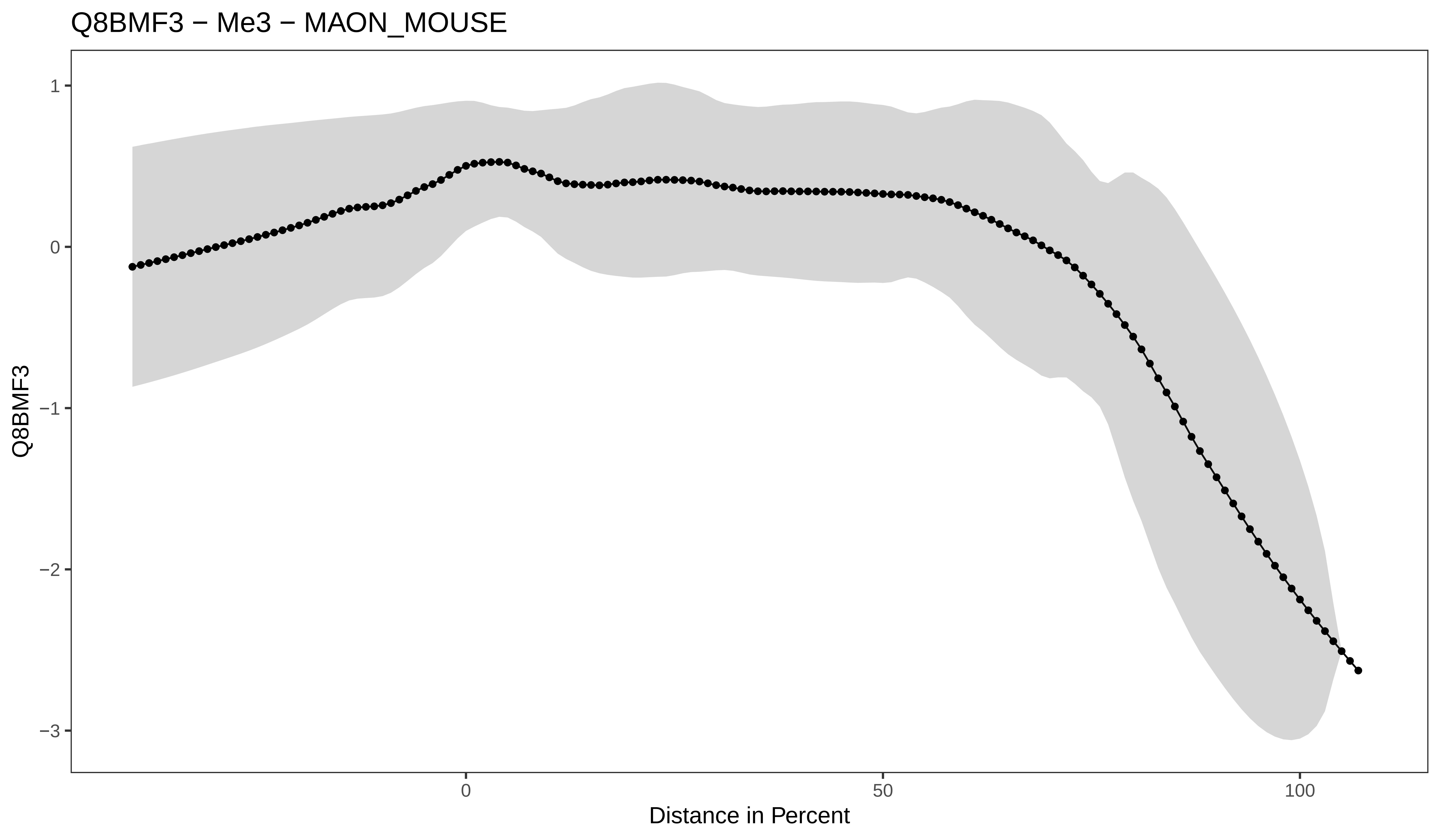

Q8BMF3 − Me3 − MAON_MOUSE
Q8BMF3
1
0
−1
−2
−3
0
50
100
Distance in Percent

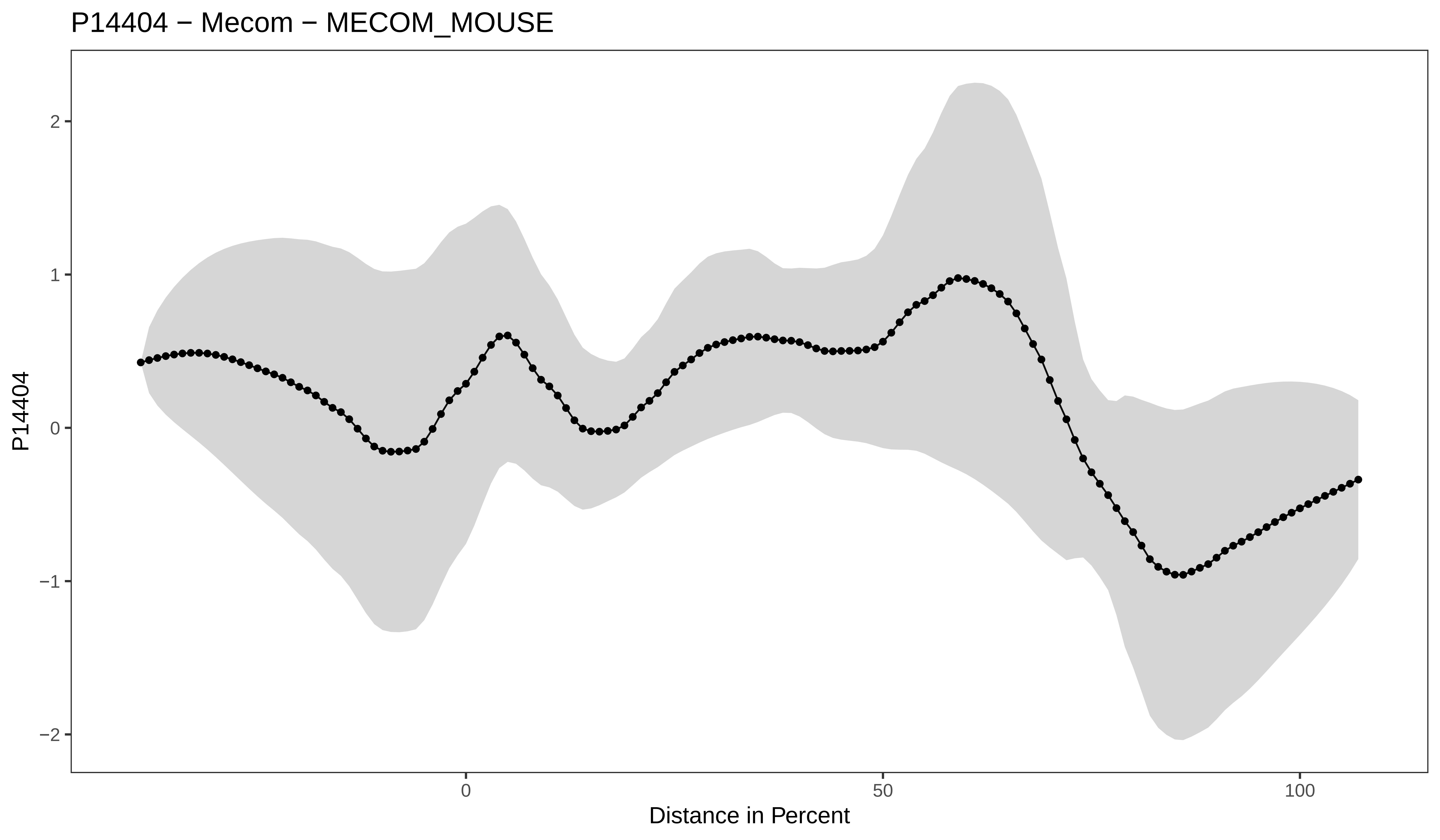

P14404 − Mecom − MECOM_MOUSE
P14404
2
1
0
−1
−2
0
50
100
Distance in Percent

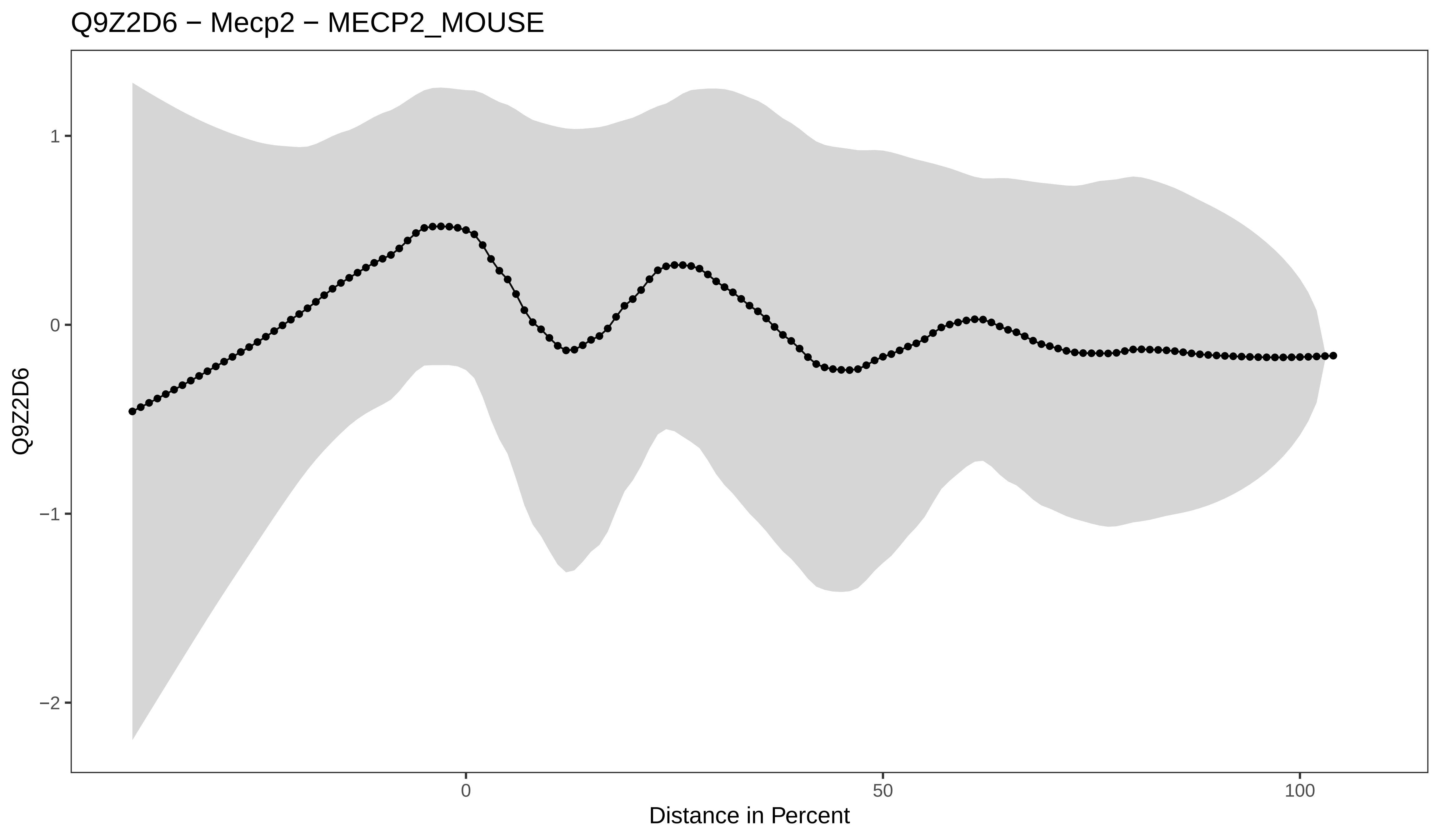

Q9Z2D6 − Mecp2 − MECP2_MOUSE
1
0
−1
−2
Q9Z2D6
0
50
100
Distance in Percent

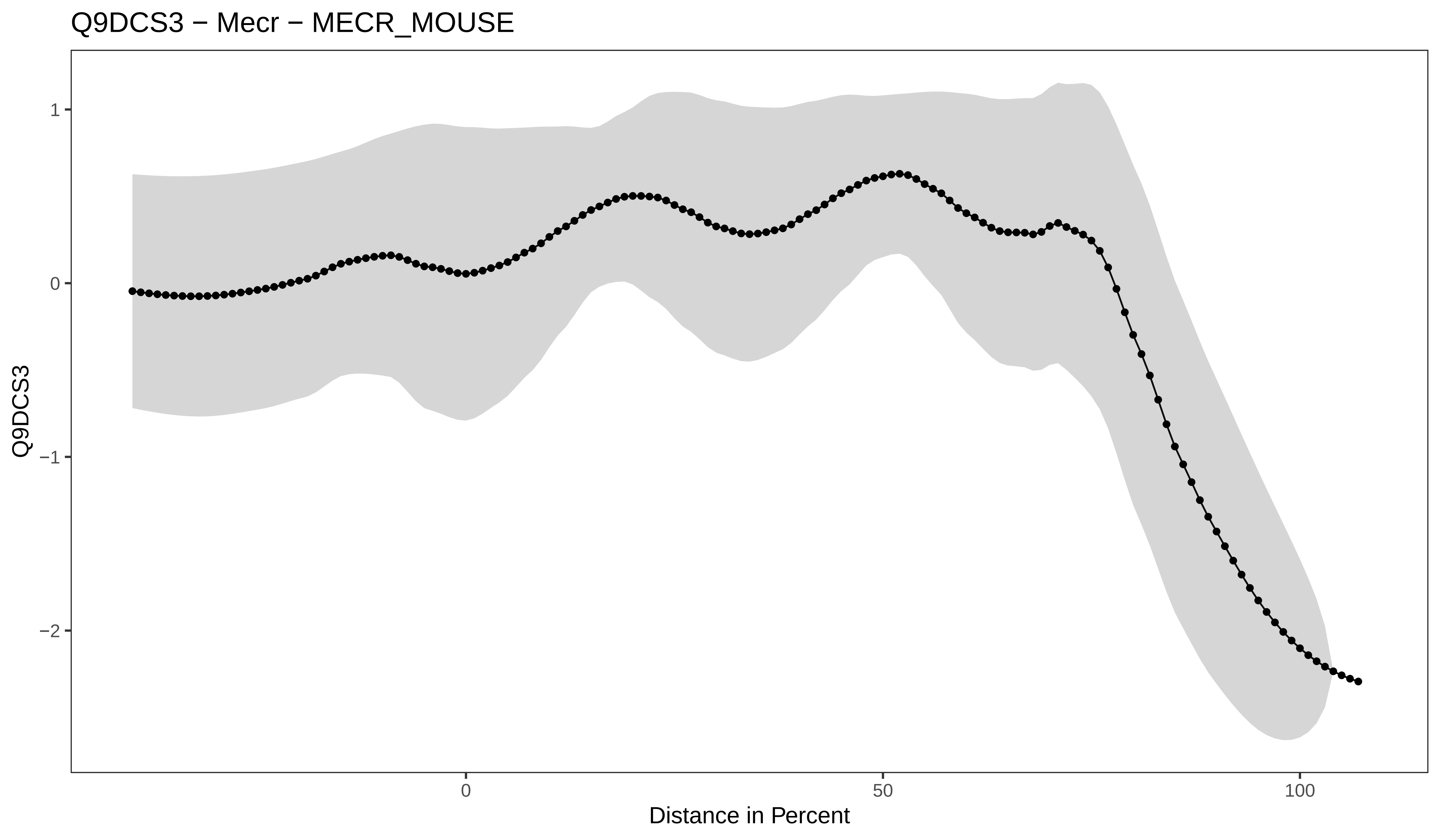
Q9DCS3 − Mecr − MECR_MOUSE
Q9DCS3
1
0
−1
−2
0
50
100
Distance in Percent

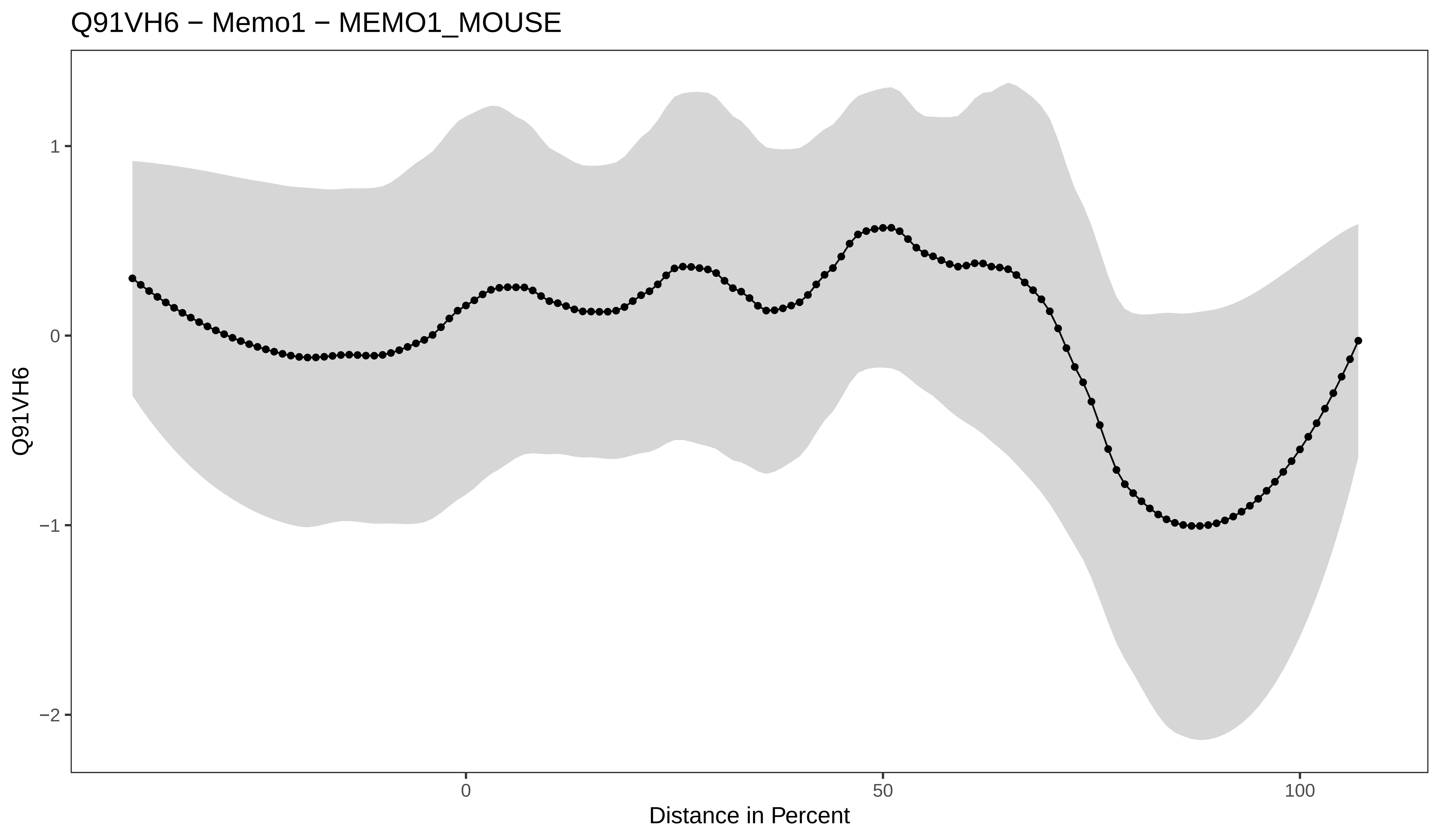

Q91VH6 − Memo1 − MEMO1_MOUSE
Q91VH6
1
0
−1
−2
0
50
100
Distance in Percent

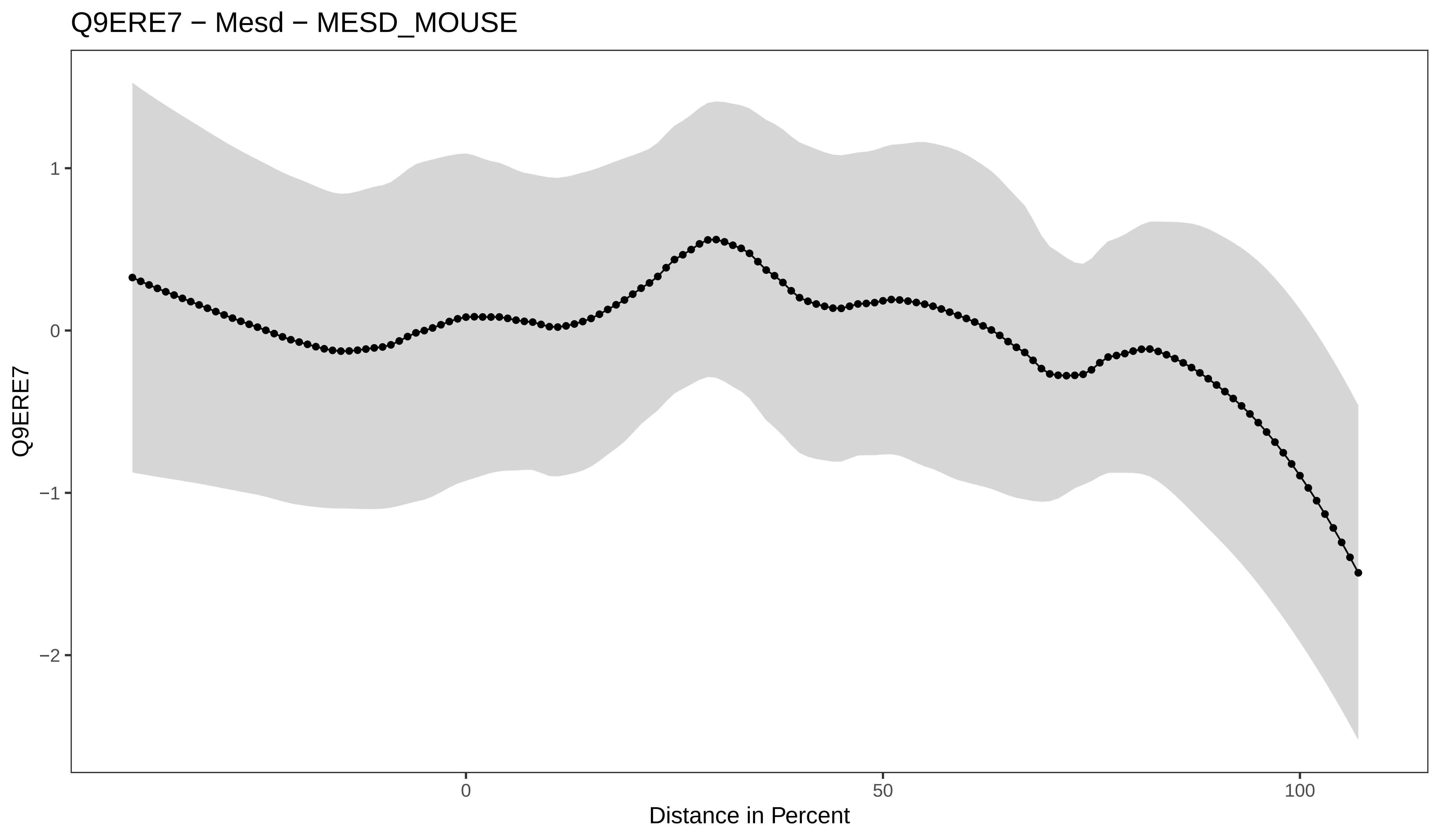

Q9ERE7 − Mesd − MESD_MOUSE
Q9ERE7
1
0
−1
−2
0
50
100
Distance in Percent

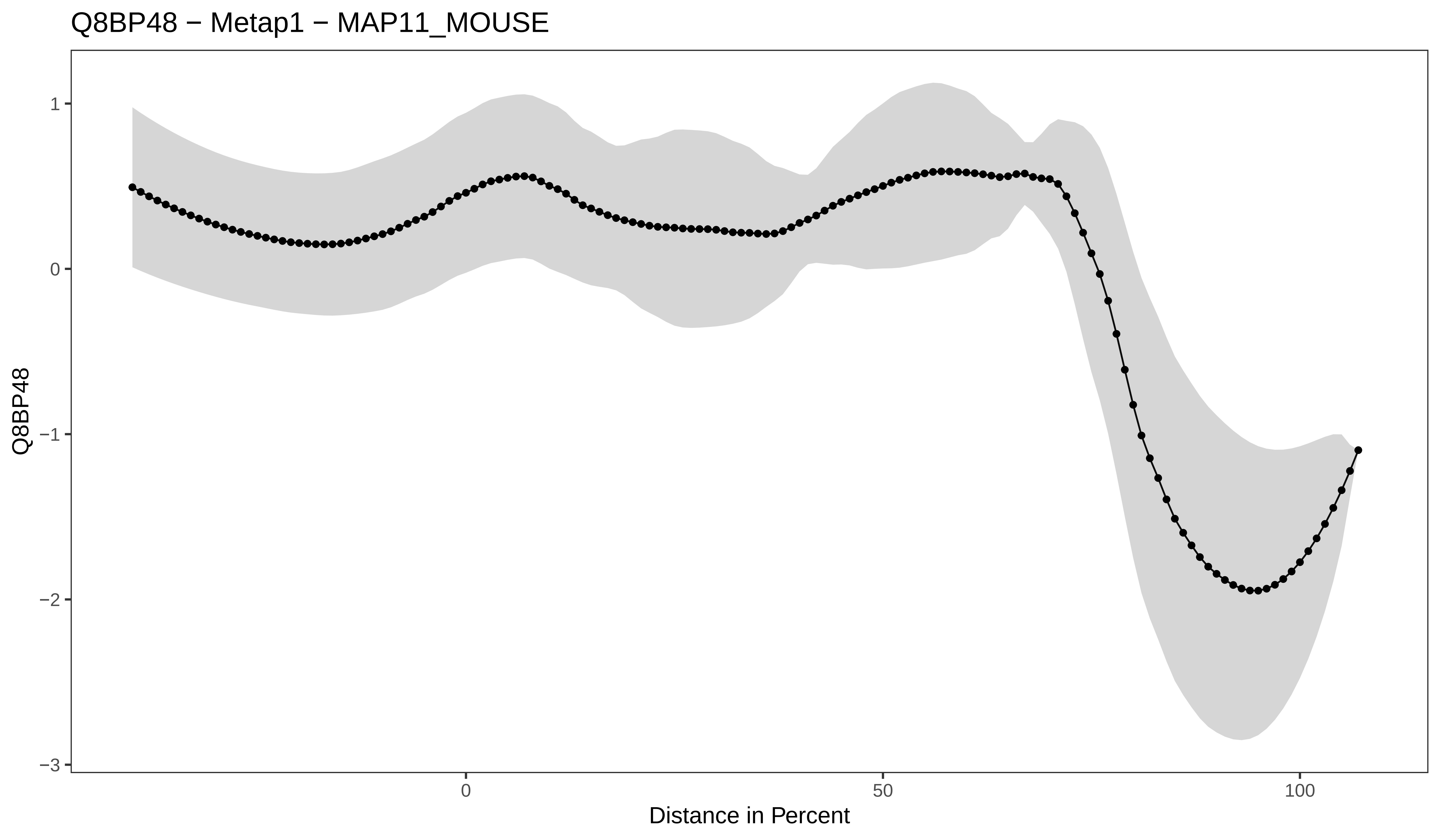
Q8BP48 − Metap1 − MAP11_MOUSE
Q8BP48
Distance in Percent
1
0
−1
−2
−3
0
50
100

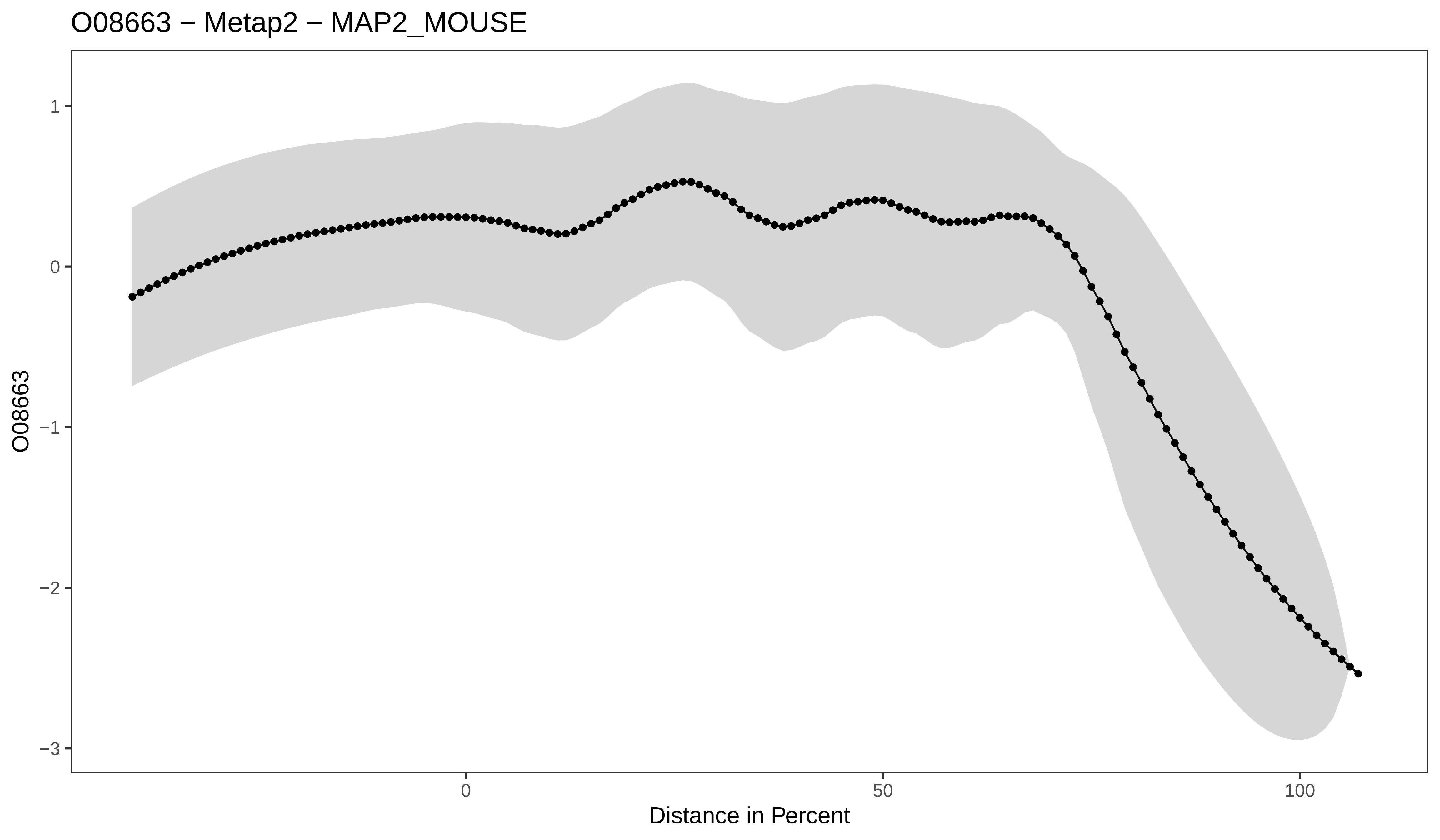

O08663 − Metap2 − MAP2_MOUSE
1
0
−1
−2
−3
O08663
0
50
100
Distance in Percent

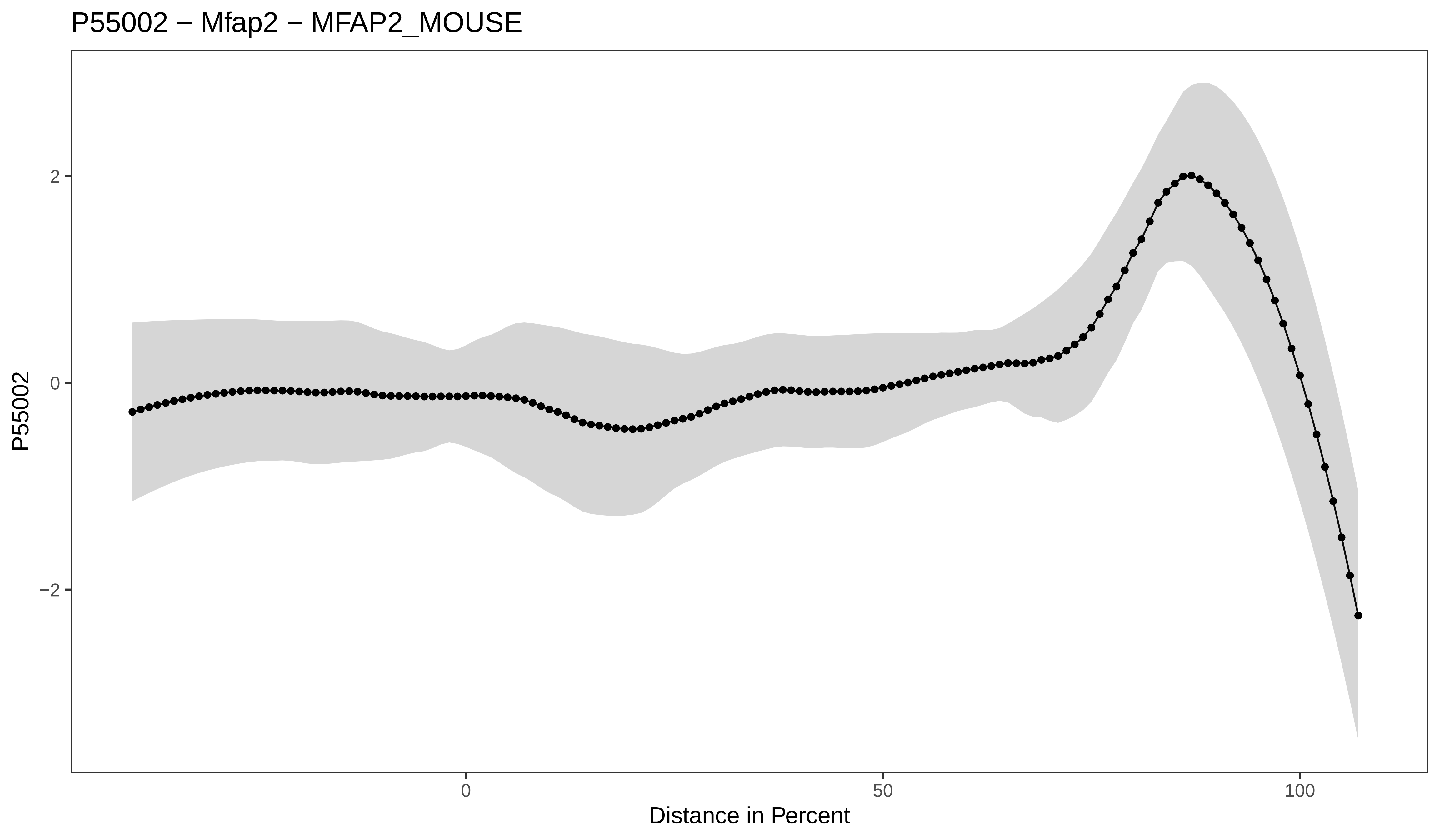
P55002 − Mfap2 − MFAP2_MOUSE
2
0
−2
P55002
0
50
100
Distance in Percent

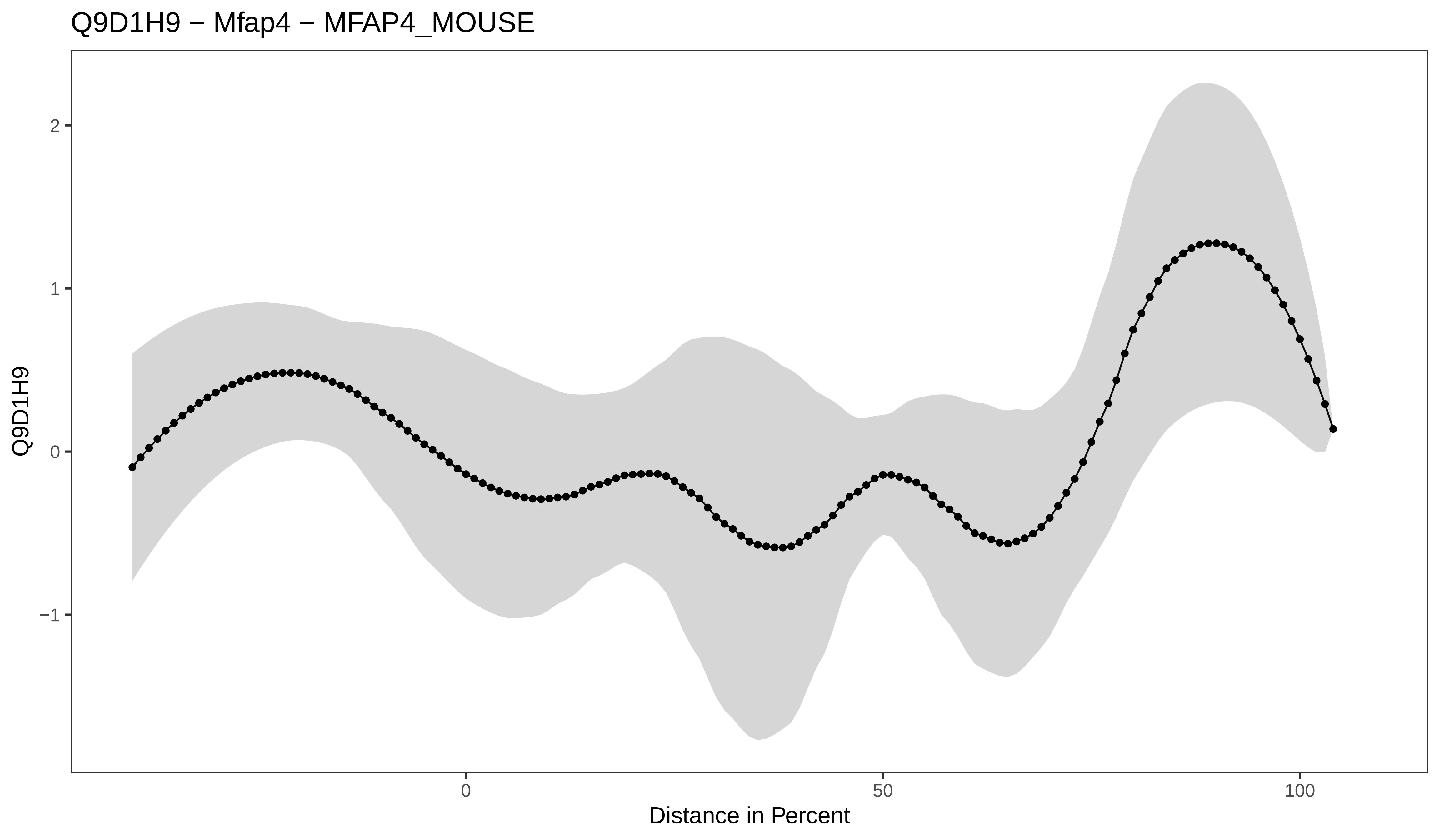
Q9D1H9 − Mfap4 − MFAP4_MOUSE
Q9D1H9
2
1
0
−1
0
50
100
Distance in Percent

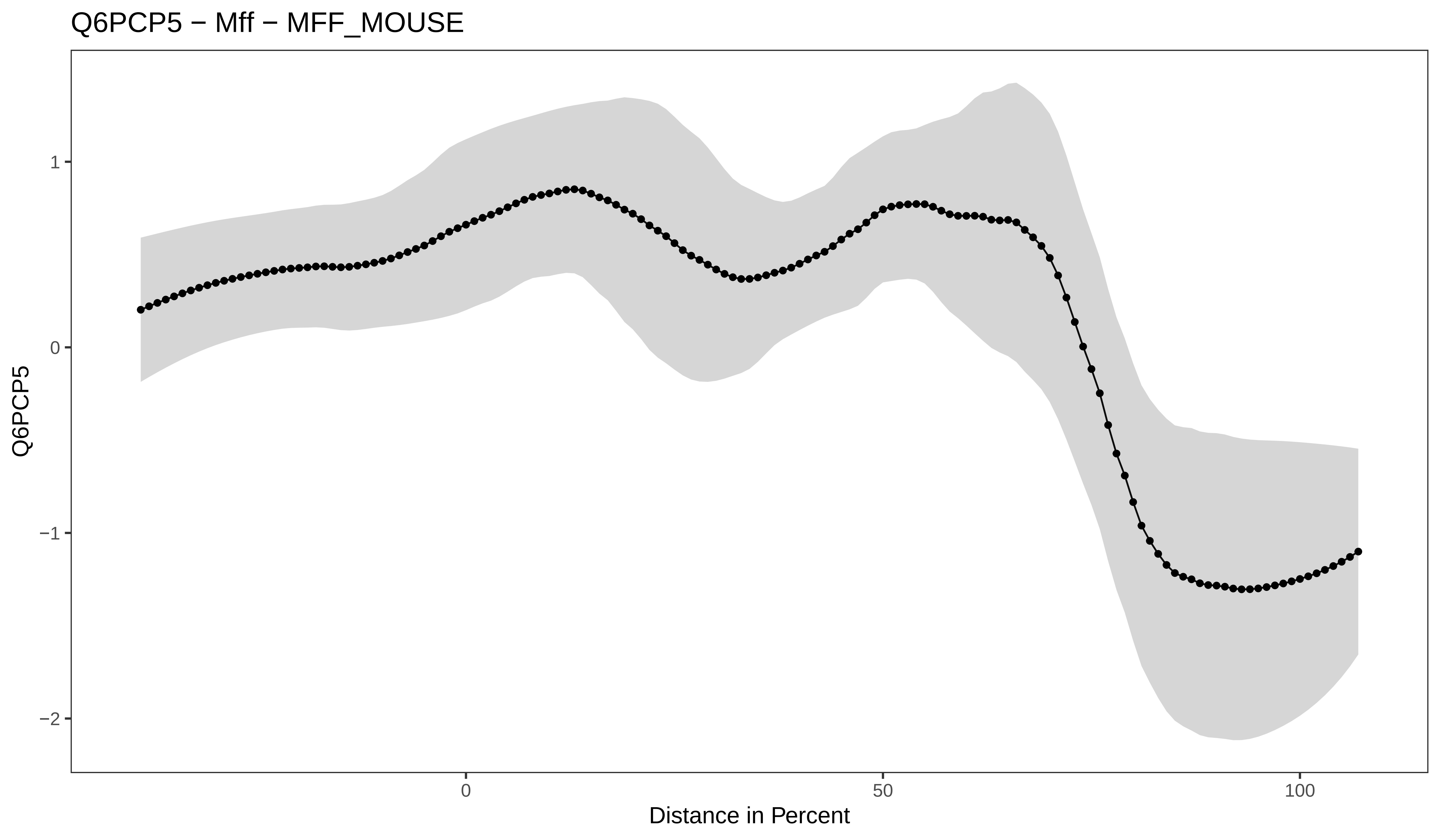

Q6PCP5 − Mff − MFF_MOUSE
1
0
−1
−2
Q6PCP5
0
50
100
Distance in Percent

P21956 − Mfge8 − MFGM_MOUSE

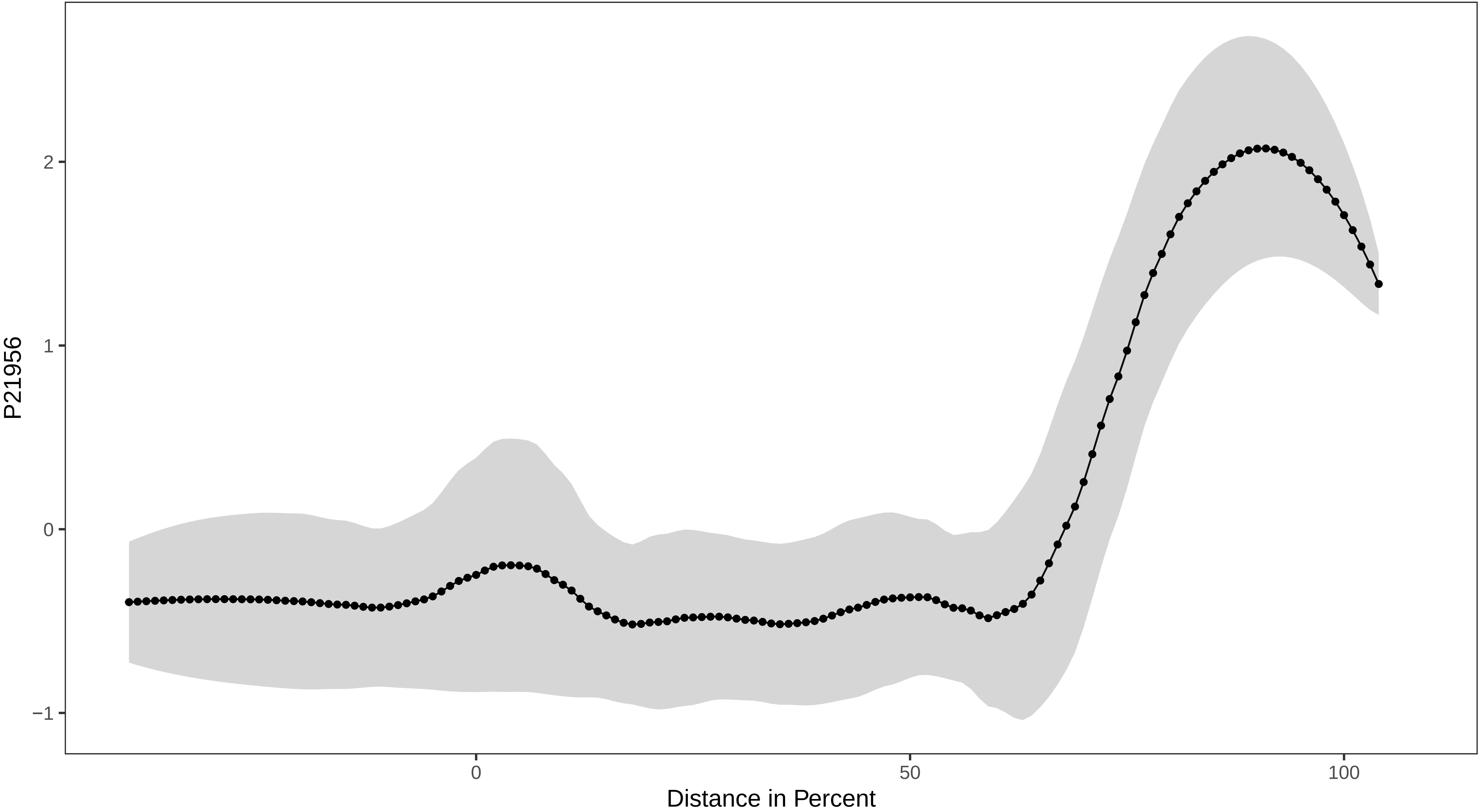

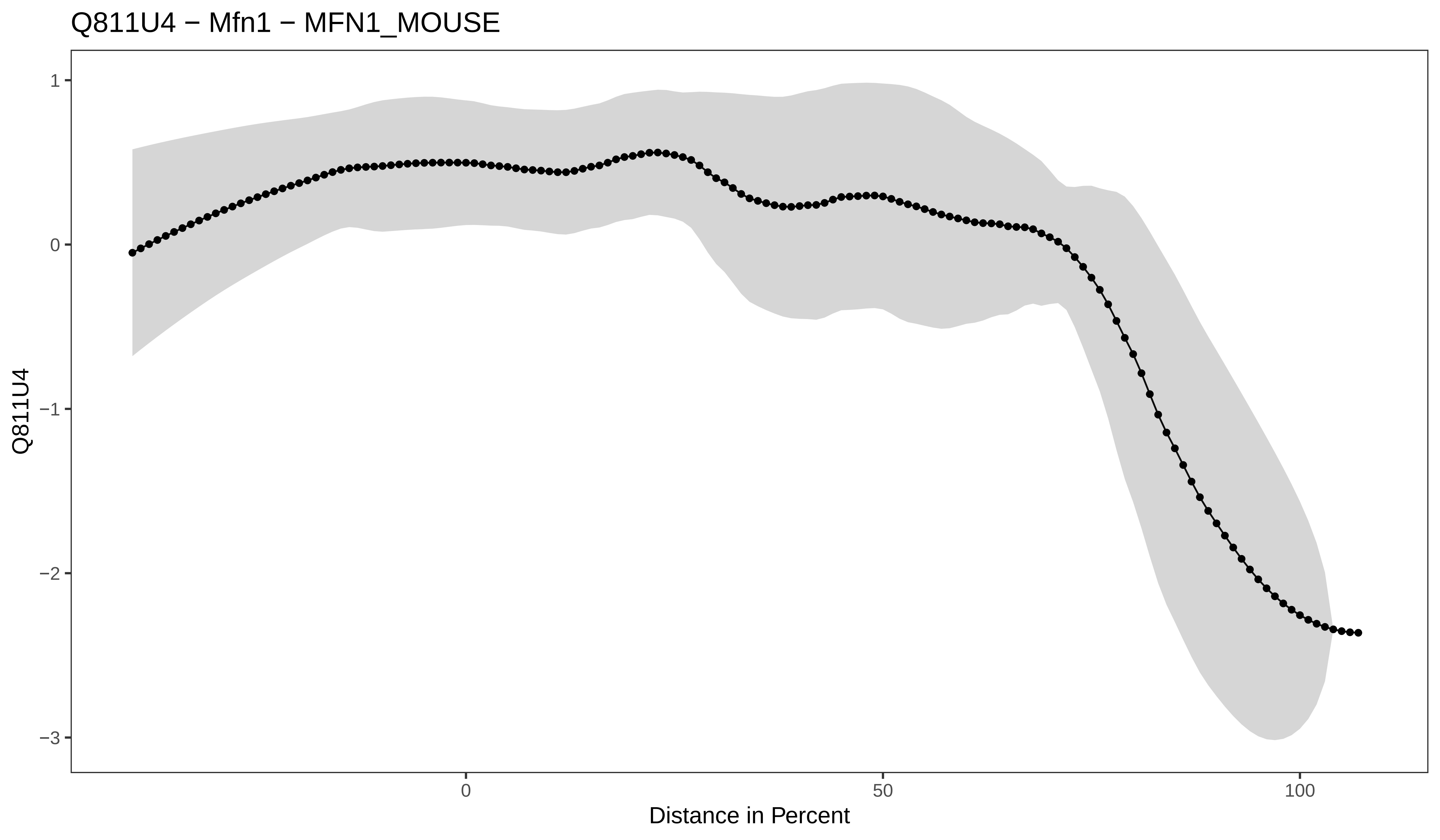
Q811U4 − Mfn1 − MFN1_MOUSE
Q811U4
Distance in Percent
1
0
−1
−2
−3
0
50
100

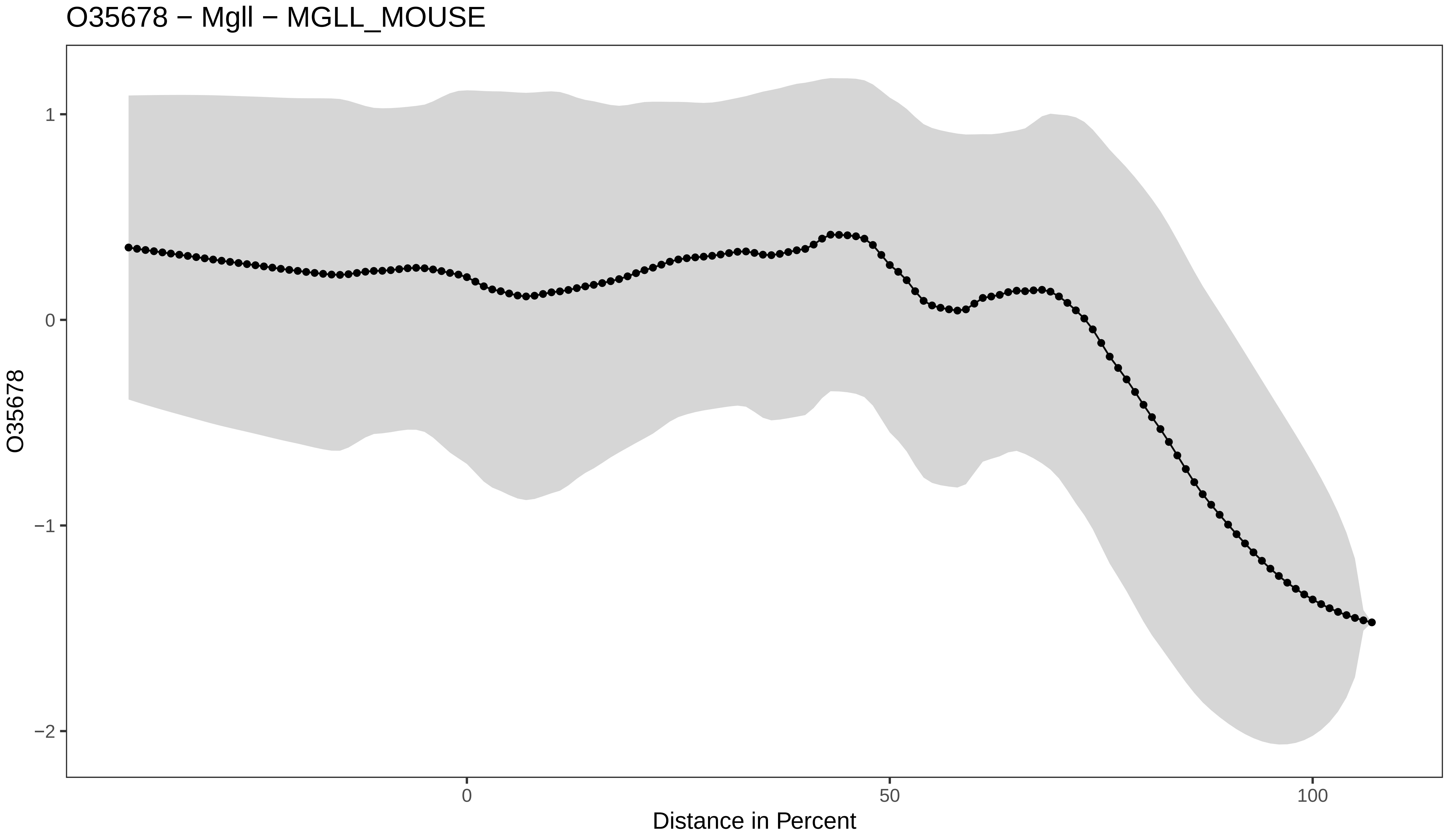

O35678 − Mgll − MGLL_MOUSE
1
0
−1
−2
O35678
0
50
100
Distance in Percent

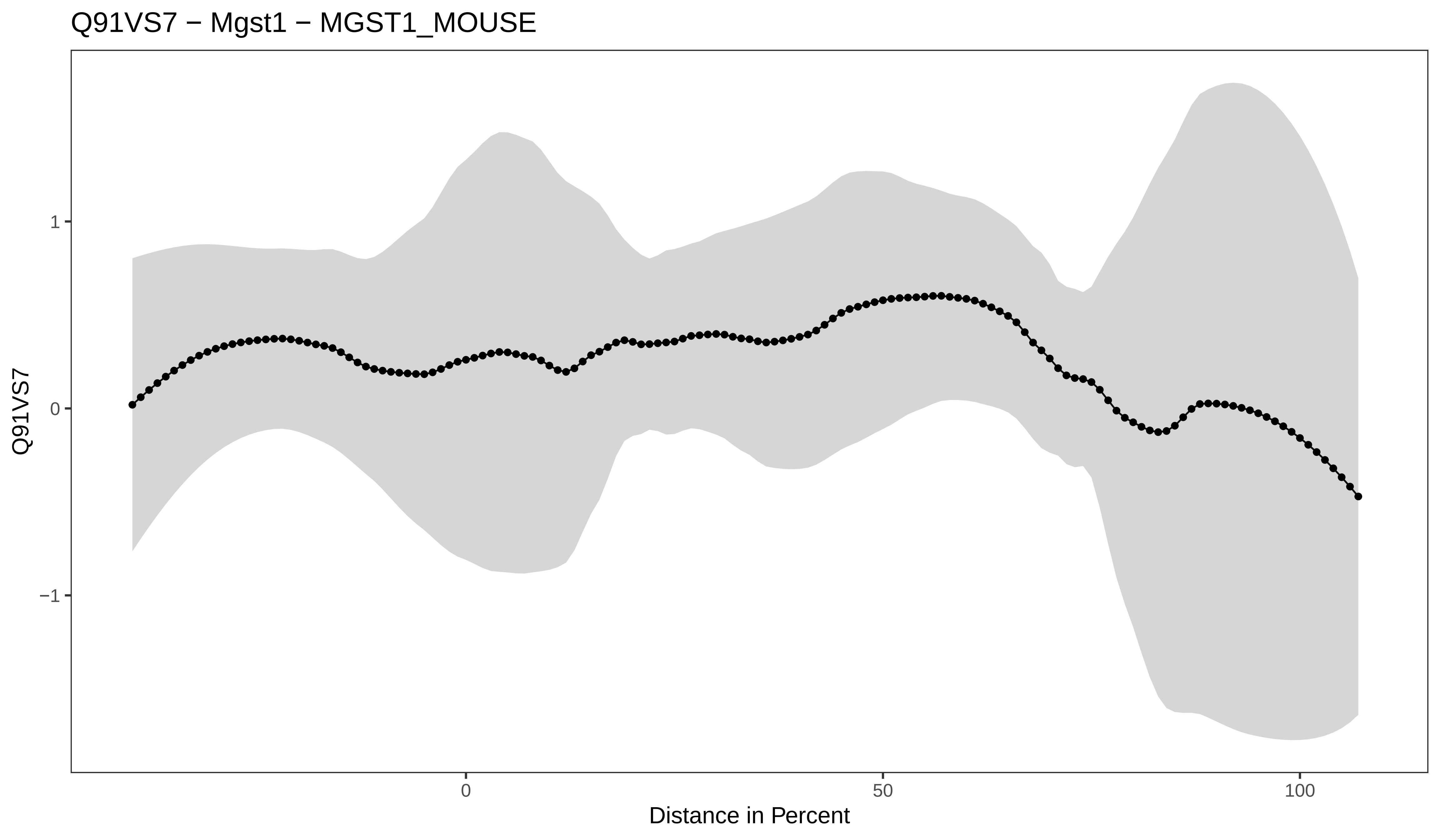

Q91VS7 − Mgst1 − MGST1_MOUSE
Q91VS7
1
0
−1
0
50
100
Distance in Percent

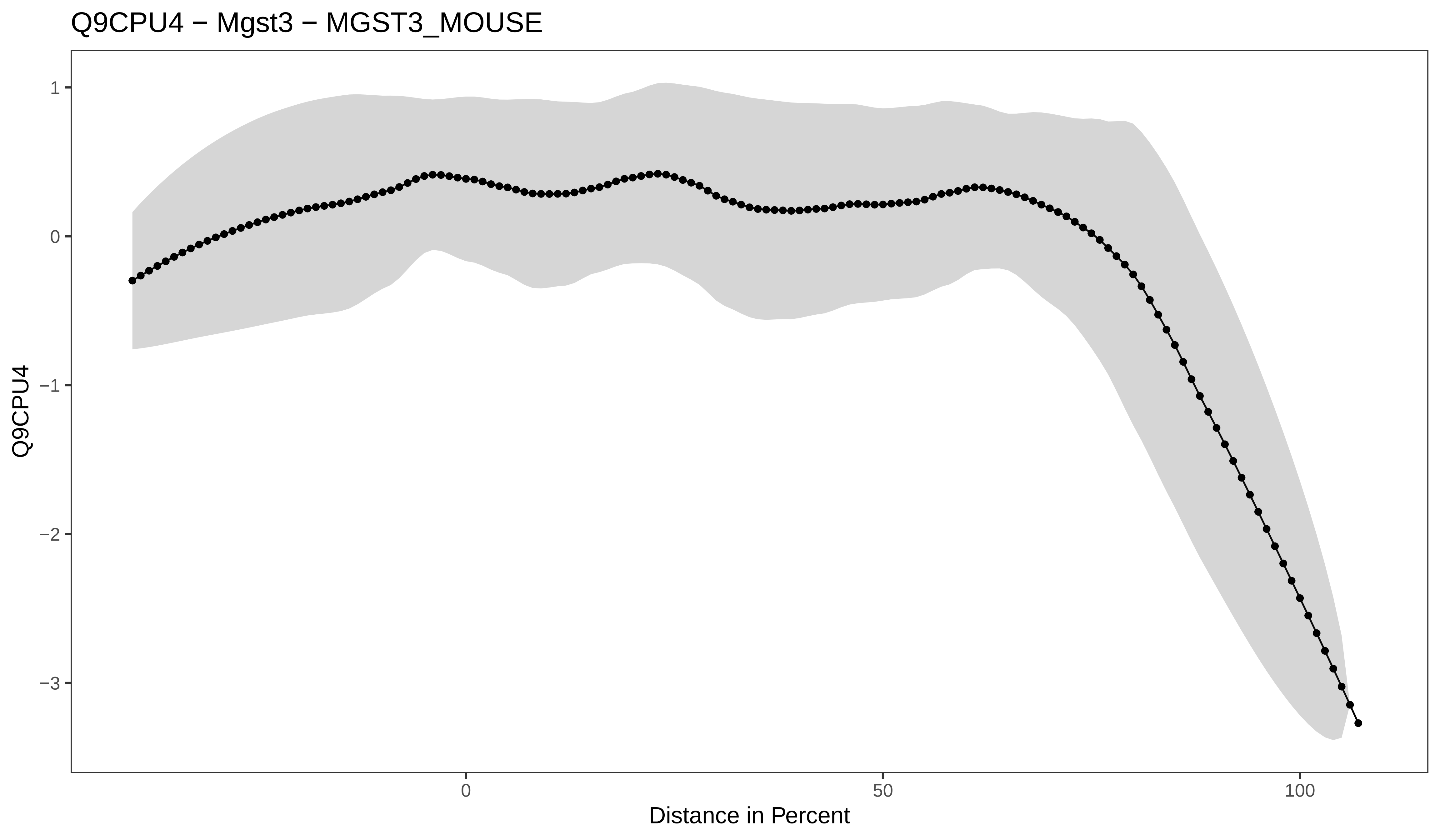

Q9CPU4 − Mgst3 − MGST3_MOUSE
Q9CPU4
1
0
−1
−2
−3
0
50
100
Distance in Percent

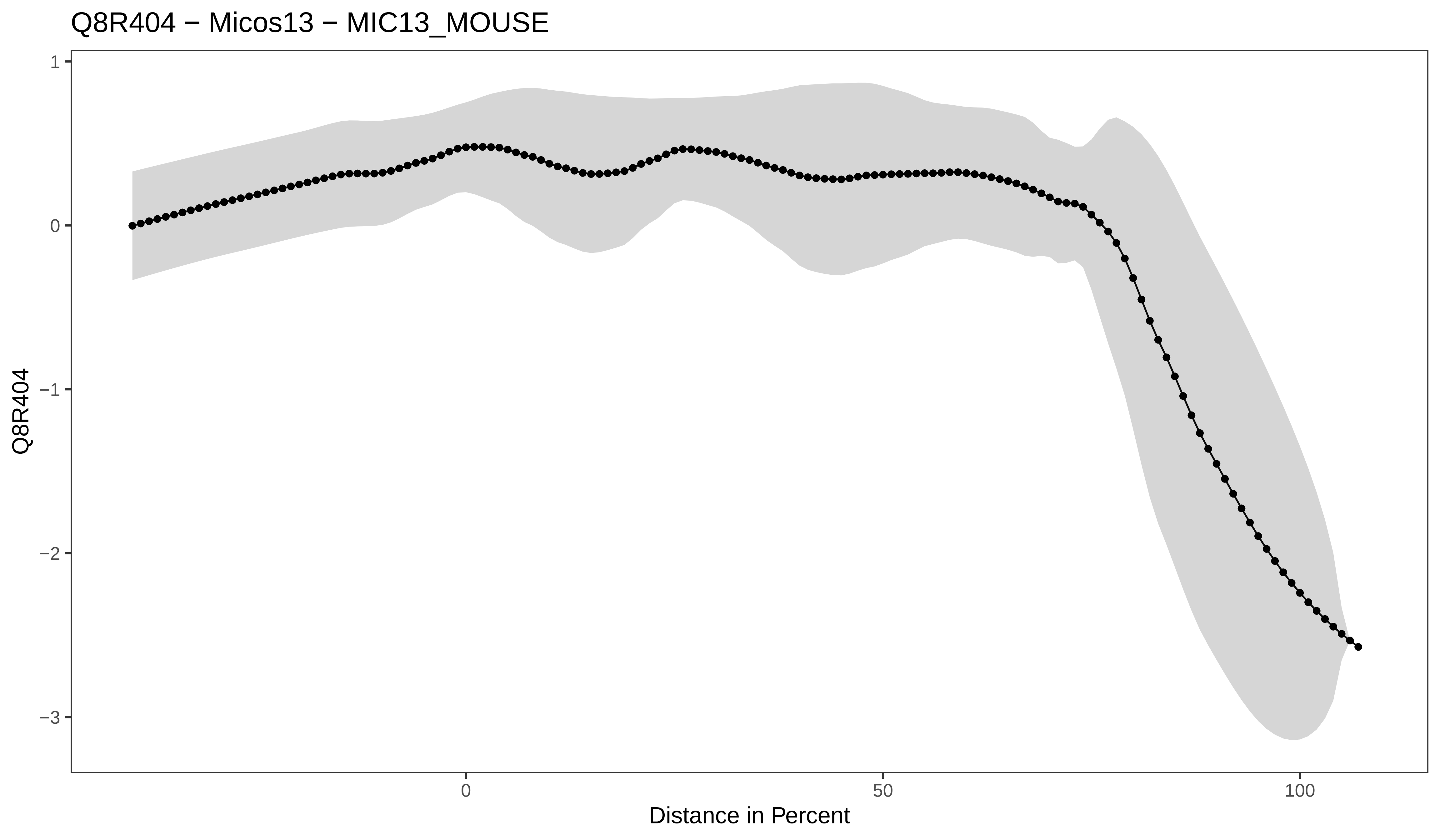

Q8R404 − Micos13 − MIC13_MOUSE
1
0
−1
−2
−3
Q8R404
0
50
100
Distance in Percent

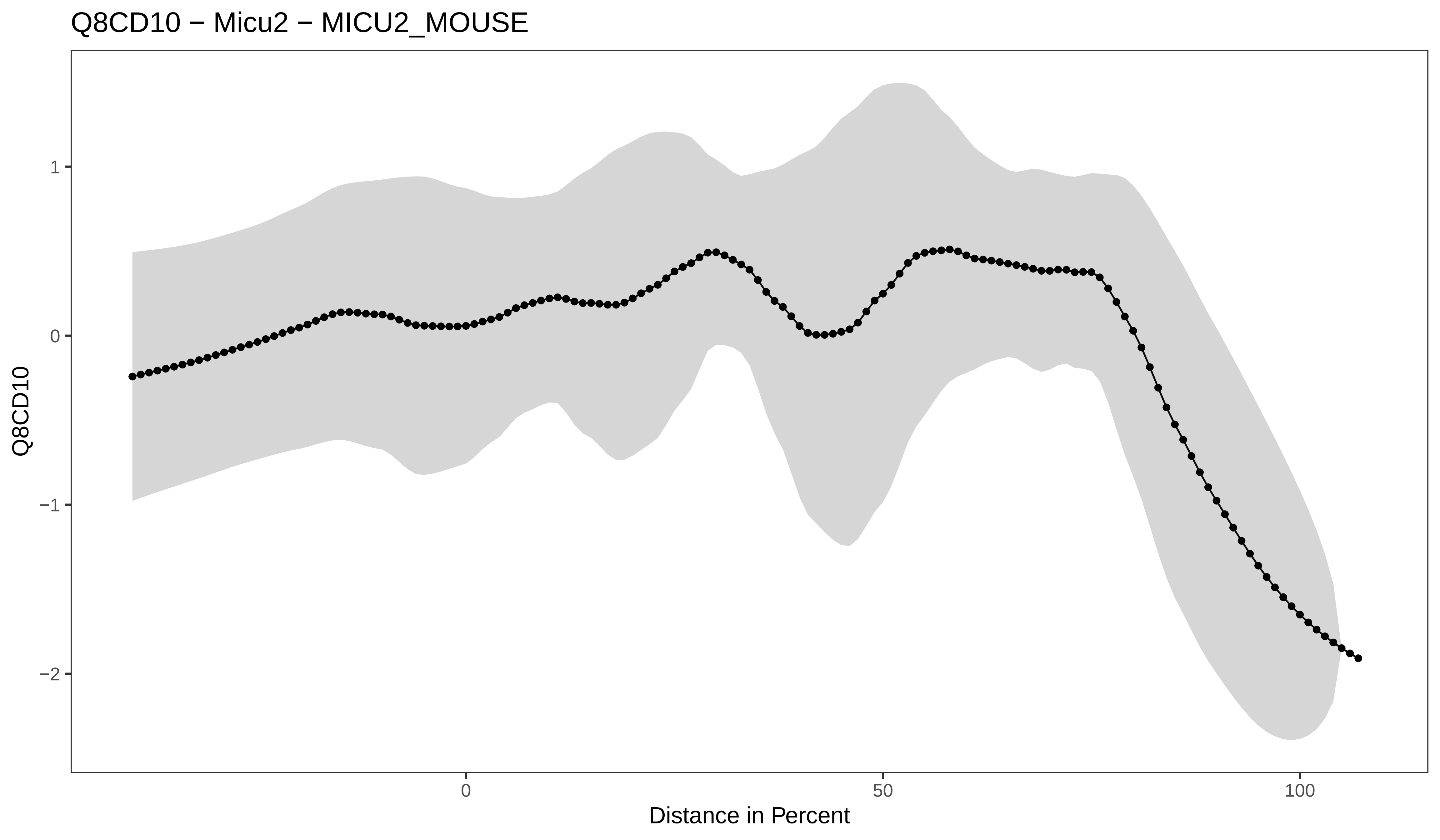

Q8CD10 − Micu2 − MICU2_MOUSE
Q8CD10
Distance in Percent
1
0
−1
−2
0
50
100

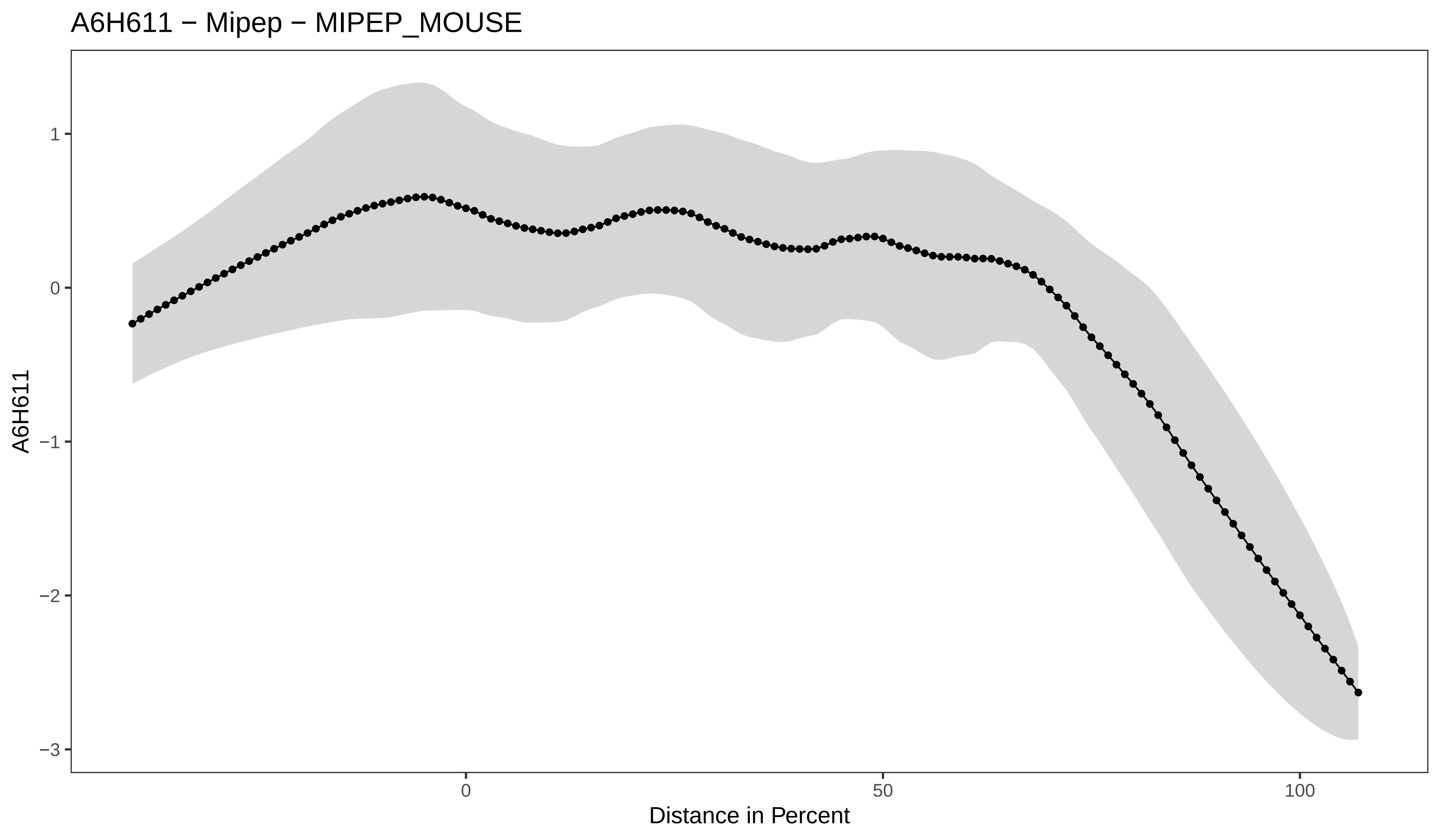
A6H611 − Mipep − MIPEP_MOUSE
A6H611
1
0
−1
−2
−3
0
50
100
Distance in Percent

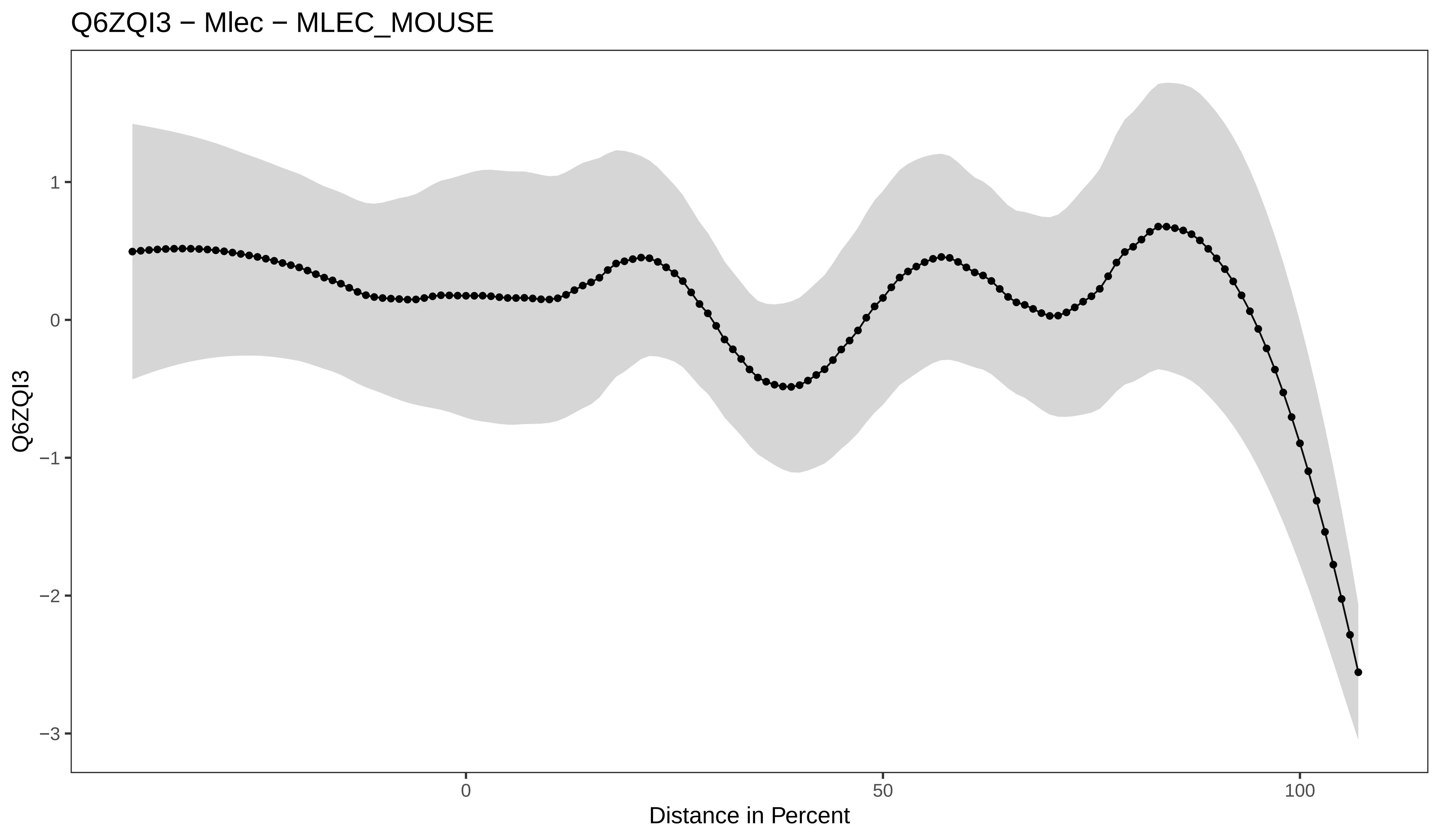

Q6ZQI3 − Mlec − MLEC_MOUSE
Q6ZQI3
1
0
−1
−2
−3
0
50
100
Distance in Percent

Q9QWV4 − Mlf1 − MLF1_MOUSE

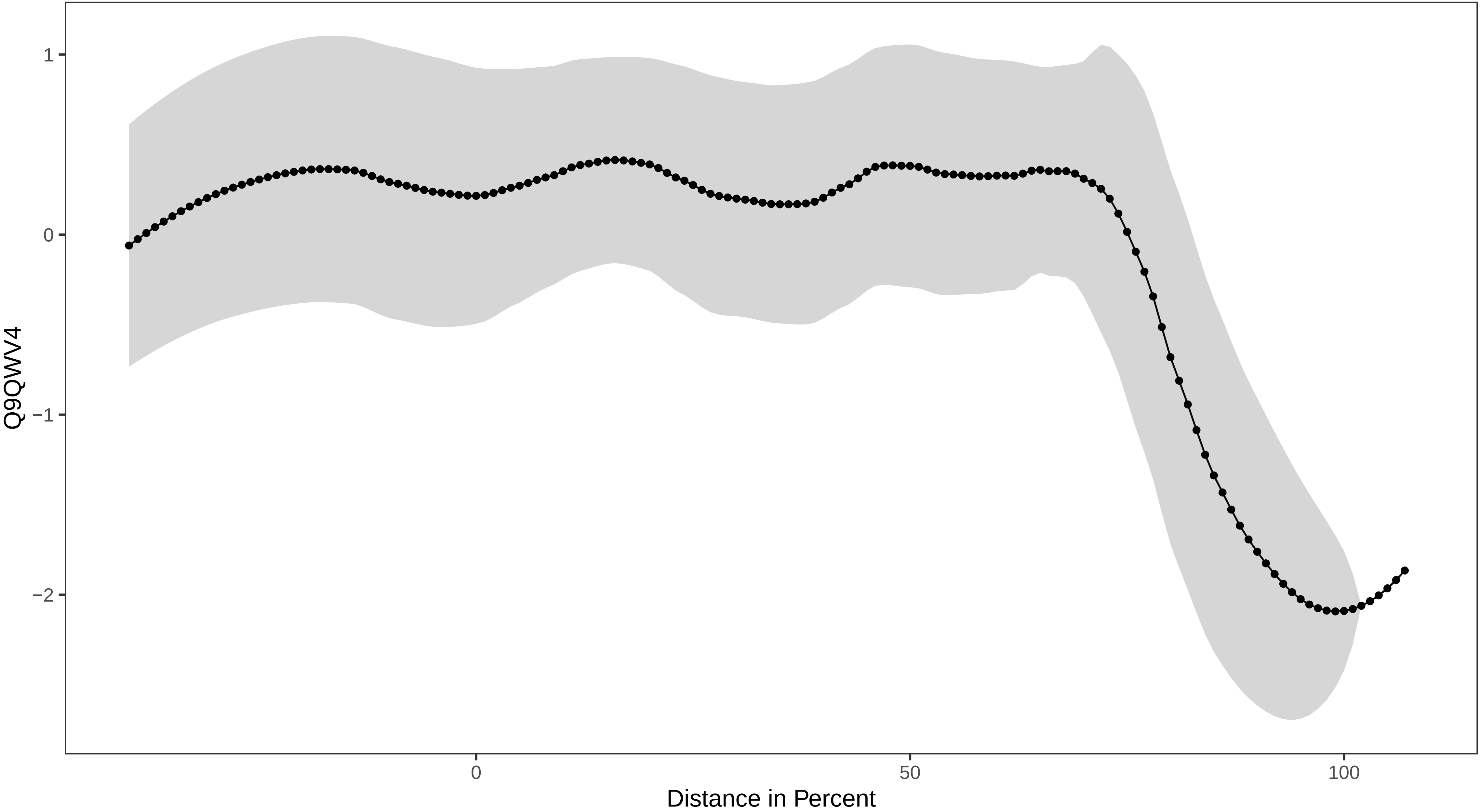

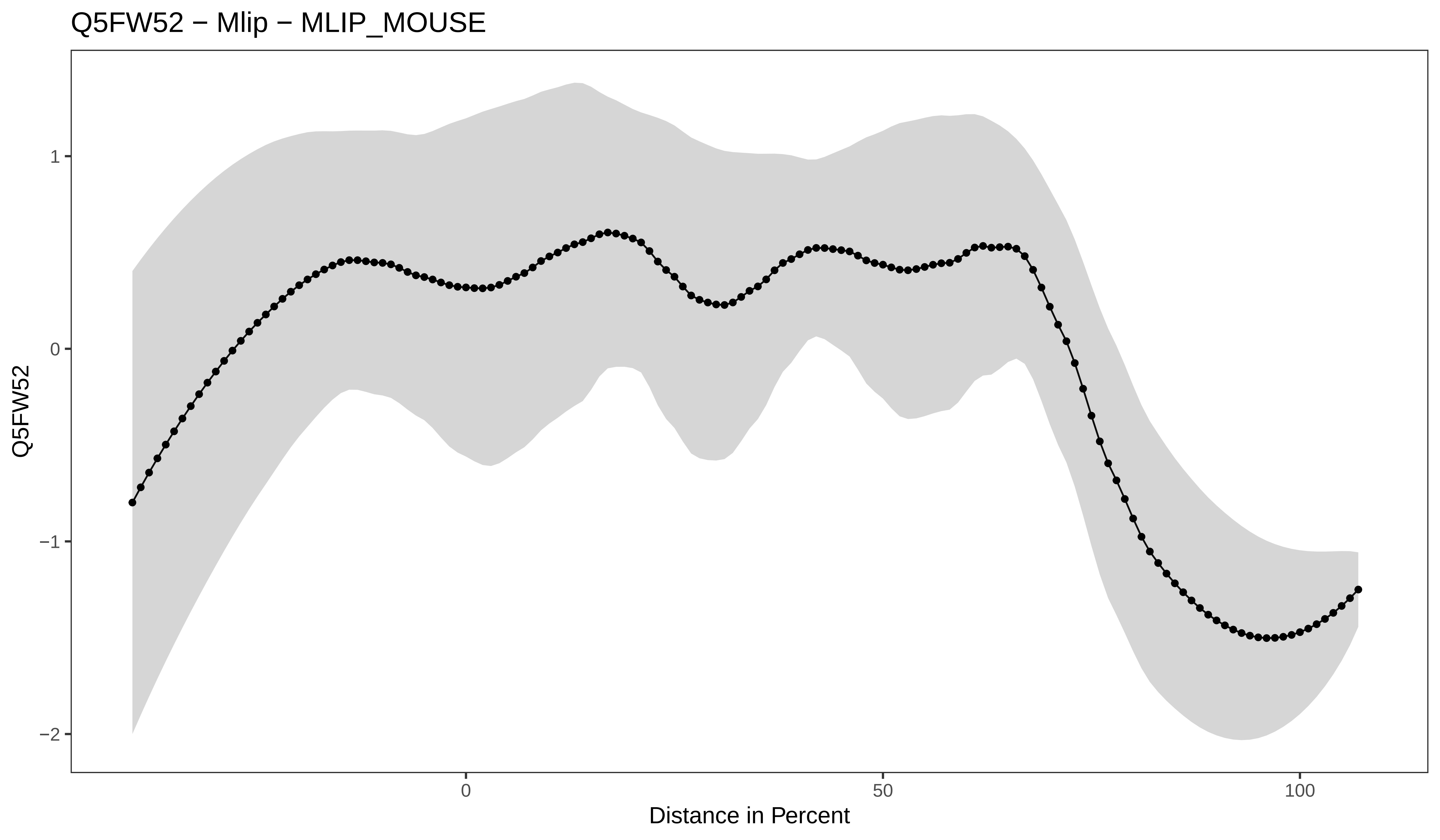

Q5FW52 − Mlip − MLIP_MOUSE
Q5FW52
1
0
−1
−2
0
50
100
Distance in Percent

Q99J39 − Mlycd − DCMC_MOUSE

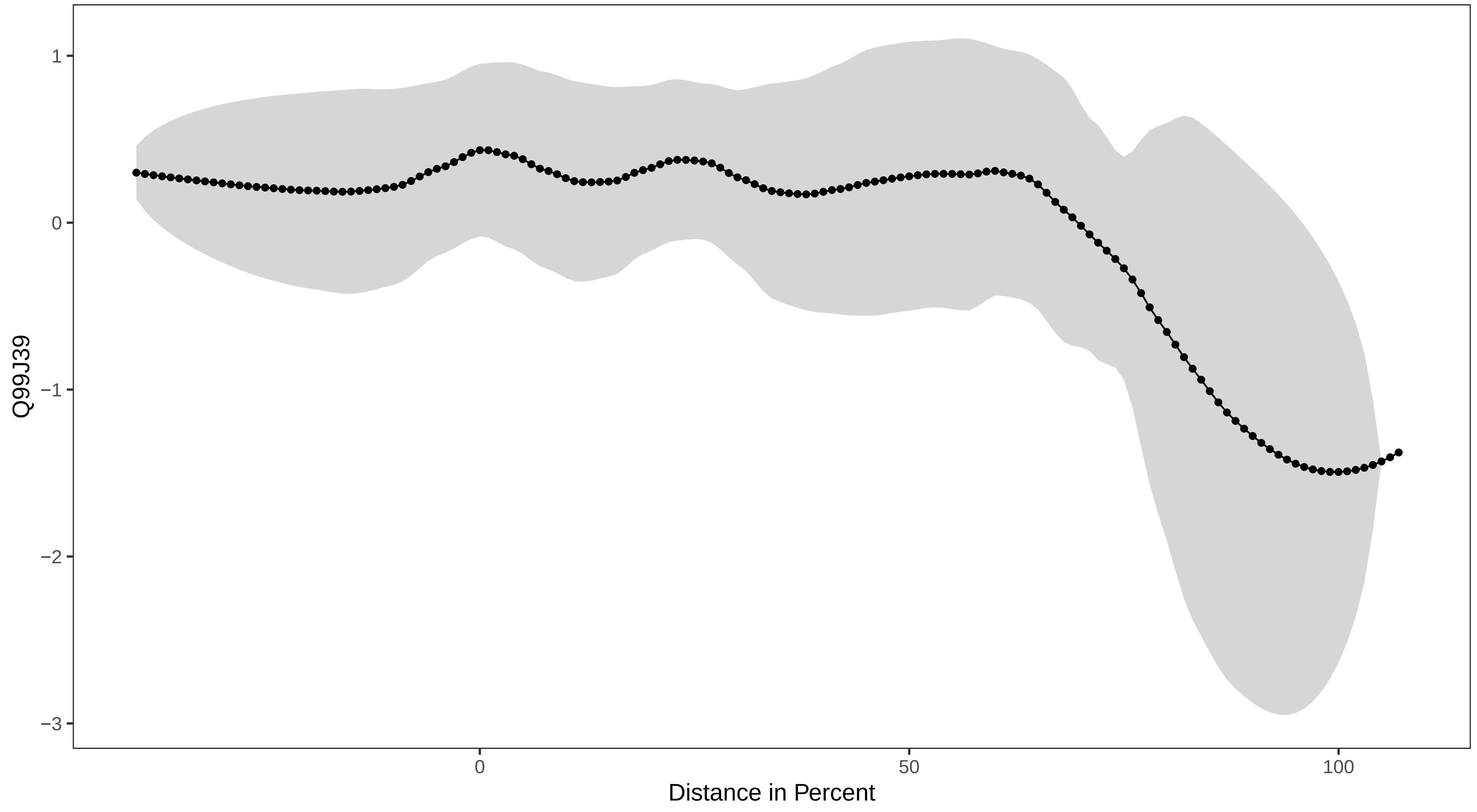

Q8C7H1 − Mmaa − MMAA_MOUSE

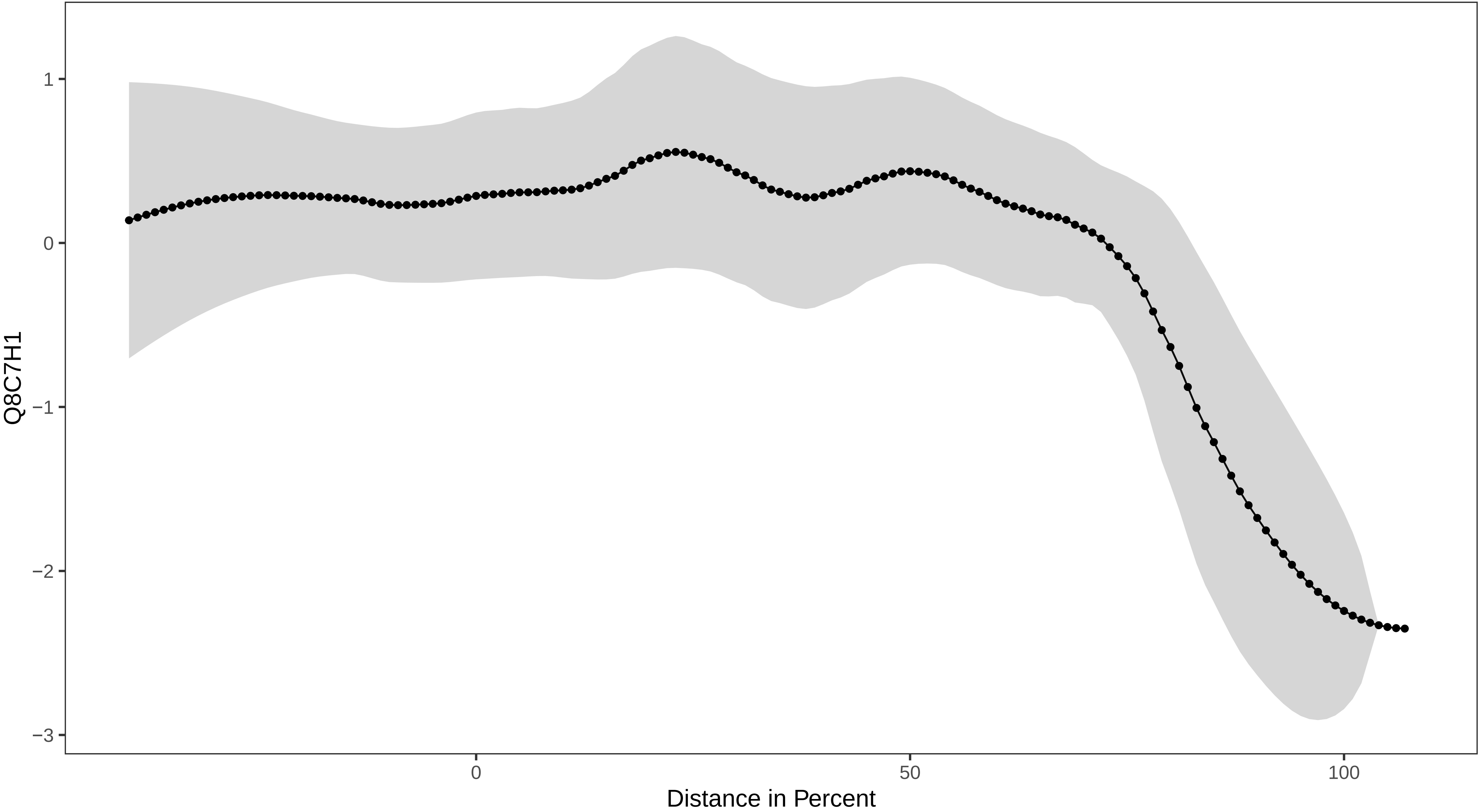

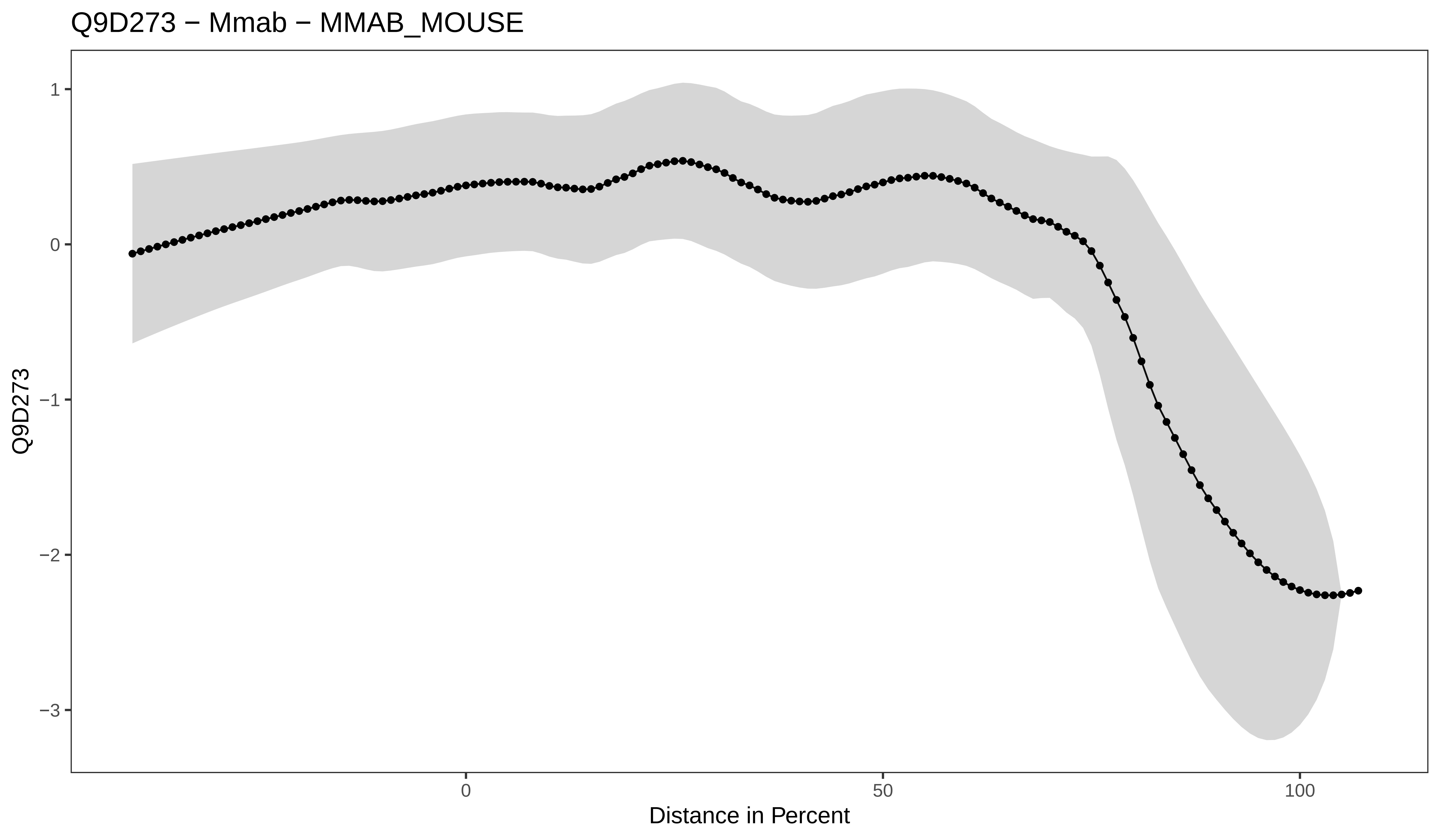

Q9D273 − Mmab − MMAB_MOUSE
1
0
−1
−2
−3
Q9D273
0
50
100
Distance in Percent

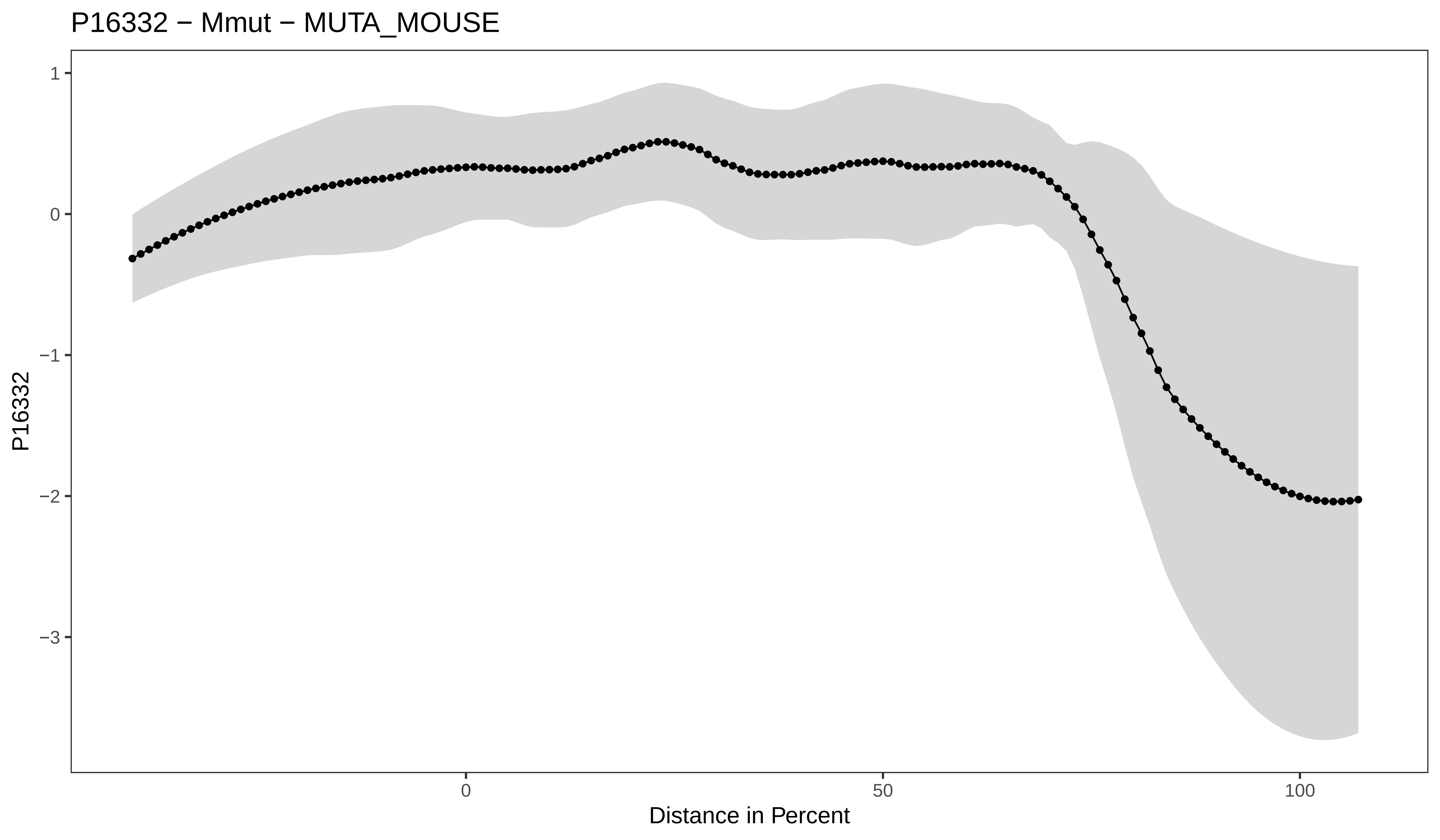
P16332 − Mmut − MUTA_MOUSE
1
0
−1
−2
−3
P16332
0
50
100
Distance in Percent

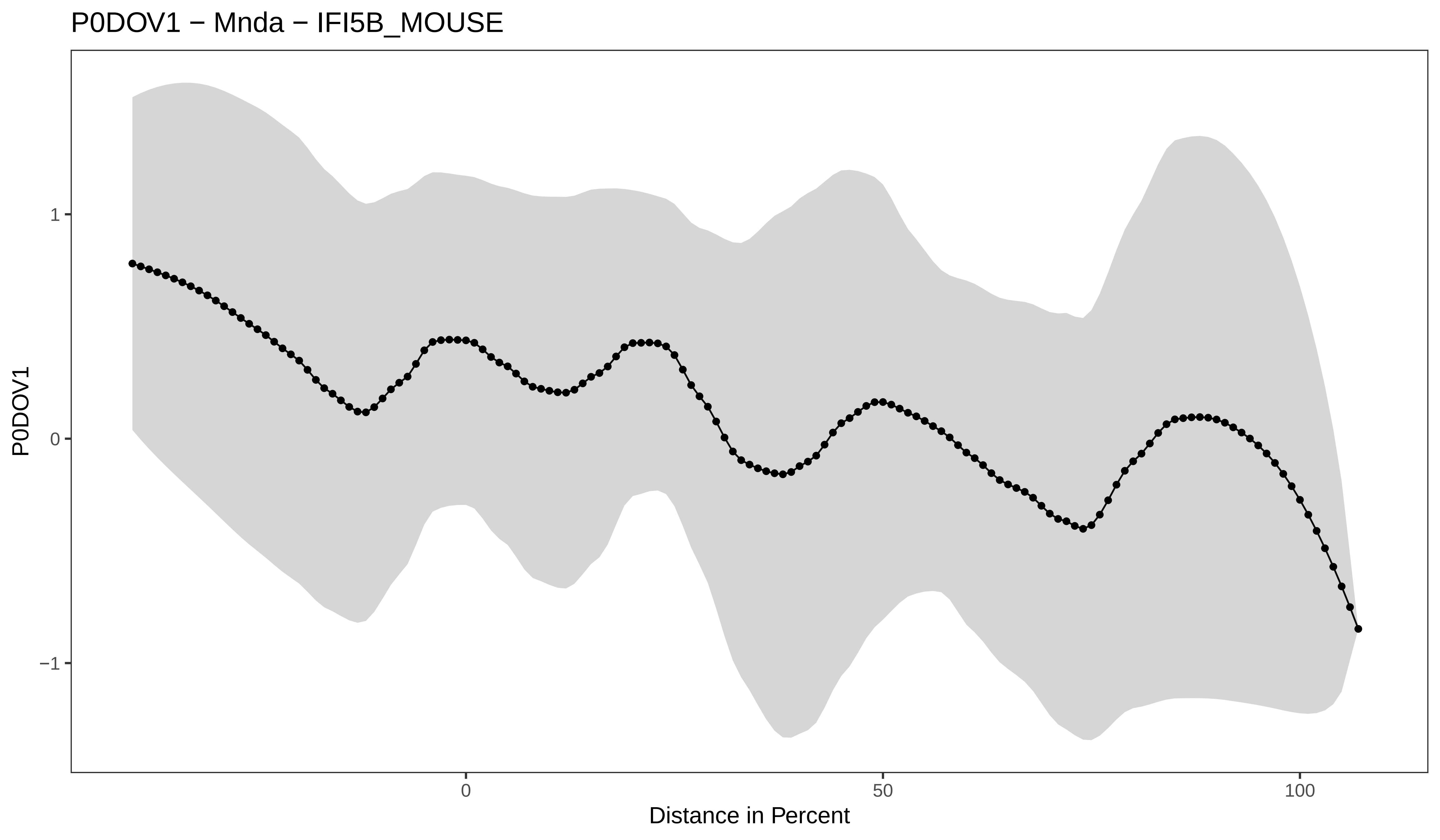
P0DOV1 − Mnda − IFI5B_MOUSE
P0DOV1
1
0
−1
0
50
100
Distance in Percent

Q8BPB0 − Mob1b − MOB1B_MOUSE

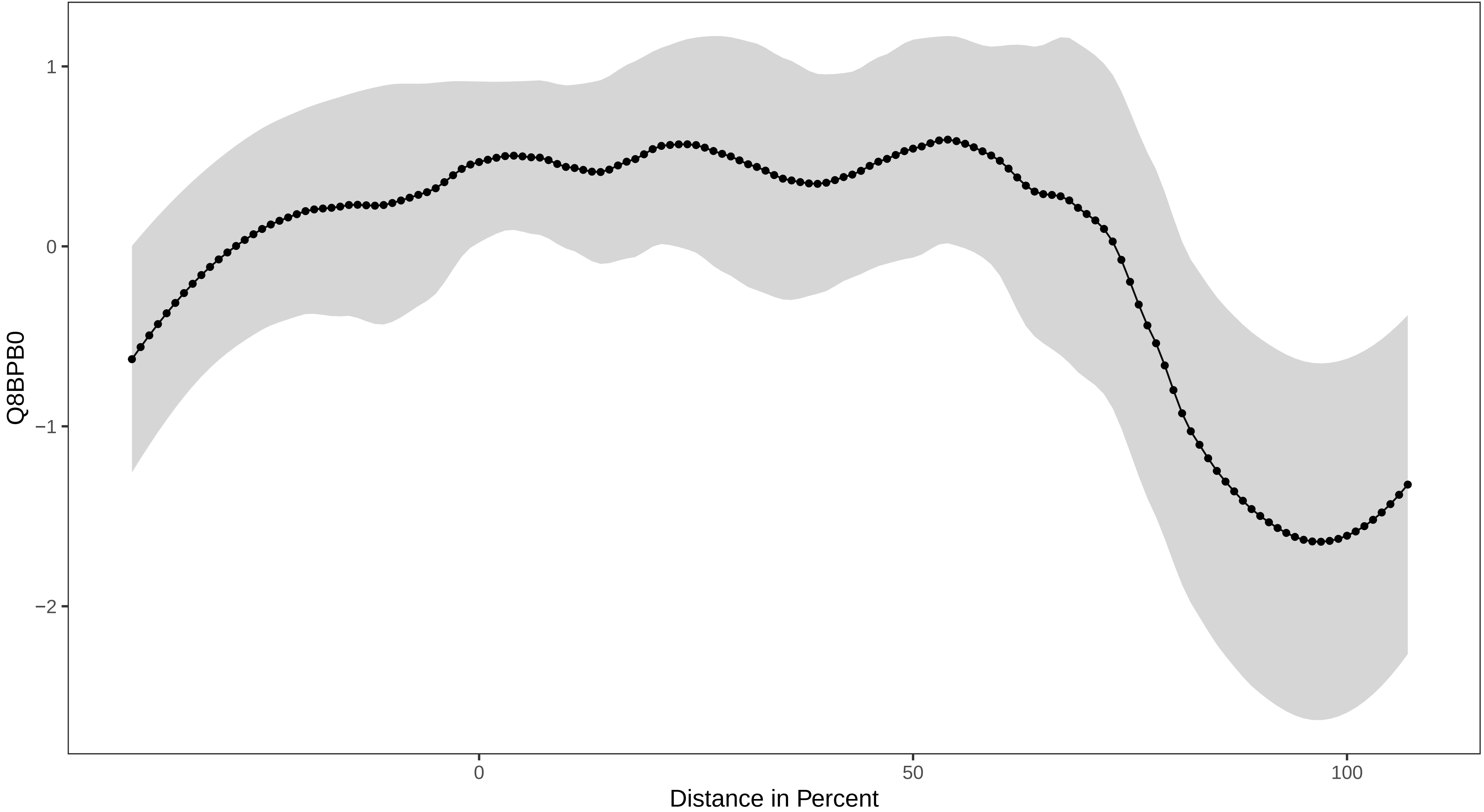

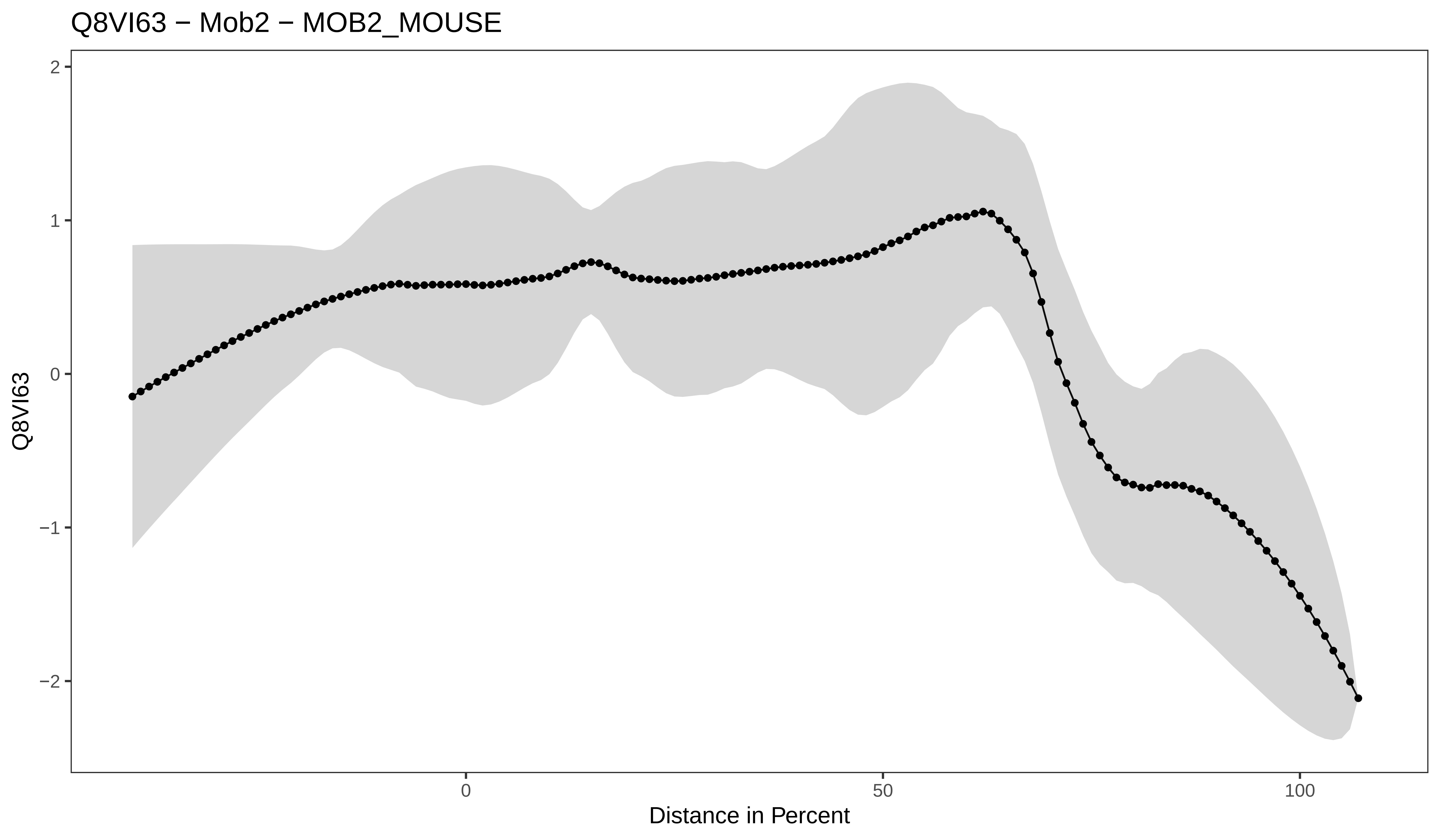

Q8VI63 − Mob2 − MOB2_MOUSE
2
1
0
−1
−2
0
50
100
Distance in Percent
Q8VI63

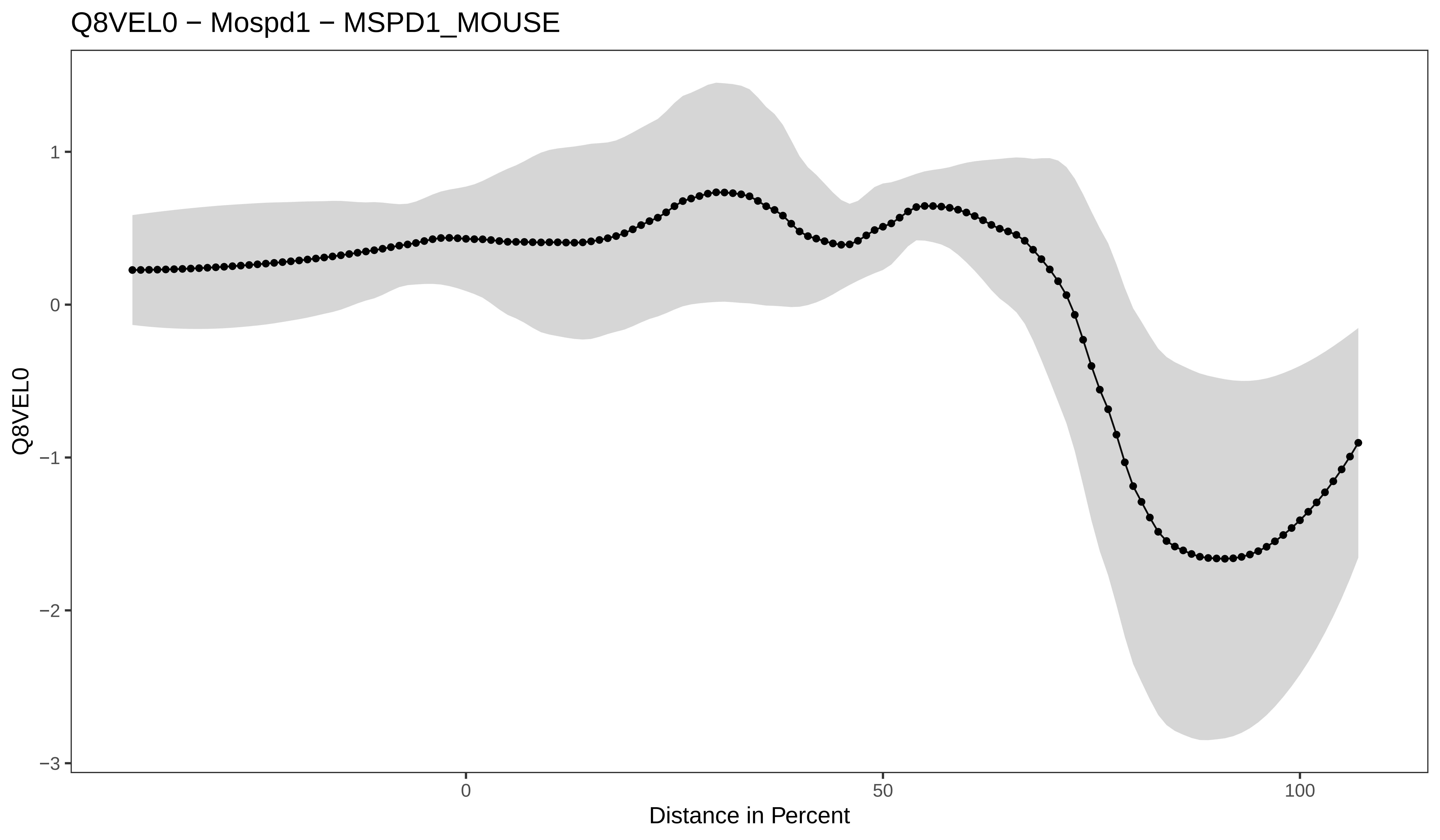

Q8VEL0 − Mospd1 − MSPD1_MOUSE
Q8VEL0
1
0
−1
−2
−3
0
50
100
Distance in Percent

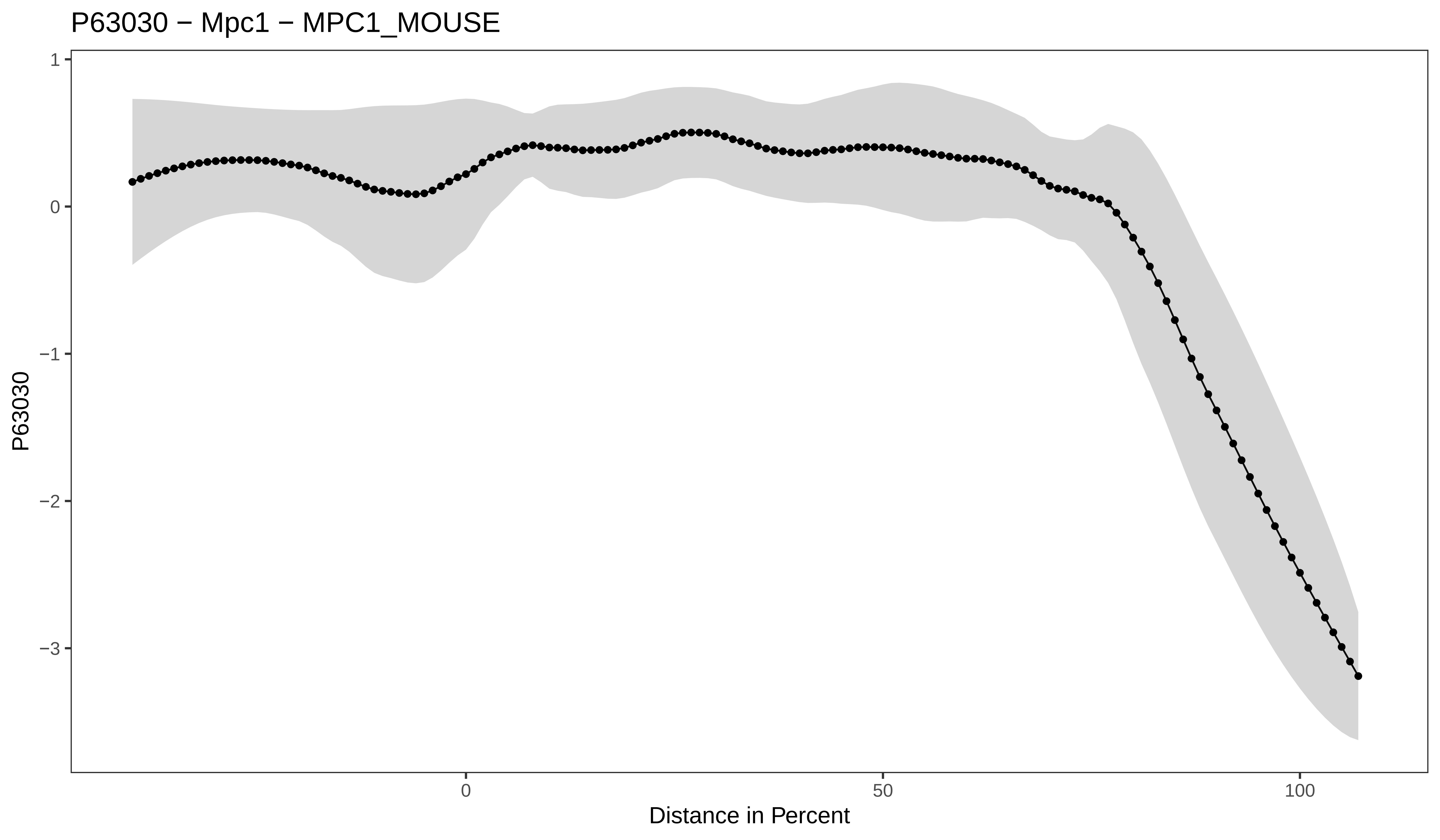
P63030 − Mpc1 − MPC1_MOUSE
1
0
−1
−2
−3
P63030
0
50
100
Distance in Percent

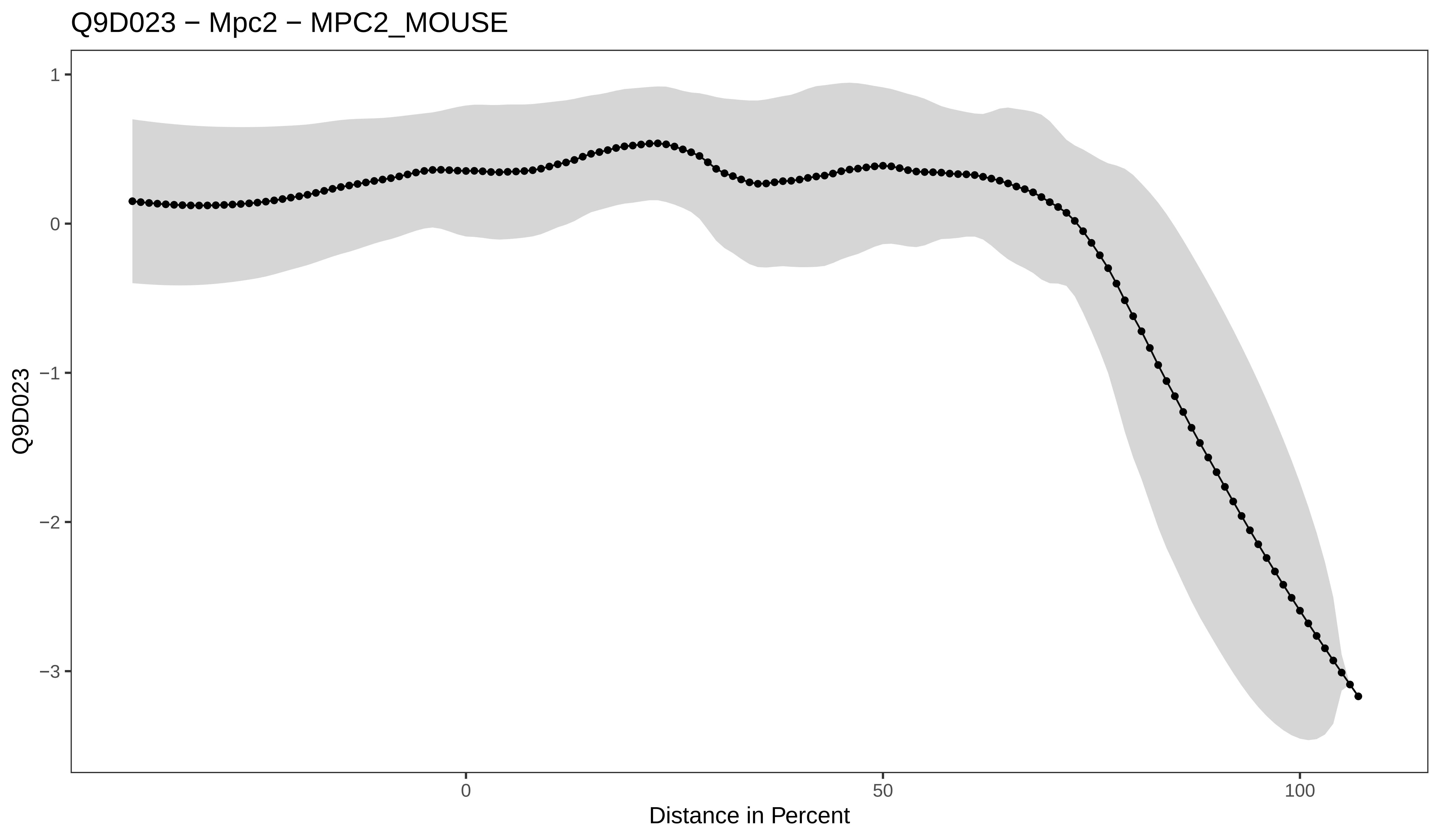

Q9D023 − Mpc2 − MPC2_MOUSE
Q9D023
Distance in Percent
1
0
−1
−2
−3
0
50
100

Q924M7 − Mpi − MPI_MOUSE

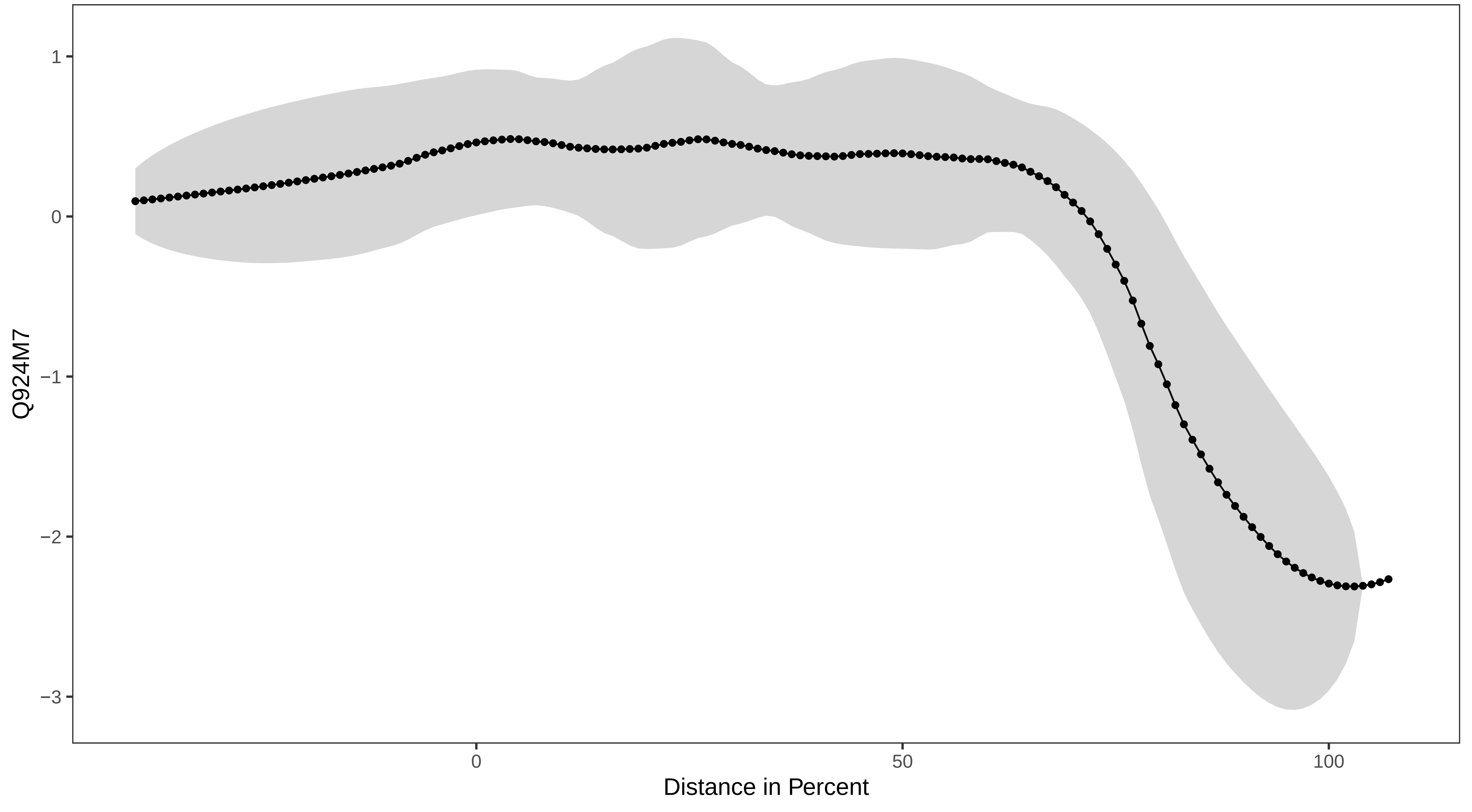

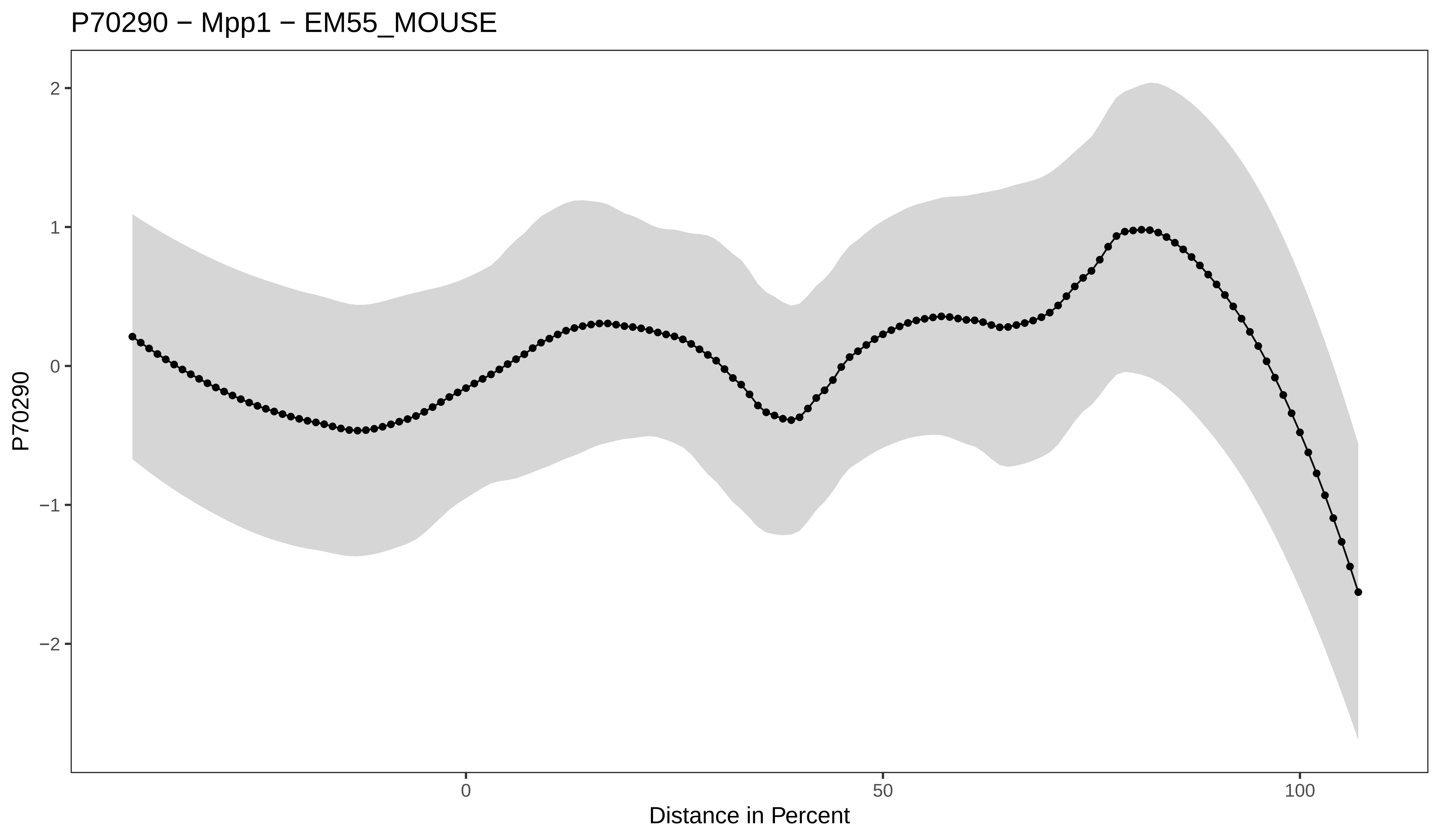
P70290 − Mpp1 − EM55_MOUSE
2
1
0
−1
−2
P70290
0
50
100
Distance in Percent

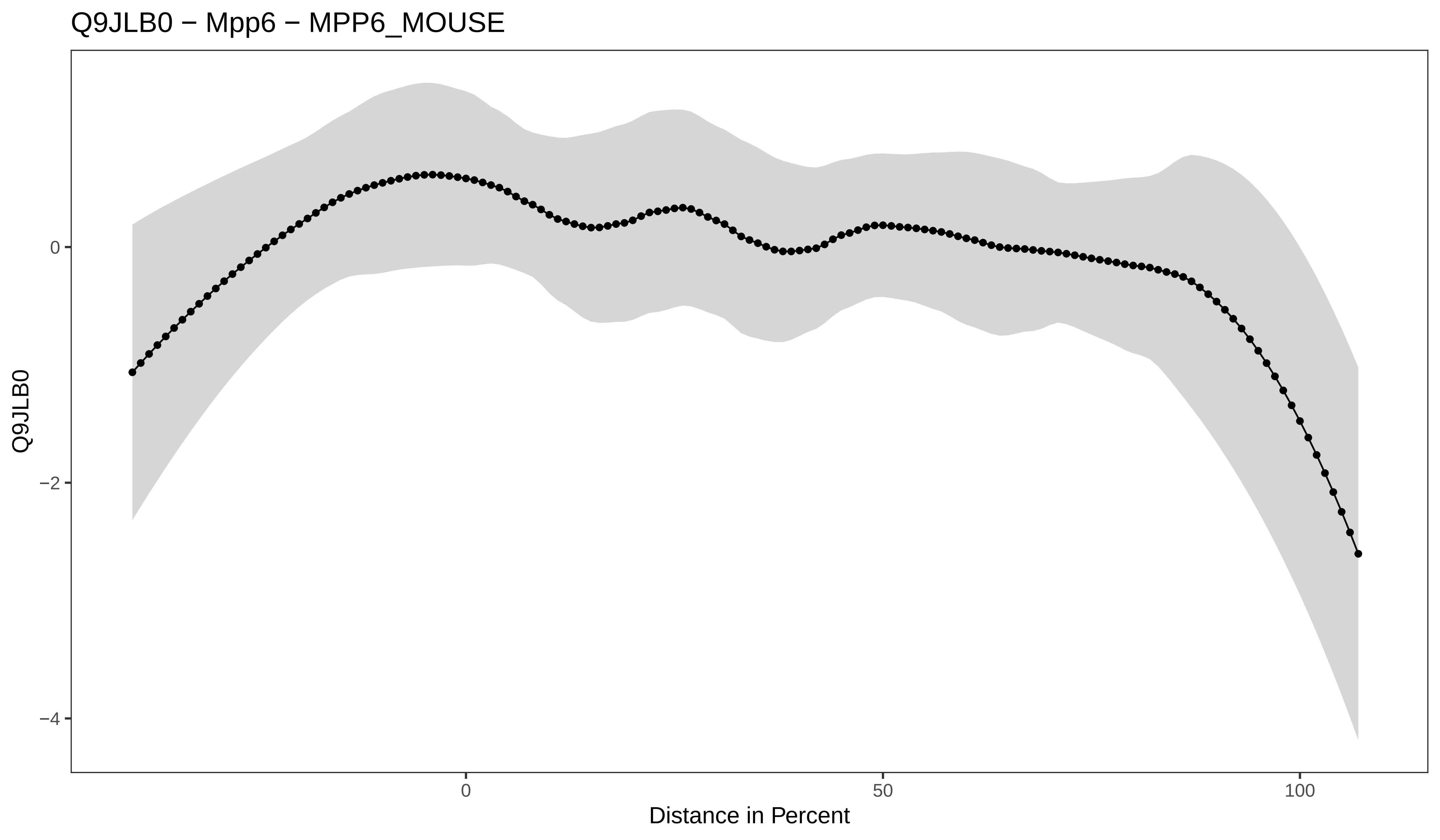
Q9JLB0 − Mpp6 − MPP6_MOUSE
Q9JLB0
0
−2
−4
0
50
100
Distance in Percent

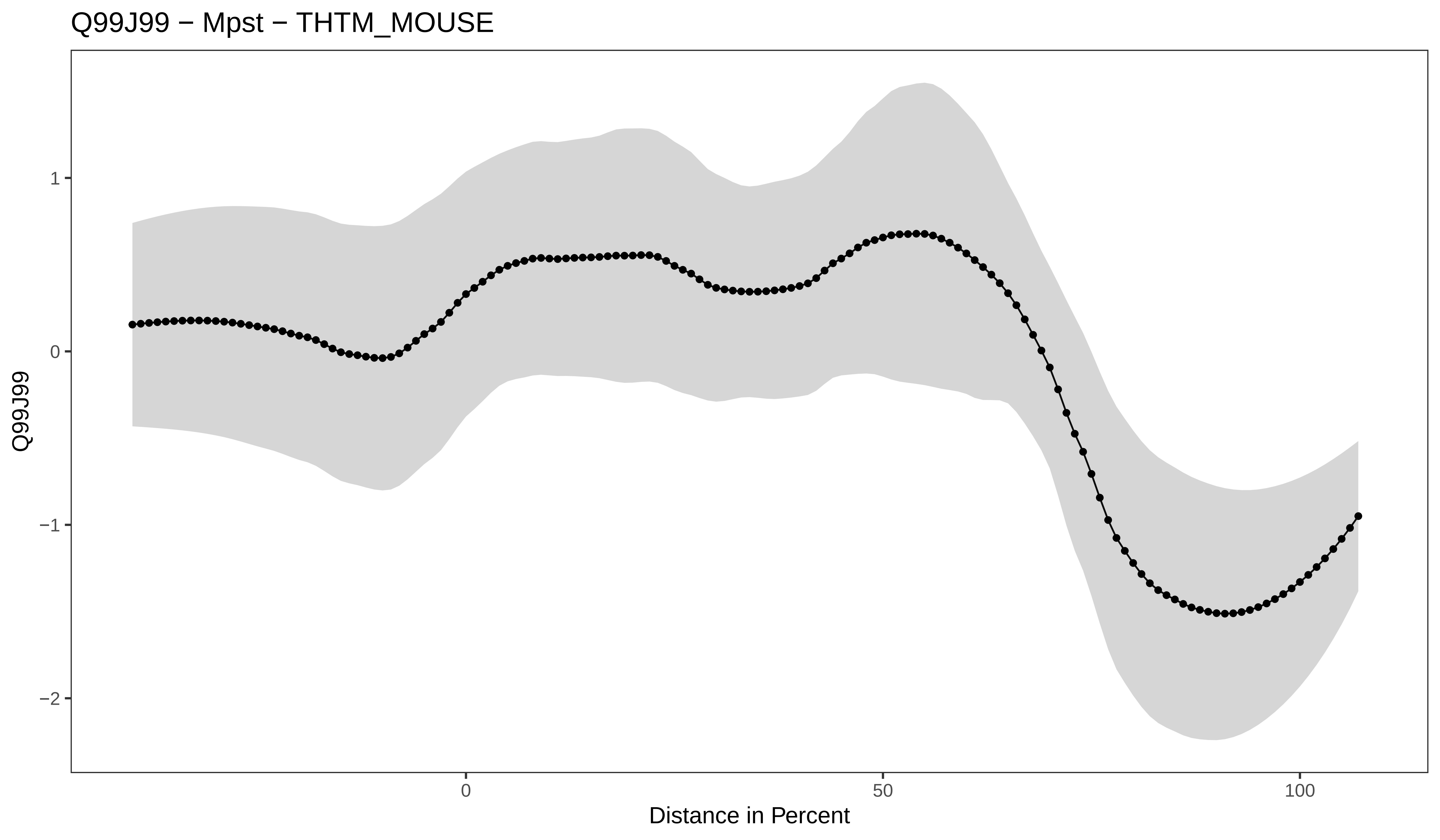

Q99J99 − Mpst − THTM_MOUSE
1
0
−1
−2
Q99J99
0
50
100
Distance in Percent

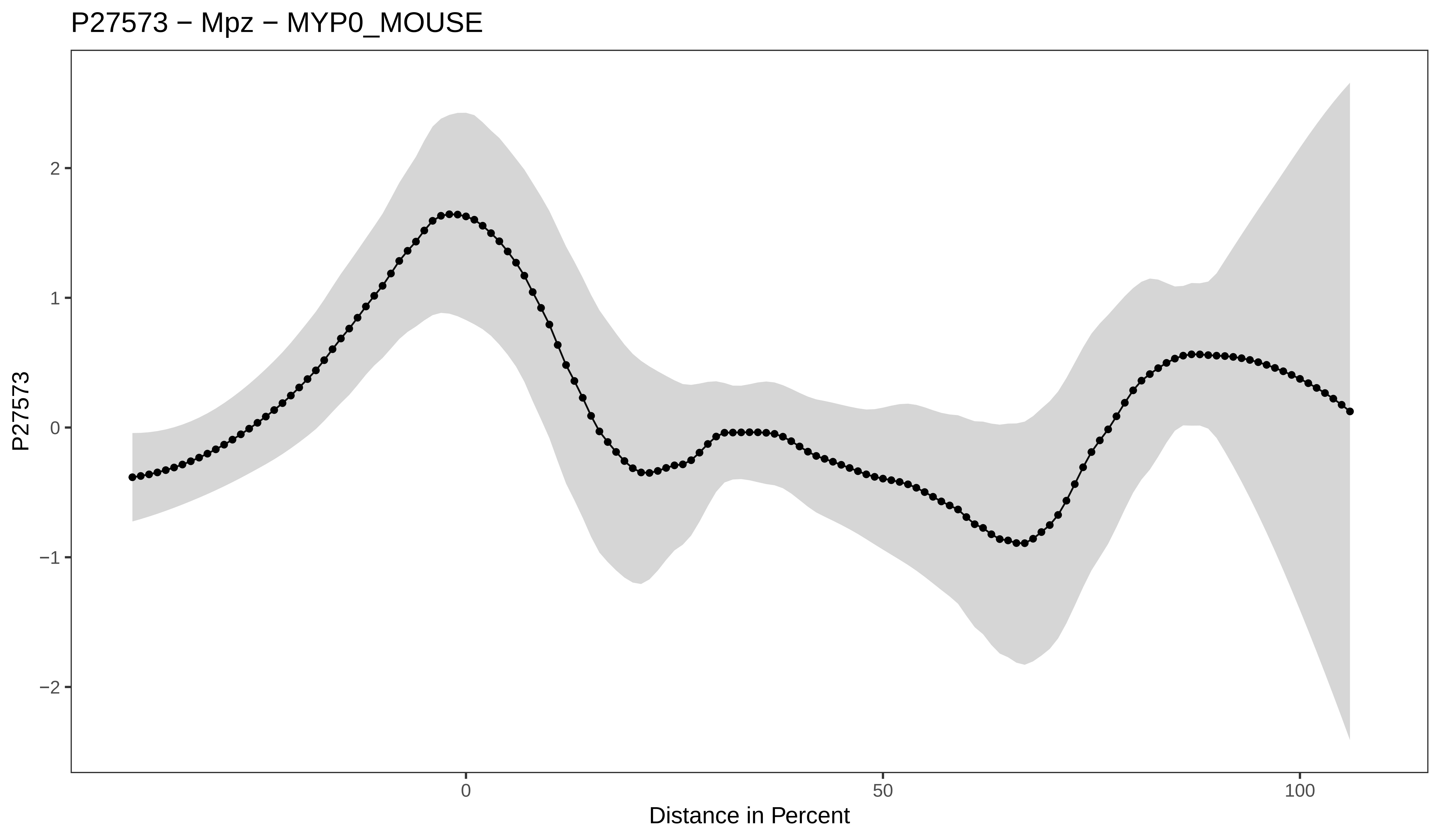

P27573 − Mpz − MYP0_MOUSE
P27573
2
1
0
−1
−2
0
50
100
Distance in Percent

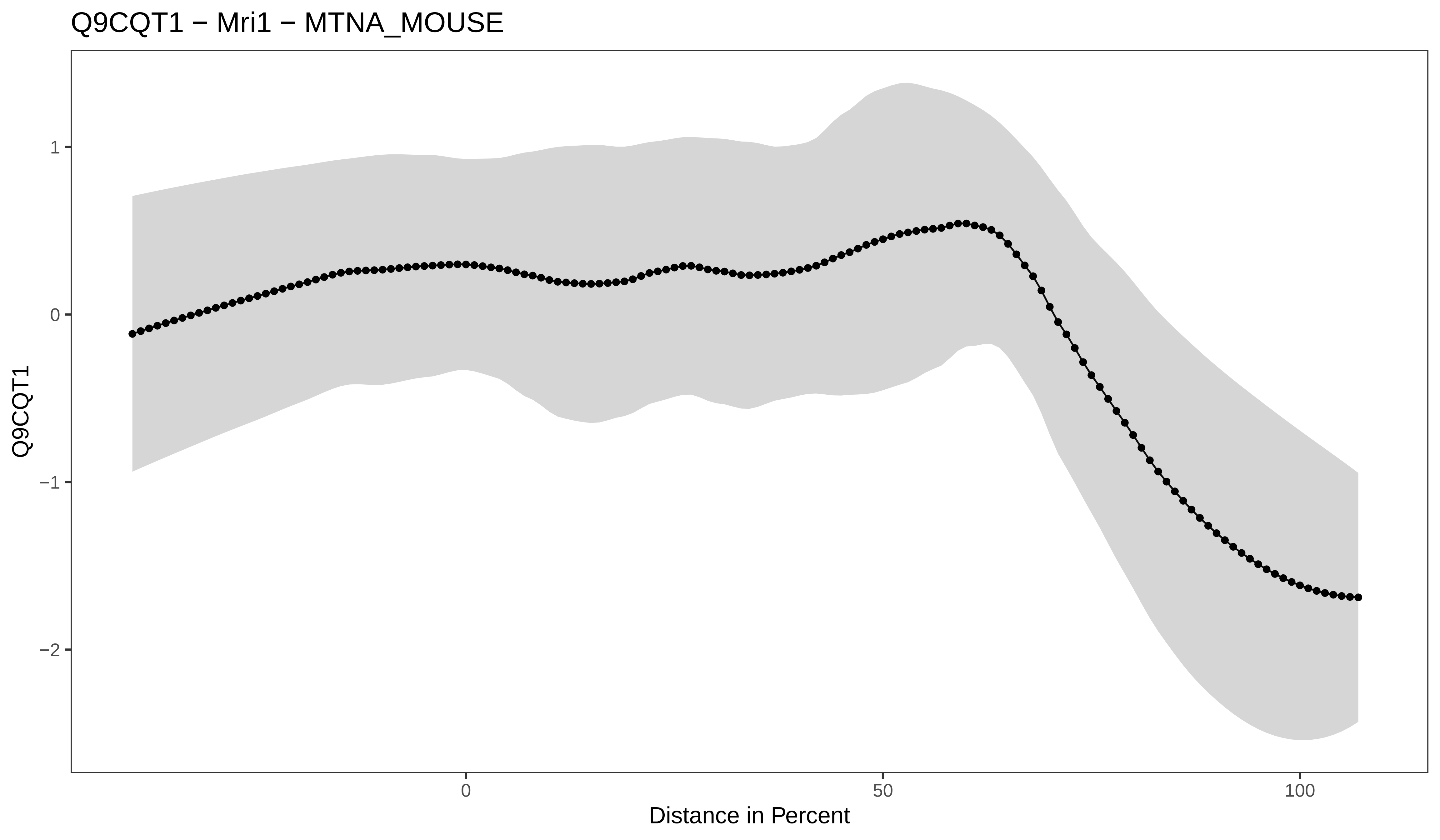
Q9CQT1 − Mri1 − MTNA_MOUSE
Q9CQT1
1
0
−1
−2
0
50
100
Distance in Percent

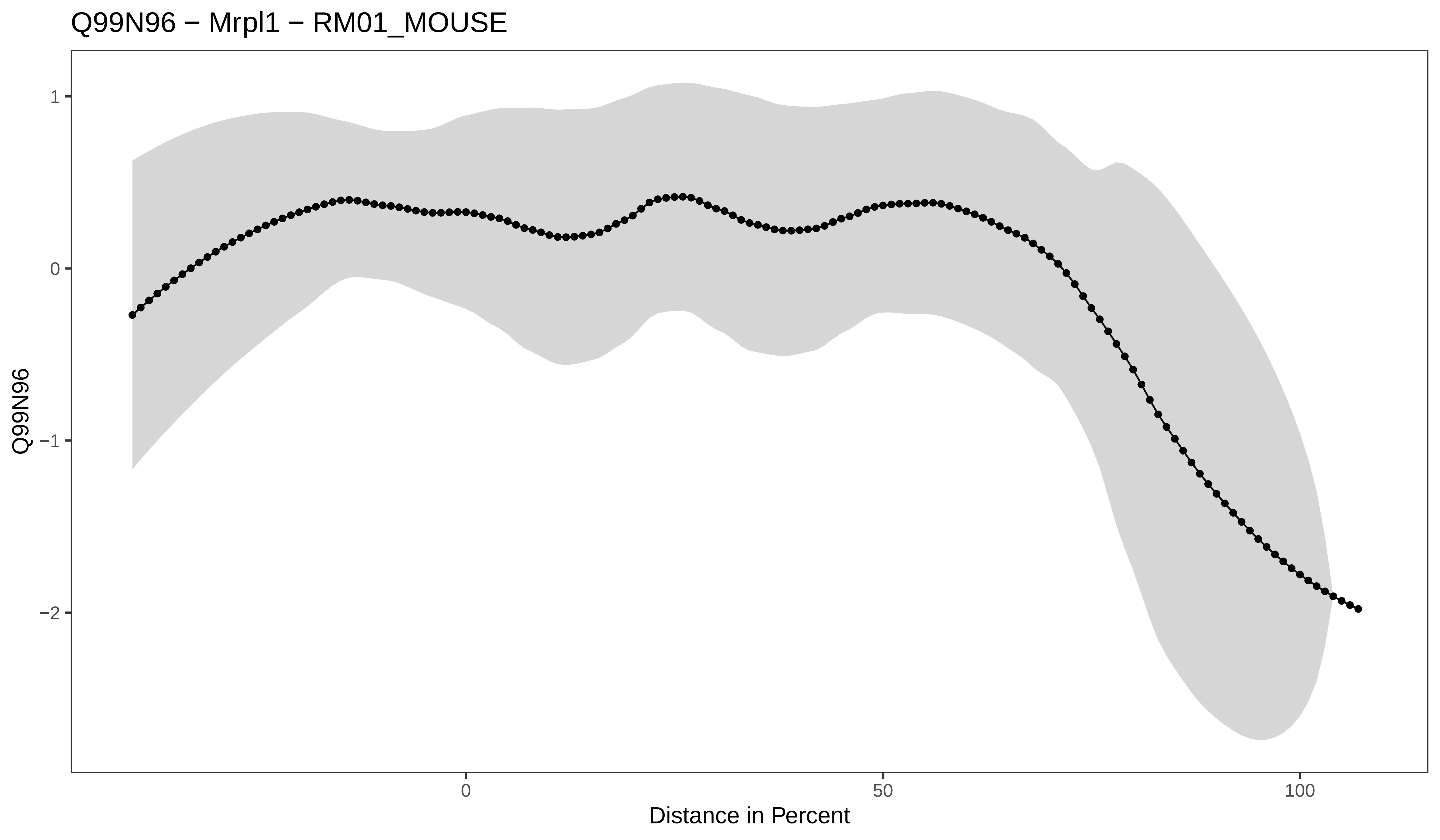

Q99N96 − Mrpl1 − RM01_MOUSE
Q99N96
1
0
−1
−2
0
50
100
Distance in Percent

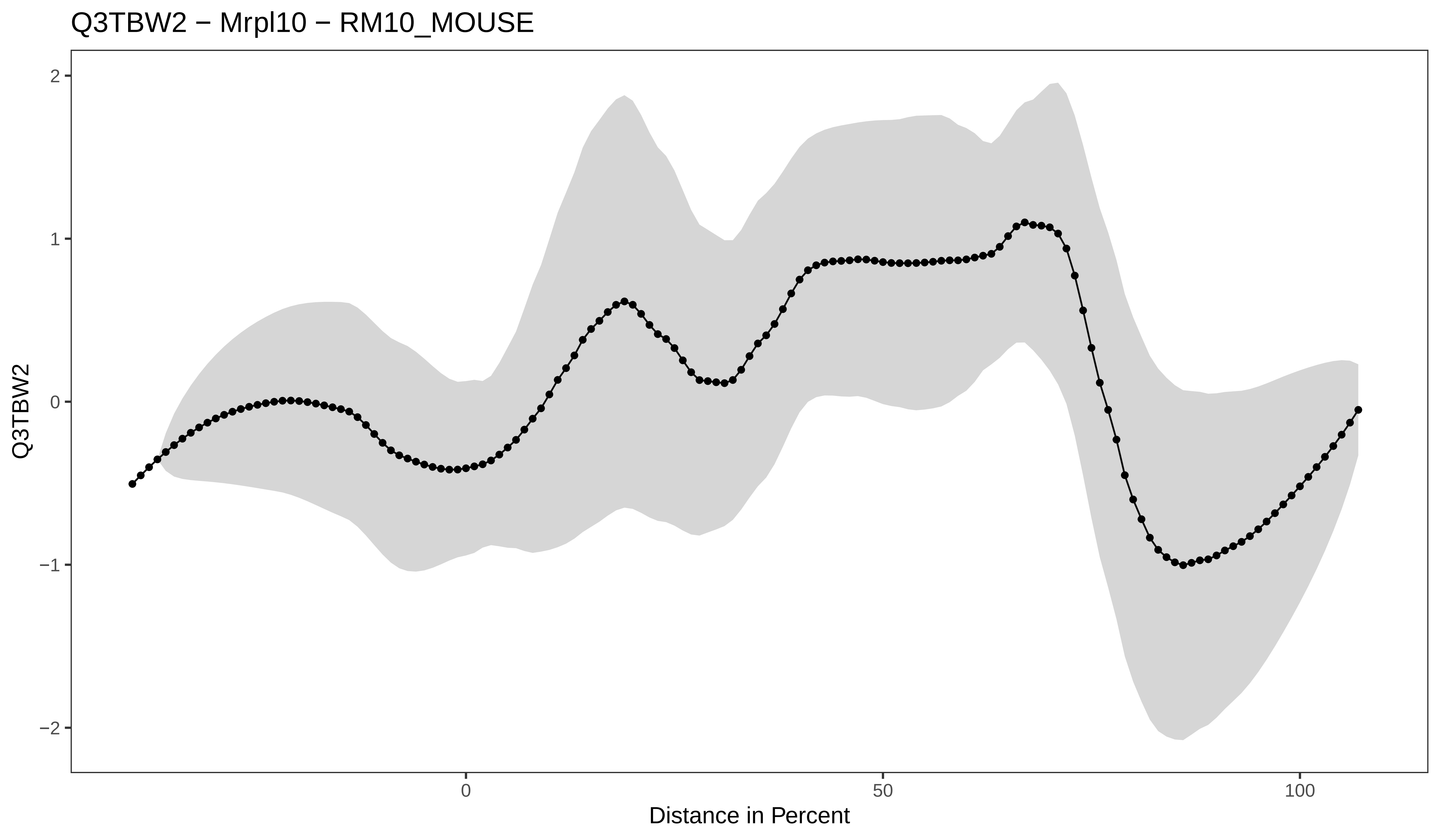

Q3TBW2 − Mrpl10 − RM10_MOUSE
Q3TBW2
2
1
0
−1
−2
0
50
100
Distance in Percent

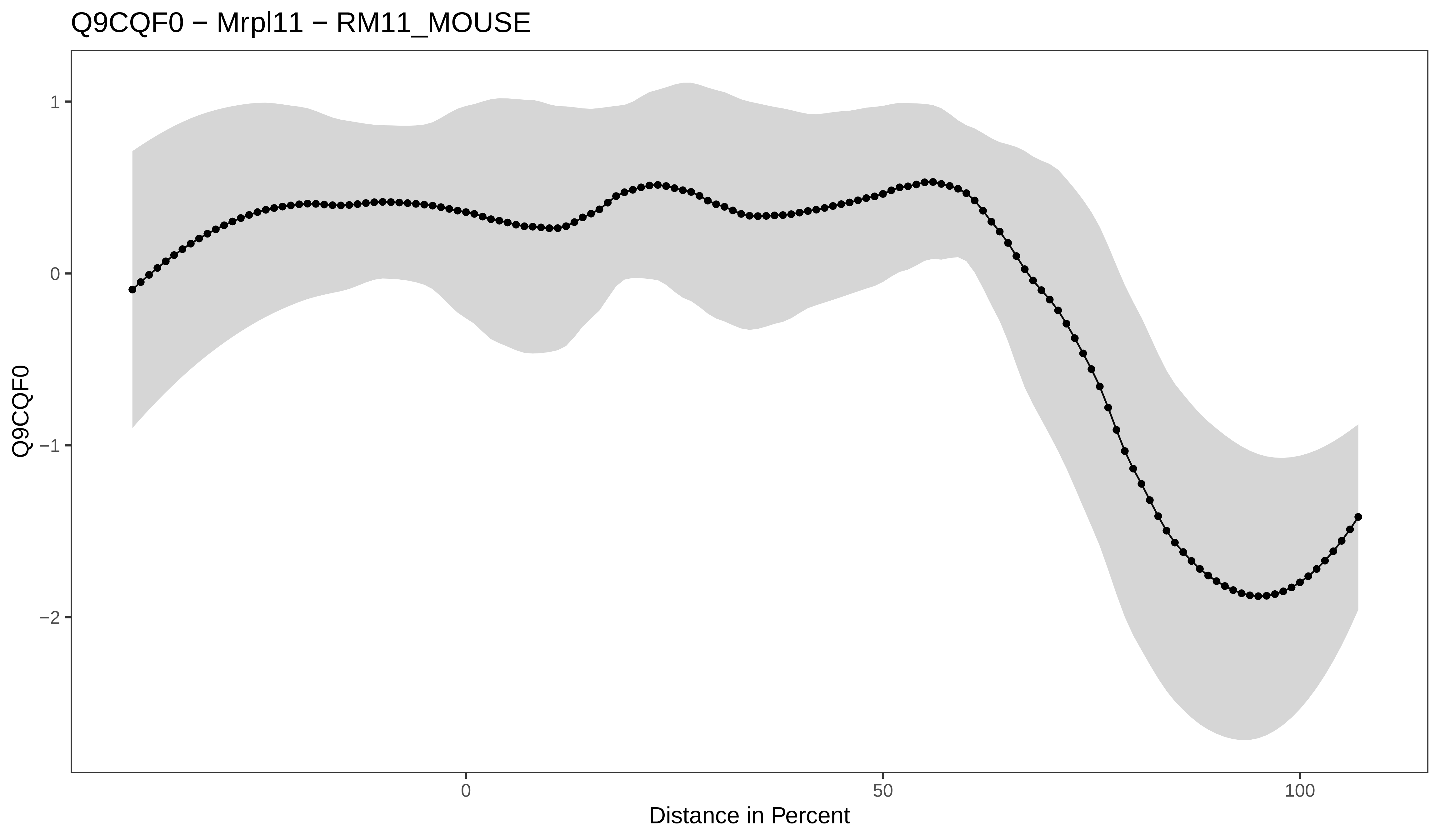
Q9CQF0 − Mrpl11 − RM11_MOUSE
Q9CQF0
1
0
−1
−2
0
50
100
Distance in Percent

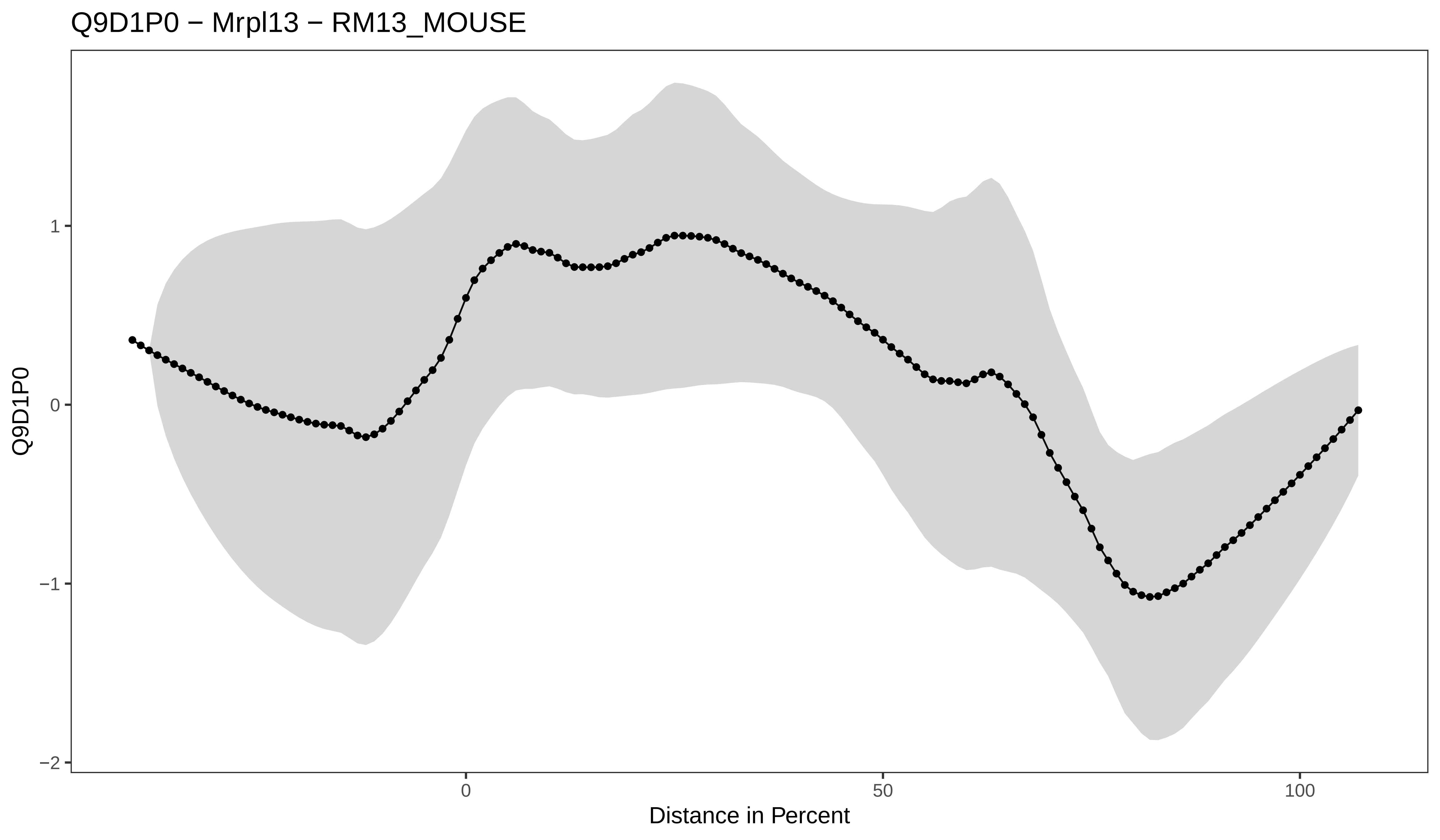

Q9D1P0 − Mrpl13 − RM13_MOUSE
Q9D1P0
1
0
−1
−2
0
50
100
Distance in Percent

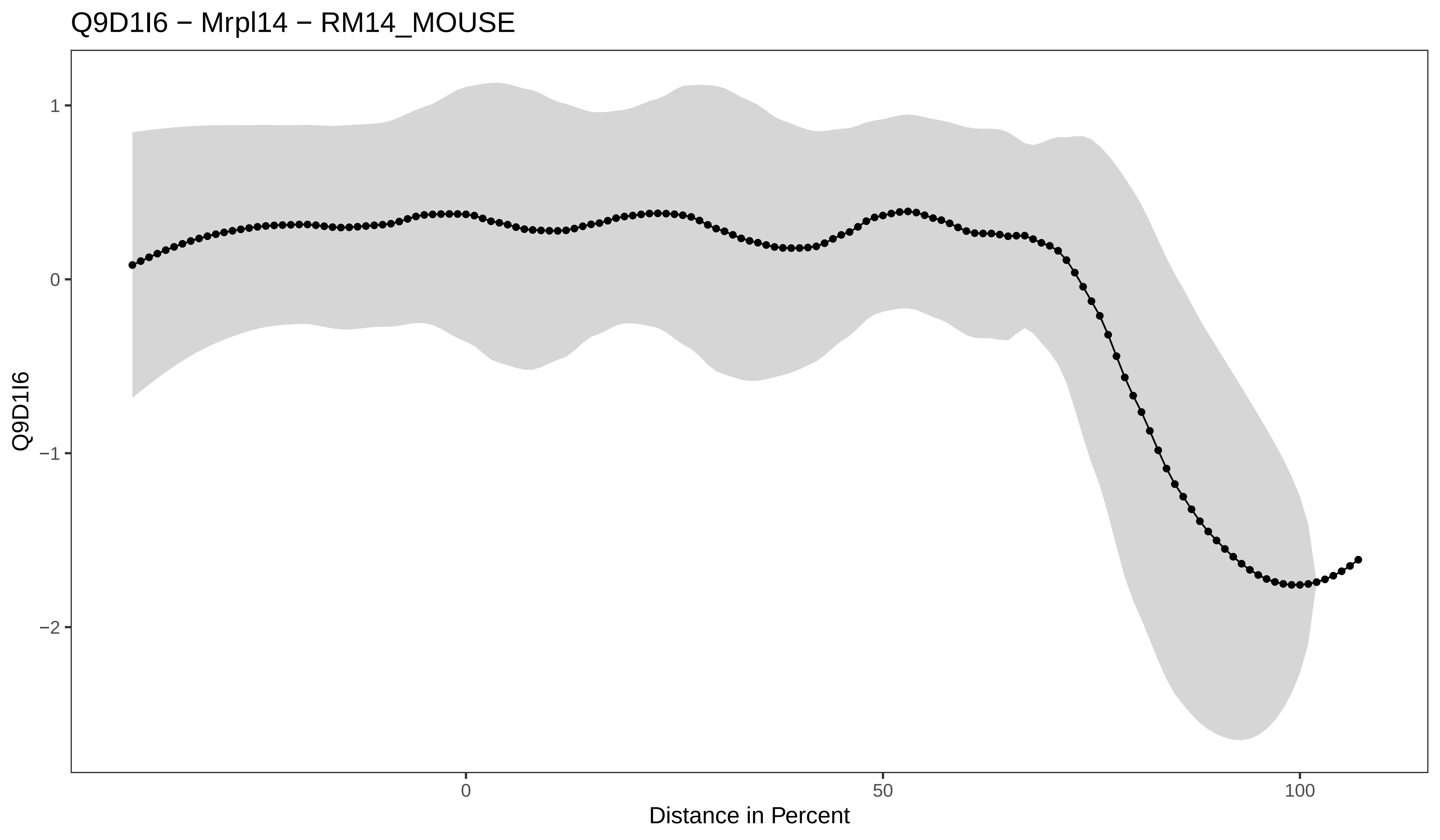

Q9D1I6 − Mrpl14 − RM14_MOUSE
1
0
−1
−2
Q9D1I6
0
50
100
Distance in Percent

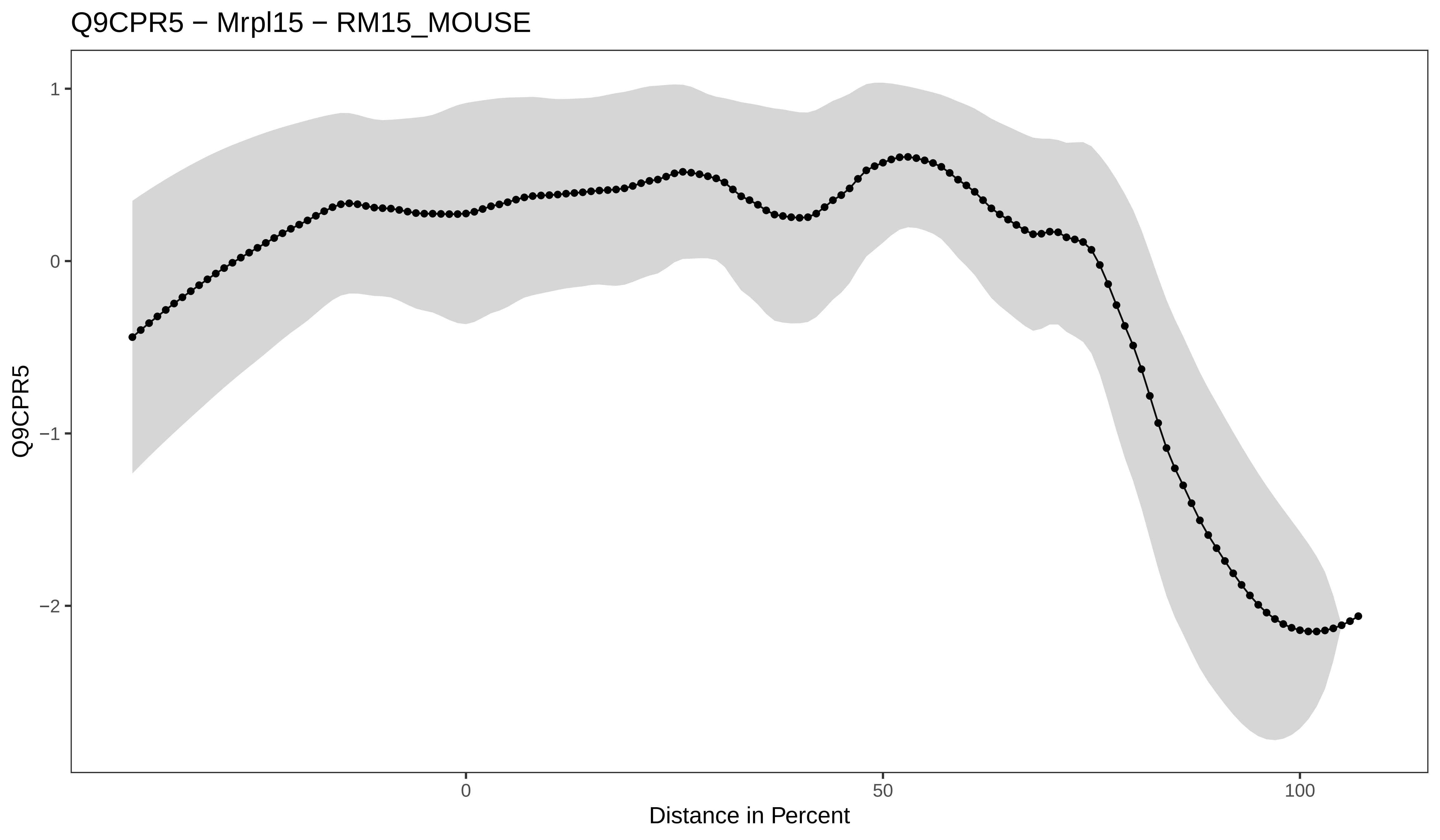

Q9CPR5 − Mrpl15 − RM15_MOUSE
Q9CPR5
Distance in Percent
1
0
−1
−2
0
50
100

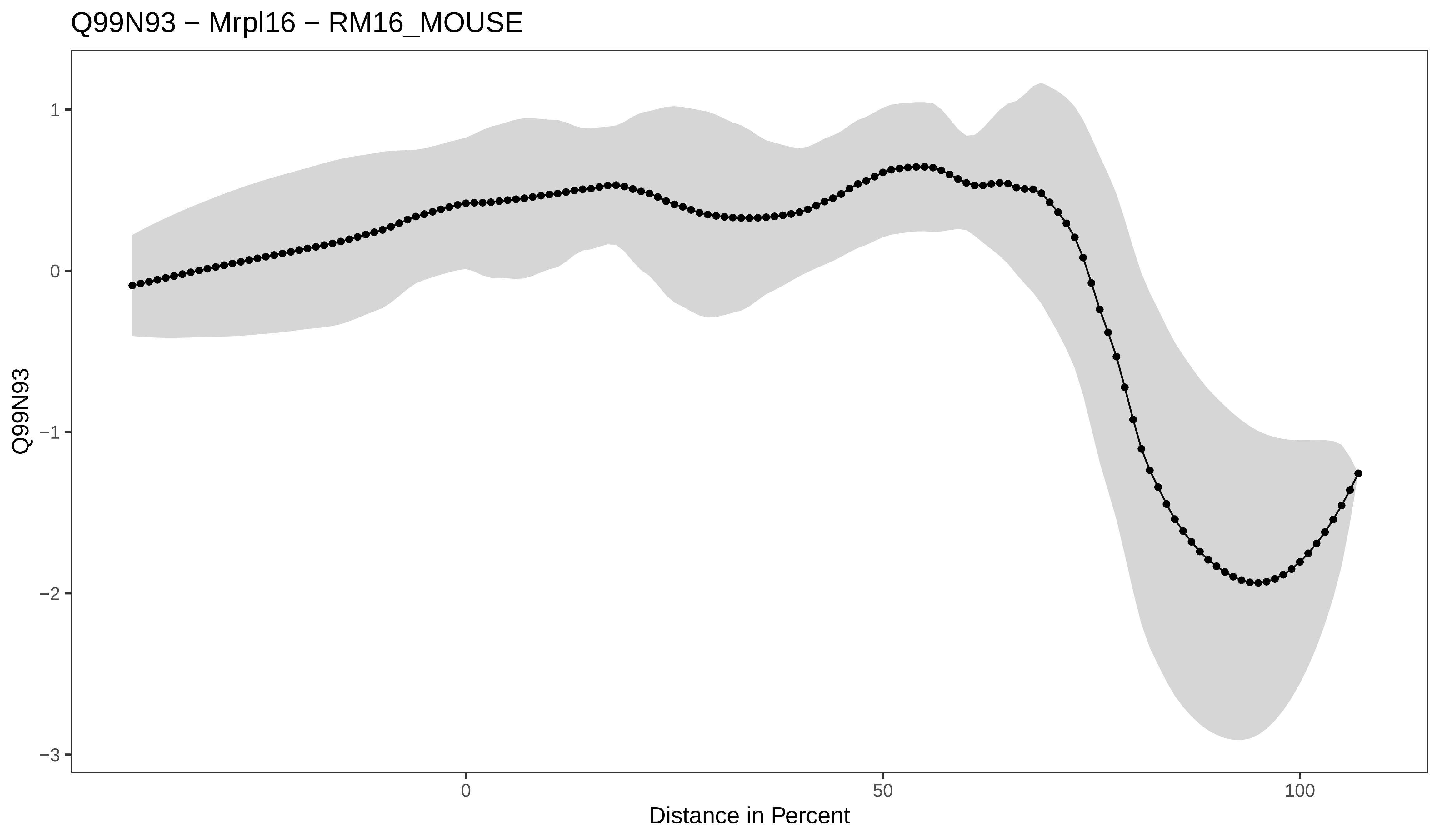
Q99N93 − Mrpl16 − RM16_MOUSE
1
0
−1
−2
−3
Q99N93
0
50
100
Distance in Percent

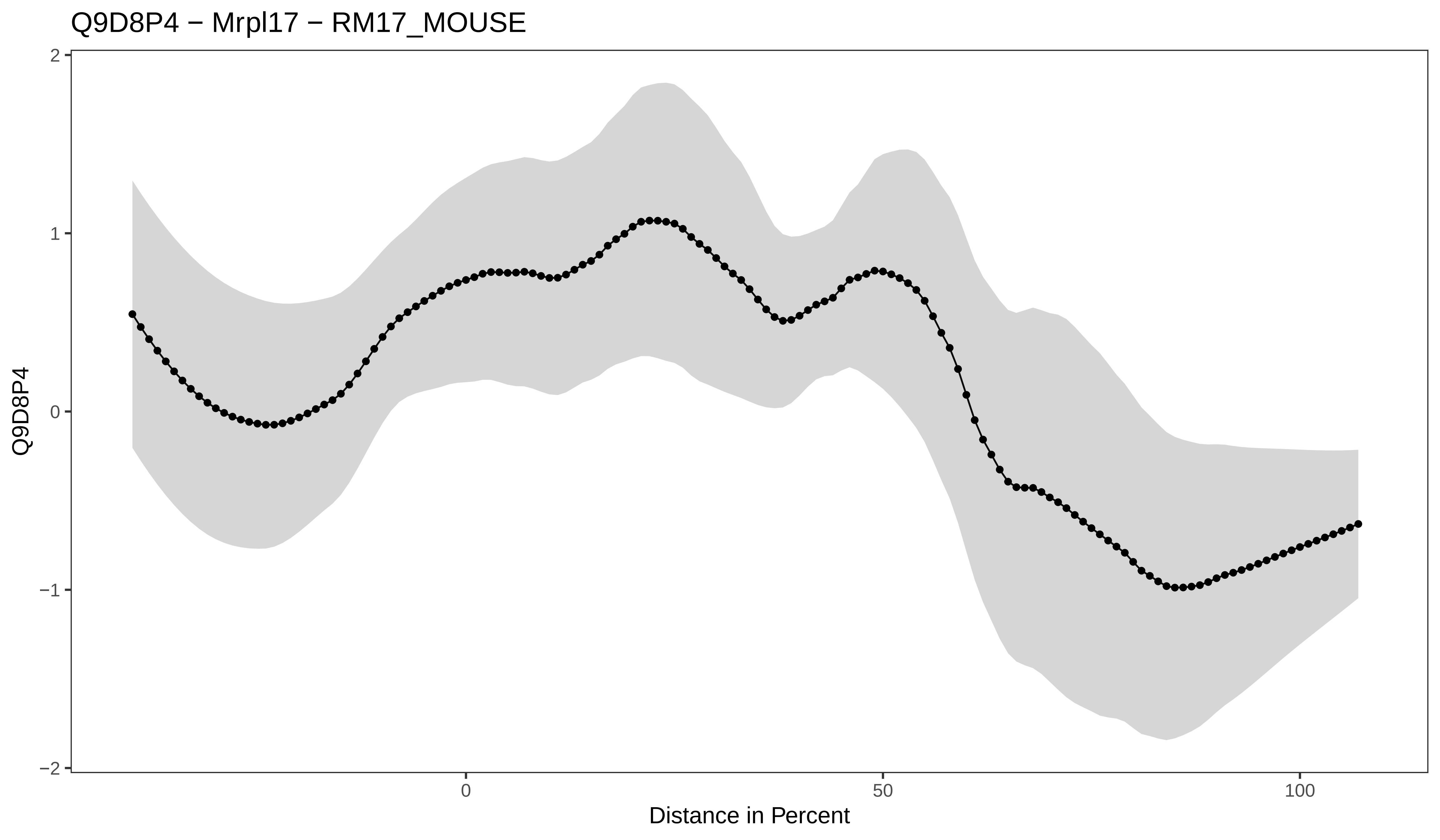

Q9D8P4 − Mrpl17 − RM17_MOUSE
2
1
0
−1
−2
Q9D8P4
0
50
100
Distance in Percent

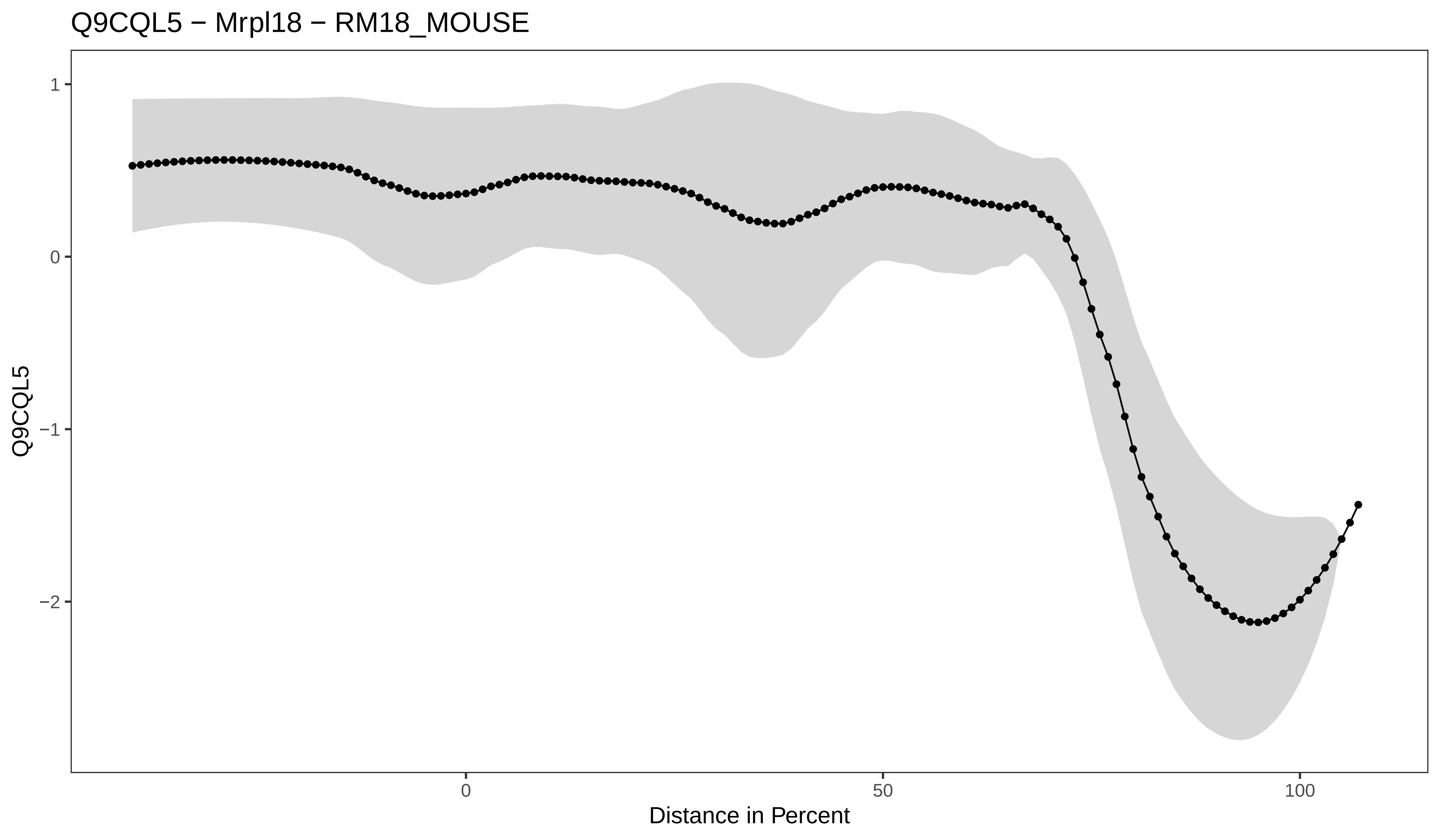

Q9CQL5 − Mrpl18 − RM18_MOUSE
1
0
−1
−2
Q9CQL5
0
50
100
Distance in Percent

Q9D338 − Mrpl19 − RM19_MOUSE

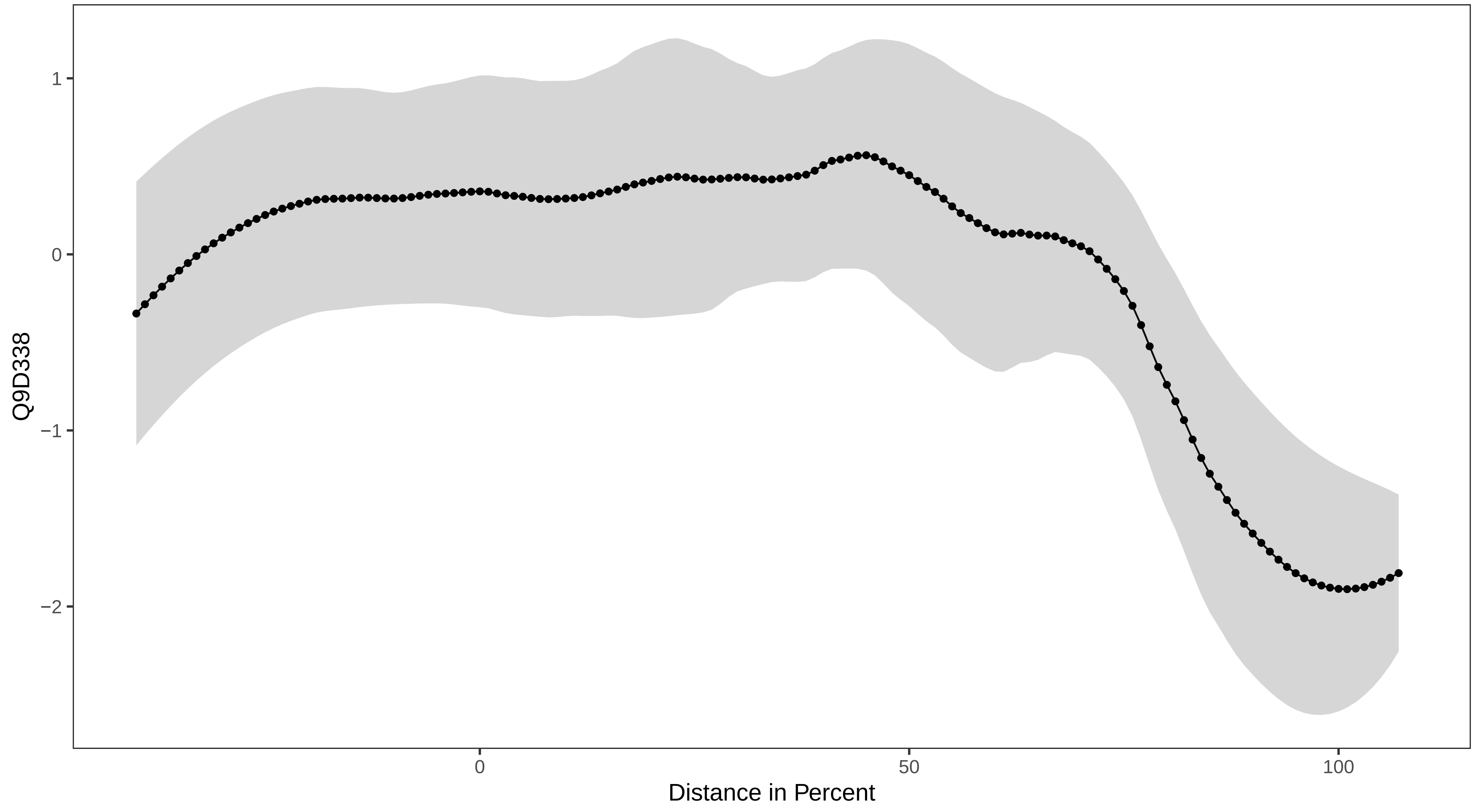

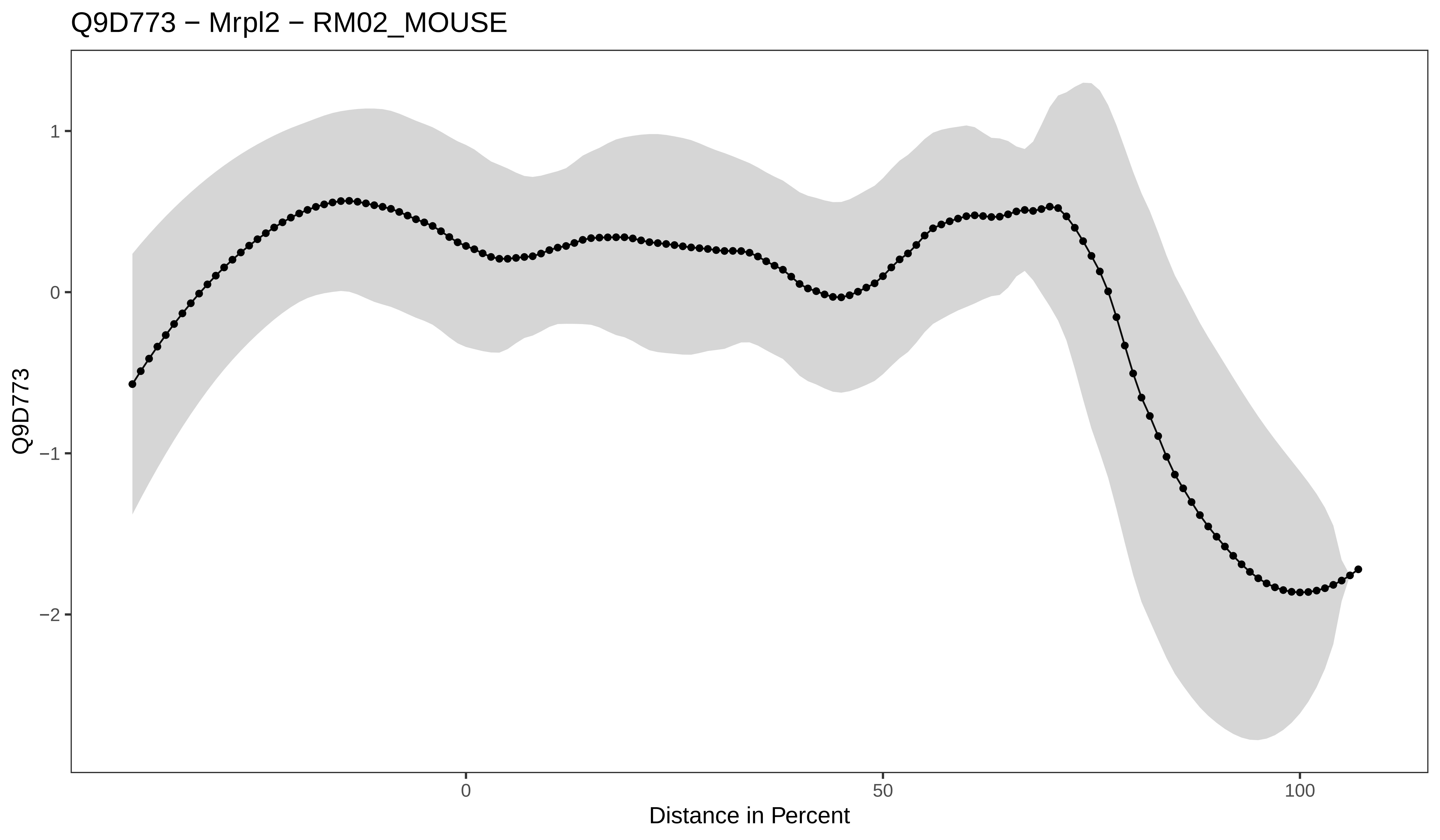
Q9D773 − Mrpl2 − RM02_MOUSE
Q9D773
1
0
−1
−2
0
50
100
Distance in Percent

Q9CQL4 − Mrpl20 − RM20_MOUSE

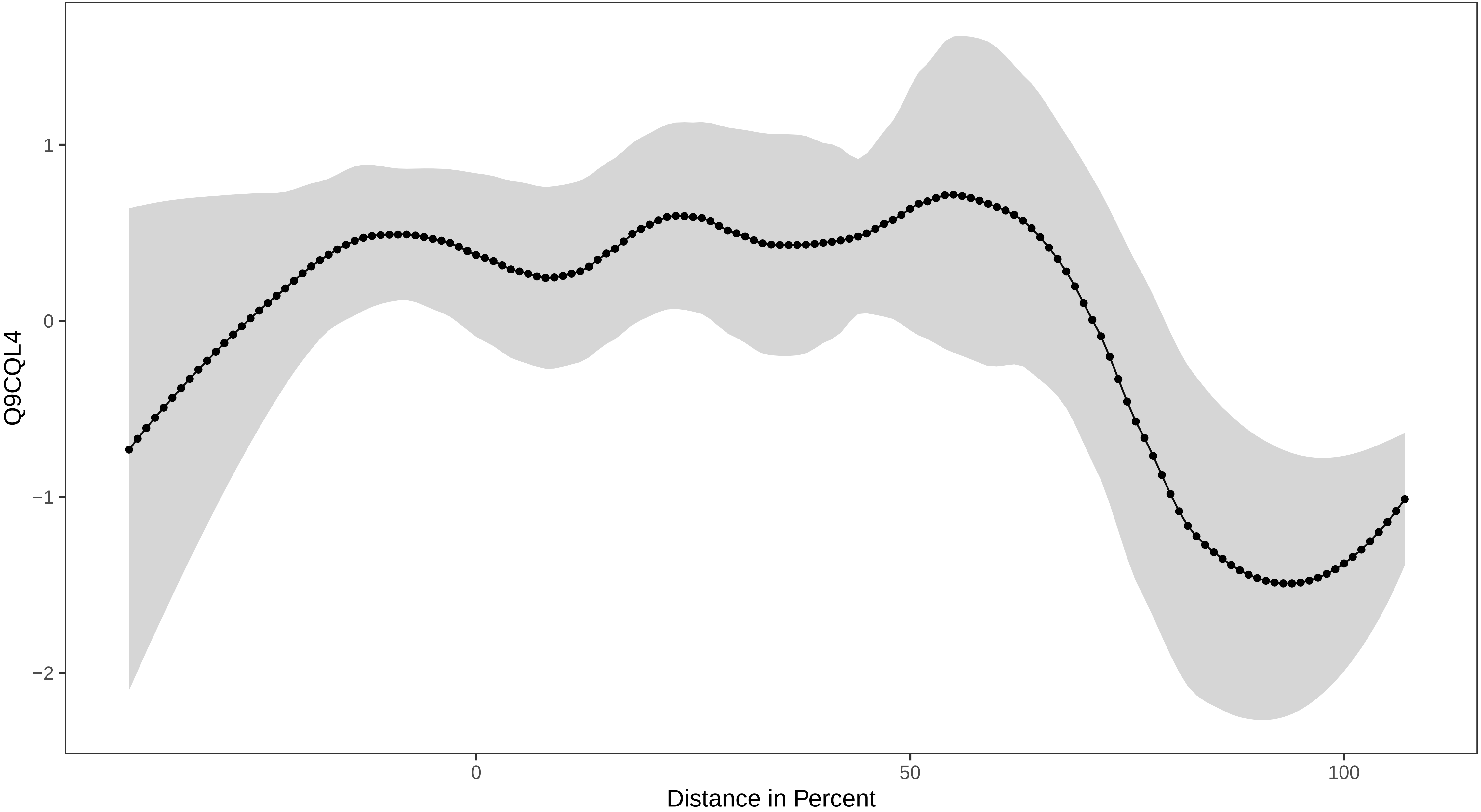

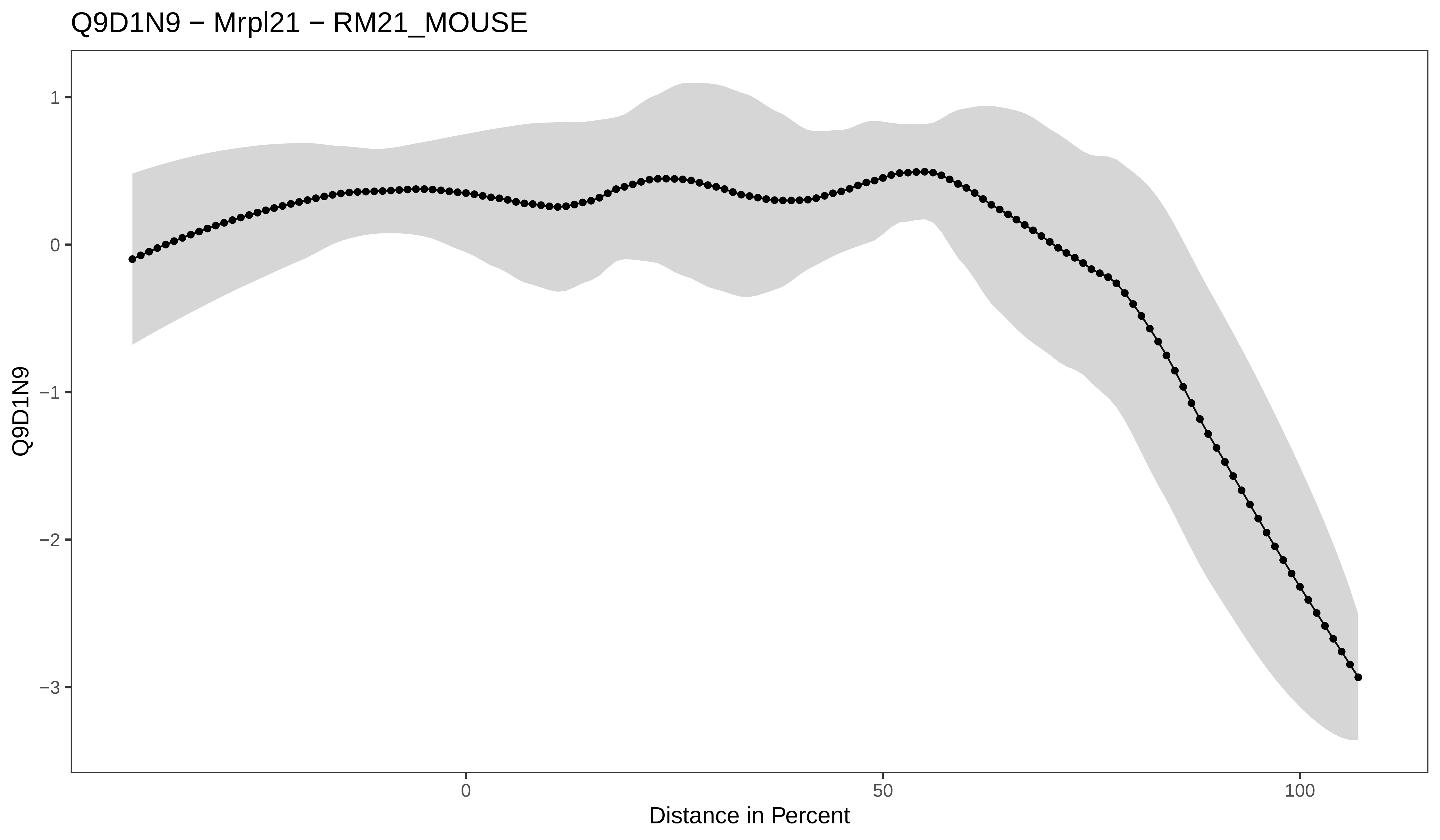

Q9D1N9 − Mrpl21 − RM21_MOUSE
1
0
−1
−2
−3
Q9D1N9
0
50
100
Distance in Percent

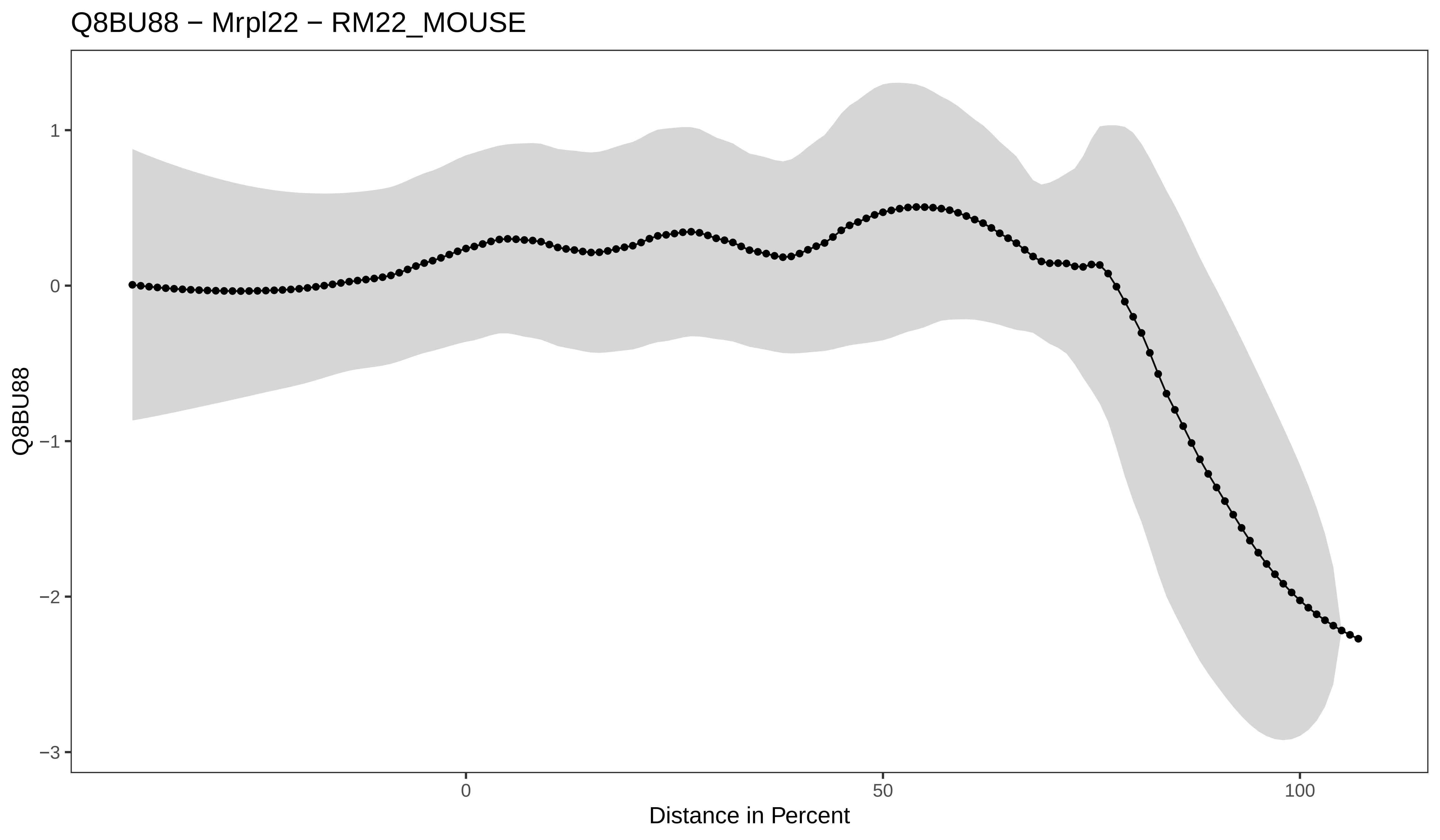

Q8BU88 − Mrpl22 − RM22_MOUSE
Q8BU88
Distance in Percent
1
0
−1
−2
−3
0
50
100

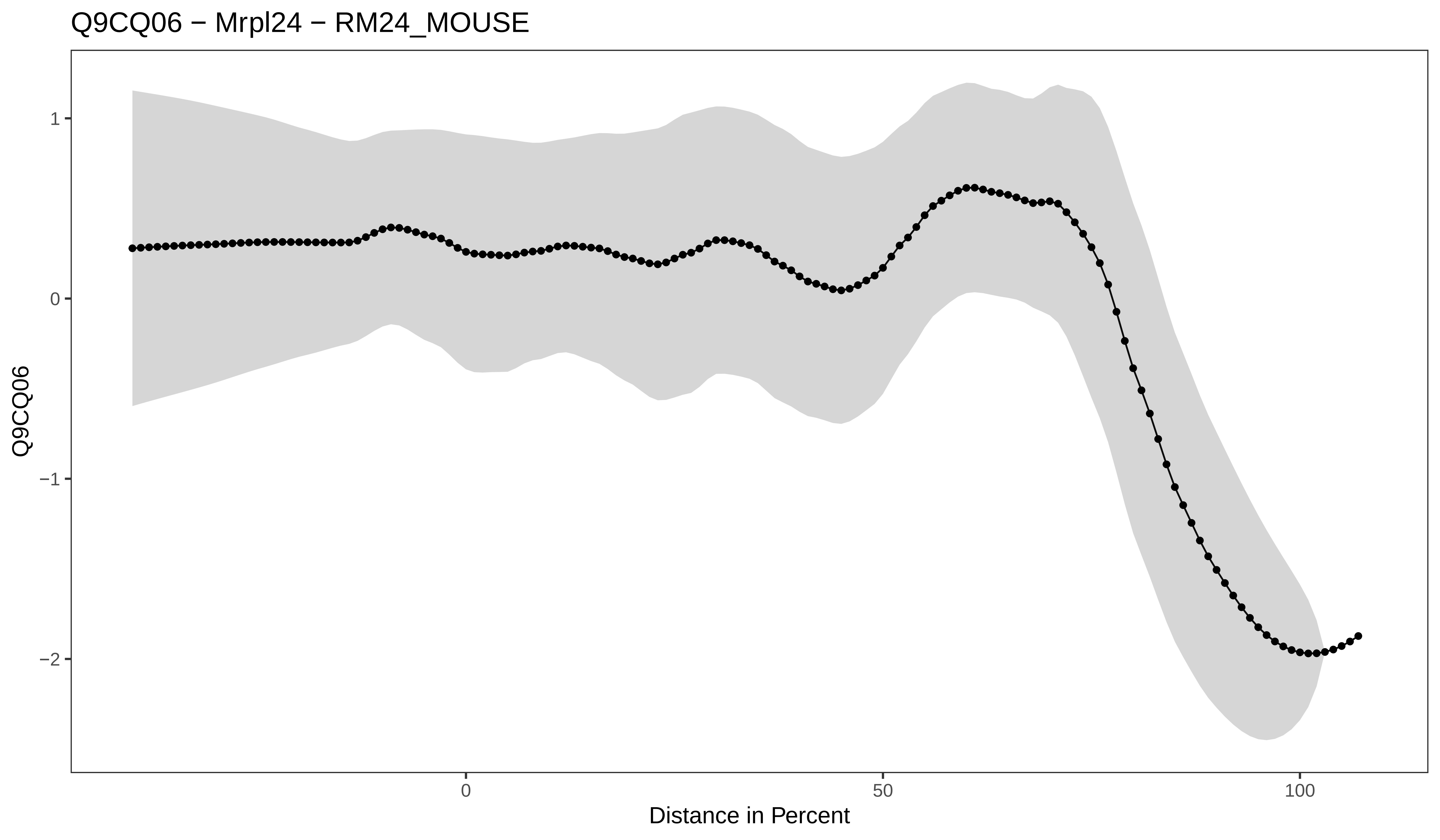

Q9CQ06 − Mrpl24 − RM24_MOUSE
Q9CQ06
1
0
−1
−2
0
50
100
Distance in Percent

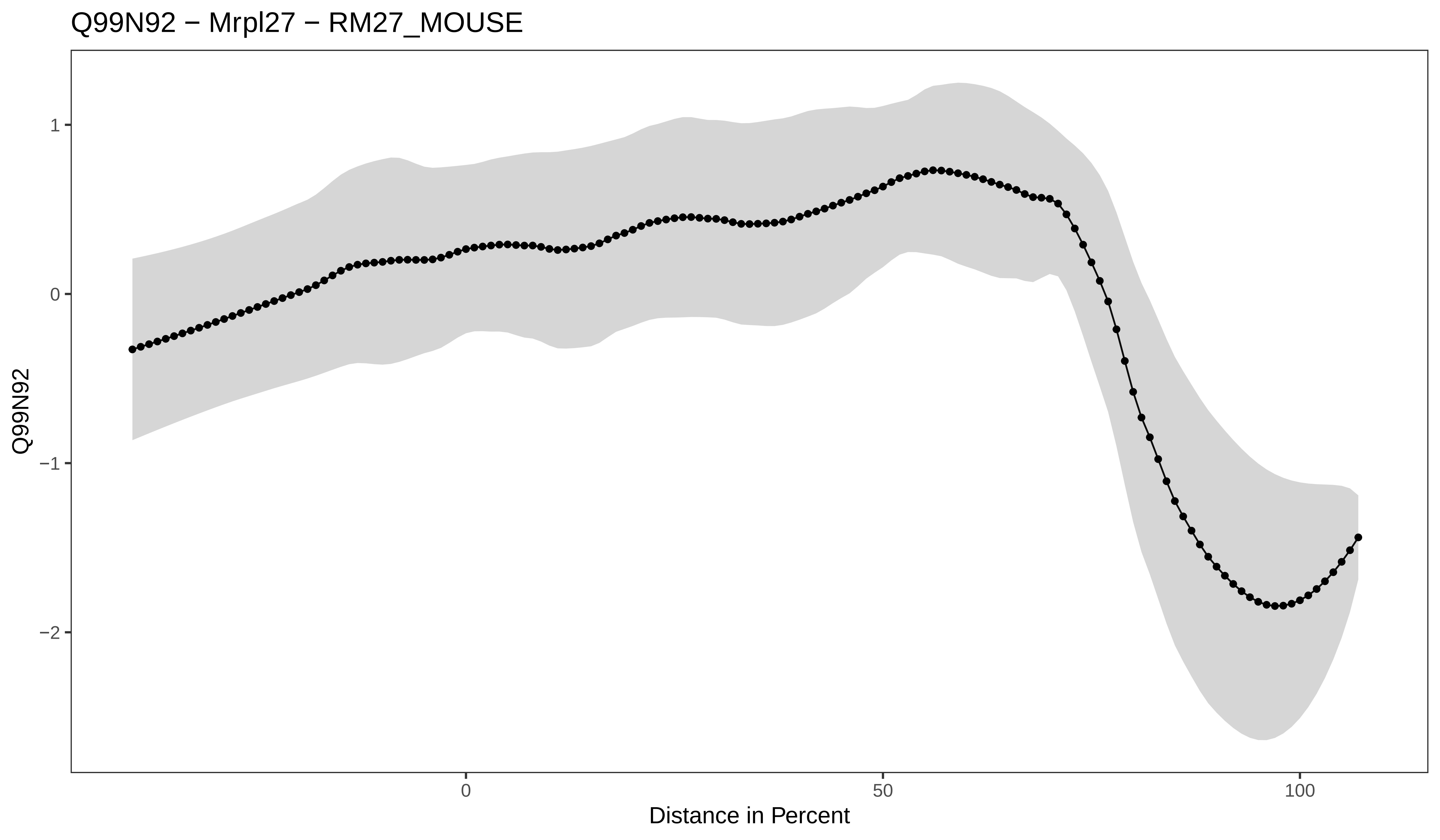

Q99N92 − Mrpl27 − RM27_MOUSE
1
0
−1
−2
Q99N92
0
50
100
Distance in Percent

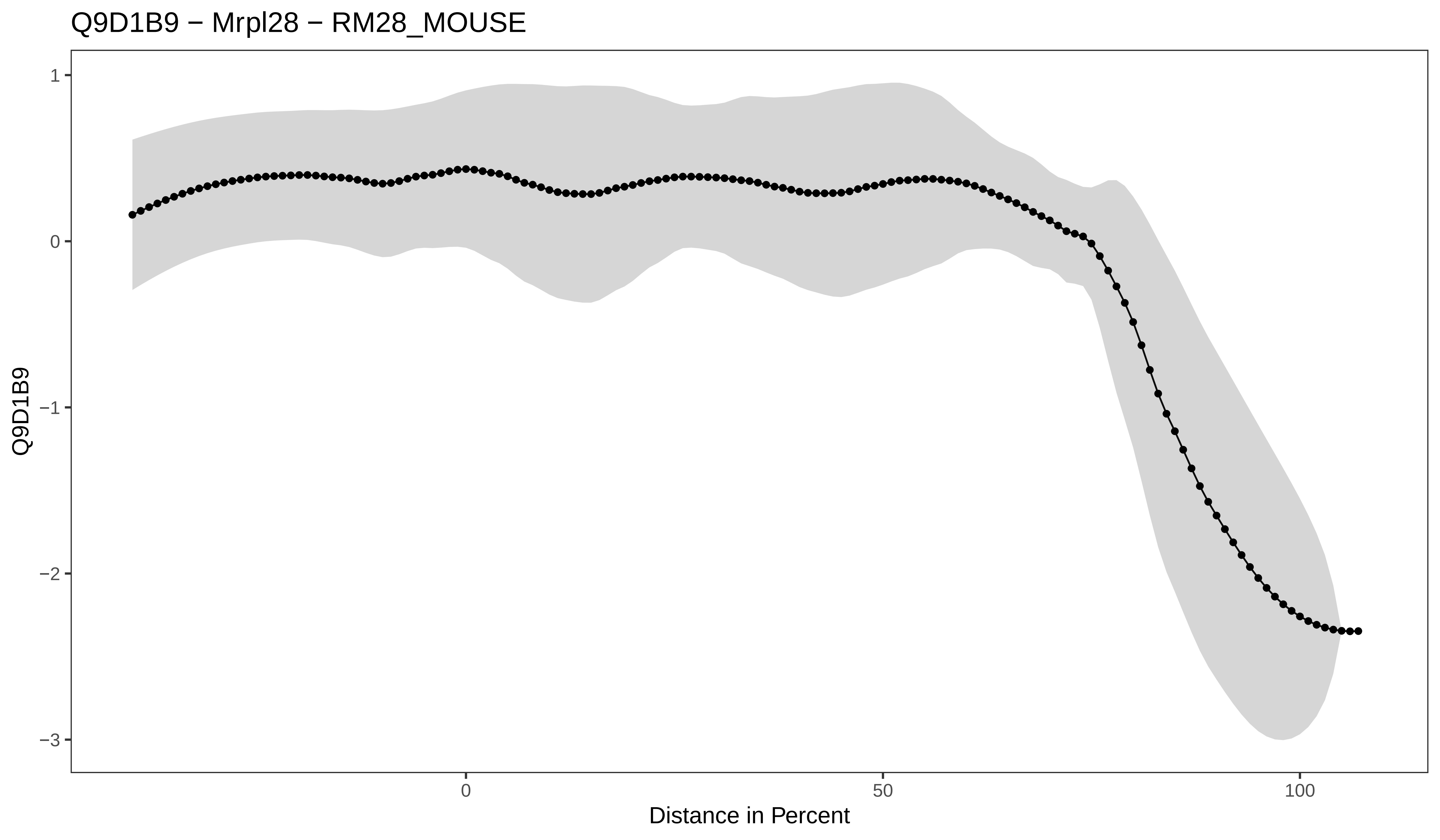
Q9D1B9 − Mrpl28 − RM28_MOUSE
Q9D1B9
1
0
−1
−2
−3
0
50
100
Distance in Percent

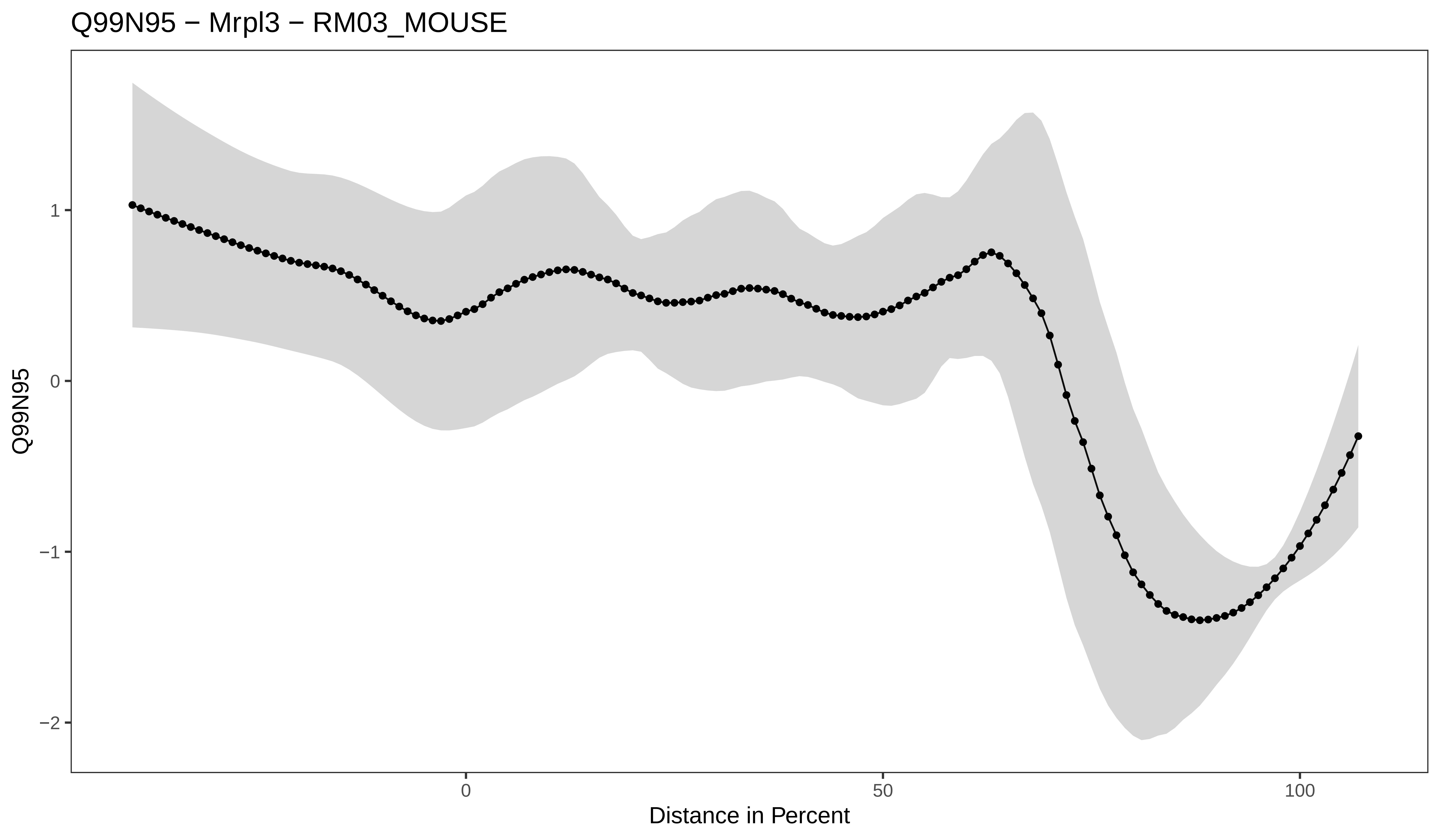

Q99N95 − Mrpl3 − RM03_MOUSE
Q99N95
1
0
−1
−2
0
50
100
Distance in Percent

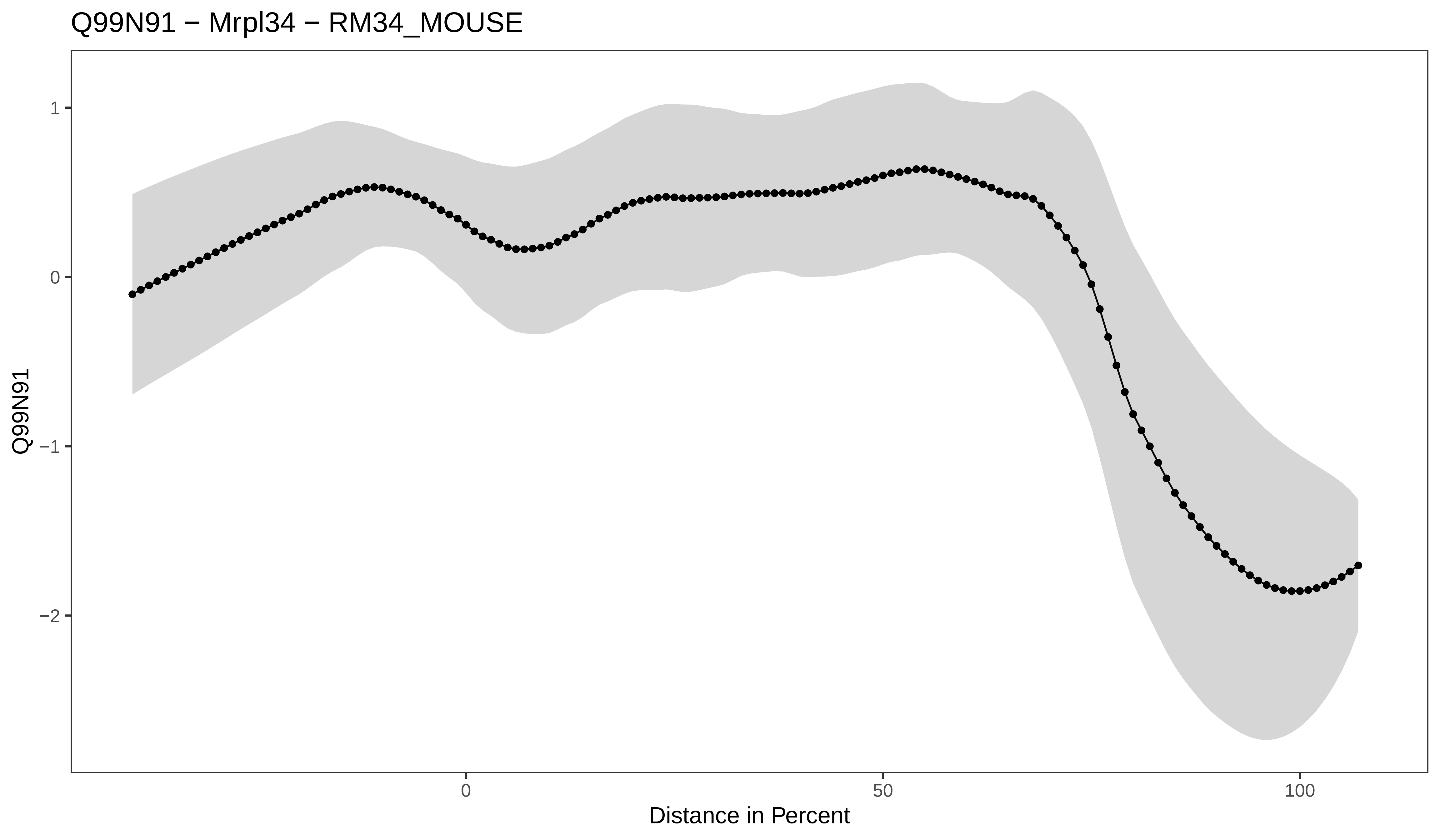
Q99N91 − Mrpl34 − RM34_MOUSE
Q99N91
1
0
−1
−2
0
50
100
Distance in Percent

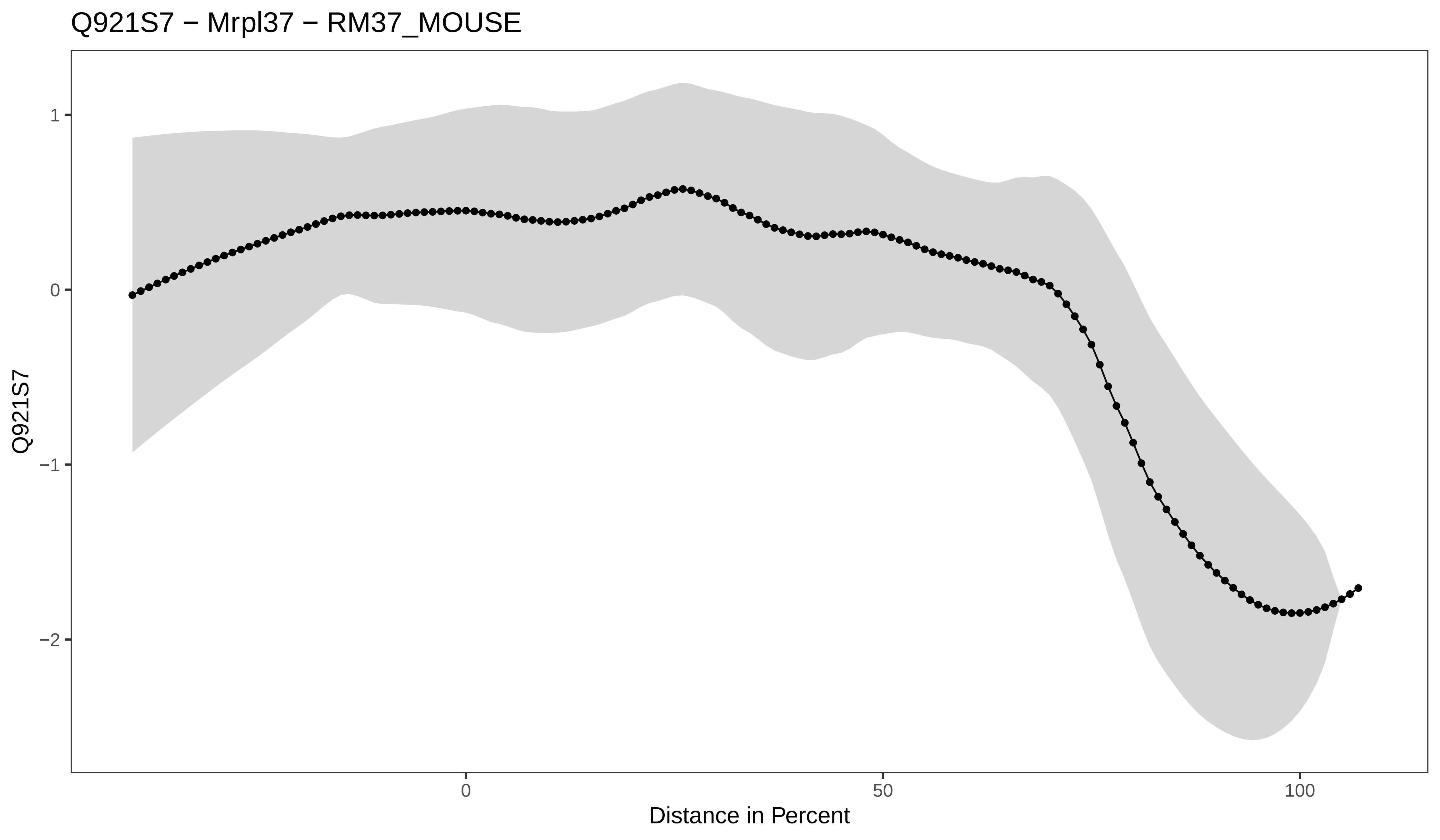

Q921S7 − Mrpl37 − RM37_MOUSE
Q921S7
1
0
−1
−2
0
50
100
Distance in Percent

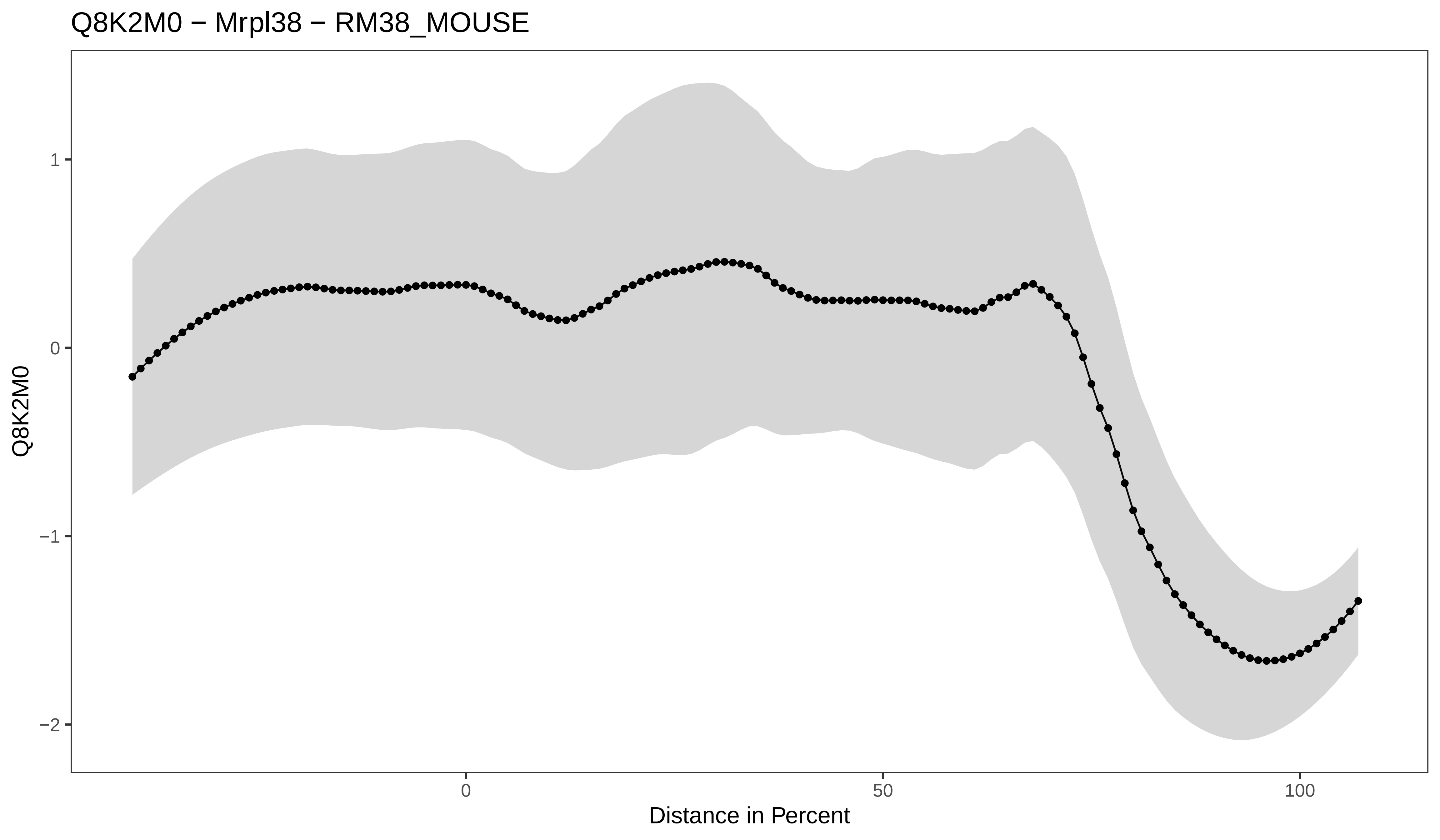
Q8K2M0 − Mrpl38 − RM38_MOUSE
Q8K2M0
Distance in Percent
1
0
−1
−2
0
50
100

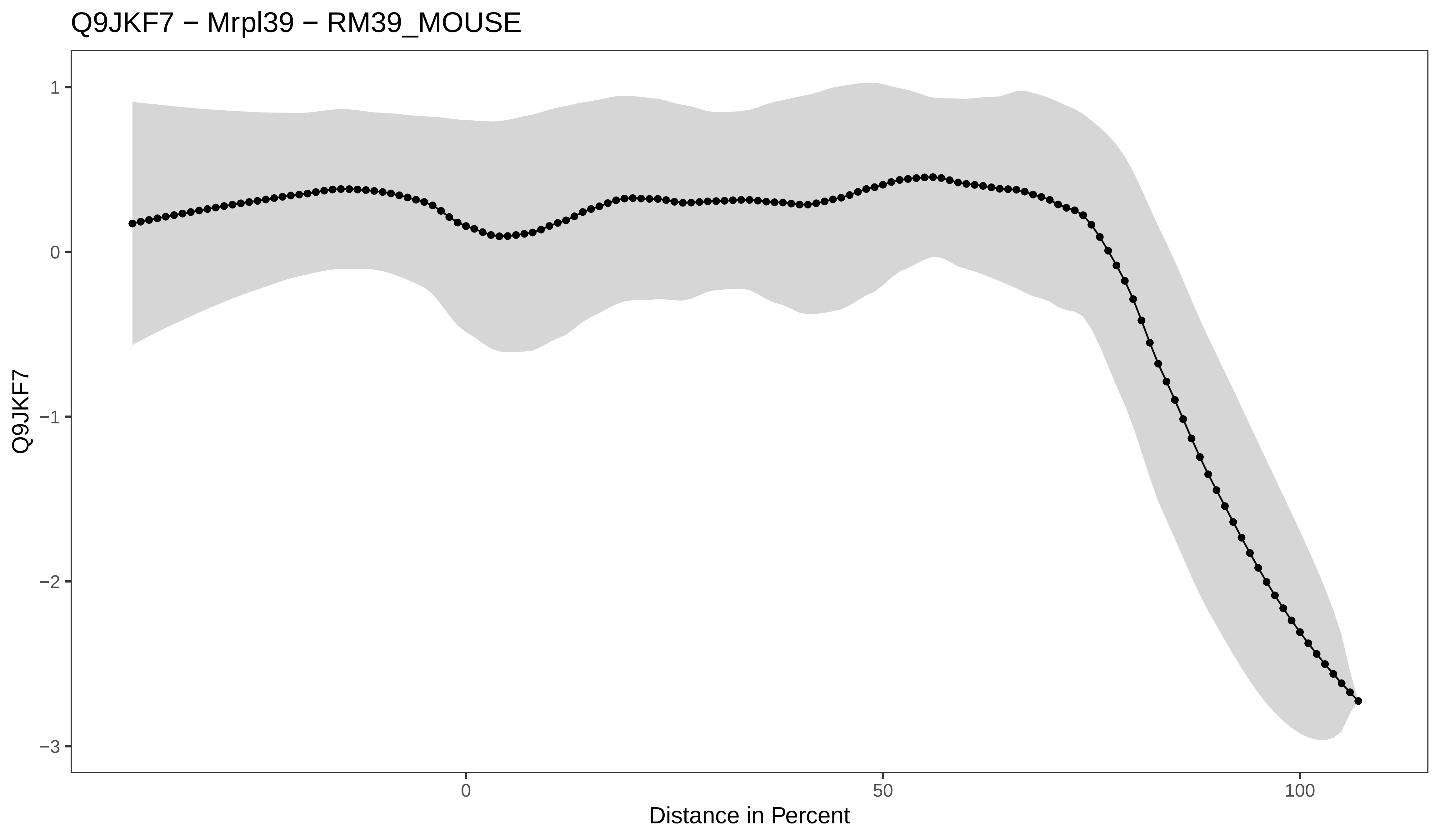

Q9JKF7 − Mrpl39 − RM39_MOUSE
1
0
−1
−2
−3
Q9JKF7
0
50
100
Distance in Percent

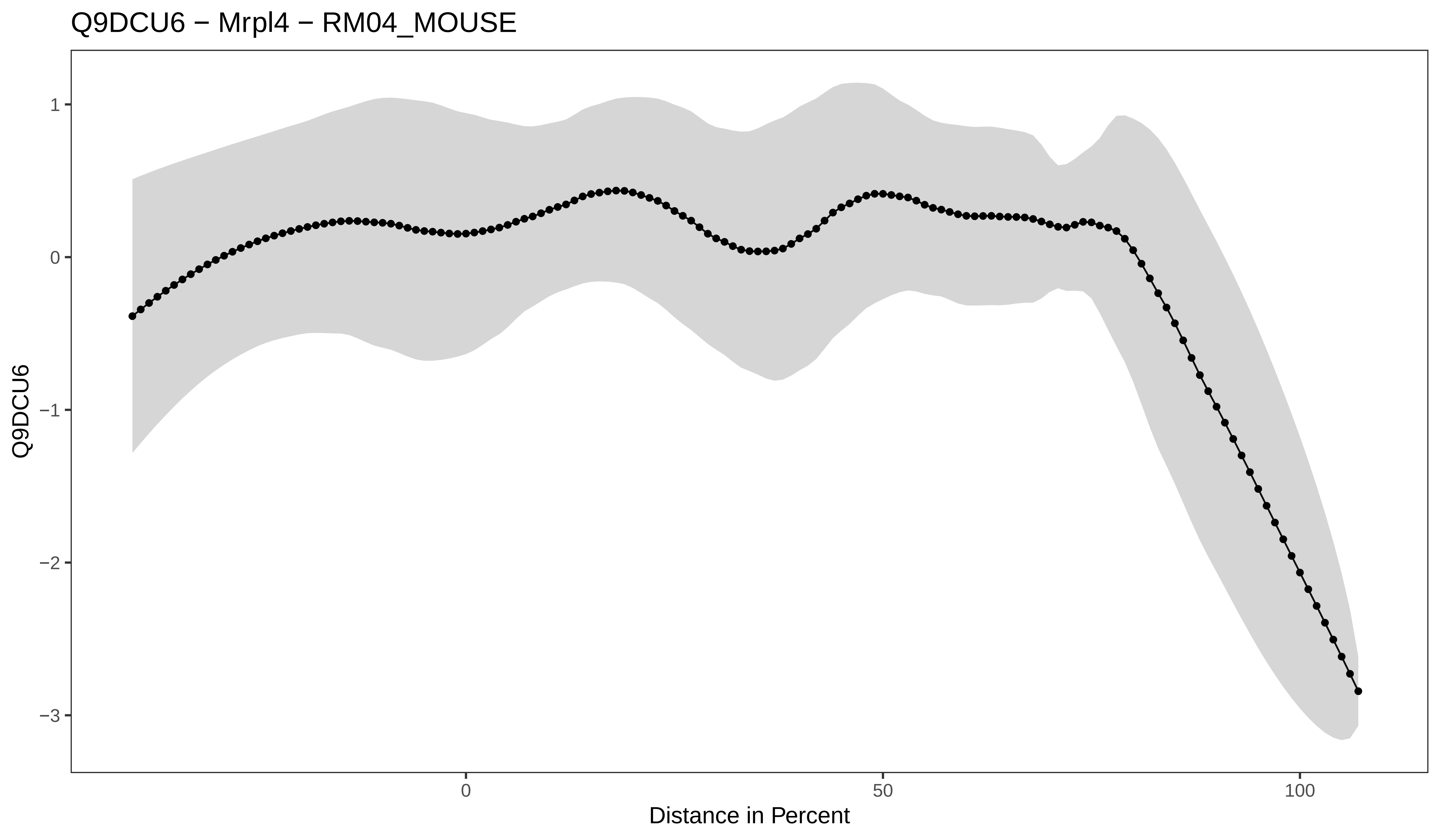

Q9DCU6 − Mrpl4 − RM04_MOUSE
Q9DCU6
Distance in Percent
1
0
−1
−2
−3
0
50
100

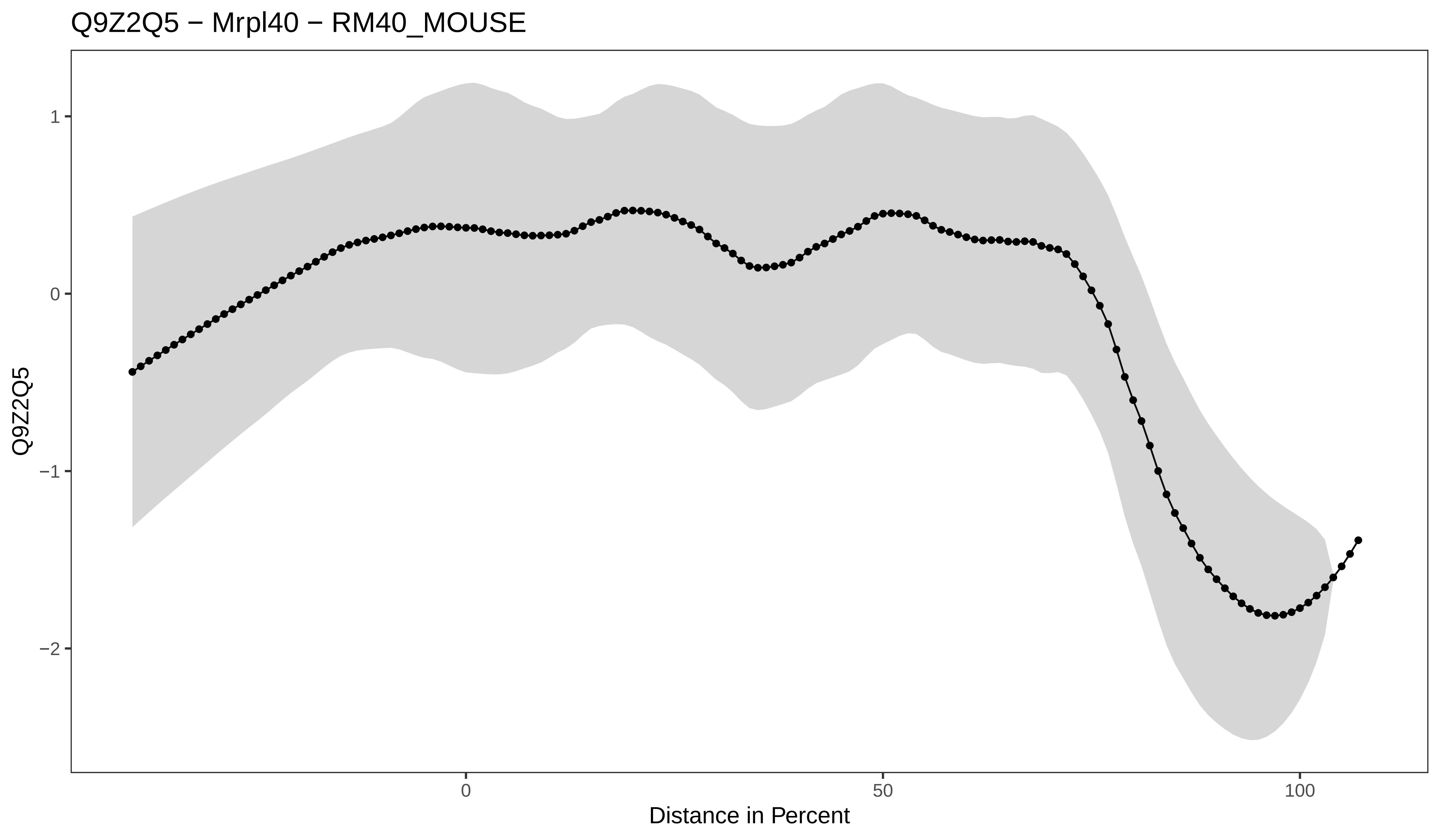
Q9Z2Q5 − Mrpl40 − RM40_MOUSE
1
0
−1
−2
Q9Z2Q5
0
50
100
Distance in Percent

Q9CQN7 − Mrpl41 − RM41_MOUSE

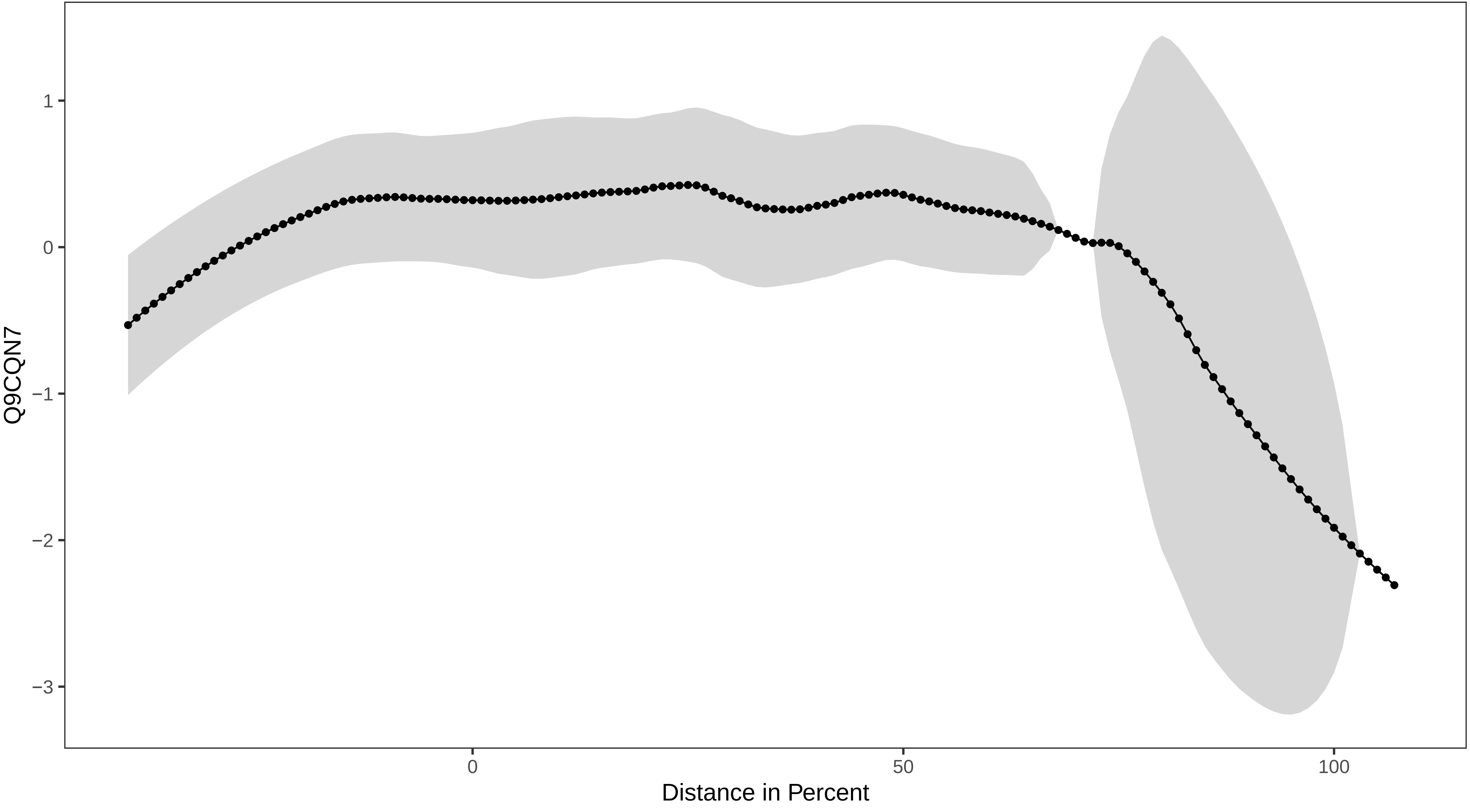

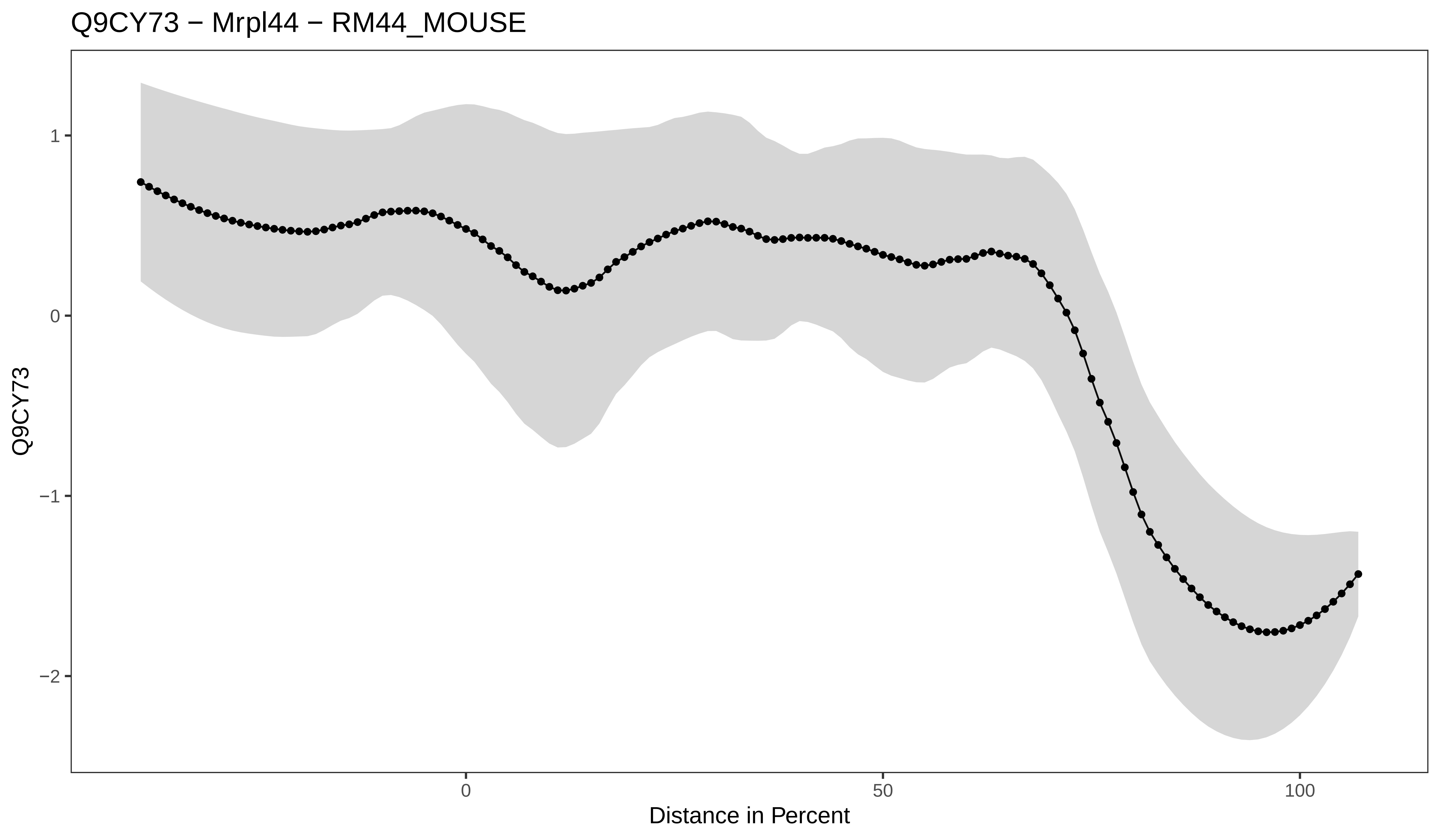
Q9CY73 − Mrpl44 − RM44_MOUSE
1
0
−1
−2
Q9CY73
0
50
100
Distance in Percent

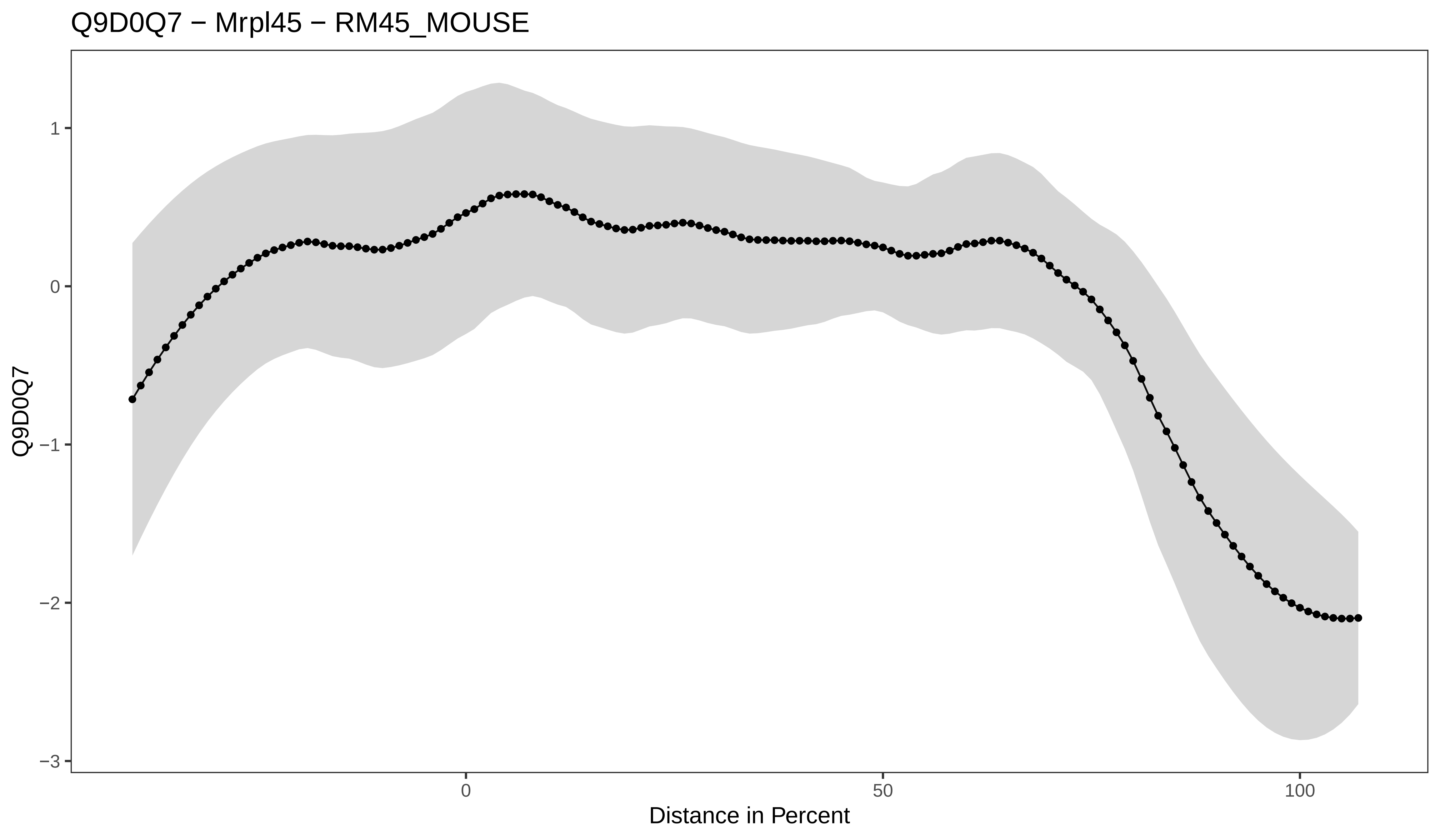
Q9D0Q7 − Mrpl45 − RM45_MOUSE
1
0
−1
−2
−3
Q9D0Q7
0
50
100
Distance in Percent

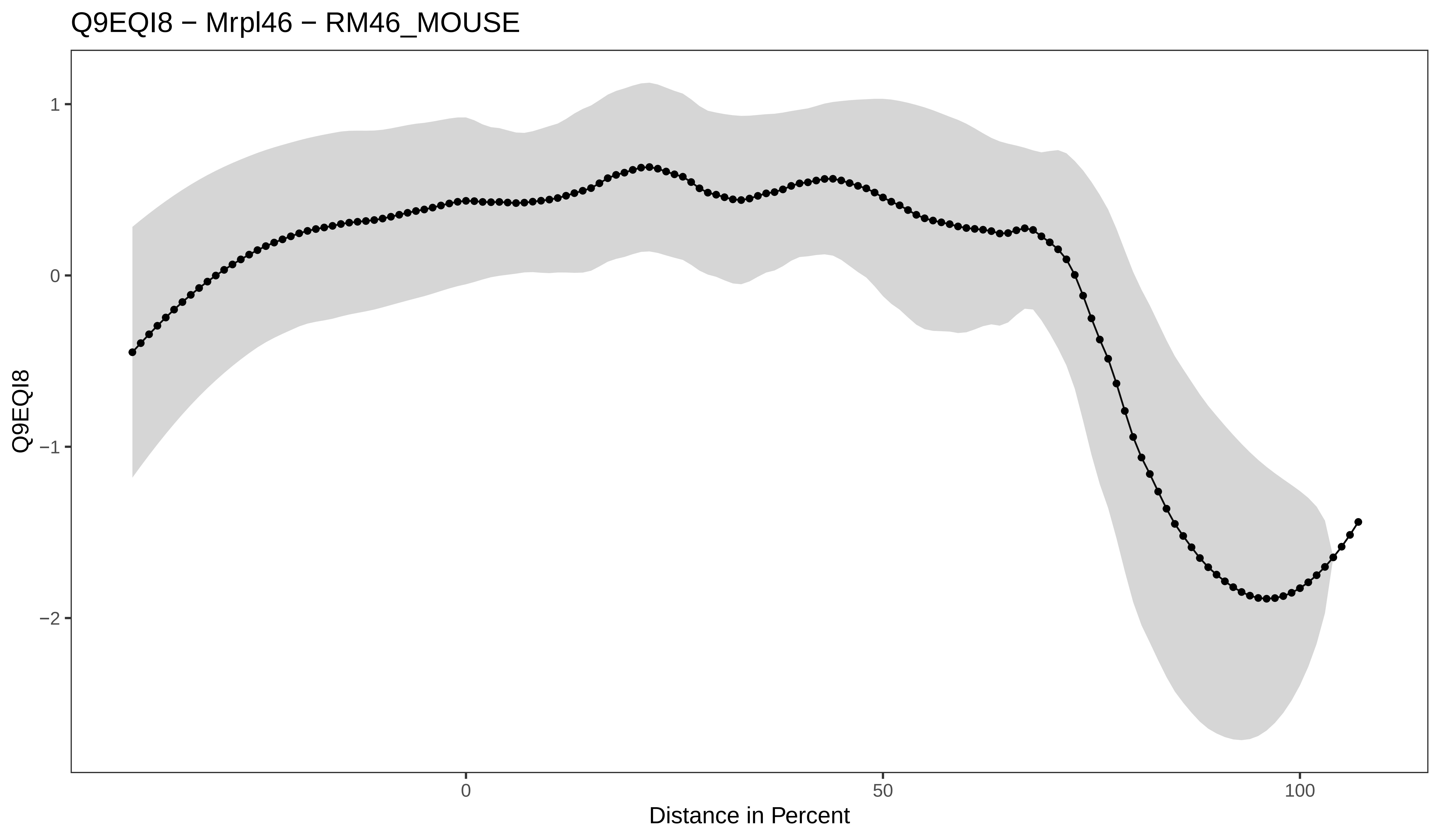

Q9EQI8 − Mrpl46 − RM46_MOUSE
Q9EQI8
1
0
−1
−2
0
50
100
Distance in Percent

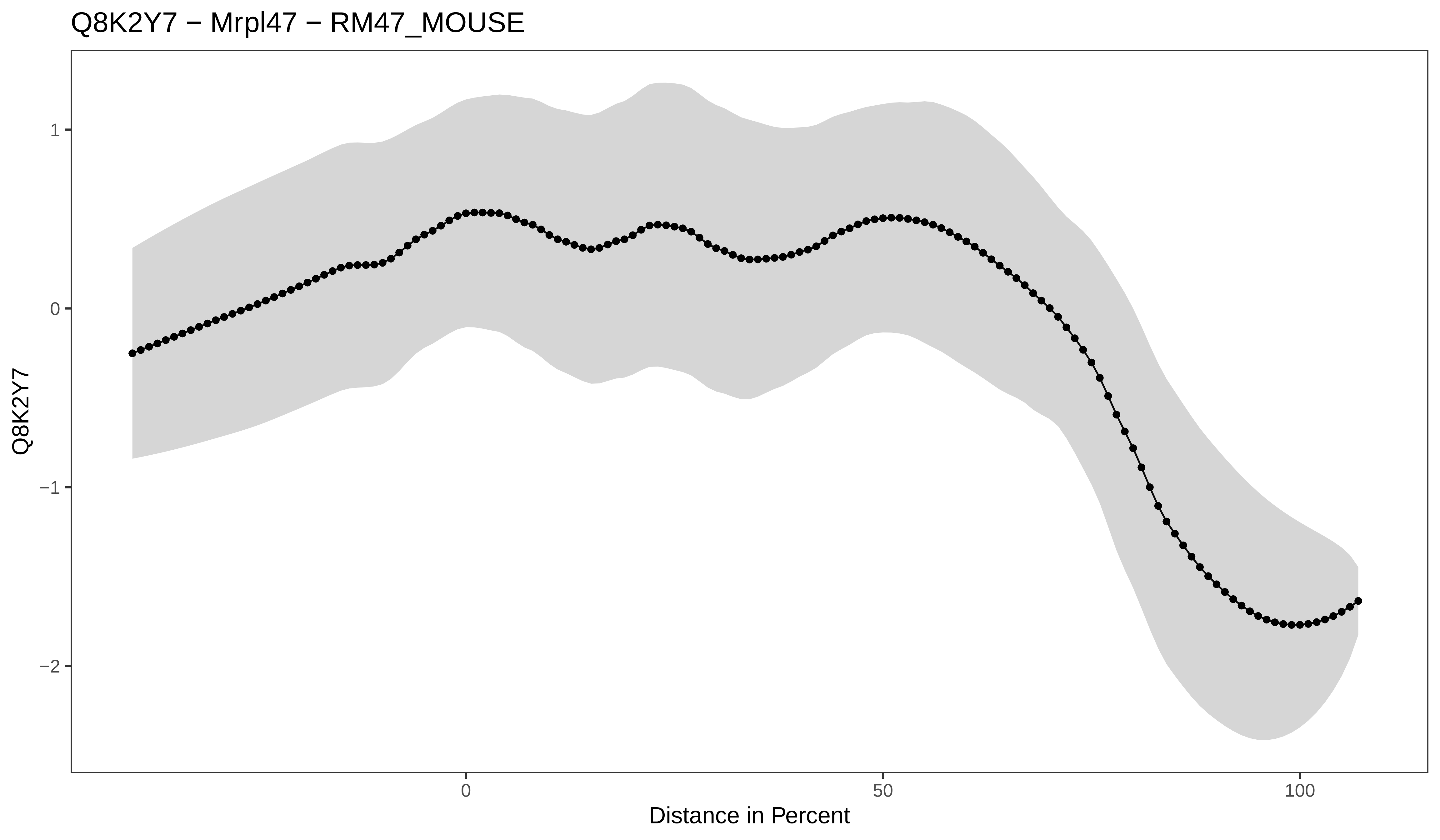
Q8K2Y7 − Mrpl47 − RM47_MOUSE
Q8K2Y7
1
0
−1
−2
0
50
100
Distance in Percent

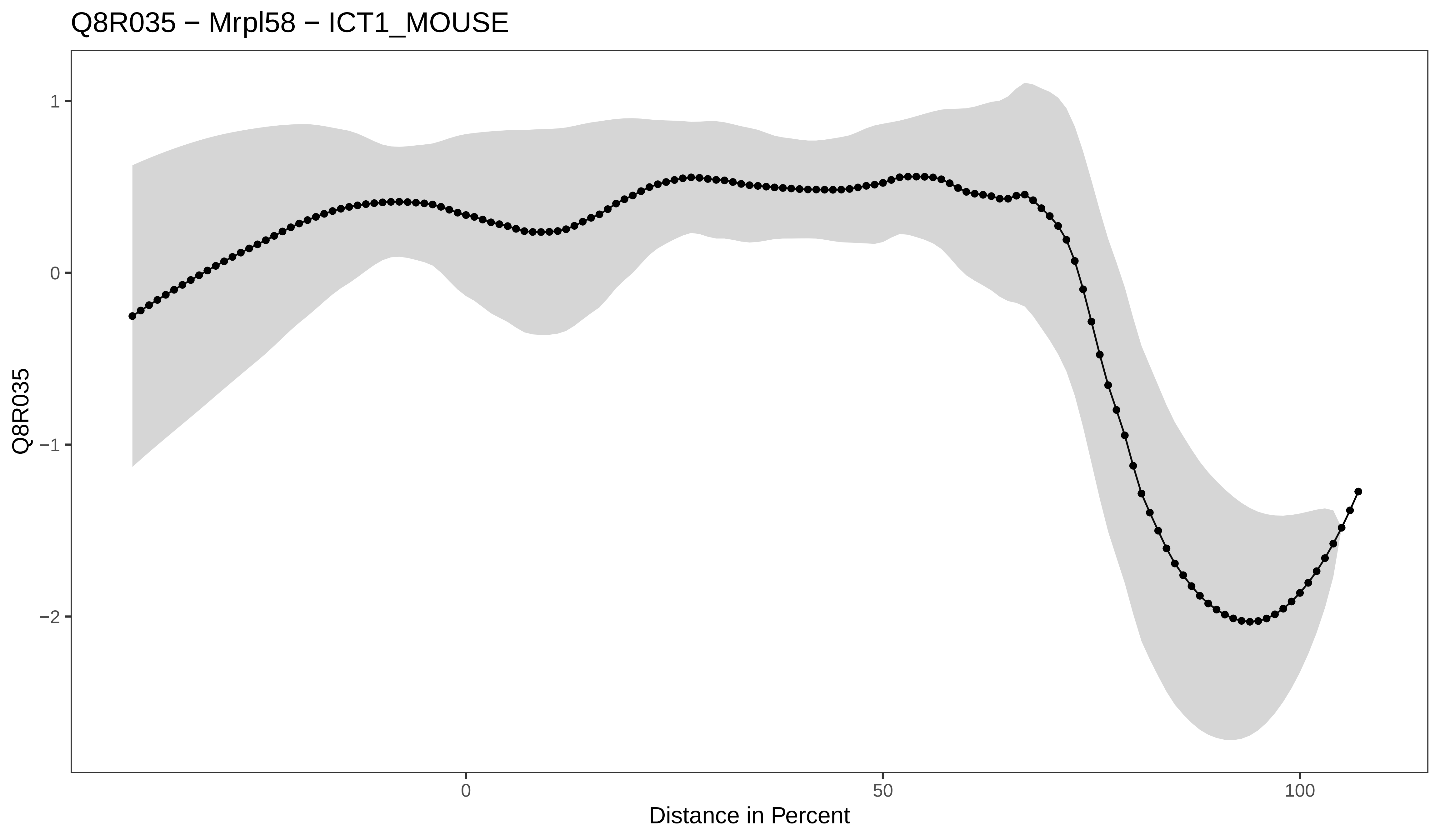

Q8R035 − Mrpl58 − ICT1_MOUSE
1
0
−1
−2
Q8R035
0
50
100
Distance in Percent

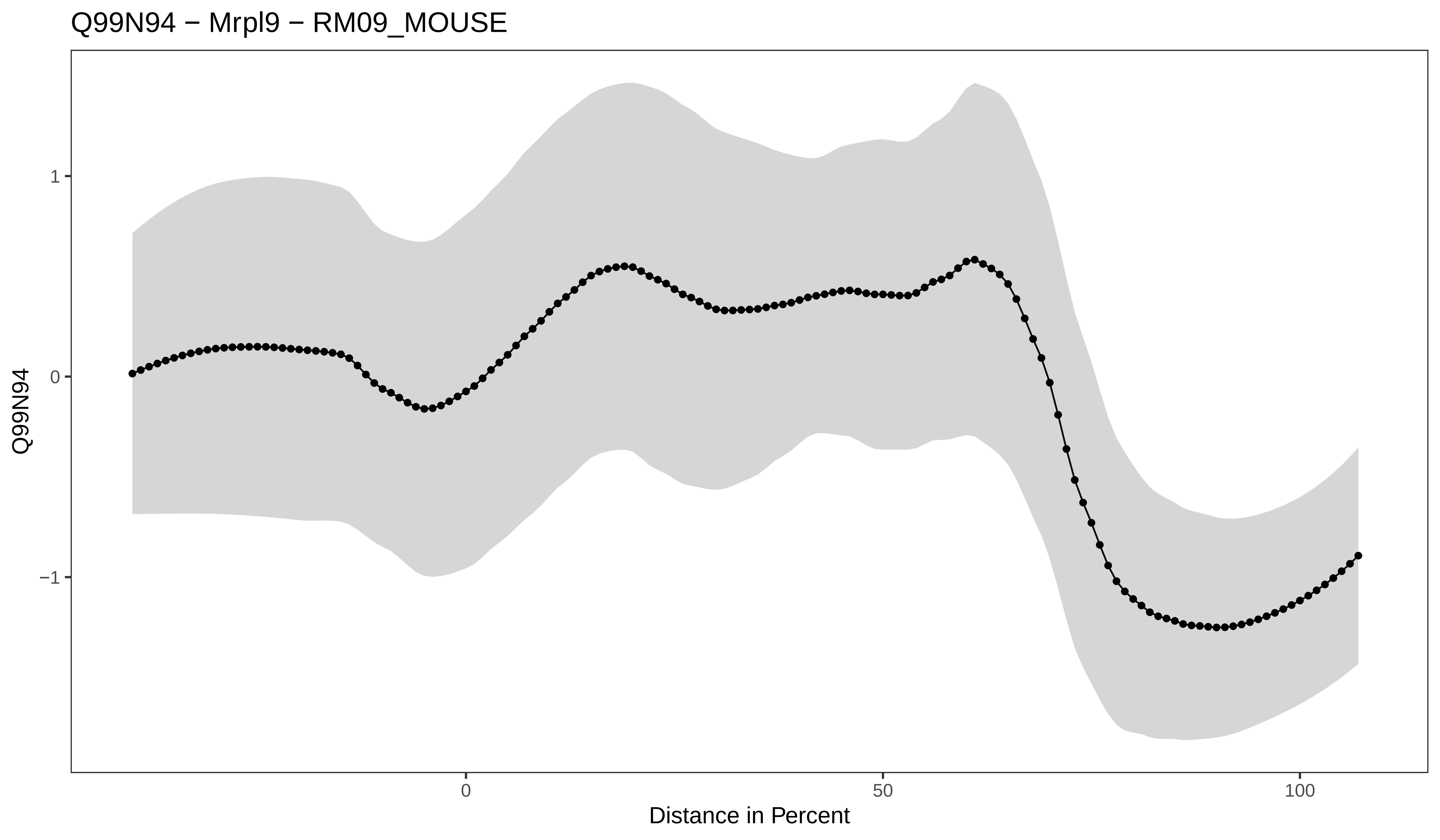
Q99N94 − Mrpl9 − RM09_MOUSE
1
0
−1
Q99N94
0
50
100
Distance in Percent

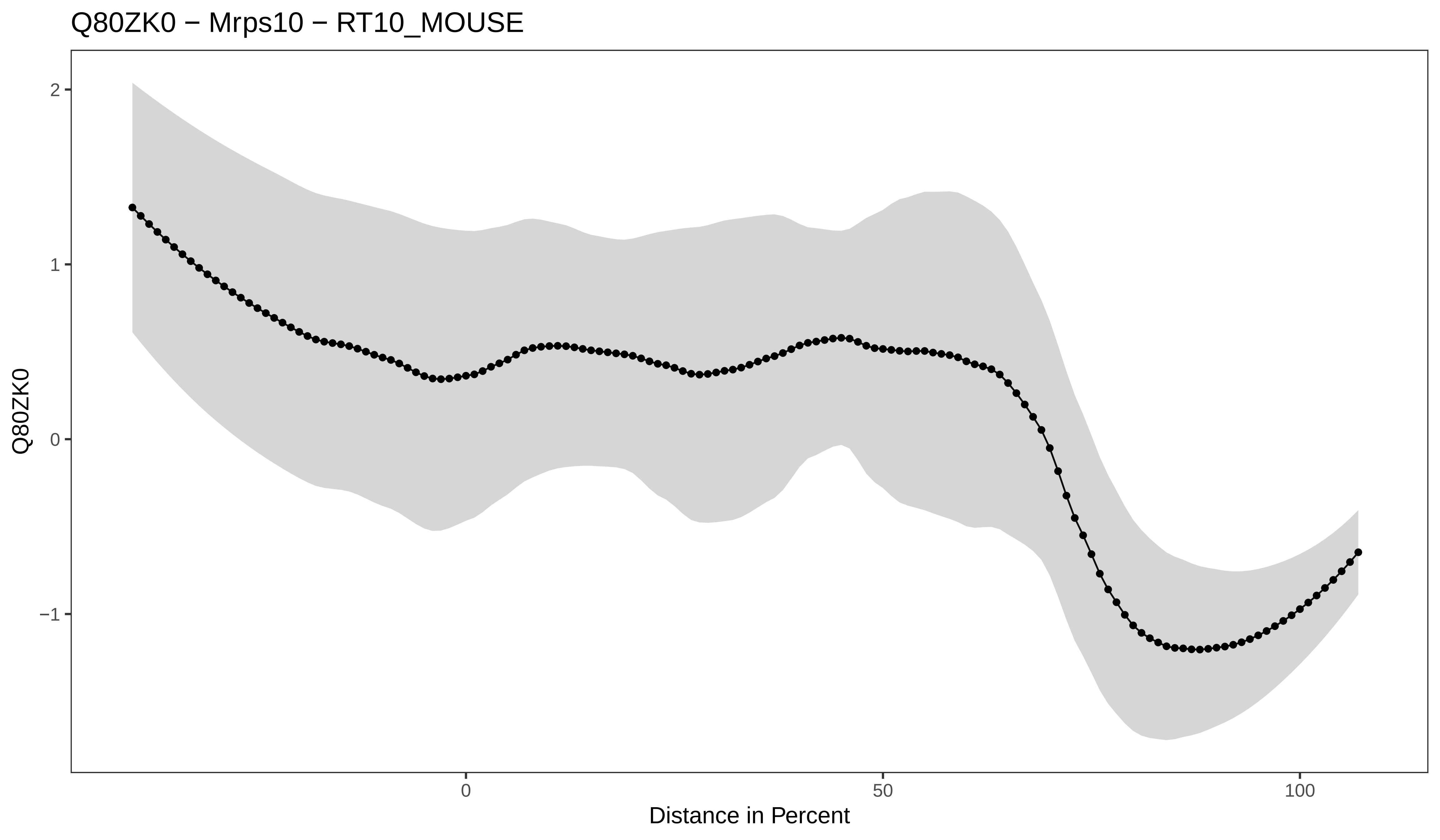

Q80ZK0 − Mrps10 − RT10_MOUSE
Q80ZK0
2
1
0
−1
0
50
100
Distance in Percent

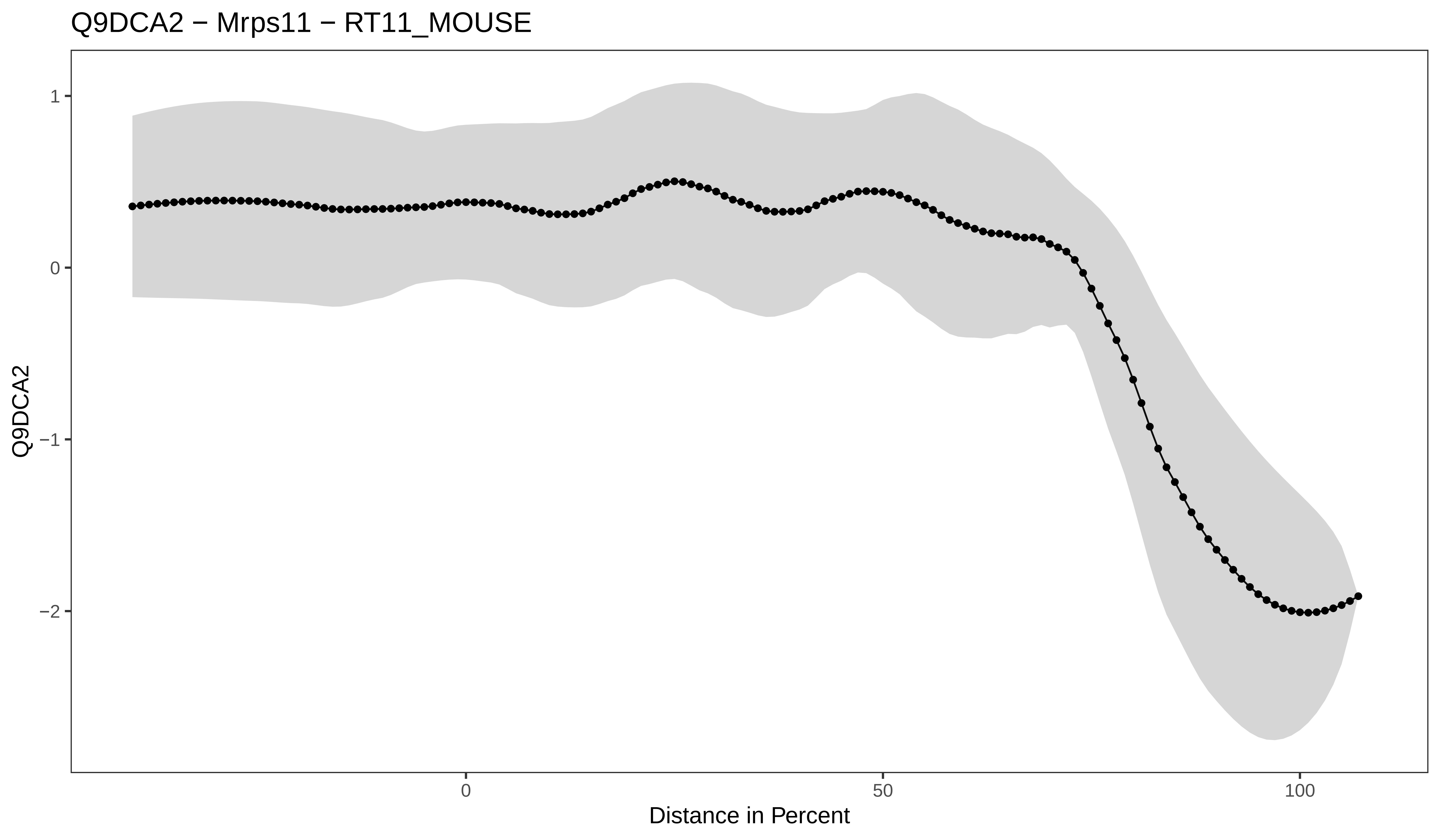

Q9DCA2 − Mrps11 − RT11_MOUSE
Q9DCA2
1
0
−1
−2
0
50
100
Distance in Percent

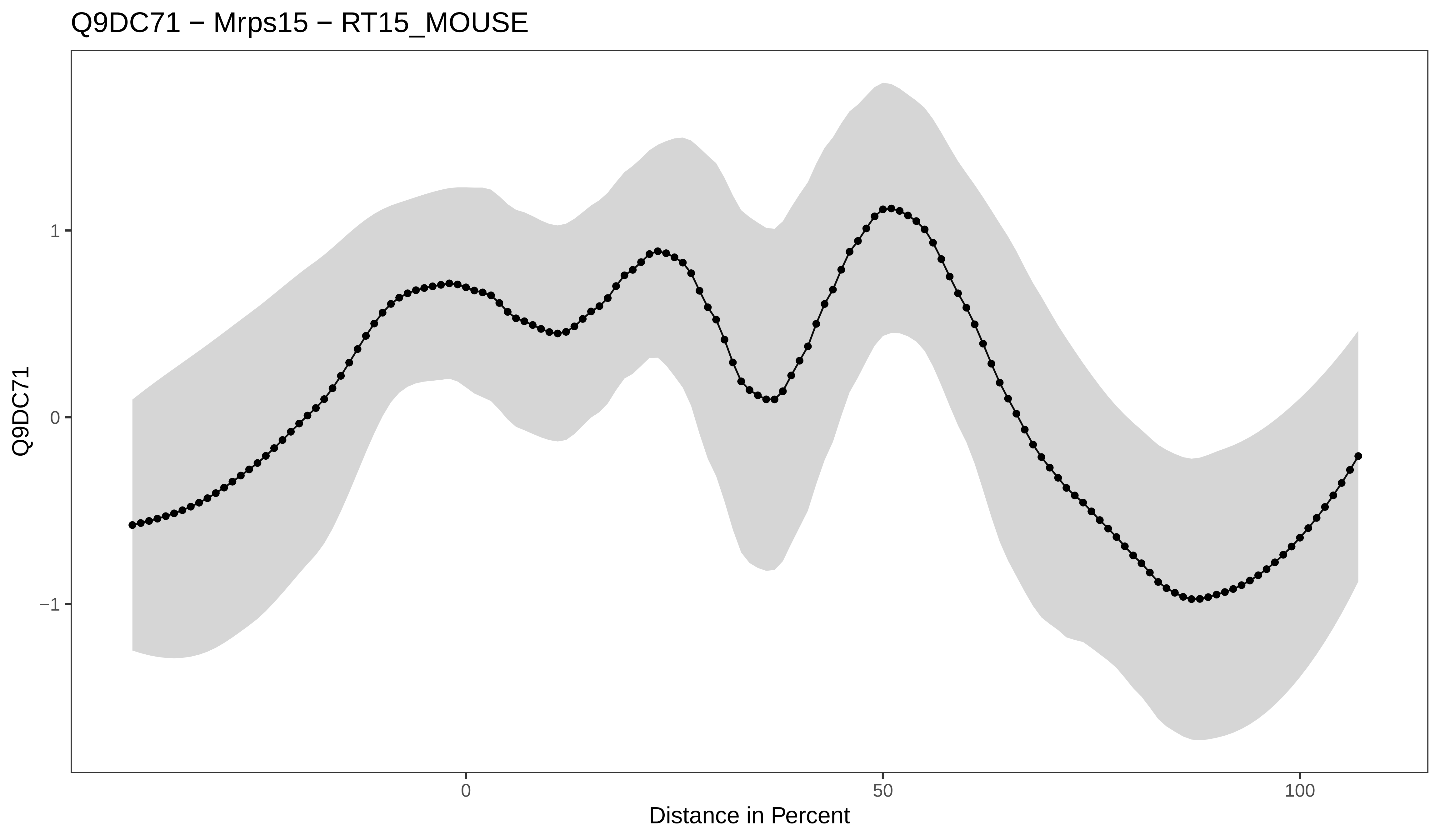

Q9DC71 − Mrps15 − RT15_MOUSE
Q9DC71
1
0
−1
0
50
100
Distance in Percent

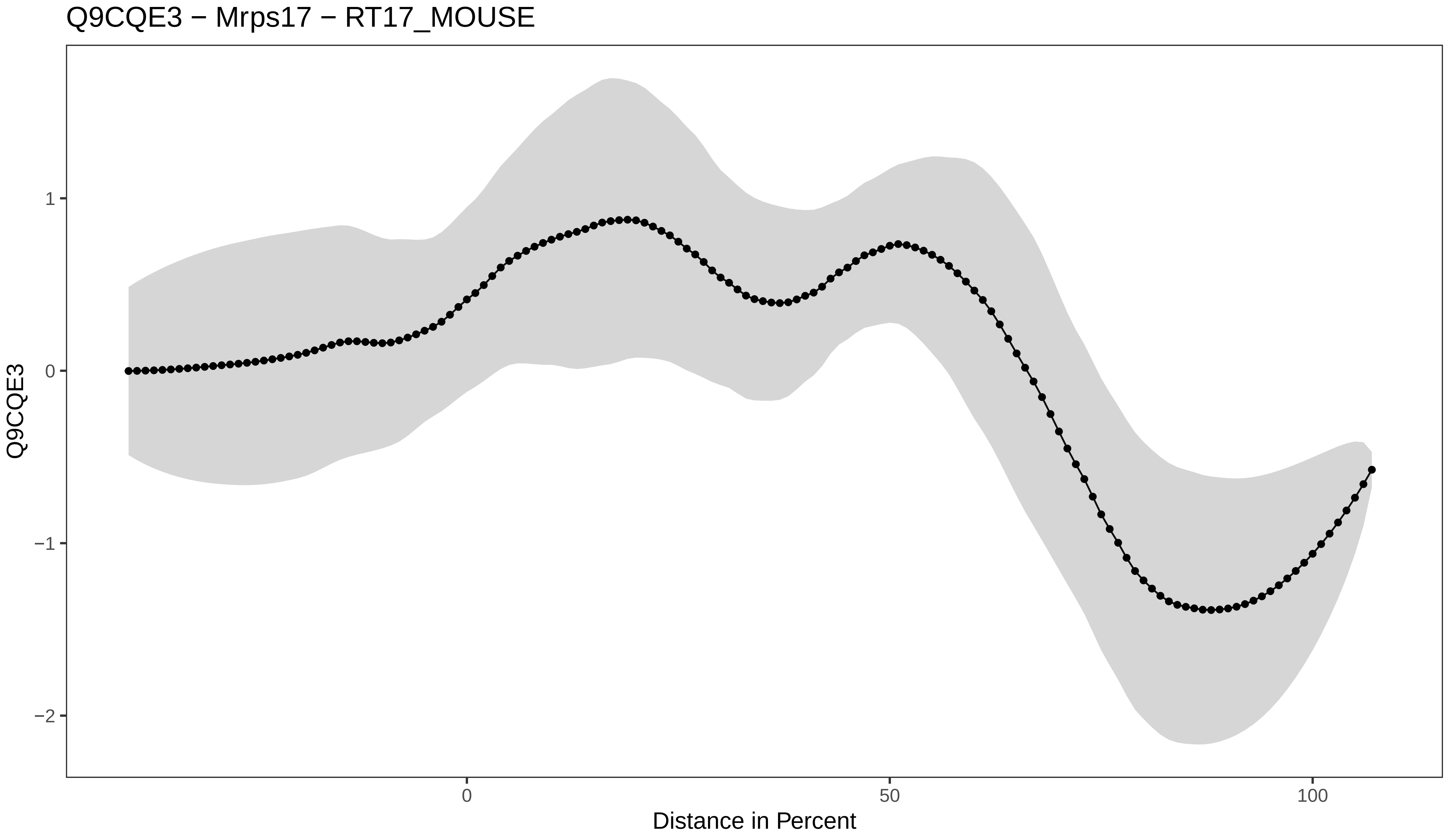

Q9CQE3 − Mrps17 − RT17_MOUSE
Q9CQE3
Distance in Percent
1
0
−1
−2
0
50
100

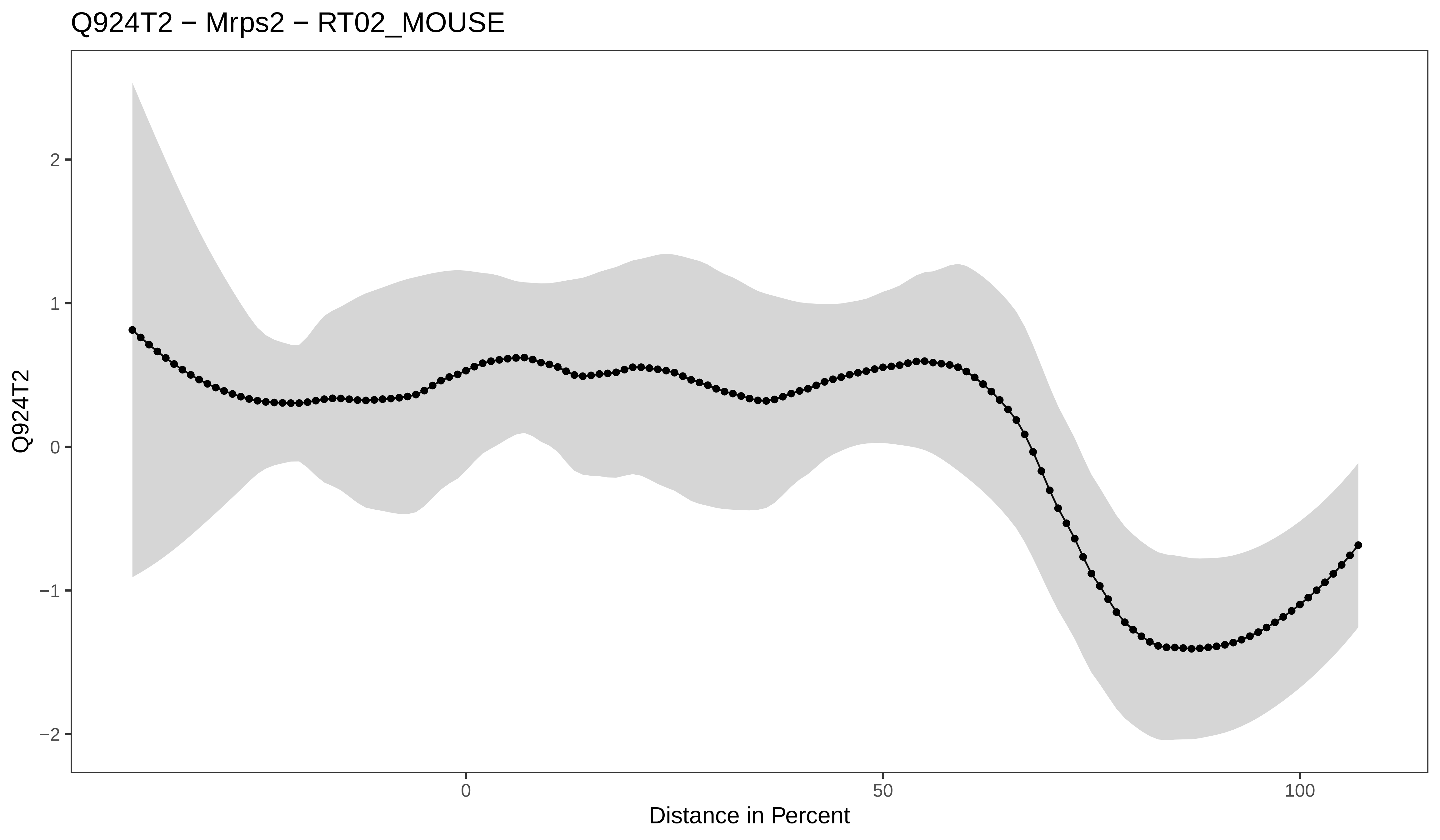
Q924T2 − Mrps2 − RT02_MOUSE
Q924T2
Distance in Percent
2
1
0
−1
−2
0
50
100

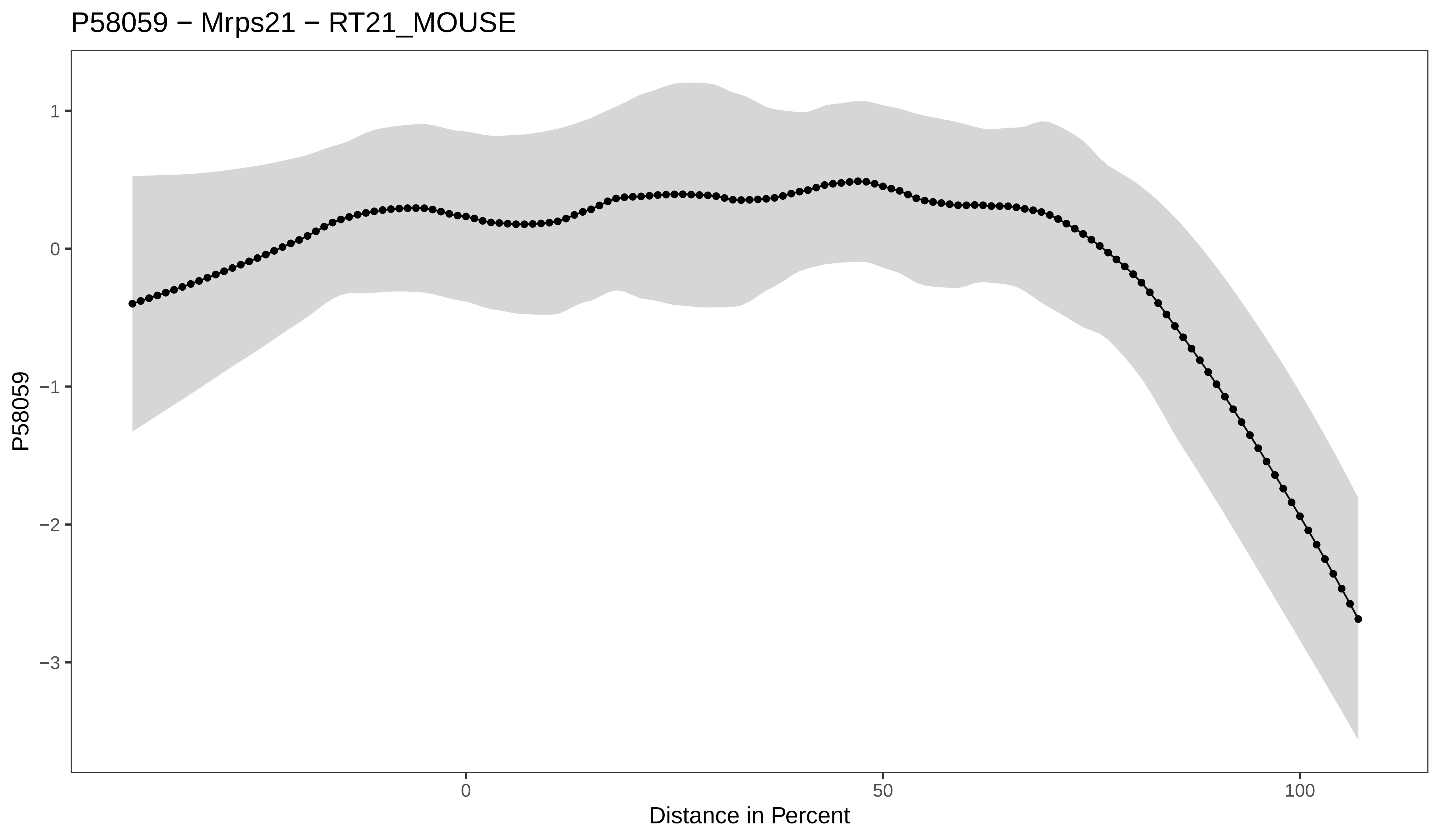
P58059 − Mrps21 − RT21_MOUSE
1
0
−1
−2
−3
P58059
0
50
100
Distance in Percent

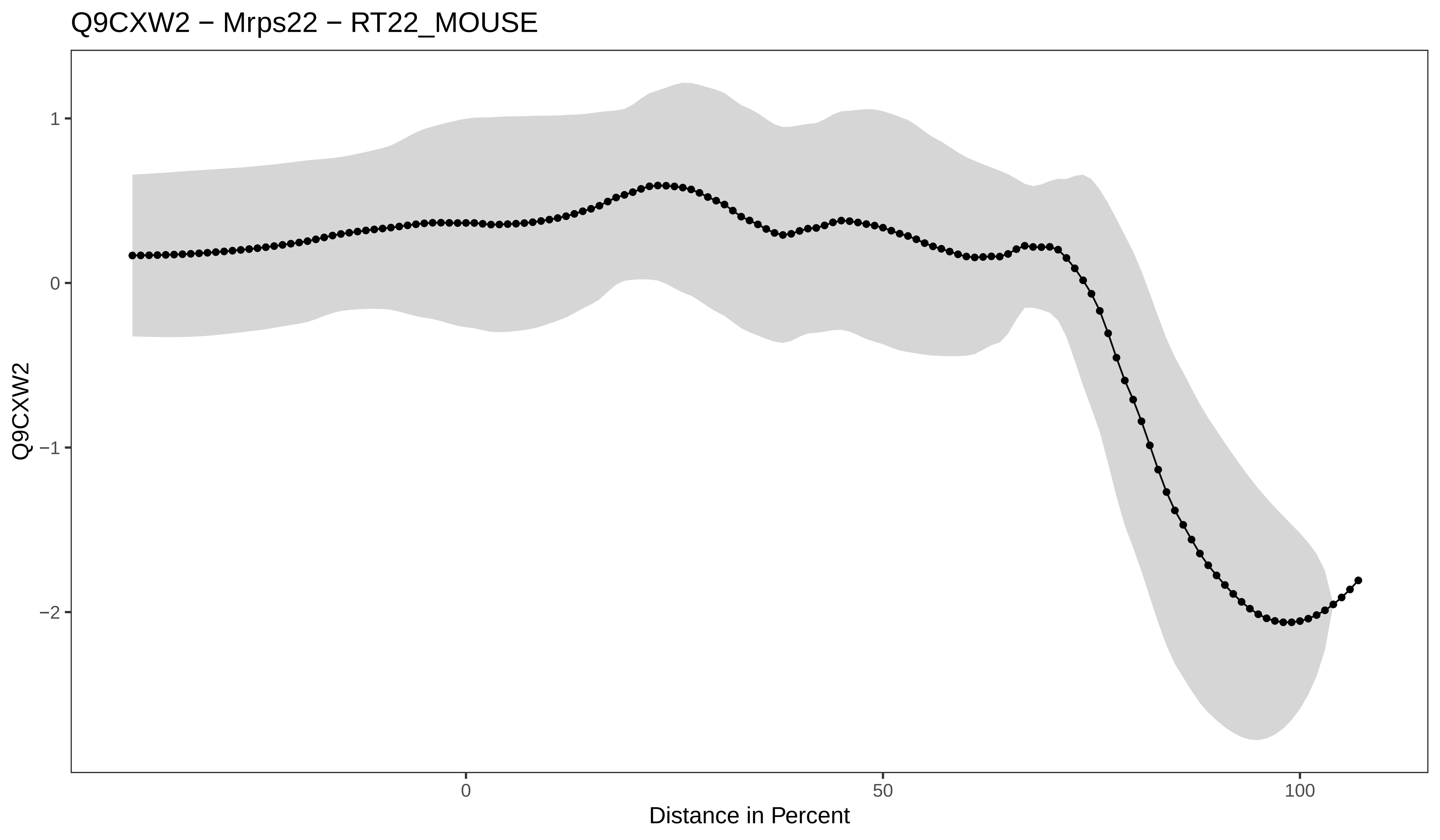
Q9CXW2 − Mrps22 − RT22_MOUSE
Q9CXW2
1
0
−1
−2
0
50
100
Distance in Percent

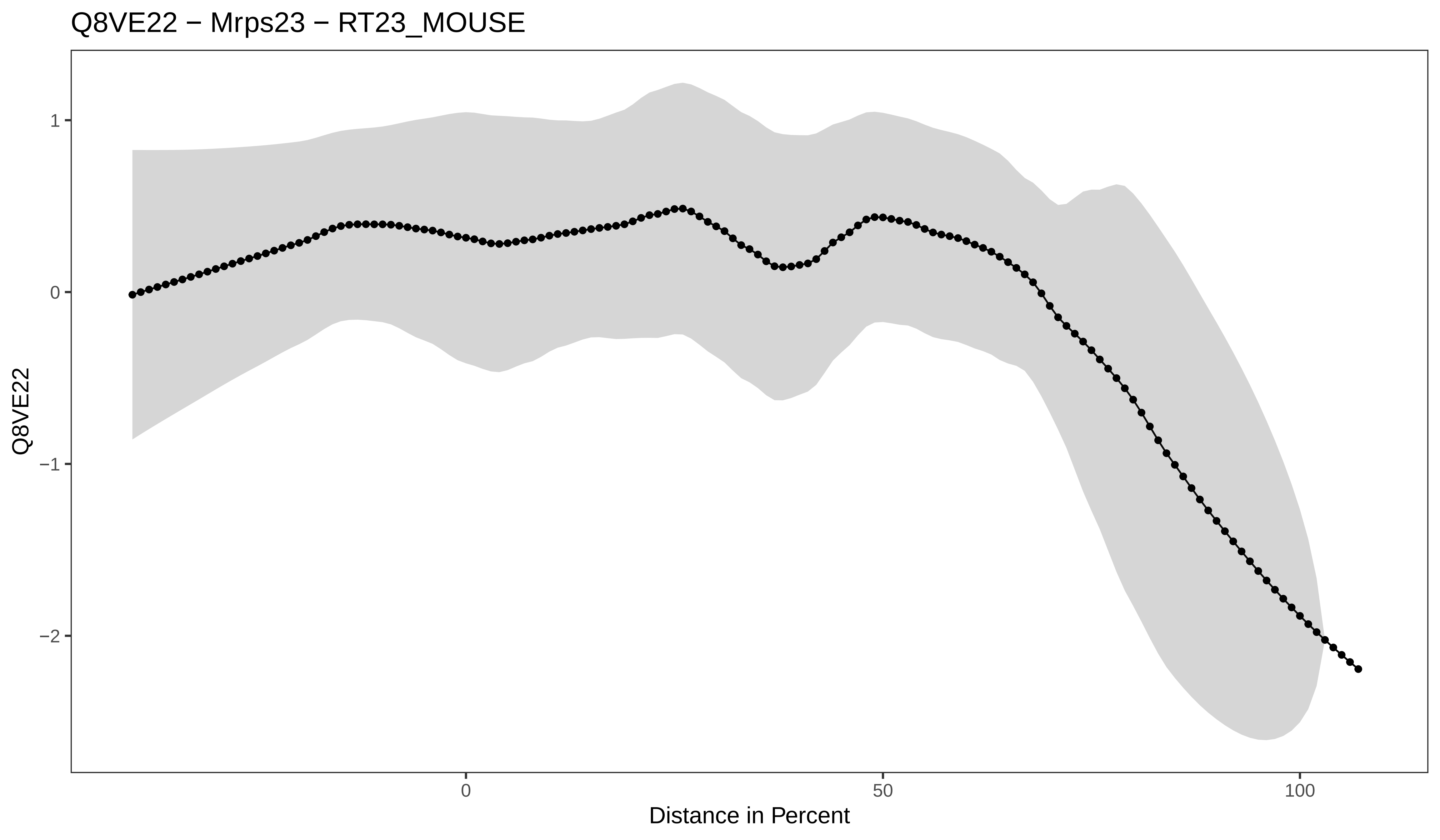

Q8VE22 − Mrps23 − RT23_MOUSE
Q8VE22
Distance in Percent
1
0
−1
−2
0
50
100

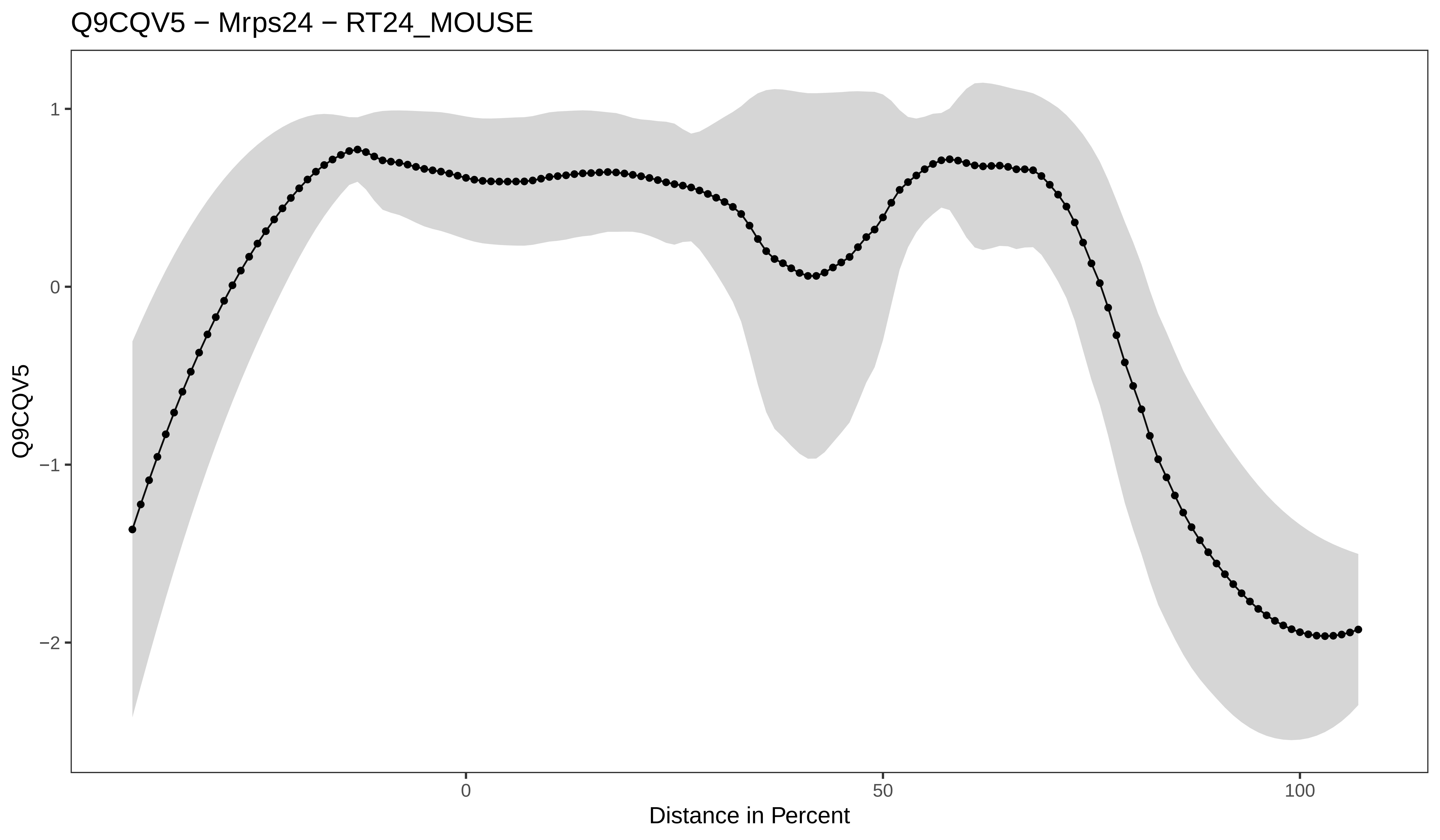
Q9CQV5 − Mrps24 − RT24_MOUSE
Q9CQV5
Distance in Percent
1
0
−1
−2
0
50
100

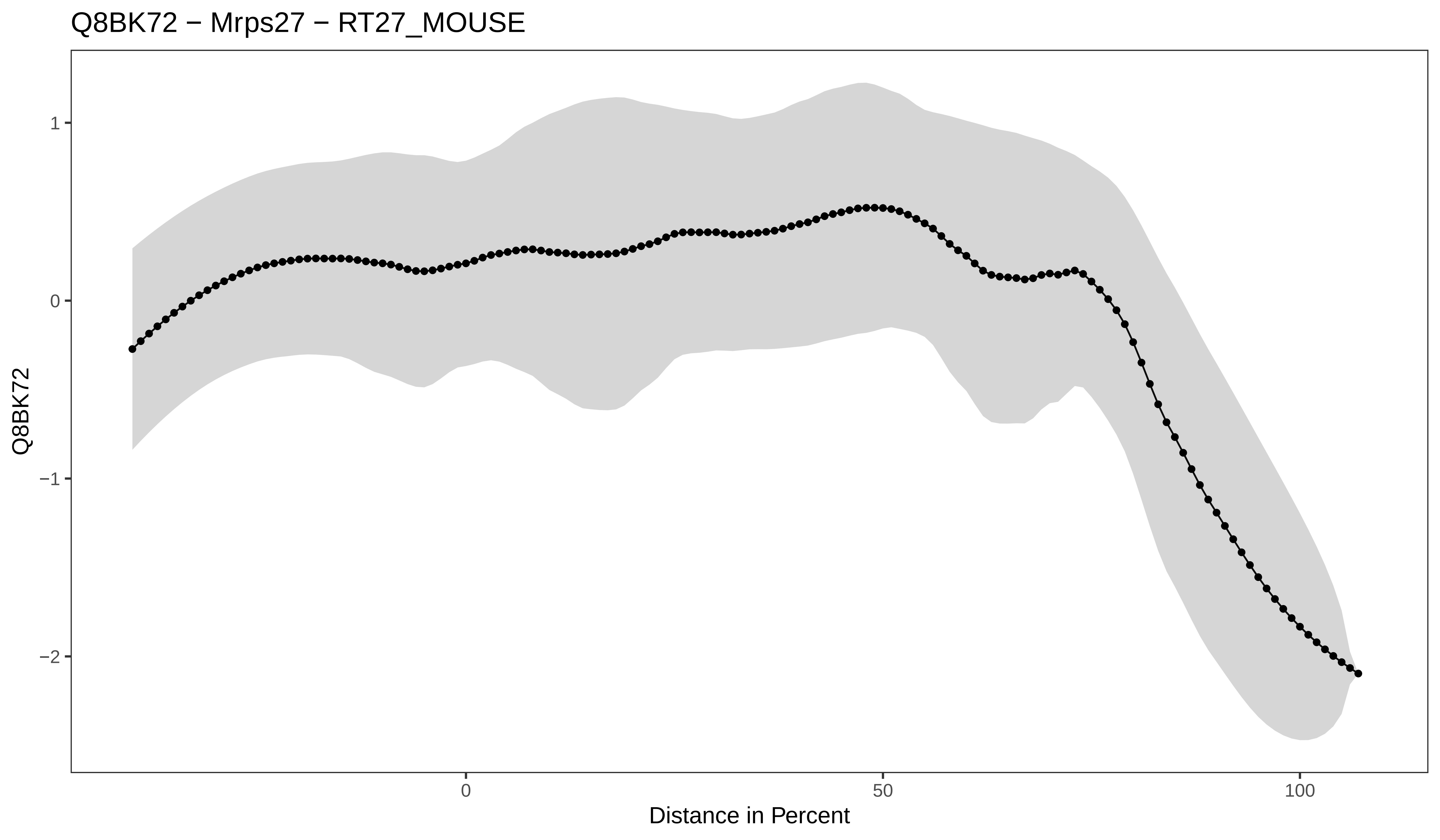

Q8BK72 − Mrps27 − RT27_MOUSE
1
0
−1
−2
Q8BK72
0
50
100
Distance in Percent

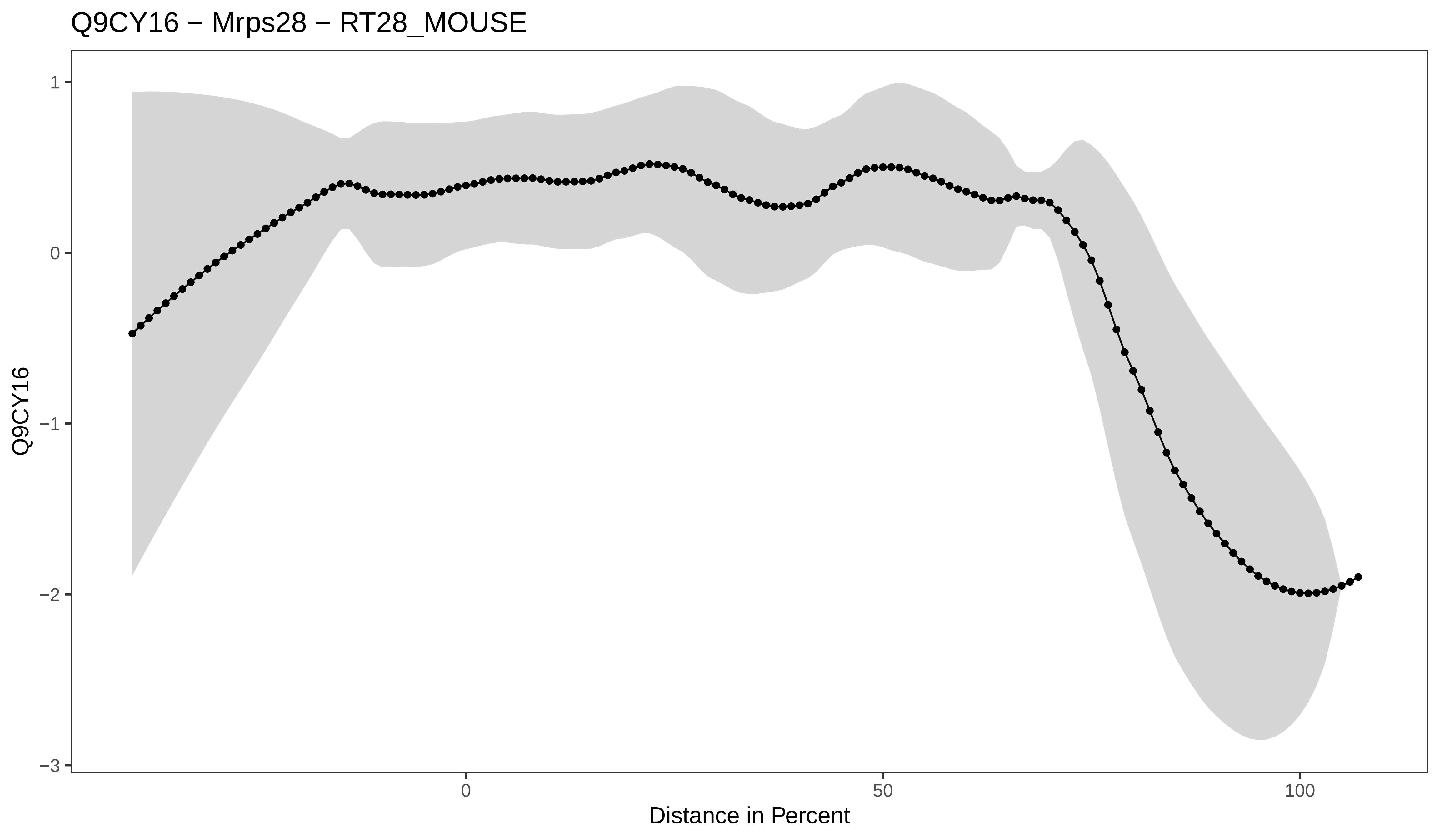

Q9CY16 − Mrps28 − RT28_MOUSE
Q9CY16
1
0
−1
−2
−3
0
50
100
Distance in Percent

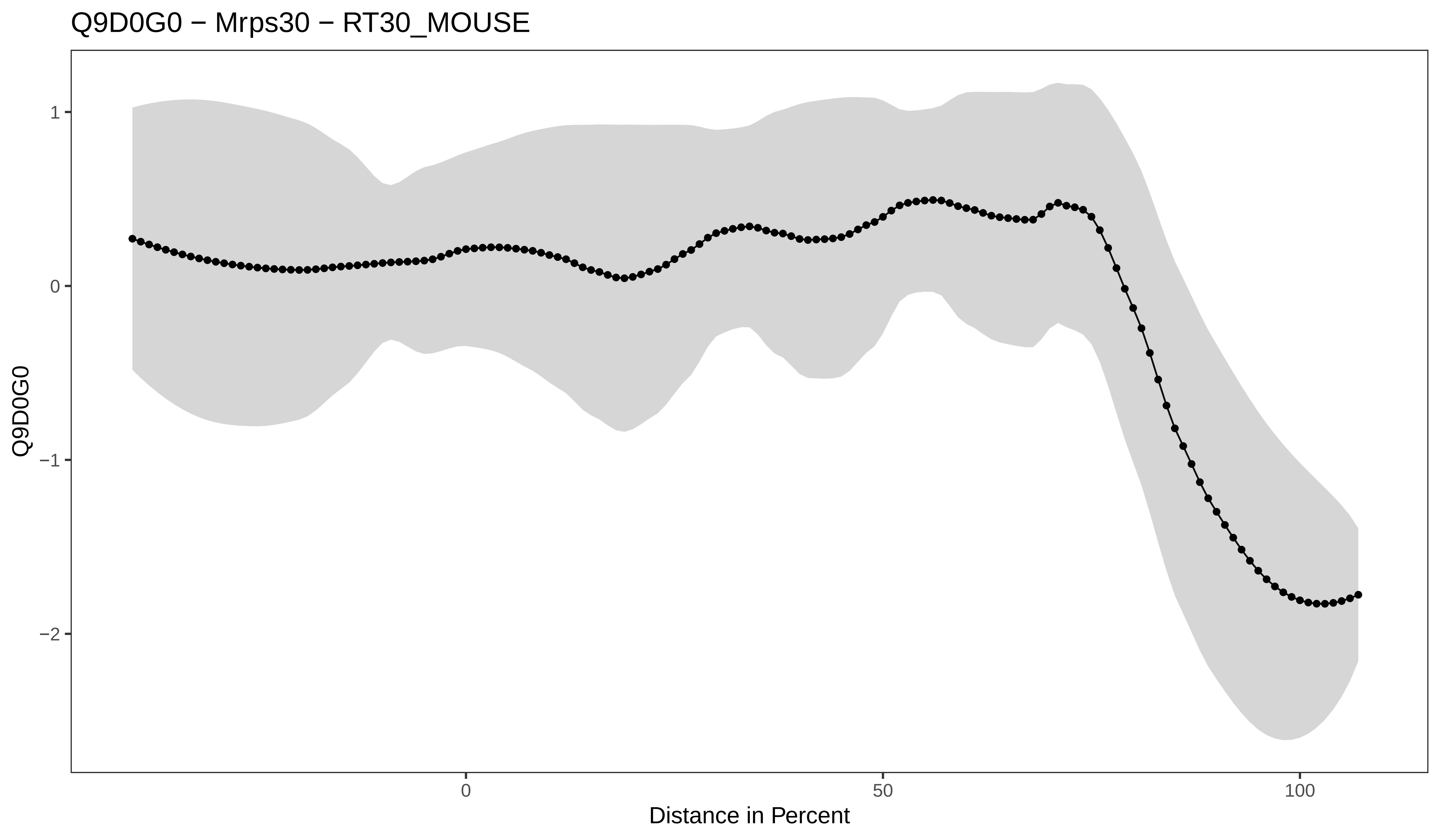

Q9D0G0 − Mrps30 − RT30_MOUSE
1
0
−1
−2
Q9D0G0
0
50
100
Distance in Percent

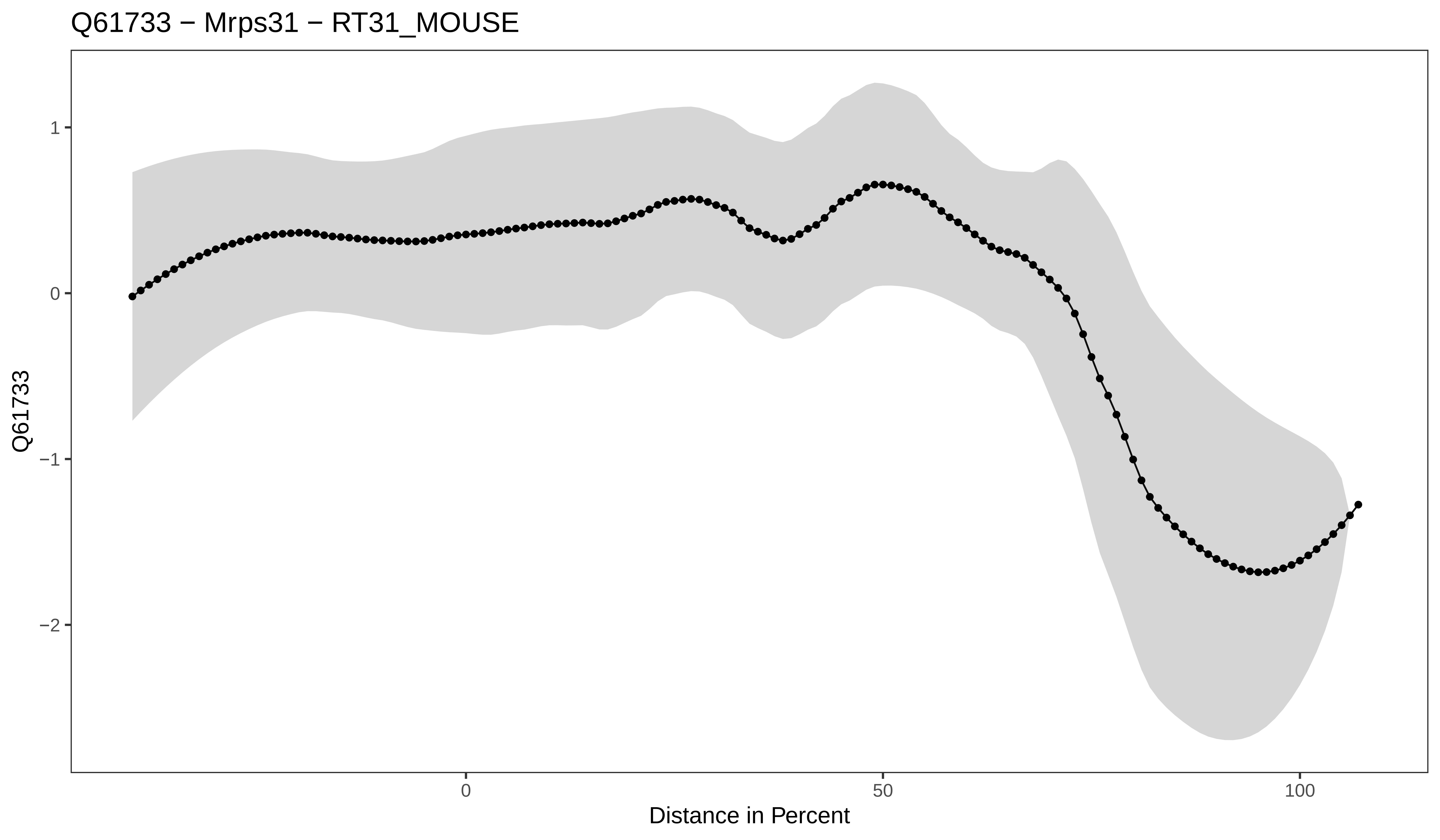

Q61733 − Mrps31 − RT31_MOUSE
Q61733
1
0
−1
−2
0
50
100
Distance in Percent

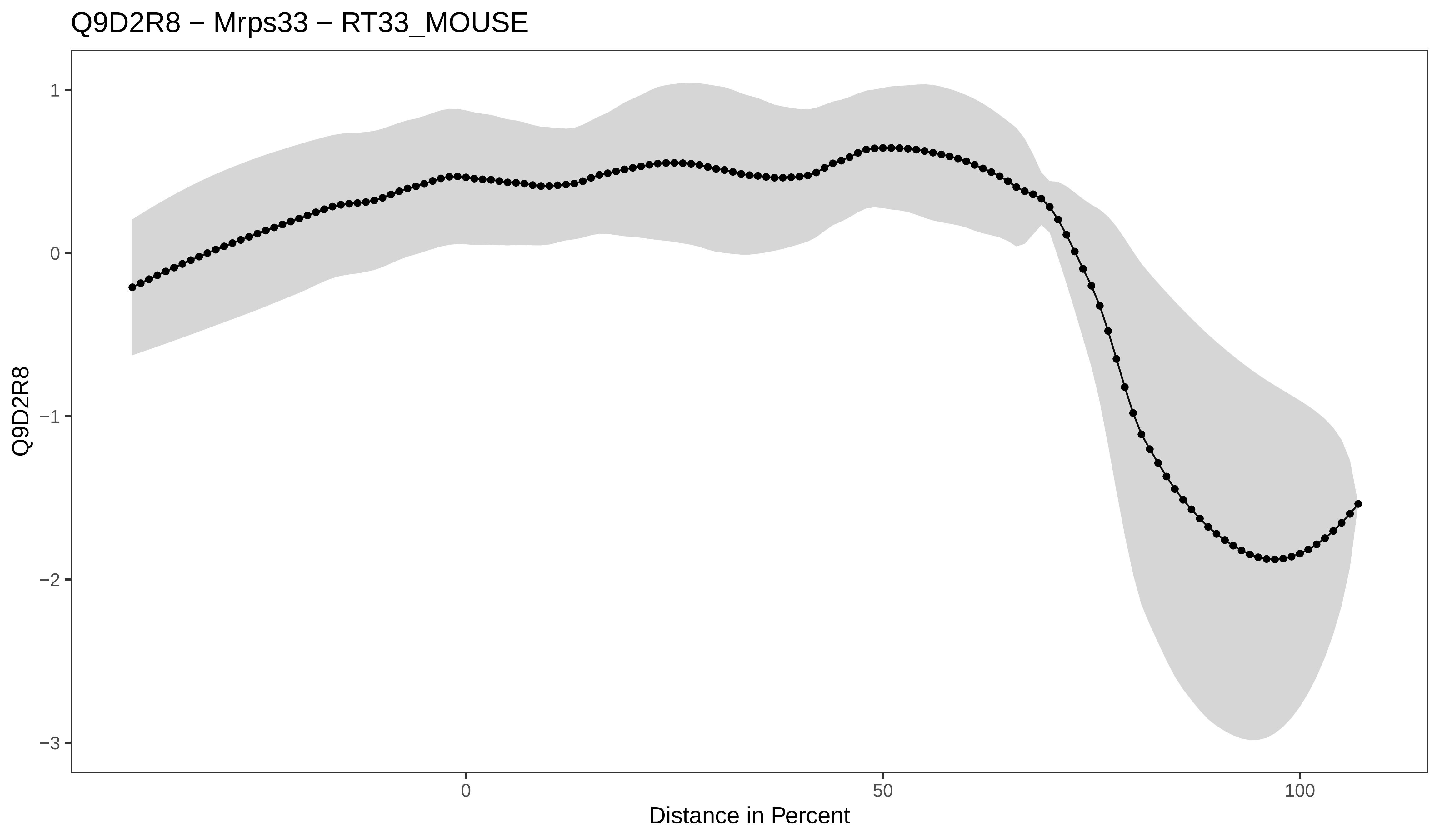

Q9D2R8 − Mrps33 − RT33_MOUSE
Q9D2R8
1
0
−1
−2
−3
0
50
100
Distance in Percent

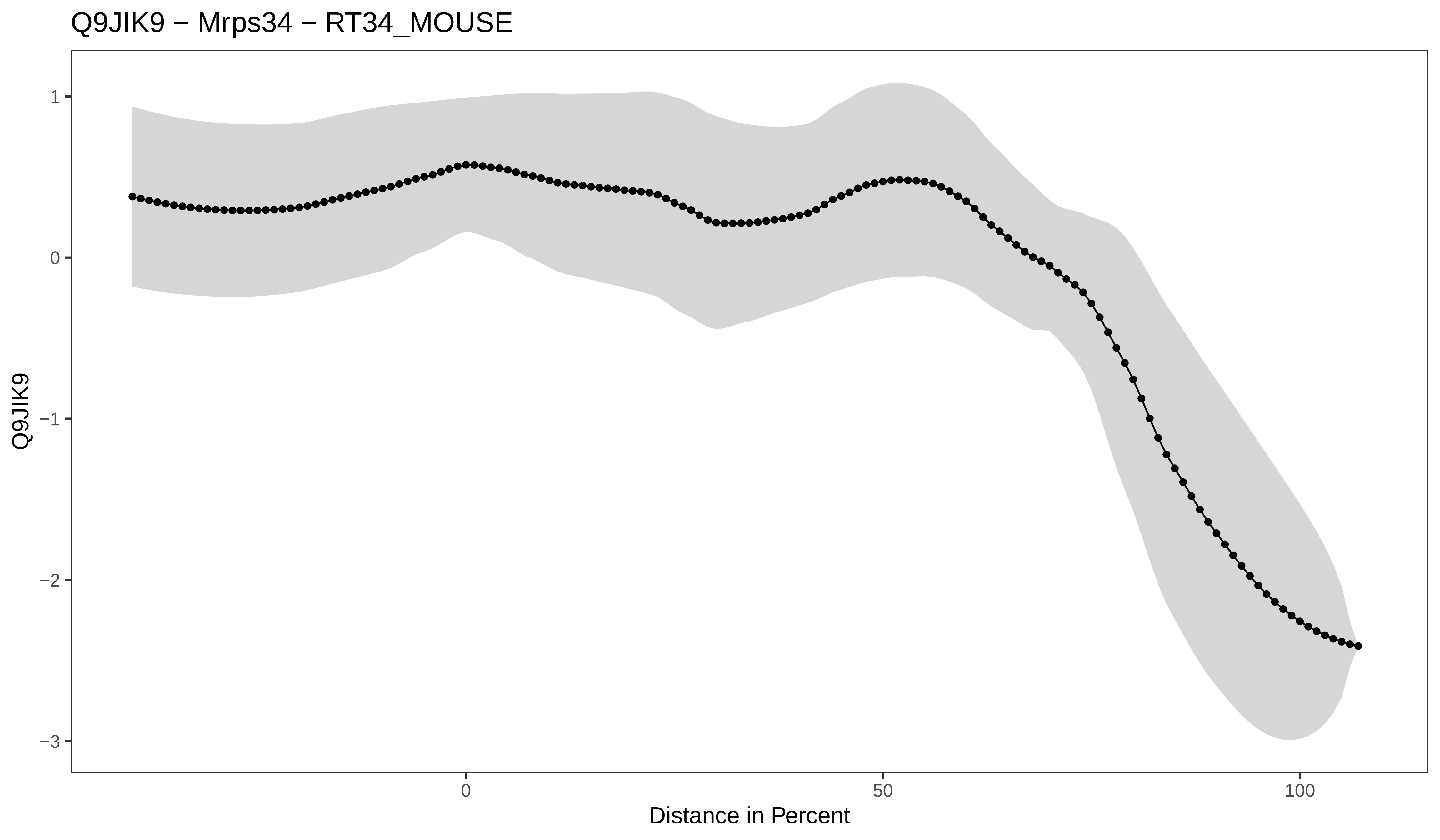
Q9JIK9 − Mrps34 − RT34_MOUSE
Q9JIK9
Distance in Percent
1
0
−1
−2
−3
0
50
100

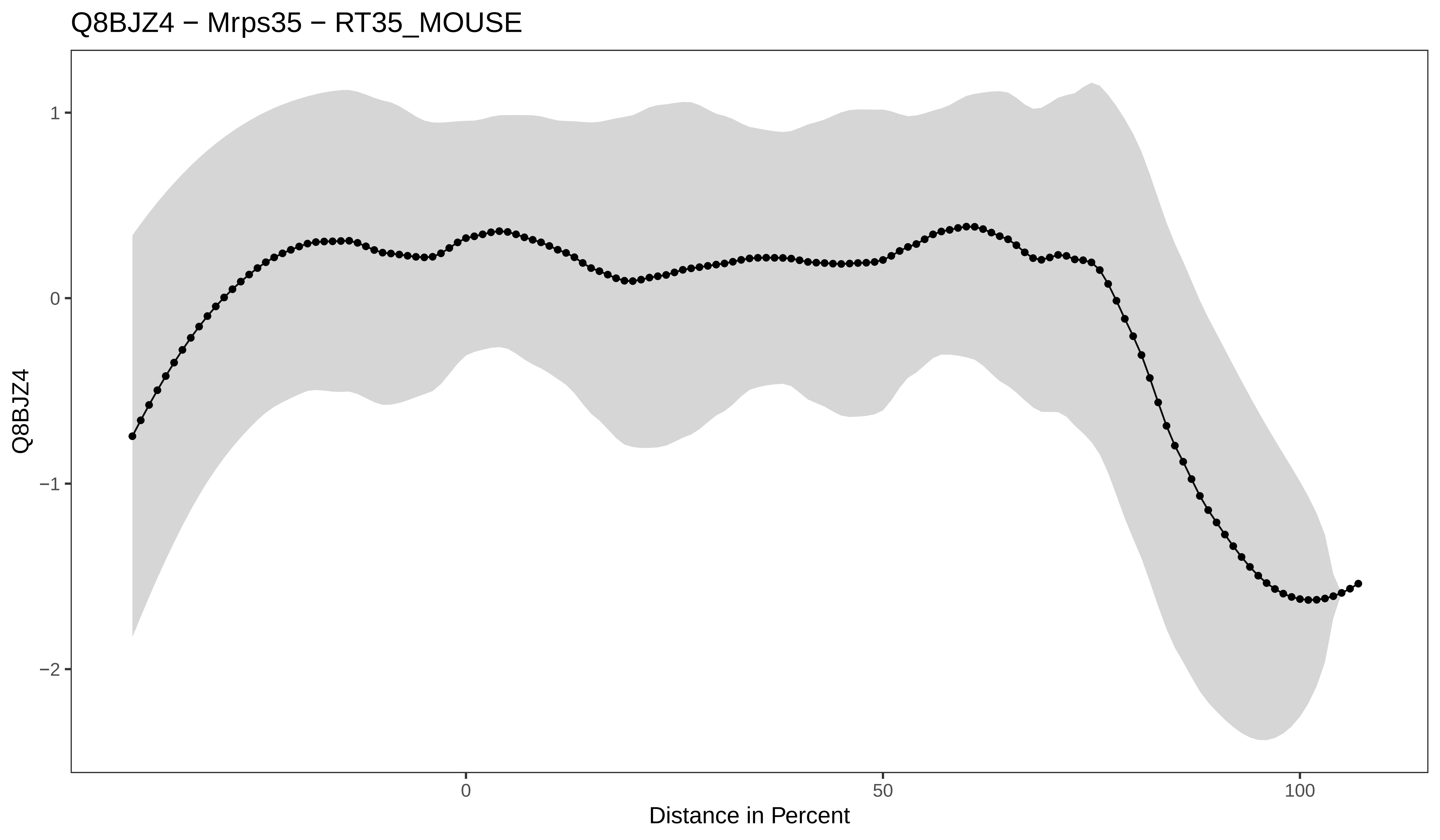
Q8BJZ4 − Mrps35 − RT35_MOUSE
Q8BJZ4
1
0
−1
−2
0
50
100
Distance in Percent

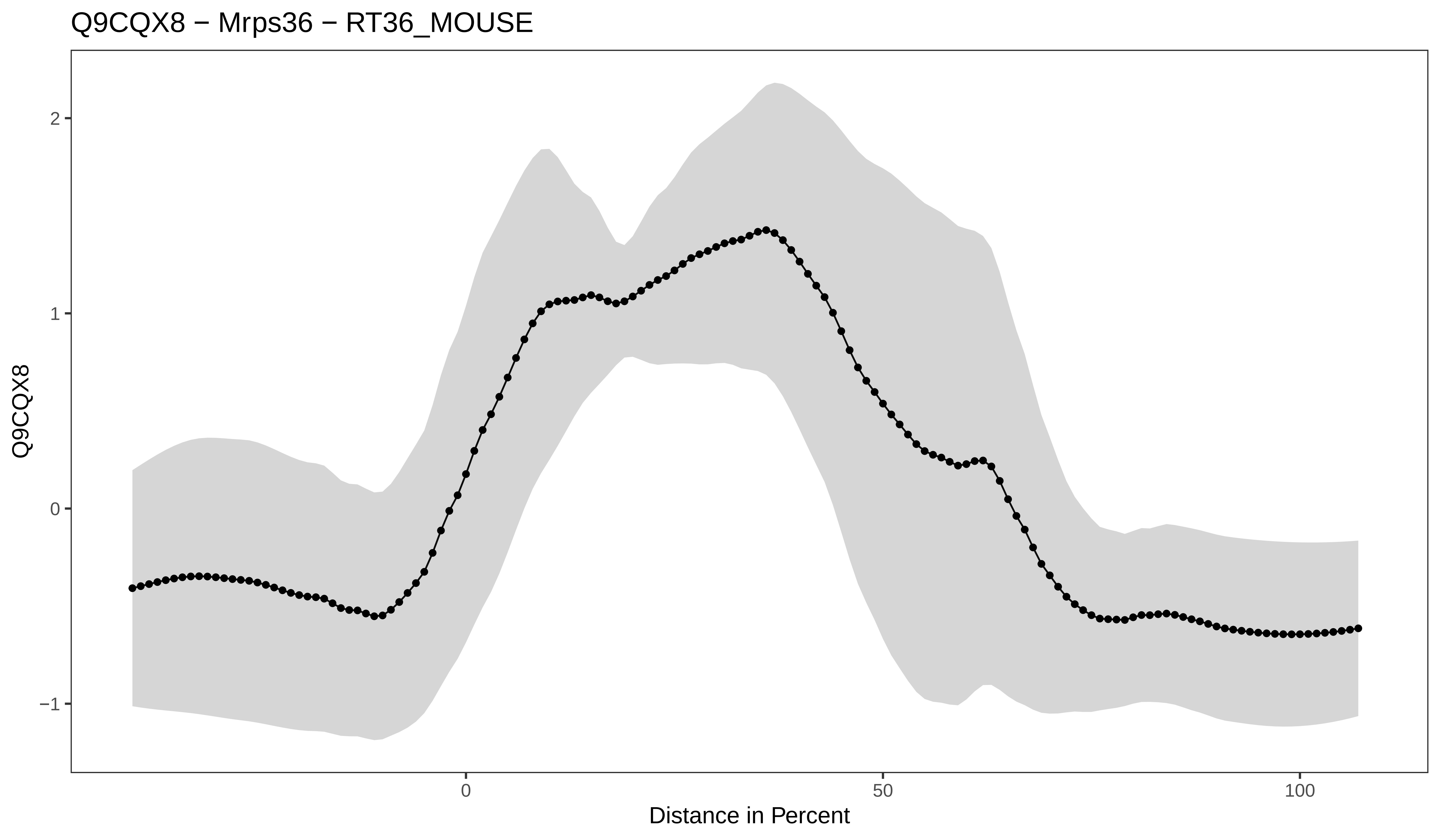
Q9CQX8 − Mrps36 − RT36_MOUSE
Q9CQX8
Distance in Percent
2
1
0
−1
0
50
100

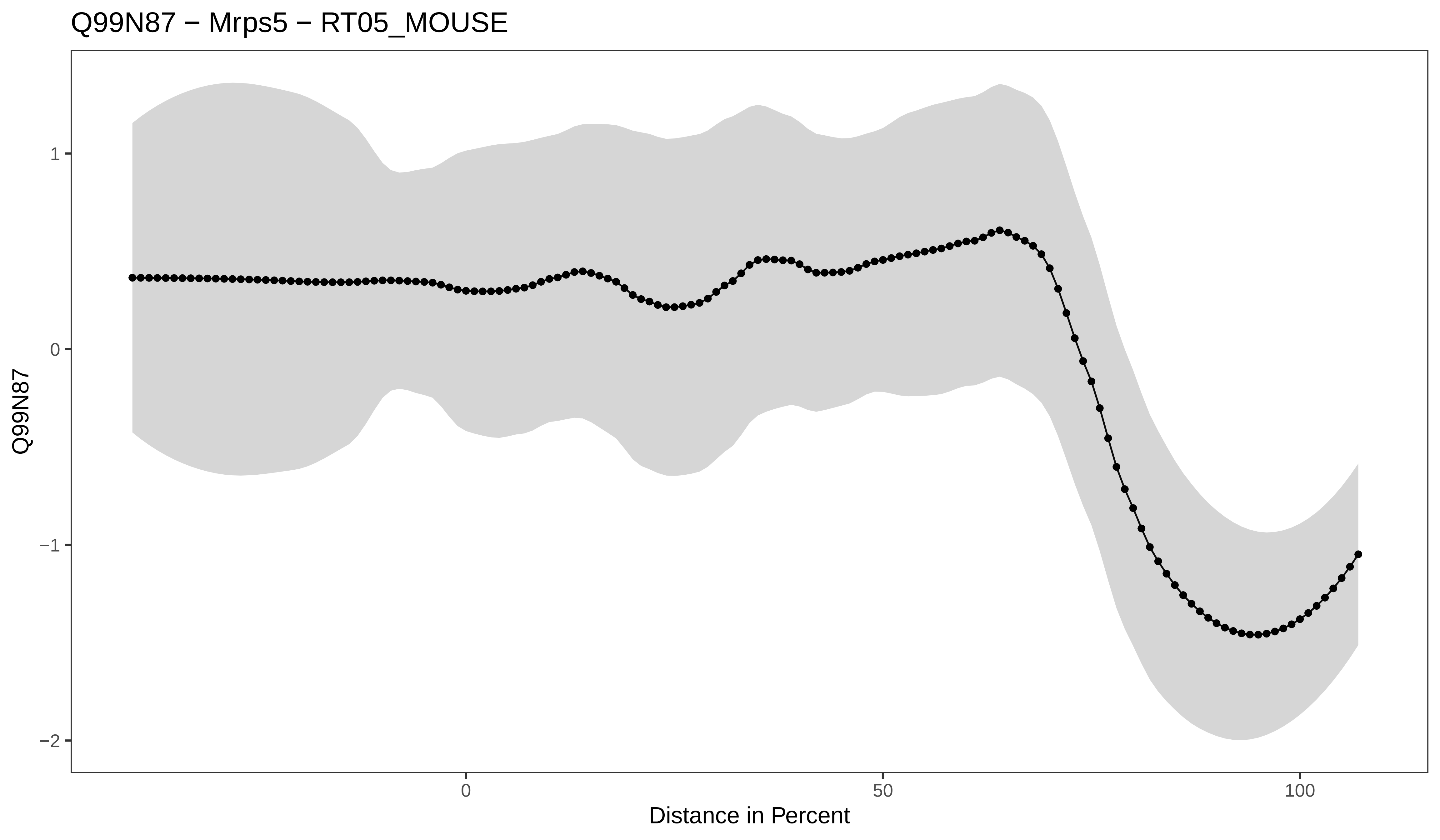

Q99N87 − Mrps5 − RT05_MOUSE
1
0
−1
−2
Q99N87
0
50
100
Distance in Percent

Q80X85 − Mrps7 − RT07_MOUSE

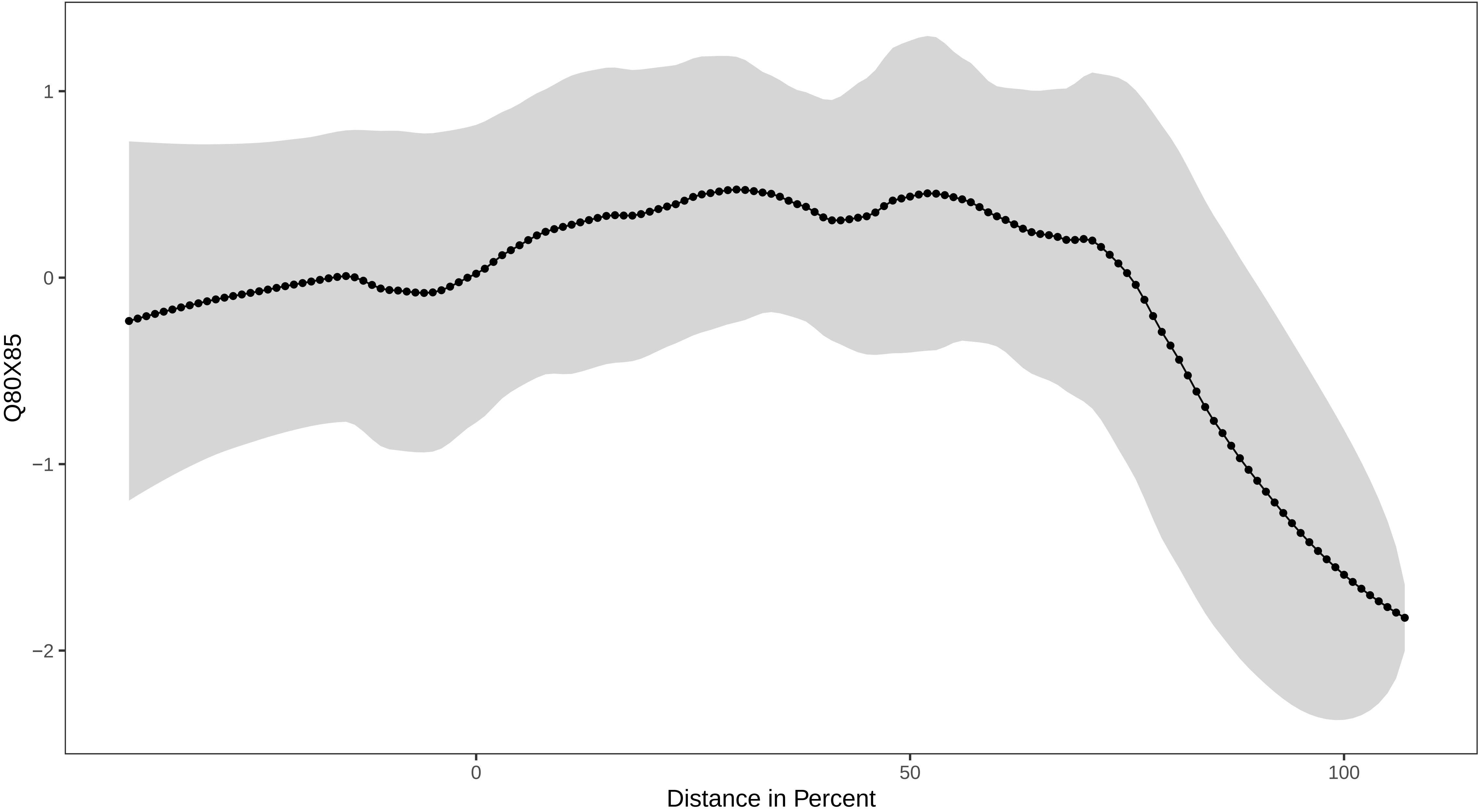

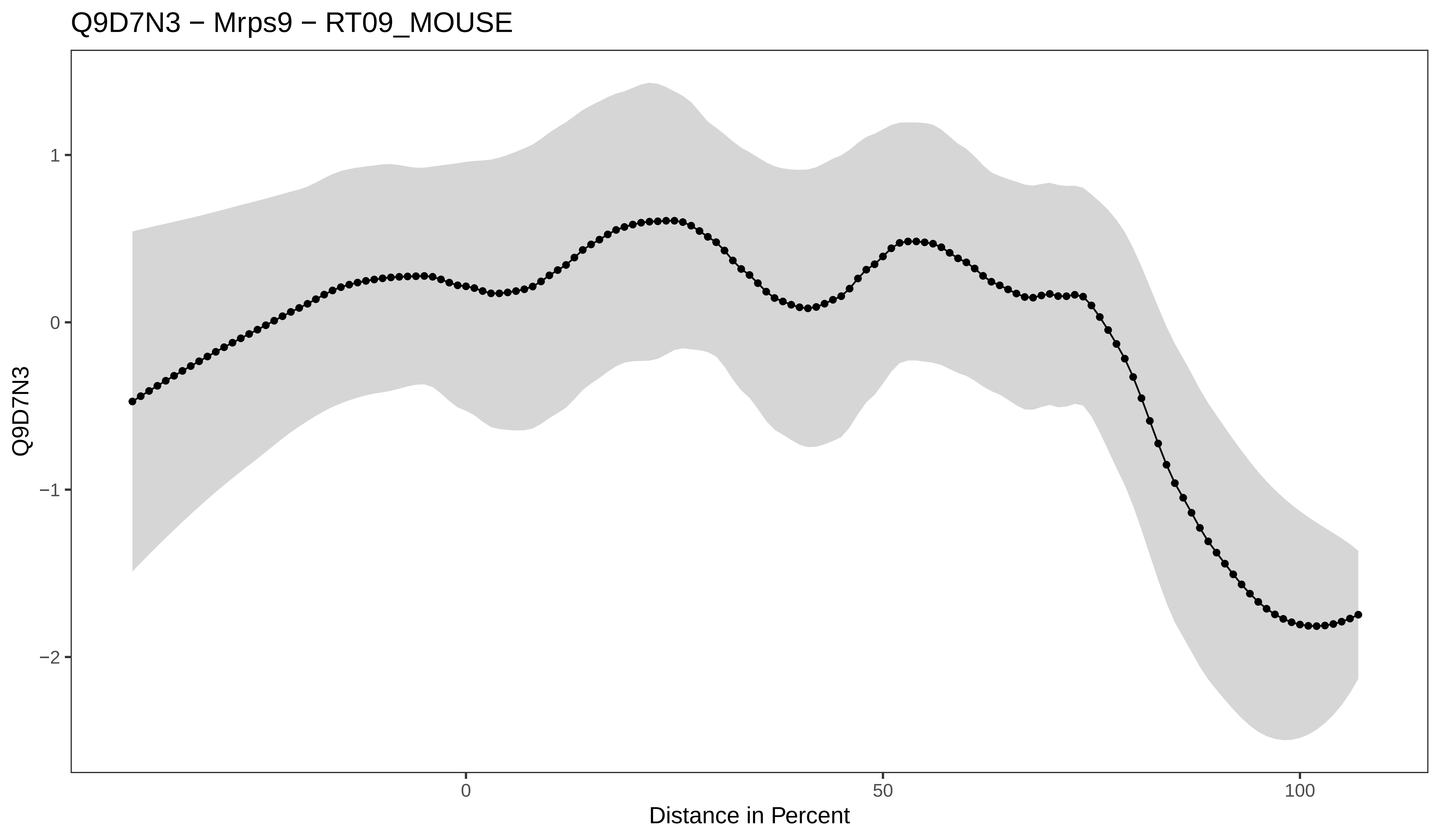
Q9D7N3 − Mrps9 − RT09_MOUSE
Q9D7N3
1
0
−1
−2
0
50
100
Distance in Percent

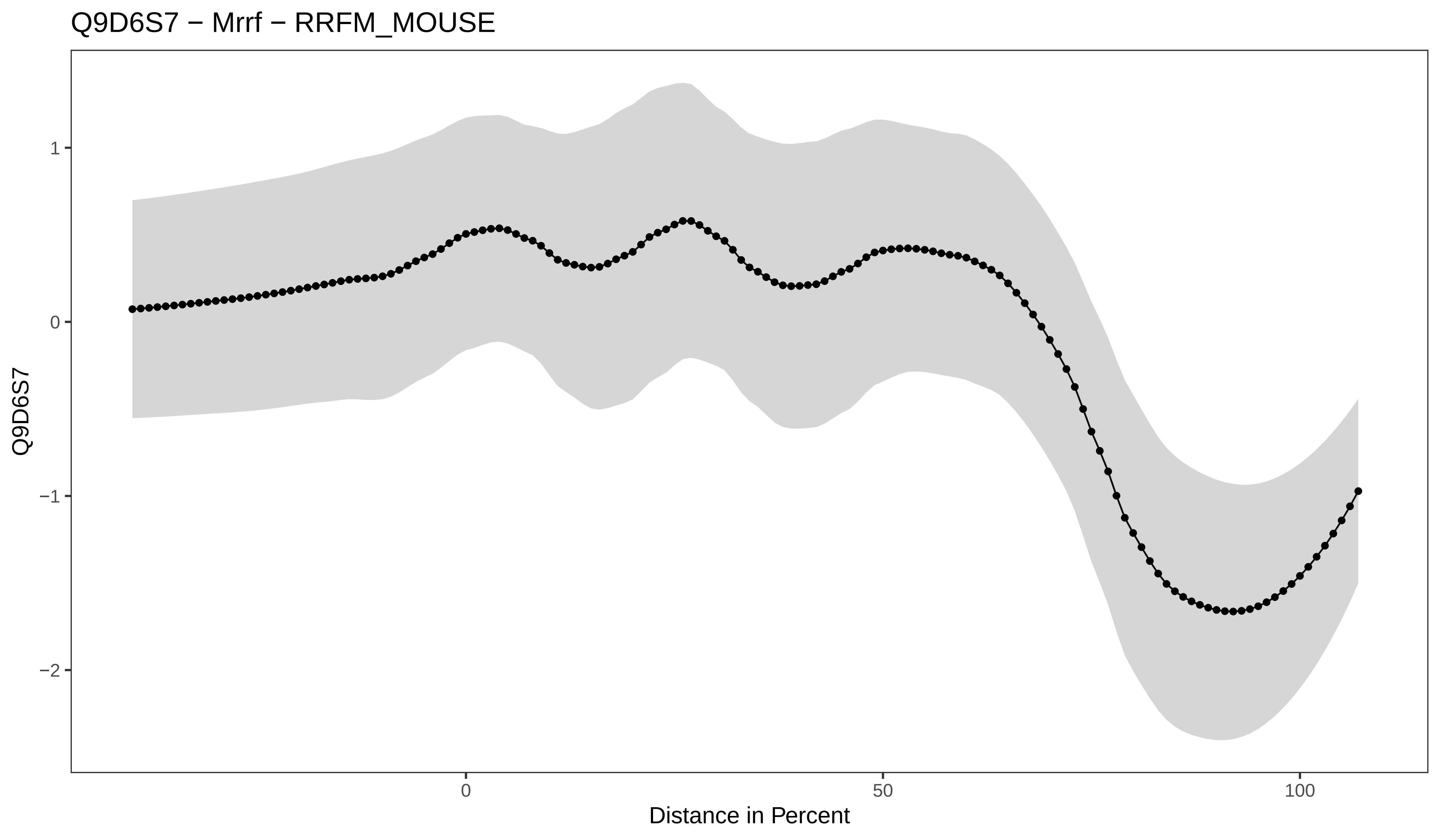
Q9D6S7 − Mrrf − RRFM_MOUSE
Q9D6S7
1
0
−1
−2
0
50
100
Distance in Percent

Q920Q6 − Msi2 − MSI2H_MOUSE

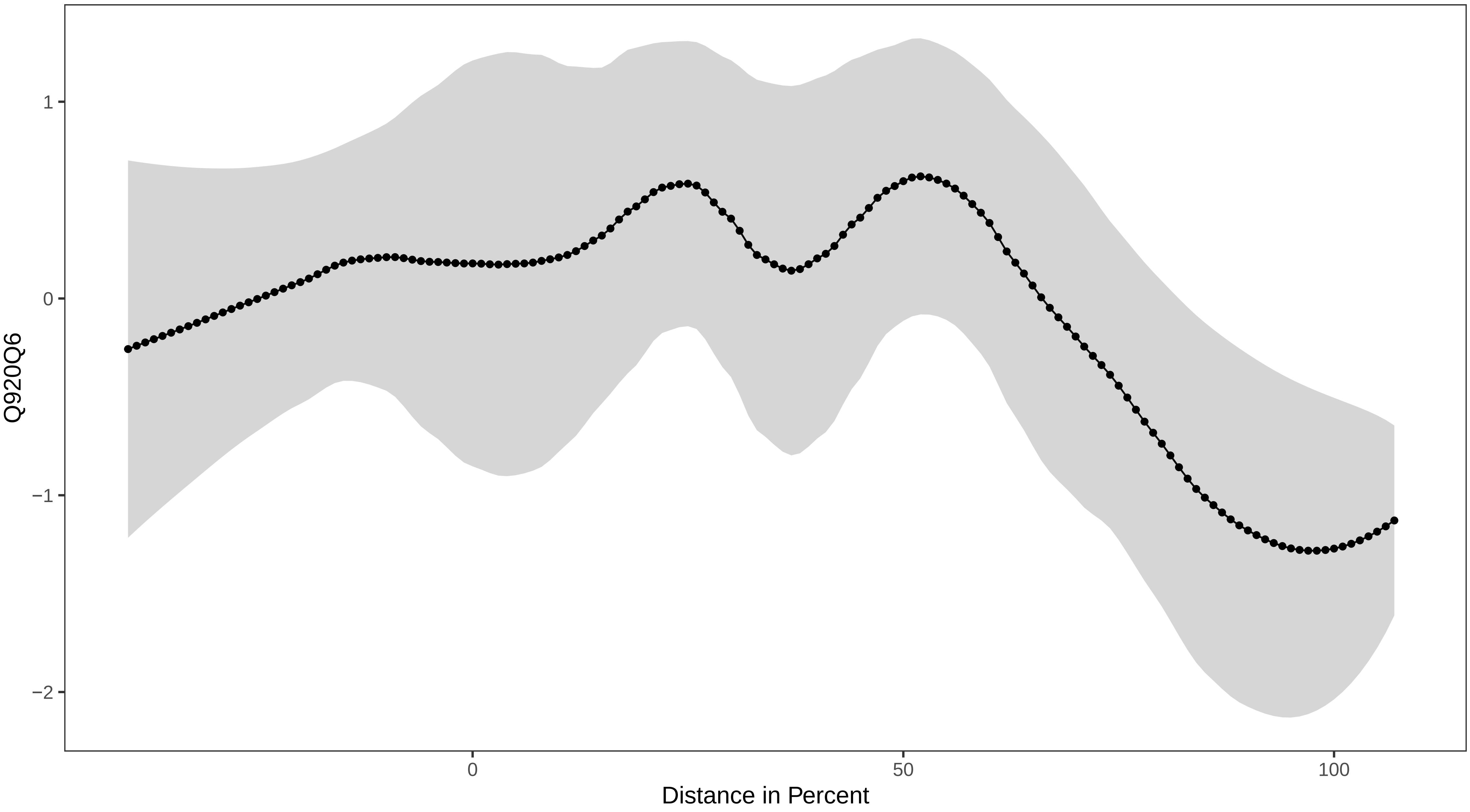

P26041 − Msn − MOES_MOUSE

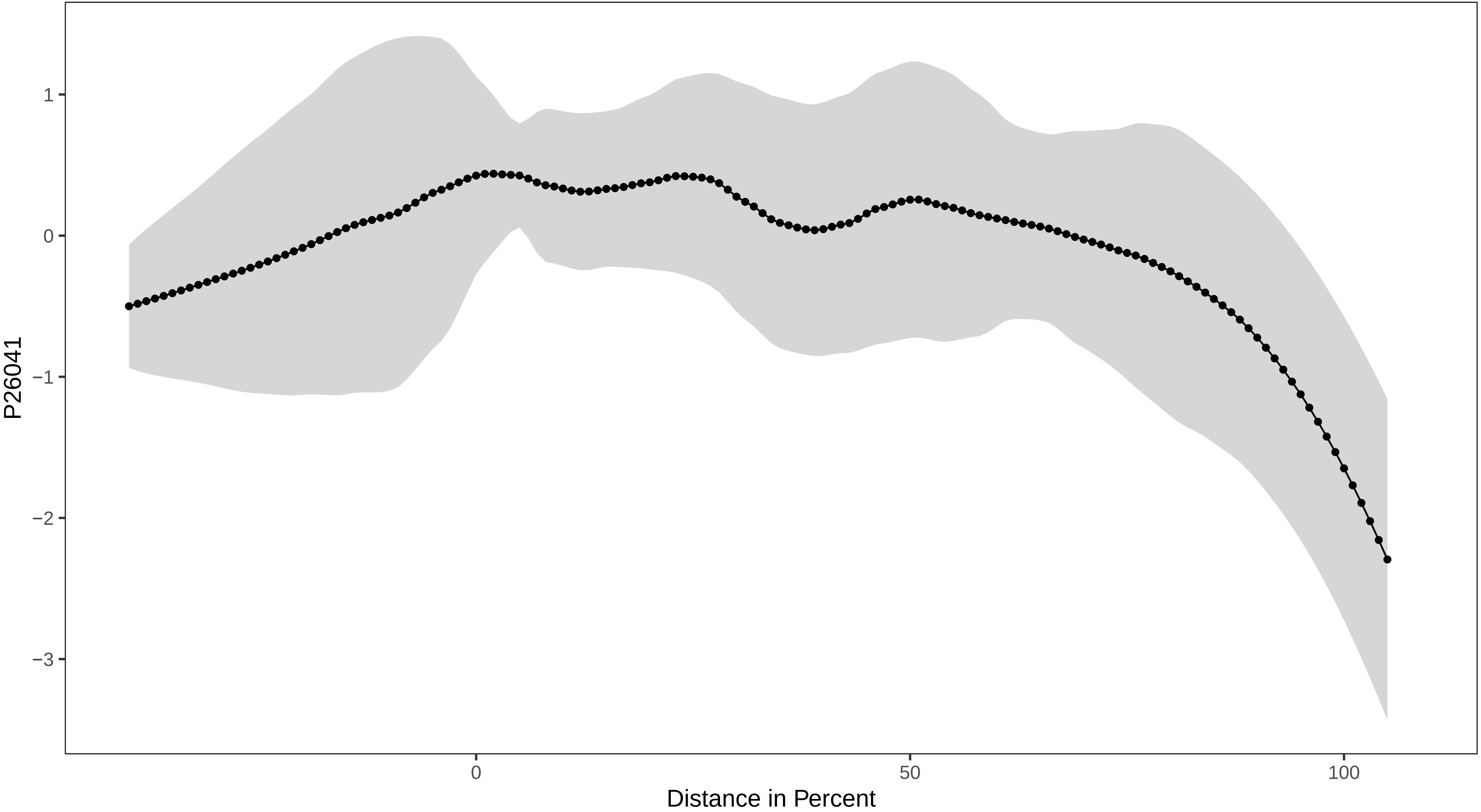

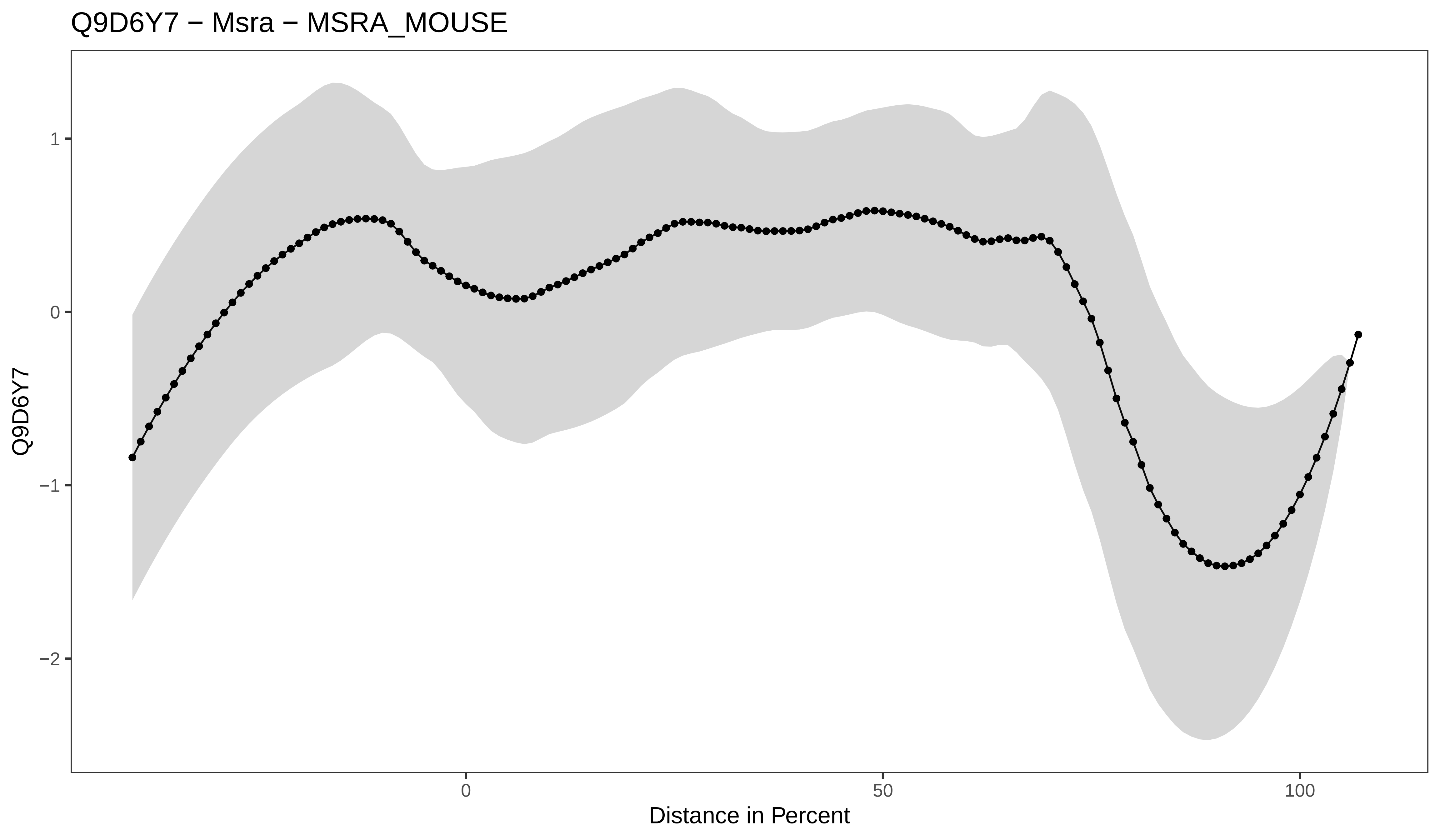

Q9D6Y7 − Msra − MSRA_MOUSE
1
0
−1
−2
Q9D6Y7
0
50
100
Distance in Percent

Q9R190 − Mta2 − MTA2_MOUSE

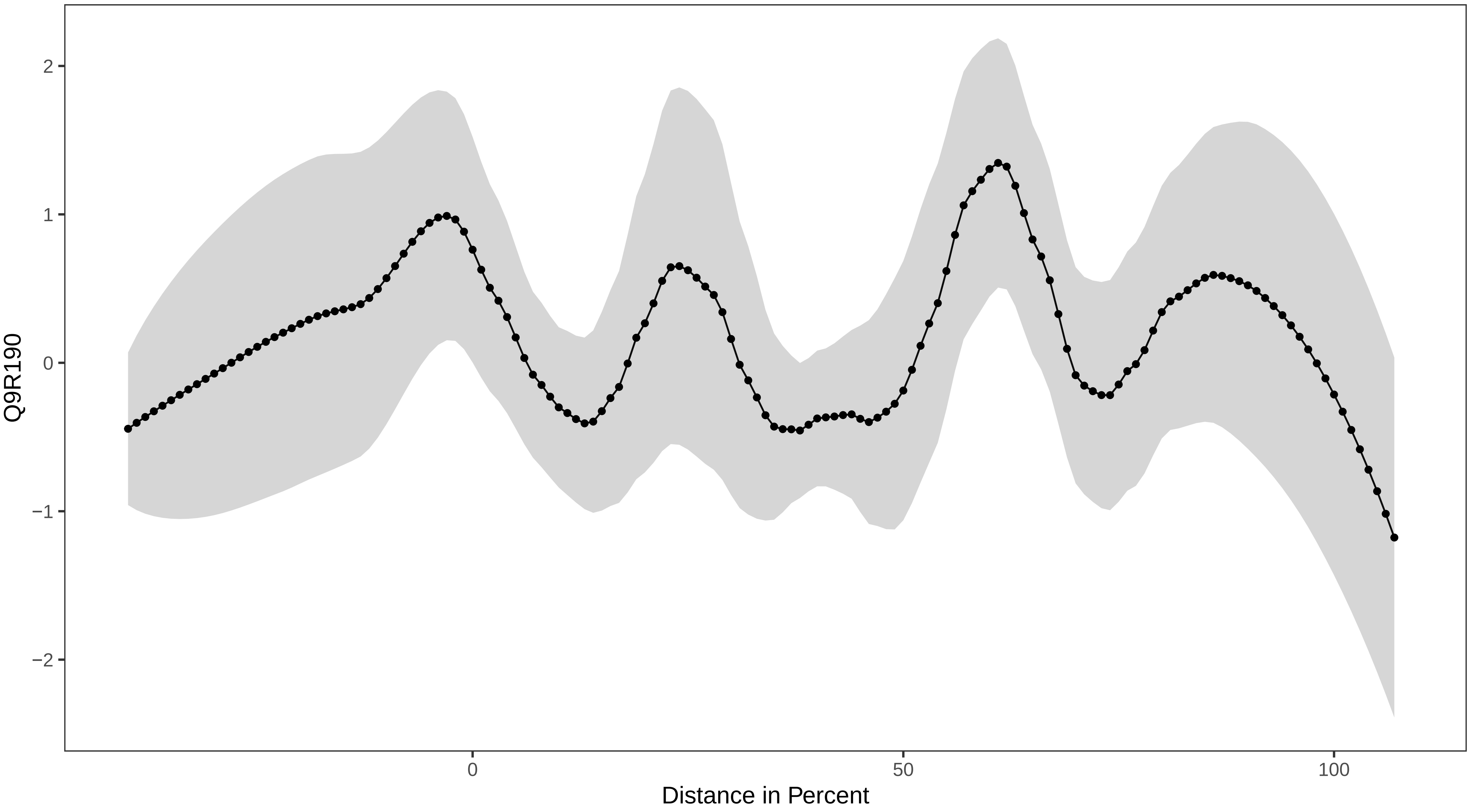

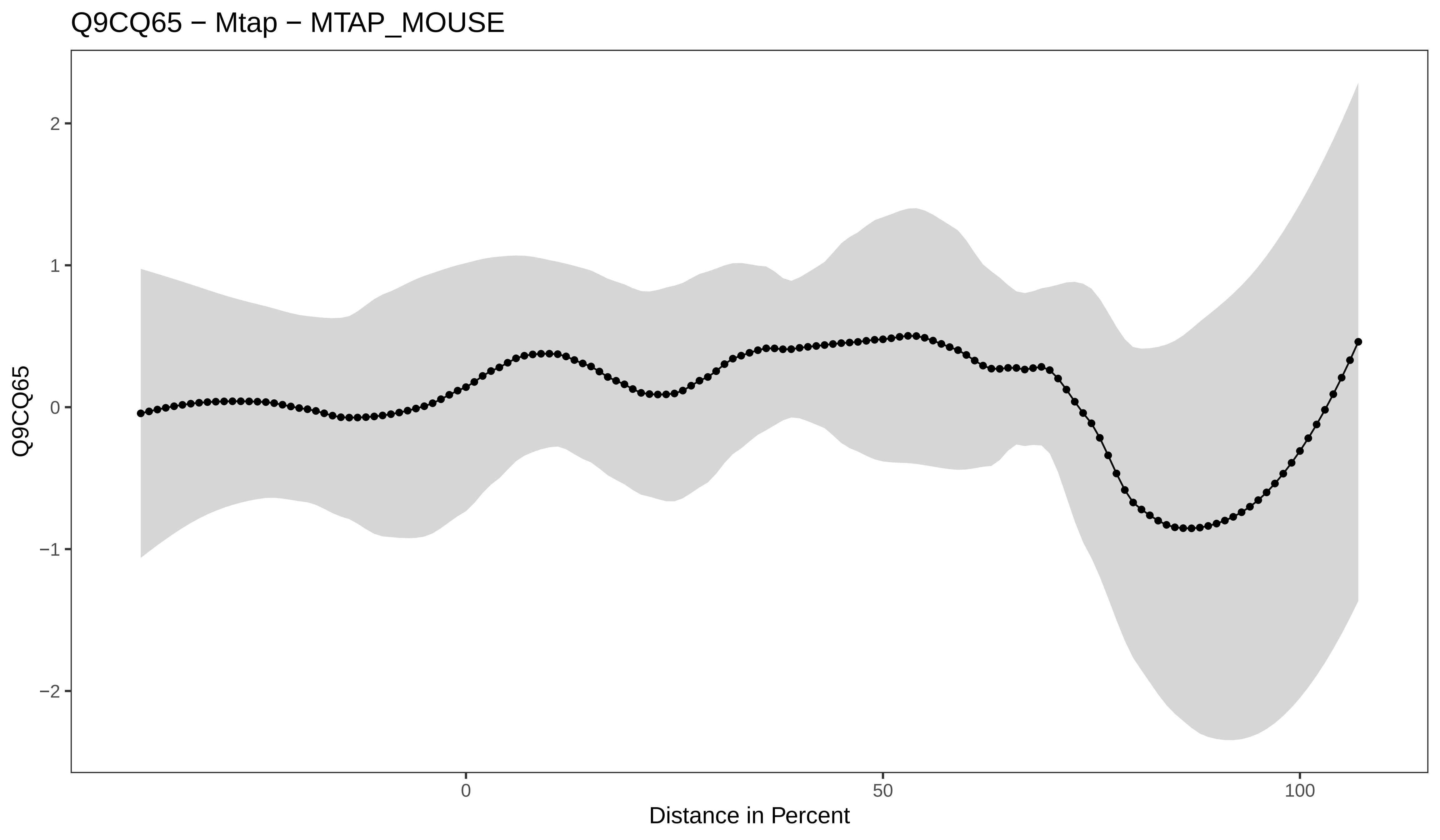

Q9CQ65 − Mtap − MTAP_MOUSE
Q9CQ65
2
1
0
−1
−2
0
50
100
Distance in Percent

P00848 − Mtatp6 − ATP6_MOUSE

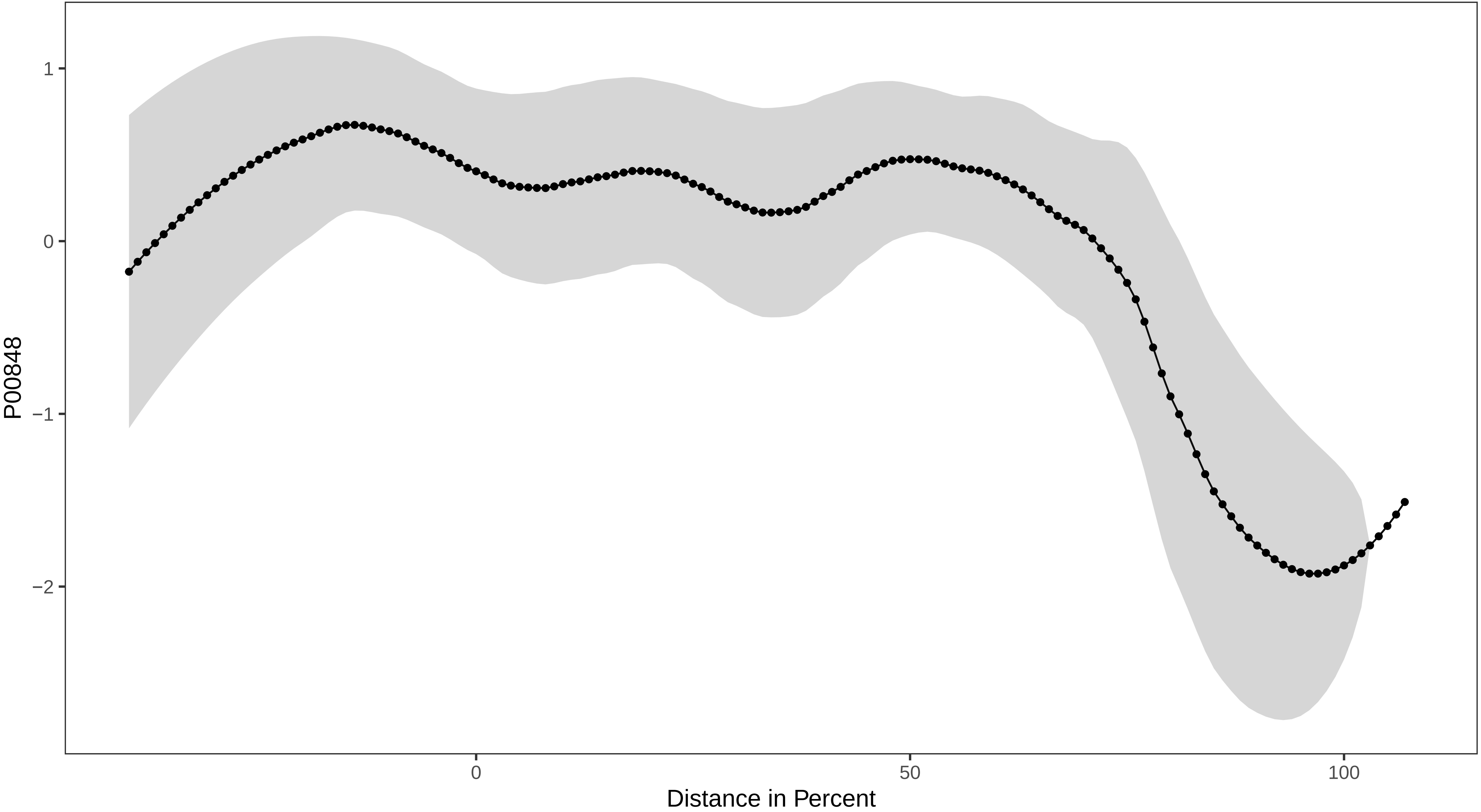

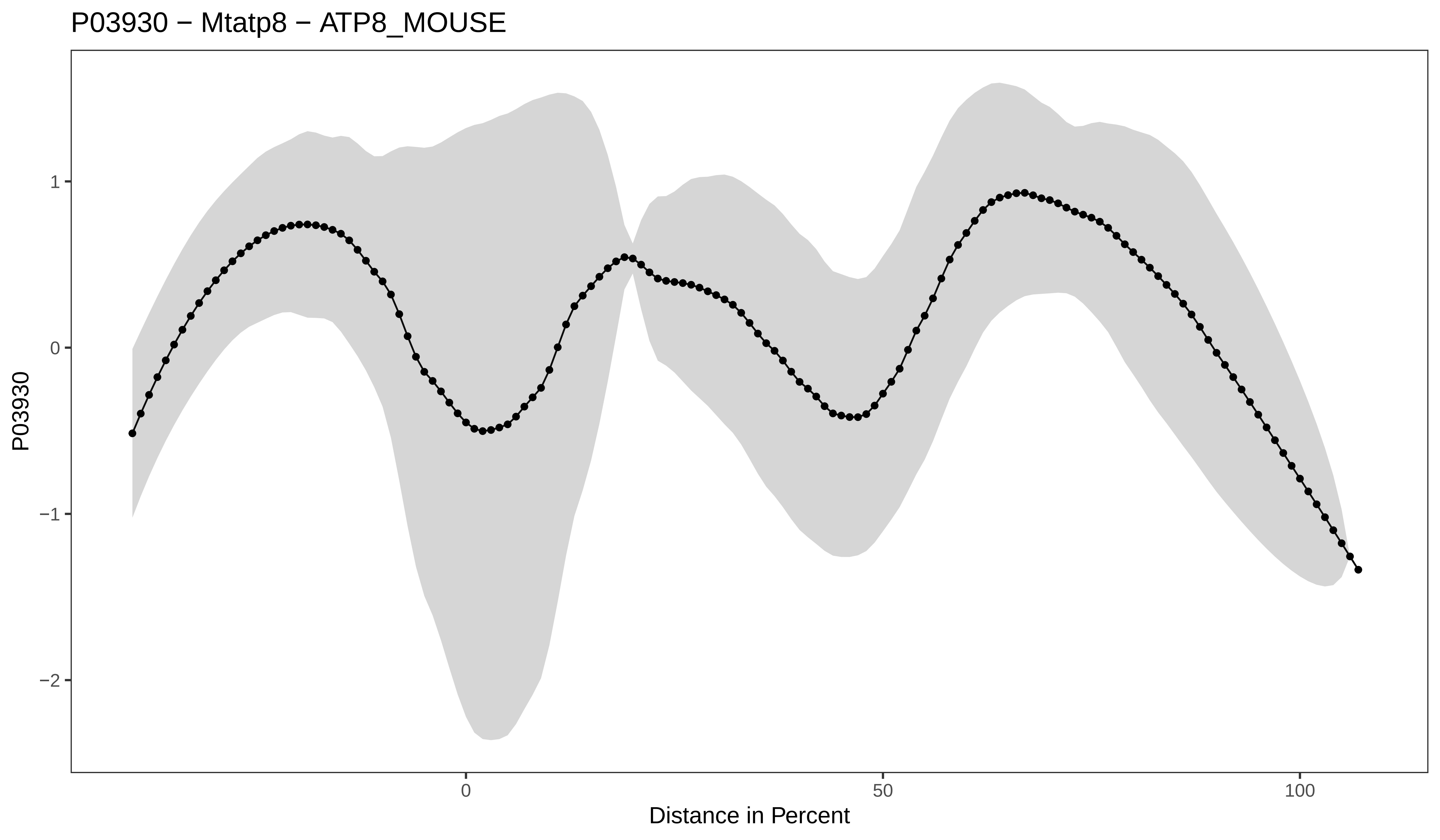
P03930 − Mtatp8 − ATP8_MOUSE
1
0
−1
−2
P03930
0
50
100
Distance in Percent

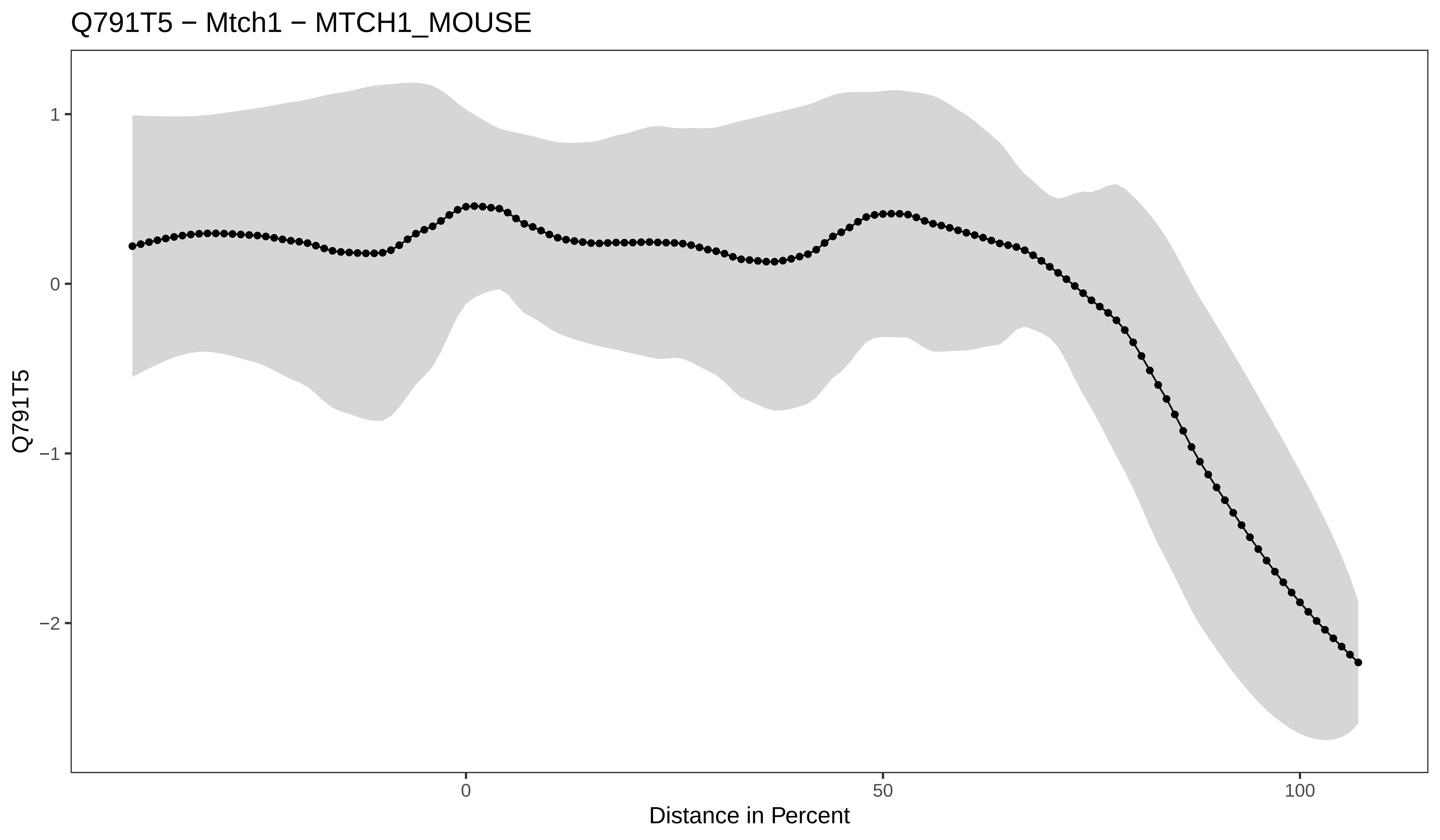
Q791T5 − Mtch1 − MTCH1_MOUSE
Q791T5
1
0
−1
−2
0
50
100
Distance in Percent

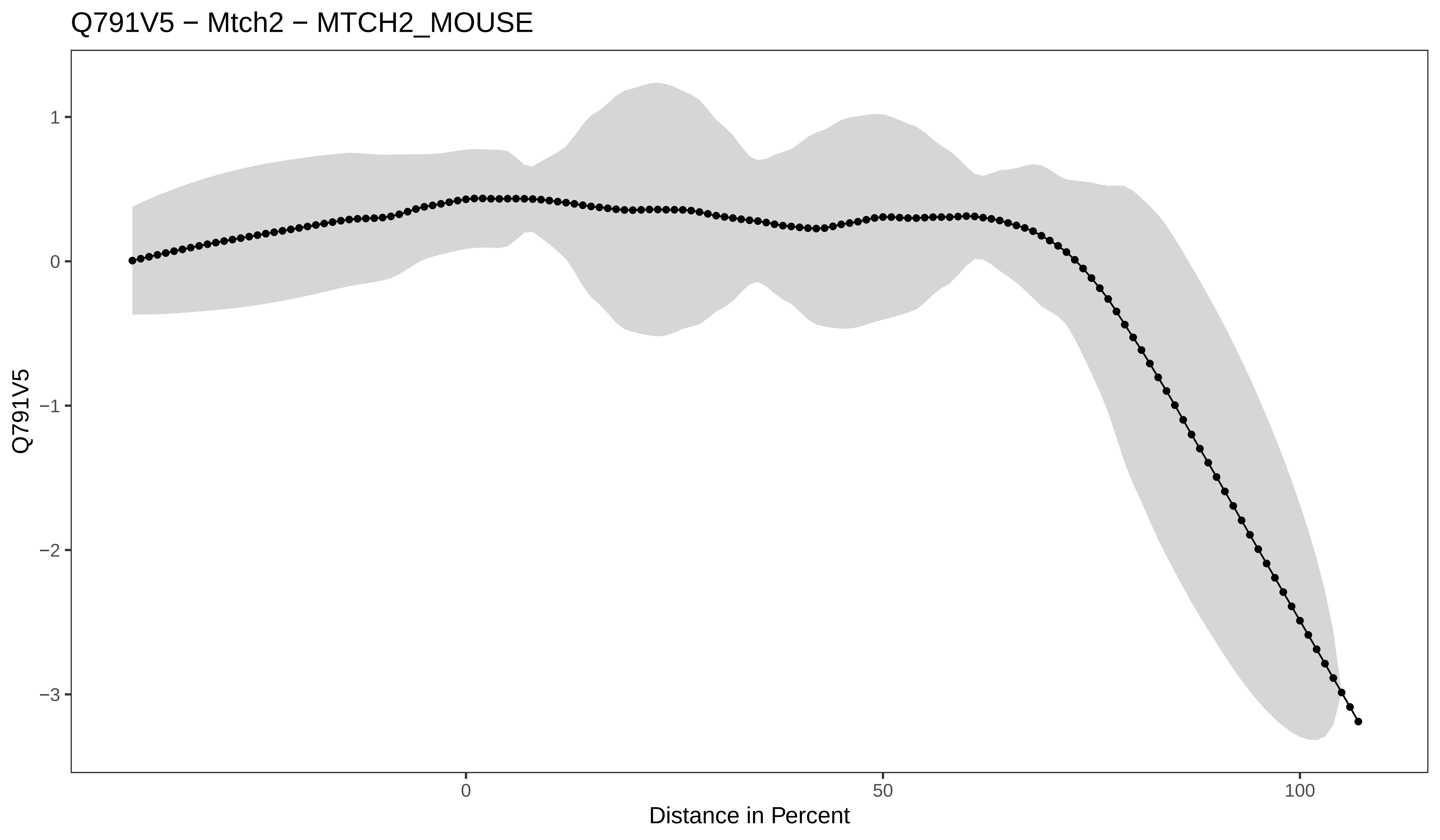
Q791V5 − Mtch2 − MTCH2_MOUSE
1
0
−1
−2
−3
Q791V5
0
50
100
Distance in Percent

P00397 − Mtco1 − COX1_MOUSE

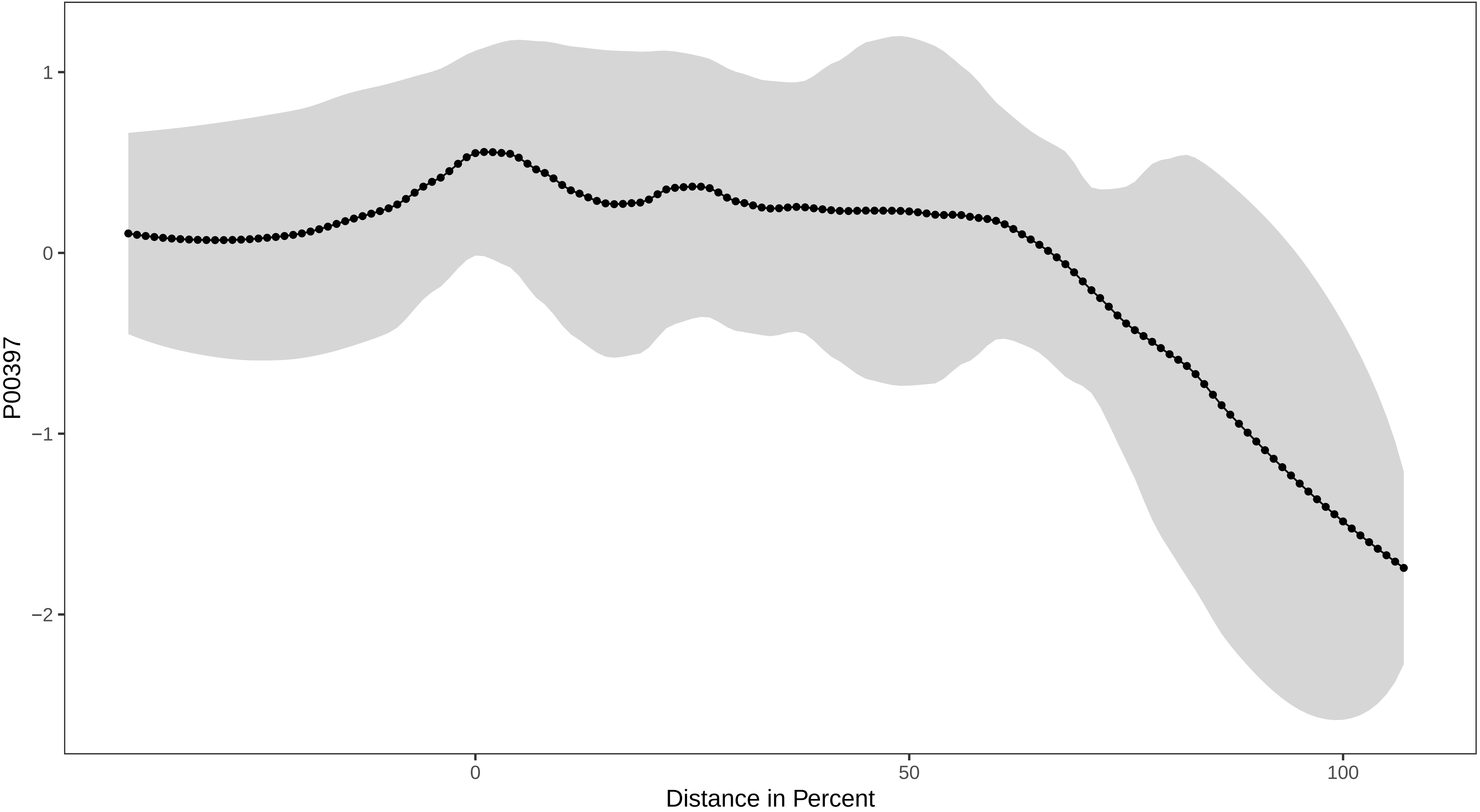

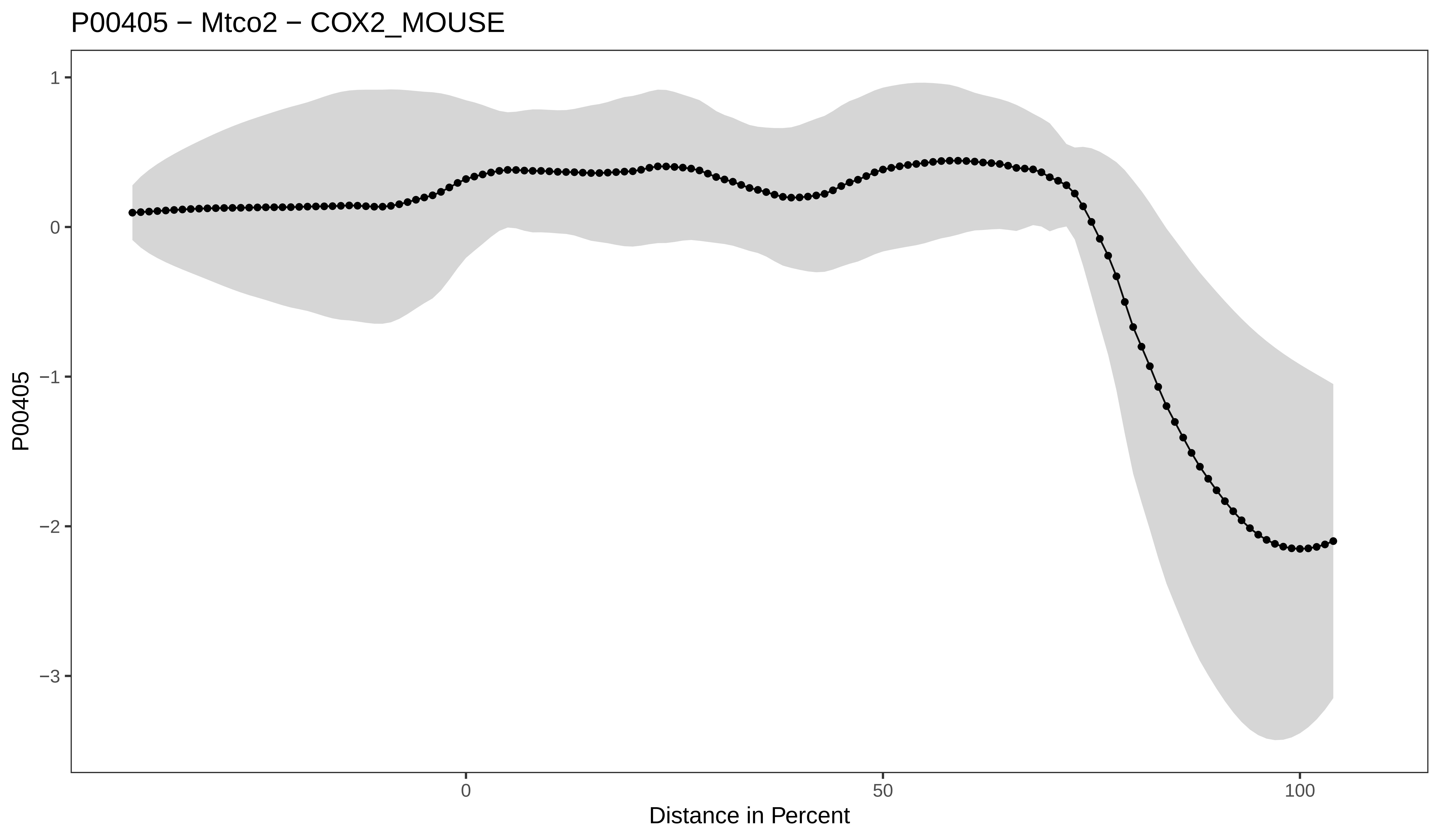

P00405 − Mtco2 − COX2_MOUSE
1
0
−1
−2
−3
P00405
0
50
100
Distance in Percent

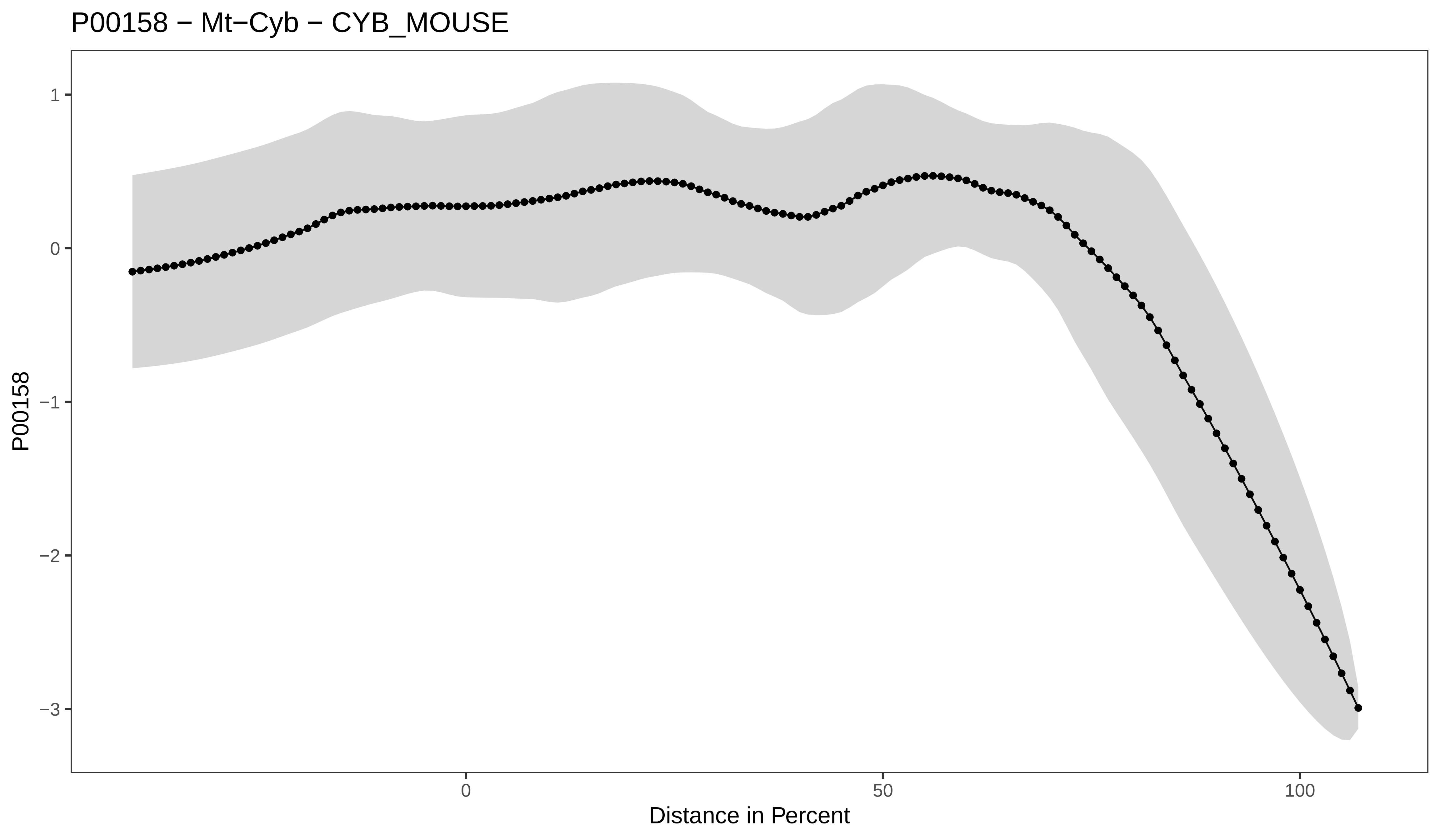
P00158 − Mt−Cyb − CYB_MOUSE
1
0
−1
−2
−3
P00158
0
50
100
Distance in Percent

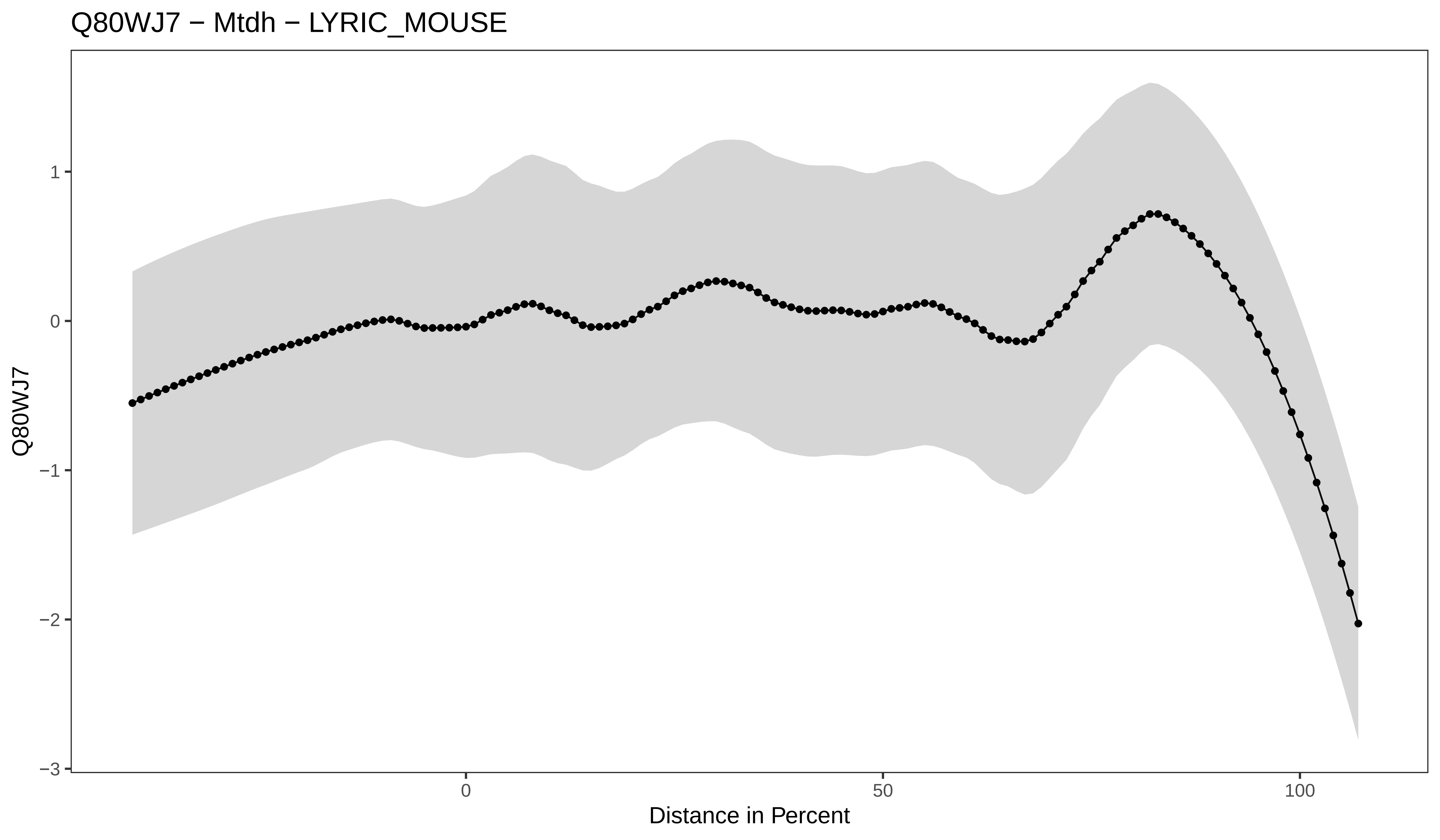

Q80WJ7 − Mtdh − LYRIC_MOUSE
Q80WJ7
Distance in Percent
1
0
−1
−2
−3
0
50
100

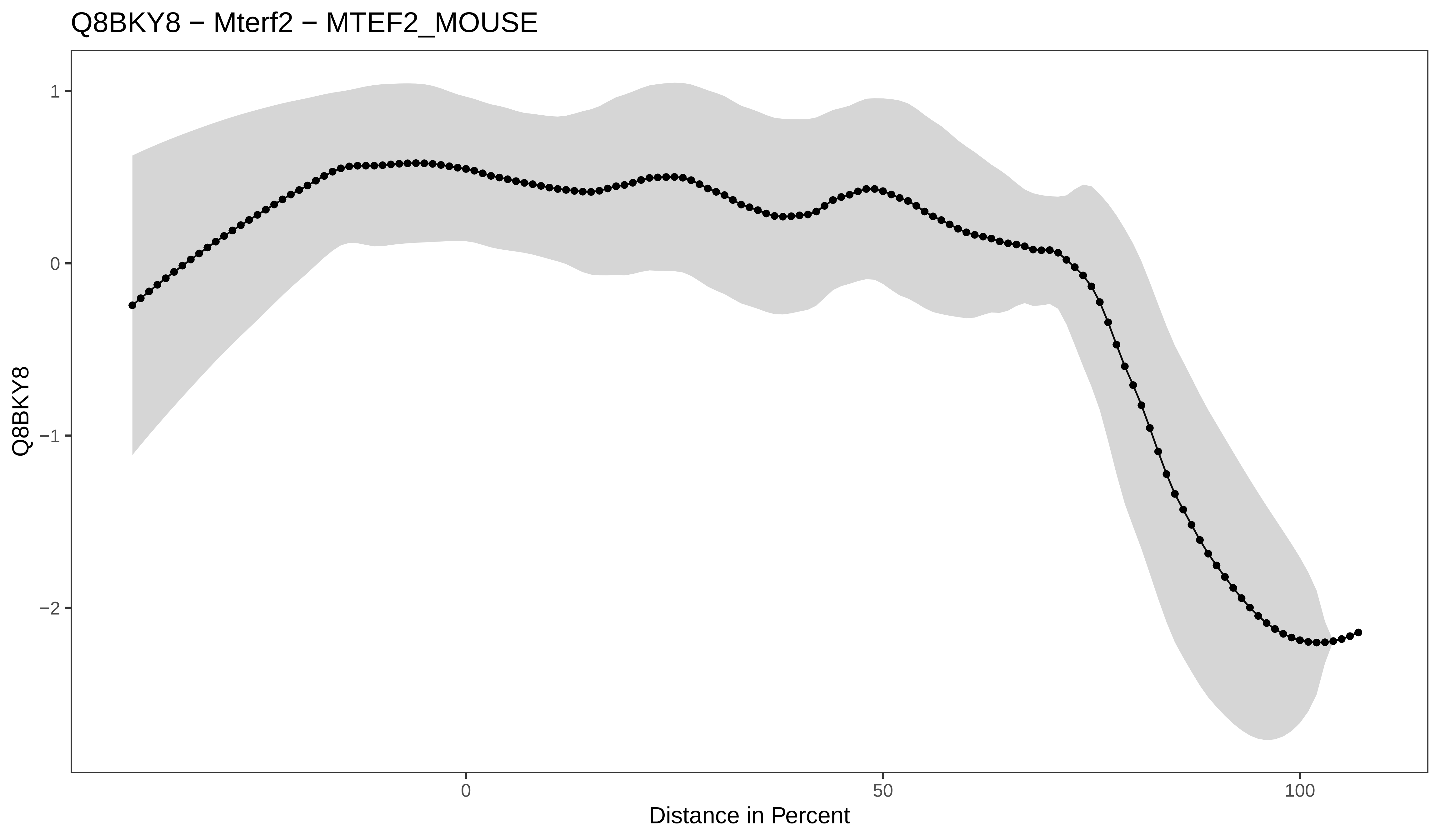

Q8BKY8 − Mterf2 − MTEF2_MOUSE
Q8BKY8
1
0
−1
−2
0
50
100
Distance in Percent

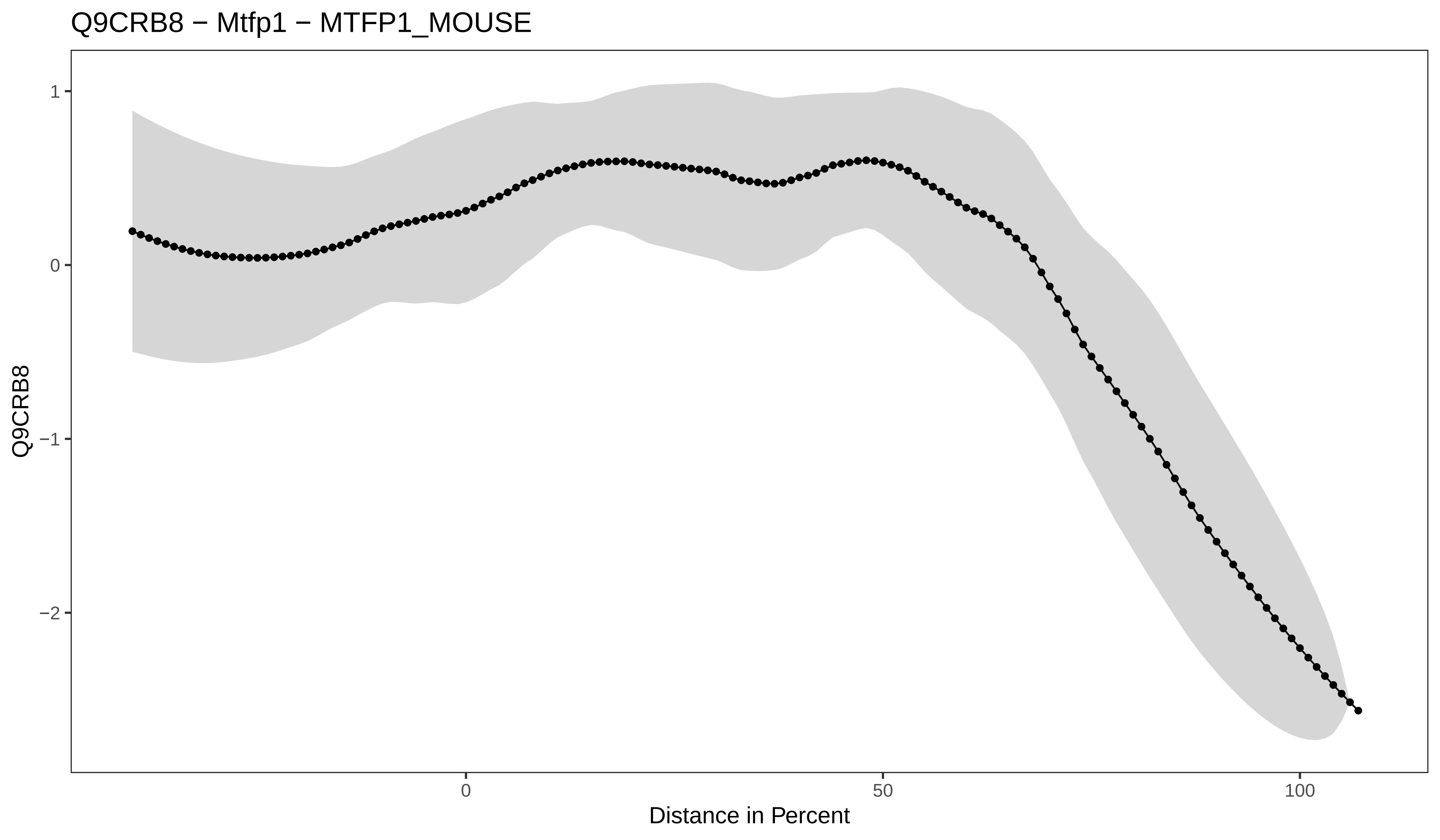

Q9CRB8 − Mtfp1 − MTFP1_MOUSE
1
0
−1
−2
Q9CRB8
0
50
100
Distance in Percent

Q922D8 − Mthfd1 − C1TC_MOUSE

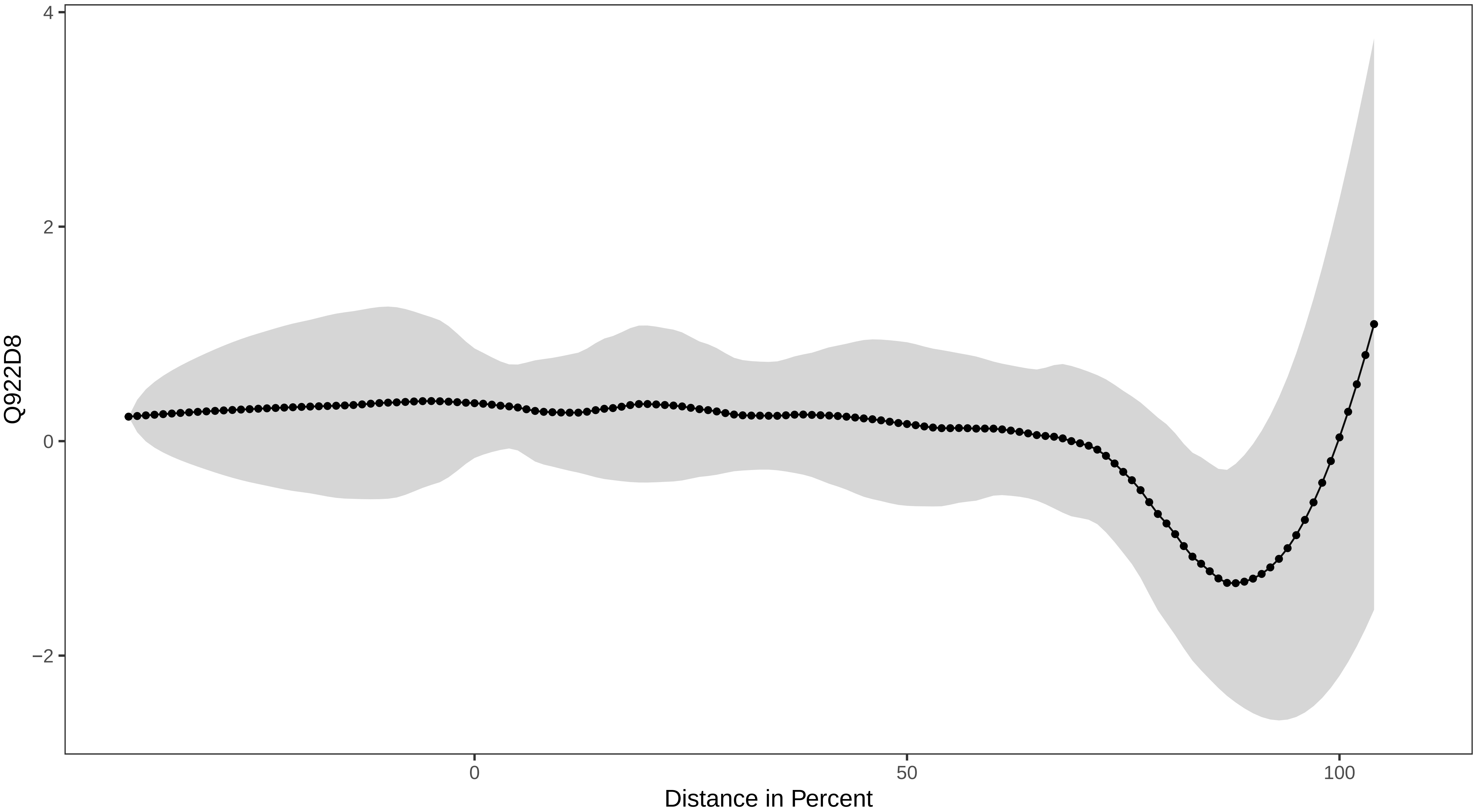

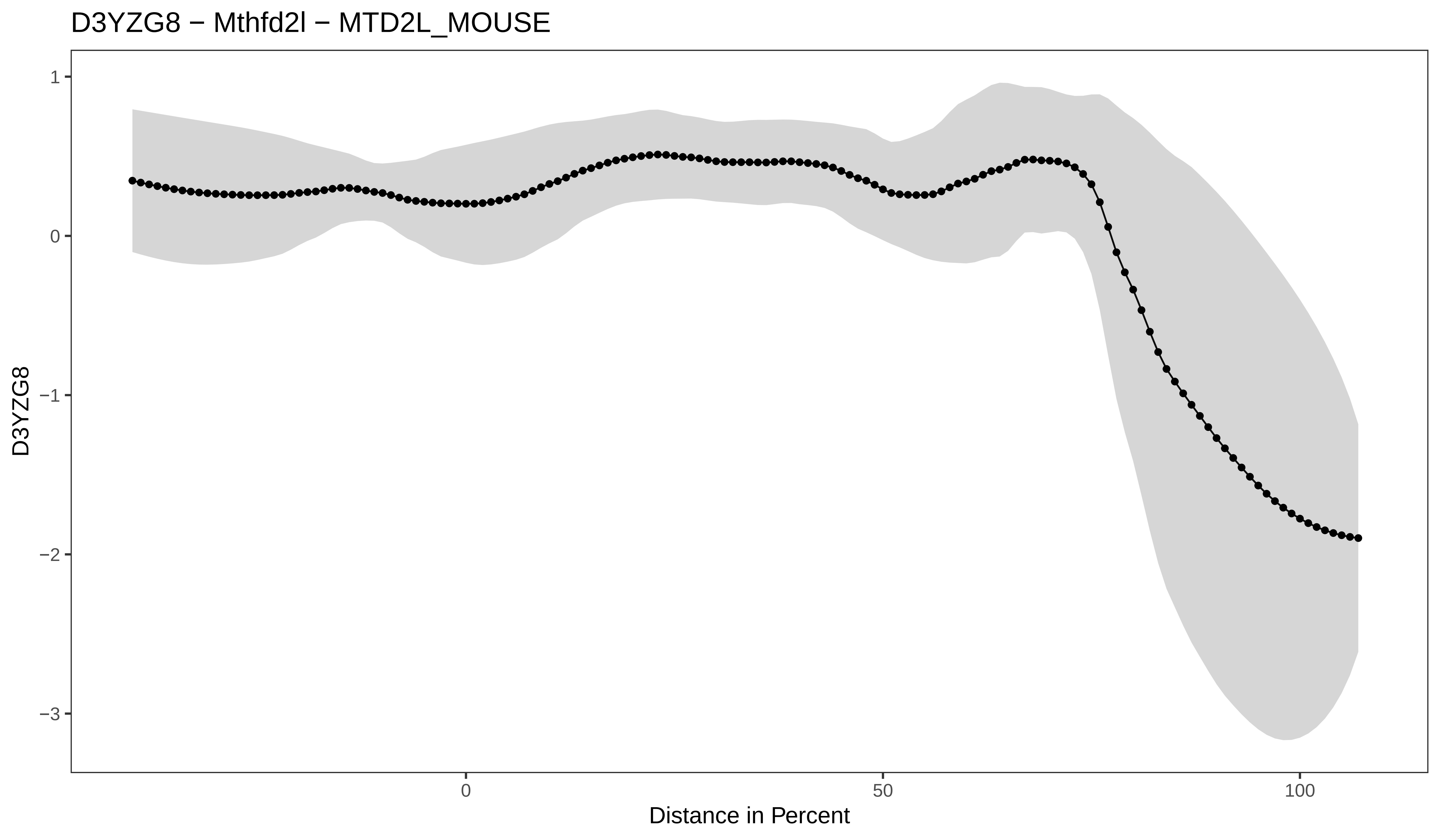

D3YZG8 − Mthfd2l − MTD2L_MOUSE
1
0
−1
−2
−3
D3YZG8
0
50
100
Distance in Percent

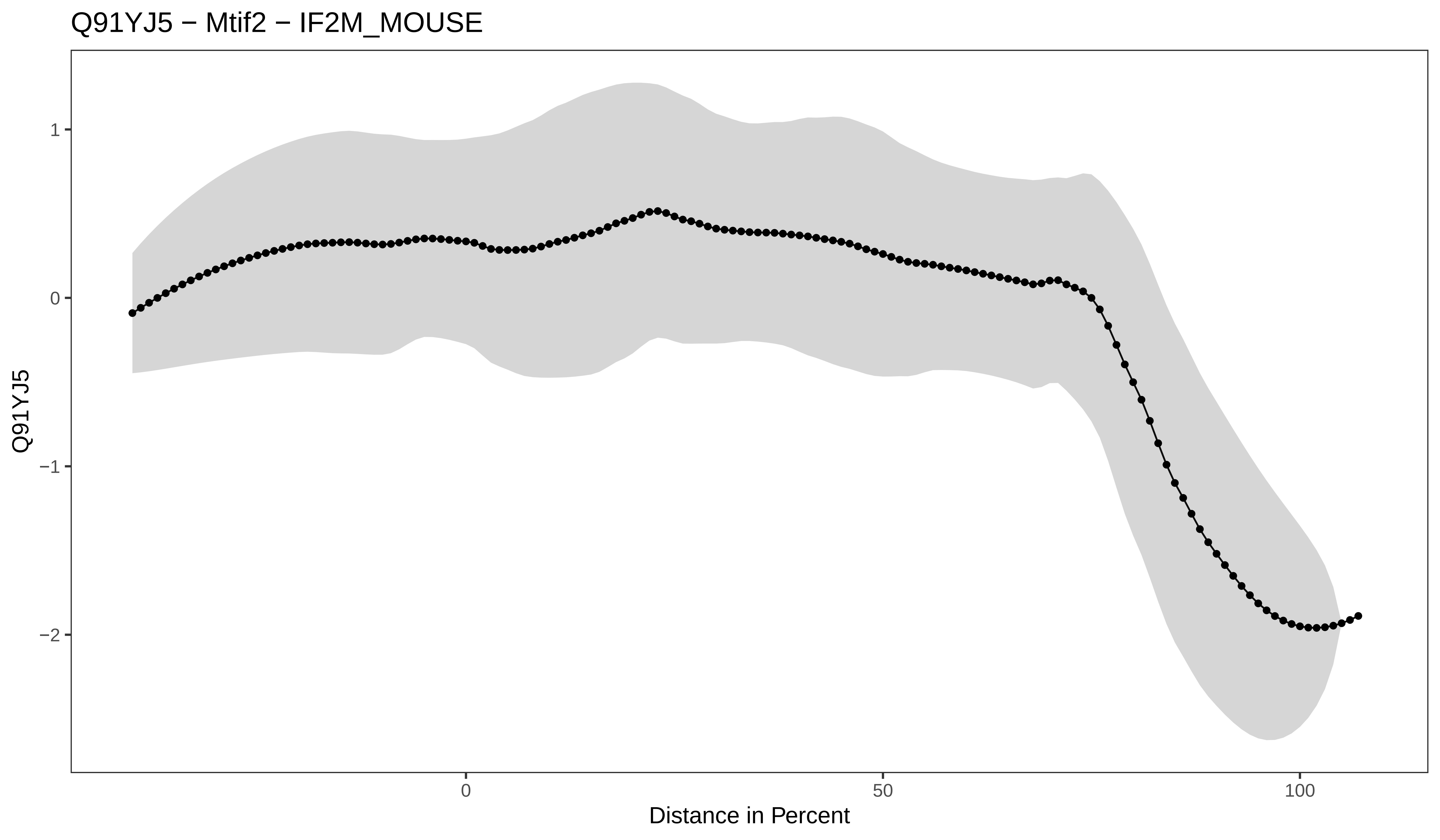

Q91YJ5 − Mtif2 − IF2M_MOUSE
1
0
−1
−2
Q91YJ5
0
50
100
Distance in Percent

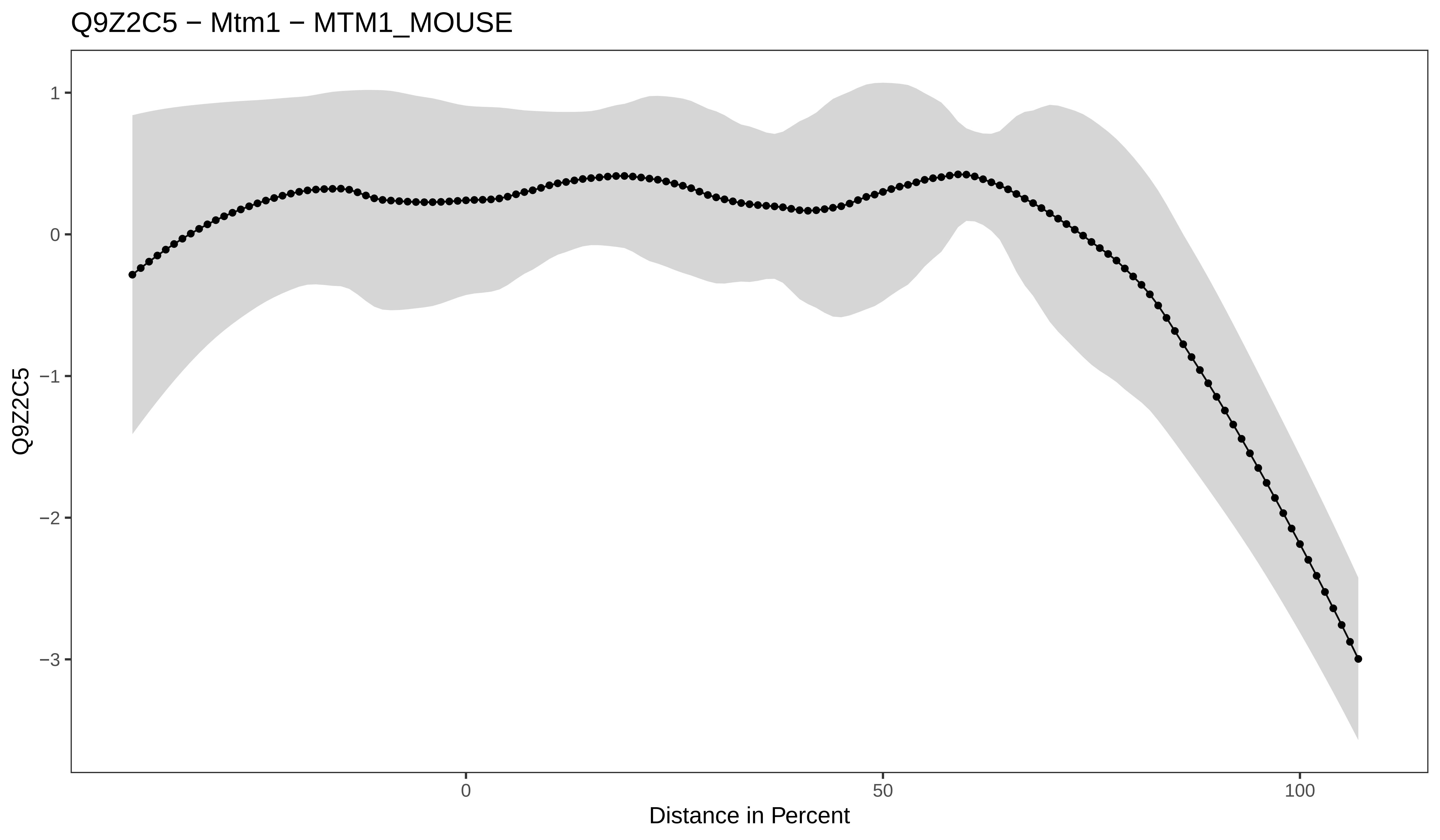
Q9Z2C5 − Mtm1 − MTM1_MOUSE
1
0
−1
−2
−3
Q9Z2C5
0
50
100
Distance in Percent

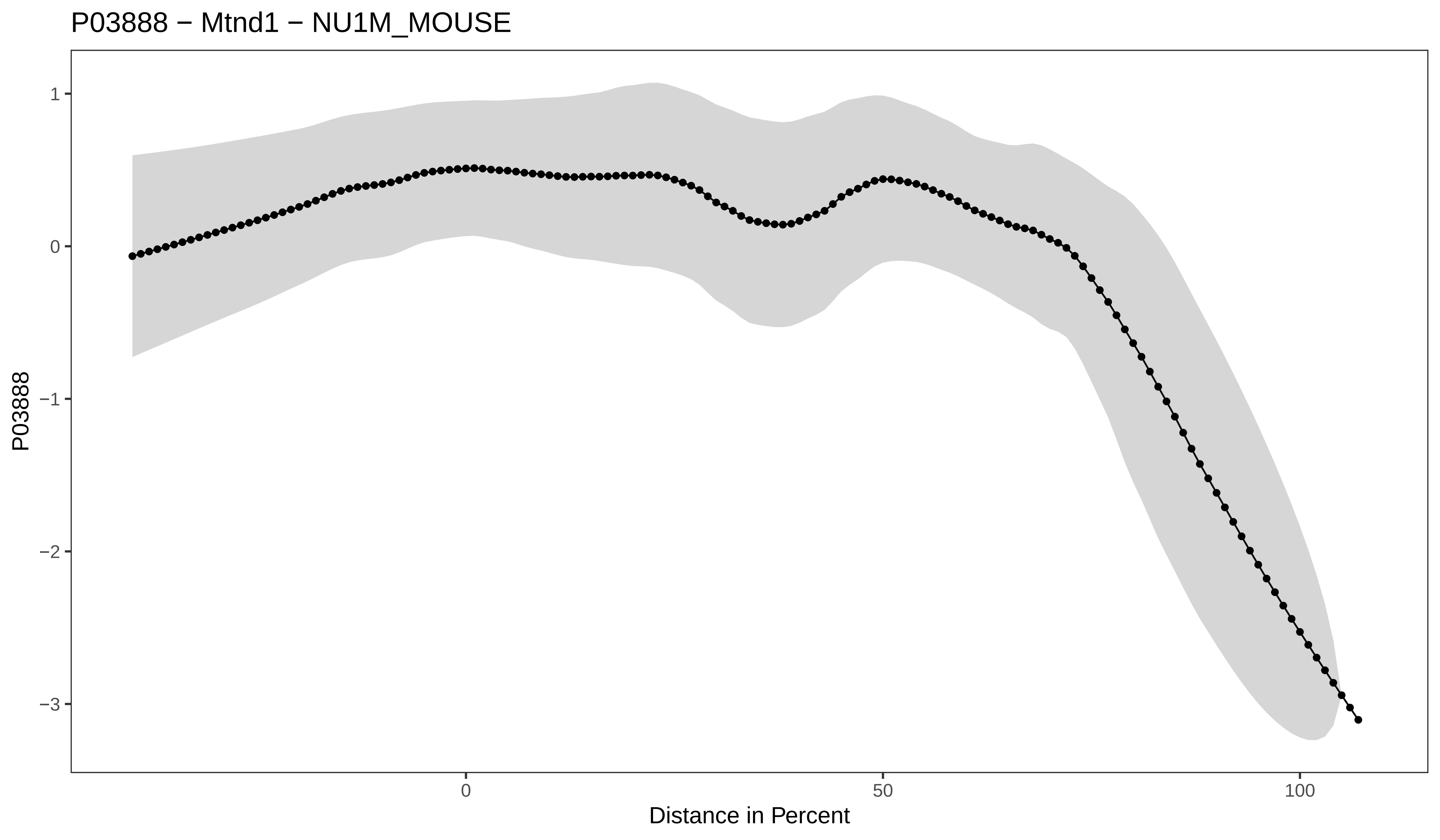

P03888 − Mtnd1 − NU1M_MOUSE
1
0
−1
−2
−3
P03888
0
50
100
Distance in Percent

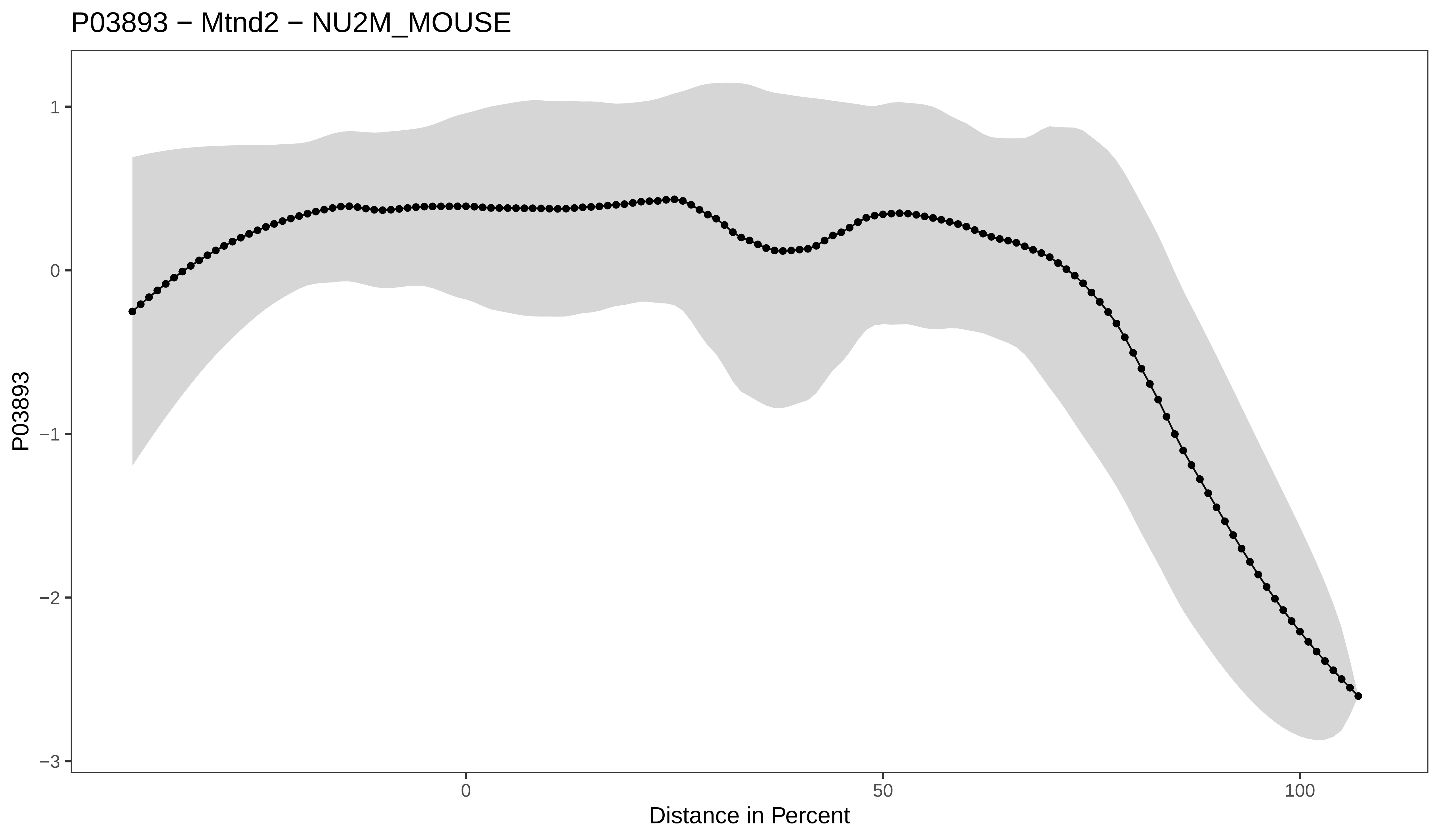
P03893 − Mtnd2 − NU2M_MOUSE
P03893
1
0
−1
−2
−3
0
50
100
Distance in Percent

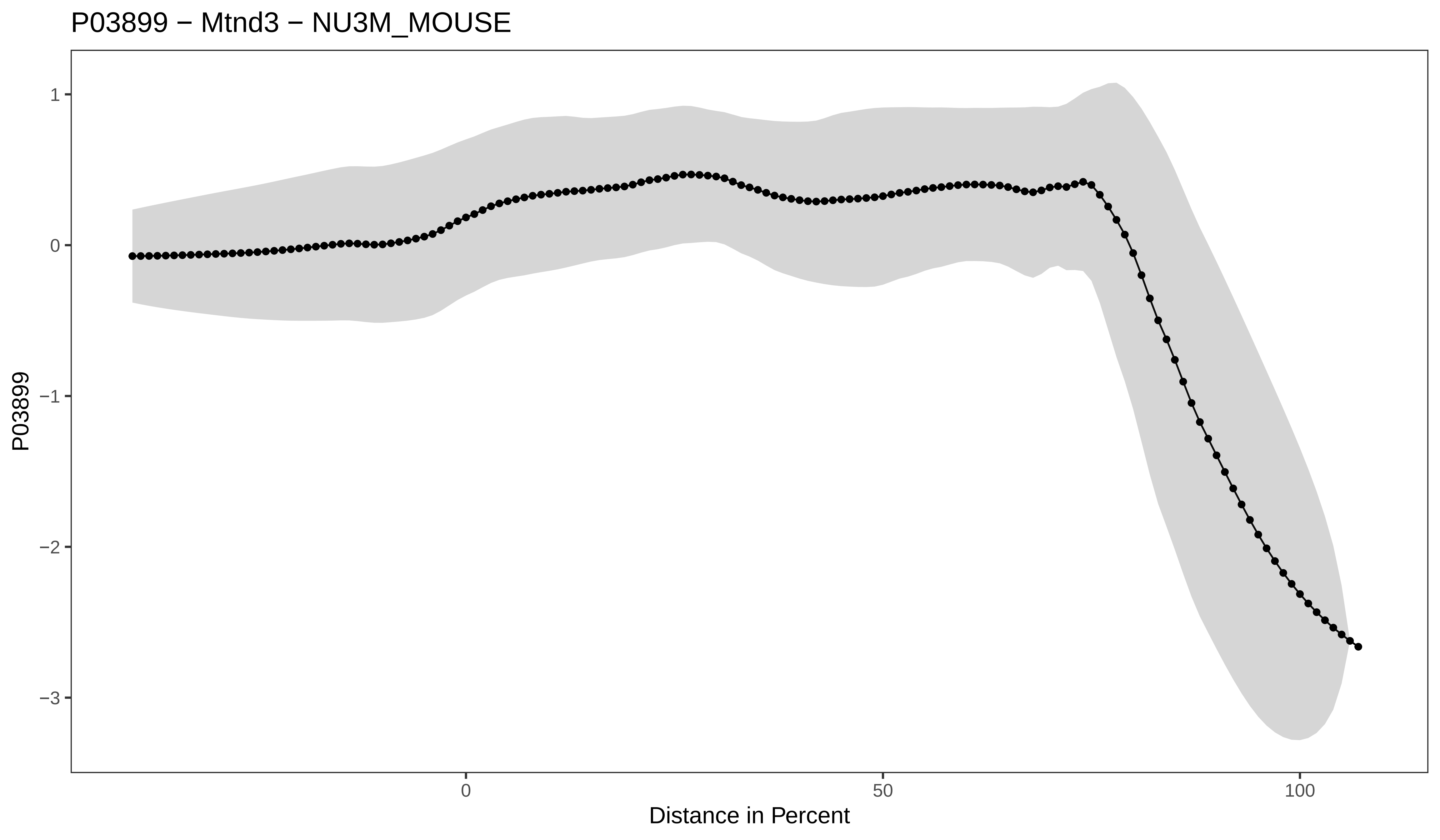

P03899 − Mtnd3 − NU3M_MOUSE
1
0
−1
−2
−3
P03899
0
50
100
Distance in Percent

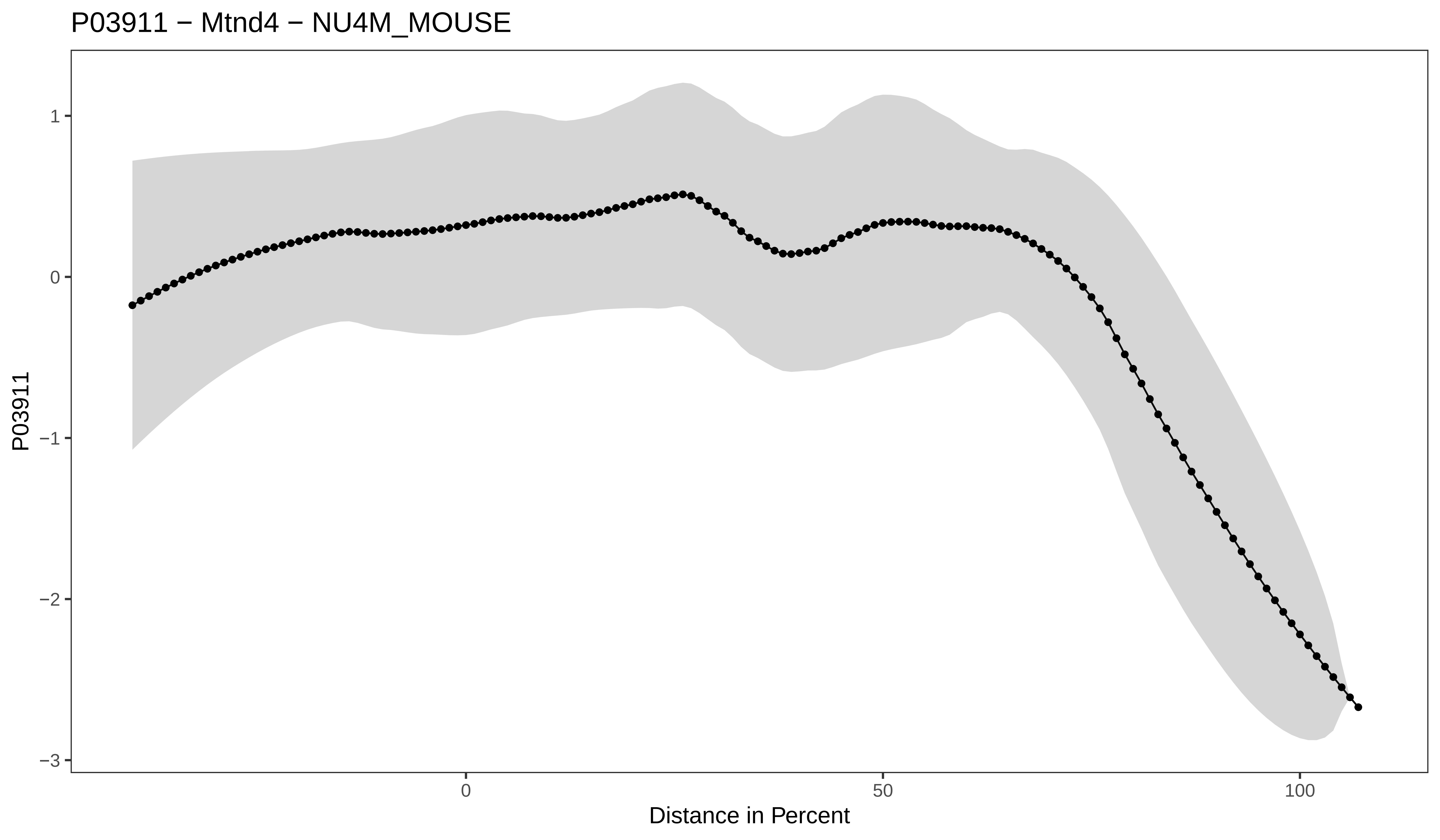

P03911 − Mtnd4 − NU4M_MOUSE
1
0
−1
−2
−3
P03911
0
50
100
Distance in Percent

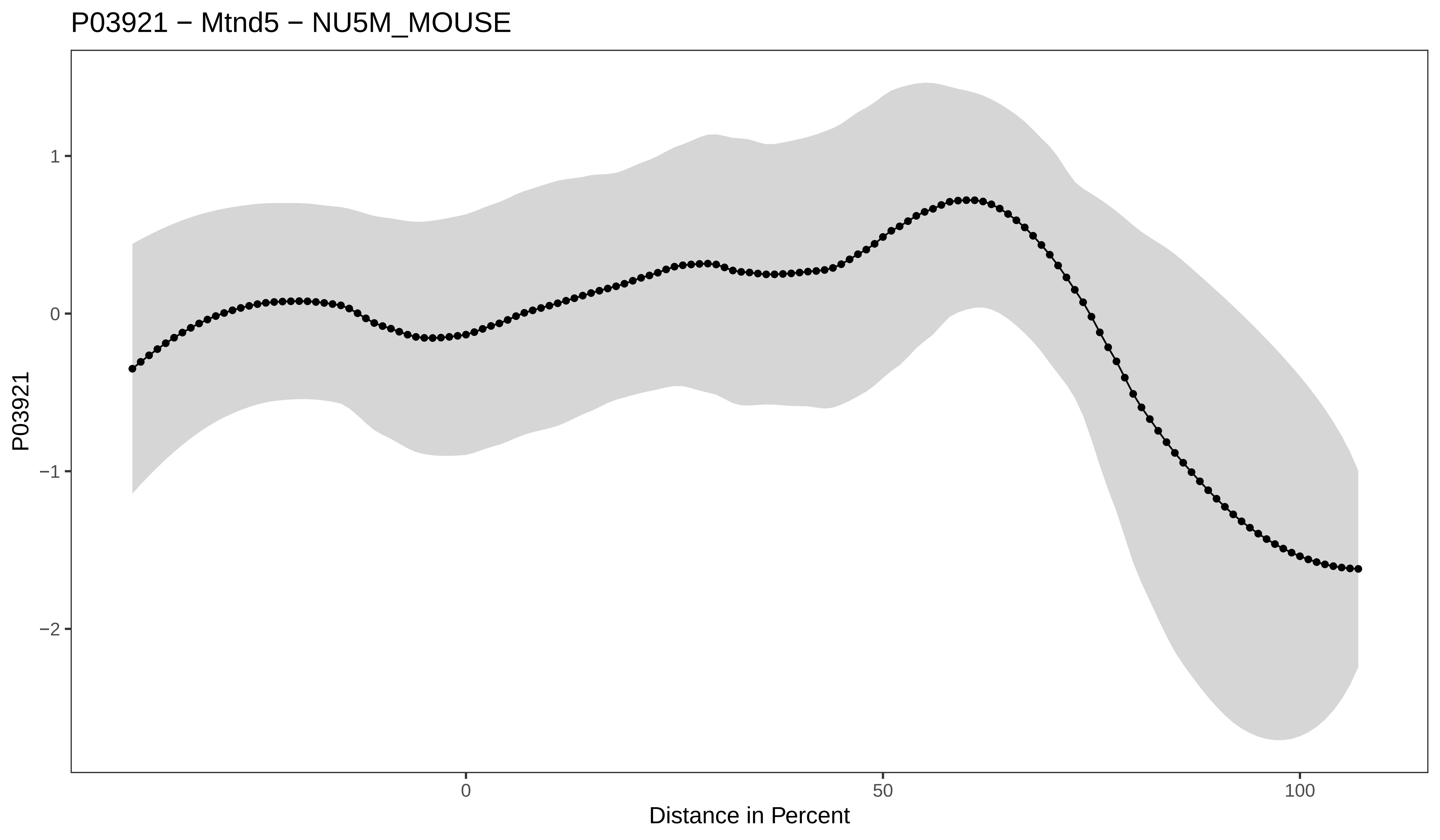

P03921 − Mtnd5 − NU5M_MOUSE
1
0
−1
−2
P03921
0
50
100
Distance in Percent

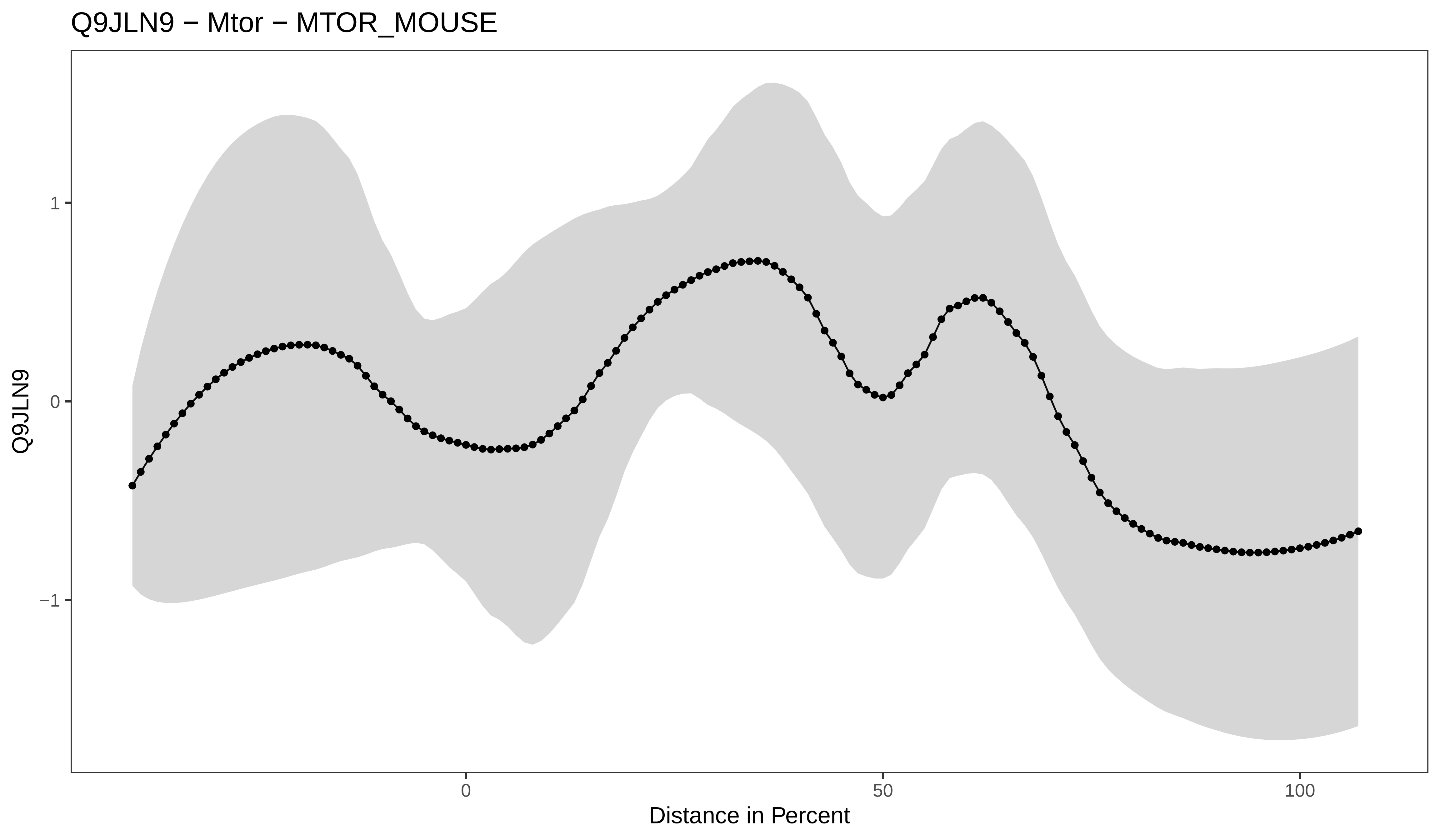
Q9JLN9 − Mtor − MTOR_MOUSE
Q9JLN9
1
0
−1
0
50
100
Distance in Percent

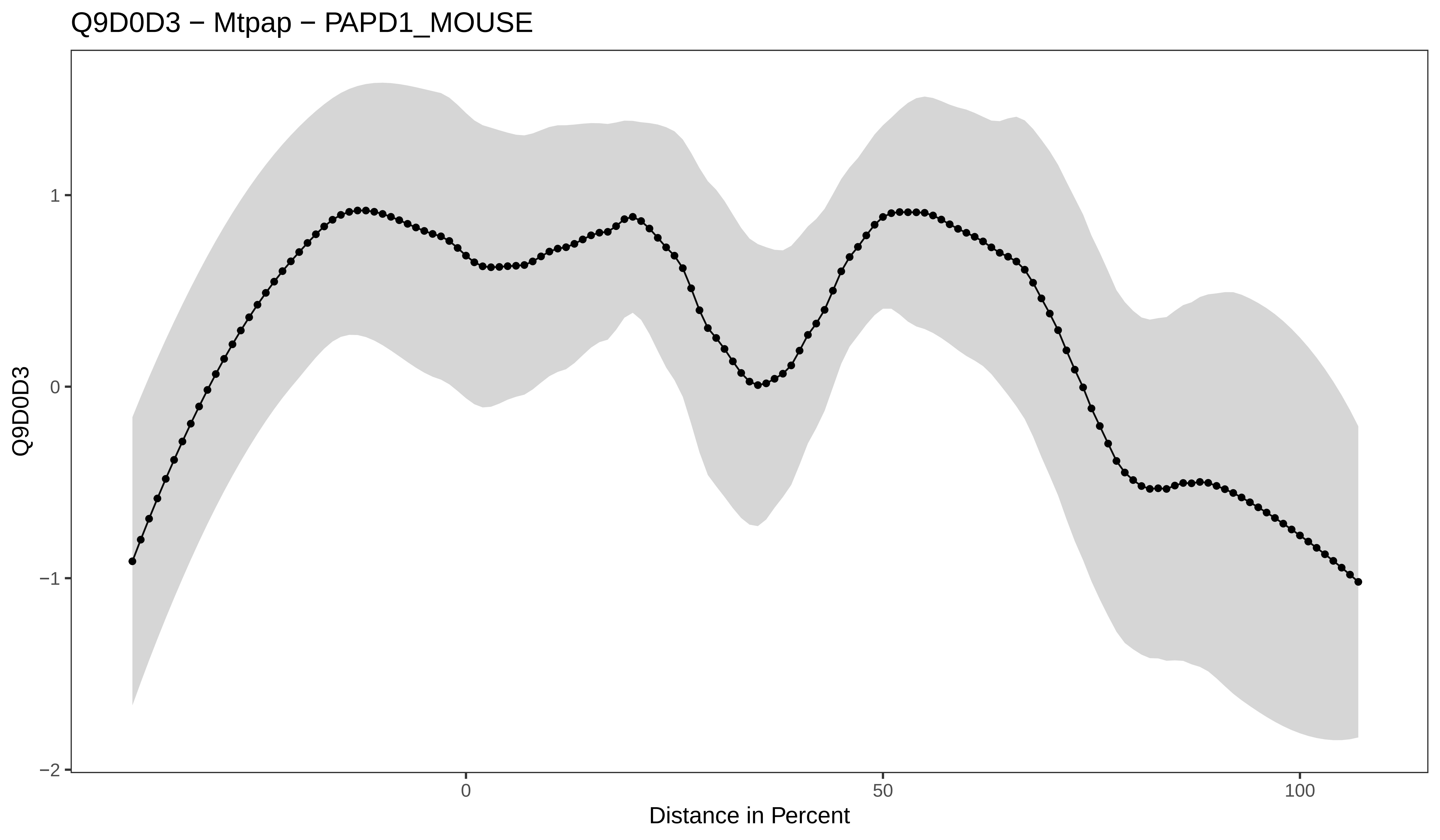
Q9D0D3 − Mtpap − PAPD1_MOUSE
Q9D0D3
1
0
−1
−2
0
50
100
Distance in Percent

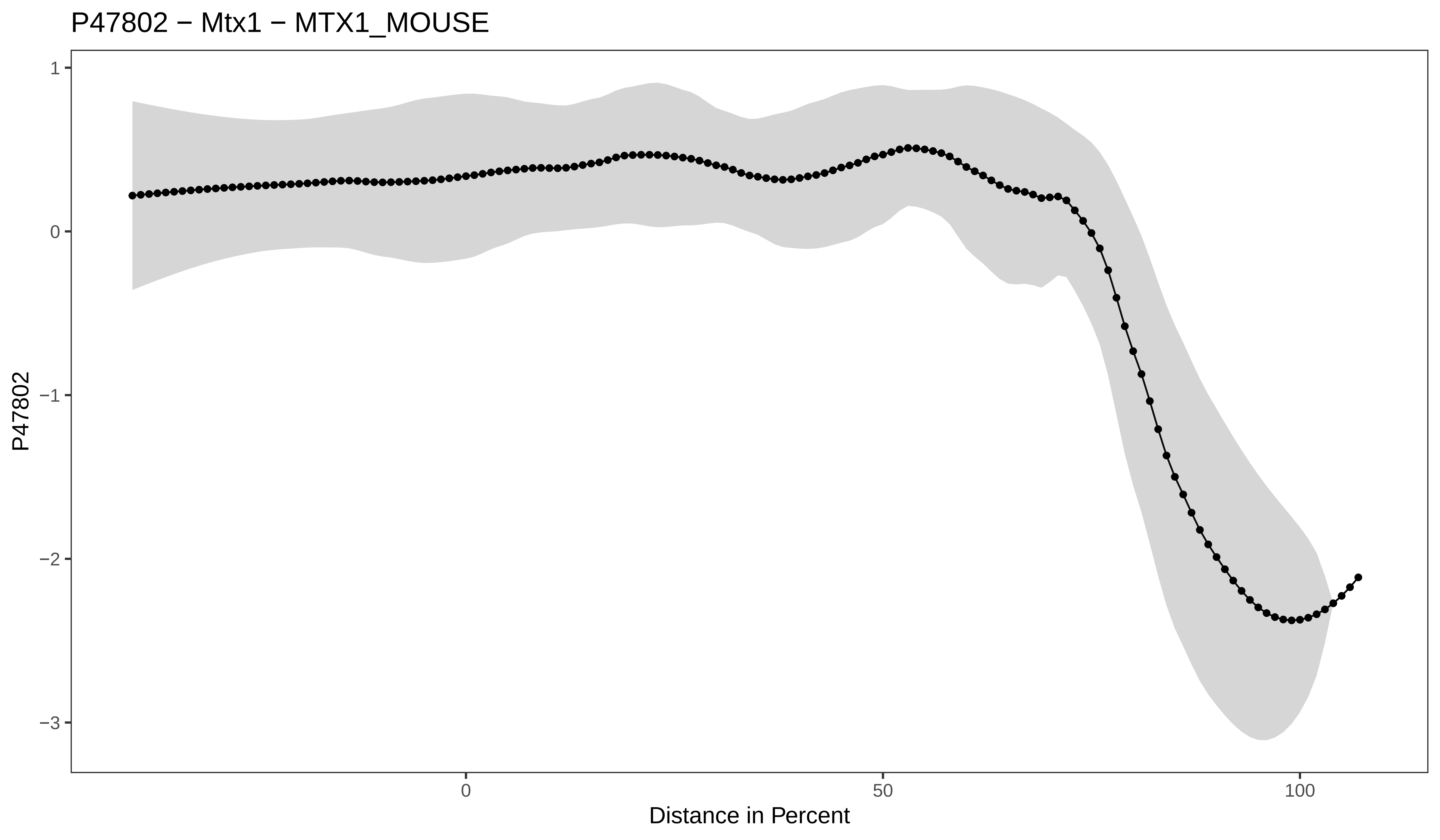

P47802 − Mtx1 − MTX1_MOUSE
1
0
−1
−2
−3
P47802
0
50
100
Distance in Percent

O88441 − Mtx2 − MTX2_MOUSE

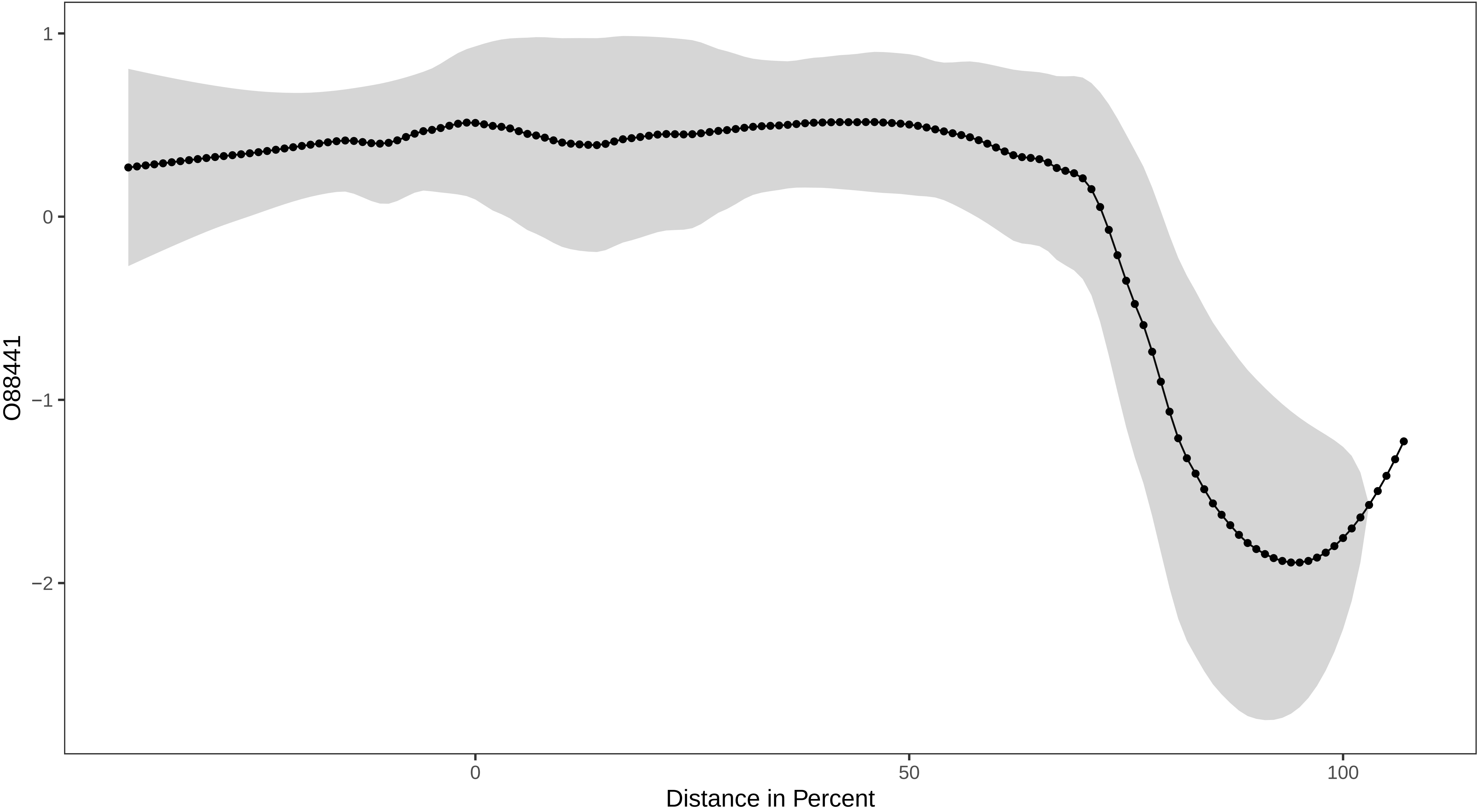

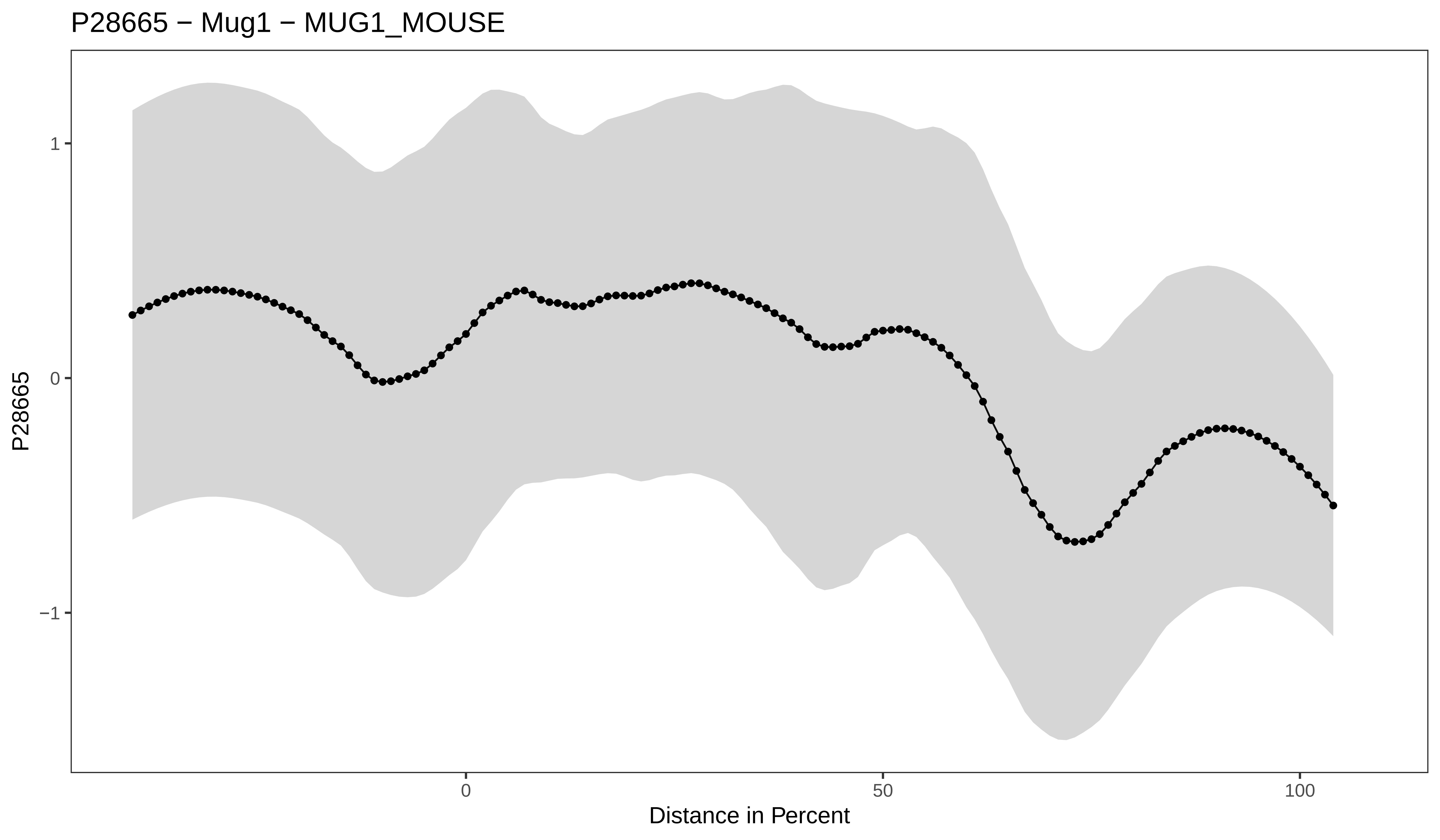
P28665 − Mug1 − MUG1_MOUSE
P28665
1
0
−1
0
50
100
Distance in Percent

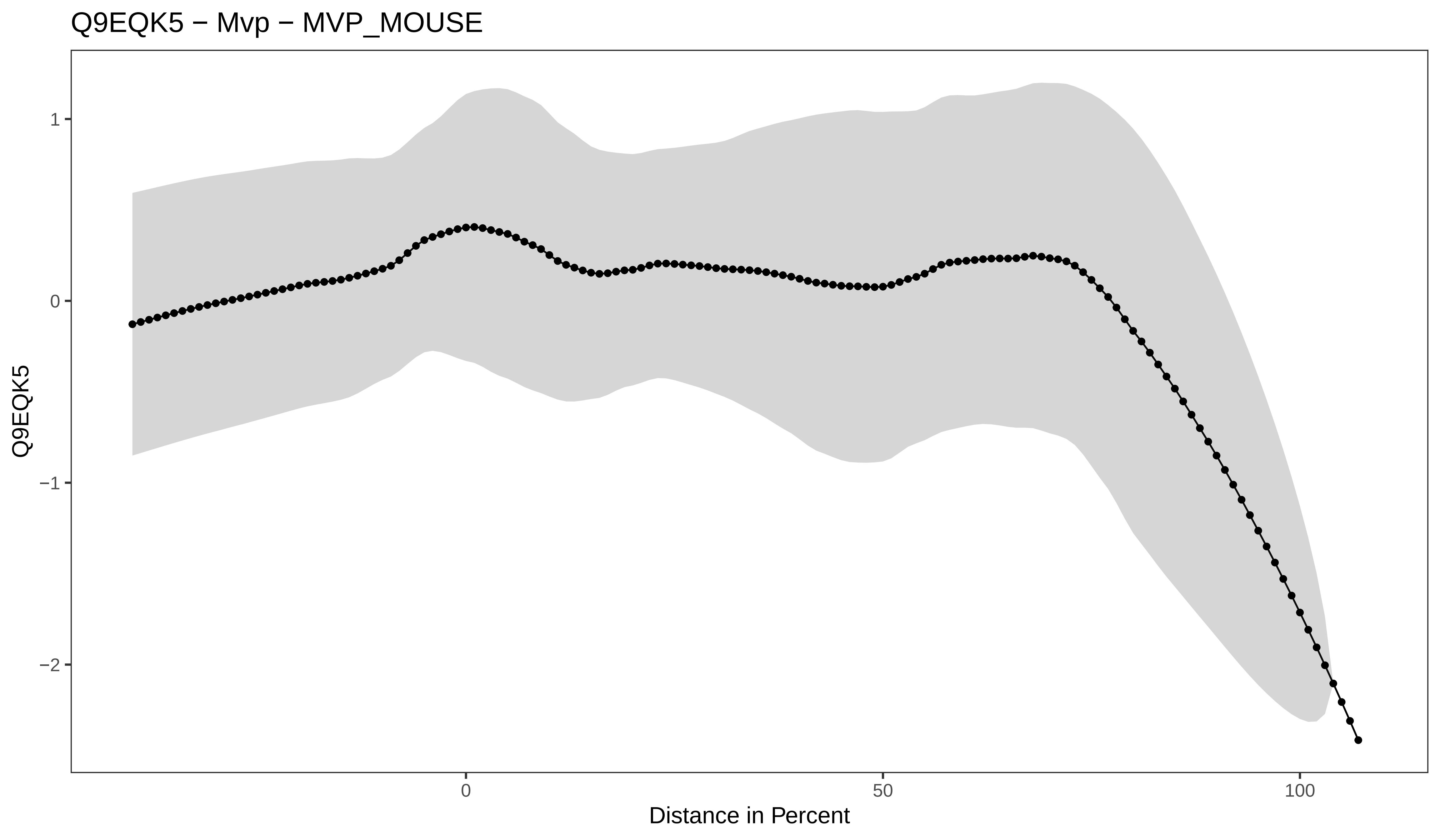
Q9EQK5 − Mvp − MVP_MOUSE
Q9EQK5
1
0
−1
−2
0
50
100
Distance in Percent

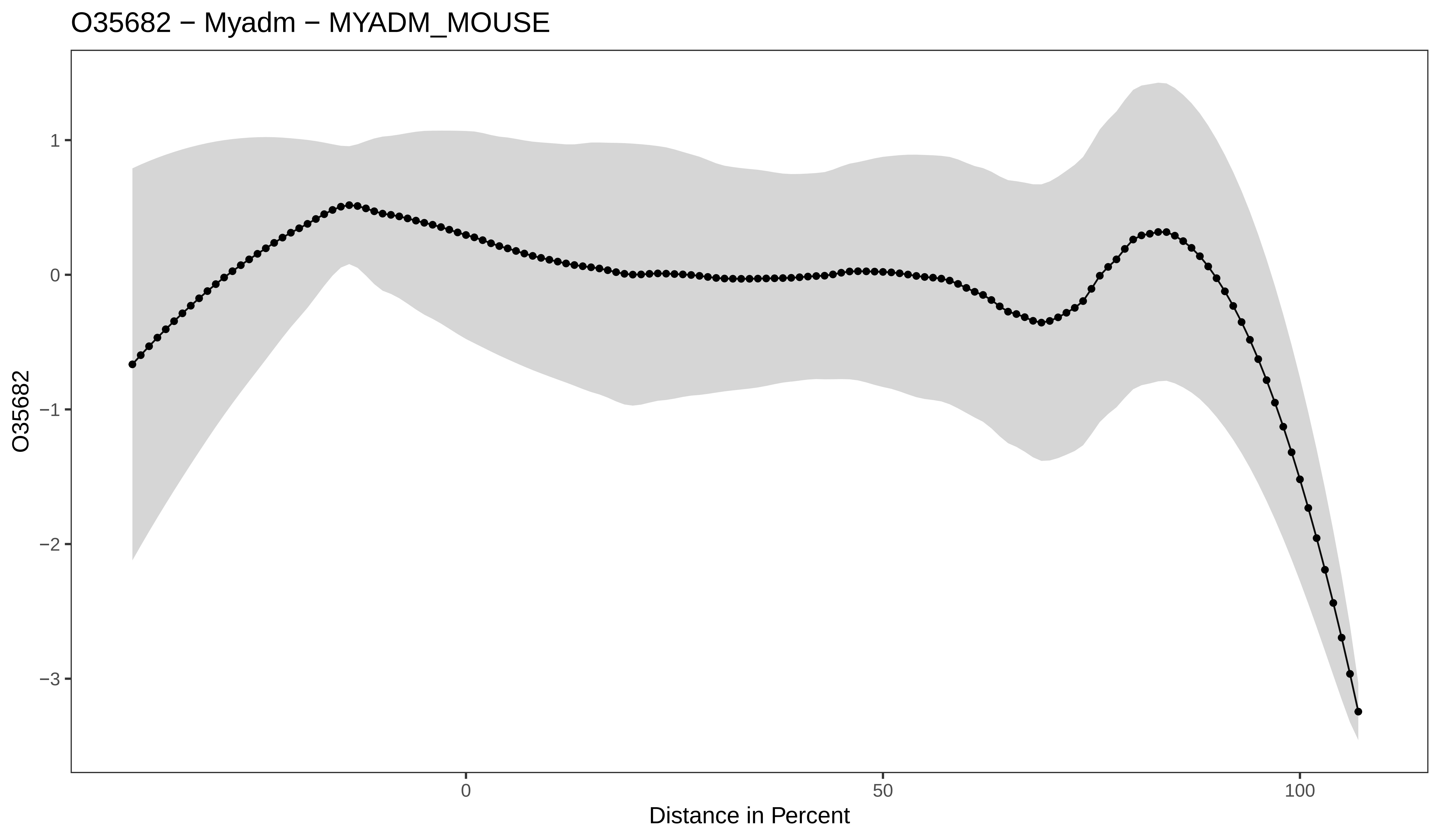

O35682 − Myadm − MYADM_MOUSE
1
0
−1
−2
−3
O35682
0
50
100
Distance in Percent

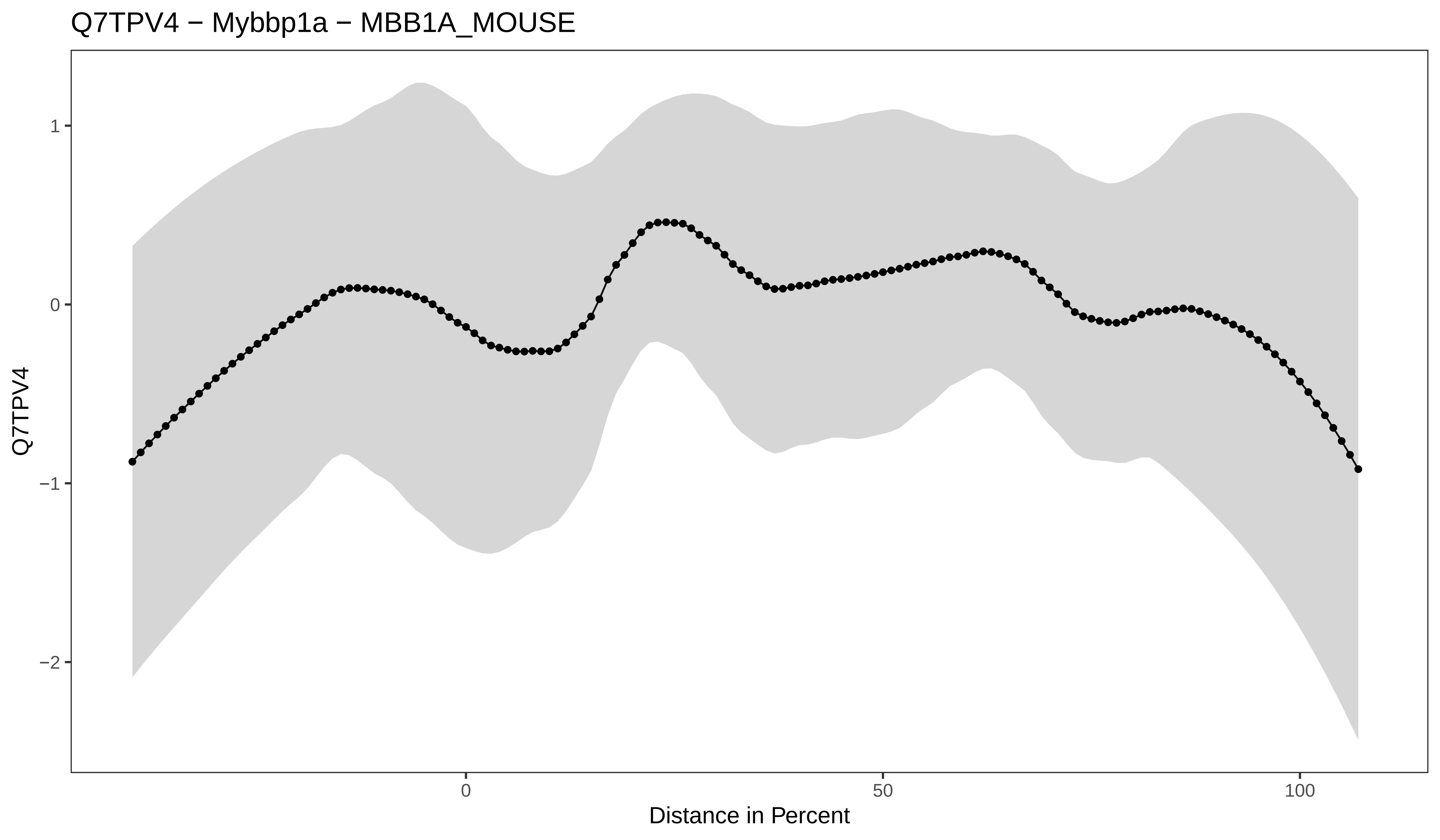
Q7TPV4 − Mybbp1a − MBB1A_MOUSE
Q7TPV4
1
0
−1
−2
0
50
100
Distance in Percent

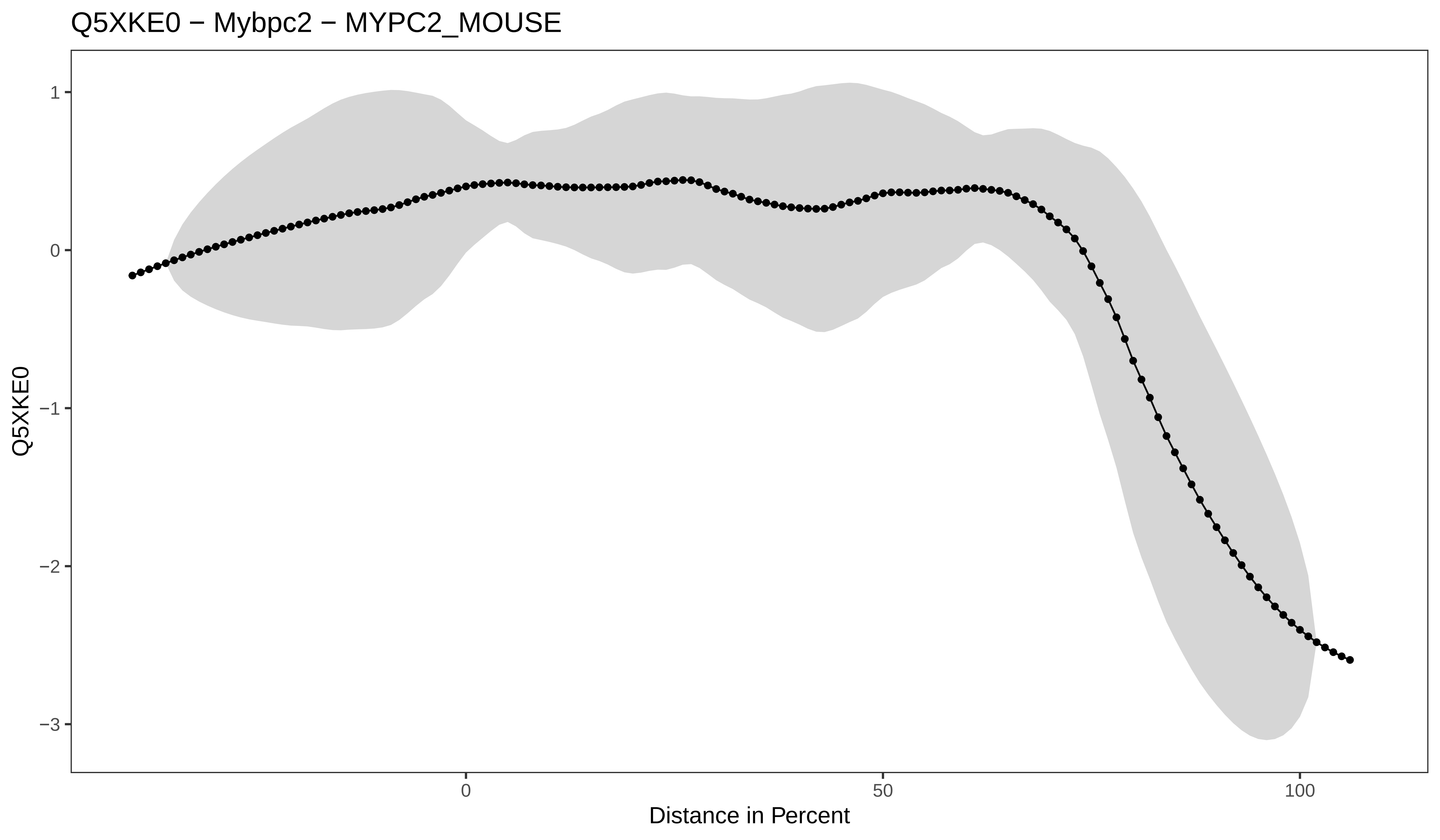

Q5XKE0 − Mybpc2 − MYPC2_MOUSE
1
0
−1
−2
−3
Q5XKE0
0
50
100
Distance in Percent

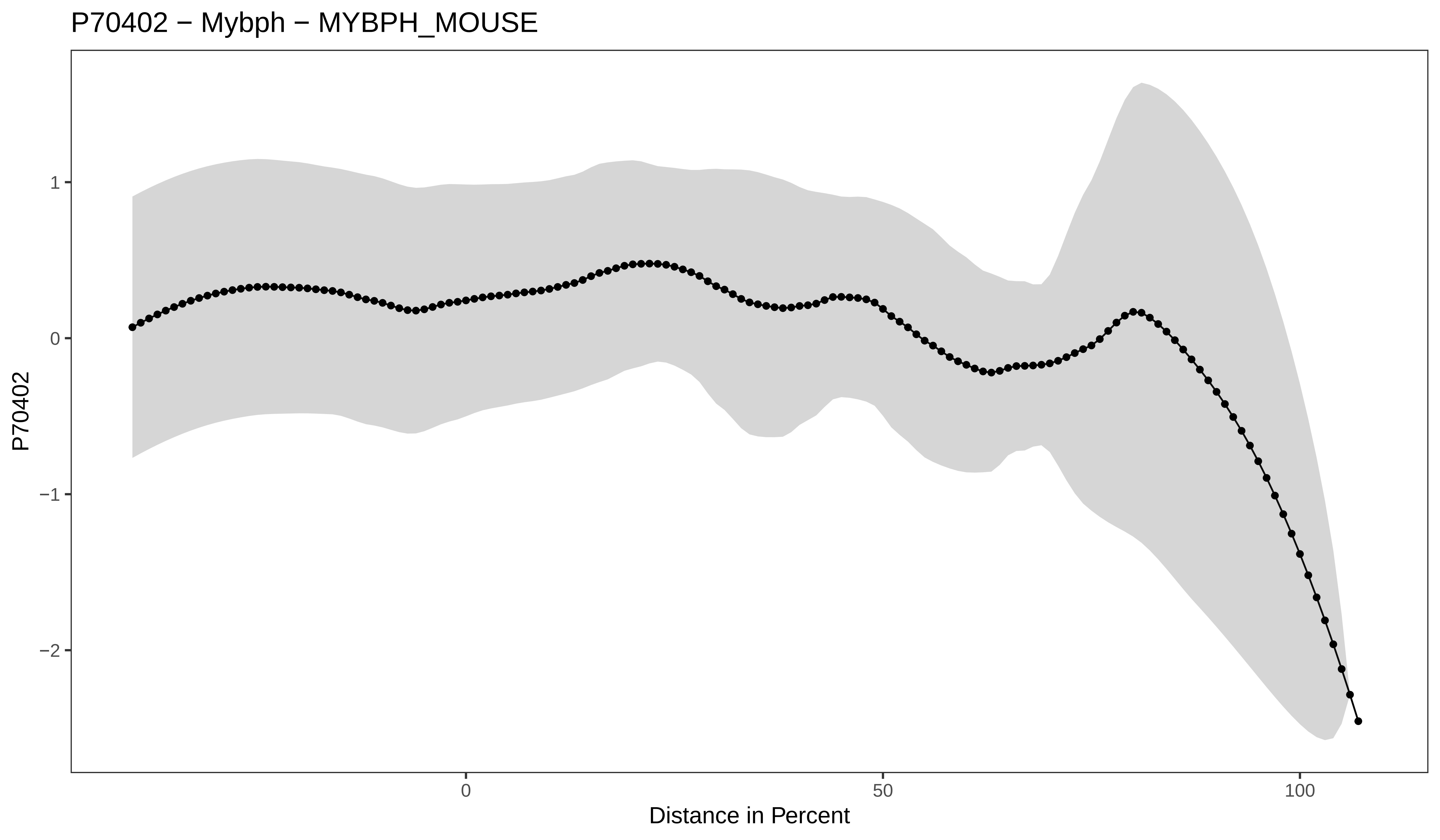
P70402 − Mybph − MYBPH_MOUSE
P70402
1
0
−1
−2
0
50
100
Distance in Percent

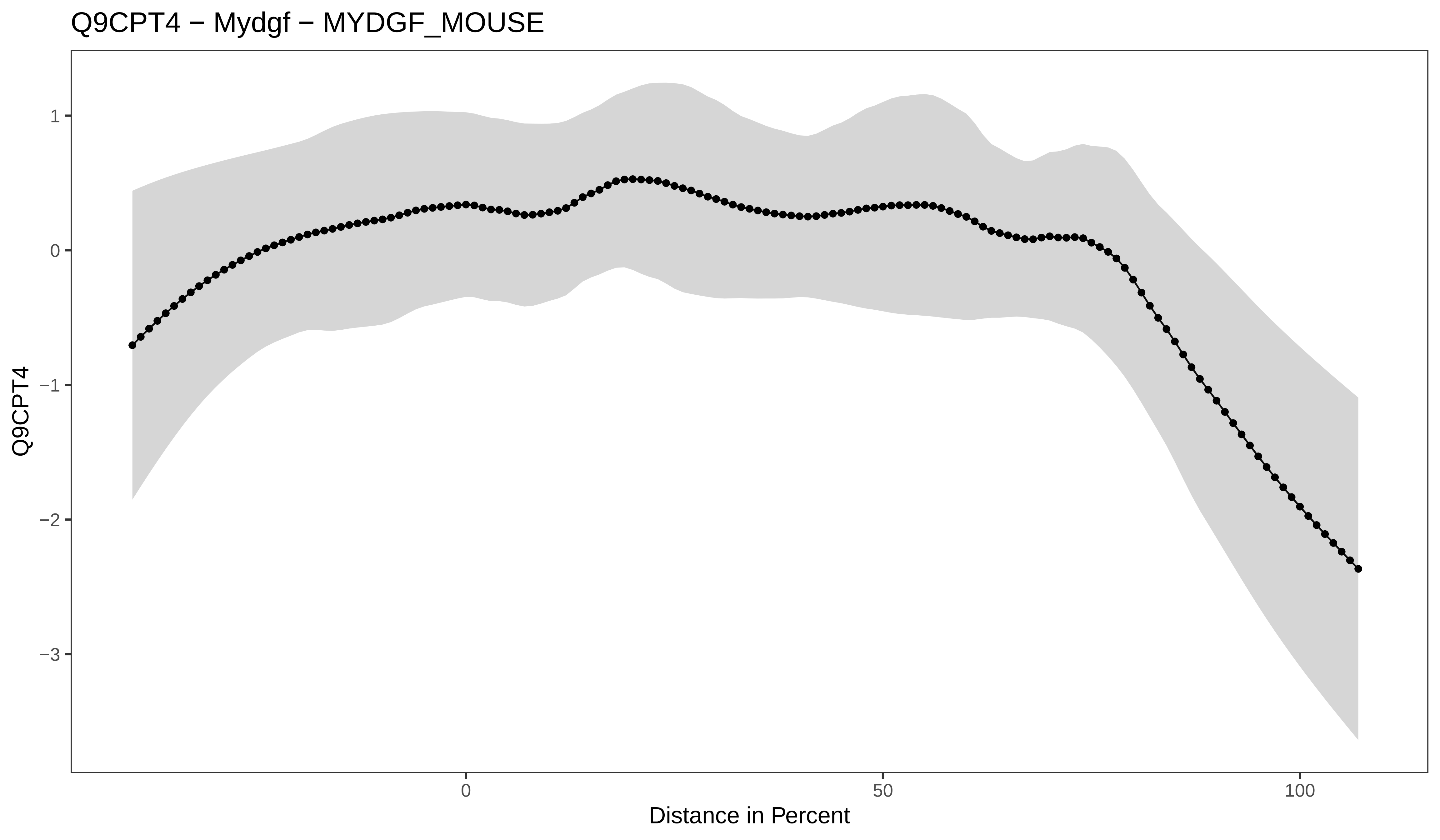

Q9CPT4 − Mydgf − MYDGF_MOUSE
Q9CPT4
Distance in Percent
1
0
−1
−2
−3
0
50
100

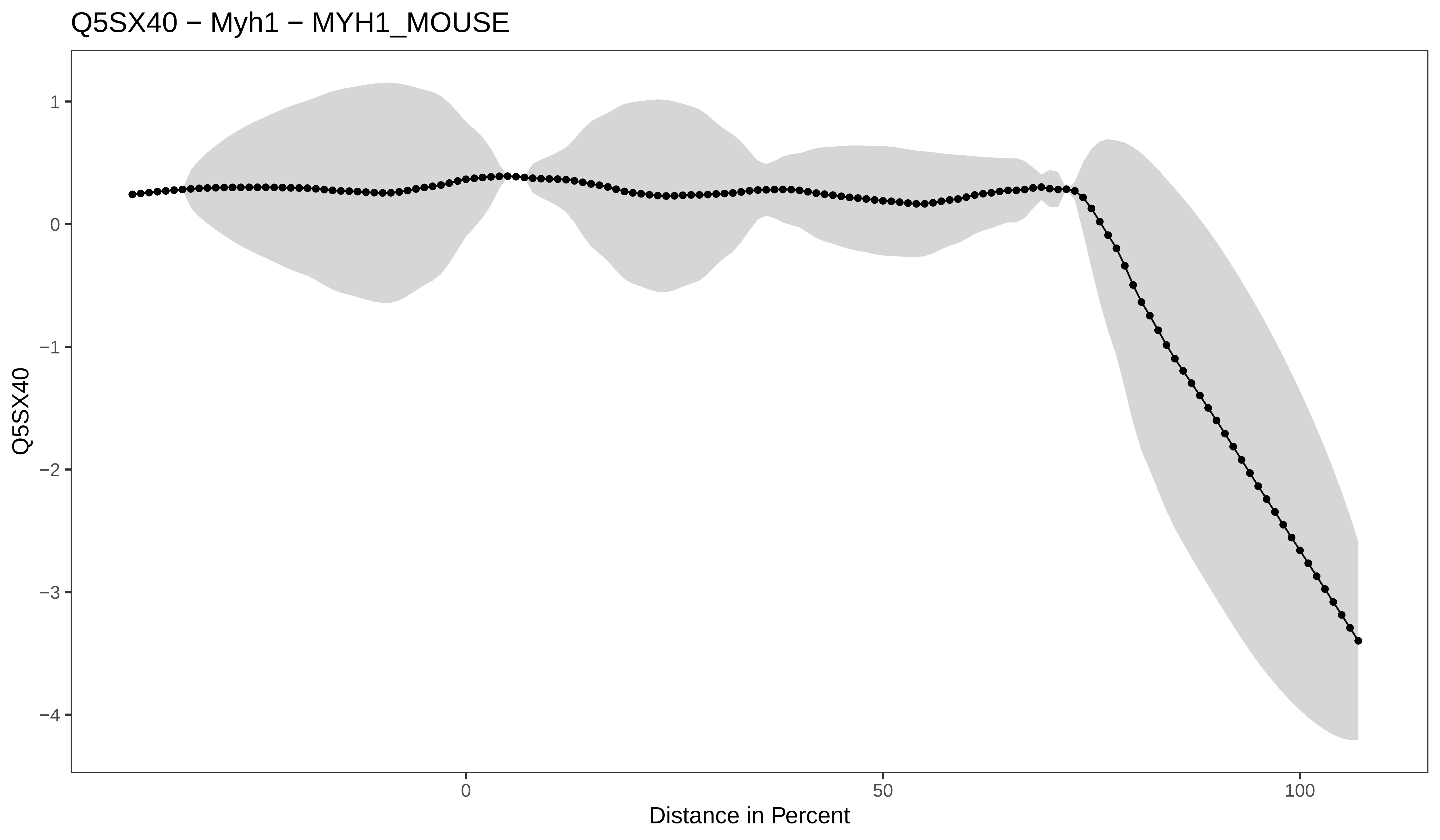

Q5SX40 − Myh1 − MYH1_MOUSE
1
0
−1
−2
−3
−4
Q5SX40
0
50
100
Distance in Percent

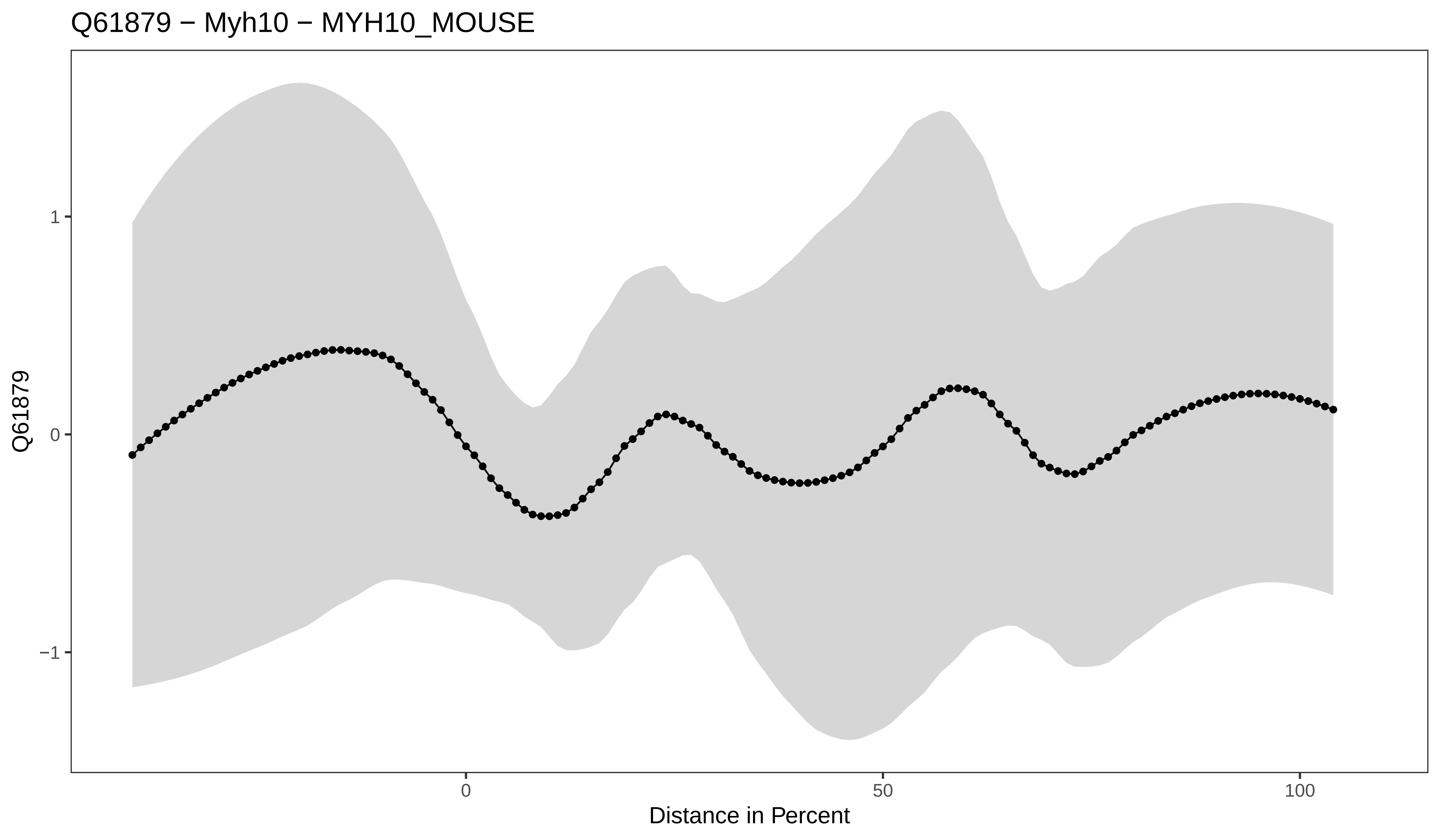
Q61879 − Myh10 − MYH10_MOUSE
Q61879
1
0
−1
0
50
100
Distance in Percent

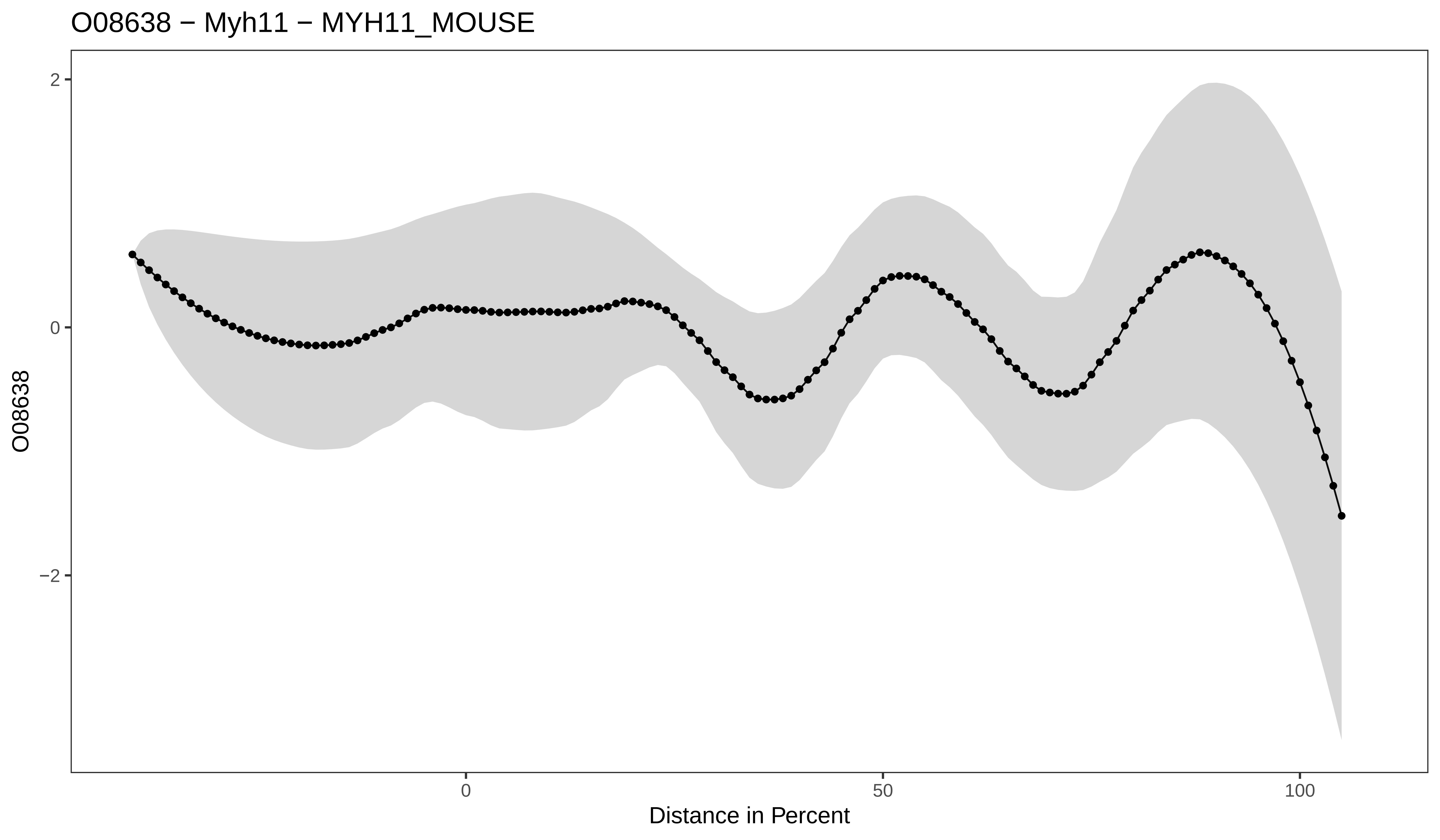
O08638 − Myh11 − MYH11_MOUSE
2
0
−2
O08638
0
50
100
Distance in Percent

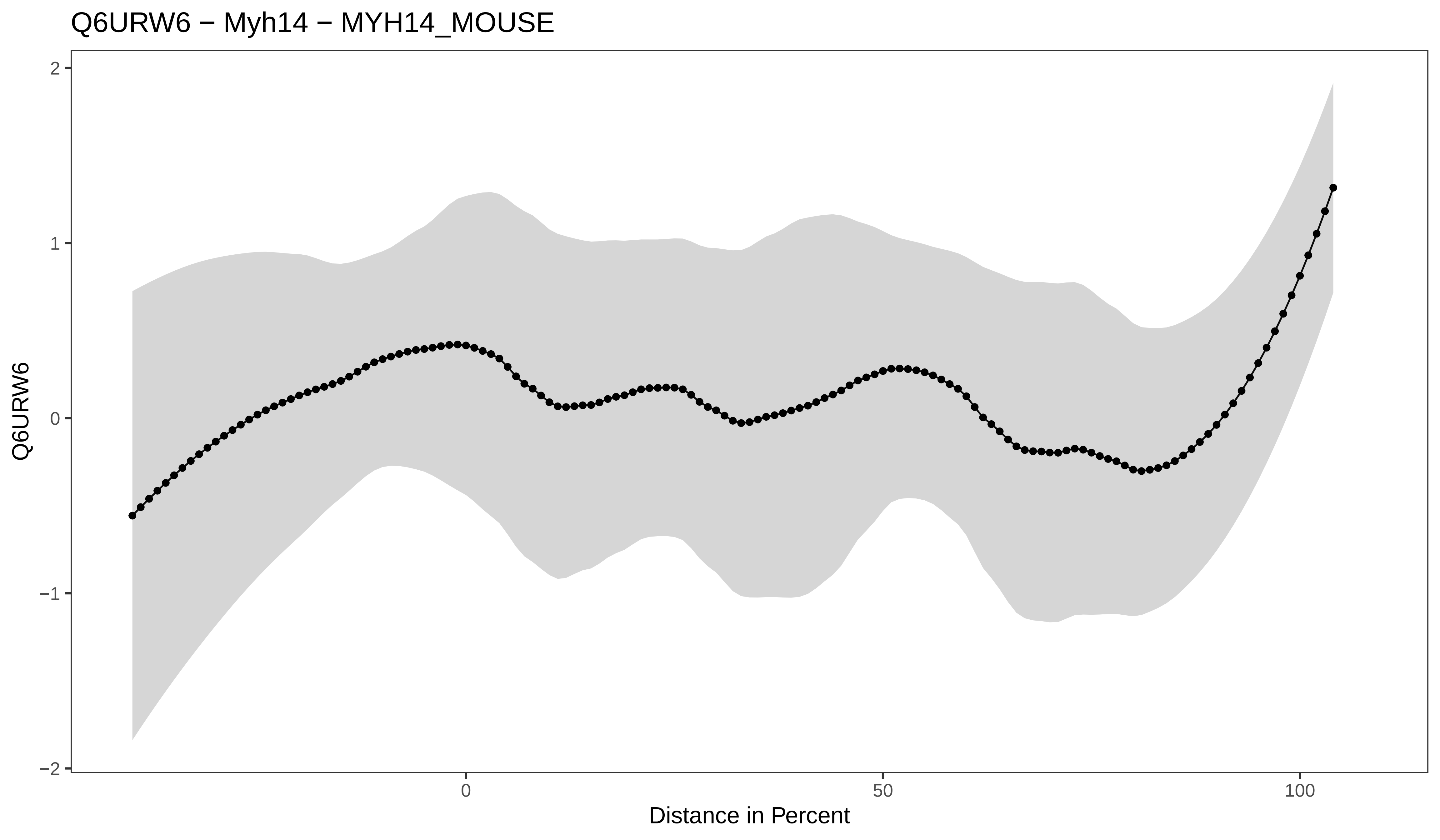
Q6URW6 − Myh14 − MYH14_MOUSE
Q6URW6
2
1
0
−1
−2
0
50
100
Distance in Percent

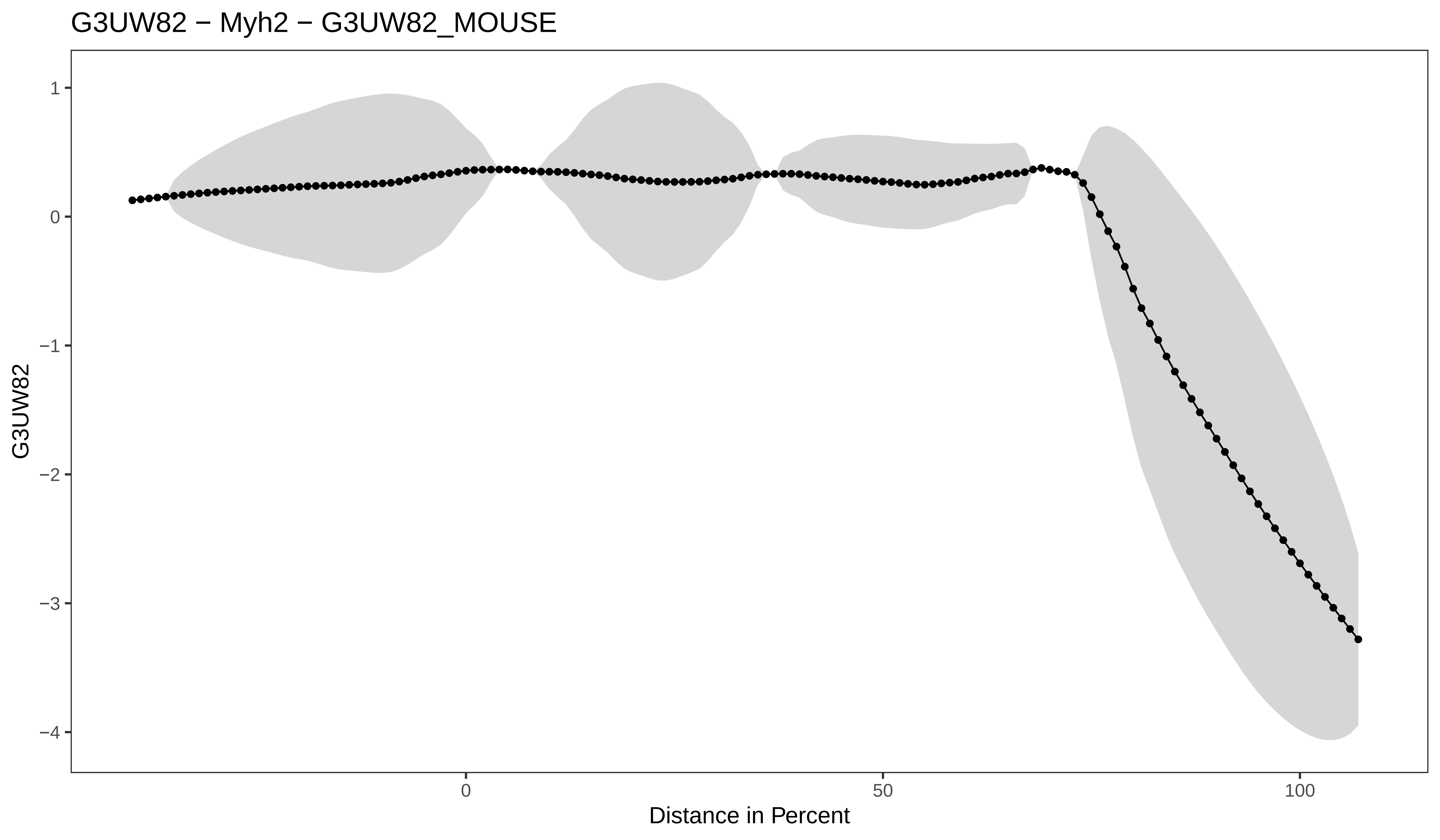

G3UW82 − Myh2 − G3UW82_MOUSE
G3UW82
Distance in Percent
1
0
−1
−2
−3
−4
0
50
100

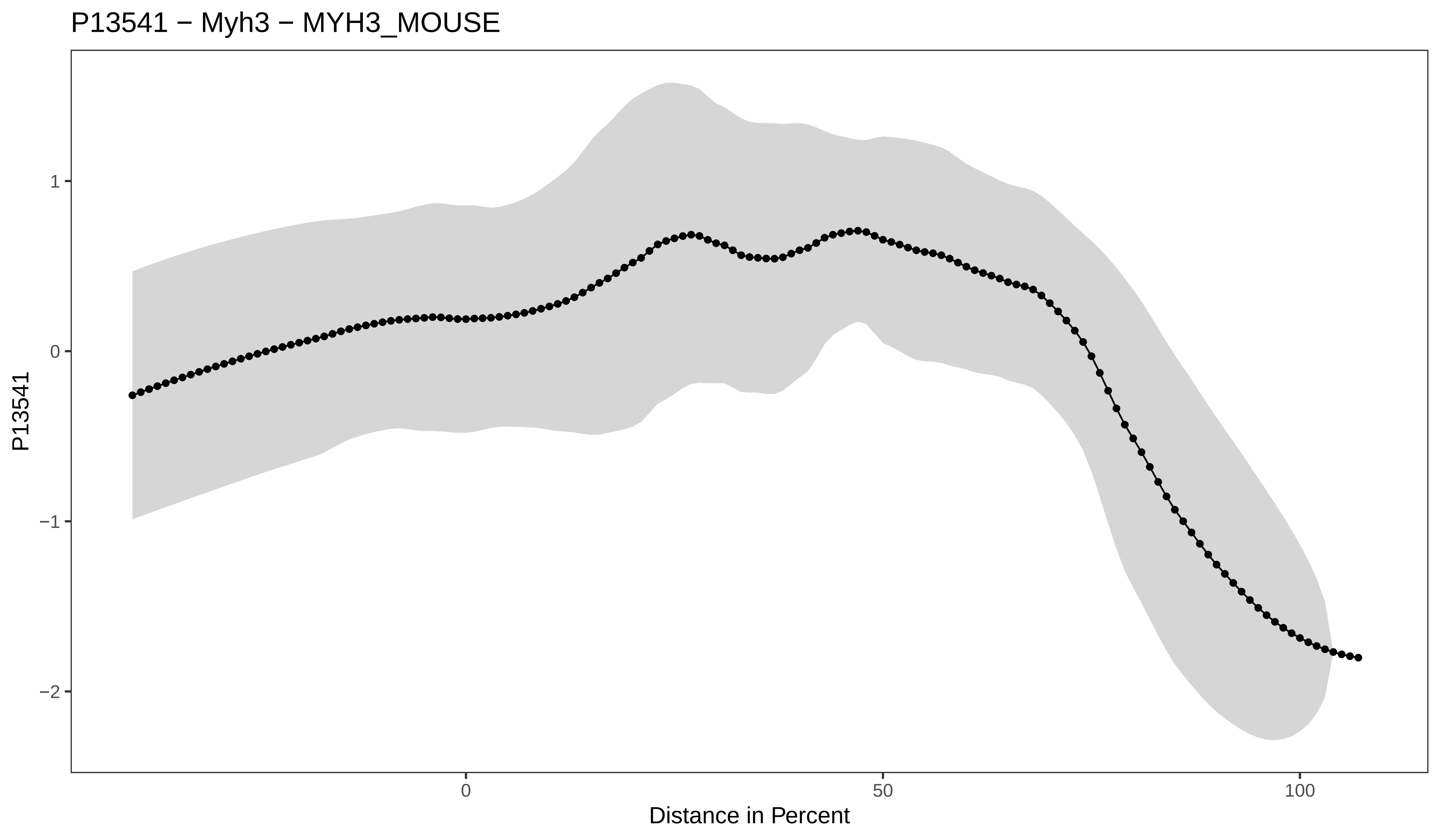

P13541 − Myh3 − MYH3_MOUSE
P13541
1
0
−1
−2
0
50
100
Distance in Percent

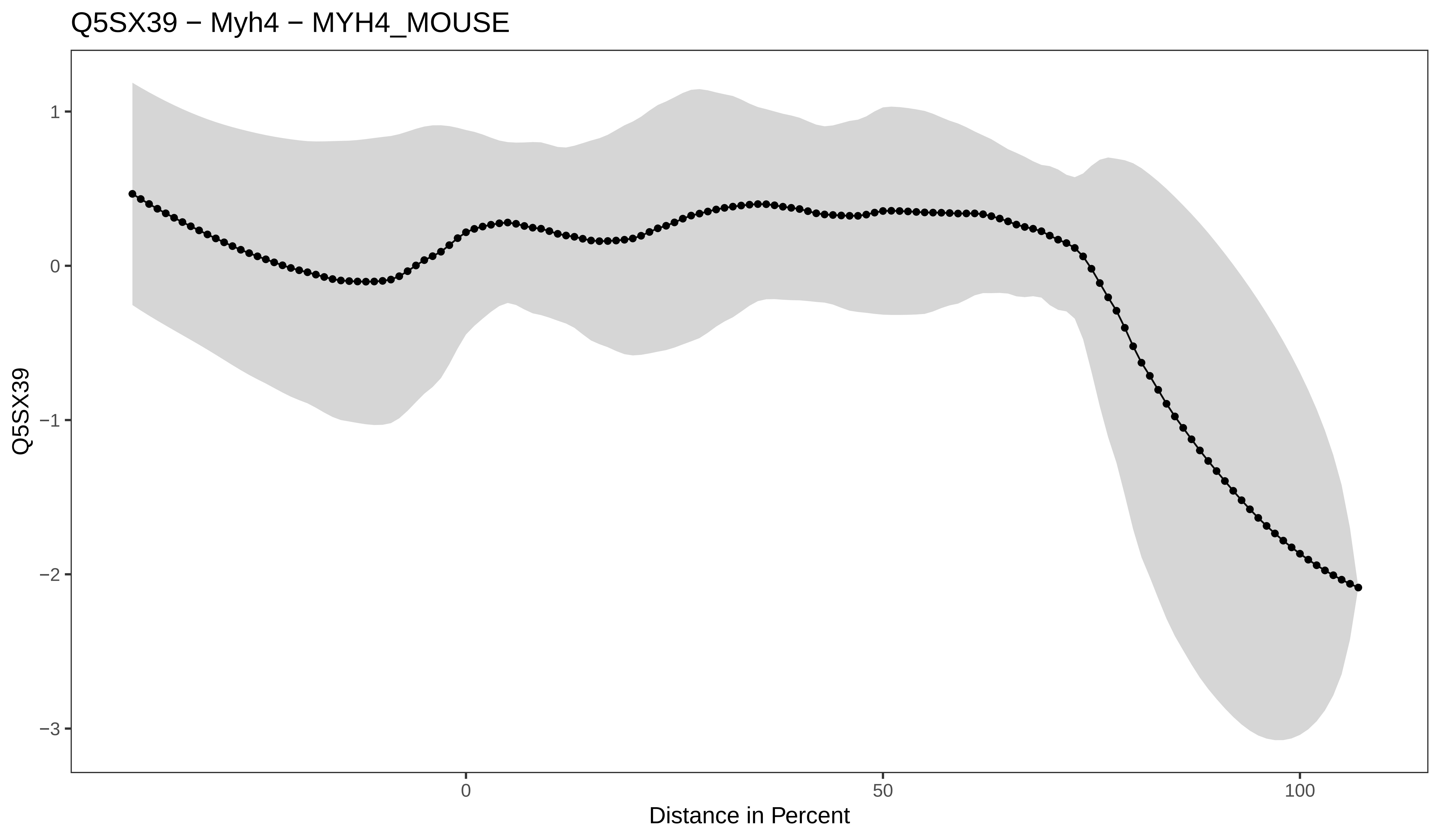

Q5SX39 − Myh4 − MYH4_MOUSE
Q5SX39
Distance in Percent
1
0
−1
−2
−3
0
50
100

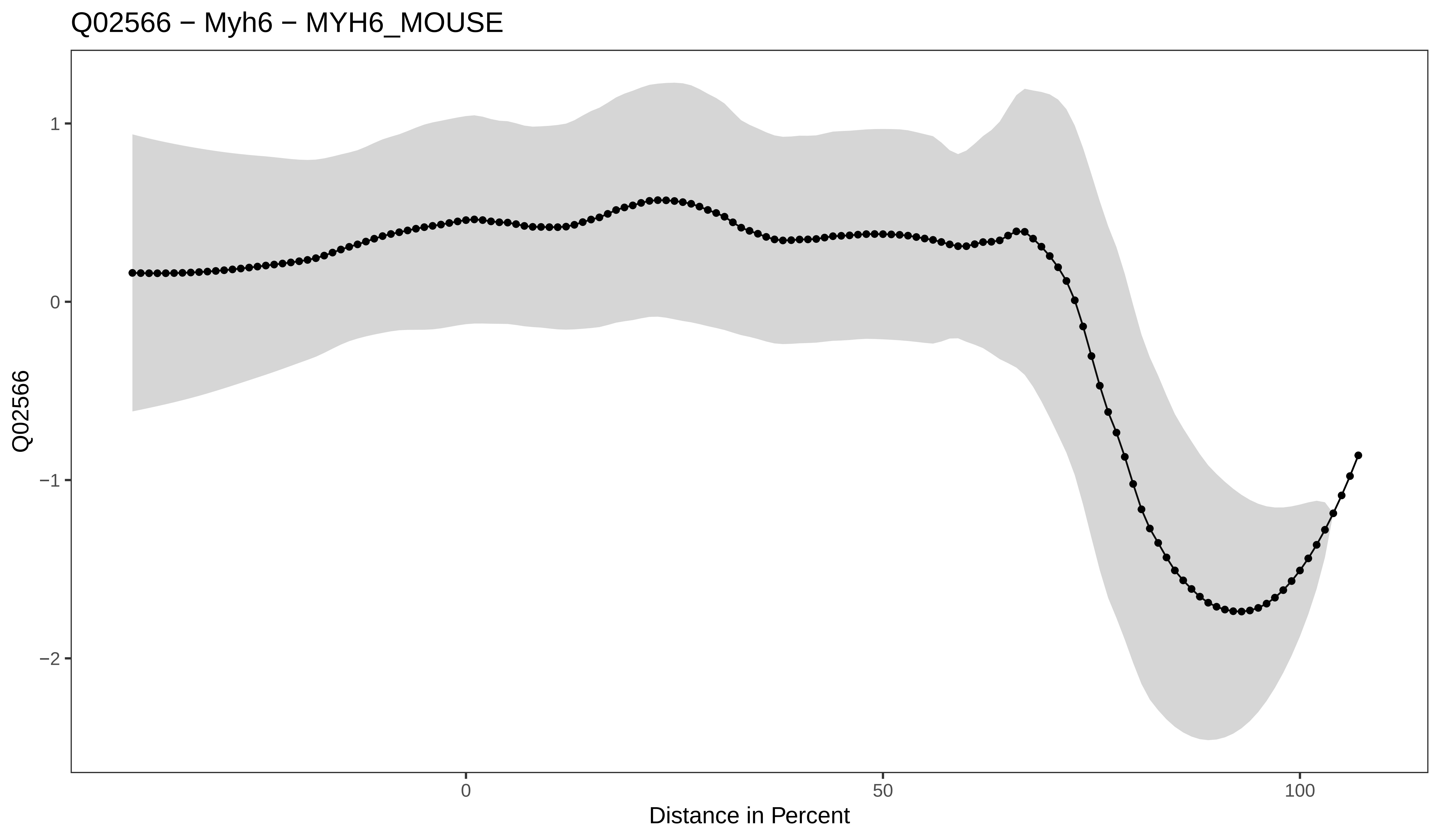
Q02566 − Myh6 − MYH6_MOUSE
1
0
−1
−2
Q02566
0
50
100
Distance in Percent

Q91Z83 − Myh7 − MYH7_MOUSE

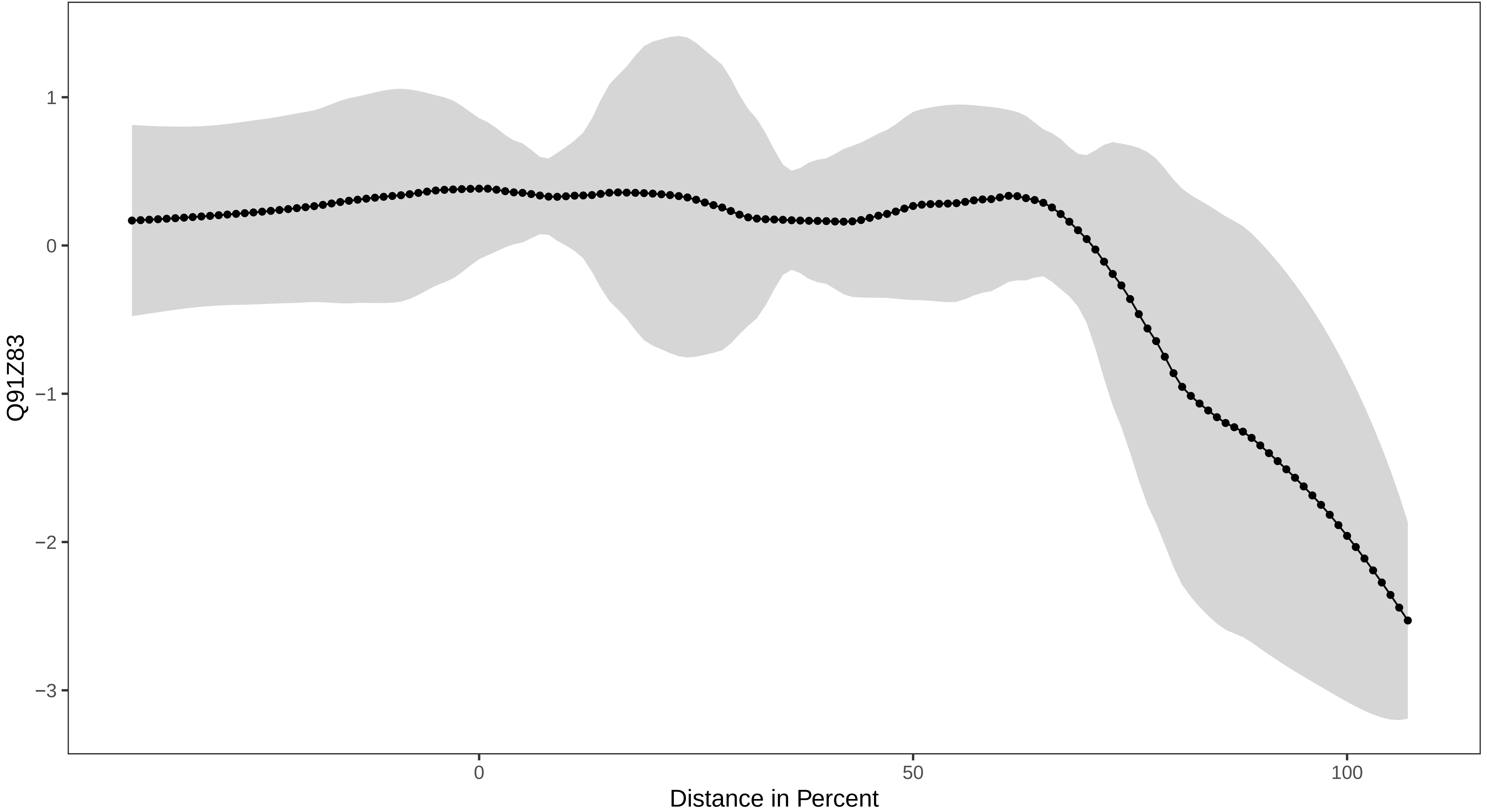

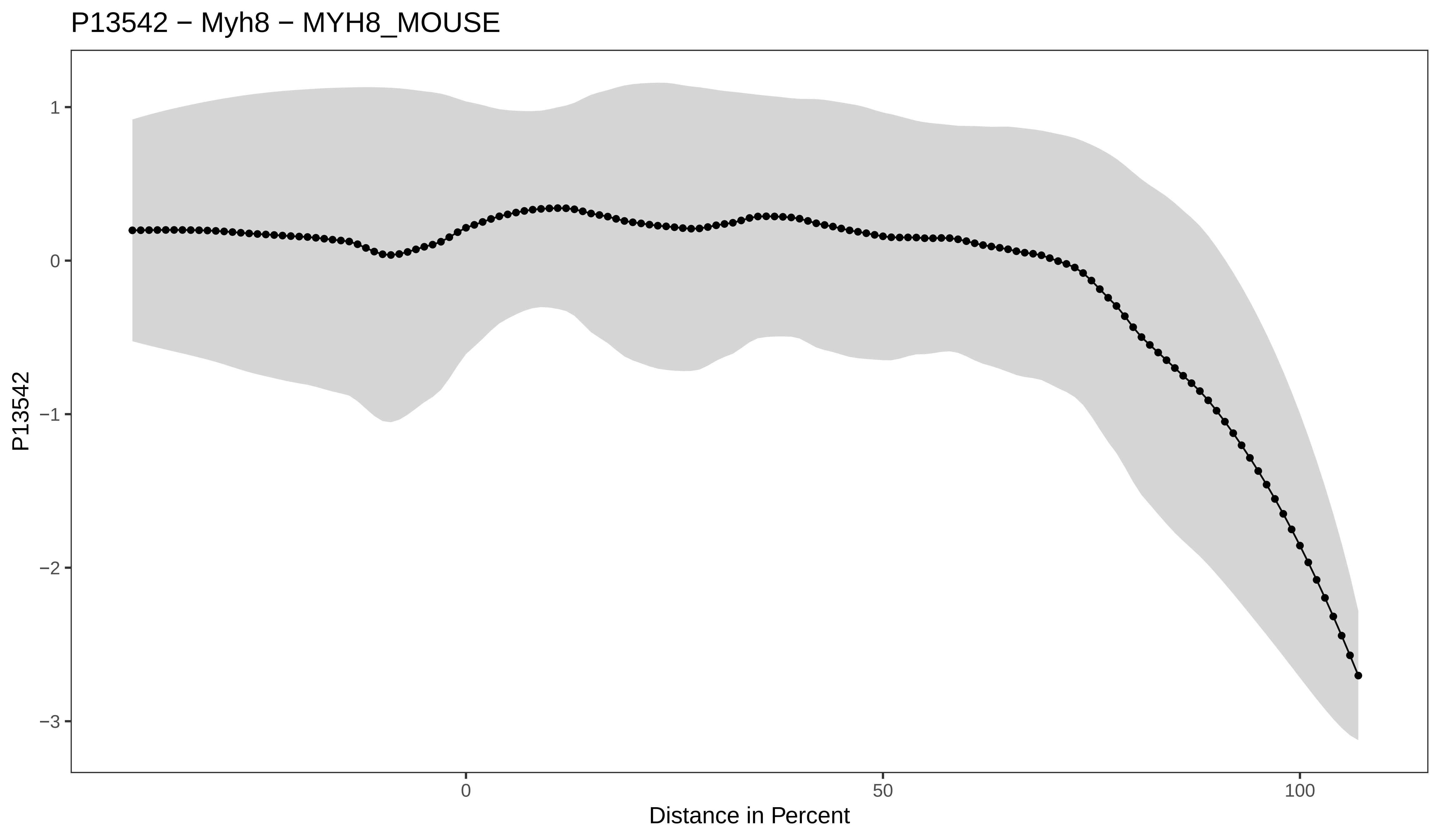
P13542 − Myh8 − MYH8_MOUSE
P13542
1
0
−1
−2
−3
0
50
100
Distance in Percent

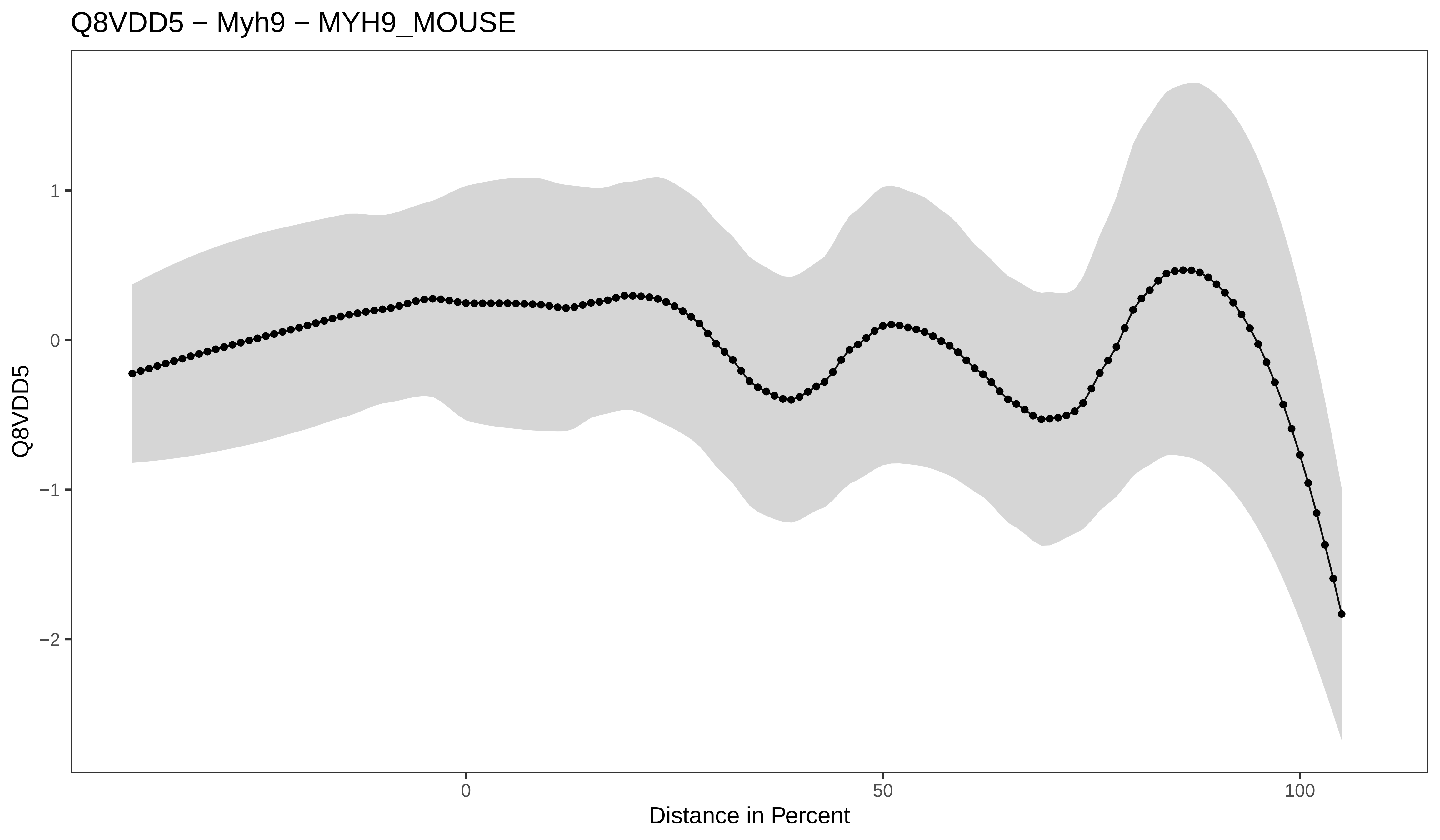
Q8VDD5 − Myh9 − MYH9_MOUSE
Q8VDD5
1
0
−1
−2
0
50
100
Distance in Percent

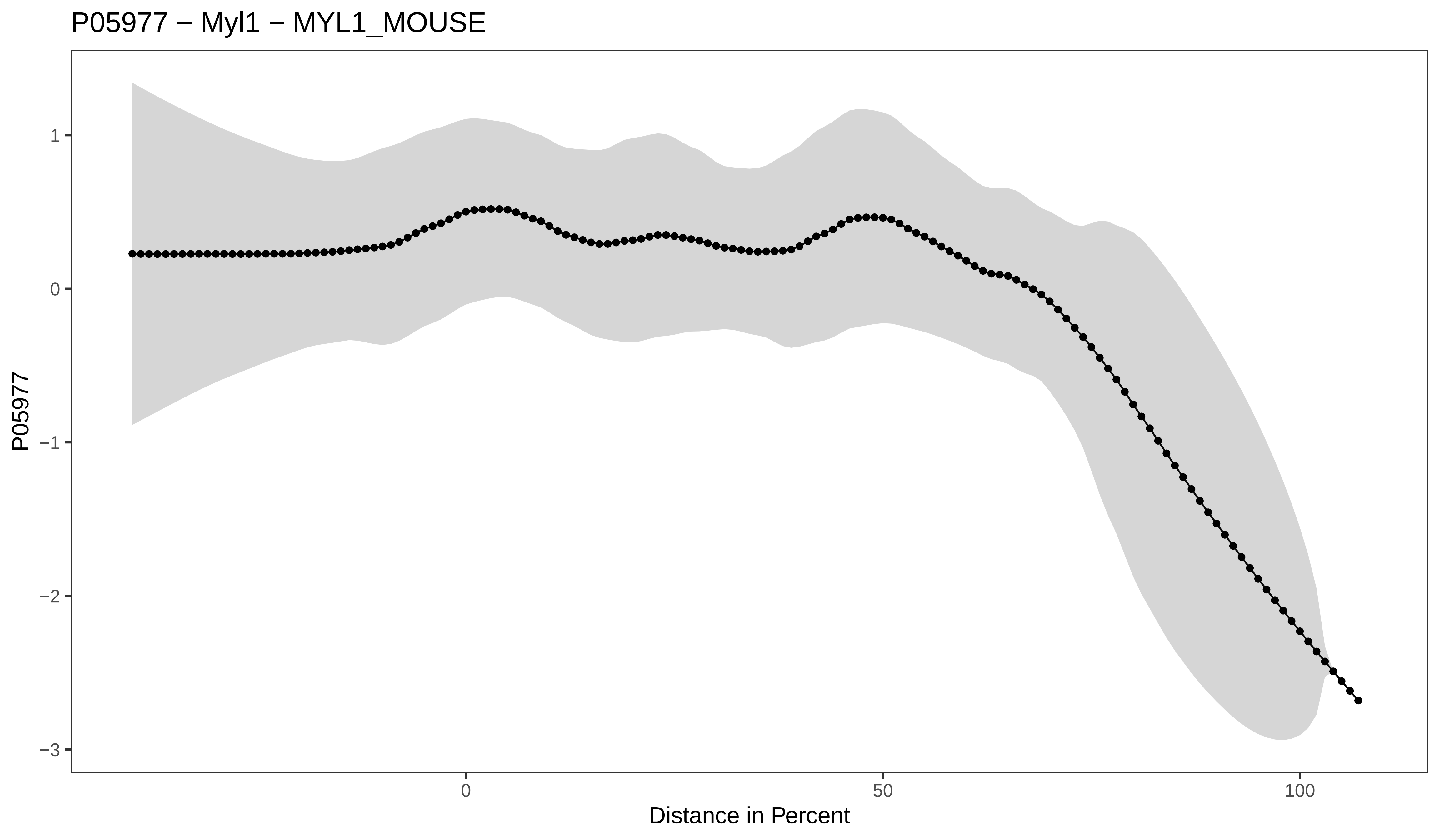

P05977 − Myl1 − MYL1_MOUSE
1
0
−1
−2
−3
P05977
0
50
100
Distance in Percent

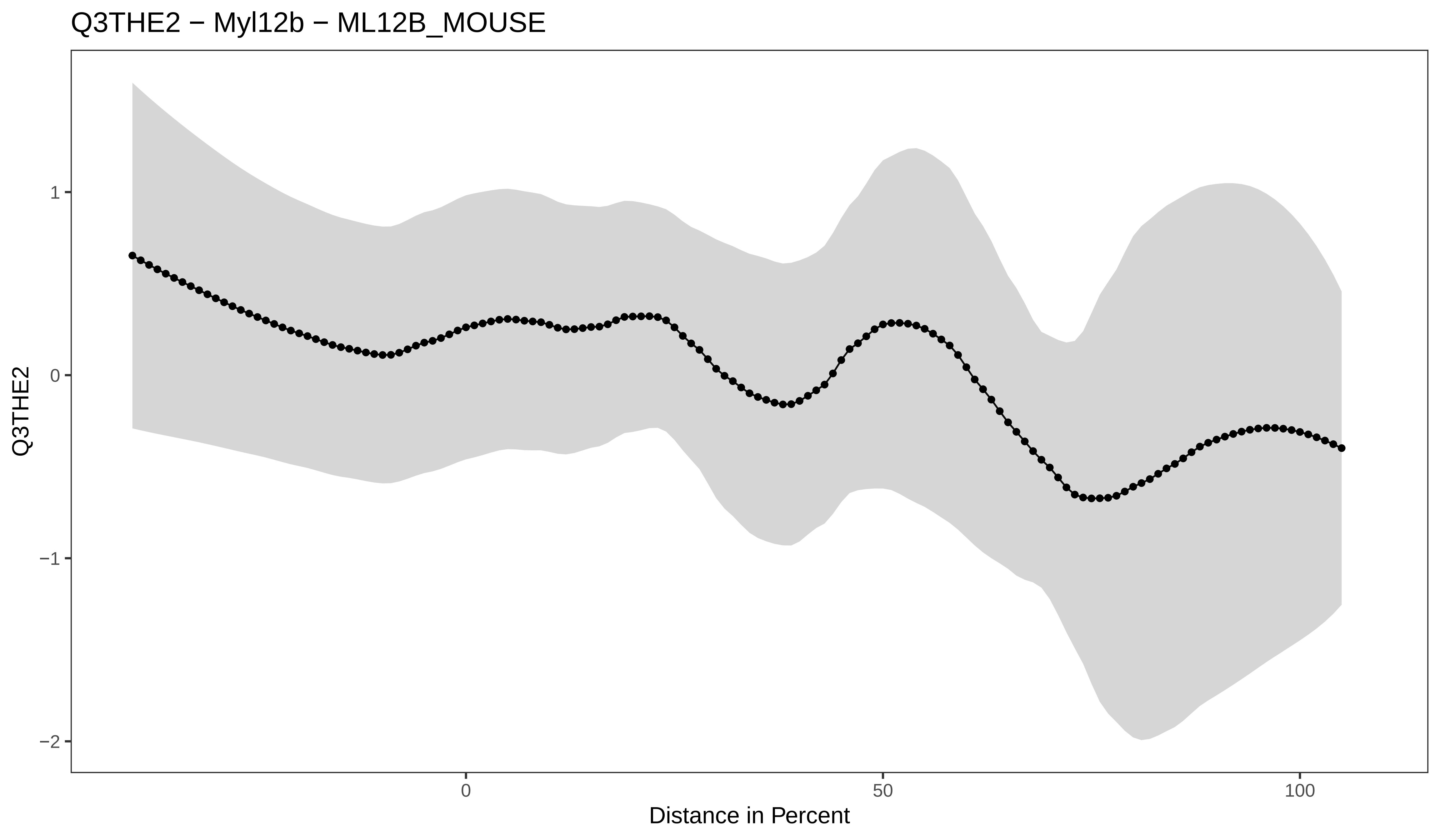

Q3THE2 − Myl12b − ML12B_MOUSE
Q3THE2
1
0
−1
−2
0
50
100
Distance in Percent

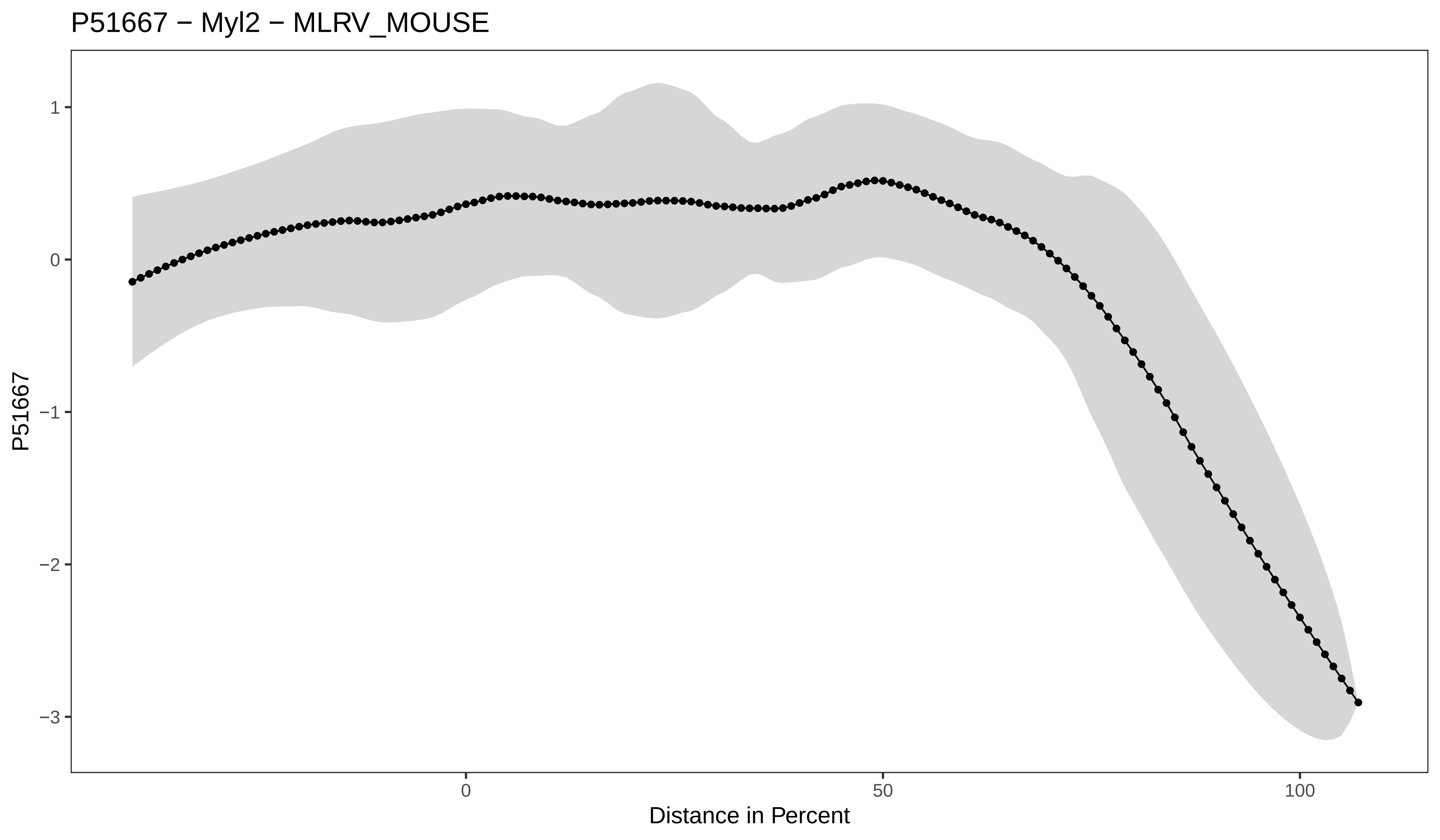

P51667 − Myl2 − MLRV_MOUSE
P51667
Distance in Percent
1
0
−1
−2
−3
0
50
100

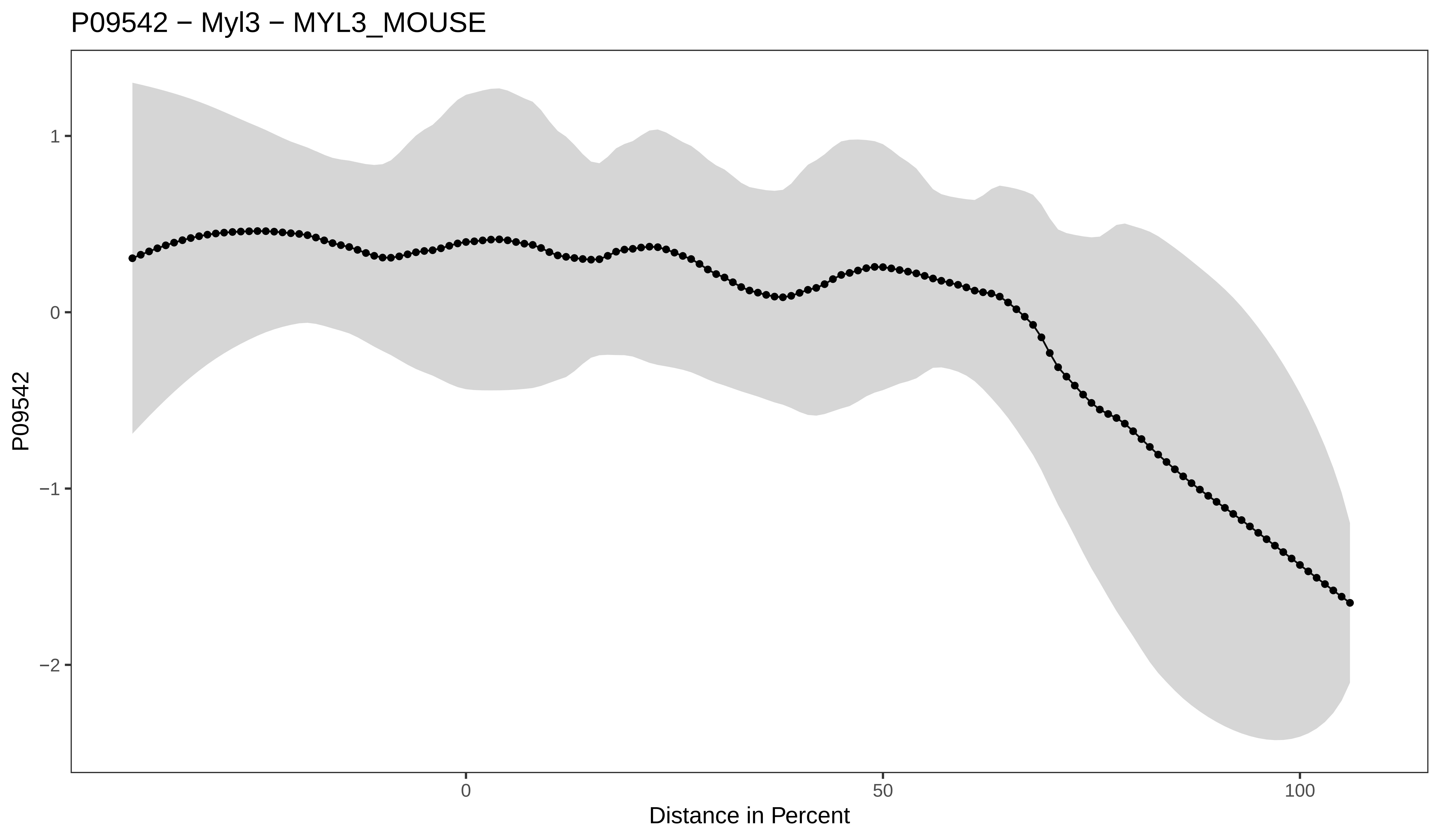
P09542 − Myl3 − MYL3_MOUSE
1
0
−1
−2
0
50
100
Distance in Percent
P09542

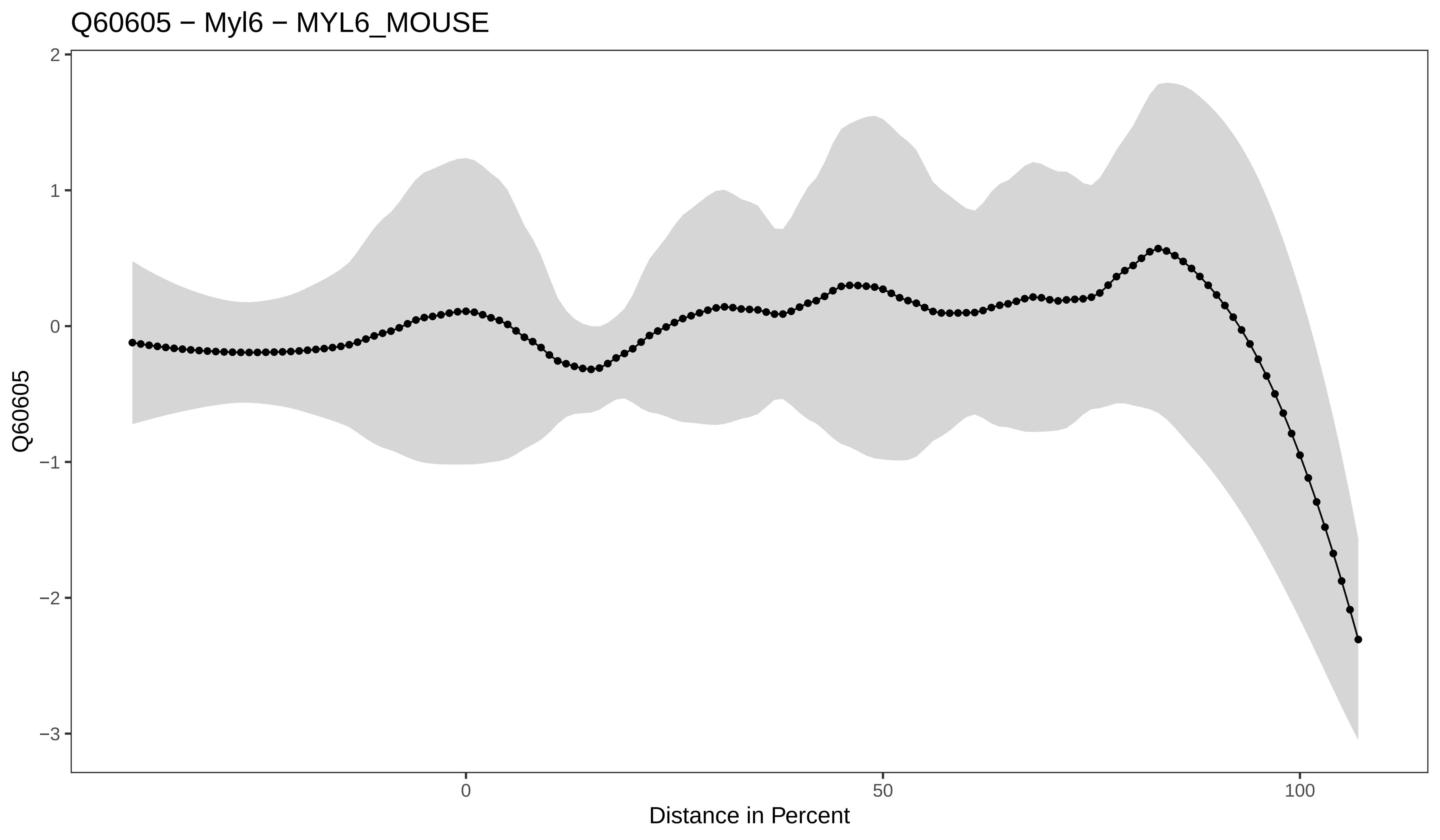

Q60605 − Myl6 − MYL6_MOUSE
Q60605
2
1
0
−1
−2
−3
0
50
100
Distance in Percent

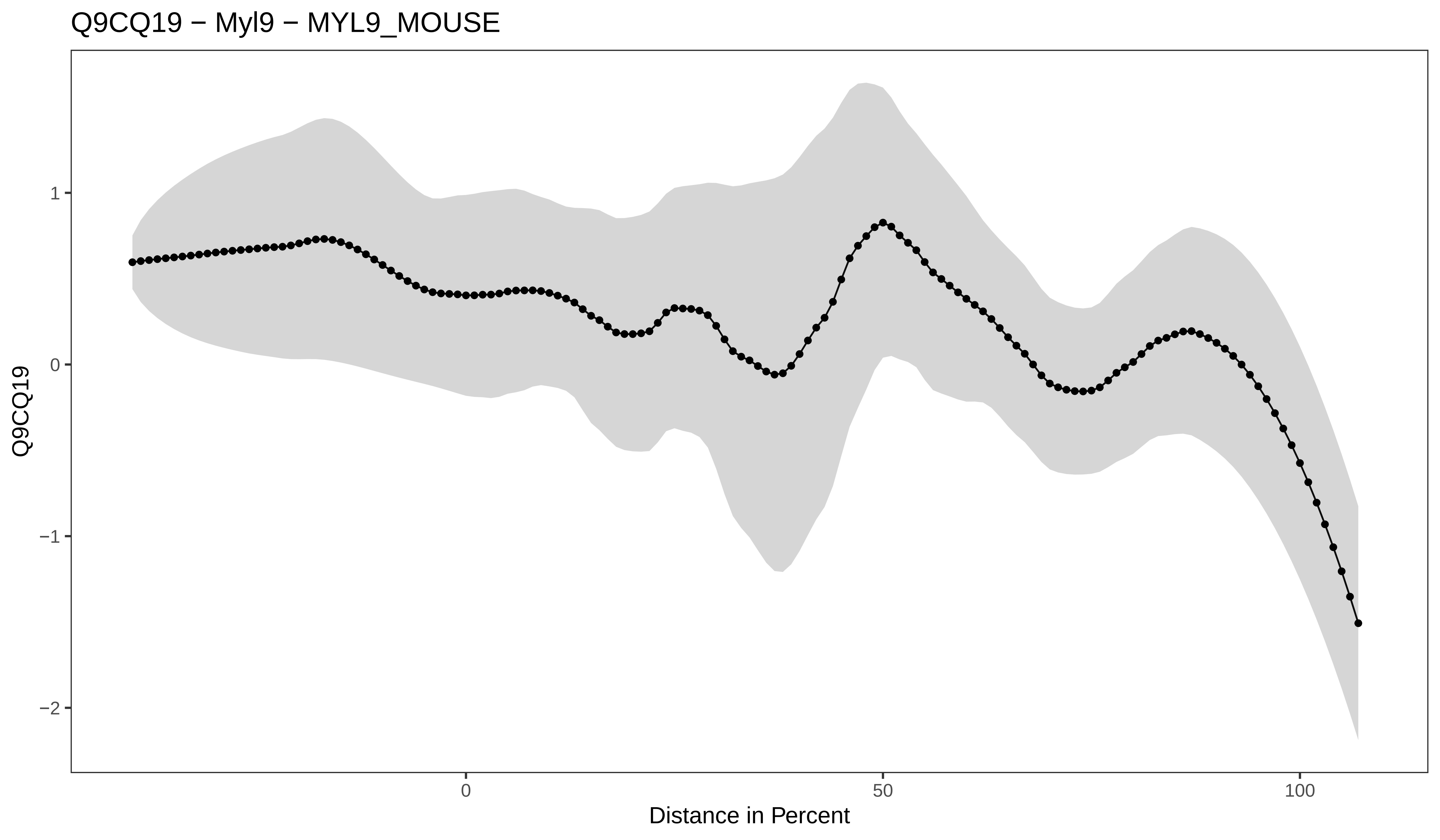
Q9CQ19 − Myl9 − MYL9_MOUSE
1
0
−1
−2
Q9CQ19
0
50
100
Distance in Percent

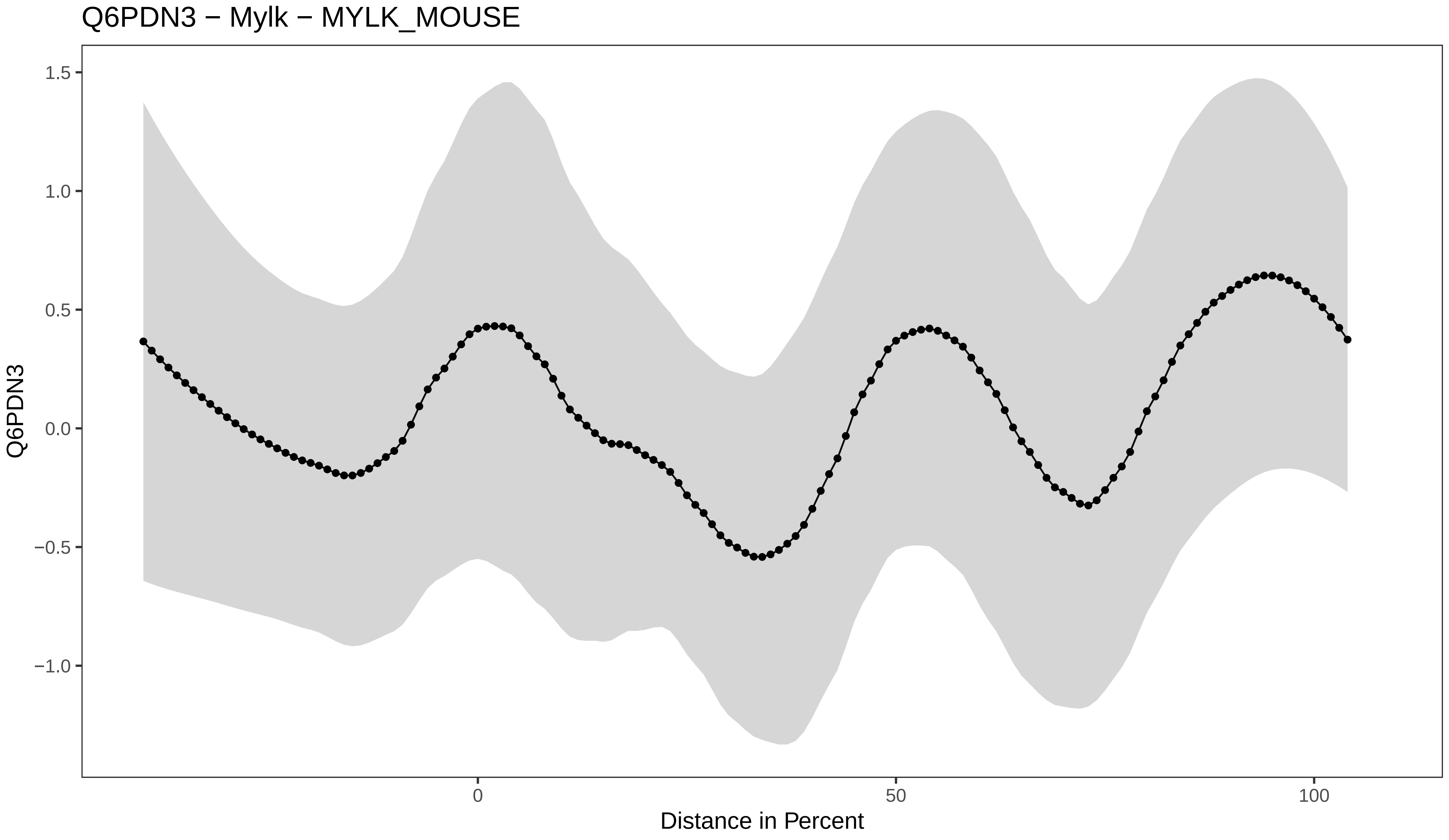

Q6PDN3 − Mylk − MYLK_MOUSE
Q6PDN3
1.5
1.0
0.5
0.0
−0.5
−1.0
0
50
100
Distance in Percent

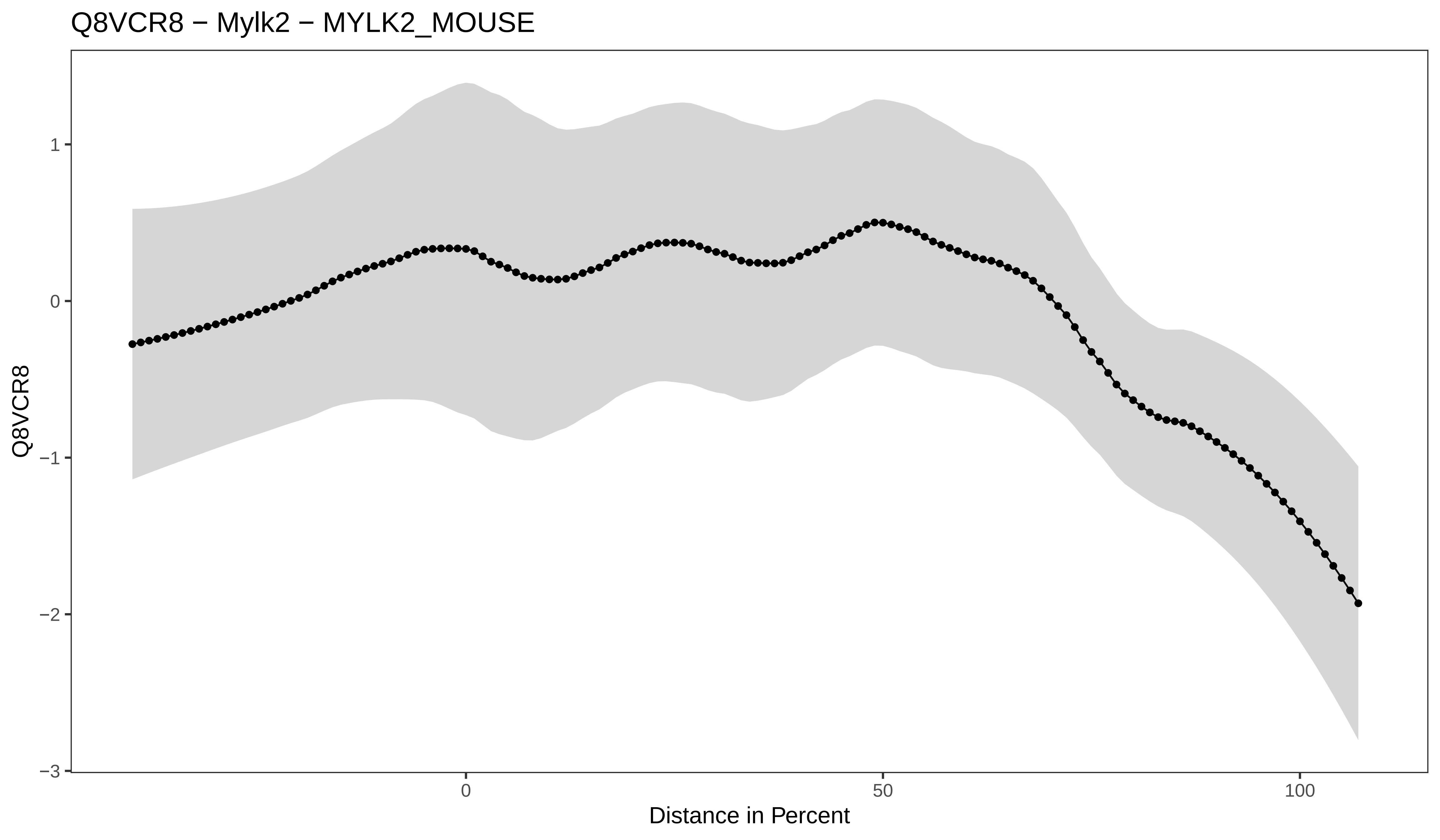
Q8VCR8 − Mylk2 − MYLK2_MOUSE
Q8VCR8
1
0
−1
−2
−3
0
50
100
Distance in Percent

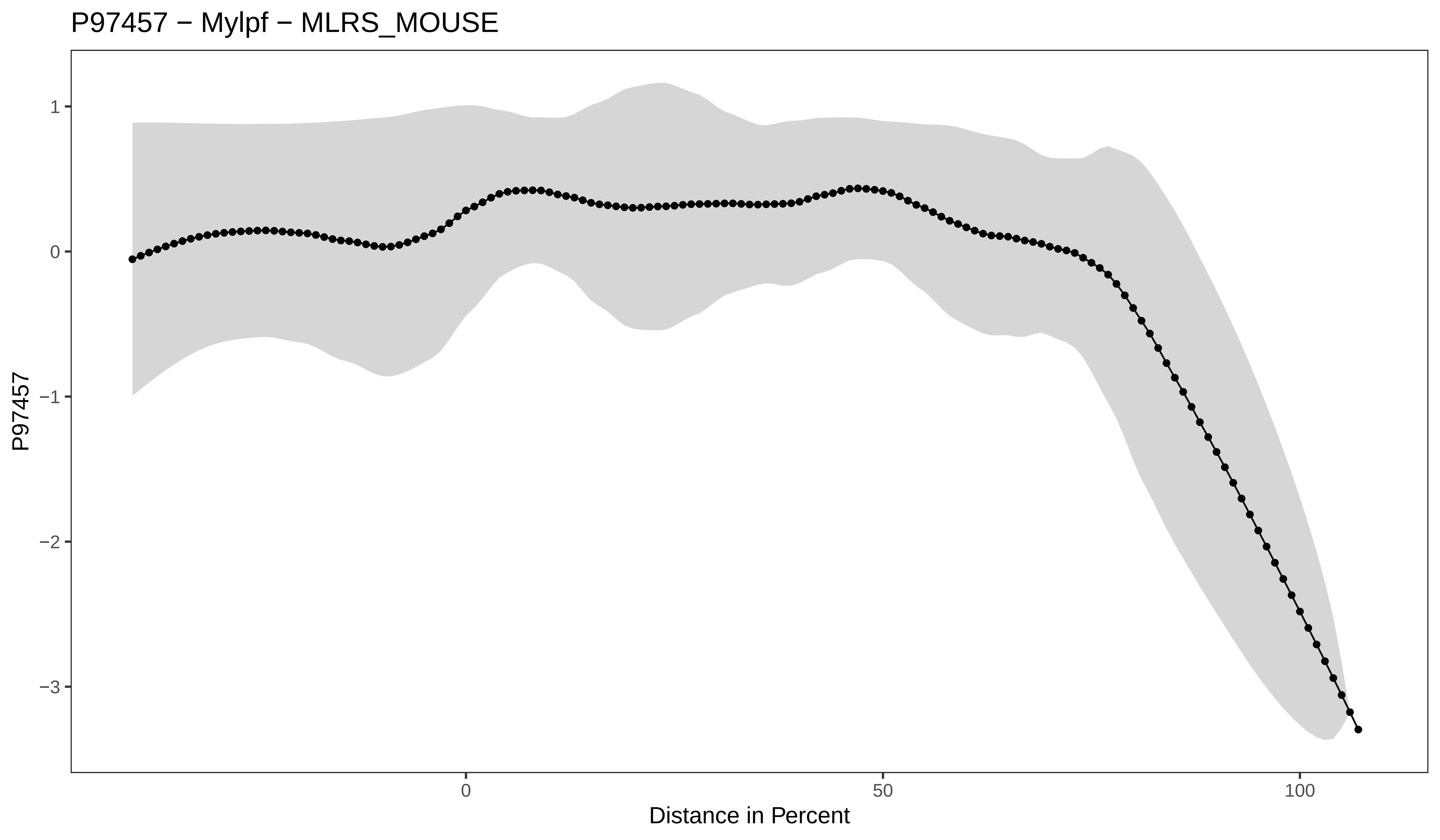

P97457 − Mylpf − MLRS_MOUSE
1
0
−1
−2
−3
P97457
0
50
100
Distance in Percent

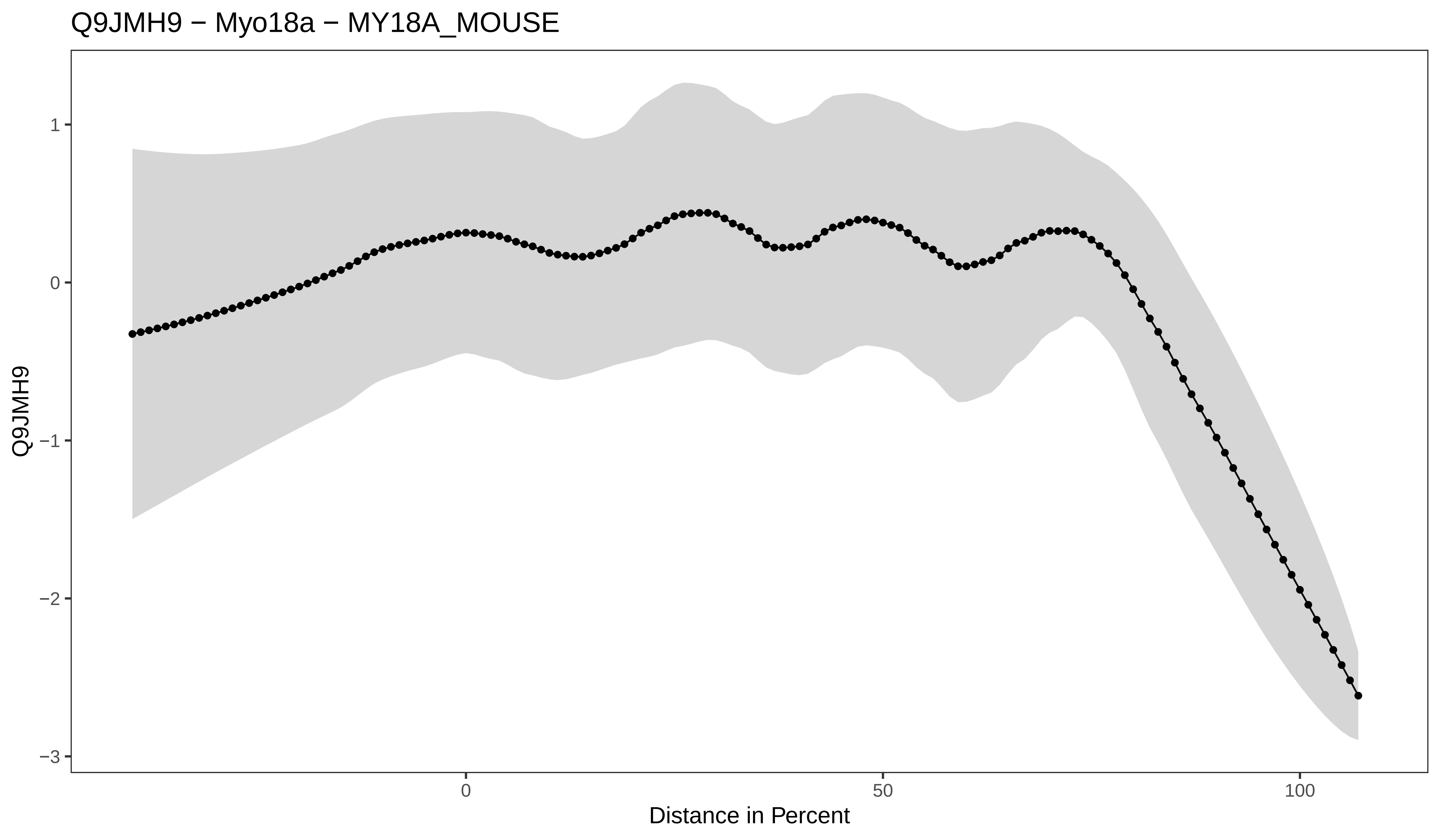
Q9JMH9 − Myo18a − MY18A_MOUSE
Q9JMH9
Distance in Percent
1
0
−1
−2
−3
0
50
100

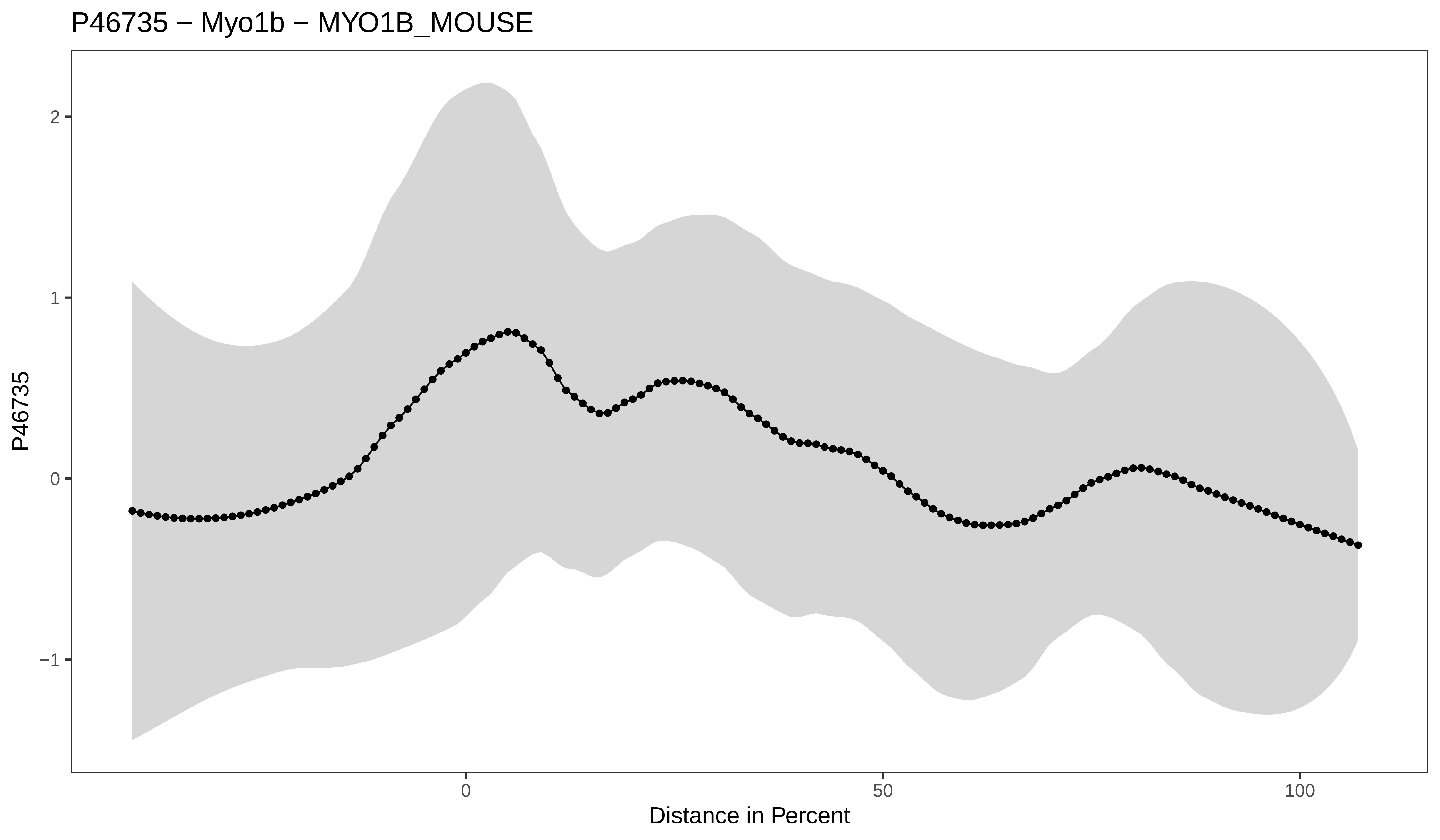

P46735 − Myo1b − MYO1B_MOUSE
P46735
Distance in Percent
2
1
0
−1
0
50
100

Q9WTI7 − Myo1c − MYO1C_MOUSE

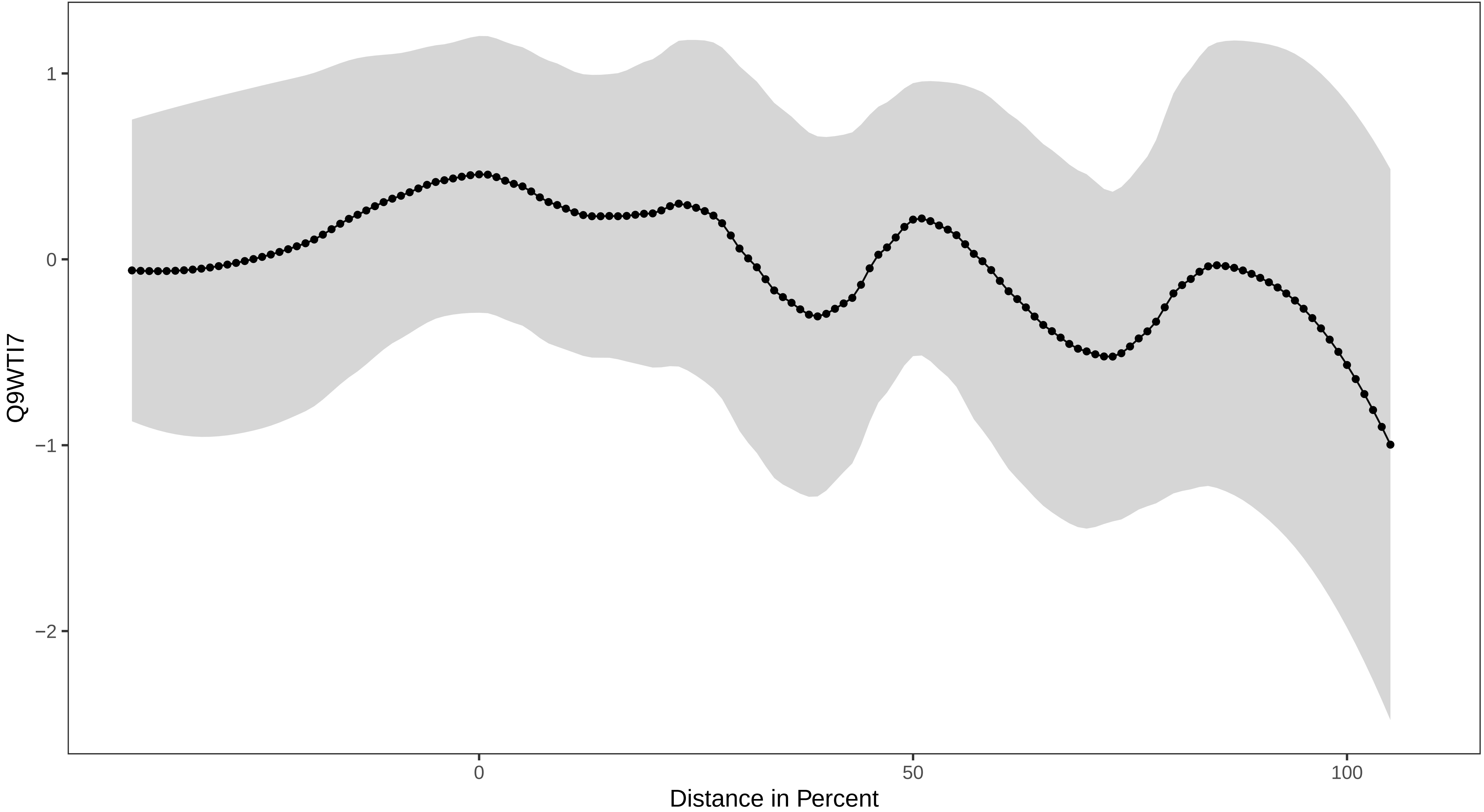

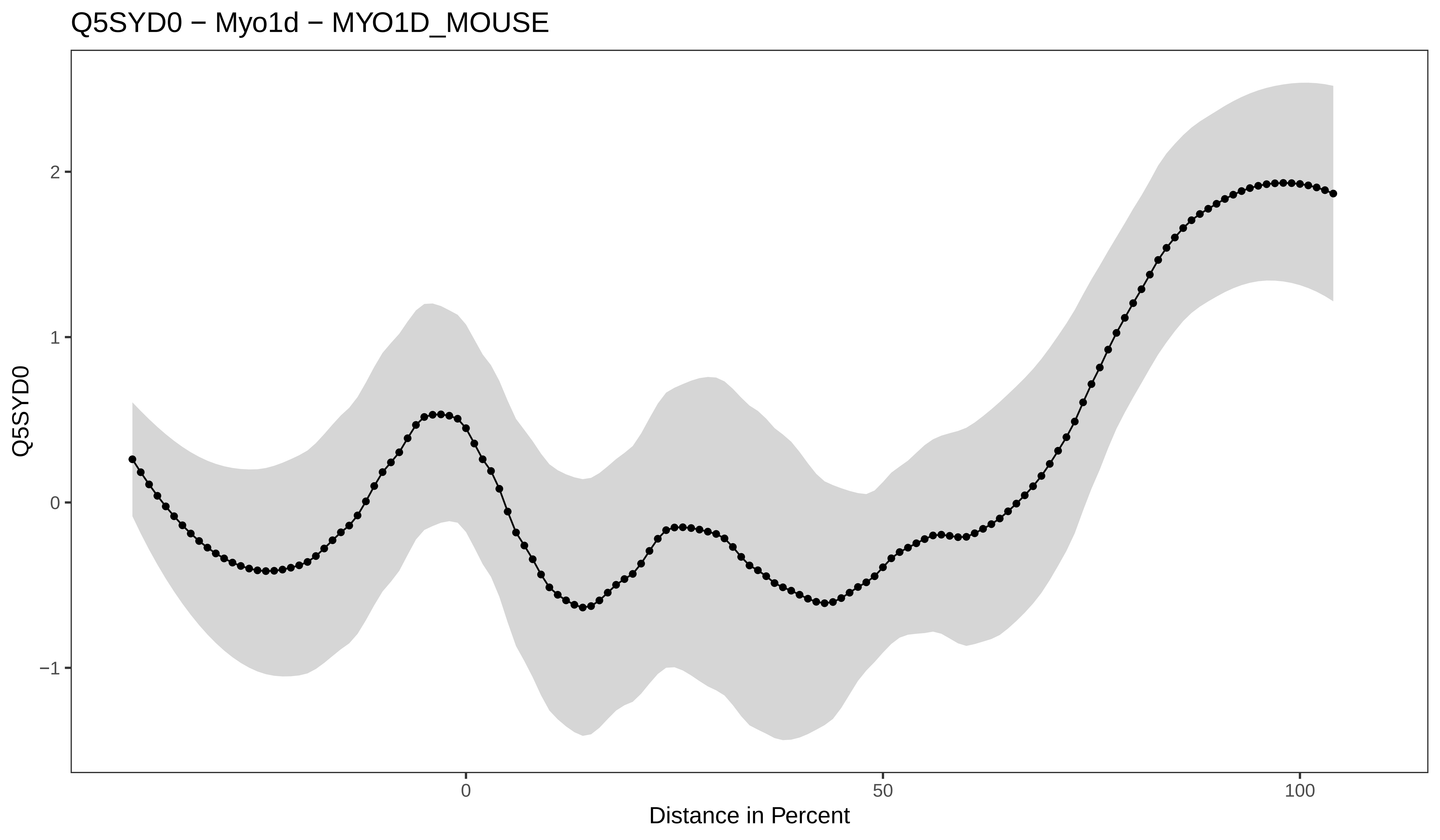

Q5SYD0 − Myo1d − MYO1D_MOUSE
Q5SYD0
Distance in Percent
2
1
0
−1
0
50
100

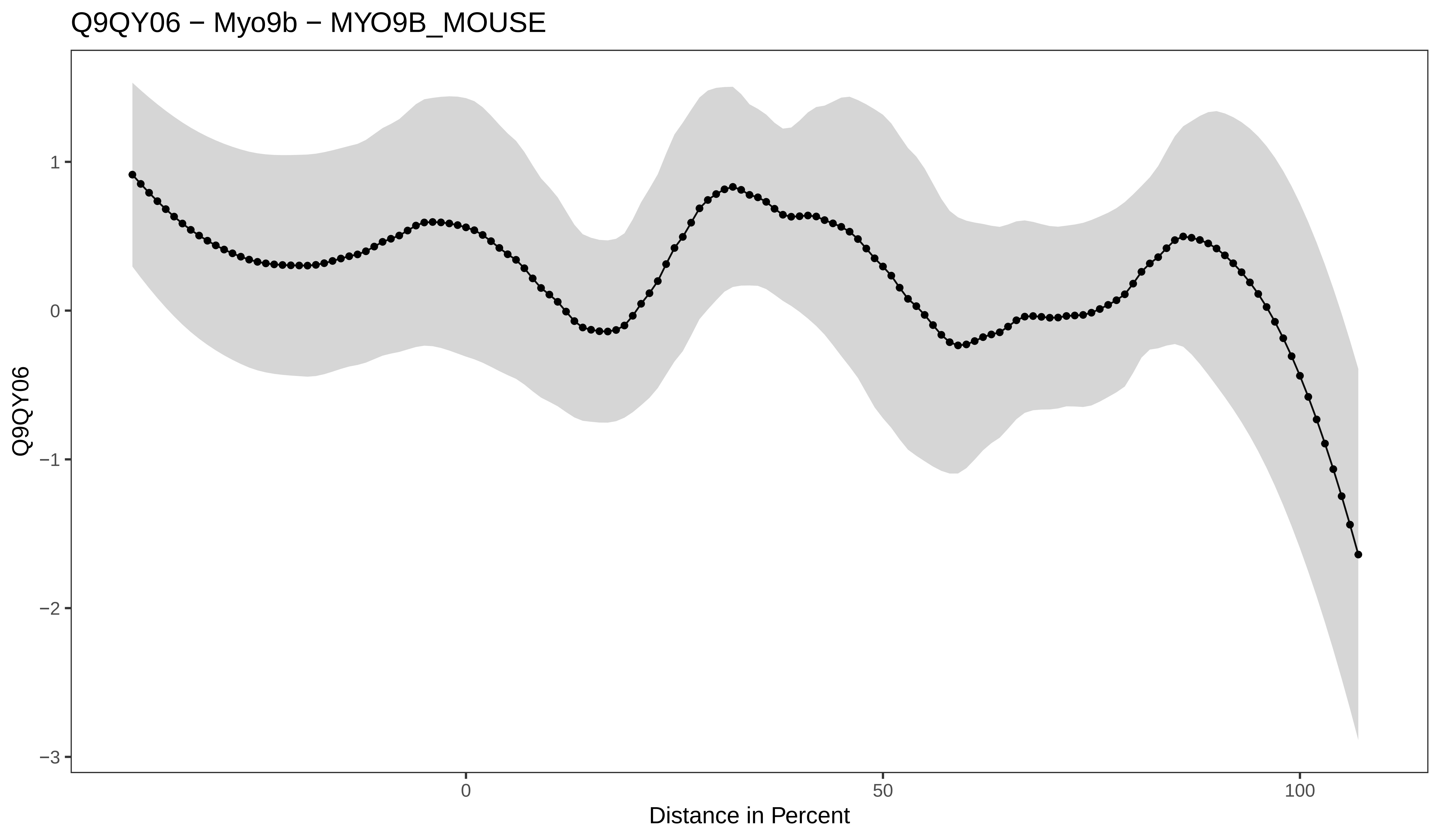

Q9QY06 − Myo9b − MYO9B_MOUSE
Q9QY06
1
0
−1
−2
−3
0
50
100
Distance in Percent

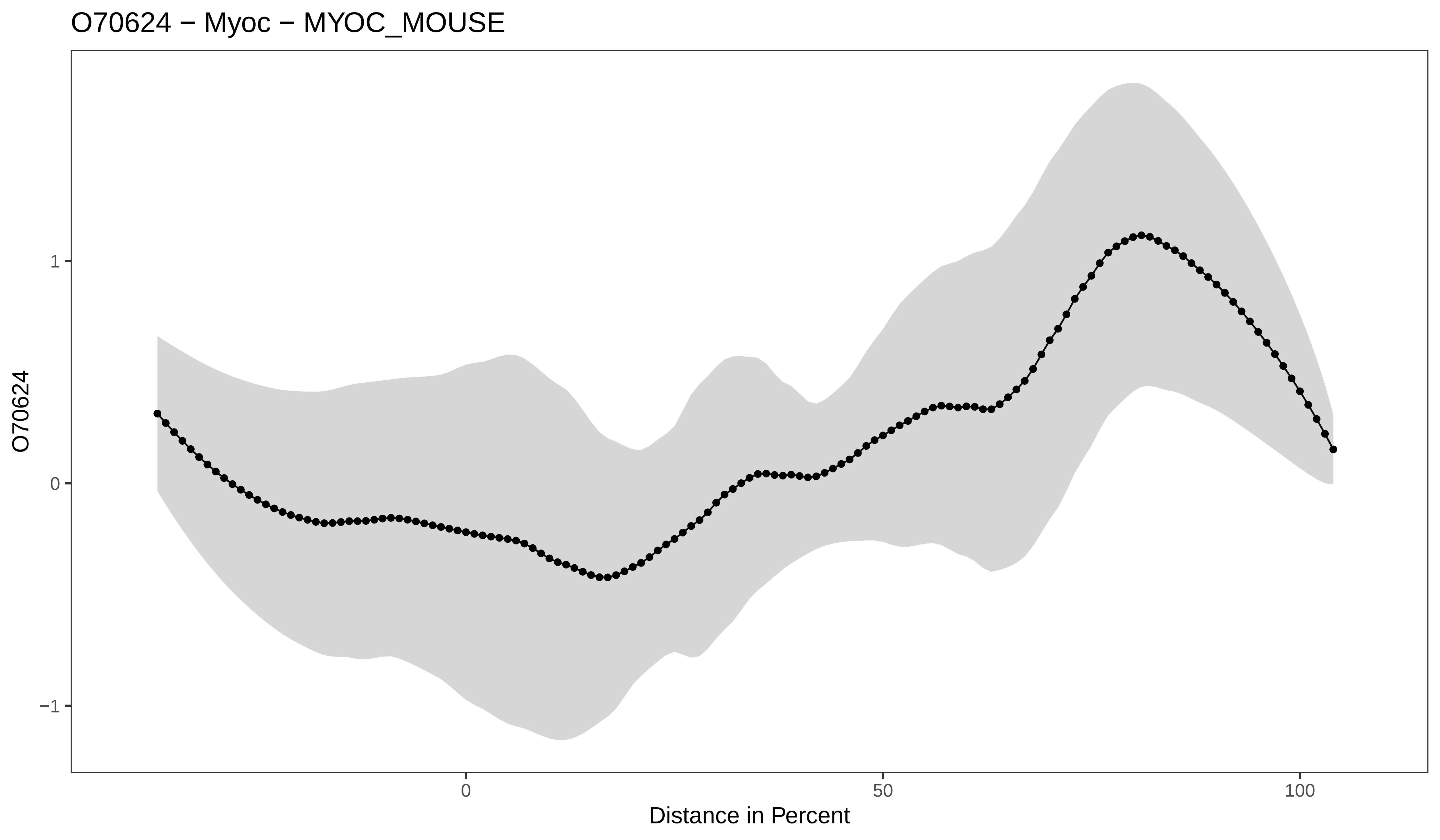
O70624 − Myoc − MYOC_MOUSE
1
0
−1
O70624
0
50
100
Distance in Percent

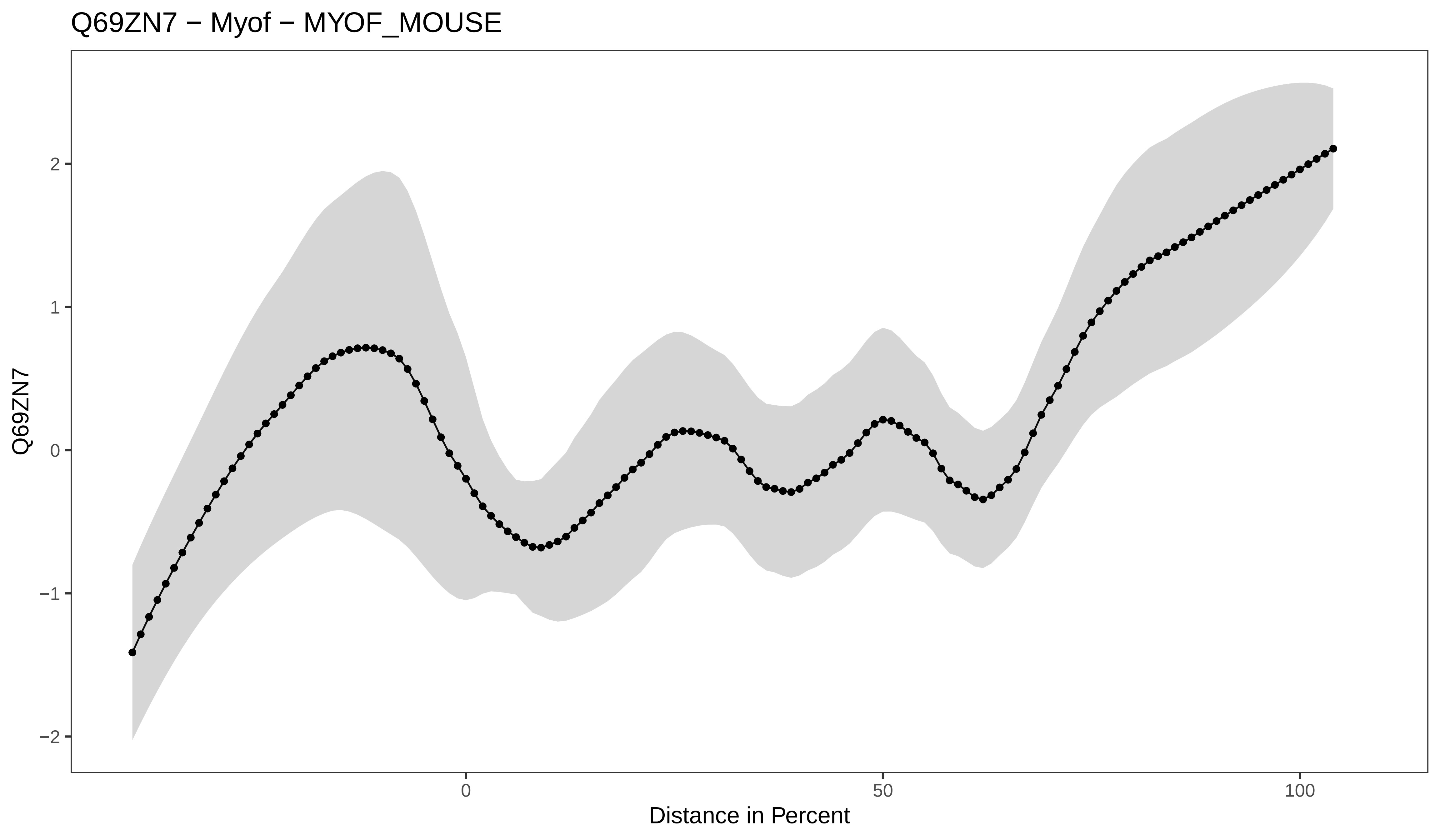
Q69ZN7 − Myof − MYOF_MOUSE
Q69ZN7
Distance in Percent
2
1
0
−1
−2
0
50
100

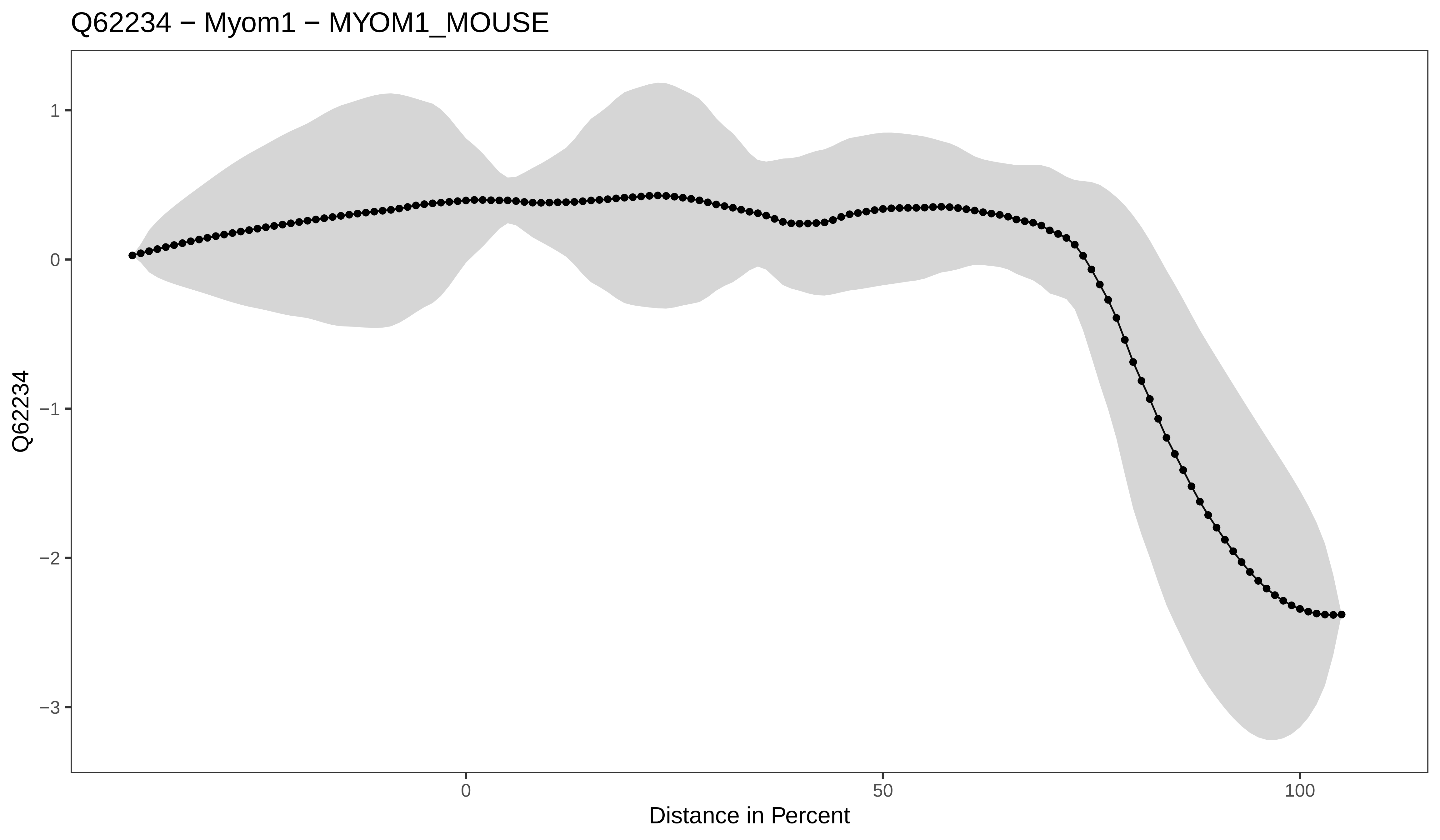
Q62234 − Myom1 − MYOM1_MOUSE
Q62234
1
0
−1
−2
−3
0
50
100
Distance in Percent

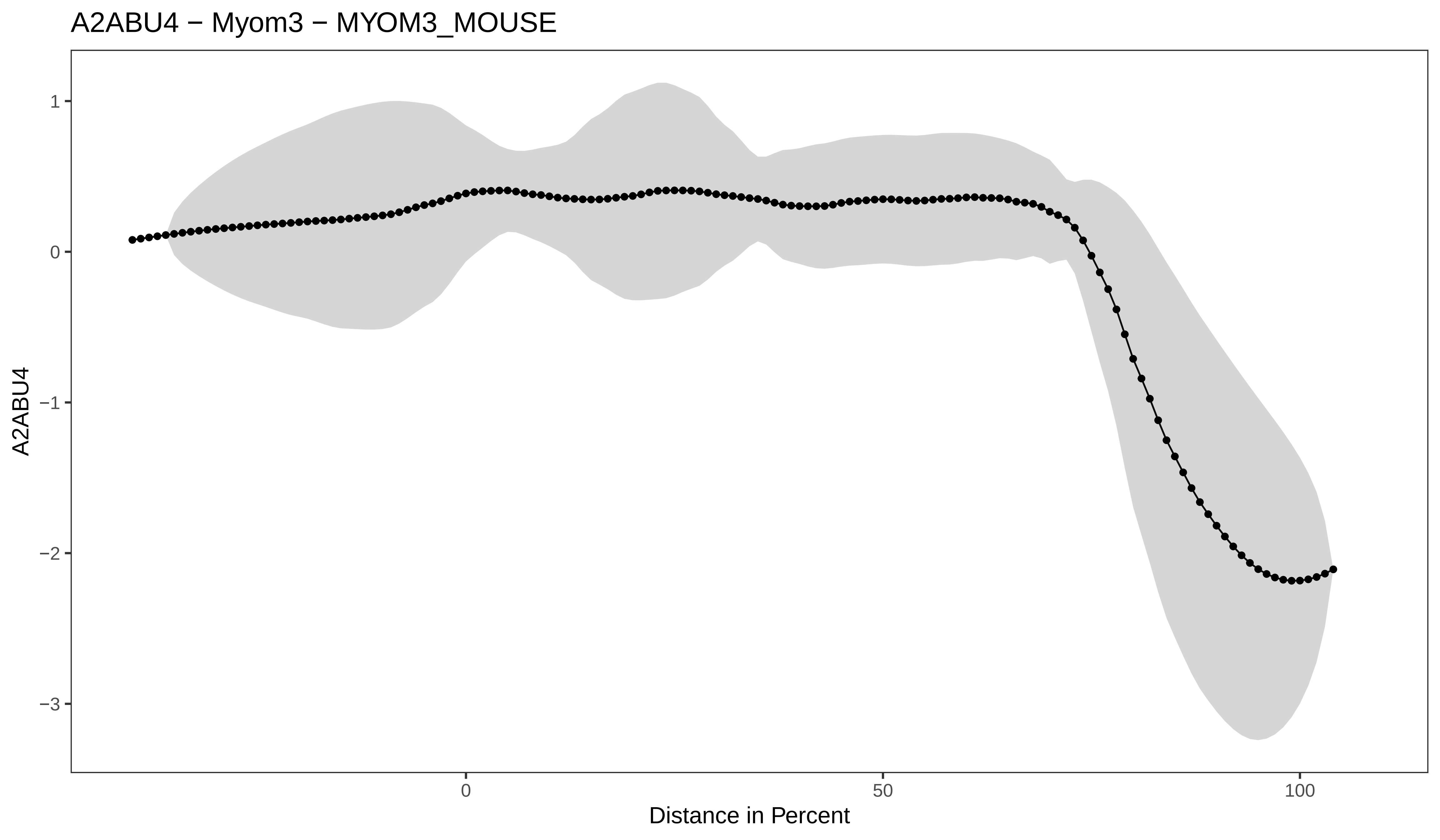
A2ABU4 − Myom3 − MYOM3_MOUSE
1
0
−1
−2
−3
A2ABU4
0
50
100
Distance in Percent

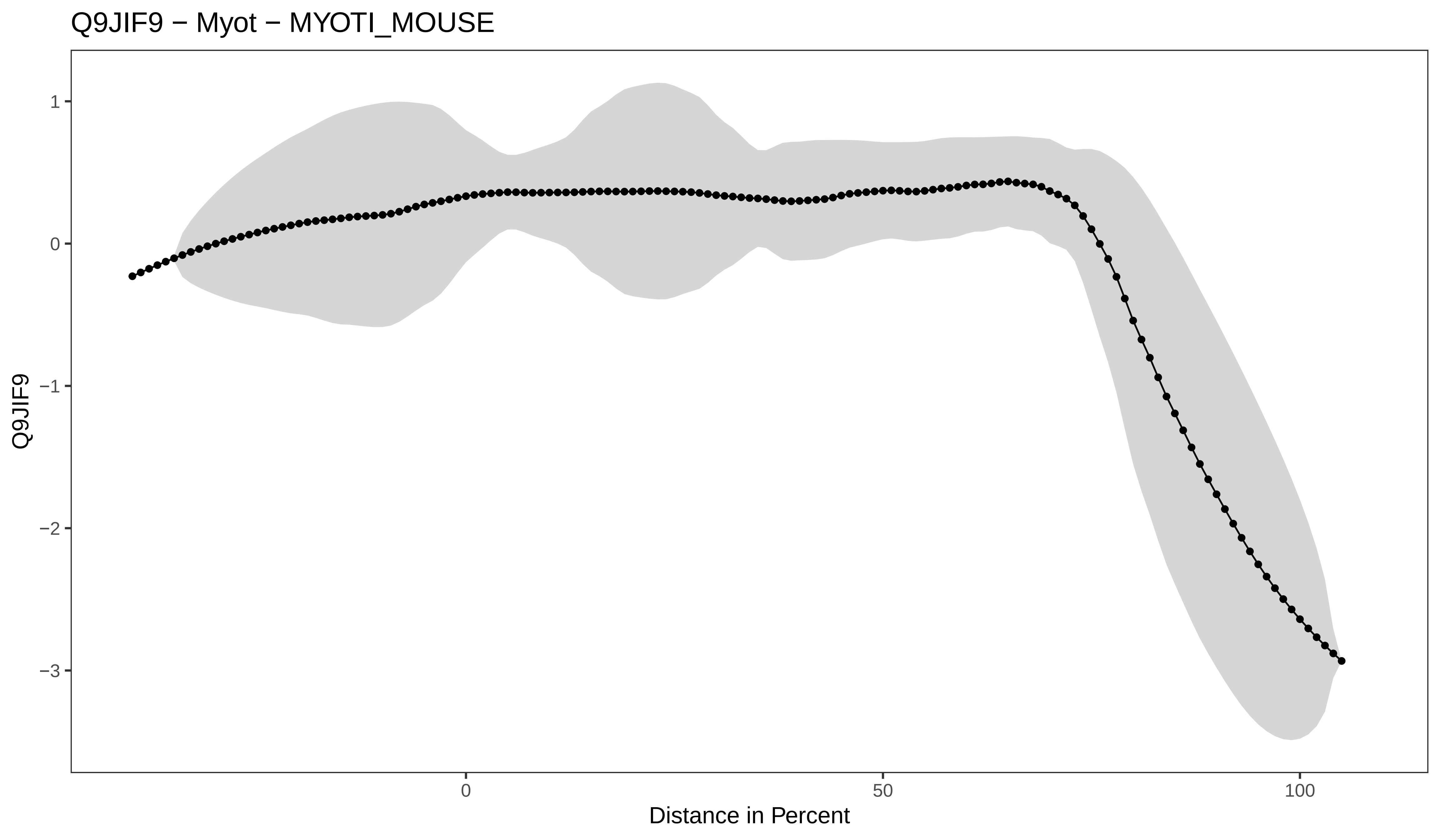
Q9JIF9 − Myot − MYOTI_MOUSE
Q9JIF9
Distance in Percent
1
0
−1
−2
−3
0
50
100

Q9JK37 − Myoz1 − MYOZ1_MOUSE

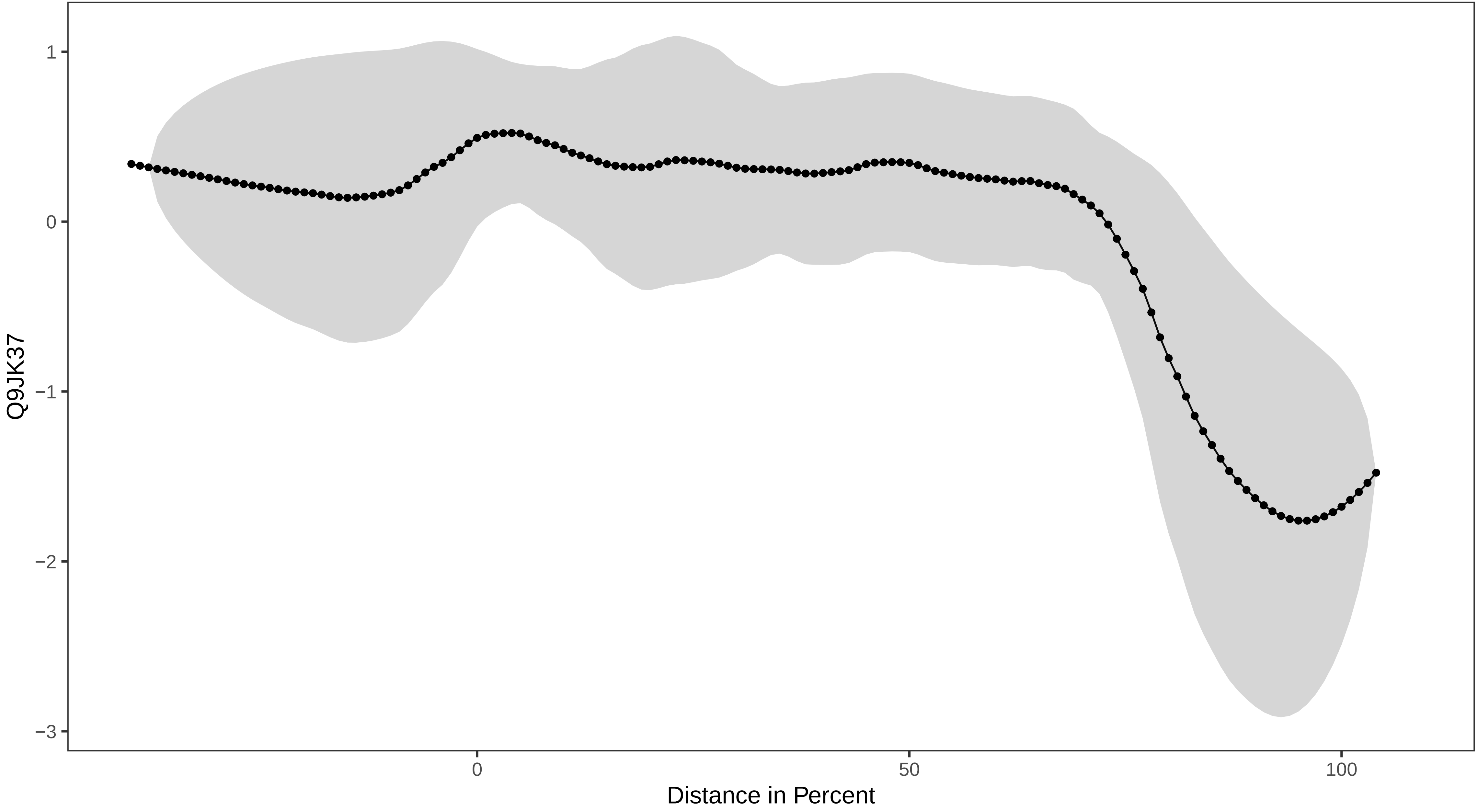

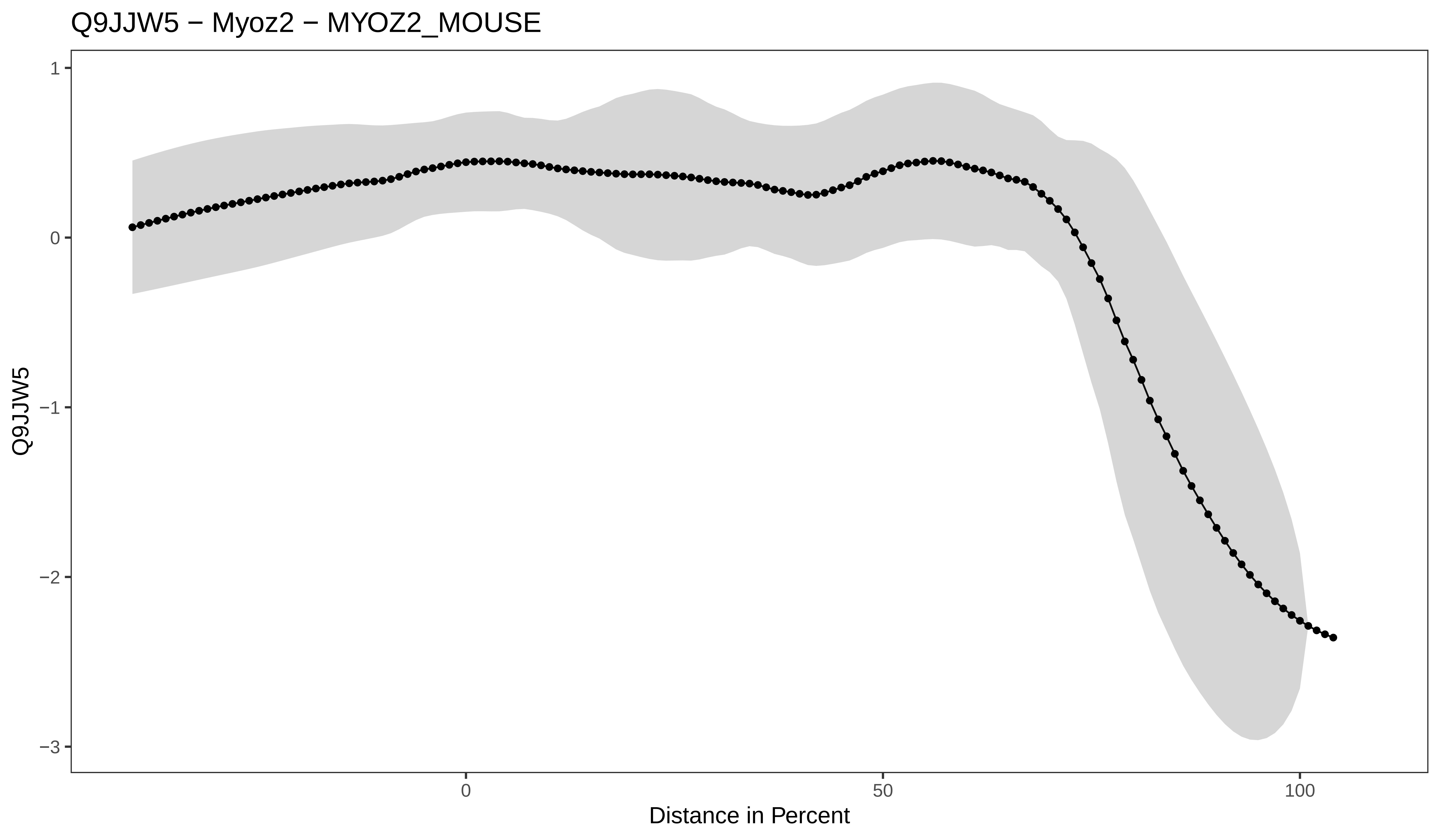
Q9JJW5 − Myoz2 − MYOZ2_MOUSE
Q9JJW5
Distance in Percent
1
0
−1
−2
−3
0
50
100

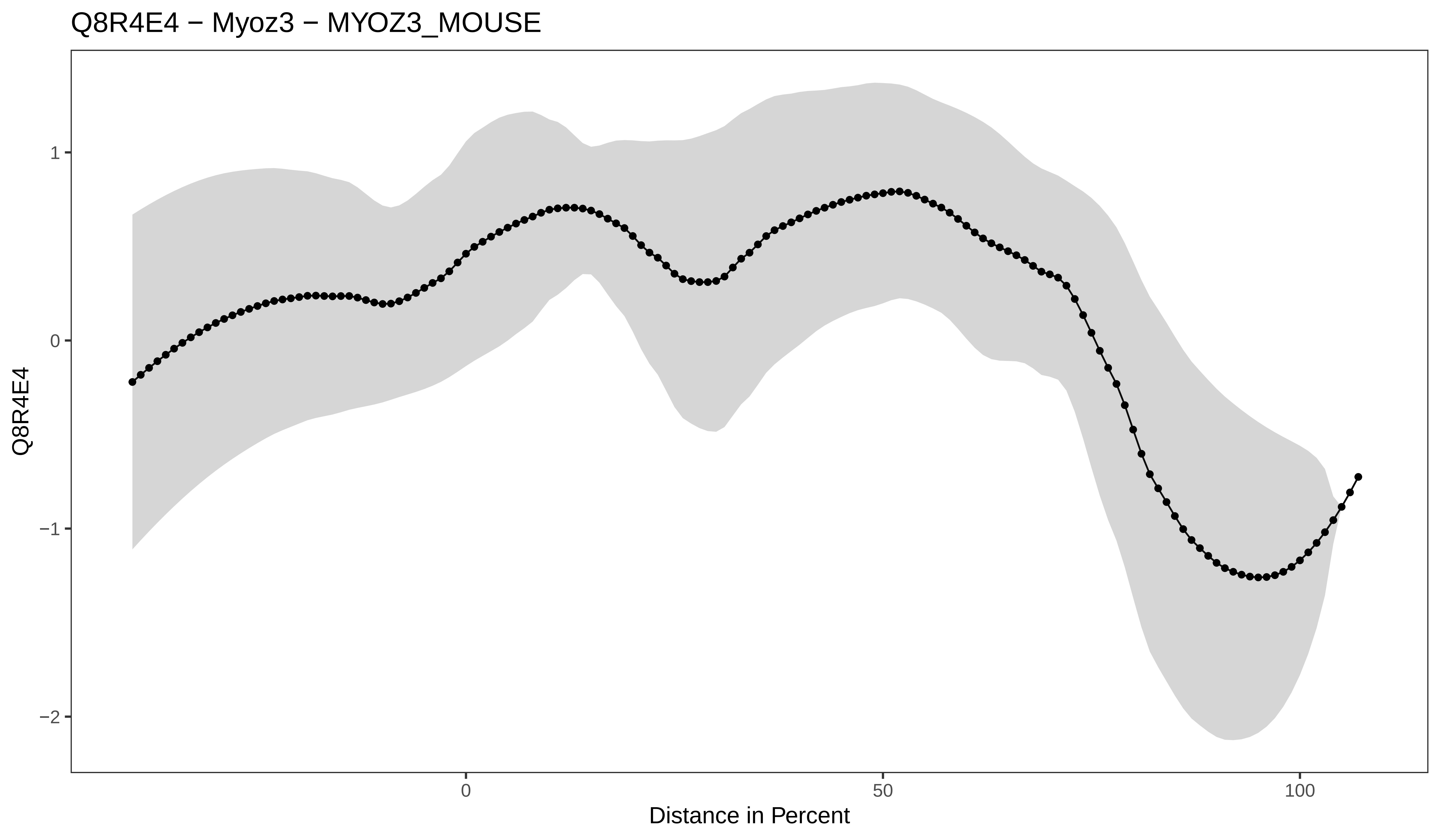

Q8R4E4 − Myoz3 − MYOZ3_MOUSE
1
0
−1
−2
Q8R4E4
0
50
100
Distance in Percent

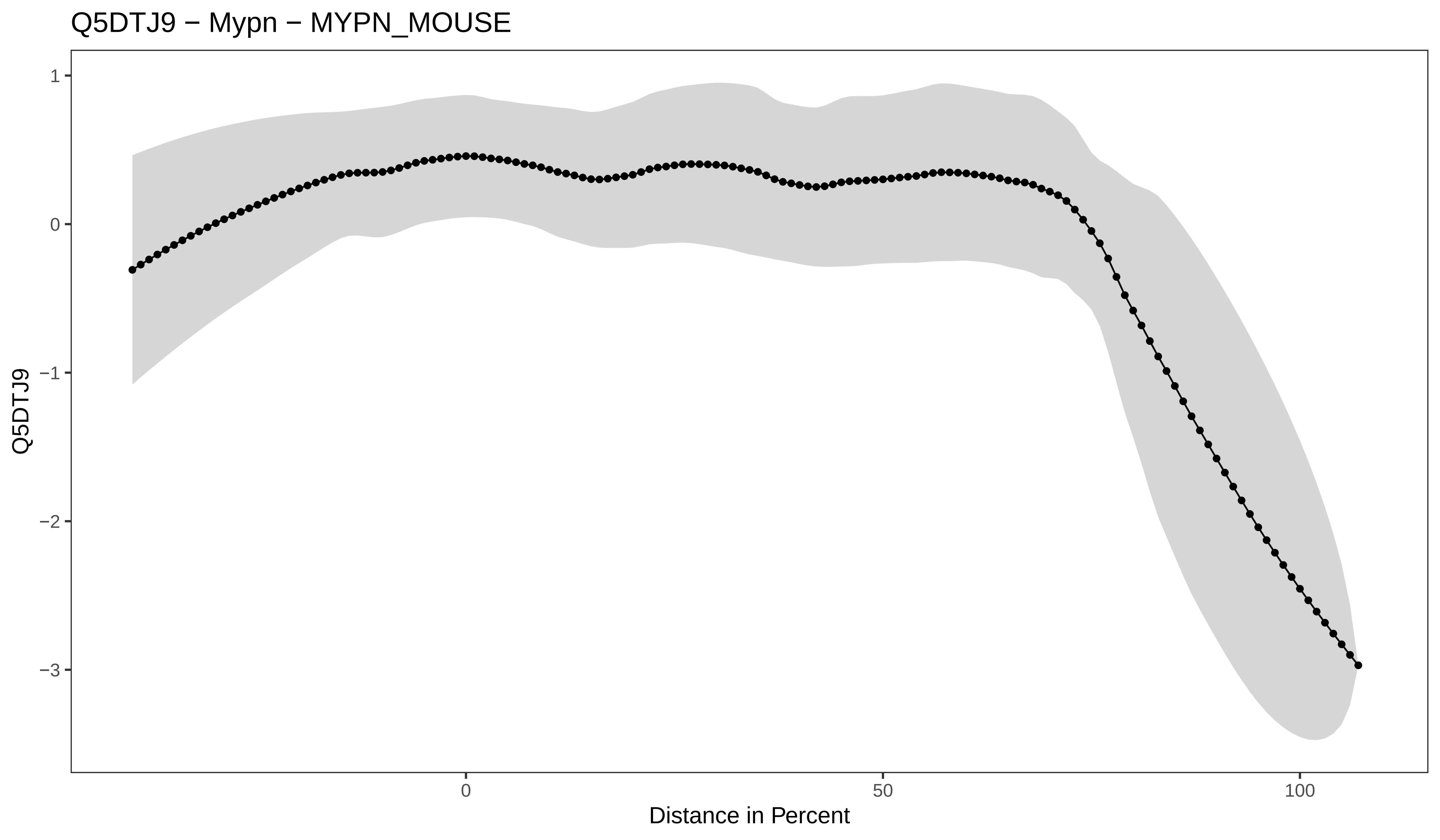

Q5DTJ9 − Mypn − MYPN_MOUSE
1
0
−1
−2
−3
Q5DTJ9
0
50
100
Distance in Percent

Q6A037 − N4bp1 − N4BP1_MOUSE

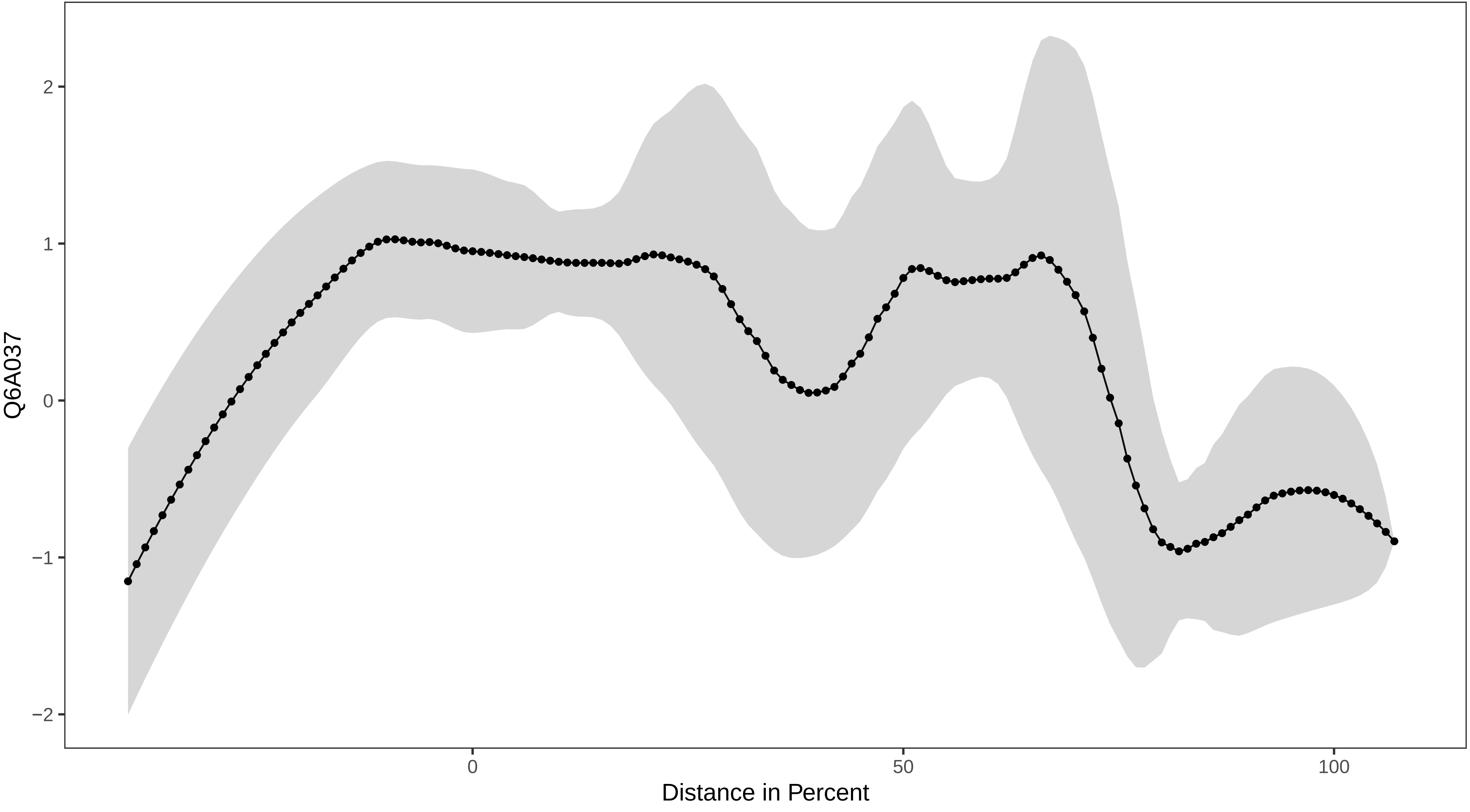

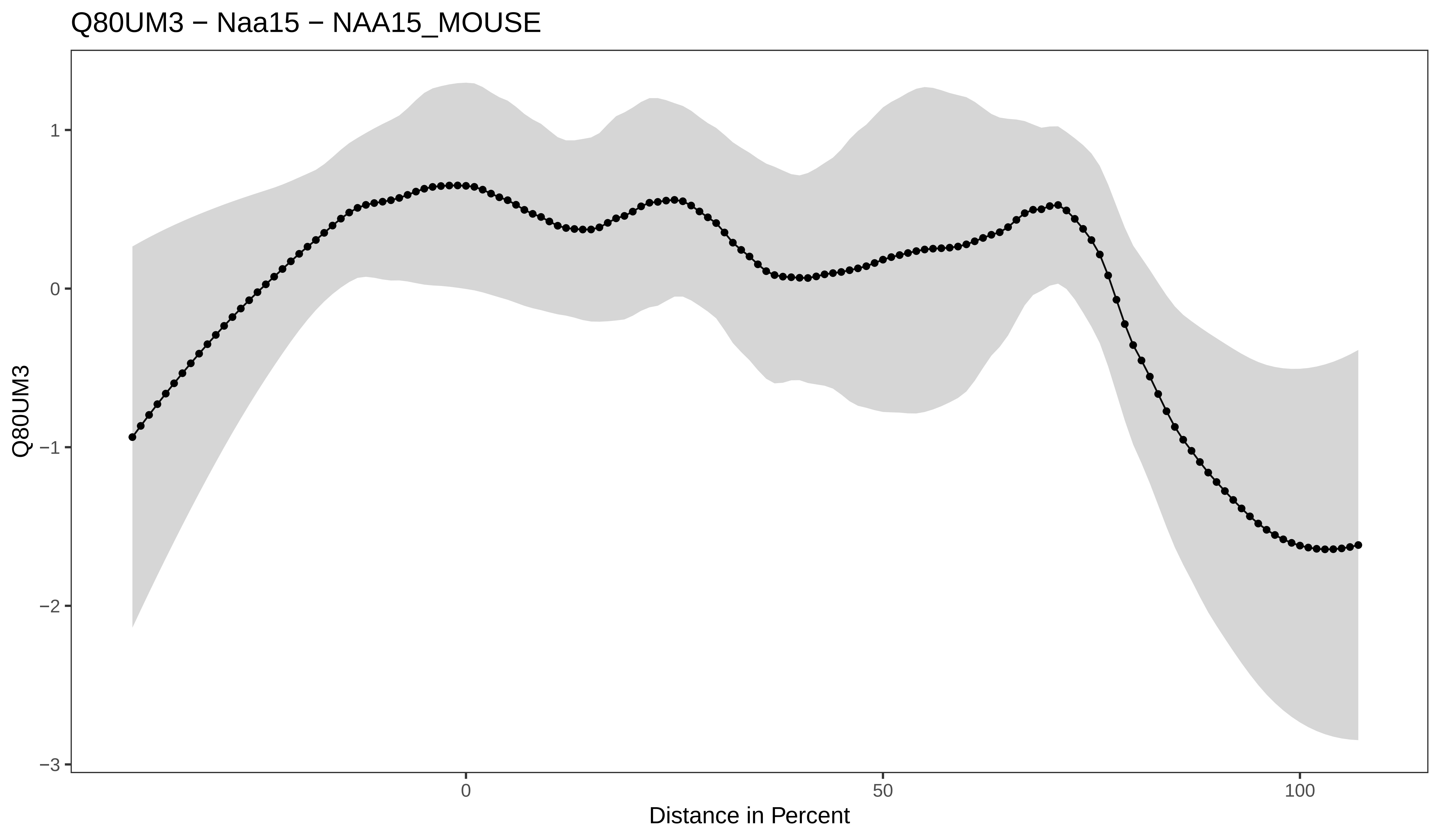

Q80UM3 − Naa15 − NAA15_MOUSE
Q80UM3
1
0
−1
−2
−3
0
50
100
Distance in Percent

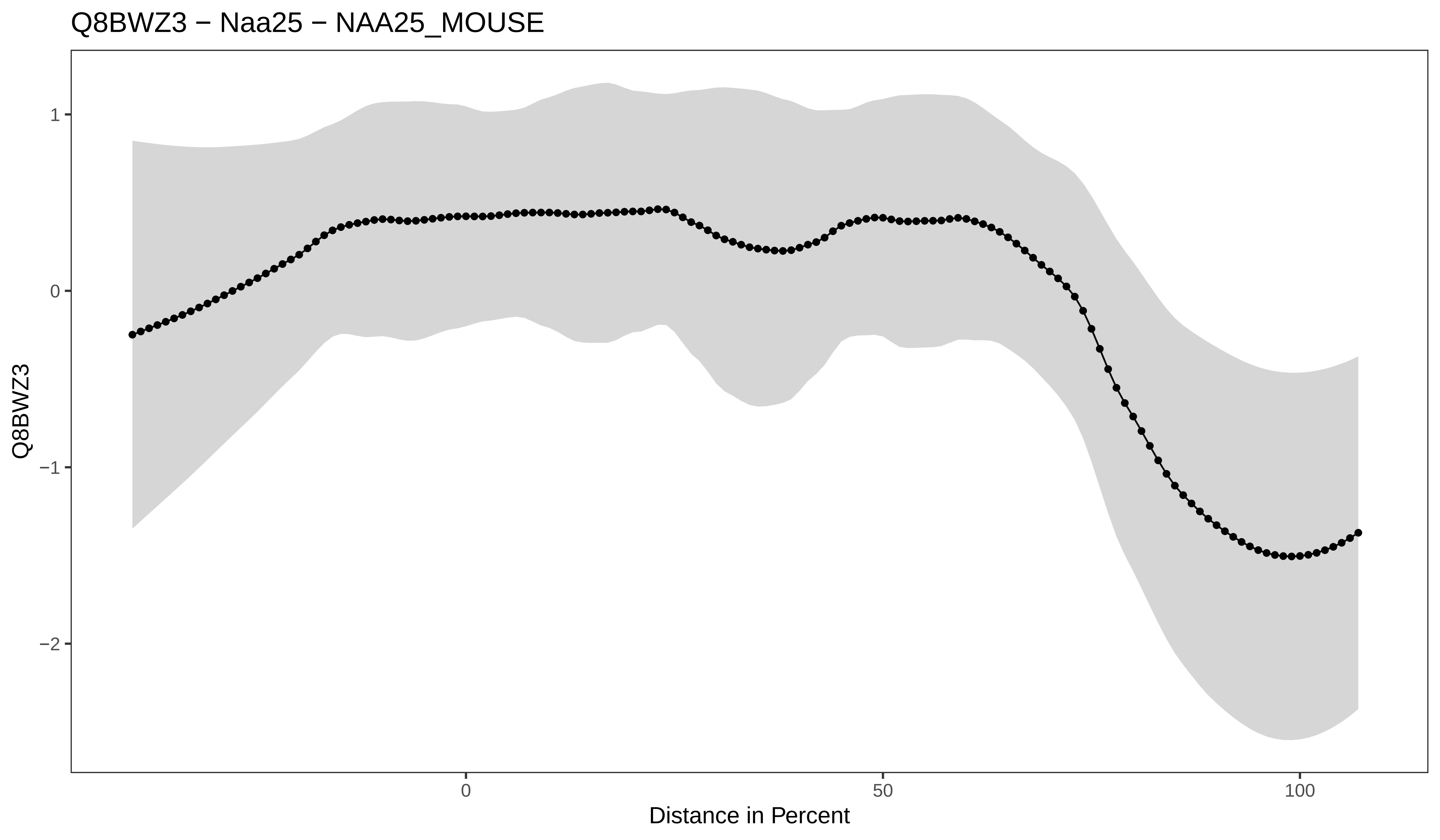

Q8BWZ3 − Naa25 − NAA25_MOUSE
Q8BWZ3
1
0
−1
−2
0
50
100
Distance in Percent

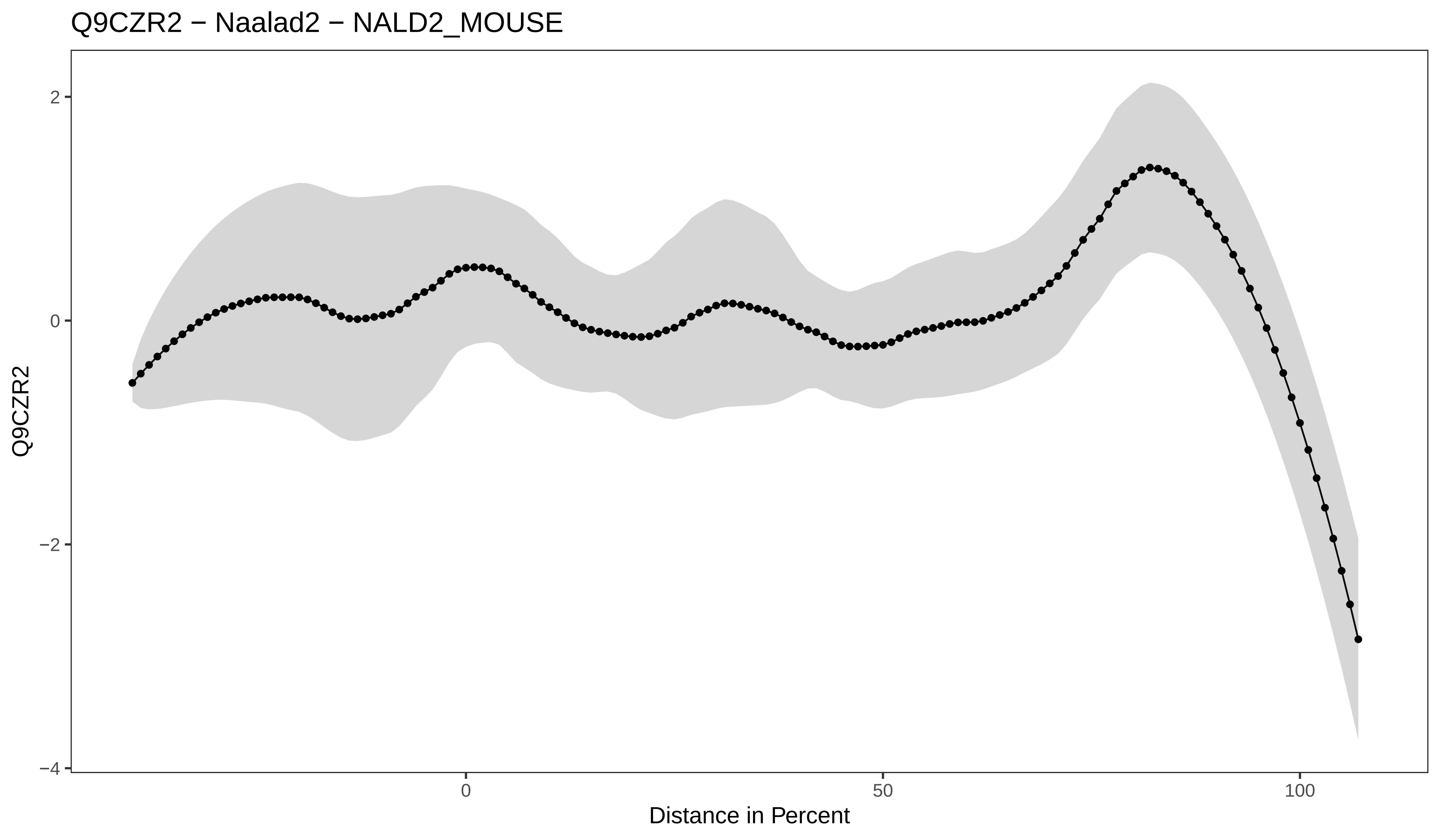

Q9CZR2 − Naalad2 − NALD2_MOUSE
2
0
−2
−4
Q9CZR2
0
50
100
Distance in Percent

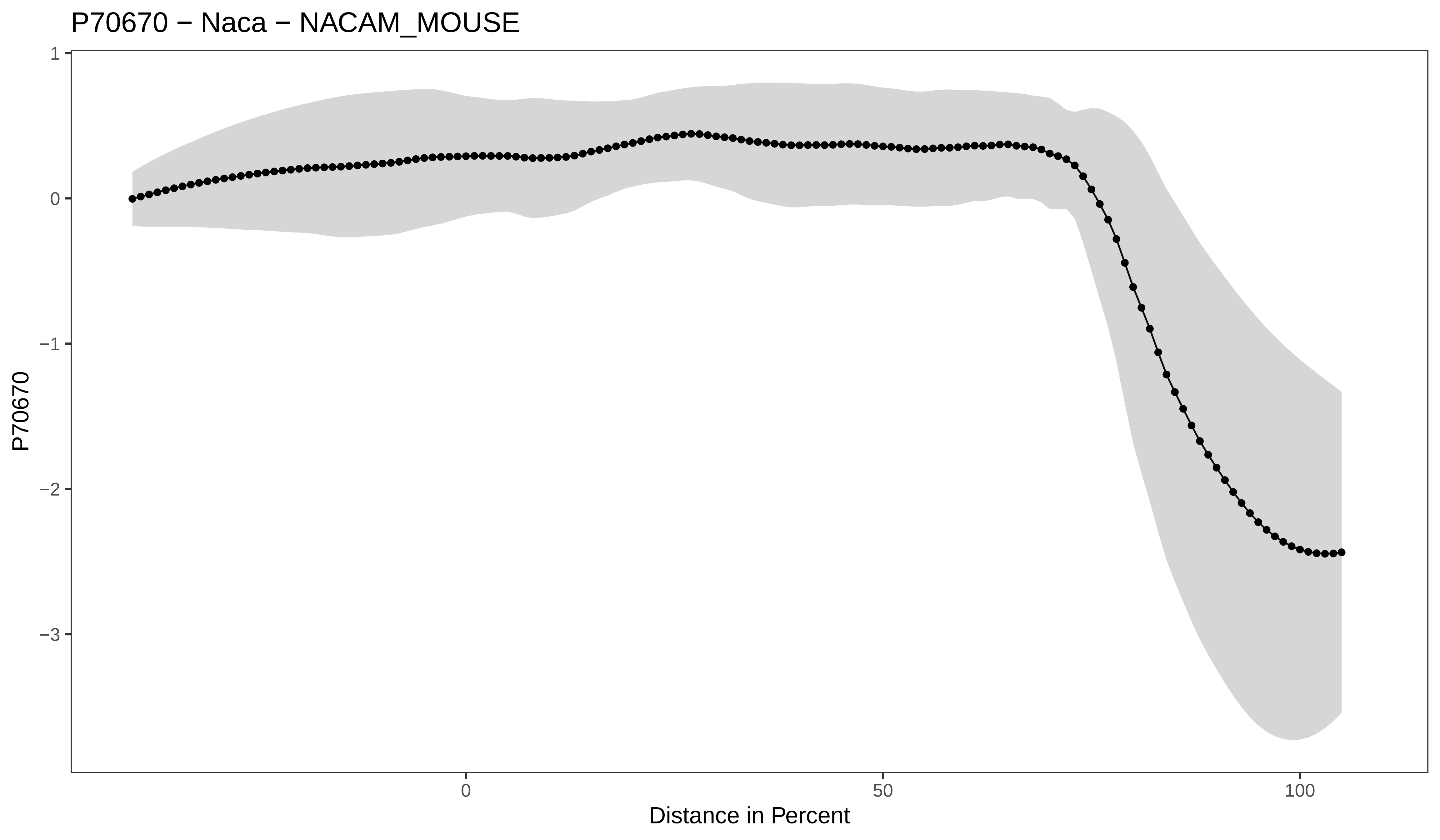
P70670 − Naca − NACAM_MOUSE
P70670
Distance in Percent
1
0
−1
−2
−3
0
50
100

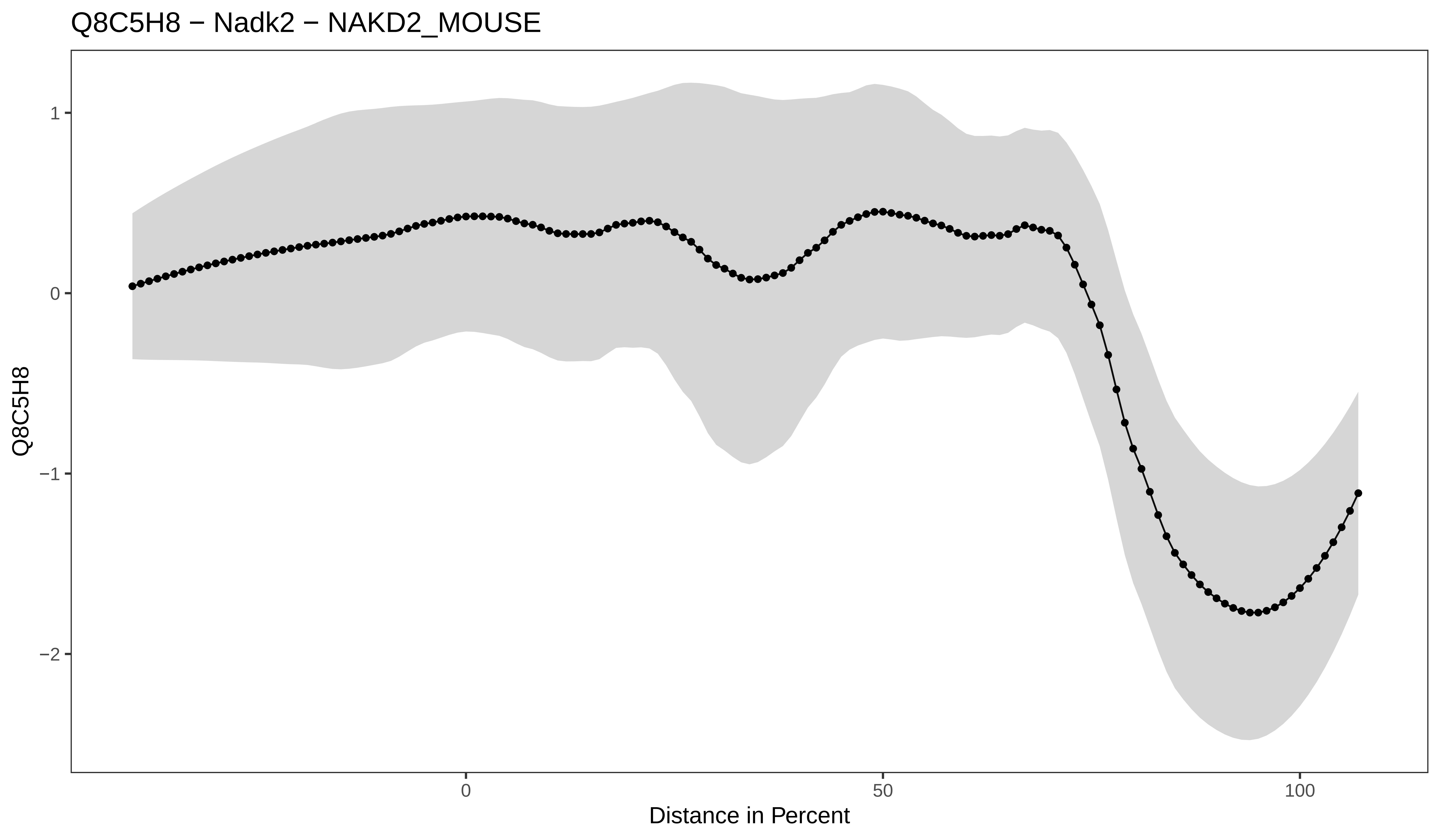
Q8C5H8 − Nadk2 − NAKD2_MOUSE
Q8C5H8
Distance in Percent
1
0
−1
−2
0
50
100

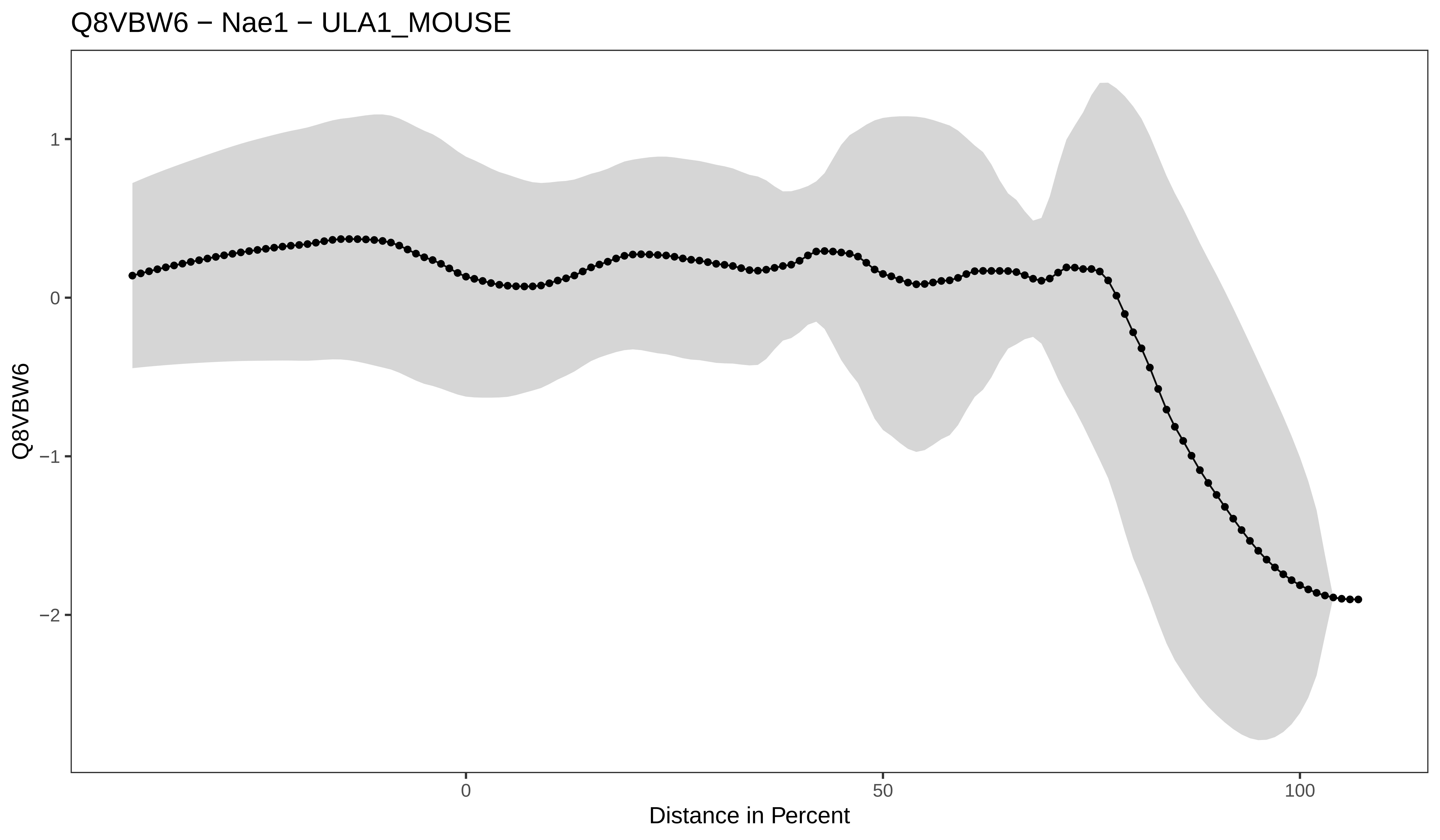

Q8VBW6 − Nae1 − ULA1_MOUSE
Q8VBW6
1
0
−1
−2
0
50
100
Distance in Percent

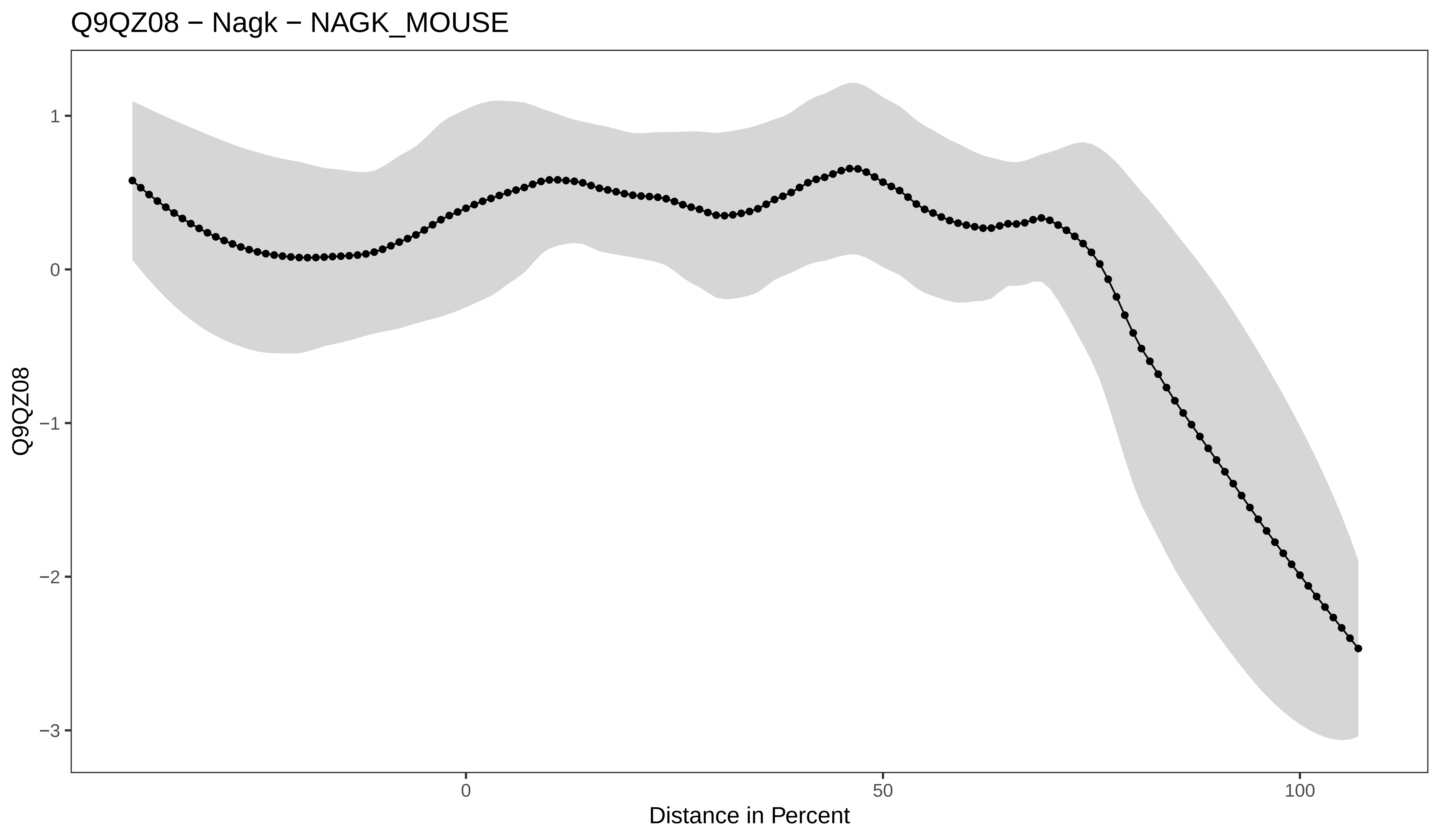
Q9QZ08 − Nagk − NAGK_MOUSE
1
0
−1
−2
−3
Q9QZ08
0
50
100
Distance in Percent

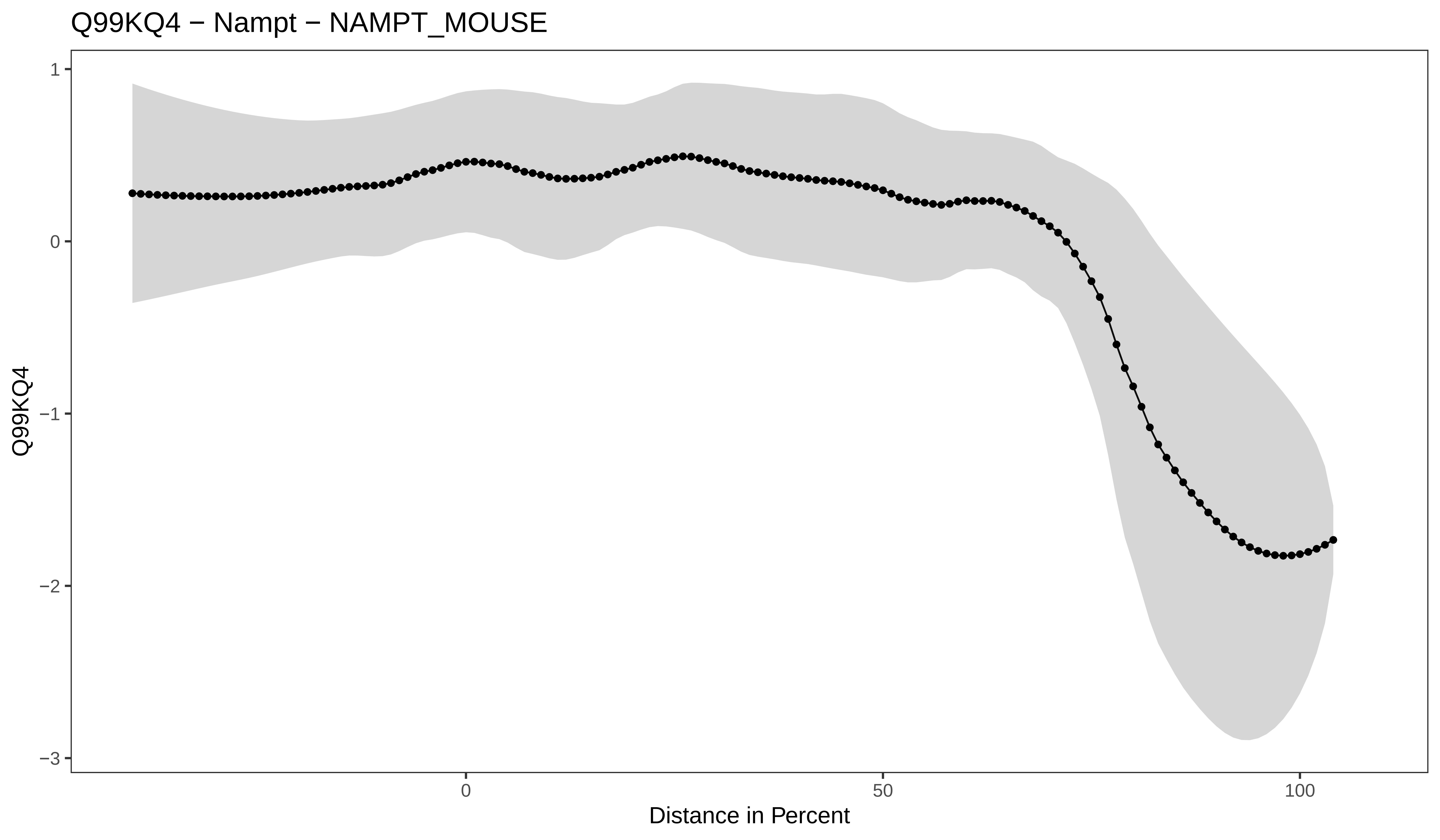

Q99KQ4 − Nampt − NAMPT_MOUSE
1
0
−1
−2
−3
Q99KQ4
0
50
100
Distance in Percent

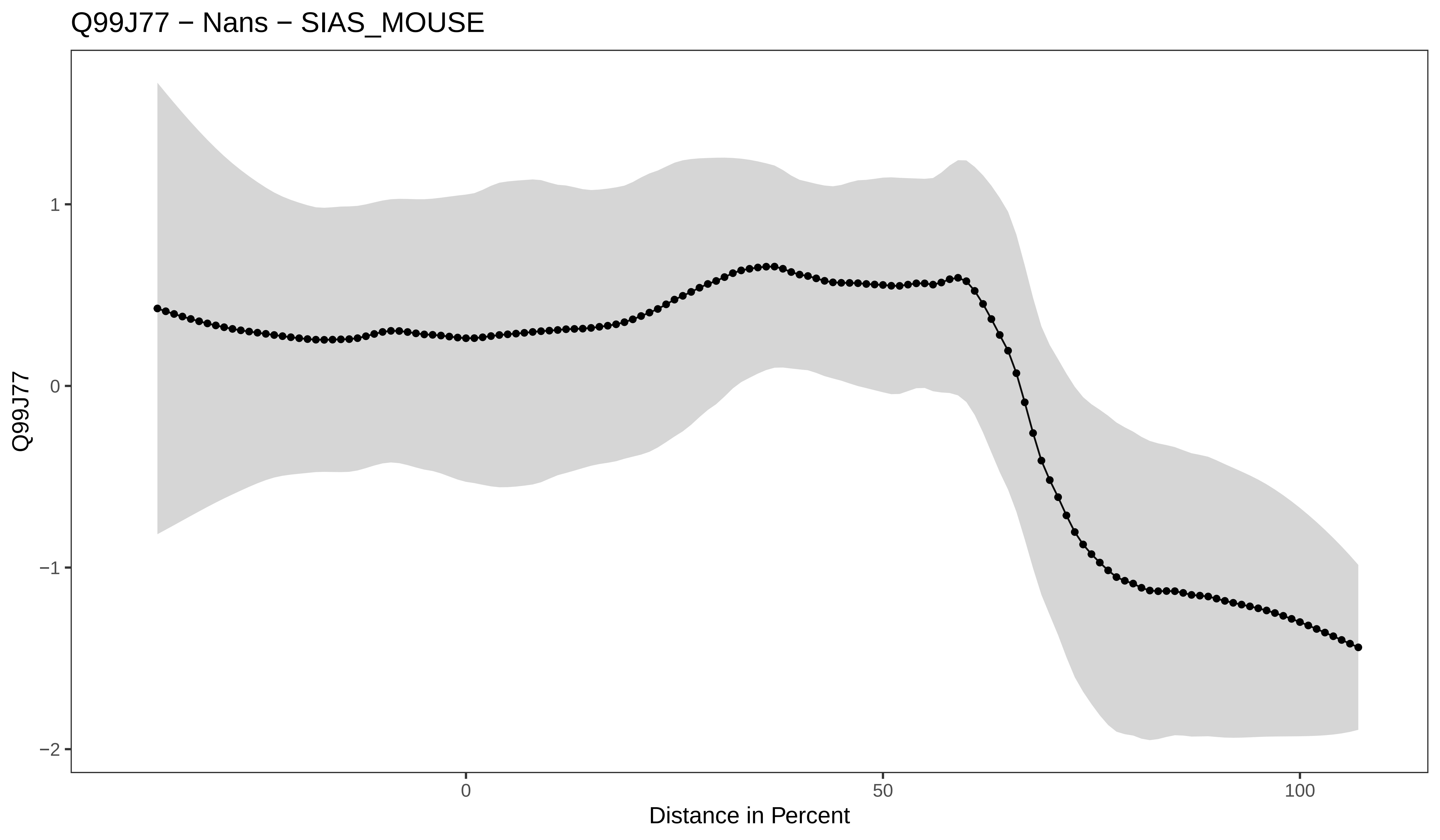

Q99J77 − Nans − SIAS_MOUSE
Q99J77
1
0
−1
−2
0
50
100
Distance in Percent

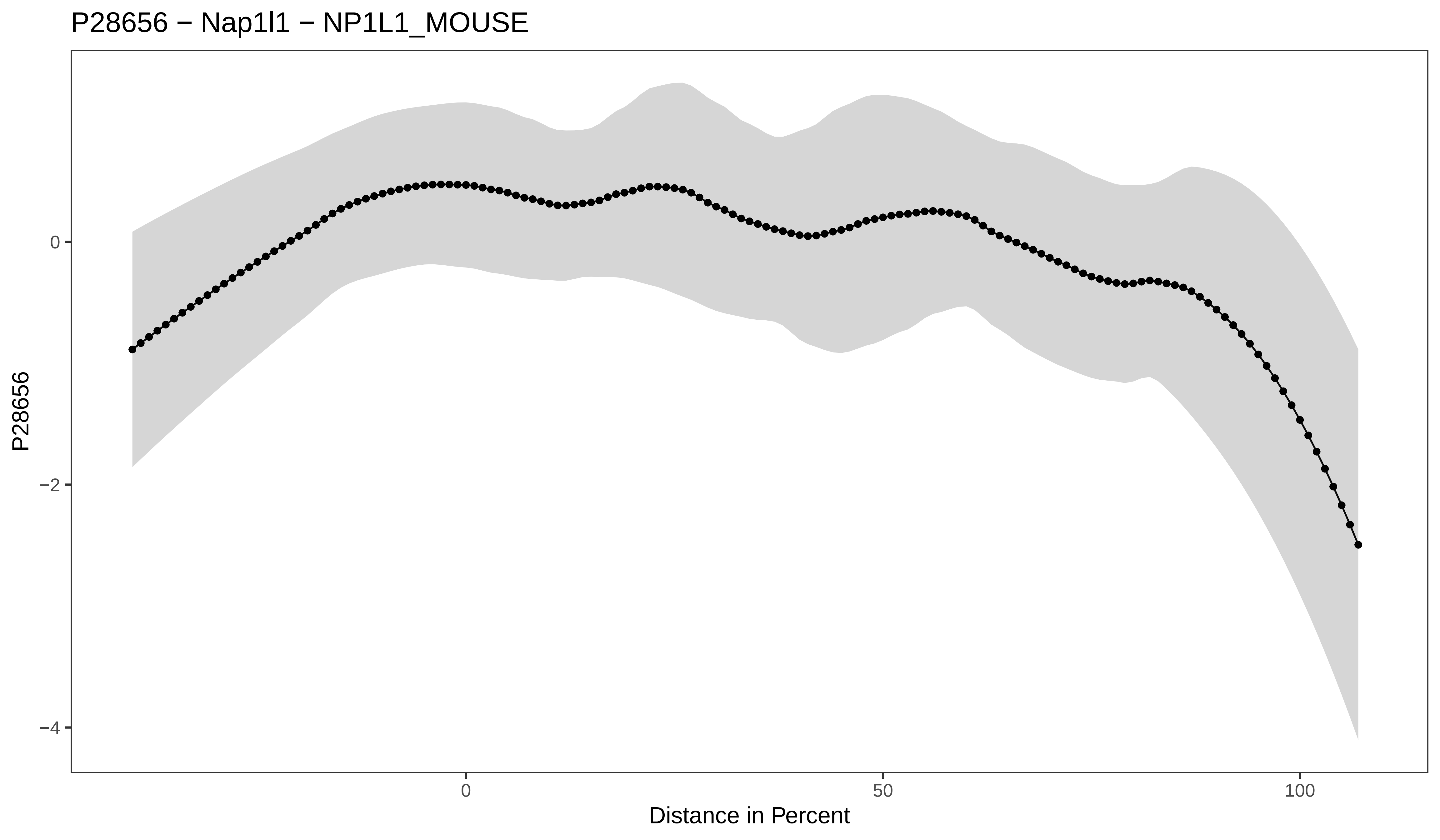

P28656 − Nap1l1 − NP1L1_MOUSE
0
−2
−4
P28656
0
50
100
Distance in Percent

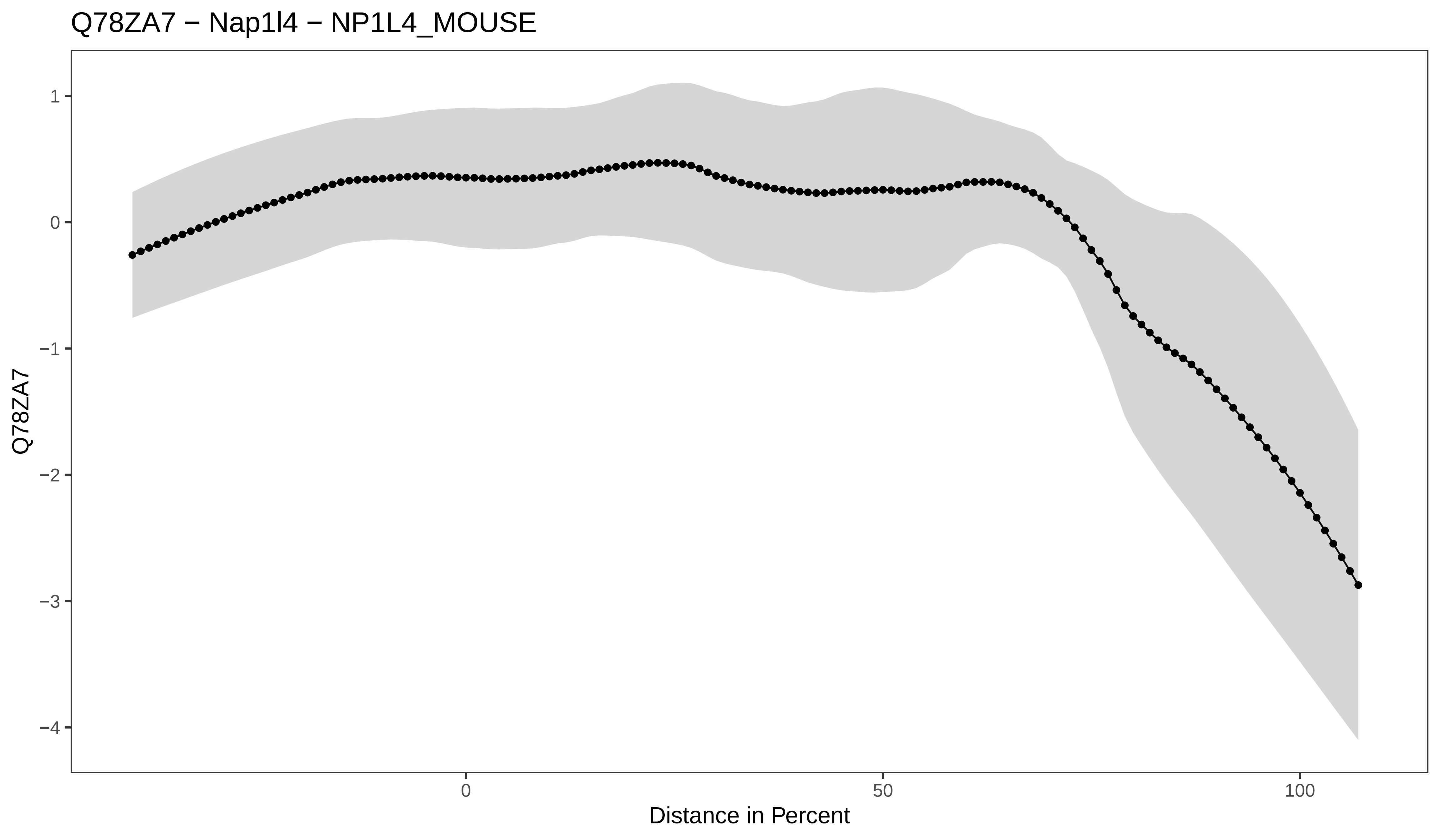
Q78ZA7 − Nap1l4 − NP1L4_MOUSE
Q78ZA7
Distance in Percent
1
0
−1
−2
−3
−4
0
50
100

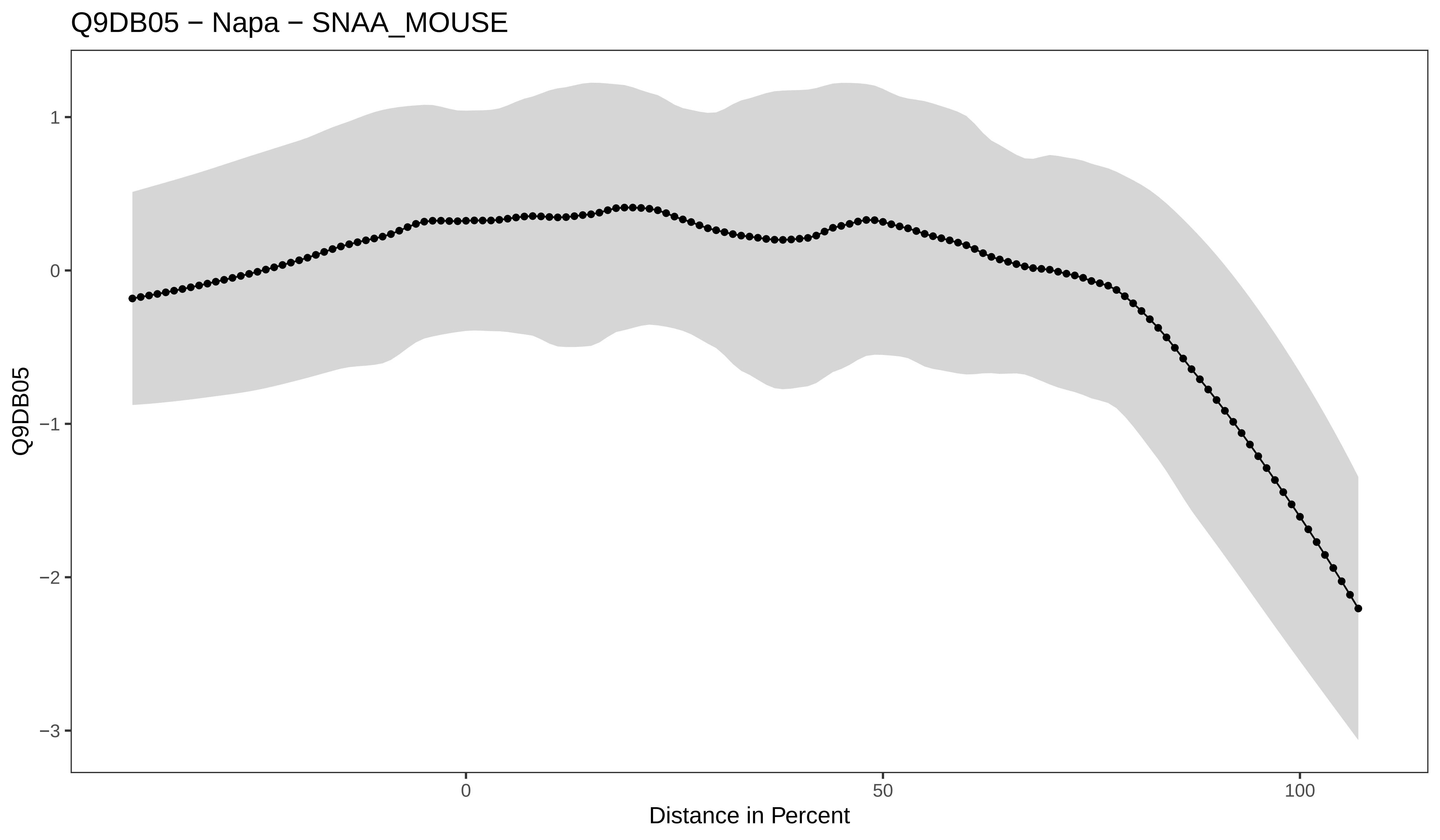
Q9DB05 − Napa − SNAA_MOUSE
Q9DB05
1
0
−1
−2
−3
0
50
100
Distance in Percent

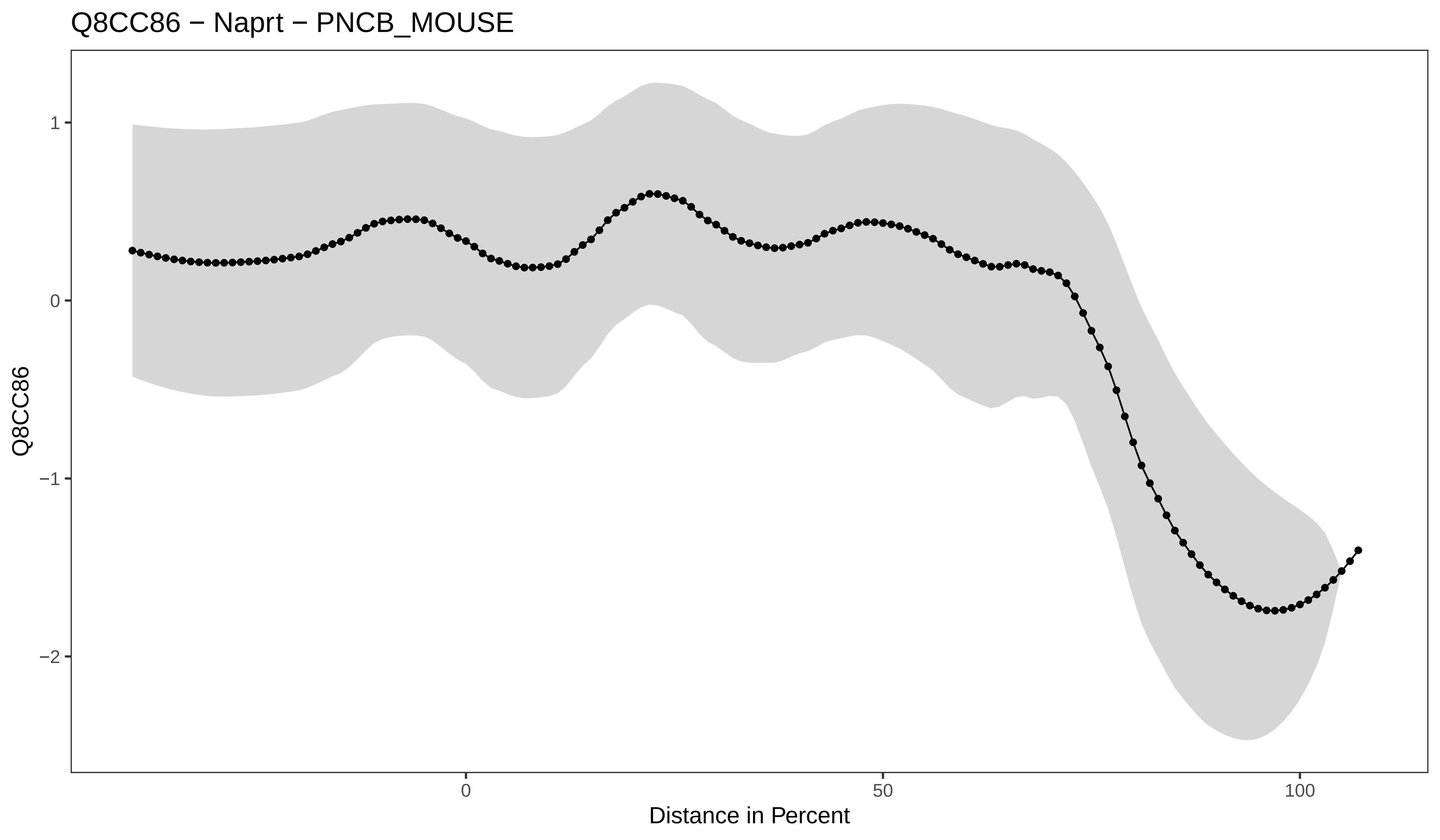

Q8CC86 − Naprt − PNCB_MOUSE
Q8CC86
1
0
−1
−2
0
50
100
Distance in Percent

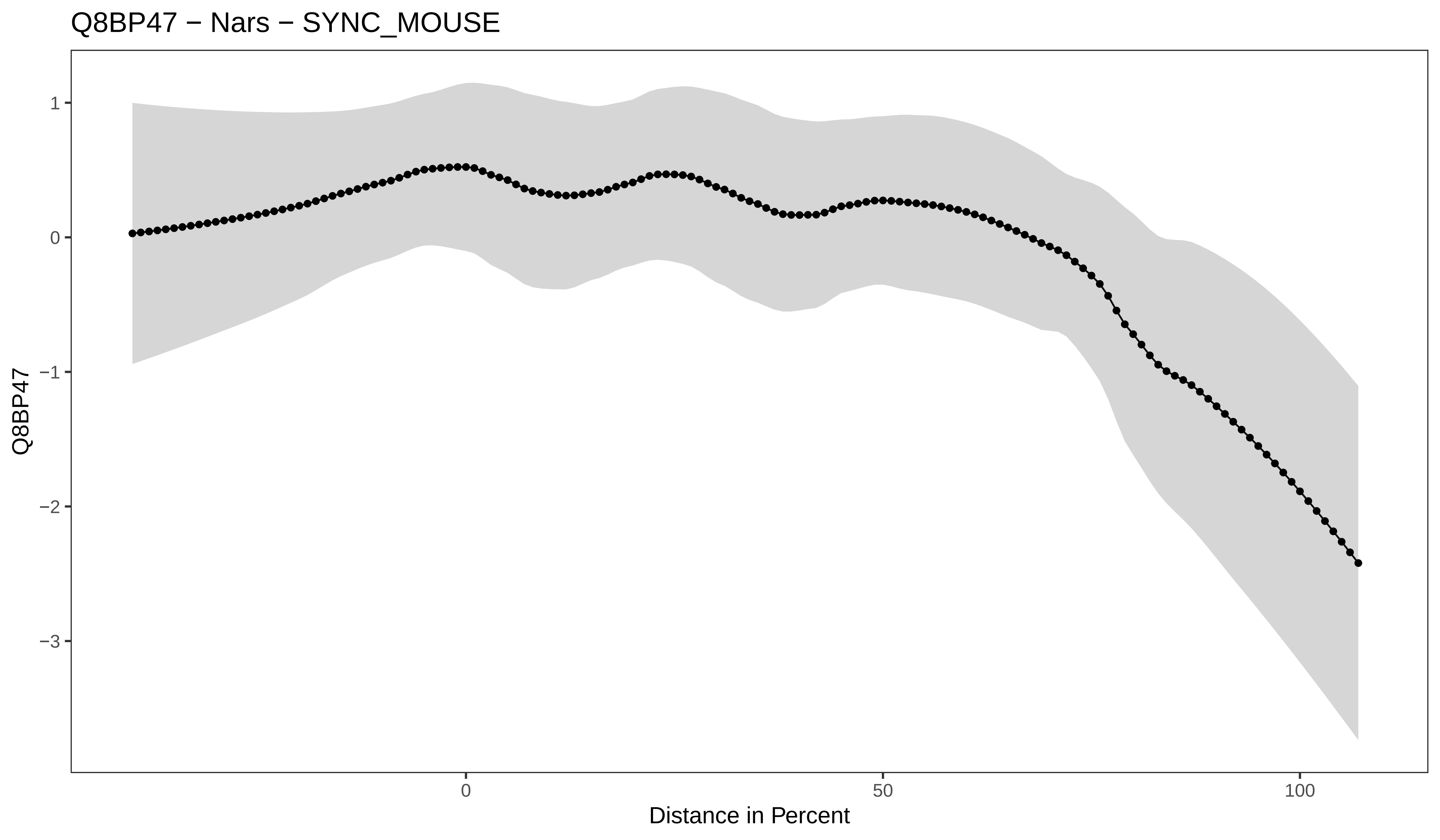

Q8BP47 − Nars − SYNC_MOUSE
Q8BP47
1
0
−1
−2
−3
0
50
100
Distance in Percent

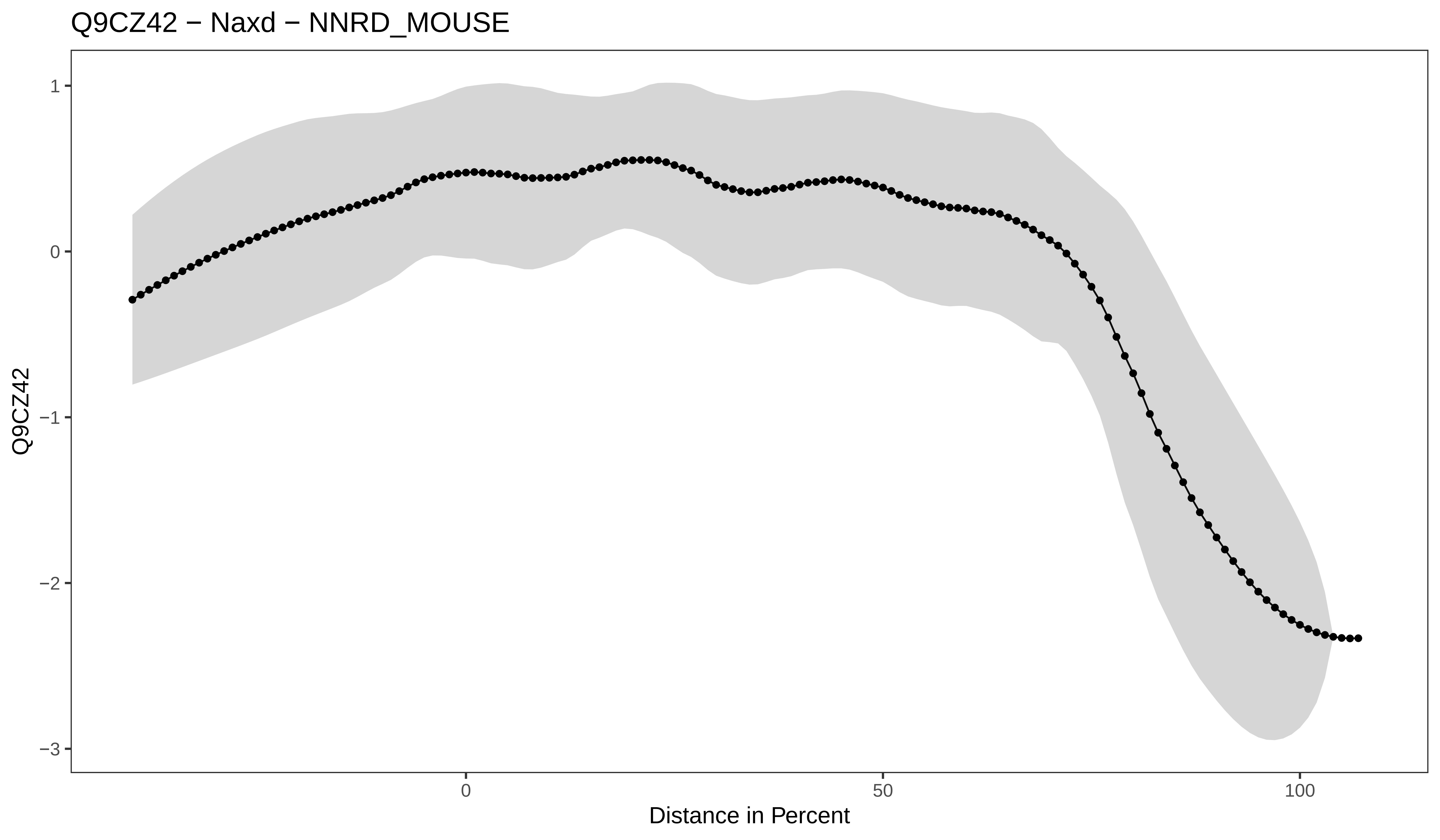

Q9CZ42 − Naxd − NNRD_MOUSE
1
0
−1
−2
−3
Q9CZ42
0
50
100
Distance in Percent

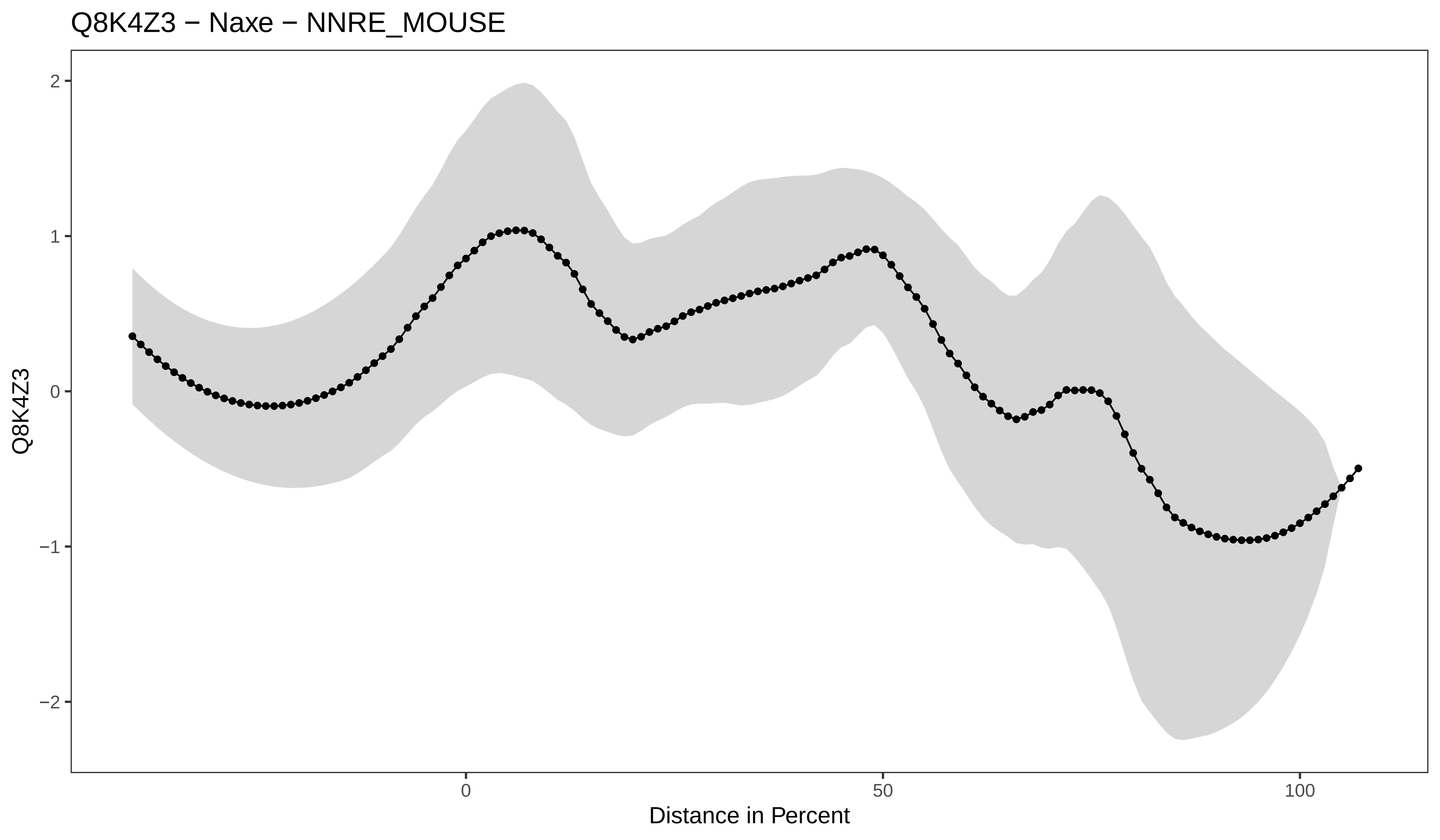
Q8K4Z3 − Naxe − NNRE_MOUSE
Q8K4Z3
Distance in Percent
2
1
0
−1
−2
0
50
100

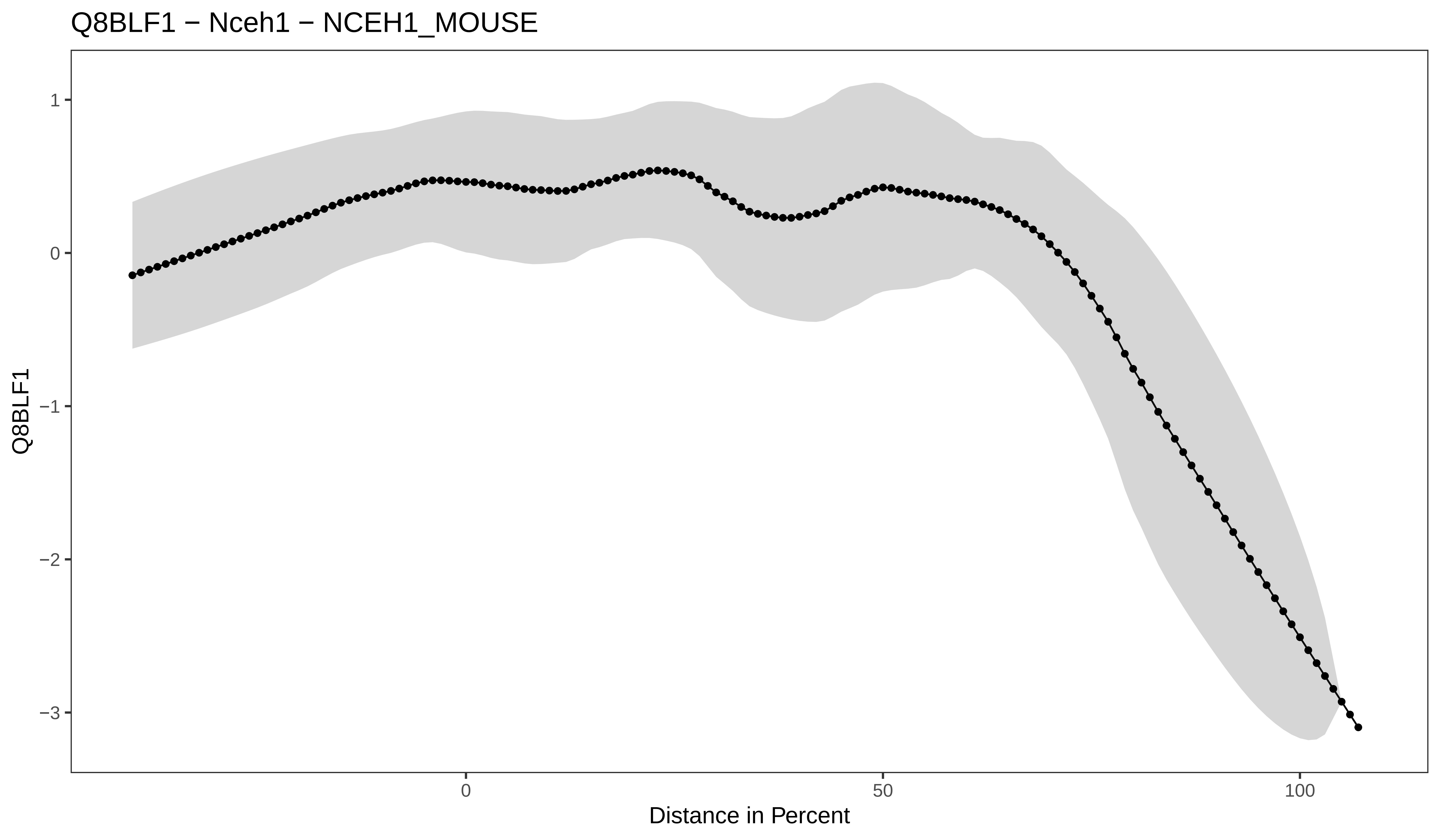

Q8BLF1 − Nceh1 − NCEH1_MOUSE
1
0
−1
−2
−3
Q8BLF1
0
50
100
Distance in Percent

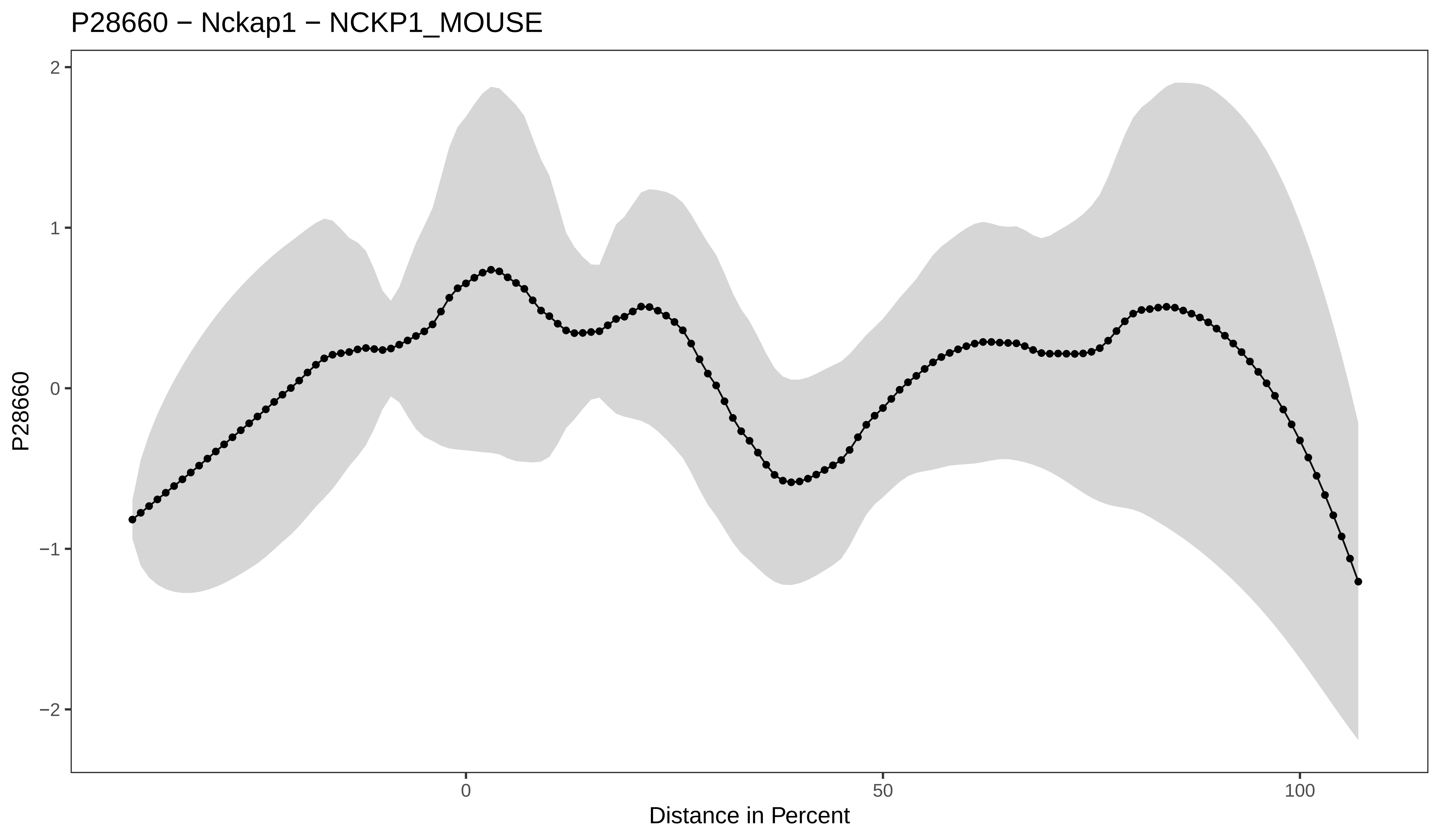
P28660 − Nckap1 − NCKP1_MOUSE
2
1
0
−1
−2
P28660
0
50
100
Distance in Percent

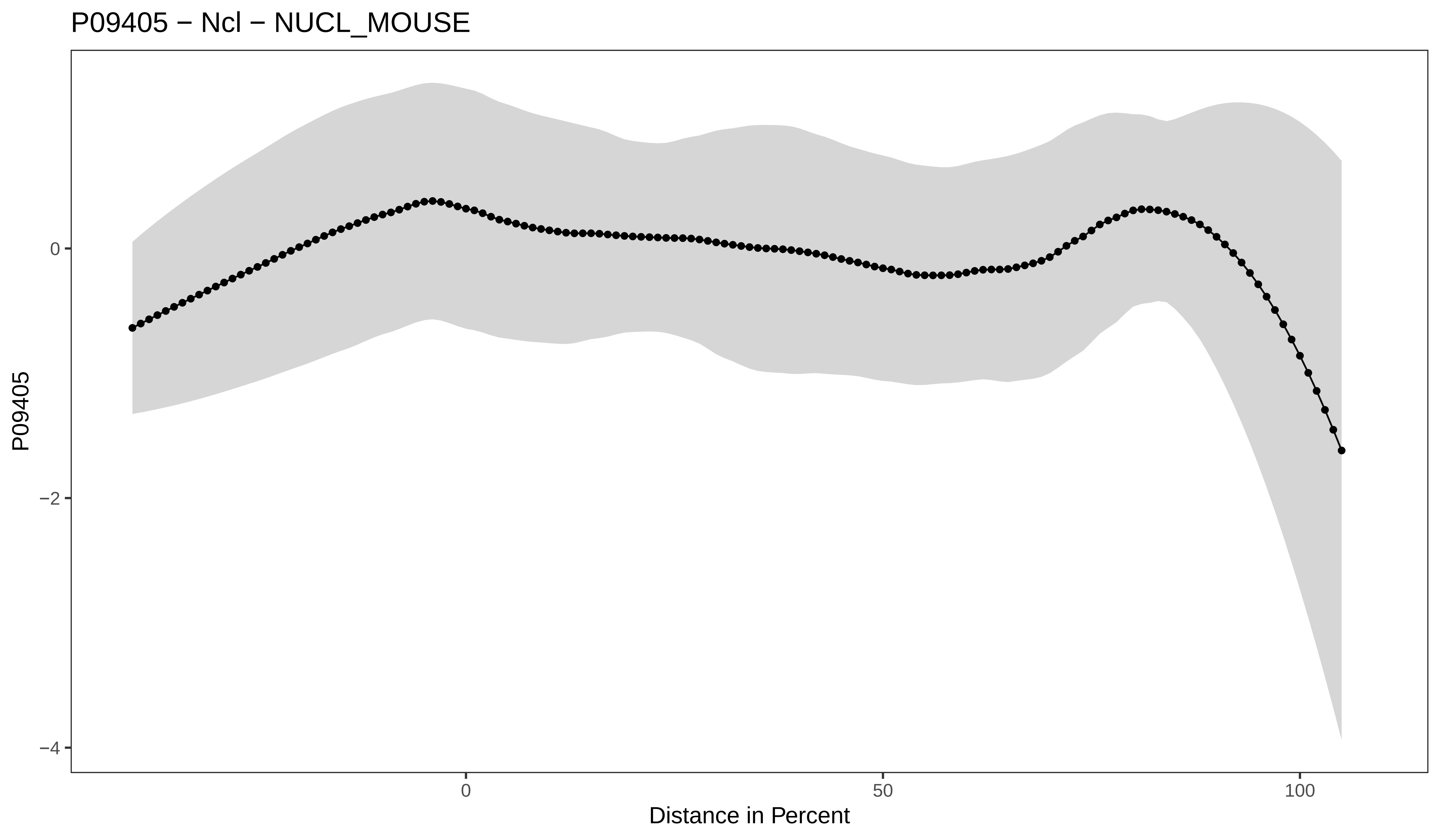

P09405 − Ncl − NUCL_MOUSE
P09405
0
−2
−4
0
50
100
Distance in Percent

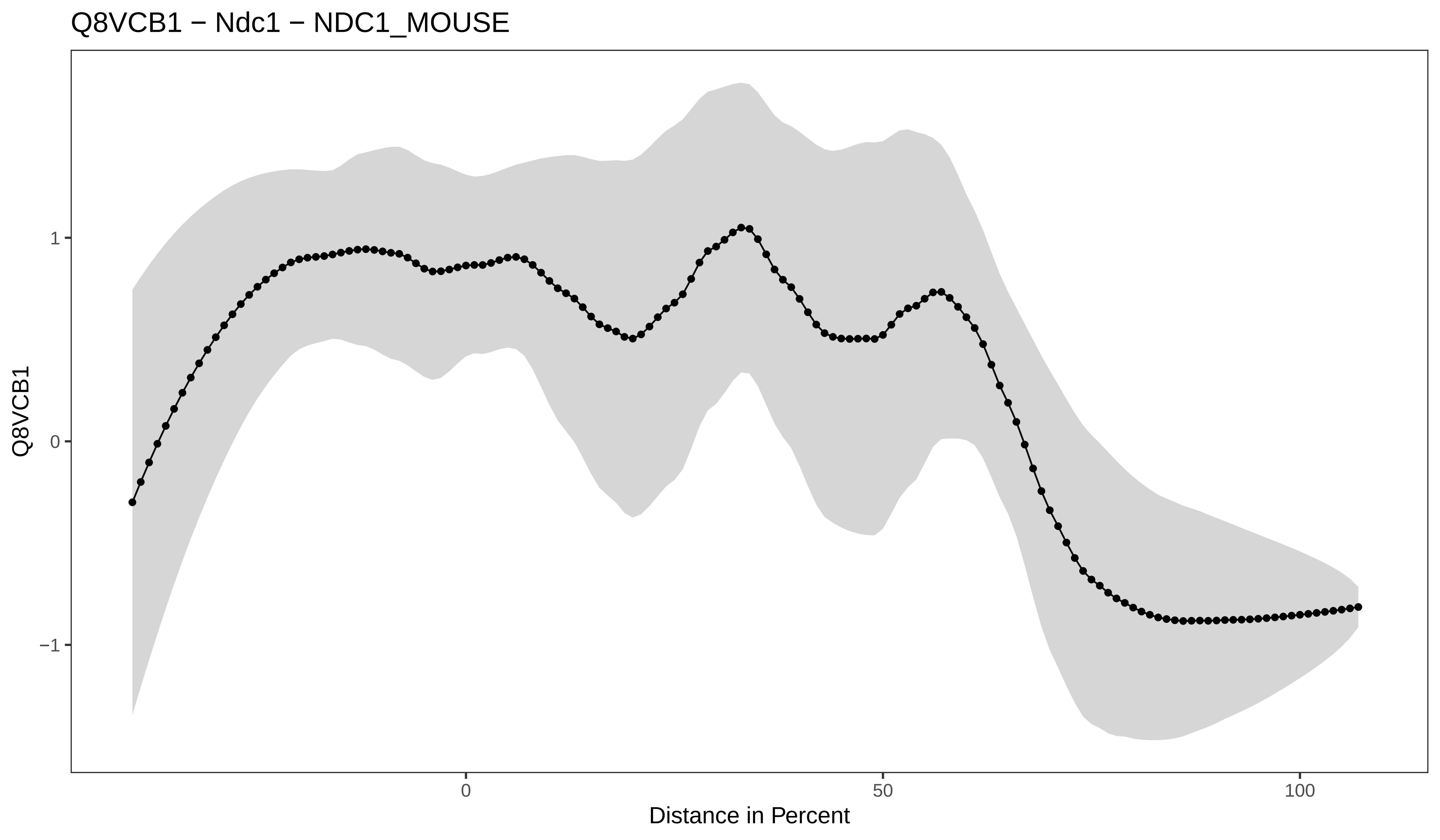

Q8VCB1 − Ndc1 − NDC1_MOUSE
Q8VCB1
1
0
−1
0
50
100
Distance in Percent

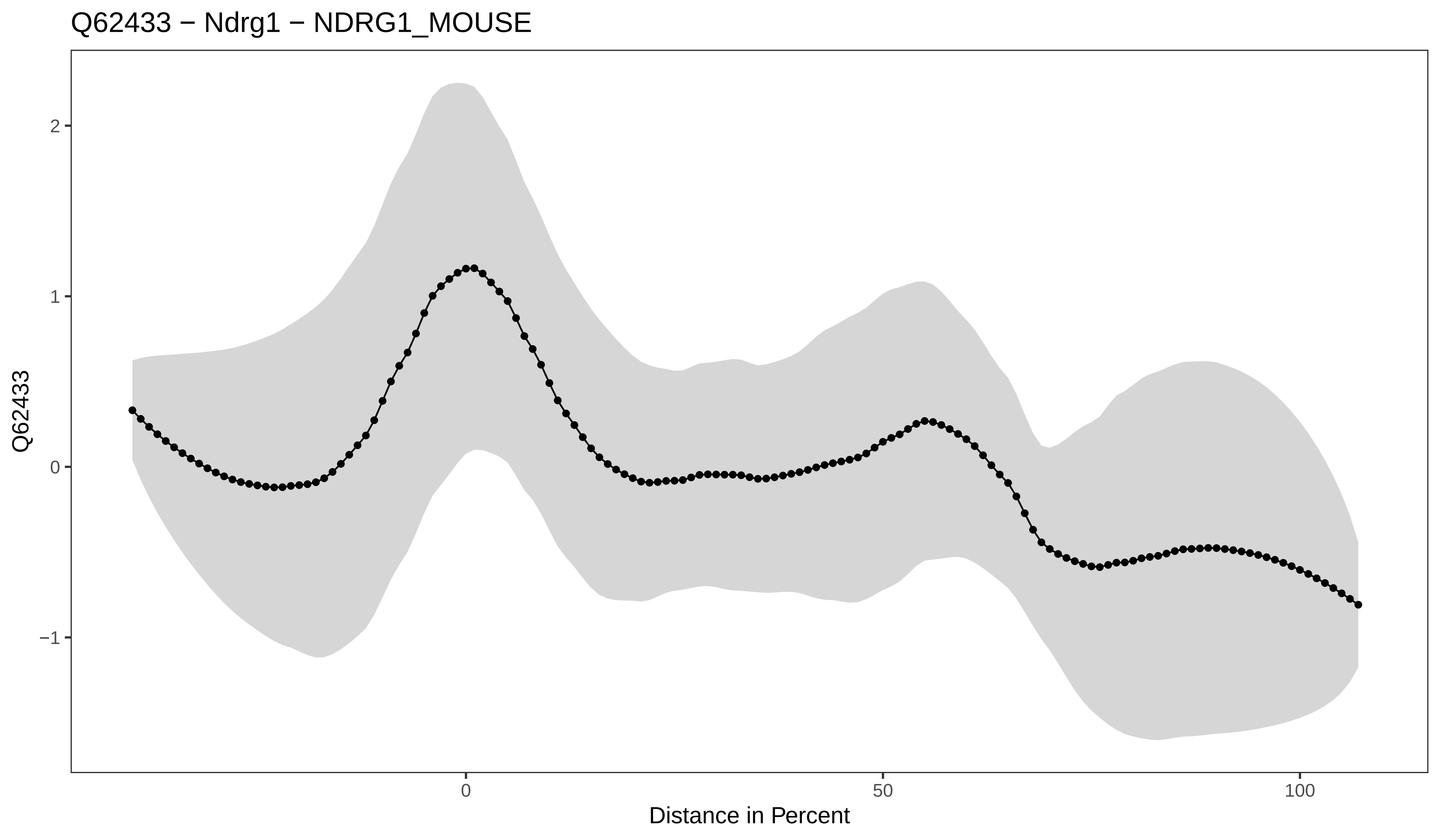

Q62433 − Ndrg1 − NDRG1_MOUSE
Q62433
2
1
0
−1
0
50
100
Distance in Percent

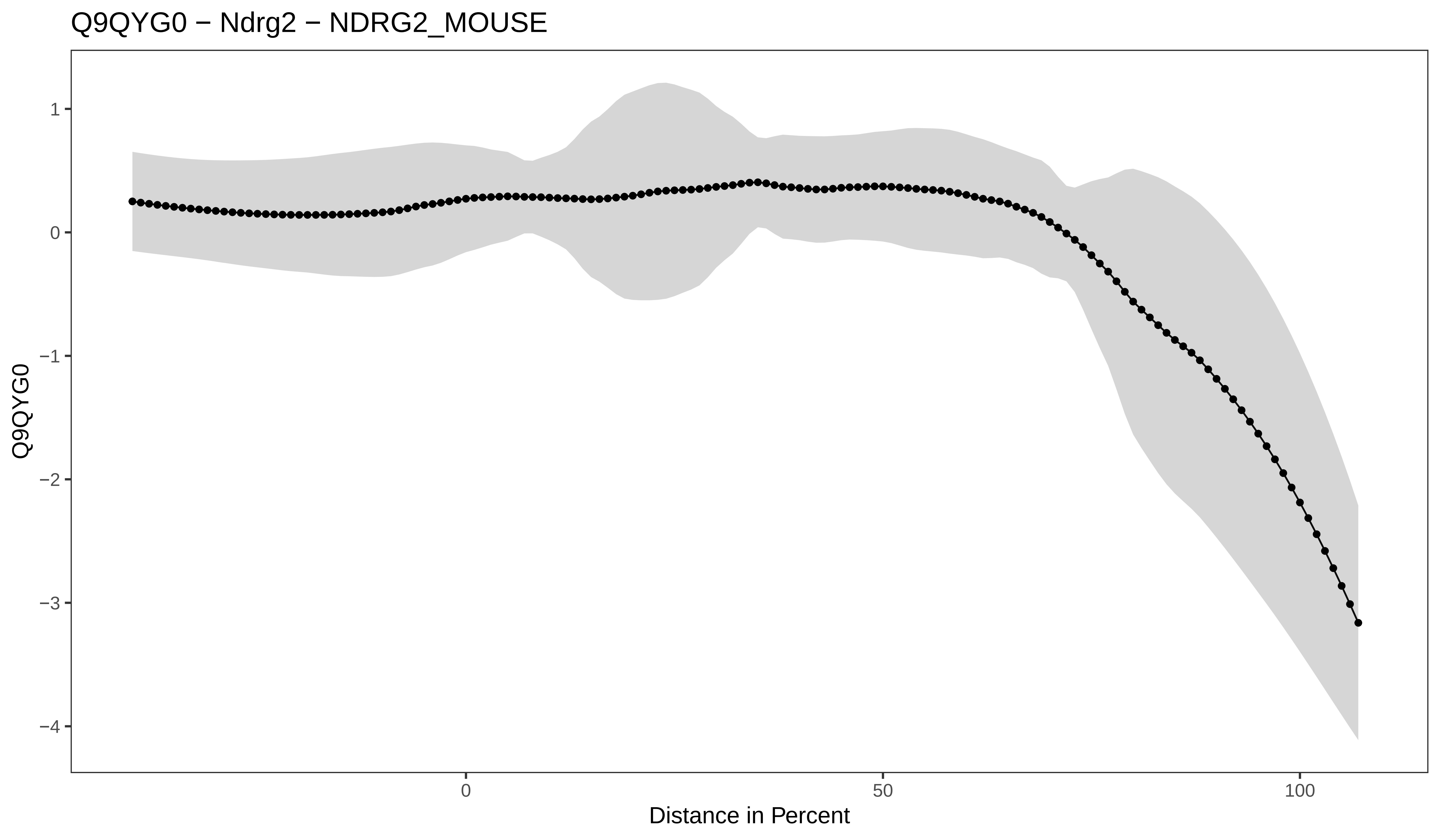
Q9QYG0 − Ndrg2 − NDRG2_MOUSE
1
0
−1
−2
−3
−4
Q9QYG0
0
50
100
Distance in Percent

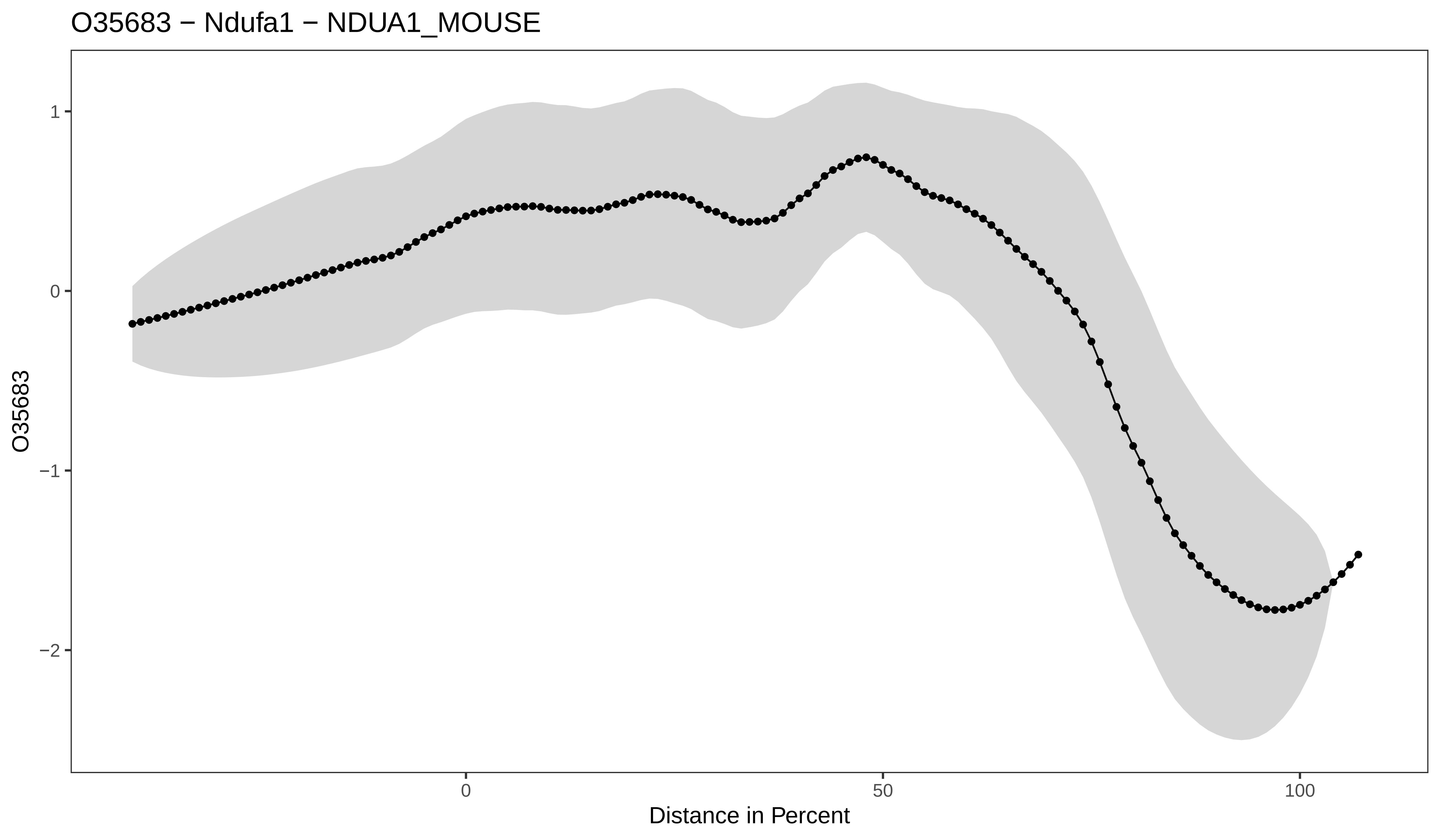

O35683 − Ndufa1 − NDUA1_MOUSE
1
0
−1
−2
O35683
0
50
100
Distance in Percent

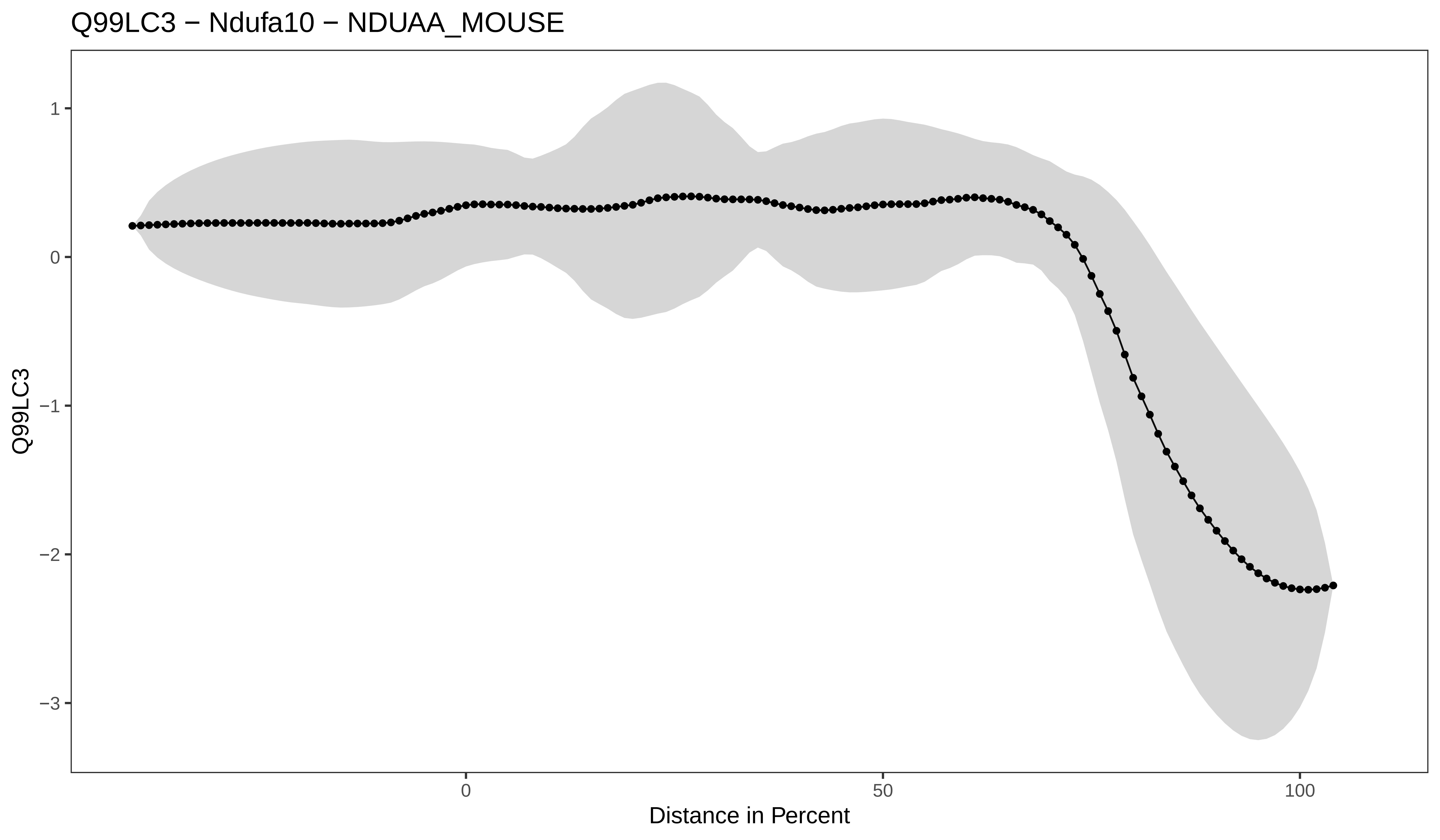
Q99LC3 − Ndufa10 − NDUAA_MOUSE
Q99LC3
Distance in Percent
1
0
−1
−2
−3
0
50
100

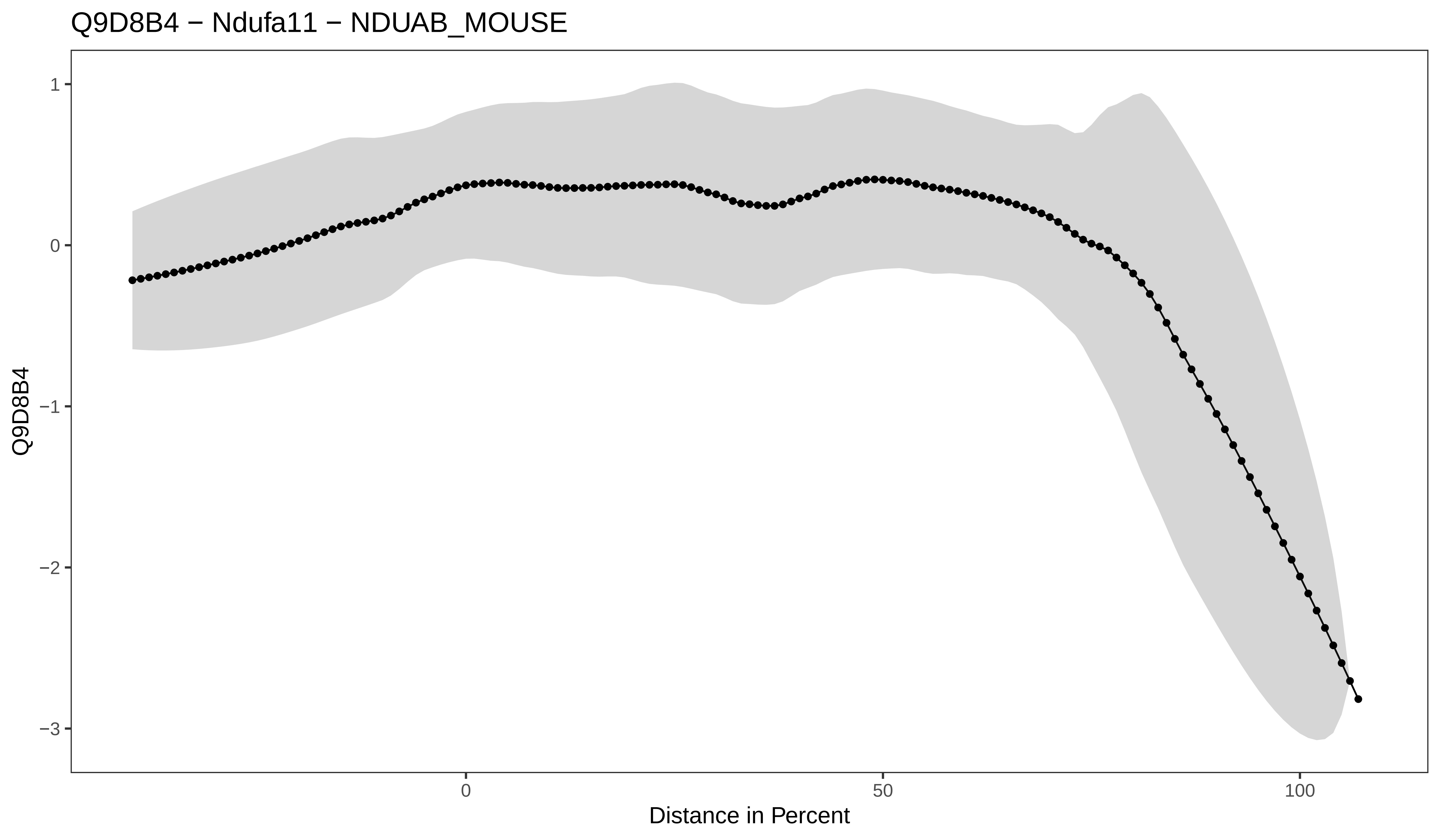
Q9D8B4 − Ndufa11 − NDUAB_MOUSE
Q9D8B4
Distance in Percent
1
0
−1
−2
−3
0
50
100

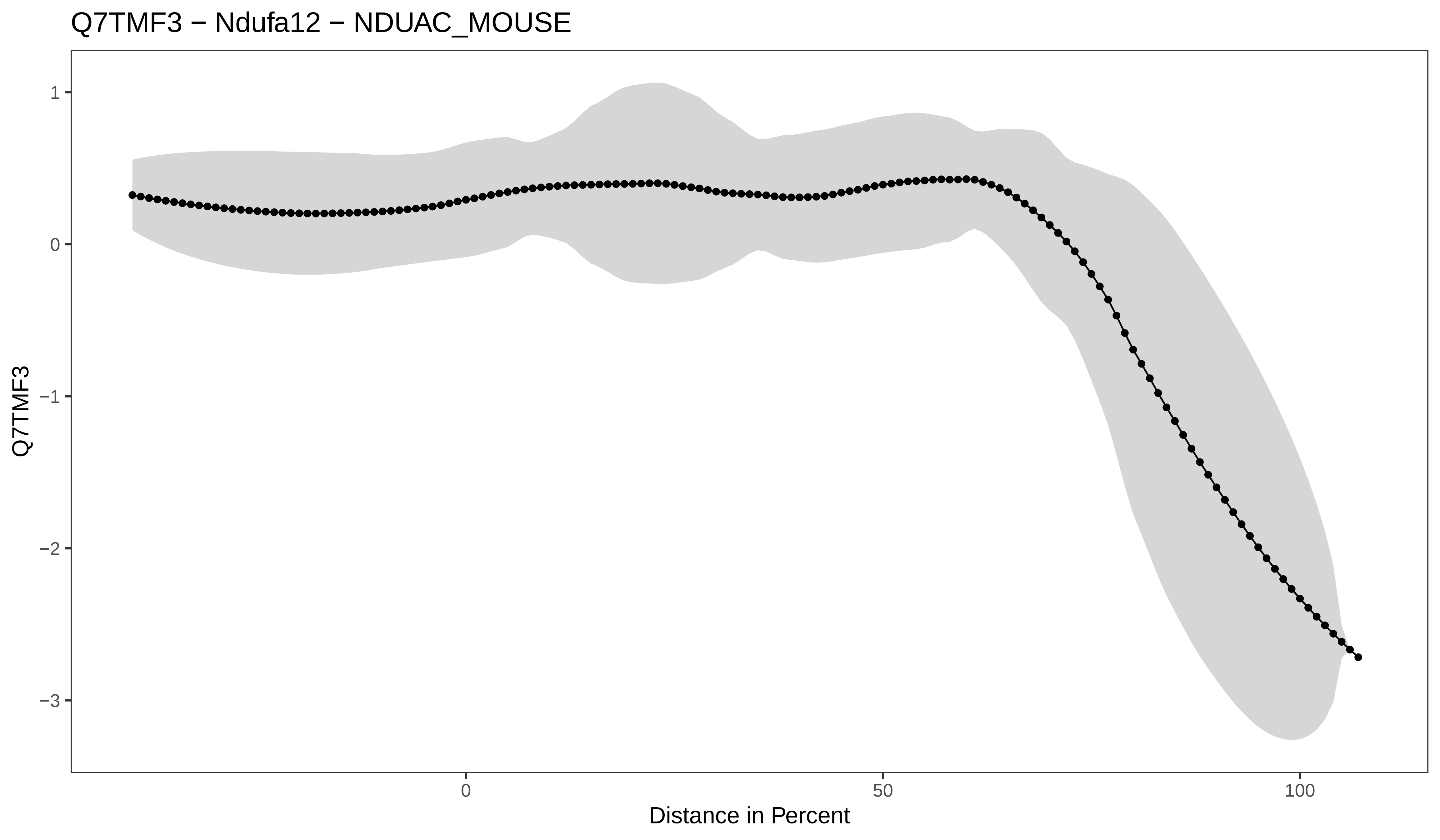

Q7TMF3 − Ndufa12 − NDUAC_MOUSE
Q7TMF3
1
0
−1
−2
−3
0
50
100
Distance in Percent

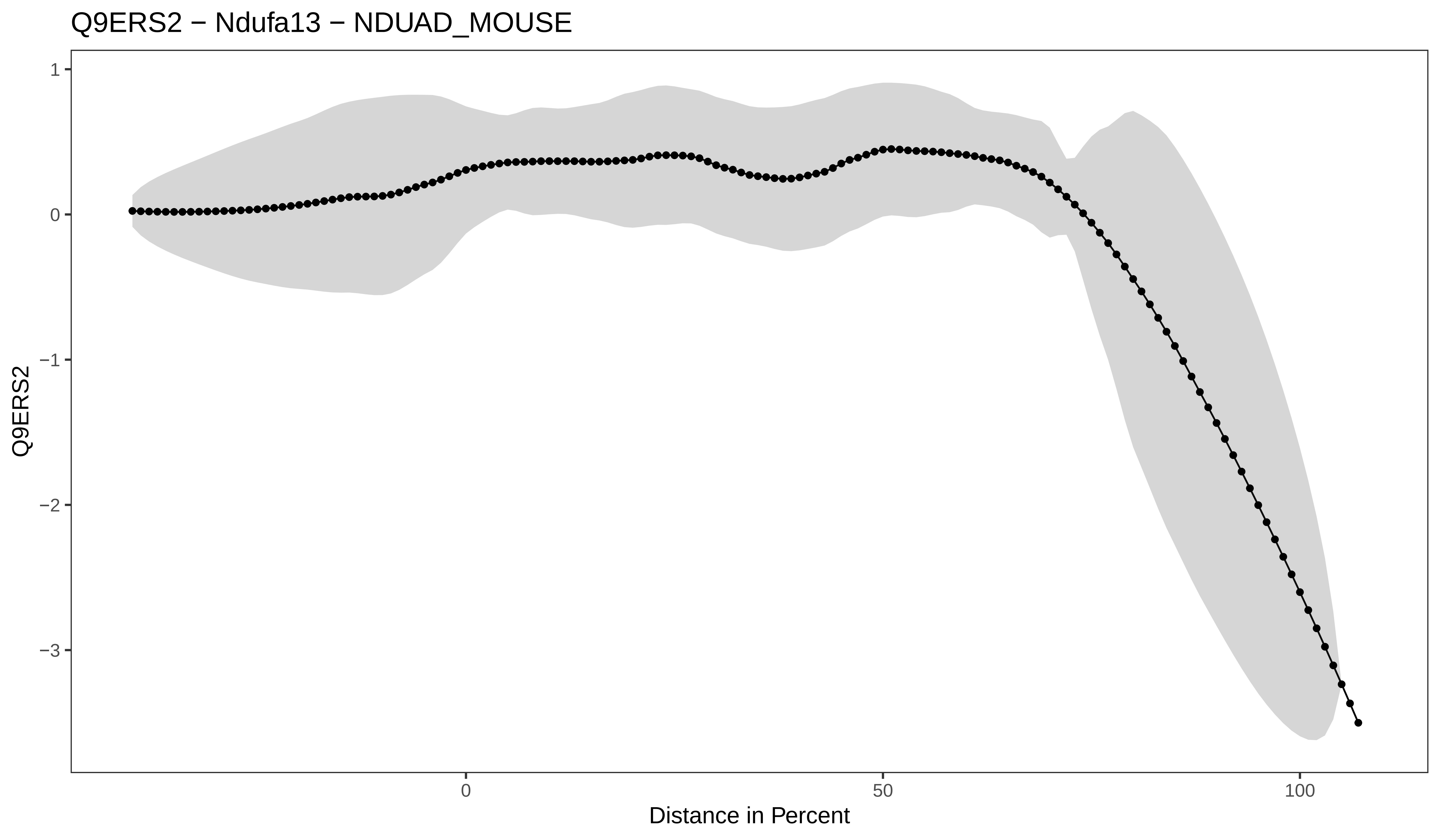

Q9ERS2 − Ndufa13 − NDUAD_MOUSE
1
0
−1
−2
−3
Q9ERS2
0
50
100
Distance in Percent

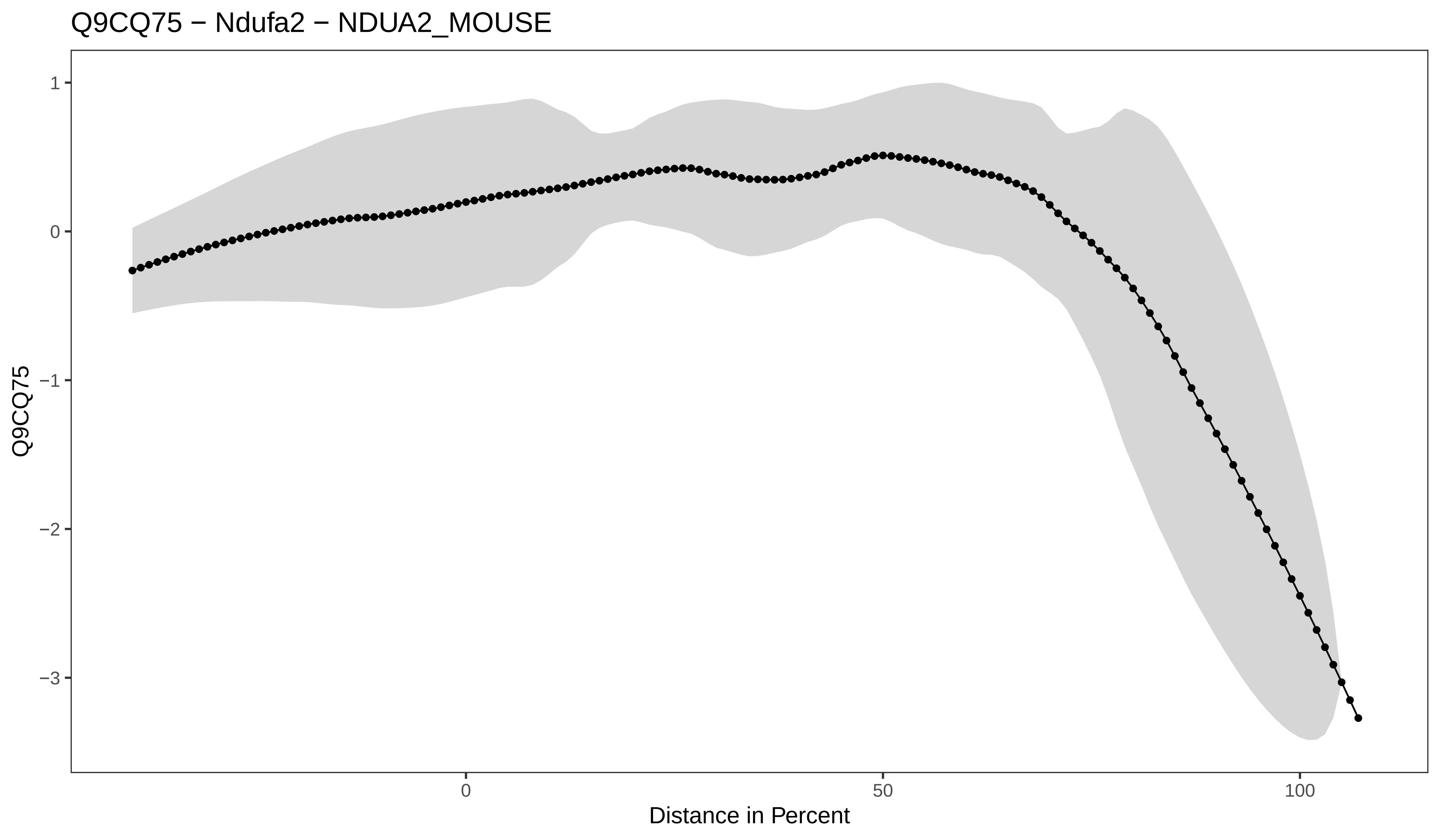
Q9CQ75 − Ndufa2 − NDUA2_MOUSE
1
0
−1
−2
−3
Q9CQ75
0
50
100
Distance in Percent

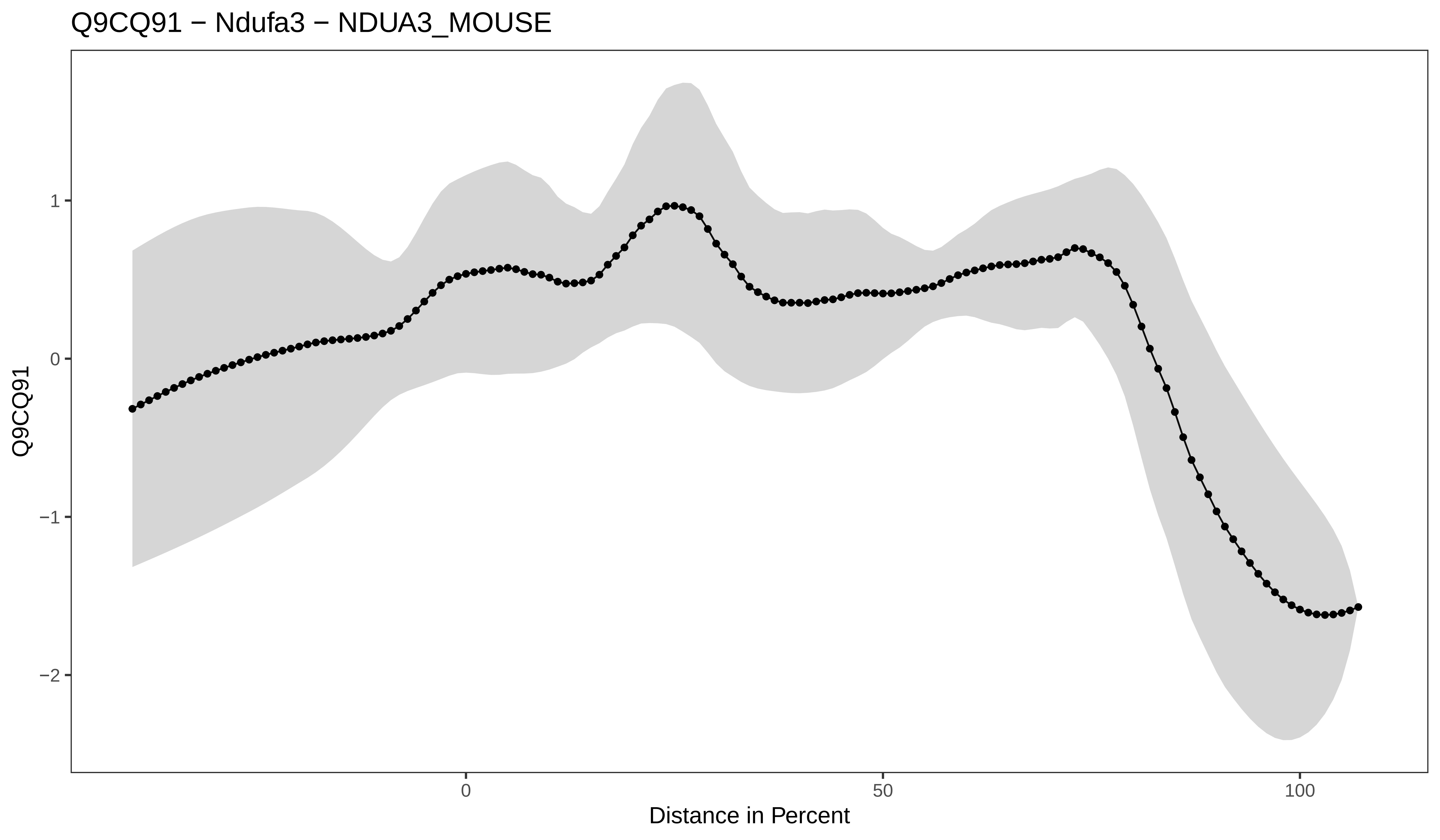

Q9CQ91 − Ndufa3 − NDUA3_MOUSE
Q9CQ91
1
0
−1
−2
0
50
100
Distance in Percent

Q62425 − Ndufa4 − NDUA4_MOUSE

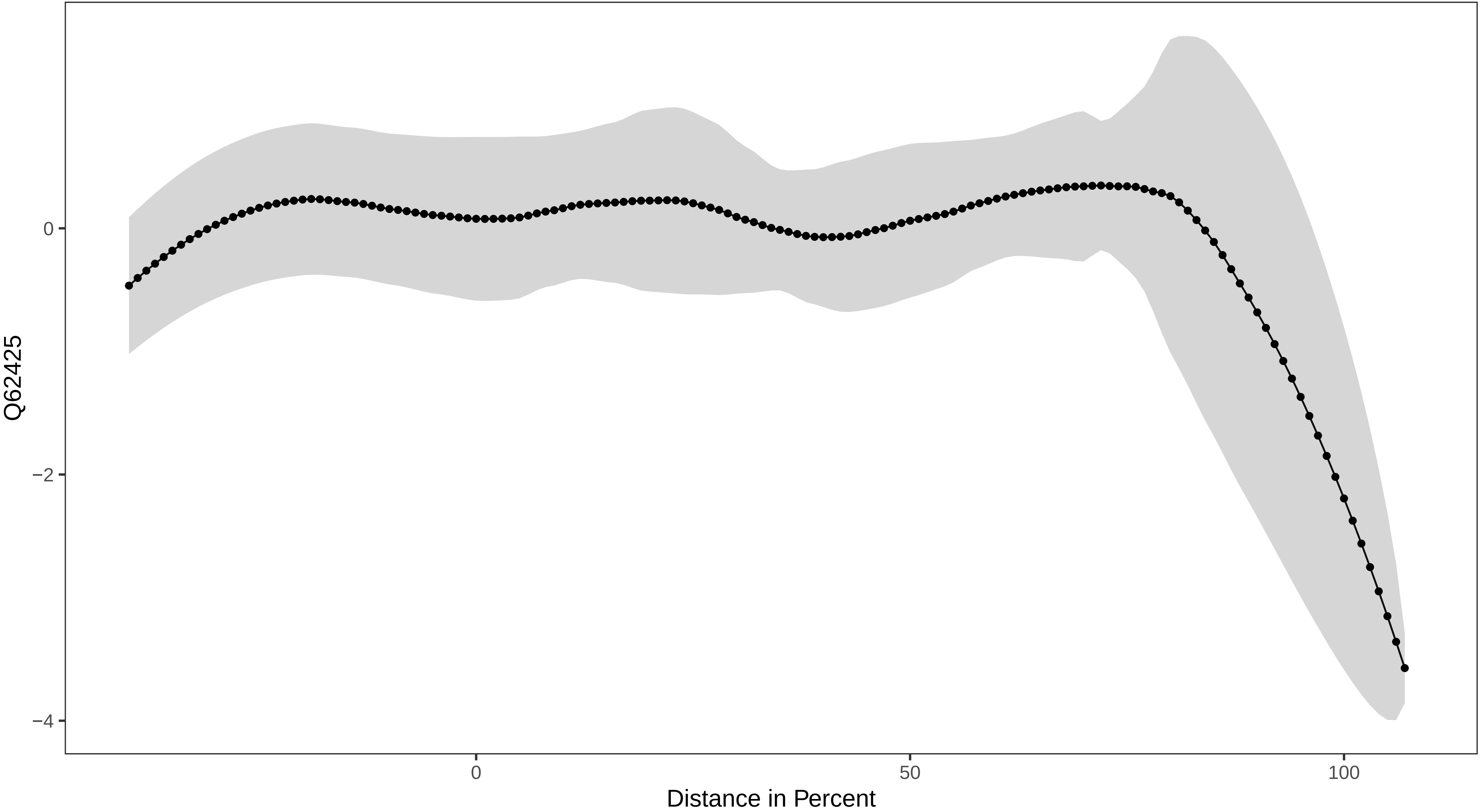

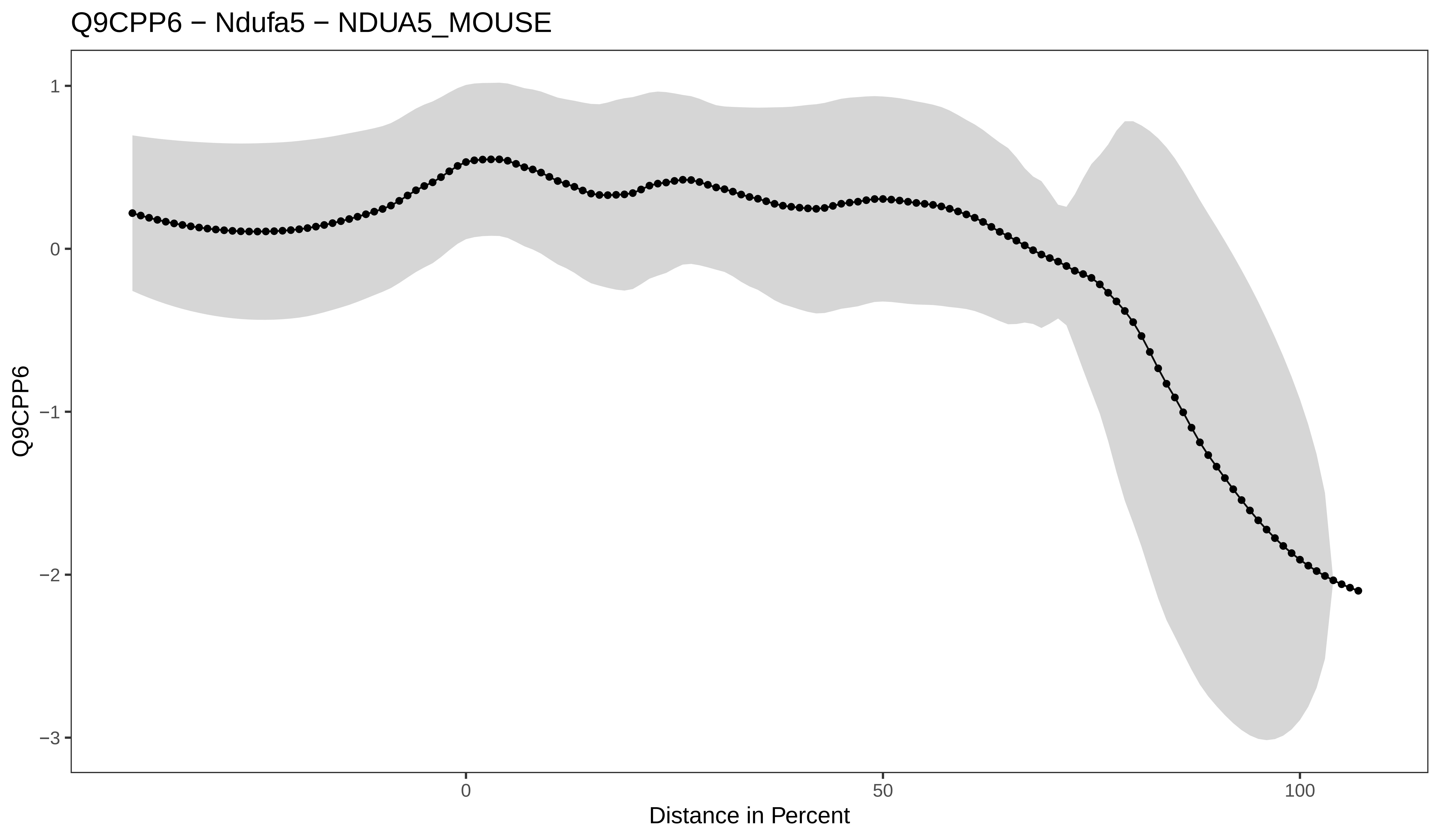
Q9CPP6 − Ndufa5 − NDUA5_MOUSE
Q9CPP6
1
0
−1
−2
−3
0
50
100
Distance in Percent

Q9CQZ5 − Ndufa6 − NDUA6_MOUSE

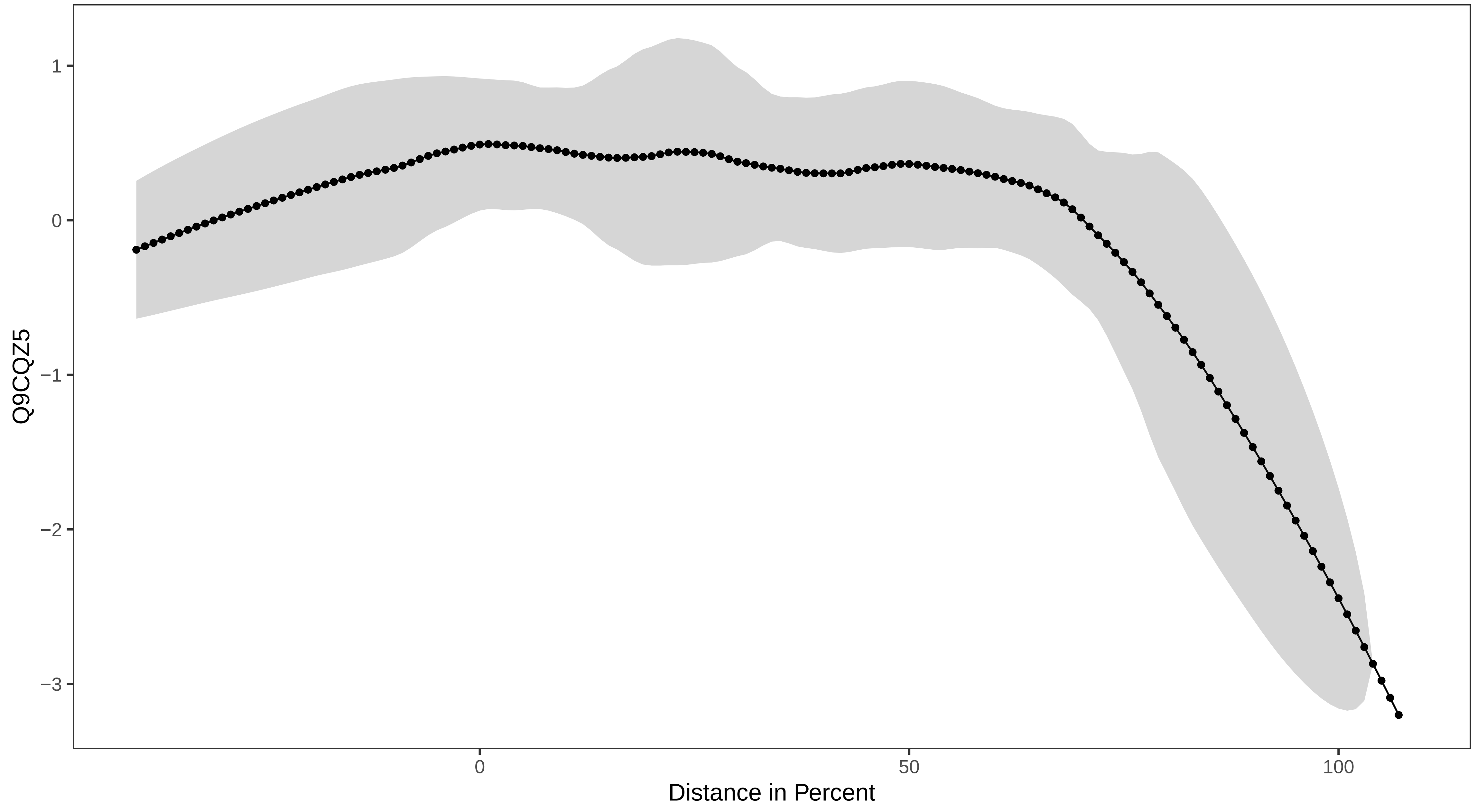

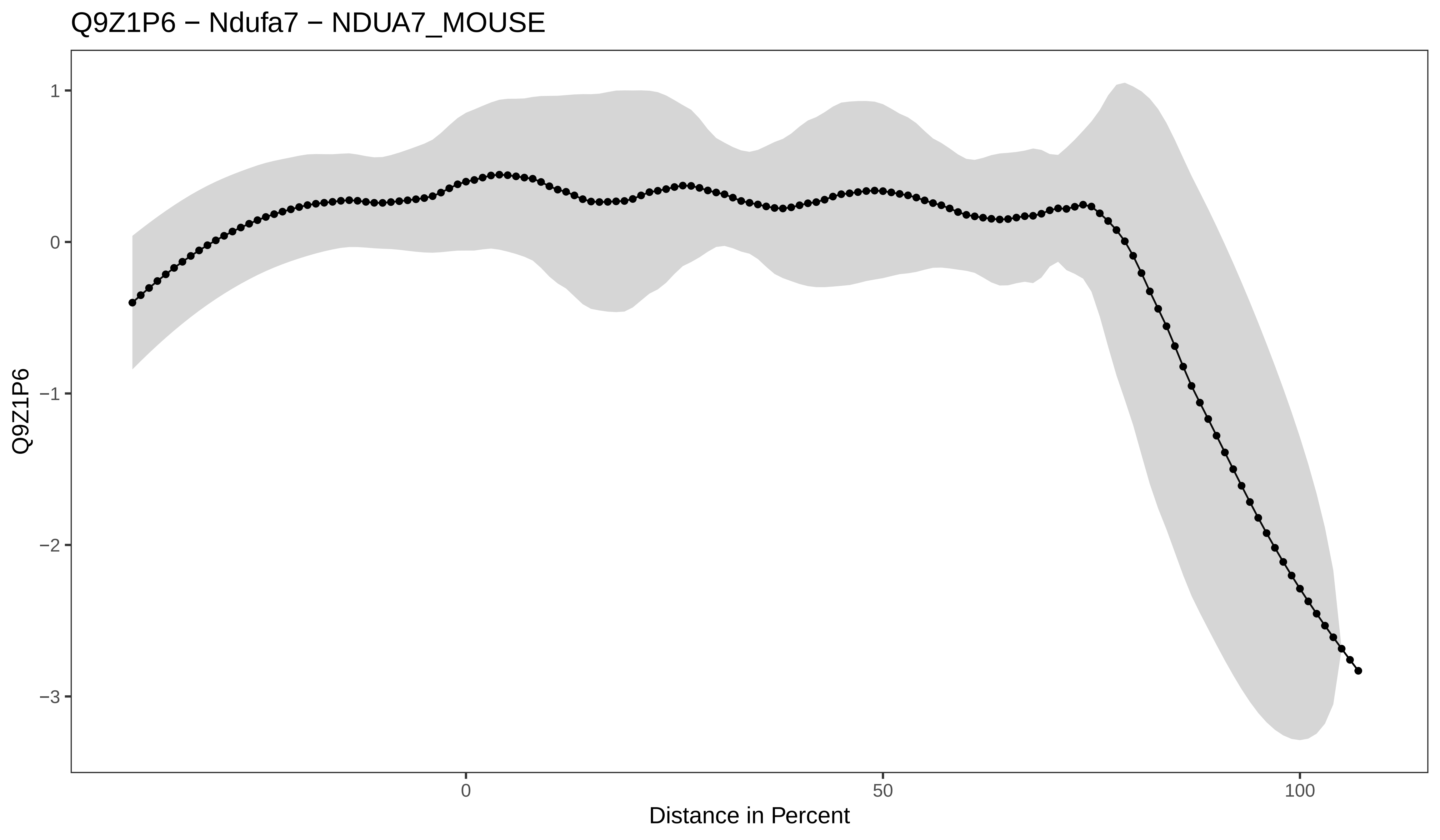
Q9Z1P6 − Ndufa7 − NDUA7_MOUSE
Q9Z1P6
Distance in Percent
1
0
−1
−2
−3
0
50
100

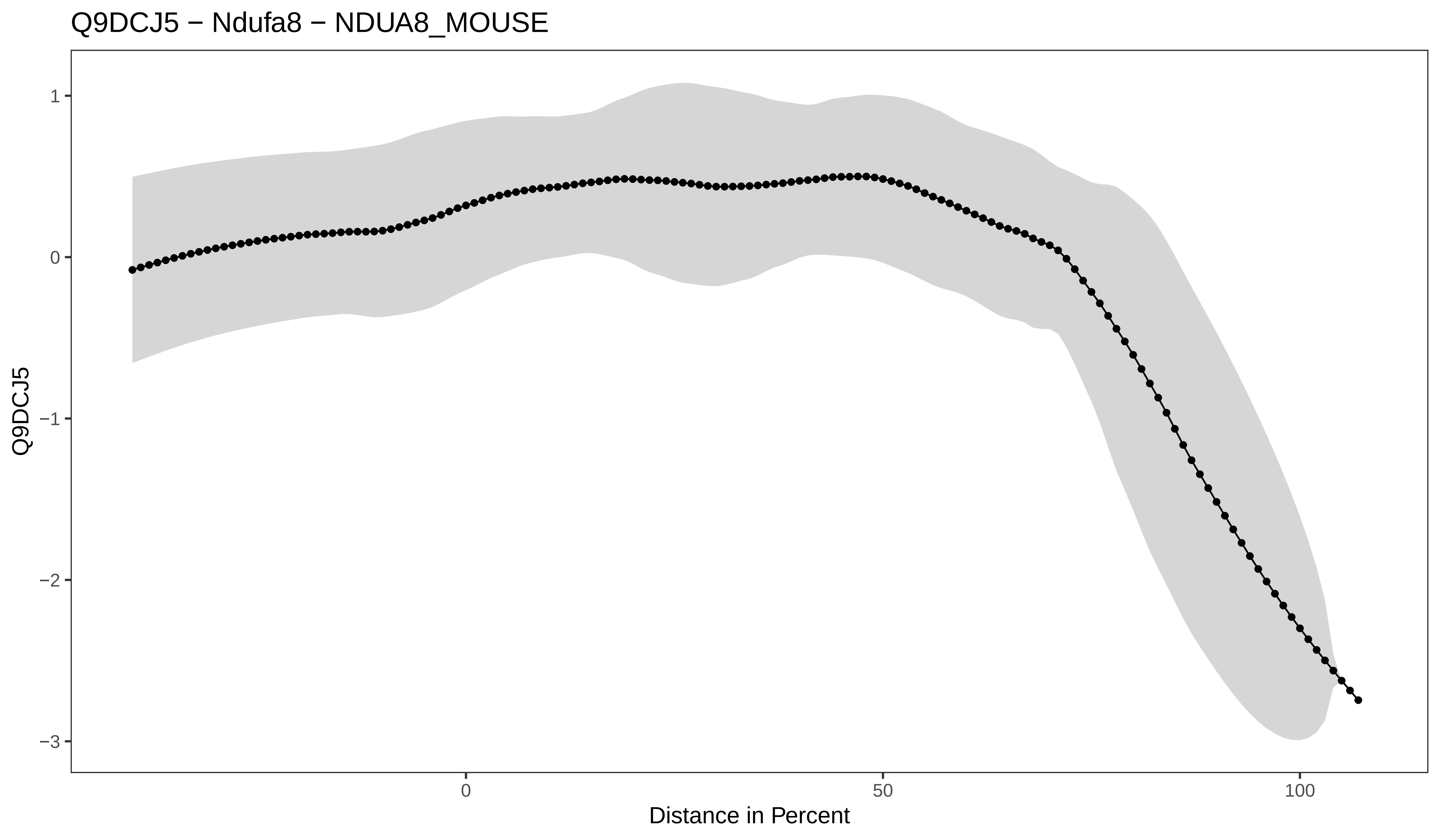

Q9DCJ5 − Ndufa8 − NDUA8_MOUSE
1
0
−1
−2
−3
Q9DCJ5
0
50
100
Distance in Percent

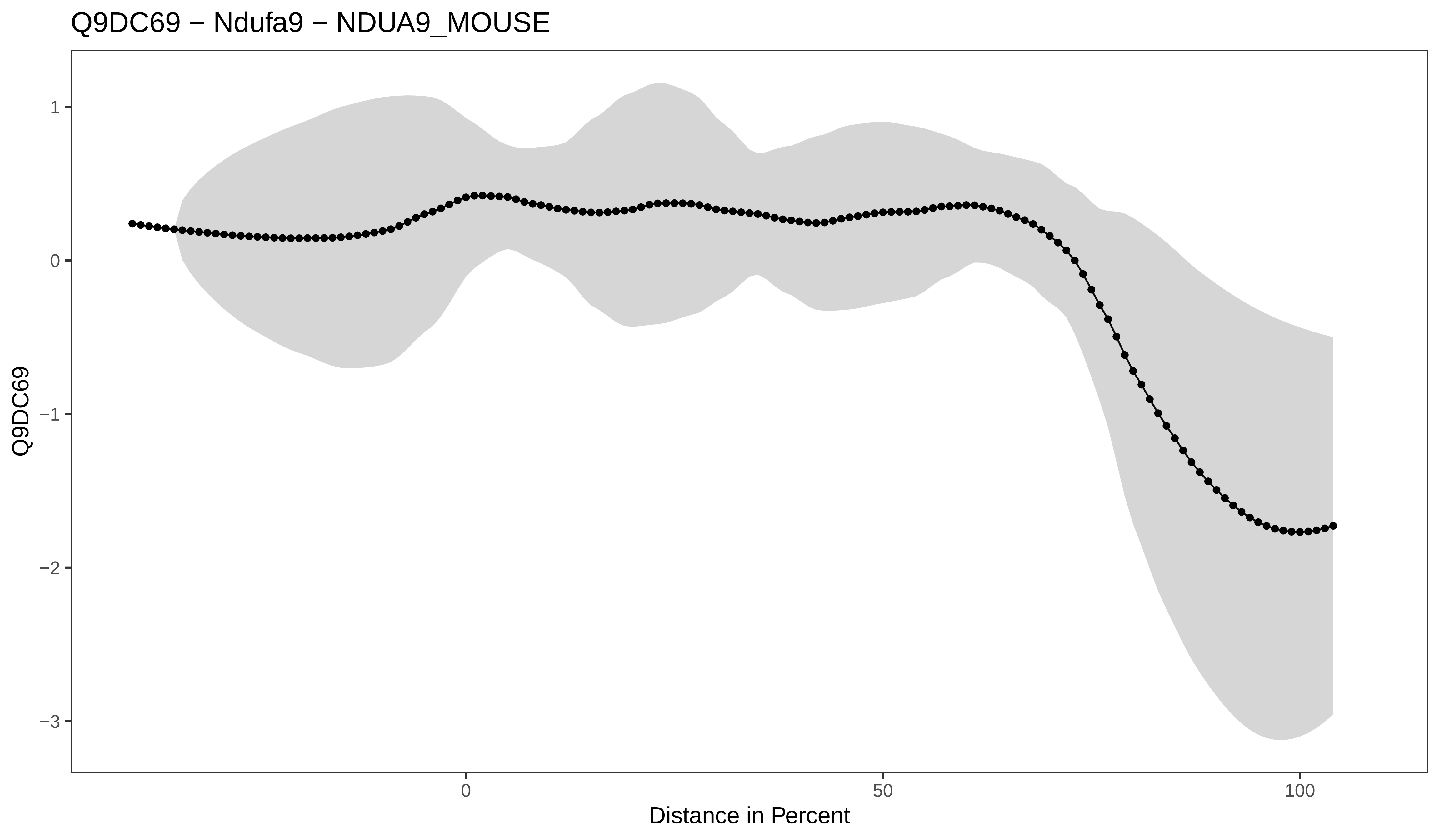

Q9DC69 − Ndufa9 − NDUA9_MOUSE
1
0
−1
−2
−3
Q9DC69
0
50
100
Distance in Percent

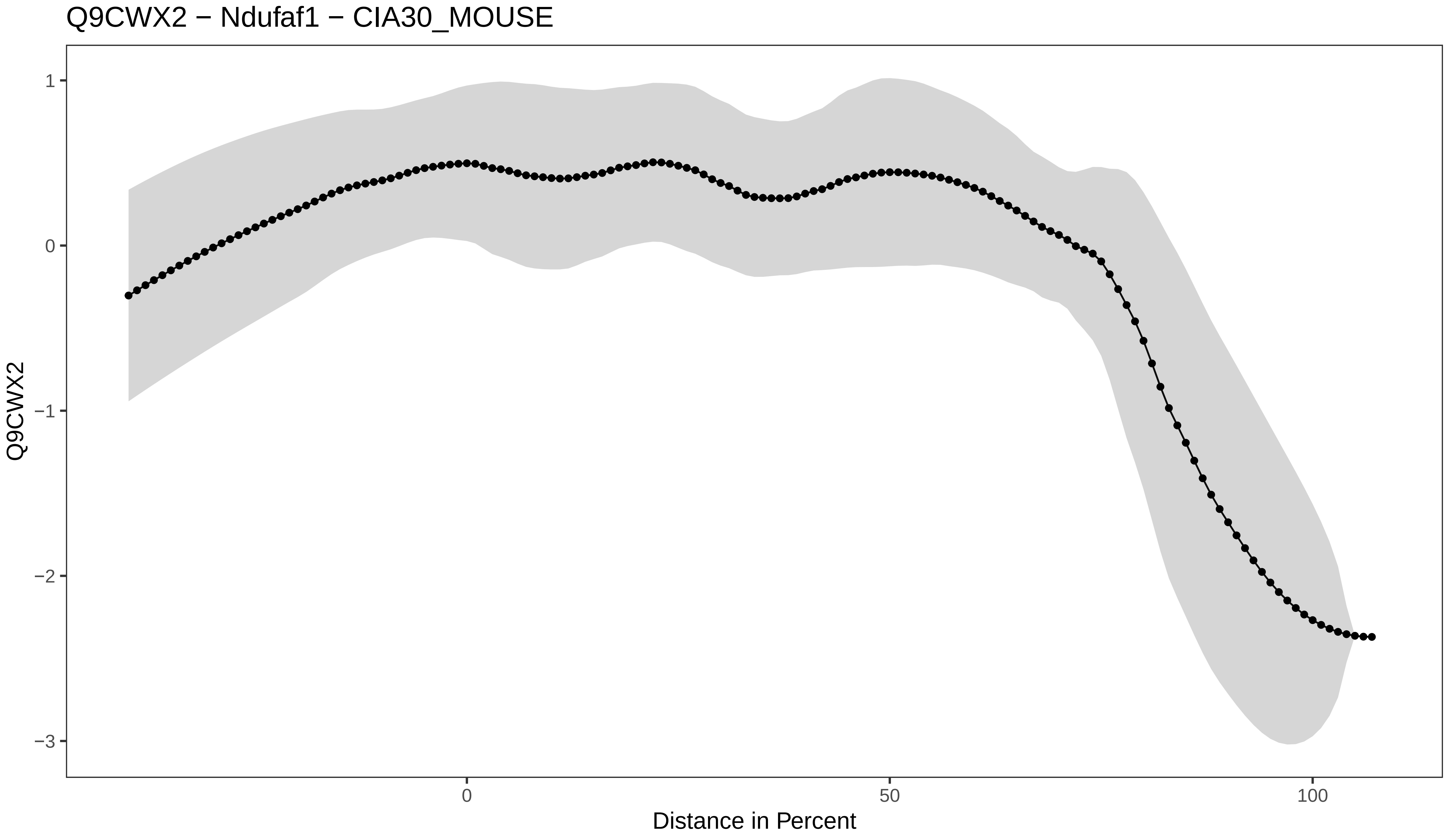
Q9CWX2 − Ndufaf1 − CIA30_MOUSE
1
0
−1
−2
−3
Q9CWX2
0
50
100
Distance in Percent

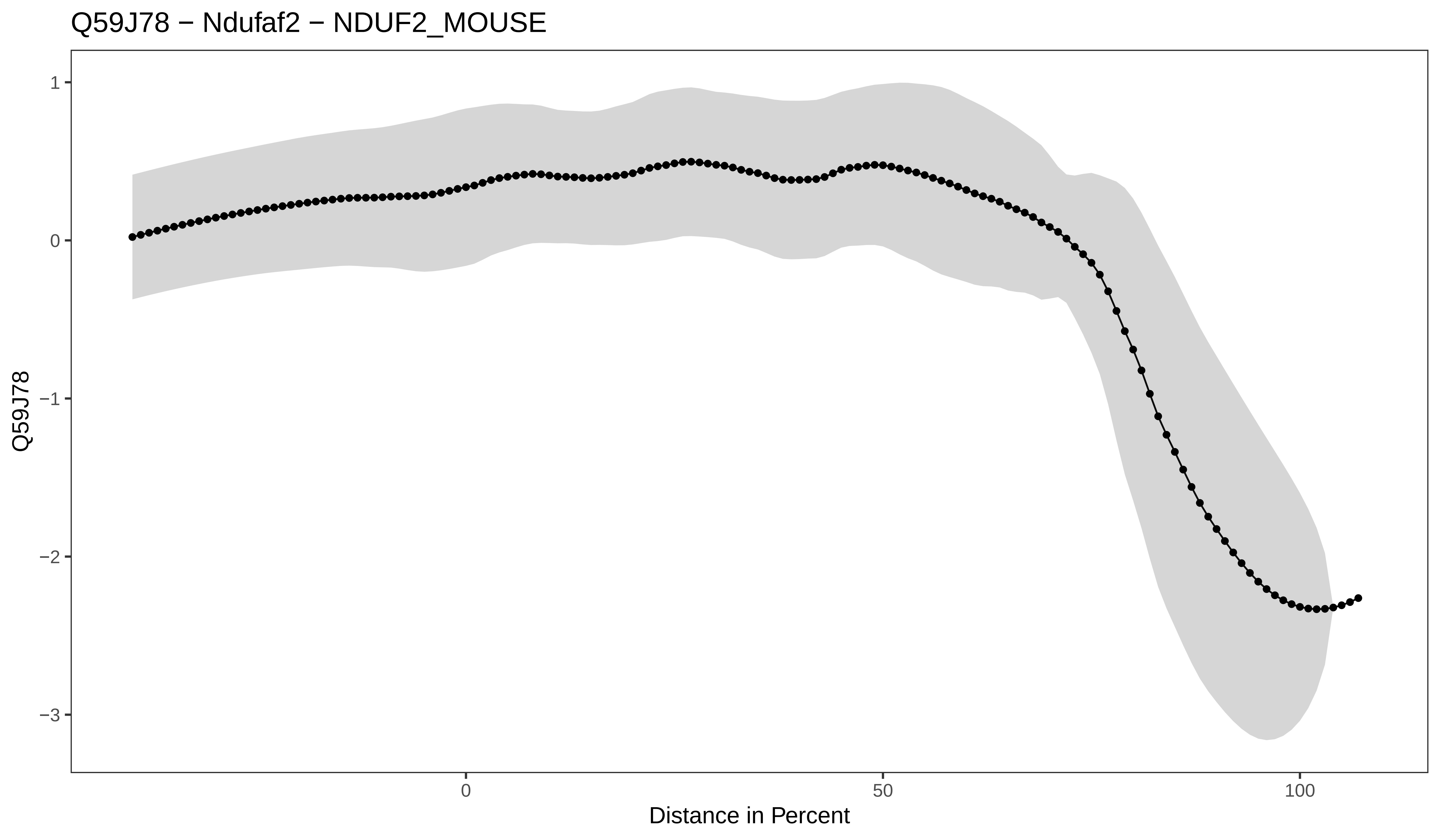

Q59J78 − Ndufaf2 − NDUF2_MOUSE
1
0
−1
−2
−3
Q59J78
0
50
100
Distance in Percent

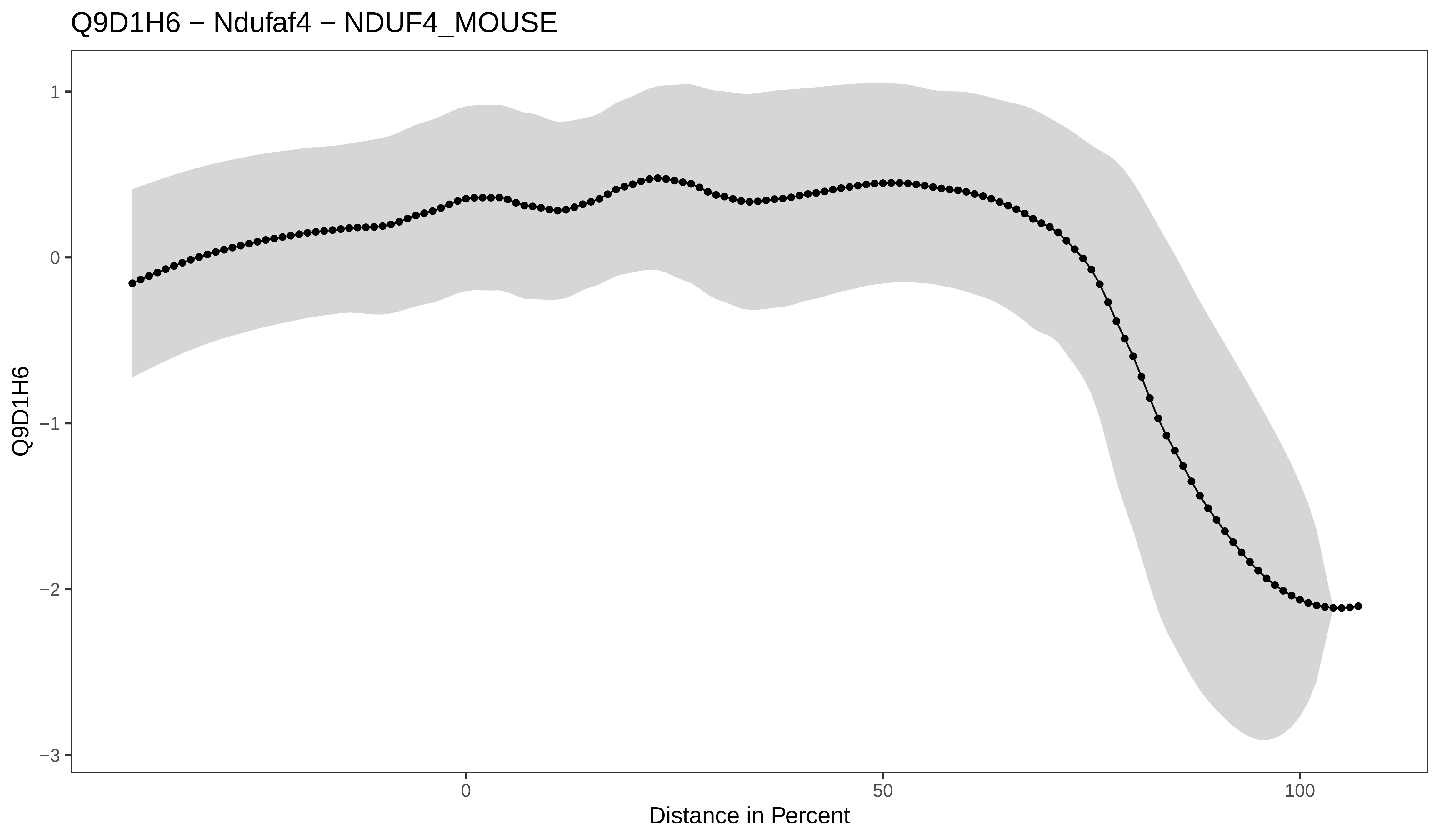

Q9D1H6 − Ndufaf4 − NDUF4_MOUSE
Q9D1H6
1
0
−1
−2
−3
0
50
100
Distance in Percent

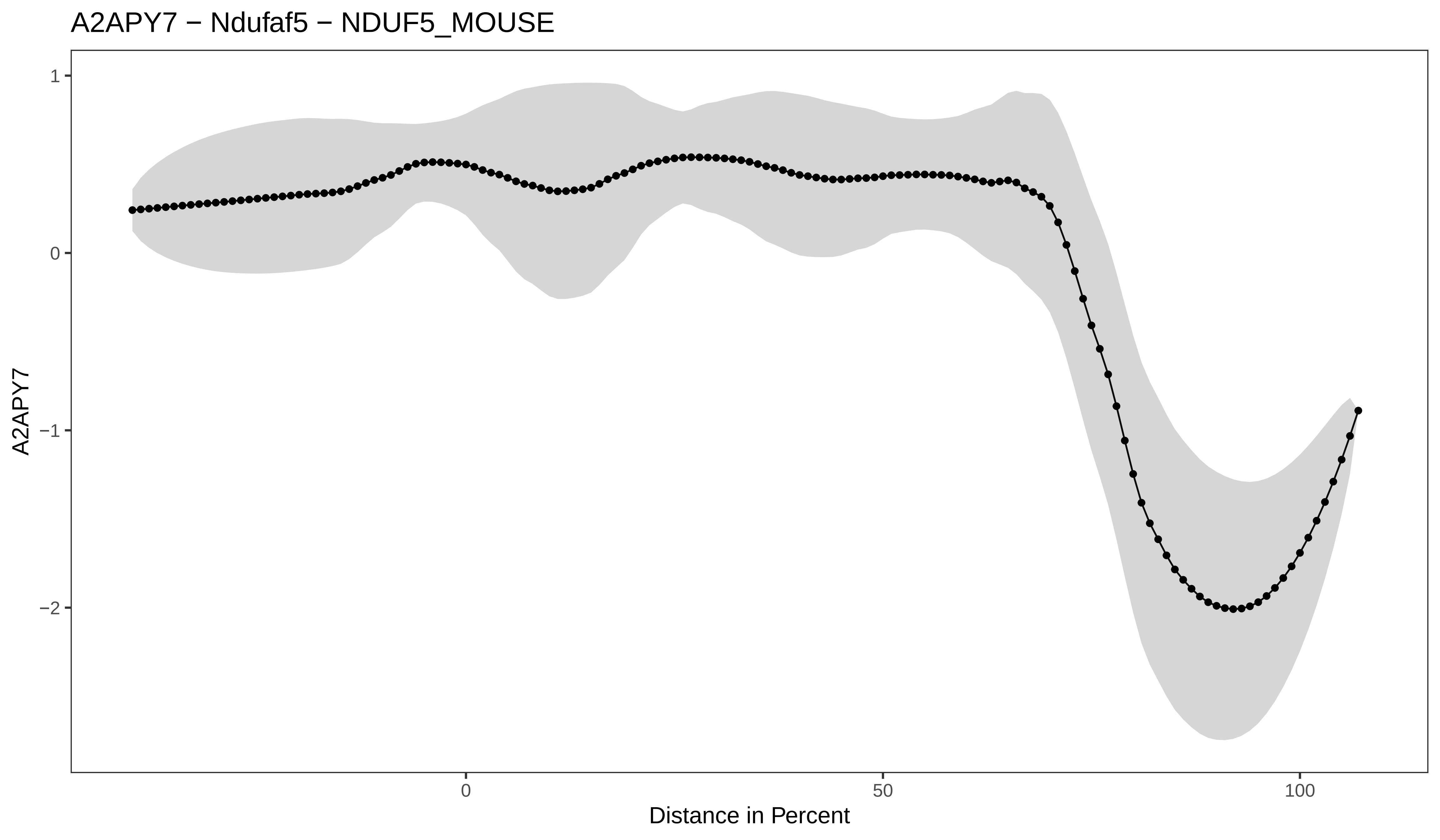

A2APY7 − Ndufaf5 − NDUF5_MOUSE
A2APY7
1
0
−1
−2
0
50
100
Distance in Percent

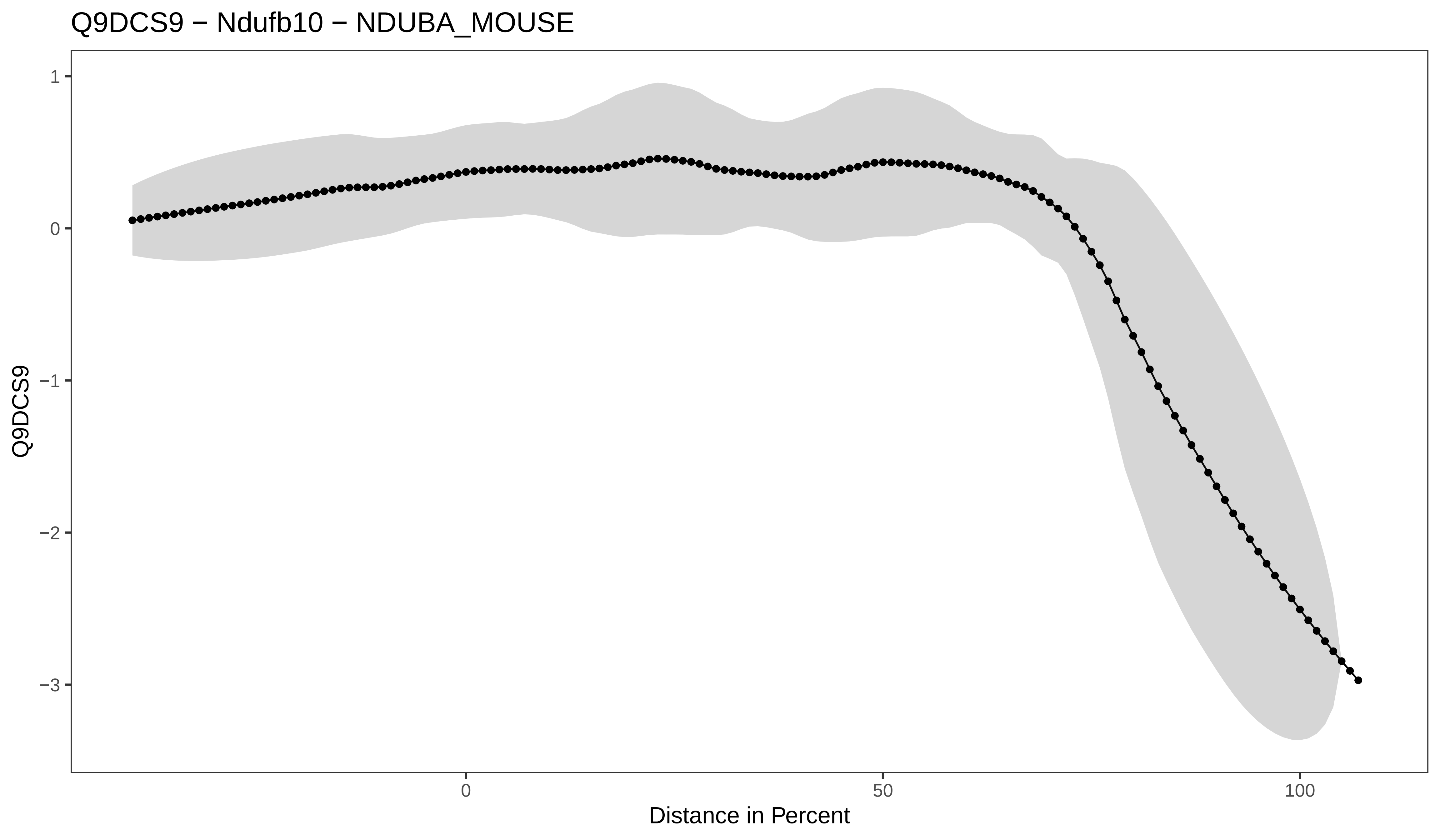
Q9DCS9 − Ndufb10 − NDUBA_MOUSE
Q9DCS9
1
0
−1
−2
−3
0
50
100
Distance in Percent

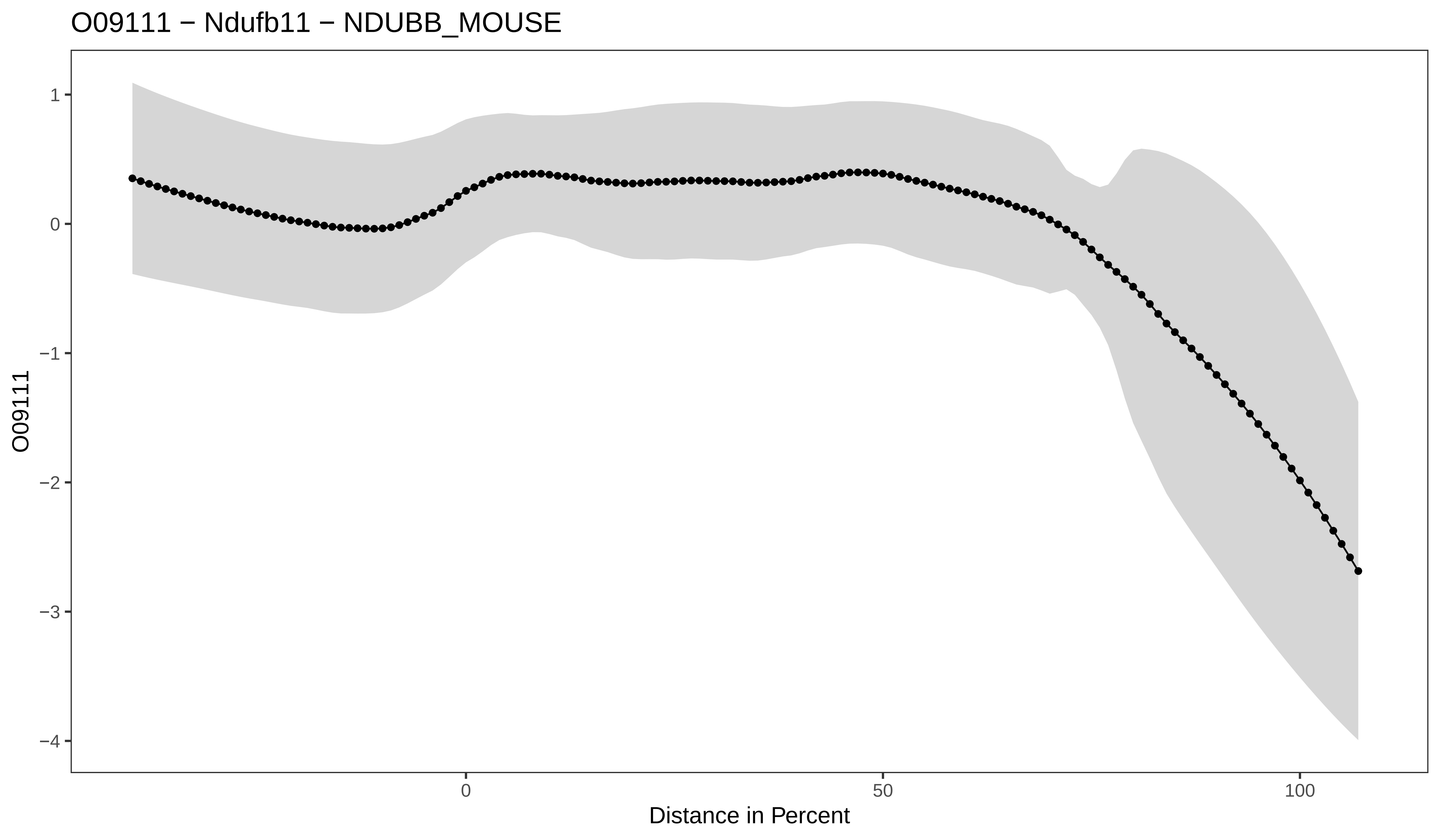

O09111 − Ndufb11 − NDUBB_MOUSE
1
0
−1
−2
−3
−4
O09111
0
50
100
Distance in Percent

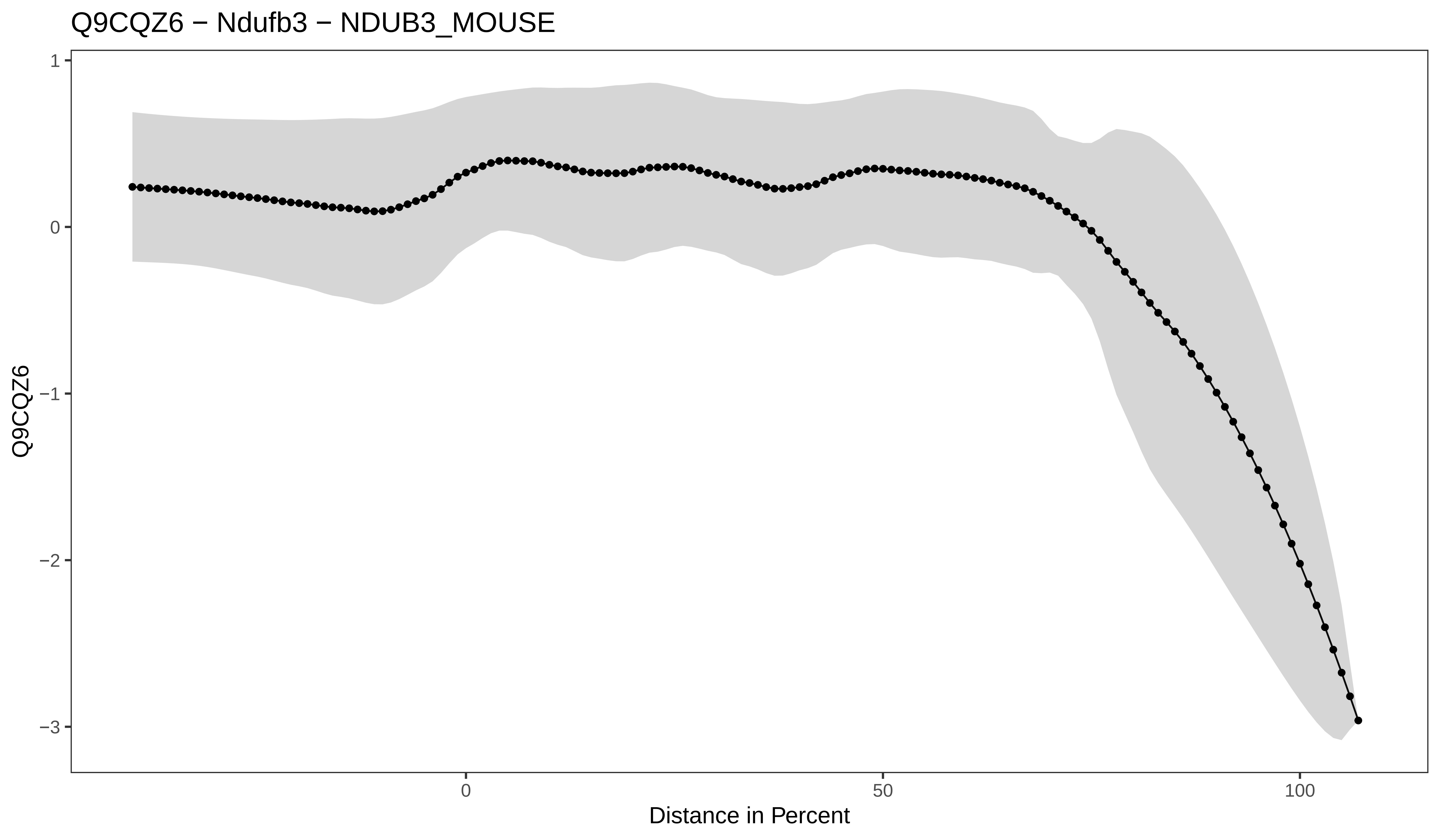

Q9CQZ6 − Ndufb3 − NDUB3_MOUSE
1
0
−1
−2
−3
Q9CQZ6
0
50
100
Distance in Percent

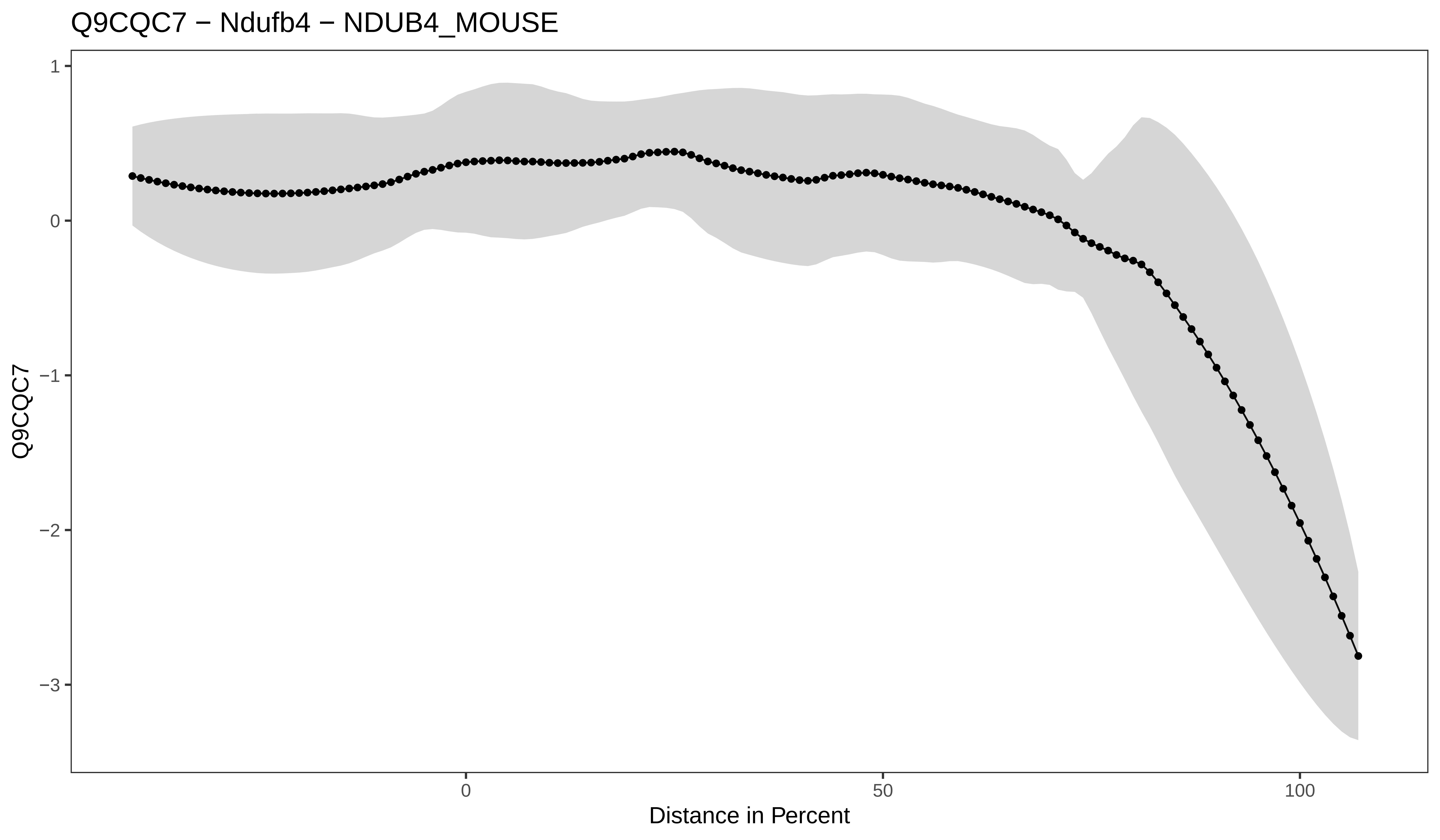

Q9CQC7 − Ndufb4 − NDUB4_MOUSE
Q9CQC7
Distance in Percent
1
0
−1
−2
−3
0
50
100

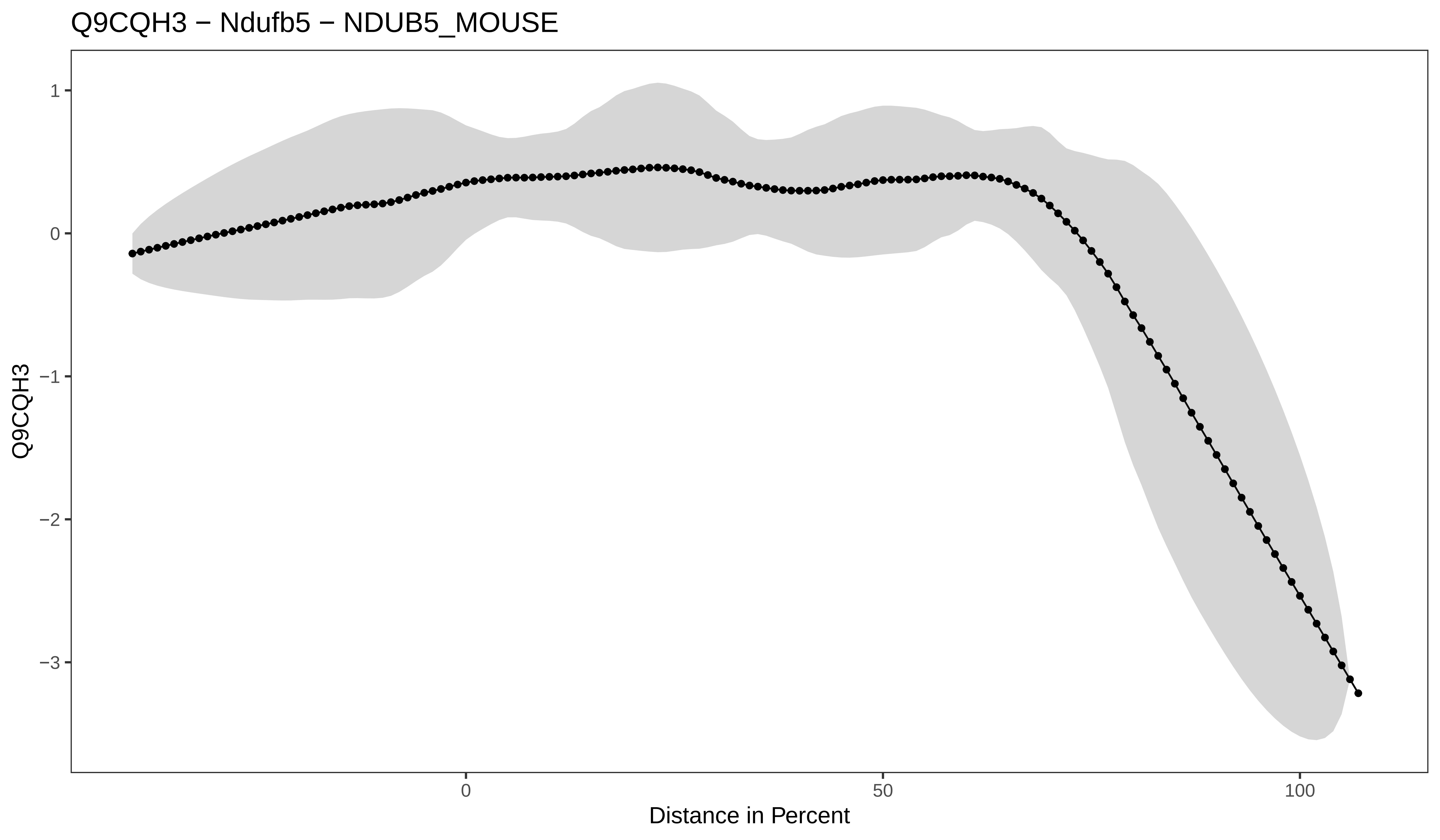

Q9CQH3 − Ndufb5 − NDUB5_MOUSE
1
0
−1
−2
−3
Q9CQH3
0
50
100
Distance in Percent

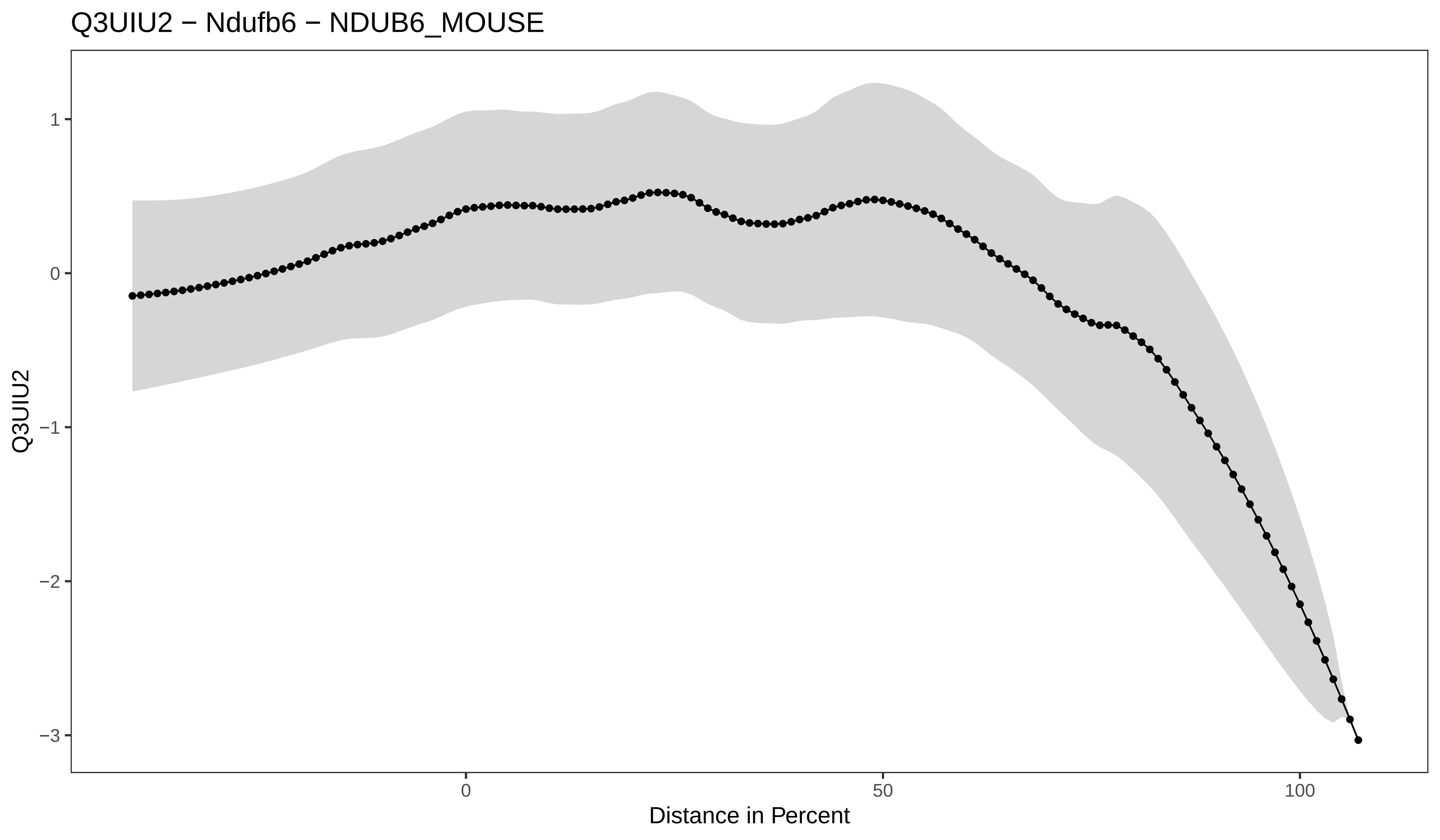

Q3UIU2 − Ndufb6 − NDUB6_MOUSE
Q3UIU2
1
0
−1
−2
−3
0
50
100
Distance in Percent

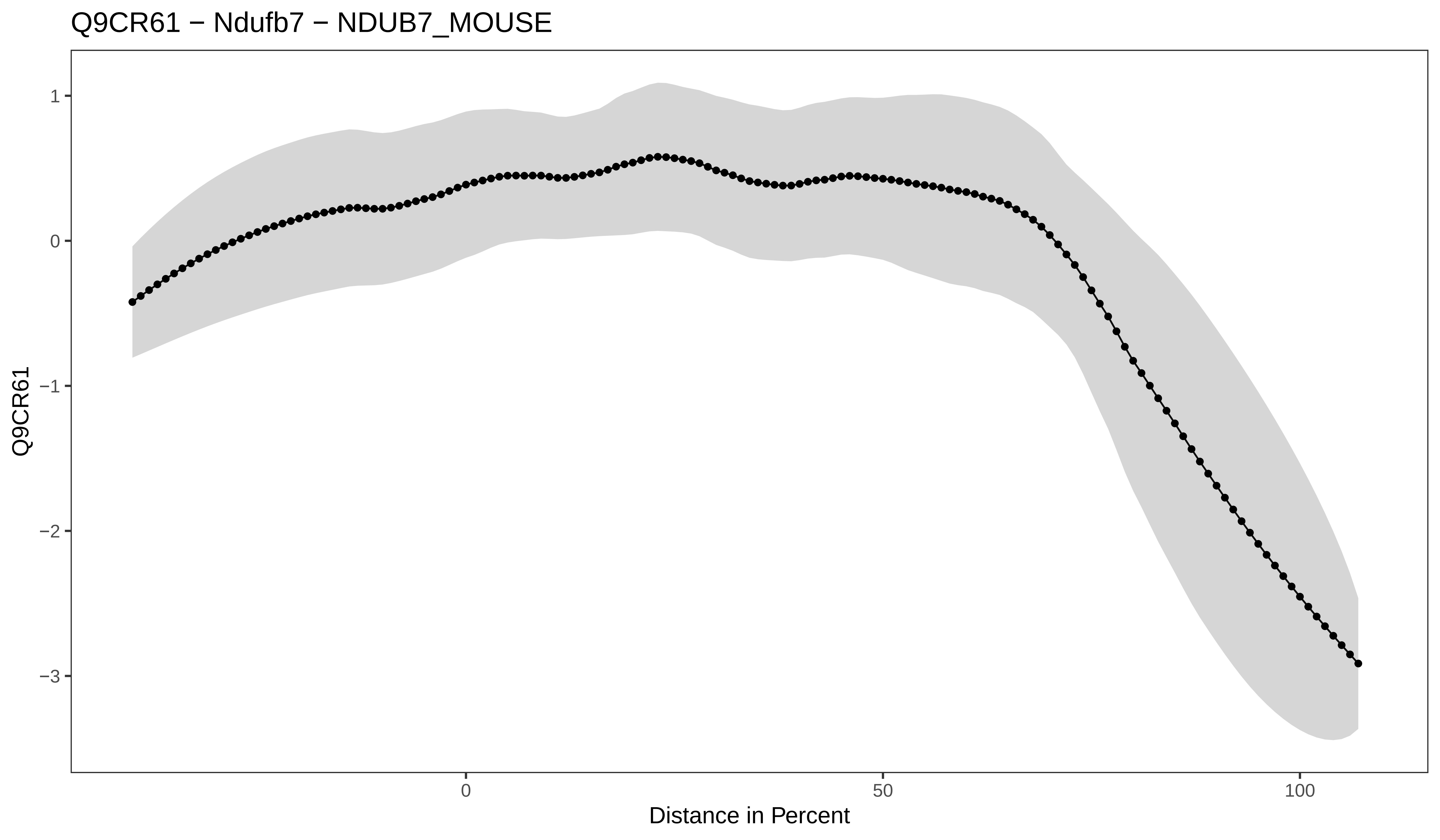

Q9CR61 − Ndufb7 − NDUB7_MOUSE
1
0
−1
−2
−3
Q9CR61
0
50
100
Distance in Percent

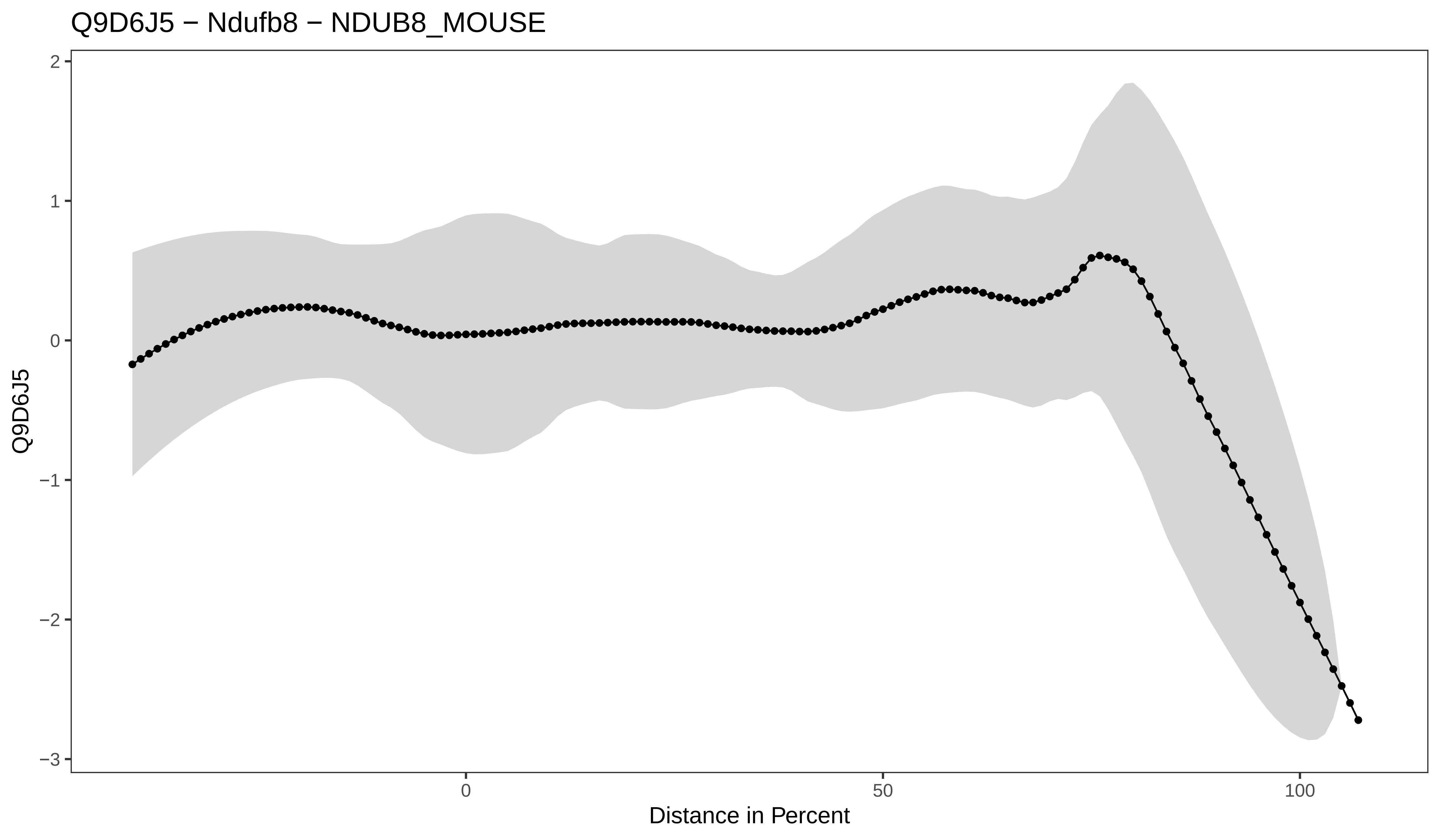

Q9D6J5 − Ndufb8 − NDUB8_MOUSE
2
1
0
−1
−2
−3
Q9D6J5
0
50
100
Distance in Percent

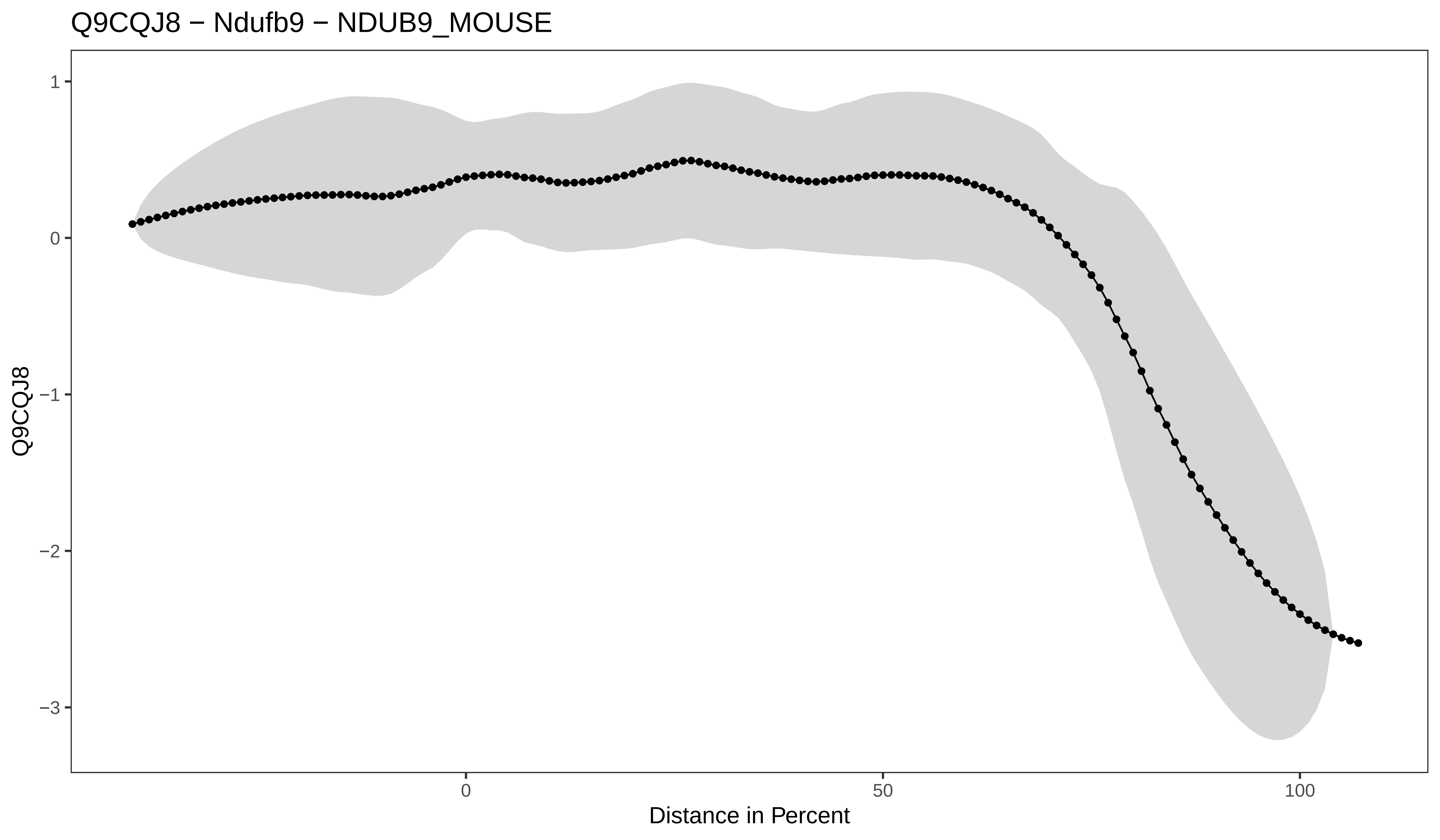
Q9CQJ8 − Ndufb9 − NDUB9_MOUSE
1
0
−1
−2
−3
Q9CQJ8
0
50
100
Distance in Percent

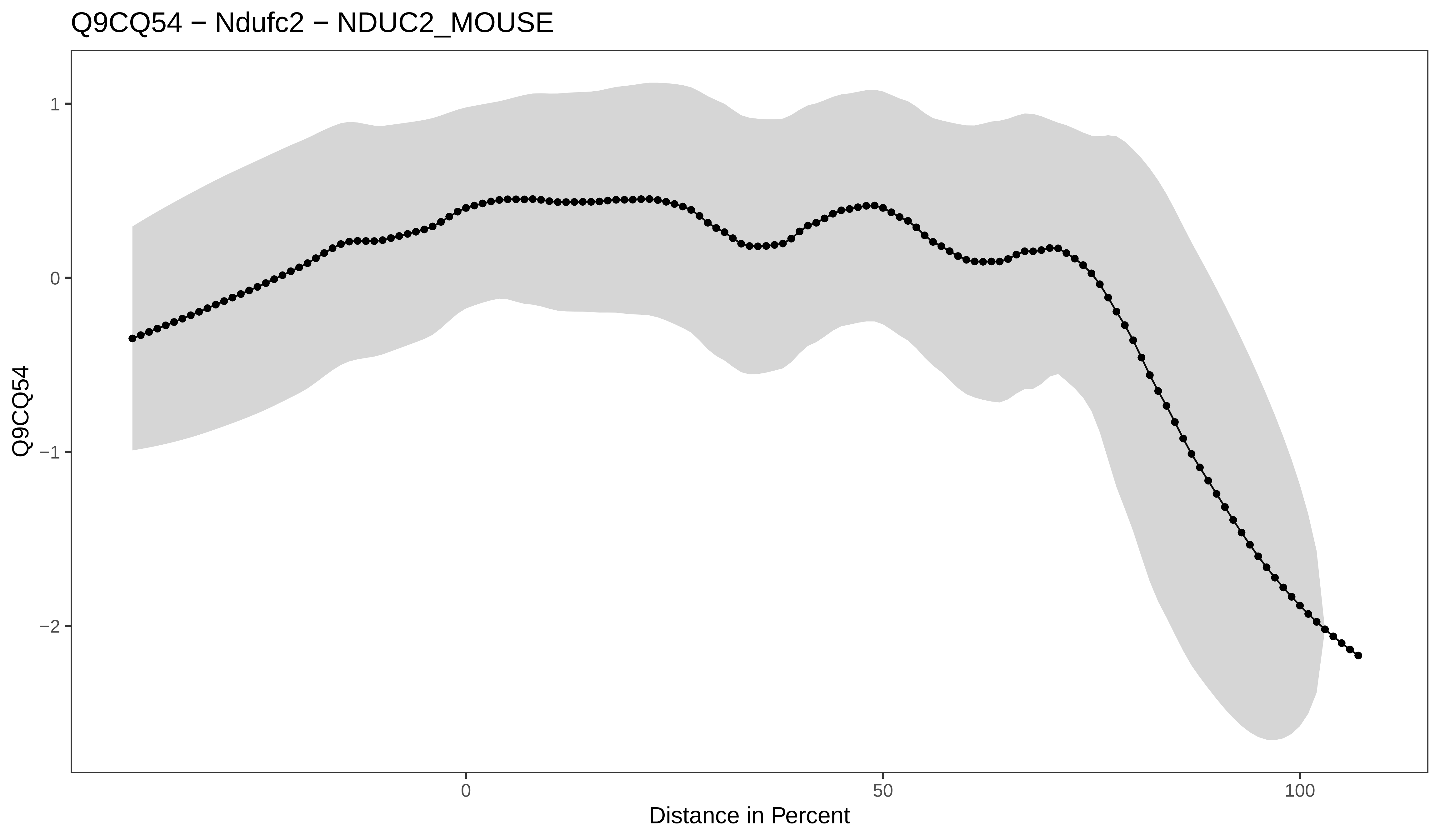

Q9CQ54 − Ndufc2 − NDUC2_MOUSE
Q9CQ54
1
0
−1
−2
0
50
100
Distance in Percent

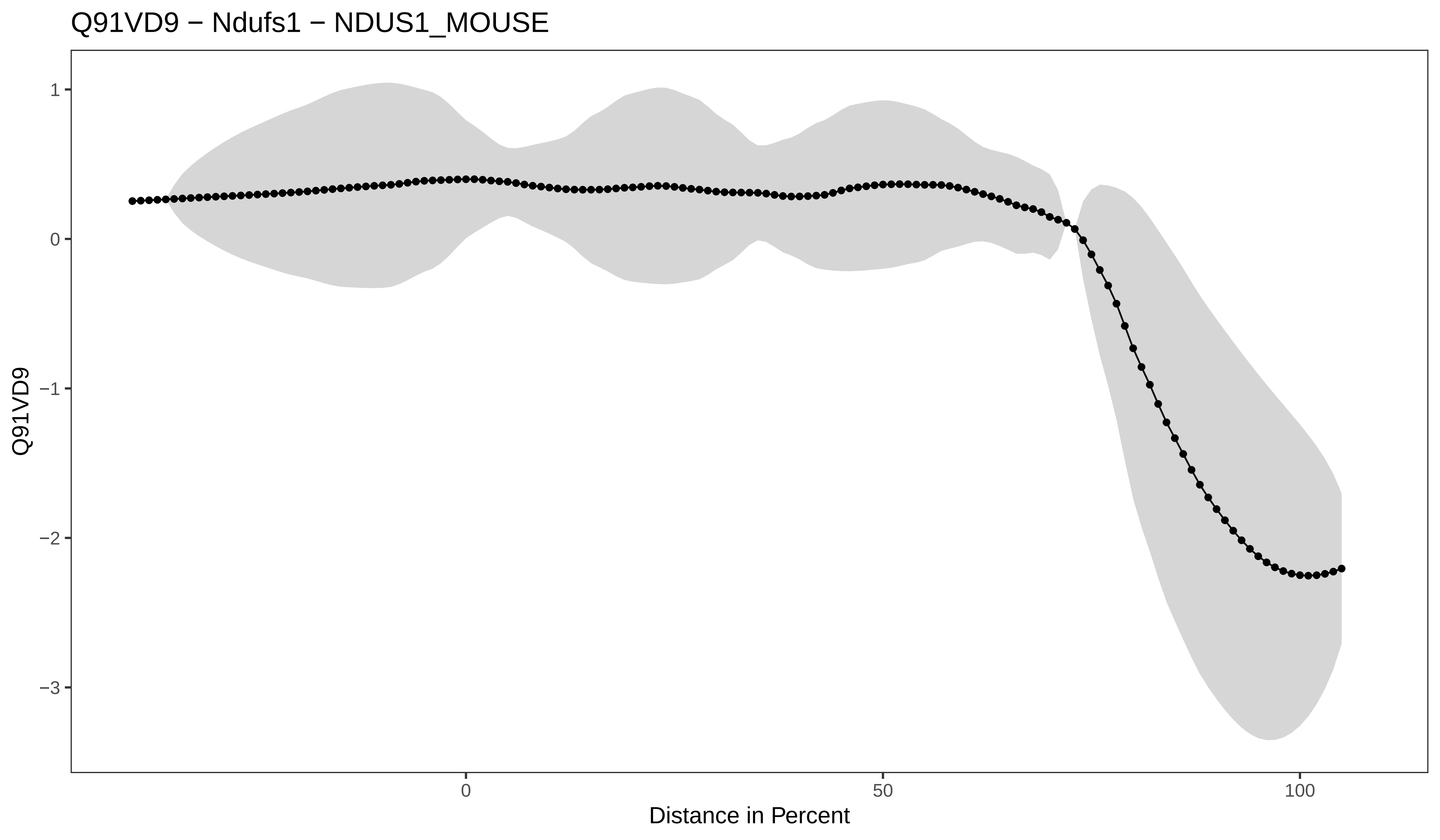

Q91VD9 − Ndufs1 − NDUS1_MOUSE
1
0
−1
−2
−3
Q91VD9
0
50
100
Distance in Percent

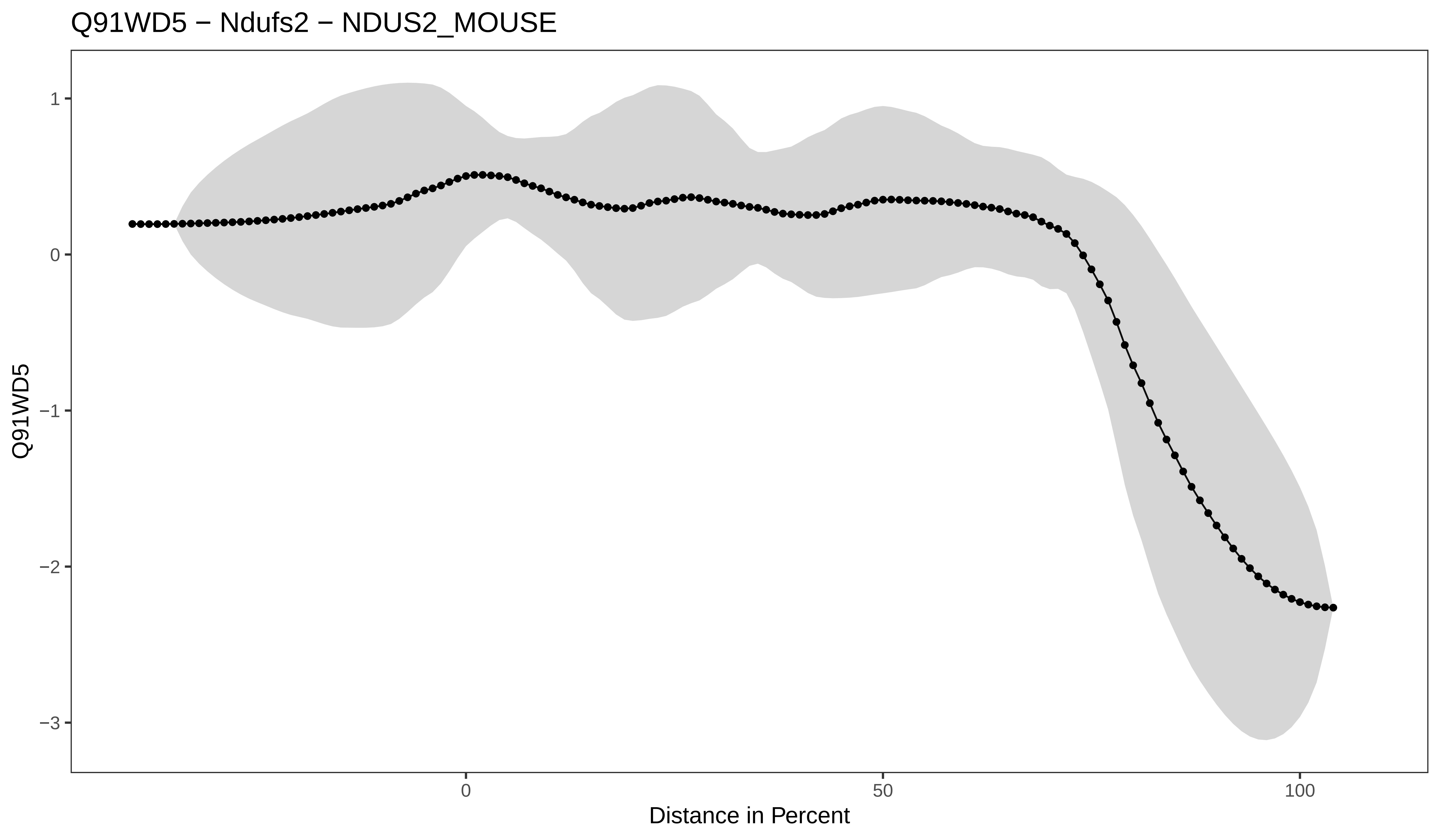

Q91WD5 − Ndufs2 − NDUS2_MOUSE
1
0
−1
−2
−3
Q91WD5
0
50
100
Distance in Percent

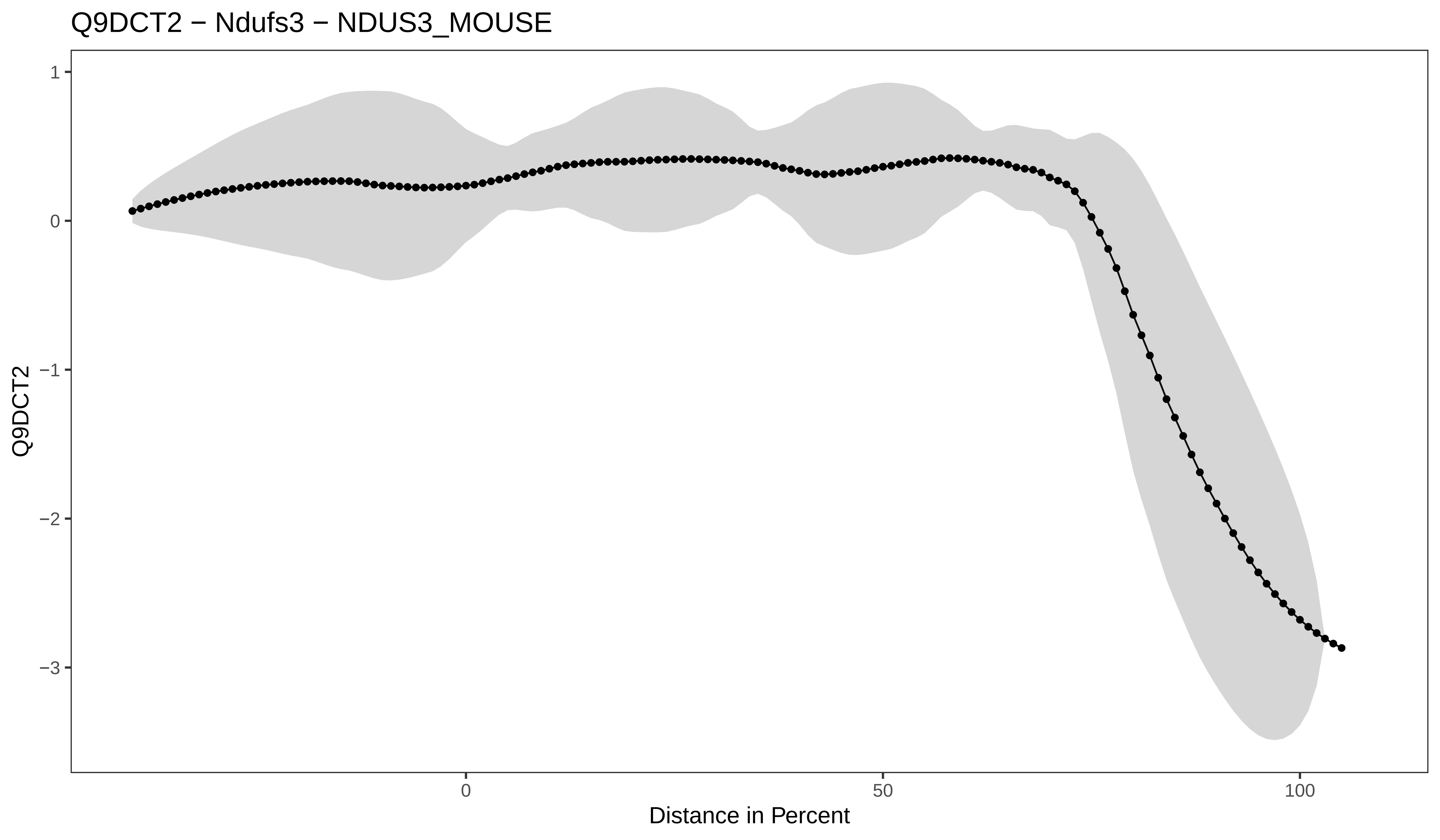
Q9DCT2 − Ndufs3 − NDUS3_MOUSE
Q9DCT2
1
0
−1
−2
−3
0
50
100
Distance in Percent

Q9CXZ1 − Ndufs4 − NDUS4_MOUSE

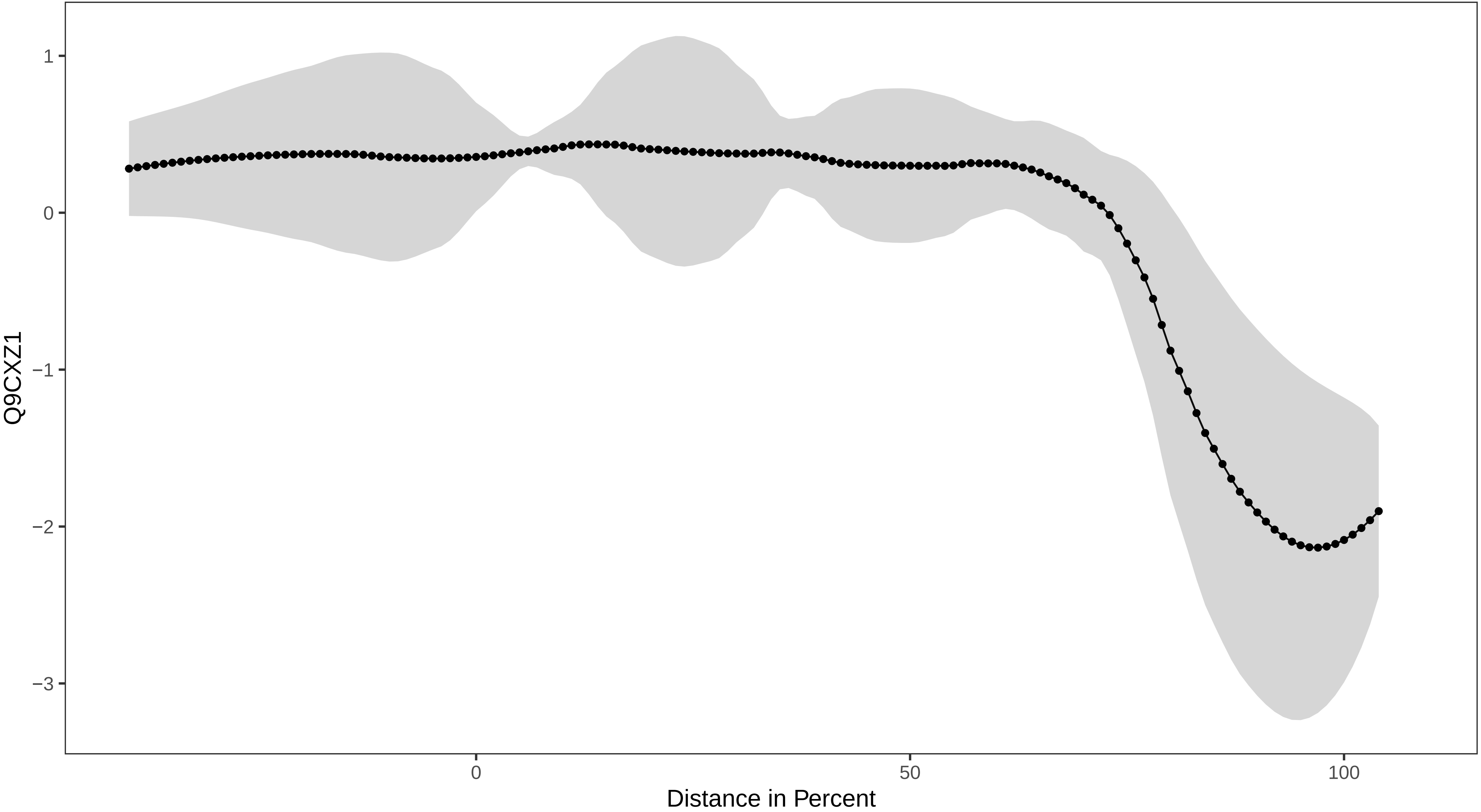

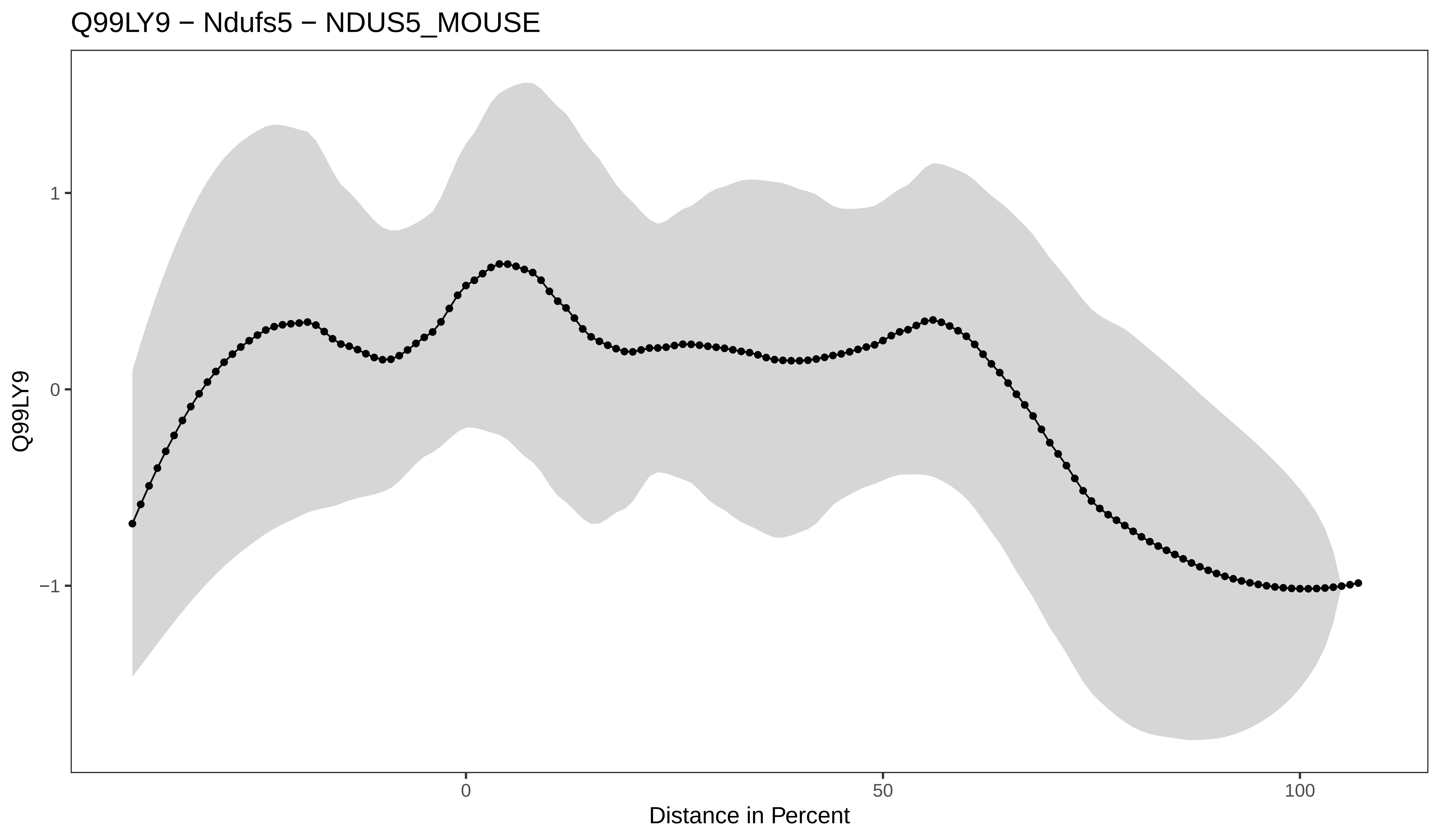

Q99LY9 − Ndufs5 − NDUS5_MOUSE
Q99LY9
1
0
−1
0
50
100
Distance in Percent

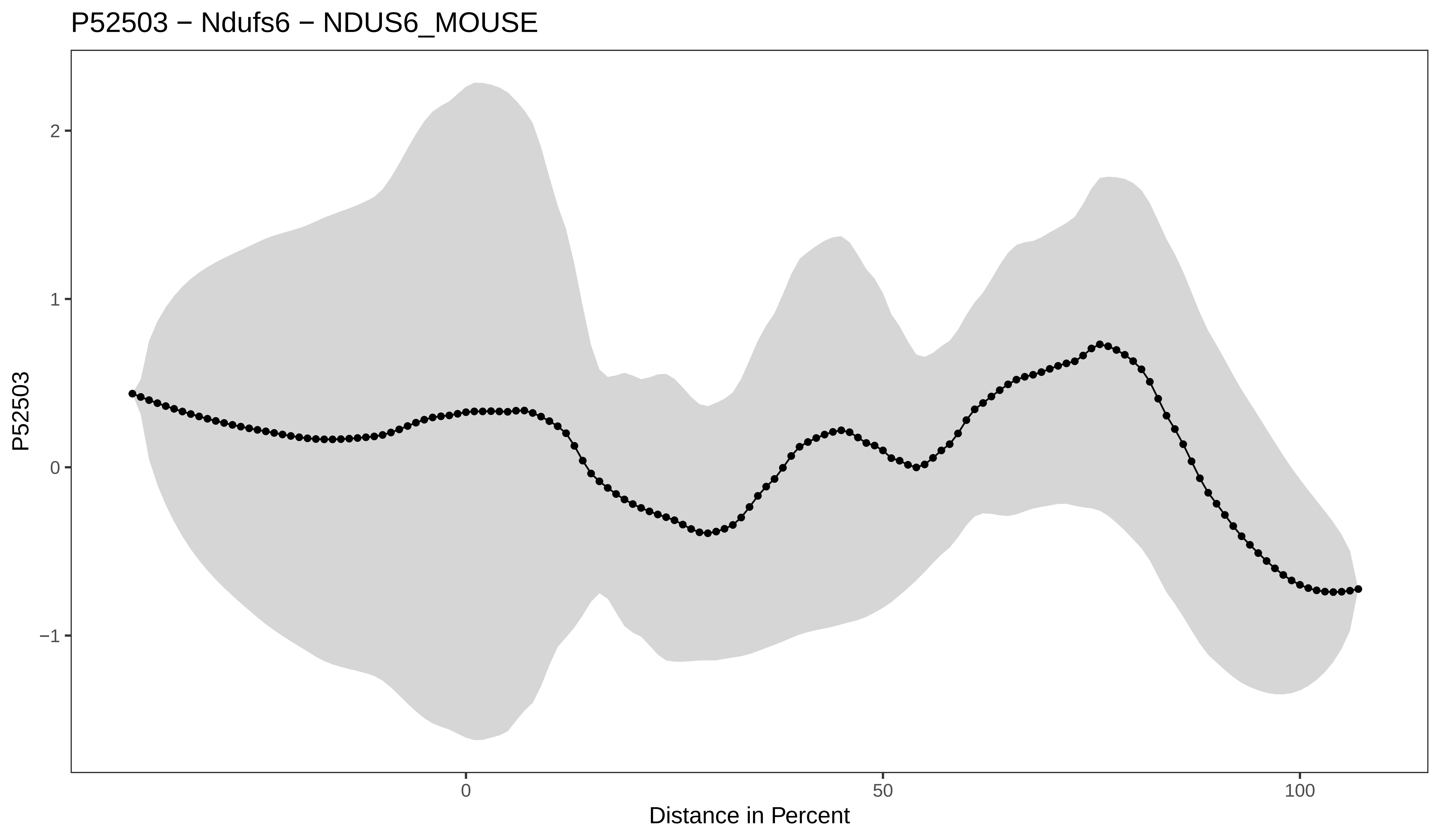

P52503 − Ndufs6 − NDUS6_MOUSE
2
1
0
−1
P52503
0
50
100
Distance in Percent

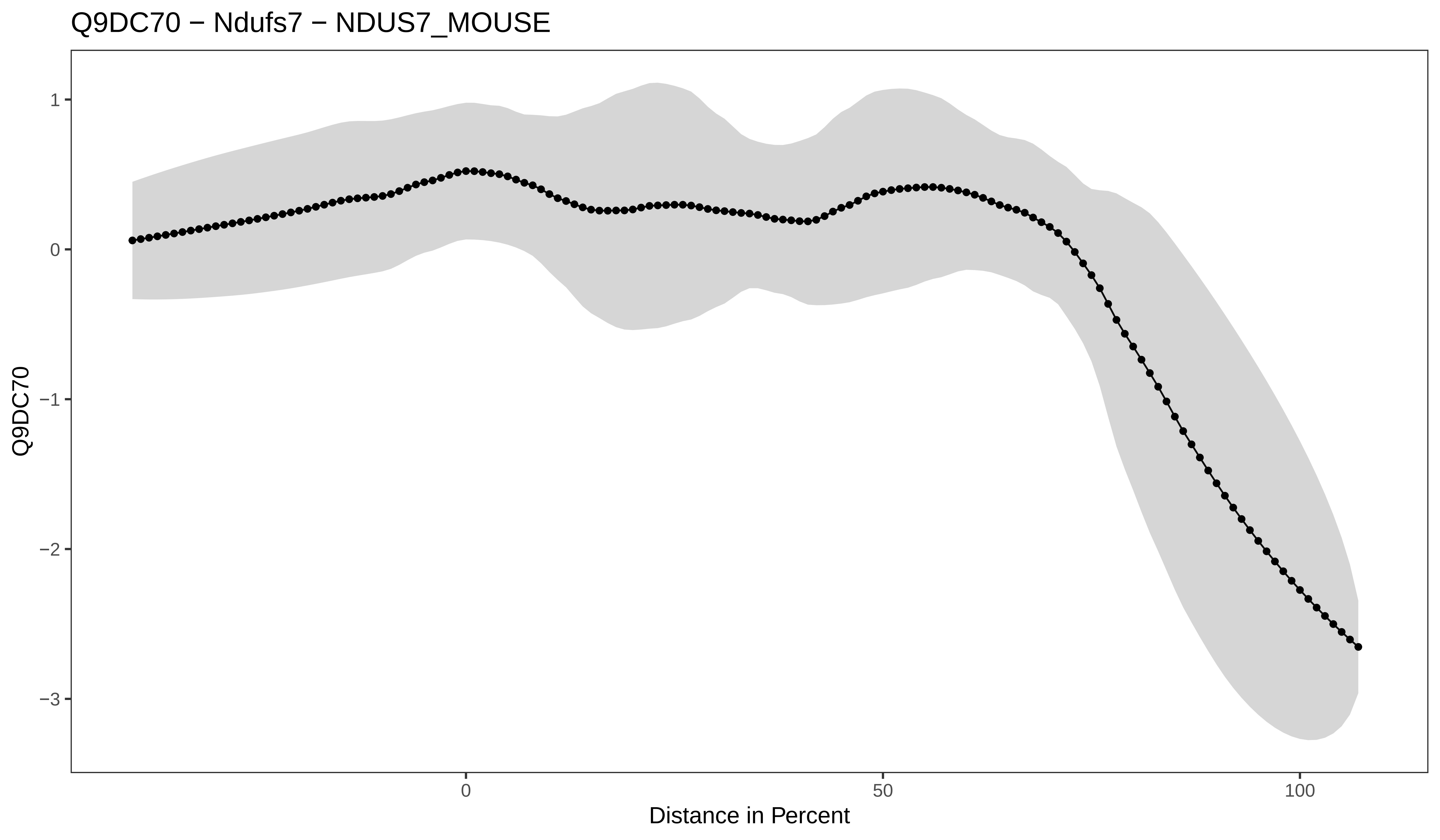
Q9DC70 − Ndufs7 − NDUS7_MOUSE
1
0
−1
−2
−3
Q9DC70
0
50
100
Distance in Percent

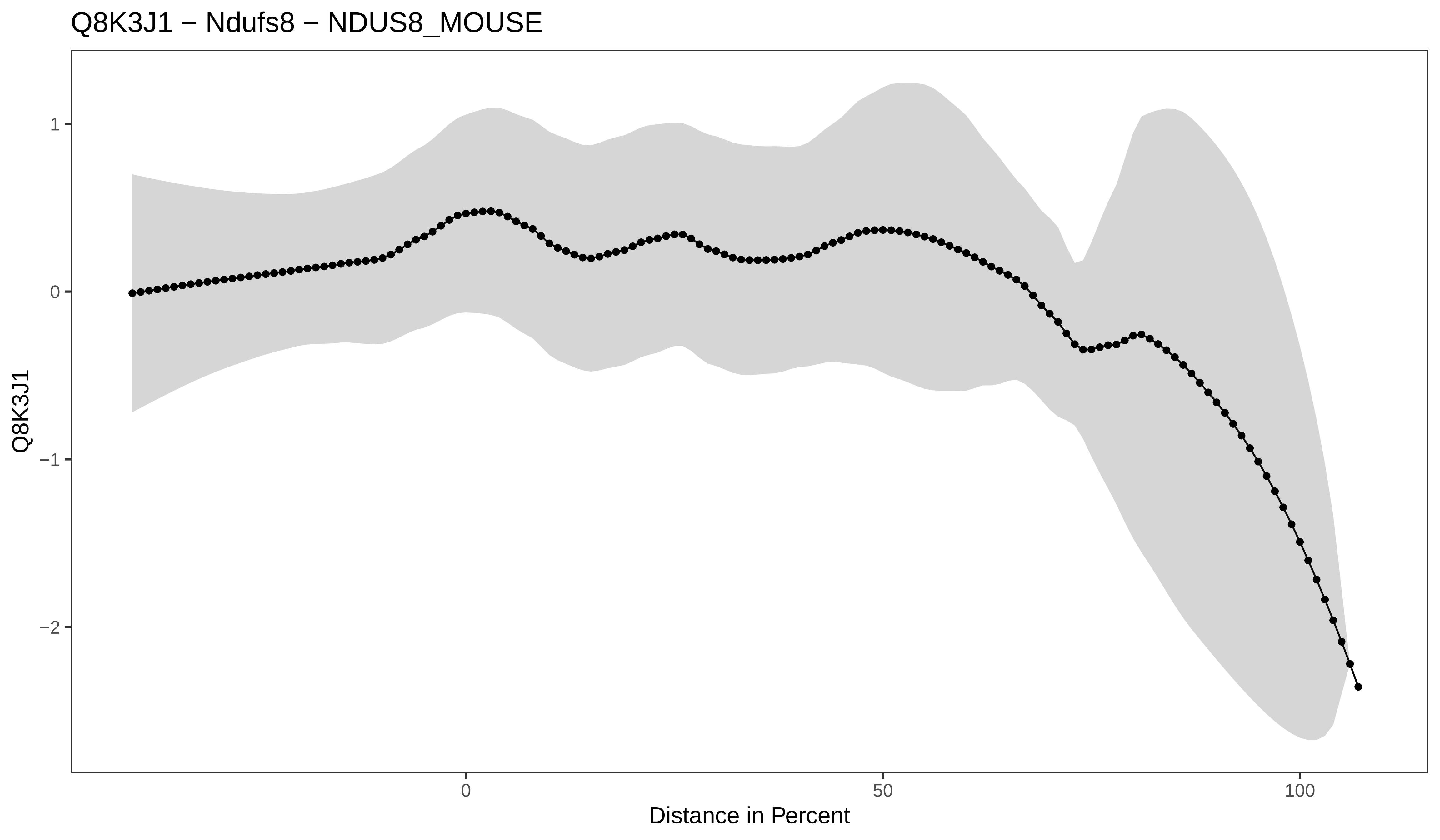

Q8K3J1 − Ndufs8 − NDUS8_MOUSE
1
0
−1
−2
Q8K3J1
0
50
100
Distance in Percent

Q91YT0 − Ndufv1 − NDUV1_MOUSE

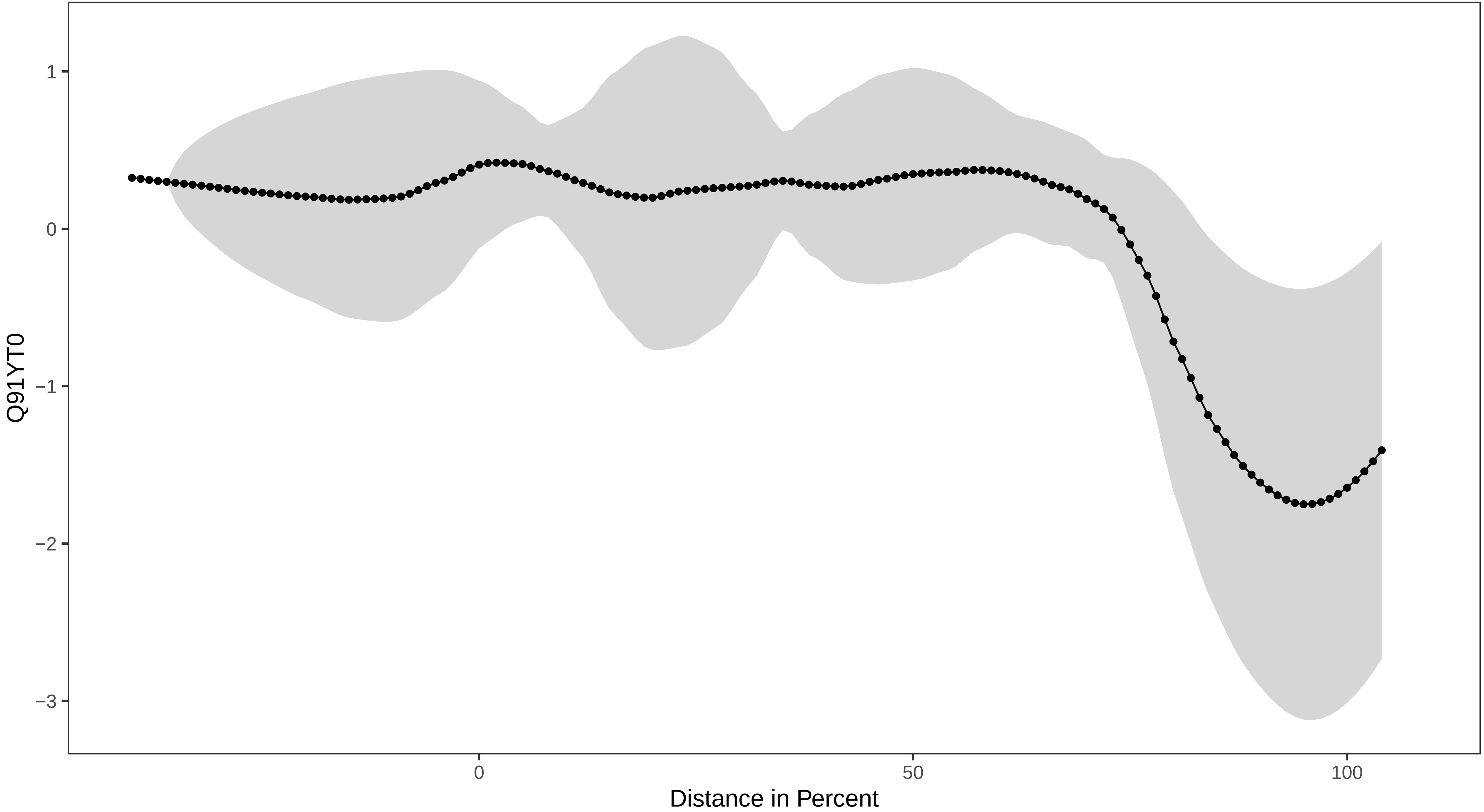

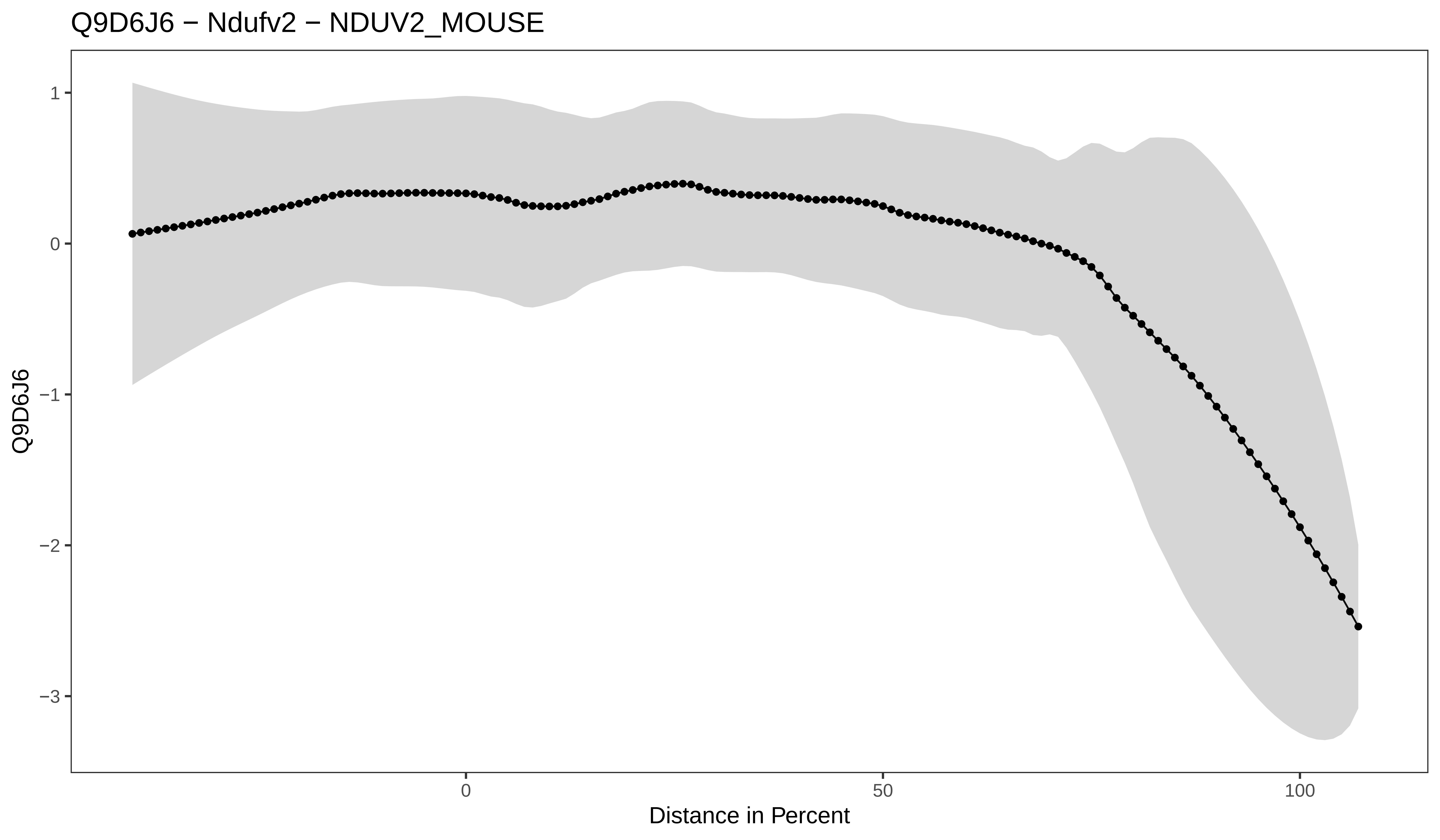

Q9D6J6 − Ndufv2 − NDUV2_MOUSE
1
0
−1
−2
−3
Q9D6J6
0
50
100
Distance in Percent

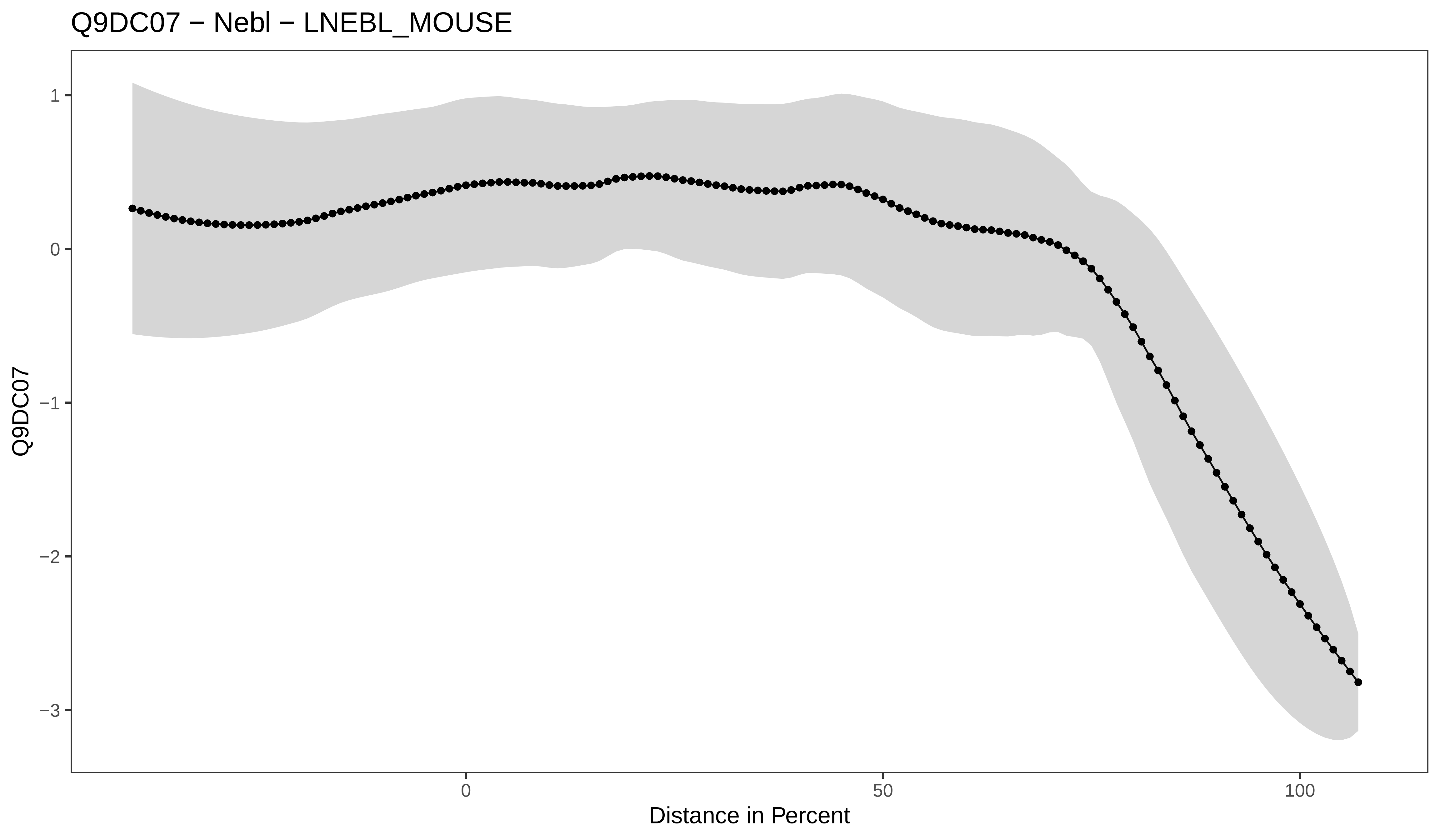

Q9DC07 − Nebl − LNEBL_MOUSE
1
0
−1
−2
−3
Q9DC07
0
50
100
Distance in Percent

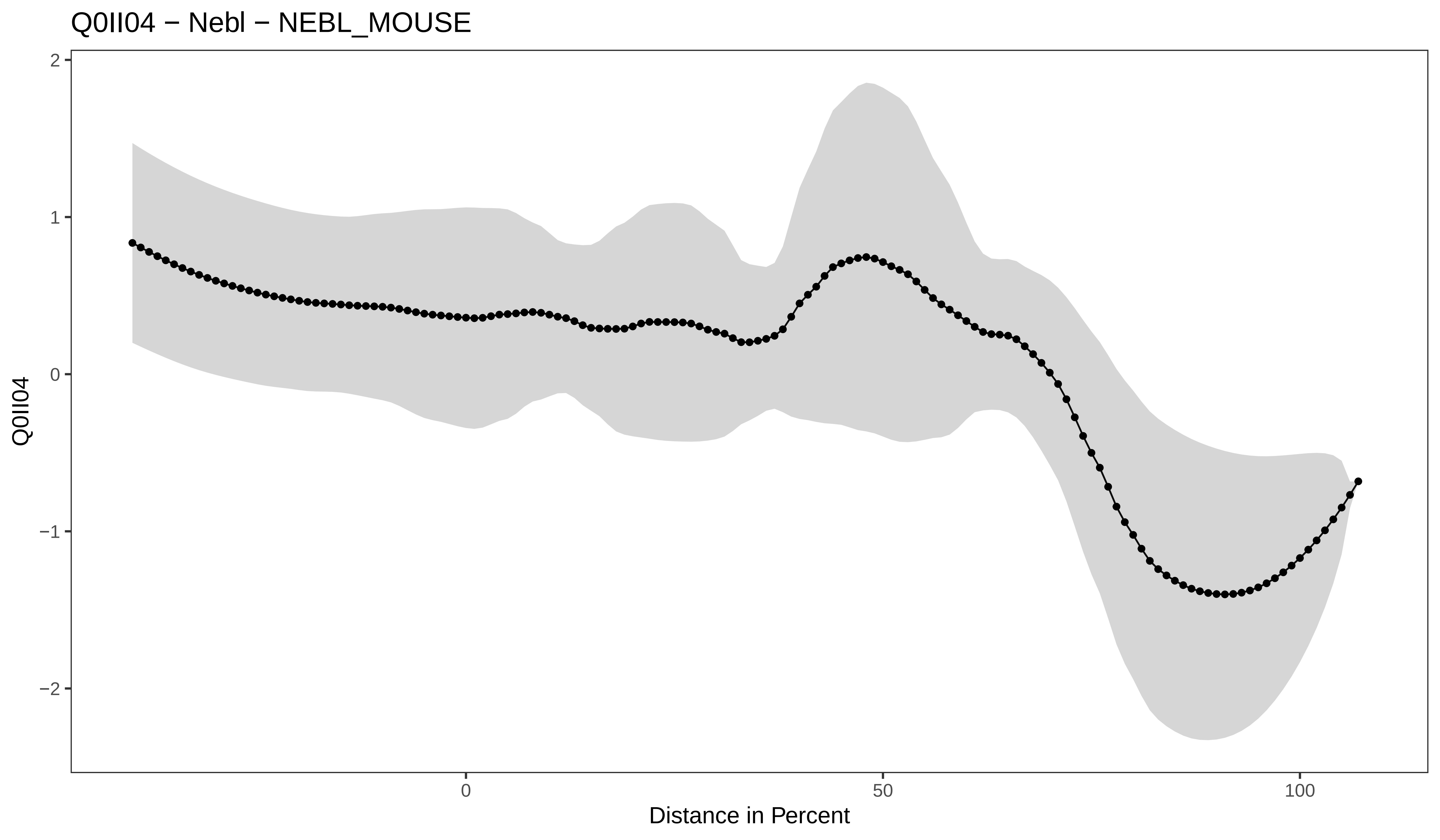

Q0II04 − Nebl − NEBL_MOUSE
Q0II04
2
1
0
−1
−2
0
50
100
Distance in Percent

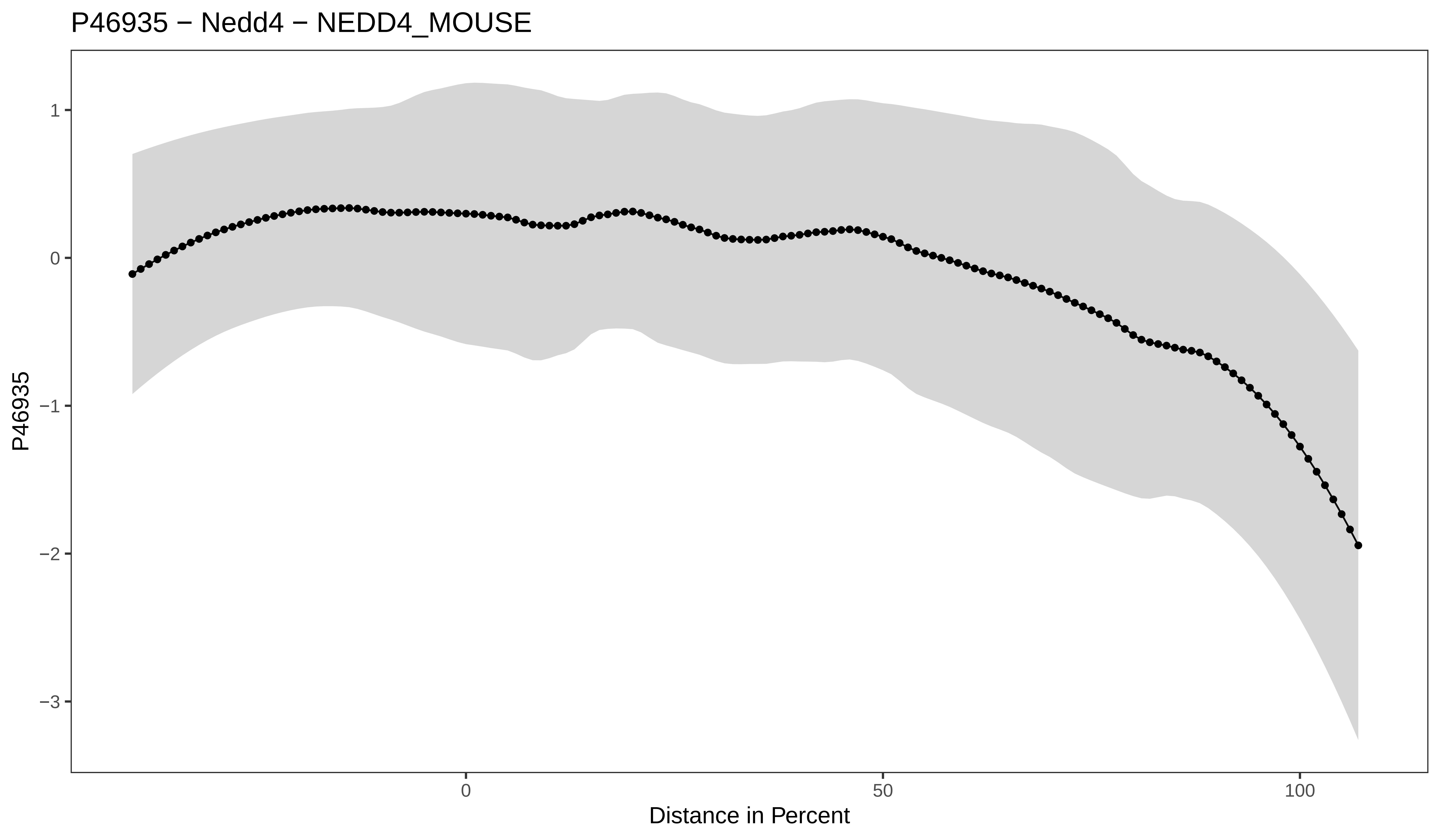

P46935 − Nedd4 − NEDD4_MOUSE
1
0
−1
−2
−3
P46935
0
50
100
Distance in Percent

P29595 − Nedd8 − NEDD8_MOUSE

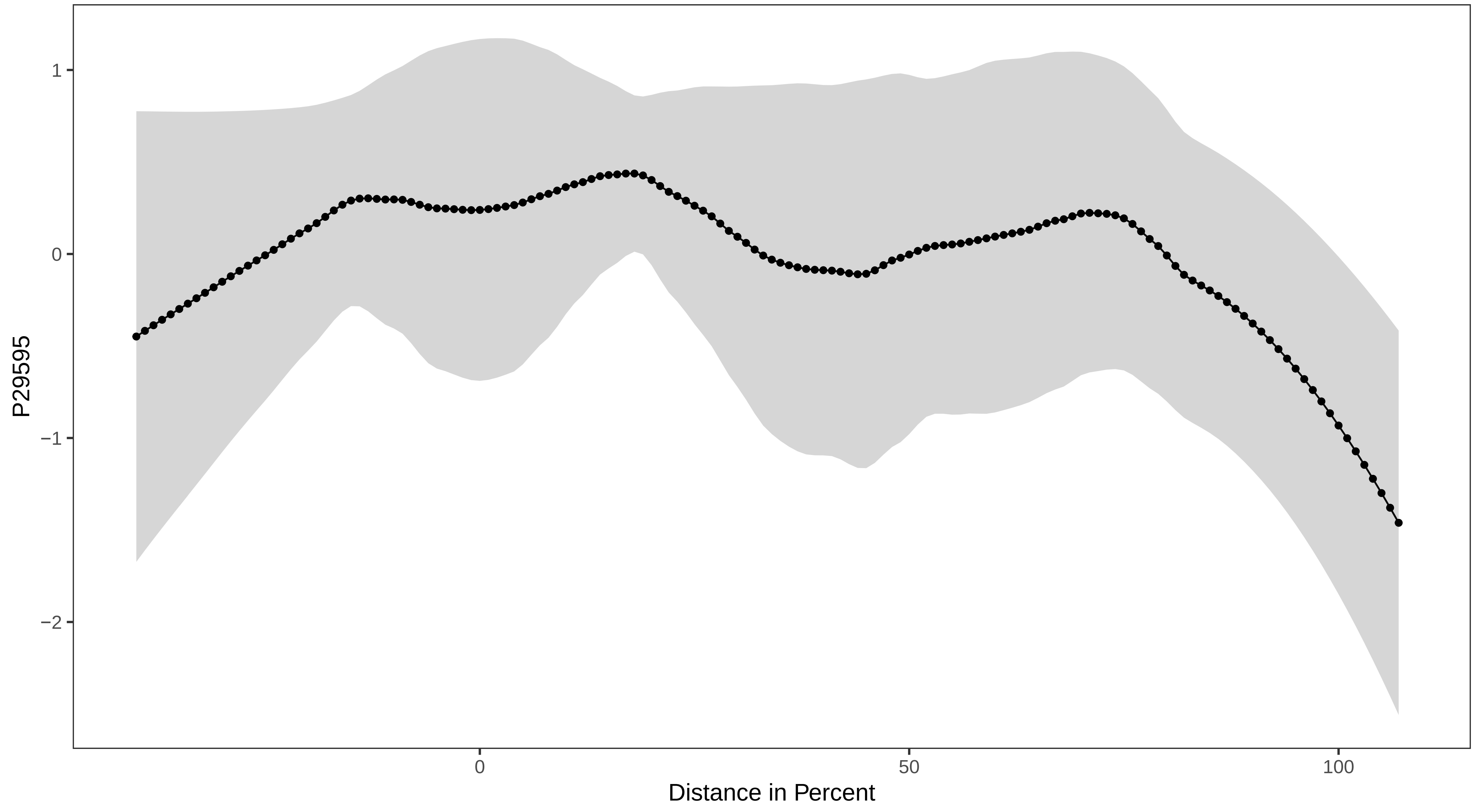

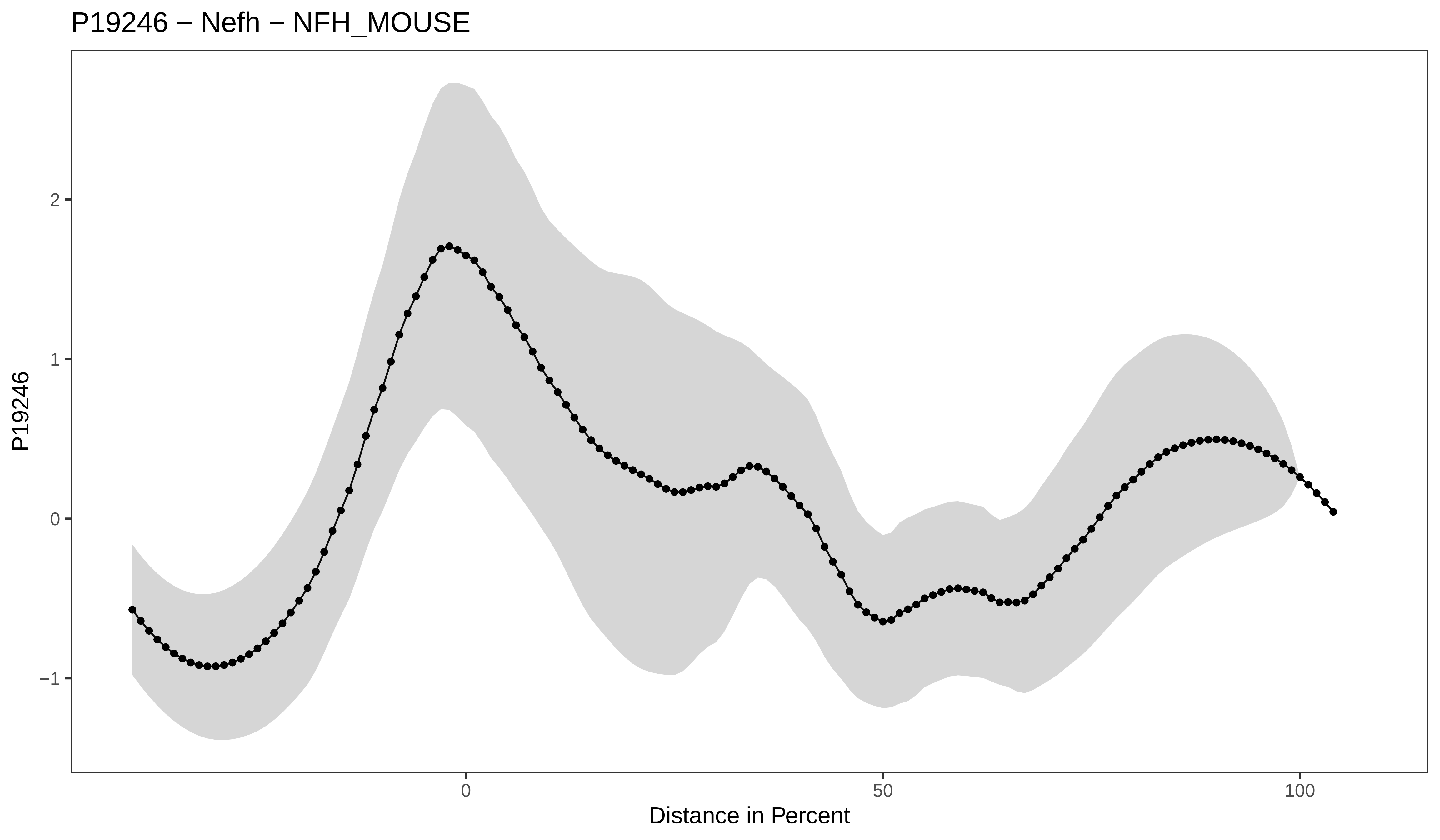
P19246 − Nefh − NFH_MOUSE
P19246
2
1
0
−1
0
50
100
Distance in Percent

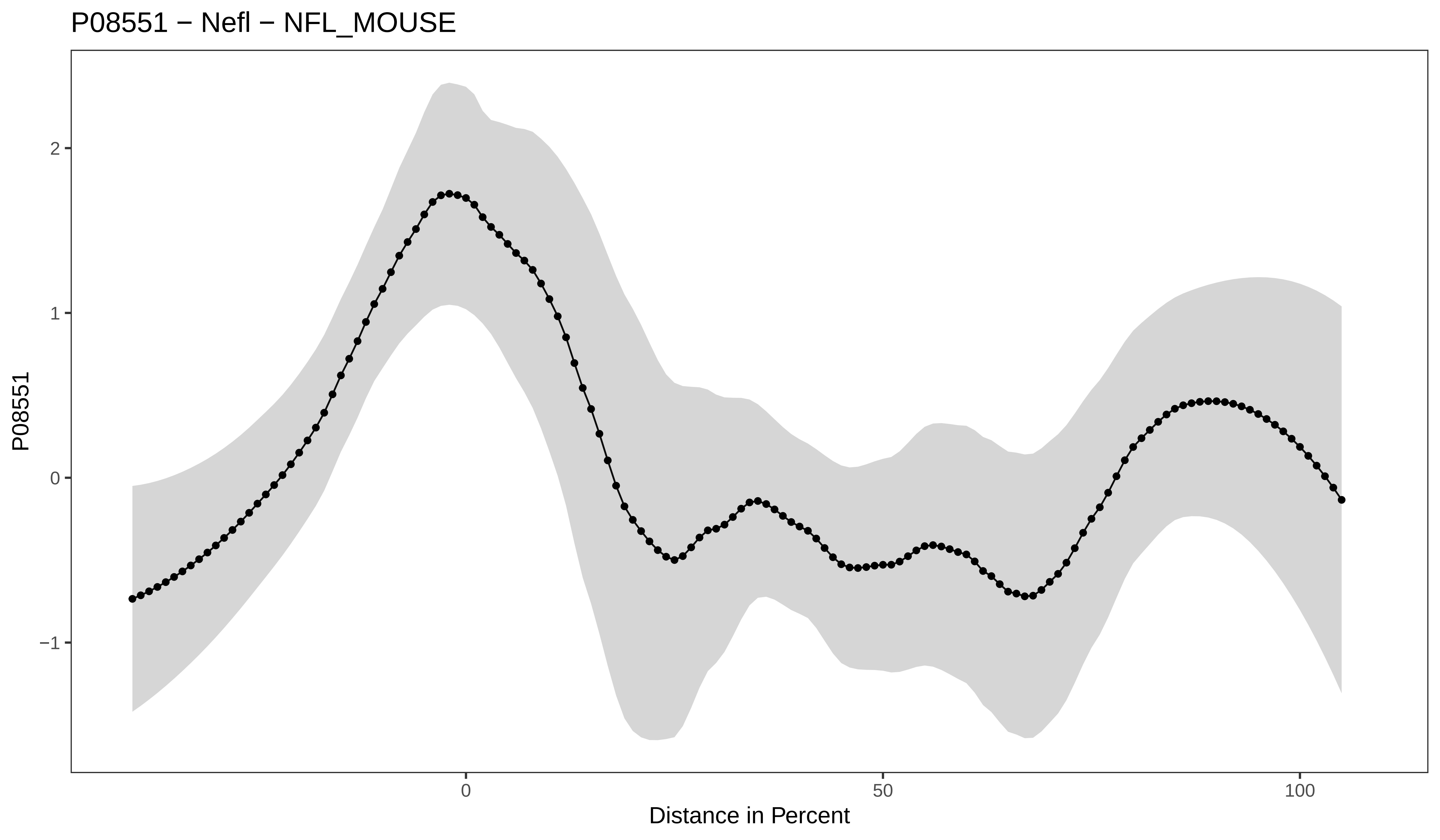

P08551 − Nefl − NFL_MOUSE
P08551
Distance in Percent
2
1
0
−1
0
50
100

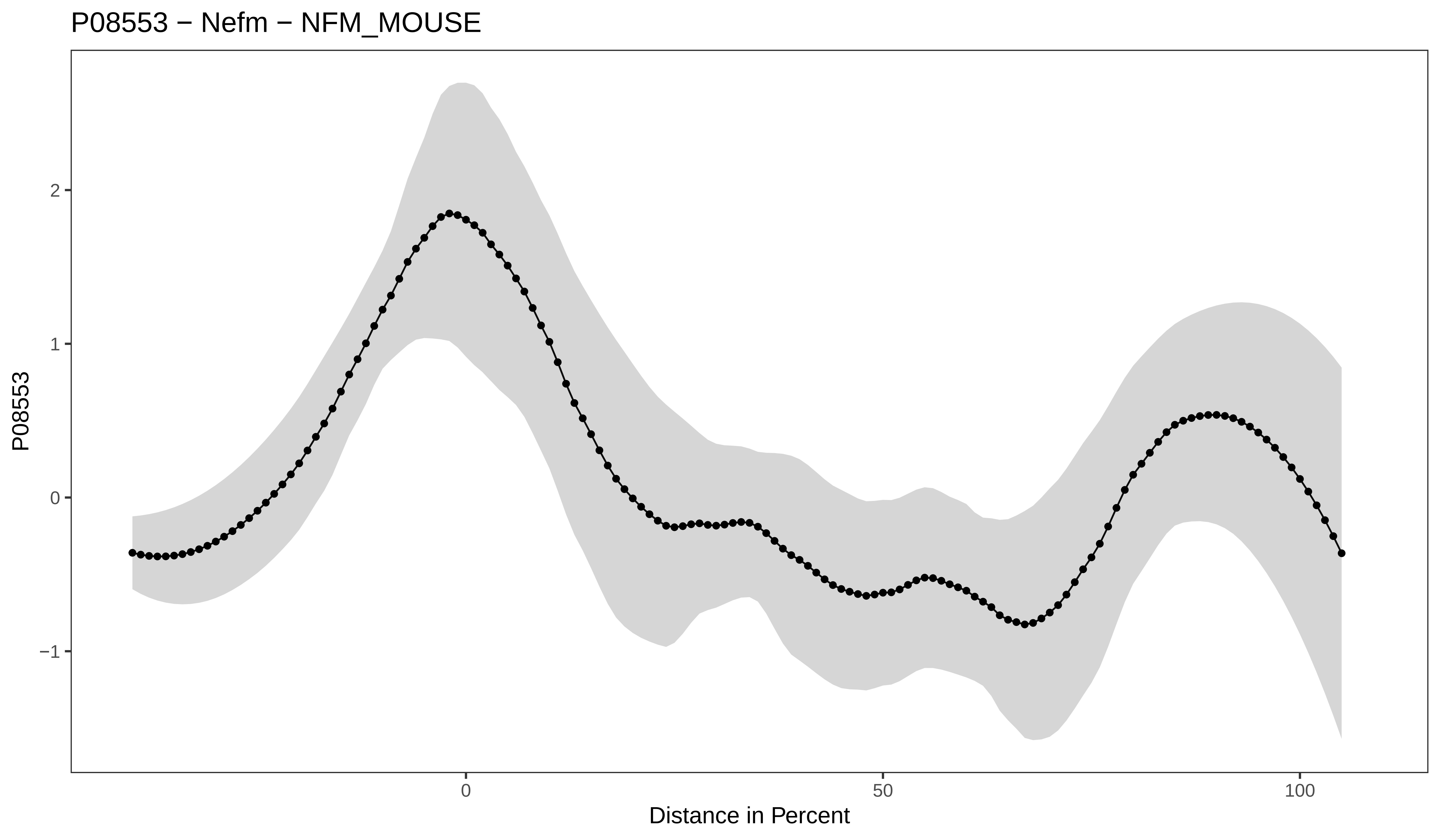

P08553 − Nefm − NFM_MOUSE
P08553
2
1
0
−1
0
50
100
Distance in Percent

Q8K1R7 − Nek9 − NEK9_MOUSE

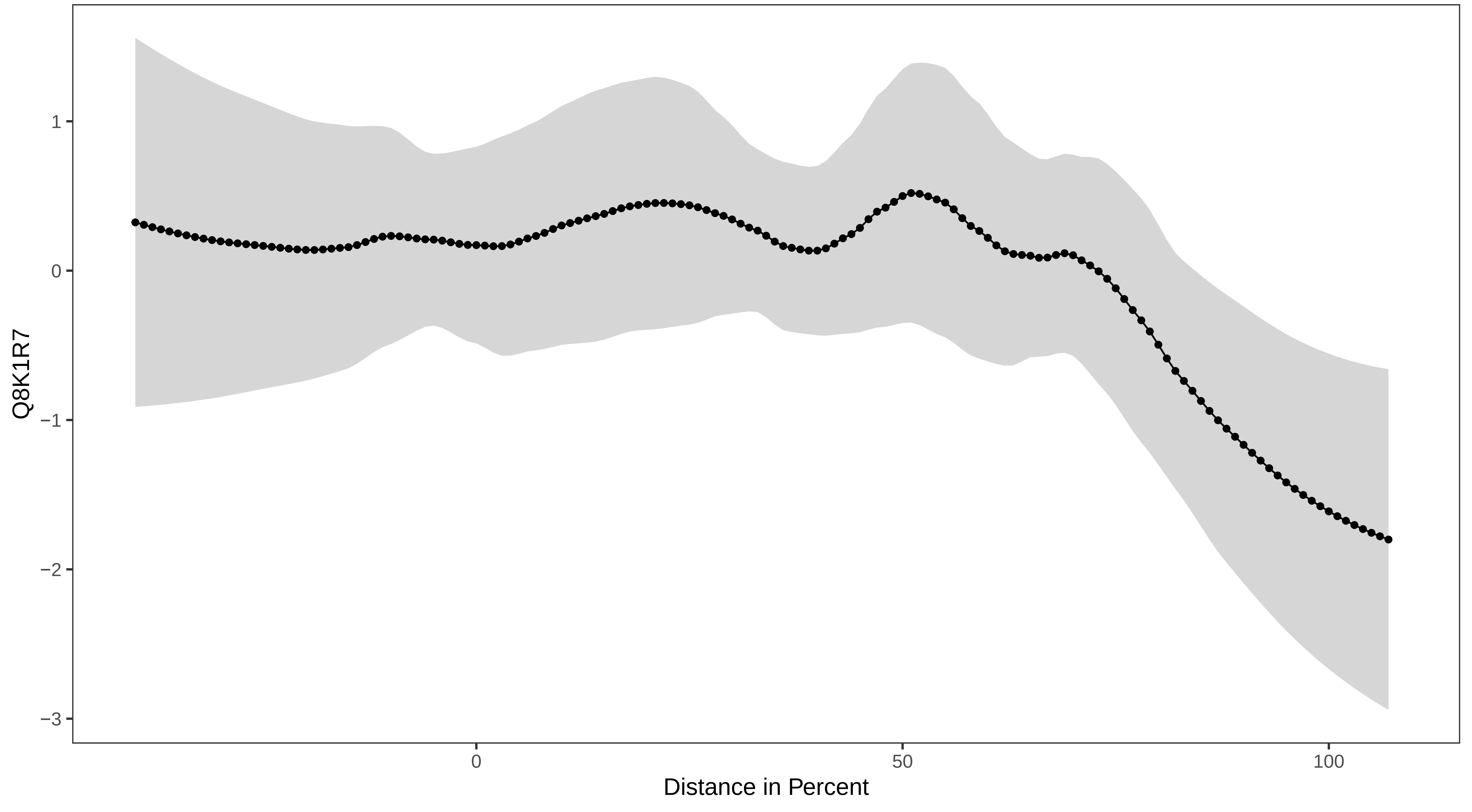

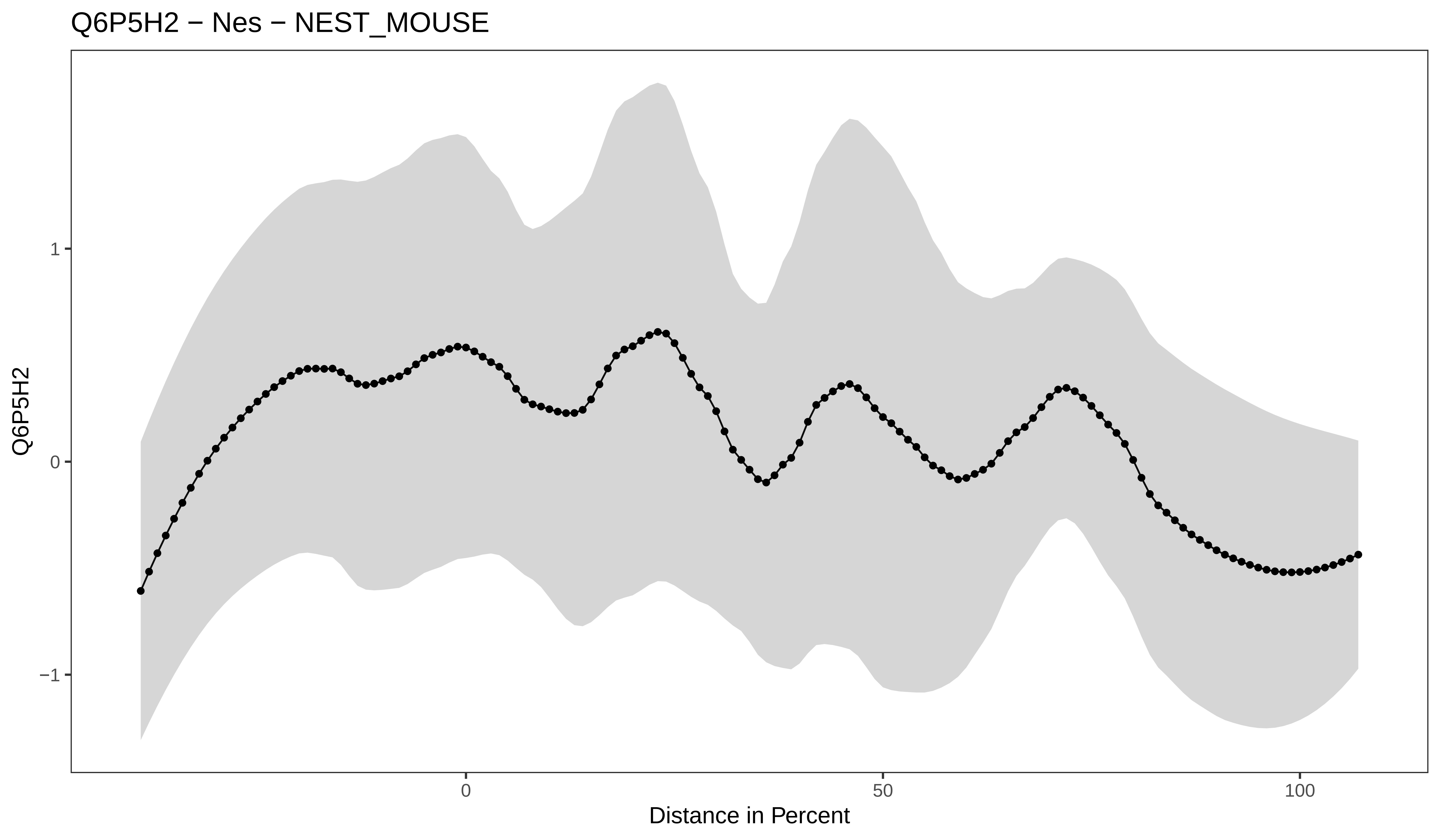

Q6P5H2 − Nes − NEST_MOUSE
Q6P5H2
1
0
−1
0
50
100
Distance in Percent

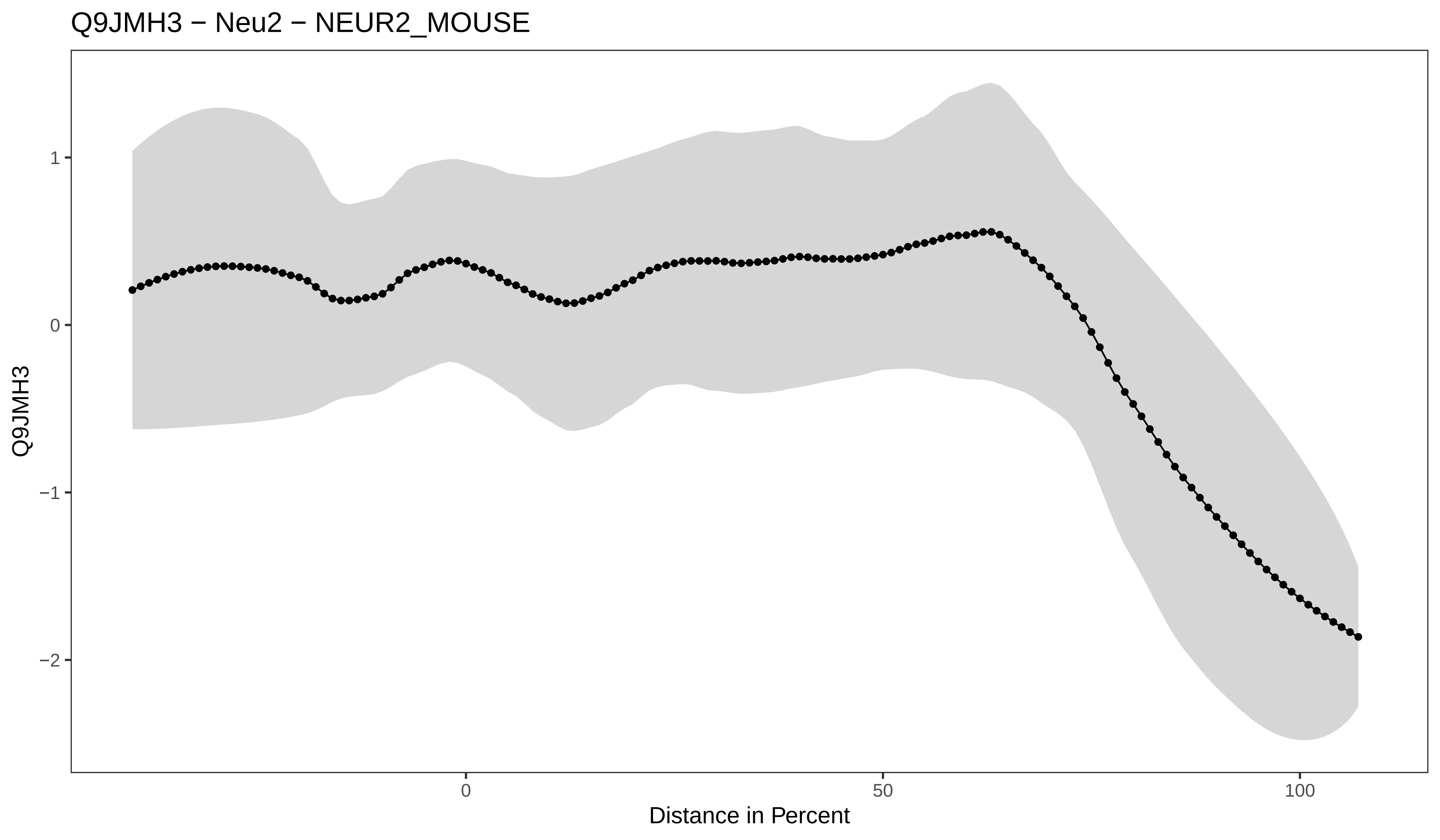
Q9JMH3 − Neu2 − NEUR2_MOUSE
Q9JMH3
1
0
−1
−2
0
50
100
Distance in Percent

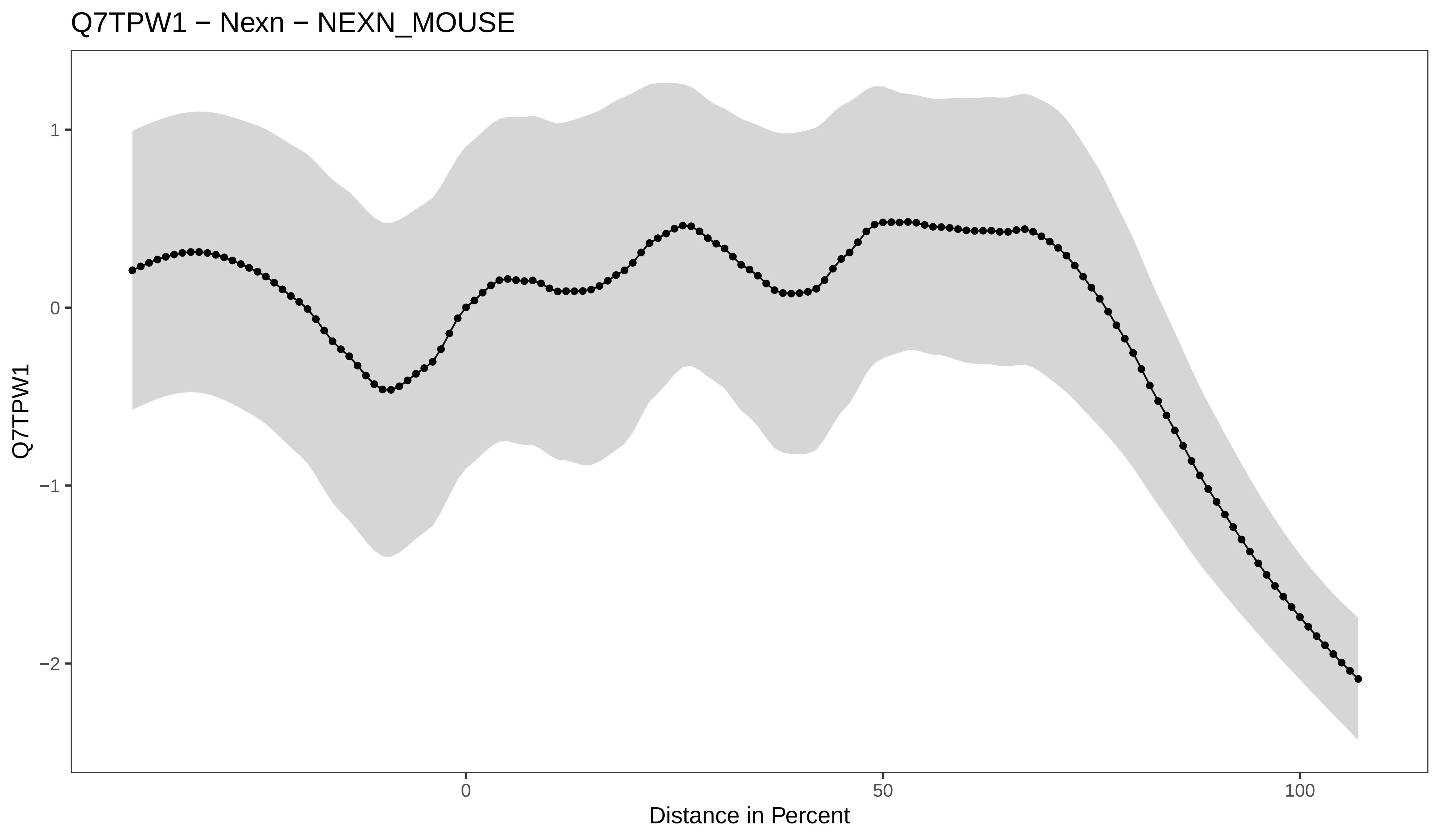

Q7TPW1 − Nexn − NEXN_MOUSE
Q7TPW1
1
0
−1
−2
0
50
100
Distance in Percent

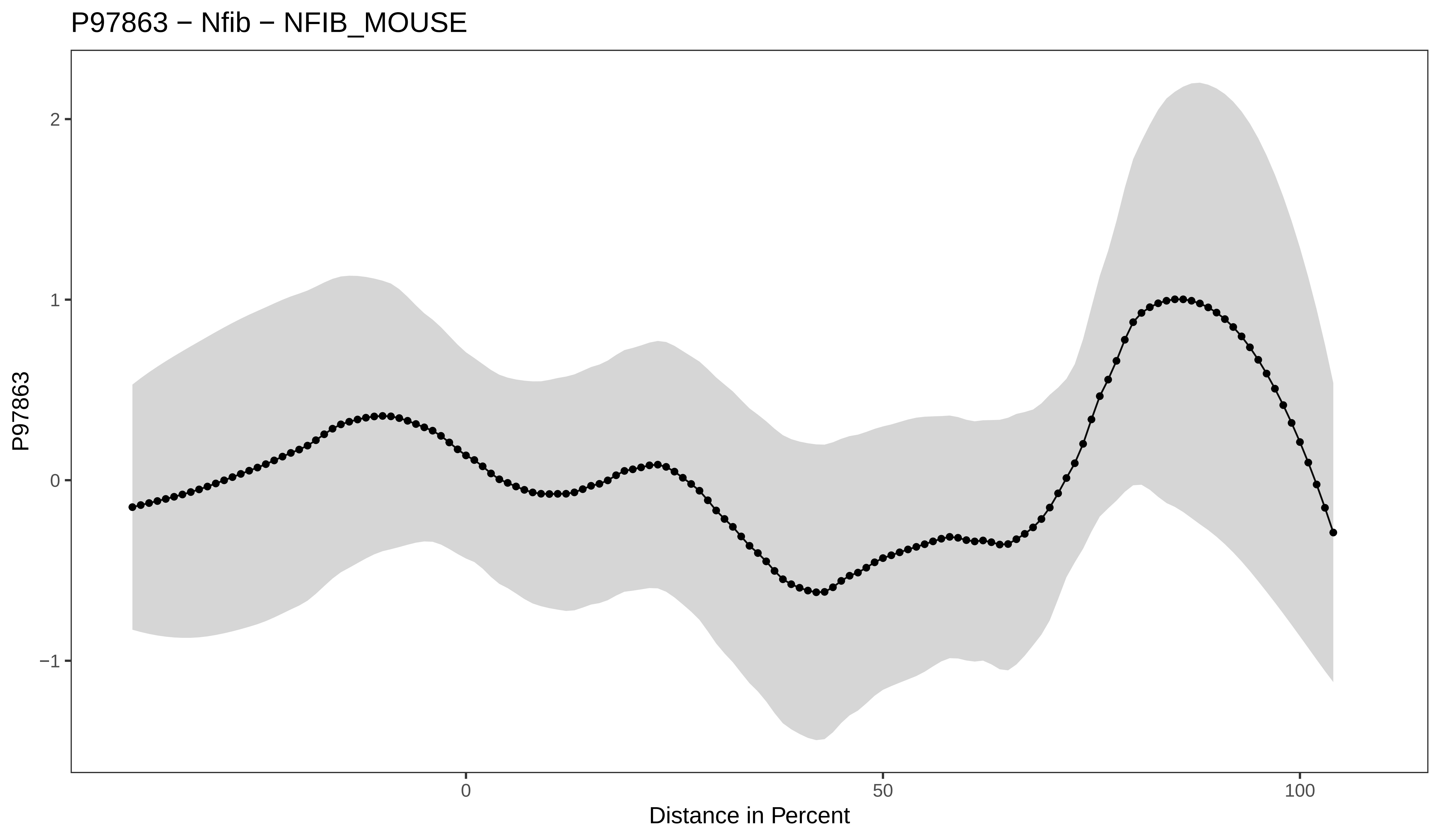

P97863 − Nfib − NFIB_MOUSE
P97863
Distance in Percent
2
1
0
−1
0
50
100

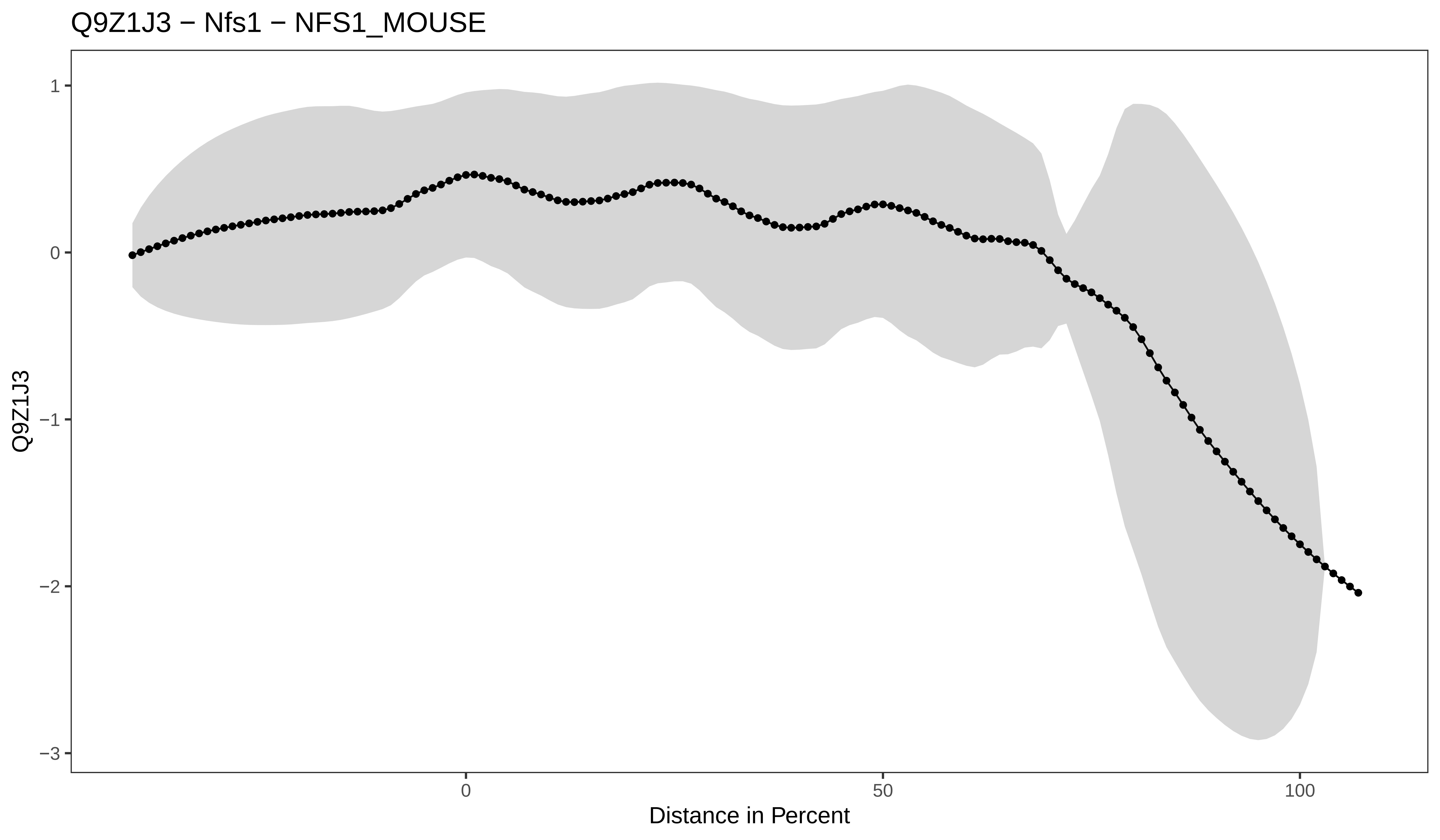

Q9Z1J3 − Nfs1 − NFS1_MOUSE
Q9Z1J3
1
0
−1
−2
−3
0
50
100
Distance in Percent

Q8BZW8 − Nhlrc2 − NHLC2_MOUSE

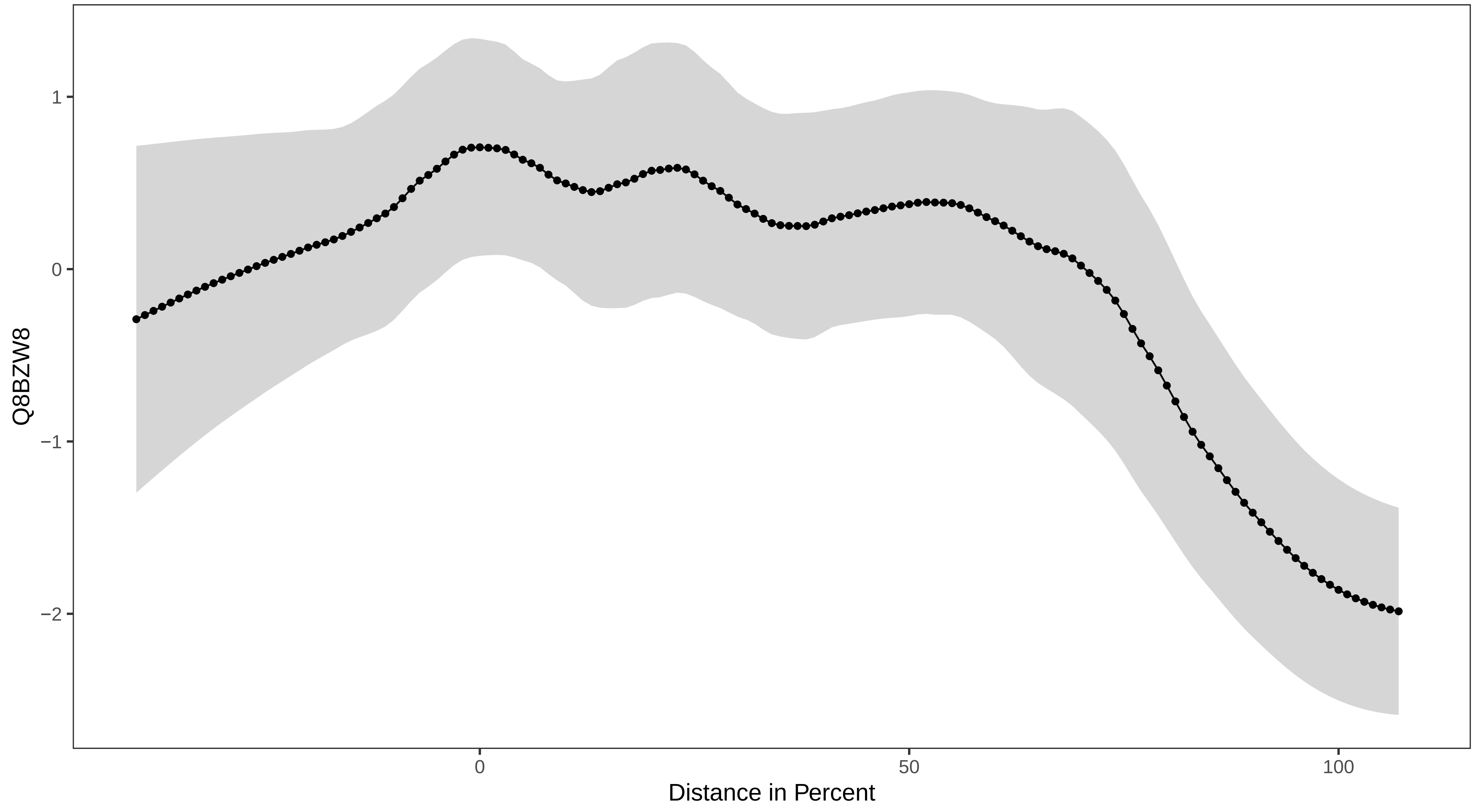

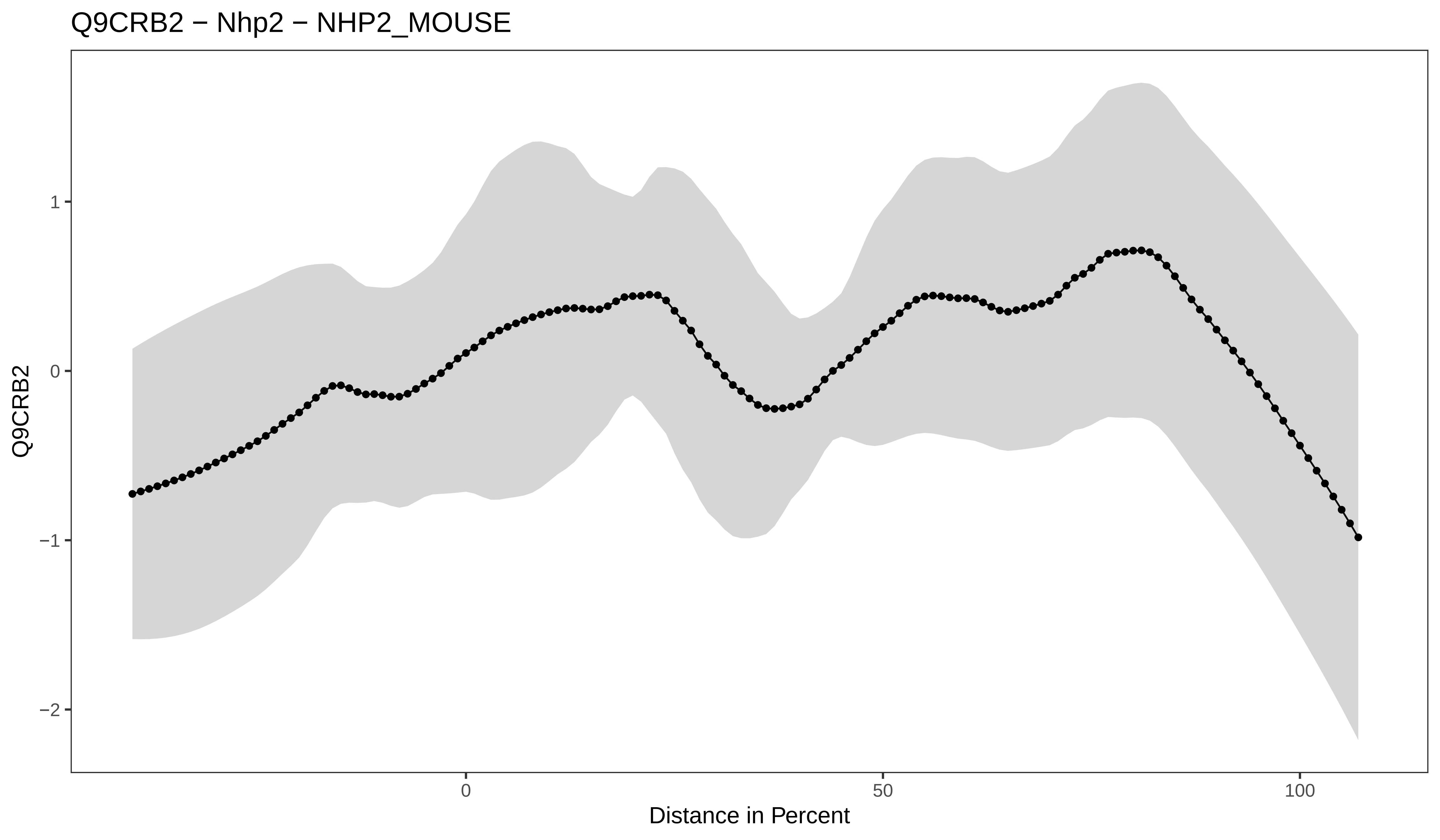

Q9CRB2 − Nhp2 − NHP2_MOUSE
Q9CRB2
1
0
−1
−2
0
50
100
Distance in Percent

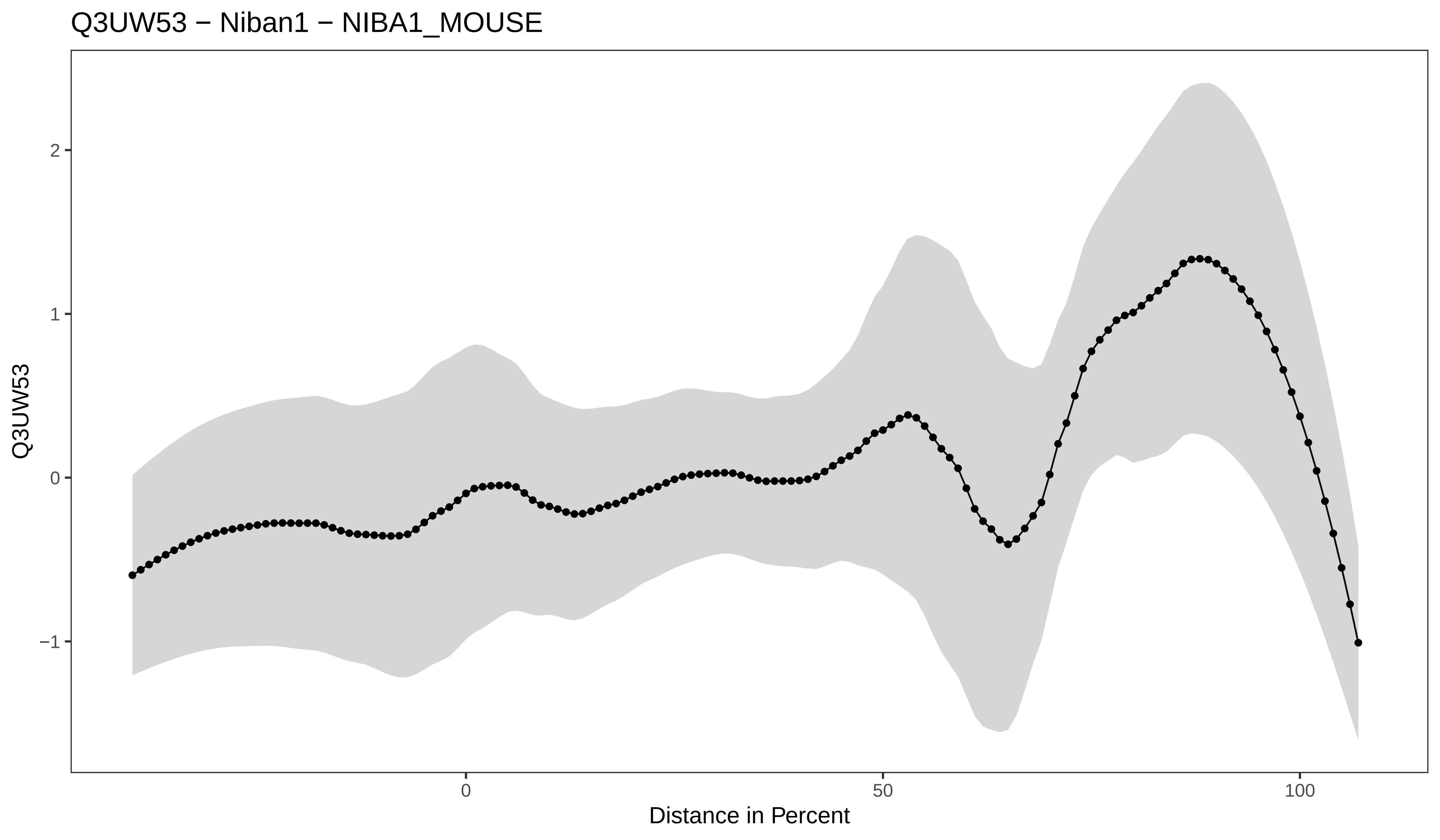
Q3UW53 − Niban1 − NIBA1_MOUSE
Q3UW53
Distance in Percent
2
1
0
−1
0
50
100

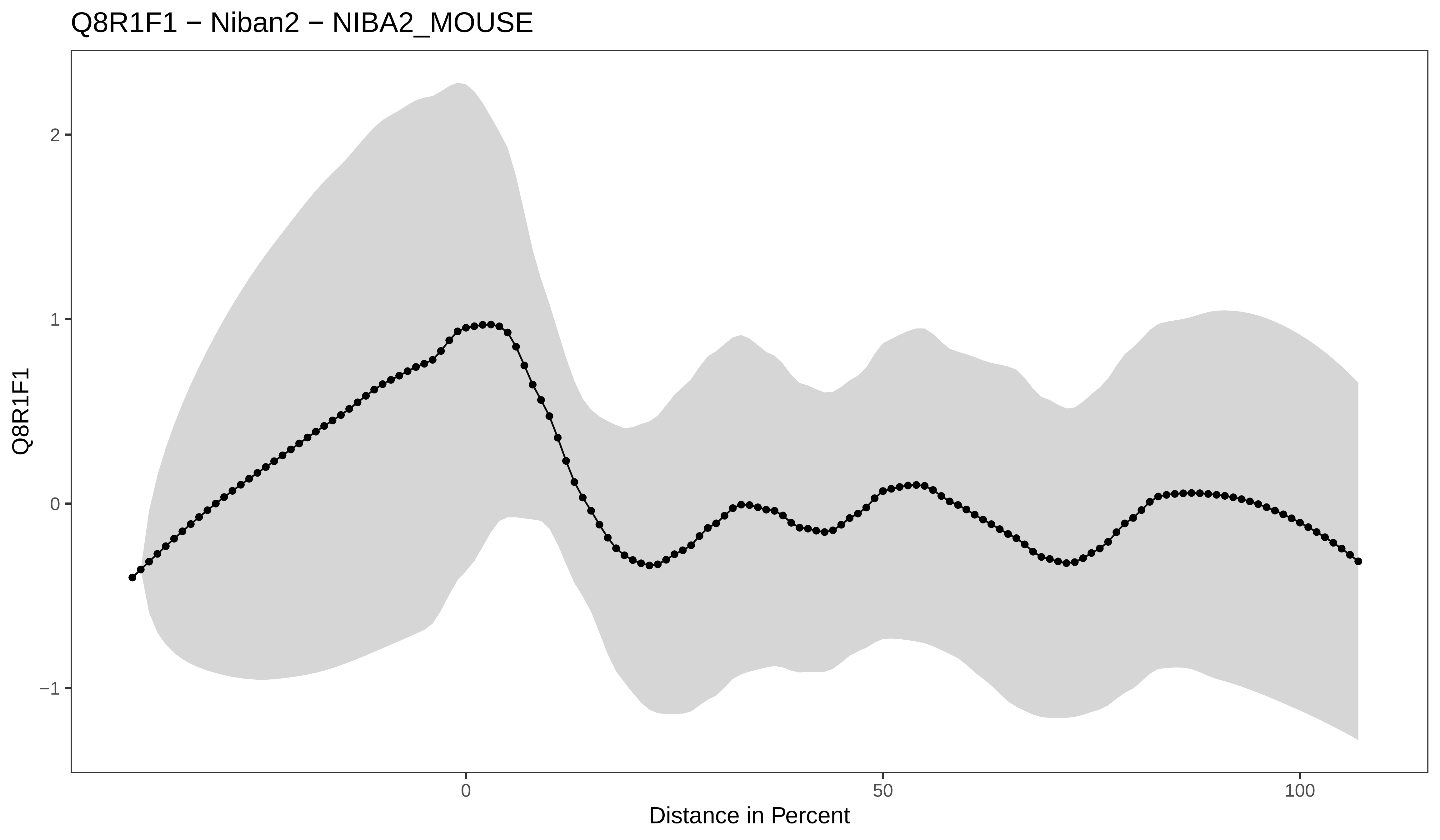
Q8R1F1 − Niban2 − NIBA2_MOUSE
Q8R1F1
2
1
0
−1
0
50
100
Distance in Percent

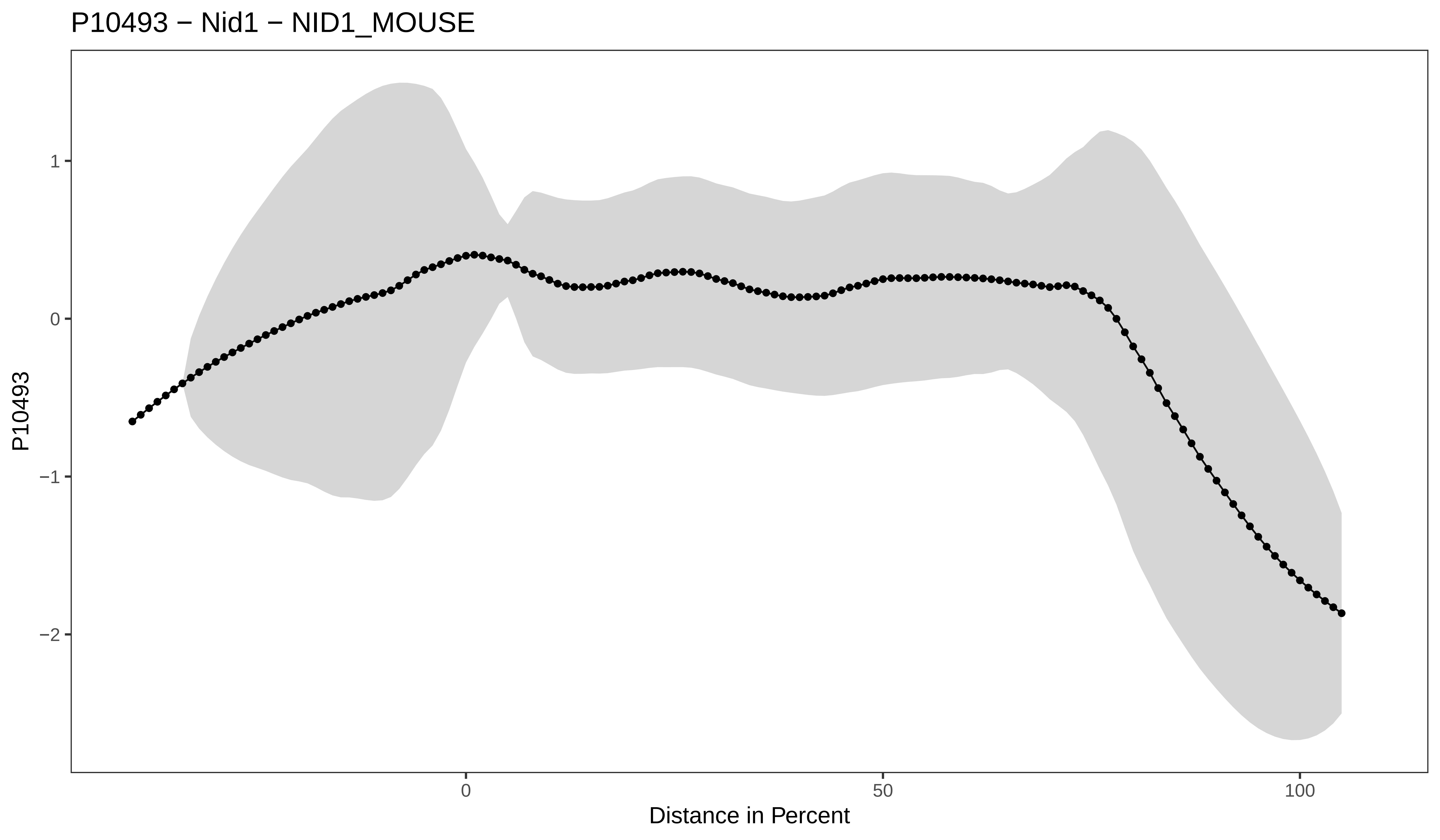
P10493 − Nid1 − NID1_MOUSE
1
0
−1
−2
P10493
0
50
100
Distance in Percent

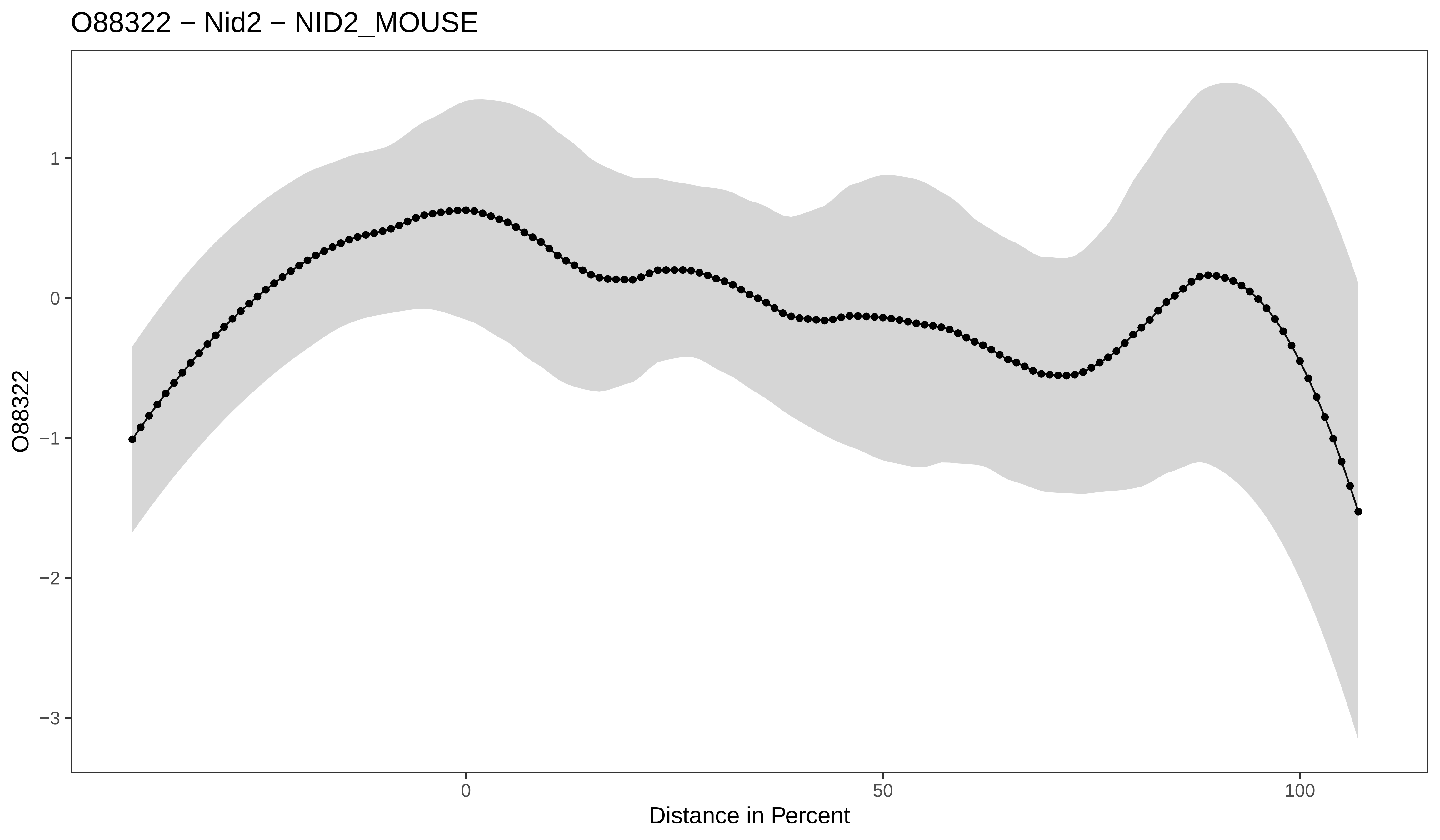
O88322 − Nid2 − NID2_MOUSE
O88322
1
0
−1
−2
−3
0
50
100
Distance in Percent

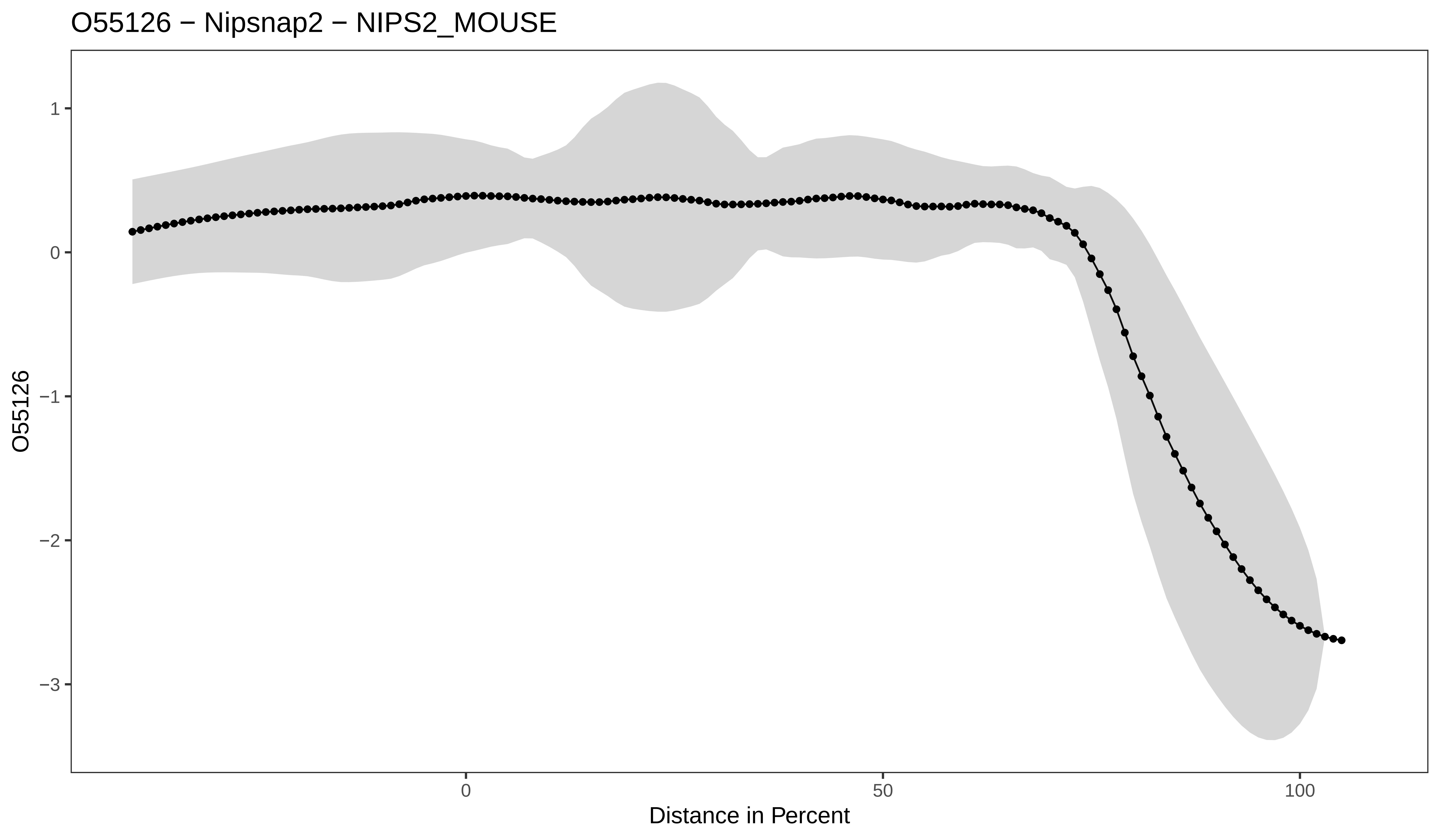
O55126 − Nipsnap2 − NIPS2_MOUSE
1
0
−1
−2
−3
O55126
0
50
100
Distance in Percent

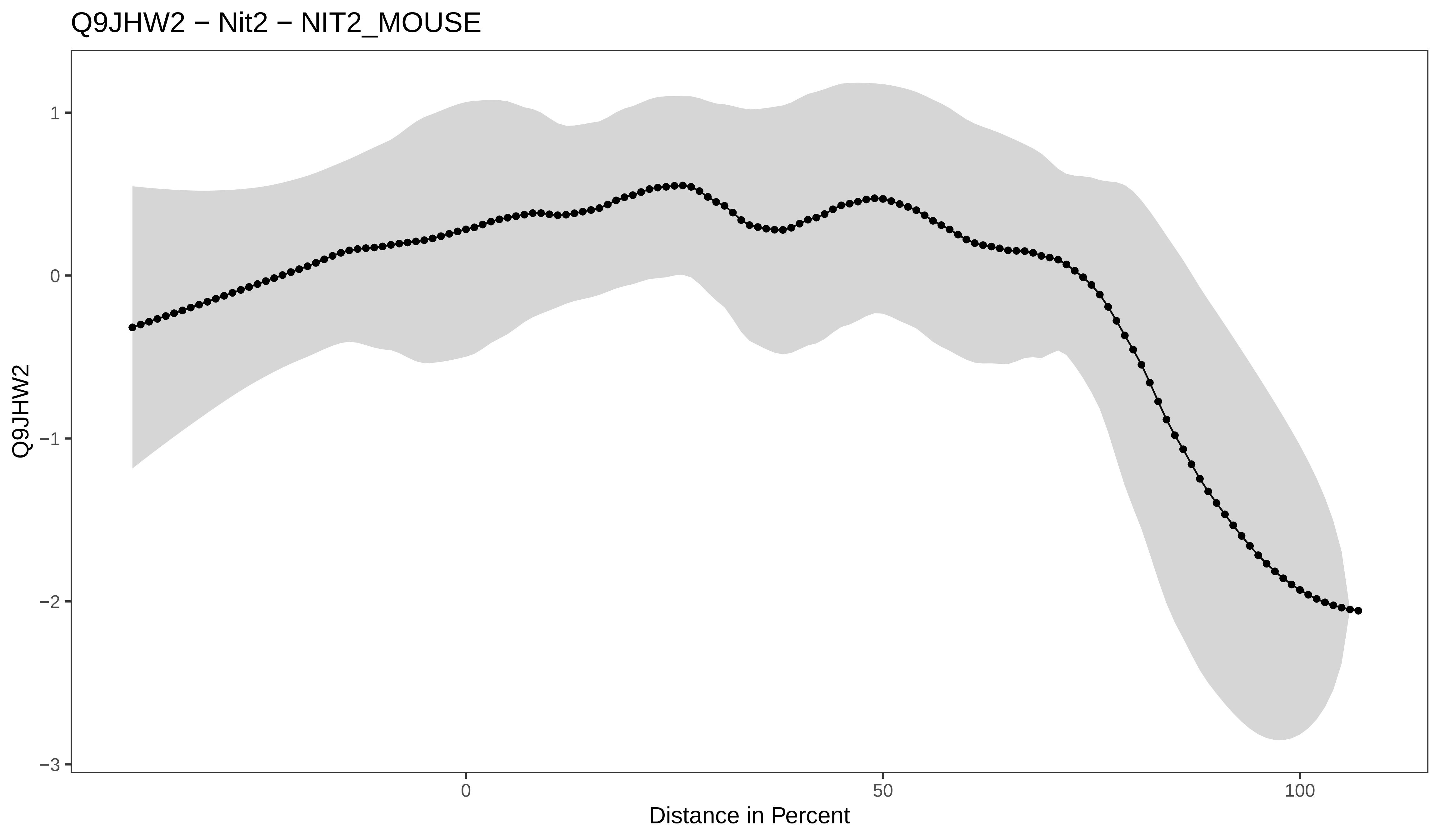

Q9JHW2 − Nit2 − NIT2_MOUSE
Q9JHW2
1
0
−1
−2
−3
0
50
100
Distance in Percent

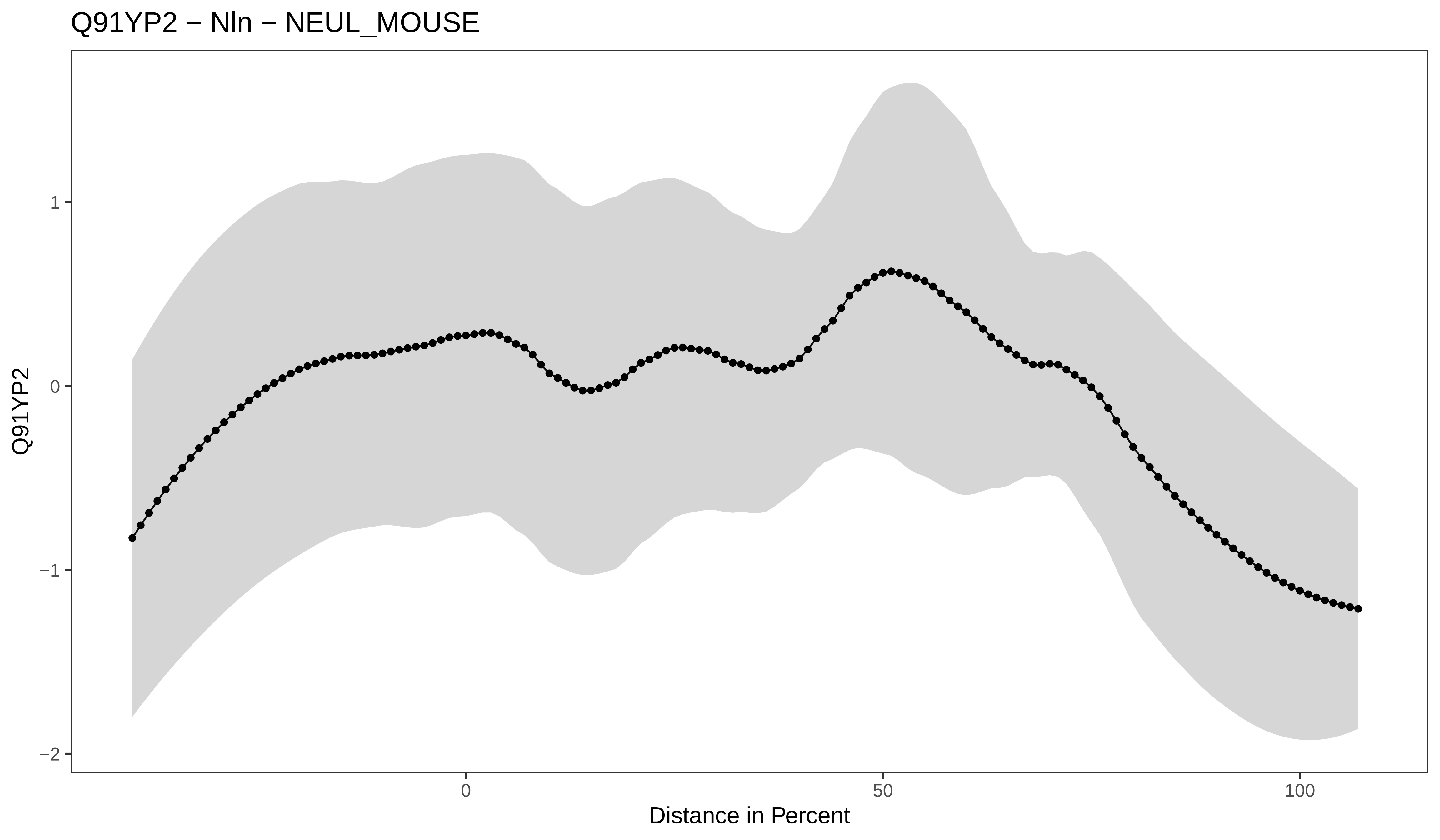
Q91YP2 − Nln − NEUL_MOUSE
Q91YP2
1
0
−1
−2
0
50
100
Distance in Percent

Q66X19 − Nlrp4e − NAL4E_MOUSE

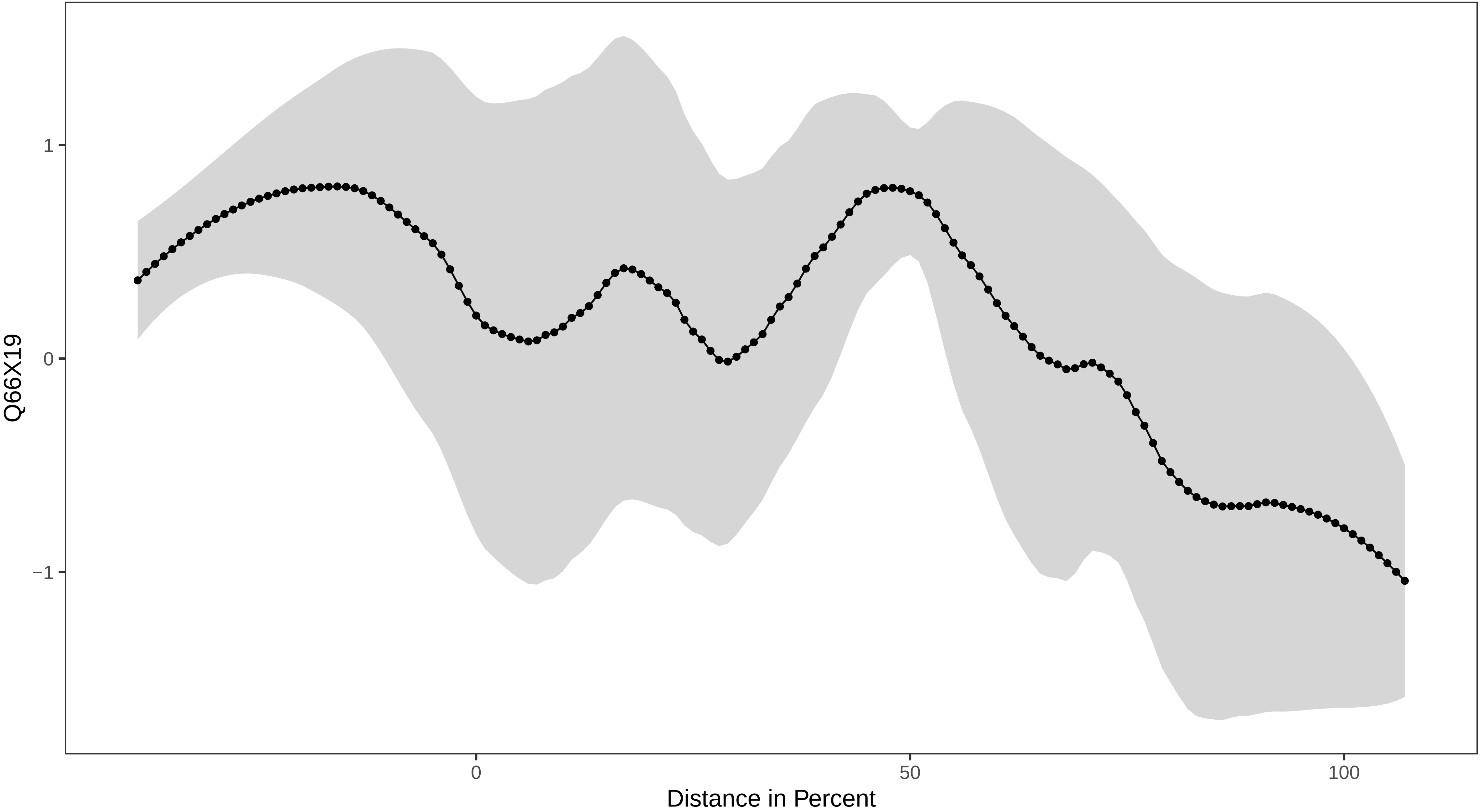

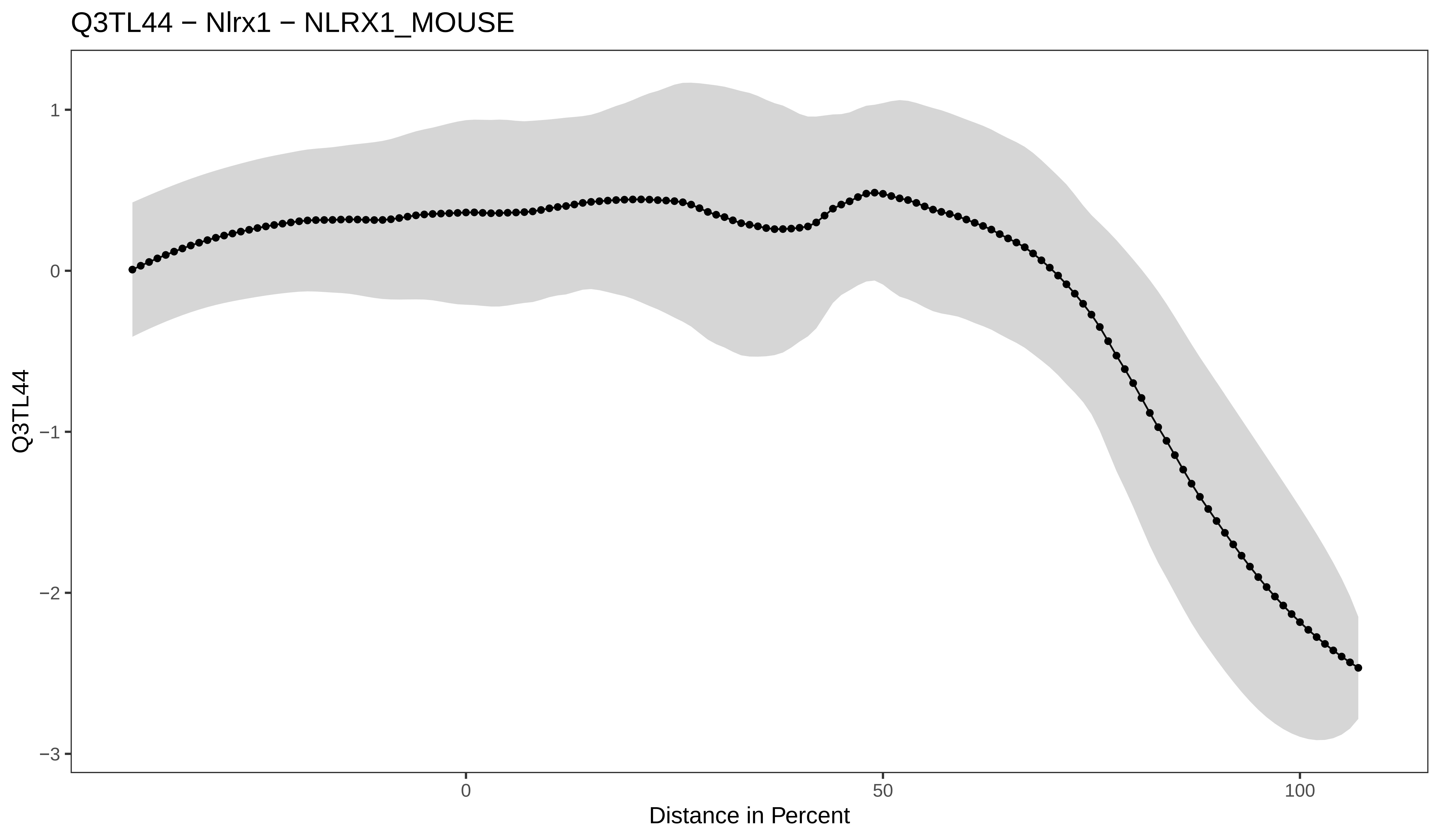

Q3TL44 − Nlrx1 − NLRX1_MOUSE
1
0
−1
−2
−3
Q3TL44
0
50
100
Distance in Percent

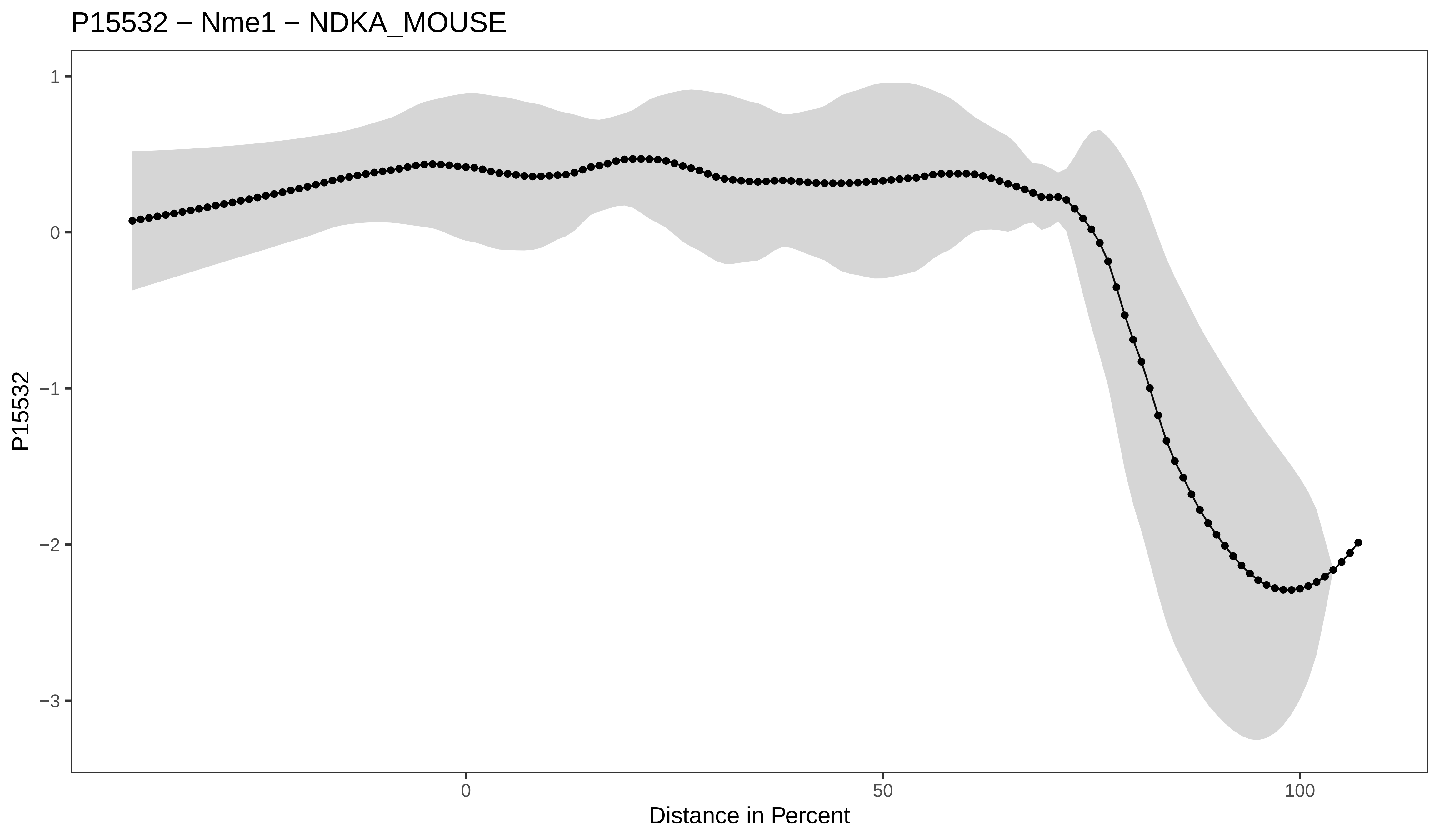

P15532 − Nme1 − NDKA_MOUSE
1
0
−1
−2
−3
P15532
0
50
100
Distance in Percent

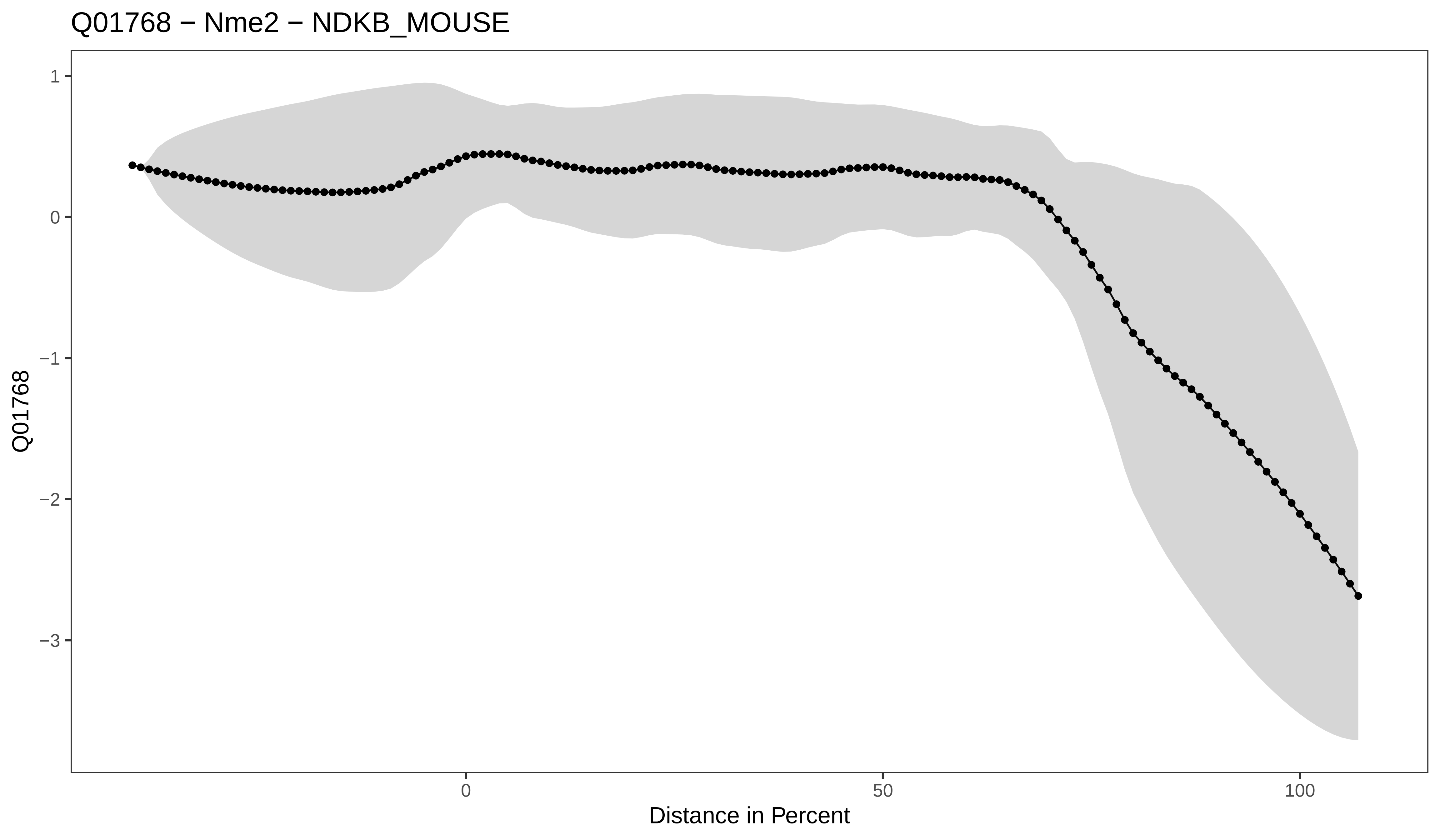
Q01768 − Nme2 − NDKB_MOUSE
1
0
−1
−2
−3
Q01768
0
50
100
Distance in Percent

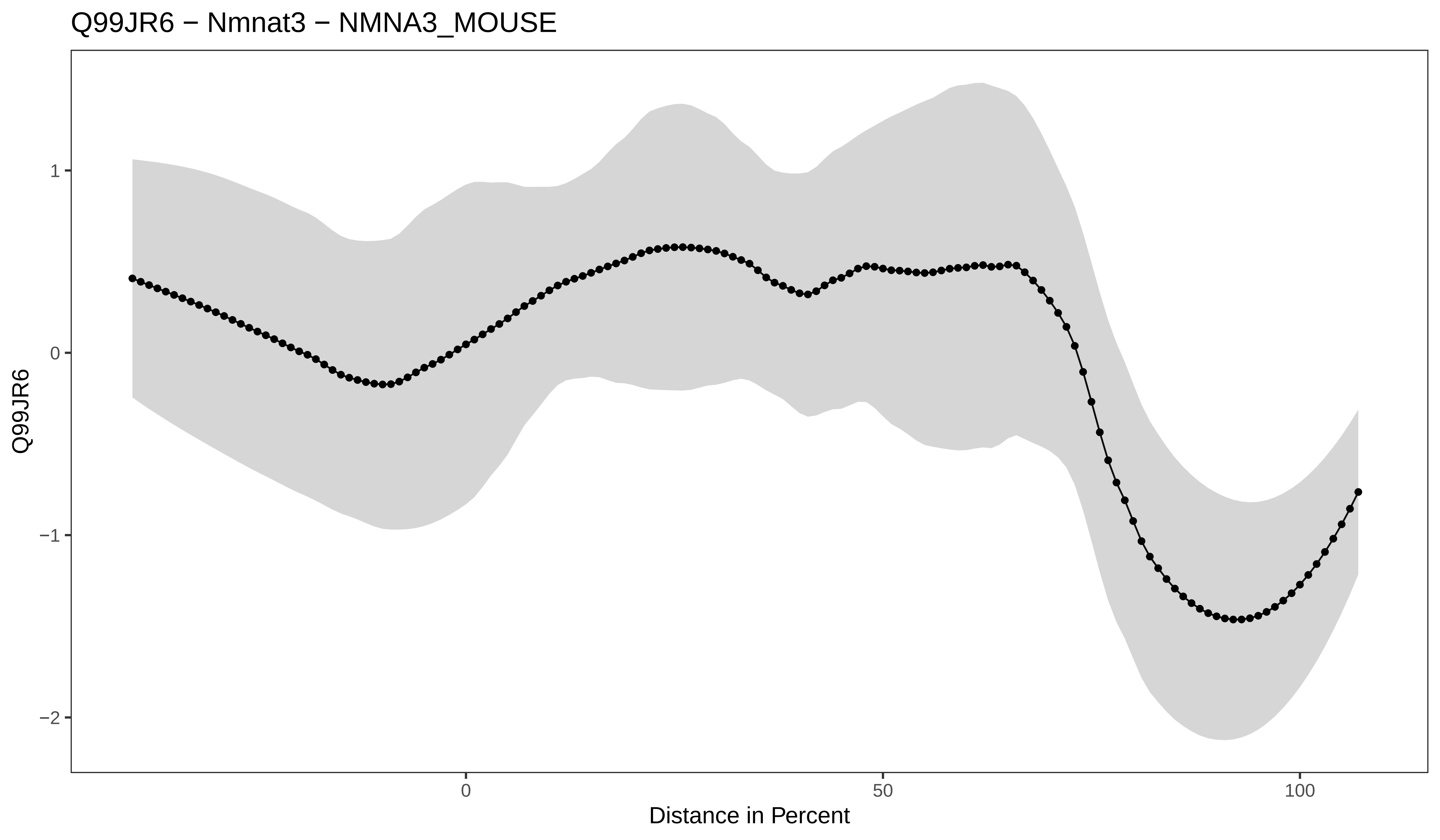

Q99JR6 − Nmnat3 − NMNA3_MOUSE
Q99JR6
Distance in Percent
1
0
−1
−2
0
50
100

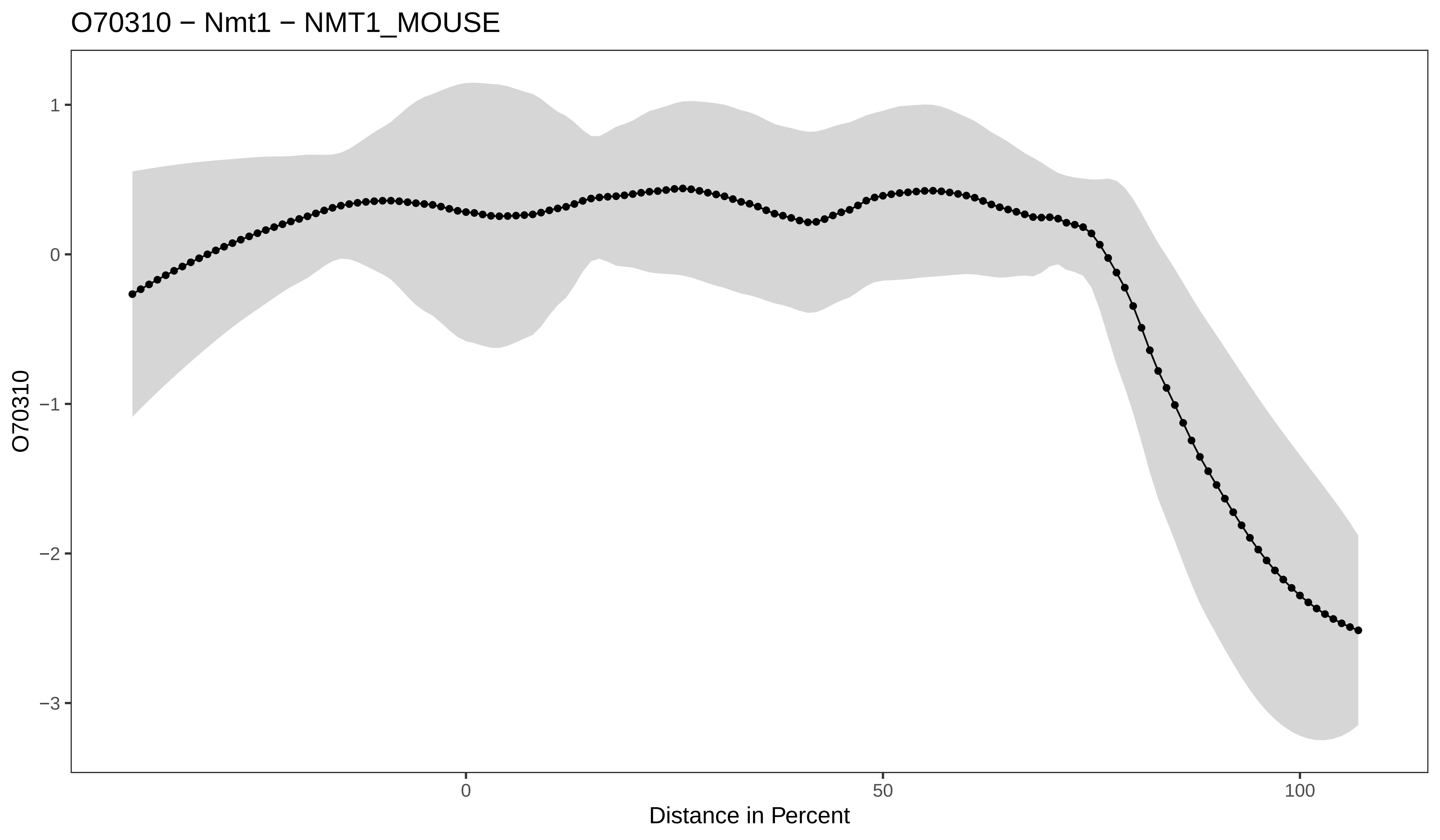
O70310 − Nmt1 − NMT1_MOUSE
O70310
1
0
−1
−2
−3
0
50
100
Distance in Percent

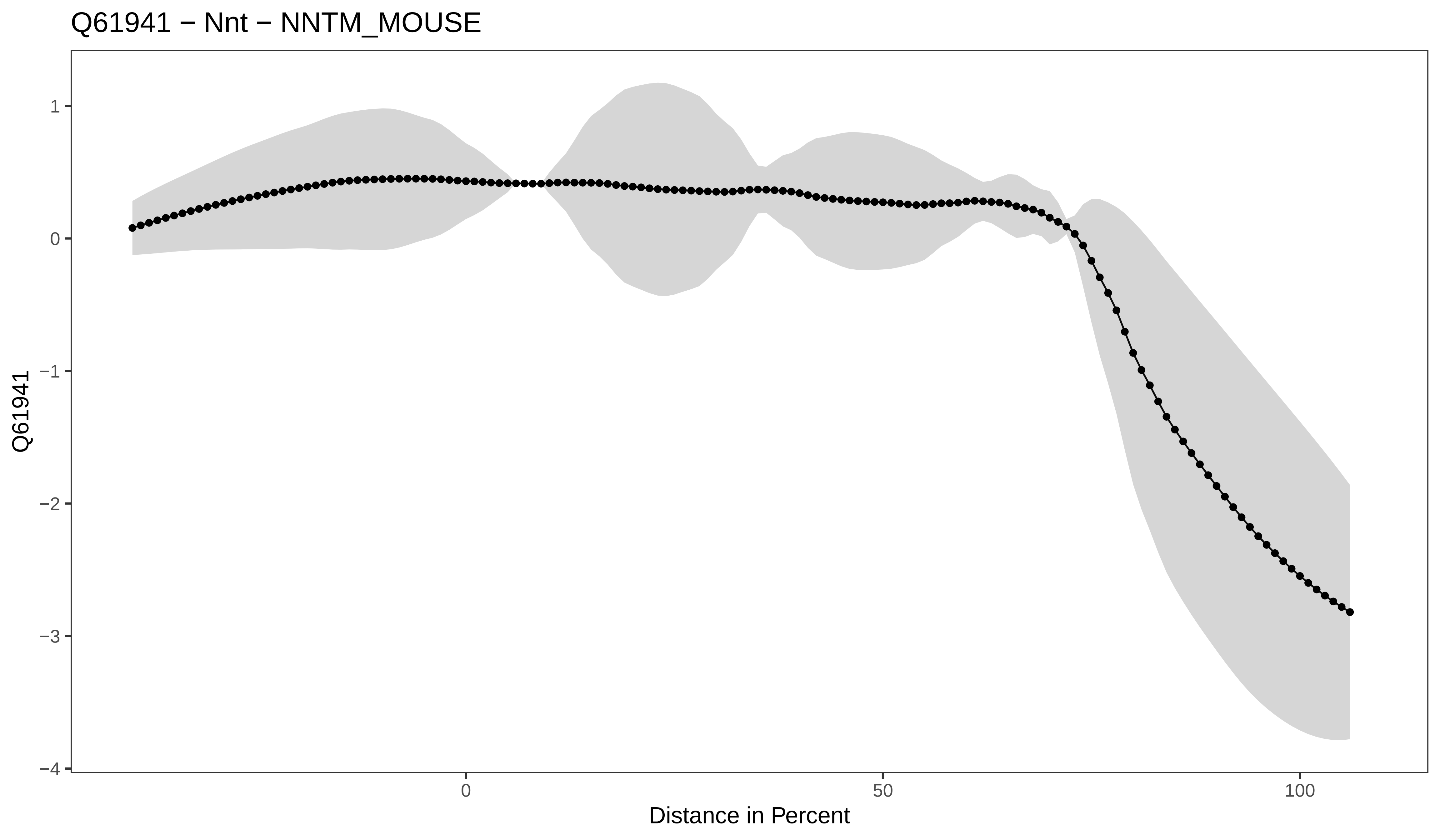

Q61941 − Nnt − NNTM_MOUSE
Q61941
Distance in Percent
1
0
−1
−2
−3
−4
0
50
100

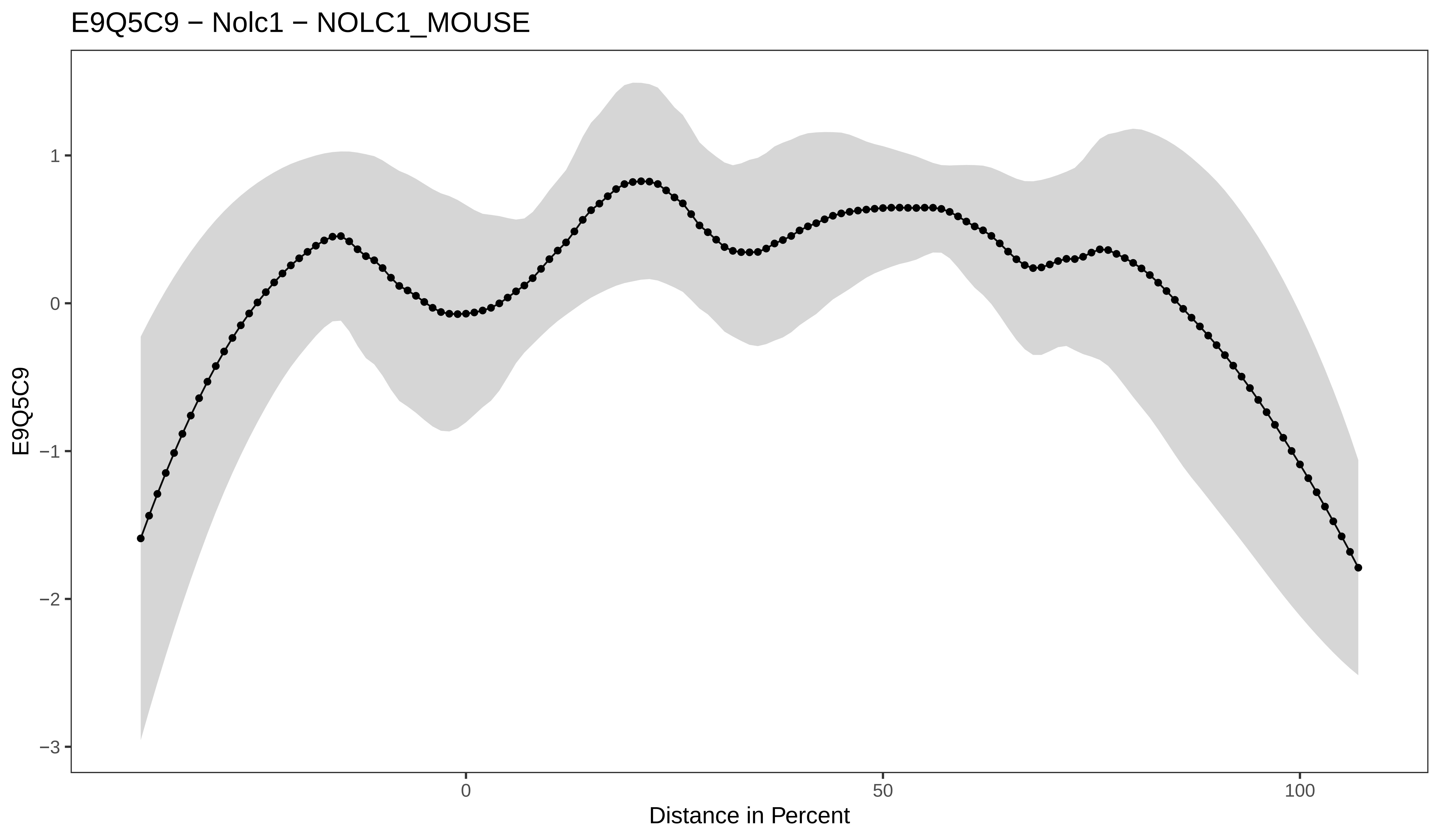
E9Q5C9 − Nolc1 − NOLC1_MOUSE
E9Q5C9
1
0
−1
−2
−3
0
50
100
Distance in Percent

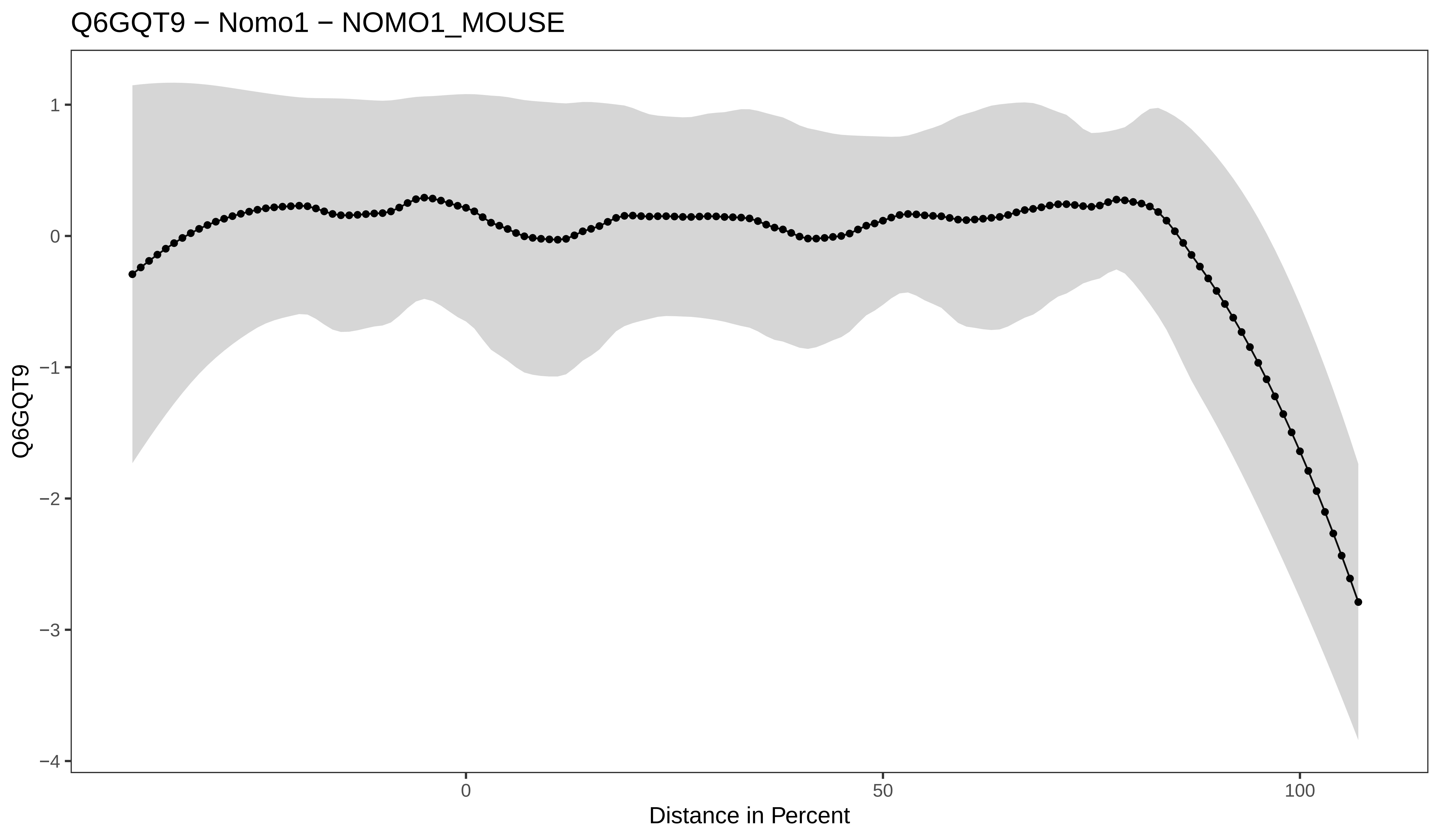

Q6GQT9 − Nomo1 − NOMO1_MOUSE
Q6GQT9
Distance in Percent
1
0
−1
−2
−3
−4
0
50
100

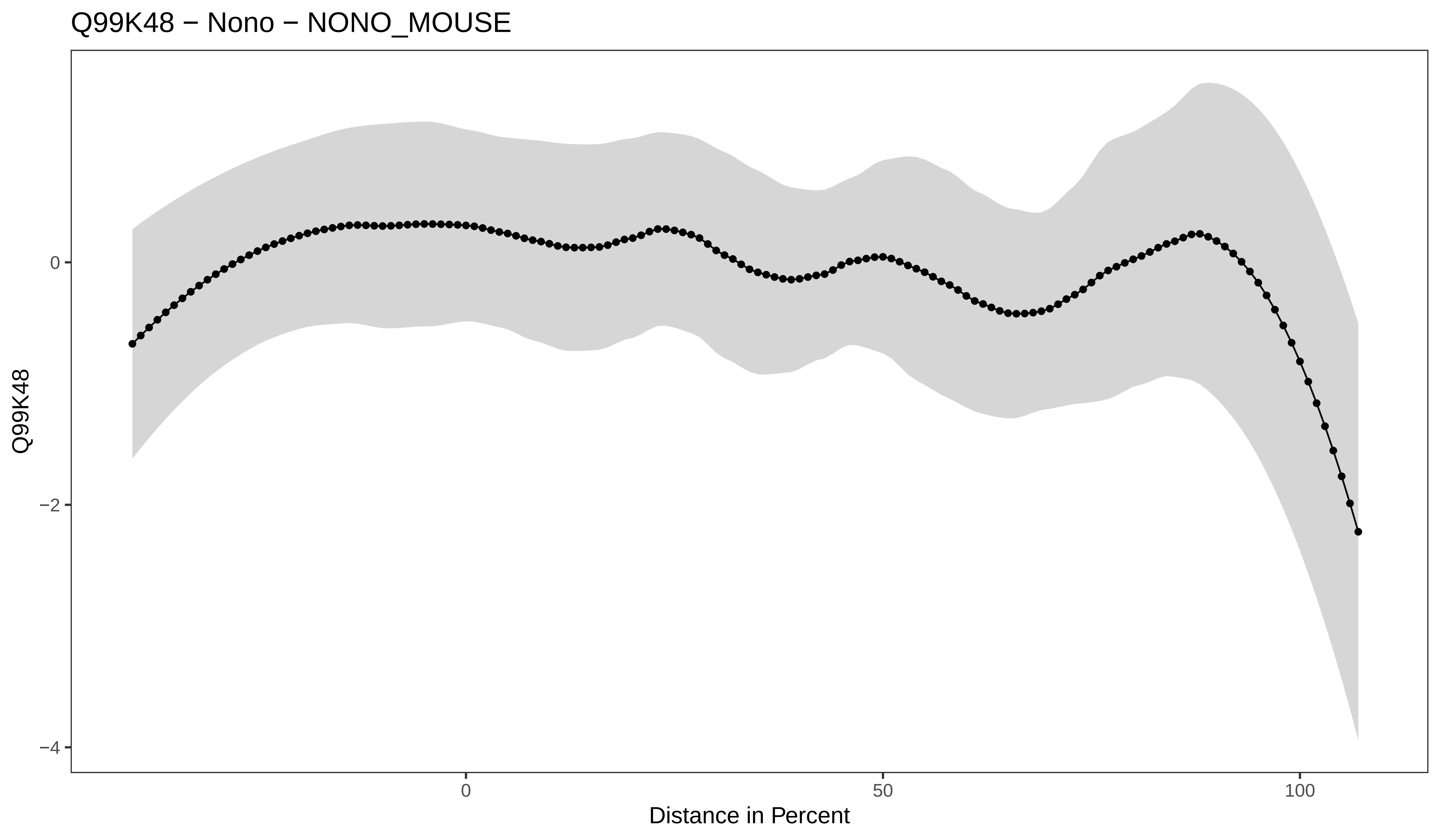

Q99K48 − Nono − NONO_MOUSE
Q99K48
0
−2
−4
0
50
100
Distance in Percent

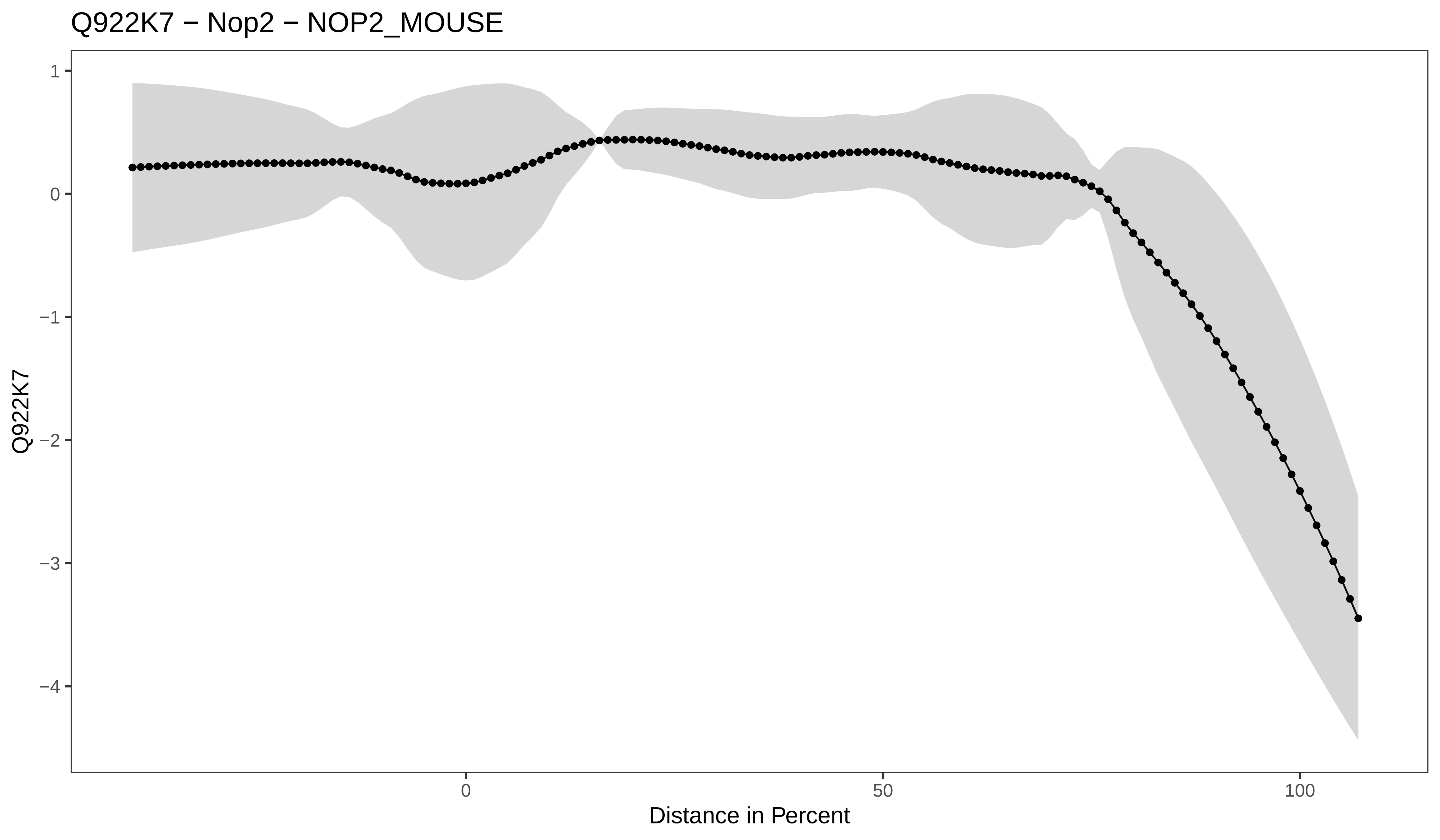

Q922K7 − Nop2 − NOP2_MOUSE
1
0
−1
−2
−3
−4
Q922K7
0
50
100
Distance in Percent

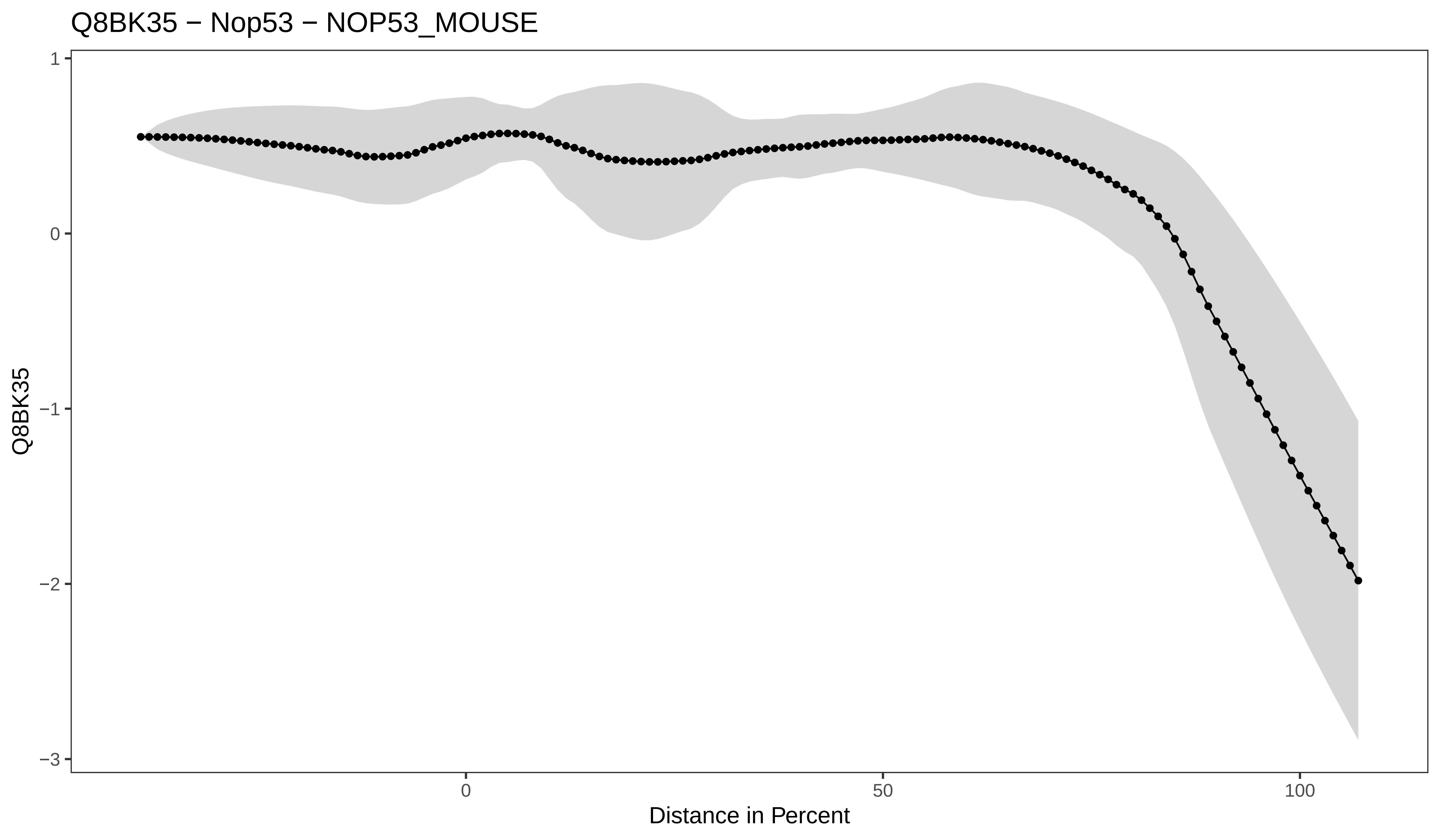

Q8BK35 − Nop53 − NOP53_MOUSE
Q8BK35
Distance in Percent
1
0
−1
−2
−3
0
50
100

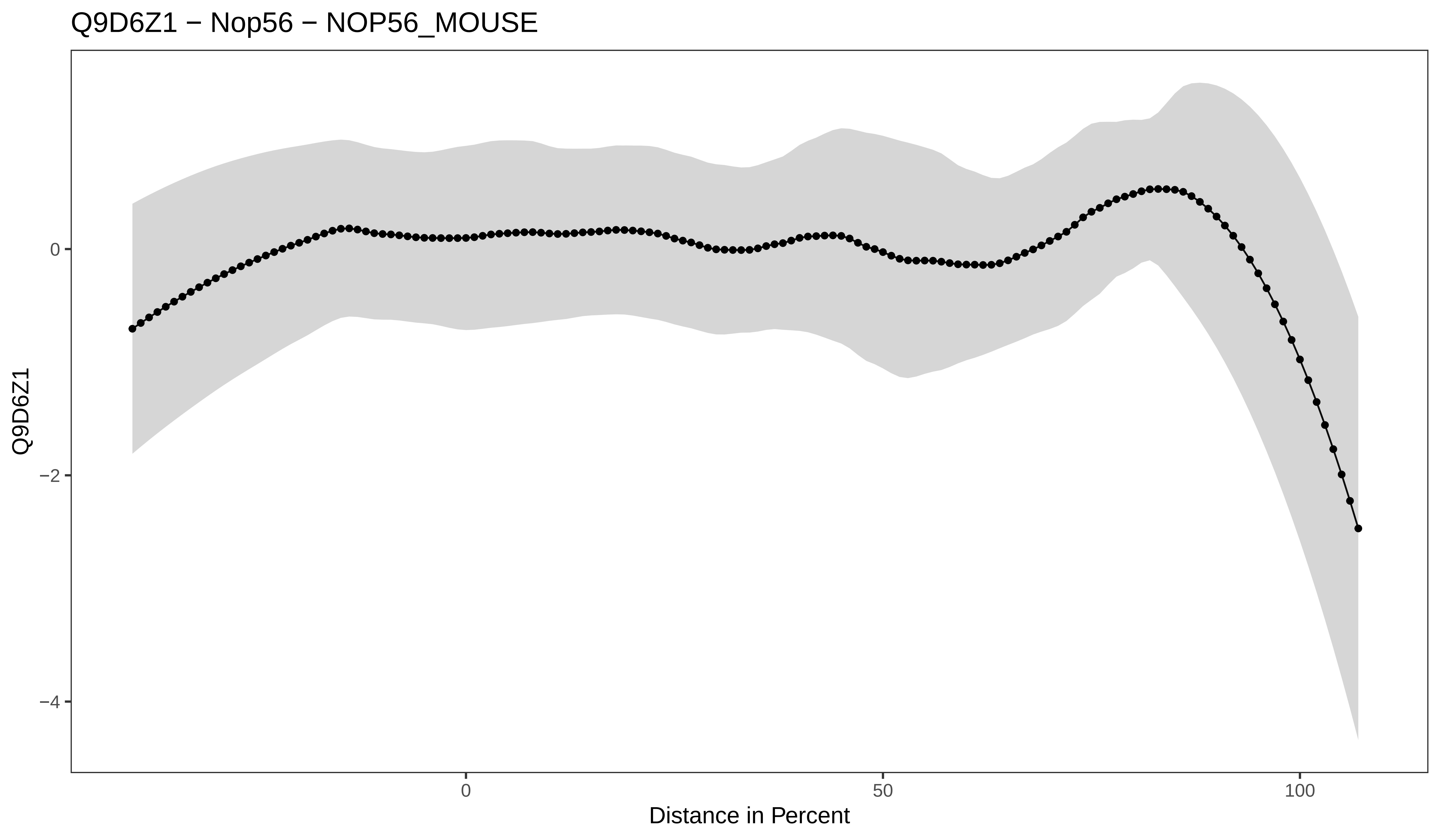
Q9D6Z1 − Nop56 − NOP56_MOUSE
Q9D6Z1
0
−2
−4
0
50
100
Distance in Percent

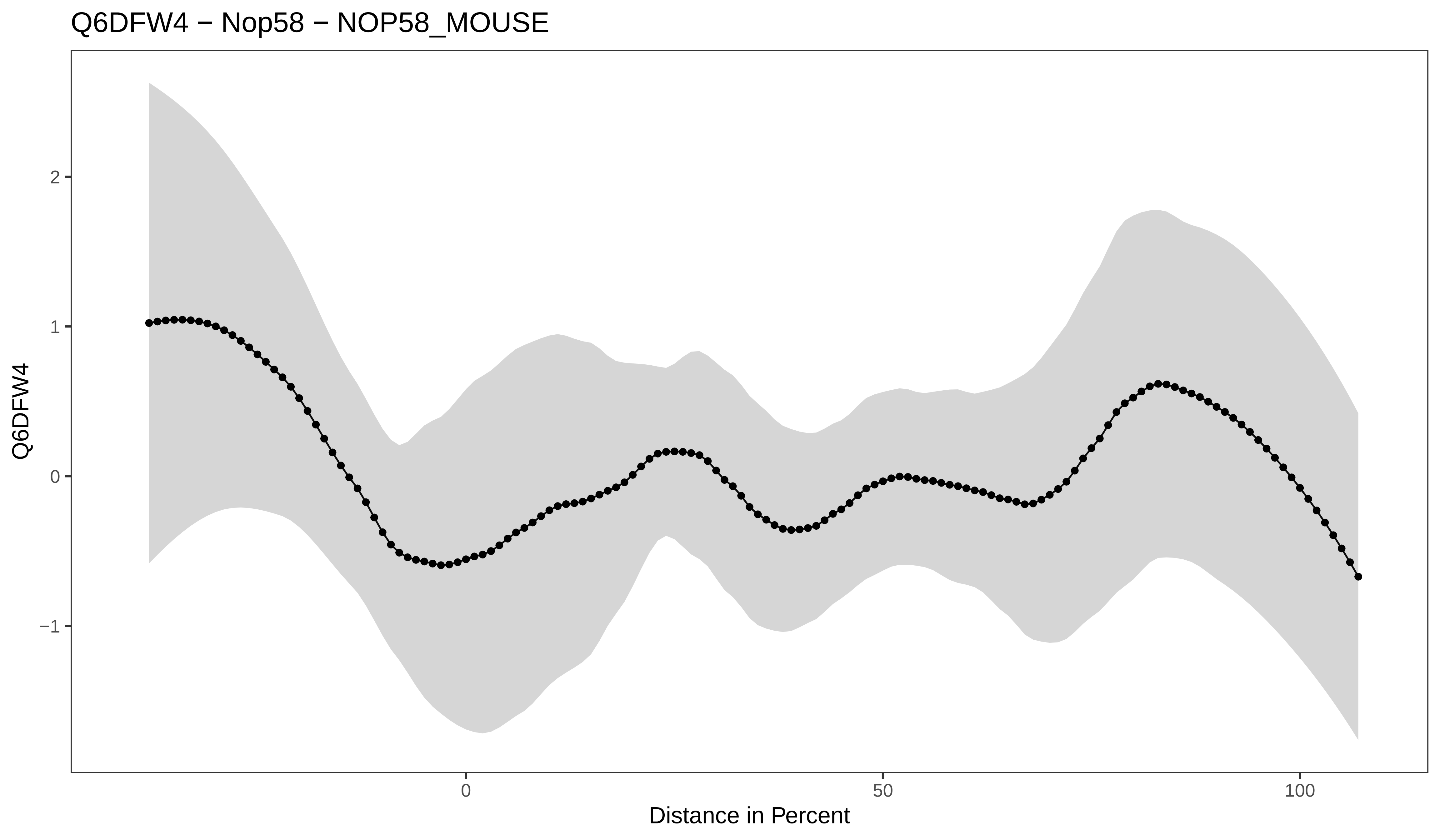

Q6DFW4 − Nop58 − NOP58_MOUSE
Q6DFW4
2
1
0
−1
0
50
100
Distance in Percent

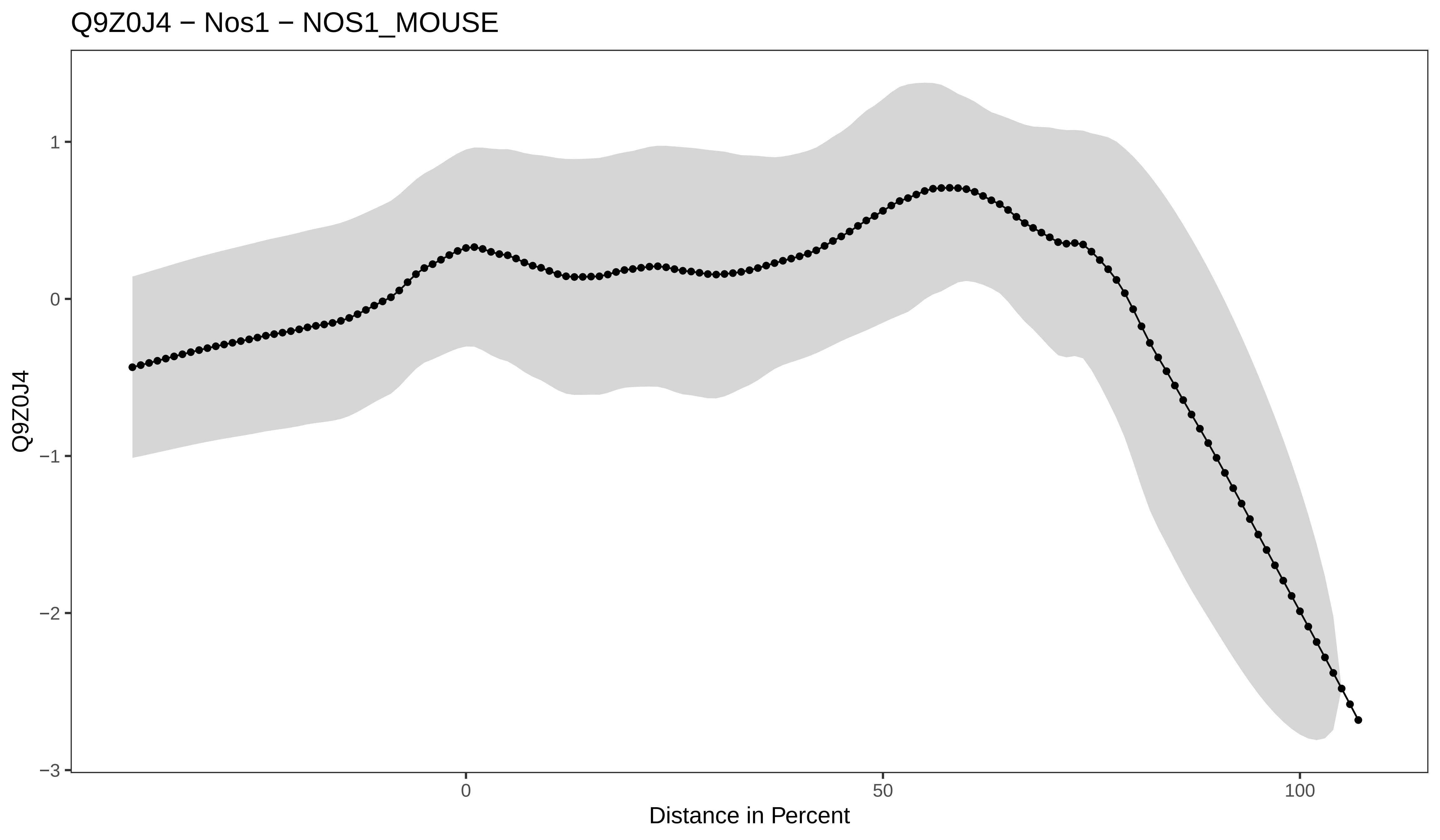
Q9Z0J4 − Nos1 − NOS1_MOUSE
Q9Z0J4
1
0
−1
−2
−3
0
50
100
Distance in Percent

Q6NSR8 − Npepl1 − PEPL1_MOUSE

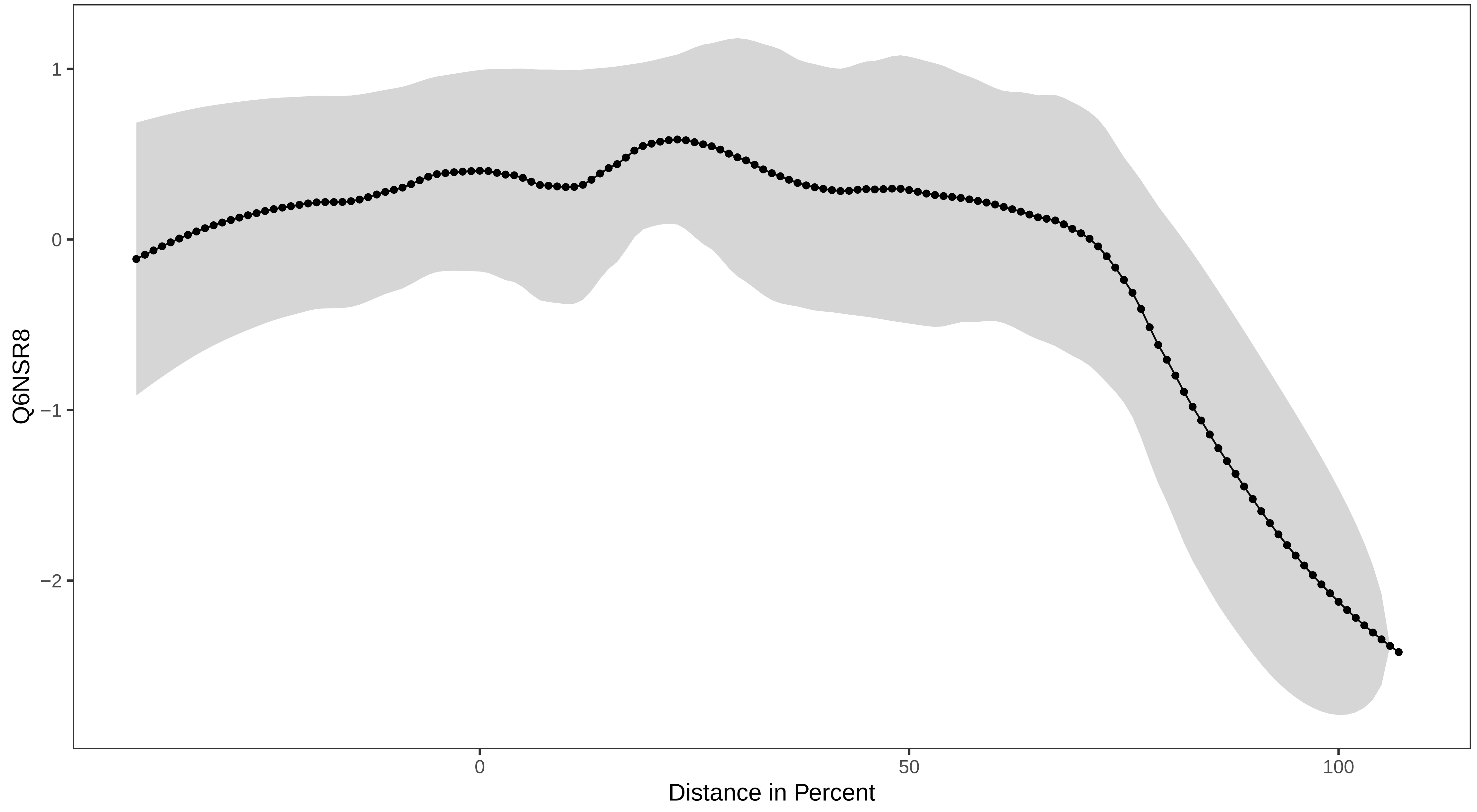

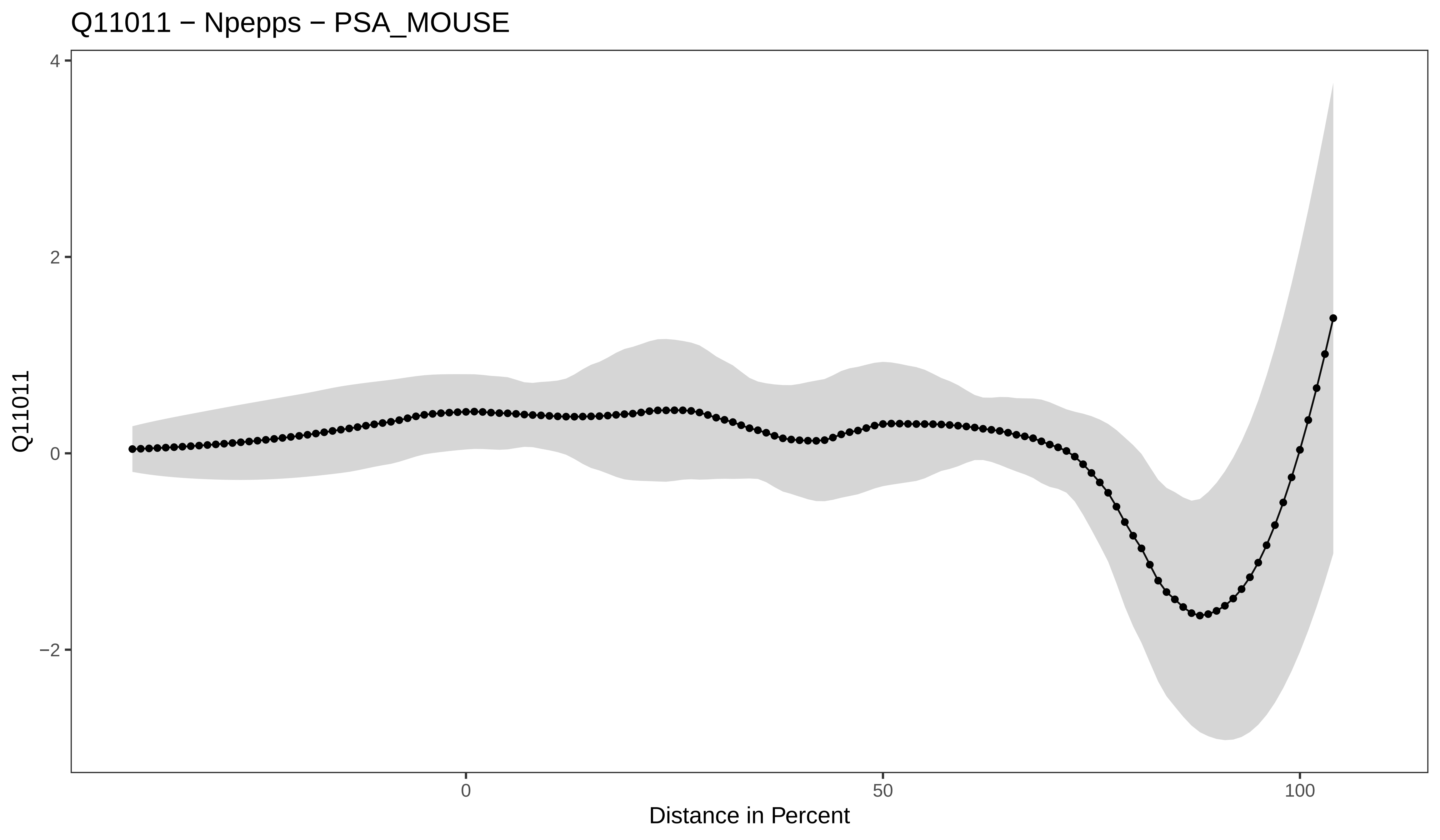

Q11011 − Npepps − PSA_MOUSE
Q11011
4
2
0
−2
0
50
100
Distance in Percent

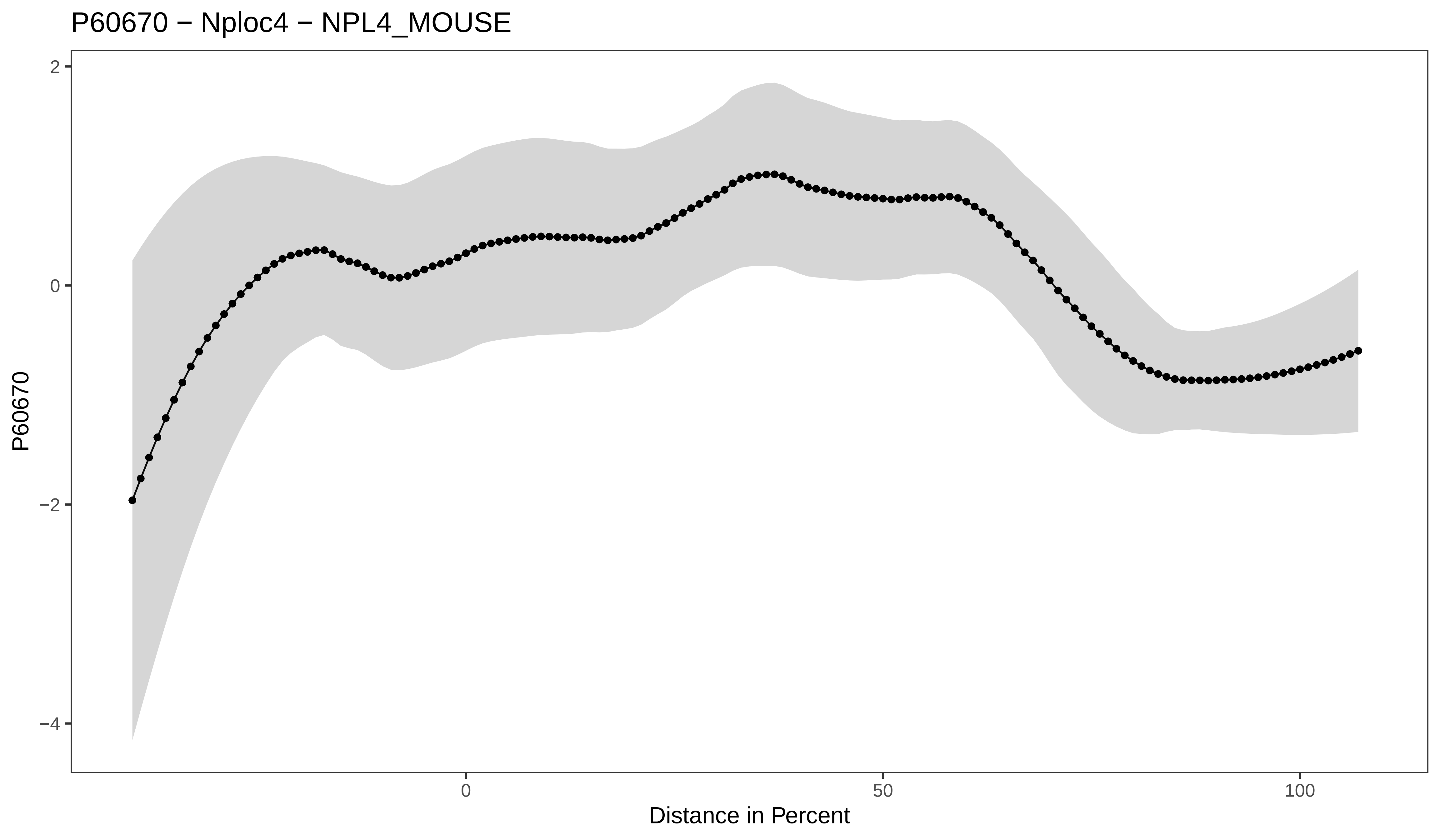

P60670 − Nploc4 − NPL4_MOUSE
P60670
2
0
−2
−4
0
50
100
Distance in Percent

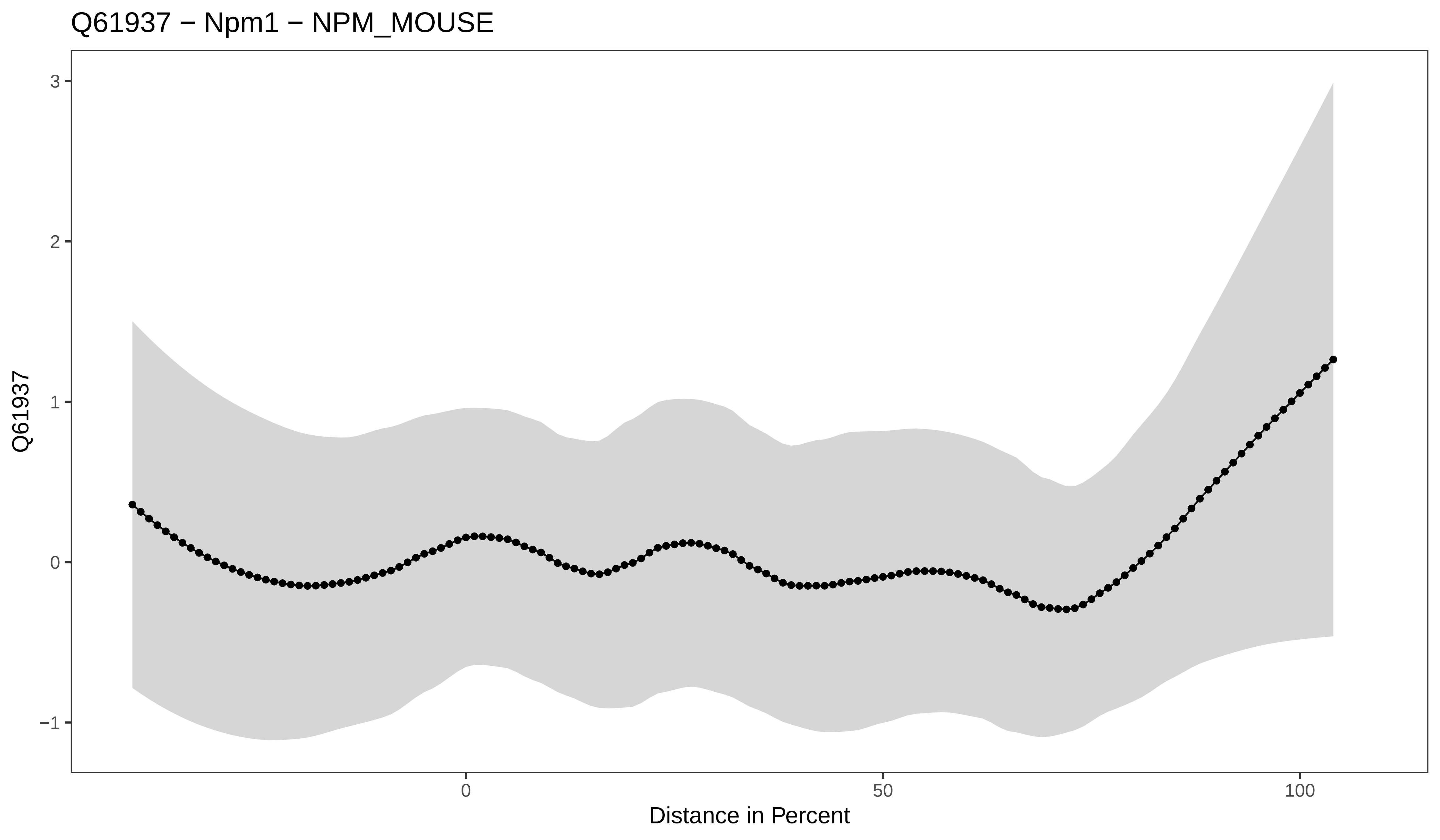

Q61937 − Npm1 − NPM_MOUSE
Q61937
Distance in Percent
3
2
1
0
−1
0
50
100

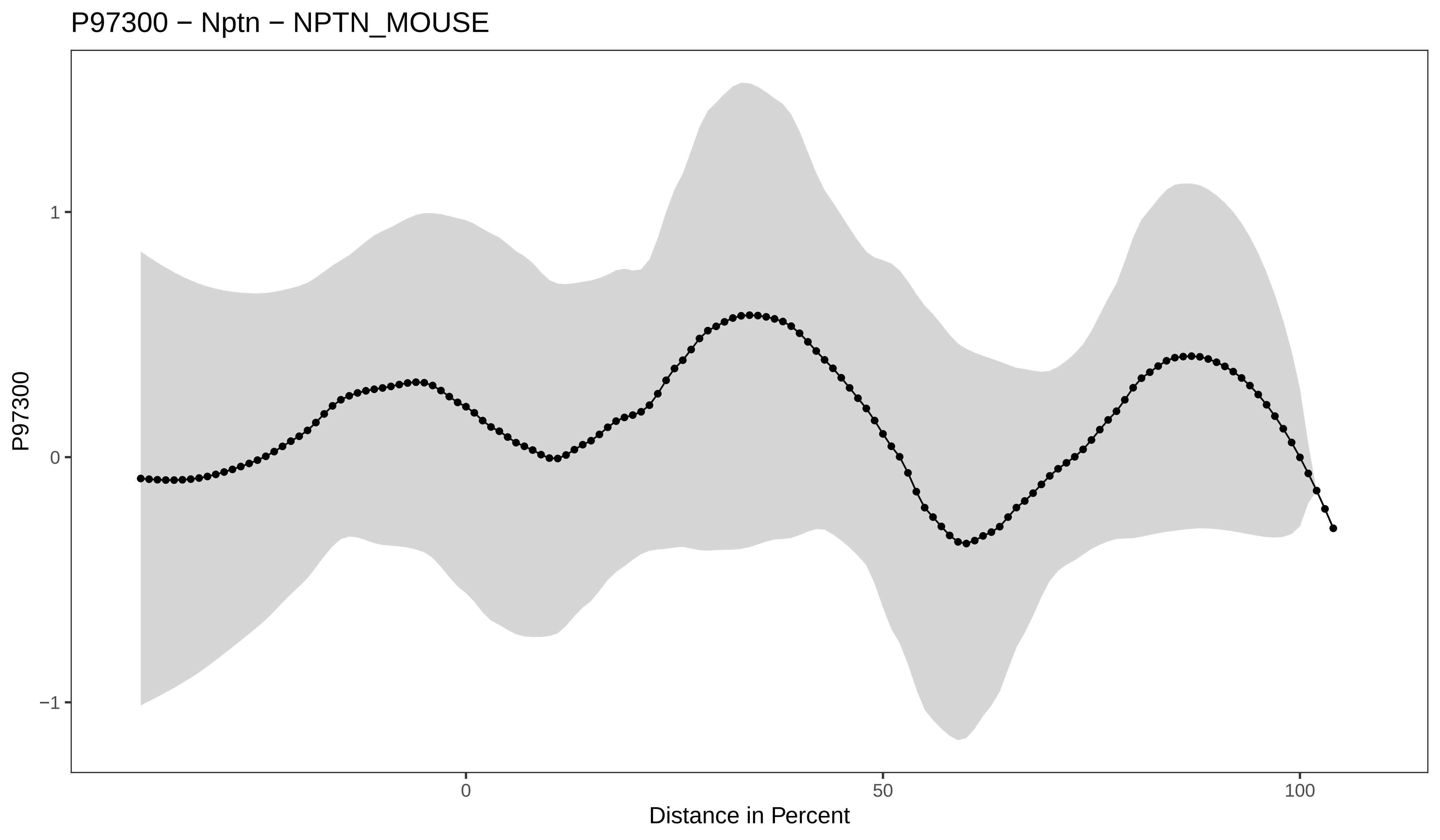

P97300 − Nptn − NPTN_MOUSE
P97300
1
0
−1
0
50
100
Distance in Percent

Q64669 − Nqo1 − NQO1_MOUSE

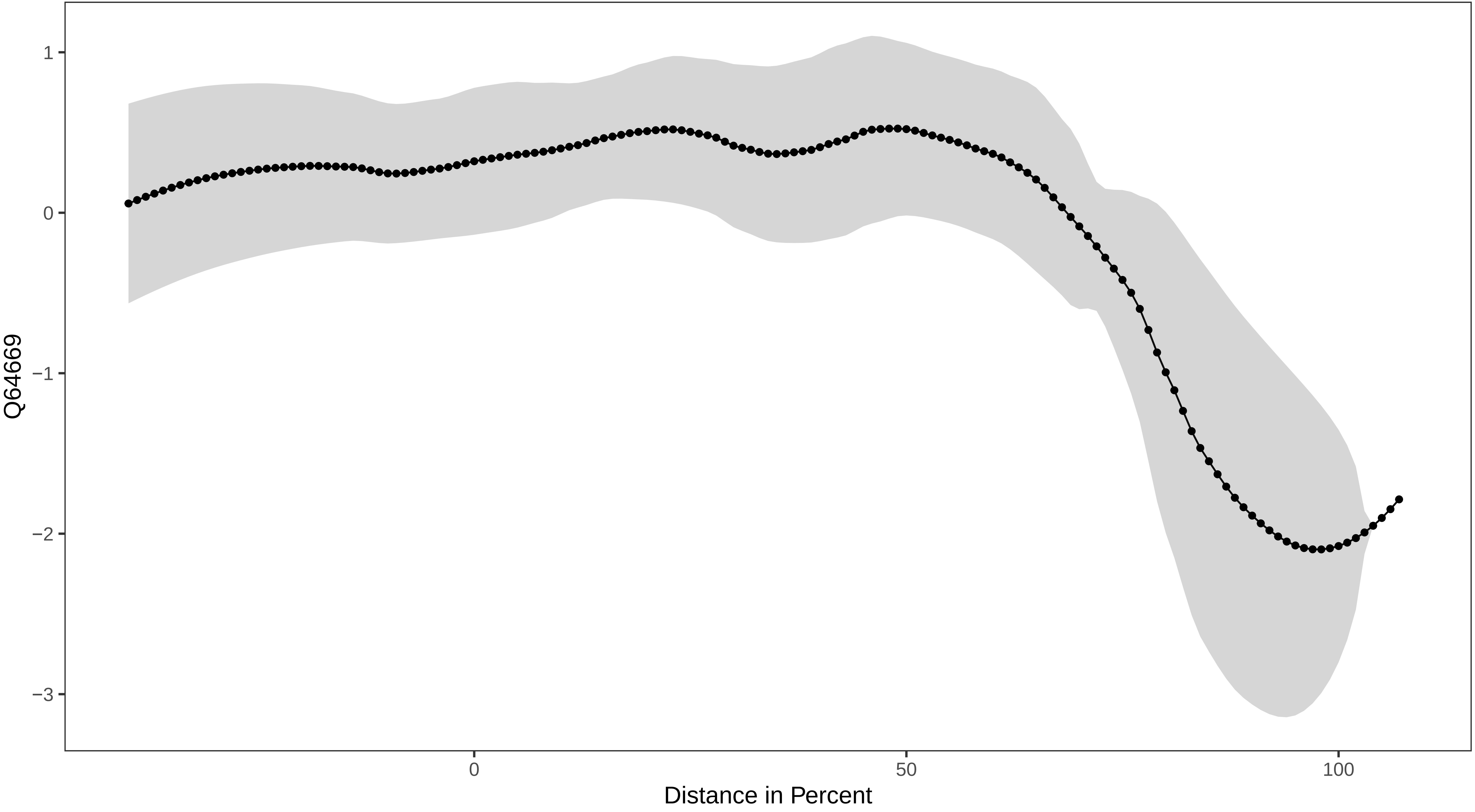

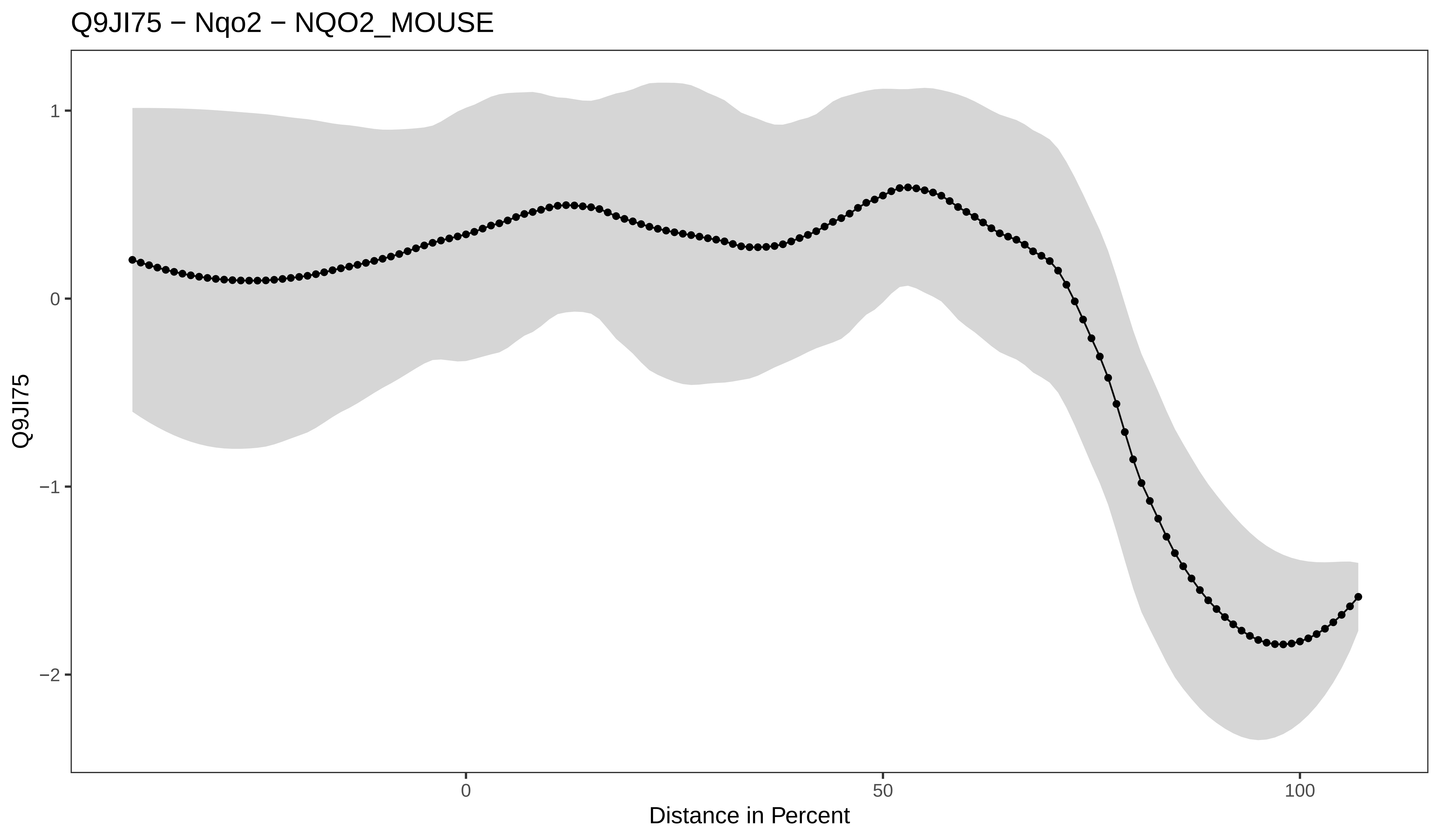
Q9JI75 − Nqo2 − NQO2_MOUSE
1
0
−1
−2
Q9JI75
0
50
100
Distance in Percent

Q80XB4 − Nrap − NRAP_MOUSE

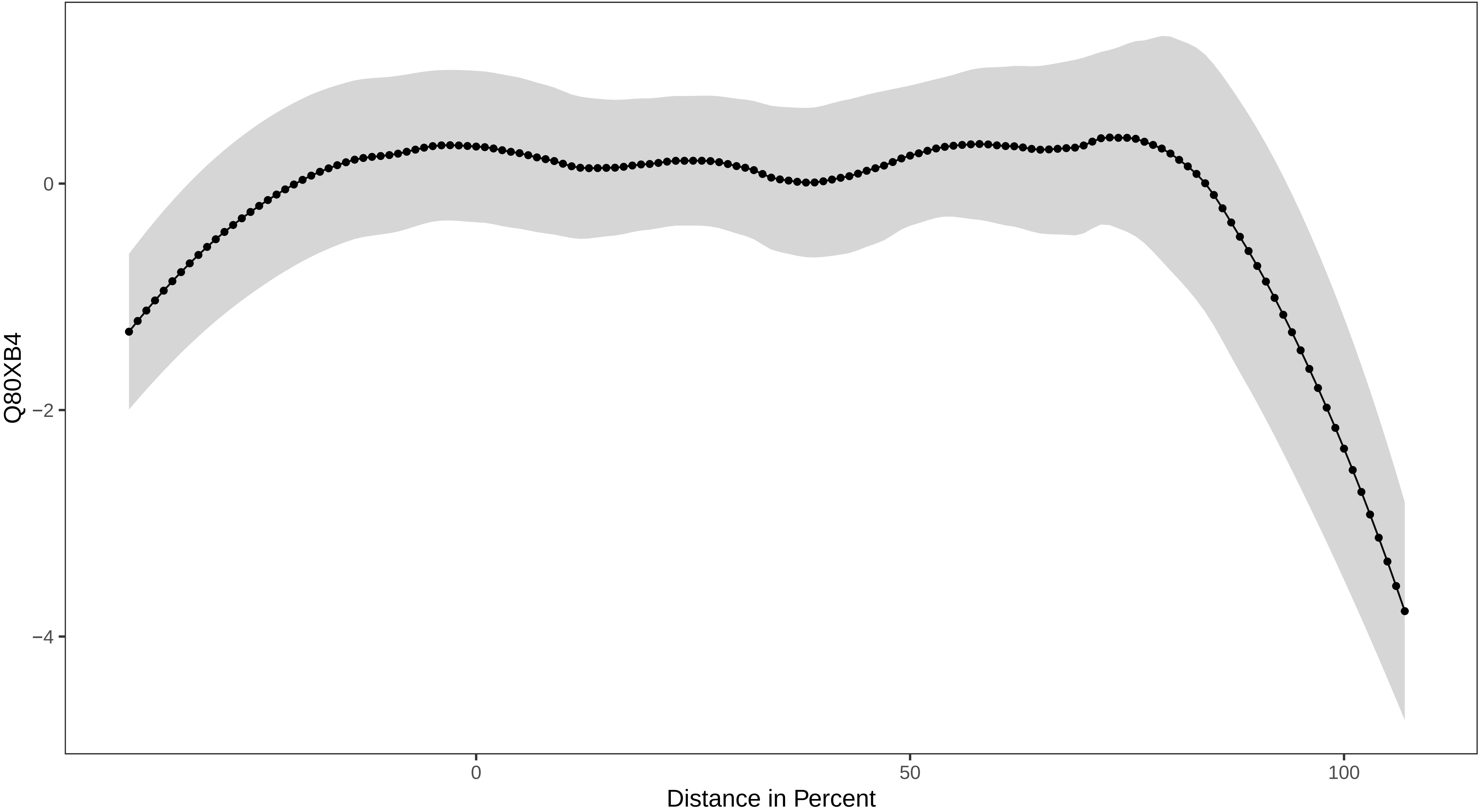

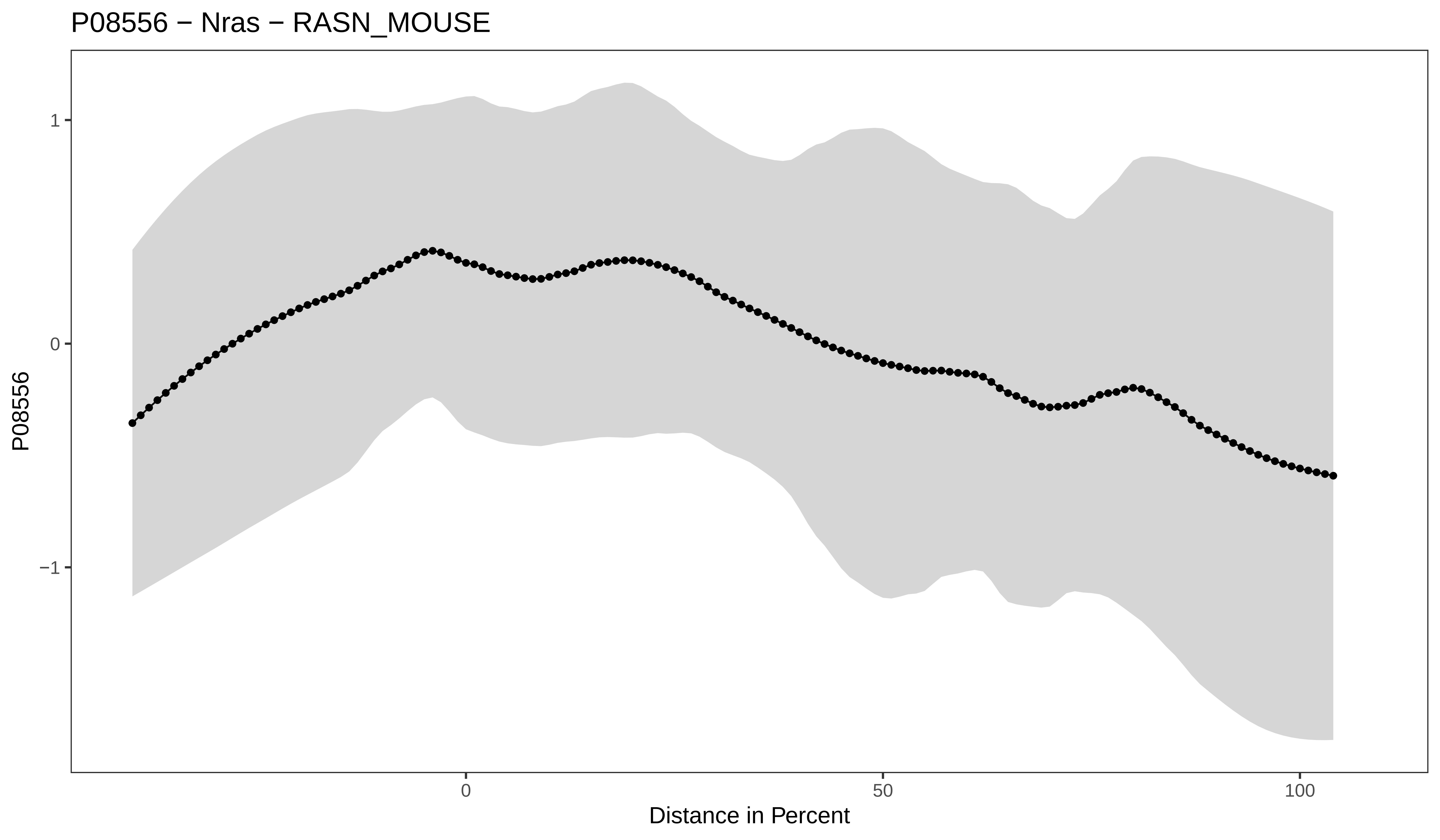

P08556 − Nras − RASN_MOUSE
1
0
−1
P08556
0
50
100
Distance in Percent

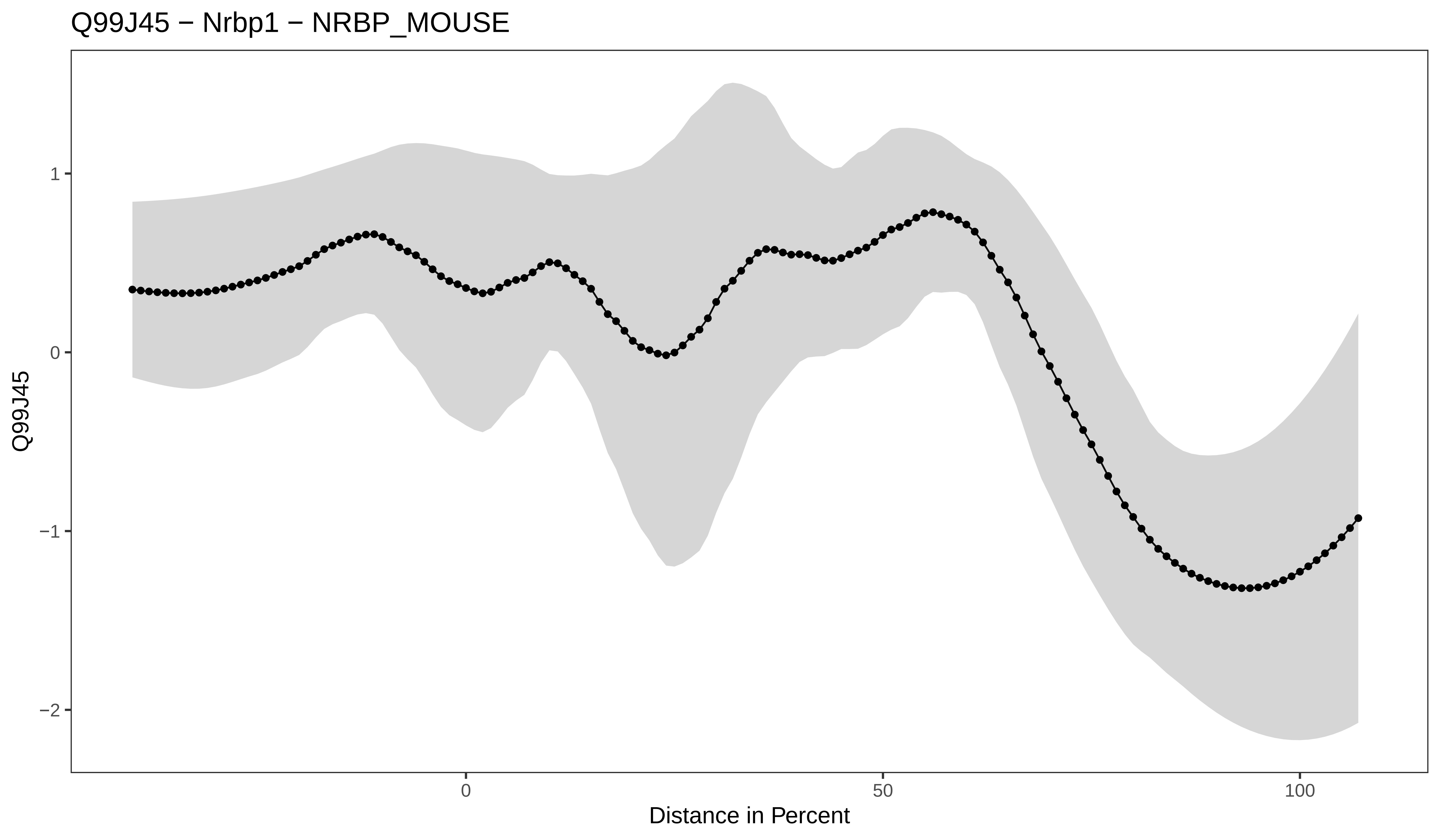

Q99J45 − Nrbp1 − NRBP_MOUSE
Q99J45
1
0
−1
−2
0
50
100
Distance in Percent

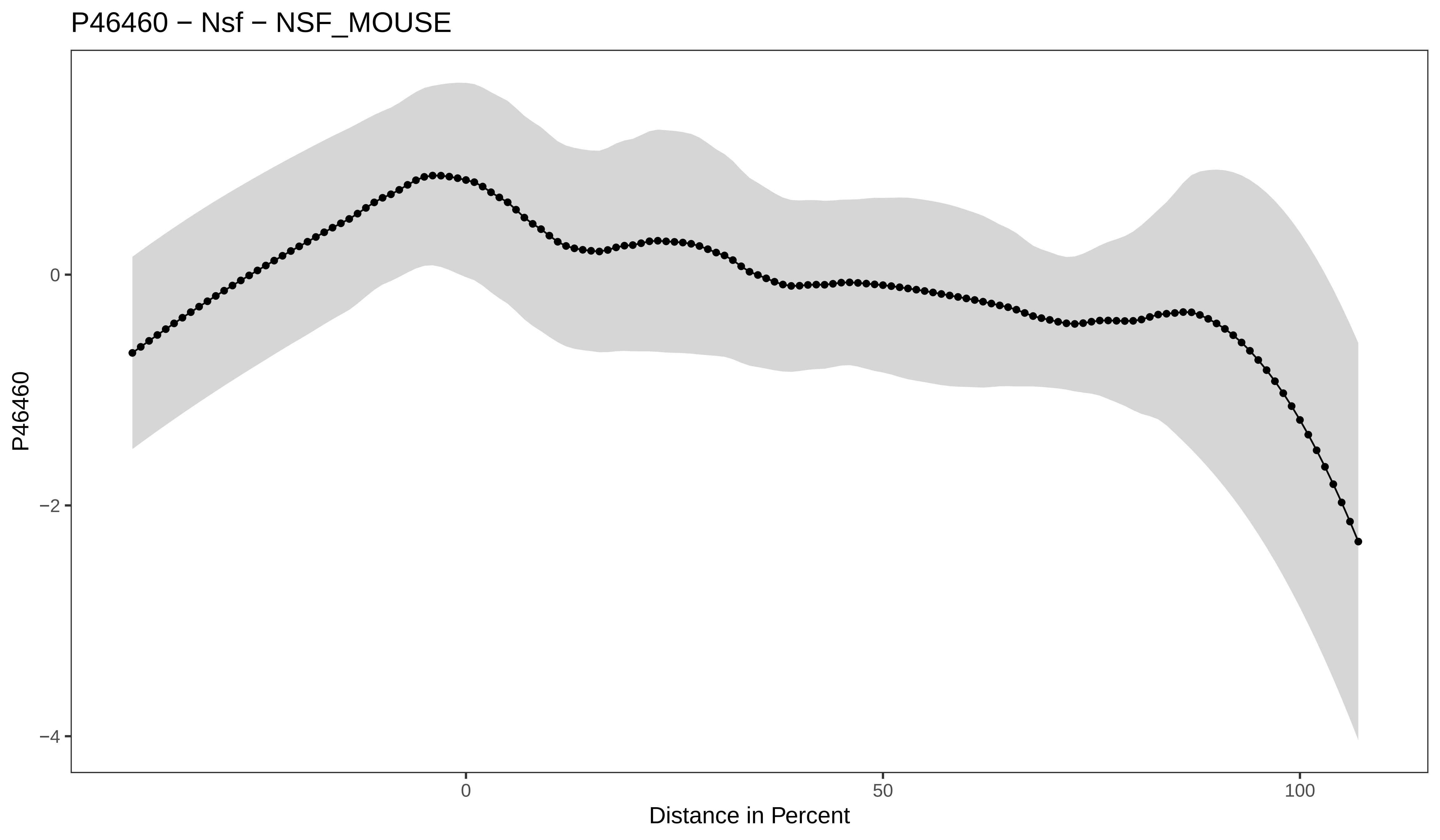

P46460 − Nsf − NSF_MOUSE
0
−2
−4
P46460
0
50
100
Distance in Percent

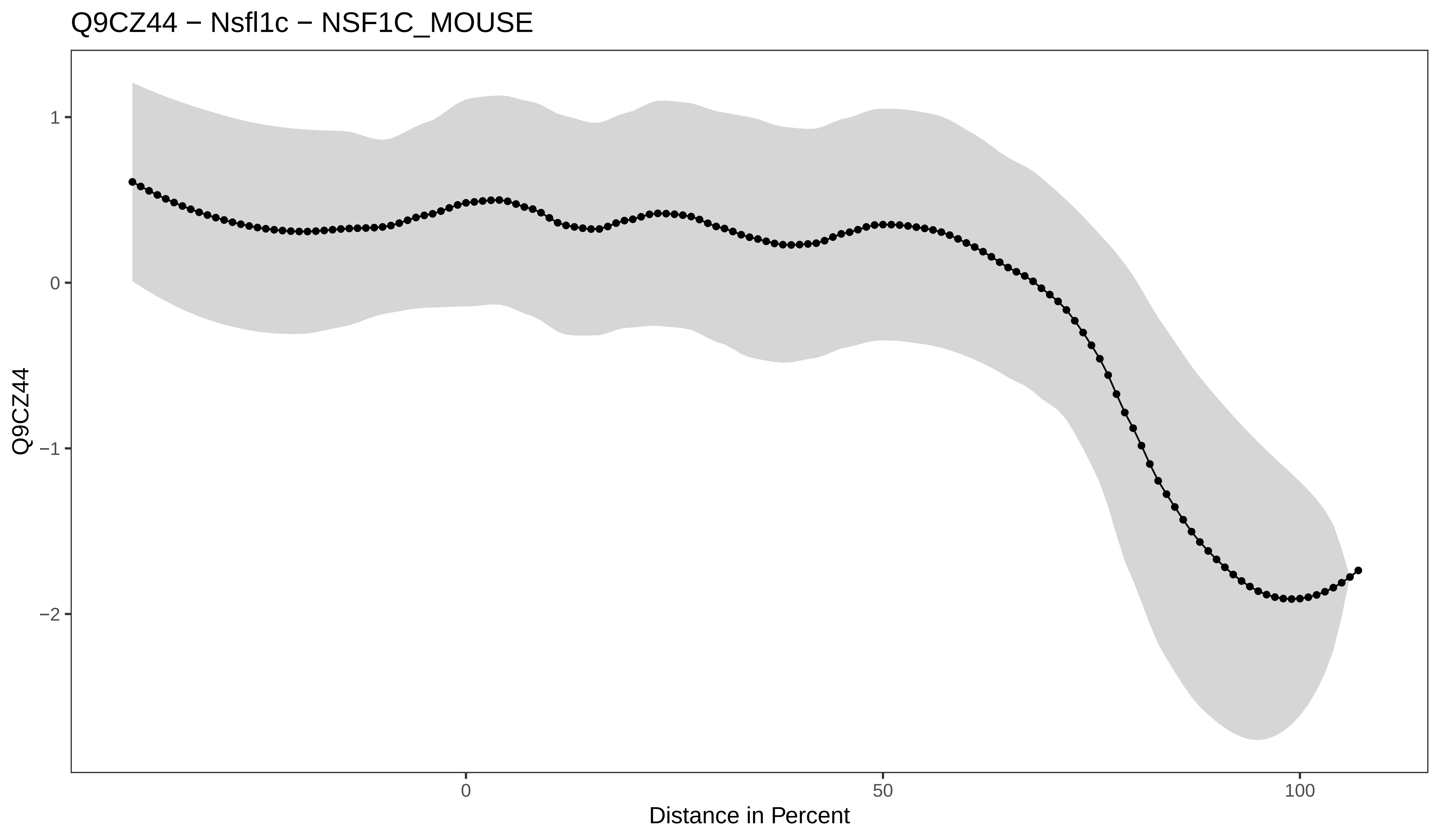

Q9CZ44 − Nsfl1c − NSF1C_MOUSE
1
0
−1
−2
Q9CZ44
0
50
100
Distance in Percent

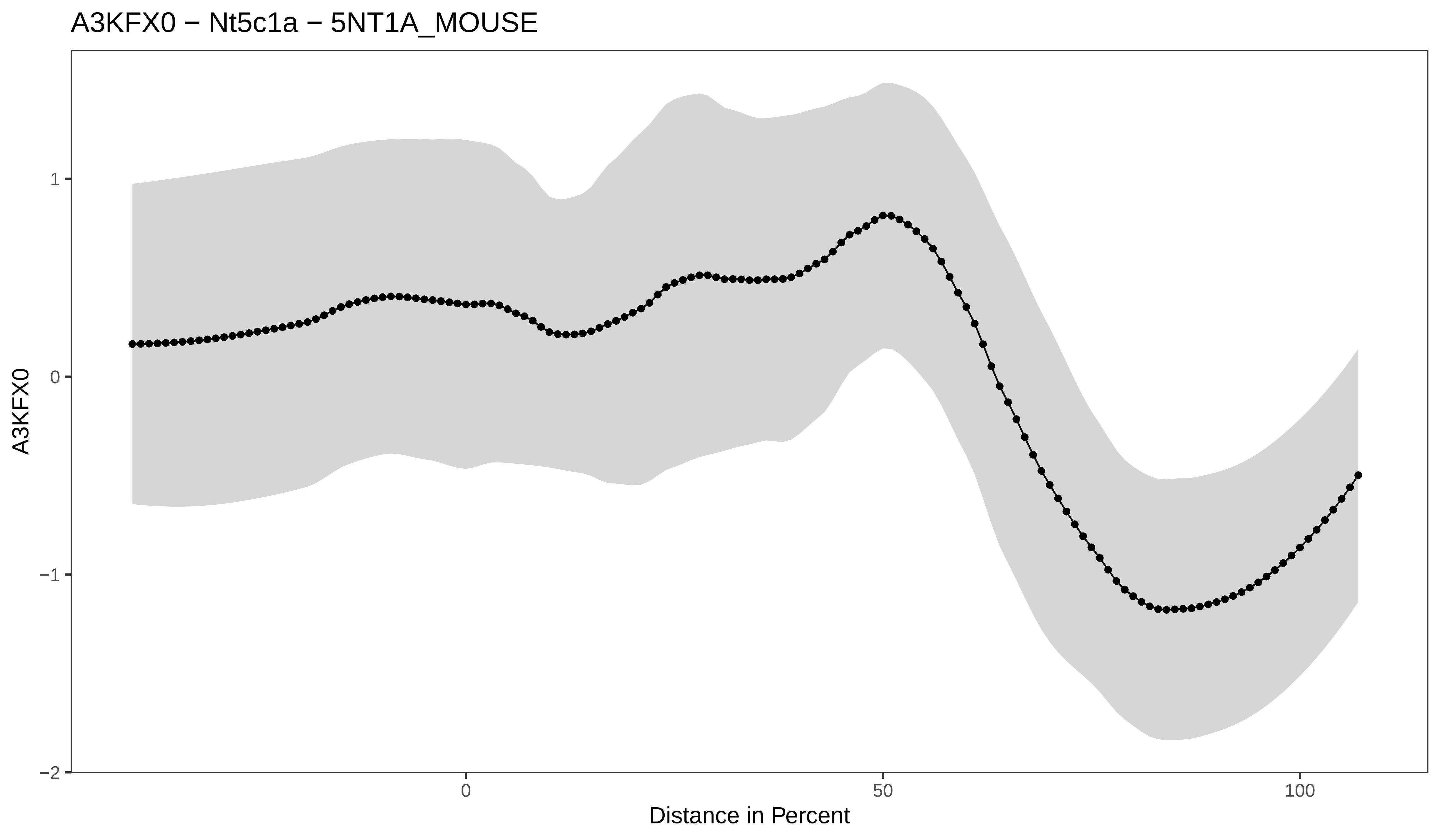
A3KFX0 − Nt5c1a − 5NT1A_MOUSE
A3KFX0
1
0
−1
−2
0
50
100
Distance in Percent

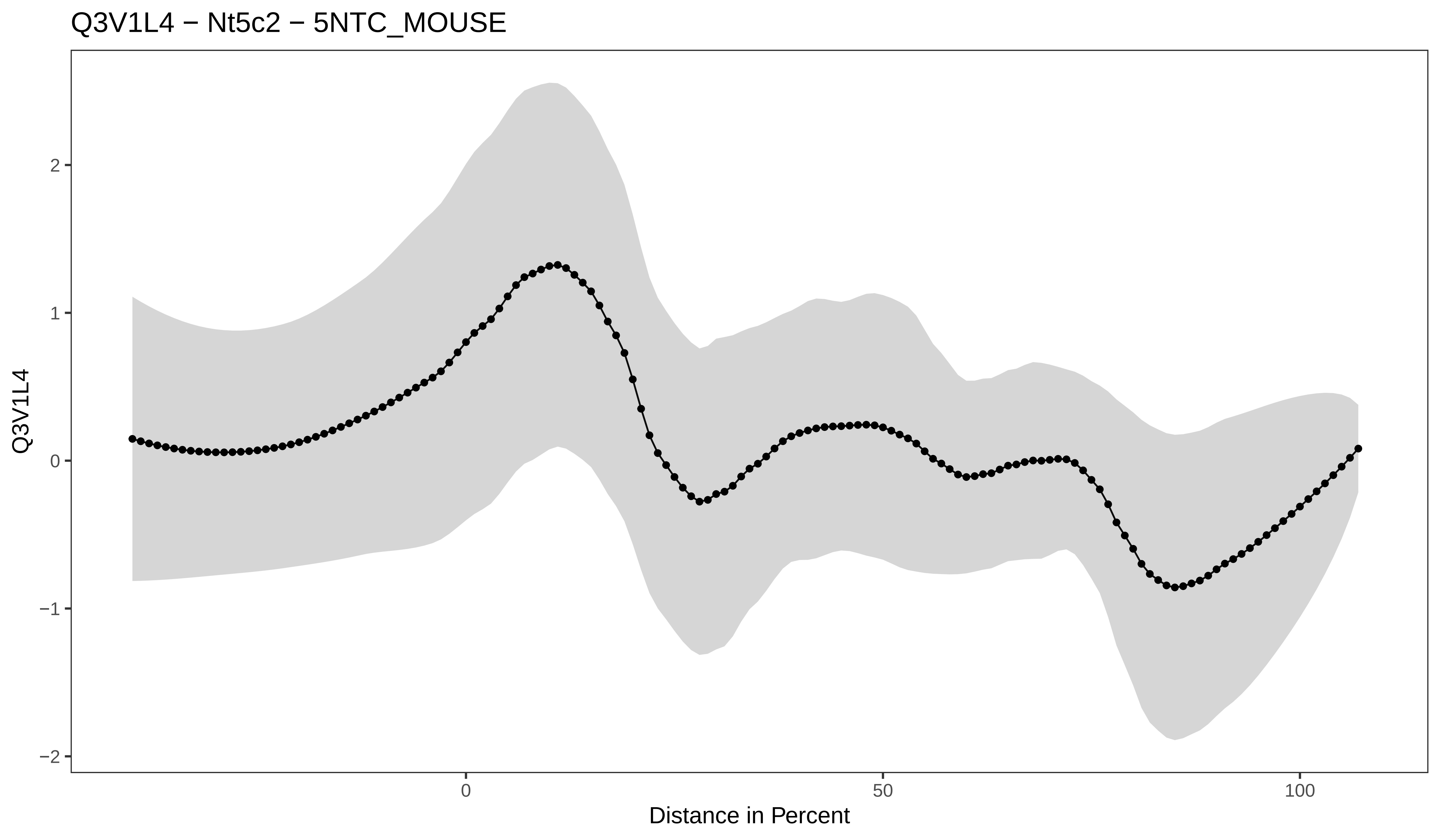

Q3V1L4 − Nt5c2 − 5NTC_MOUSE
Q3V1L4
Distance in Percent
2
1
0
−1
−2
0
50
100

Q9D020 − Nt5c3a − 5NT3A_MOUSE

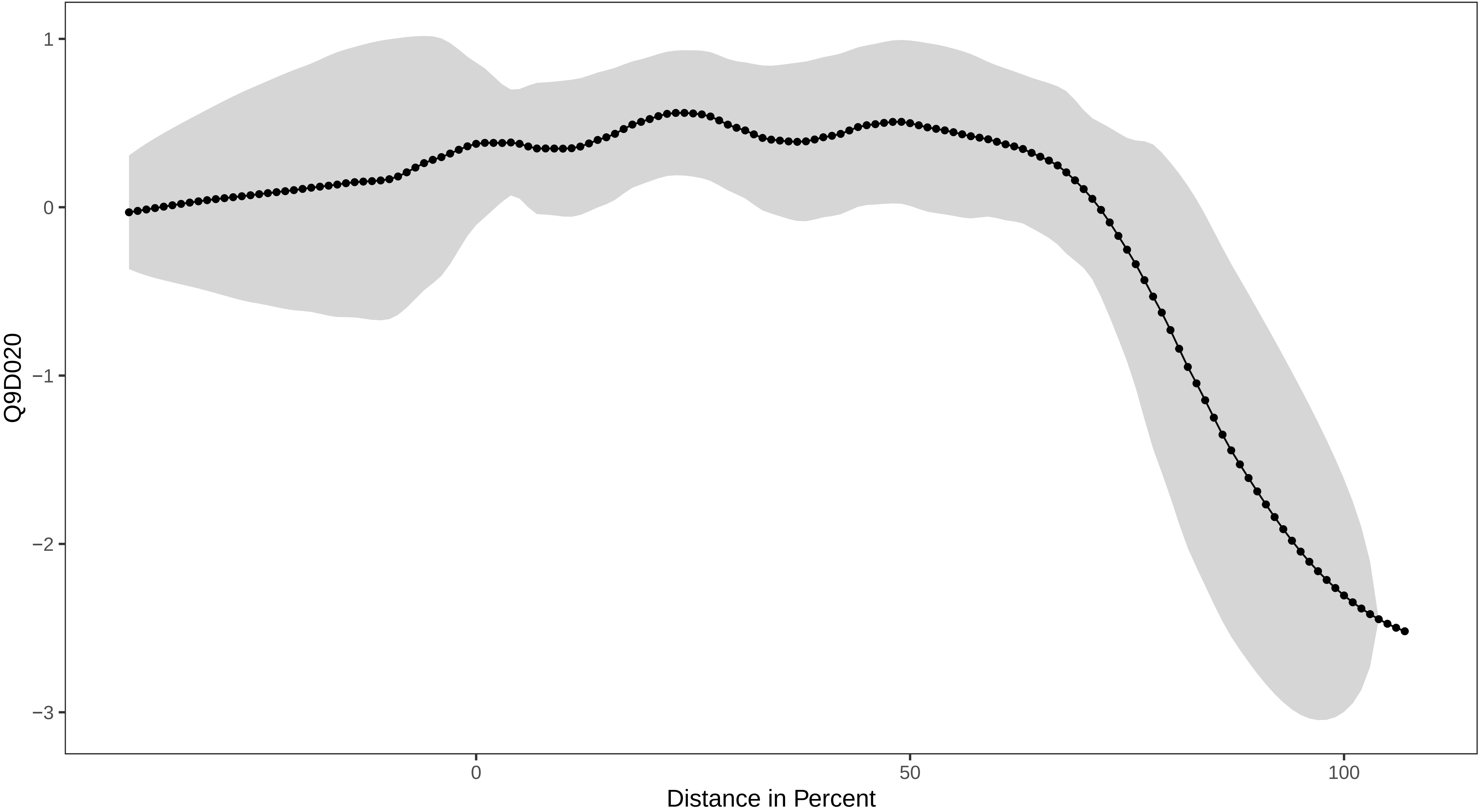

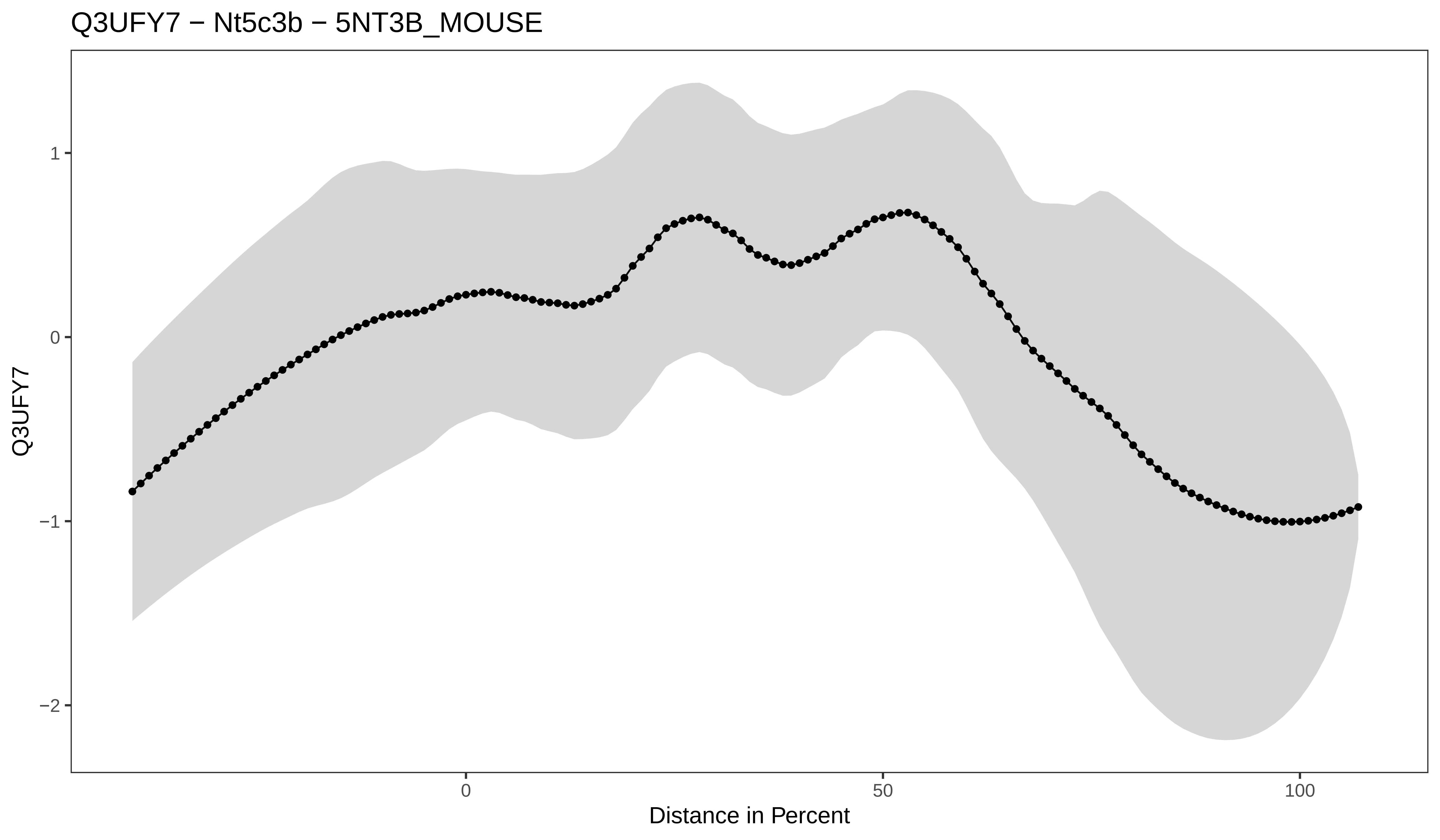

Q3UFY7 − Nt5c3b − 5NT3B_MOUSE
Q3UFY7
Distance in Percent
1
0
−1
−2
0
50
100

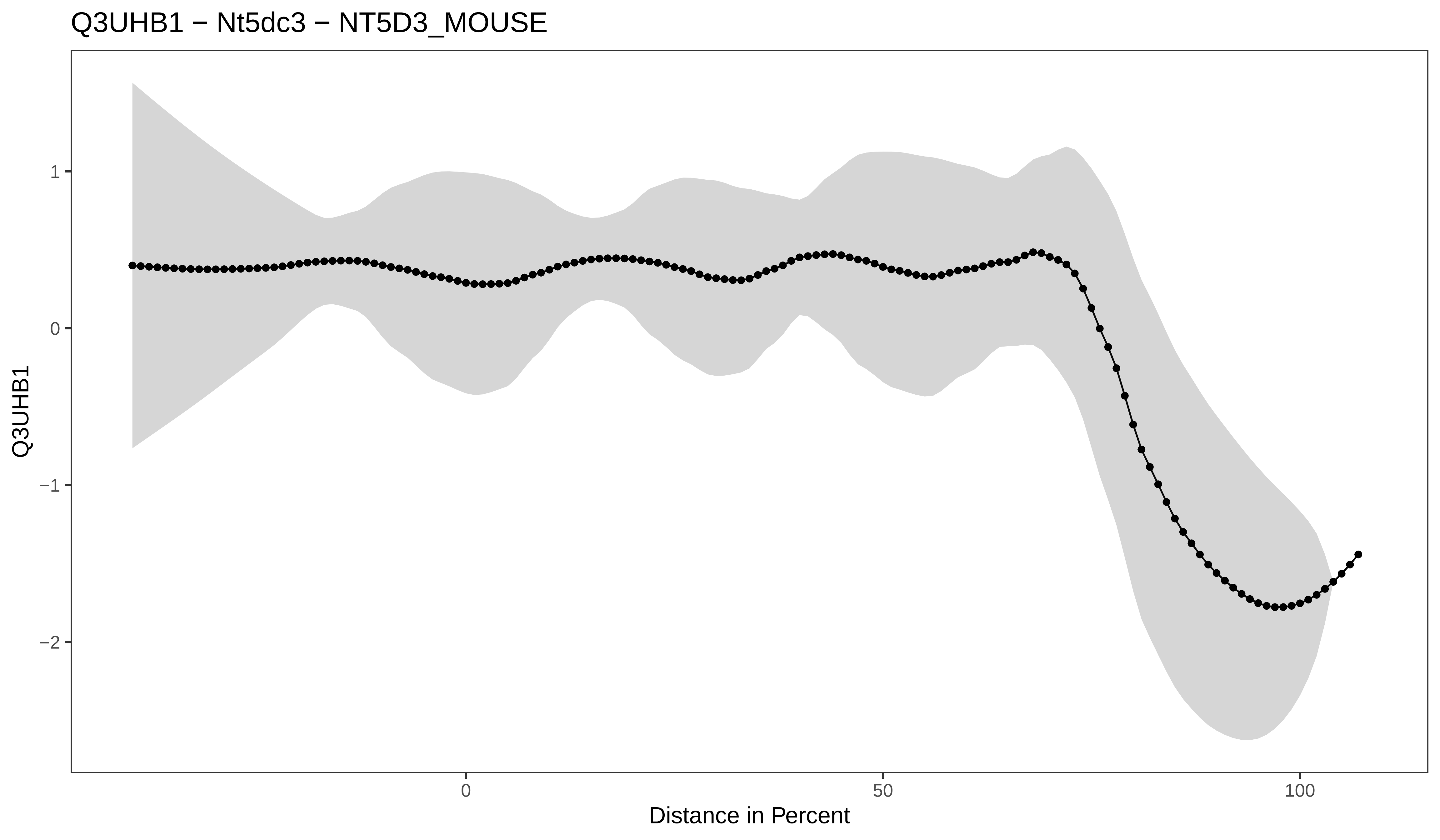

Q3UHB1 − Nt5dc3 − NT5D3_MOUSE
Q3UHB1
1
0
−1
−2
0
50
100
Distance in Percent

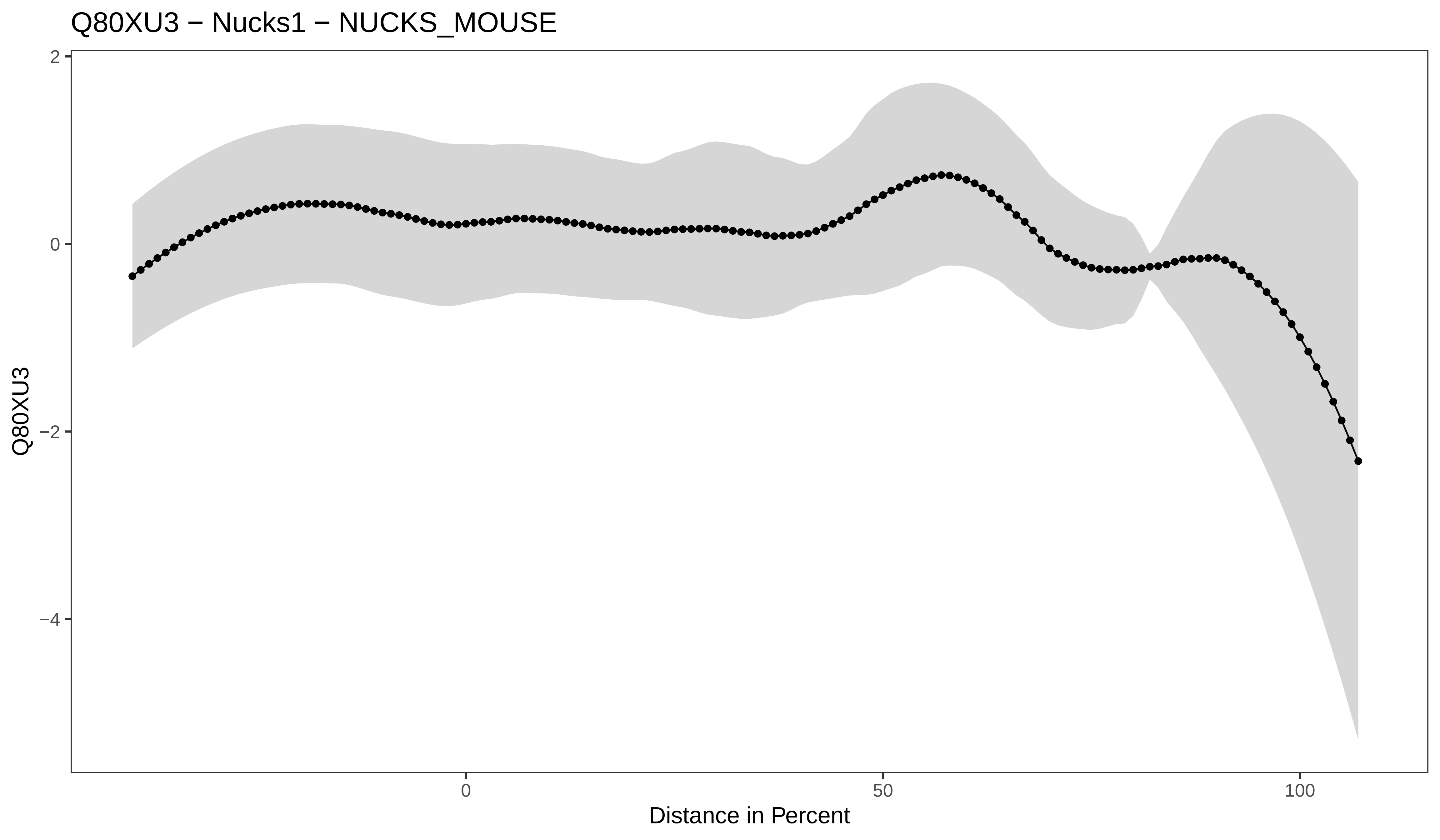

Q80XU3 − Nucks1 − NUCKS_MOUSE
Q80XU3
2
0
−2
−4
0
50
100
Distance in Percent

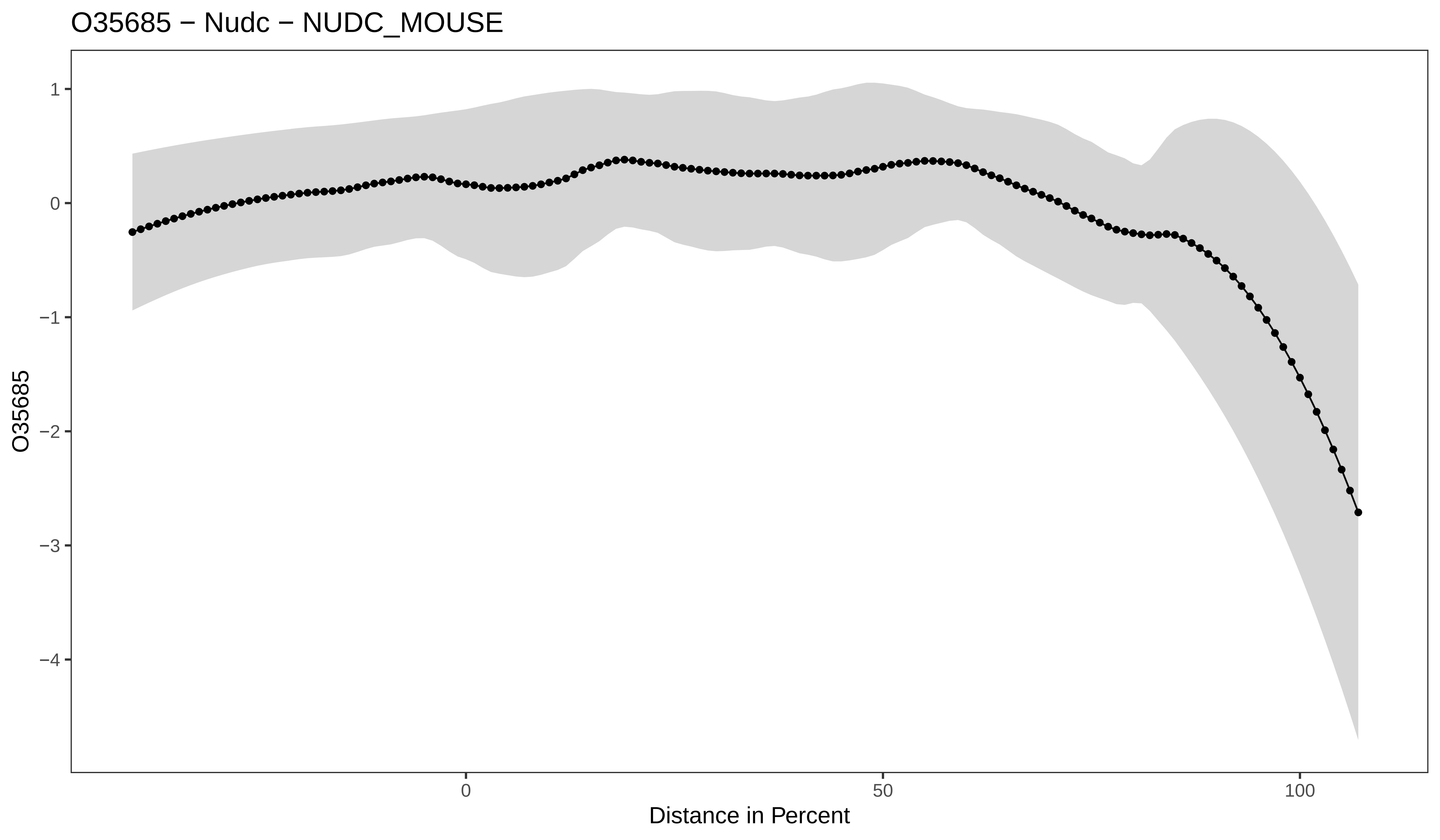

O35685 − Nudc − NUDC_MOUSE
1
0
−1
−2
−3
−4
O35685
0
50
100
Distance in Percent

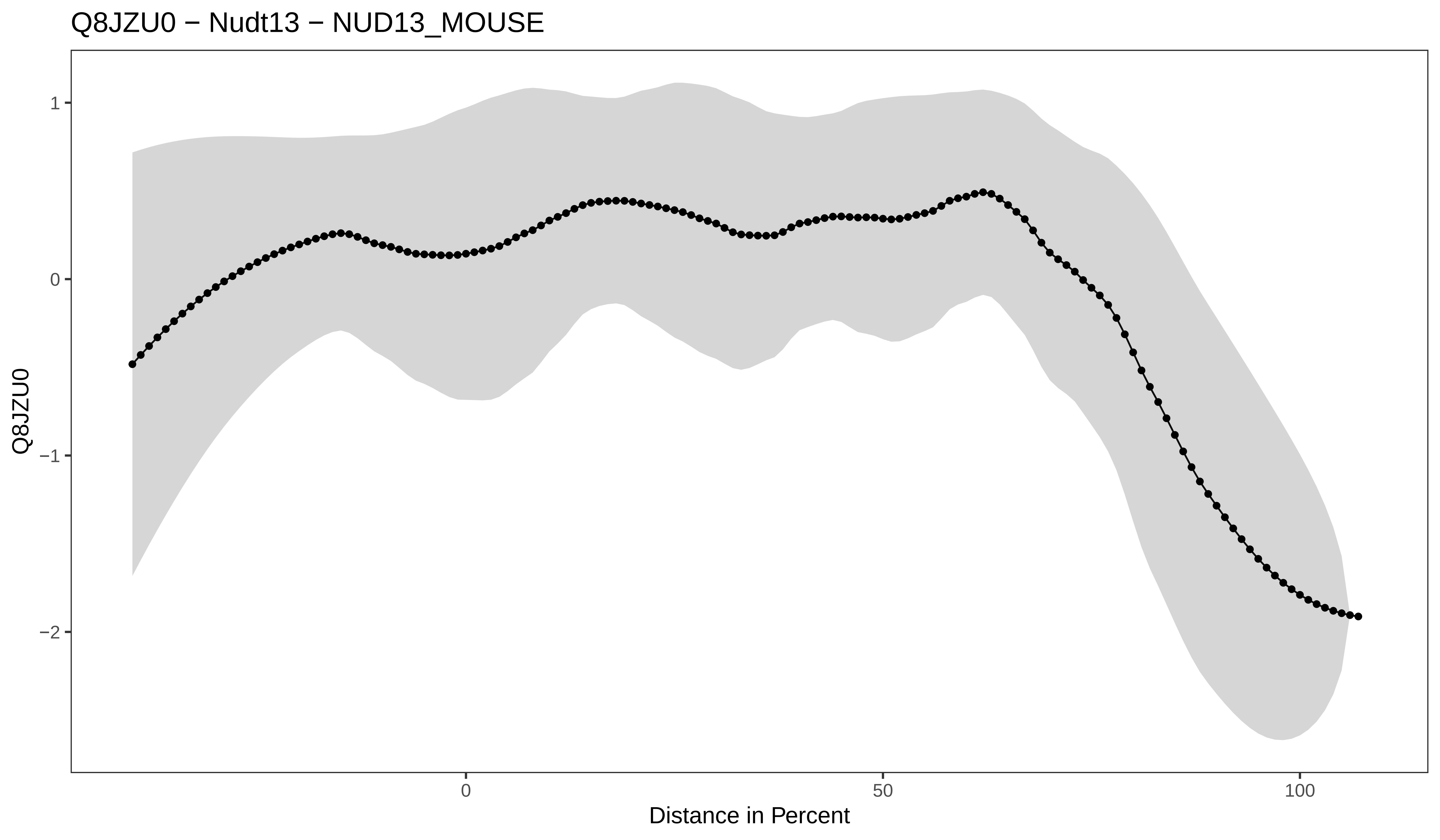

Q8JZU0 − Nudt13 − NUD13_MOUSE
1
0
−1
−2
Q8JZU0
0
50
100
Distance in Percent

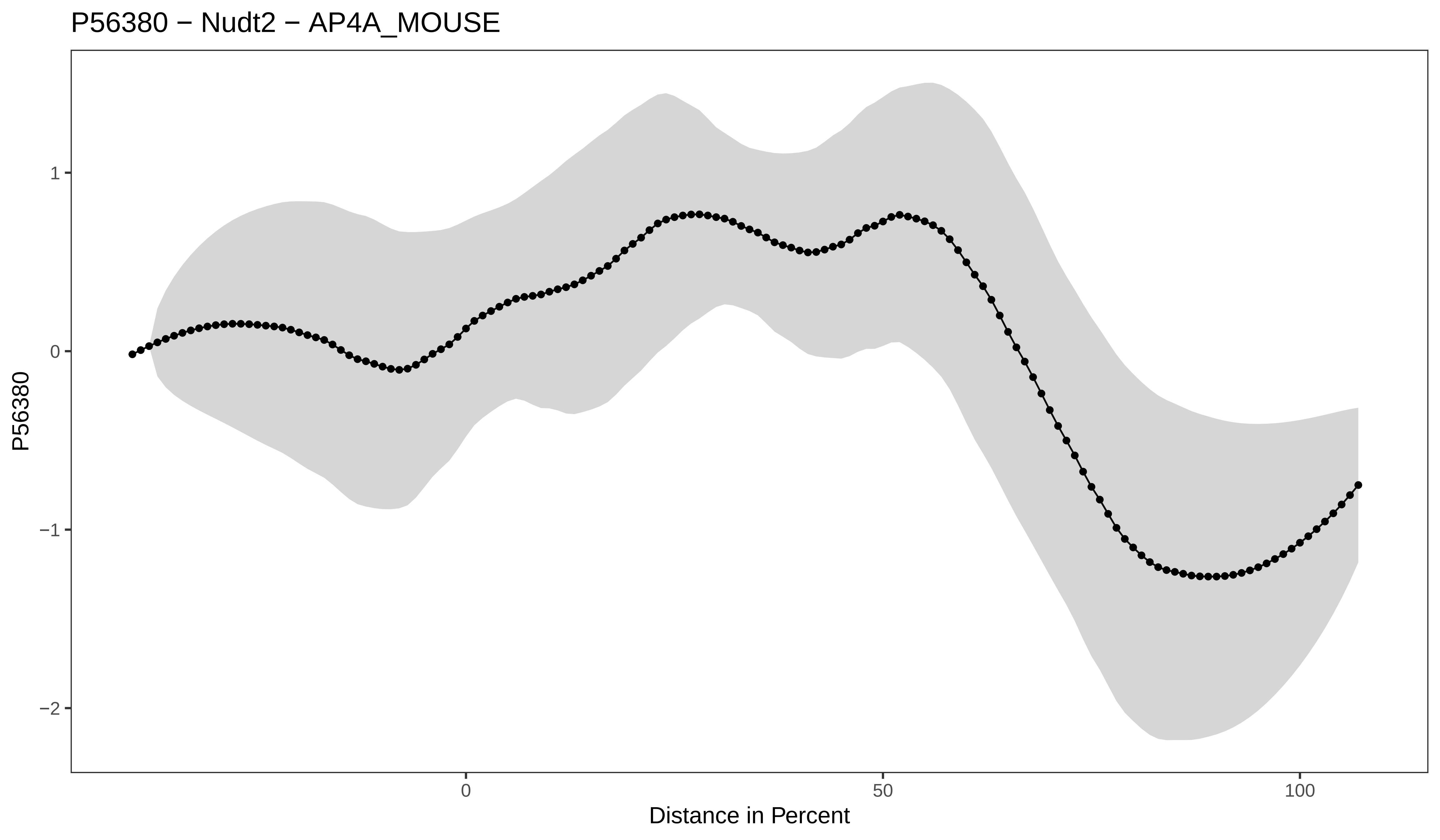

P56380 − Nudt2 − AP4A_MOUSE
1
0
−1
−2
P56380
0
50
100
Distance in Percent

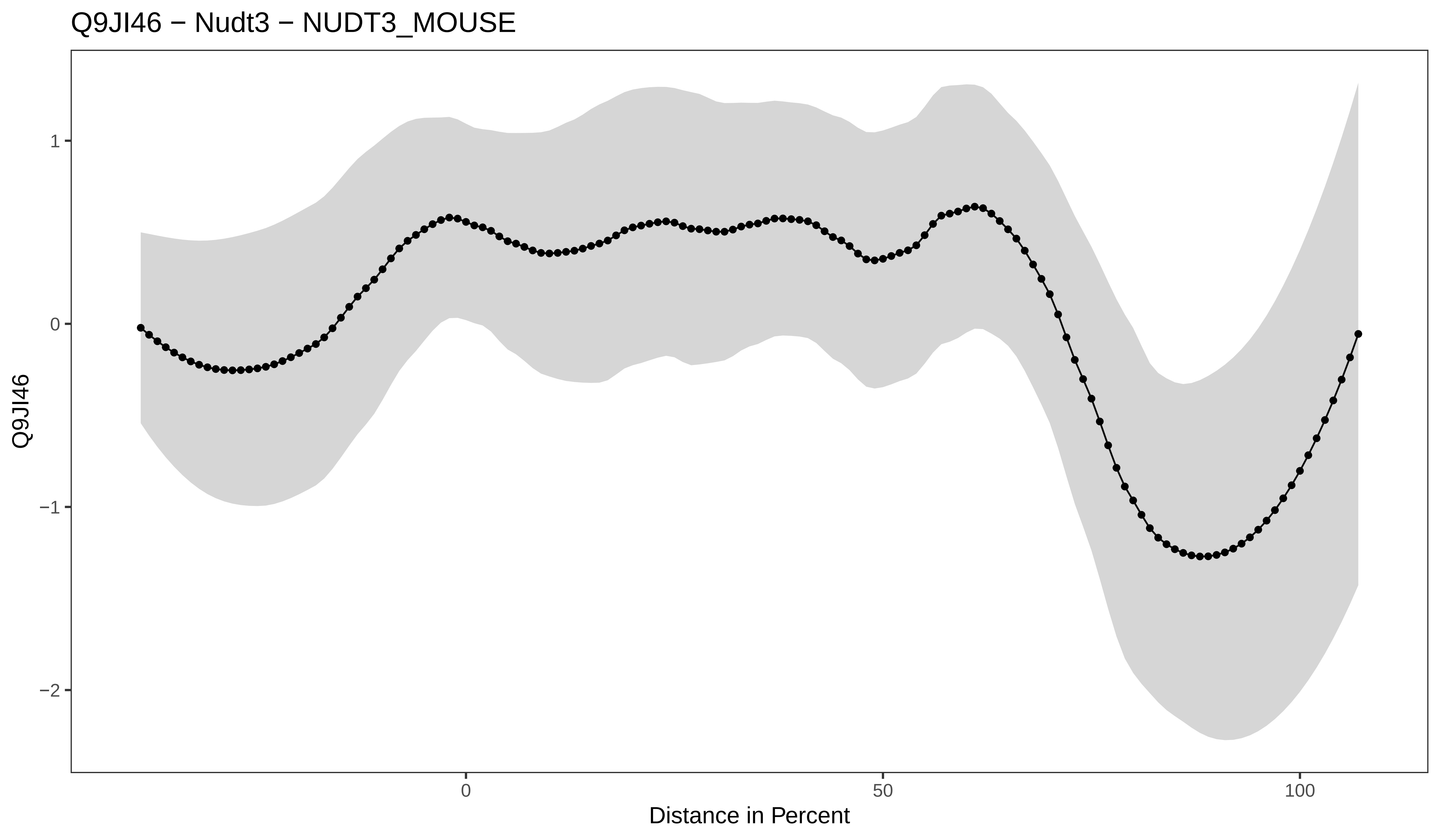

Q9JI46 − Nudt3 − NUDT3_MOUSE
1
0
−1
−2
Q9JI46
0
50
100
Distance in Percent

Q9JKX6 − Nudt5 − NUDT5_MOUSE

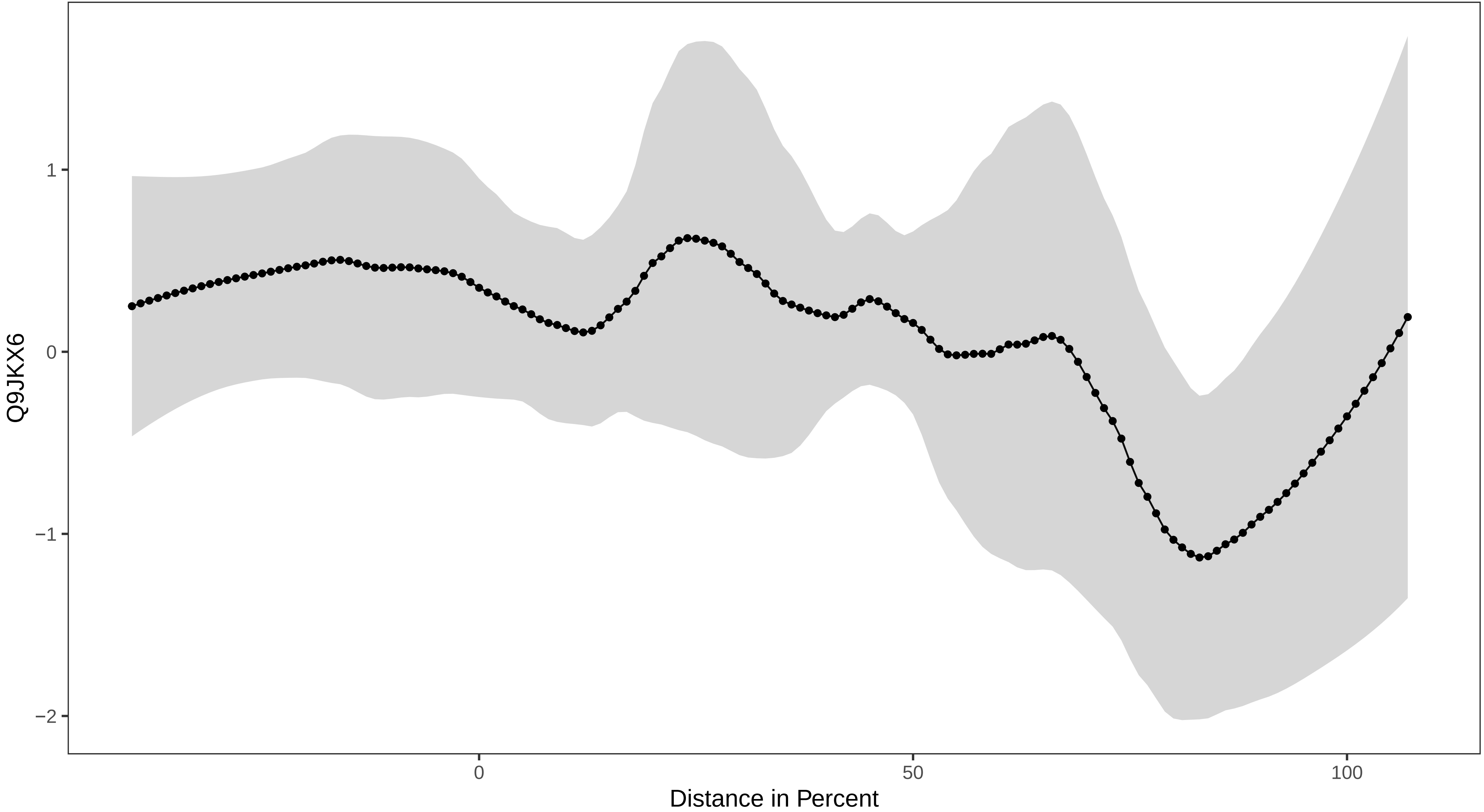

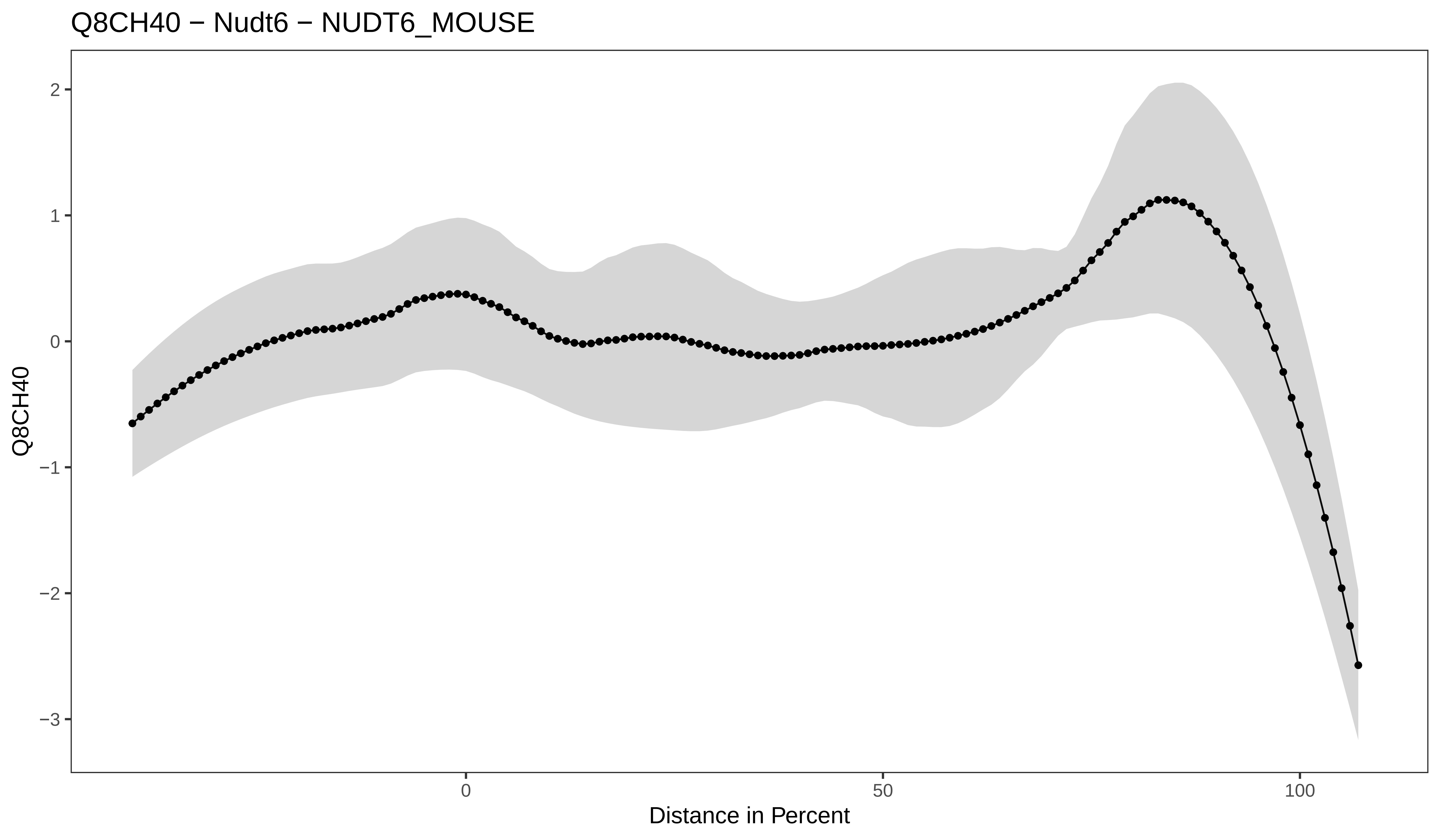

Q8CH40 − Nudt6 − NUDT6_MOUSE
Q8CH40
2
1
0
−1
−2
−3
0
50
100
Distance in Percent

Q99P30 − Nudt7 − NUDT7_MOUSE

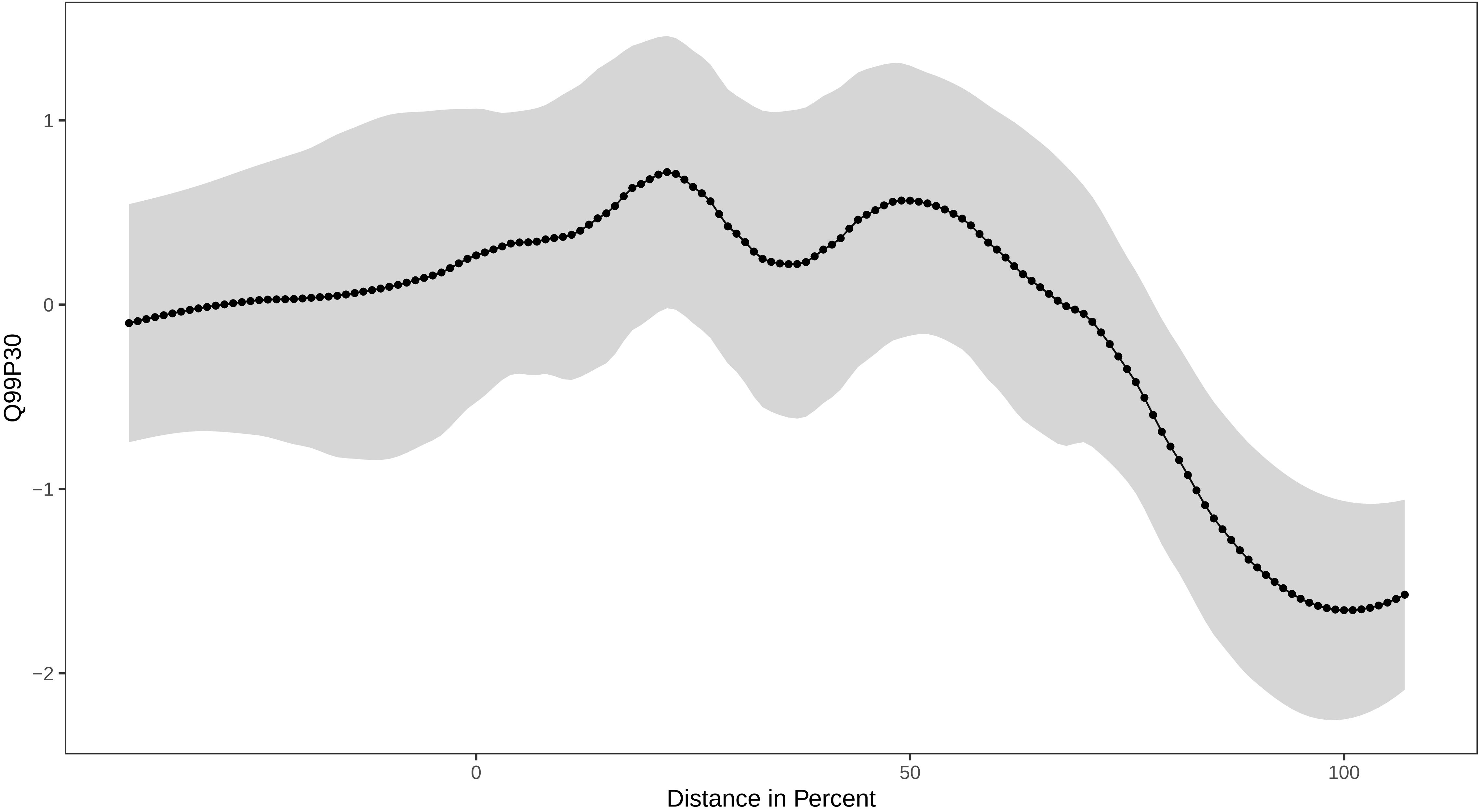

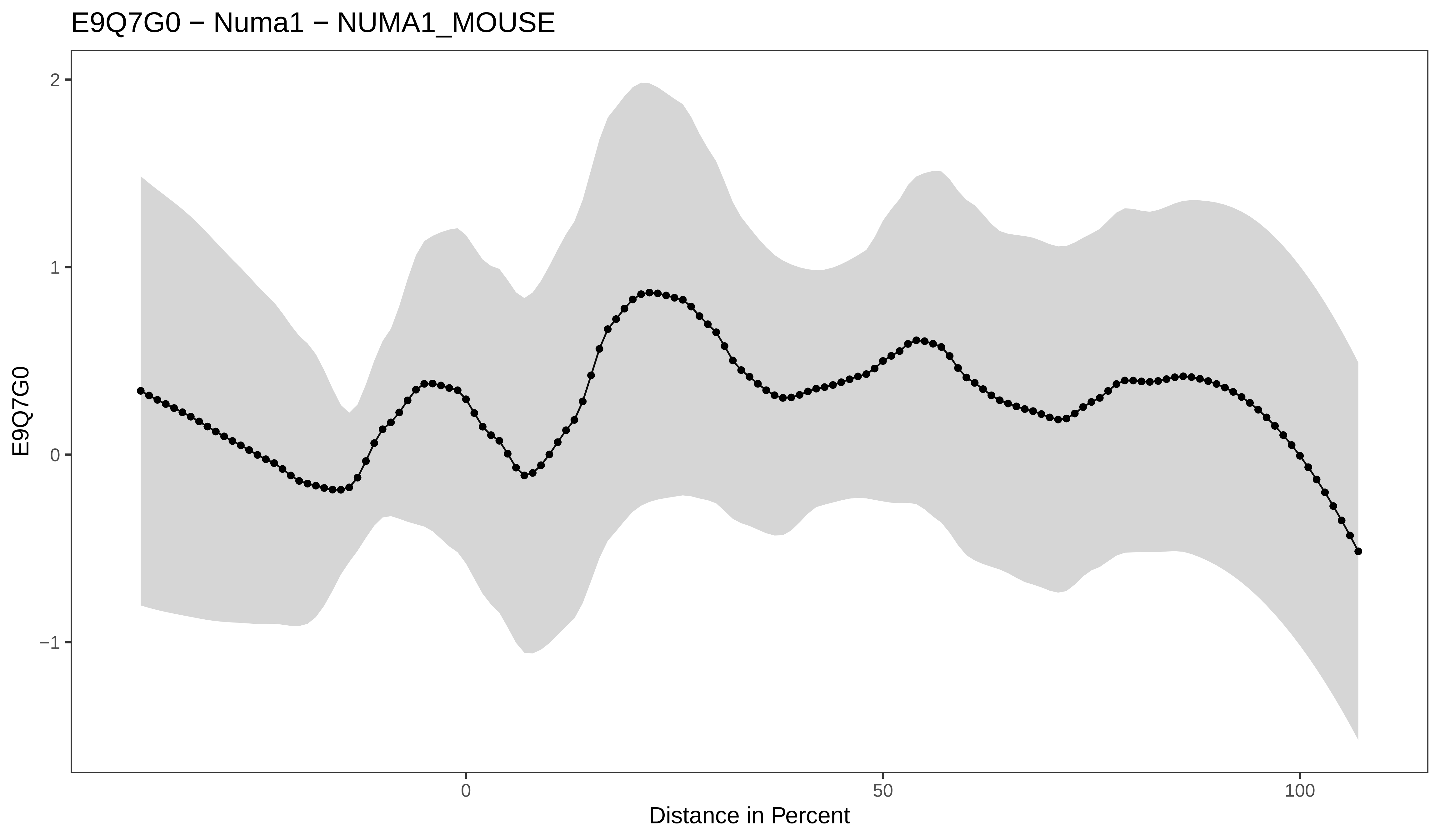

E9Q7G0 − Numa1 − NUMA1_MOUSE
E9Q7G0
2
1
0
−1
0
50
100
Distance in Percent

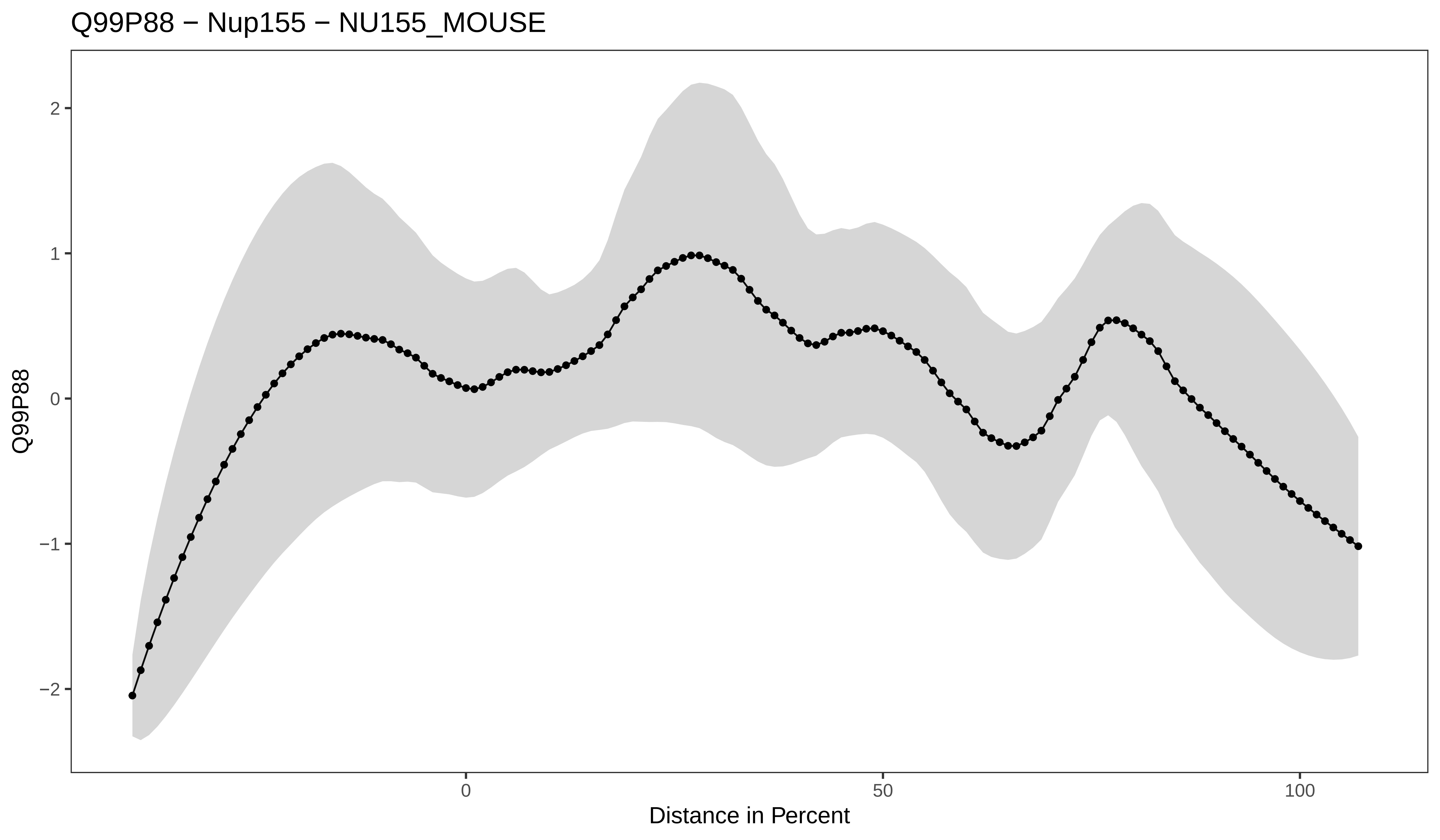

Q99P88 − Nup155 − NU155_MOUSE
Q99P88
2
1
0
−1
−2
0
50
100
Distance in Percent

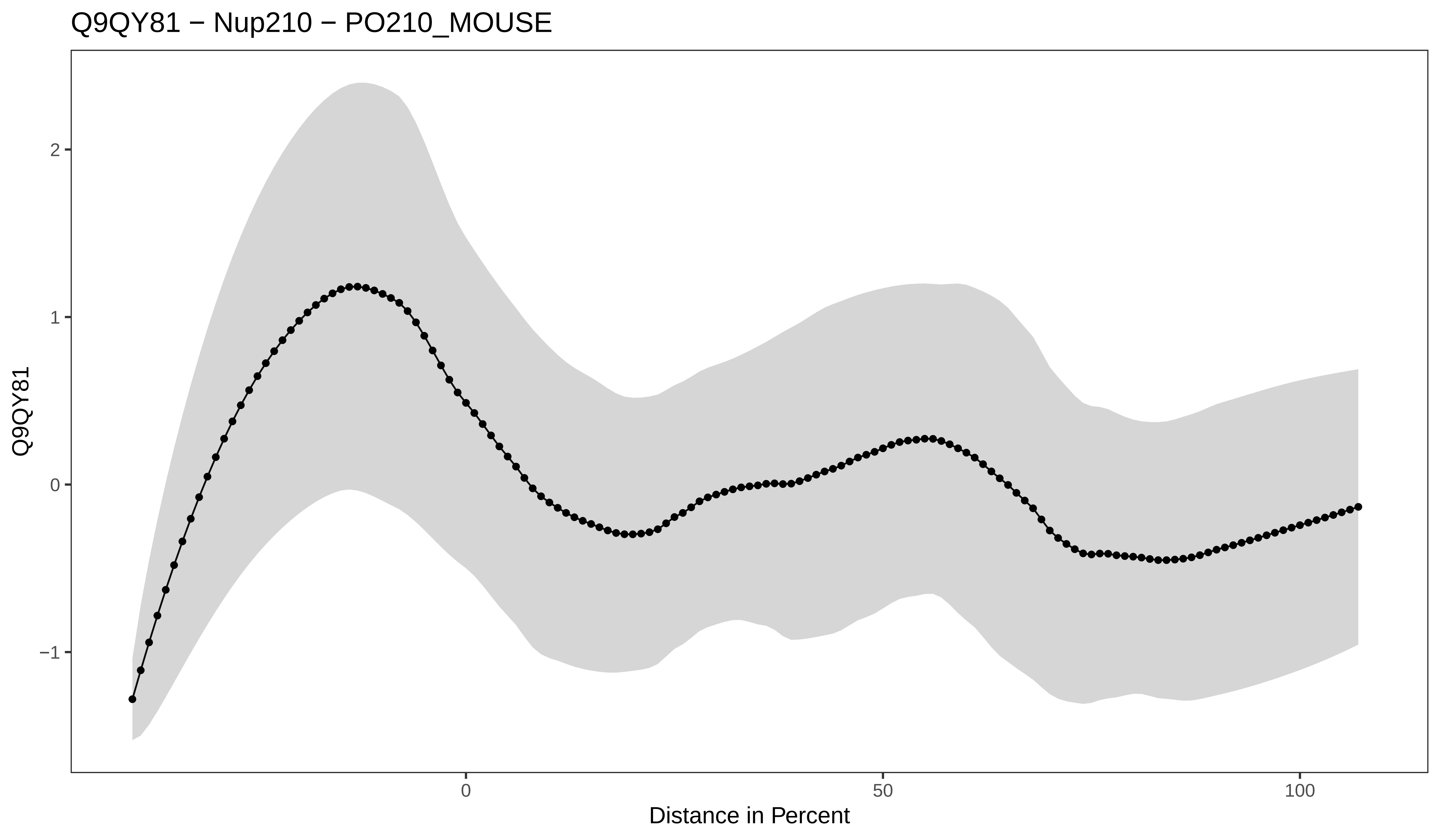

Q9QY81 − Nup210 − PO210_MOUSE
Q9QY81
2
1
0
−1
0
50
100
Distance in Percent

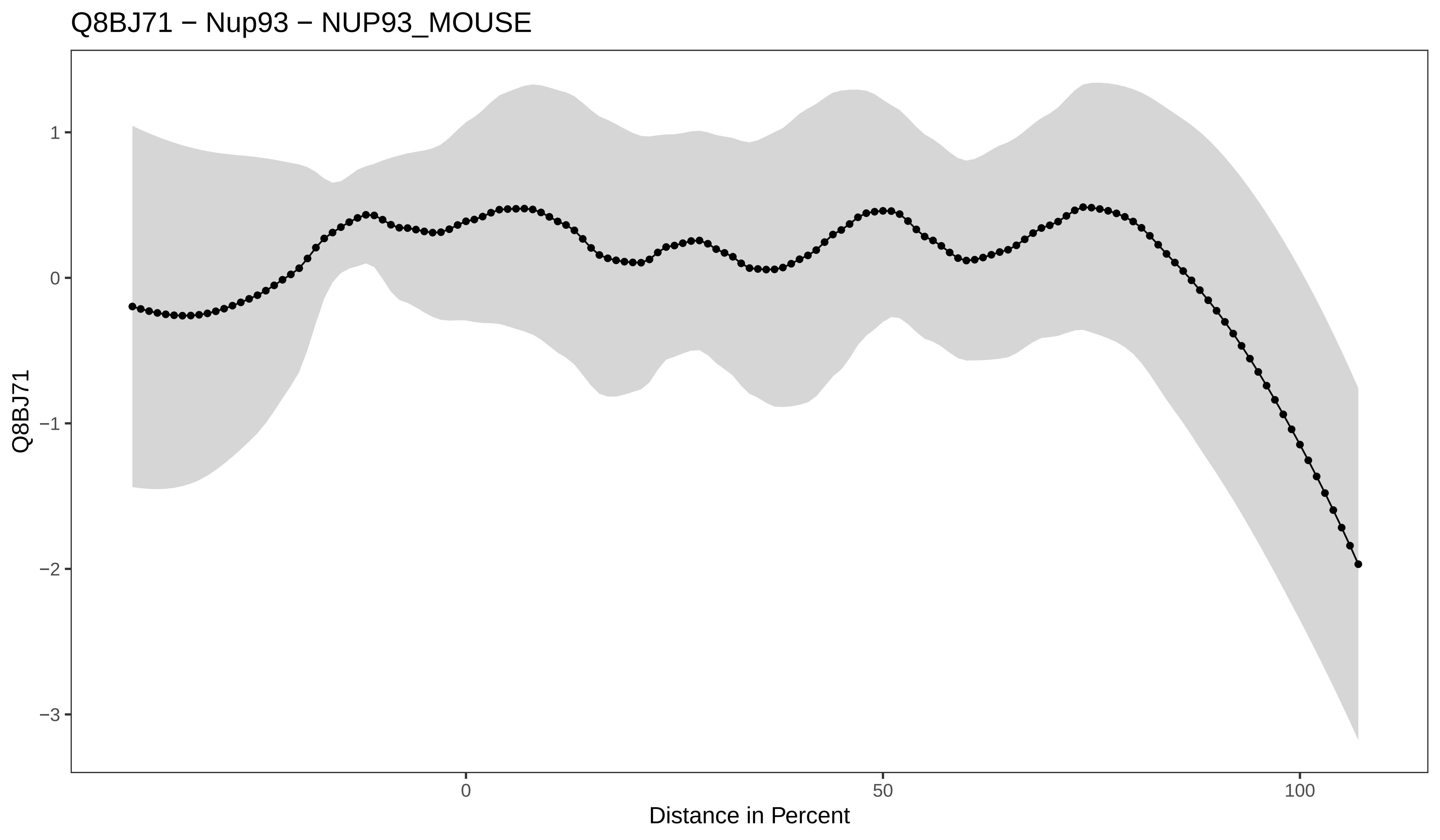

Q8BJ71 − Nup93 − NUP93_MOUSE
1
0
−1
−2
−3
Q8BJ71
0
50
100
Distance in Percent

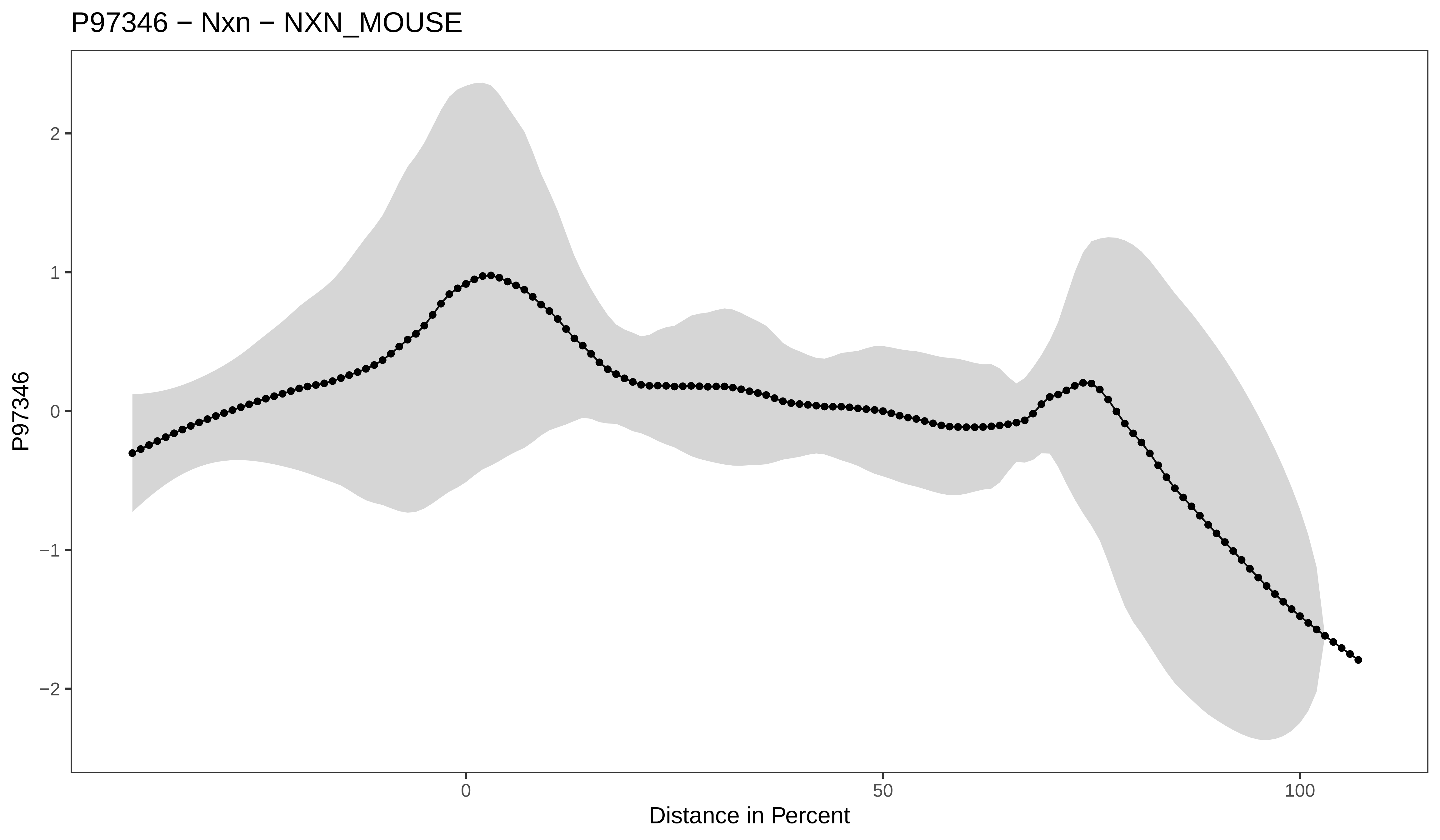
P97346 − Nxn − NXN_MOUSE
P97346
2
1
0
−1
−2
0
50
100
Distance in Percent

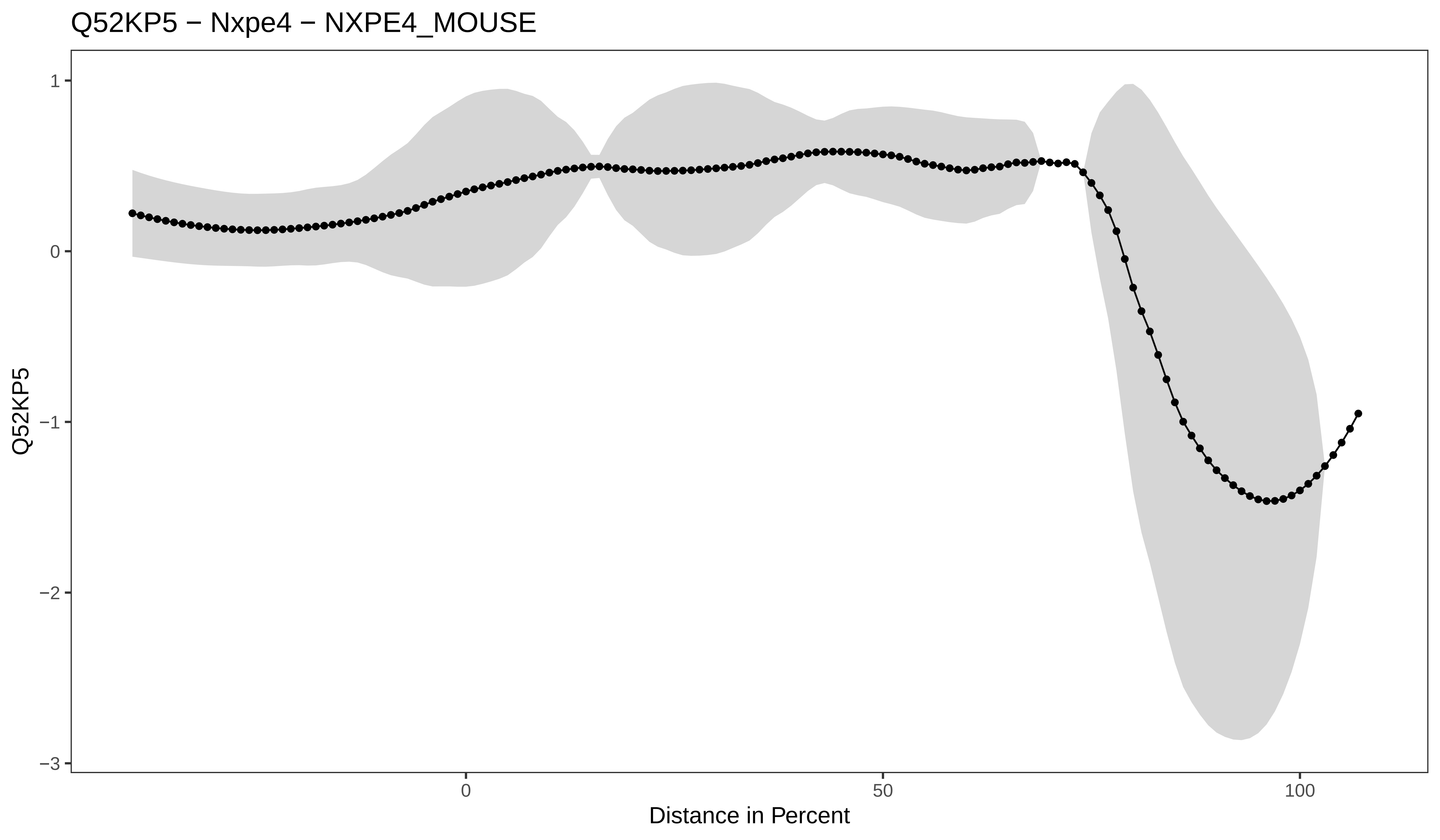
Q52KP5 − Nxpe4 − NXPE4_MOUSE
1
0
−1
−2
−3
Q52KP5
0
50
100
Distance in Percent

P29758 − Oat − OAT_MOUSE

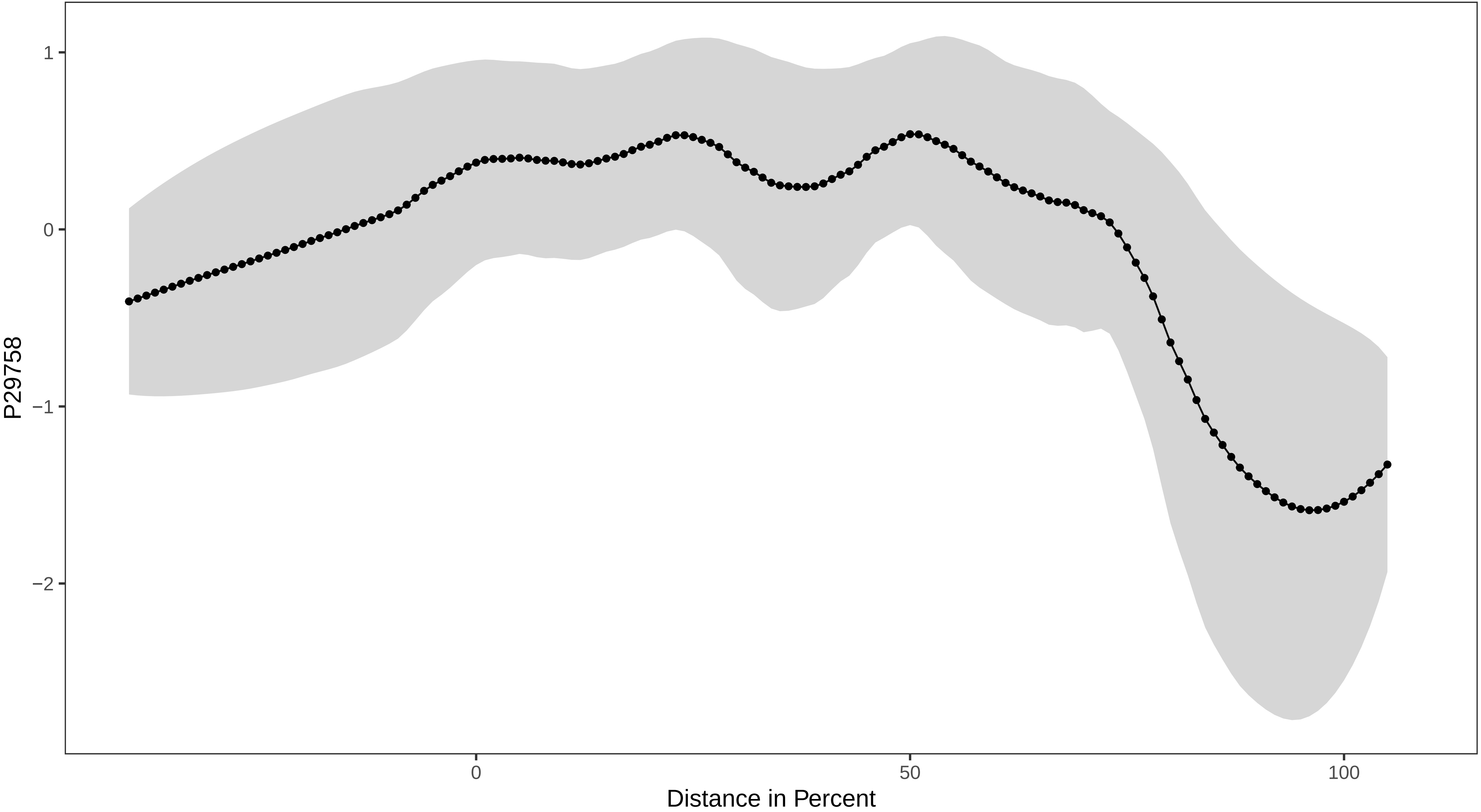

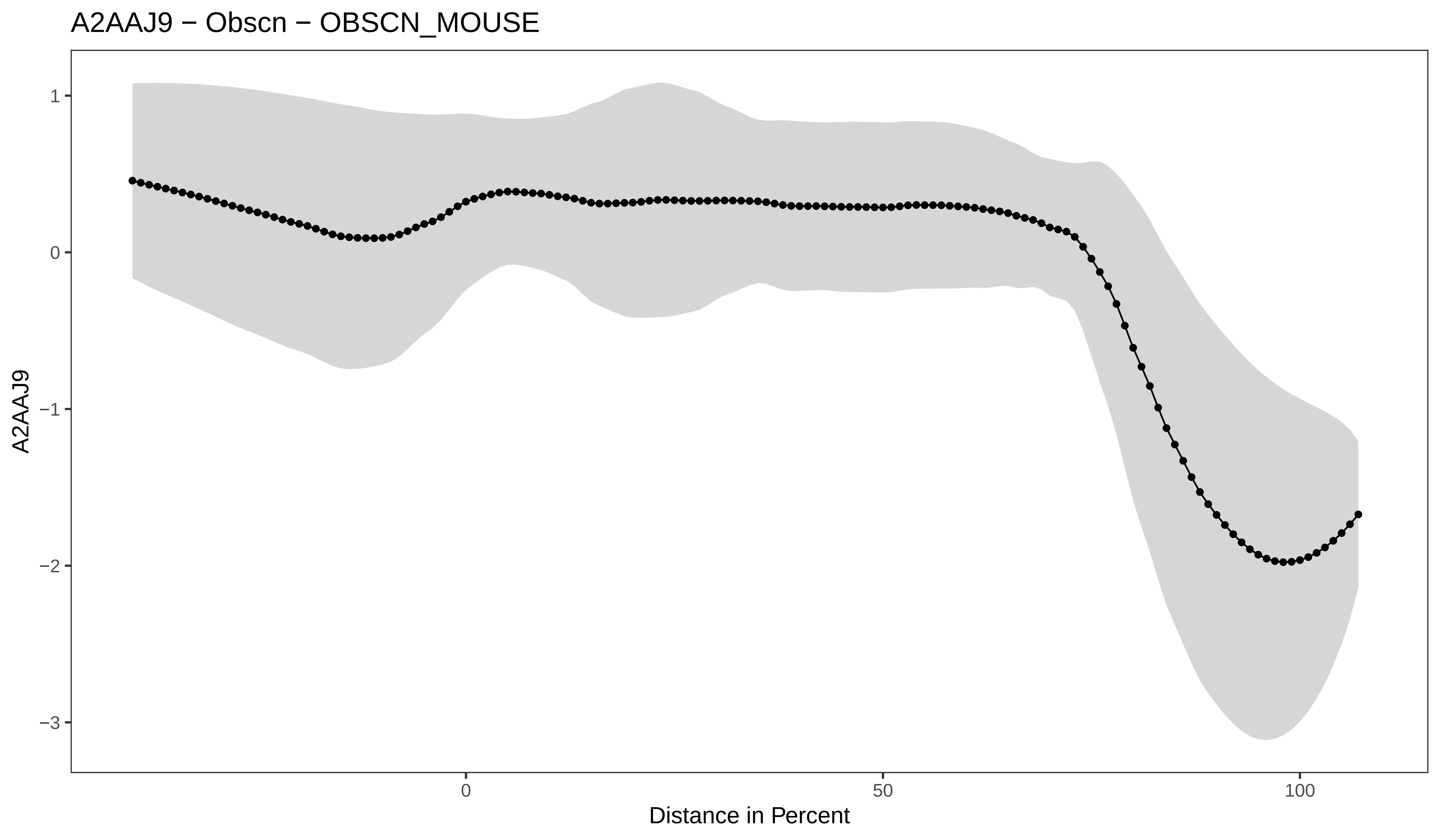

A2AAJ9 − Obscn − OBSCN_MOUSE
A2AAJ9
1
0
−1
−2
−3
0
50
100
Distance in Percent

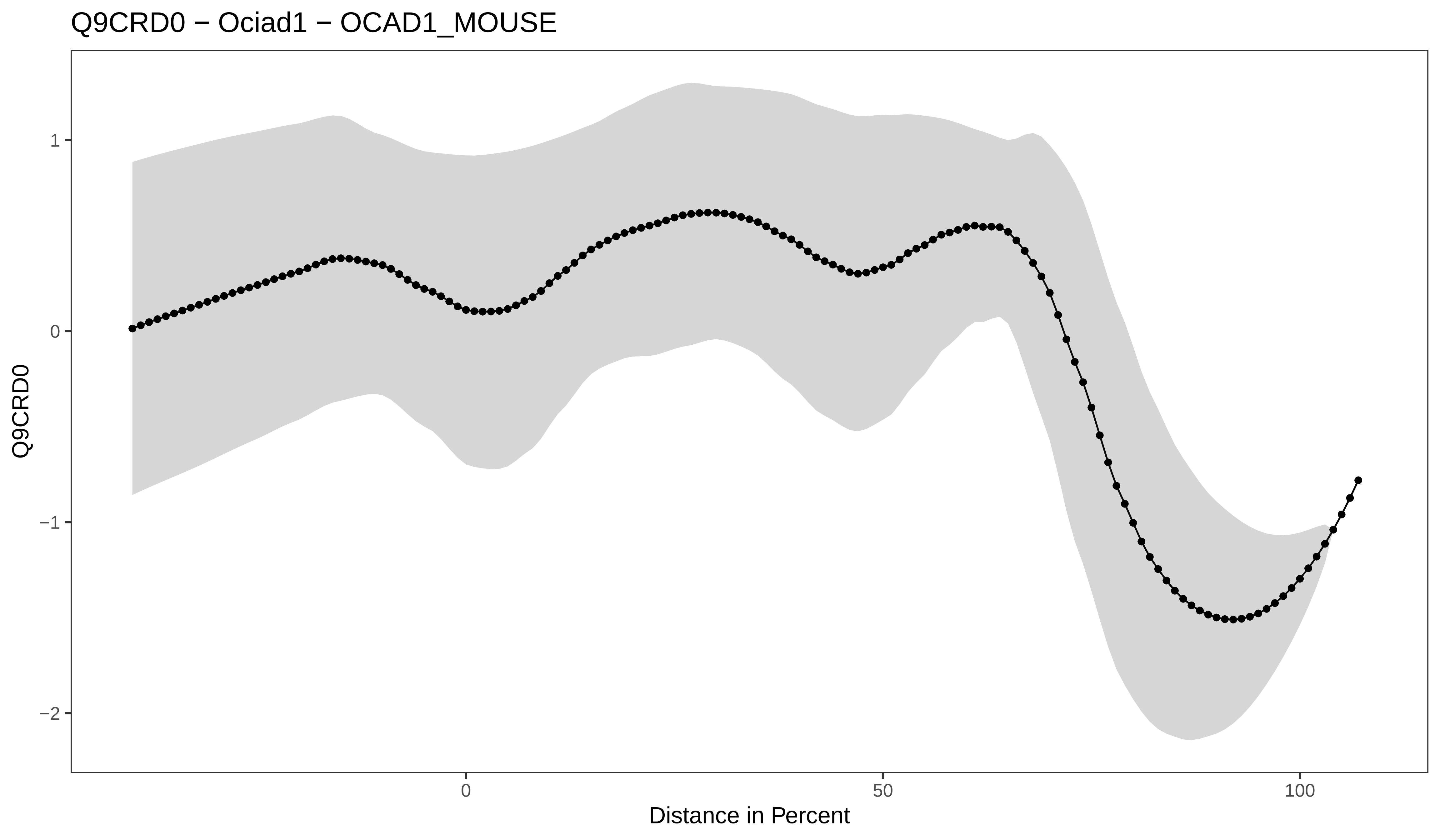

Q9CRD0 − Ociad1 − OCAD1_MOUSE
Q9CRD0
1
0
−1
−2
0
50
100
Distance in Percent

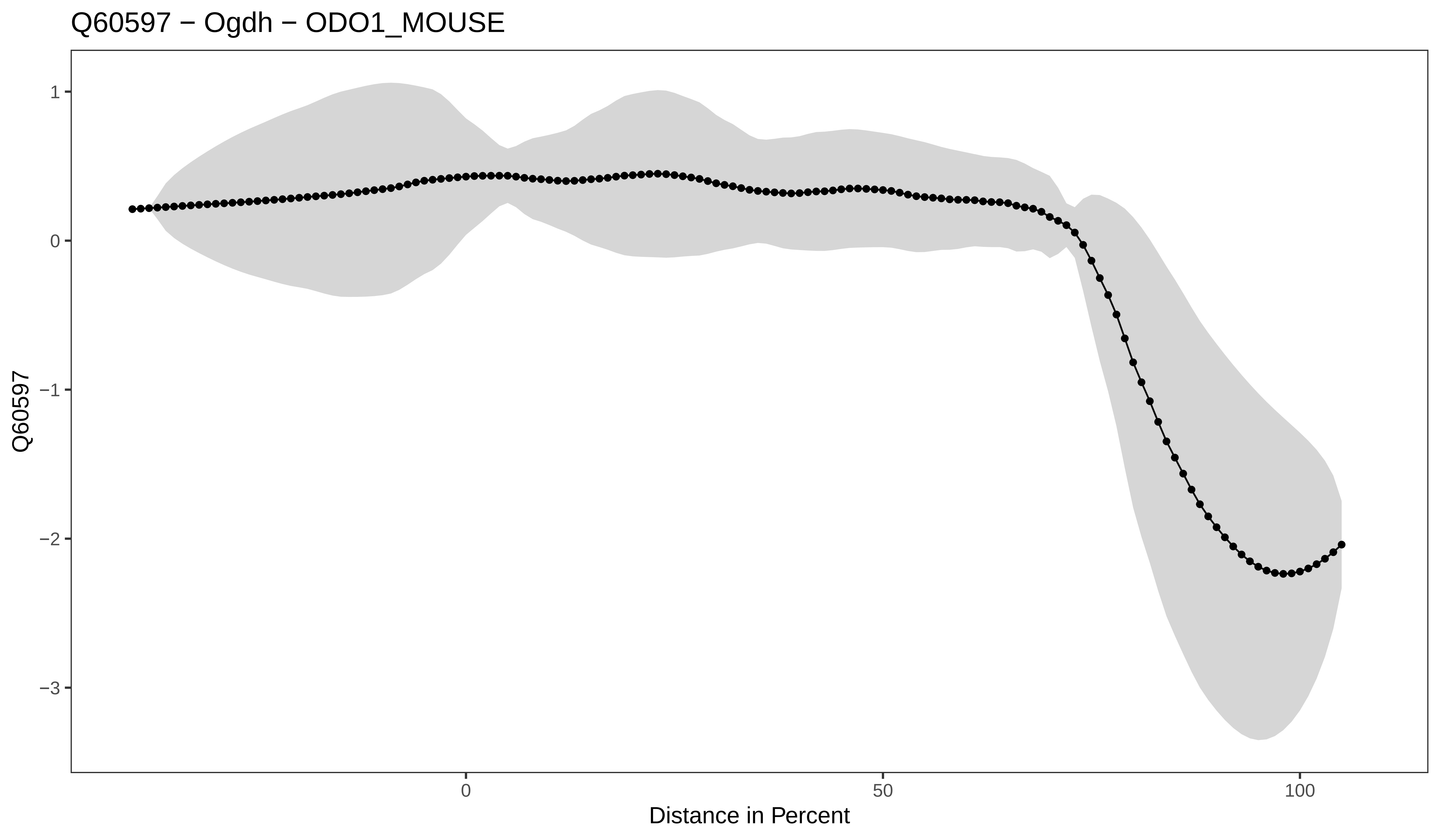

Q60597 − Ogdh − ODO1_MOUSE
1
0
−1
−2
−3
Q60597
0
50
100
Distance in Percent

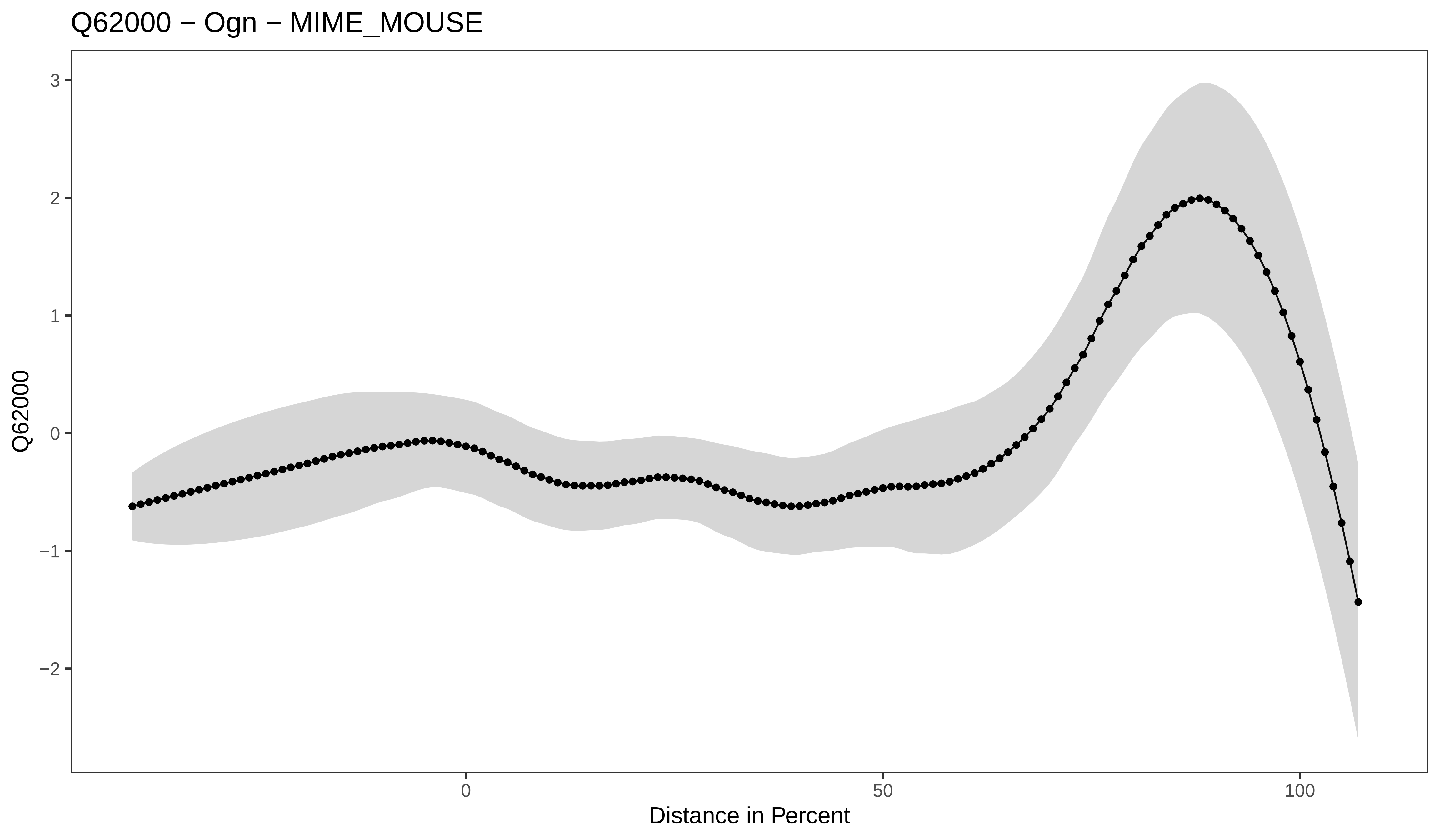

Q62000 − Ogn − MIME_MOUSE
Q62000
3
2
1
0
−1
−2
0
50
100
Distance in Percent

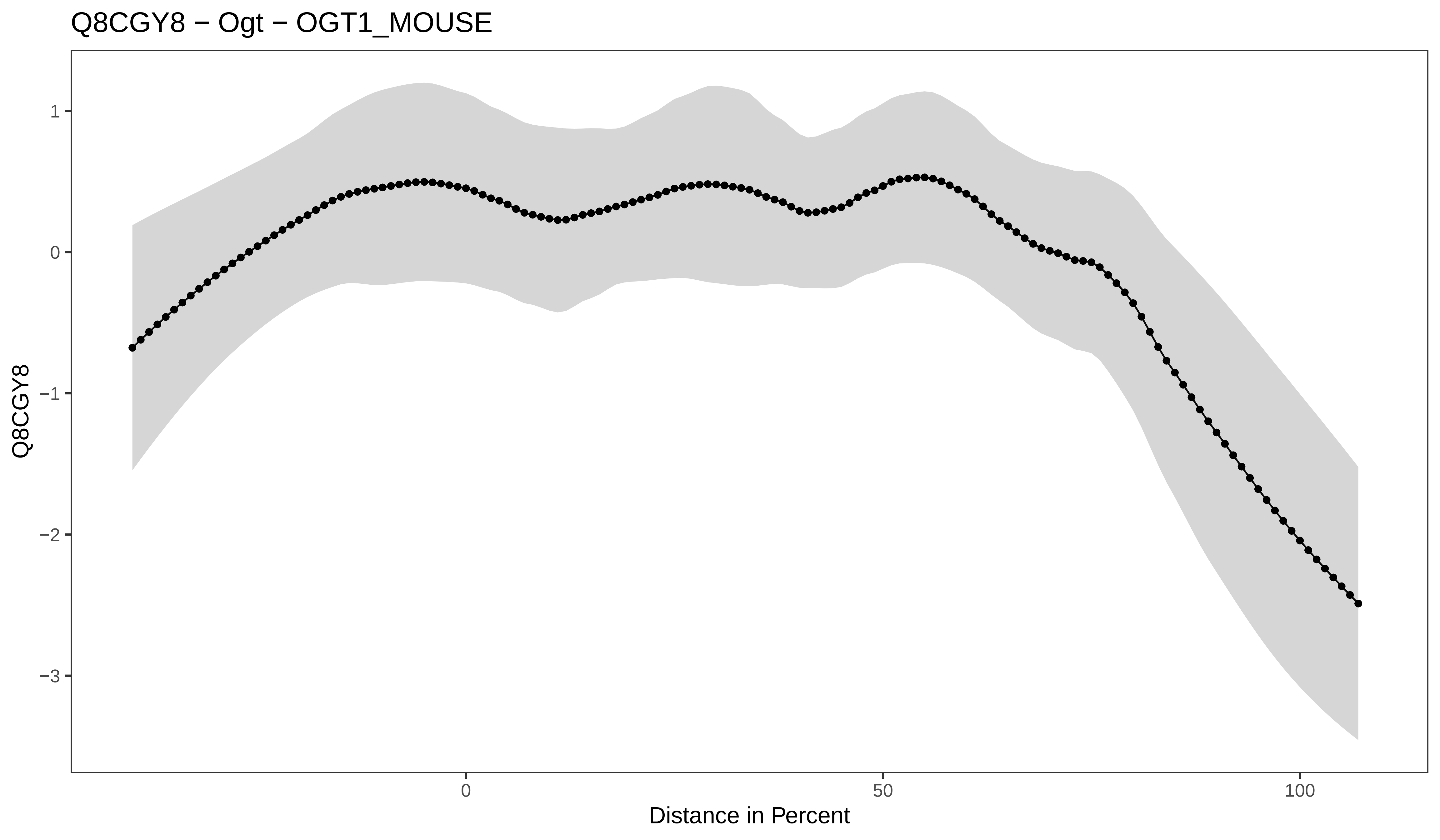
Q8CGY8 − Ogt − OGT1_MOUSE
Q8CGY8
1
0
−1
−2
−3
0
50
100
Distance in Percent

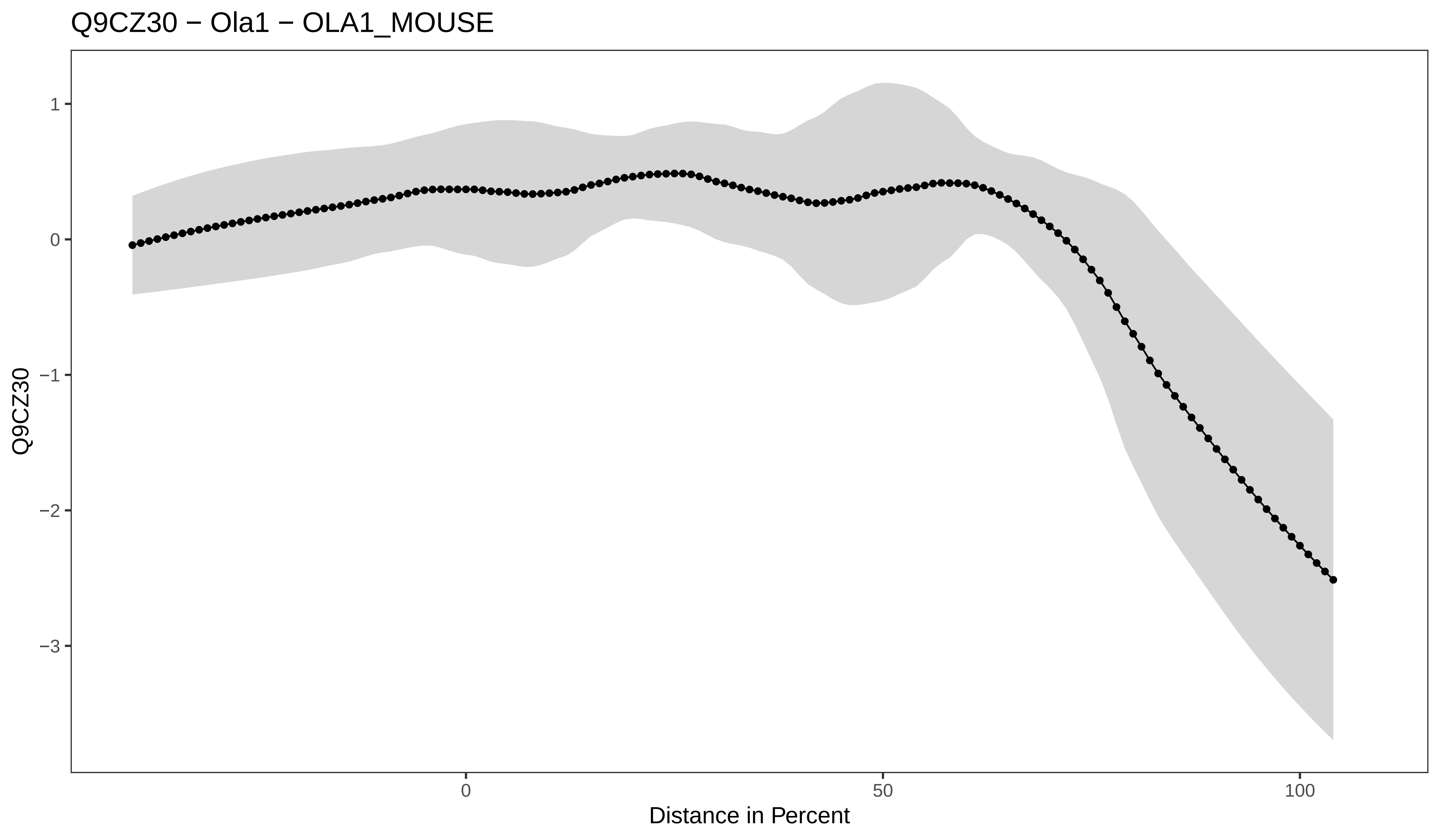

Q9CZ30 − Ola1 − OLA1_MOUSE
1
0
−1
−2
−3
Q9CZ30
0
50
100
Distance in Percent

Q9D8H7 − Oma1 − OMA1_MOUSE

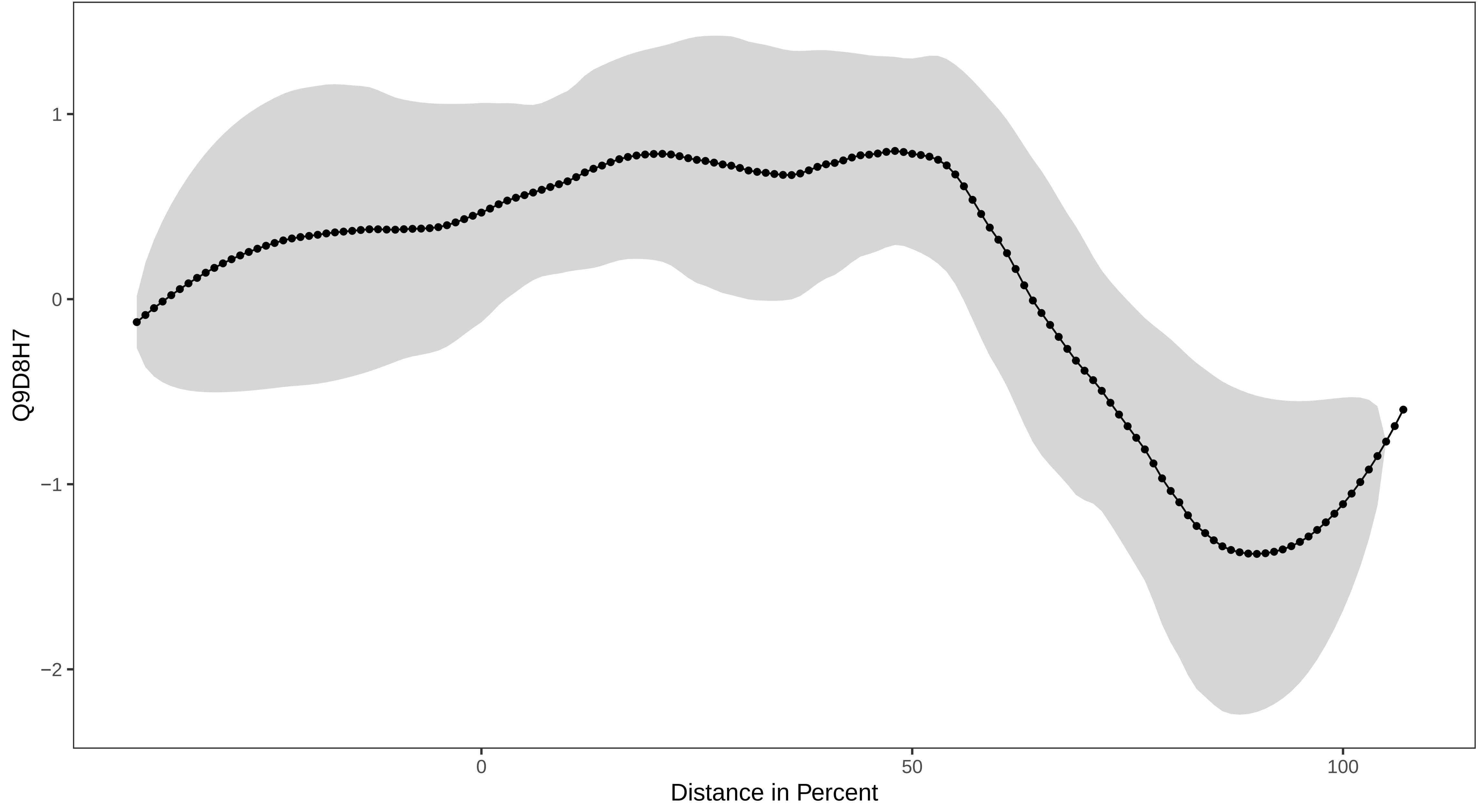

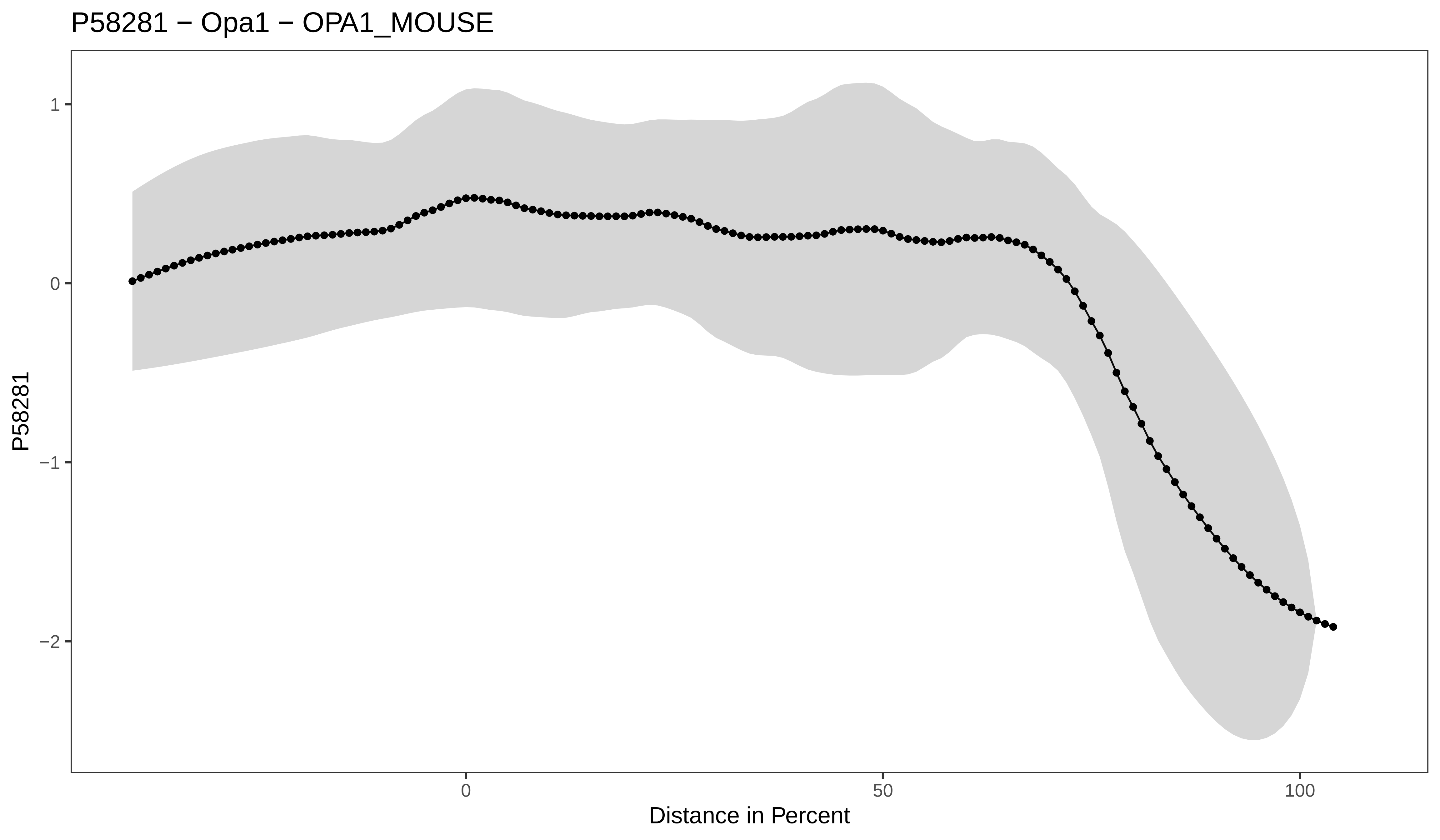

P58281 − Opa1 − OPA1_MOUSE
P58281
1
0
−1
−2
0
50
100
Distance in Percent

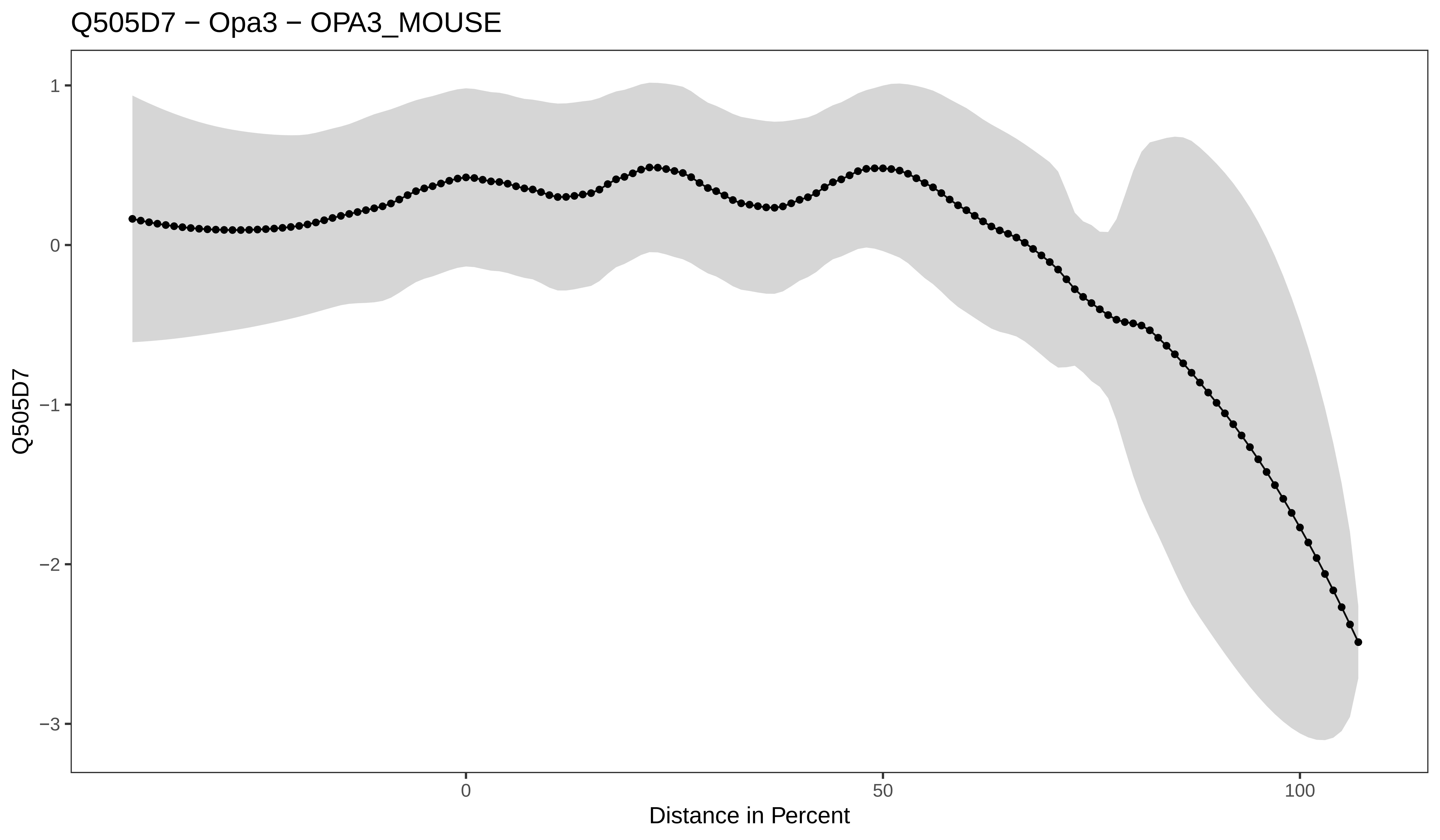

Q505D7 − Opa3 − OPA3_MOUSE
1
0
−1
−2
−3
Q505D7
0
50
100
Distance in Percent

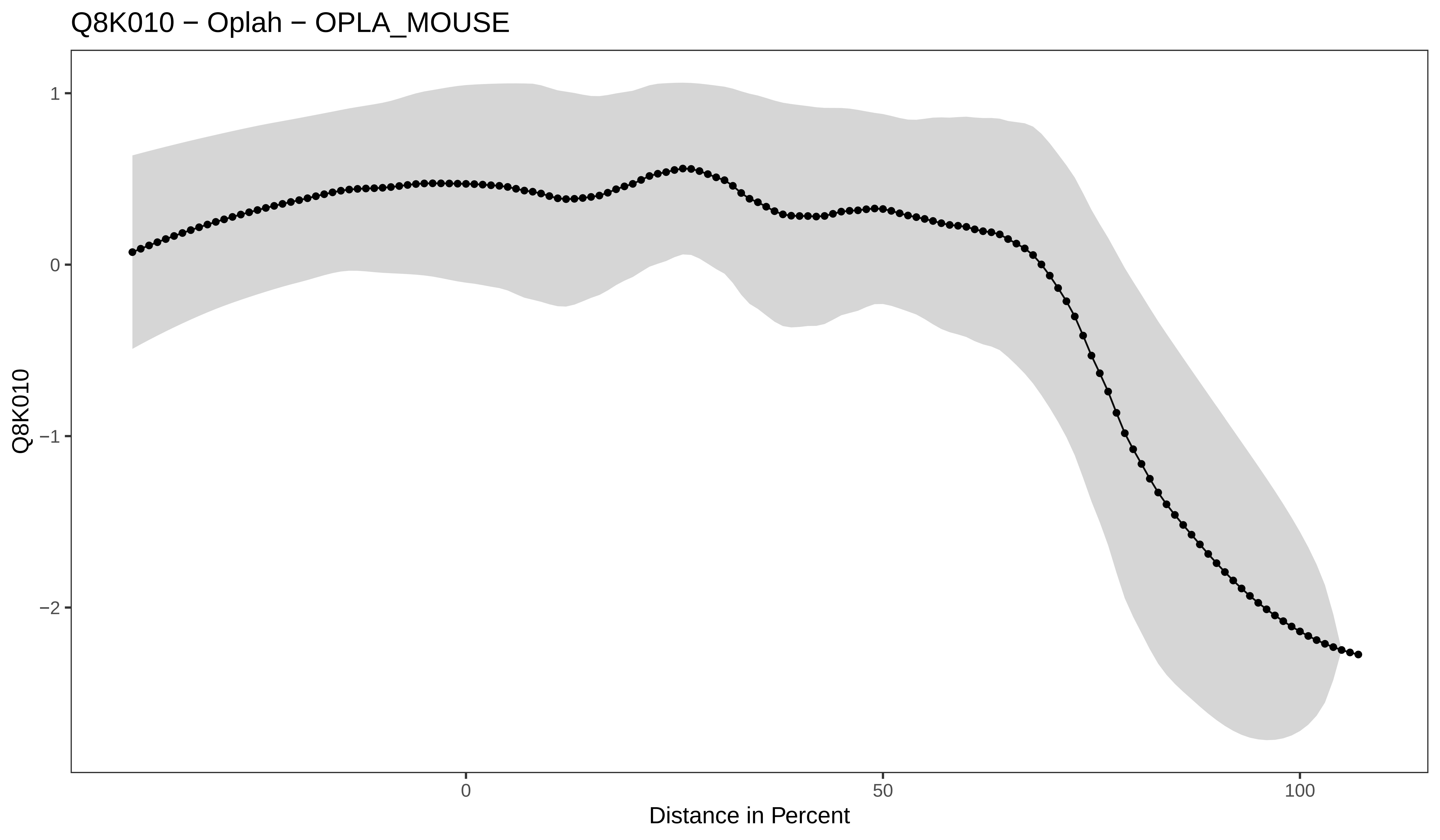

Q8K010 − Oplah − OPLA_MOUSE
Q8K010
1
0
−1
−2
0
50
100
Distance in Percent

Q60590 − Orm1 − A1AG1_MOUSE

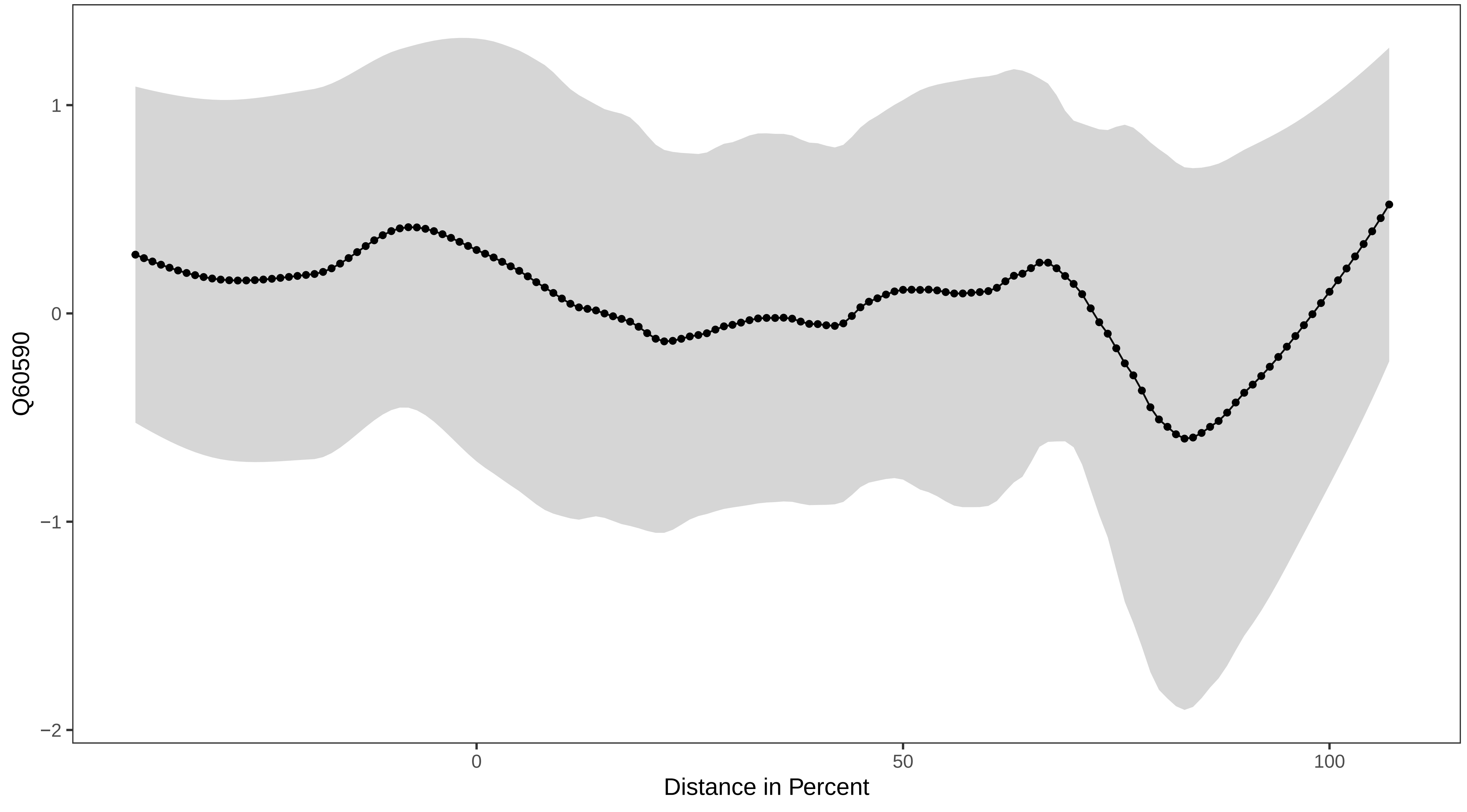

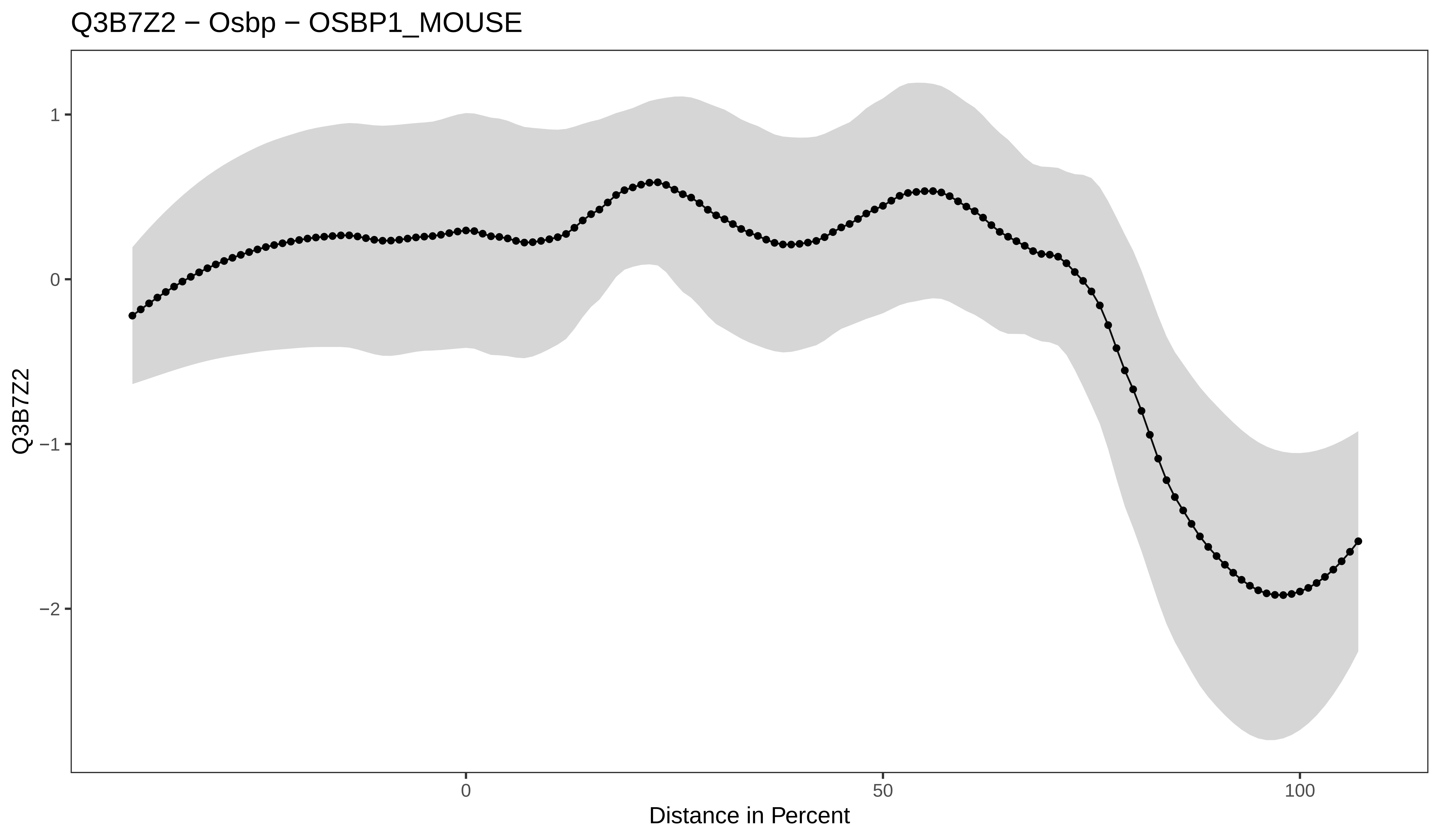
Q3B7Z2 − Osbp − OSBP1_MOUSE
1
0
−1
−2
Q3B7Z2
0
50
100
Distance in Percent

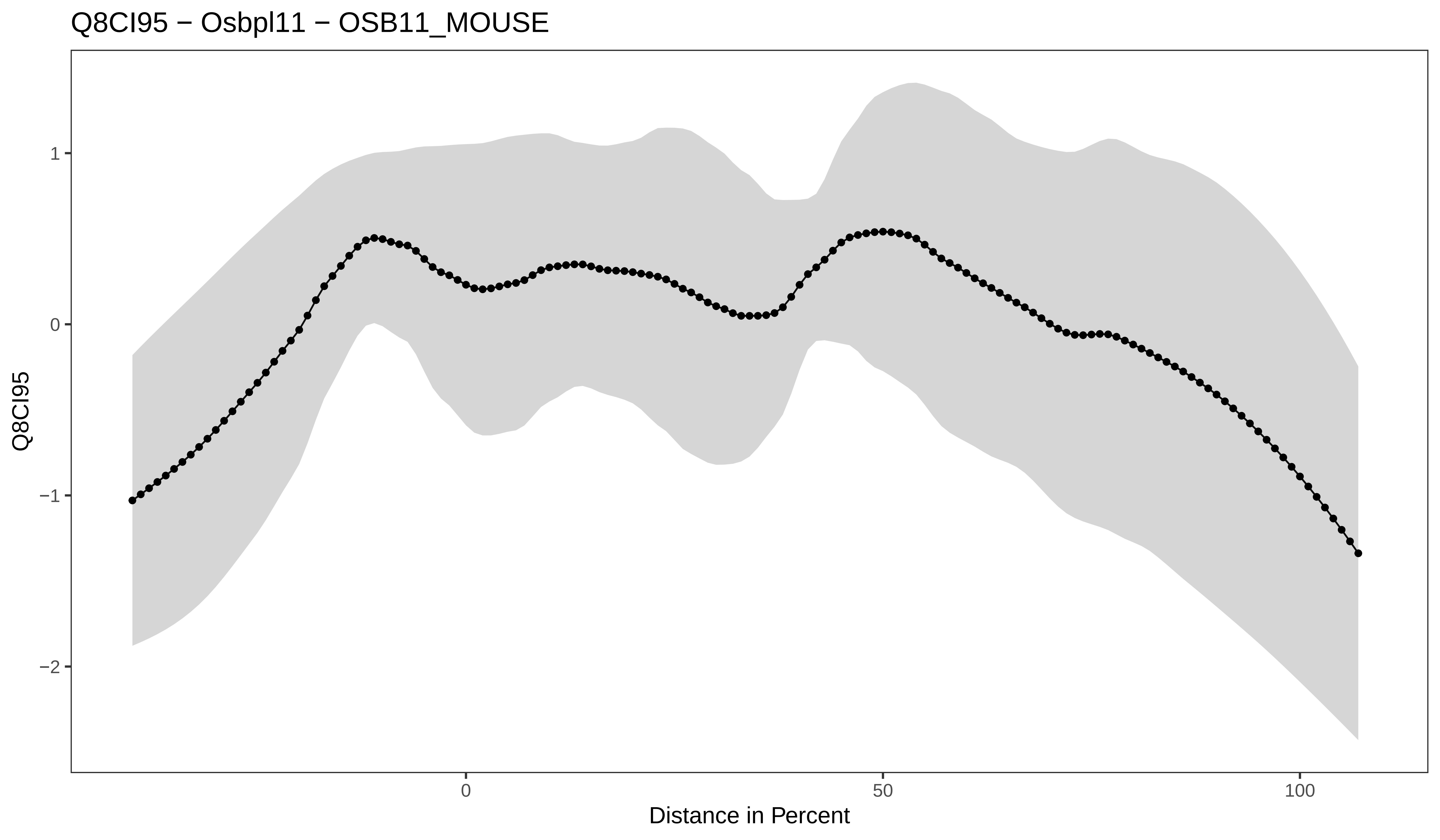

Q8CI95 − Osbpl11 − OSB11_MOUSE
Q8CI95
1
0
−1
−2
0
50
100
Distance in Percent

Q91XL9 − Osbpl1a − OSBL1_MOUSE

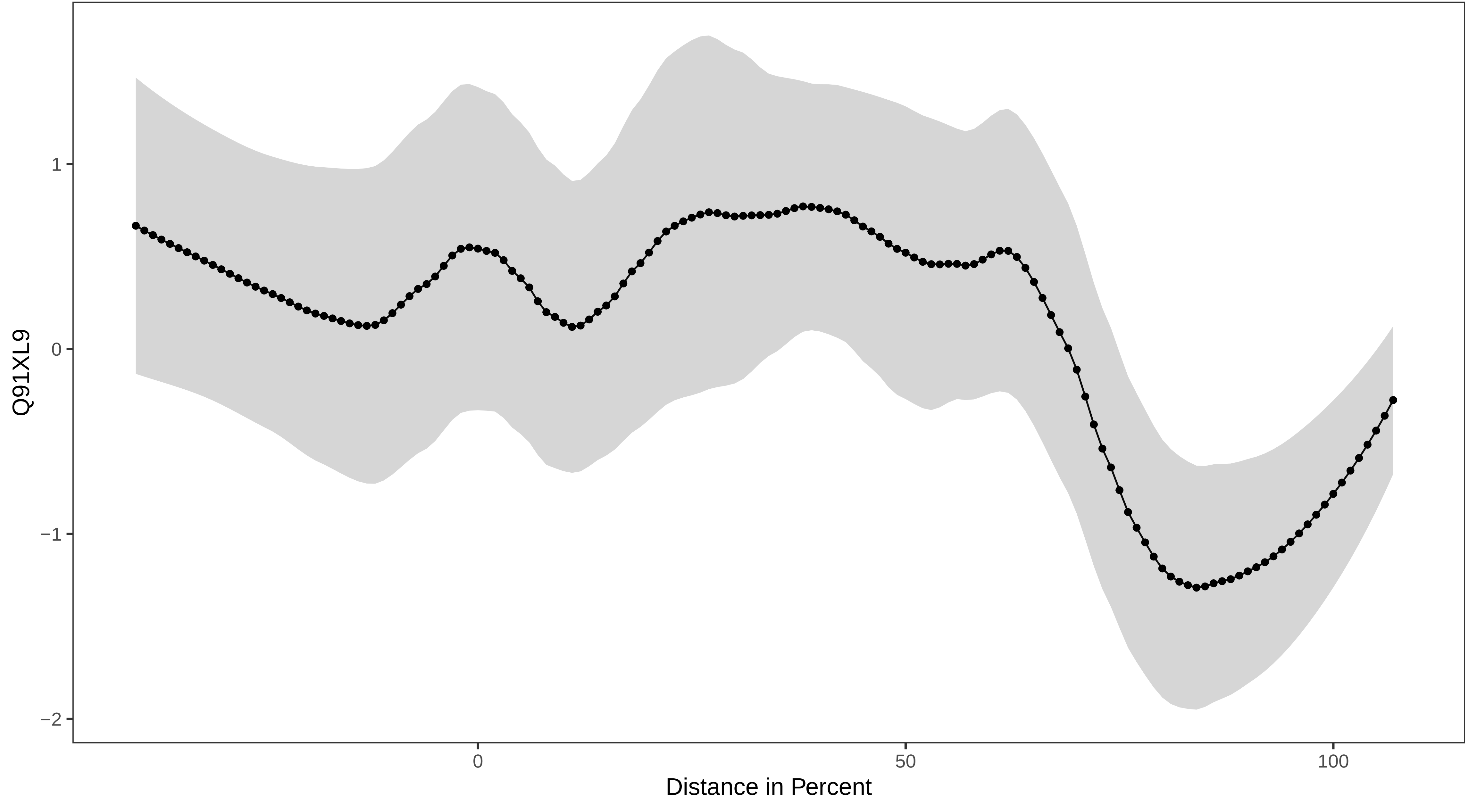

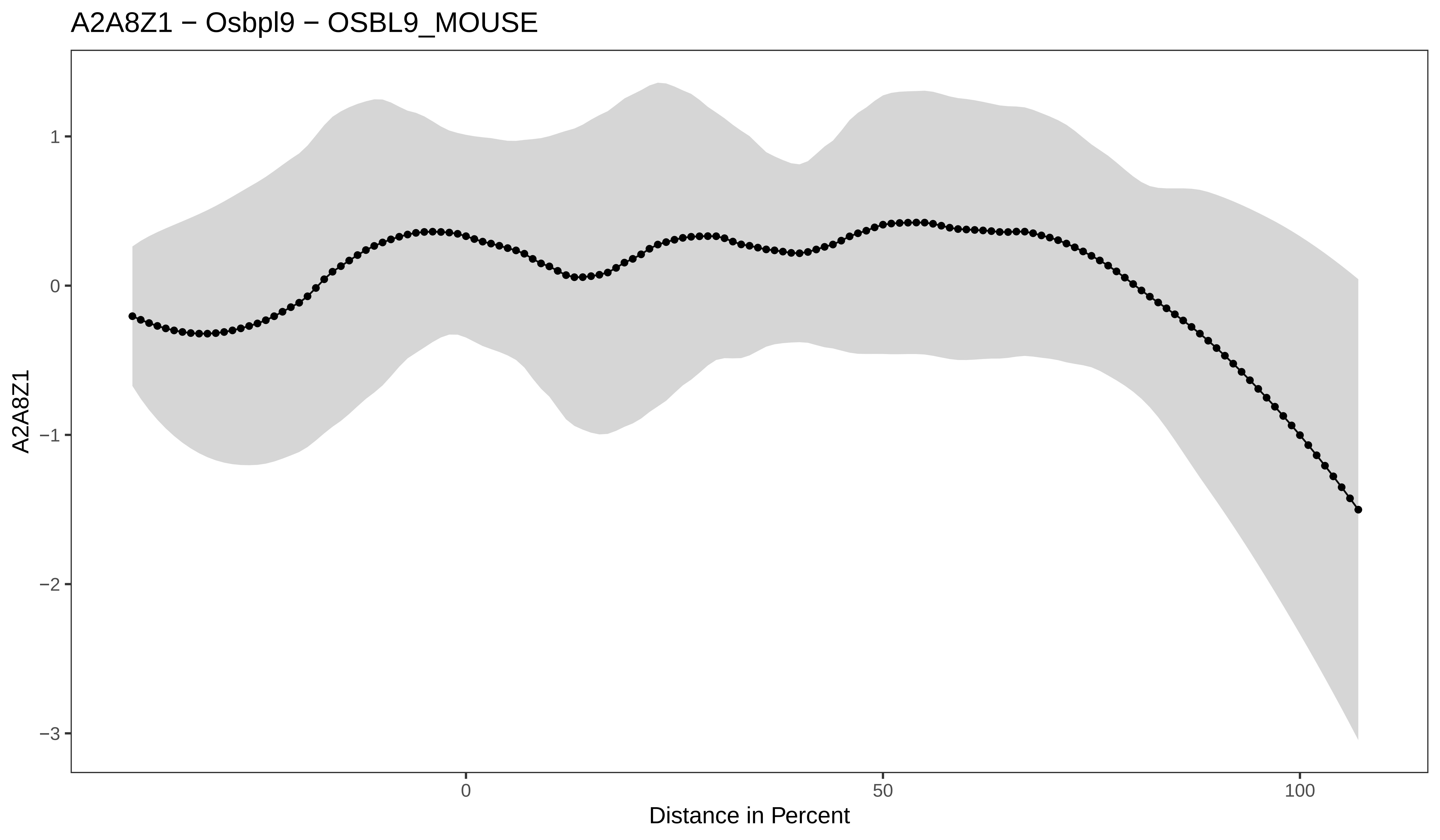

A2A8Z1 − Osbpl9 − OSBL9_MOUSE
A2A8Z1
Distance in Percent
1
0
−1
−2
−3
0
50
100

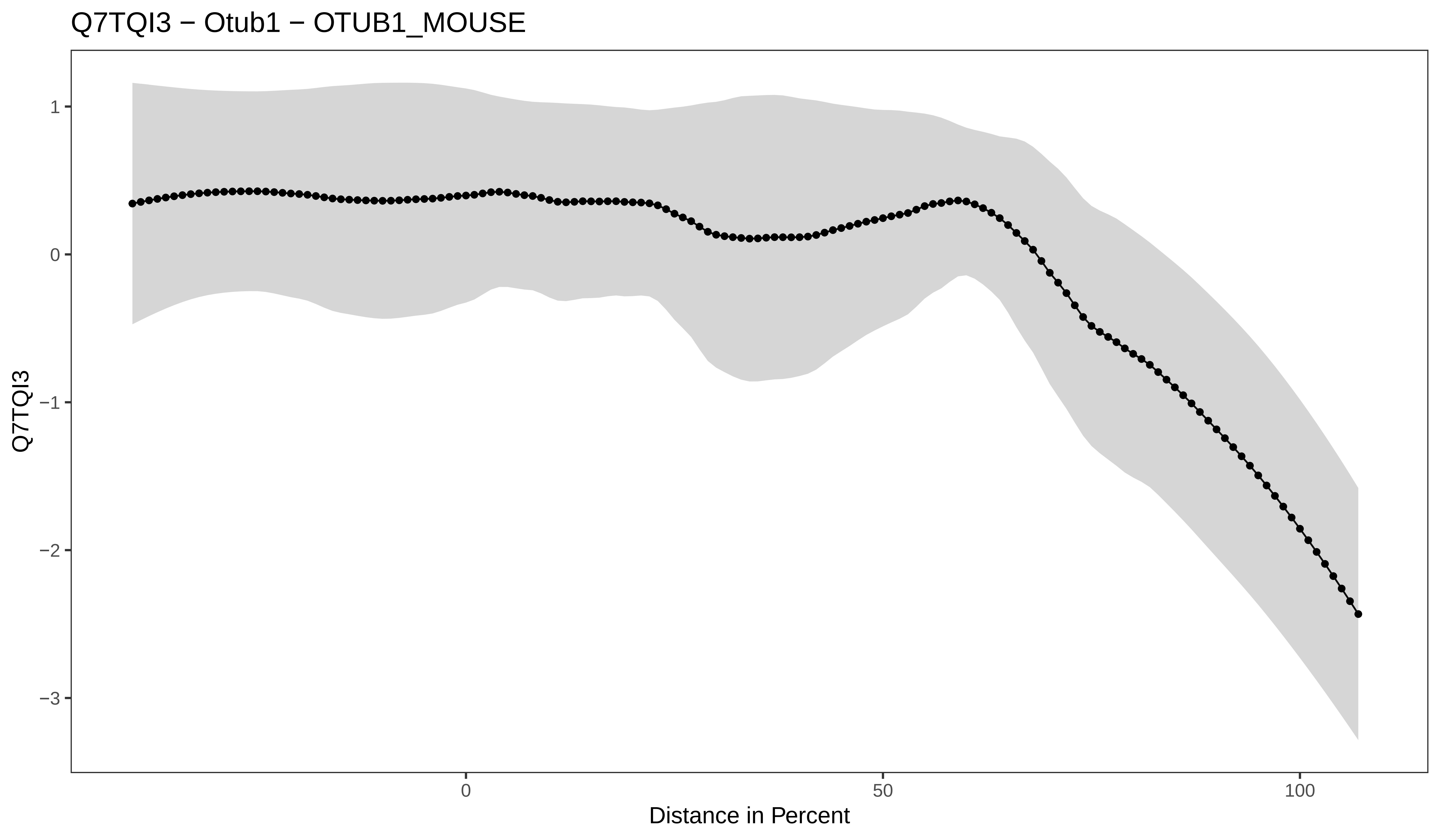

Q7TQI3 − Otub1 − OTUB1_MOUSE
1
0
−1
−2
−3
Q7TQI3
0
50
100
Distance in Percent

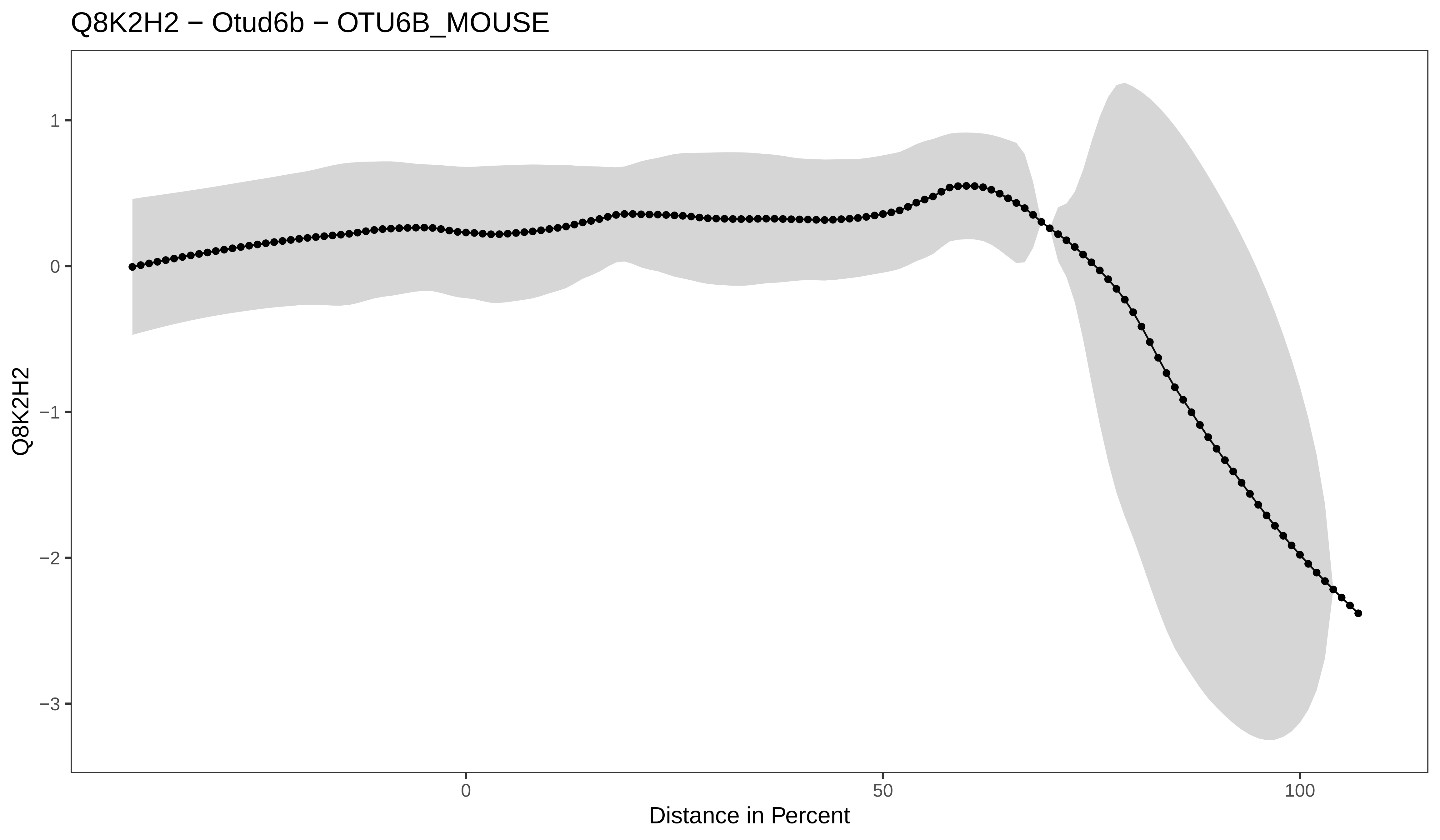

Q8K2H2 − Otud6b − OTU6B_MOUSE
1
0
−1
−2
−3
Q8K2H2
0
50
100
Distance in Percent

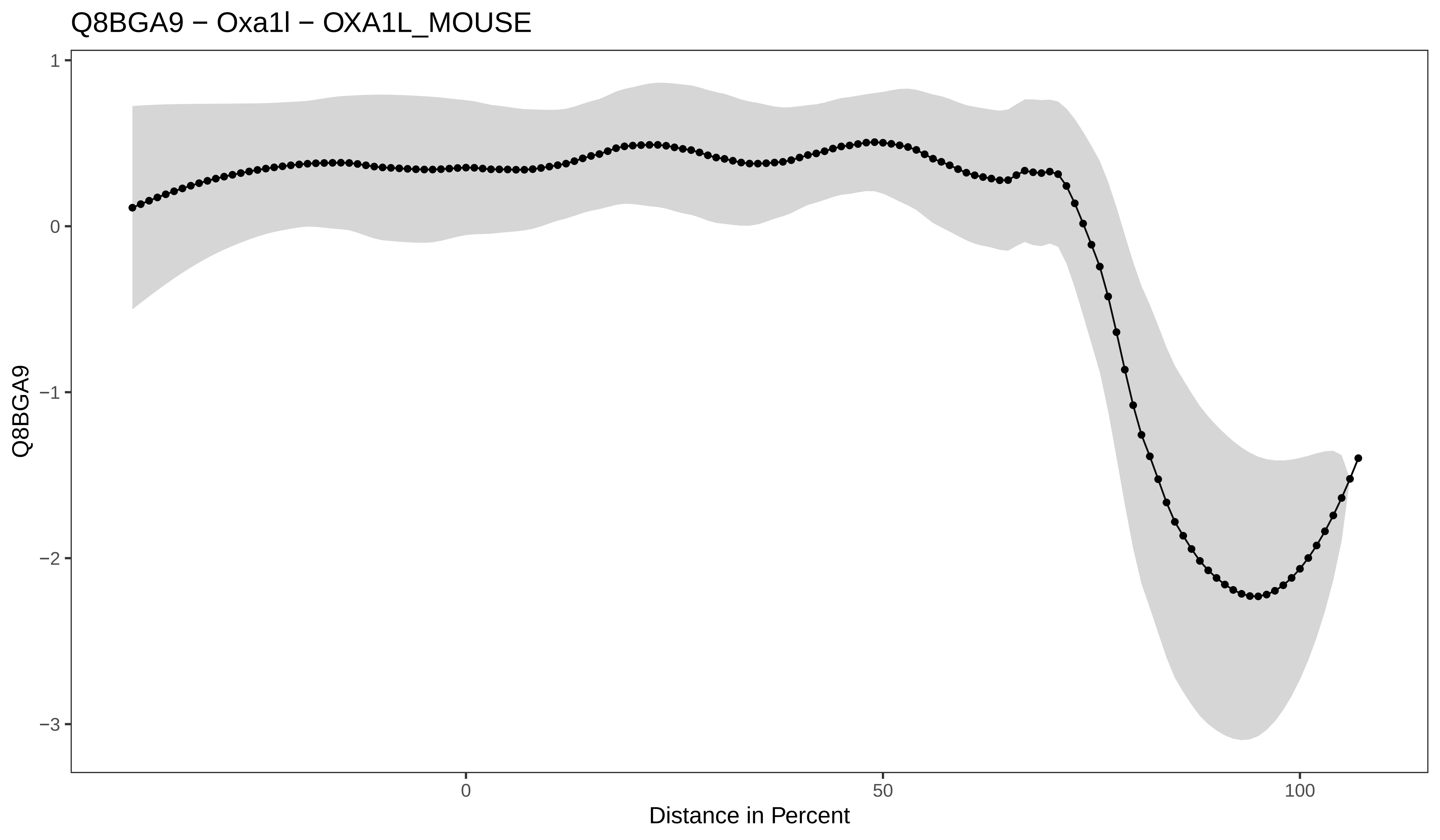

Q8BGA9 − Oxa1l − OXA1L_MOUSE
Q8BGA9
1
0
−1
−2
−3
0
50
100
Distance in Percent

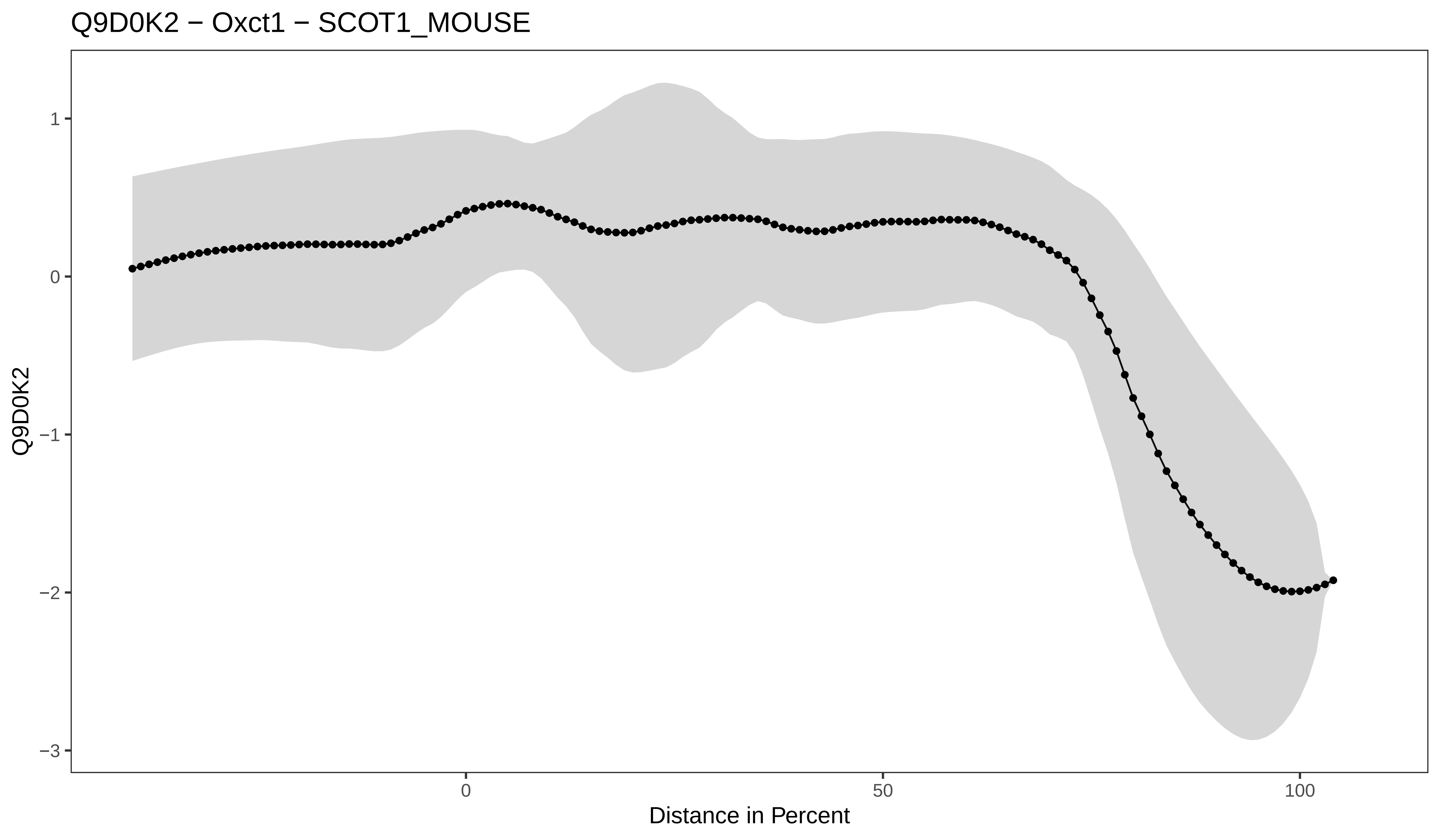

Q9D0K2 − Oxct1 − SCOT1_MOUSE
Q9D0K2
Distance in Percent
1
0
−1
−2
−3
0
50
100

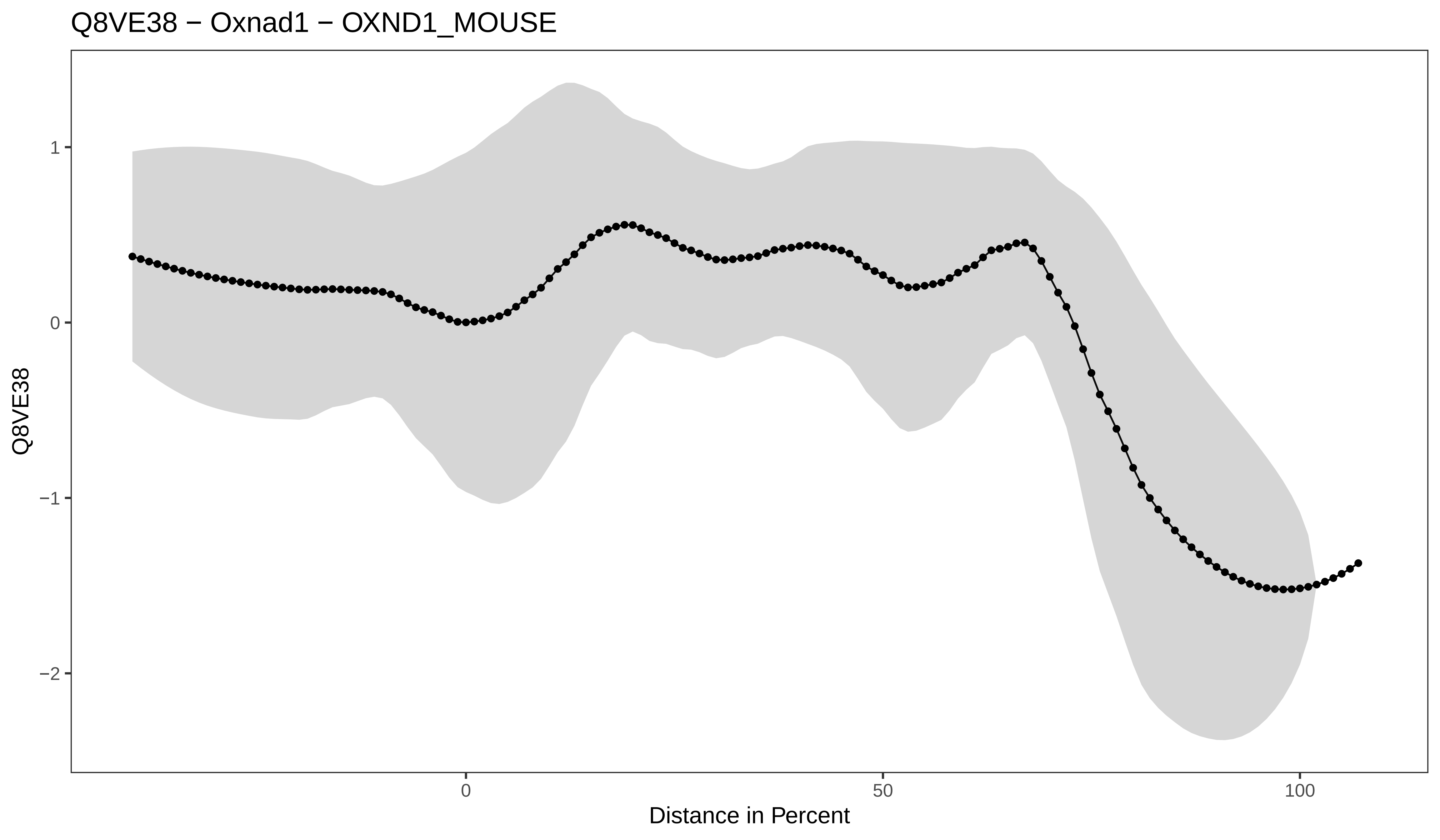

Q8VE38 − Oxnad1 − OXND1_MOUSE
Q8VE38
1
0
−1
−2
0
50
100
Distance in Percent

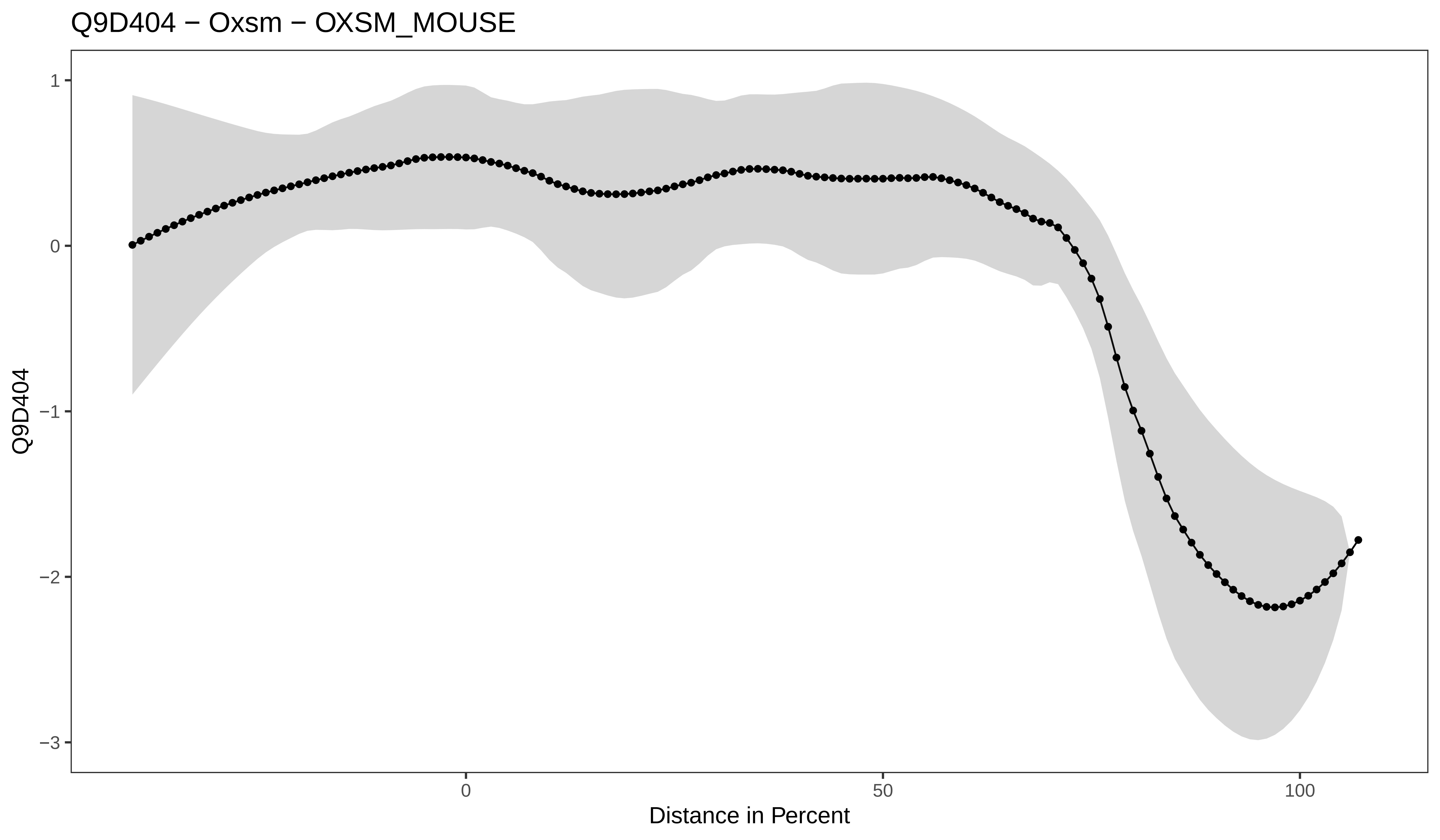
Q9D404 − Oxsm − OXSM_MOUSE
Q9D404
Distance in Percent
1
0
−1
−2
−3
0
50
100

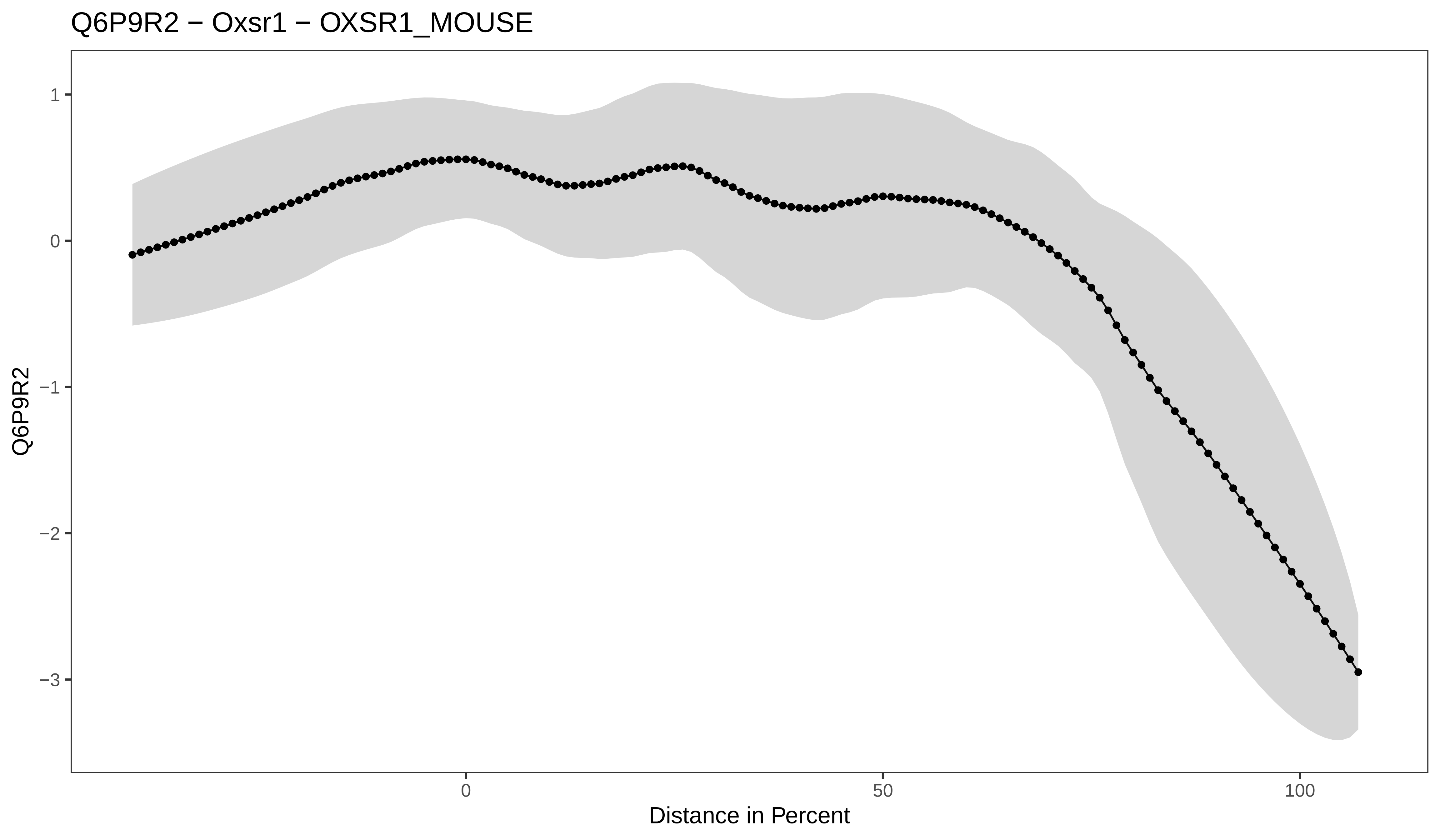

Q6P9R2 − Oxsr1 − OXSR1_MOUSE
Q6P9R2
1
0
−1
−2
−3
0
50
100
Distance in Percent

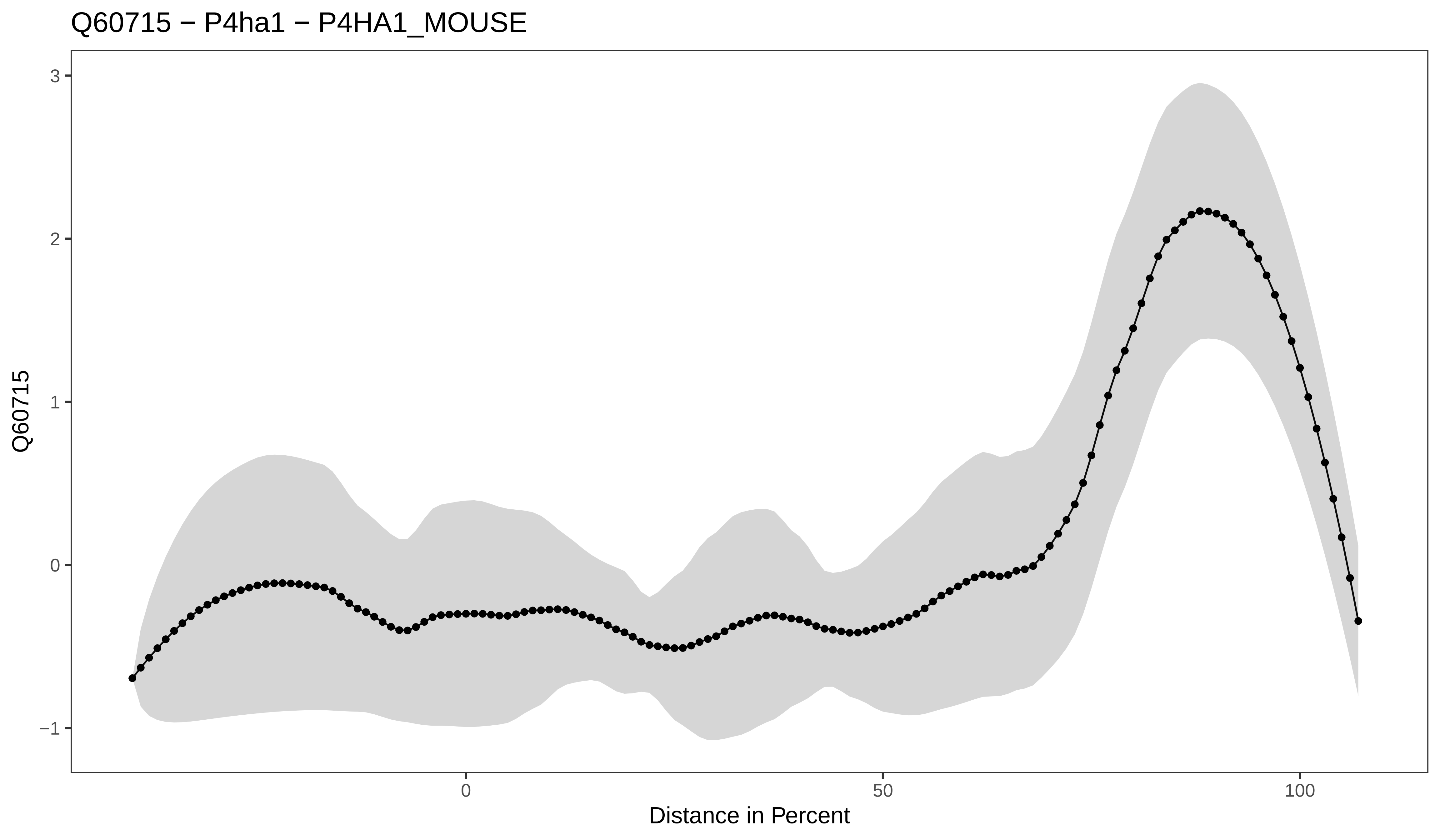

Q60715 − P4ha1 − P4HA1_MOUSE
Q60715
3
2
1
0
−1
0
50
100
Distance in Percent

P09103 − P4hb − PDIA1_MOUSE

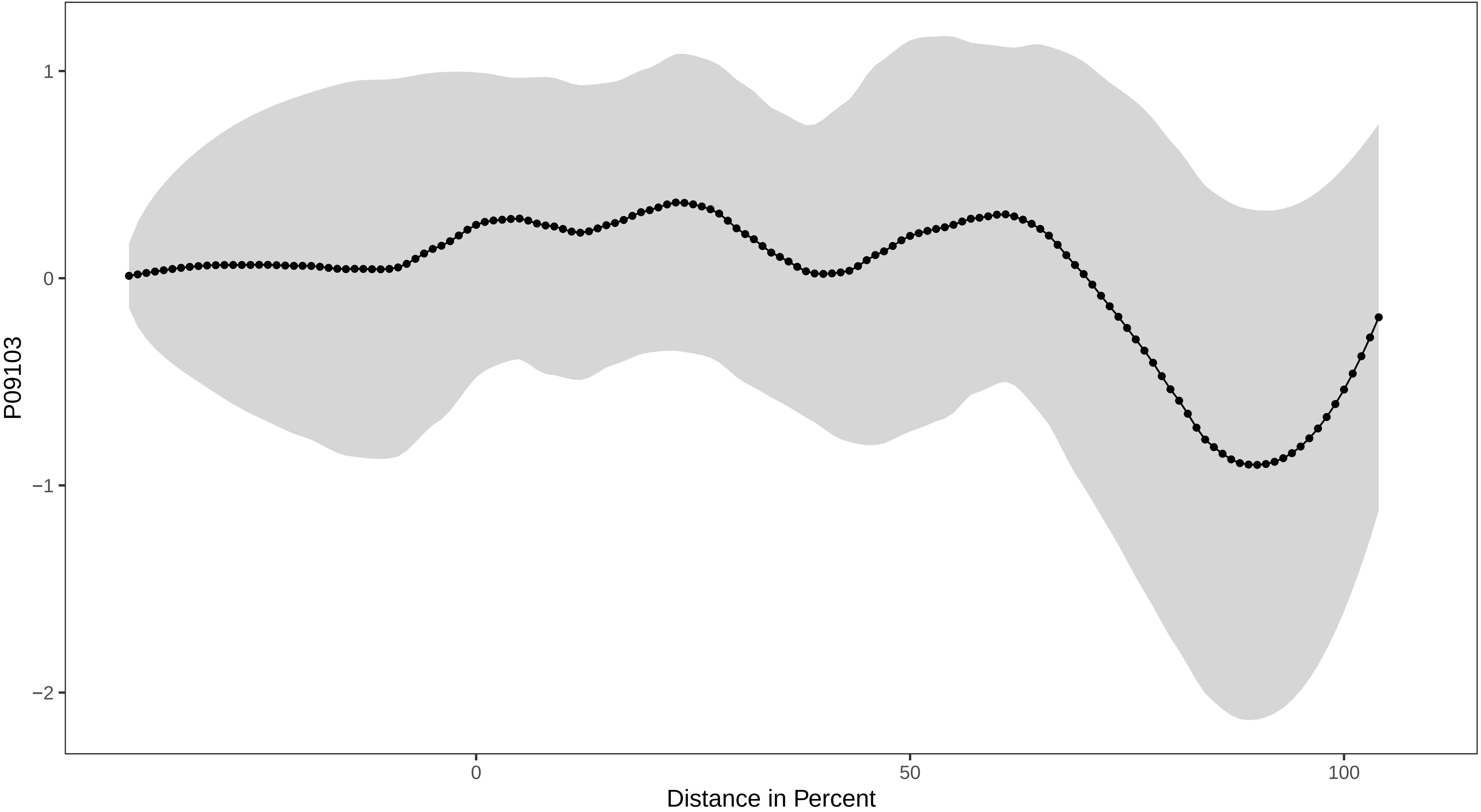

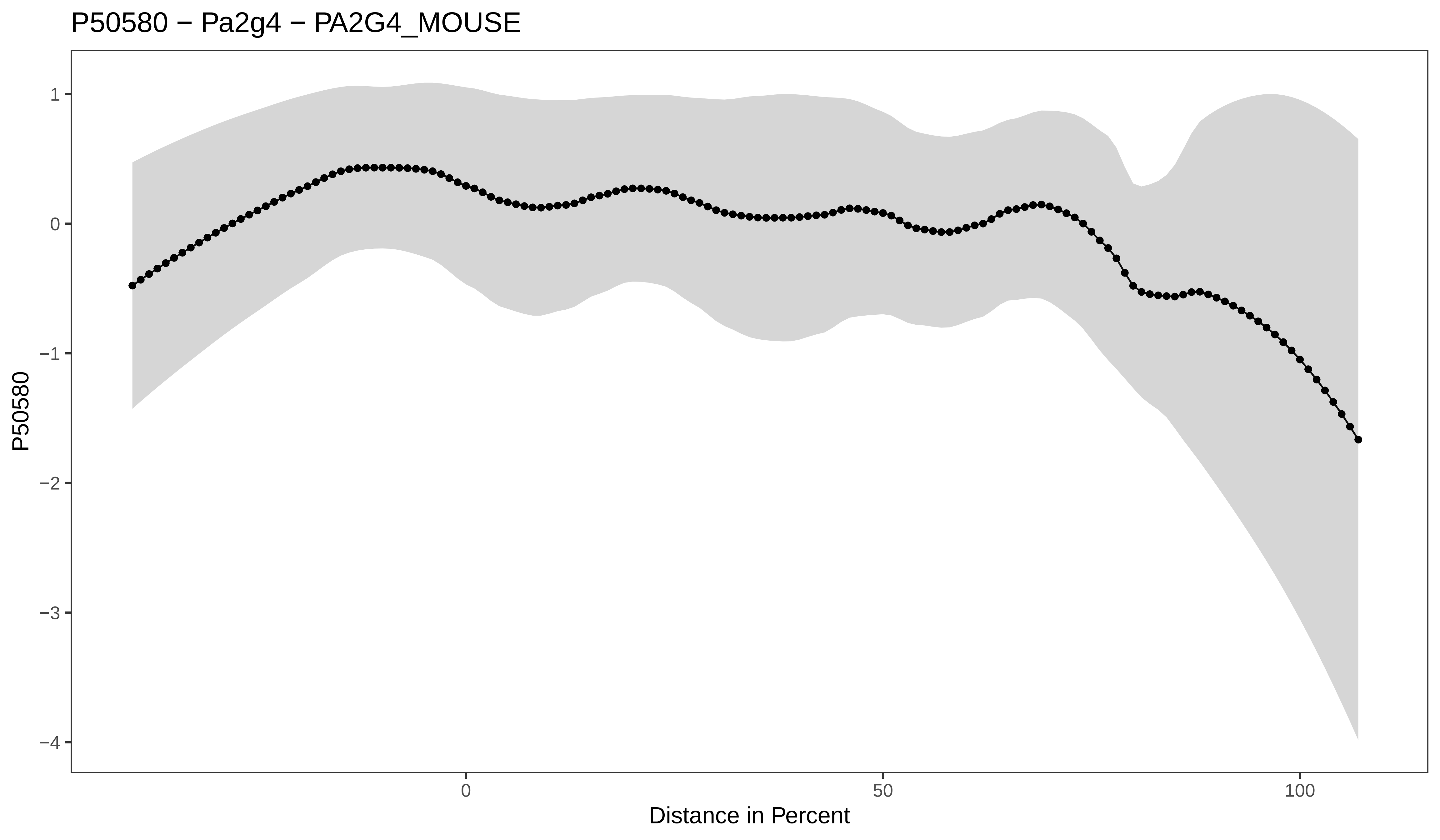

P50580 − Pa2g4 − PA2G4_MOUSE
P50580
1
0
−1
−2
−3
−4
0
50
100
Distance in Percent

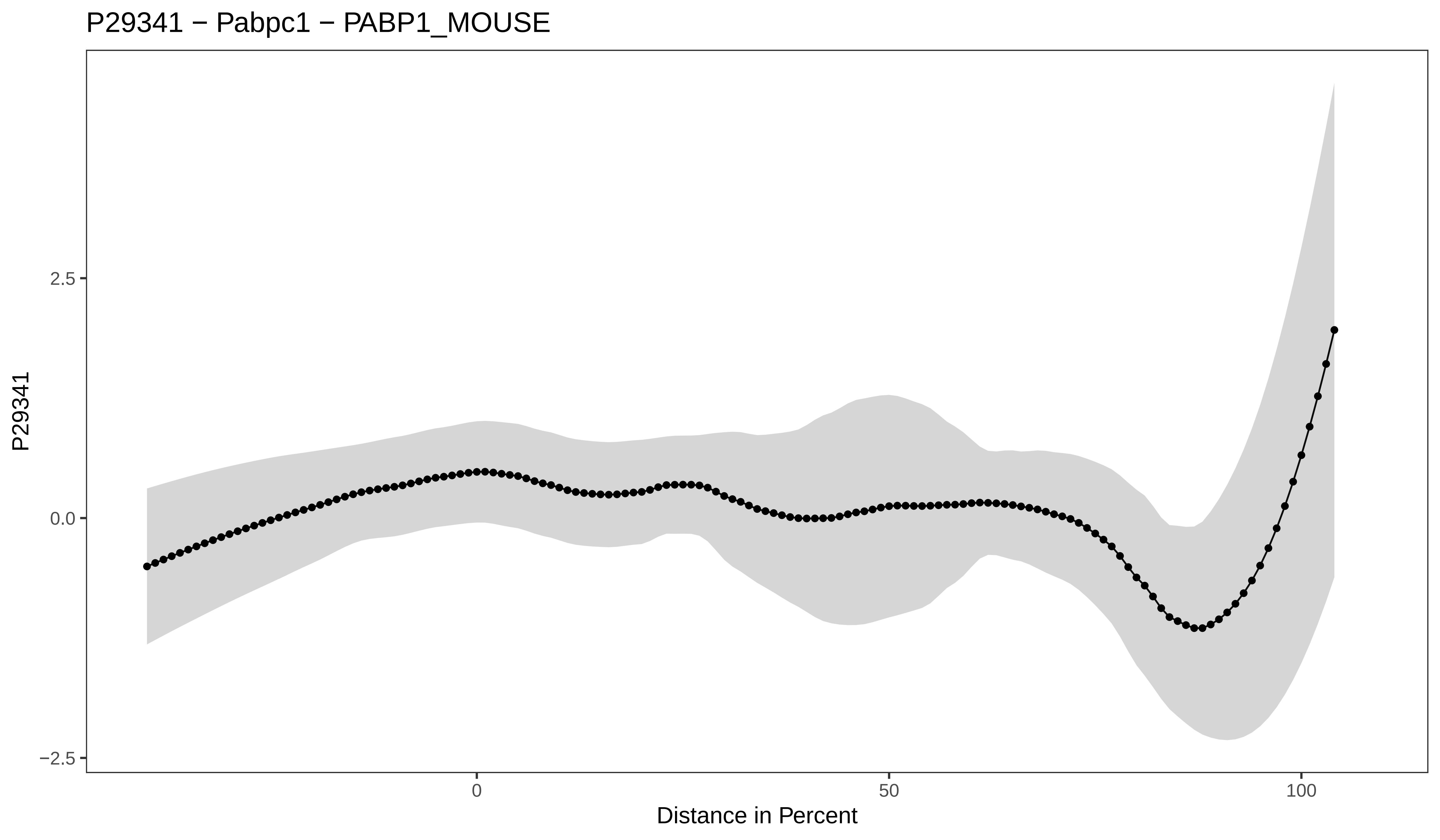
P29341 − Pabpc1 − PABP1_MOUSE
P29341
2.5
0.0
−2.5
0
50
100
Distance in Percent

Q8CCS6 − Pabpn1 − PABP2_MOUSE

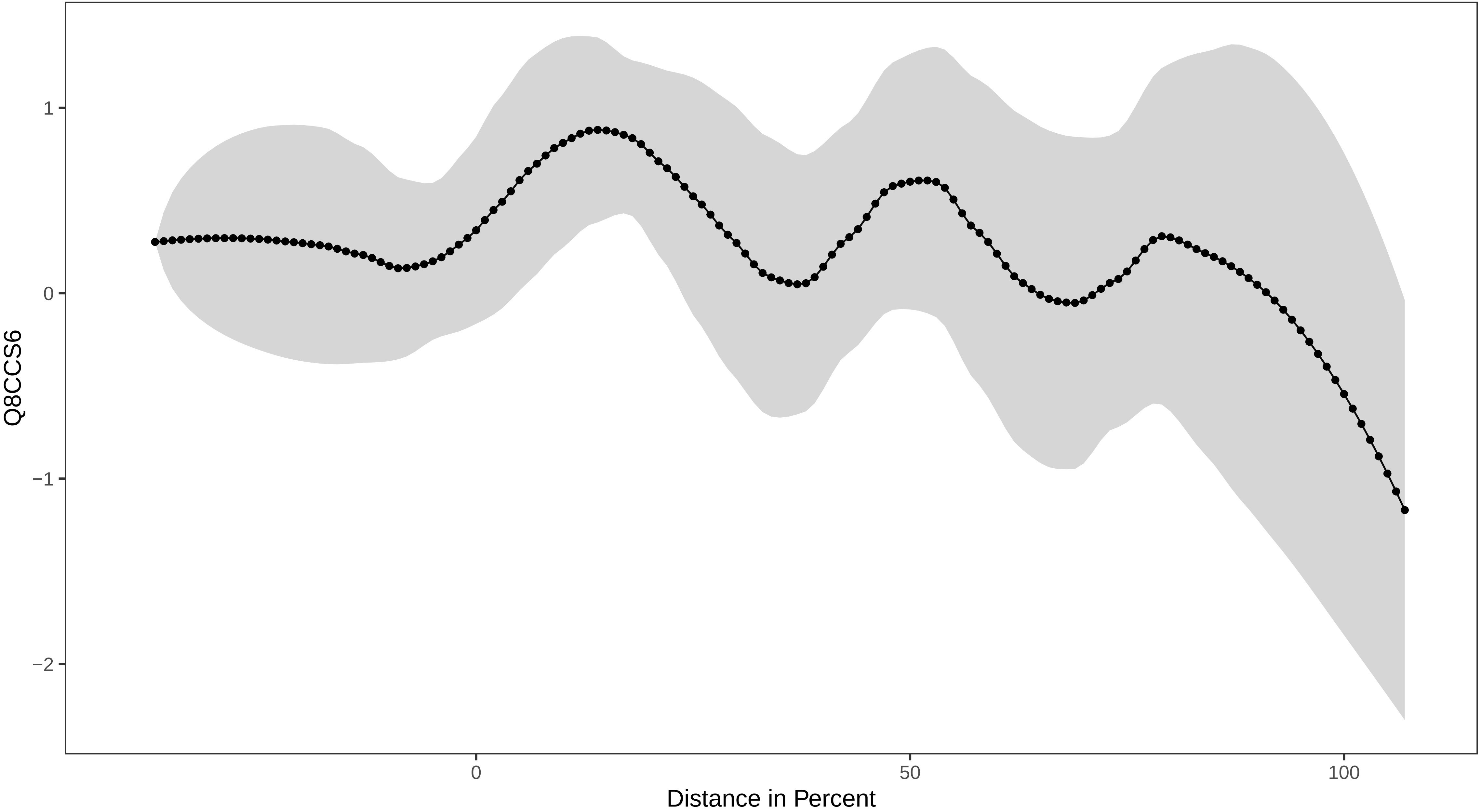

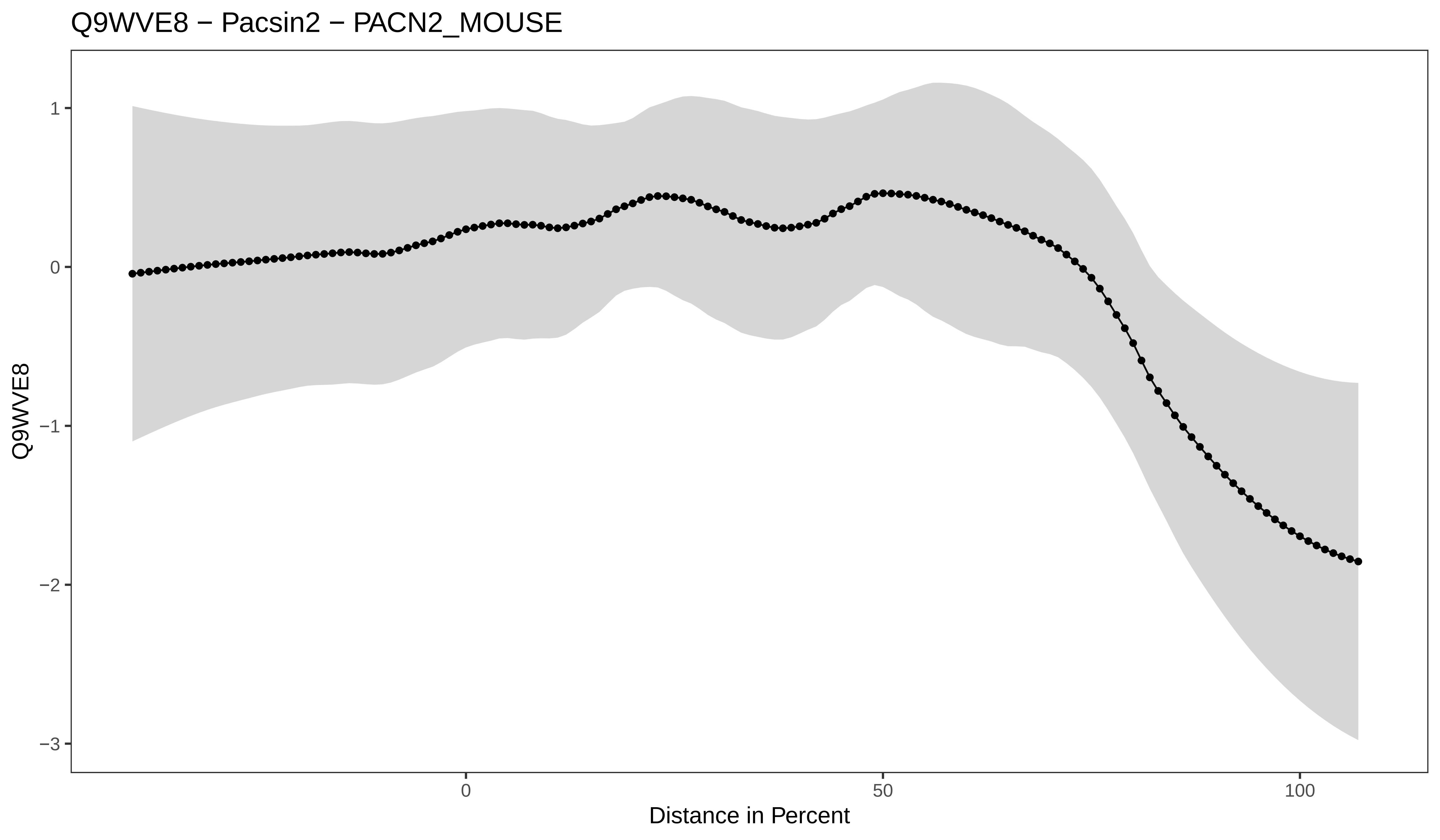

Q9WVE8 − Pacsin2 − PACN2_MOUSE
Q9WVE8
1
0
−1
−2
−3
0
50
100
Distance in Percent

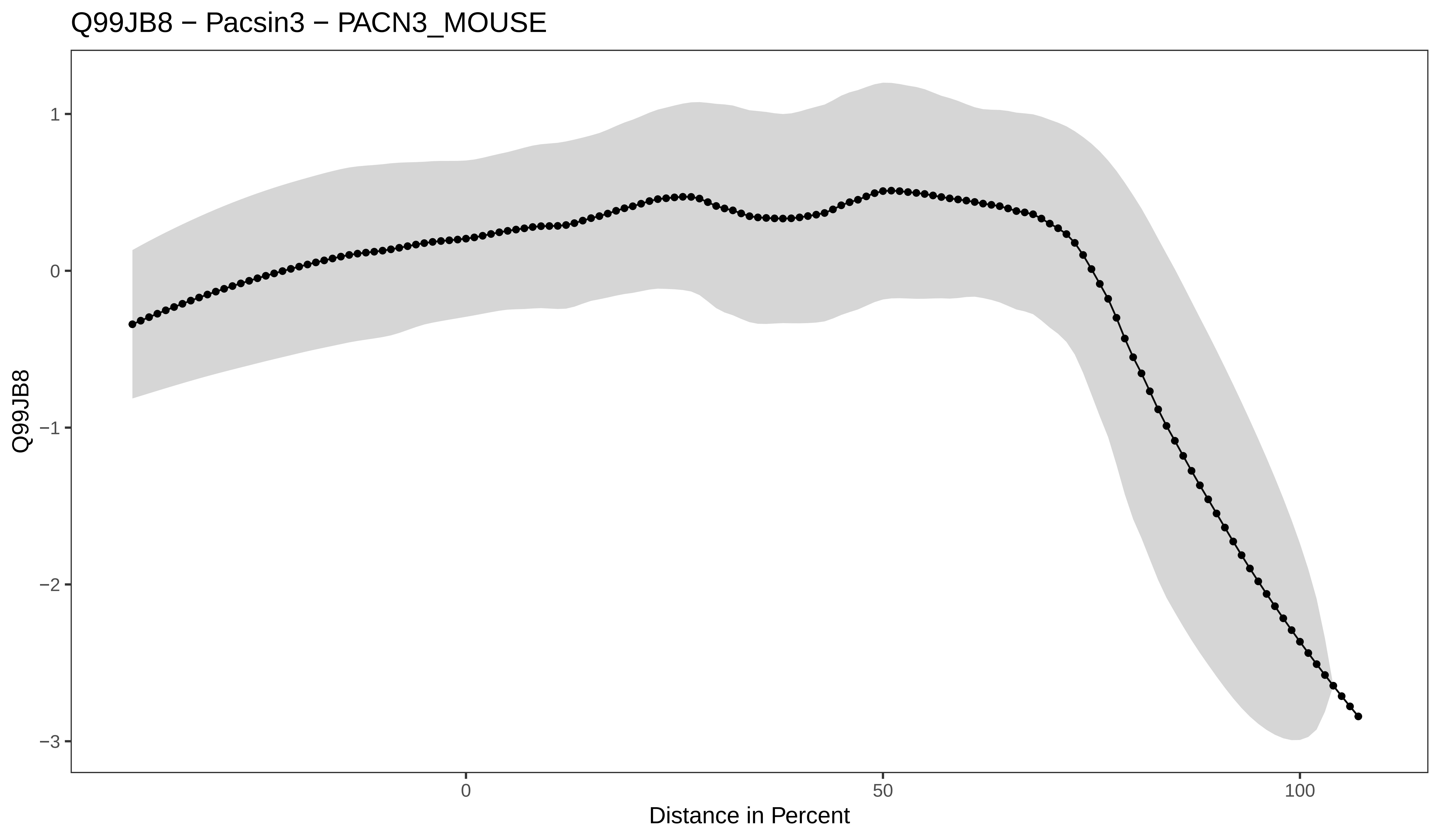

Q99JB8 − Pacsin3 − PACN3_MOUSE
Q99JB8
1
0
−1
−2
−3
0
50
100
Distance in Percent

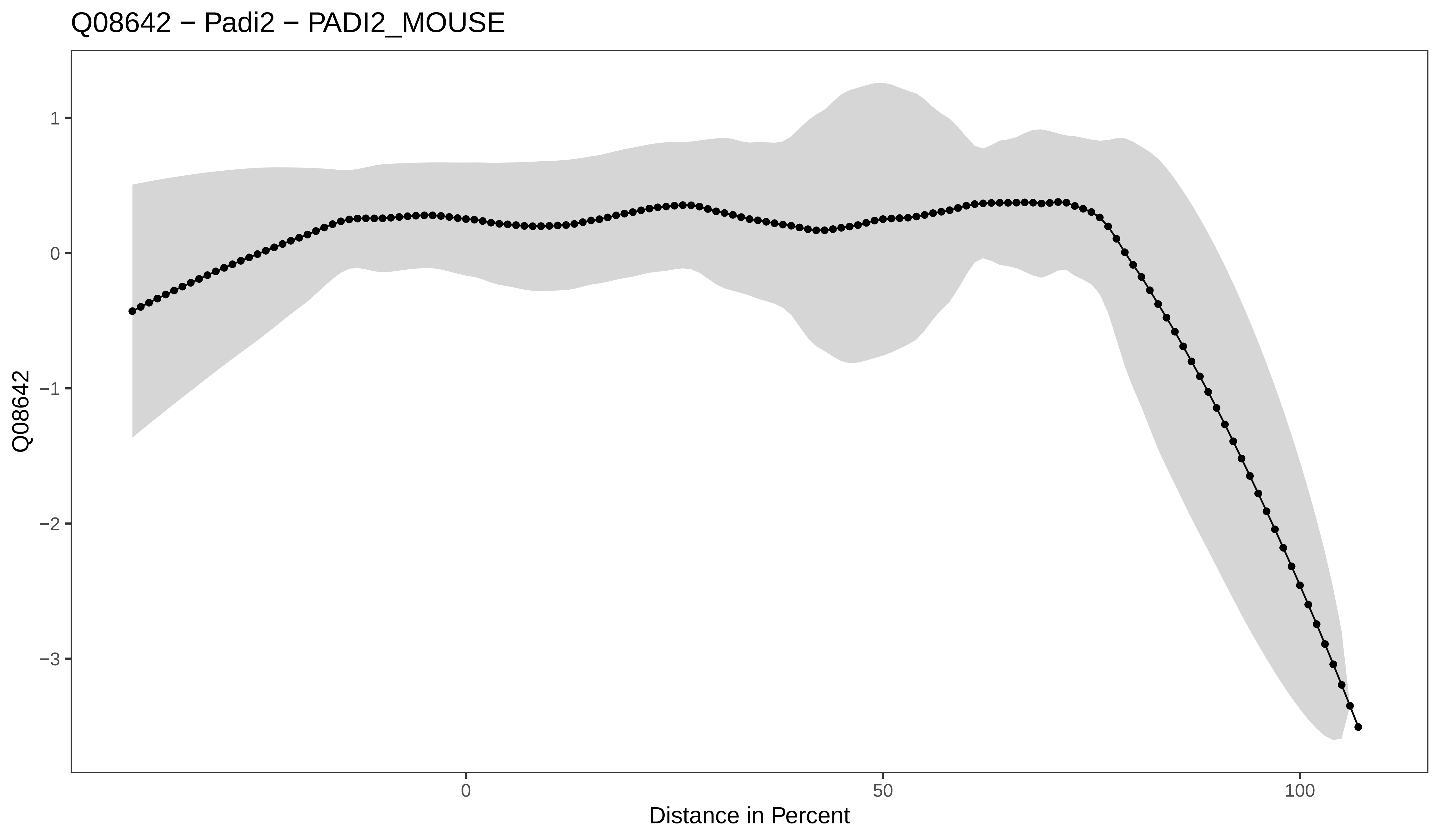
Q08642 − Padi2 − PADI2_MOUSE
1
0
−1
−2
−3
Q08642
0
50
100
Distance in Percent

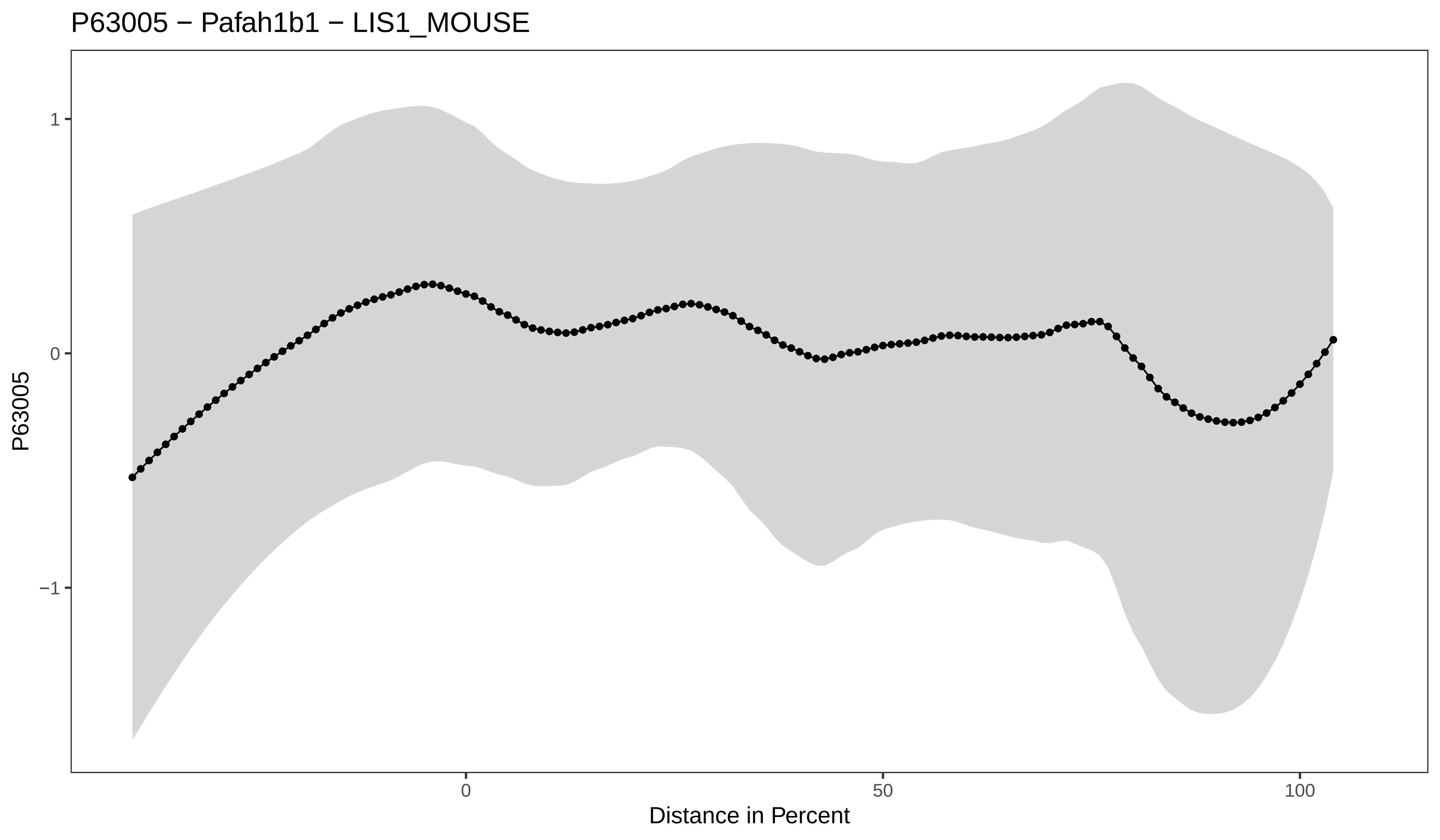

P63005 − Pafah1b1 − LIS1_MOUSE
1
0
−1
P63005
0
50
100
Distance in Percent

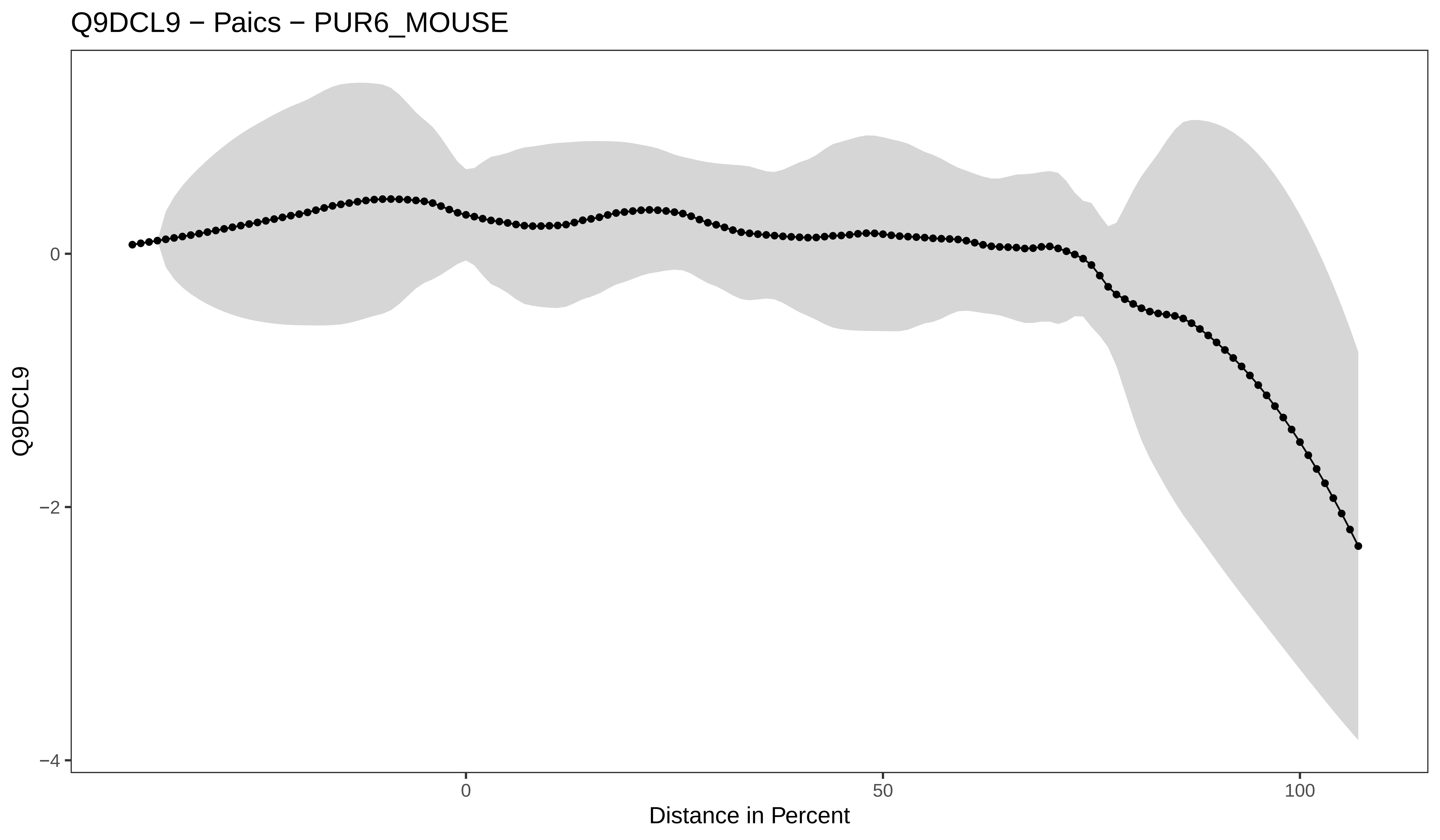
Q9DCL9 − Paics − PUR6_MOUSE
Q9DCL9
0
−2
−4
0
50
100
Distance in Percent

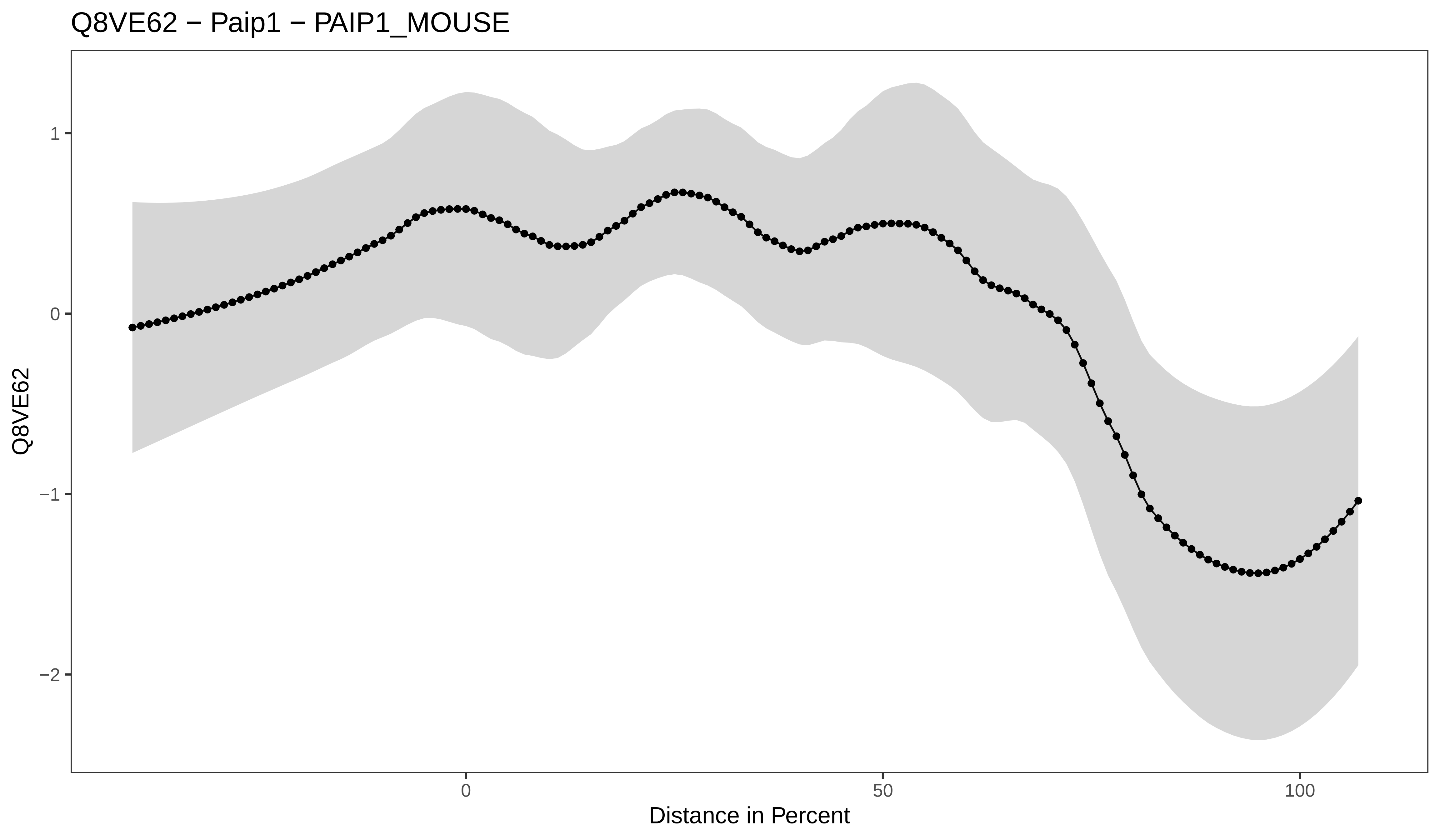

Q8VE62 − Paip1 − PAIP1_MOUSE
Q8VE62
1
0
−1
−2
0
50
100
Distance in Percent

Q8CIN4 − Pak2 − PAK2_MOUSE

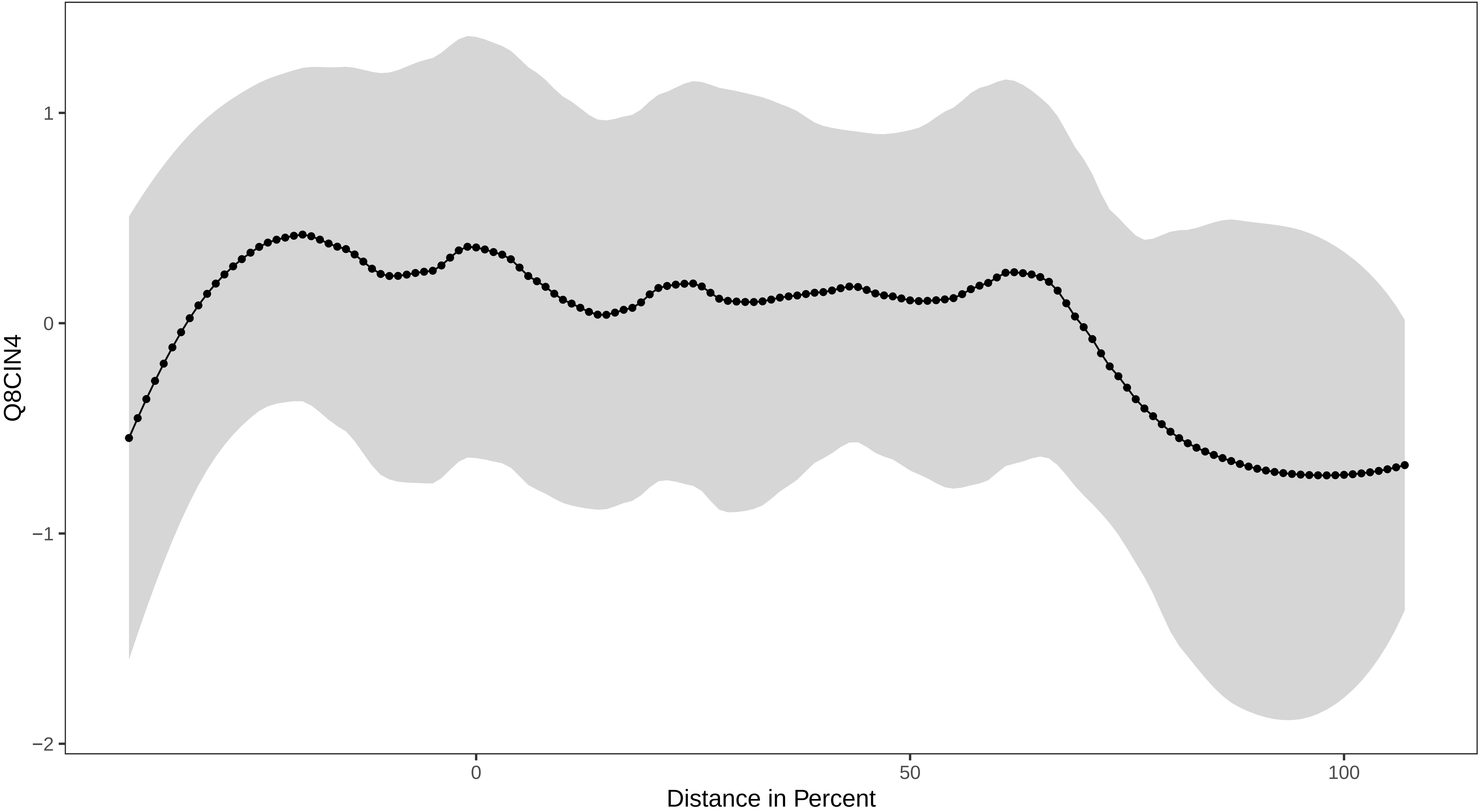

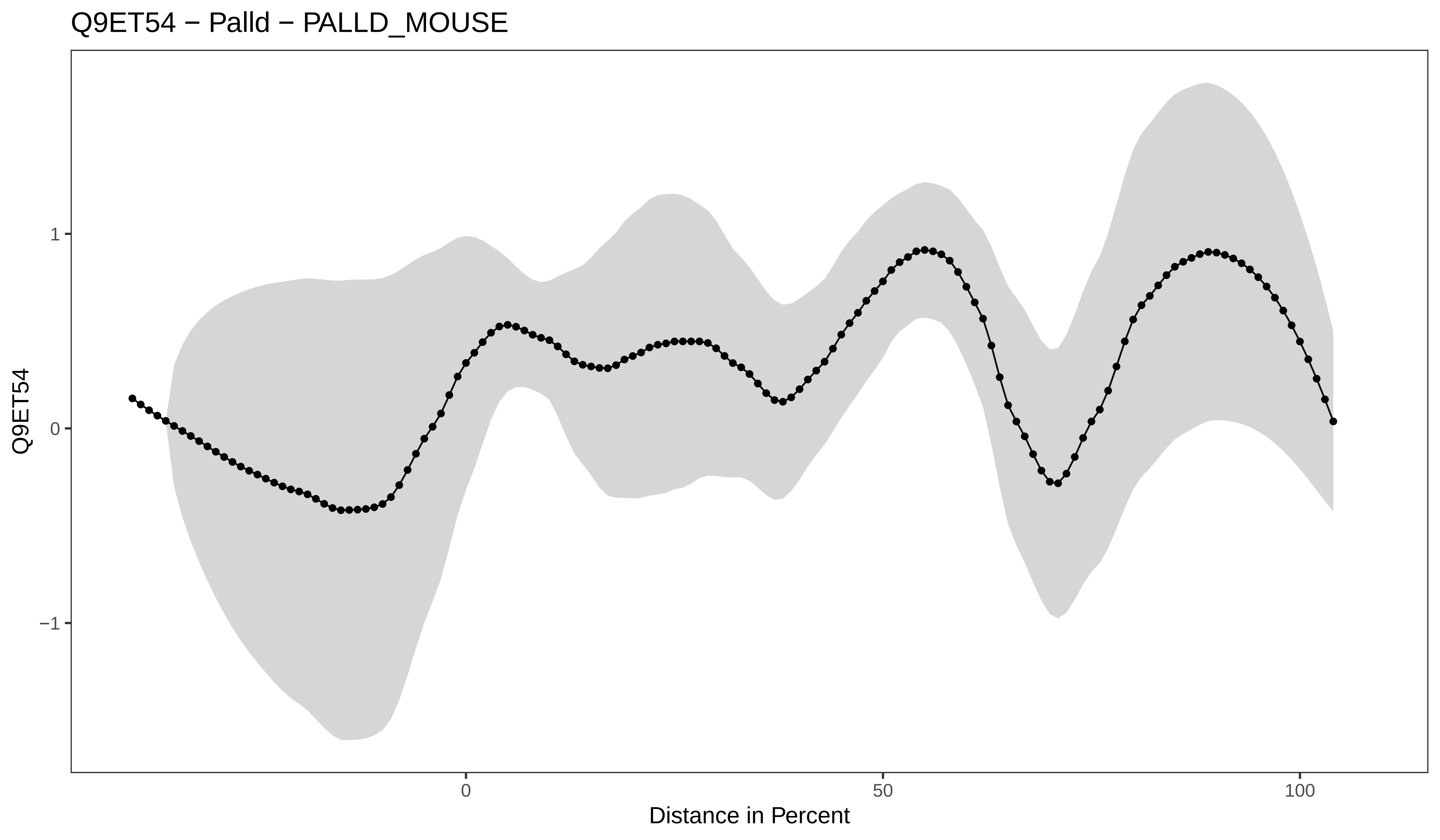

Q9ET54 − Palld − PALLD_MOUSE
Q9ET54
1
0
−1
0
50
100
Distance in Percent

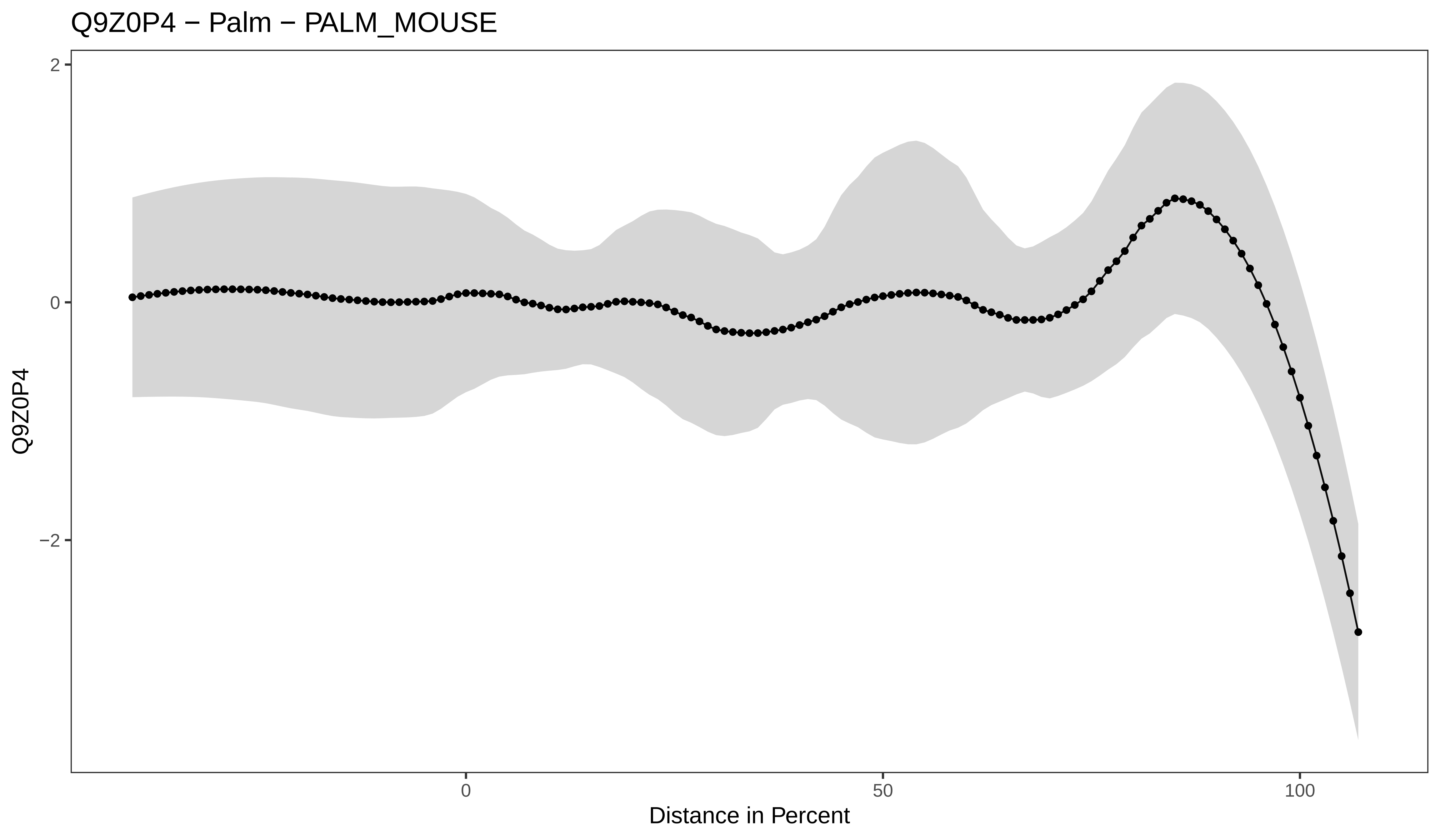
Q9Z0P4 − Palm − PALM_MOUSE
2
0
−2
Q9Z0P4
0
50
100
Distance in Percent

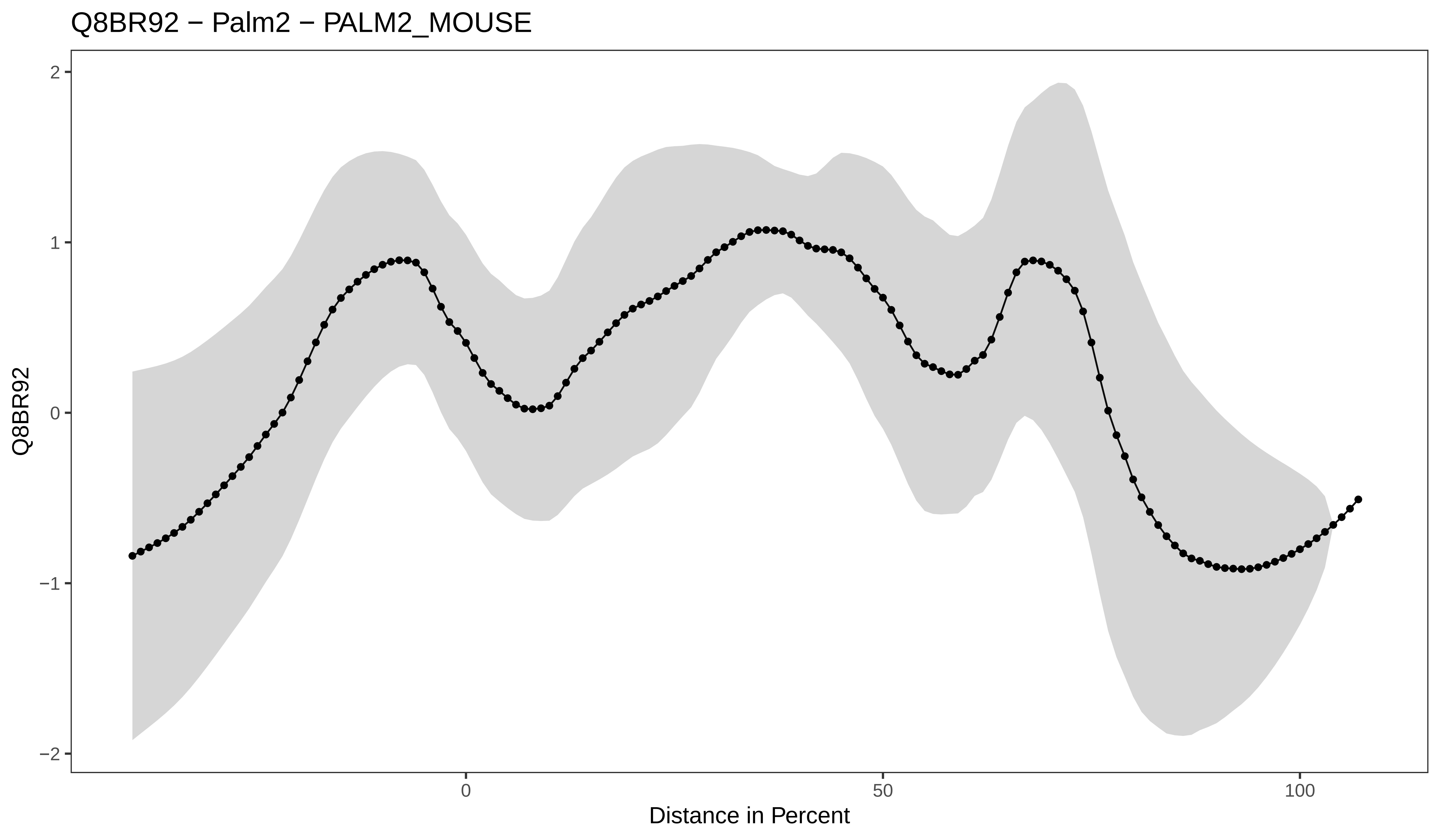
Q8BR92 − Palm2 − PALM2_MOUSE
Q8BR92
2
1
0
−1
−2
0
50
100
Distance in Percent

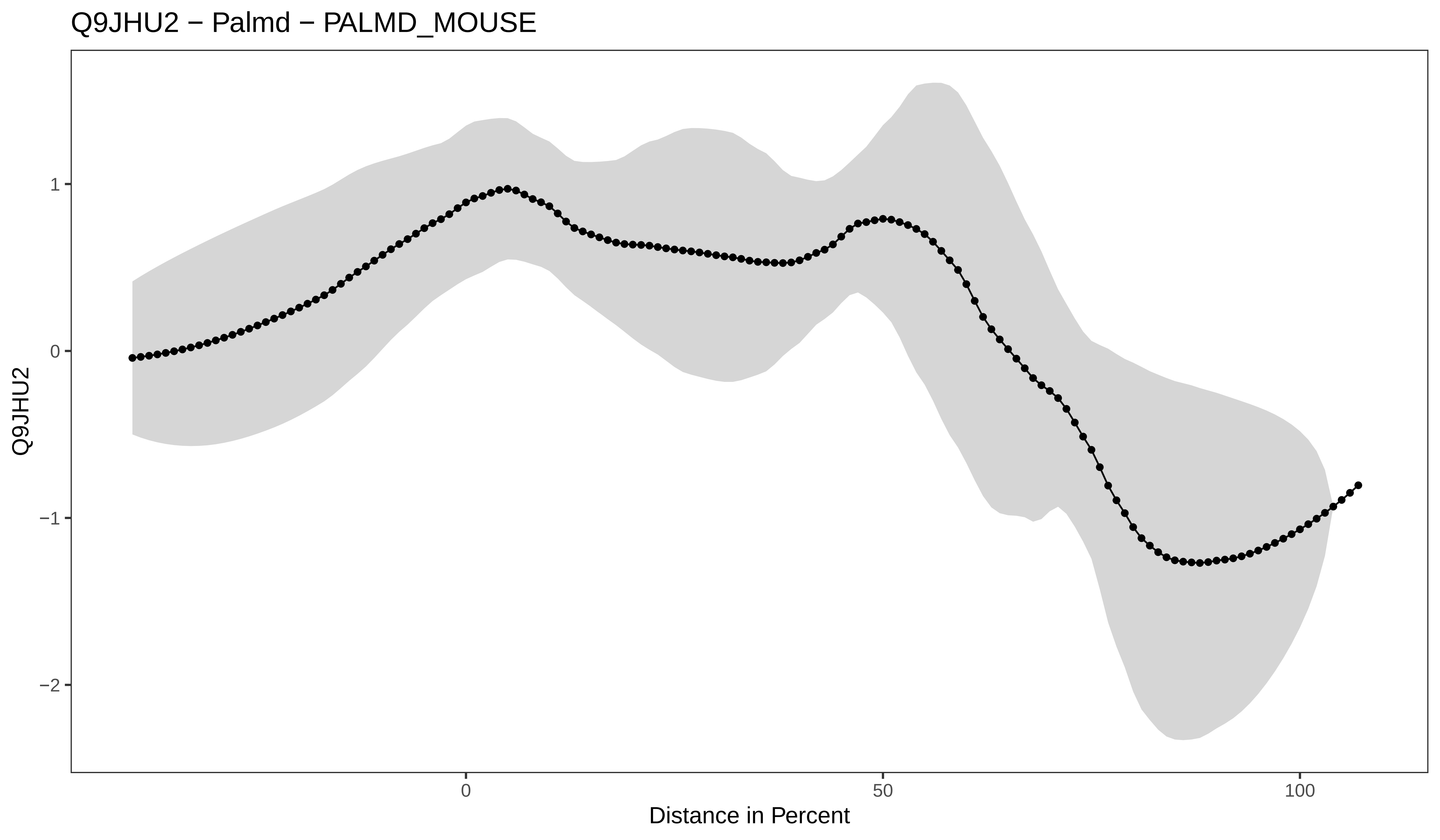

Q9JHU2 − Palmd − PALMD_MOUSE
1
0
−1
−2
Q9JHU2
0
50
100
Distance in Percent

Q80YV4 − Pank4 − PANK4_MOUSE

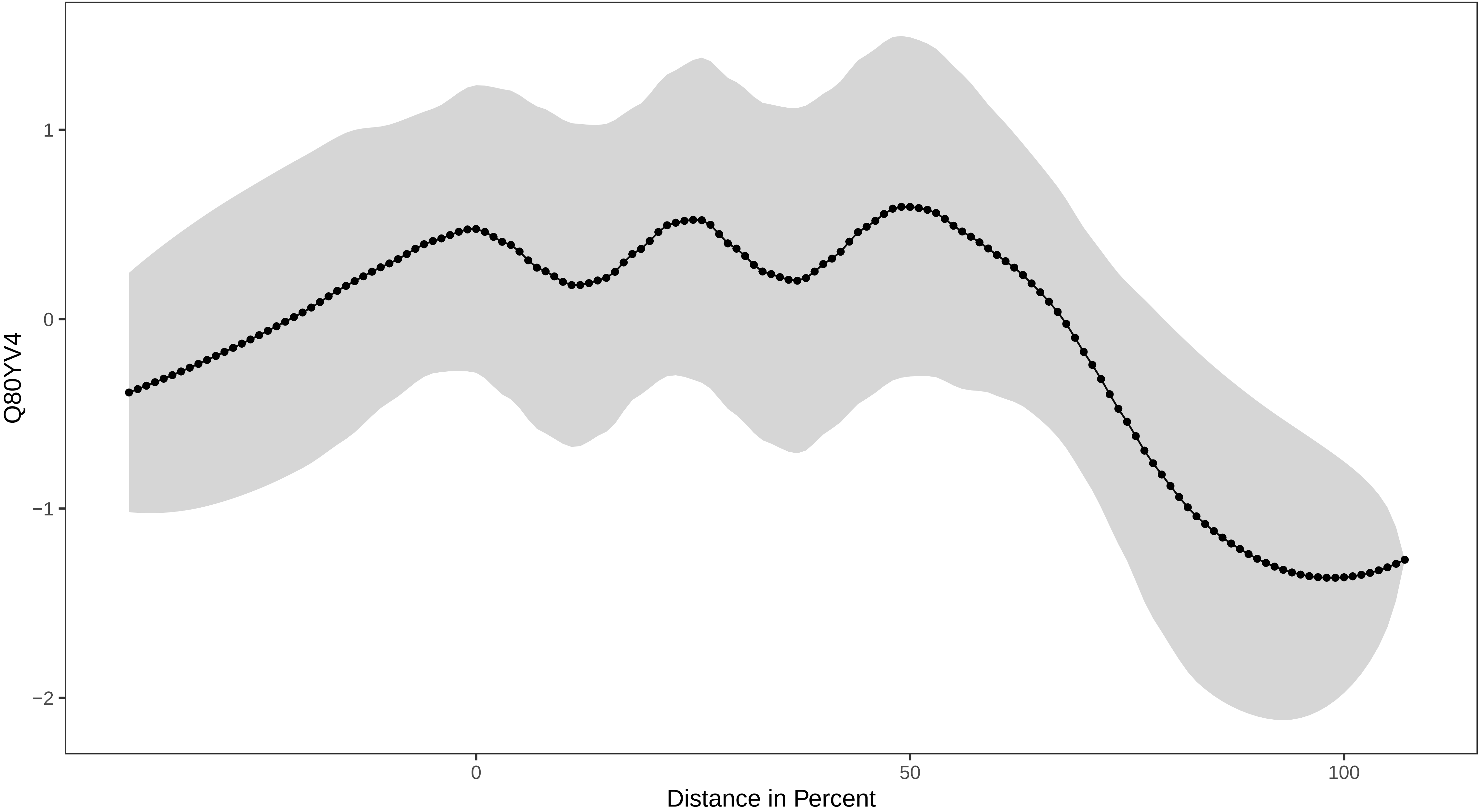

Q99LX0 − Park7 − PARK7_MOUSE

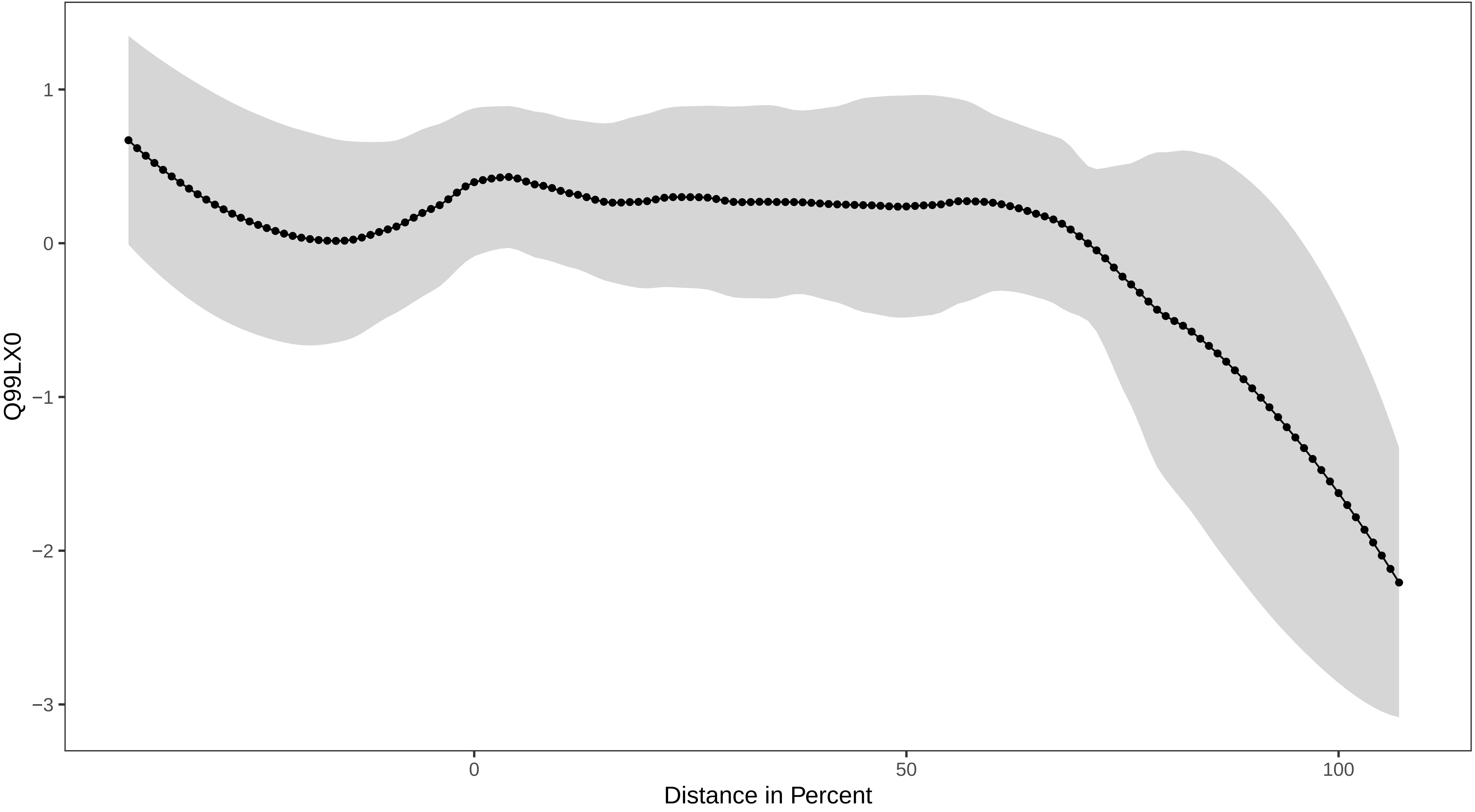

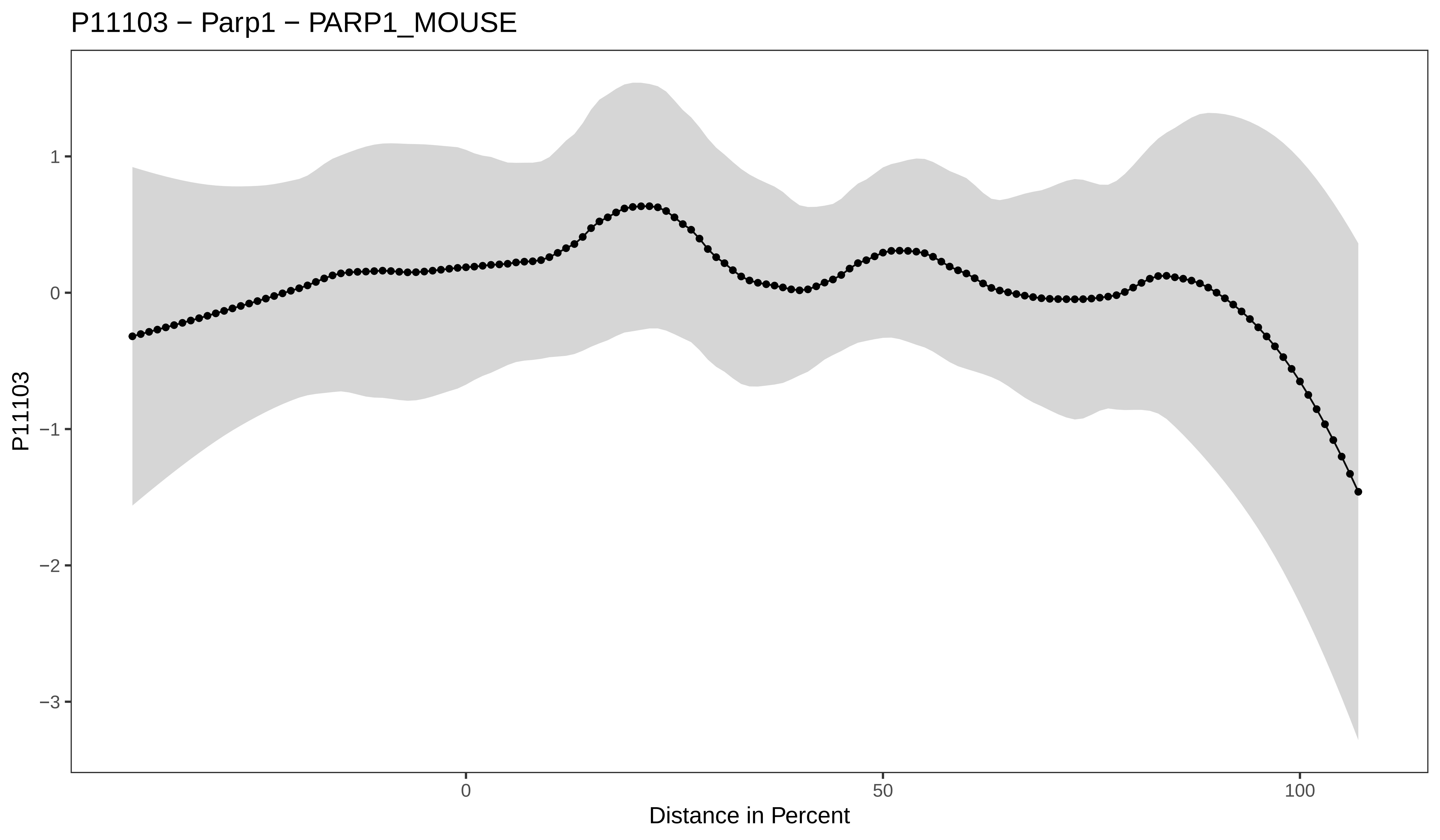
P11103 − Parp1 − PARP1_MOUSE
1
0
−1
−2
−3
P11103
0
50
100
Distance in Percent

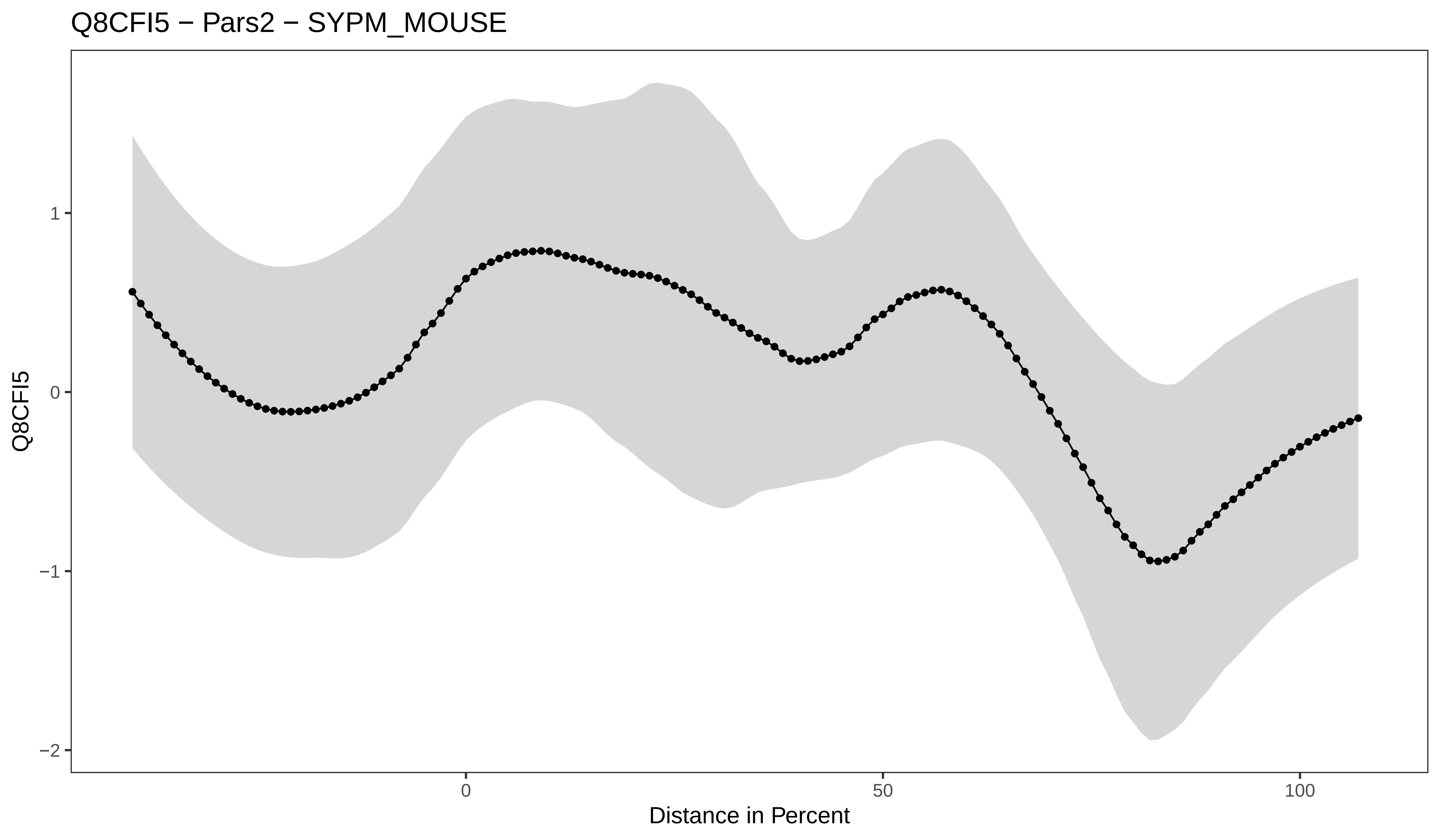
Q8CFI5 − Pars2 − SYPM_MOUSE
Q8CFI5
1
0
−1
−2
0
50
100
Distance in Percent

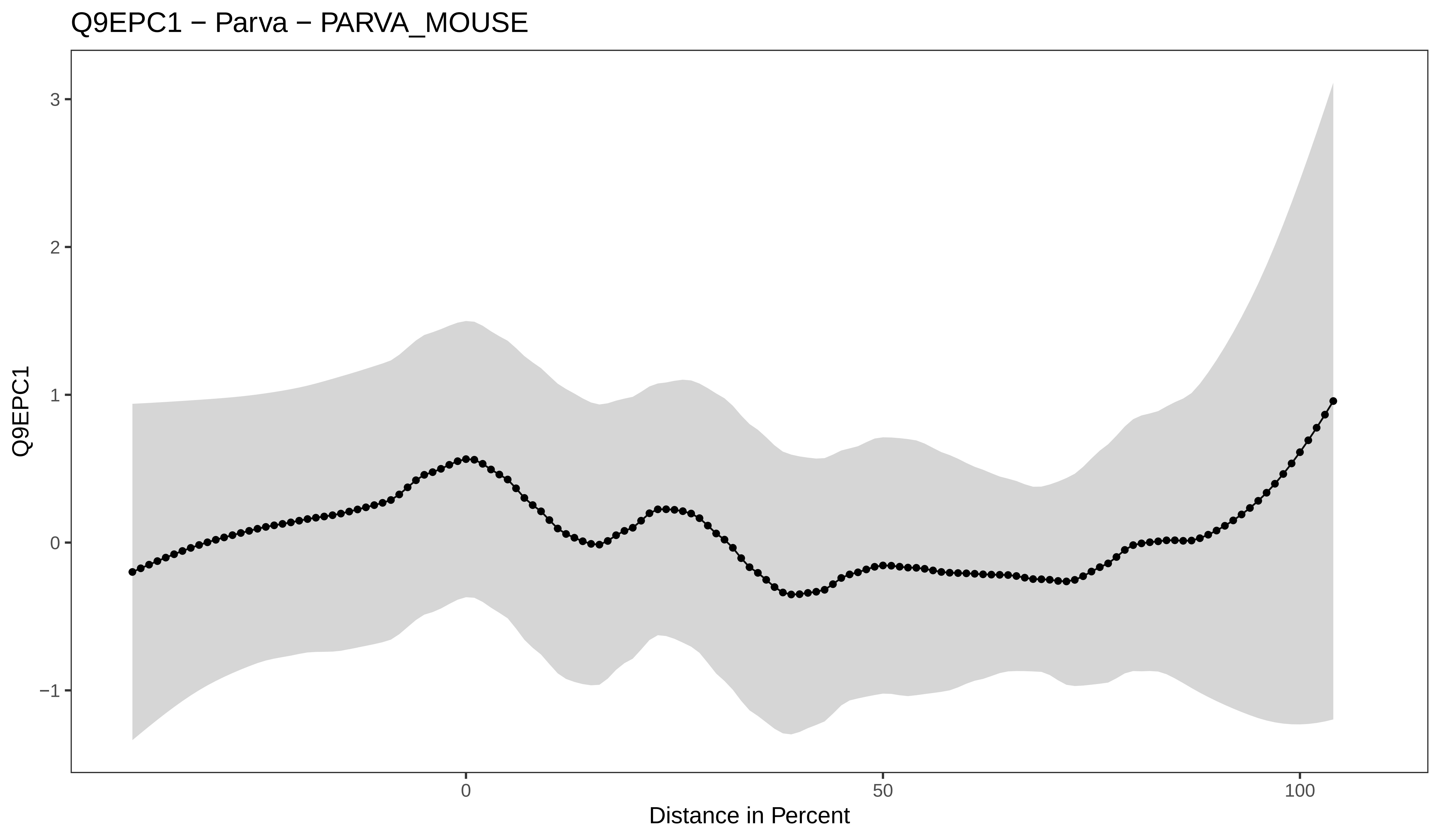
Q9EPC1 − Parva − PARVA_MOUSE
Q9EPC1
Distance in Percent
3
2
1
0
−1
0
50
100

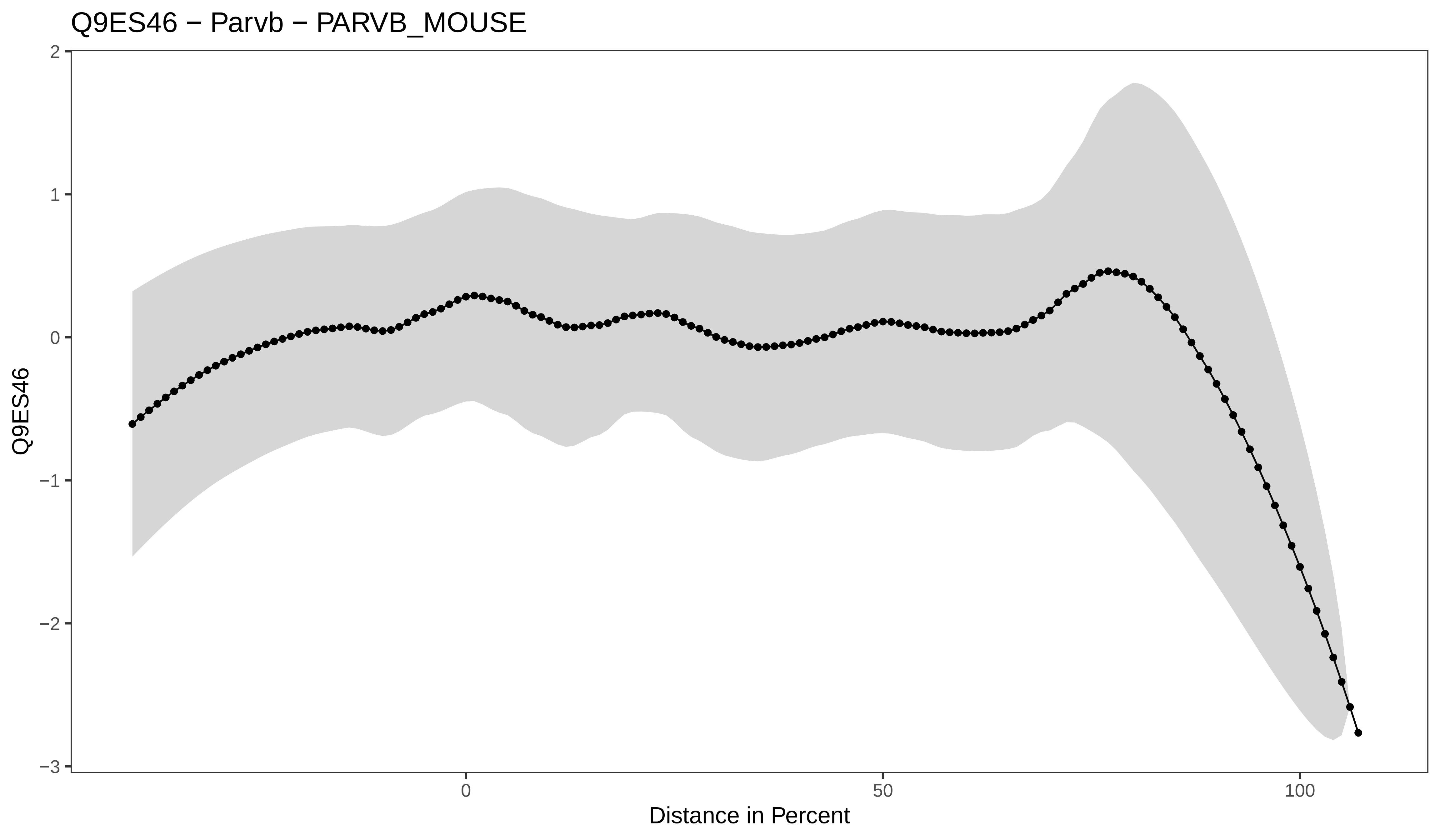

Q9ES46 − Parvb − PARVB_MOUSE
2
1
0
−1
−2
−3
Q9ES46
0
50
100
Distance in Percent

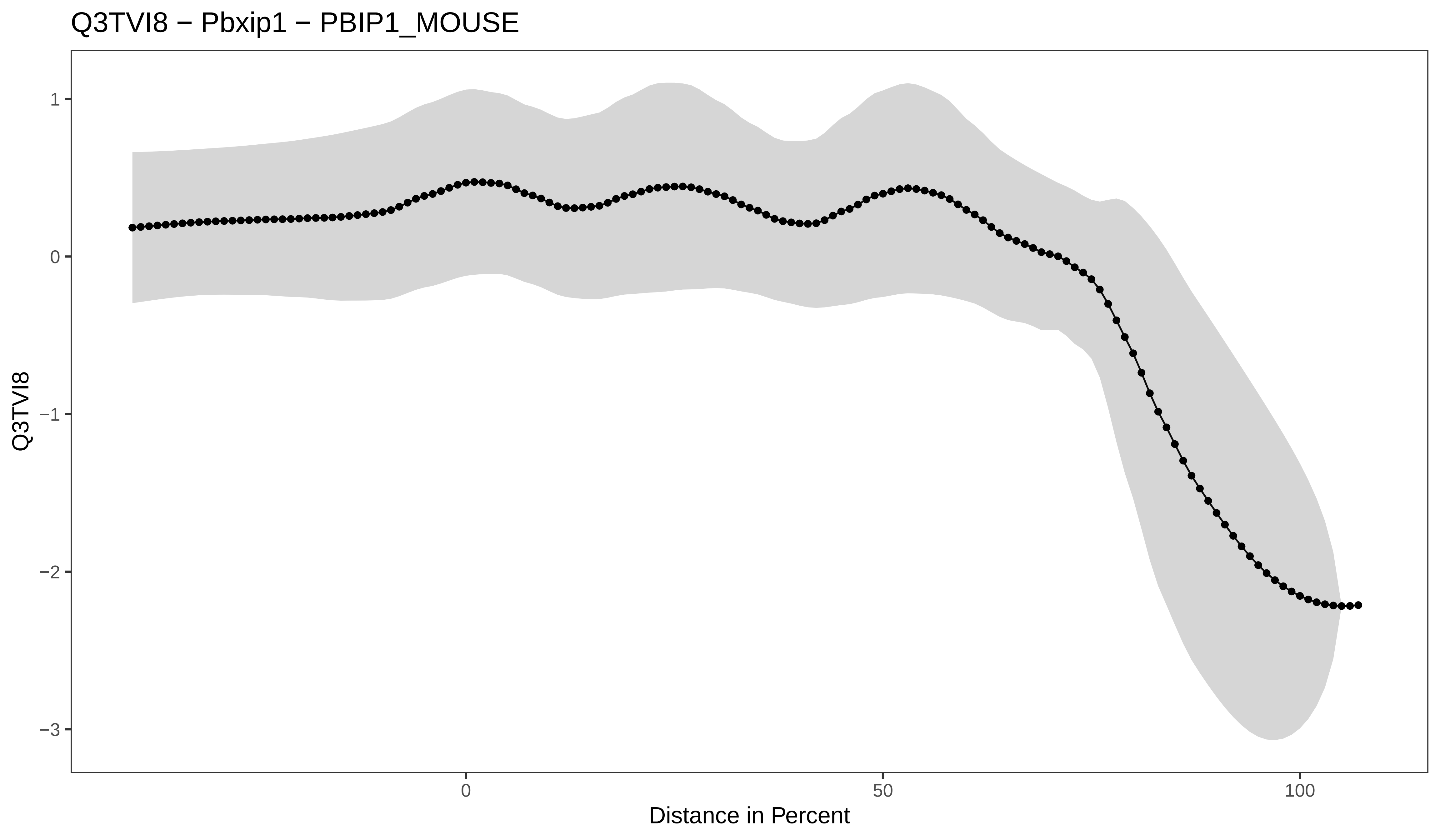
Q3TVI8 − Pbxip1 − PBIP1_MOUSE
Q3TVI8
Distance in Percent
1
0
−1
−2
−3
0
50
100

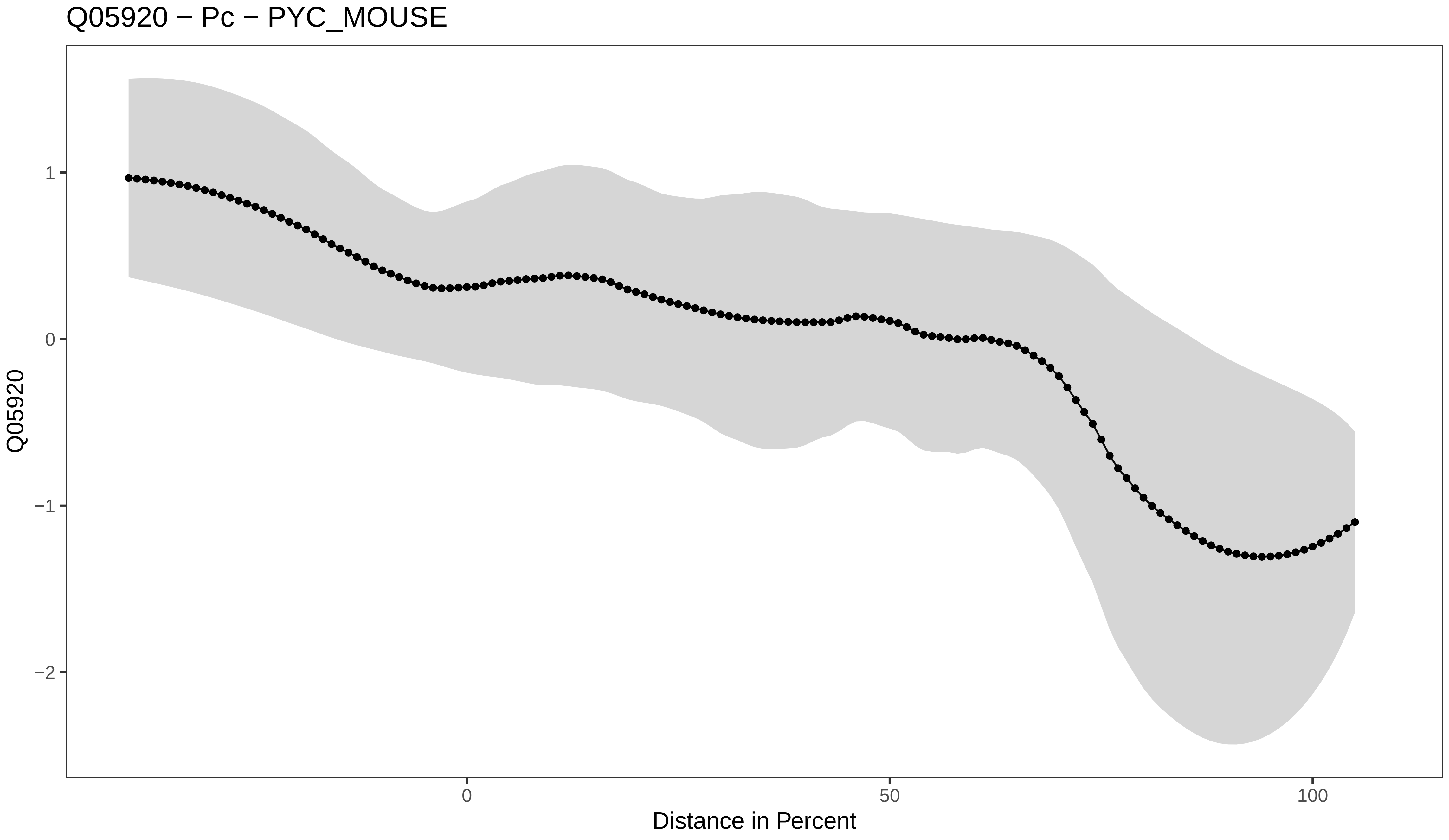

Q05920 − Pc − PYC_MOUSE
Q05920
1
0
−1
−2
0
50
100
Distance in Percent

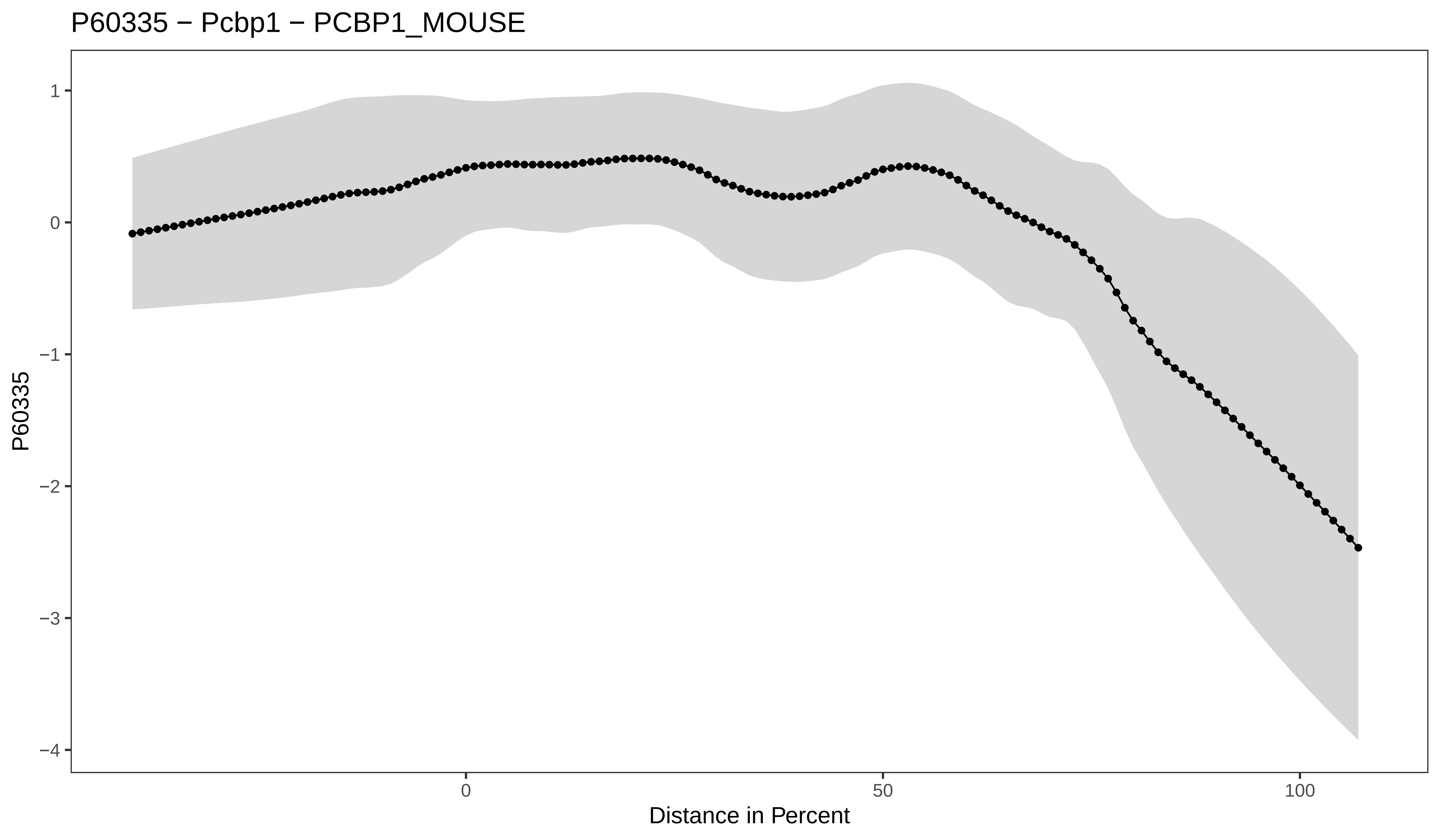

P60335 − Pcbp1 − PCBP1_MOUSE
1
0
−1
−2
−3
−4
P60335
0
50
100
Distance in Percent

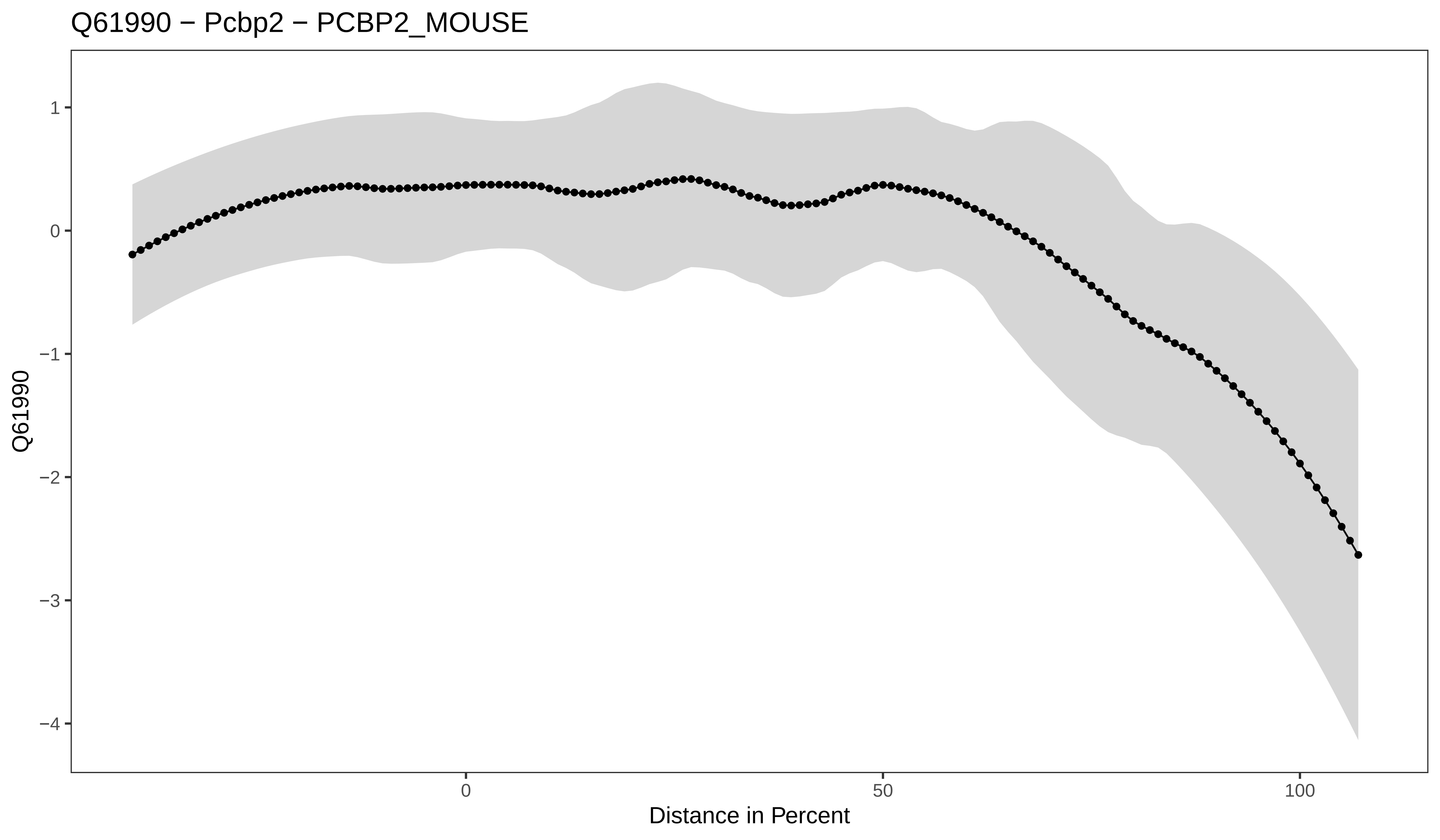

Q61990 − Pcbp2 − PCBP2_MOUSE
1
0
−1
−2
−3
−4
Q61990
0
50
100
Distance in Percent

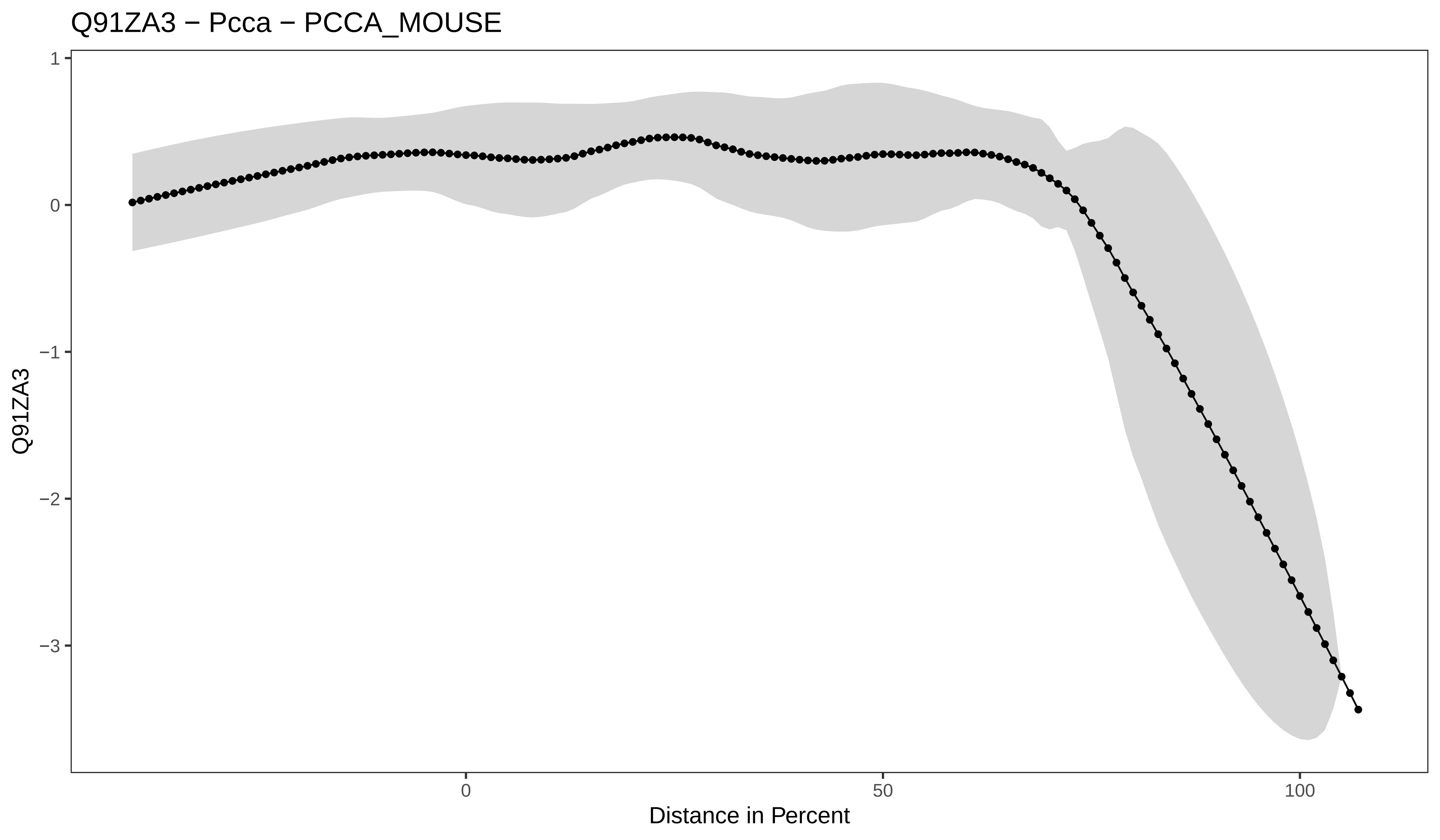

Q91ZA3 − Pcca − PCCA_MOUSE
1
0
−1
−2
−3
Q91ZA3
0
50
100
Distance in Percent

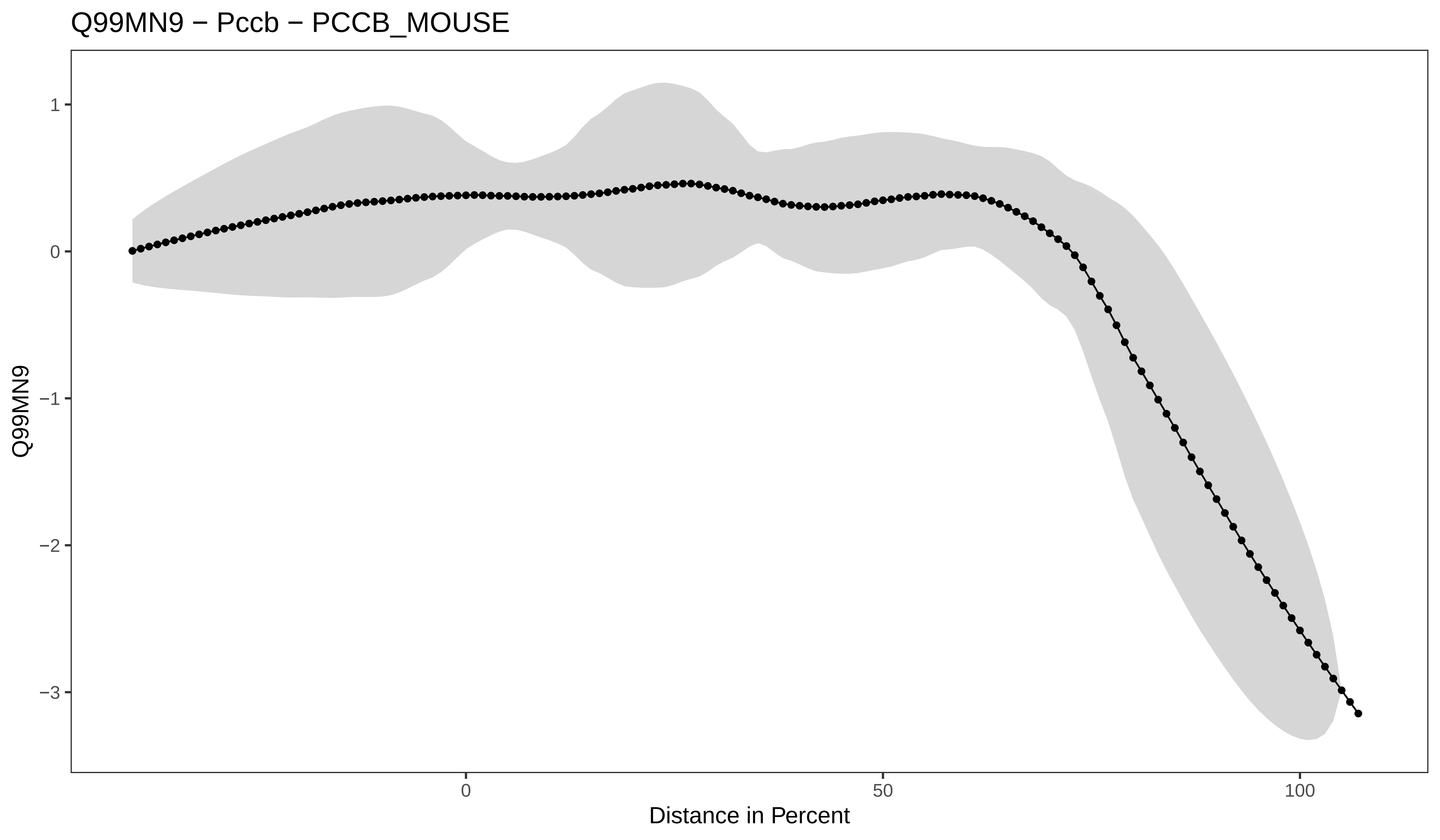

Q99MN9 − Pccb − PCCB_MOUSE
1
0
−1
−2
−3
Q99MN9
0
50
100
Distance in Percent

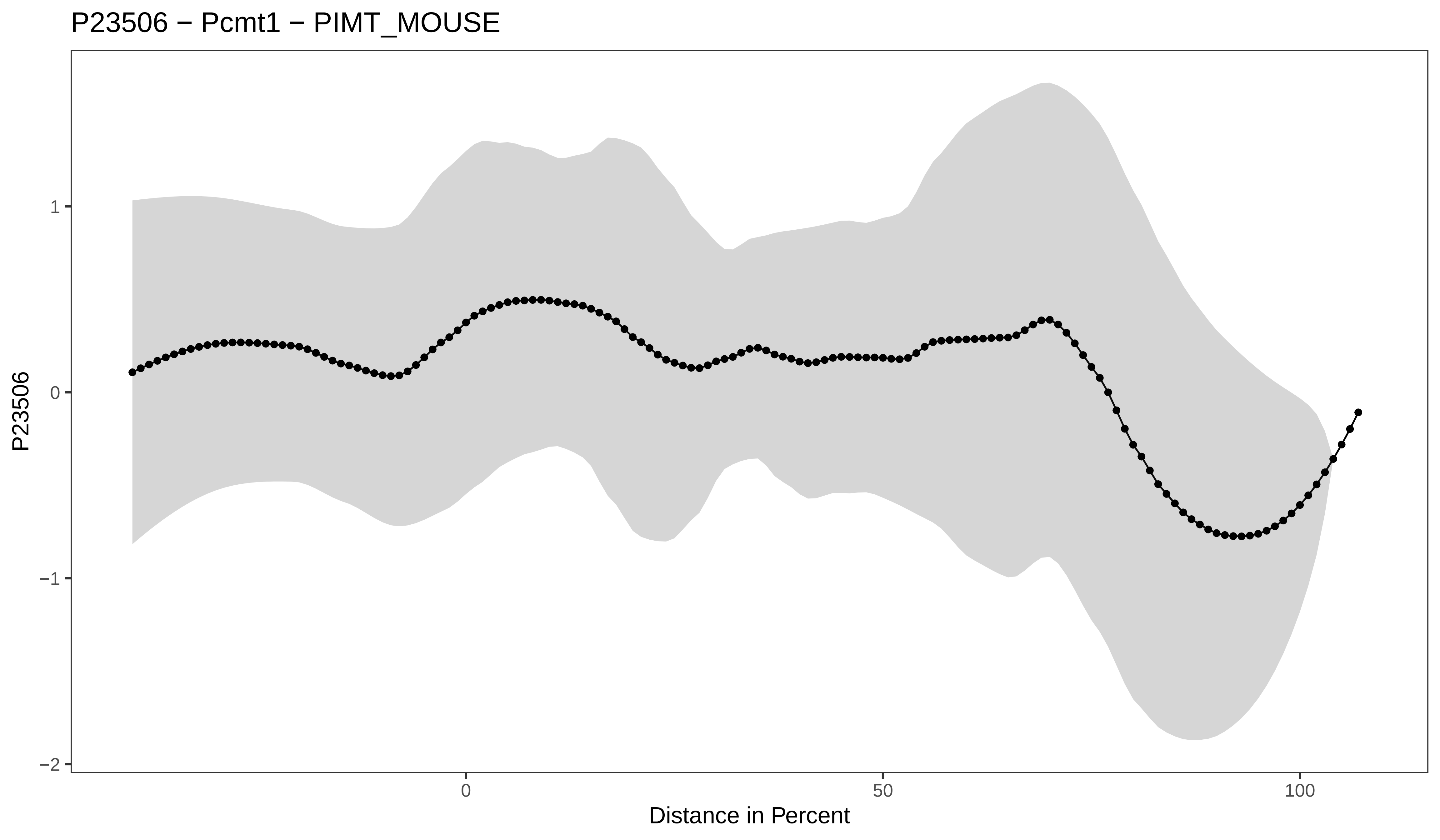

P23506 − Pcmt1 − PIMT_MOUSE
P23506
1
0
−1
−2
0
50
100
Distance in Percent

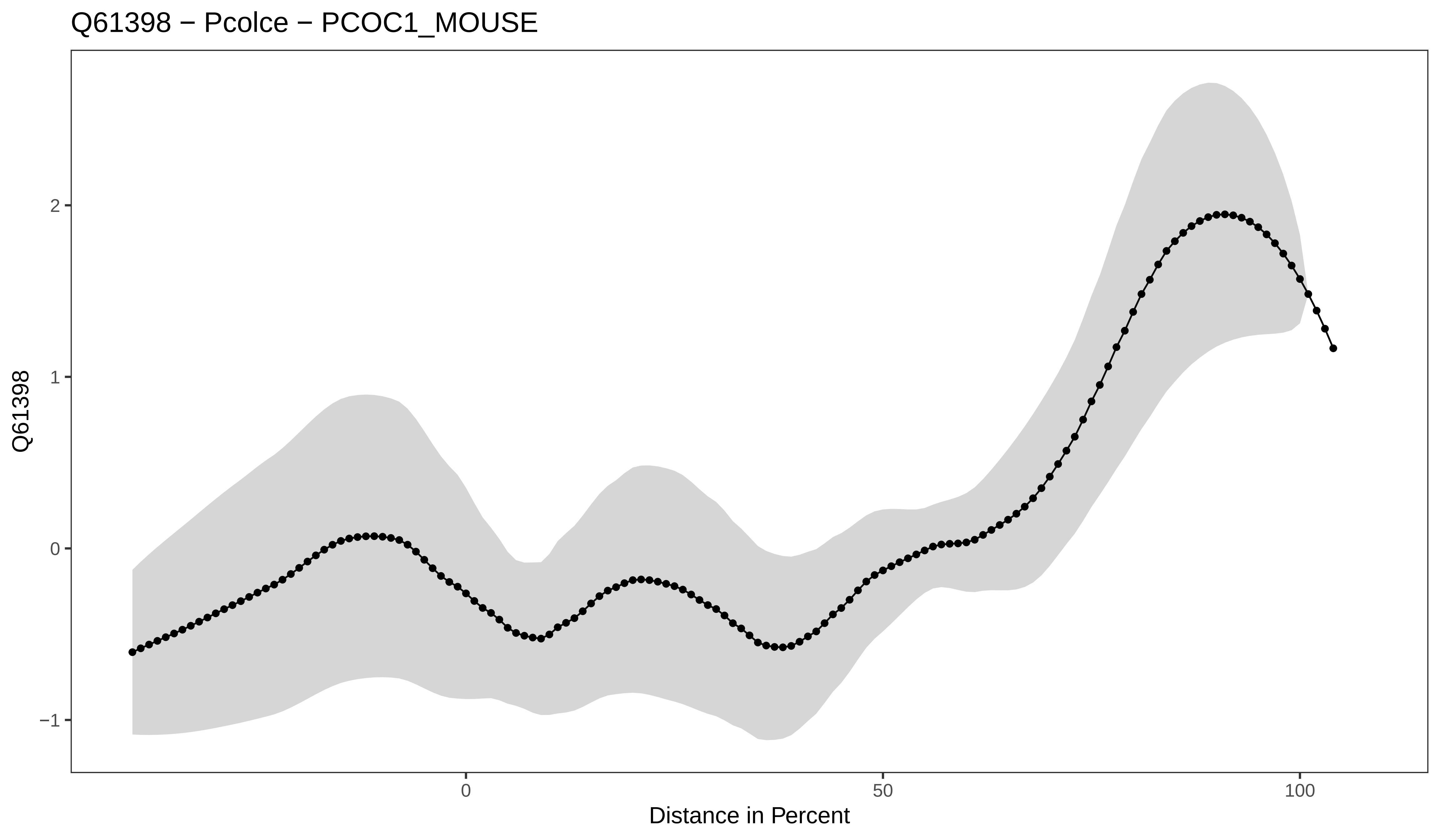
Q61398 − Pcolce − PCOC1_MOUSE
Q61398
2
1
0
−1
0
50
100
Distance in Percent

Q9CQF9 − Pcyox1 − PCYOX_MOUSE

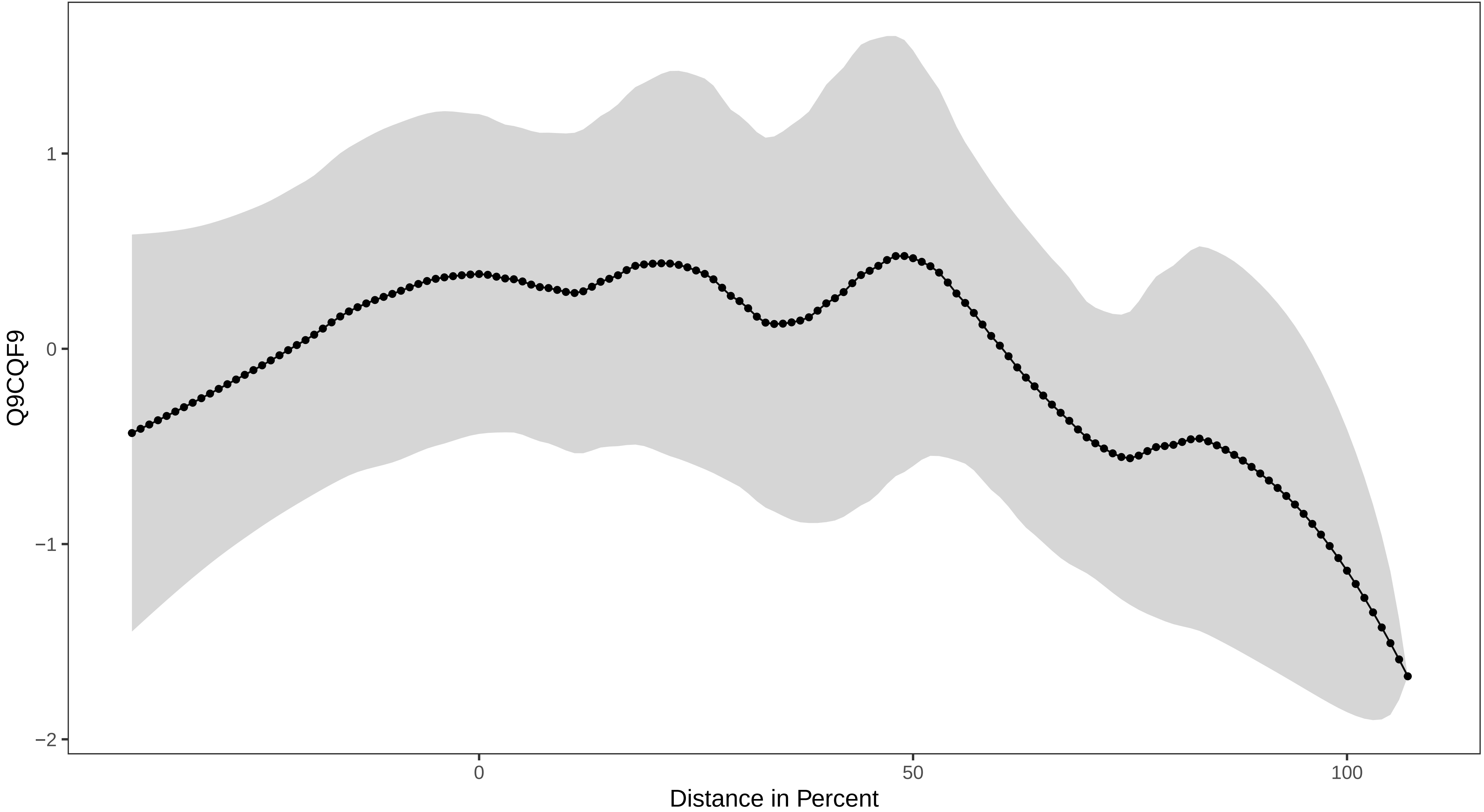

P49586 − Pcyt1a − PCY1A_MOUSE

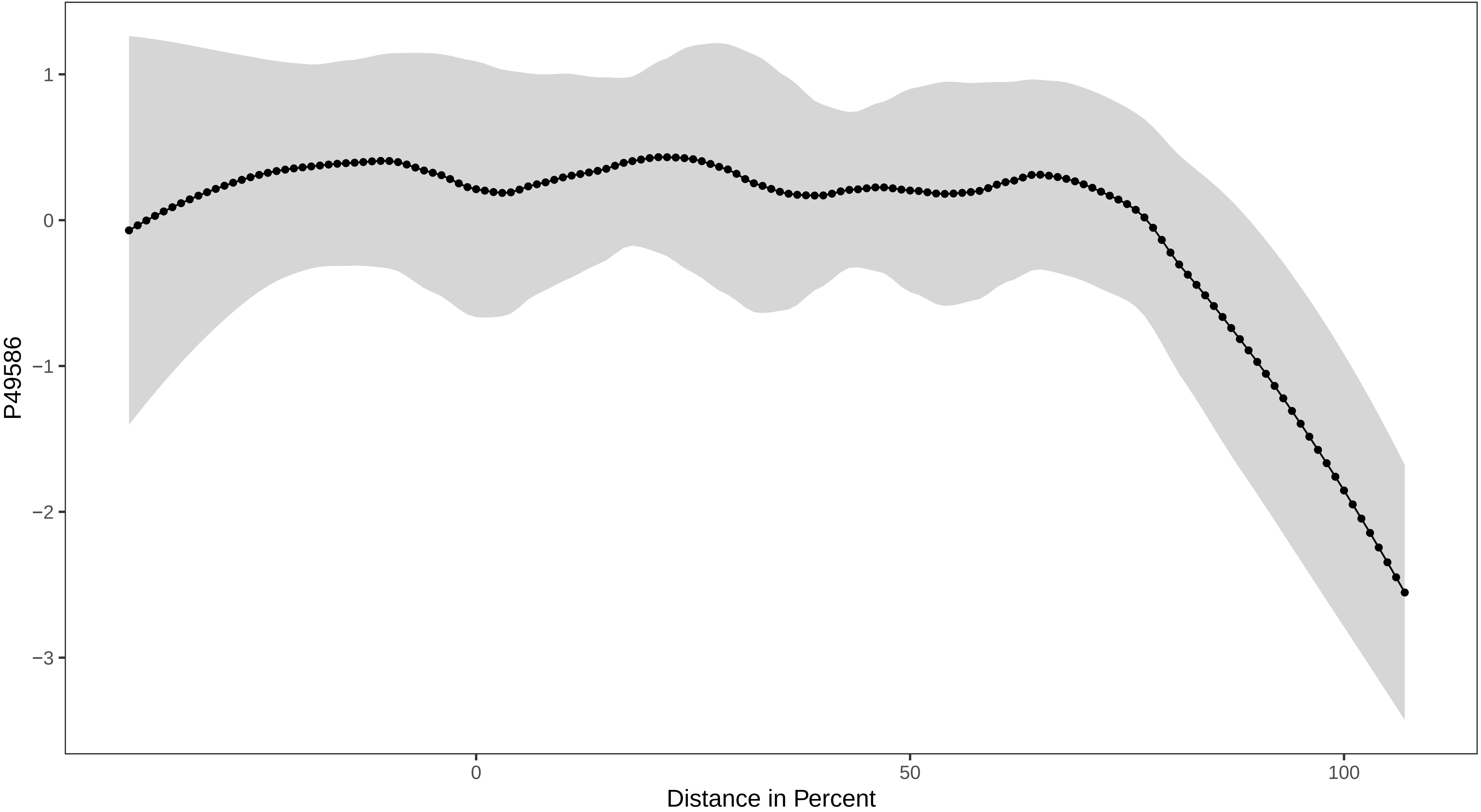

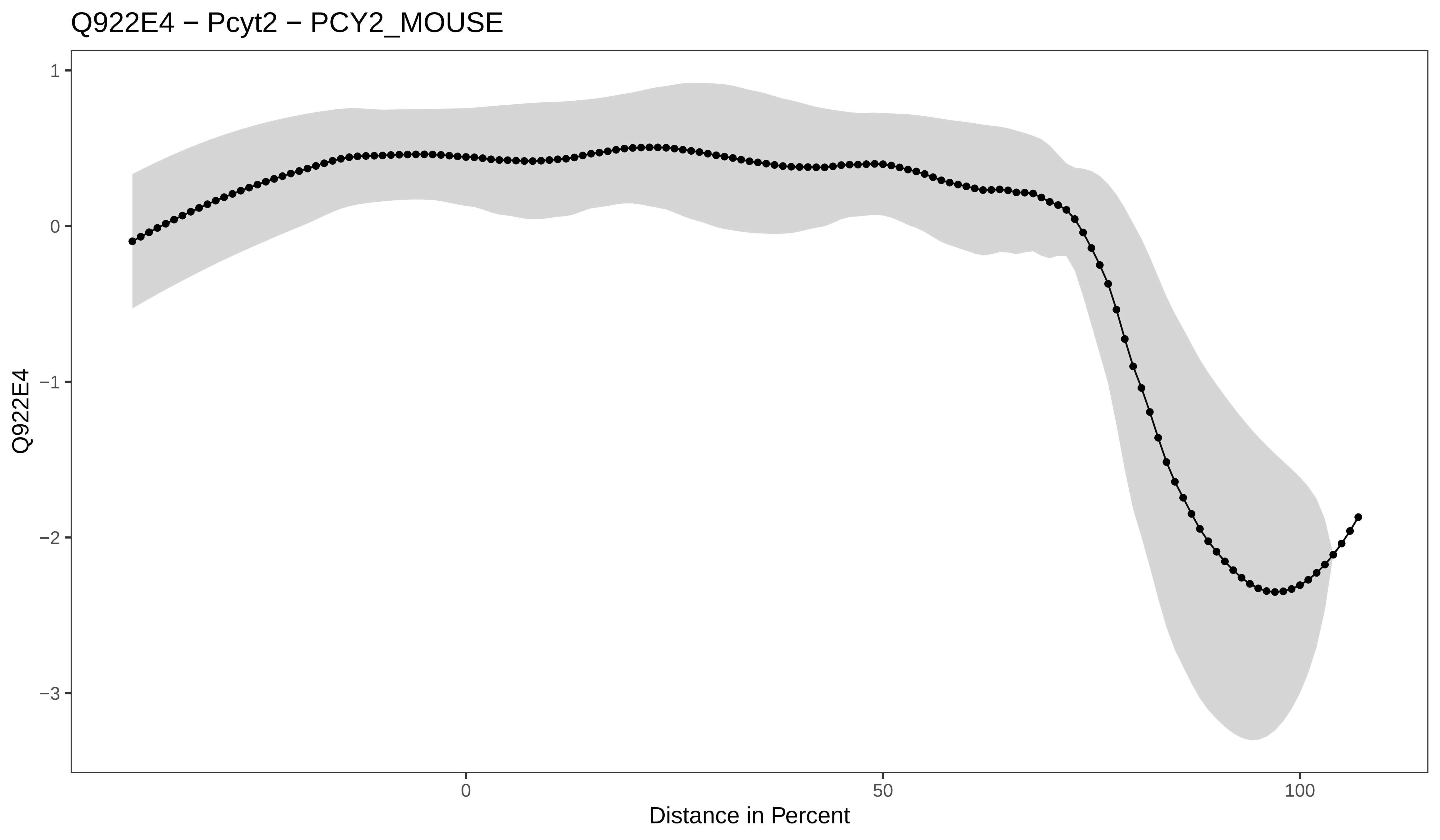

Q922E4 − Pcyt2 − PCY2_MOUSE
Q922E4
1
0
−1
−2
−3
0
50
100
Distance in Percent

Q3UHX2 − Pdap1 − HAP28_MOUSE

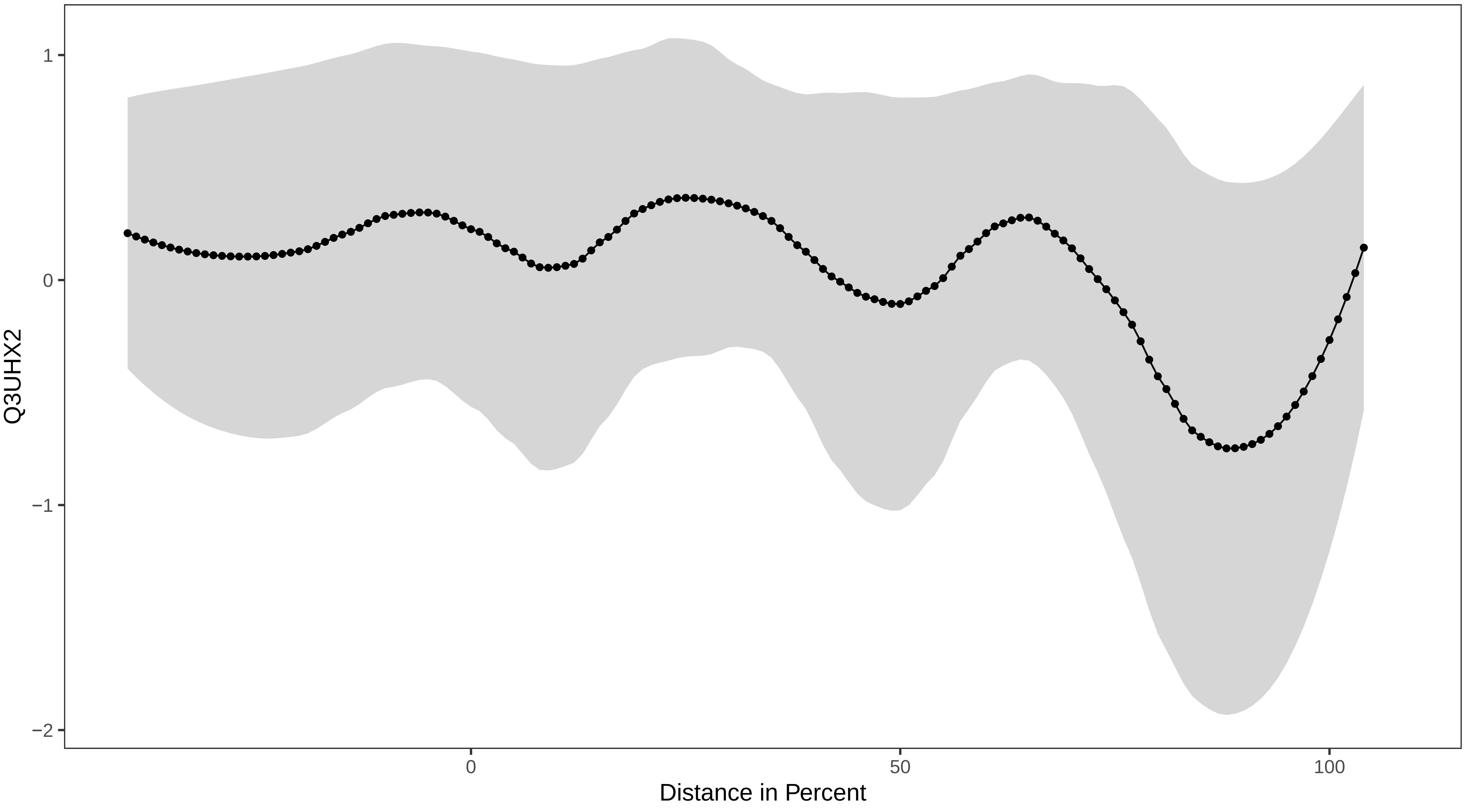

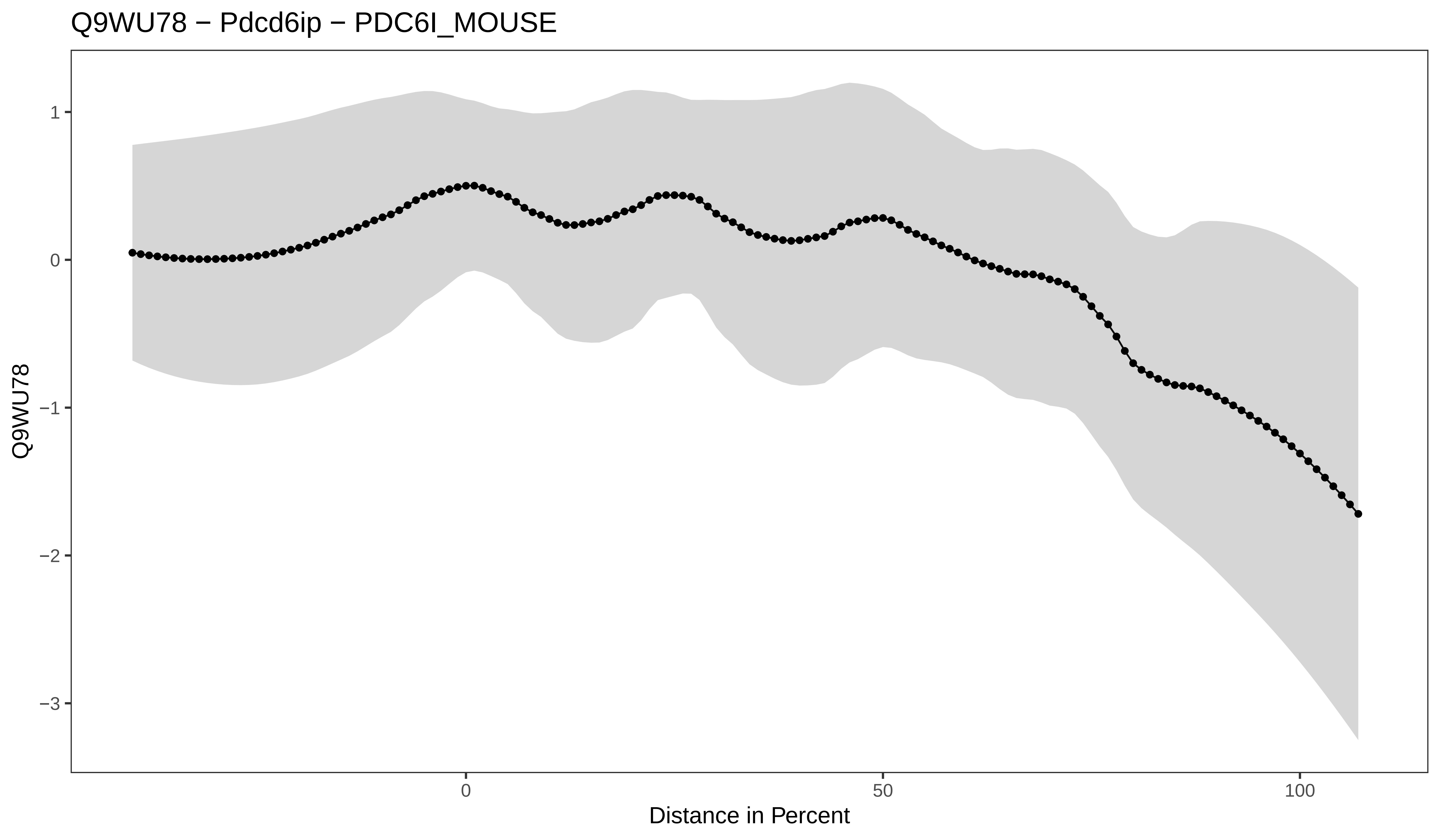
Q9WU78 − Pdcd6ip − PDC6I_MOUSE
Q9WU78
1
0
−1
−2
−3
0
50
100
Distance in Percent

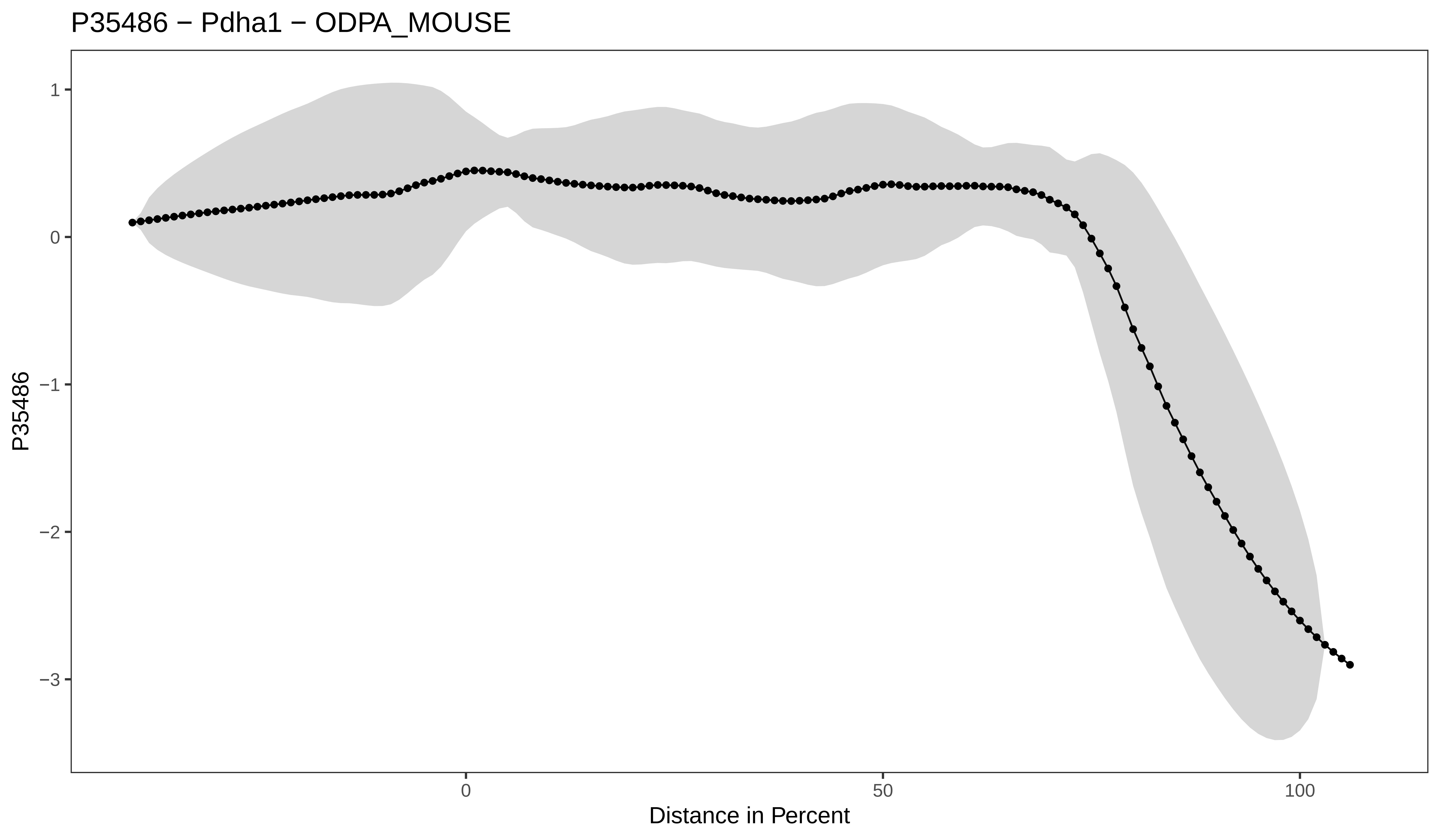

P35486 − Pdha1 − ODPA_MOUSE
1
0
−1
−2
−3
P35486
0
50
100
Distance in Percent

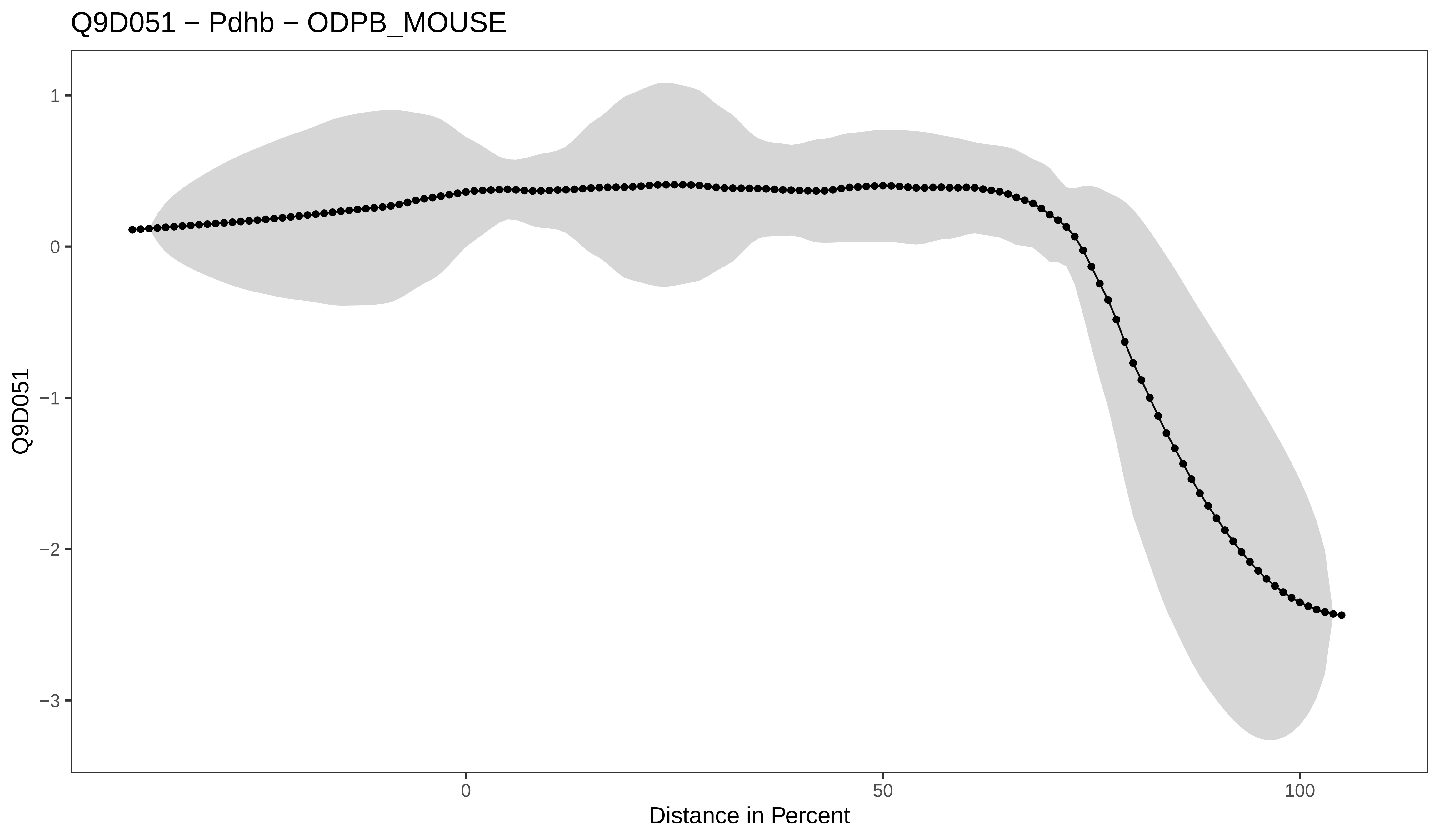
Q9D051 − Pdhb − ODPB_MOUSE
Q9D051
1
0
−1
−2
−3
0
50
100
Distance in Percent

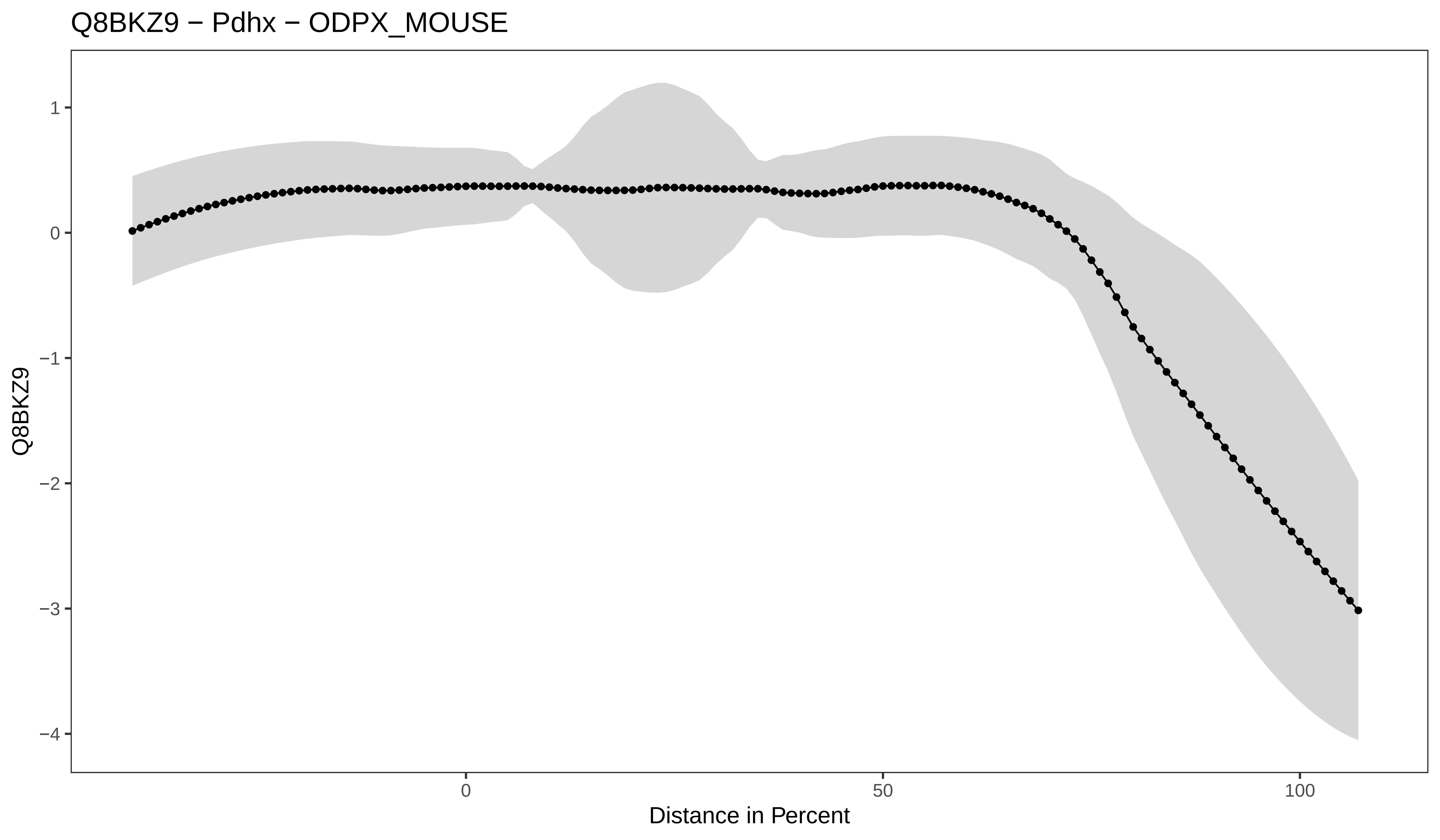
Q8BKZ9 − Pdhx − ODPX_MOUSE
Q8BKZ9
1
0
−1
−2
−3
−4
0
50
100
Distance in Percent

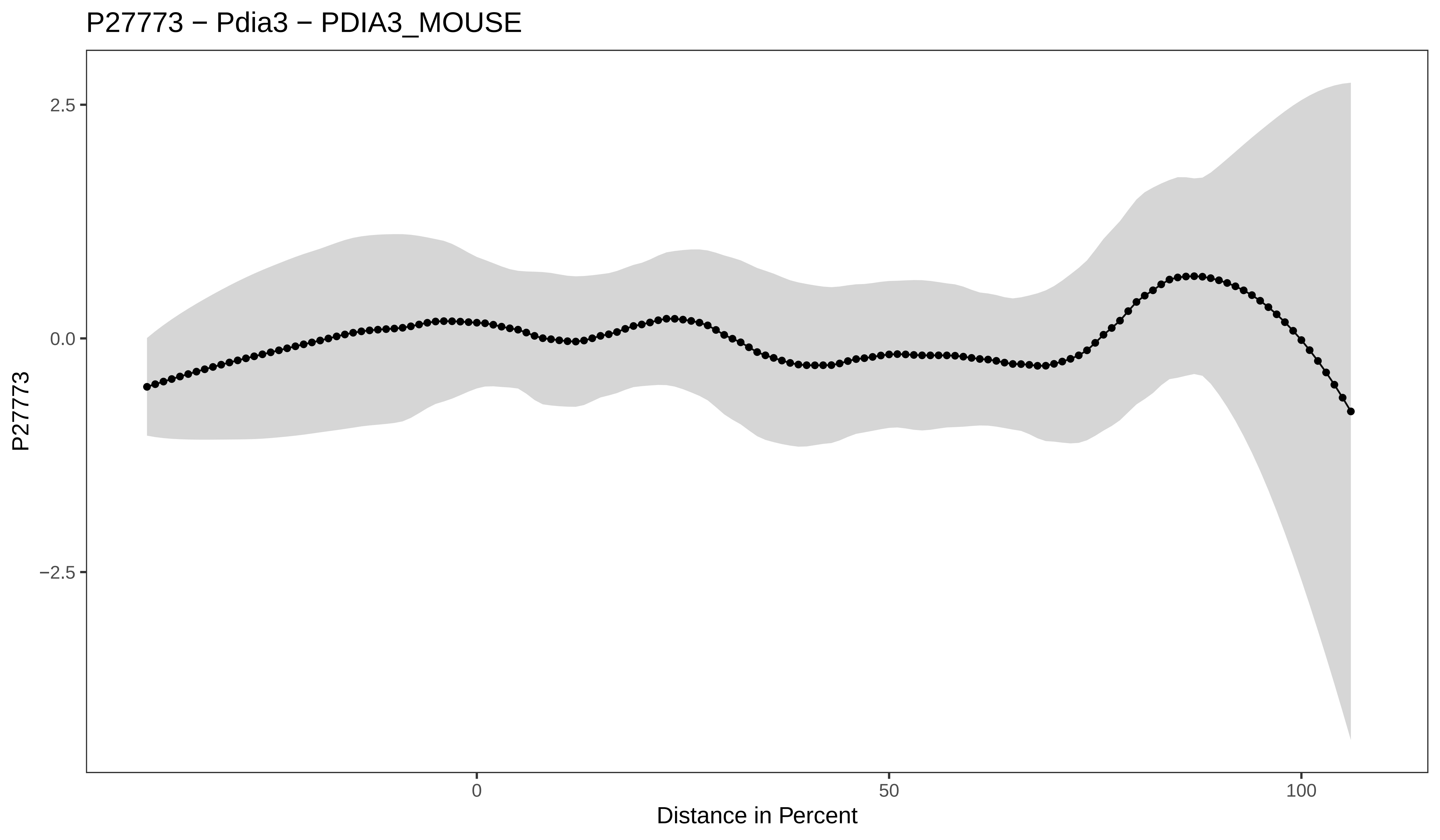

P27773 − Pdia3 − PDIA3_MOUSE
2.5
0.0
−2.5
P27773
0
50
100
Distance in Percent

P08003 − Pdia4 − PDIA4_MOUSE

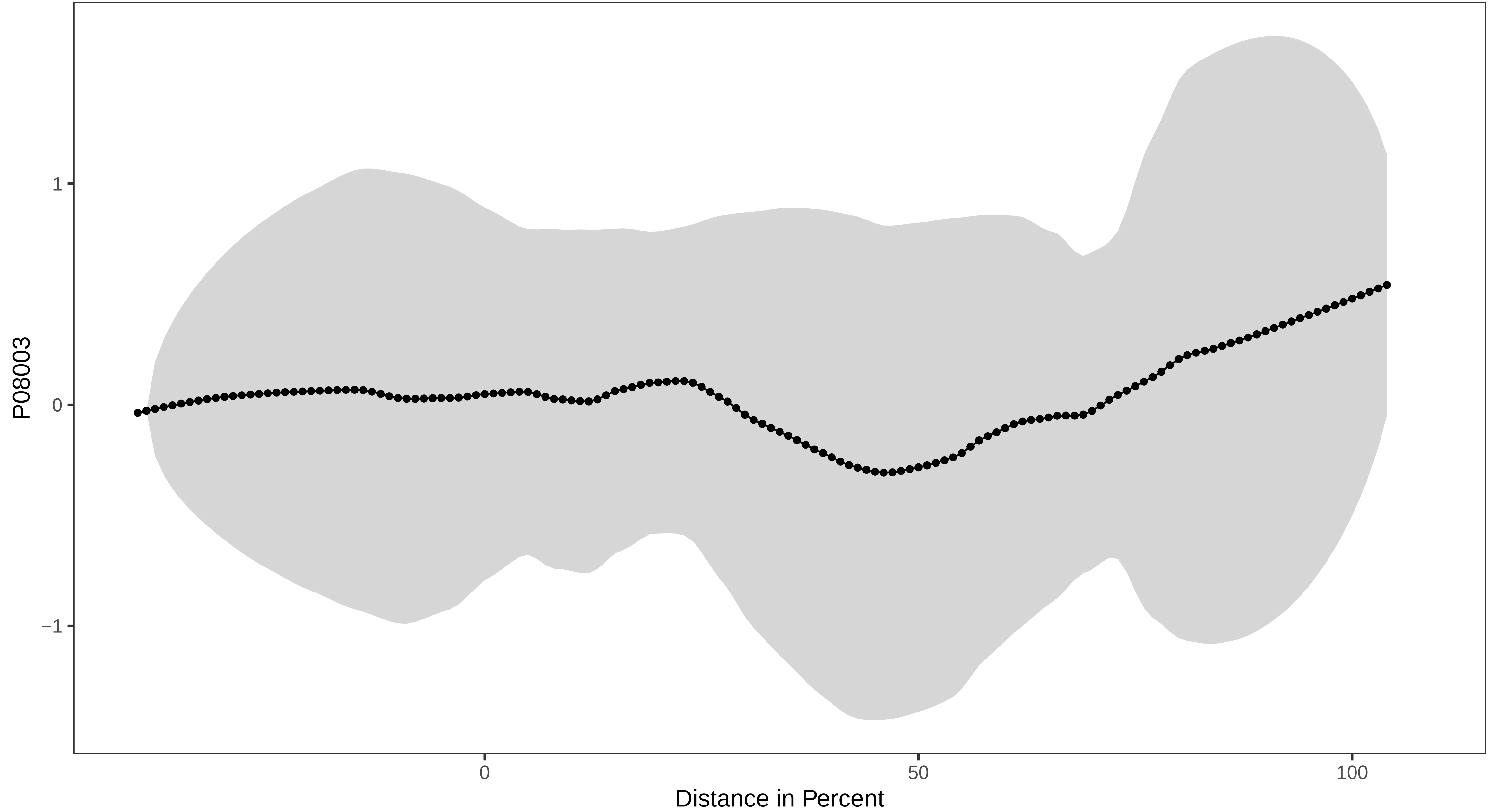

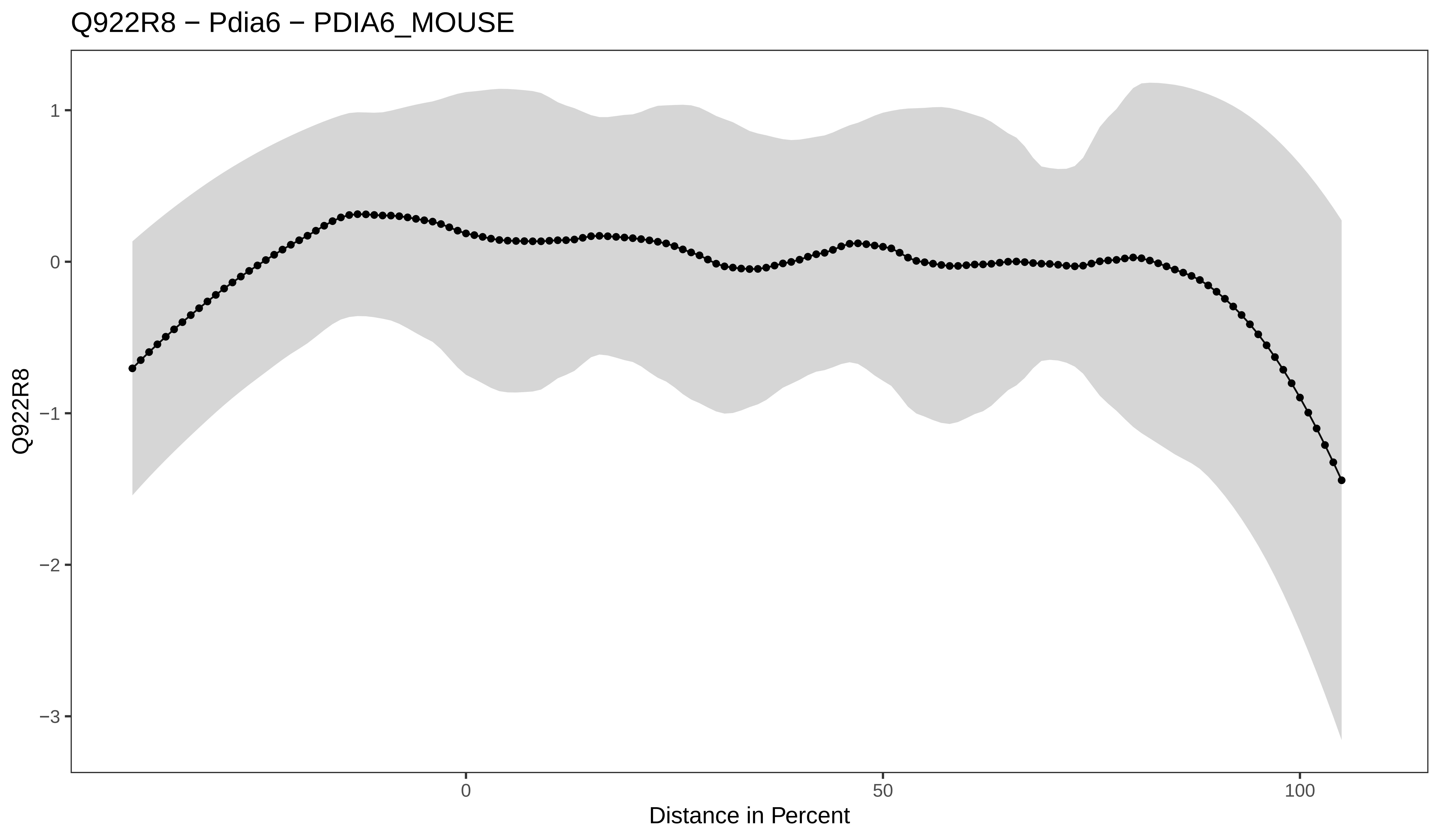
Q922R8 − Pdia6 − PDIA6_MOUSE
1
0
−1
−2
−3
Q922R8
0
50
100
Distance in Percent

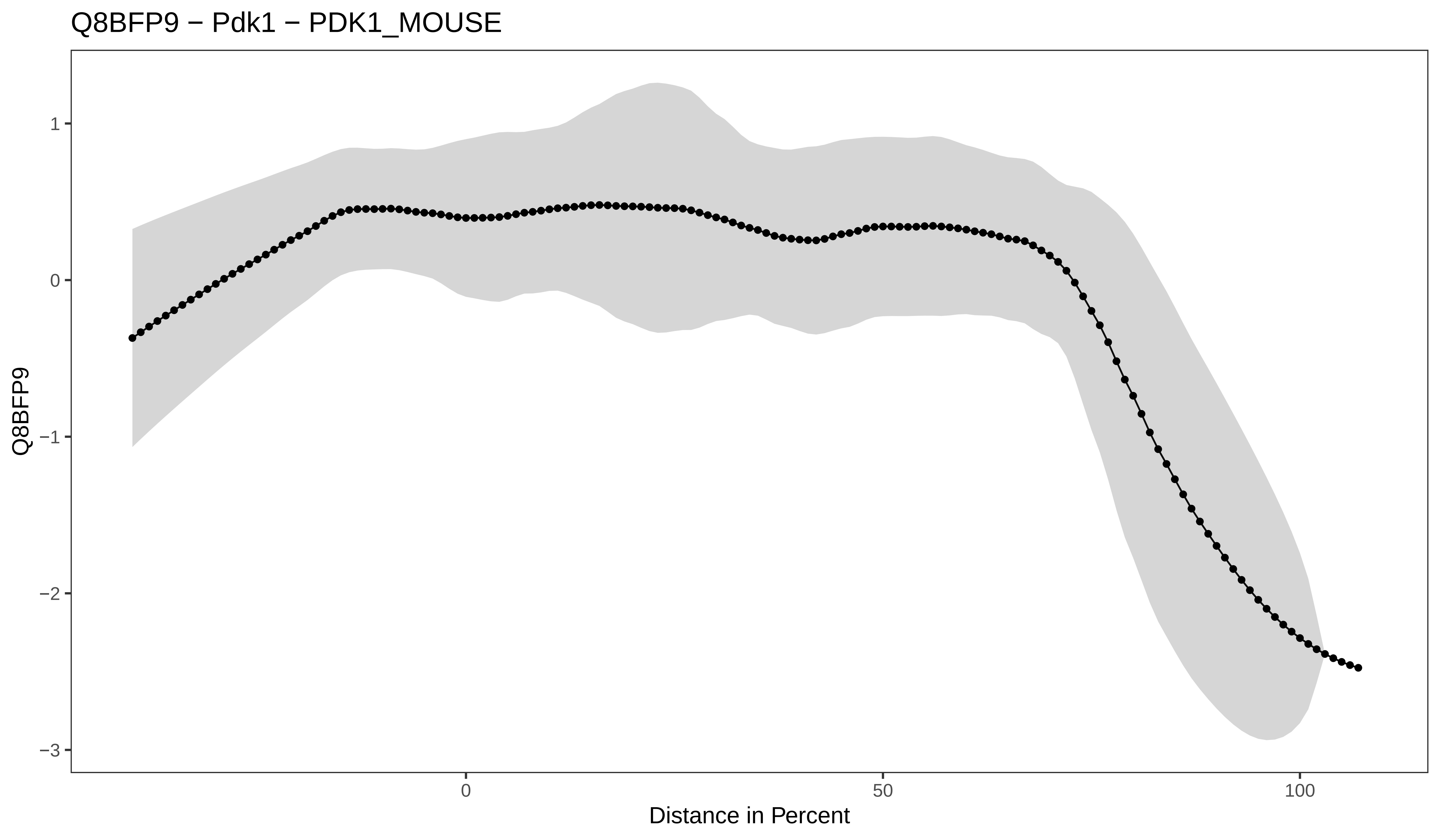
Q8BFP9 − Pdk1 − PDK1_MOUSE
1
0
−1
−2
−3
Q8BFP9
0
50
100
Distance in Percent

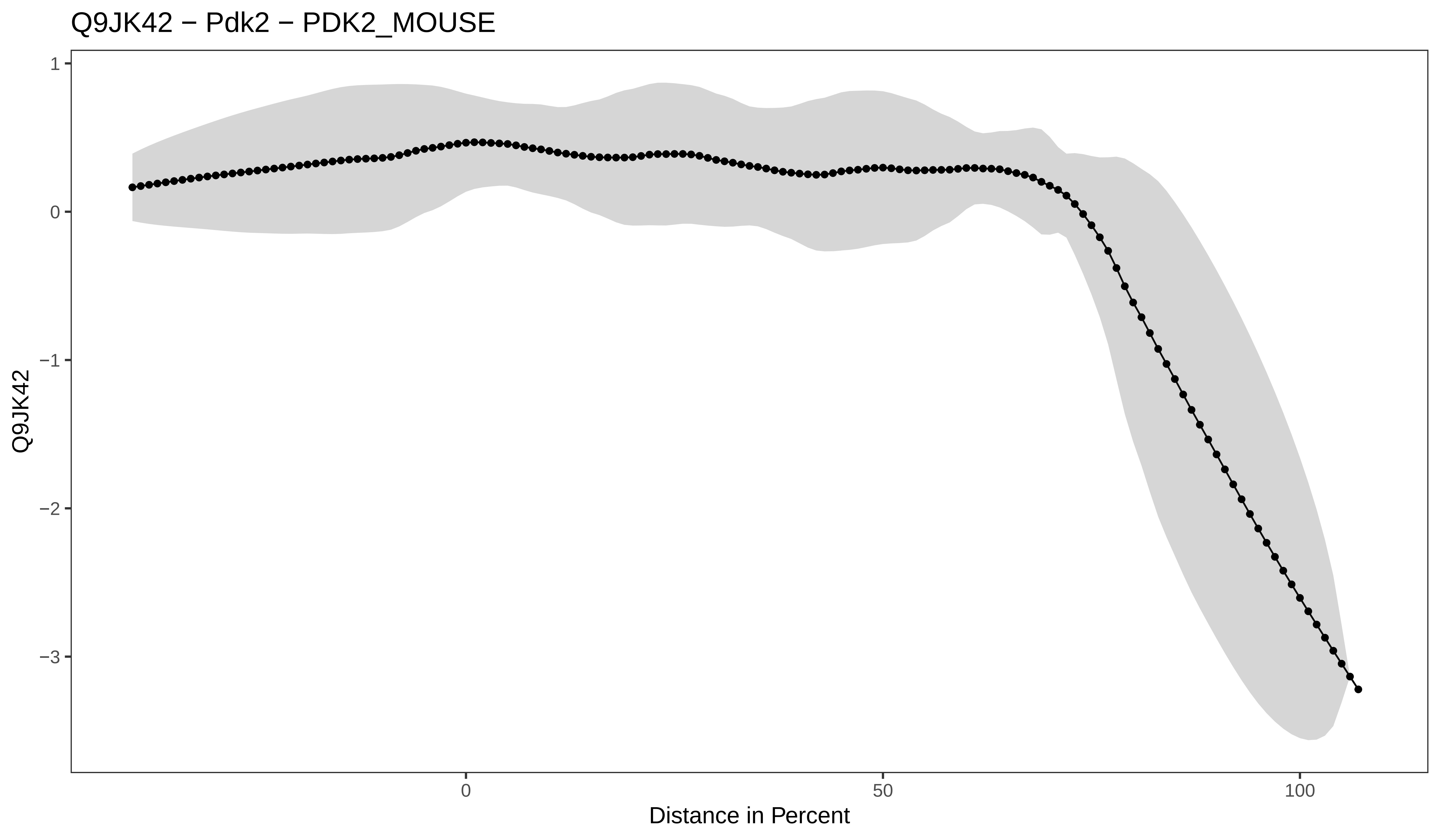

Q9JK42 − Pdk2 − PDK2_MOUSE
1
0
−1
−2
−3
Q9JK42
0
50
100
Distance in Percent

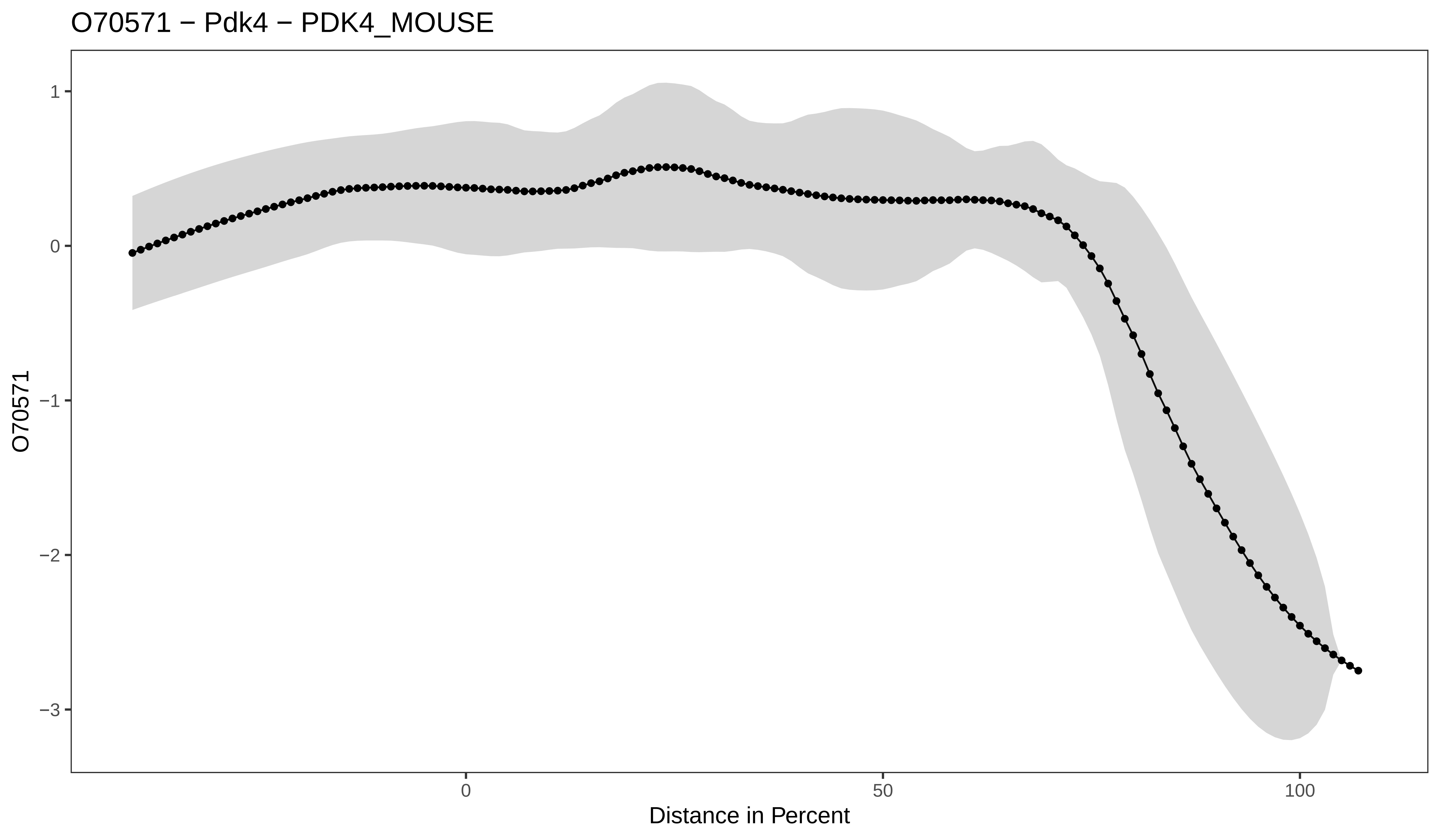

O70571 − Pdk4 − PDK4_MOUSE
1
0
−1
−2
−3
O70571
0
50
100
Distance in Percent

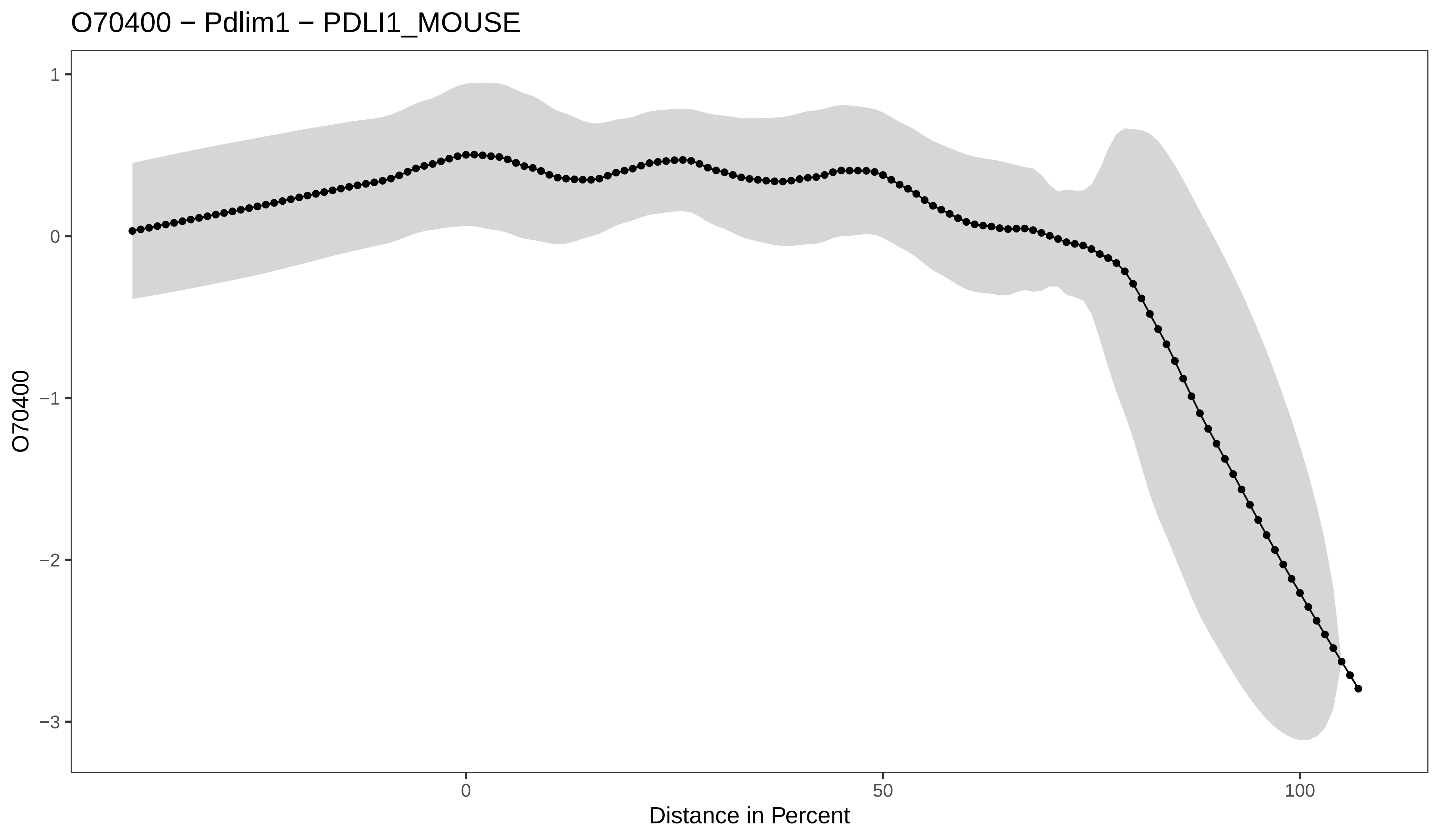

O70400 − Pdlim1 − PDLI1_MOUSE
1
0
−1
−2
−3
O70400
0
50
100
Distance in Percent

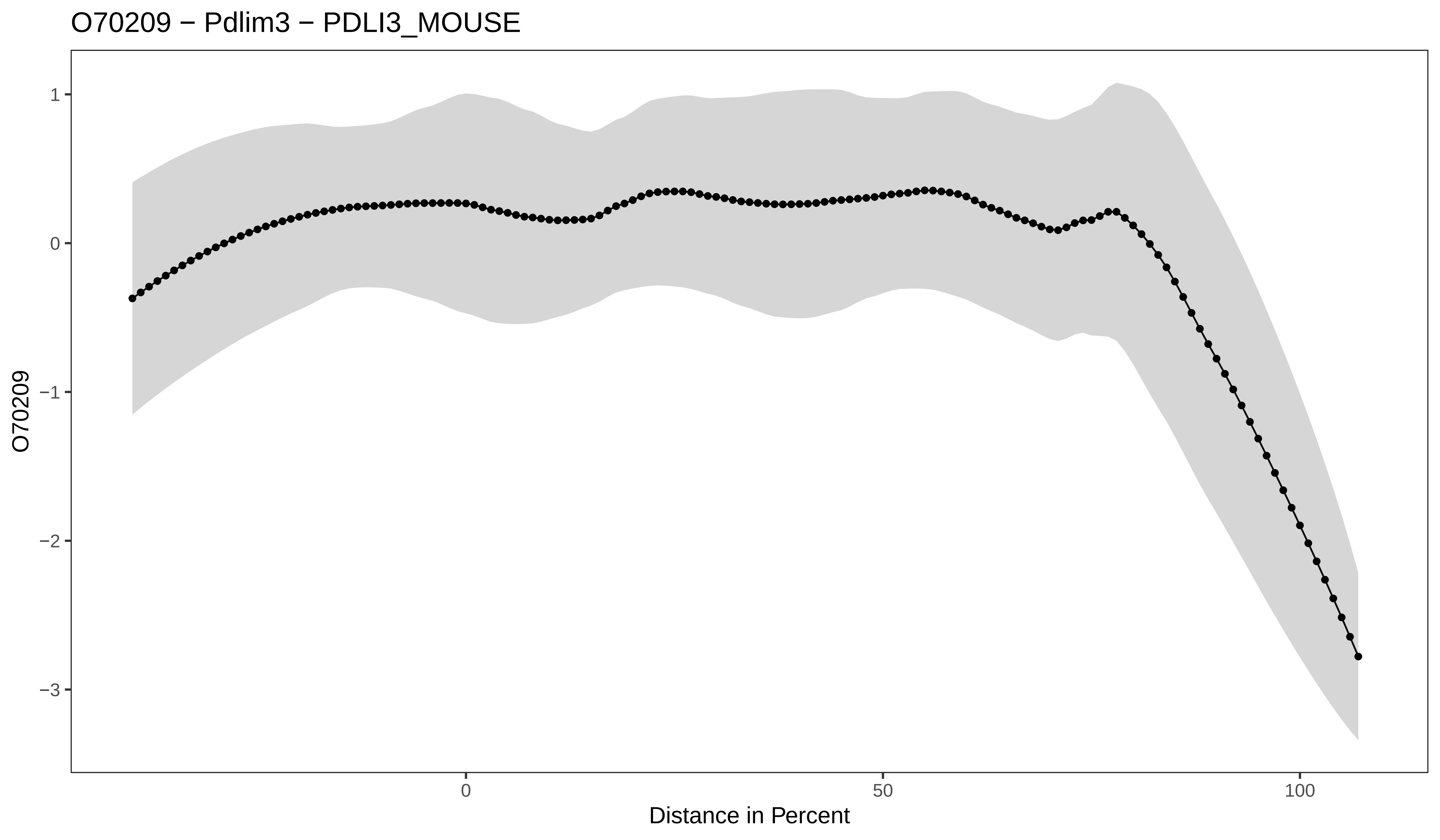

O70209 − Pdlim3 − PDLI3_MOUSE
1
0
−1
−2
−3
O70209
0
50
100
Distance in Percent

Q8CI51 − Pdlim5 − PDLI5_MOUSE

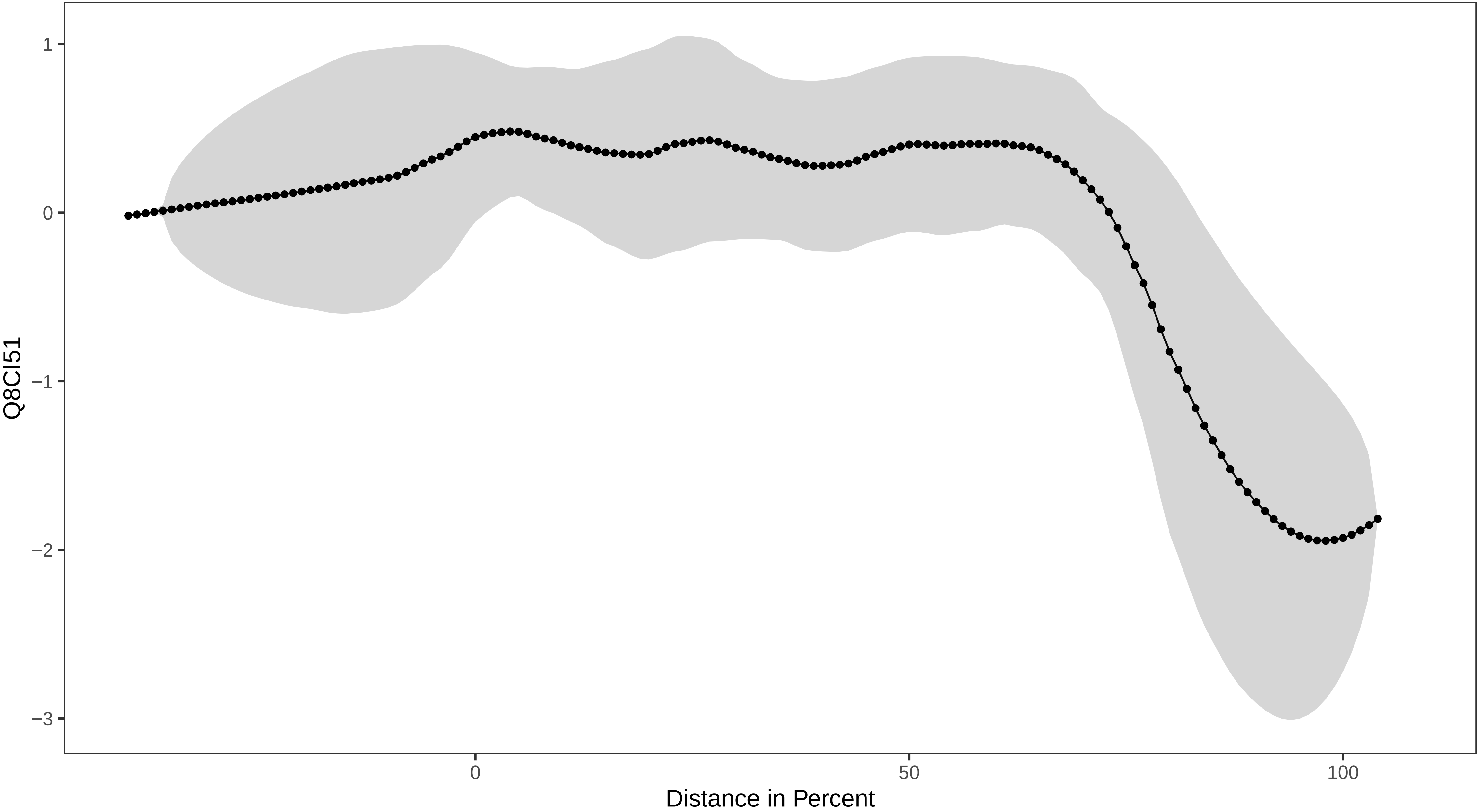

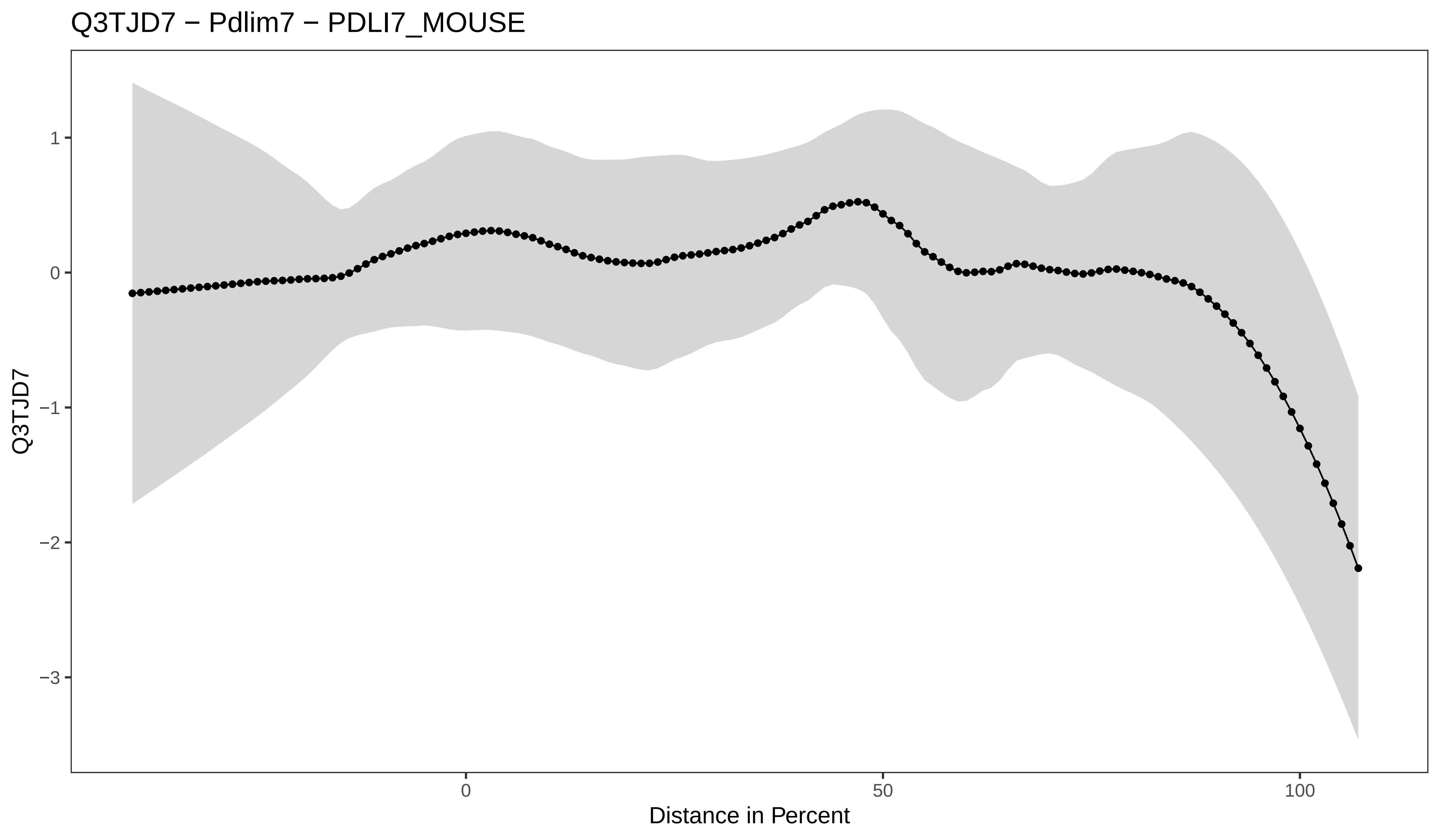
Q3TJD7 − Pdlim7 − PDLI7_MOUSE
Q3TJD7
1
0
−1
−2
−3
0
50
100
Distance in Percent

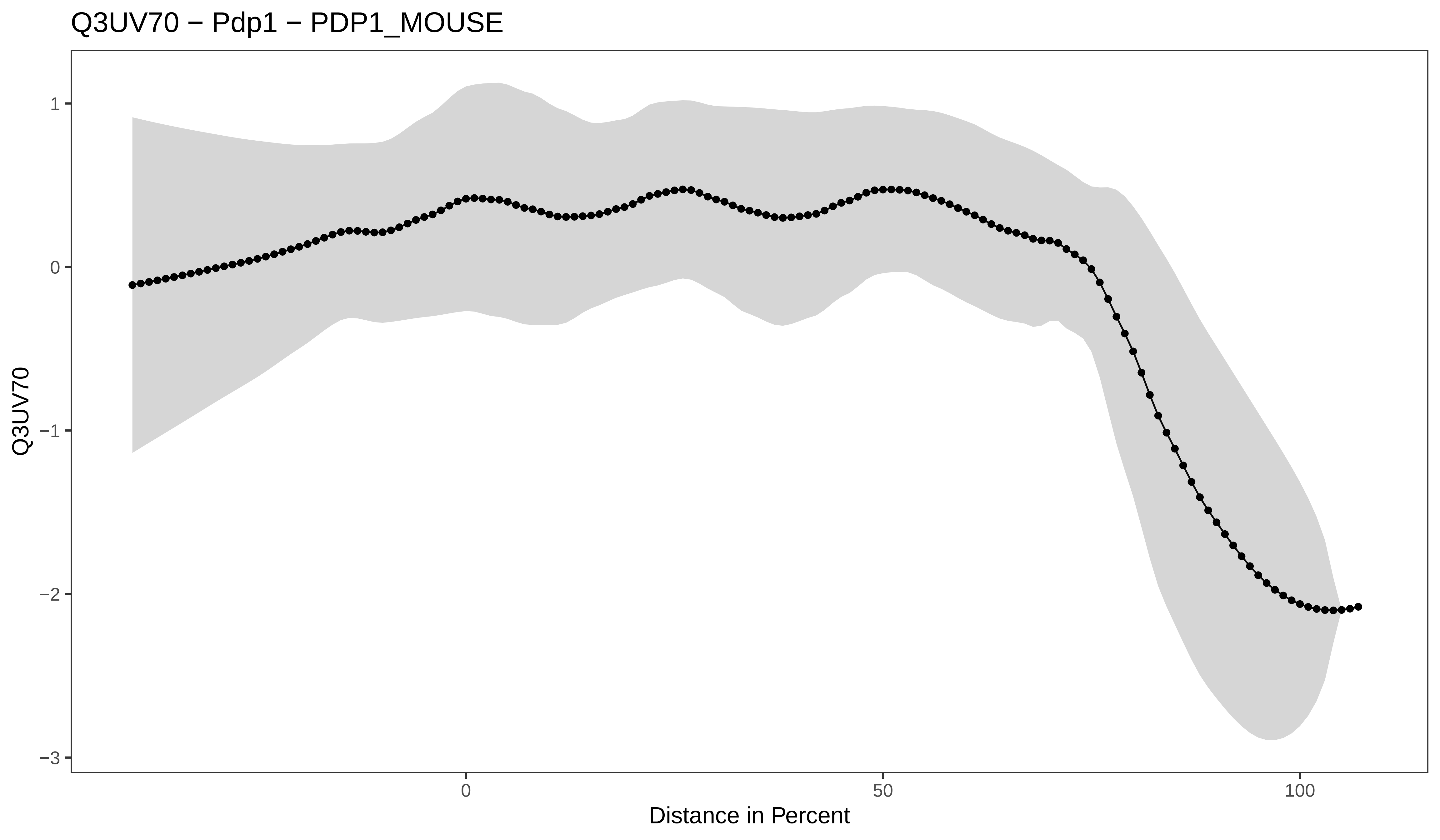
Q3UV70 − Pdp1 − PDP1_MOUSE
Q3UV70
1
0
−1
−2
−3
0
50
100
Distance in Percent

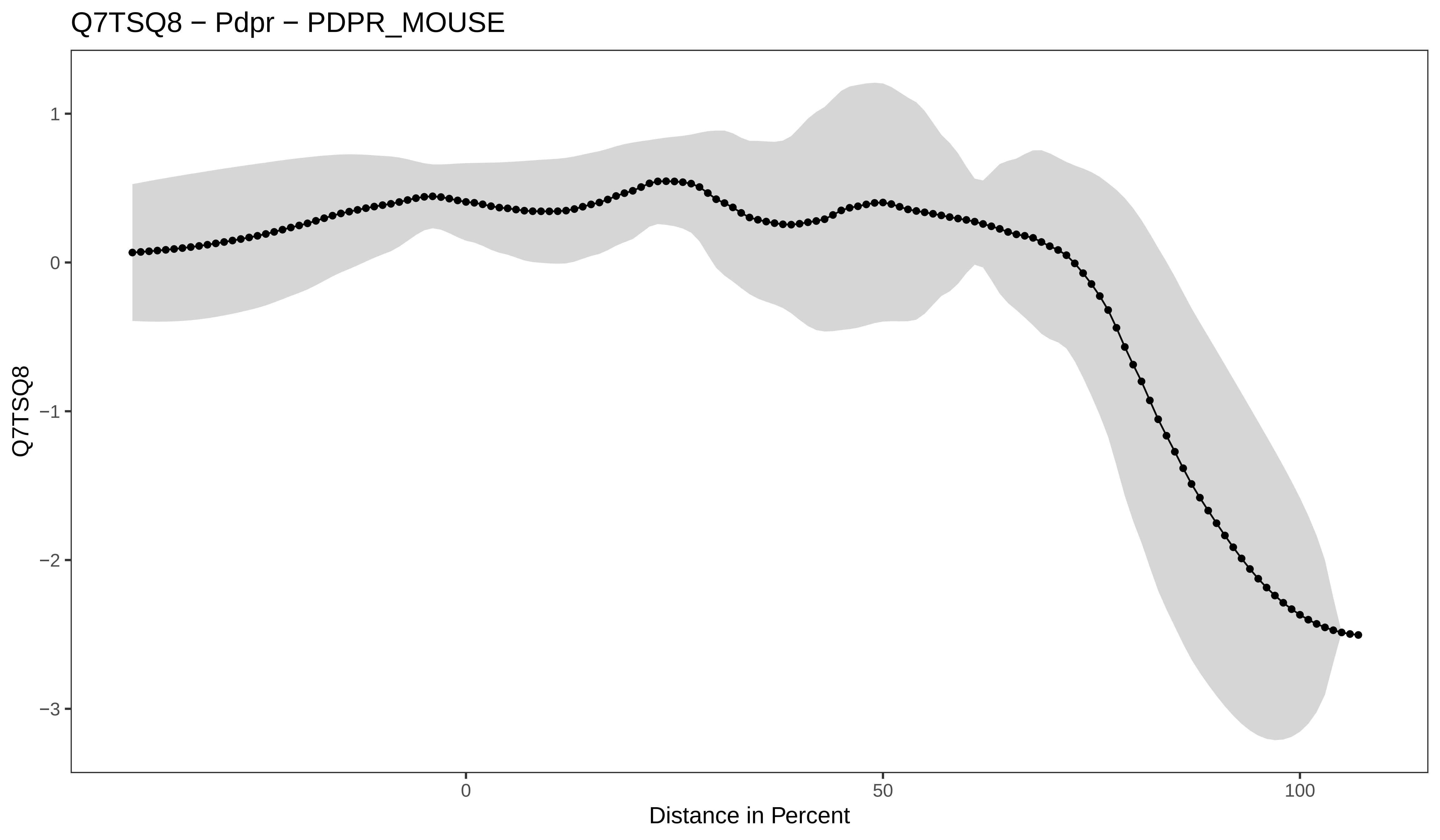

Q7TSQ8 − Pdpr − PDPR_MOUSE
1
0
−1
−2
−3
Q7TSQ8
0
50
100
Distance in Percent

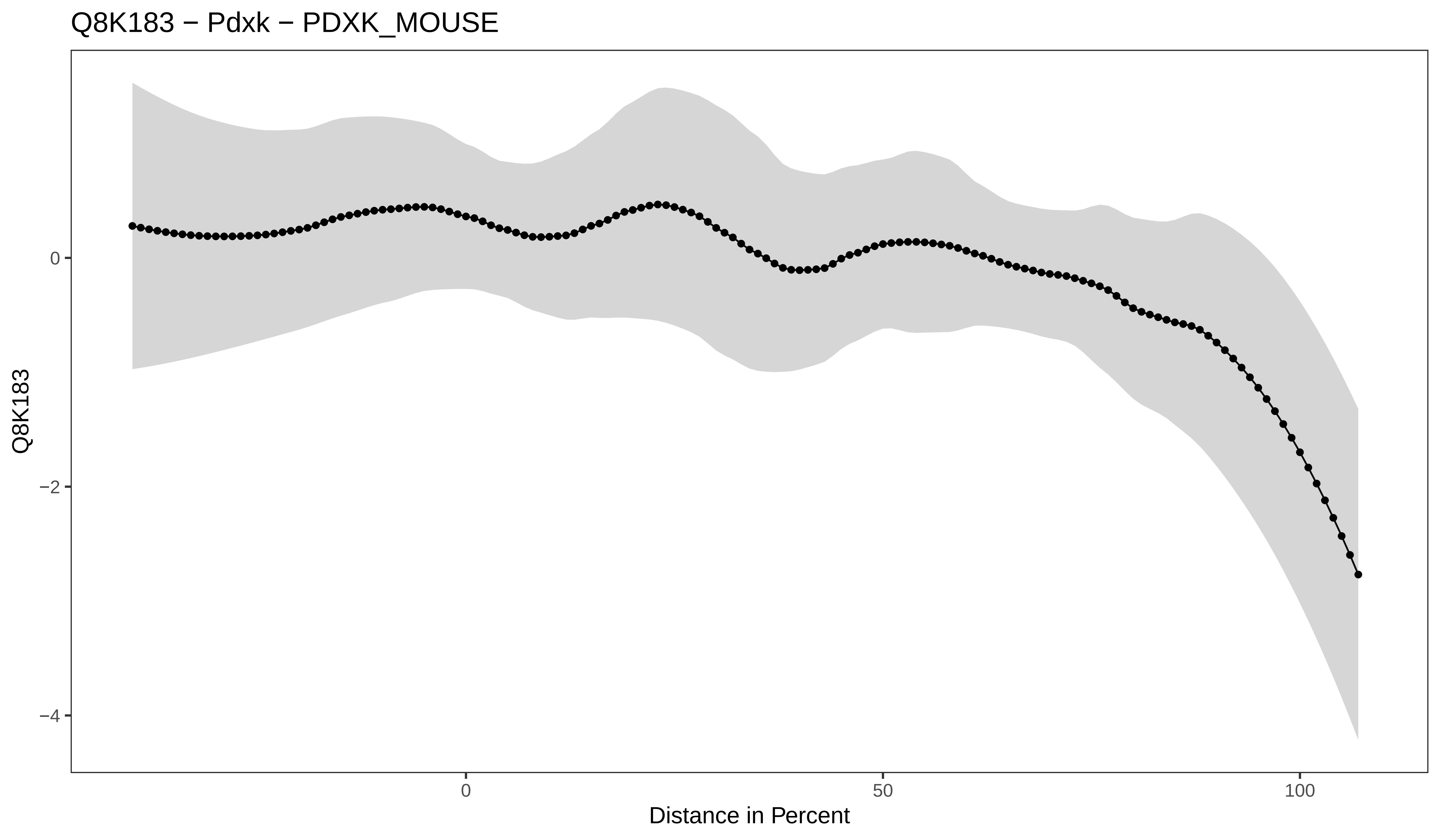

Q8K183 − Pdxk − PDXK_MOUSE
0
−2
−4
Q8K183
0
50
100
Distance in Percent

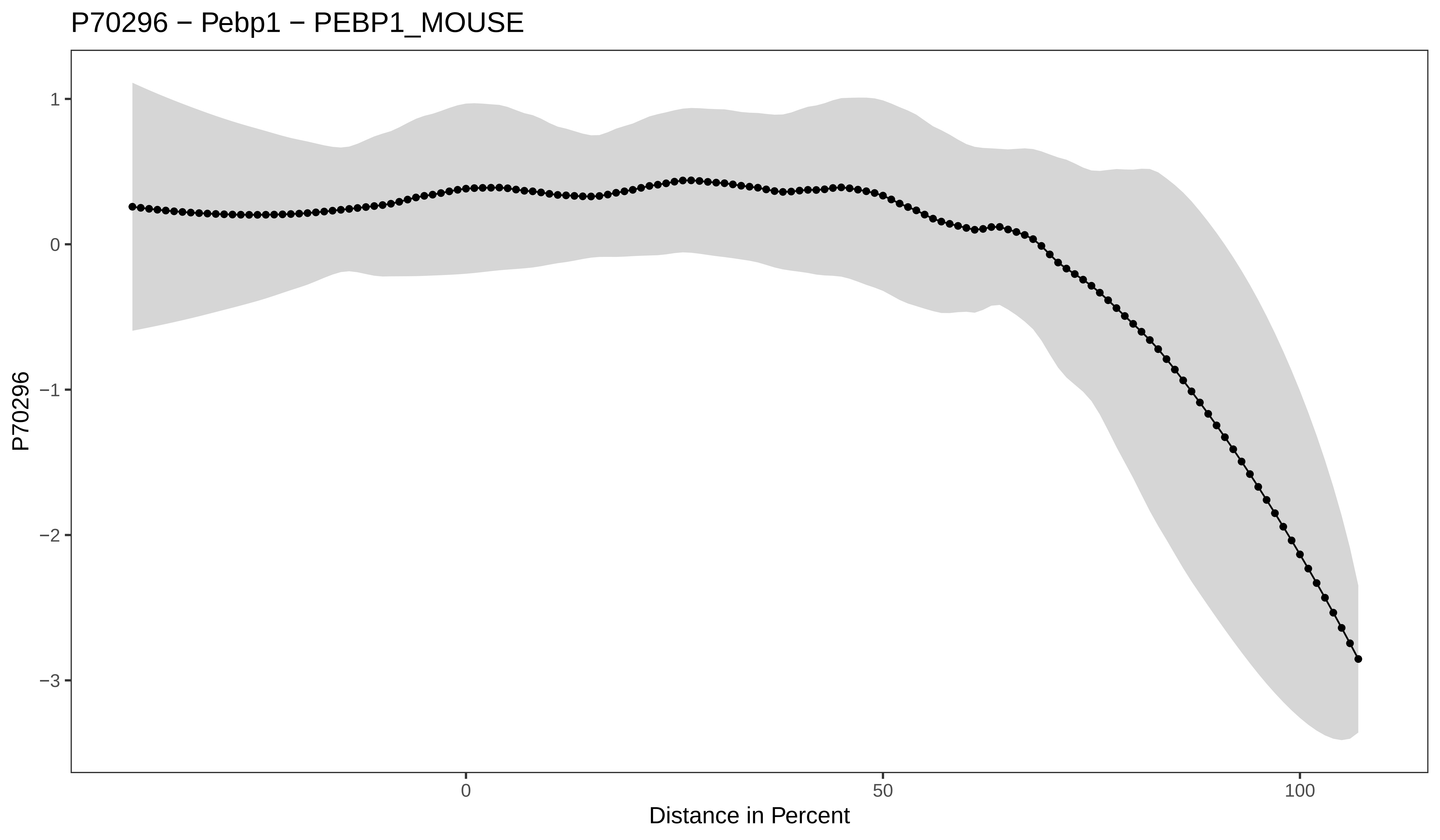
P70296 − Pebp1 − PEBP1_MOUSE
P70296
Distance in Percent
1
0
−1
−2
−3
0
50
100

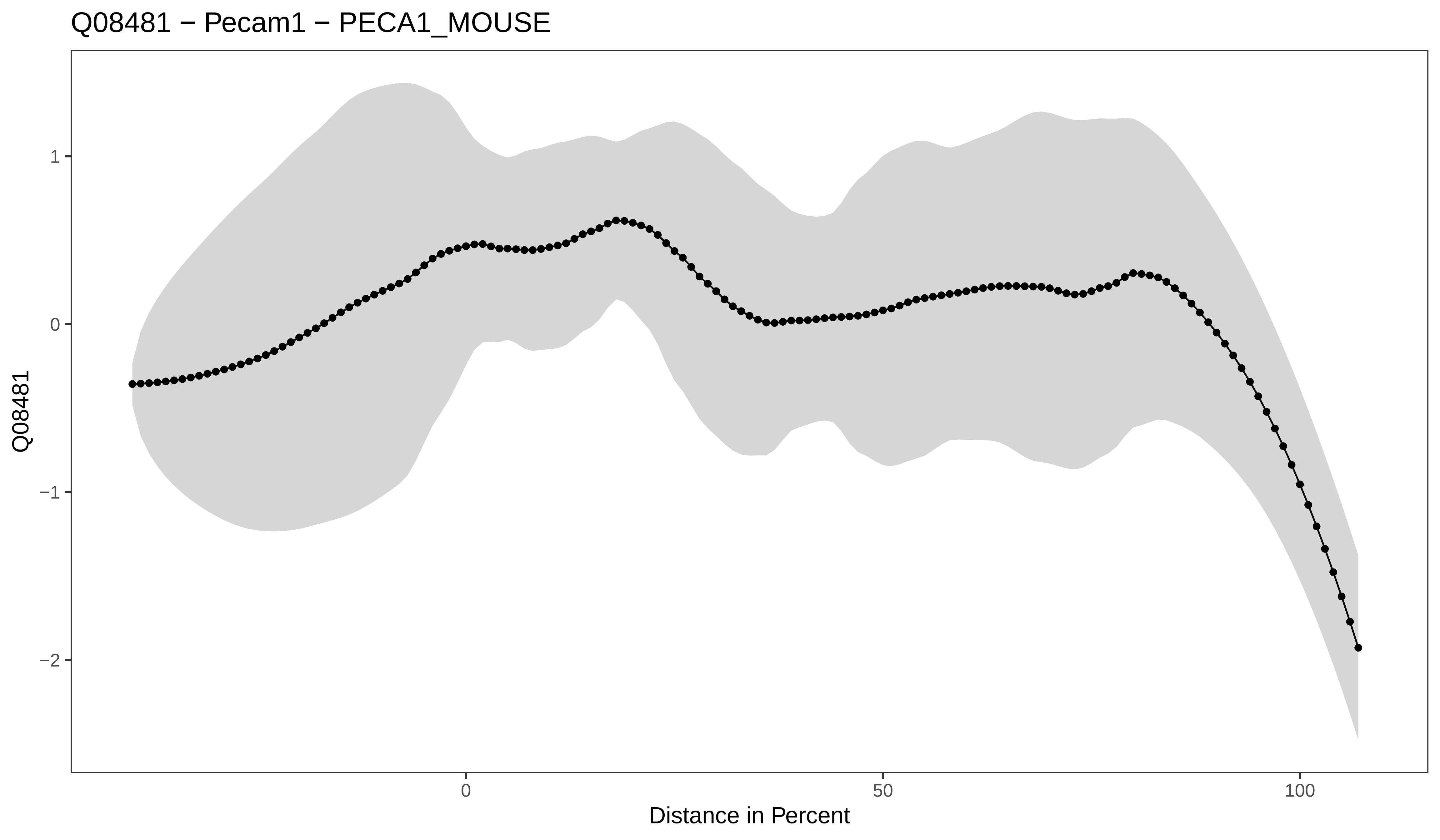

Q08481 − Pecam1 − PECA1_MOUSE
Q08481
1
0
−1
−2
0
50
100
Distance in Percent

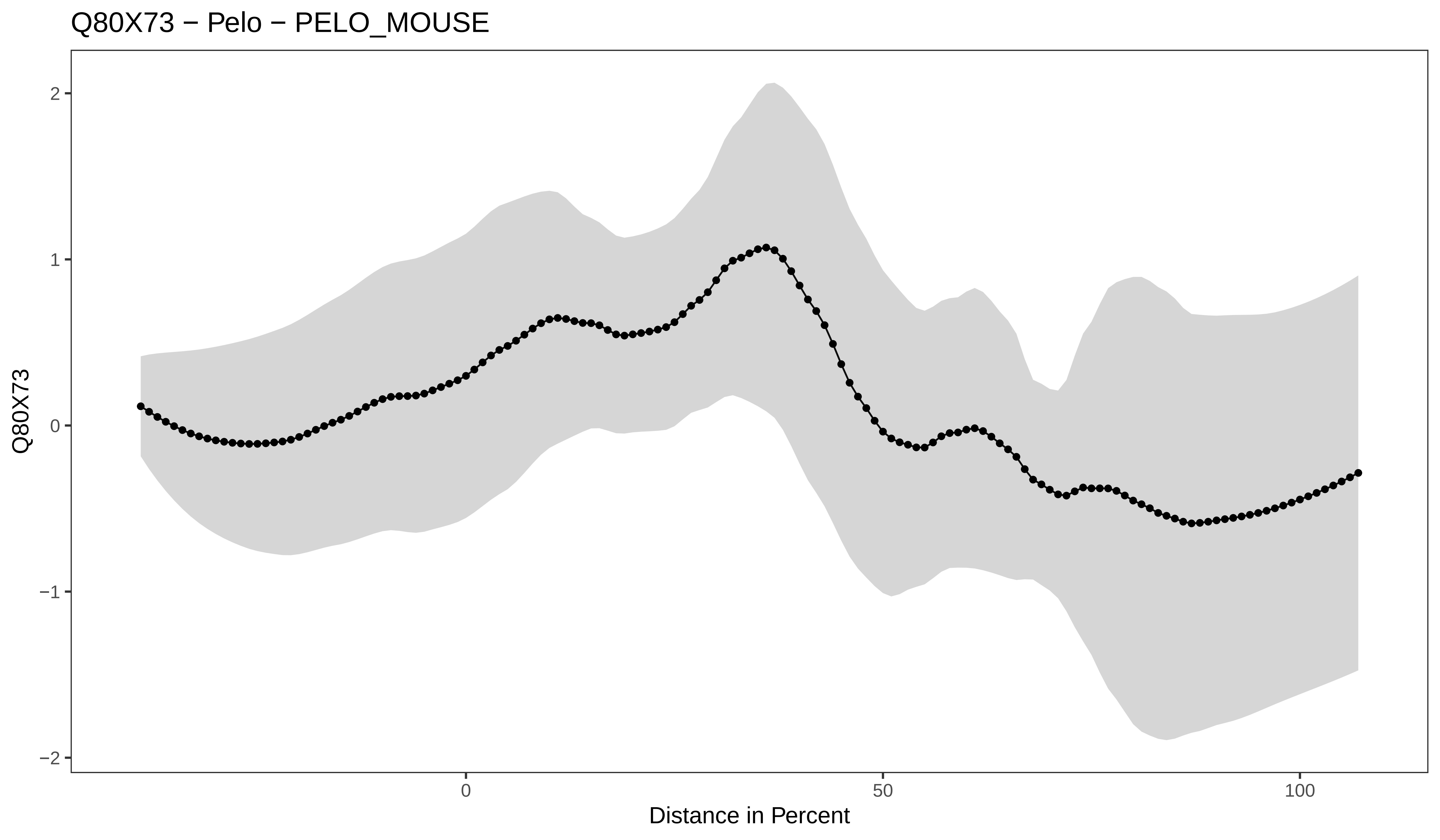

Q80X73 − Pelo − PELO_MOUSE
Q80X73
2
1
0
−1
−2
0
50
100
Distance in Percent

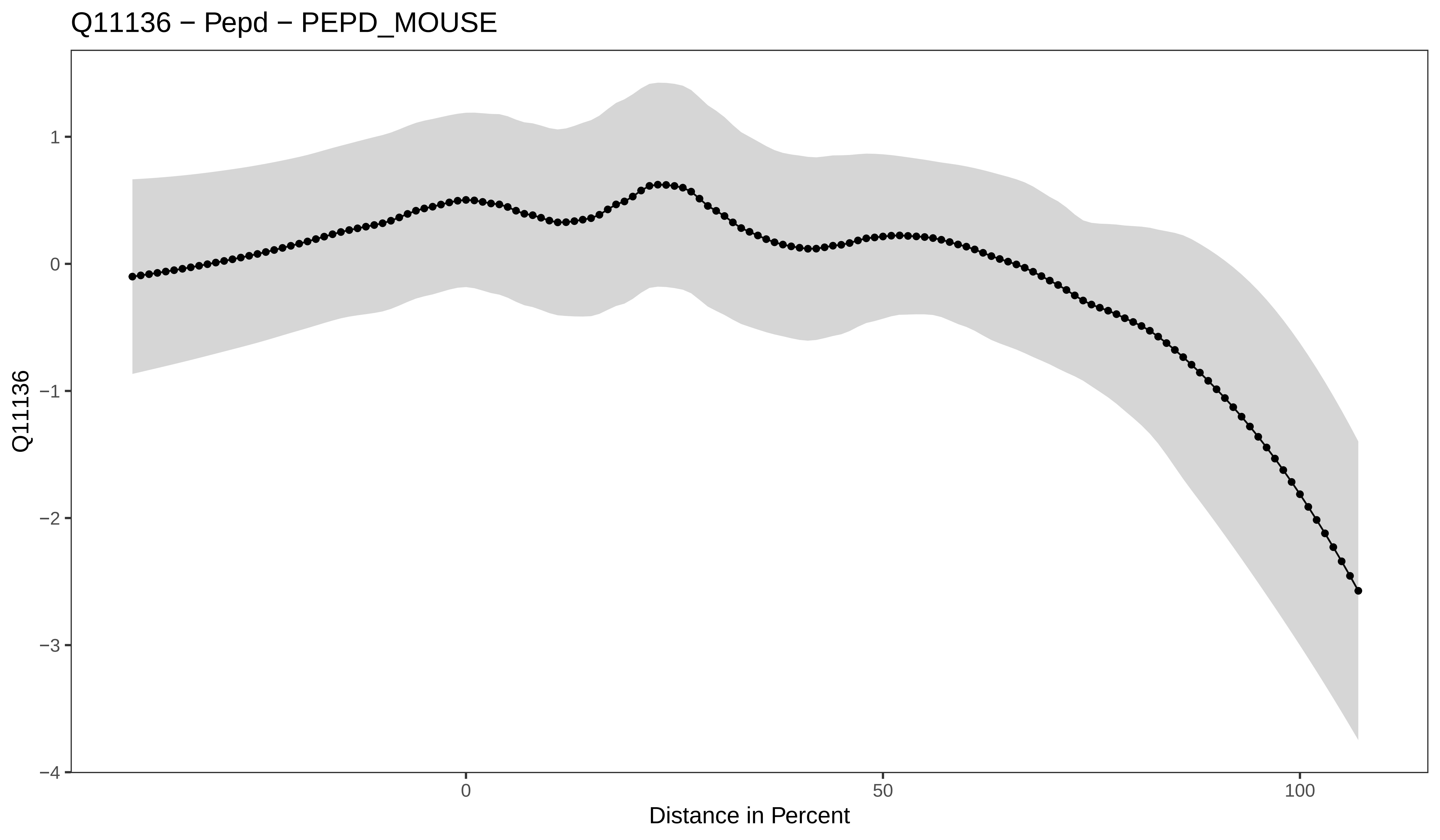
Q11136 − Pepd − PEPD_MOUSE
Q11136
1
0
−1
−2
−3
−4
0
50
100
Distance in Percent

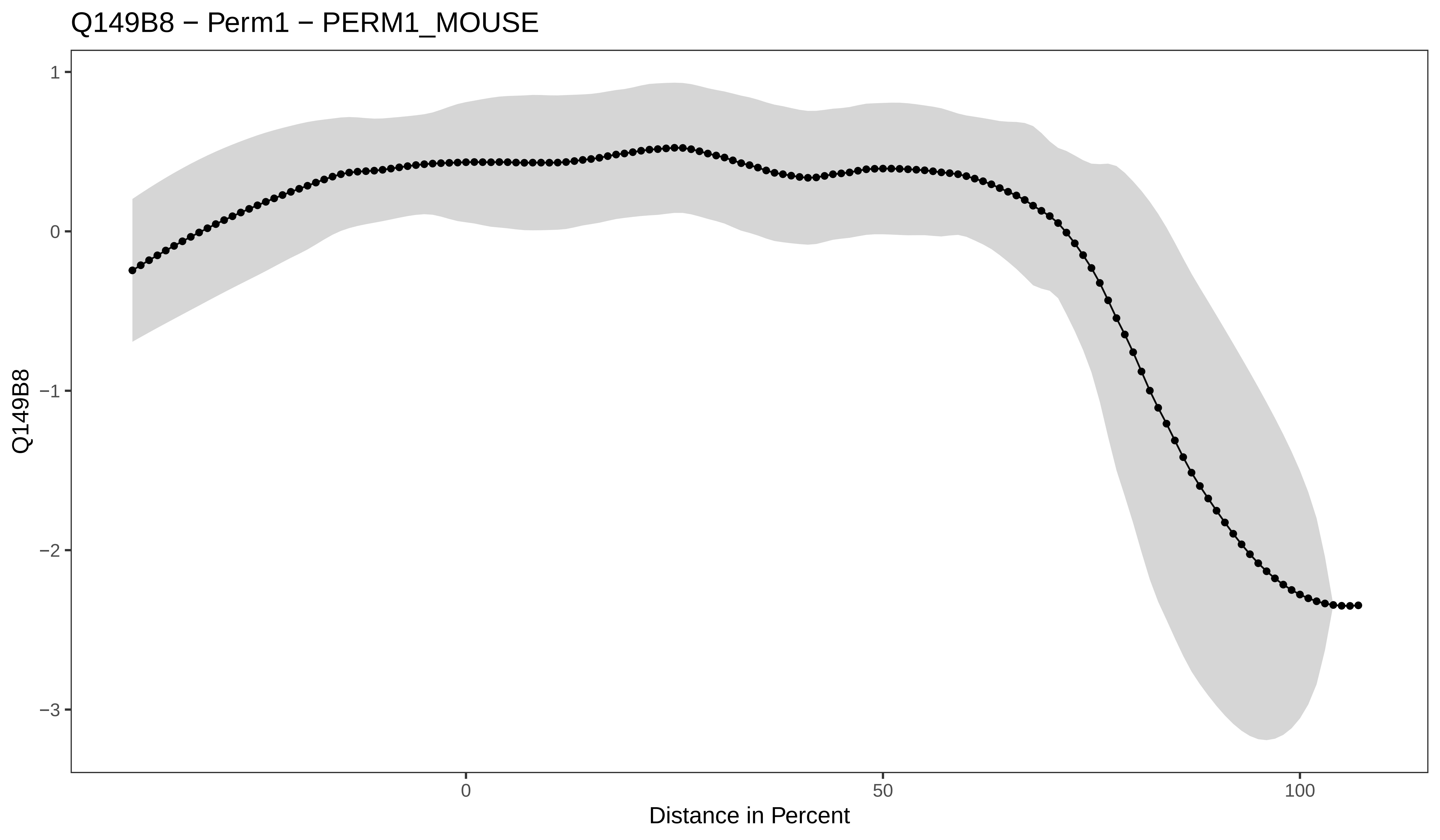

Q149B8 − Perm1 − PERM1_MOUSE
1
0
−1
−2
−3
Q149B8
0
50
100
Distance in Percent

P70266 − Pfkfb1 − F261_MOUSE

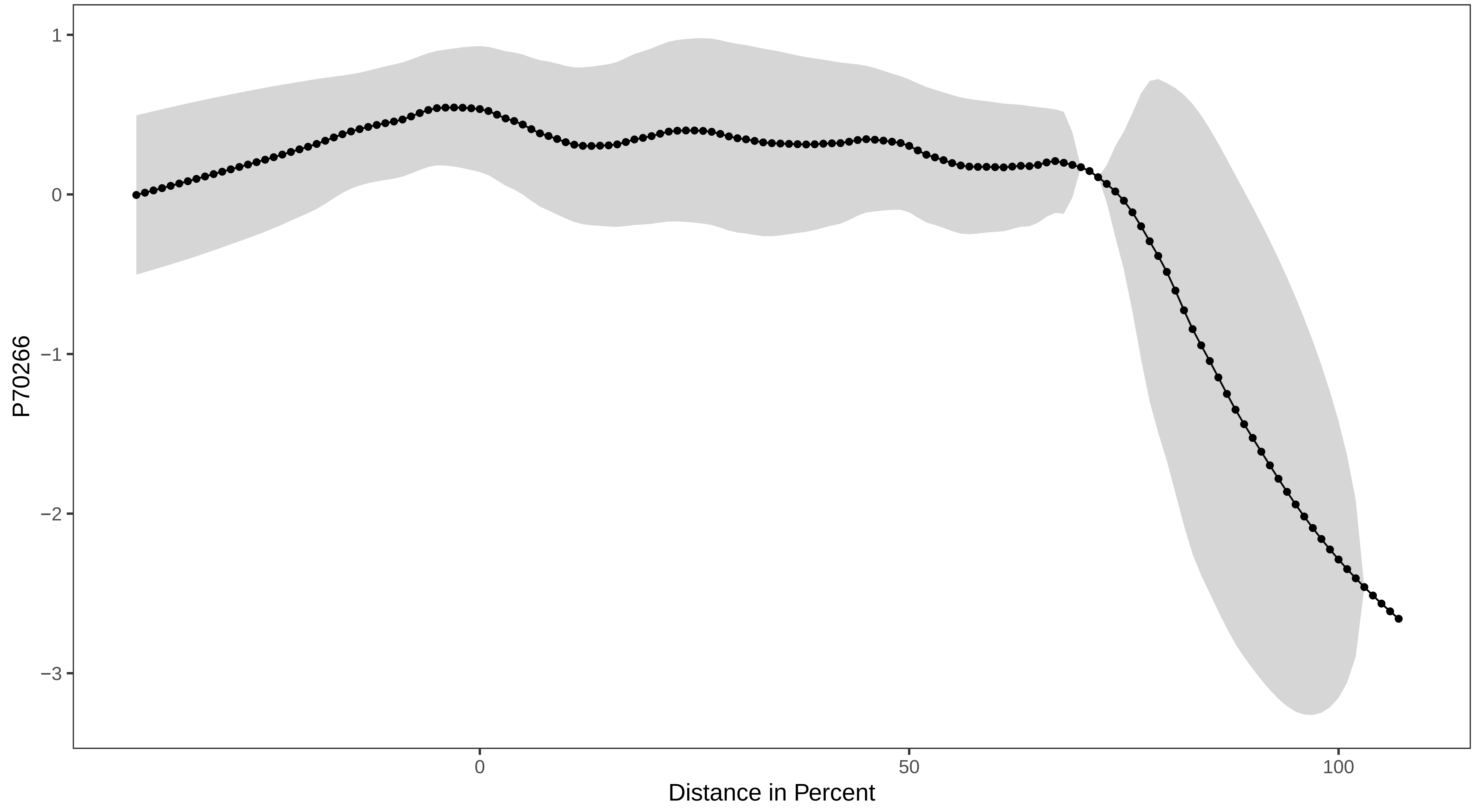

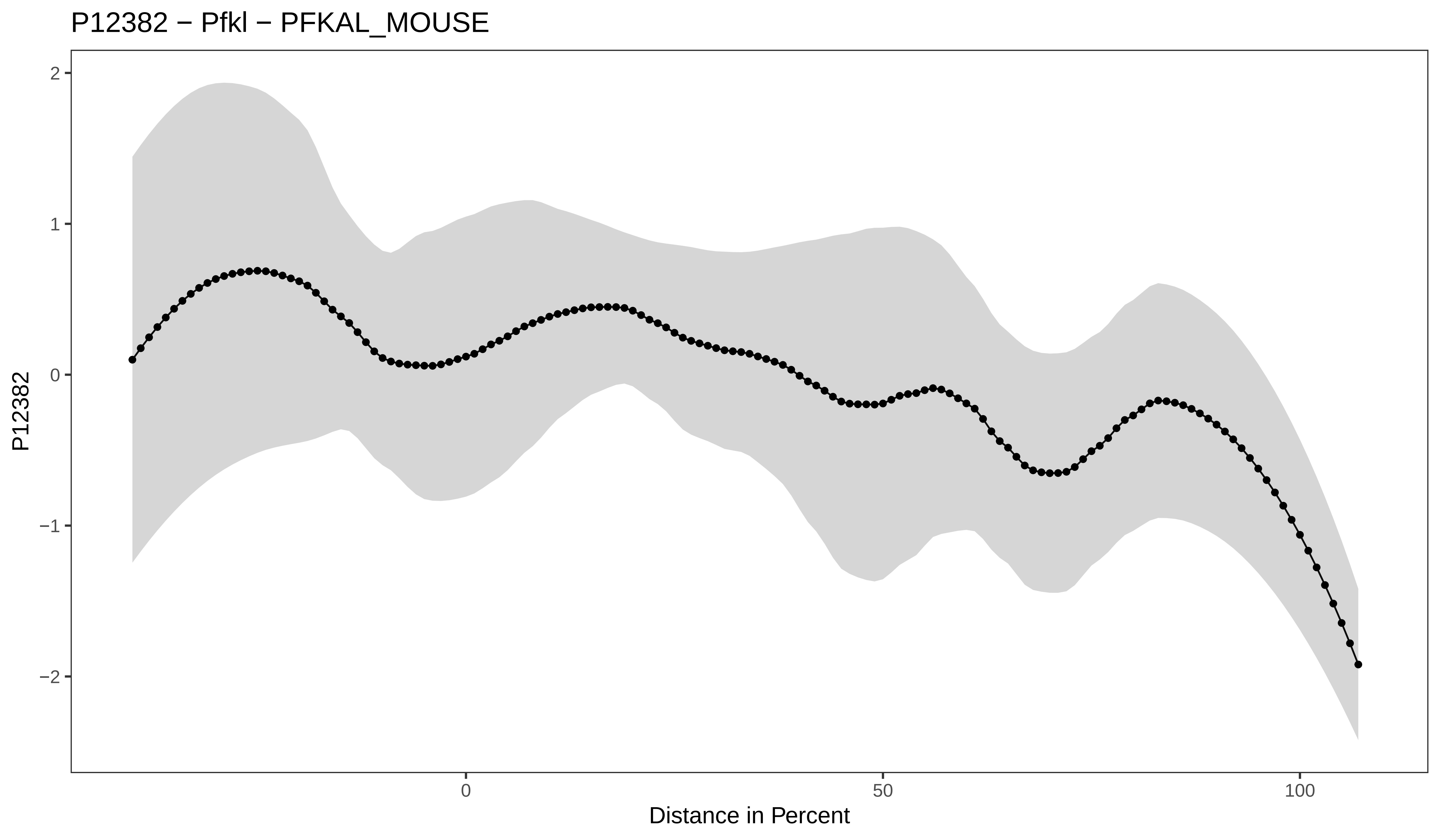
P12382 − Pfkl − PFKAL_MOUSE
P12382
Distance in Percent
2
1
0
−1
−2
0
50
100

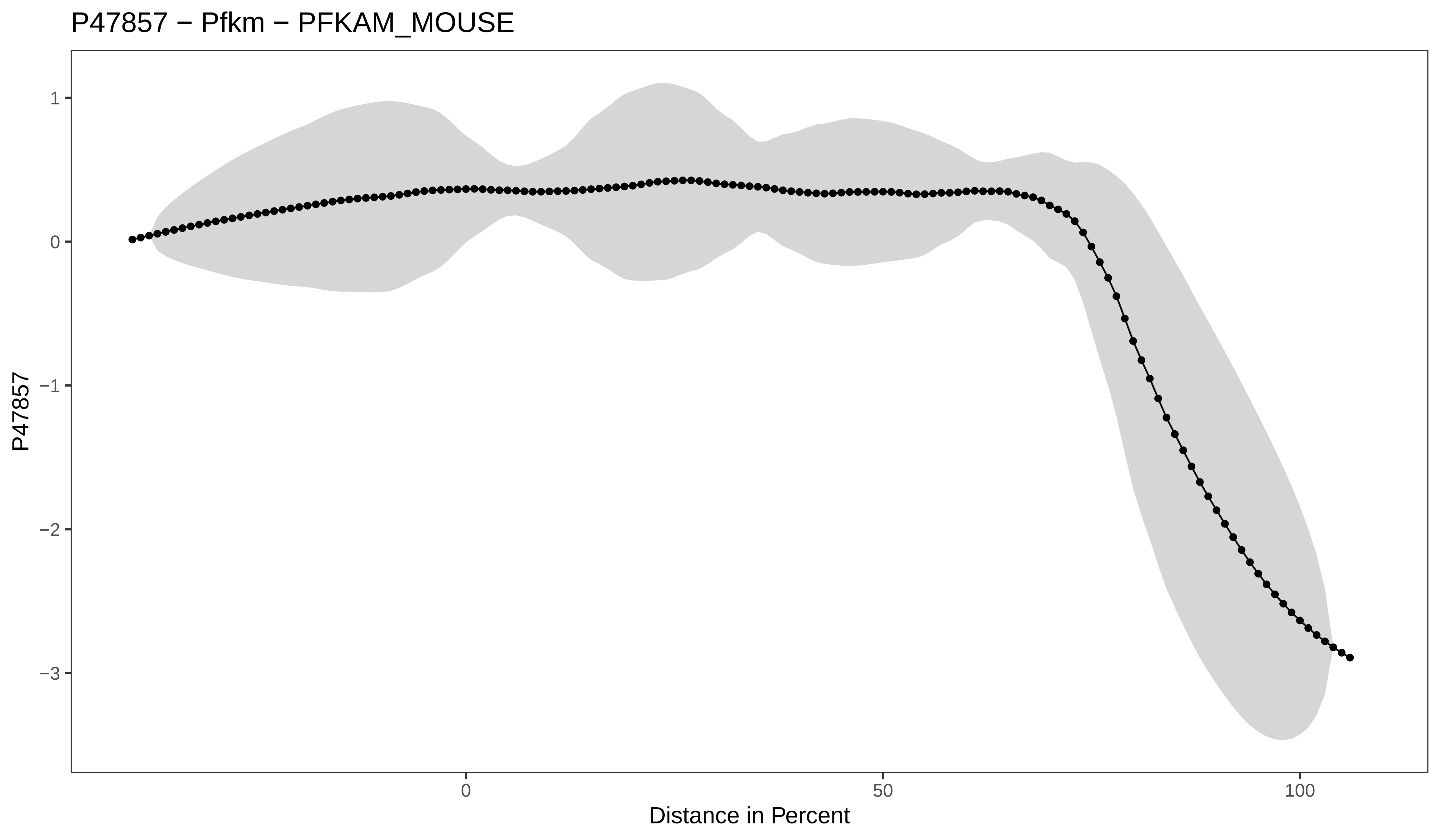

P47857 − Pfkm − PFKAM_MOUSE
P47857
Distance in Percent
1
0
−1
−2
−3
0
50
100

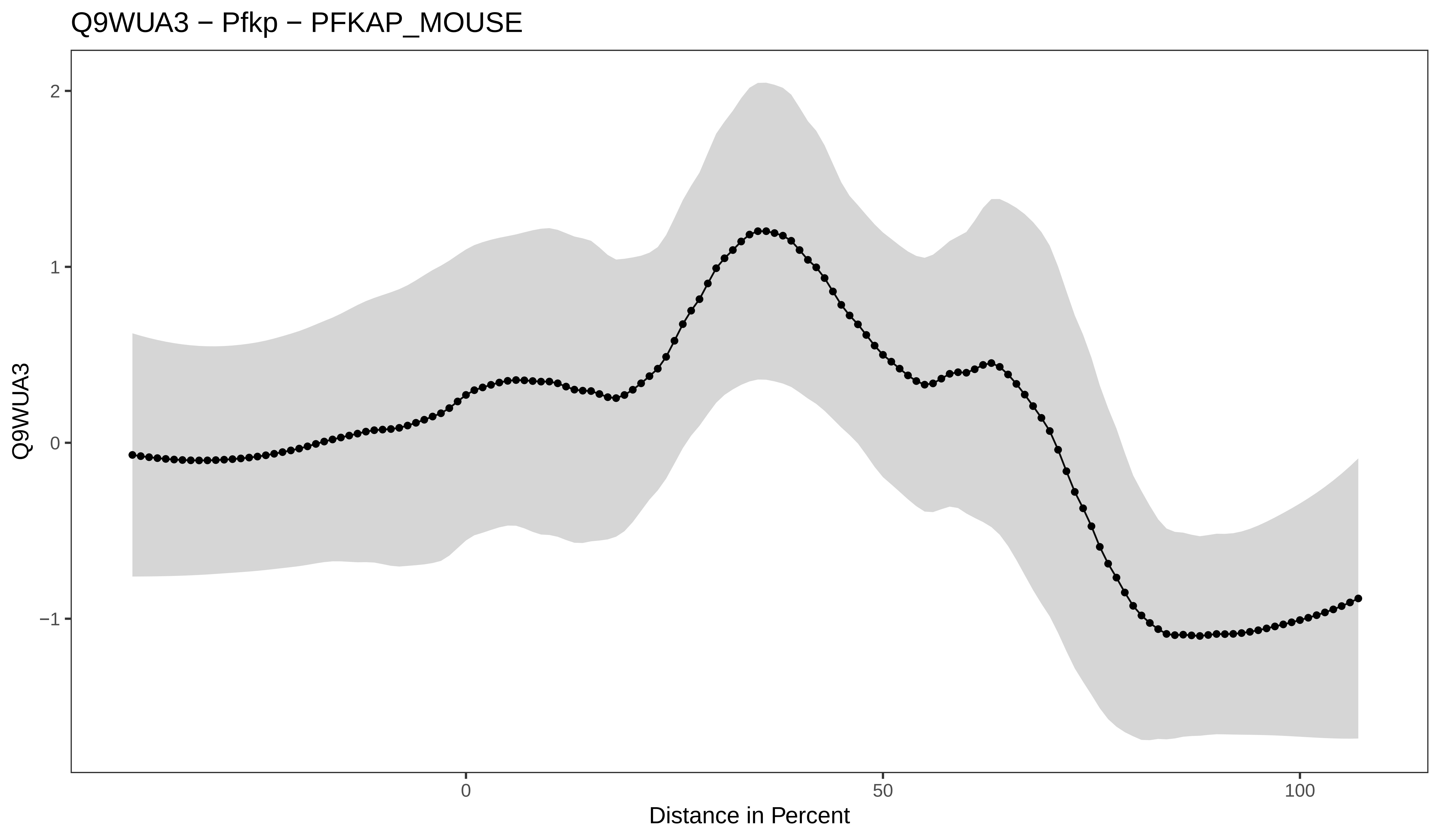

Q9WUA3 − Pfkp − PFKAP_MOUSE
Q9WUA3
2
1
0
−1
0
50
100
Distance in Percent

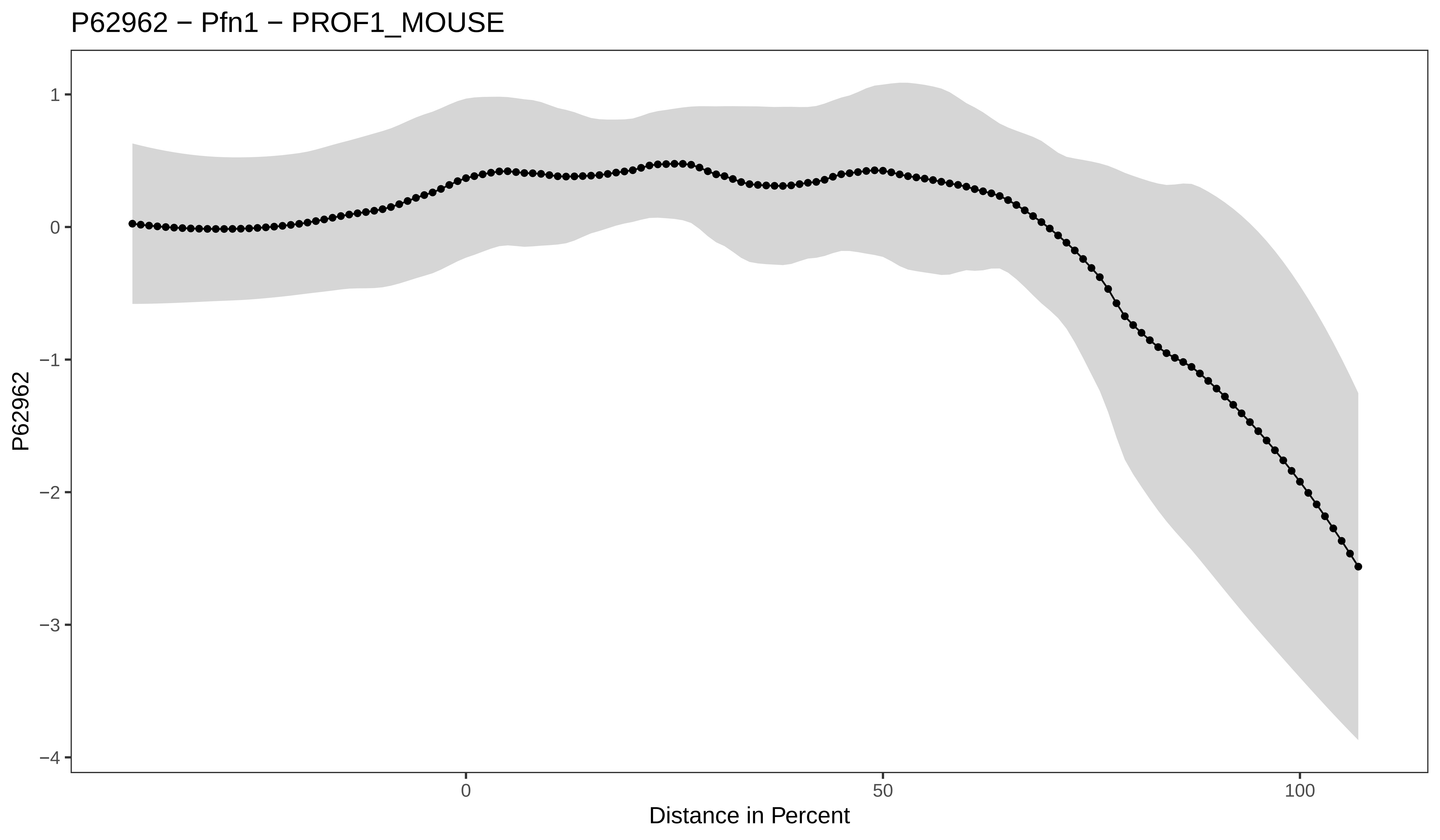

P62962 − Pfn1 − PROF1_MOUSE
1
0
−1
−2
−3
−4
P62962
0
50
100
Distance in Percent

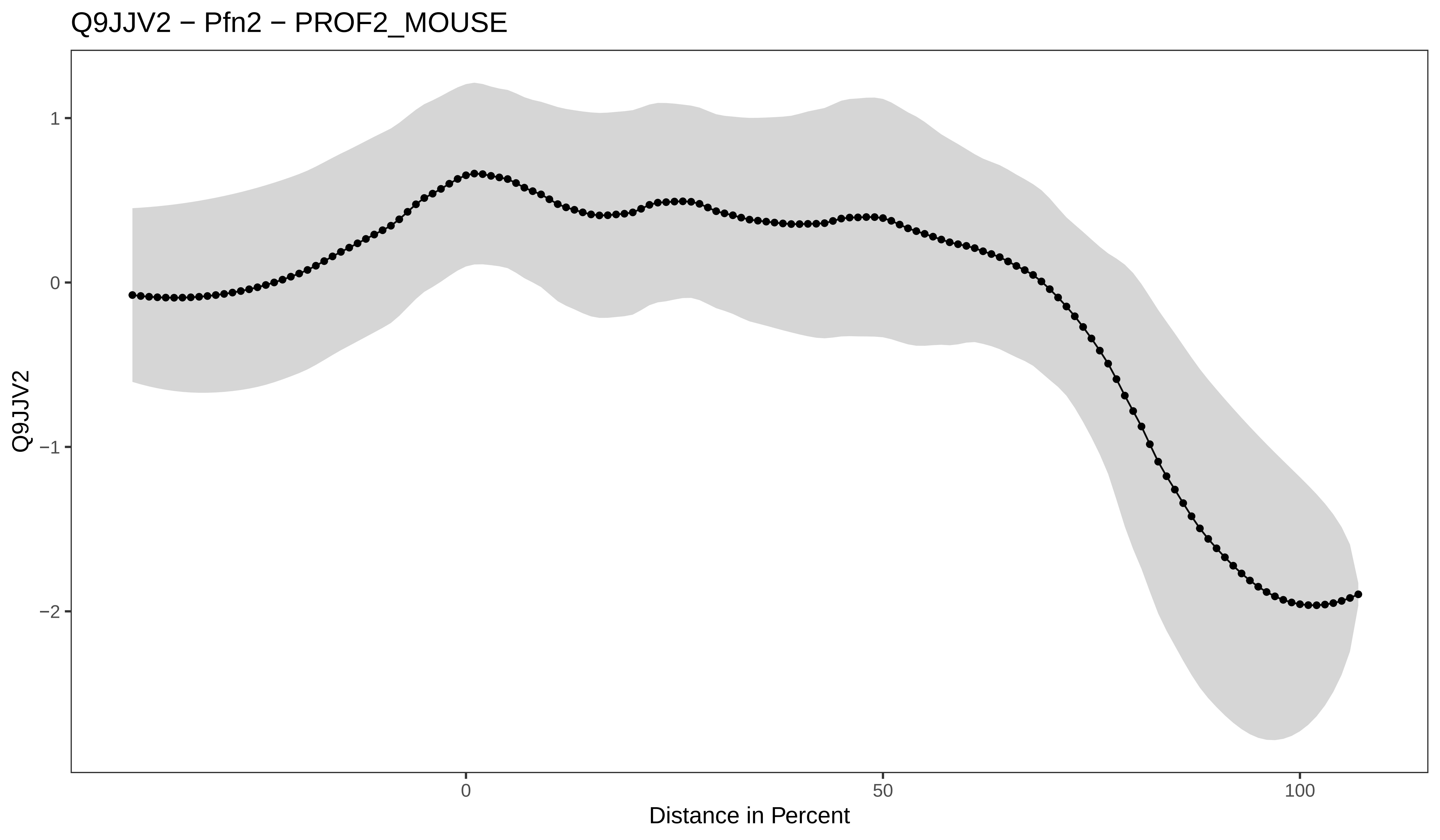

Q9JJV2 − Pfn2 − PROF2_MOUSE
1
0
−1
−2
Q9JJV2
0
50
100
Distance in Percent

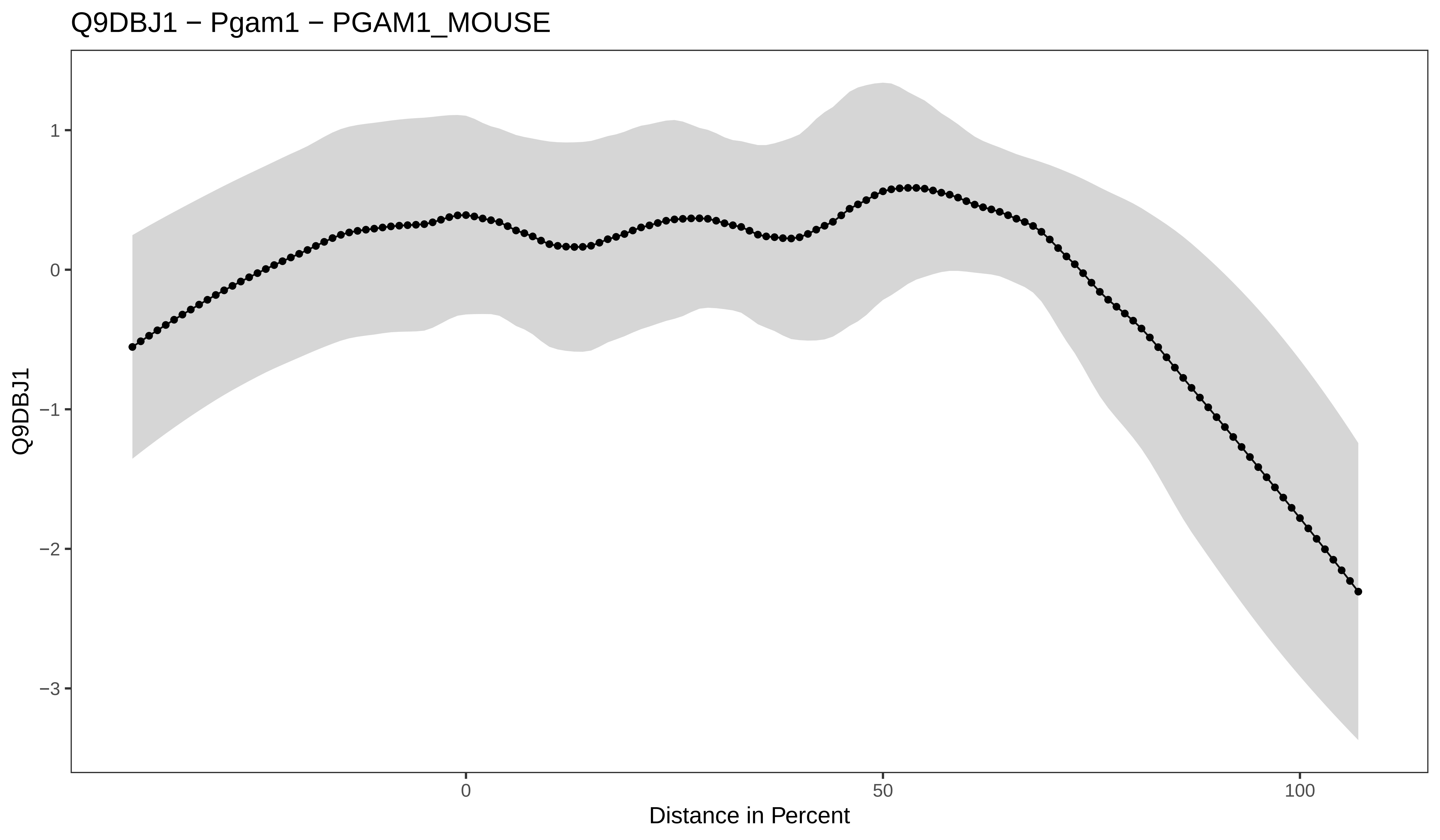
Q9DBJ1 − Pgam1 − PGAM1_MOUSE
1
0
−1
−2
−3
Q9DBJ1
0
50
100
Distance in Percent

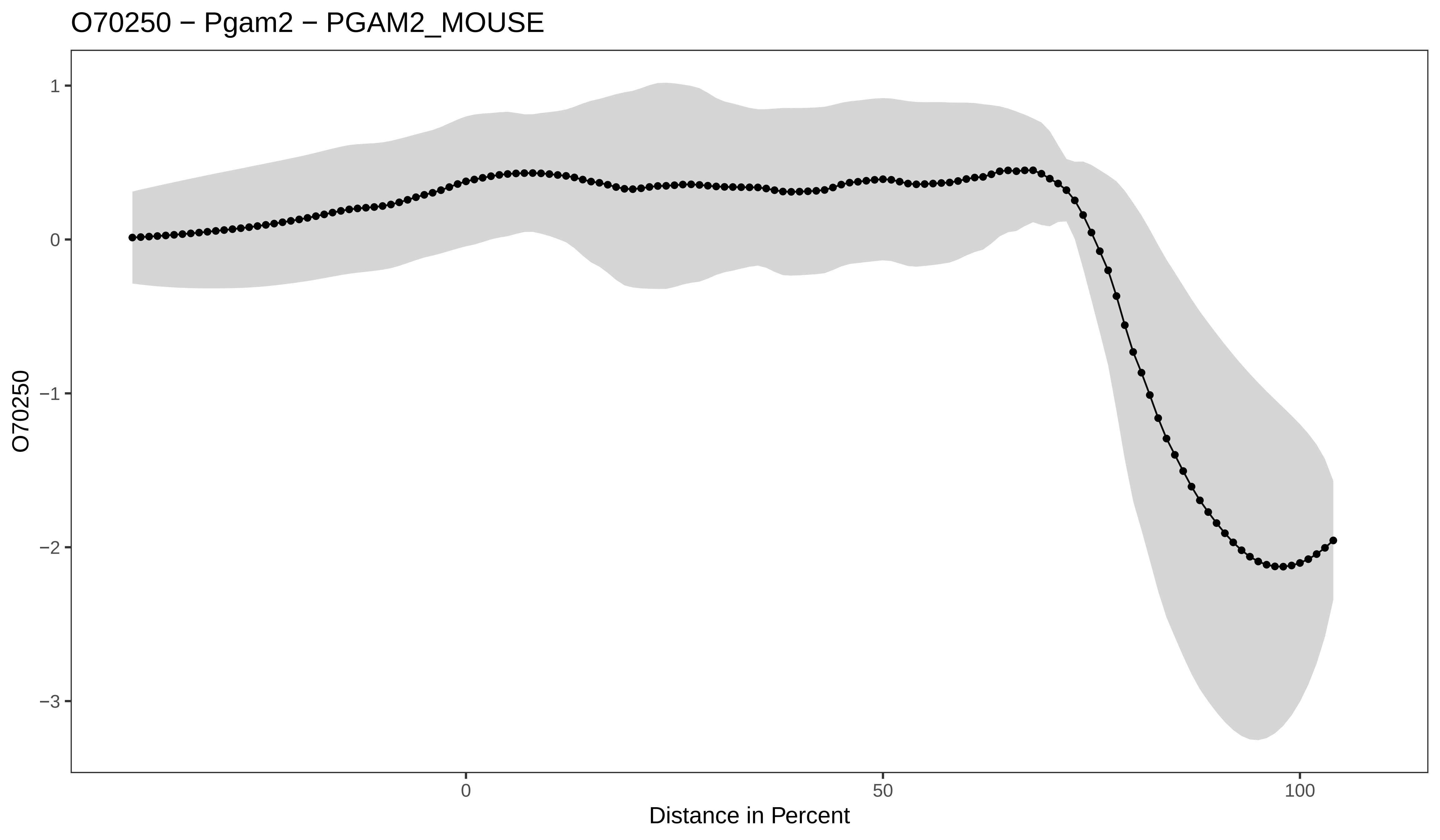

O70250 − Pgam2 − PGAM2_MOUSE
1
0
−1
−2
−3
O70250
0
50
100
Distance in Percent

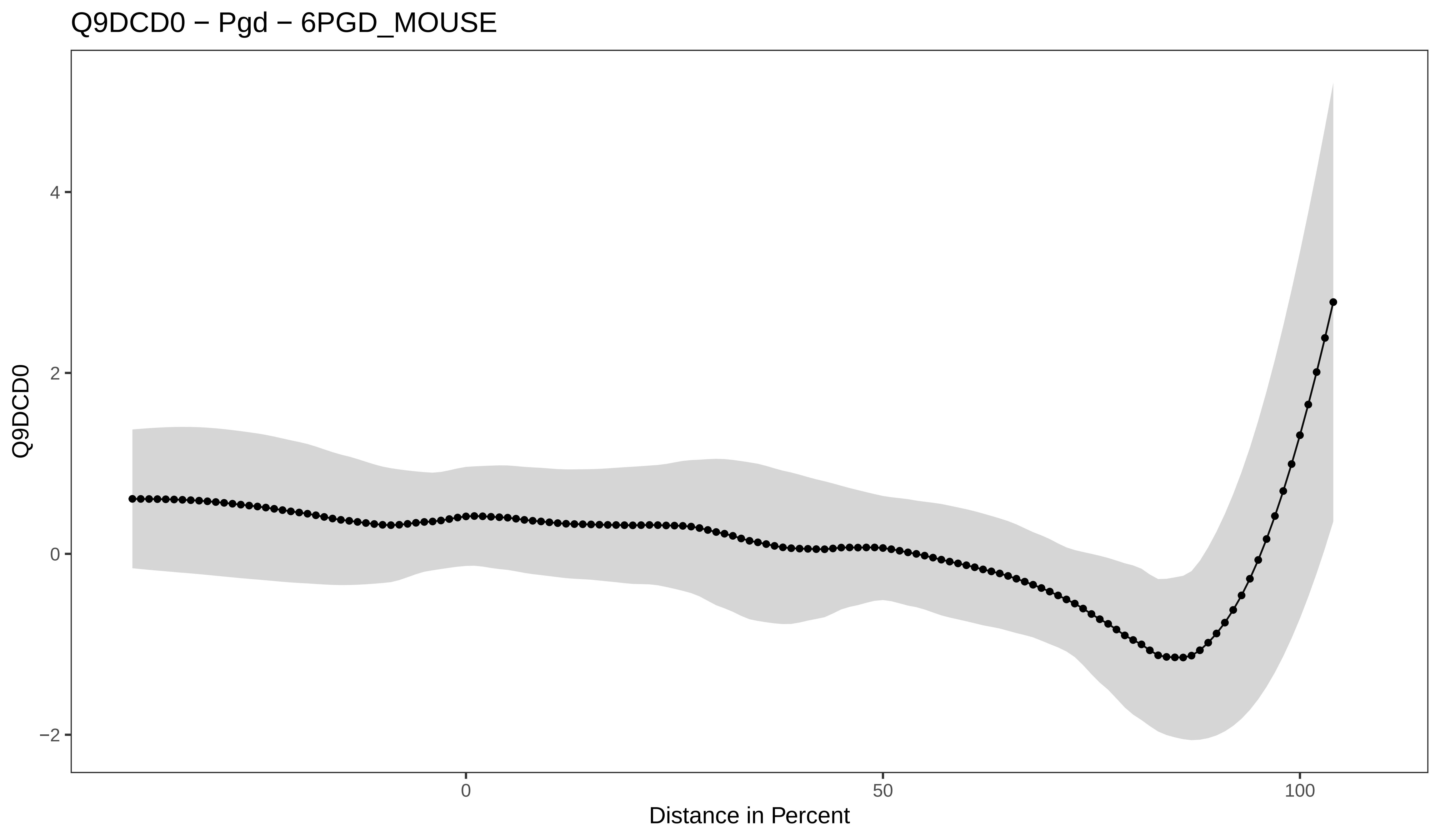

Q9DCD0 − Pgd − 6PGD_MOUSE
Q9DCD0
4
2
0
−2
0
50
100
Distance in Percent

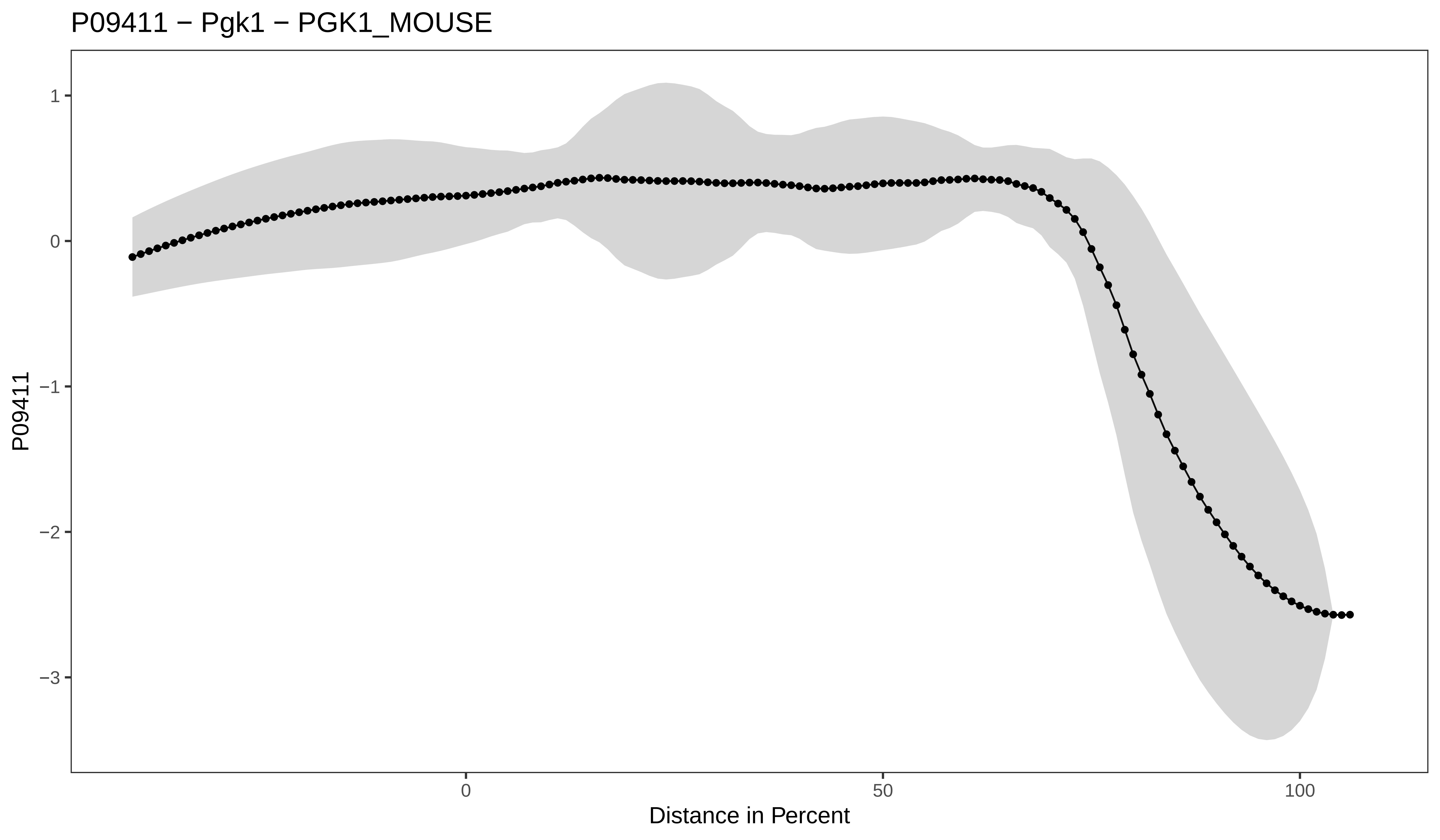
P09411 − Pgk1 − PGK1_MOUSE
P09411
1
0
−1
−2
−3
0
50
100
Distance in Percent

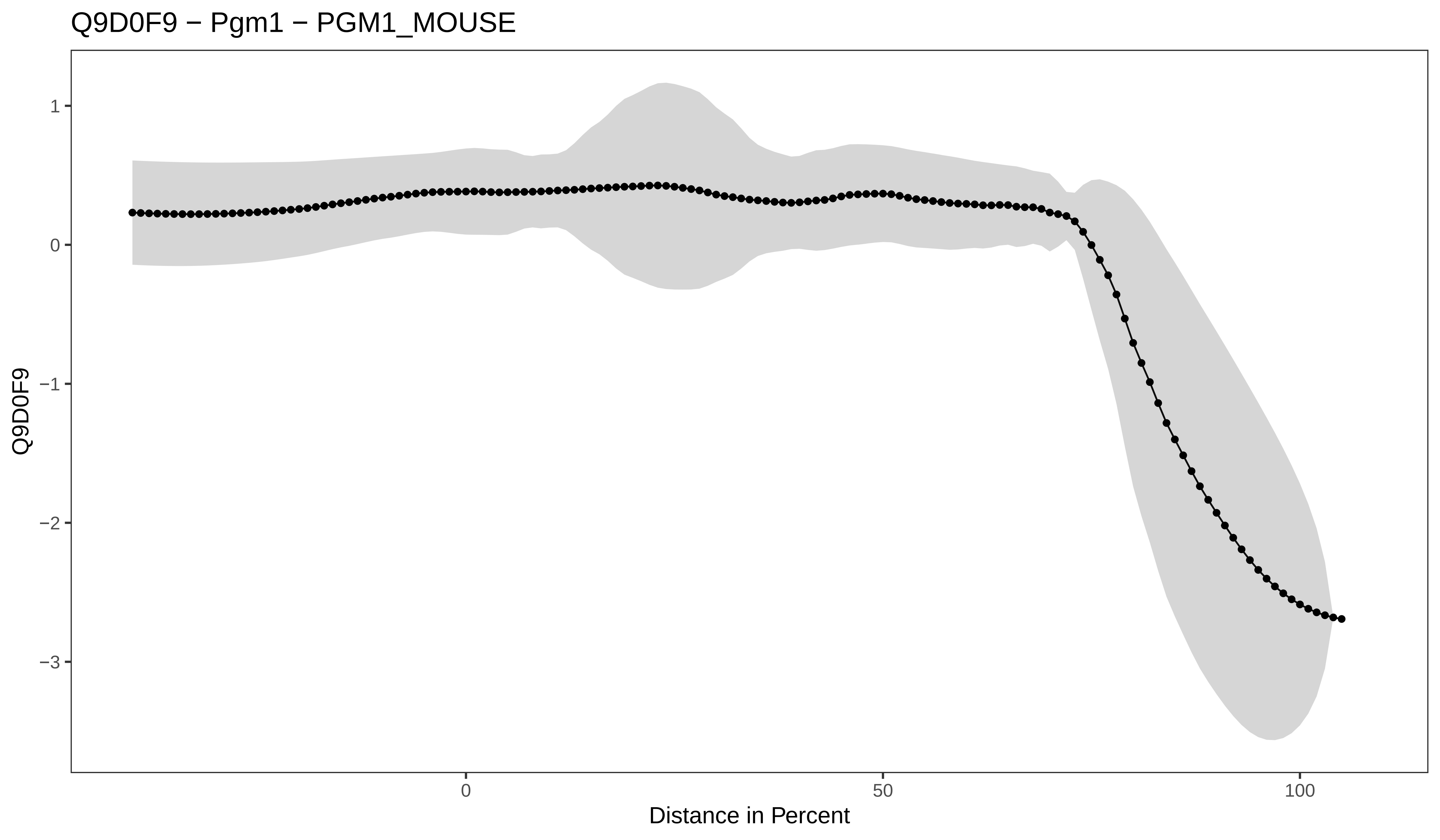

Q9D0F9 − Pgm1 − PGM1_MOUSE
Q9D0F9
1
0
−1
−2
−3
0
50
100
Distance in Percent

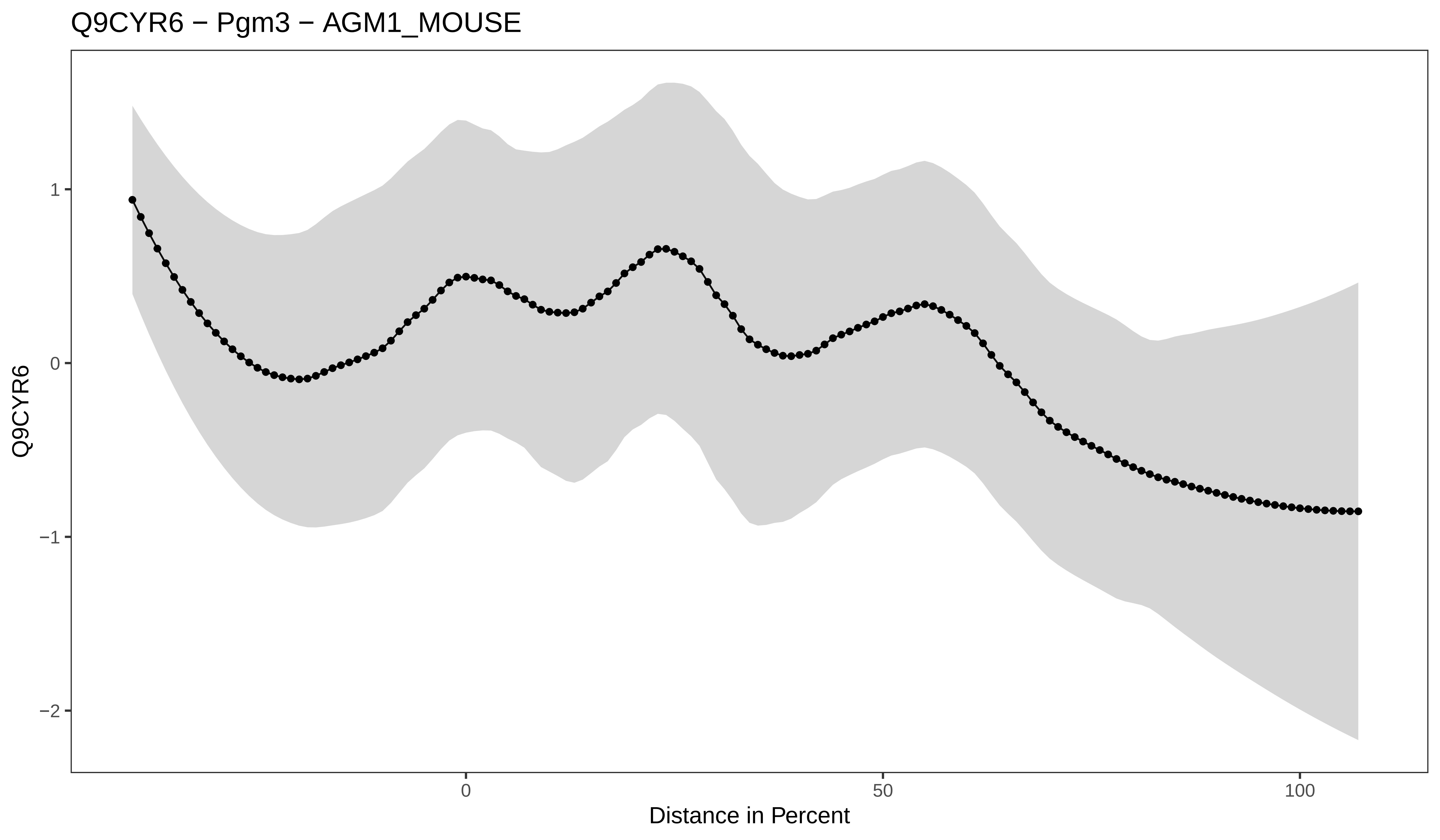
Q9CYR6 − Pgm3 − AGM1_MOUSE
Q9CYR6
1
0
−1
−2
0
50
100
Distance in Percent

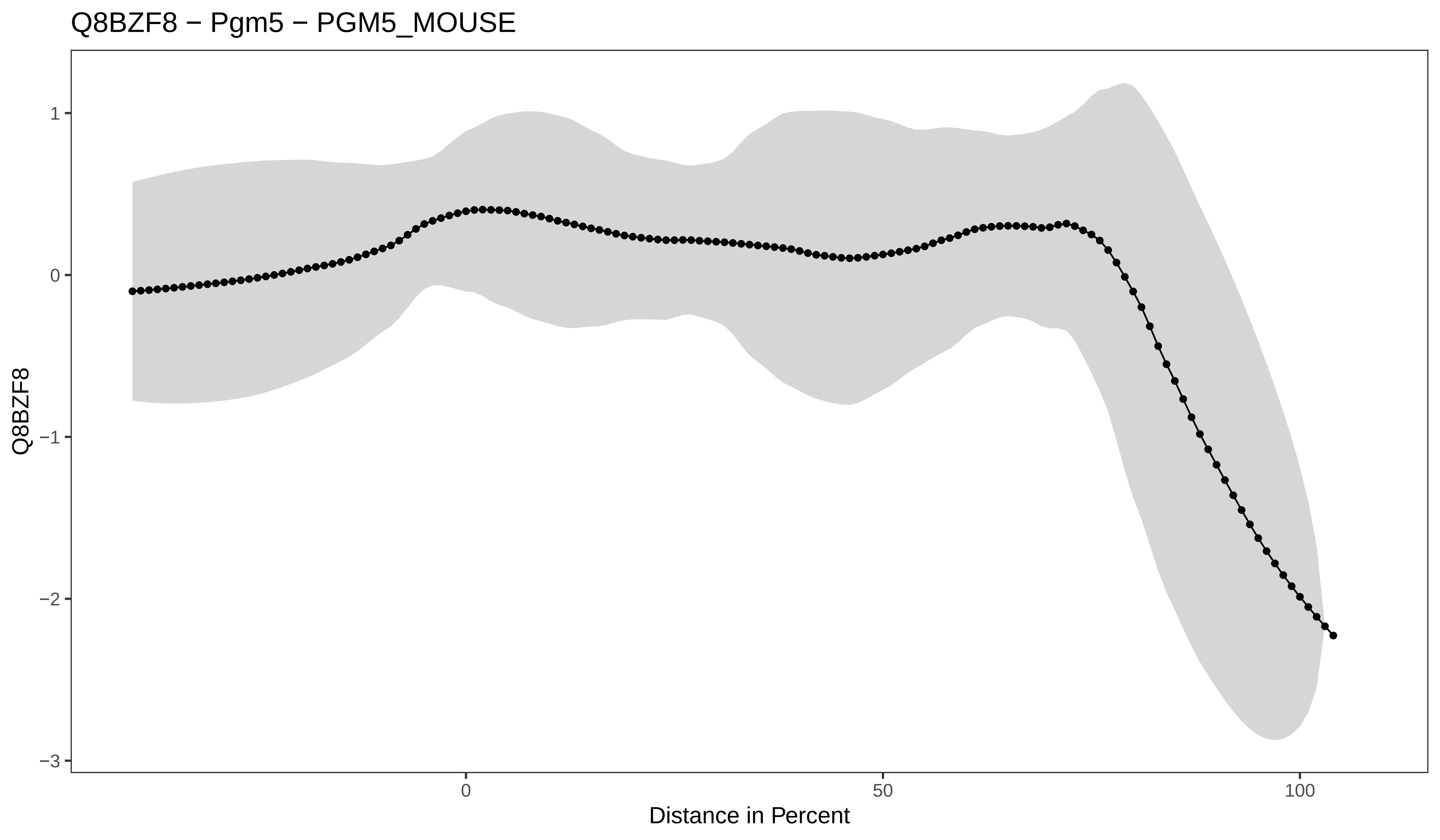

Q8BZF8 − Pgm5 − PGM5_MOUSE
1
0
−1
−2
−3
Q8BZF8
0
50
100
Distance in Percent

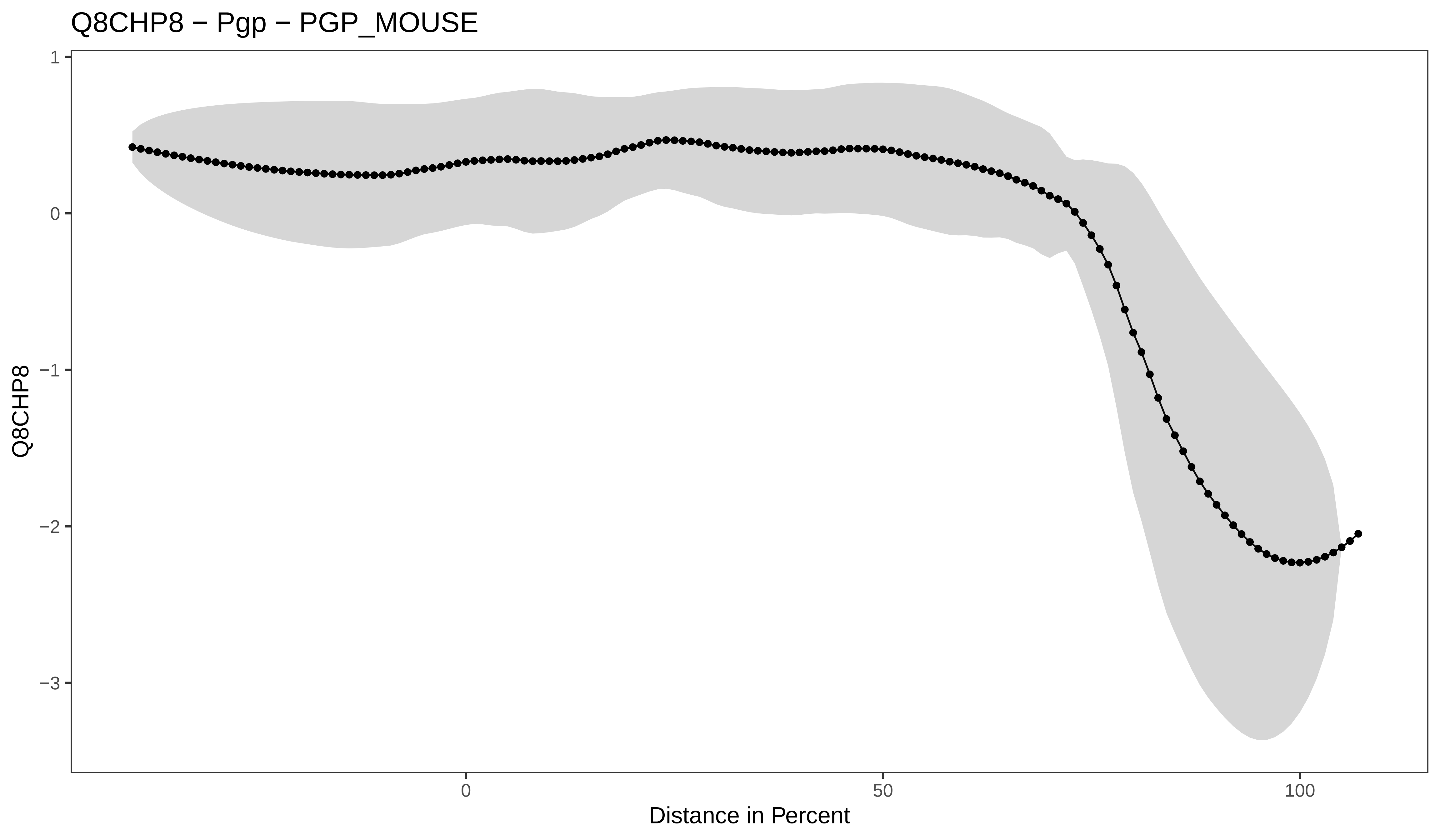
Q8CHP8 − Pgp − PGP_MOUSE
1
0
−1
−2
−3
Q8CHP8
0
50
100
Distance in Percent

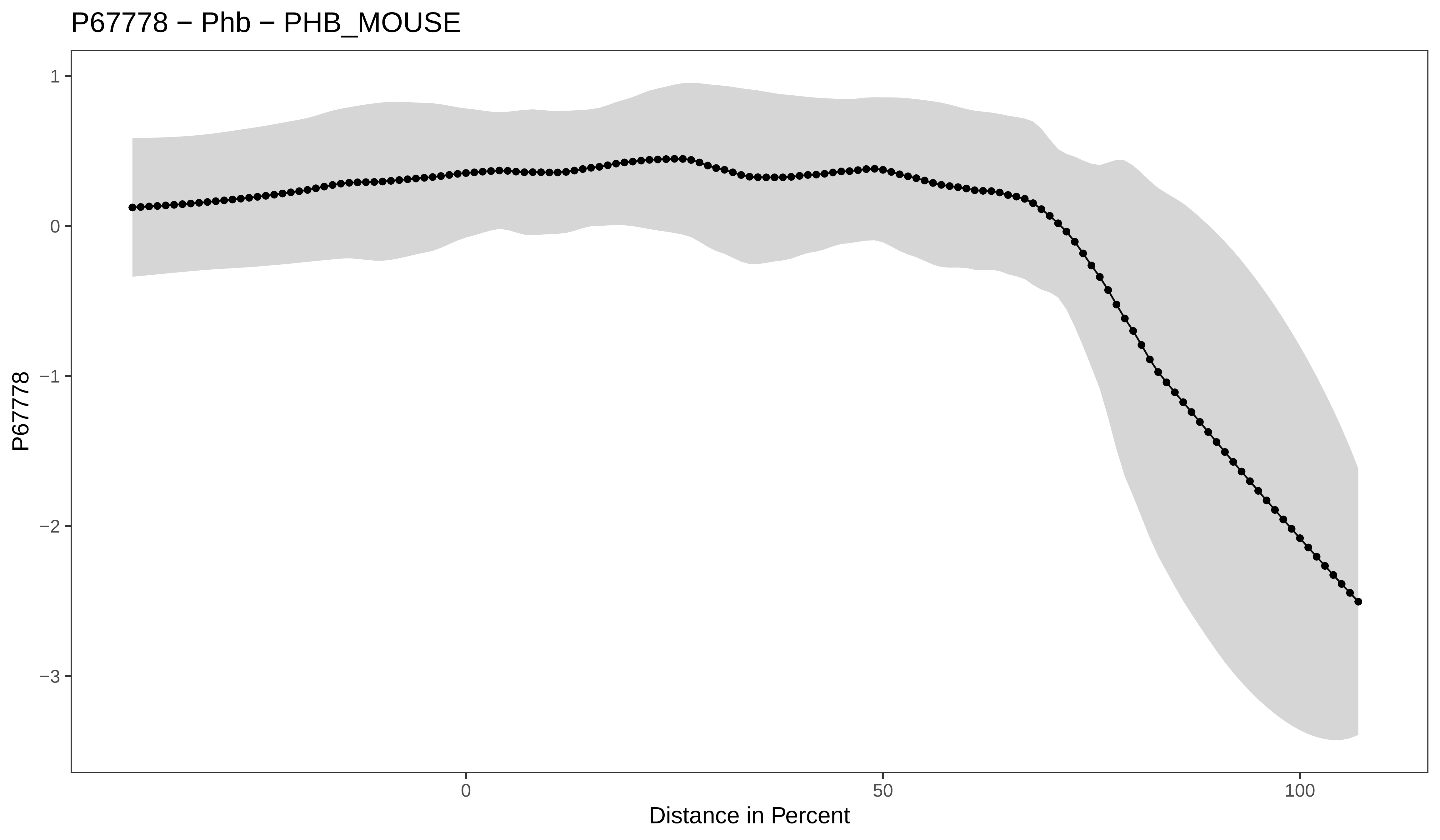

P67778 − Phb − PHB_MOUSE
P67778
1
0
−1
−2
−3
0
50
100
Distance in Percent

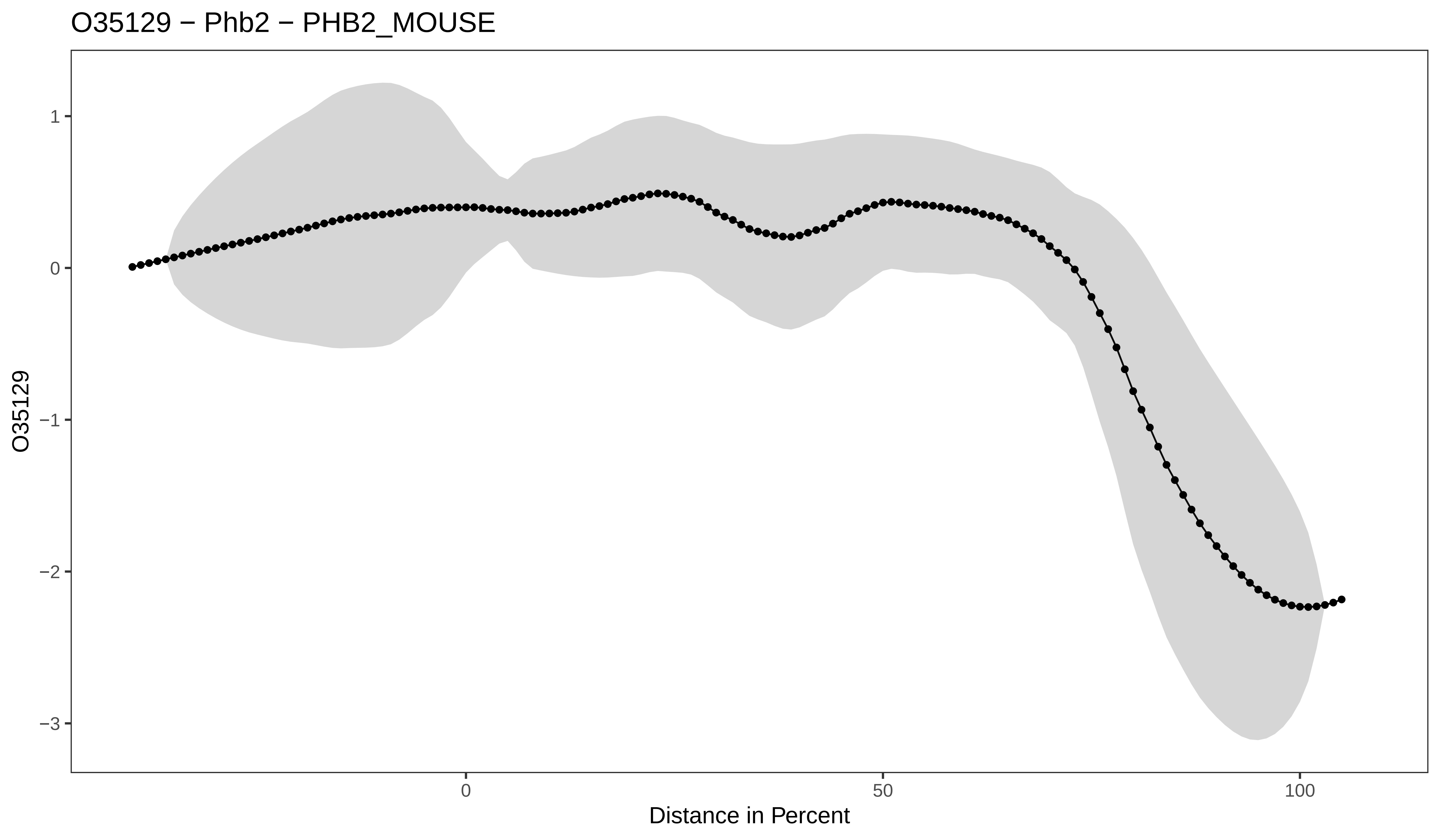

O35129 − Phb2 − PHB2_MOUSE
O35129
Distance in Percent
1
0
−1
−2
−3
0
50
100

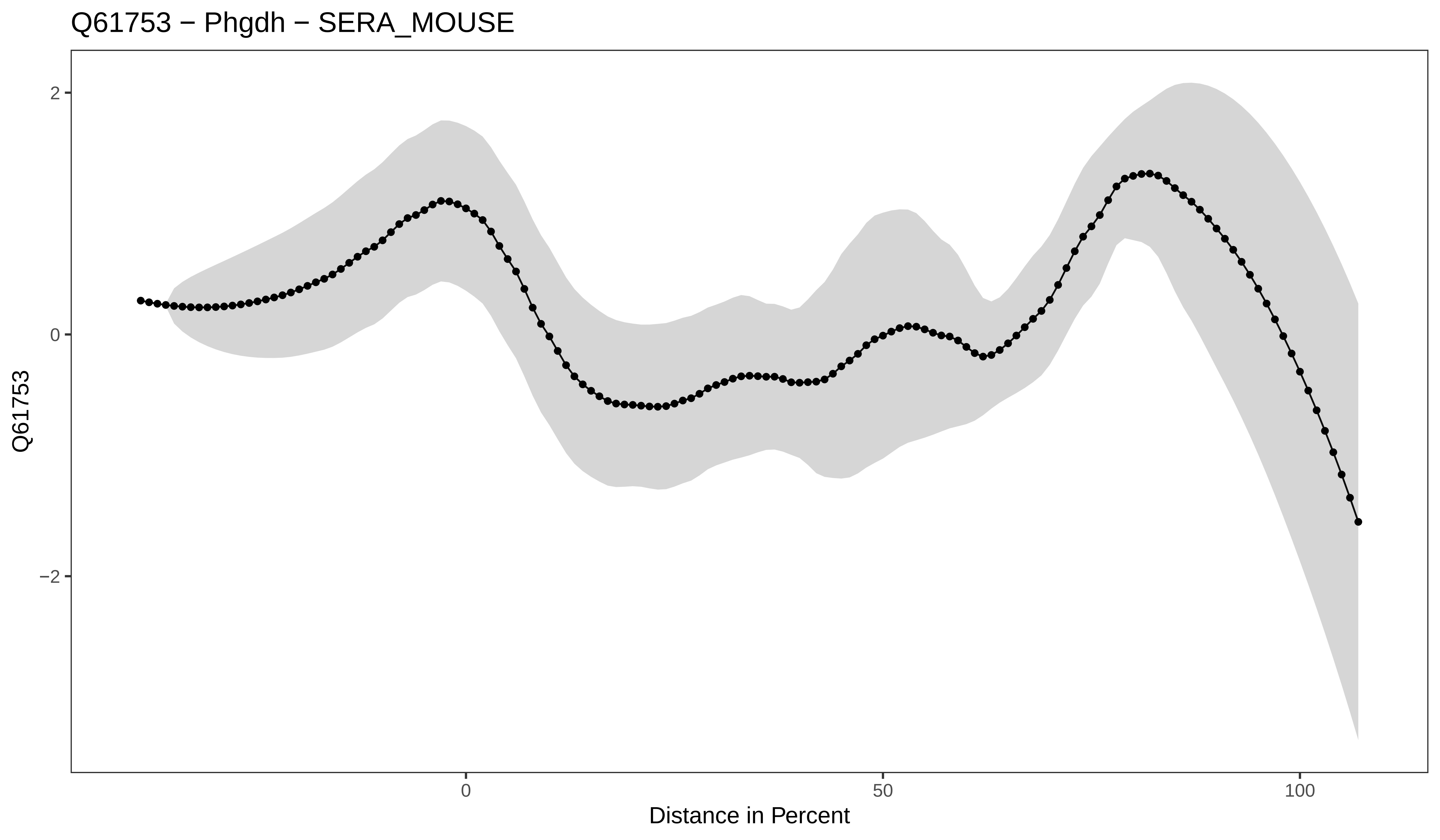

Q61753 − Phgdh − SERA_MOUSE
2
0
−2
Q61753
0
50
100
Distance in Percent

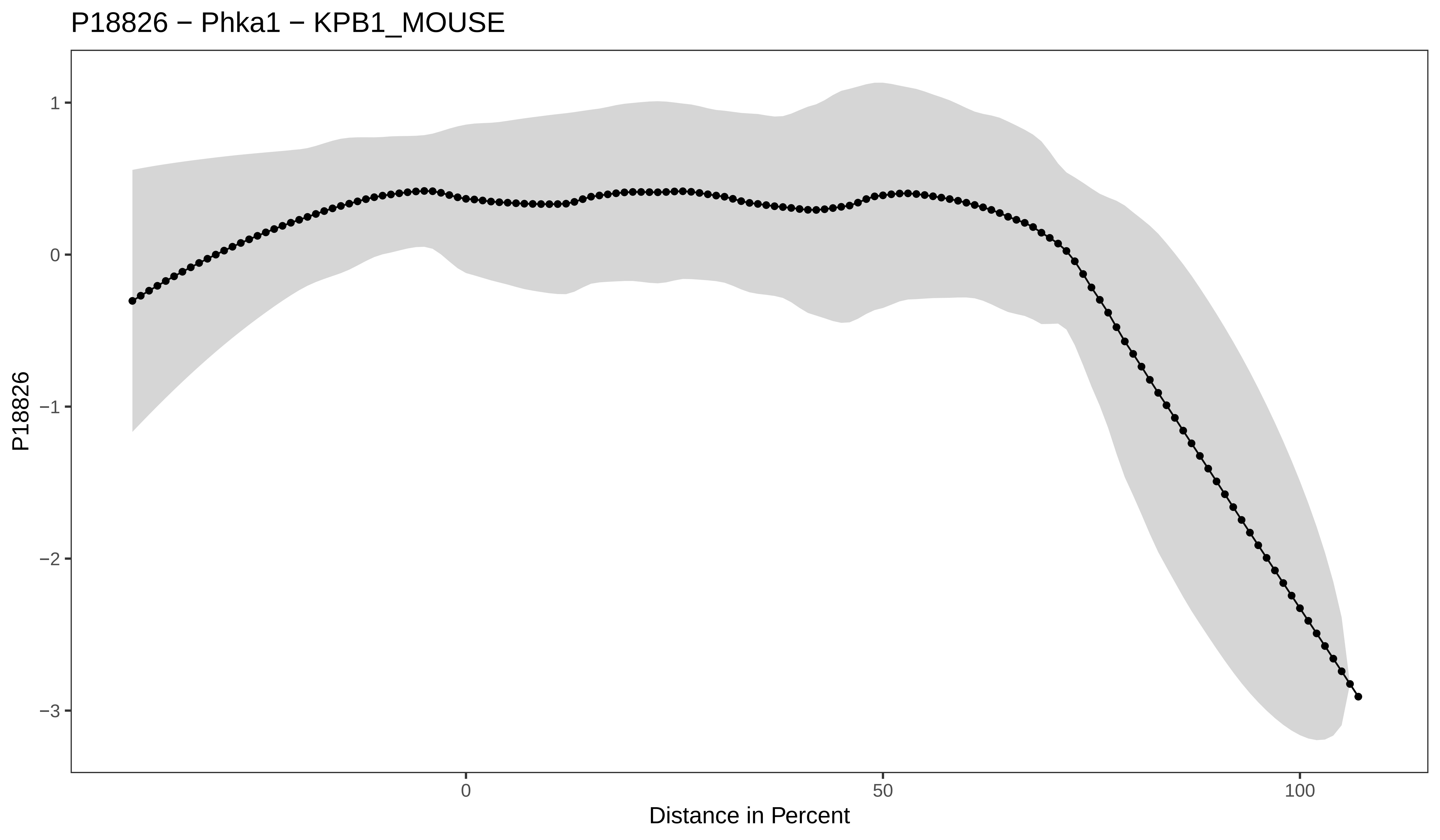

P18826 − Phka1 − KPB1_MOUSE
1
0
−1
−2
−3
P18826
0
50
100
Distance in Percent

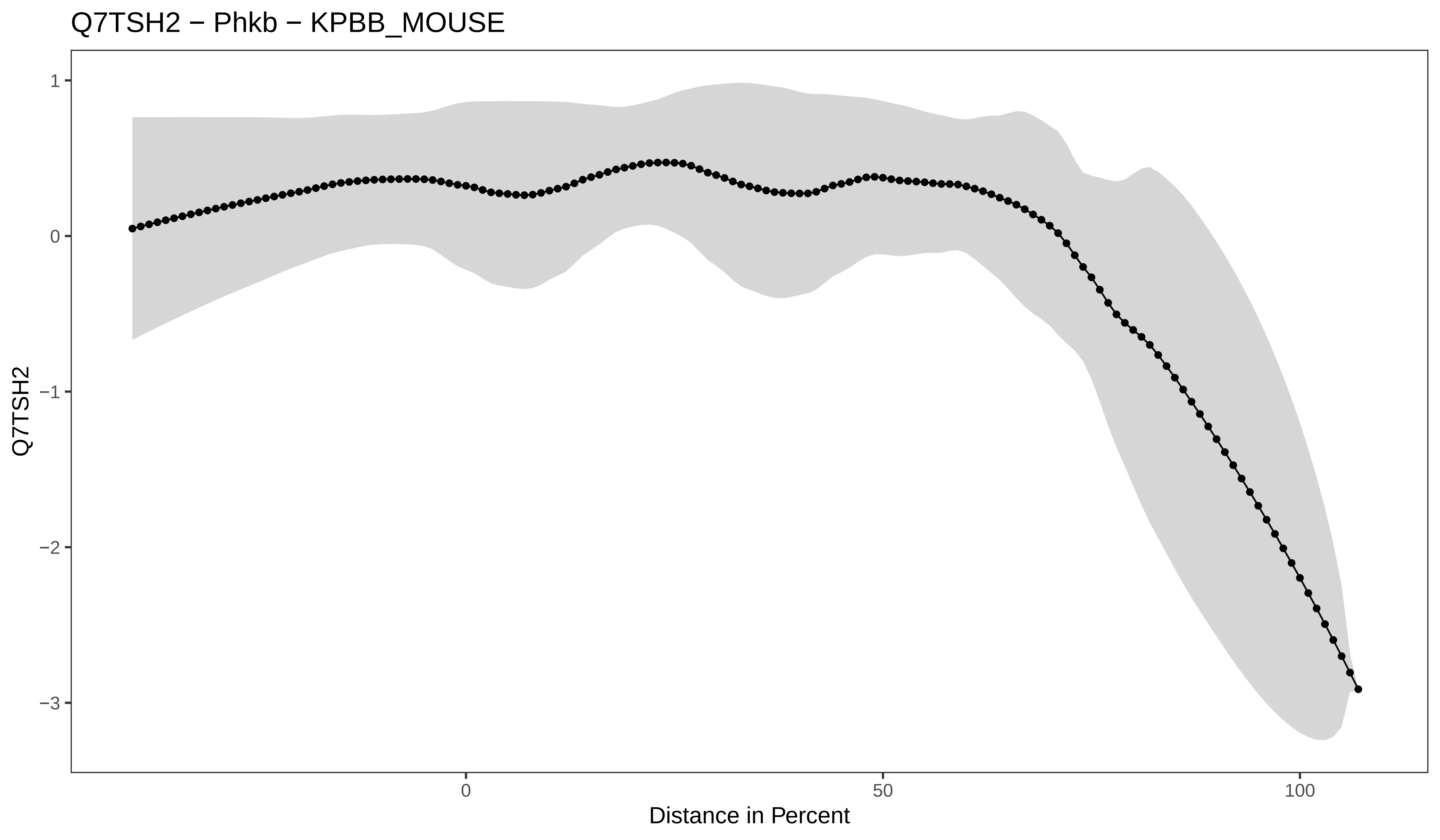
Q7TSH2 − Phkb − KPBB_MOUSE
1
0
−1
−2
−3
Q7TSH2
0
50
100
Distance in Percent

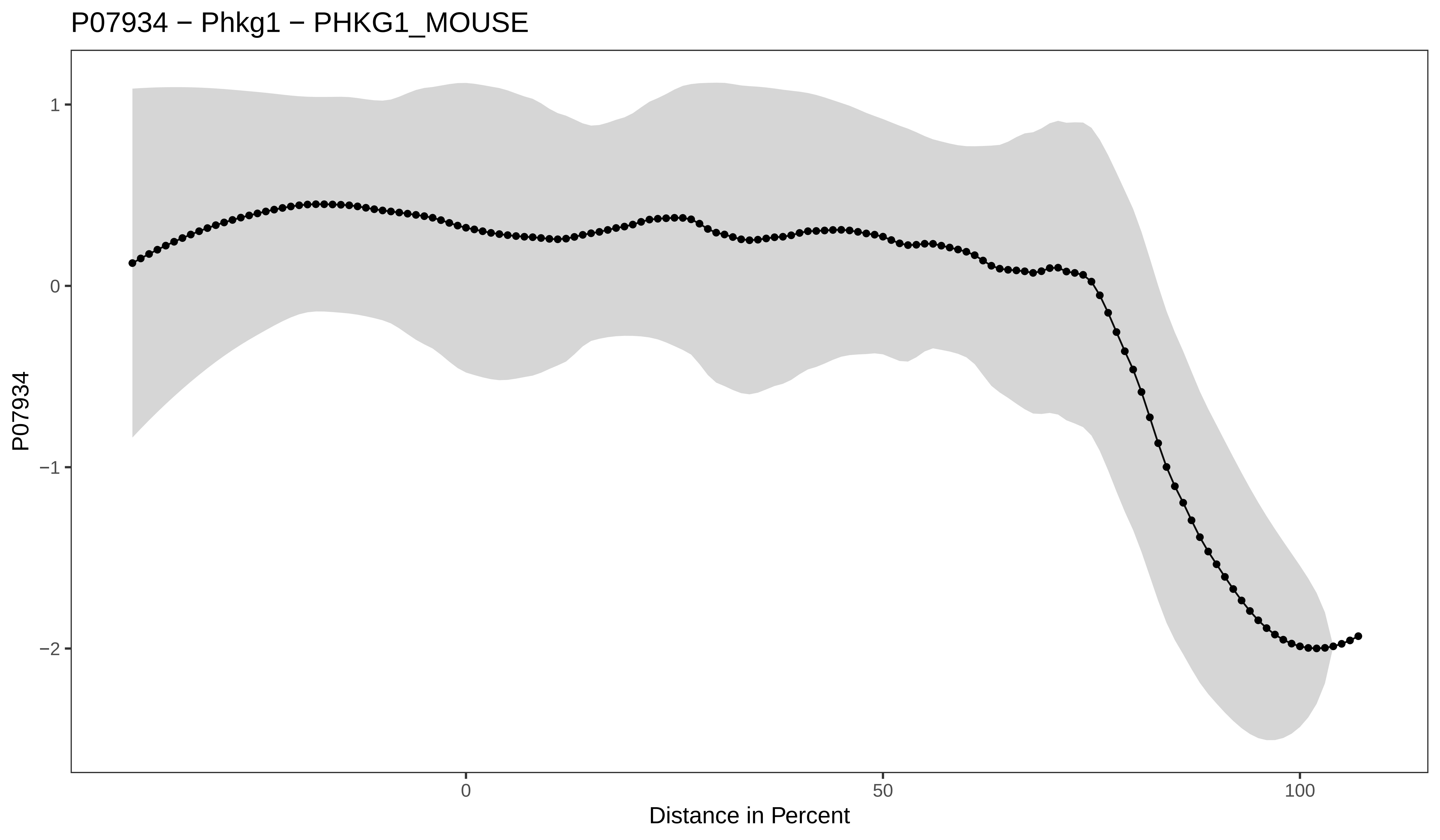

P07934 − Phkg1 − PHKG1_MOUSE
1
0
−1
−2
P07934
0
50
100
Distance in Percent

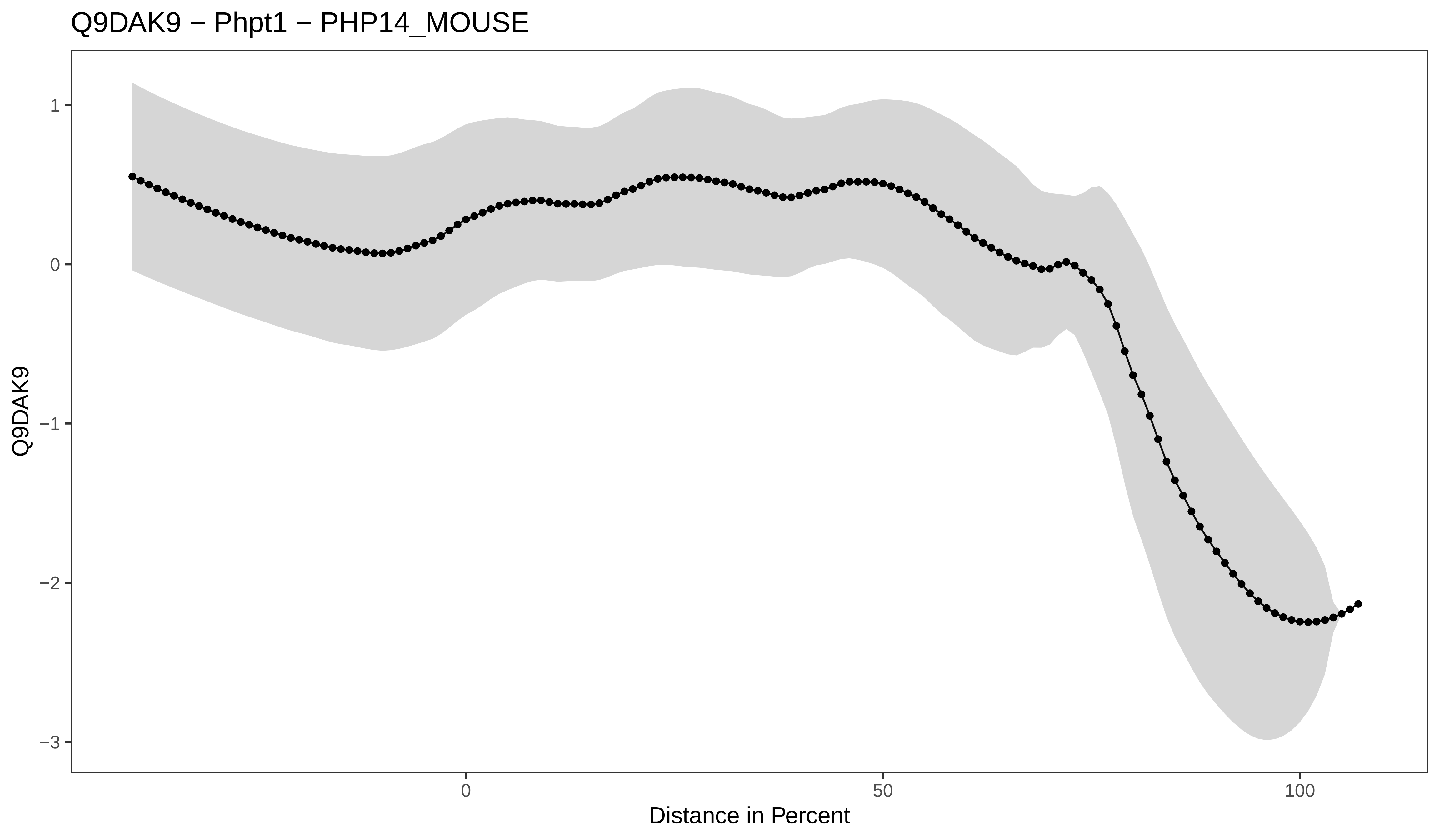

Q9DAK9 − Phpt1 − PHP14_MOUSE
1
0
−1
−2
−3
Q9DAK9
0
50
100
Distance in Percent

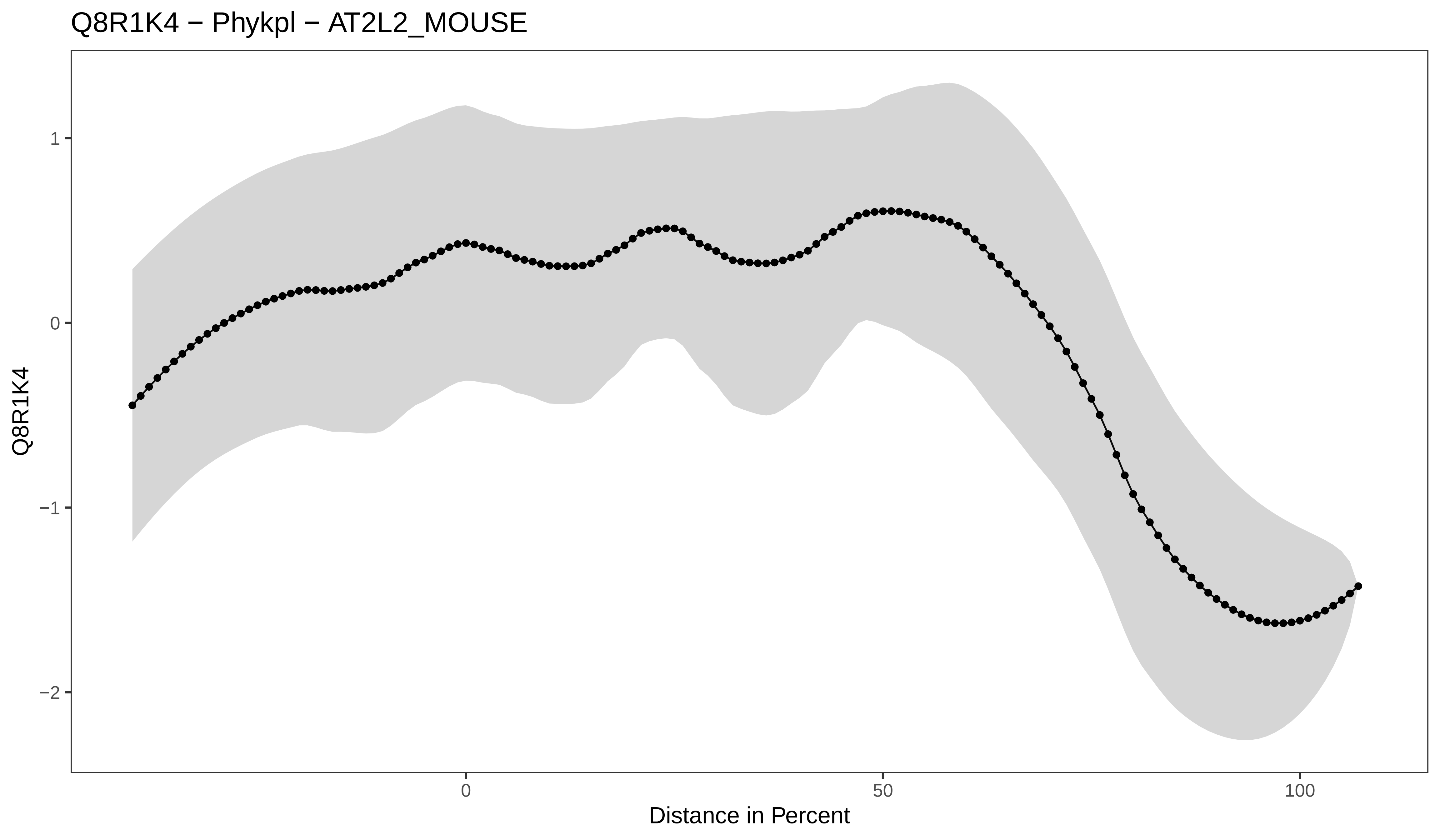

Q8R1K4 − Phykpl − AT2L2_MOUSE
Q8R1K4
Distance in Percent
1
0
−1
−2
0
50
100

E9Q3L2 − Pi4ka − PI4KA_MOUSE

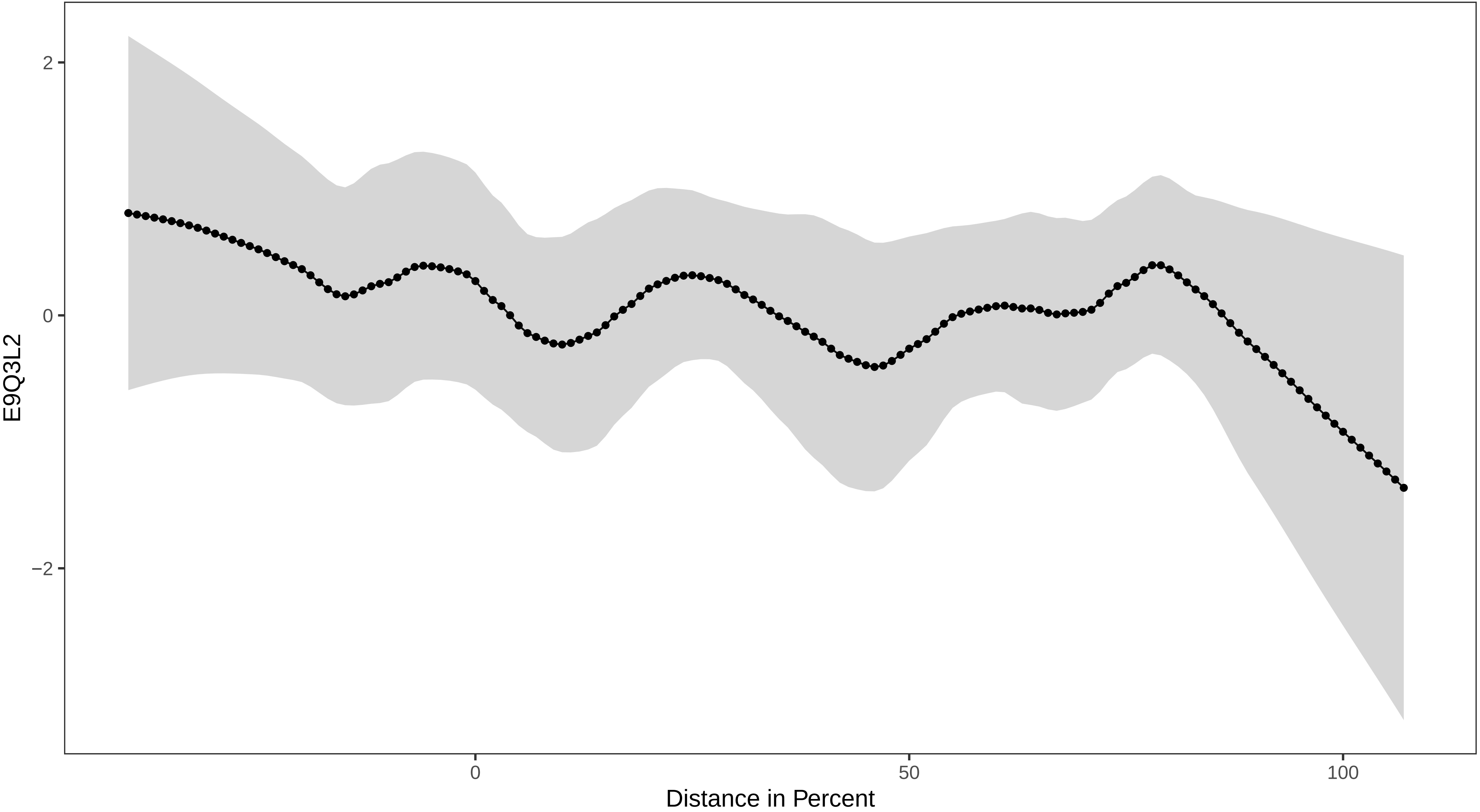

Q7M6Y3 − Picalm − PICAL_MOUSE

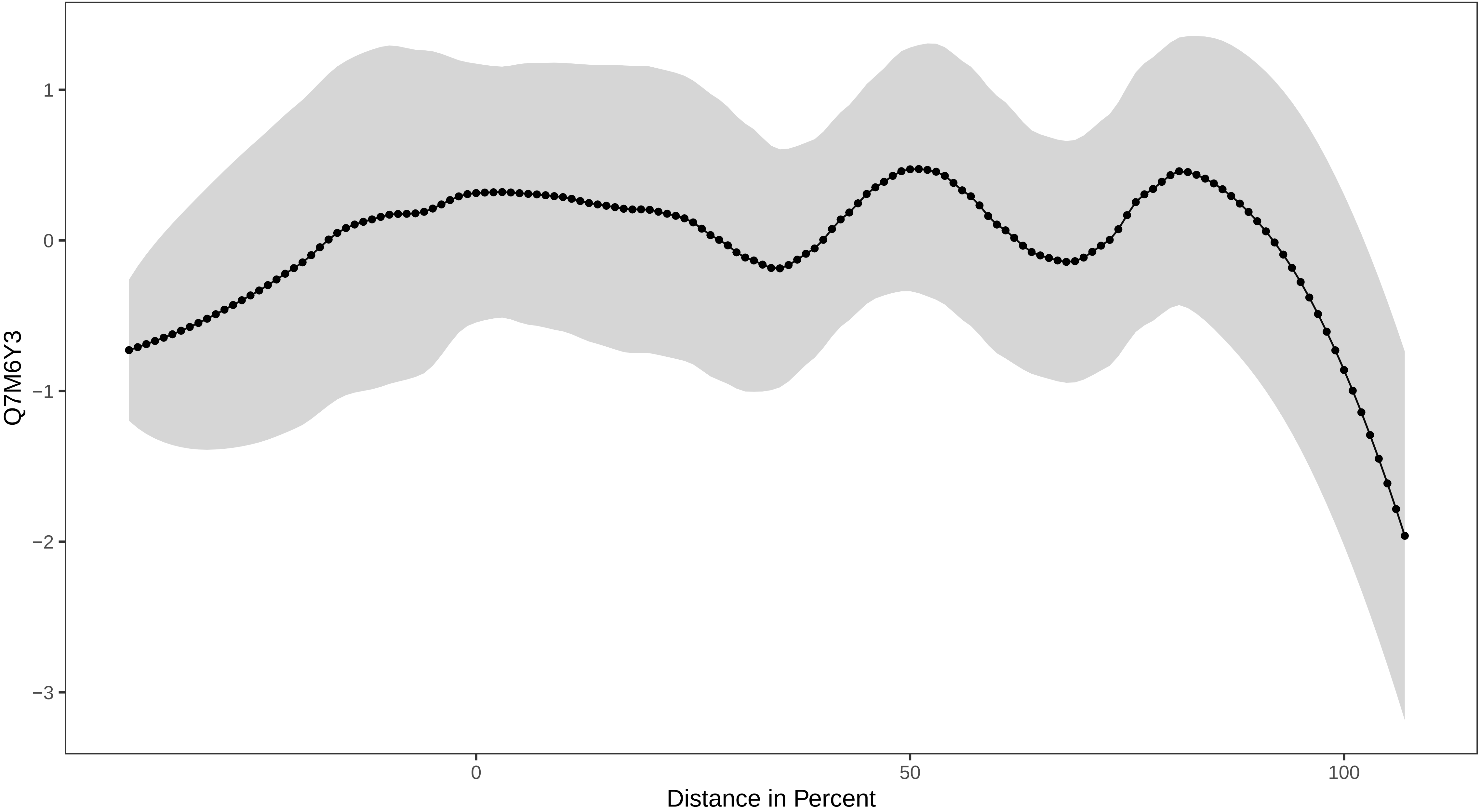

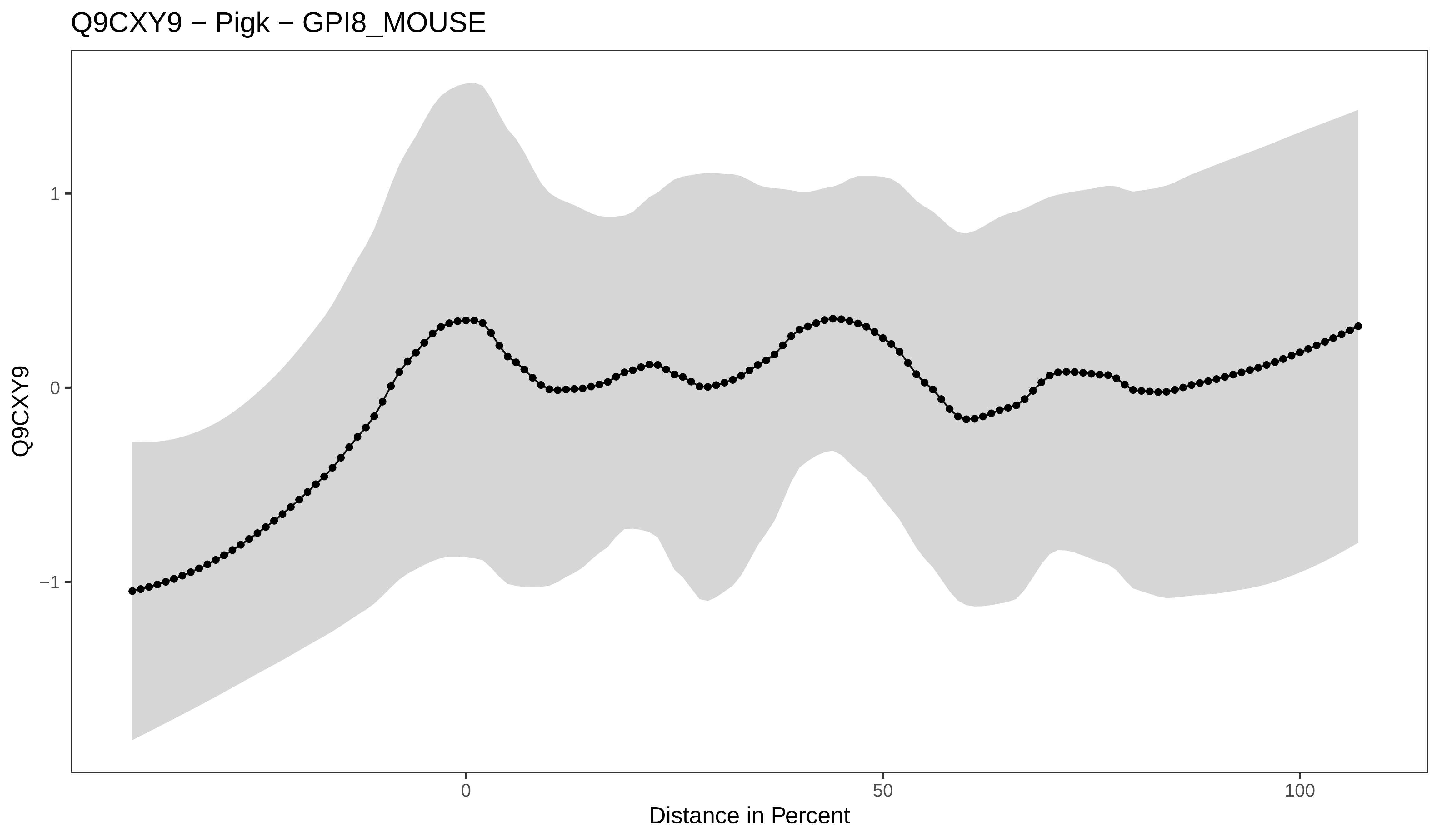

Q9CXY9 − Pigk − GPI8_MOUSE
Q9CXY9
1
0
−1
0
50
100
Distance in Percent

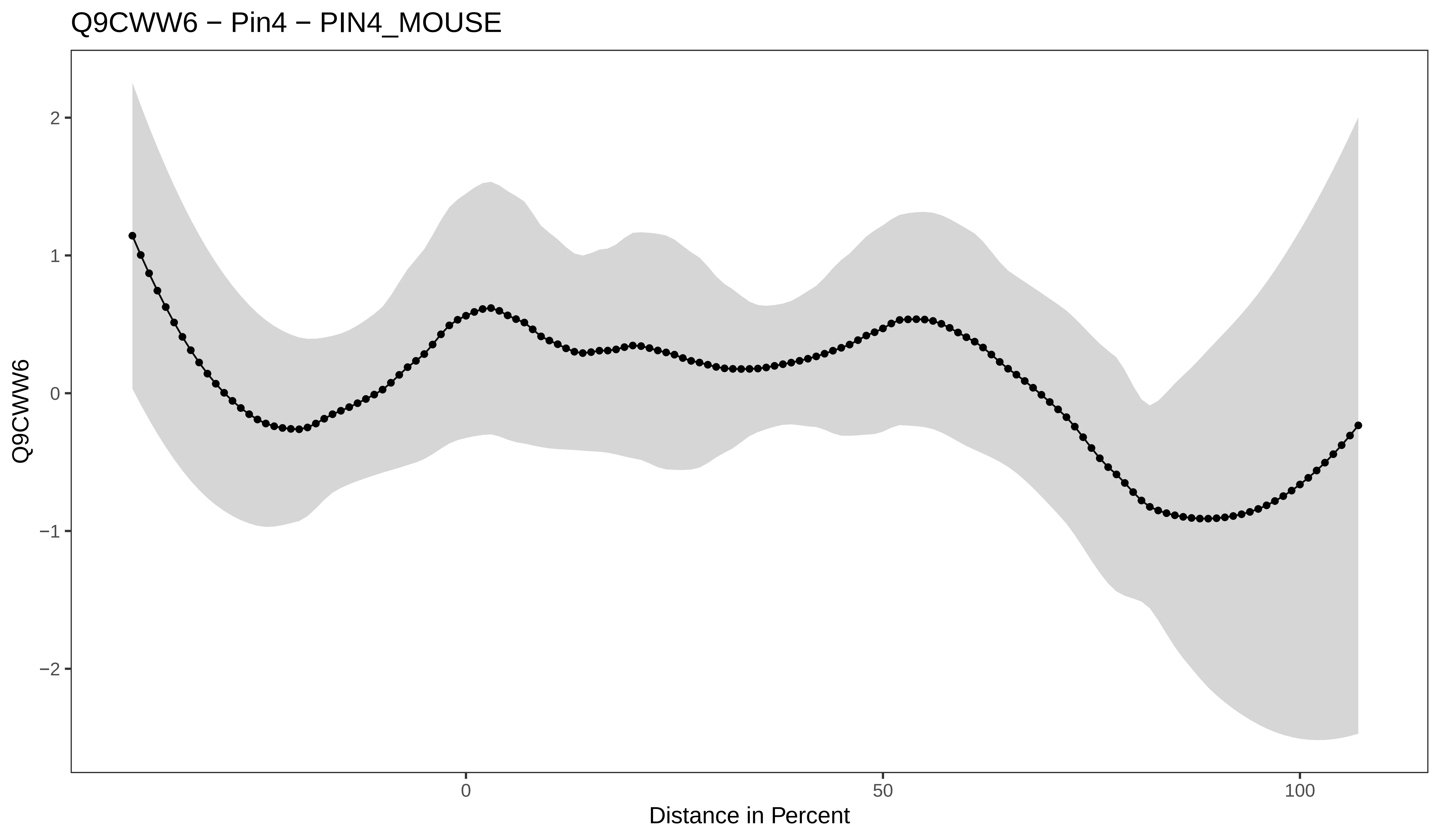

Q9CWW6 − Pin4 − PIN4_MOUSE
Q9CWW6
Distance in Percent
2
1
0
−1
−2
0
50
100

Q80XI4 − Pip4k2b − PI42B_MOUSE

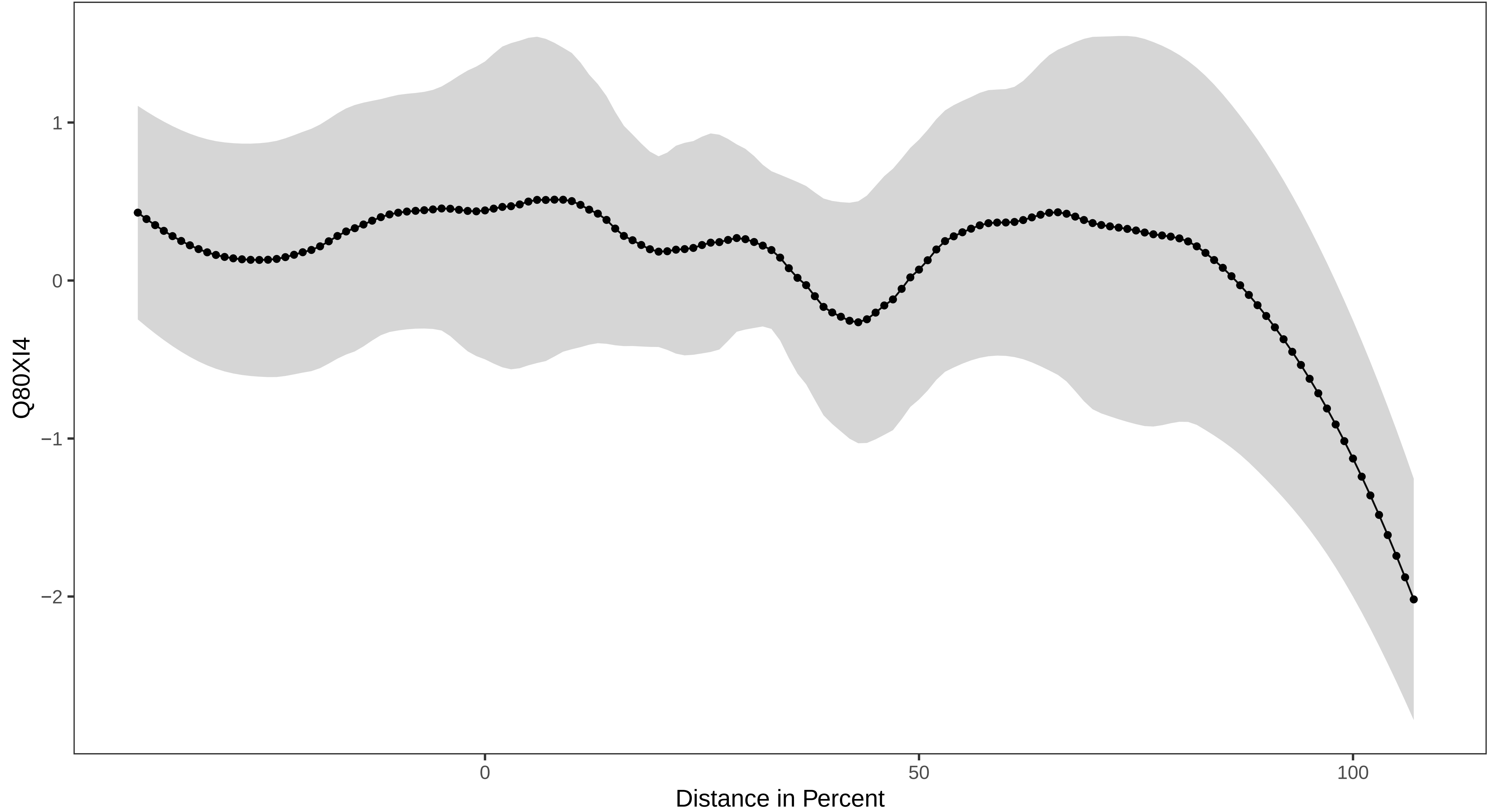

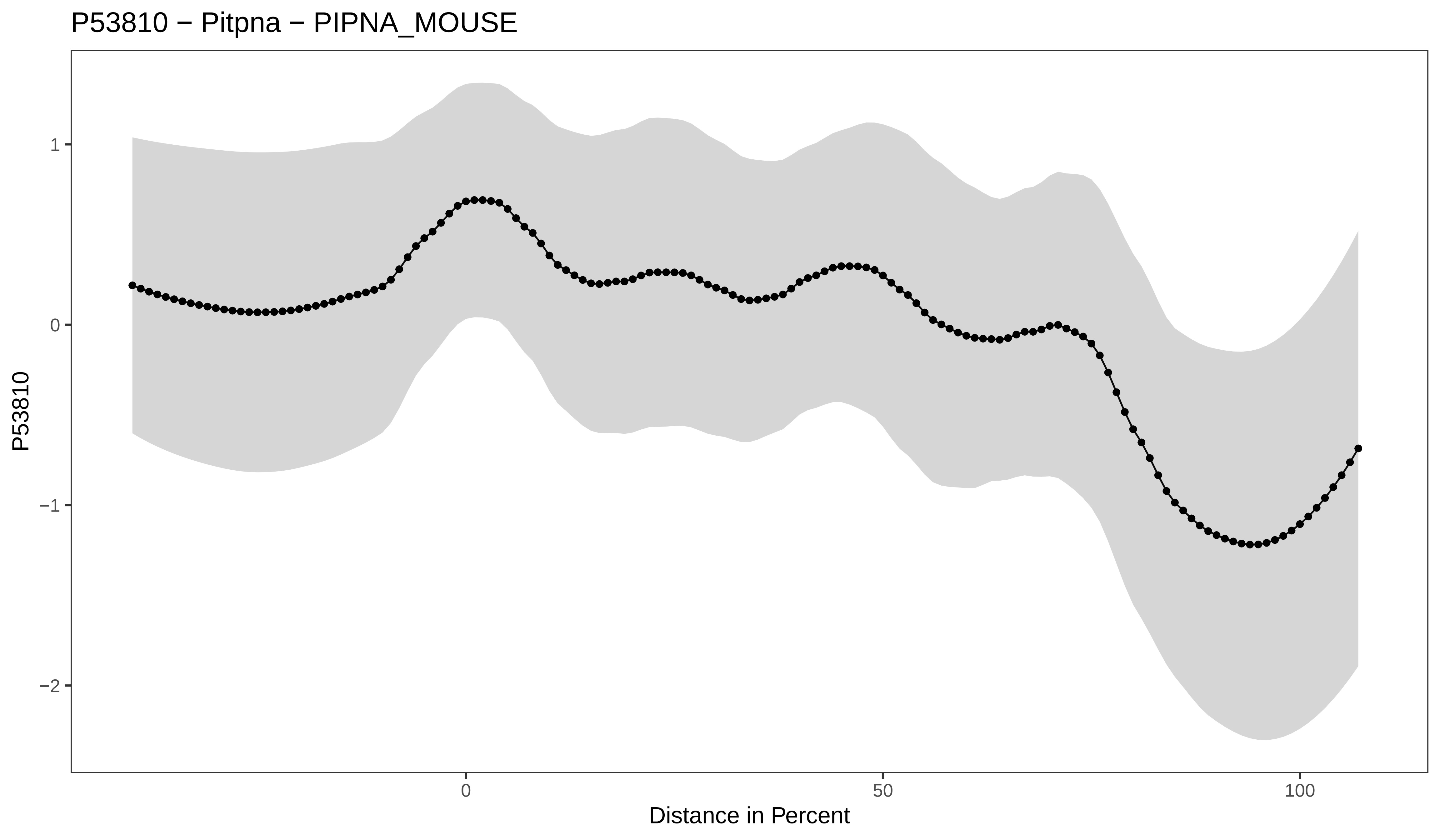

P53810 − Pitpna − PIPNA_MOUSE
1
0
−1
−2
P53810
0
50
100
Distance in Percent

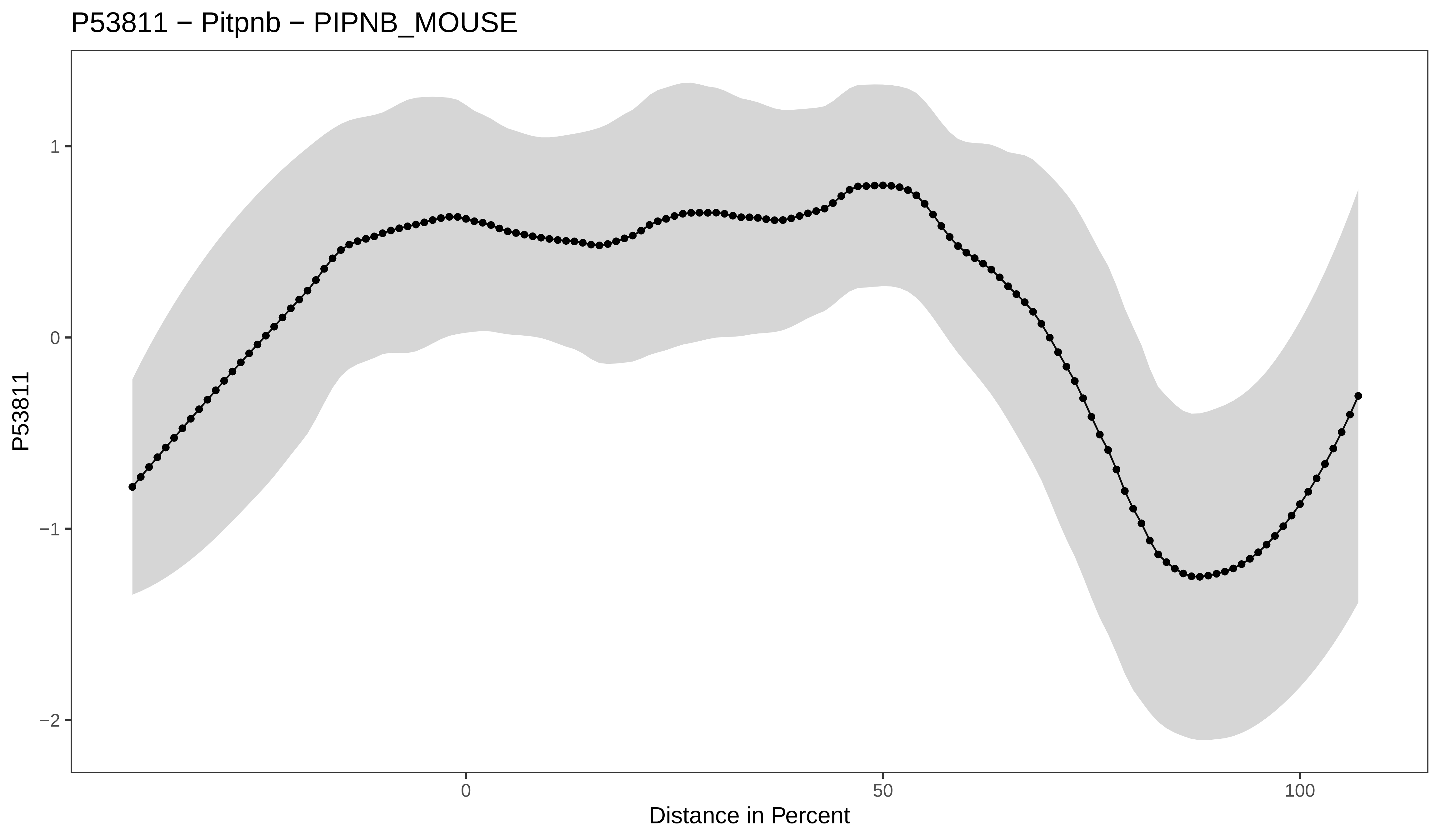

P53811 − Pitpnb − PIPNB_MOUSE
1
0
−1
−2
P53811
0
50
100
Distance in Percent

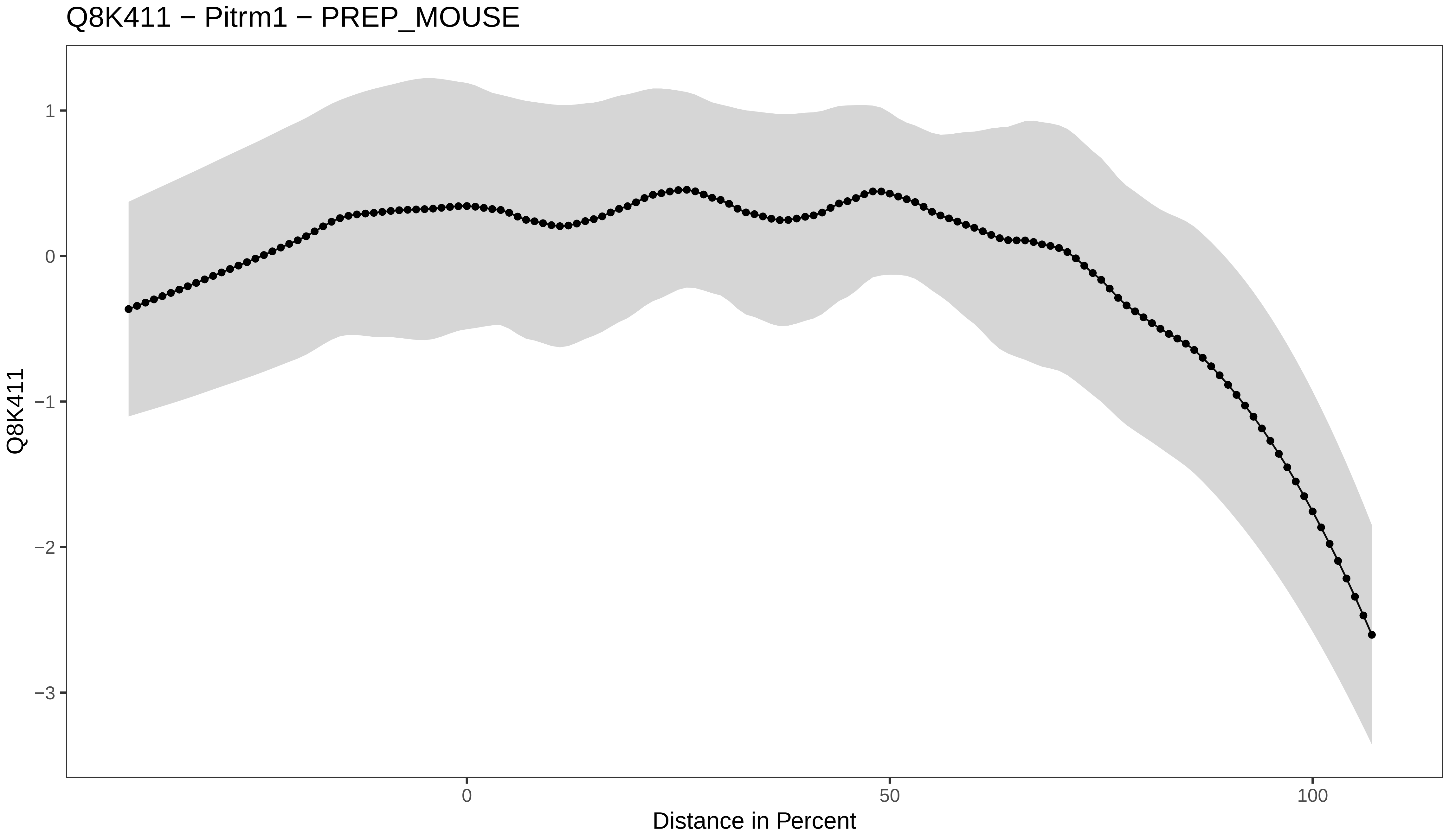

Q8K411 − Pitrm1 − PREP_MOUSE
Q8K411
1
0
−1
−2
−3
0
50
100
Distance in Percent

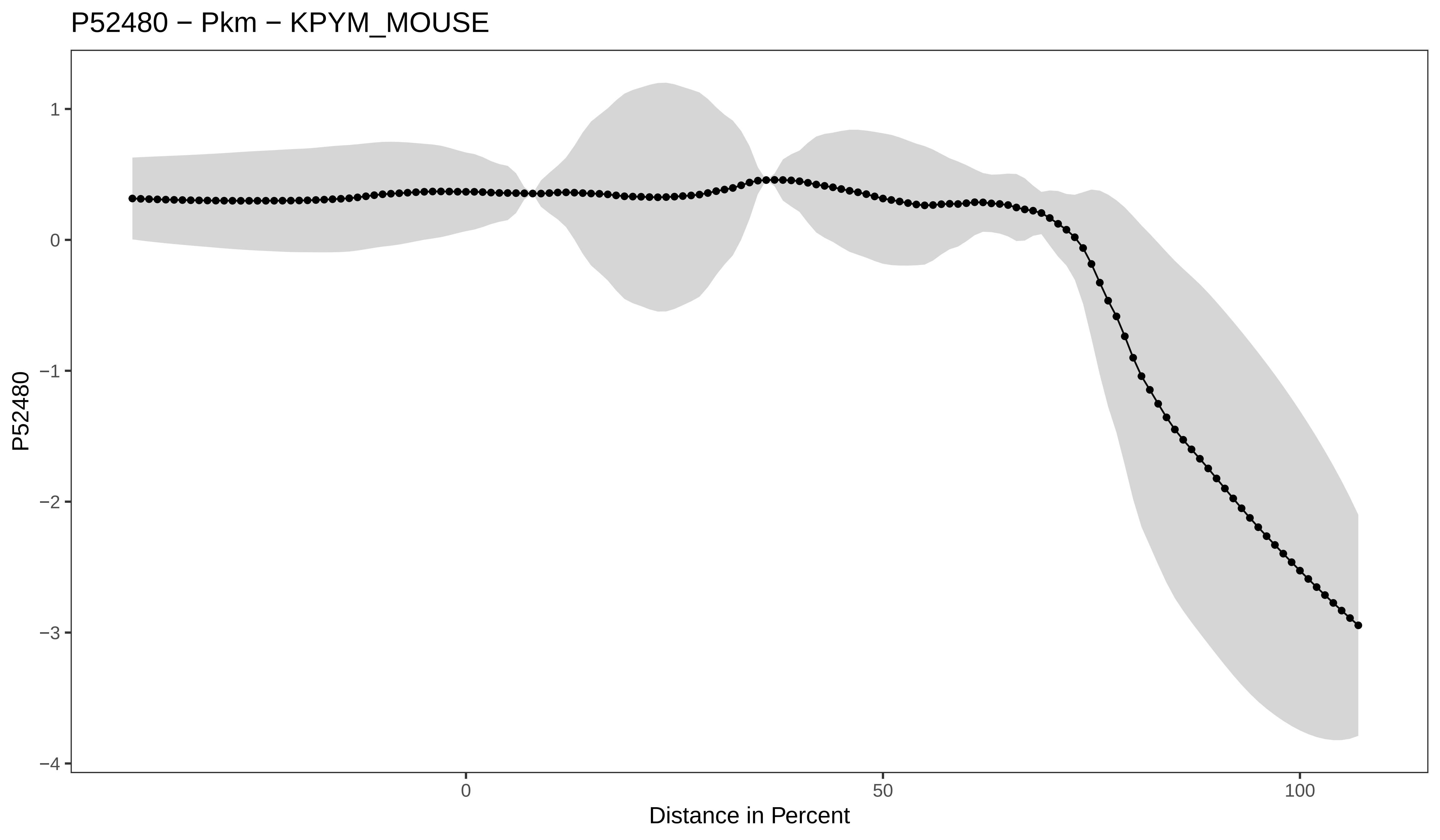

P52480 − Pkm − KPYM_MOUSE
1
0
−1
−2
−3
−4
P52480
0
50
100
Distance in Percent

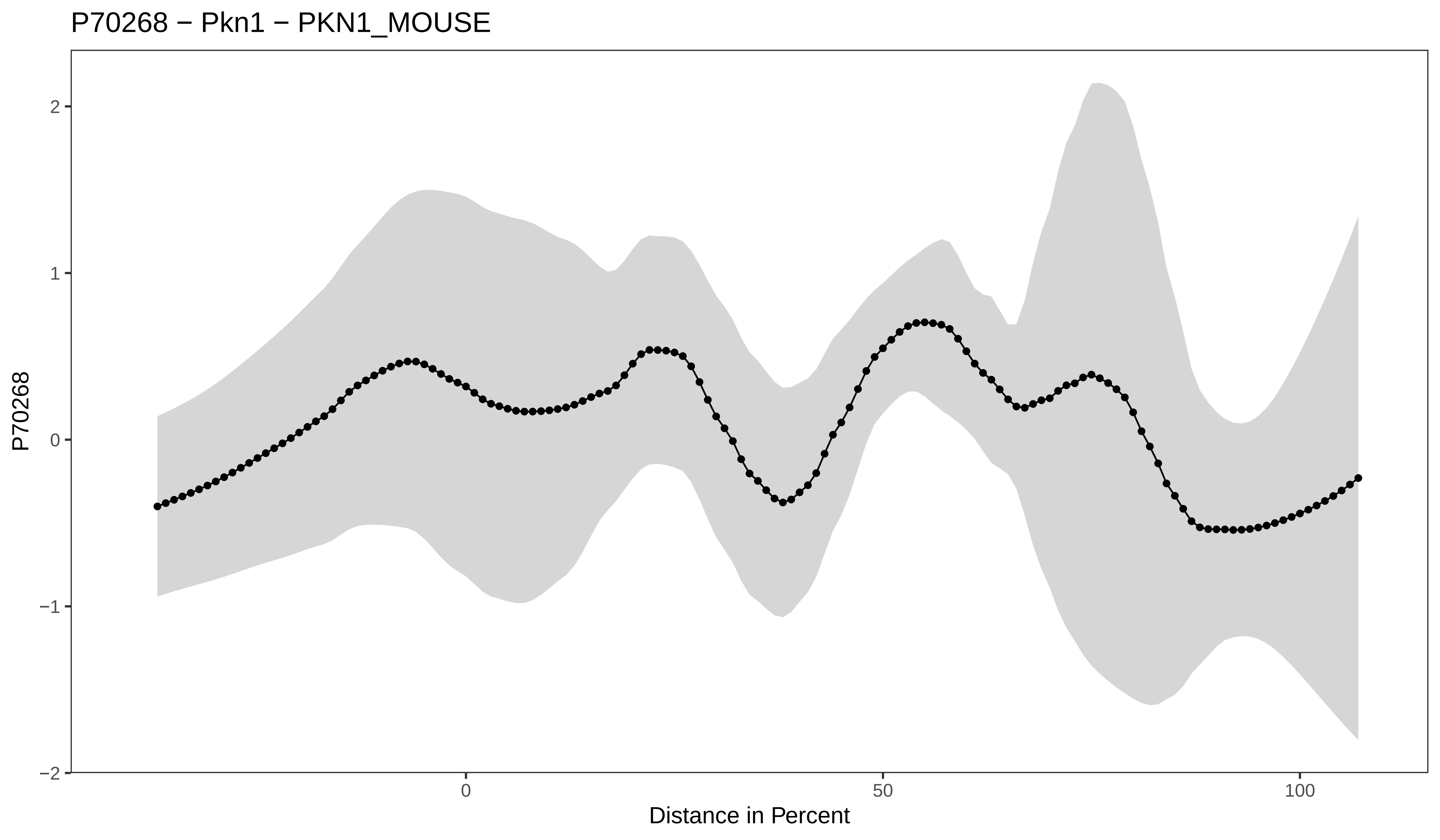
P70268 − Pkn1 − PKN1_MOUSE
P70268
2
1
0
−1
−2
0
50
100
Distance in Percent

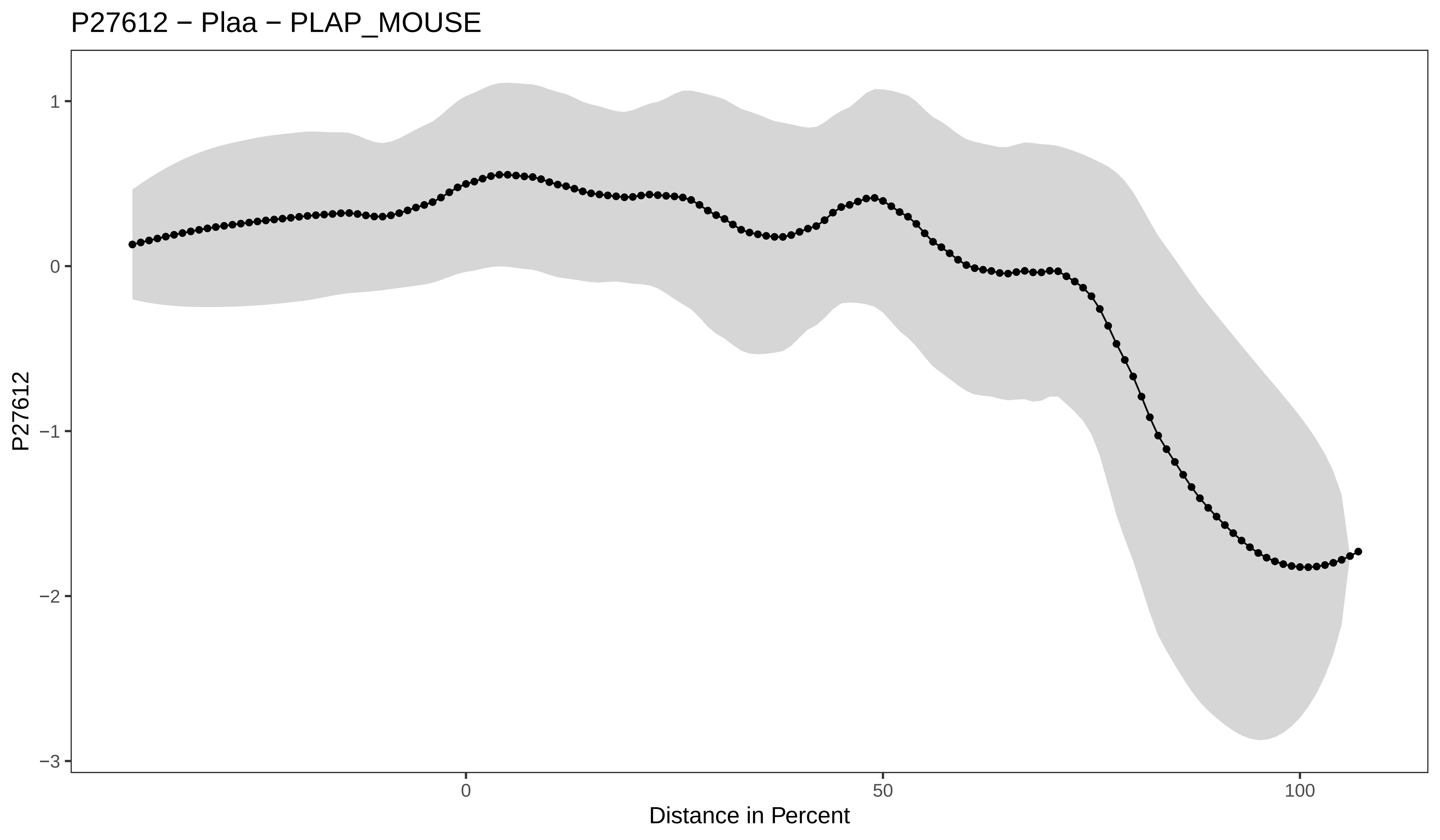

P27612 − Plaa − PLAP_MOUSE
P27612
1
0
−1
−2
−3
0
50
100
Distance in Percent

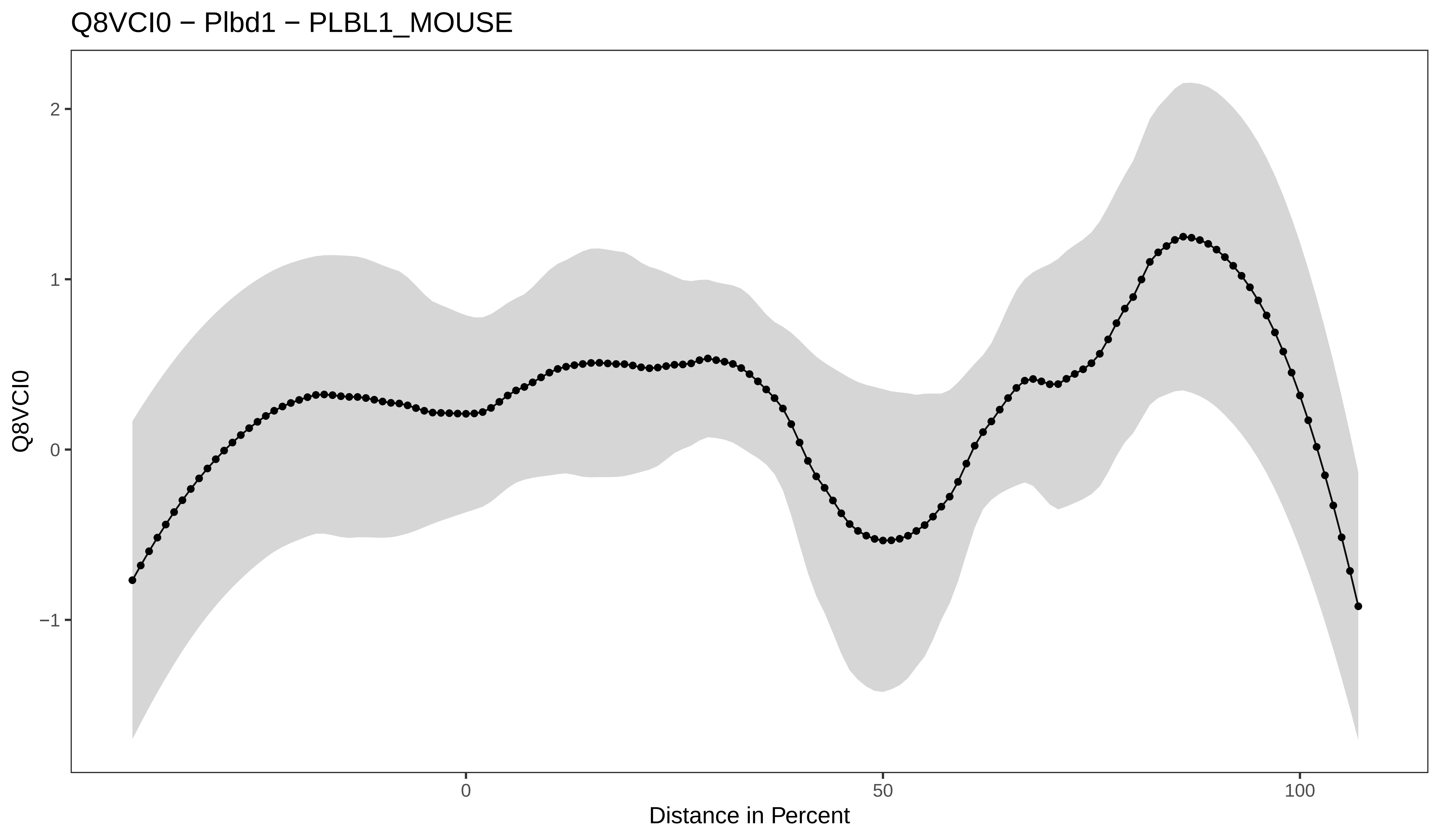

Q8VCI0 − Plbd1 − PLBL1_MOUSE
Q8VCI0
2
1
0
−1
0
50
100
Distance in Percent

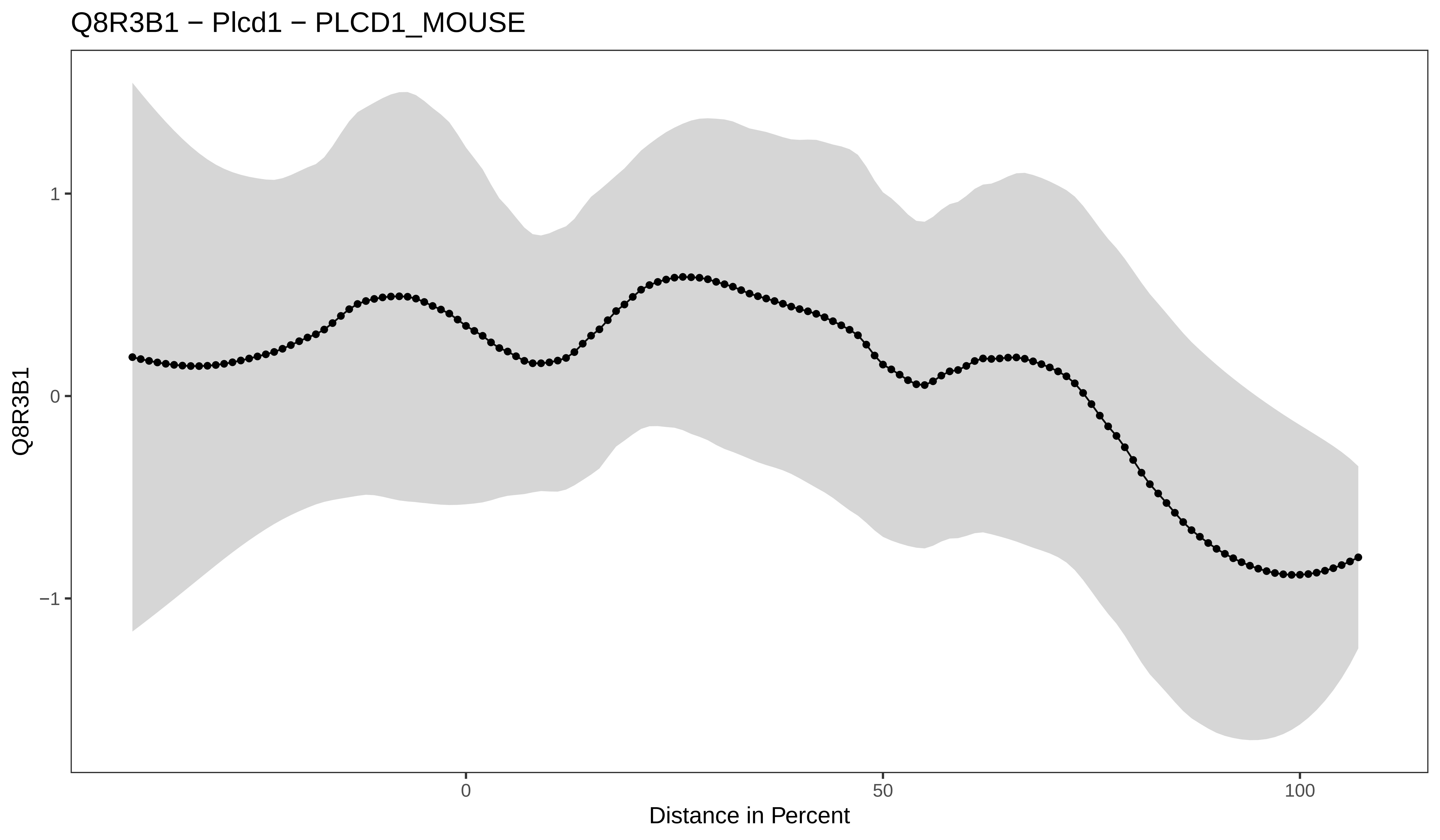
Q8R3B1 − Plcd1 − PLCD1_MOUSE
Q8R3B1
1
0
−1
0
50
100
Distance in Percent

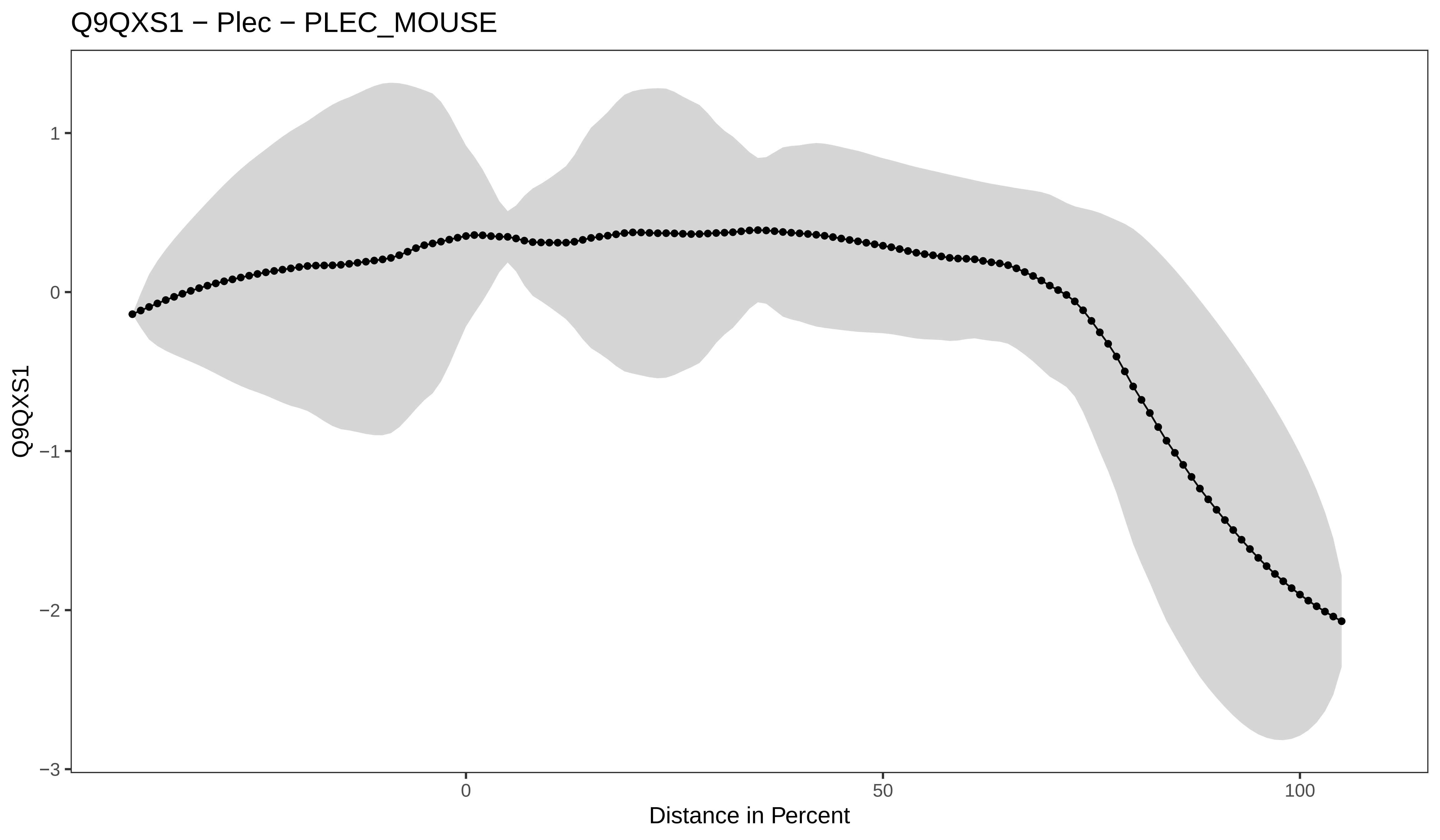

Q9QXS1 − Plec − PLEC_MOUSE
Q9QXS1
1
0
−1
−2
−3
0
50
100
Distance in Percent

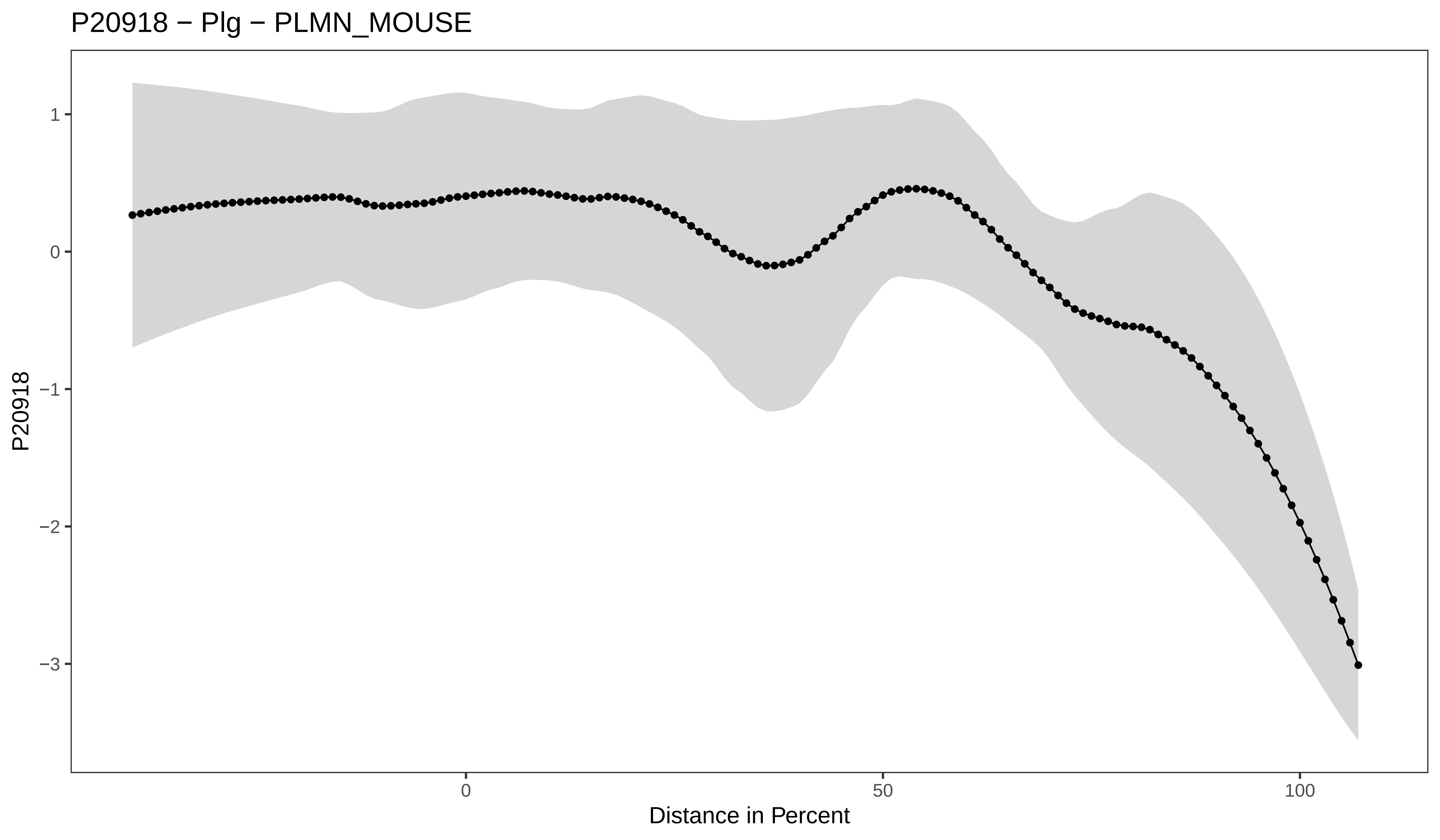

P20918 − Plg − PLMN_MOUSE
1
0
−1
−2
−3
P20918
0
50
100
Distance in Percent

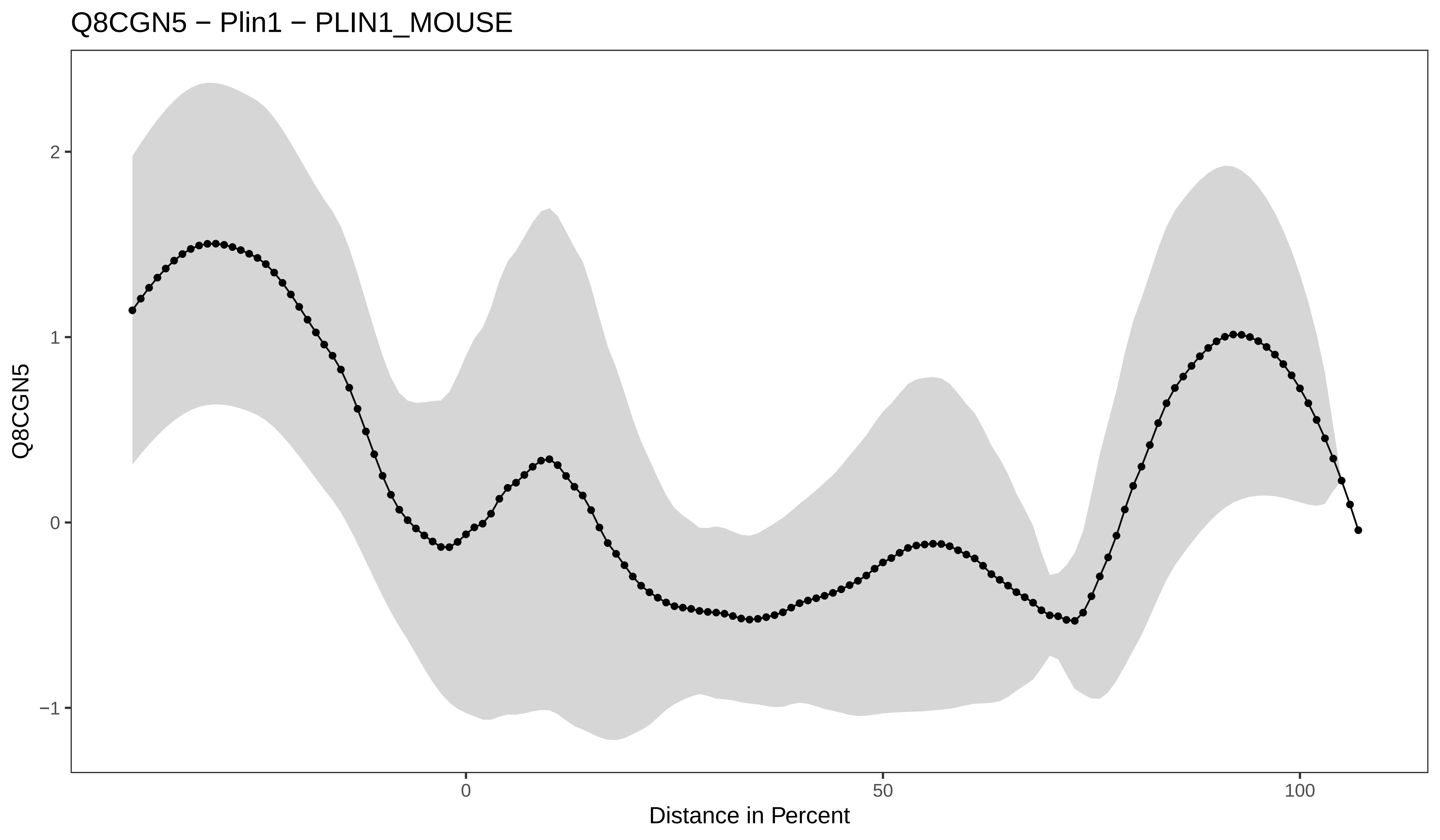

Q8CGN5 − Plin1 − PLIN1_MOUSE
Q8CGN5
2
1
0
−1
0
50
100
Distance in Percent

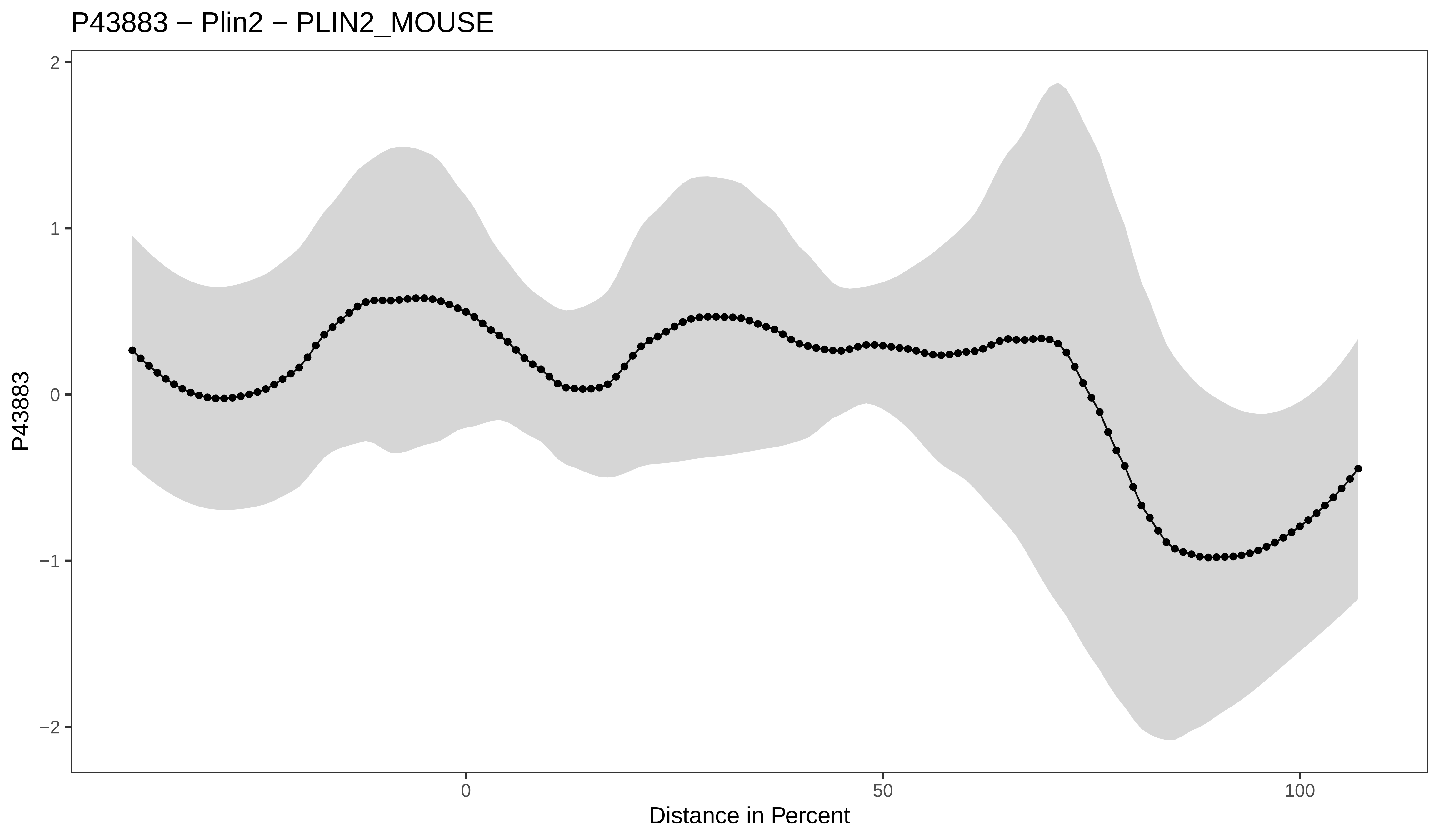

P43883 − Plin2 − PLIN2_MOUSE
P43883
2
1
0
−1
−2
0
50
100
Distance in Percent

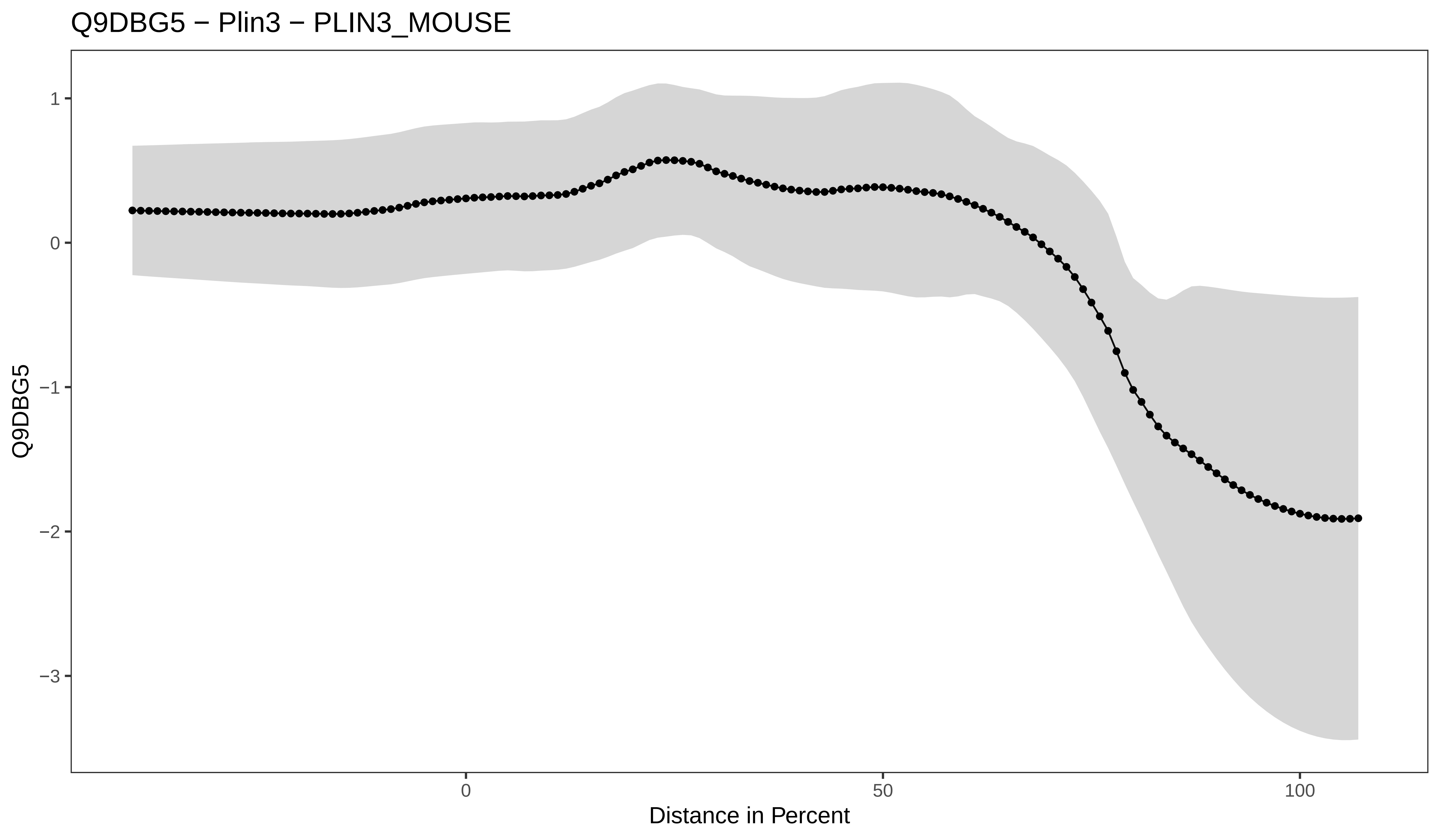

Q9DBG5 − Plin3 − PLIN3_MOUSE
1
0
−1
−2
−3
Q9DBG5
0
50
100
Distance in Percent

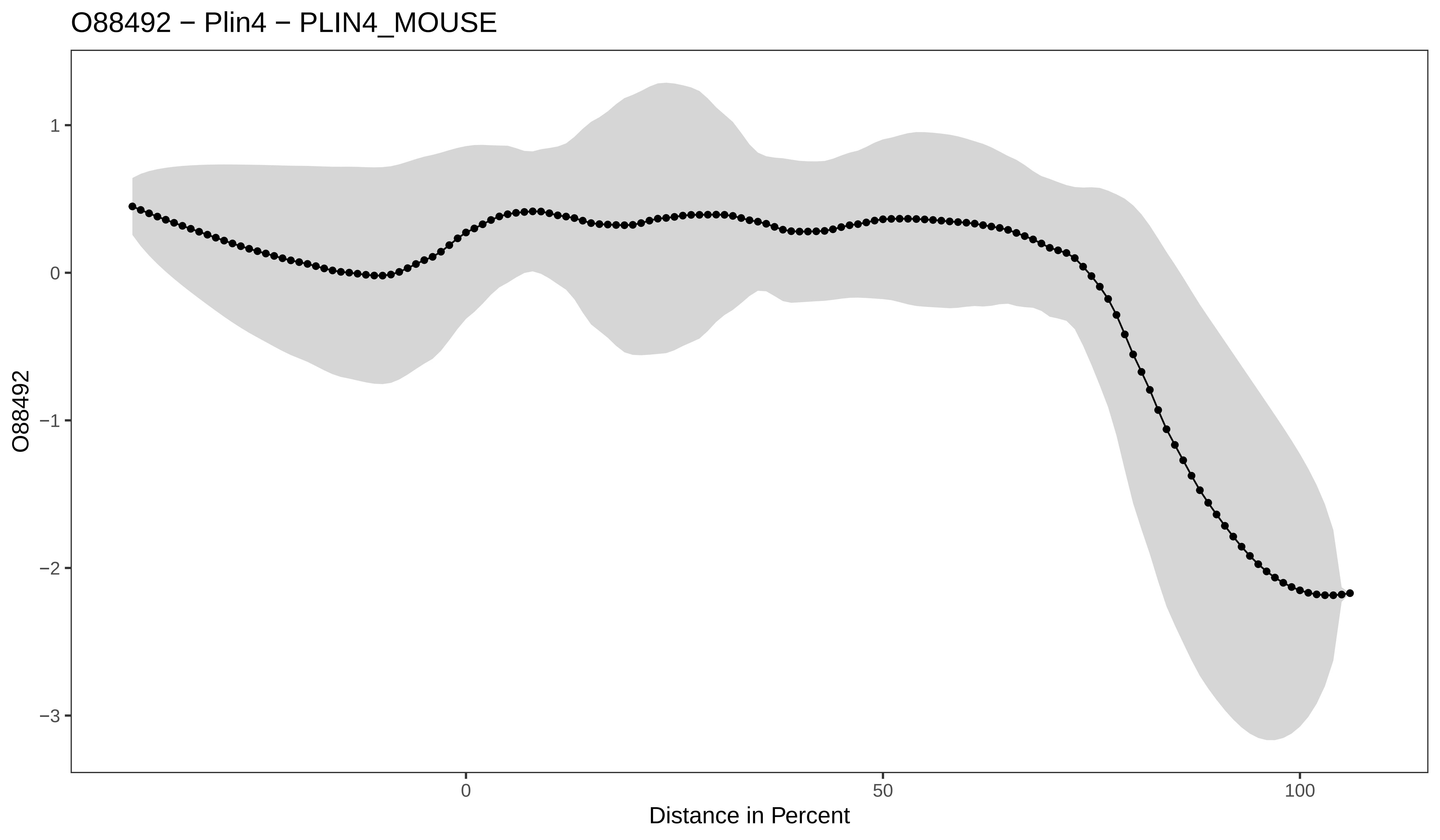

O88492 − Plin4 − PLIN4_MOUSE
O88492
1
0
−1
−2
−3
0
50
100
Distance in Percent

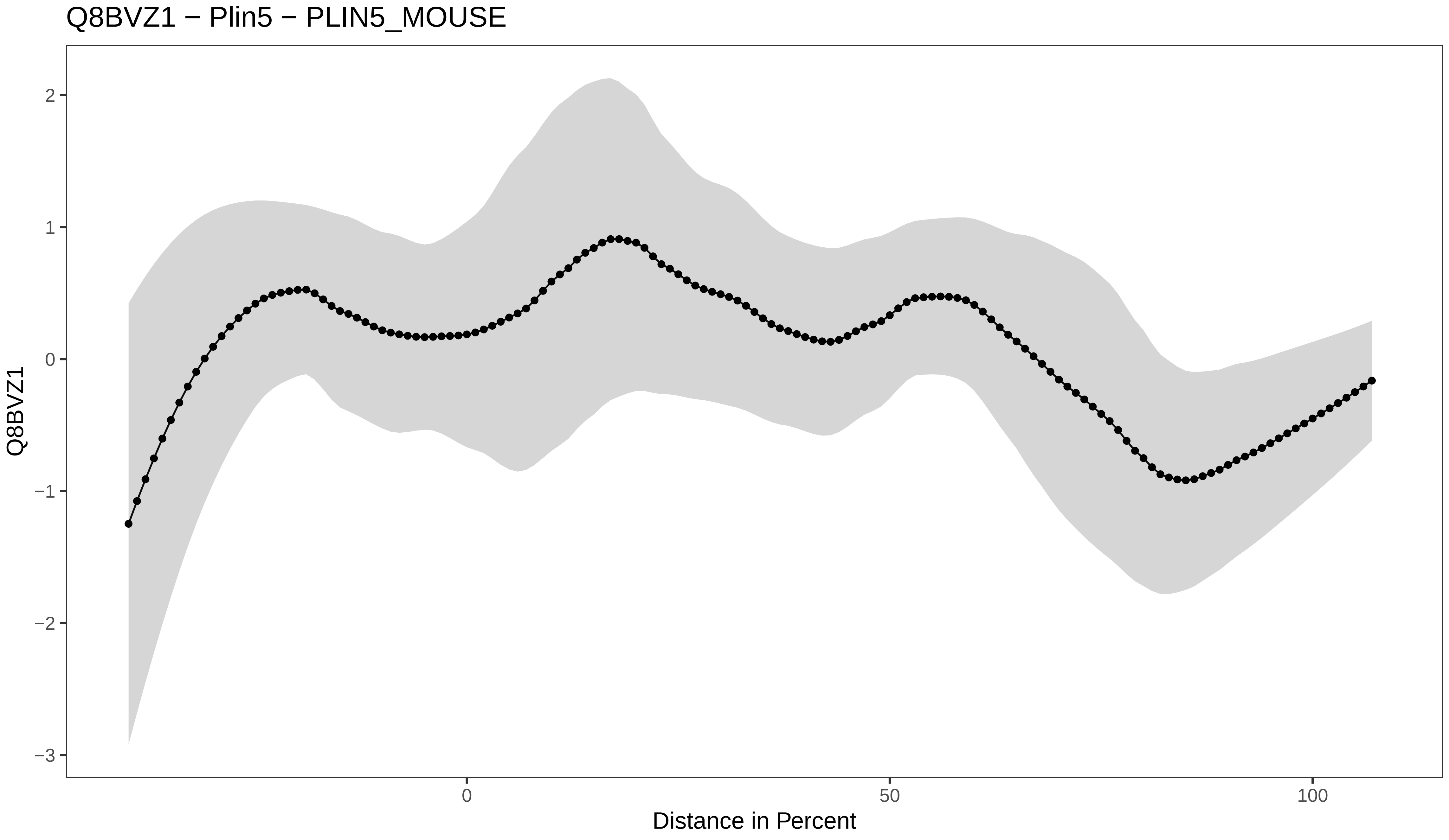

Q8BVZ1 − Plin5 − PLIN5_MOUSE
Q8BVZ1
2
1
0
−1
−2
−3
0
50
100
Distance in Percent

Q9R0E2 − Plod1 − PLOD1_MOUSE

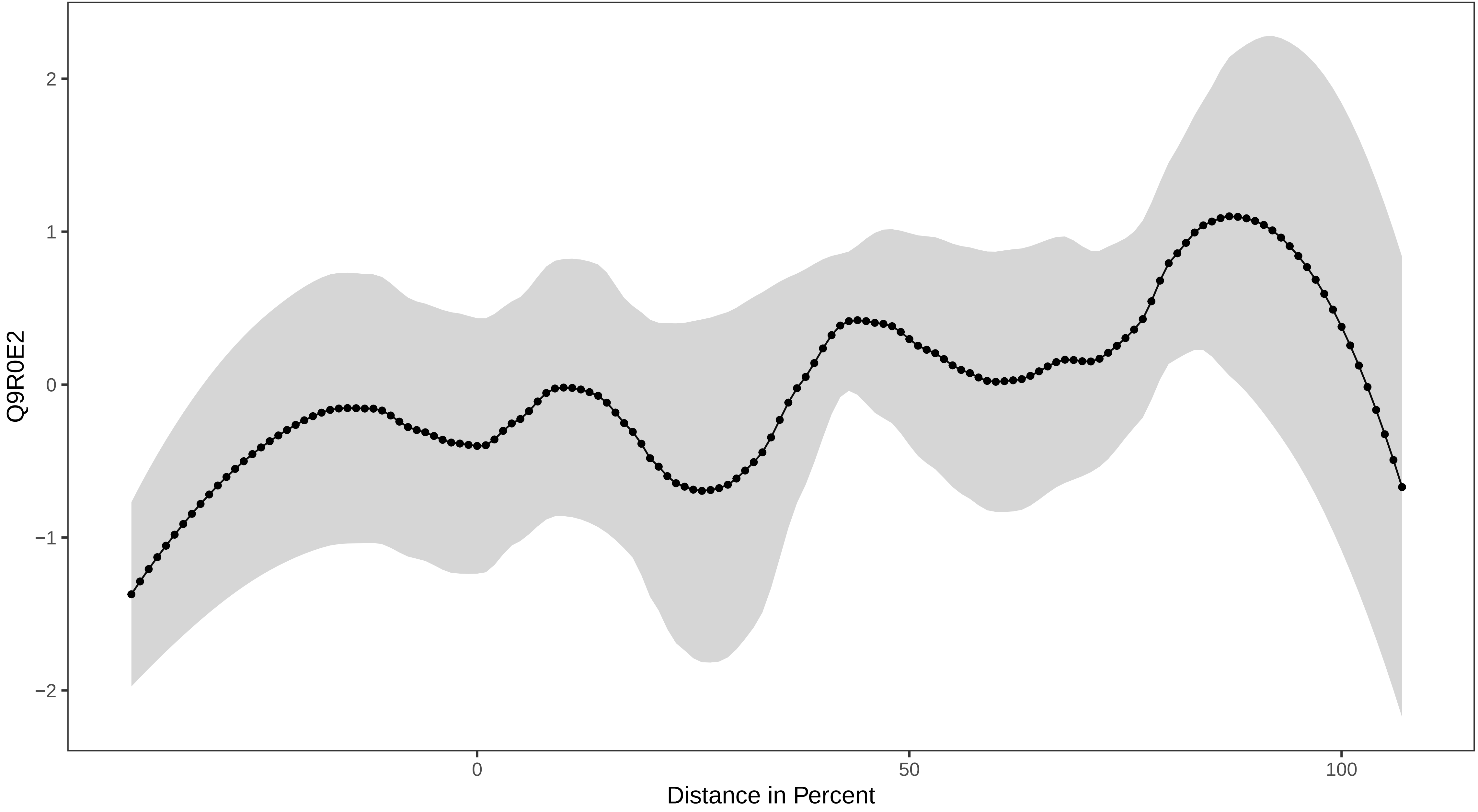

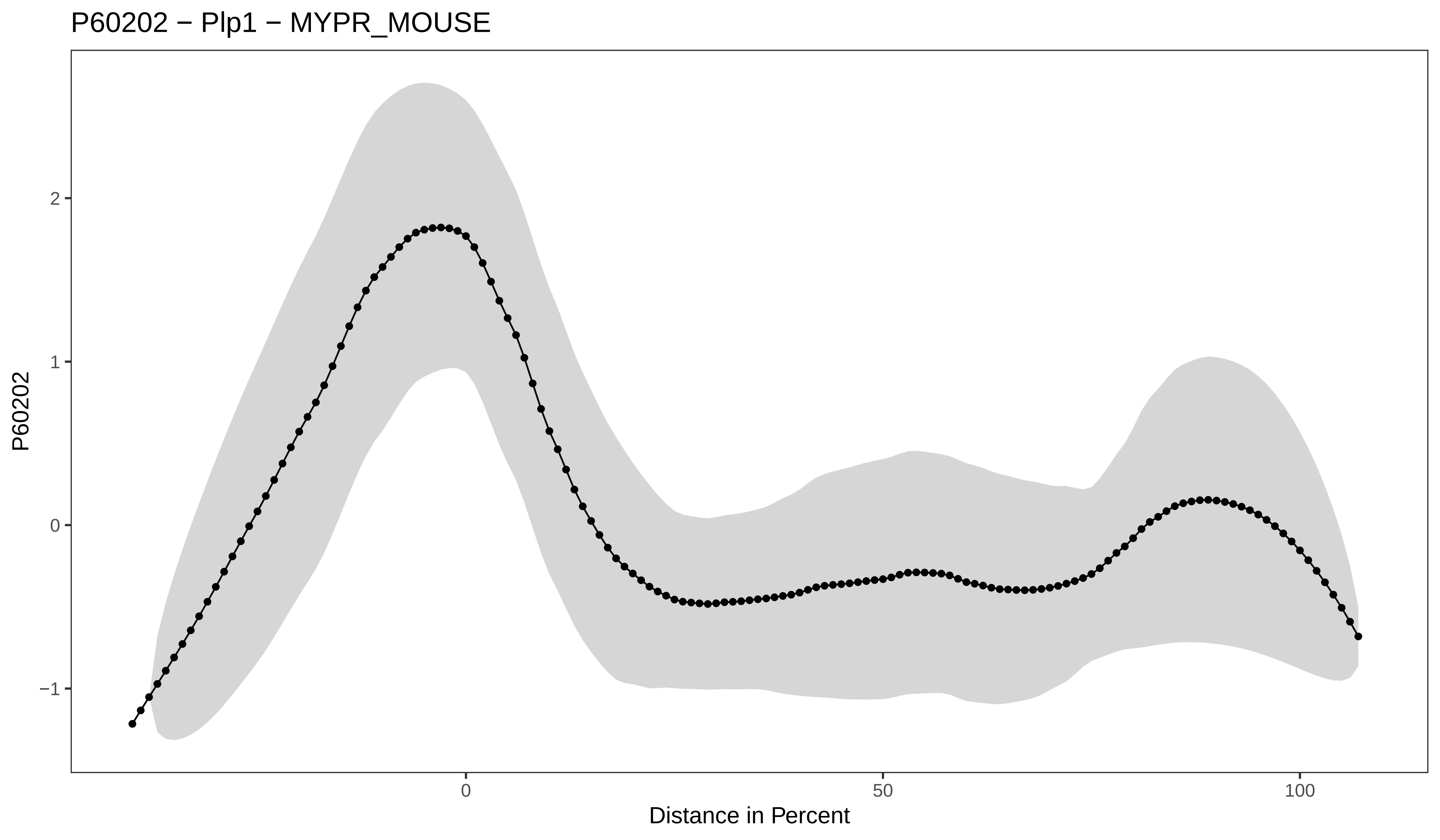

P60202 − Plp1 − MYPR_MOUSE
P60202
2
1
0
−1
0
50
100
Distance in Percent

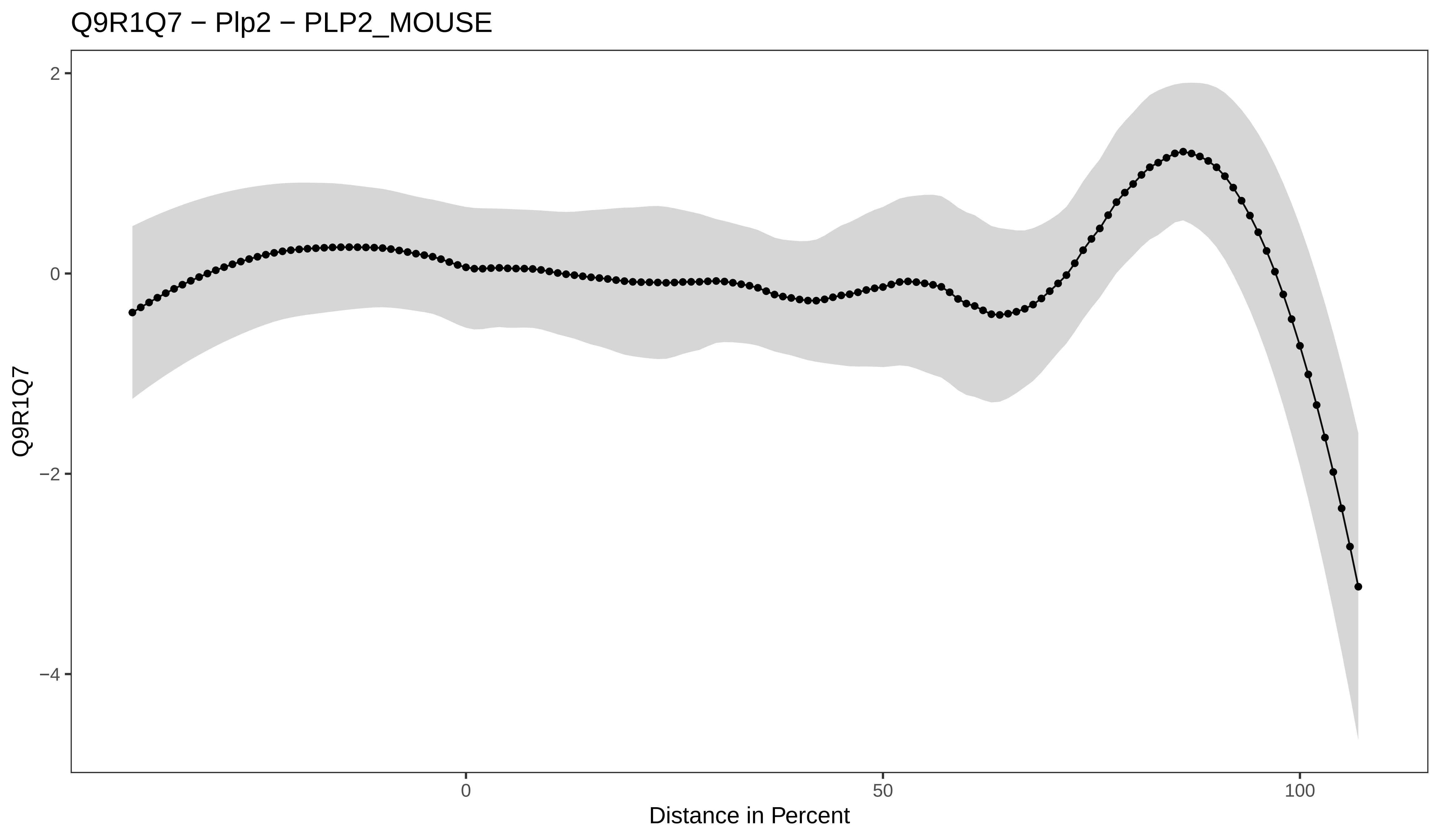

Q9R1Q7 − Plp2 − PLP2_MOUSE
2
0
−2
−4
Q9R1Q7
0
50
100
Distance in Percent

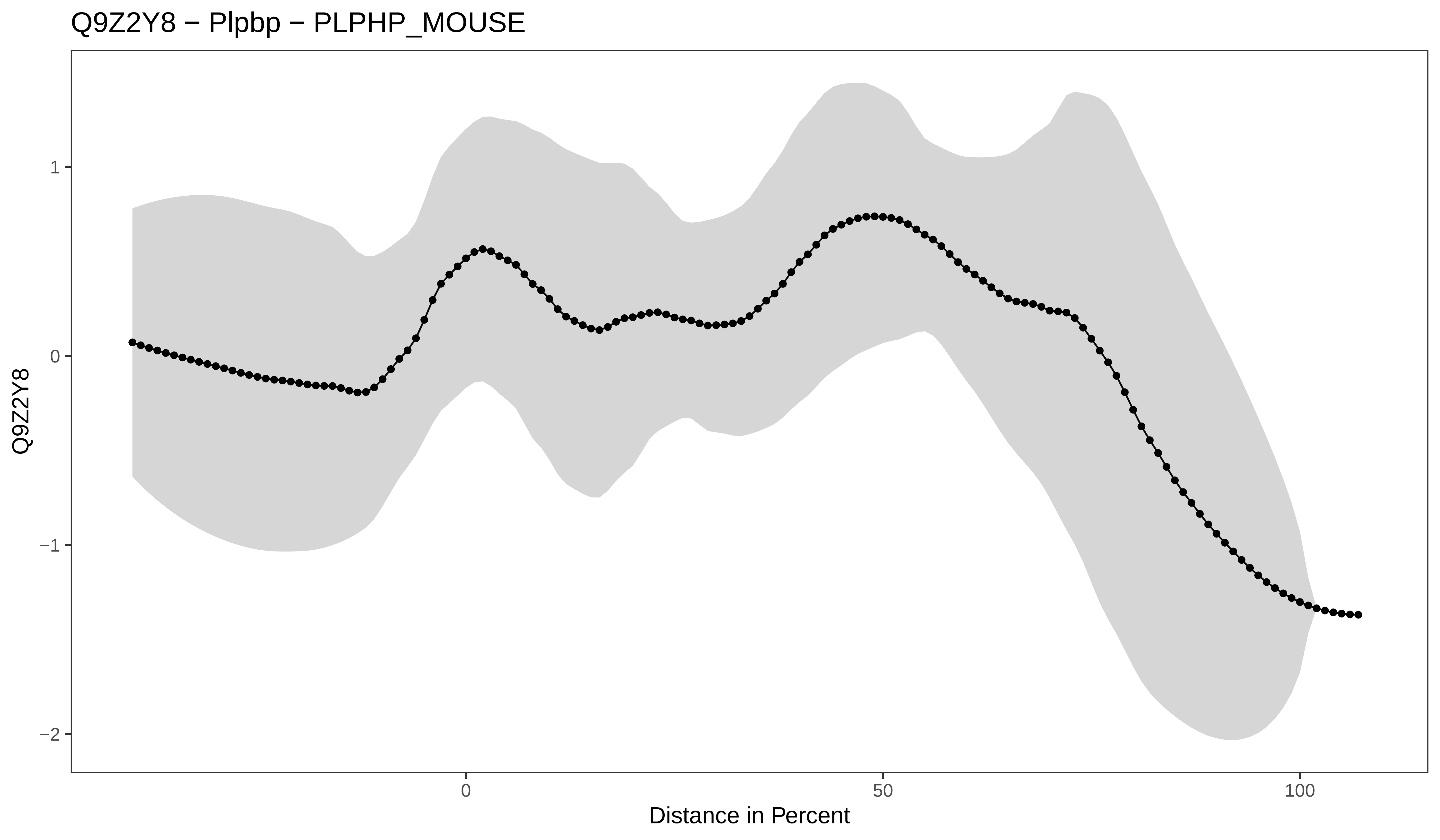

Q9Z2Y8 − Plpbp − PLPHP_MOUSE
Q9Z2Y8
1
0
−1
−2
0
50
100
Distance in Percent

Q99JY8 − Plpp3 − PLPP3_MOUSE

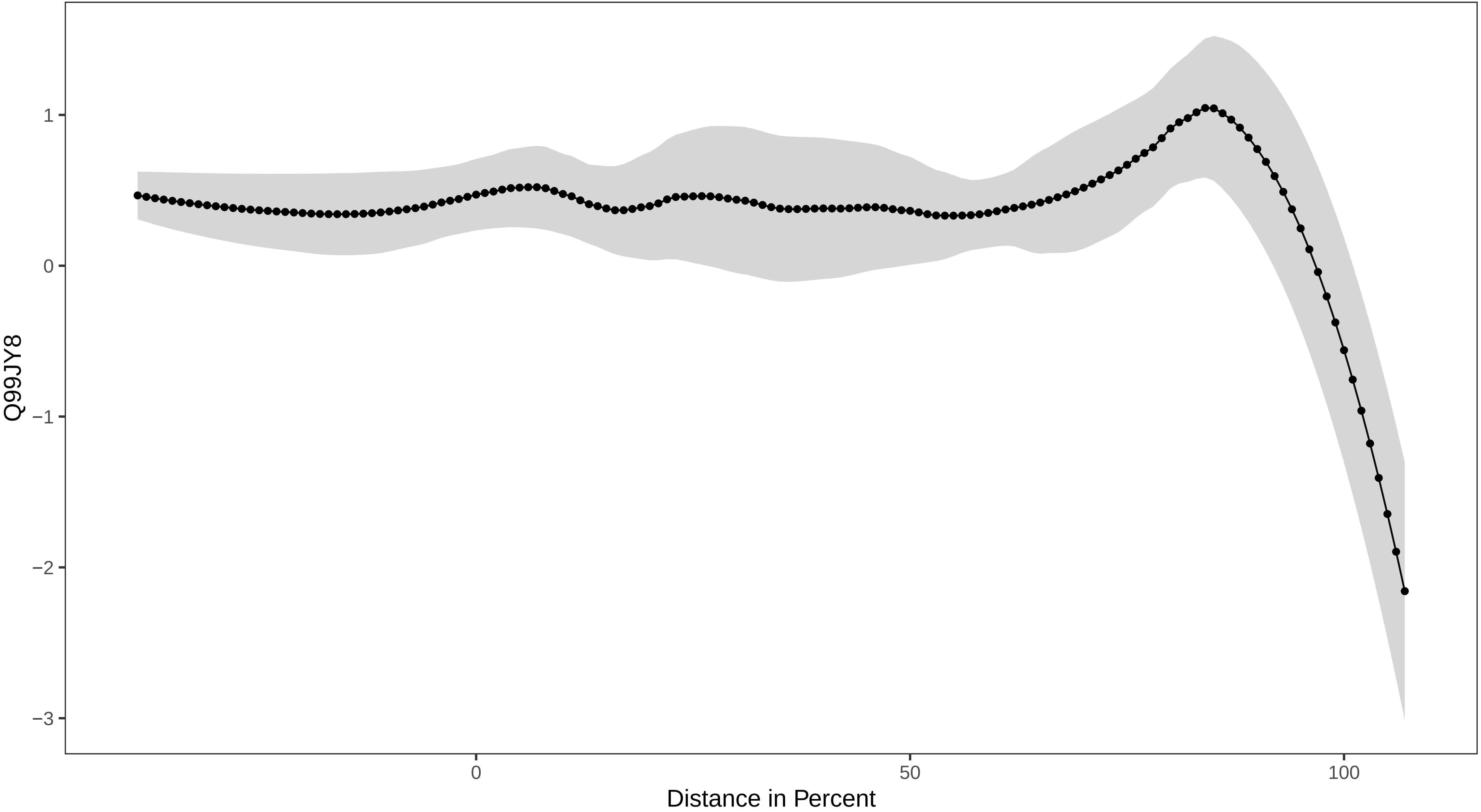

Q99K51 − Pls3 − PLST_MOUSE

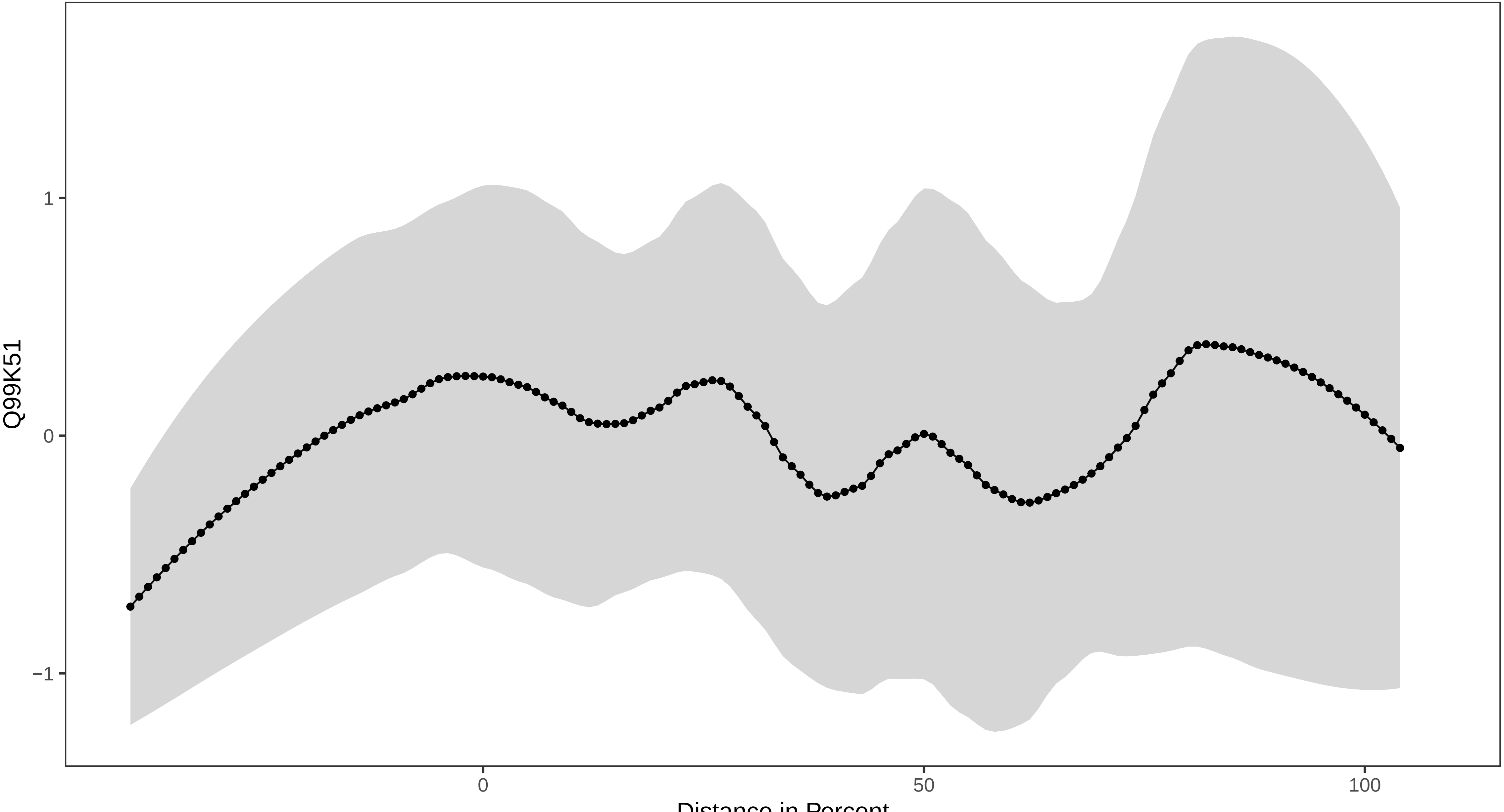

Q9DC11 − Plxdc2 − PXDC2_MOUSE

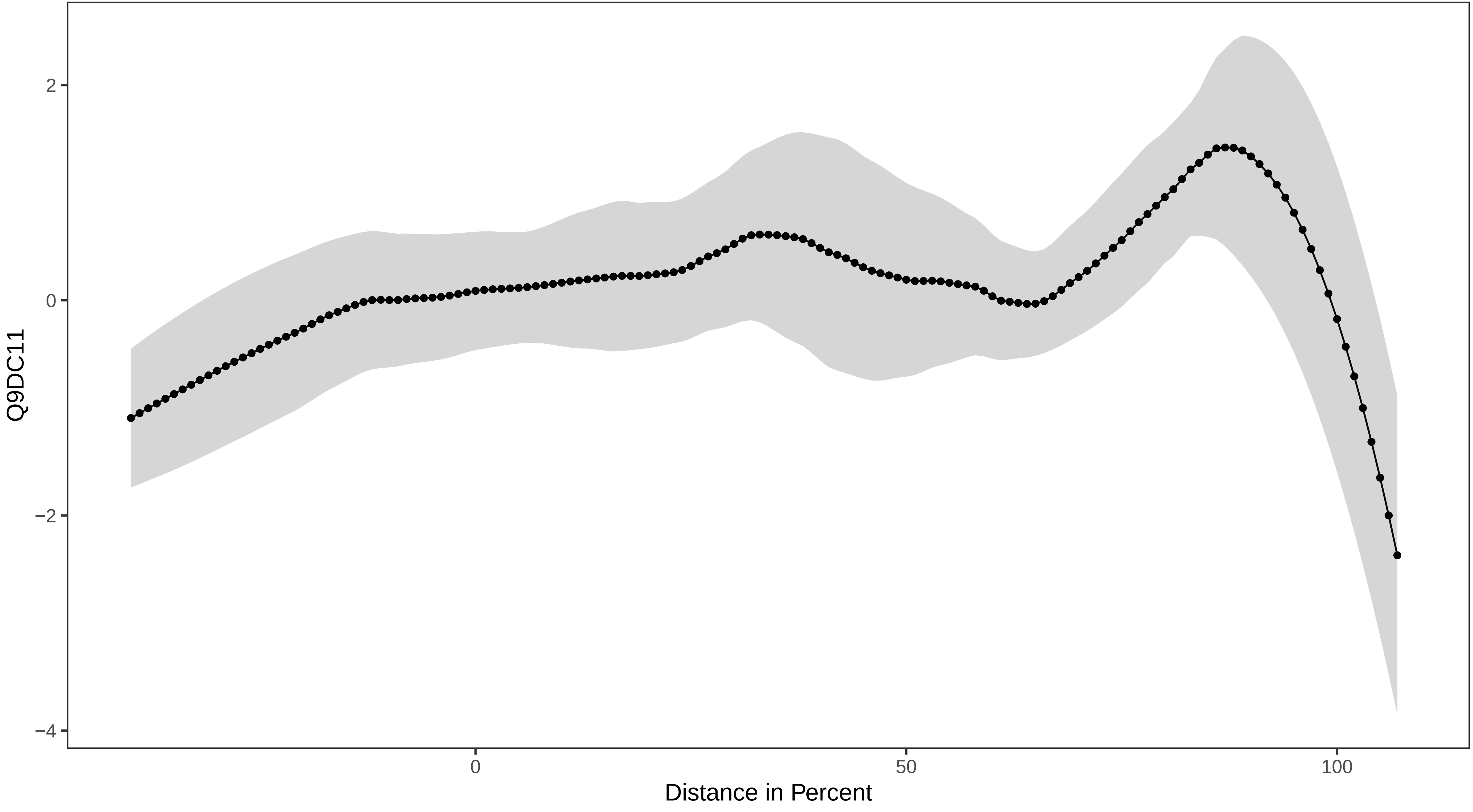

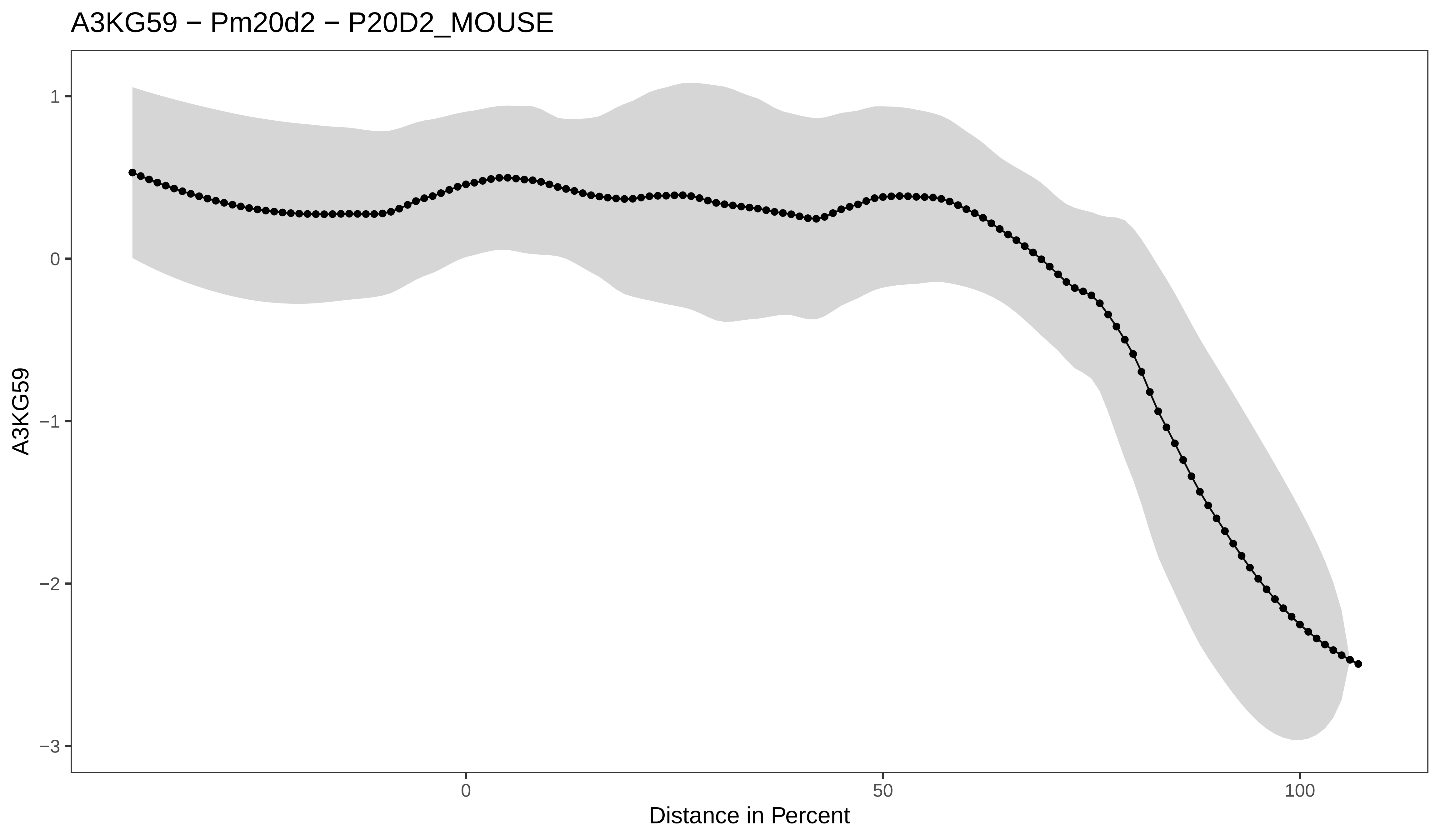

A3KG59 − Pm20d2 − P20D2_MOUSE
A3KG59
1
0
−1
−2
−3
0
50
100
Distance in Percent

Q9WVQ0 − Pmfbp1 − PMFBP_MOUSE

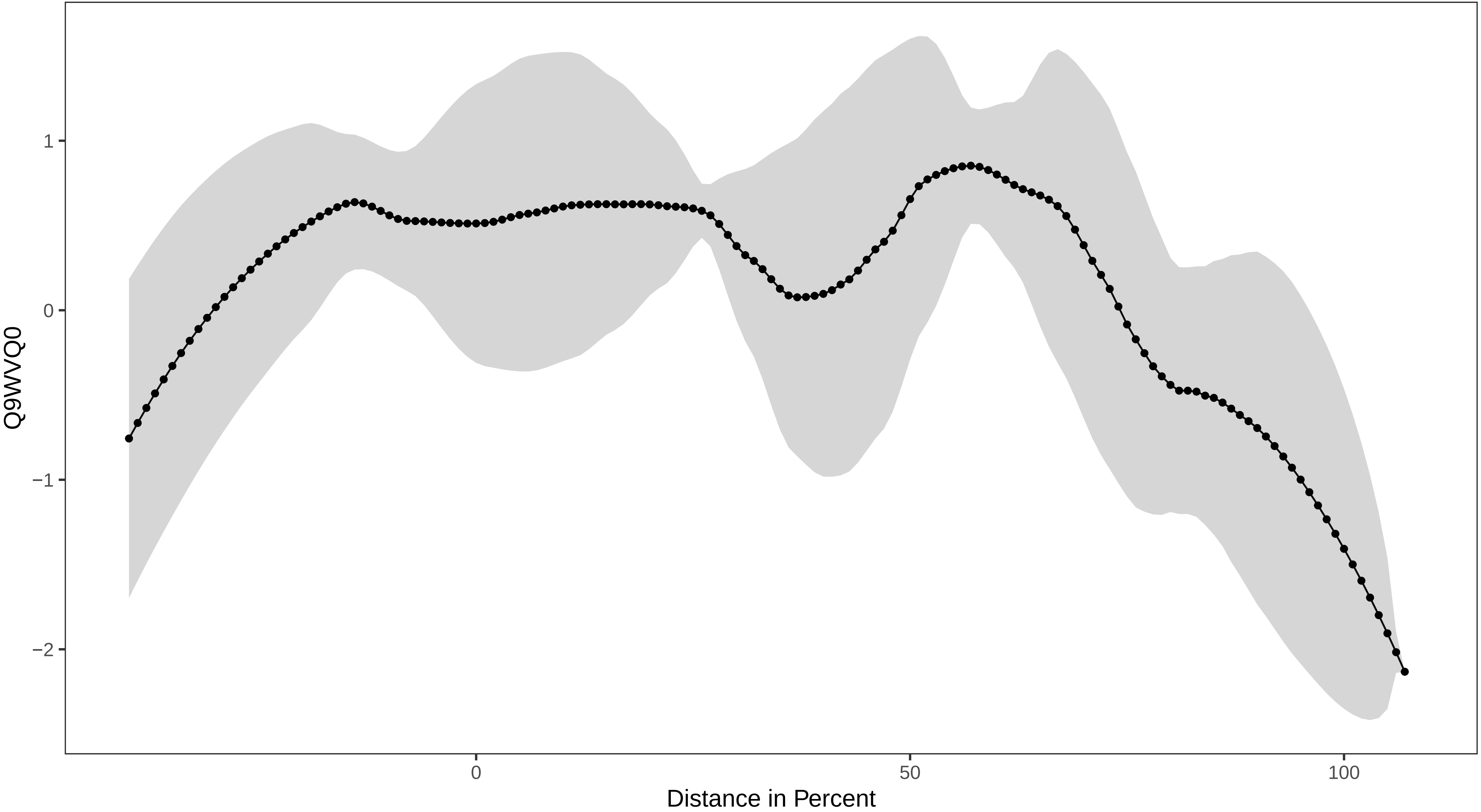

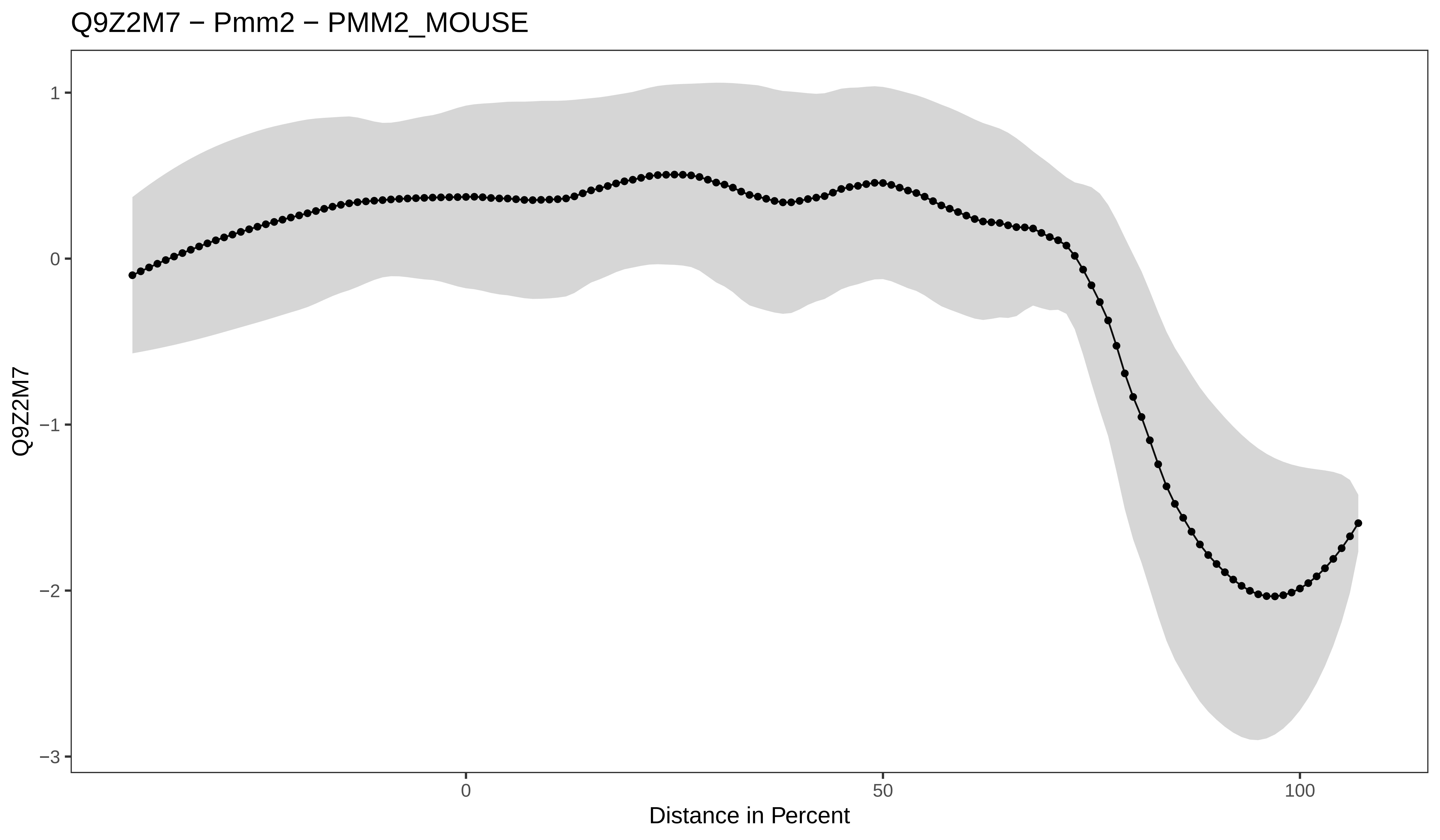

Q9Z2M7 − Pmm2 − PMM2_MOUSE
1
0
−1
−2
−3
Q9Z2M7
0
50
100
Distance in Percent

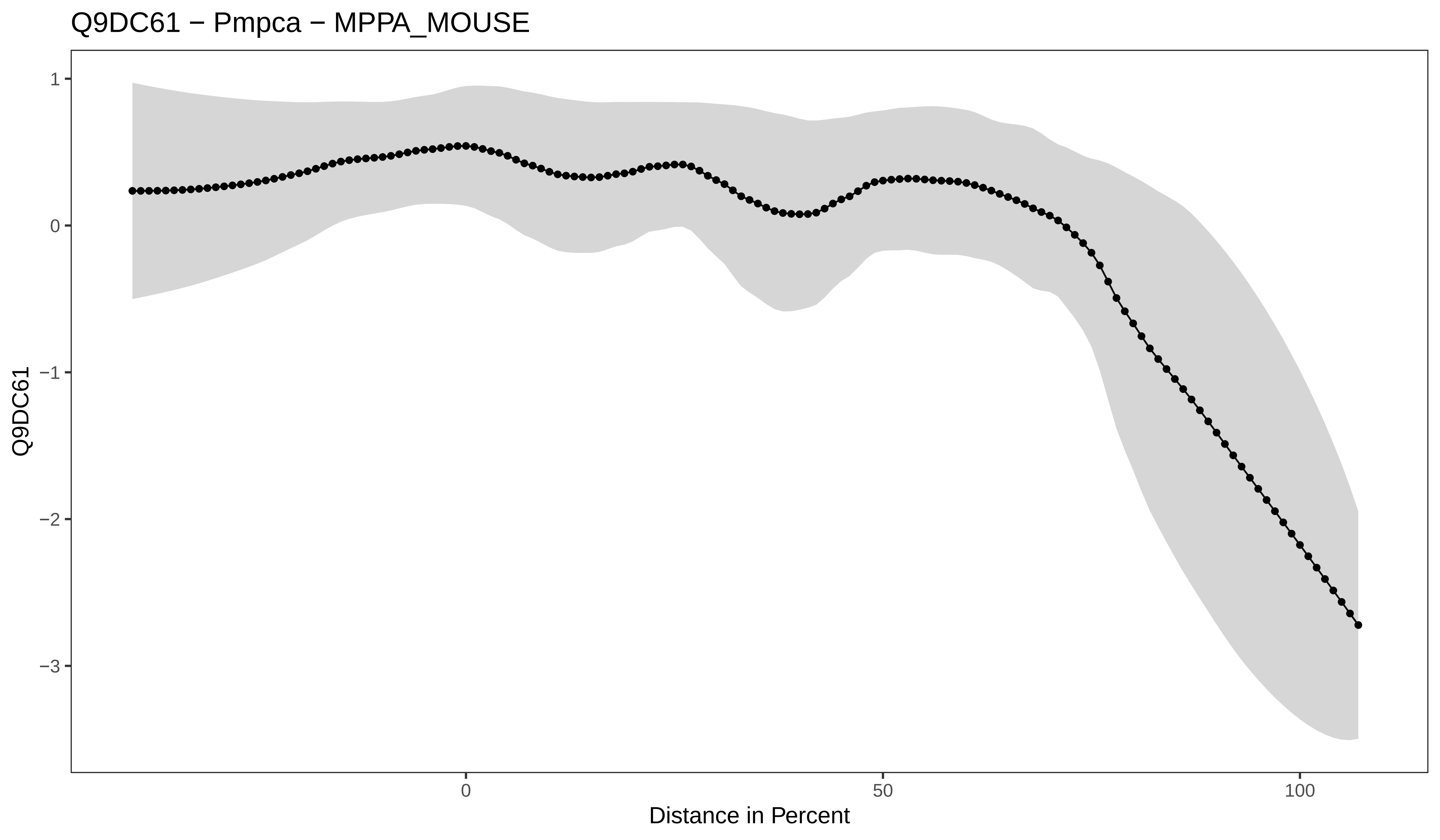

Q9DC61 − Pmpca − MPPA_MOUSE
Q9DC61
Distance in Percent
1
0
−1
−2
−3
0
50
100

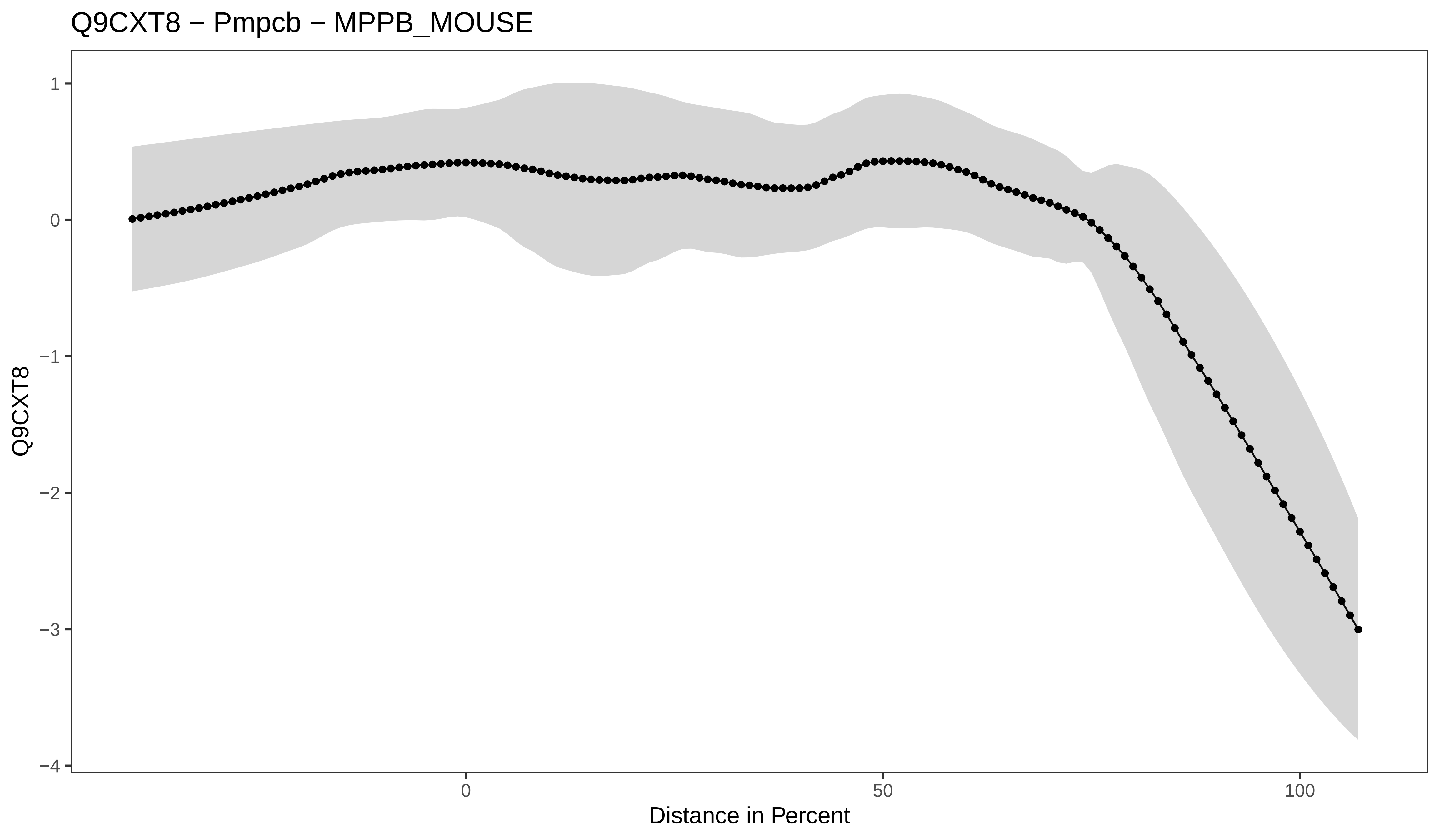

Q9CXT8 − Pmpcb − MPPB_MOUSE
1
0
−1
−2
−3
−4
Q9CXT8
0
50
100
Distance in Percent

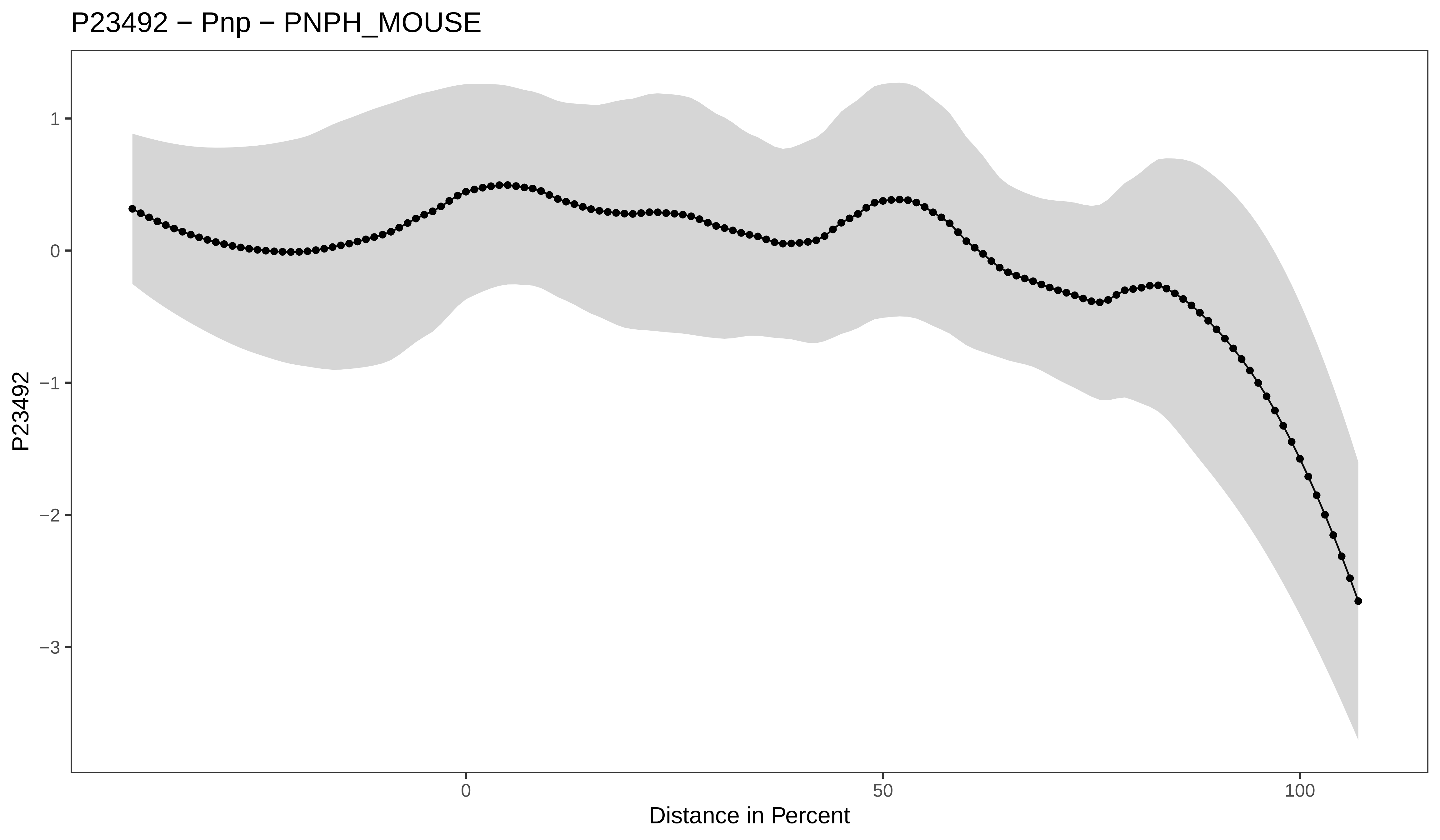

P23492 − Pnp − PNPH_MOUSE
1
0
−1
−2
−3
P23492
0
50
100
Distance in Percent

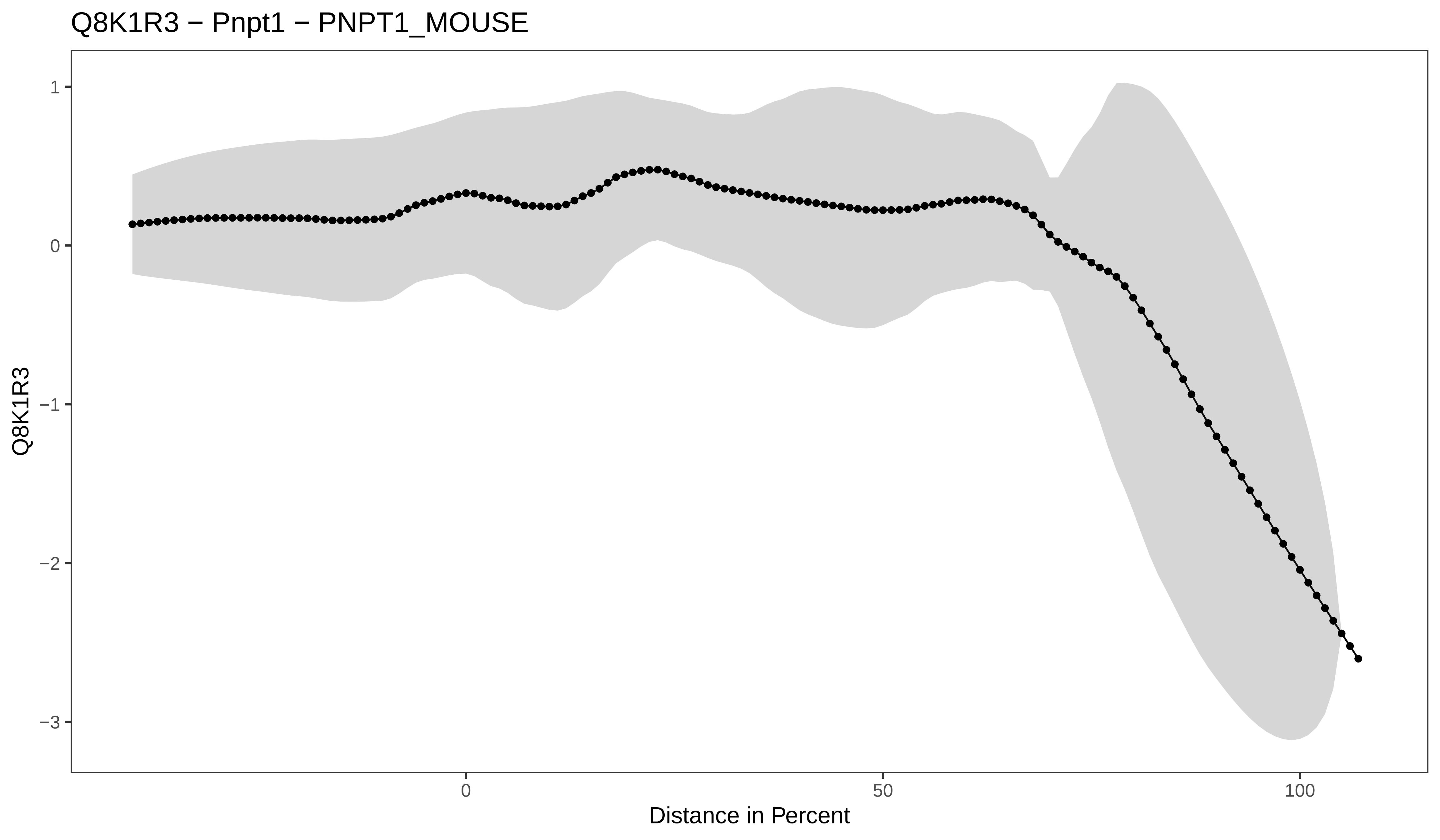

Q8K1R3 − Pnpt1 − PNPT1_MOUSE
1
0
−1
−2
−3
Q8K1R3
0
50
100
Distance in Percent

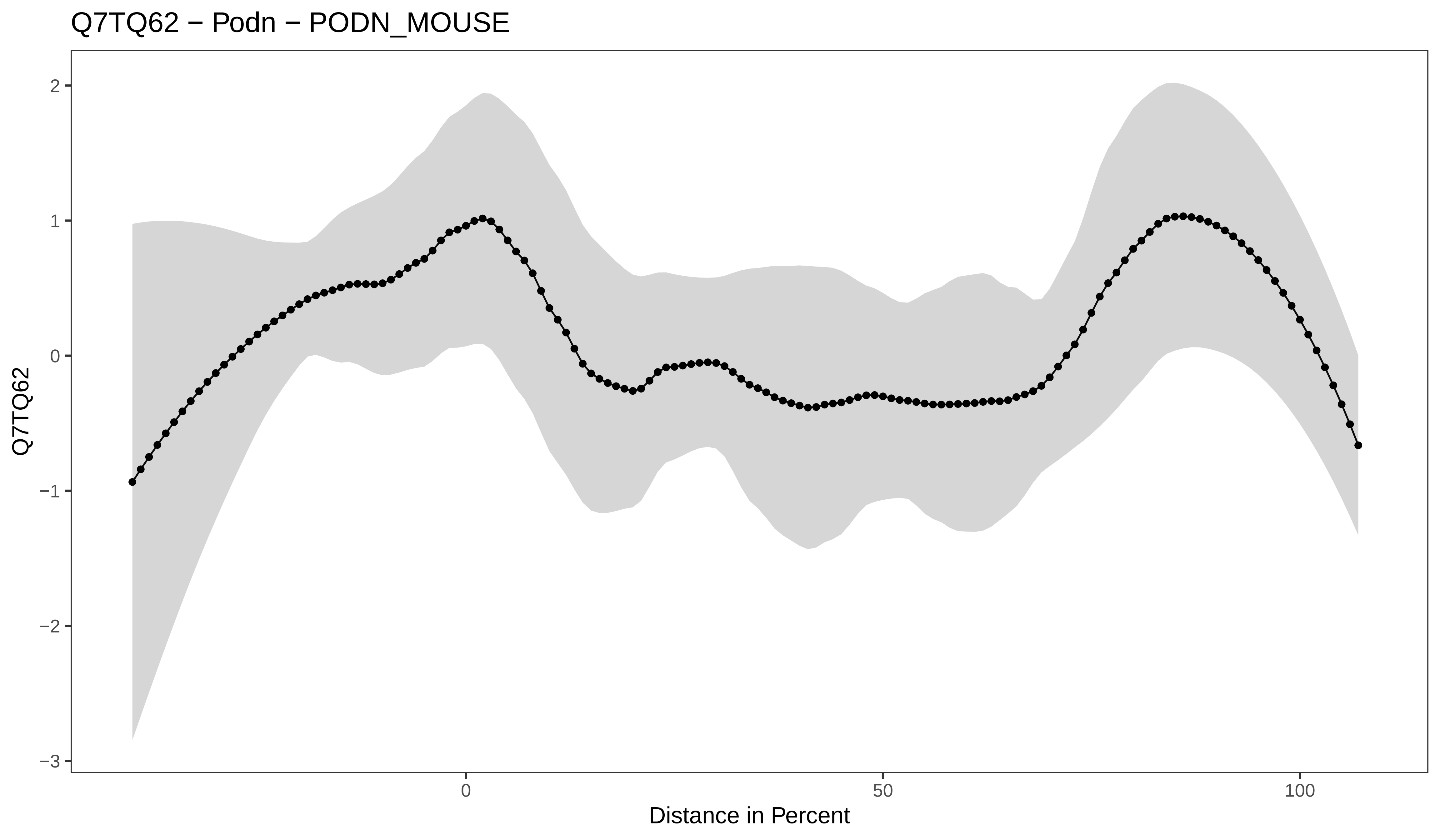
Q7TQ62 − Podn − PODN_MOUSE
2
1
0
−1
−2
−3
Q7TQ62
0
50
100
Distance in Percent

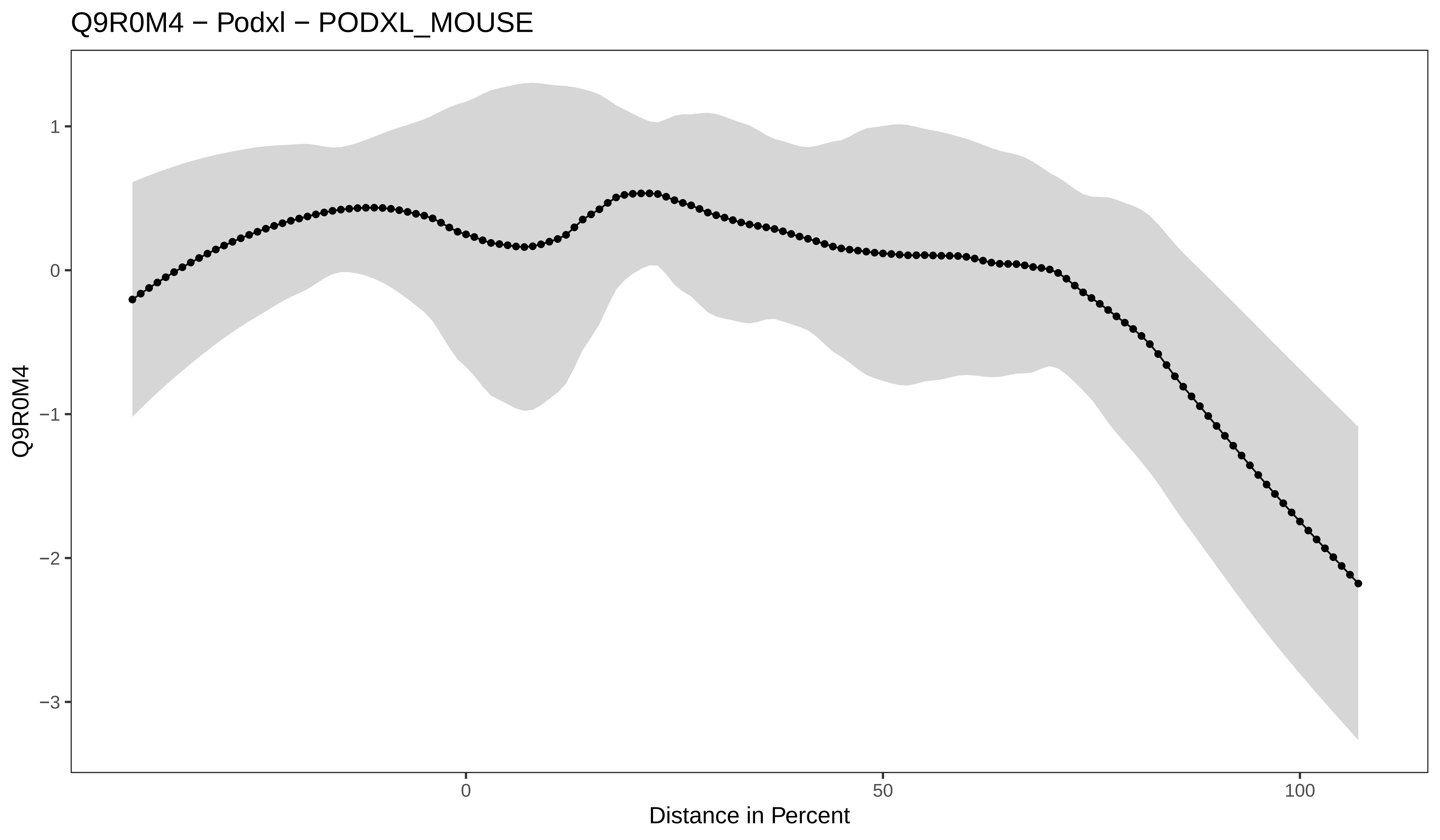
Q9R0M4 − Podxl − PODXL_MOUSE
Q9R0M4
Distance in Percent
1
0
−1
−2
−3
0
50
100

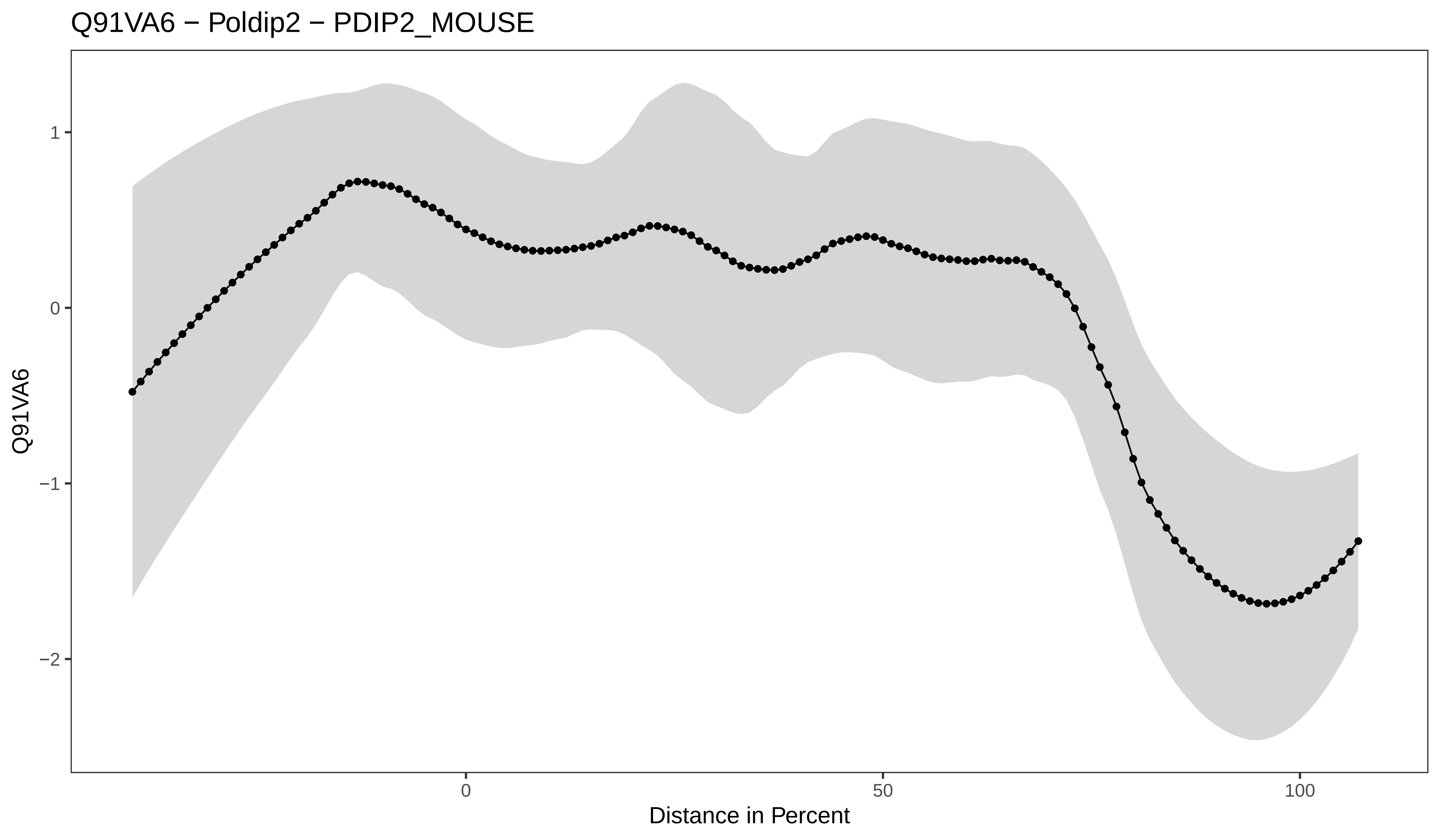

Q91VA6 − Poldip2 − PDIP2_MOUSE
Q91VA6
1
0
−1
−2
0
50
100
Distance in Percent

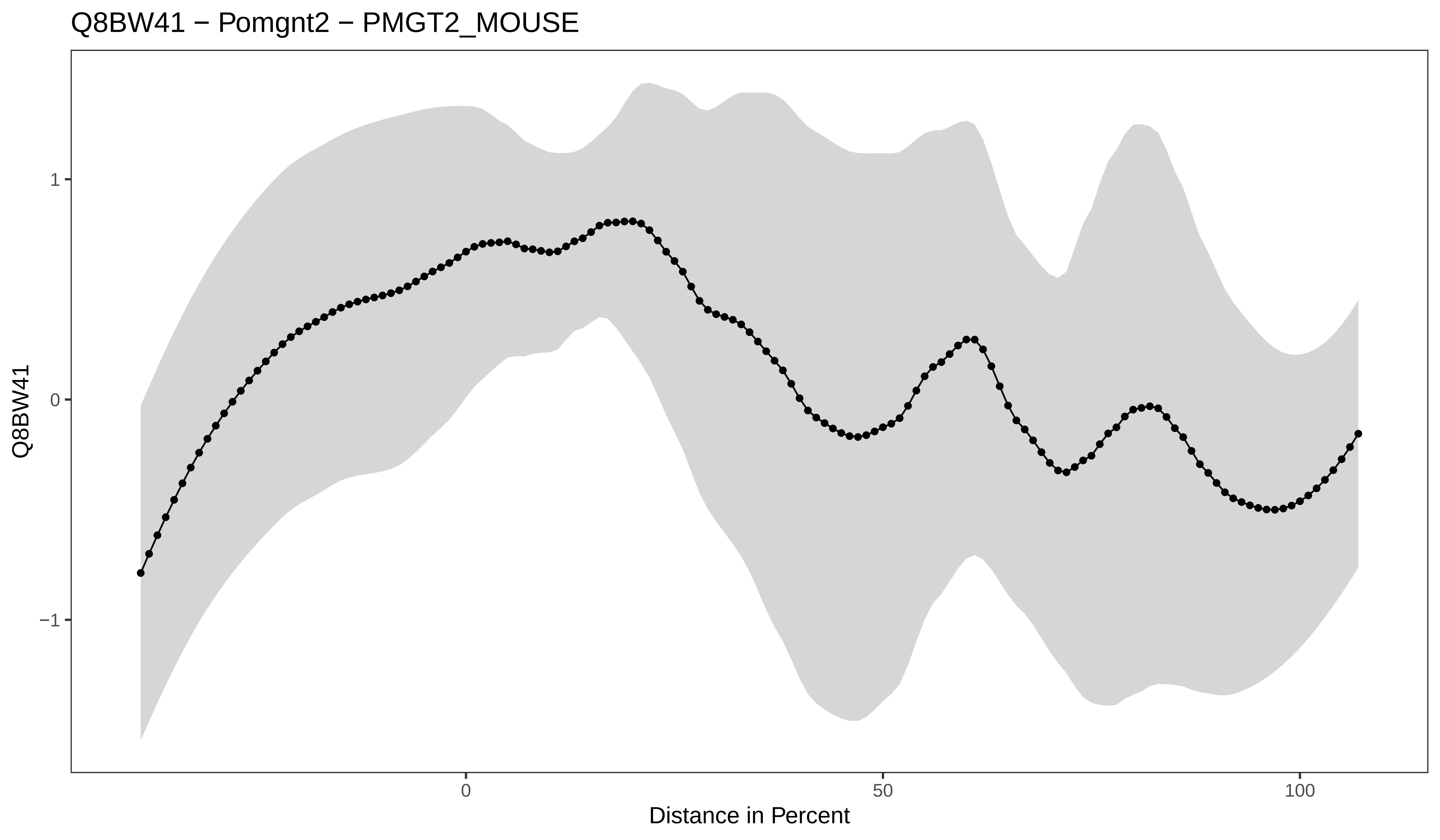

Q8BW41 − Pomgnt2 − PMGT2_MOUSE
1
0
−1
Q8BW41
0
50
100
Distance in Percent

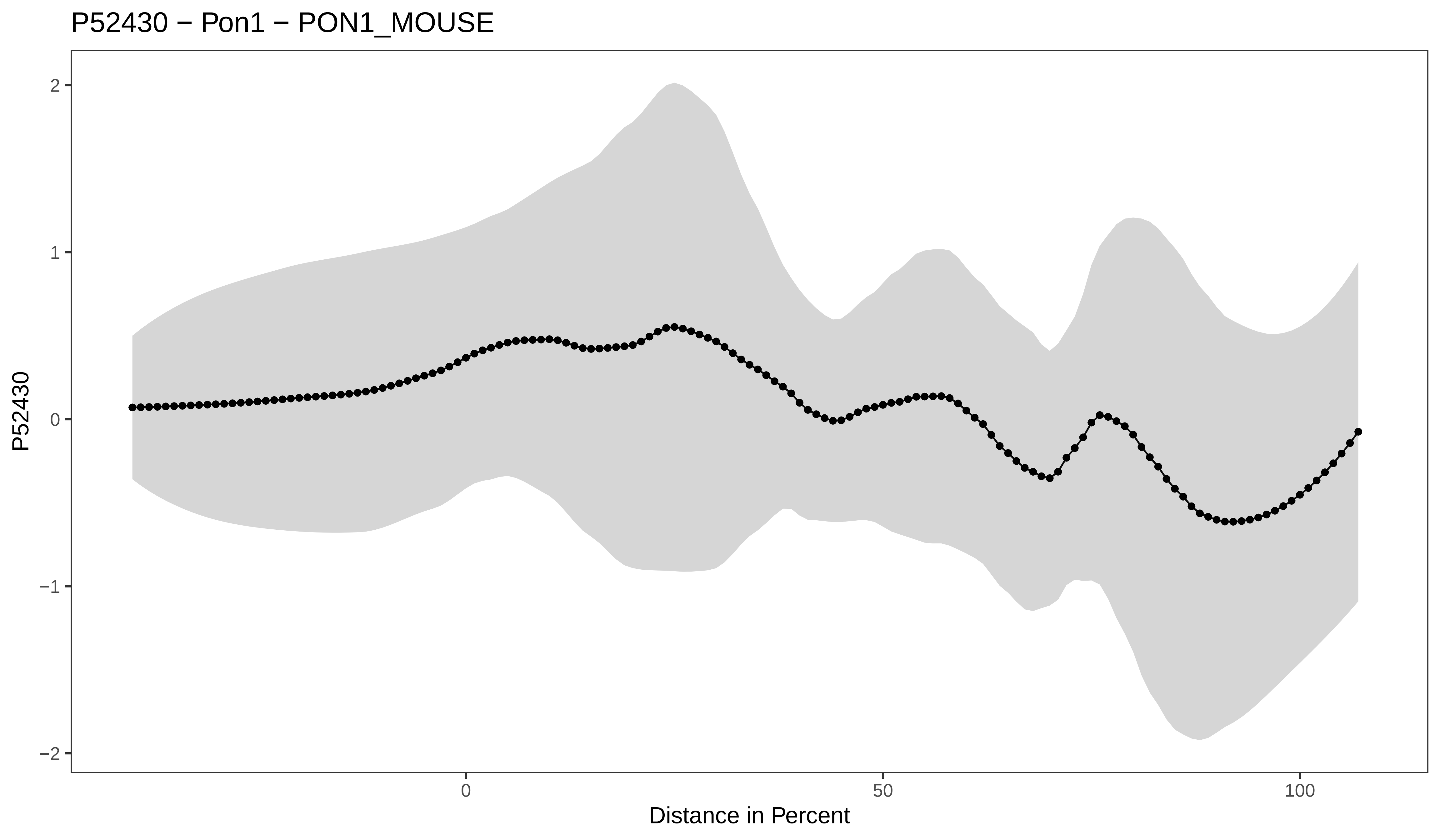

P52430 − Pon1 − PON1_MOUSE
P52430
2
1
0
−1
−2
0
50
100
Distance in Percent

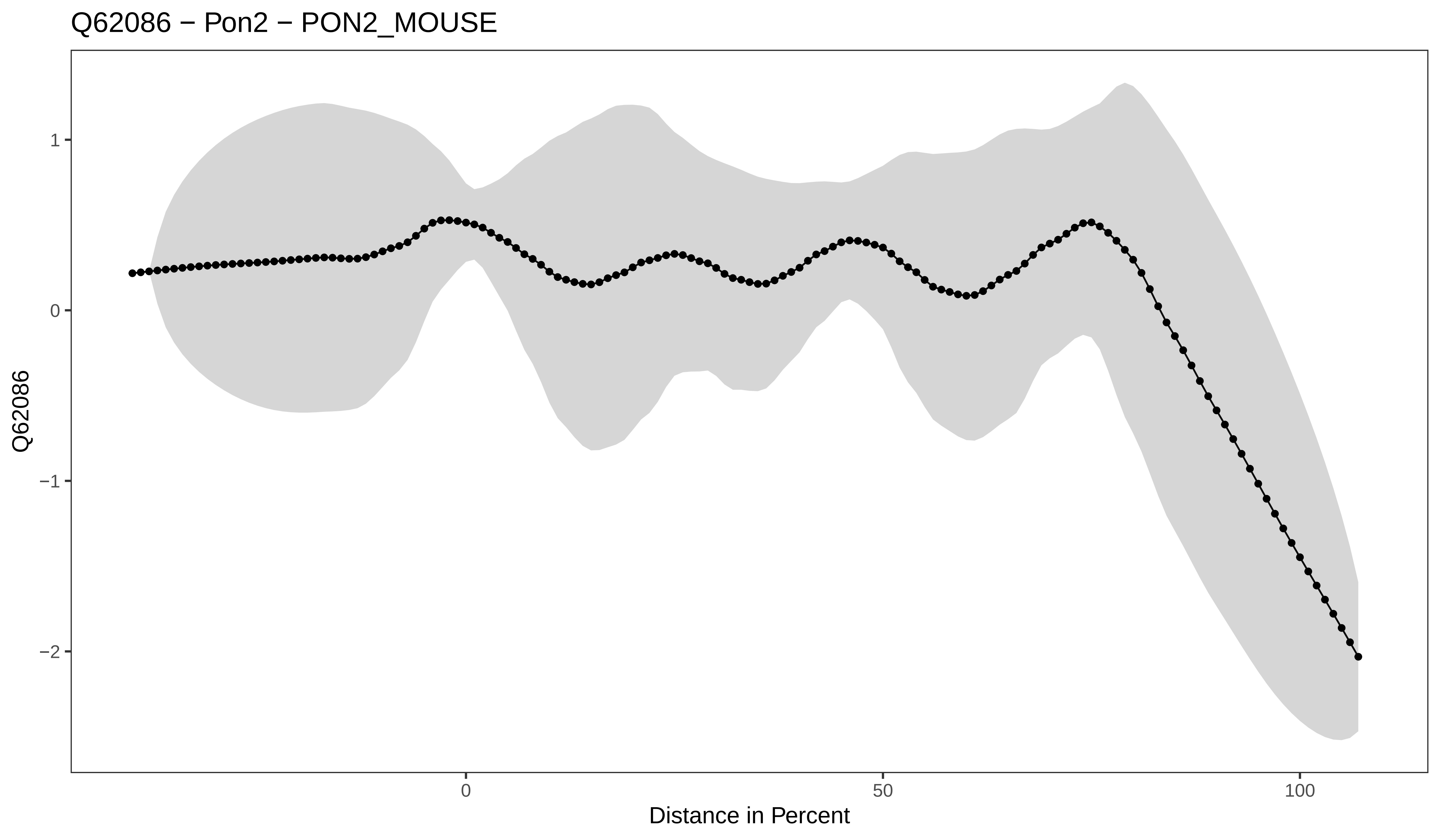

Q62086 − Pon2 − PON2_MOUSE
Q62086
Distance in Percent
1
0
−1
−2
0
50
100

Q62087 − Pon3 − PON3_MOUSE

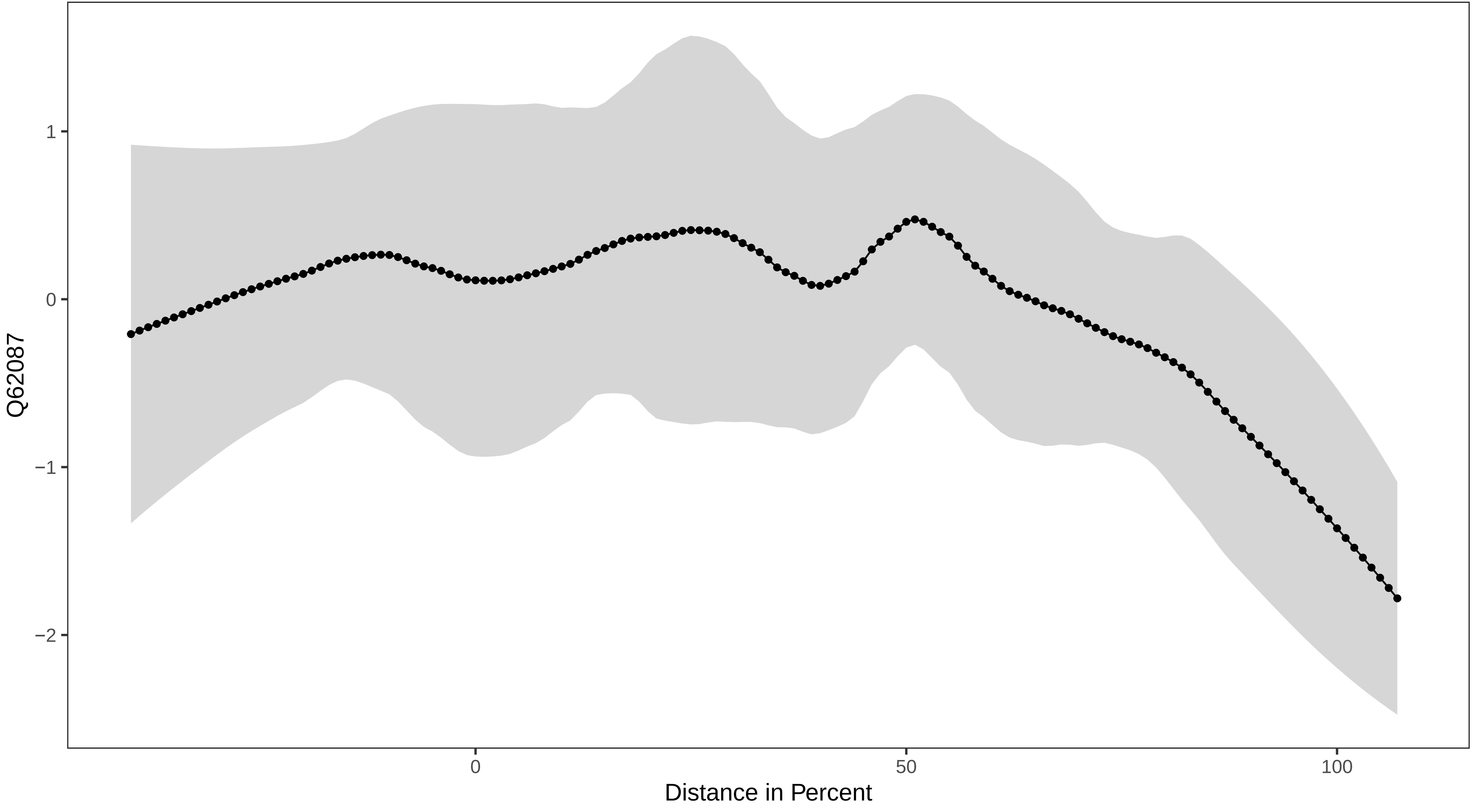

P37040 − Por − NCPR_MOUSE

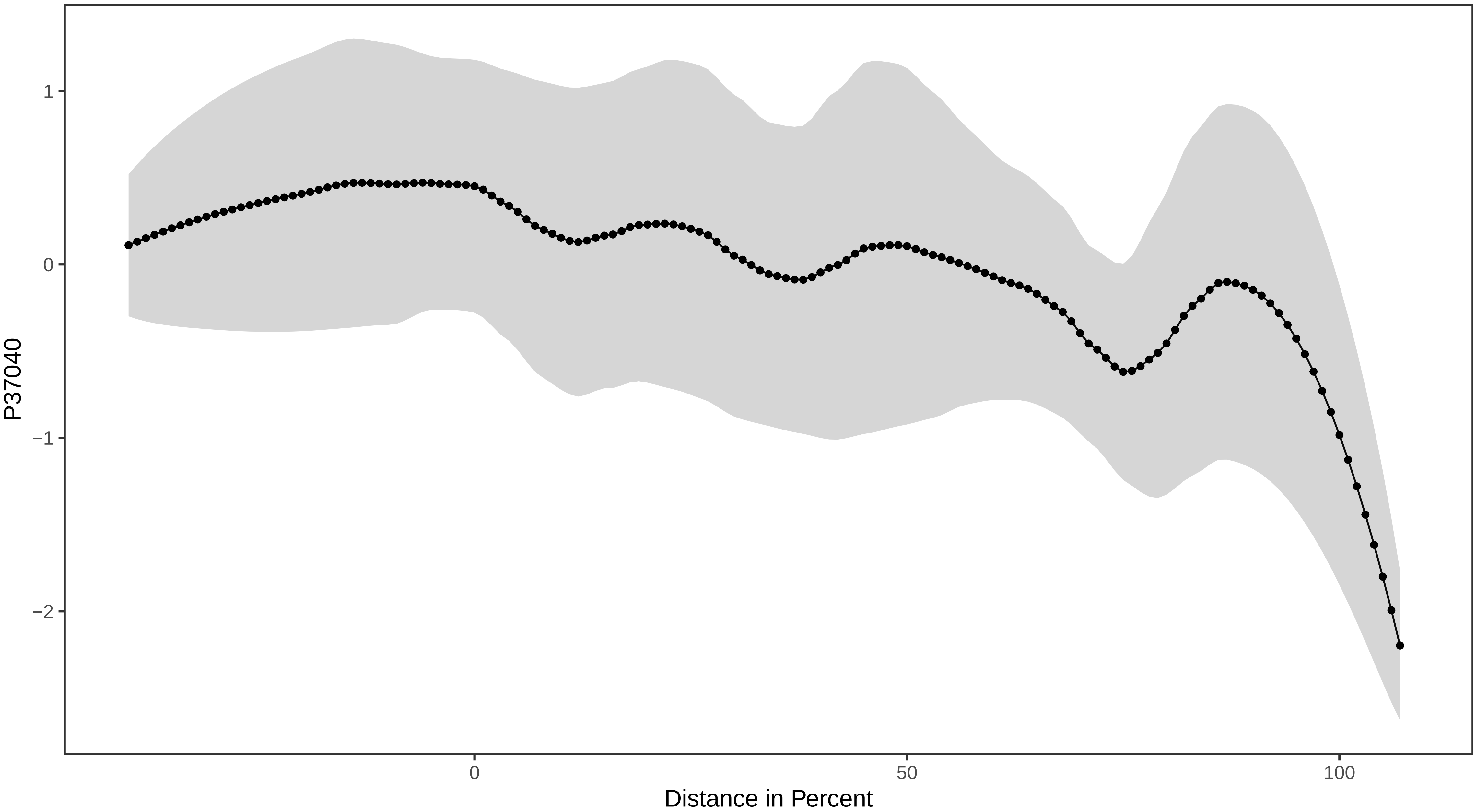

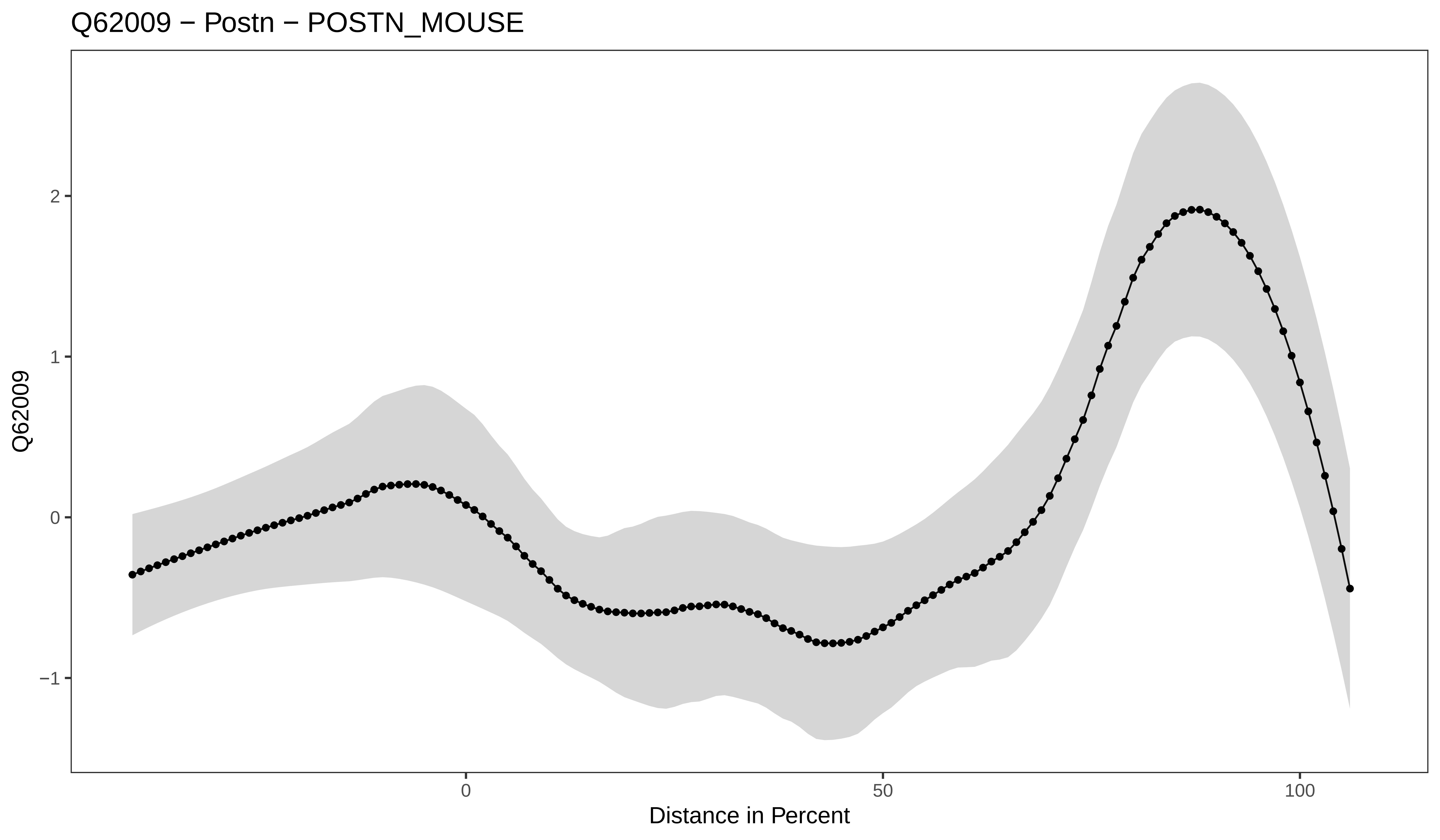

Q62009 − Postn − POSTN_MOUSE
Q62009
2
1
0
−1
0
50
100
Distance in Percent

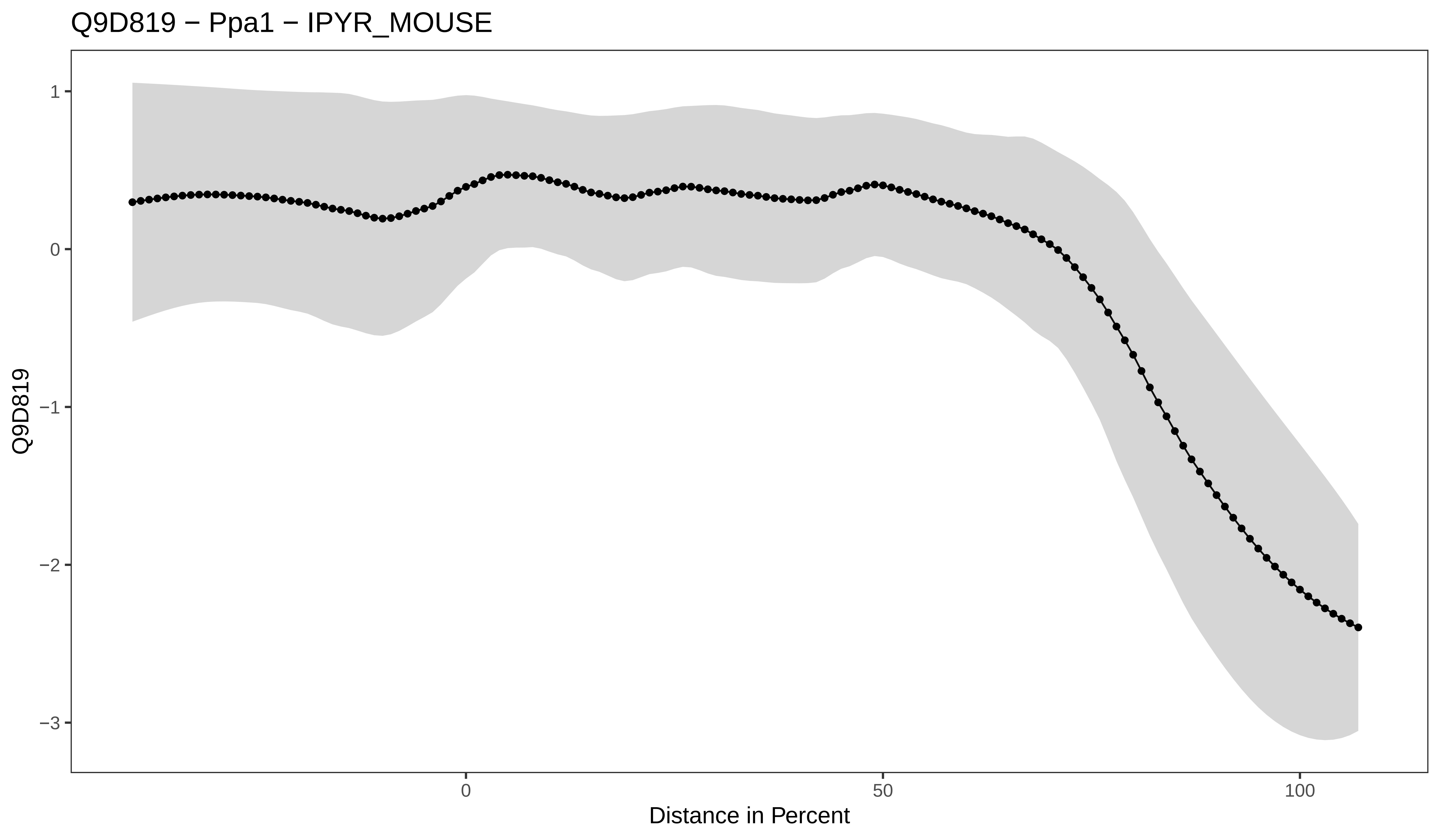

Q9D819 − Ppa1 − IPYR_MOUSE
1
0
−1
−2
−3
Q9D819
0
50
100
Distance in Percent

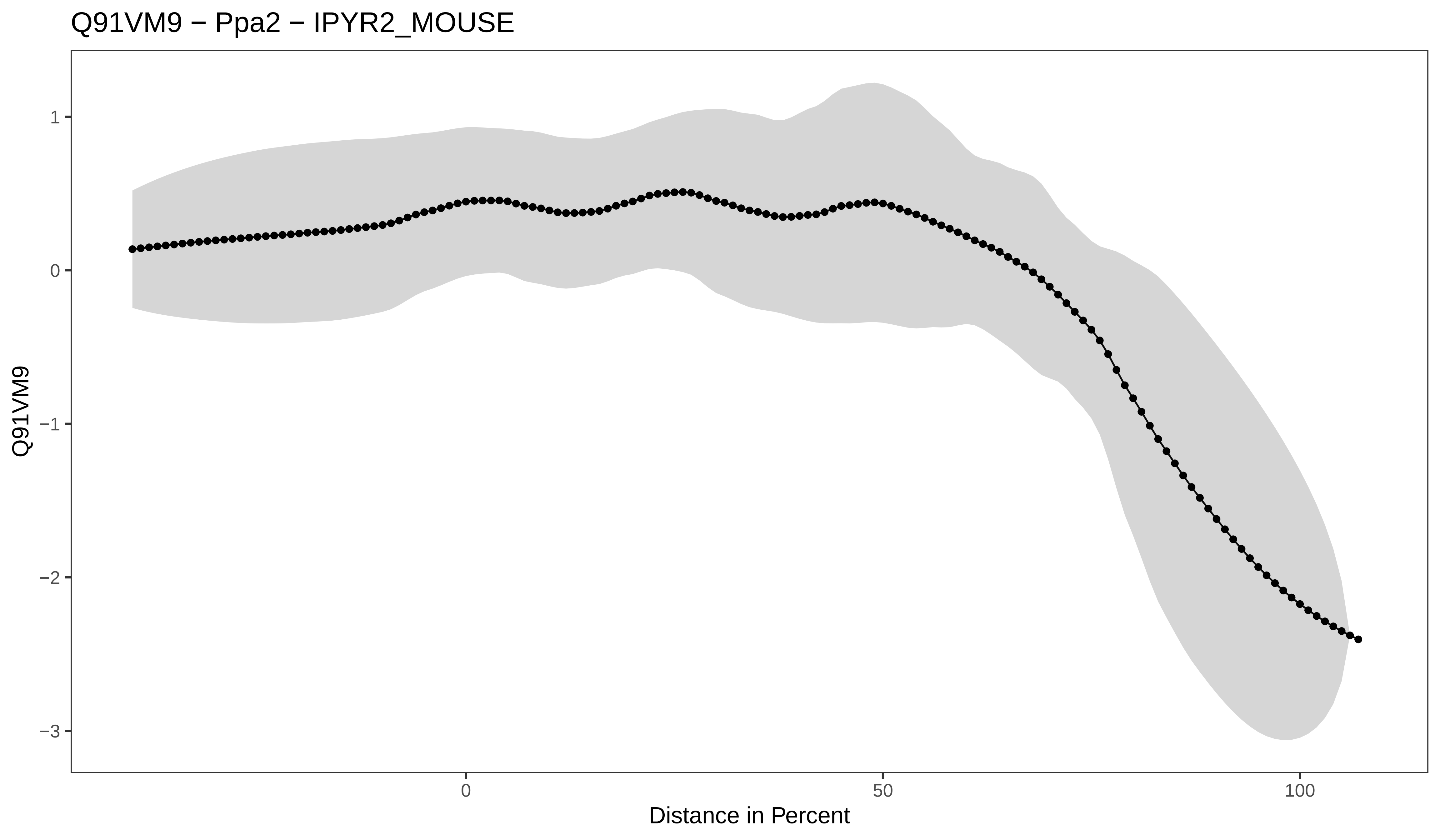

Q91VM9 − Ppa2 − IPYR2_MOUSE
Q91VM9
1
0
−1
−2
−3
0
50
100
Distance in Percent

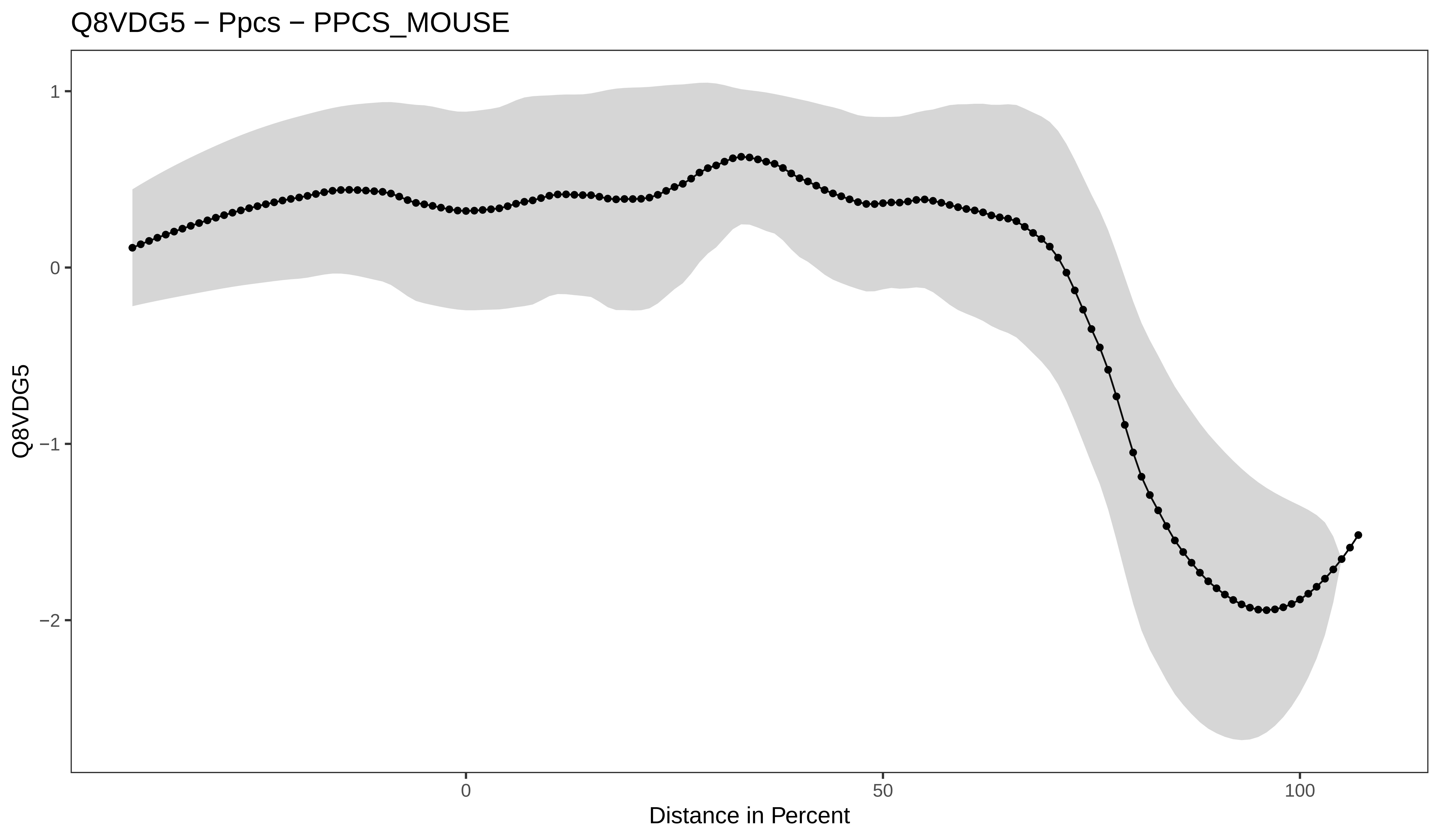

Q8VDG5 − Ppcs − PPCS_MOUSE
Q8VDG5
1
0
−1
−2
0
50
100
Distance in Percent

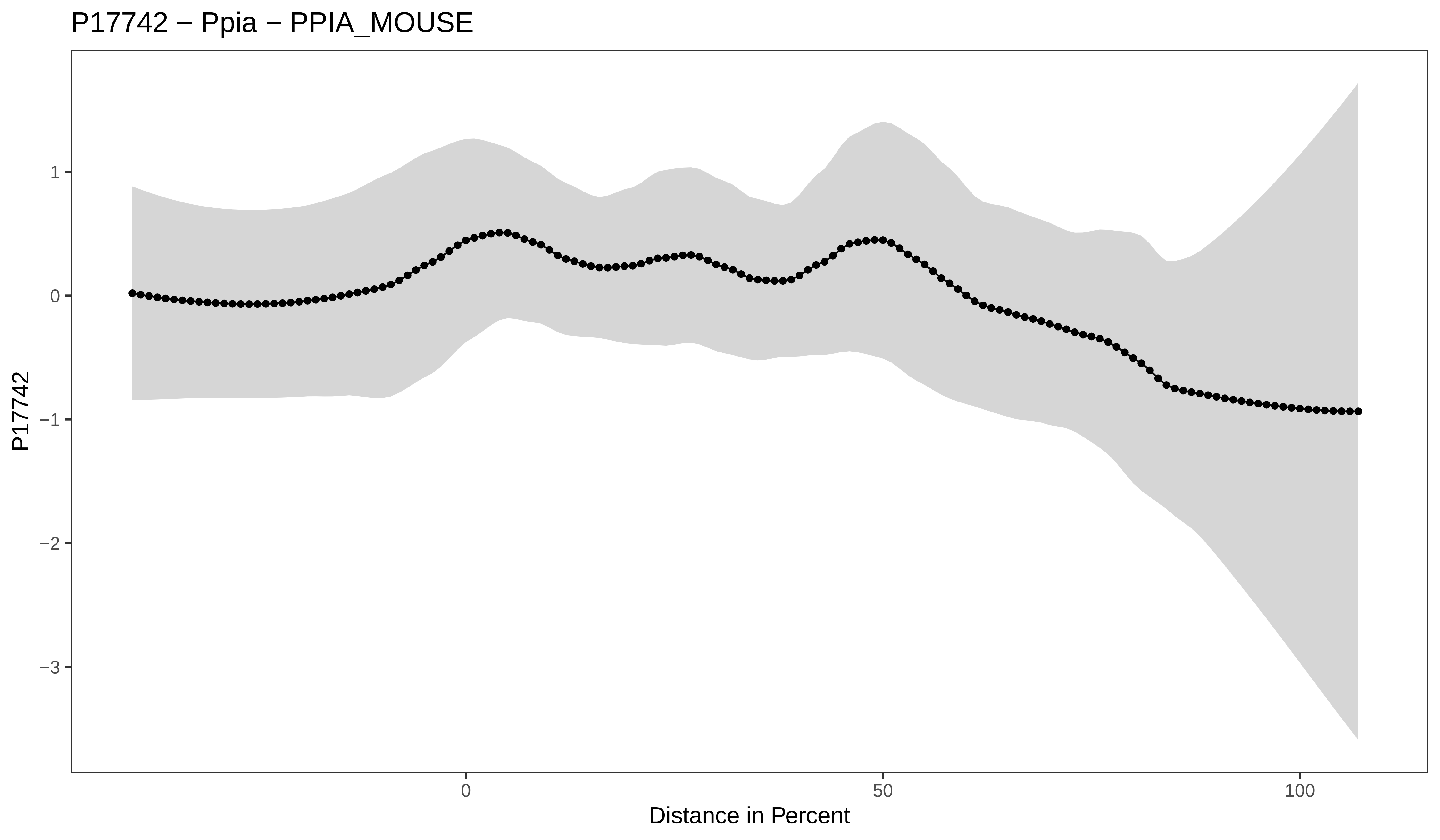

P17742 − Ppia − PPIA_MOUSE
1
0
−1
−2
−3
P17742
0
50
100
Distance in Percent

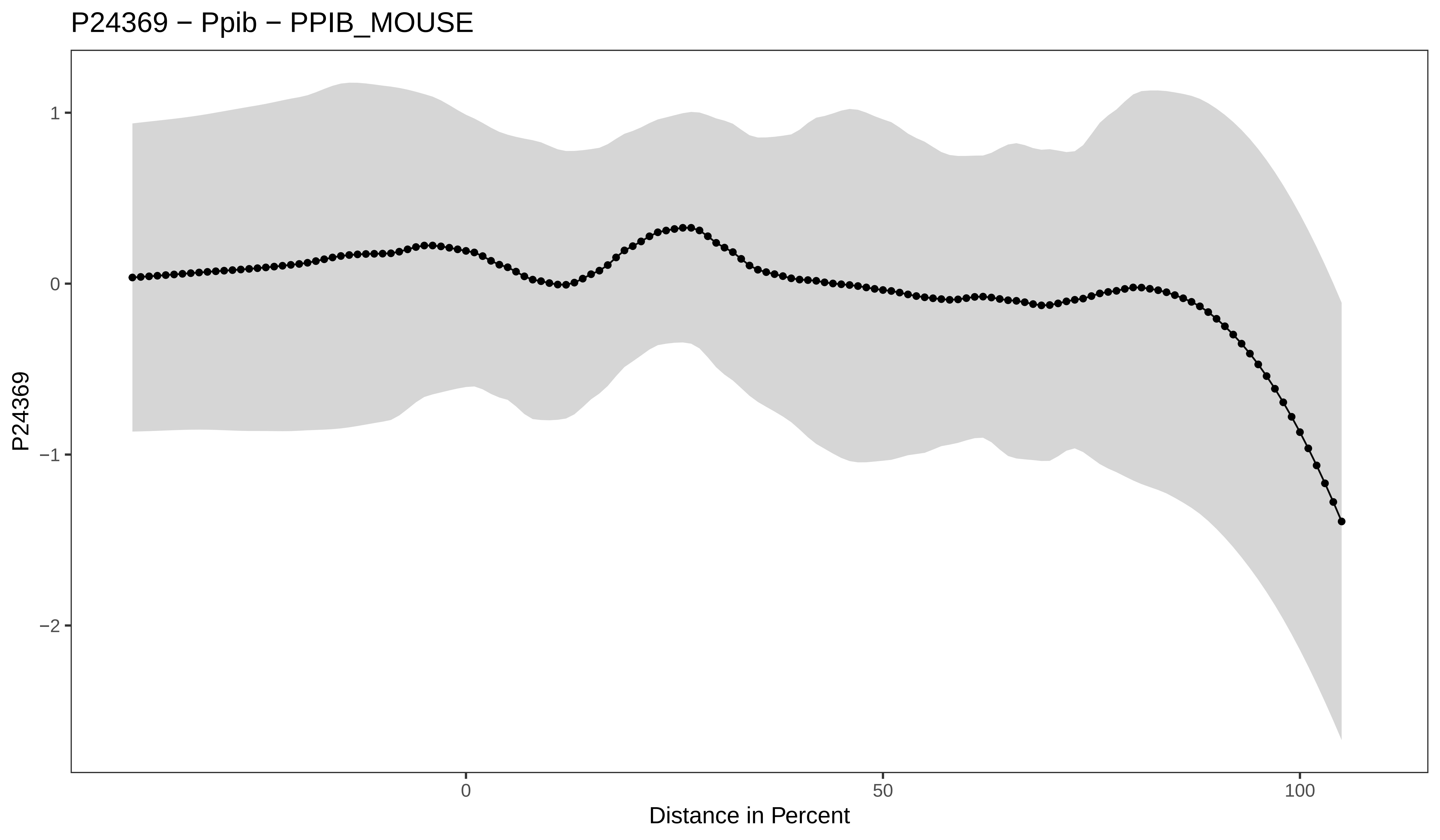
P24369 − Ppib − PPIB_MOUSE
1
0
−1
−2
P24369
0
50
100
Distance in Percent

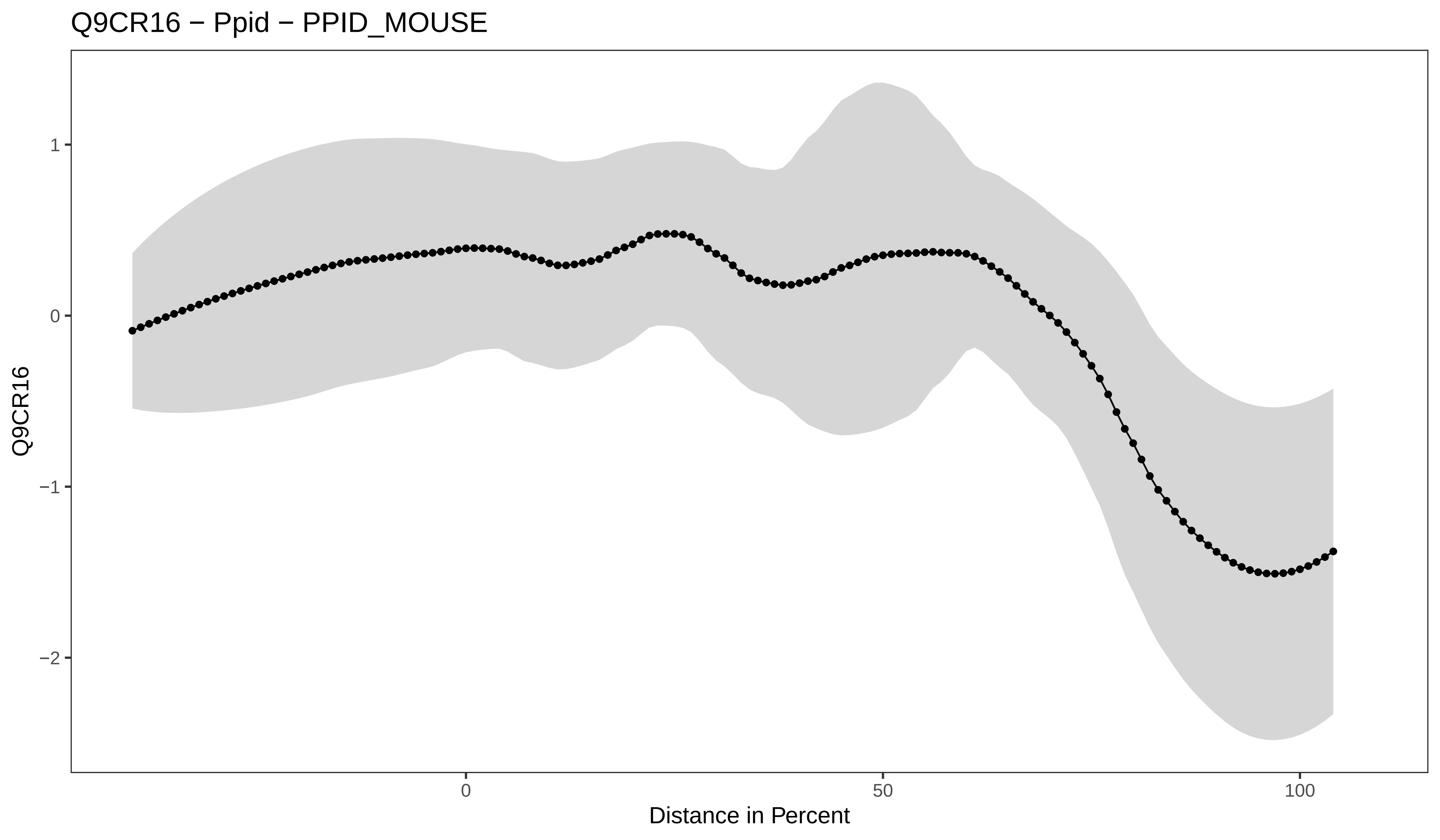

Q9CR16 − Ppid − PPID_MOUSE
1
0
−1
−2
Q9CR16
0
50
100
Distance in Percent

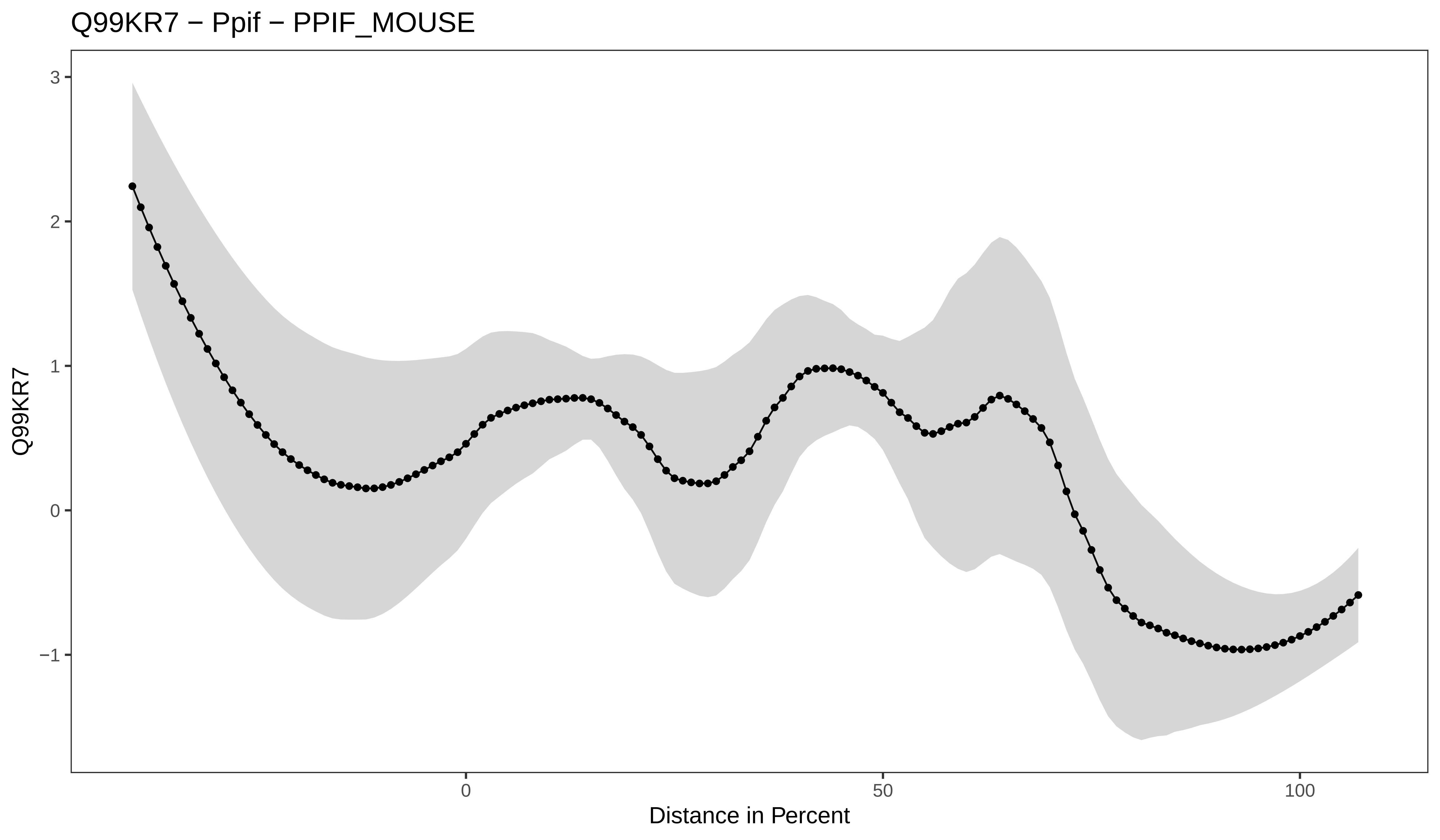

Q99KR7 − Ppif − PPIF_MOUSE
Q99KR7
3
2
1
0
−1
0
50
100
Distance in Percent

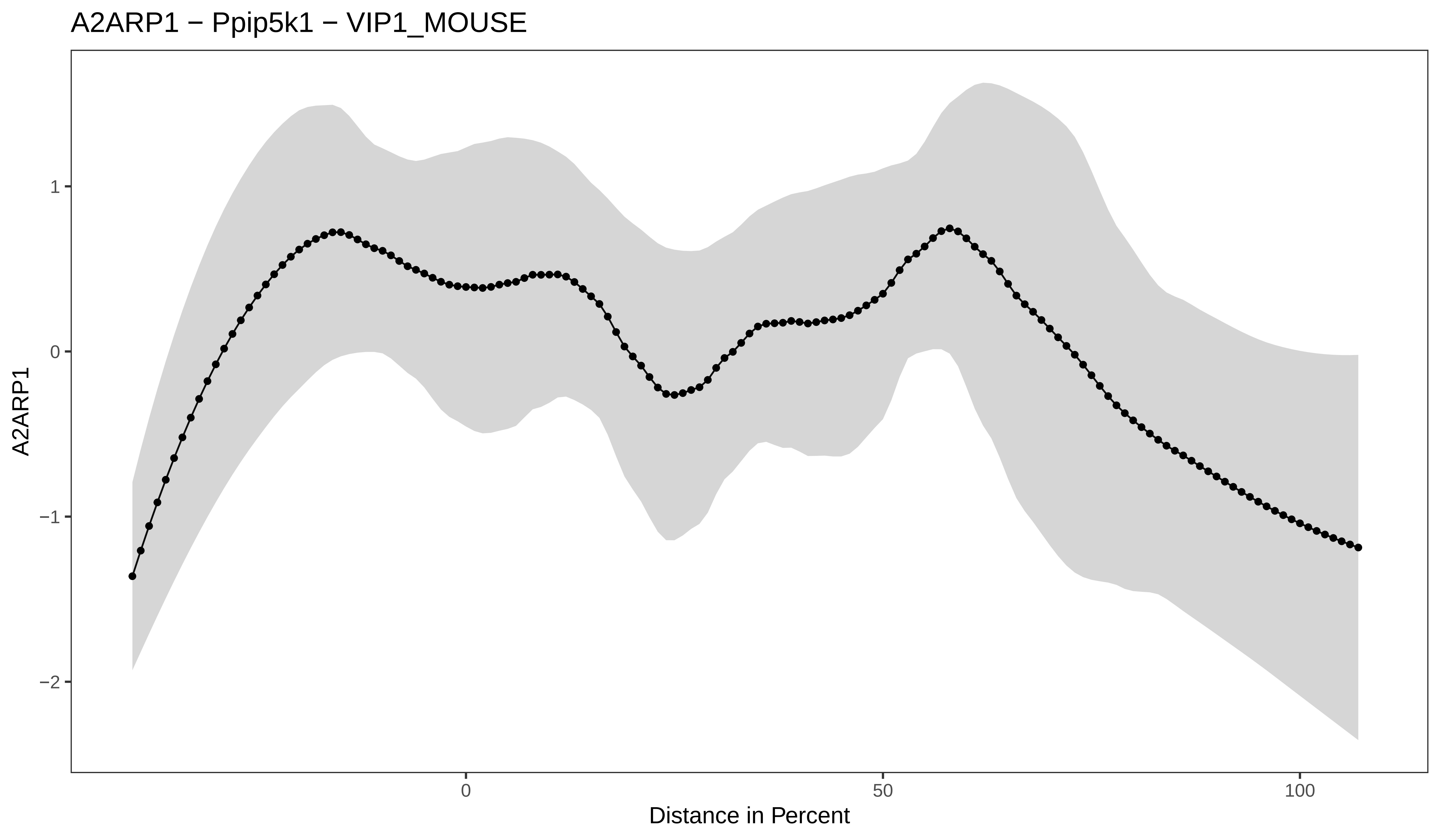
A2ARP1 − Ppip5k1 − VIP1_MOUSE
A2ARP1
1
0
−1
−2
0
50
100
Distance in Percent

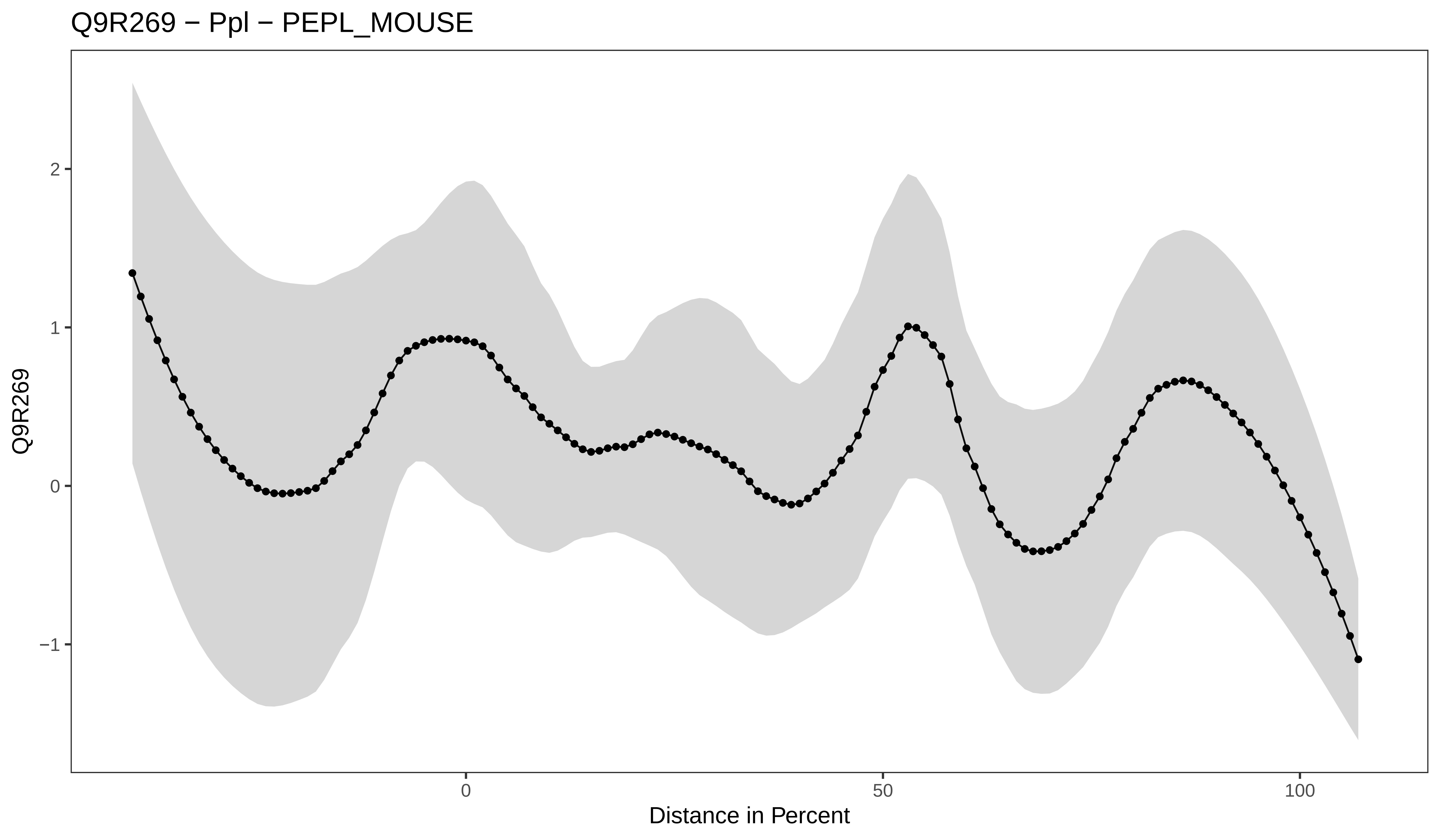
Q9R269 − Ppl − PEPL_MOUSE
Q9R269
Distance in Percent
2
1
0
−1
0
50
100

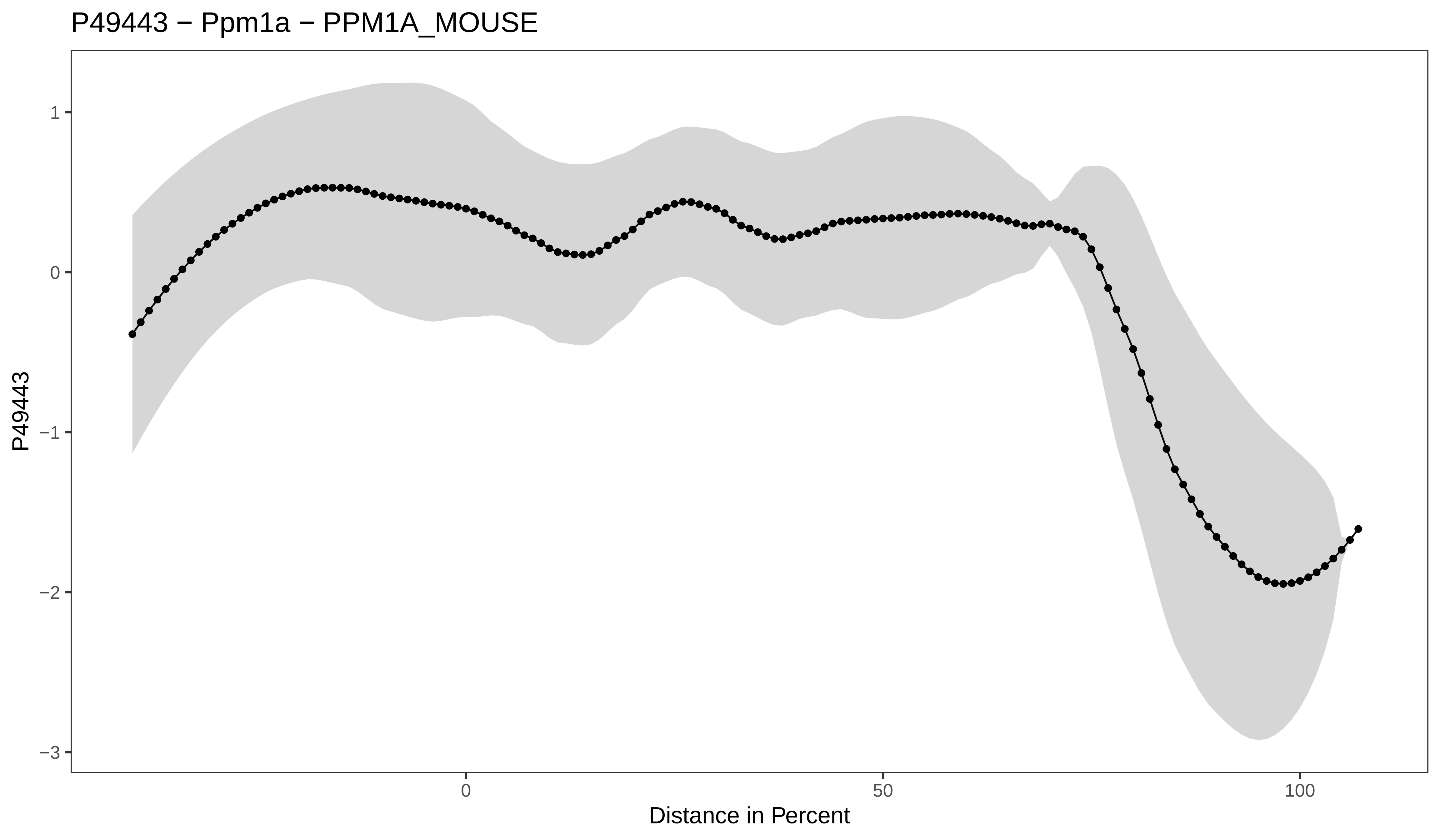
P49443 − Ppm1a − PPM1A_MOUSE
P49443
Distance in Percent
1
0
−1
−2
−3
0
50
100

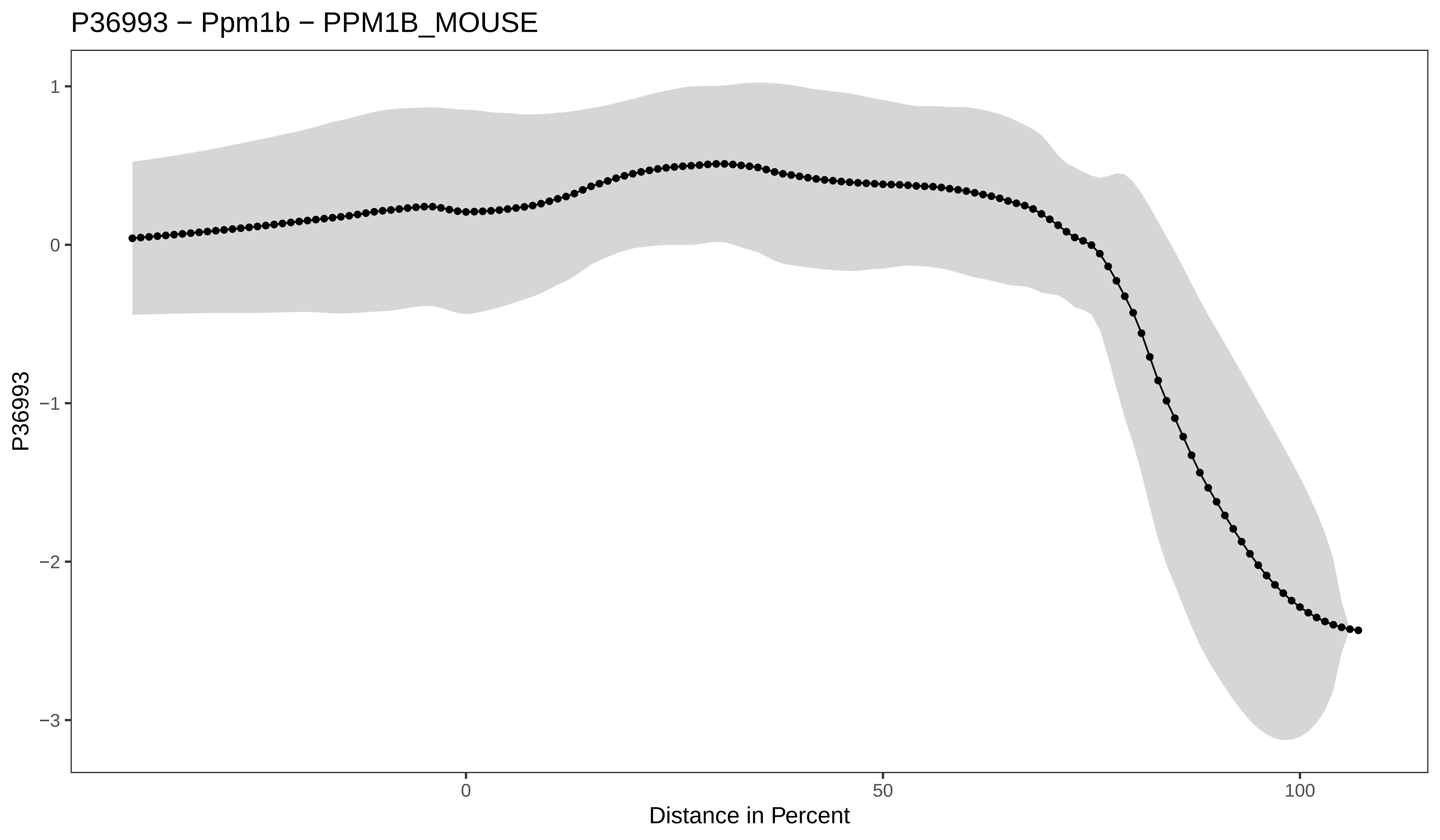

P36993 − Ppm1b − PPM1B_MOUSE
1
0
−1
−2
−3
P36993
0
50
100
Distance in Percent

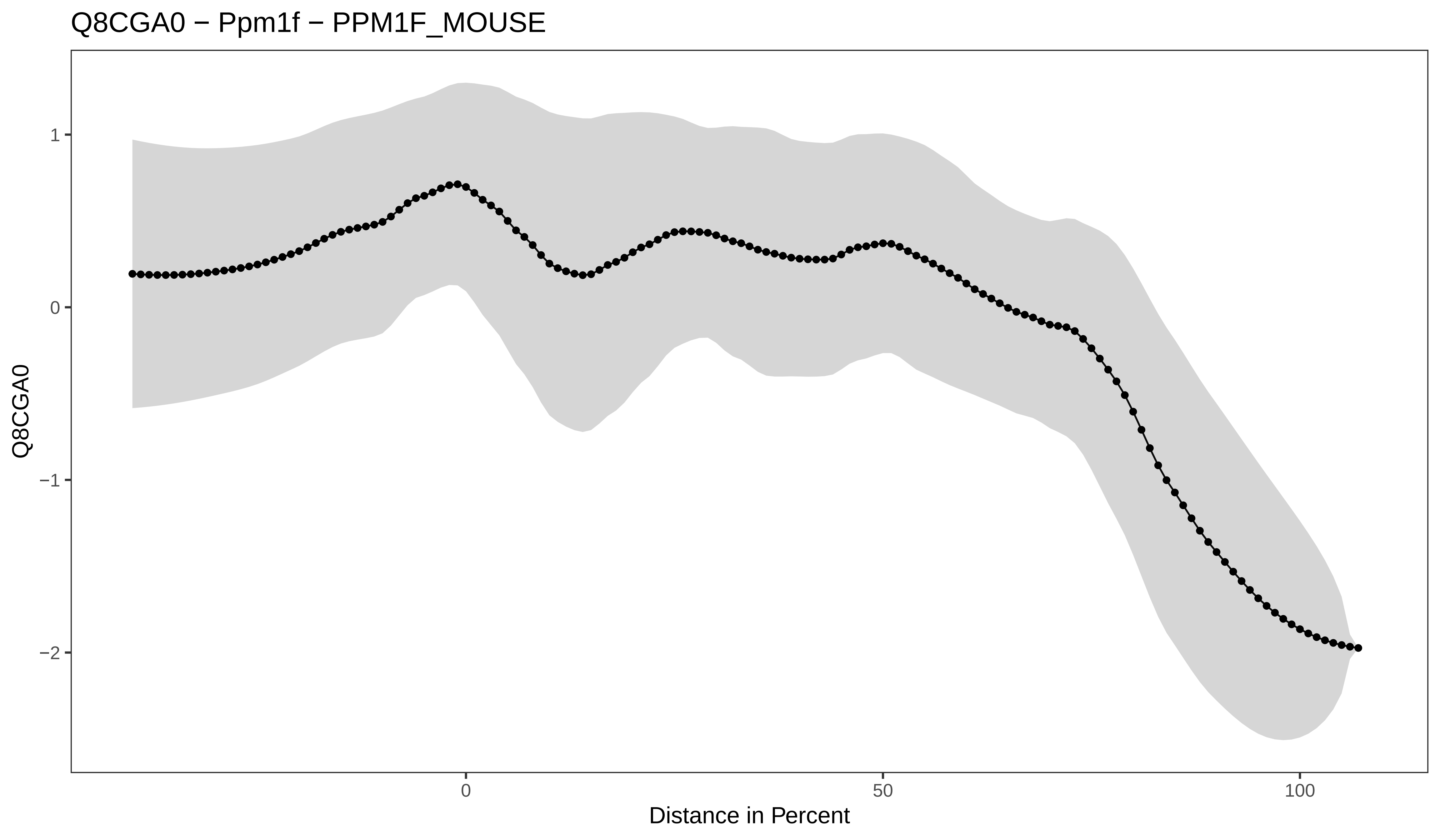

Q8CGA0 − Ppm1f − PPM1F_MOUSE
Q8CGA0
1
0
−1
−2
0
50
100
Distance in Percent

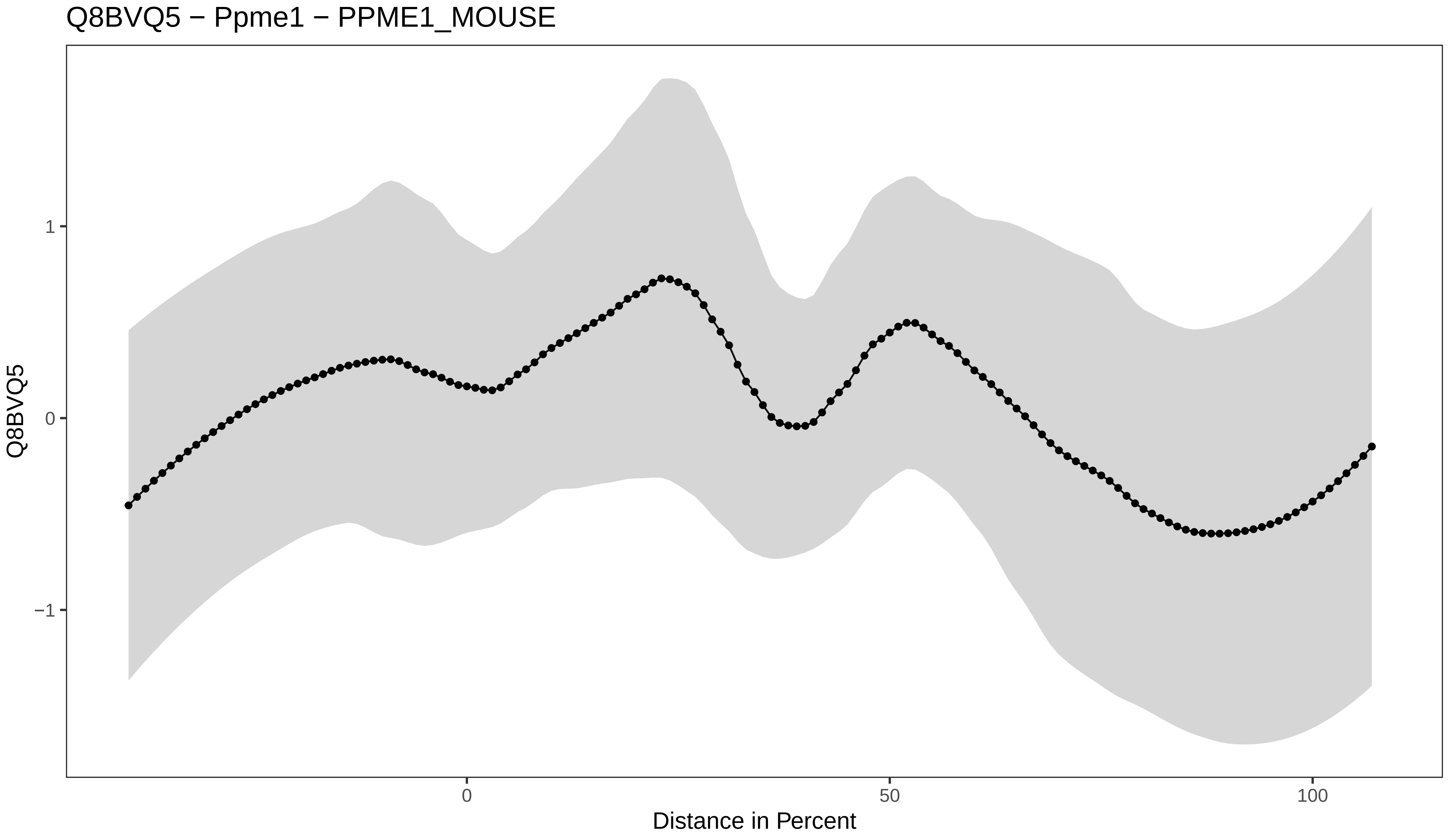

Q8BVQ5 − Ppme1 − PPME1_MOUSE
Q8BVQ5
1
0
−1
0
50
100
Distance in Percent

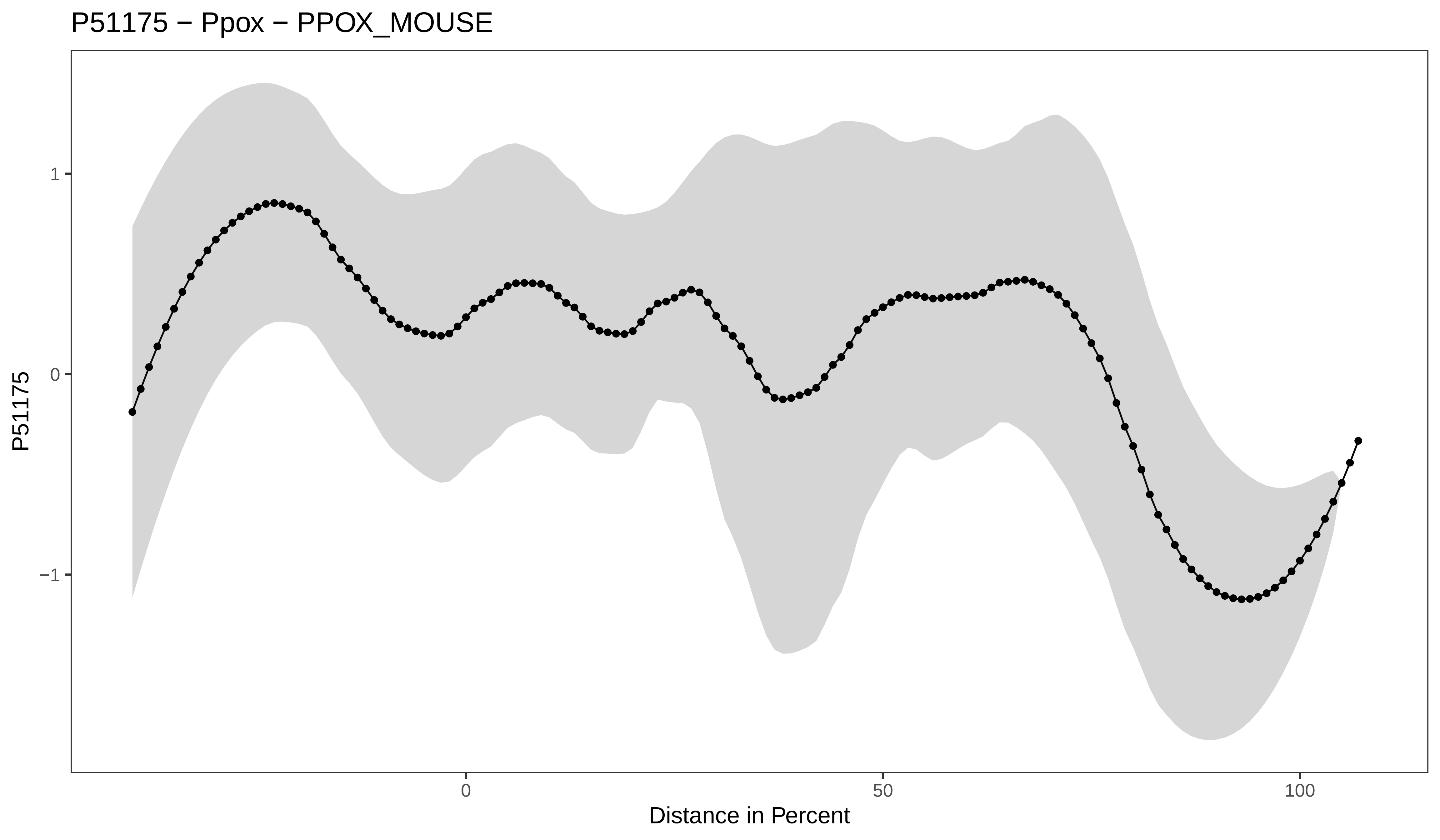

P51175 − Ppox − PPOX_MOUSE
P51175
Distance in Percent
1
0
−1
0
50
100

P62137 − Ppp1ca − PP1A_MOUSE

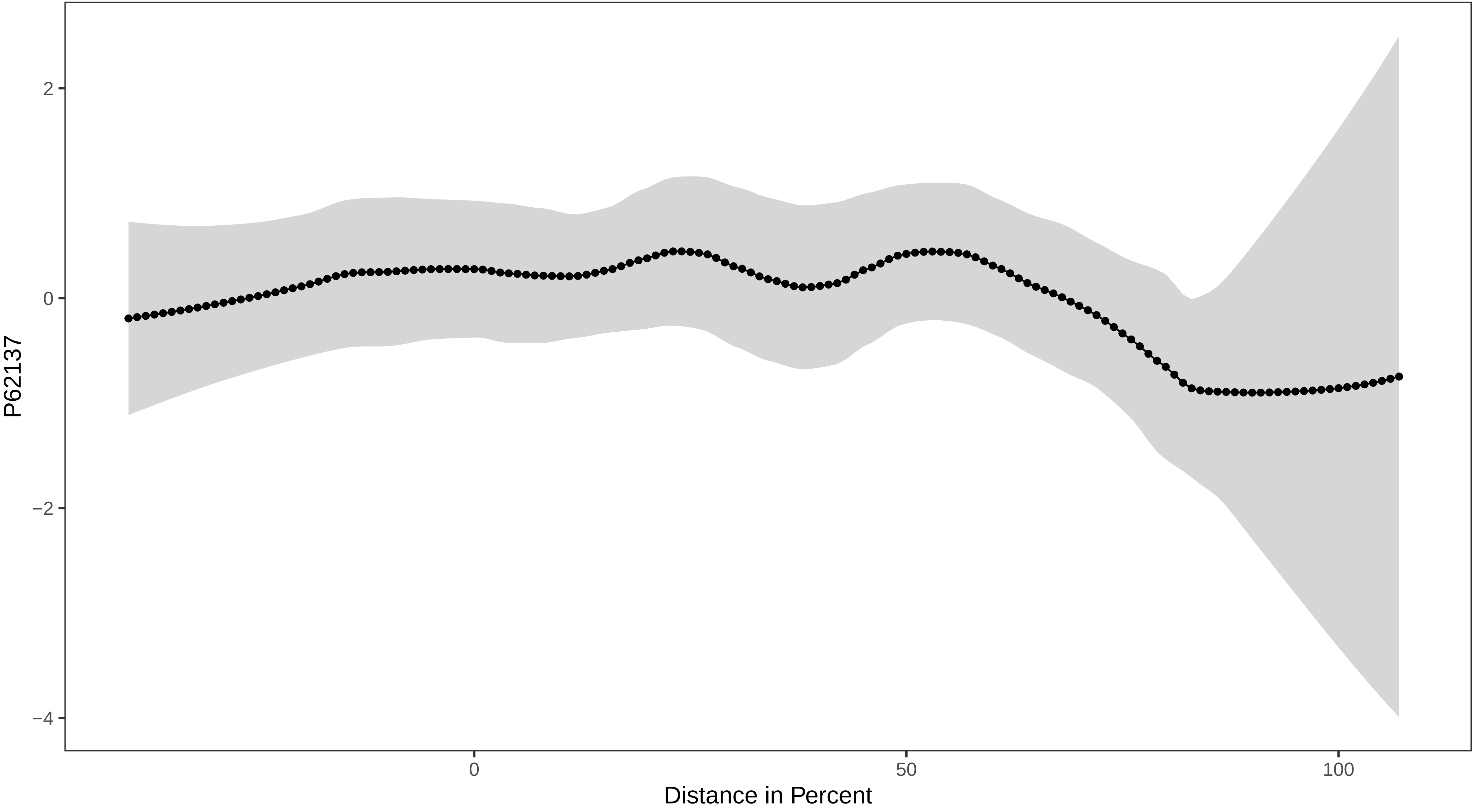

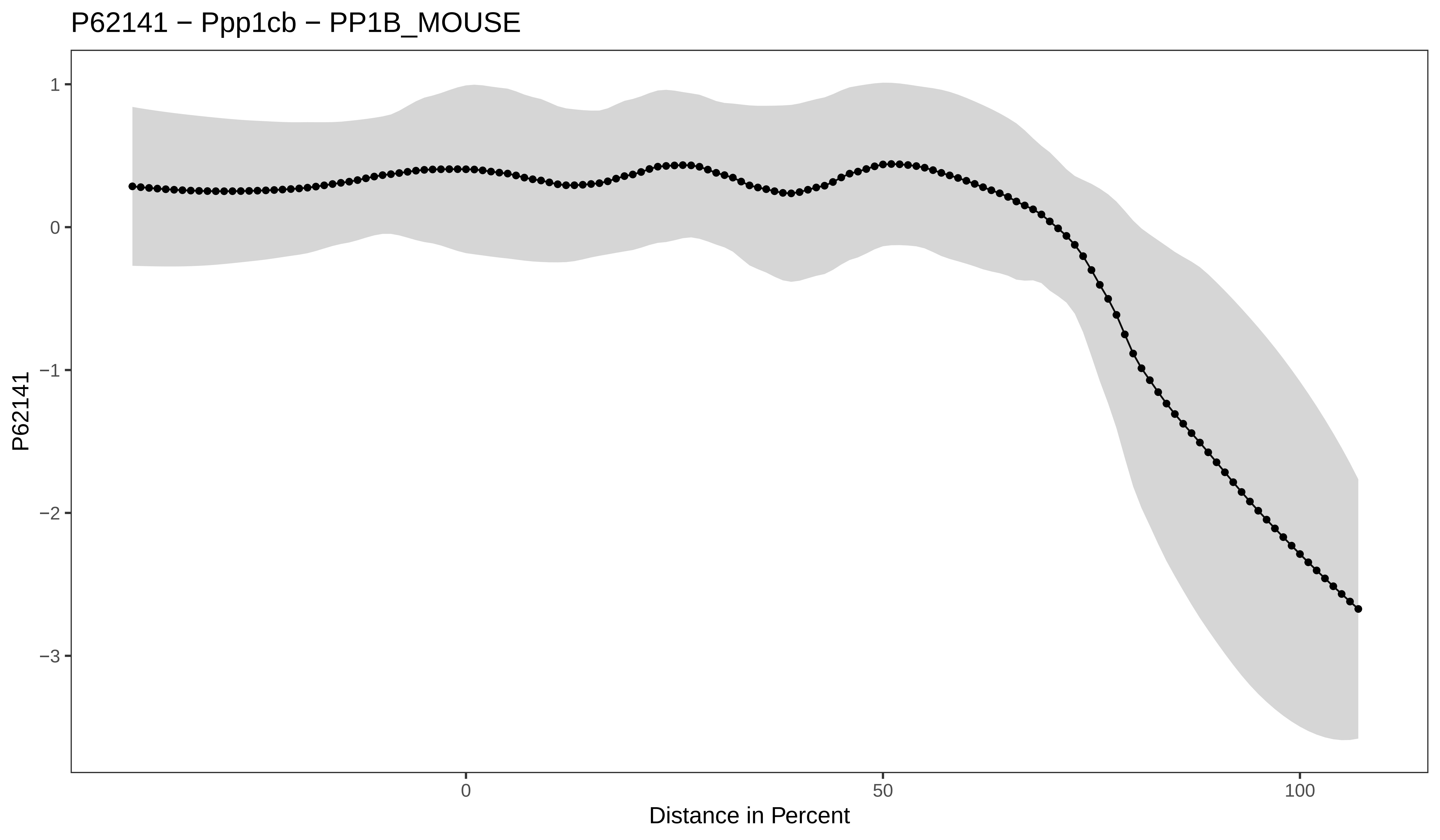

P62141 − Ppp1cb − PP1B_MOUSE
1
0
−1
−2
−3
P62141
0
50
100
Distance in Percent

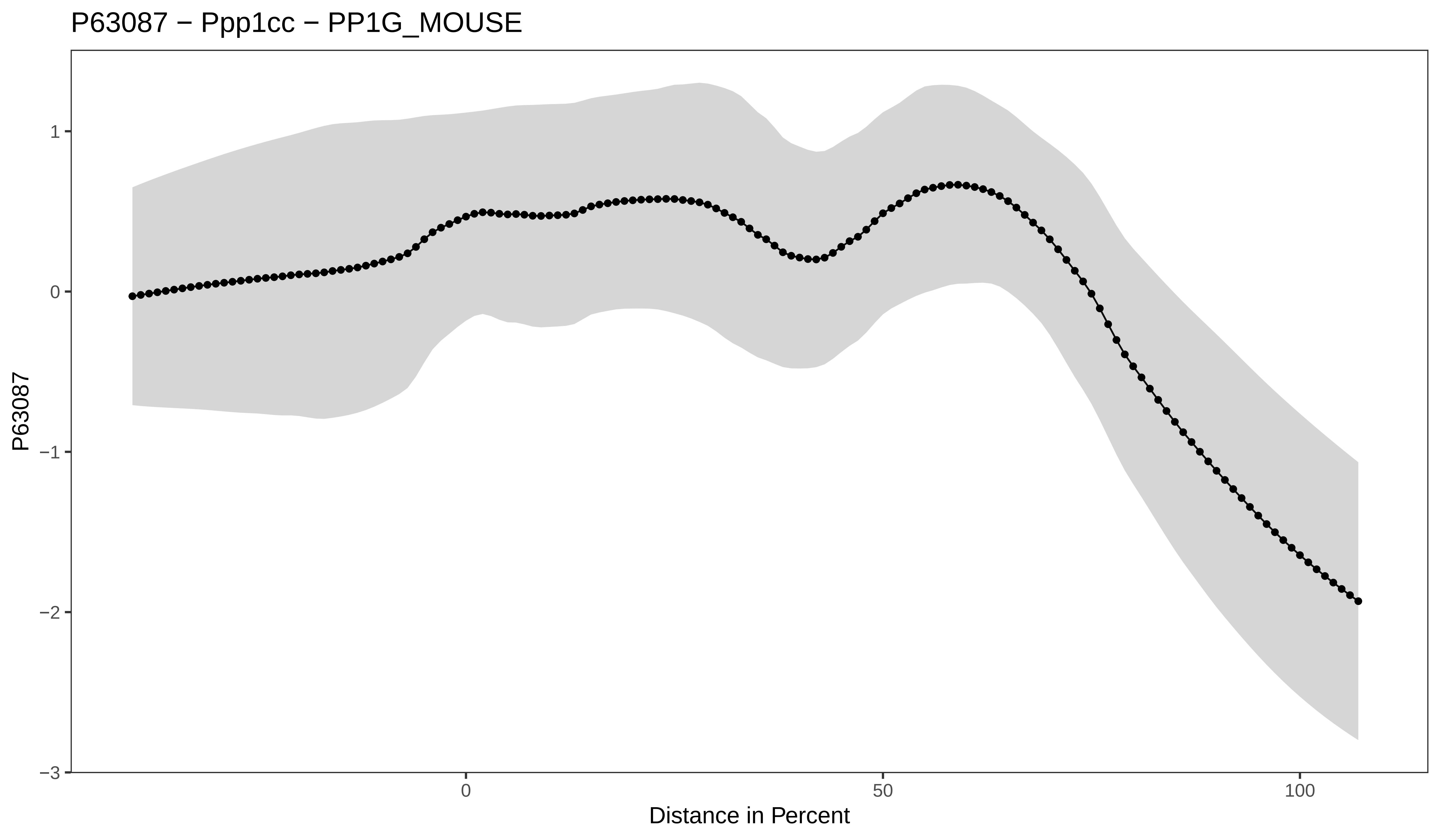

P63087 − Ppp1cc − PP1G_MOUSE
P63087
Distance in Percent
1
0
−1
−2
−3
0
50
100

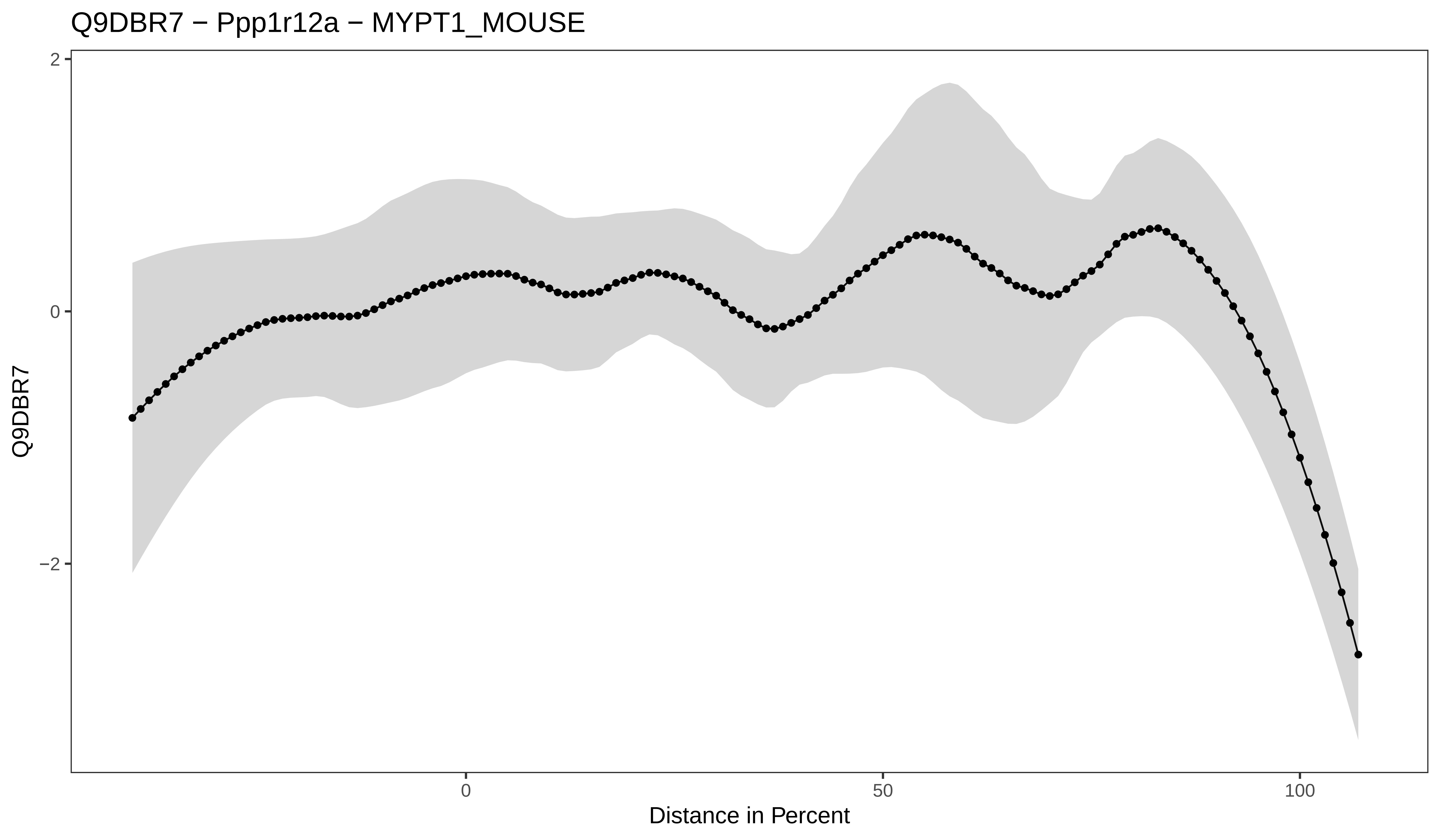

Q9DBR7 − Ppp1r12a − MYPT1_MOUSE
2
0
−2
Q9DBR7
0
50
100
Distance in Percent

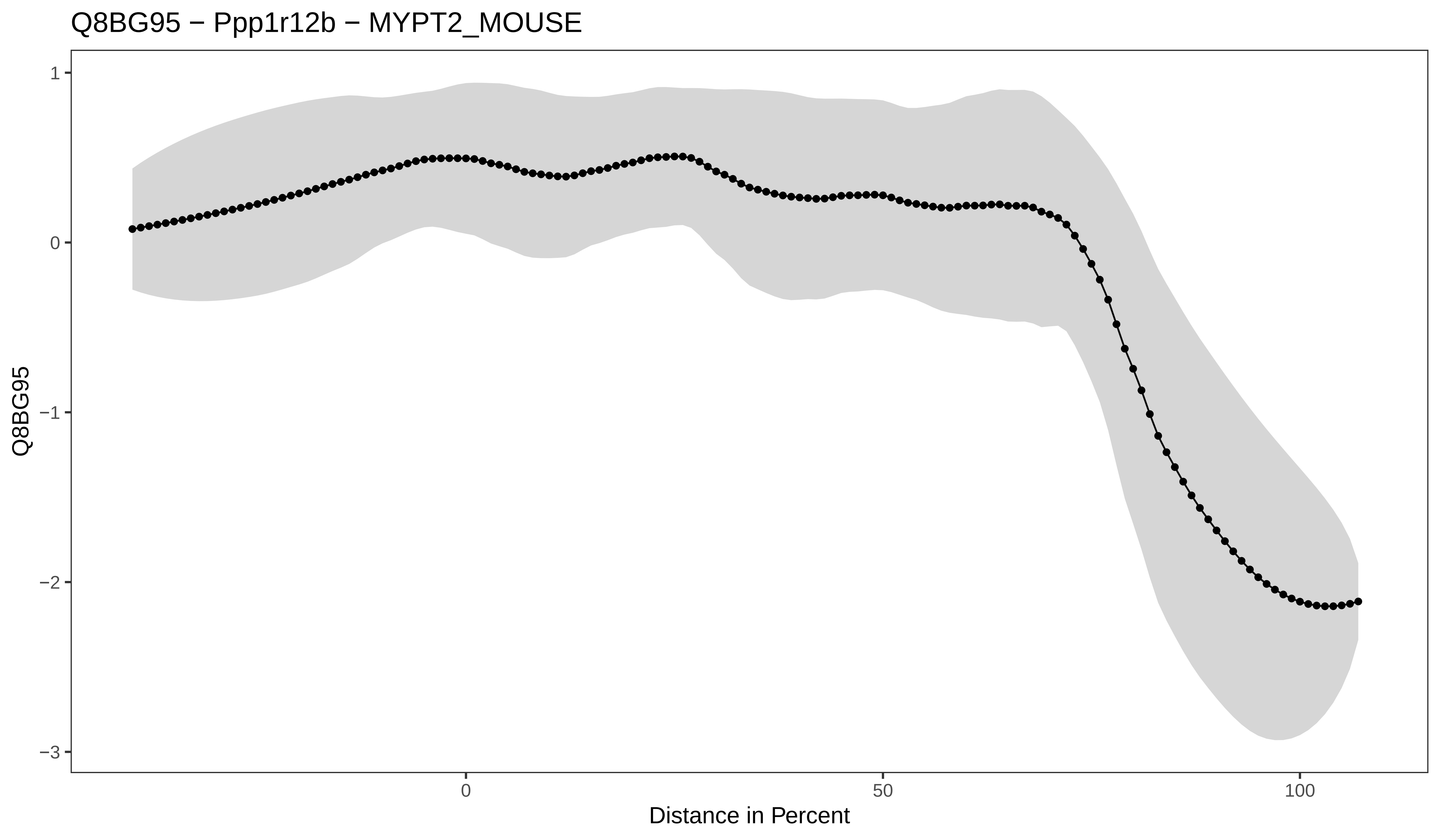
Q8BG95 − Ppp1r12b − MYPT2_MOUSE
Q8BG95
Distance in Percent
1
0
−1
−2
−3
0
50
100

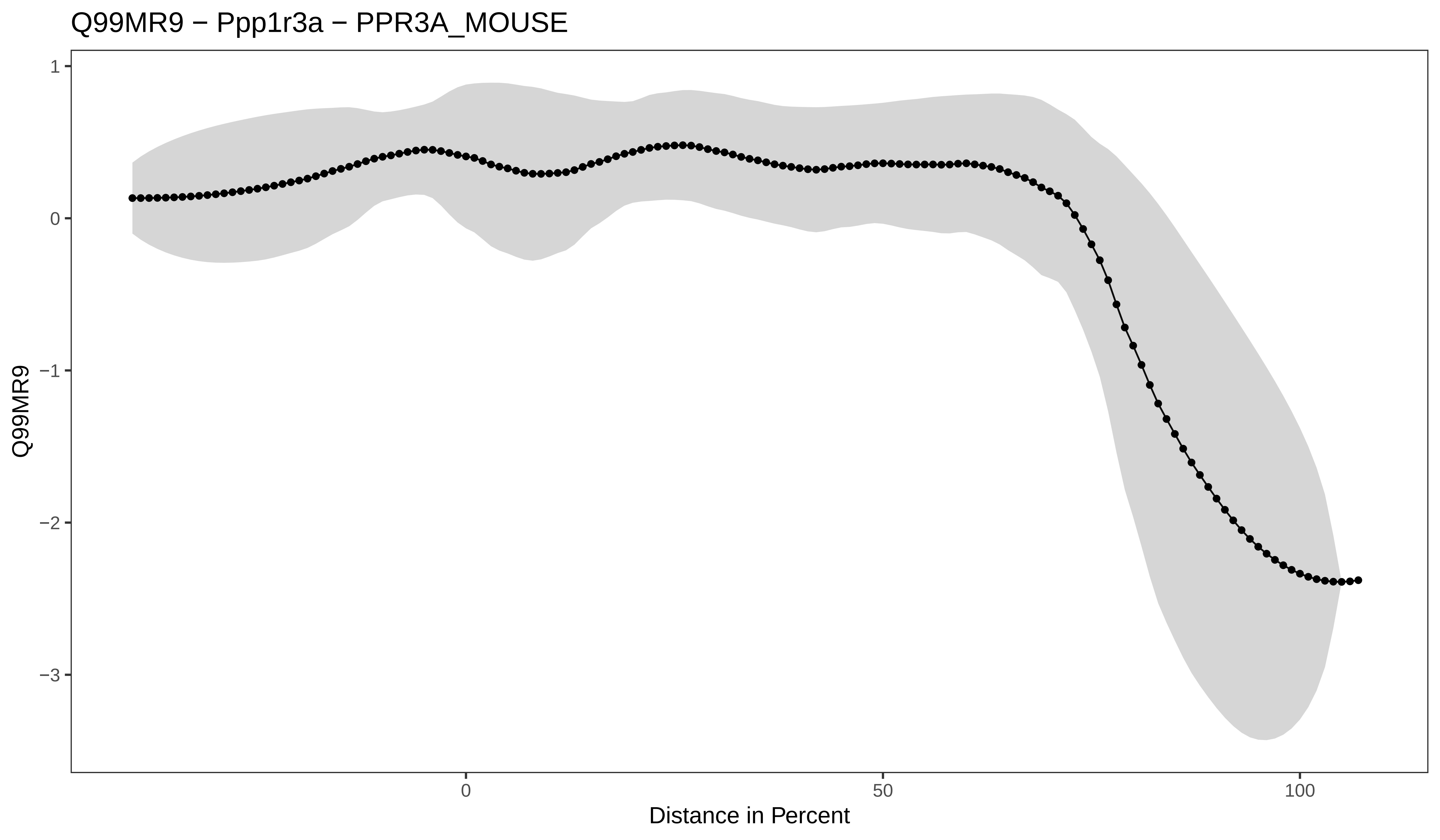

Q99MR9 − Ppp1r3a − PPR3A_MOUSE
1
0
−1
−2
−3
Q99MR9
0
50
100
Distance in Percent

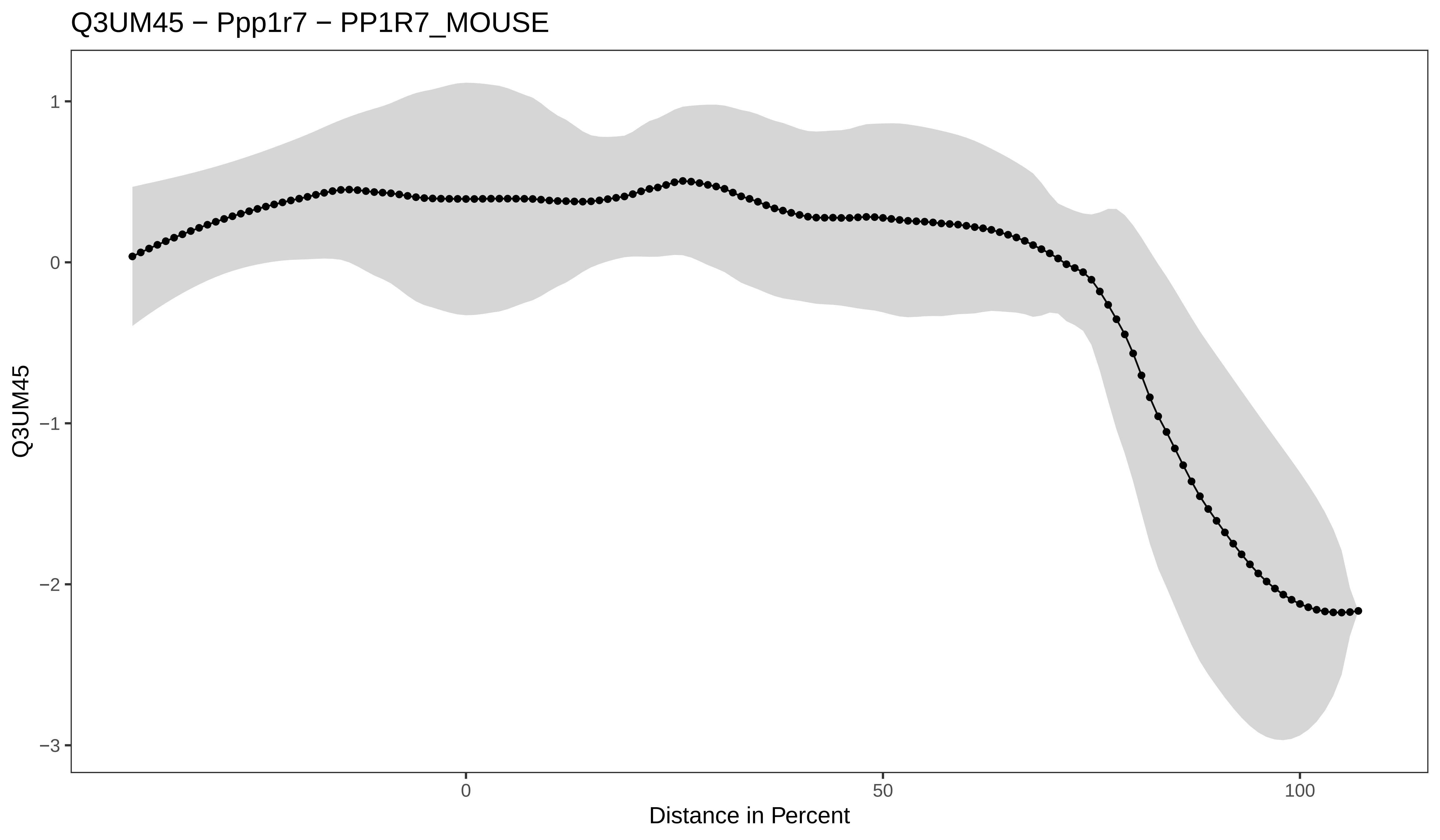
Q3UM45 − Ppp1r7 − PP1R7_MOUSE
1
0
−1
−2
−3
Q3UM45
0
50
100
Distance in Percent

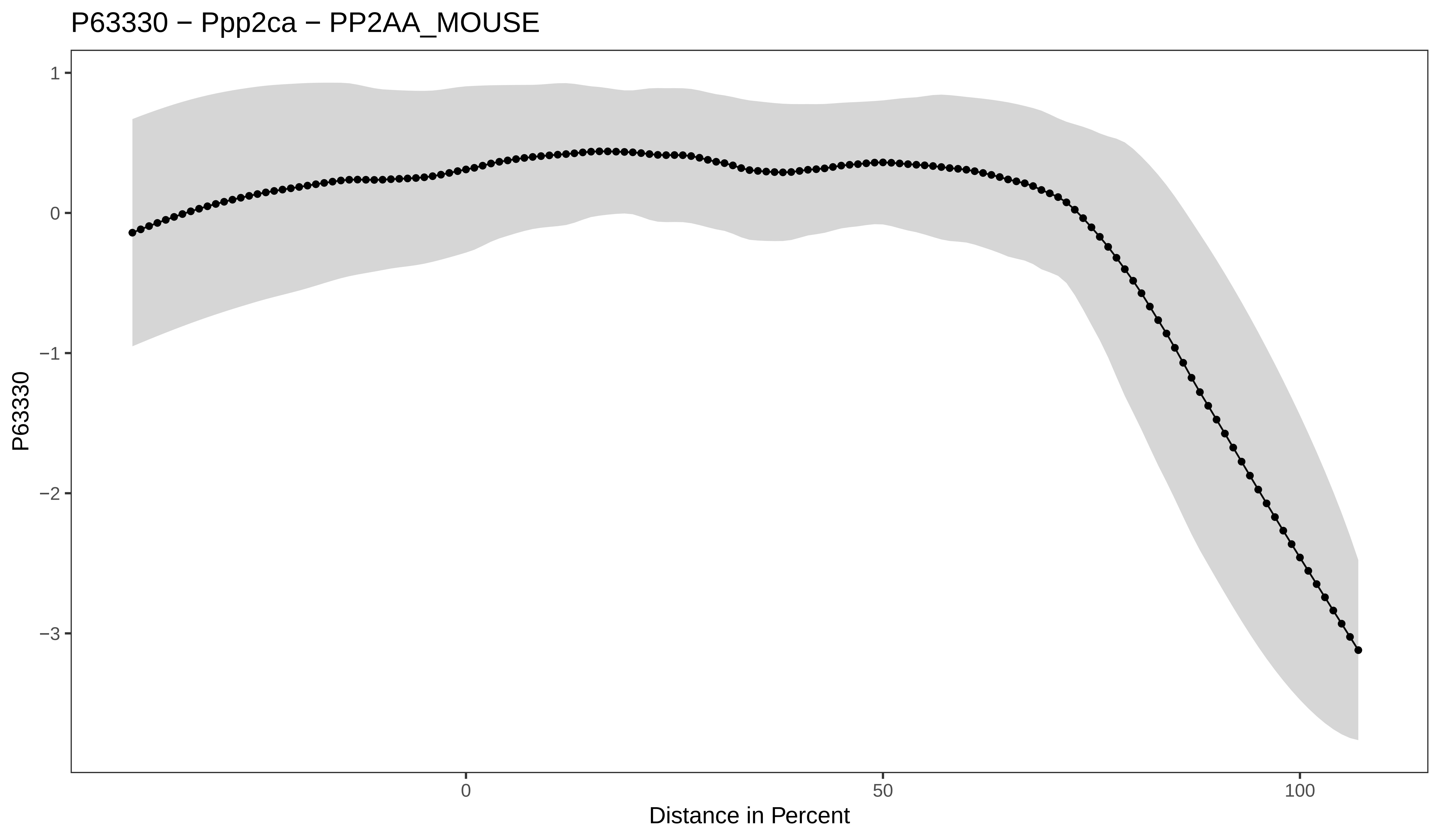
P63330 − Ppp2ca − PP2AA_MOUSE
1
0
−1
−2
−3
P63330
0
50
100
Distance in Percent

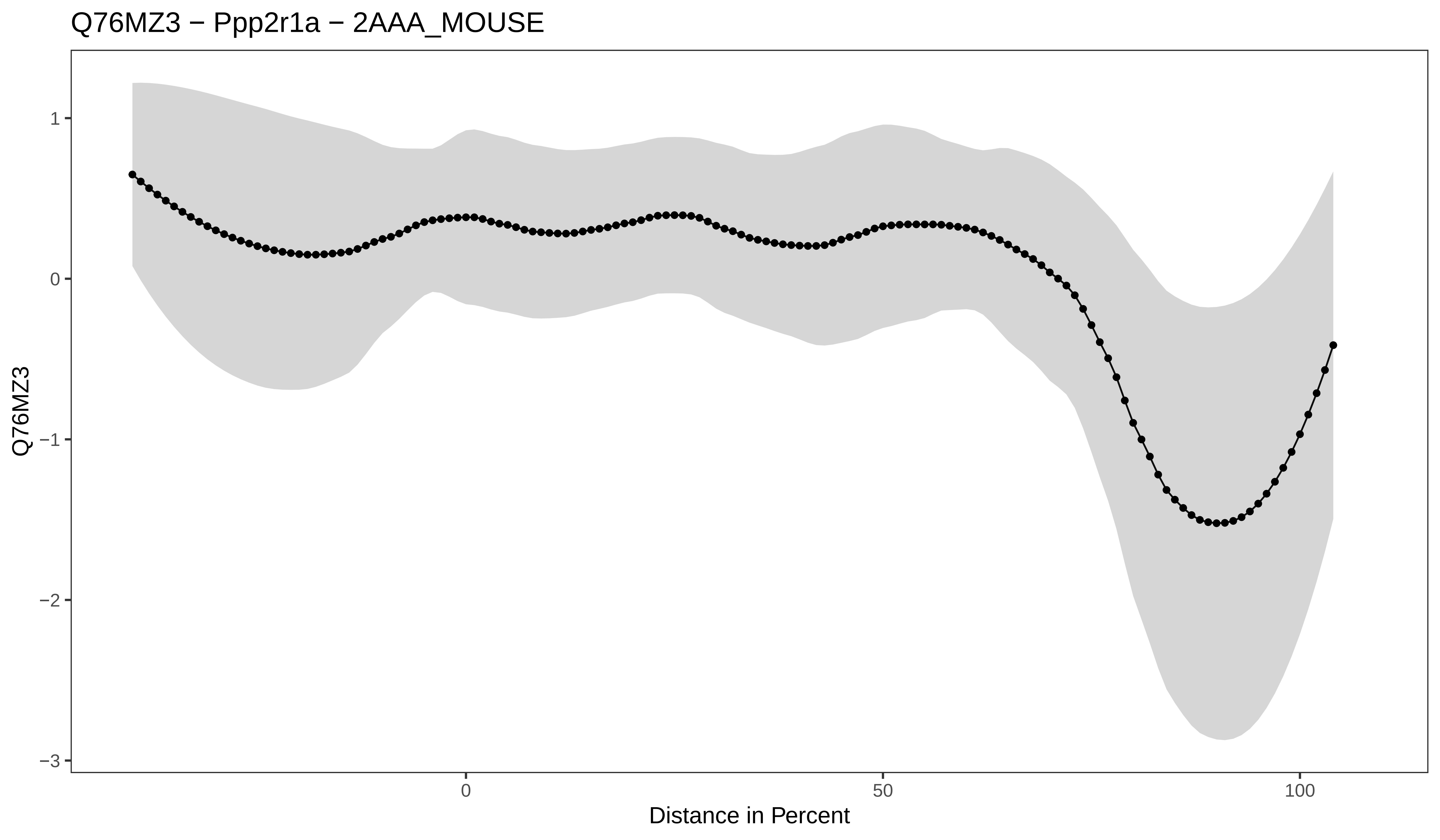
Q76MZ3 − Ppp2r1a − 2AAA_MOUSE
Q76MZ3
Distance in Percent
1
0
−1
−2
−3
0
50
100

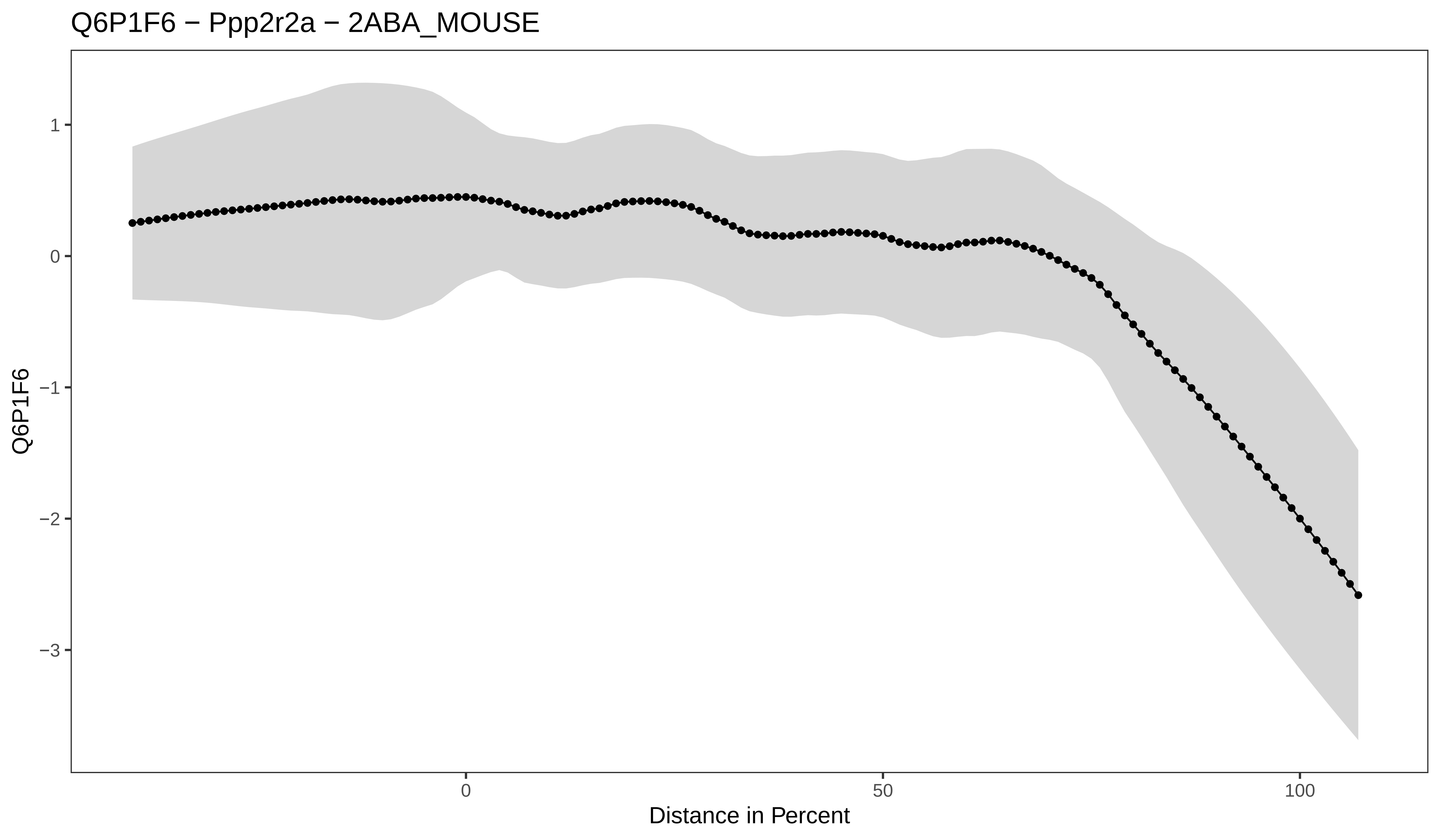

Q6P1F6 − Ppp2r2a − 2ABA_MOUSE
Q6P1F6
Distance in Percent
1
0
−1
−2
−3
0
50
100

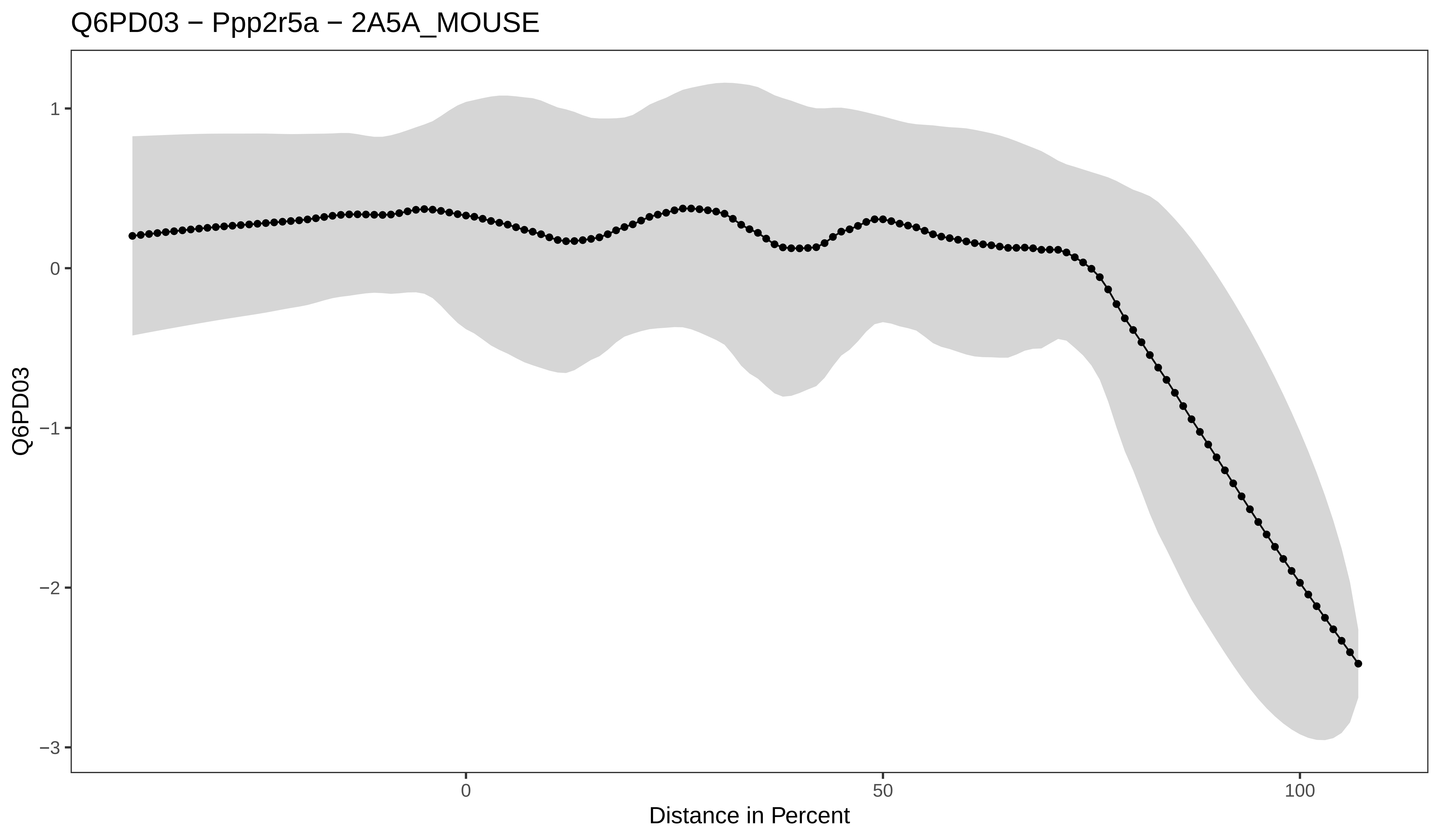

Q6PD03 − Ppp2r5a − 2A5A_MOUSE
Q6PD03
1
0
−1
−2
−3
0
50
100
Distance in Percent

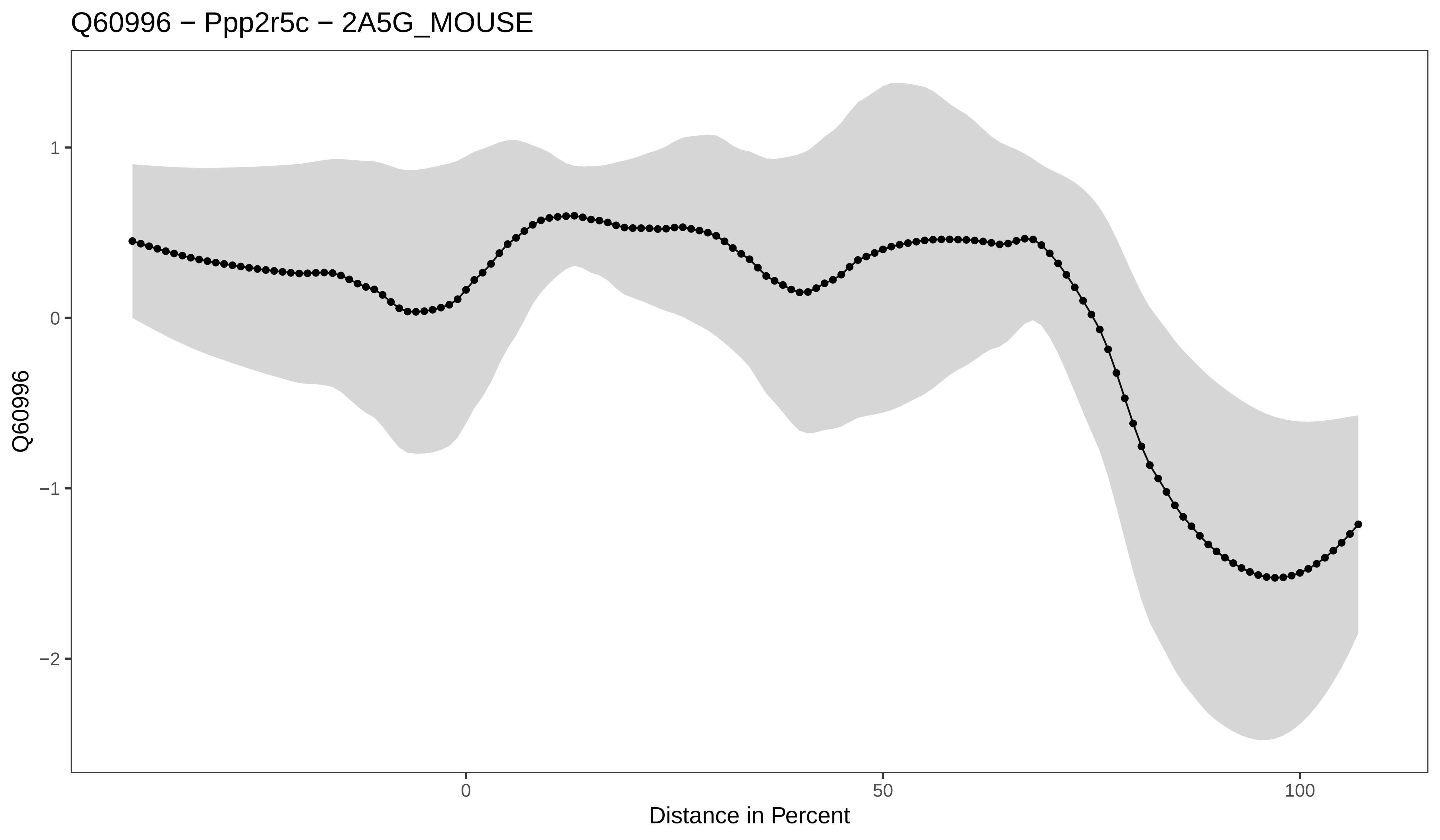

Q60996 − Ppp2r5c − 2A5G_MOUSE
Q60996
1
0
−1
−2
0
50
100
Distance in Percent

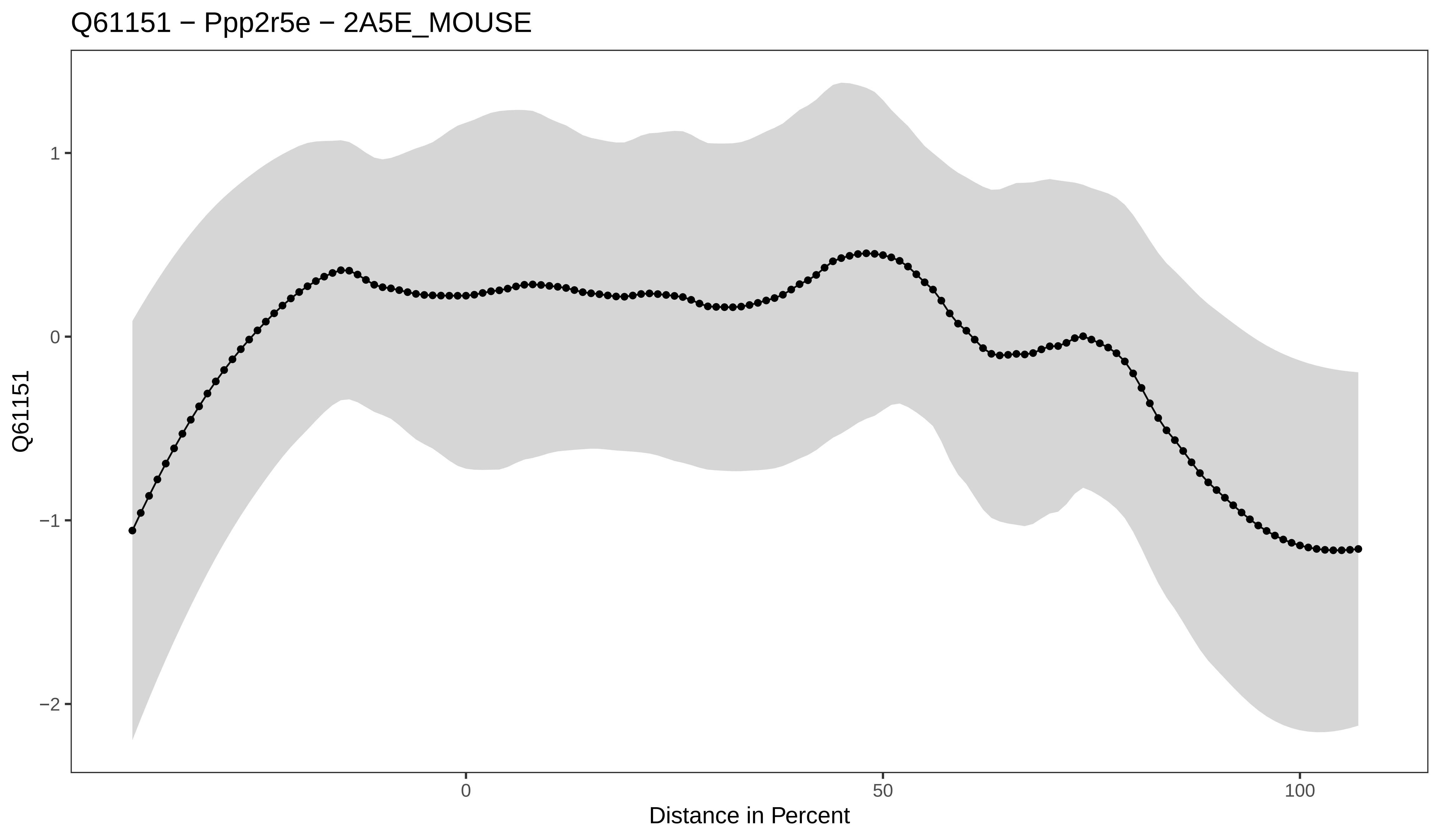

Q61151 − Ppp2r5e − 2A5E_MOUSE
Q61151
1
0
−1
−2
0
50
100
Distance in Percent

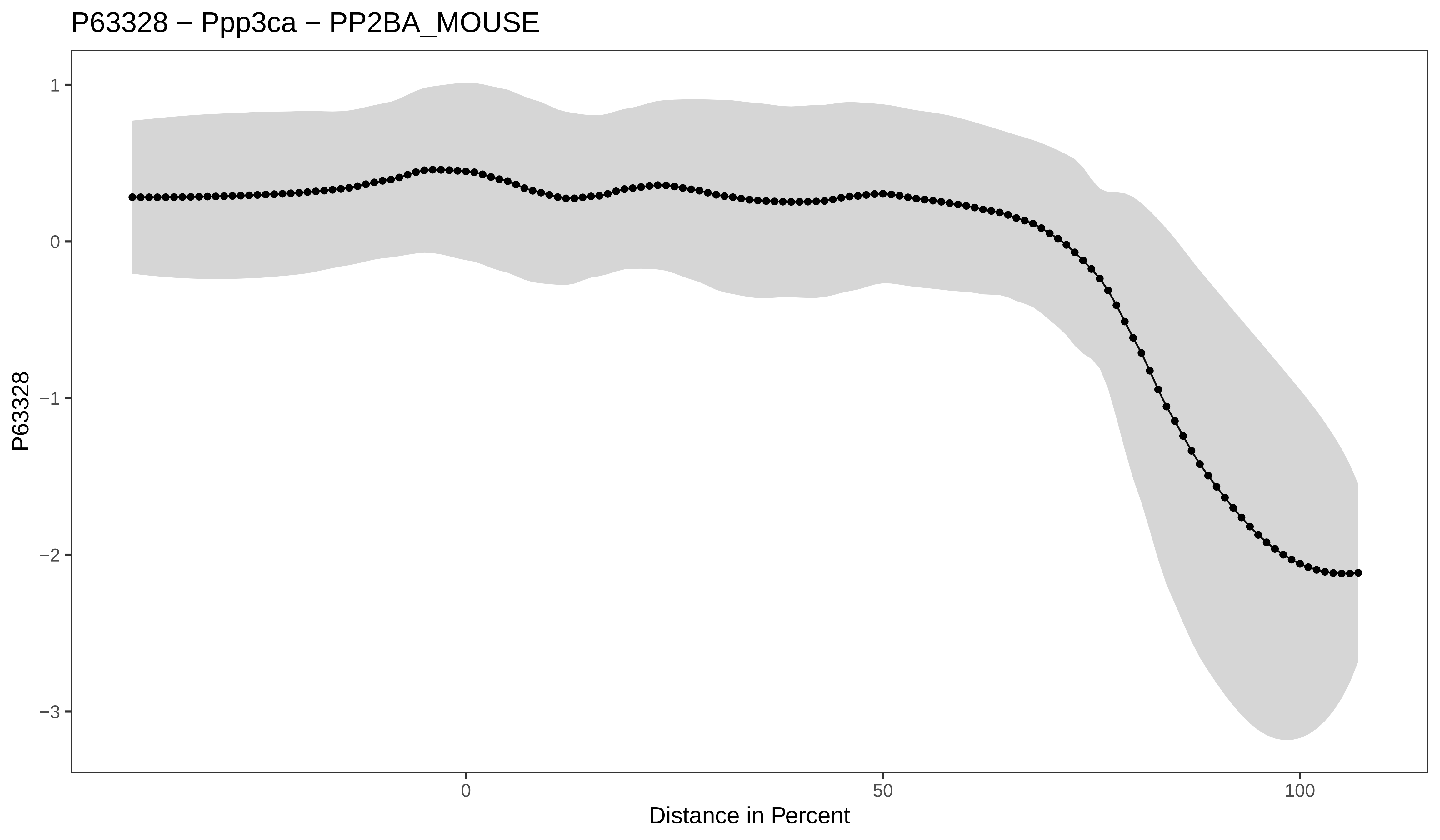

P63328 − Ppp3ca − PP2BA_MOUSE
P63328
Distance in Percent
1
0
−1
−2
−3
0
50
100

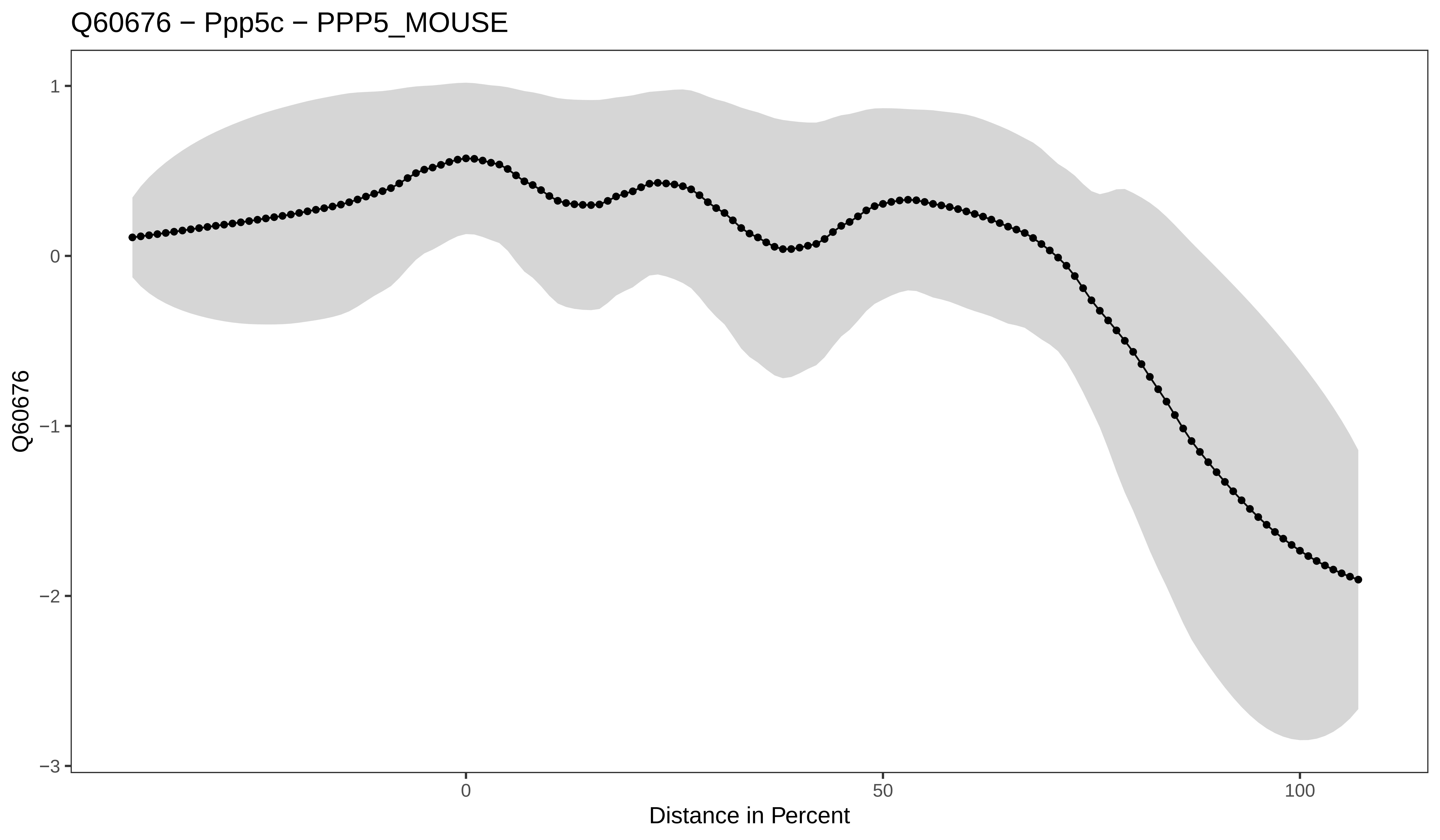

Q60676 − Ppp5c − PPP5_MOUSE
Q60676
Distance in Percent
1
0
−1
−2
−3
0
50
100

Q9CQR6 − Ppp6c − PPP6_MOUSE

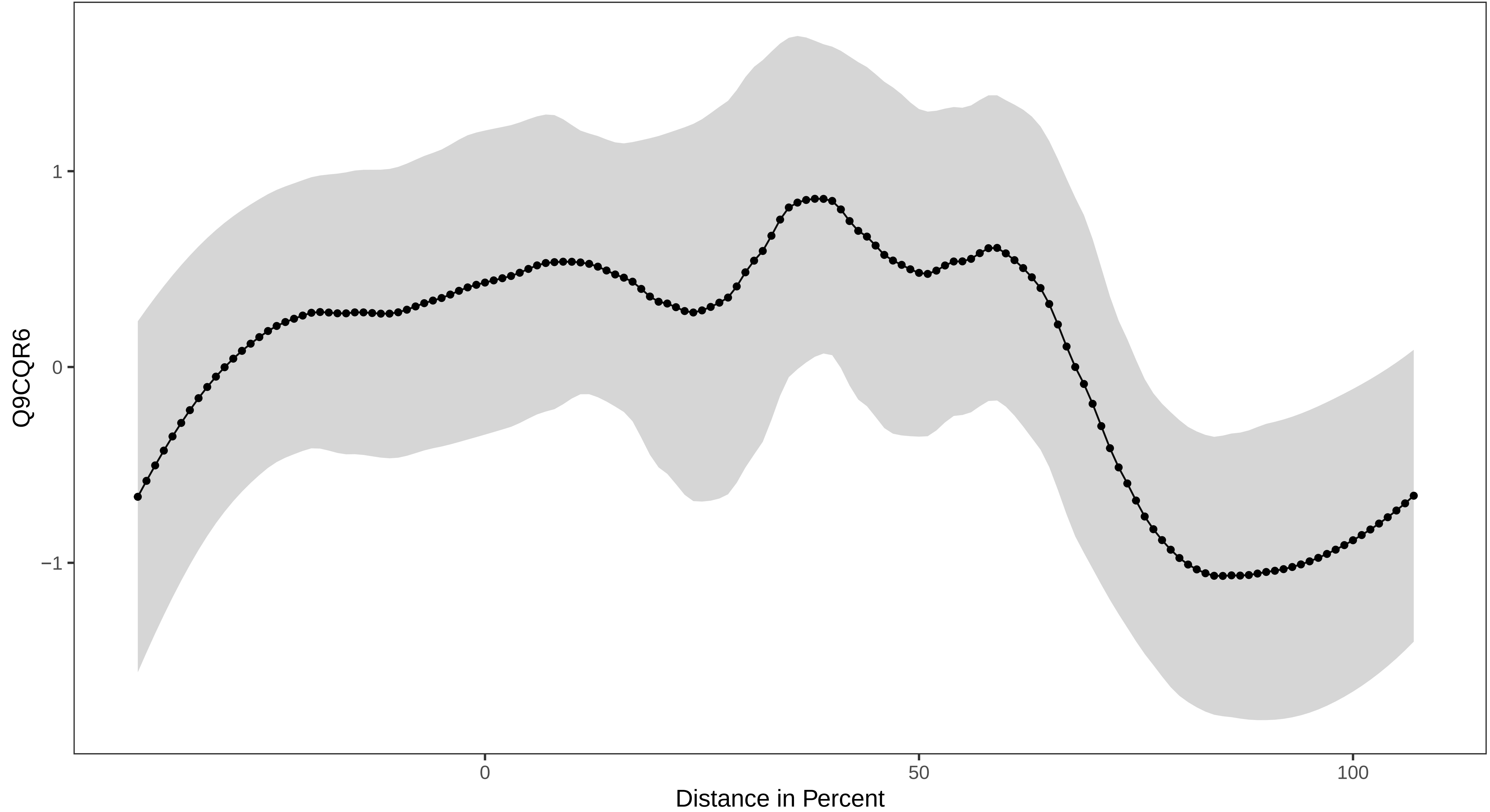

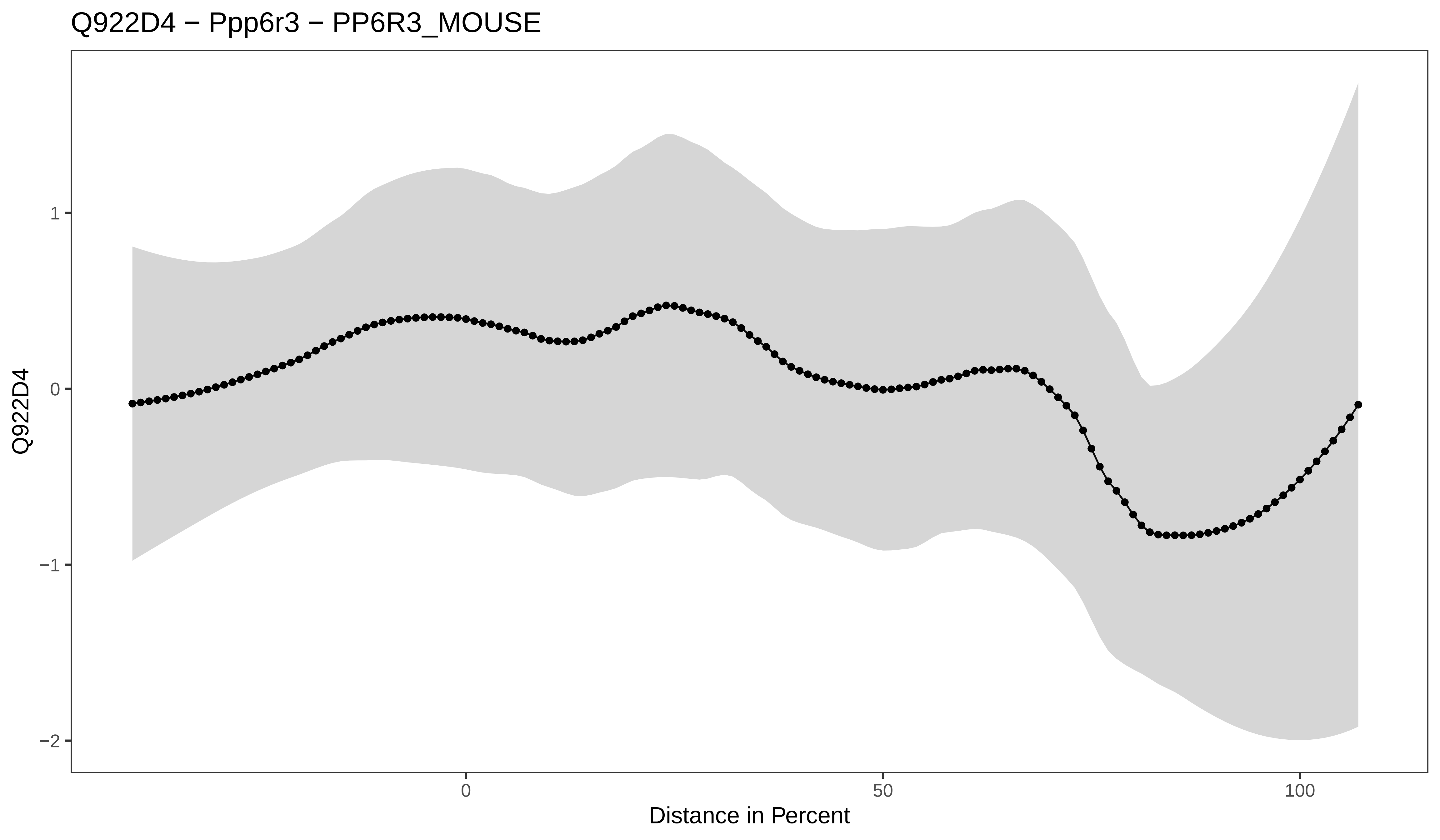

Q922D4 − Ppp6r3 − PP6R3_MOUSE
Q922D4
1
0
−1
−2
0
50
100
Distance in Percent

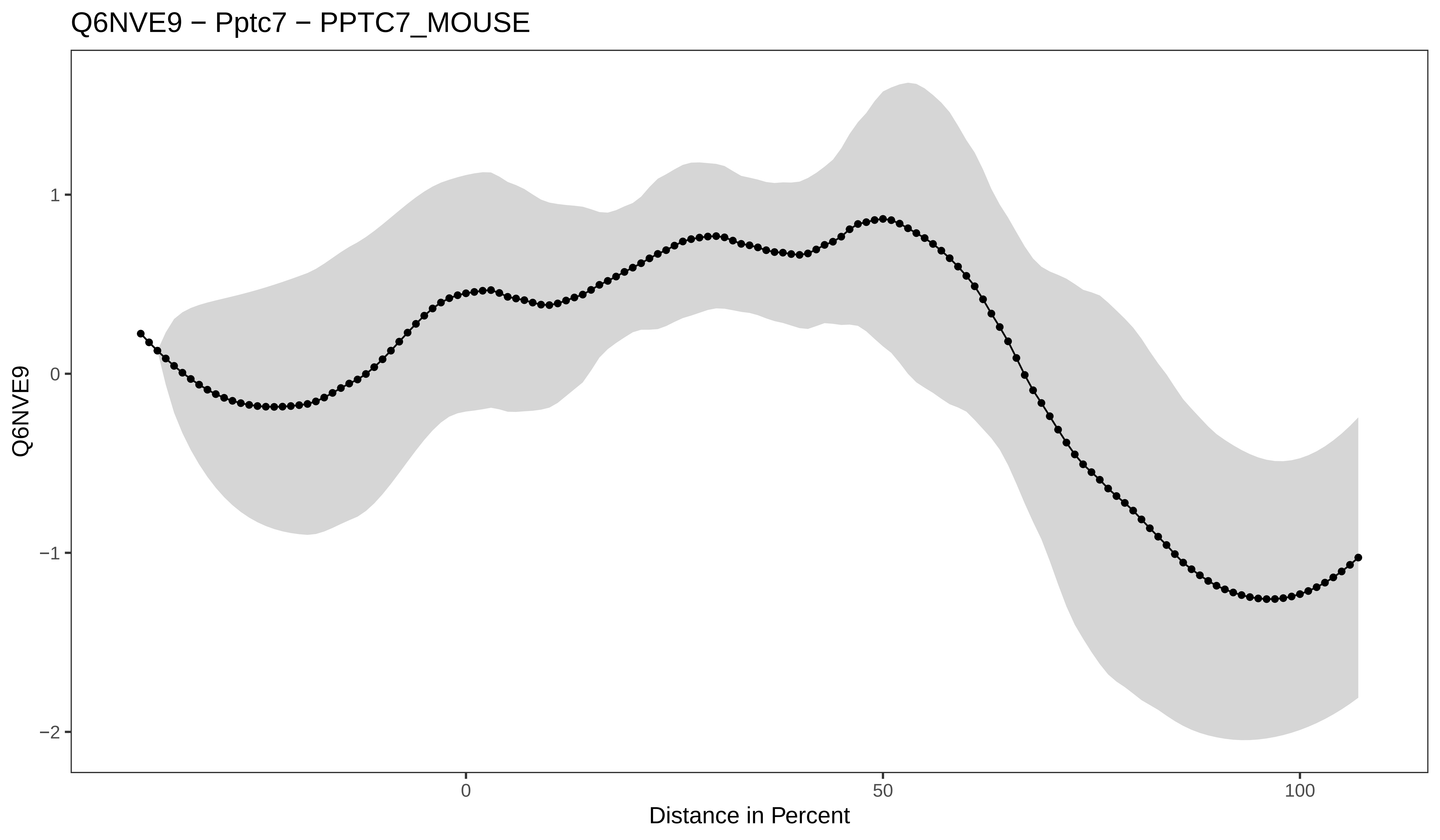

Q6NVE9 − Pptc7 − PPTC7_MOUSE
Q6NVE9
1
0
−1
−2
0
50
100
Distance in Percent

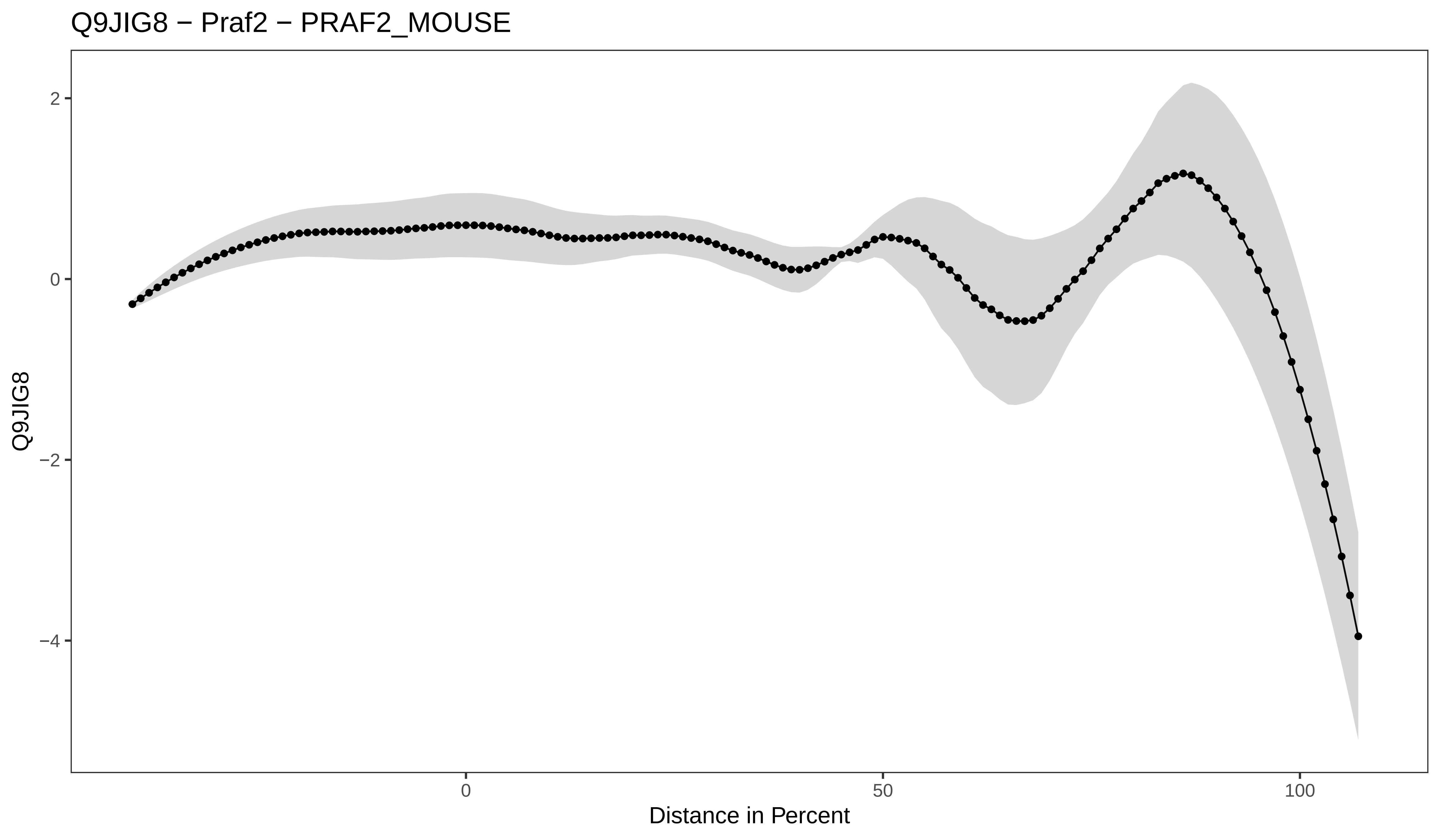

Q9JIG8 − Praf2 − PRAF2_MOUSE
Q9JIG8
2
0
−2
−4
0
50
100
Distance in Percent

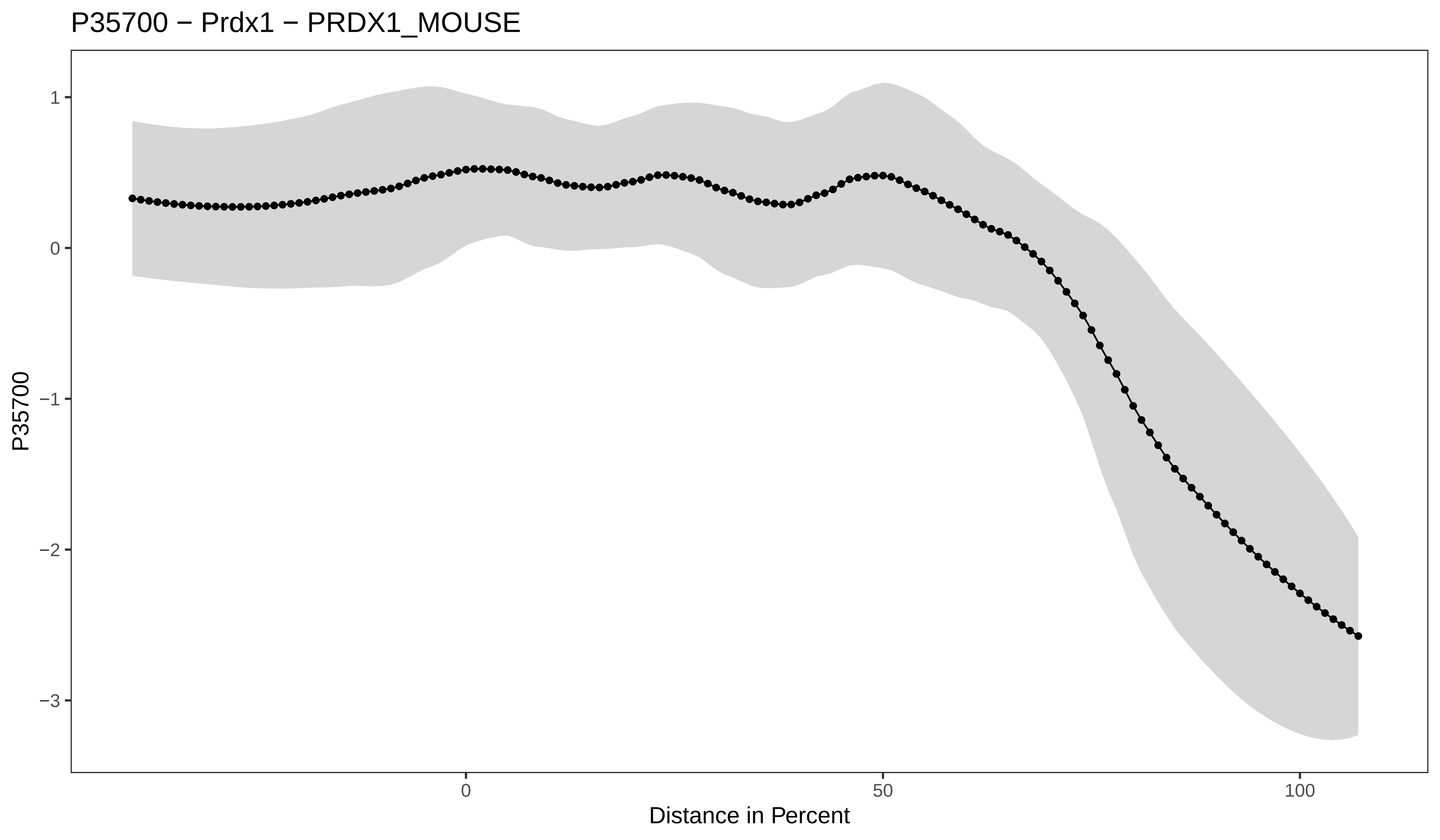

P35700 − Prdx1 − PRDX1_MOUSE
1
0
−1
−2
−3
P35700
0
50
100
Distance in Percent

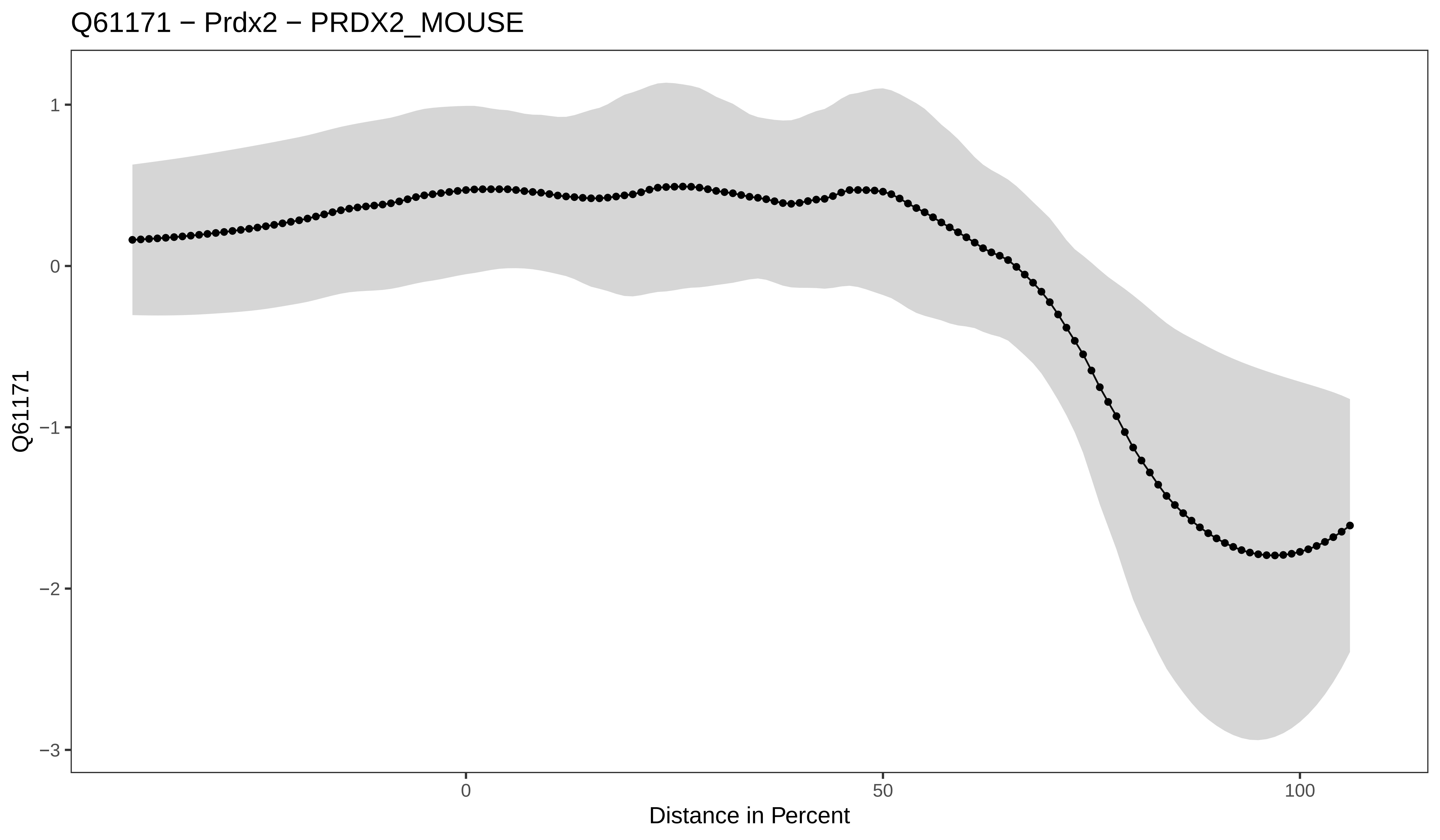

Q61171 − Prdx2 − PRDX2_MOUSE
1
0
−1
−2
−3
Q61171
0
50
100
Distance in Percent

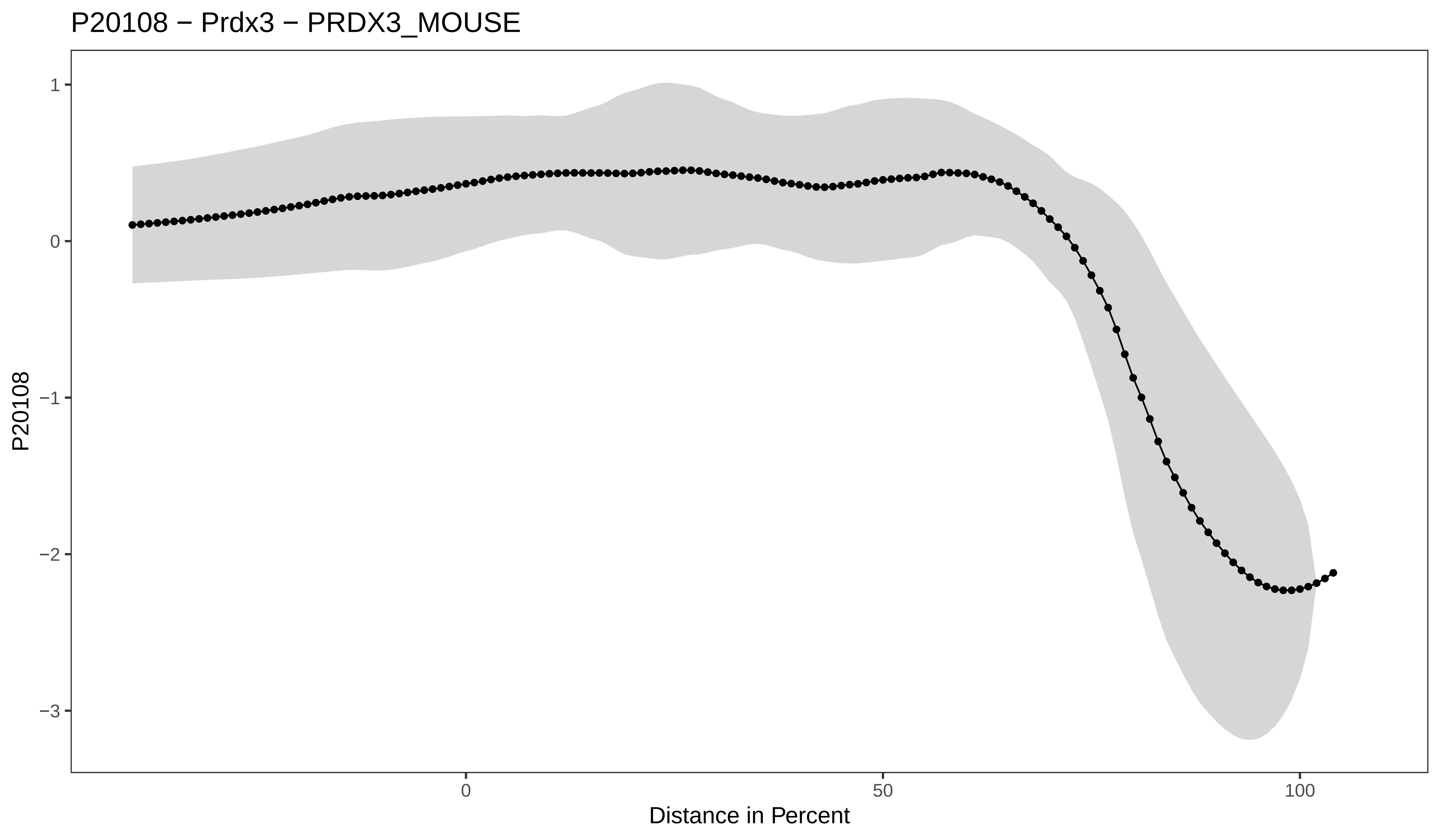
P20108 − Prdx3 − PRDX3_MOUSE
P20108
Distance in Percent
1
0
−1
−2
−3
0
50
100

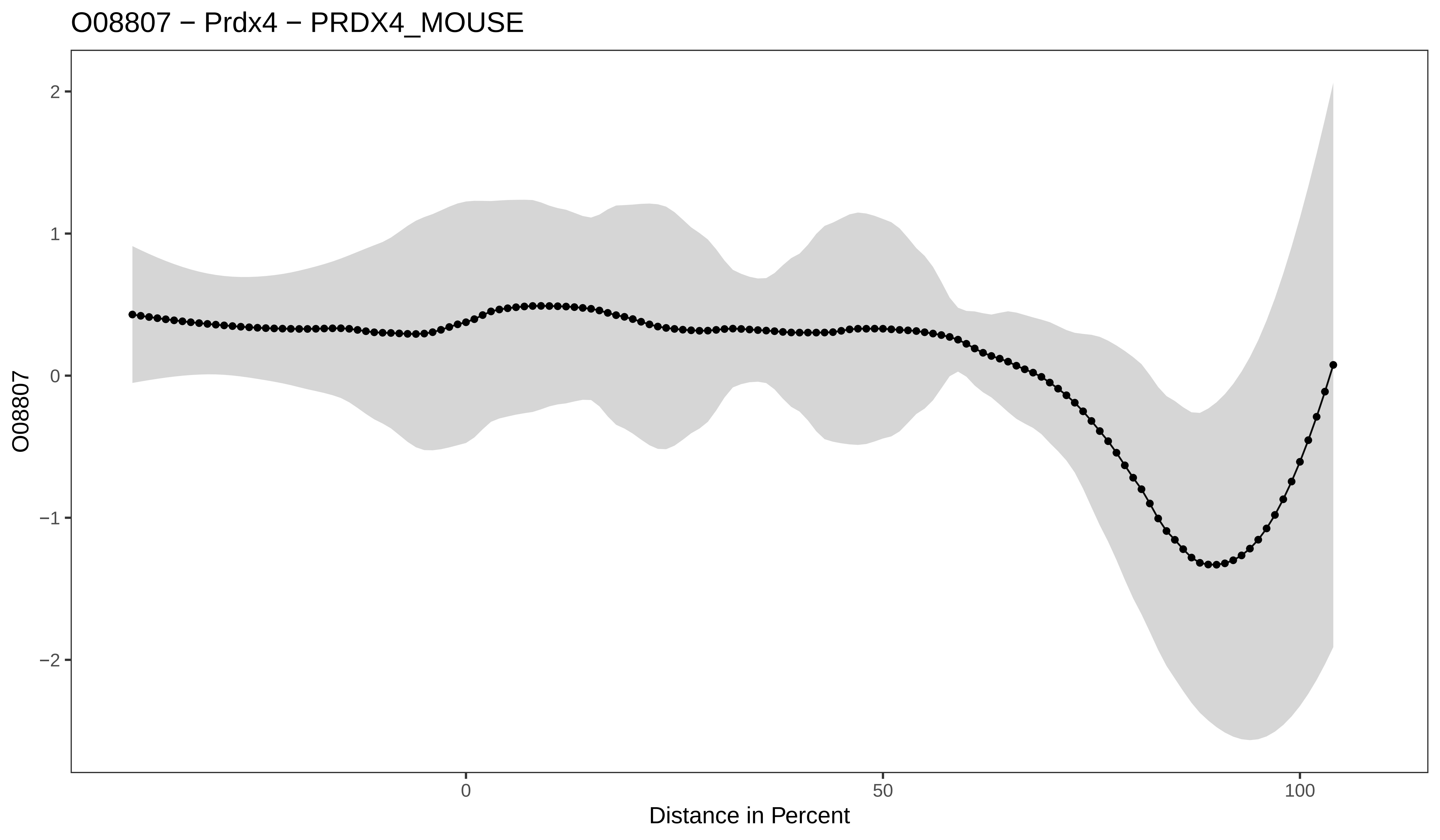

O08807 − Prdx4 − PRDX4_MOUSE
2
1
0
−1
−2
O08807
0
50
100
Distance in Percent

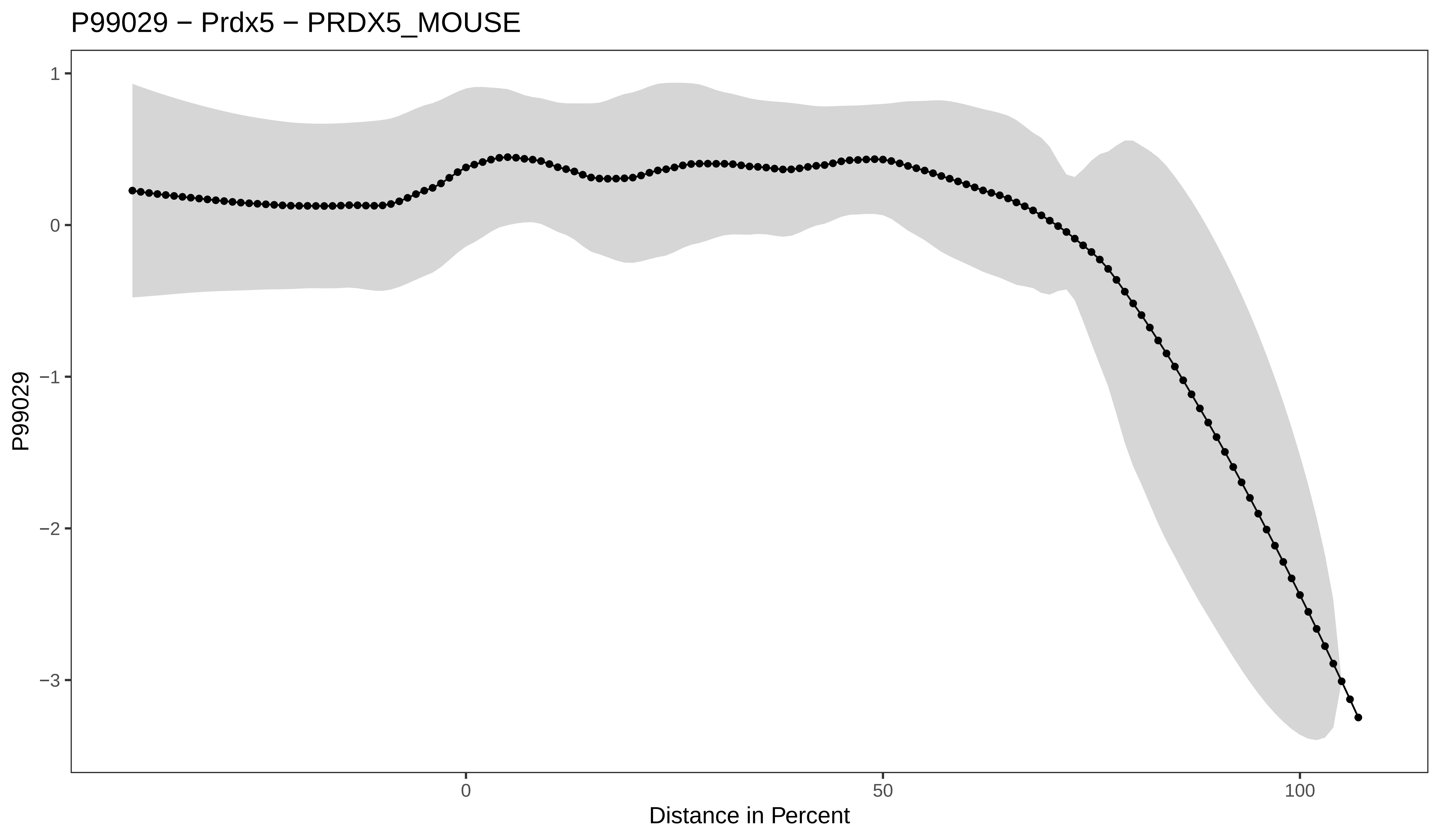

P99029 − Prdx5 − PRDX5_MOUSE
P99029
Distance in Percent
1
0
−1
−2
−3
0
50
100

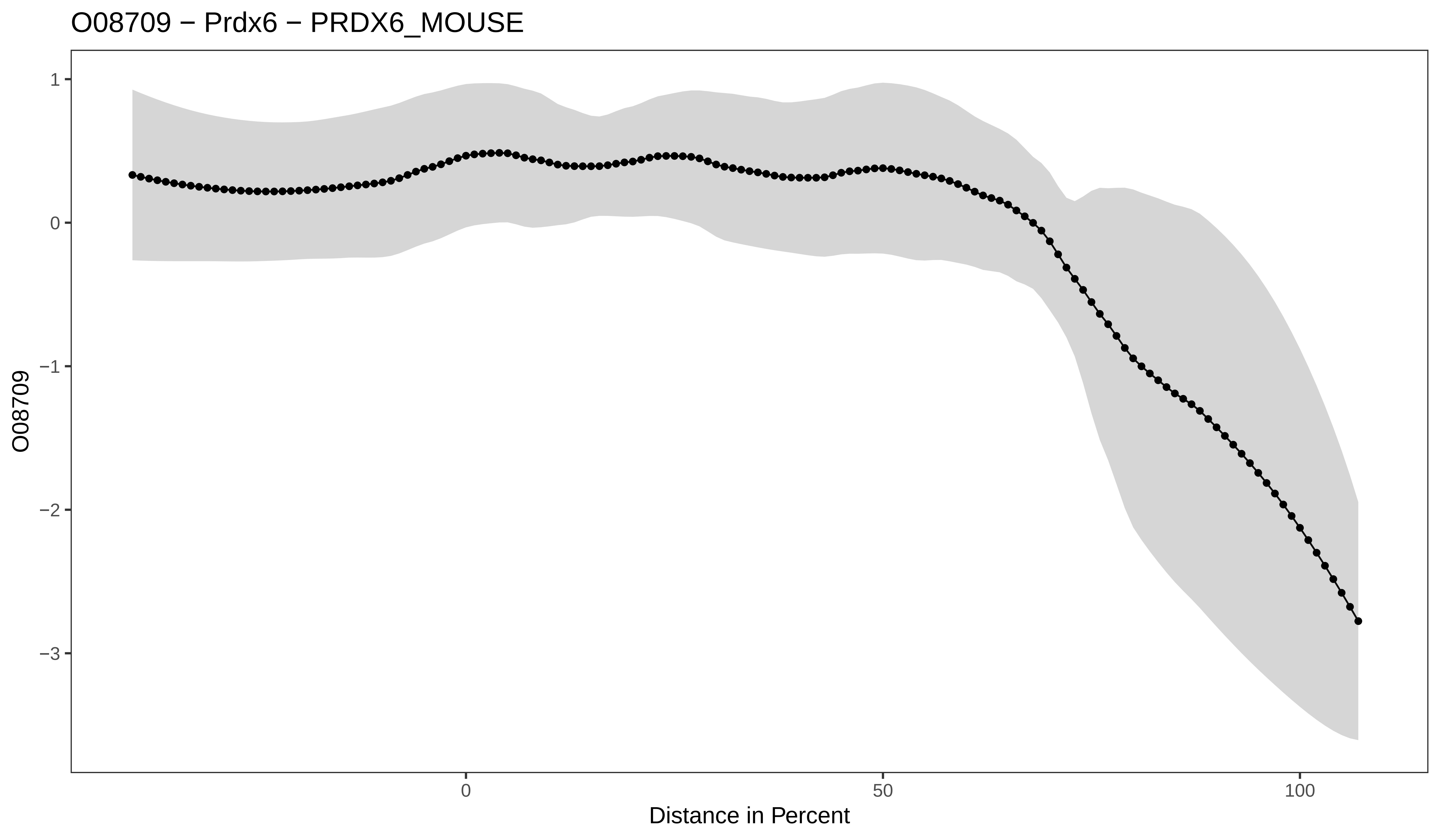
O08709 − Prdx6 − PRDX6_MOUSE
1
0
−1
−2
−3
O08709
0
50
100
Distance in Percent

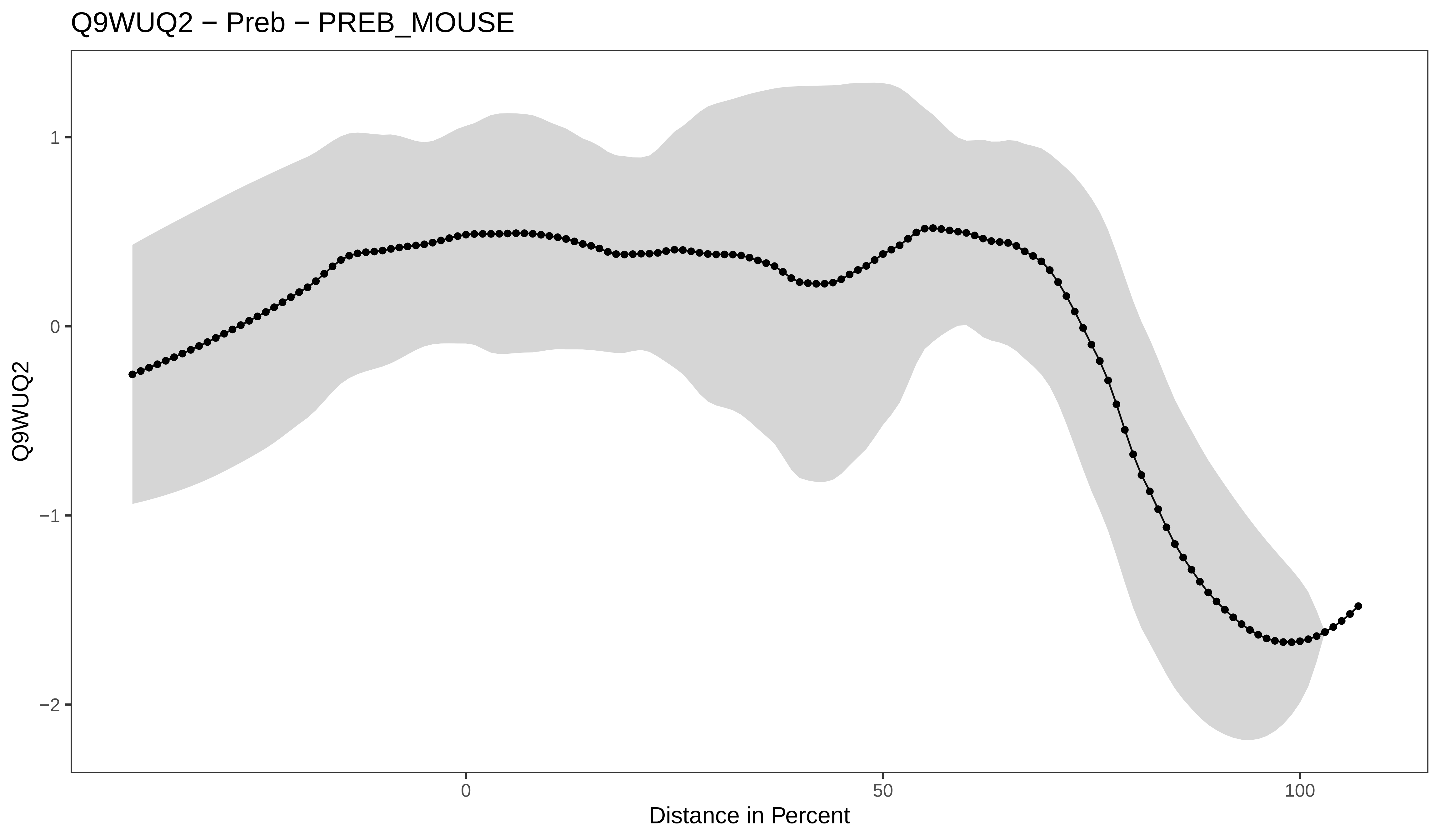

Q9WUQ2 − Preb − PREB_MOUSE
Q9WUQ2
Distance in Percent
1
0
−1
−2
0
50
100

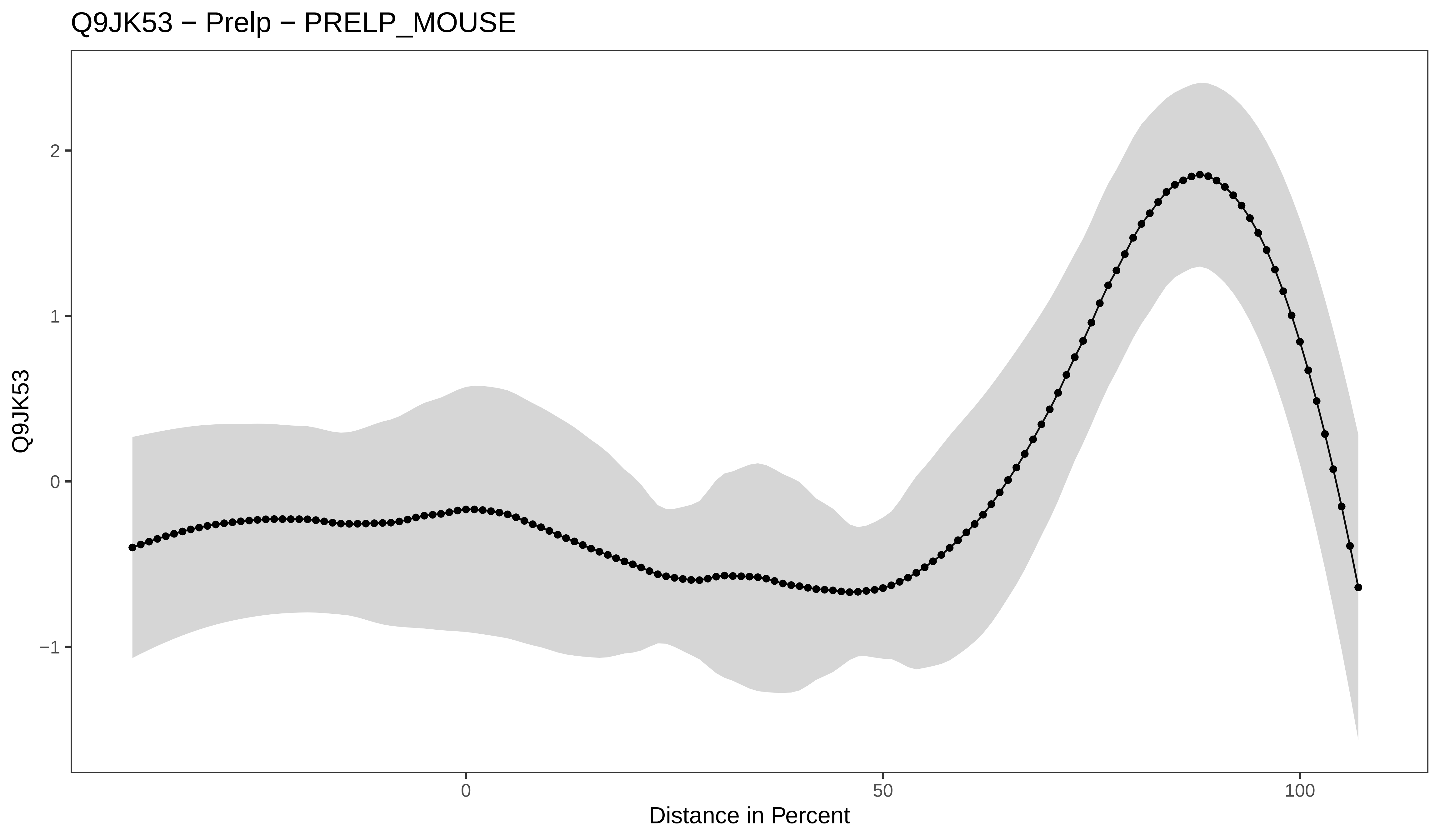
Q9JK53 − Prelp − PRELP_MOUSE
Q9JK53
Distance in Percent
2
1
0
−1
0
50
100

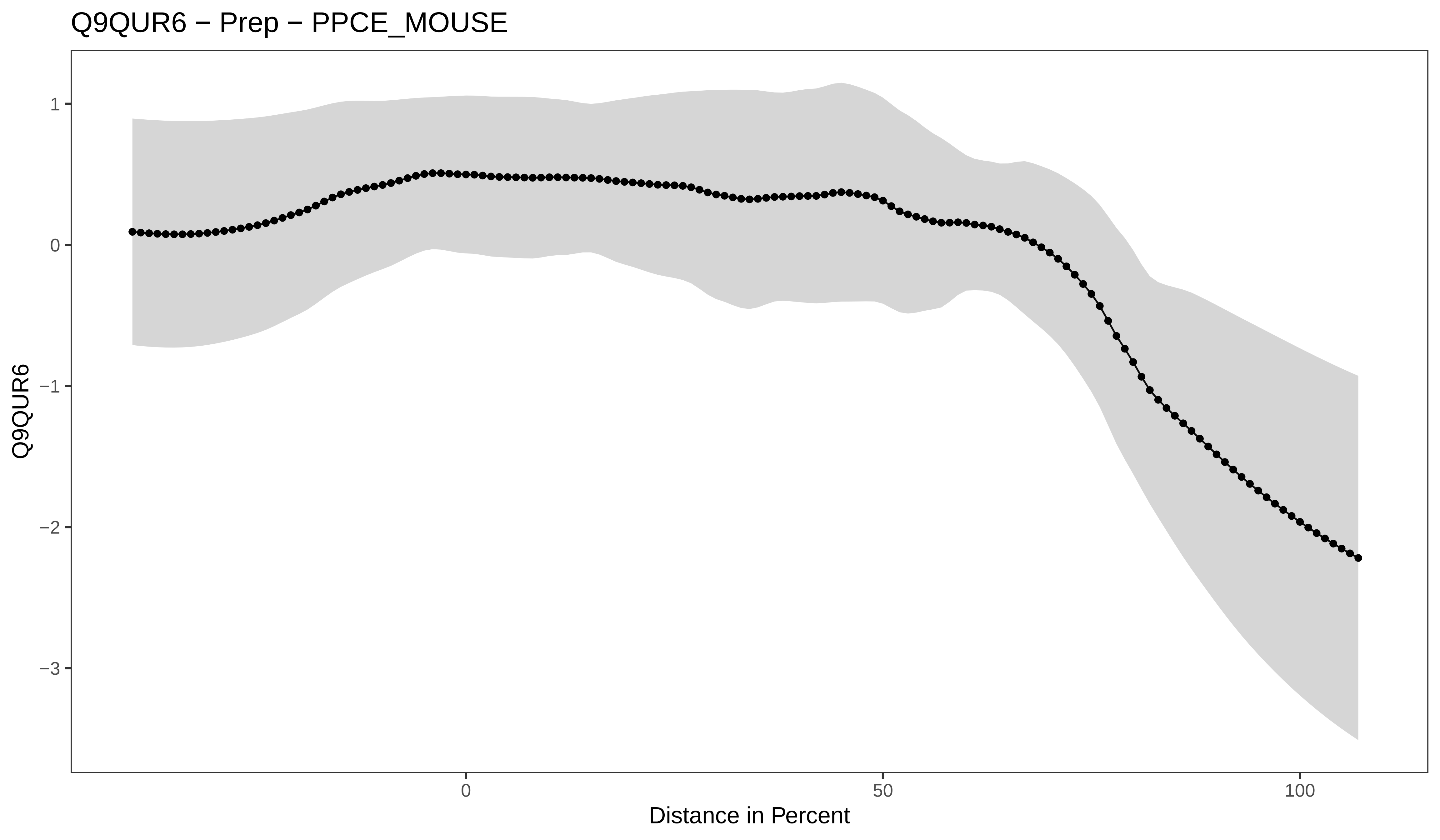
Q9QUR6 − Prep − PPCE_MOUSE
1
0
−1
−2
−3
Q9QUR6
0
50
100
Distance in Percent

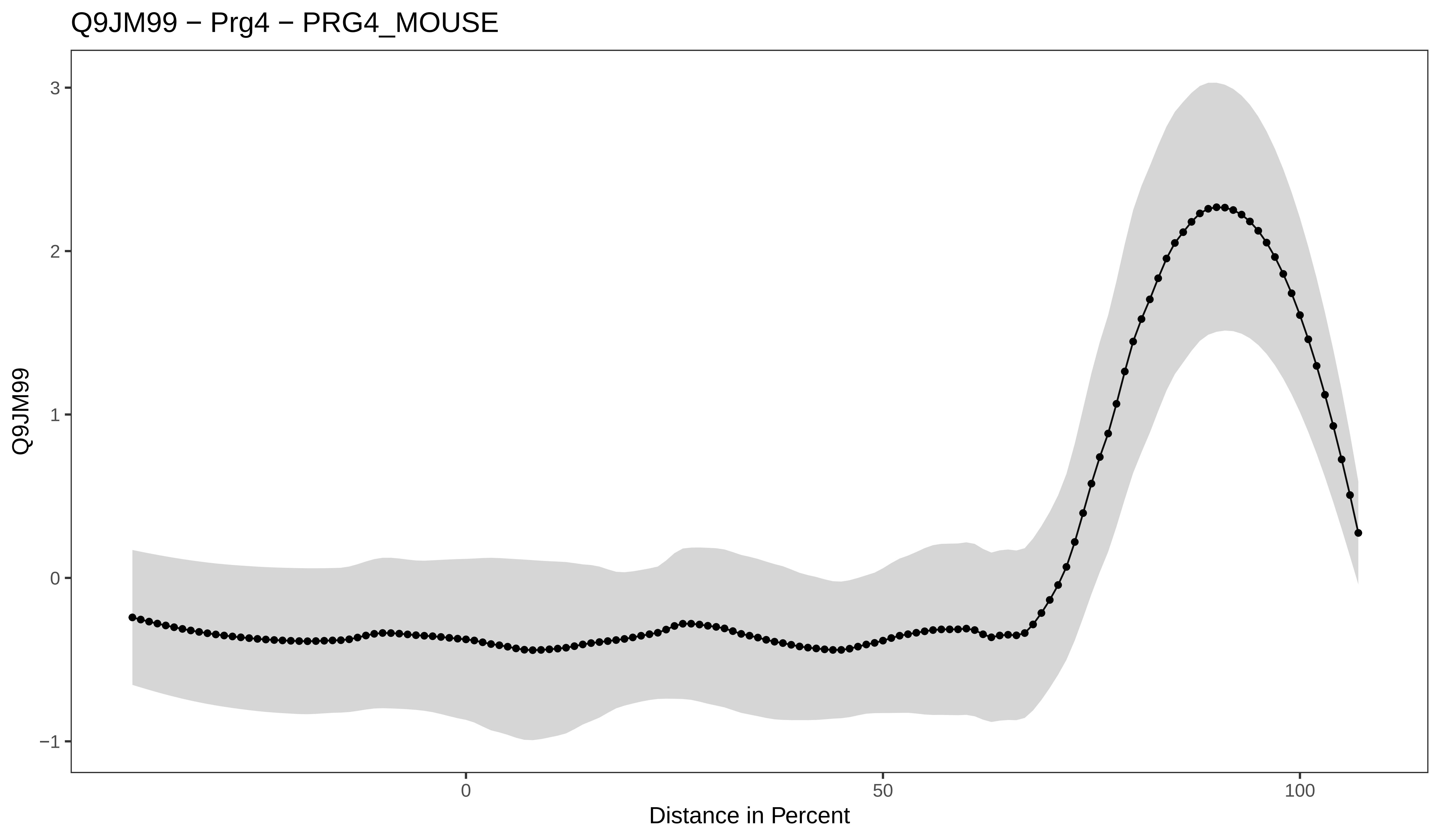
Q9JM99 − Prg4 − PRG4_MOUSE
3
2
1
0
−1
Q9JM99
0
50
100
Distance in Percent

Q5EG47 − Prkaa1 − AAPK1_MOUSE

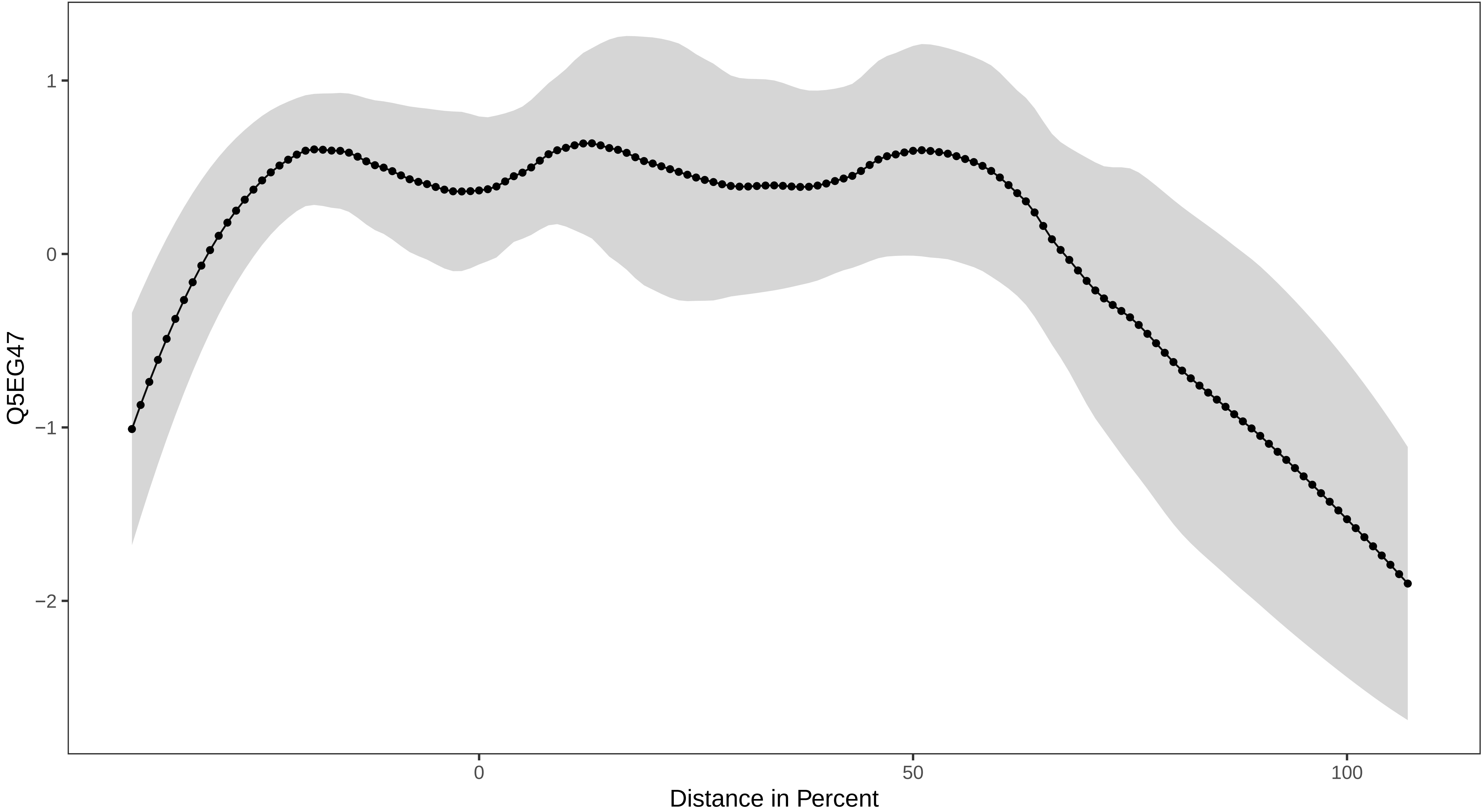

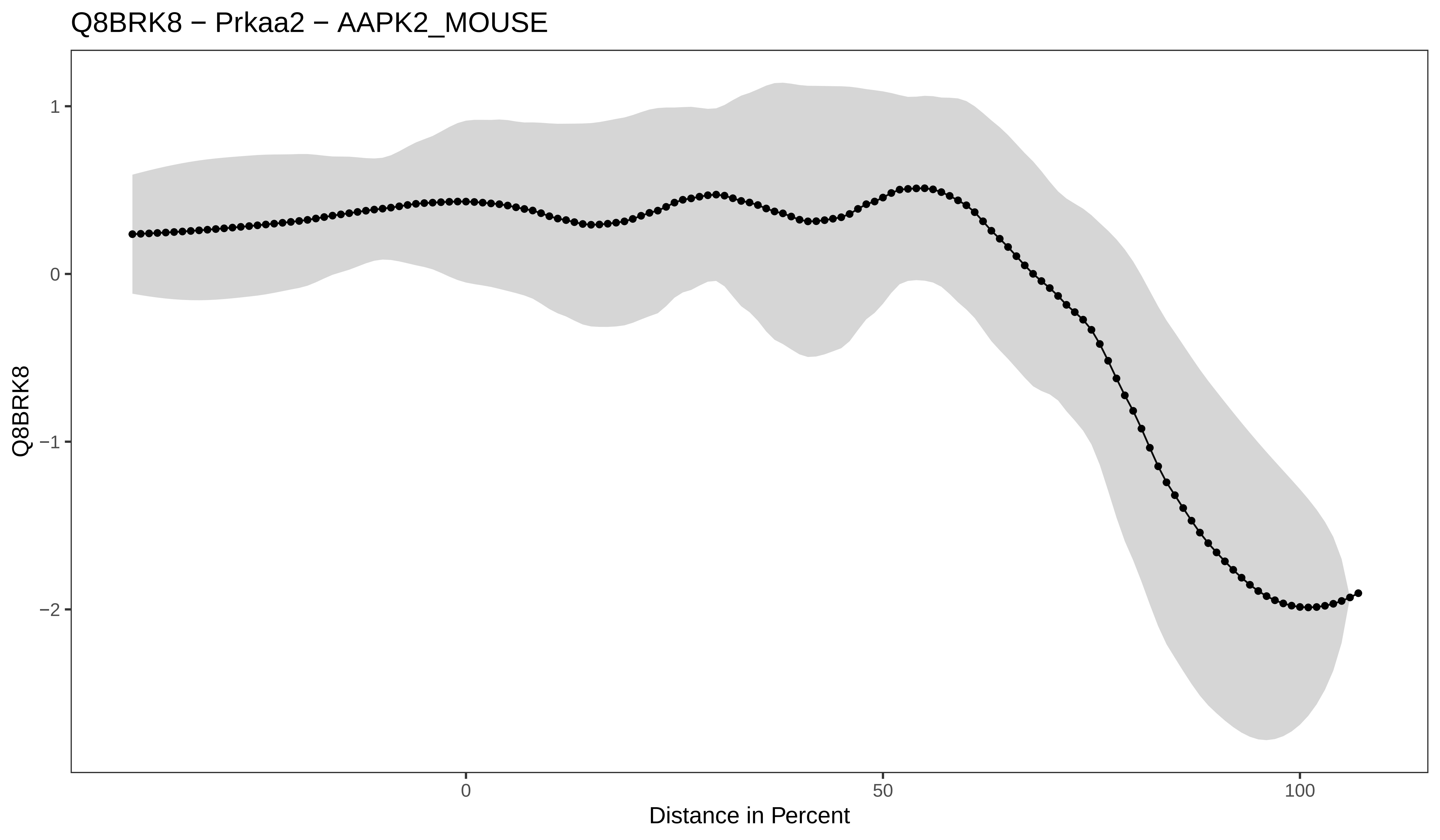

Q8BRK8 − Prkaa2 − AAPK2_MOUSE
1
0
−1
−2
Q8BRK8
0
50
100
Distance in Percent

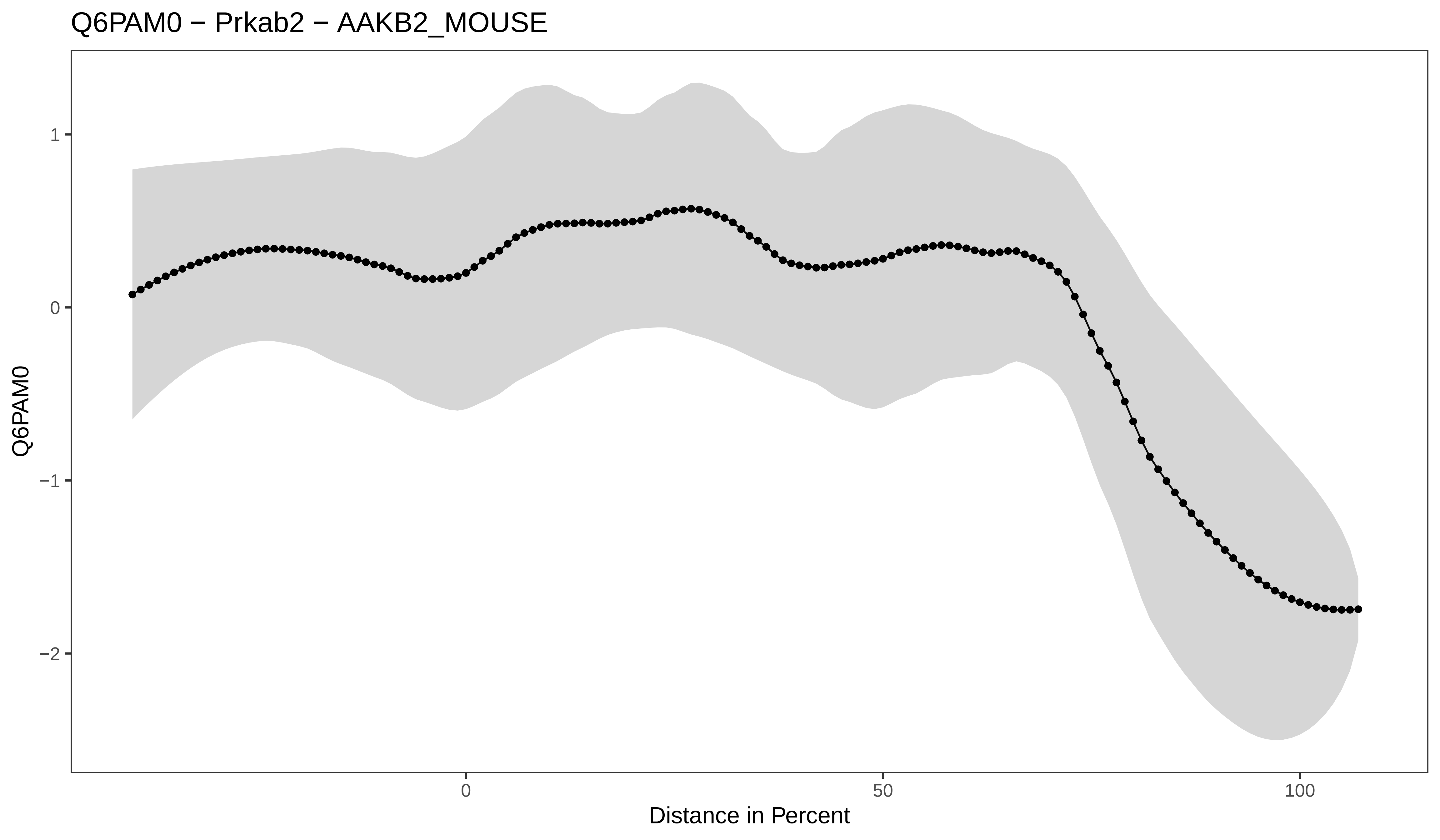

Q6PAM0 − Prkab2 − AAKB2_MOUSE
Q6PAM0
1
0
−1
−2
0
50
100
Distance in Percent

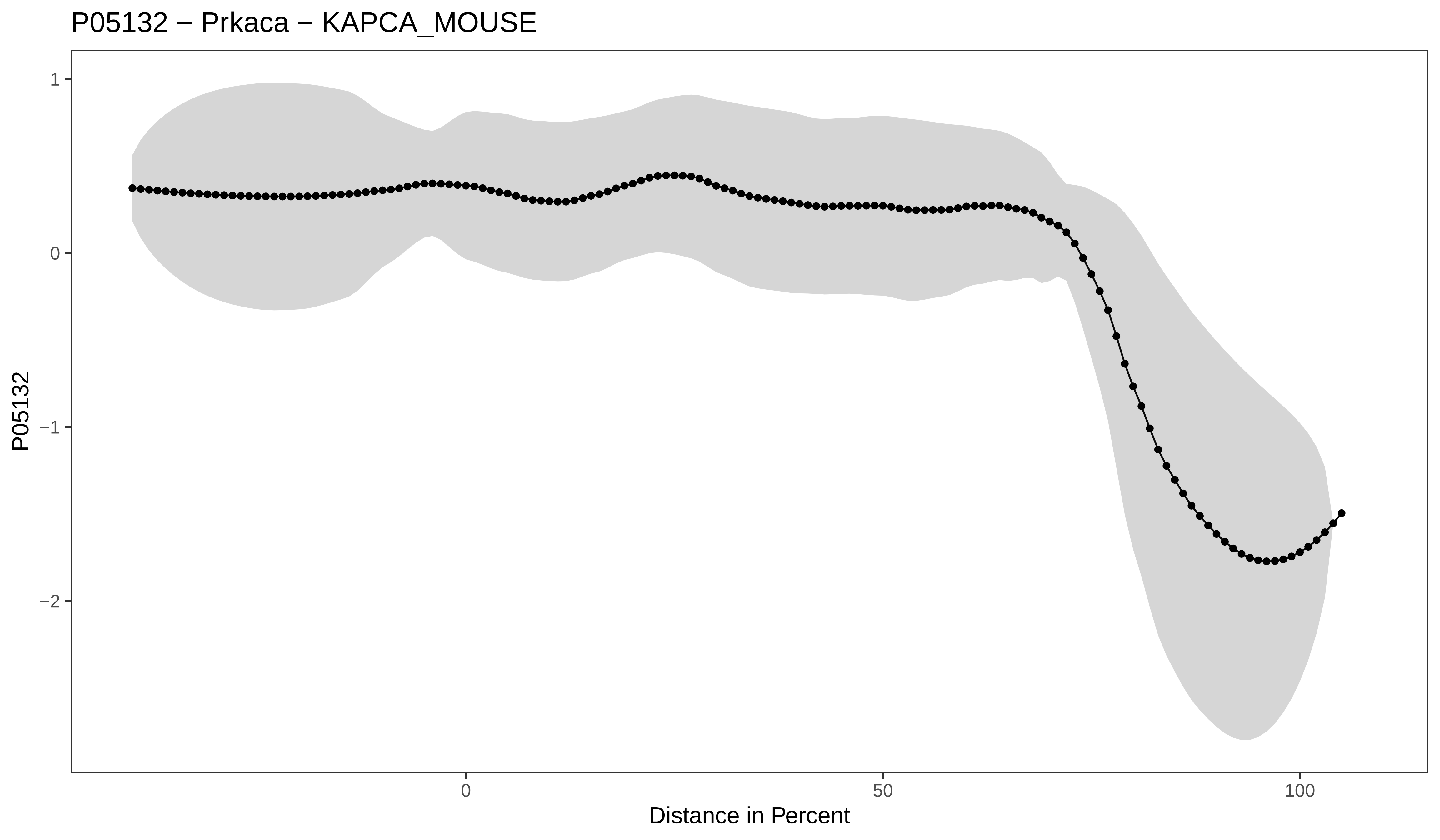
P05132 − Prkaca − KAPCA_MOUSE
1
0
−1
−2
P05132
0
50
100
Distance in Percent

P68181 − Prkacb − KAPCB_MOUSE

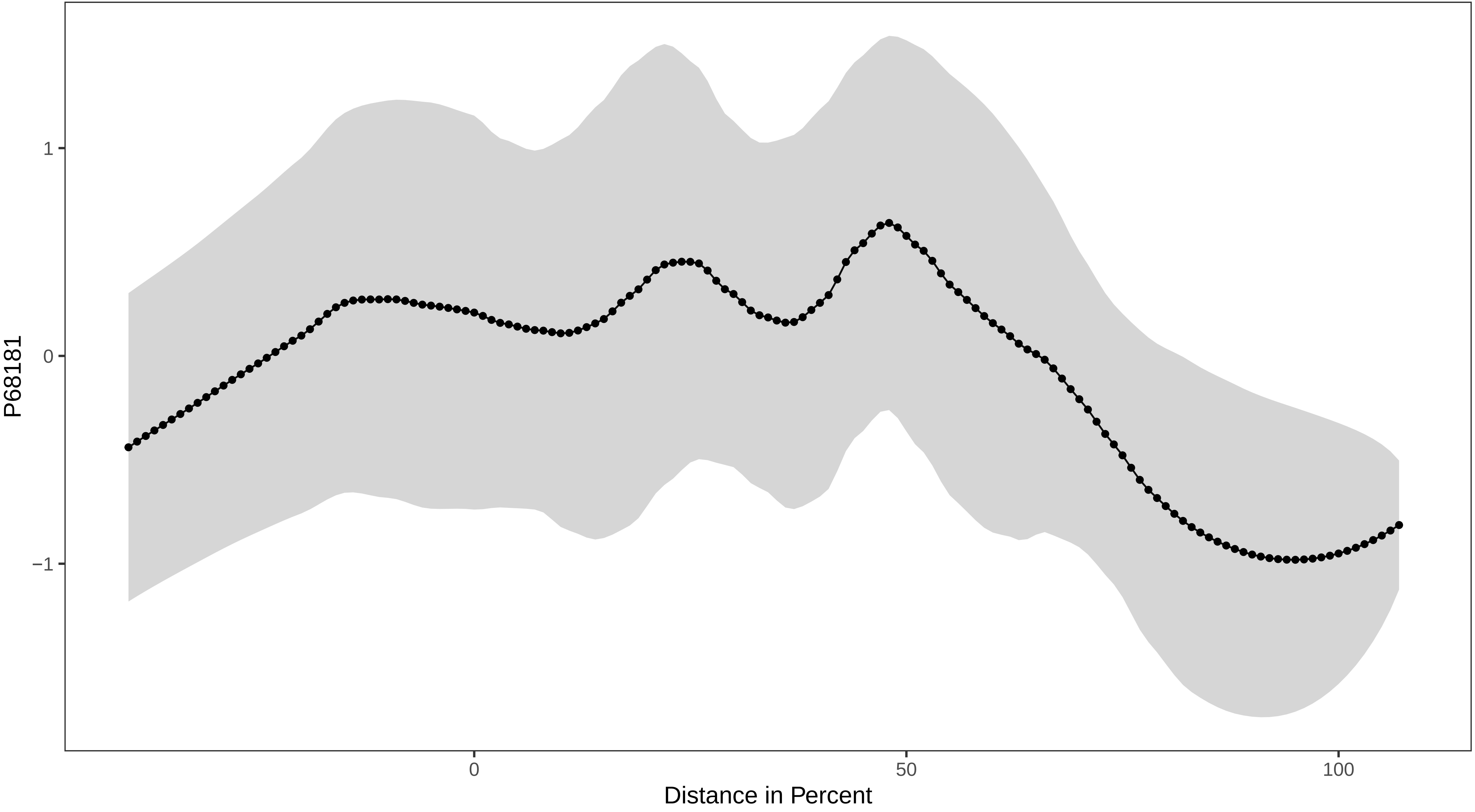

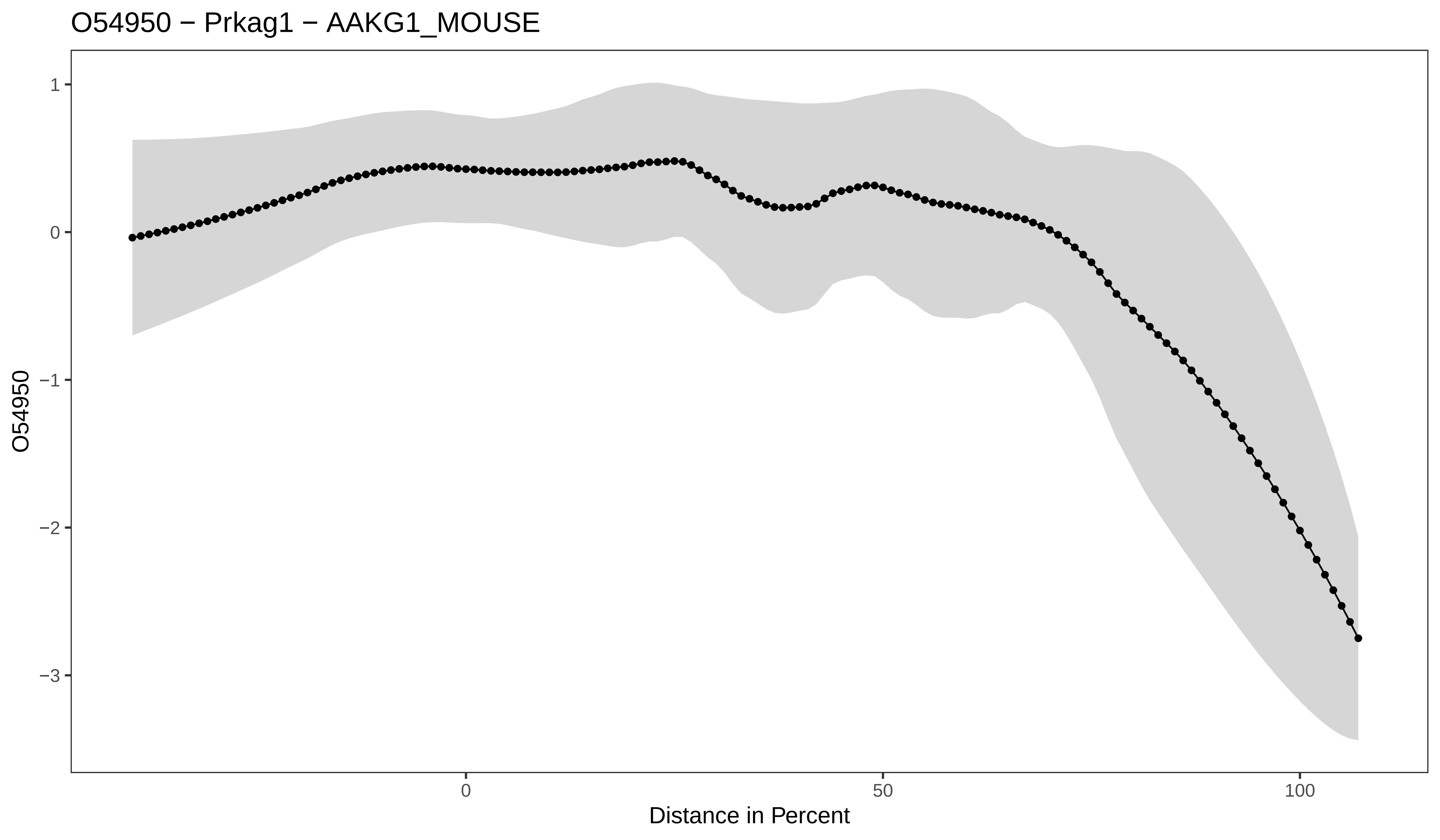
O54950 − Prkag1 − AAKG1_MOUSE
O54950
Distance in Percent
1
0
−1
−2
−3
0
50
100

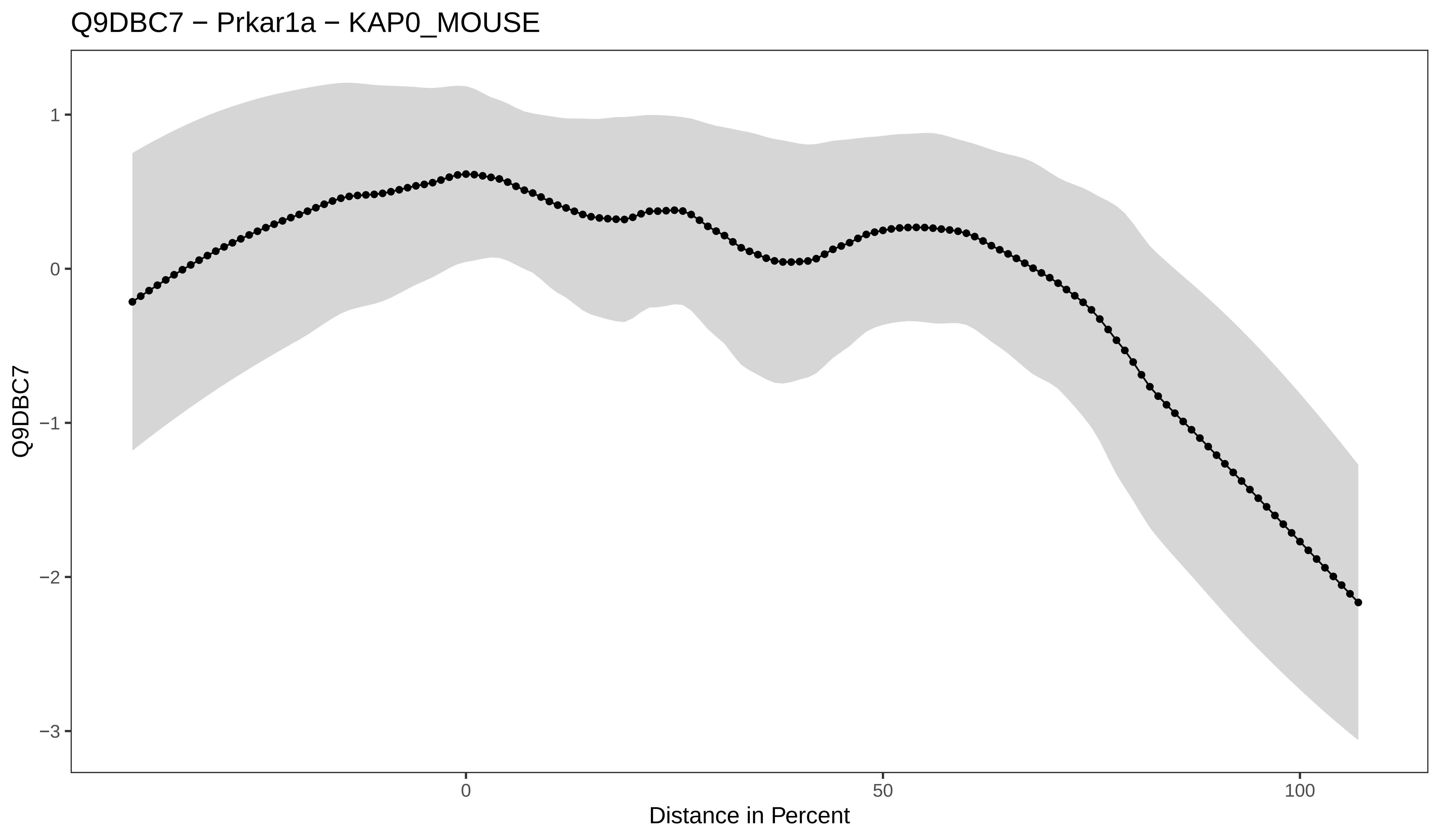
Q9DBC7 − Prkar1a − KAP0_MOUSE
Q9DBC7
1
0
−1
−2
−3
0
50
100
Distance in Percent

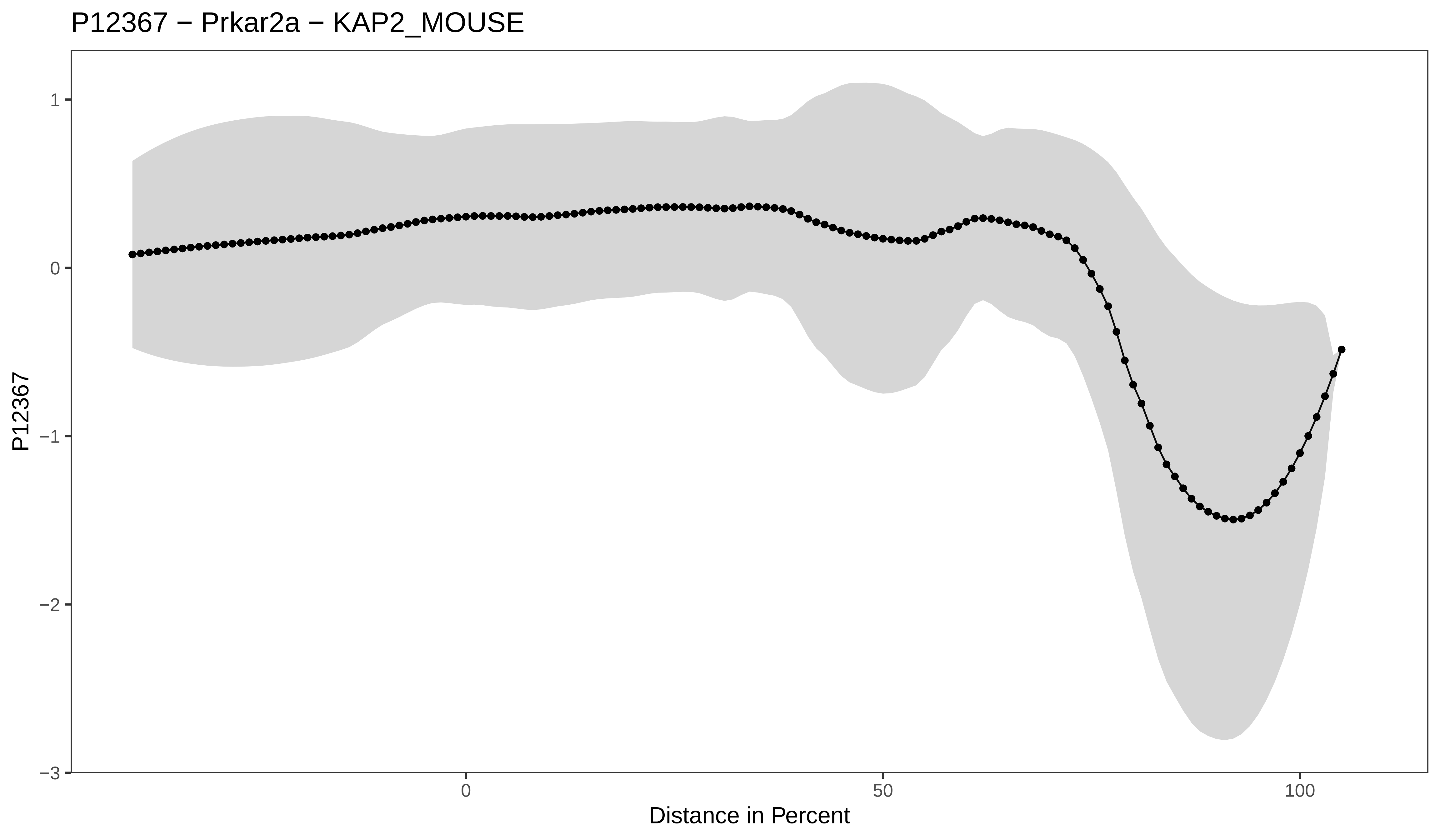

P12367 − Prkar2a − KAP2_MOUSE
P12367
Distance in Percent
1
0
−1
−2
−3
0
50
100

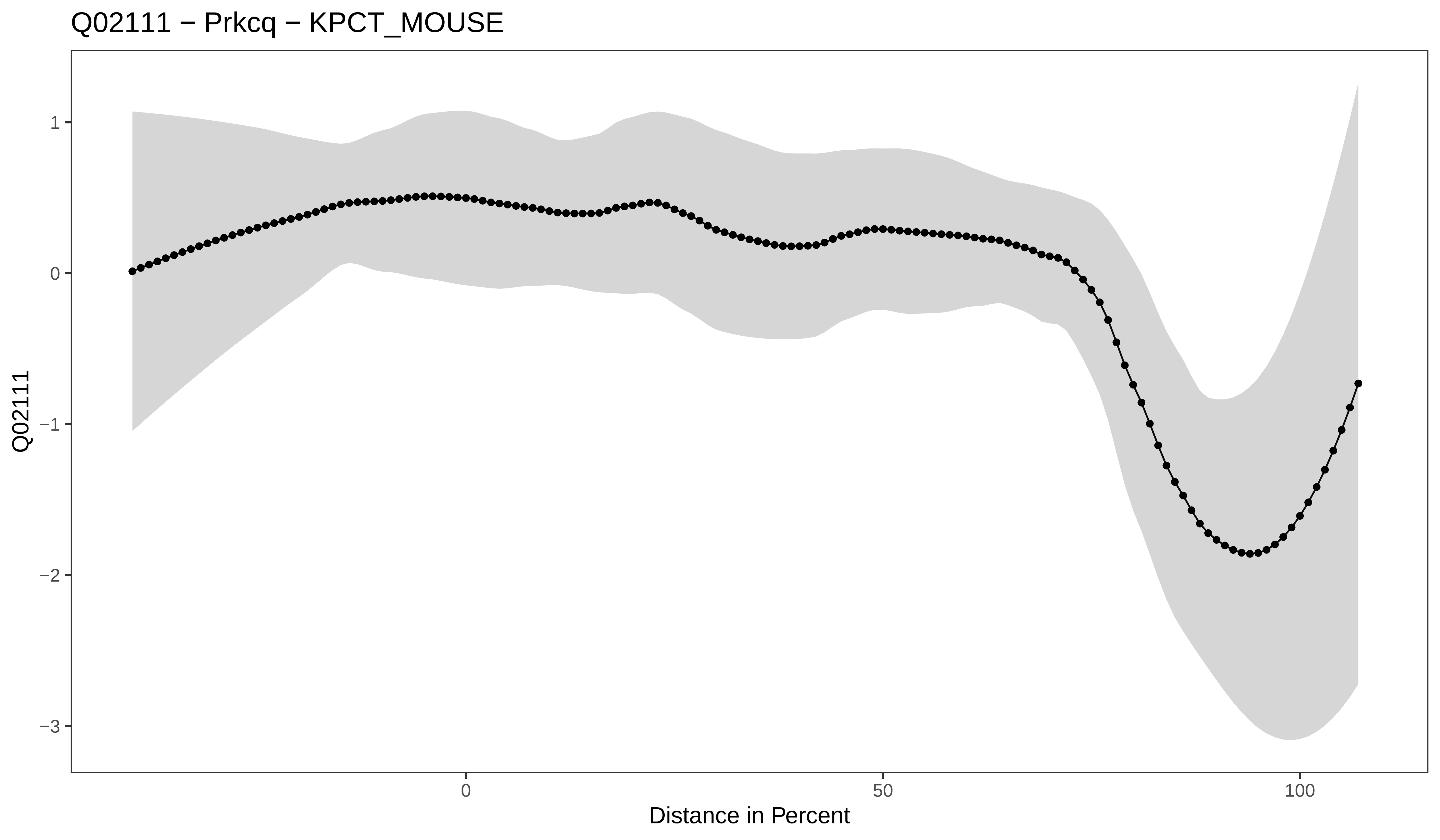
Q02111 − Prkcq − KPCT_MOUSE
1
0
−1
−2
−3
Q02111
0
50
100
Distance in Percent

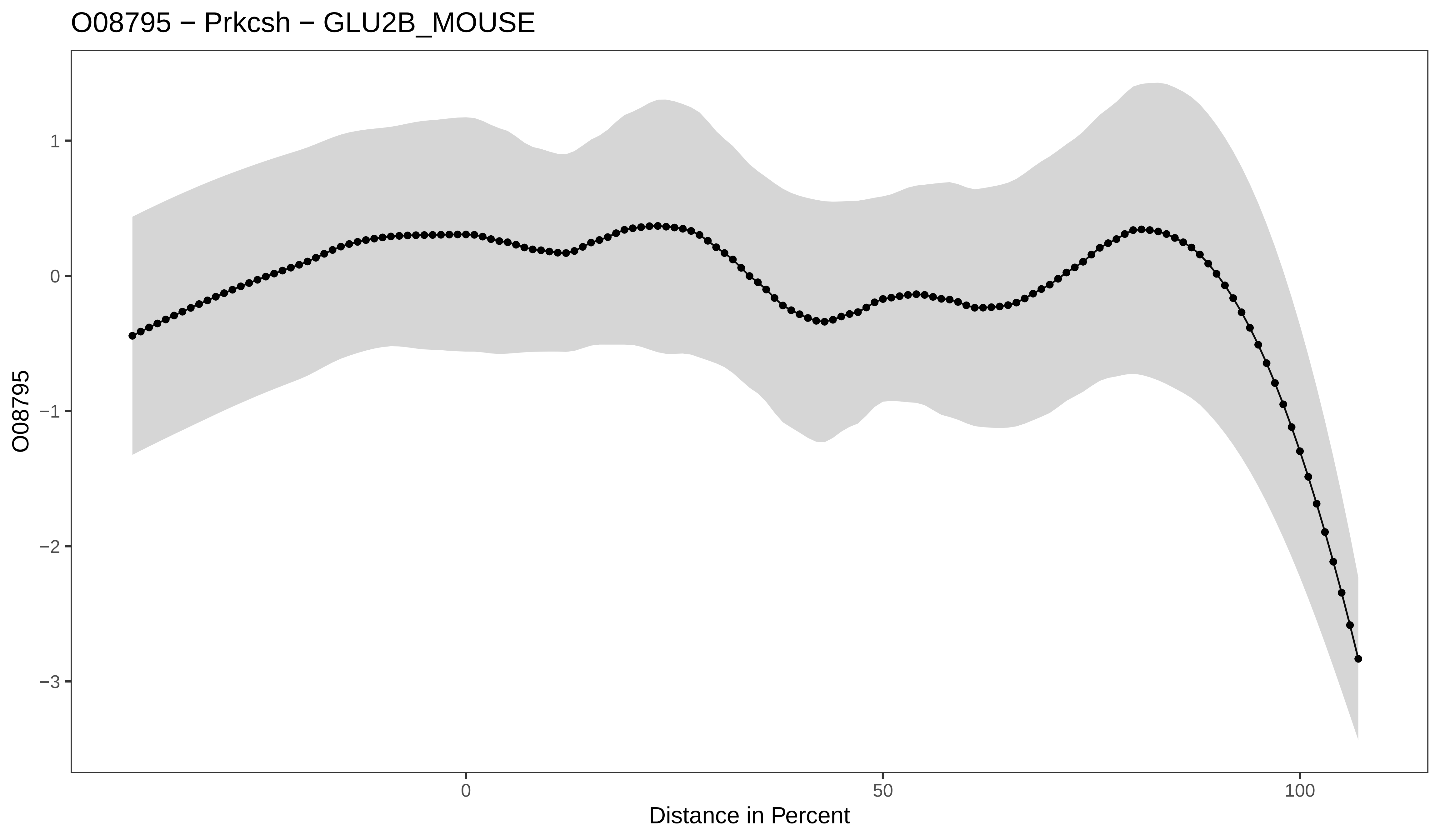

O08795 − Prkcsh − GLU2B_MOUSE
O08795
1
0
−1
−2
−3
0
50
100
Distance in Percent

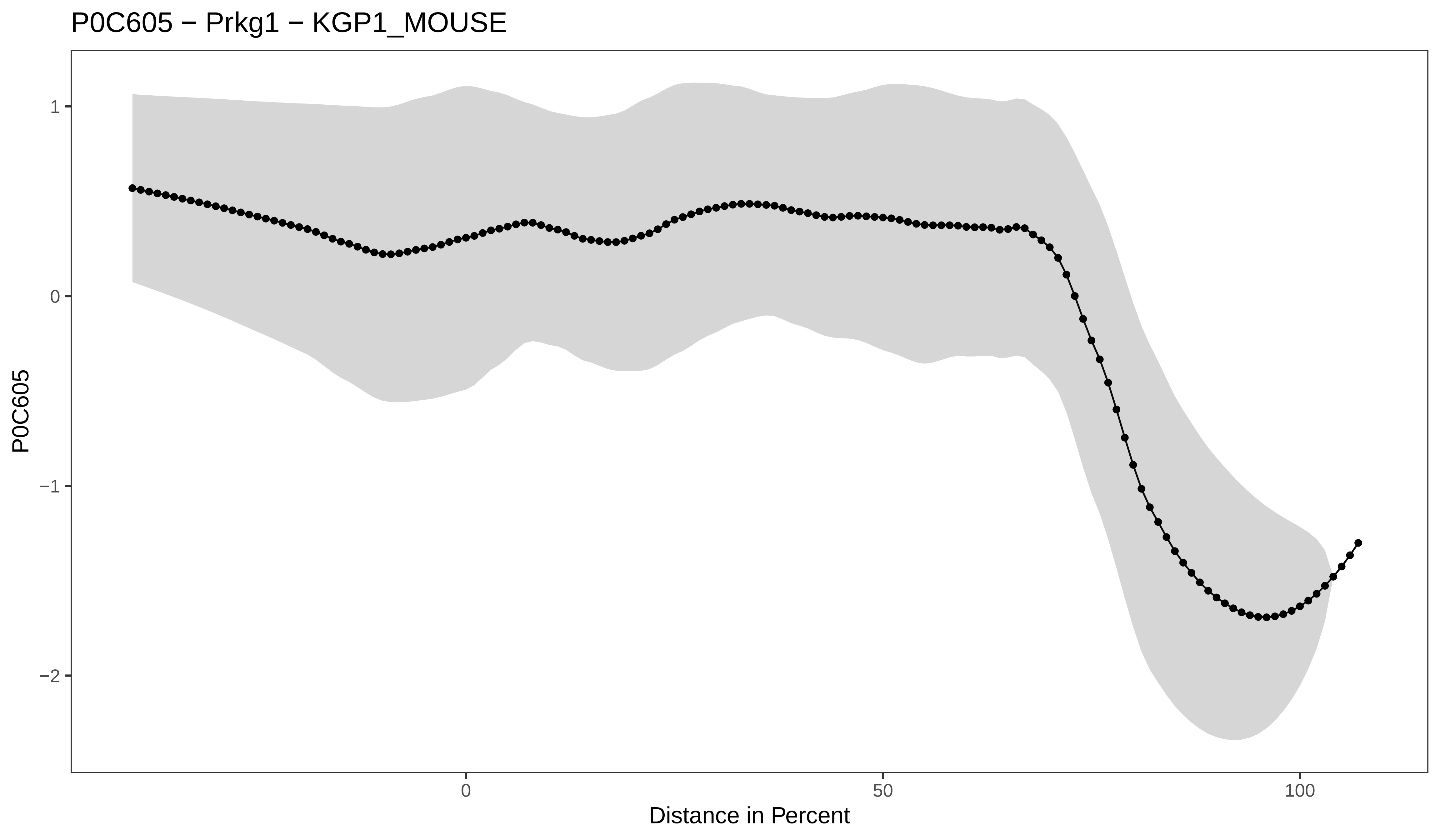

P0C605 − Prkg1 − KGP1_MOUSE
1
0
−1
−2
P0C605
0
50
100
Distance in Percent

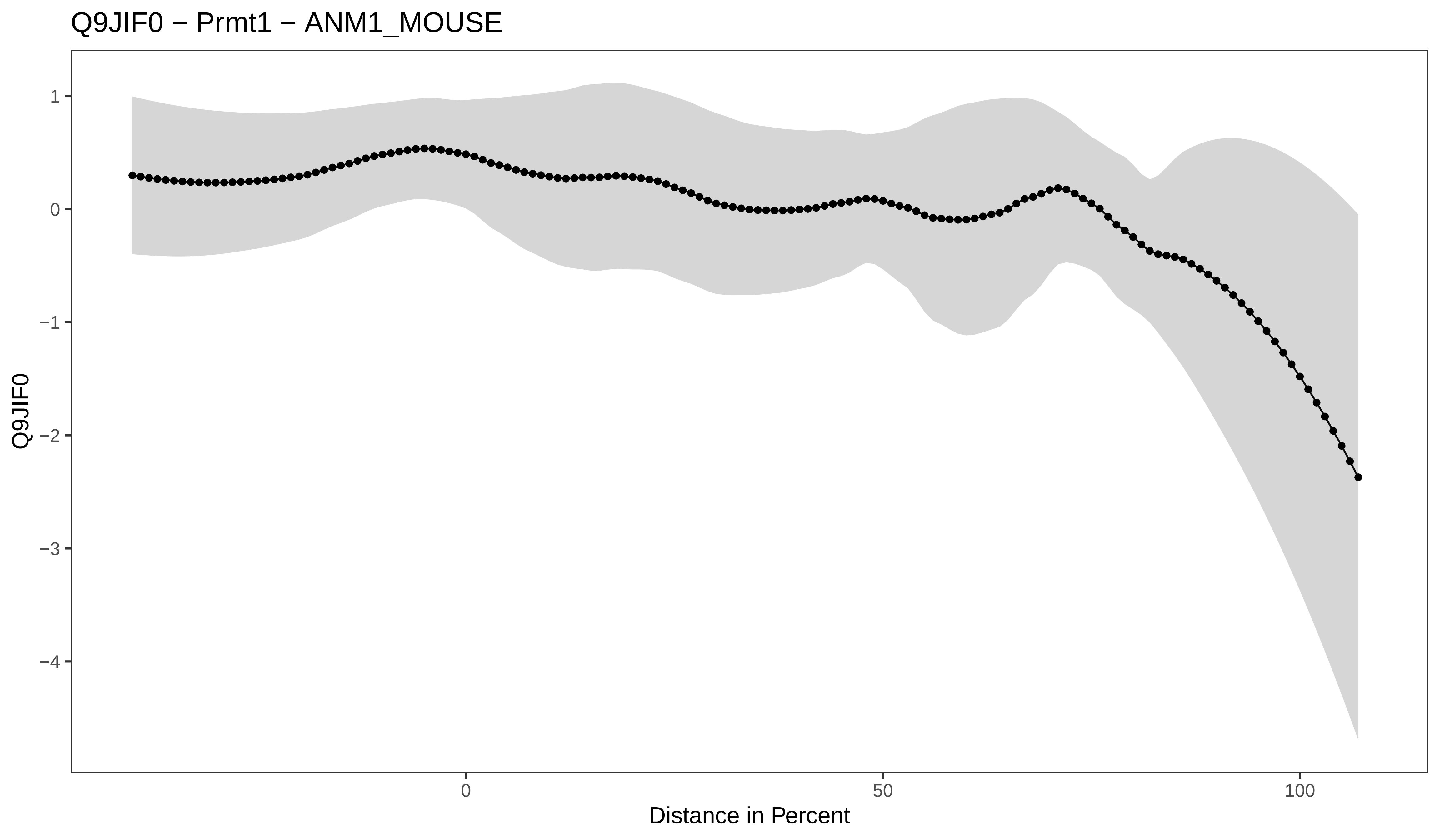
Q9JIF0 − Prmt1 − ANM1_MOUSE
1
0
−1
−2
−3
−4
Q9JIF0
0
50
100
Distance in Percent

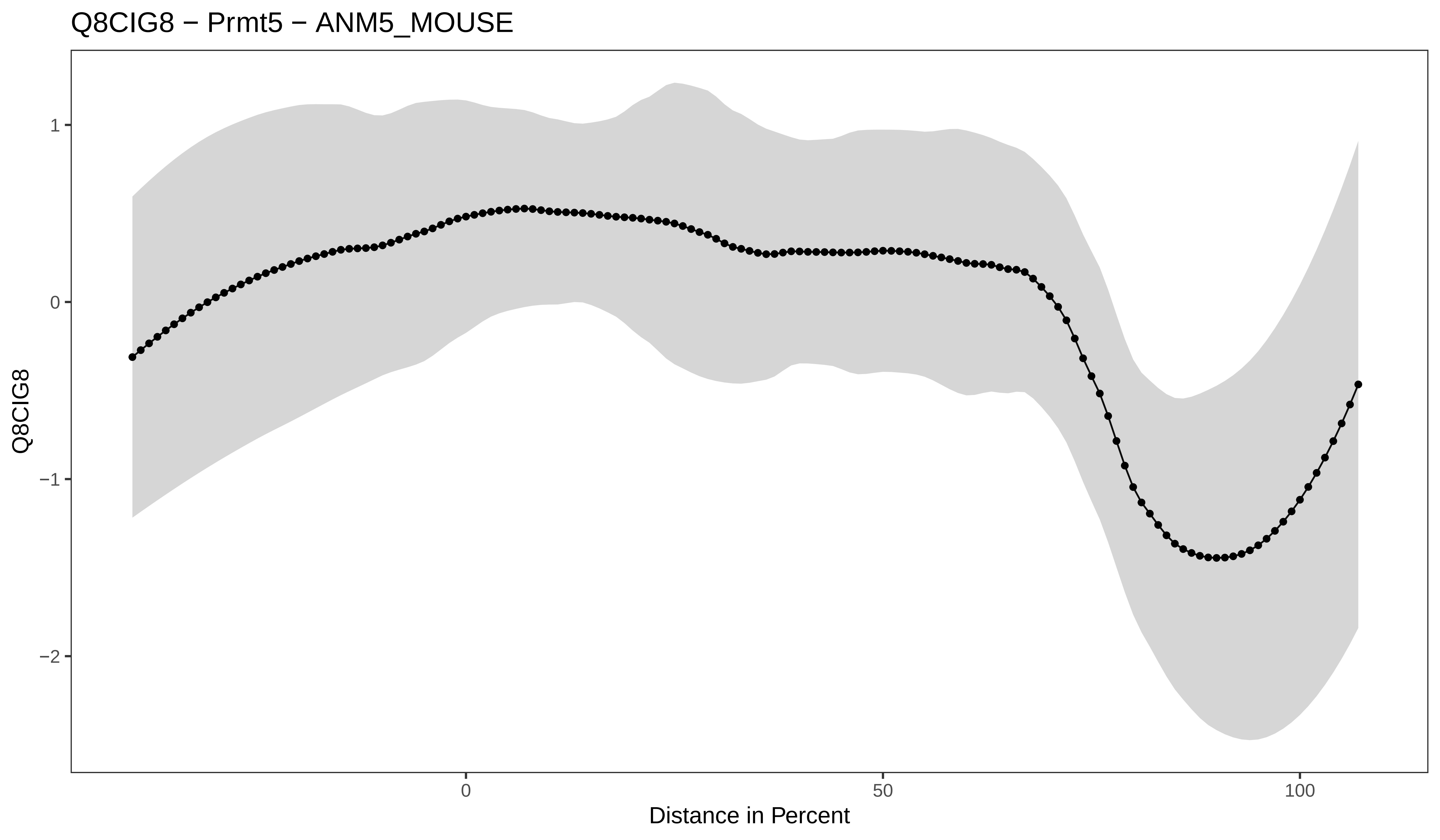

Q8CIG8 − Prmt5 − ANM5_MOUSE
Q8CIG8
1
0
−1
−2
0
50
100
Distance in Percent

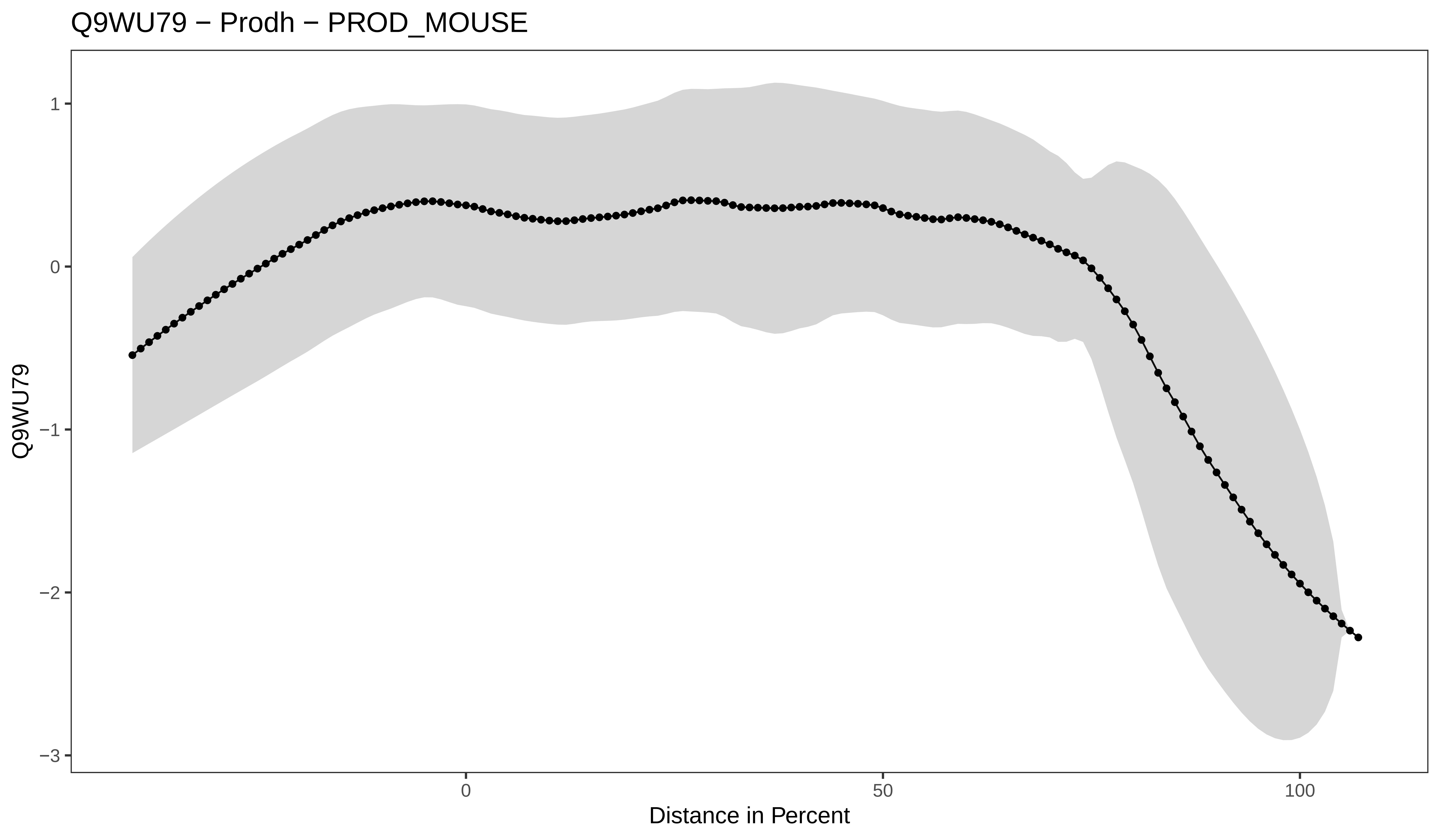

Q9WU79 − Prodh − PROD_MOUSE
1
0
−1
−2
−3
Q9WU79
0
50
100
Distance in Percent

Q99KP6 − Prpf19 − PRP19_MOUSE

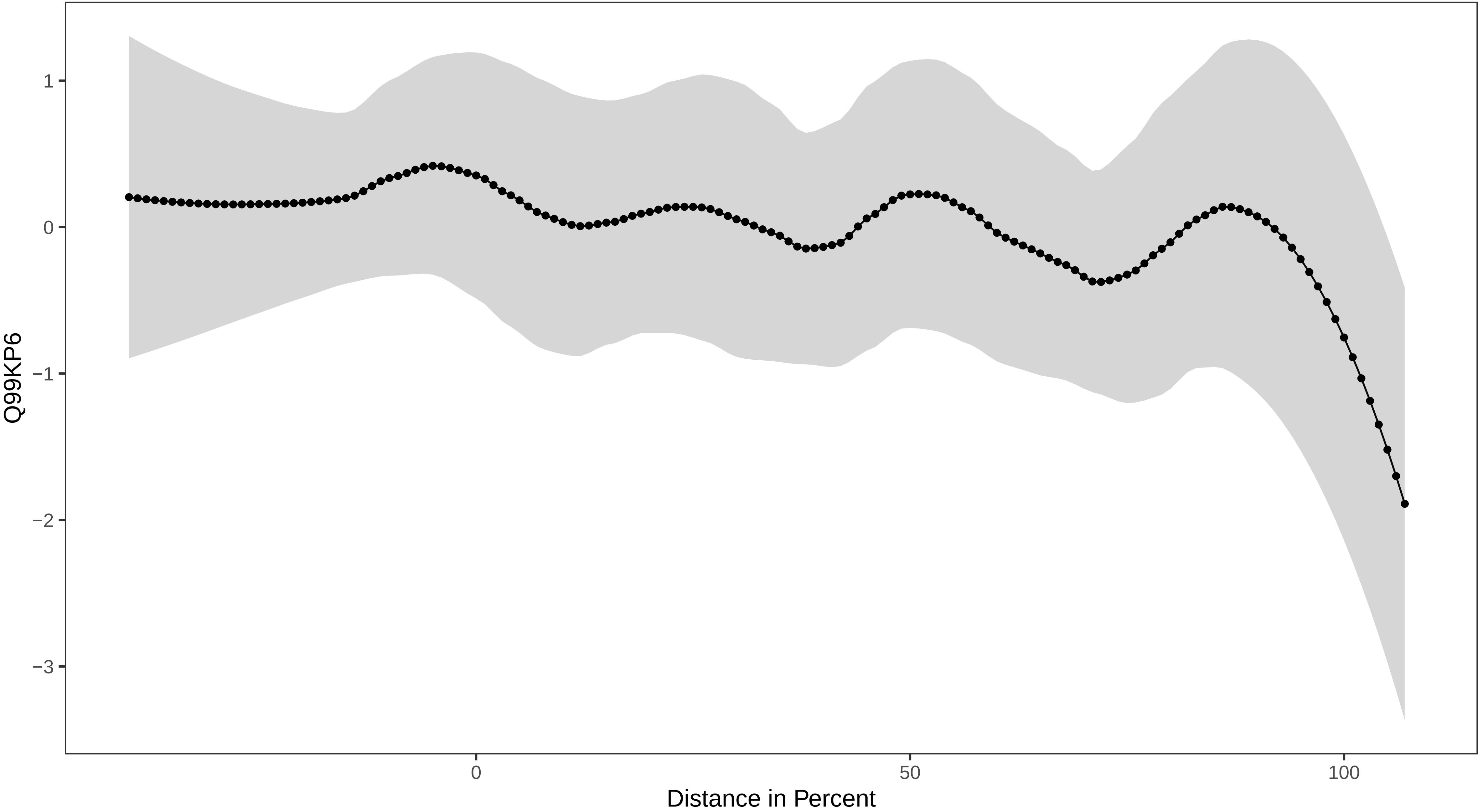

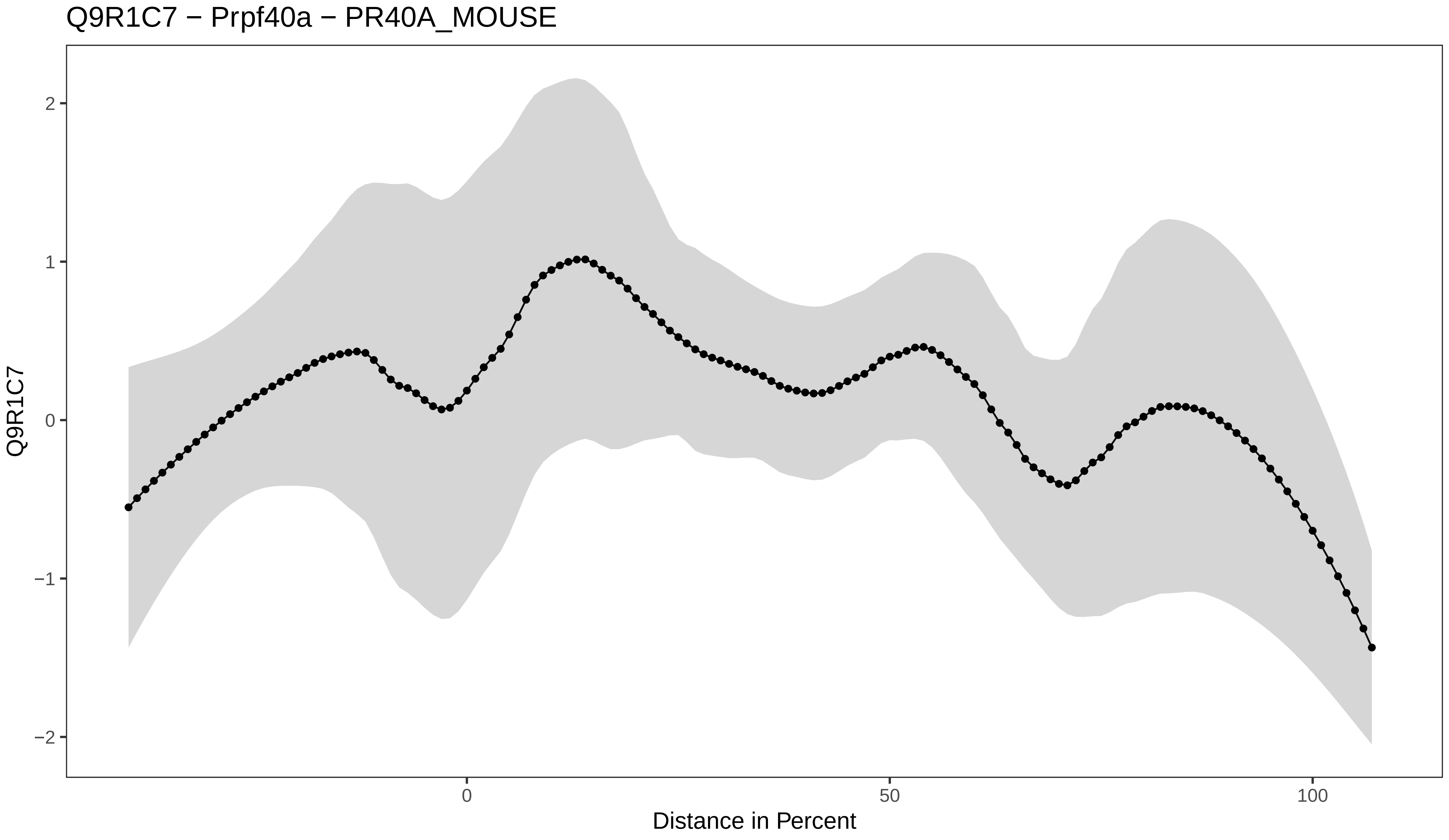

Q9R1C7 − Prpf40a − PR40A_MOUSE
Q9R1C7
Distance in Percent
2
1
0
−1
−2
0
50
100

Q99PV0 − Prpf8 − PRP8_MOUSE

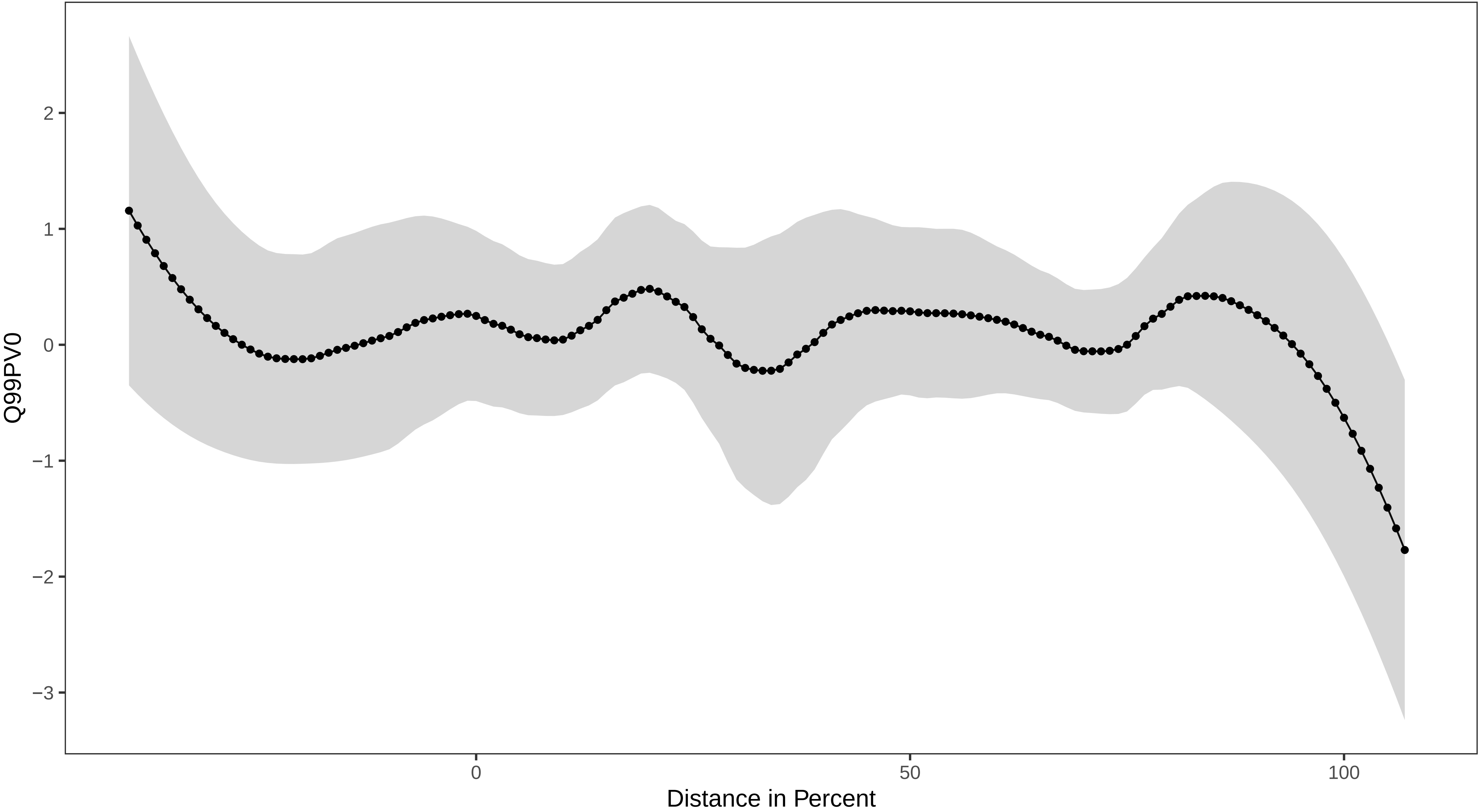

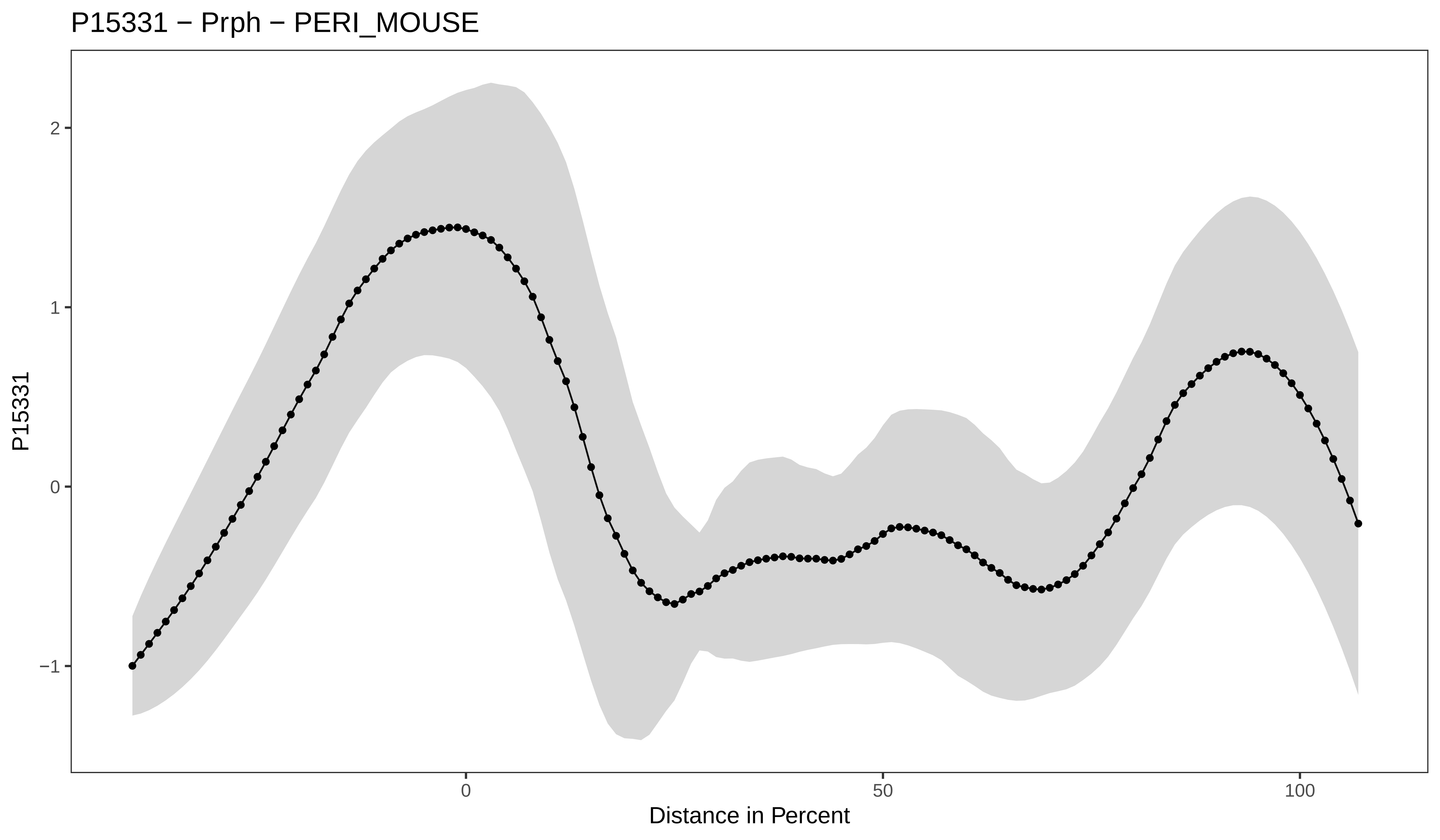

P15331 − Prph − PERI_MOUSE
P15331
2
1
0
−1
0
50
100
Distance in Percent

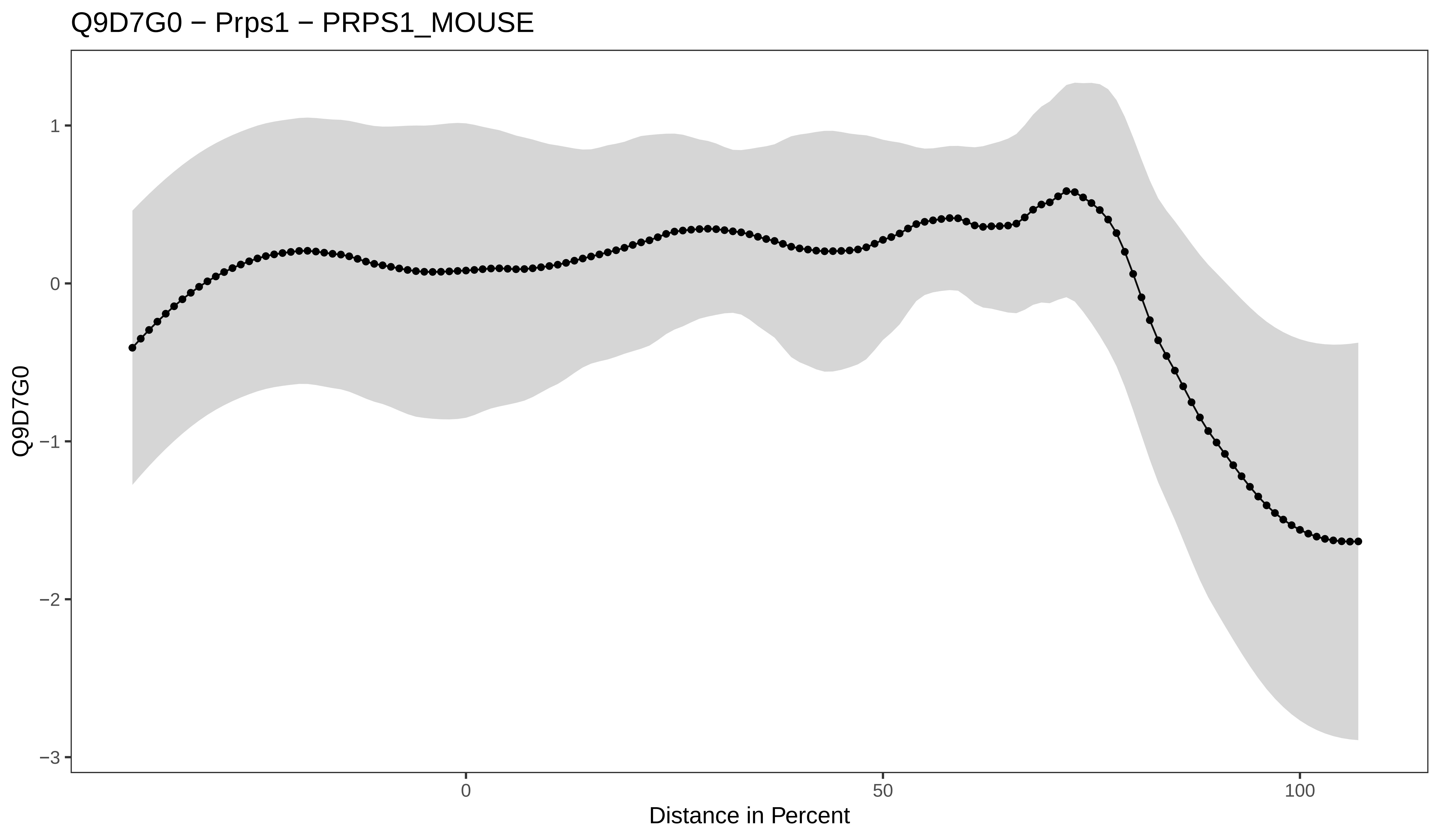

Q9D7G0 − Prps1 − PRPS1_MOUSE
Q9D7G0
Distance in Percent
1
0
−1
−2
−3
0
50
100

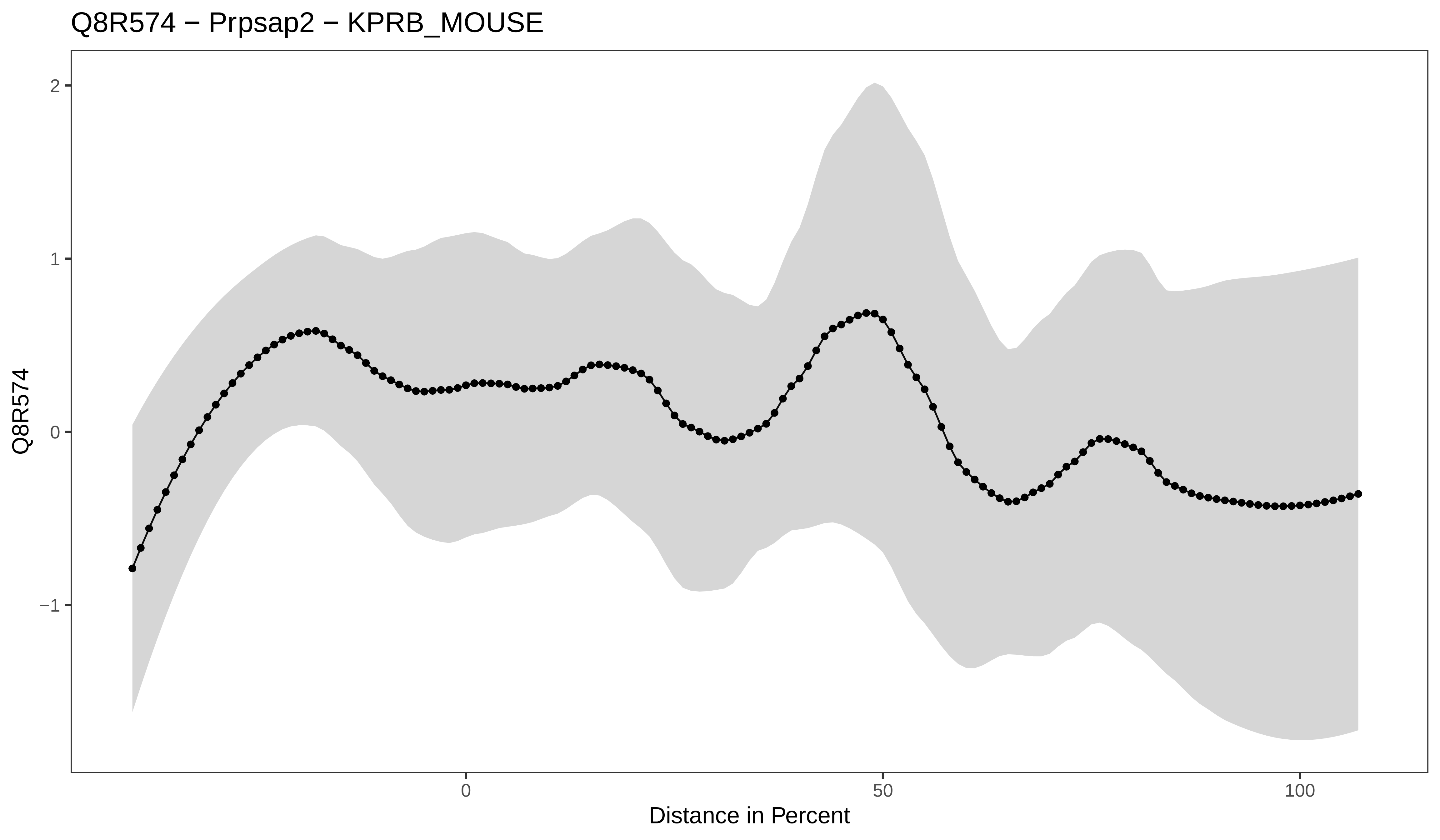
Q8R574 − Prpsap2 − KPRB_MOUSE
Q8R574
2
1
0
−1
0
50
100
Distance in Percent

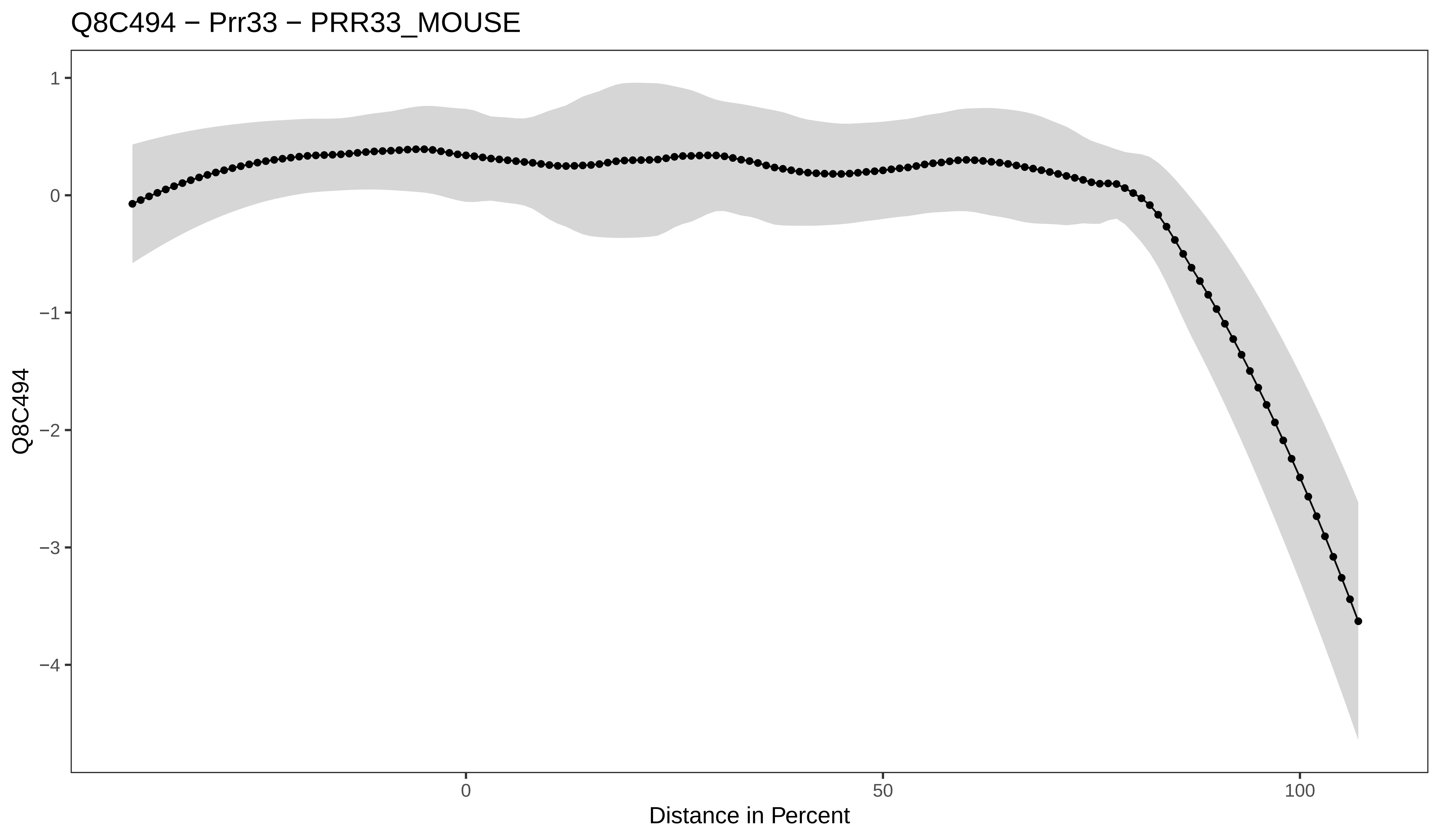

Q8C494 − Prr33 − PRR33_MOUSE
1
0
−1
−2
−3
−4
Q8C494
0
50
100
Distance in Percent

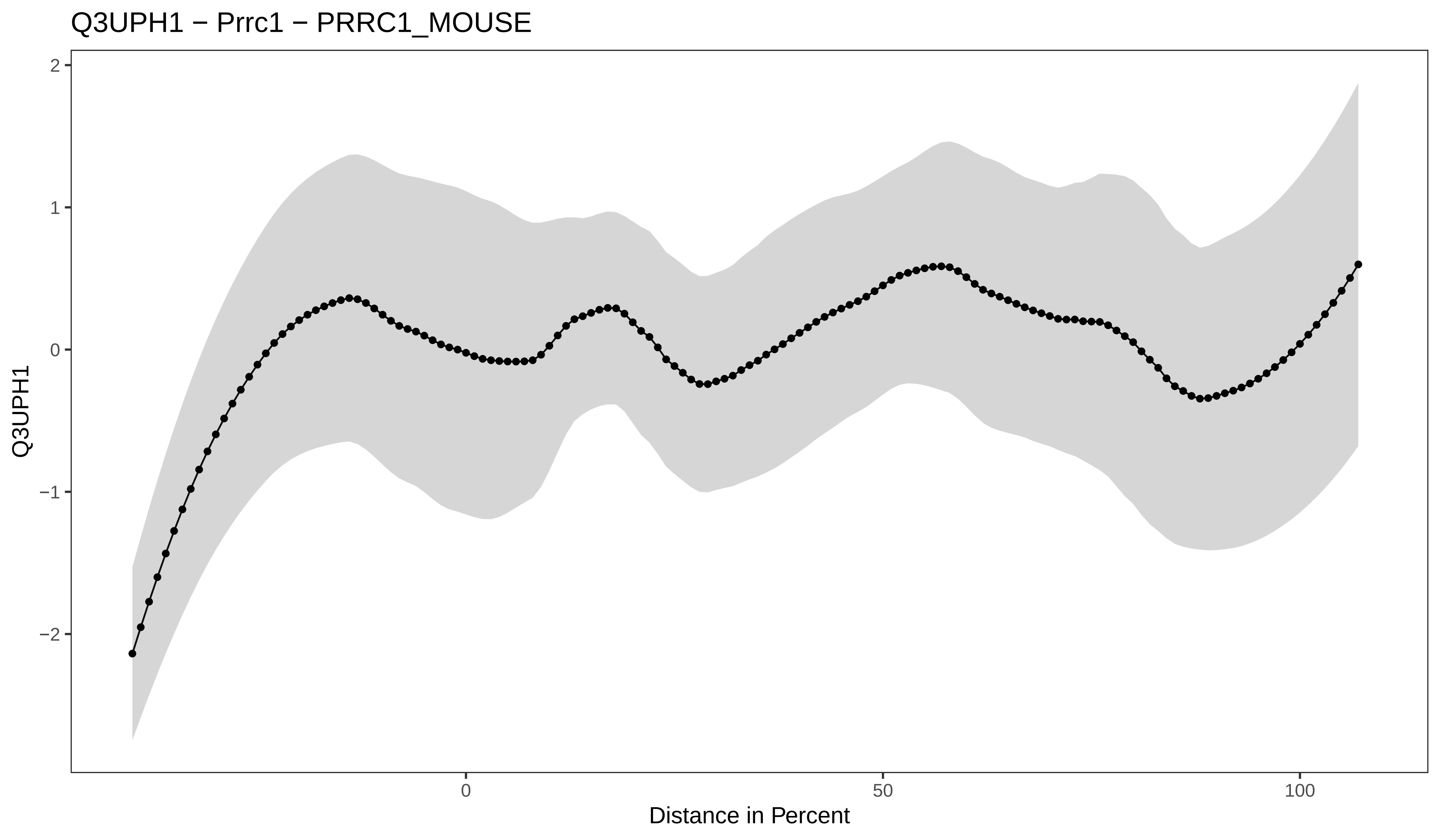

Q3UPH1 − Prrc1 − PRRC1_MOUSE
2
1
0
−1
−2
Q3UPH1
0
50
100
Distance in Percent

Q8BIW1 − Prune1 − PRUN1_MOUSE

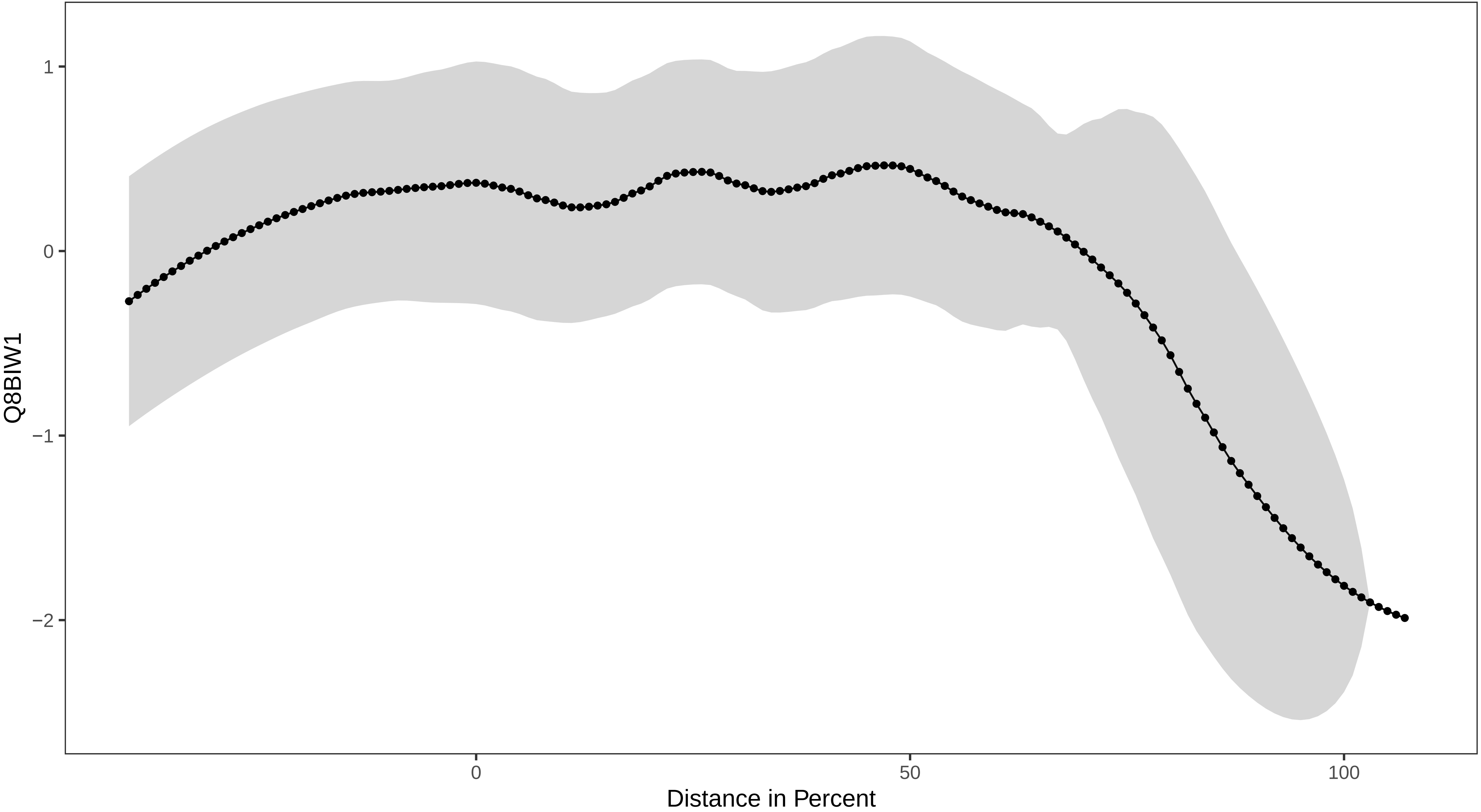

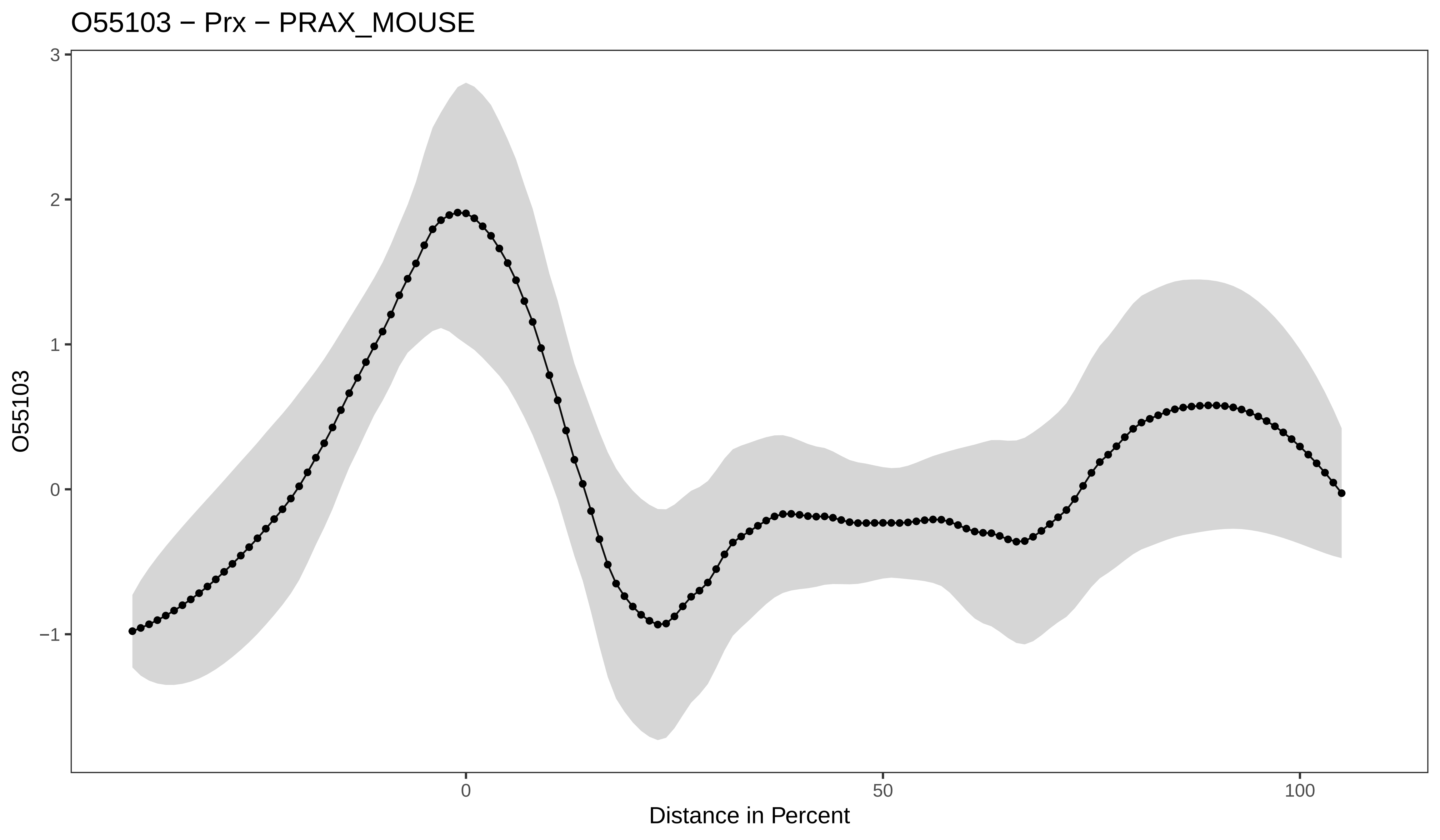
O55103 − Prx − PRAX_MOUSE
O55103
3
2
1
0
−1
0
50
100
Distance in Percent

Q9CYH2 − Prxl2a − PXL2A_MOUSE

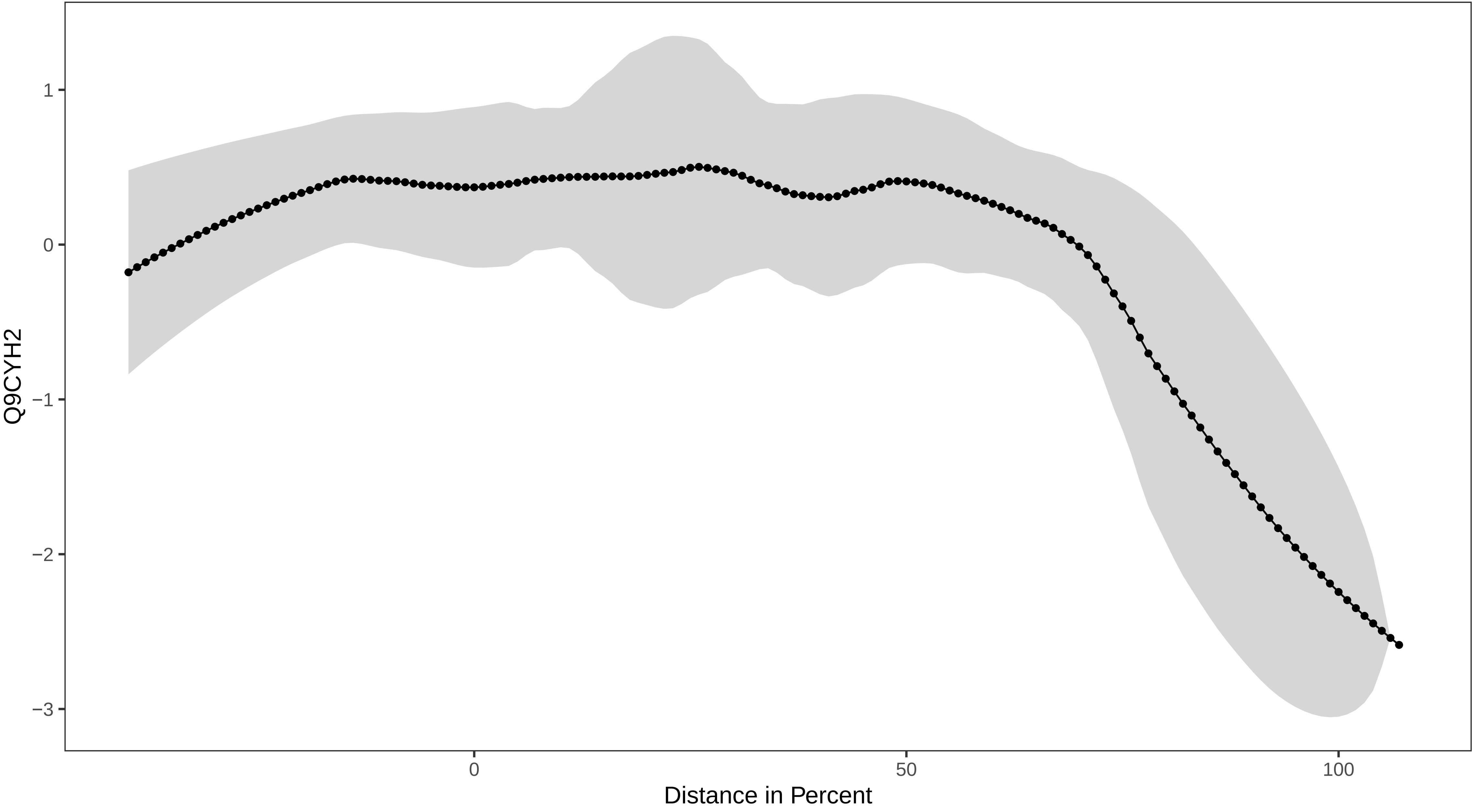

Q9DB60 − Prxl2b − PXL2B_MOUSE

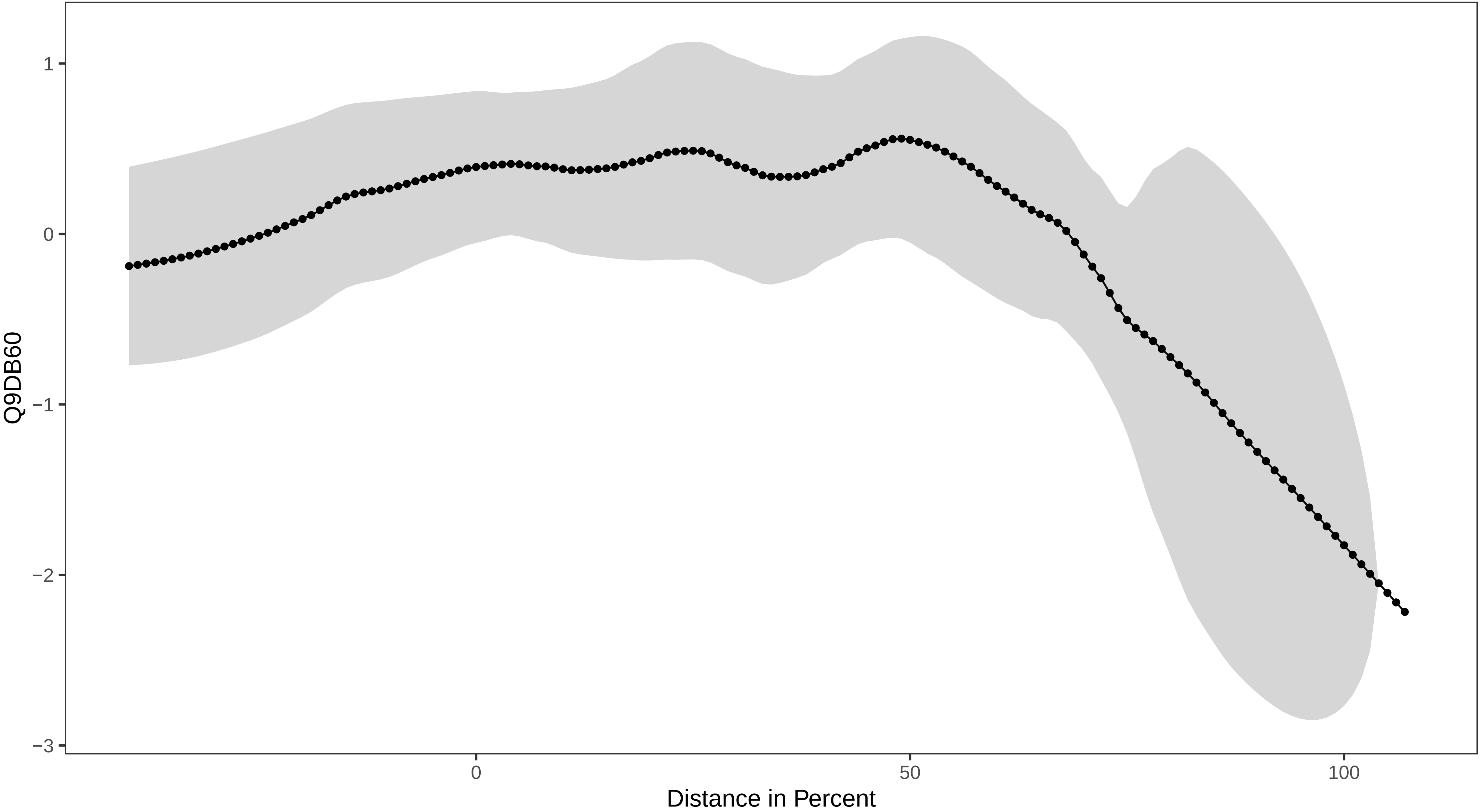

Q61207 − Psap − SAP_MOUSE

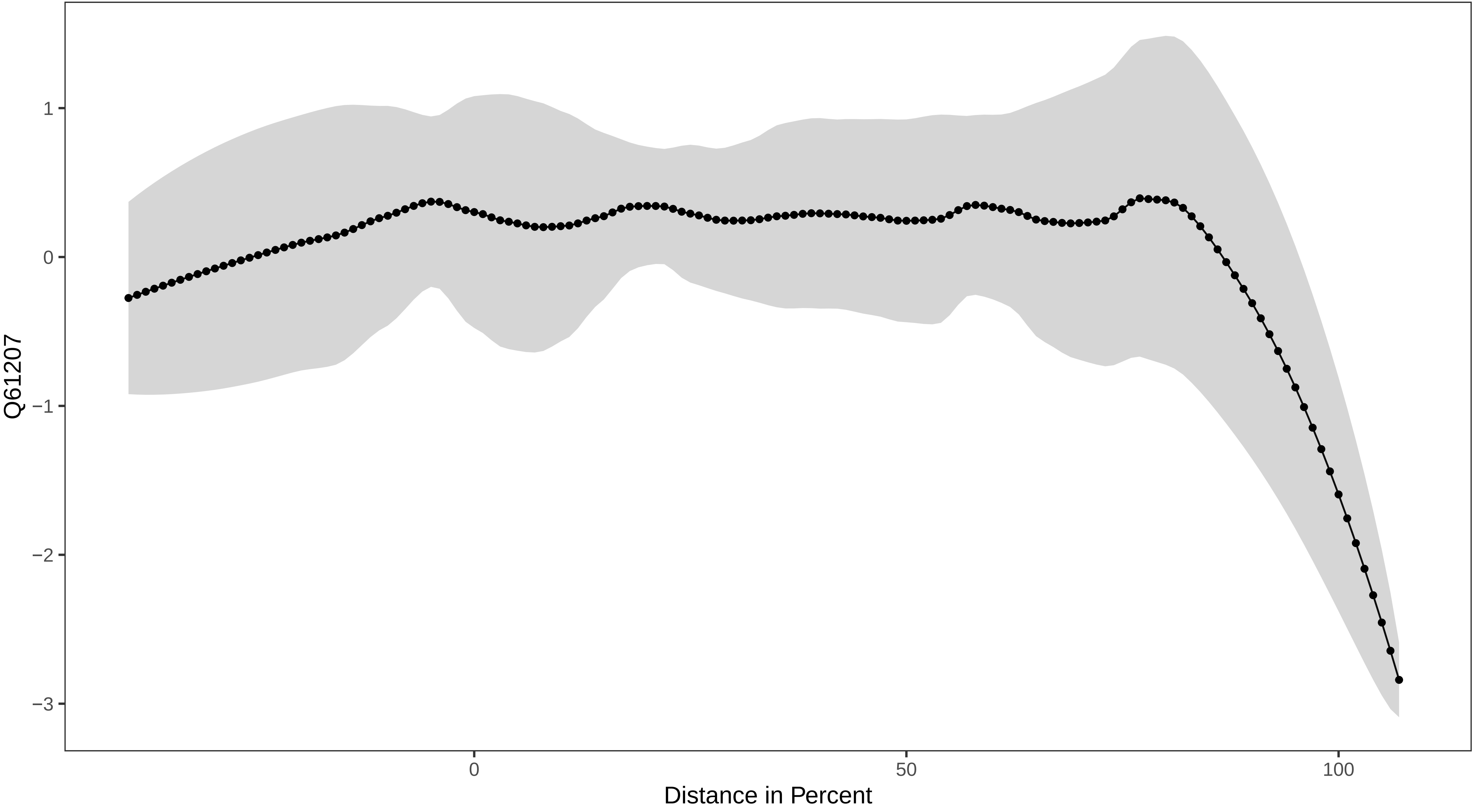

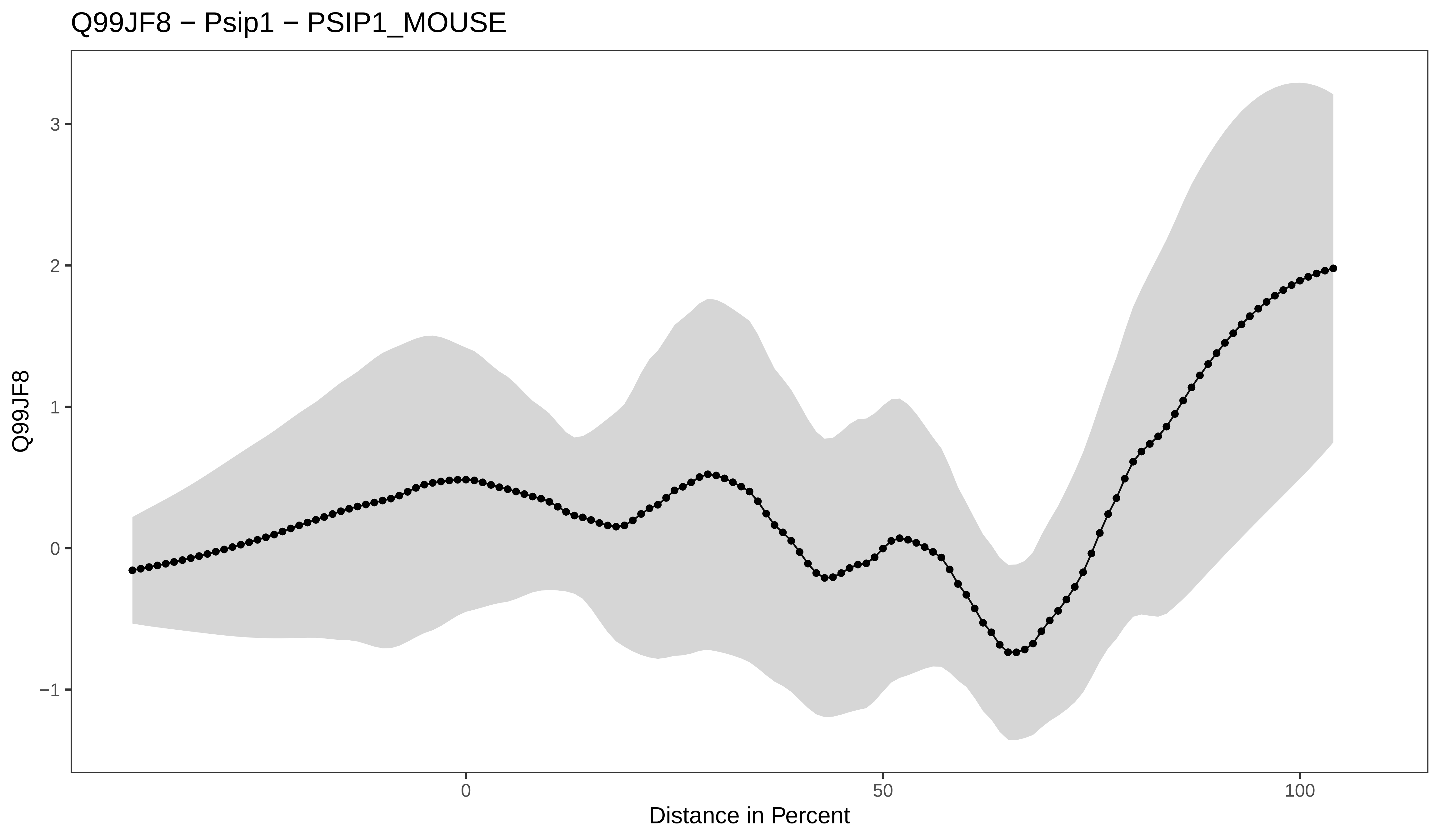

Q99JF8 − Psip1 − PSIP1_MOUSE
Q99JF8
3
2
1
0
−1
0
50
100
Distance in Percent

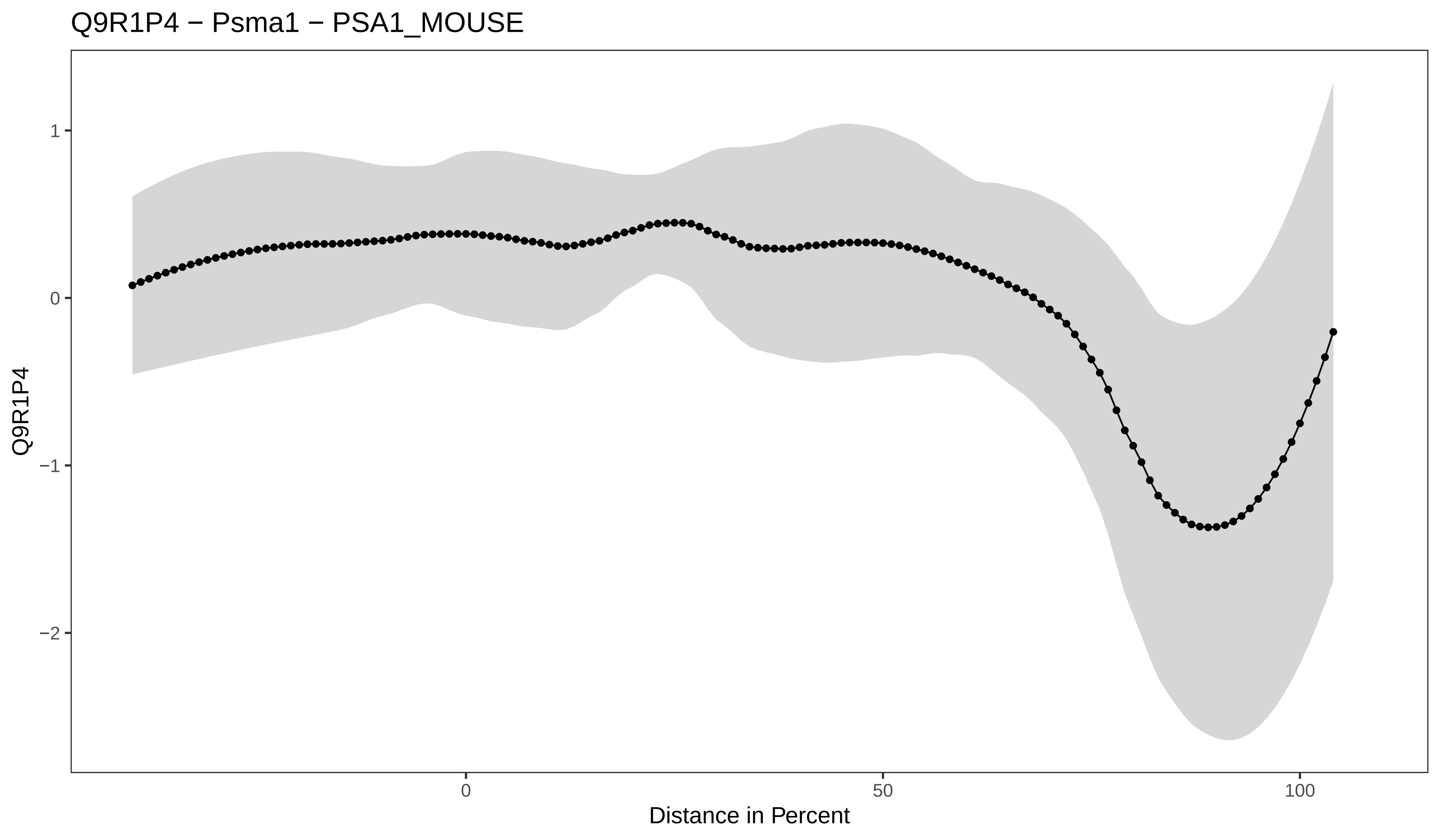

Q9R1P4 − Psma1 − PSA1_MOUSE
Q9R1P4
Distance in Percent
1
0
−1
−2
0
50
100

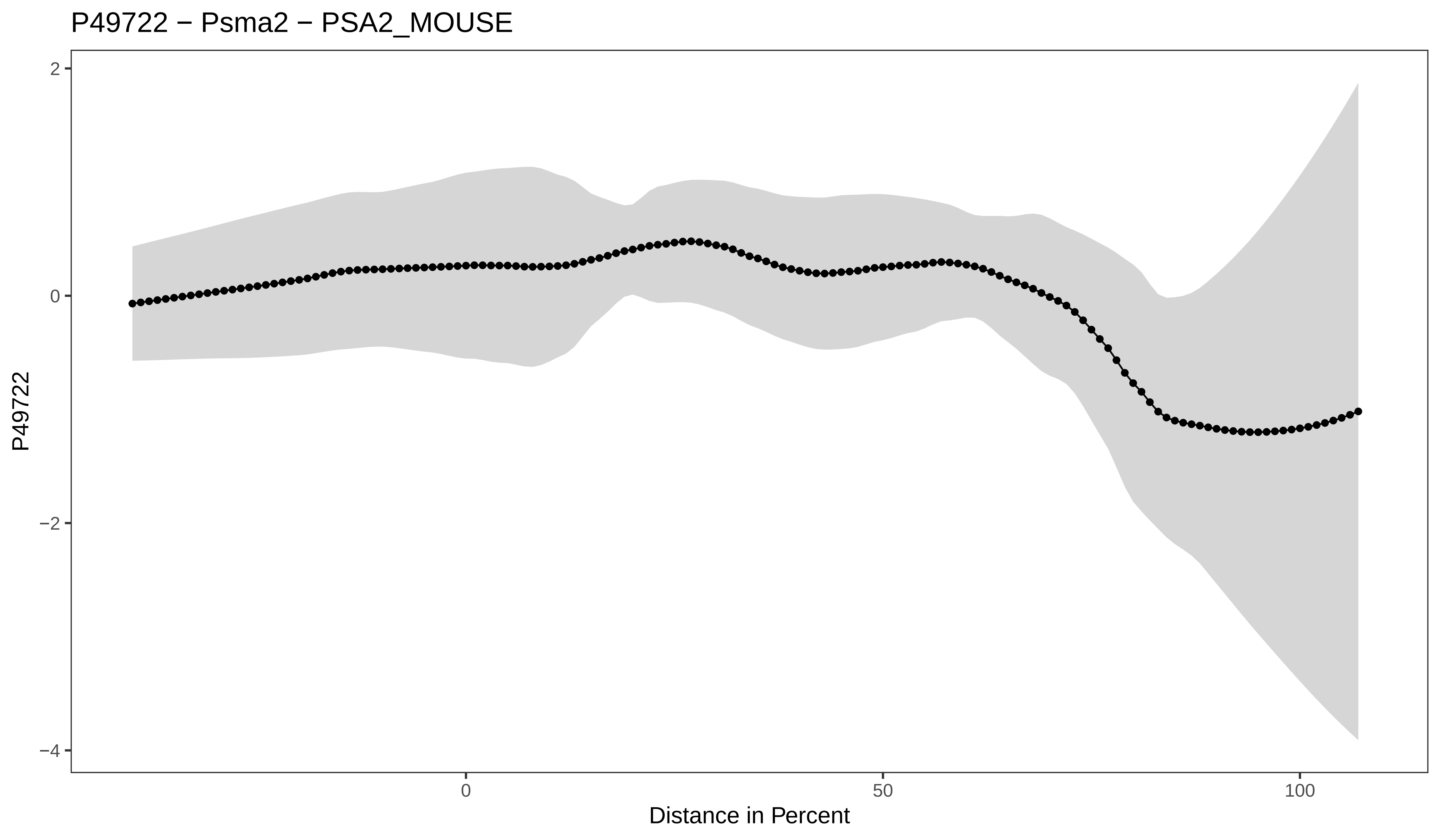

P49722 − Psma2 − PSA2_MOUSE
2
0
−2
−4
P49722
0
50
100
Distance in Percent

O70435 − Psma3 − PSA3_MOUSE

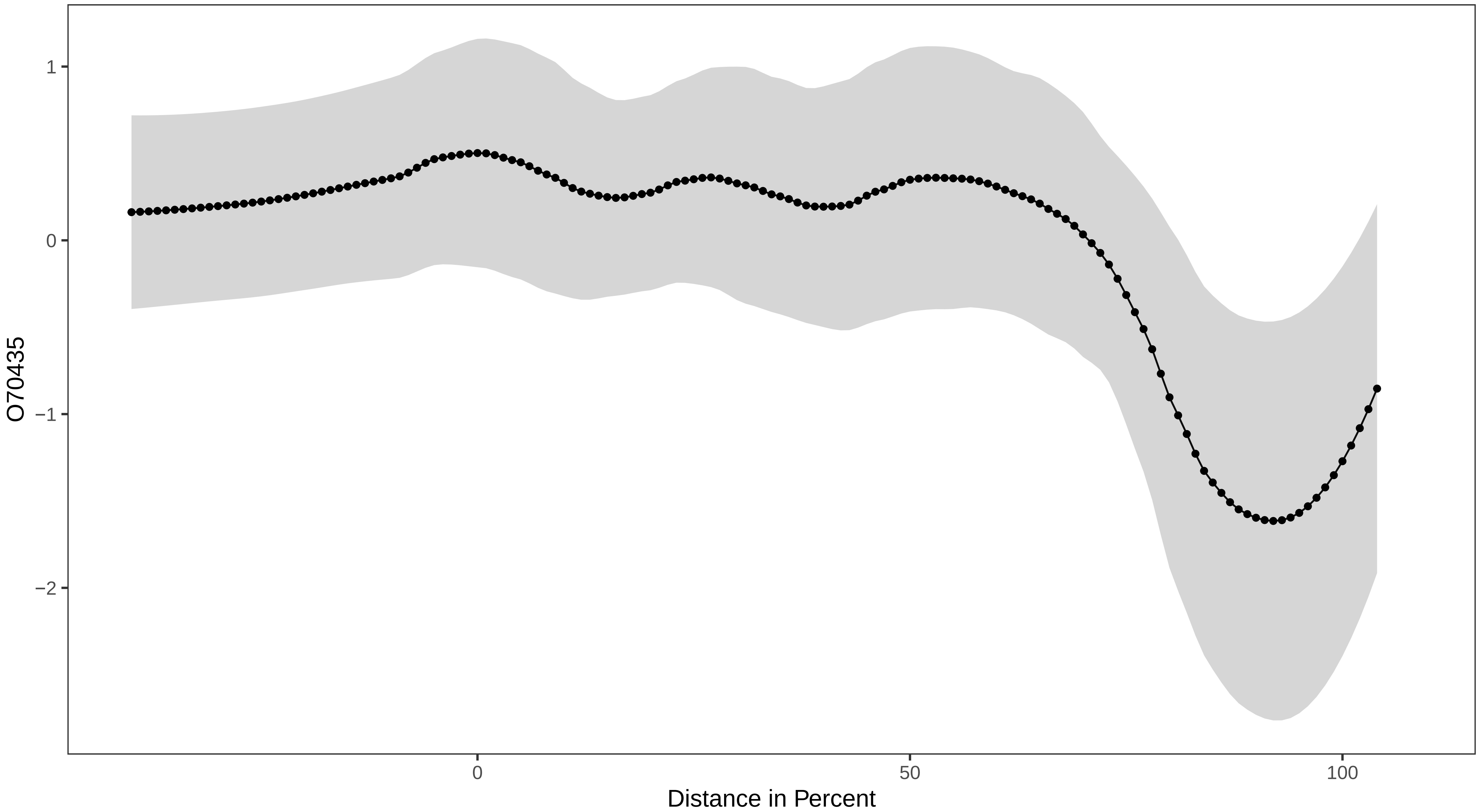

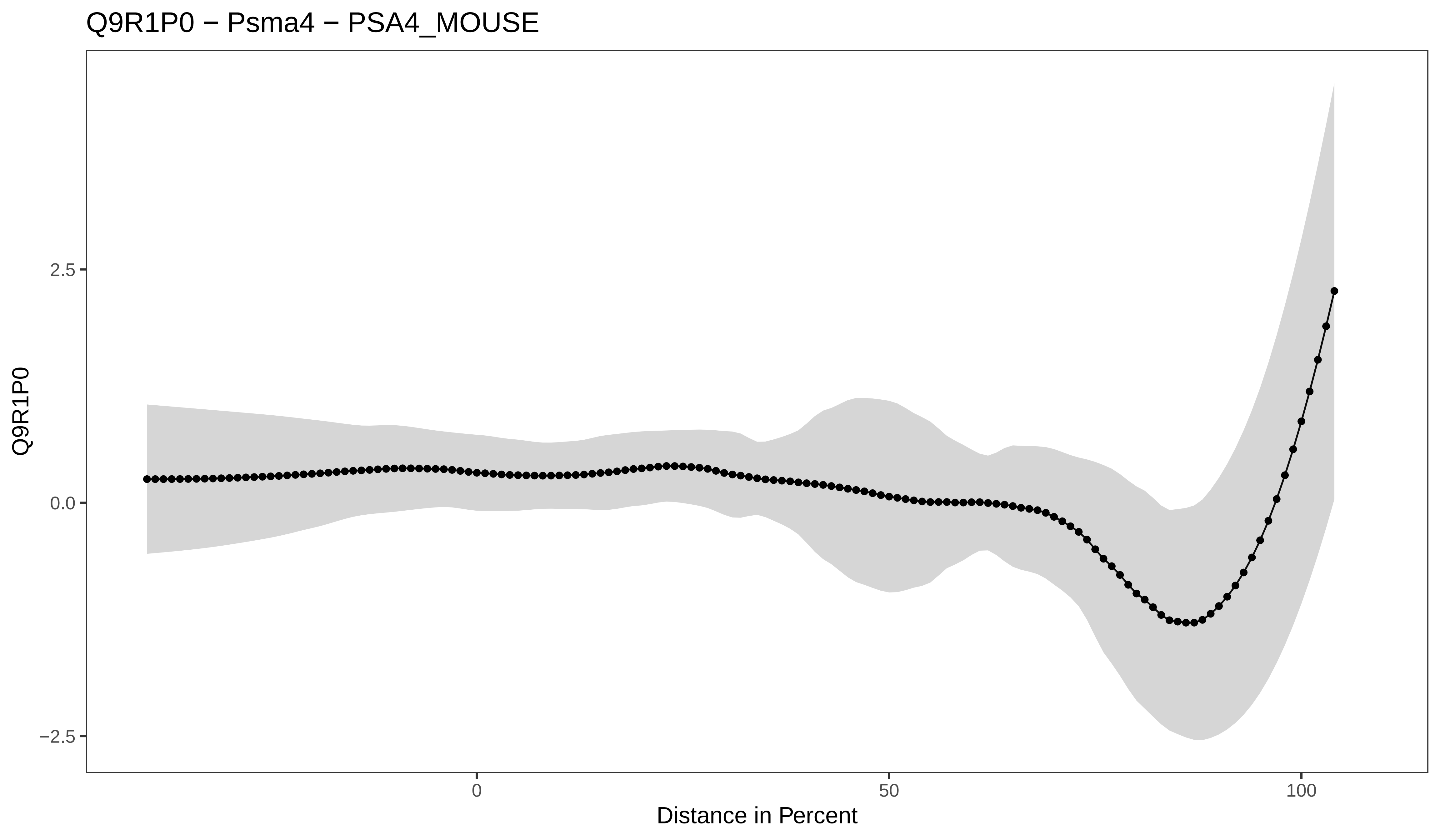

Q9R1P0 − Psma4 − PSA4_MOUSE
Q9R1P0
2.5
0.0
−2.5
0
50
100
Distance in Percent

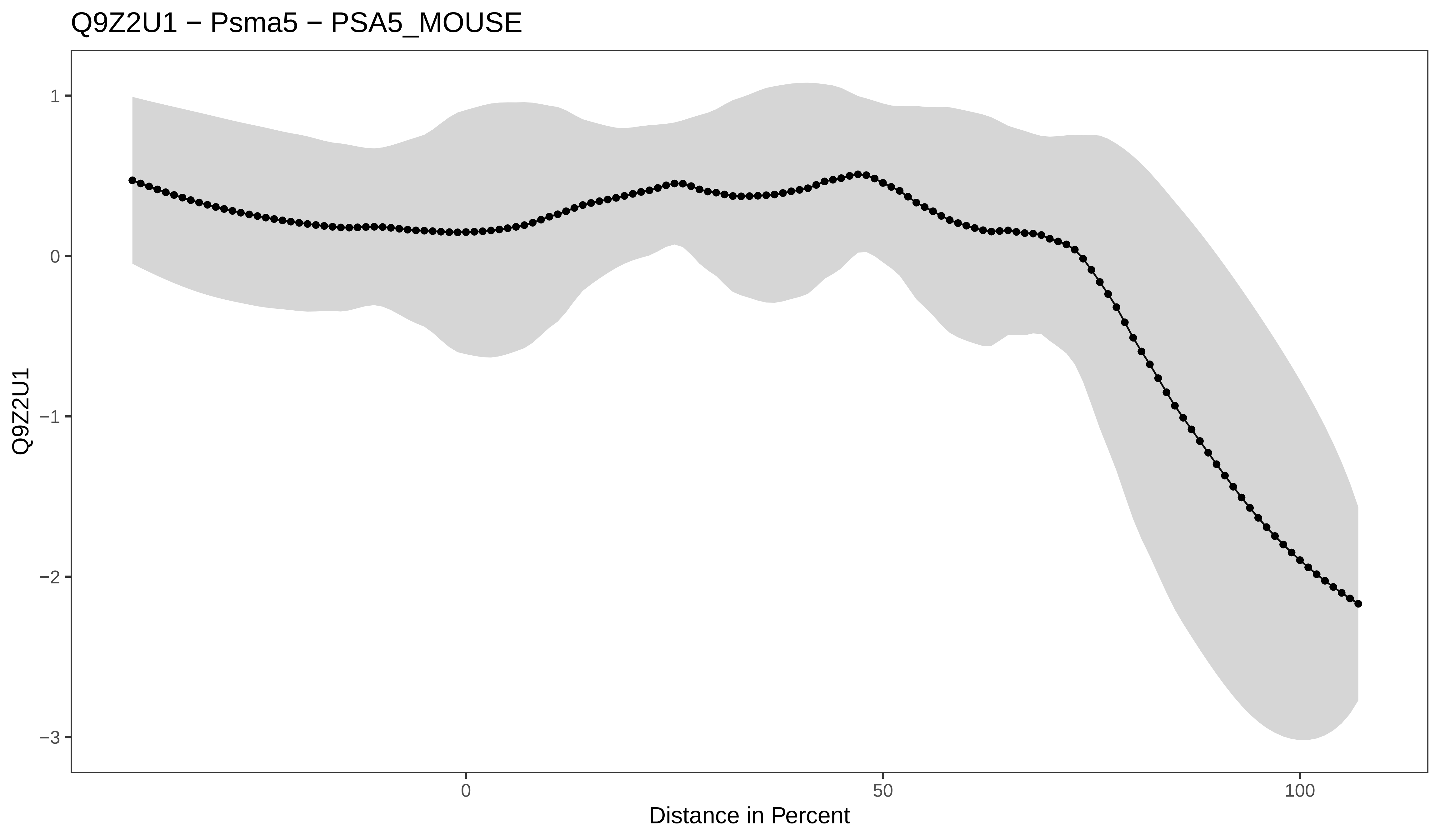

Q9Z2U1 − Psma5 − PSA5_MOUSE
Q9Z2U1
1
0
−1
−2
−3
0
50
100
Distance in Percent

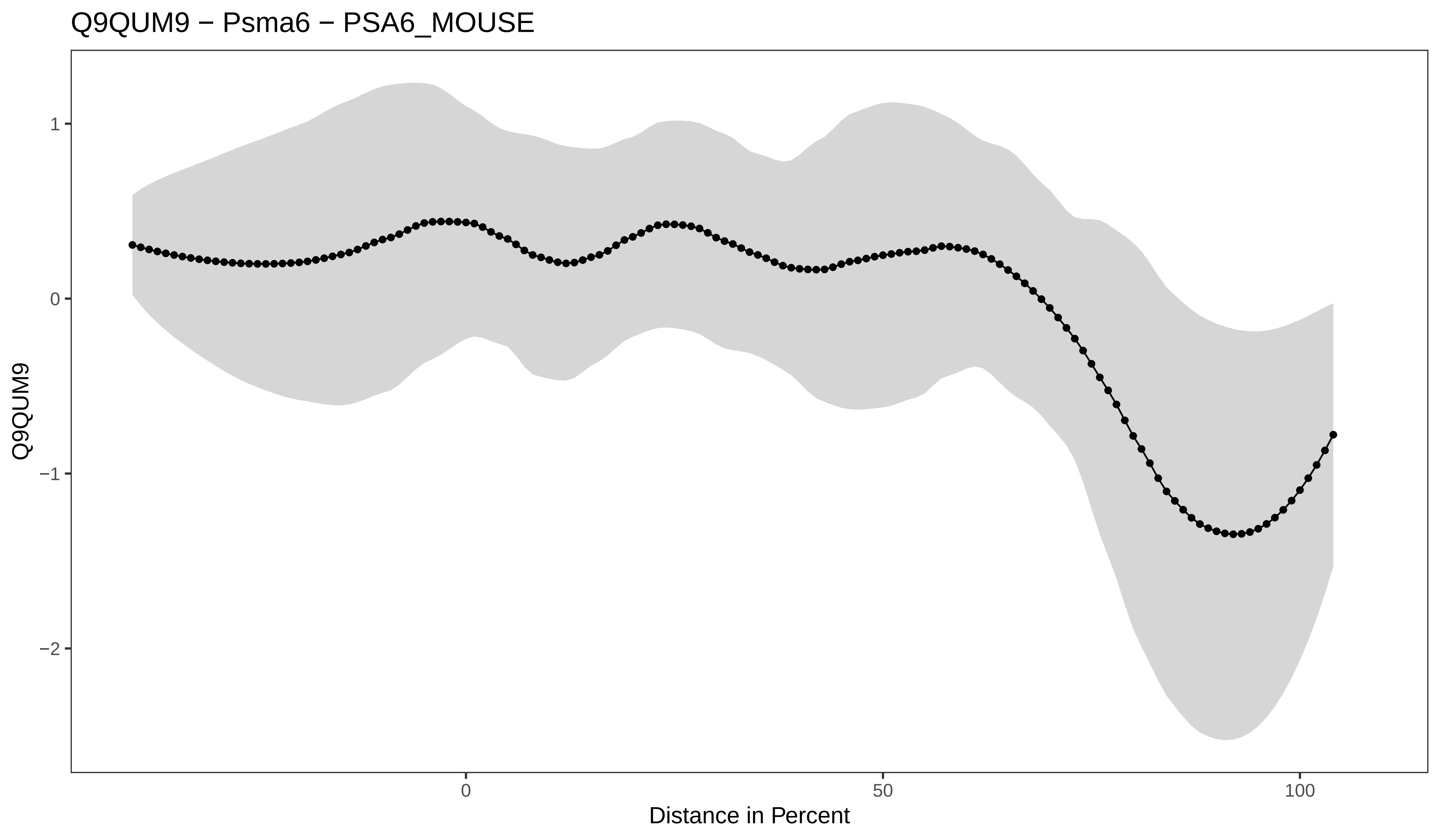

Q9QUM9 − Psma6 − PSA6_MOUSE
1
0
−1
−2
Q9QUM9
0
50
100
Distance in Percent

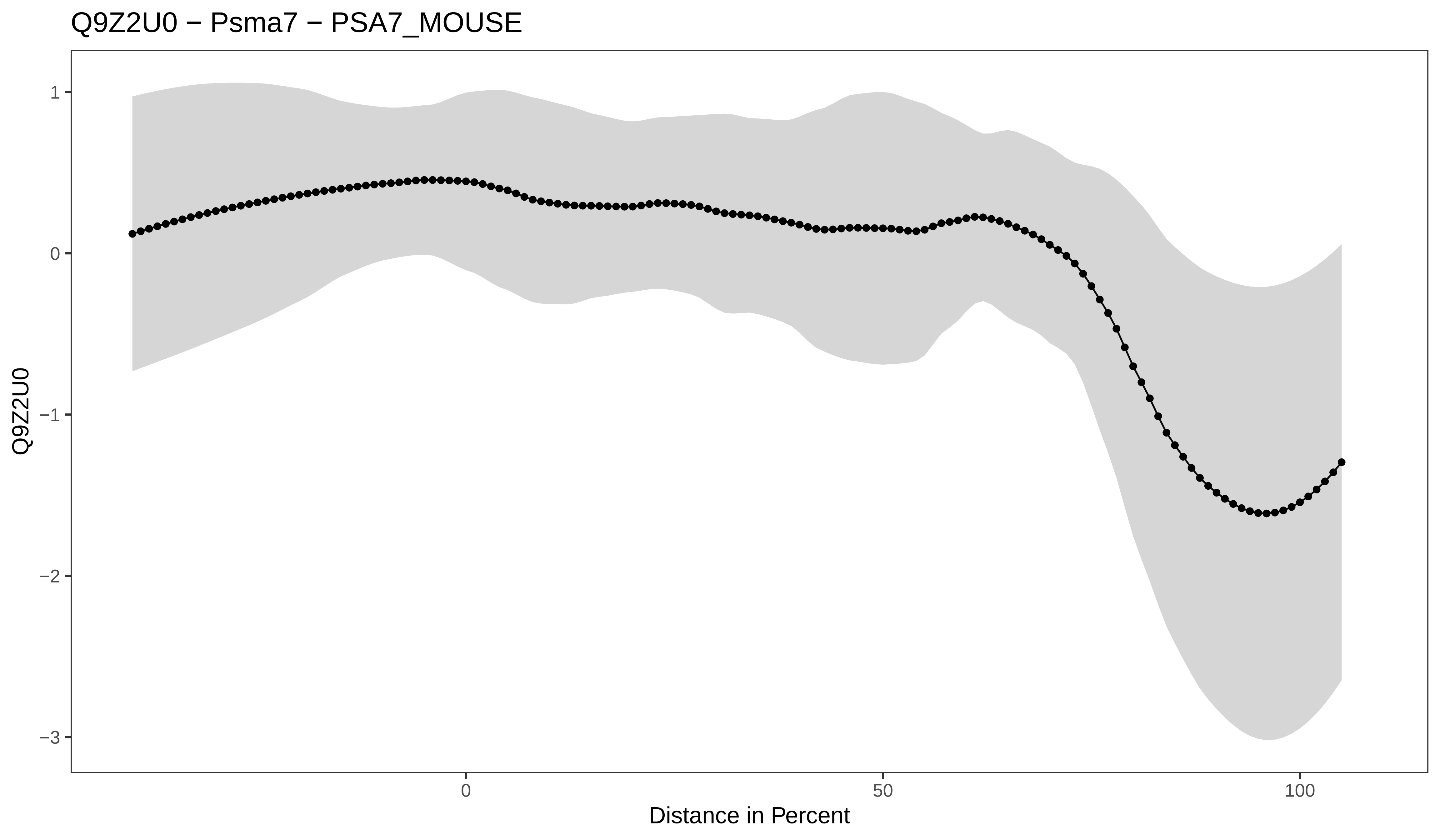

Q9Z2U0 − Psma7 − PSA7_MOUSE
1
0
−1
−2
−3
Q9Z2U0
0
50
100
Distance in Percent

O09061 − Psmb1 − PSB1_MOUSE

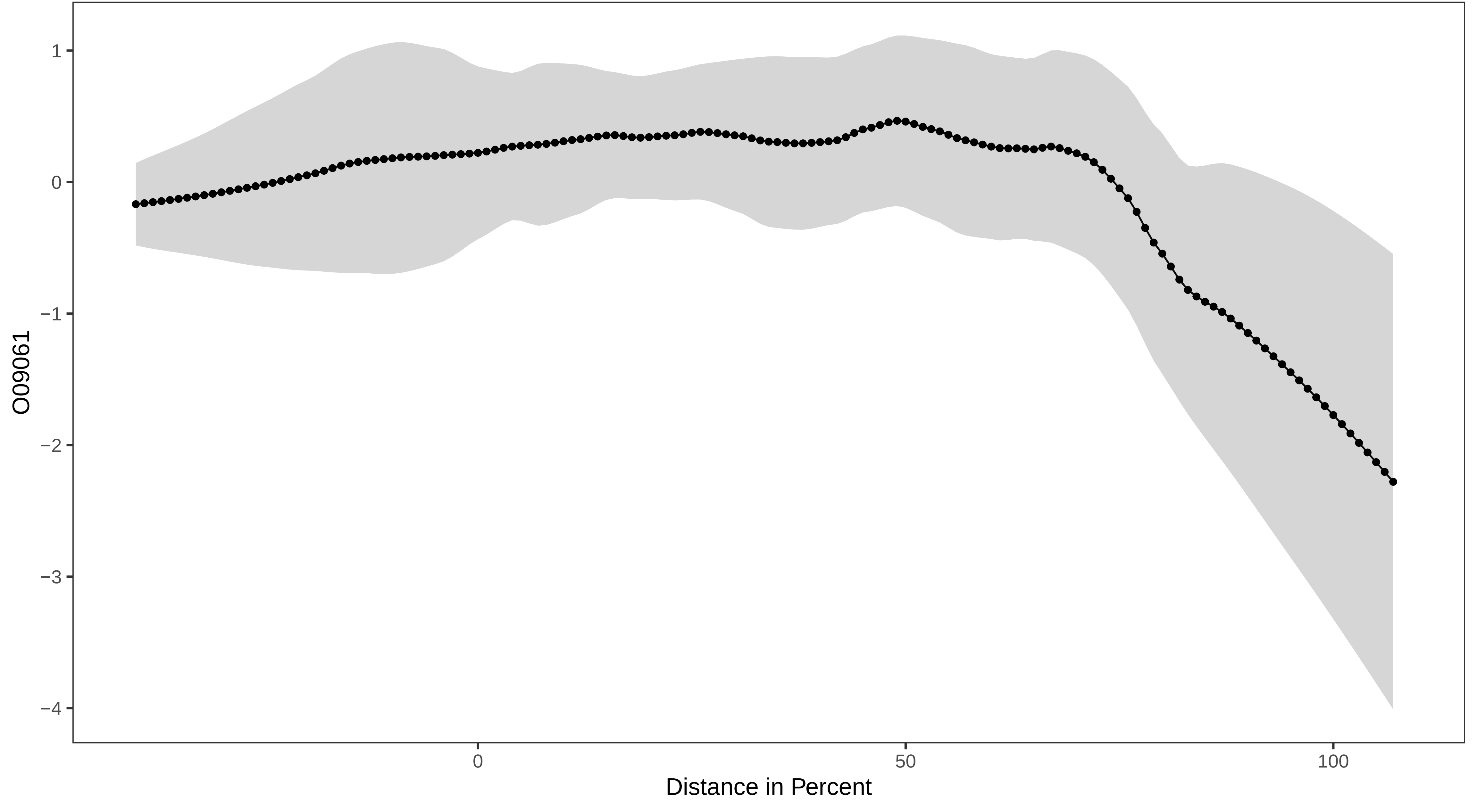

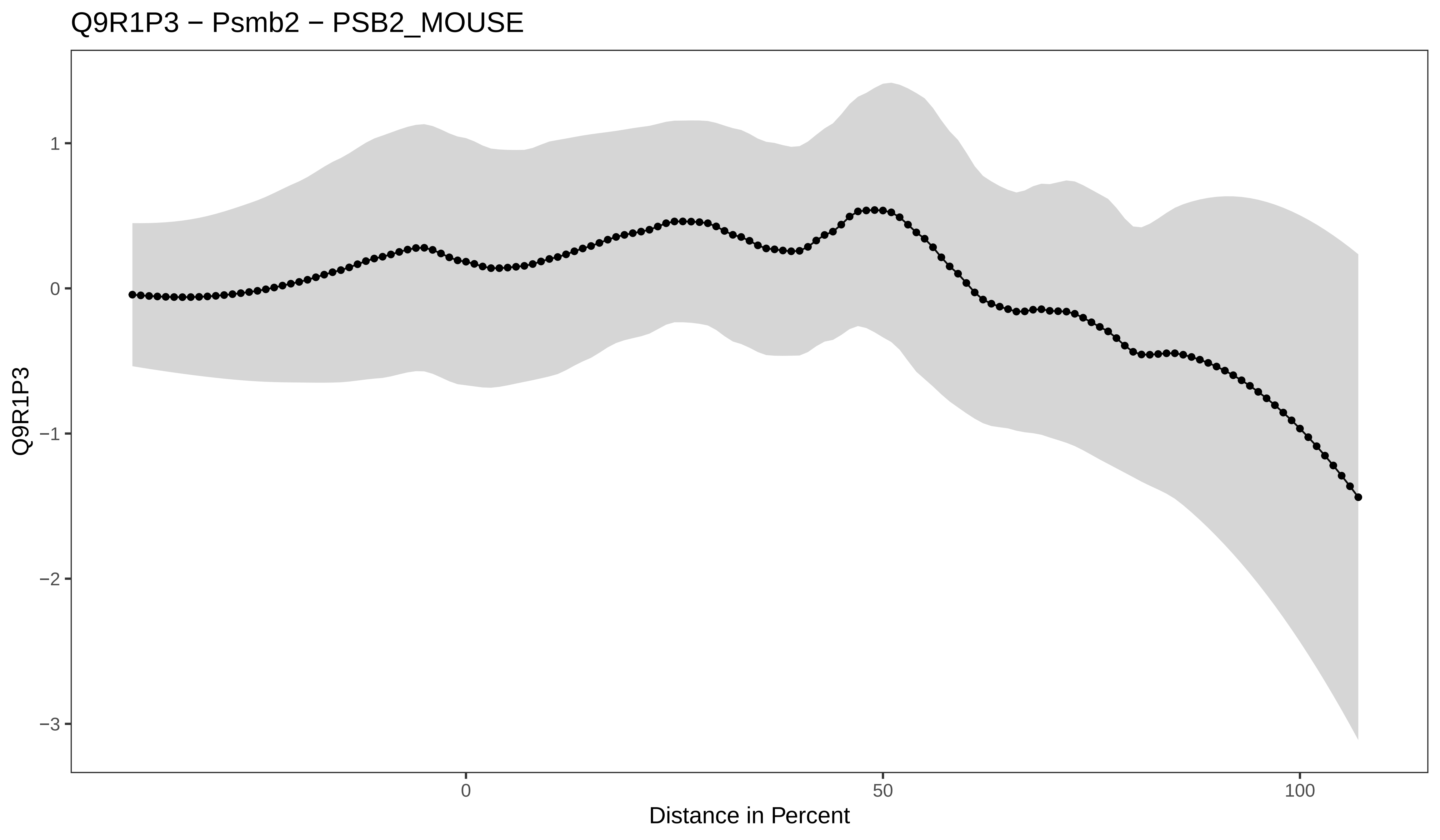

Q9R1P3 − Psmb2 − PSB2_MOUSE
Q9R1P3
Distance in Percent
1
0
−1
−2
−3
0
50
100

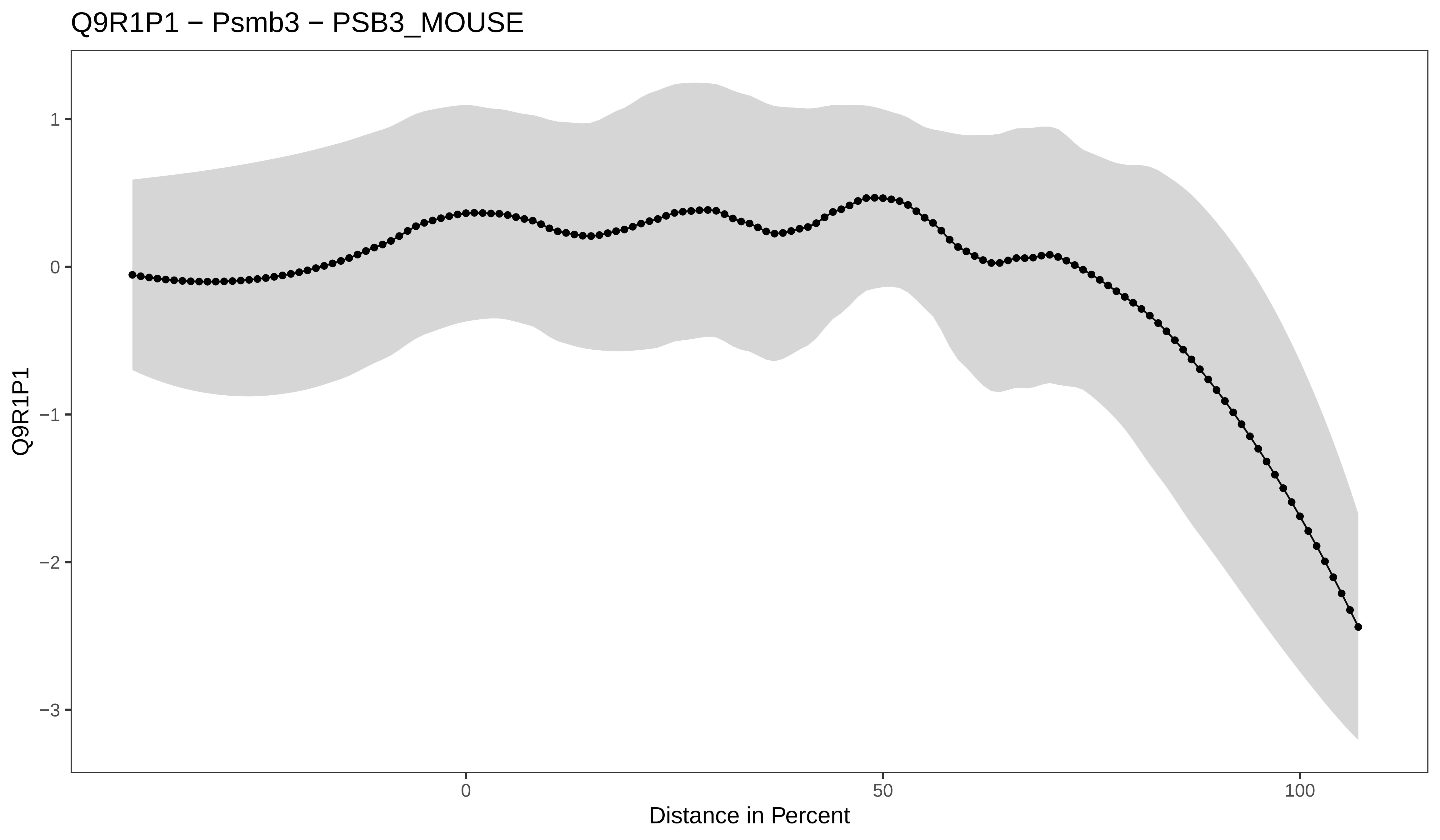

Q9R1P1 − Psmb3 − PSB3_MOUSE
Q9R1P1
1
0
−1
−2
−3
0
50
100
Distance in Percent

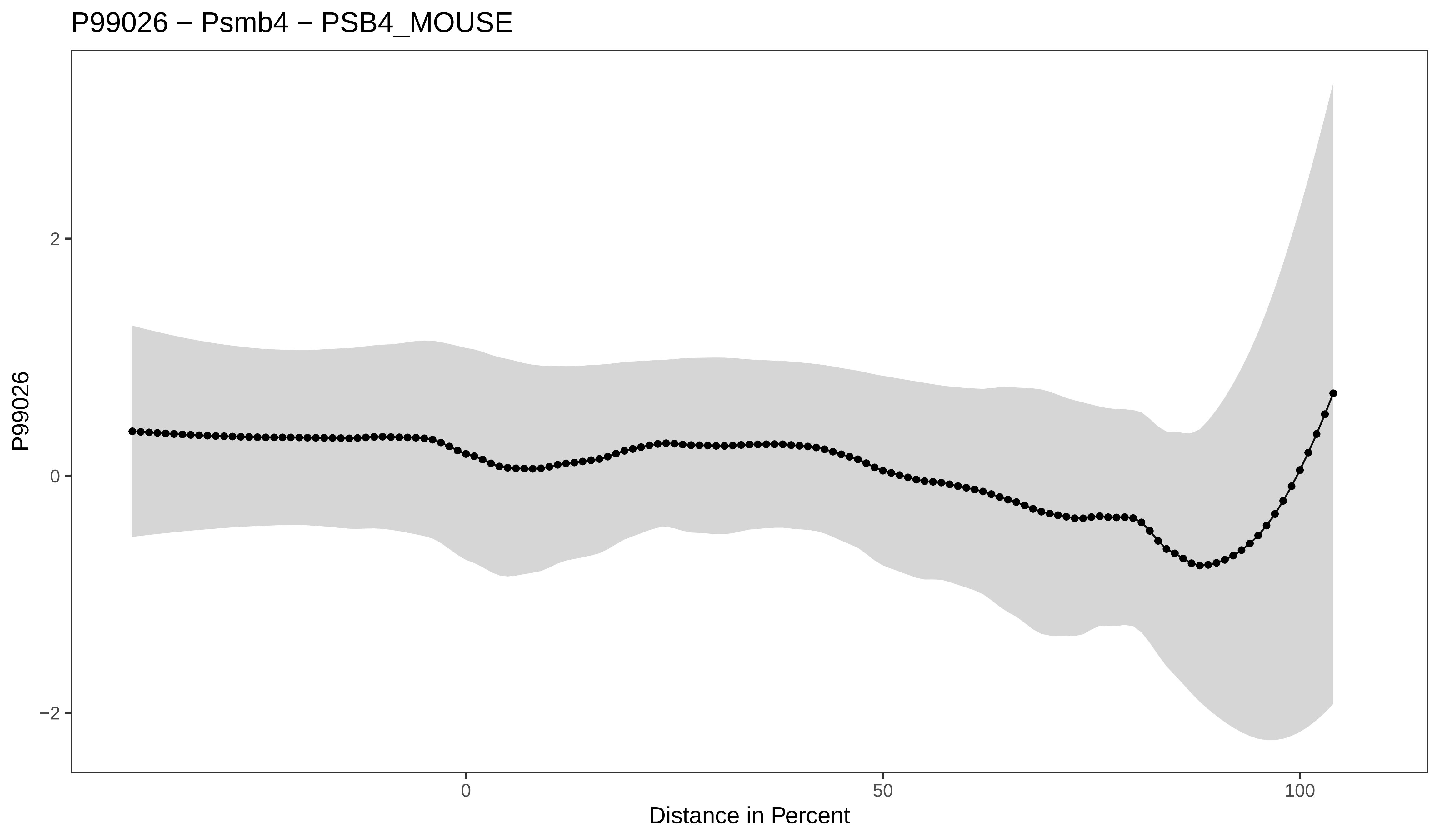
P99026 − Psmb4 − PSB4_MOUSE
P99026
2
0
−2
0
50
100
Distance in Percent

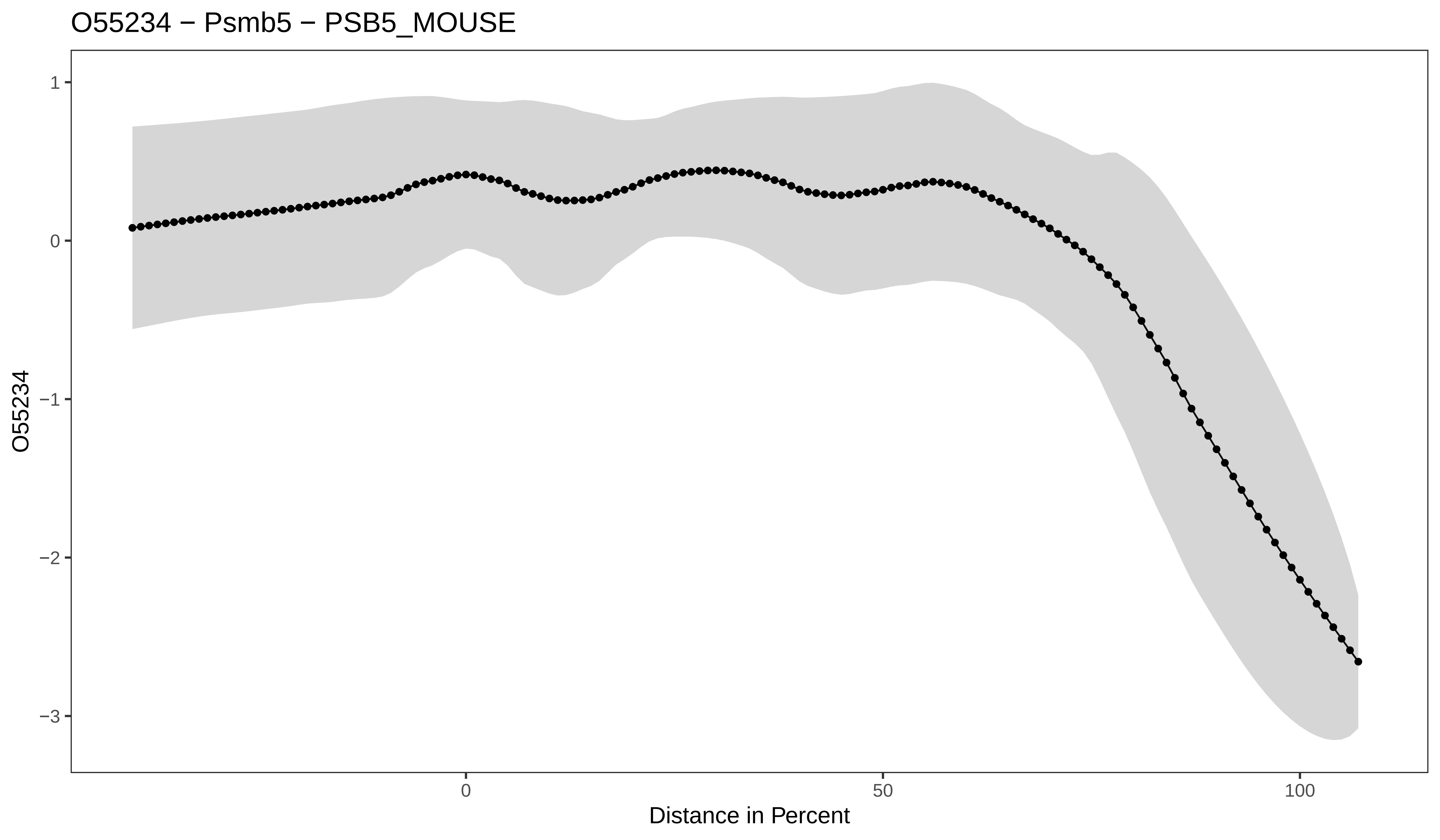

O55234 − Psmb5 − PSB5_MOUSE
1
0
−1
−2
−3
O55234
0
50
100
Distance in Percent

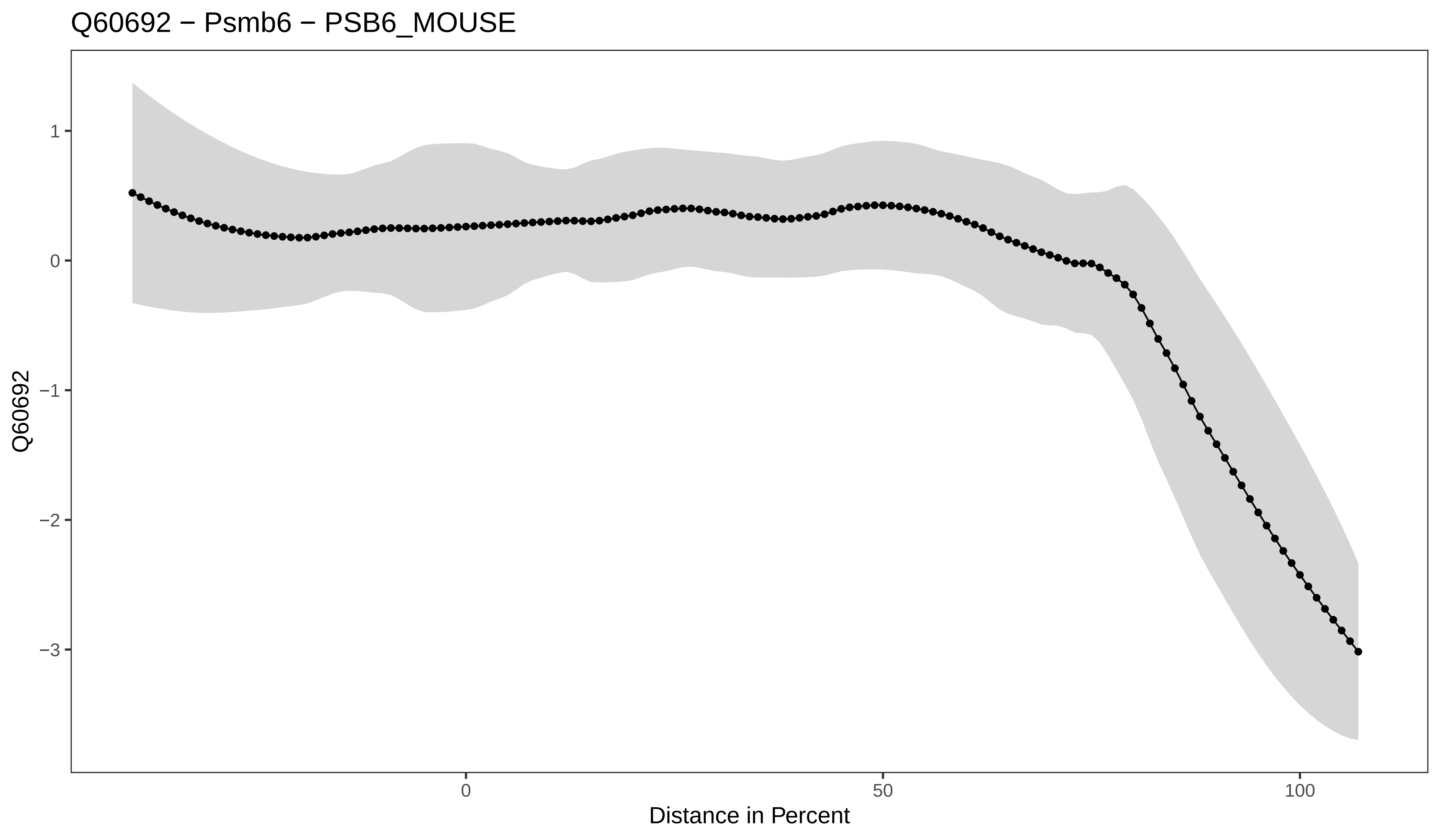

Q60692 − Psmb6 − PSB6_MOUSE
1
0
−1
−2
−3
Q60692
0
50
100
Distance in Percent

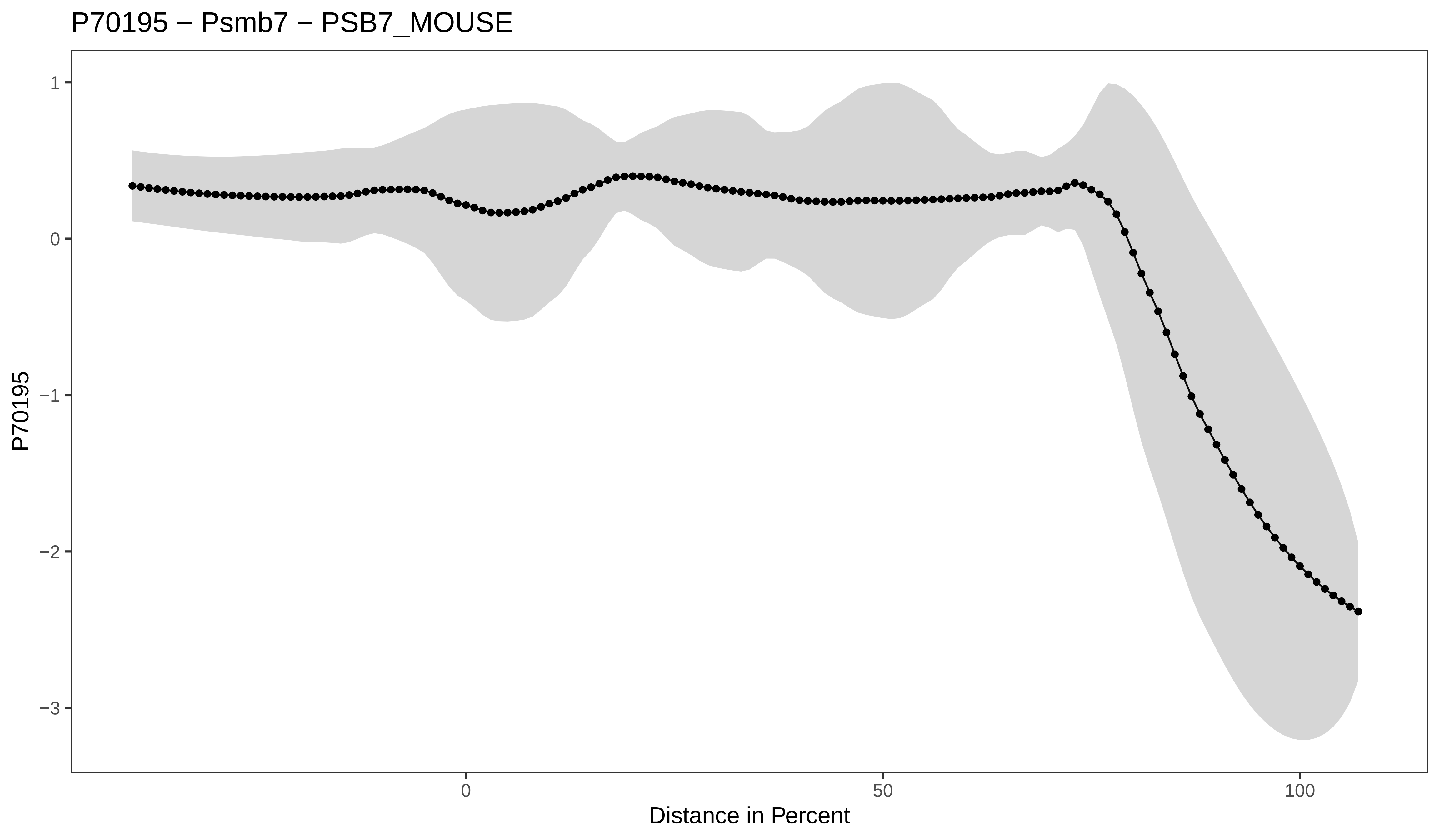

P70195 − Psmb7 − PSB7_MOUSE
1
0
−1
−2
−3
P70195
0
50
100
Distance in Percent

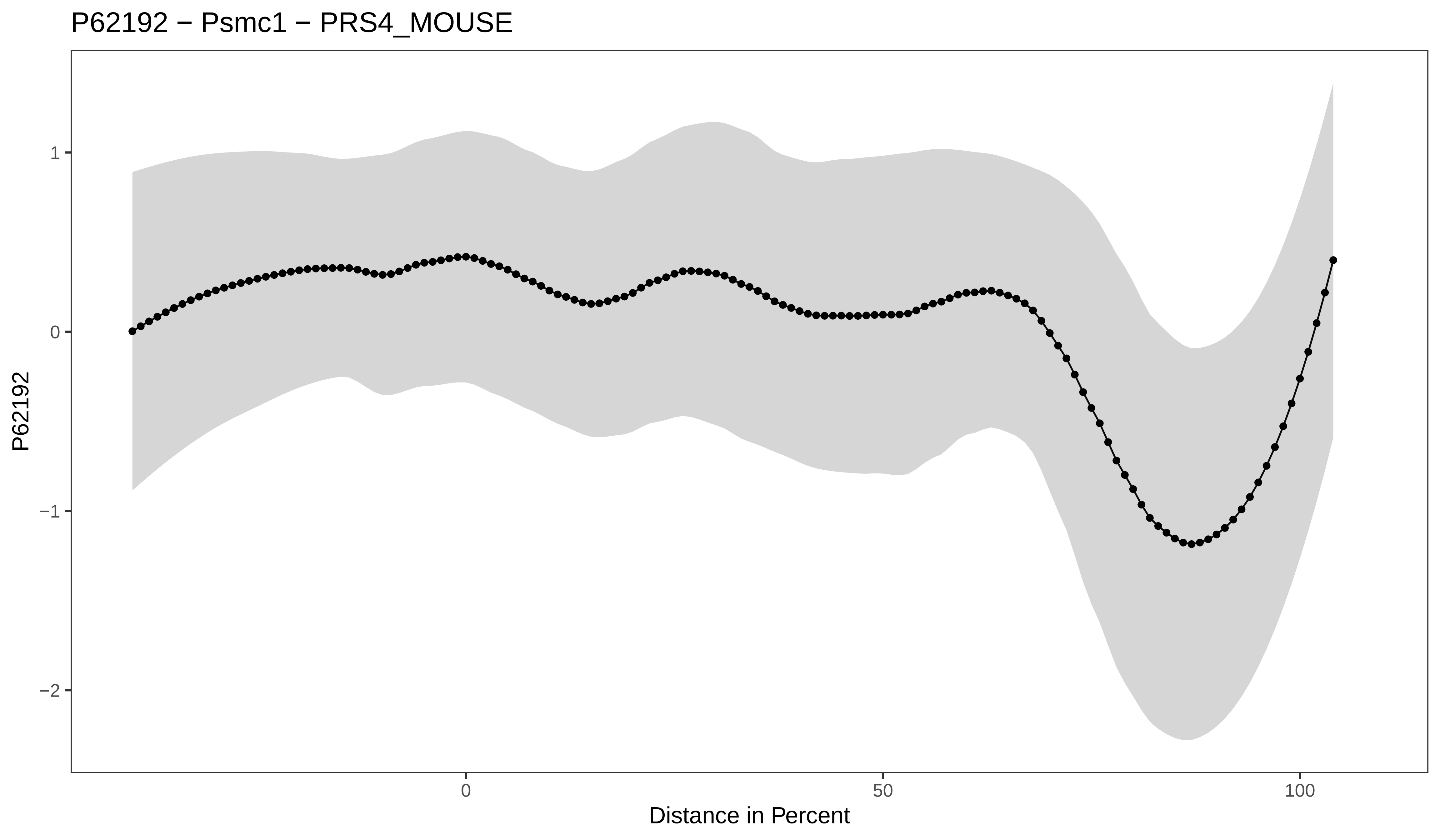
P62192 − Psmc1 − PRS4_MOUSE
P62192
Distance in Percent
1
0
−1
−2
0
50
100

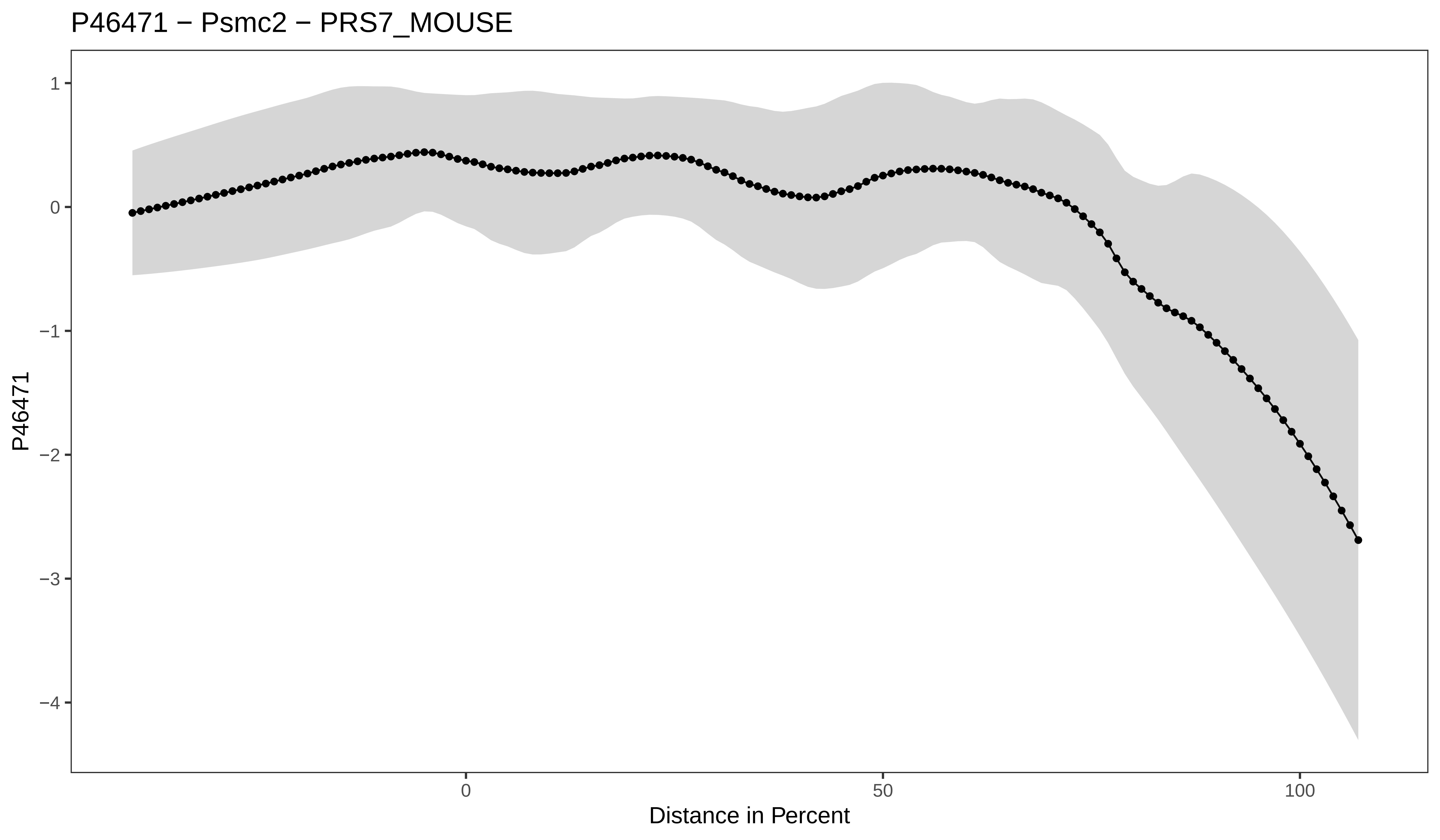

P46471 − Psmc2 − PRS7_MOUSE
1
0
−1
−2
−3
−4
P46471
0
50
100
Distance in Percent

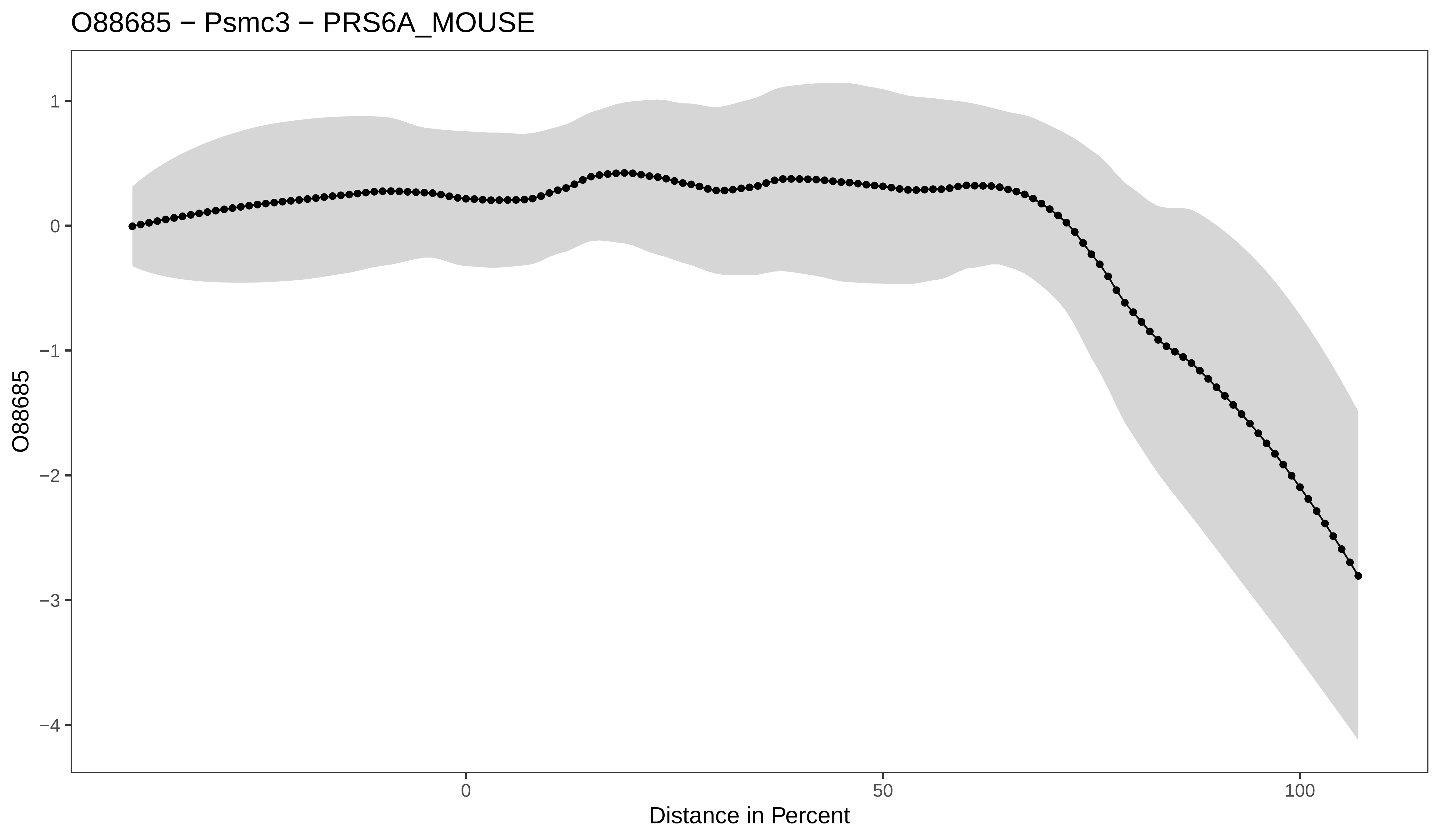

O88685 − Psmc3 − PRS6A_MOUSE
O88685
1
0
−1
−2
−3
−4
0
50
100
Distance in Percent

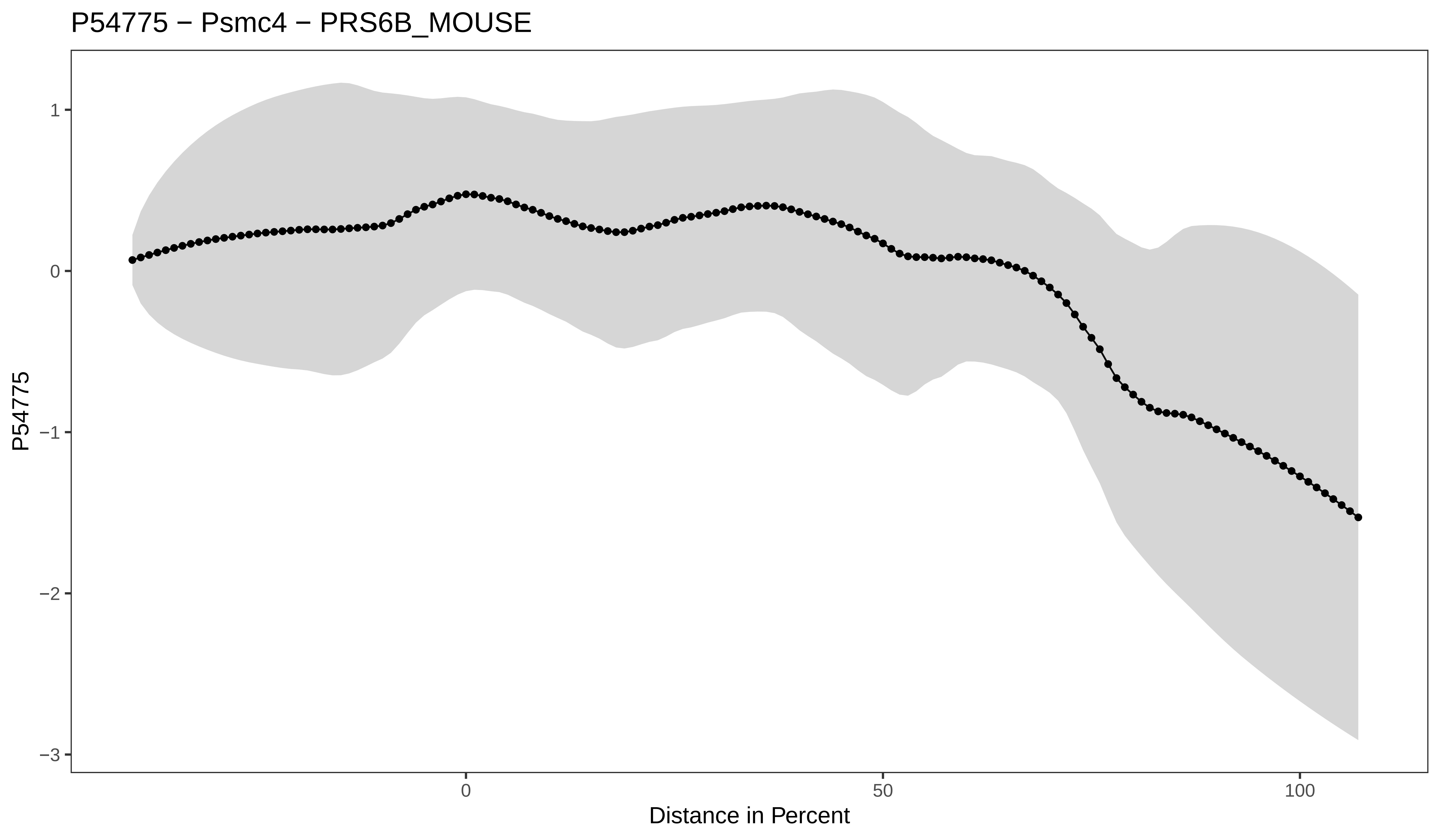

P54775 − Psmc4 − PRS6B_MOUSE
1
0
−1
−2
−3
P54775
0
50
100
Distance in Percent

P62196 − Psmc5 − PRS8_MOUSE

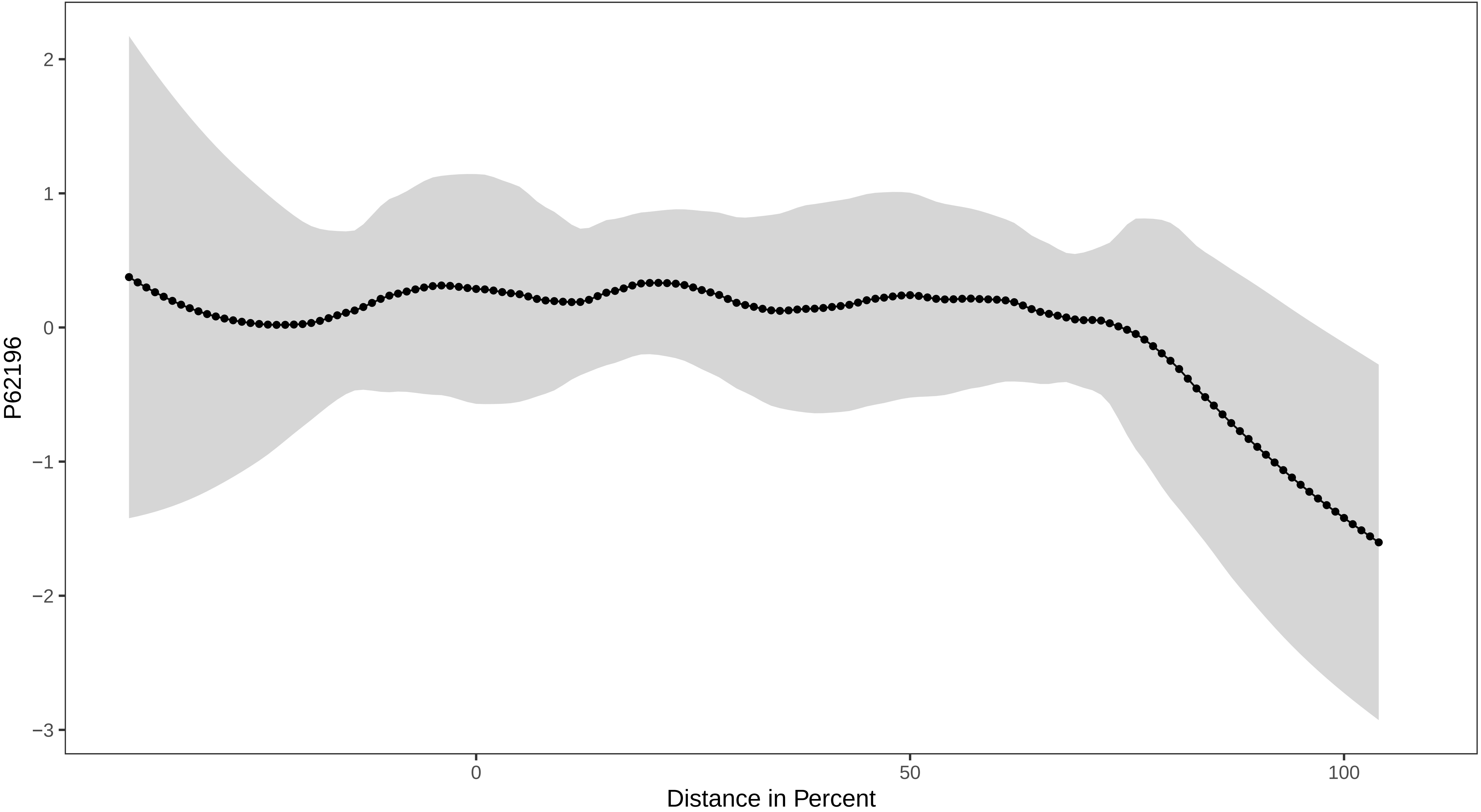

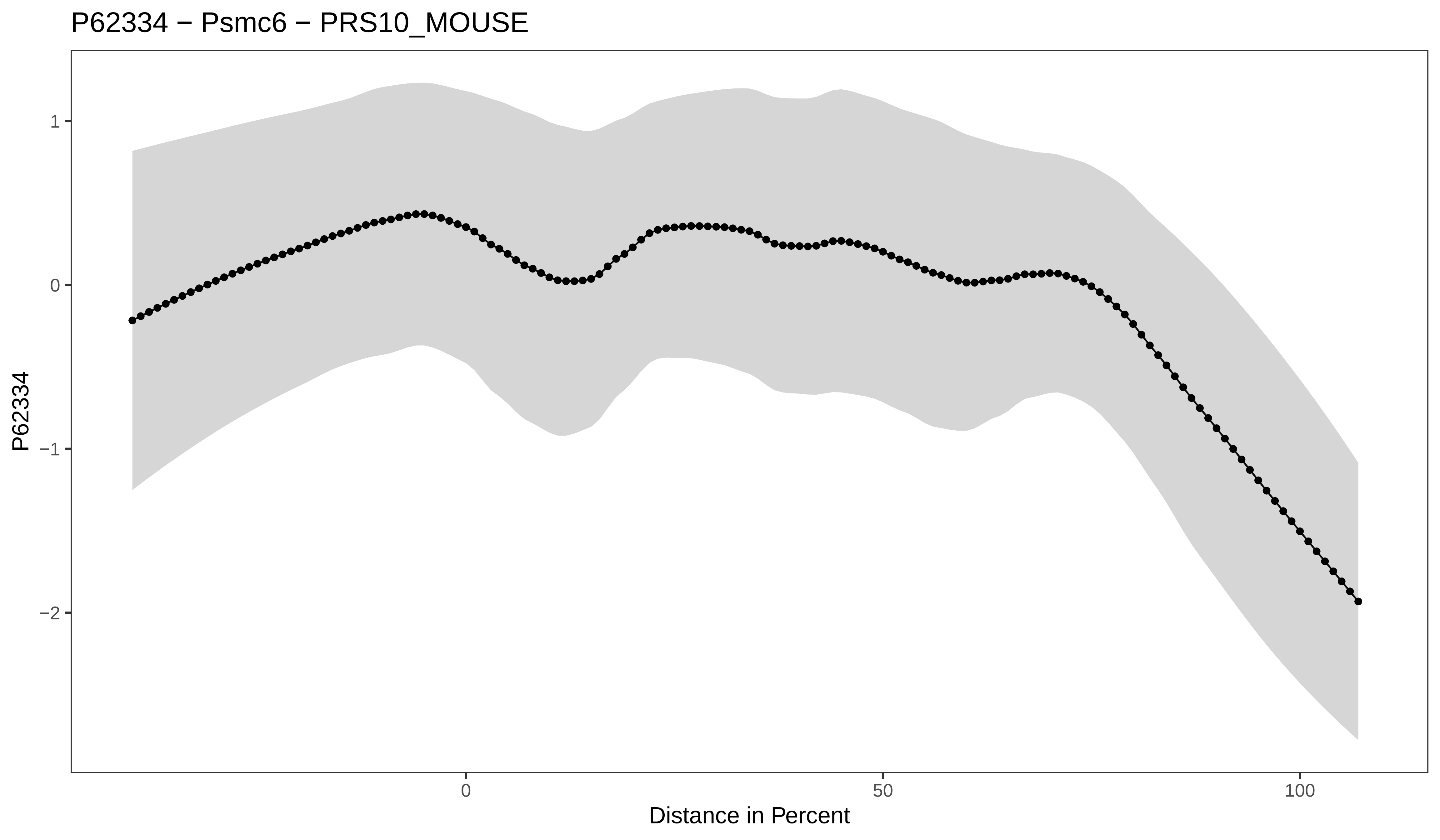

P62334 − Psmc6 − PRS10_MOUSE
1
0
−1
−2
P62334
0
50
100
Distance in Percent

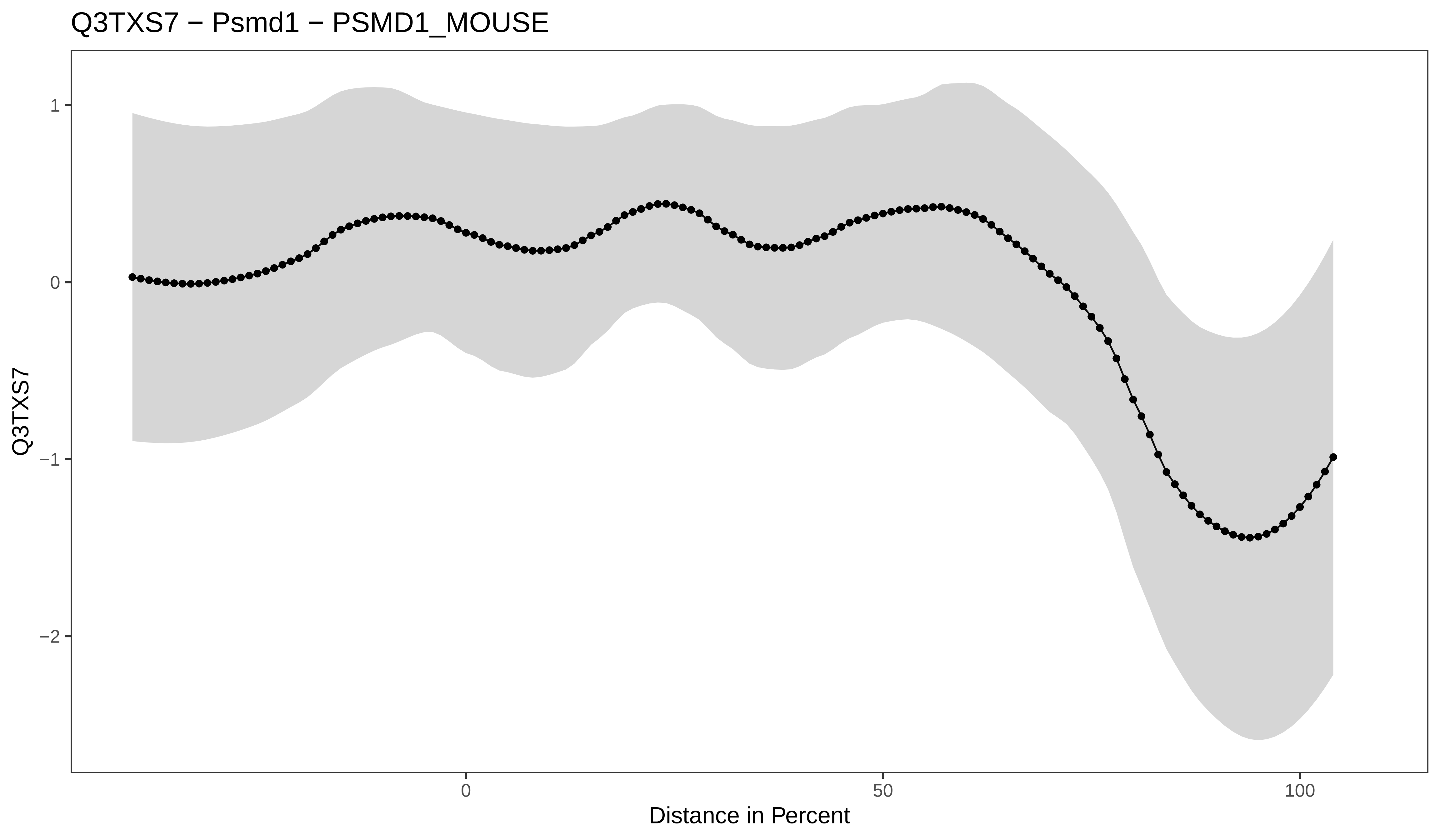
Q3TXS7 − Psmd1 − PSMD1_MOUSE
1
0
−1
−2
Q3TXS7
0
50
100
Distance in Percent

Q8BG32 − Psmd11 − PSD11_MOUSE

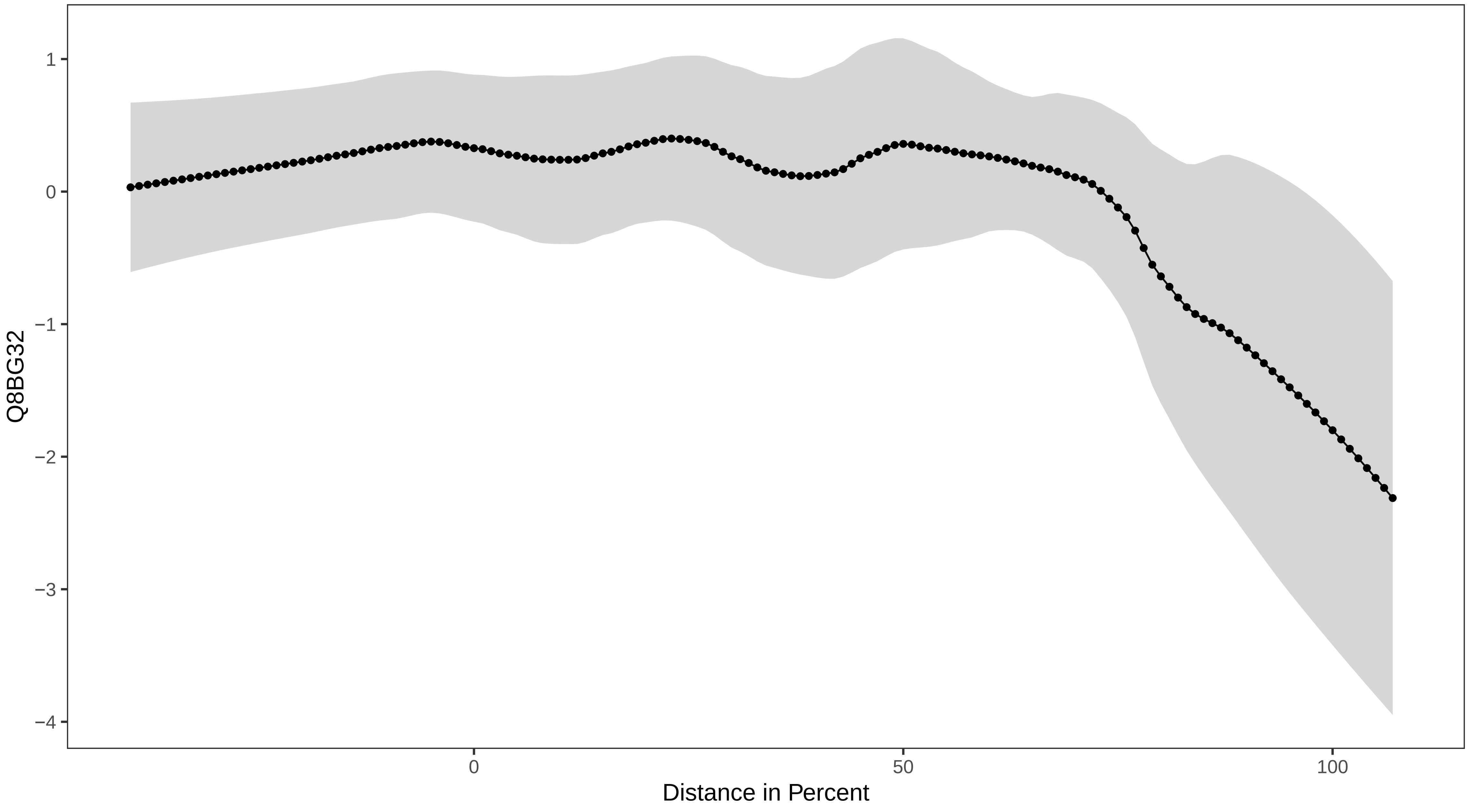

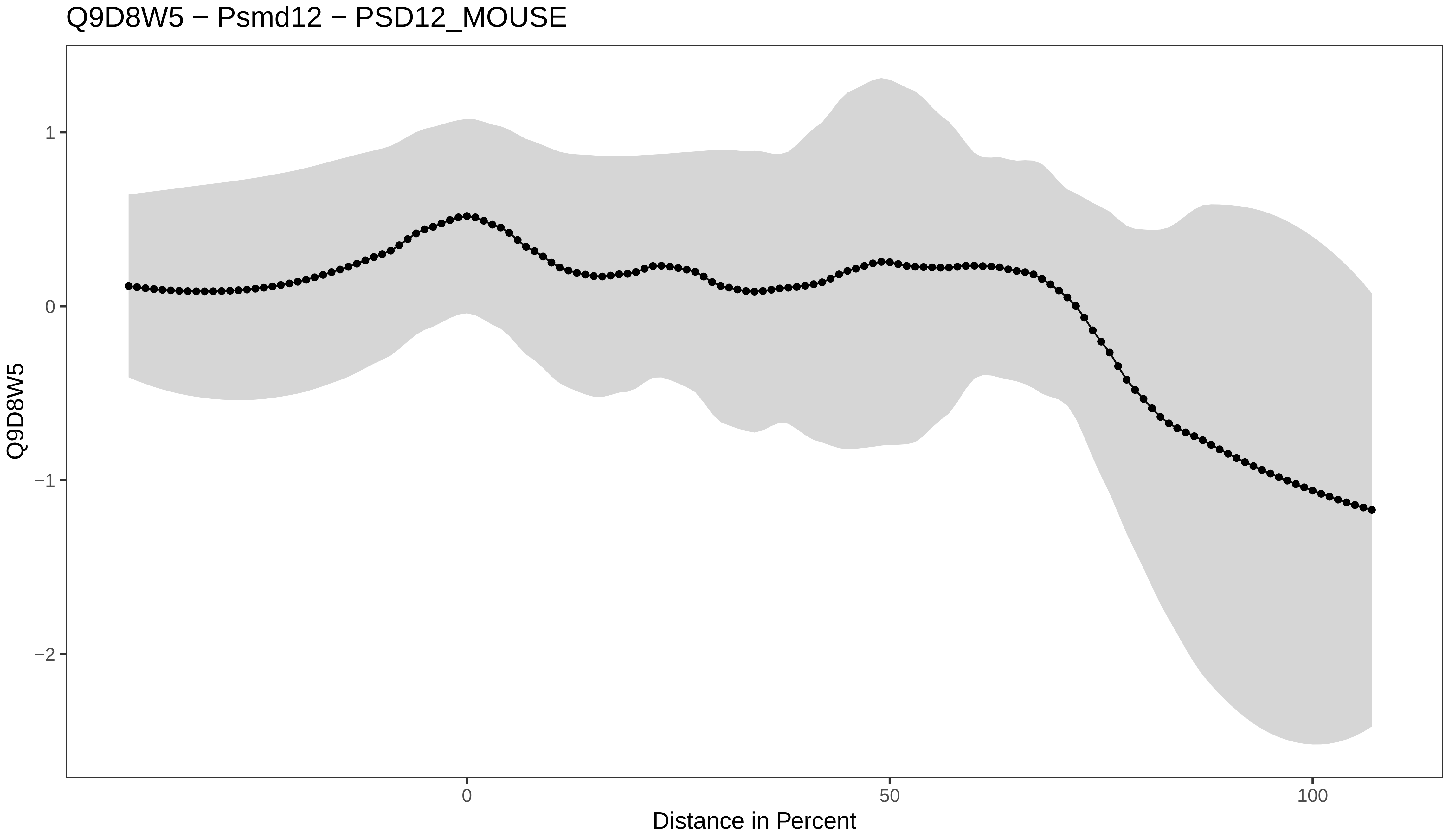

Q9D8W5 − Psmd12 − PSD12_MOUSE
Q9D8W5
1
0
−1
−2
0
50
100
Distance in Percent

Q9WVJ2 − Psmd13 − PSD13_MOUSE

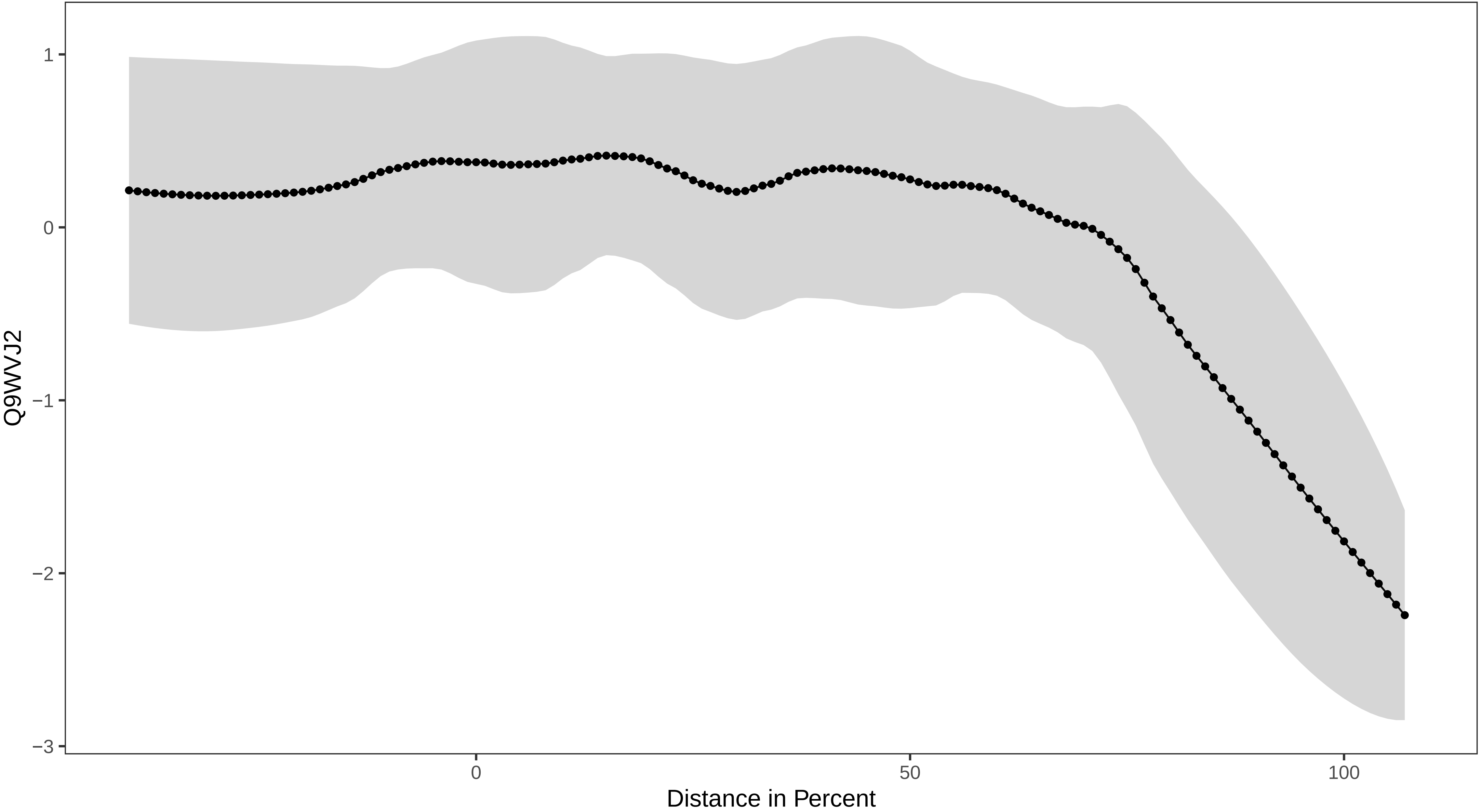

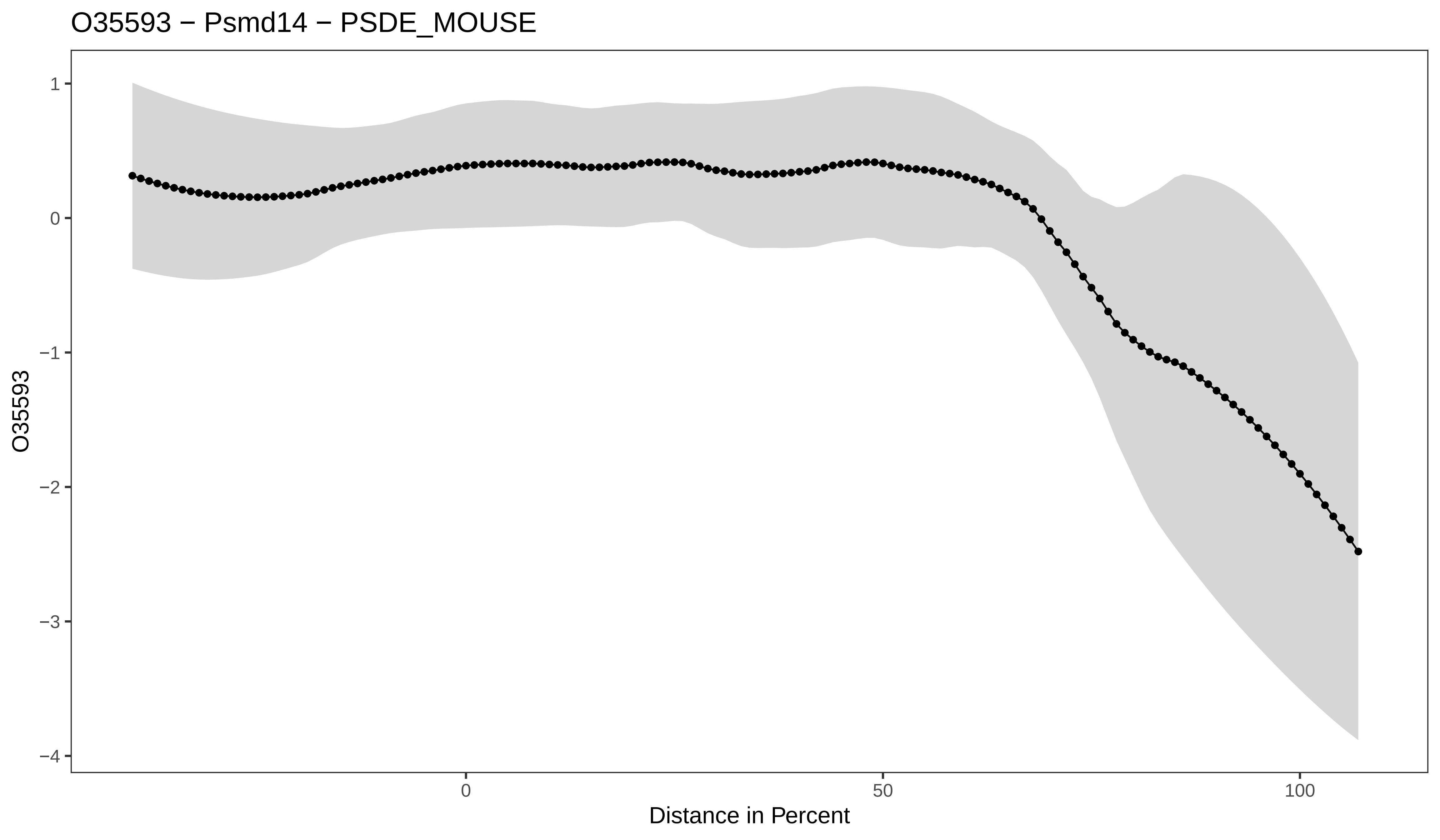

O35593 − Psmd14 − PSDE_MOUSE
1
0
−1
−2
−3
−4
O35593
0
50
100
Distance in Percent

Q8VDM4 − Psmd2 − PSMD2_MOUSE

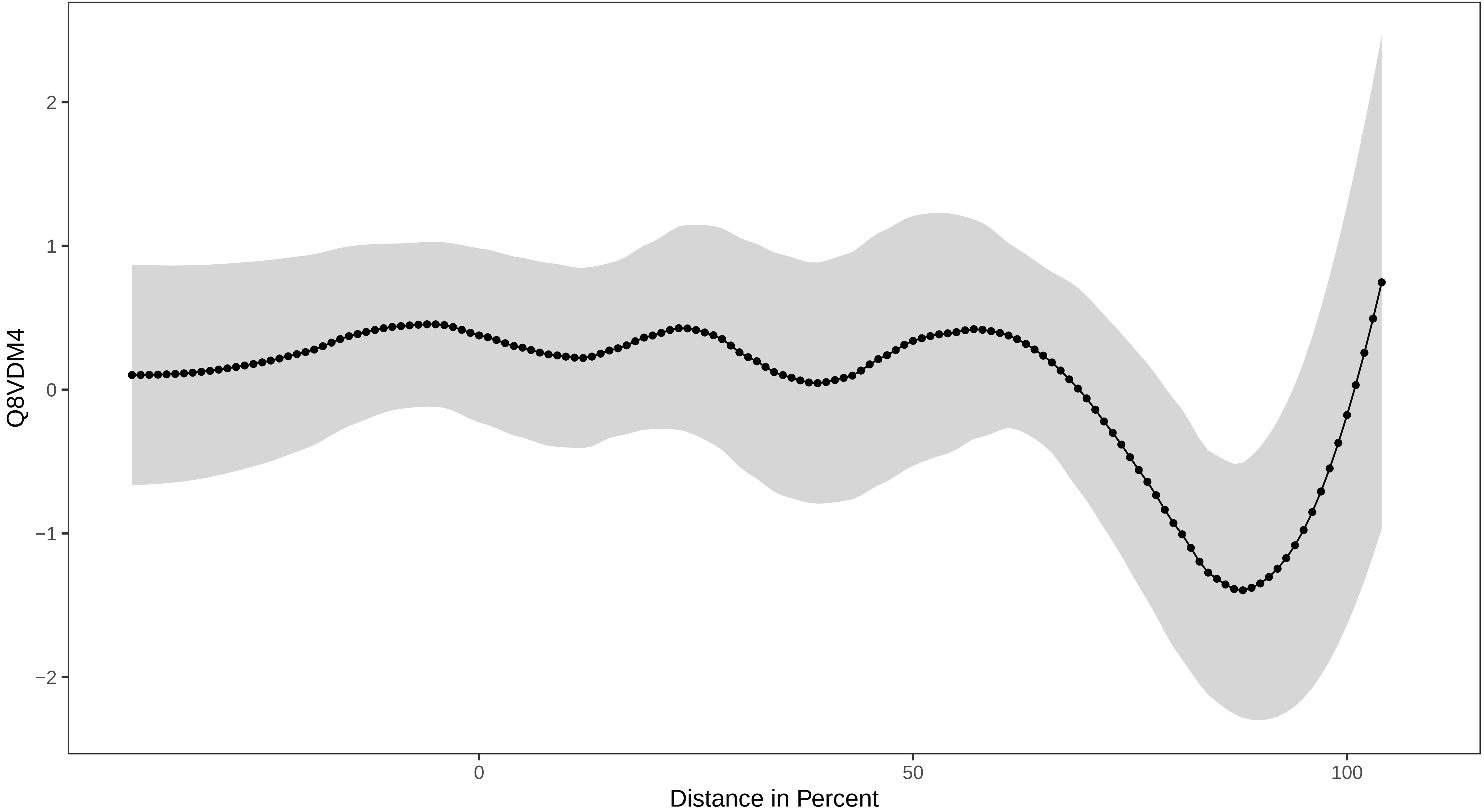

P14685 − Psmd3 − PSMD3_MOUSE

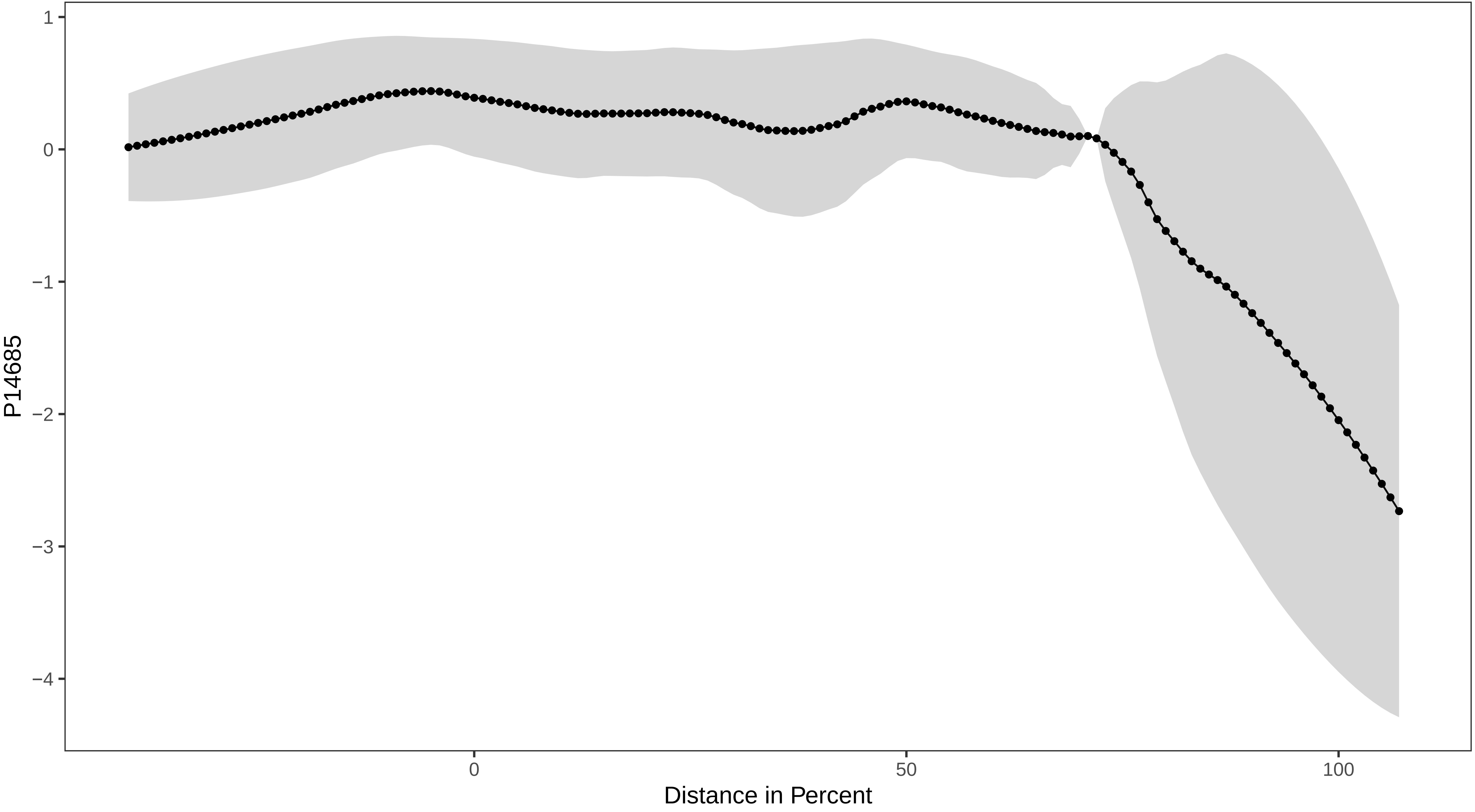

O35226 − Psmd4 − PSMD4_MOUSE

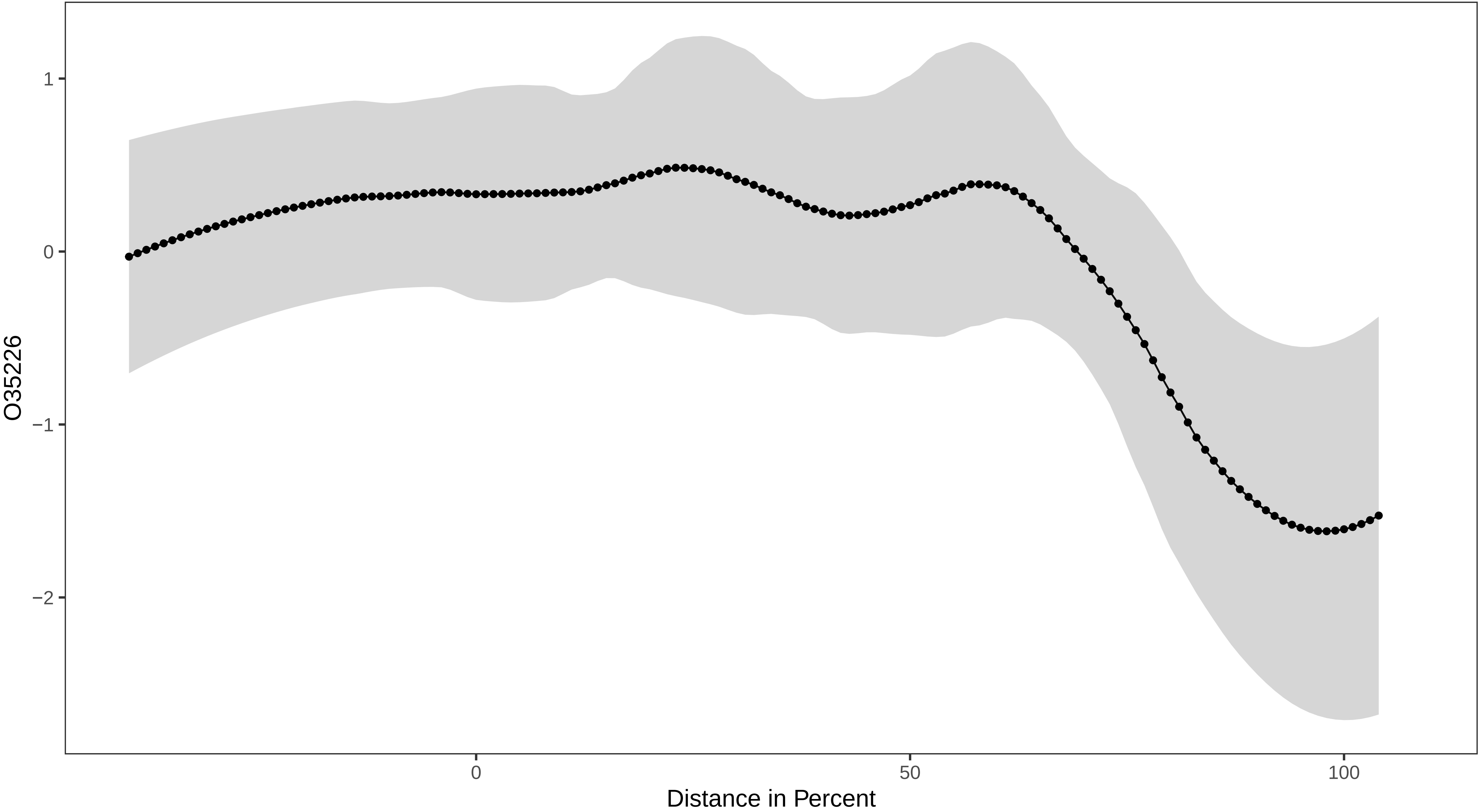

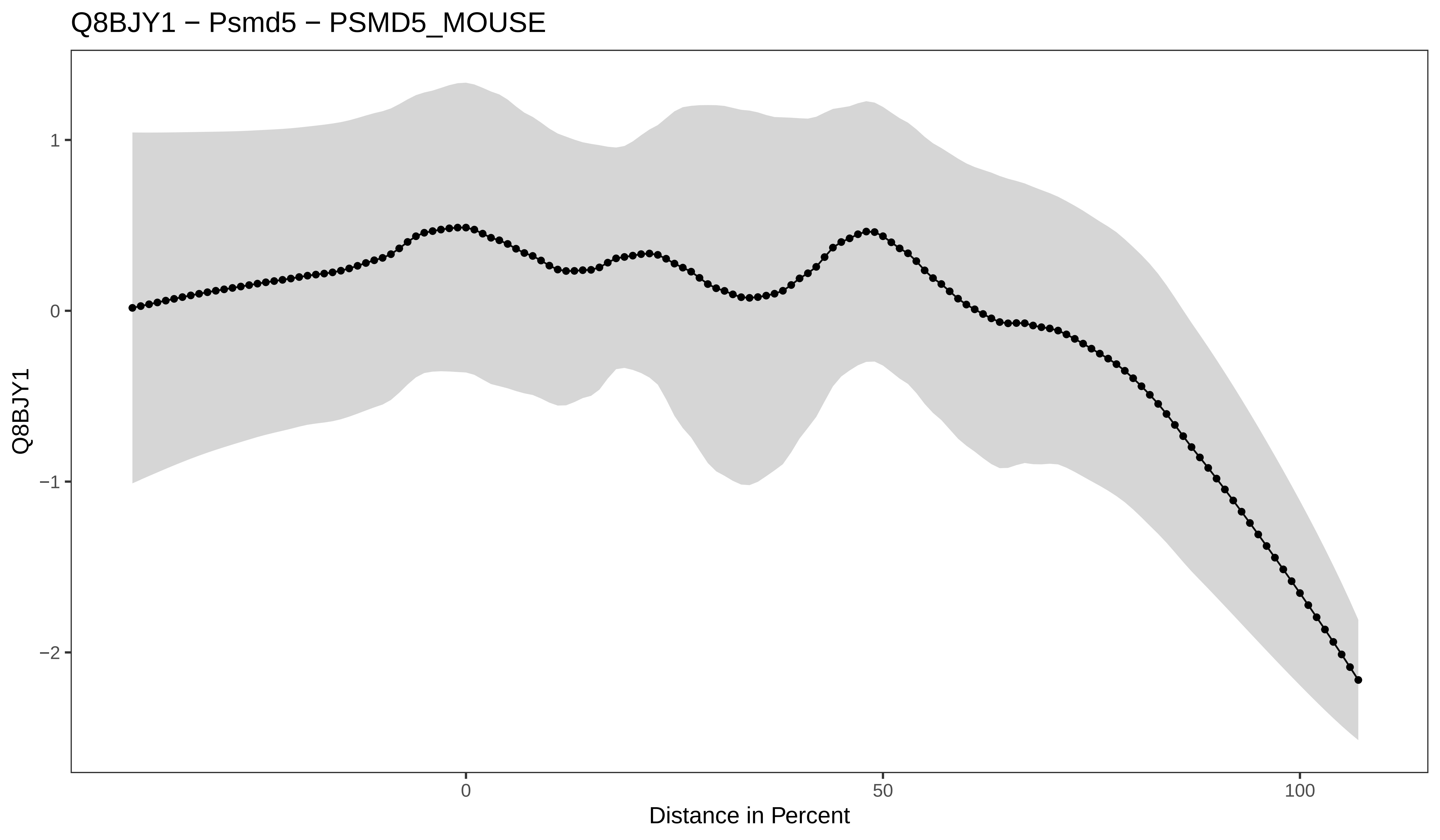

Q8BJY1 − Psmd5 − PSMD5_MOUSE
Q8BJY1
1
0
−1
−2
0
50
100
Distance in Percent

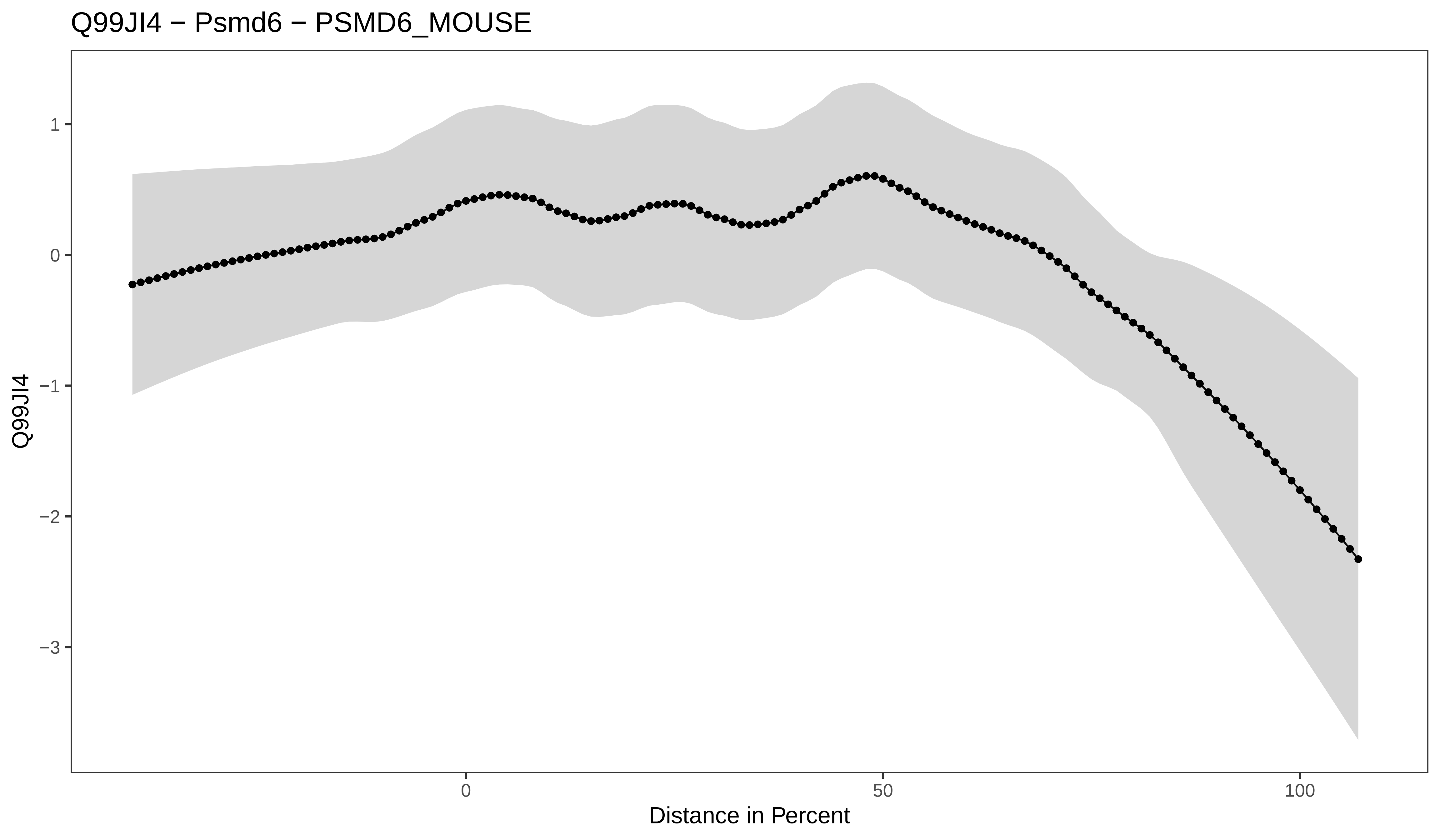

Q99JI4 − Psmd6 − PSMD6_MOUSE
Q99JI4
1
0
−1
−2
−3
0
50
100
Distance in Percent

P26516 − Psmd7 − PSMD7_MOUSE

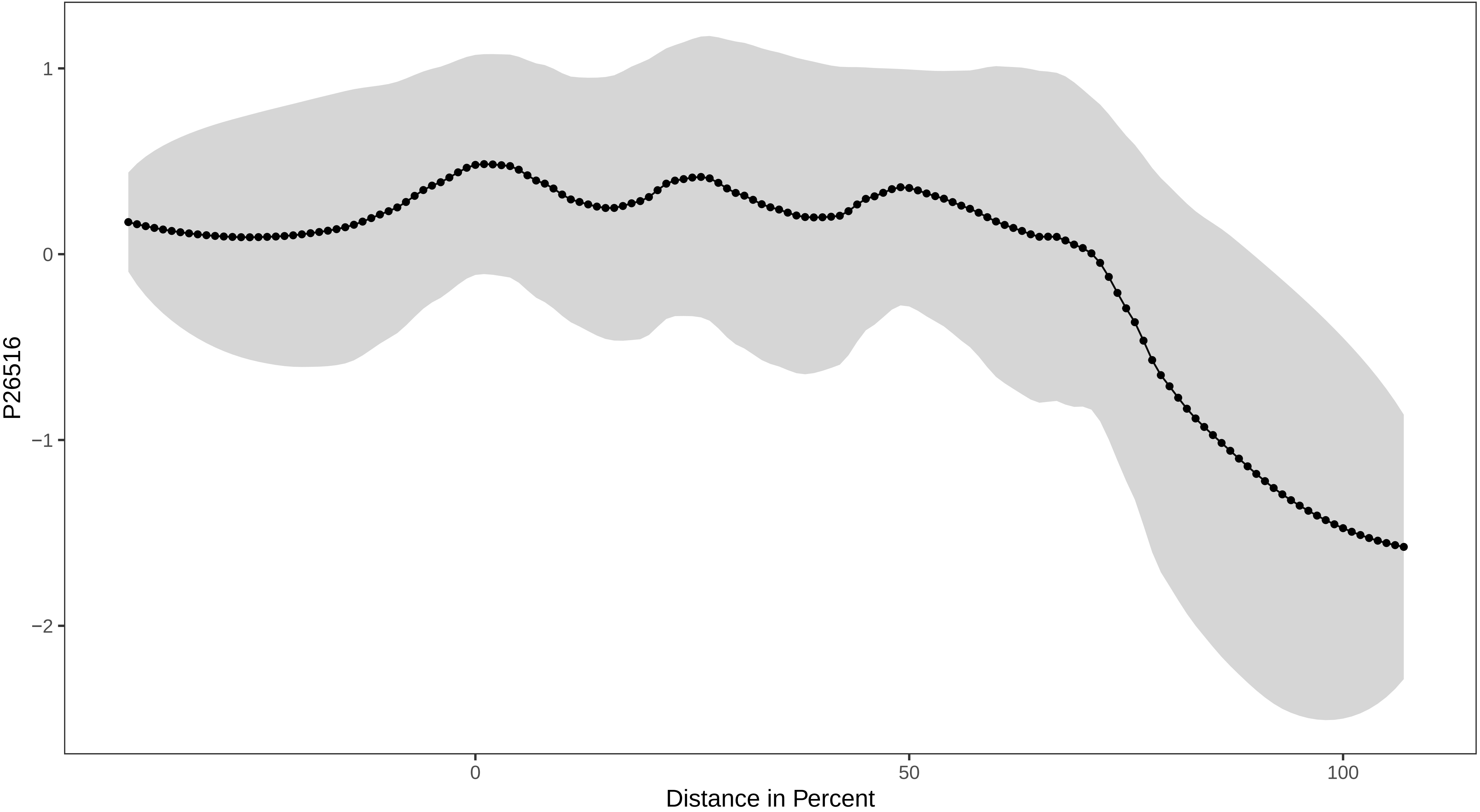

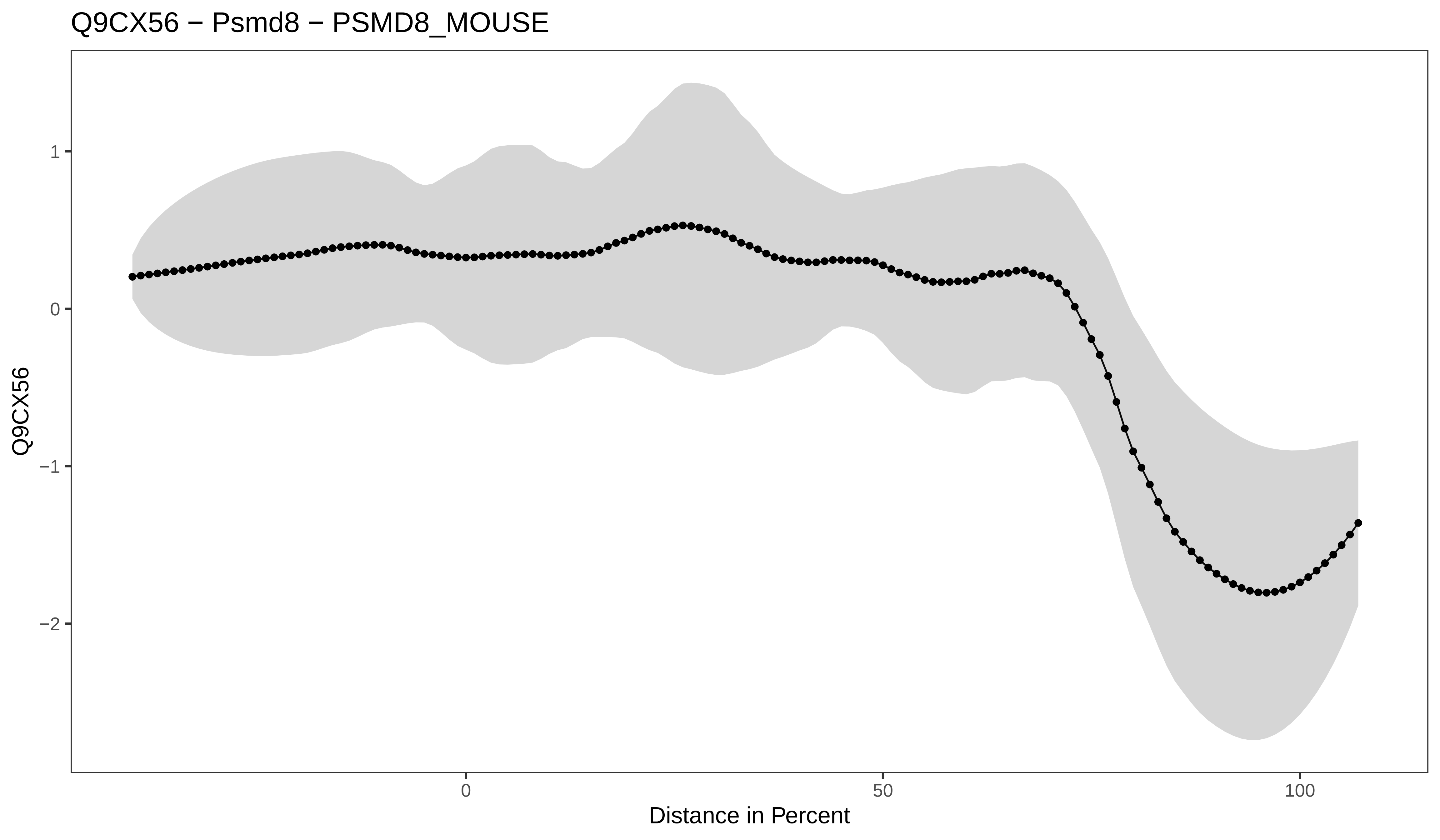
Q9CX56 − Psmd8 − PSMD8_MOUSE
Q9CX56
1
0
−1
−2
0
50
100
Distance in Percent

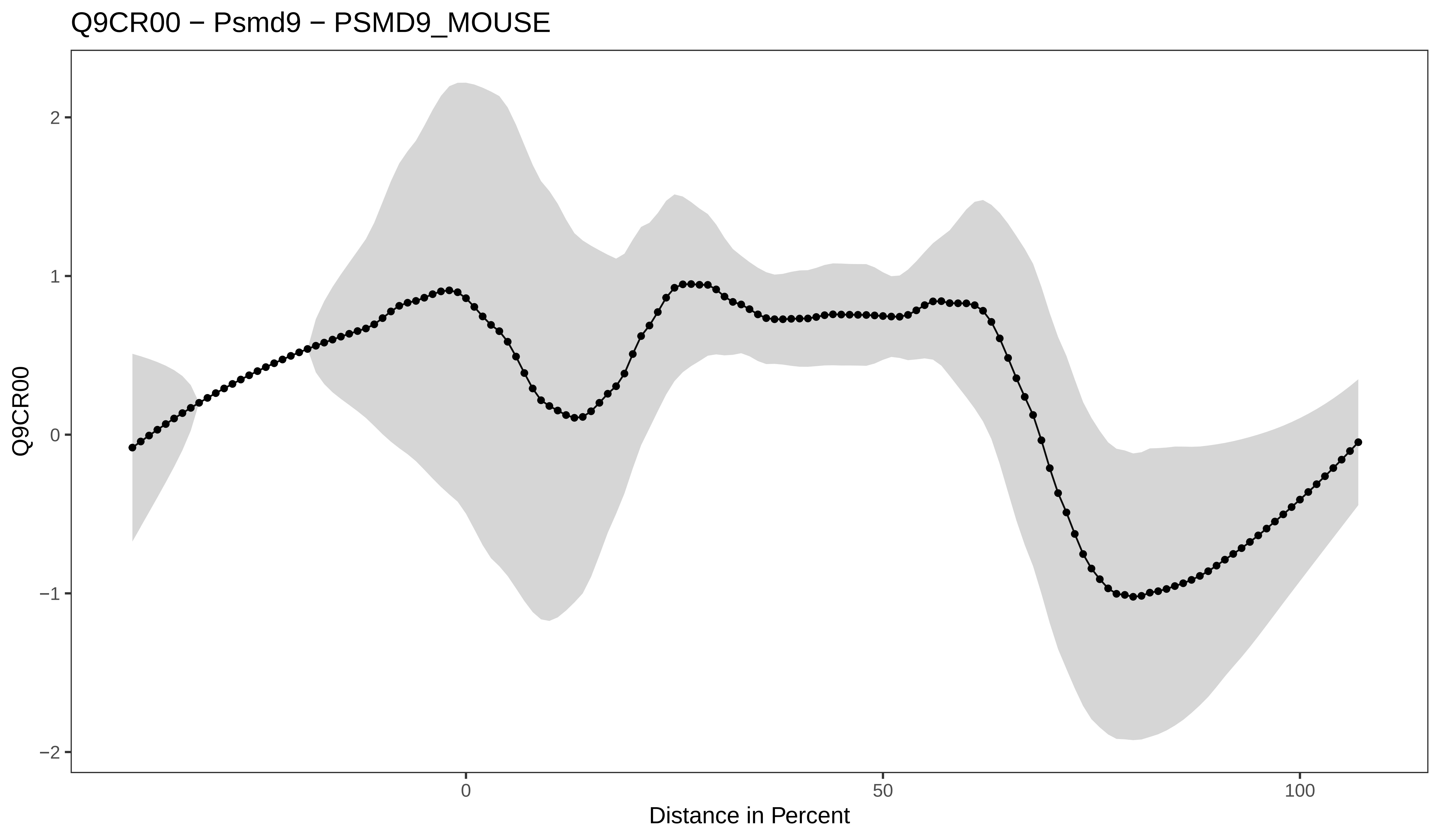
Q9CR00 − Psmd9 − PSMD9_MOUSE
Q9CR00
Distance in Percent
2
1
0
−1
−2
0
50
100

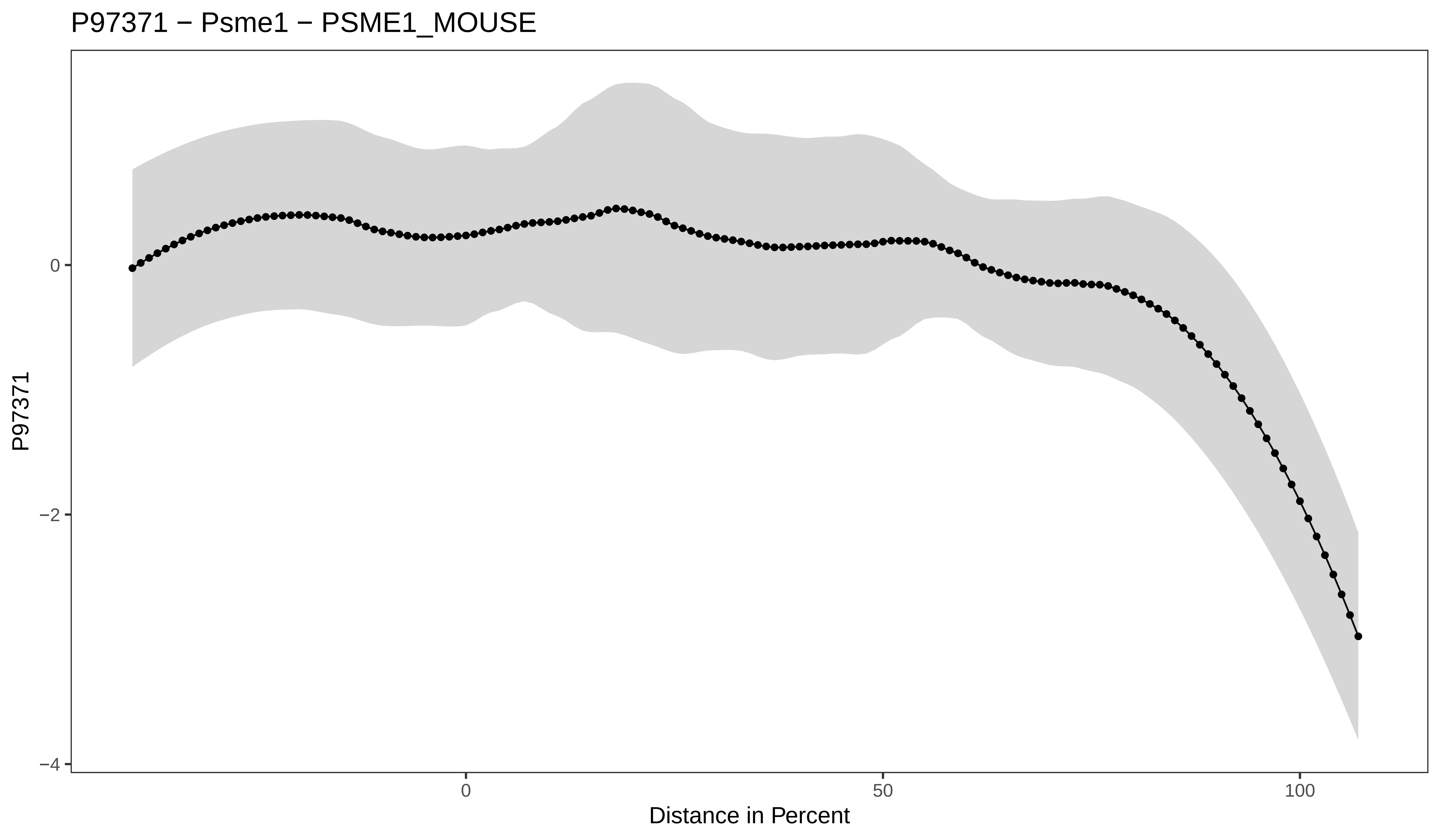

P97371 − Psme1 − PSME1_MOUSE
P97371
0
−2
−4
0
50
100
Distance in Percent

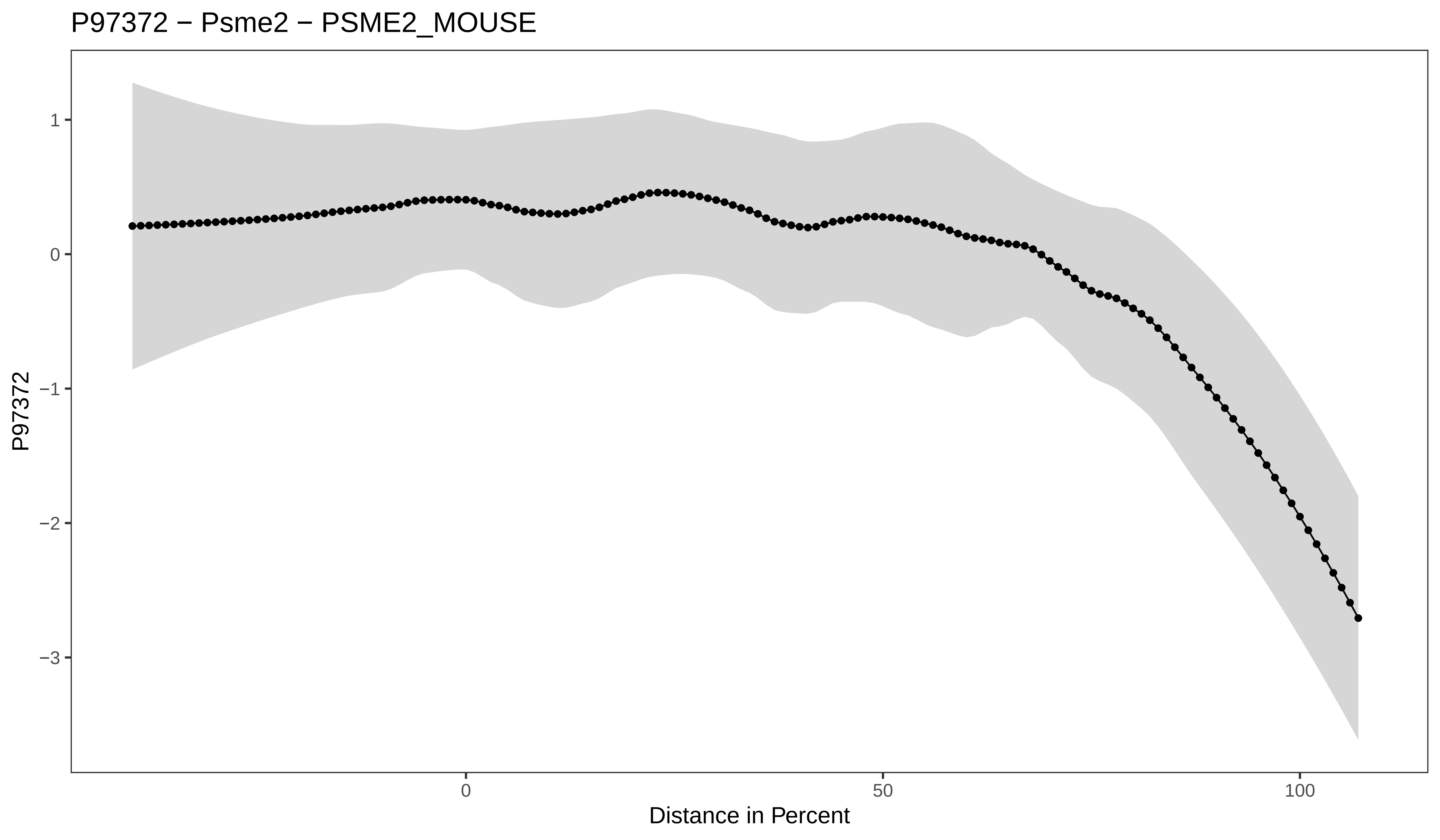
P97372 − Psme2 − PSME2_MOUSE
P97372
1
0
−1
−2
−3
0
50
100
Distance in Percent

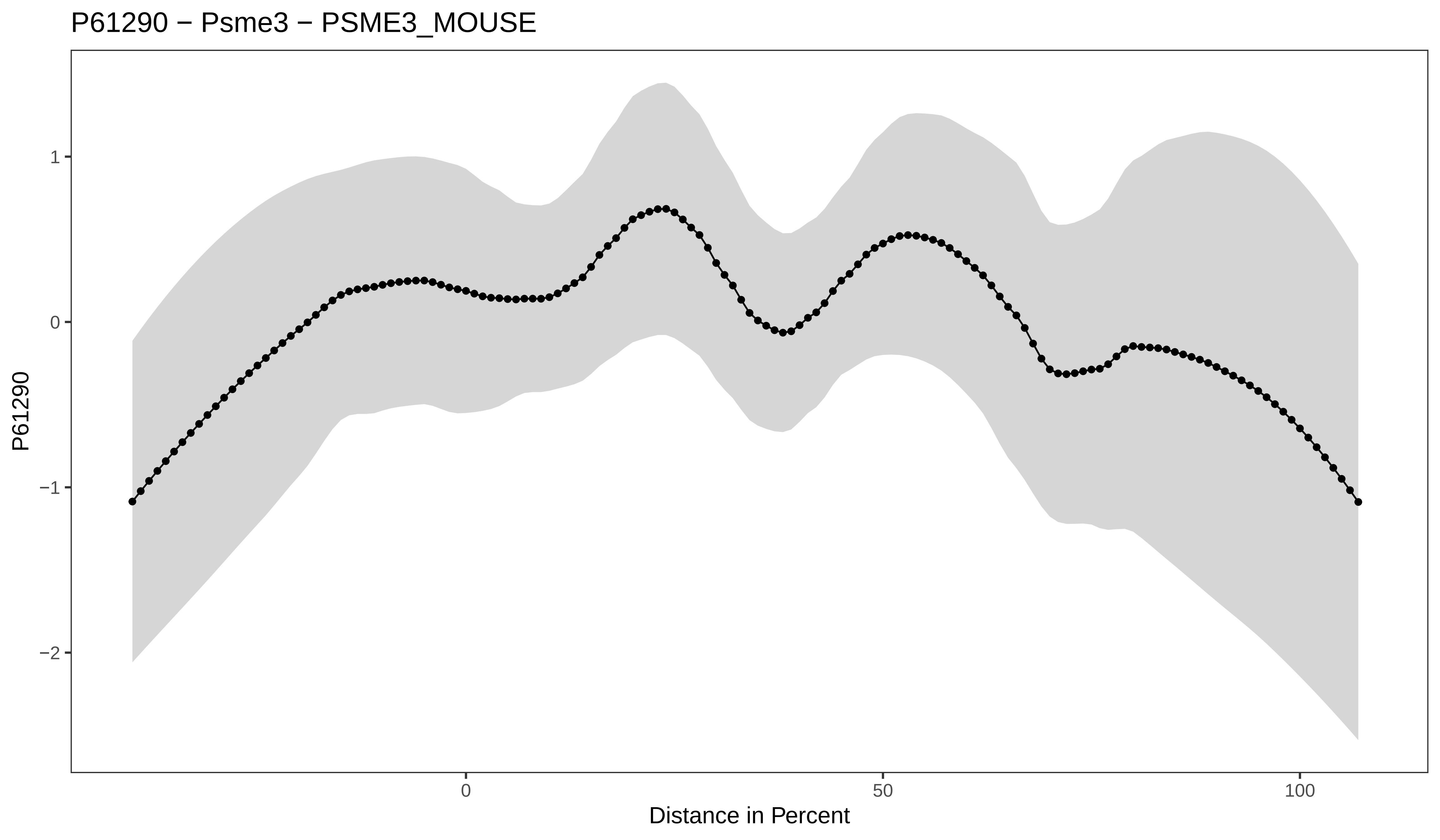
P61290 − Psme3 − PSME3_MOUSE
P61290
Distance in Percent
1
0
−1
−2
0
50
100

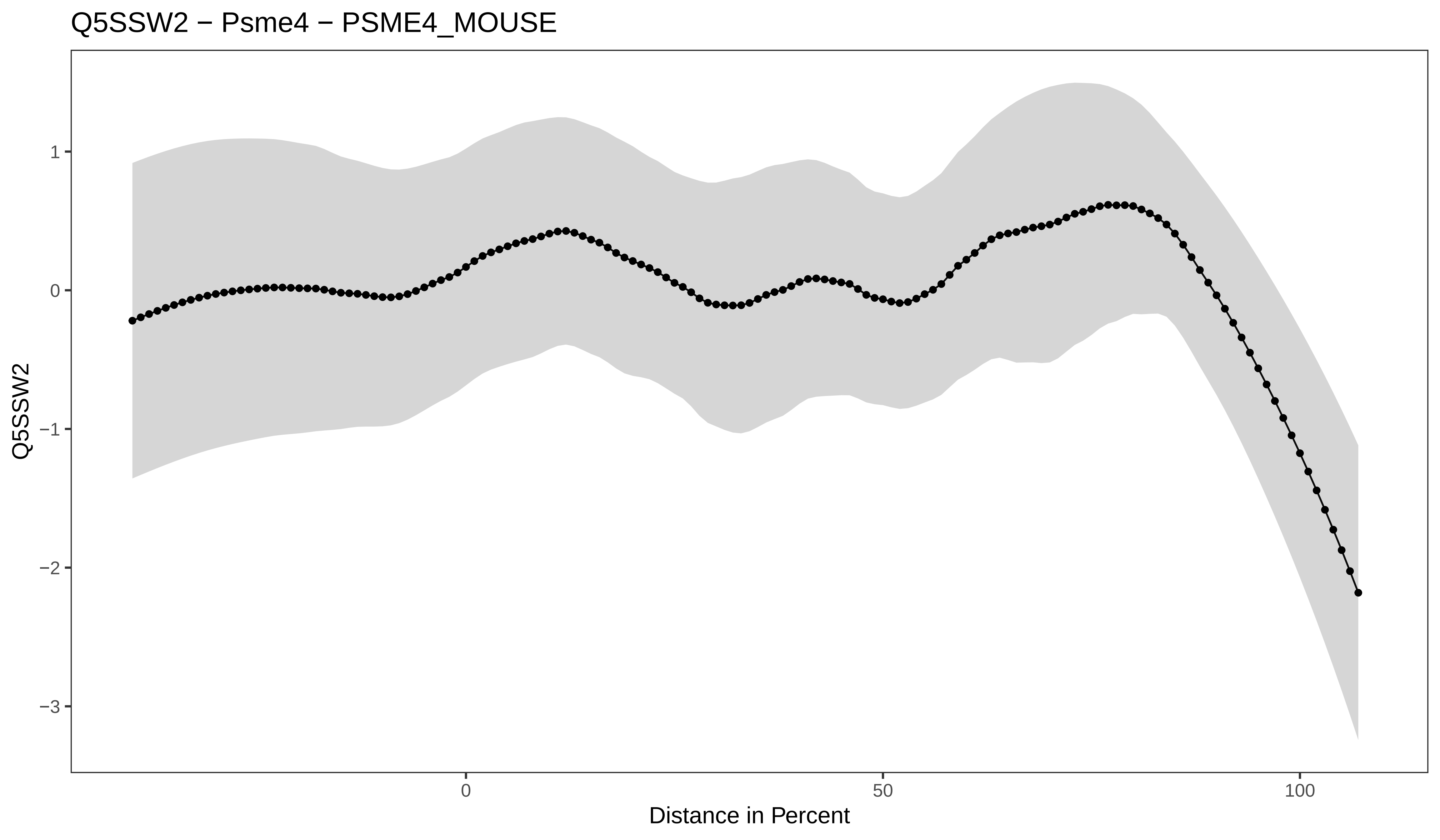

Q5SSW2 − Psme4 − PSME4_MOUSE
Q5SSW2
1
0
−1
−2
−3
0
50
100
Distance in Percent

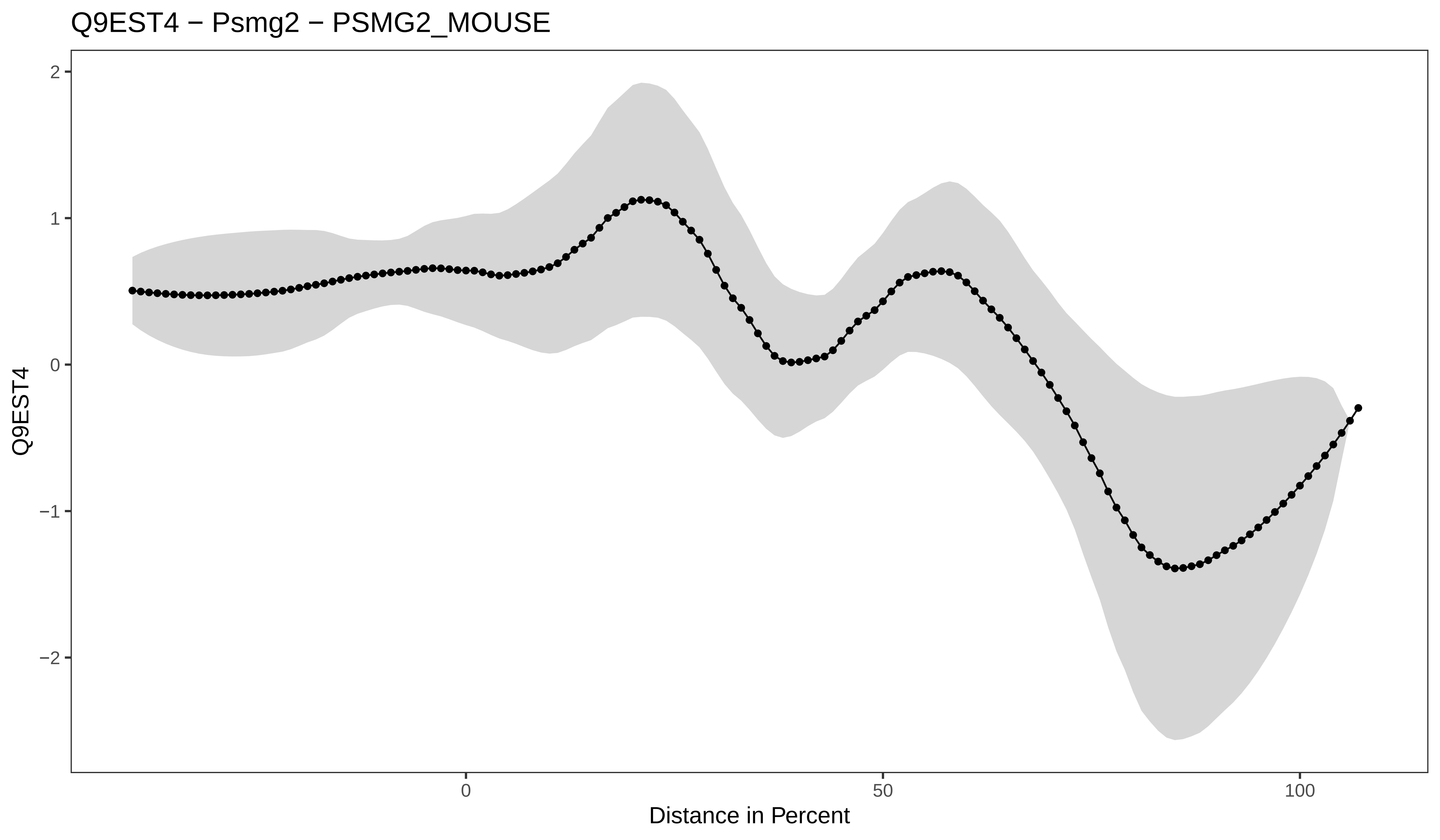

Q9EST4 − Psmg2 − PSMG2_MOUSE
2
1
0
−1
−2
Q9EST4
0
50
100
Distance in Percent

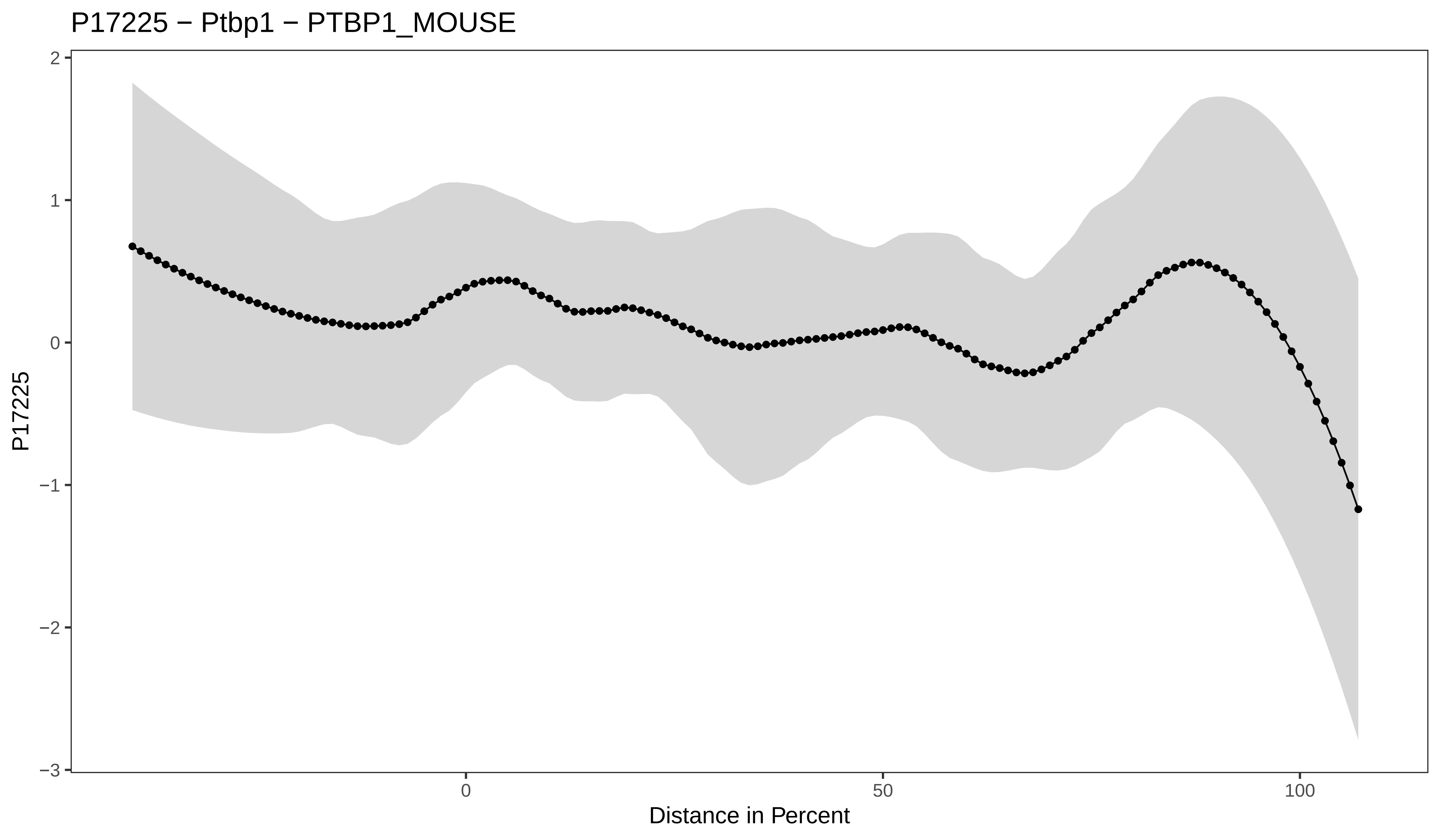

P17225 − Ptbp1 − PTBP1_MOUSE
2
1
0
−1
−2
−3
P17225
0
50
100
Distance in Percent

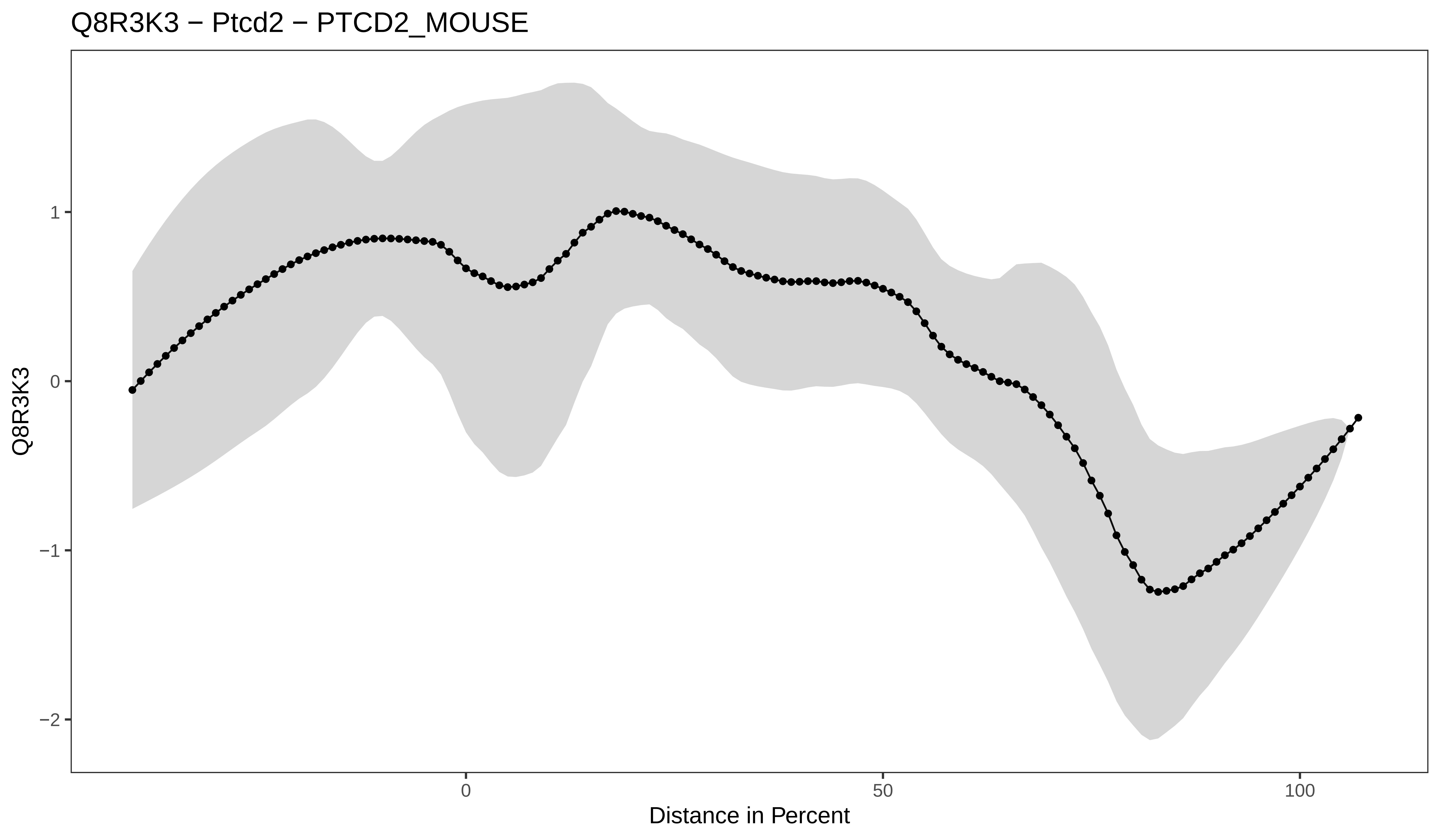

Q8R3K3 − Ptcd2 − PTCD2_MOUSE
1
0
−1
−2
Q8R3K3
0
50
100
Distance in Percent

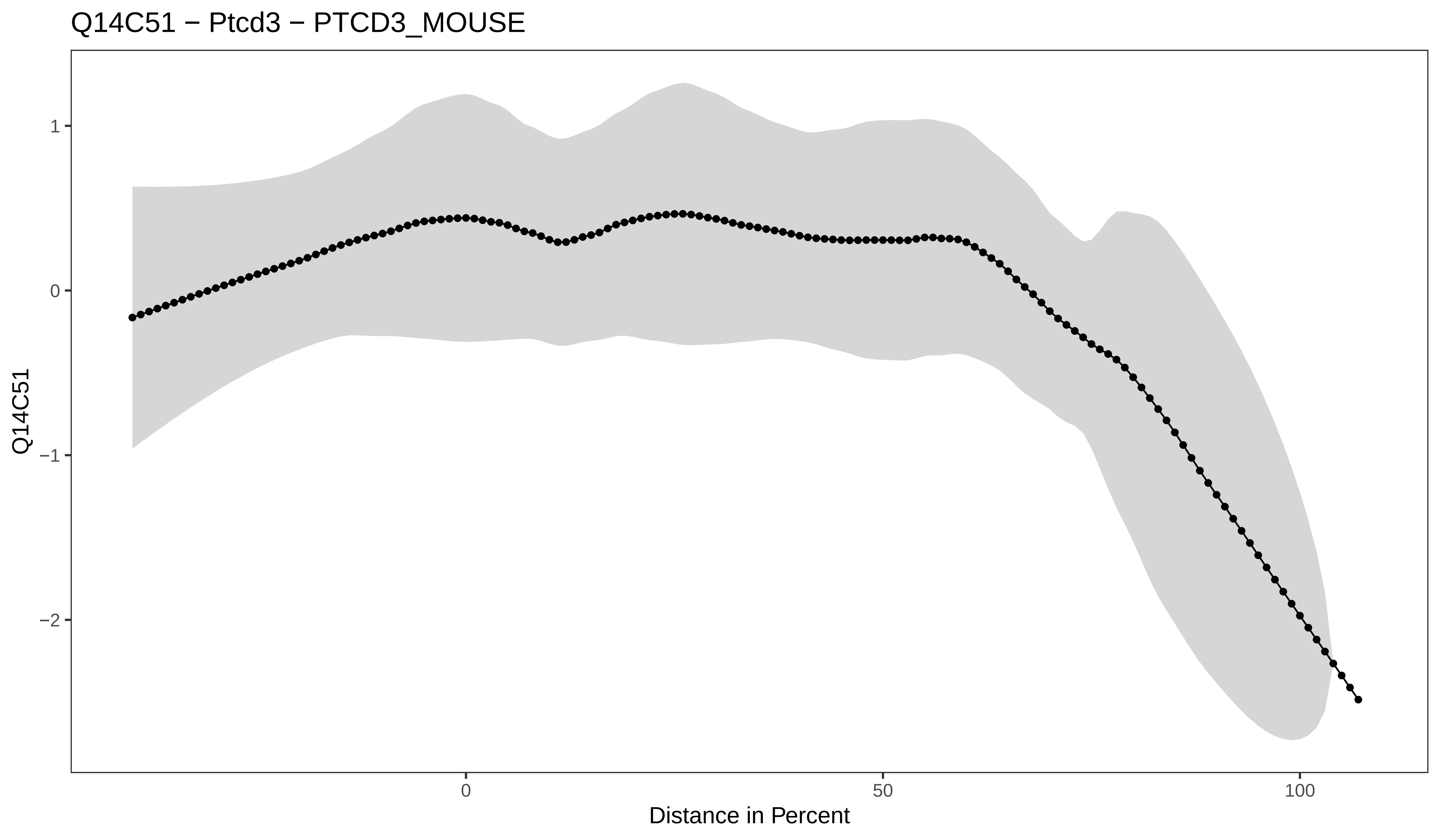

Q14C51 − Ptcd3 − PTCD3_MOUSE
1
0
−1
−2
Q14C51
0
50
100
Distance in Percent

Q60866 − Pter − PTER_MOUSE

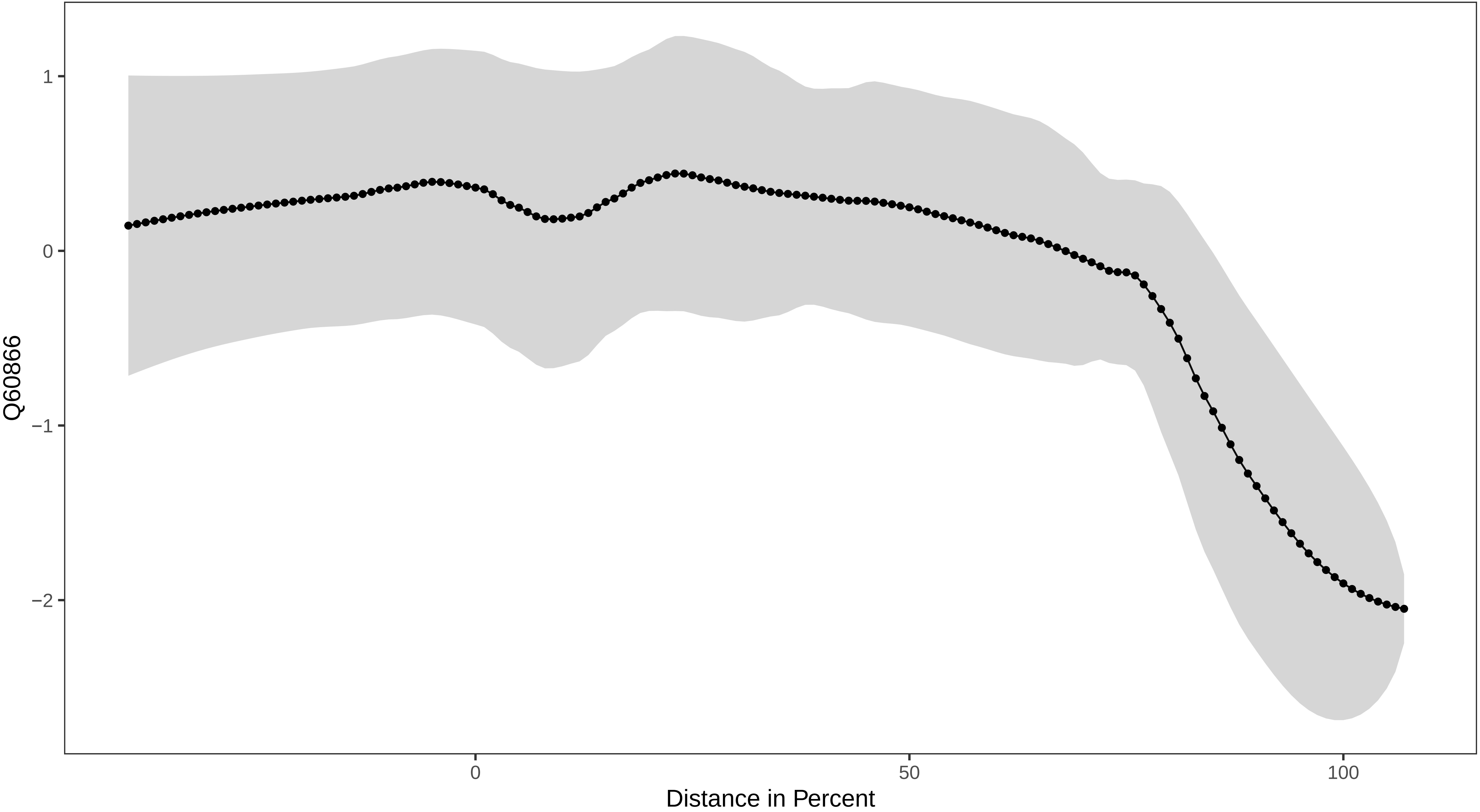

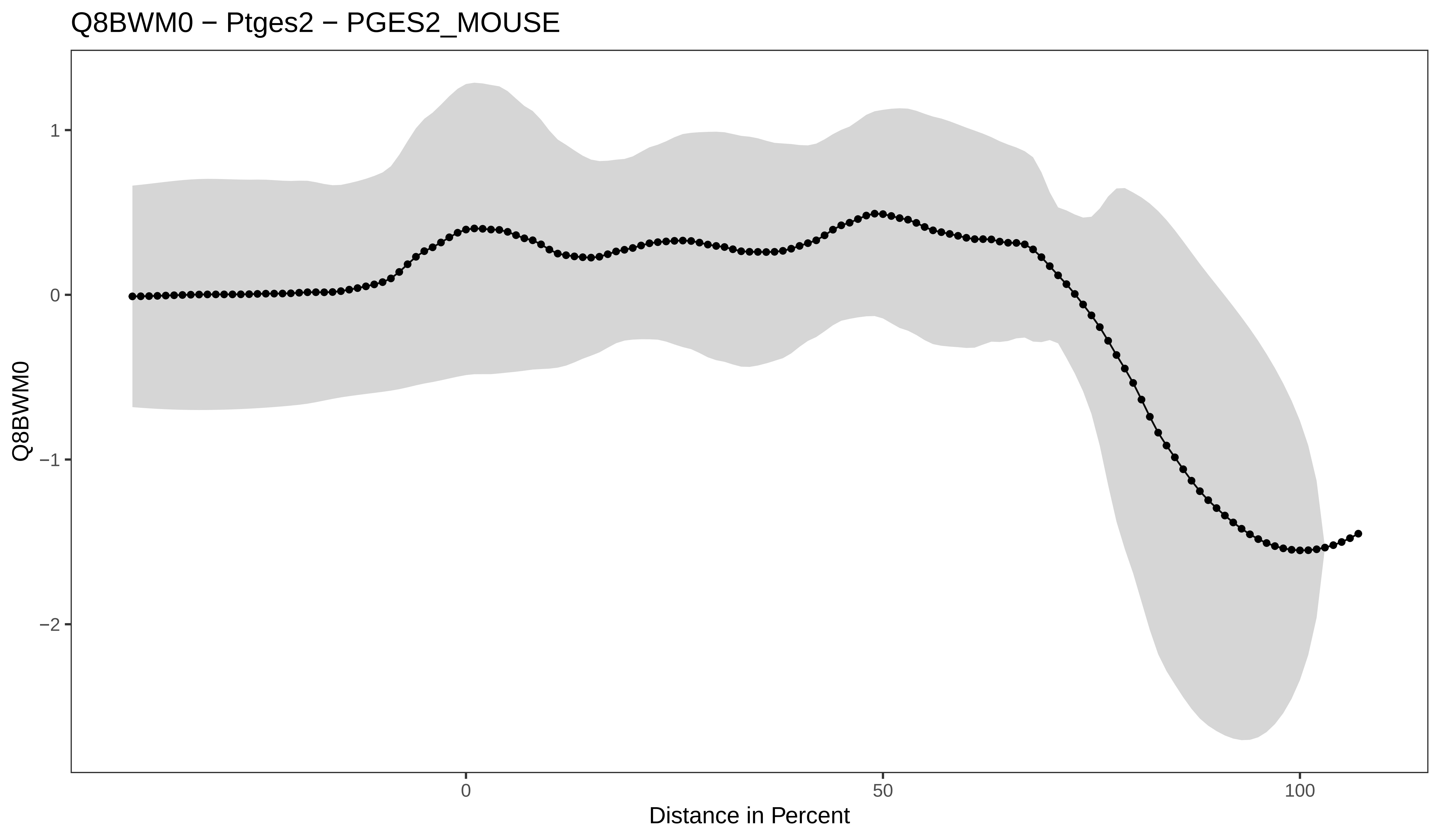

Q8BWM0 − Ptges2 − PGES2_MOUSE
Q8BWM0
1
0
−1
−2
0
50
100
Distance in Percent

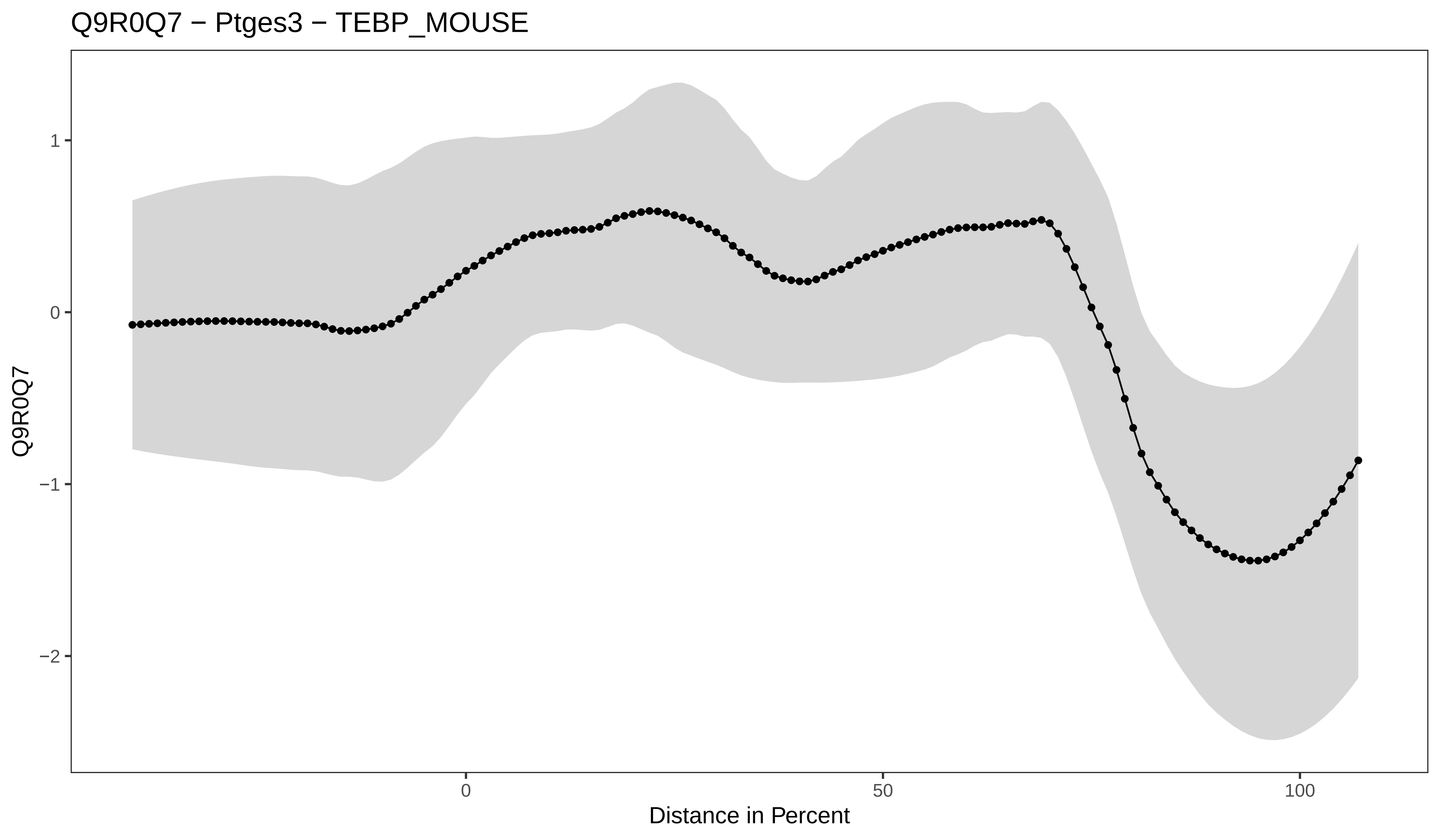
Q9R0Q7 − Ptges3 − TEBP_MOUSE
1
0
−1
−2
Q9R0Q7
0
50
100
Distance in Percent

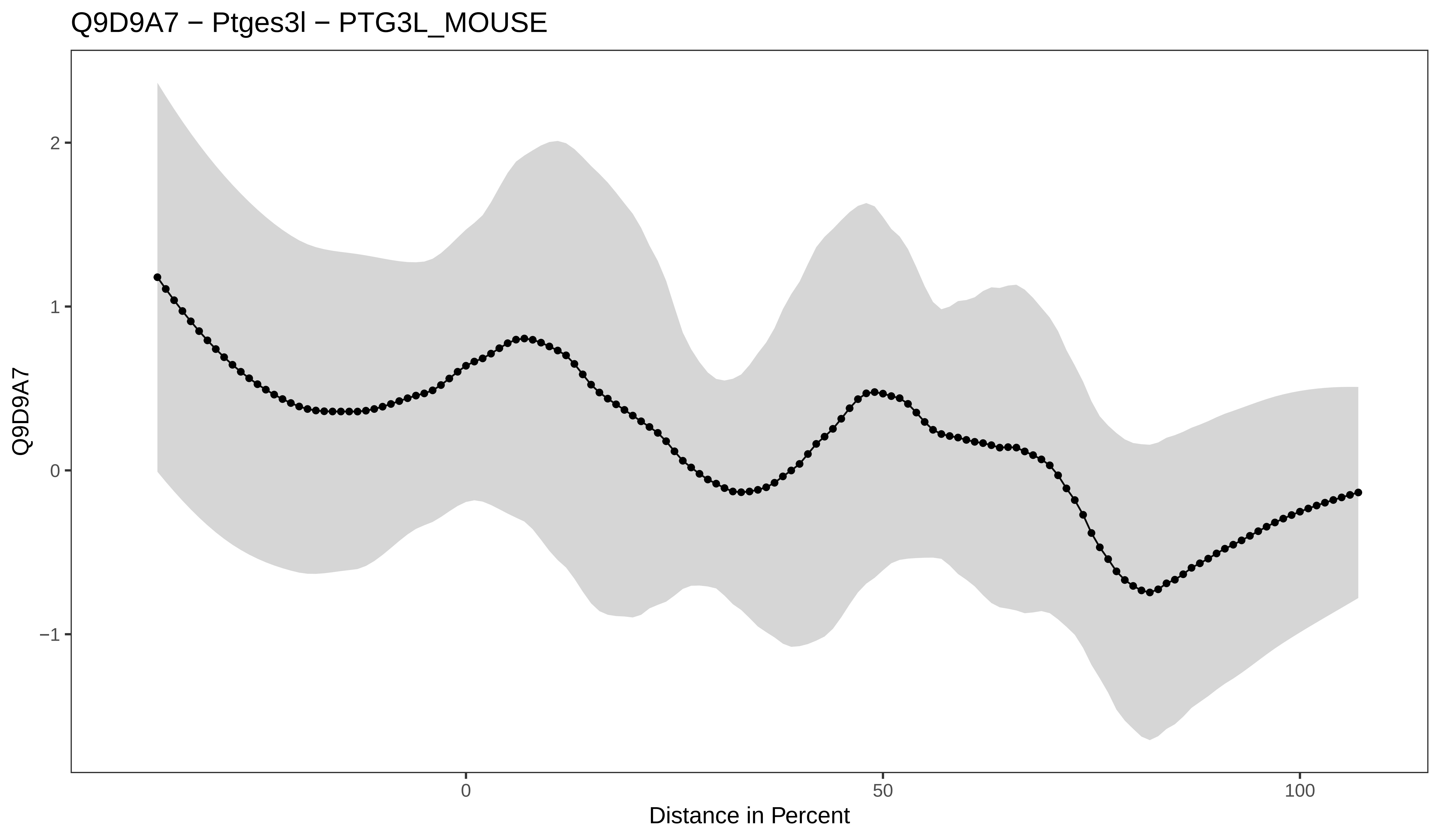

Q9D9A7 − Ptges3l − PTG3L_MOUSE
Q9D9A7
2
1
0
−1
0
50
100
Distance in Percent

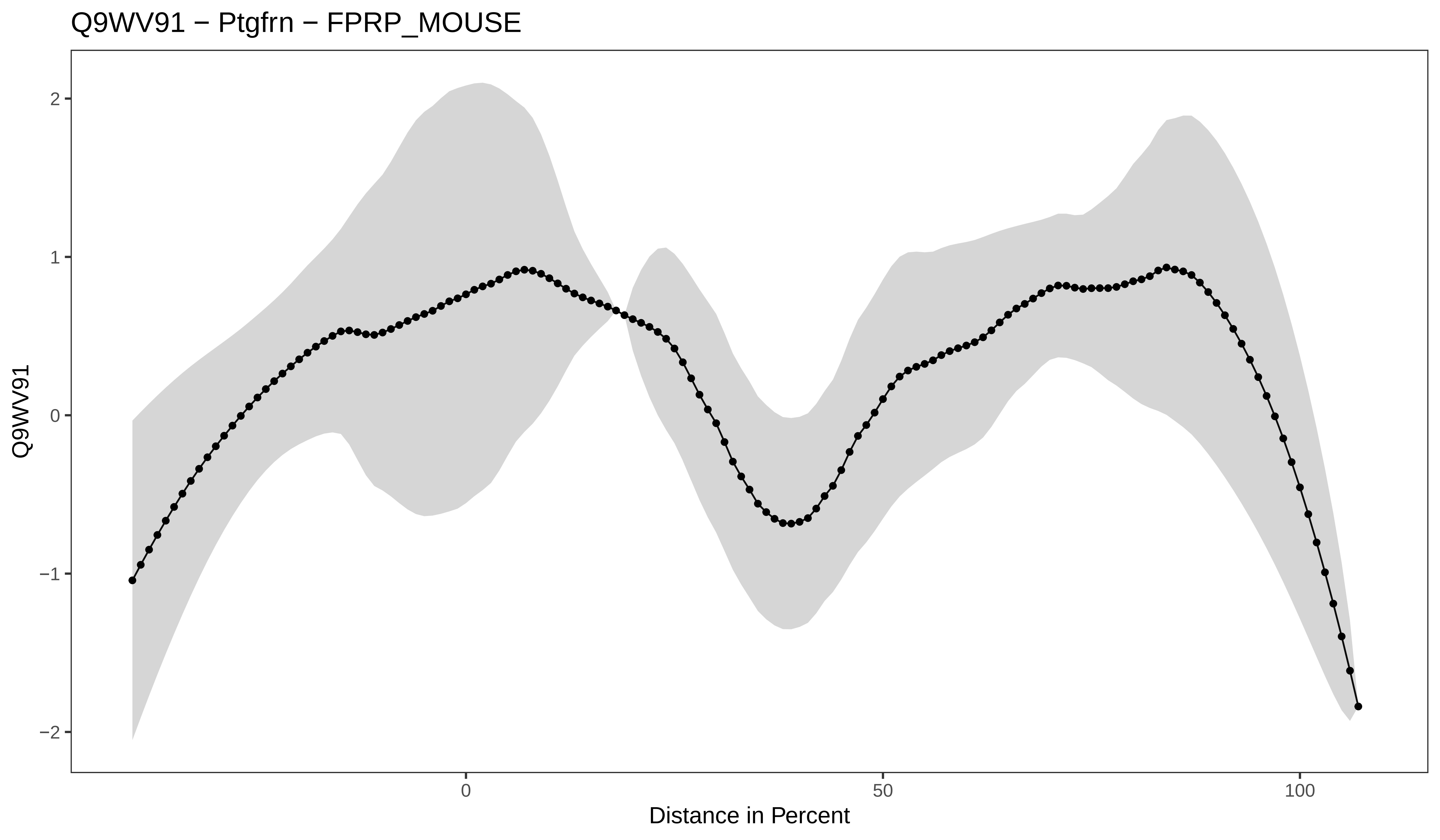
Q9WV91 − Ptgfrn − FPRP_MOUSE
Q9WV91
2
1
0
−1
−2
0
50
100
Distance in Percent

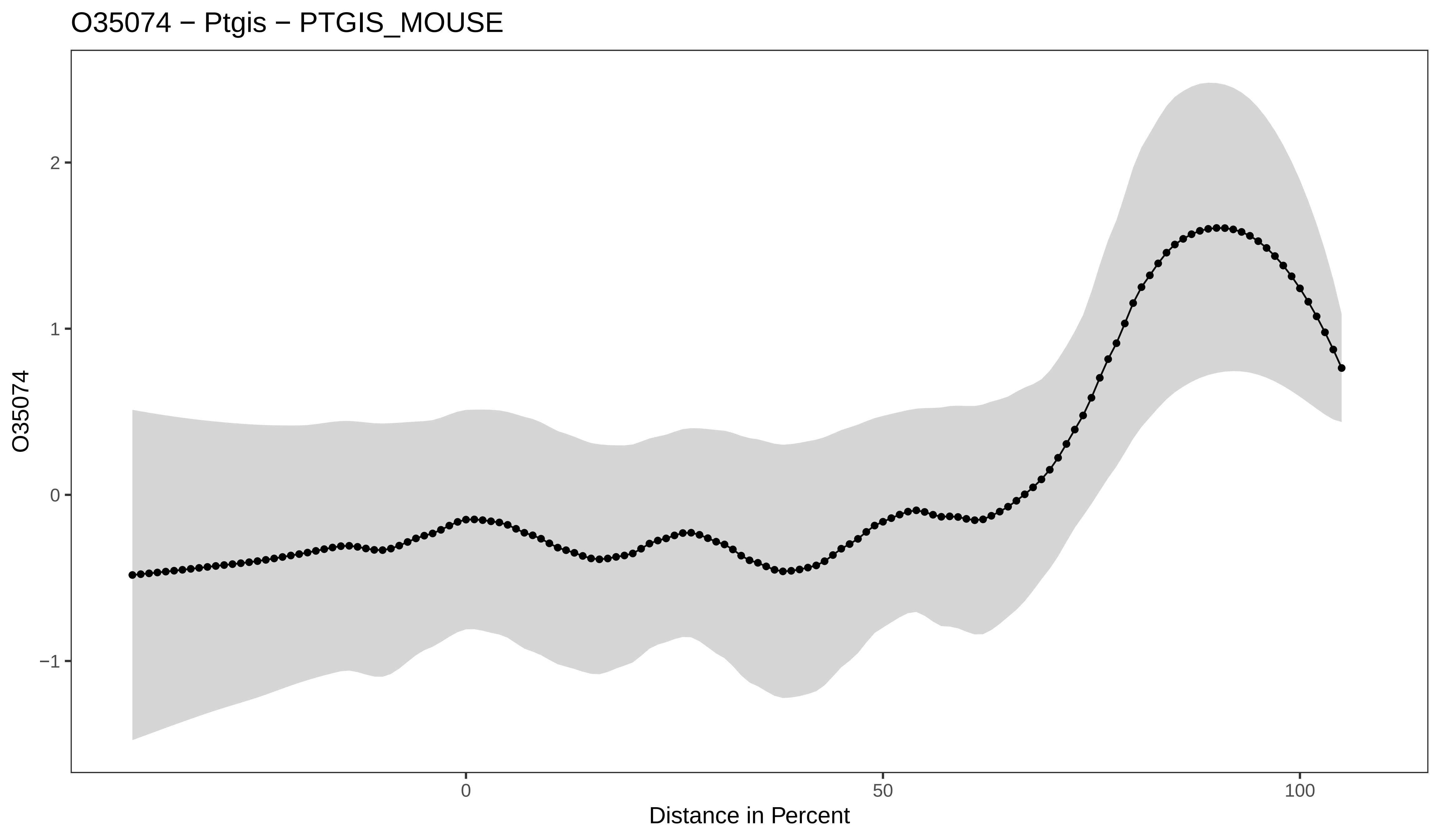
O35074 − Ptgis − PTGIS_MOUSE
O35074
Distance in Percent
2
1
0
−1
0
50
100

Q8VDQ1 − Ptgr2 − PTGR2_MOUSE

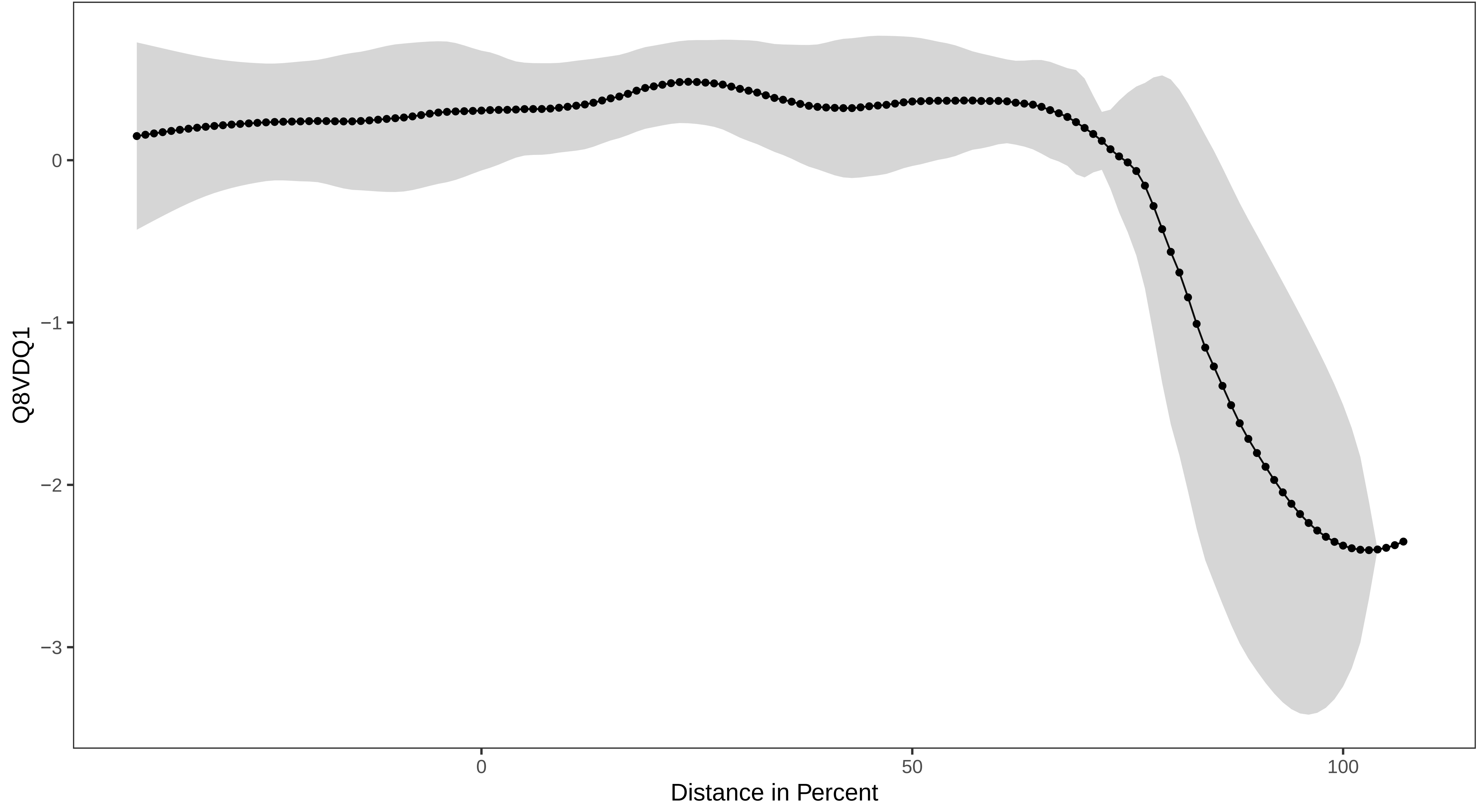

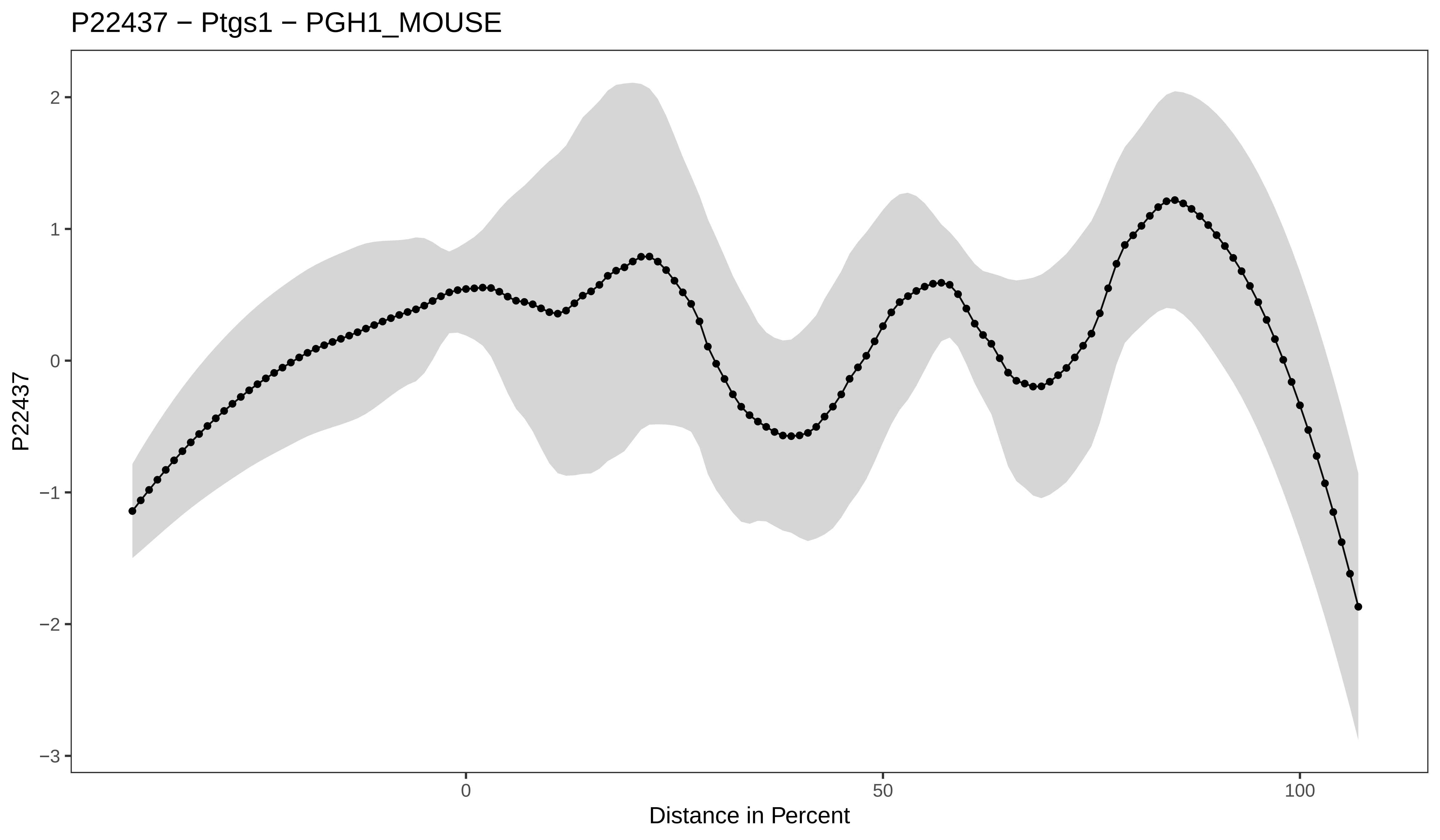

P22437 − Ptgs1 − PGH1_MOUSE
P22437
2
1
0
−1
−2
−3
0
50
100
Distance in Percent

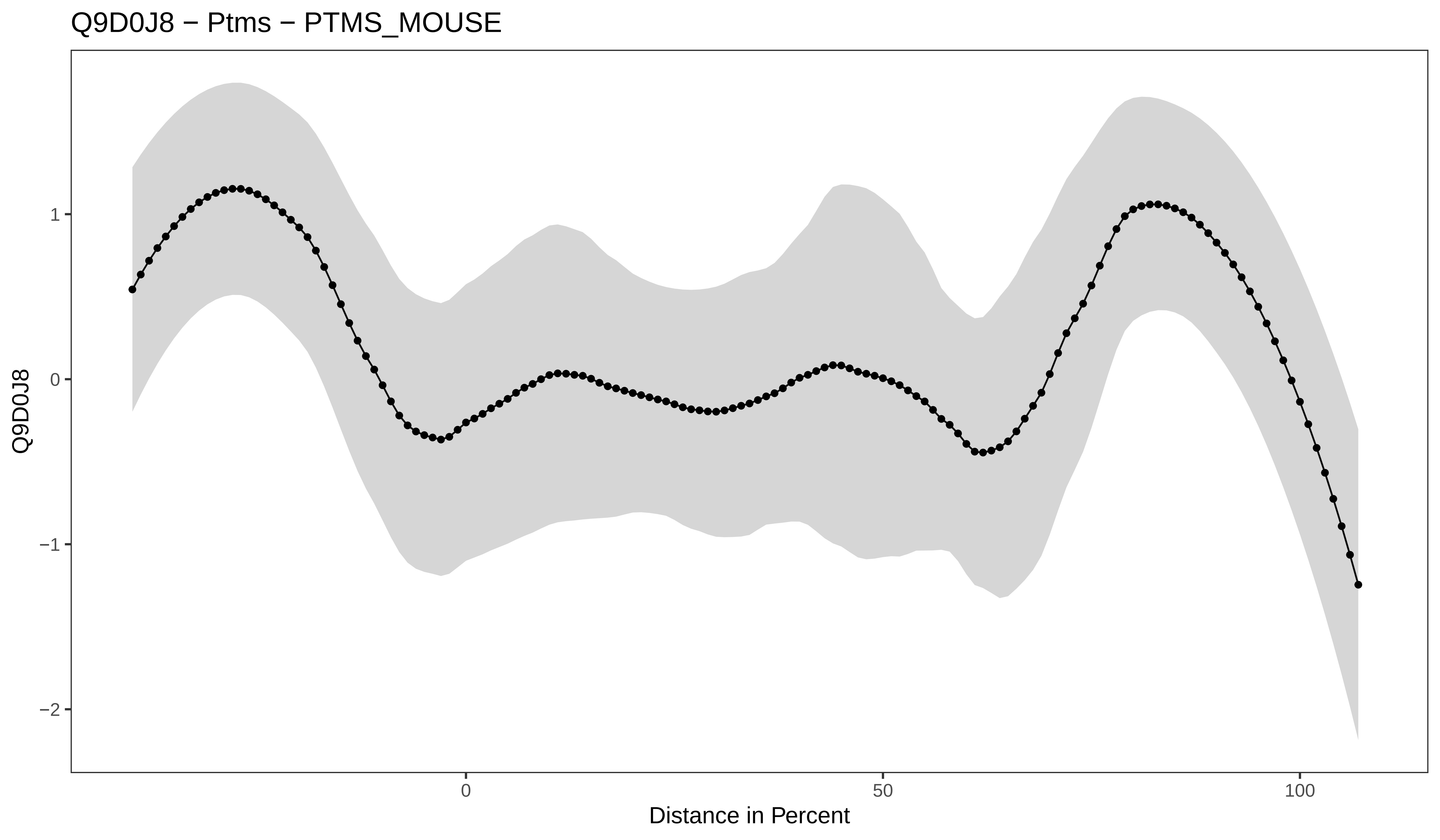

Q9D0J8 − Ptms − PTMS_MOUSE
Q9D0J8
1
0
−1
−2
0
50
100
Distance in Percent

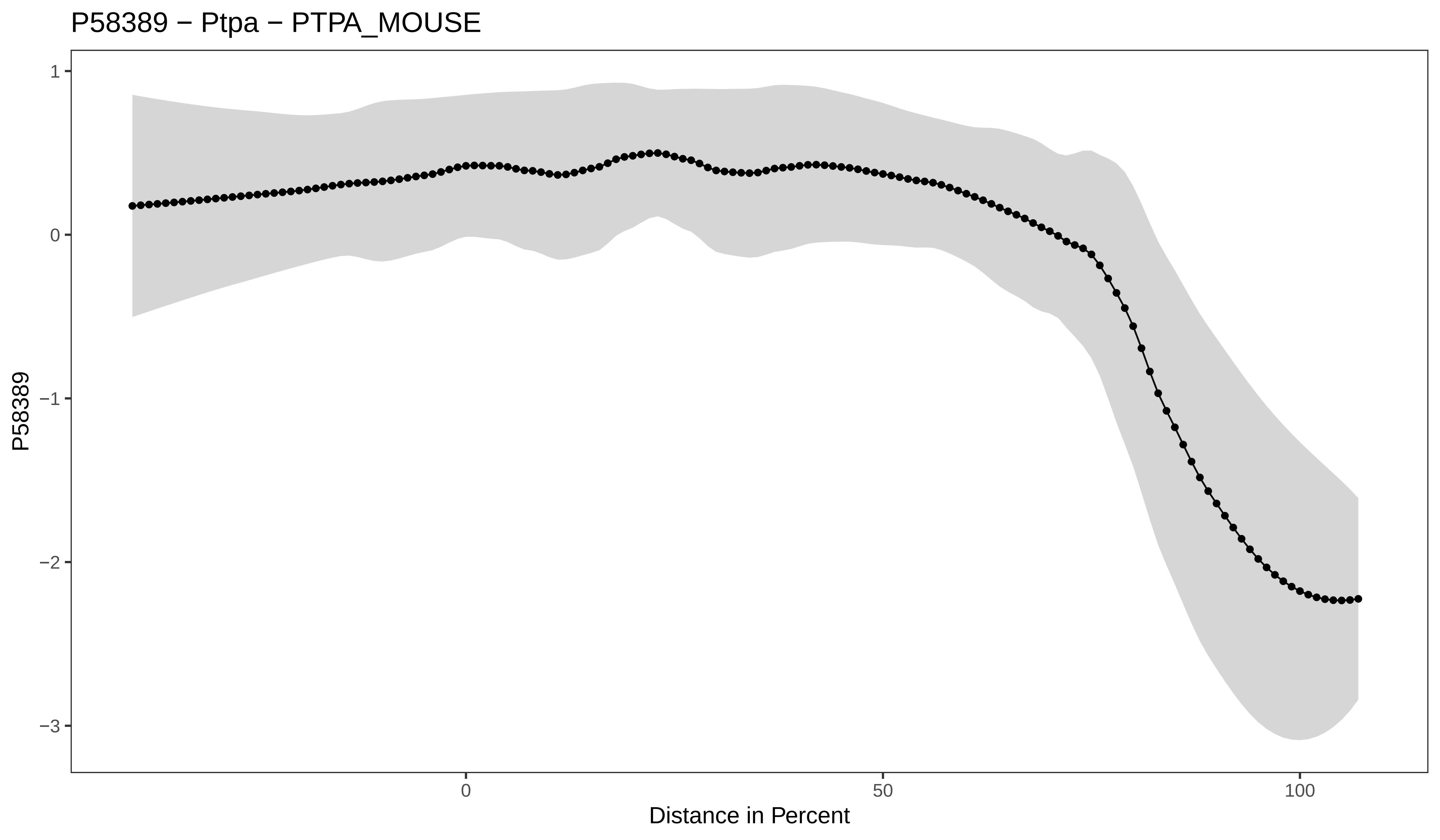

P58389 − Ptpa − PTPA_MOUSE
1
0
−1
−2
−3
P58389
0
50
100
Distance in Percent

Q66GT5 − Ptpmt1 − PTPM1_MOUSE

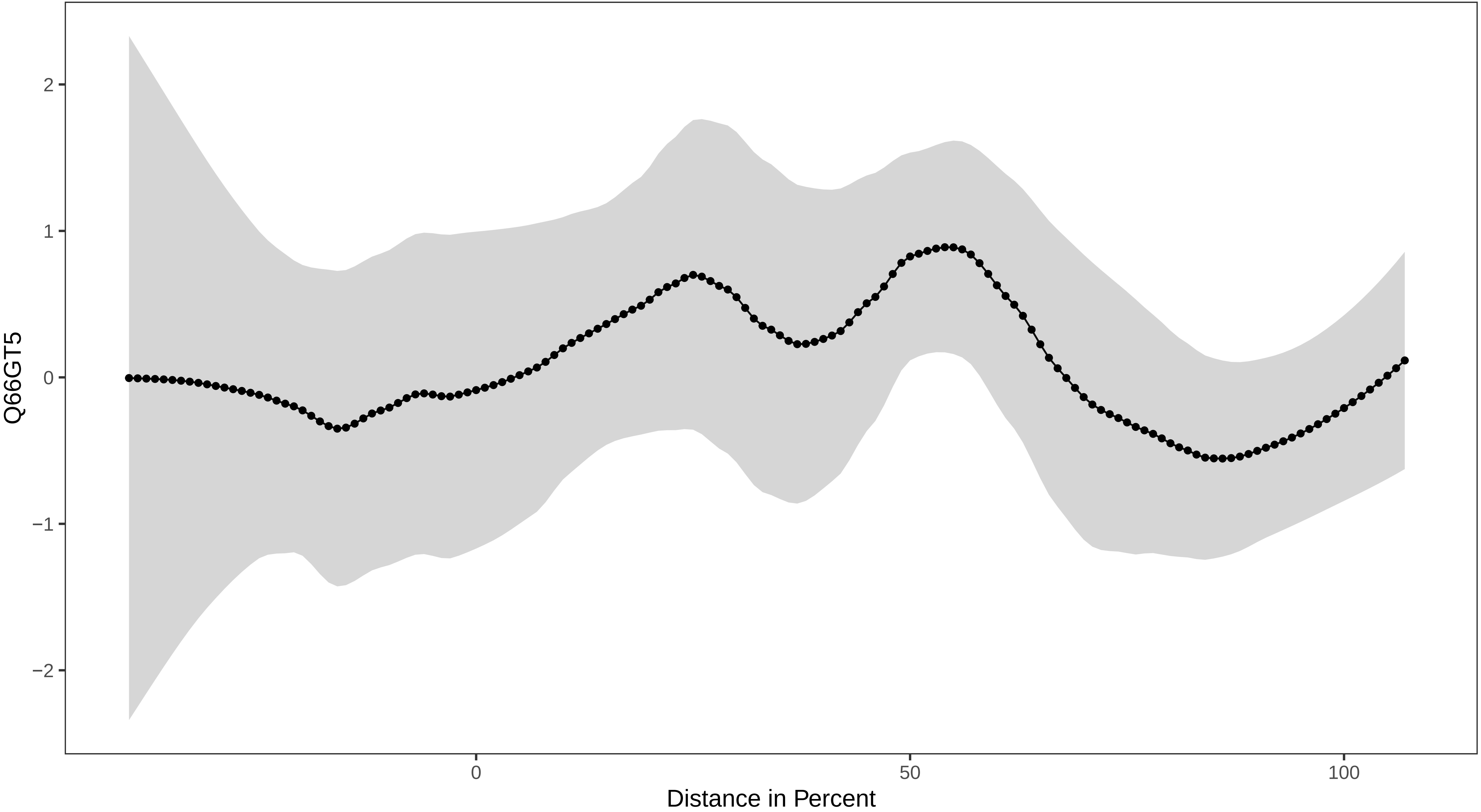

P35235 − Ptpn11 − PTN11_MOUSE

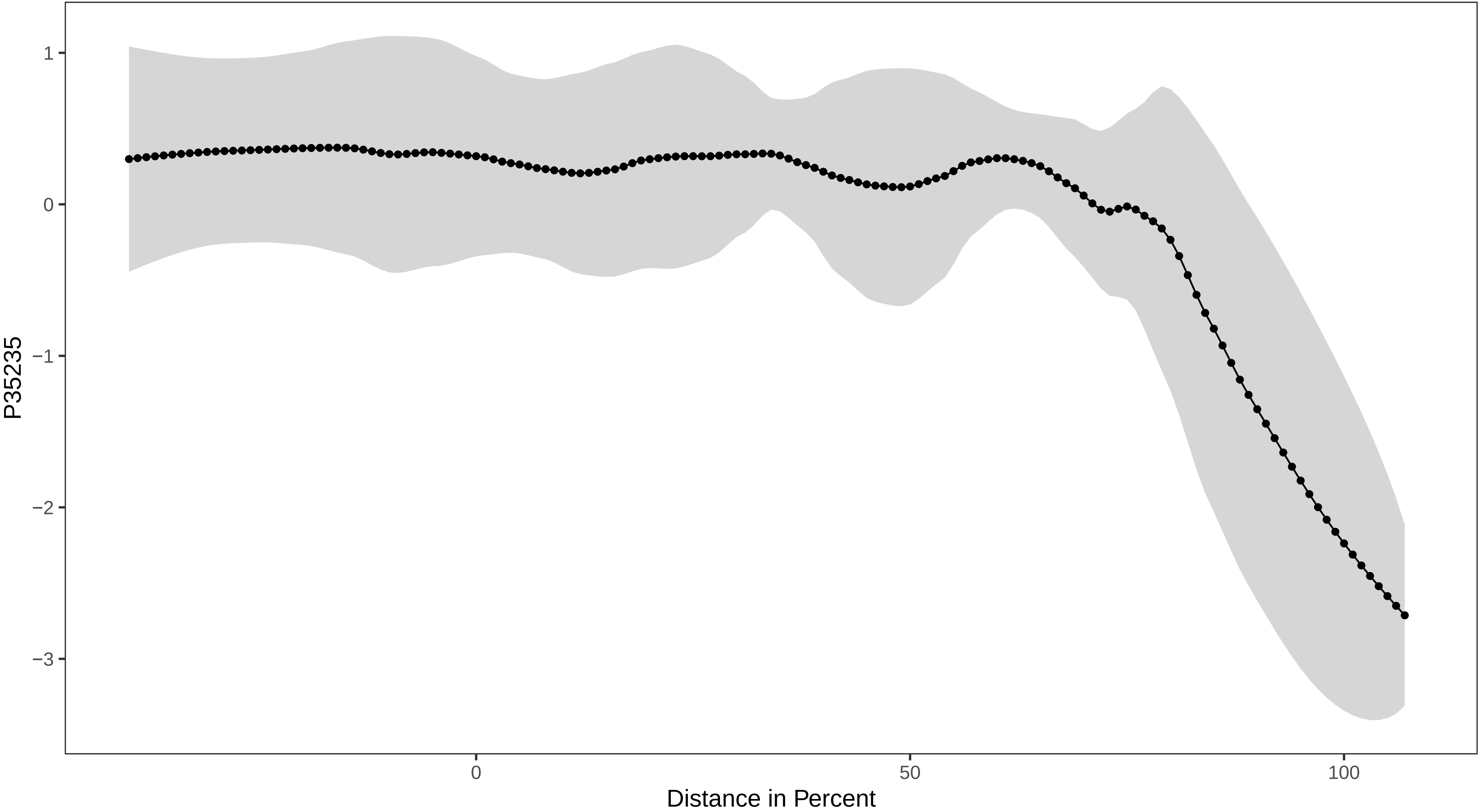

Q64455 − Ptprj − PTPRJ_MOUSE

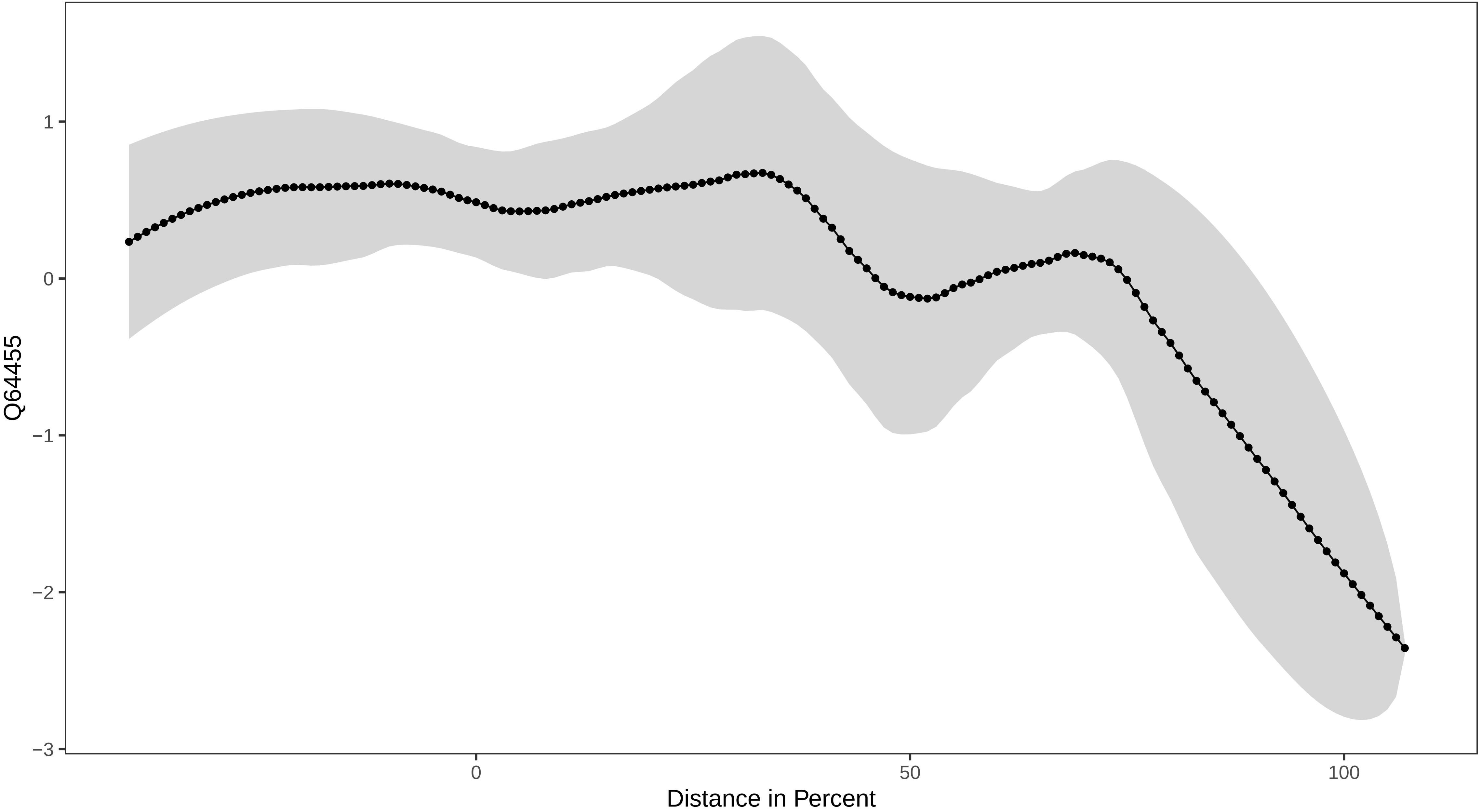

Q8R2Y8 − Ptrh2 − PTH2_MOUSE

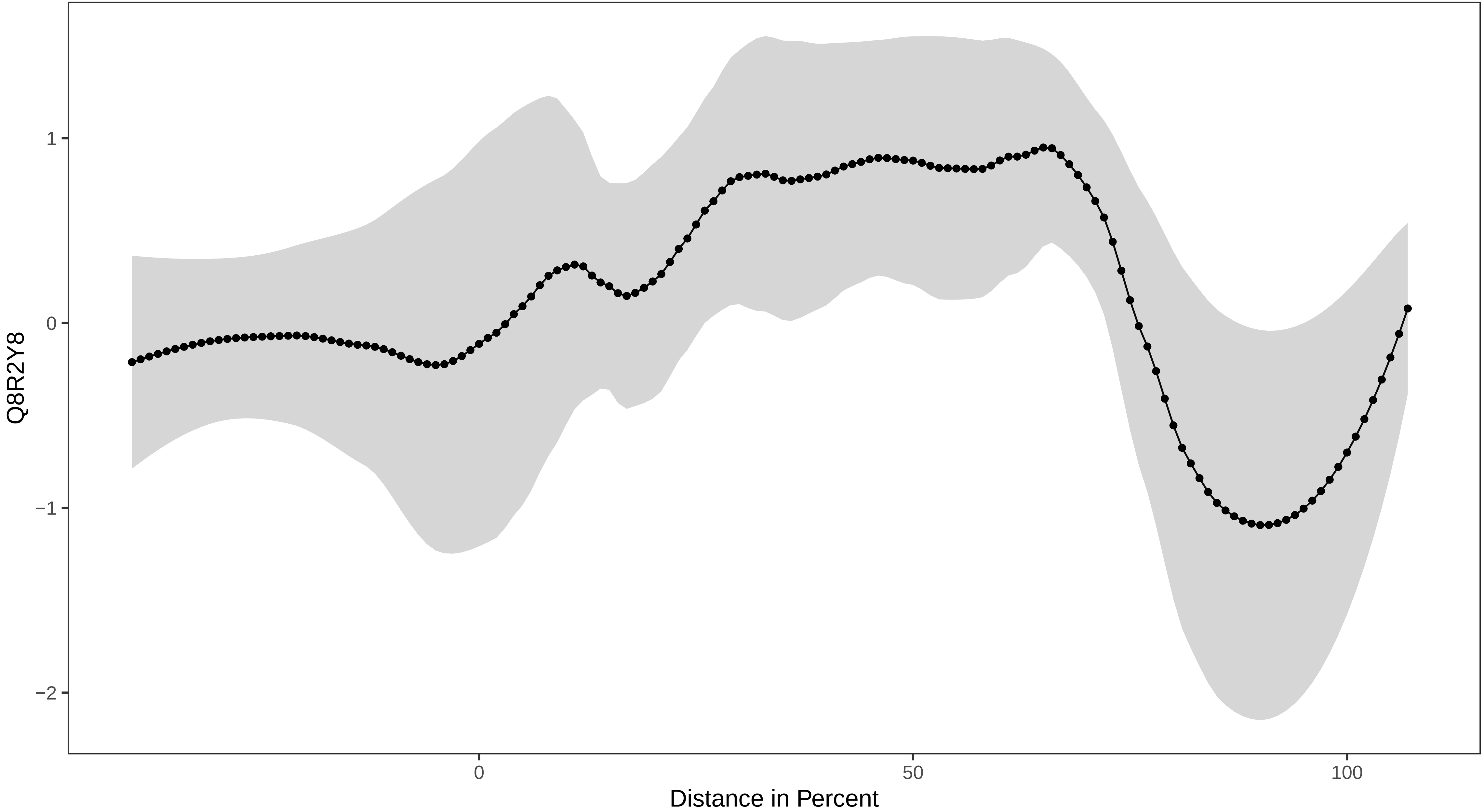

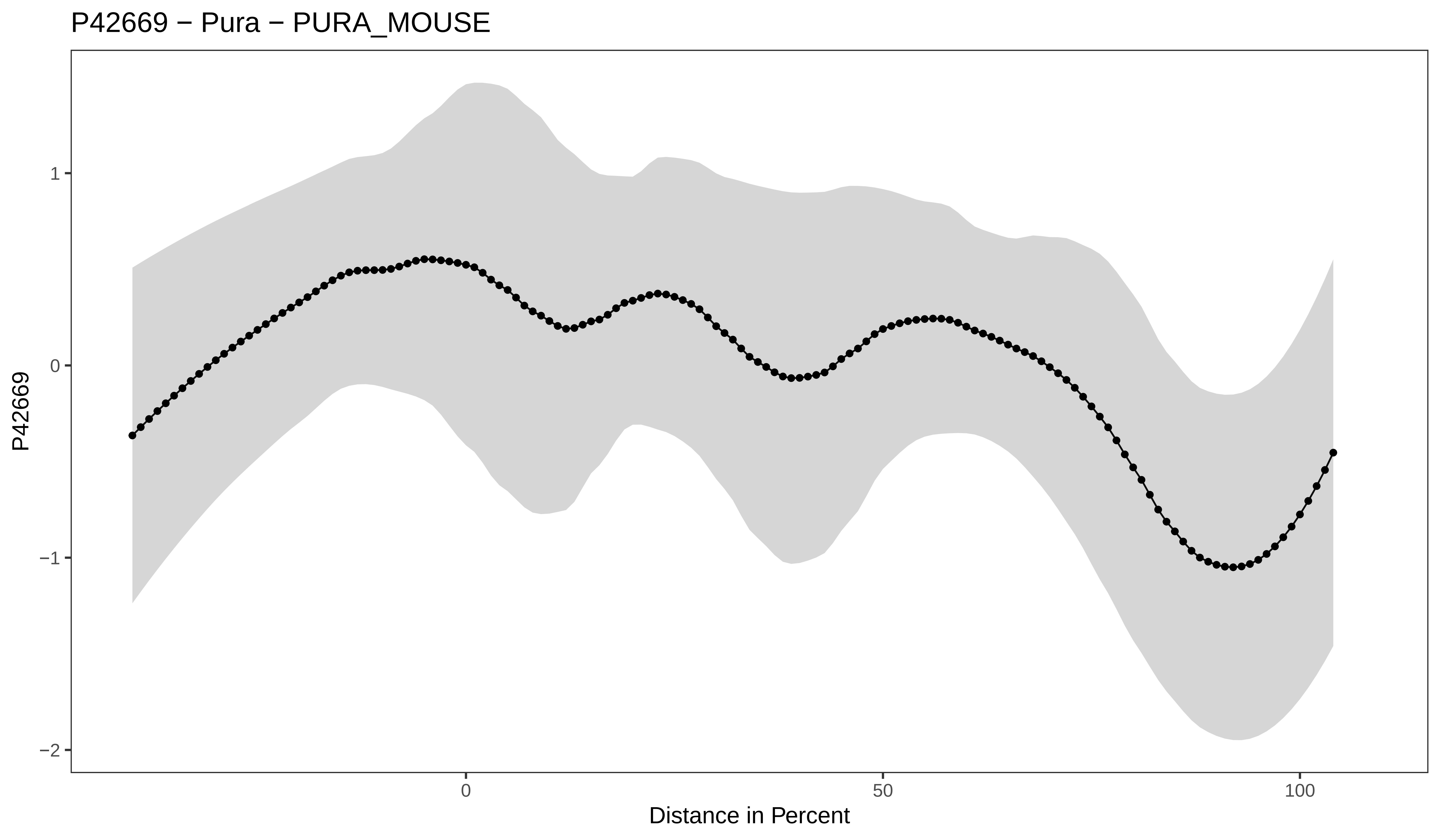
P42669 − Pura − PURA_MOUSE
P42669
1
0
−1
−2
0
50
100
Distance in Percent

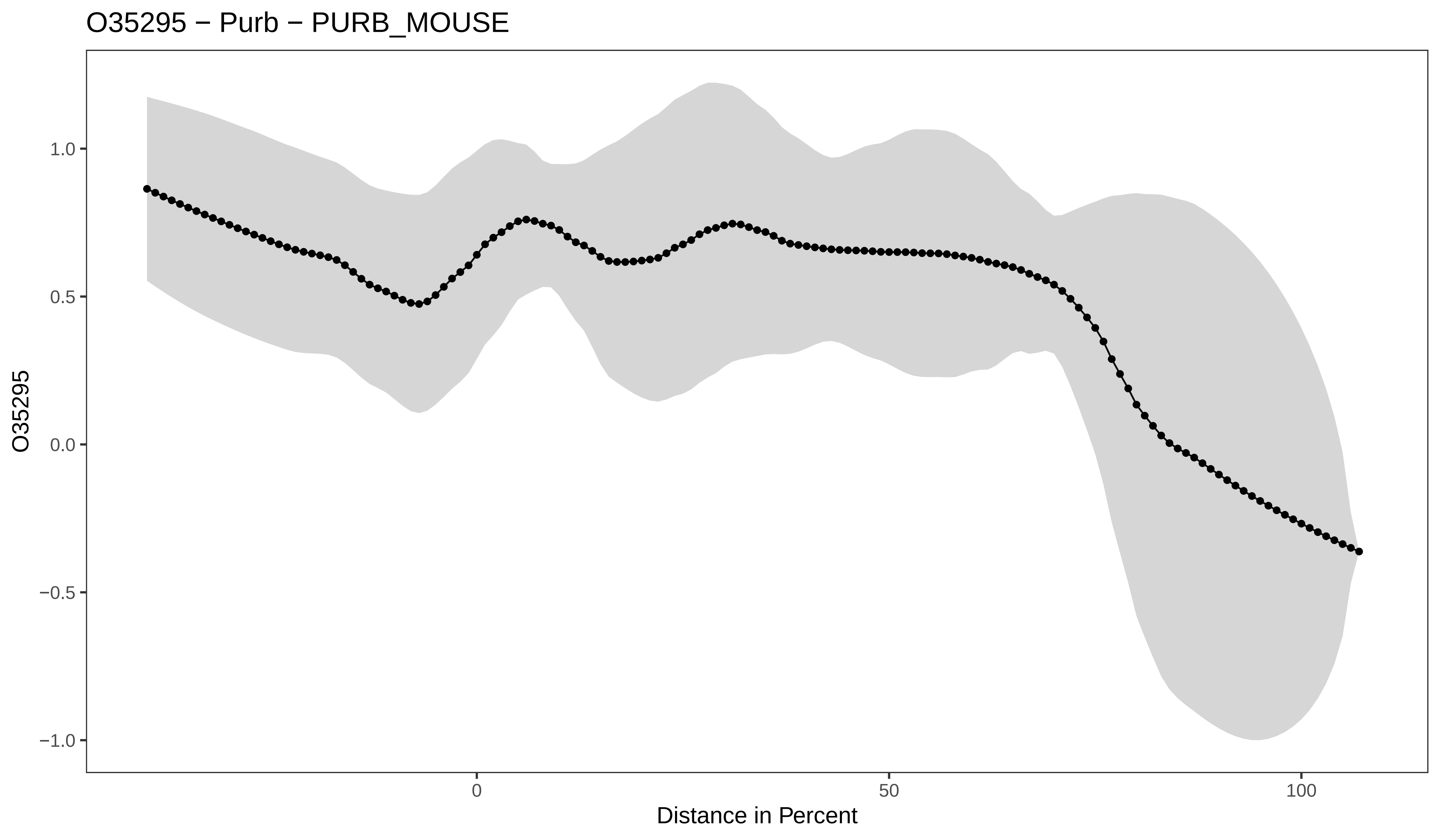

O35295 − Purb − PURB_MOUSE
O35295
1.0
0.5
0.0
−0.5
−1.0
0
50
100
Distance in Percent

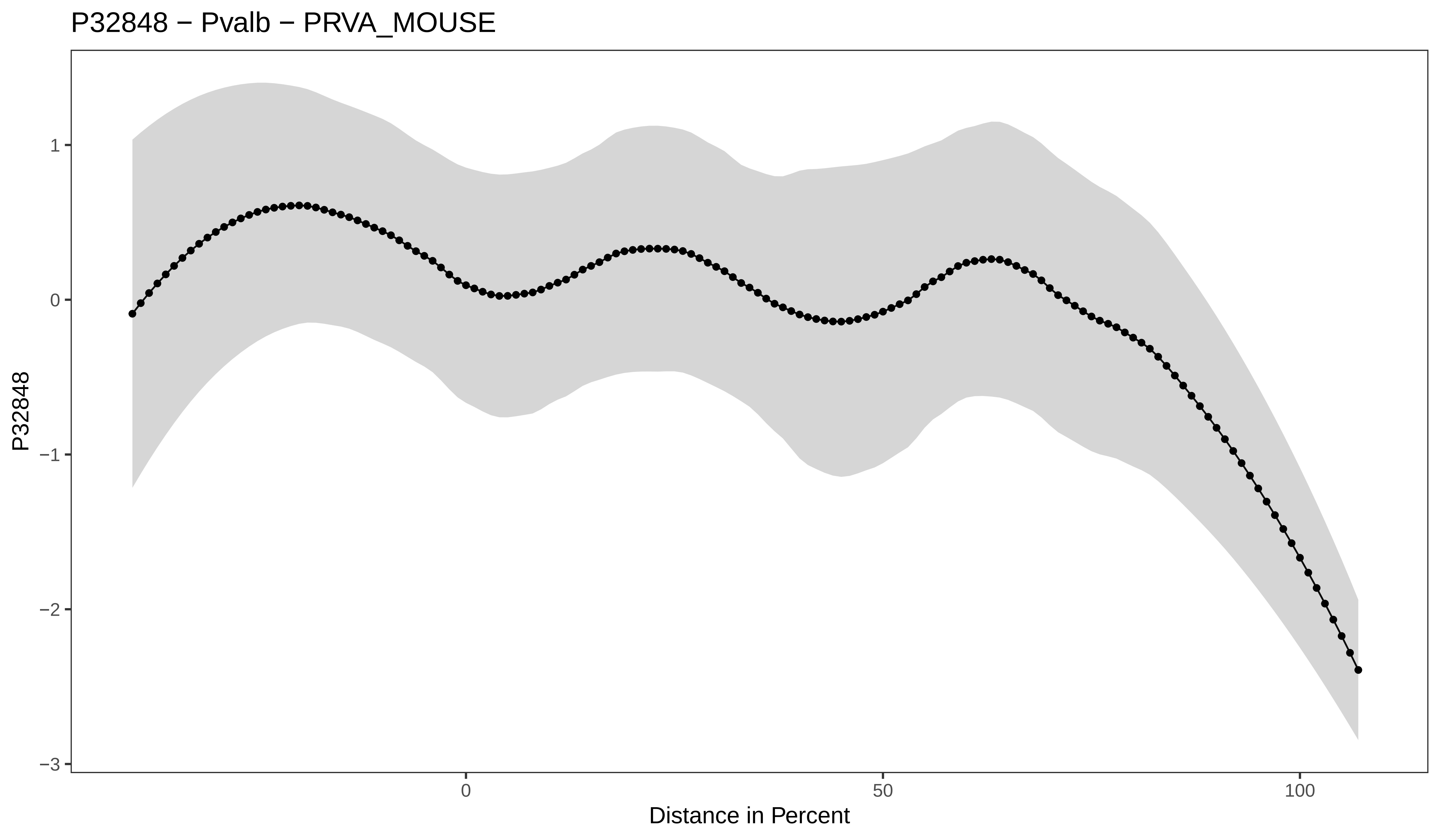

P32848 − Pvalb − PRVA_MOUSE
P32848
1
0
−1
−2
−3
0
50
100
Distance in Percent

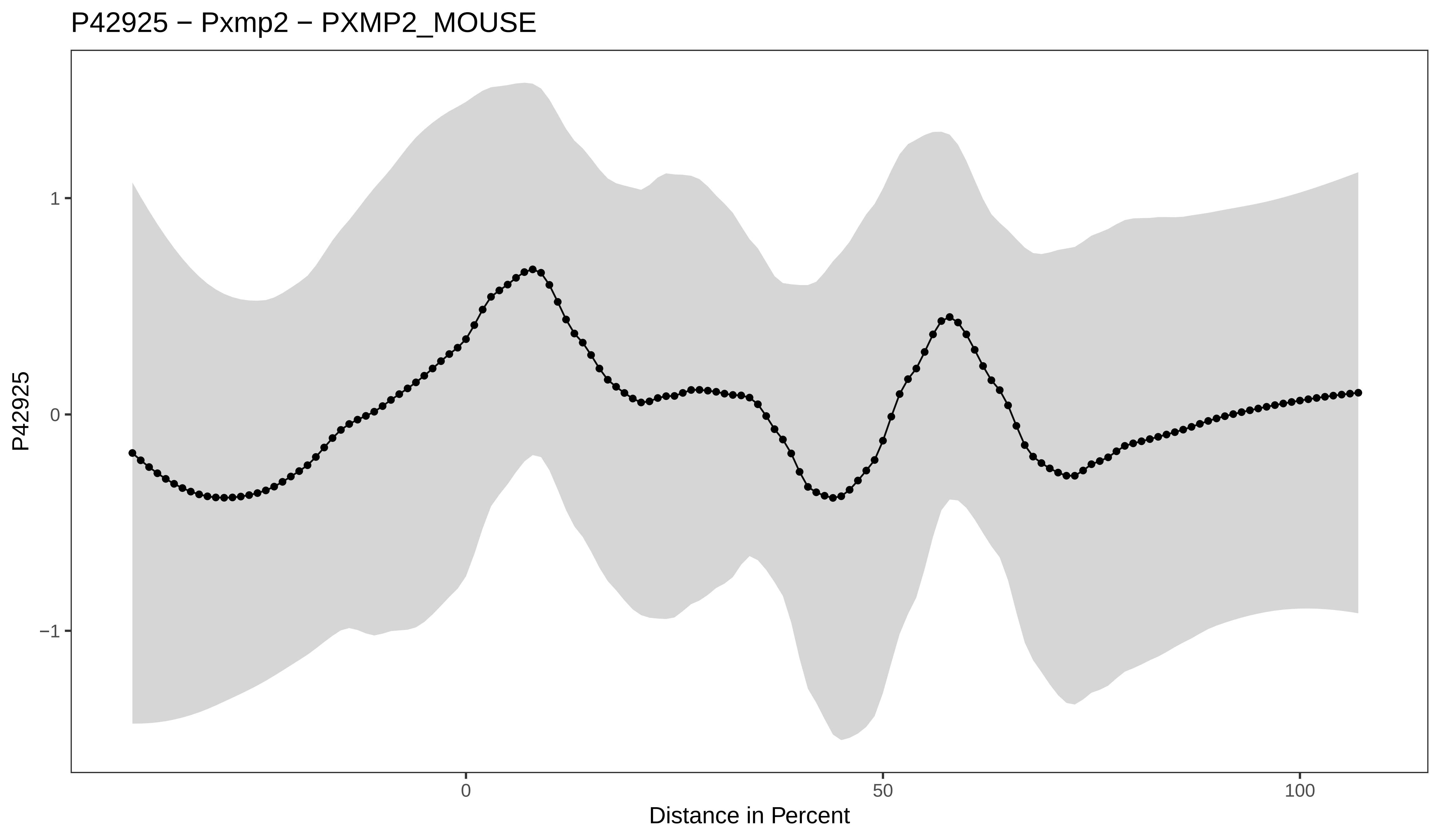

P42925 − Pxmp2 − PXMP2_MOUSE
P42925
1
0
−1
0
50
100
Distance in Percent

Q8CI94 − Pygb − PYGB_MOUSE

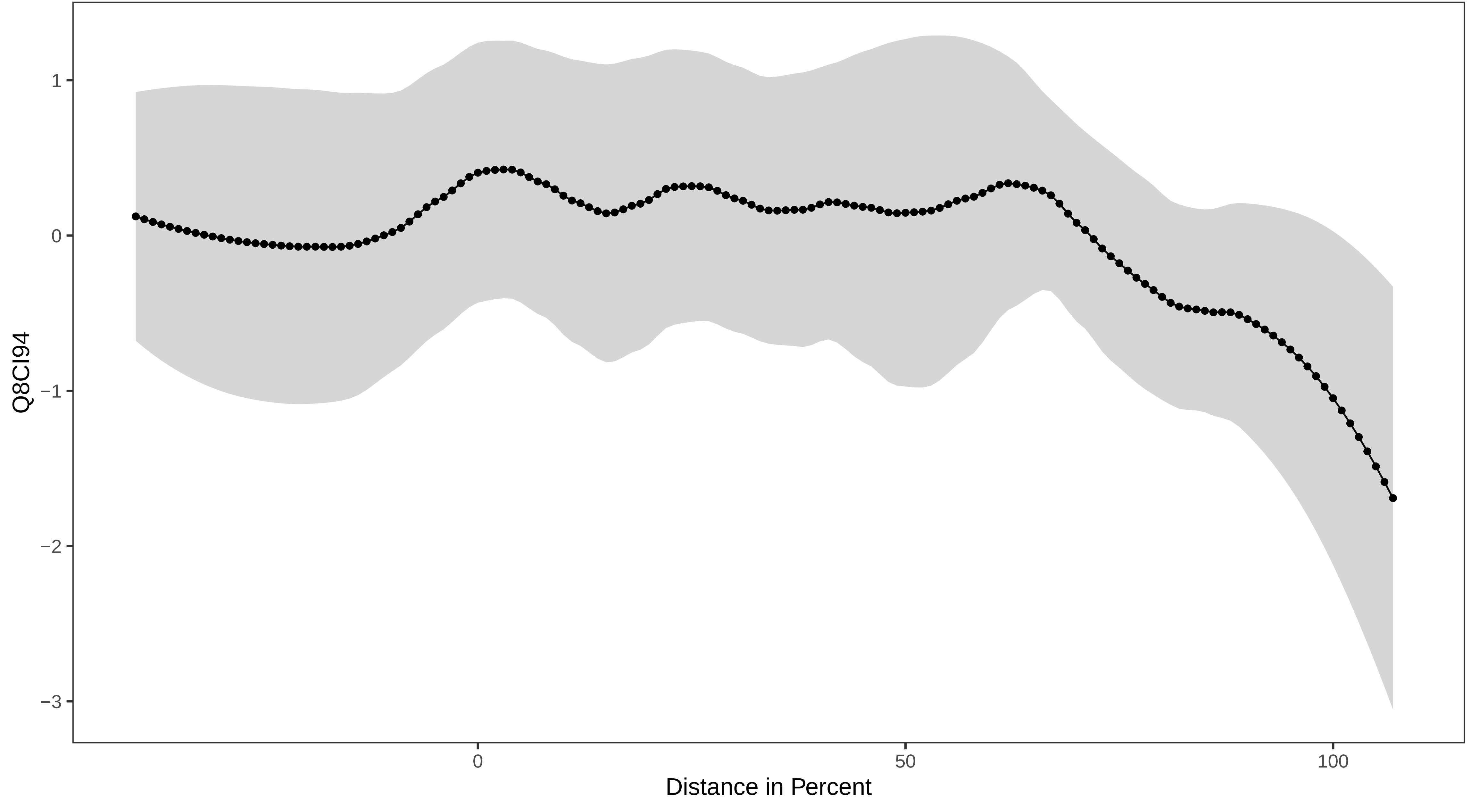

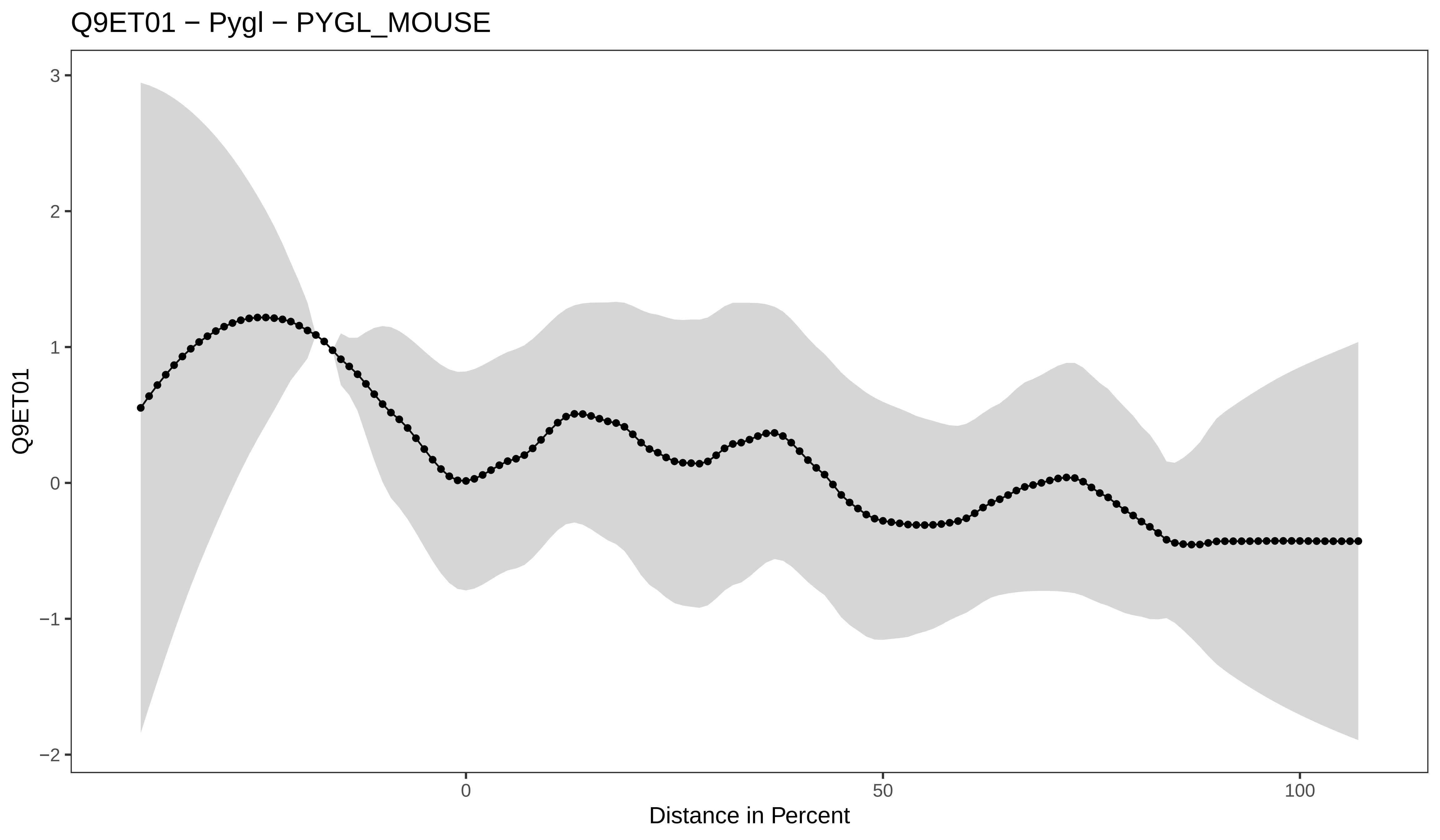

Q9ET01 − Pygl − PYGL_MOUSE
Q9ET01
Distance in Percent
3
2
1
0
−1
−2
0
50
100

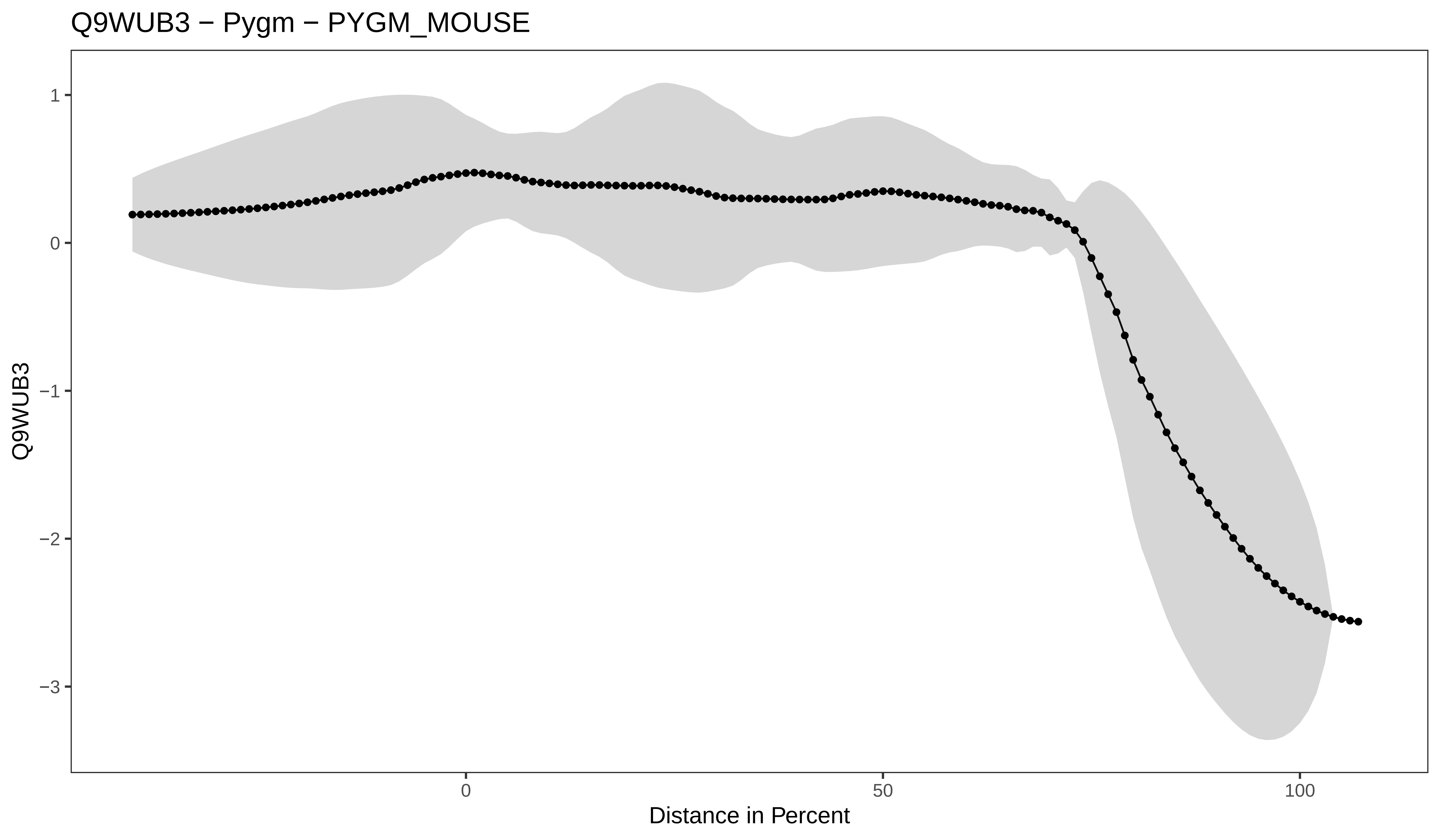

Q9WUB3 − Pygm − PYGM_MOUSE
1
0
−1
−2
−3
Q9WUB3
0
50
100
Distance in Percent

Q61838 − Pzp − PZP_MOUSE

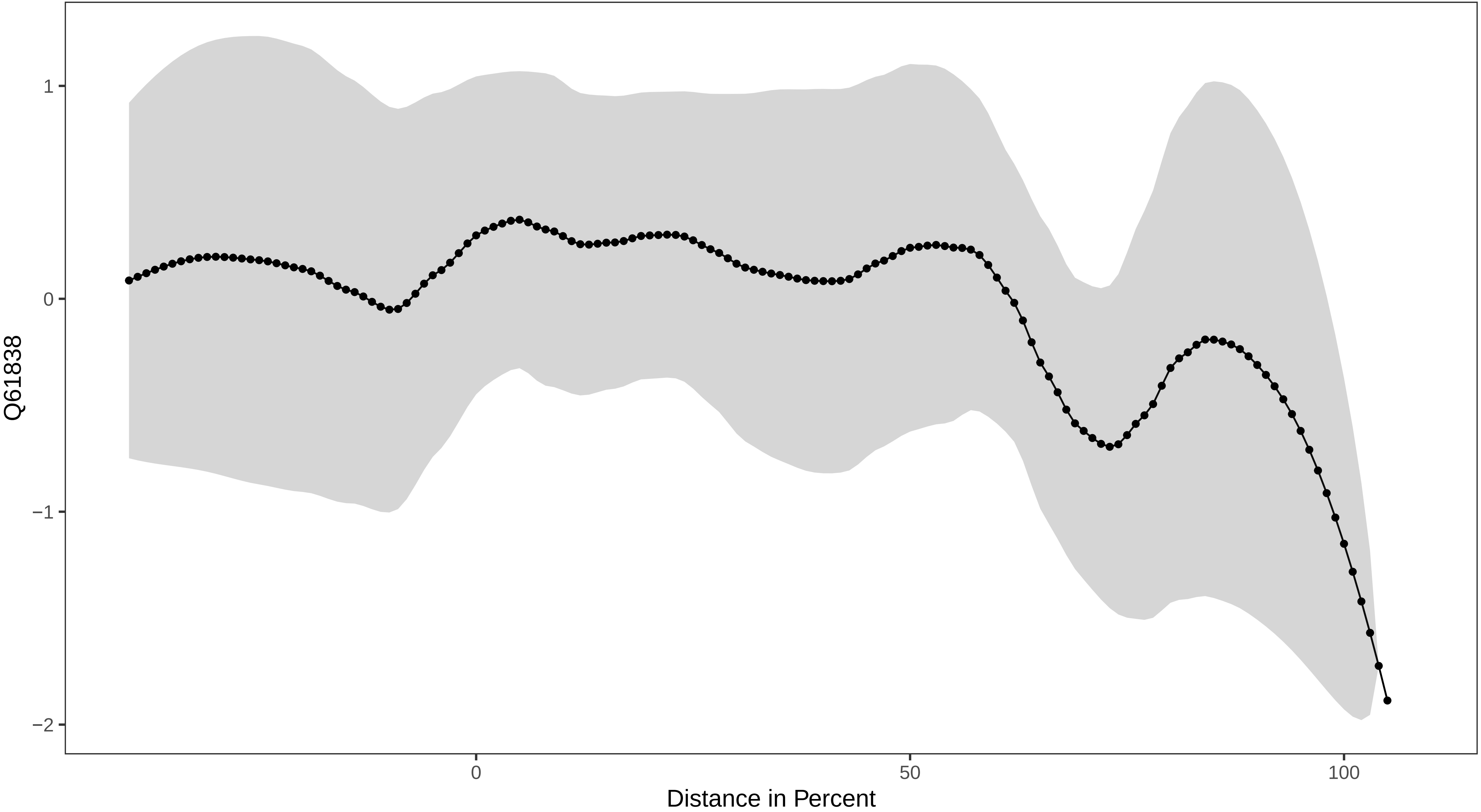

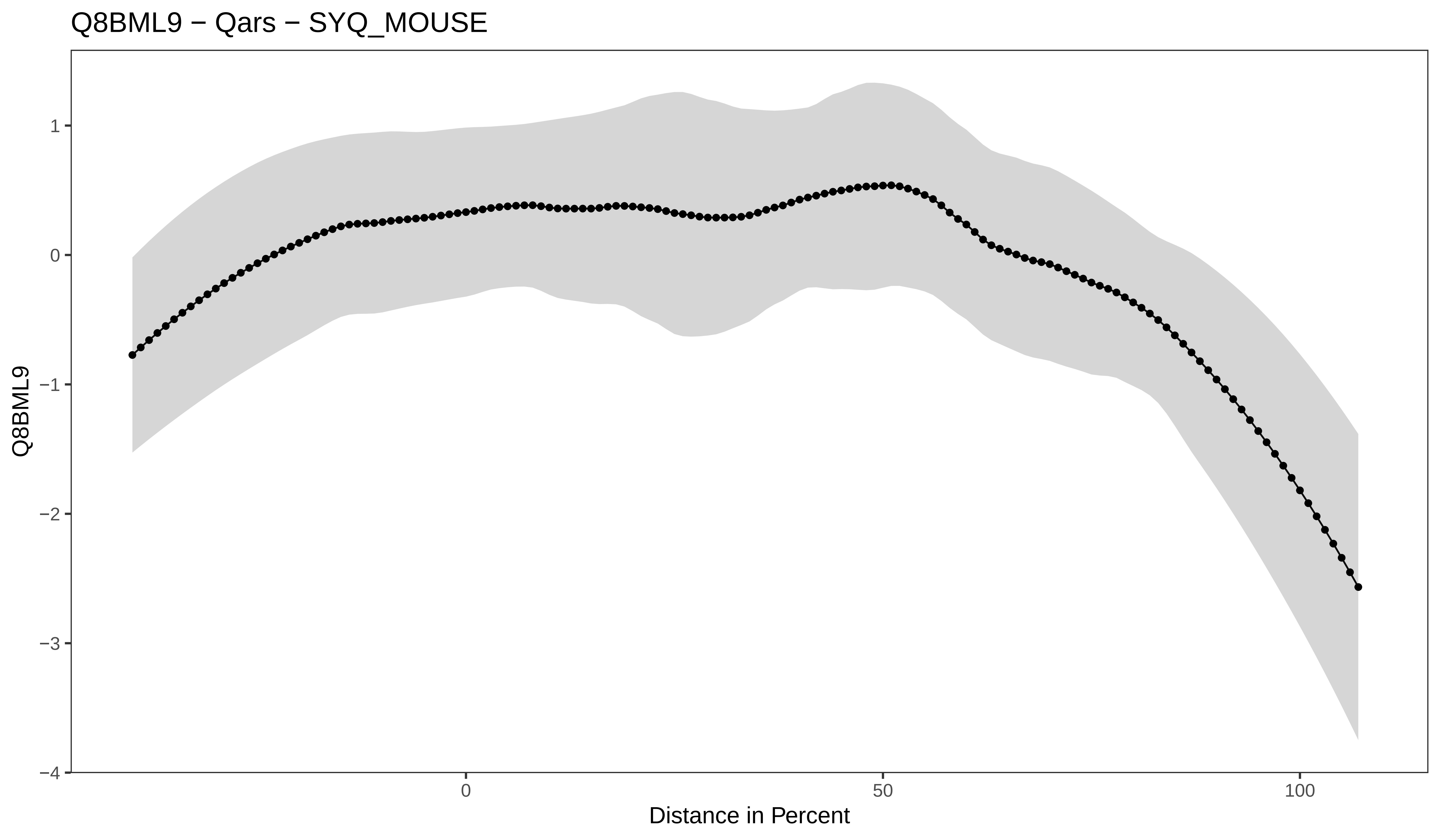

Q8BML9 − Qars − SYQ_MOUSE
Q8BML9
1
0
−1
−2
−3
−4
0
50
100
Distance in Percent

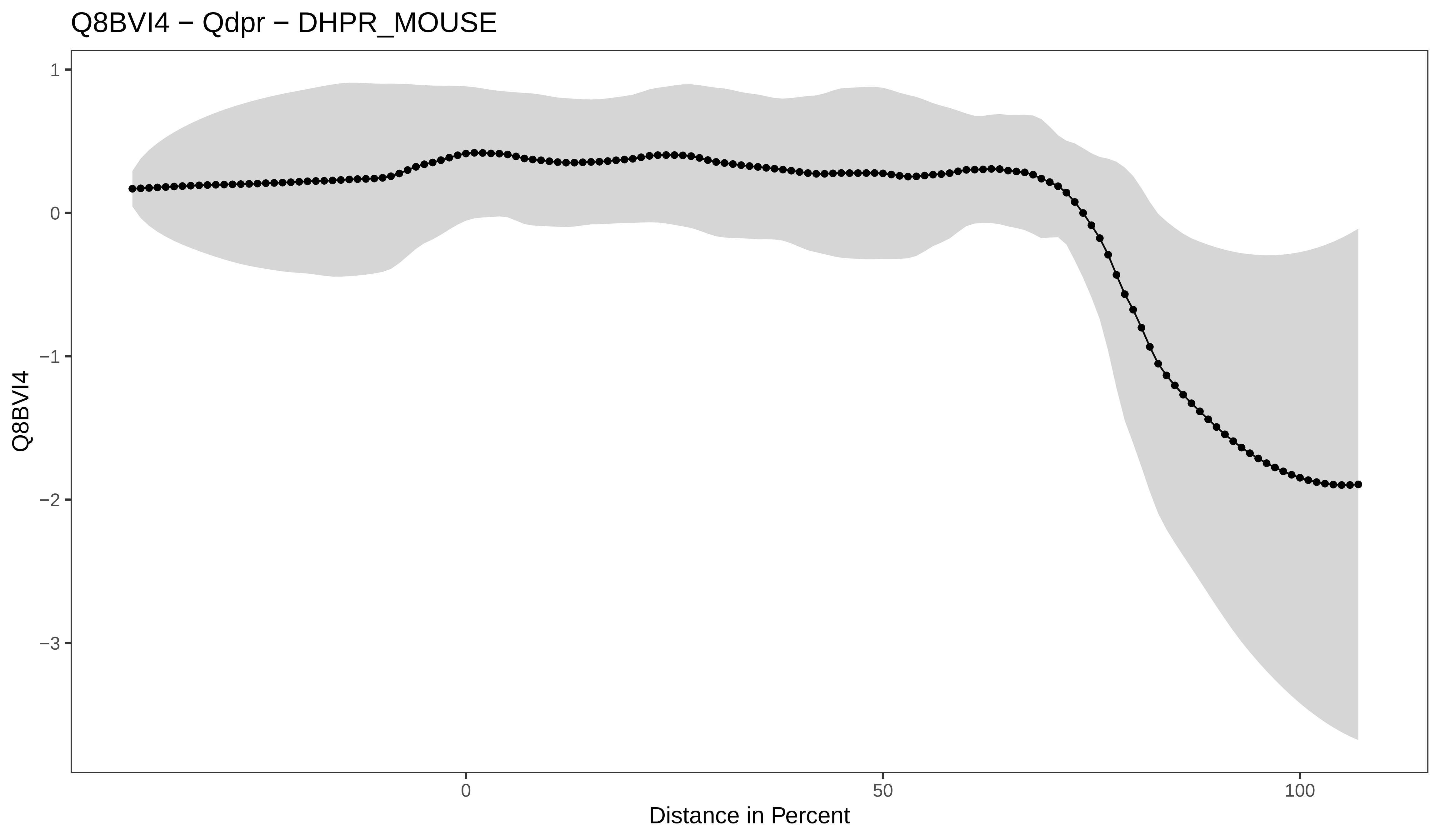
Q8BVI4 − Qdpr − DHPR_MOUSE
Q8BVI4
1
0
−1
−2
−3
0
50
100
Distance in Percent

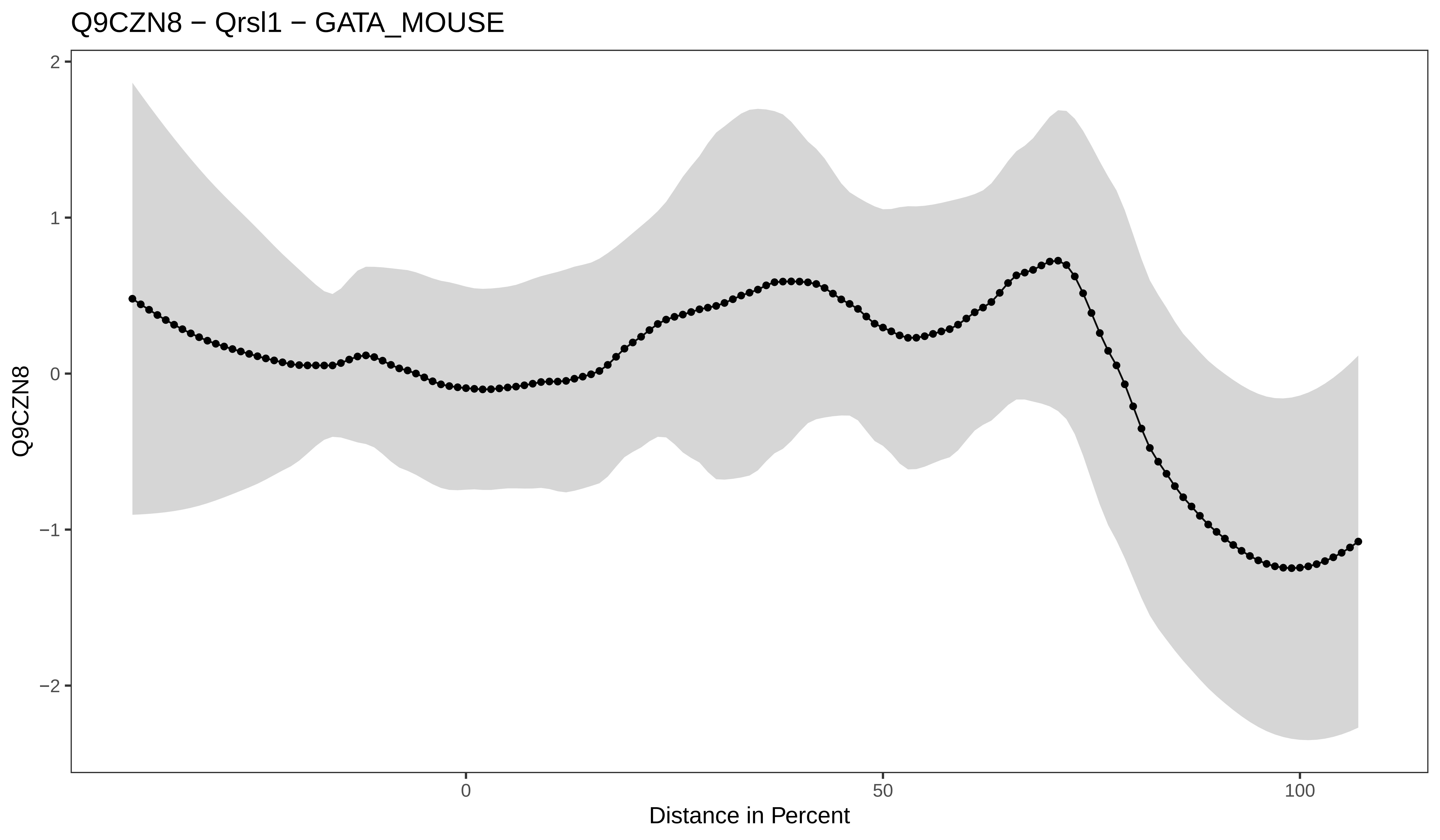

Q9CZN8 − Qrsl1 − GATA_MOUSE
2
1
0
−1
−2
Q9CZN8
0
50
100
Distance in Percent

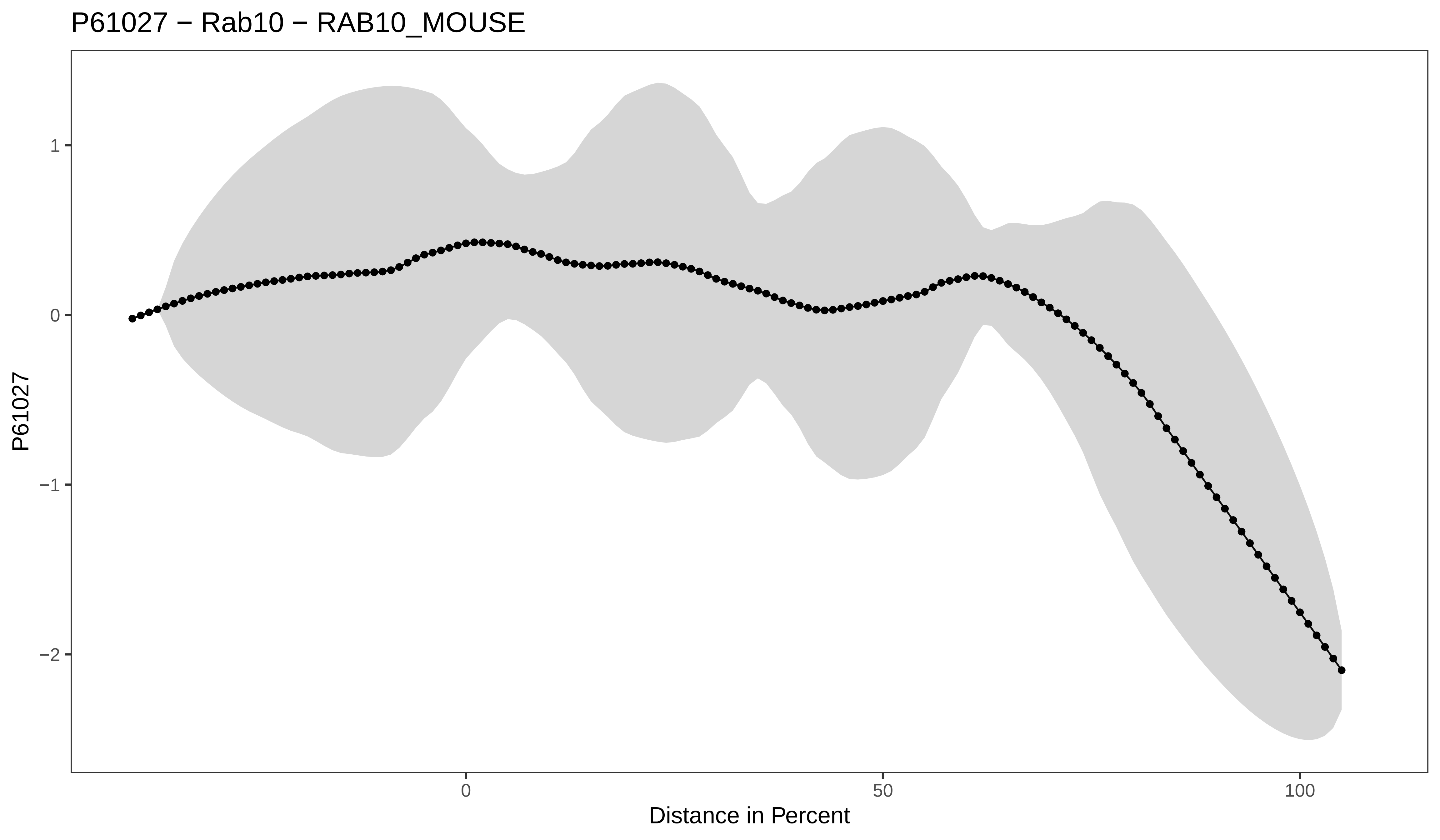
P61027 − Rab10 − RAB10_MOUSE
1
0
−1
−2
P61027
0
50
100
Distance in Percent

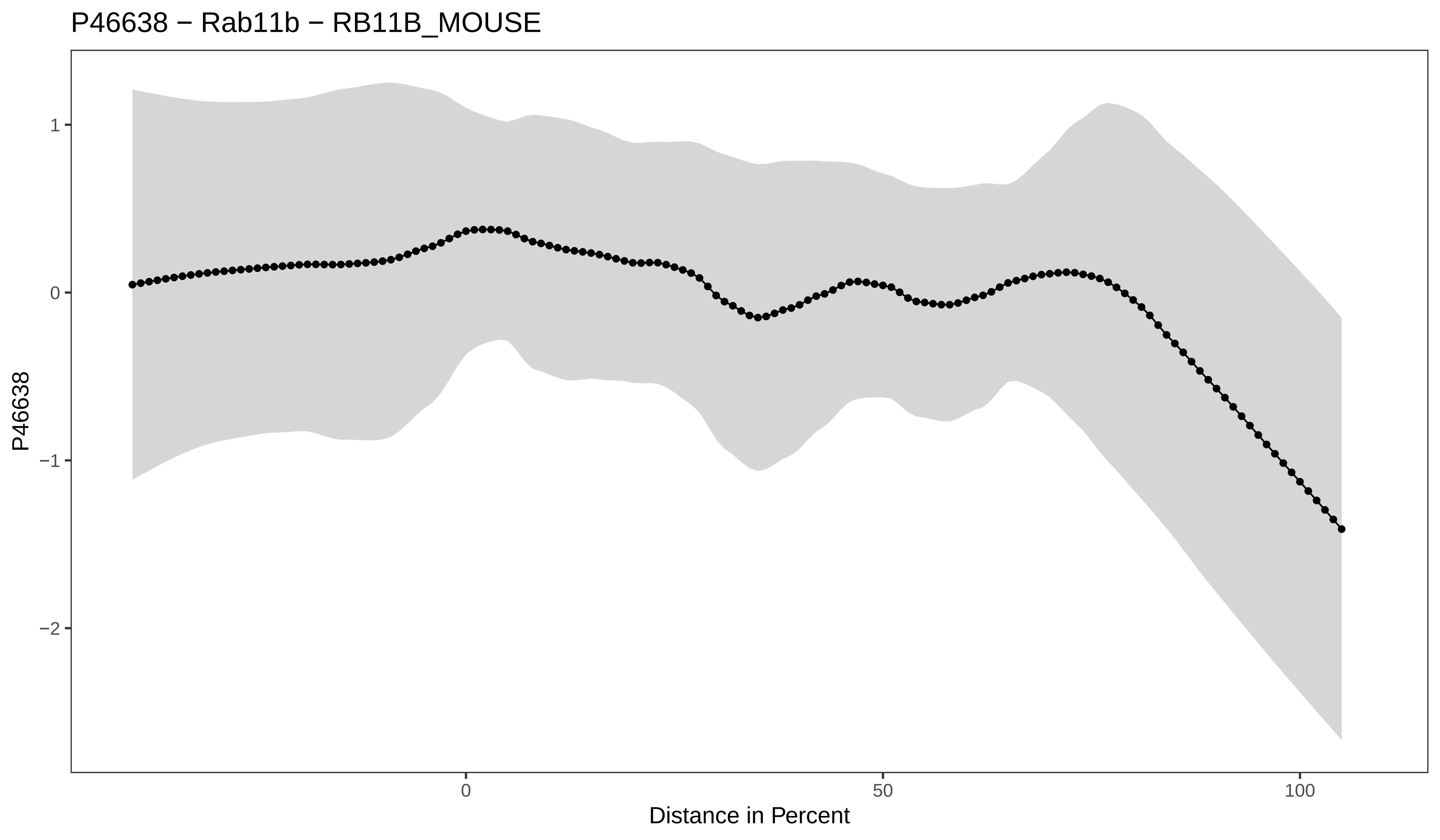

P46638 − Rab11b − RB11B_MOUSE
1
0
−1
−2
P46638
0
50
100
Distance in Percent

P35283 − Rab12 − RAB12_MOUSE

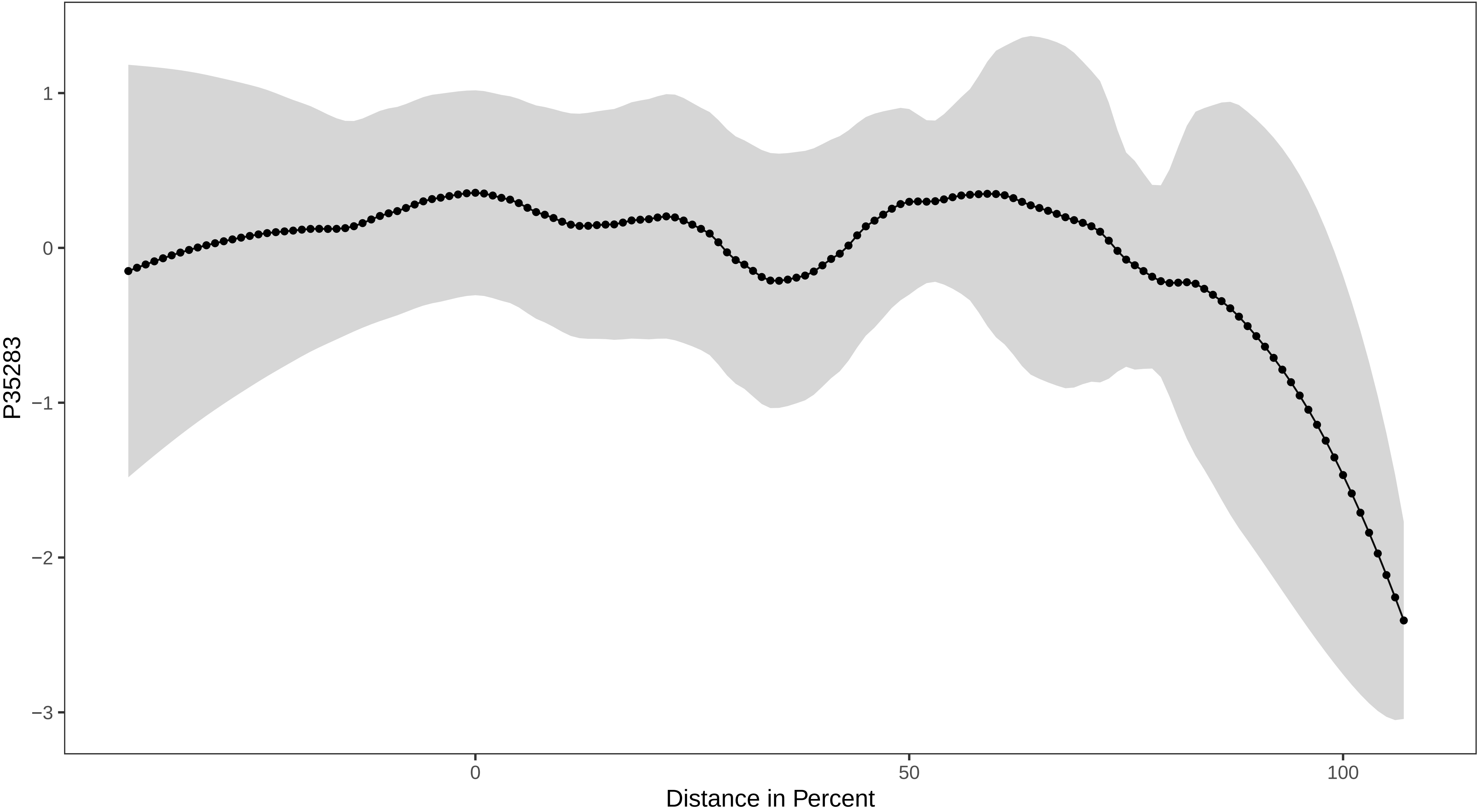

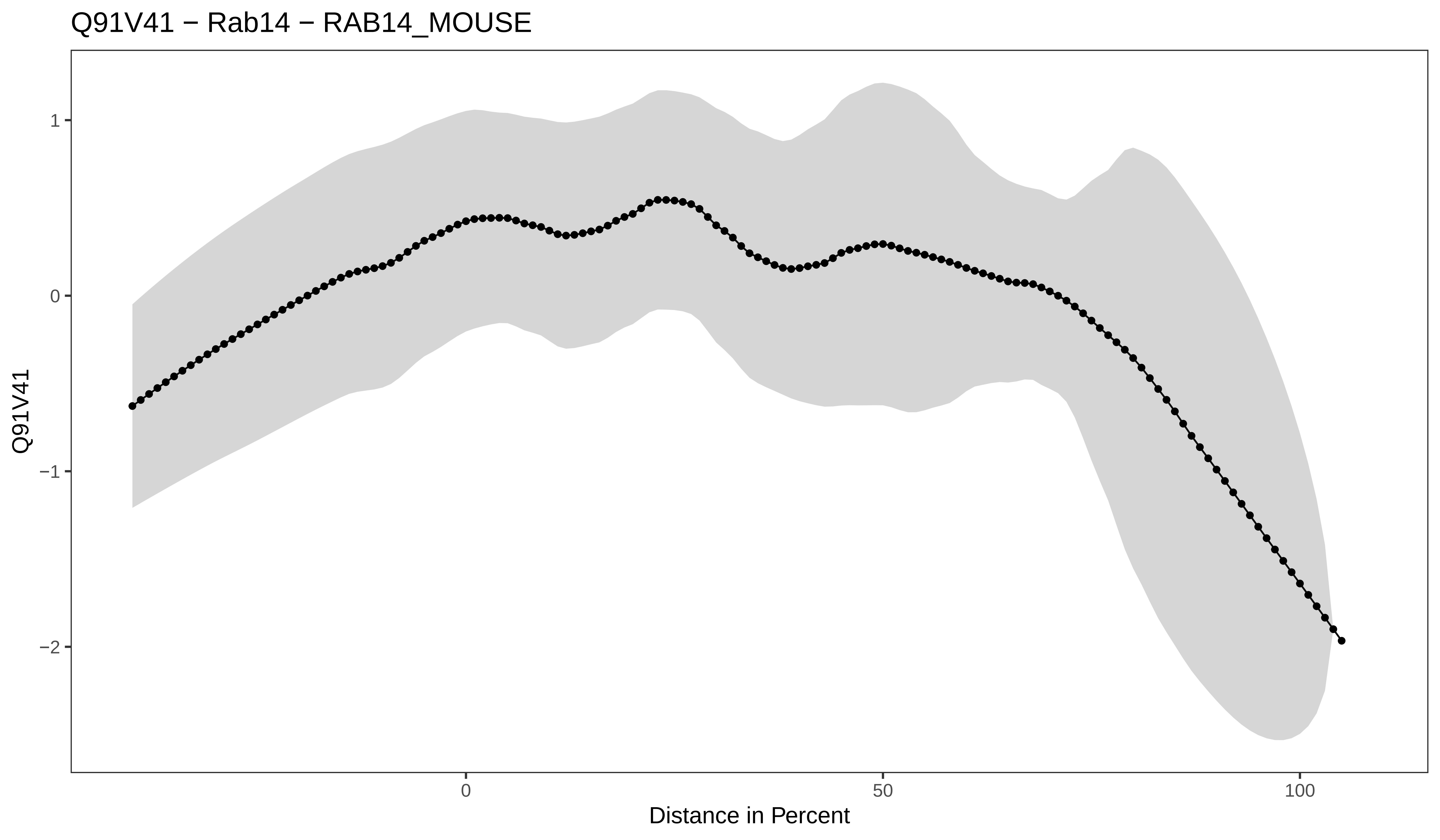
Q91V41 − Rab14 − RAB14_MOUSE
Q91V41
1
0
−1
−2
0
50
100
Distance in Percent

P35293 − Rab18 − RAB18_MOUSE

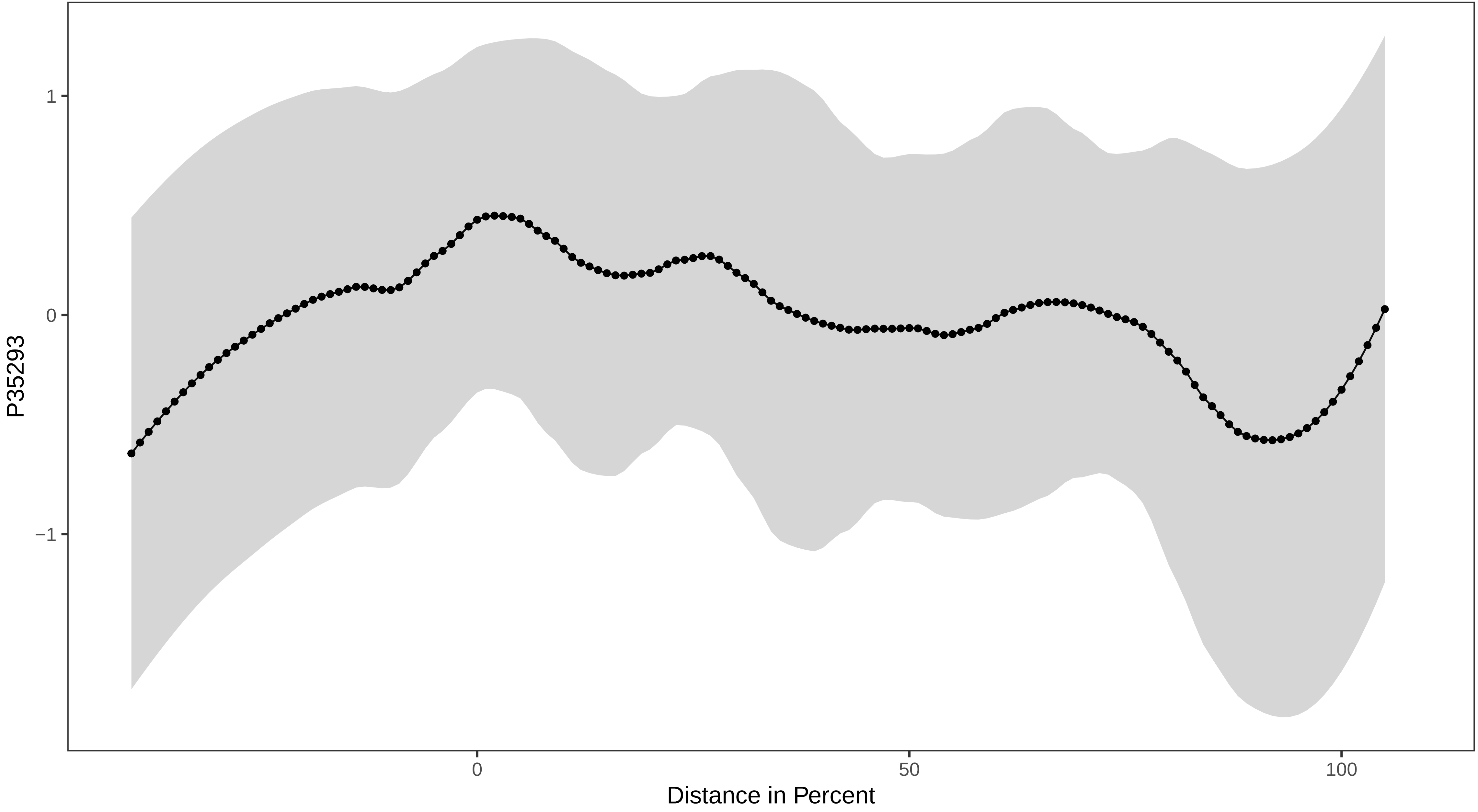

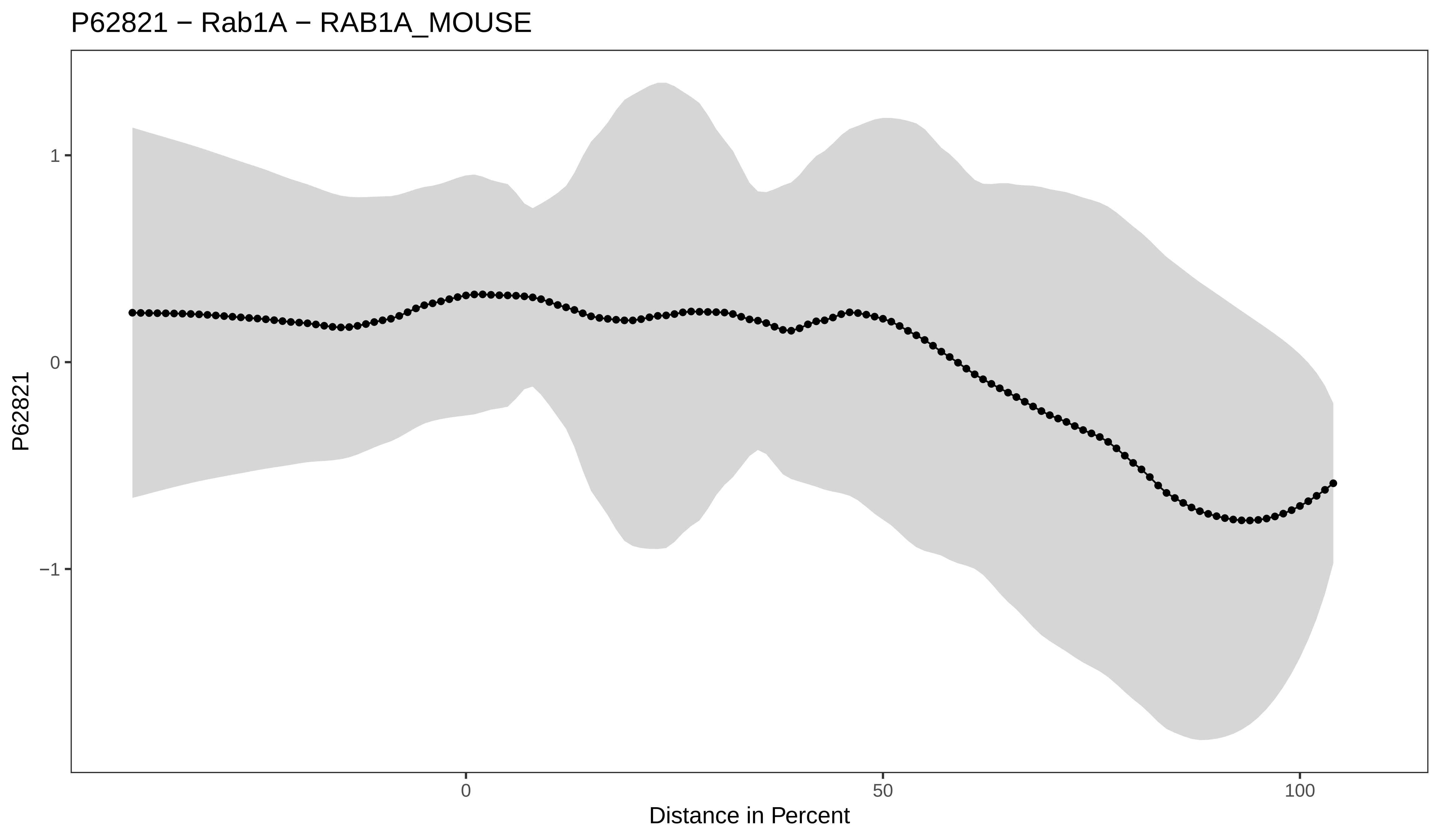

P62821 − Rab1A − RAB1A_MOUSE
1
0
−1
P62821
0
50
100
Distance in Percent

Q9D1G1 − Rab1b − RAB1B_MOUSE

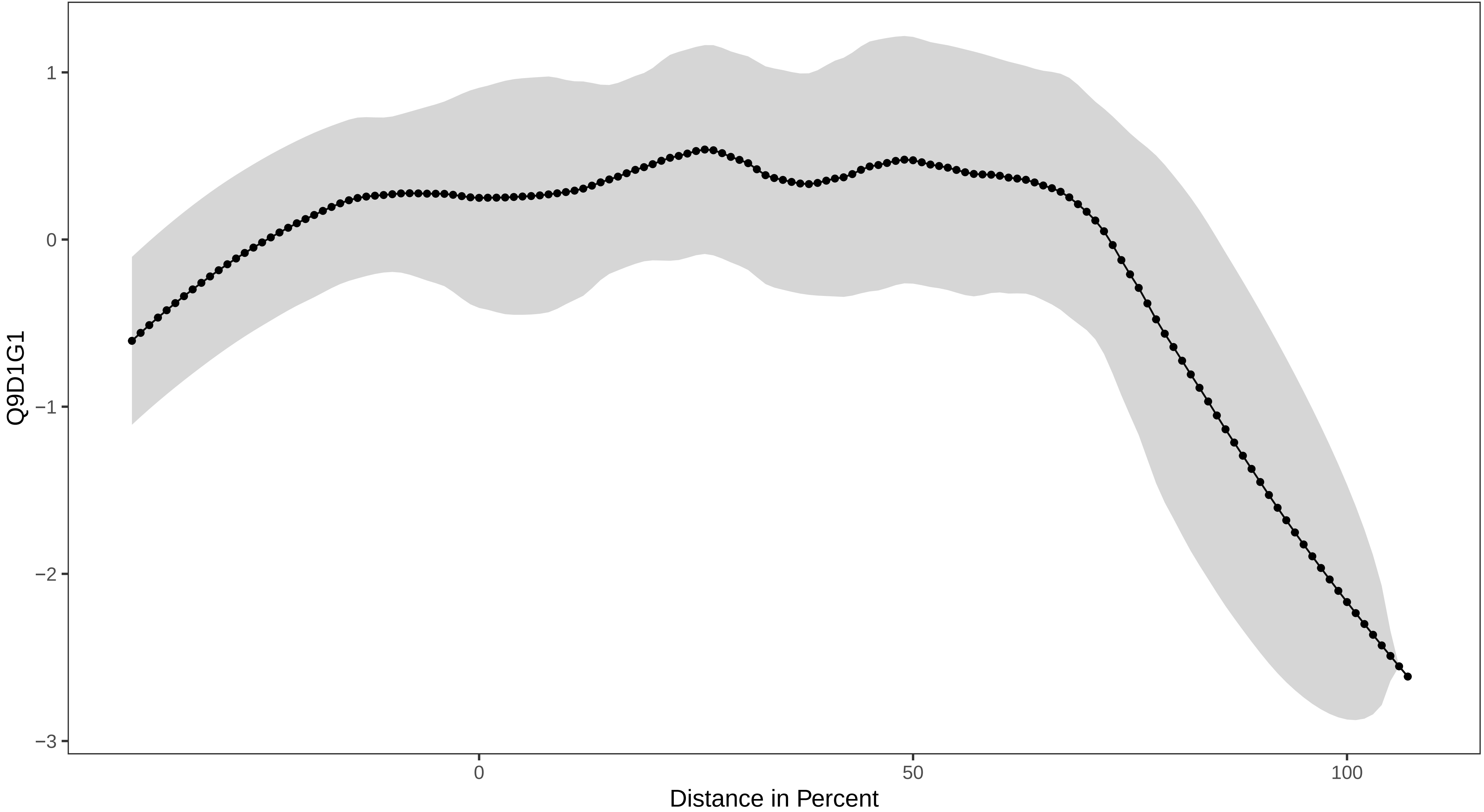

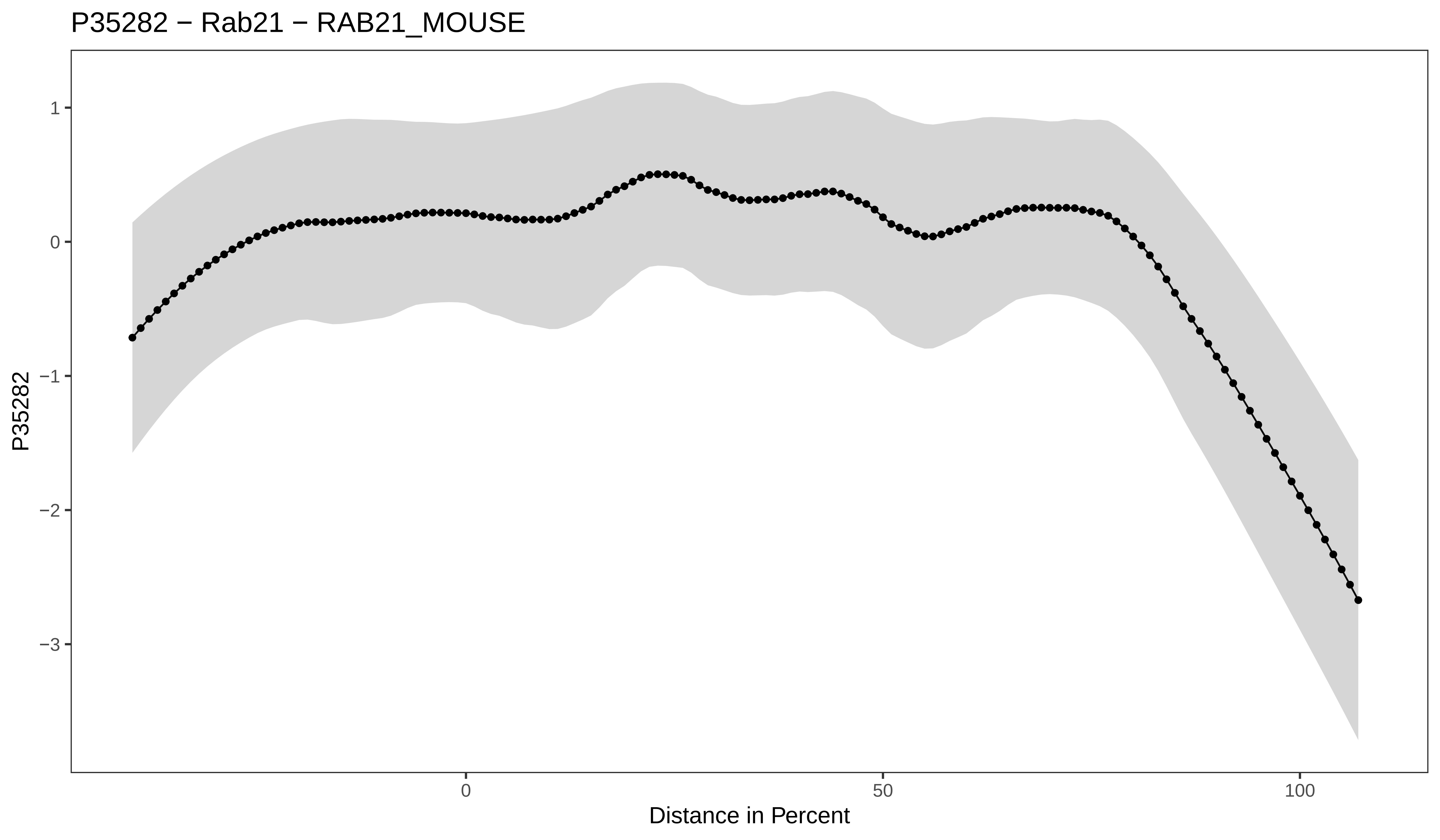

P35282 − Rab21 − RAB21_MOUSE
1
0
−1
−2
−3
P35282
0
50
100
Distance in Percent

P35285 − Rab22a − RB22A_MOUSE

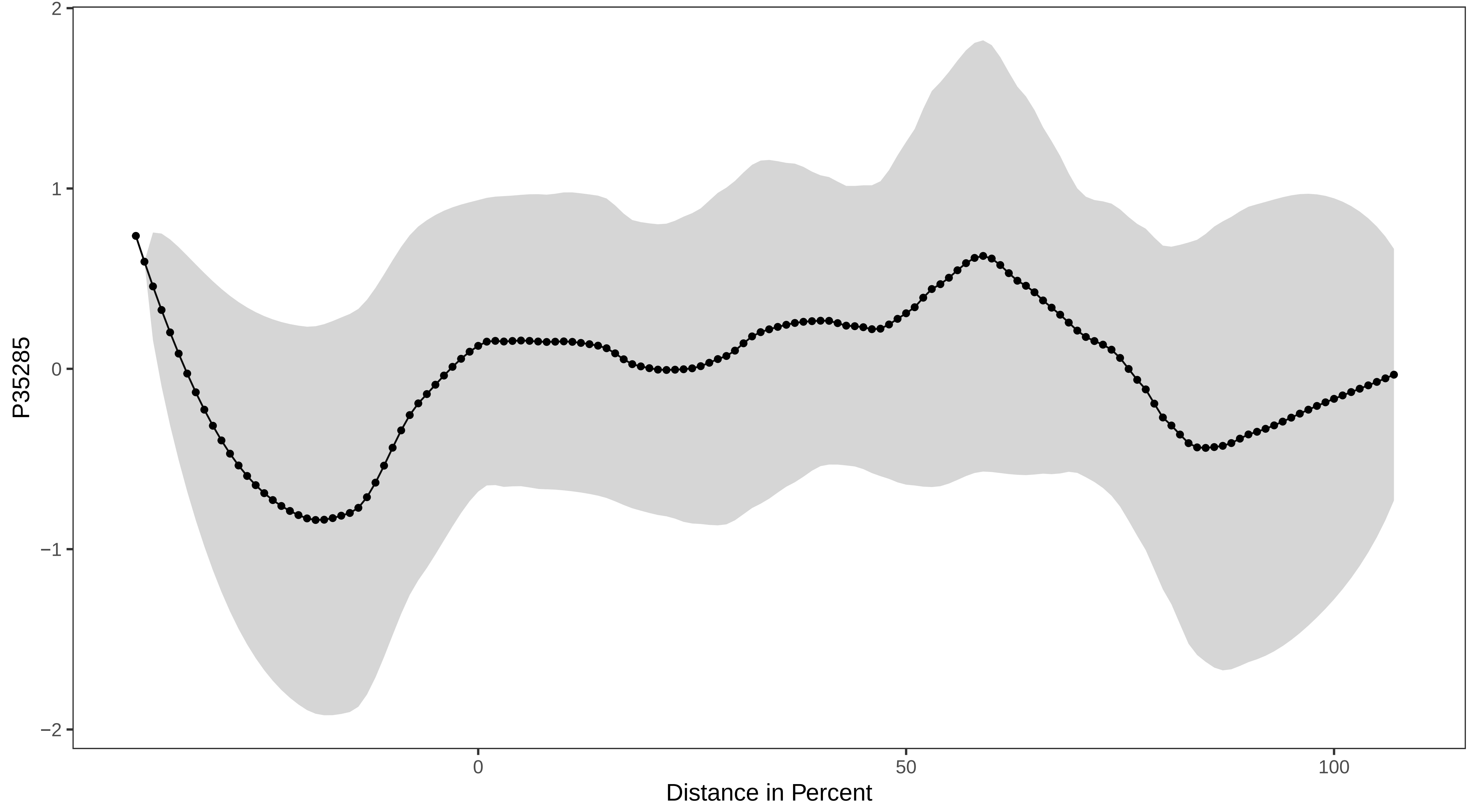

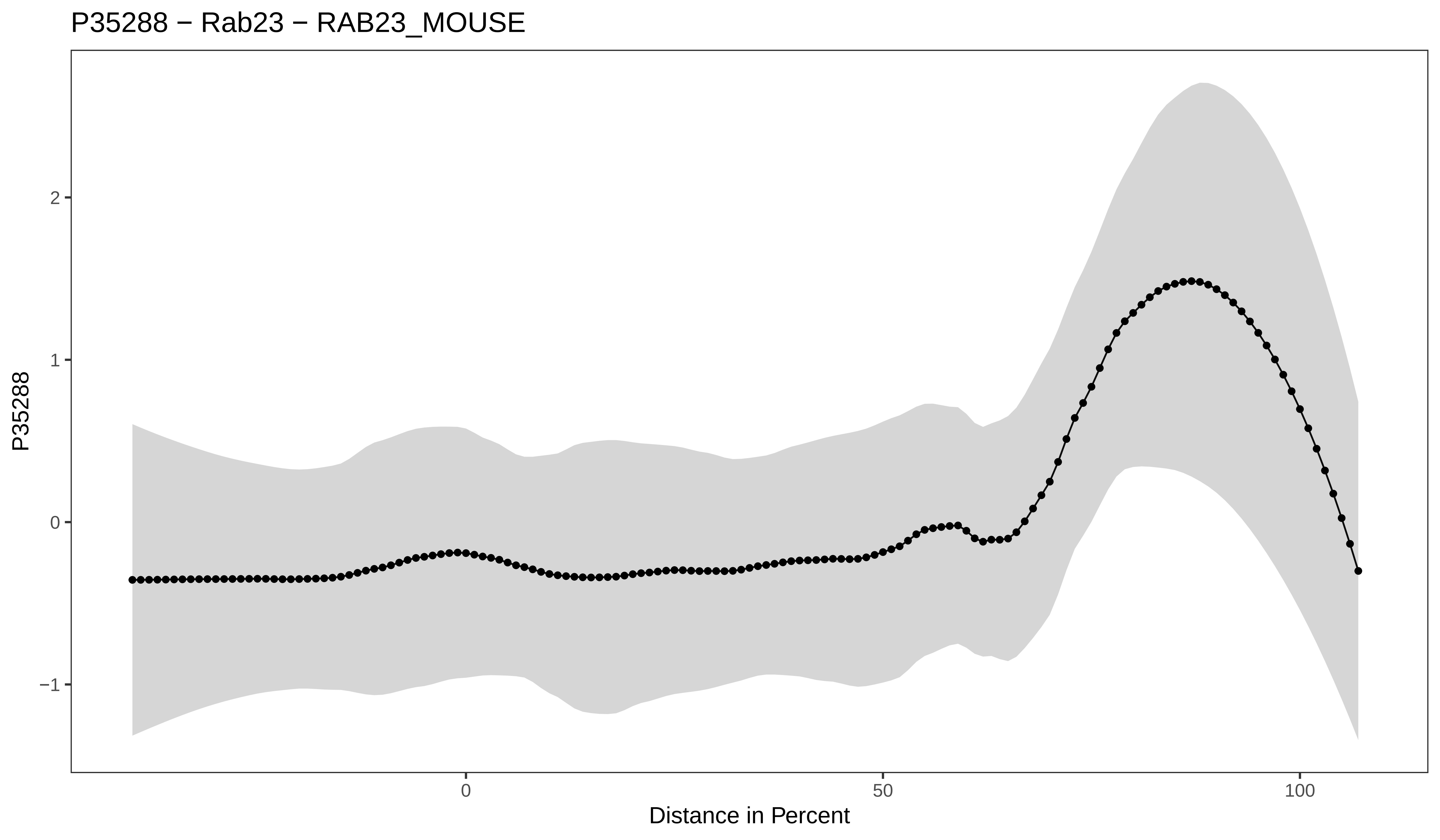
P35288 − Rab23 − RAB23_MOUSE
P35288
2
1
0
−1
0
50
100
Distance in Percent

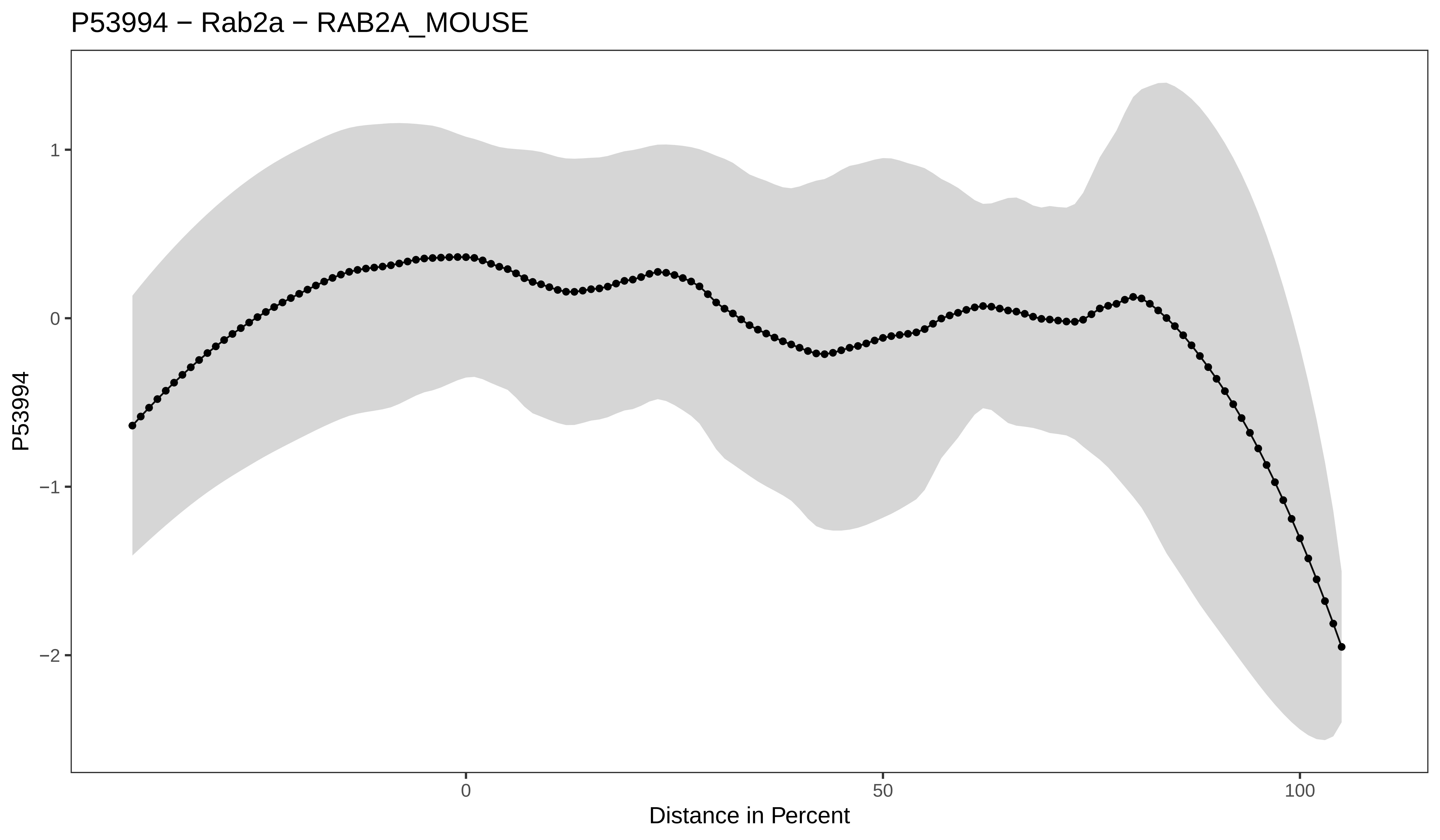
P53994 − Rab2a − RAB2A_MOUSE
P53994
1
0
−1
−2
0
50
100
Distance in Percent

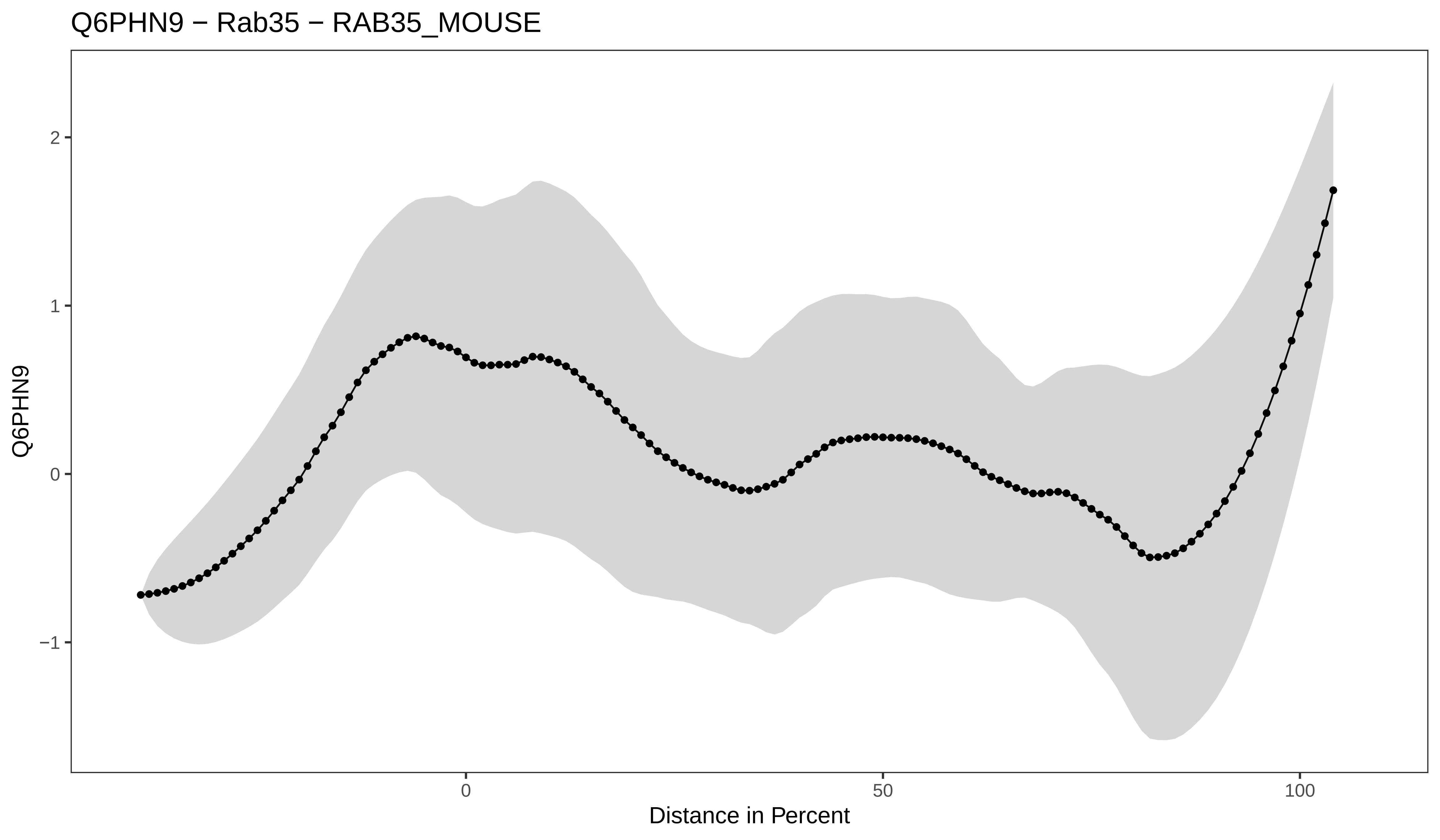

Q6PHN9 − Rab35 − RAB35_MOUSE
Q6PHN9
2
1
0
−1
0
50
100
Distance in Percent

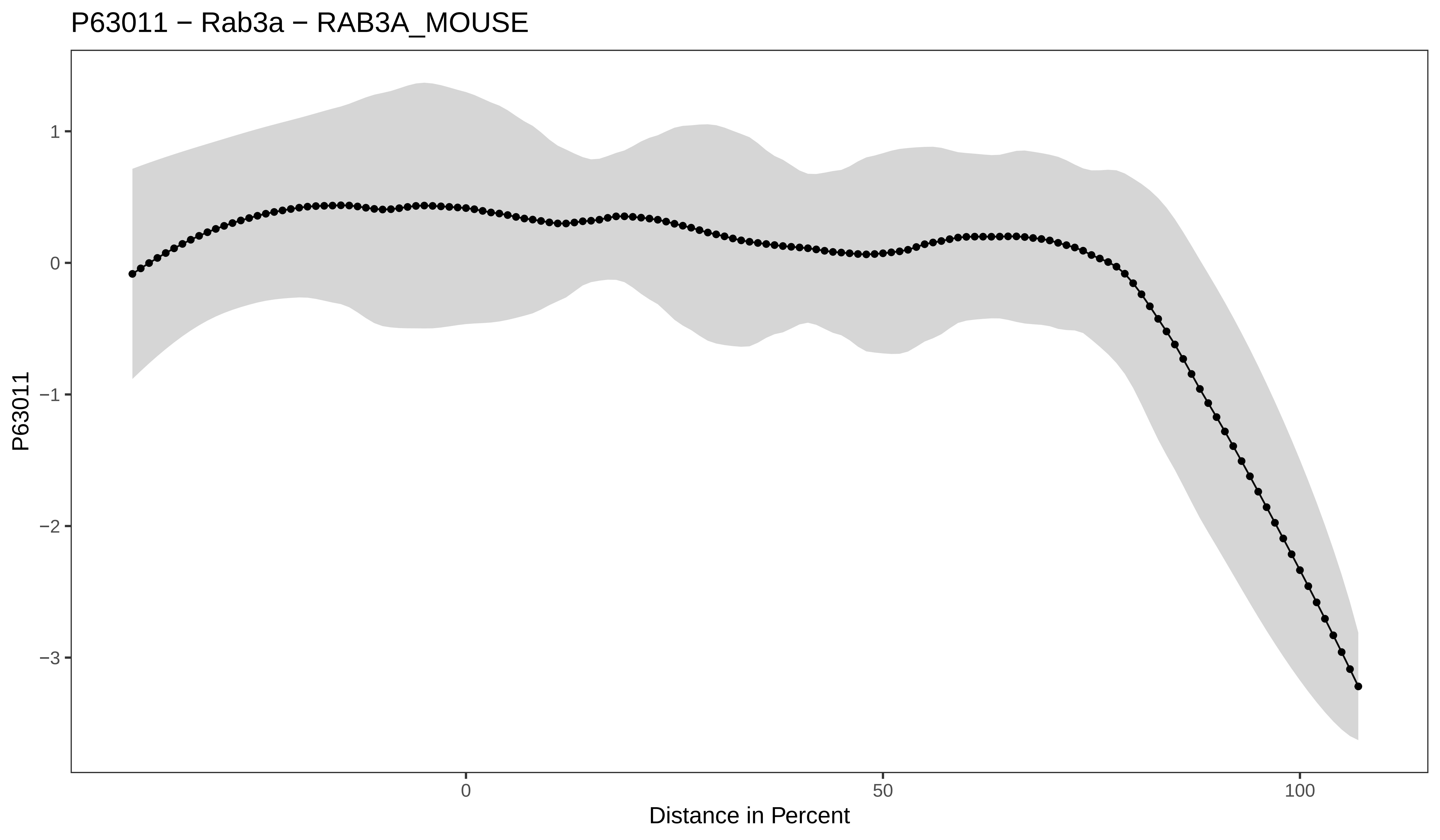

P63011 − Rab3a − RAB3A_MOUSE
1
0
−1
−2
−3
P63011
0
50
100
Distance in Percent

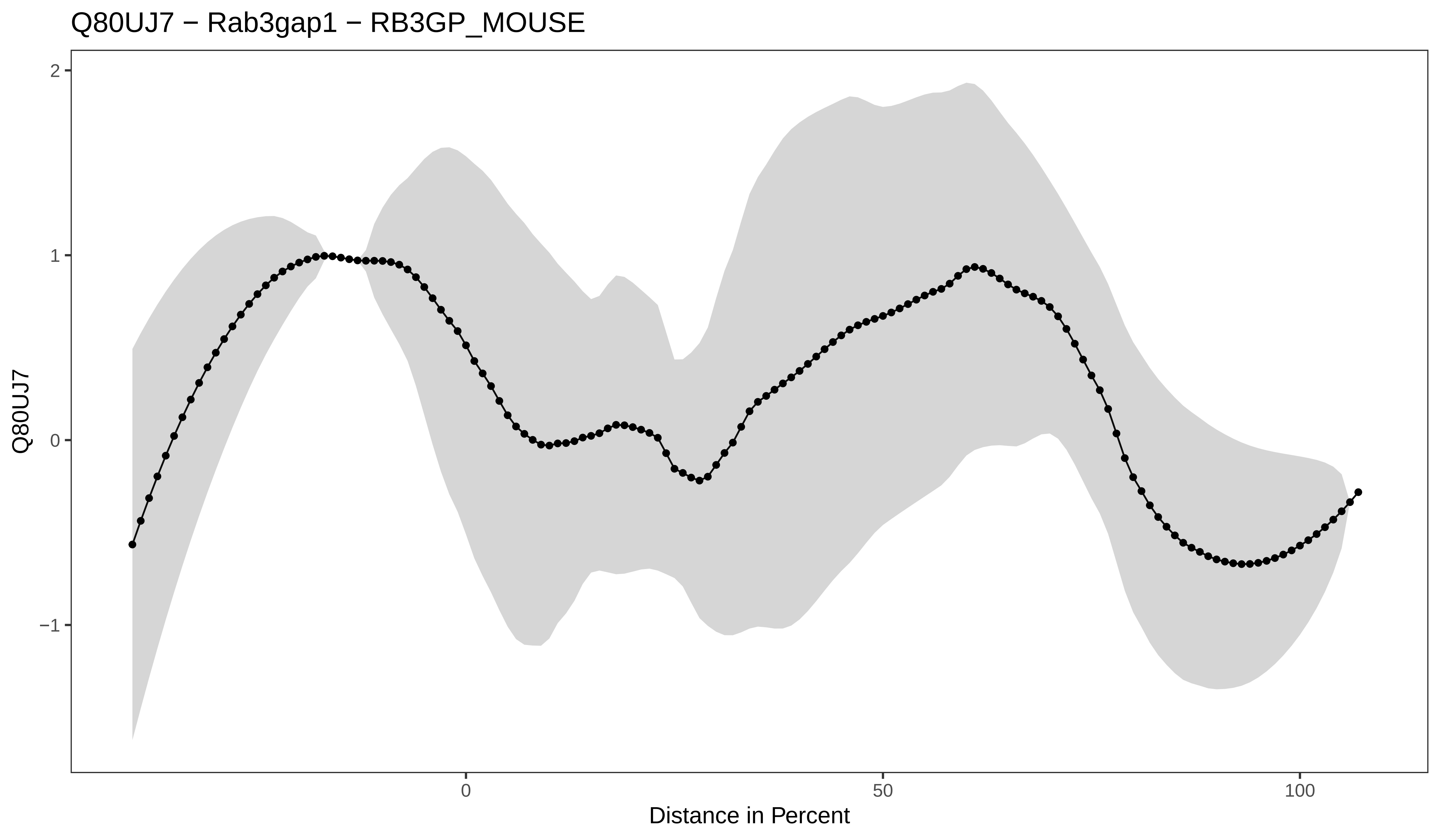
Q80UJ7 − Rab3gap1 − RB3GP_MOUSE
Q80UJ7
2
1
0
−1
0
50
100
Distance in Percent

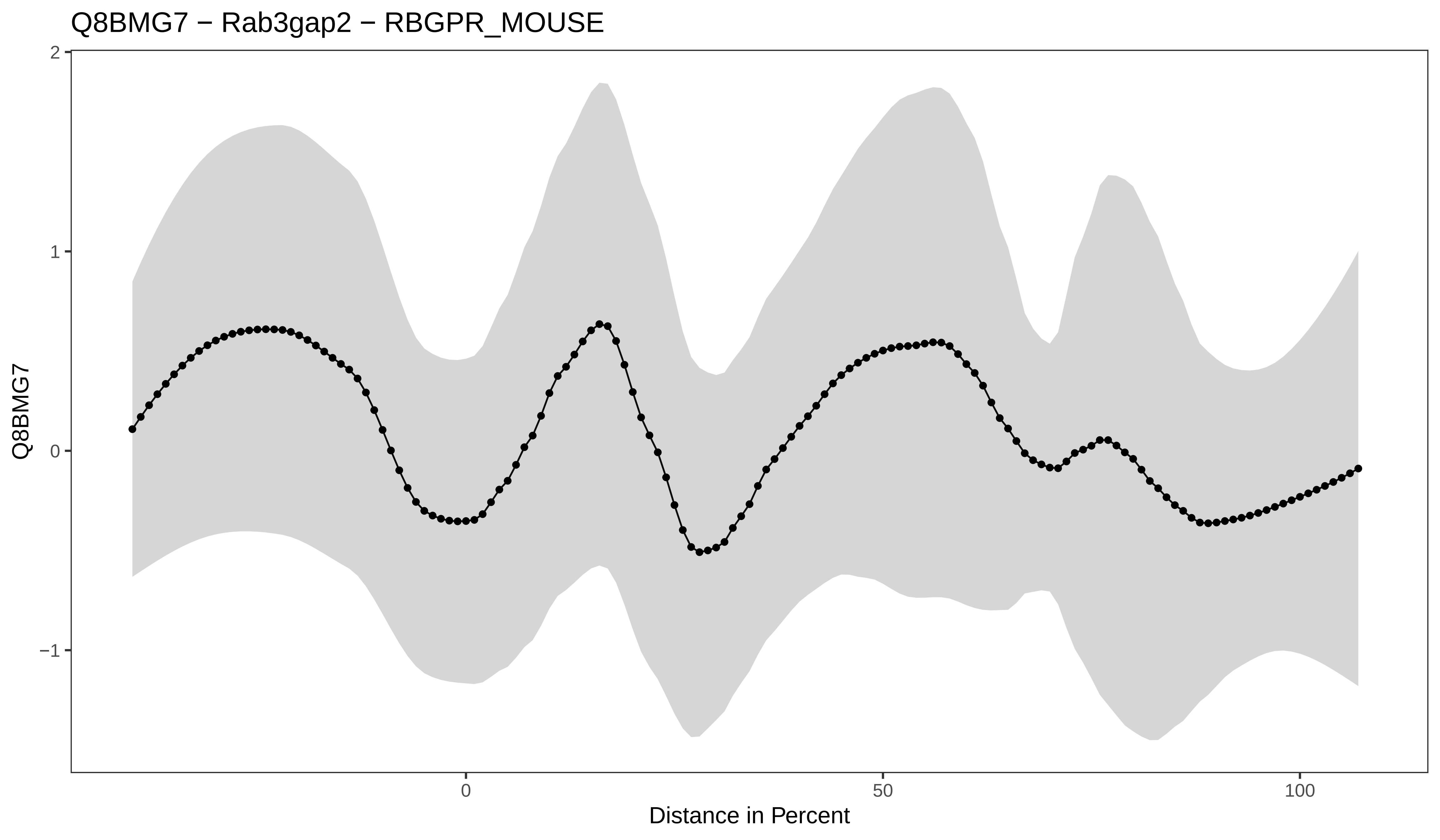

Q8BMG7 − Rab3gap2 − RBGPR_MOUSE
Q8BMG7
Distance in Percent
2
1
0
−1
0
50
100

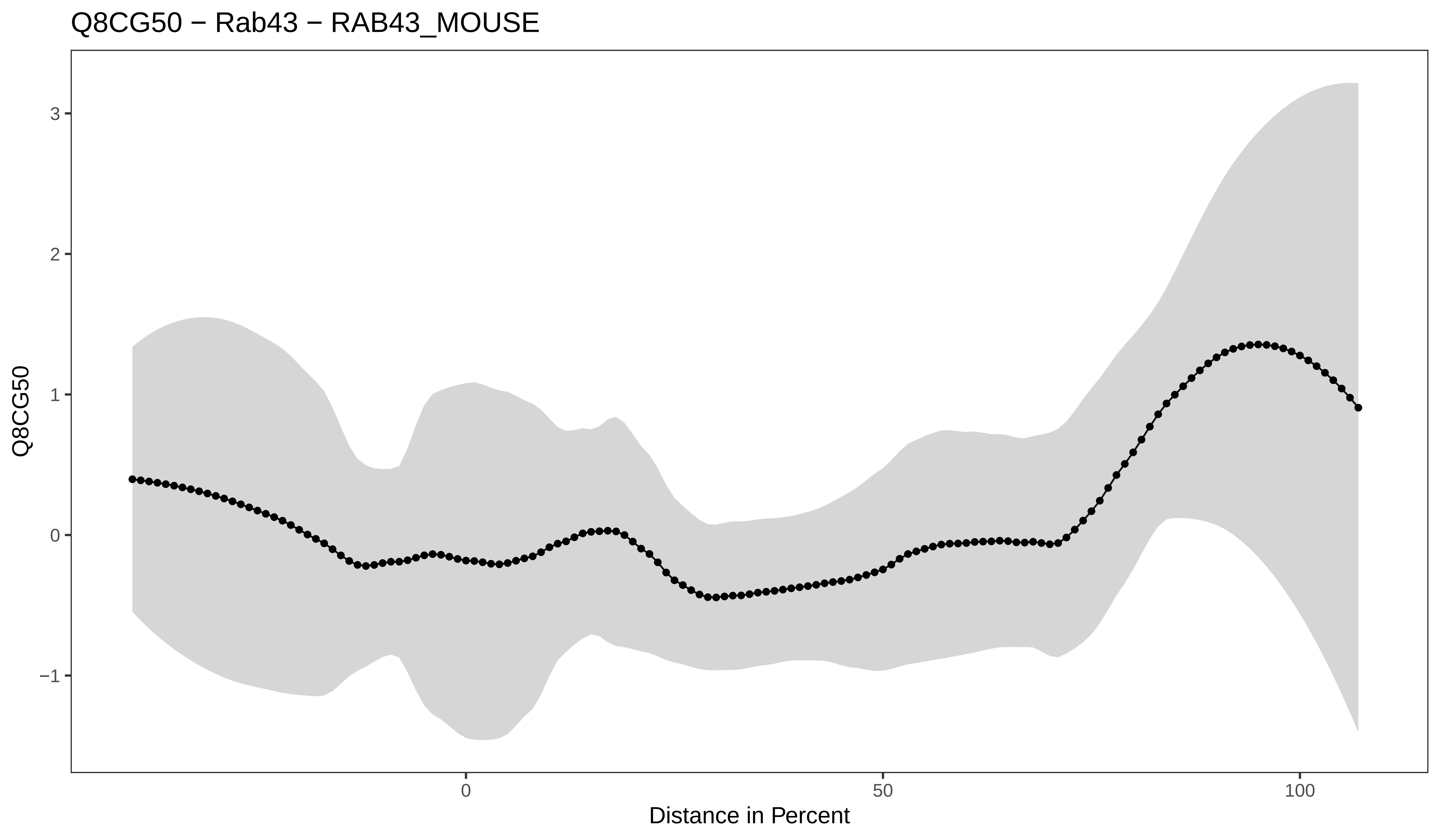

Q8CG50 − Rab43 − RAB43_MOUSE
3
2
1
0
−1
Q8CG50
0
50
100
Distance in Percent

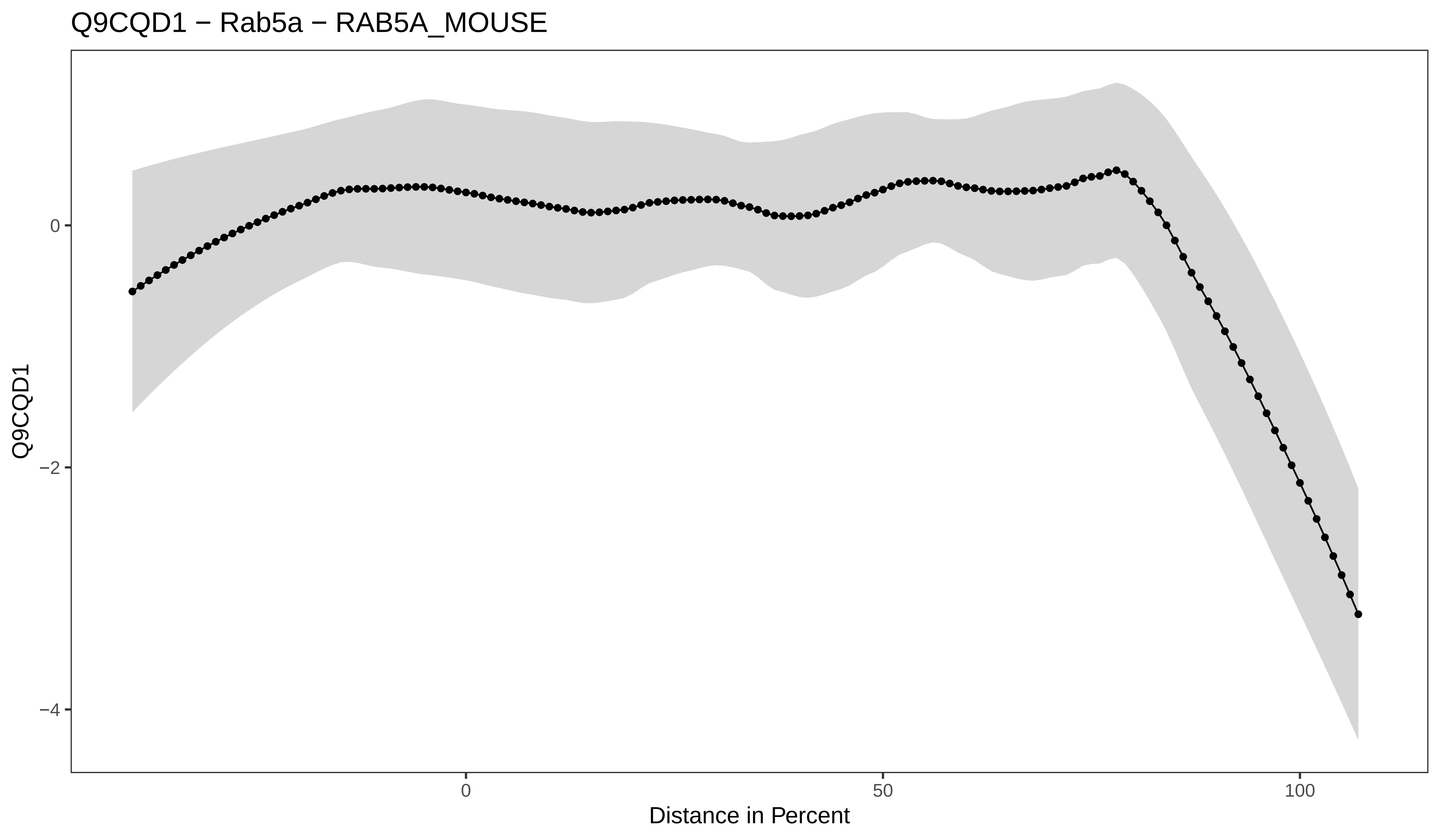
Q9CQD1 − Rab5a − RAB5A_MOUSE
Q9CQD1
0
−2
−4
0
50
100
Distance in Percent

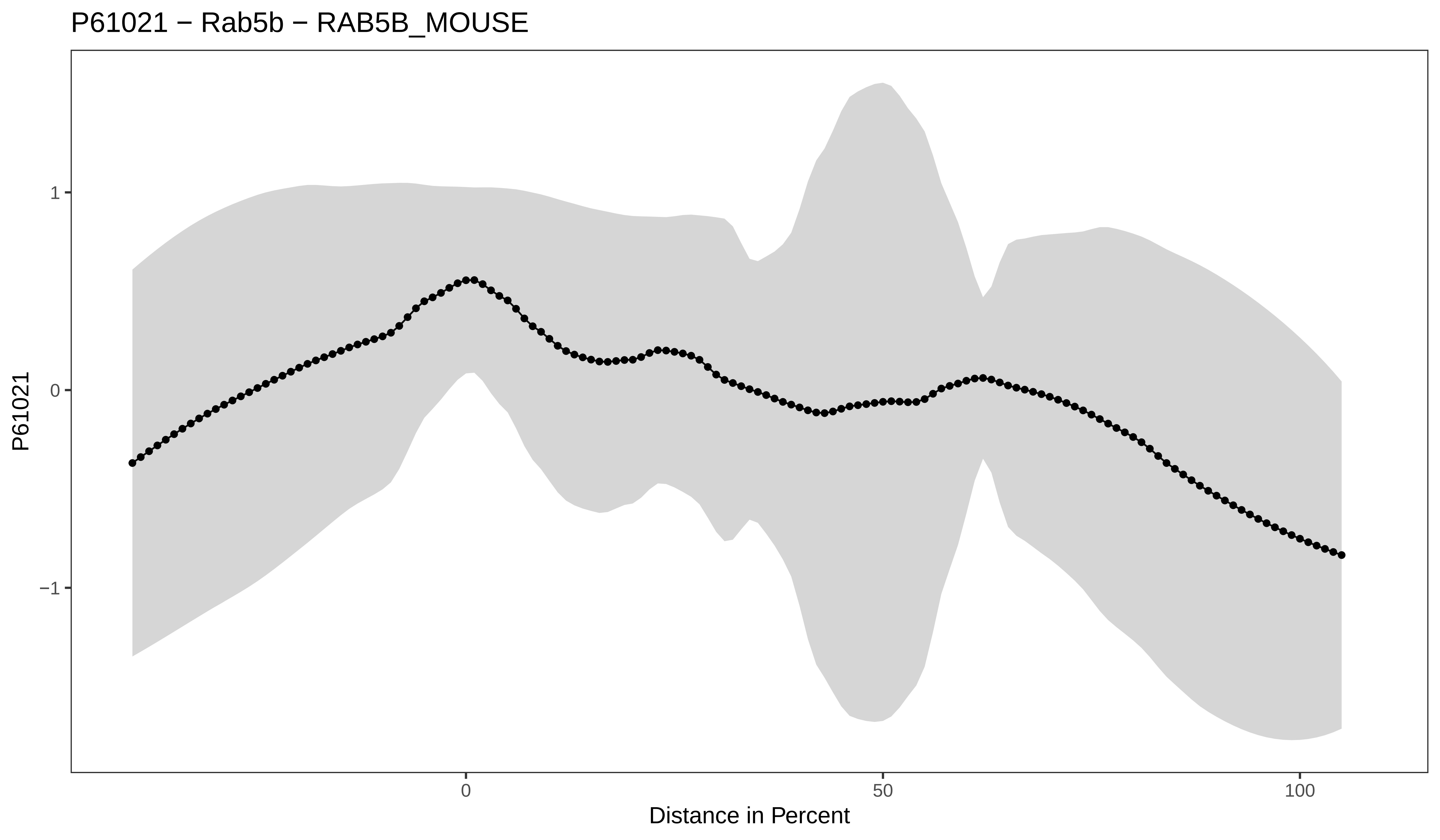

P61021 − Rab5b − RAB5B_MOUSE
P61021
1
0
−1
0
50
100
Distance in Percent

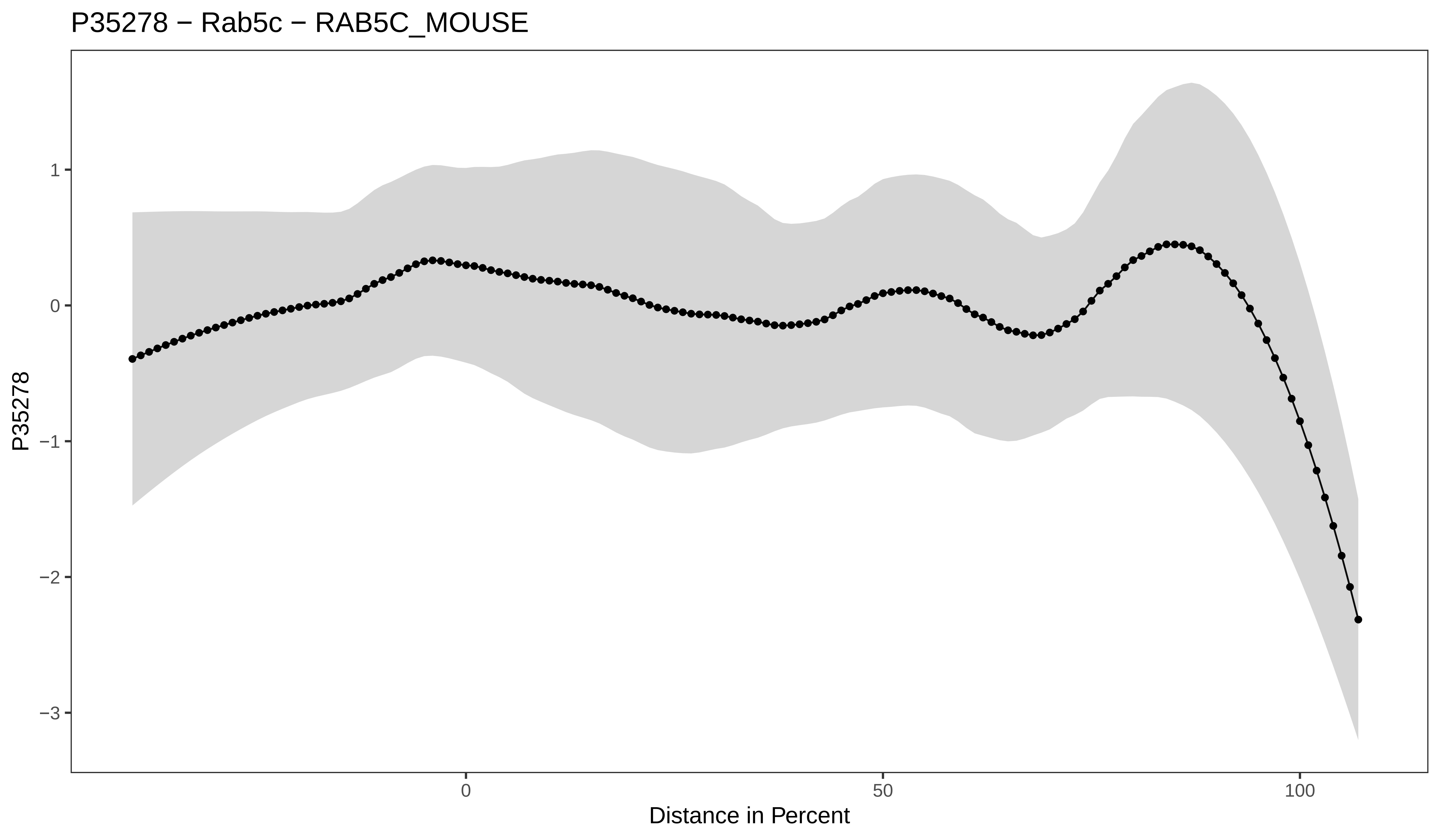

P35278 − Rab5c − RAB5C_MOUSE
P35278
1
0
−1
−2
−3
0
50
100
Distance in Percent

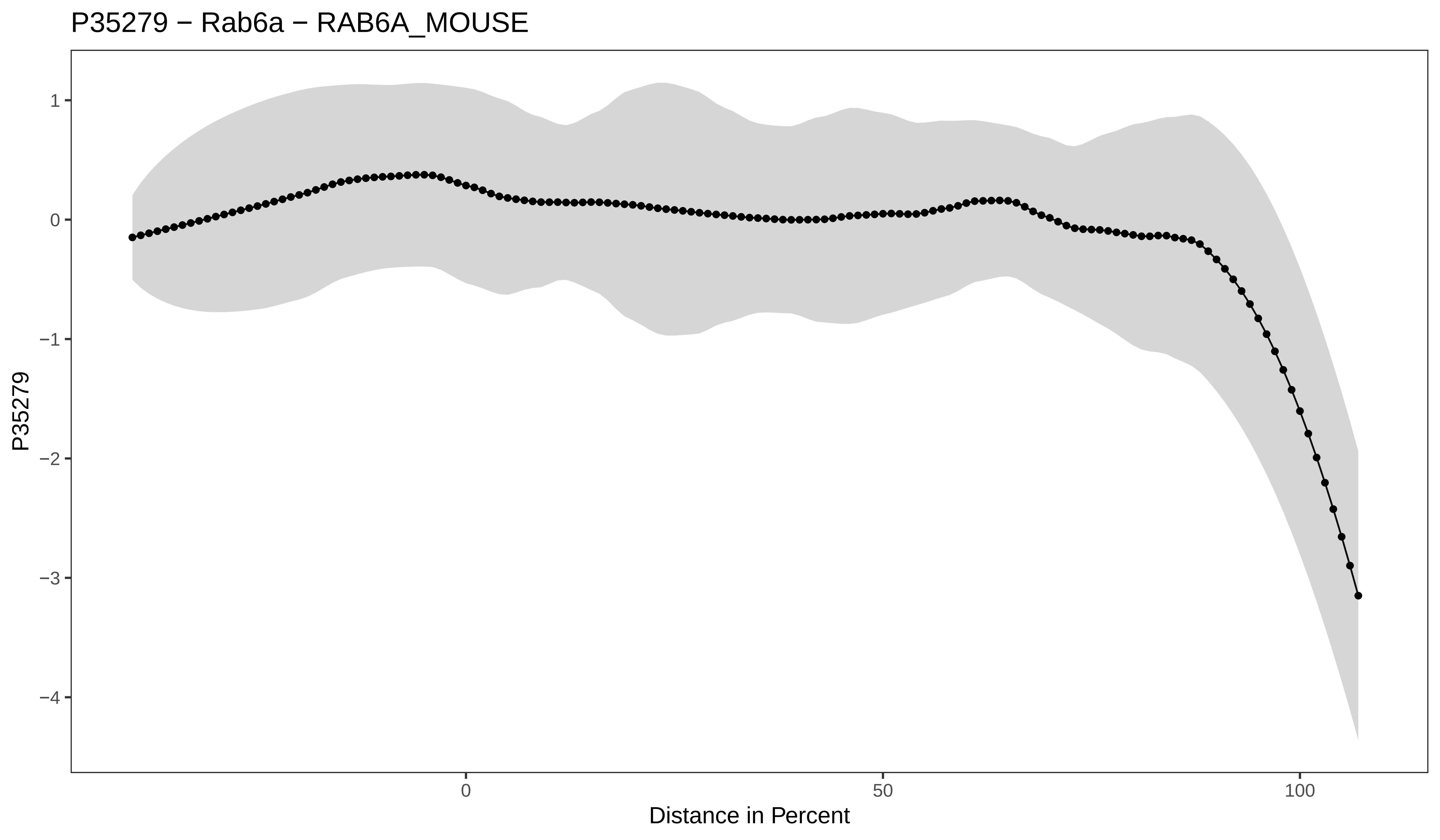

P35279 − Rab6a − RAB6A_MOUSE
1
0
−1
−2
−3
−4
P35279
0
50
100
Distance in Percent

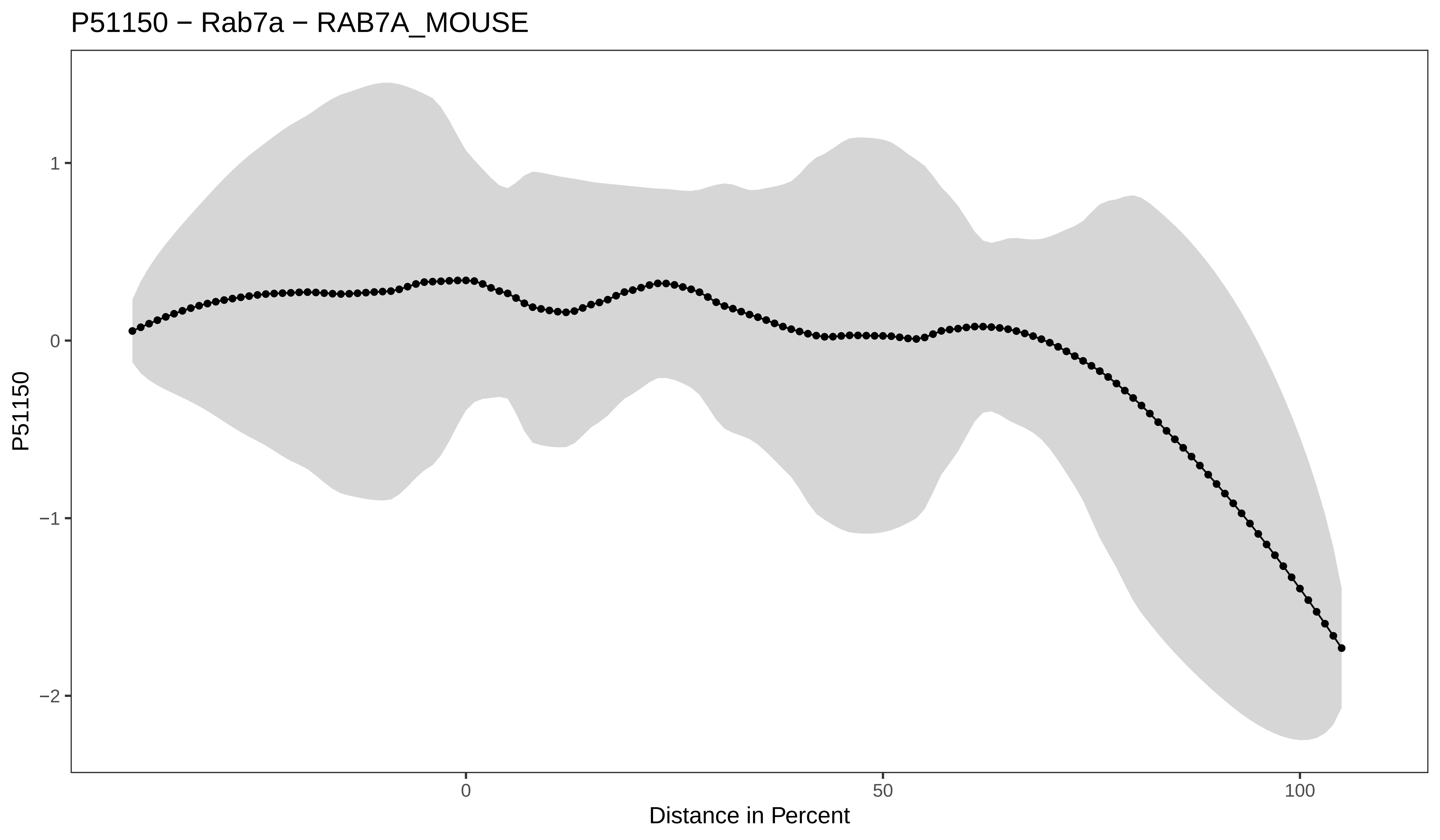
P51150 − Rab7a − RAB7A_MOUSE
1
0
−1
−2
P51150
0
50
100
Distance in Percent

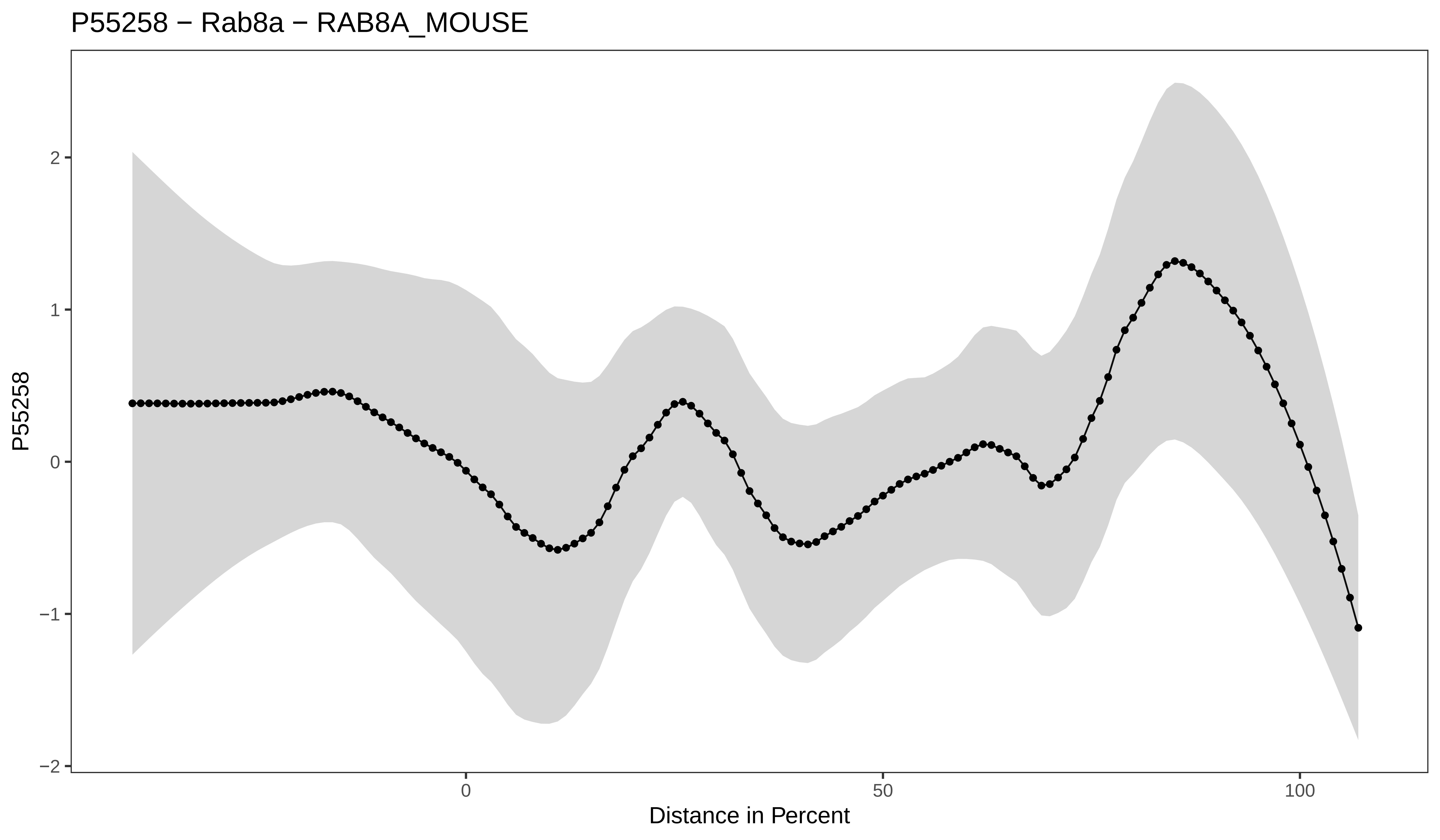

P55258 − Rab8a − RAB8A_MOUSE
P55258
2
1
0
−1
−2
0
50
100
Distance in Percent

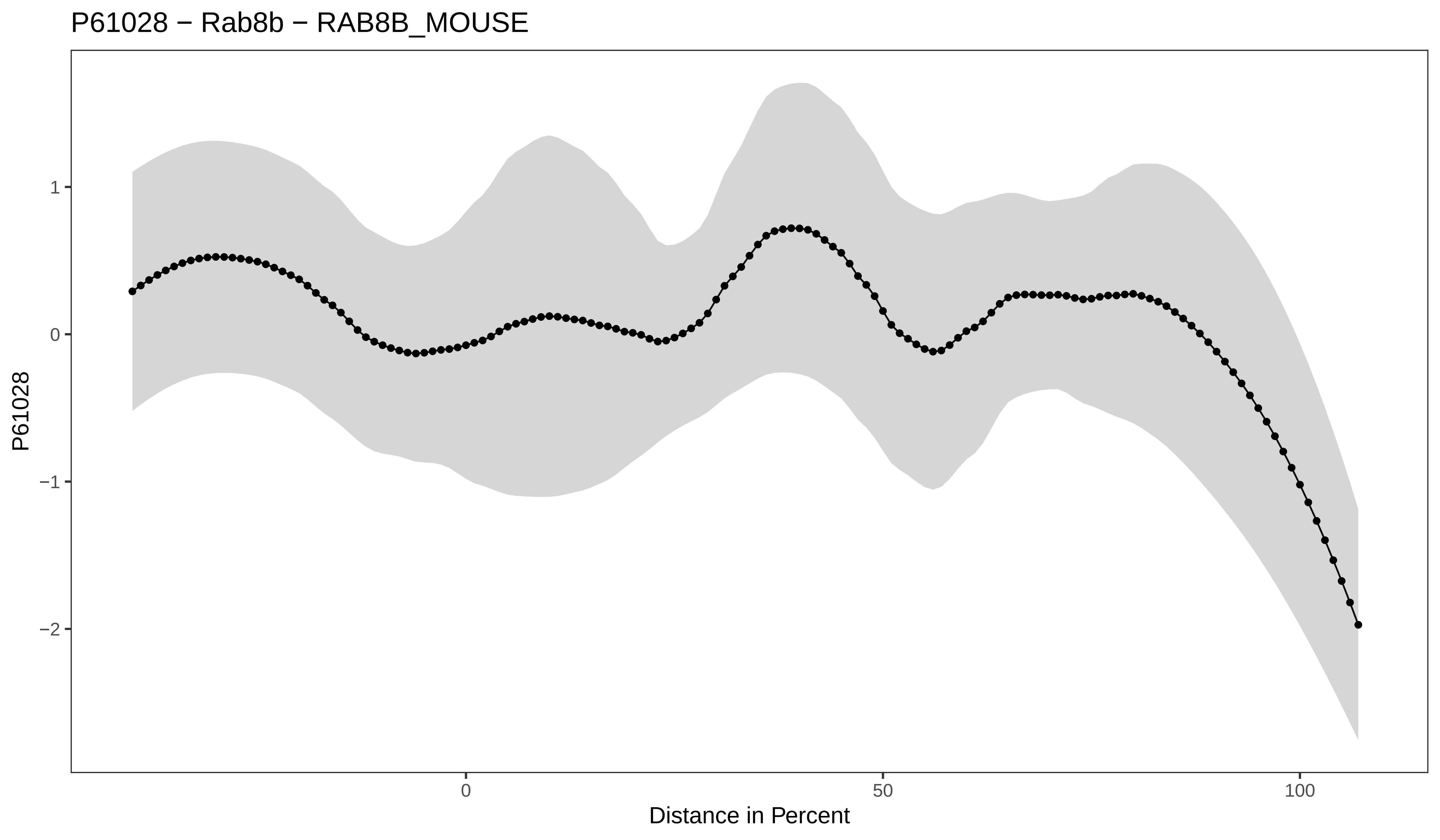

P61028 − Rab8b − RAB8B_MOUSE
1
0
−1
−2
P61028
0
50
100
Distance in Percent

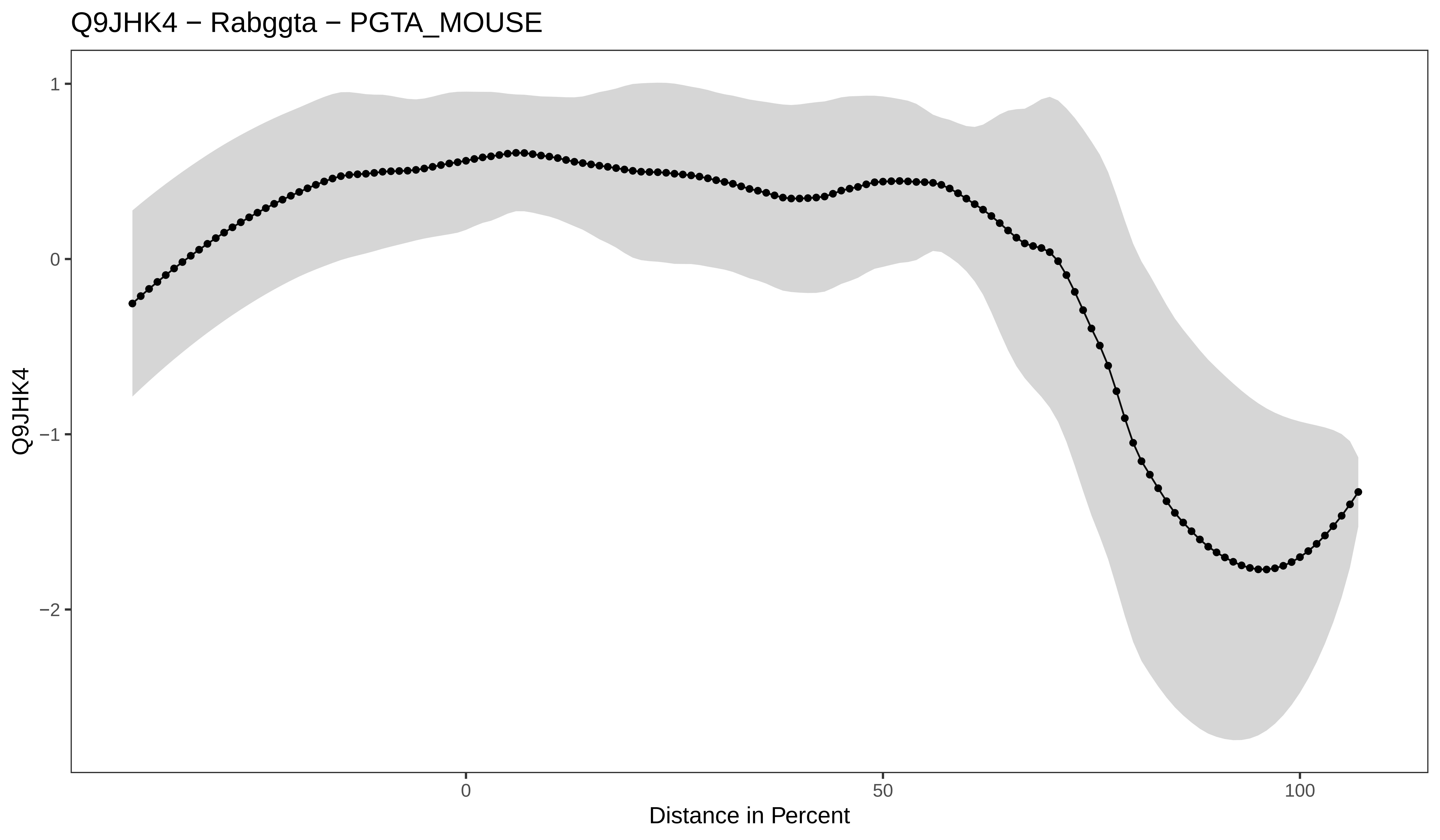
Q9JHK4 − Rabggta − PGTA_MOUSE
Q9JHK4
1
0
−1
−2
0
50
100
Distance in Percent

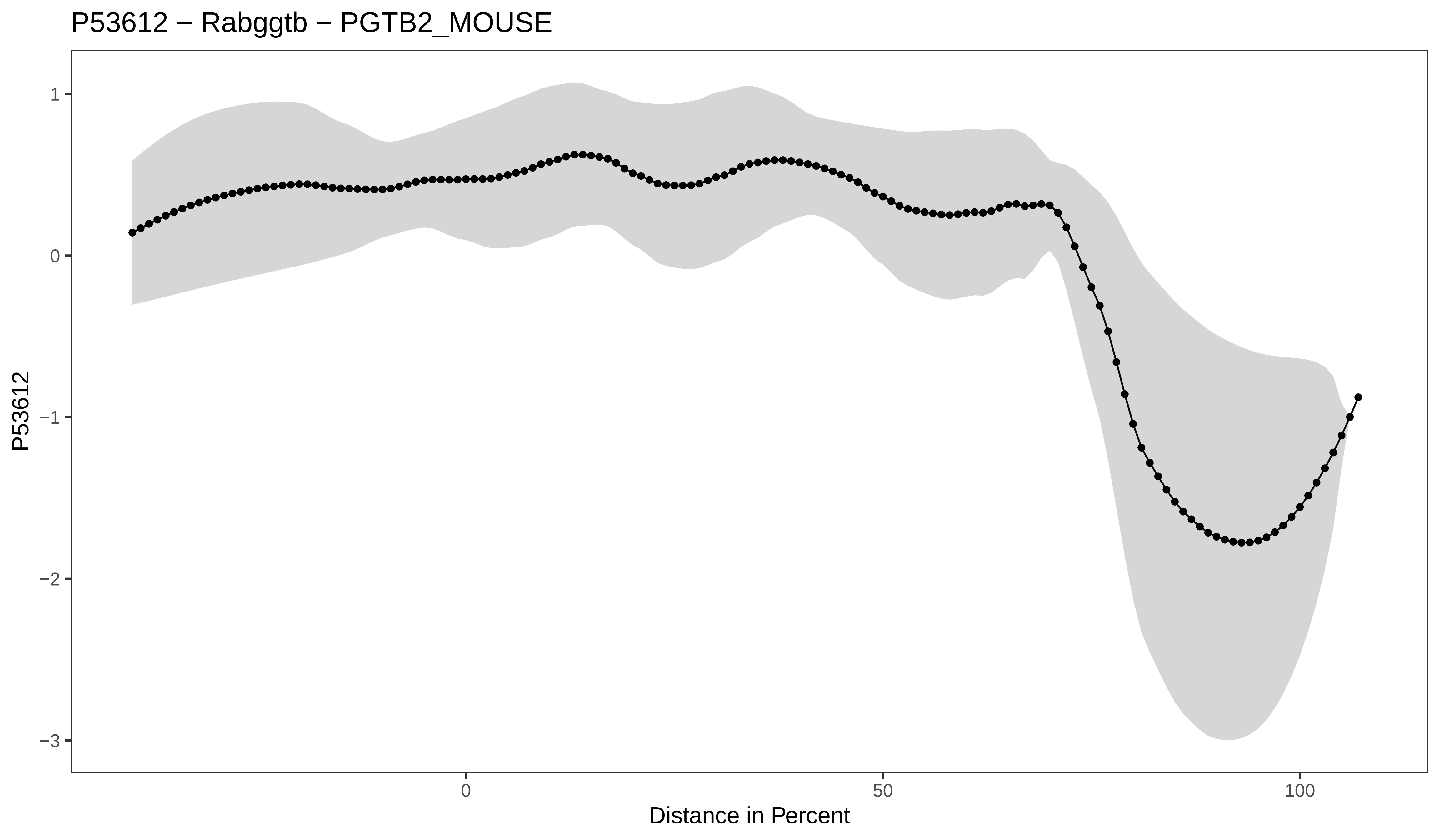

P53612 − Rabggtb − PGTB2_MOUSE
P53612
1
0
−1
−2
−3
0
50
100
Distance in Percent

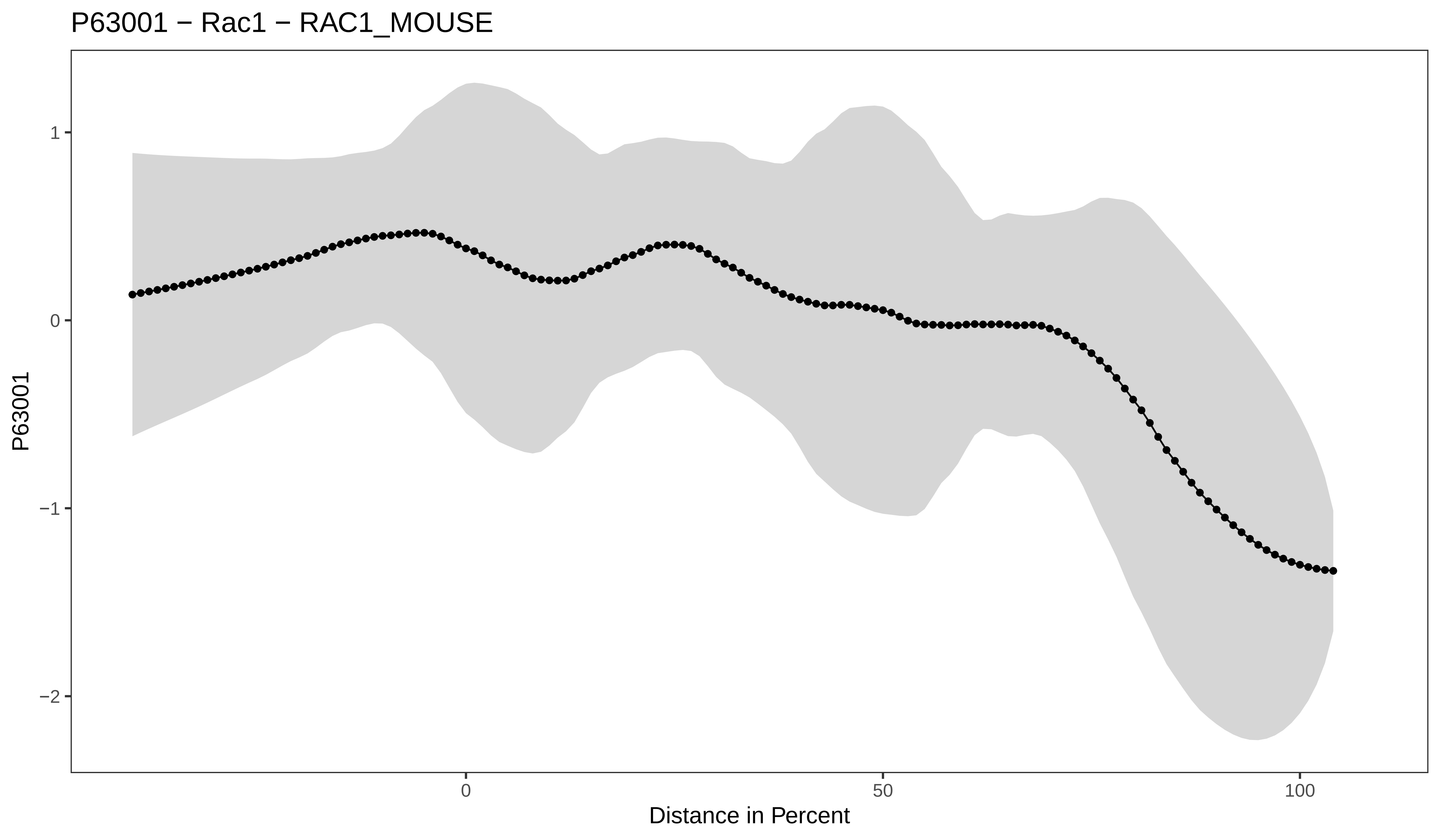

P63001 − Rac1 − RAC1_MOUSE
1
0
−1
−2
P63001
0
50
100
Distance in Percent

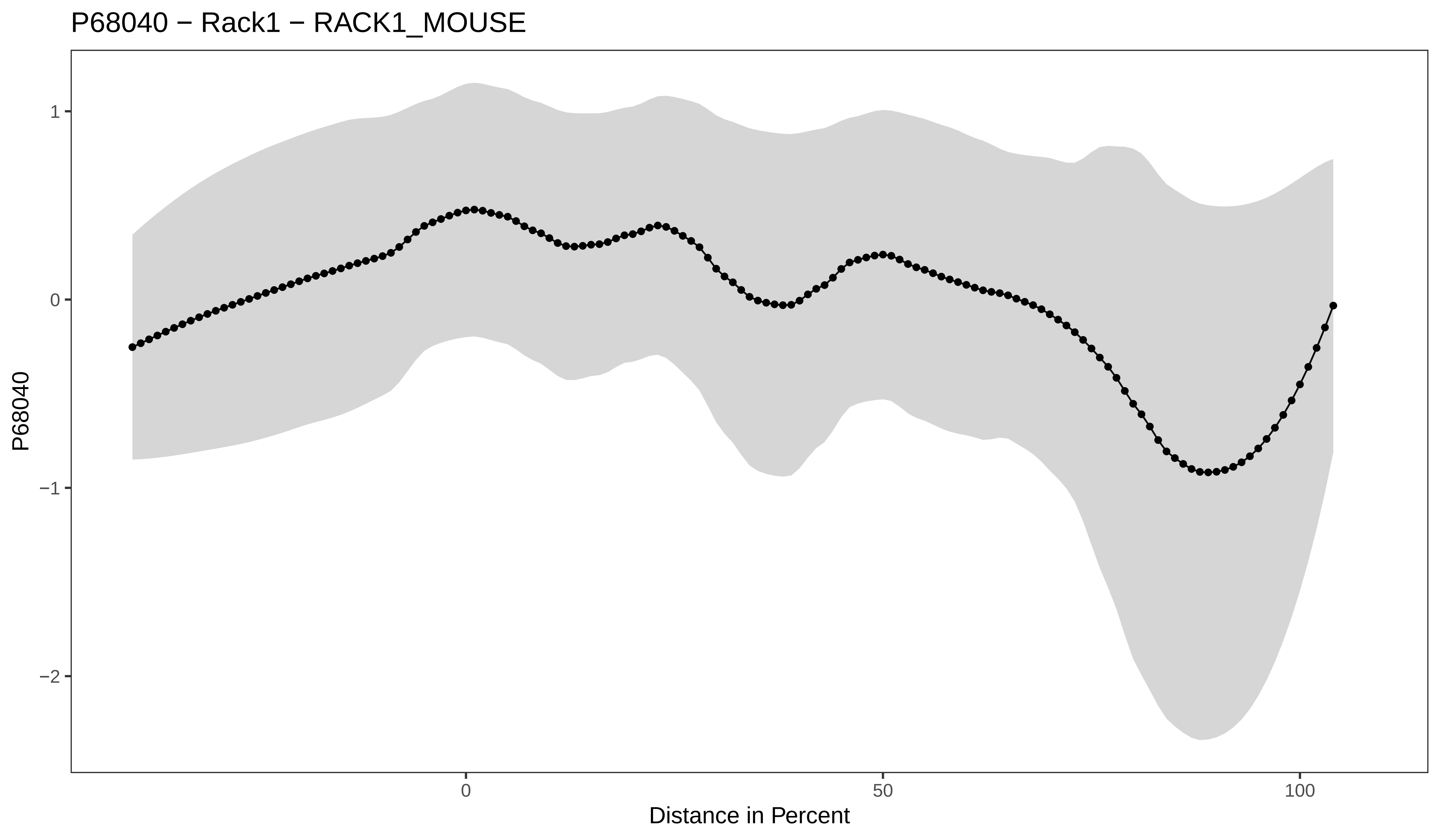

P68040 − Rack1 − RACK1_MOUSE
P68040
1
0
−1
−2
0
50
100
Distance in Percent

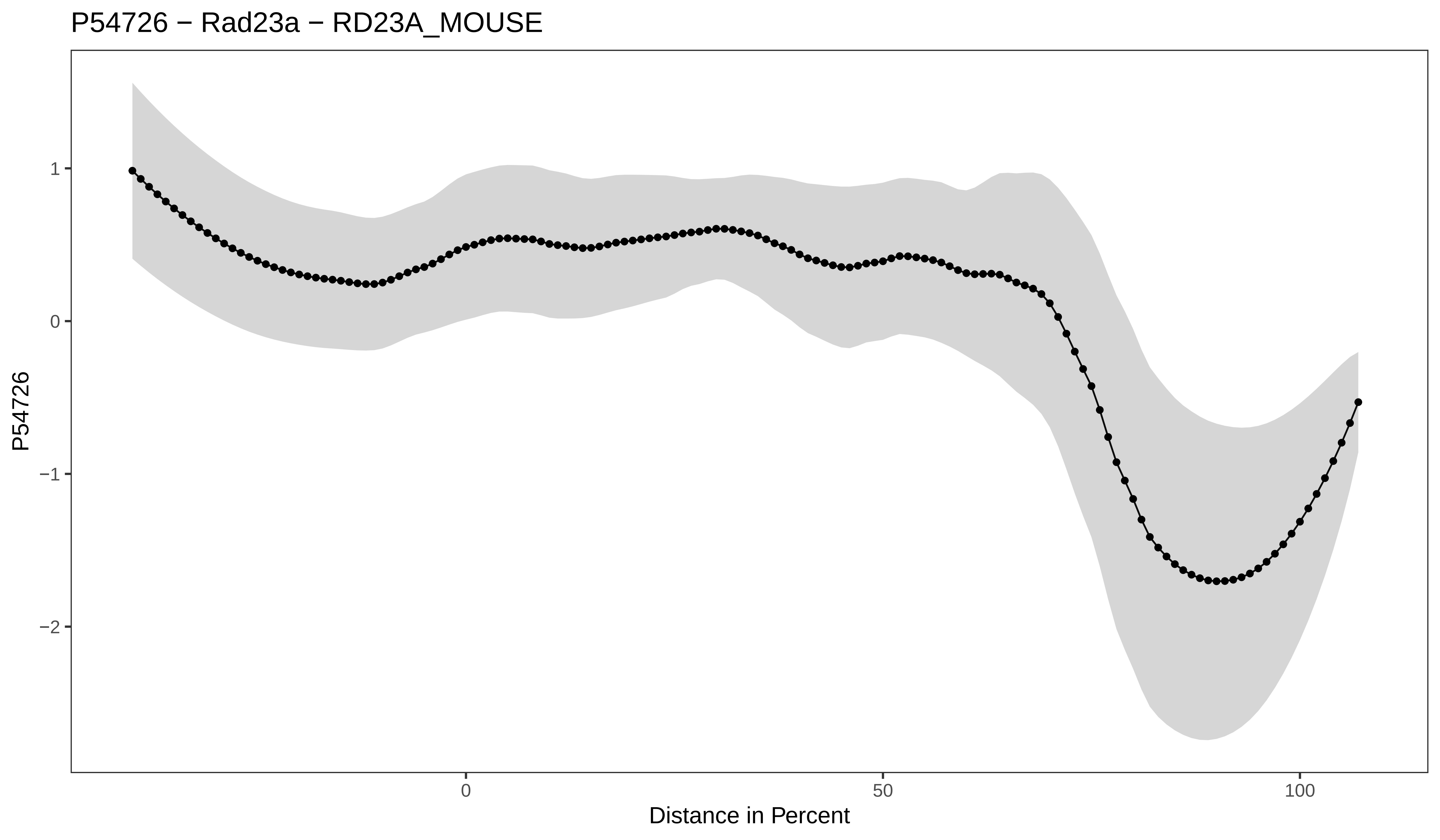
P54726 − Rad23a − RD23A_MOUSE
P54726
1
0
−1
−2
0
50
100
Distance in Percent

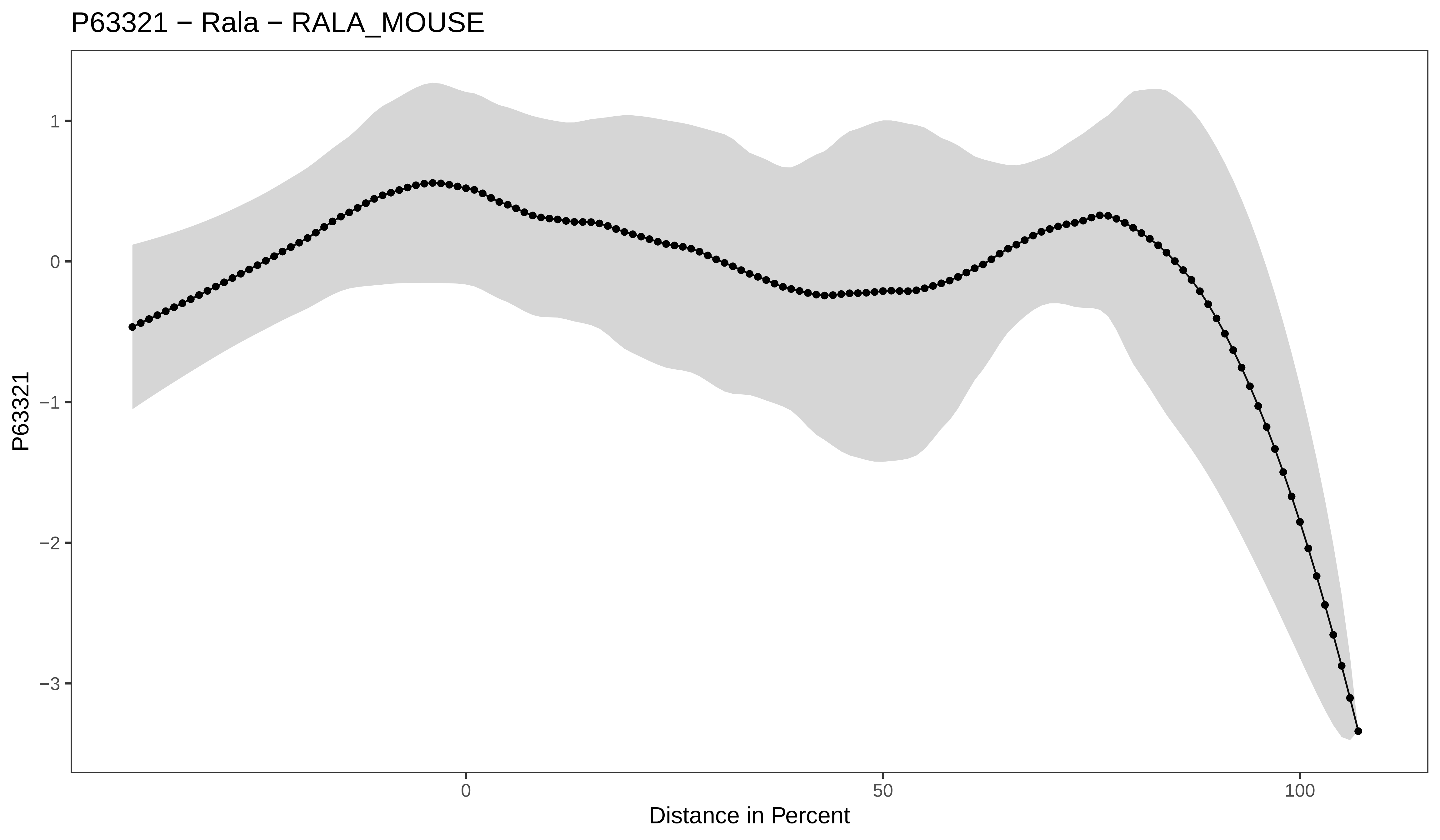

P63321 − Rala − RALA_MOUSE
P63321
1
0
−1
−2
−3
0
50
100
Distance in Percent

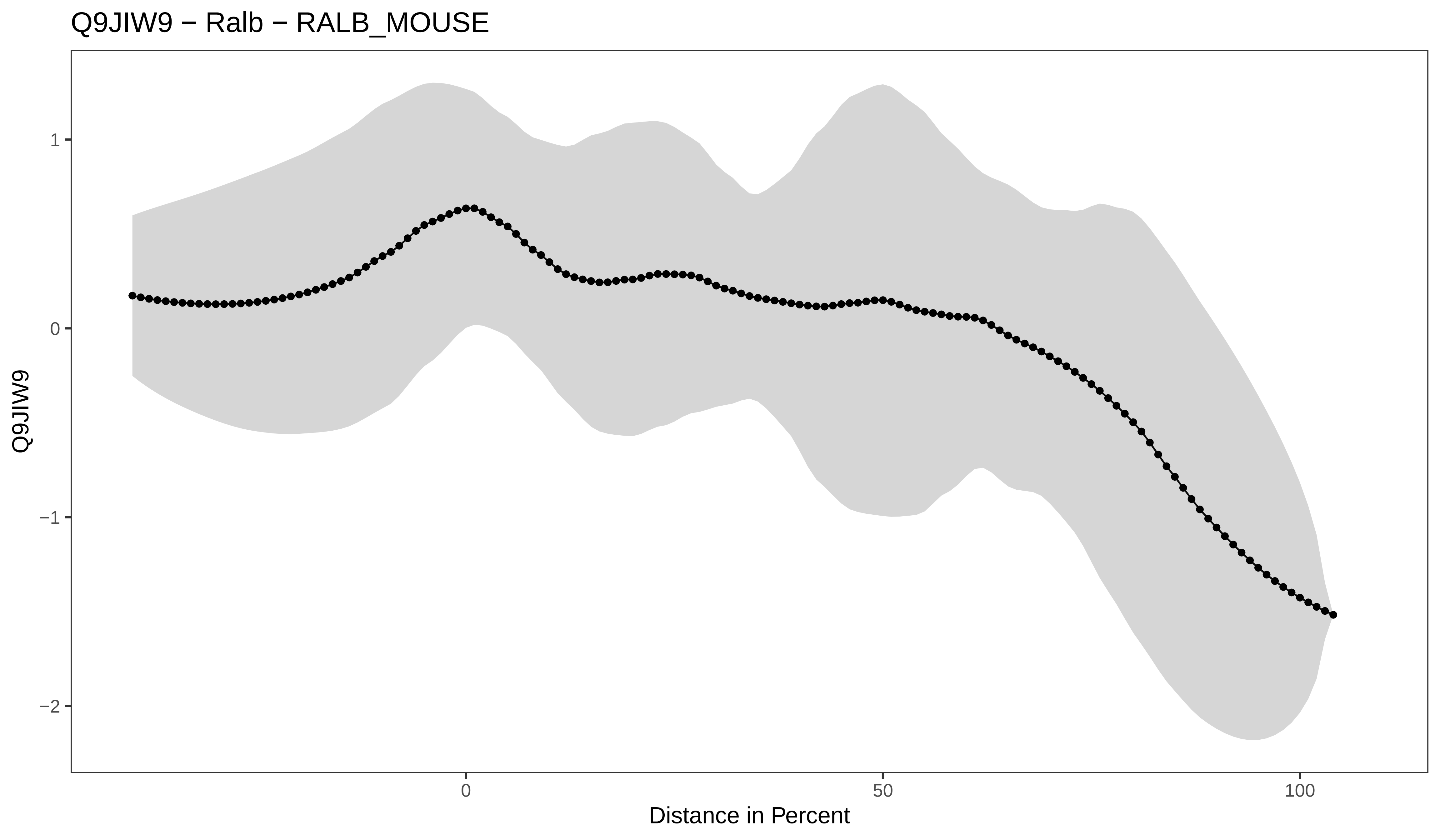

Q9JIW9 − Ralb − RALB_MOUSE
1
0
−1
−2
Q9JIW9
0
50
100
Distance in Percent

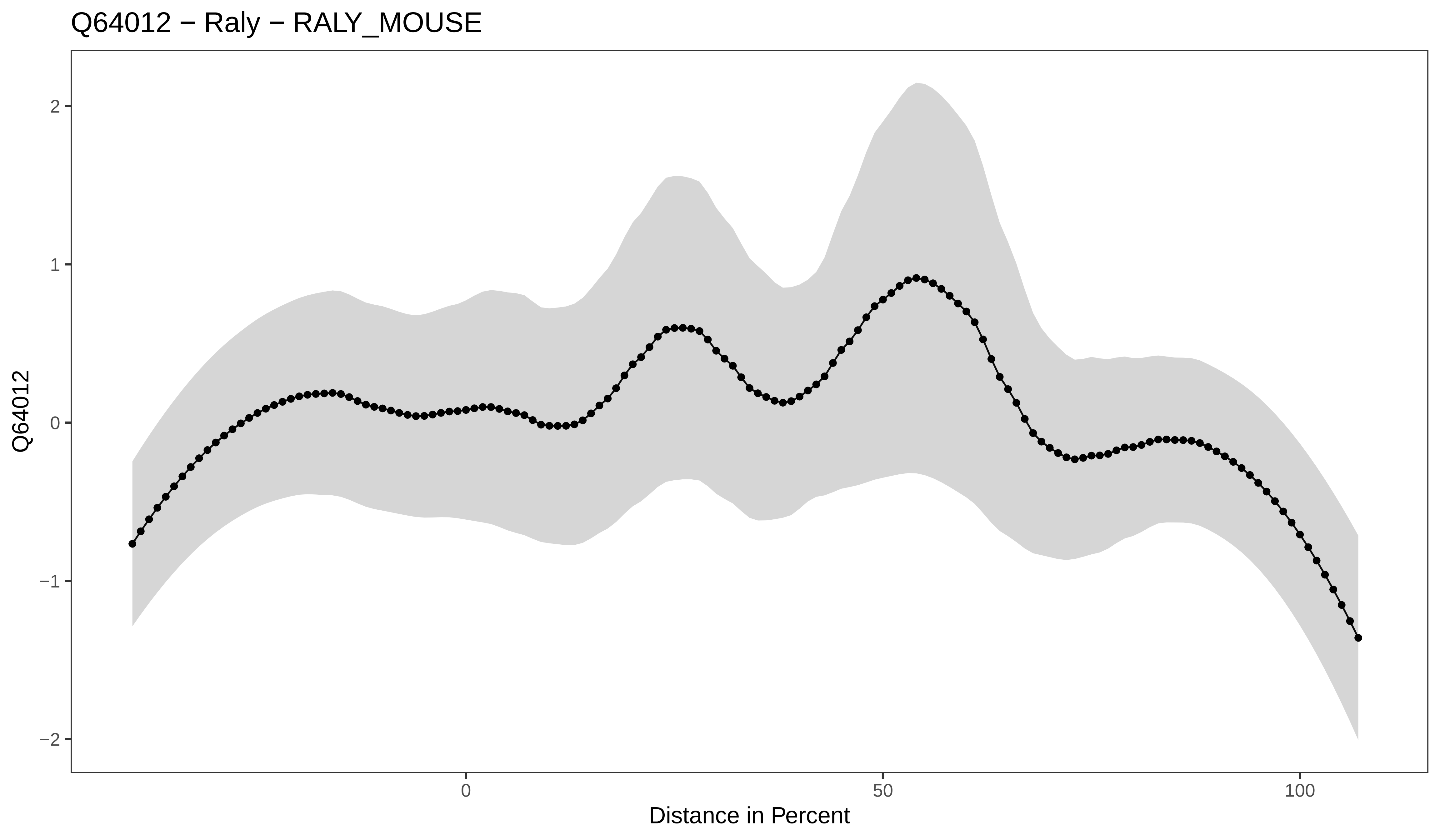

Q64012 − Raly − RALY_MOUSE
Q64012
2
1
0
−1
−2
0
50
100
Distance in Percent

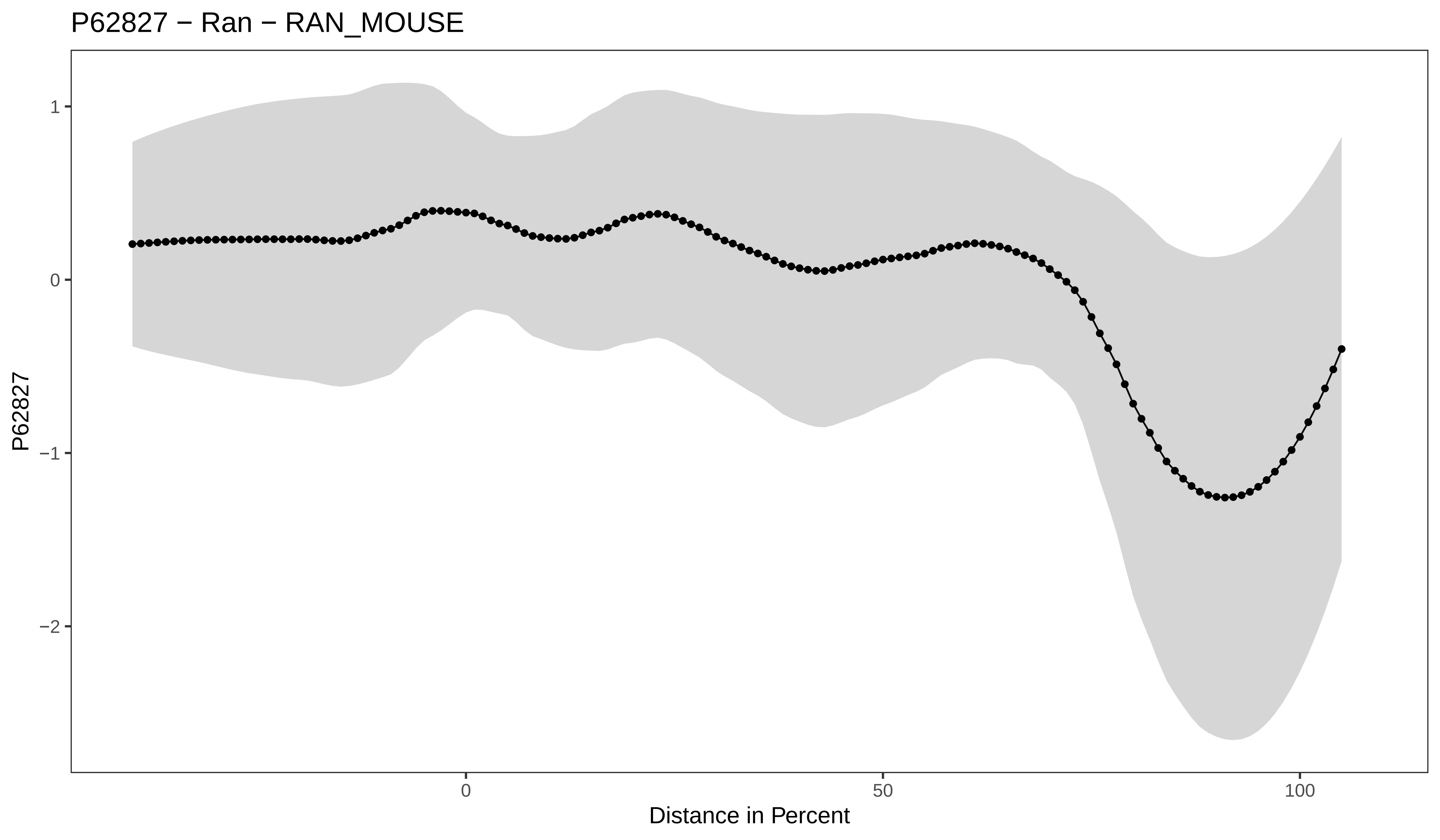

P62827 − Ran − RAN_MOUSE
P62827
1
0
−1
−2
0
50
100
Distance in Percent

Q9CT10 − Ranbp3 − RANB3_MOUSE

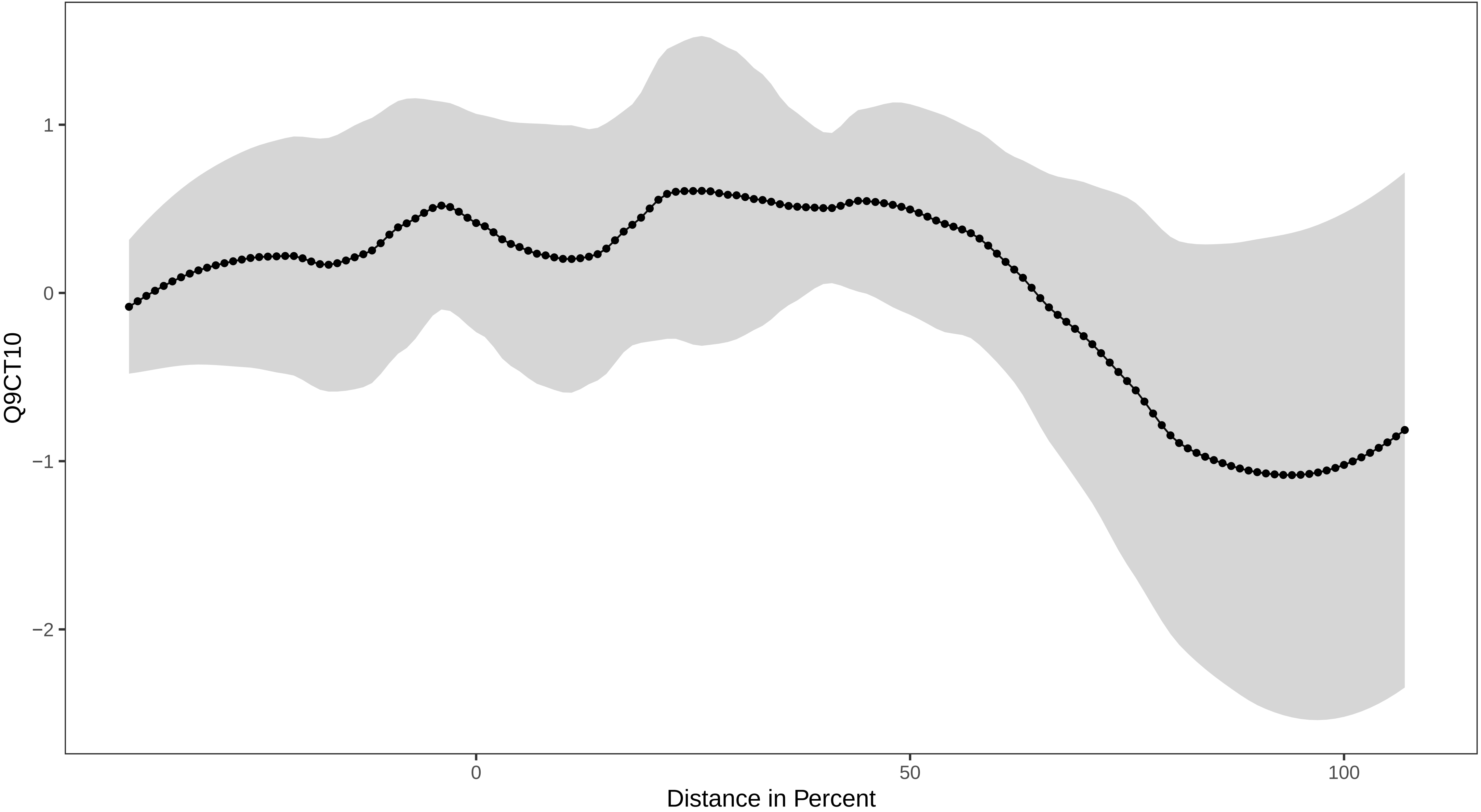

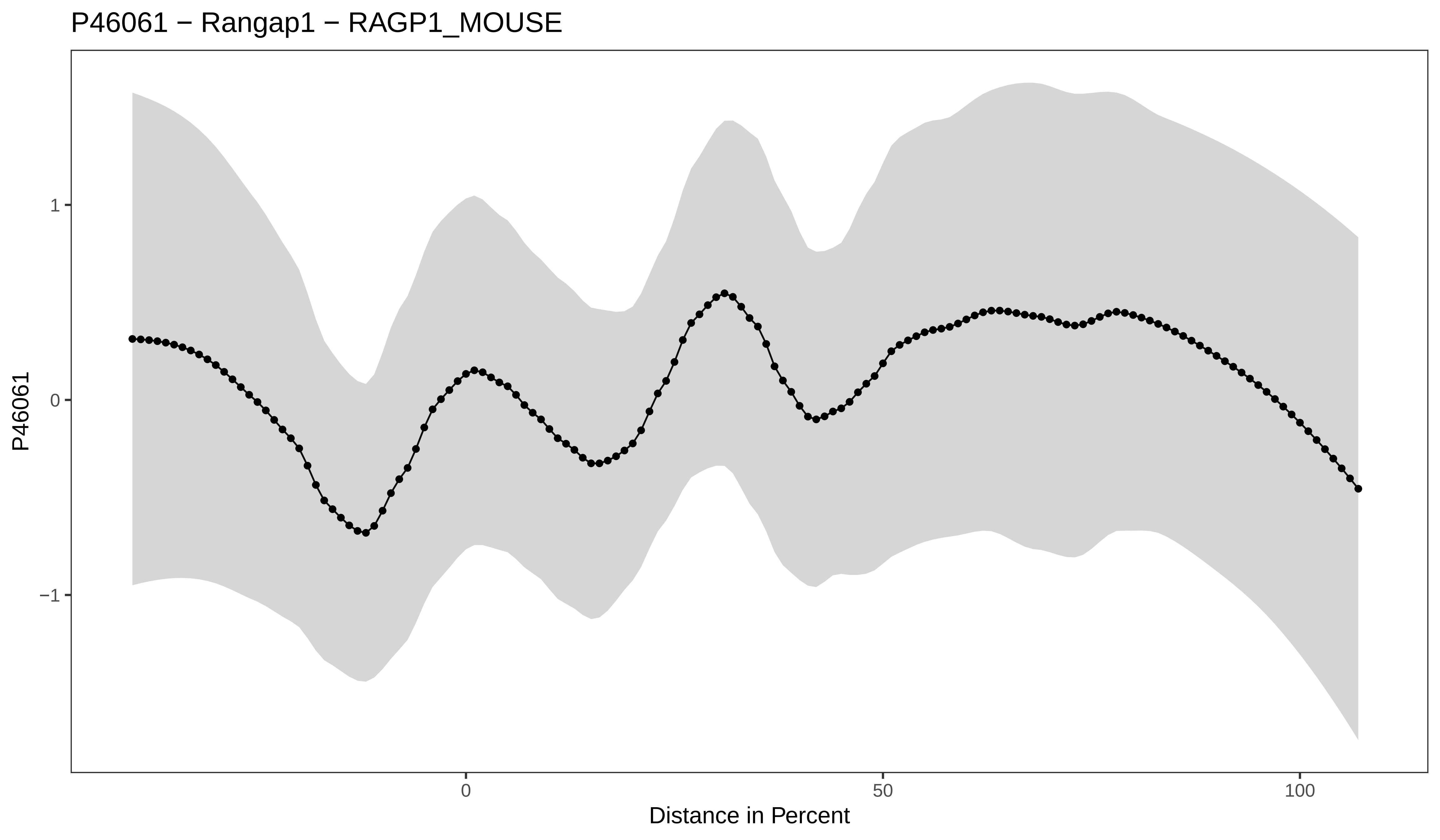

P46061 − Rangap1 − RAGP1_MOUSE
P46061
1
0
−1
0
50
100
Distance in Percent

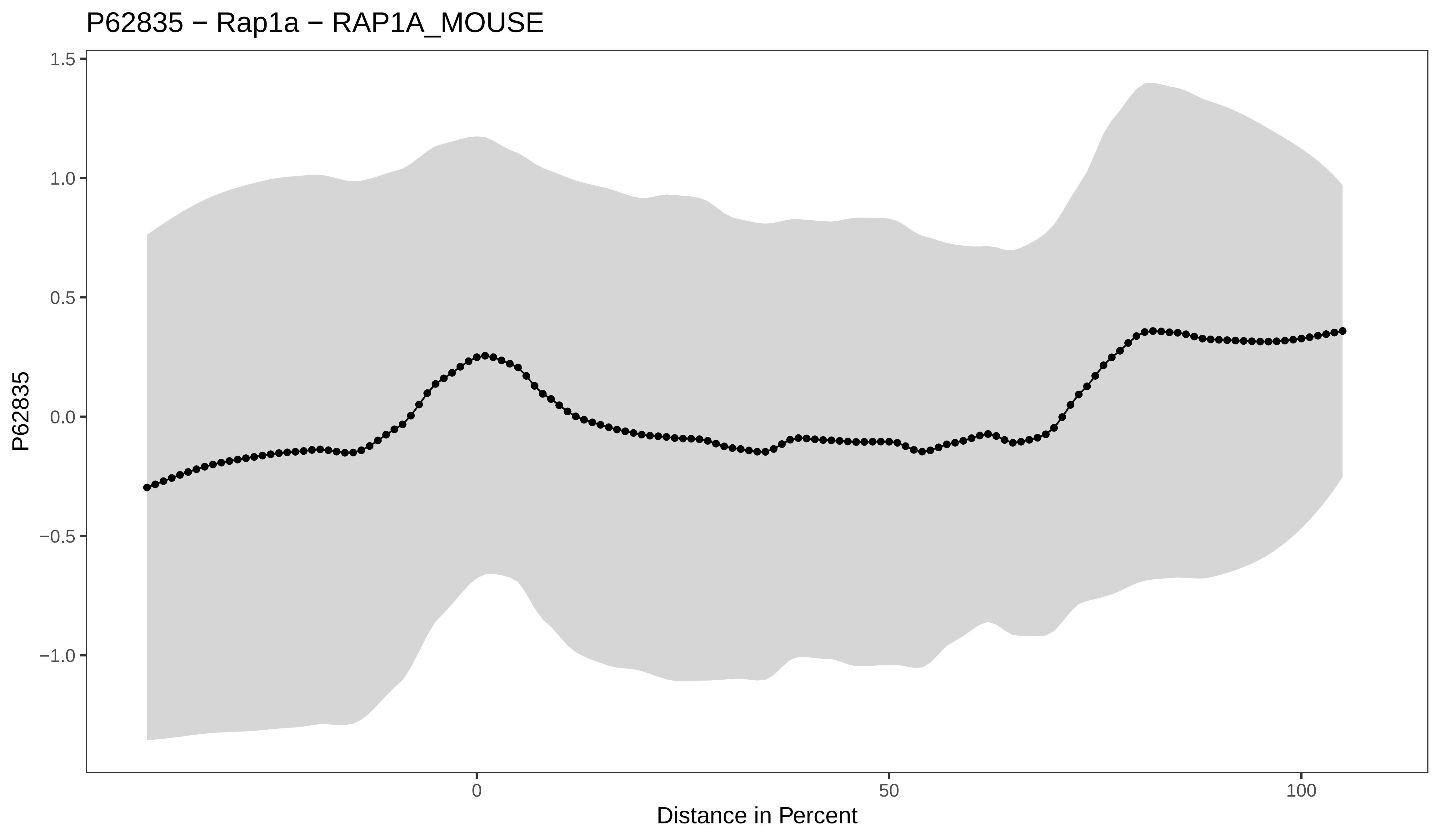
P62835 − Rap1a − RAP1A_MOUSE
P62835
Distance in Percent
1.5
1.0
0.5
0.0
−0.5
−1.0
0
50
100

Q99JI6 − Rap1b − RAP1B_MOUSE

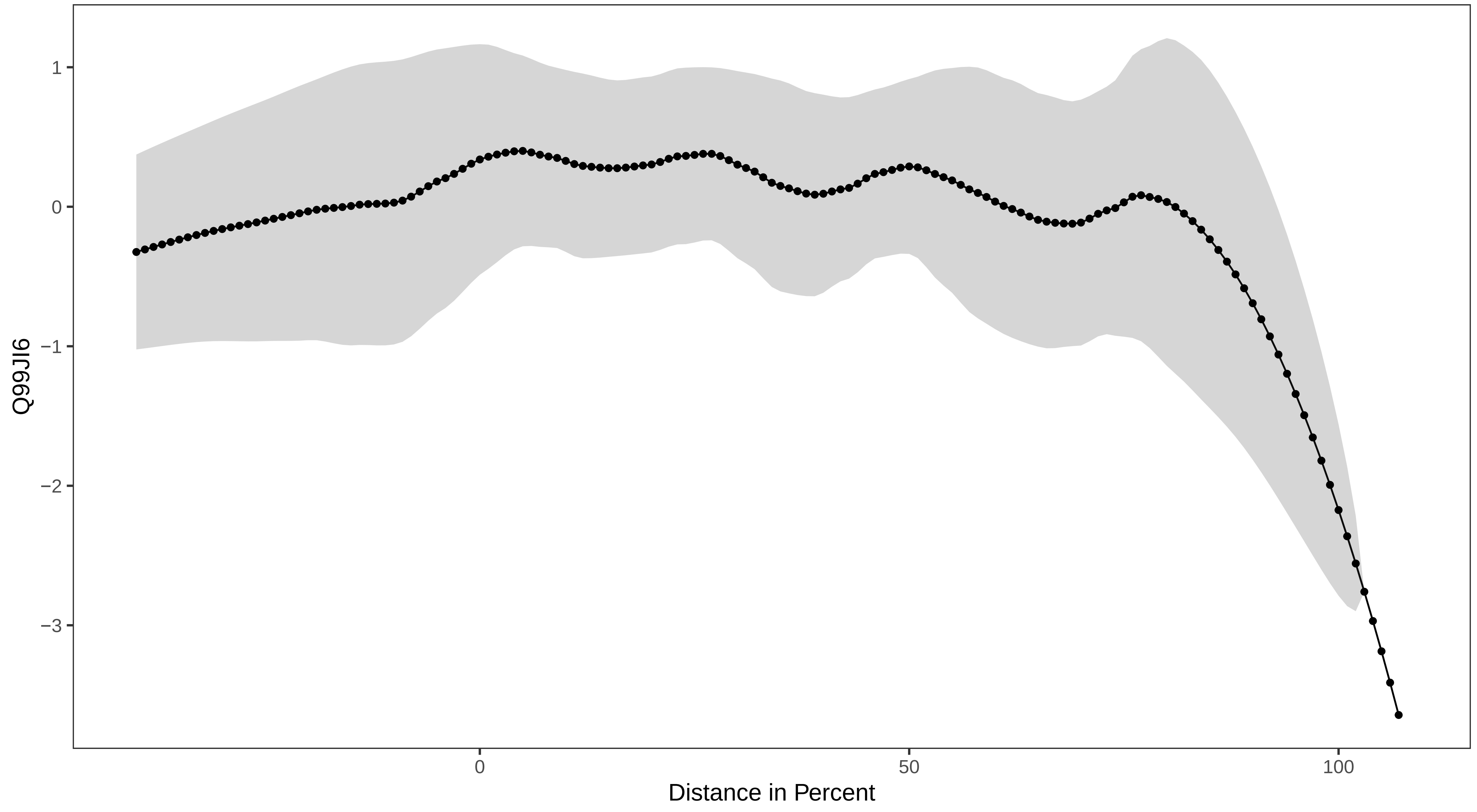

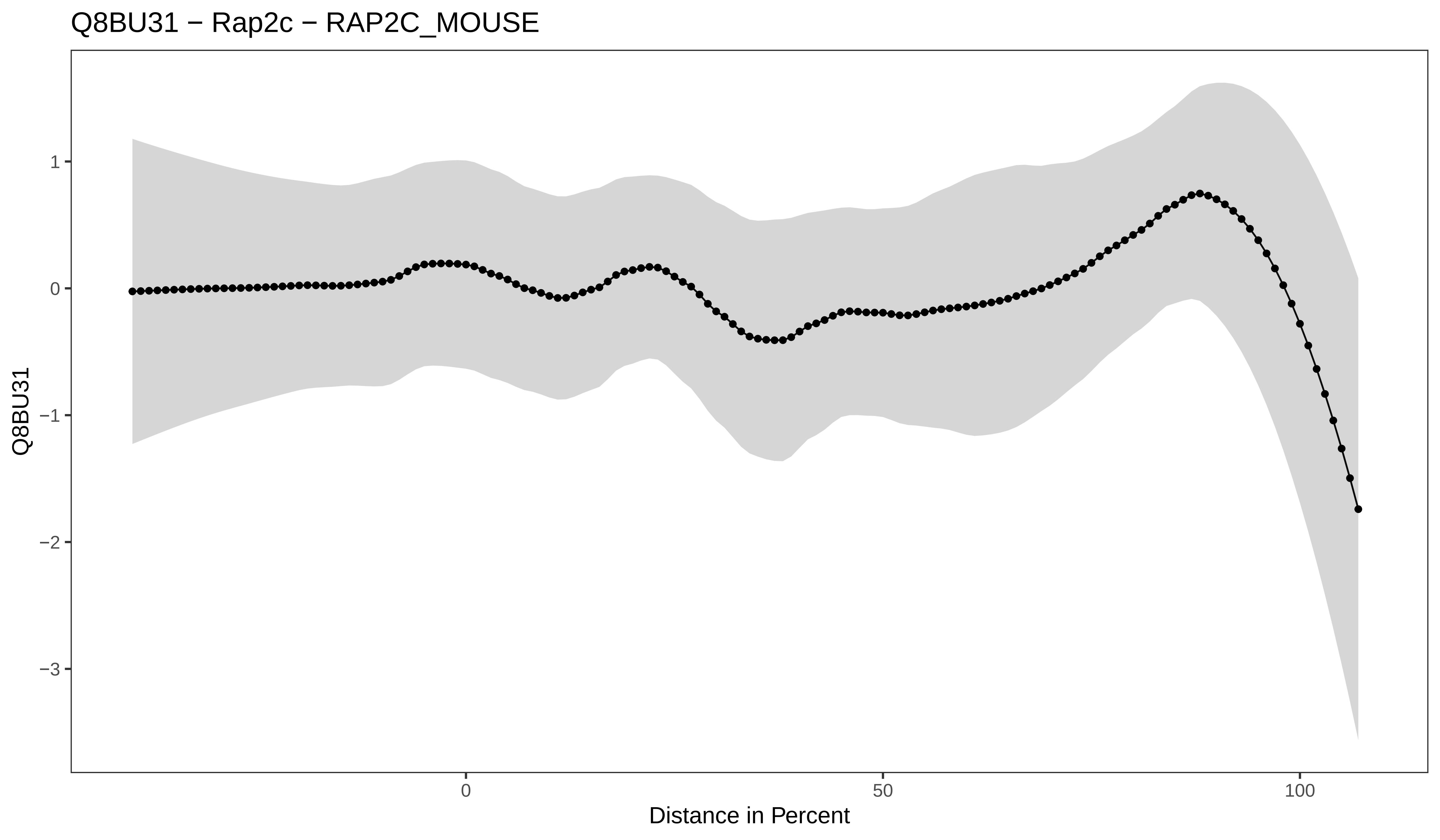

Q8BU31 − Rap2c − RAP2C_MOUSE
Q8BU31
1
0
−1
−2
−3
0
50
100
Distance in Percent

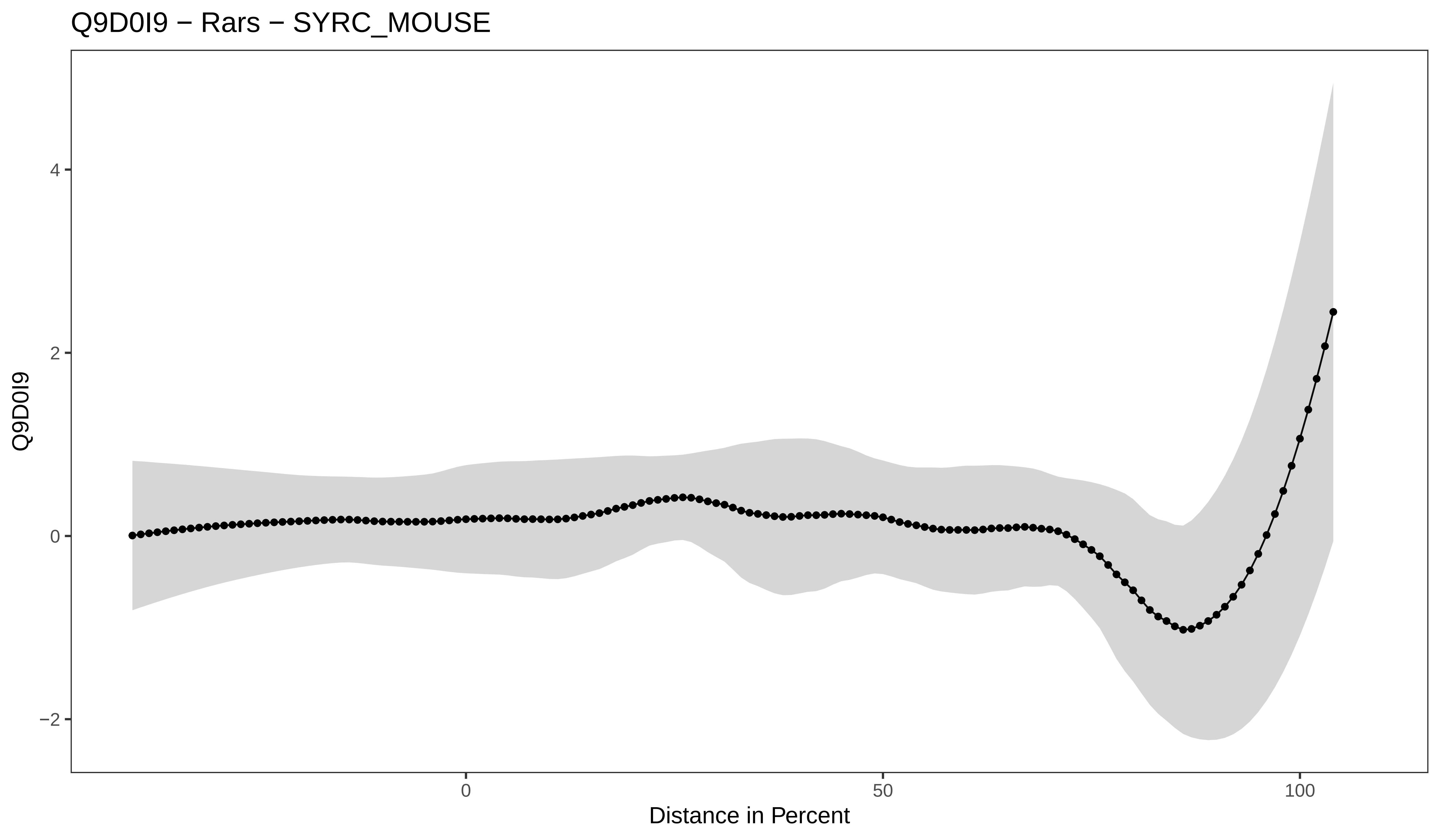
Q9D0I9 − Rars − SYRC_MOUSE
Q9D0I9
4
2
0
−2
0
50
100
Distance in Percent

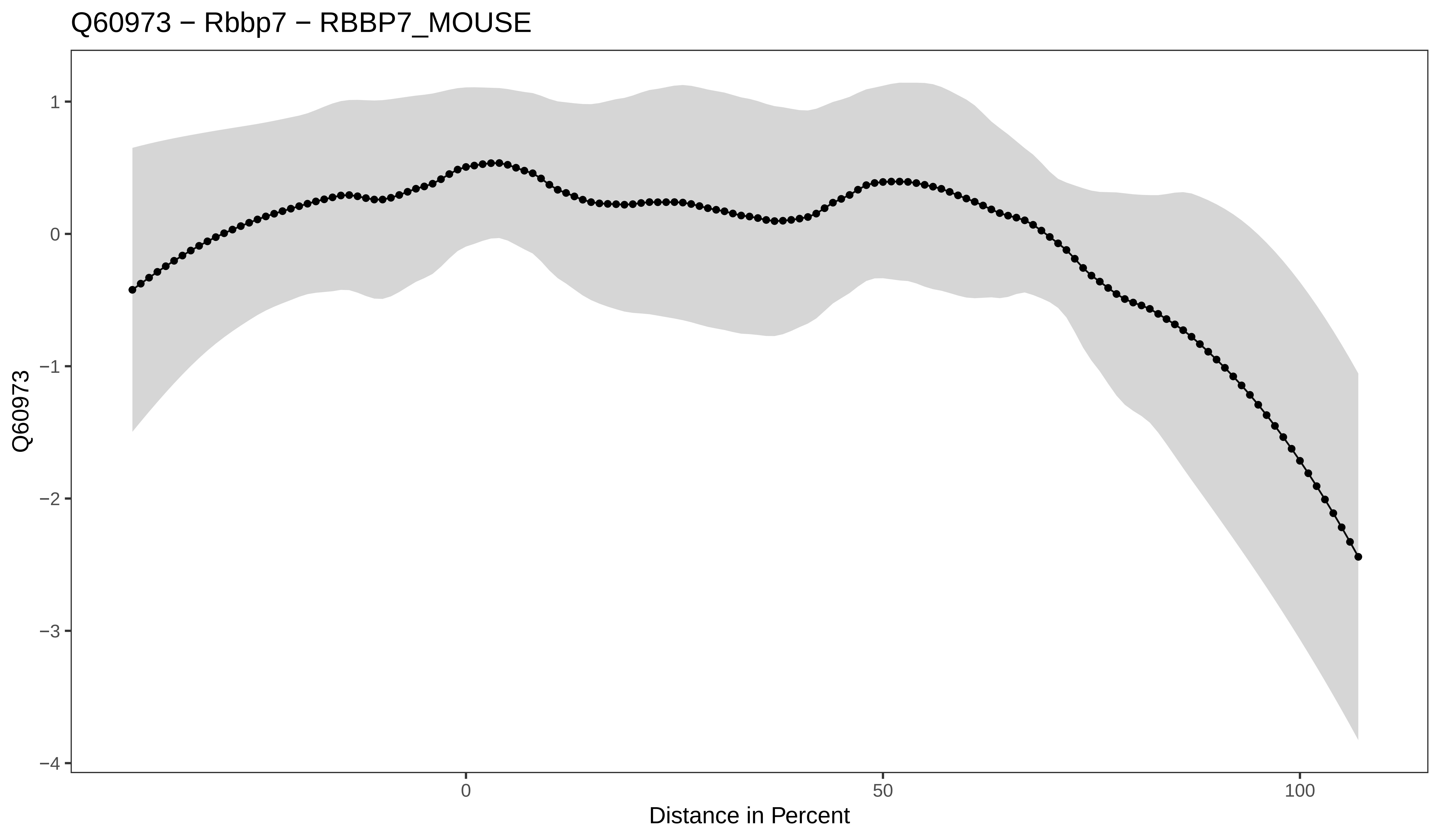
Q60973 − Rbbp7 − RBBP7_MOUSE
Q60973
Distance in Percent
1
0
−1
−2
−3
−4
0
50
100

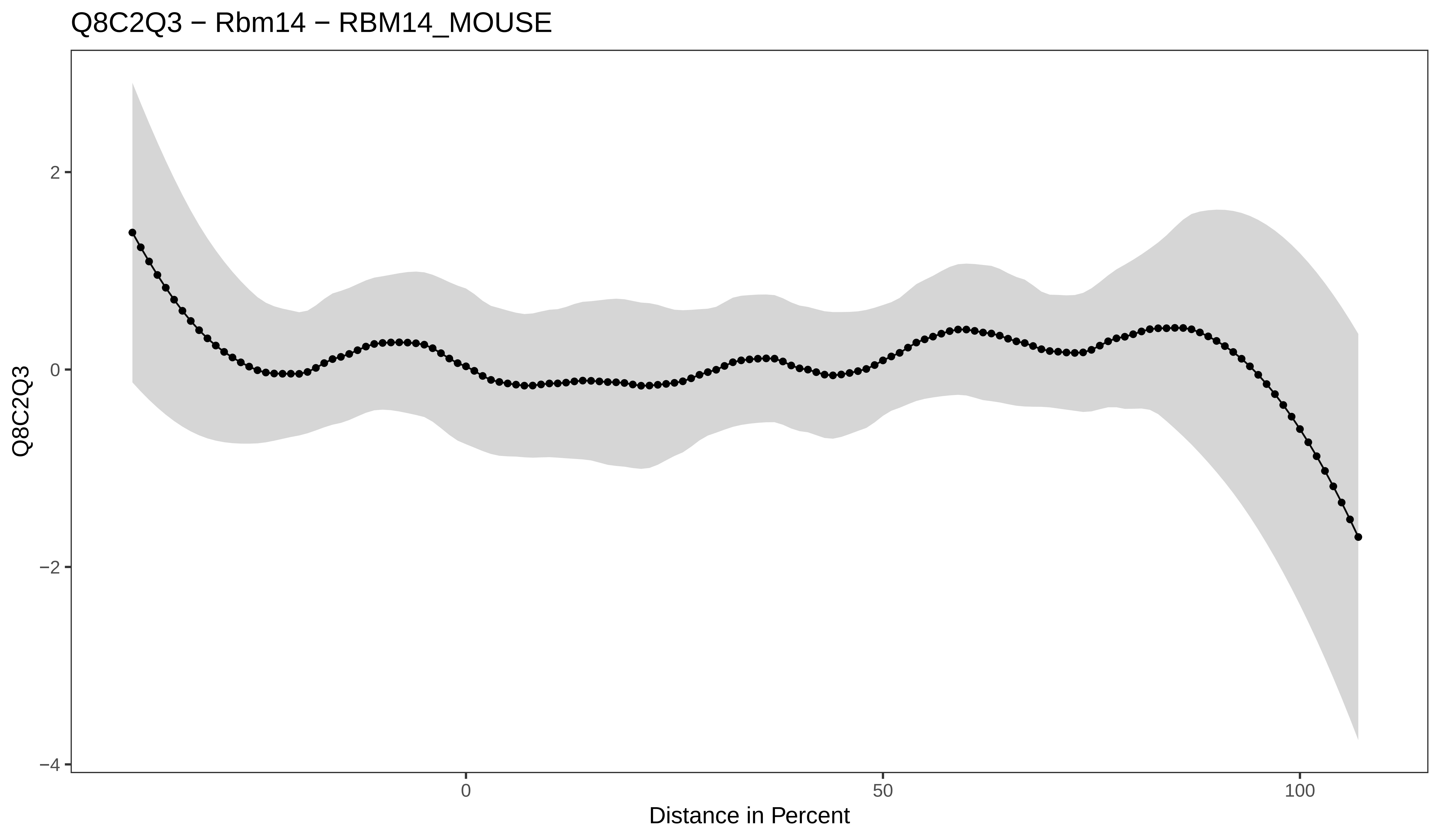
Q8C2Q3 − Rbm14 − RBM14_MOUSE
Q8C2Q3
2
0
−2
−4
0
50
100
Distance in Percent

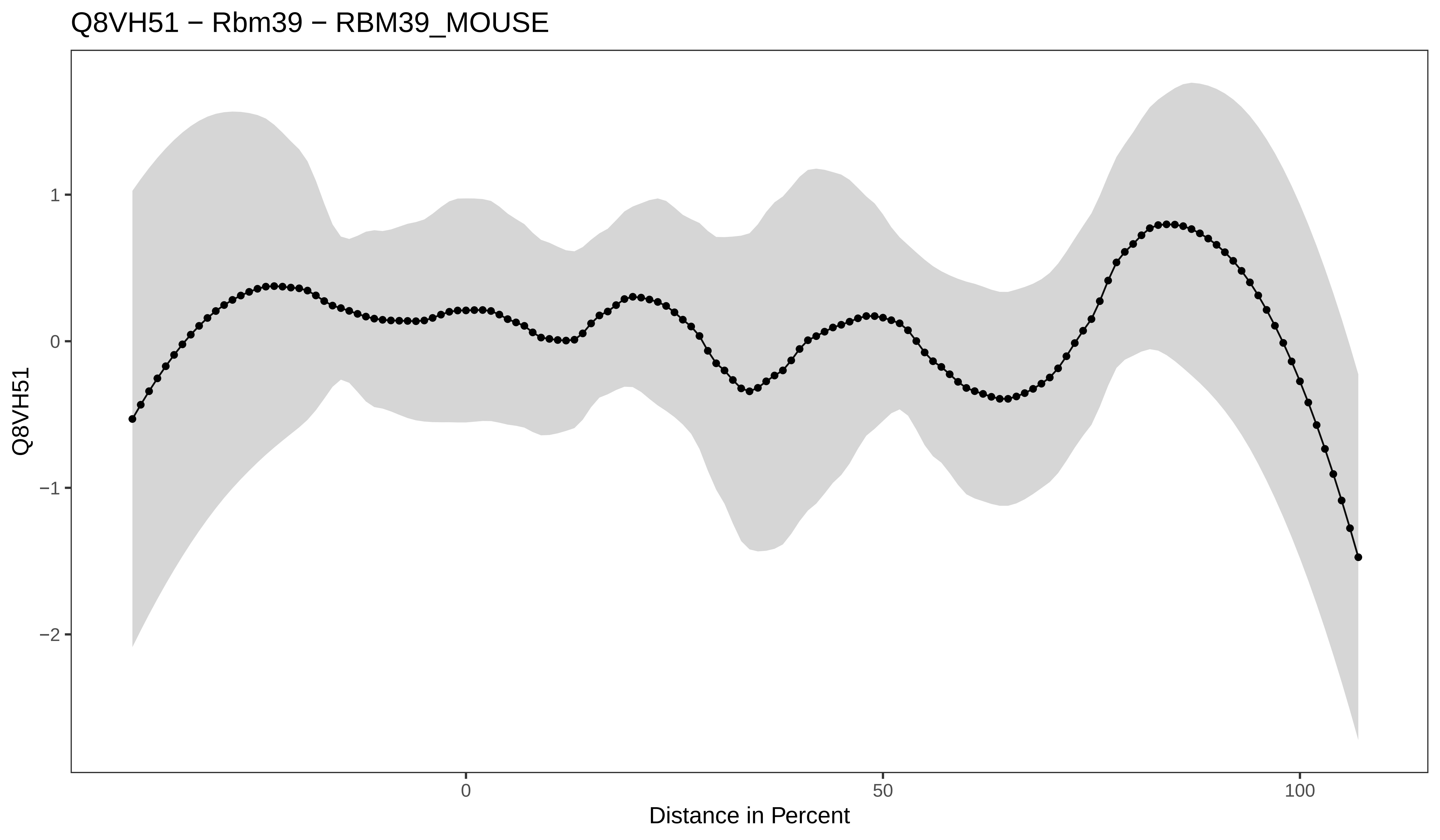

Q8VH51 − Rbm39 − RBM39_MOUSE
1
0
−1
−2
Q8VH51
0
50
100
Distance in Percent

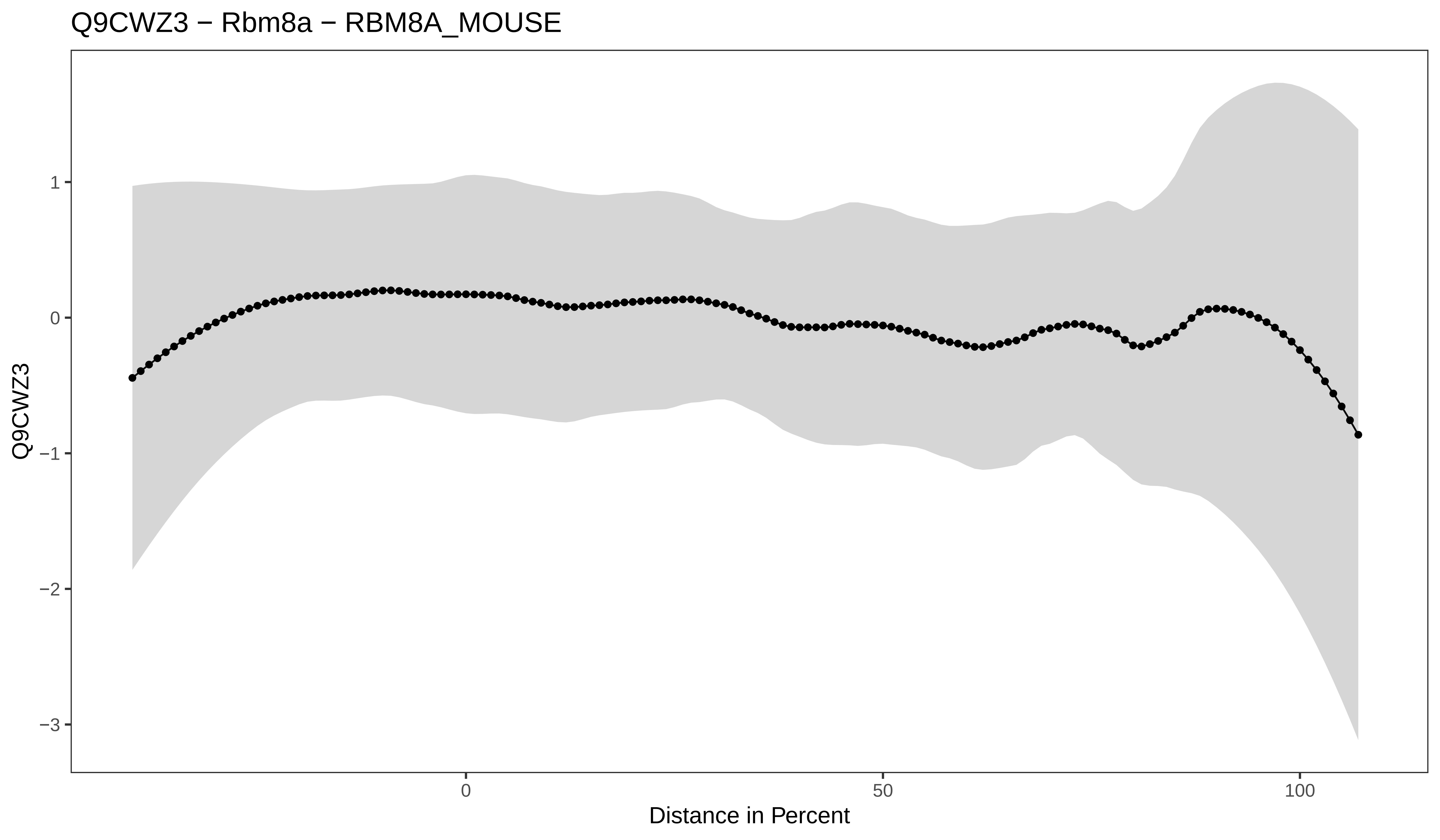
Q9CWZ3 − Rbm8a − RBM8A_MOUSE
1
0
−1
−2
−3
Q9CWZ3
0
50
100
Distance in Percent

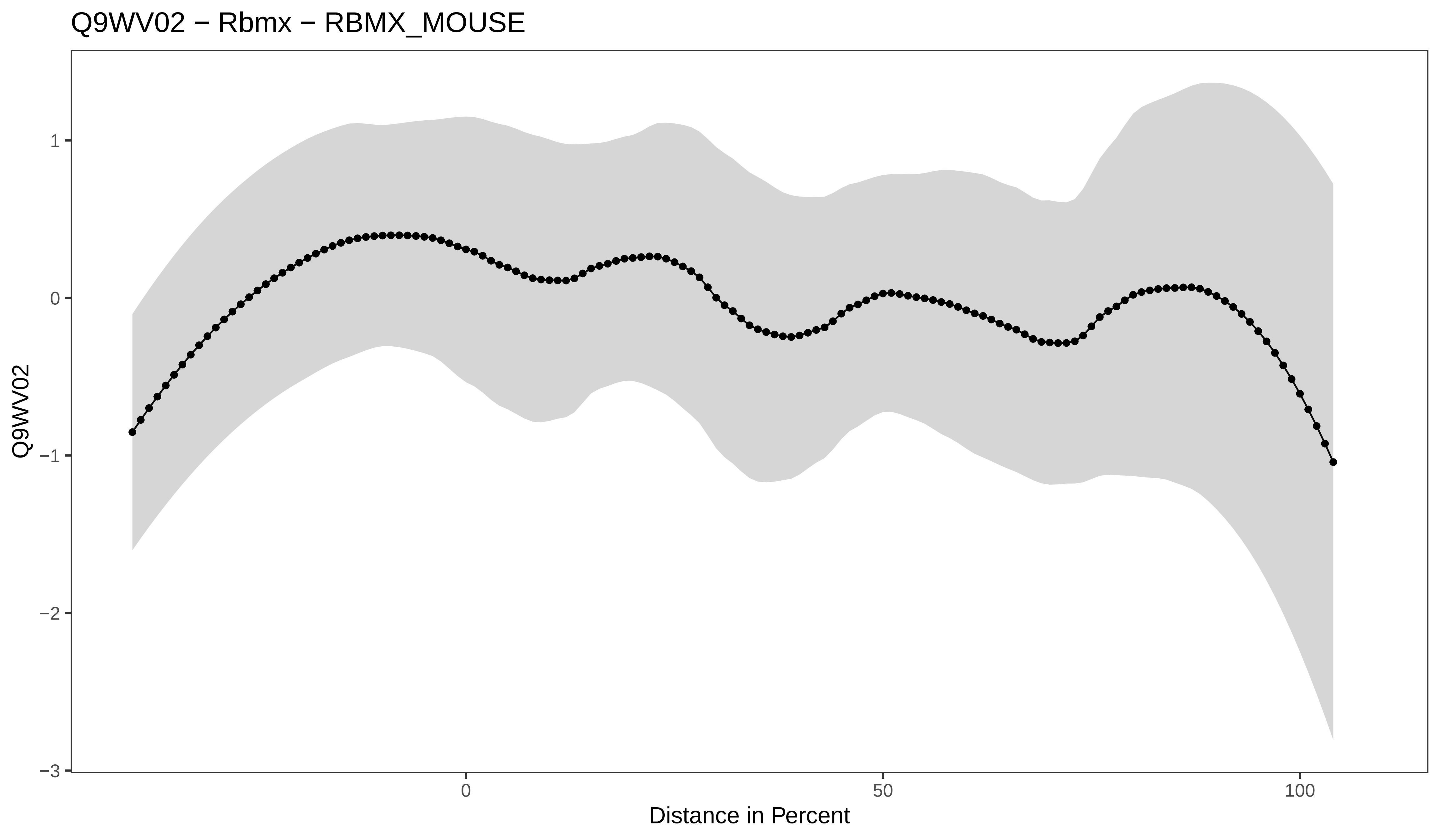
Q9WV02 − Rbmx − RBMX_MOUSE
Q9WV02
1
0
−1
−2
−3
0
50
100
Distance in Percent

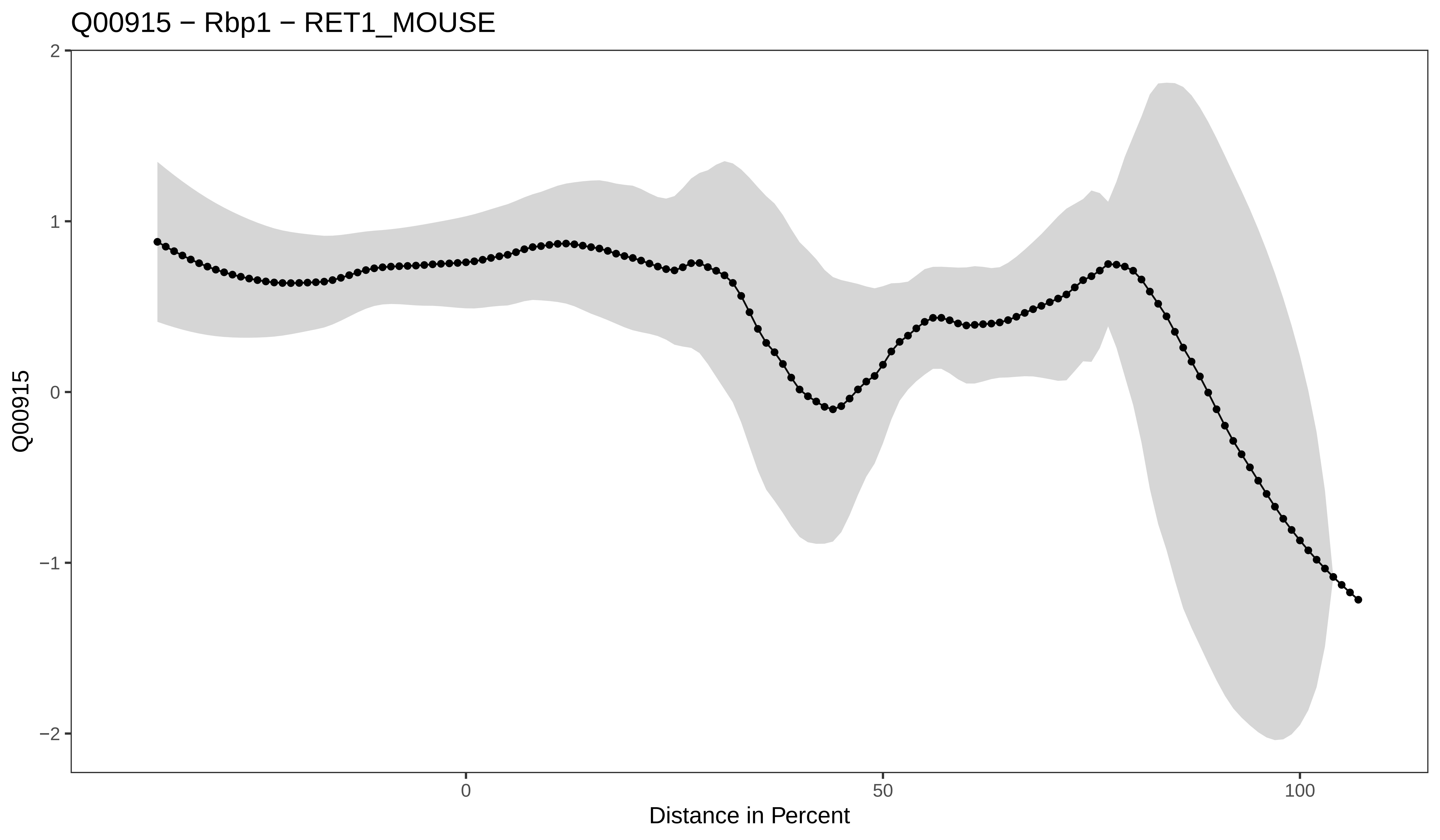

Q00915 − Rbp1 − RET1_MOUSE
2
1
0
−1
−2
Q00915
0
50
100
Distance in Percent

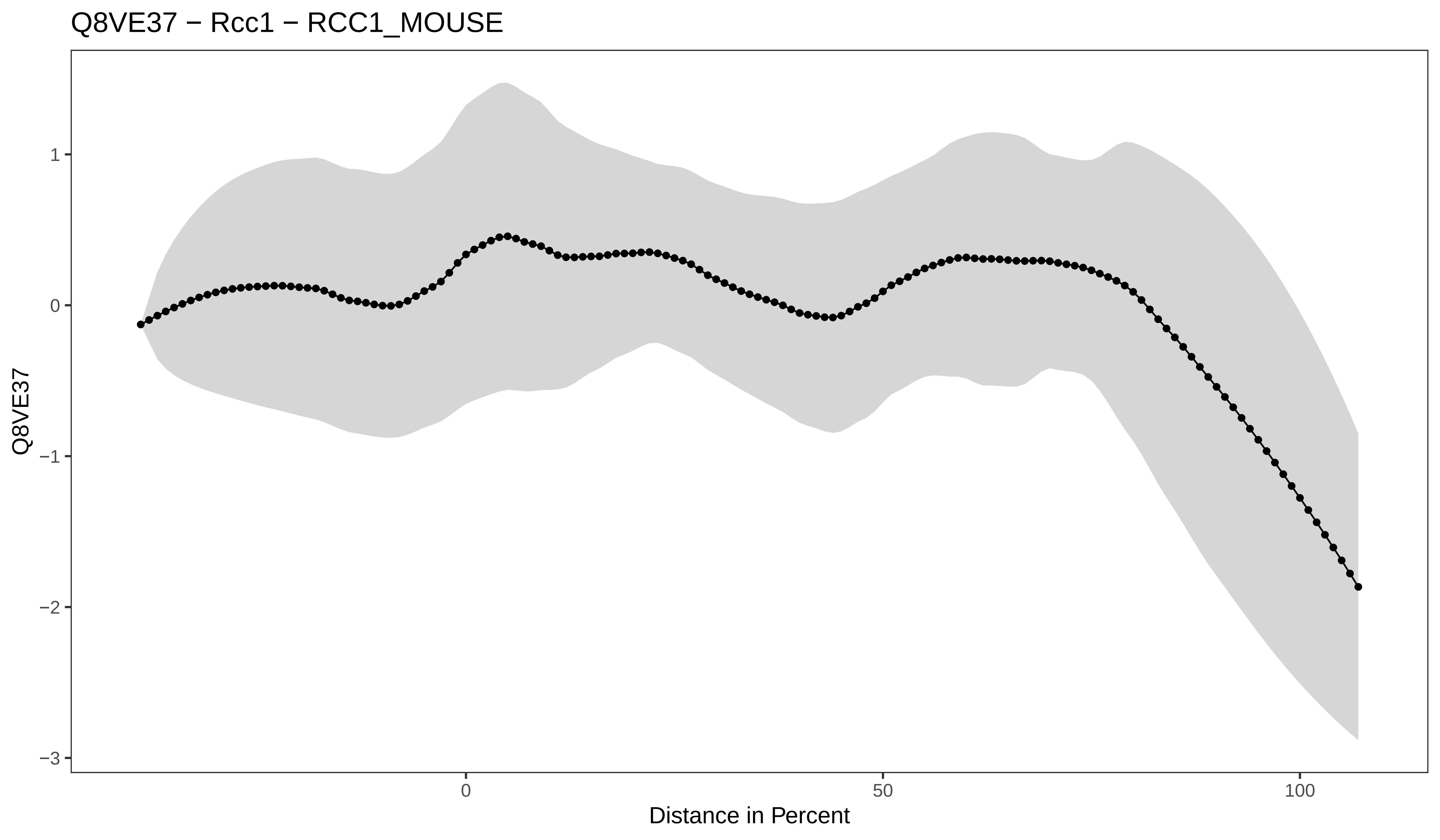

Q8VE37 − Rcc1 − RCC1_MOUSE
Q8VE37
1
0
−1
−2
−3
0
50
100
Distance in Percent

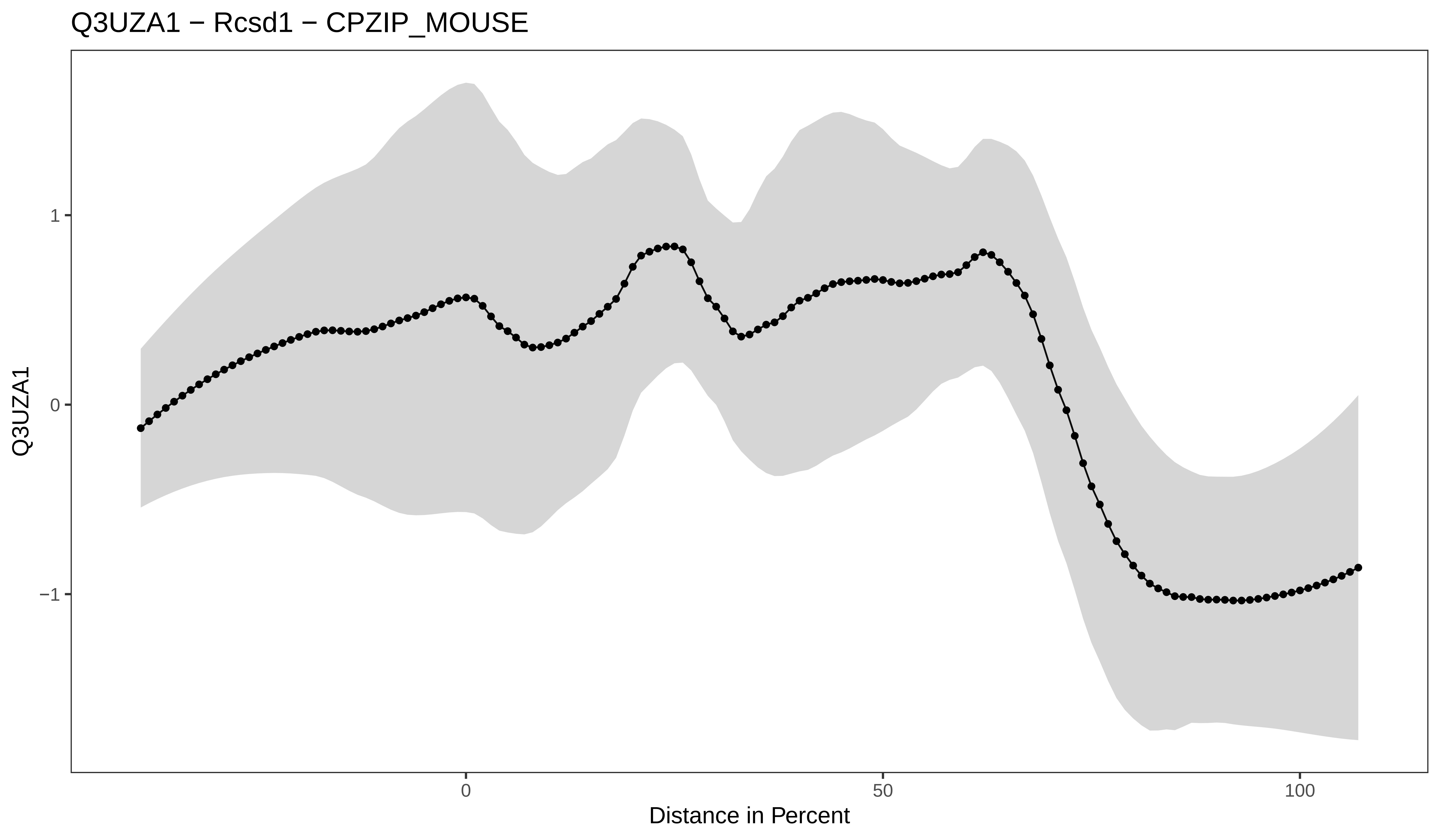

Q3UZA1 − Rcsd1 − CPZIP_MOUSE
Q3UZA1
1
0
−1
0
50
100
Distance in Percent

Q8CEE7 − Rdh13 − RDH13_MOUSE

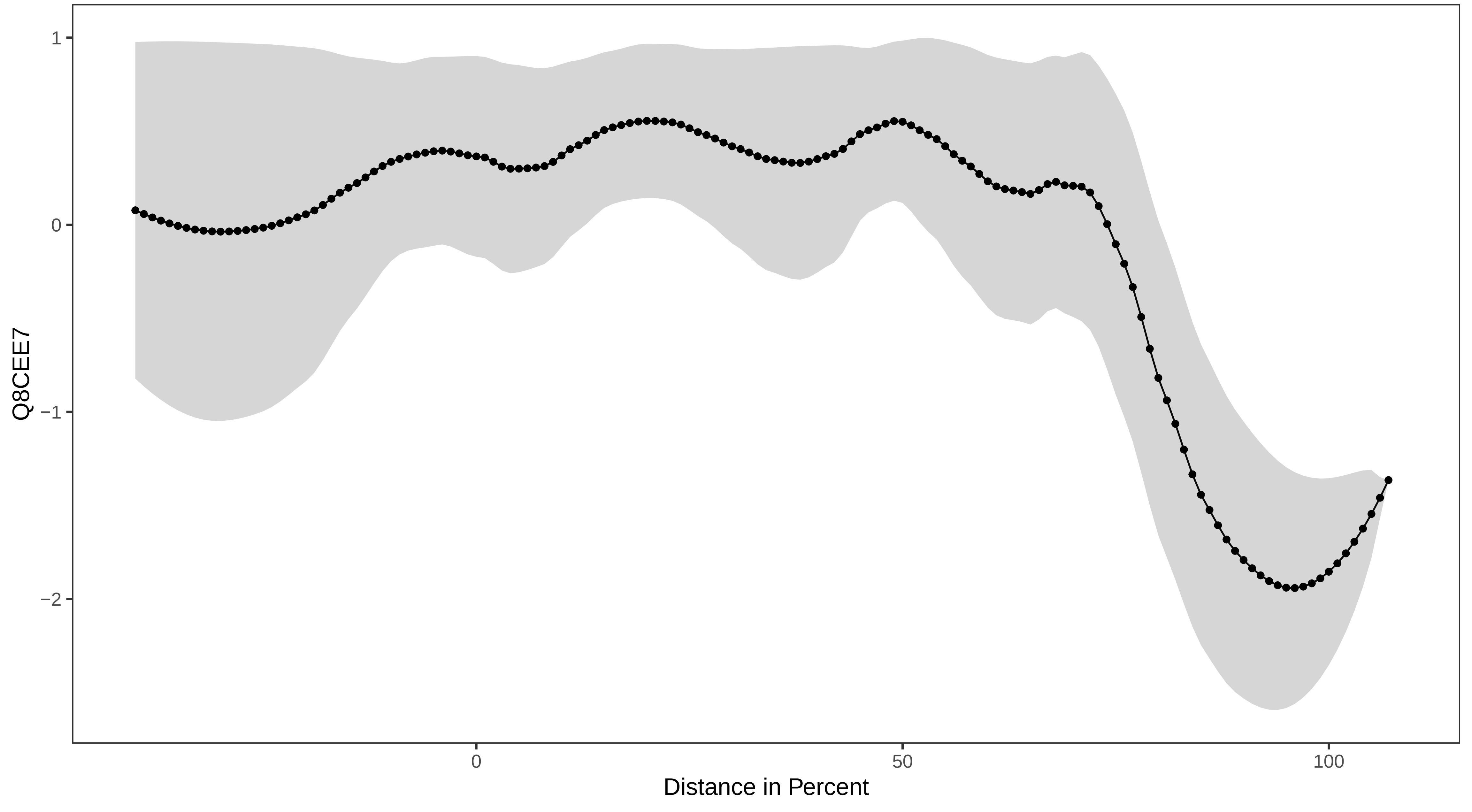

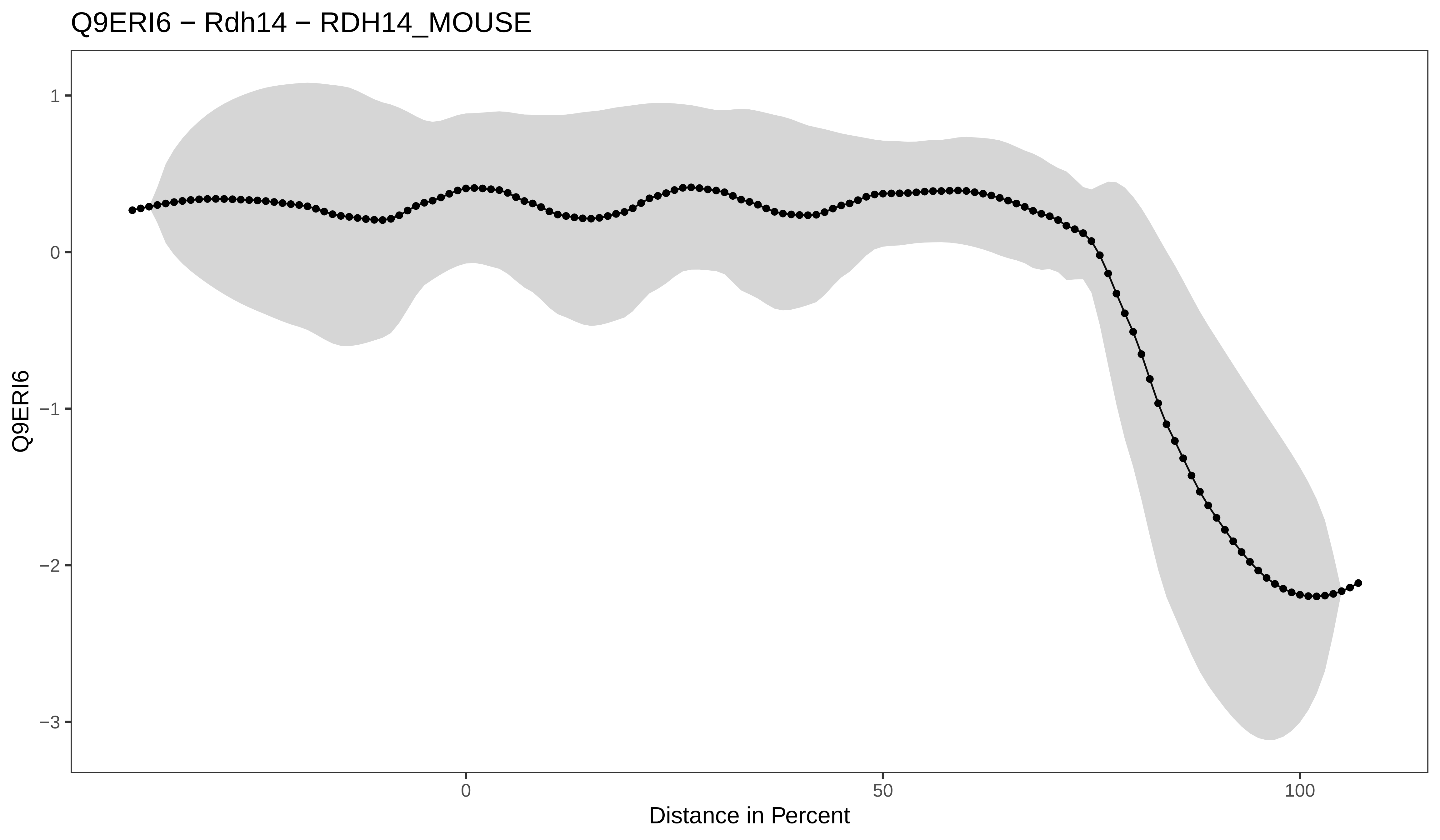
Q9ERI6 − Rdh14 − RDH14_MOUSE
1
0
−1
−2
−3
Q9ERI6
0
50
100
Distance in Percent

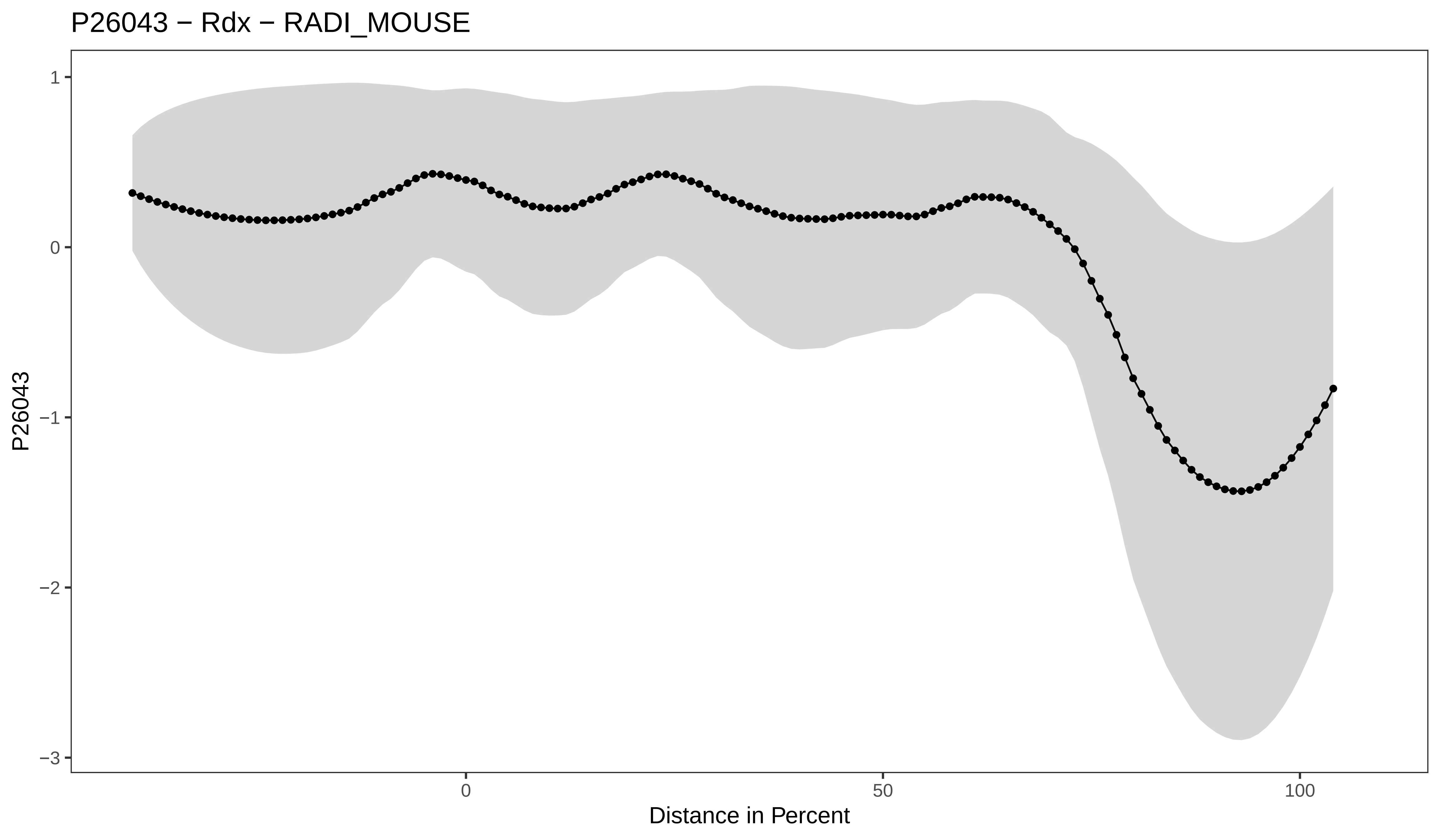

P26043 − Rdx − RADI_MOUSE
P26043
Distance in Percent
1
0
−1
−2
−3
0
50
100

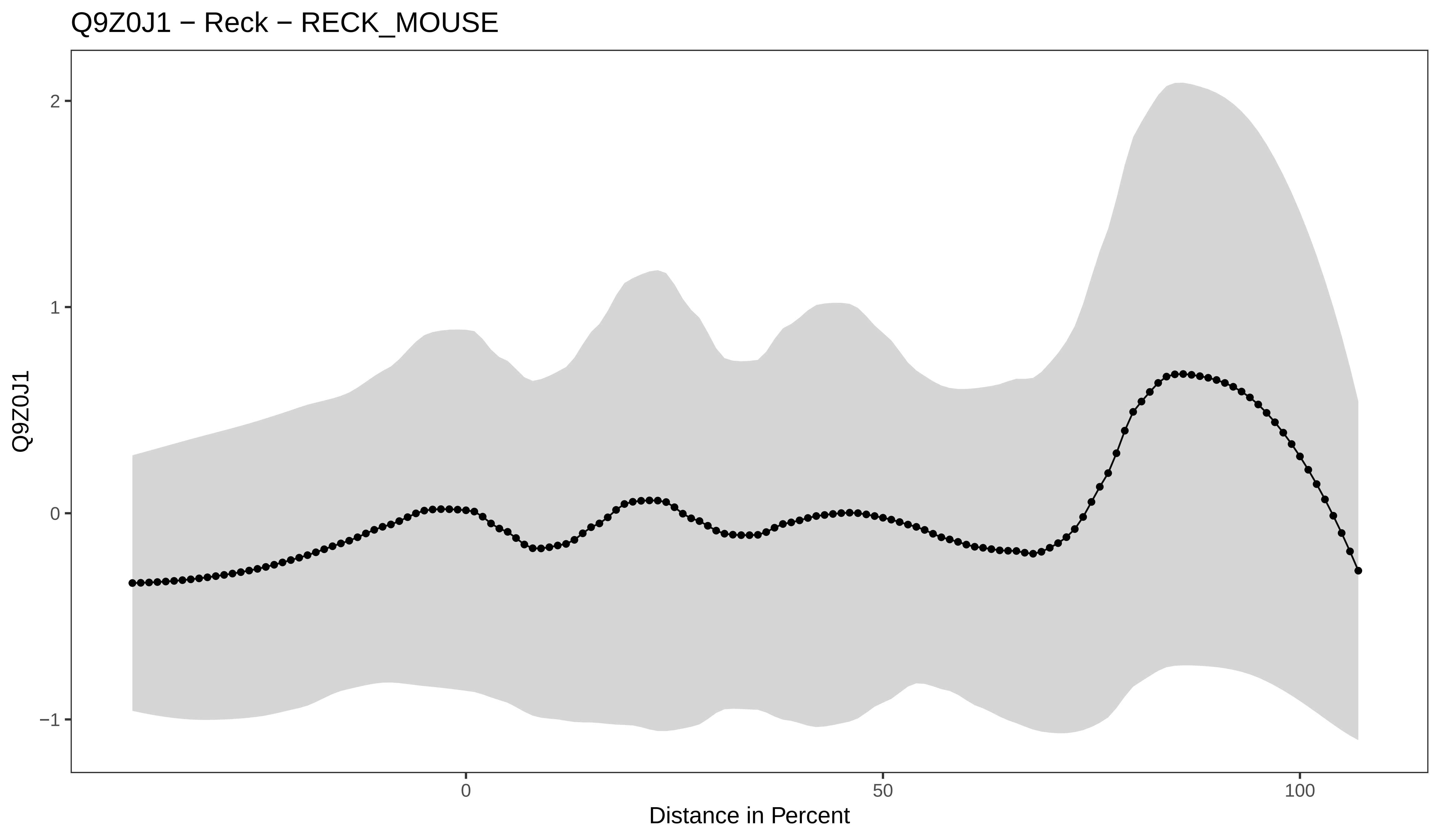

Q9Z0J1 − Reck − RECK_MOUSE
Q9Z0J1
2
1
0
−1
0
50
100
Distance in Percent

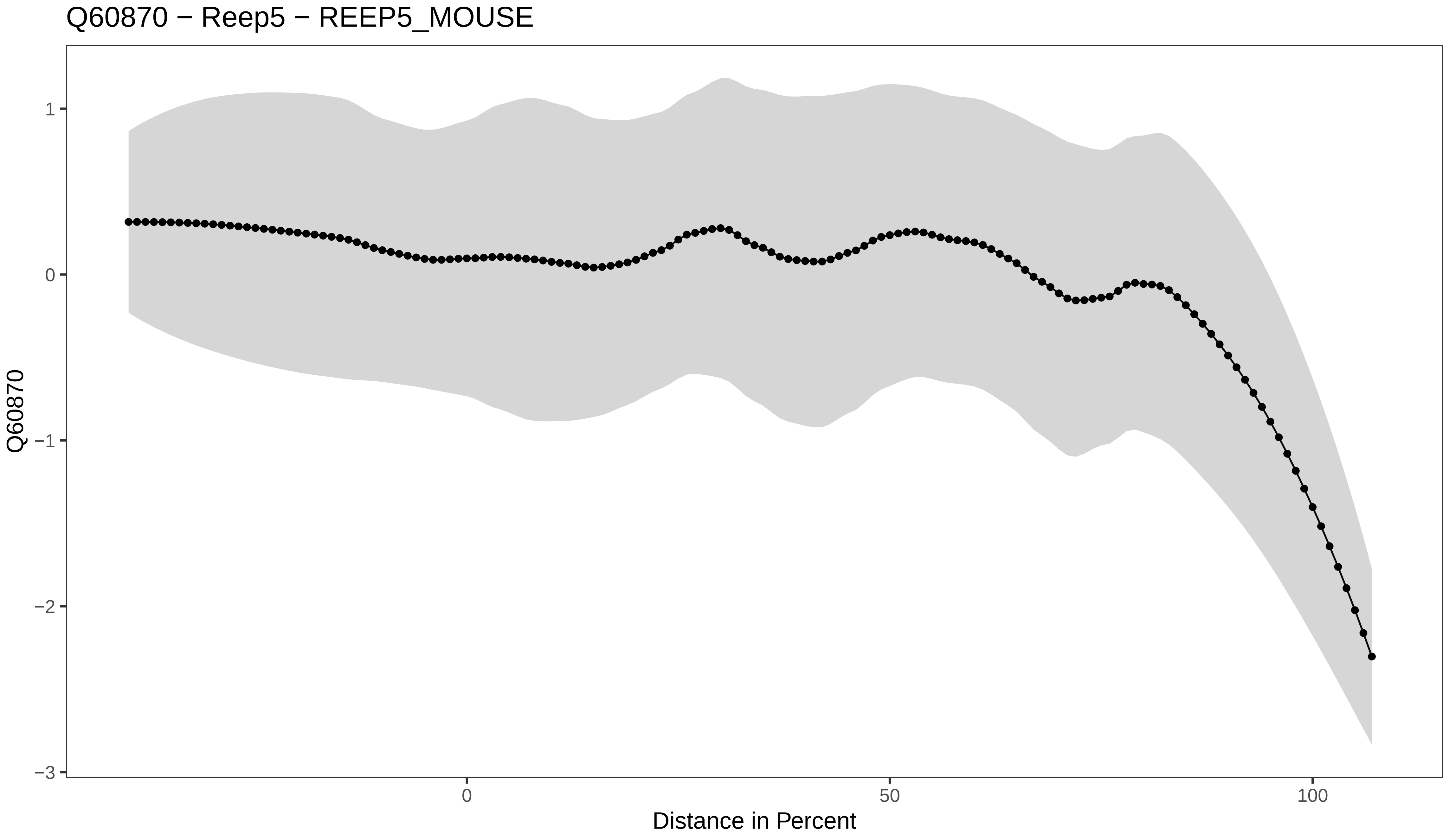

Q60870 − Reep5 − REEP5_MOUSE
Q60870
Distance in Percent
1
0
−1
−2
−3
0
50
100

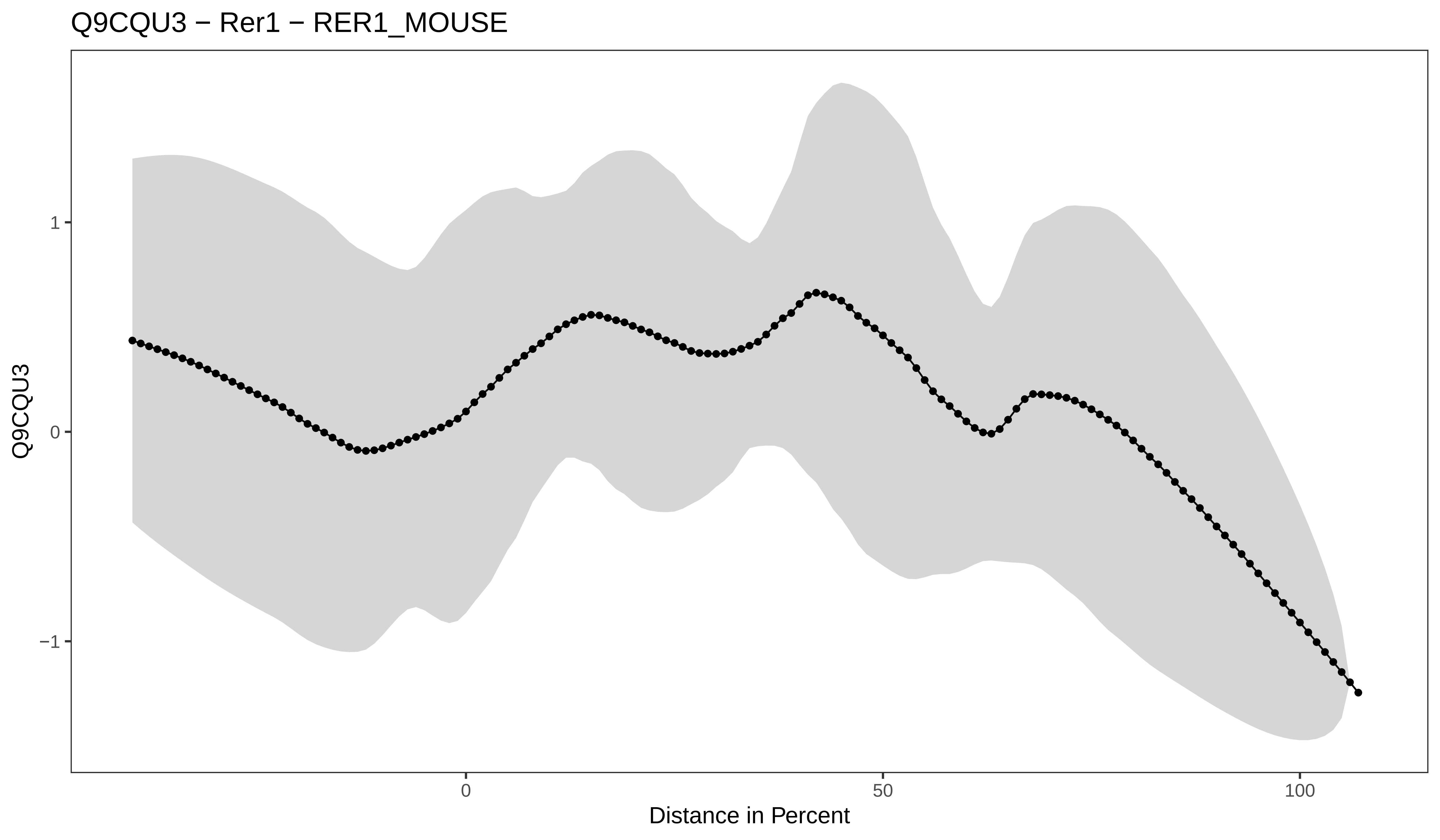

Q9CQU3 − Rer1 − RER1_MOUSE
1
0
−1
Q9CQU3
0
50
100
Distance in Percent

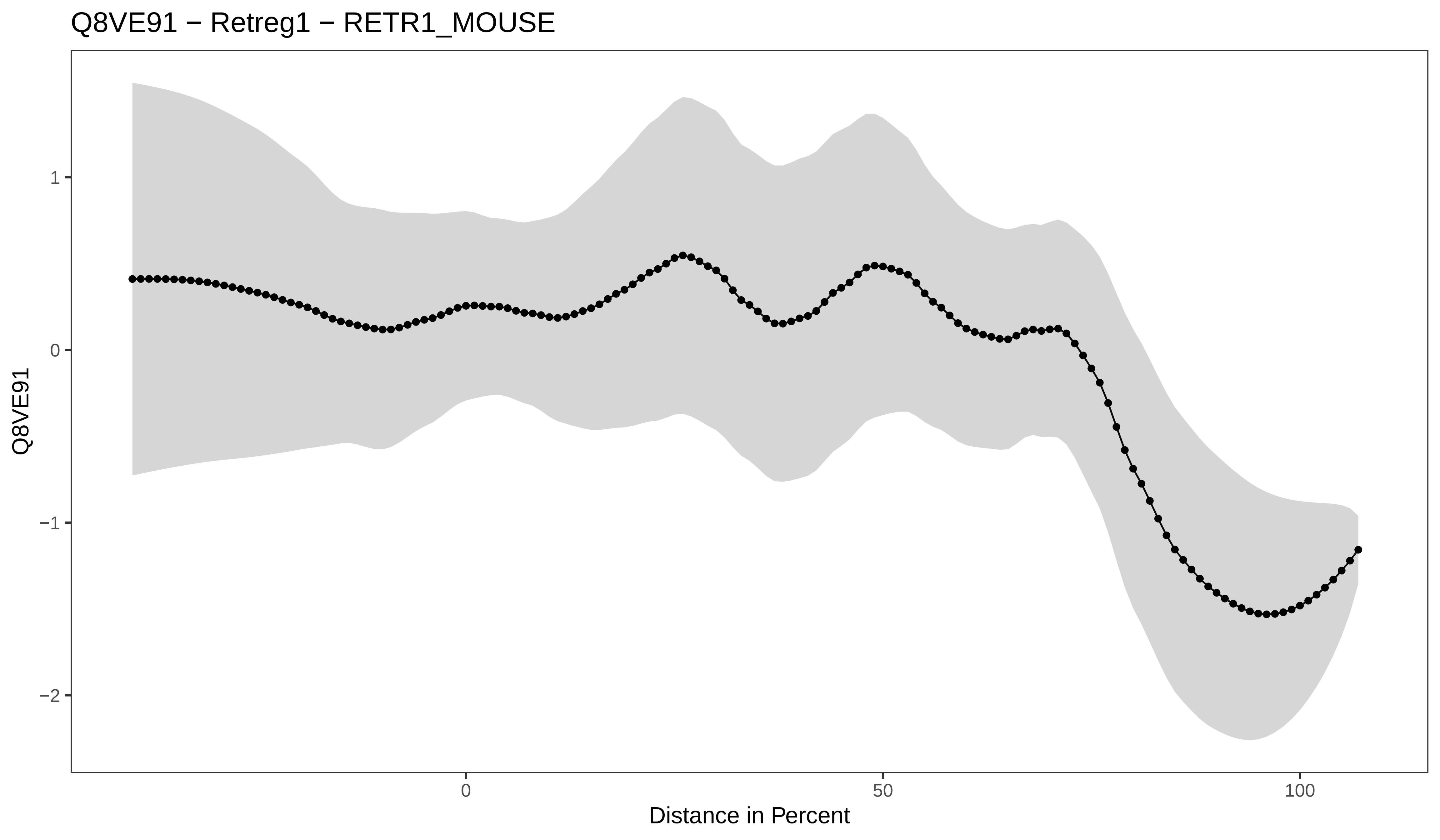
Q8VE91 − Retreg1 − RETR1_MOUSE
1
0
−1
−2
Q8VE91
0
50
100
Distance in Percent

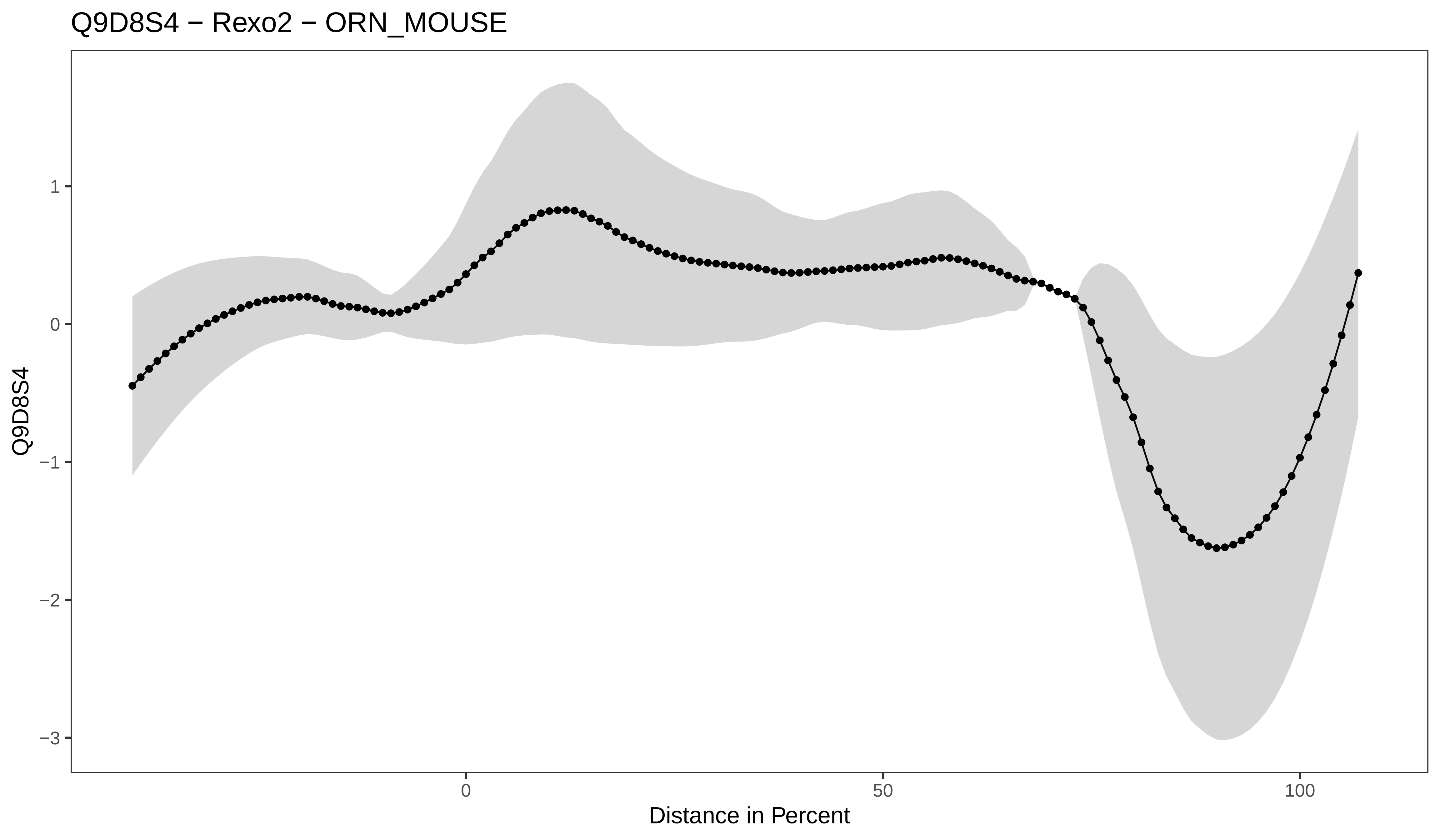

Q9D8S4 − Rexo2 − ORN_MOUSE
Q9D8S4
1
0
−1
−2
−3
0
50
100
Distance in Percent

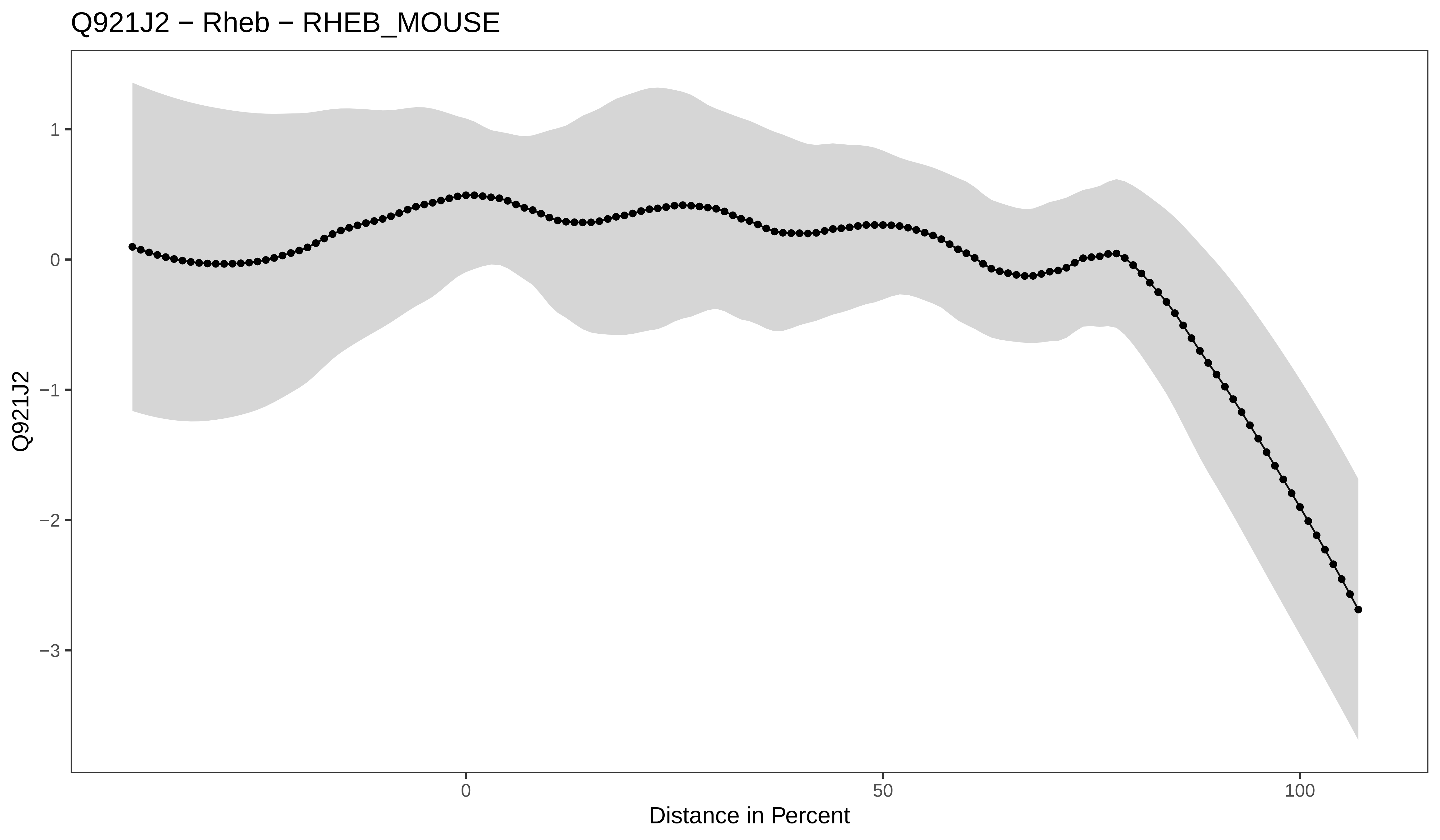
Q921J2 − Rheb − RHEB_MOUSE
Q921J2
1
0
−1
−2
−3
0
50
100
Distance in Percent

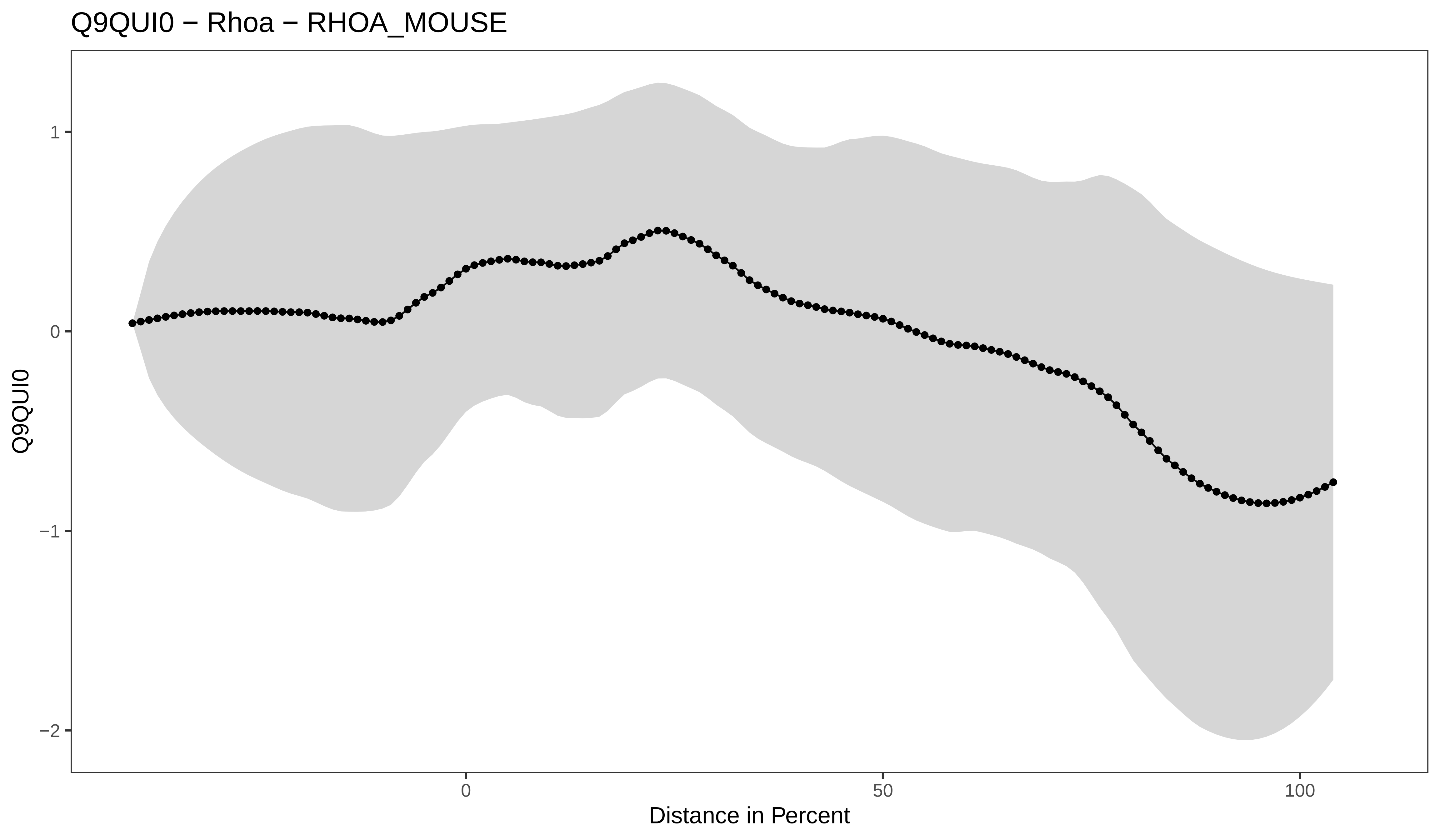

Q9QUI0 − Rhoa − RHOA_MOUSE
Q9QUI0
1
0
−1
−2
0
50
100
Distance in Percent

P84096 − Rhog − RHOG_MOUSE

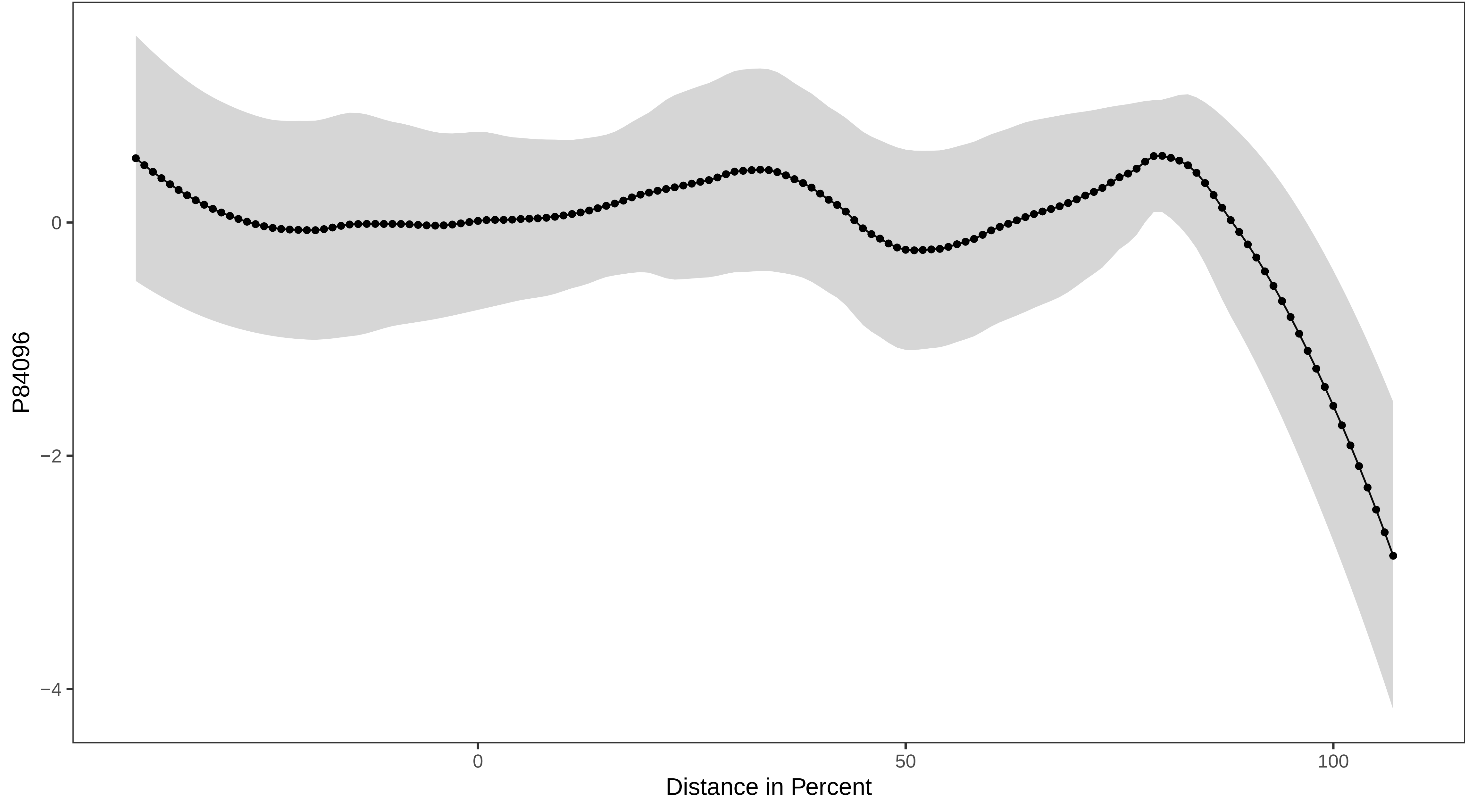

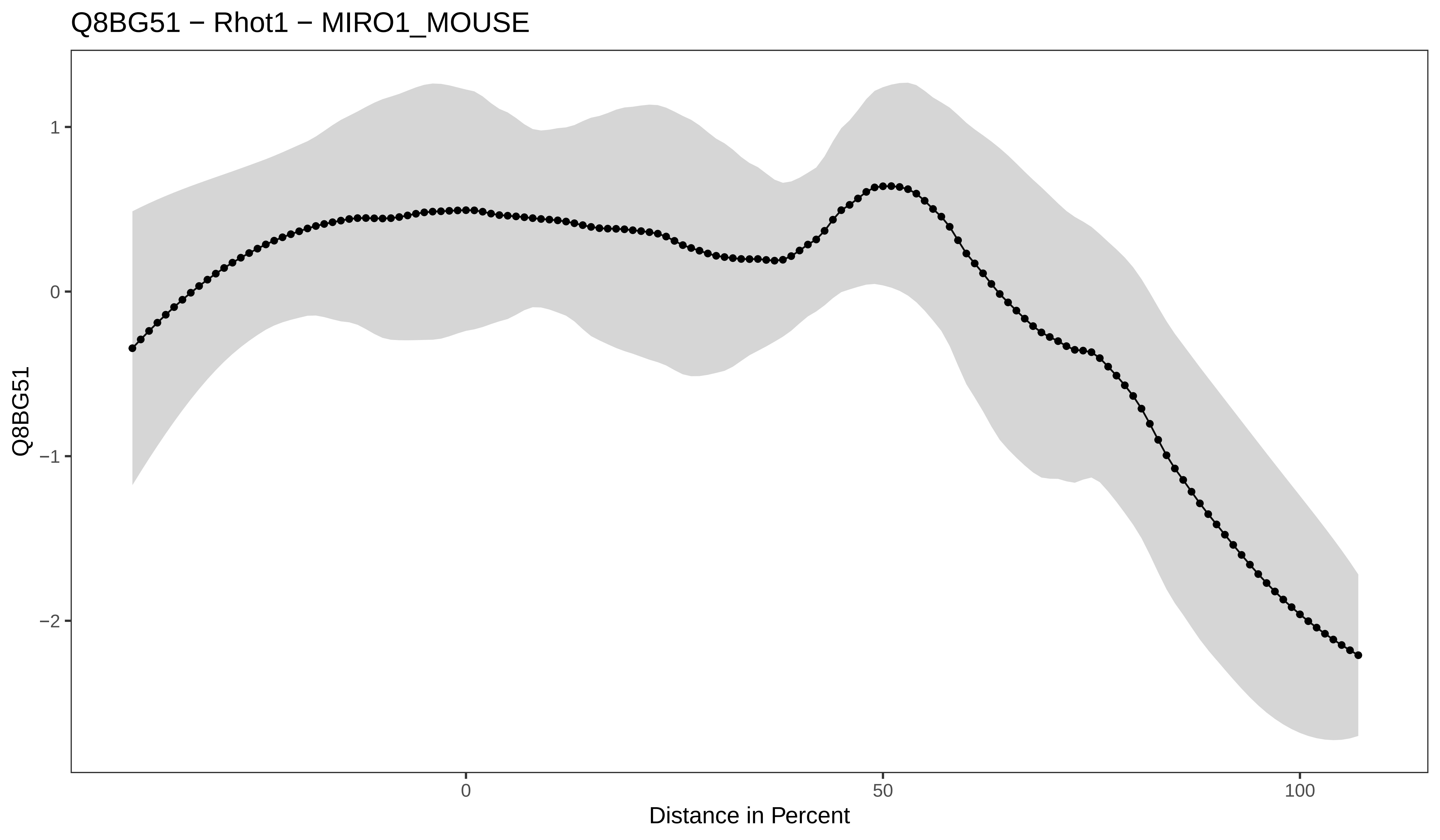

Q8BG51 − Rhot1 − MIRO1_MOUSE
1
0
−1
−2
Q8BG51
0
50
100
Distance in Percent

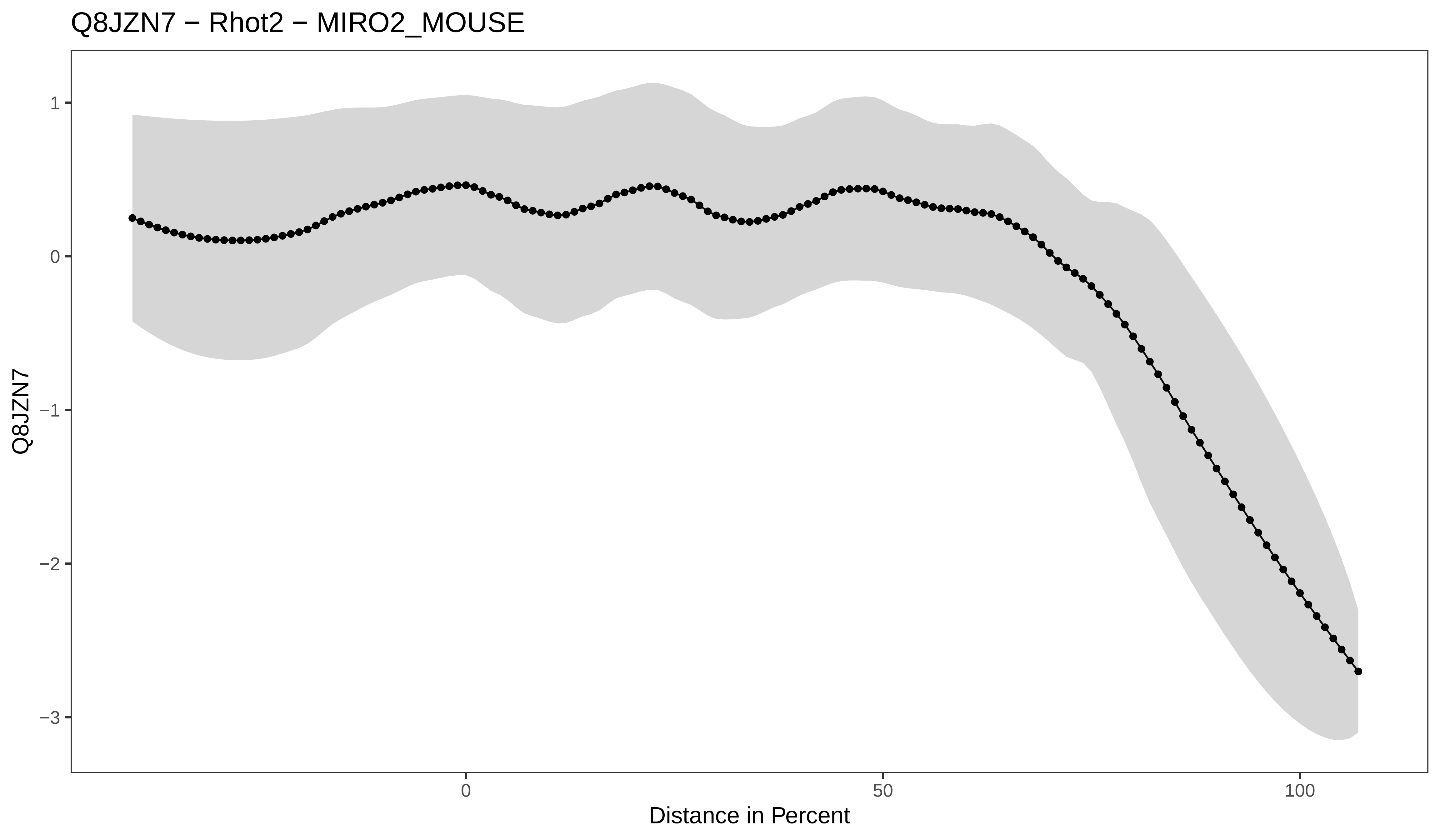

Q8JZN7 − Rhot2 − MIRO2_MOUSE
Q8JZN7
1
0
−1
−2
−3
0
50
100
Distance in Percent

Q5ND29 − Rilp − RILP_MOUSE

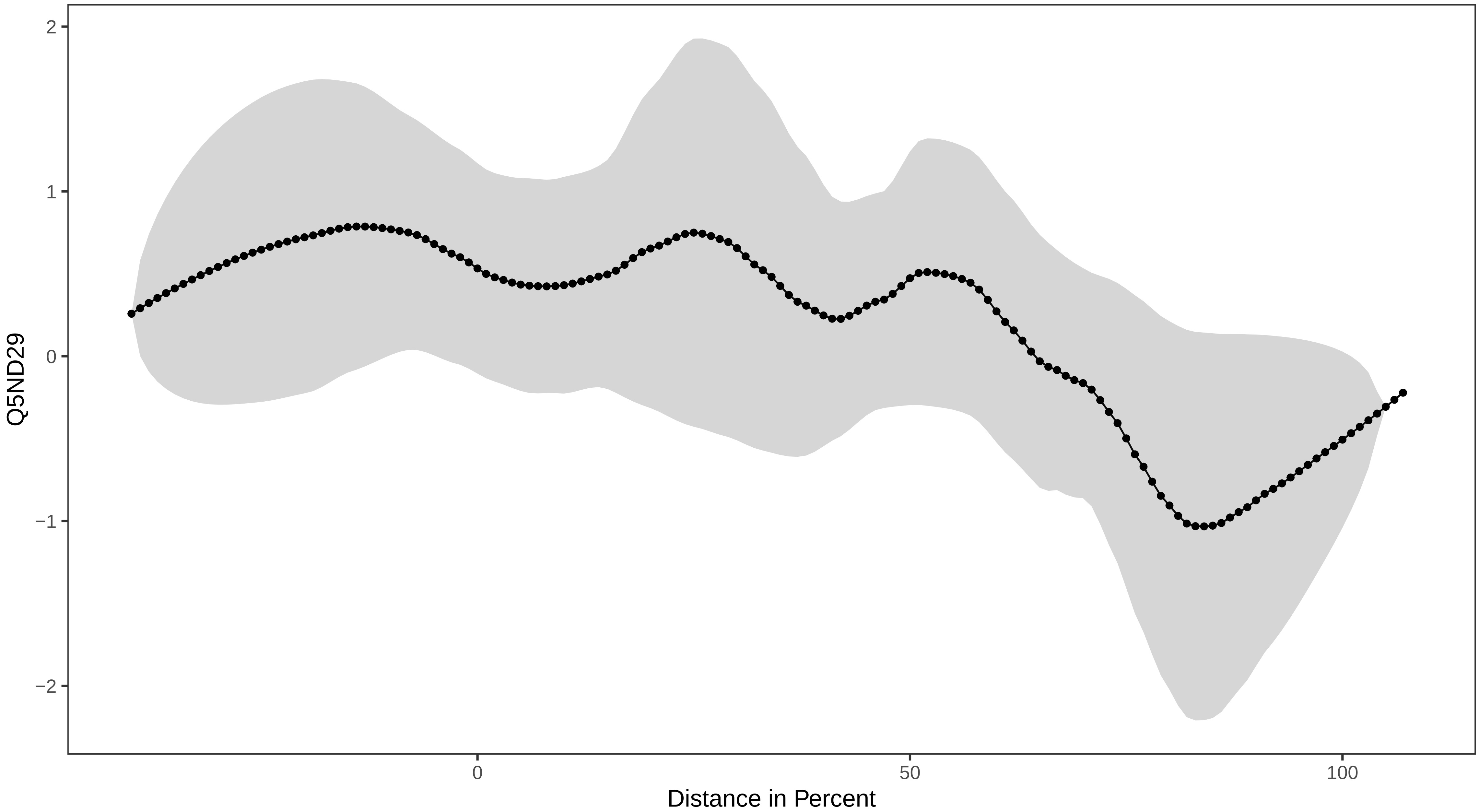

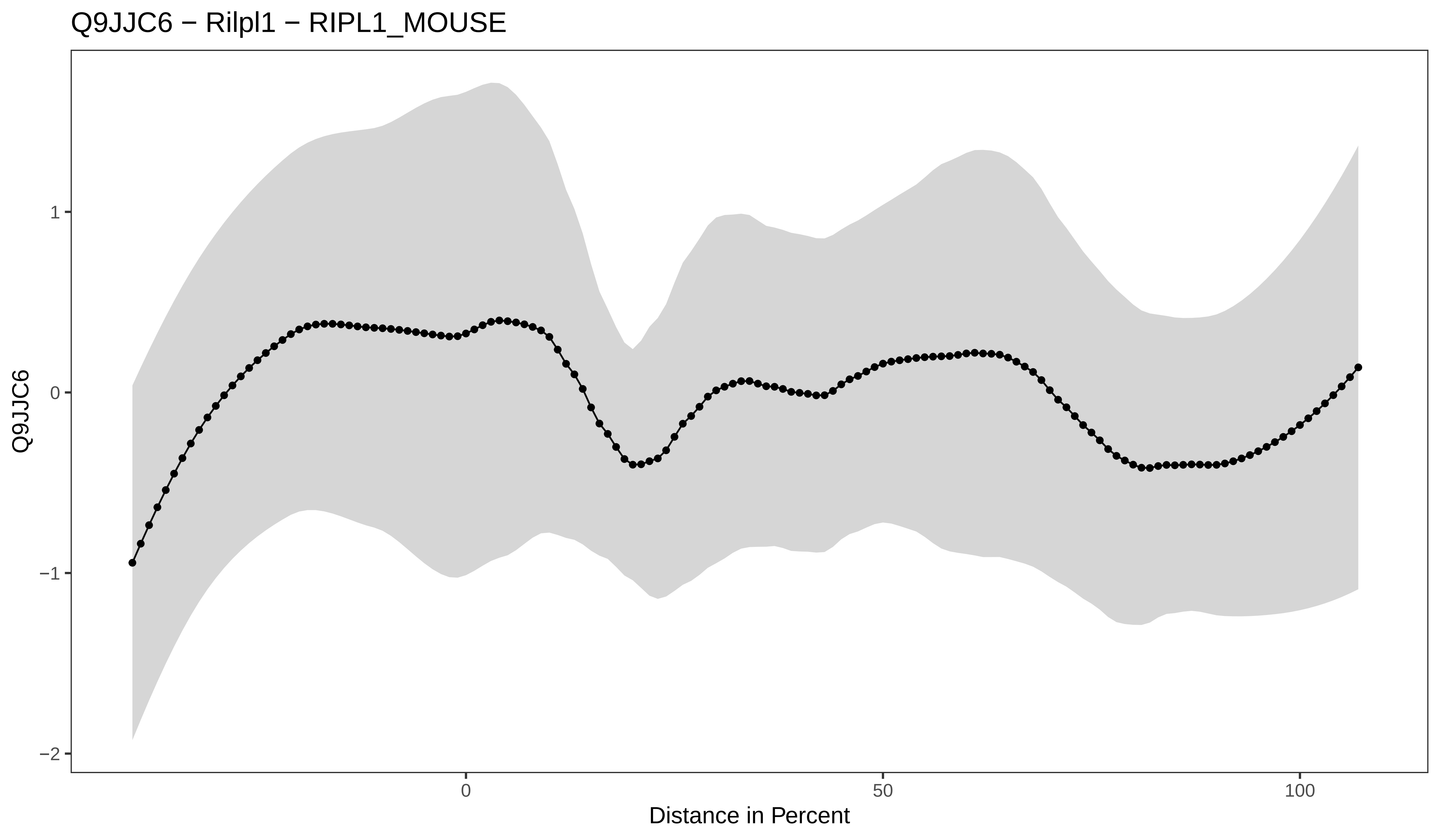

Q9JJC6 − Rilpl1 − RIPL1_MOUSE
Q9JJC6
1
0
−1
−2
0
50
100
Distance in Percent

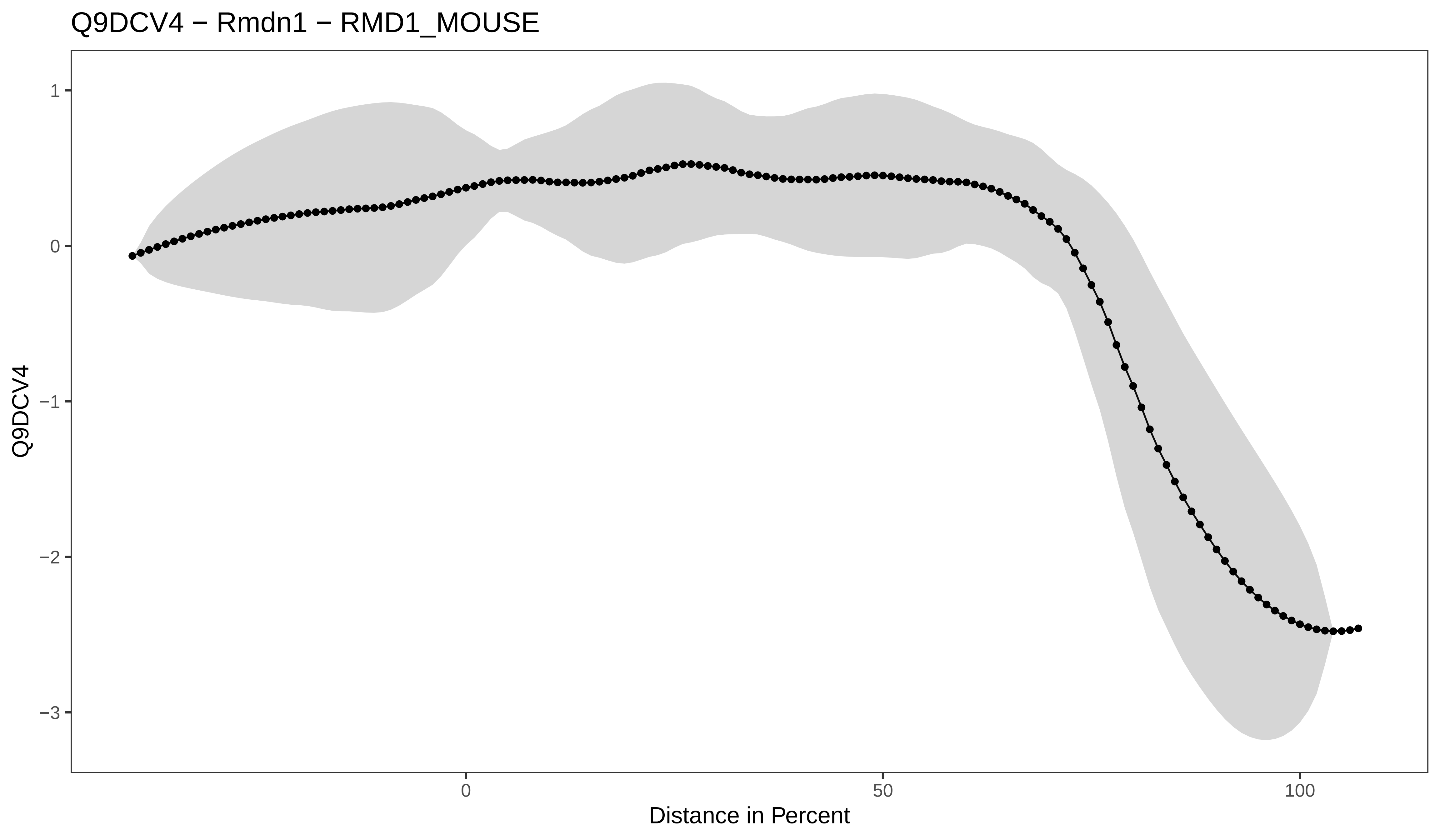

Q9DCV4 − Rmdn1 − RMD1_MOUSE
1
0
−1
−2
−3
Q9DCV4
0
50
100
Distance in Percent

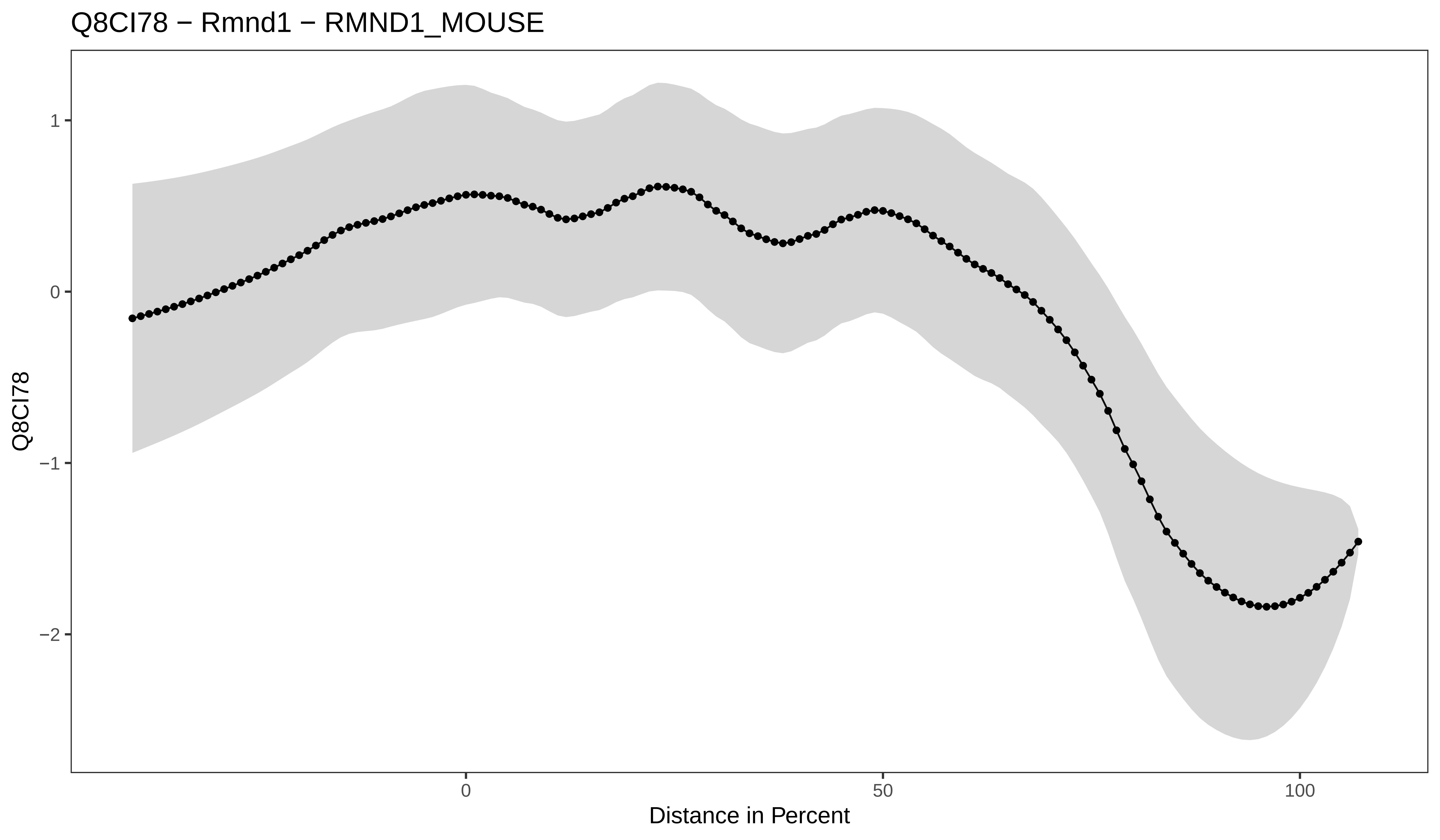

Q8CI78 − Rmnd1 − RMND1_MOUSE
1
0
−1
−2
Q8CI78
0
50
100
Distance in Percent

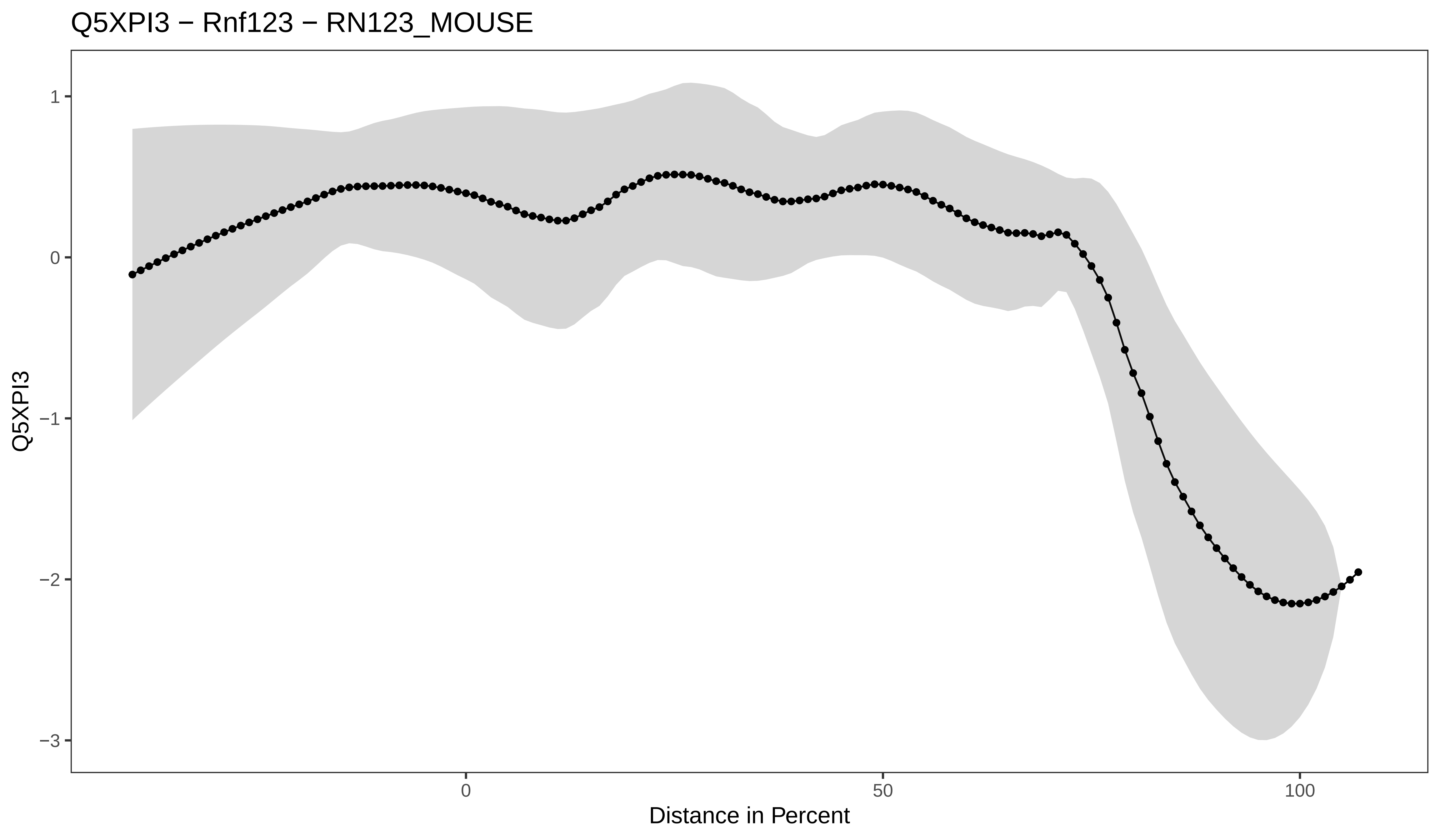

Q5XPI3 − Rnf123 − RN123_MOUSE
Q5XPI3
1
0
−1
−2
−3
0
50
100
Distance in Percent

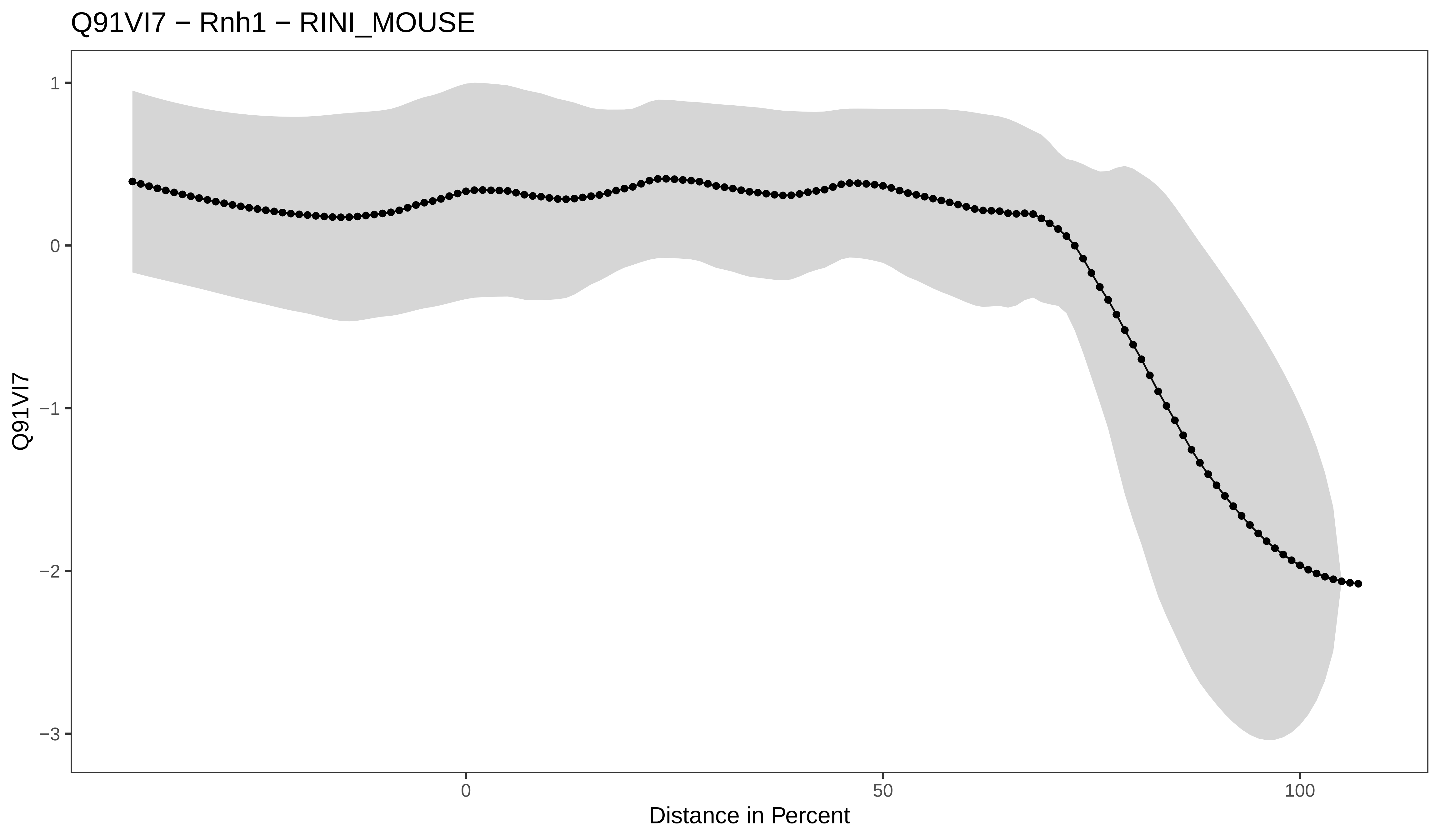

Q91VI7 − Rnh1 − RINI_MOUSE
Q91VI7
1
0
−1
−2
−3
0
50
100
Distance in Percent

Q8VCT3 − Rnpep − AMPB_MOUSE

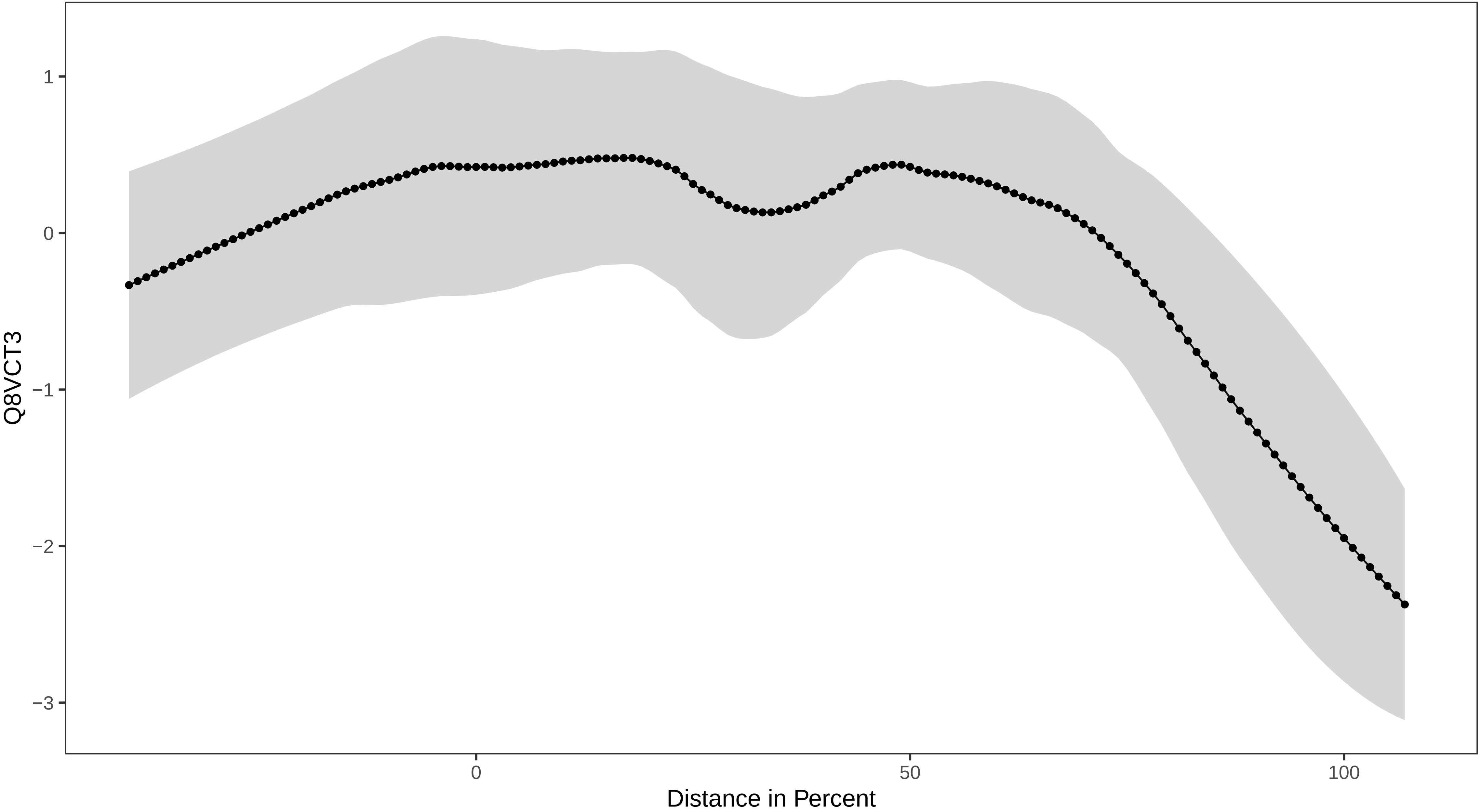

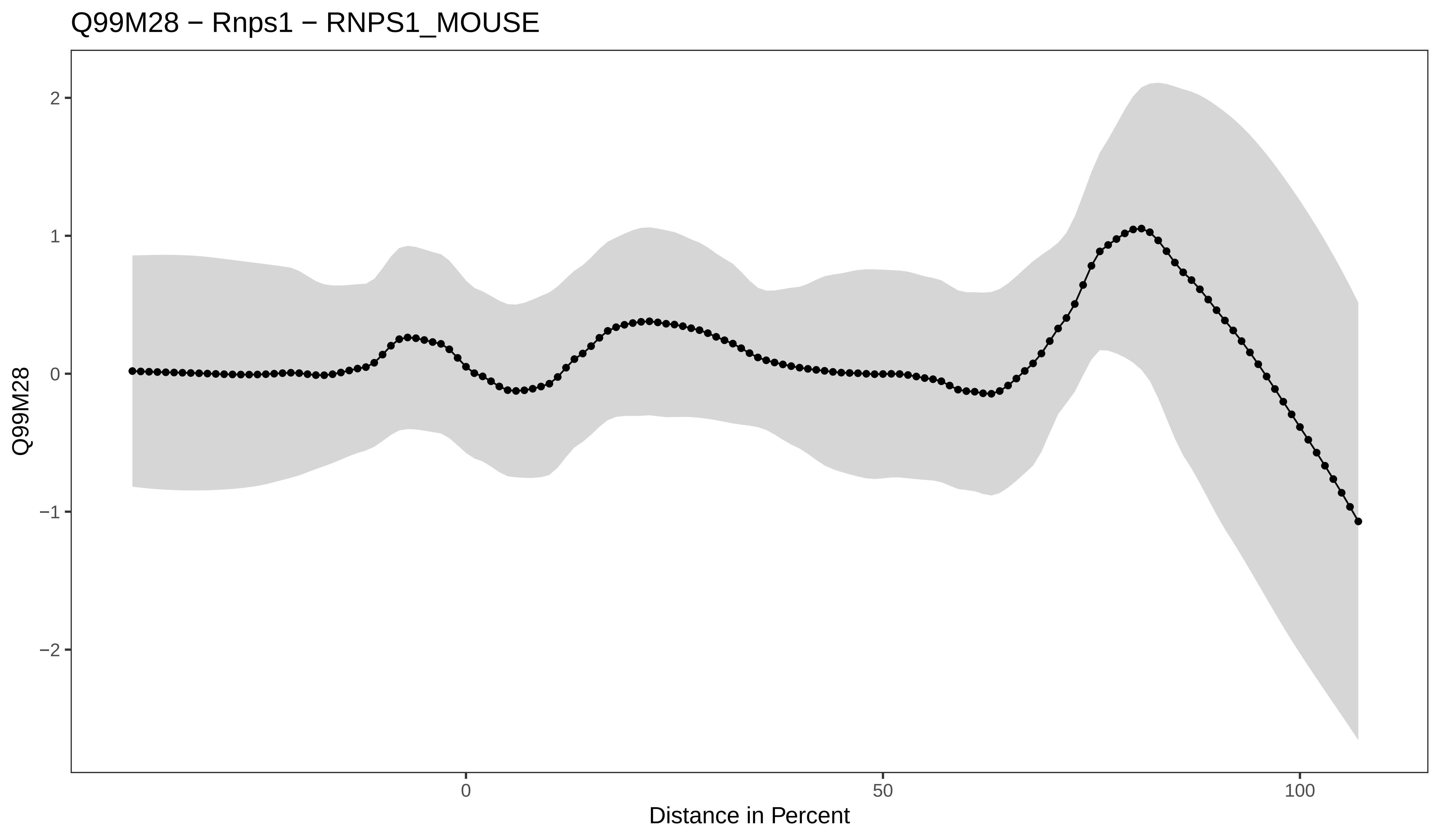

Q99M28 − Rnps1 − RNPS1_MOUSE
Q99M28
2
1
0
−1
−2
0
50
100
Distance in Percent

P70336 − Rock2 − ROCK2_MOUSE

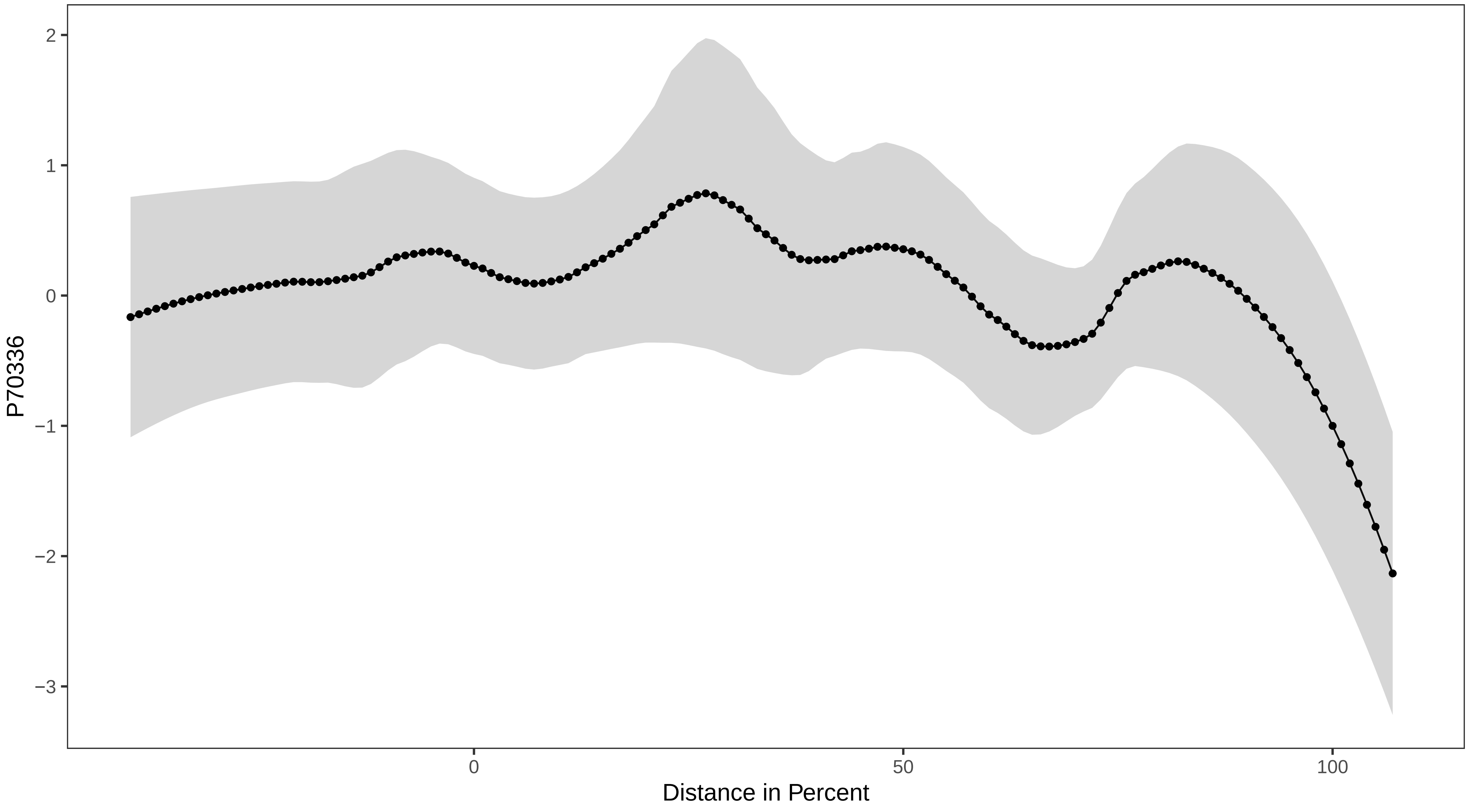

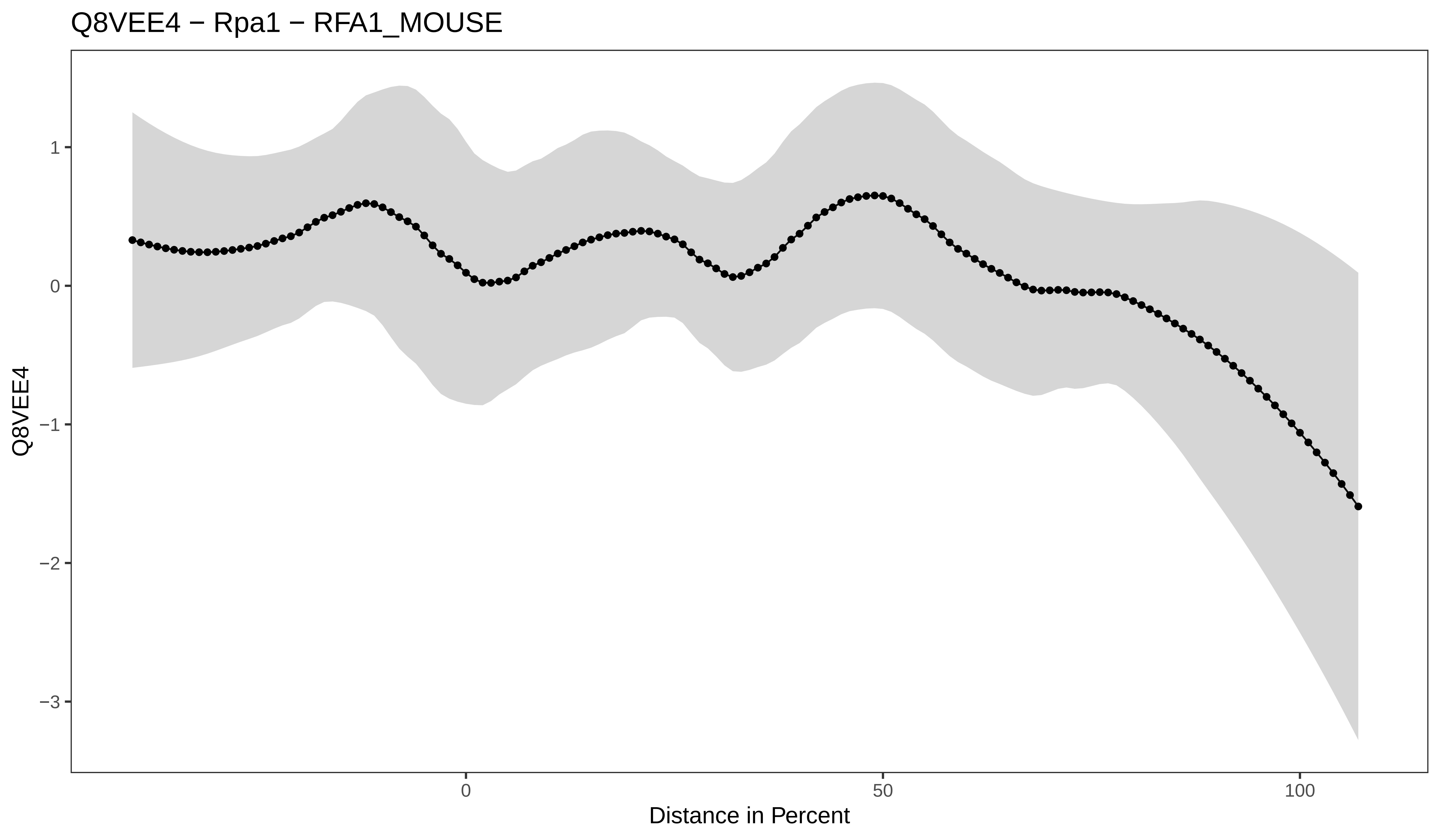

Q8VEE4 − Rpa1 − RFA1_MOUSE
Q8VEE4
1
0
−1
−2
−3
0
50
100
Distance in Percent

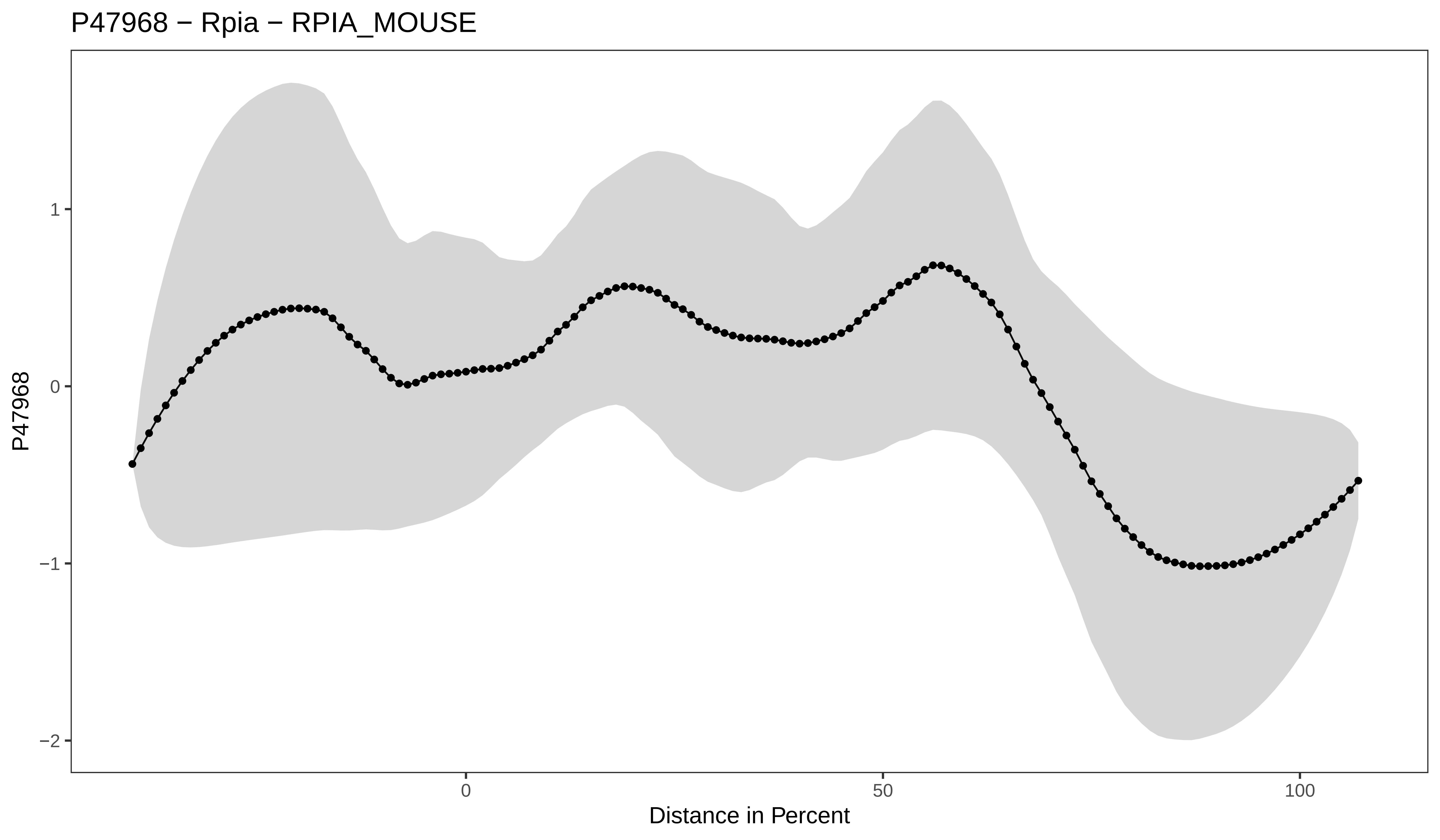

P47968 − Rpia − RPIA_MOUSE
P47968
1
0
−1
−2
0
50
100
Distance in Percent

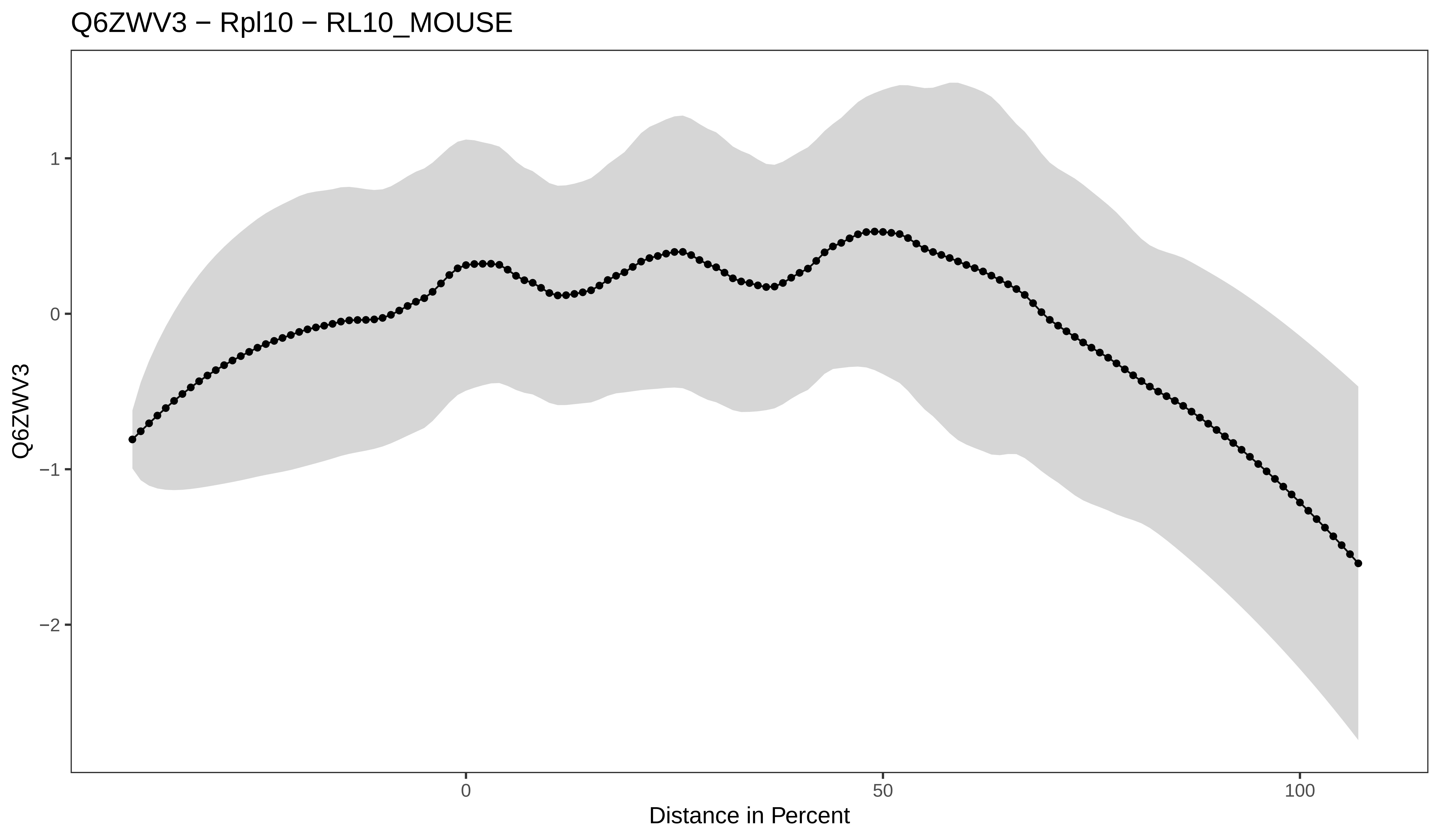

Q6ZWV3 − Rpl10 − RL10_MOUSE
1
0
−1
−2
Q6ZWV3
0
50
100
Distance in Percent

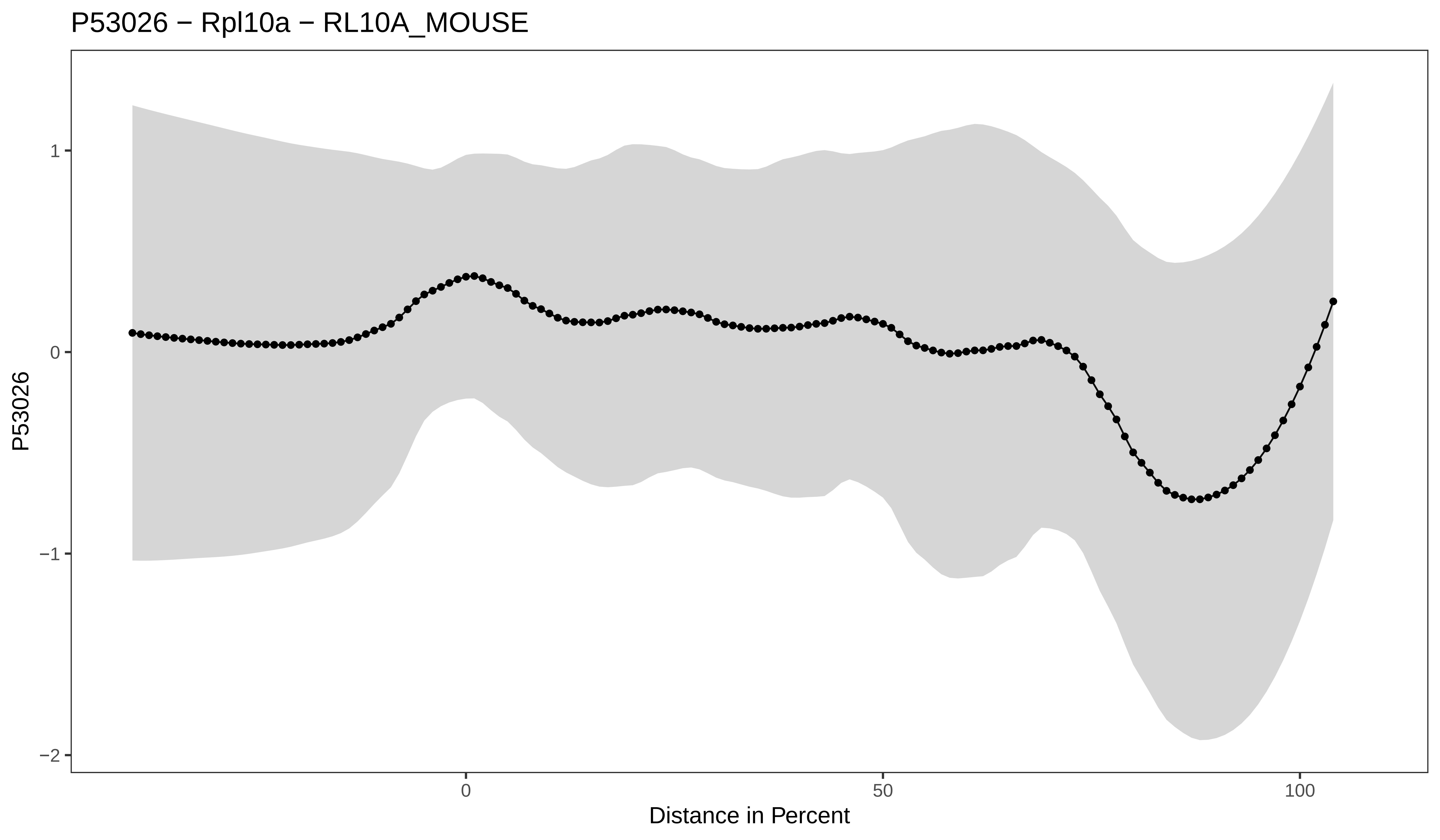
P53026 − Rpl10a − RL10A_MOUSE
P53026
1
0
−1
−2
0
50
100
Distance in Percent

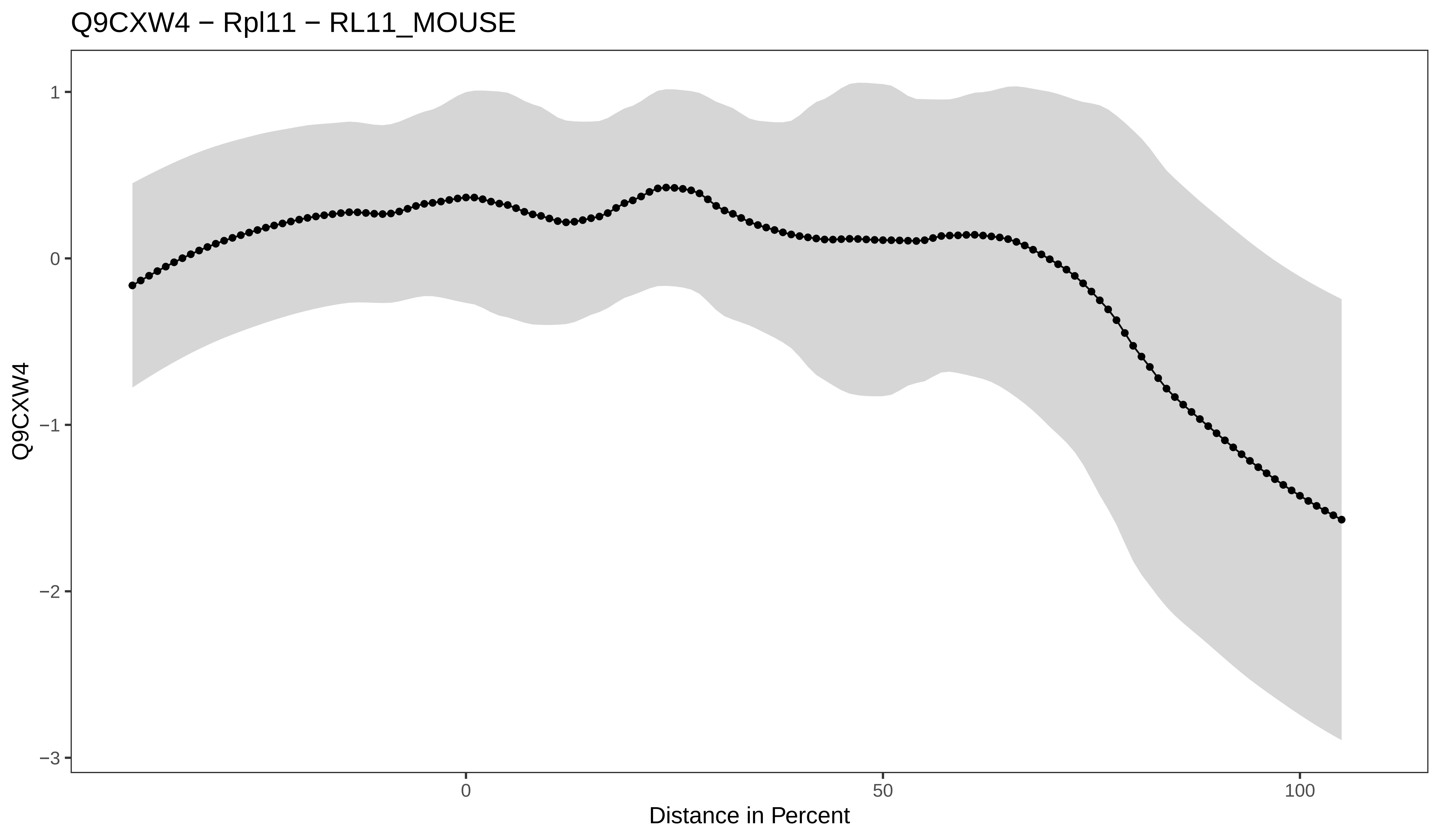

Q9CXW4 − Rpl11 − RL11_MOUSE
1
0
−1
−2
−3
Q9CXW4
0
50
100
Distance in Percent

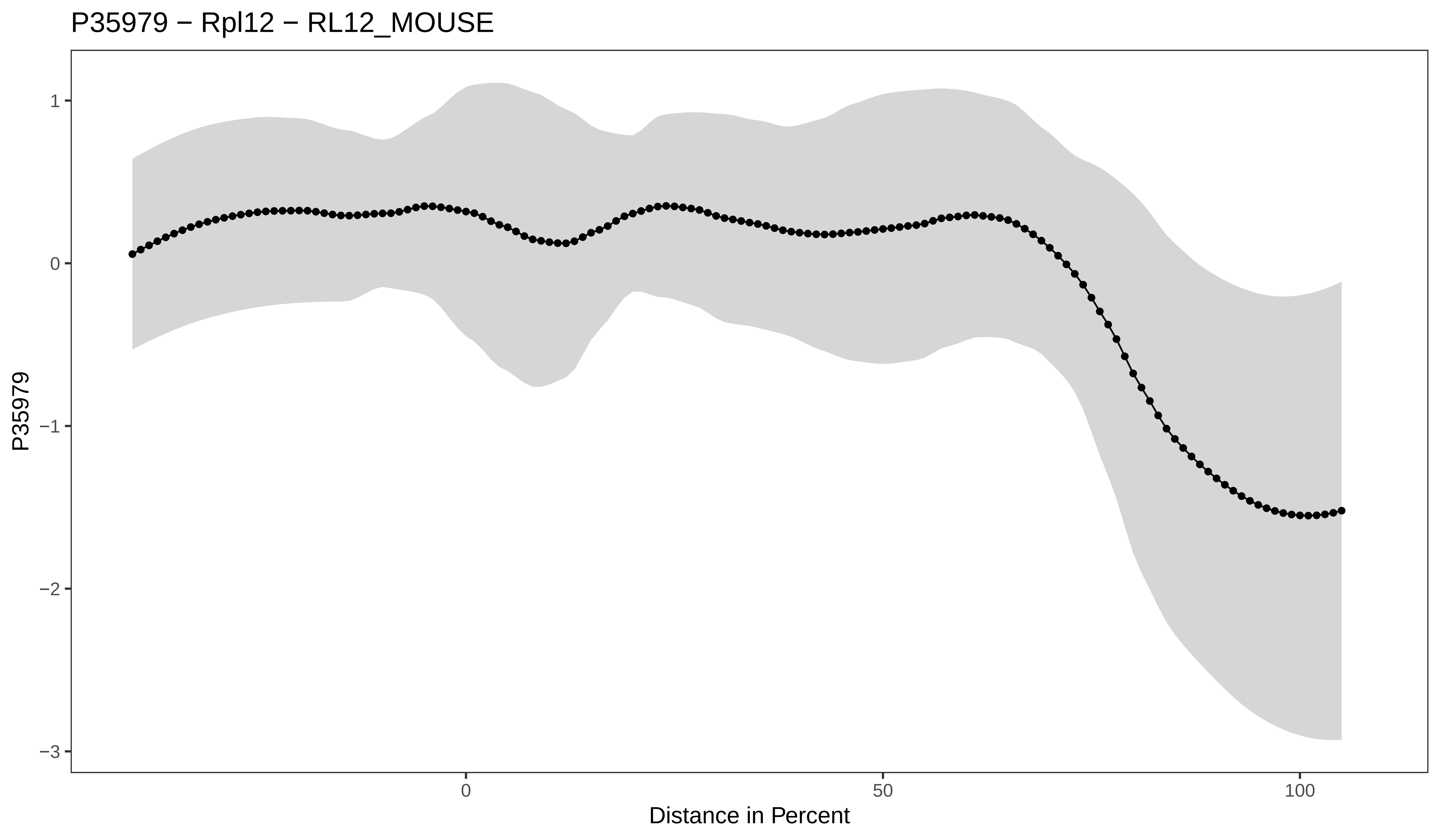

P35979 − Rpl12 − RL12_MOUSE
P35979
1
0
−1
−2
−3
0
50
100
Distance in Percent

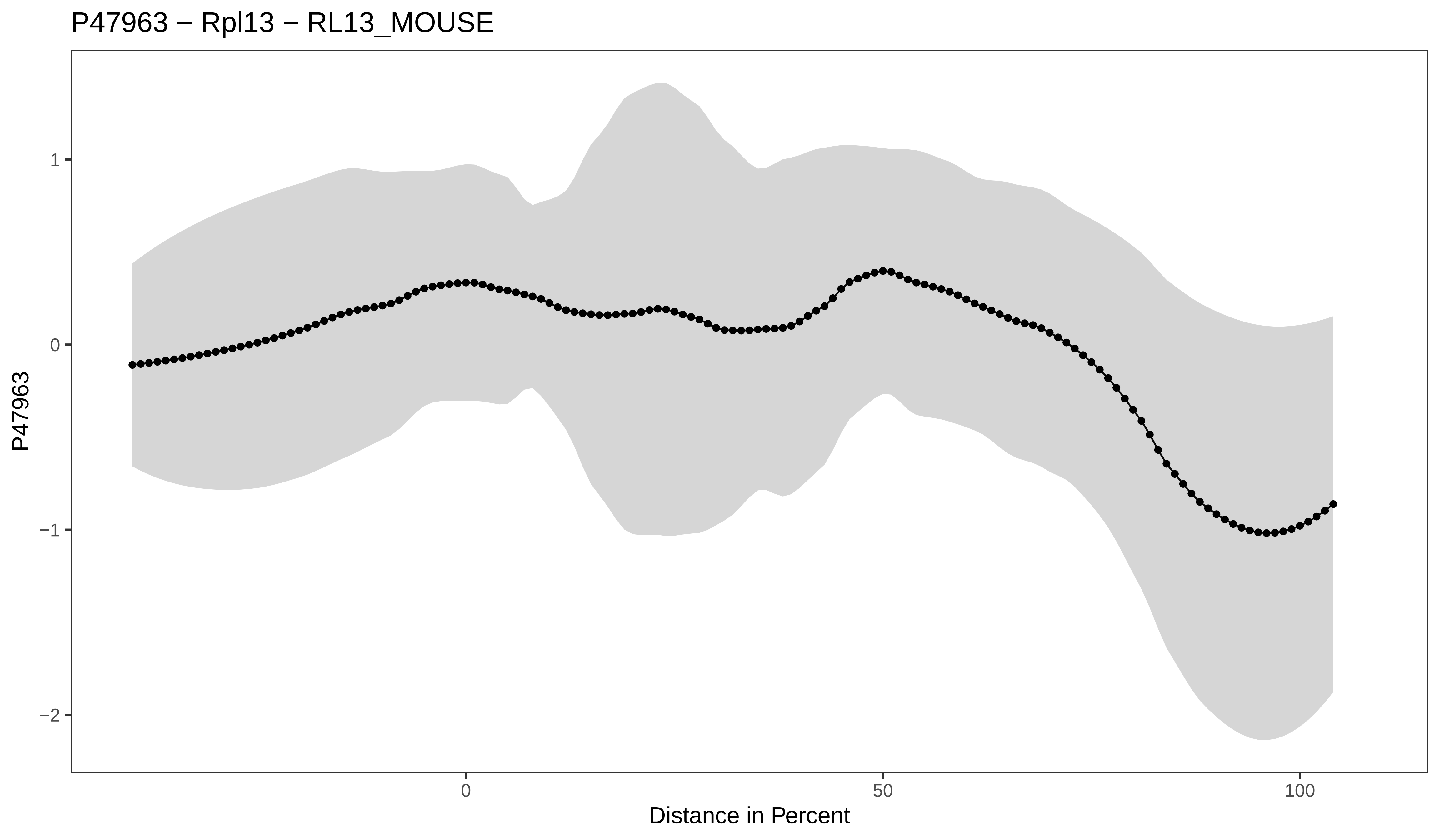

P47963 − Rpl13 − RL13_MOUSE
P47963
1
0
−1
−2
0
50
100
Distance in Percent

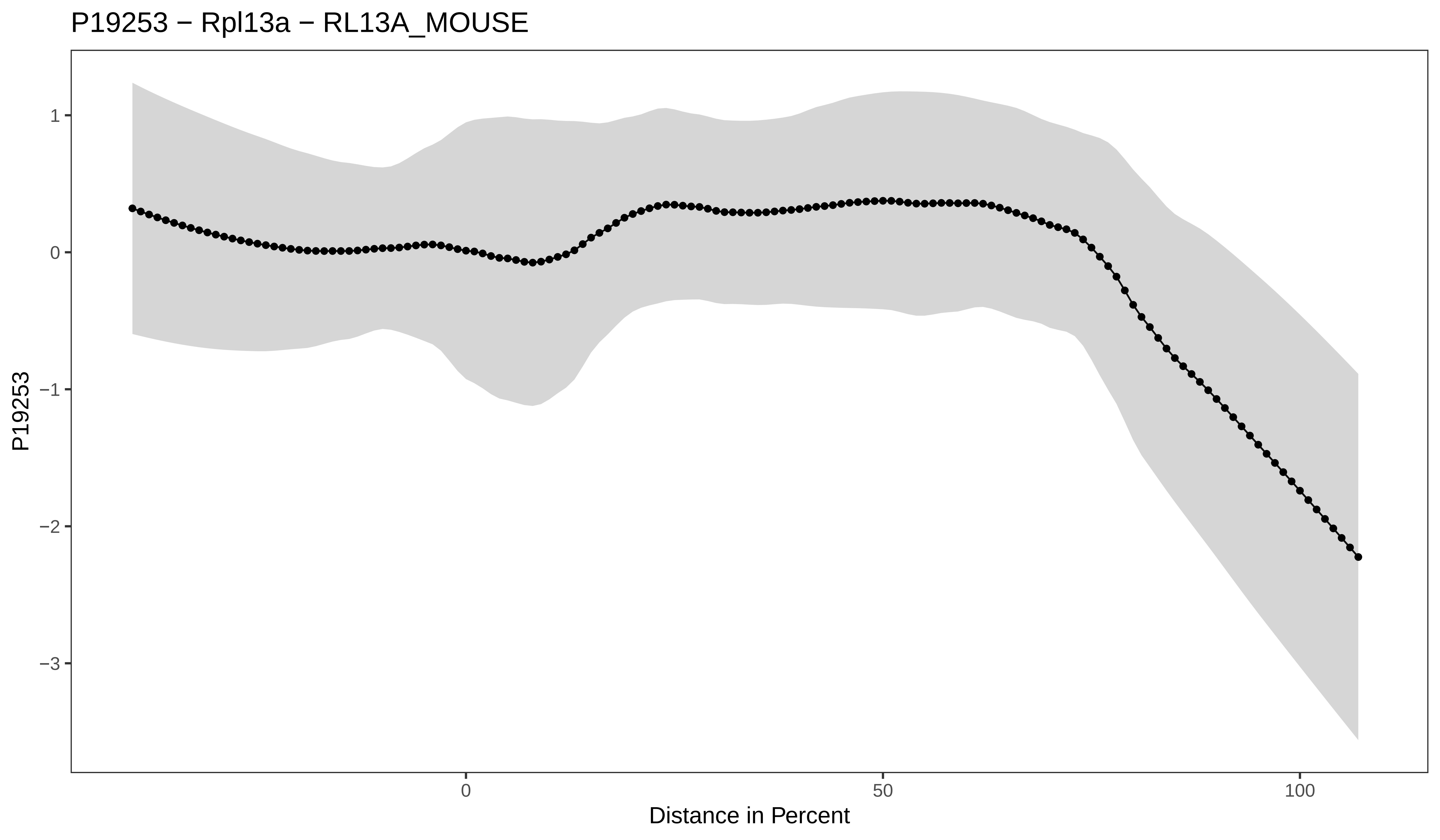
P19253 − Rpl13a − RL13A_MOUSE
P19253
1
0
−1
−2
−3
0
50
100
Distance in Percent

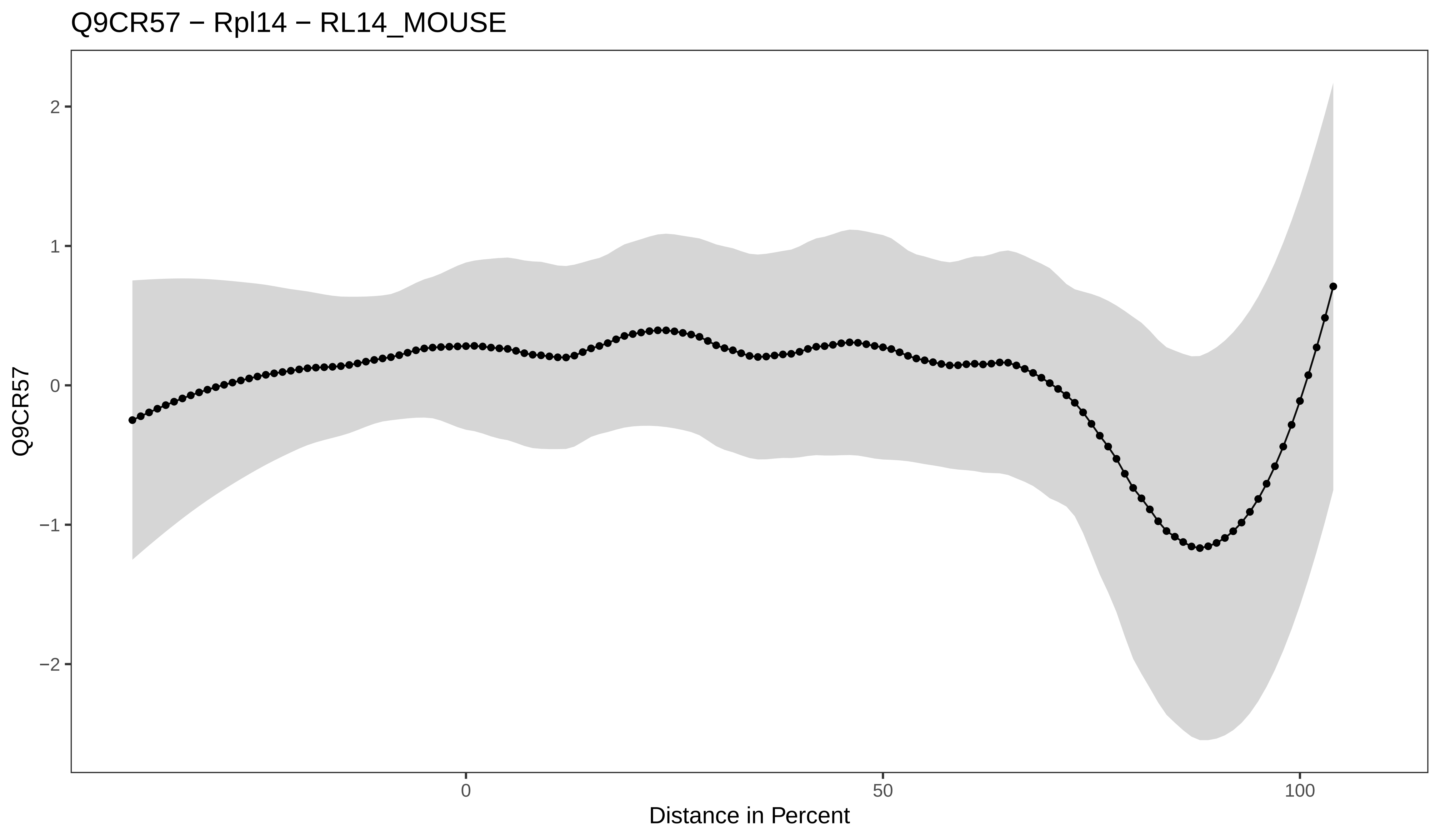

Q9CR57 − Rpl14 − RL14_MOUSE
Q9CR57
Distance in Percent
2
1
0
−1
−2
0
50
100

Q9CZM2 − Rpl15 − RL15_MOUSE

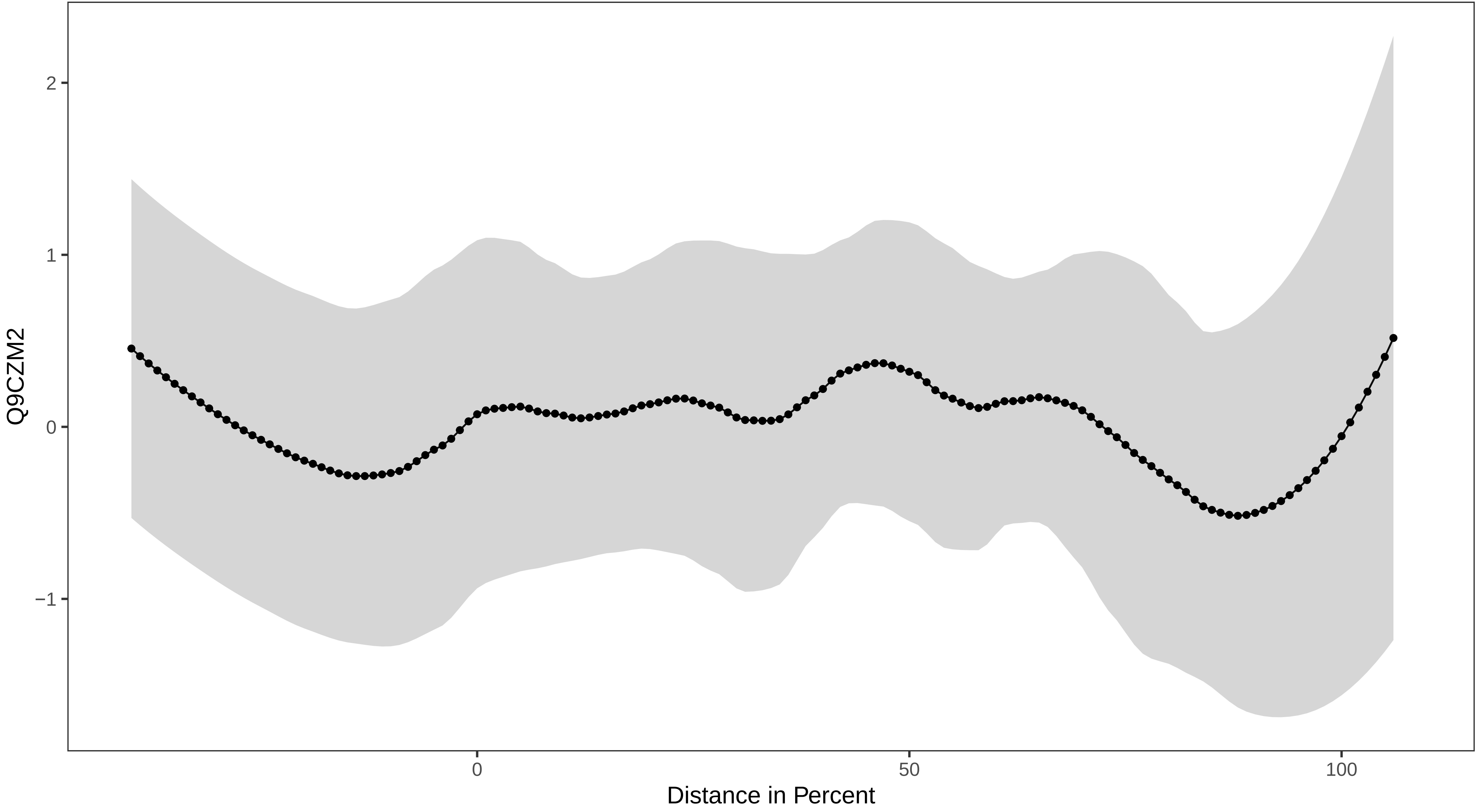

Q9CPR4 − Rpl17 − RL17_MOUSE

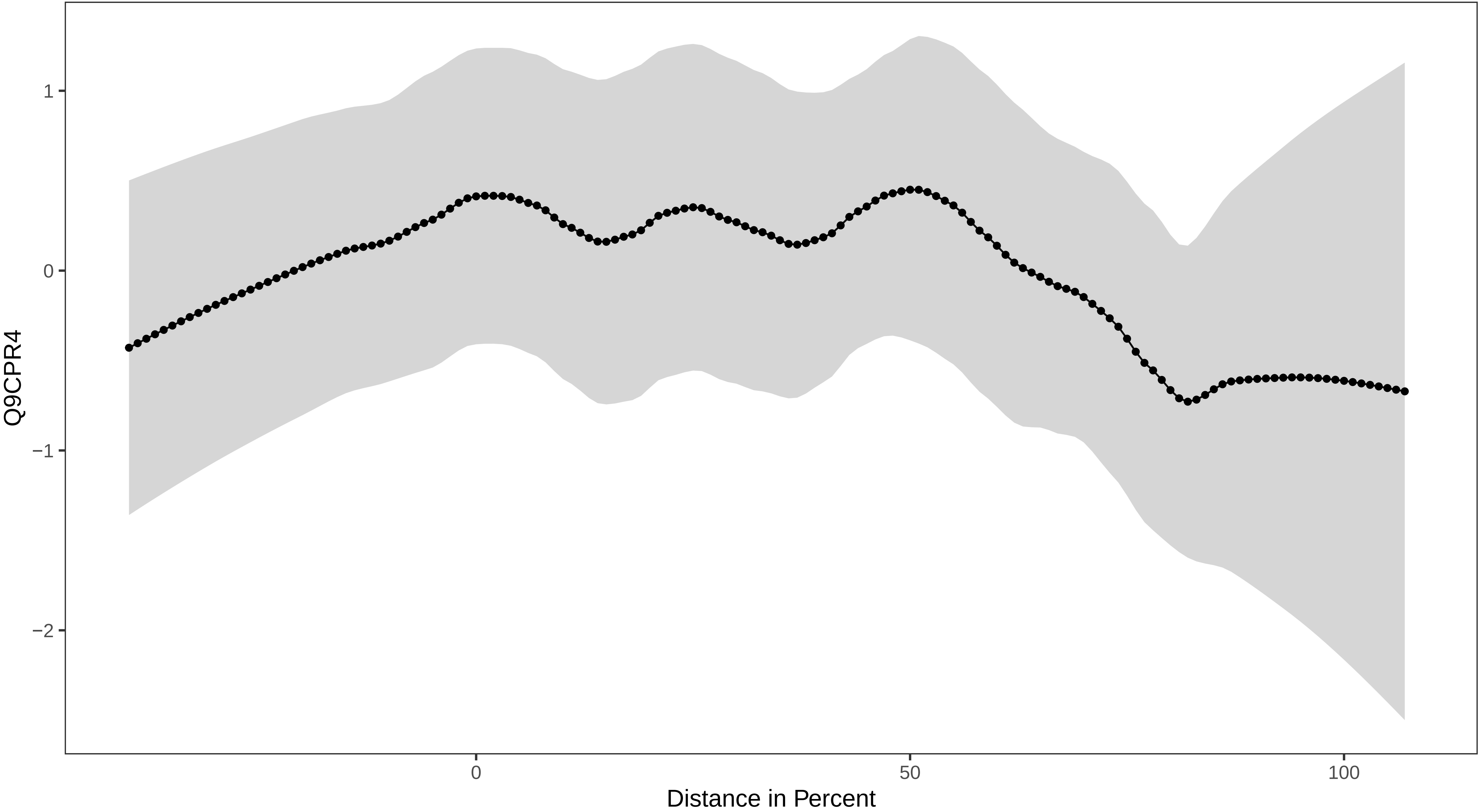

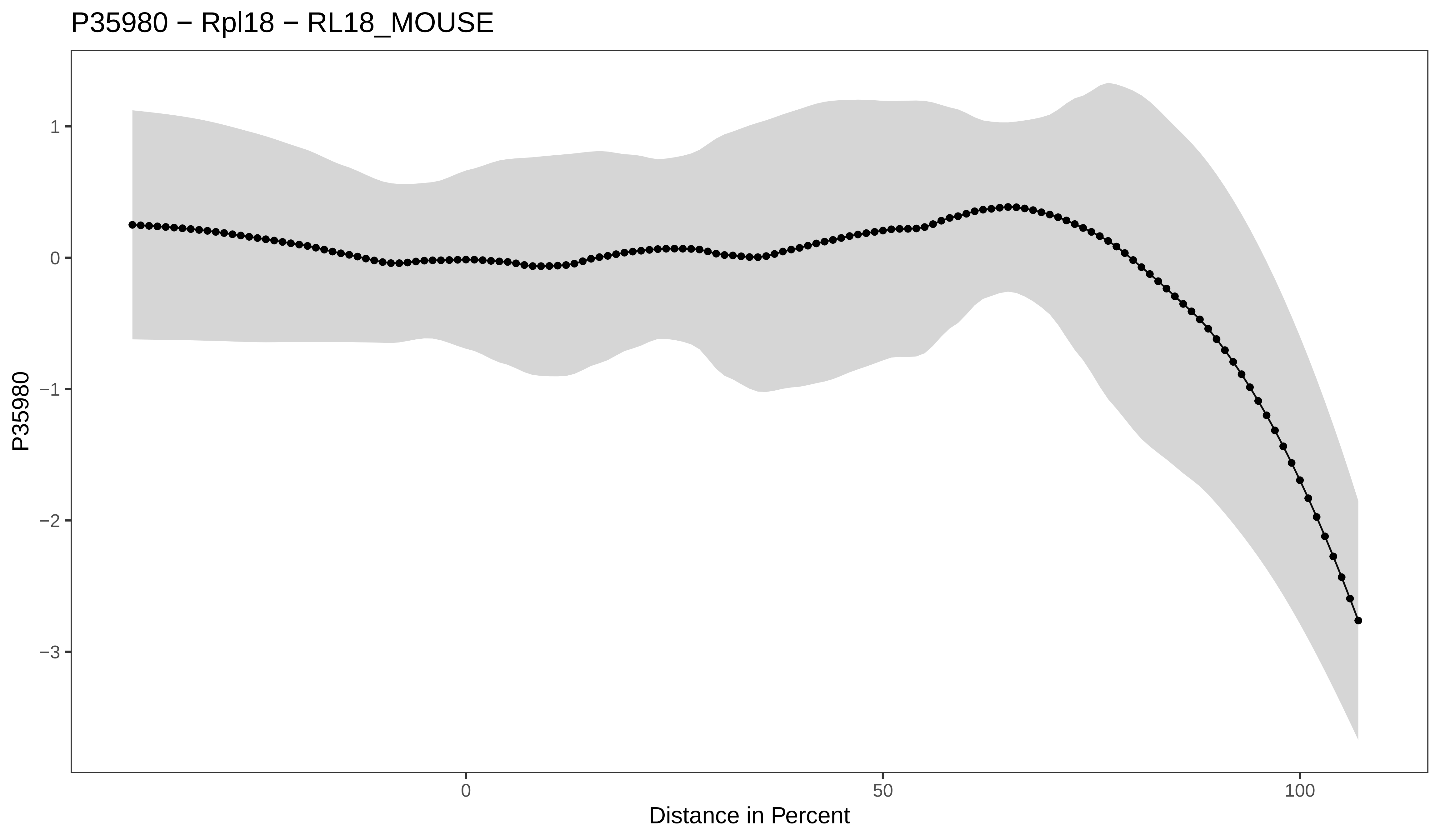
P35980 − Rpl18 − RL18_MOUSE
P35980
1
0
−1
−2
−3
0
50
100
Distance in Percent

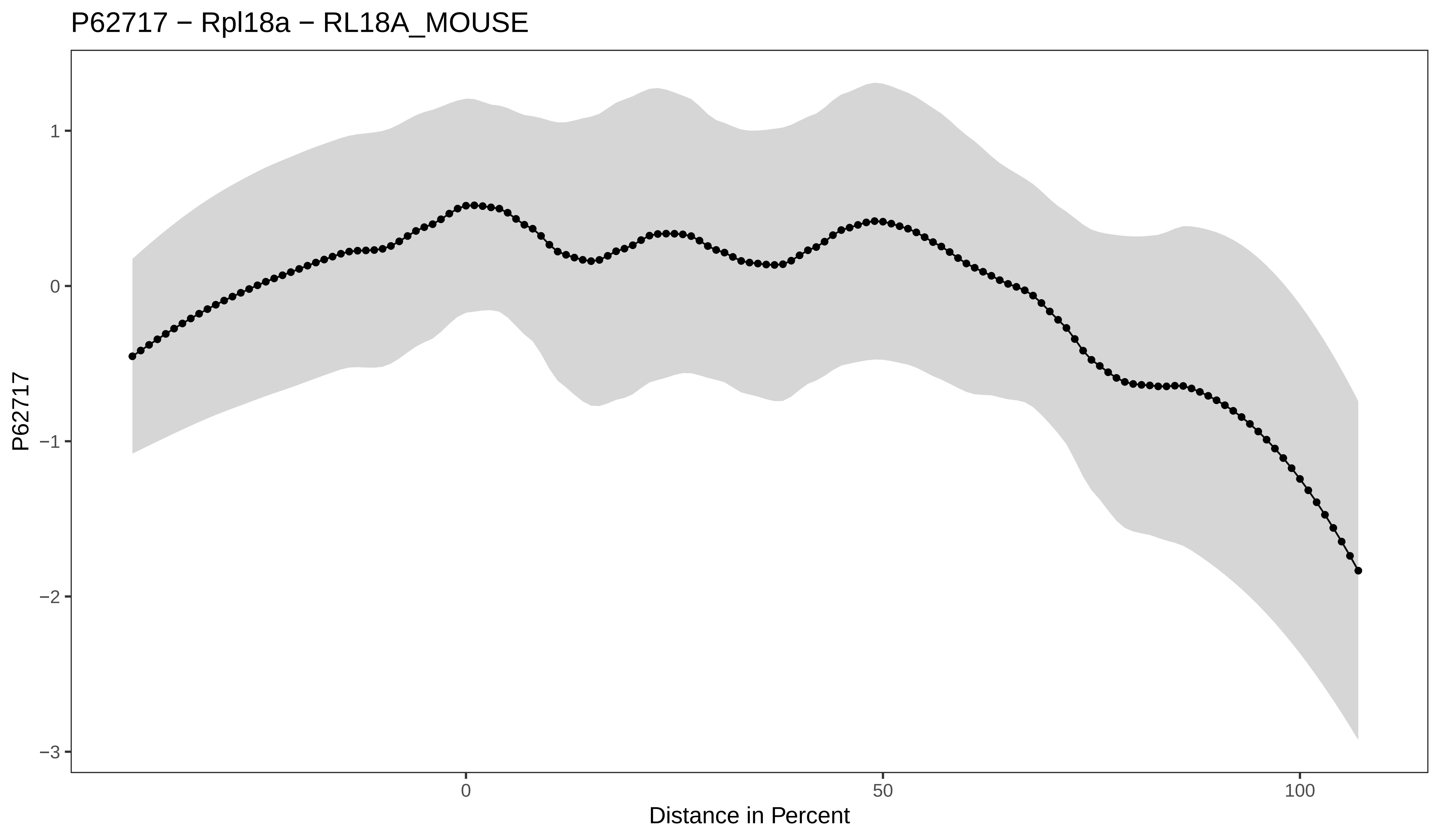

P62717 − Rpl18a − RL18A_MOUSE
P62717
Distance in Percent
1
0
−1
−2
−3
0
50
100

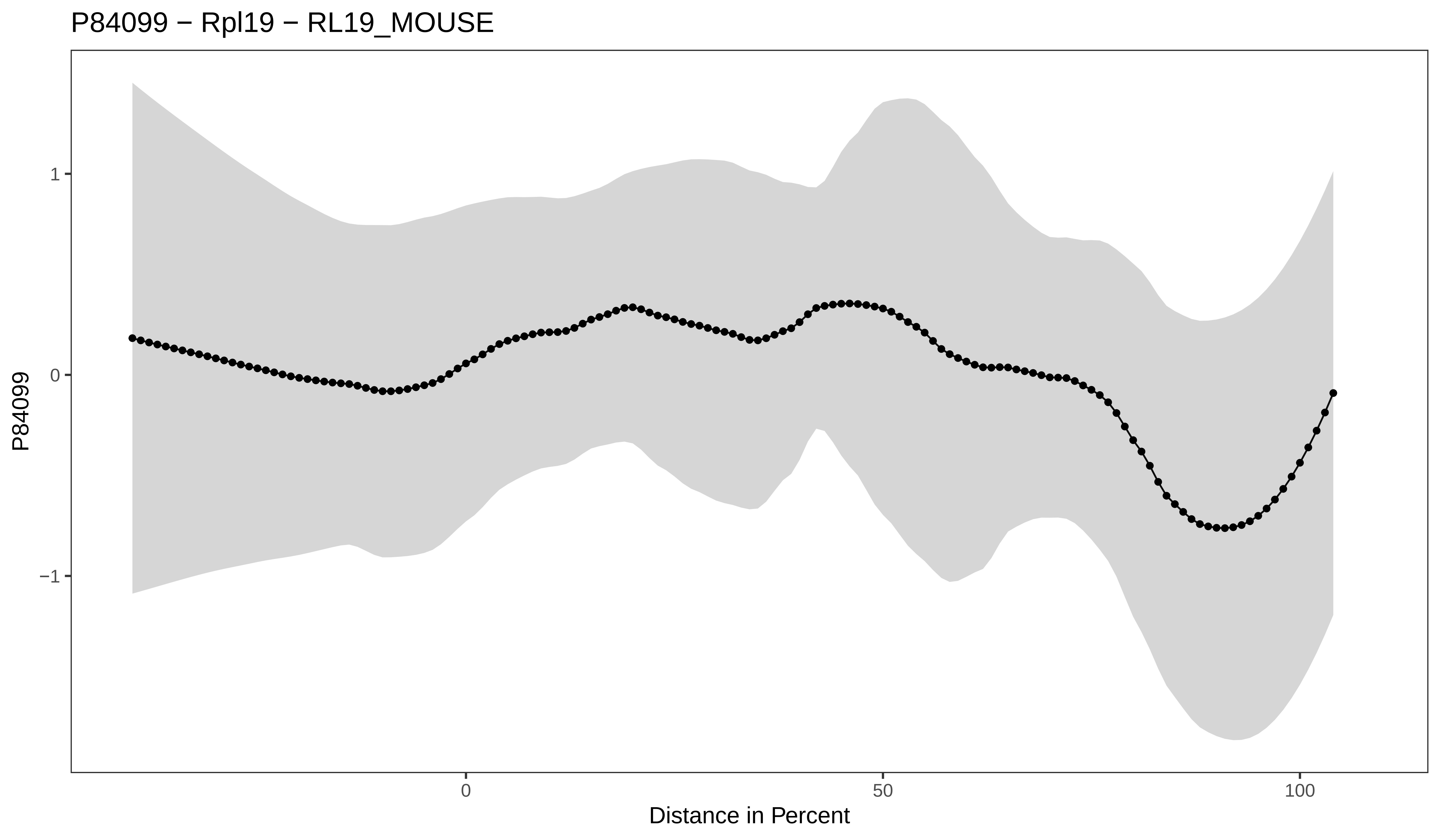

P84099 − Rpl19 − RL19_MOUSE
1
0
−1
P84099
0
50
100
Distance in Percent

O09167 − Rpl21 − RL21_MOUSE

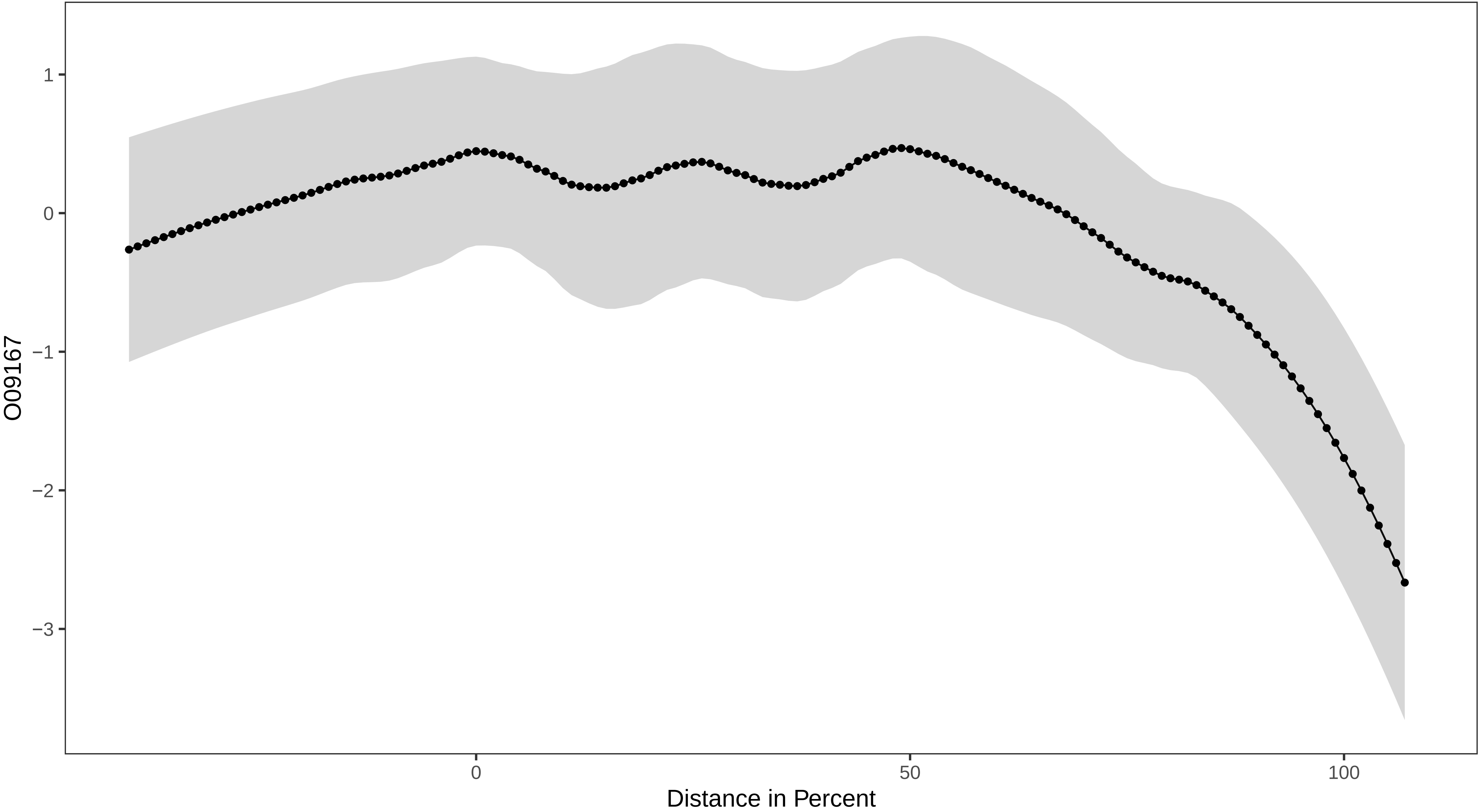

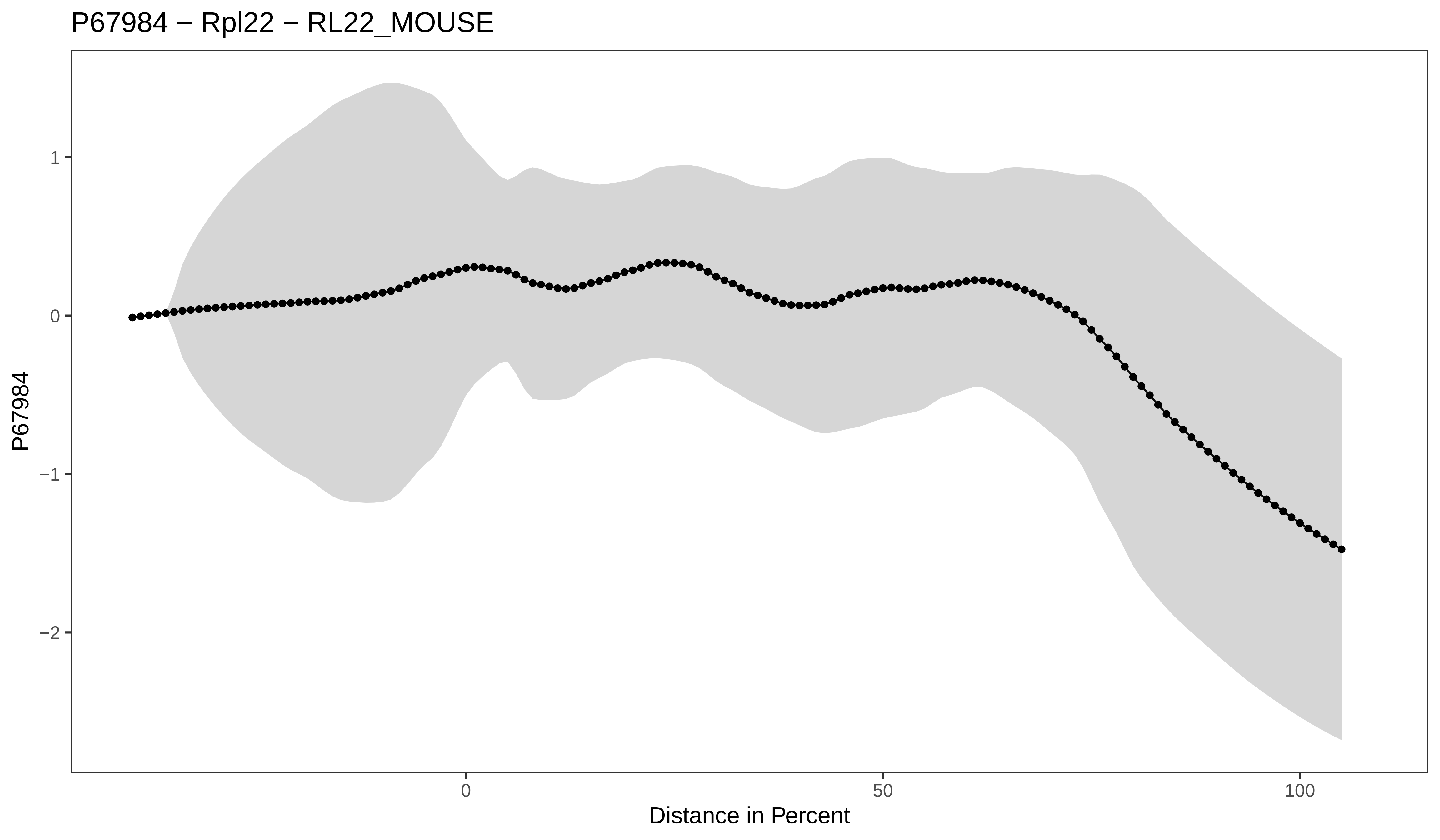
P67984 − Rpl22 − RL22_MOUSE
P67984
Distance in Percent
1
0
−1
−2
0
50
100

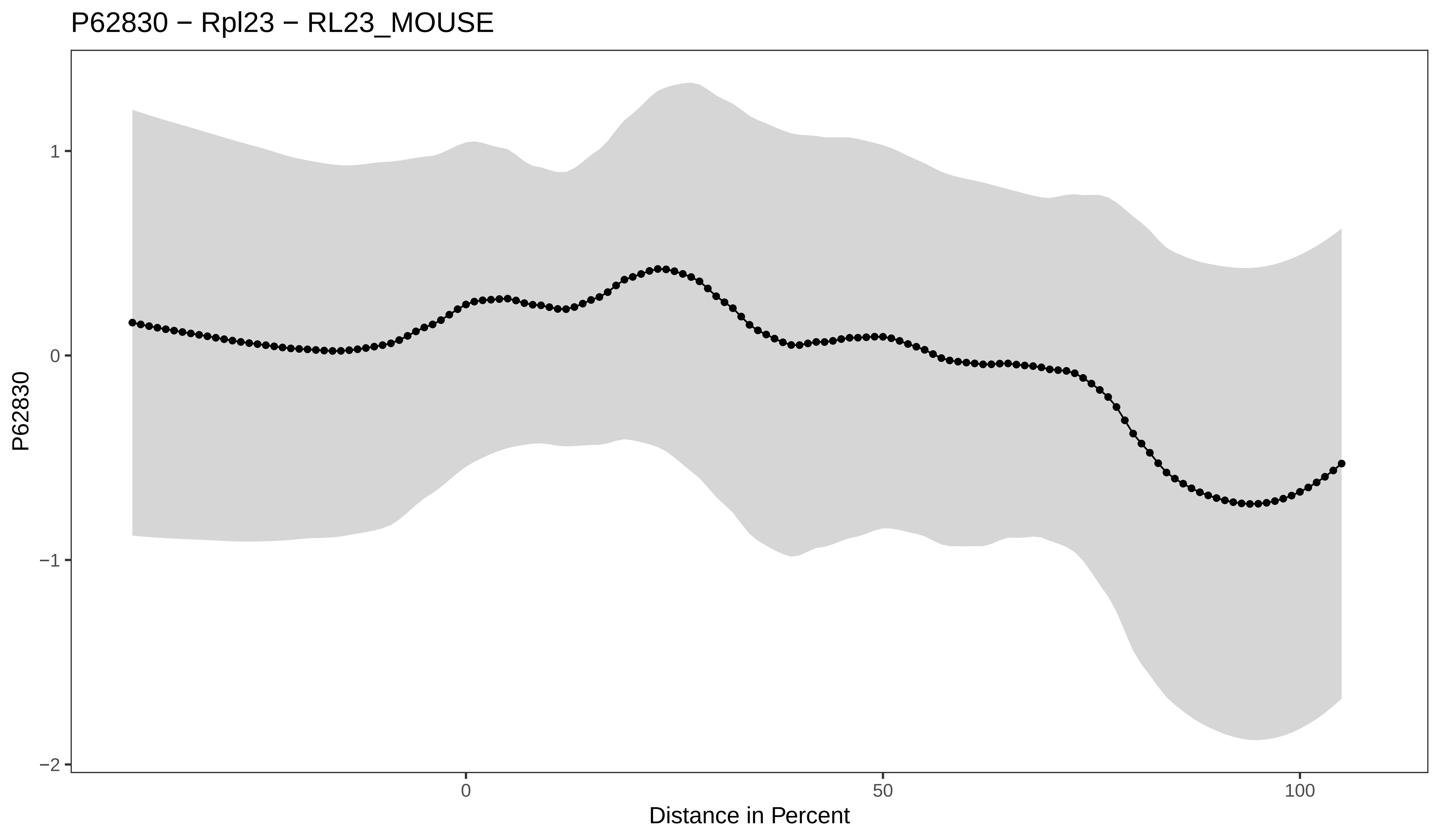

P62830 − Rpl23 − RL23_MOUSE
1
0
−1
−2
P62830
0
50
100
Distance in Percent

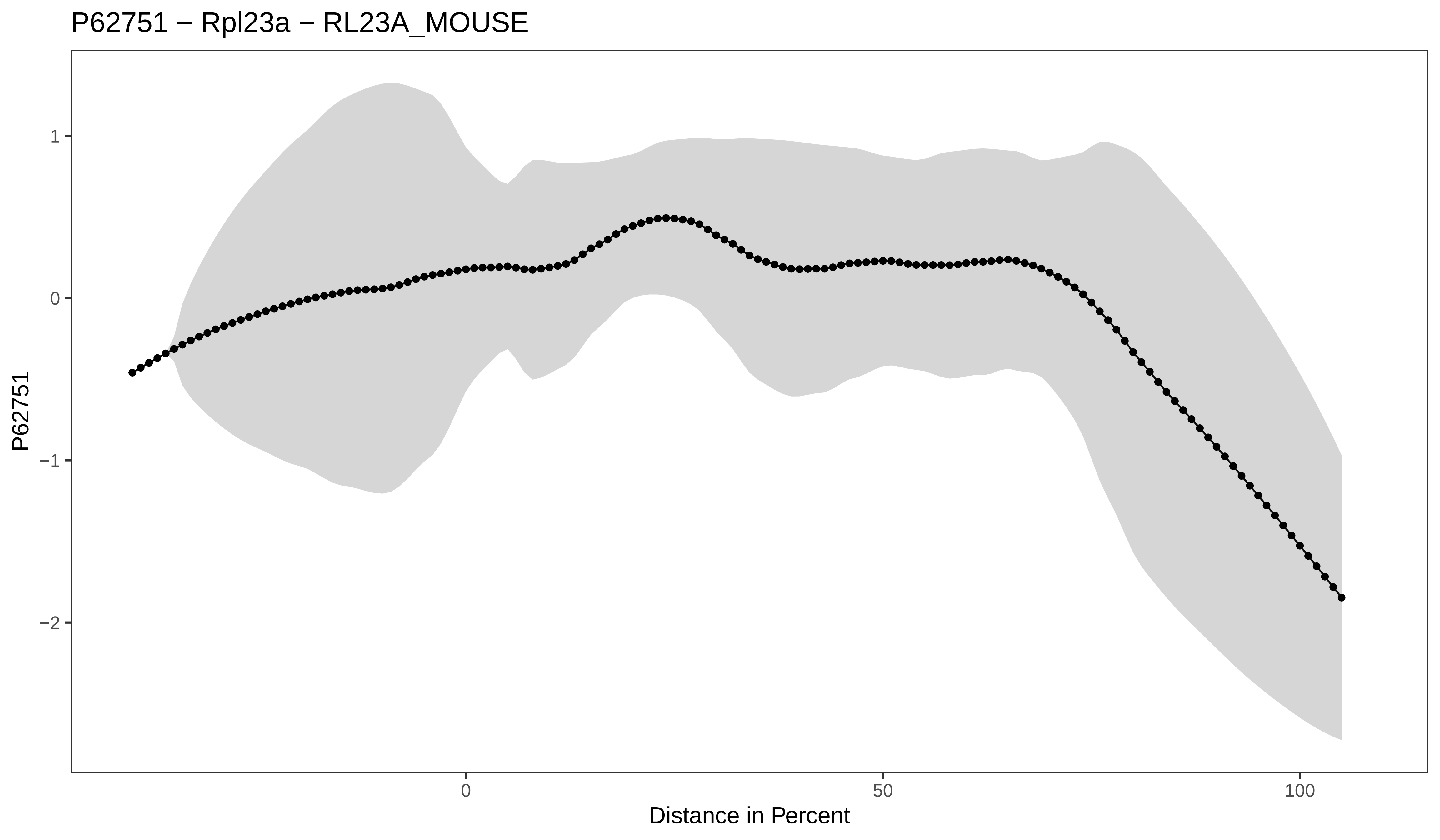

P62751 − Rpl23a − RL23A_MOUSE
P62751
Distance in Percent
1
0
−1
−2
0
50
100

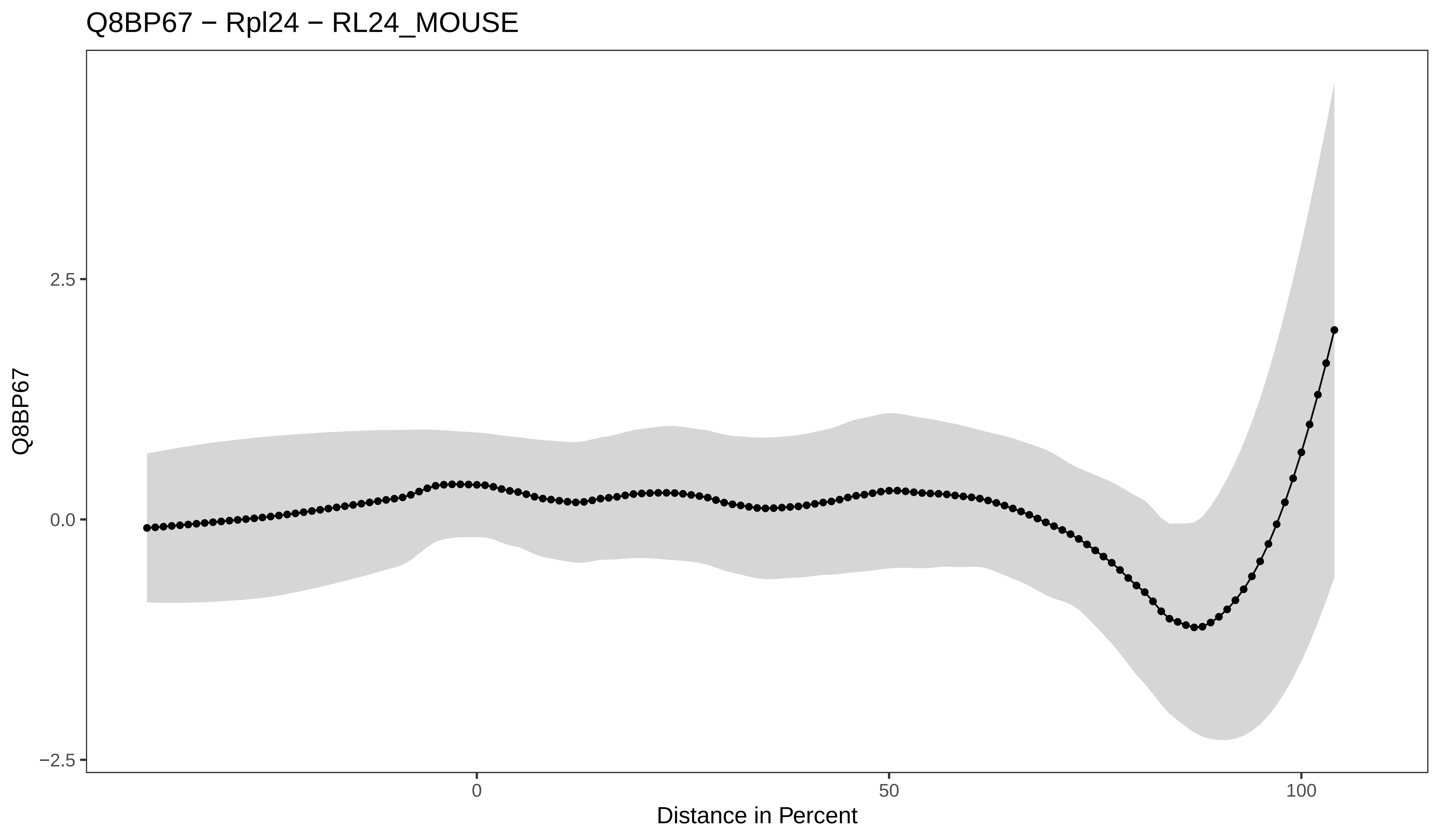
Q8BP67 − Rpl24 − RL24_MOUSE
Q8BP67
2.5
0.0
−2.5
0
50
100
Distance in Percent

P61255 − Rpl26 − RL26_MOUSE

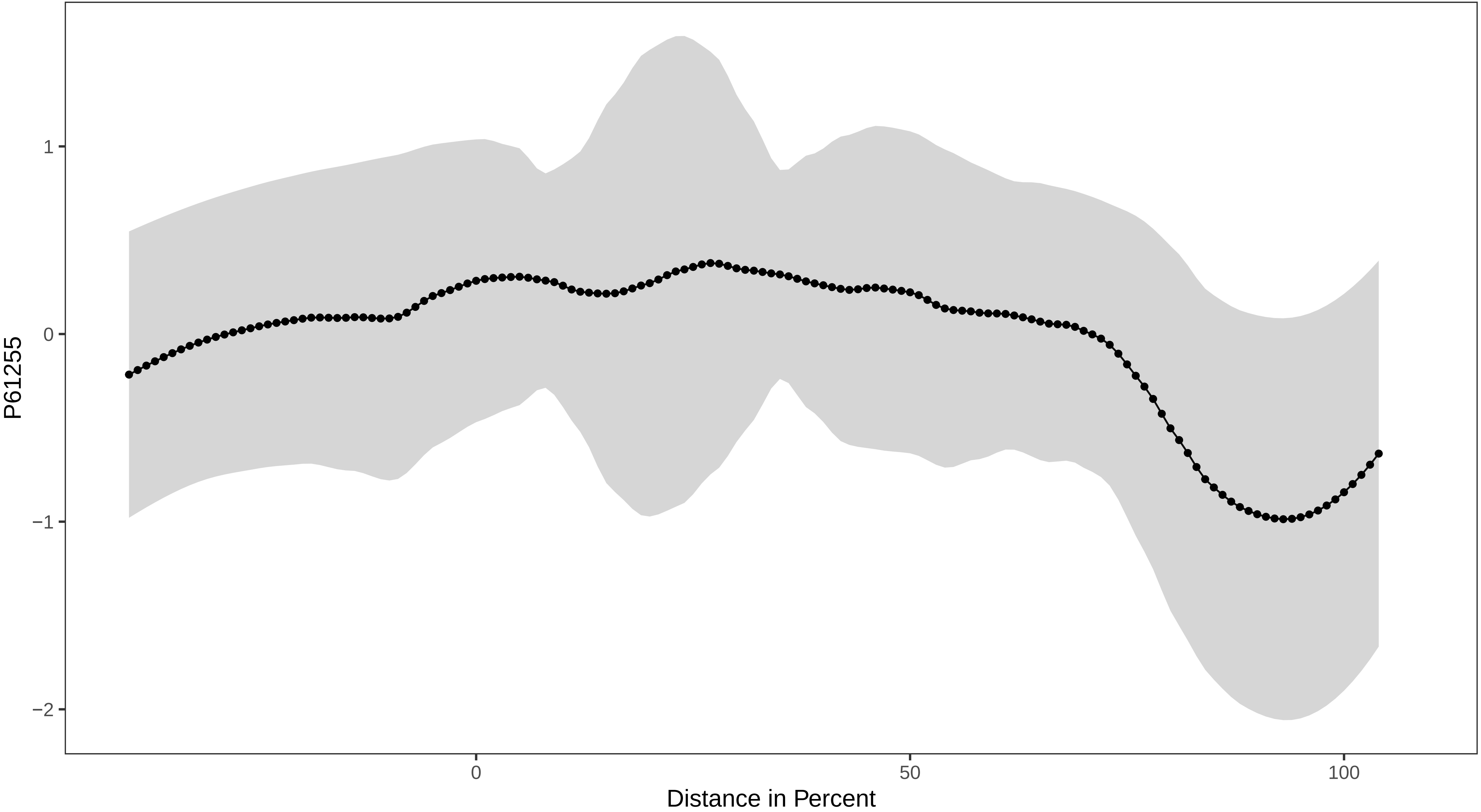

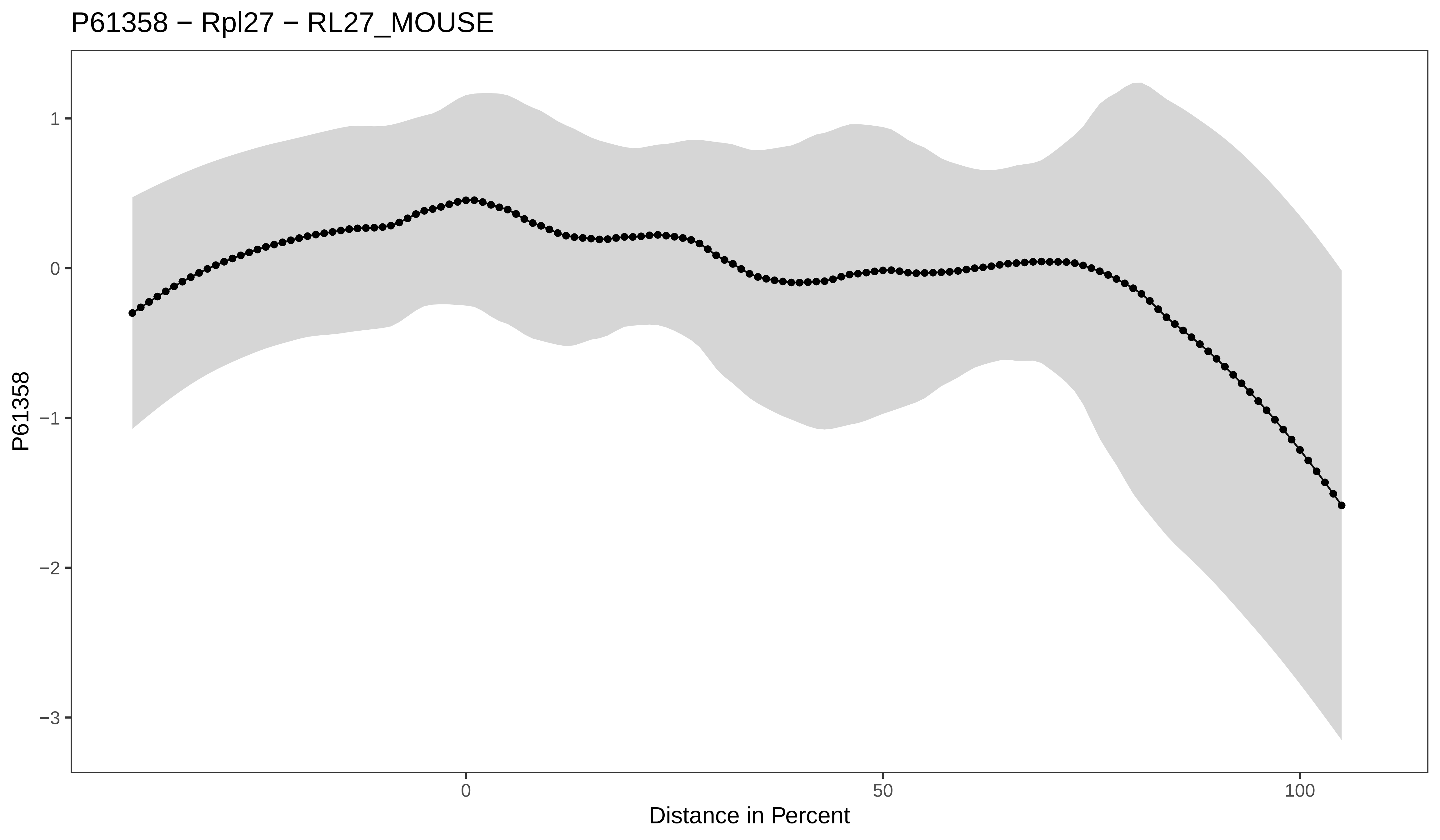

P61358 − Rpl27 − RL27_MOUSE
1
0
−1
−2
−3
P61358
0
50
100
Distance in Percent

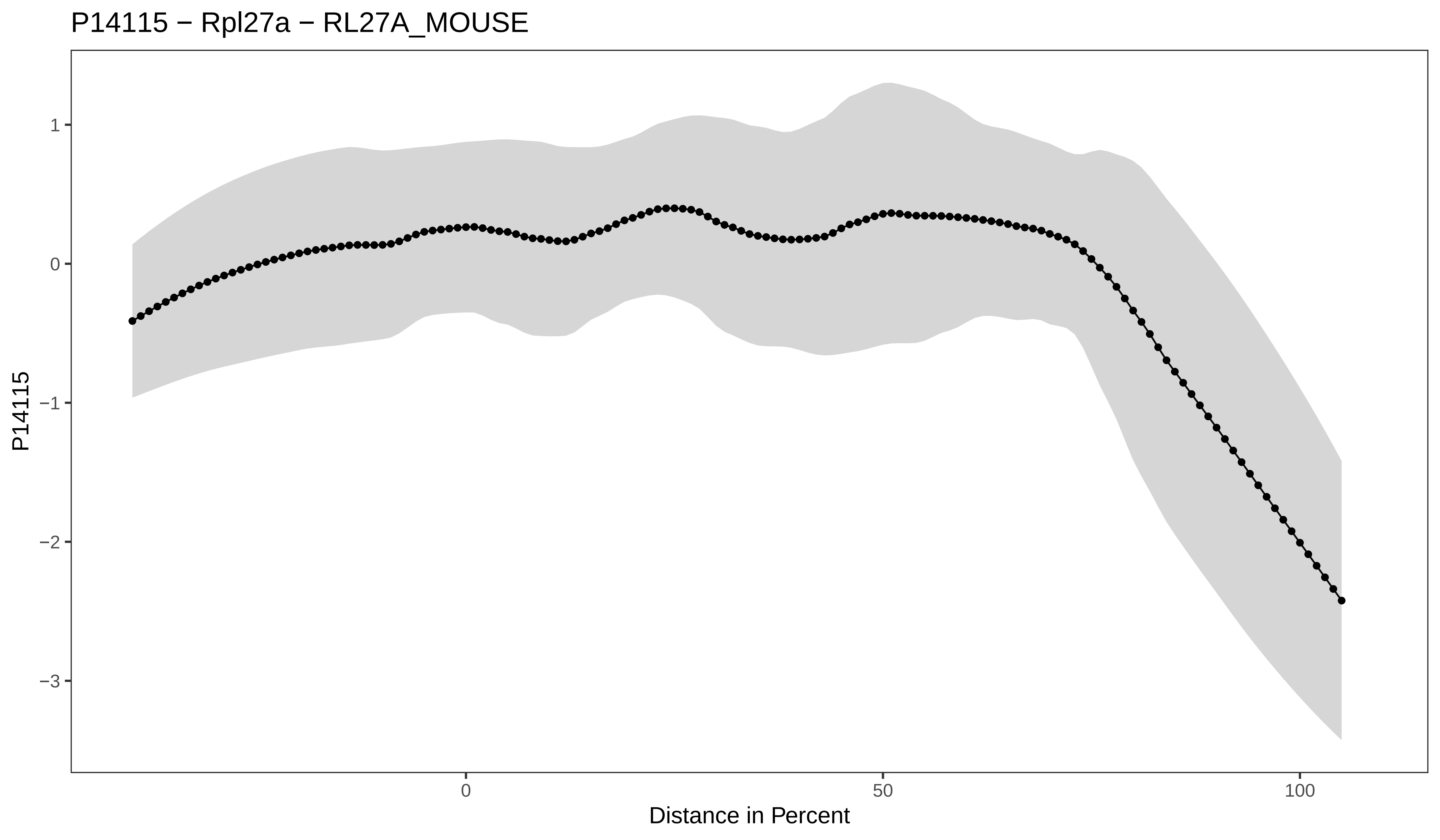

P14115 − Rpl27a − RL27A_MOUSE
P14115
Distance in Percent
1
0
−1
−2
−3
0
50
100

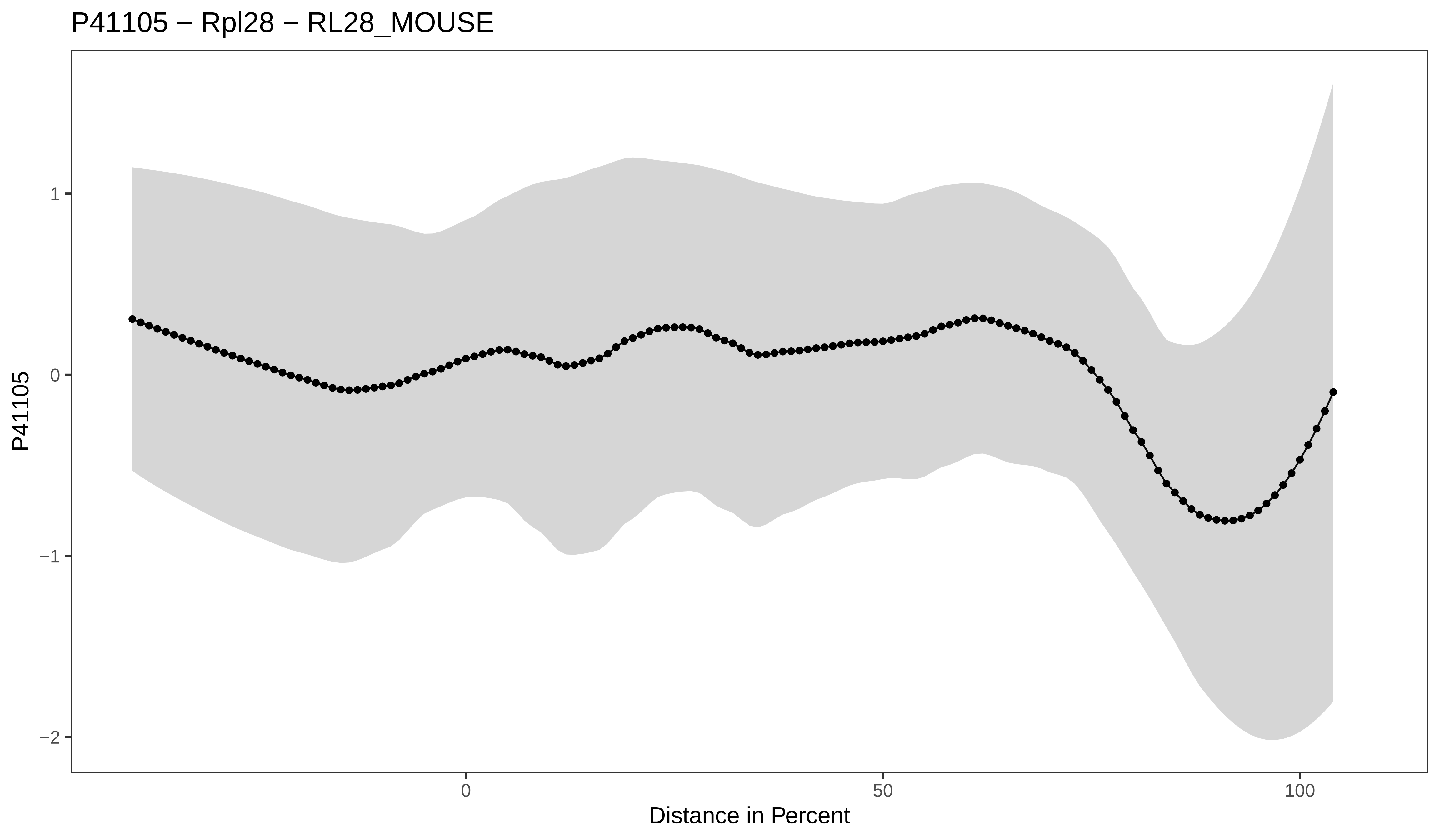
P41105 − Rpl28 − RL28_MOUSE
P41105
1
0
−1
−2
0
50
100
Distance in Percent

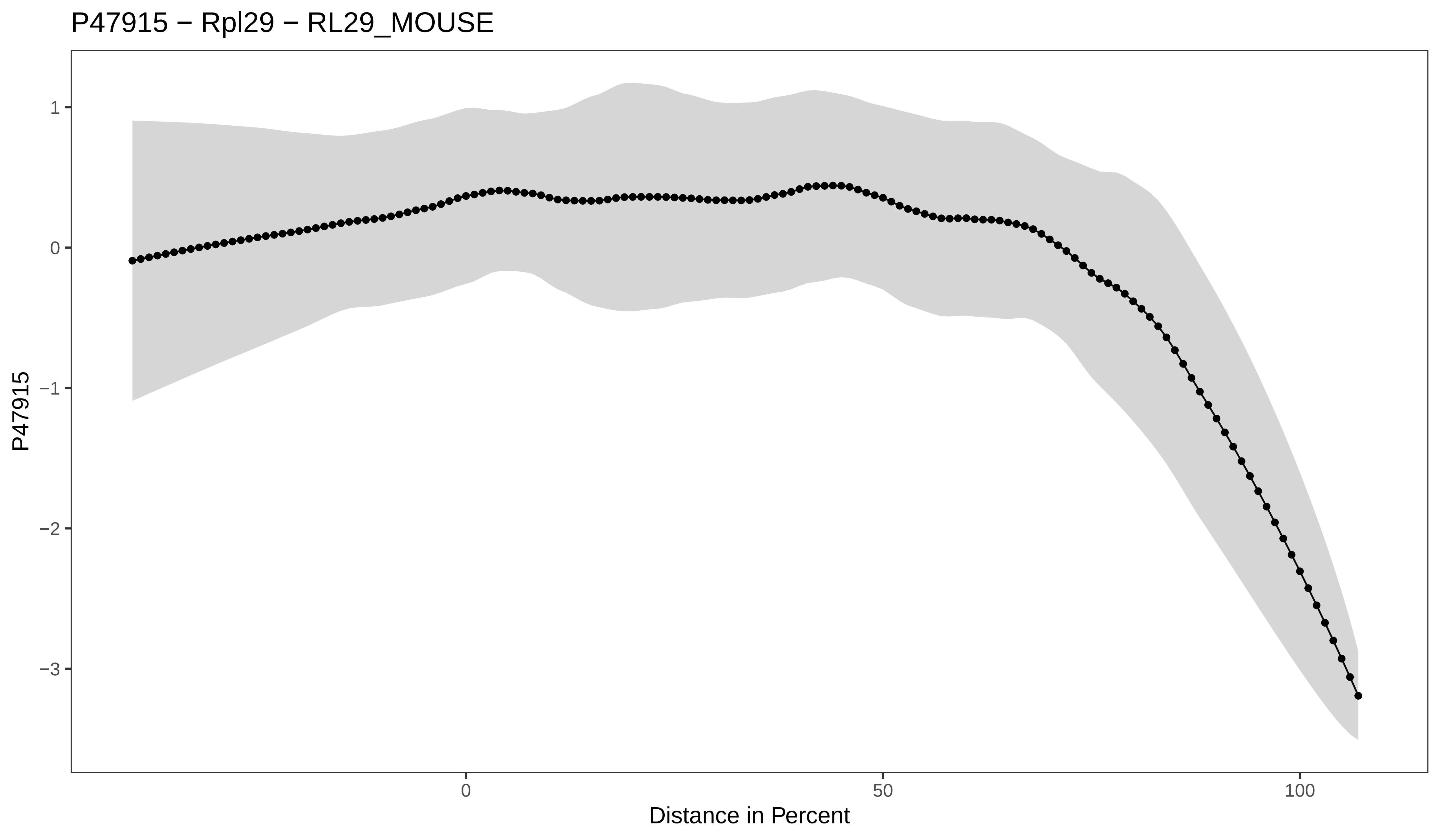

P47915 − Rpl29 − RL29_MOUSE
1
0
−1
−2
−3
P47915
0
50
100
Distance in Percent

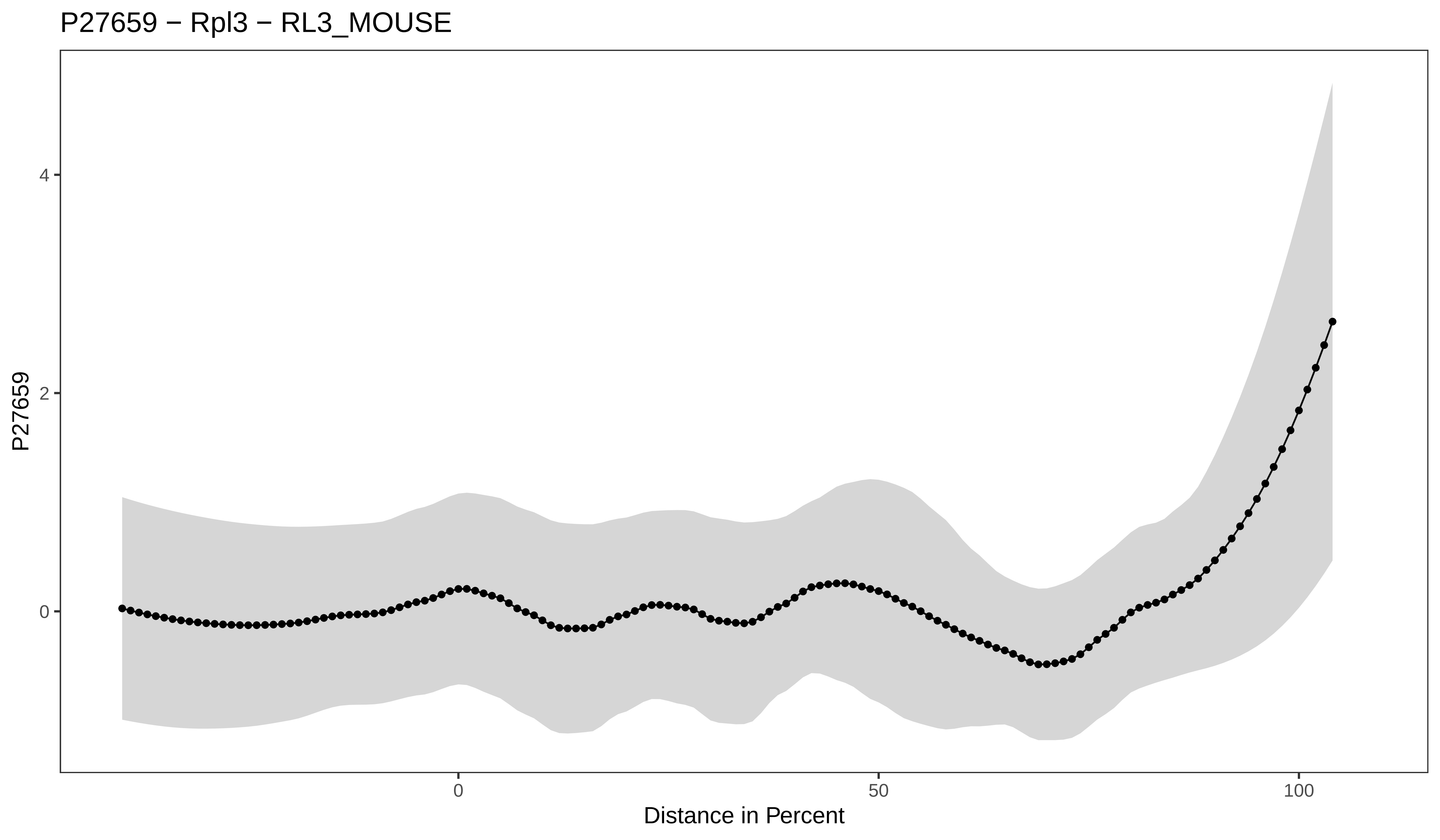
P27659 − Rpl3 − RL3_MOUSE
4
2
0
P27659
0
50
100
Distance in Percent

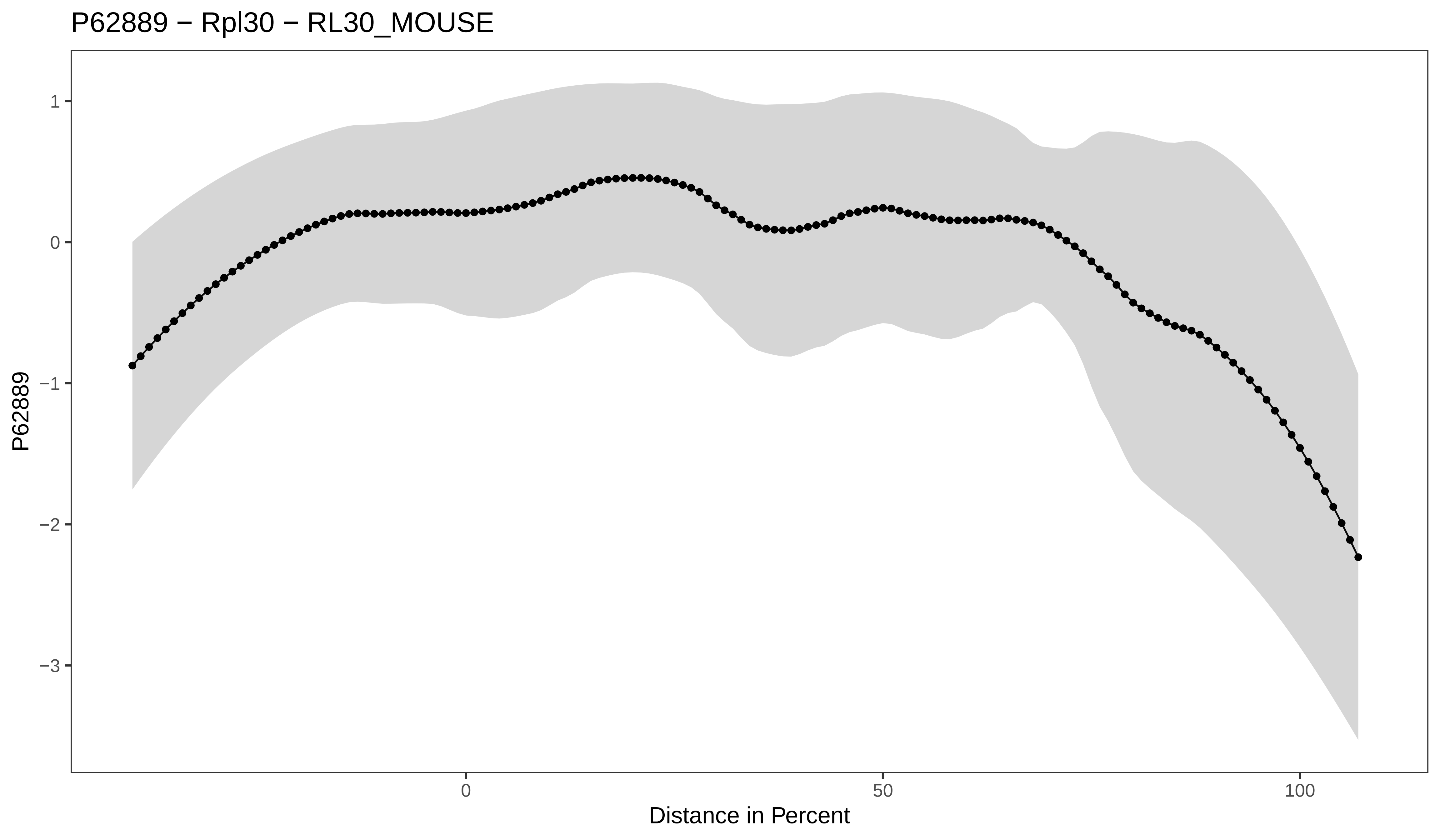
P62889 − Rpl30 − RL30_MOUSE
1
0
−1
−2
−3
P62889
0
50
100
Distance in Percent

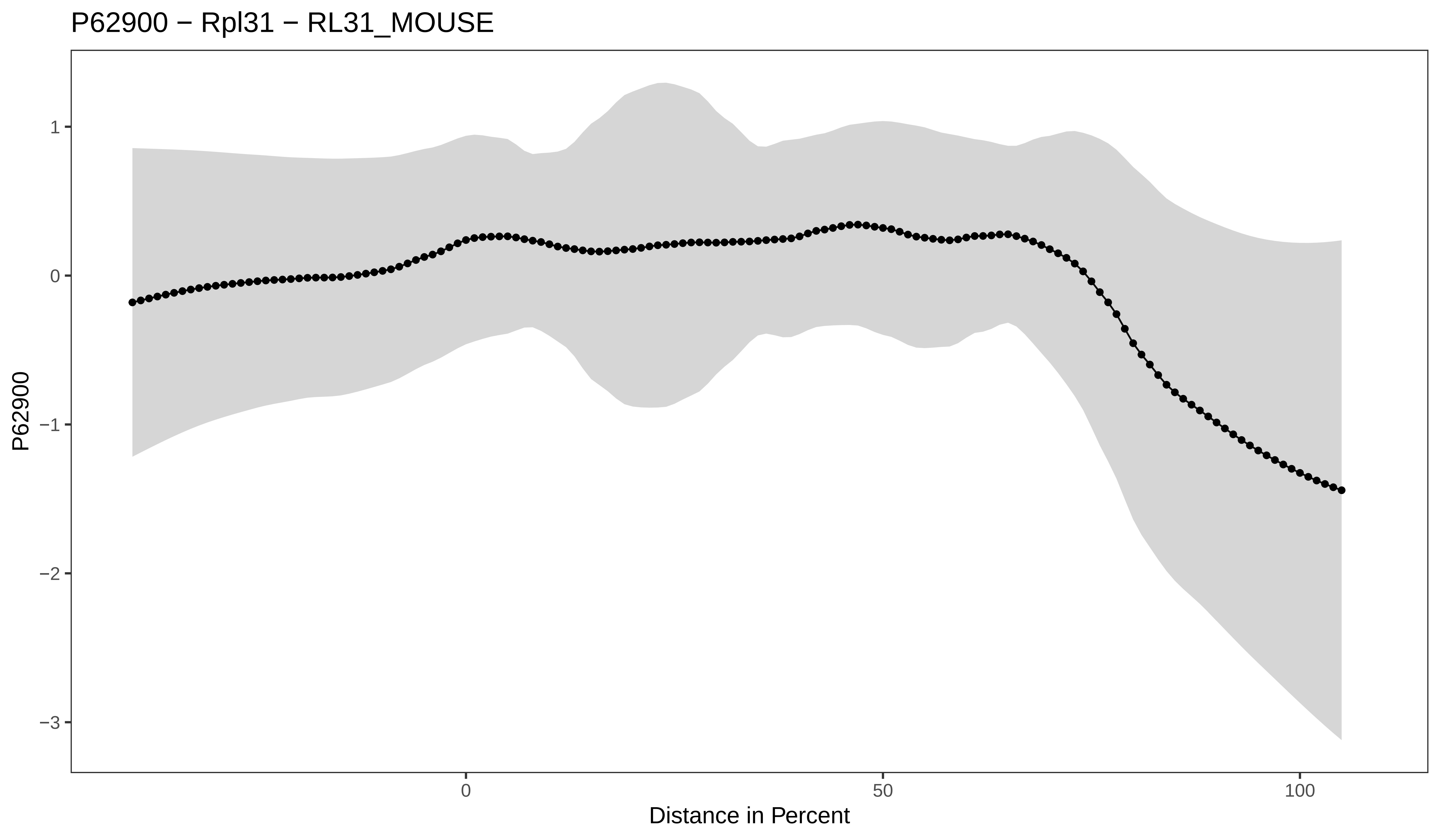

P62900 − Rpl31 − RL31_MOUSE
1
0
−1
−2
−3
P62900
0
50
100
Distance in Percent

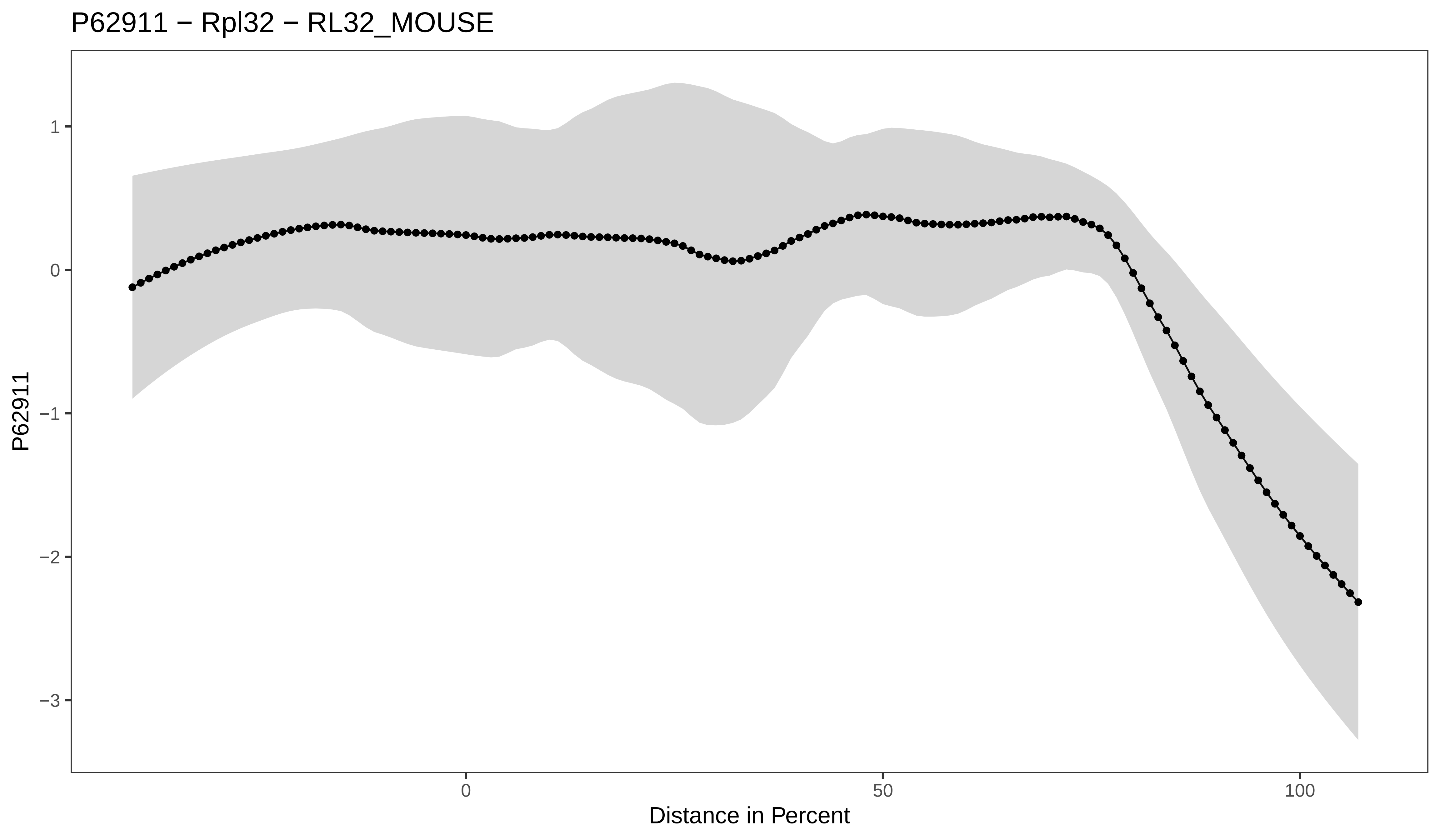
P62911 − Rpl32 − RL32_MOUSE
P62911
1
0
−1
−2
−3
0
50
100
Distance in Percent

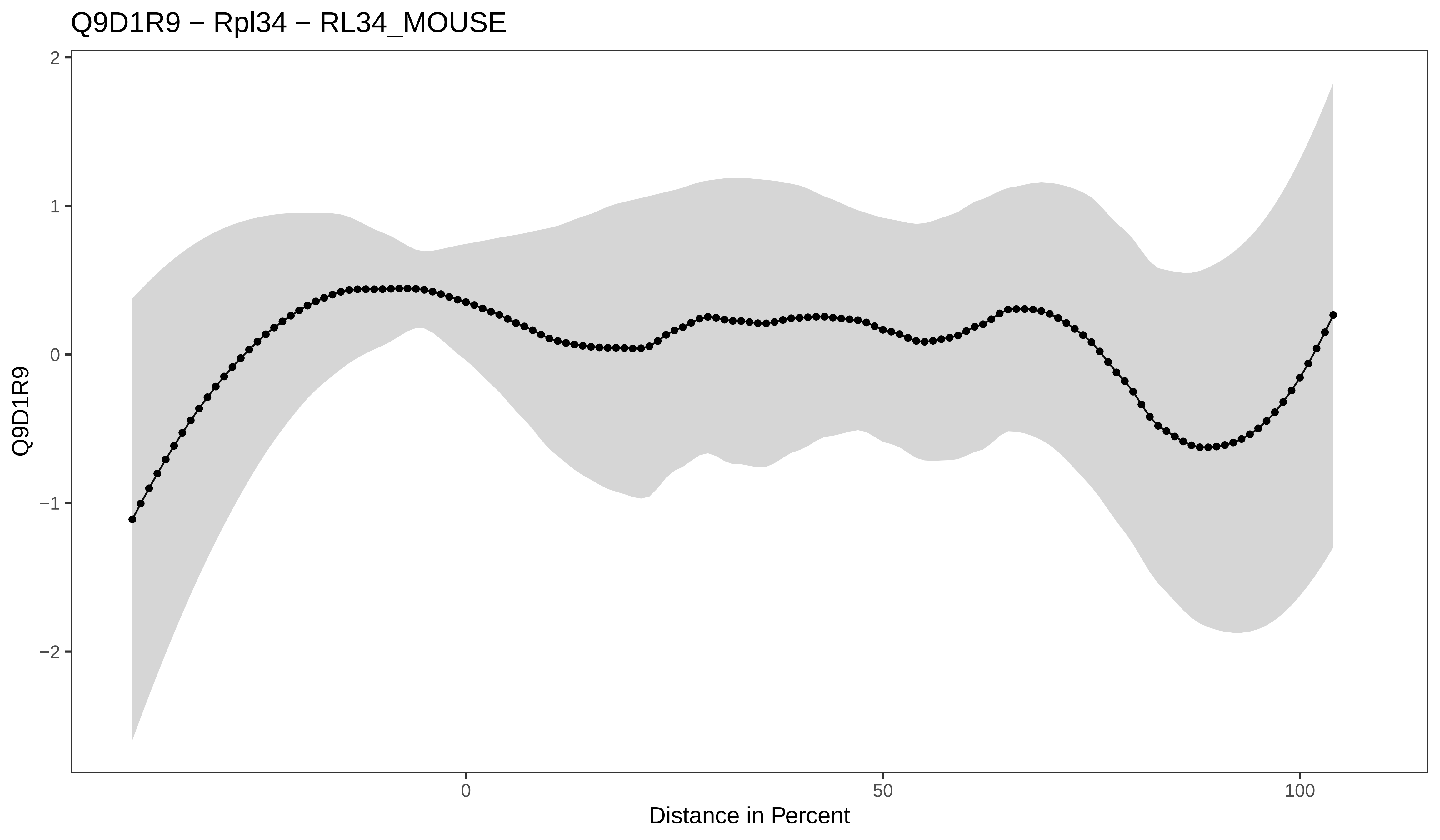
Q9D1R9 − Rpl34 − RL34_MOUSE
Q9D1R9
Distance in Percent
2
1
0
−1
−2
0
50
100

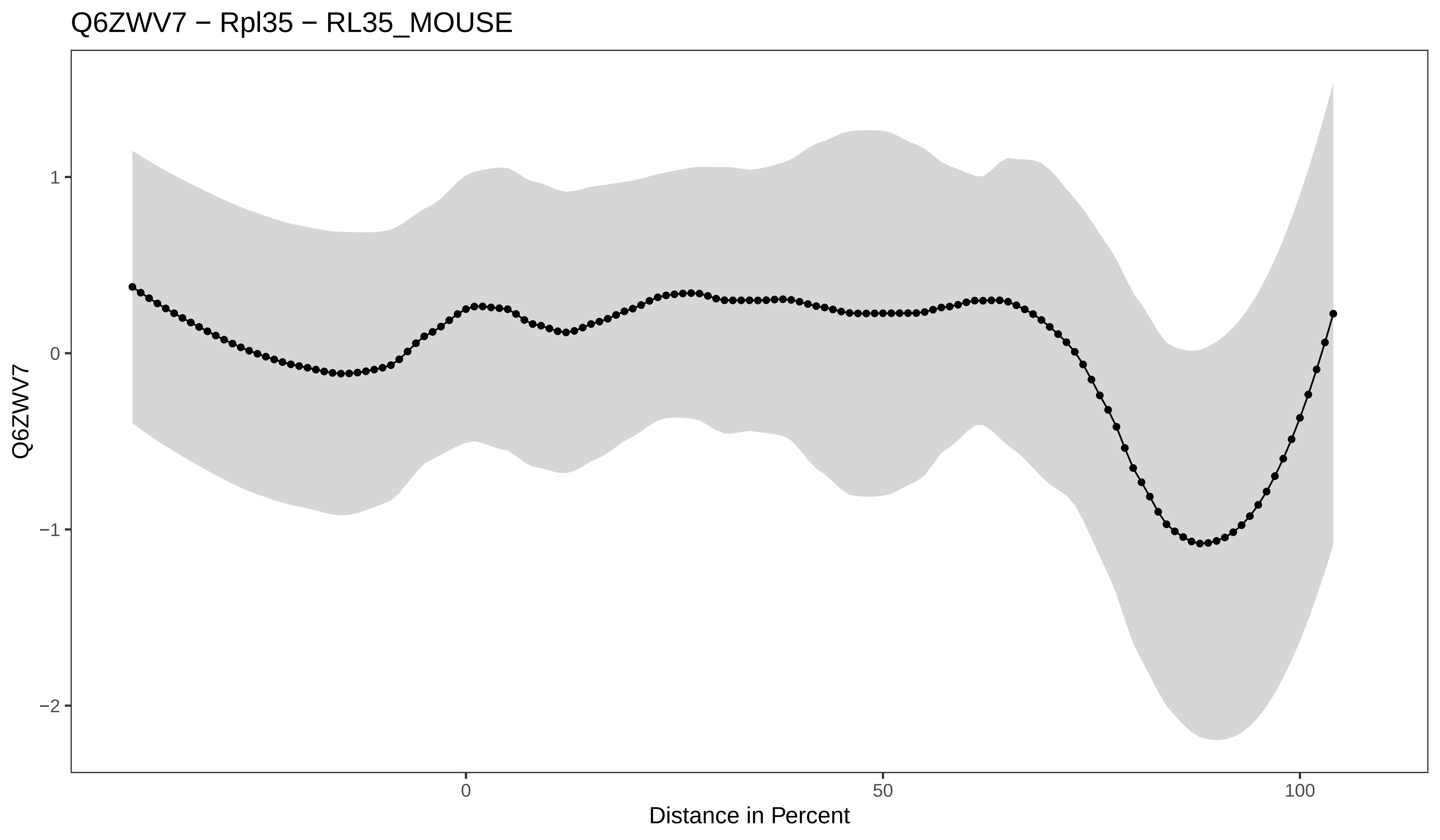

Q6ZWV7 − Rpl35 − RL35_MOUSE
Q6ZWV7
1
0
−1
−2
0
50
100
Distance in Percent

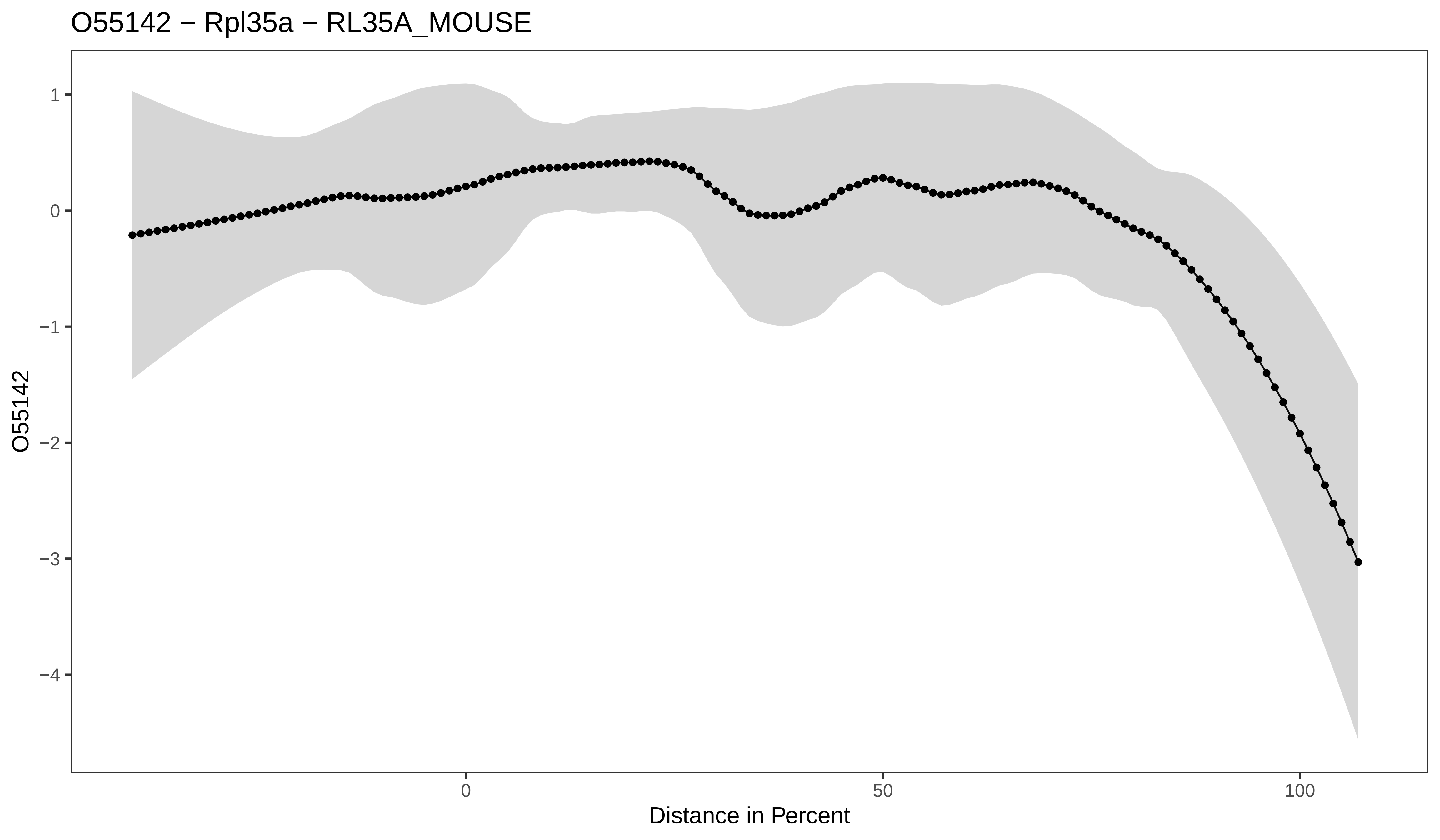

O55142 − Rpl35a − RL35A_MOUSE
O55142
1
0
−1
−2
−3
−4
0
50
100
Distance in Percent

P47964 − Rpl36 − RL36_MOUSE

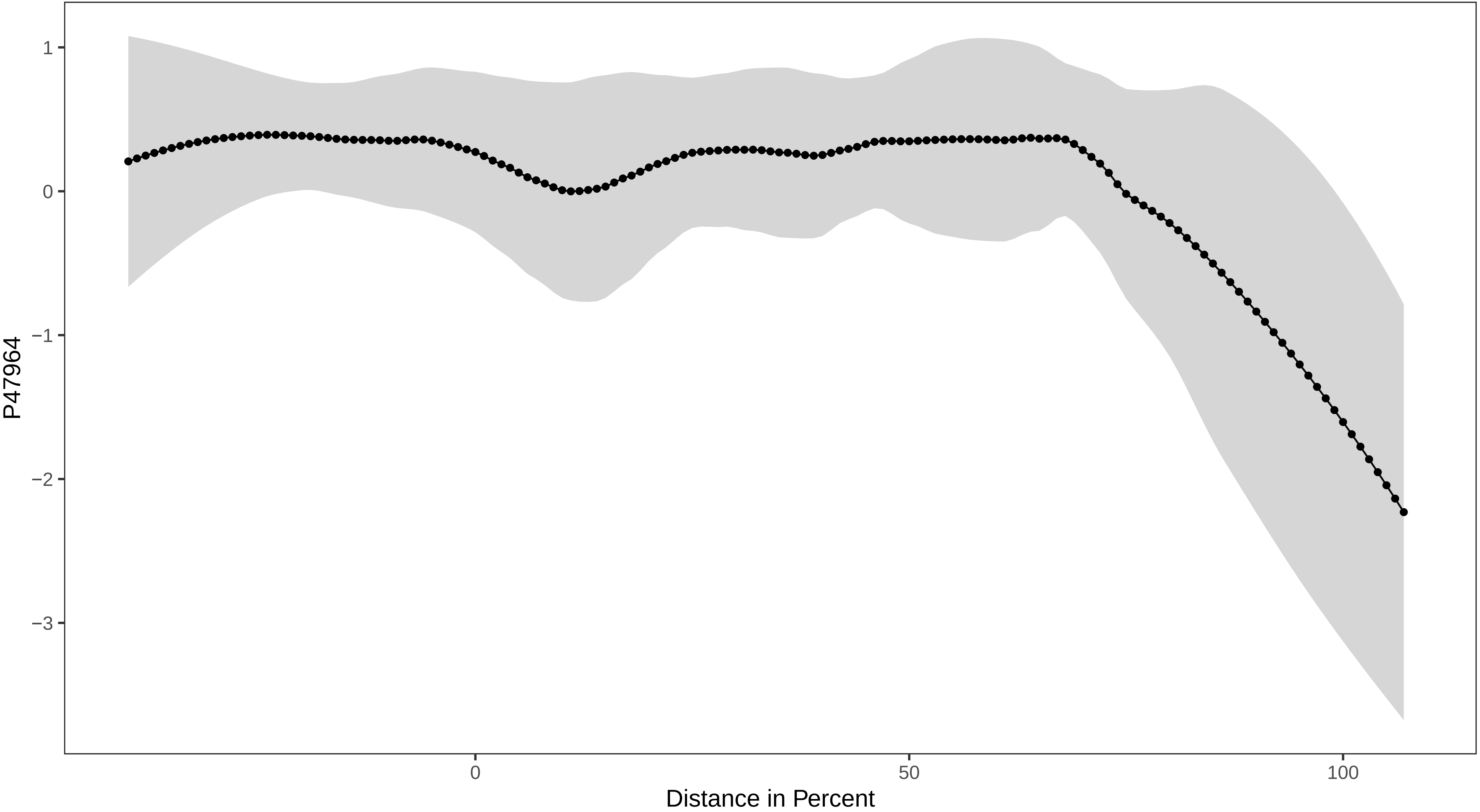

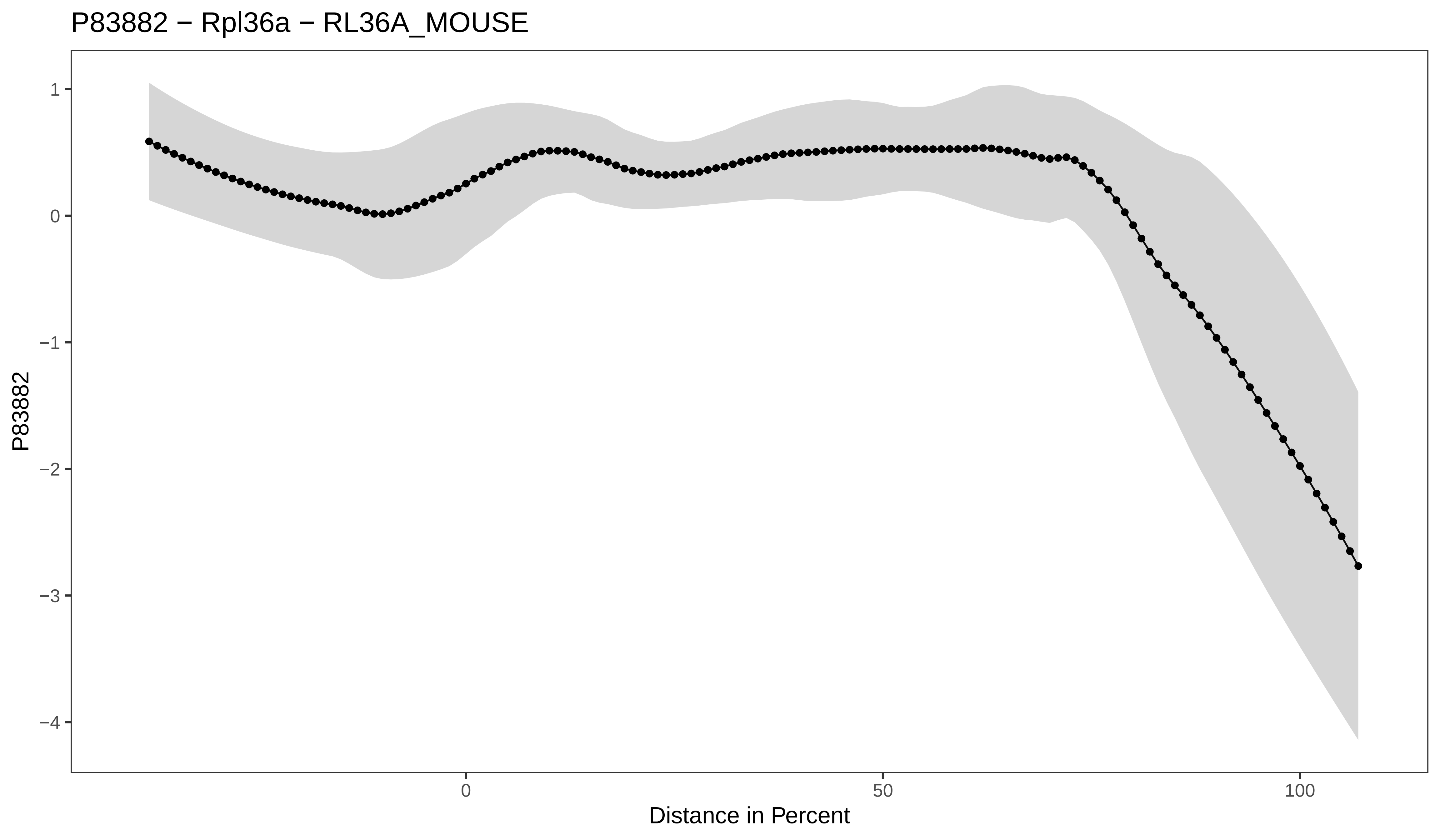

P83882 − Rpl36a − RL36A_MOUSE
1
0
−1
−2
−3
−4
P83882
0
50
100
Distance in Percent

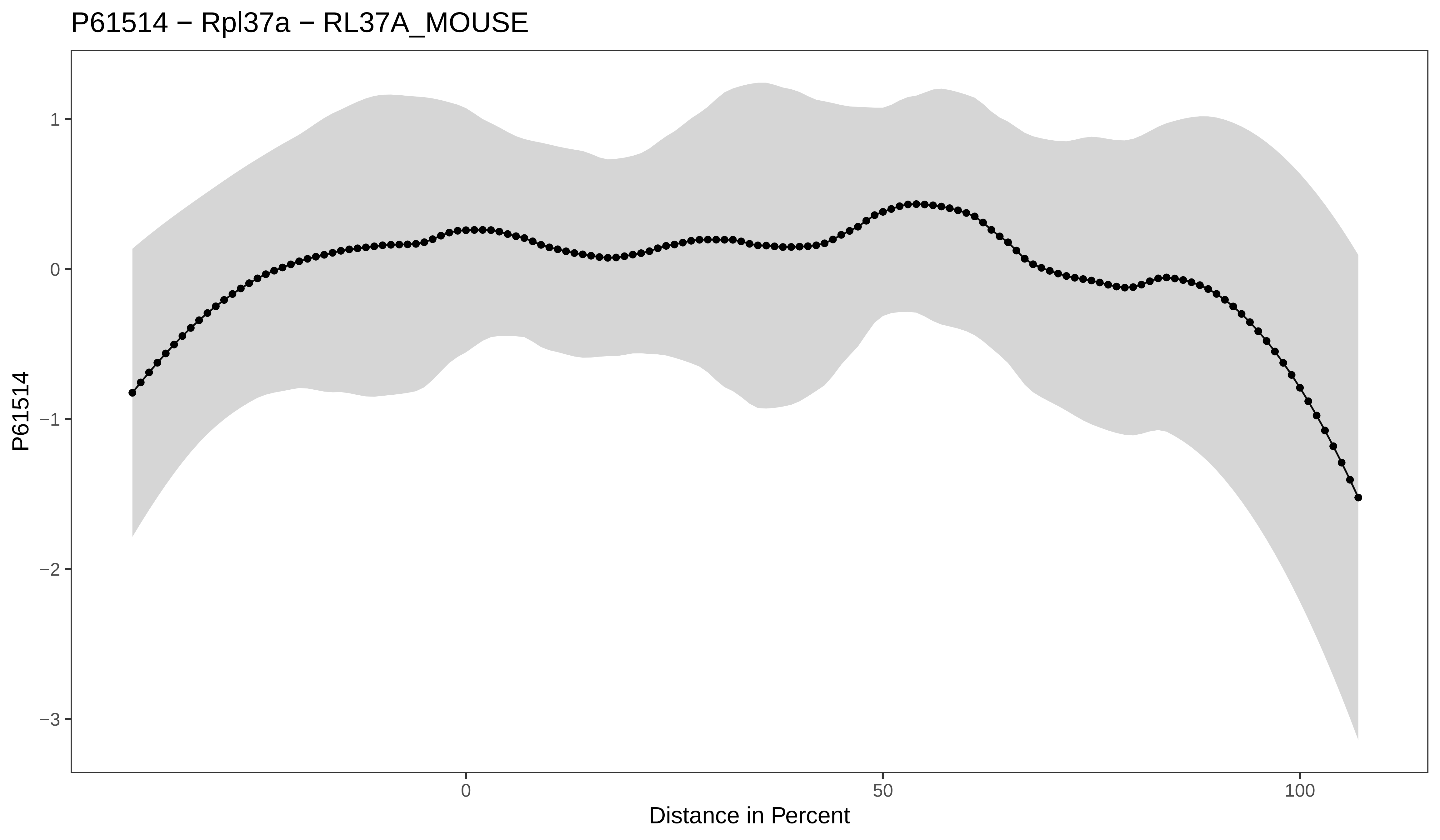
P61514 − Rpl37a − RL37A_MOUSE
P61514
Distance in Percent
1
0
−1
−2
−3
0
50
100

Q9JJI8 − Rpl38 − RL38_MOUSE

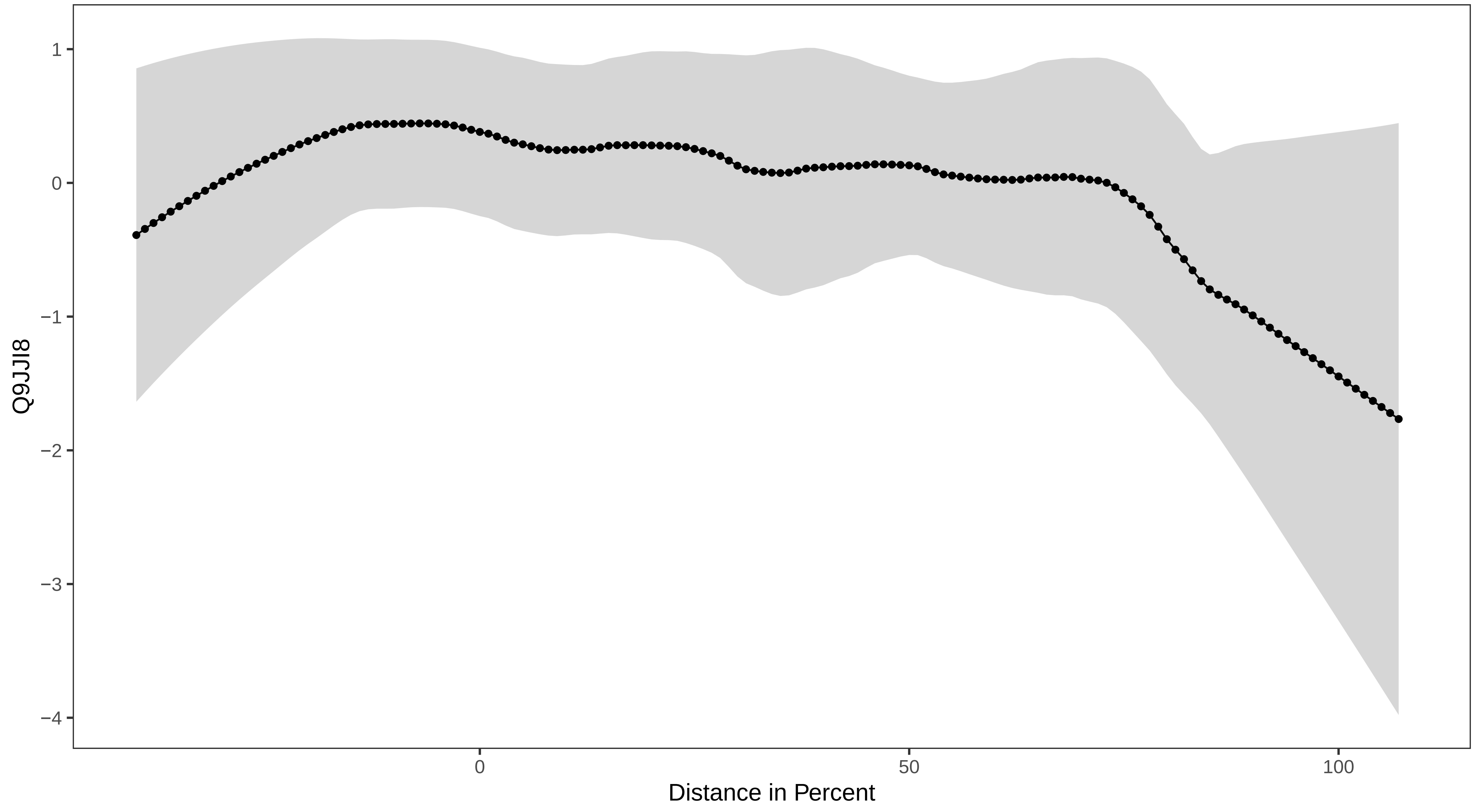

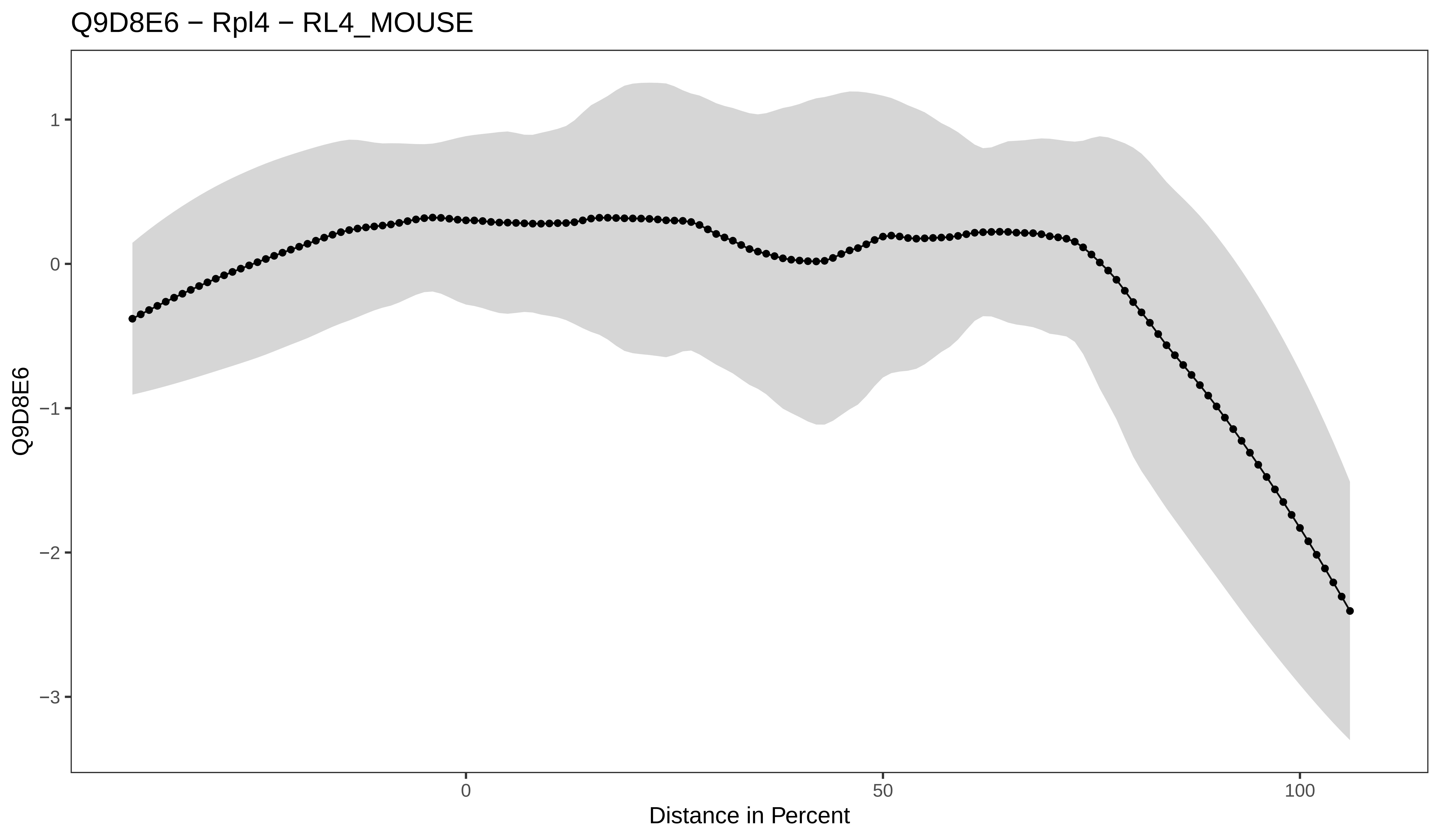

Q9D8E6 − Rpl4 − RL4_MOUSE
Q9D8E6
1
0
−1
−2
−3
0
50
100
Distance in Percent

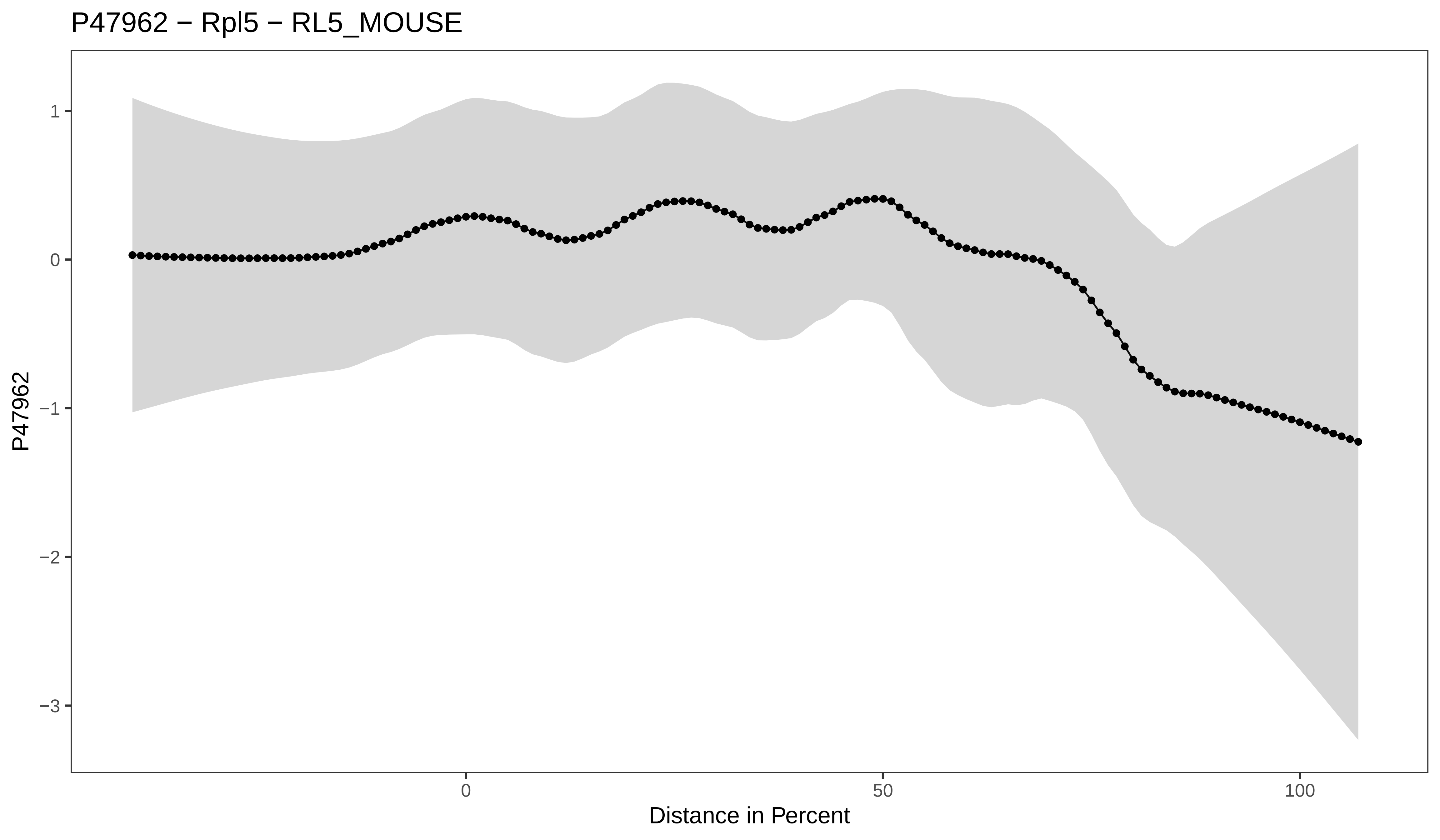

P47962 − Rpl5 − RL5_MOUSE
P47962
1
0
−1
−2
−3
0
50
100
Distance in Percent

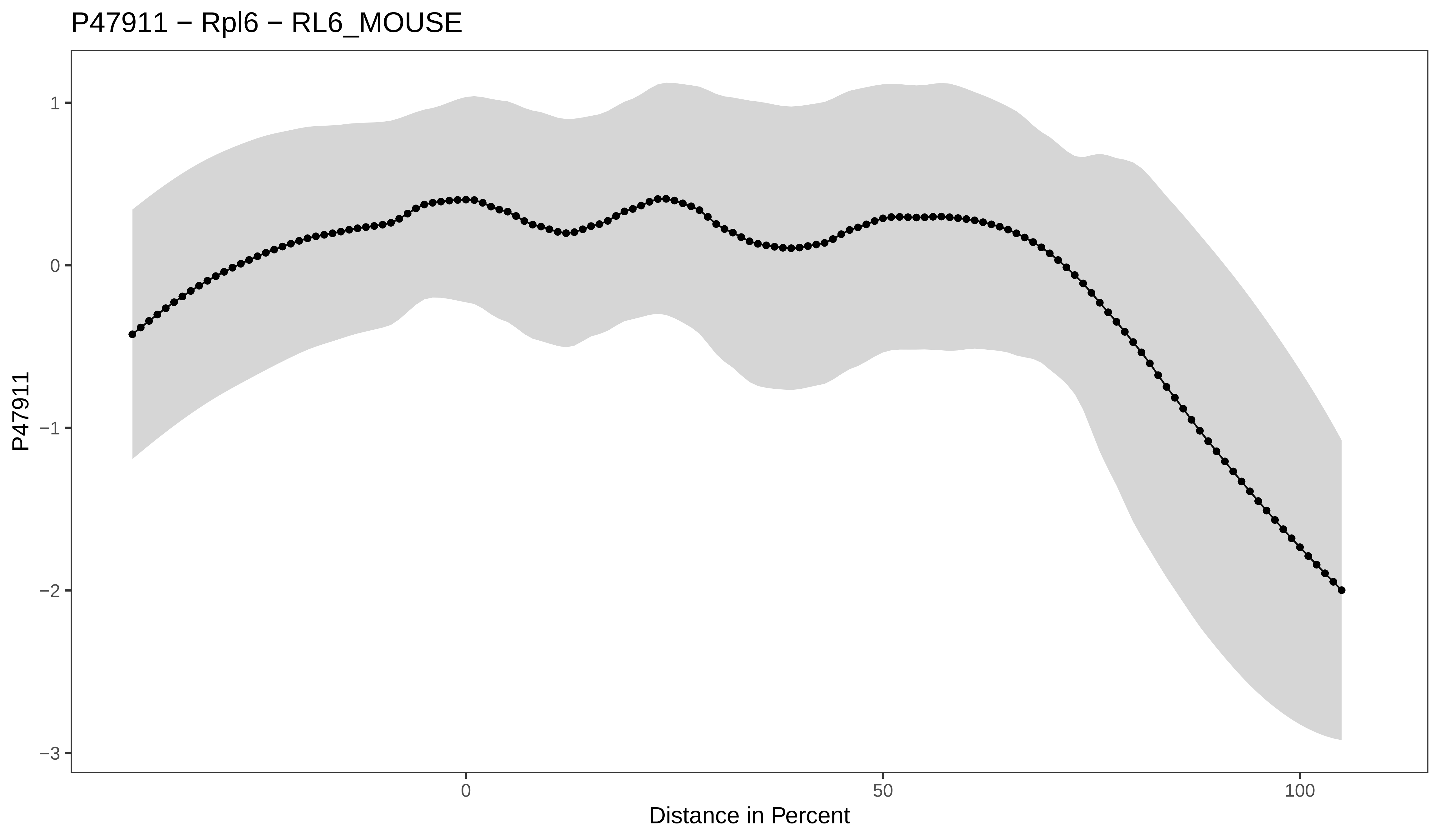

P47911 − Rpl6 − RL6_MOUSE
1
0
−1
−2
−3
P47911
0
50
100
Distance in Percent

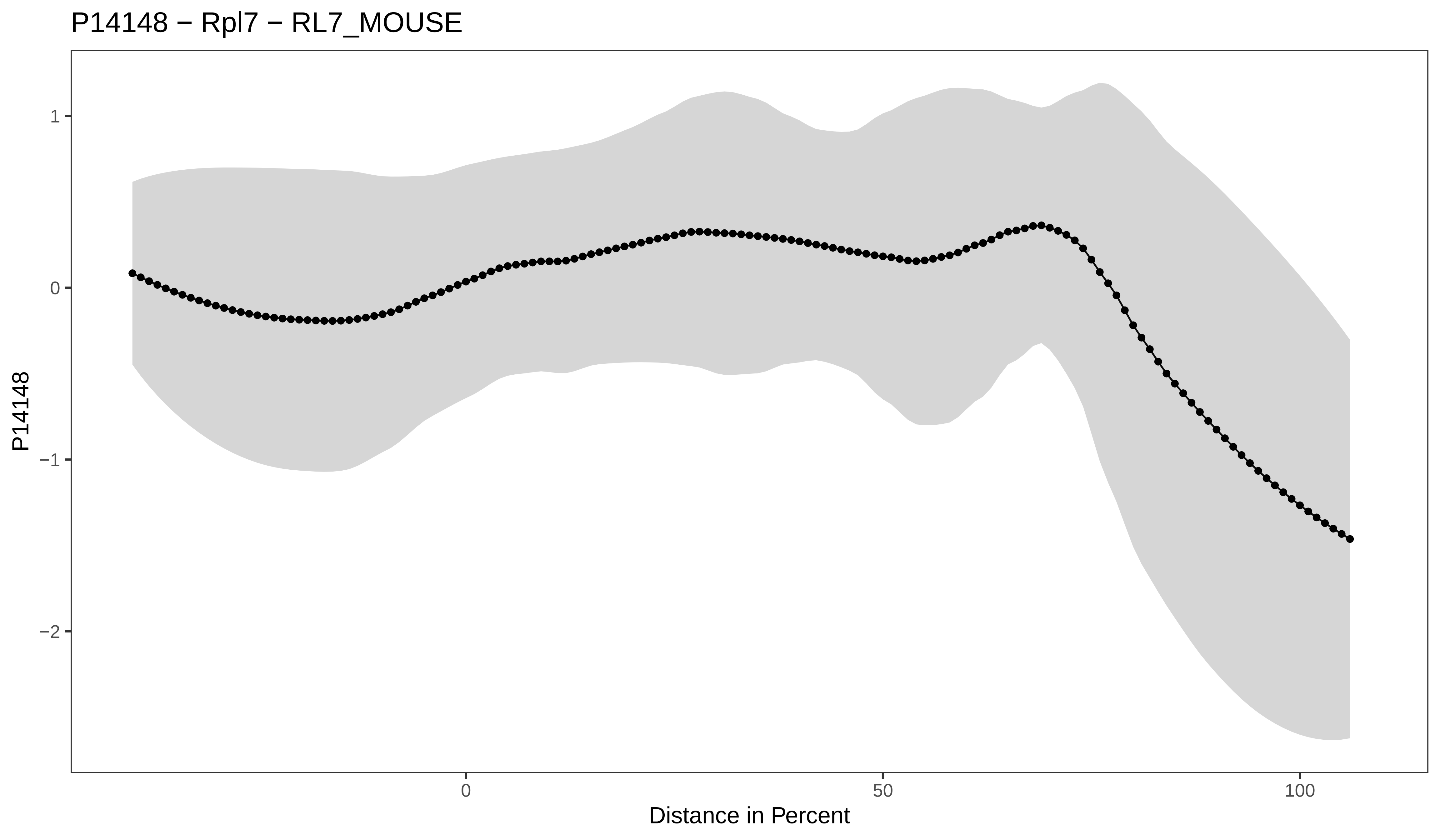
P14148 − Rpl7 − RL7_MOUSE
1
0
−1
−2
P14148
0
50
100
Distance in Percent

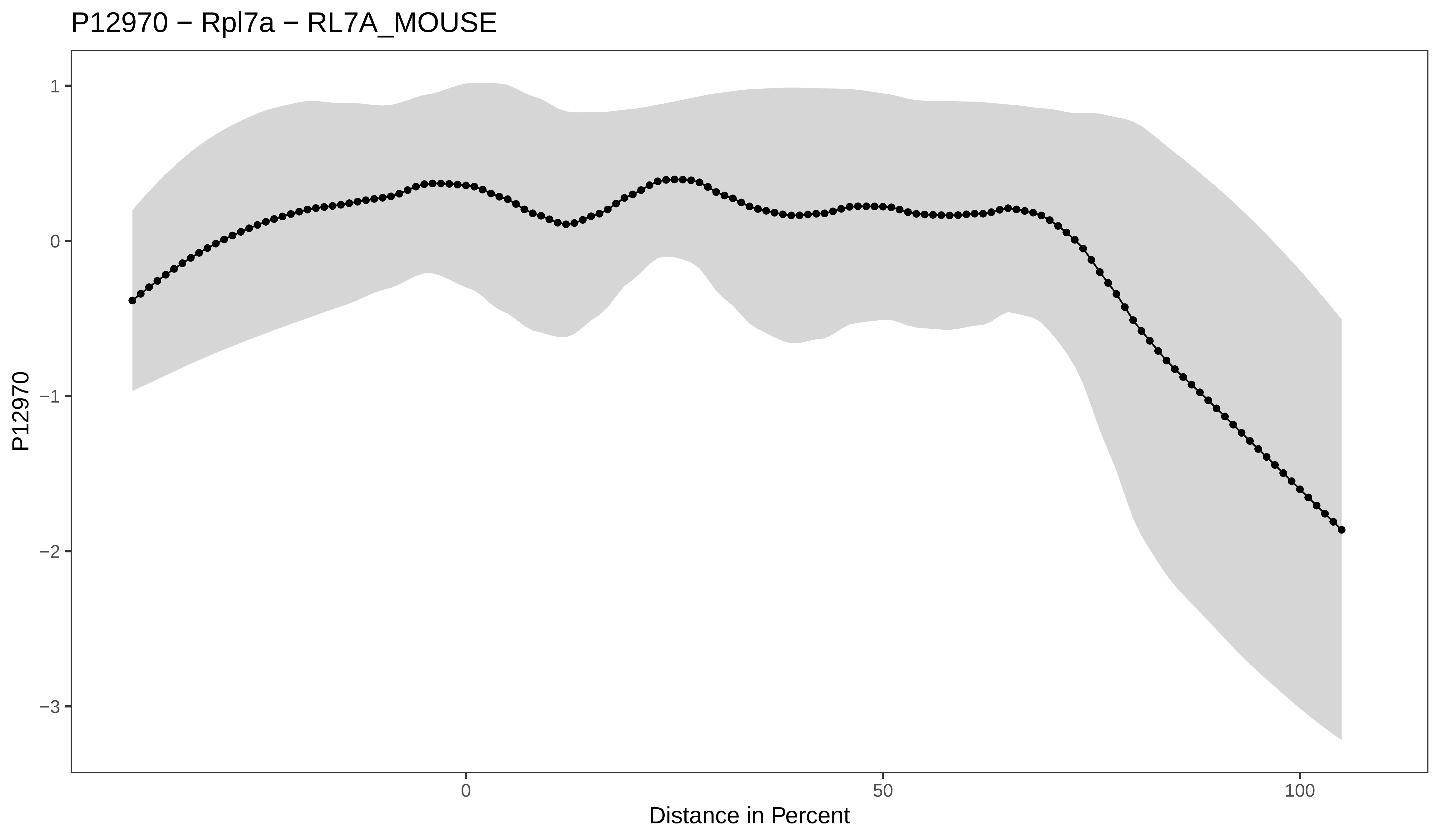

P12970 − Rpl7a − RL7A_MOUSE
P12970
1
0
−1
−2
−3
0
50
100
Distance in Percent

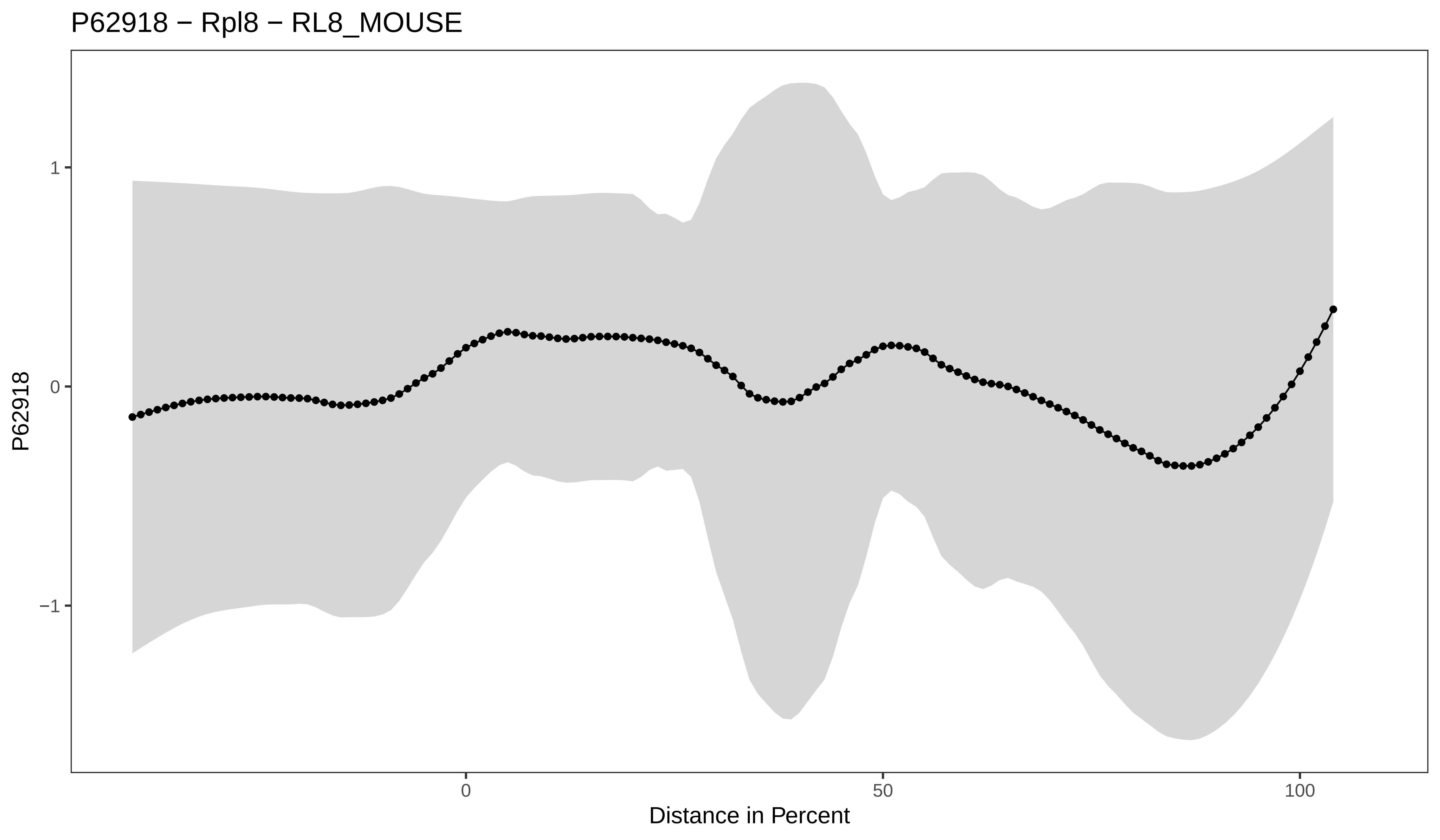
P62918 − Rpl8 − RL8_MOUSE
1
0
−1
P62918
0
50
100
Distance in Percent

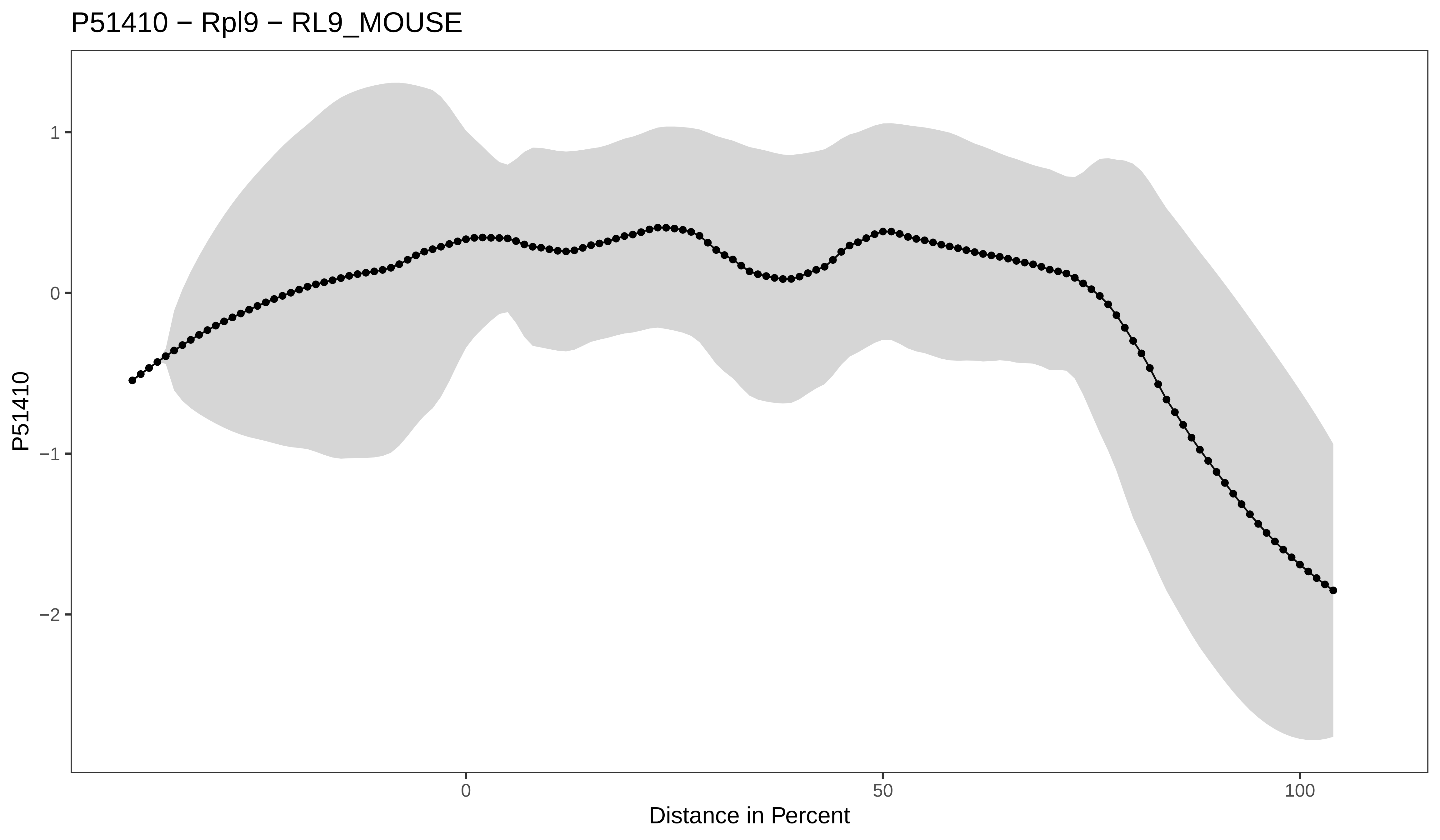

P51410 − Rpl9 − RL9_MOUSE
P51410
1
0
−1
−2
0
50
100
Distance in Percent

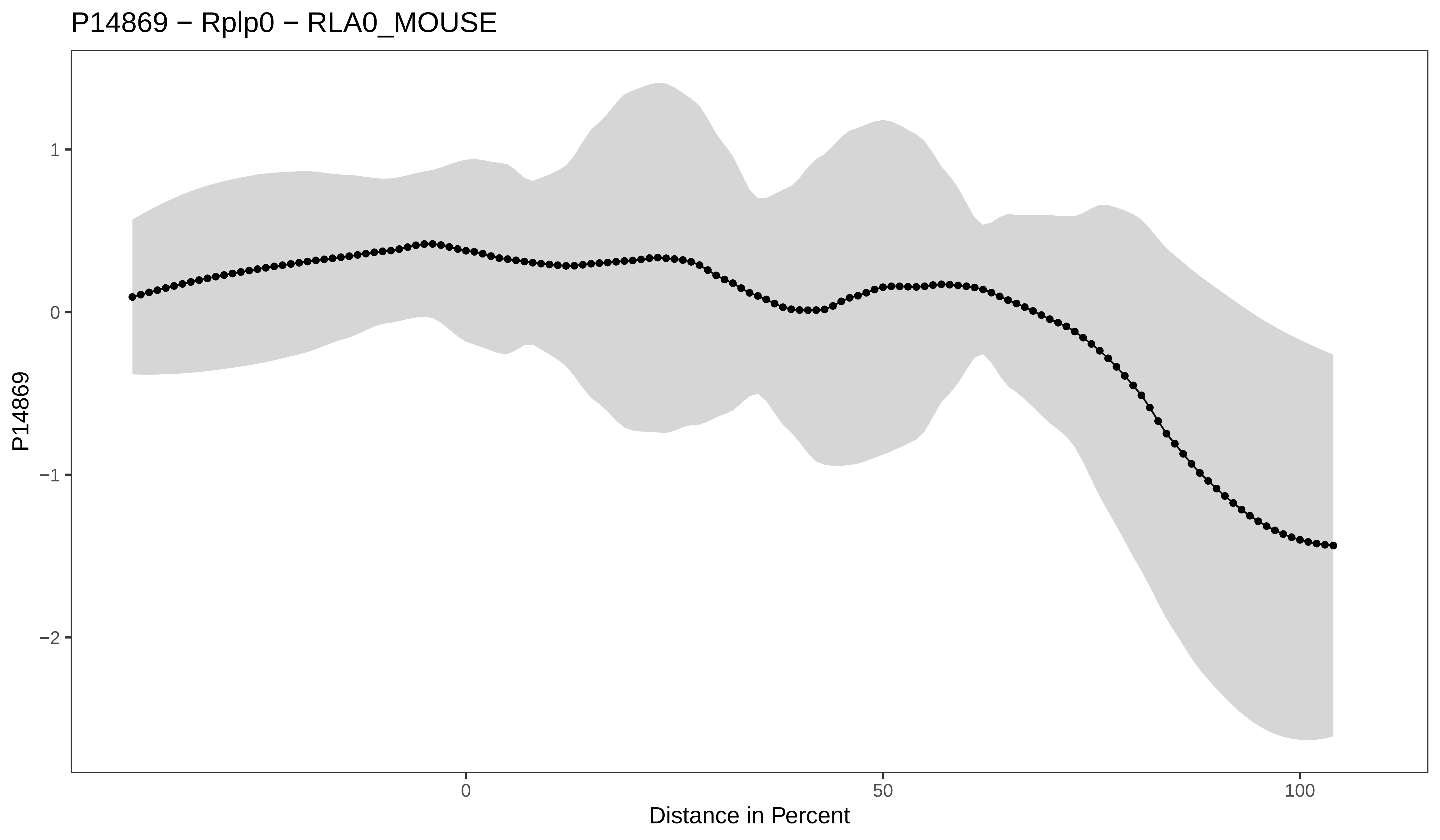

P14869 − Rplp0 − RLA0_MOUSE
P14869
1
0
−1
−2
0
50
100
Distance in Percent

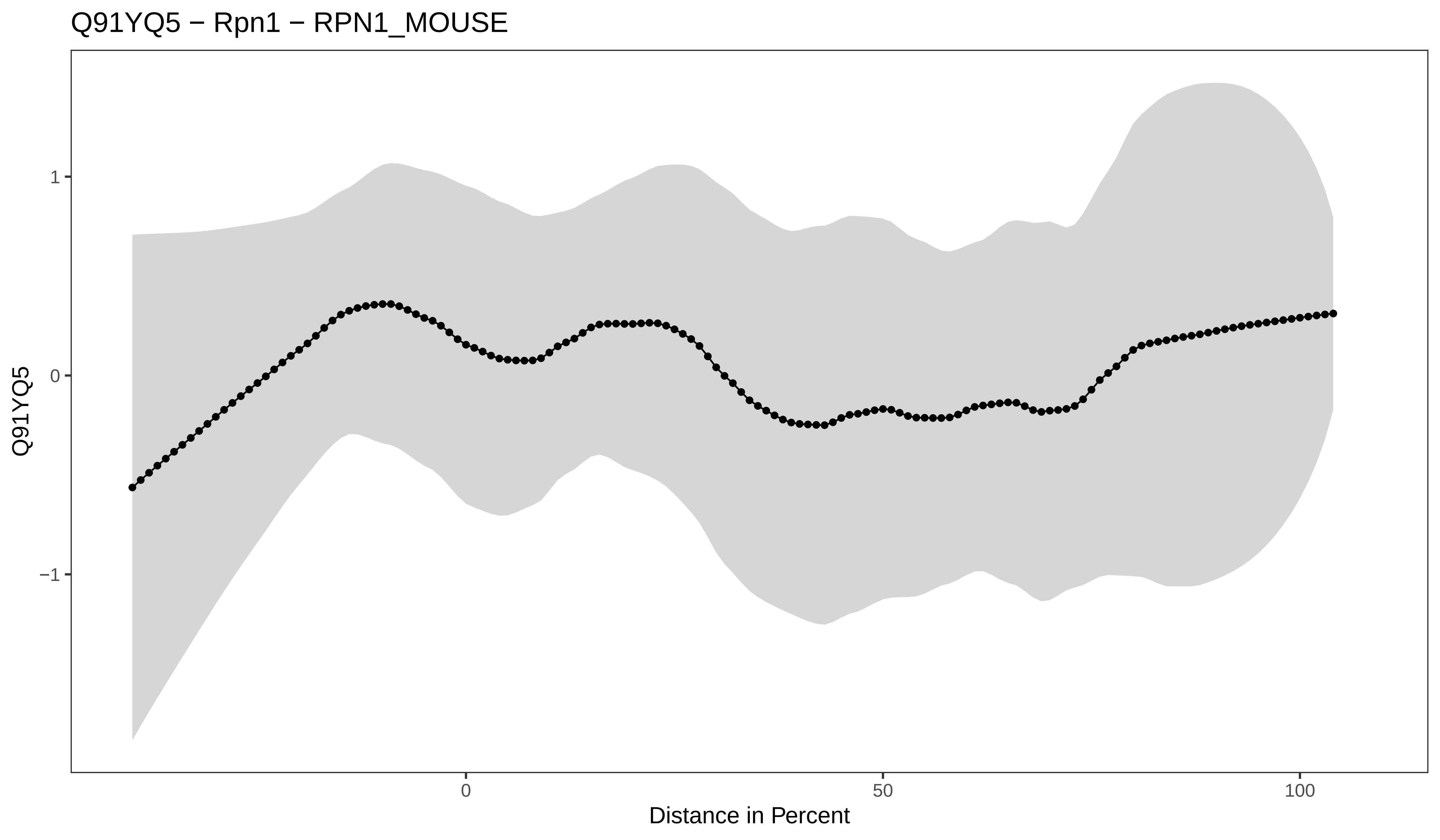
Q91YQ5 − Rpn1 − RPN1_MOUSE
Q91YQ5
1
0
−1
0
50
100
Distance in Percent

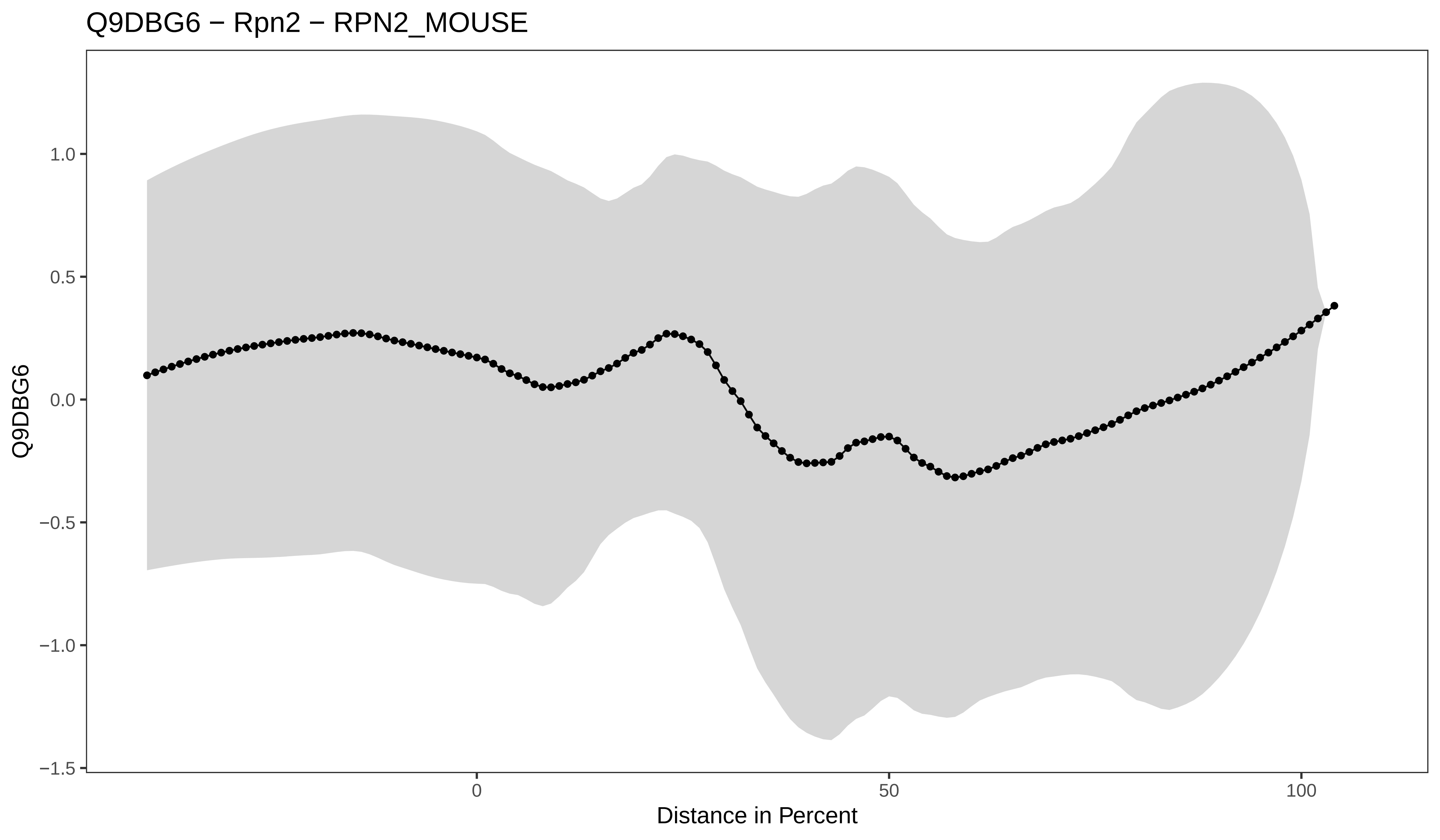
Q9DBG6 − Rpn2 − RPN2_MOUSE
Q9DBG6
1.0
0.5
0.0
−0.5
−1.0
−1.5
0
50
100
Distance in Percent

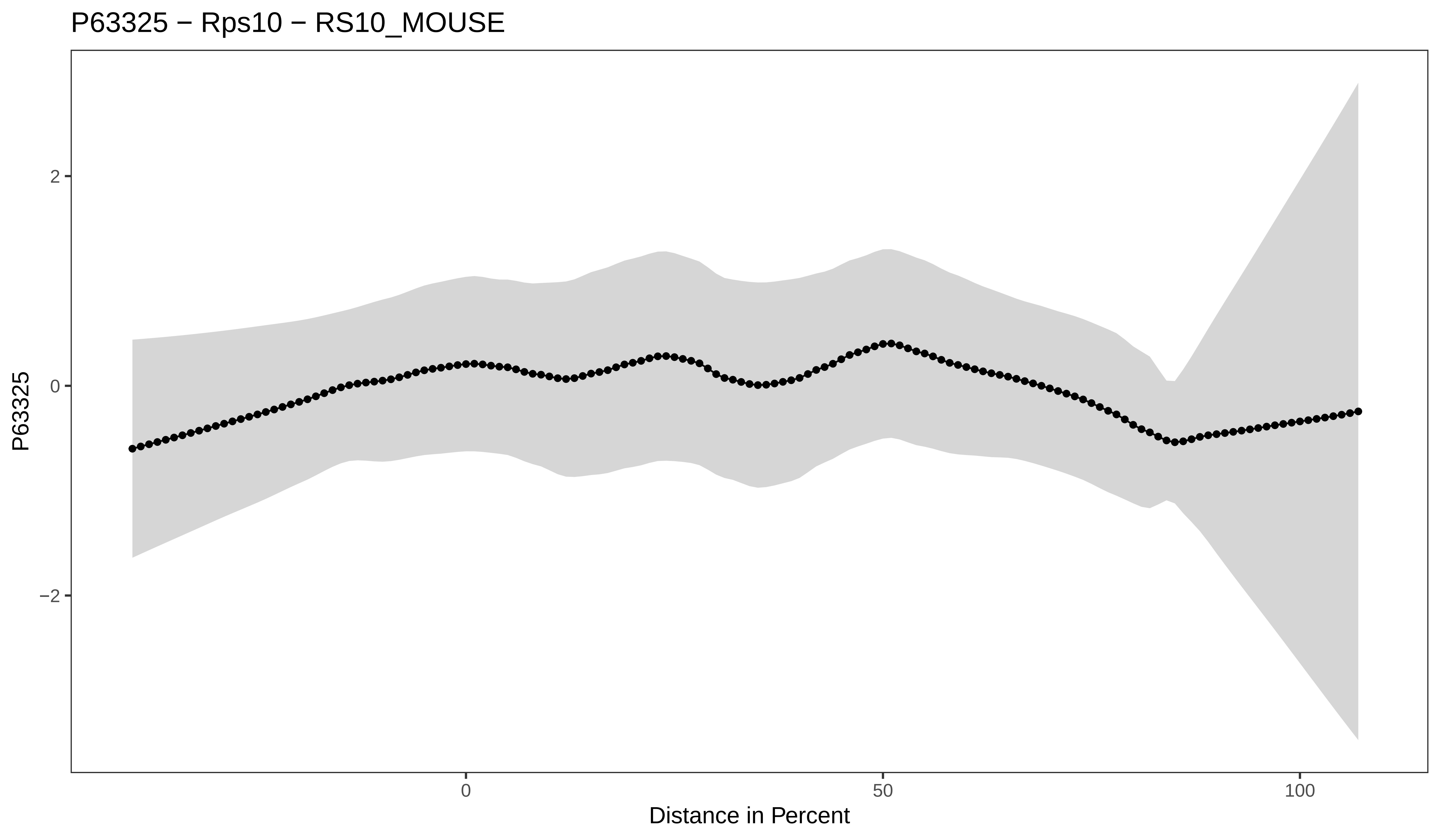

P63325 − Rps10 − RS10_MOUSE
P63325
2
0
−2
0
50
100
Distance in Percent

P62281 − Rps11 − RS11_MOUSE

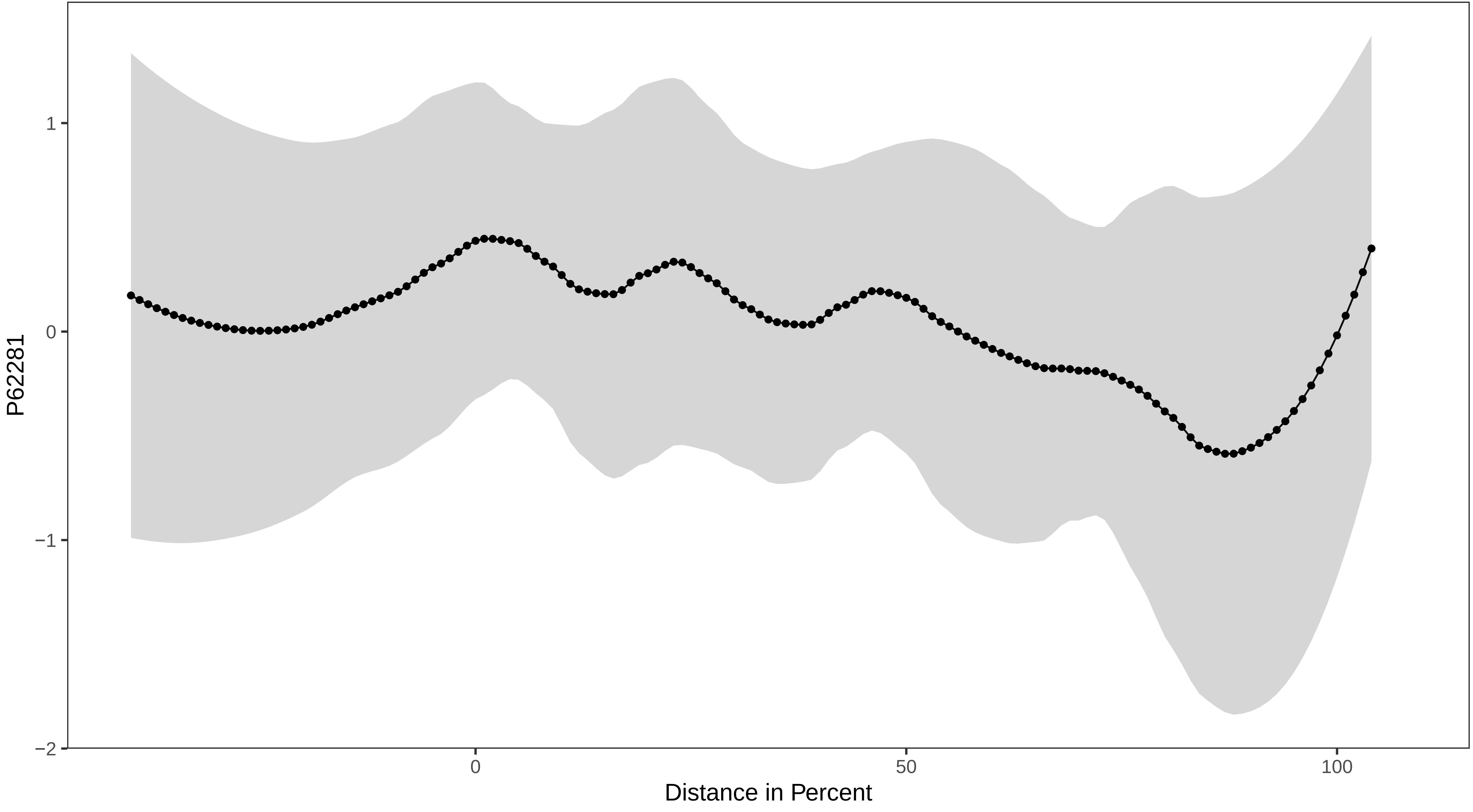

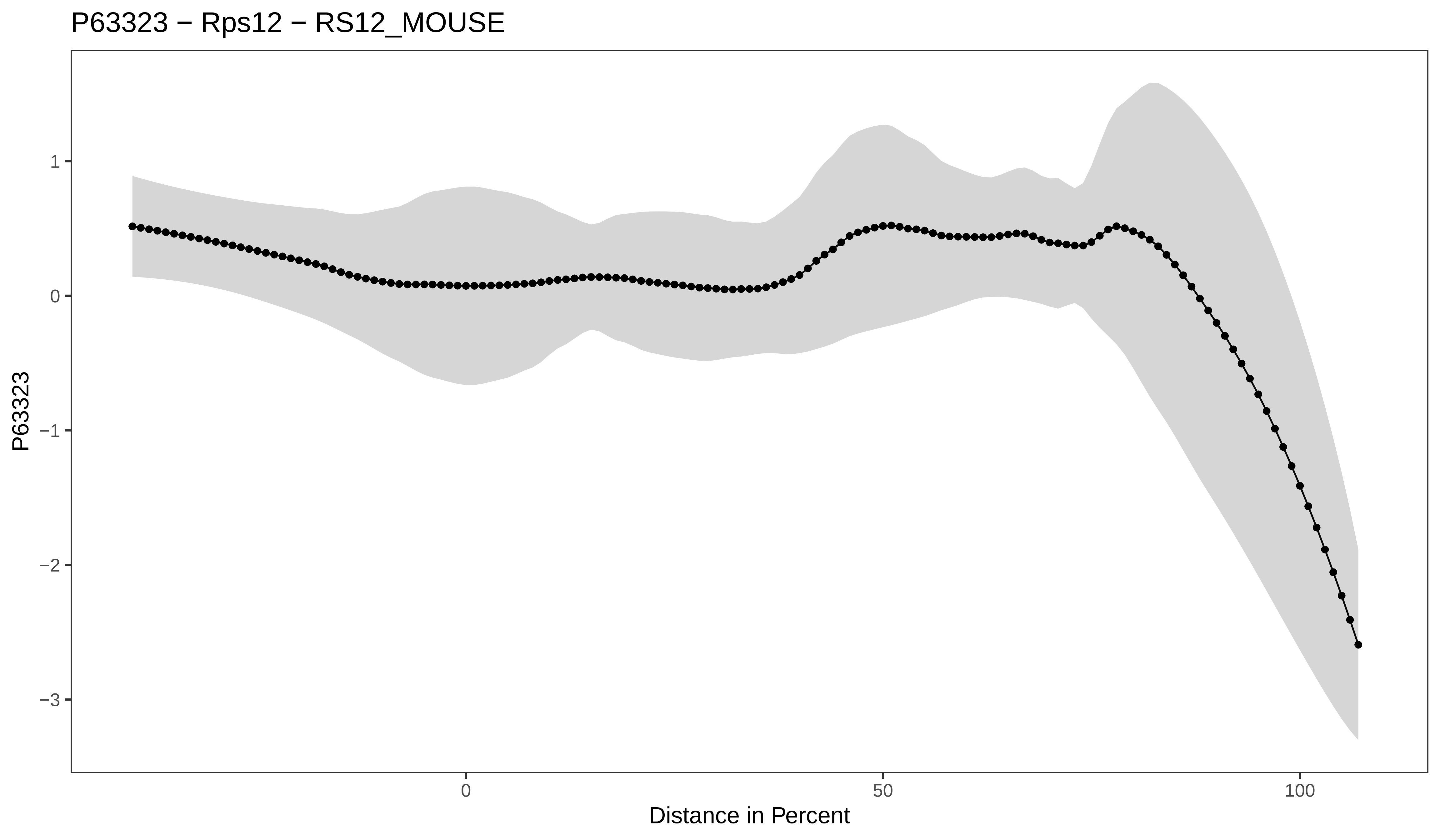
P63323 − Rps12 − RS12_MOUSE
1
0
−1
−2
−3
P63323
0
50
100
Distance in Percent

P62301 − Rps13 − RS13_MOUSE

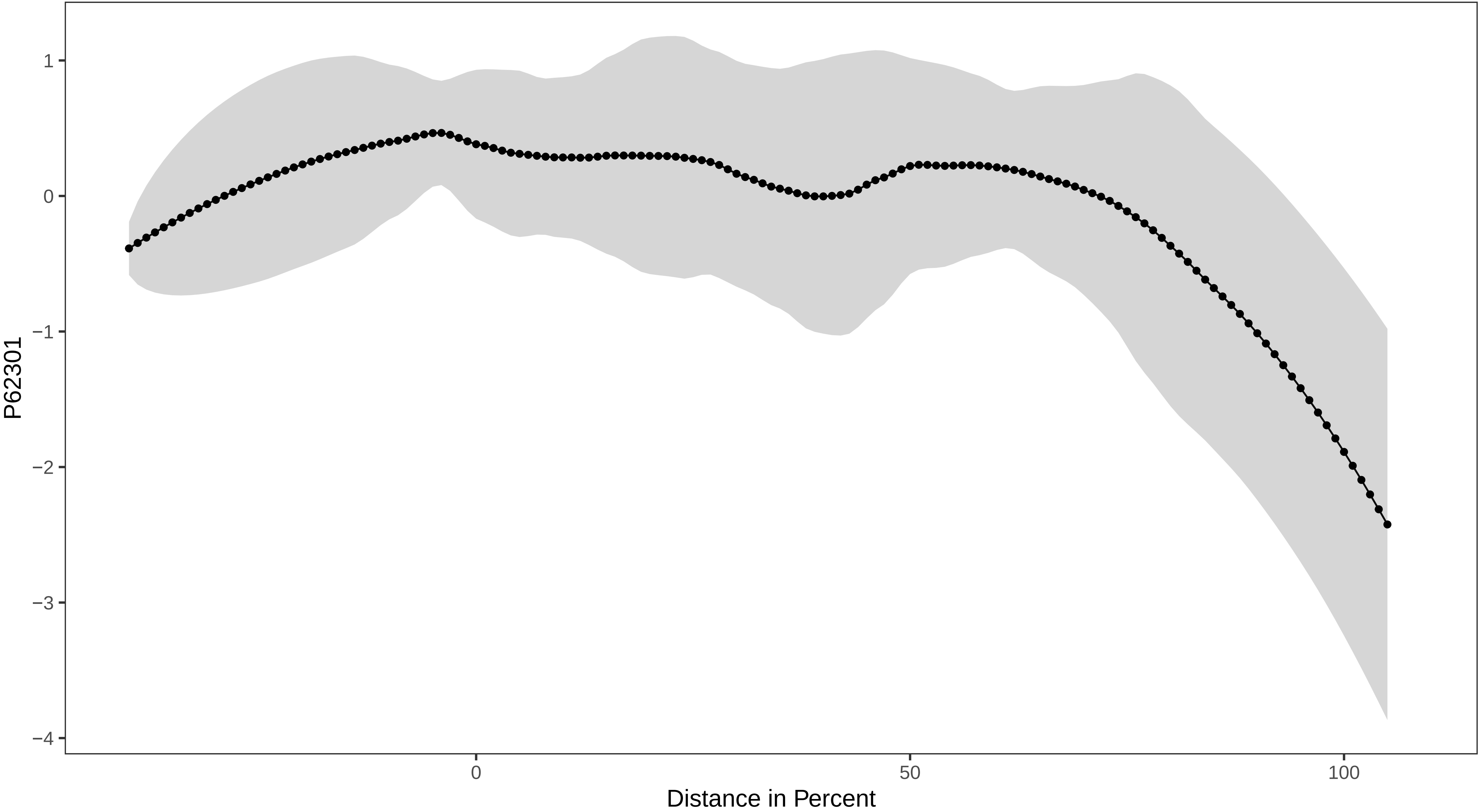

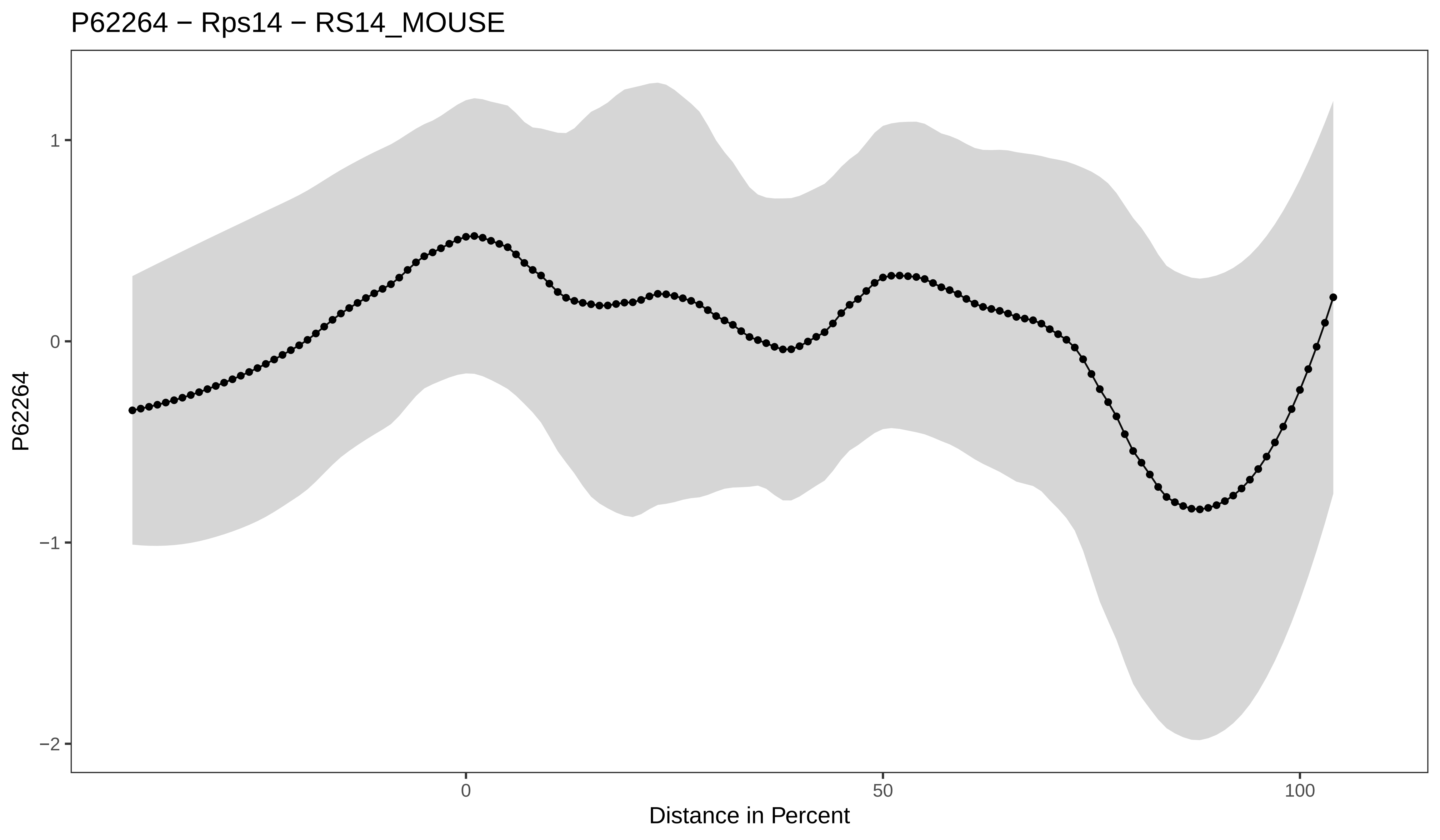

P62264 − Rps14 − RS14_MOUSE
P62264
1
0
−1
−2
0
50
100
Distance in Percent

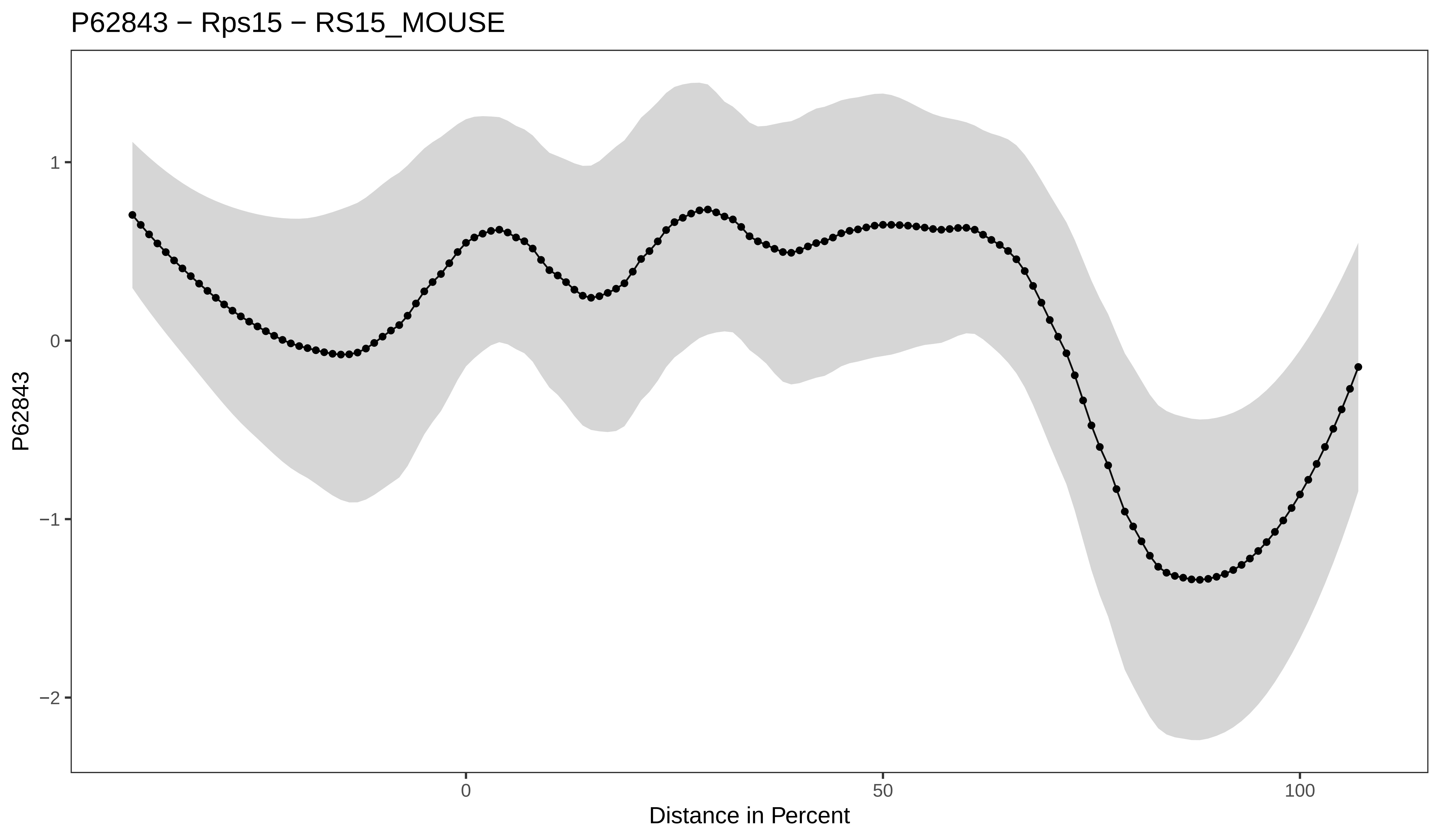

P62843 − Rps15 − RS15_MOUSE
P62843
1
0
−1
−2
0
50
100
Distance in Percent

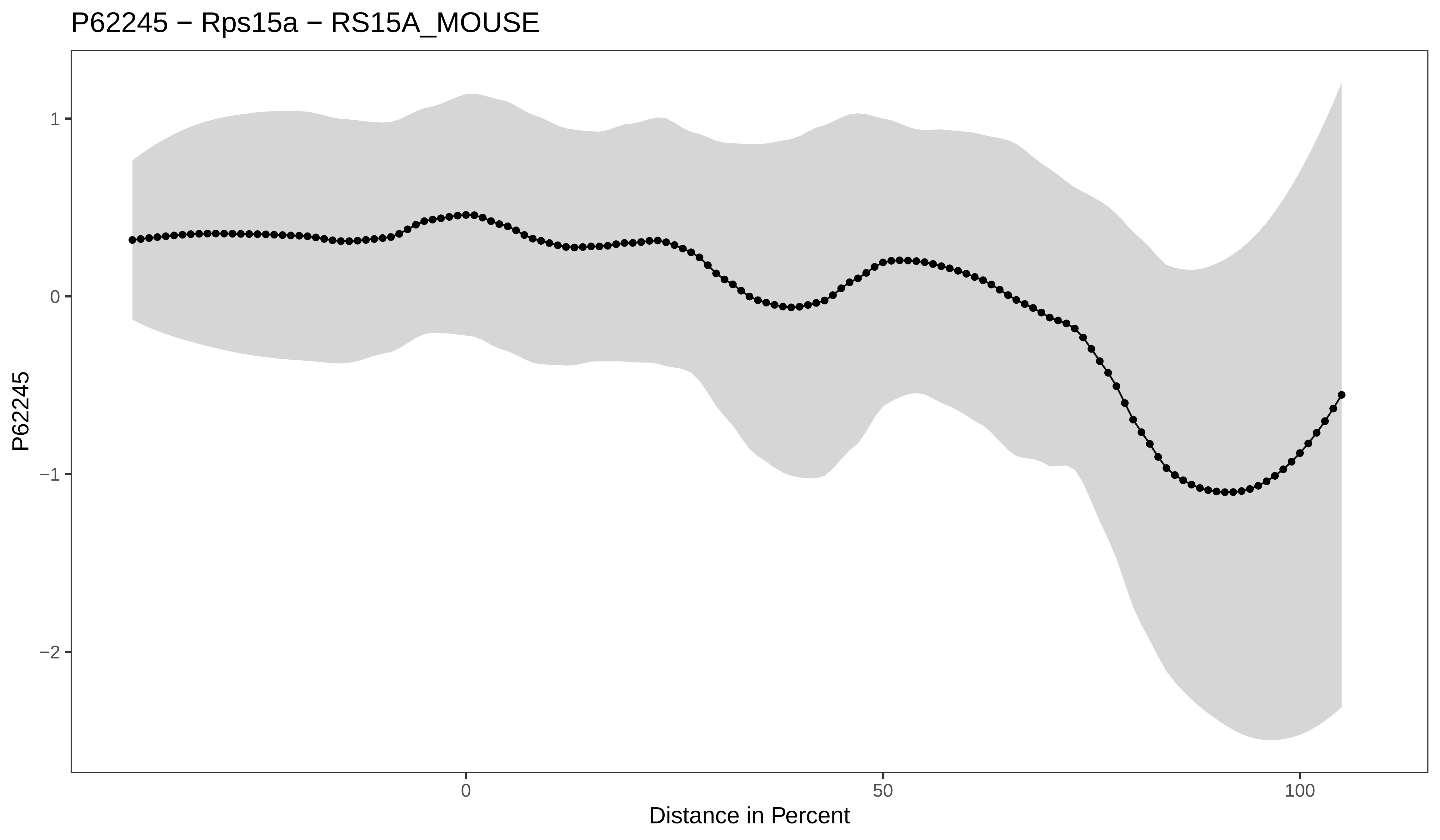

P62245 − Rps15a − RS15A_MOUSE
1
0
−1
−2
0
50
100
P62245
Distance in Percent

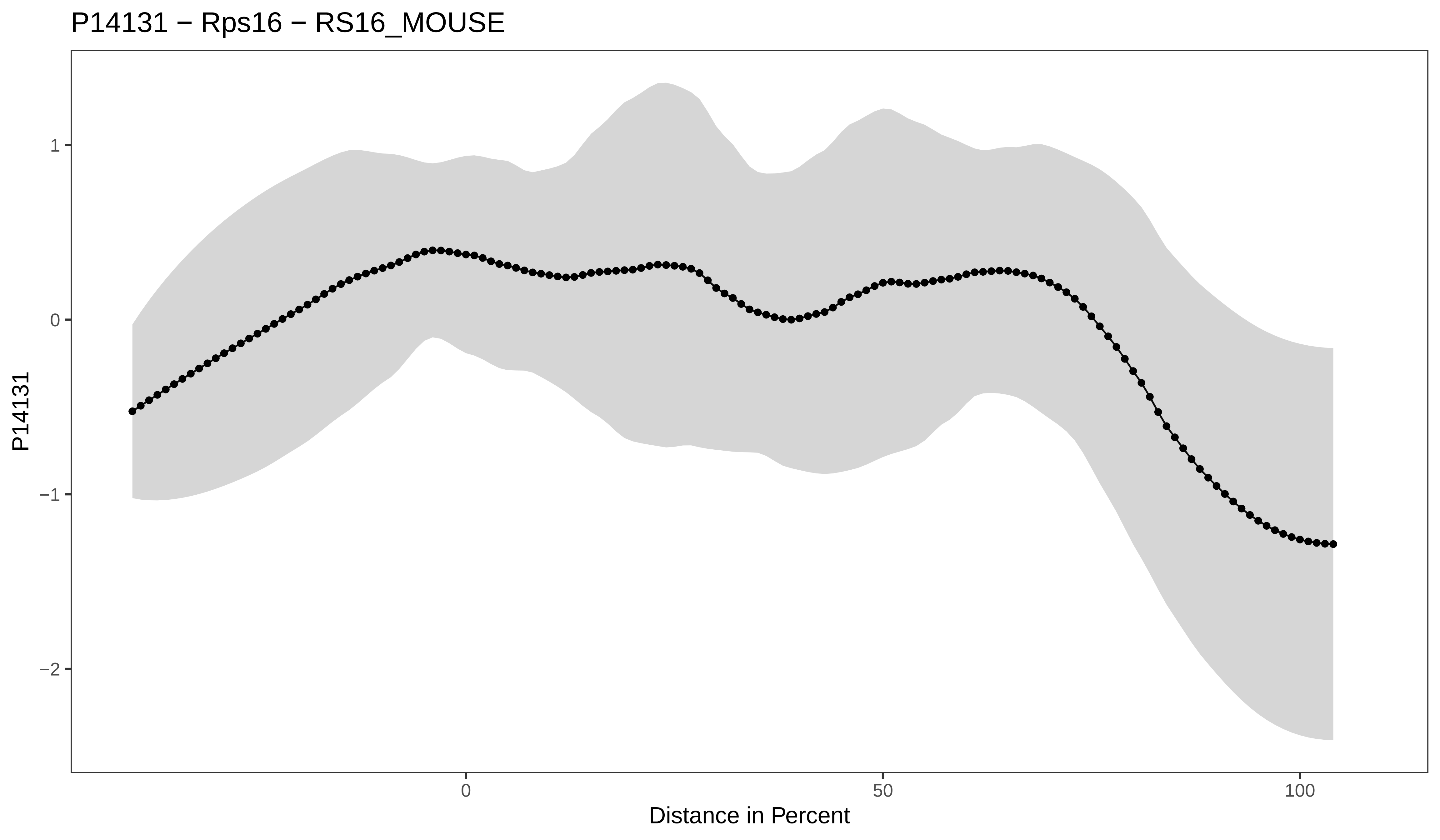
P14131 − Rps16 − RS16_MOUSE
1
0
−1
−2
P14131
0
50
100
Distance in Percent

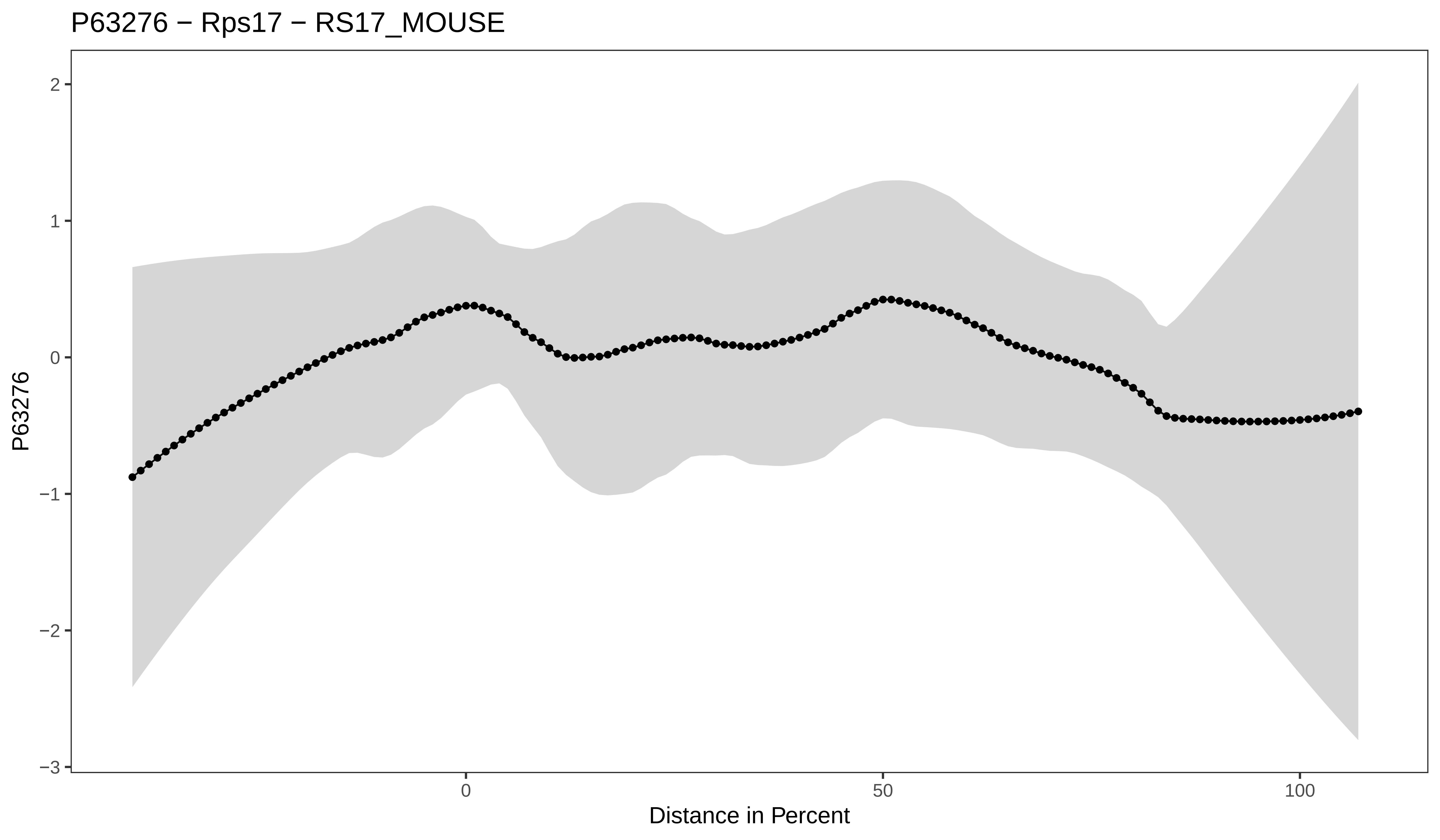

P63276 − Rps17 − RS17_MOUSE
2
1
0
−1
−2
−3
P63276
0
50
100
Distance in Percent

P62270 − Rps18 − RS18_MOUSE

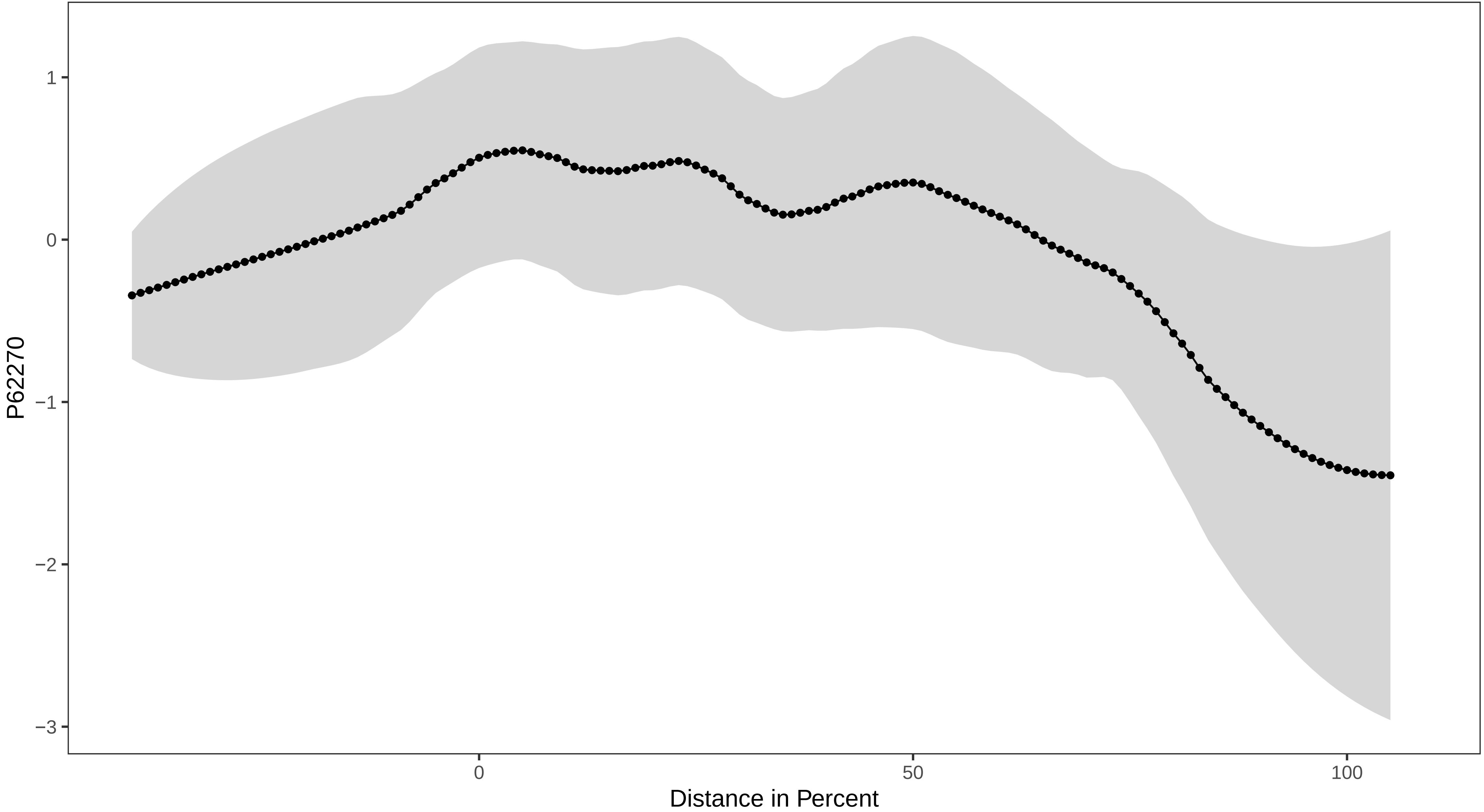

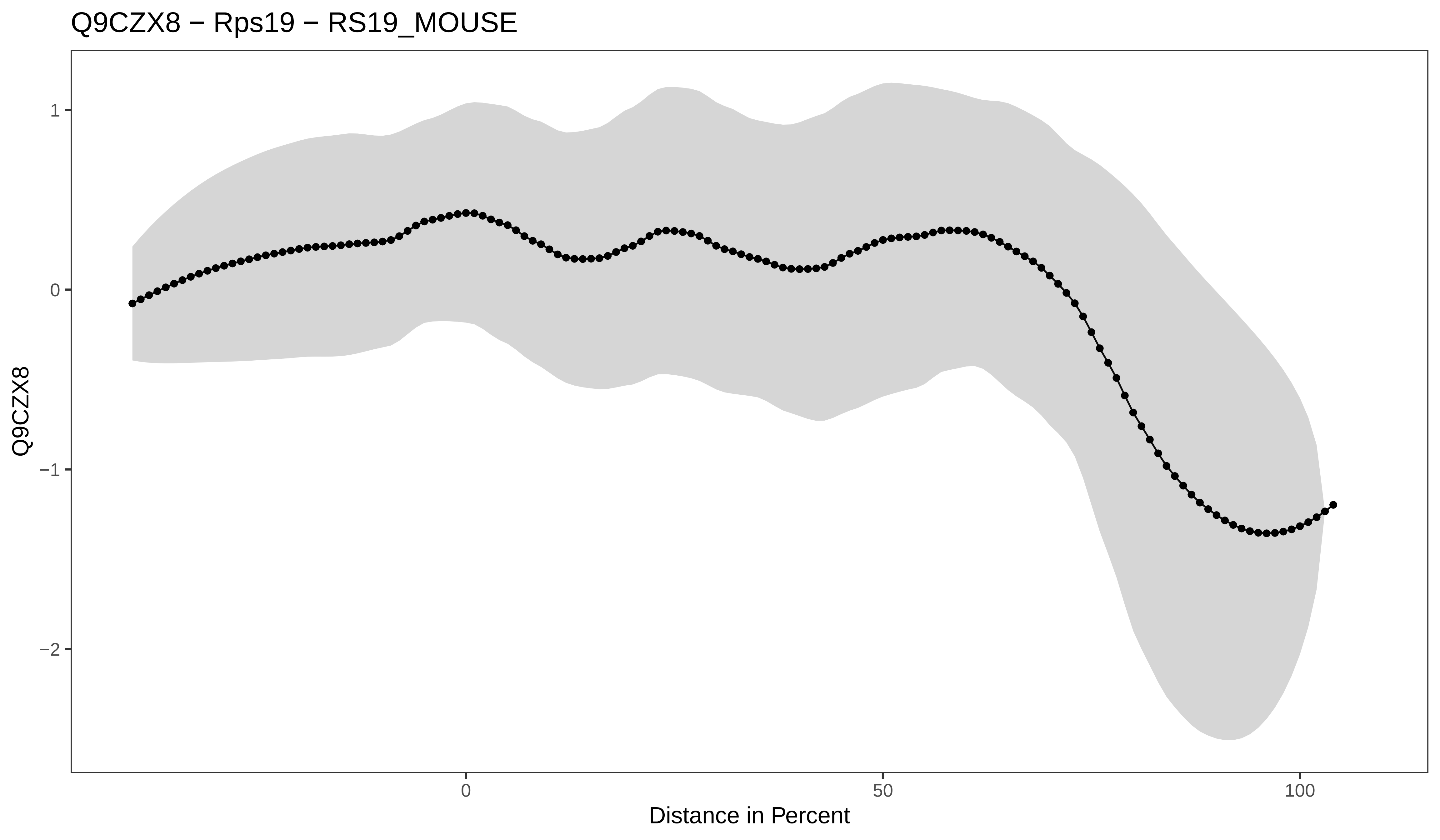

Q9CZX8 − Rps19 − RS19_MOUSE
1
0
−1
−2
Q9CZX8
0
50
100
Distance in Percent

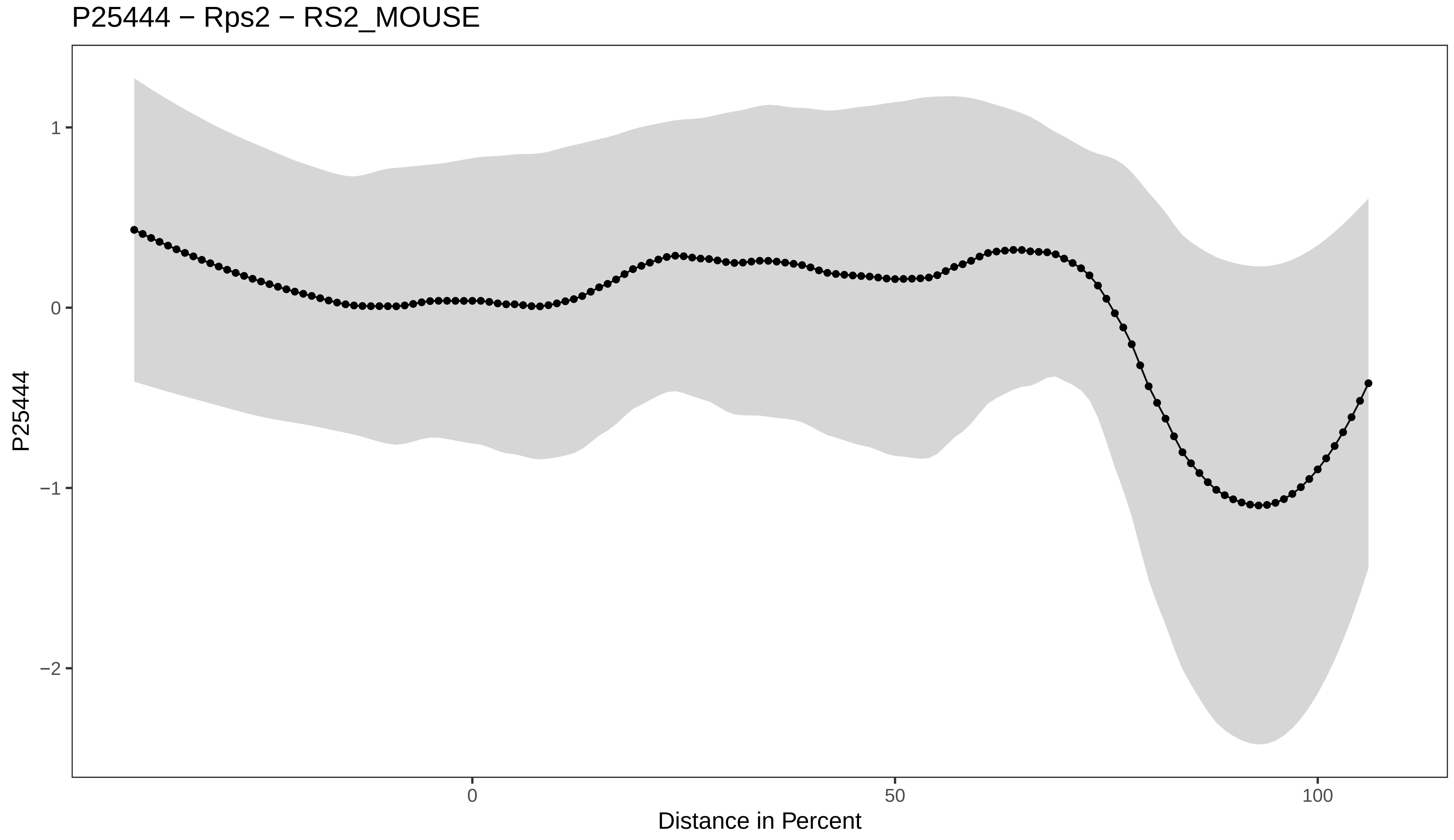
P25444 − Rps2 − RS2_MOUSE
P25444
1
0
−1
−2
0
50
100
Distance in Percent

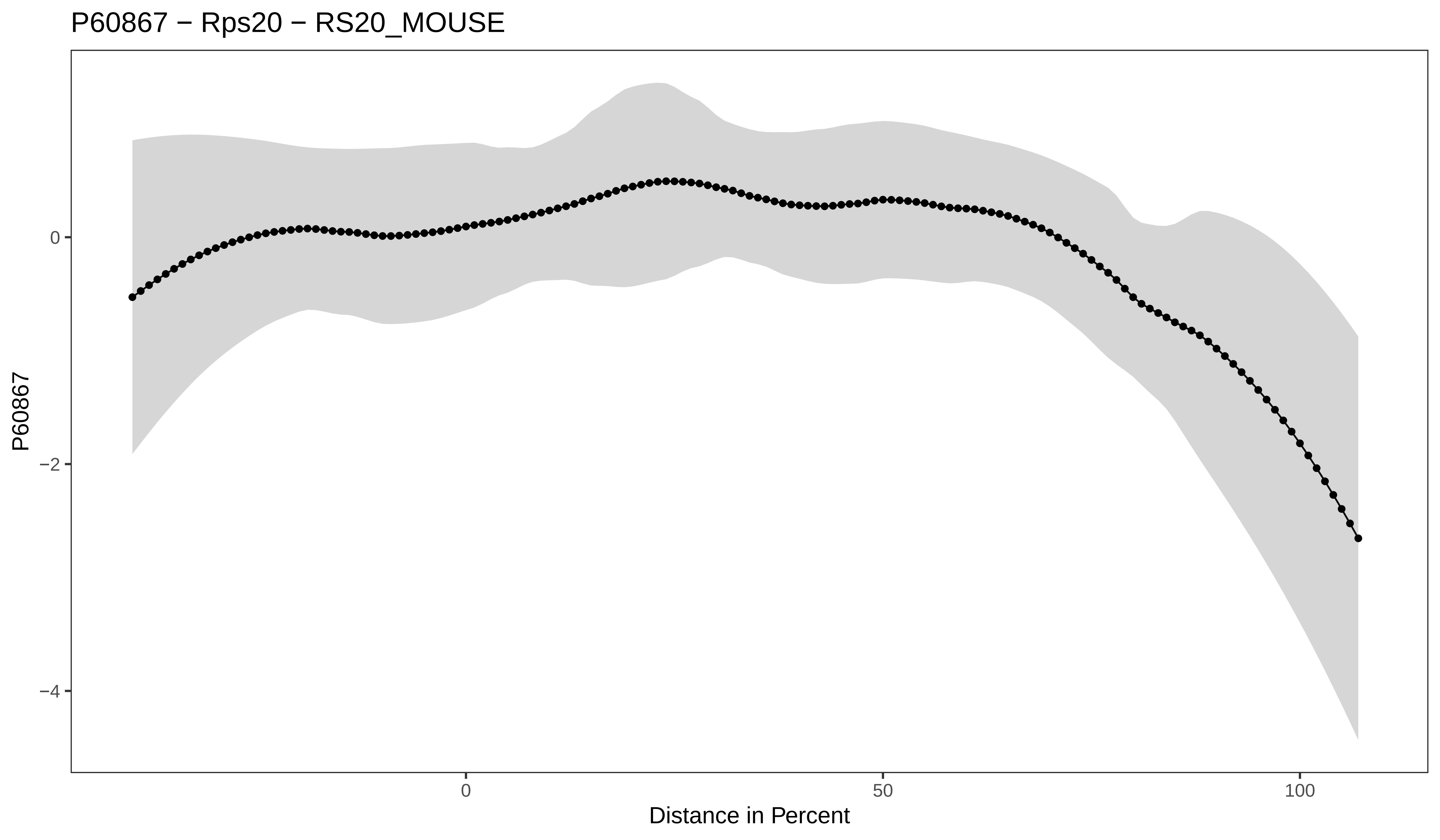

P60867 − Rps20 − RS20_MOUSE
0
−2
−4
P60867
0
50
100
Distance in Percent

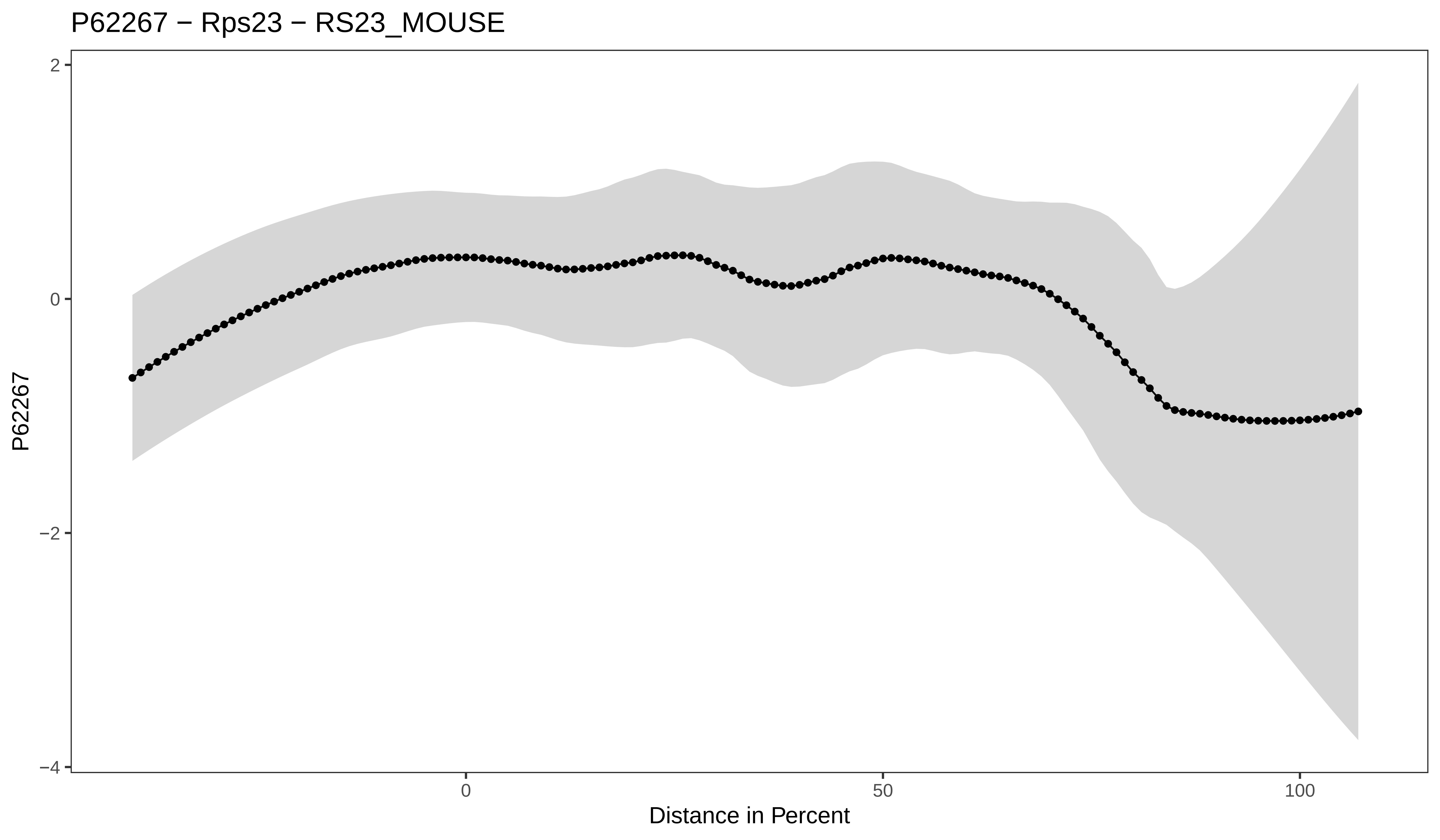
P62267 − Rps23 − RS23_MOUSE
2
0
−2
−4
P62267
0
50
100
Distance in Percent

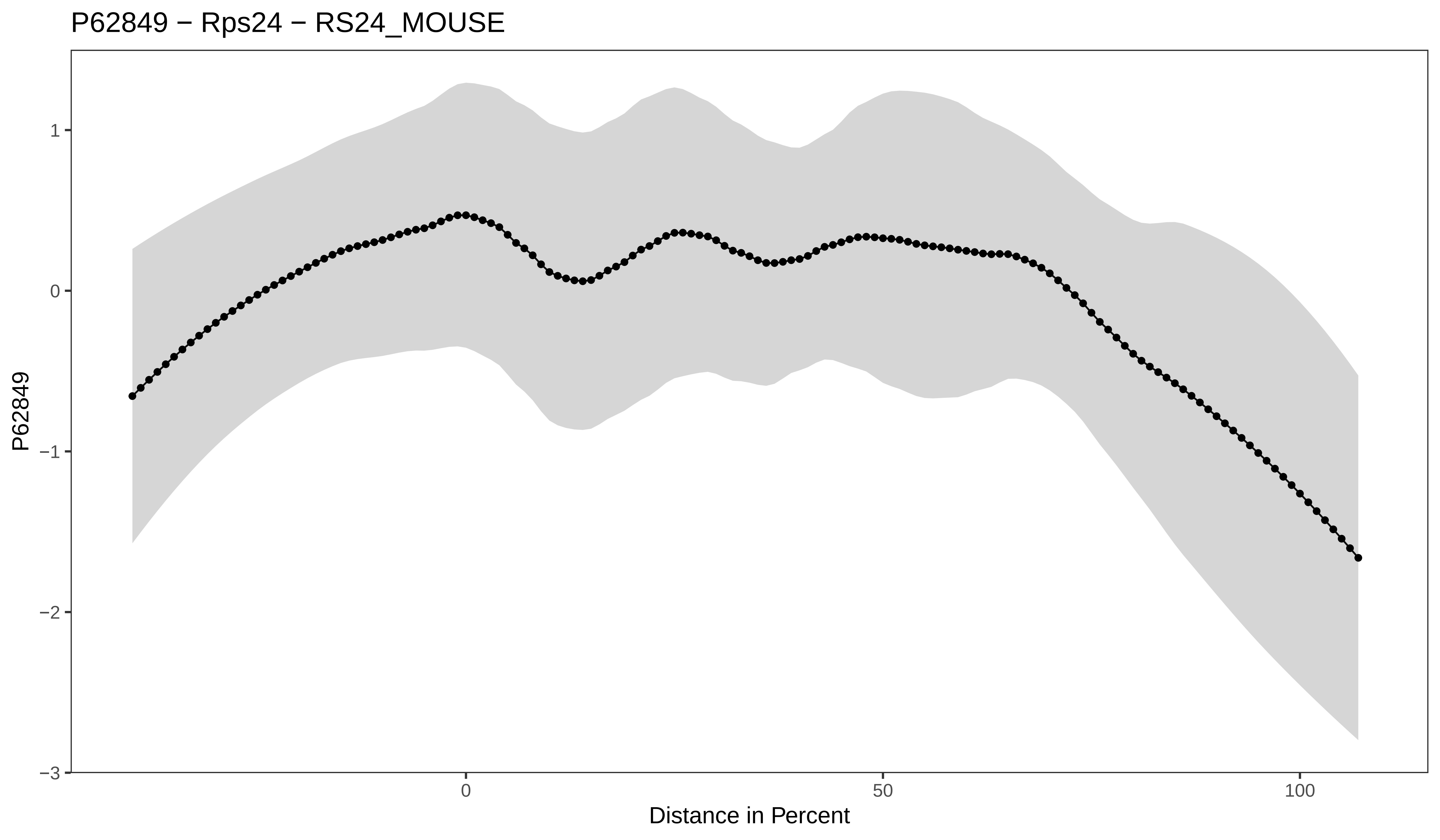

P62849 − Rps24 − RS24_MOUSE
1
0
−1
−2
−3
P62849
0
50
100
Distance in Percent

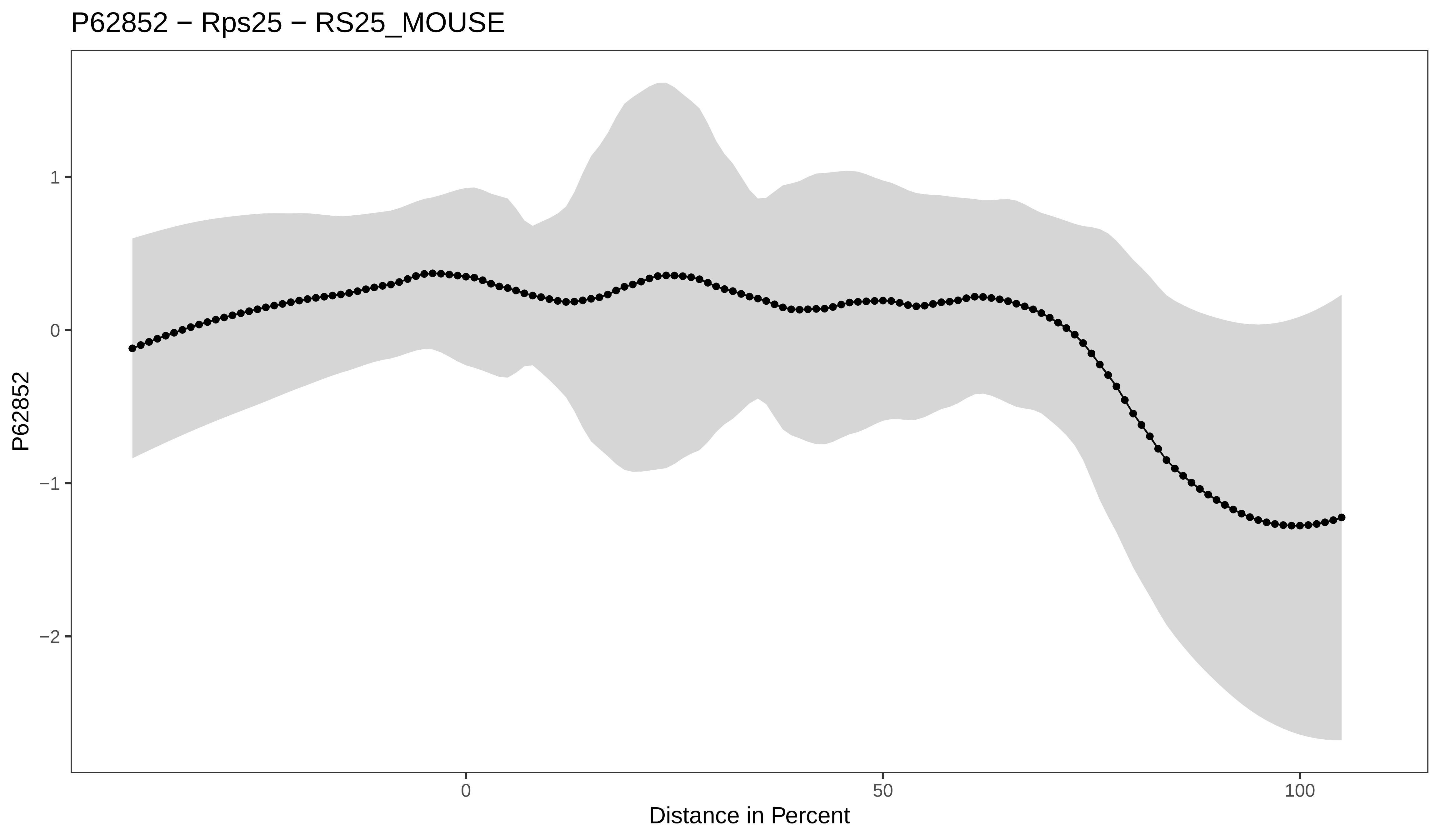
P62852 − Rps25 − RS25_MOUSE
P62852
1
0
−1
−2
0
50
100
Distance in Percent

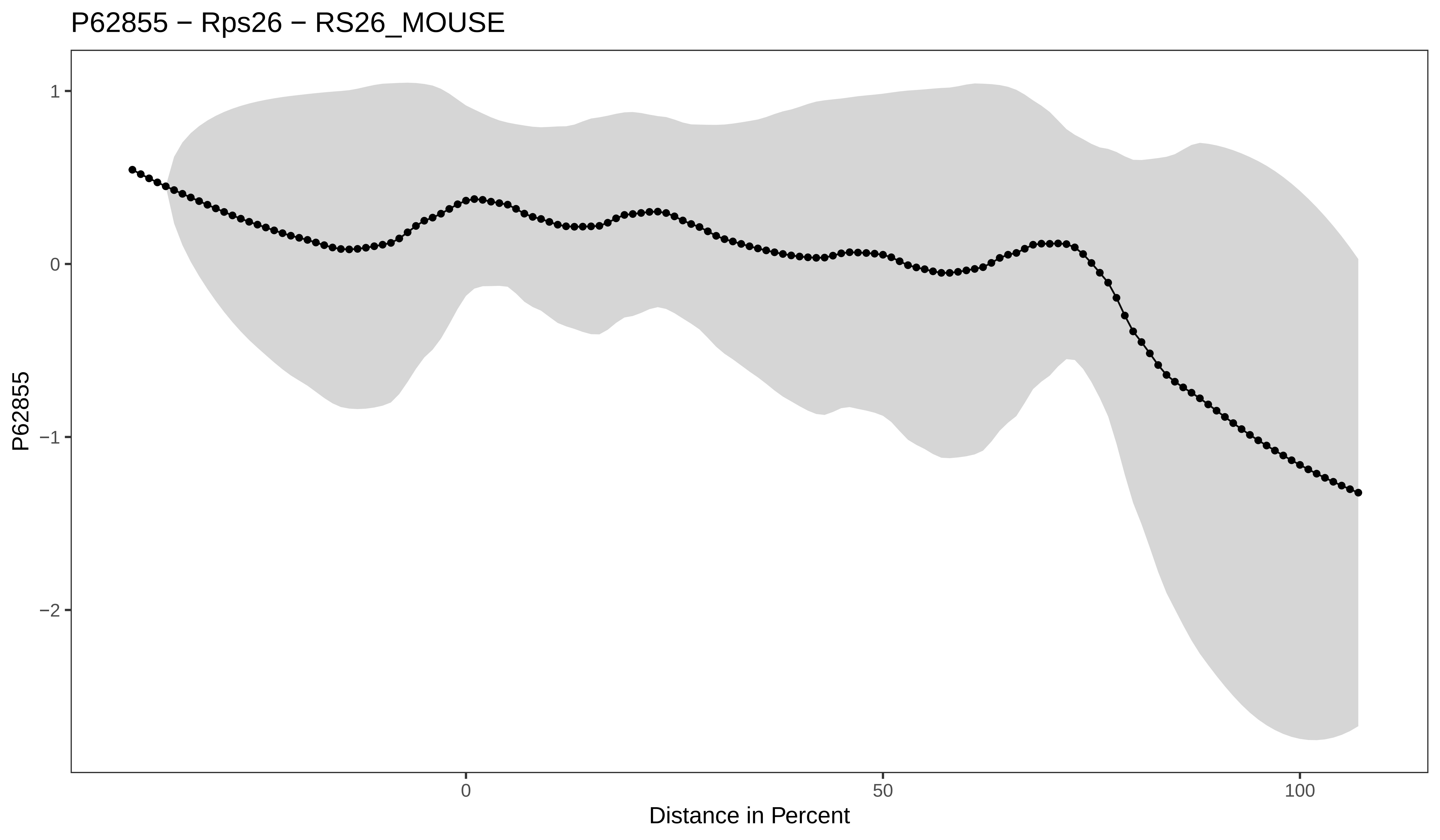

P62855 − Rps26 − RS26_MOUSE
1
0
−1
−2
P62855
0
50
100
Distance in Percent

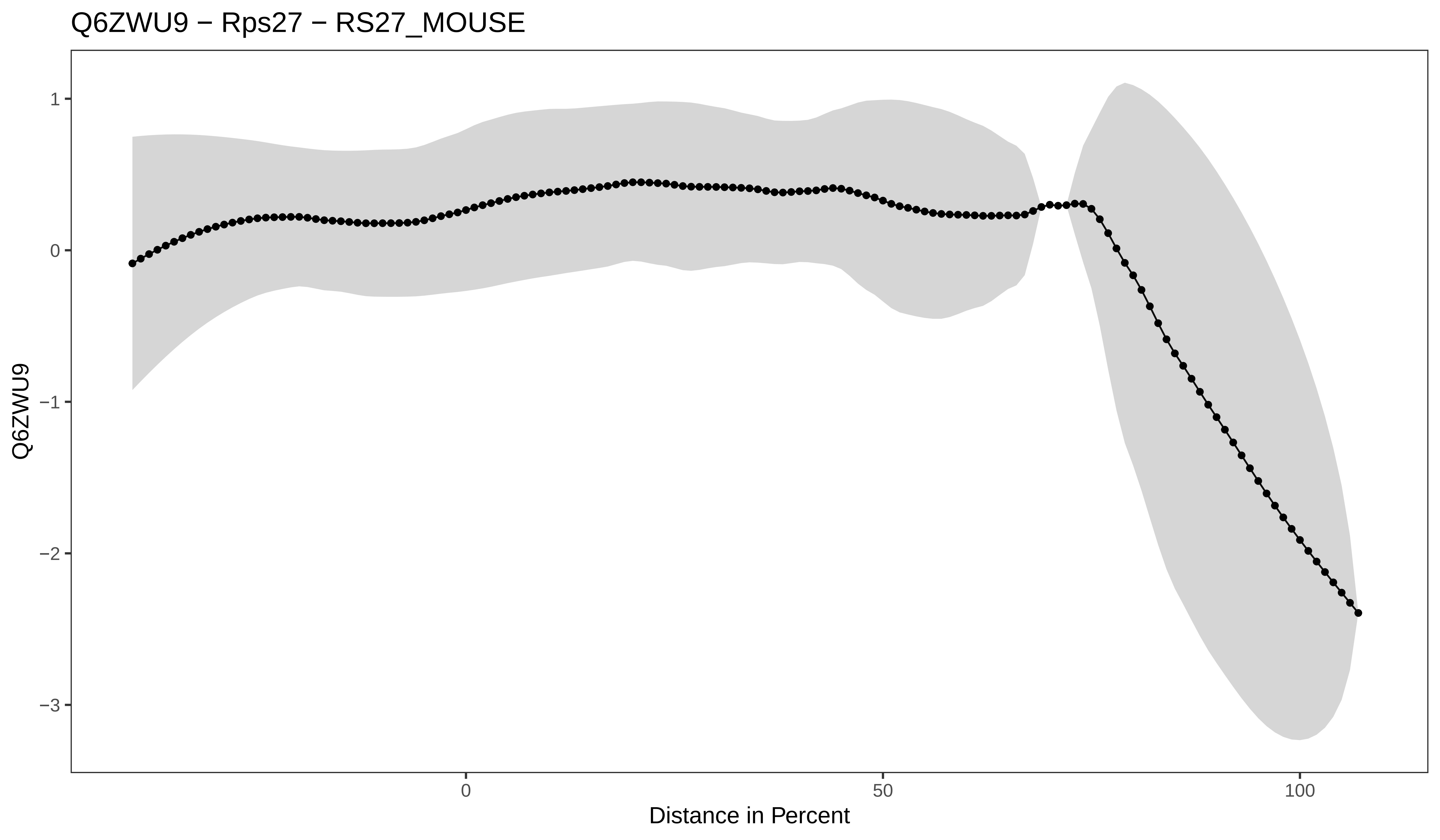
Q6ZWU9 − Rps27 − RS27_MOUSE
Q6ZWU9
1
0
−1
−2
−3
0
50
100
Distance in Percent

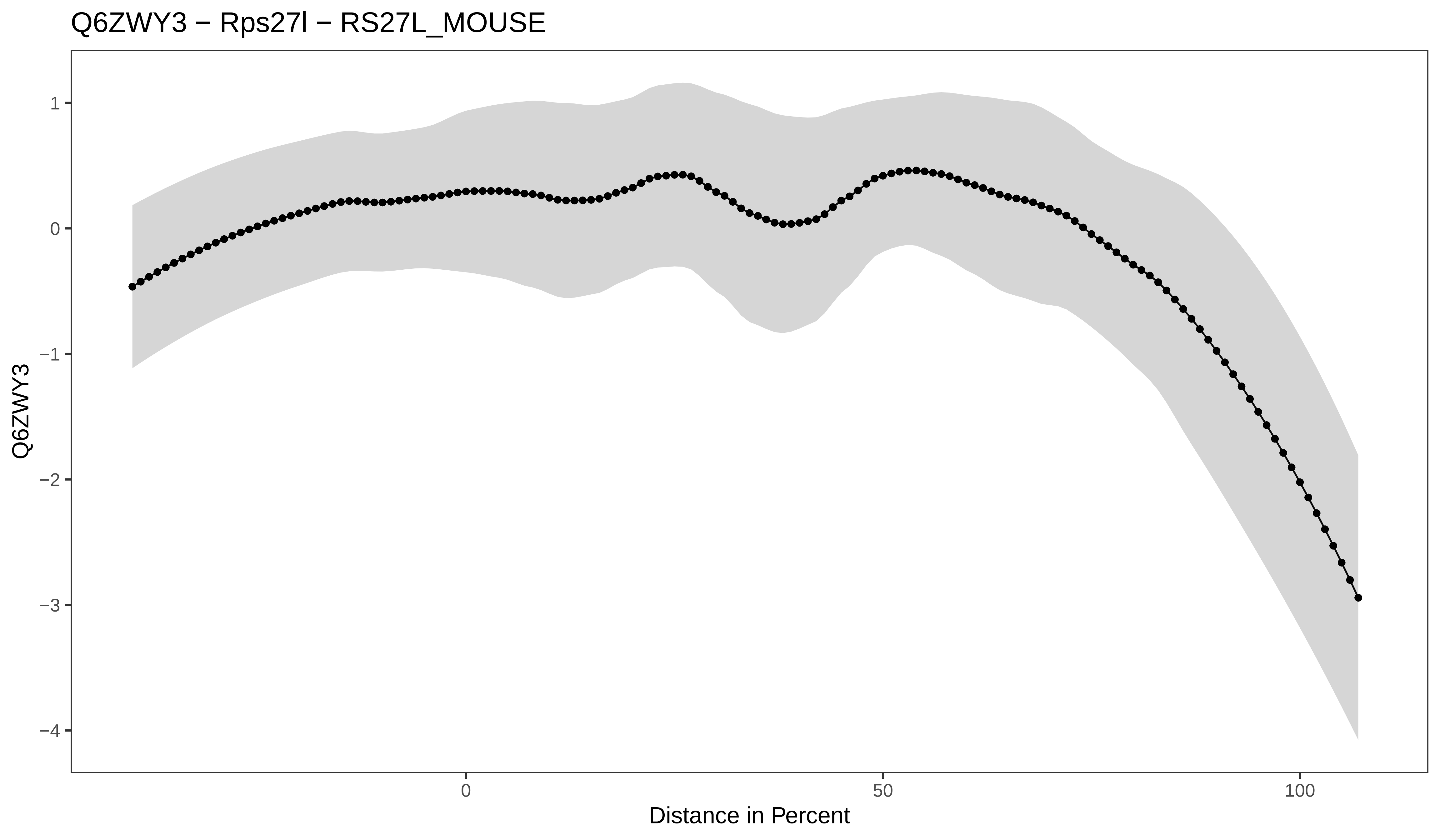

Q6ZWY3 − Rps27l − RS27L_MOUSE
Q6ZWY3
Distance in Percent
1
0
−1
−2
−3
−4
0
50
100

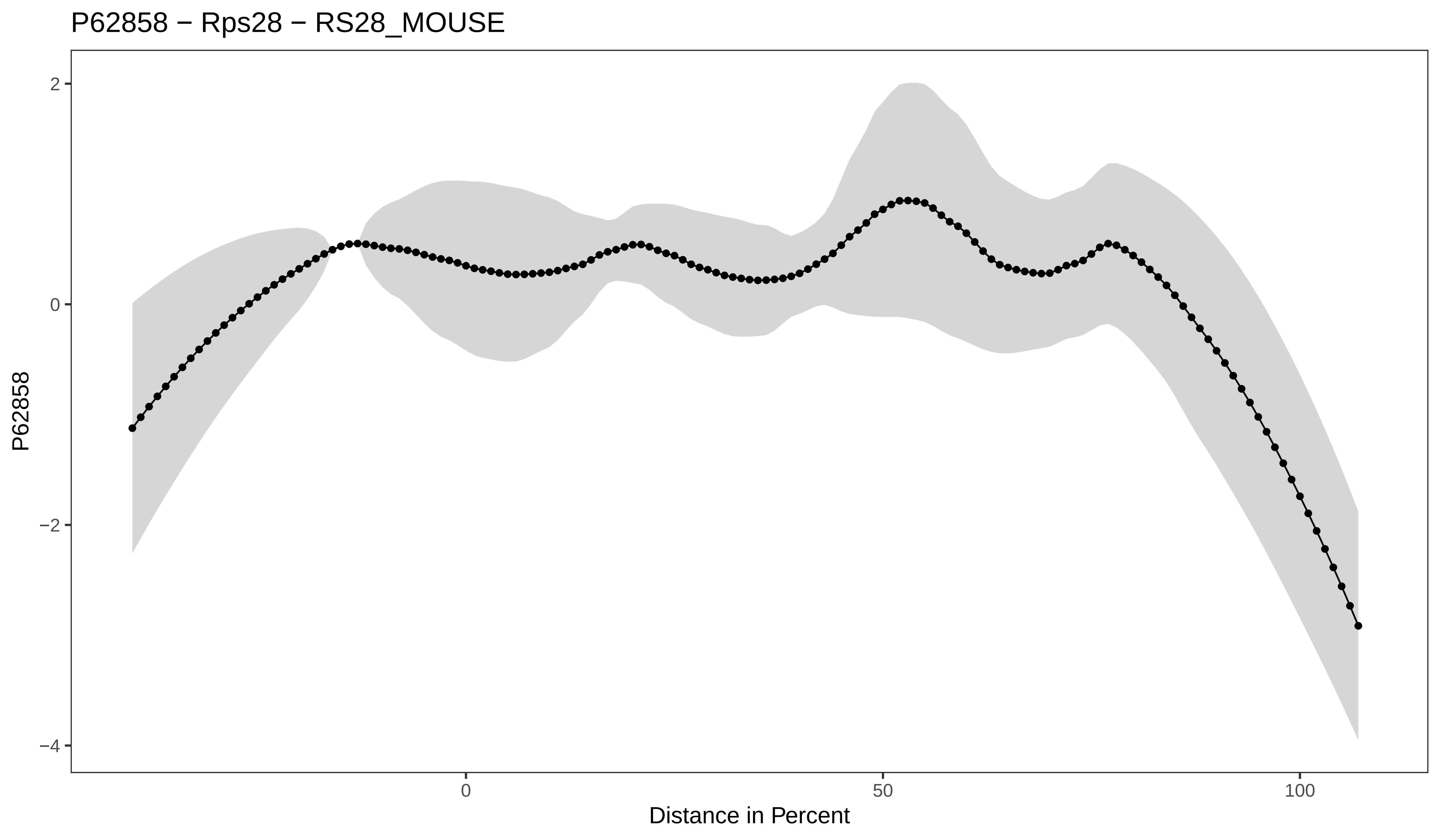
P62858 − Rps28 − RS28_MOUSE
P62858
Distance in Percent
2
0
−2
−4
0
50
100

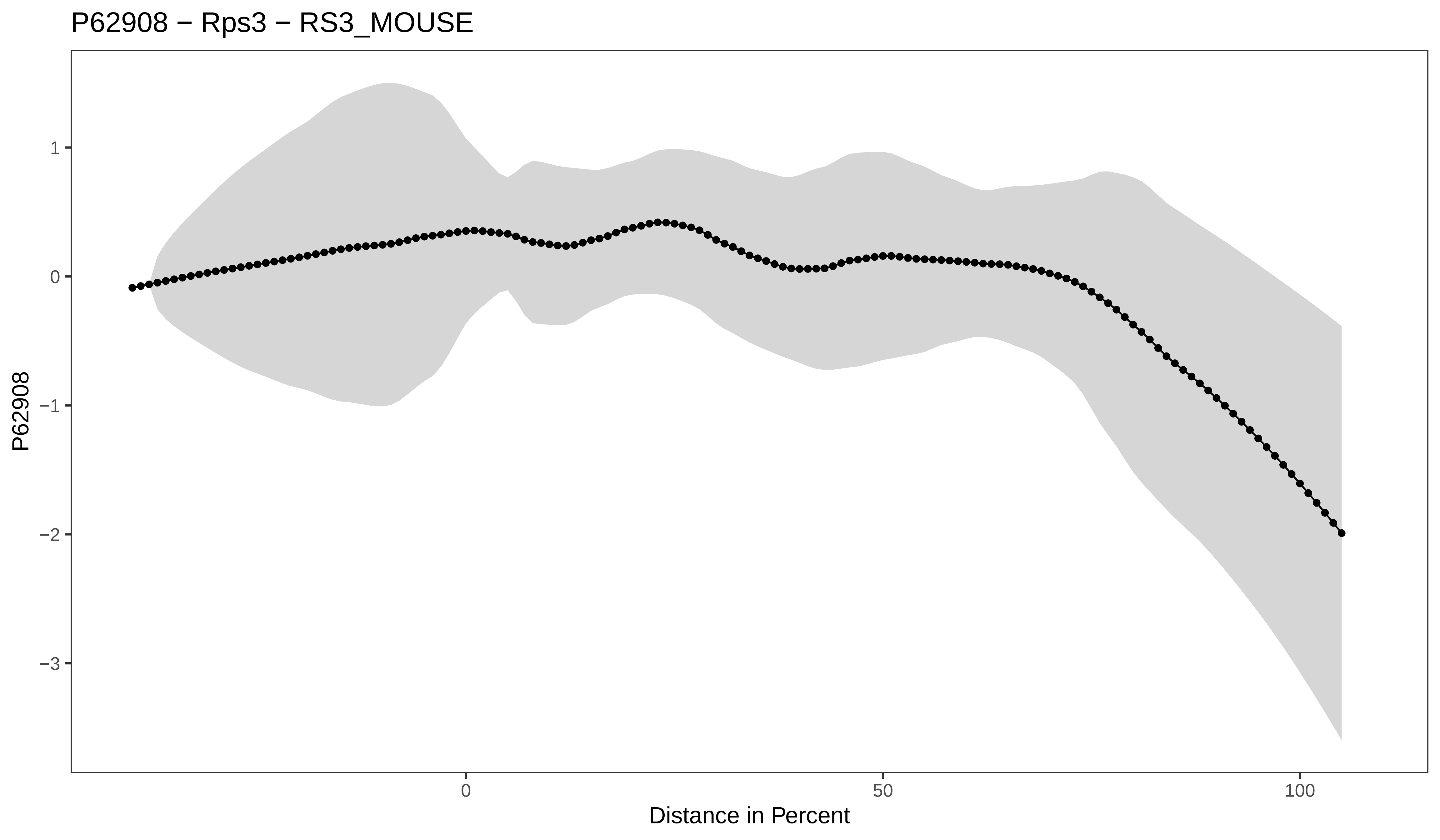

P62908 − Rps3 − RS3_MOUSE
1
0
−1
−2
−3
P62908
0
50
100
Distance in Percent

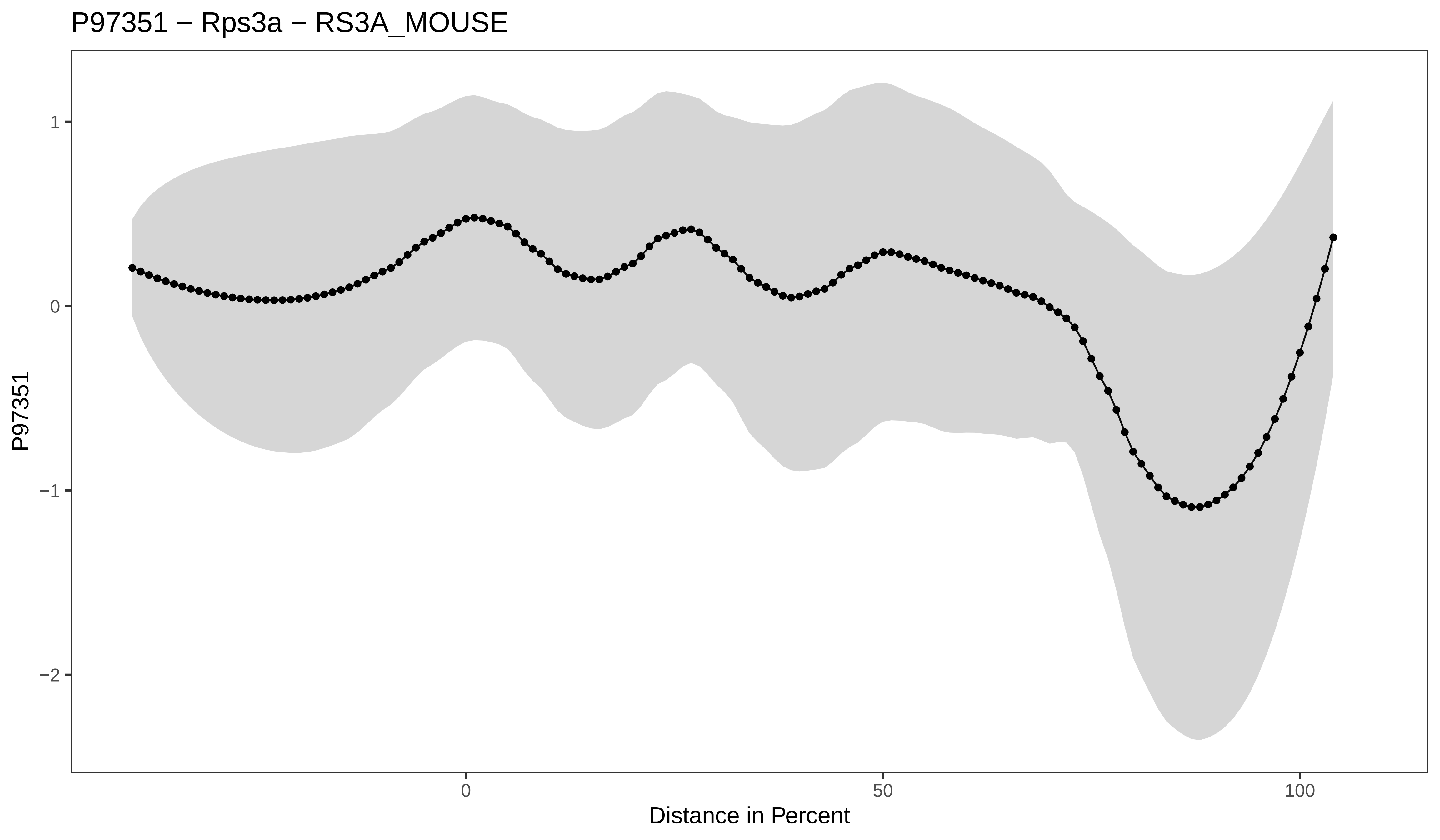

P97351 − Rps3a − RS3A_MOUSE
P97351
Distance in Percent
1
0
−1
−2
0
50
100

P62702 − Rps4x − RS4X_MOUSE

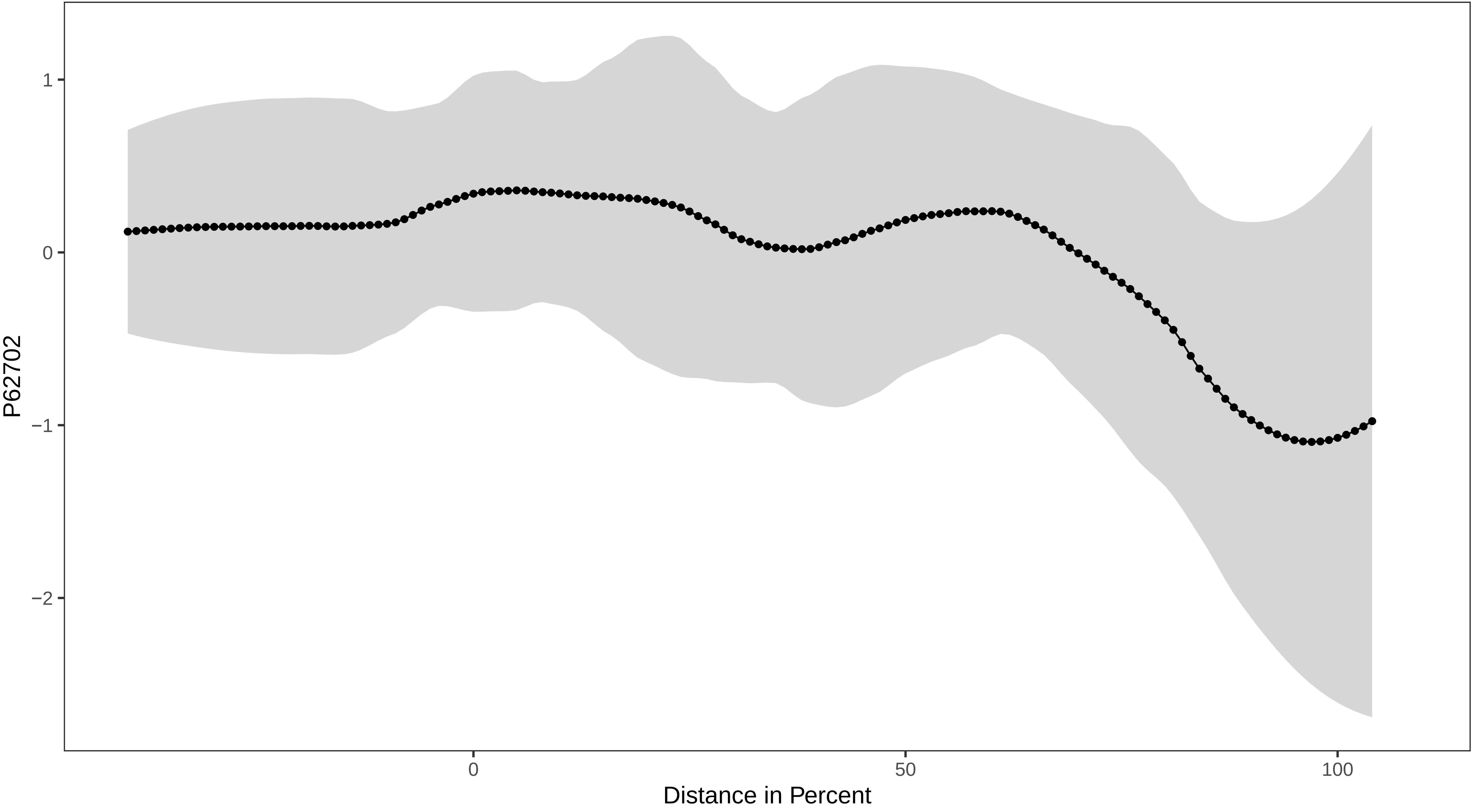

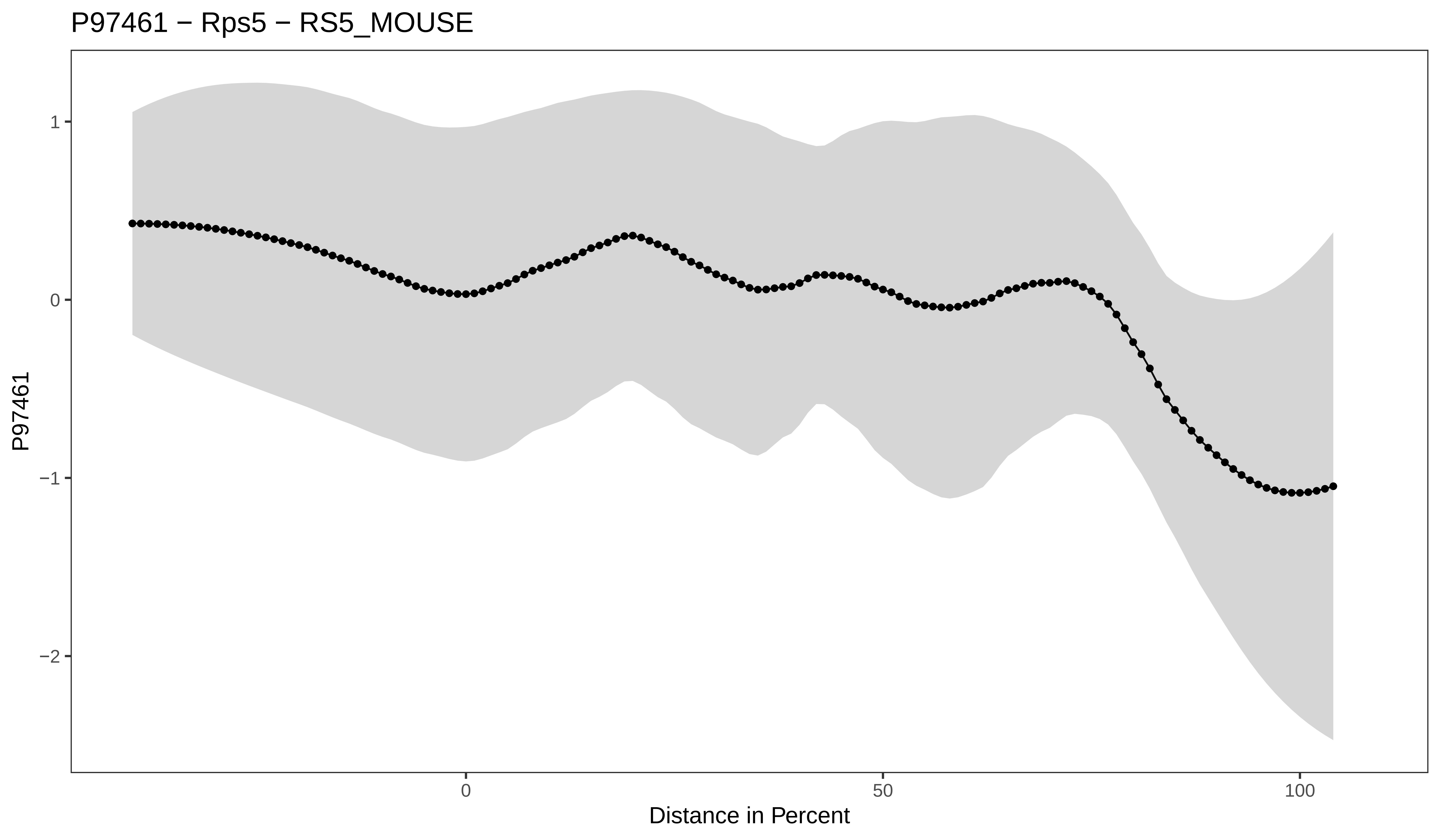

P97461 − Rps5 − RS5_MOUSE
P97461
1
0
−1
−2
0
50
100
Distance in Percent

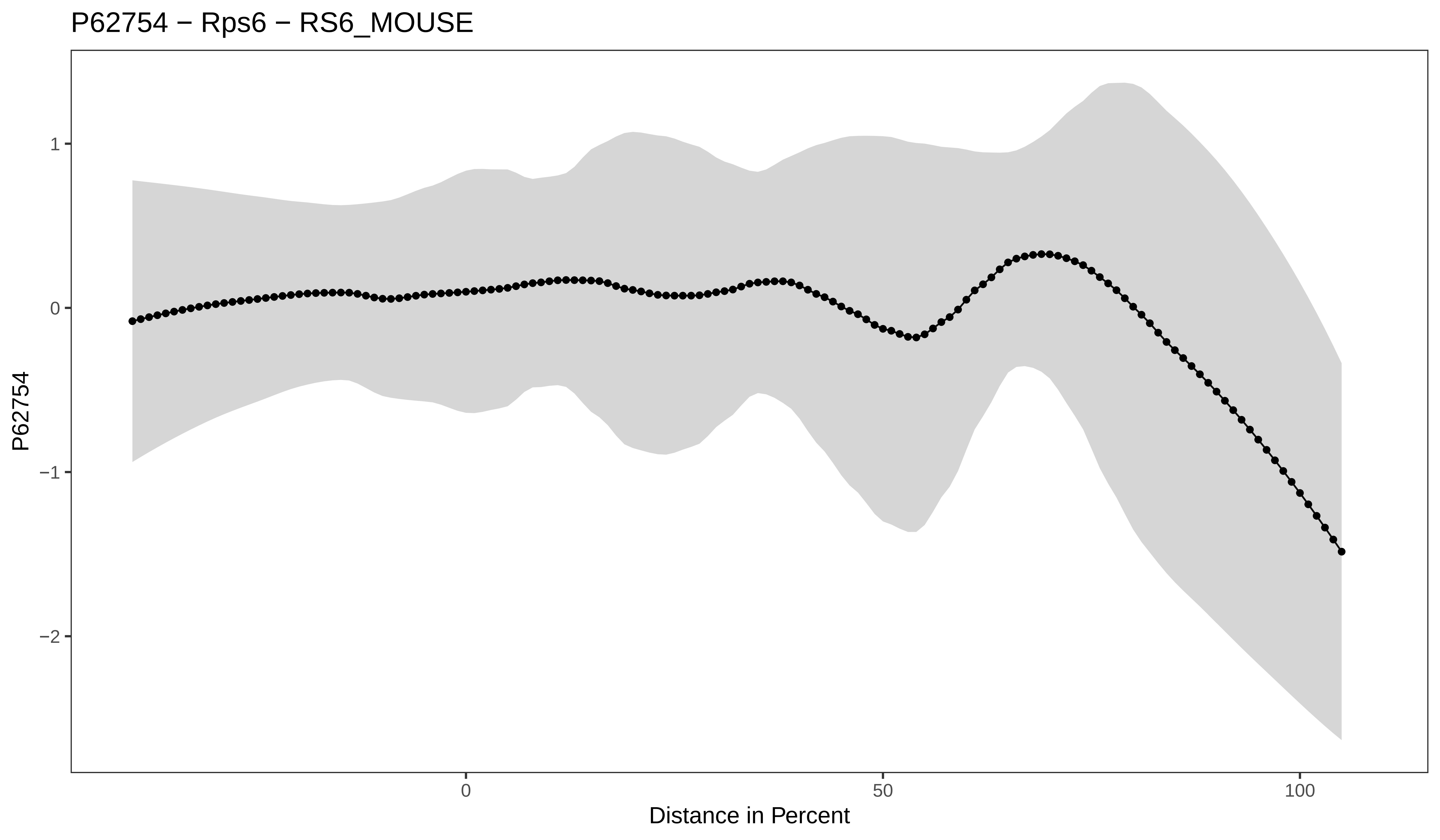
P62754 − Rps6 − RS6_MOUSE
1
0
−1
−2
P62754
0
50
100
Distance in Percent

P18654 − Rps6ka3 − KS6A3_MOUSE

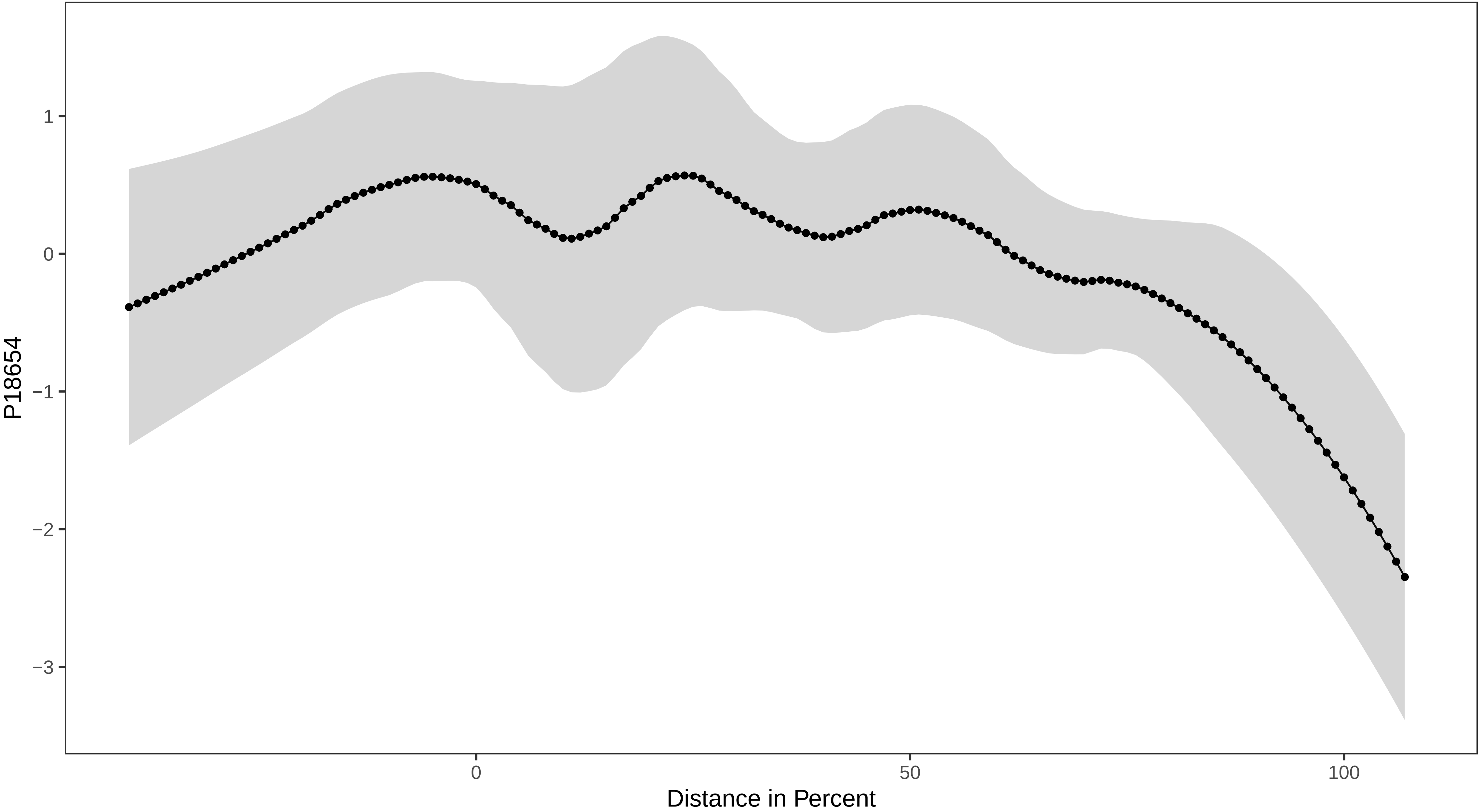

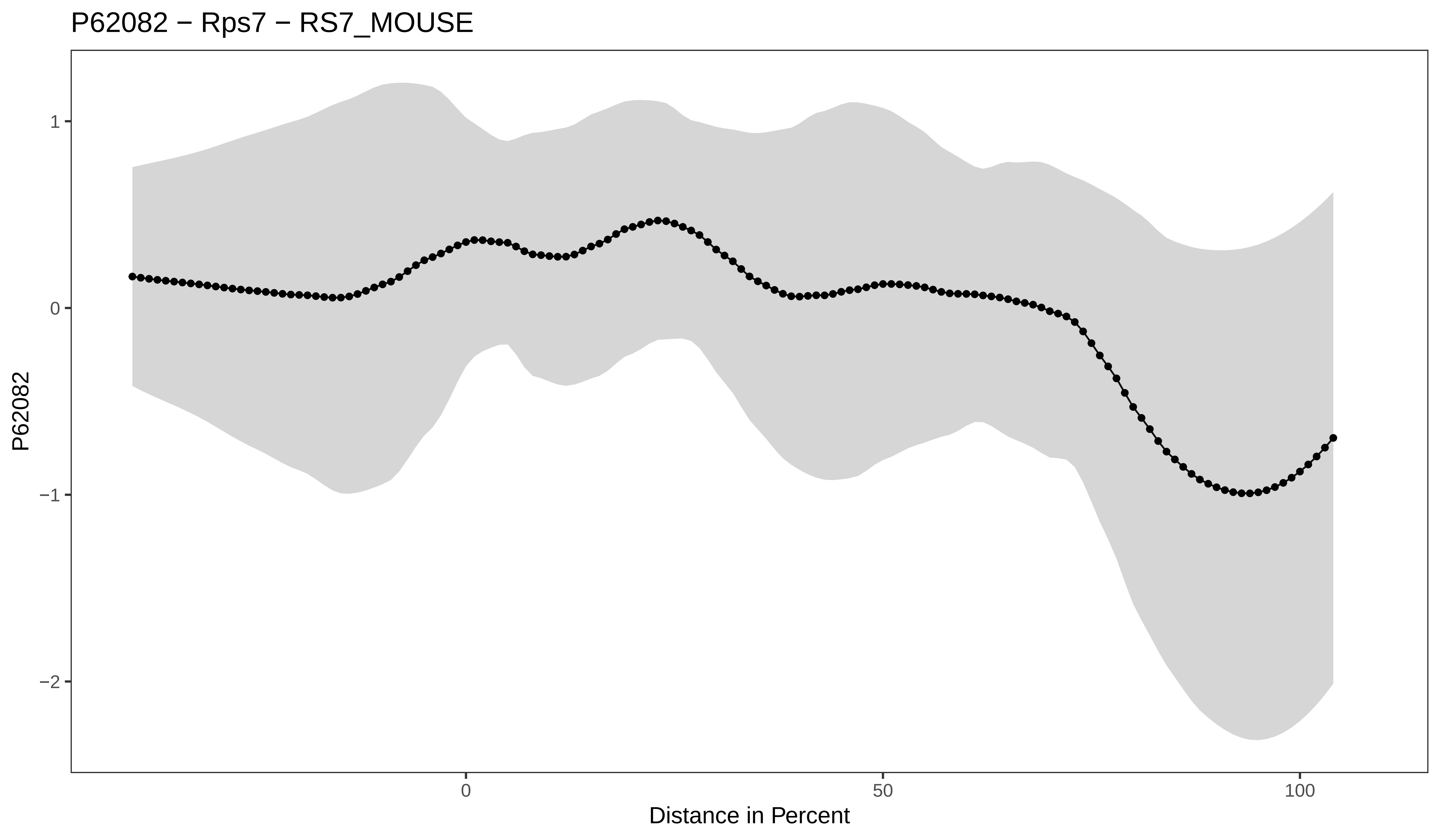
P62082 − Rps7 − RS7_MOUSE
1
0
−1
−2
P62082
0
50
100
Distance in Percent

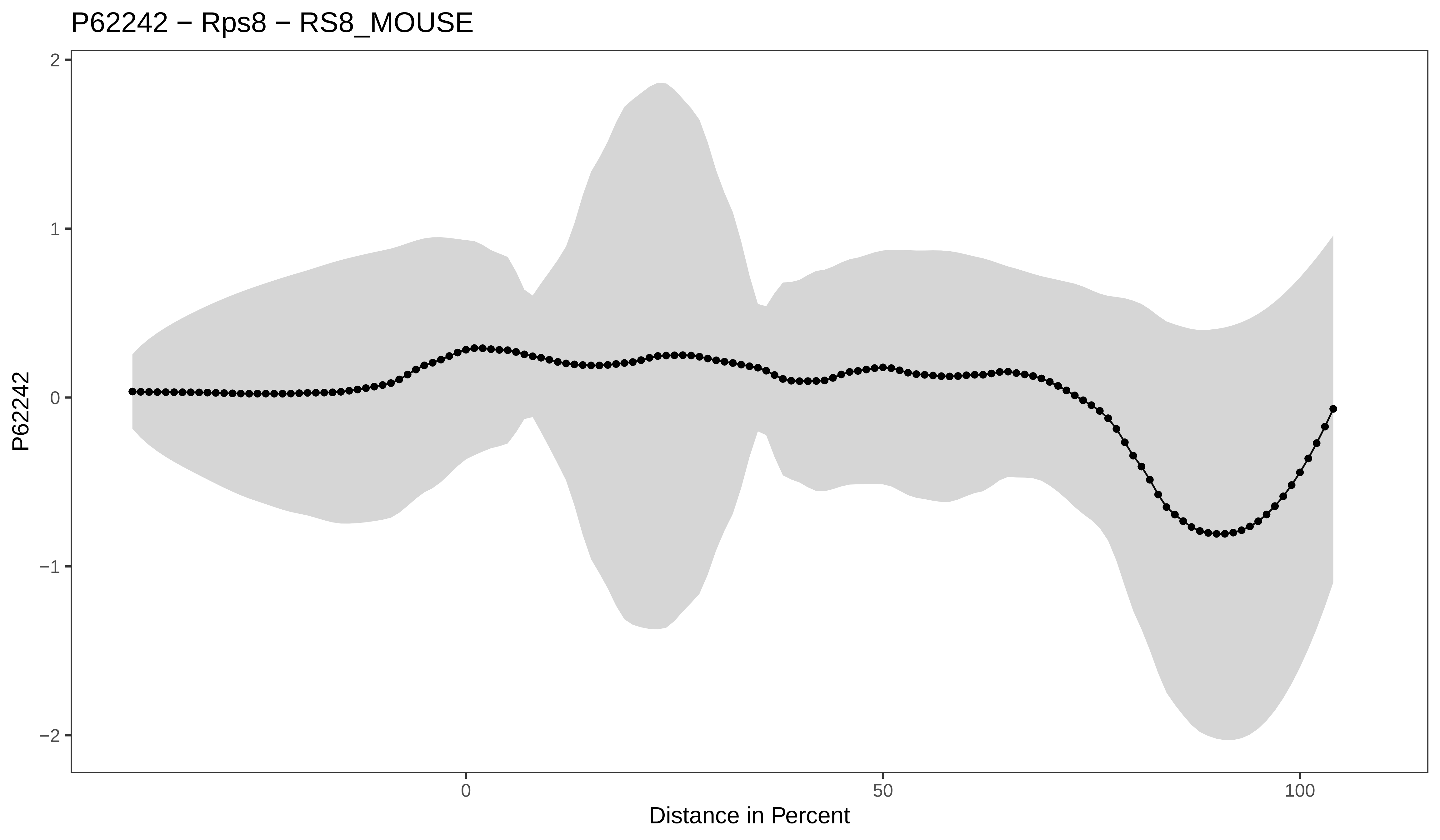

P62242 − Rps8 − RS8_MOUSE
2
1
0
−1
−2
P62242
0
50
100
Distance in Percent

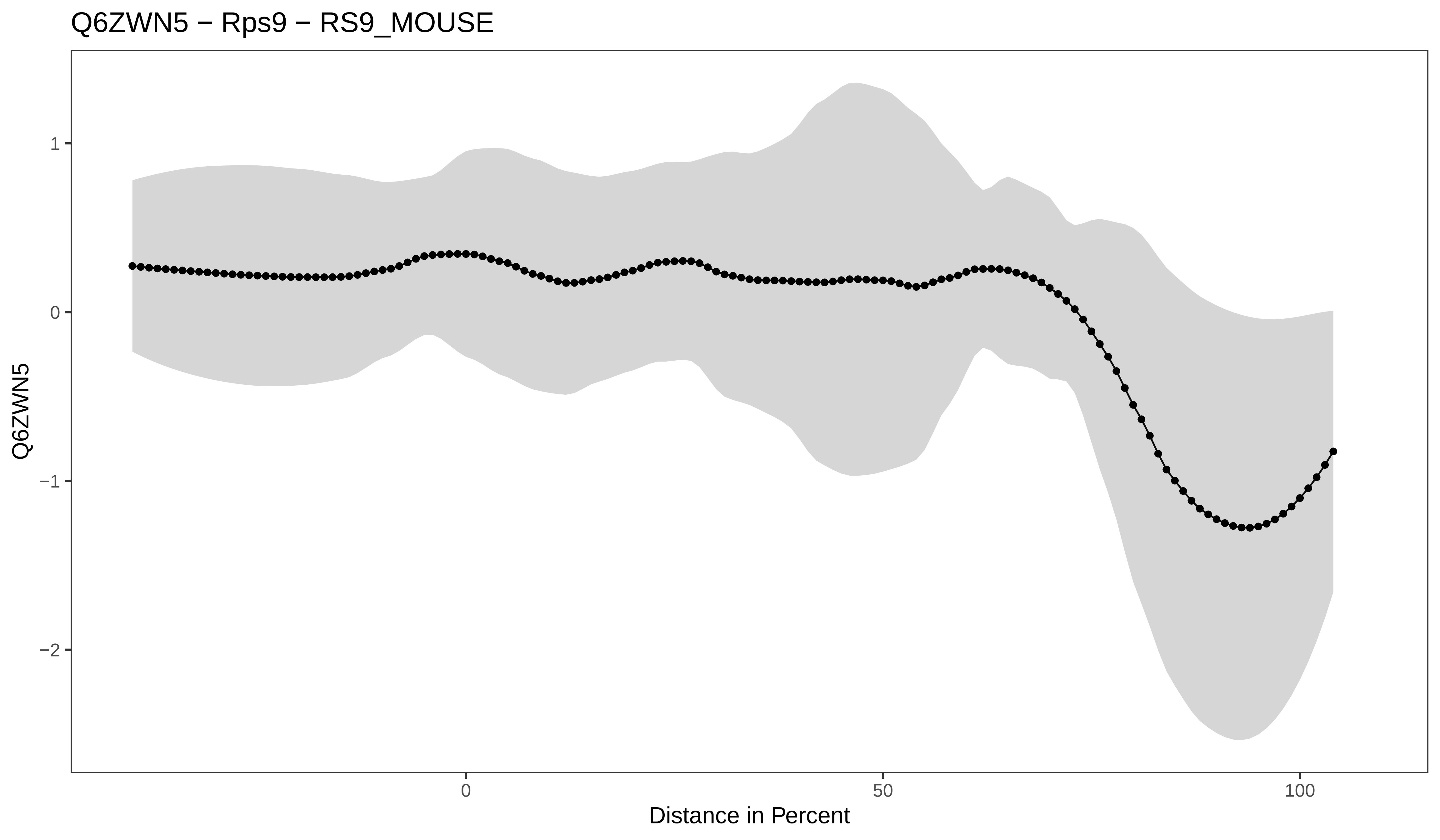

Q6ZWN5 − Rps9 − RS9_MOUSE
Q6ZWN5
1
0
−1
−2
0
50
100
Distance in Percent

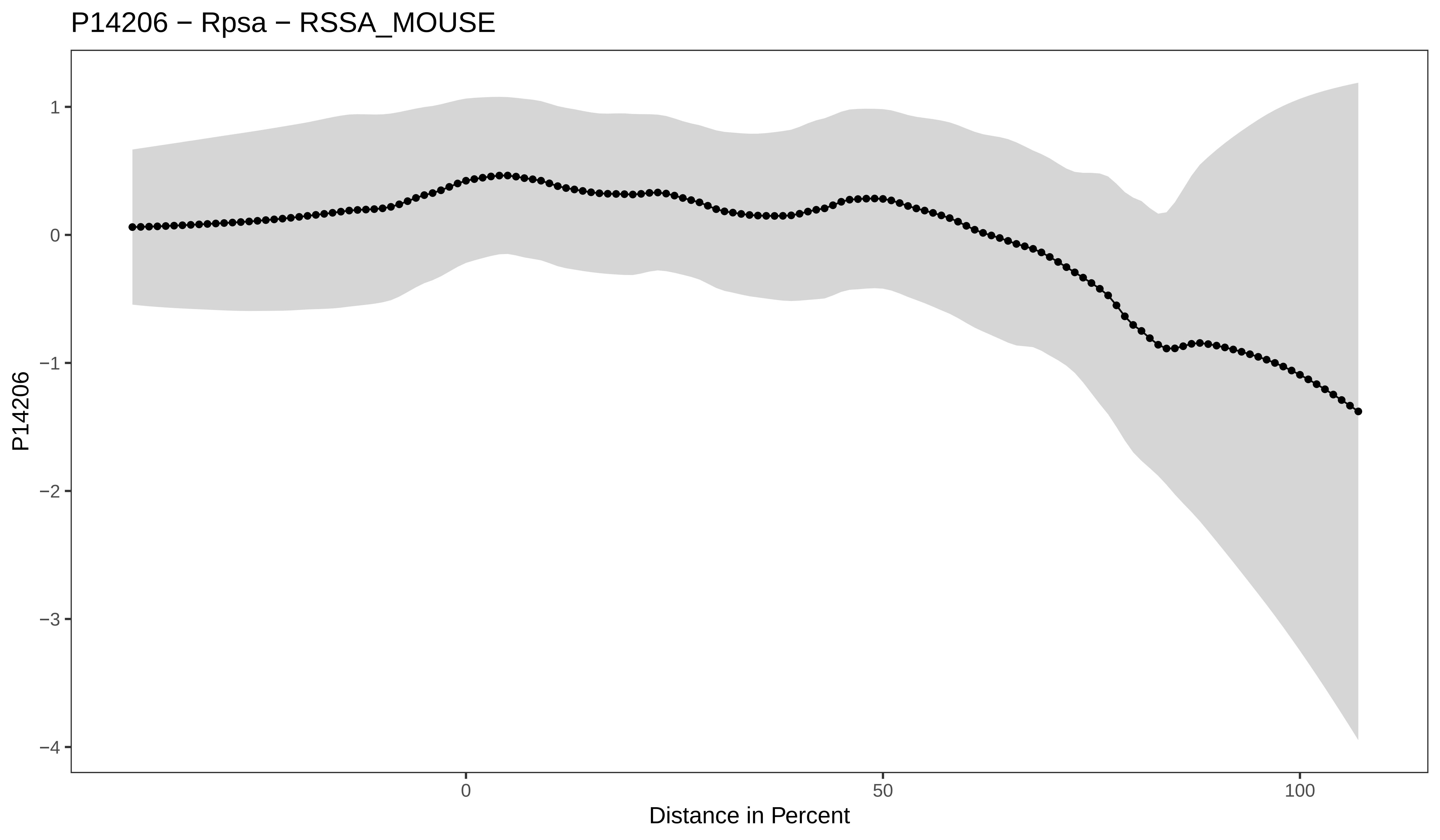

P14206 − Rpsa − RSSA_MOUSE
1
0
−1
−2
−3
−4
P14206
0
50
100
Distance in Percent

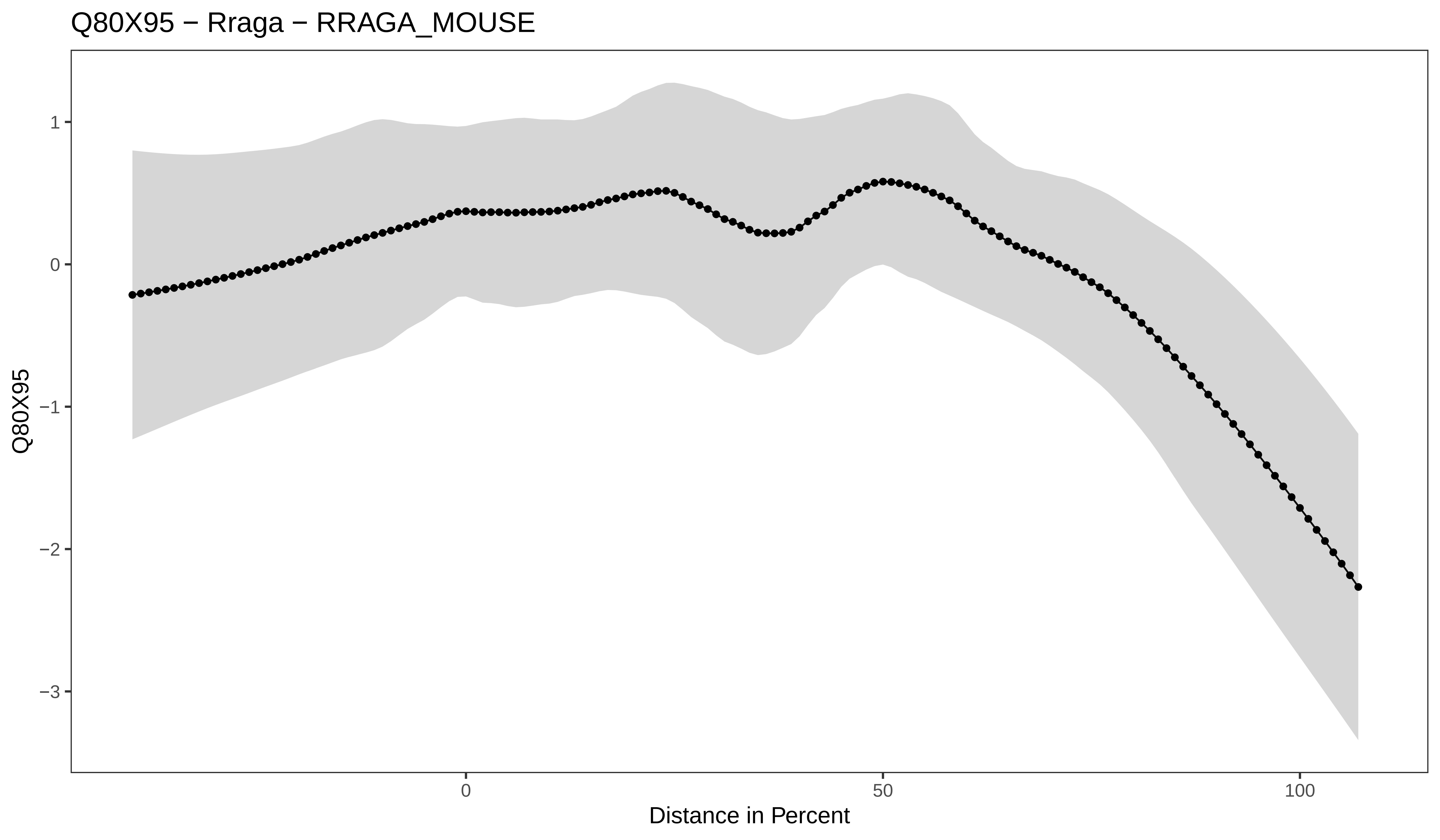

Q80X95 − Rraga − RRAGA_MOUSE
1
0
−1
−2
−3
Q80X95
0
50
100
Distance in Percent

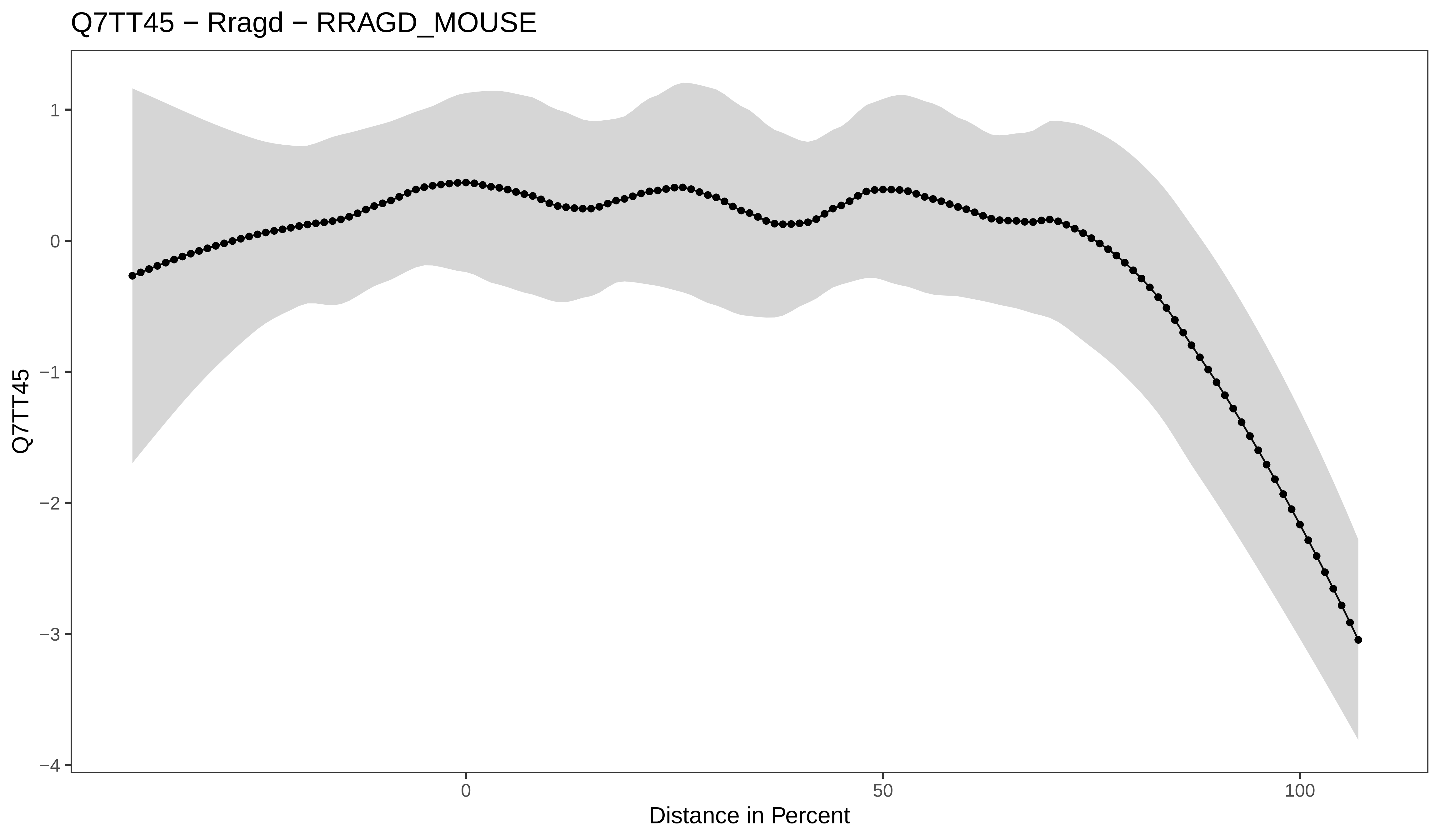

Q7TT45 − Rragd − RRAGD_MOUSE
Q7TT45
1
0
−1
−2
−3
−4
0
50
100
Distance in Percent

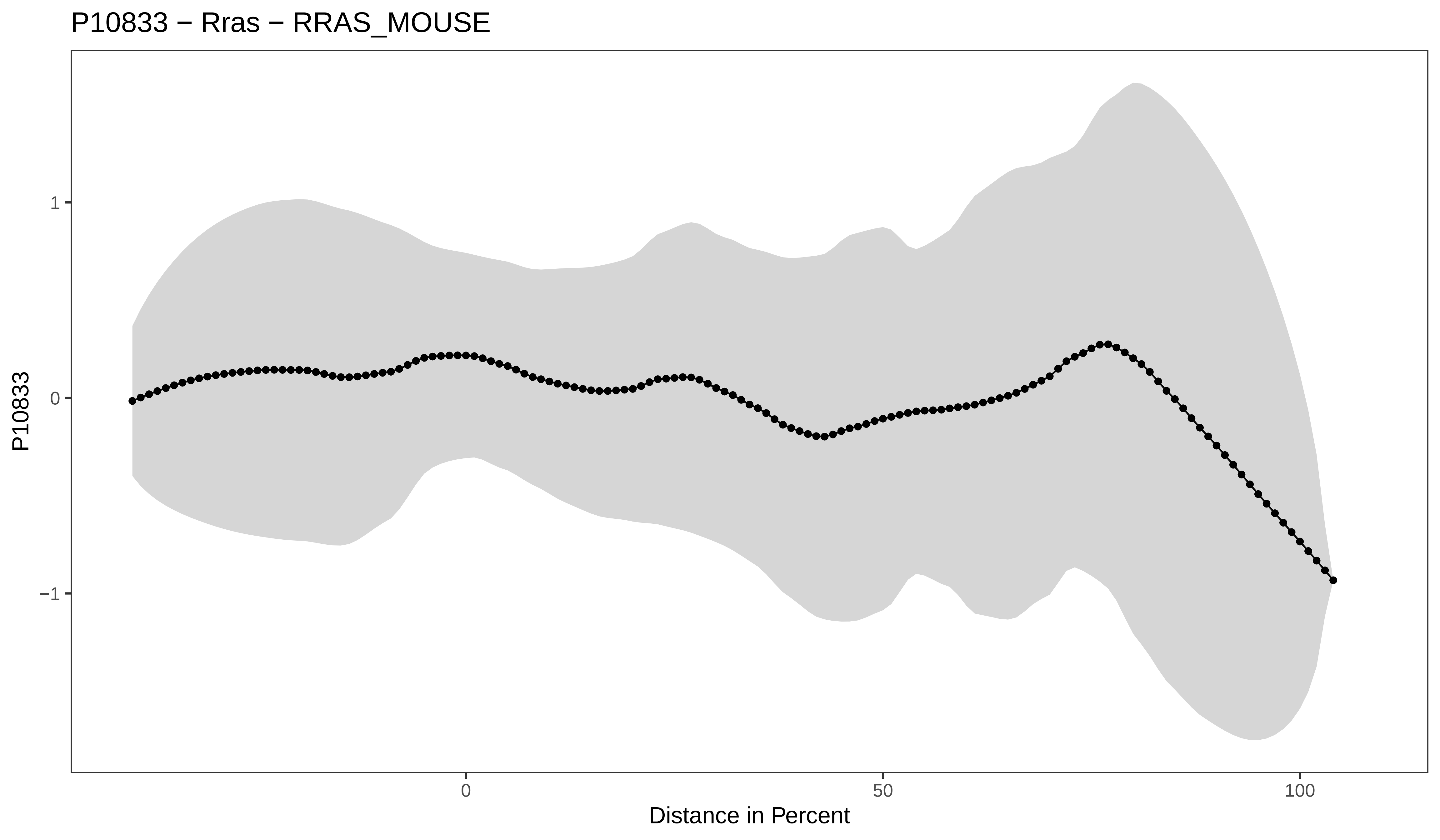
P10833 − Rras − RRAS_MOUSE
P10833
1
0
−1
0
50
100
Distance in Percent

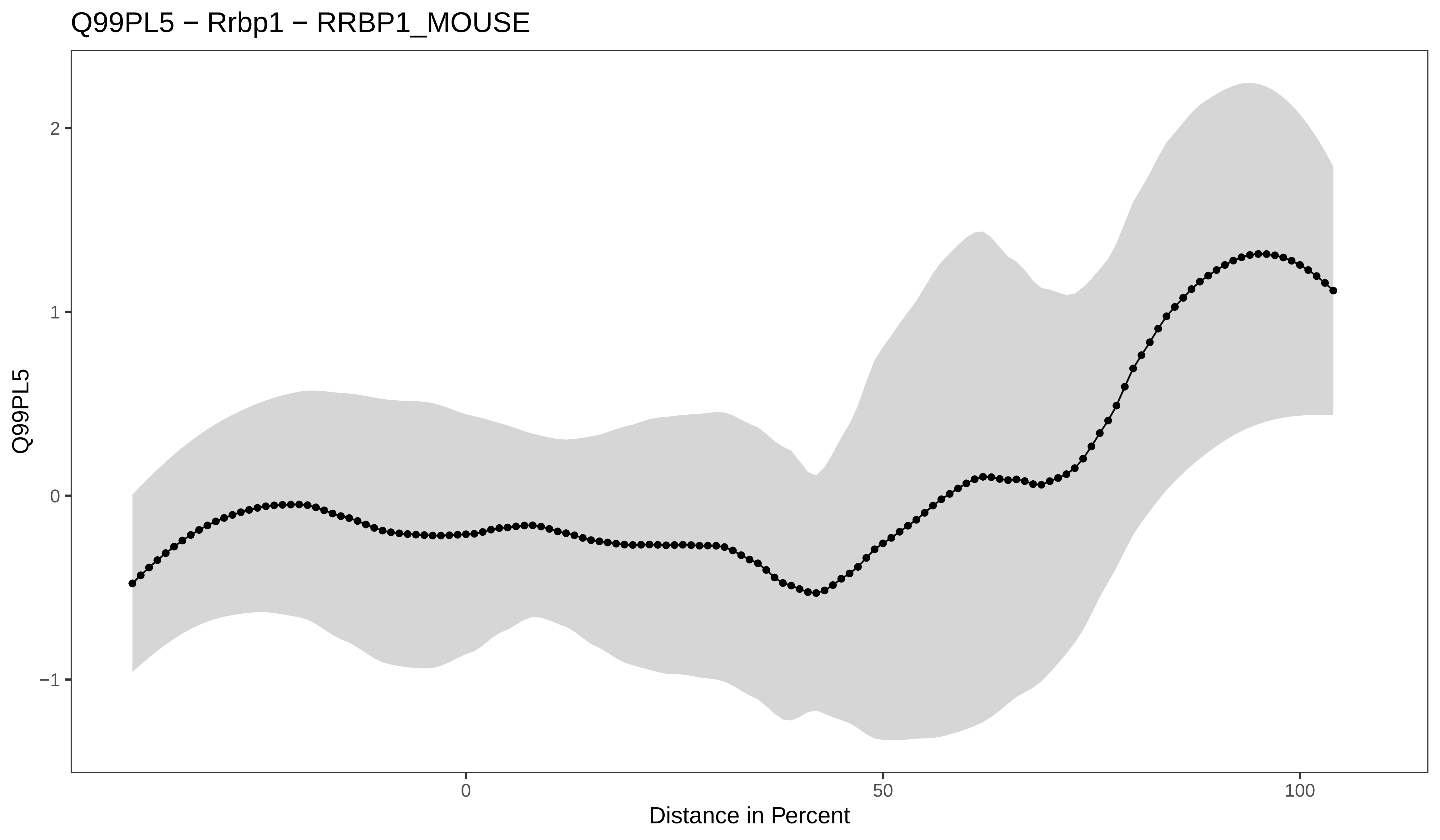
Q99PL5 − Rrbp1 − RRBP1_MOUSE
Q99PL5
2
1
0
−1
0
50
100
Distance in Percent

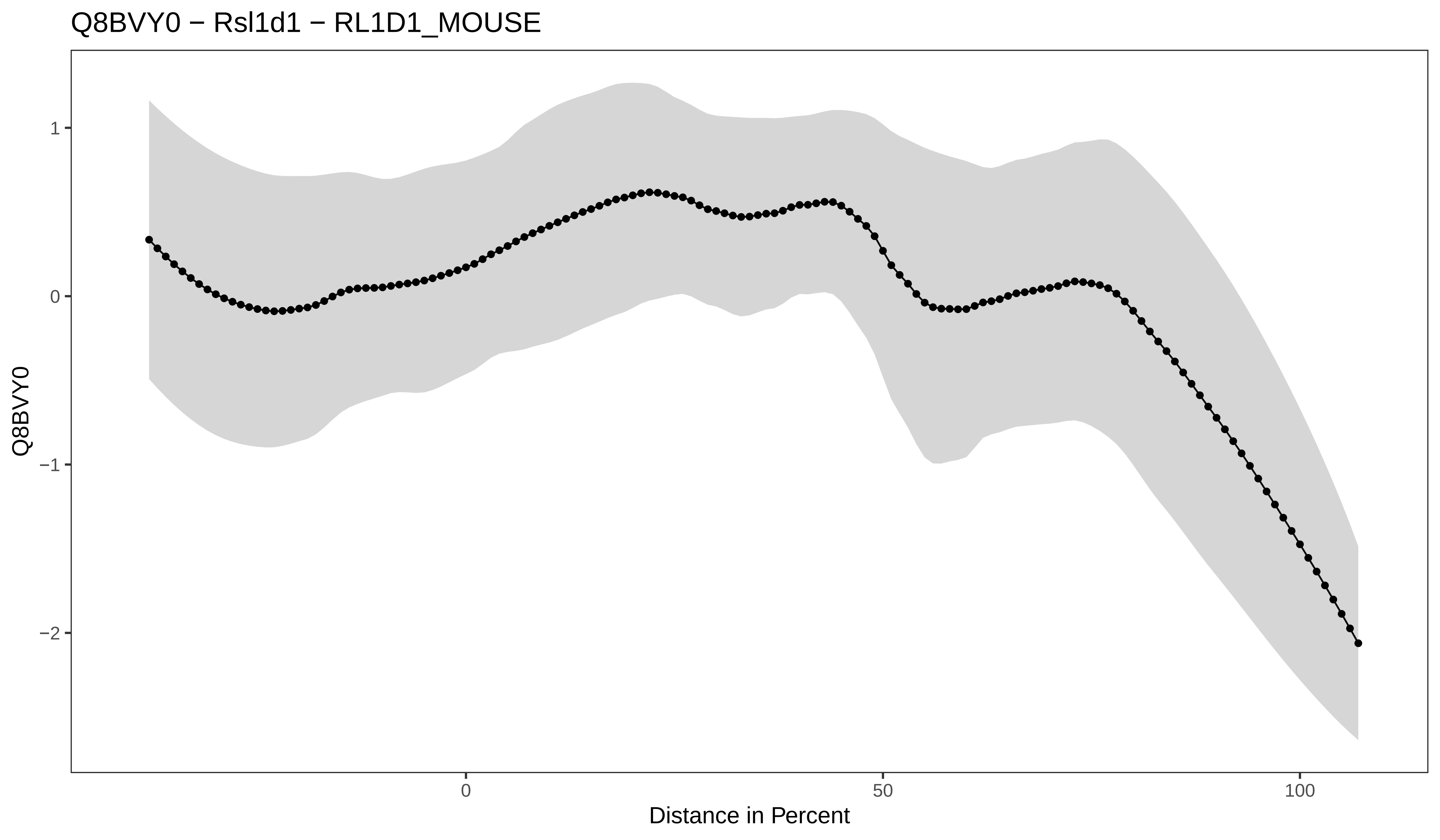
Q8BVY0 − Rsl1d1 − RL1D1_MOUSE
Q8BVY0
1
0
−1
−2
0
50
100
Distance in Percent

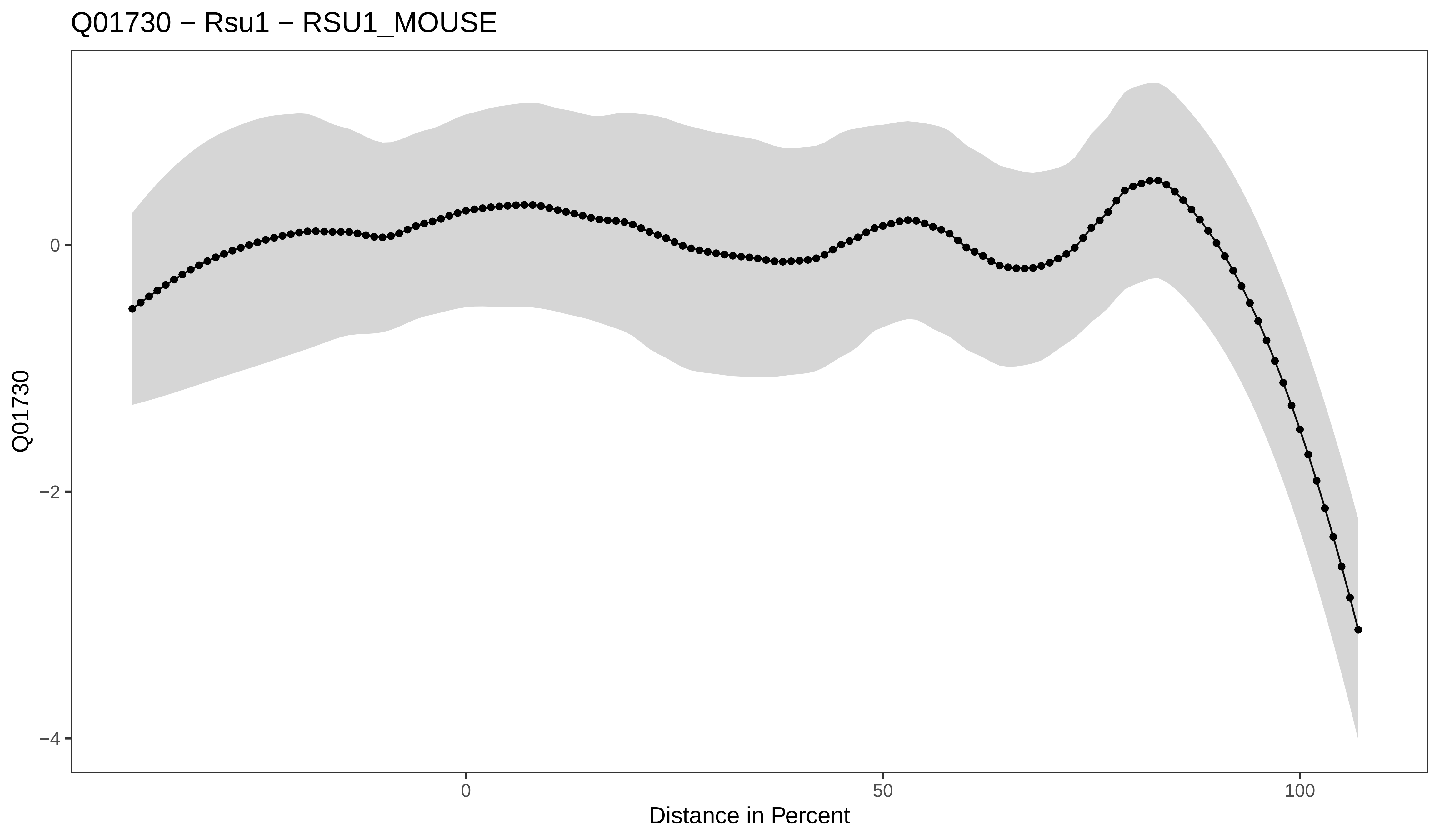

Q01730 − Rsu1 − RSU1_MOUSE
Q01730
0
−2
−4
0
50
100
Distance in Percent

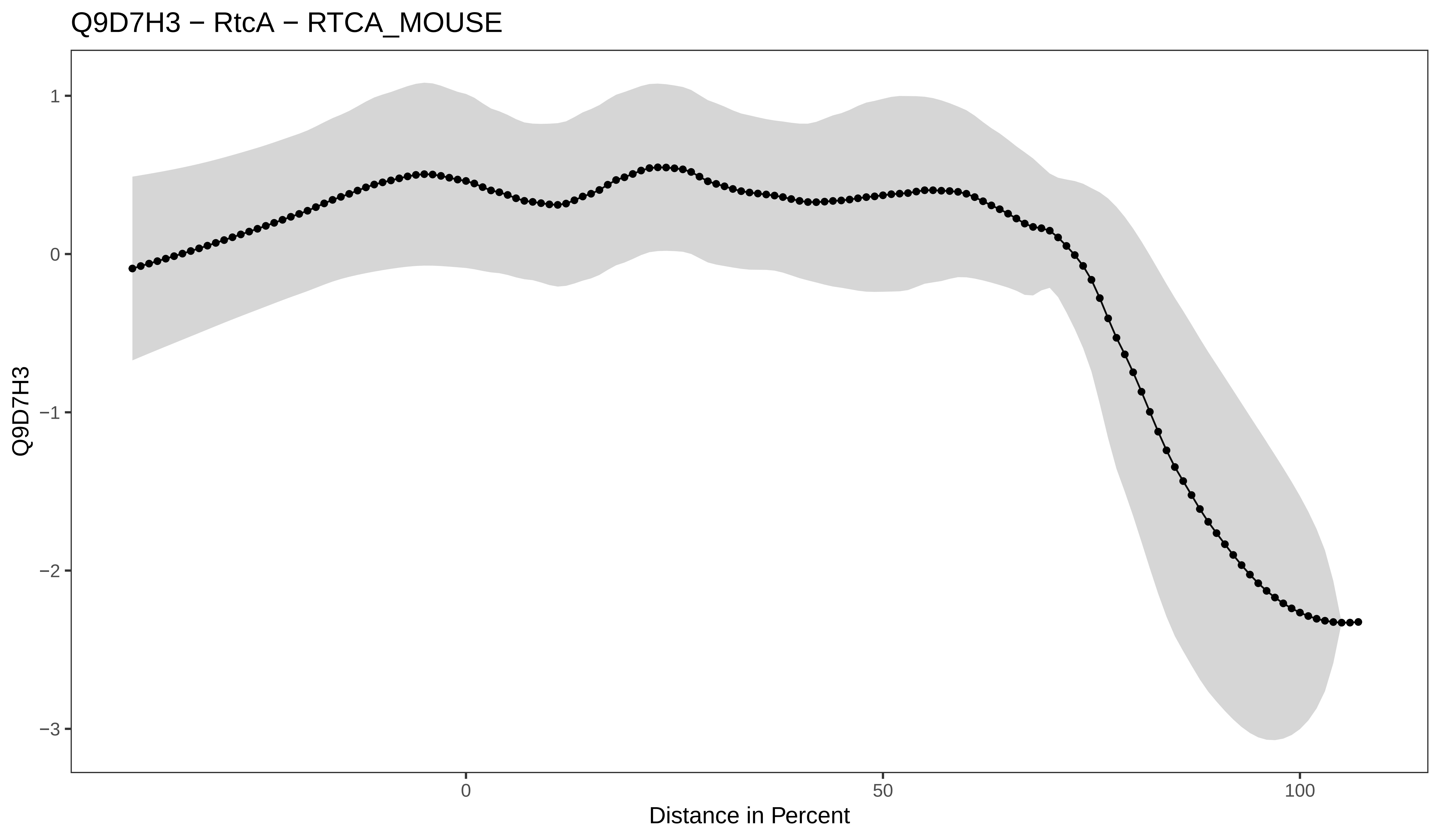

Q9D7H3 − RtcA − RTCA_MOUSE
Q9D7H3
1
0
−1
−2
−3
0
50
100
Distance in Percent

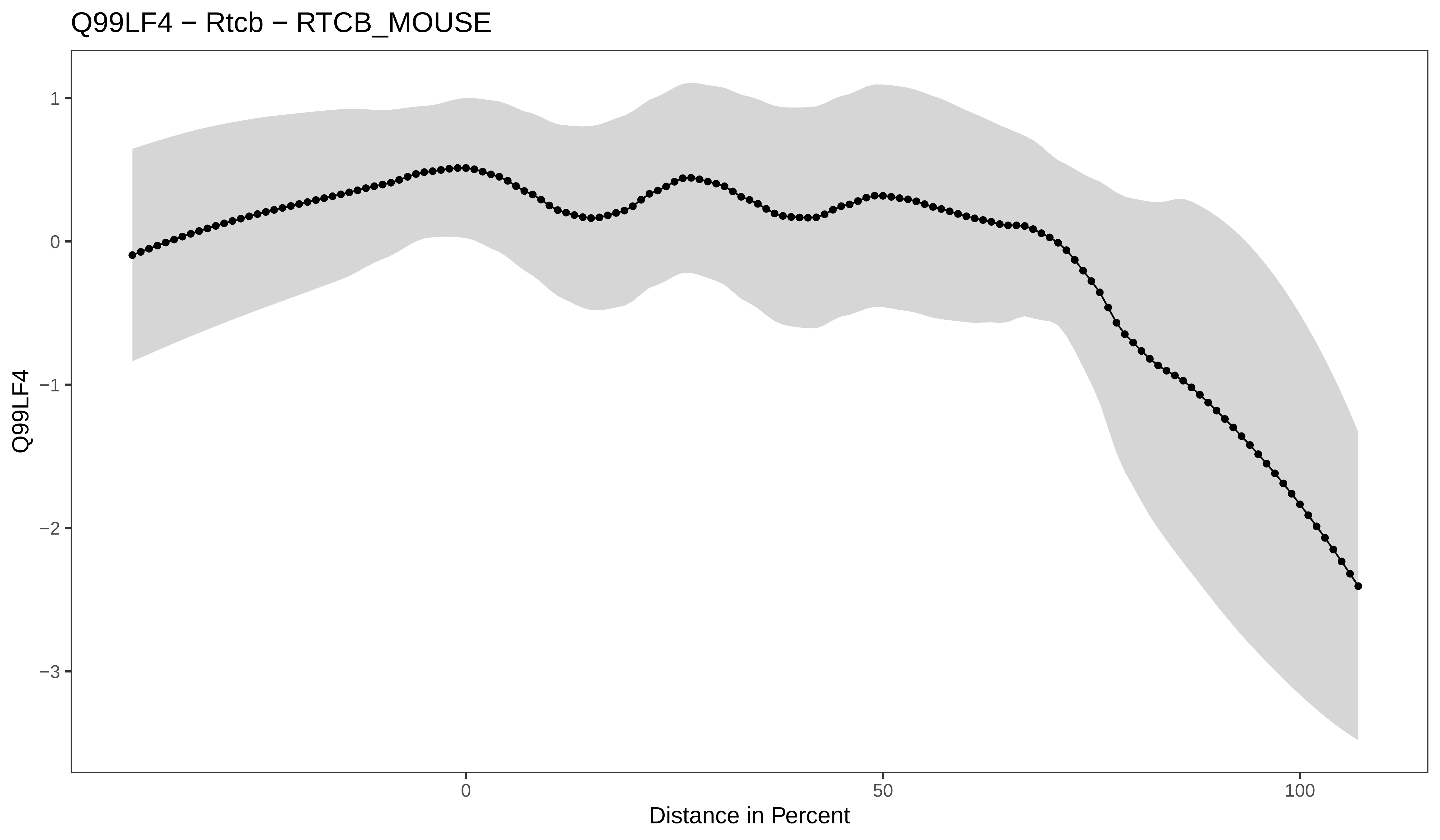

Q99LF4 − Rtcb − RTCB_MOUSE
Q99LF4
Distance in Percent
1
0
−1
−2
−3
0
50
100

O70622 − Rtn2 − RTN2_MOUSE

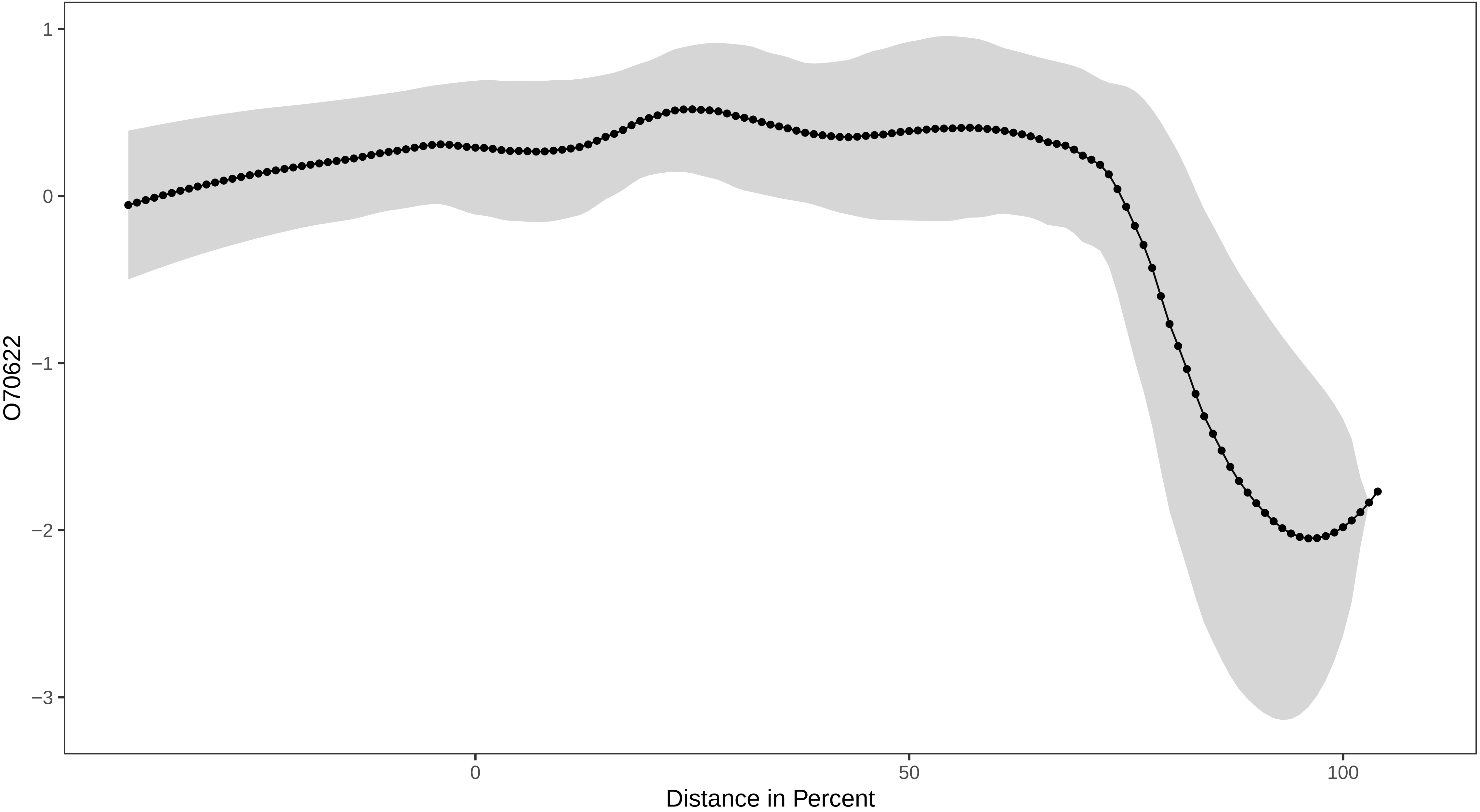

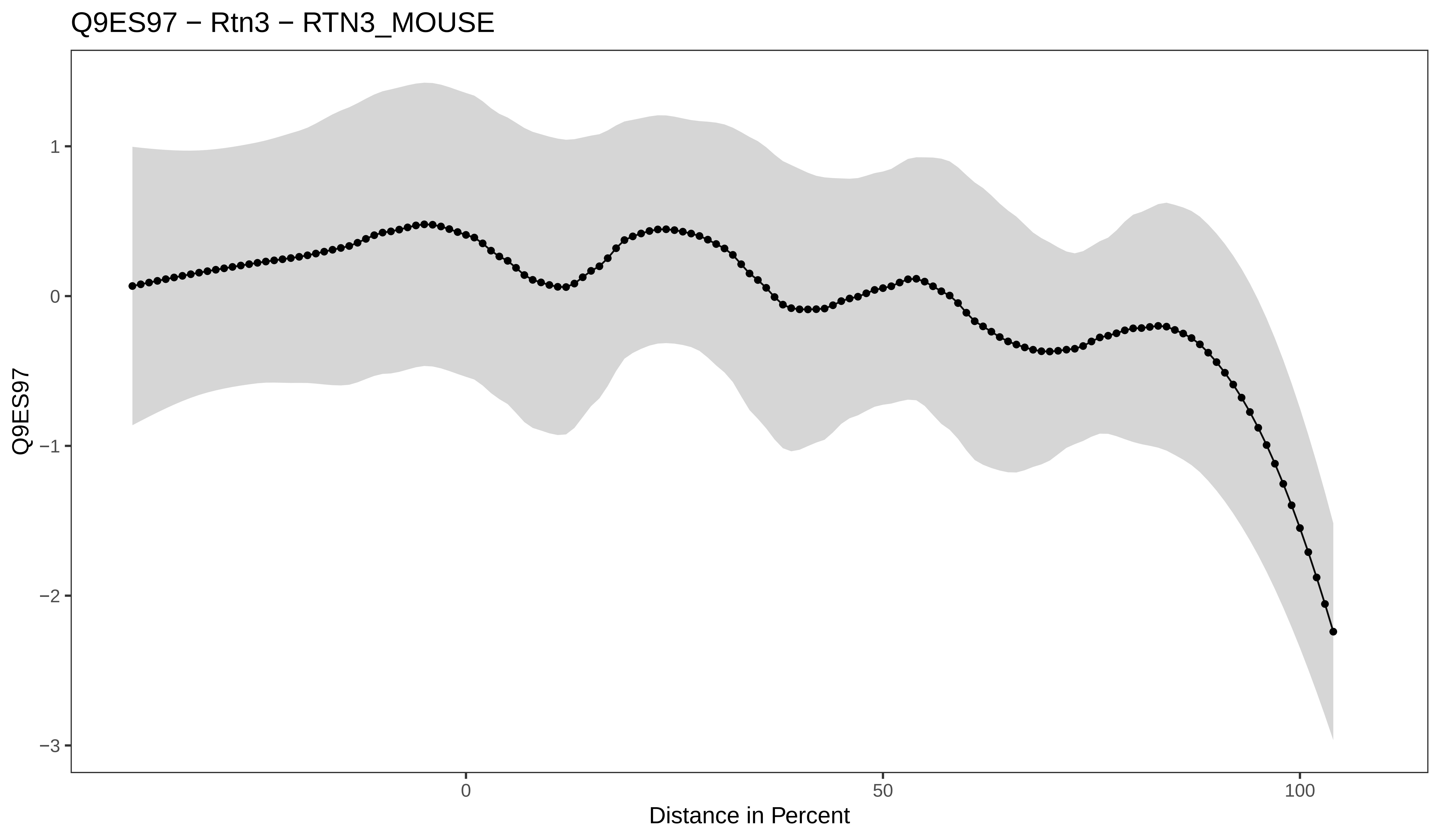

Q9ES97 − Rtn3 − RTN3_MOUSE
Q9ES97
Distance in Percent
1
0
−1
−2
−3
0
50
100

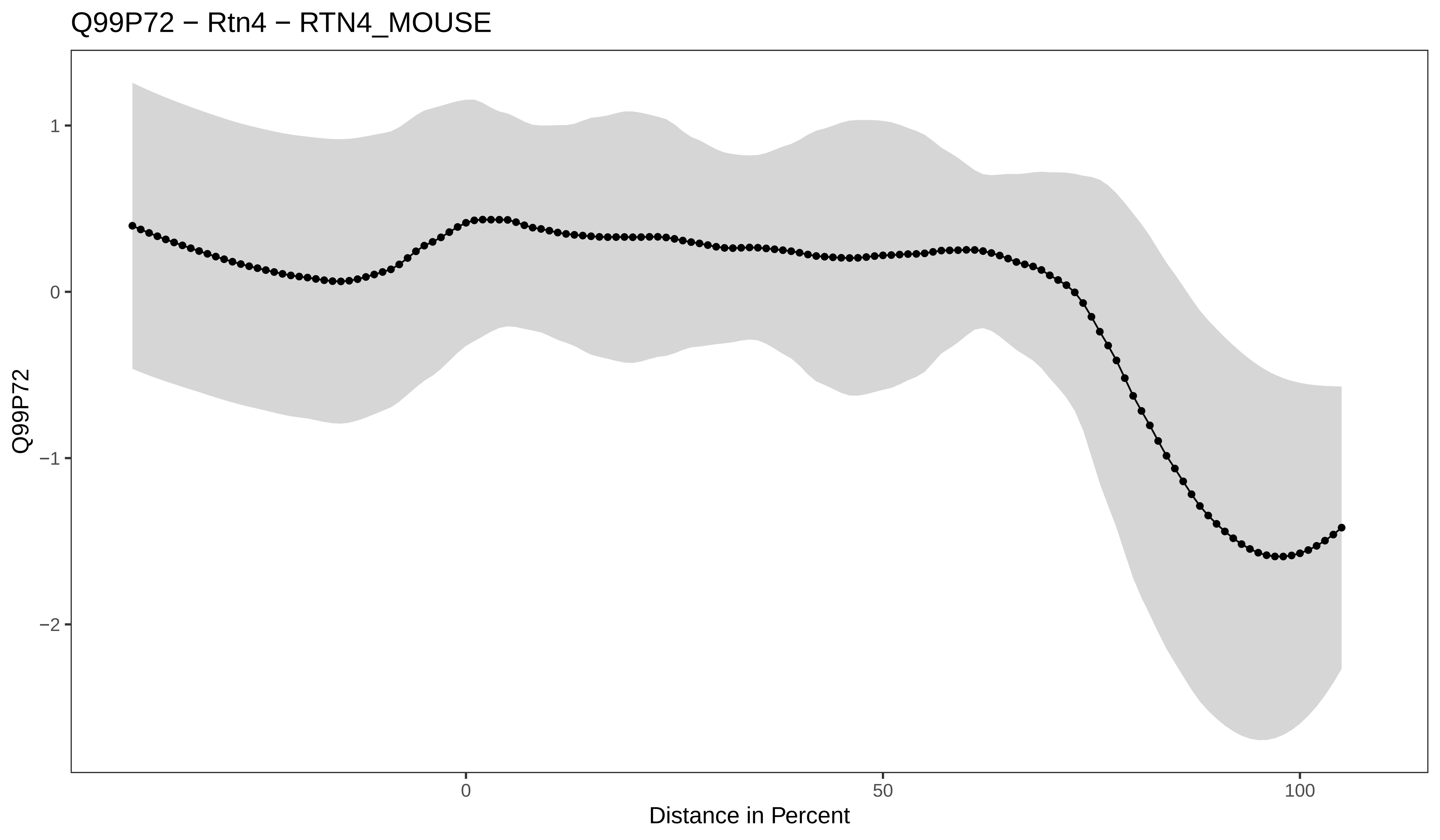

Q99P72 − Rtn4 − RTN4_MOUSE
Q99P72
Distance in Percent
1
0
−1
−2
0
50
100

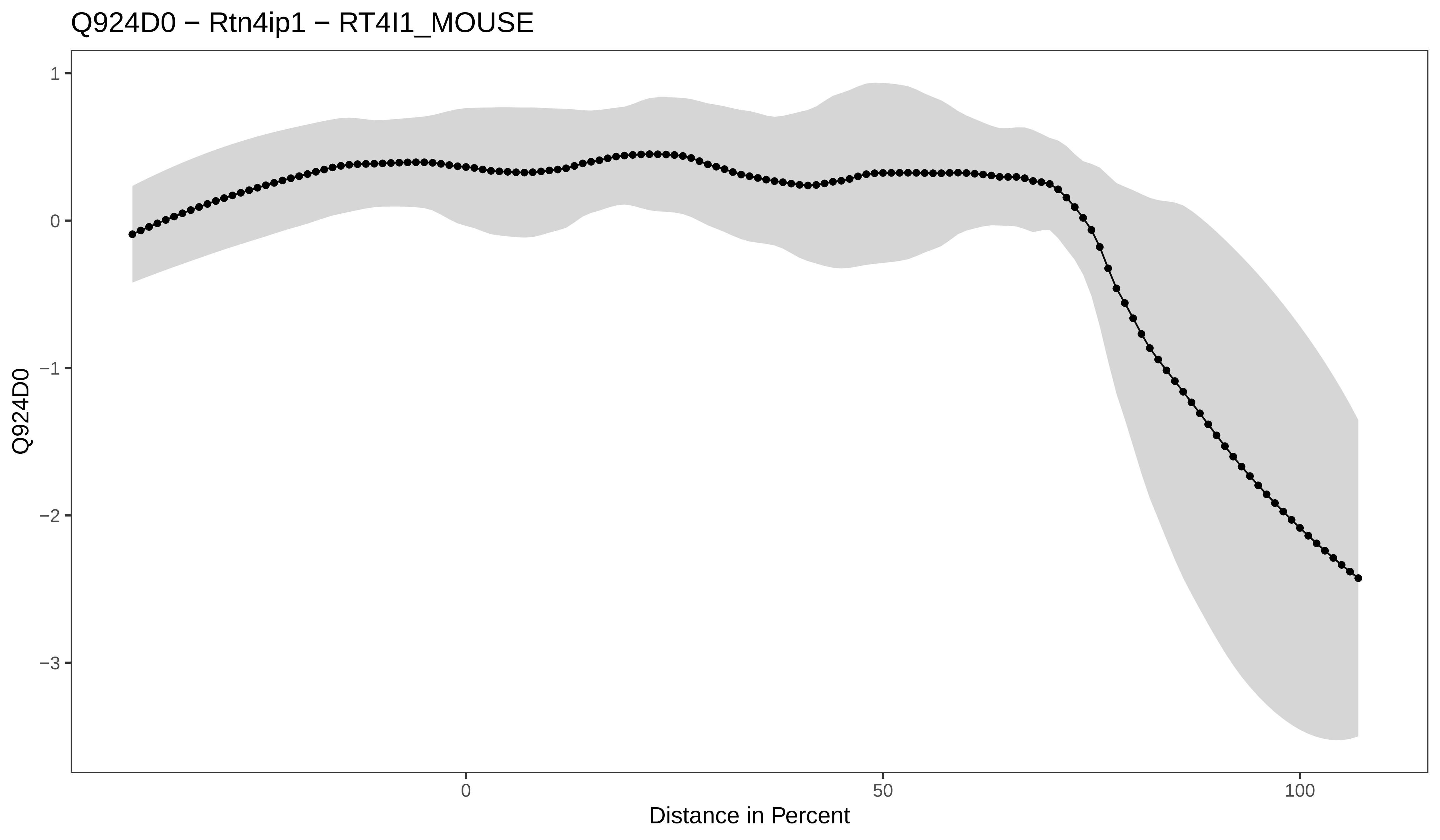
Q924D0 − Rtn4ip1 − RT4I1_MOUSE
1
0
−1
−2
−3
Q924D0
0
50
100
Distance in Percent

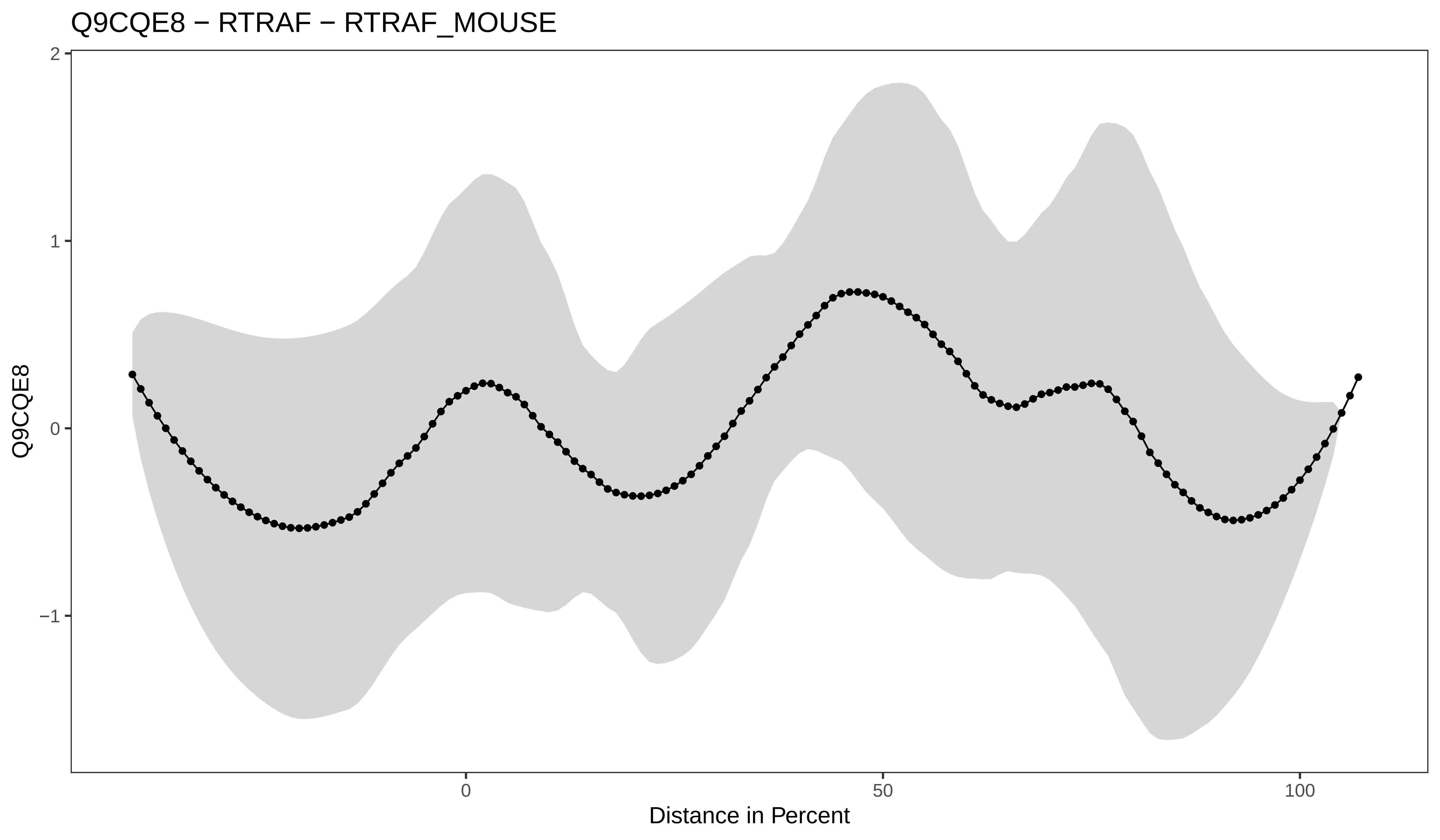

Q9CQE8 − RTRAF − RTRAF_MOUSE
Q9CQE8
2
1
0
−1
0
50
100
Distance in Percent

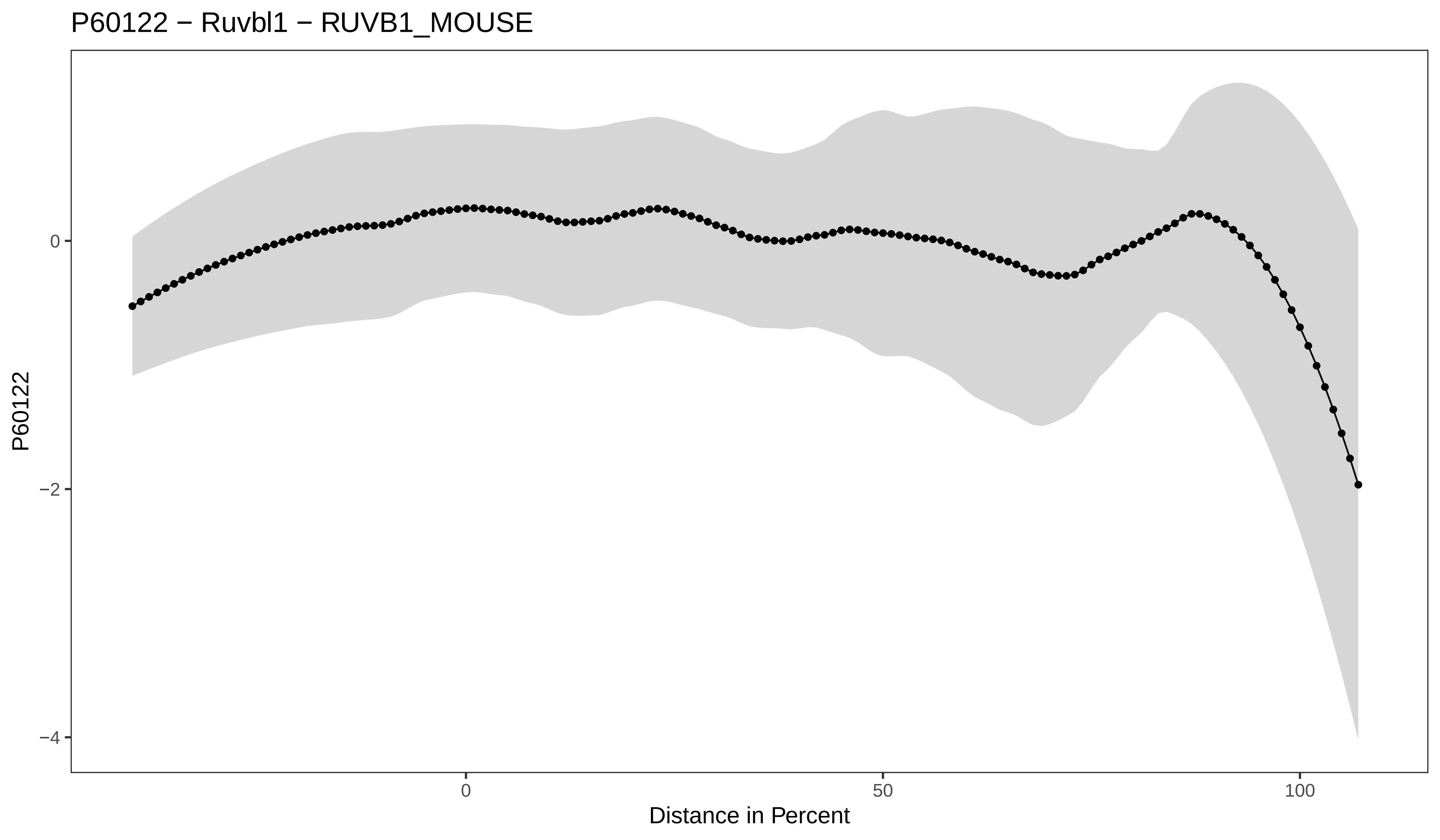

P60122 − Ruvbl1 − RUVB1_MOUSE
P60122
0
−2
−4
0
50
100
Distance in Percent

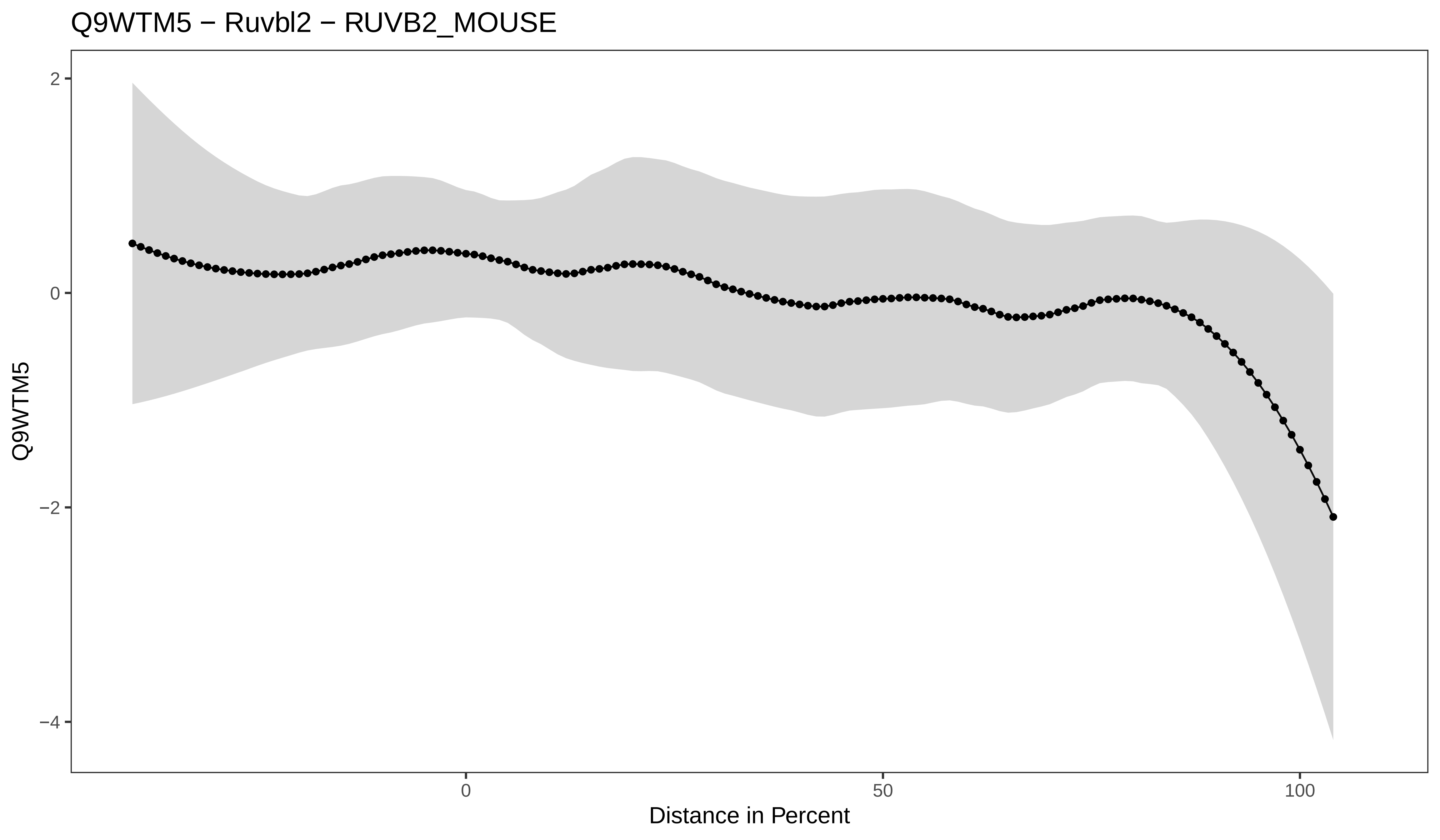

Q9WTM5 − Ruvbl2 − RUVB2_MOUSE
Q9WTM5
Distance in Percent
2
0
−2
−4
0
50
100

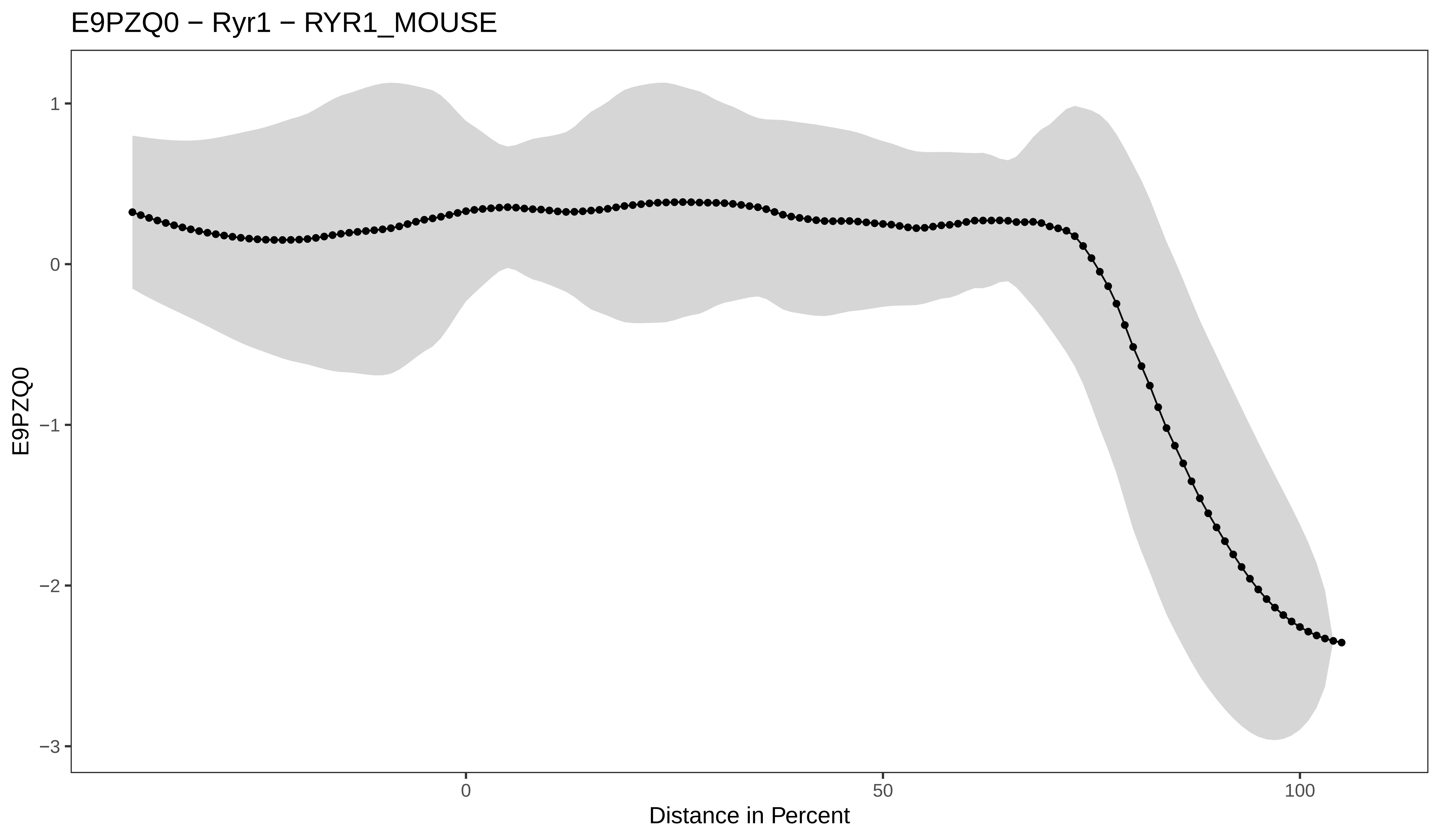

E9PZQ0 − Ryr1 − RYR1_MOUSE
1
0
−1
−2
−3
E9PZQ0
0
50
100
Distance in Percent

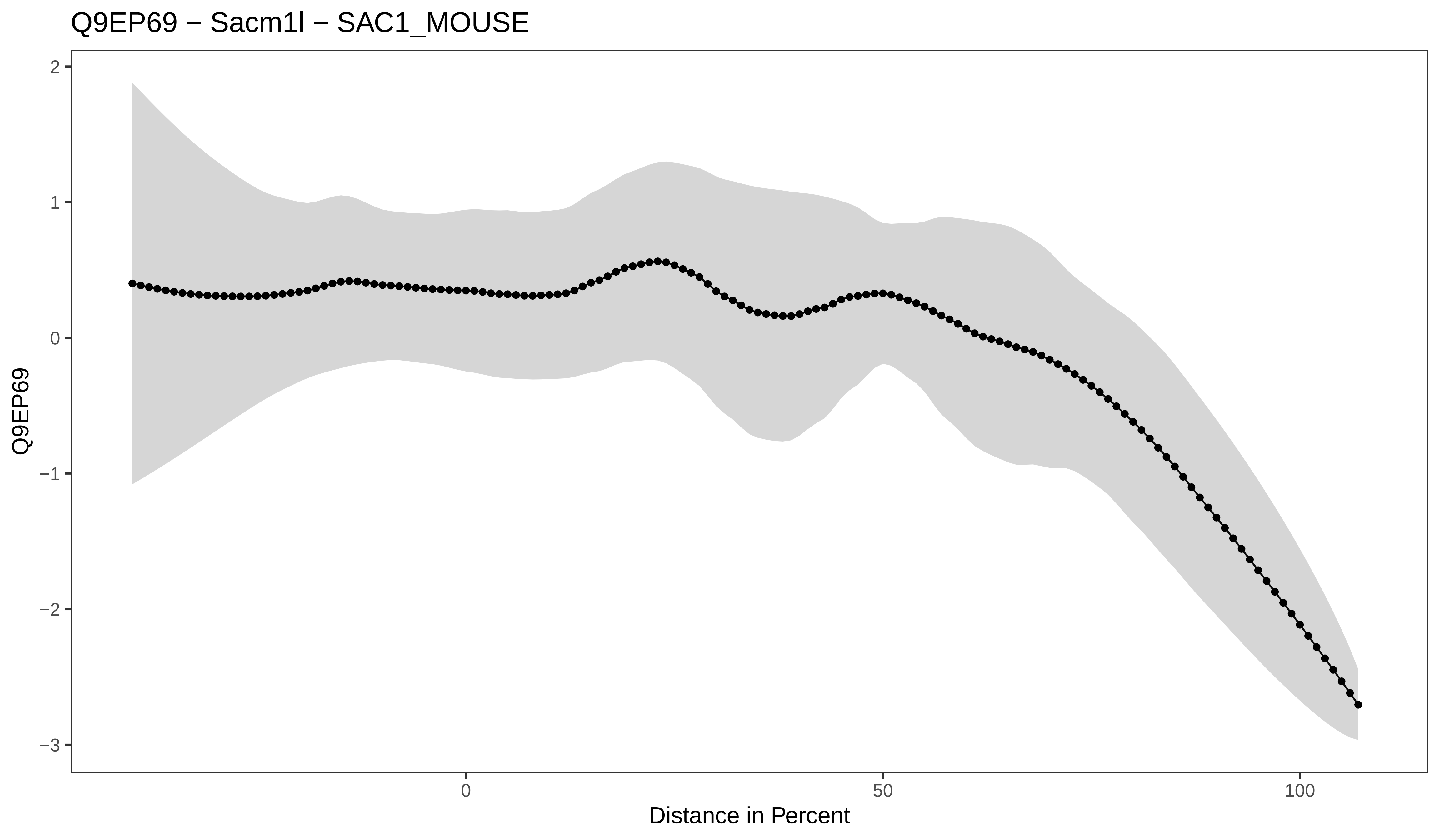

Q9EP69 − Sacm1l − SAC1_MOUSE
2
1
0
−1
−2
−3
Q9EP69
0
50
100
Distance in Percent

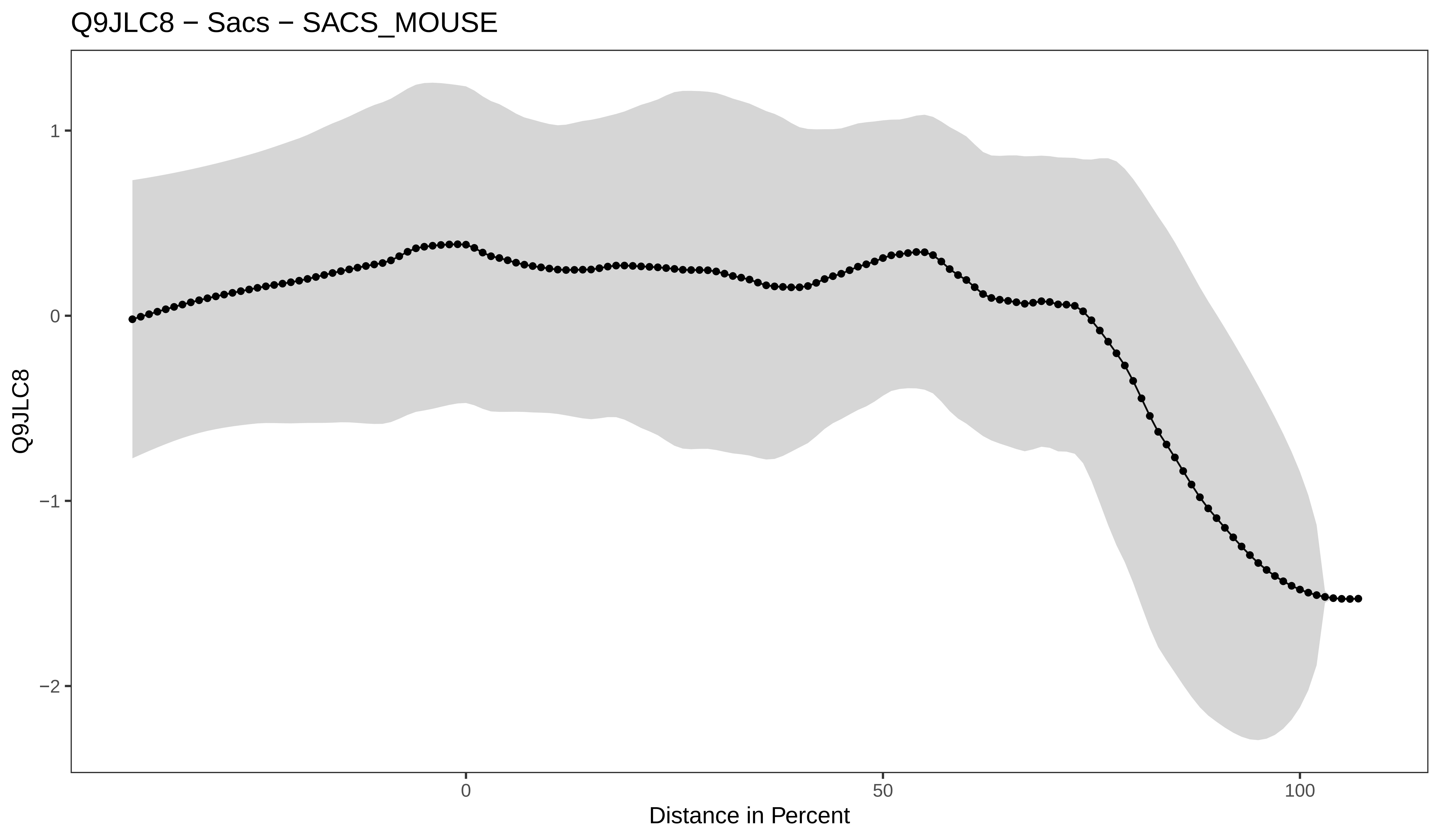

Q9JLC8 − Sacs − SACS_MOUSE
Q9JLC8
Distance in Percent
1
0
−1
−2
0
50
100

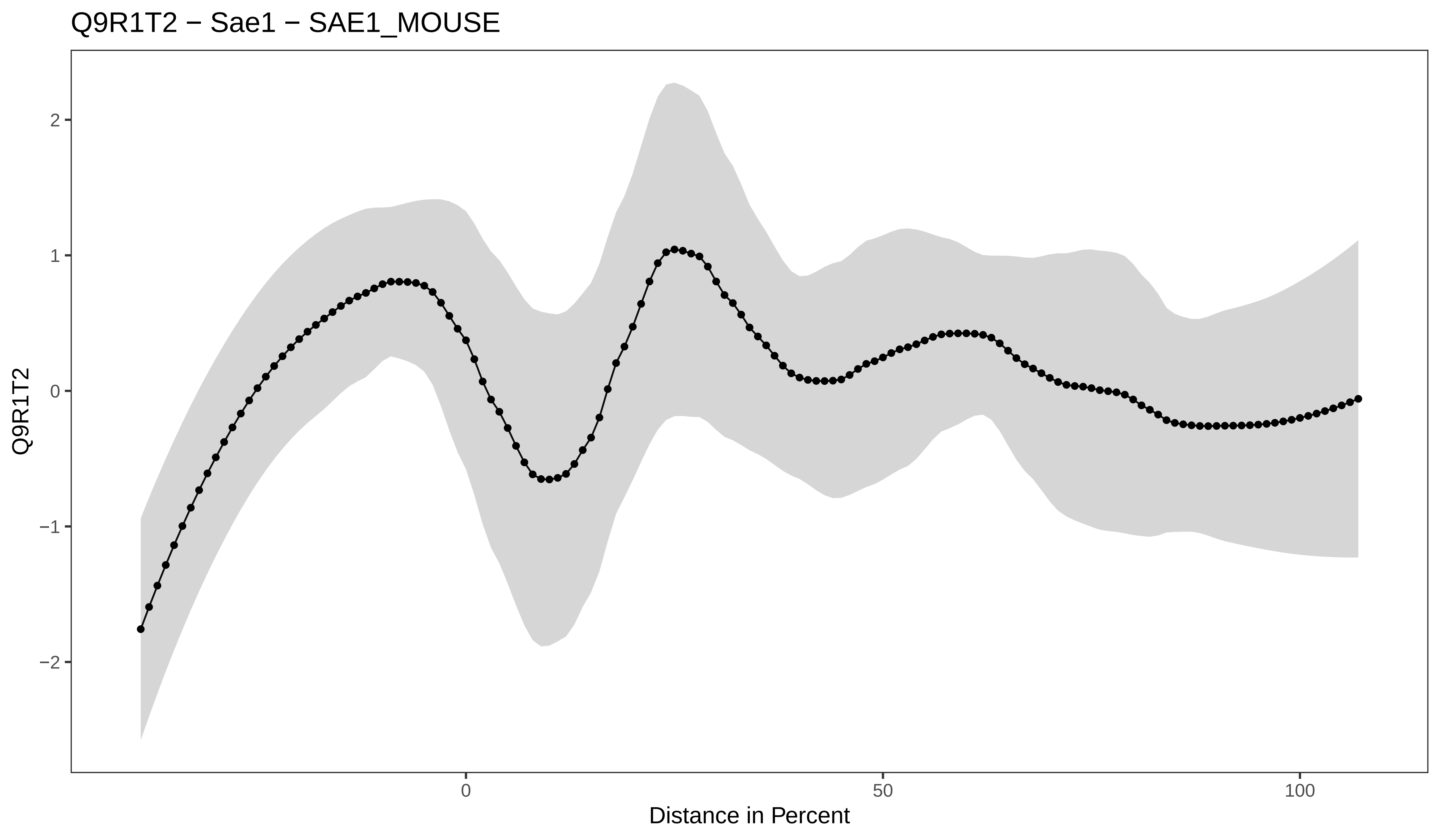
Q9R1T2 − Sae1 − SAE1_MOUSE
Q9R1T2
2
1
0
−1
−2
0
50
100
Distance in Percent

D3YXK2 − Safb − SAFB1_MOUSE

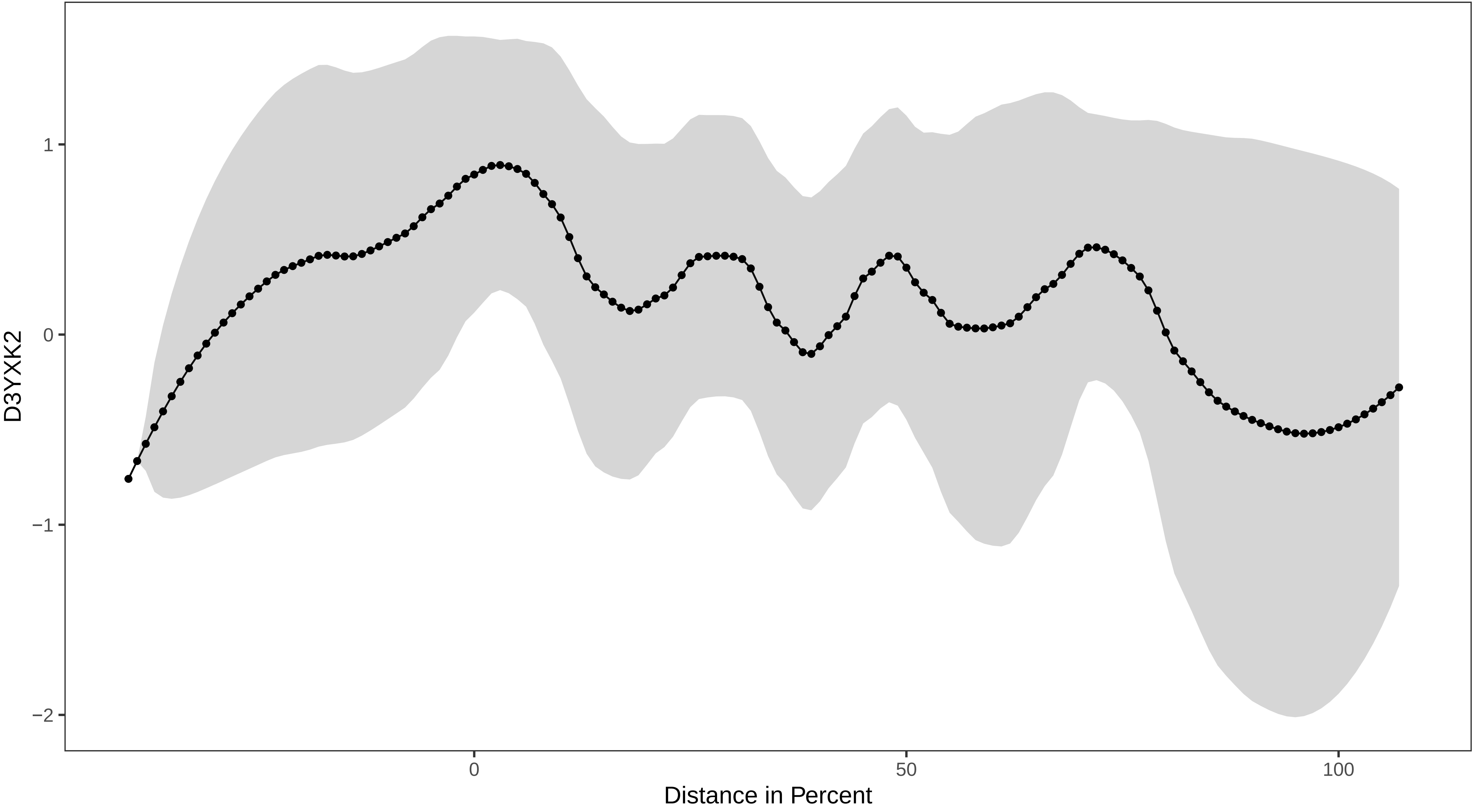

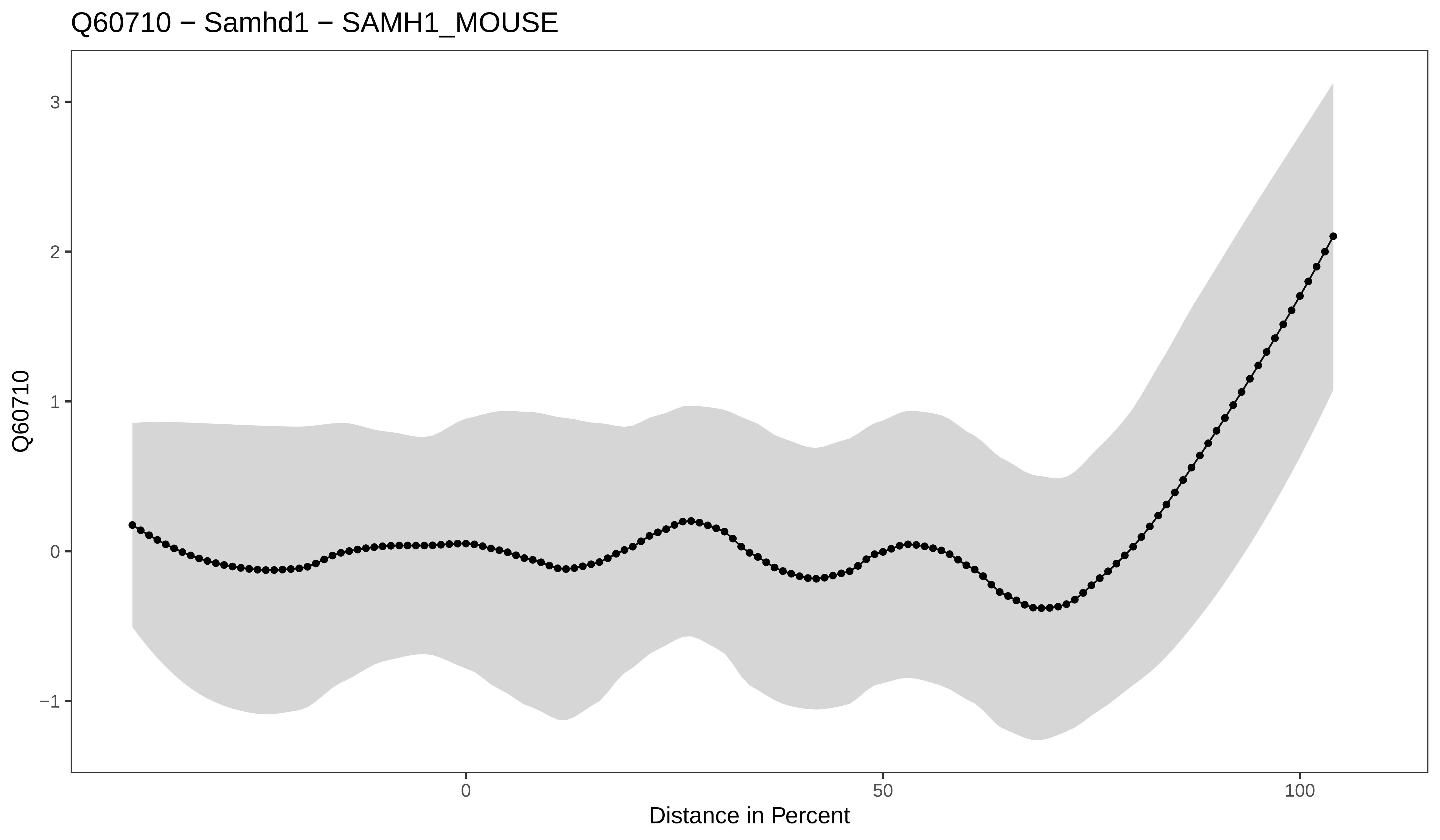

Q60710 − Samhd1 − SAMH1_MOUSE
Q60710
3
2
1
0
−1
0
50
100
Distance in Percent

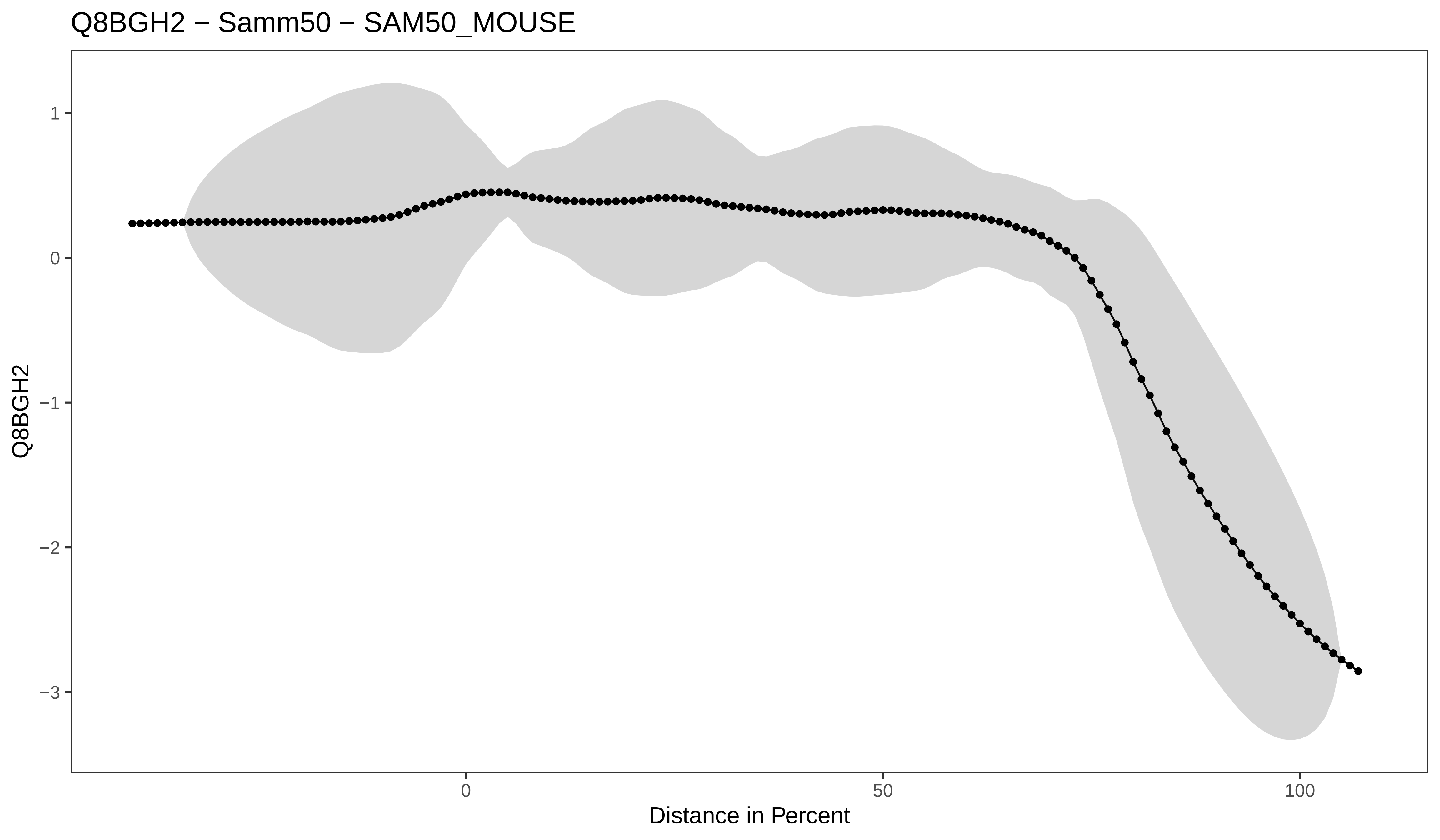
Q8BGH2 − Samm50 − SAM50_MOUSE
Q8BGH2
1
0
−1
−2
−3
0
50
100
Distance in Percent

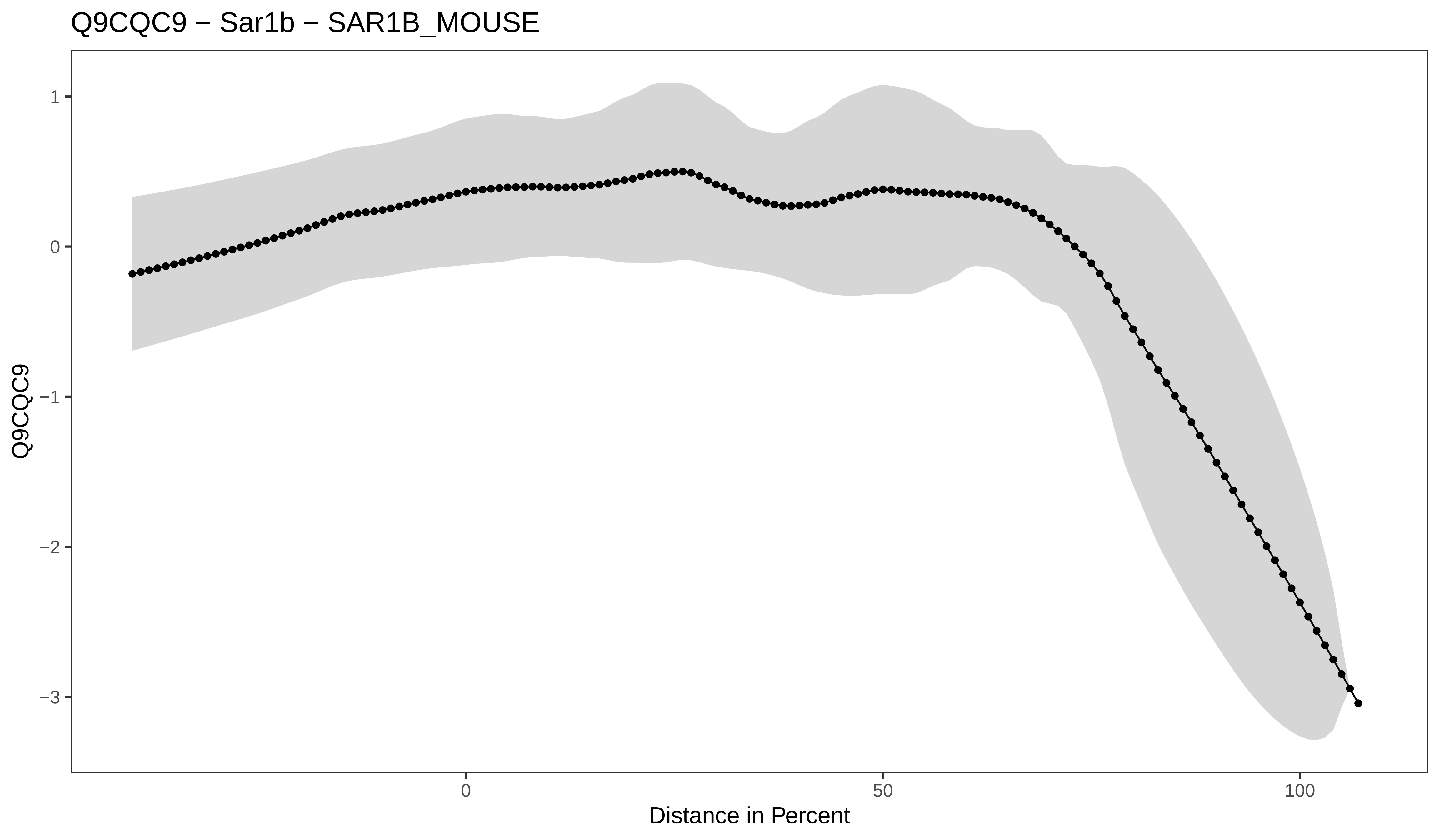

Q9CQC9 − Sar1b − SAR1B_MOUSE
1
0
−1
−2
−3
Q9CQC9
0
50
100
Distance in Percent

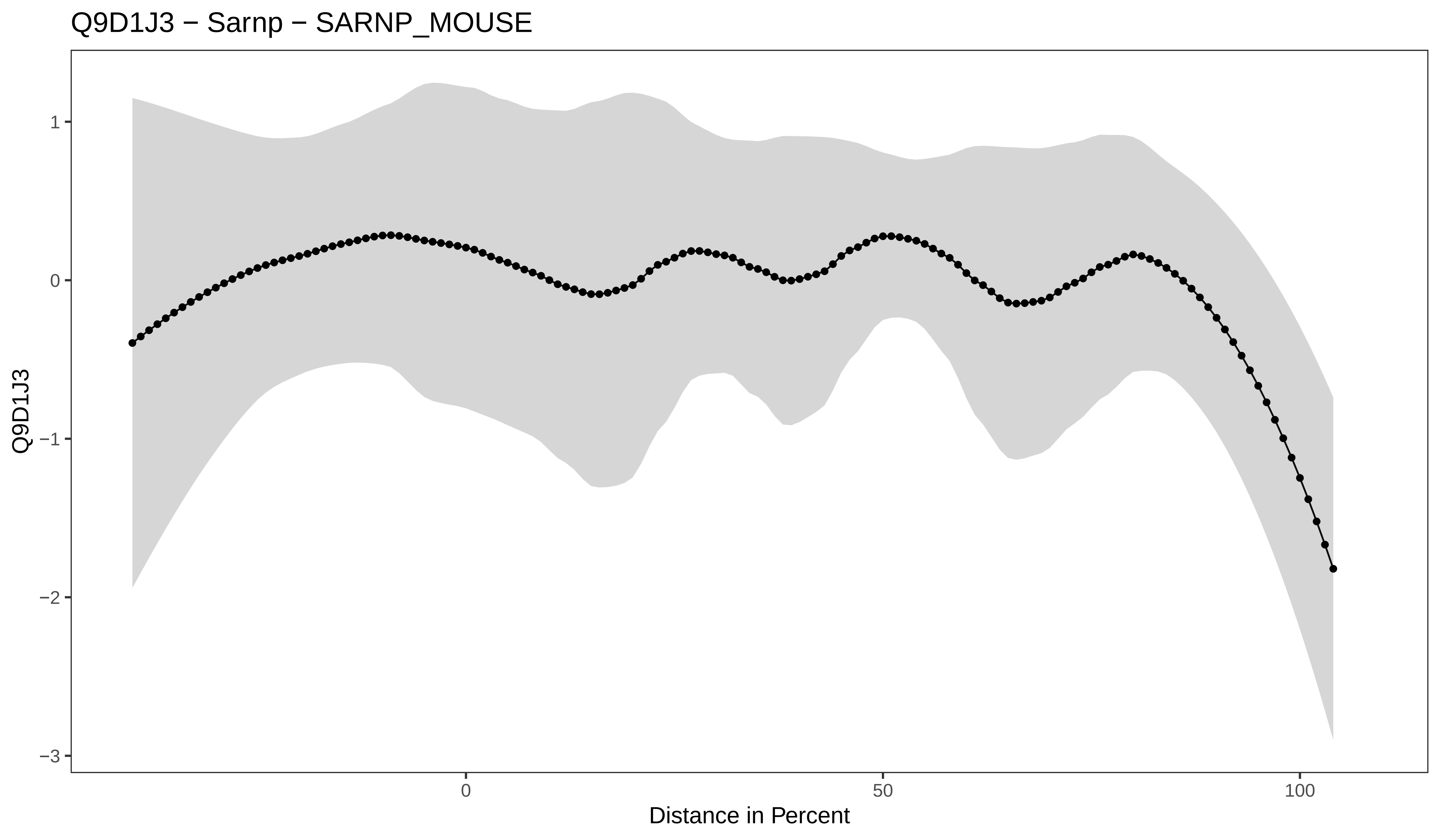

Q9D1J3 − Sarnp − SARNP_MOUSE
Q9D1J3
1
0
−1
−2
−3
0
50
100
Distance in Percent

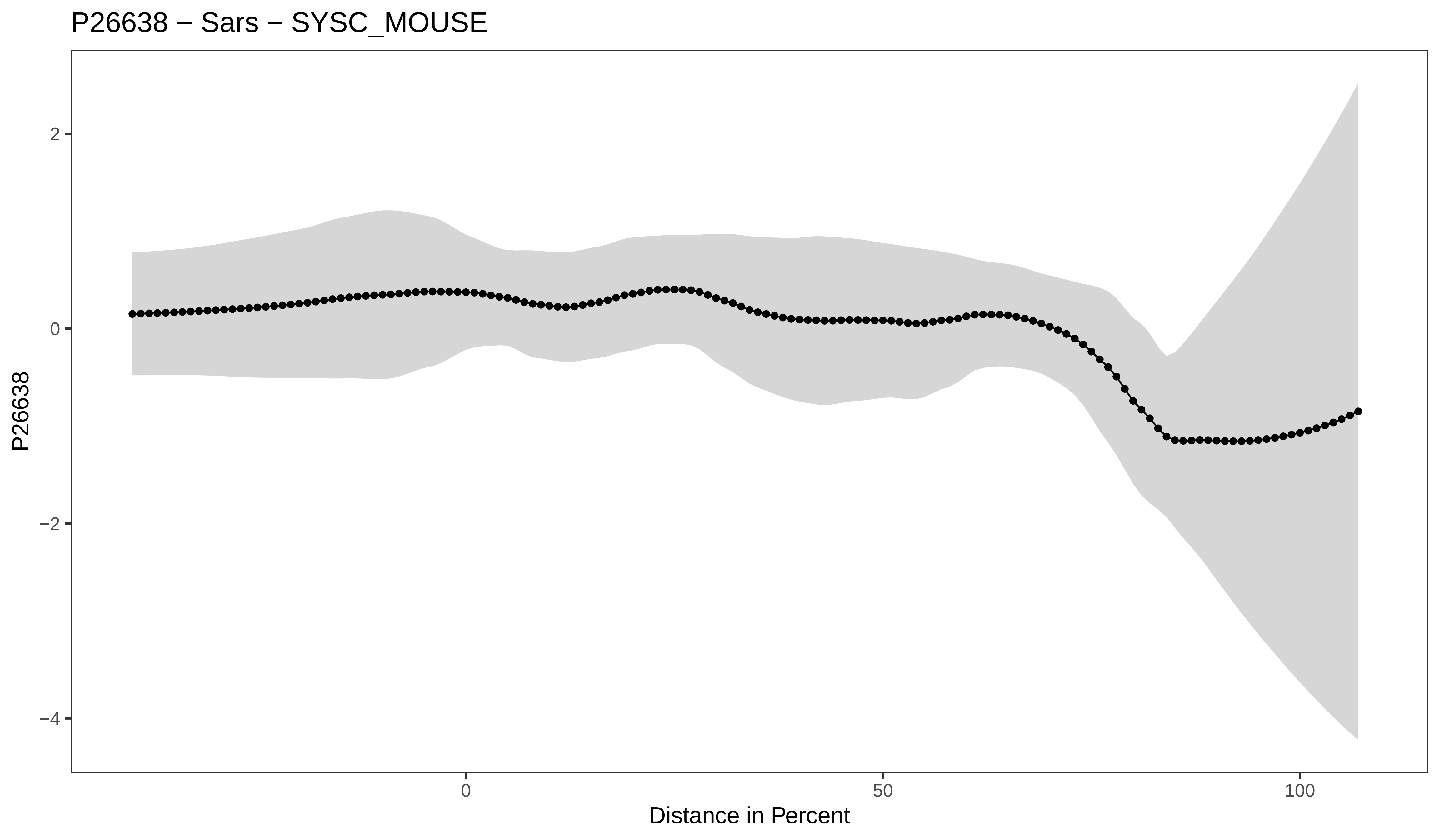

P26638 − Sars − SYSC_MOUSE
2
0
−2
−4
P26638
0
50
100
Distance in Percent

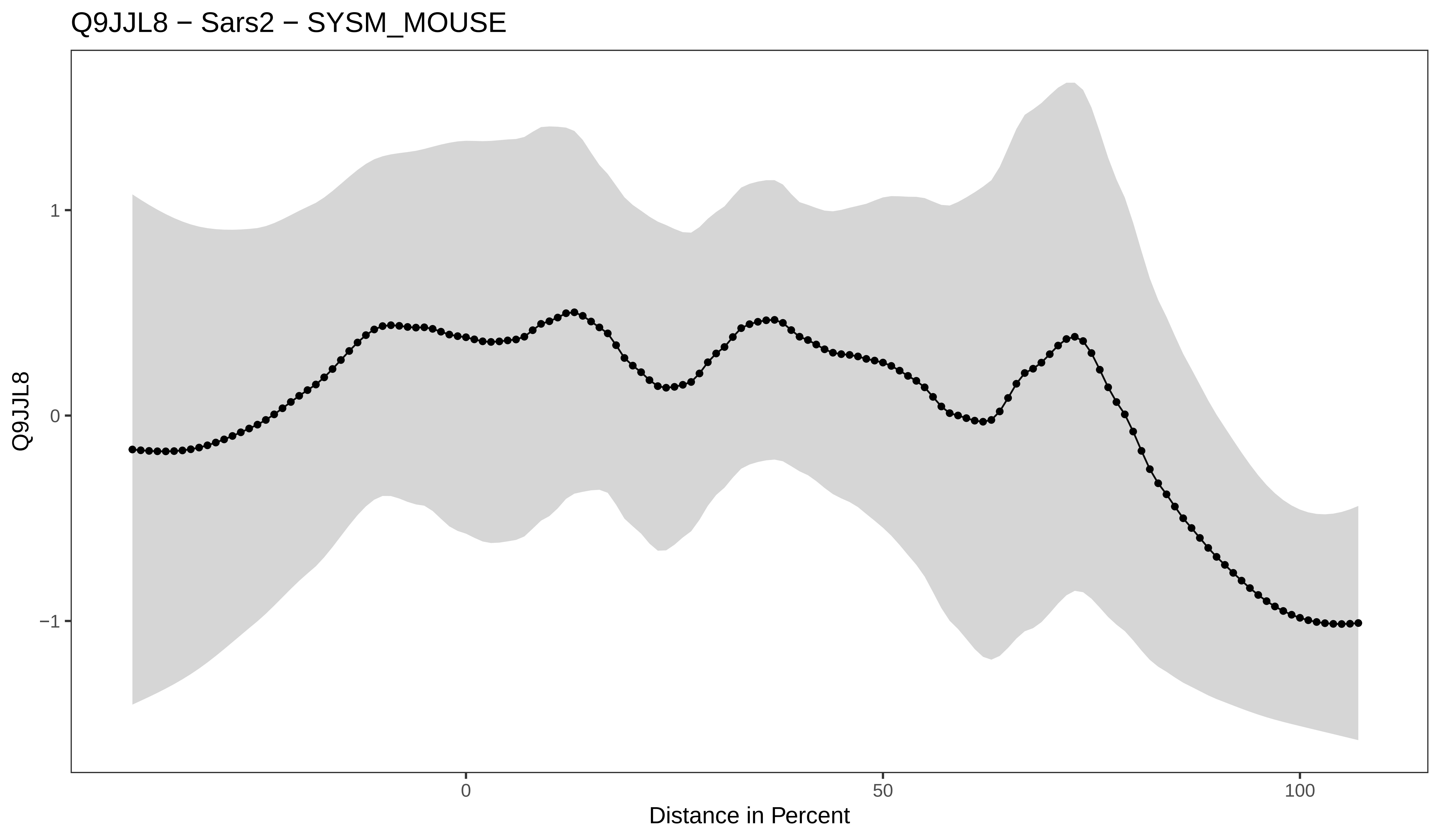
Q9JJL8 − Sars2 − SYSM_MOUSE
Q9JJL8
1
0
−1
0
50
100
Distance in Percent

P0C5K1 − Sbk2 − SBK2_MOUSE

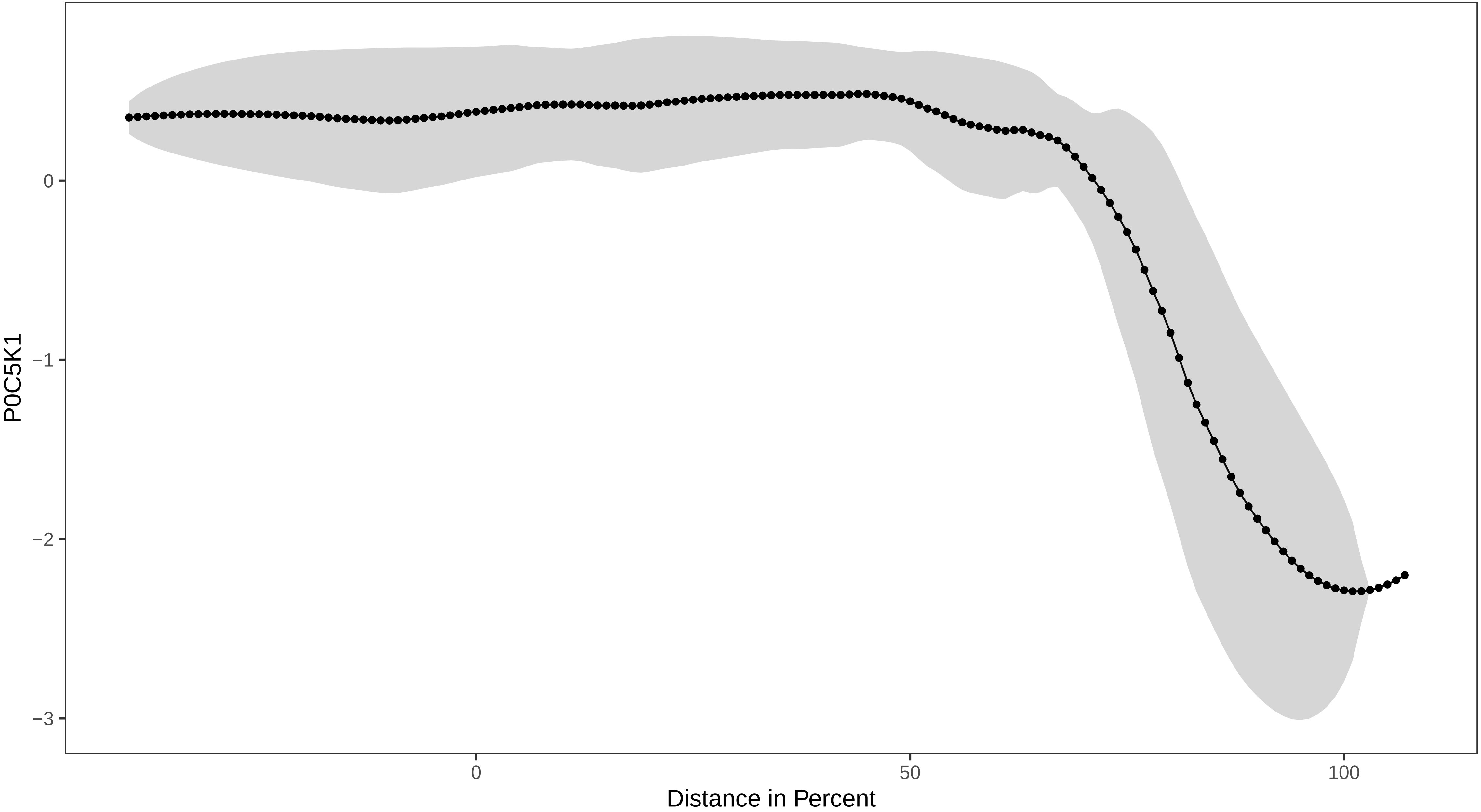

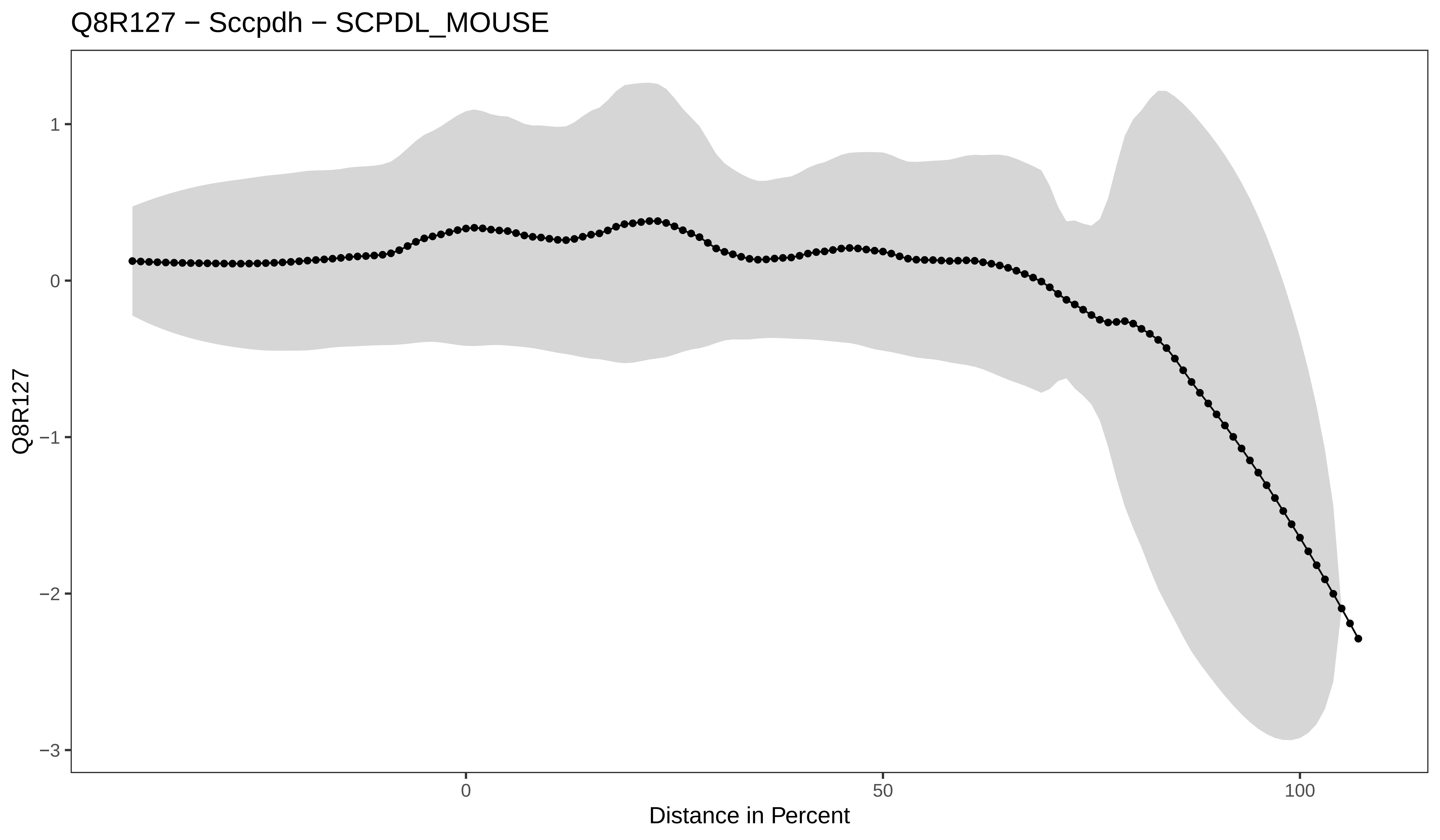

Q8R127 − Sccpdh − SCPDL_MOUSE
Q8R127
1
0
−1
−2
−3
0
50
100
Distance in Percent

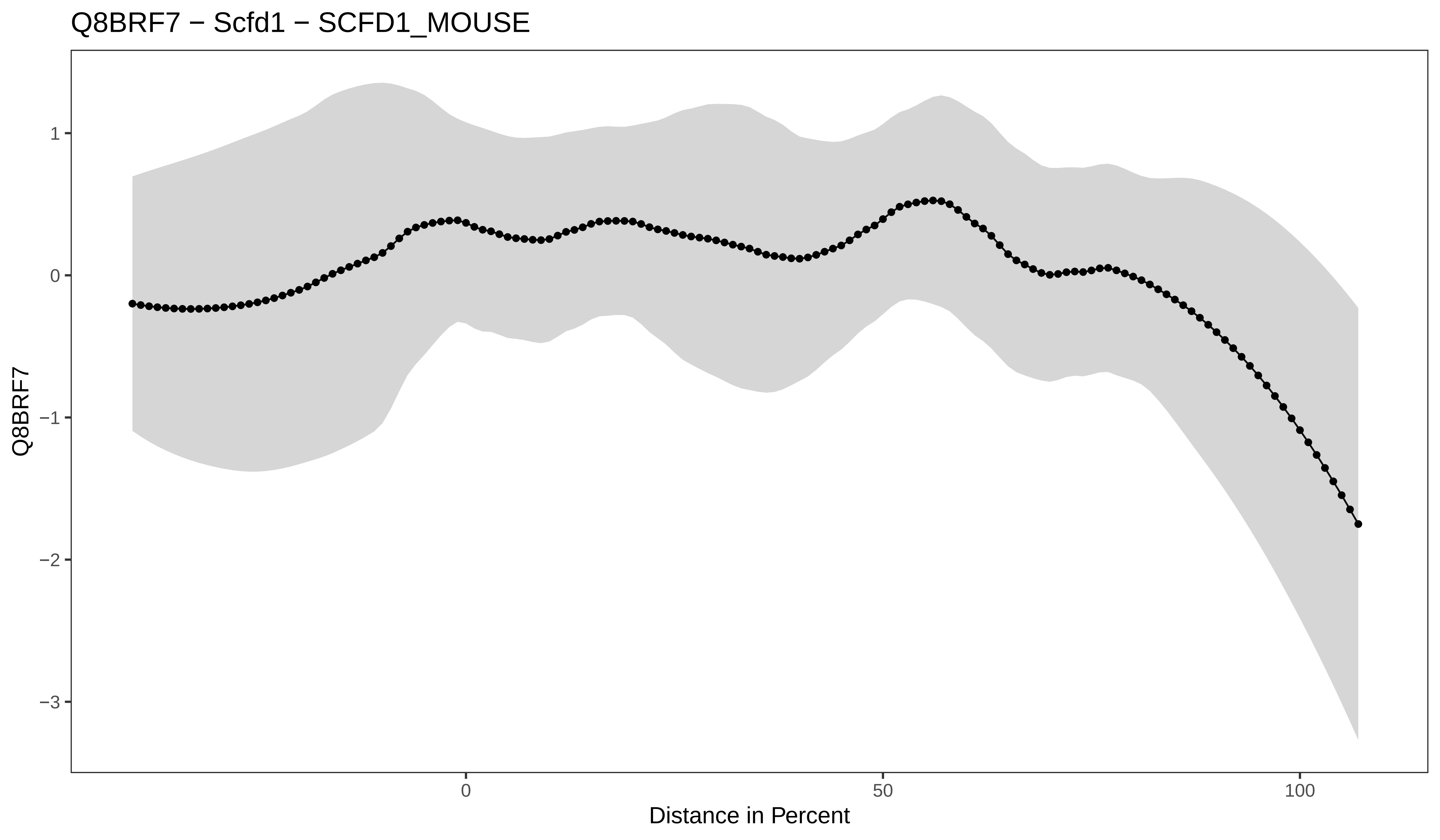
Q8BRF7 − Scfd1 − SCFD1_MOUSE
Q8BRF7
Distance in Percent
1
0
−1
−2
−3
0
50
100

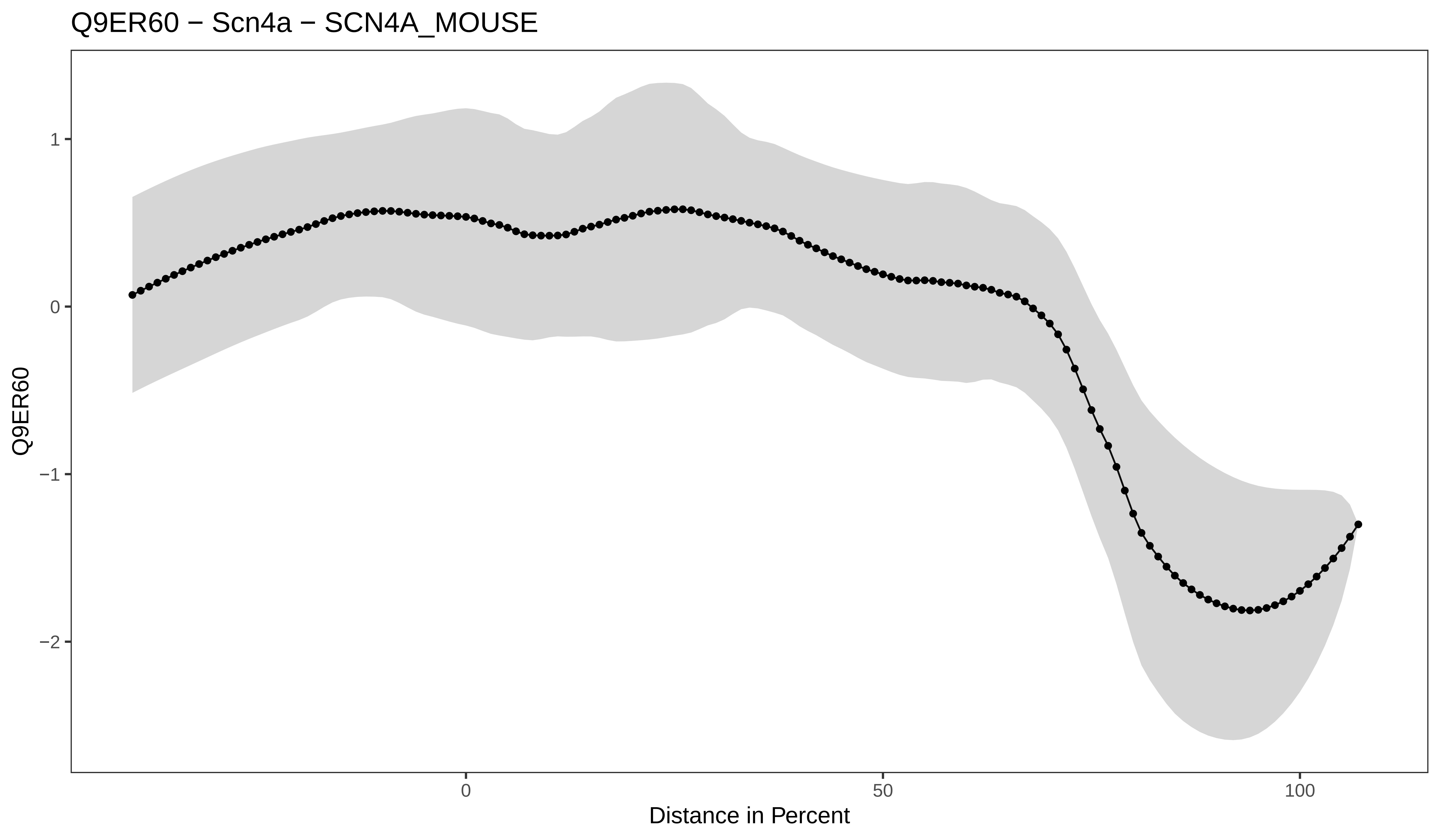
Q9ER60 − Scn4a − SCN4A_MOUSE
Q9ER60
1
0
−1
−2
0
50
100
Distance in Percent

Q7M729 − Scn4b − SCN4B_MOUSE

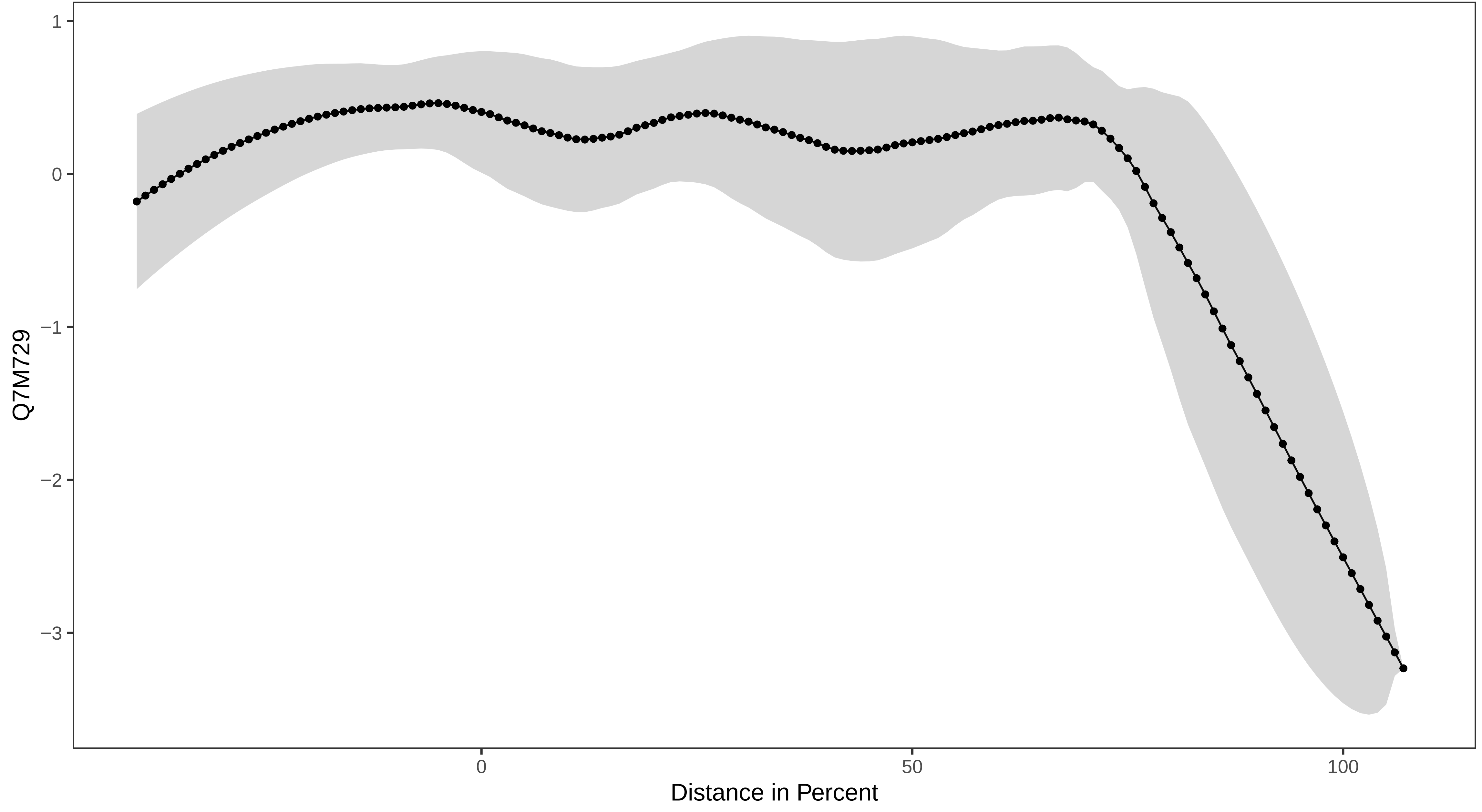

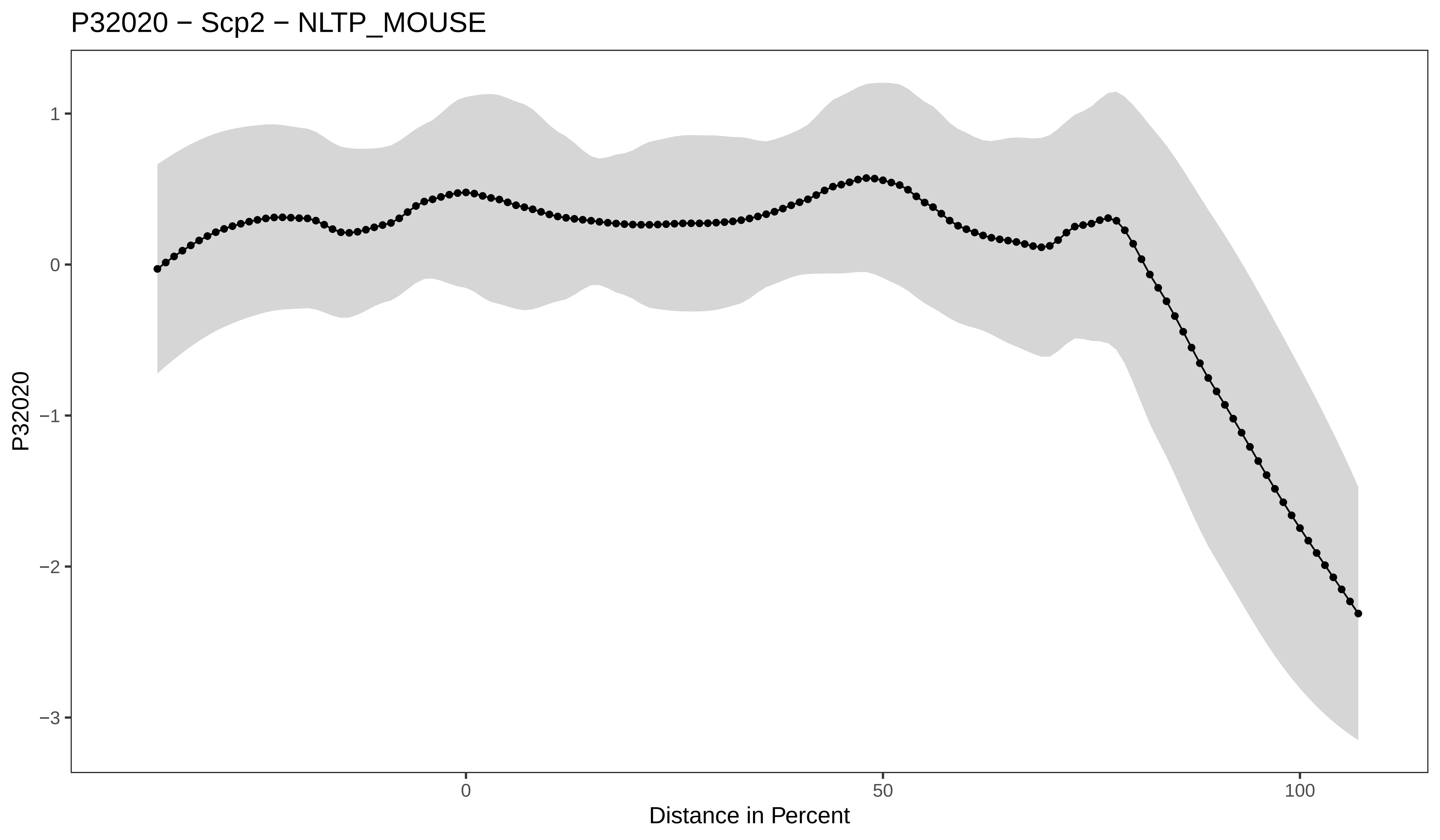

P32020 − Scp2 − NLTP_MOUSE
P32020
Distance in Percent
1
0
−1
−2
−3
0
50
100

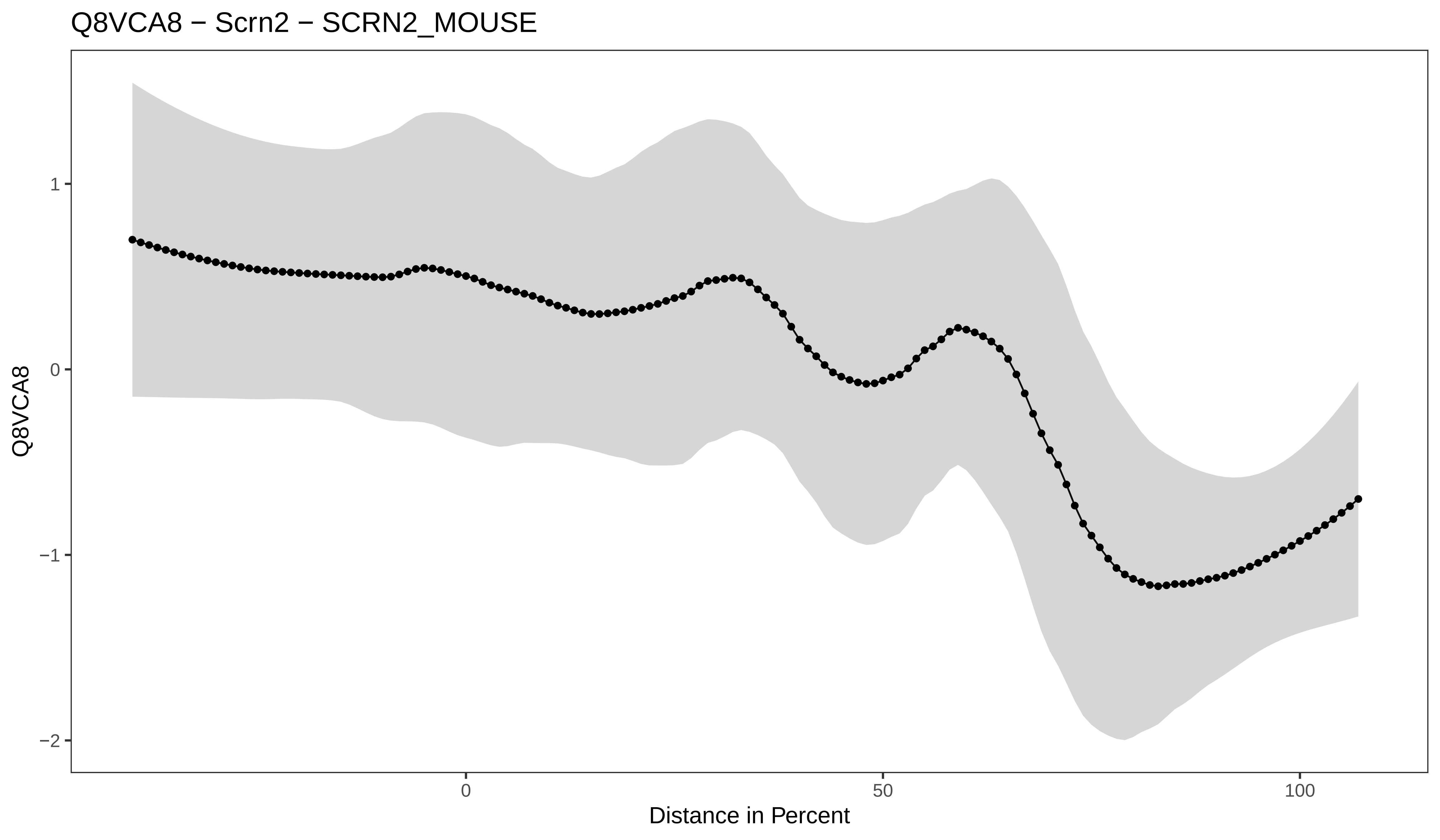
Q8VCA8 − Scrn2 − SCRN2_MOUSE
Q8VCA8
1
0
−1
−2
0
50
100
Distance in Percent

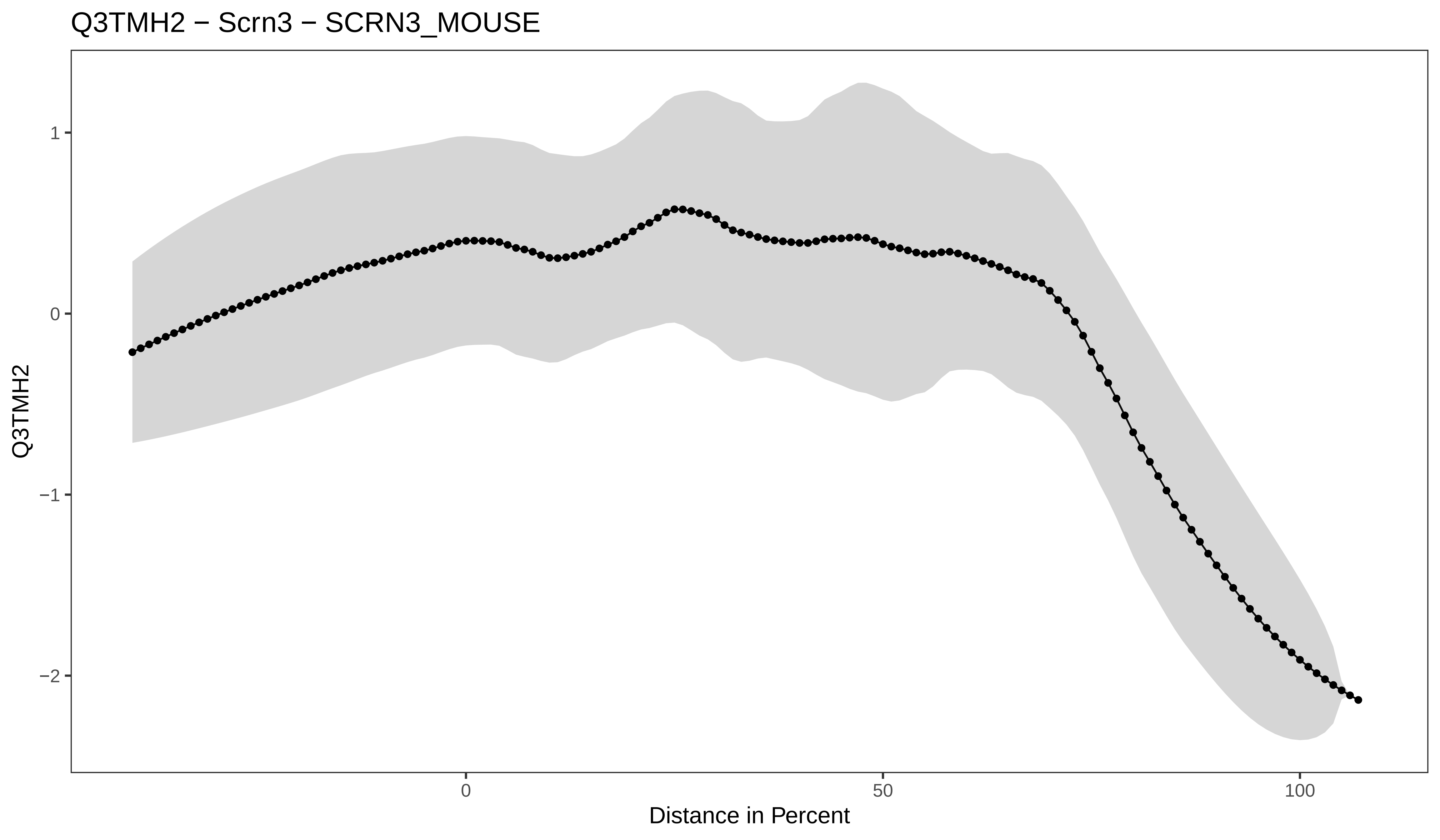

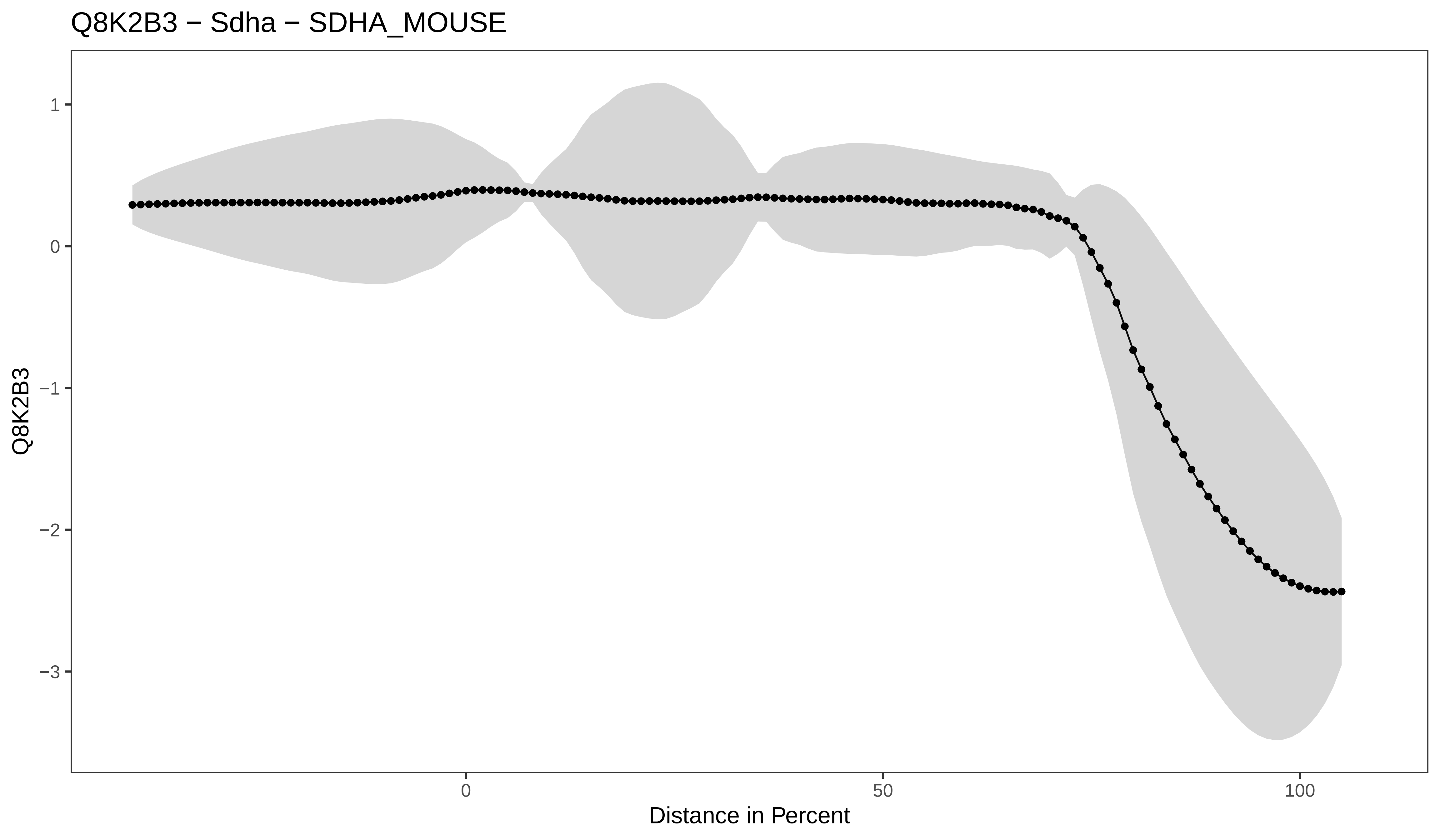

Q8K2B3 − Sdha − SDHA_MOUSE
1
0
−1
−2
−3
Q8K2B3
0
50
100
Distance in Percent

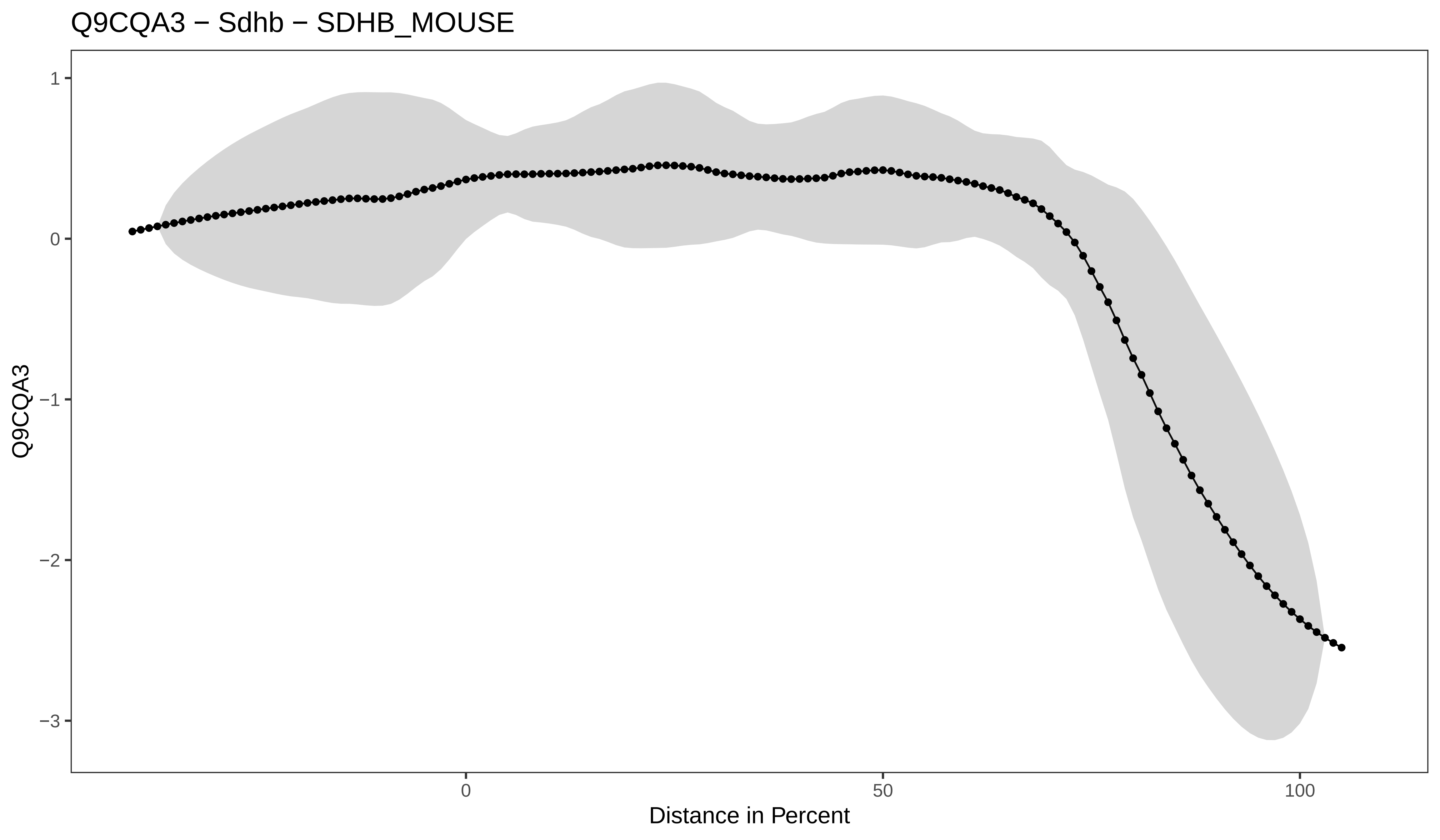
Q9CQA3 − Sdhb − SDHB_MOUSE
1
0
−1
−2
−3
Q9CQA3
0
50
100
Distance in Percent

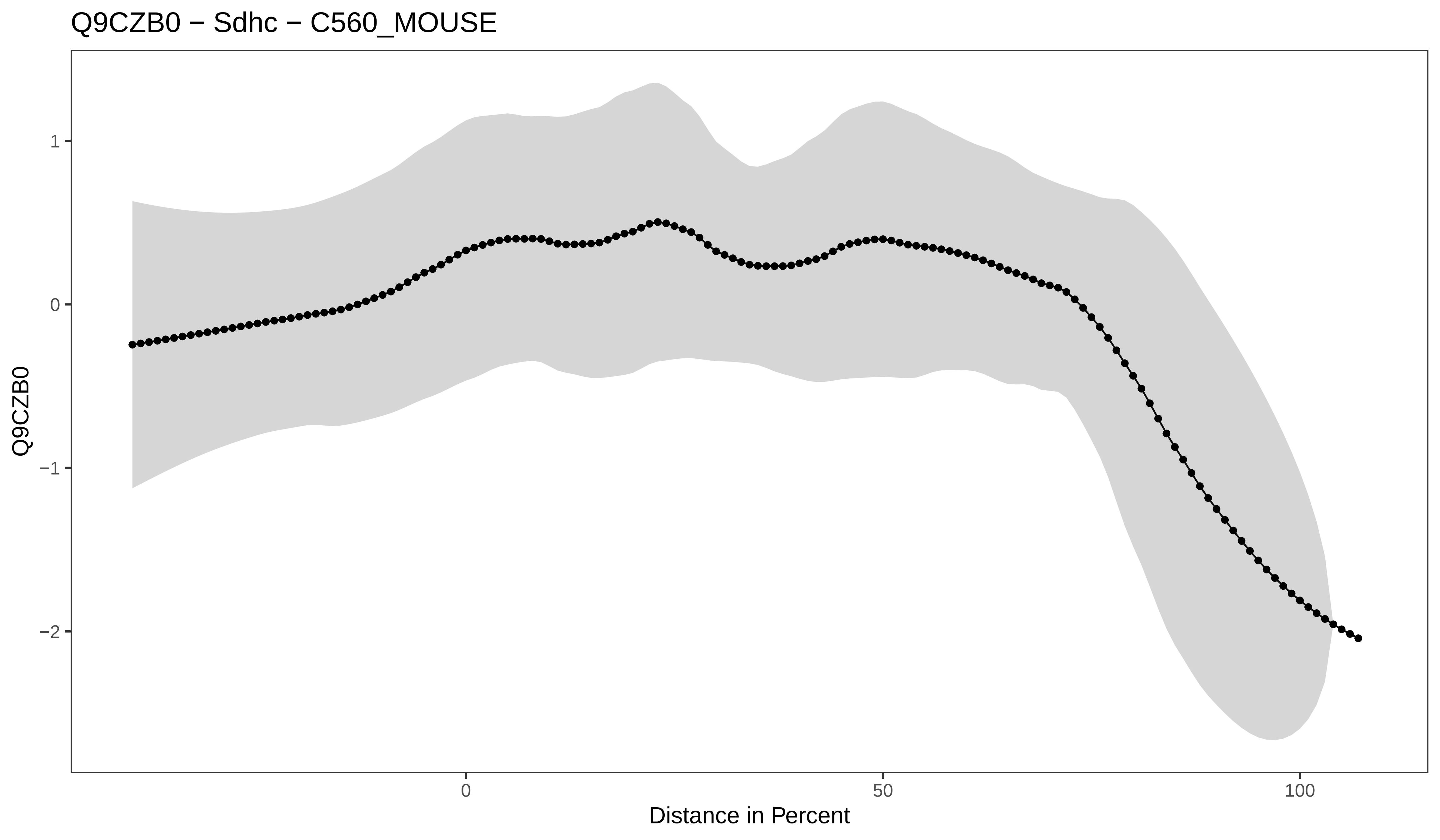

Q9CZB0 − Sdhc − C560_MOUSE
Q9CZB0
1
0
−1
−2
0
50
100
Distance in Percent

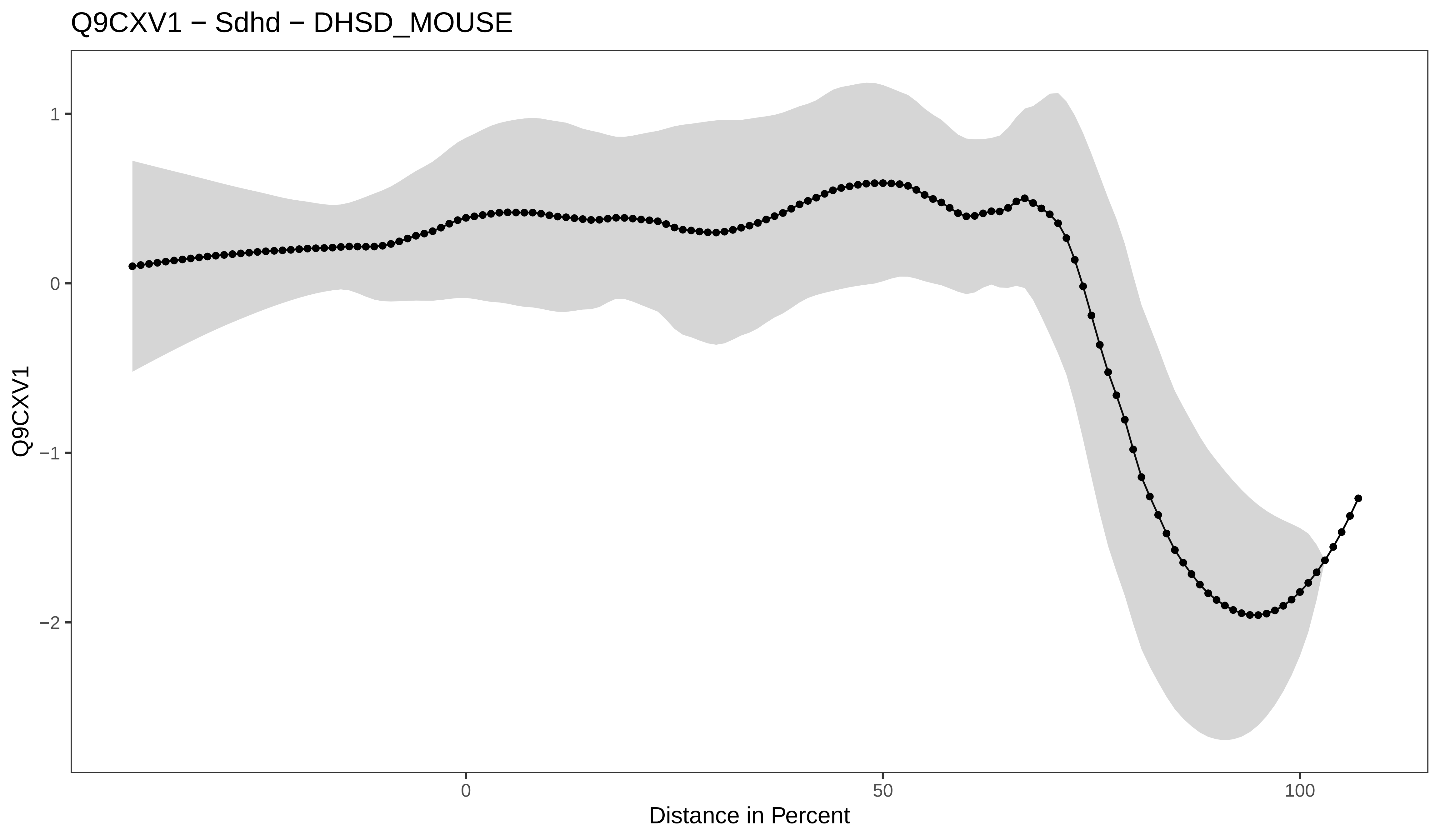

Q9CXV1 − Sdhd − DHSD_MOUSE
Q9CXV1
Distance in Percent
1
0
−1
−2
0
50
100

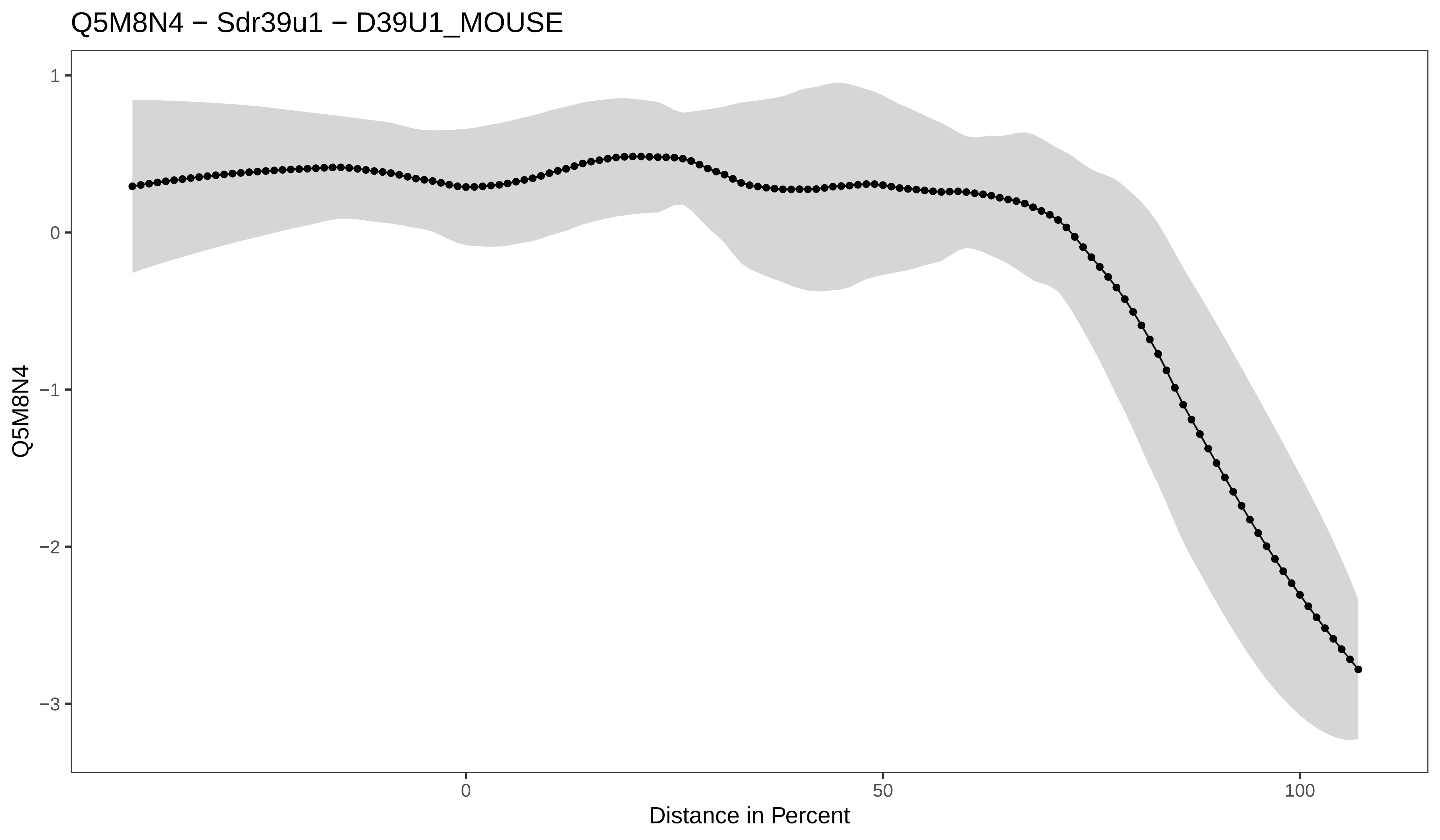

Q5M8N4 − Sdr39u1 − D39U1_MOUSE
Q5M8N4
1
0
−1
−2
−3
0
50
100
Distance in Percent

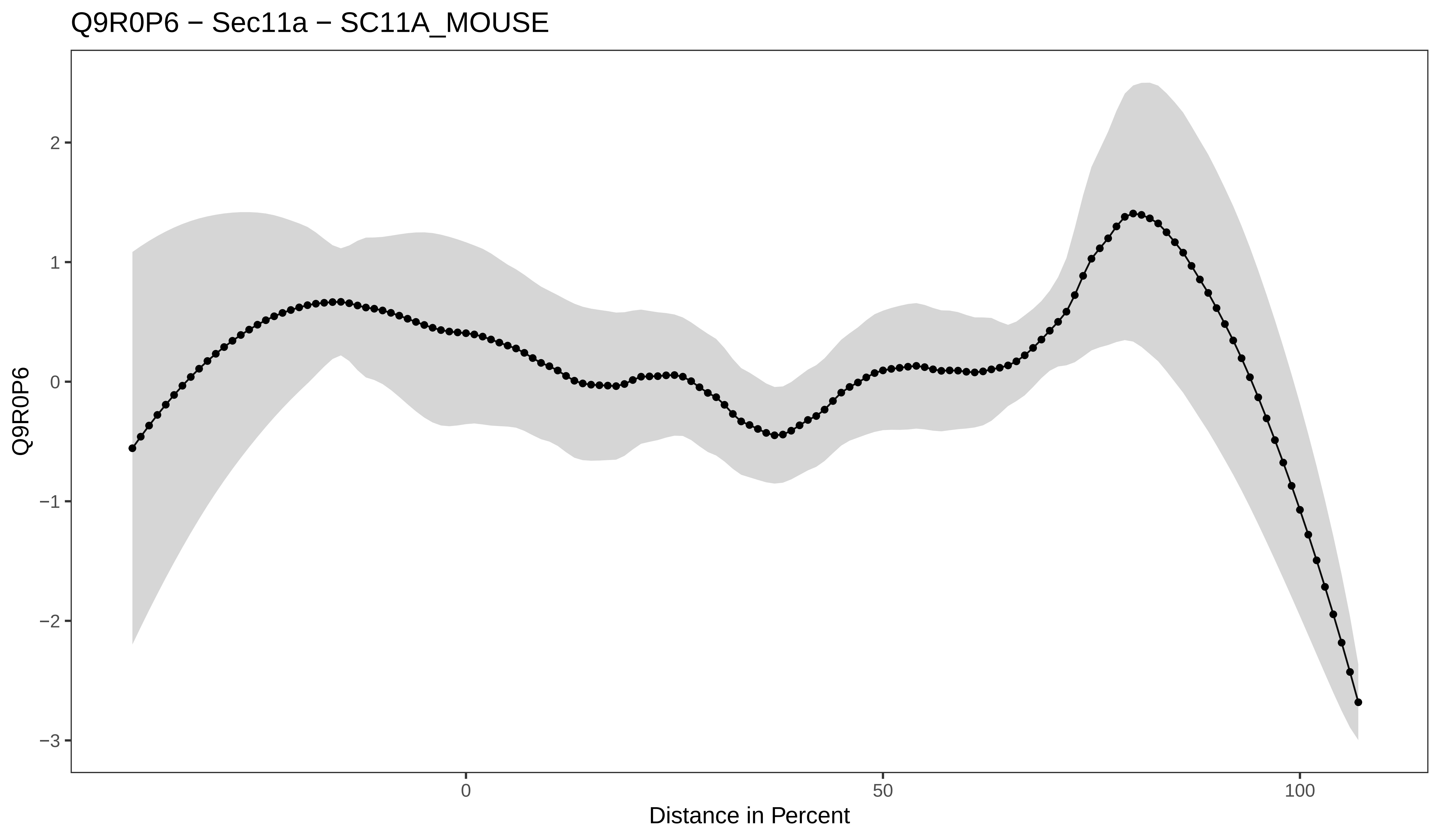
Q9R0P6 − Sec11a − SC11A_MOUSE
Q9R0P6
2
1
0
−1
−2
−3
0
50
100
Distance in Percent

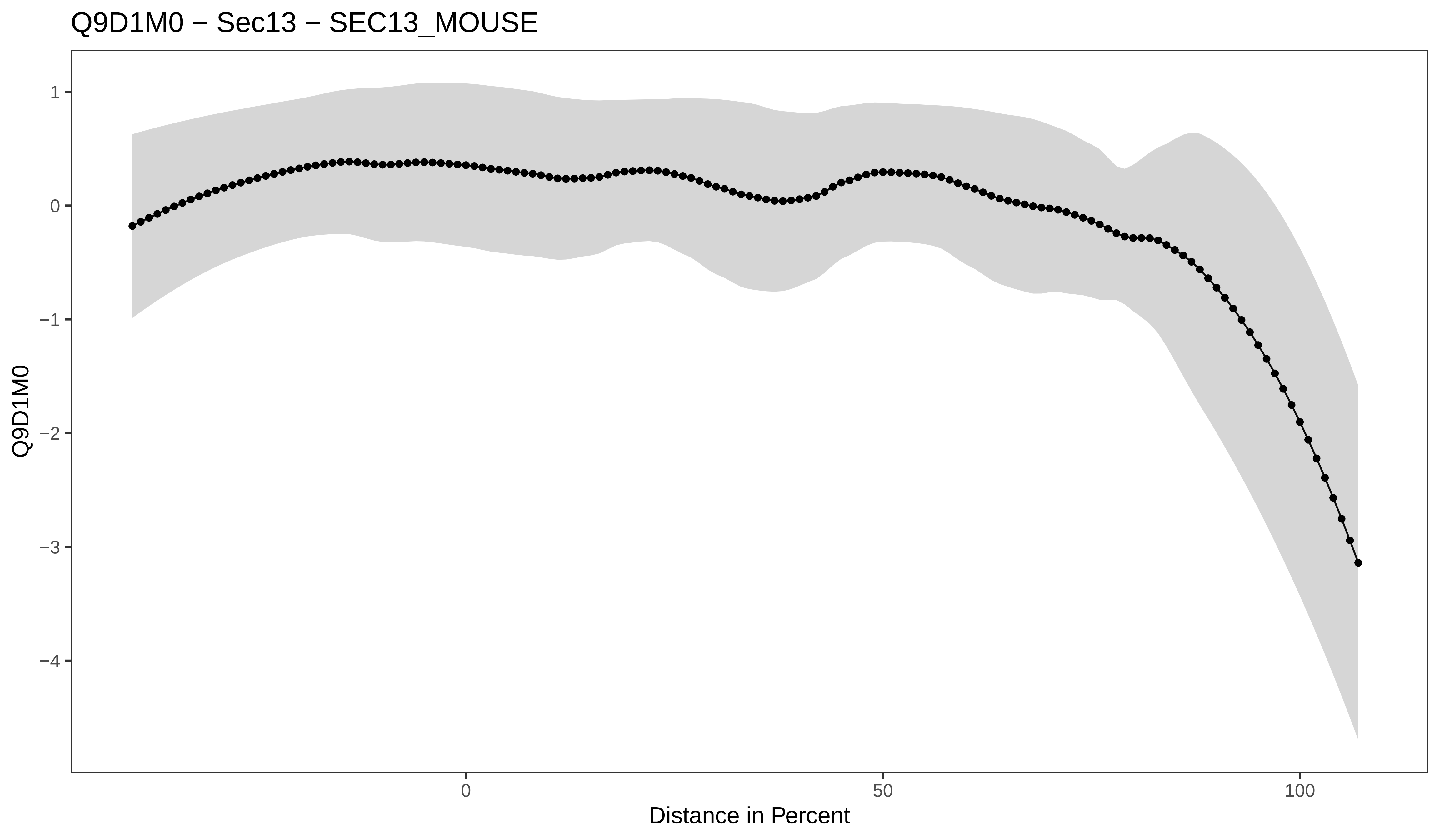

Q9D1M0 − Sec13 − SEC13_MOUSE
1
0
−1
−2
−3
−4
Q9D1M0
0
50
100
Distance in Percent

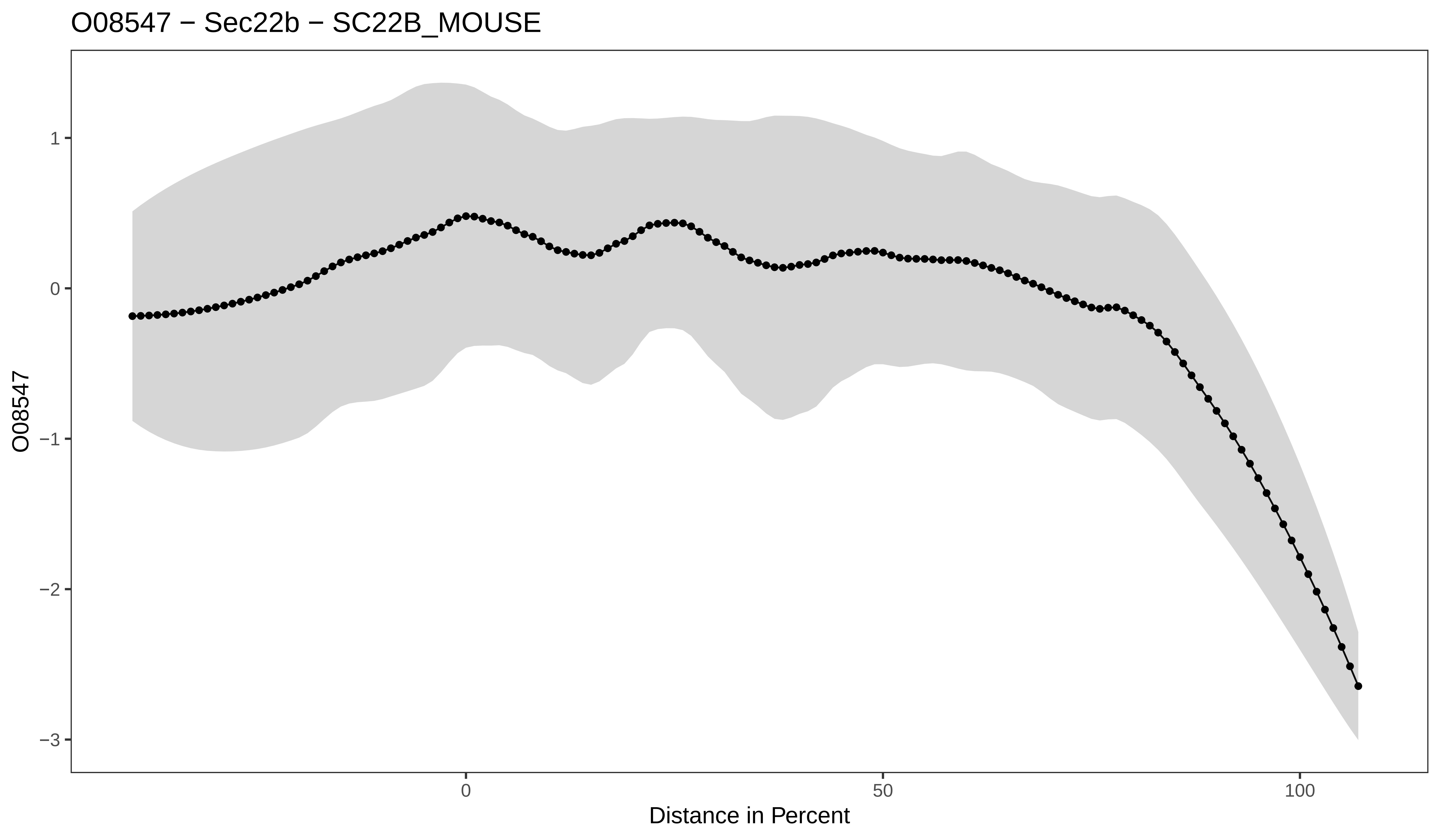
O08547 − Sec22b − SC22B_MOUSE
1
0
−1
−2
−3
O08547
0
50
100
Distance in Percent

Q01405 − Sec23a − SC23A_MOUSE

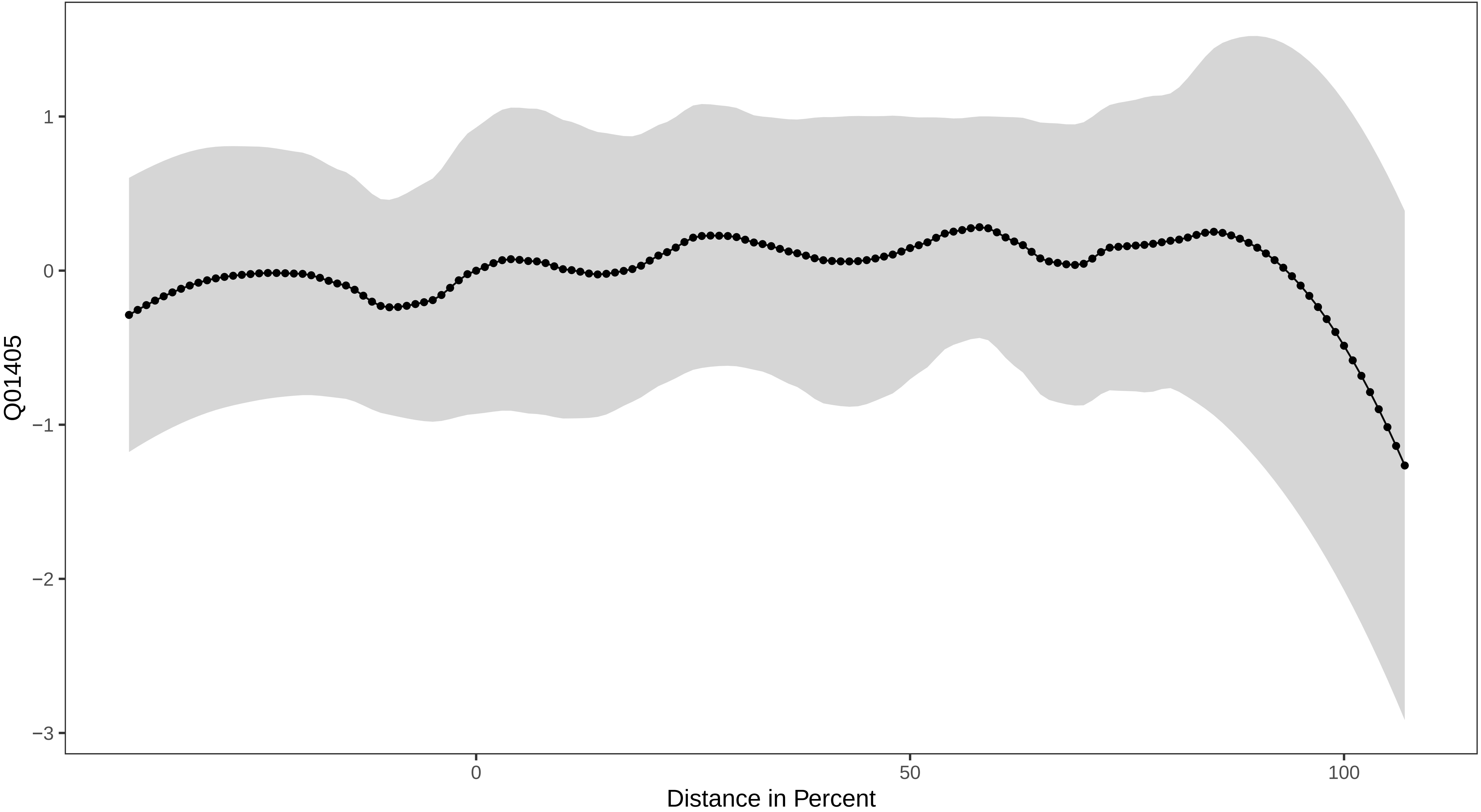

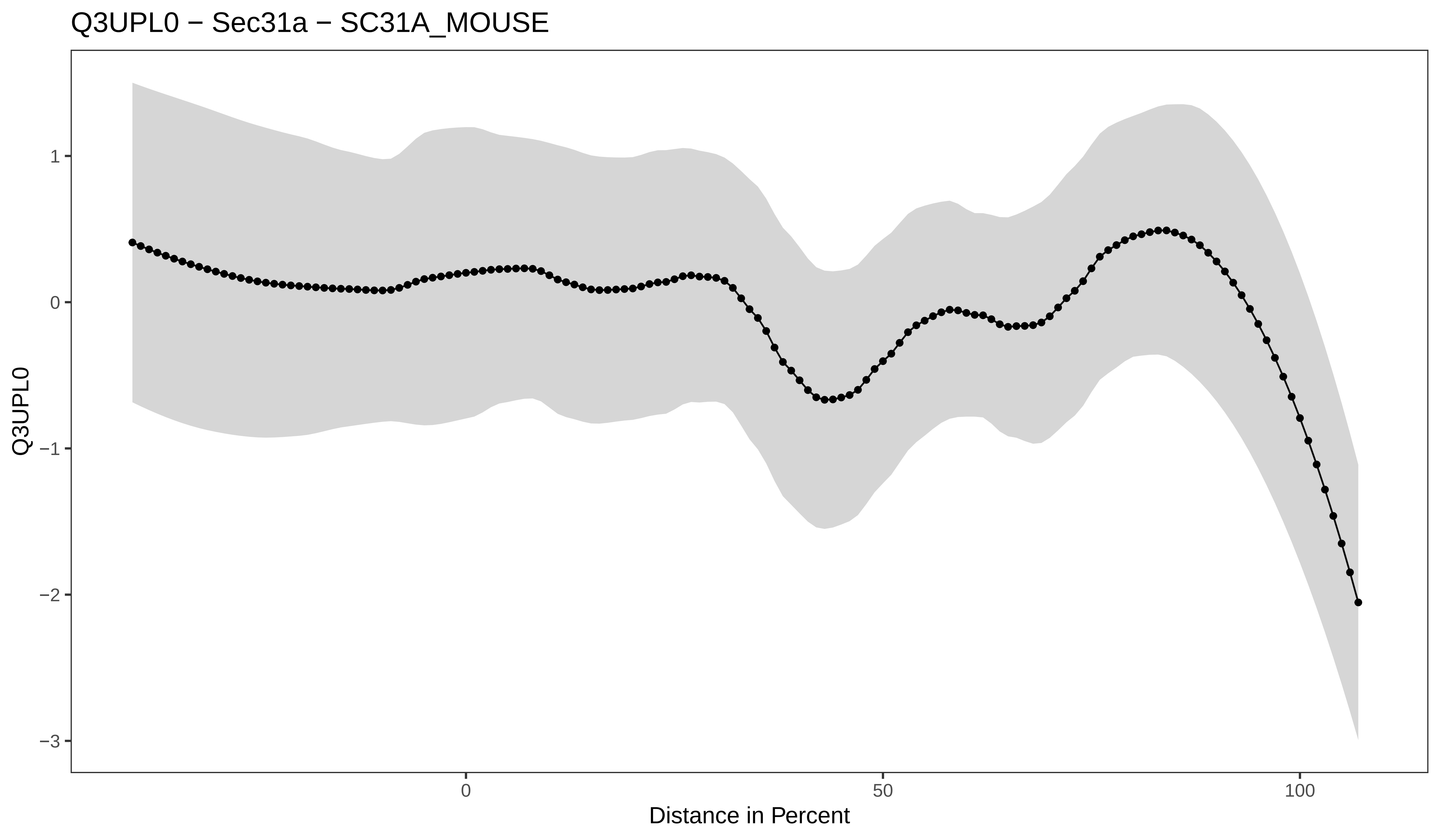
Q3UPL0 − Sec31a − SC31A_MOUSE
Q3UPL0
1
0
−1
−2
−3
0
50
100
Distance in Percent

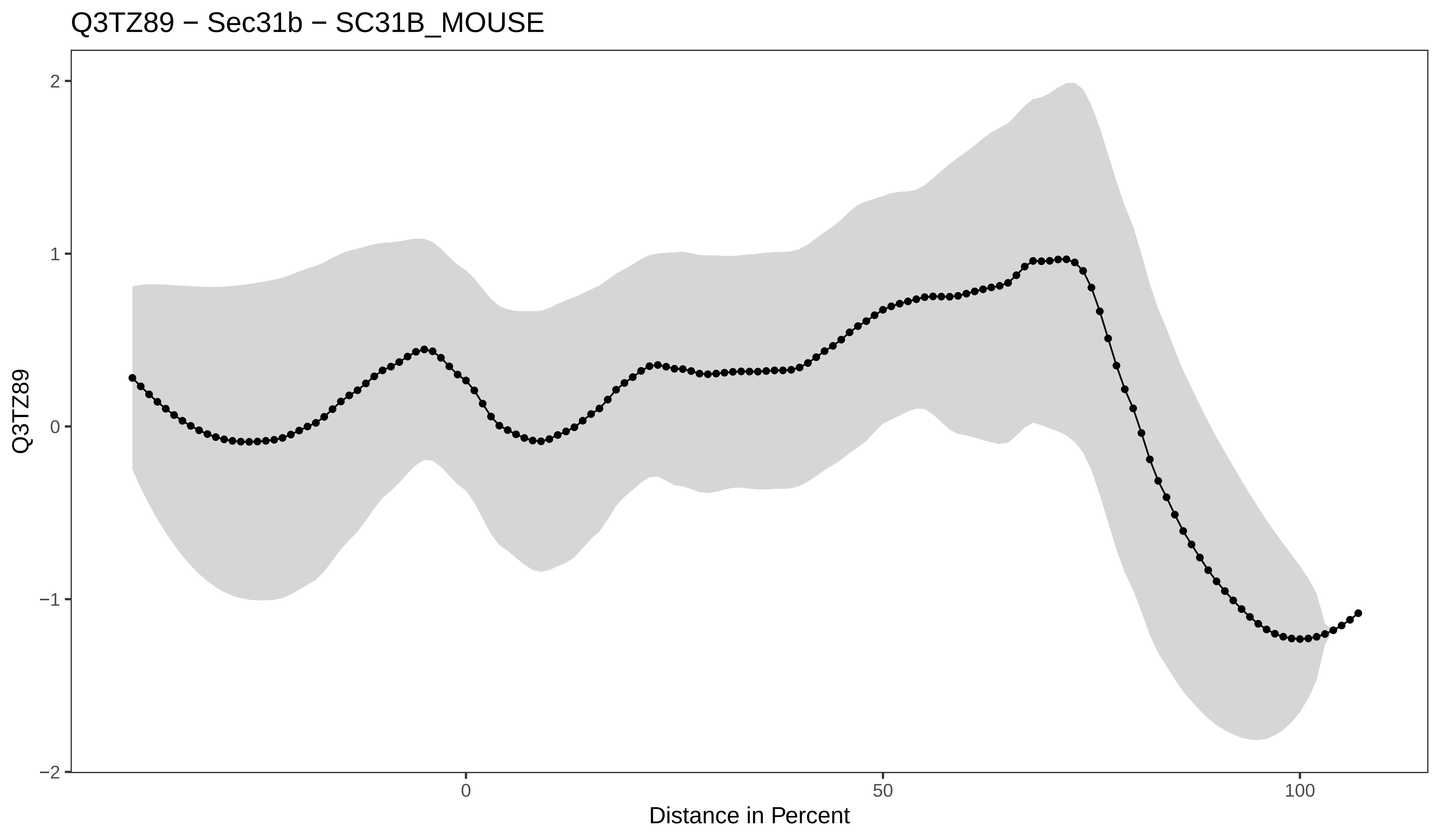
Q3TZ89 − Sec31b − SC31B_MOUSE
Q3TZ89
2
1
0
−1
−2
0
50
100
Distance in Percent

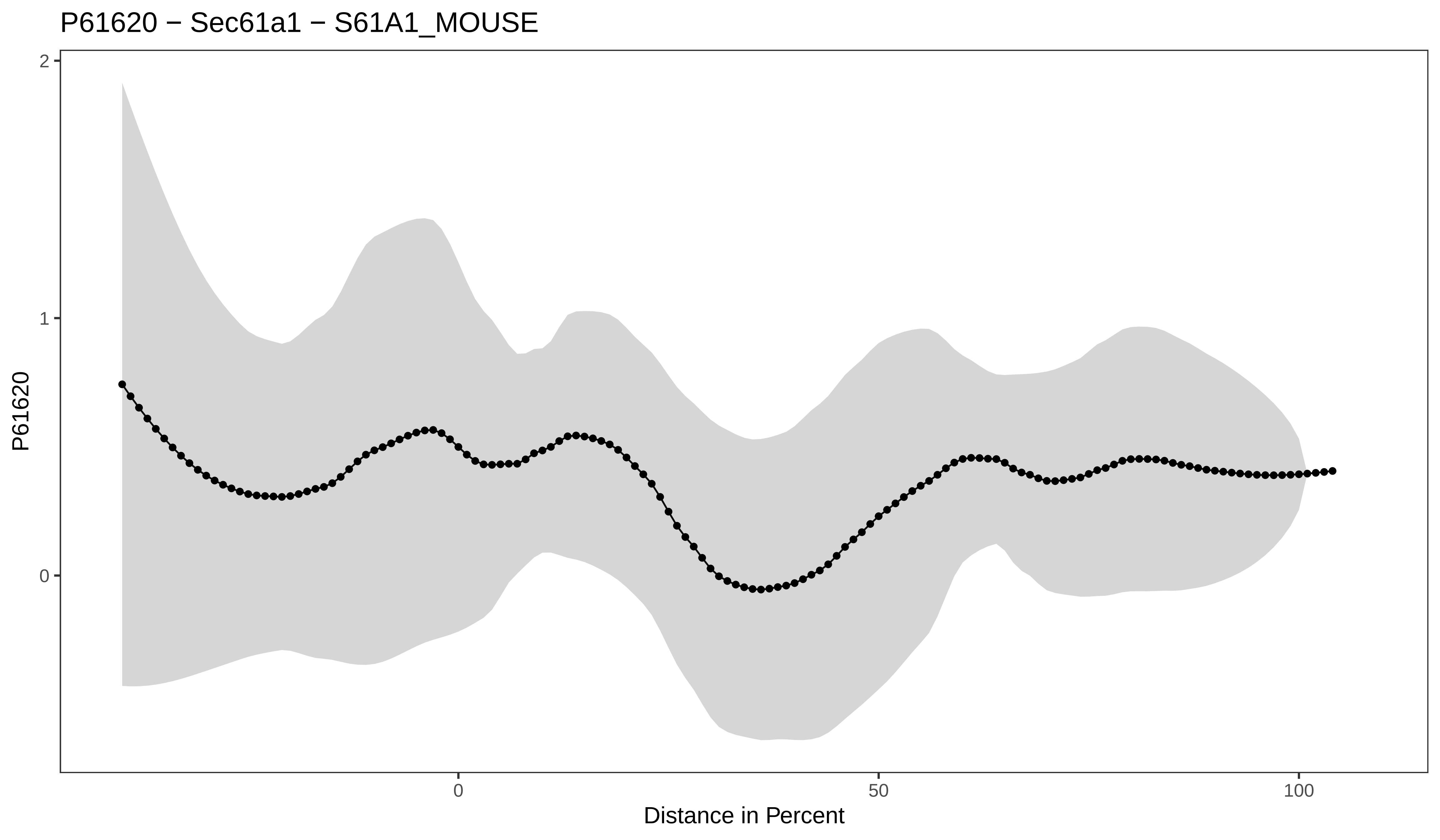

P61620 − Sec61a1 − S61A1_MOUSE
2
1
0
P61620
0
50
100
Distance in Percent

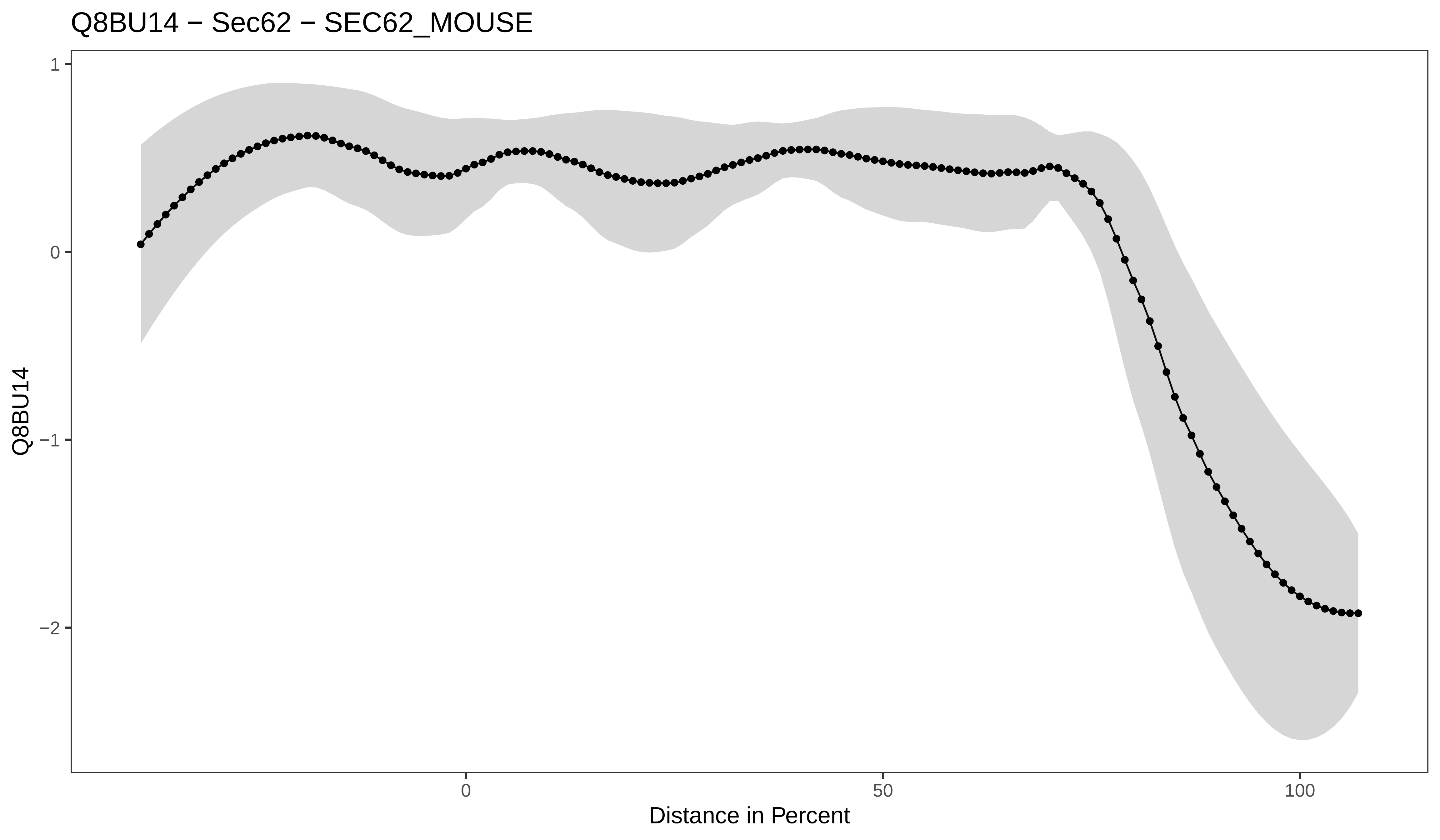

Q8BU14 − Sec62 − SEC62_MOUSE
1
0
−1
−2
Q8BU14
0
50
100
Distance in Percent

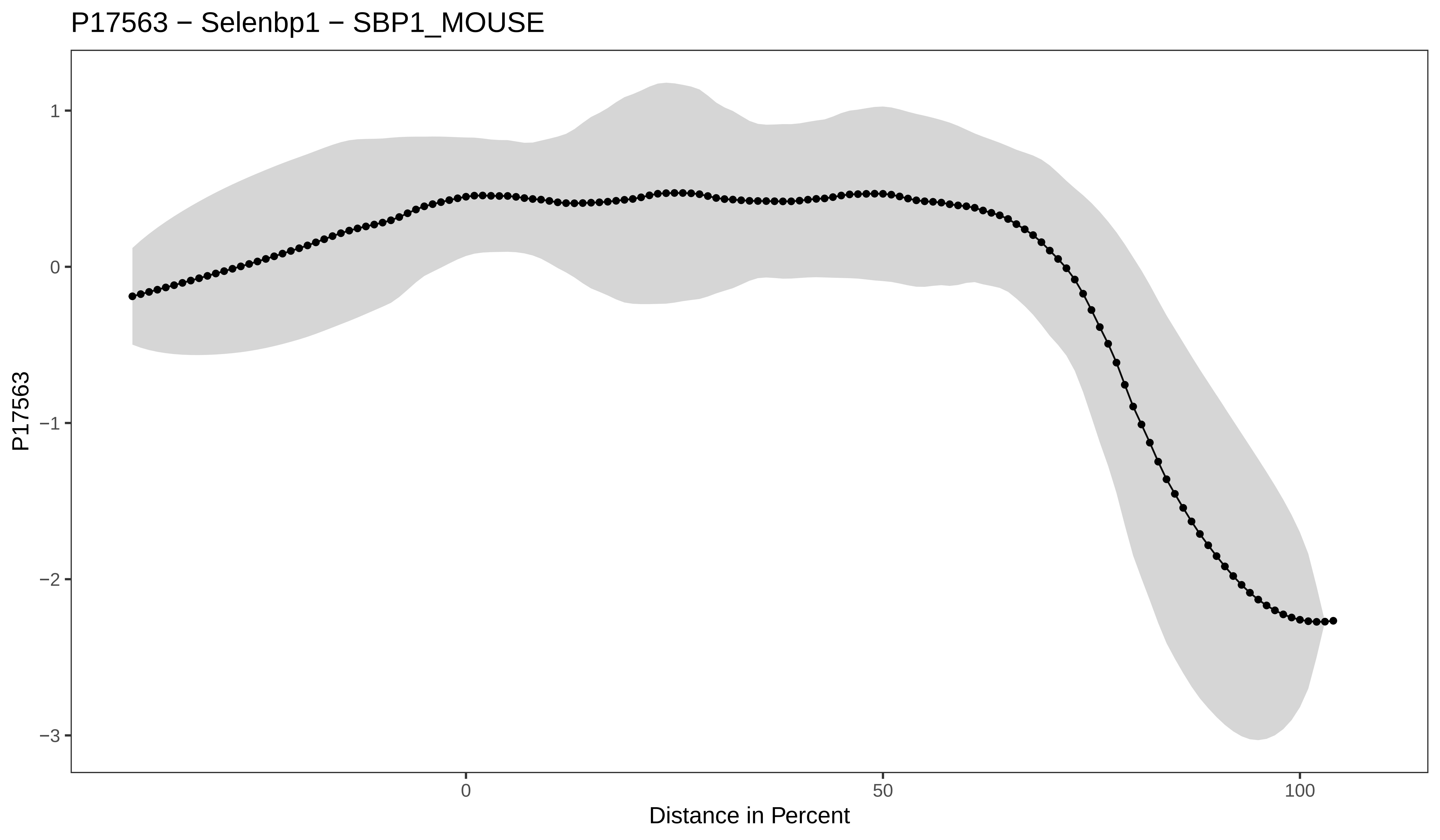

P17563 − Selenbp1 − SBP1_MOUSE
1
0
−1
−2
−3
P17563
0
50
100
Distance in Percent

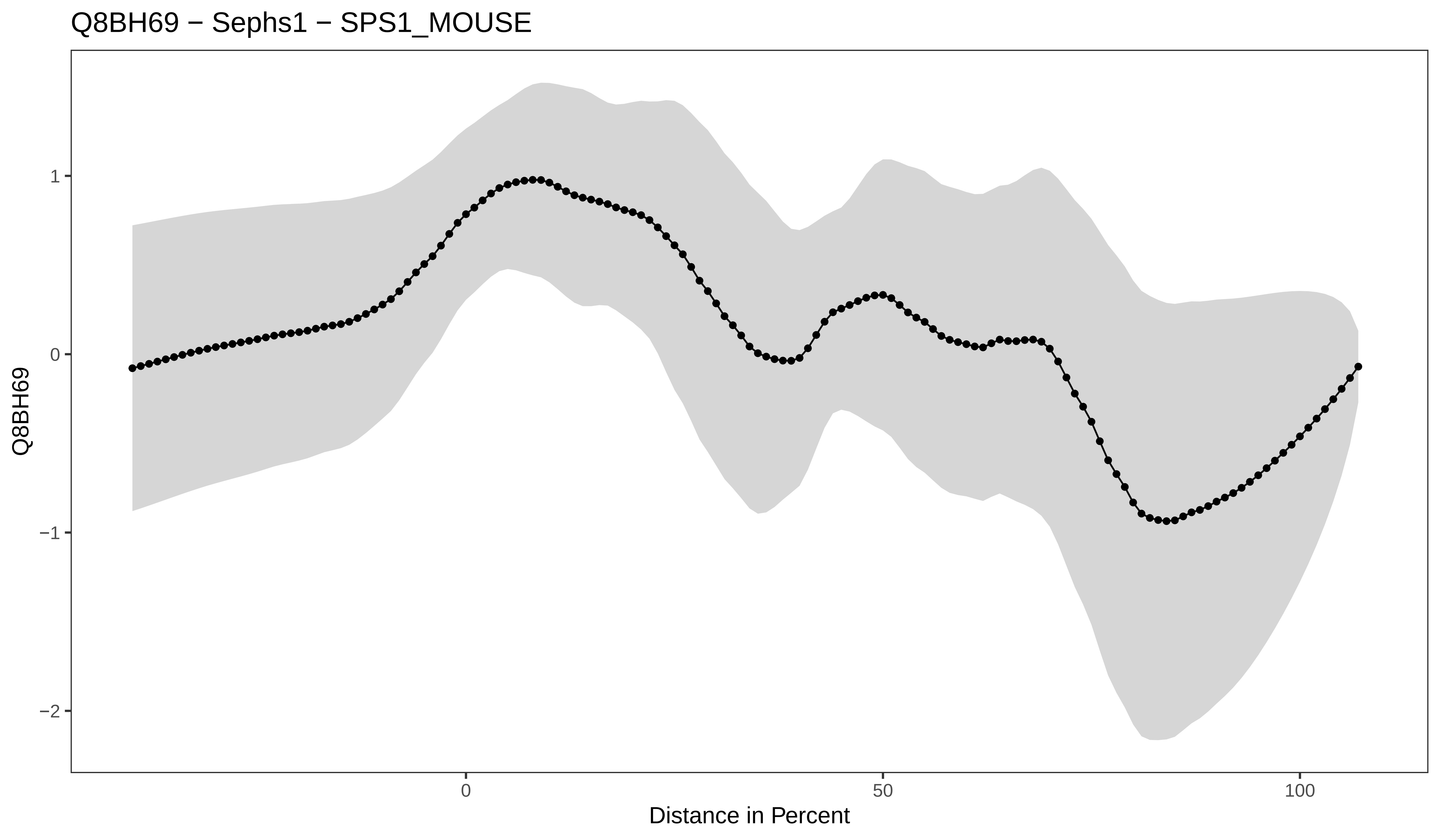

Q8BH69 − Sephs1 − SPS1_MOUSE
Q8BH69
1
0
−1
−2
0
50
100
Distance in Percent

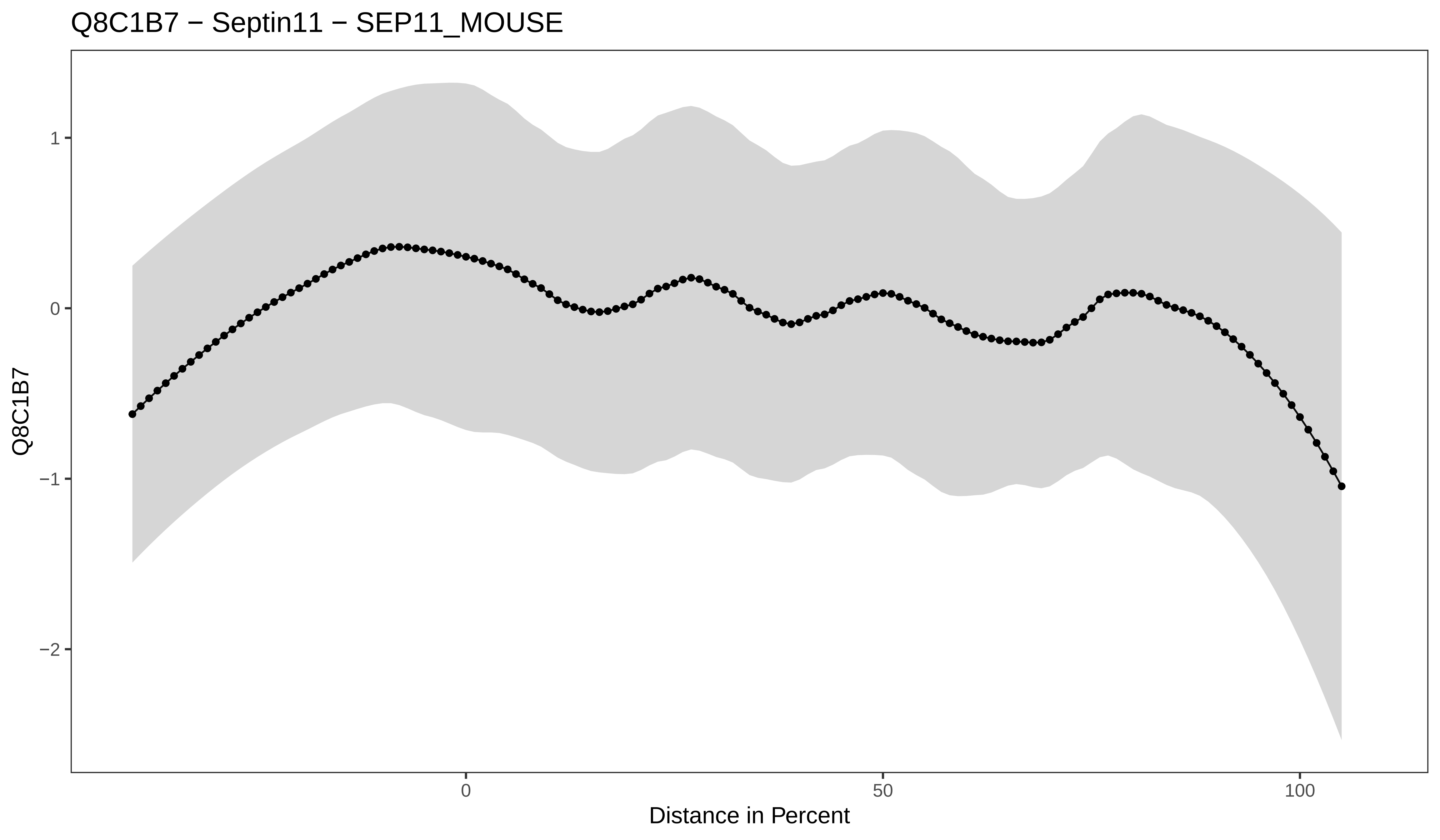

Q8C1B7 − Septin11 − SEP11_MOUSE
Q8C1B7
1
0
−1
−2
0
50
100
Distance in Percent

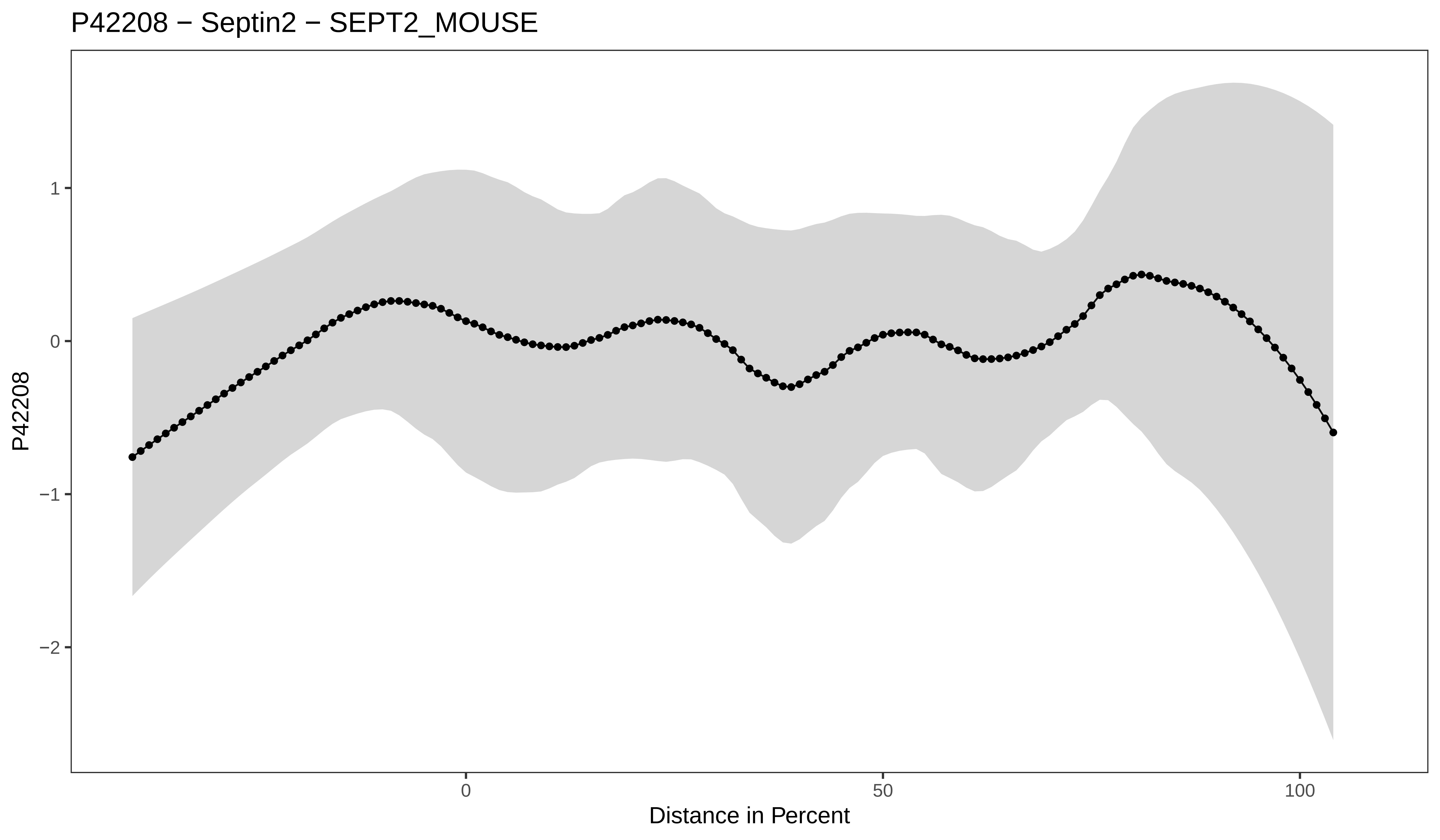
P42208 − Septin2 − SEPT2_MOUSE
1
0
−1
−2
P42208
0
50
100
Distance in Percent

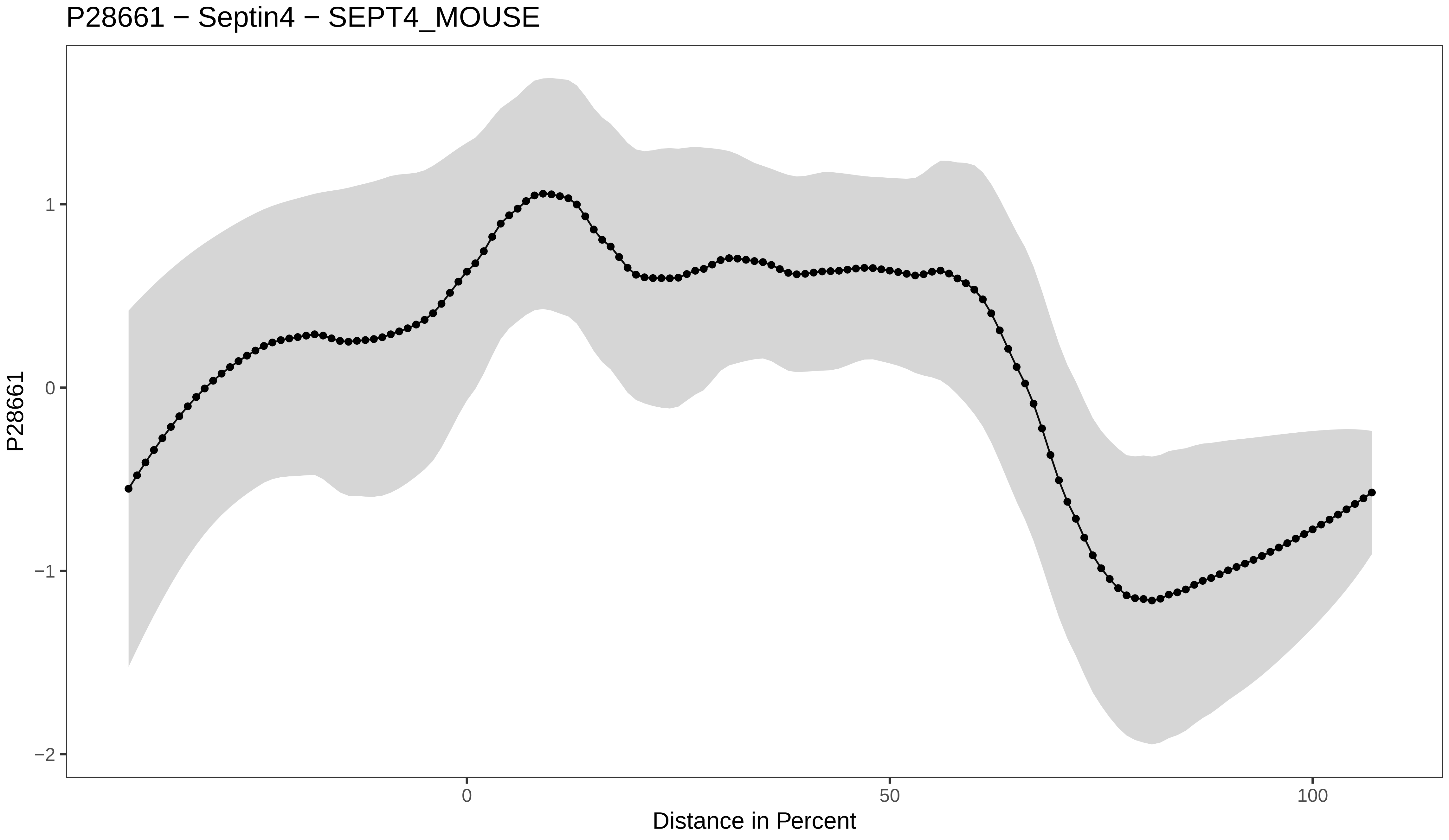

P28661 − Septin4 − SEPT4_MOUSE
P28661
1
0
−1
−2
0
50
100
Distance in Percent

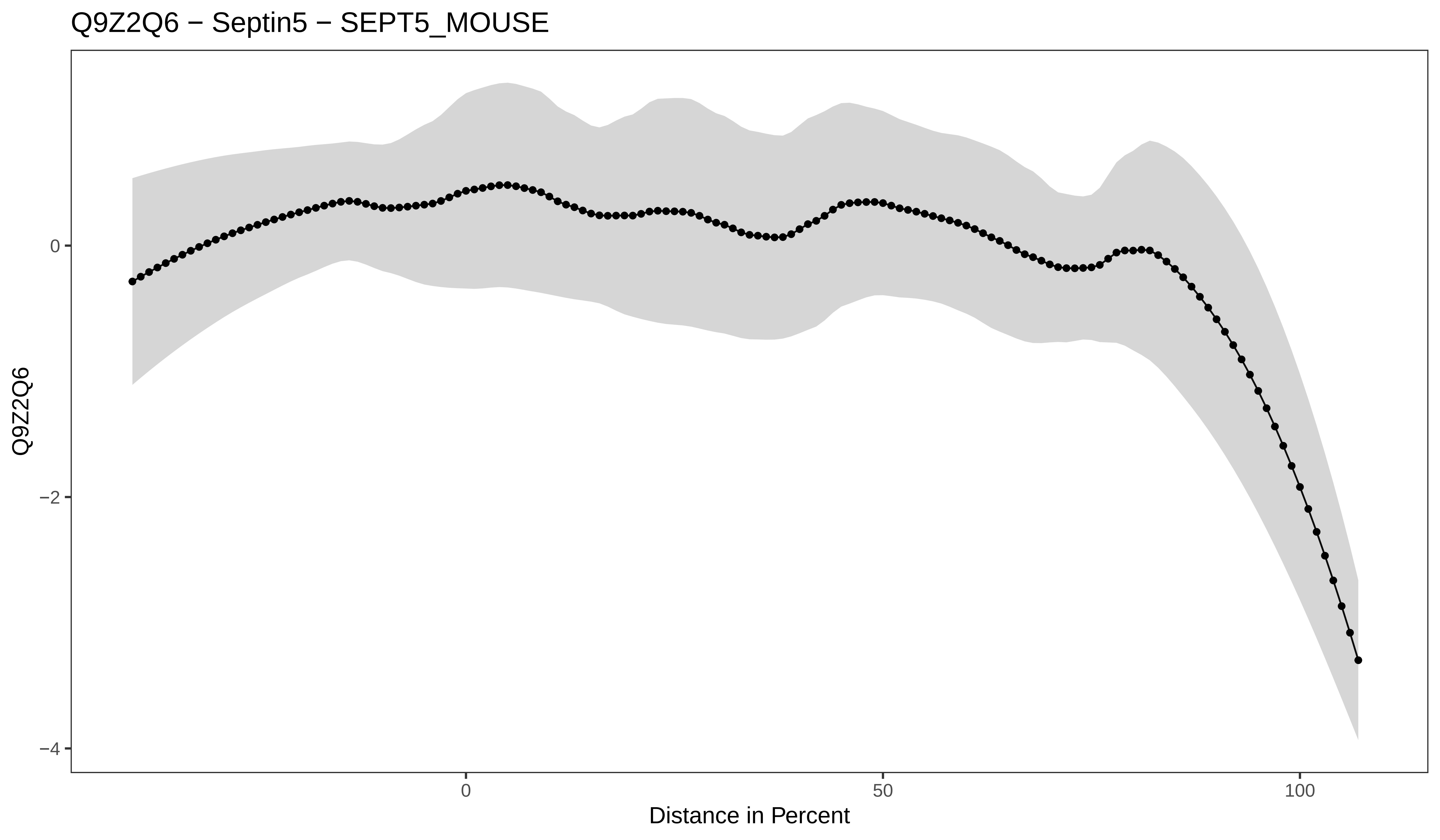

Q9Z2Q6 − Septin5 − SEPT5_MOUSE
Q9Z2Q6
0
−2
−4
0
50
100
Distance in Percent

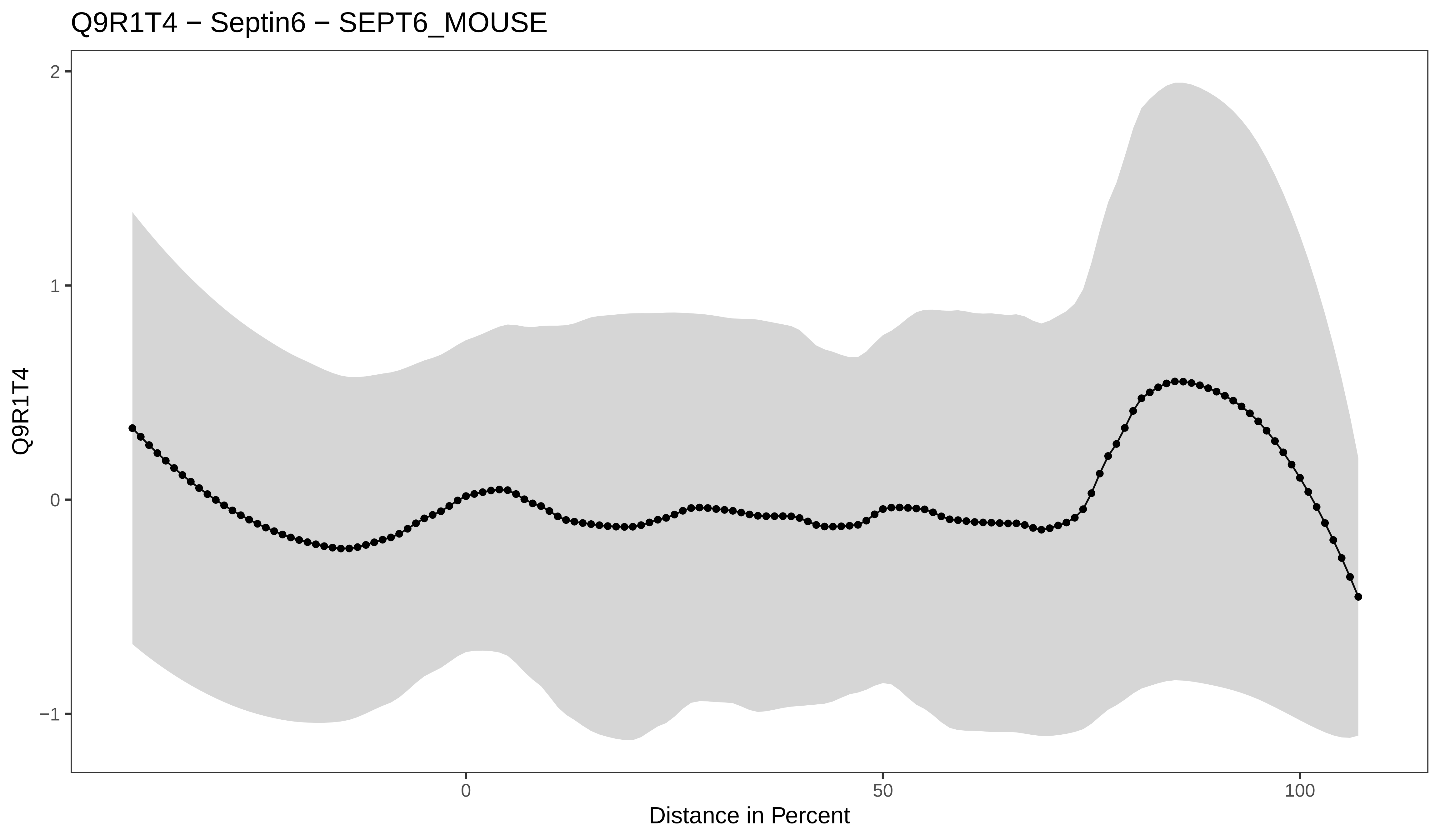

Q9R1T4 − Septin6 − SEPT6_MOUSE
2
1
0
−1
Q9R1T4
0
50
100
Distance in Percent

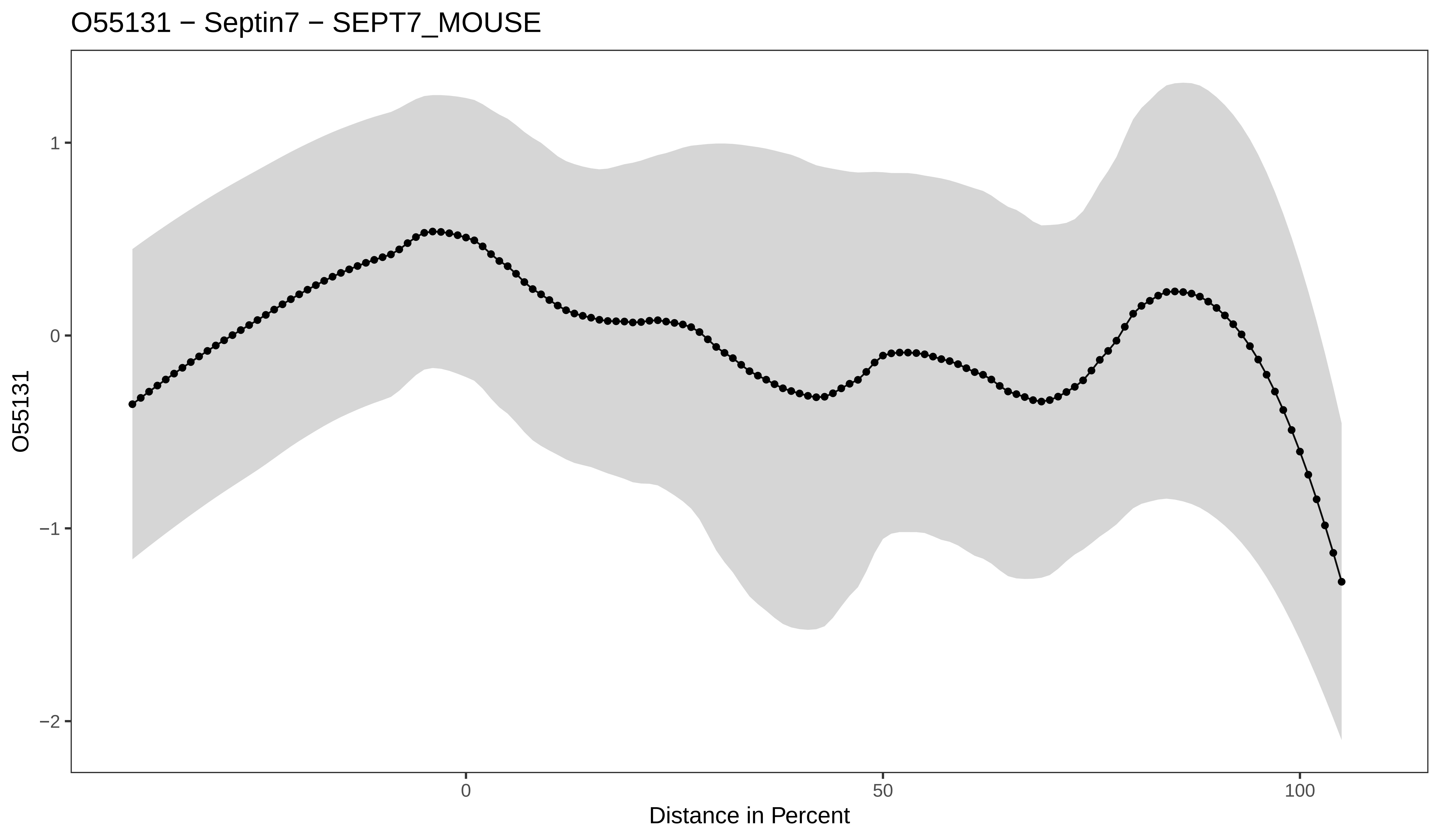

O55131 − Septin7 − SEPT7_MOUSE
O55131
1
0
−1
−2
0
50
100
Distance in Percent

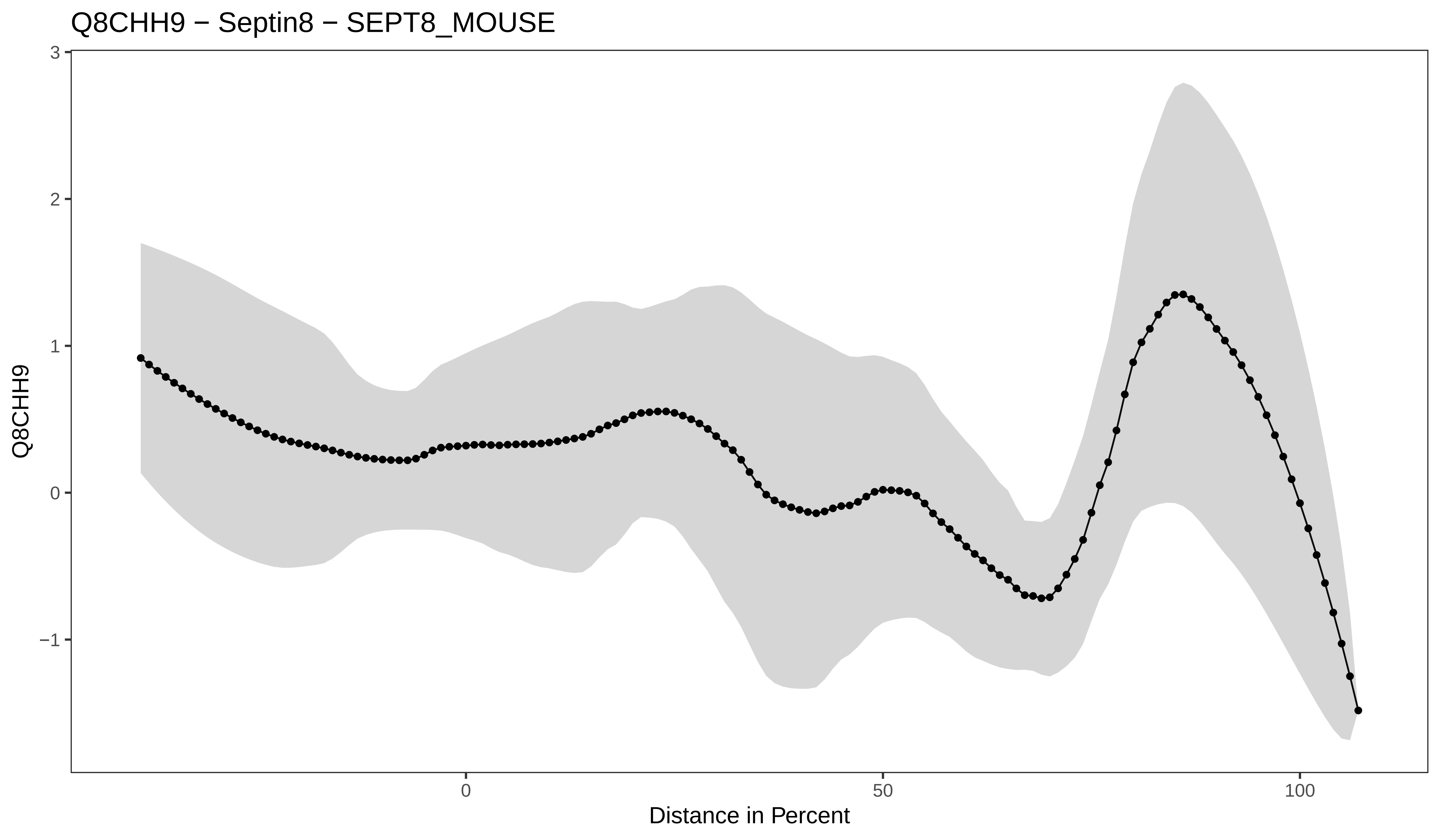

Q8CHH9 − Septin8 − SEPT8_MOUSE
3
2
1
0
−1
Q8CHH9
0
50
100
Distance in Percent

Q80UG5 − Septin9 − SEPT9_MOUSE

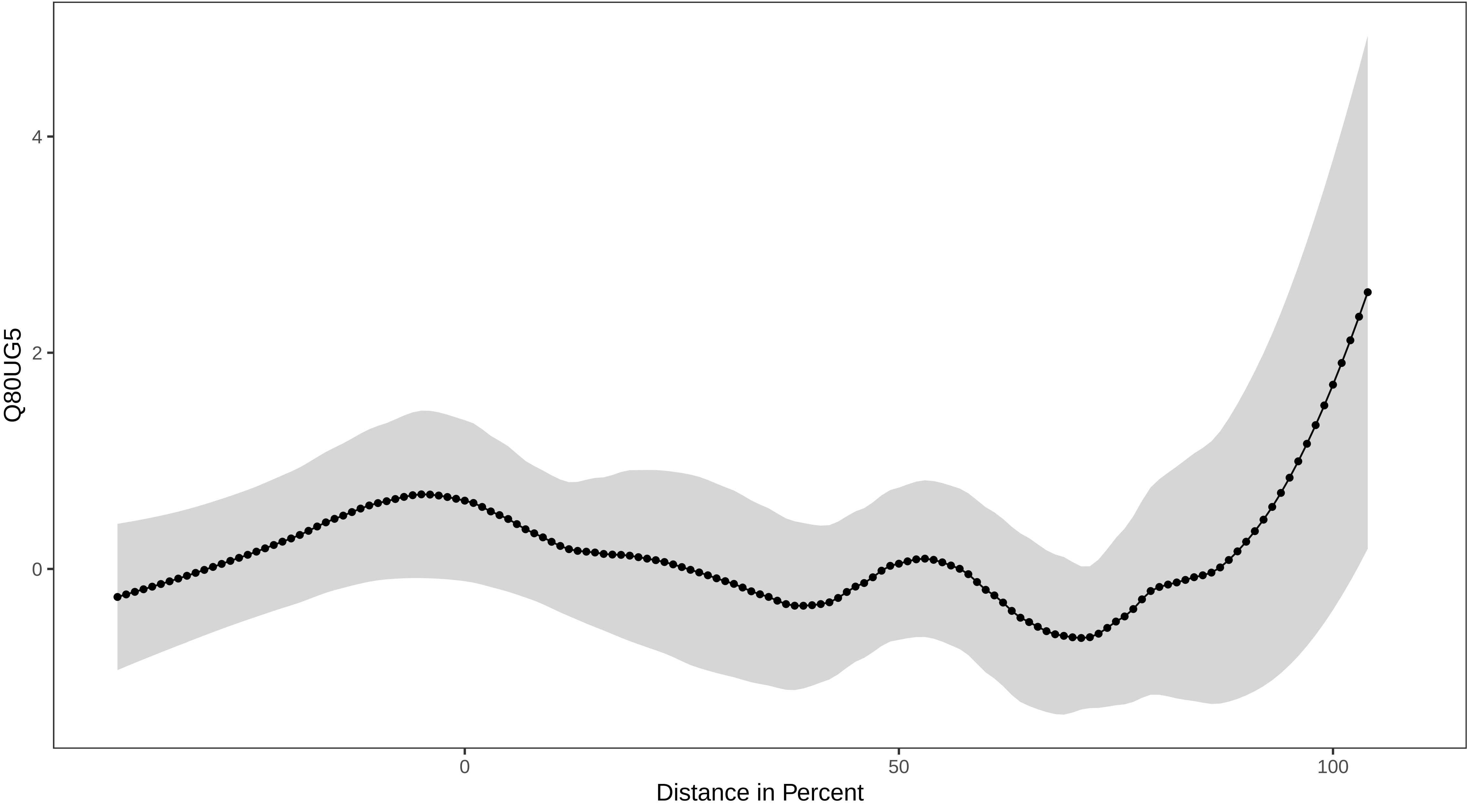

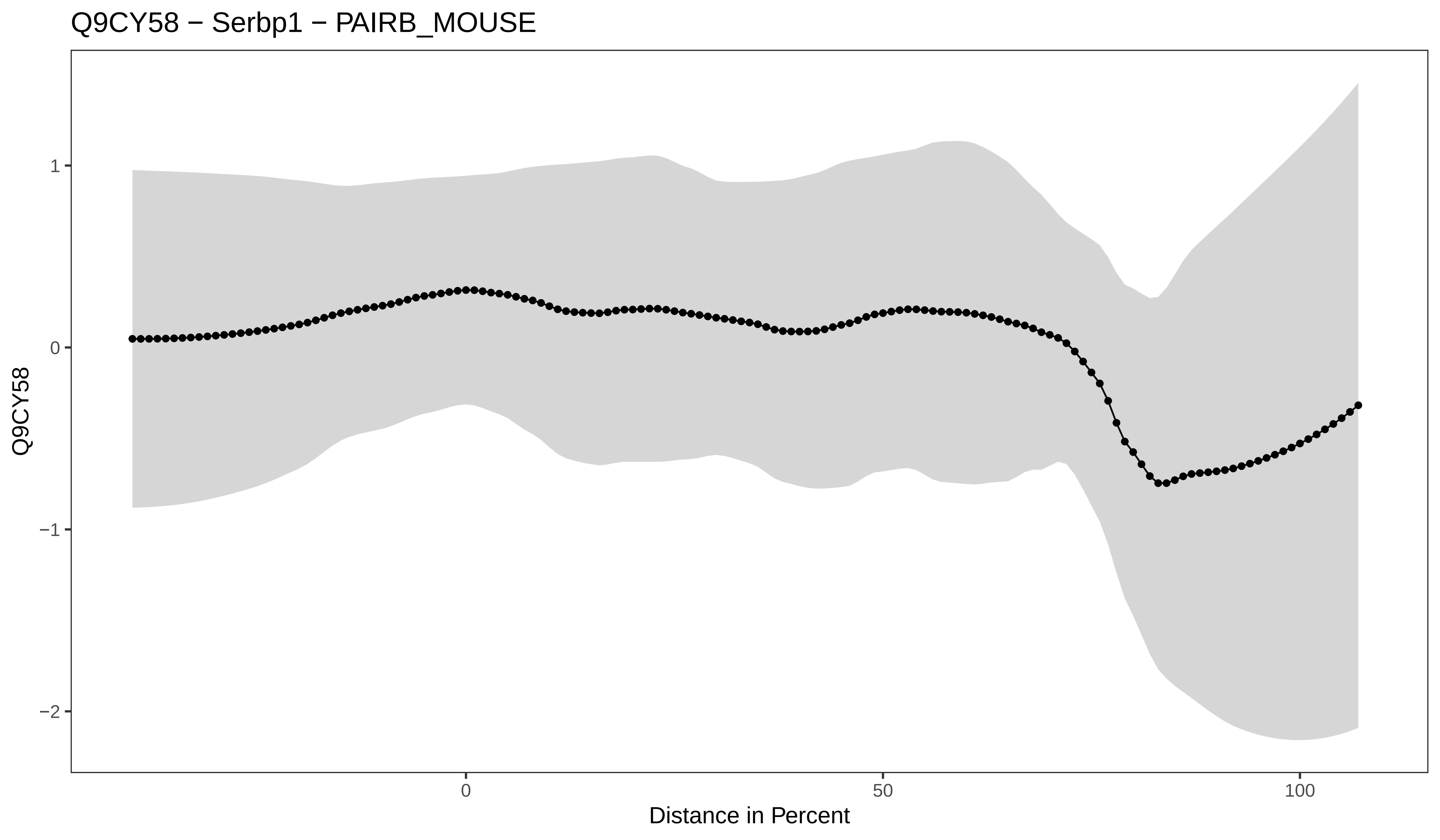
Q9CY58 − Serbp1 − PAIRB_MOUSE
Q9CY58
1
0
−1
−2
0
50
100
Distance in Percent

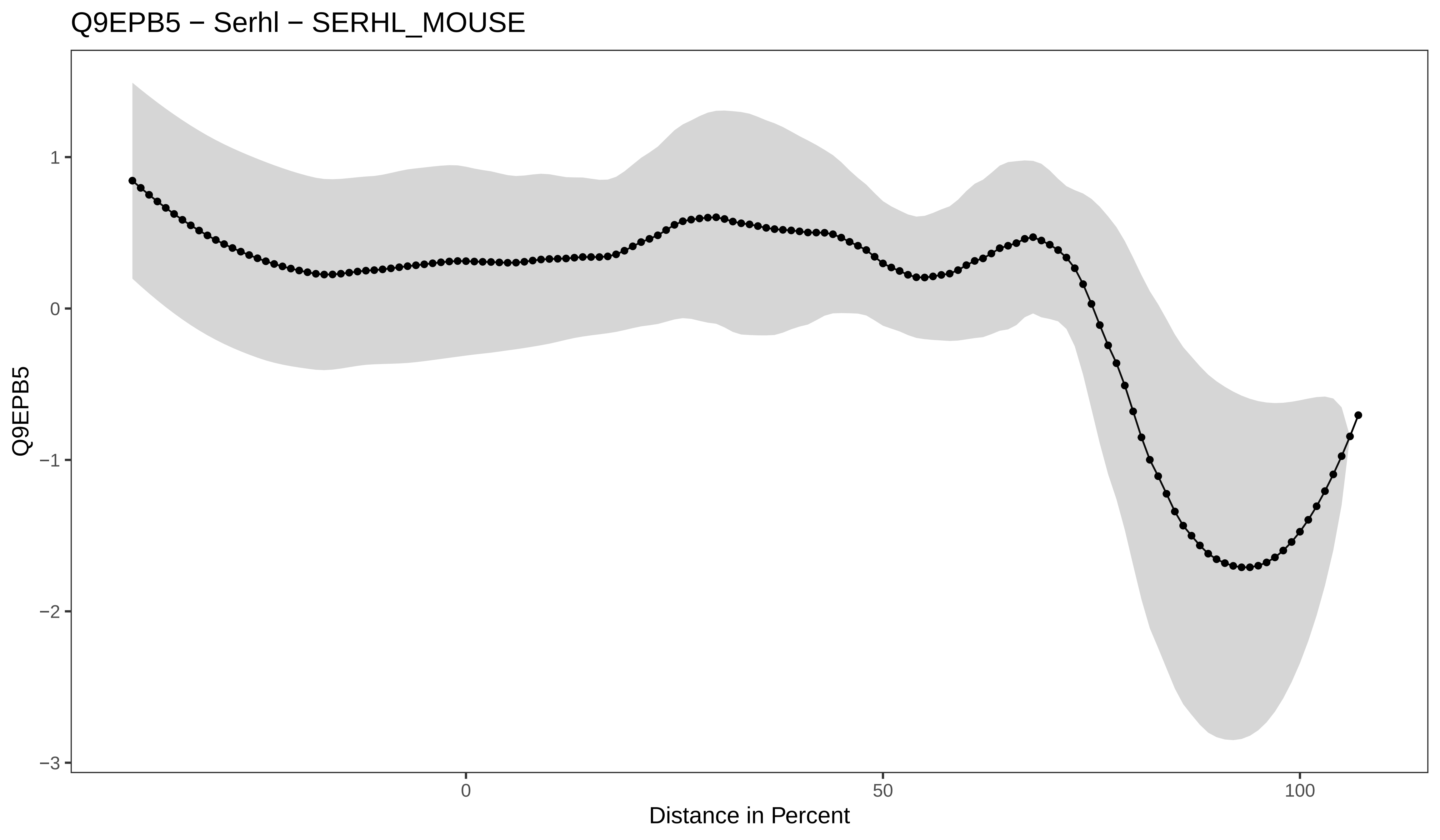

Q9EPB5 − Serhl − SERHL_MOUSE
1
0
−1
−2
−3
Q9EPB5
0
50
100
Distance in Percent

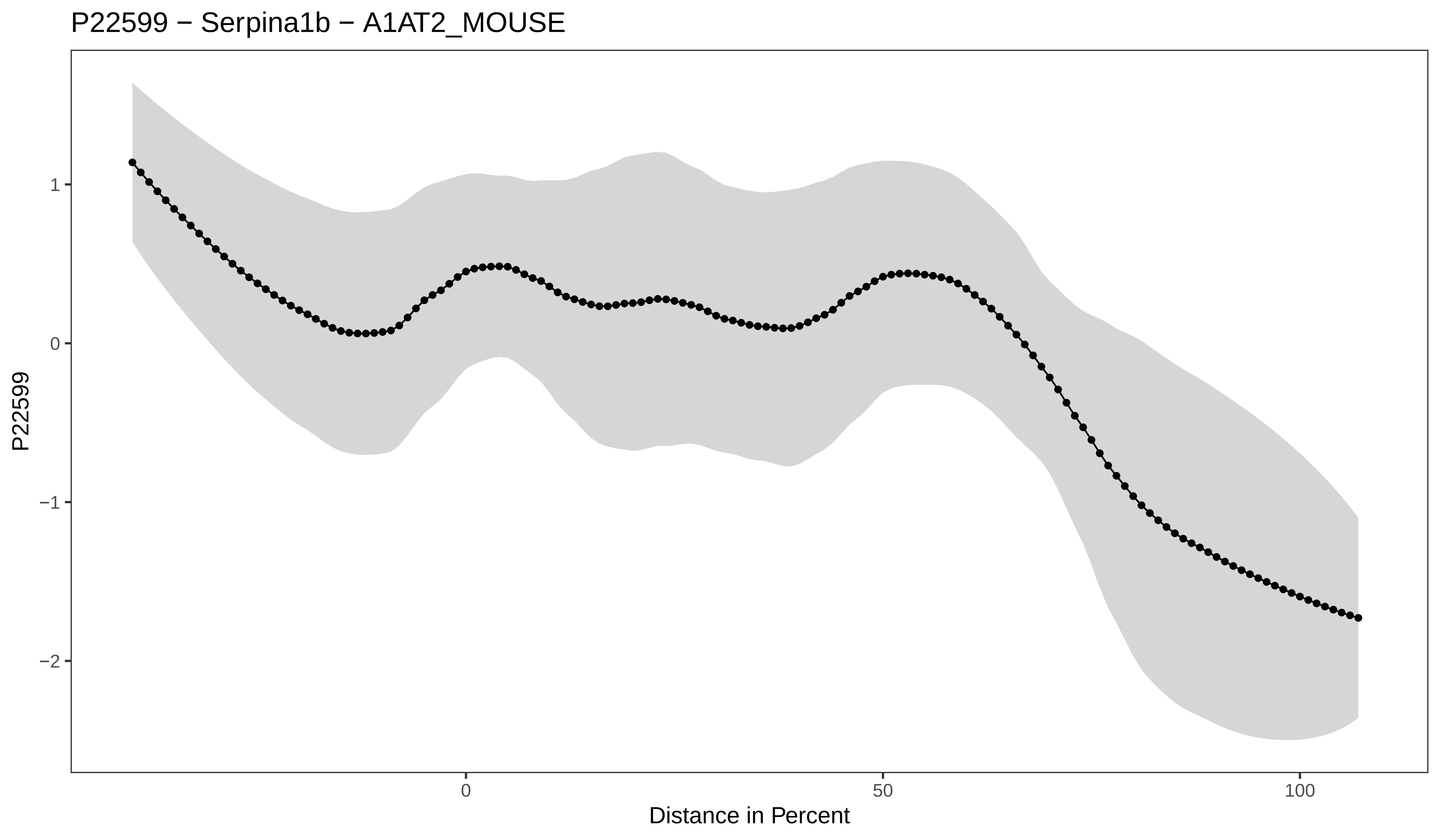
P22599 − Serpina1b − A1AT2_MOUSE
P22599
1
0
−1
−2
0
50
100
Distance in Percent

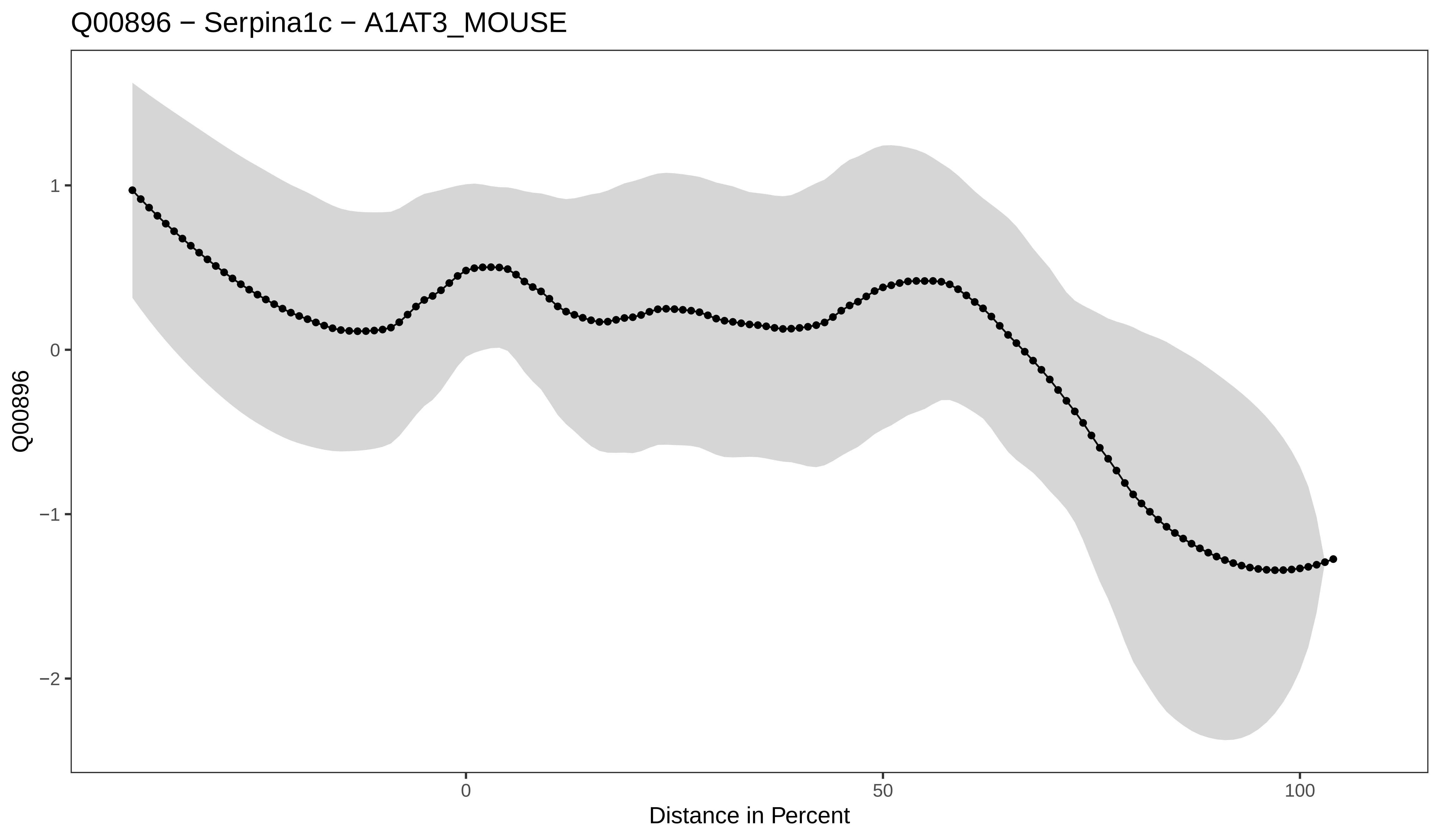
Q00896 − Serpina1c − A1AT3_MOUSE
Q00896
1
0
−1
−2
0
50
100
Distance in Percent

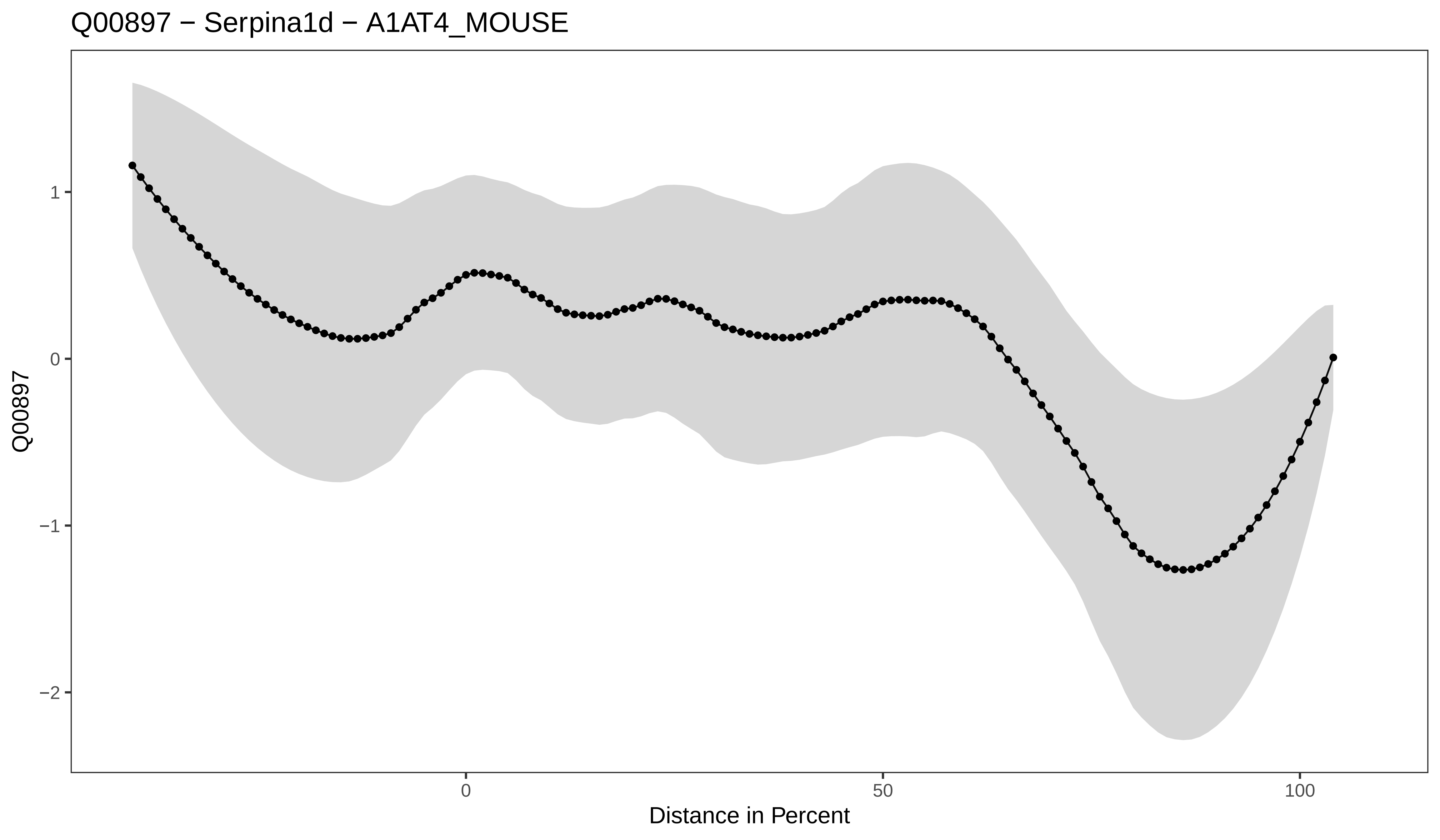

Q00897 − Serpina1d − A1AT4_MOUSE
Q00897
1
0
−1
−2
0
50
100
Distance in Percent

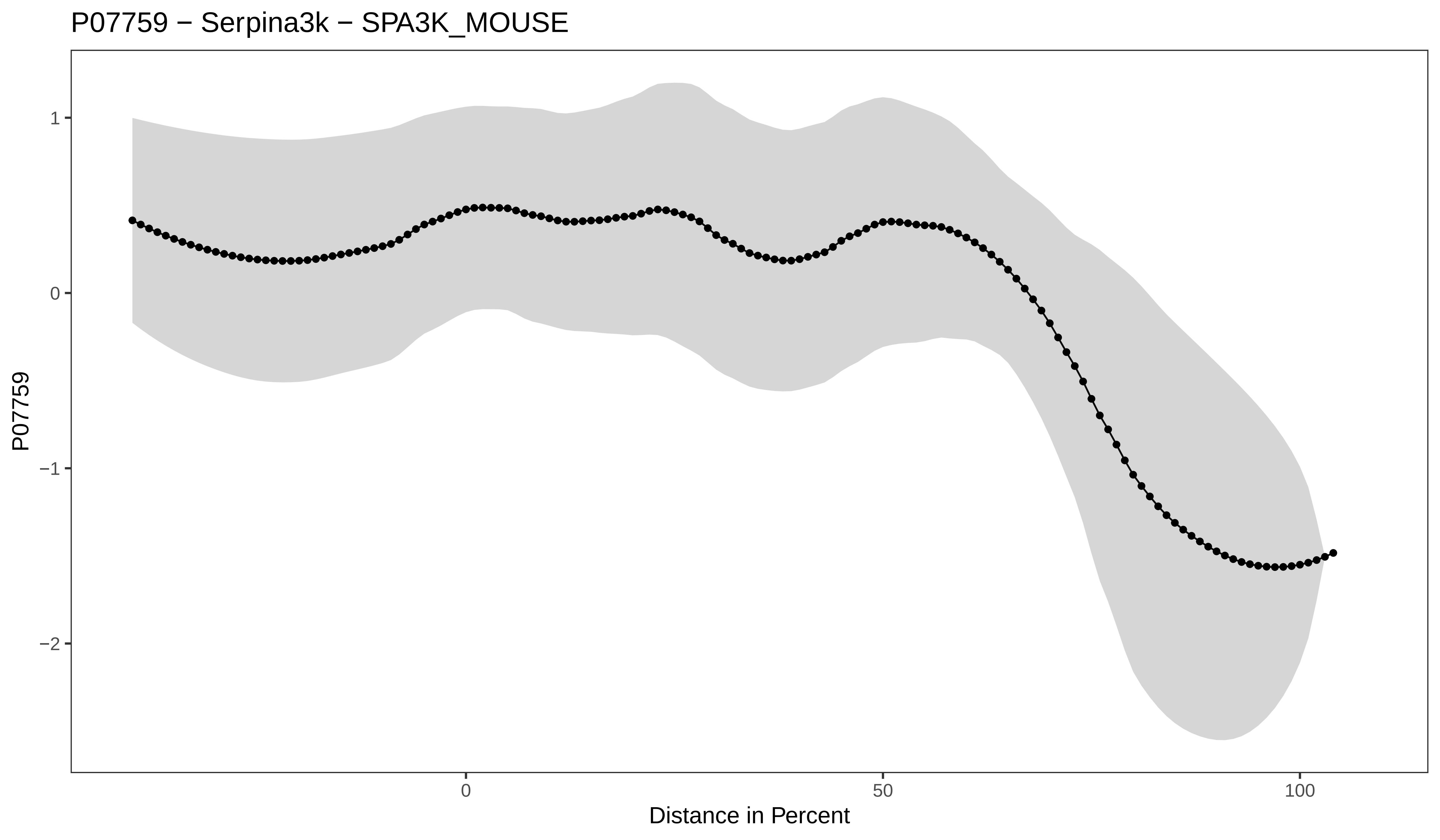

P07759 − Serpina3k − SPA3K_MOUSE
1
0
−1
−2
P07759
0
50
100
Distance in Percent

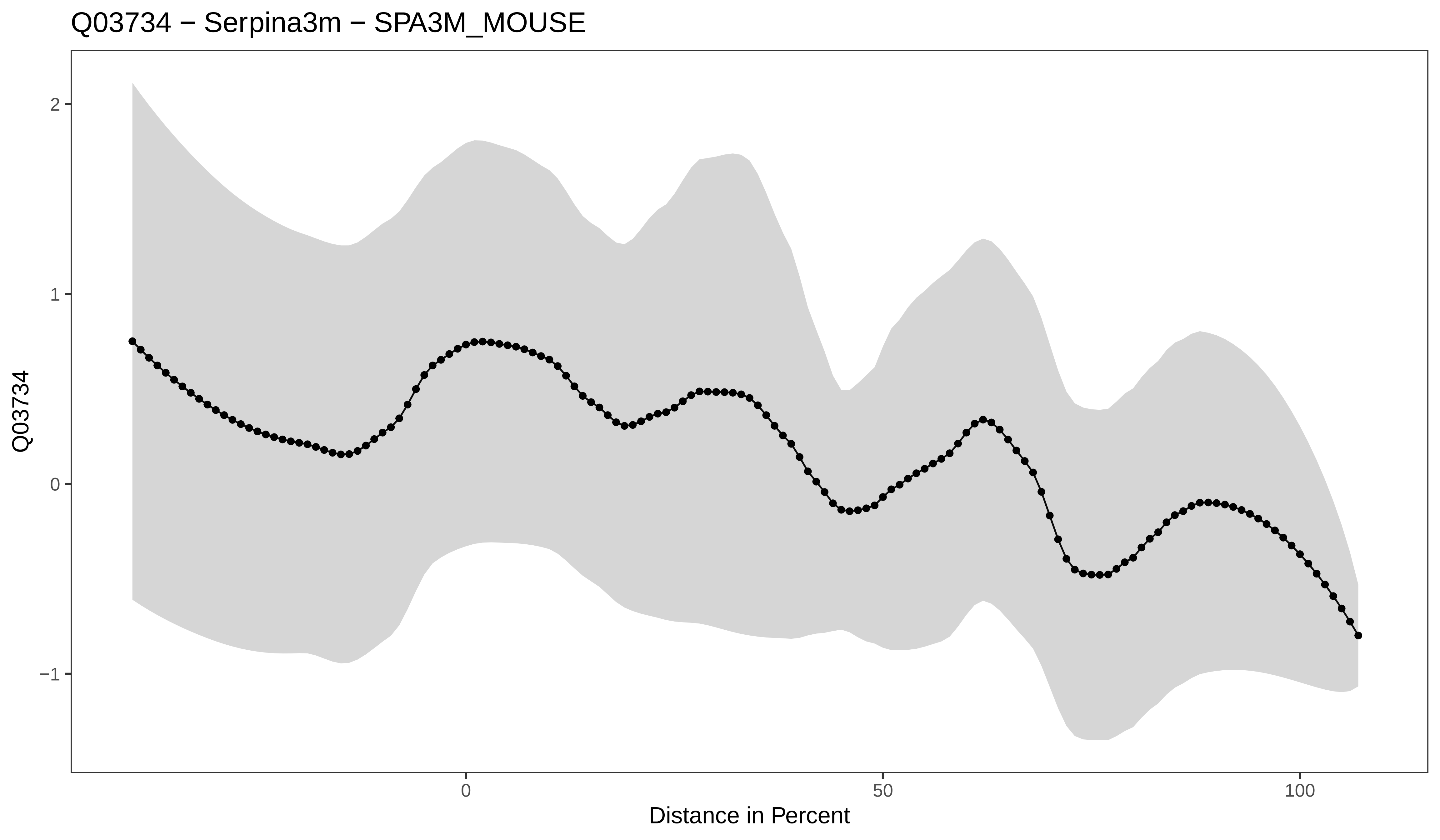
Q03734 − Serpina3m − SPA3M_MOUSE
Q03734
2
1
0
−1
0
50
100
Distance in Percent

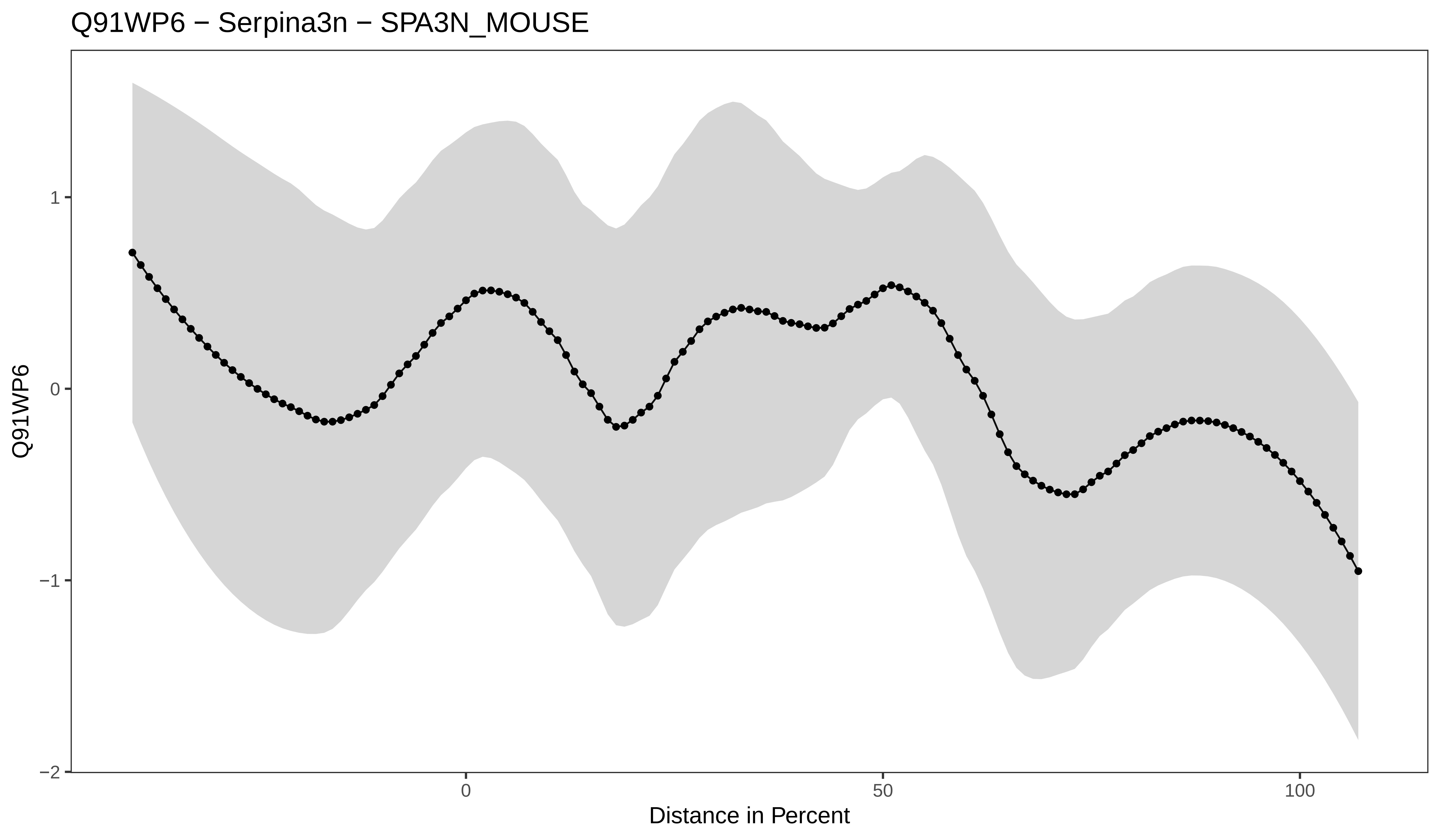

Q91WP6 − Serpina3n − SPA3N_MOUSE
Q91WP6
1
0
−1
−2
0
50
100
Distance in Percent

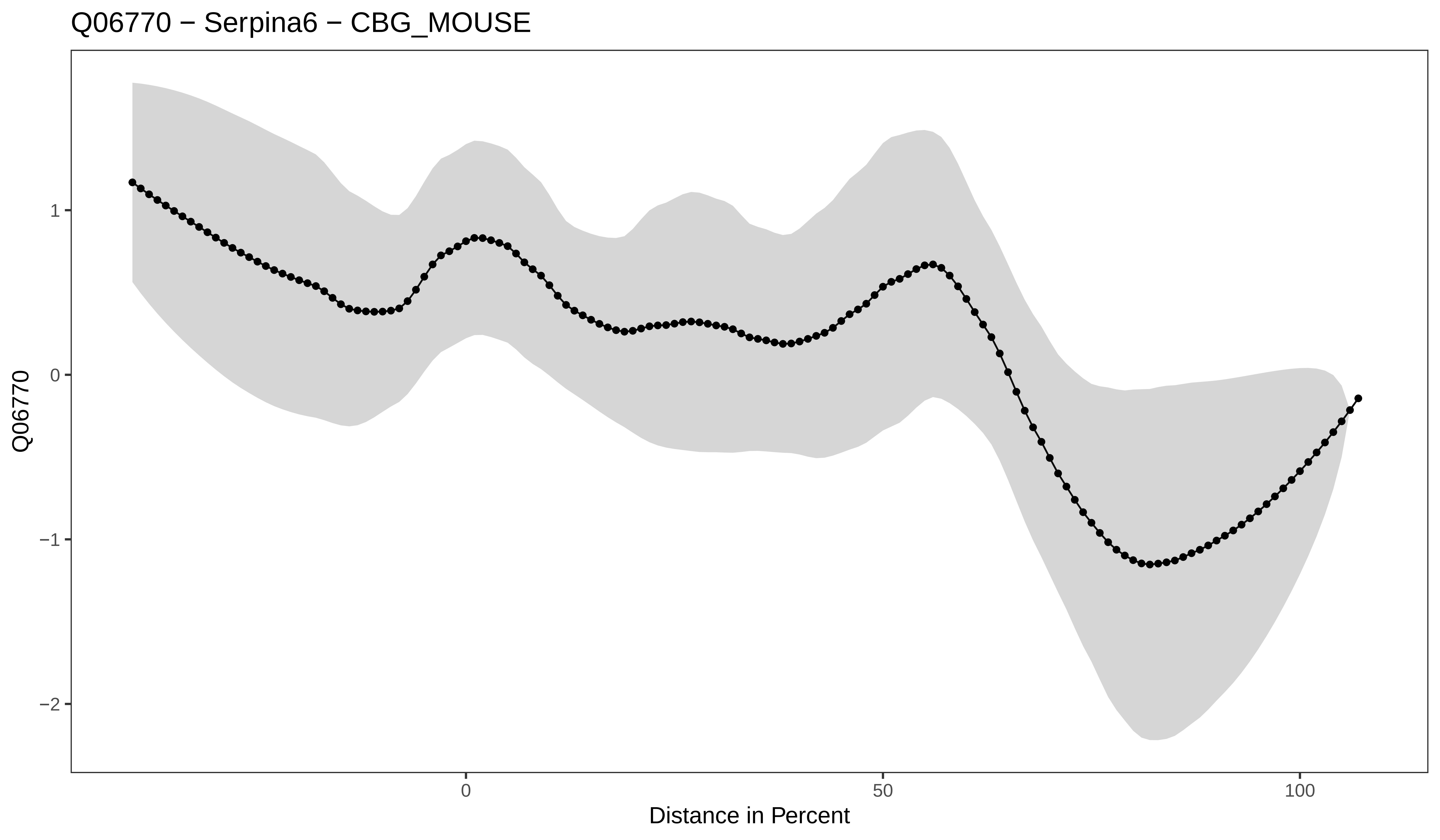

Q06770 − Serpina6 − CBG_MOUSE
Q06770
1
0
−1
−2
0
50
100
Distance in Percent

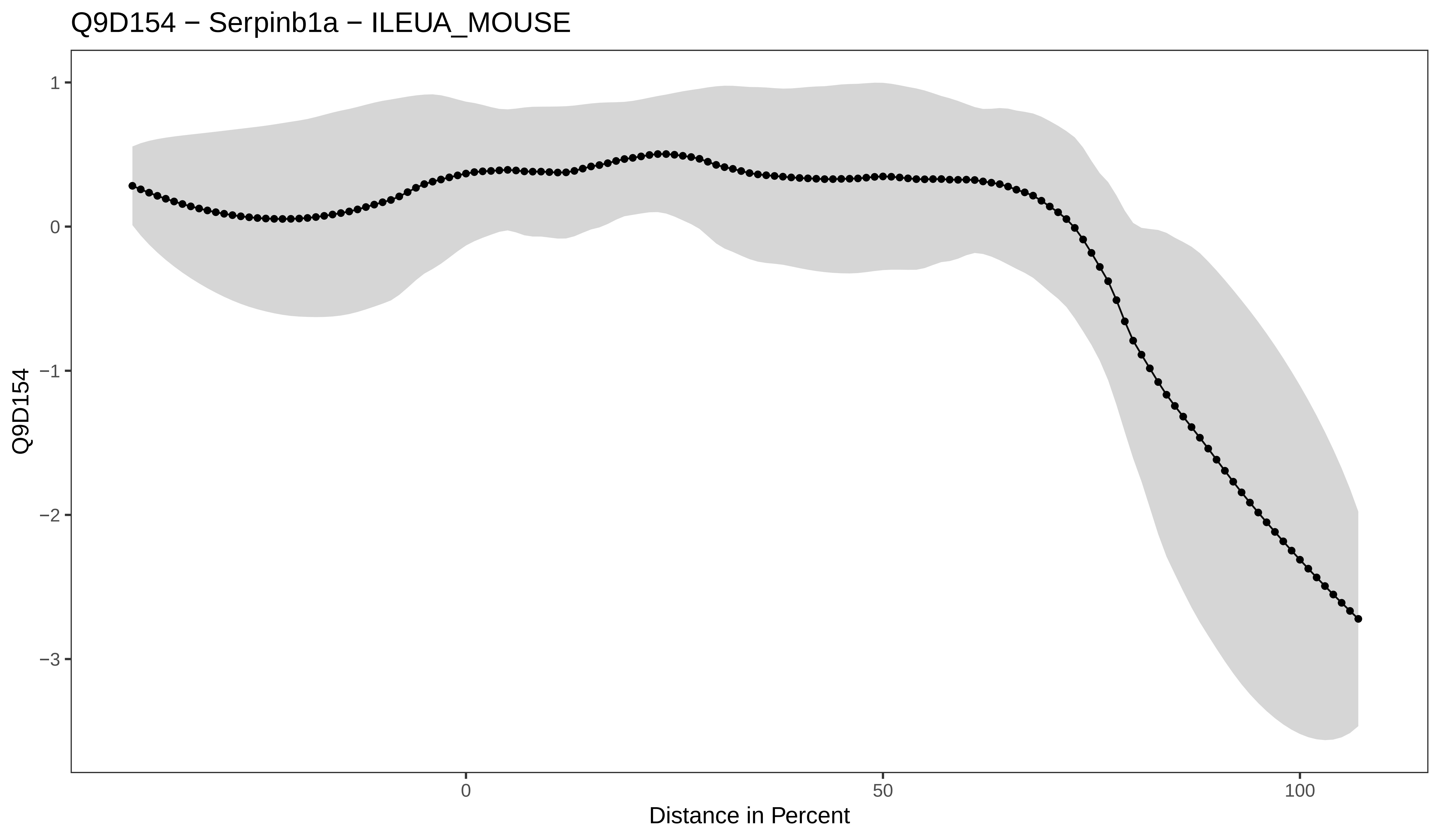
Q9D154 − Serpinb1a − ILEUA_MOUSE
Q9D154
1
0
−1
−2
−3
0
50
100
Distance in Percent

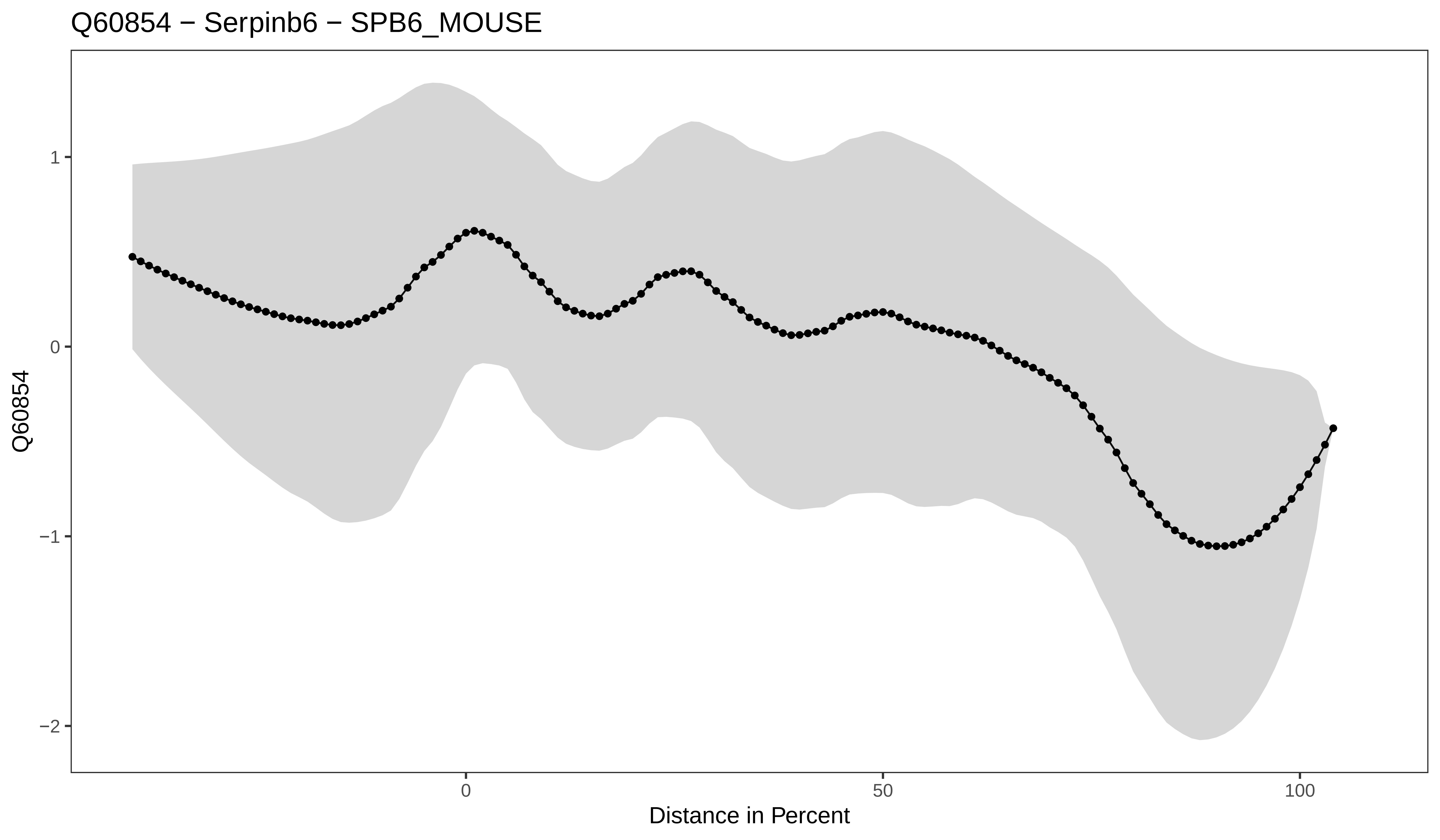
Q60854 − Serpinb6 − SPB6_MOUSE
Q60854
1
0
−1
−2
0
50
100
Distance in Percent

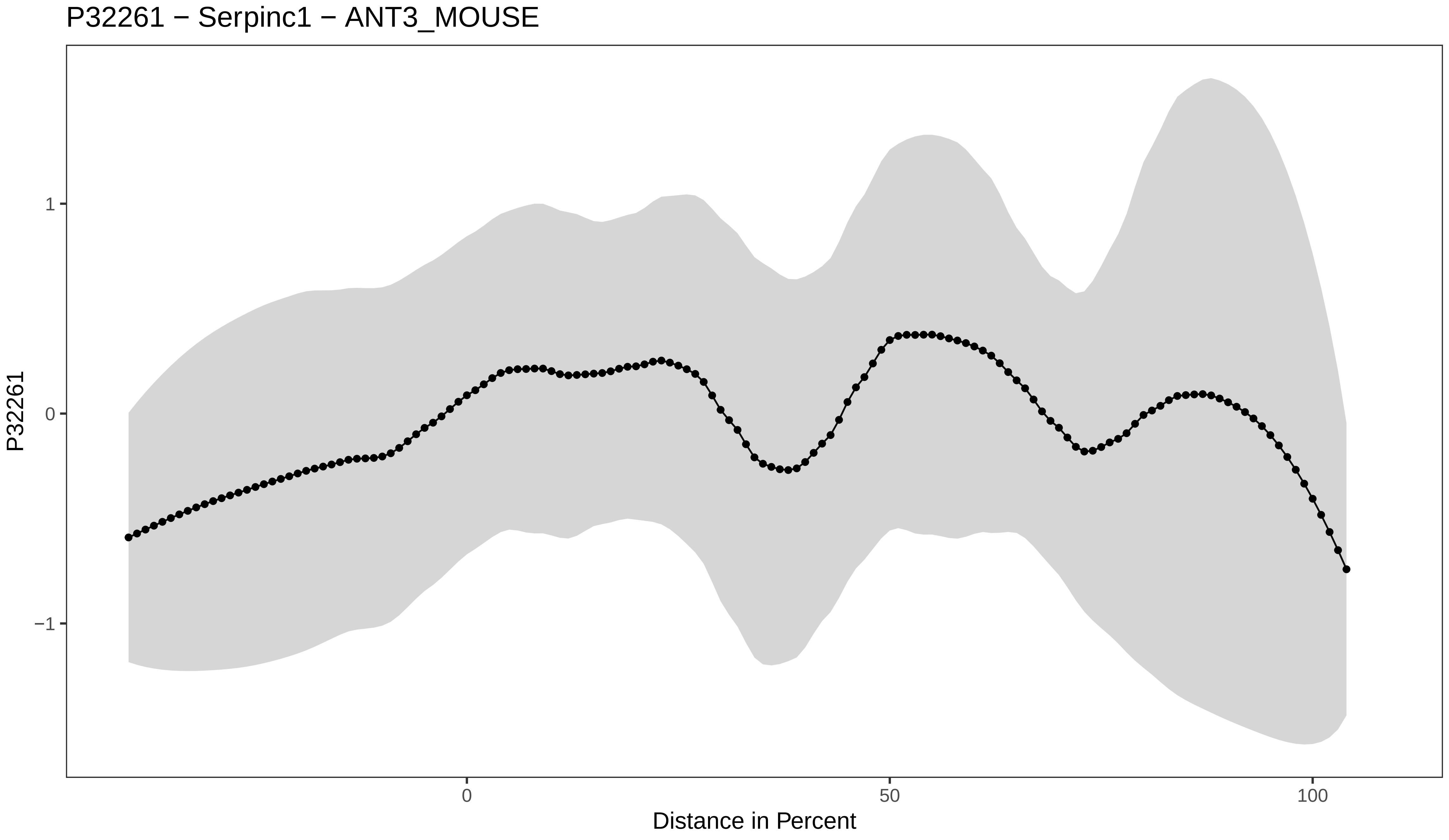
P32261 − Serpinc1 − ANT3_MOUSE
P32261
1
0
−1
0
50
100
Distance in Percent

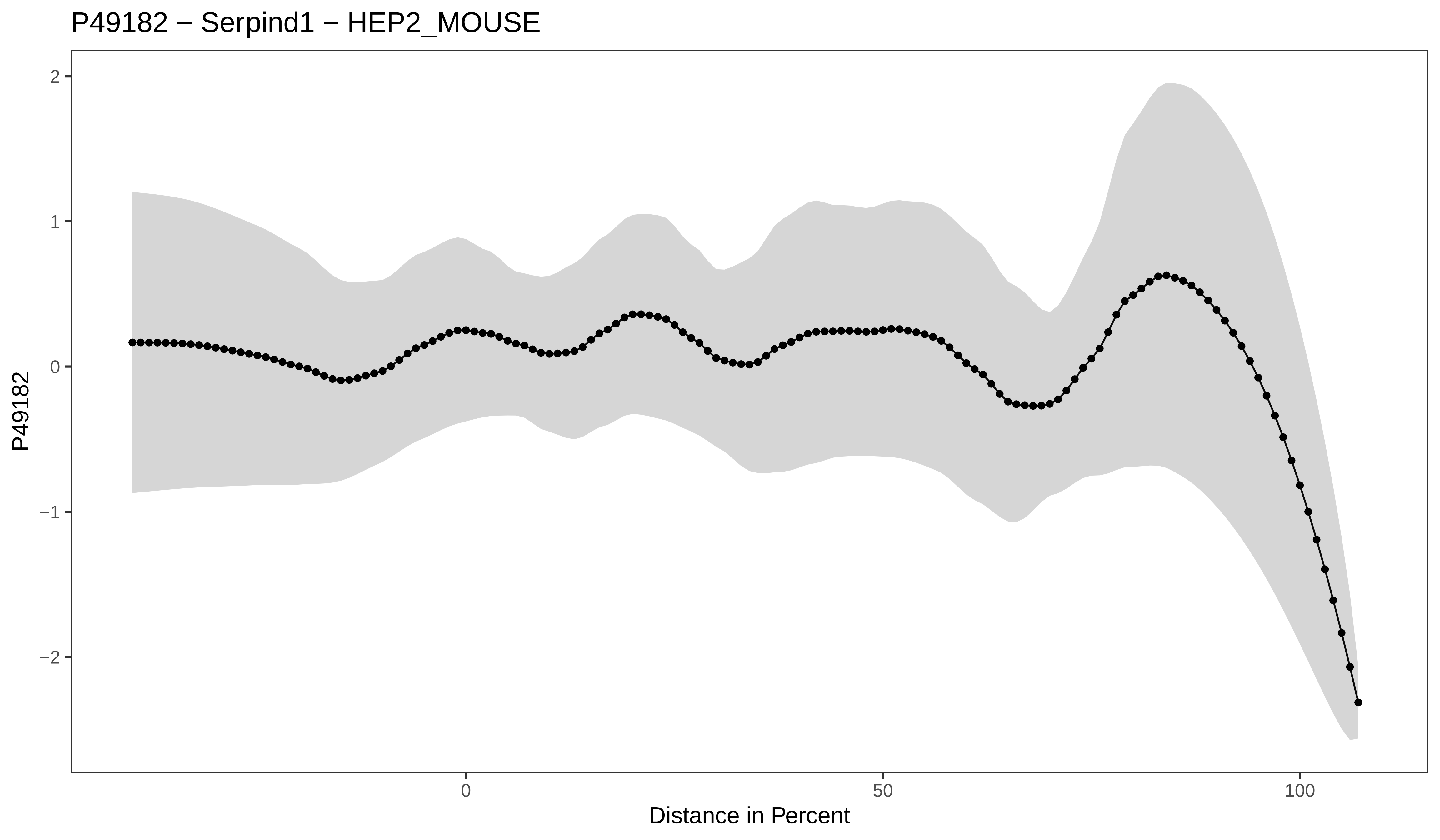
P49182 − Serpind1 − HEP2_MOUSE
P49182
2
1
0
−1
−2
0
50
100
Distance in Percent

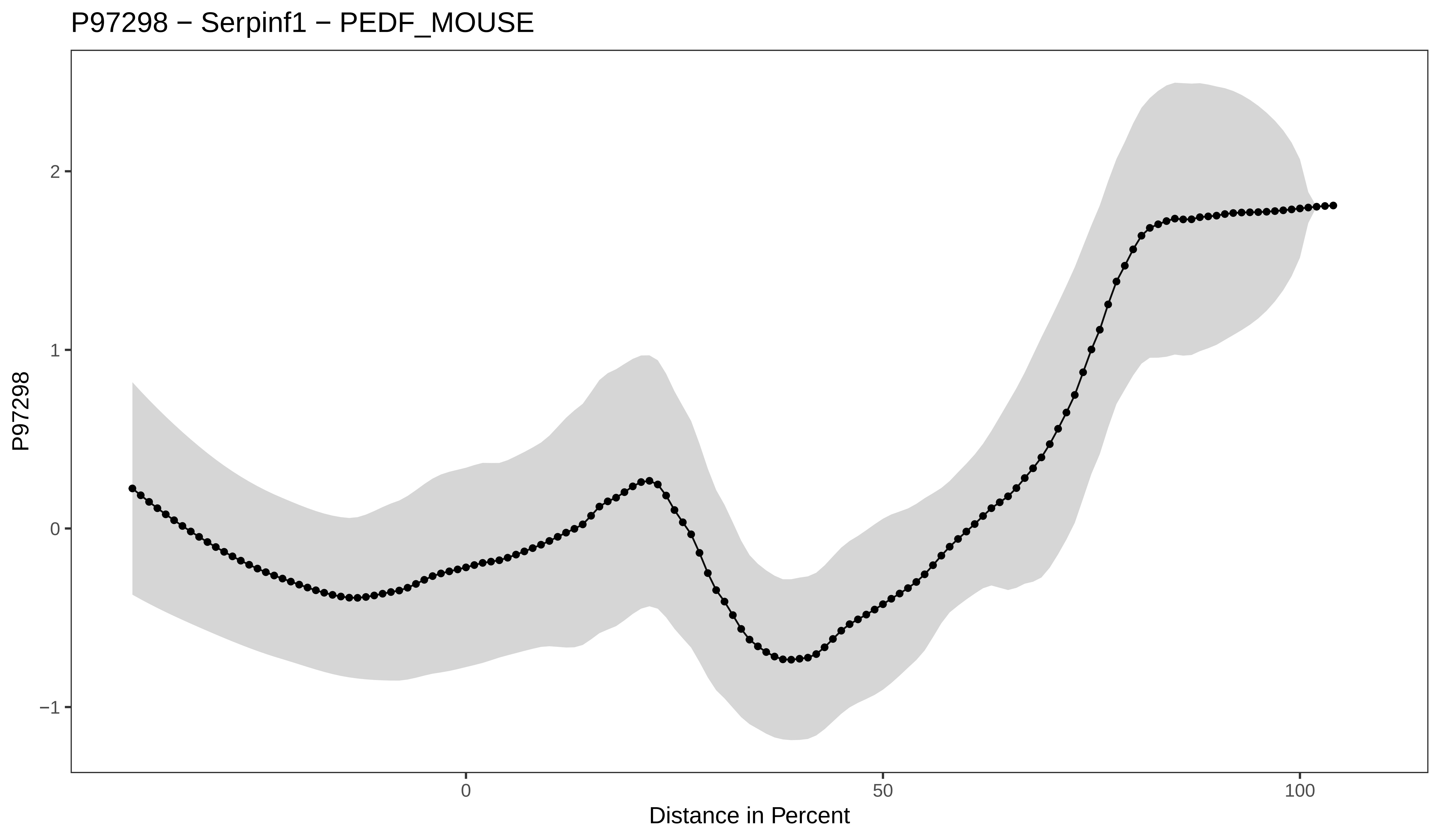

P97298 − Serpinf1 − PEDF_MOUSE
P97298
2
1
0
−1
0
50
100
Distance in Percent

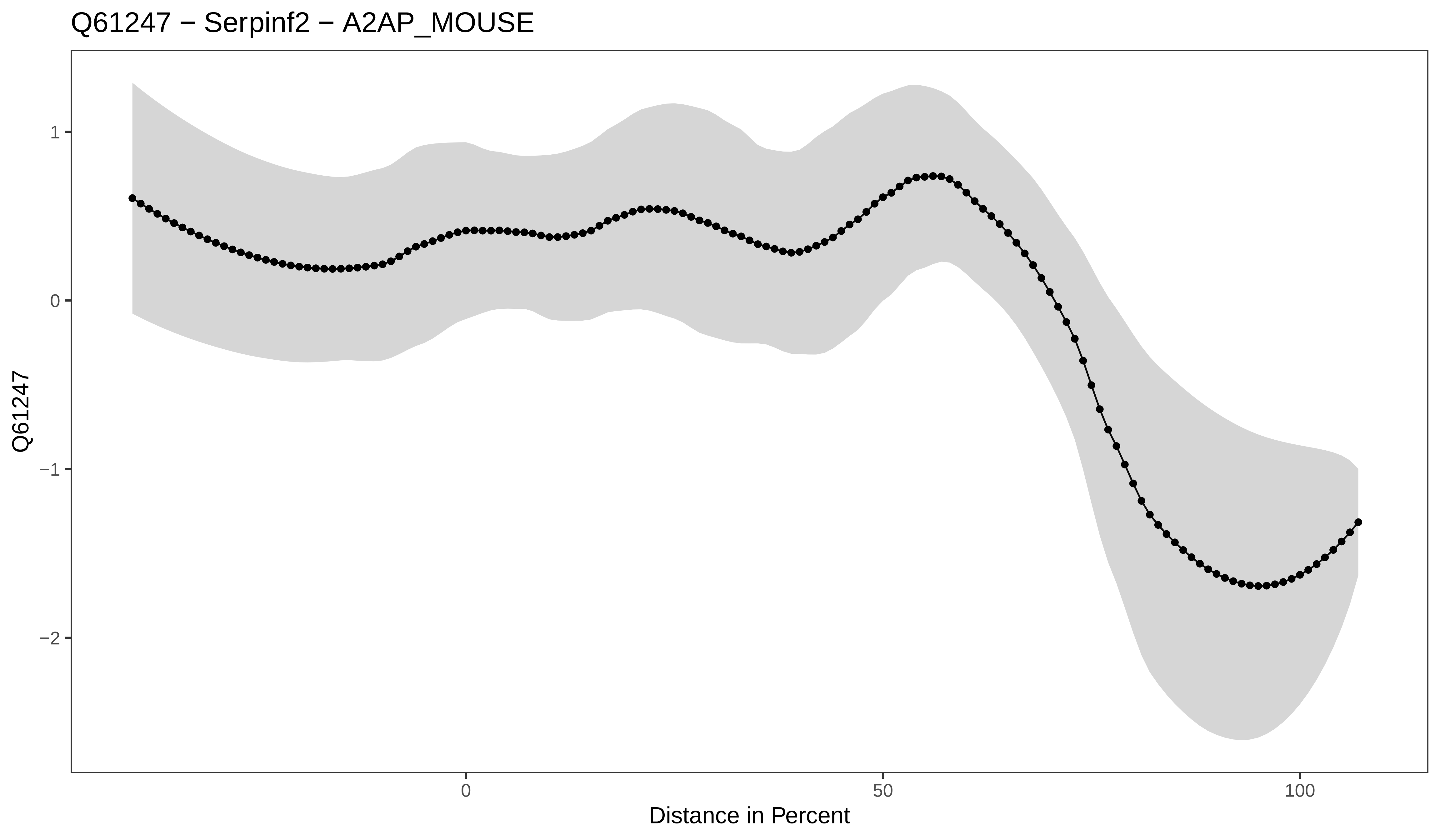

Q61247 − Serpinf2 − A2AP_MOUSE
Q61247
1
0
−1
−2
0
50
100
Distance in Percent

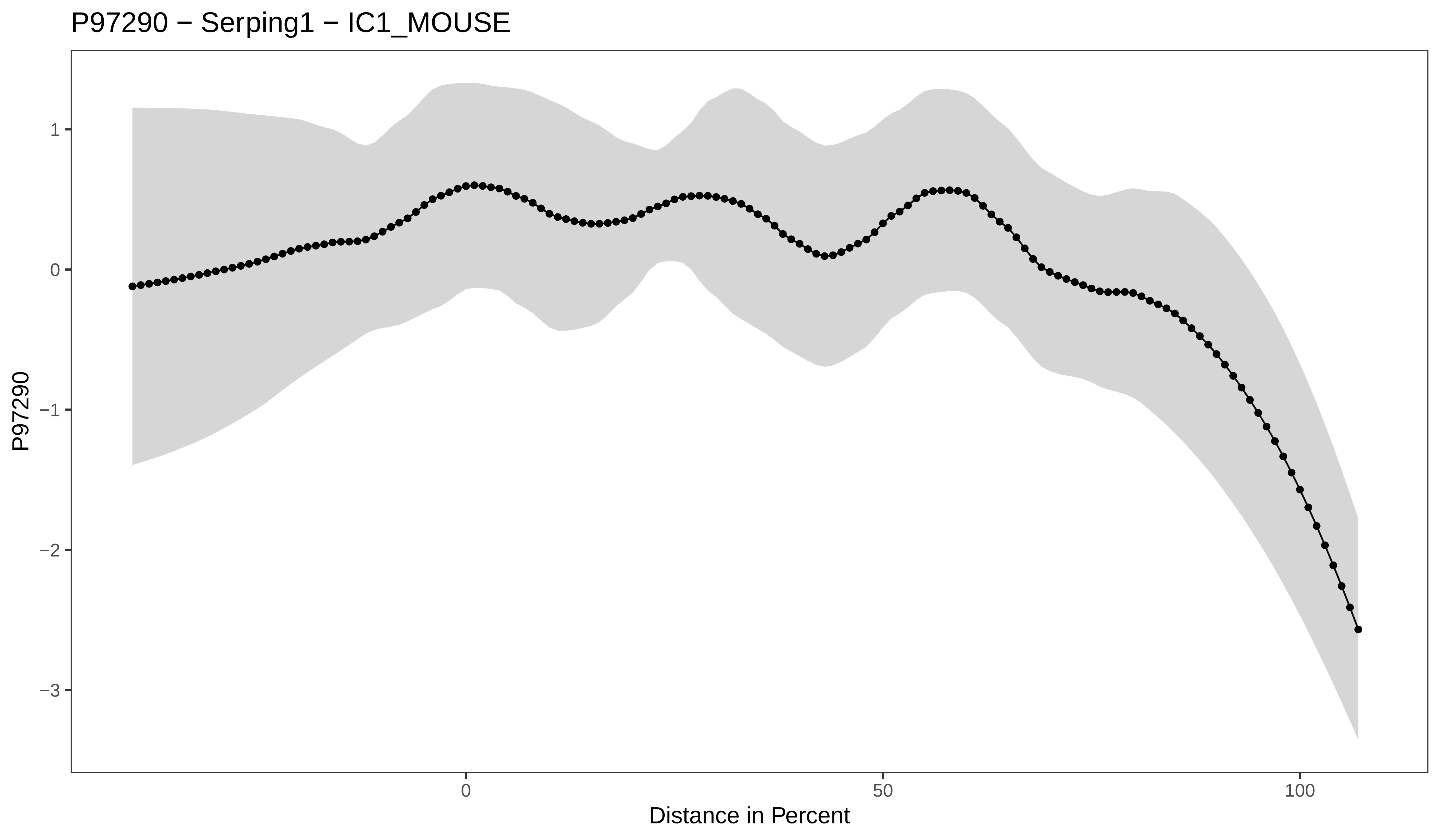
P97290 − Serping1 − IC1_MOUSE
P97290
1
0
−1
−2
−3
0
50
100
Distance in Percent

P19324 − Serpinh1 − SERPH_MOUSE

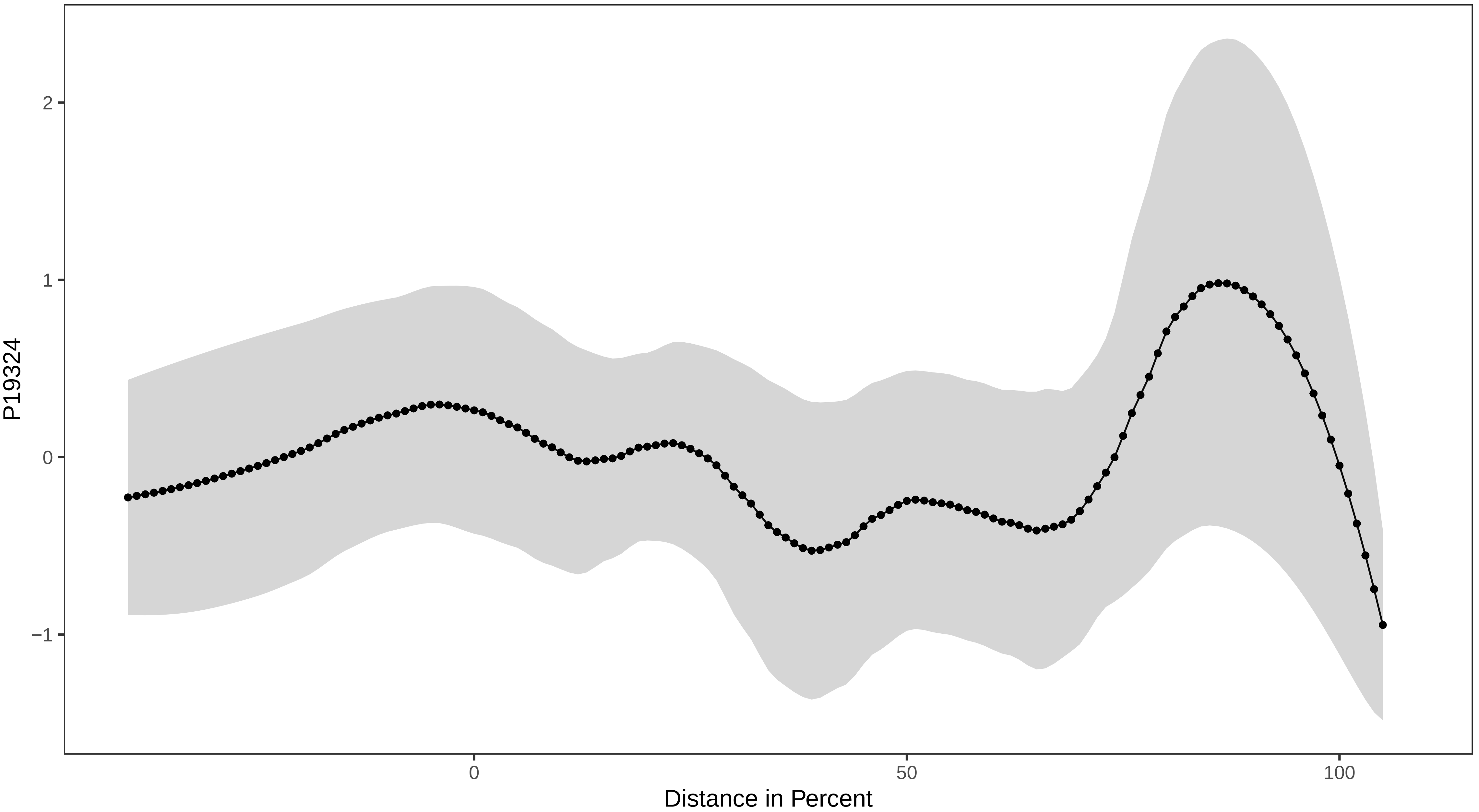

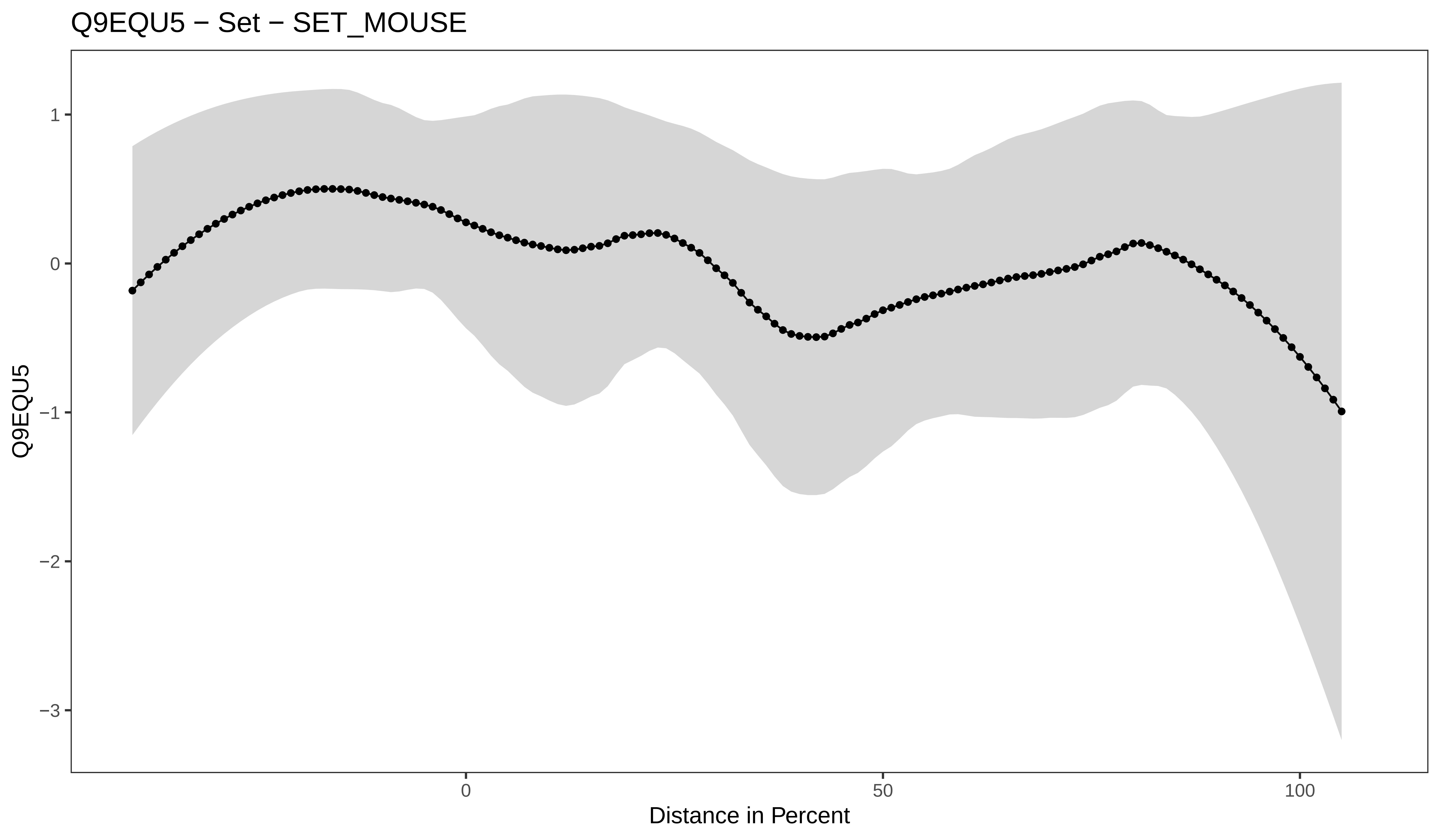

Q9EQU5 − Set − SET_MOUSE
1
0
−1
−2
−3
Q9EQU5
0
50
100
Distance in Percent

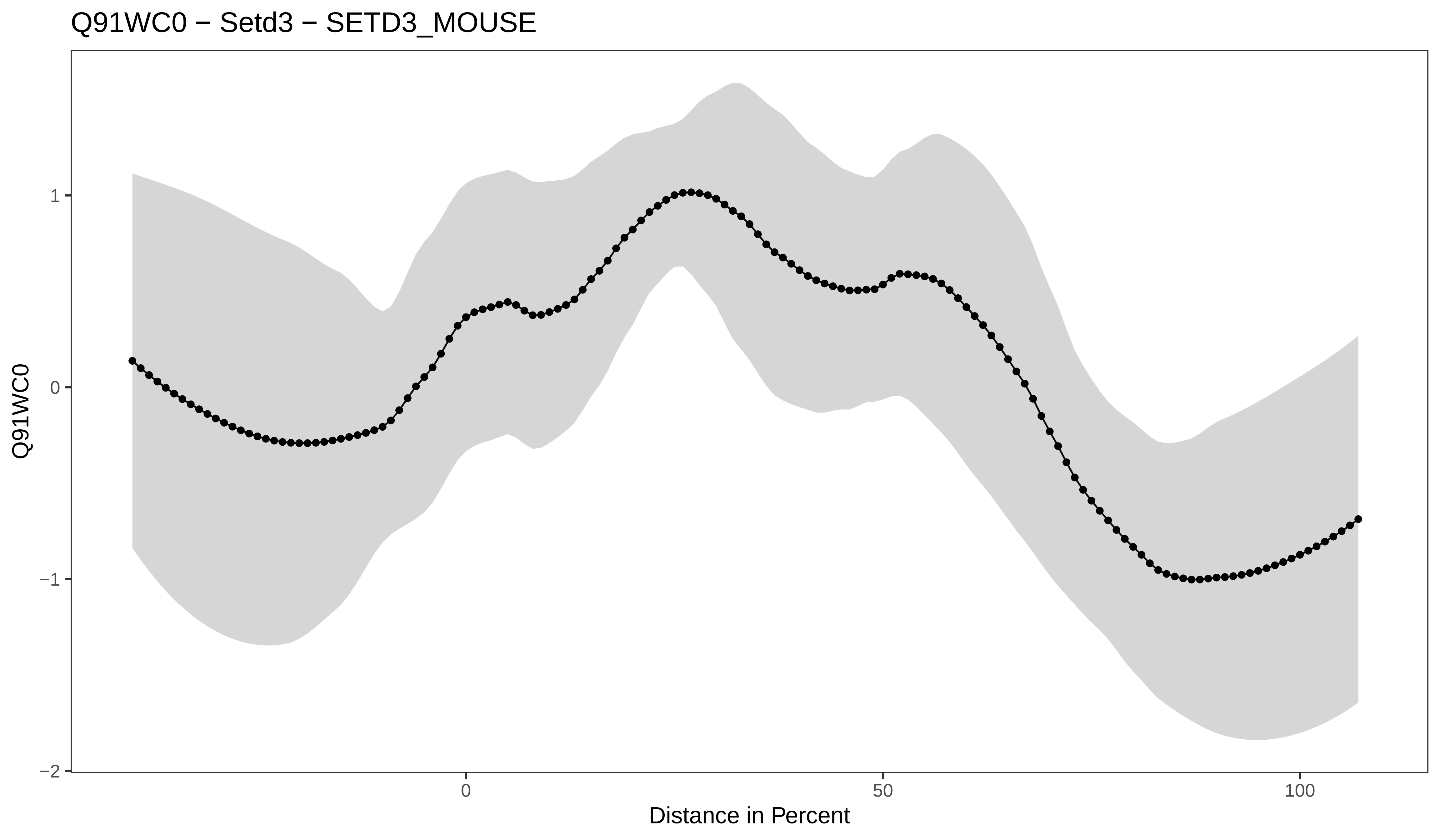
Q91WC0 − Setd3 − SETD3_MOUSE
Q91WC0
1
0
−1
−2
0
50
100
Distance in Percent

Q8K4Z5 − Sf3a1 − SF3A1_MOUSE

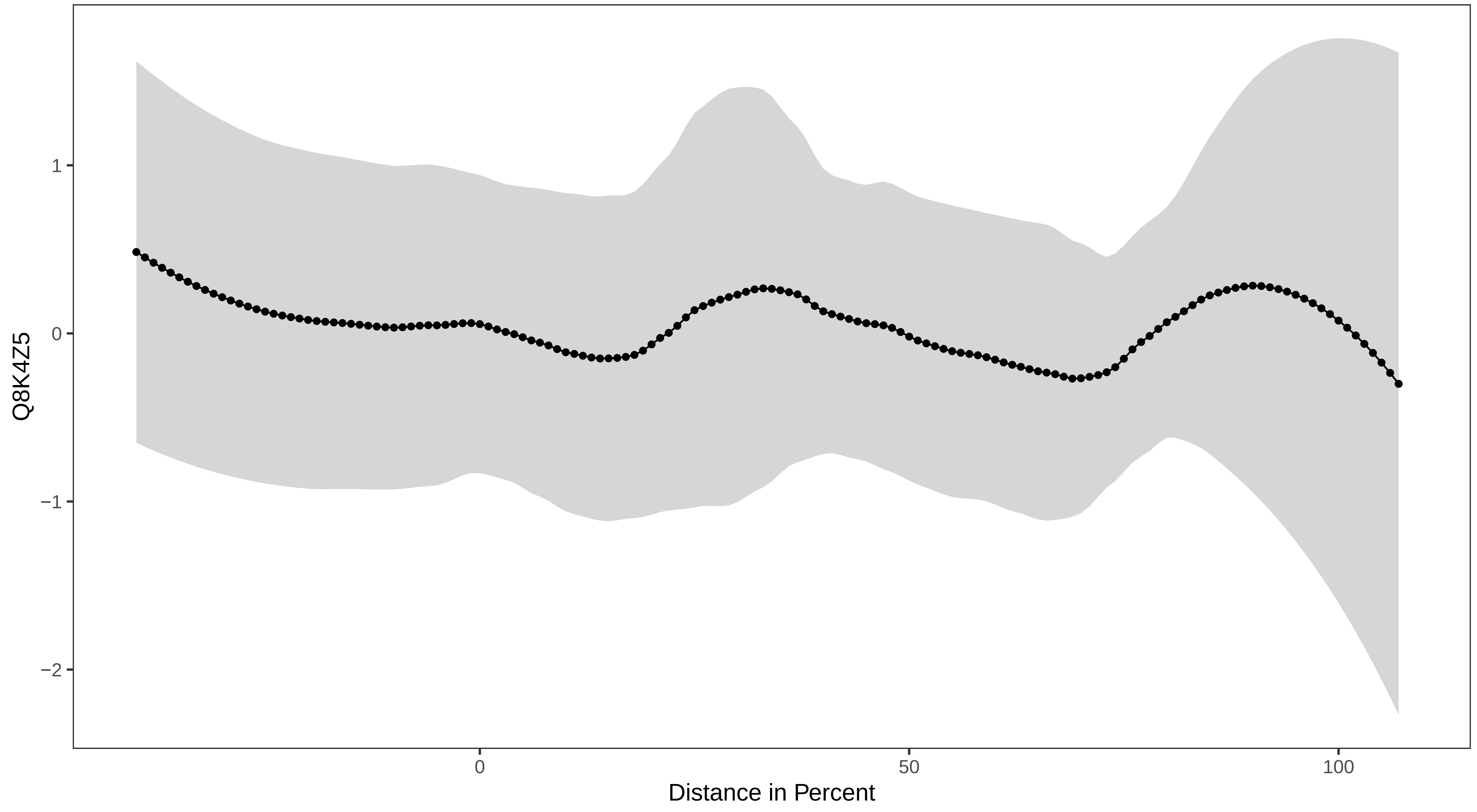

Q9D554 − Sf3a3 − SF3A3_MOUSE

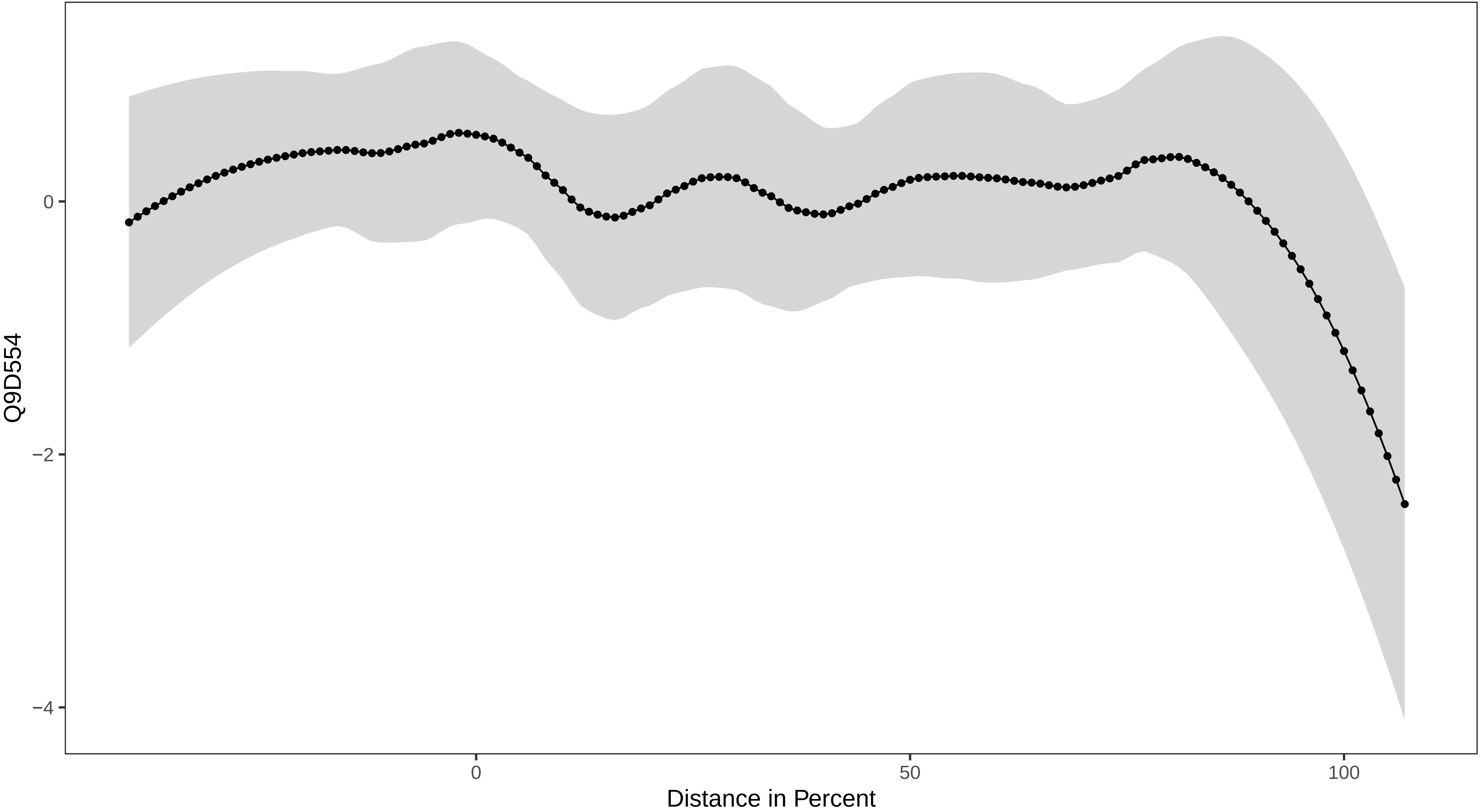

Q99NB9 − Sf3b1 − SF3B1_MOUSE

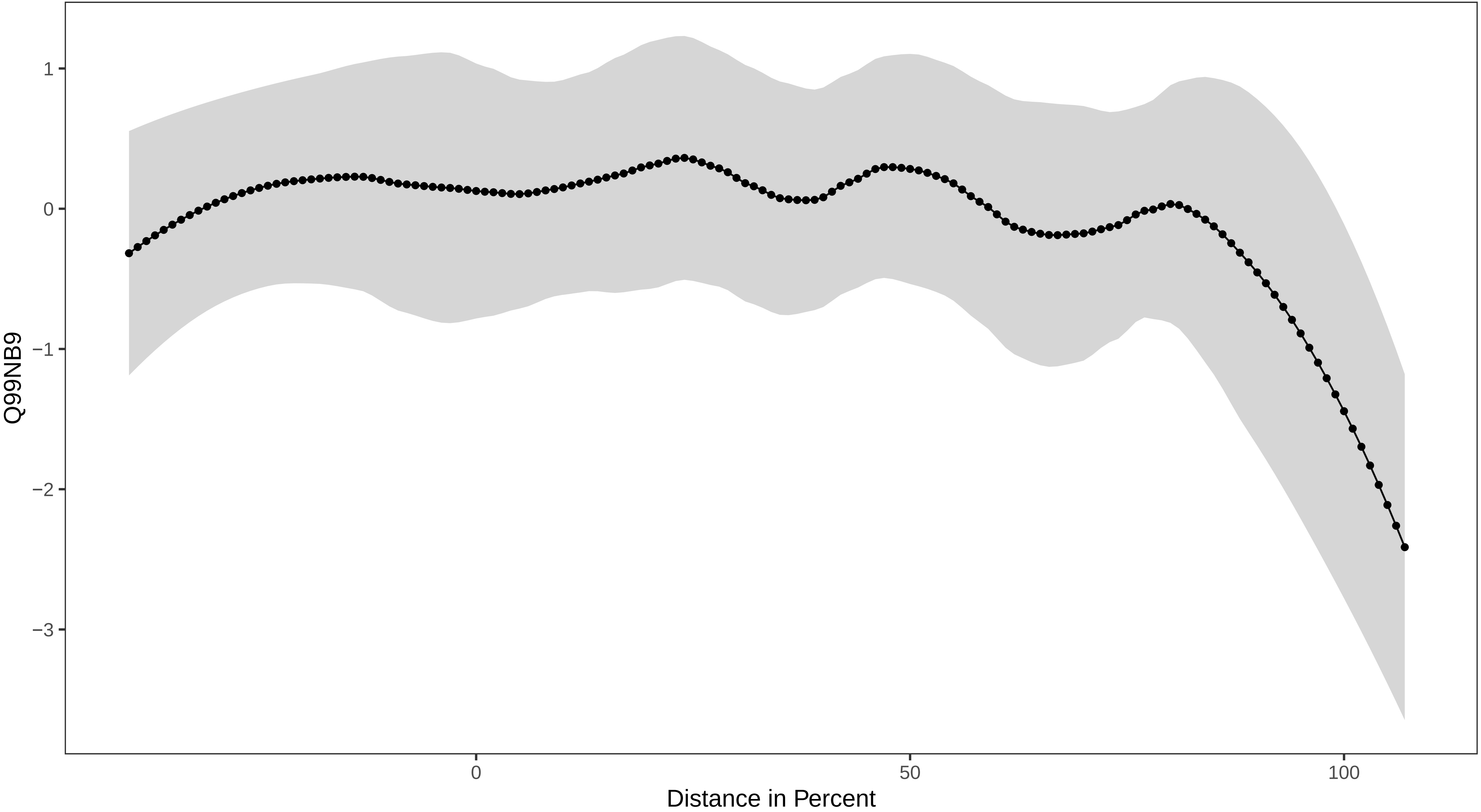

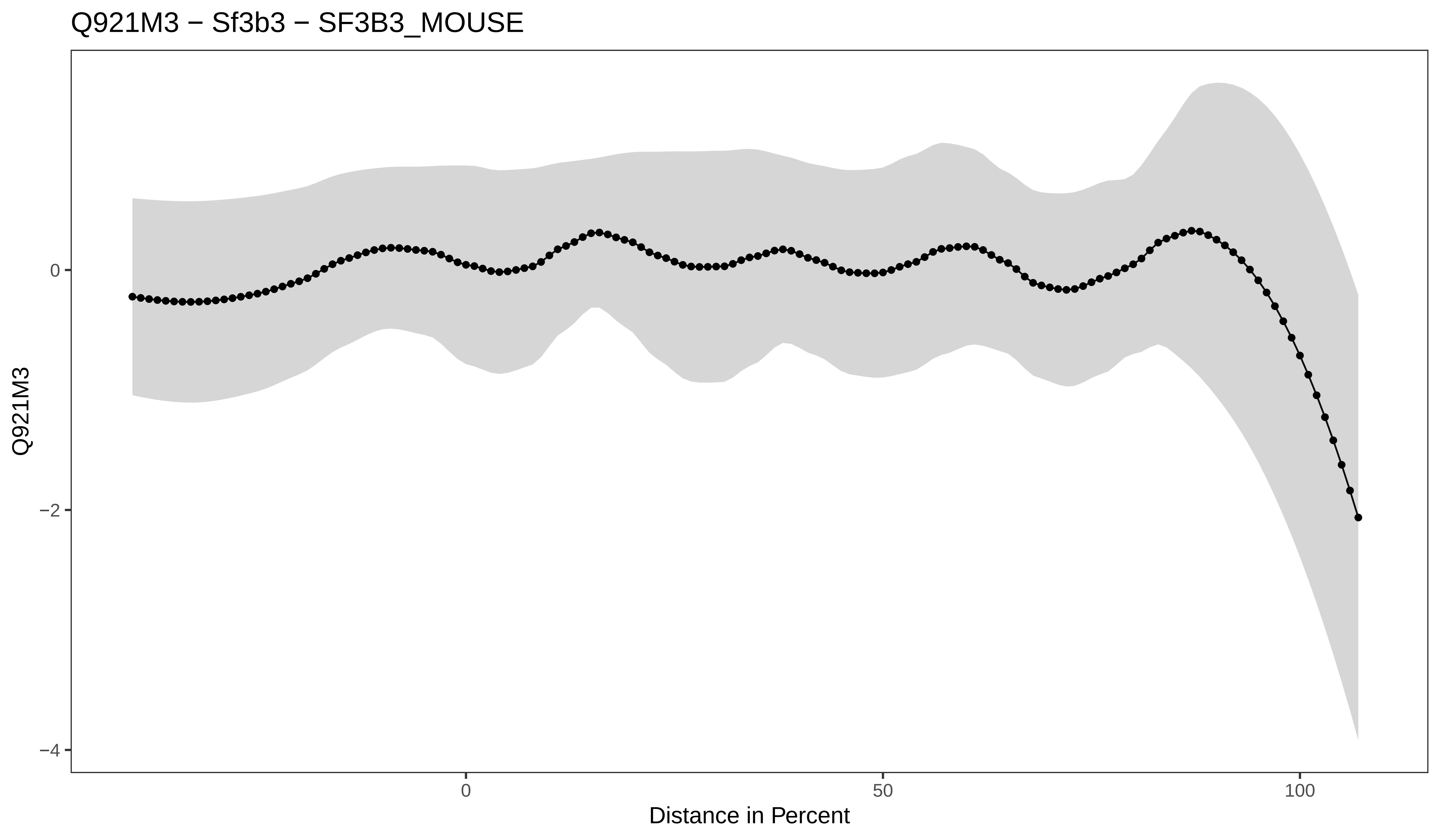

Q921M3 − Sf3b3 − SF3B3_MOUSE
Q921M3
0
−2
−4
0
50
100
Distance in Percent

Q8VIJ6 − Sfpq − SFPQ_MOUSE

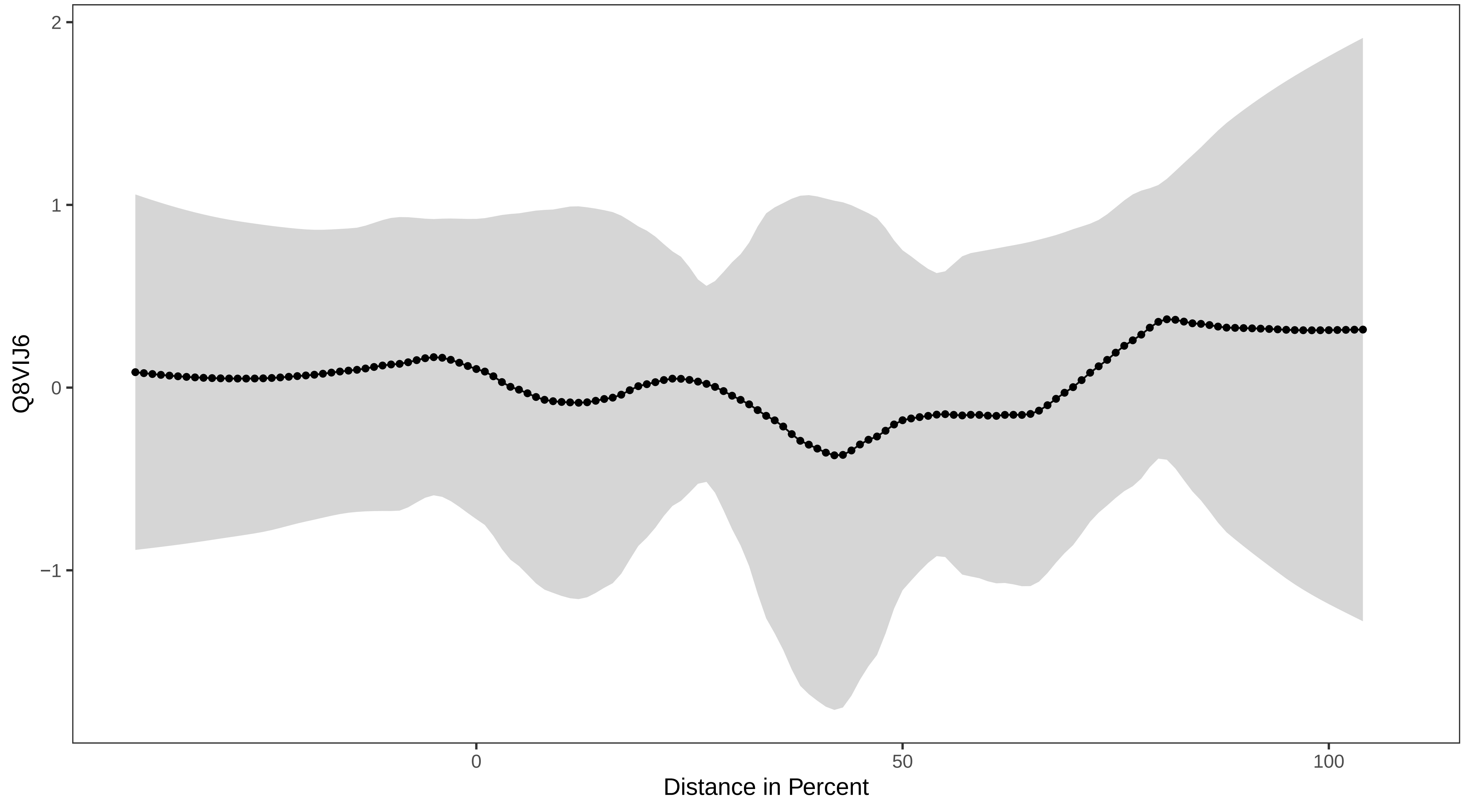

Q91V61 − Sfxn3 − SFXN3_MOUSE

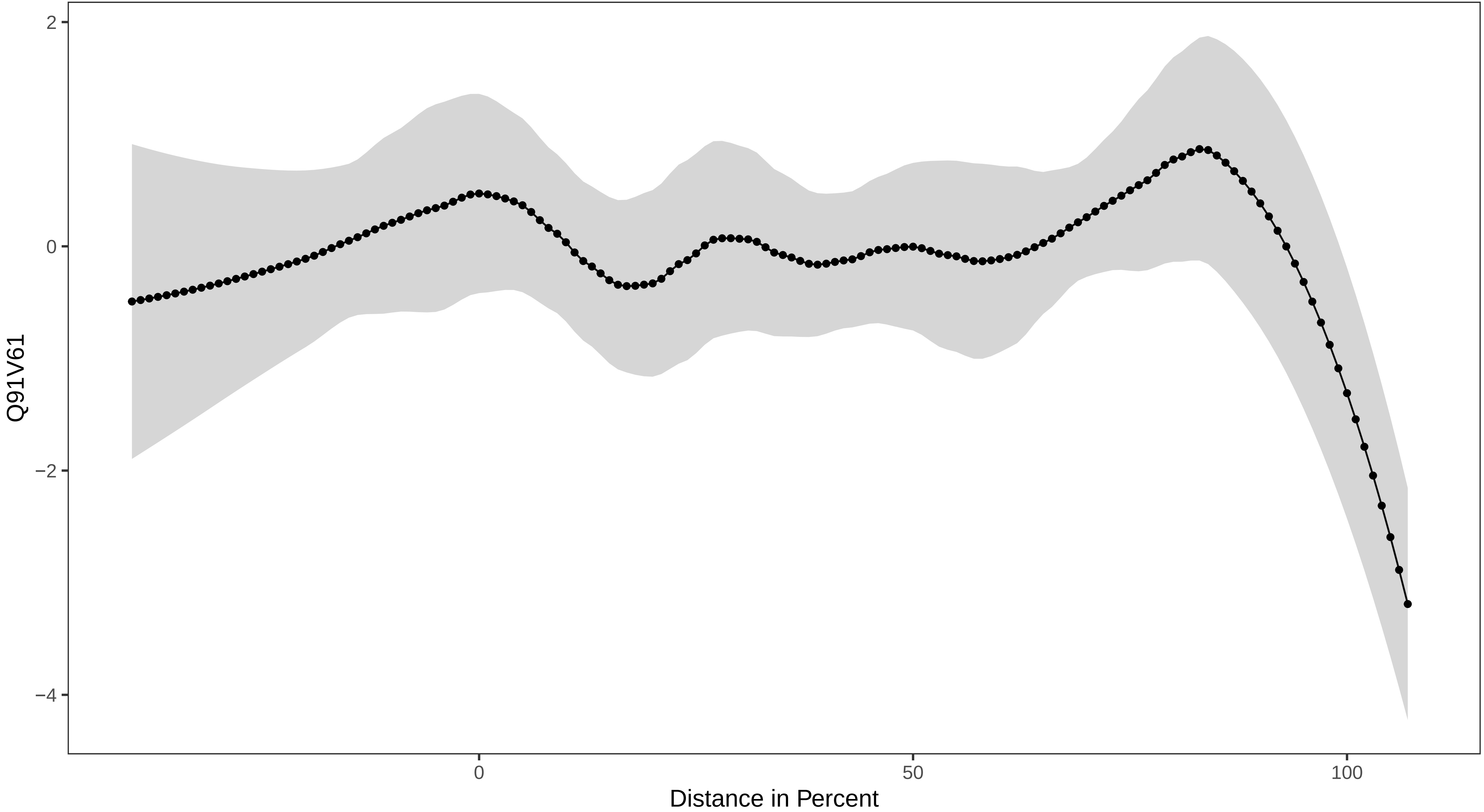

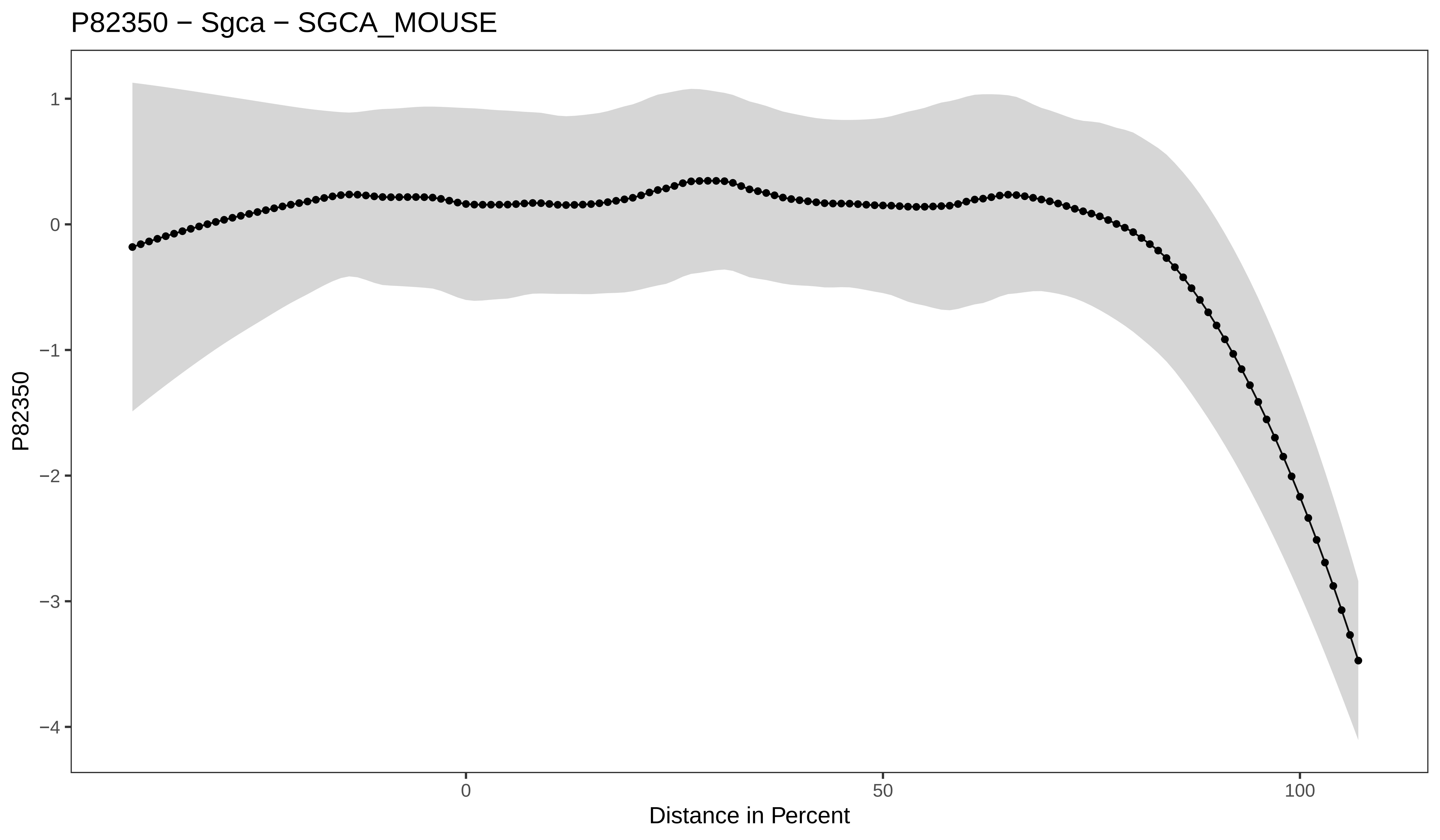
P82350 − Sgca − SGCA_MOUSE
P82350
Distance in Percent
1
0
−1
−2
−3
−4
0
50
100

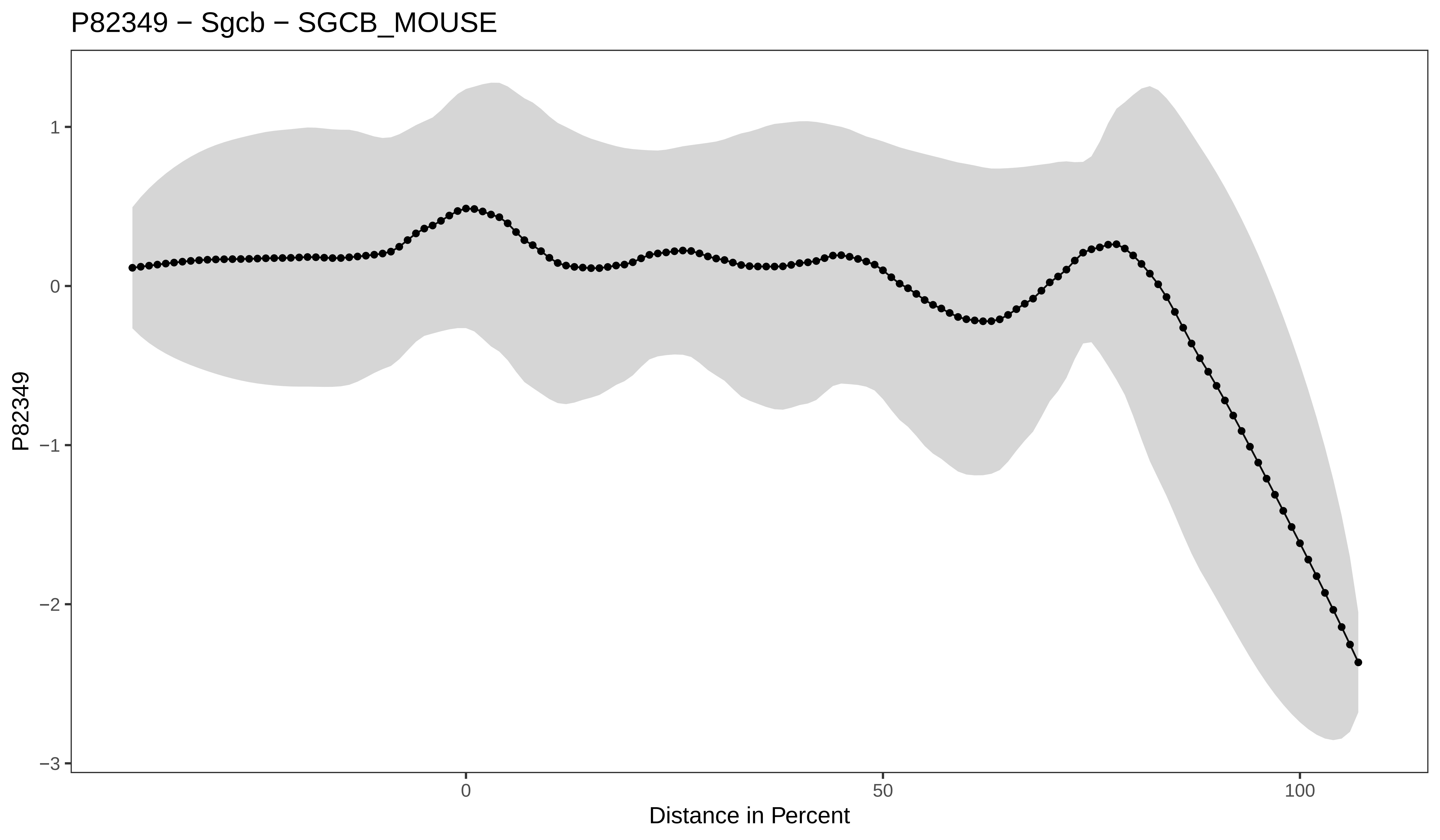
P82349 − Sgcb − SGCB_MOUSE
P82349
1
0
−1
−2
−3
0
50
100
Distance in Percent

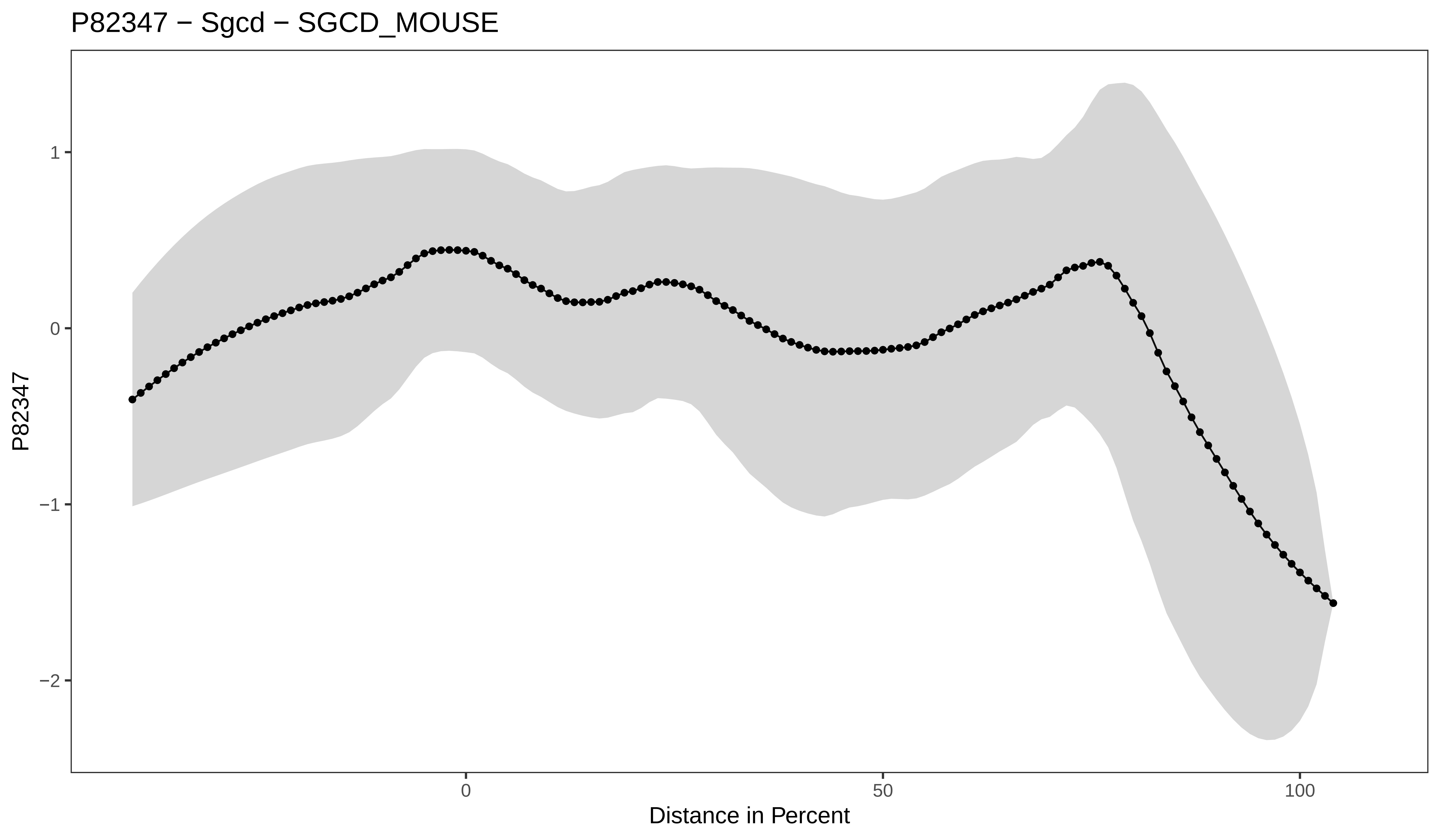
P82347 − Sgcd − SGCD_MOUSE
P82347
1
0
−1
−2
0
50
100
Distance in Percent

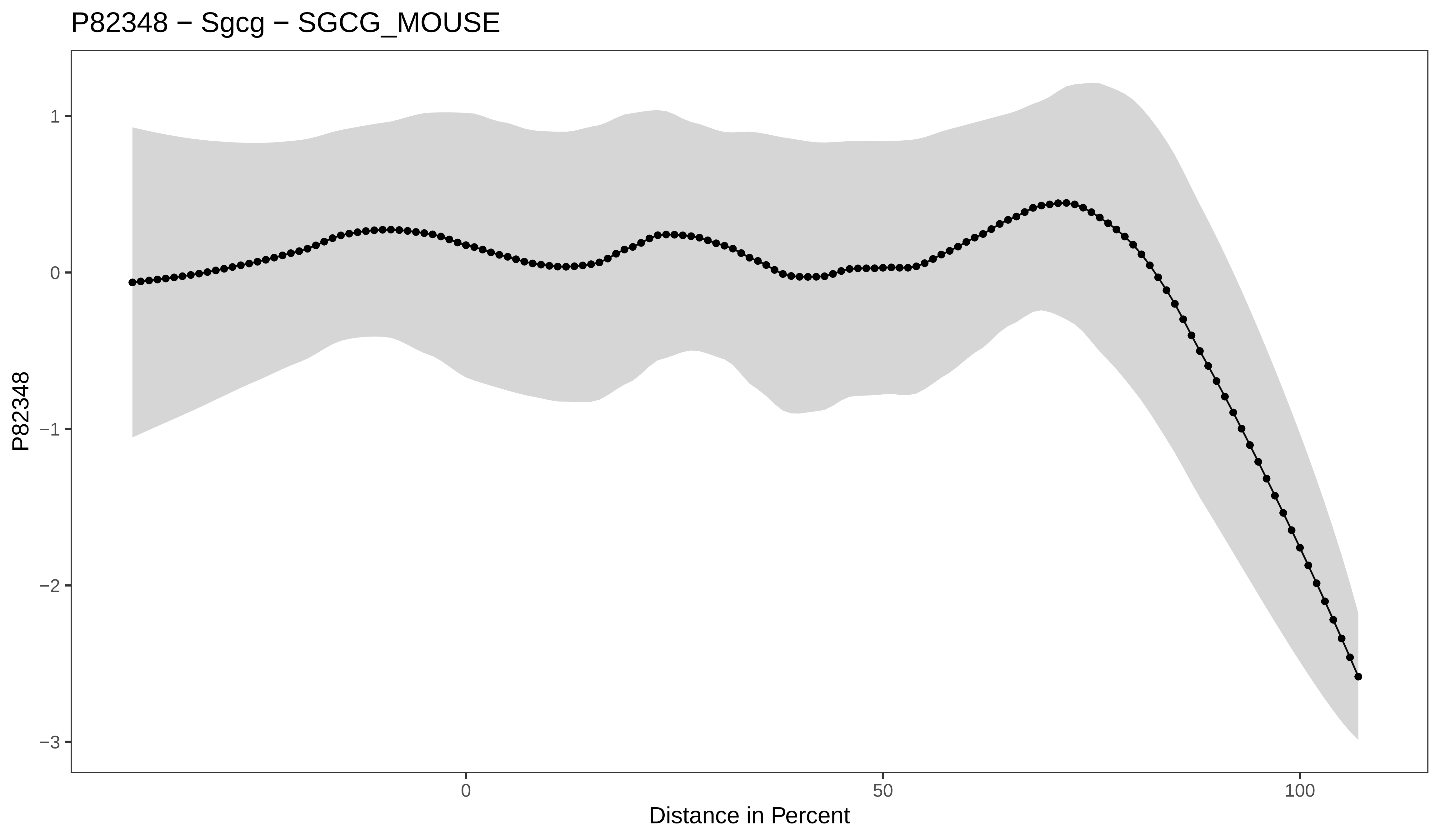

P82348 − Sgcg − SGCG_MOUSE
1
0
−1
−2
−3
P82348
0
50
100
Distance in Percent

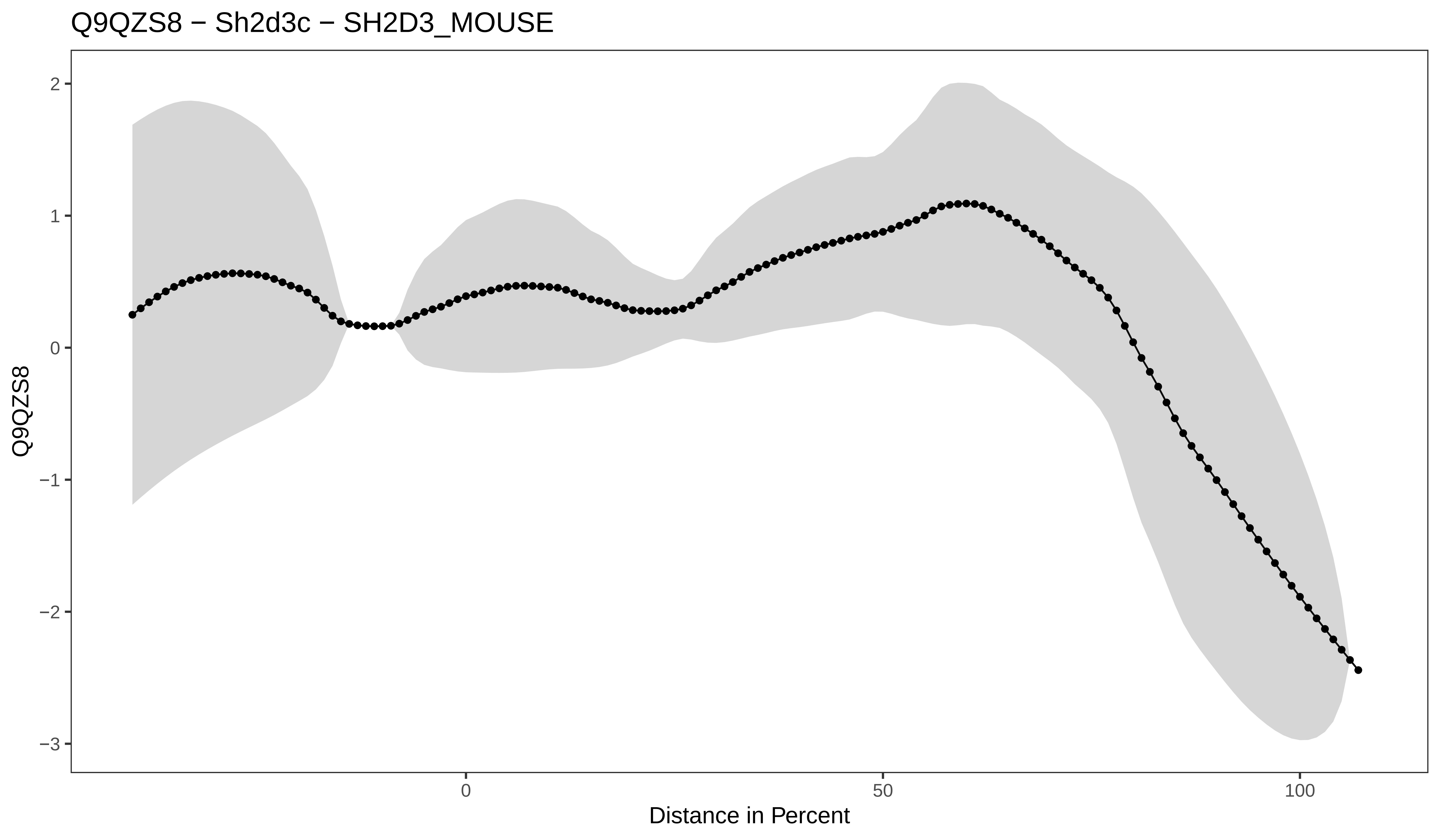
Q9QZS8 − Sh2d3c − SH2D3_MOUSE
Q9QZS8
Distance in Percent
2
1
0
−1
−2
−3
0
50
100

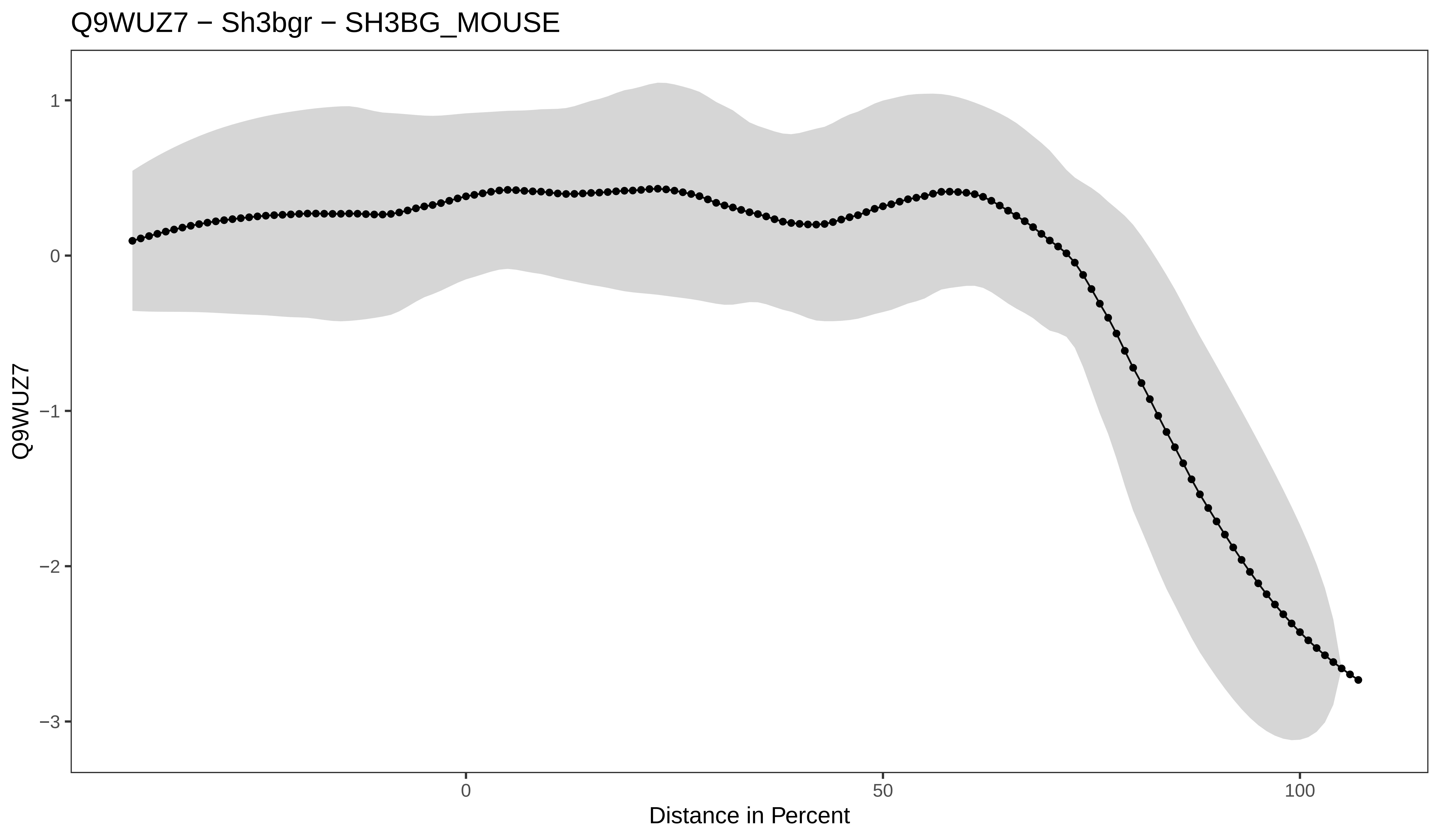

Q9WUZ7 − Sh3bgr − SH3BG_MOUSE
1
0
−1
−2
−3
Q9WUZ7
0
50
100
Distance in Percent

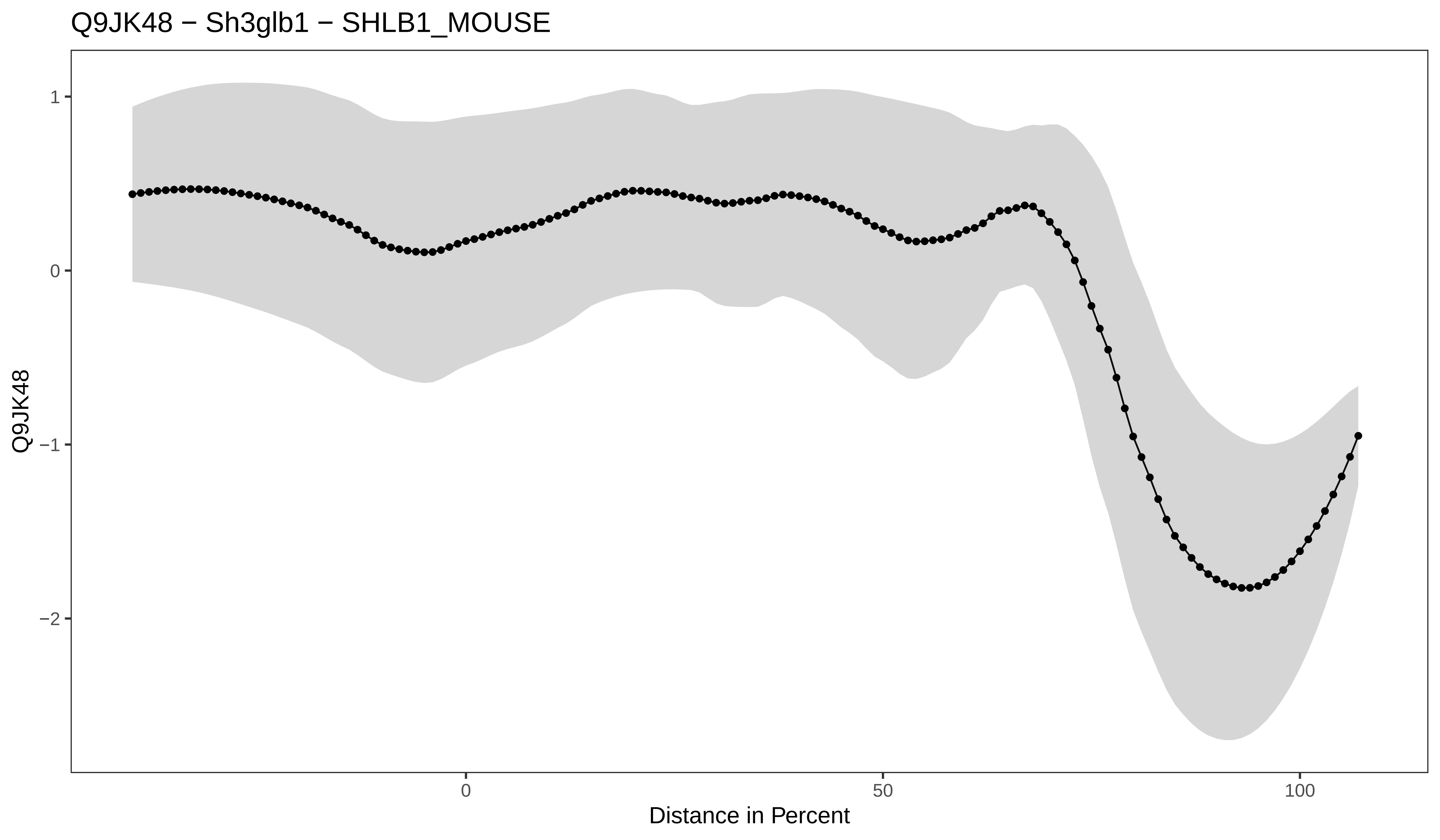
Q9JK48 − Sh3glb1 − SHLB1_MOUSE
Q9JK48
Distance in Percent
1
0
−1
−2
0
50
100

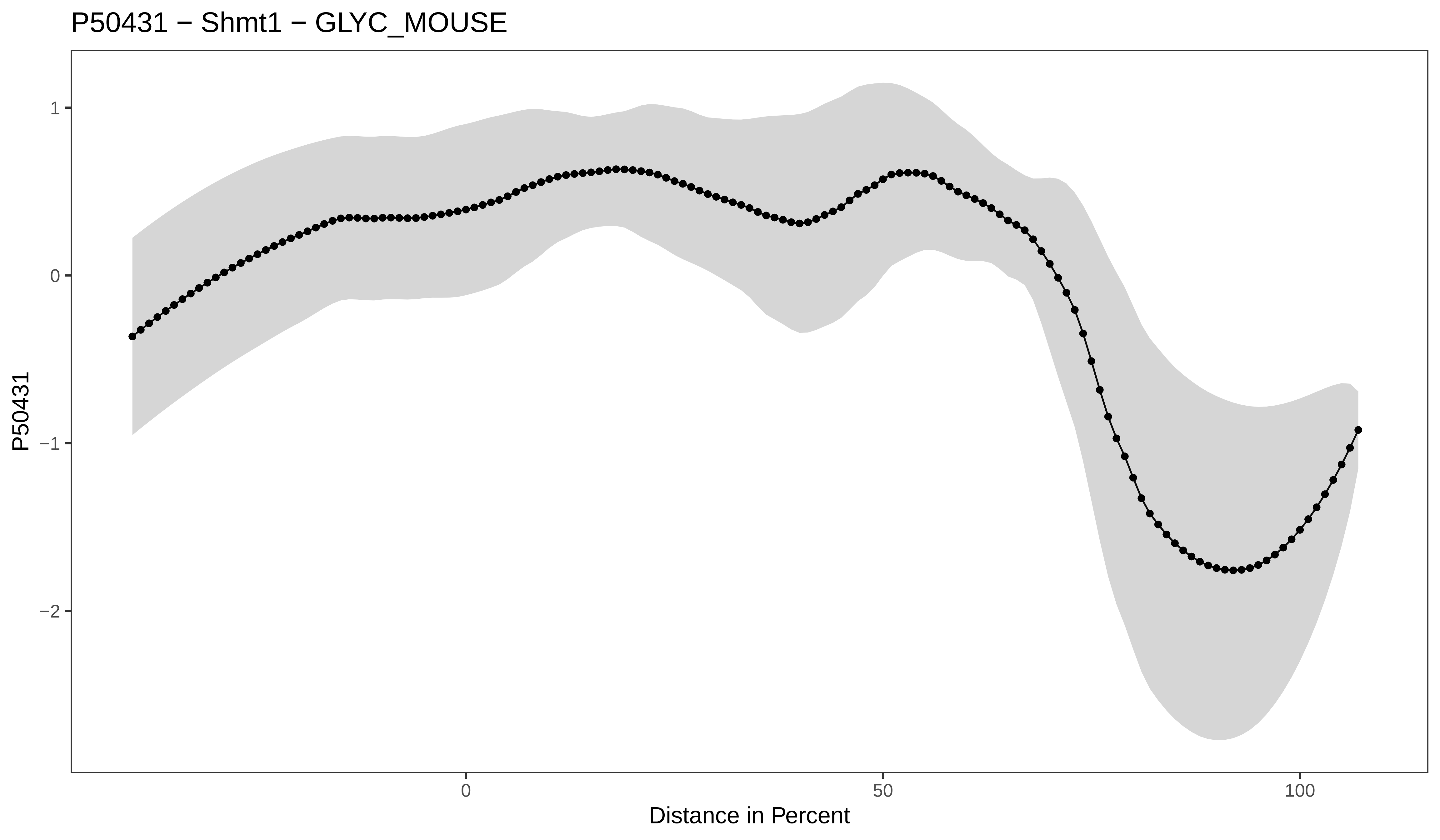

P50431 − Shmt1 − GLYC_MOUSE
1
0
−1
−2
P50431
0
50
100
Distance in Percent

Q9CZN7 − Shmt2 − GLYM_MOUSE

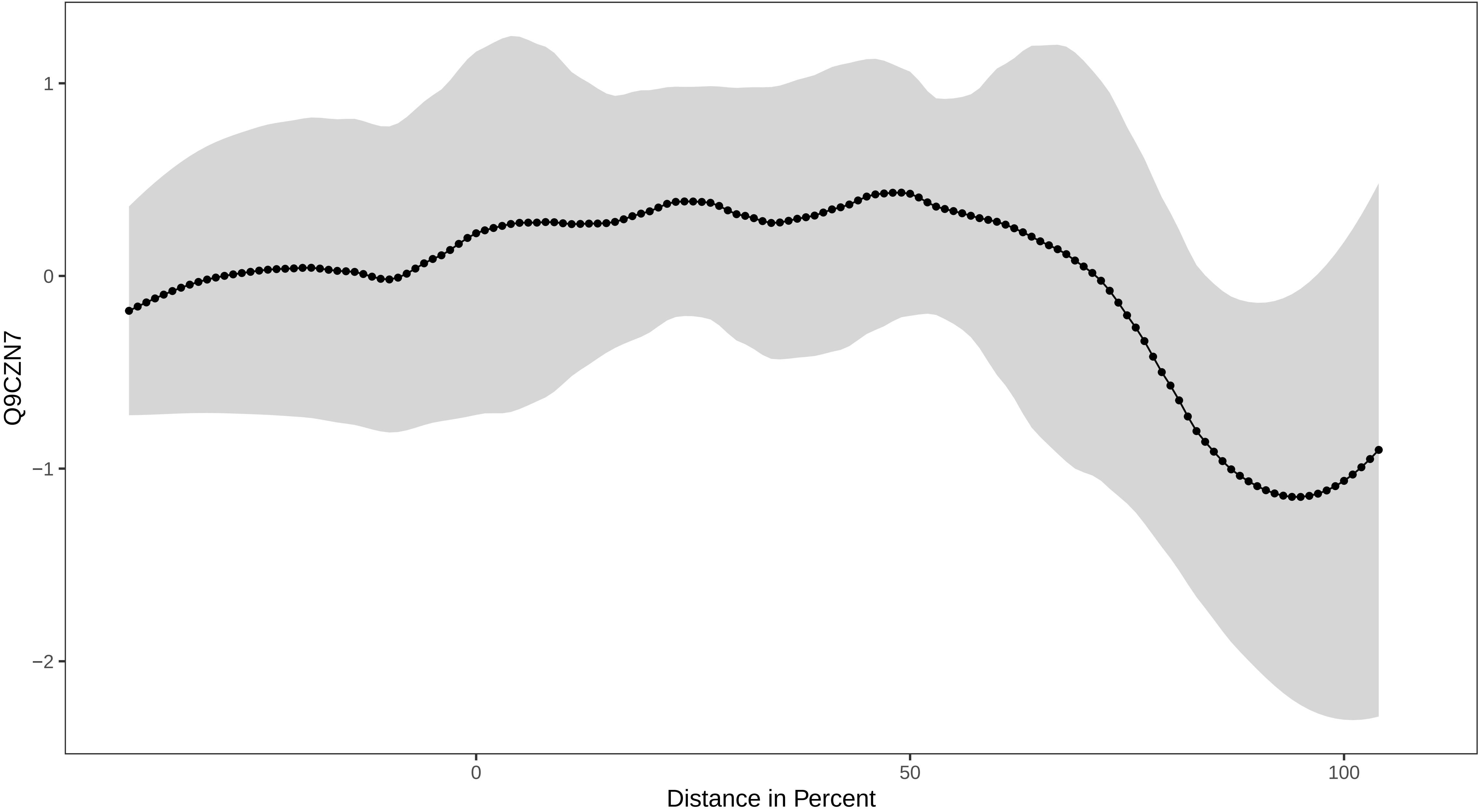

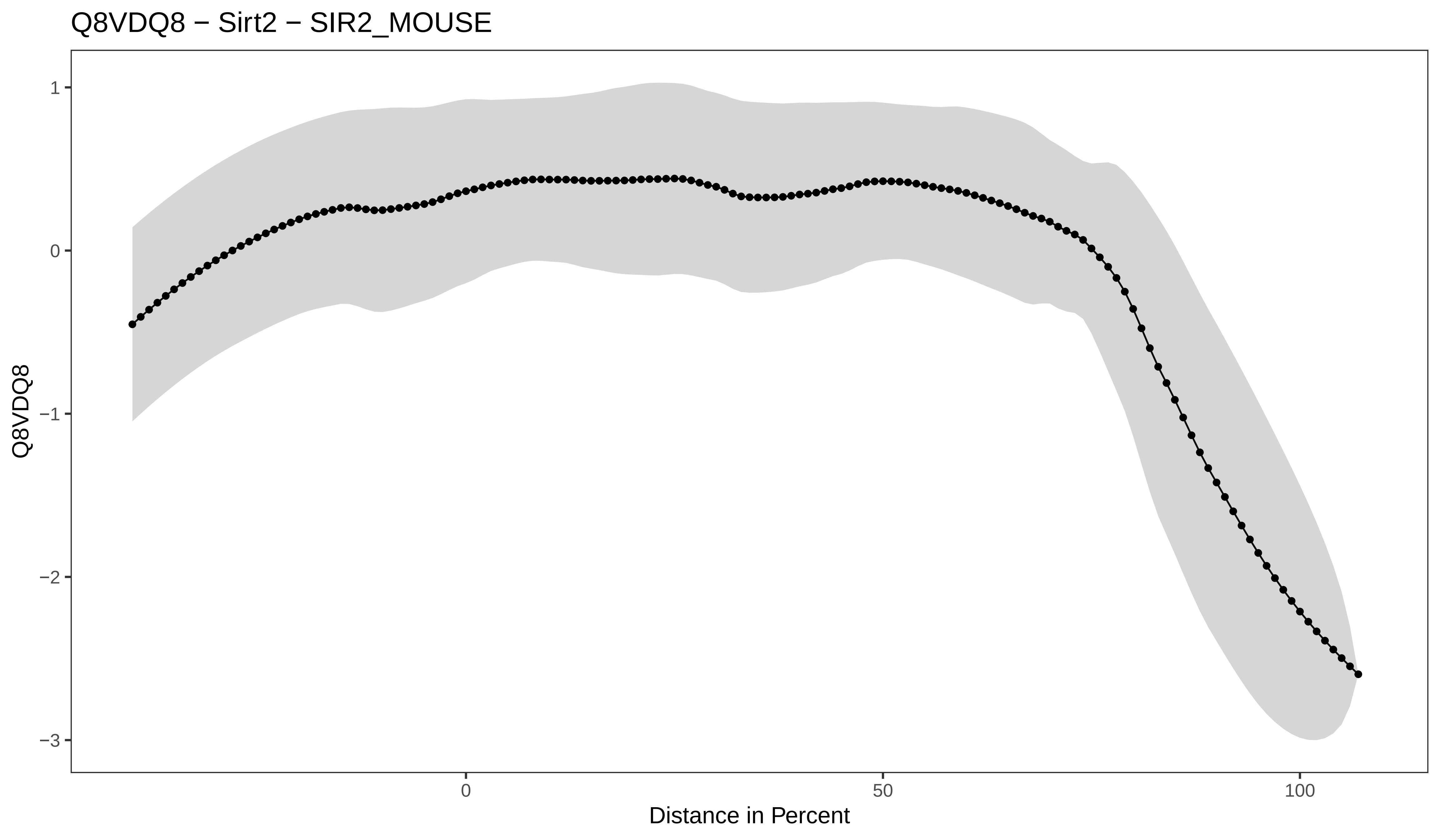

Q8VDQ8 − Sirt2 − SIR2_MOUSE
1
0
−1
−2
−3
Q8VDQ8
0
50
100
Distance in Percent

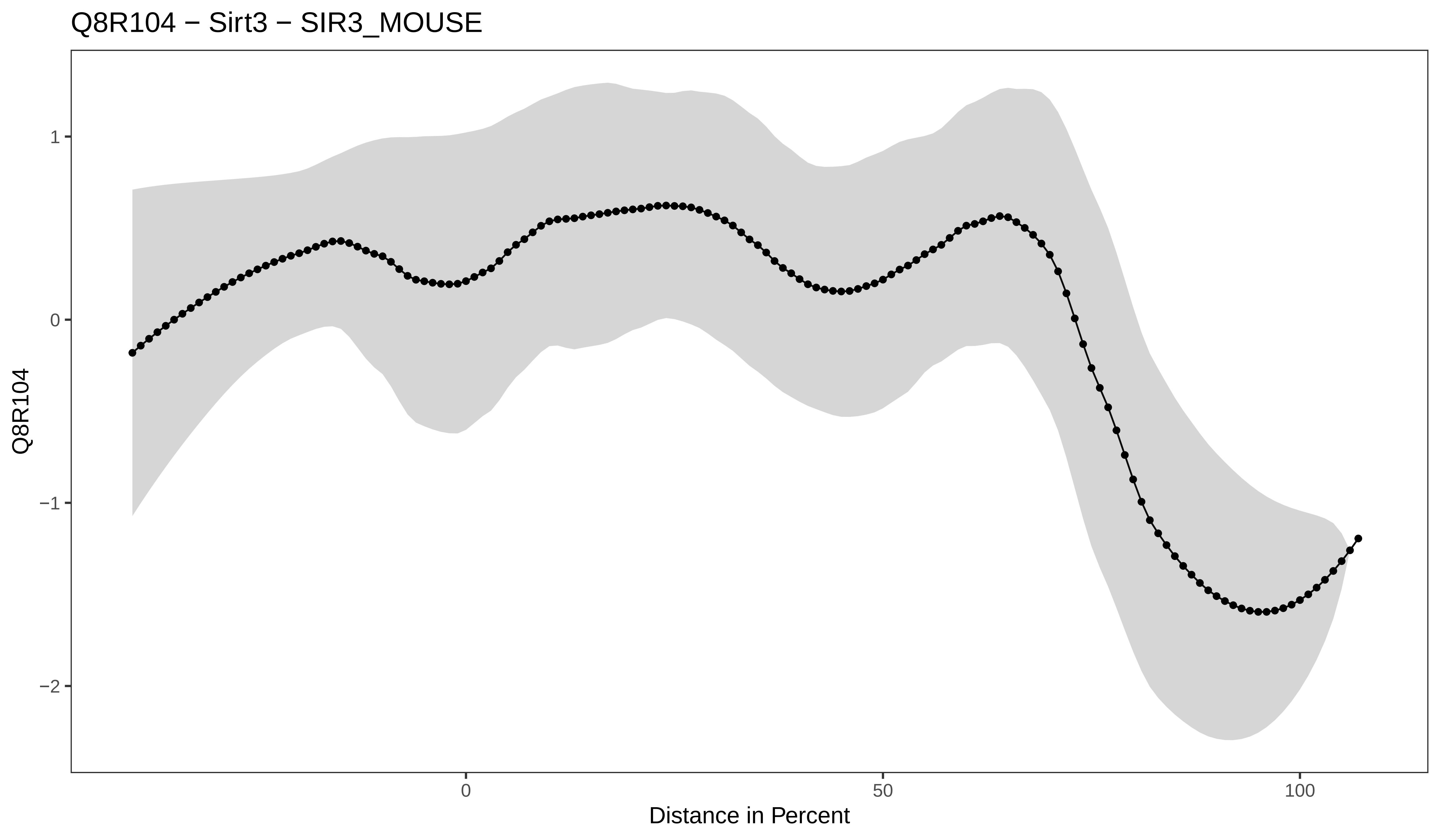

Q8R104 − Sirt3 − SIR3_MOUSE
1
0
−1
−2
Q8R104
0
50
100
Distance in Percent

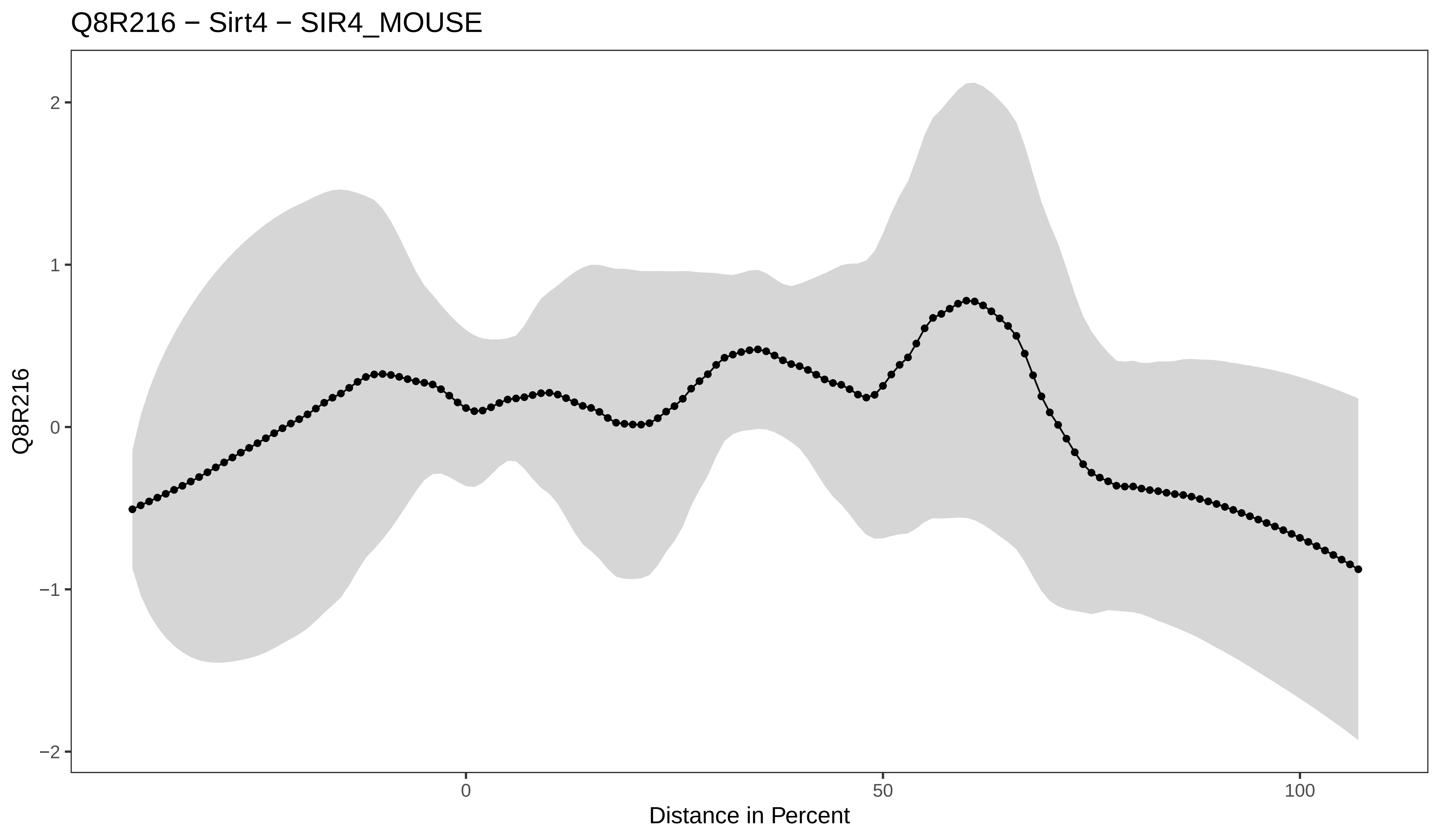
Q8R216 − Sirt4 − SIR4_MOUSE
2
1
0
−1
−2
Q8R216
0
50
100
Distance in Percent

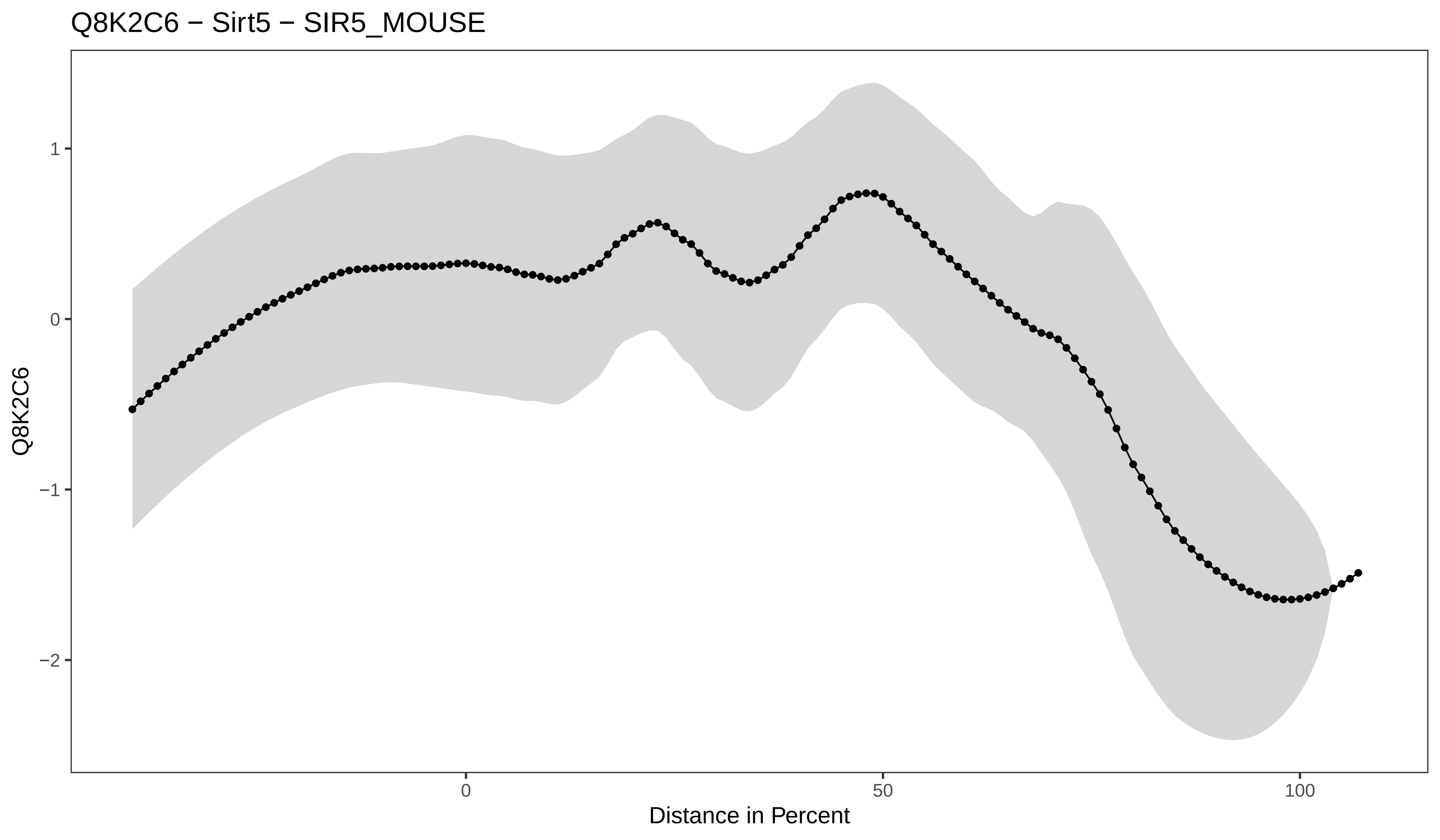

Q8K2C6 − Sirt5 − SIR5_MOUSE
Q8K2C6
Distance in Percent
1
0
−1
−2
0
50
100

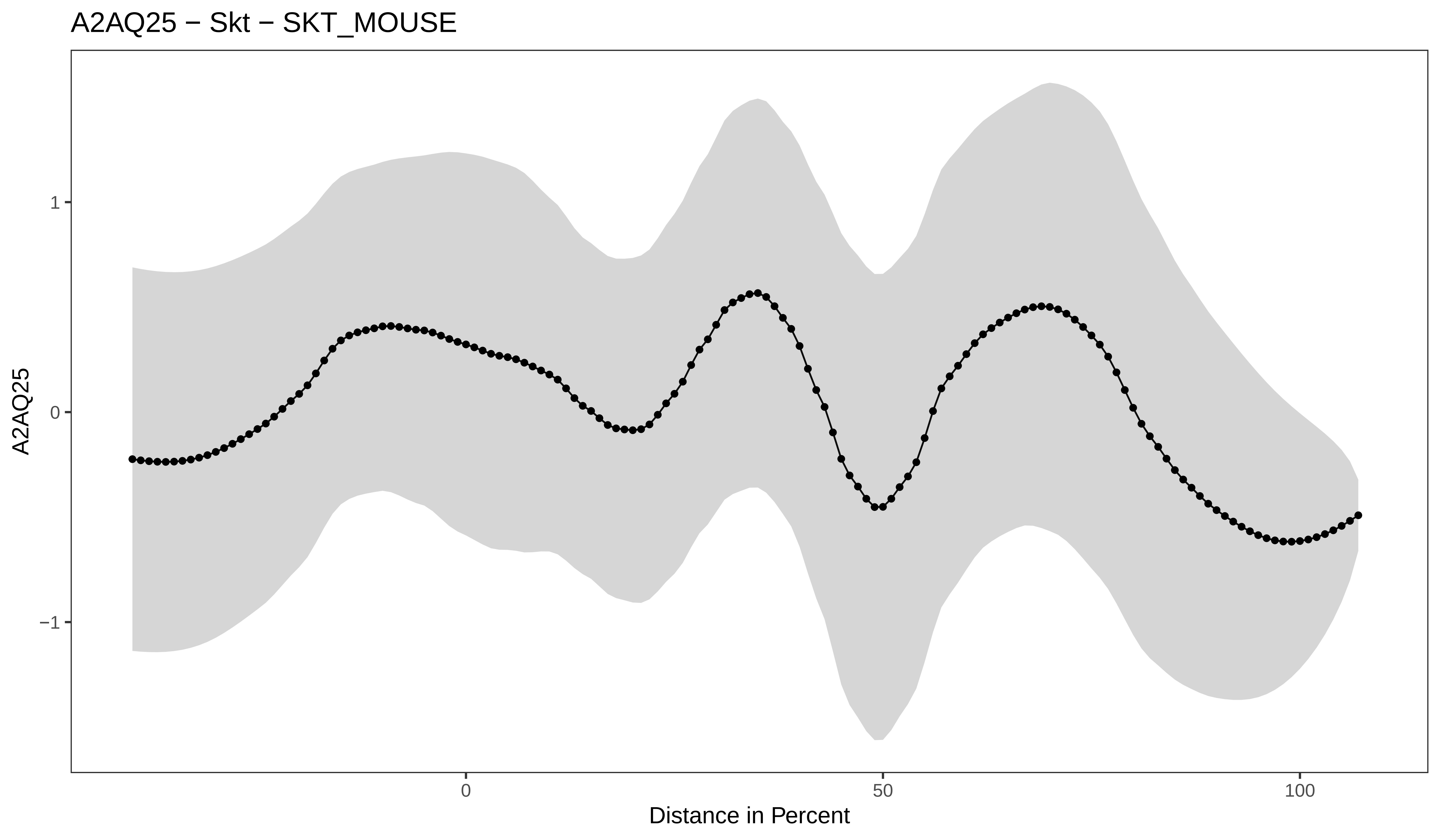
A2AQ25 − Skt − SKT_MOUSE
A2AQ25
1
0
−1
0
50
100
Distance in Percent

P55012 − Slc12a2 − S12A2_MOUSE

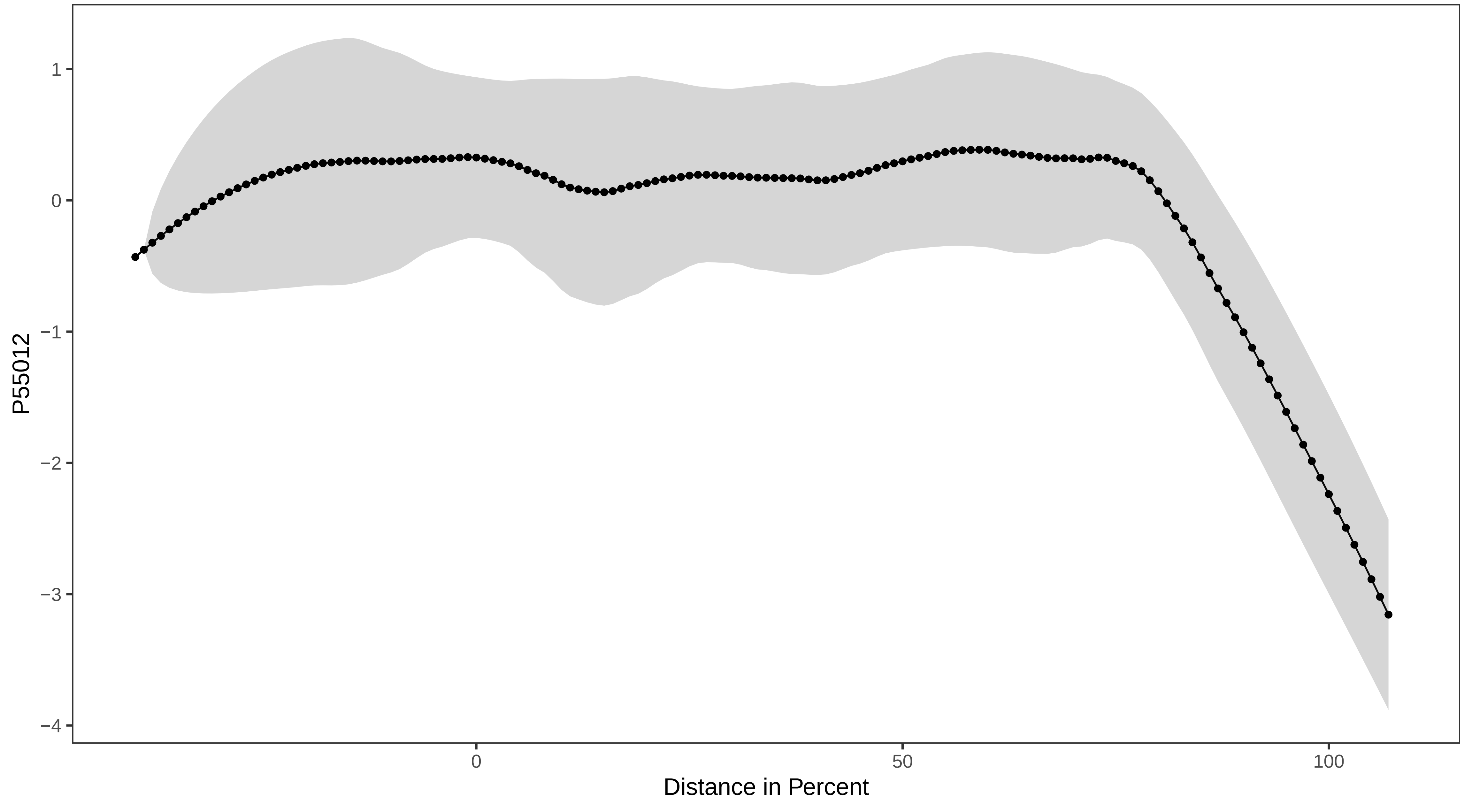

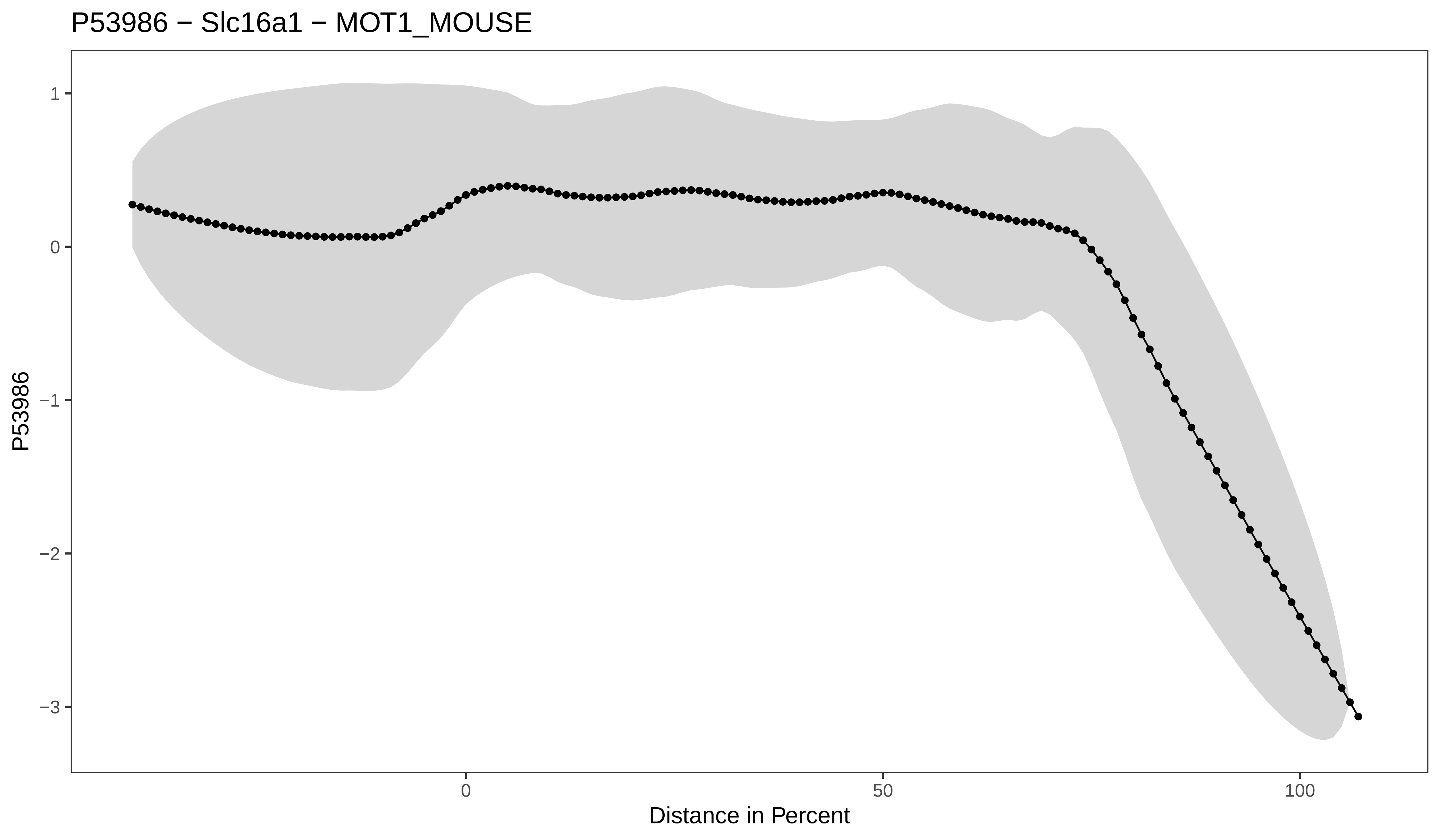

P53986 − Slc16a1 − MOT1_MOUSE
1
0
−1
−2
−3
P53986
0
50
100
Distance in Percent

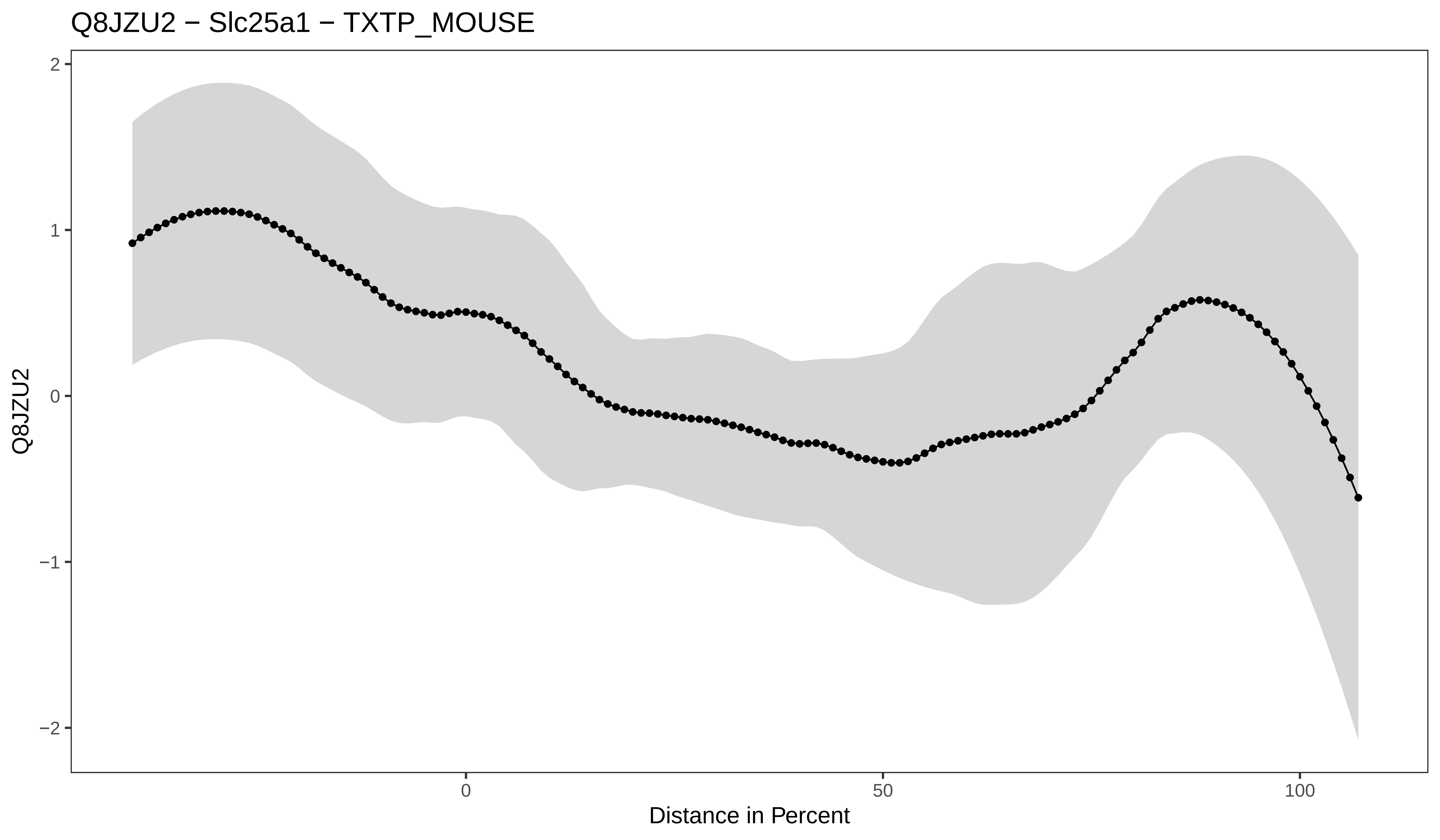
Q8JZU2 − Slc25a1 − TXTP_MOUSE
2
1
0
−1
−2
Q8JZU2
0
50
100
Distance in Percent

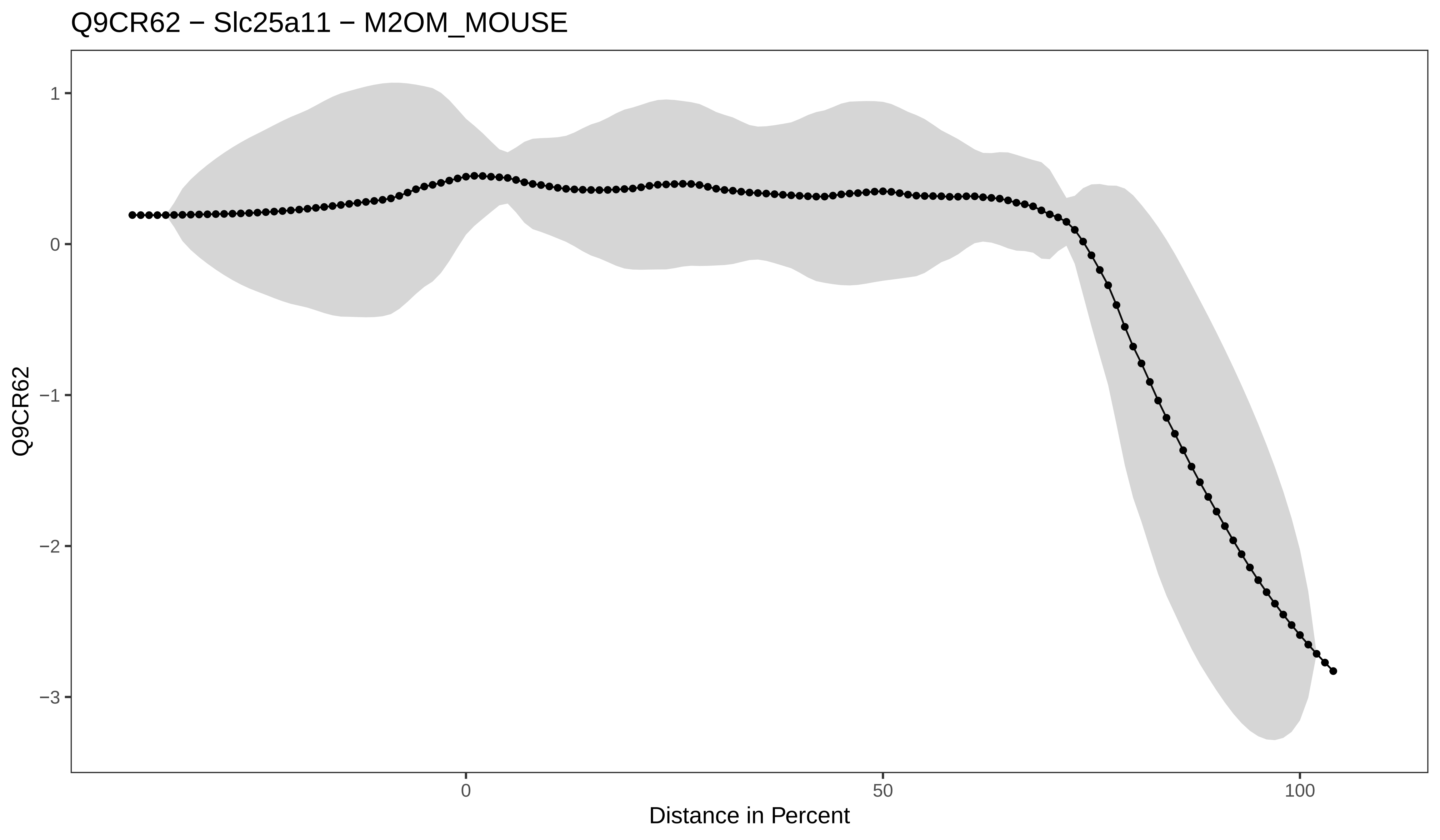

Q9CR62 − Slc25a11 − M2OM_MOUSE
Q9CR62
1
0
−1
−2
−3
0
50
100
Distance in Percent

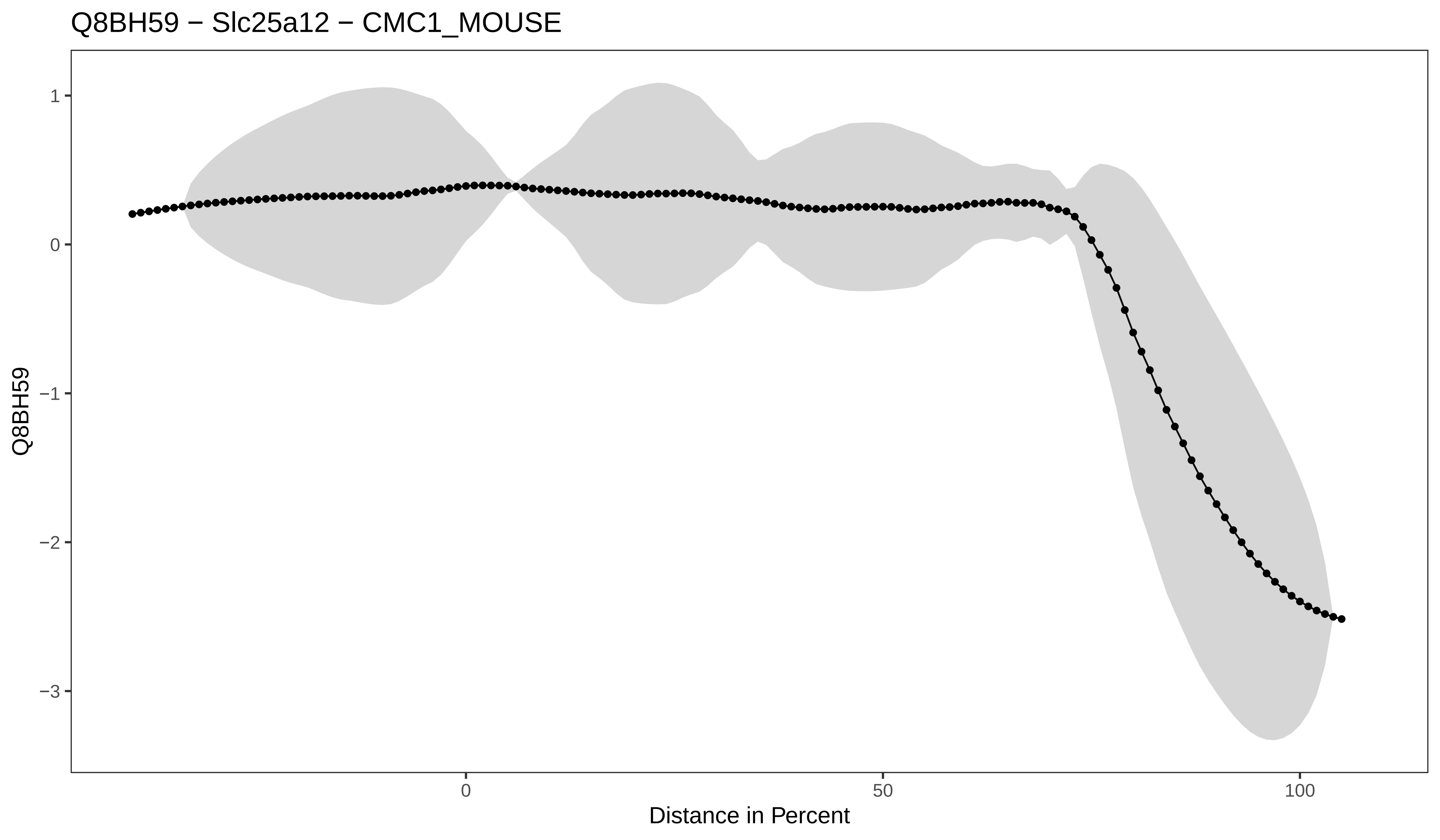
Q8BH59 − Slc25a12 − CMC1_MOUSE
1
0
−1
−2
−3
Q8BH59
0
50
100
Distance in Percent

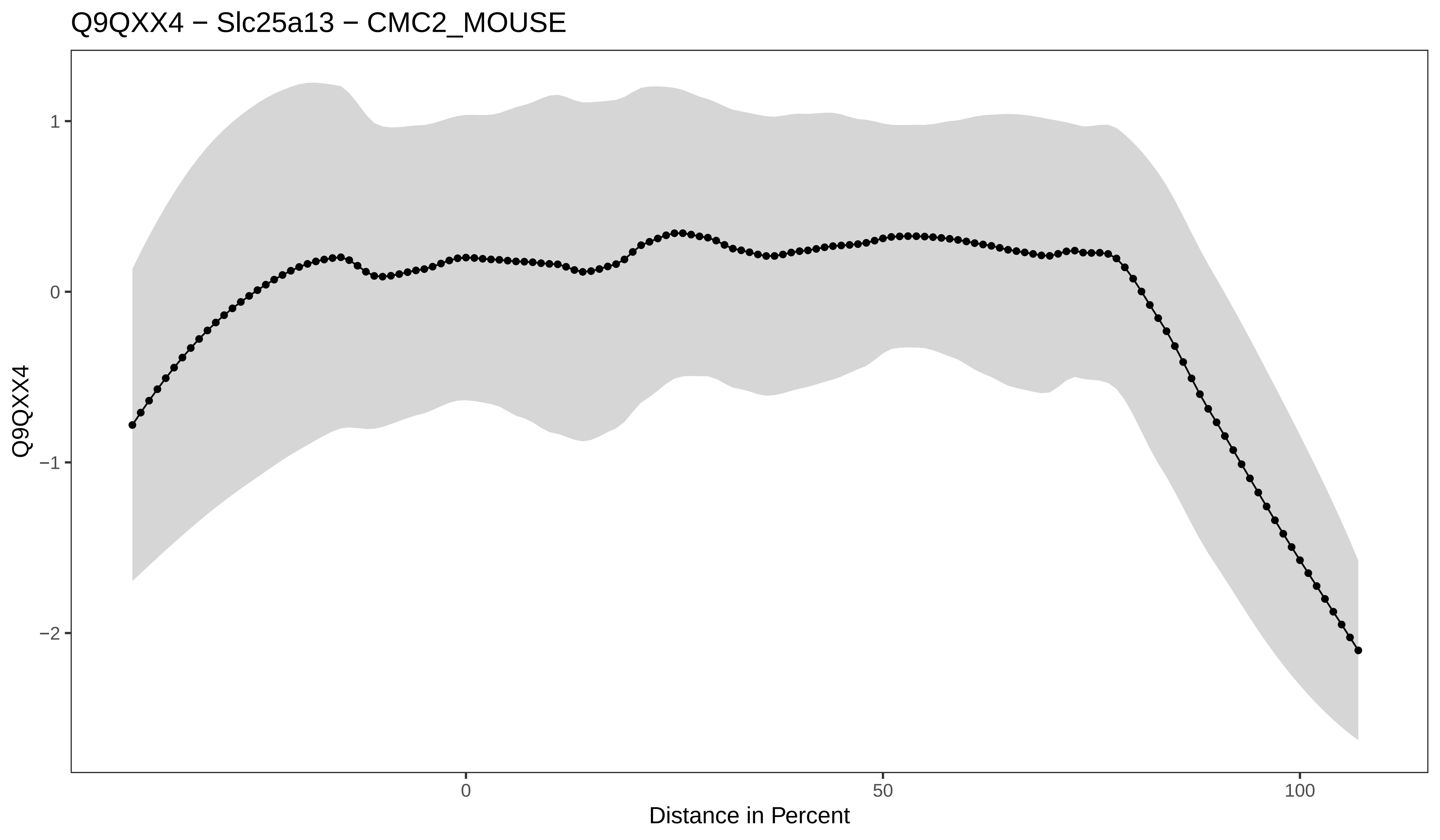
Q9QXX4 − Slc25a13 − CMC2_MOUSE
Q9QXX4
Distance in Percent
1
0
−1
−2
0
50
100

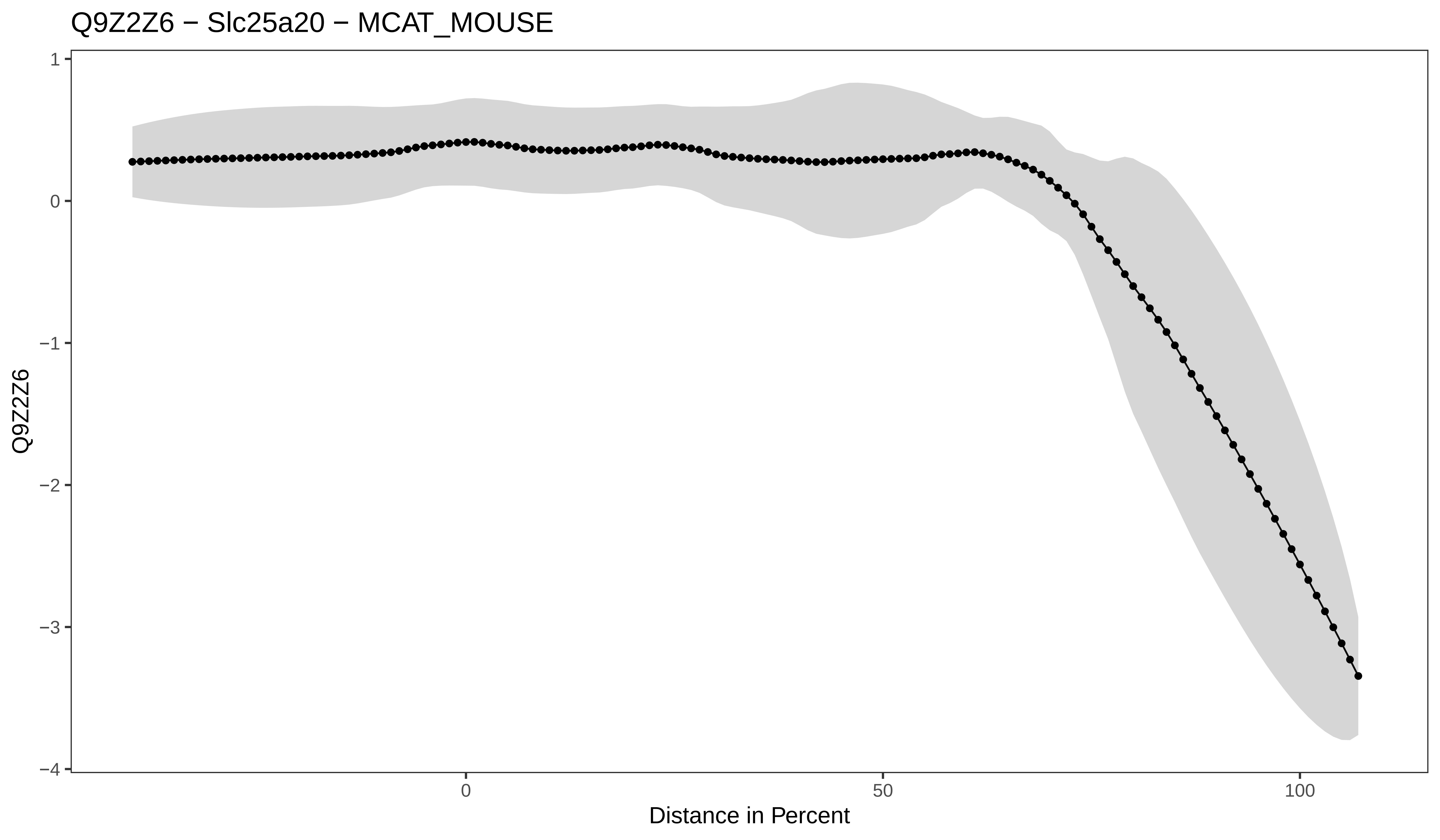

Q9Z2Z6 − Slc25a20 − MCAT_MOUSE
1
0
−1
−2
−3
−4
Q9Z2Z6
0
50
100
Distance in Percent

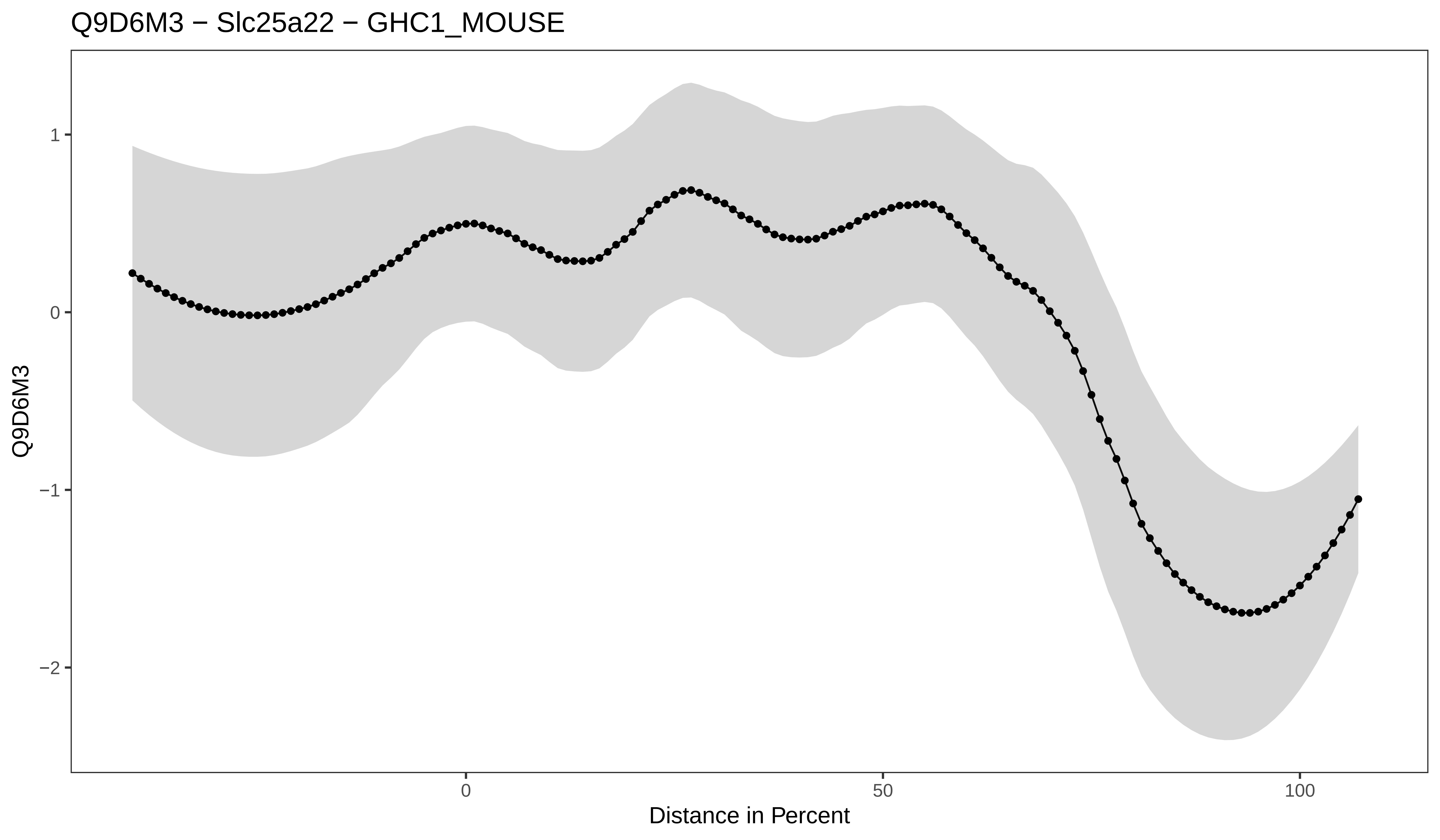
Q9D6M3 − Slc25a22 − GHC1_MOUSE
Q9D6M3
1
0
−1
−2
0
50
100
Distance in Percent

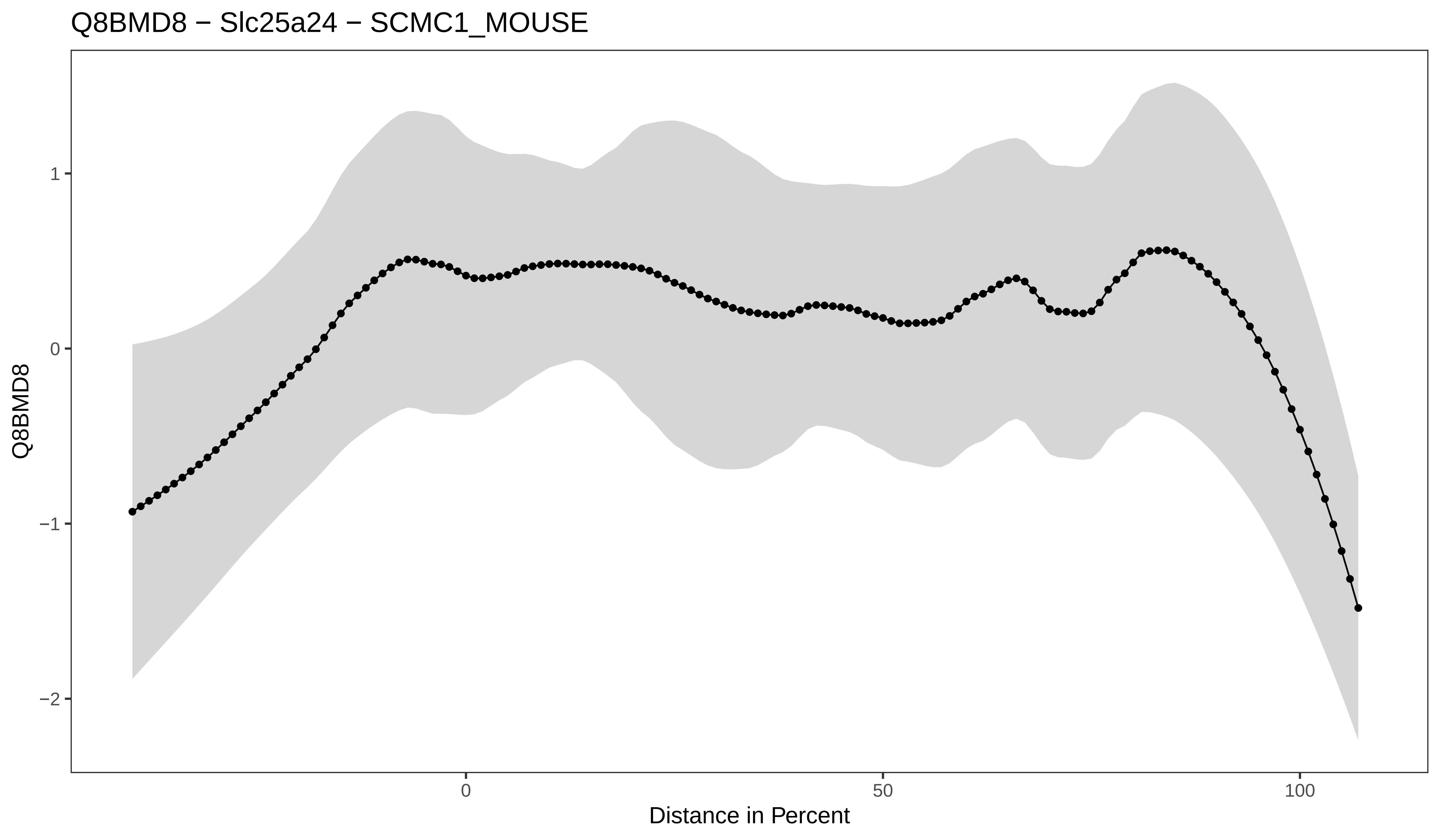

Q8BMD8 − Slc25a24 − SCMC1_MOUSE
Q8BMD8
1
0
−1
−2
0
50
100
Distance in Percent

Q8VEM8 − Slc25a3 − MPCP_MOUSE

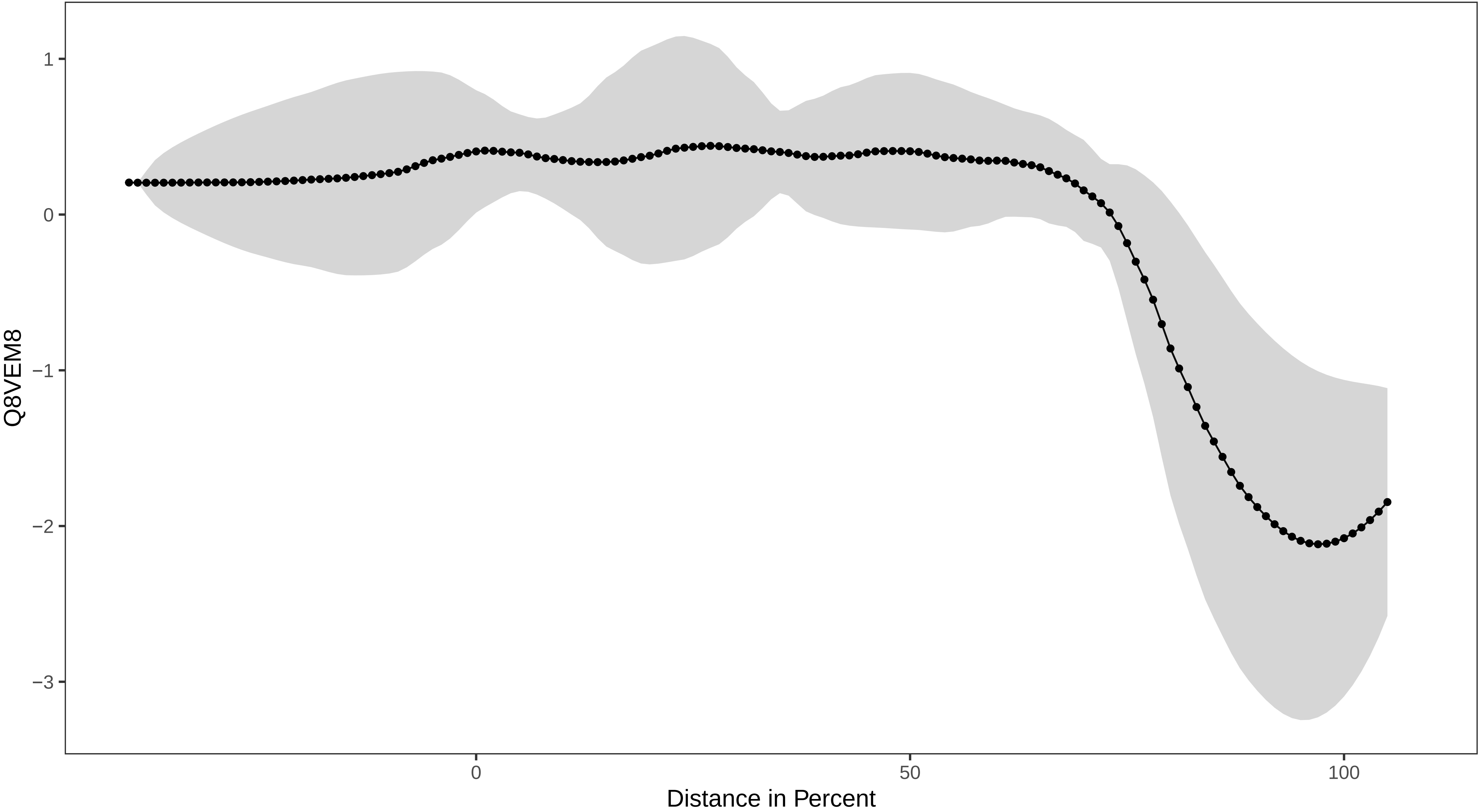

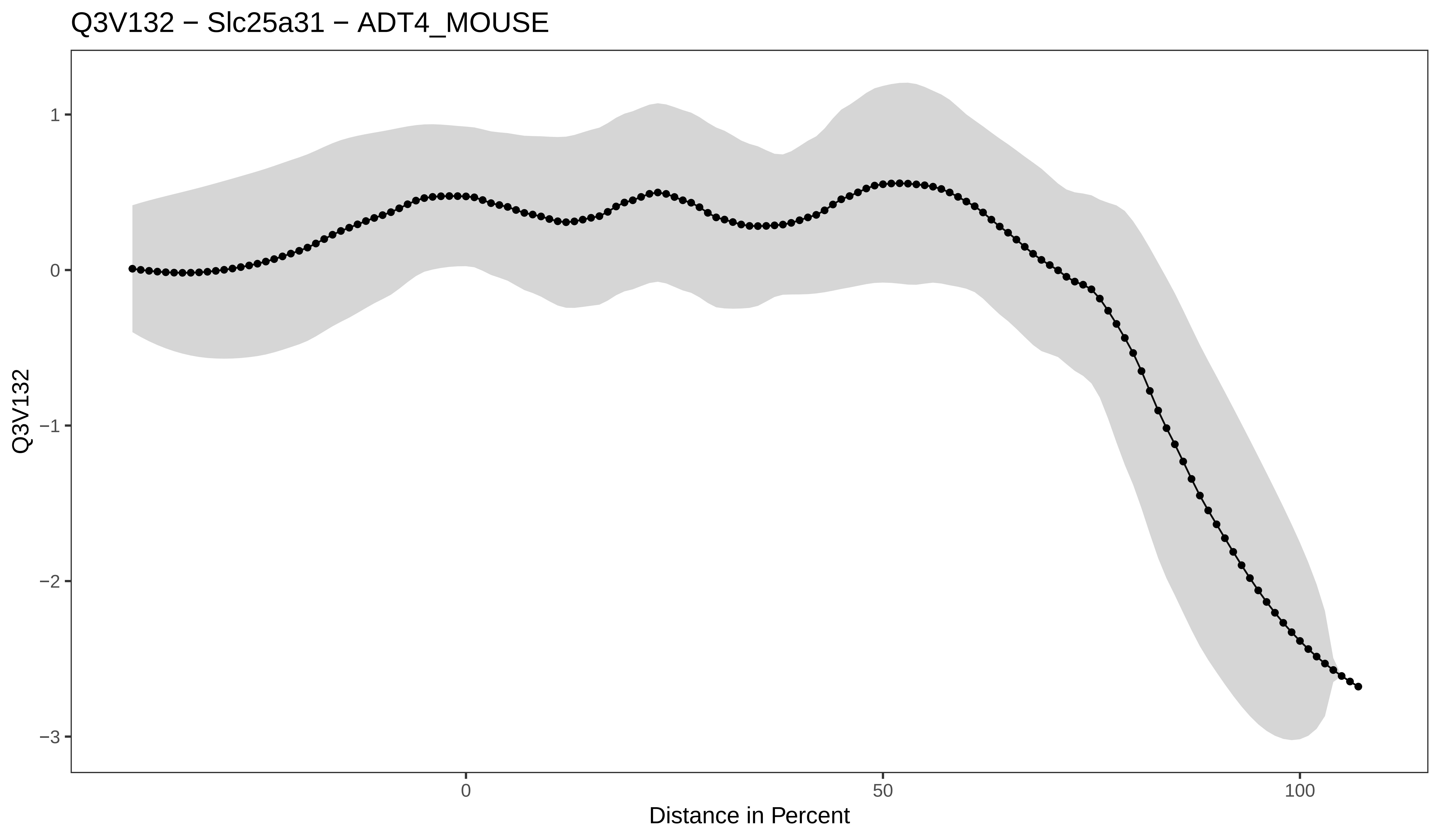

Q3V132 − Slc25a31 − ADT4_MOUSE
1
0
−1
−2
−3
Q3V132
0
50
100
Distance in Percent

Q5SWT3 − Slc25a35 − S2535_MOUSE

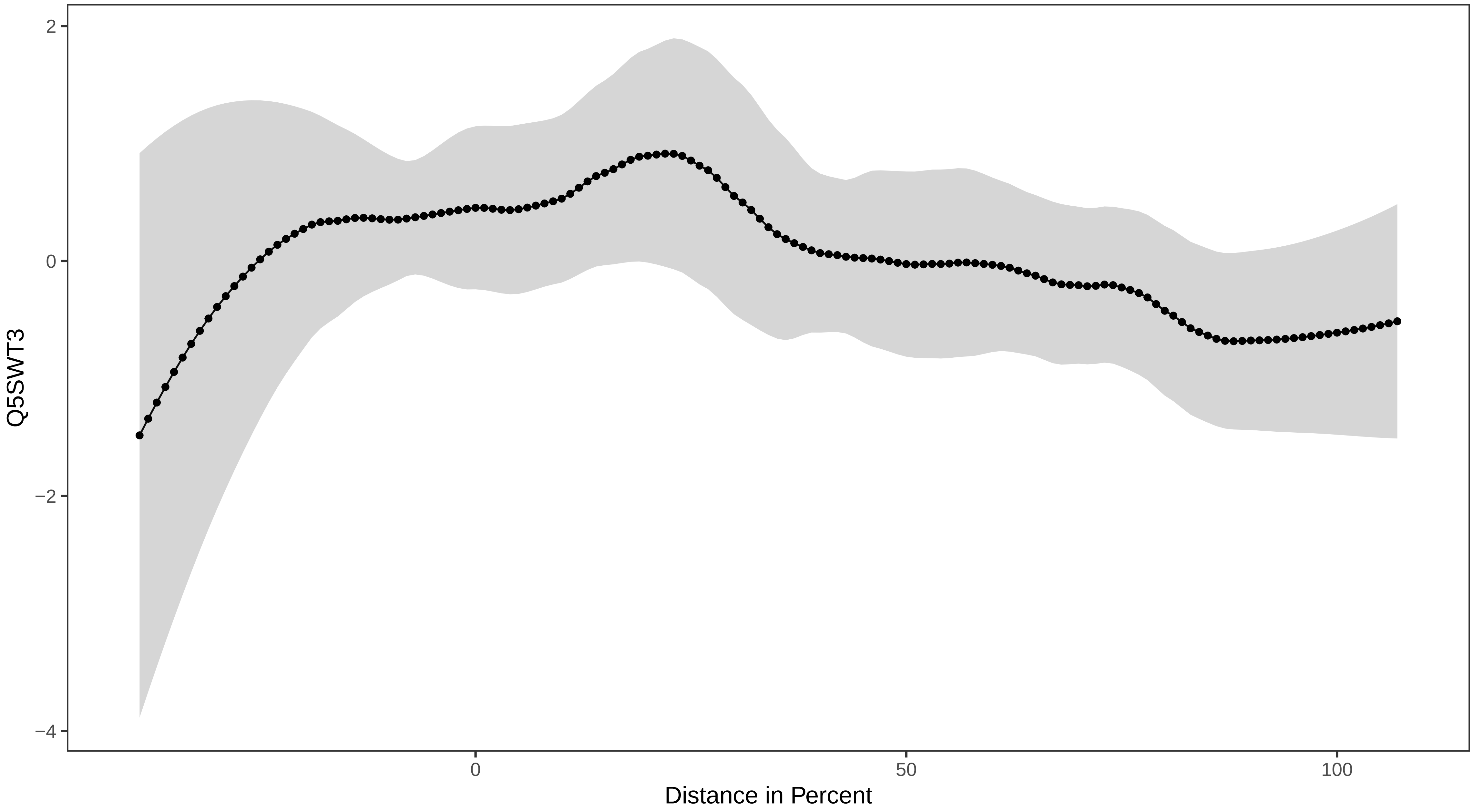

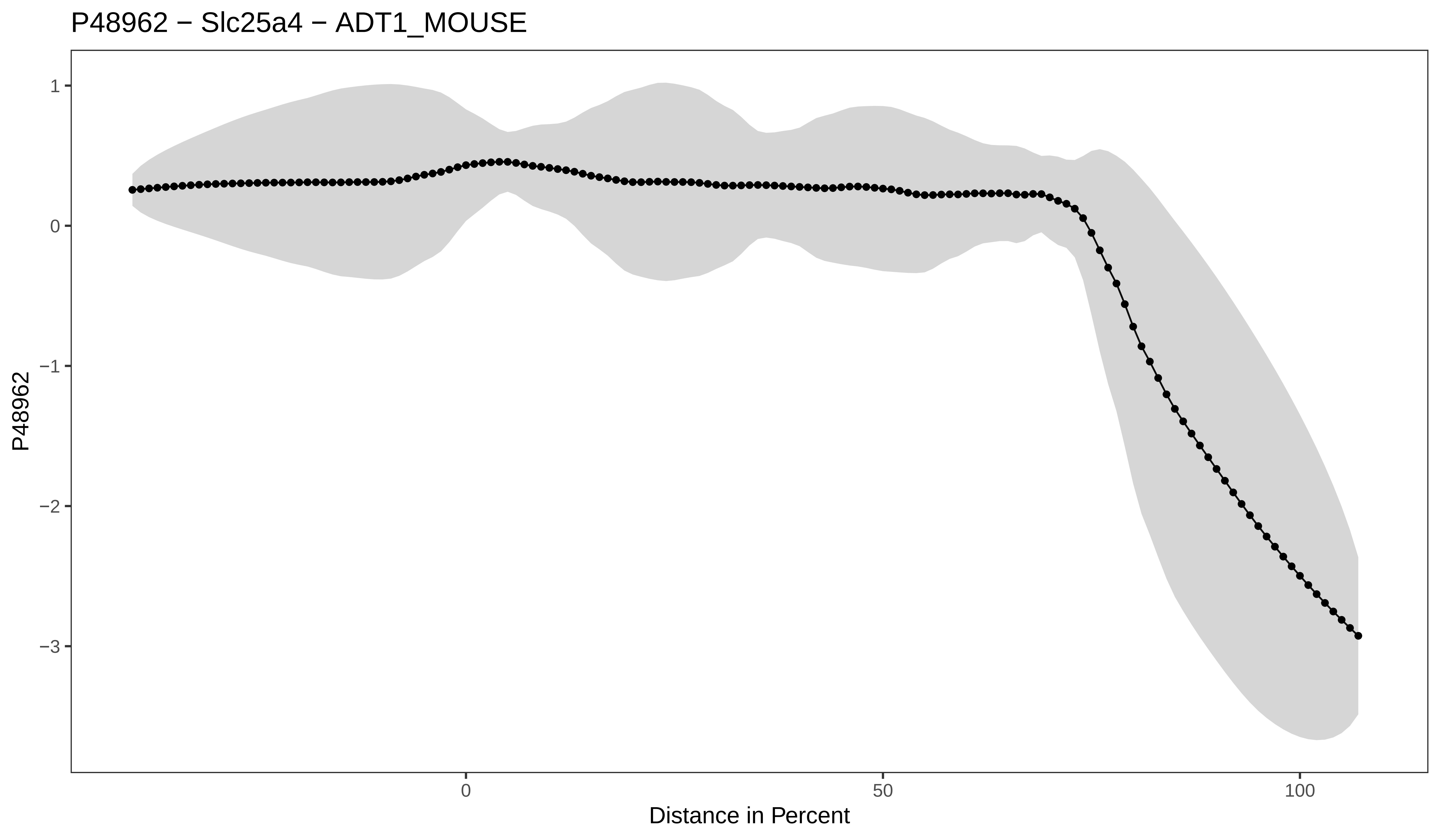

P48962 − Slc25a4 − ADT1_MOUSE
P48962
1
0
−1
−2
−3
0
50
100
Distance in Percent

Q8R0Y8 − Slc25a42 − S2542_MOUSE

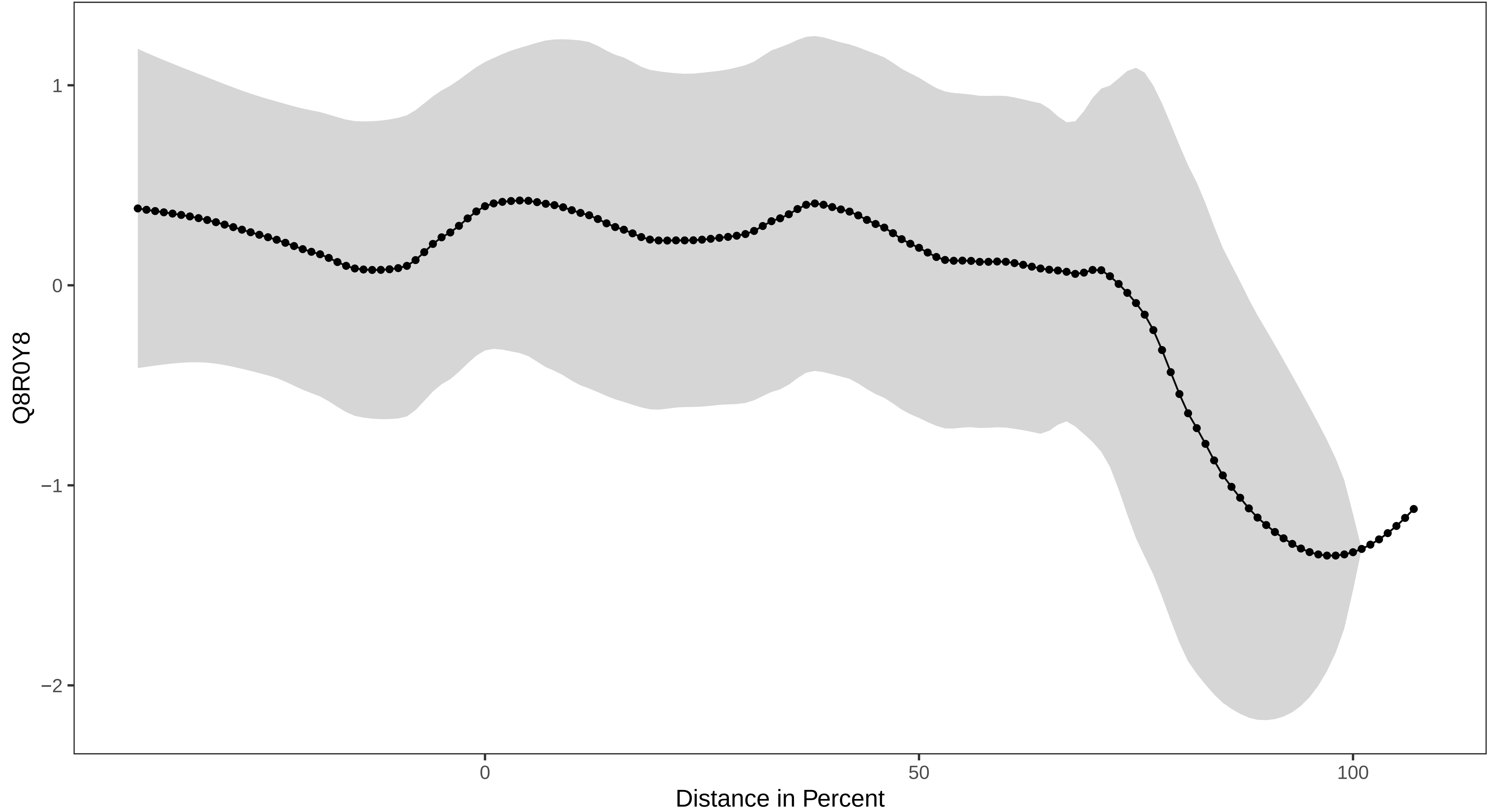

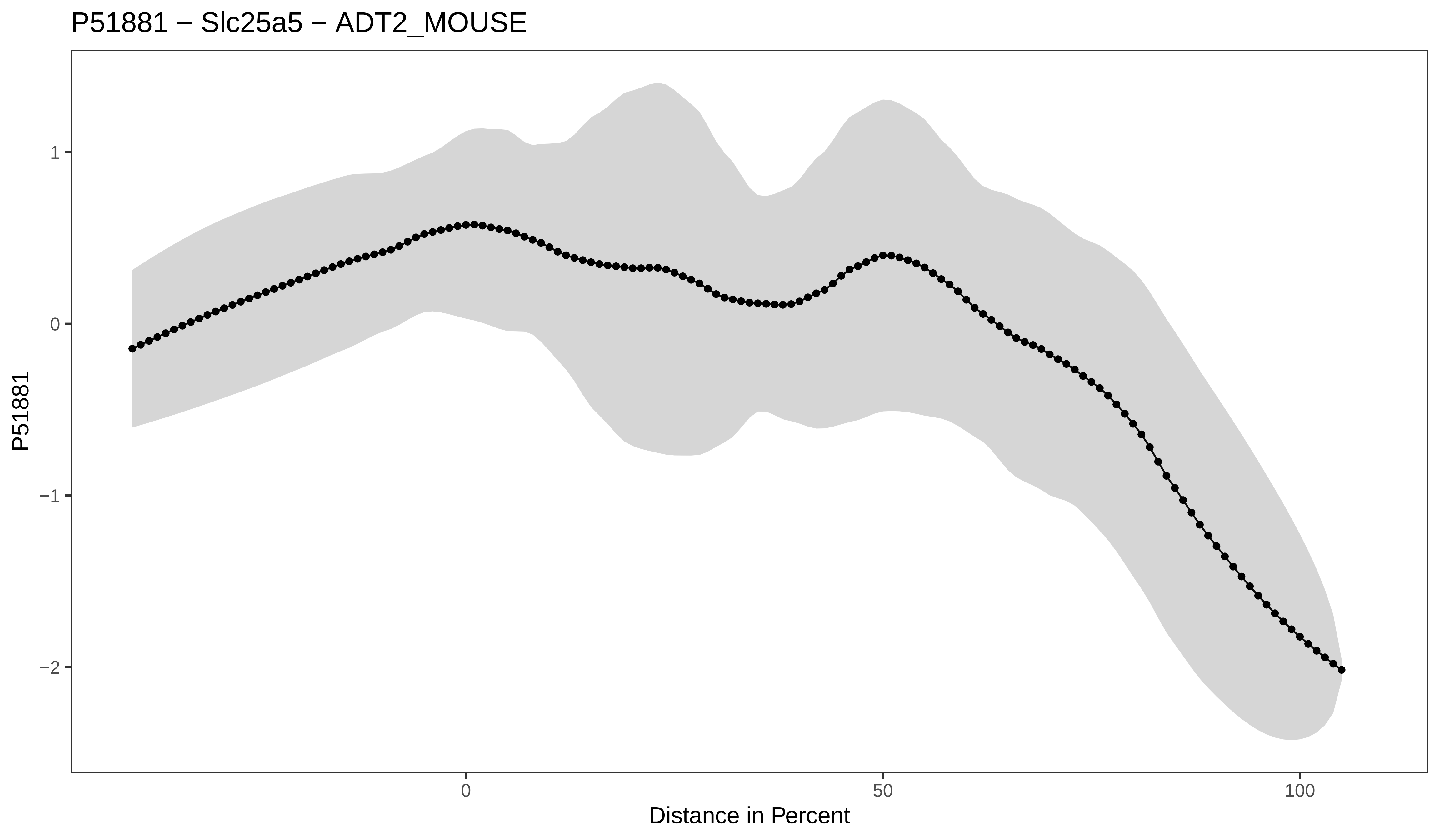

P51881 − Slc25a5 − ADT2_MOUSE
P51881
1
0
−1
−2
0
50
100
Distance in Percent

Q5HZI9 − Slc25a51 − S2551_MOUSE

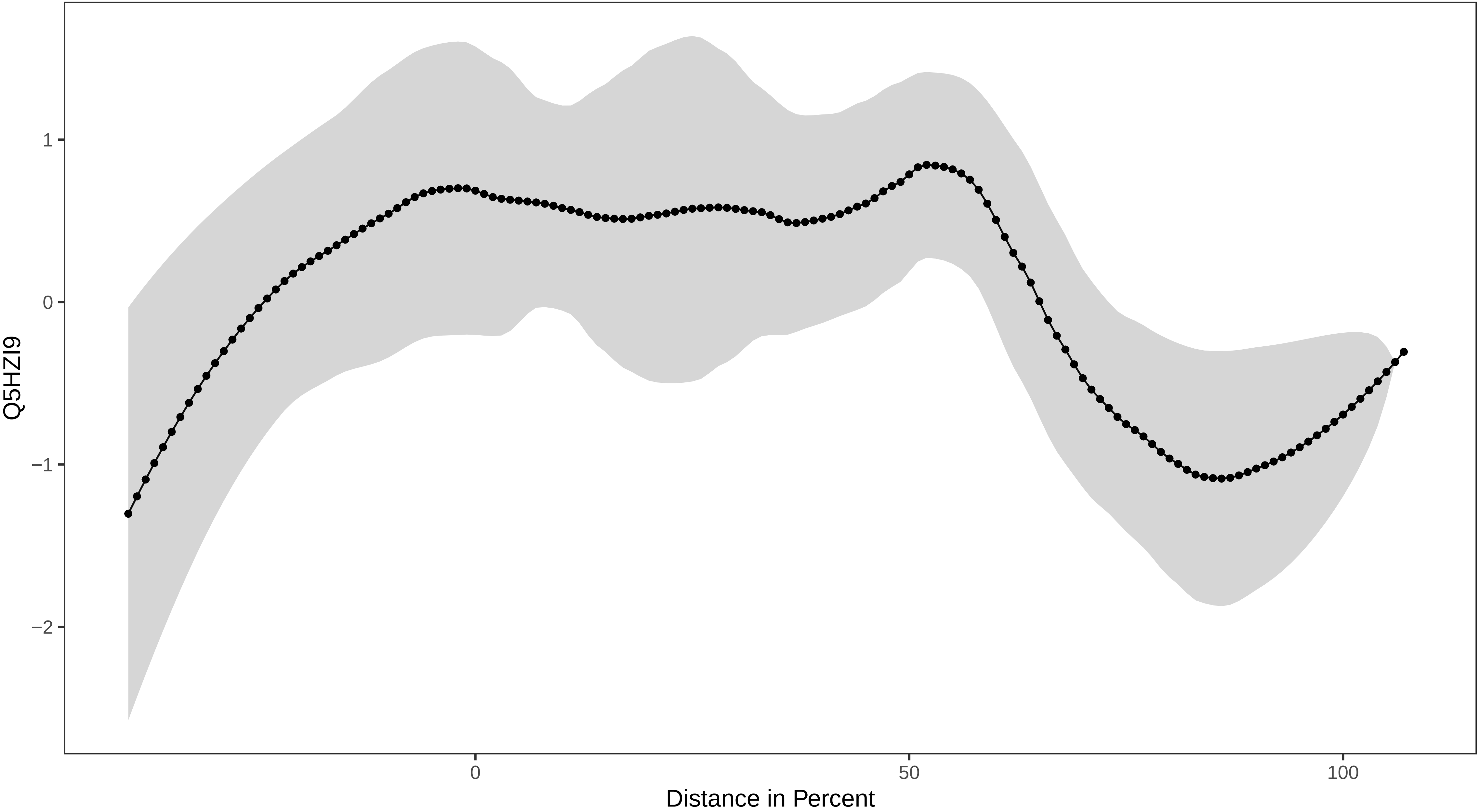

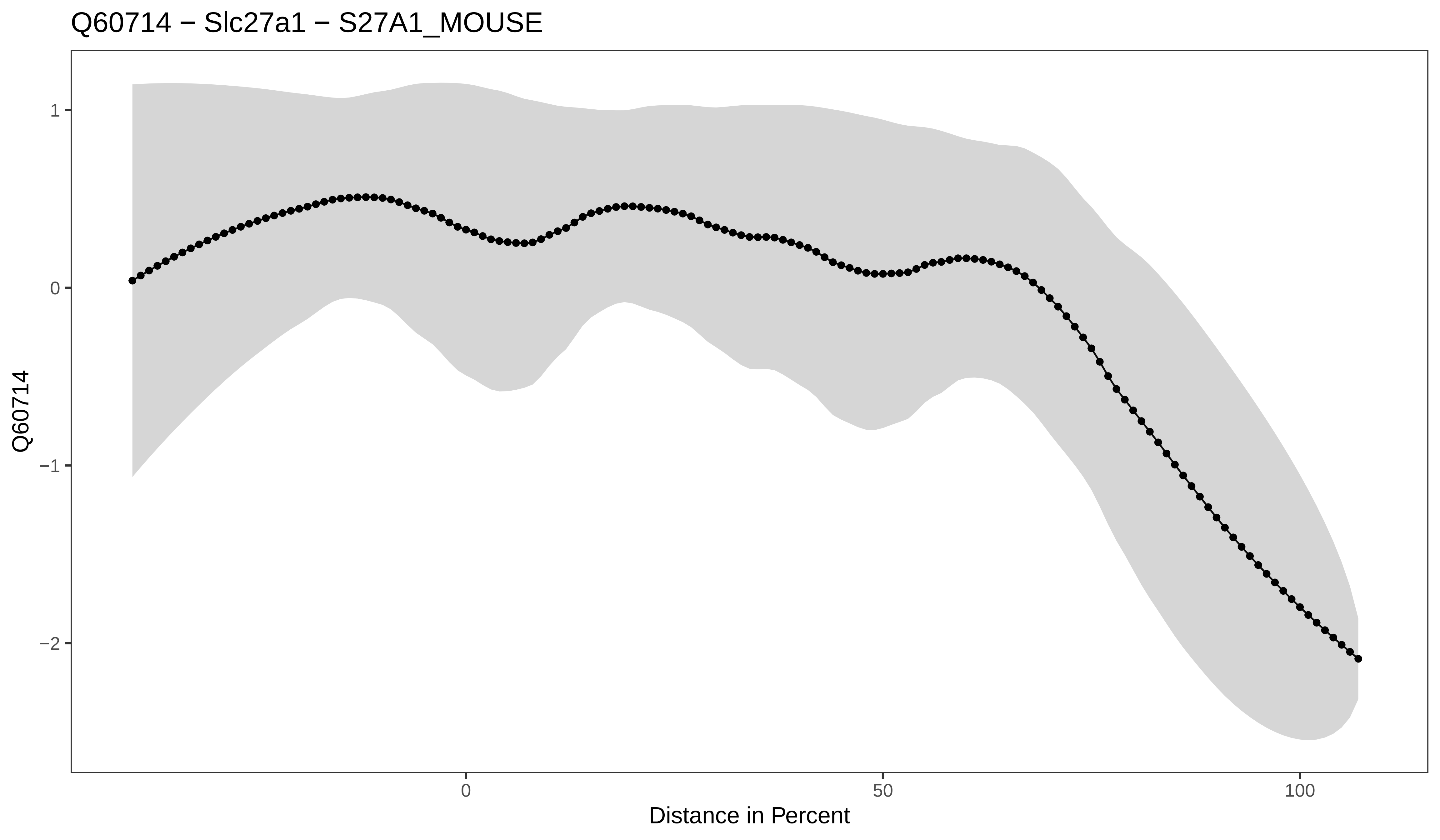
Q60714 − Slc27a1 − S27A1_MOUSE
Q60714
1
0
−1
−2
0
50
100
Distance in Percent

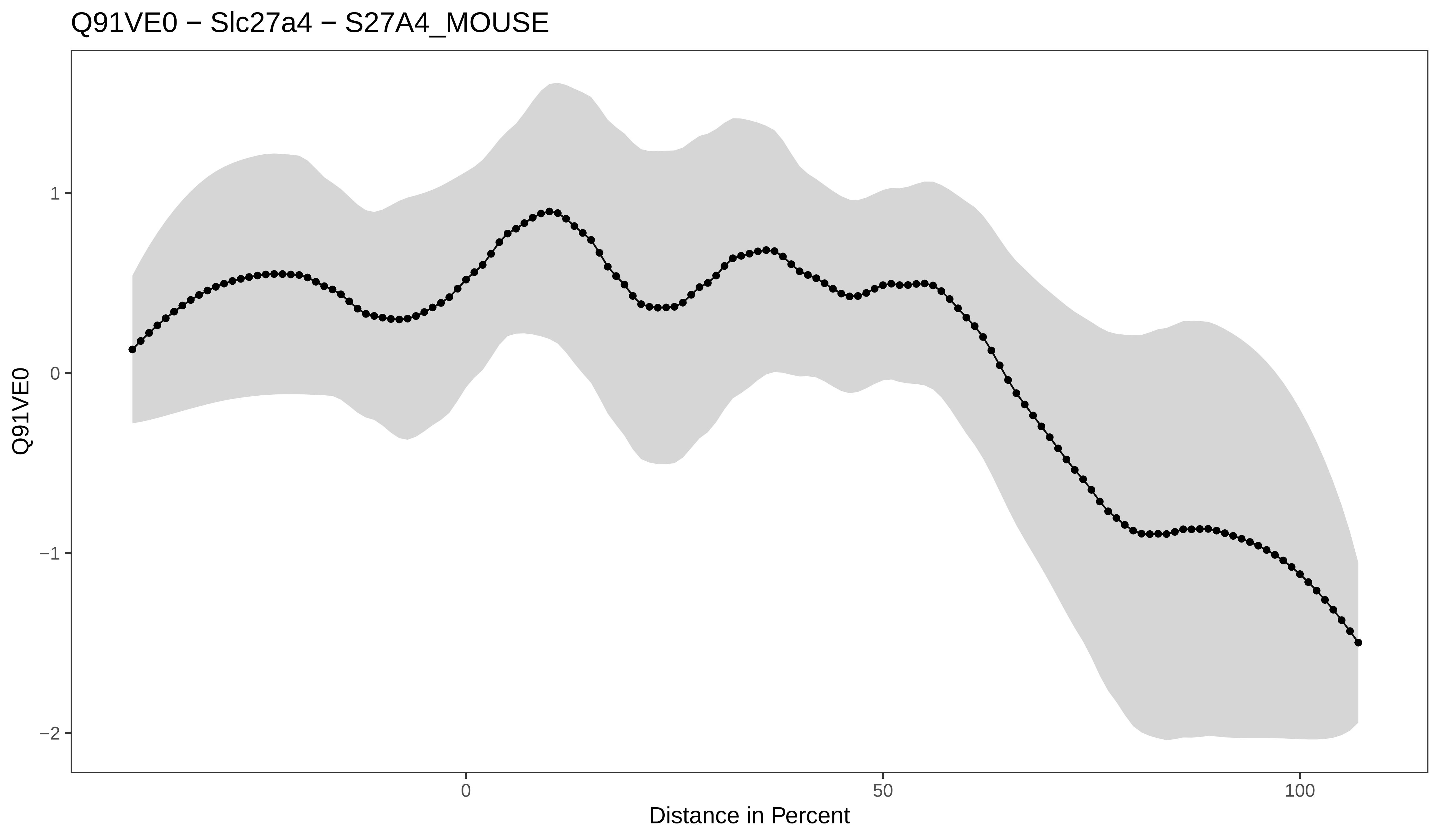

Q91VE0 − Slc27a4 − S27A4_MOUSE
1
0
−1
−2
Q91VE0
0
50
100
Distance in Percent

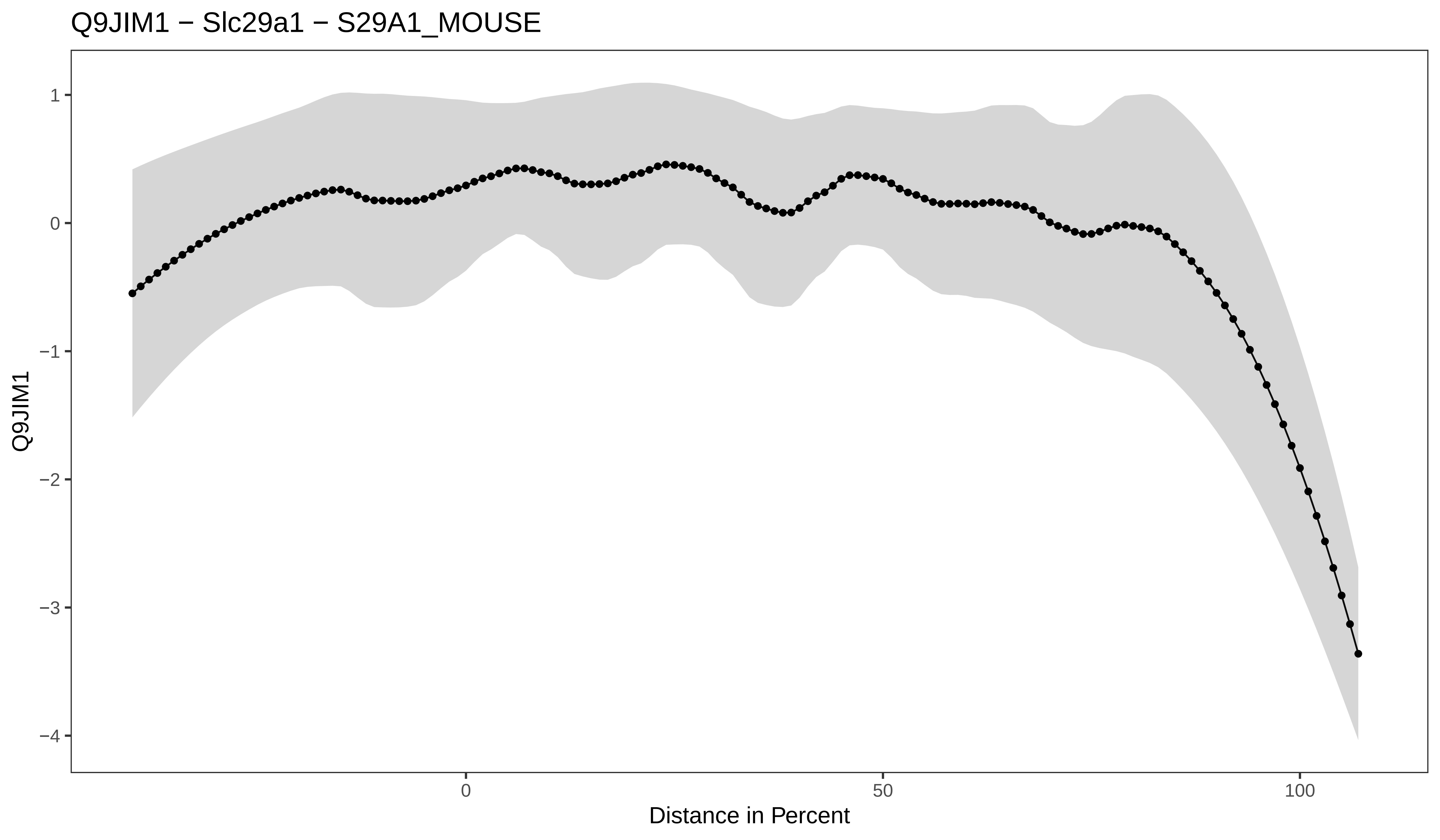

Q9JIM1 − Slc29a1 − S29A1_MOUSE
Q9JIM1
1
0
−1
−2
−3
−4
0
50
100
Distance in Percent

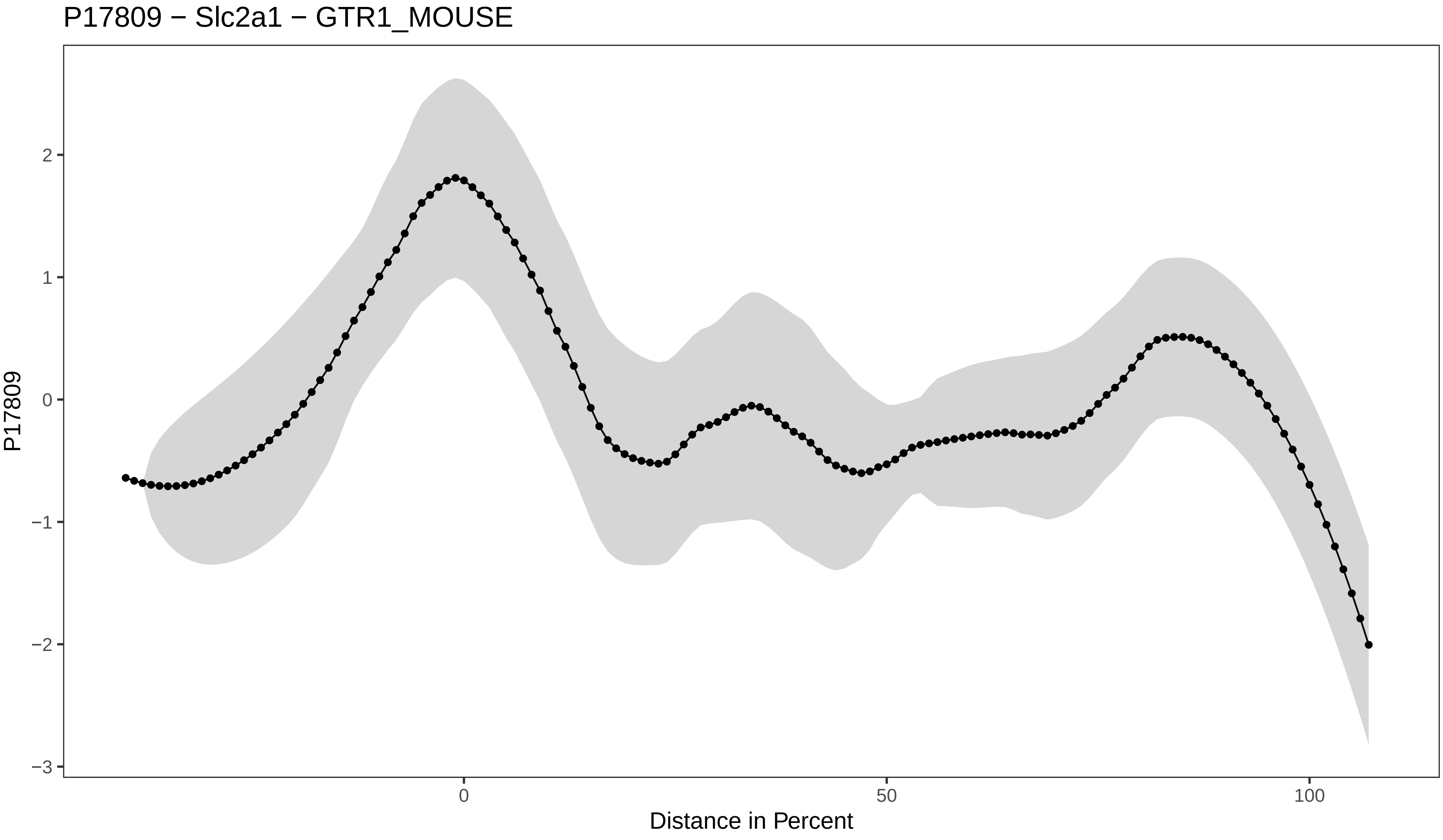

P17809 − Slc2a1 − GTR1_MOUSE
P17809
2
1
0
−1
−2
−3
0
50
100
Distance in Percent

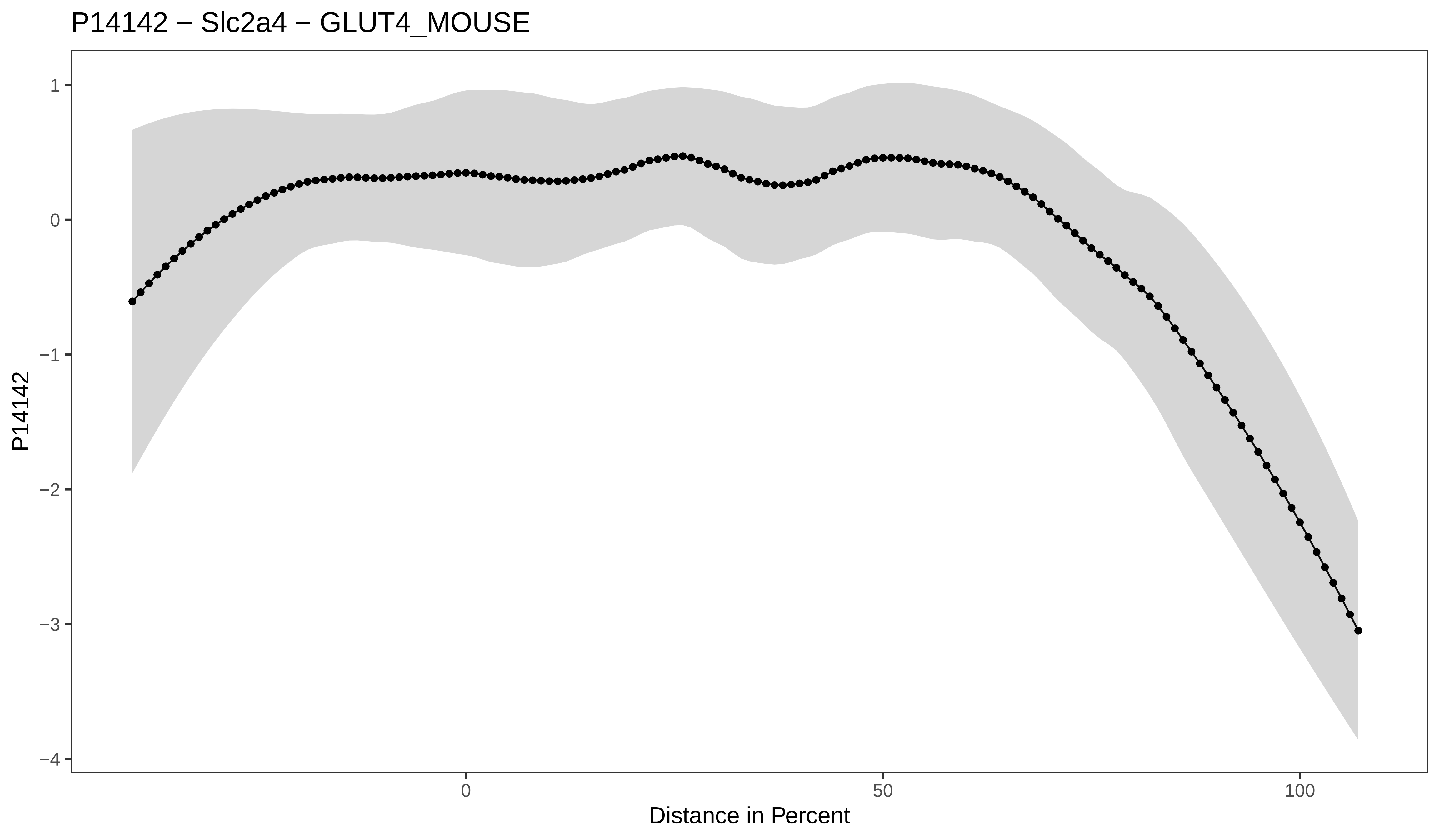

P14142 − Slc2a4 − GLUT4_MOUSE
1
0
−1
−2
−3
−4
P14142
0
50
100
Distance in Percent

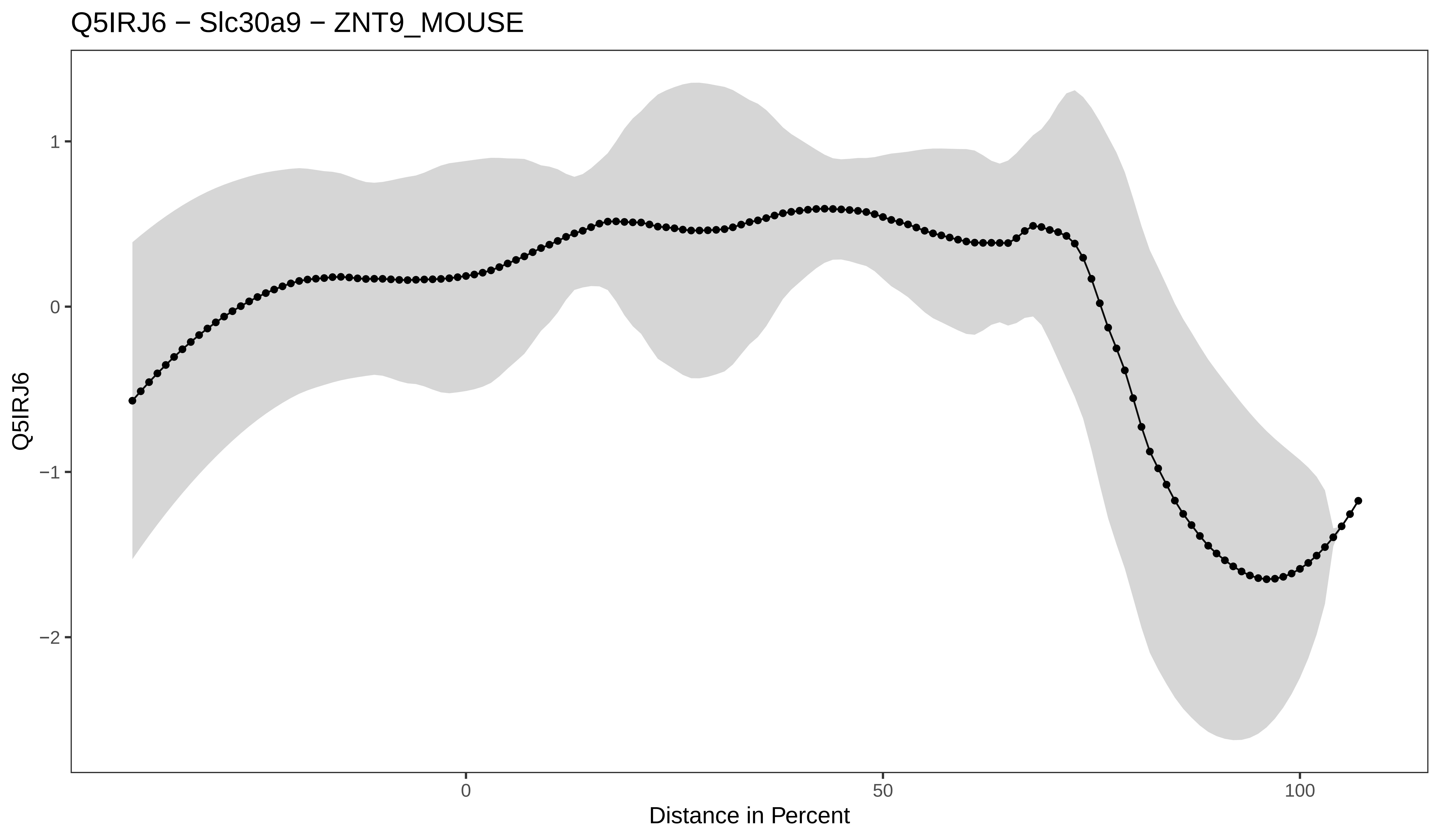

Q5IRJ6 − Slc30a9 − ZNT9_MOUSE
Q5IRJ6
1
0
−1
−2
0
50
100
Distance in Percent

P10852 − Slc3a2 − 4F2_MOUSE

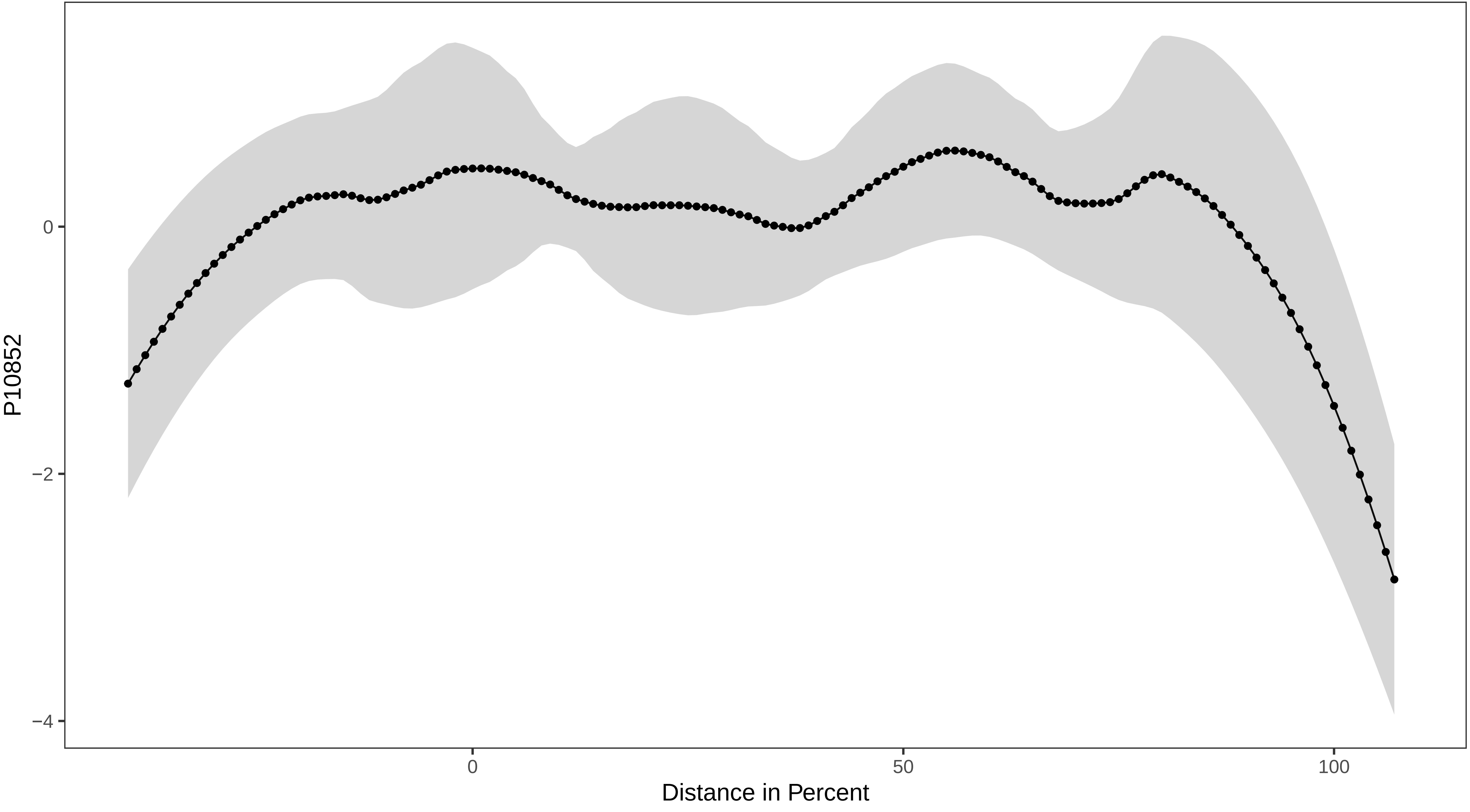

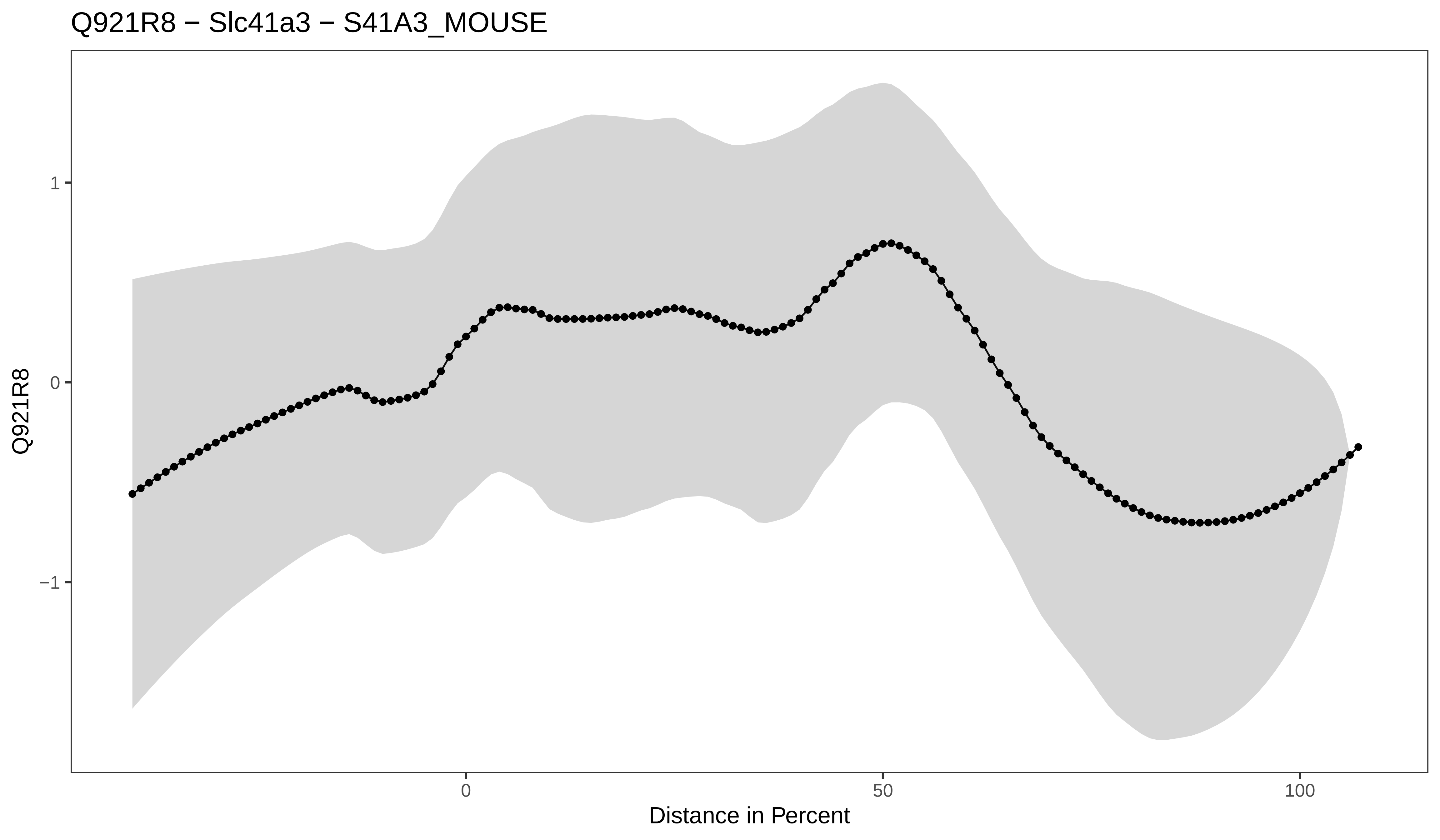

Q921R8 − Slc41a3 − S41A3_MOUSE
Q921R8
1
0
−1
0
50
100
Distance in Percent

Q6X893 − Slc44a1 − CTL1_MOUSE

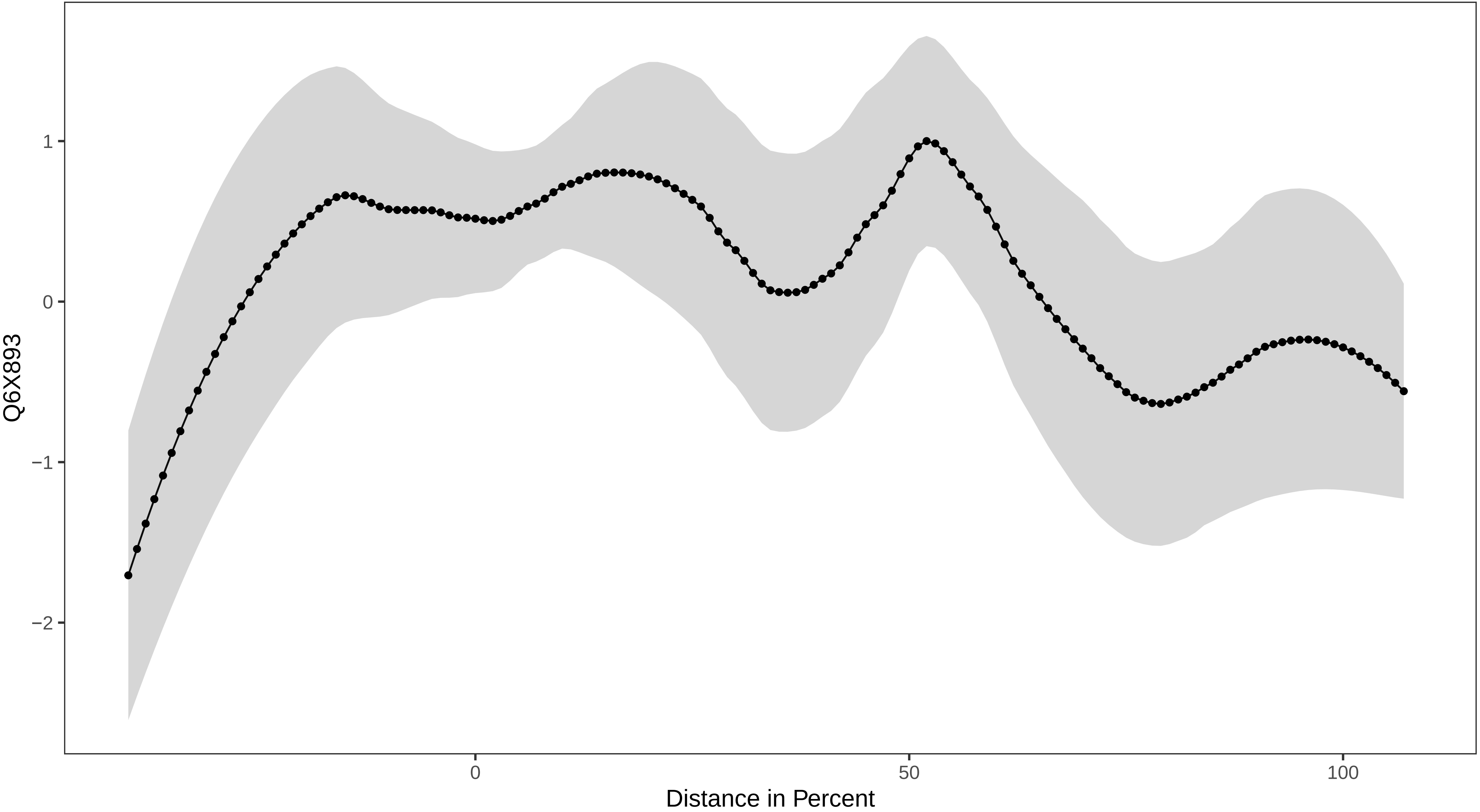

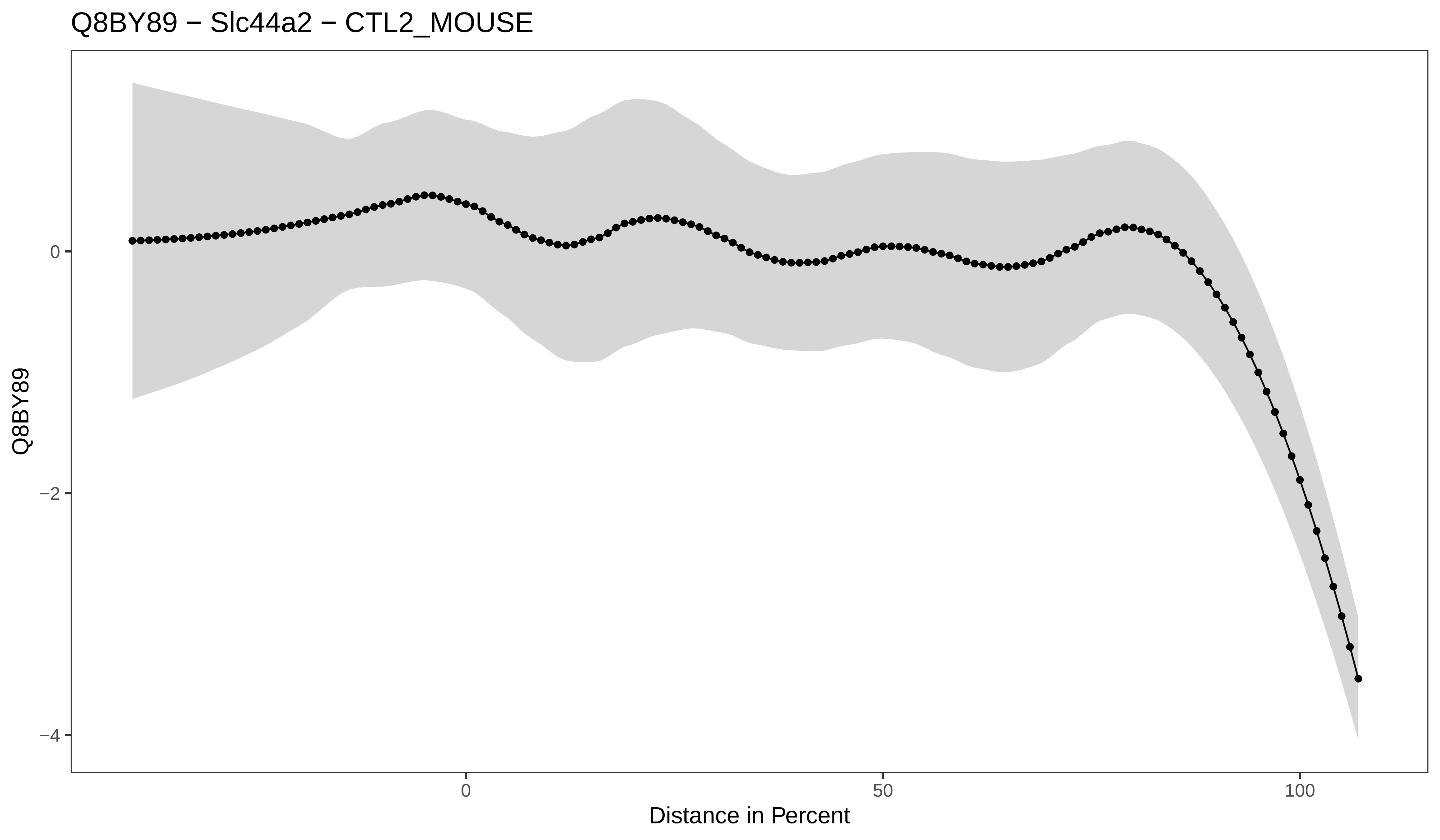

Q8BY89 − Slc44a2 − CTL2_MOUSE
Q8BY89
0
−2
−4
0
50
100
Distance in Percent

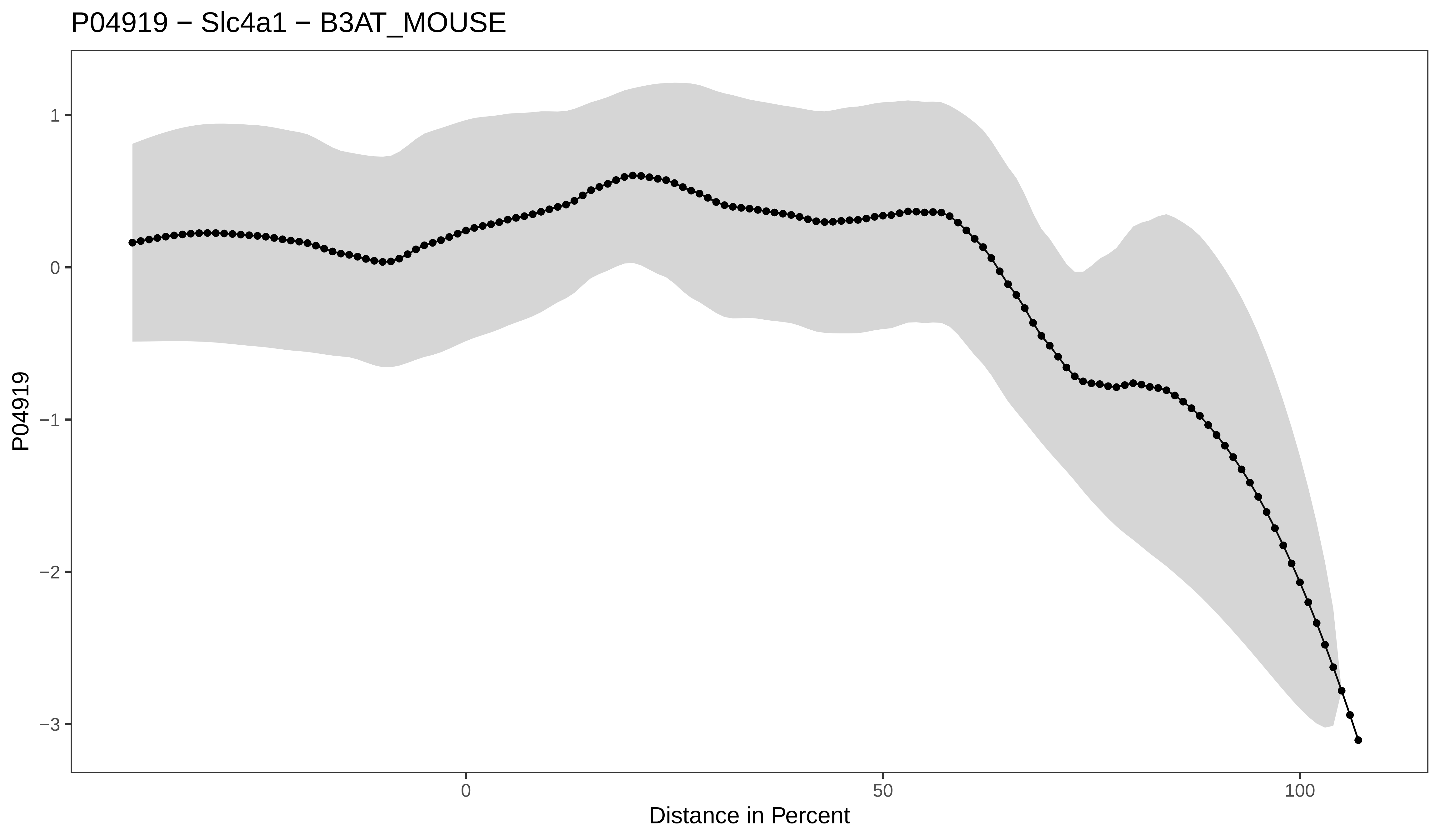

P04919 − Slc4a1 − B3AT_MOUSE
P04919
Distance in Percent
1
0
−1
−2
−3
0
50
100

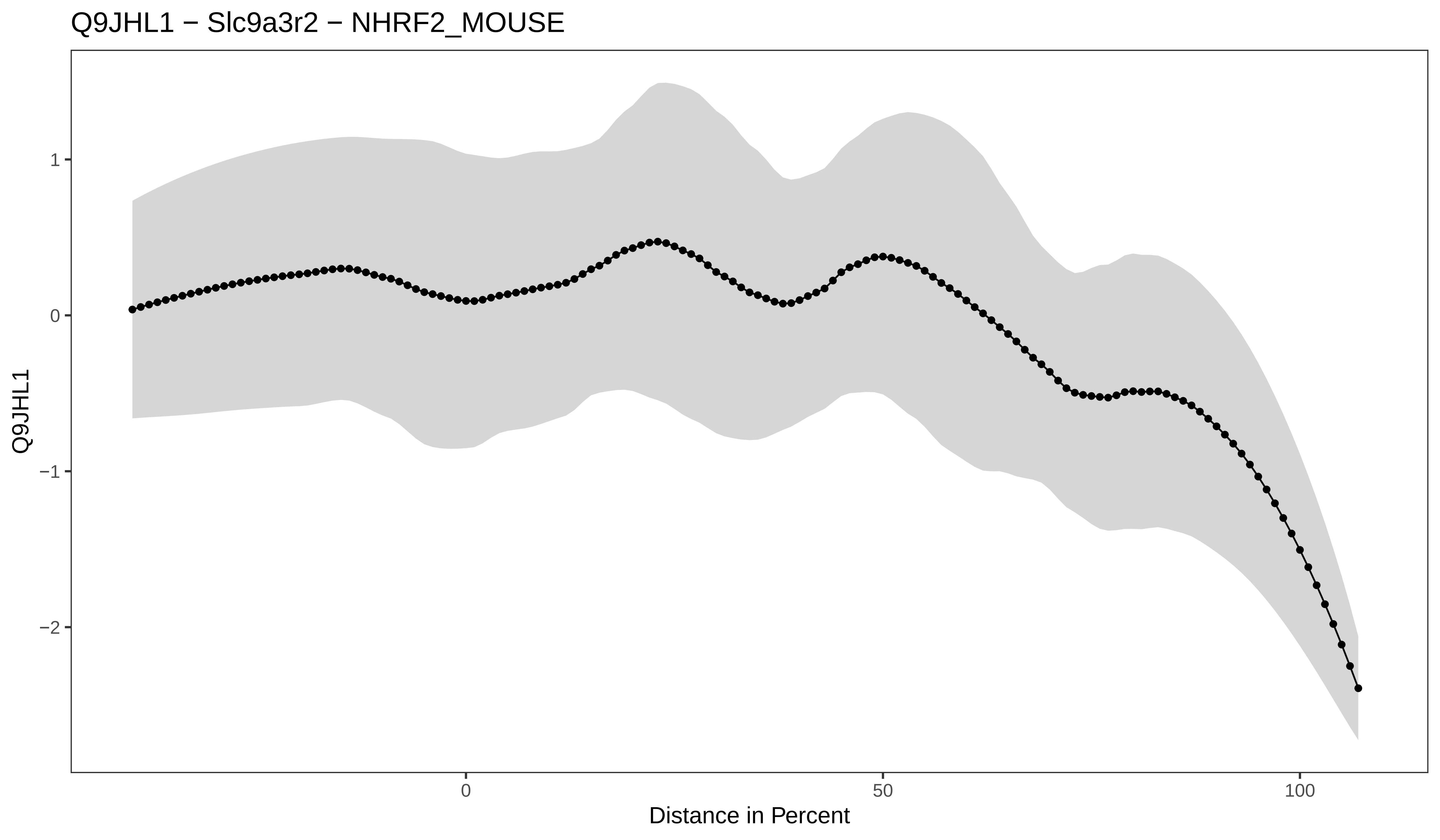

Q9JHL1 − Slc9a3r2 − NHRF2_MOUSE
1
0
−1
−2
Q9JHL1
0
50
100
Distance in Percent

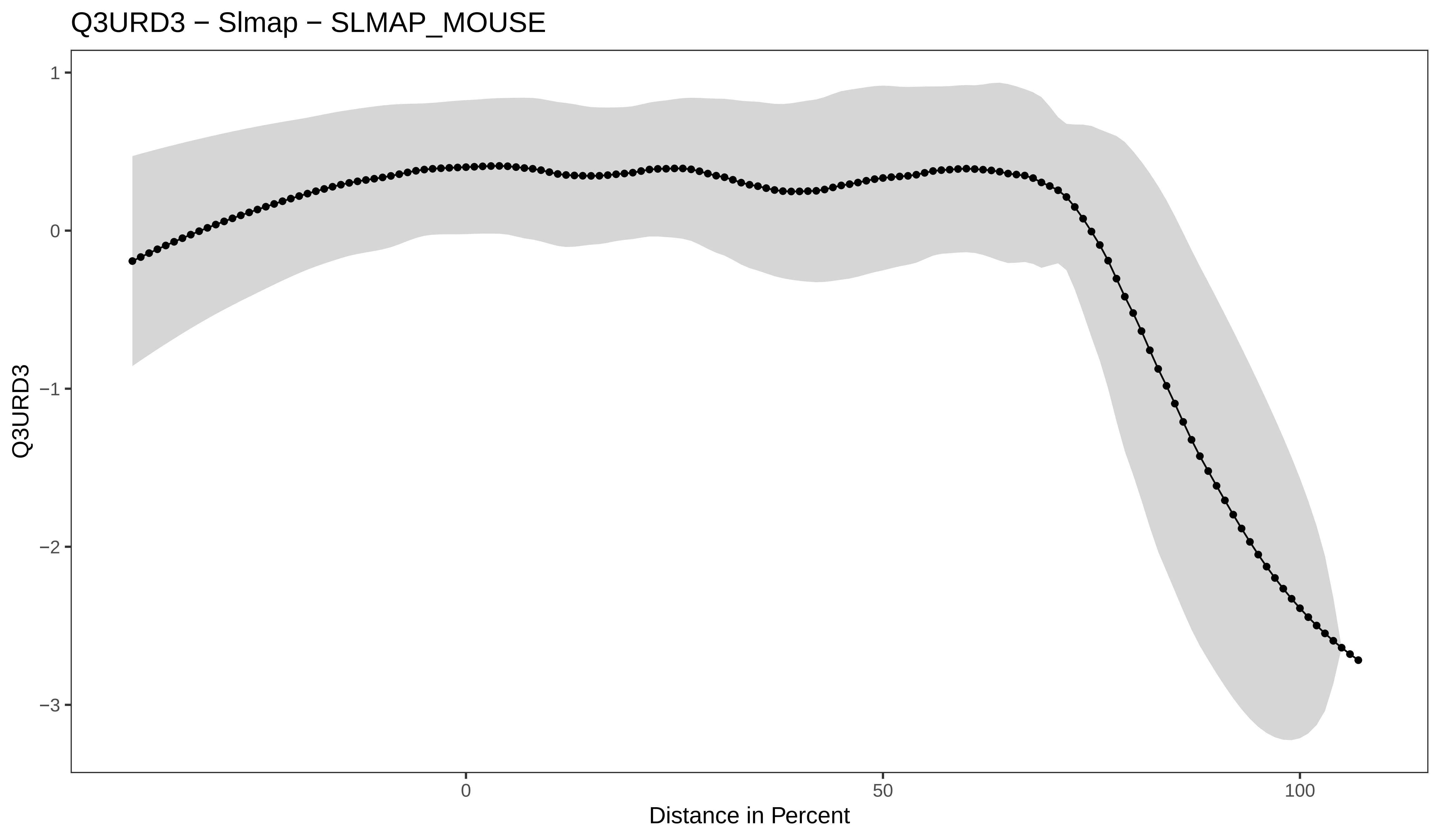
Q3URD3 − Slmap − SLMAP_MOUSE
1
0
−1
−2
−3
Q3URD3
0
50
100
Distance in Percent

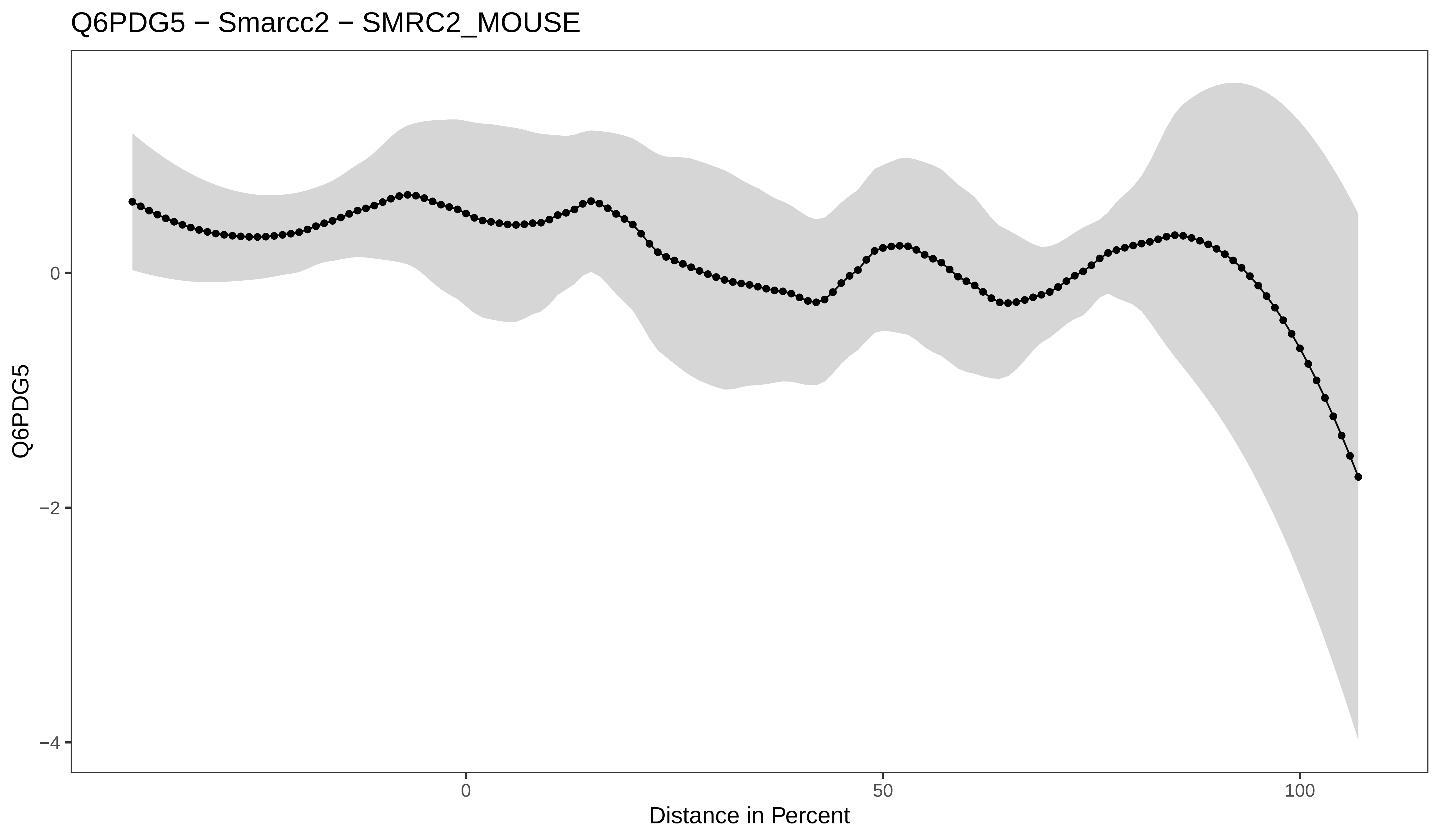

Q6PDG5 − Smarcc2 − SMRC2_MOUSE
0
−2
−4
Q6PDG5
0
50
100
Distance in Percent

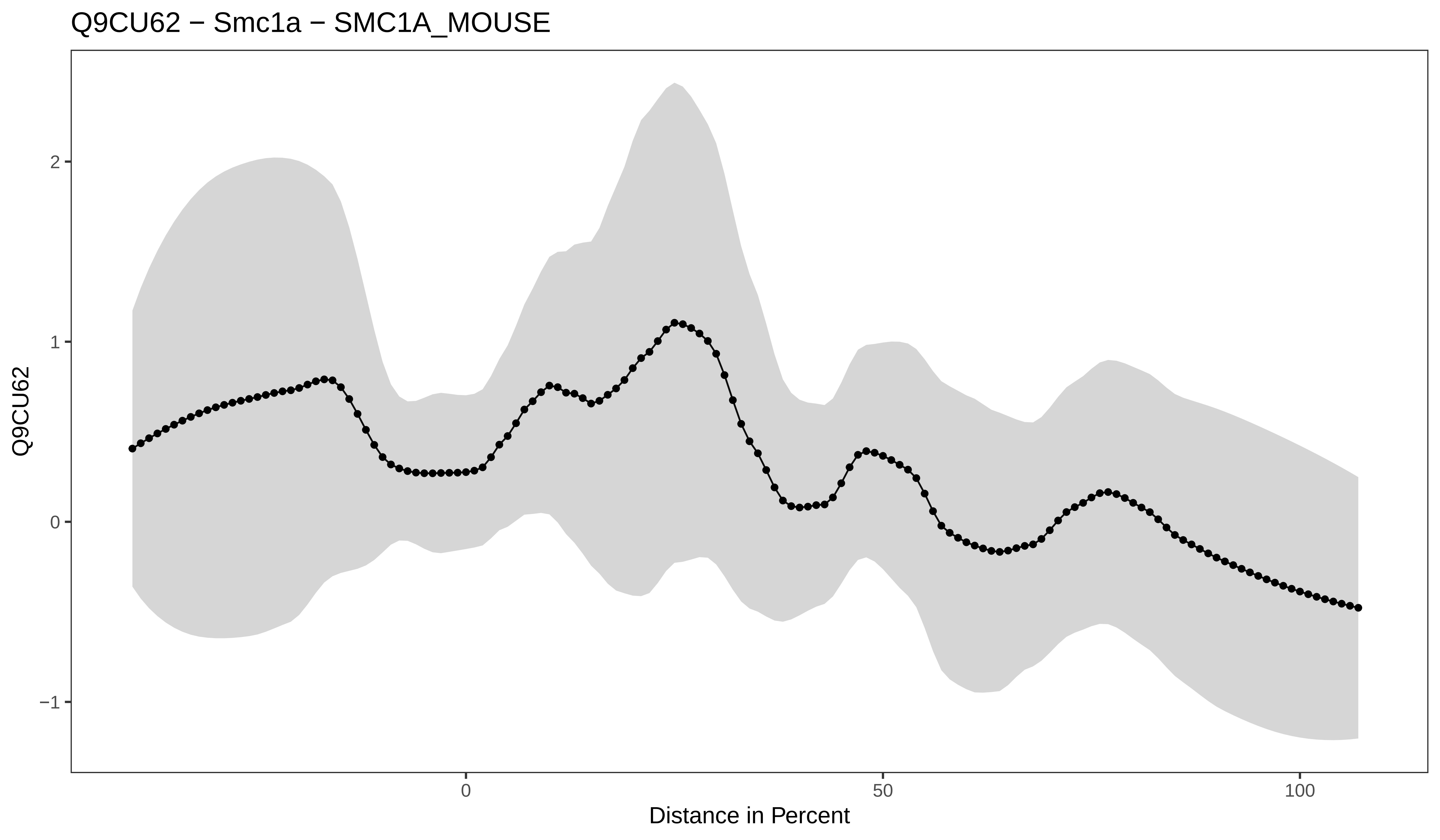

Q9CU62 − Smc1a − SMC1A_MOUSE
Q9CU62
2
1
0
−1
0
50
100
Distance in Percent

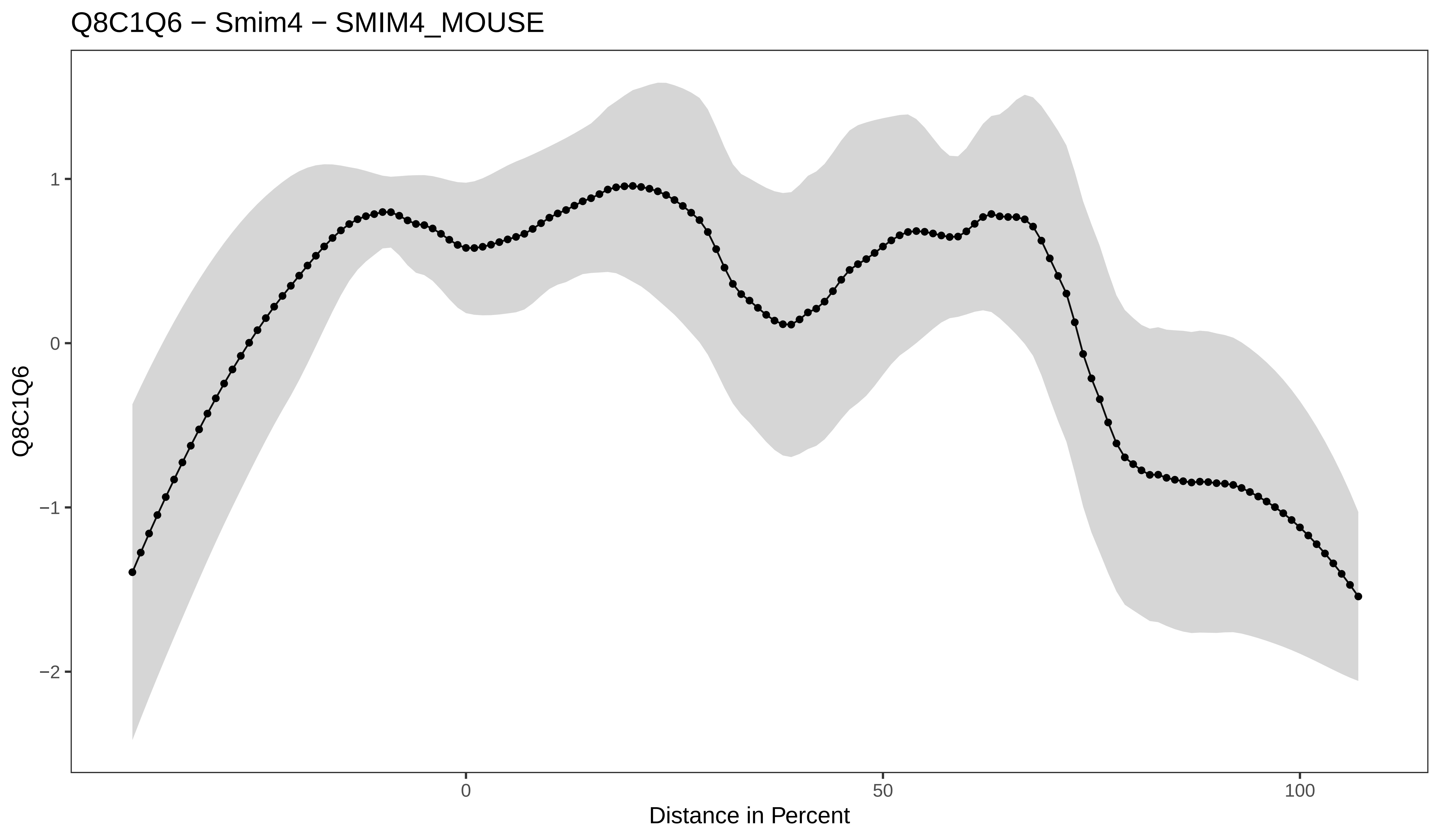
Q8C1Q6 − Smim4 − SMIM4_MOUSE
1
0
−1
−2
Q8C1Q6
0
50
100
Distance in Percent

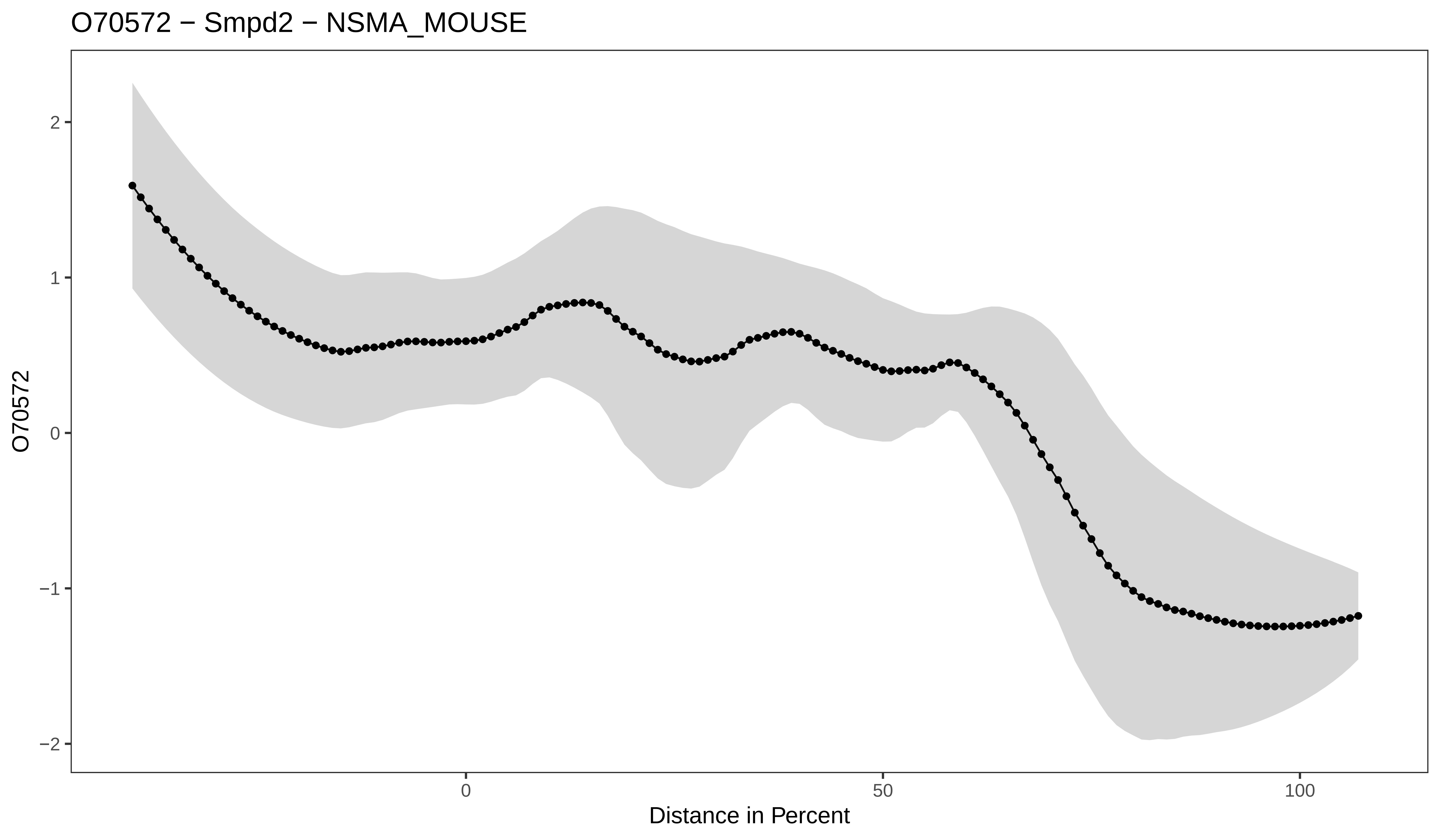

O70572 − Smpd2 − NSMA_MOUSE
O70572
2
1
0
−1
−2
0
50
100
Distance in Percent

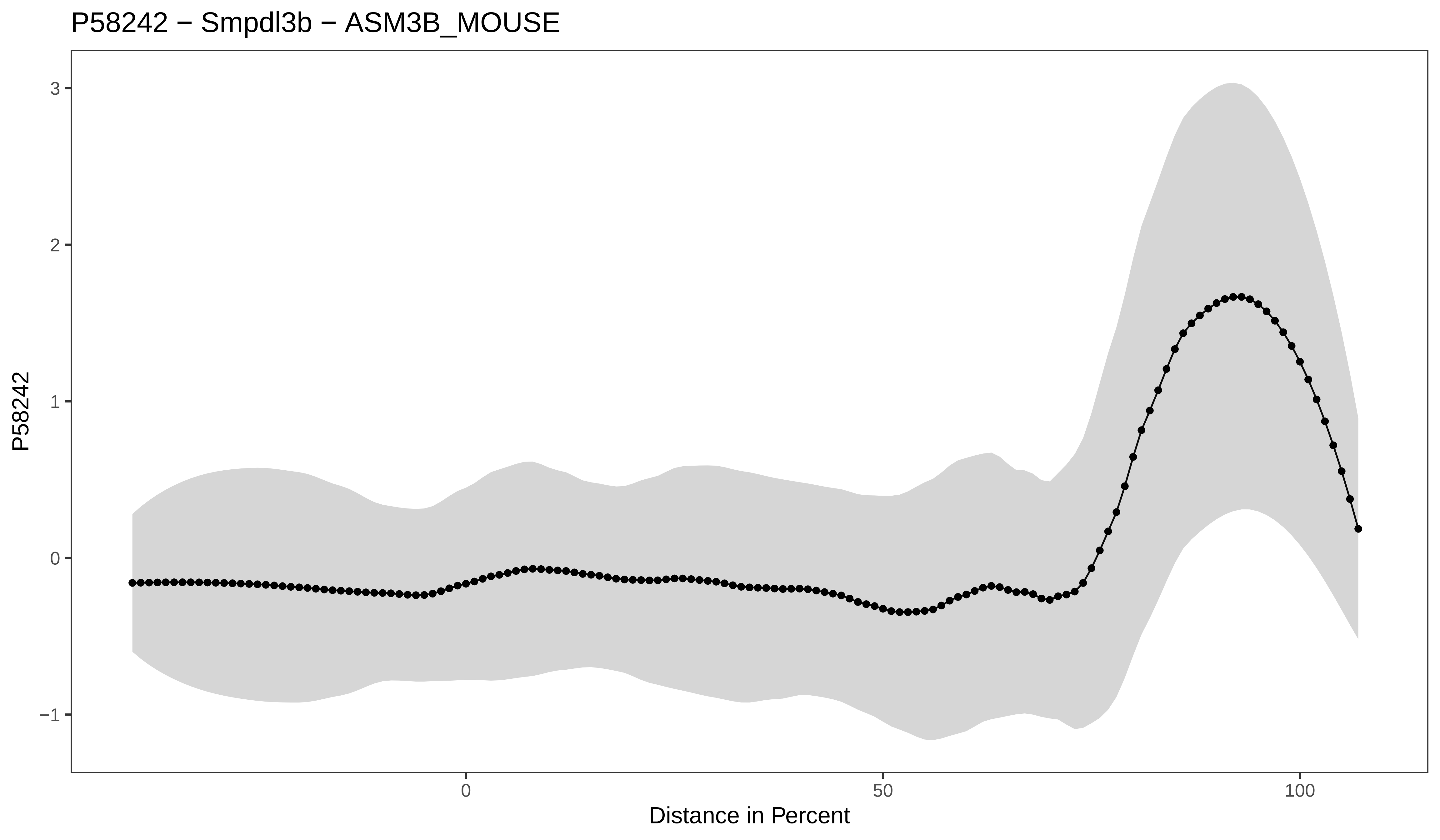

P58242 − Smpdl3b − ASM3B_MOUSE
P58242
Distance in Percent
3
2
1
0
−1
0
50
100

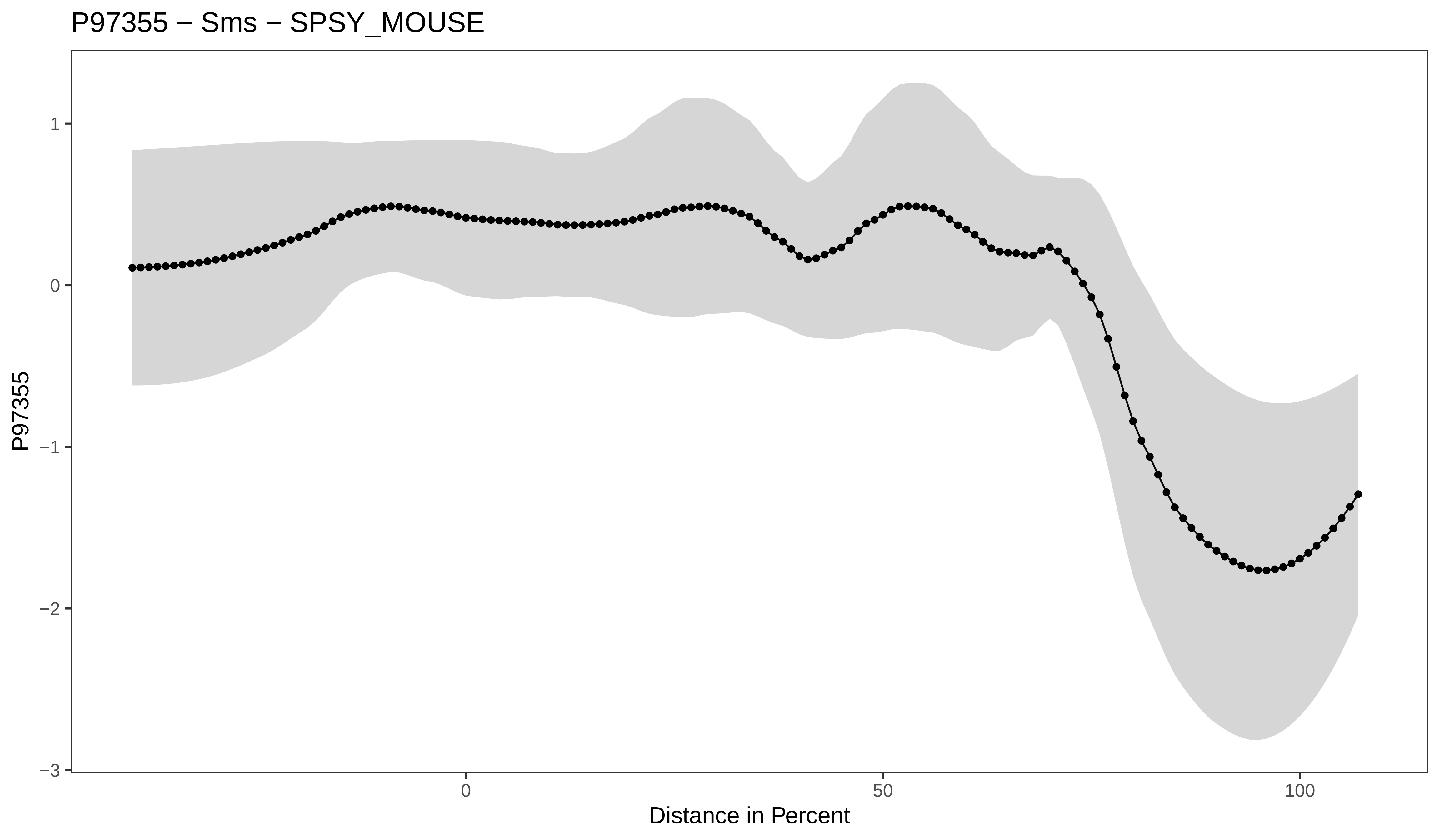

P97355 − Sms − SPSY_MOUSE
1
0
−1
−2
−3
P97355
0
50
100
Distance in Percent

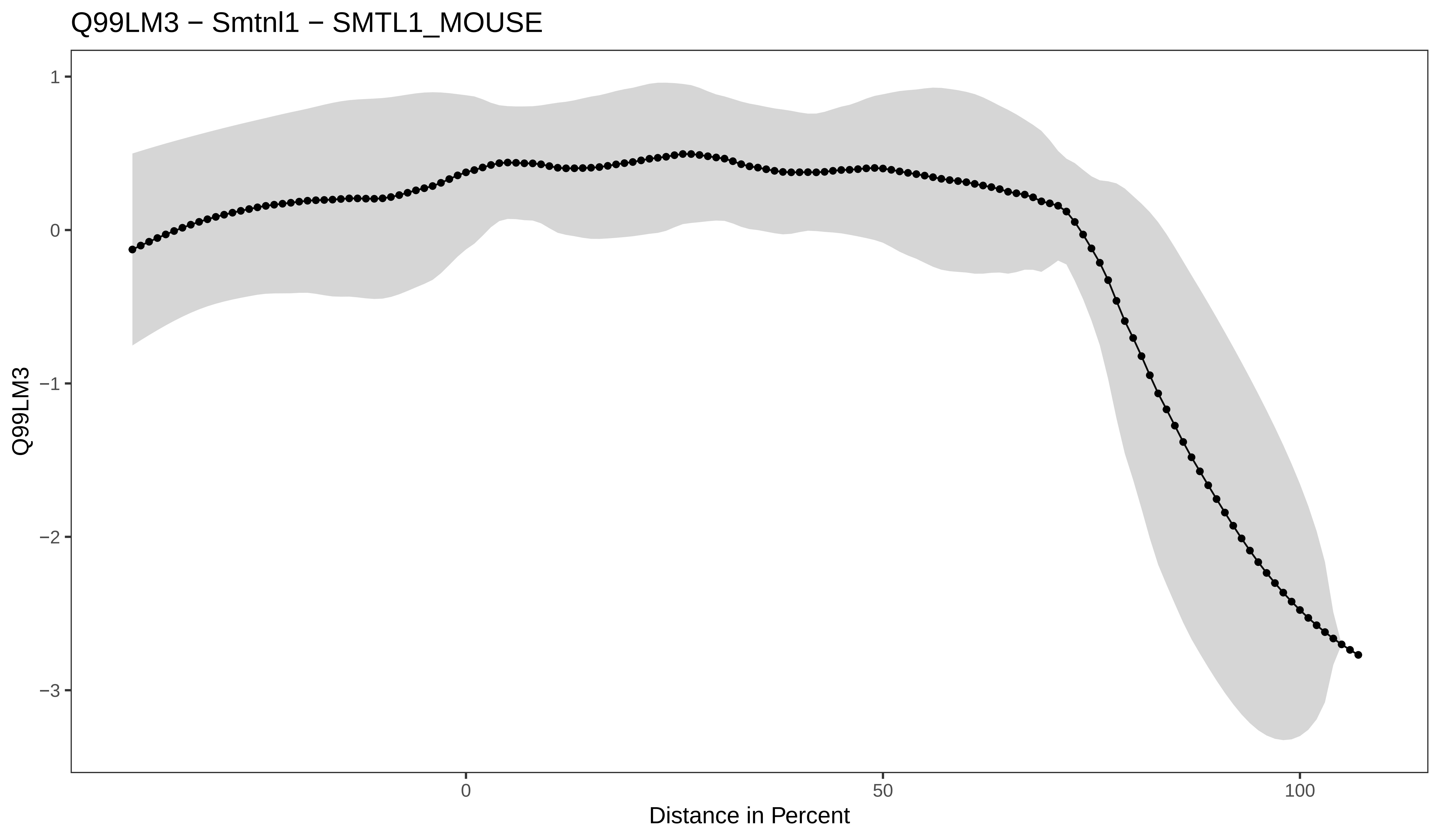
Q99LM3 − Smtnl1 − SMTL1_MOUSE
1
0
−1
−2
−3
Q99LM3
0
50
100
Distance in Percent

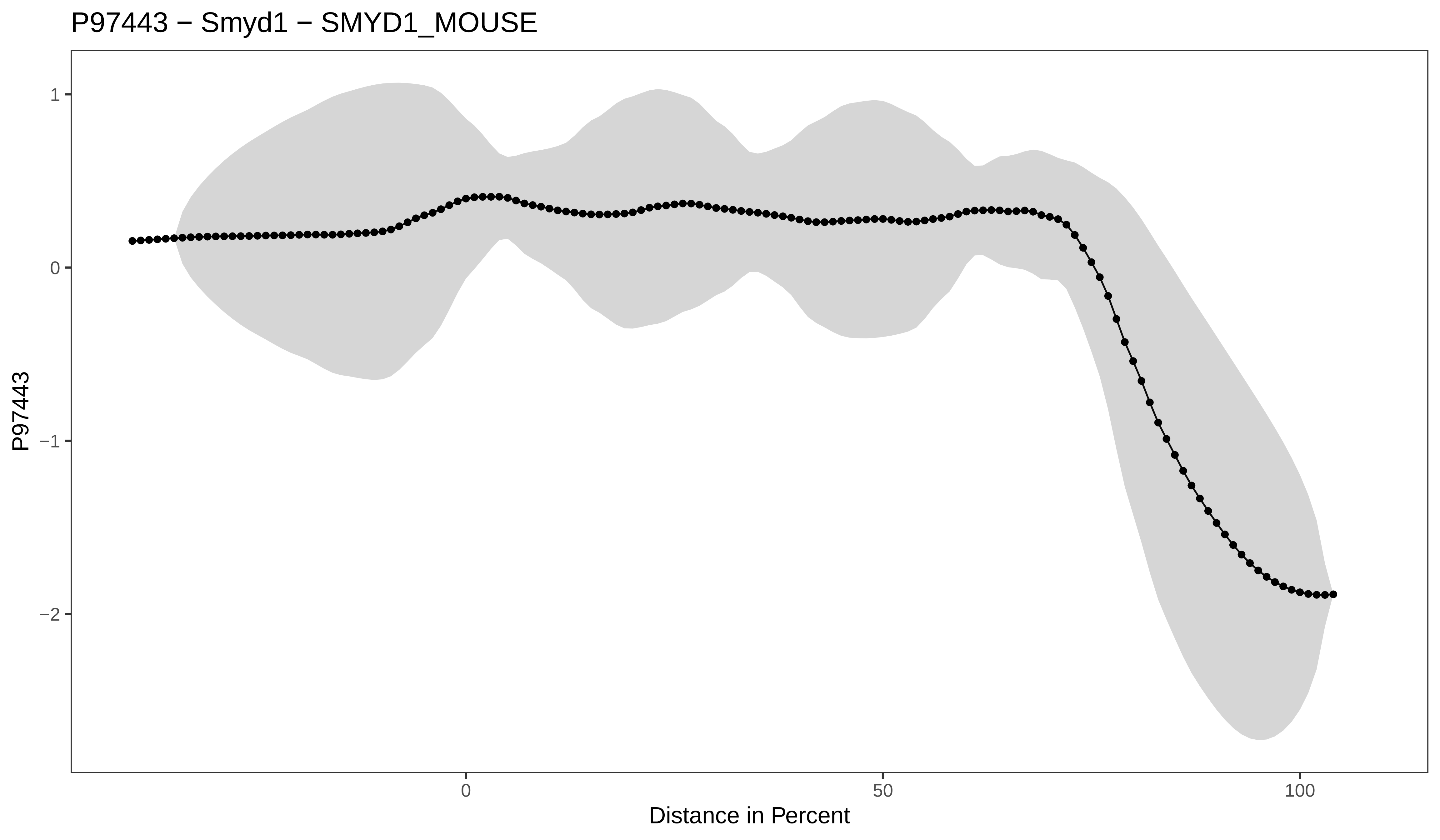

P97443 − Smyd1 − SMYD1_MOUSE
1
0
−1
−2
P97443
0
50
100
Distance in Percent

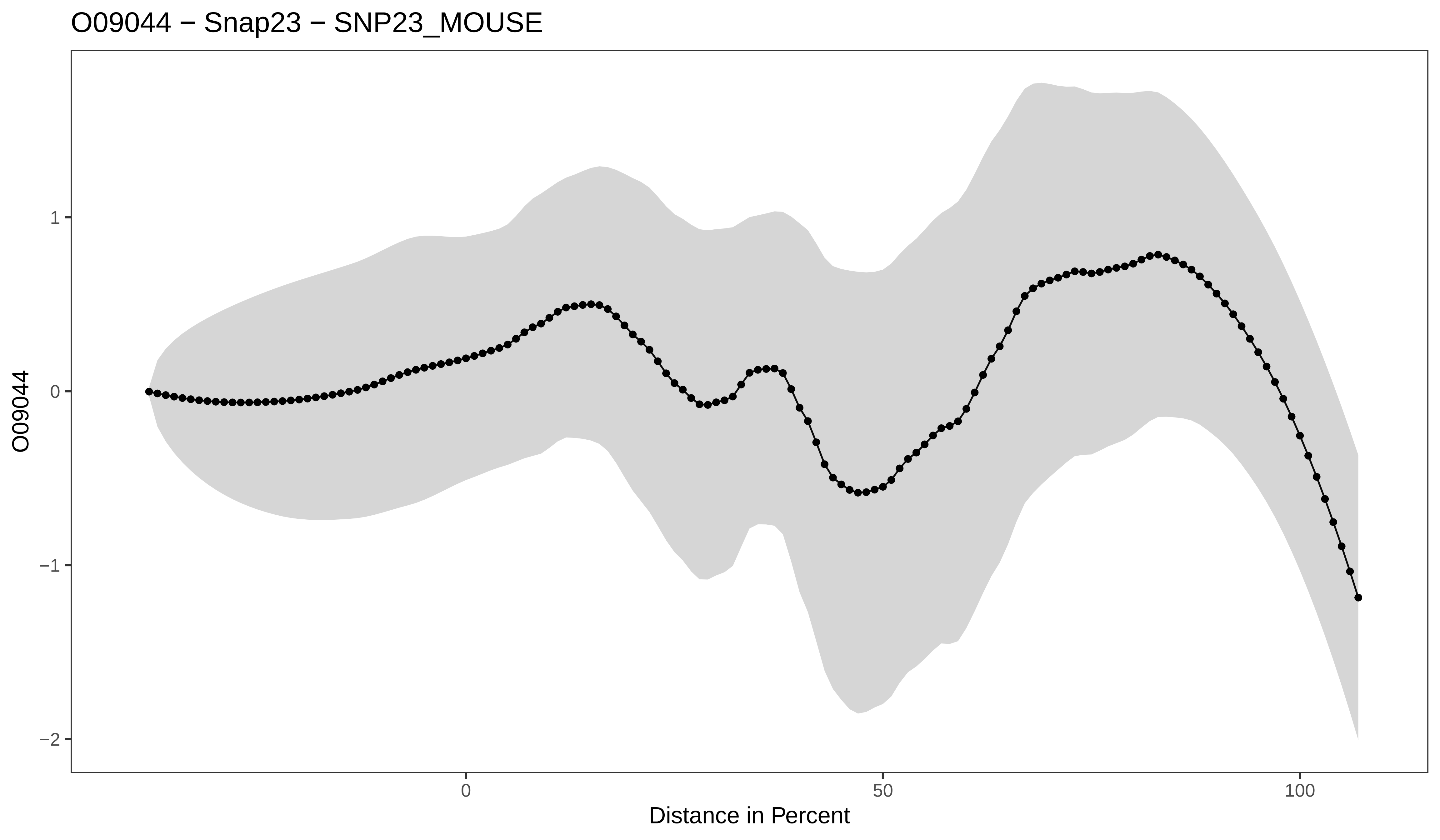
O09044 − Snap23 − SNP23_MOUSE
O09044
1
0
−1
−2
0
50
100
Distance in Percent

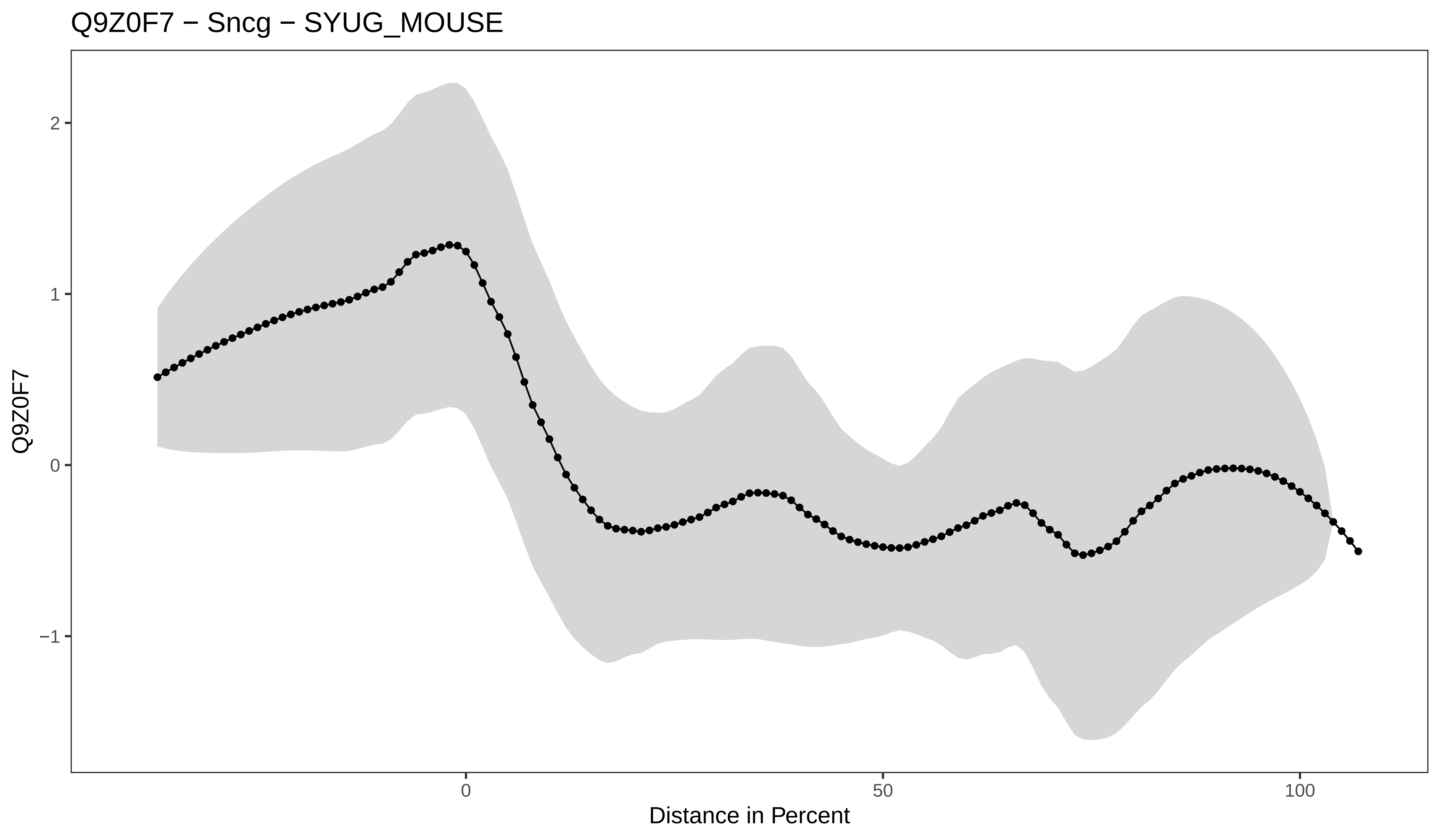
Q9Z0F7 − Sncg − SYUG_MOUSE
Q9Z0F7
2
1
0
−1
0
50
100
Distance in Percent

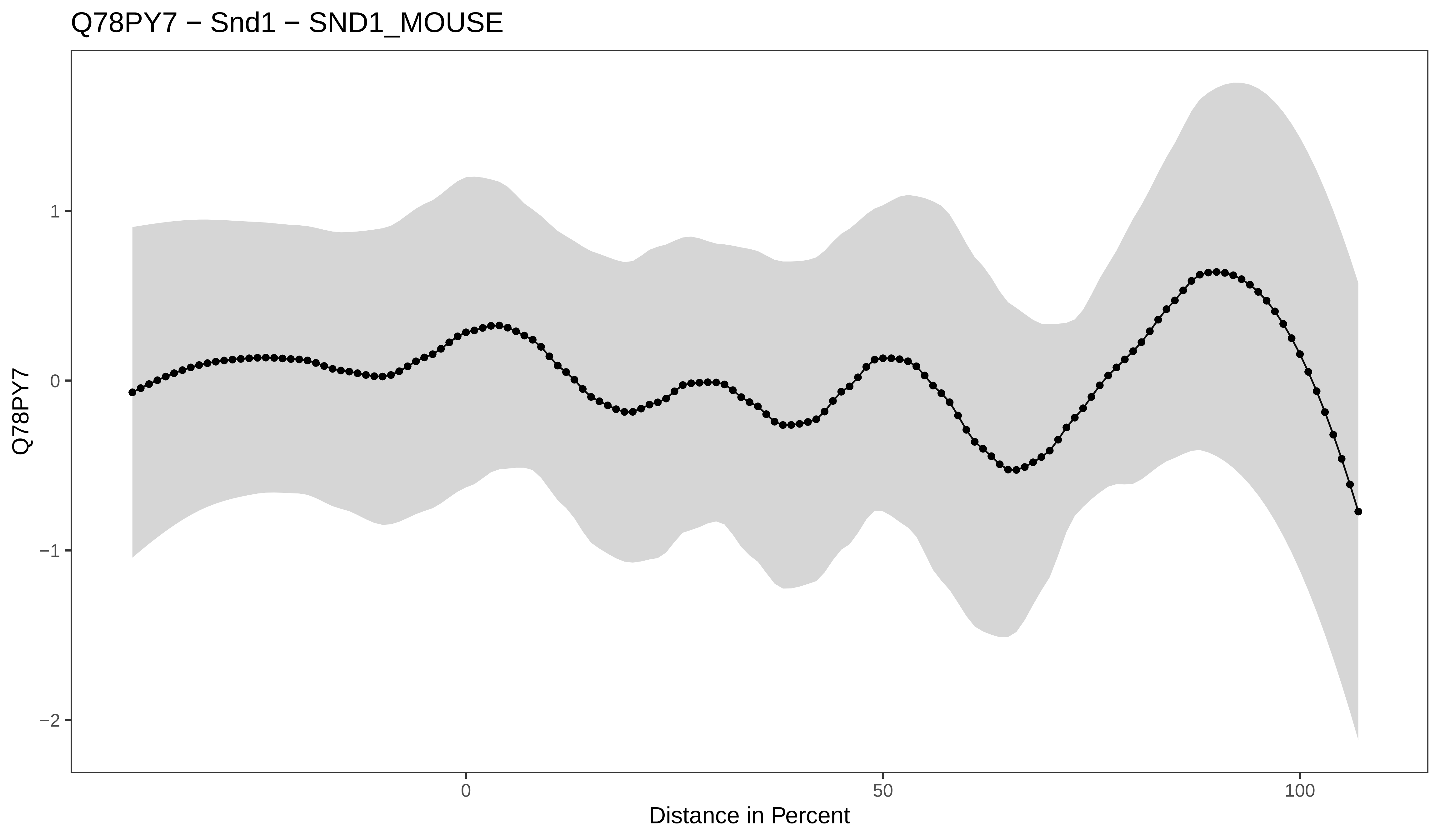
Q78PY7 − Snd1 − SND1_MOUSE
Q78PY7
1
0
−1
−2
0
50
100
Distance in Percent

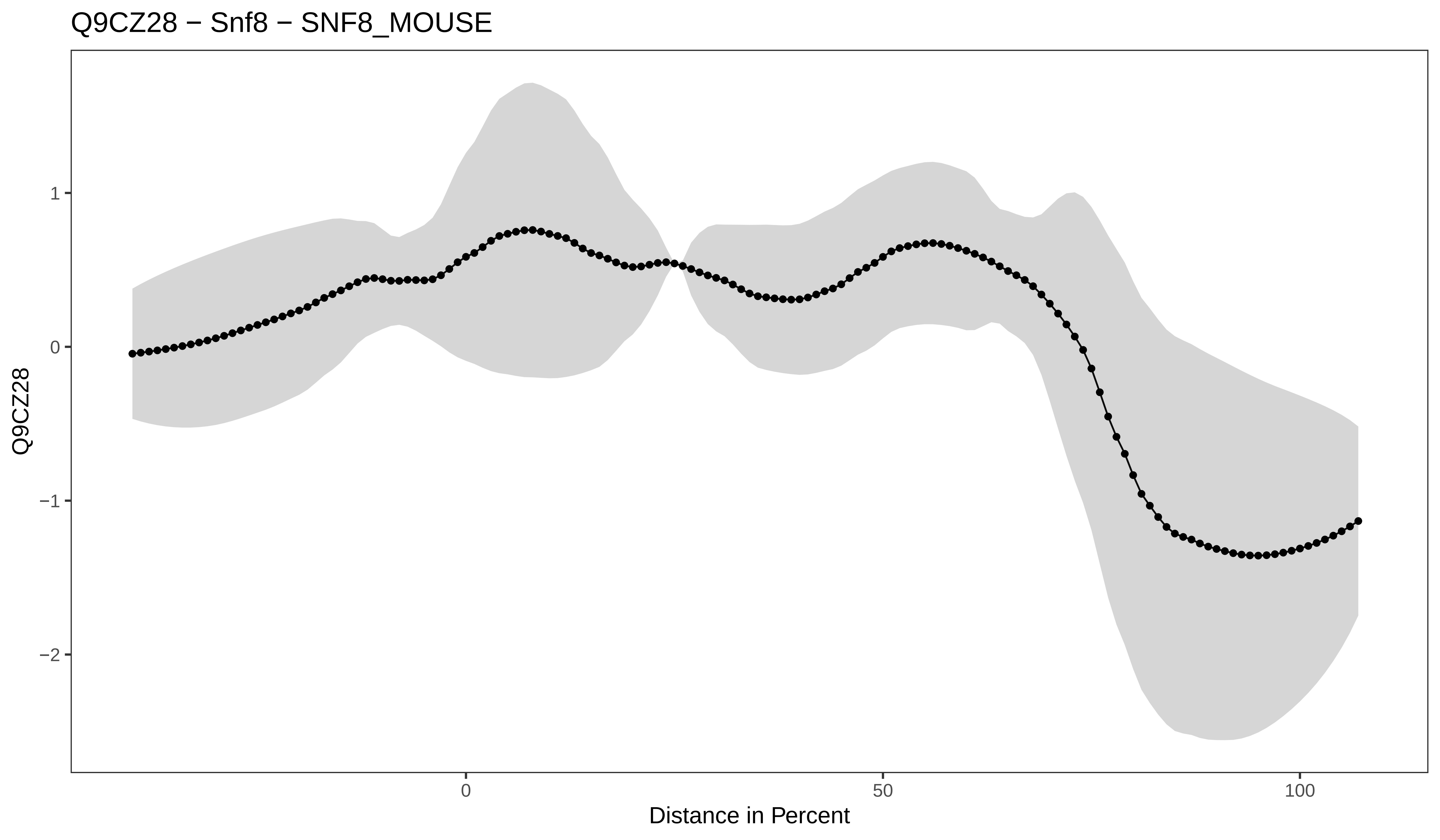
Q9CZ28 − Snf8 − SNF8_MOUSE
1
0
−1
−2
Q9CZ28
0
50
100
Distance in Percent

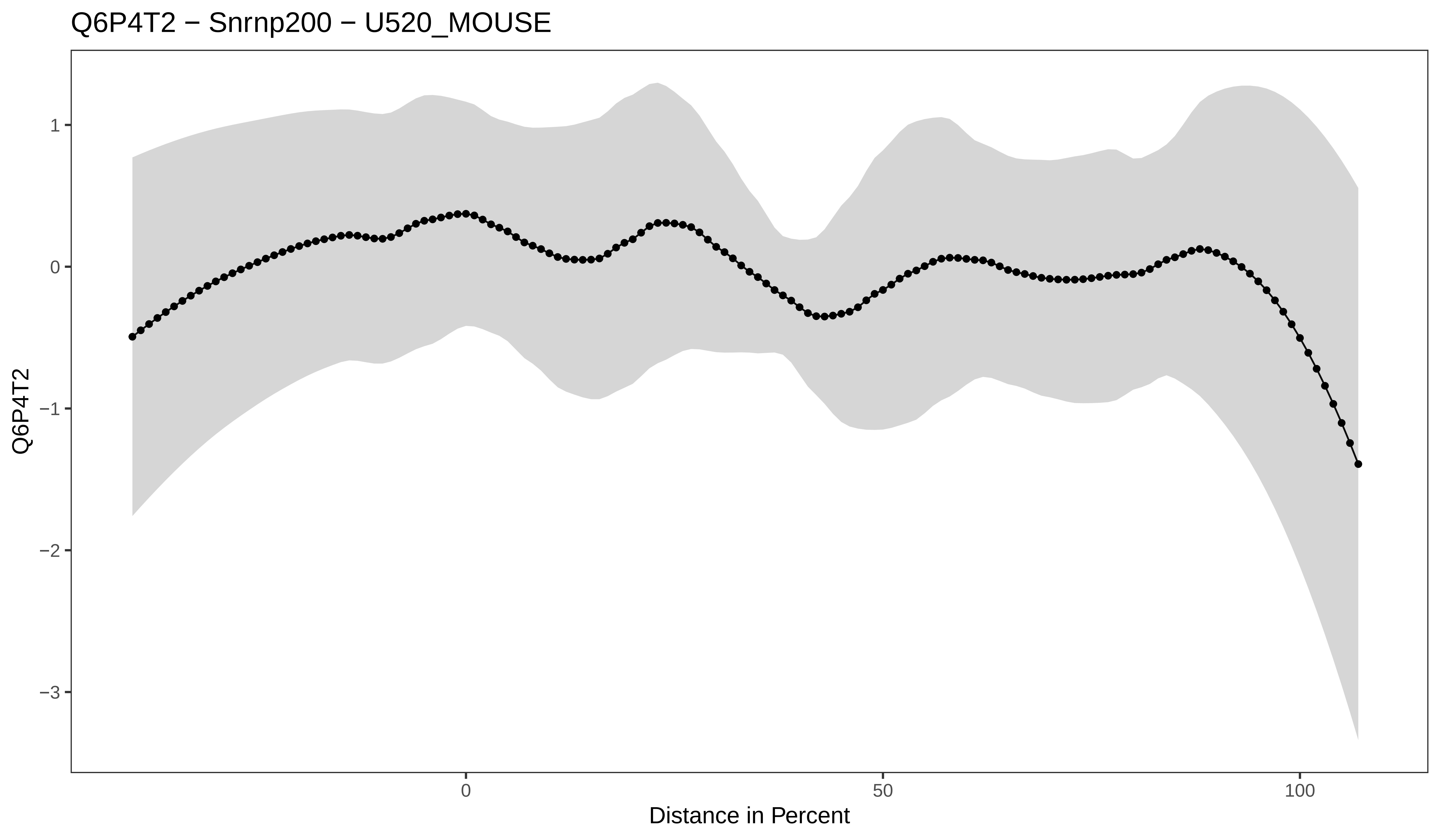

Q6P4T2 − Snrnp200 − U520_MOUSE
Q6P4T2
Distance in Percent
1
0
−1
−2
−3
0
50
100

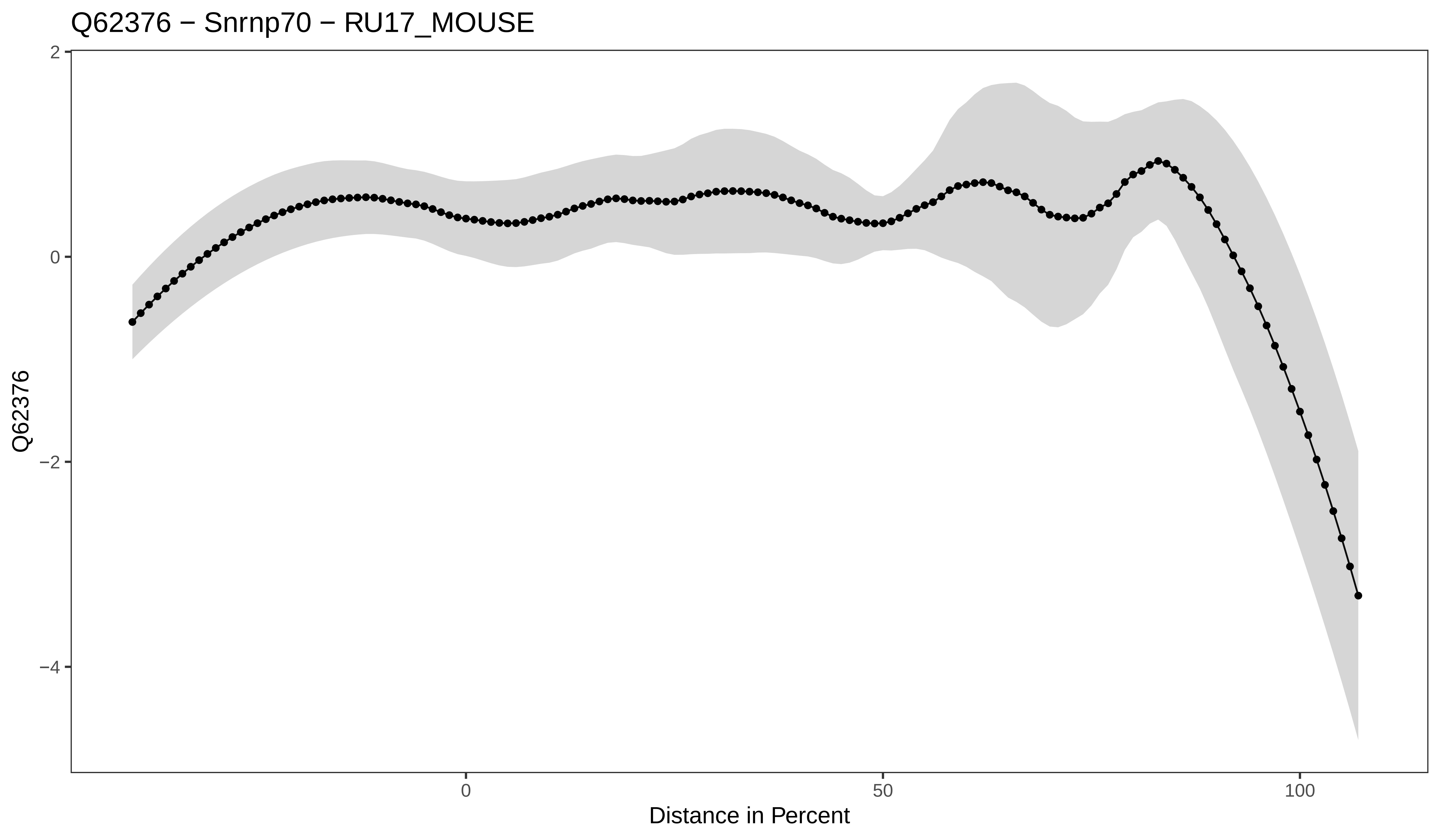

Q62376 − Snrnp70 − RU17_MOUSE
2
0
−2
−4
Q62376
0
50
100
Distance in Percent

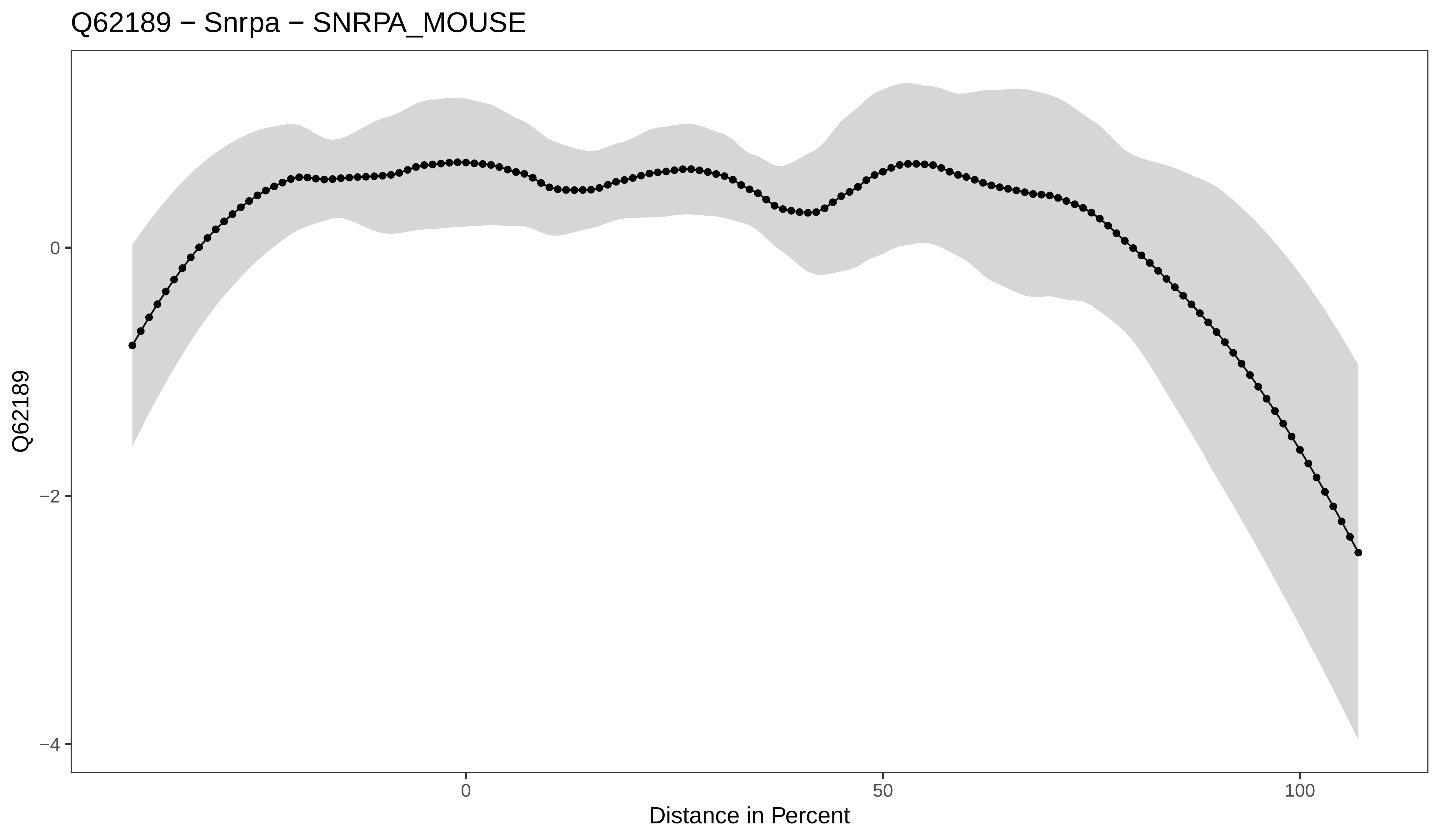

Q62189 − Snrpa − SNRPA_MOUSE
Q62189
0
−2
−4
0
50
100
Distance in Percent

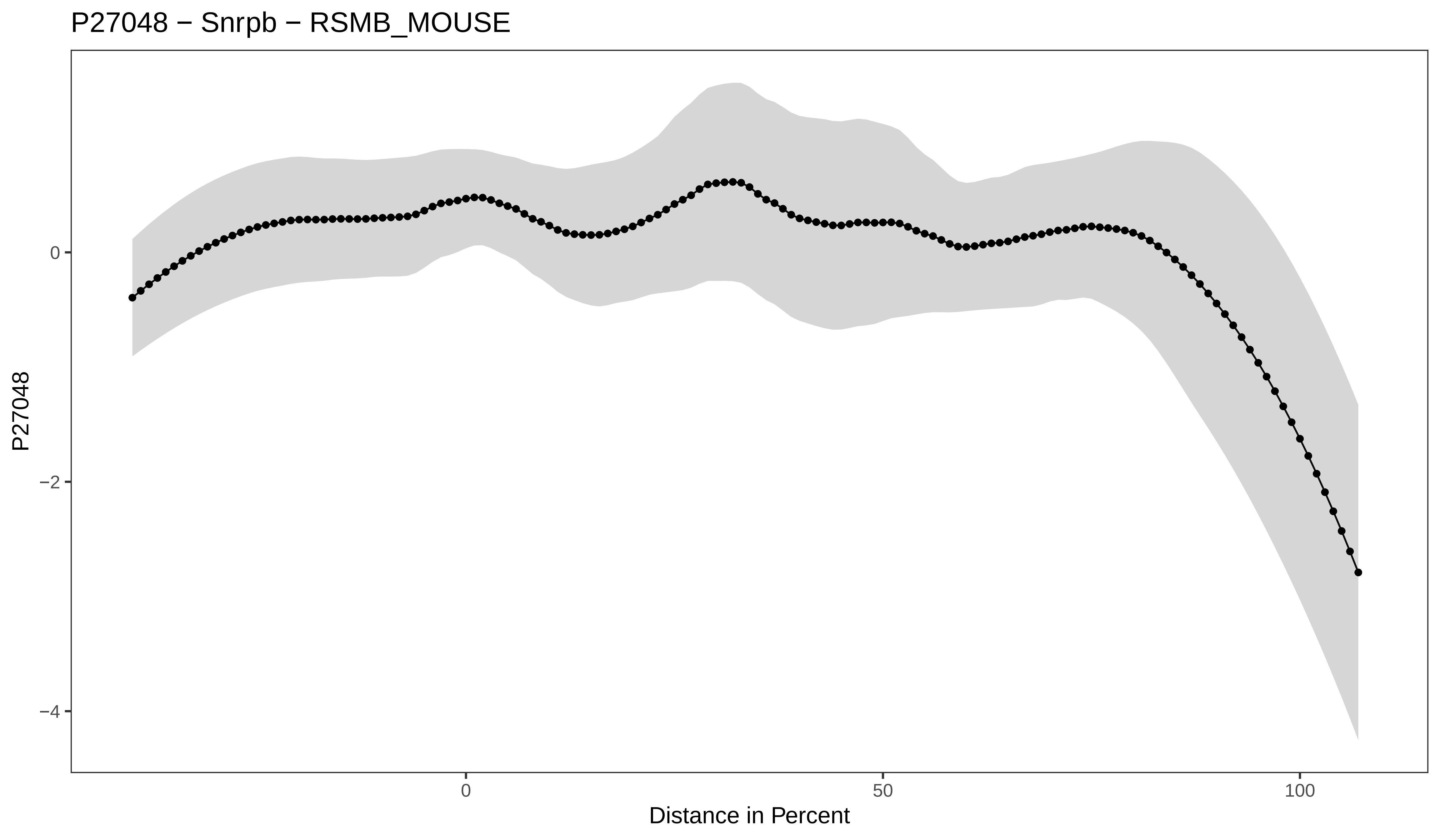
P27048 − Snrpb − RSMB_MOUSE
P27048
0
−2
−4
0
50
100
Distance in Percent

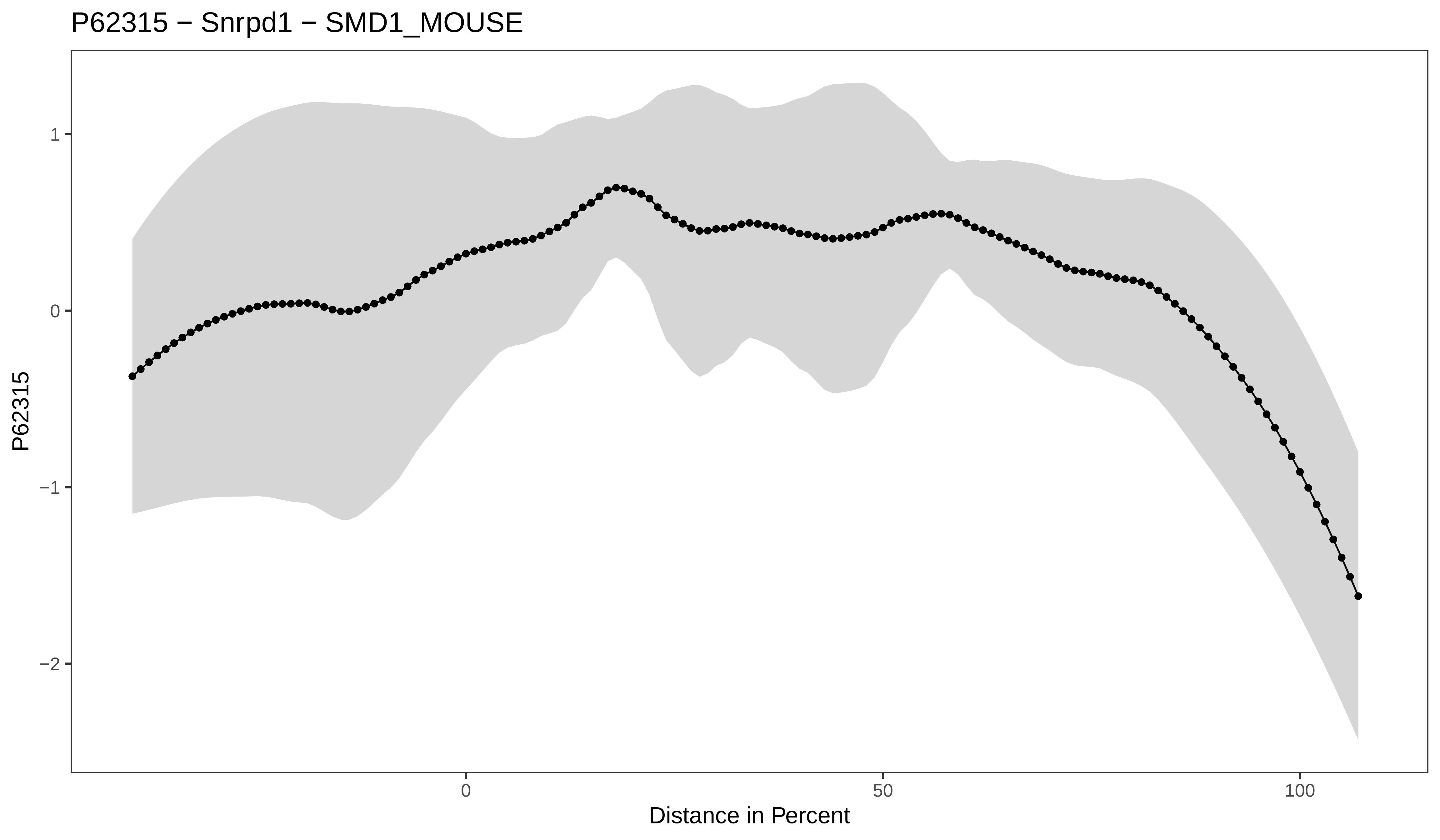

P62315 − Snrpd1 − SMD1_MOUSE
1
0
−1
−2
P62315
0
50
100
Distance in Percent

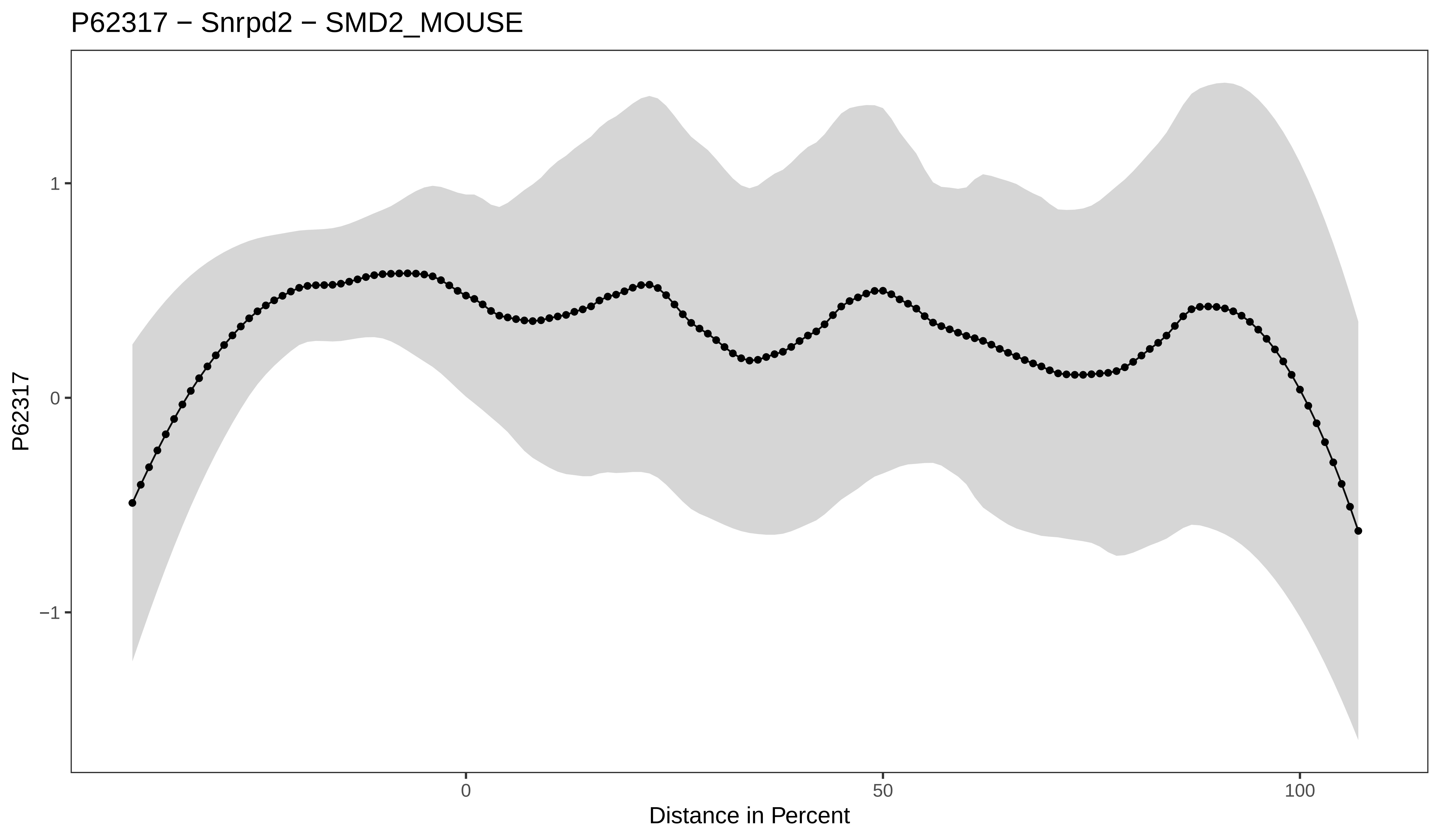
P62317 − Snrpd2 − SMD2_MOUSE
P62317
1
0
−1
0
50
100
Distance in Percent

P62320 − Snrpd3 − SMD3_MOUSE

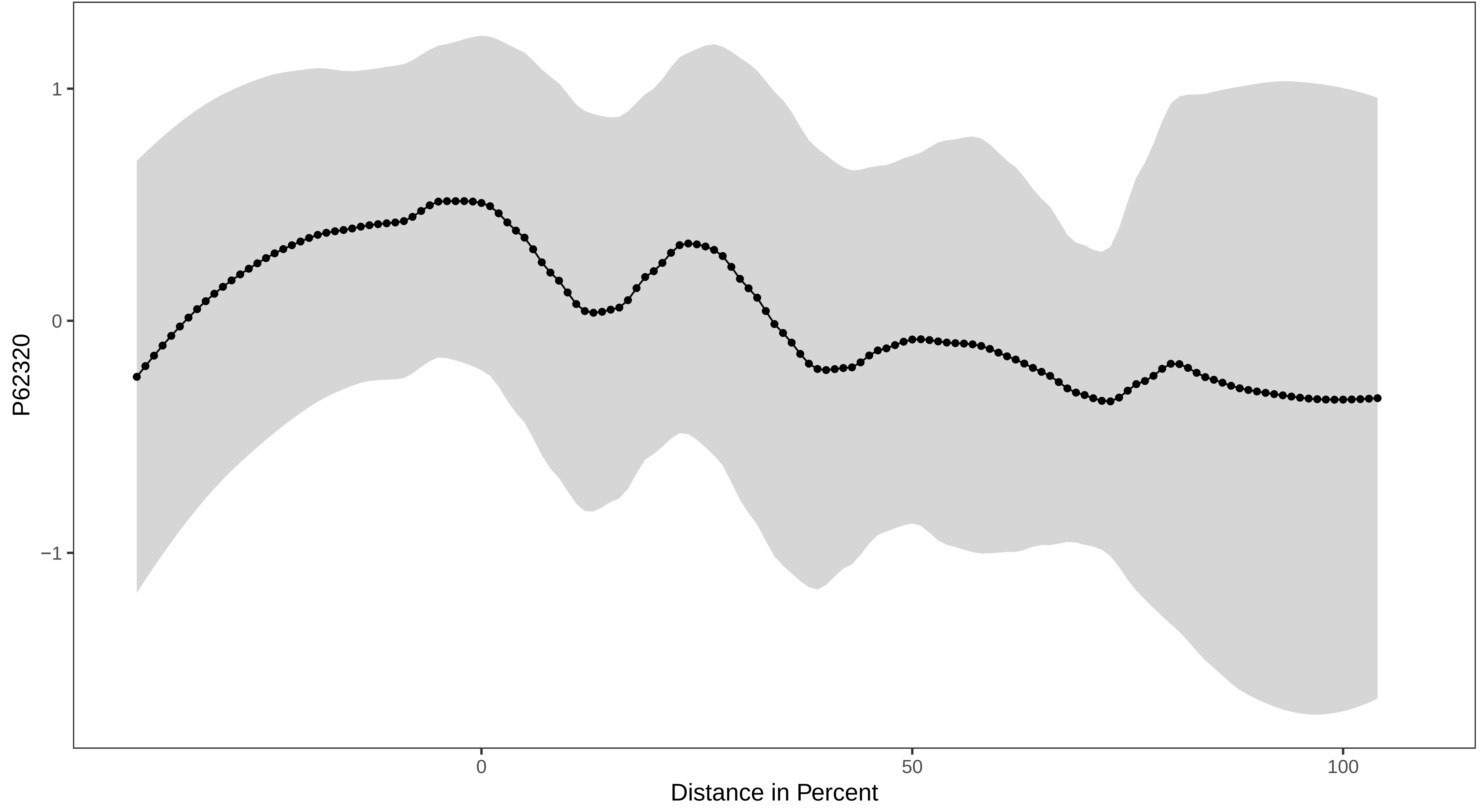

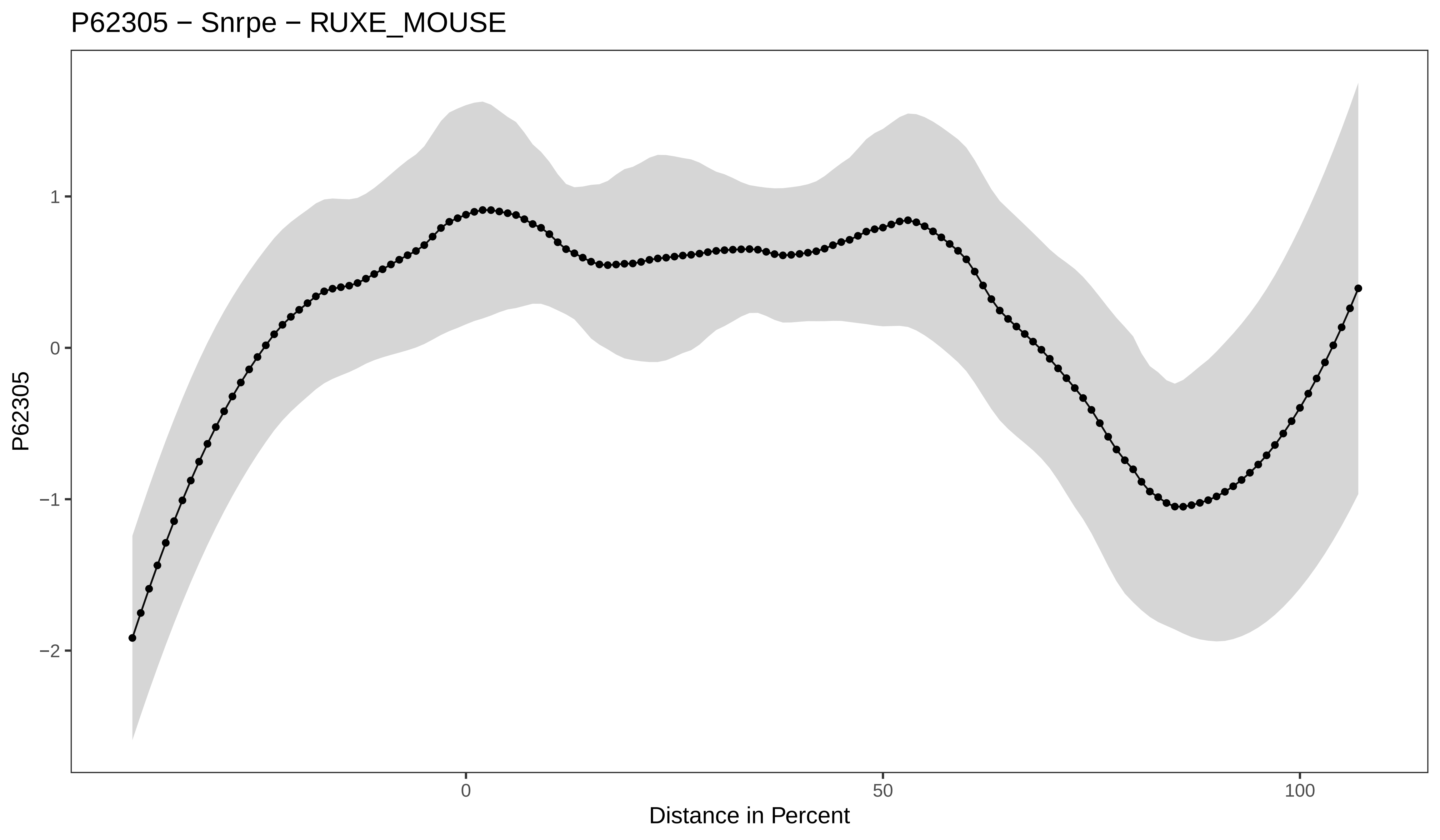
P62305 − Snrpe − RUXE_MOUSE
1
0
−1
−2
P62305
0
50
100
Distance in Percent

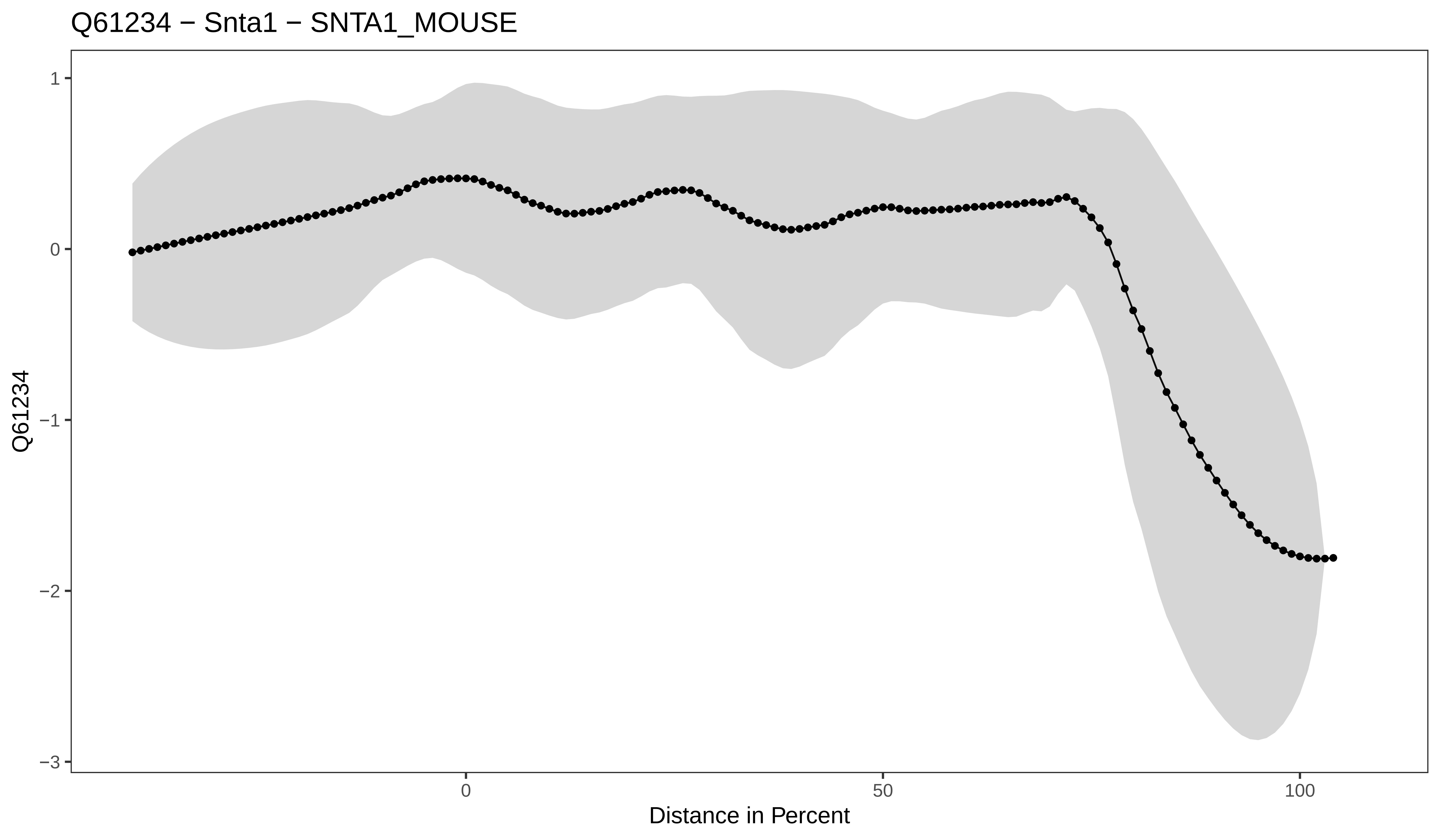
Q61234 − Snta1 − SNTA1_MOUSE
Q61234
1
0
−1
−2
−3
0
50
100
Distance in Percent

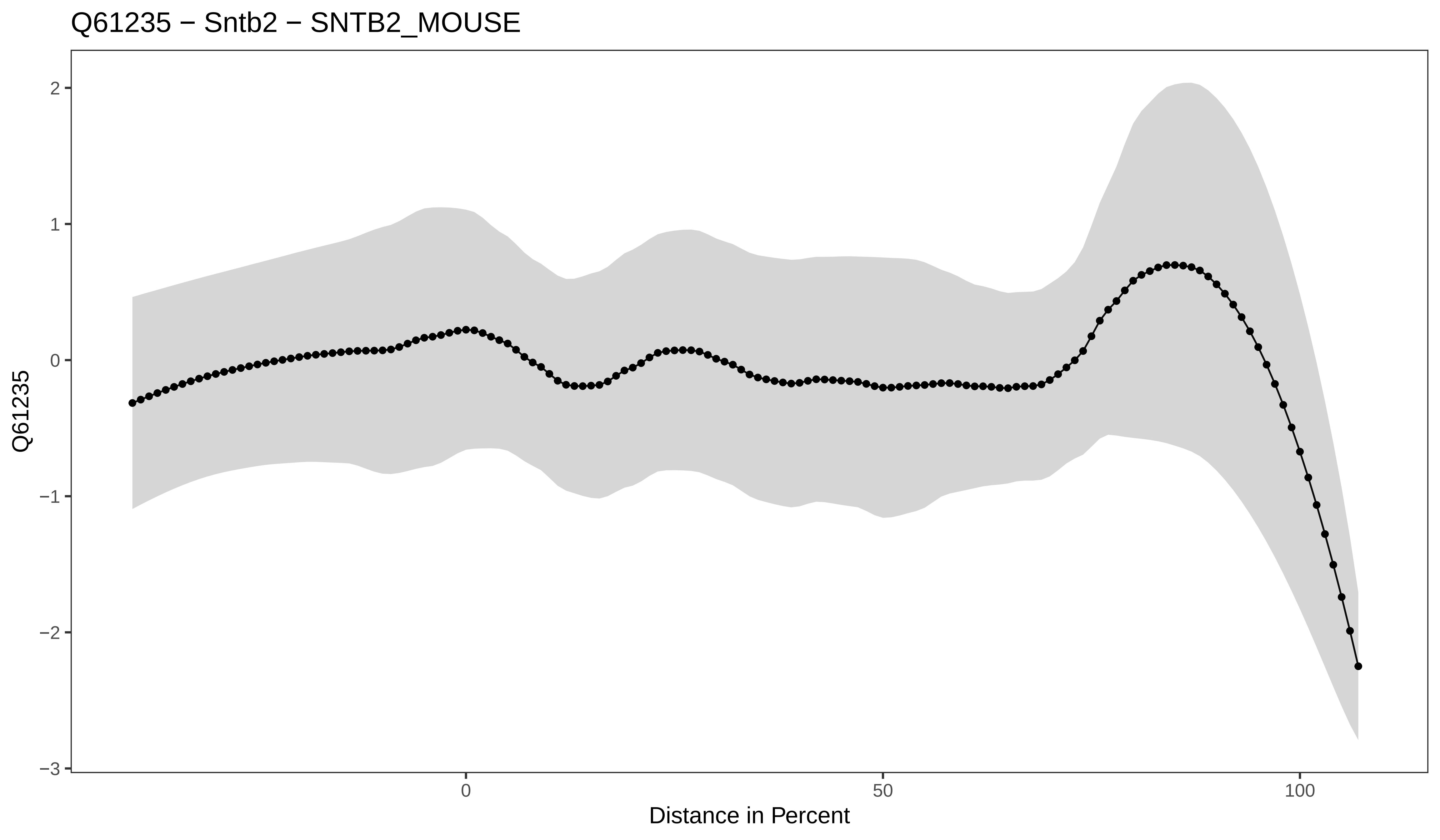
Q61235 − Sntb2 − SNTB2_MOUSE
Q61235
Distance in Percent
2
1
0
−1
−2
−3
0
50
100

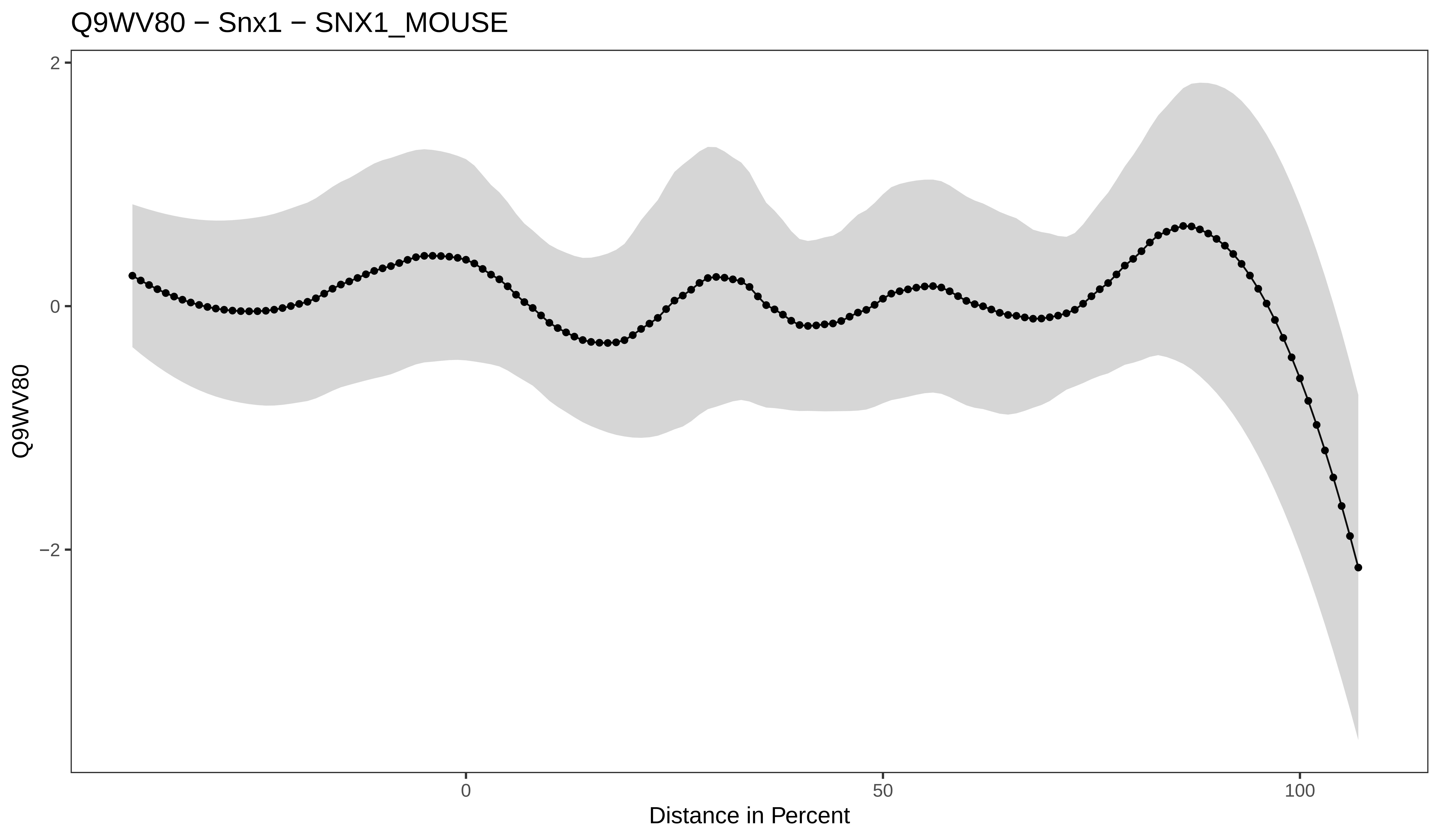

Q9WV80 − Snx1 − SNX1_MOUSE
2
0
−2
Q9WV80
0
50
100
Distance in Percent

Q9CWK8 − Snx2 − SNX2_MOUSE

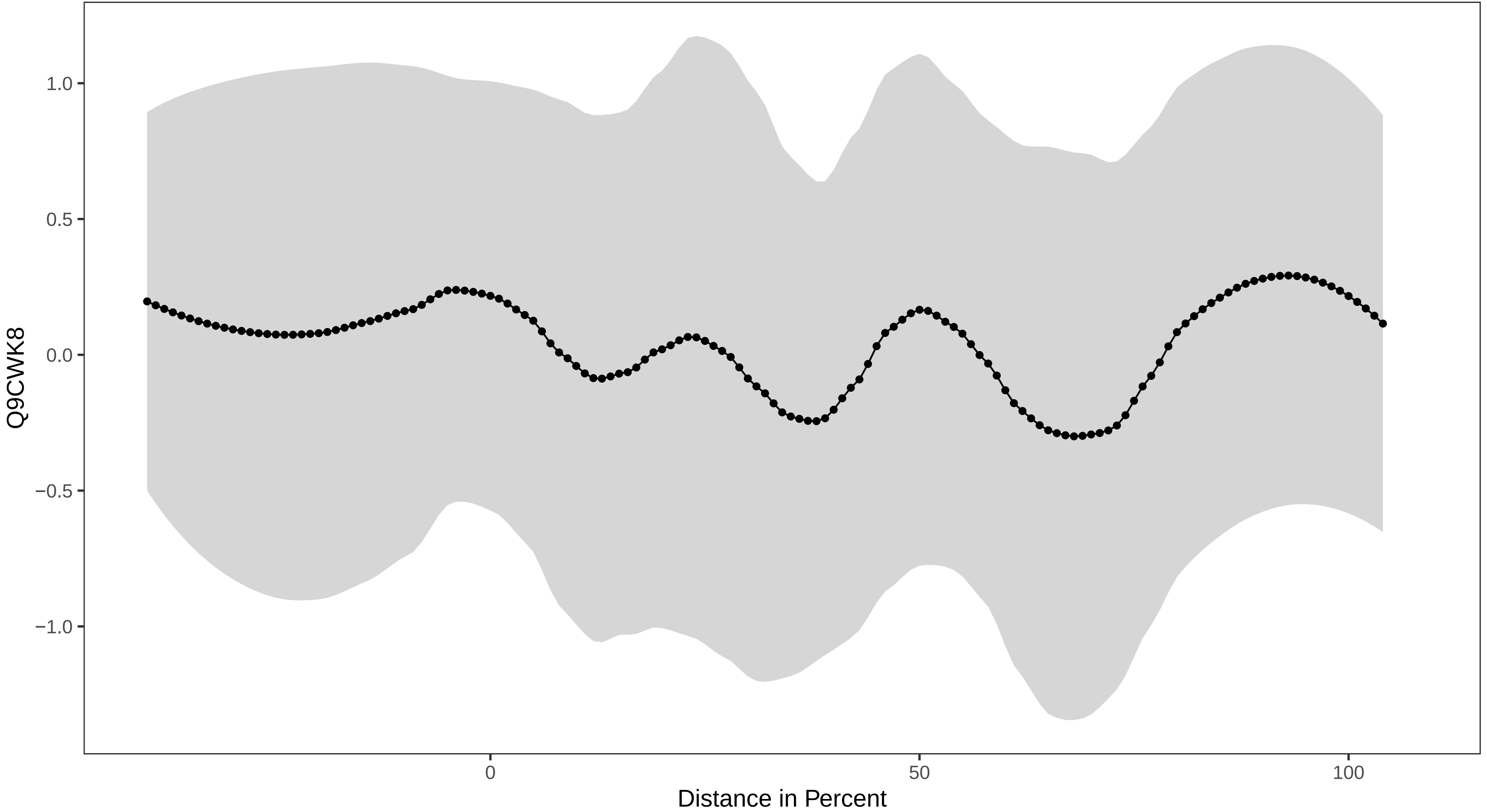

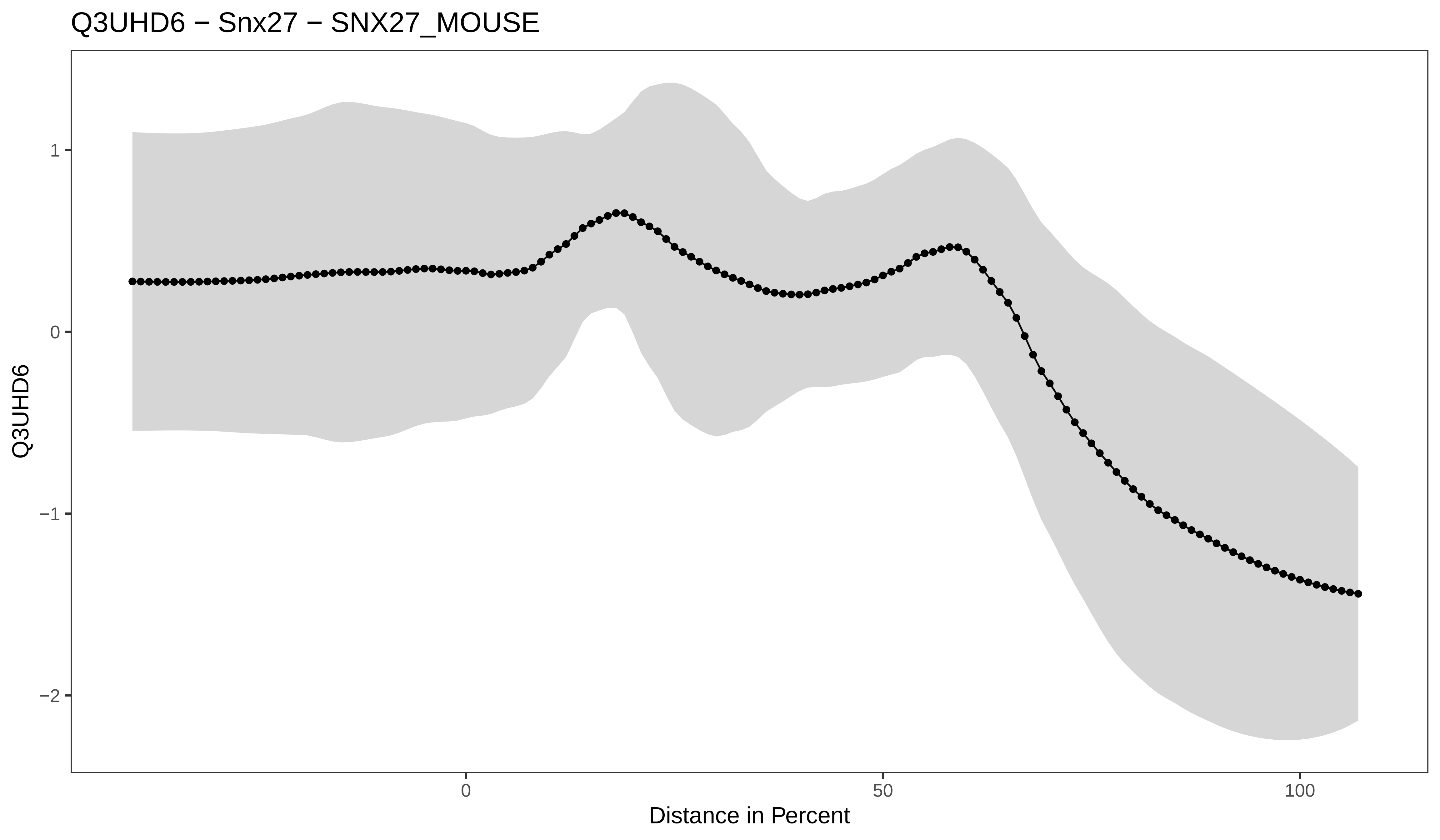
Q3UHD6 − Snx27 − SNX27_MOUSE
Q3UHD6
1
0
−1
−2
0
50
100
Distance in Percent

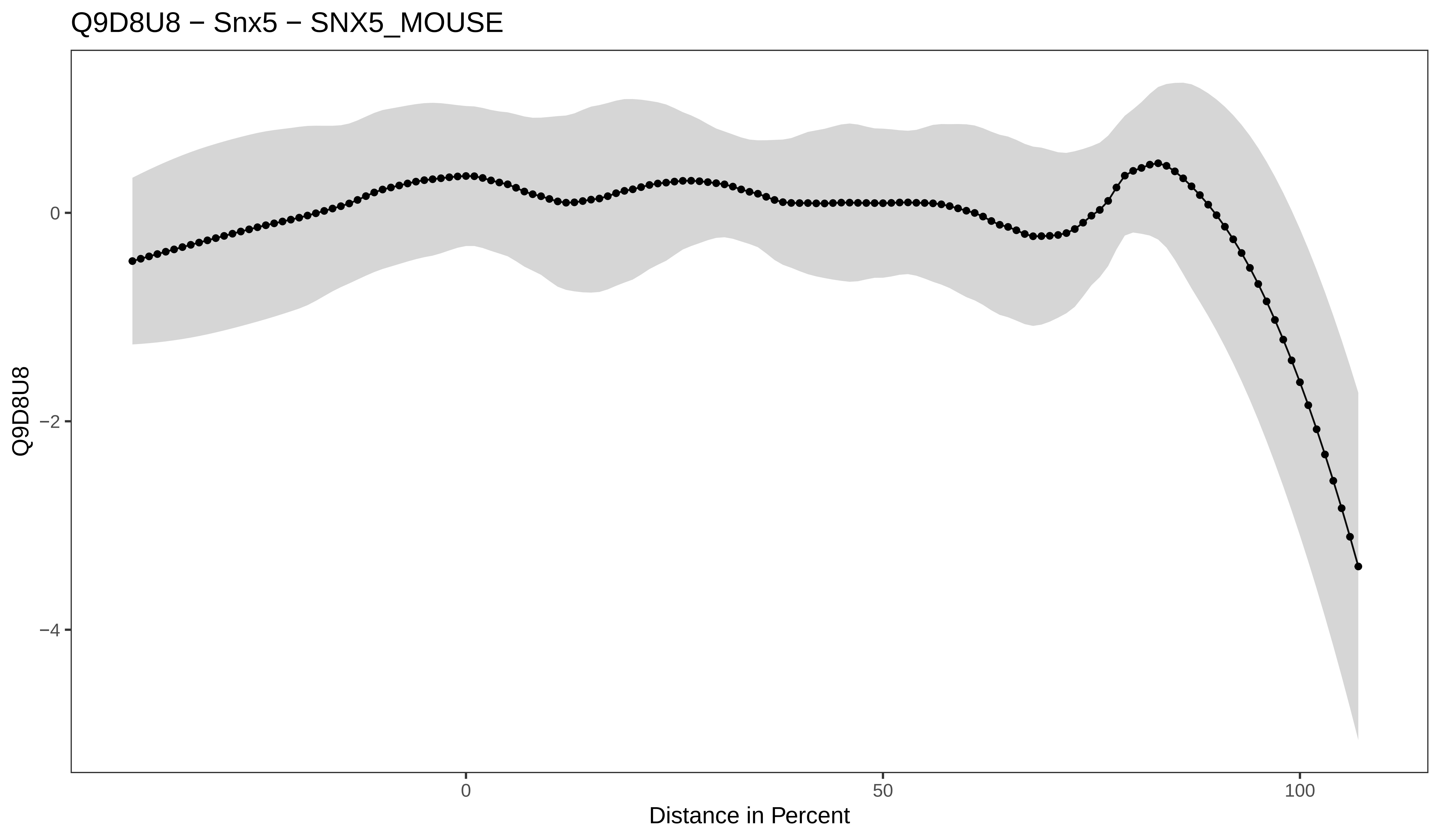
Q9D8U8 − Snx5 − SNX5_MOUSE
0
−2
−4
Q9D8U8
0
50
100
Distance in Percent

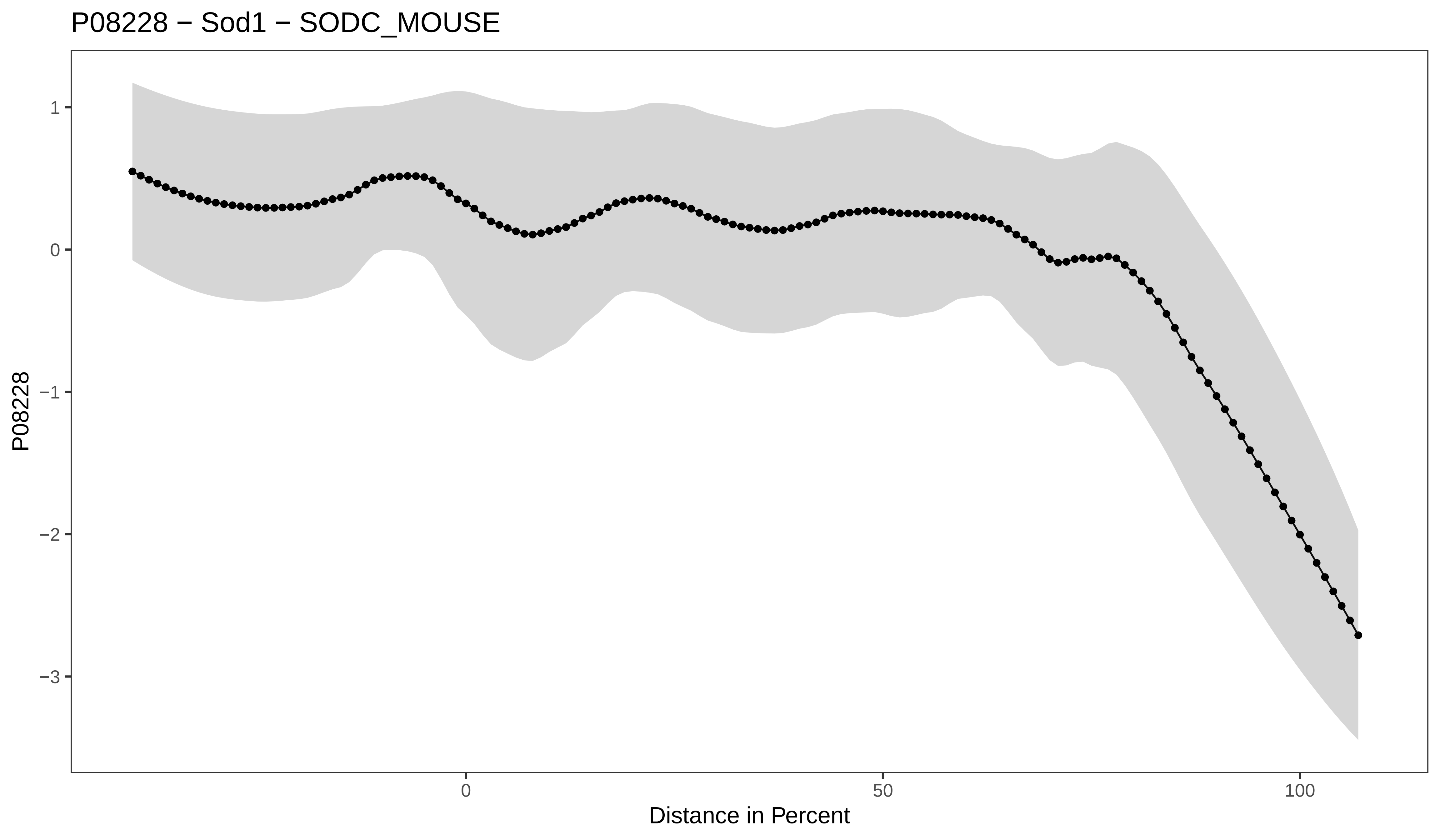

P08228 − Sod1 − SODC_MOUSE
1
0
−1
−2
−3
P08228
0
50
100
Distance in Percent

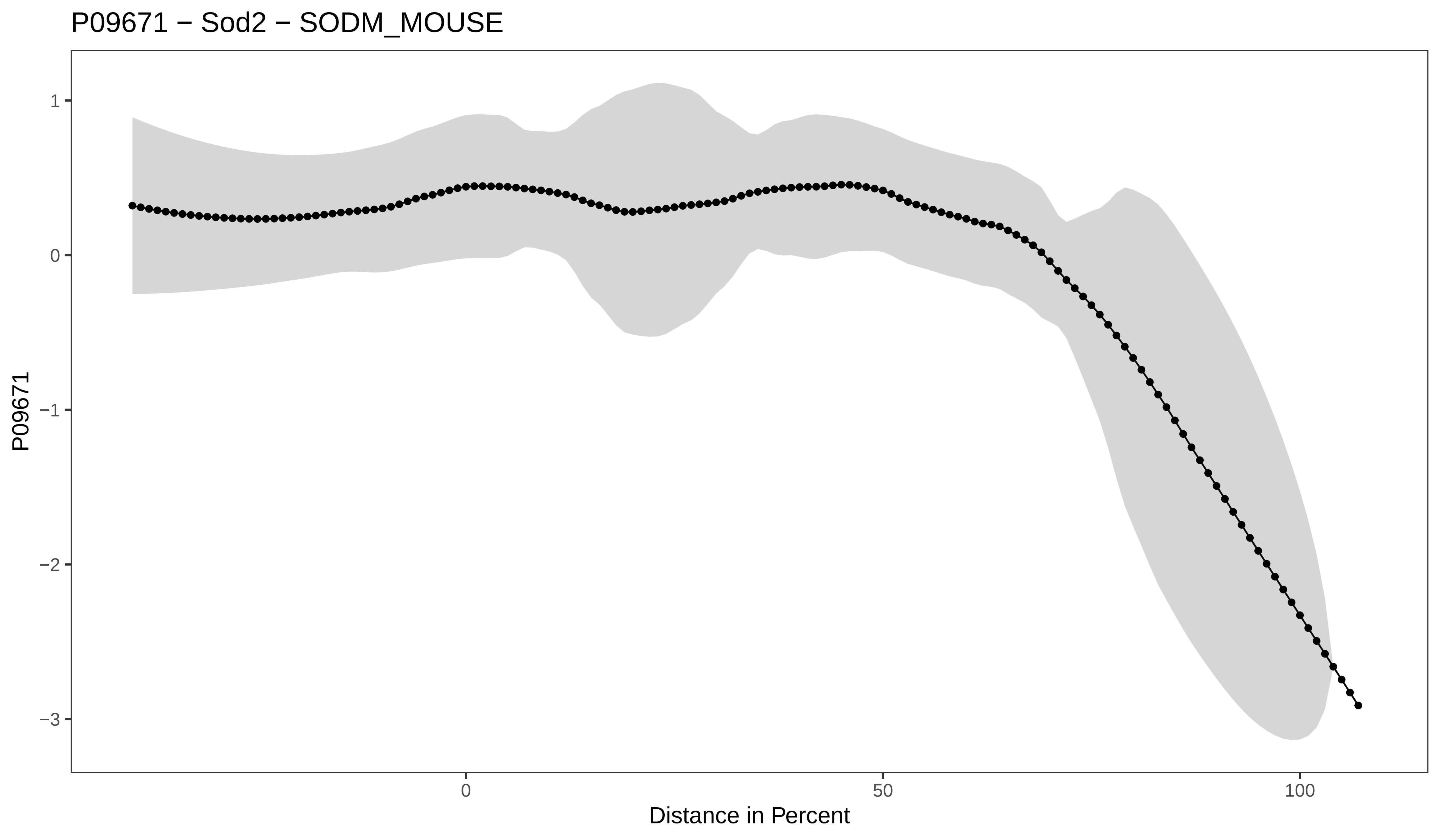
P09671 − Sod2 − SODM_MOUSE
1
0
−1
−2
−3
P09671
0
50
100
Distance in Percent

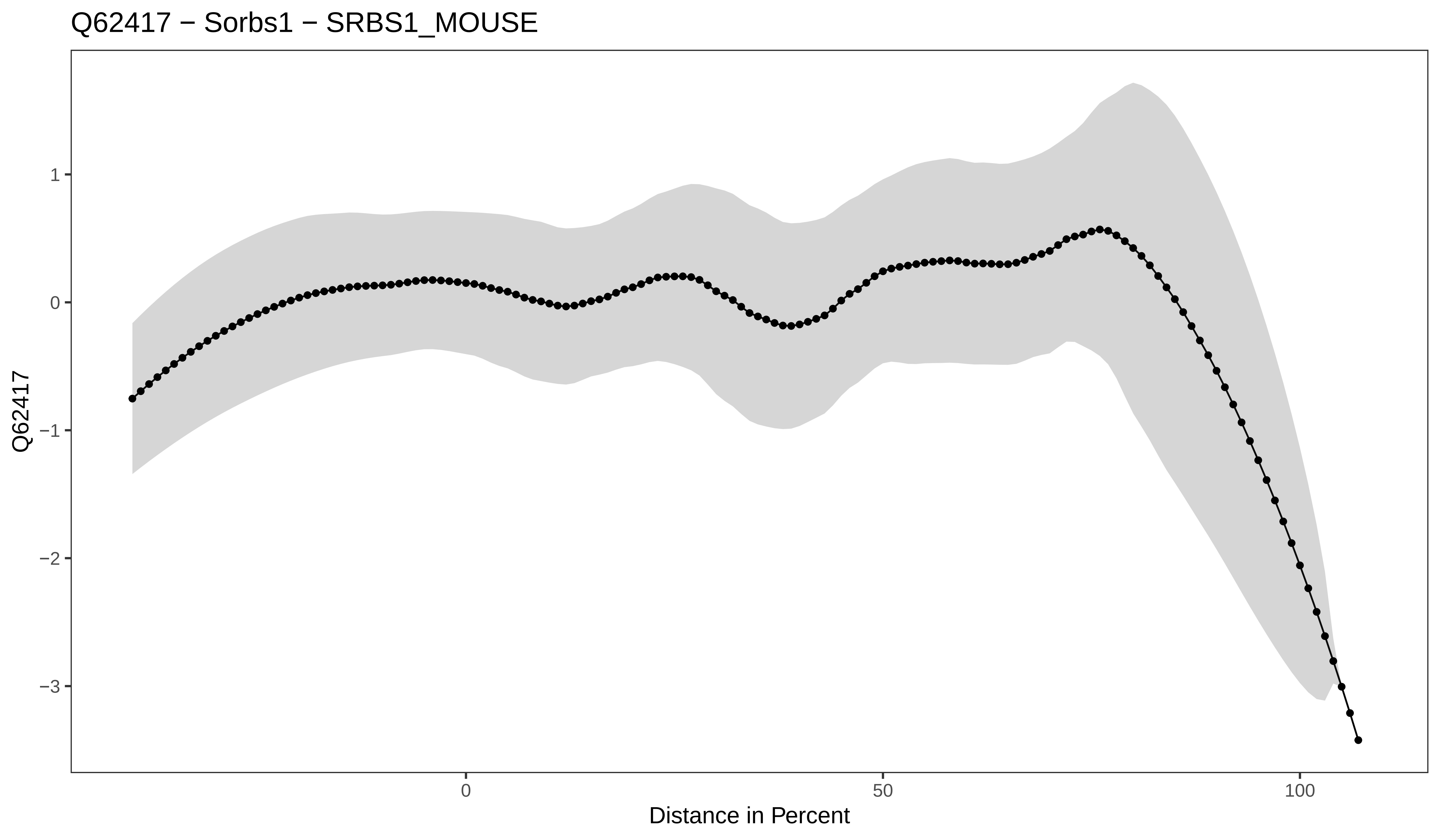
Q62417 − Sorbs1 − SRBS1_MOUSE
Q62417
1
0
−1
−2
−3
0
50
100
Distance in Percent

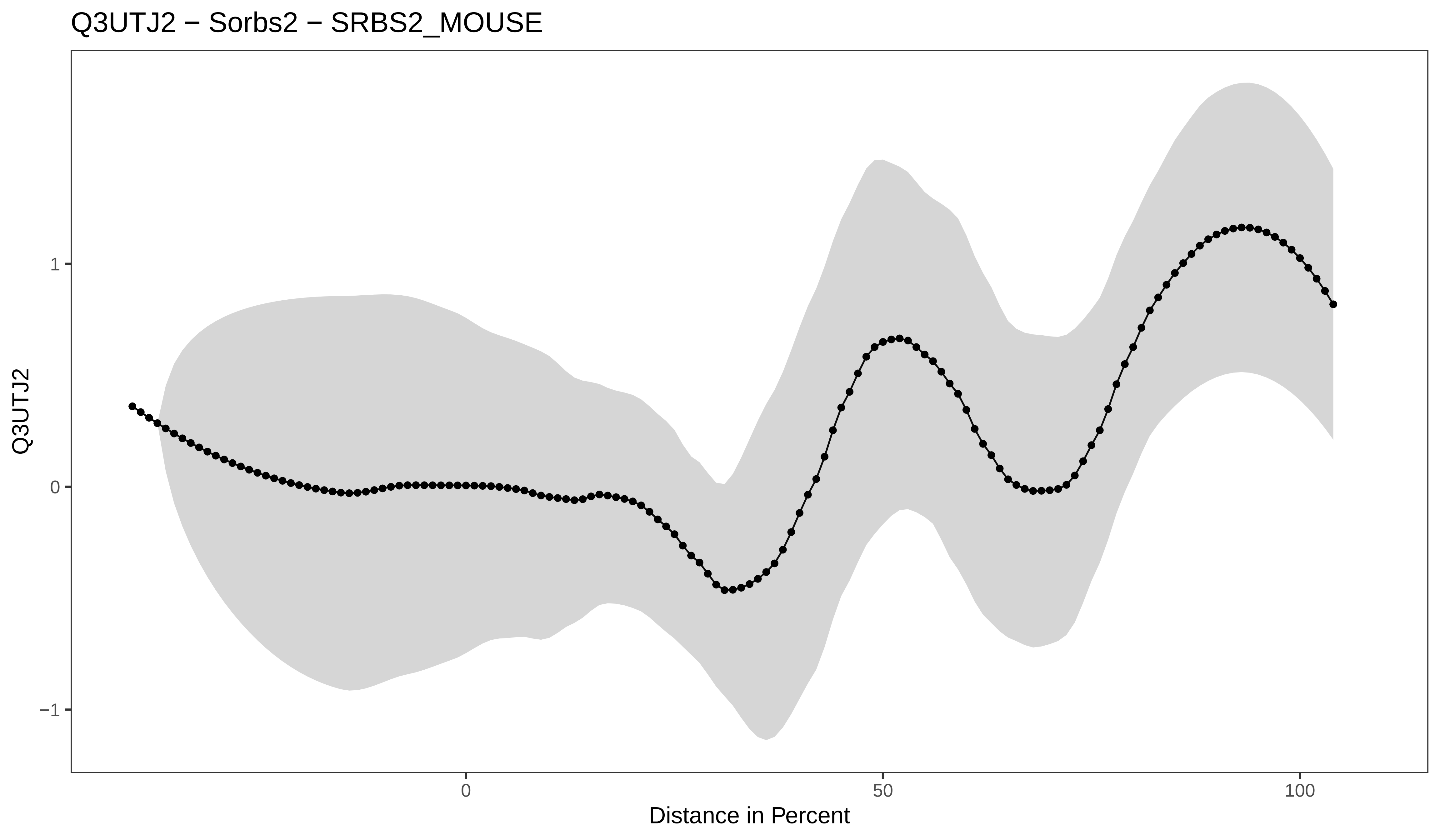
Q3UTJ2 − Sorbs2 − SRBS2_MOUSE
Q3UTJ2
1
0
−1
0
50
100
Distance in Percent

Q9R1Z8 − Sorbs3 − VINEX_MOUSE

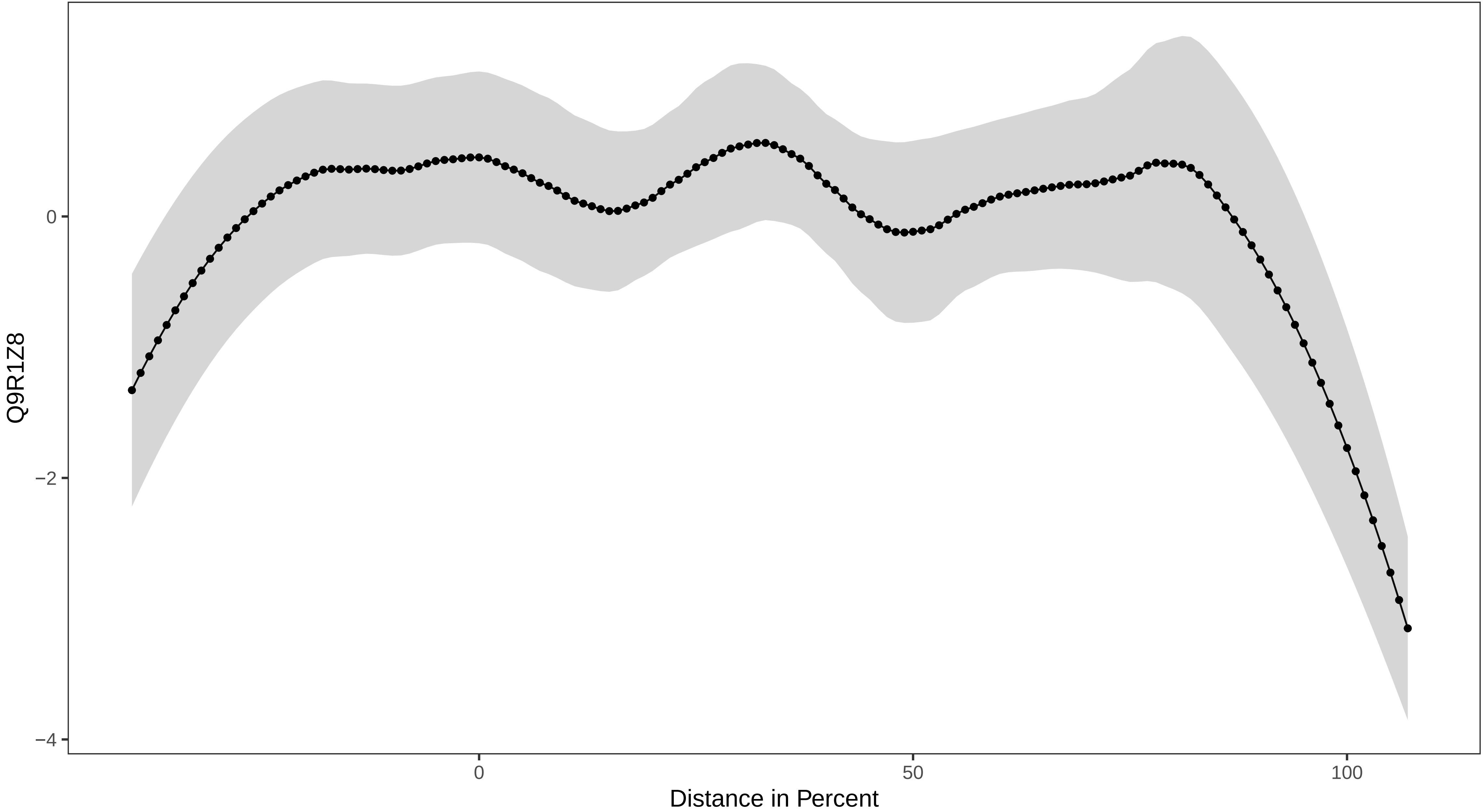

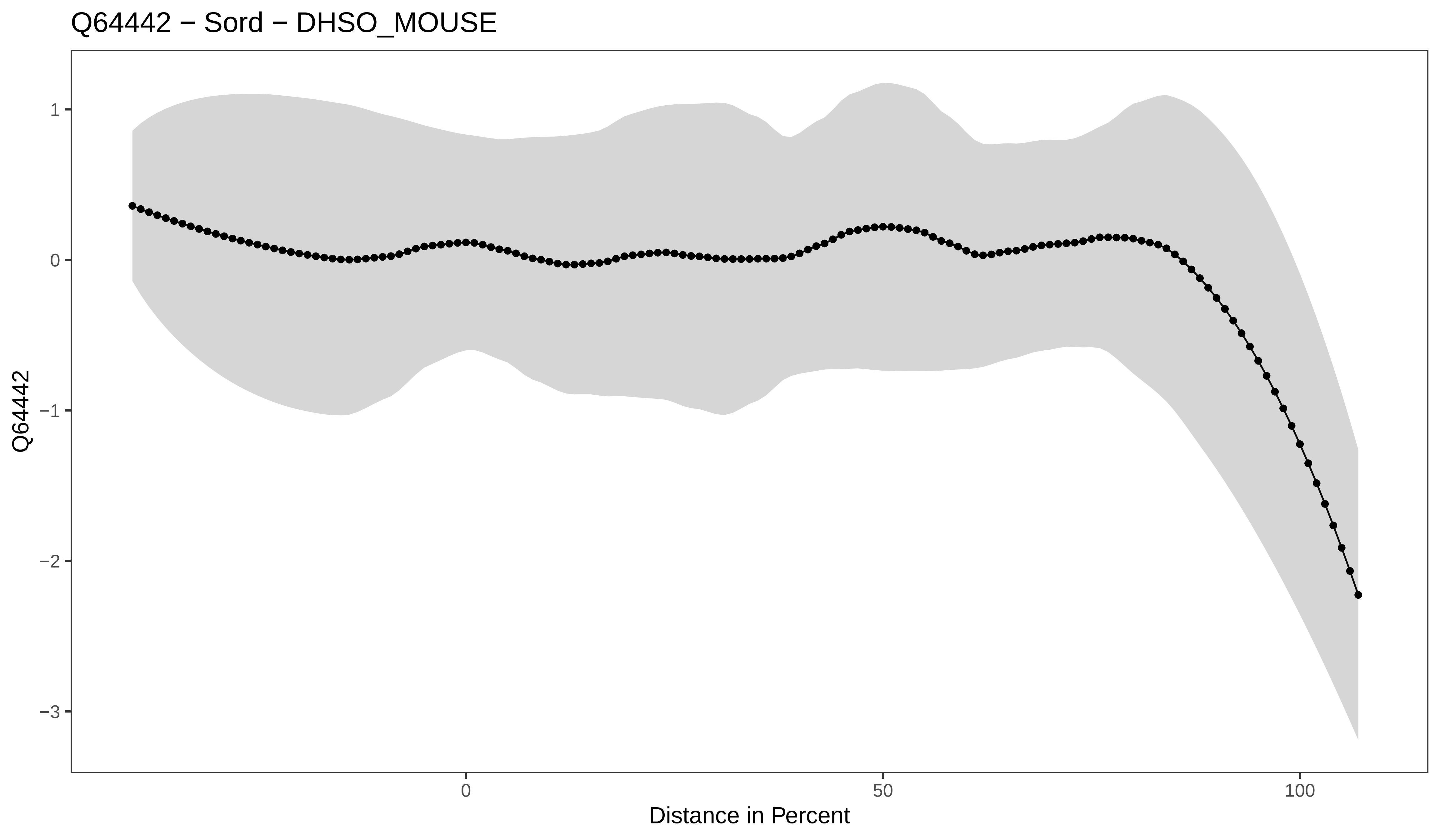

Q64442 − Sord − DHSO_MOUSE
1
0
−1
−2
−3
Q64442
0
50
100
Distance in Percent

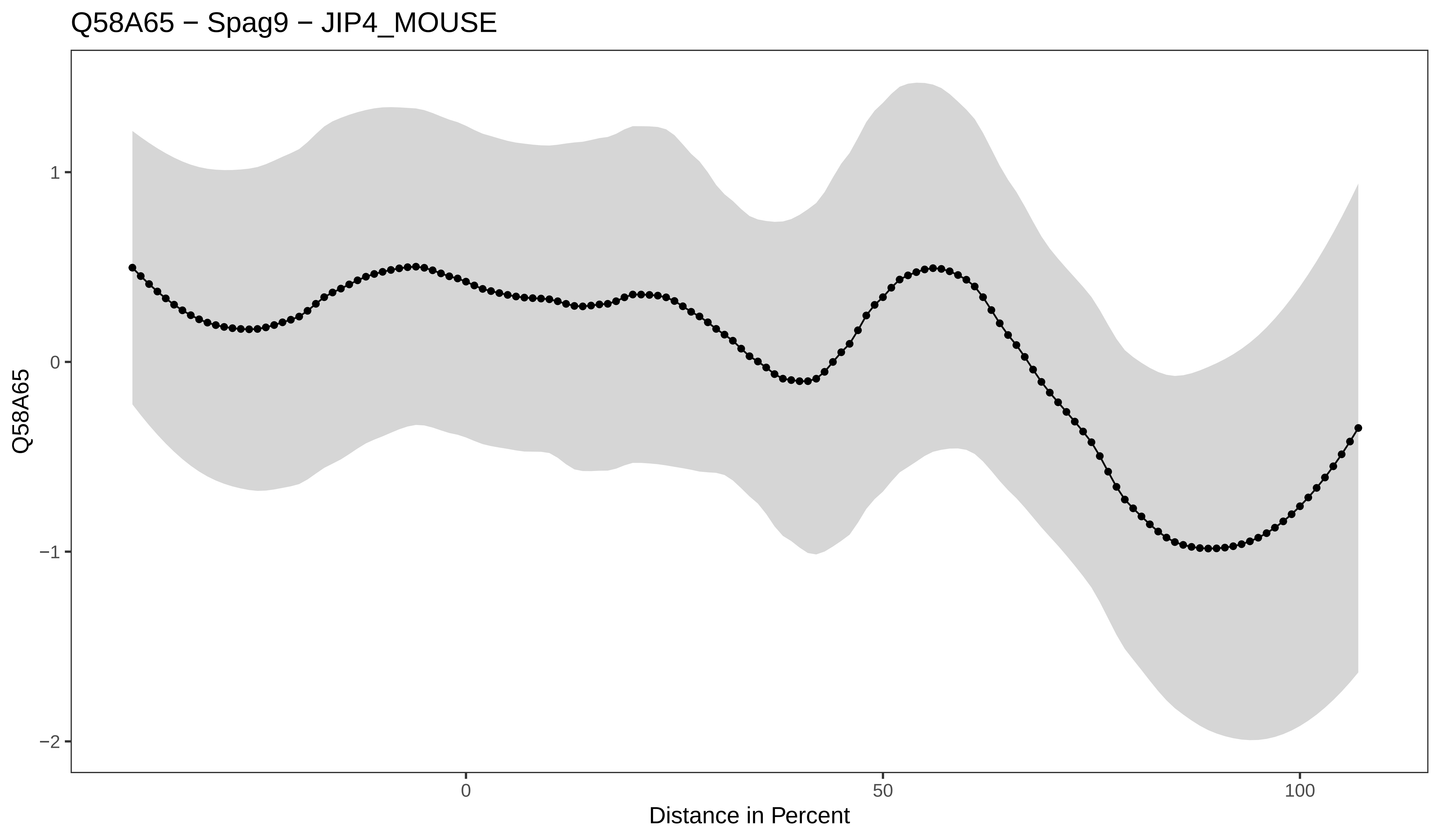

Q58A65 − Spag9 − JIP4_MOUSE
Q58A65
1
0
−1
−2
0
50
100
Distance in Percent

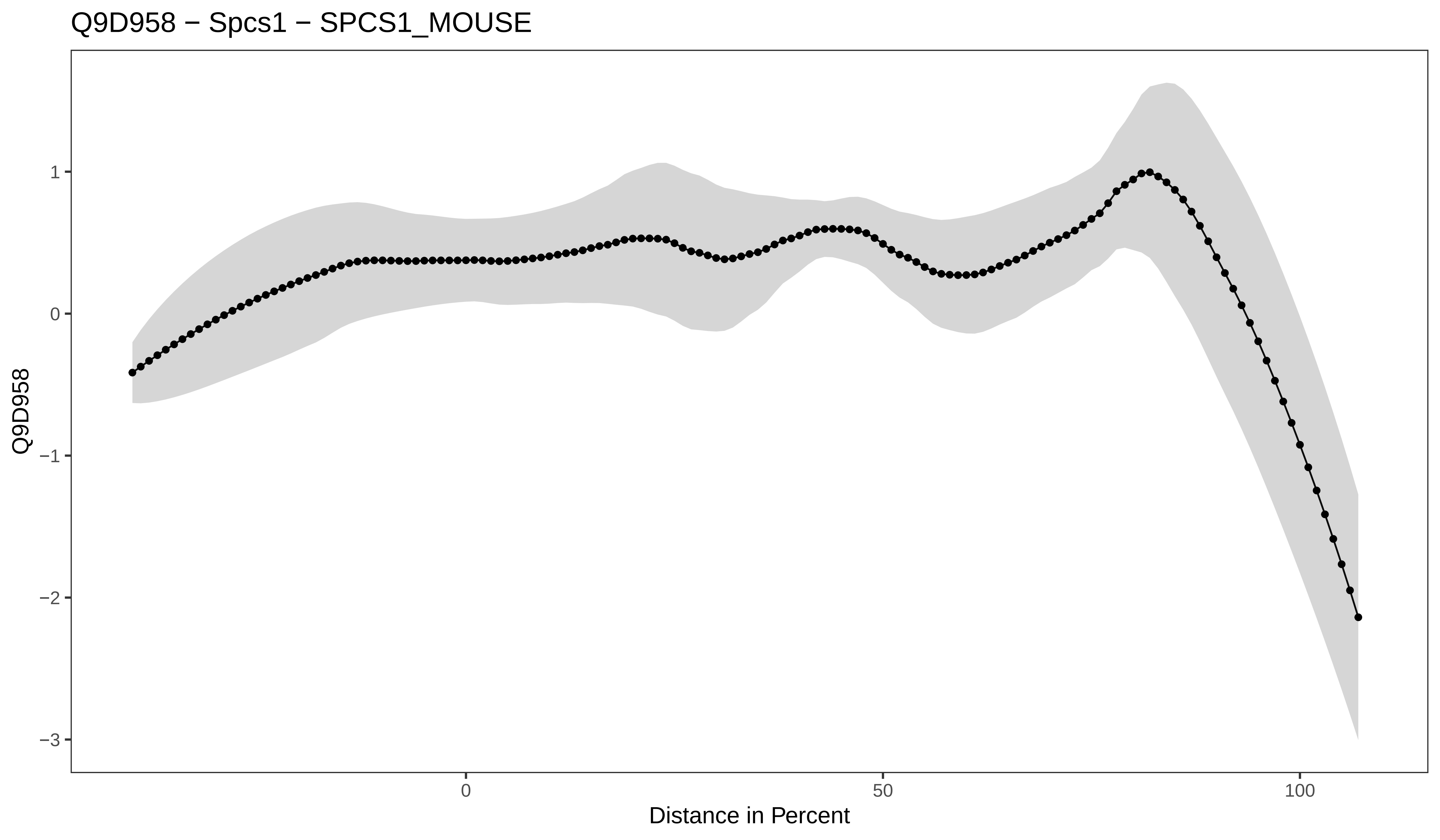

Q9D958 − Spcs1 − SPCS1_MOUSE
Q9D958
1
0
−1
−2
−3
0
50
100
Distance in Percent

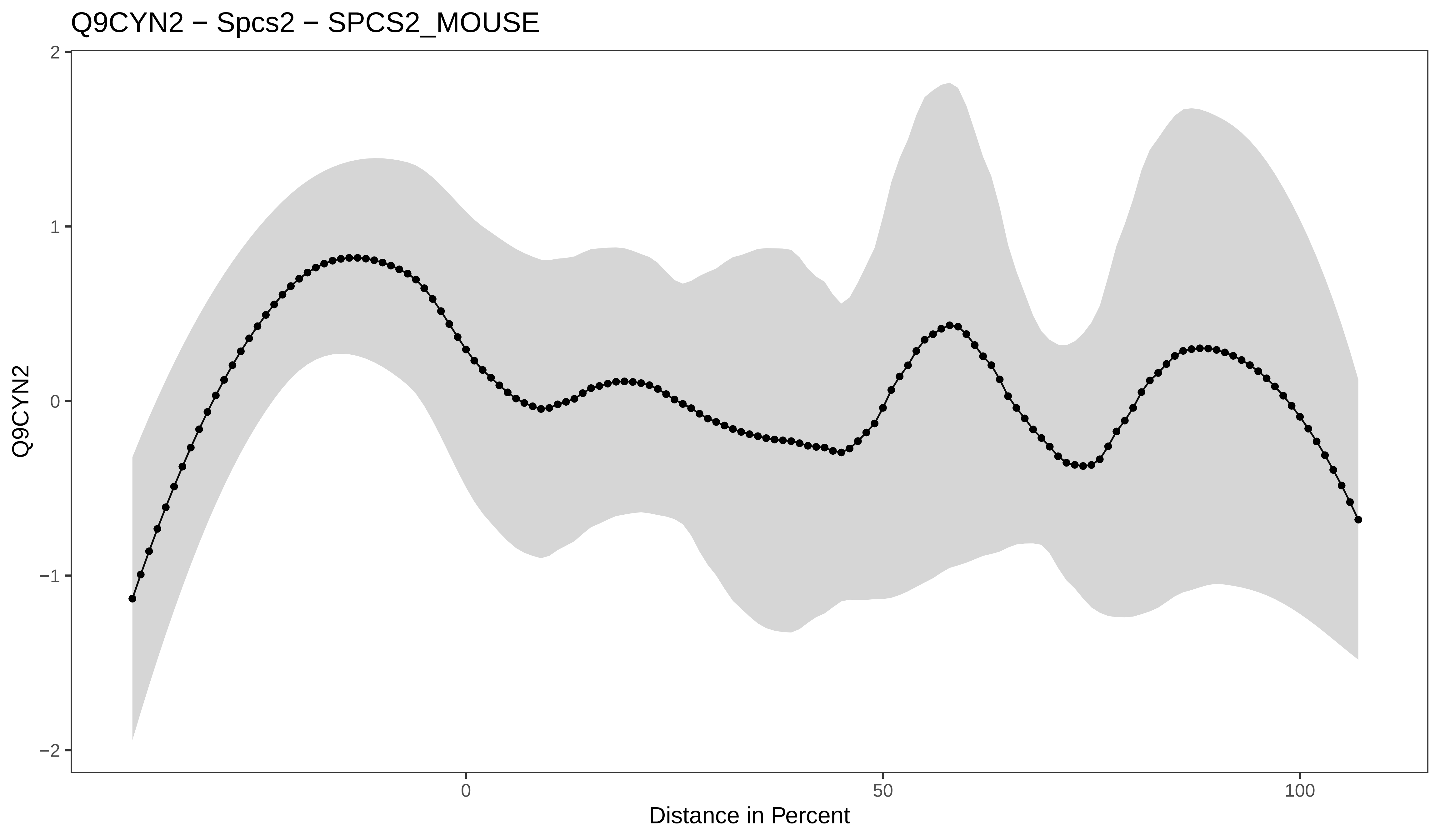
Q9CYN2 − Spcs2 − SPCS2_MOUSE
Q9CYN2
2
1
0
−1
−2
0
50
100
Distance in Percent

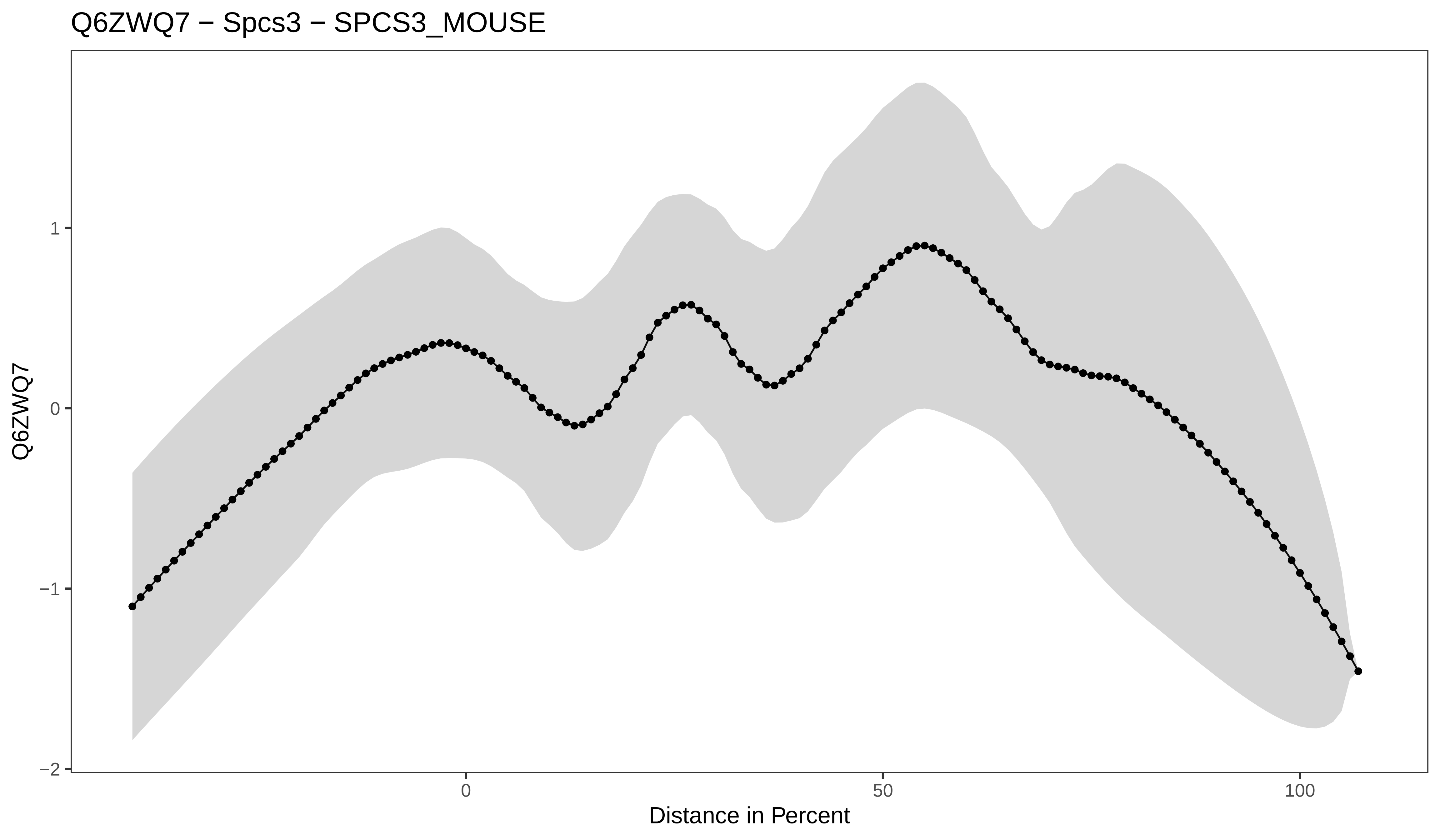

Q6ZWQ7 − Spcs3 − SPCS3_MOUSE
Q6ZWQ7
1
0
−1
−2
0
50
100
Distance in Percent

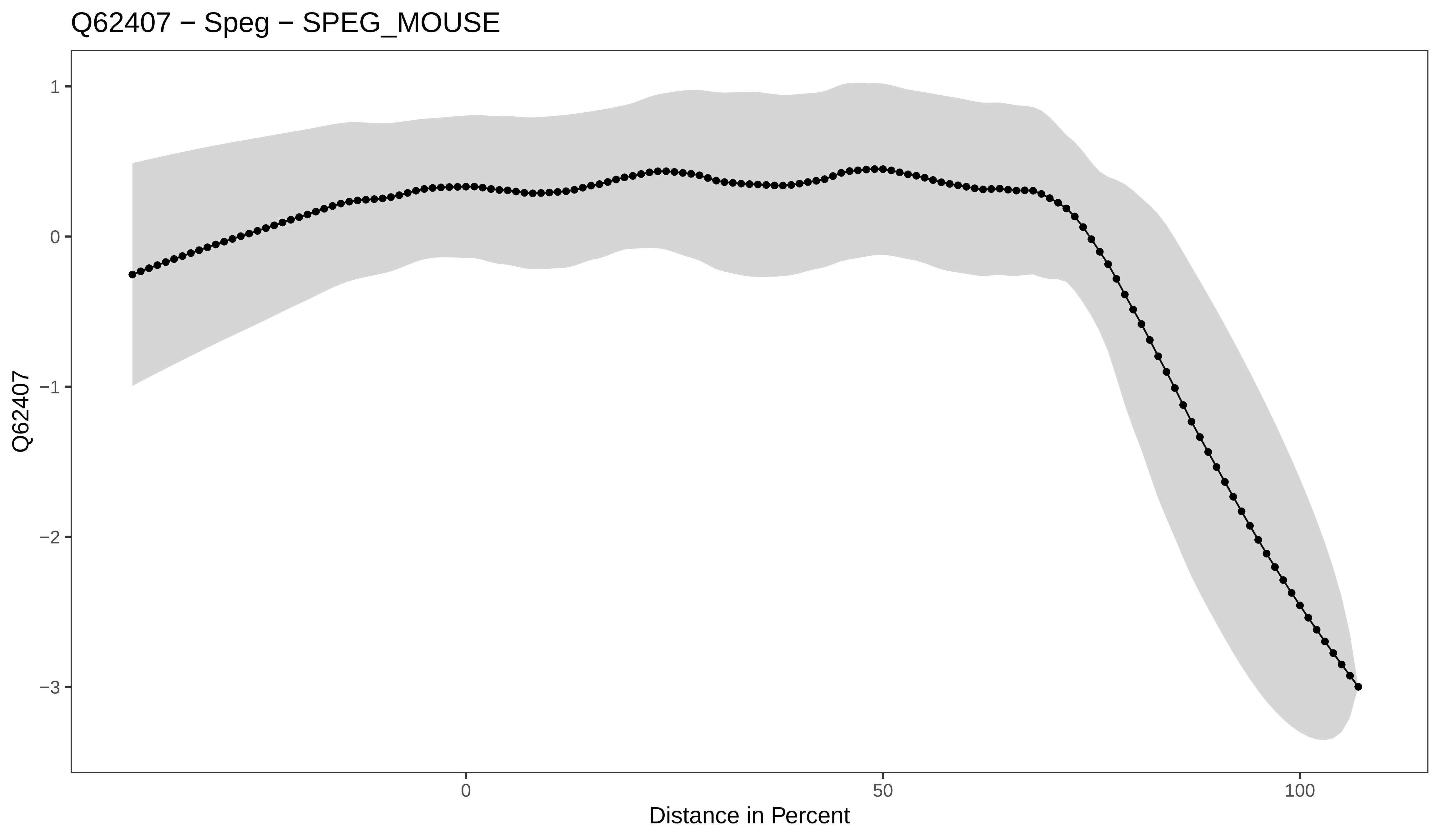

Q62407 − Speg − SPEG_MOUSE
1
0
−1
−2
−3
Q62407
0
50
100
Distance in Percent

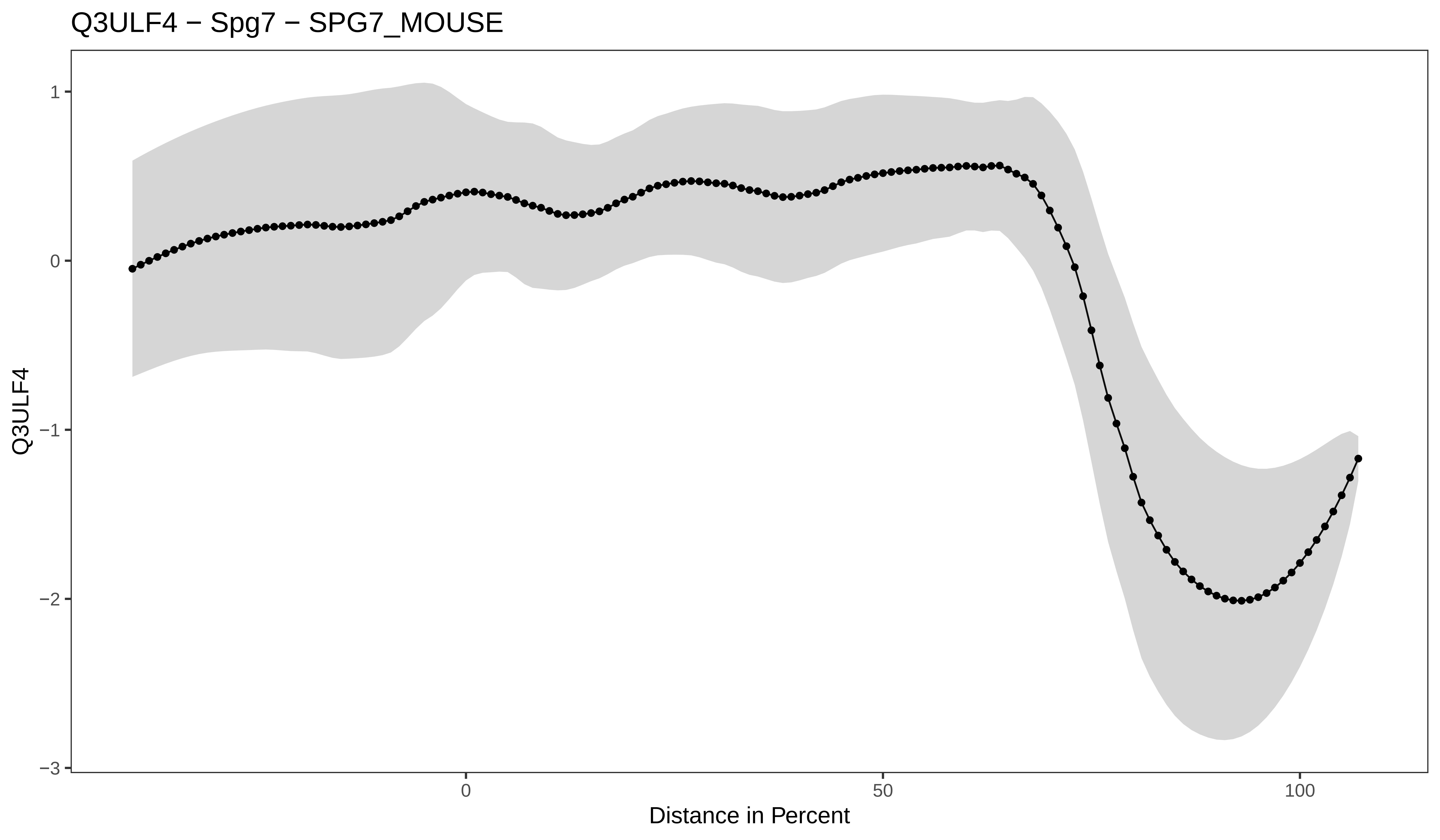
Q3ULF4 − Spg7 − SPG7_MOUSE
1
0
−1
−2
−3
Q3ULF4
0
50
100
Distance in Percent

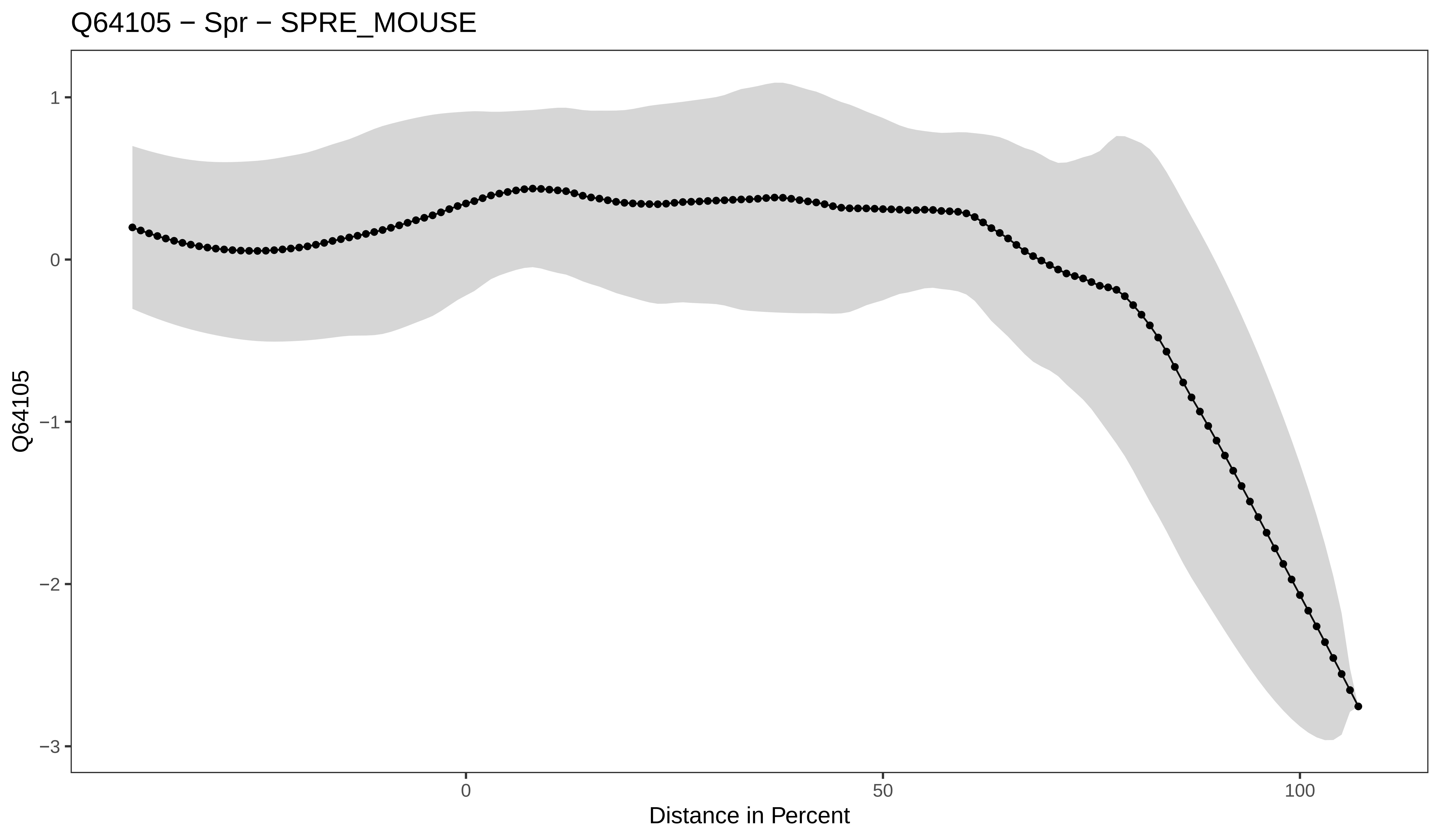

Q64105 − Spr − SPRE_MOUSE
Q64105
Distance in Percent
1
0
−1
−2
−3
0
50
100

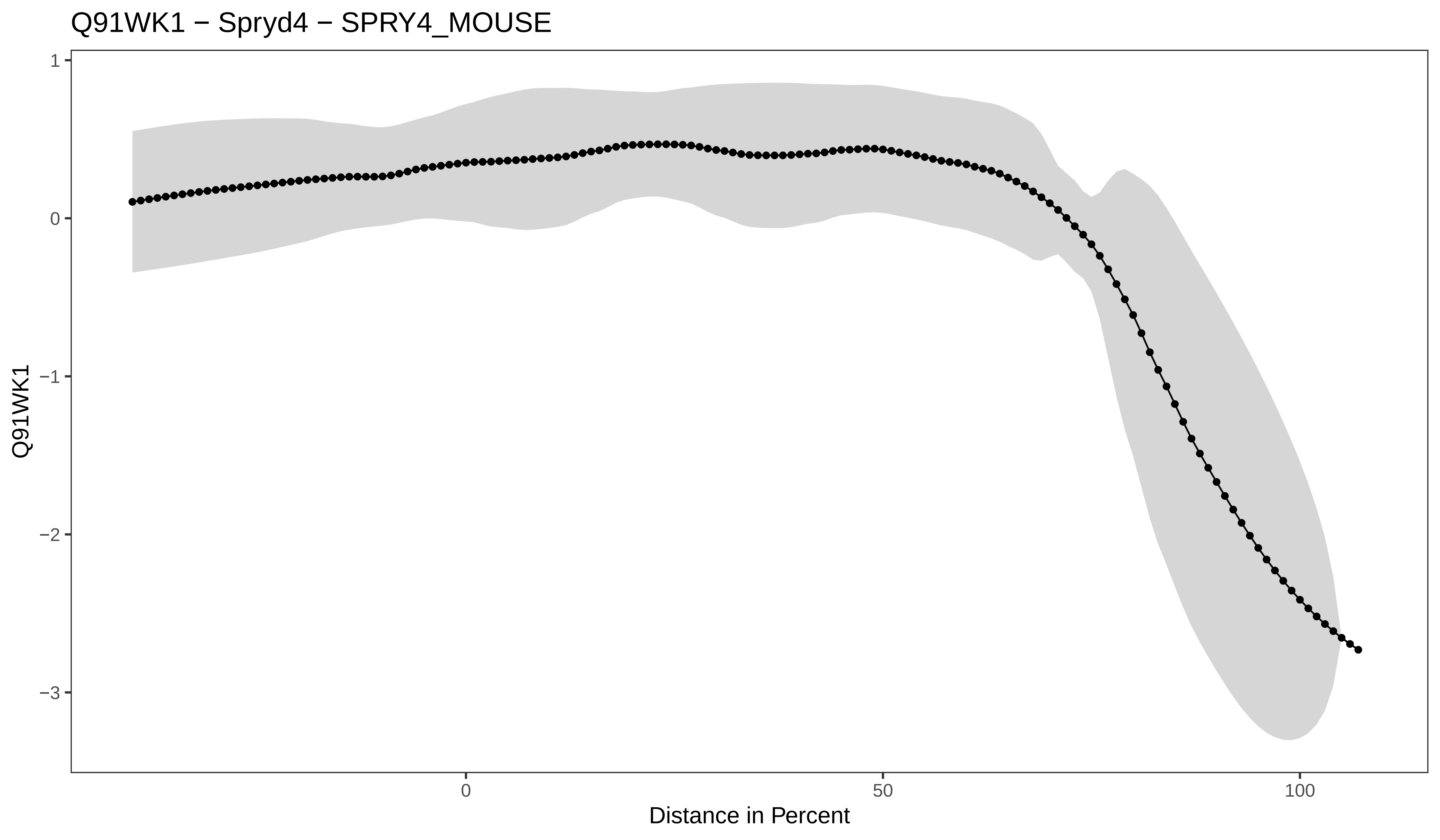

Q91WK1 − Spryd4 − SPRY4_MOUSE
1
0
−1
−2
−3
Q91WK1
0
50
100
Distance in Percent

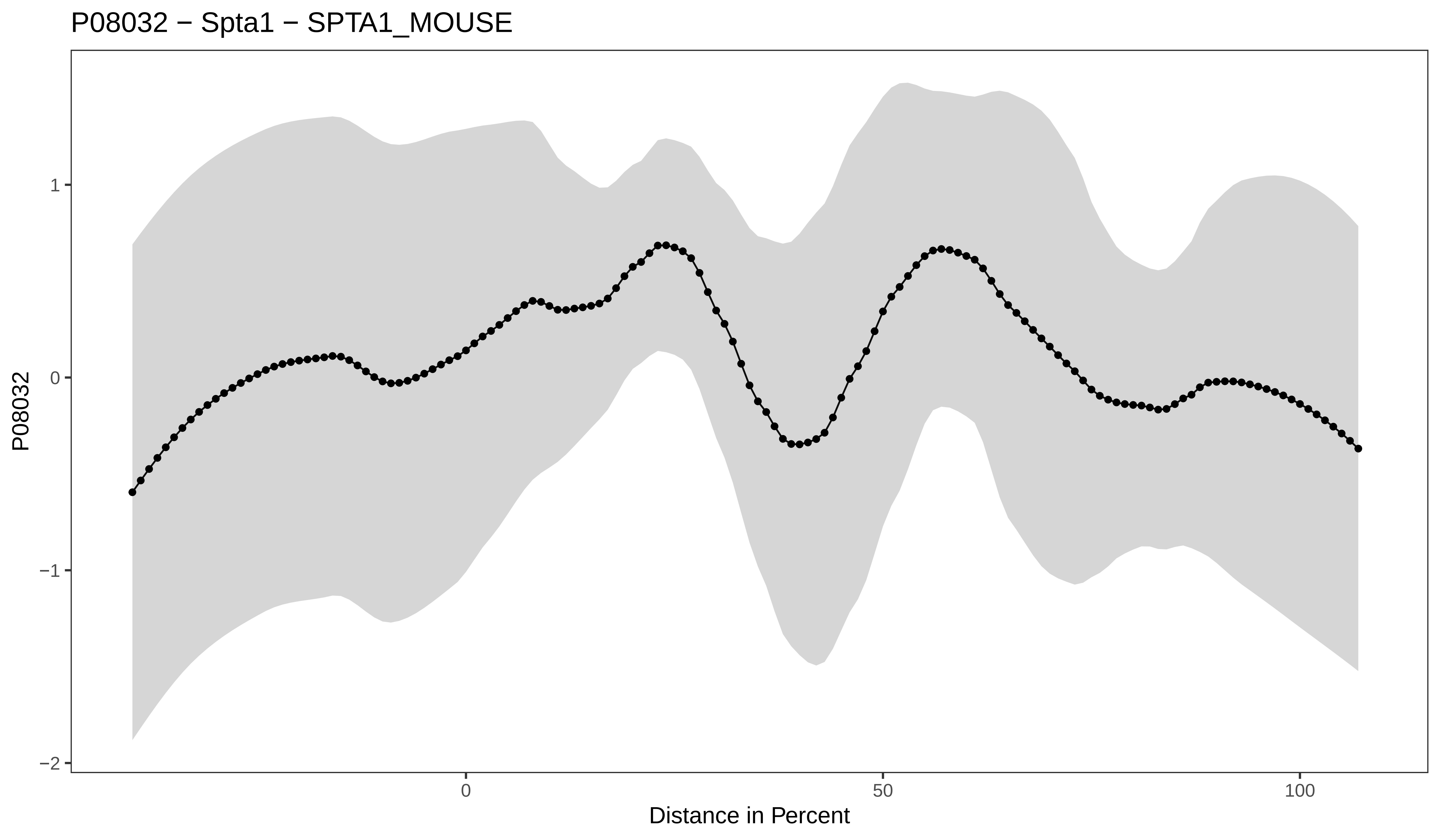
P08032 − Spta1 − SPTA1_MOUSE
P08032
1
0
−1
−2
0
50
100
Distance in Percent

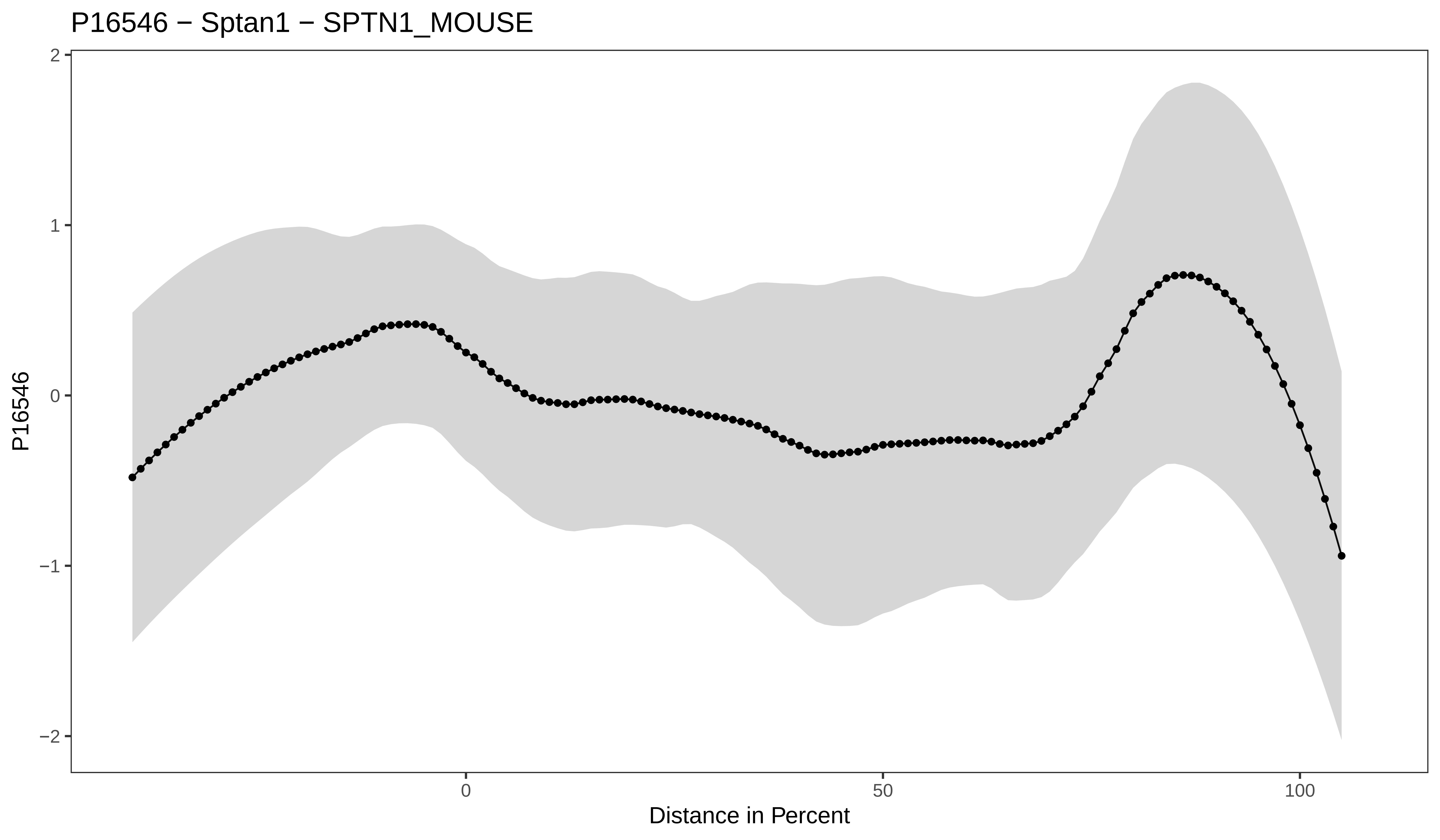

P16546 − Sptan1 − SPTN1_MOUSE
P16546
Distance in Percent
2
1
0
−1
−2
0
50
100

P15508 − Sptb − SPTB1_MOUSE

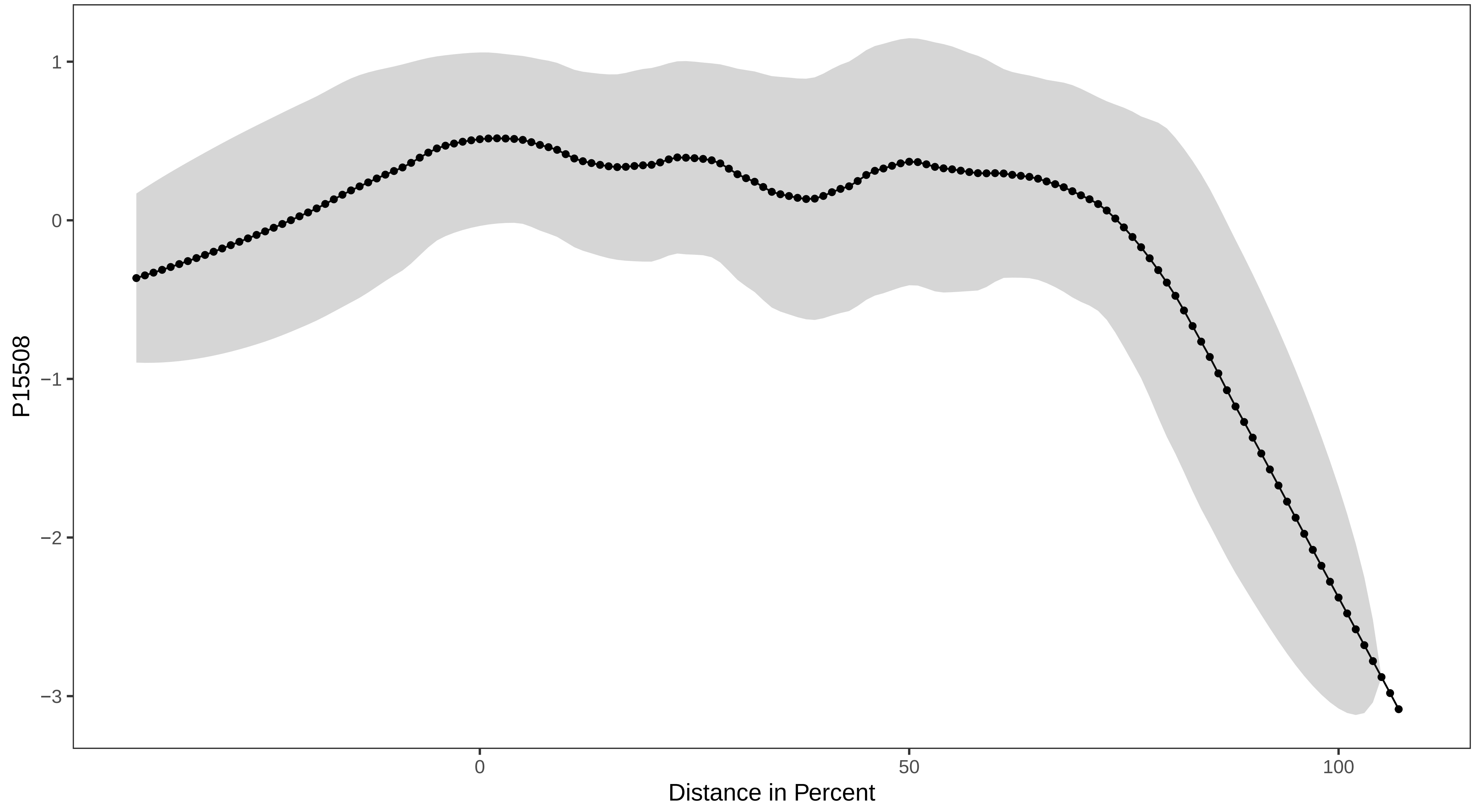

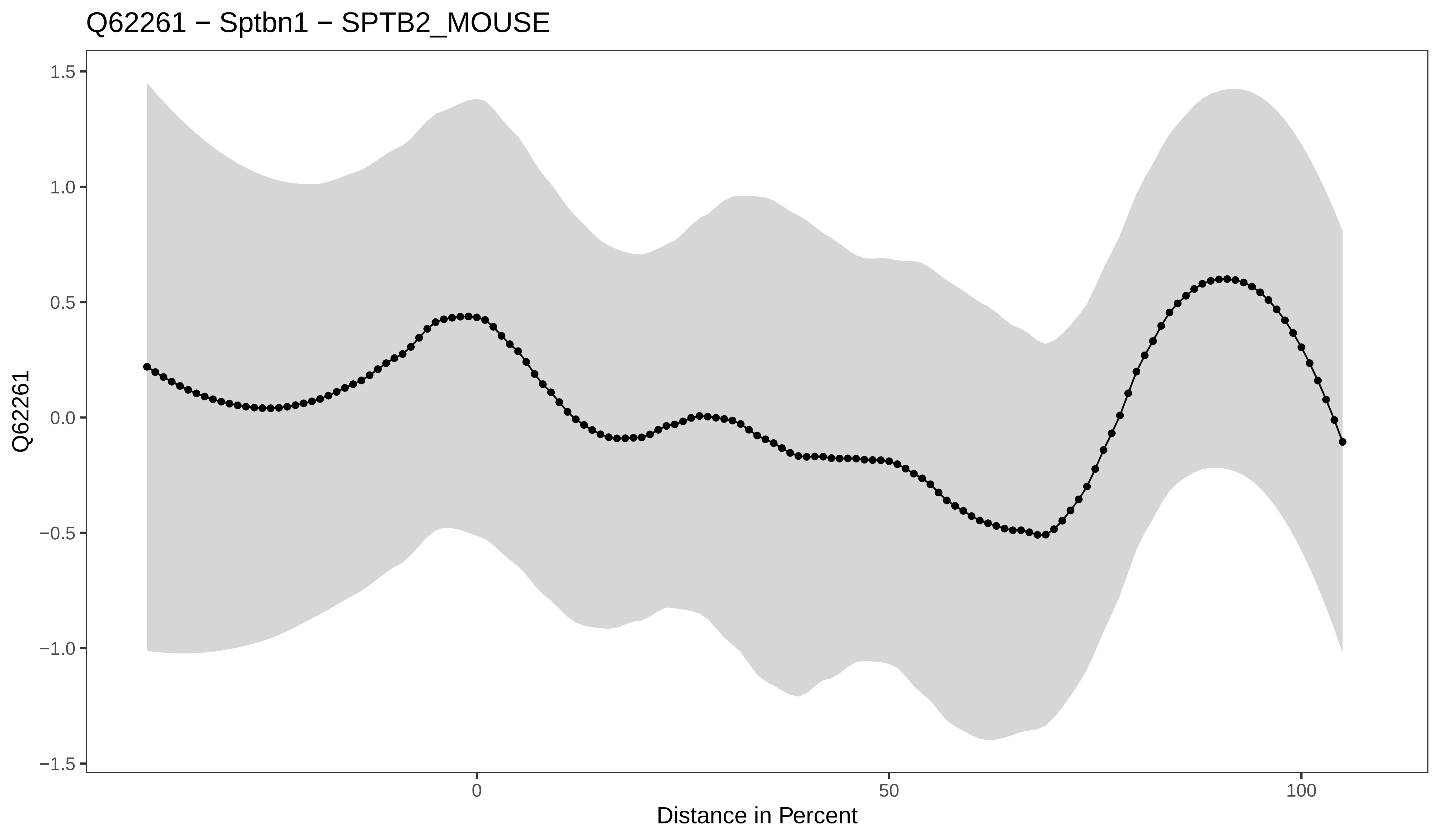
Q62261 − Sptbn1 − SPTB2_MOUSE
Q62261
1.5
1.0
0.5
0.0
−0.5
−1.0
−1.5
0
50
100
Distance in Percent

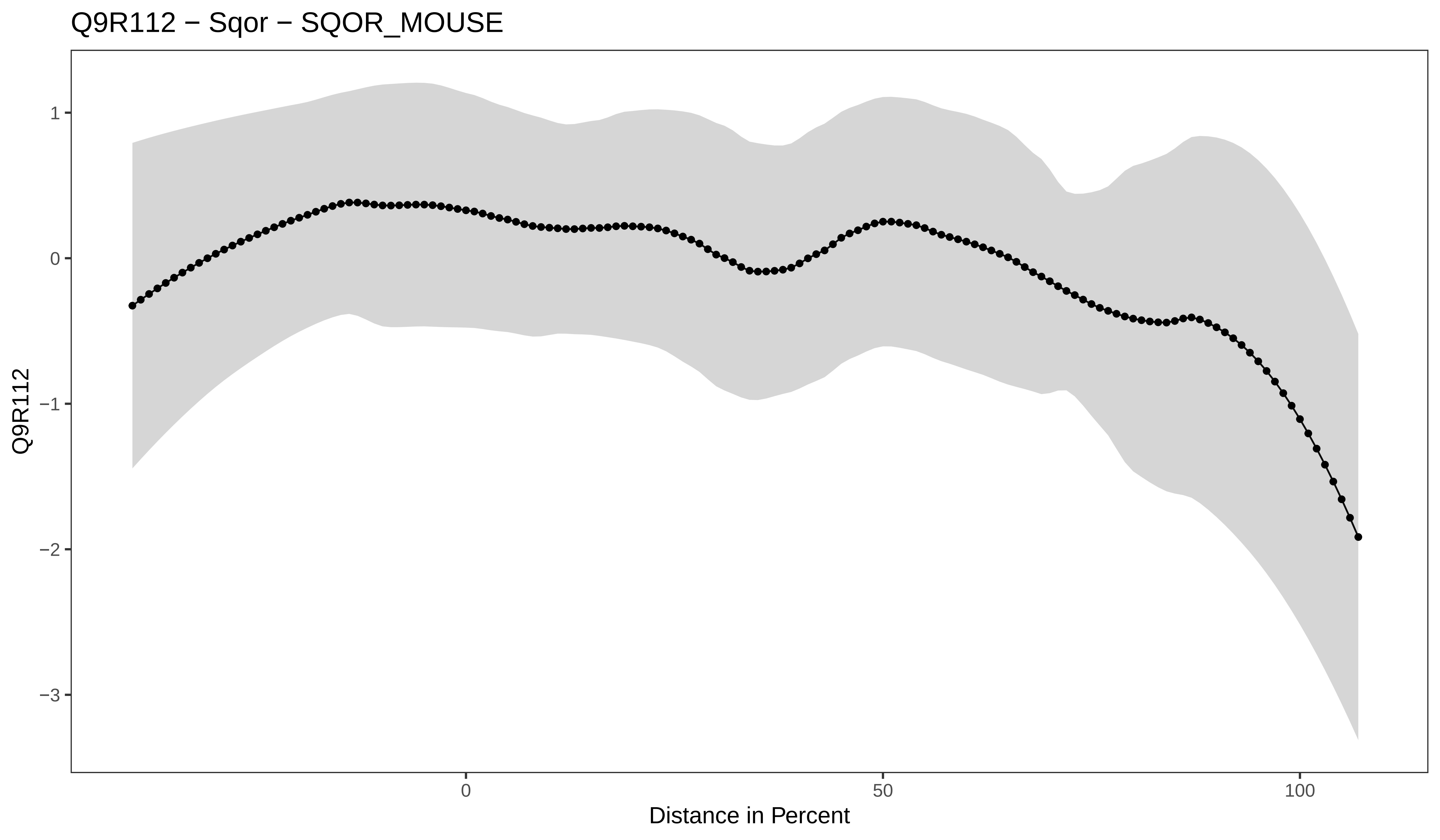

Q9R112 − Sqor − SQOR_MOUSE
1
0
−1
−2
−3
Q9R112
0
50
100
Distance in Percent

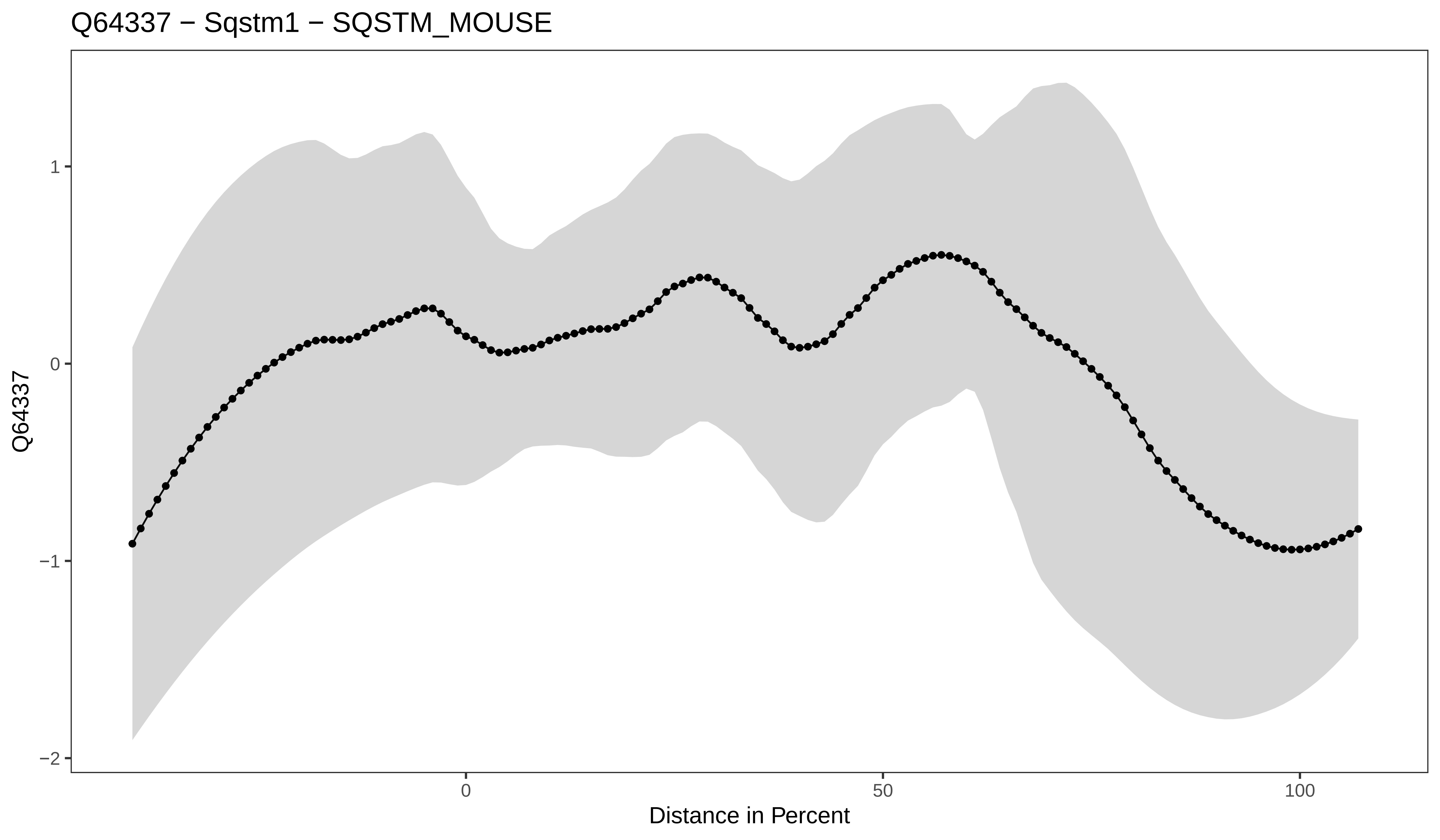

Q64337 − Sqstm1 − SQSTM_MOUSE
1
0
−1
−2
Q64337
0
50
100
Distance in Percent

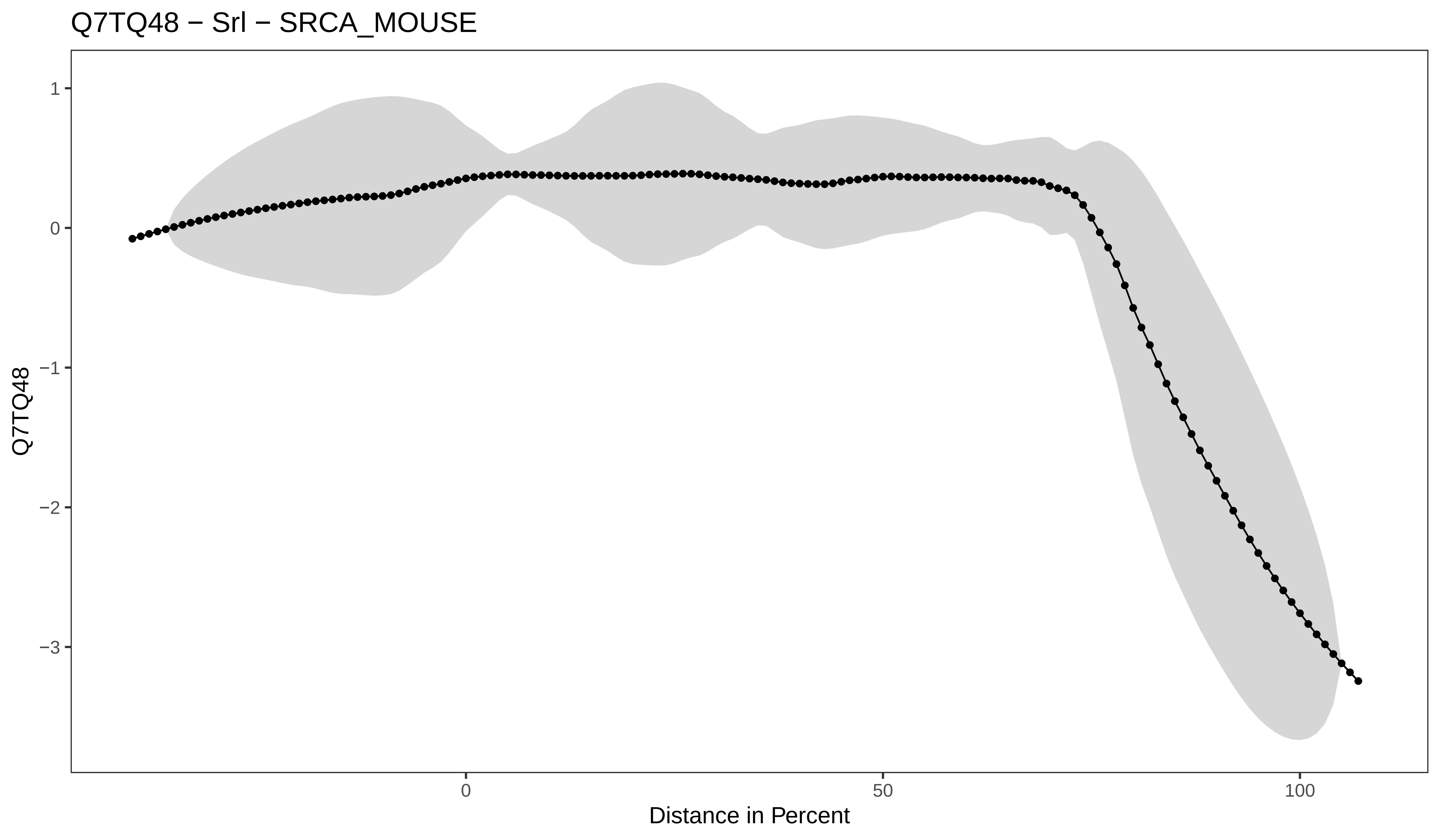

Q7TQ48 − Srl − SRCA_MOUSE
1
0
−1
−2
−3
Q7TQ48
0
50
100
Distance in Percent

Q64674 − Srm − SPEE_MOUSE

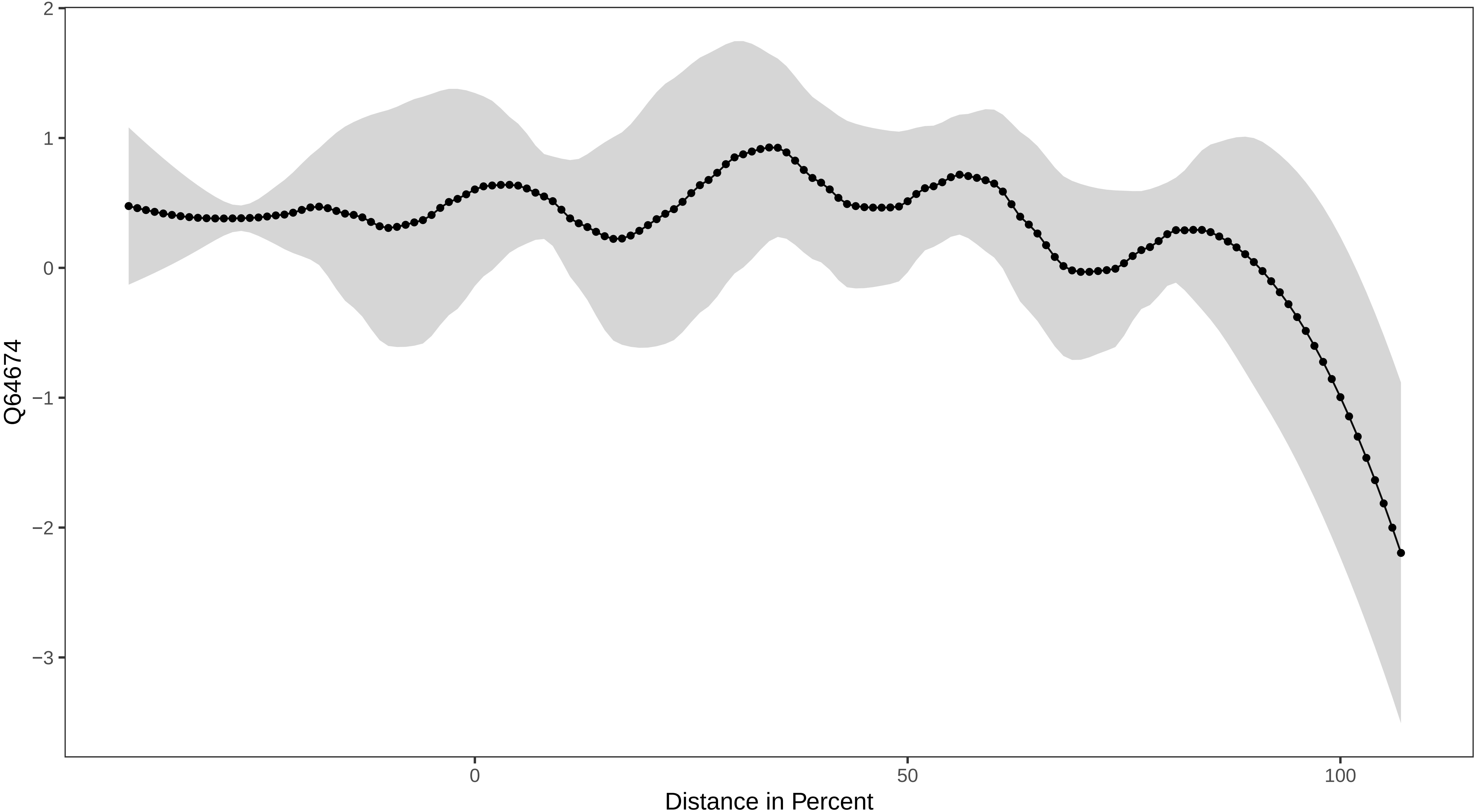

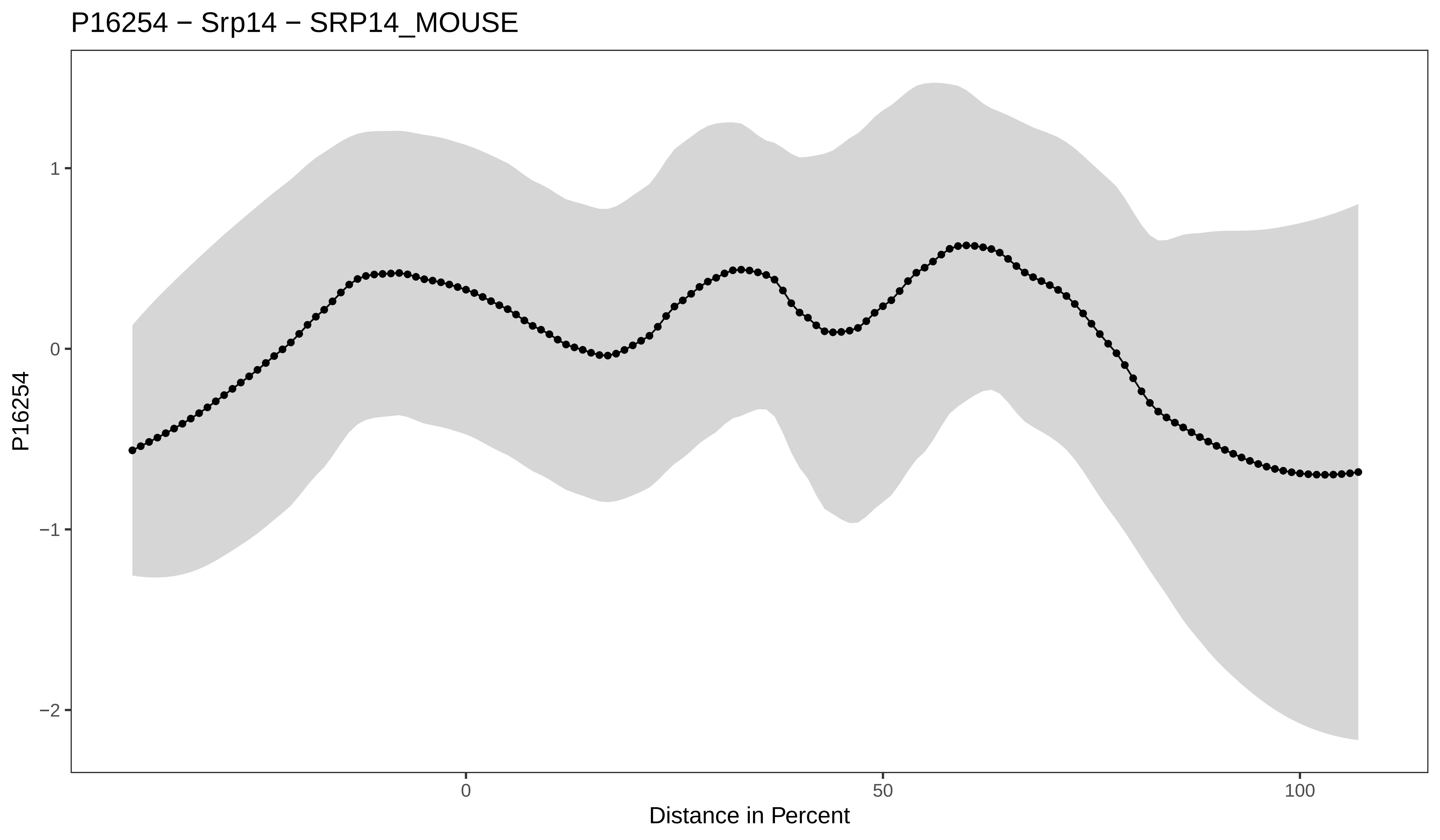

P16254 − Srp14 − SRP14_MOUSE
1
0
−1
−2
P16254
0
50
100
Distance in Percent

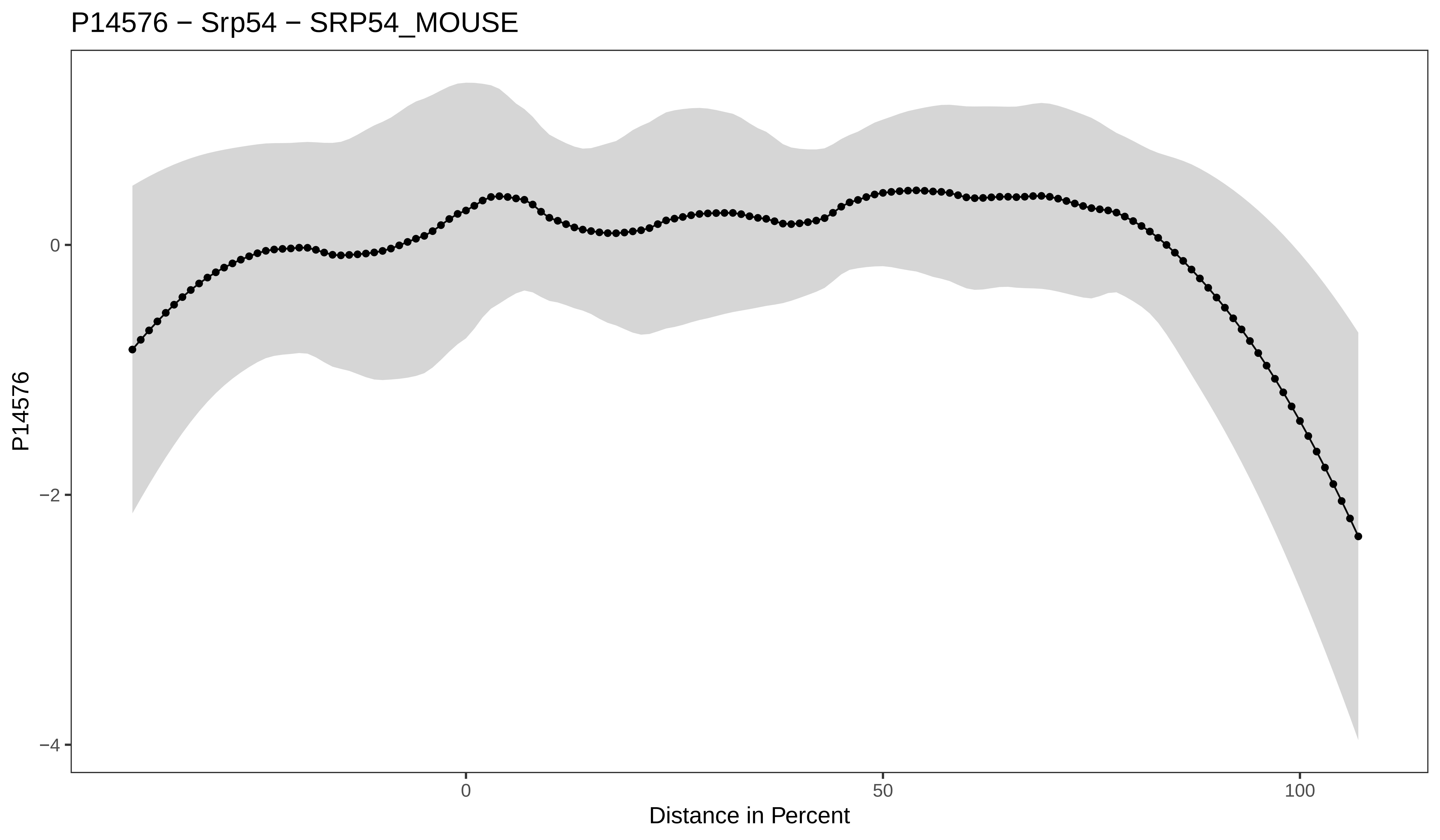
P14576 − Srp54 − SRP54_MOUSE
0
−2
−4
P14576
0
50
100
Distance in Percent

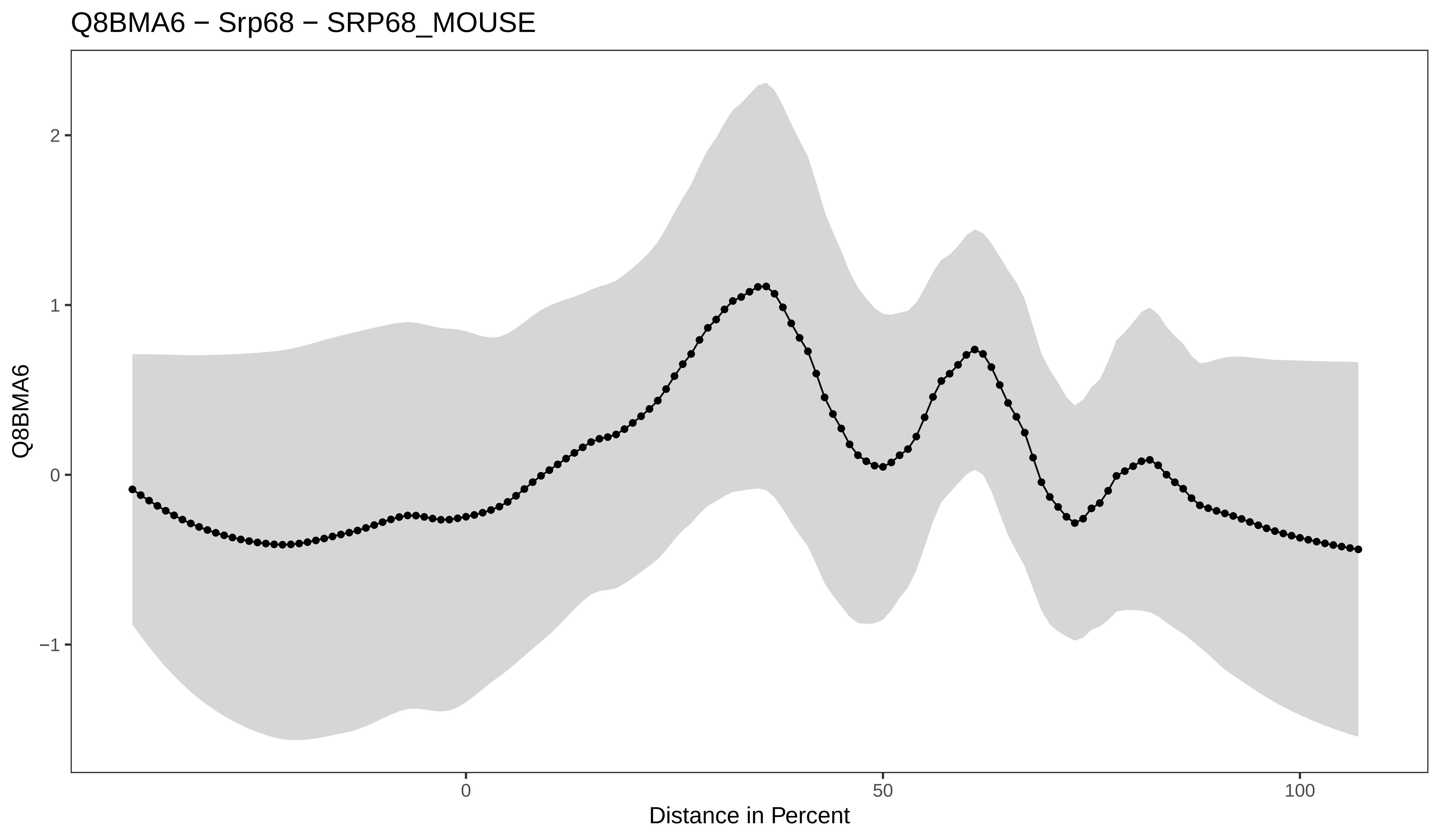

Q8BMA6 − Srp68 − SRP68_MOUSE
Q8BMA6
2
1
0
−1
0
50
100
Distance in Percent

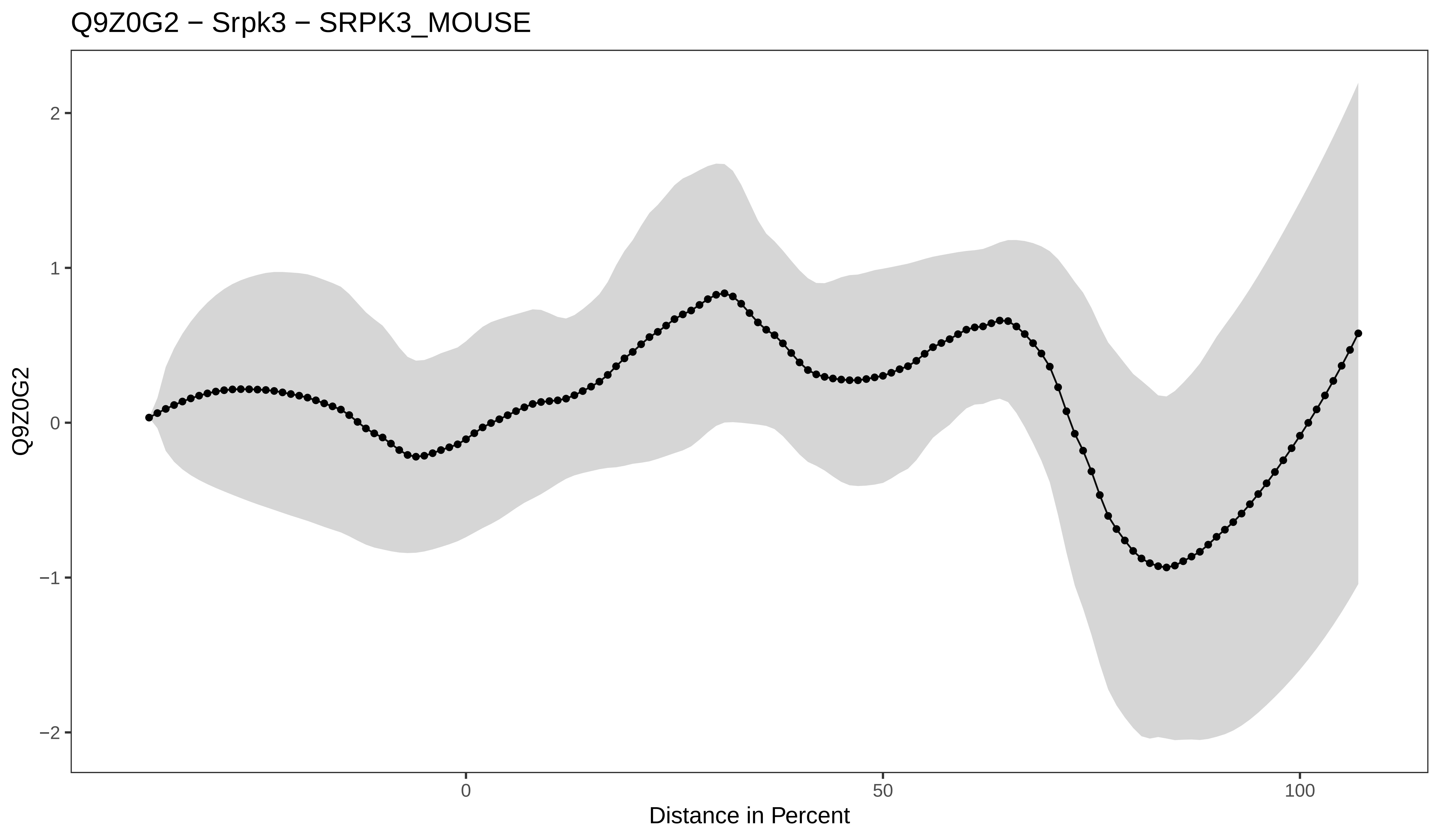

Q9Z0G2 − Srpk3 − SRPK3_MOUSE
Q9Z0G2
2
1
0
−1
−2
0
50
100
Distance in Percent

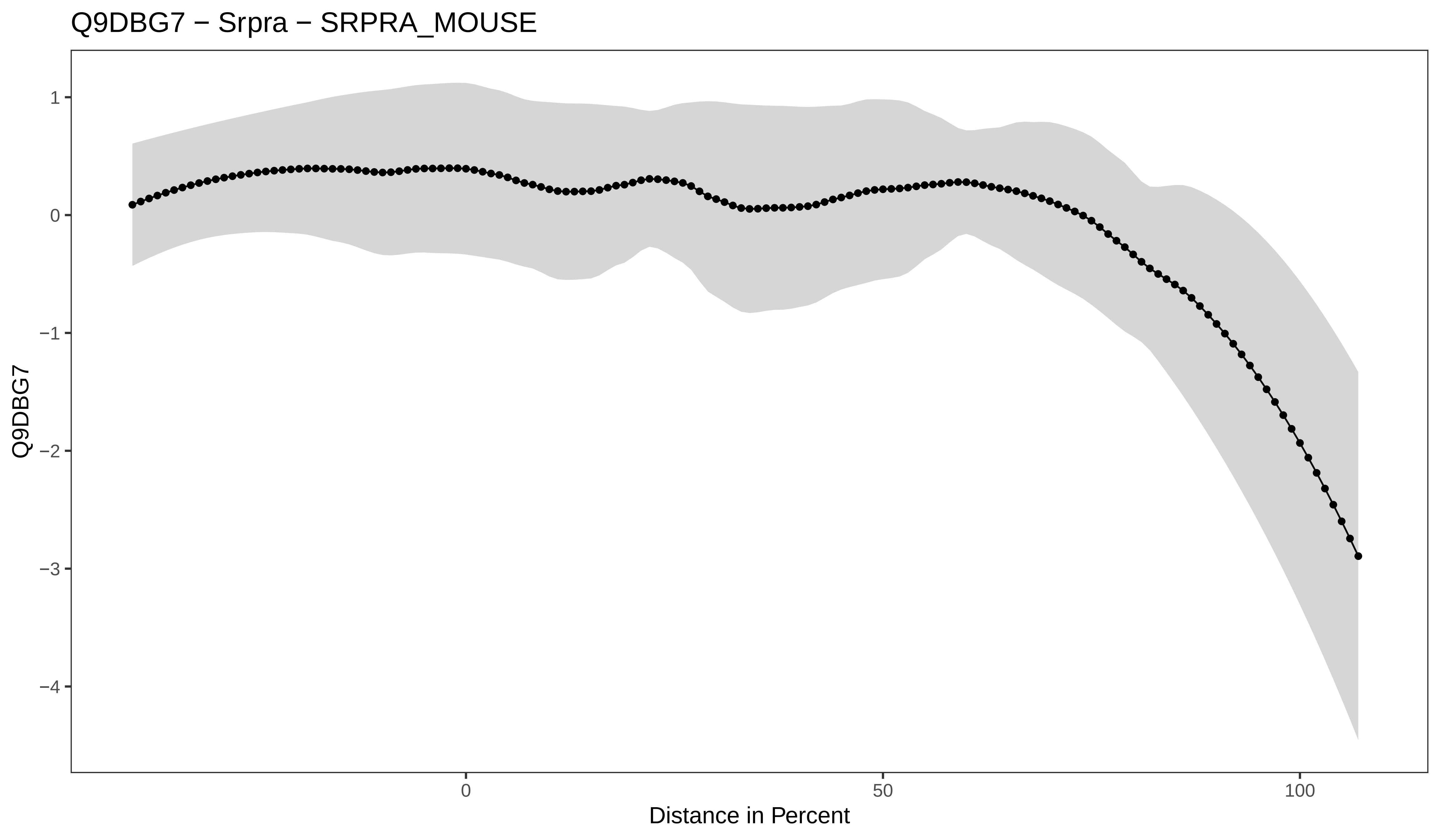
Q9DBG7 − Srpra − SRPRA_MOUSE
1
0
−1
−2
−3
−4
Q9DBG7
0
50
100
Distance in Percent

P47758 − Srprb − SRPRB_MOUSE

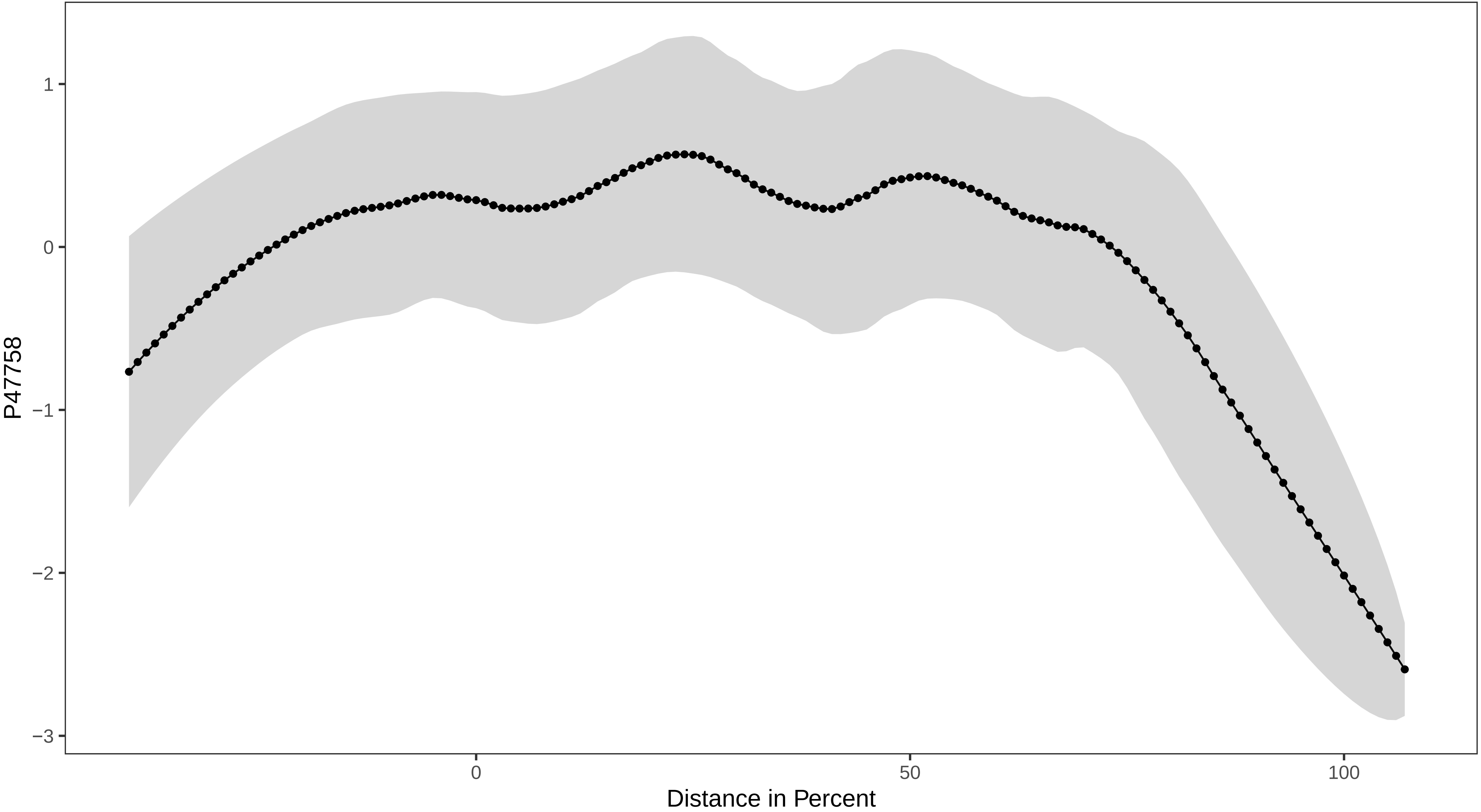

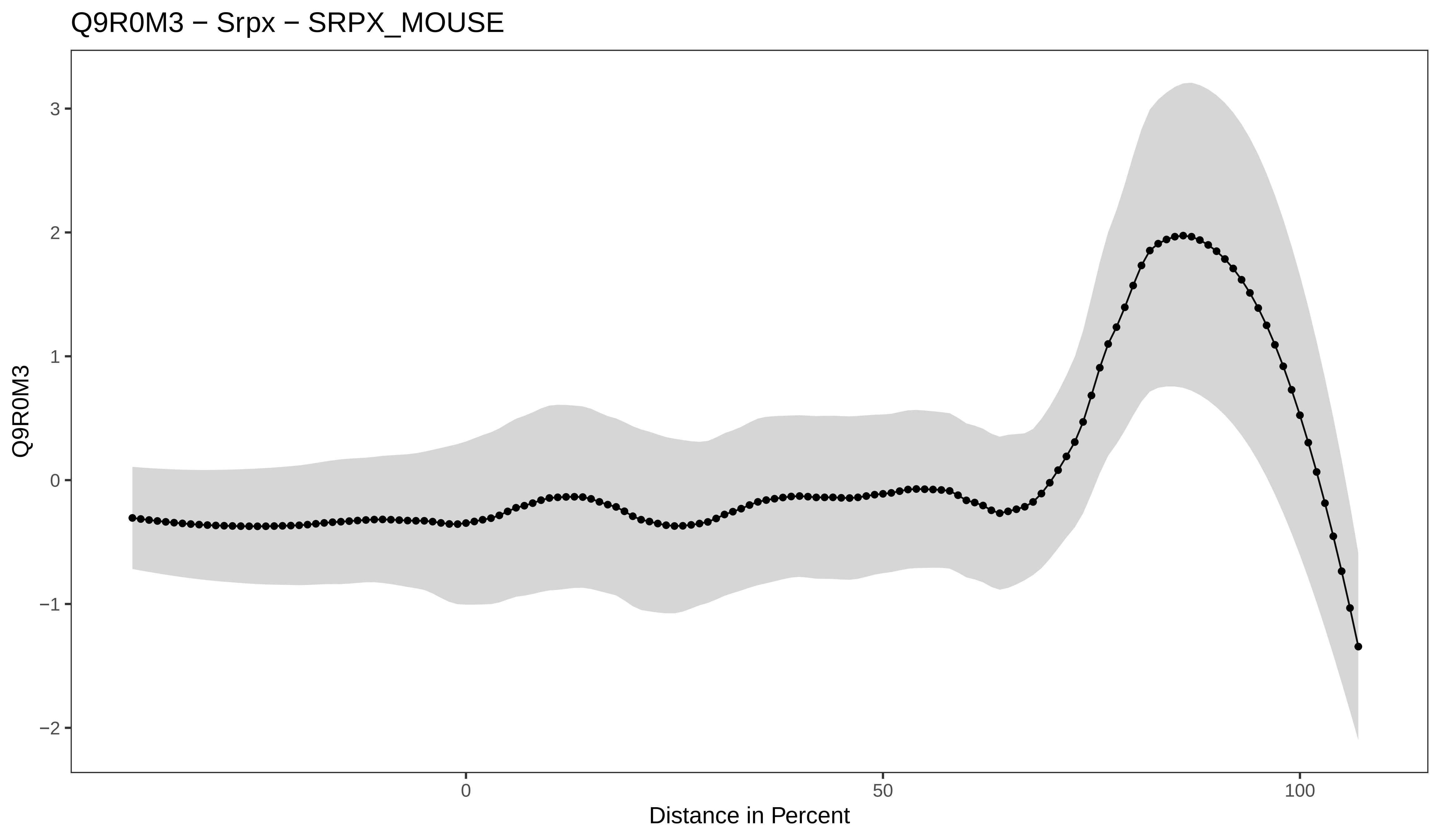
Q9R0M3 − Srpx − SRPX_MOUSE
Q9R0M3
Distance in Percent
3
2
1
0
−1
−2
0
50
100

Q8R054 − Srpx2 − SRPX2_MOUSE

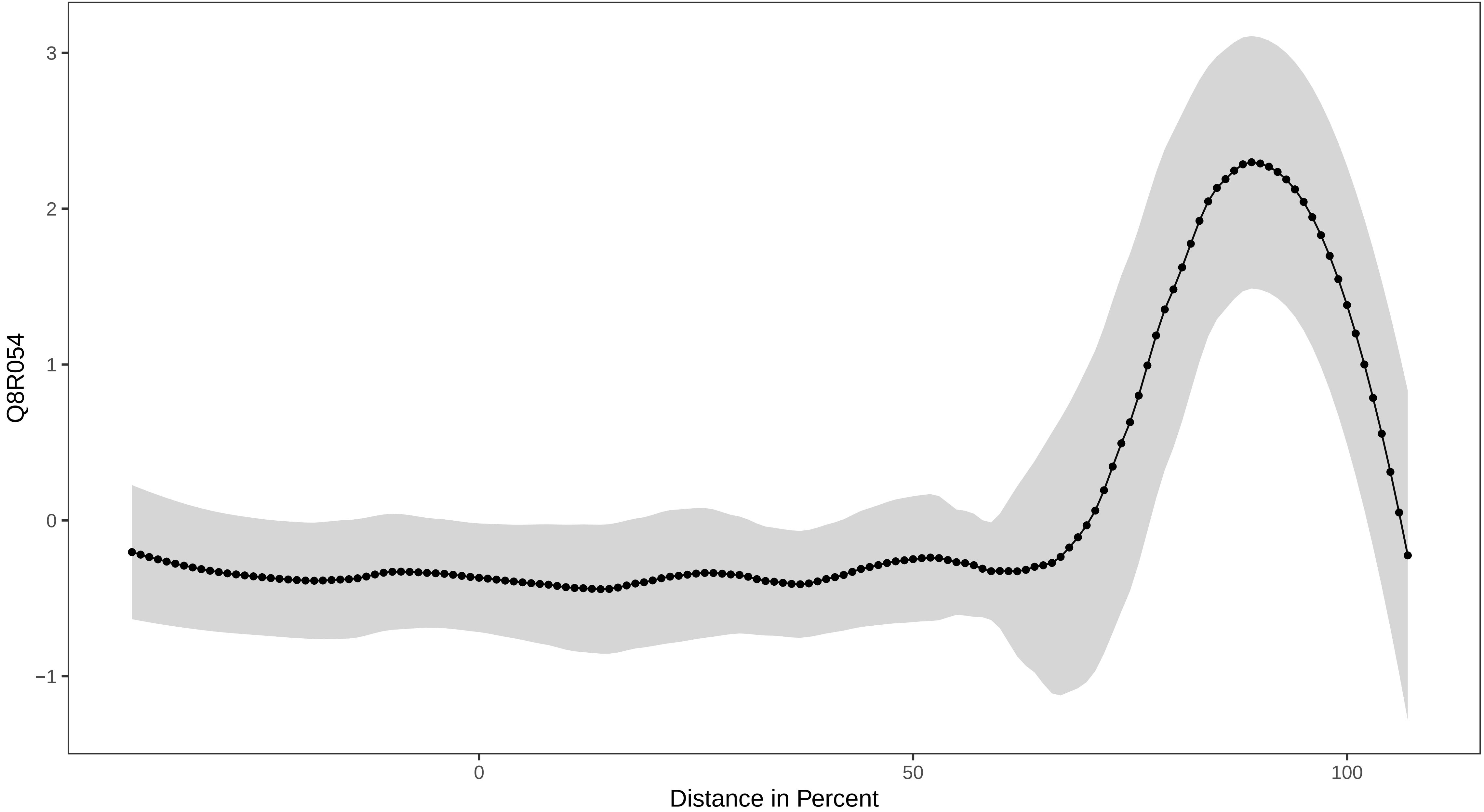

Q8K2M3 − Srrd − SRR1L_MOUSE

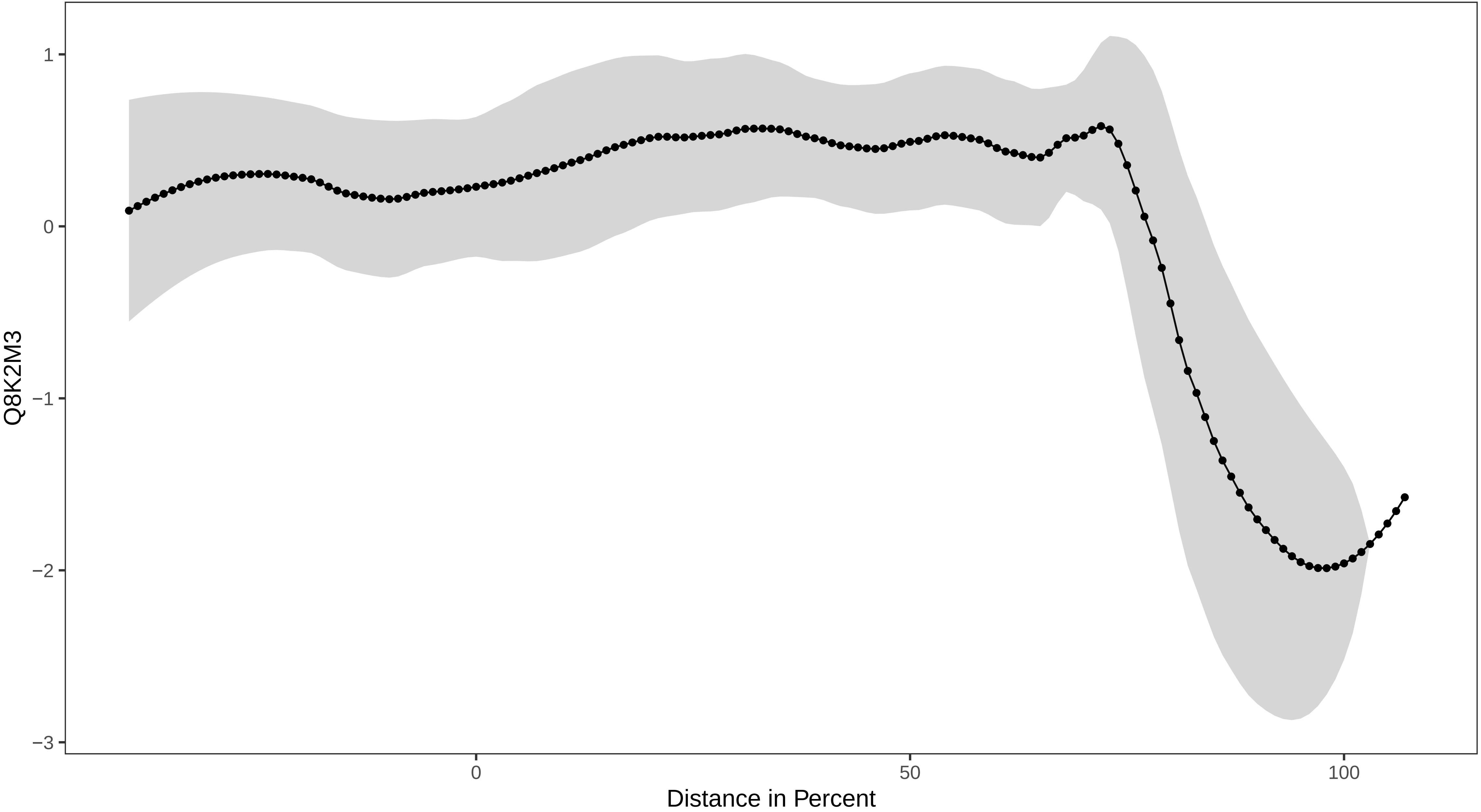

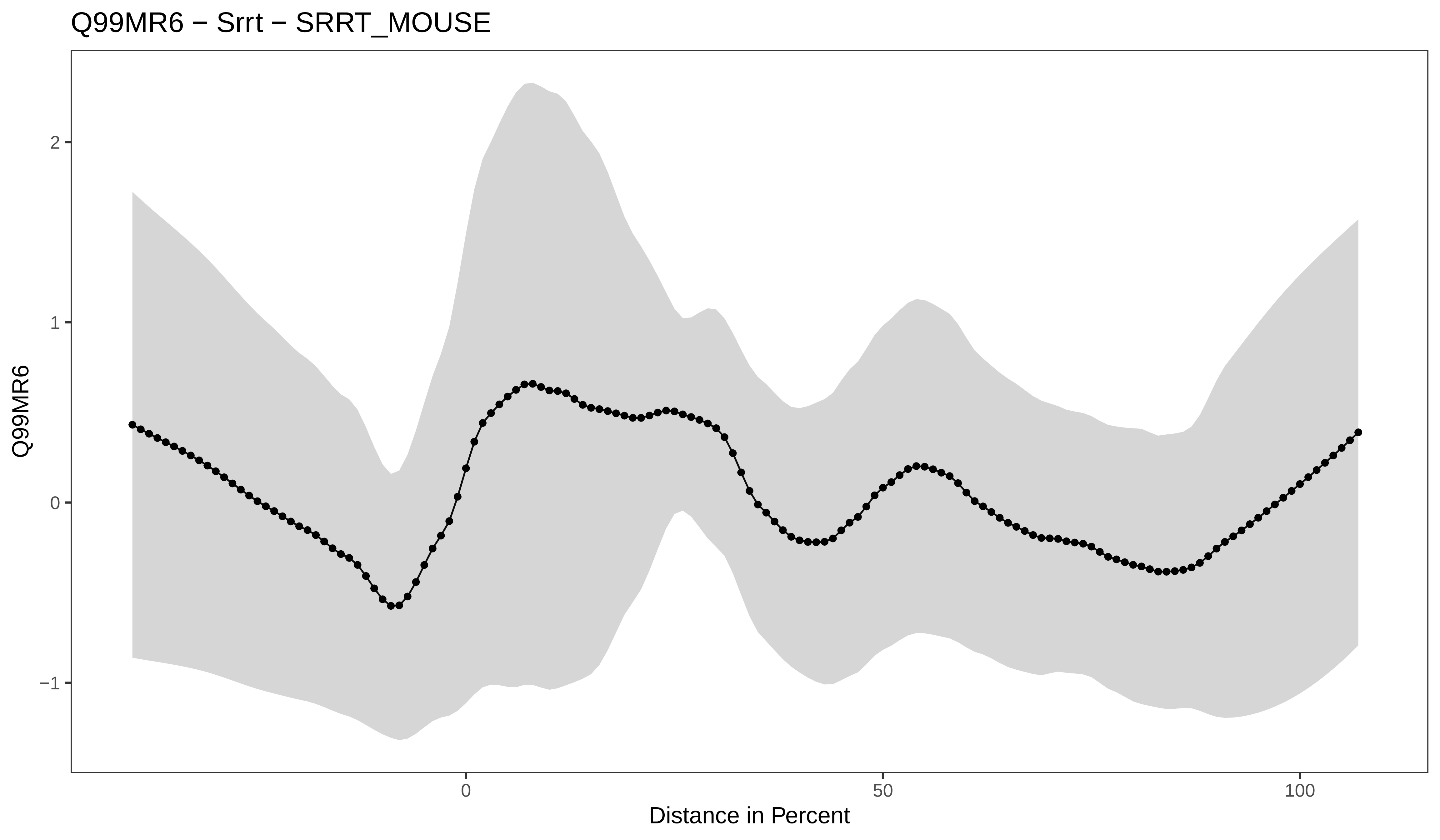

Q99MR6 − Srrt − SRRT_MOUSE
Q99MR6
Distance in Percent
2
1
0
−1
0
50
100

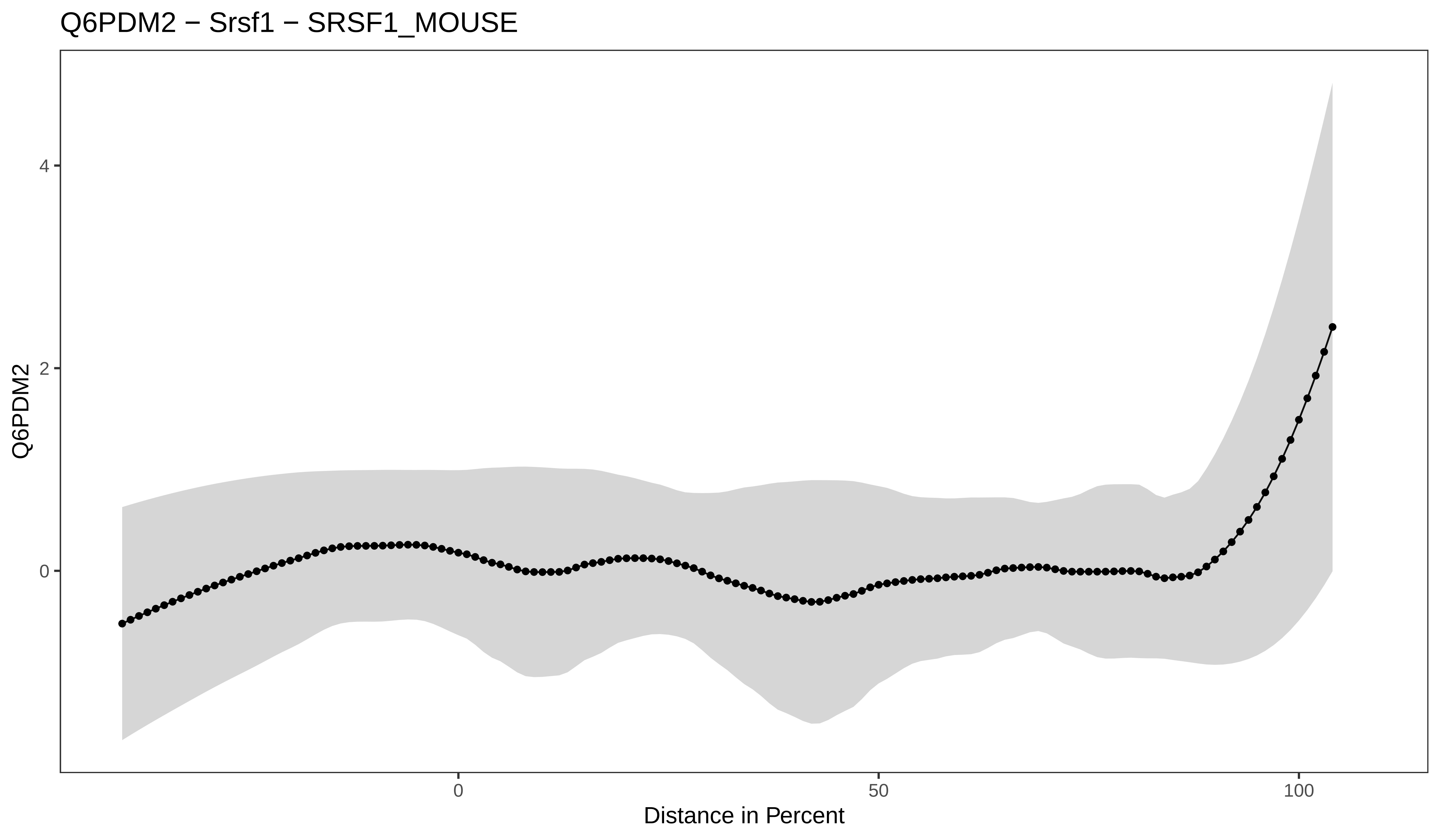

Q6PDM2 − Srsf1 − SRSF1_MOUSE
Q6PDM2
Distance in Percent
4
2
0
0
50
100

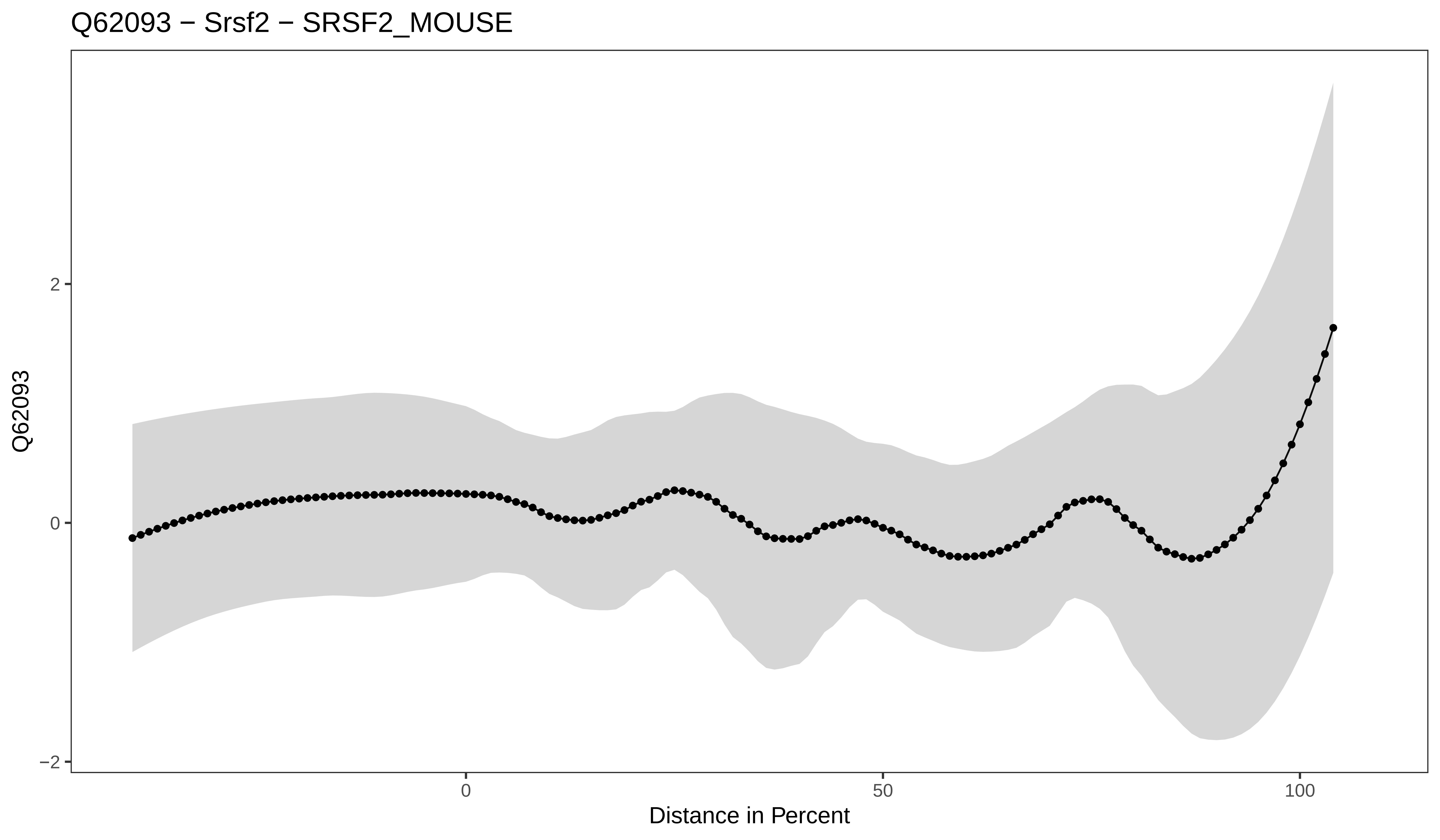
Q62093 − Srsf2 − SRSF2_MOUSE
Q62093
2
0
−2
0
50
100
Distance in Percent

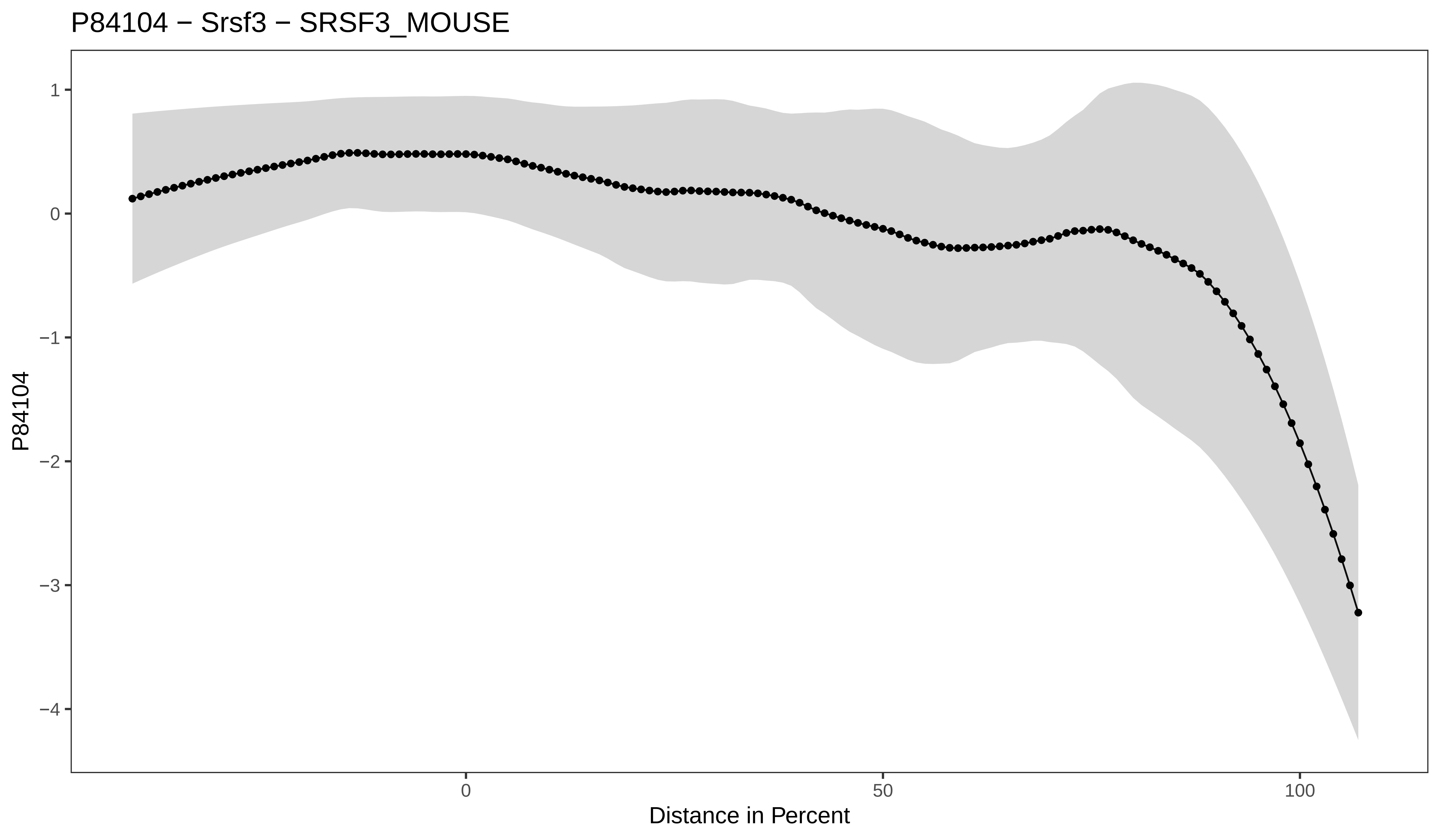

P84104 − Srsf3 − SRSF3_MOUSE
1
0
−1
−2
−3
−4
P84104
0
50
100
Distance in Percent

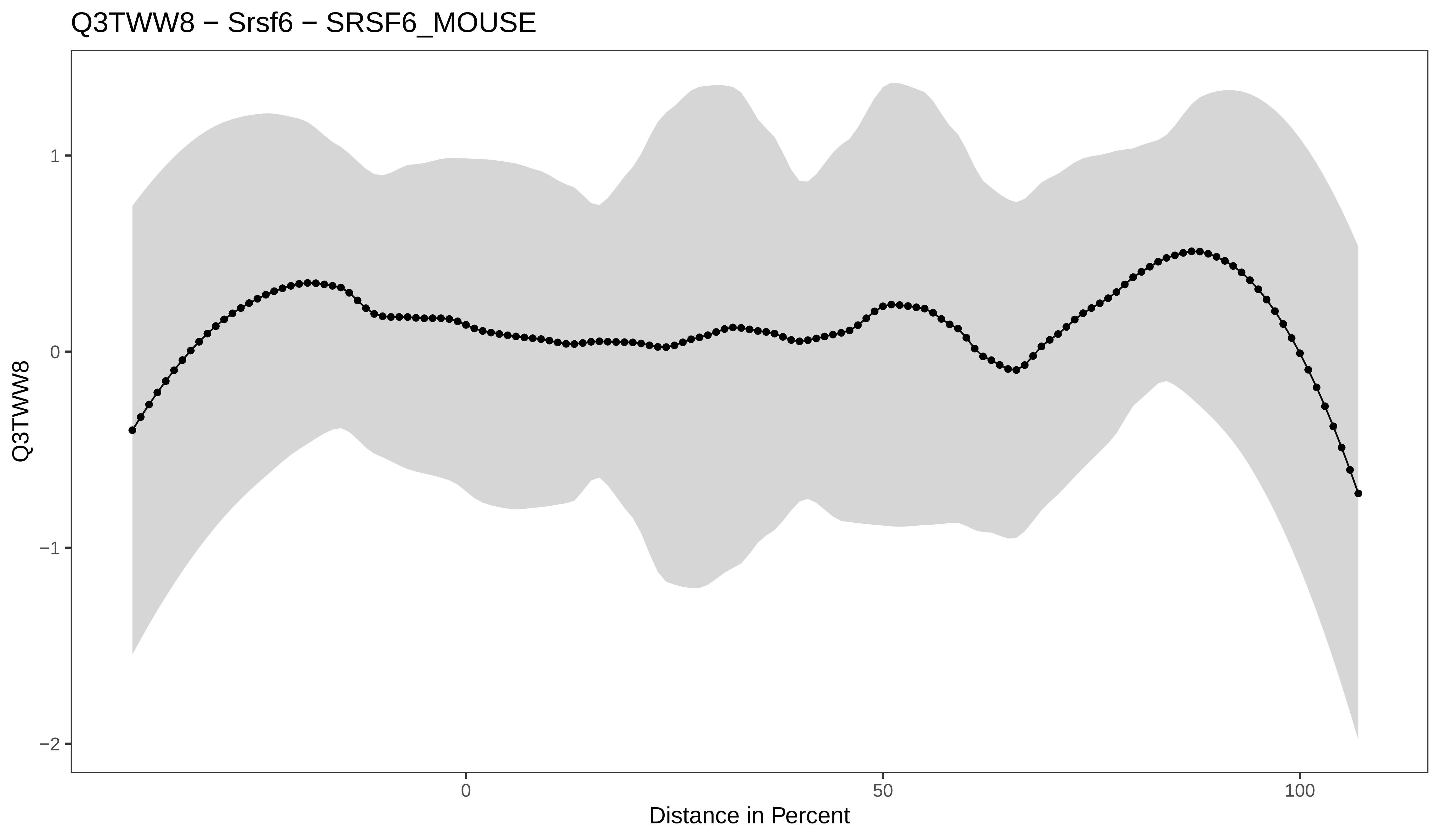

Q3TWW8 − Srsf6 − SRSF6_MOUSE
Q3TWW8
1
0
−1
−2
0
50
100
Distance in Percent

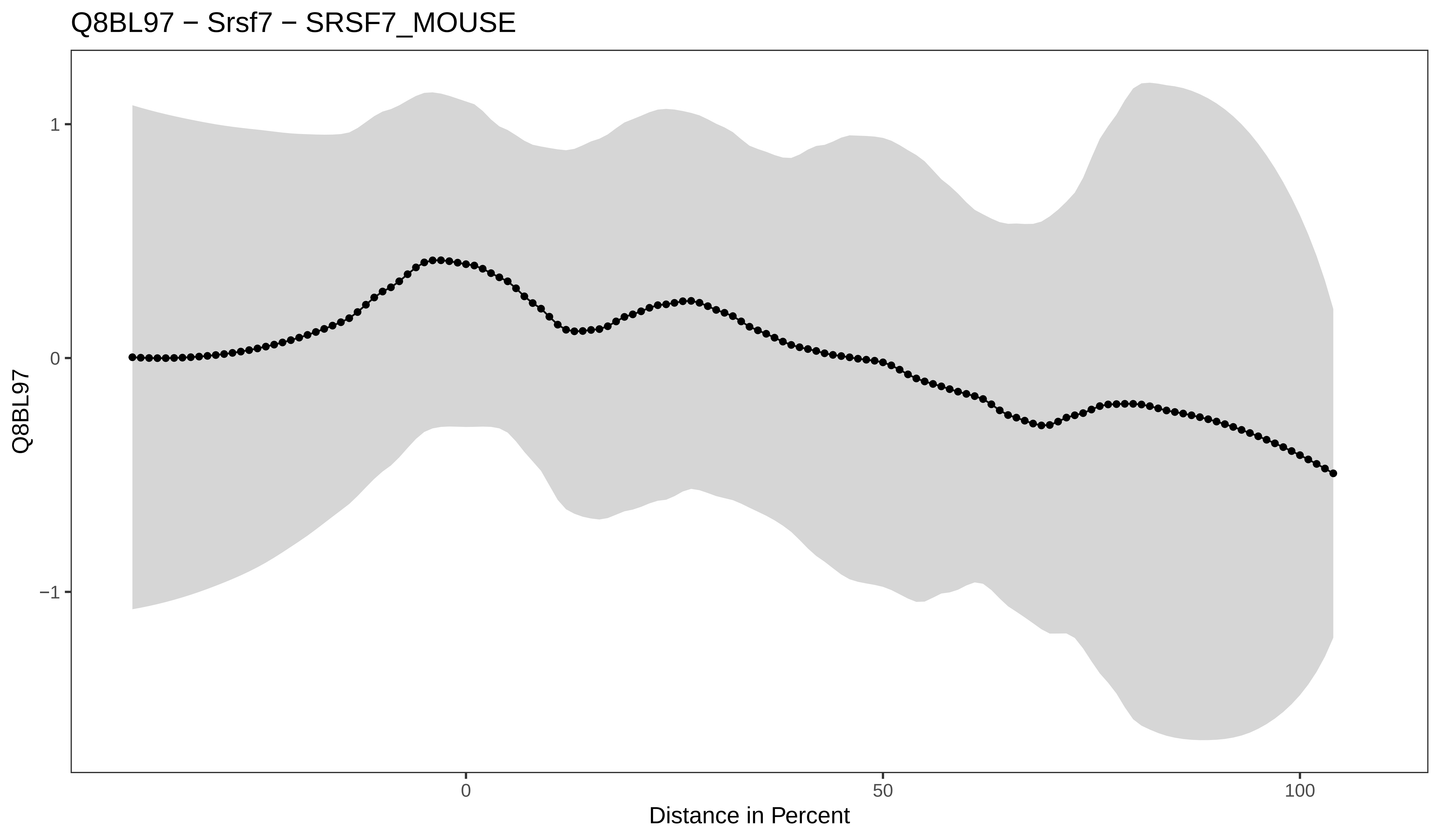
Q8BL97 − Srsf7 − SRSF7_MOUSE
1
0
−1
Q8BL97
0
50
100
Distance in Percent

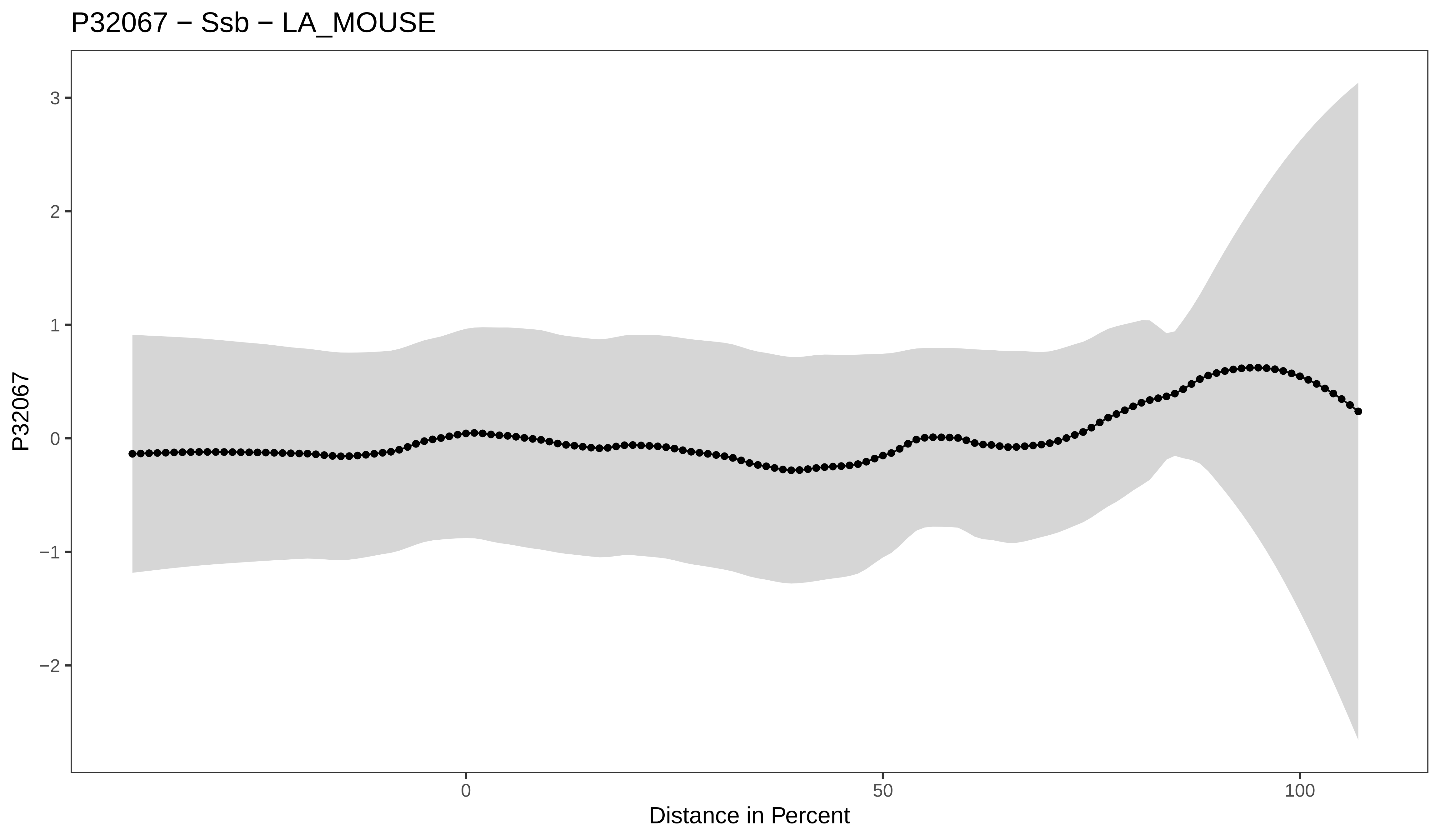

P32067 − Ssb − LA_MOUSE
P32067
3
2
1
0
−1
−2
0
50
100
Distance in Percent

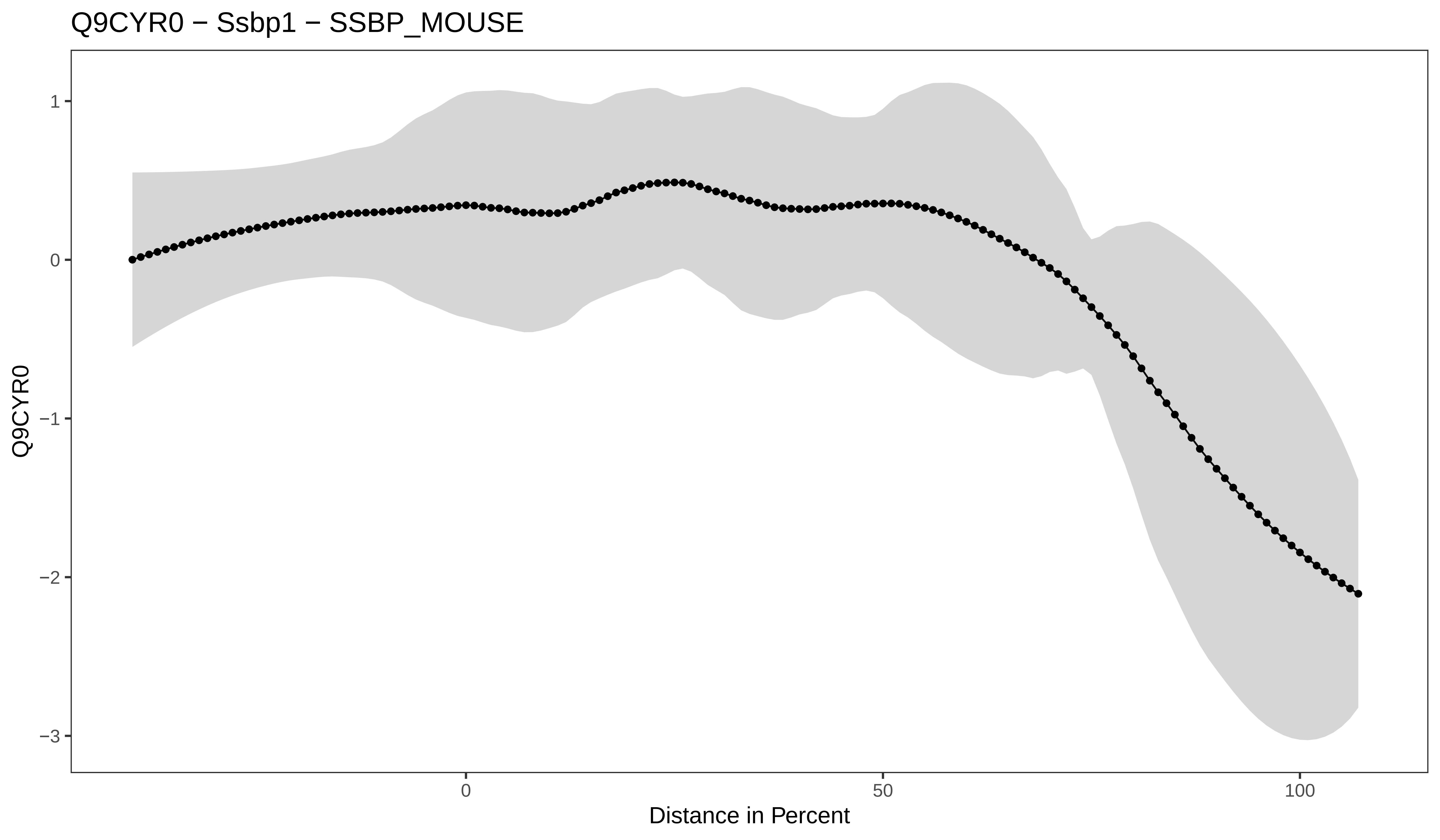

Q9CYR0 − Ssbp1 − SSBP_MOUSE
1
0
−1
−2
−3
Q9CYR0
0
50
100
Distance in Percent

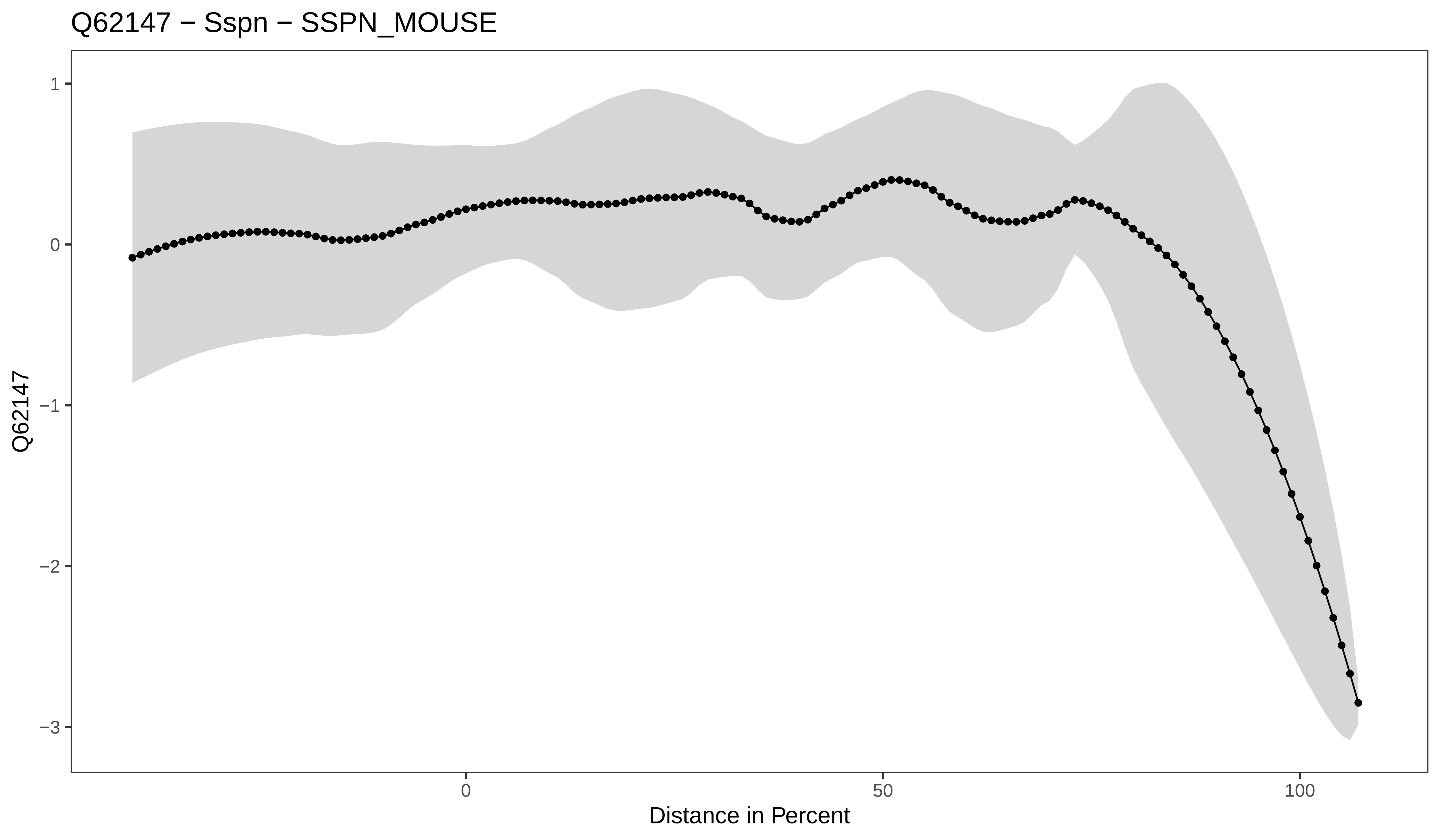

Q62147 − Sspn − SSPN_MOUSE
1
0
−1
−2
−3
Q62147
0
50
100
Distance in Percent

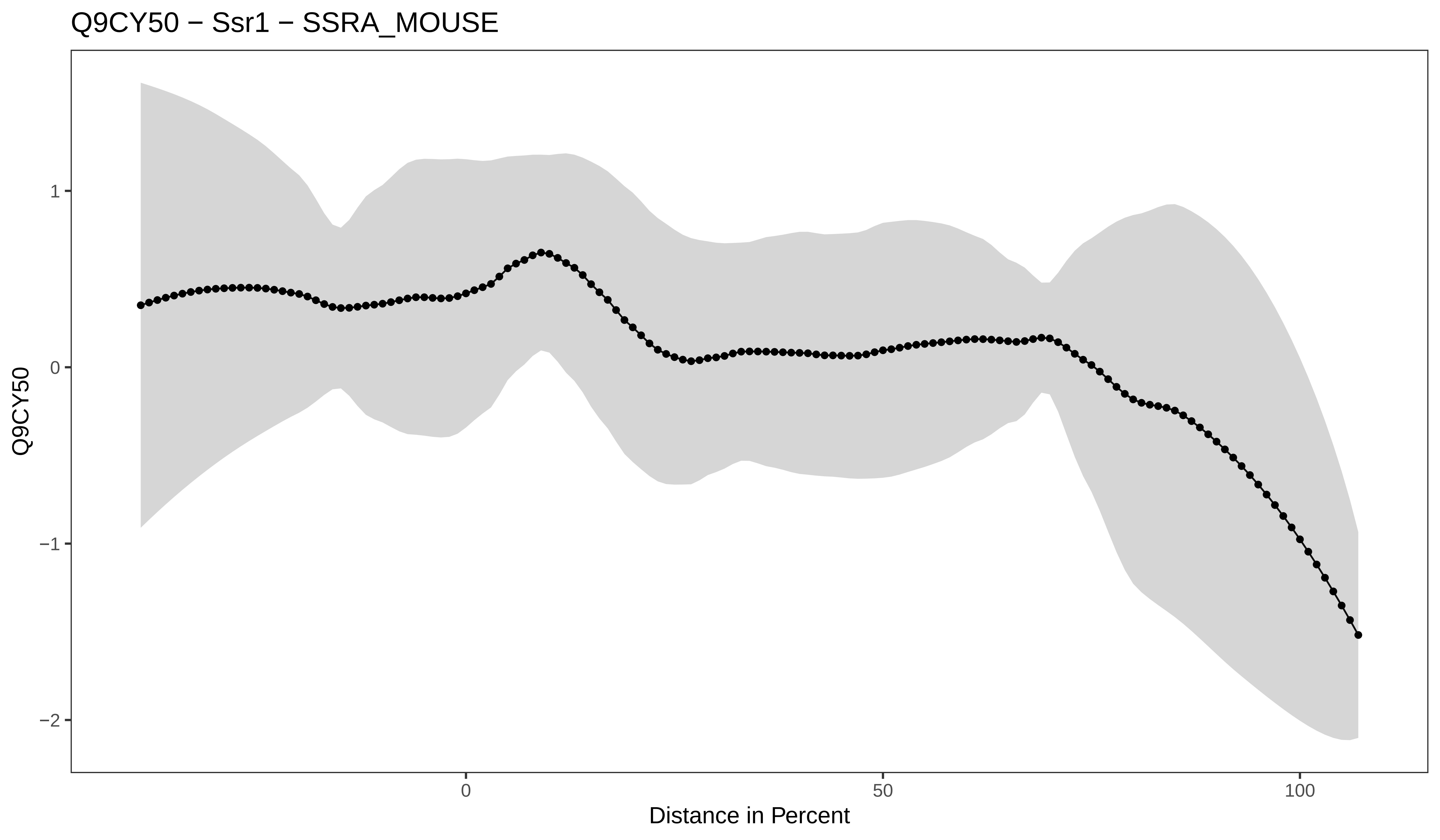
Q9CY50 − Ssr1 − SSRA_MOUSE
Q9CY50
1
0
−1
−2
0
50
100
Distance in Percent

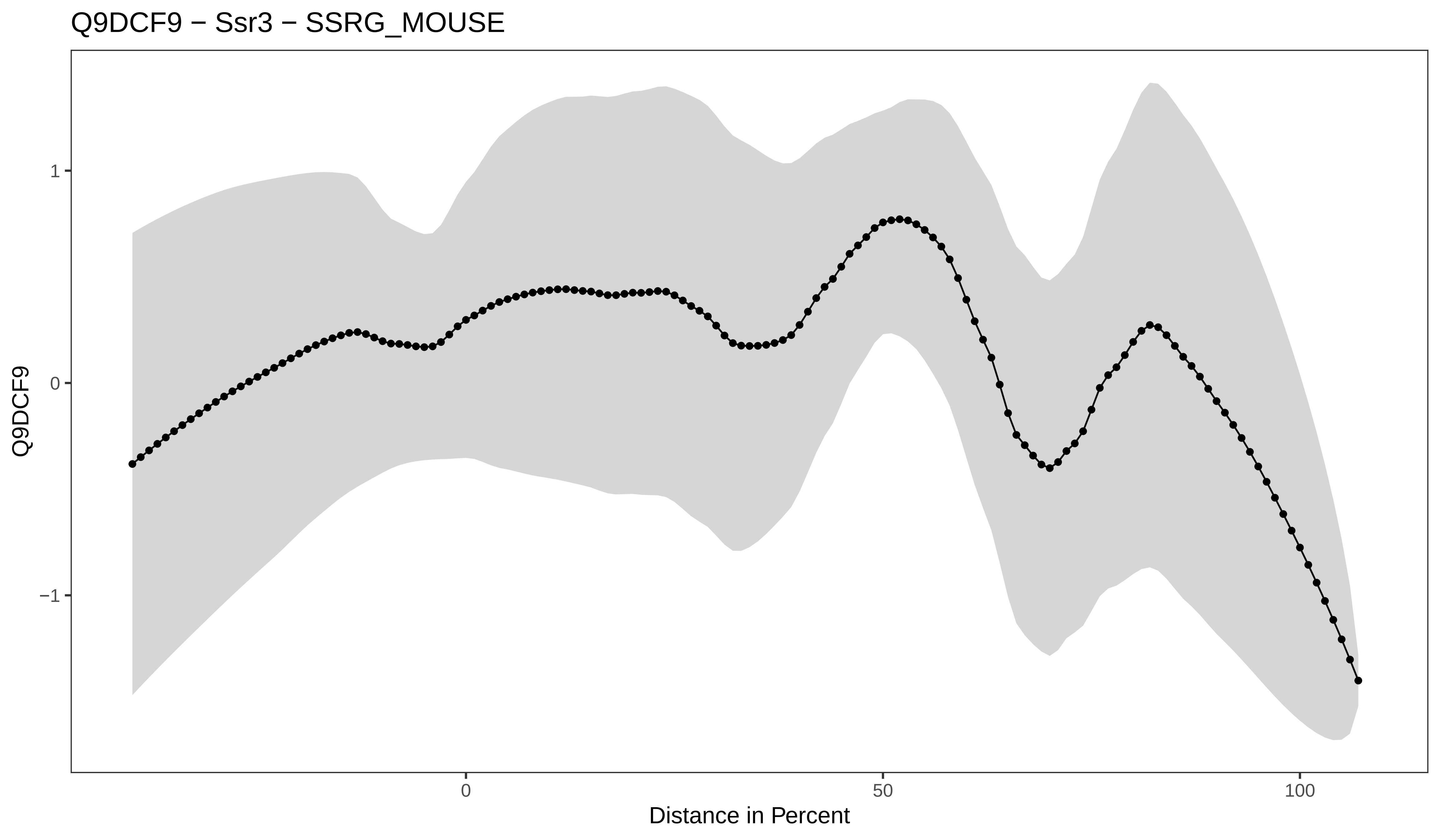

Q9DCF9 − Ssr3 − SSRG_MOUSE
Q9DCF9
1
0
−1
0
50
100
Distance in Percent

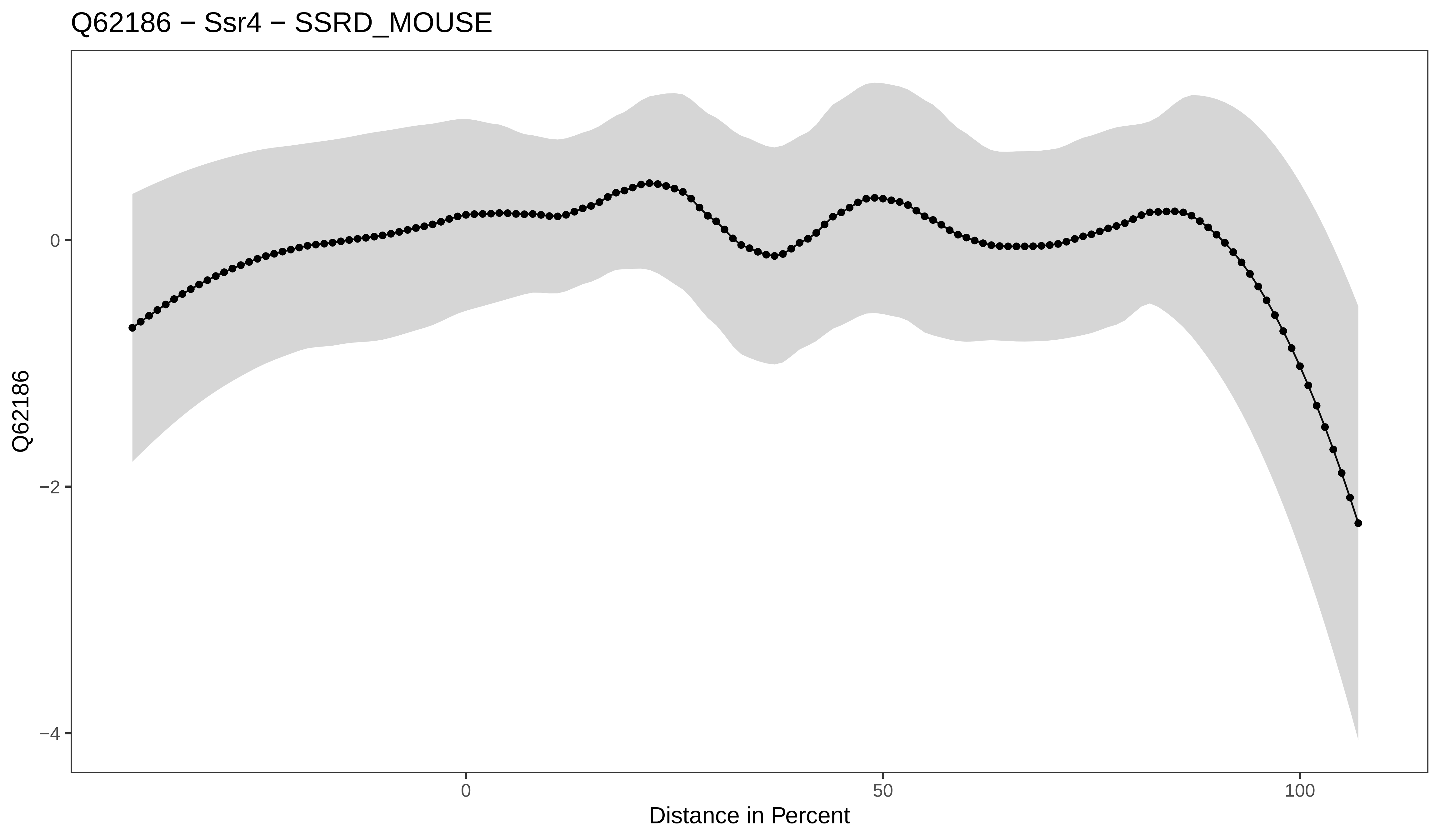

Q62186 − Ssr4 − SSRD_MOUSE
Q62186
0
−2
−4
0
50
100
Distance in Percent

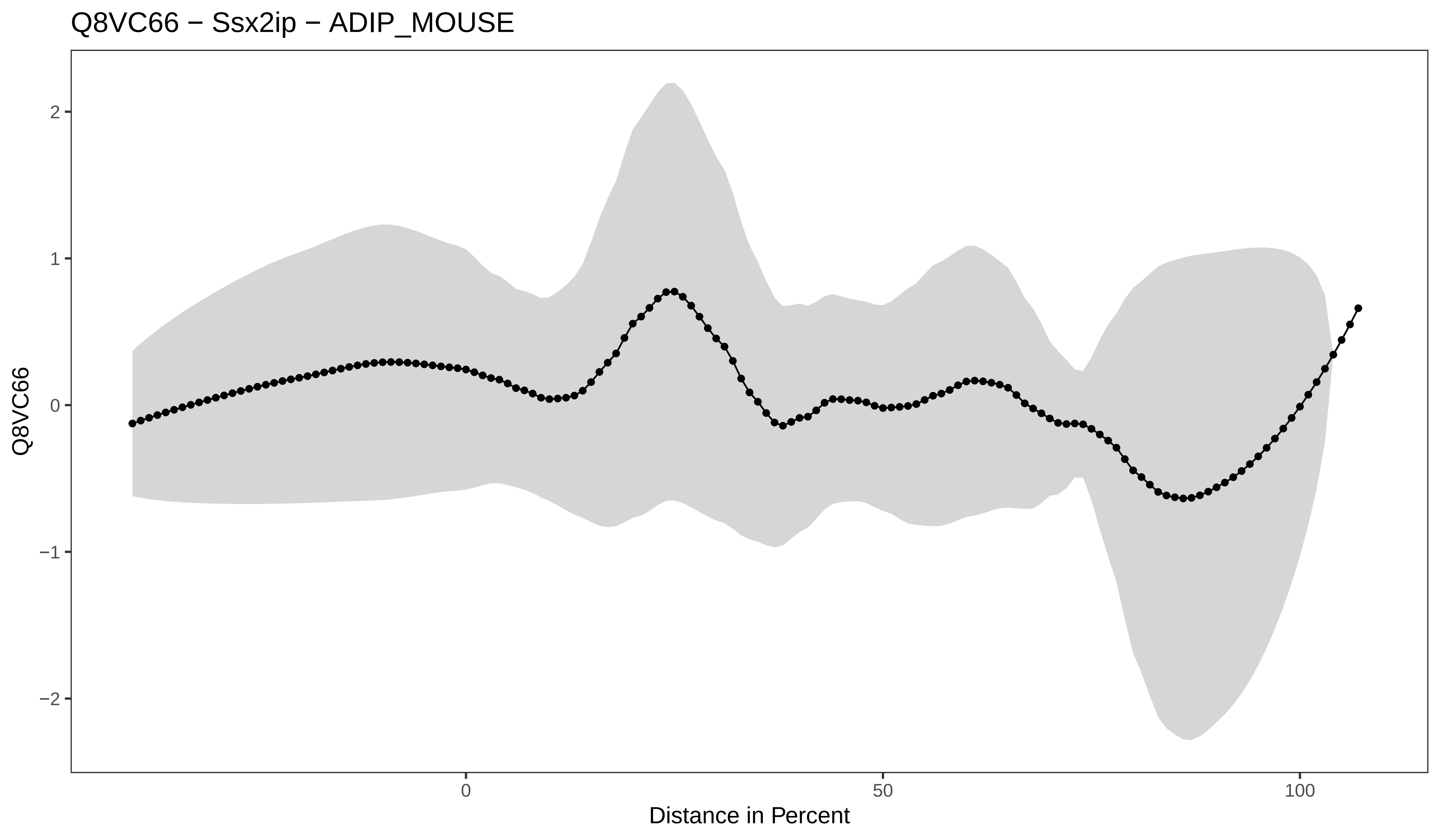

Q8VC66 − Ssx2ip − ADIP_MOUSE
Q8VC66
2
1
0
−1
−2
0
50
100
Distance in Percent

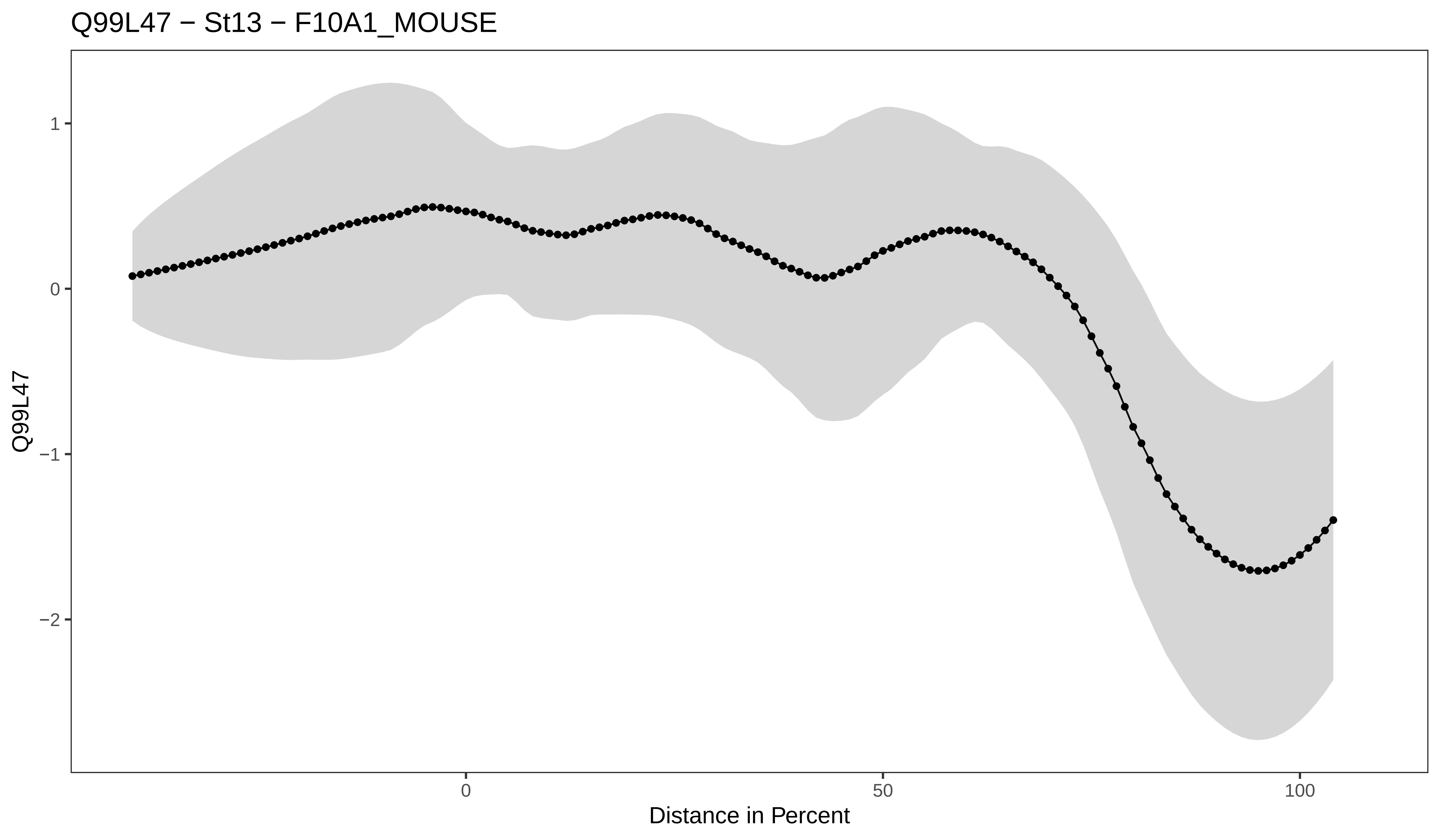

Q99L47 − St13 − F10A1_MOUSE
Q99L47
1
0
−1
−2
0
50
100
Distance in Percent

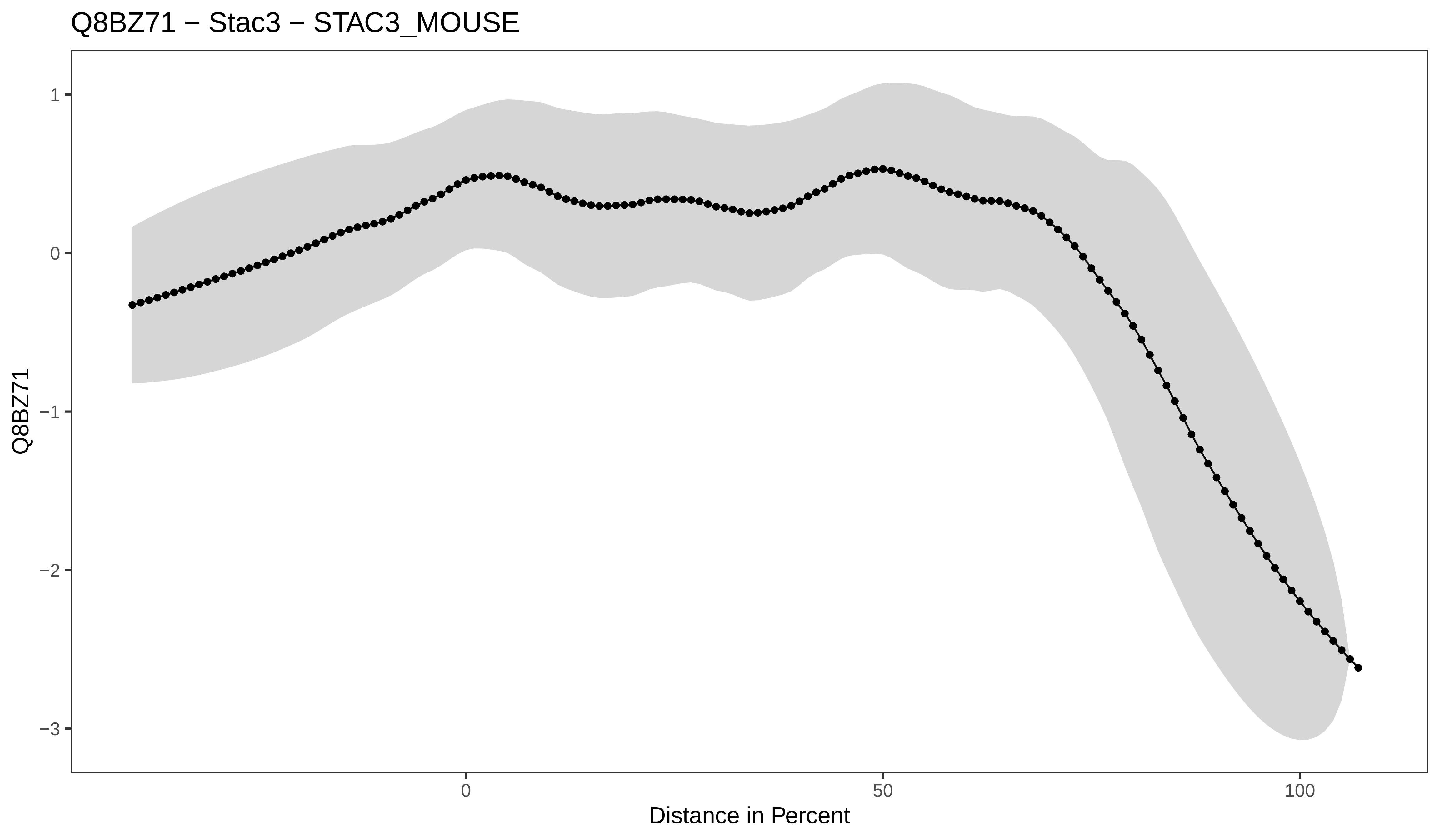
Q8BZ71 − Stac3 − STAC3_MOUSE
1
0
−1
−2
−3
Q8BZ71
0
50
100
Distance in Percent

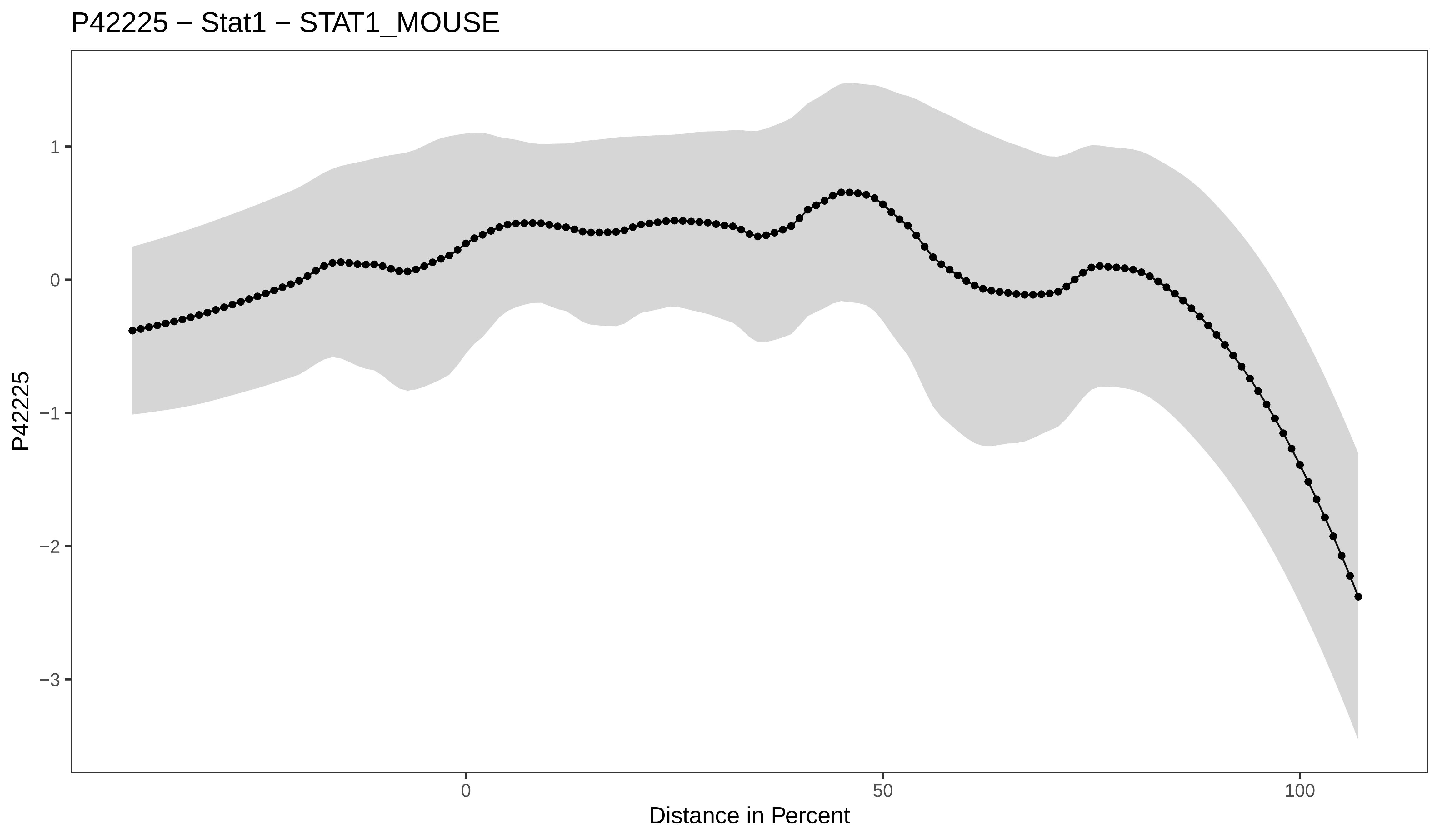

P42225 − Stat1 − STAT1_MOUSE
P42225
Distance in Percent
1
0
−1
−2
−3
0
50
100

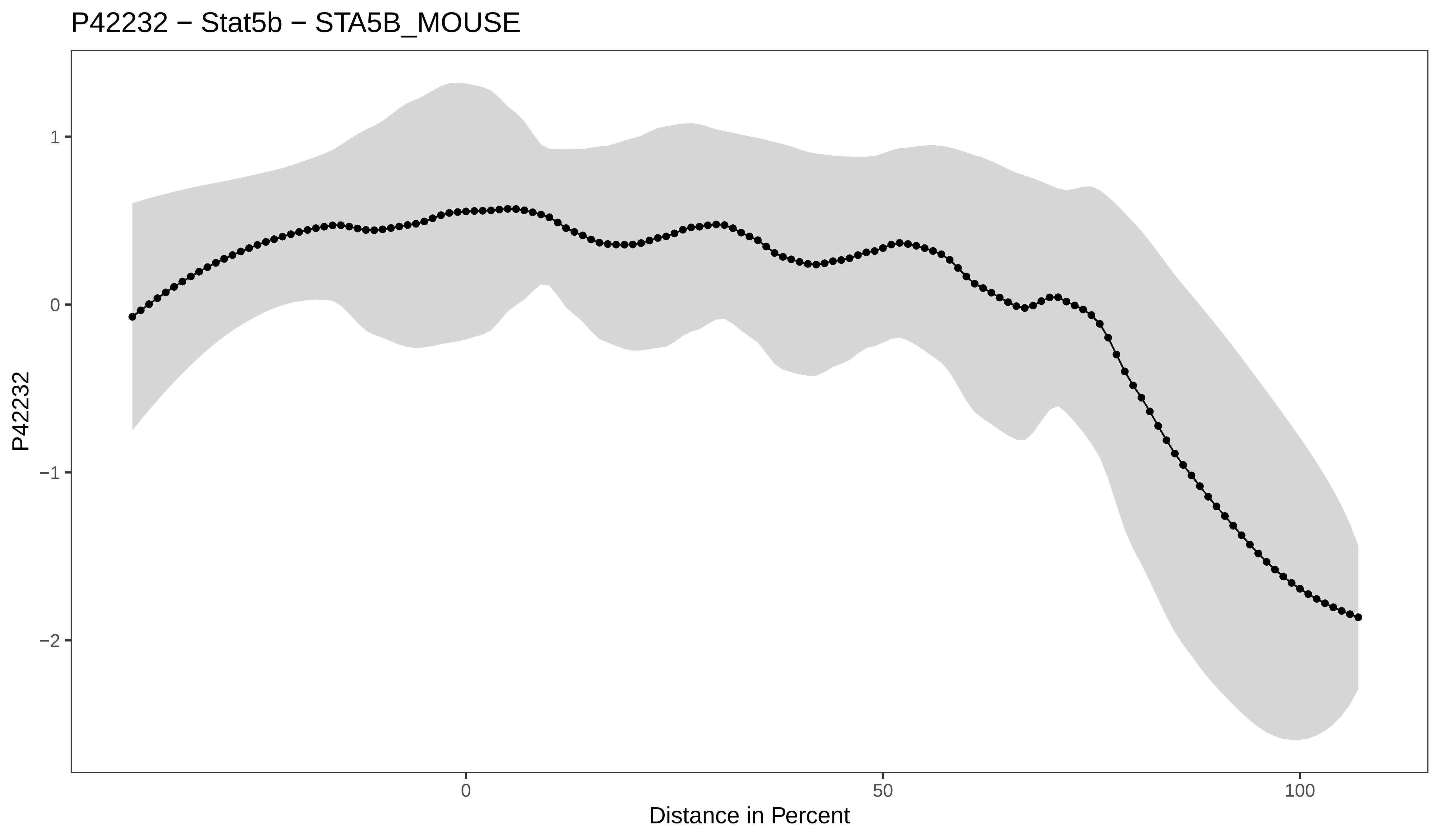

P42232 − Stat5b − STA5B_MOUSE
1
0
−1
−2
P42232
0
50
100
Distance in Percent

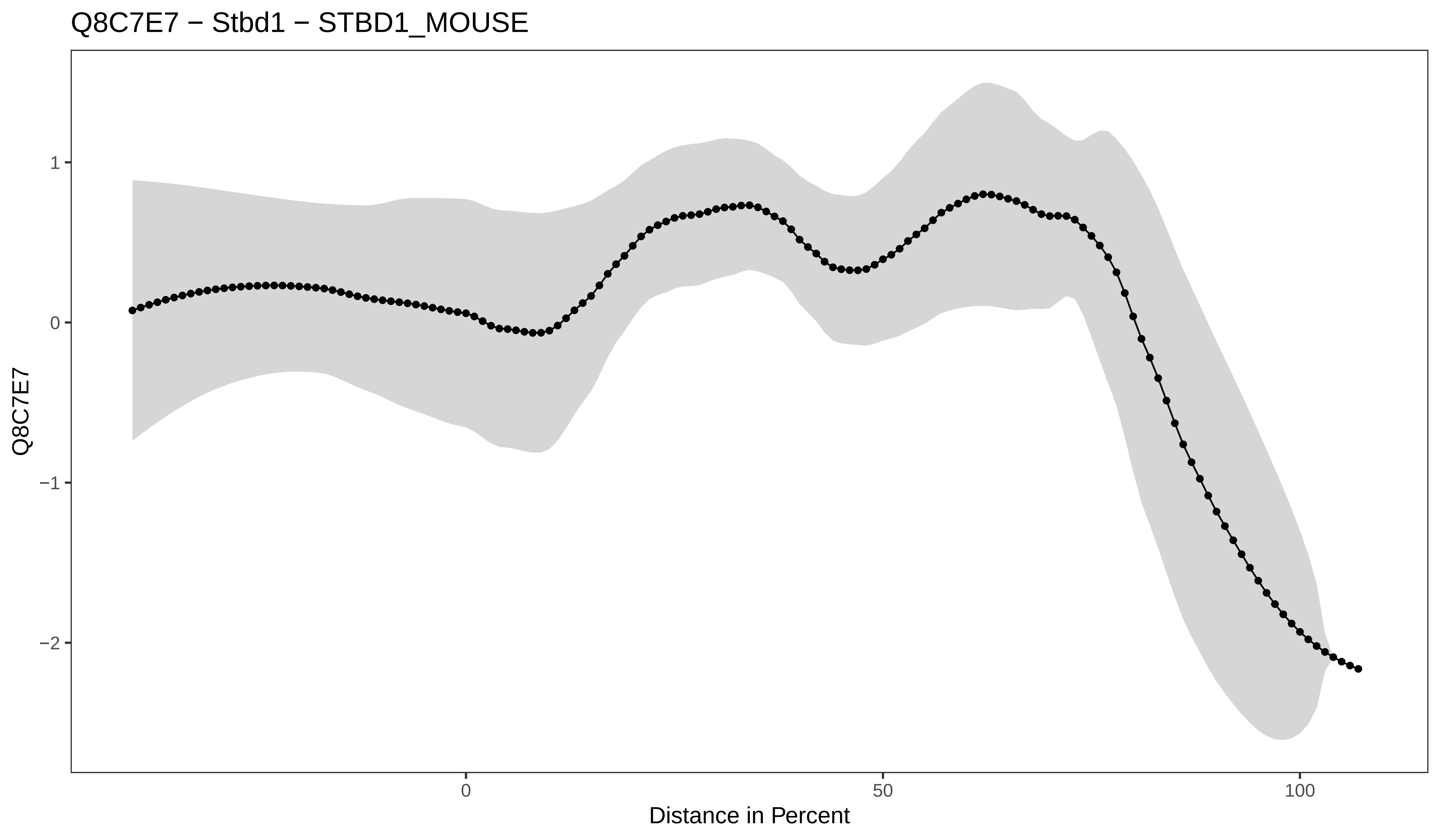
Q8C7E7 − Stbd1 − STBD1_MOUSE
Q8C7E7
1
0
−1
−2
0
50
100
Distance in Percent

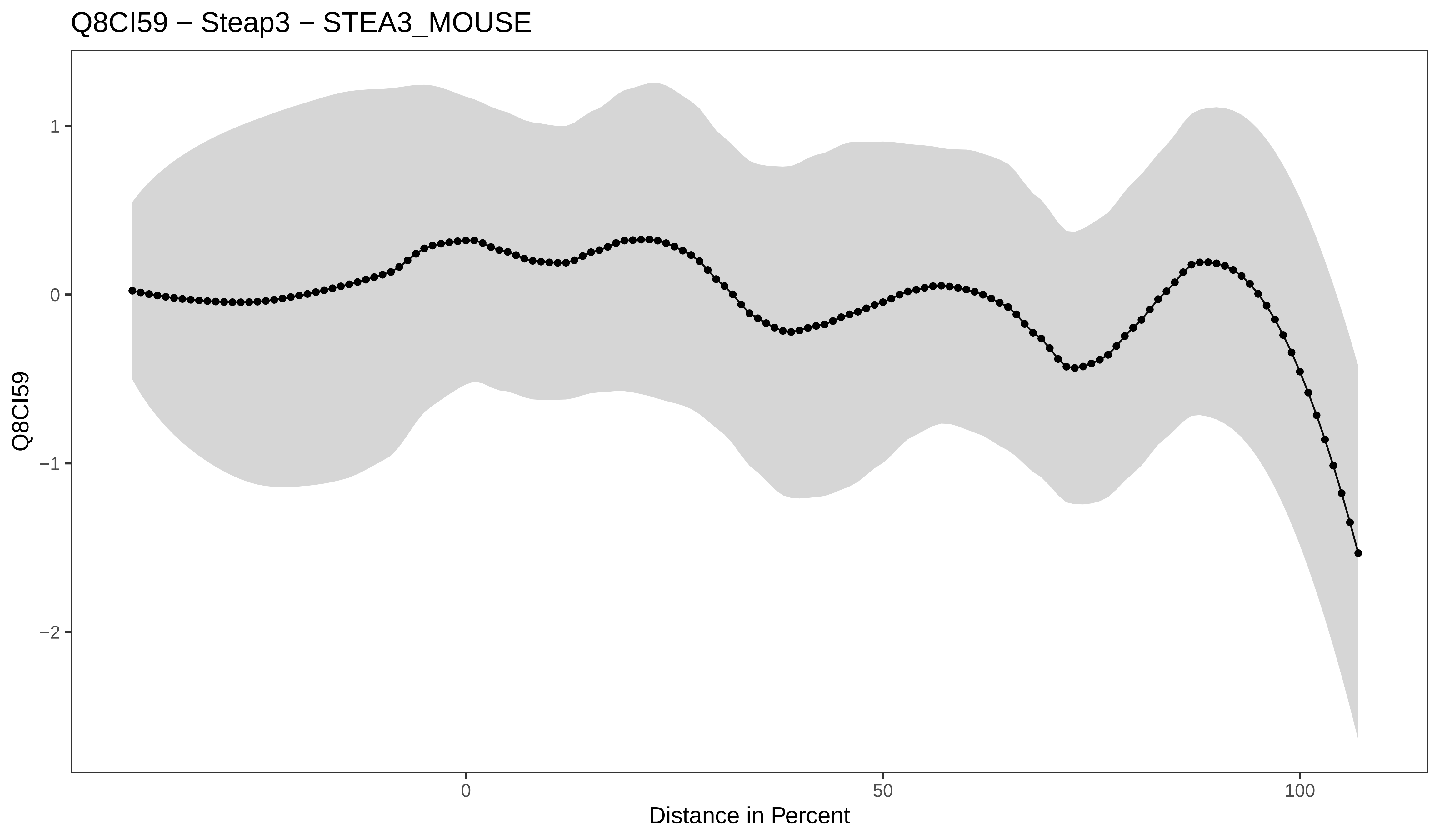

Q8CI59 − Steap3 − STEA3_MOUSE
Q8CI59
1
0
−1
−2
0
50
100
Distance in Percent

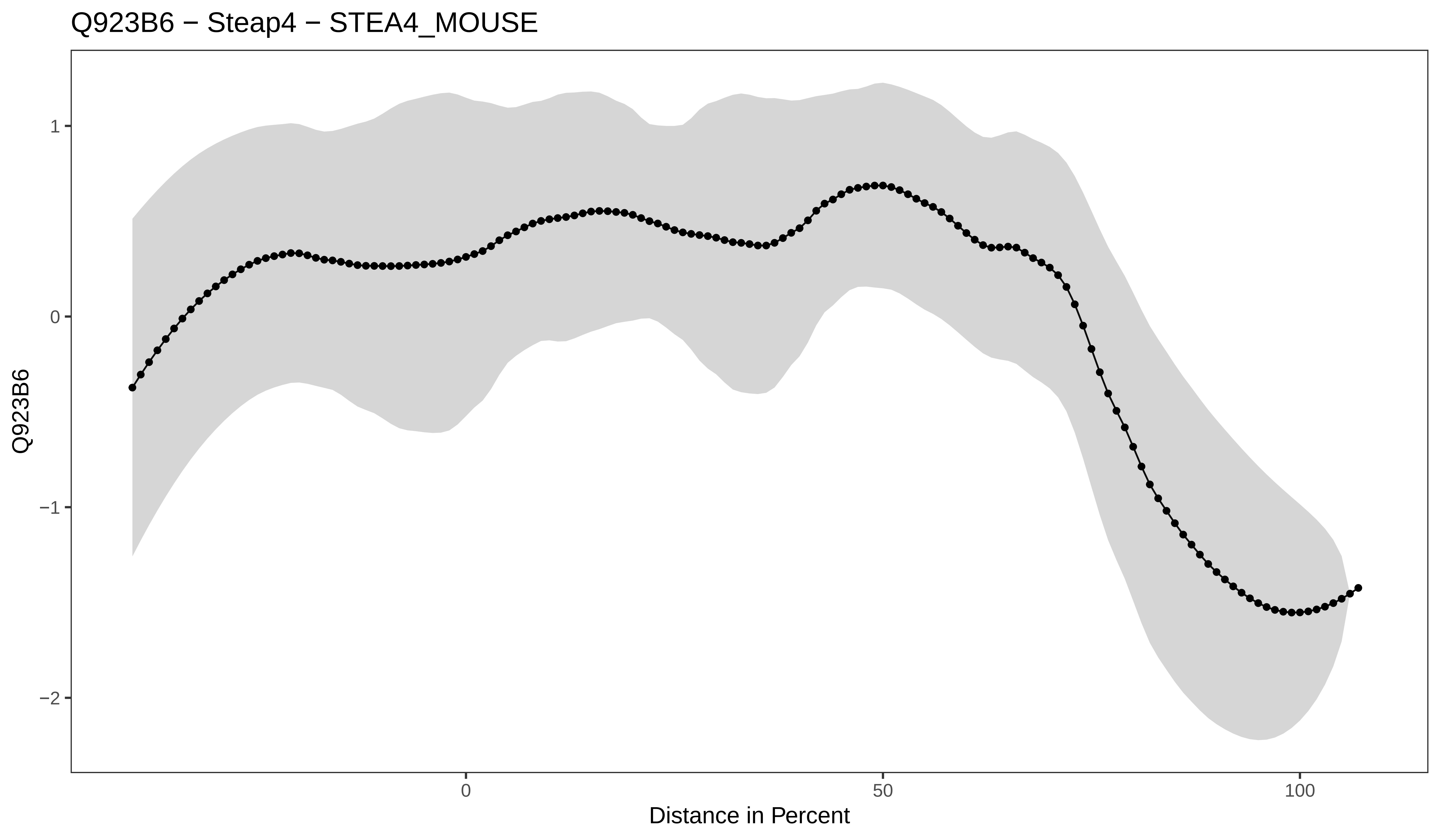
Q923B6 − Steap4 − STEA4_MOUSE
Q923B6
1
0
−1
−2
0
50
100
Distance in Percent

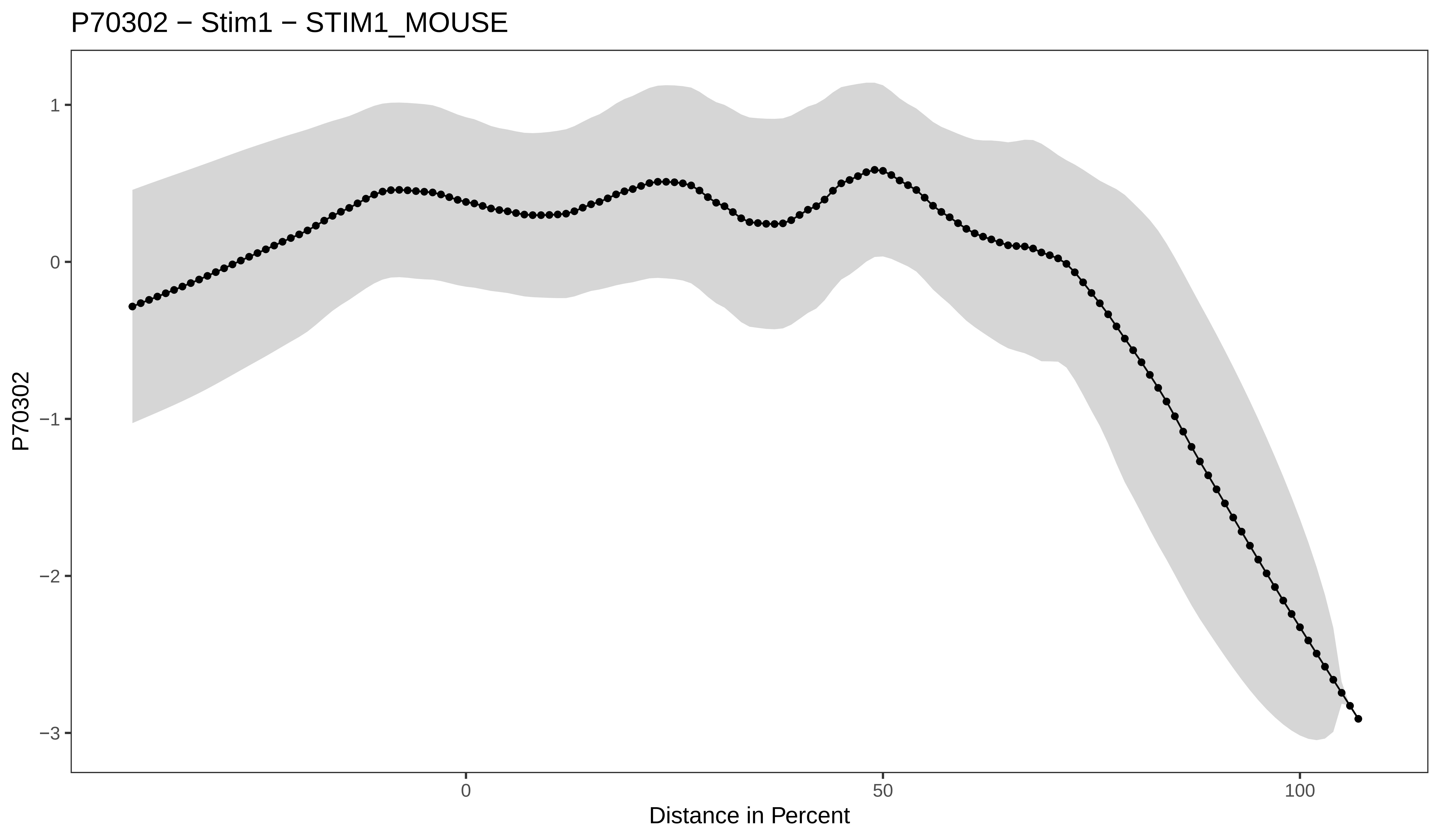

P70302 − Stim1 − STIM1_MOUSE
1
0
−1
−2
−3
P70302
0
50
100
Distance in Percent

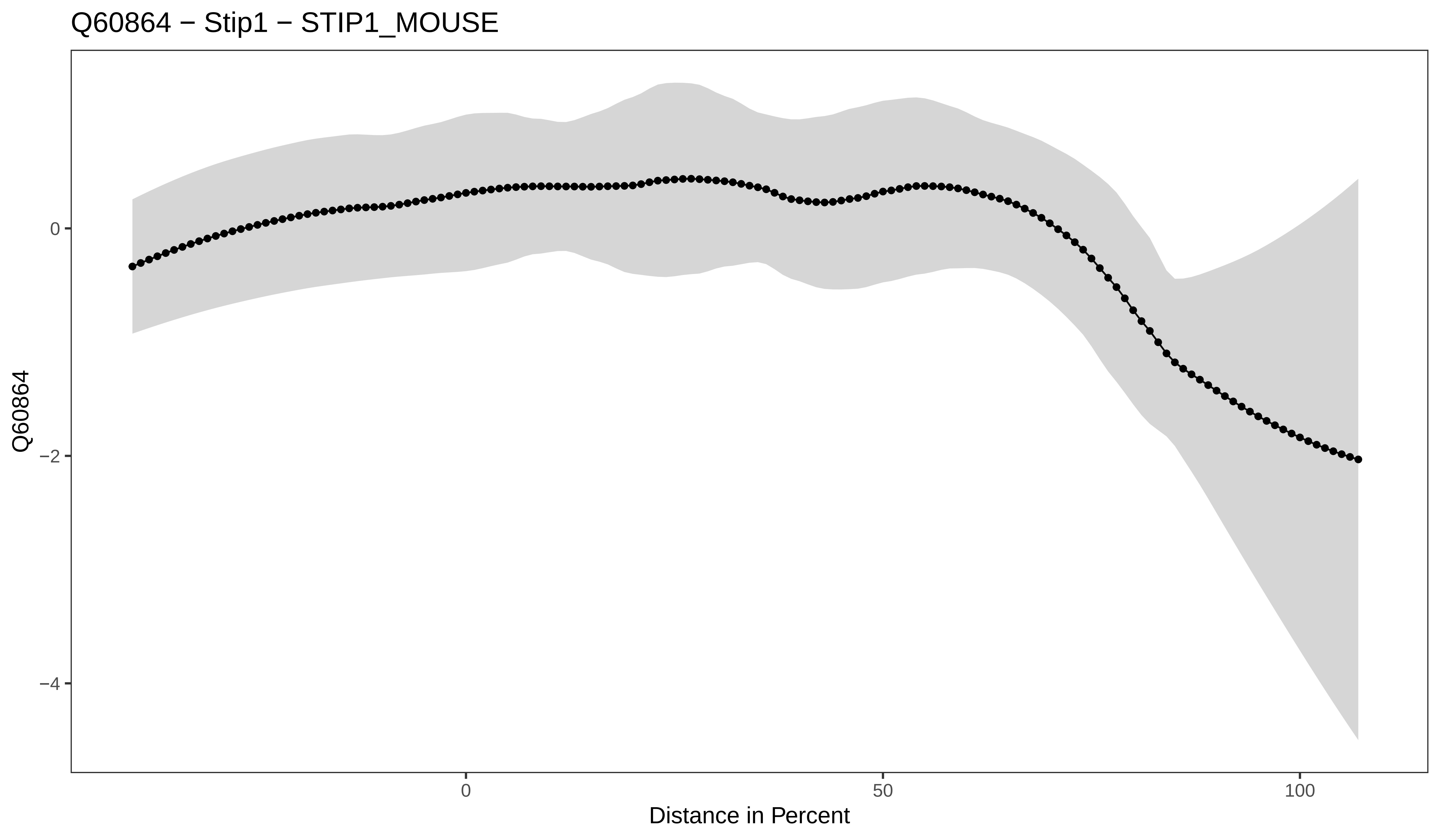
Q60864 − Stip1 − STIP1_MOUSE
Q60864
0
−2
−4
0
50
100
Distance in Percent

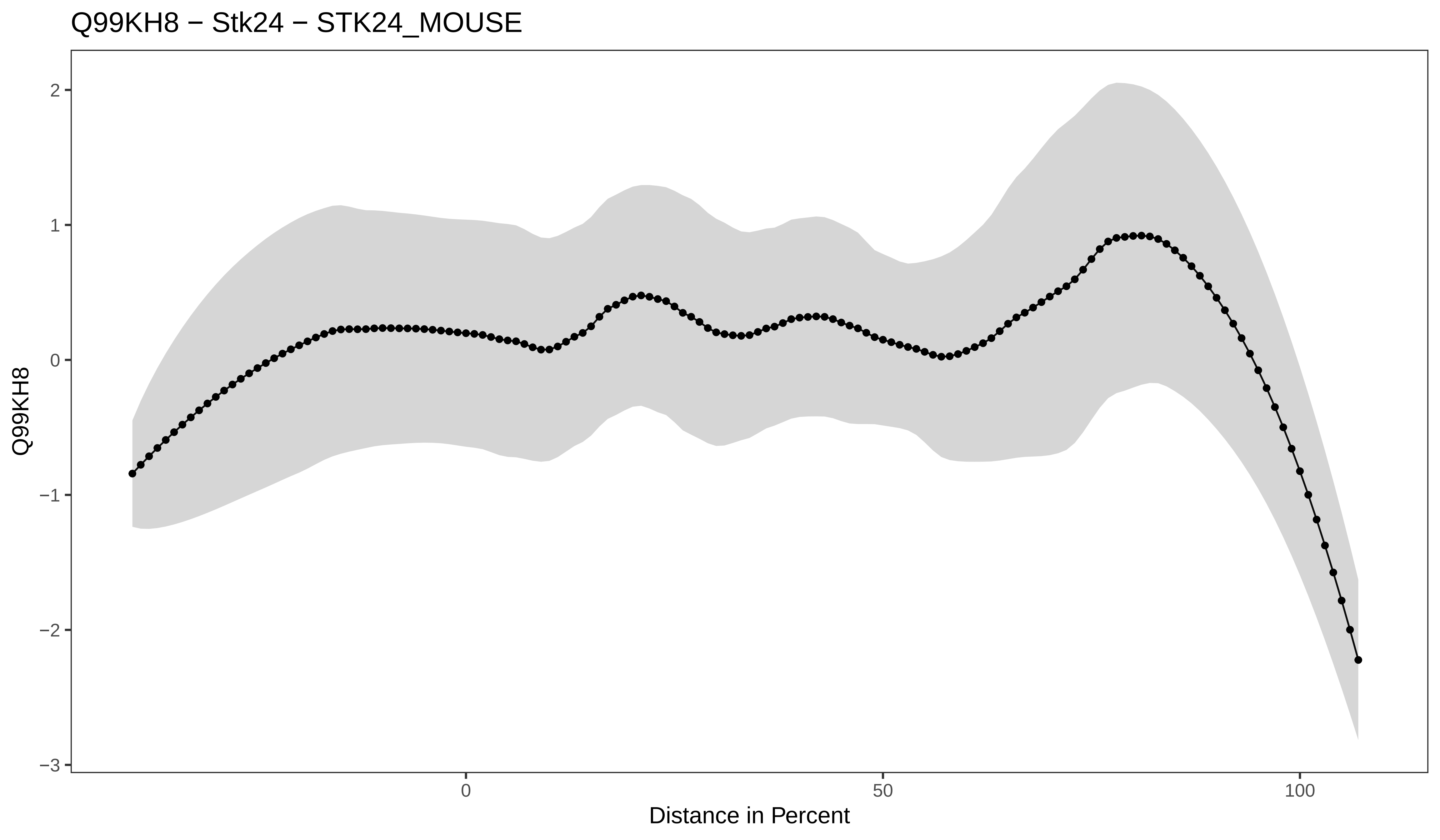

Q99KH8 − Stk24 − STK24_MOUSE
Q99KH8
Distance in Percent
2
1
0
−1
−2
−3
0
50
100

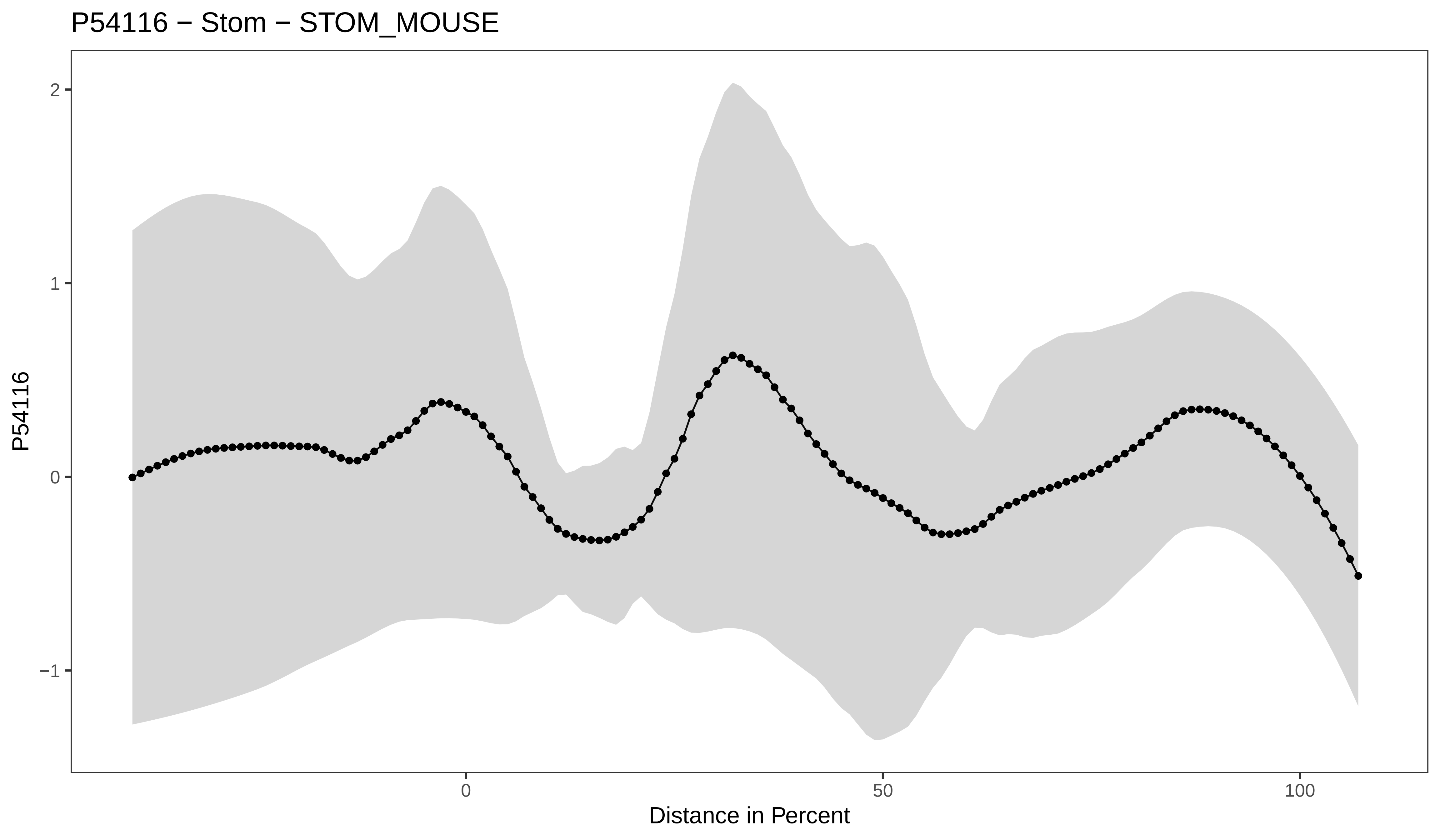

P54116 − Stom − STOM_MOUSE
P54116
2
1
0
−1
0
50
100
Distance in Percent

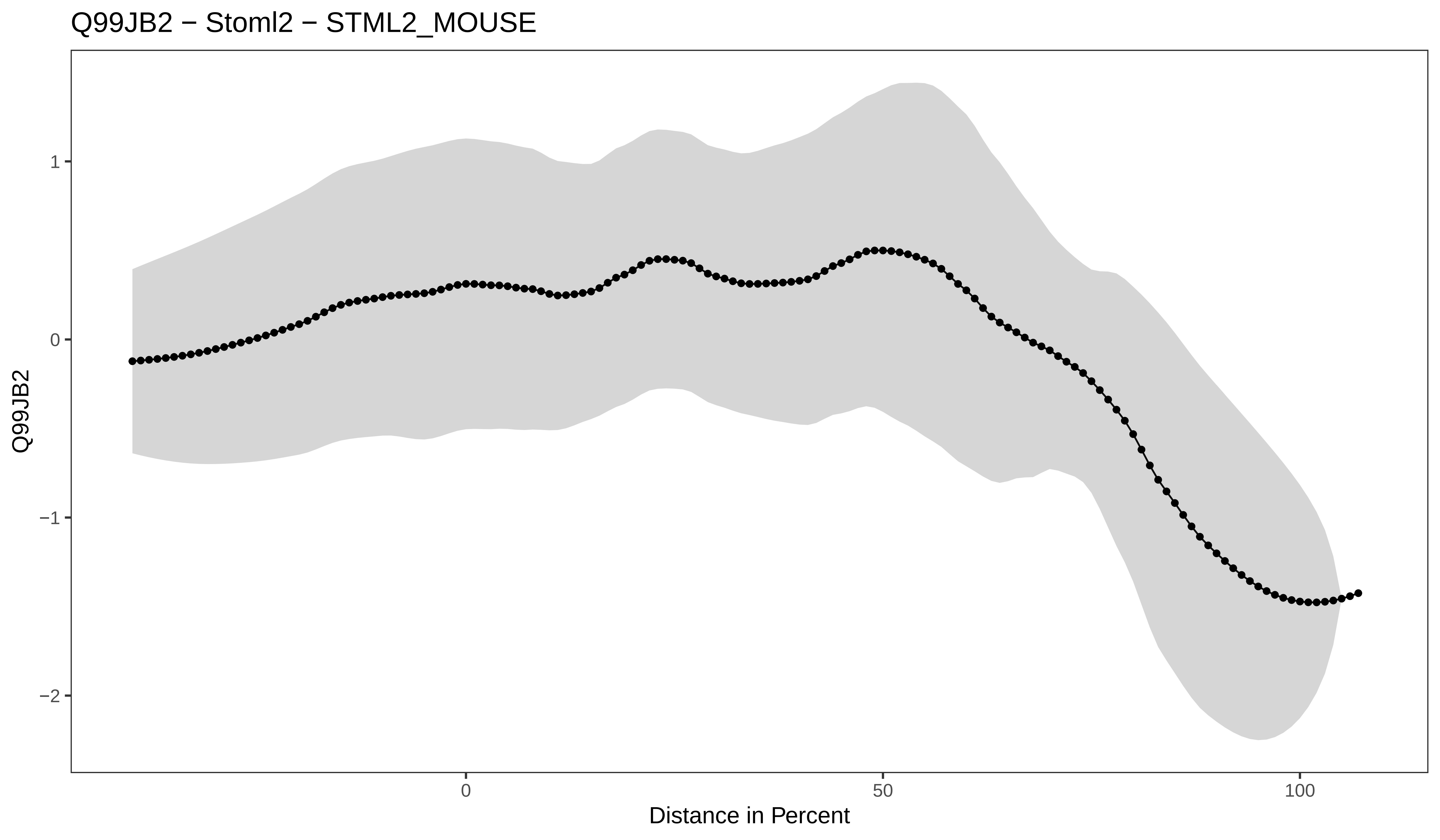
Q99JB2 − Stoml2 − STML2_MOUSE
Q99JB2
1
0
−1
−2
0
50
100
Distance in Percent

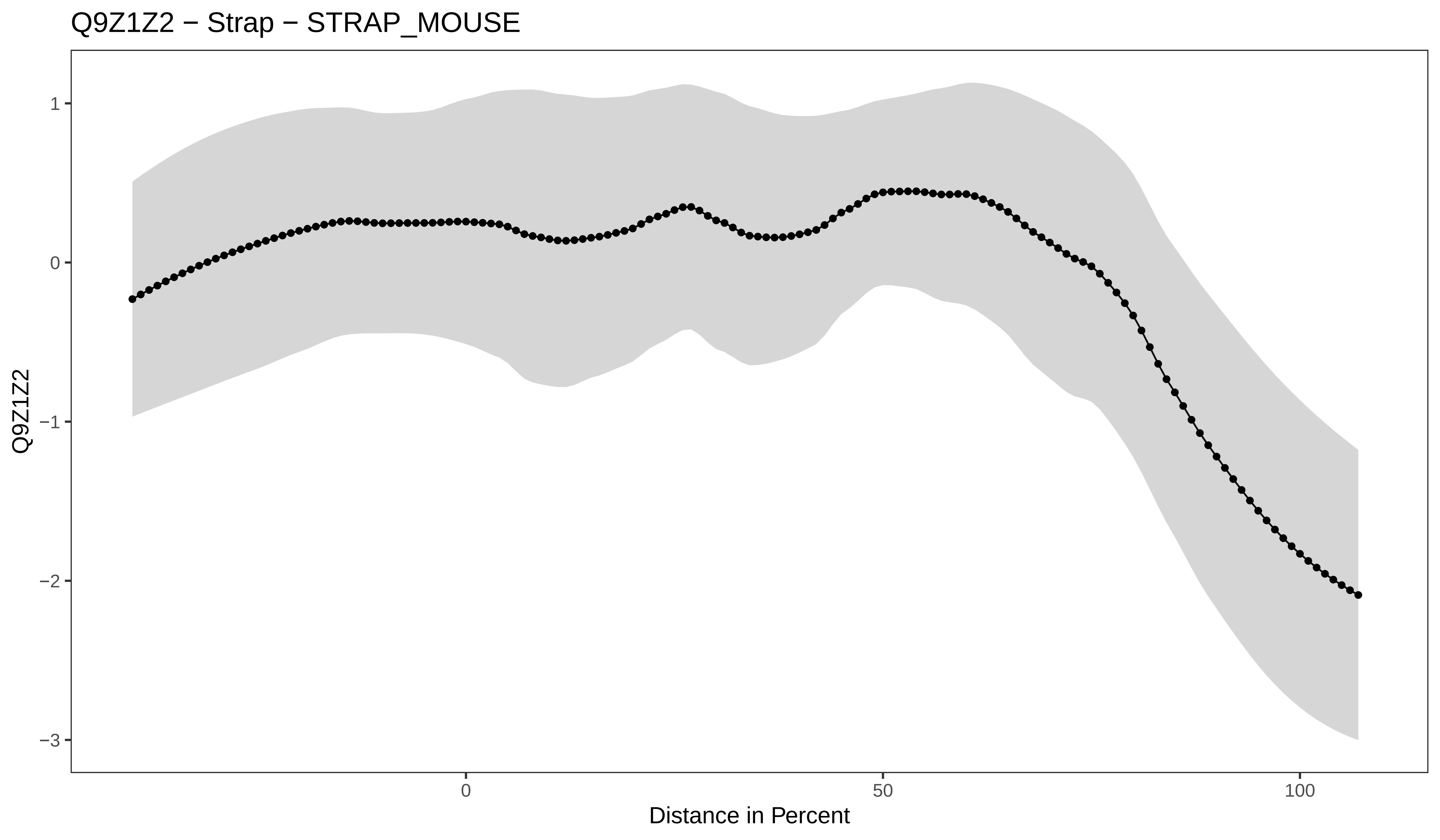

Q9Z1Z2 − Strap − STRAP_MOUSE
1
0
−1
−2
−3
Q9Z1Z2
0
50
100
Distance in Percent

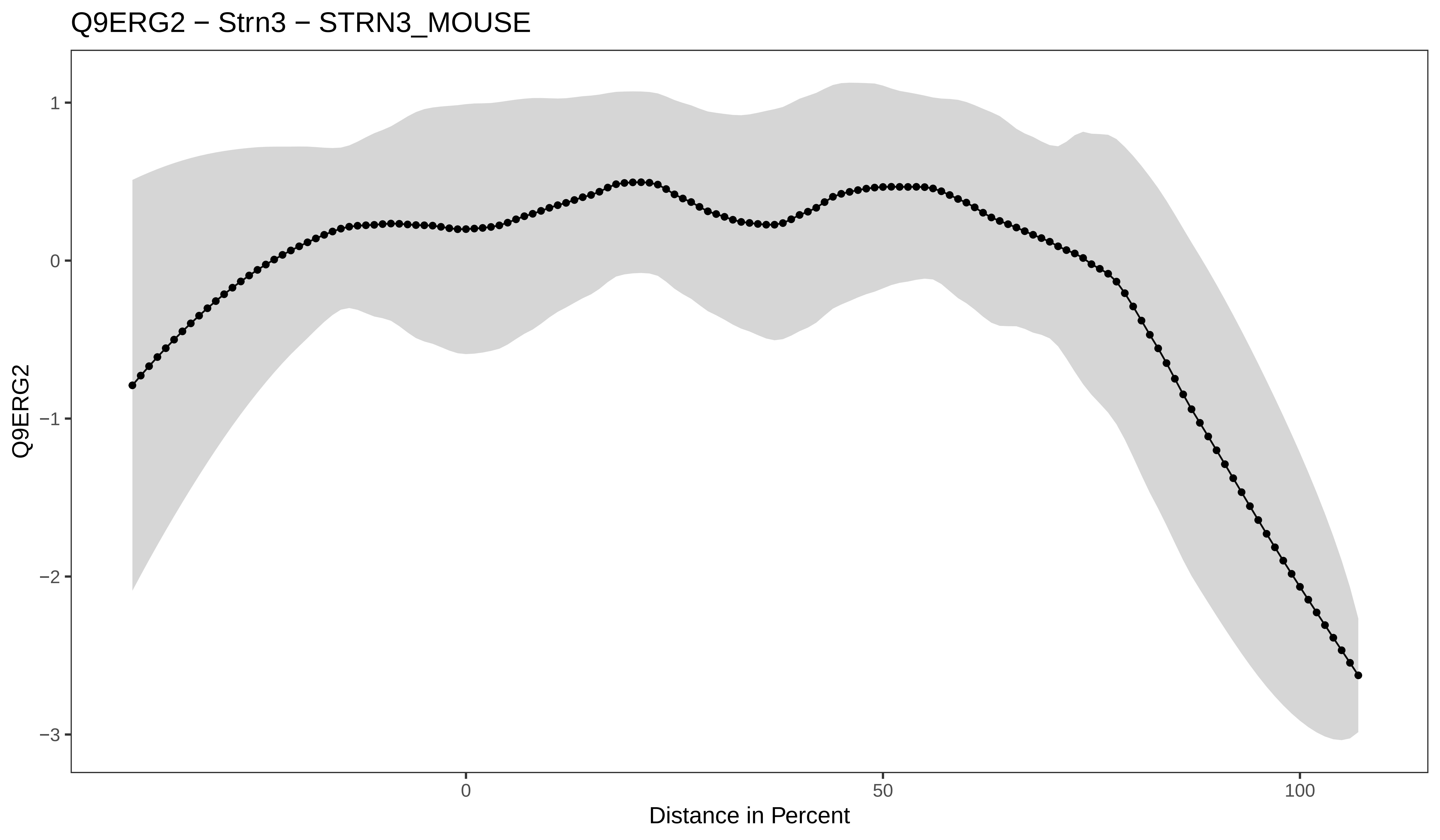

Q9ERG2 − Strn3 − STRN3_MOUSE
1
0
−1
−2
−3
Q9ERG2
0
50
100
Distance in Percent

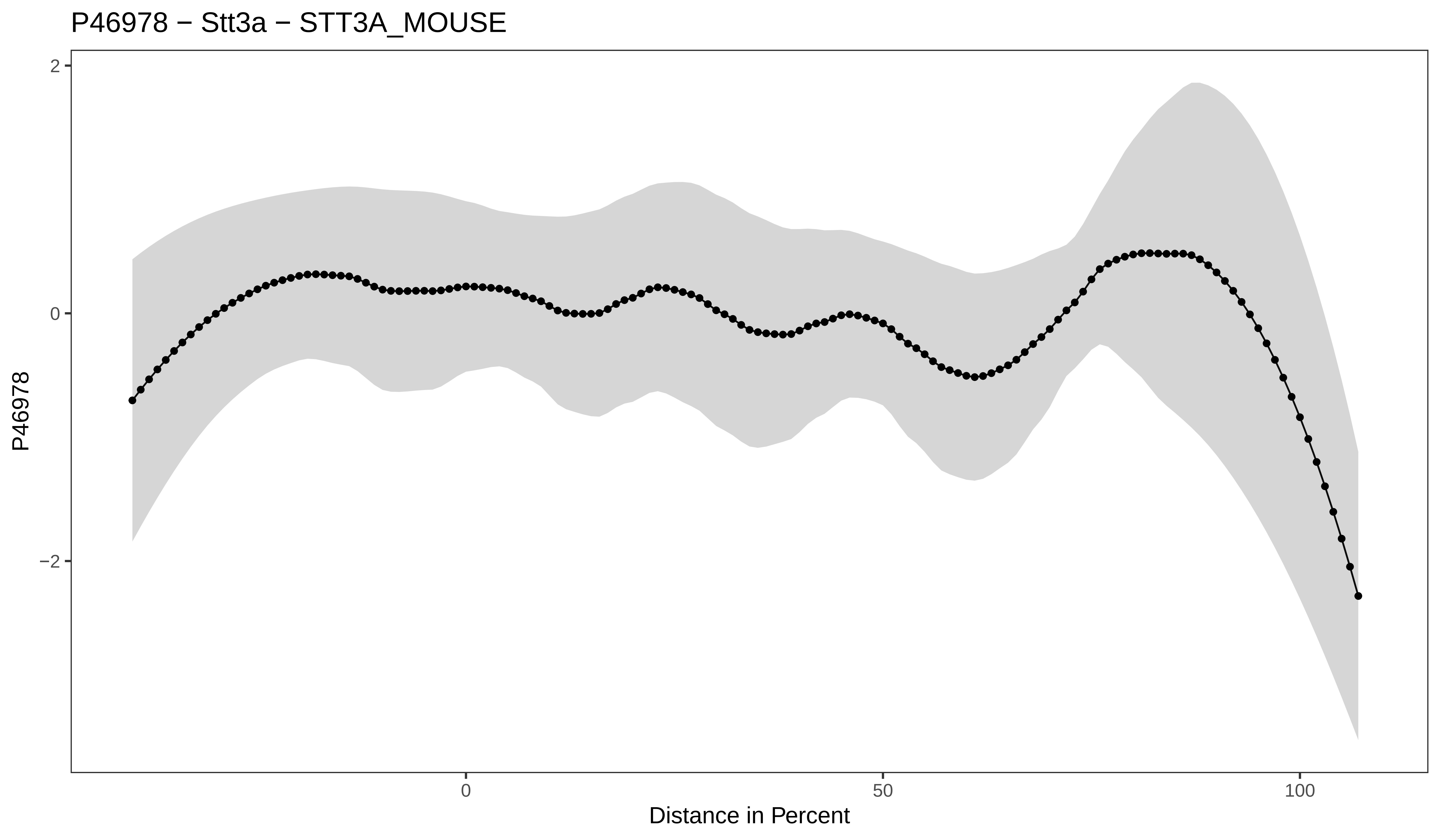

P46978 − Stt3a − STT3A_MOUSE
2
0
−2
P46978
0
50
100
Distance in Percent

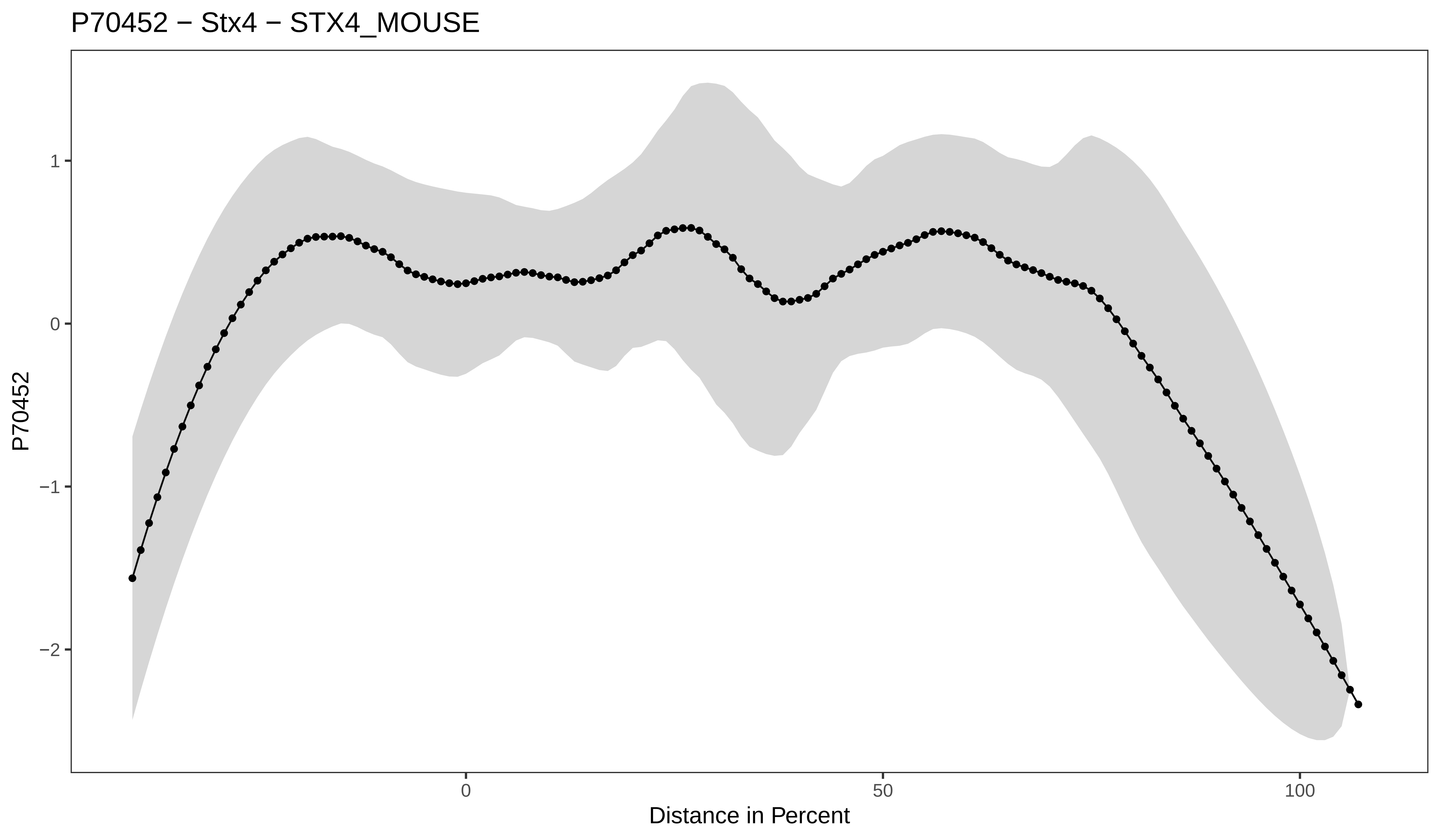

P70452 − Stx4 − STX4_MOUSE
P70452
1
0
−1
−2
0
50
100
Distance in Percent

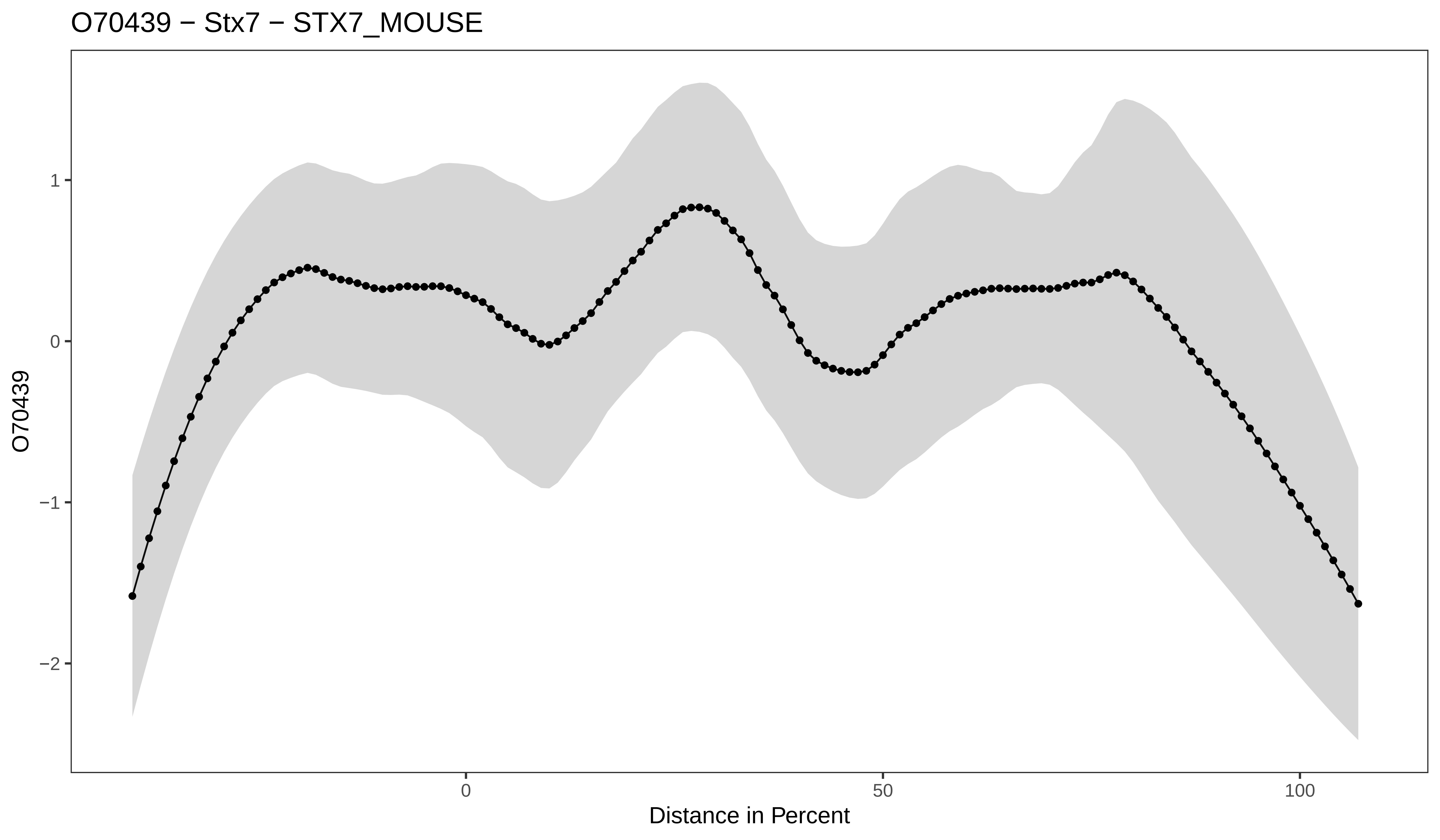
O70439 − Stx7 − STX7_MOUSE
O70439
1
0
−1
−2
0
50
100
Distance in Percent

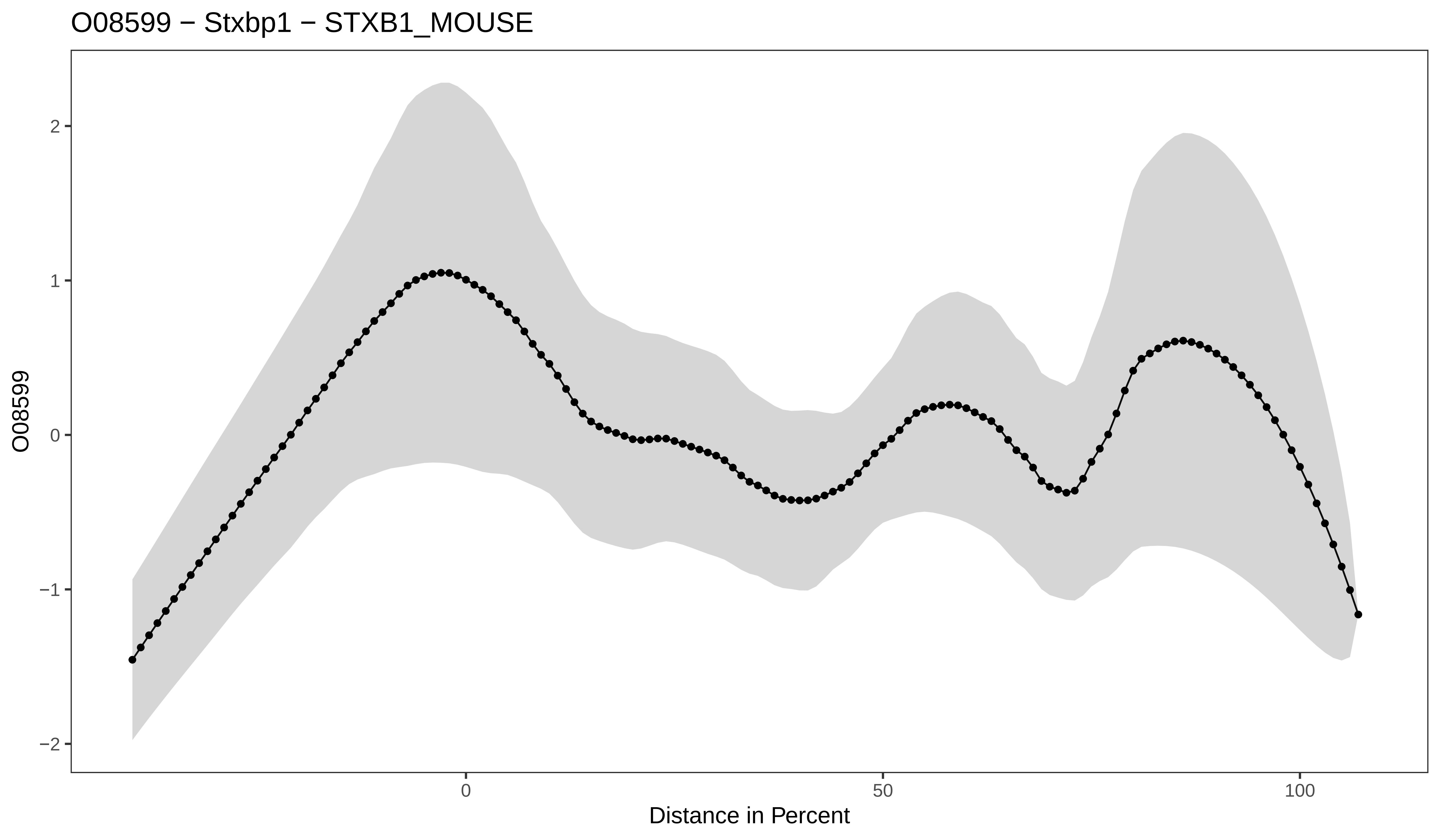
O08599 − Stxbp1 − STXB1_MOUSE
O08599
Distance in Percent
2
1
0
−1
−2
0
50
100

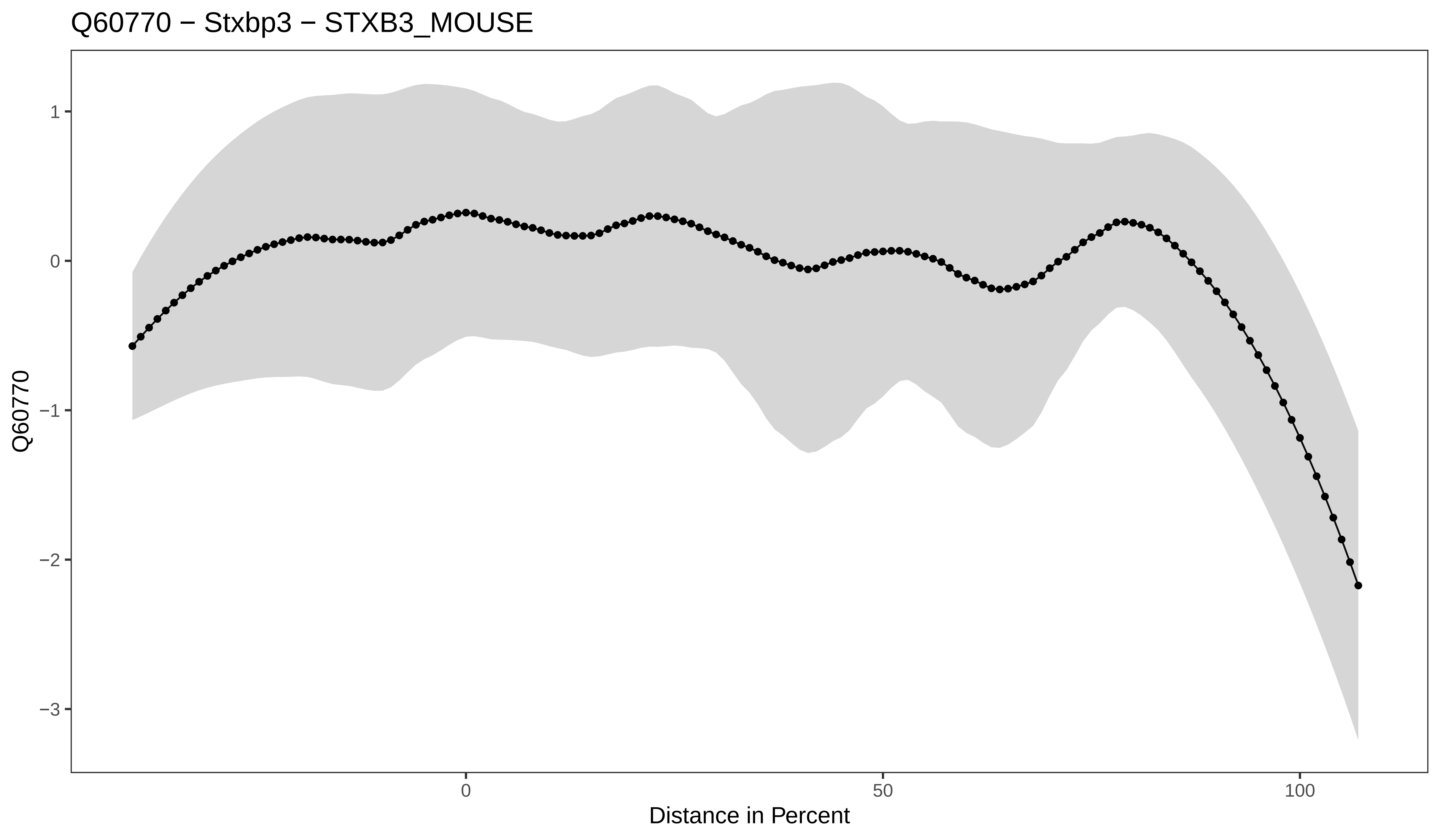

Q60770 − Stxbp3 − STXB3_MOUSE
Q60770
1
0
−1
−2
−3
0
50
100
Distance in Percent

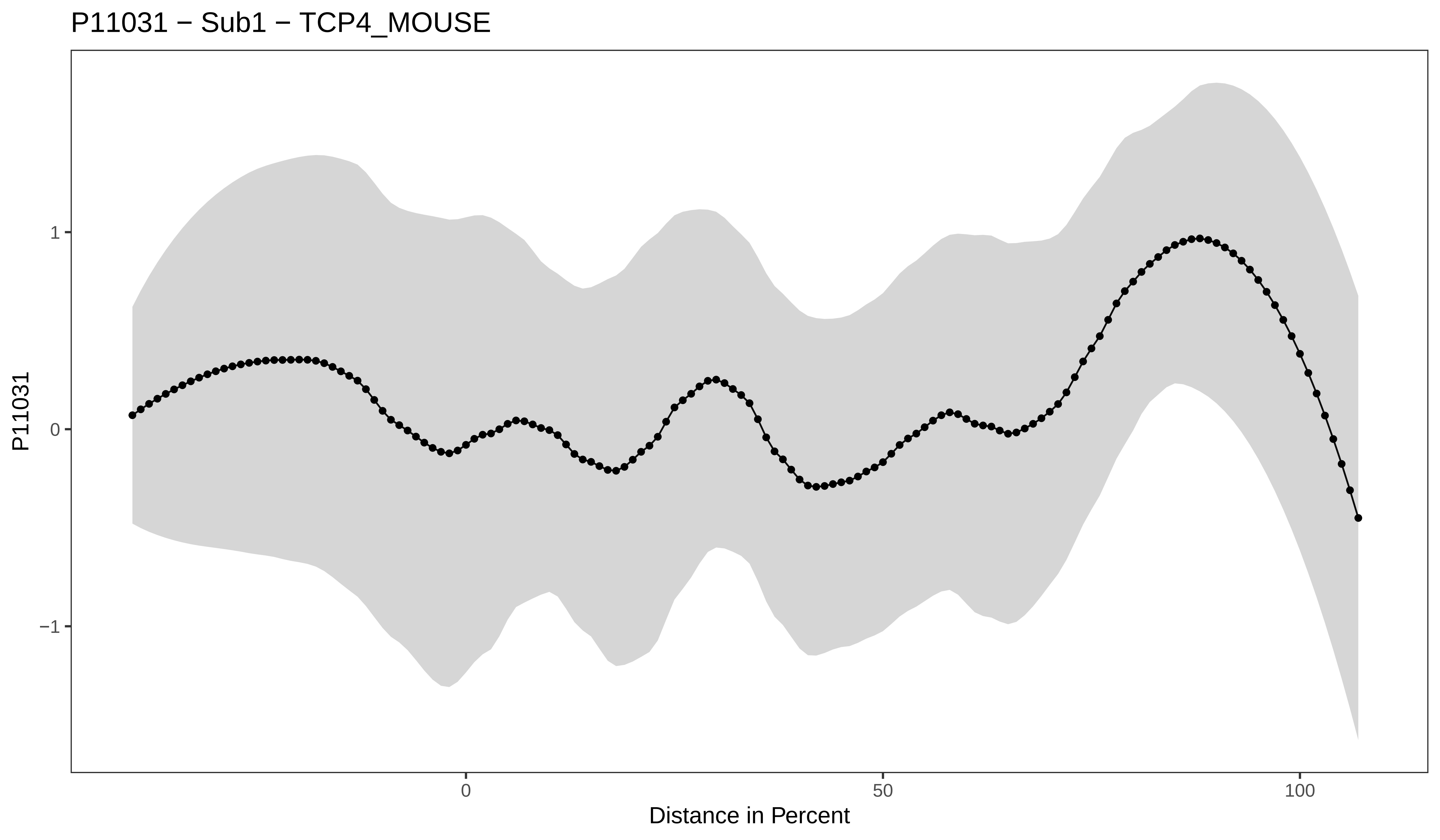
P11031 − Sub1 − TCP4_MOUSE
P11031
1
0
−1
0
50
100
Distance in Percent

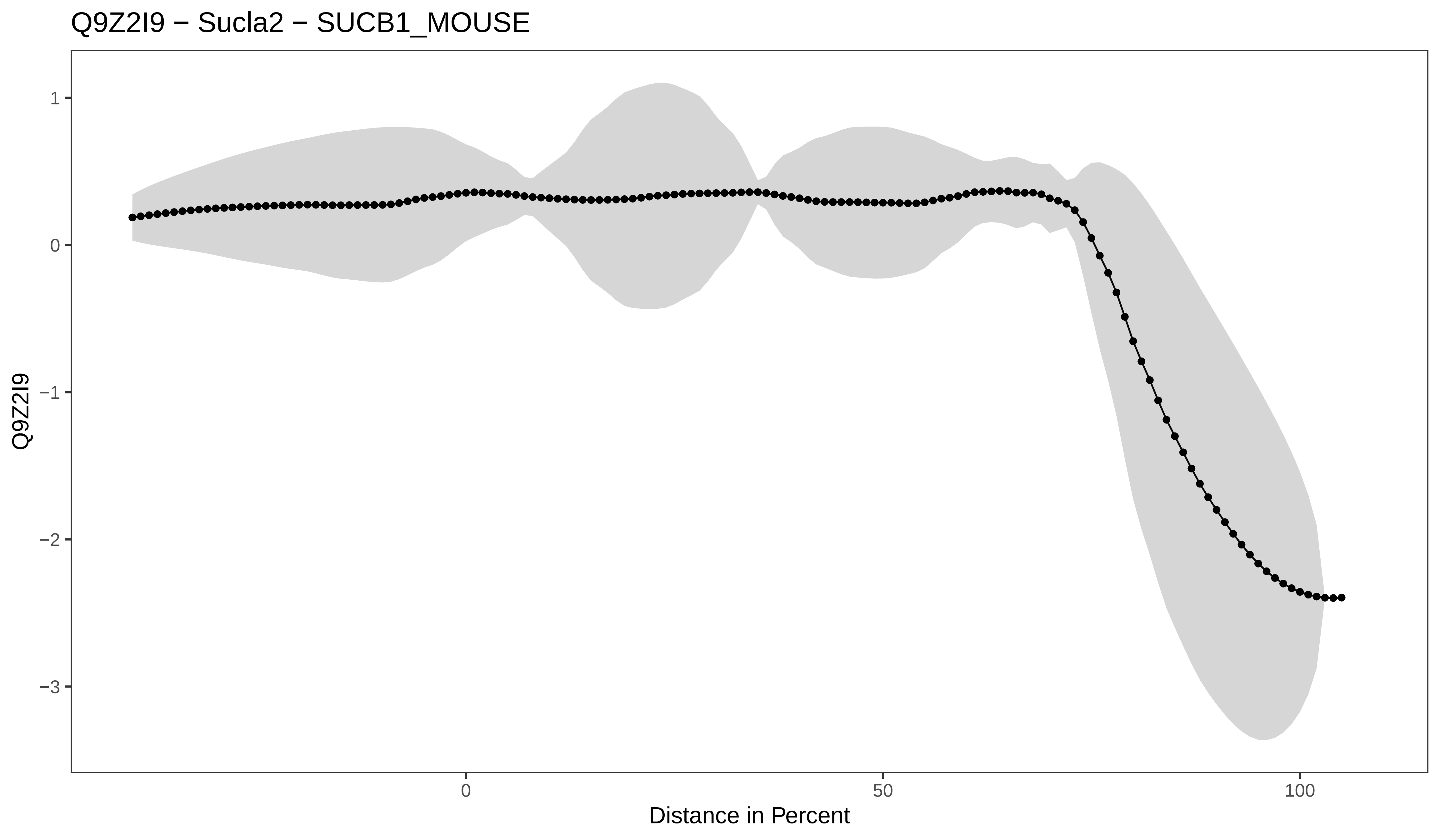

Q9Z2I9 − Sucla2 − SUCB1_MOUSE
Q9Z2I9
1
0
−1
−2
−3
0
50
100
Distance in Percent

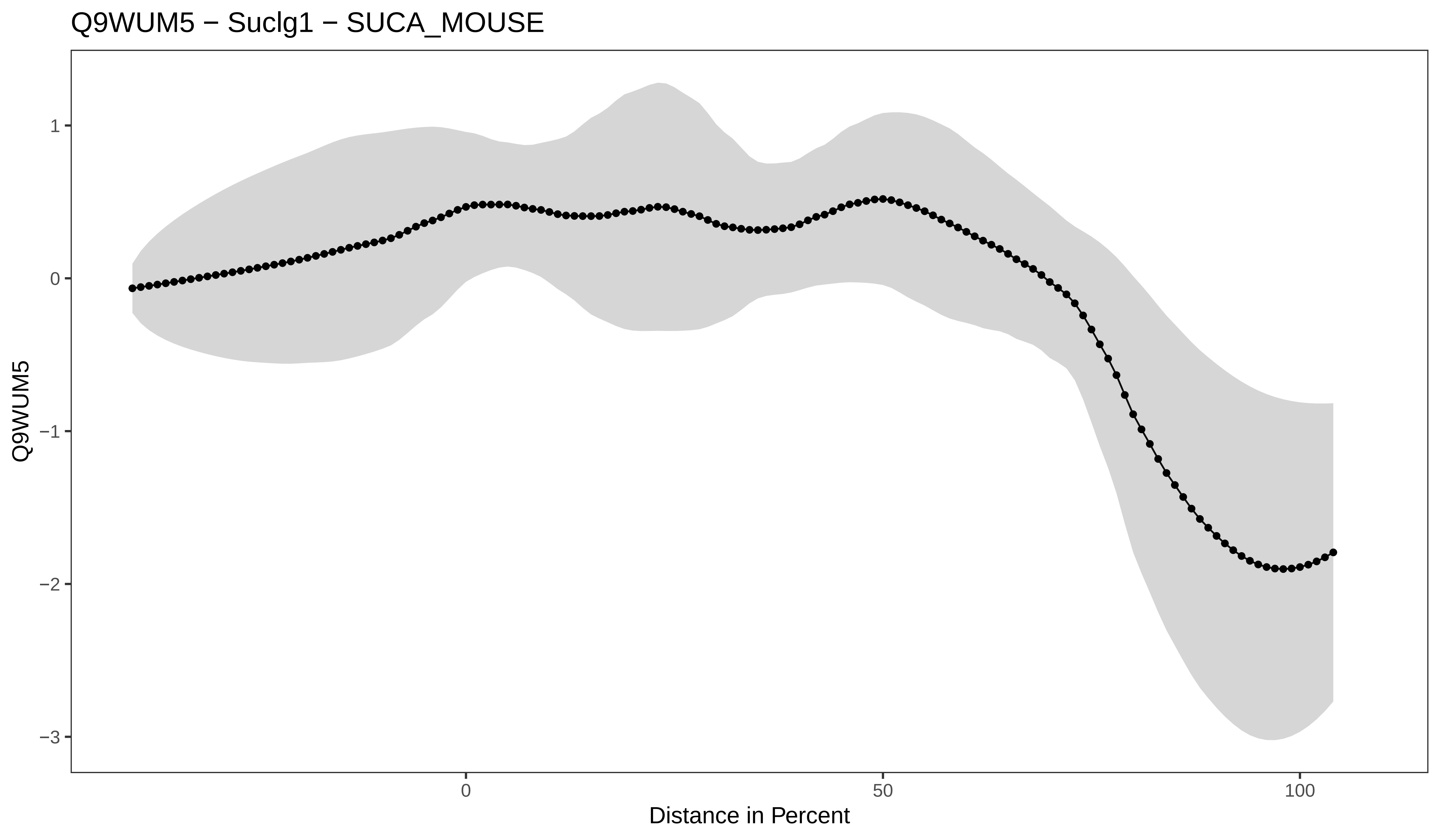

Q9WUM5 − Suclg1 − SUCA_MOUSE
1
0
−1
−2
−3
Q9WUM5
0
50
100
Distance in Percent

Q9Z2I8 − Suclg2 − SUCB2_MOUSE

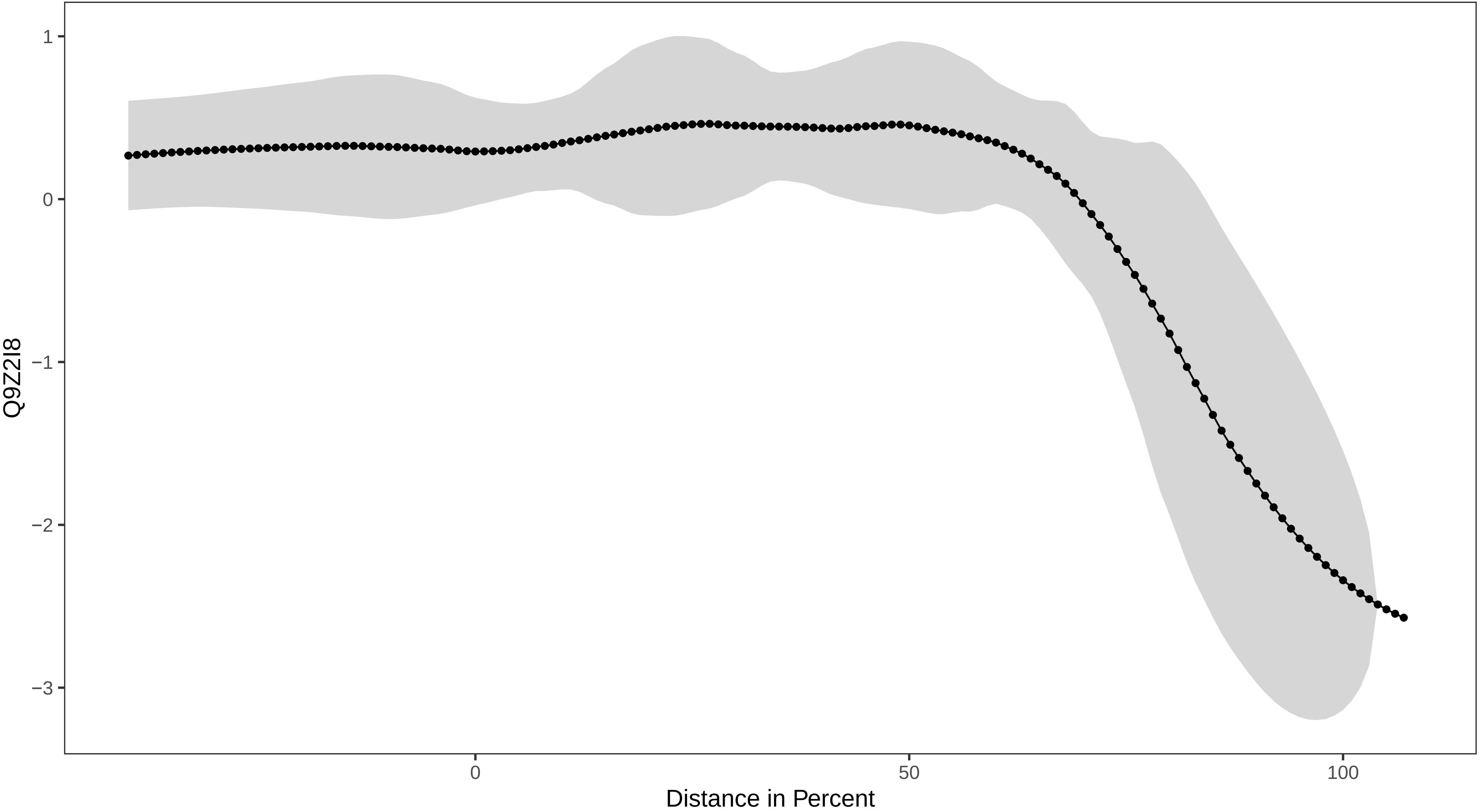

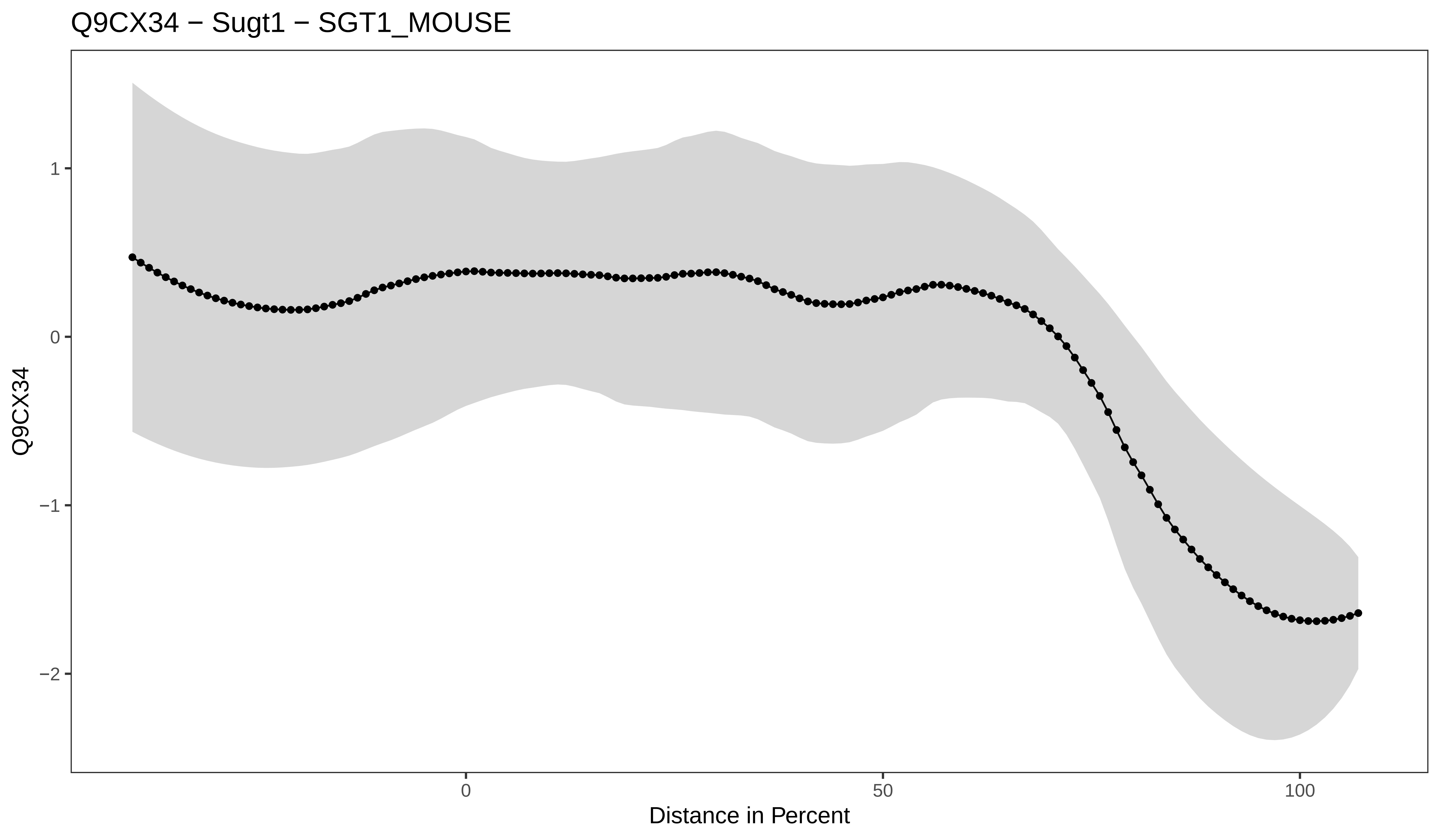
Q9CX34 − Sugt1 − SGT1_MOUSE
Q9CX34
1
0
−1
−2
0
50
100
Distance in Percent

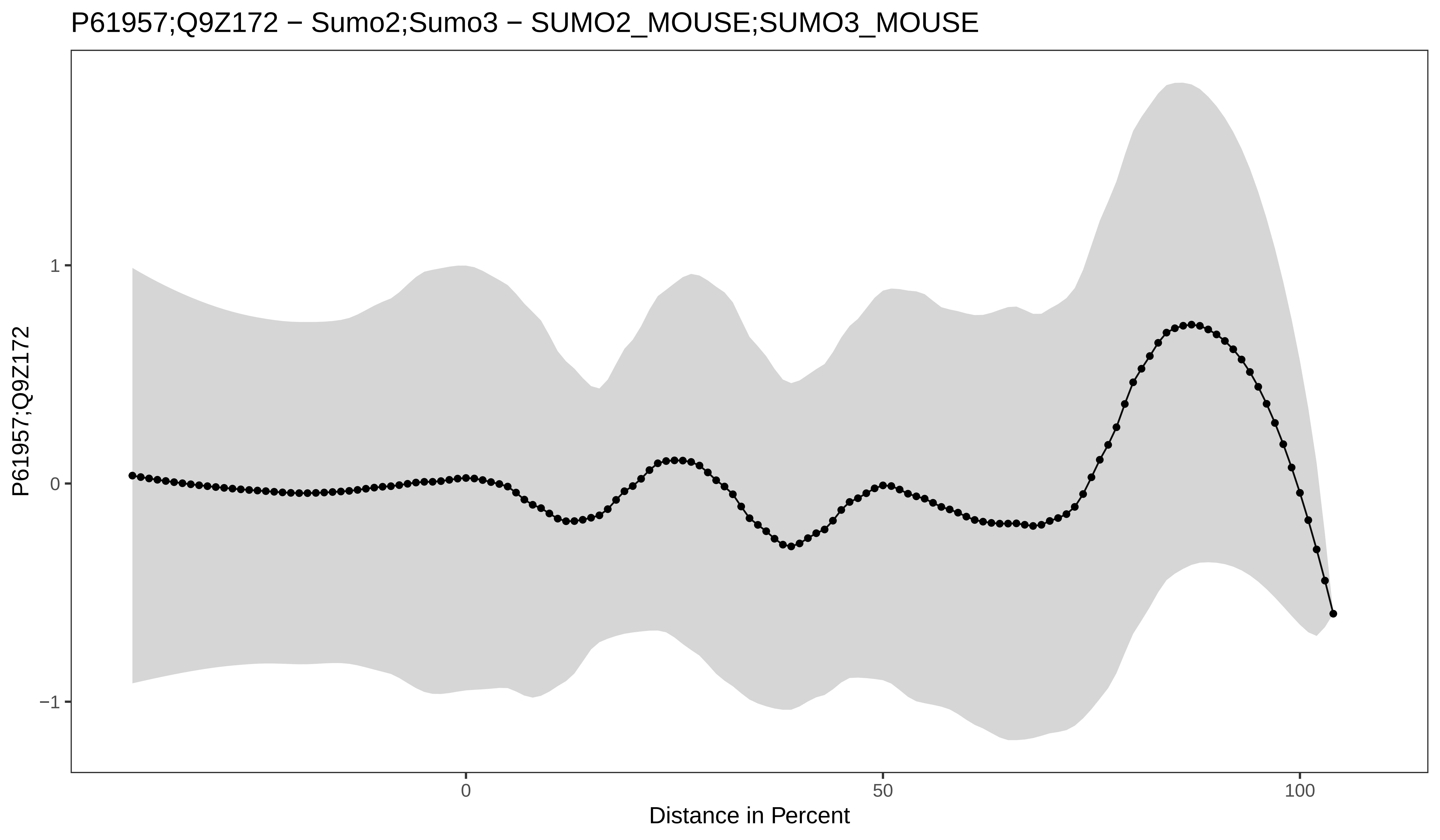

P61957;Q9Z172 − Sumo2;Sumo3 − SUMO2_MOUSE;SUMO3_MOUSE
P61957;Q9Z172
1
0
−1
0
50
100
Distance in Percent

Q8BJS4 − Sun2 − SUN2_MOUSE

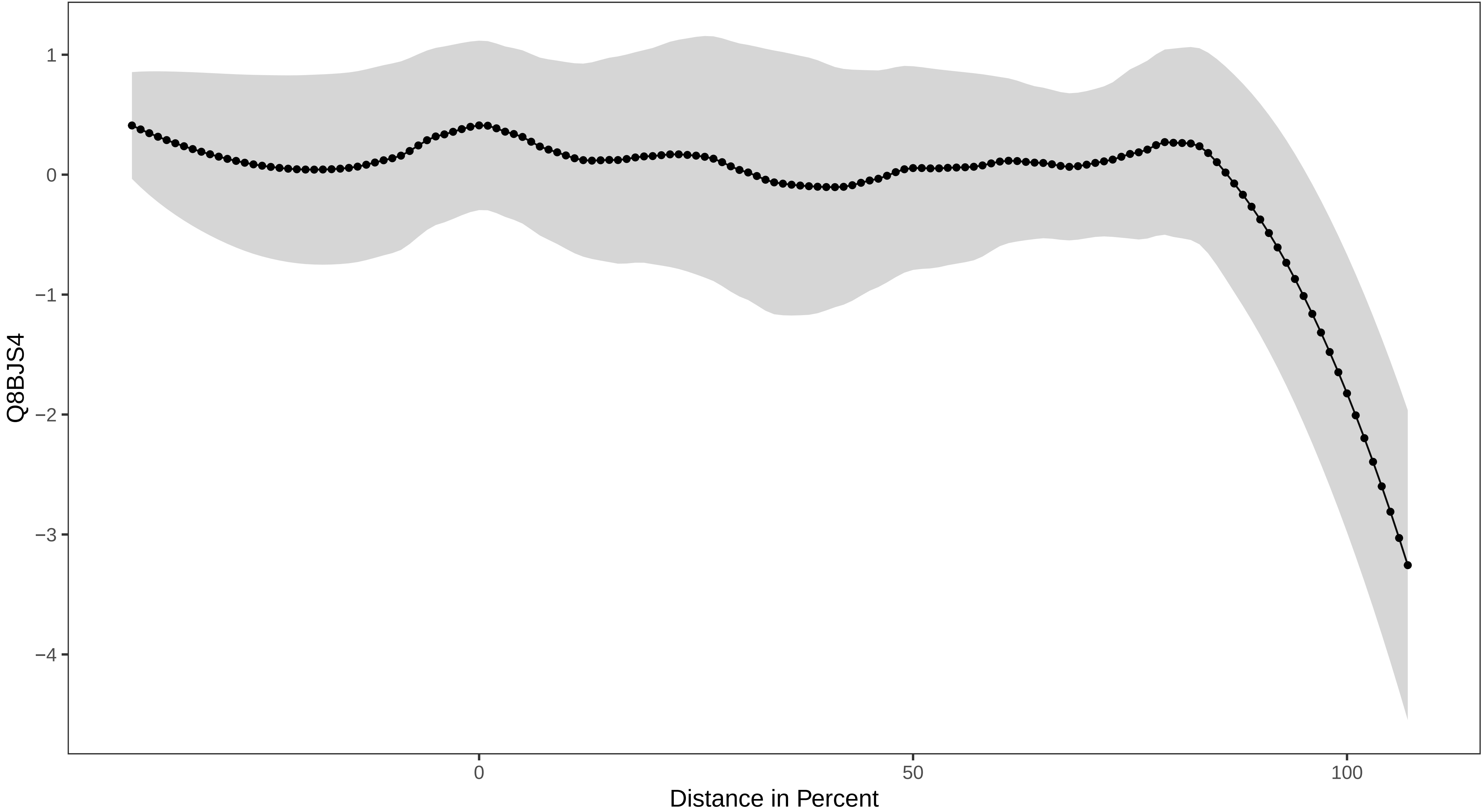

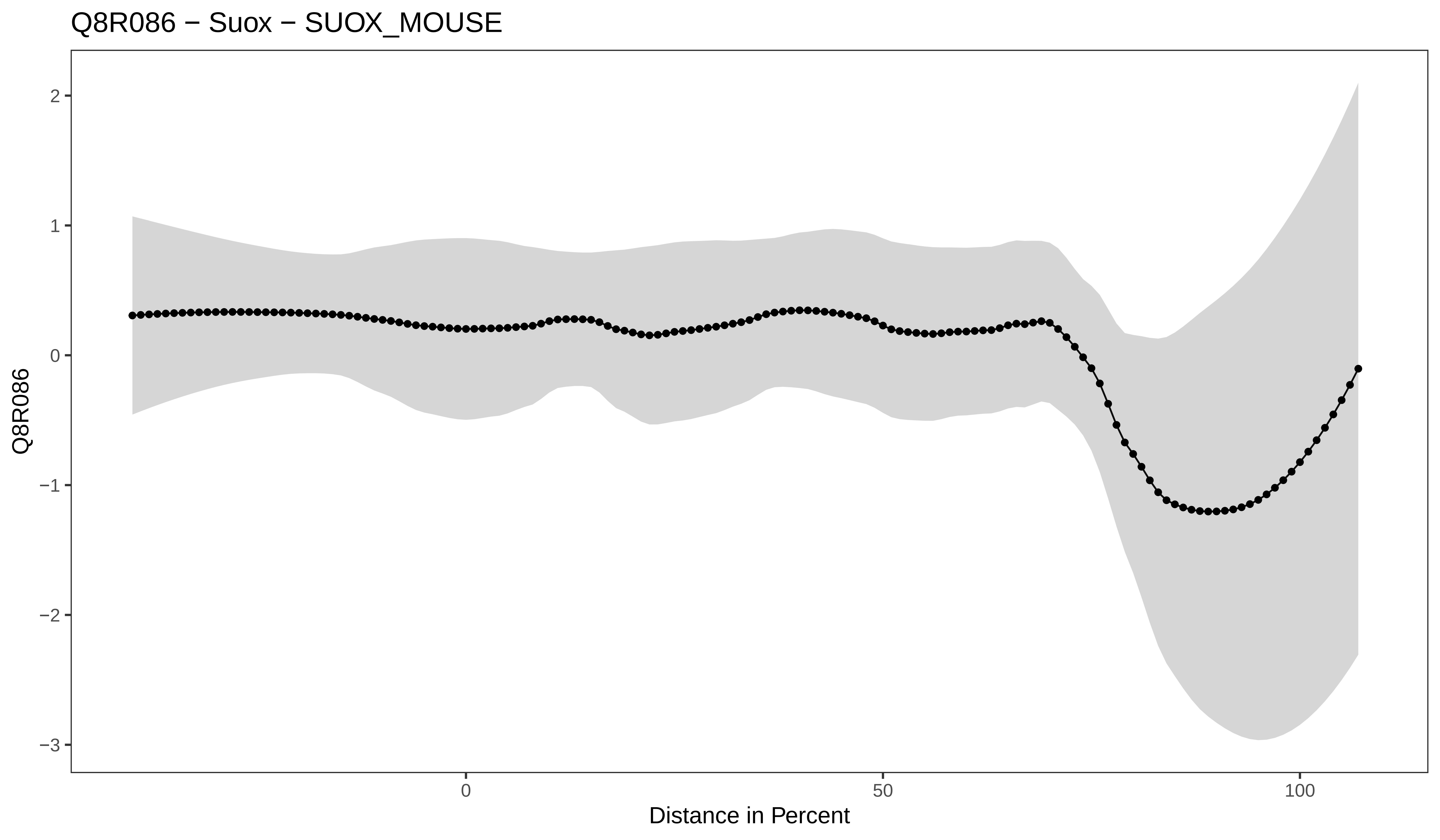

Q8R086 − Suox − SUOX_MOUSE
Q8R086
Distance in Percent
2
1
0
−1
−2
−3
0
50
100

Q80YD1 − Supv3l1 − SUV3_MOUSE

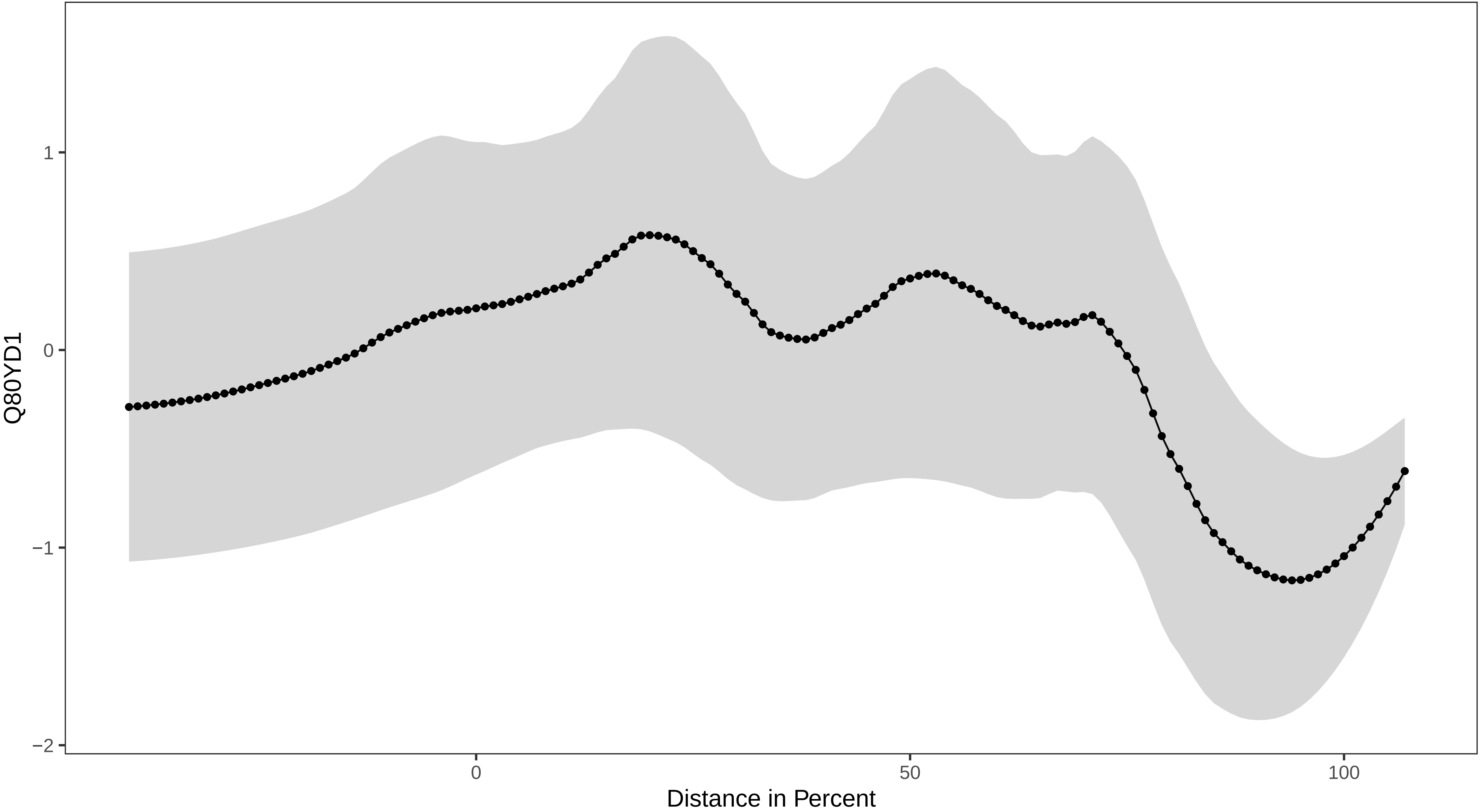

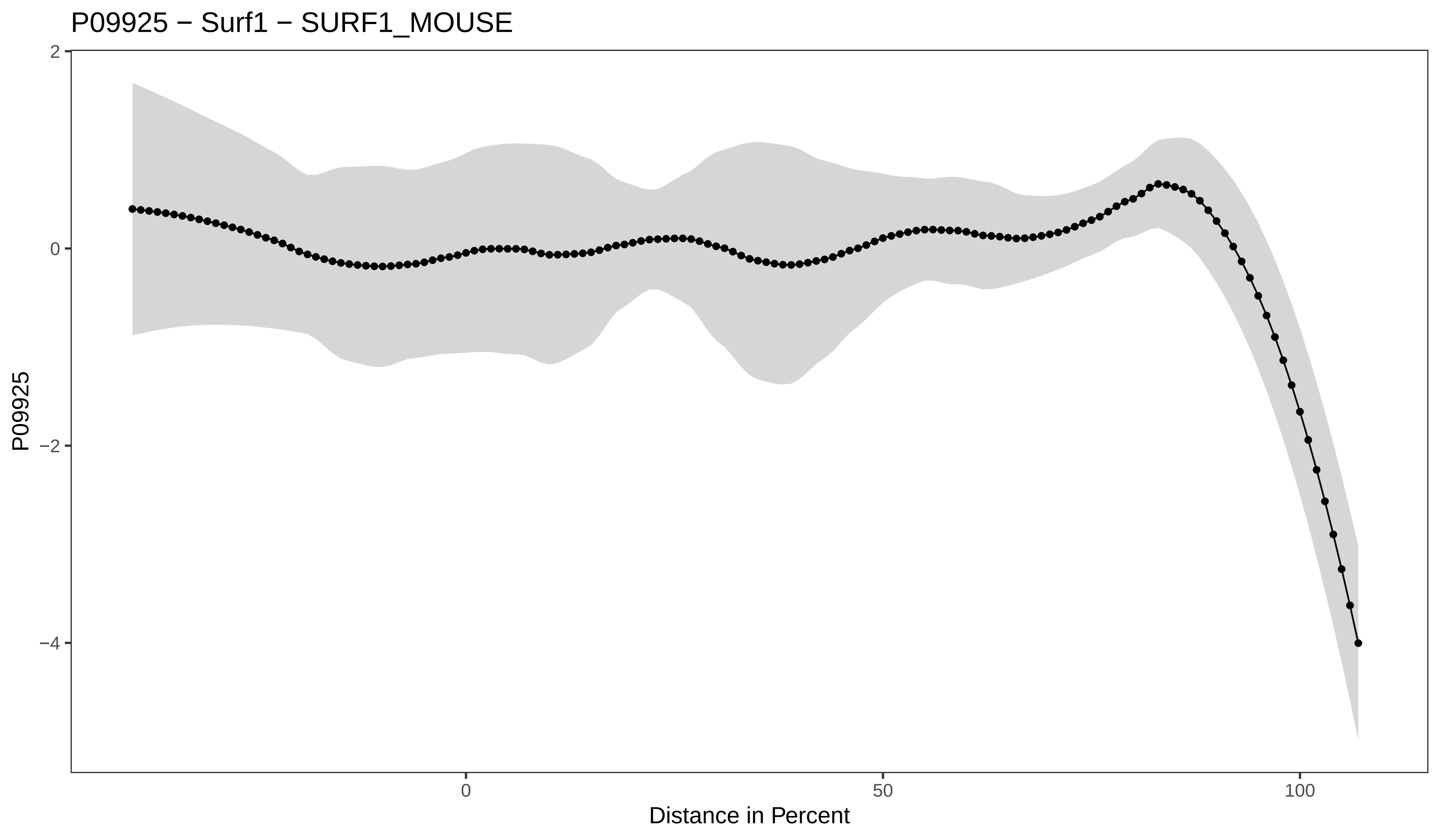

P09925 − Surf1 − SURF1_MOUSE
P09925
2
0
−2
−4
0
50
100
Distance in Percent

Q64310 − Surf4 − SURF4_MOUSE

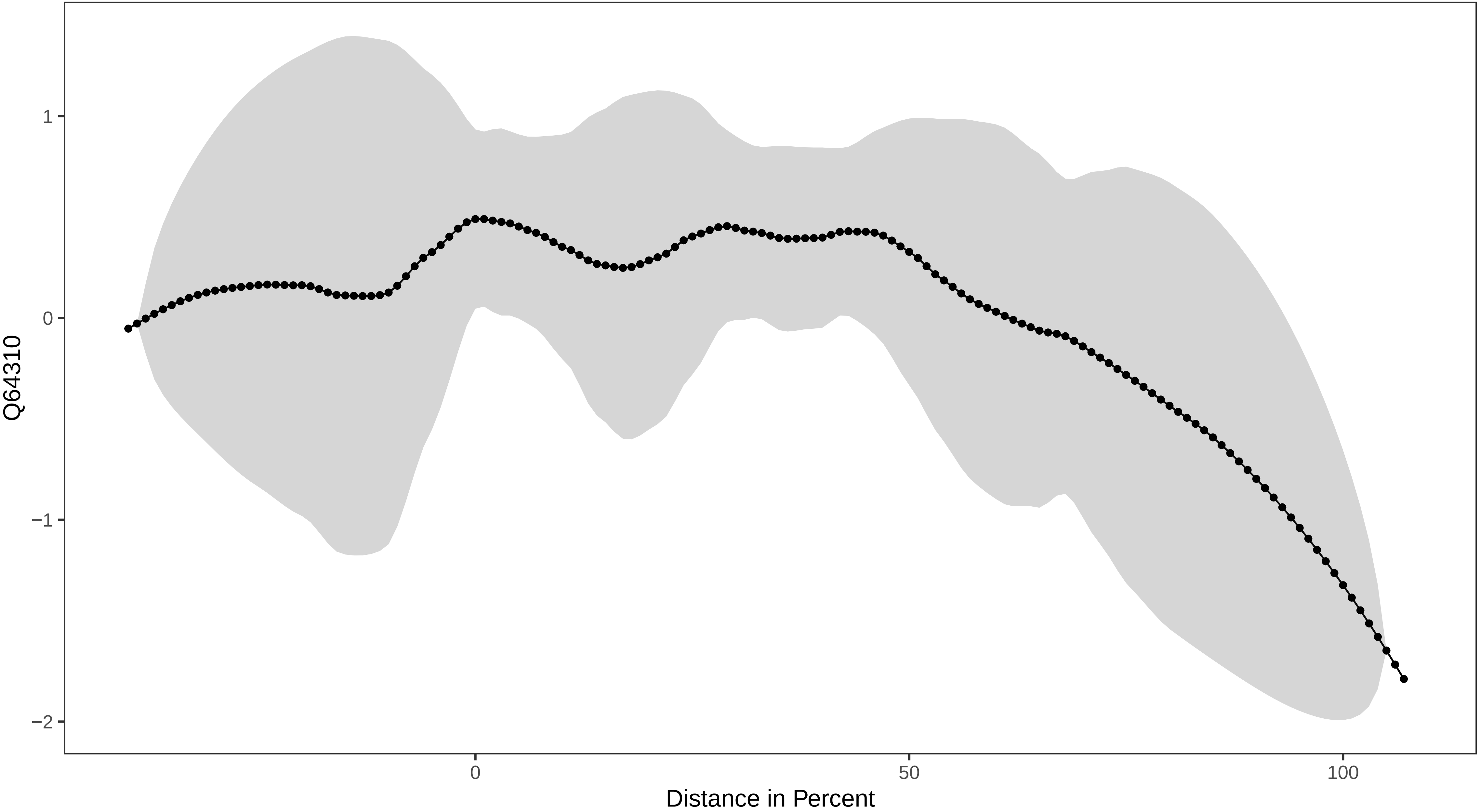

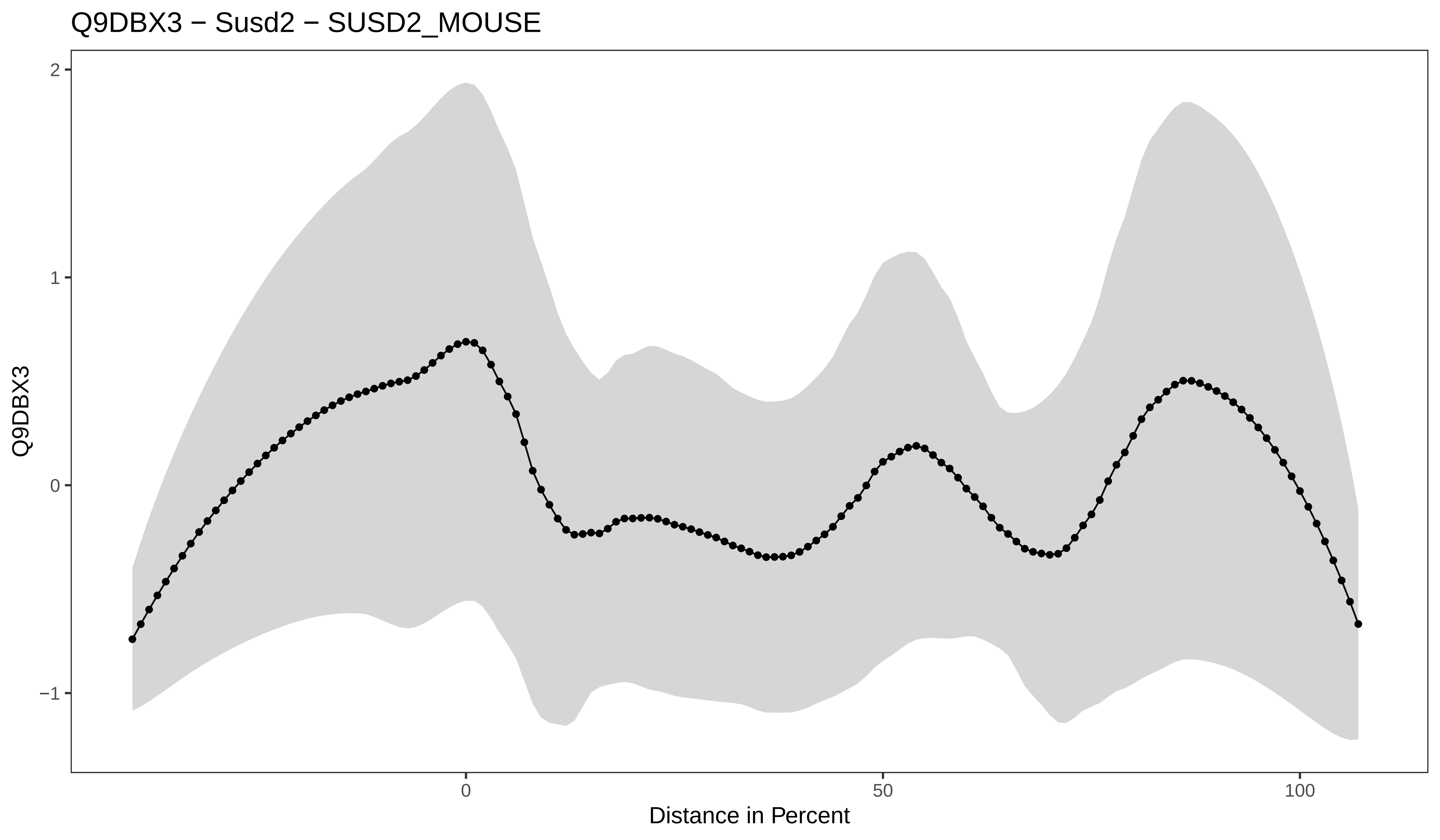
Q9DBX3 − Susd2 − SUSD2_MOUSE
Q9DBX3
Distance in Percent
2
1
0
−1
0
50
100

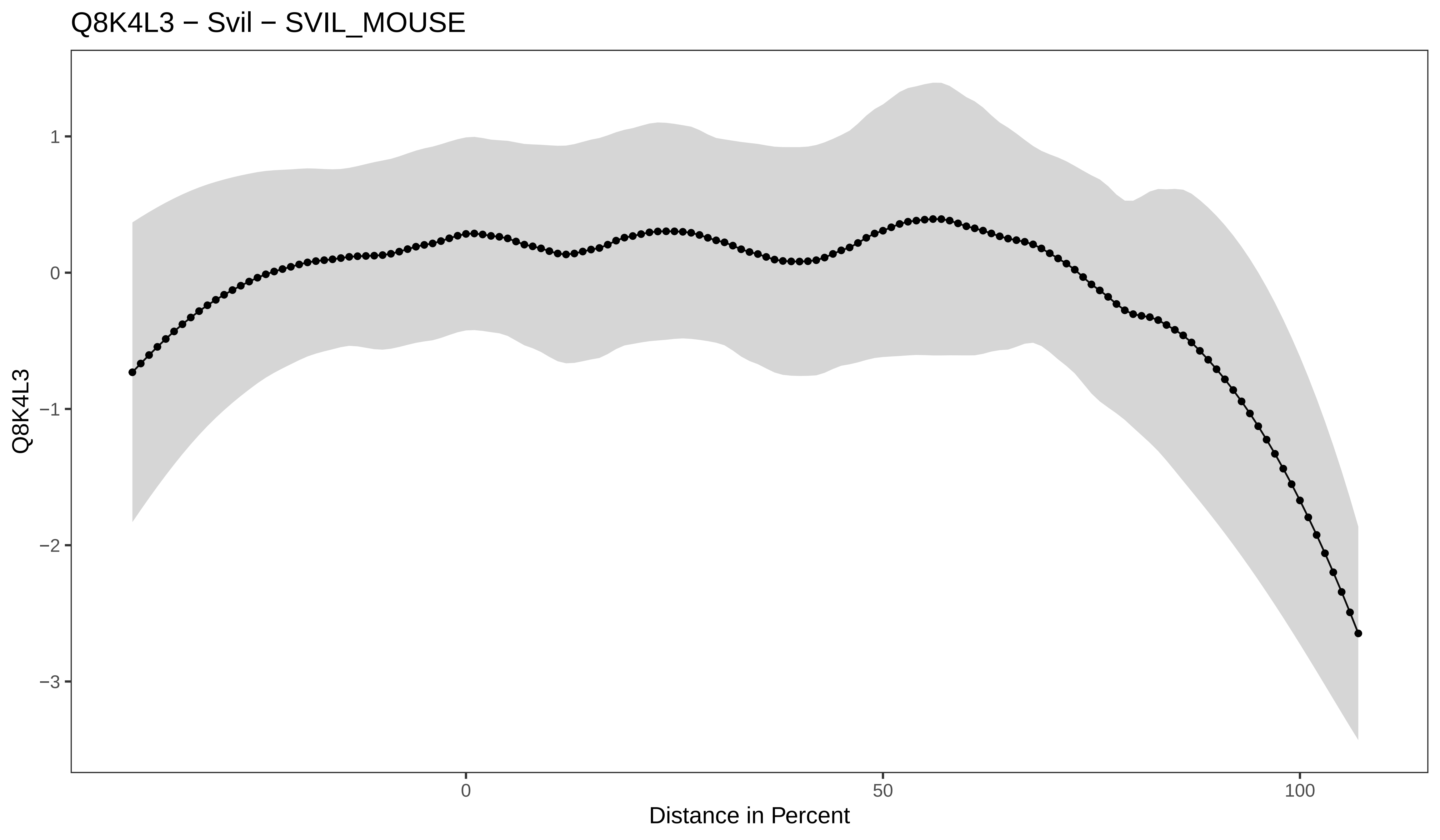

Q8K4L3 − Svil − SVIL_MOUSE
1
0
−1
−2
−3
Q8K4L3
0
50
100
Distance in Percent

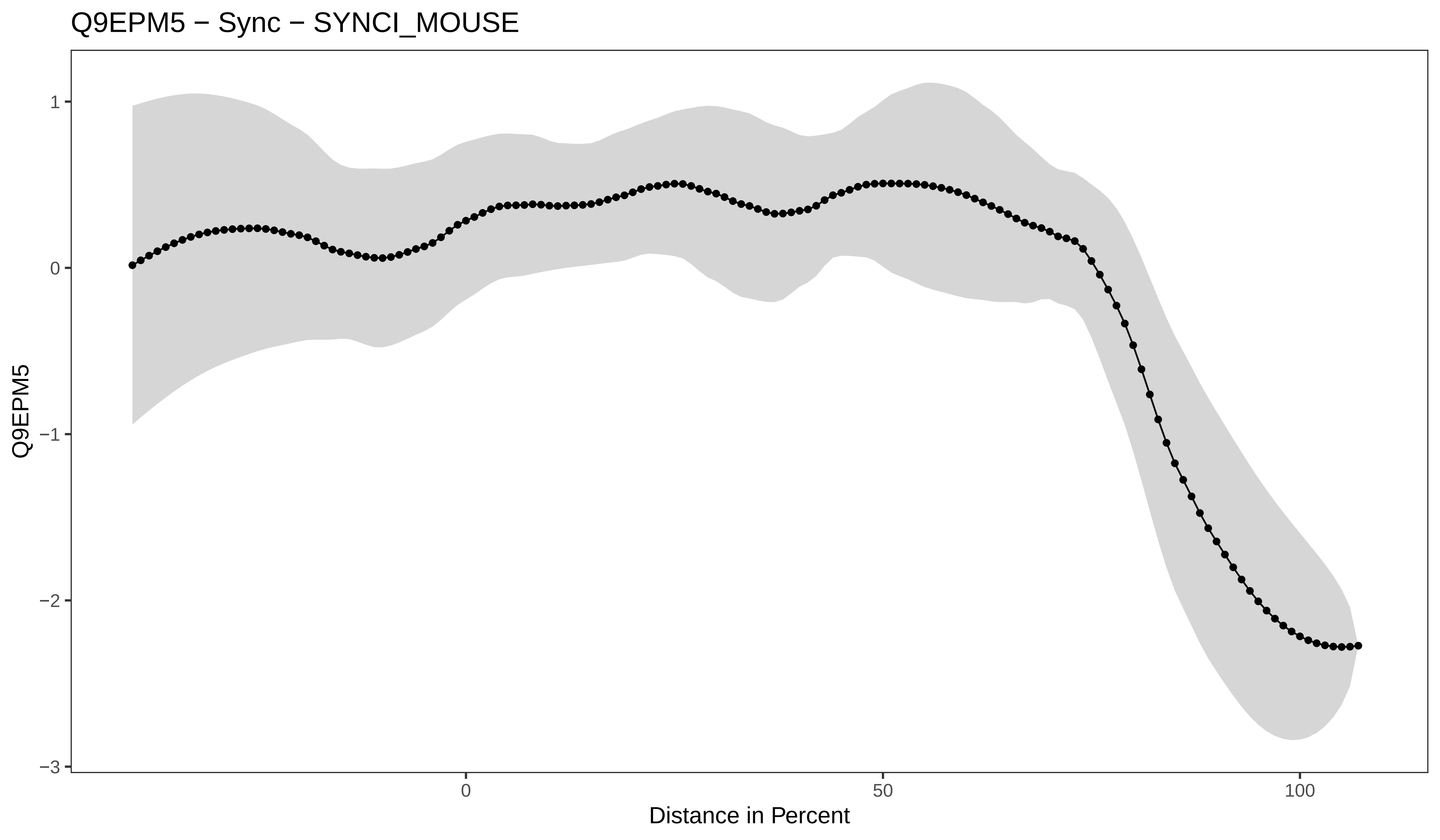

Q9EPM5 − Sync − SYNCI_MOUSE
Q9EPM5
1
0
−1
−2
−3
0
50
100
Distance in Percent

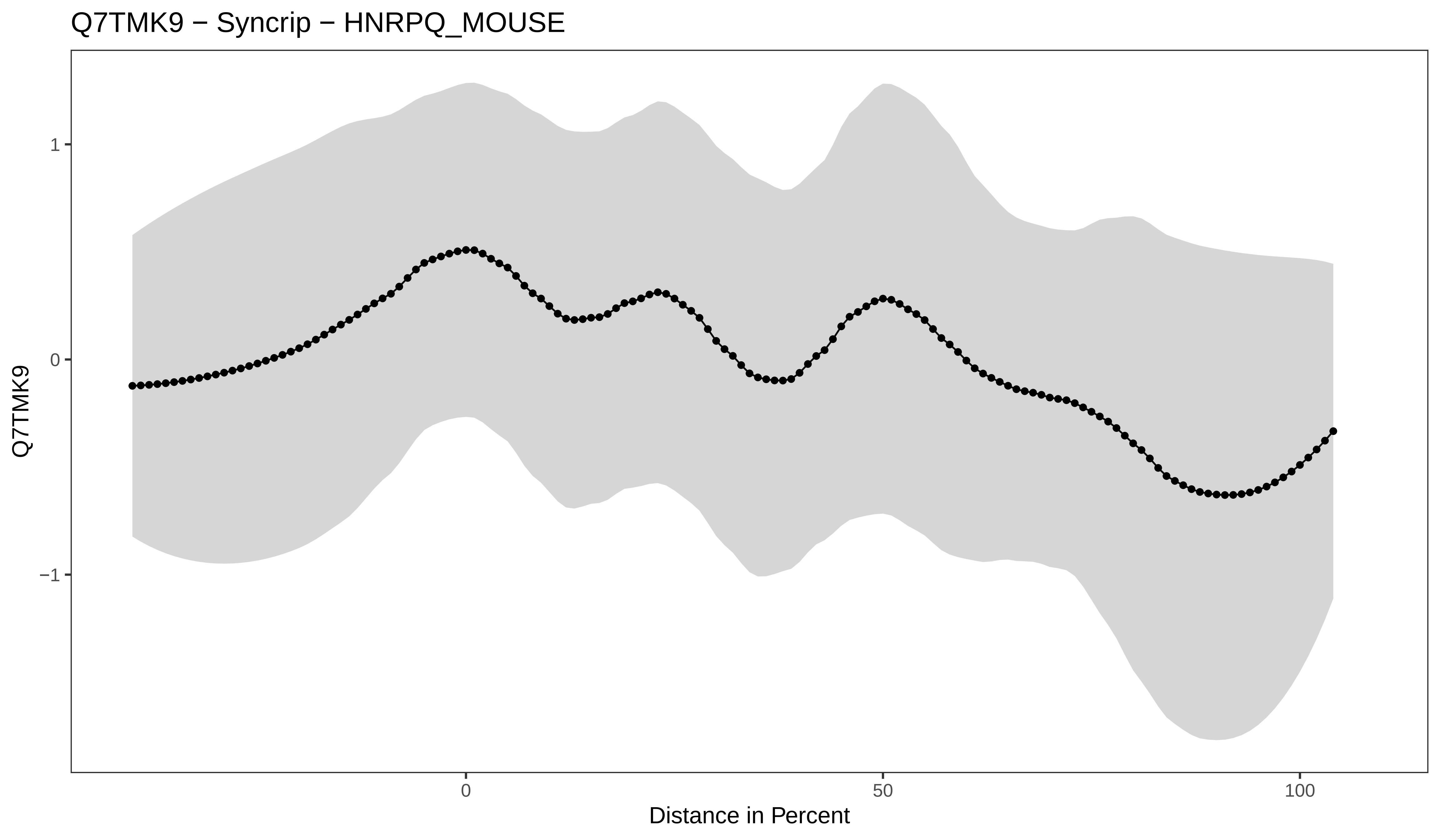

Q7TMK9 − Syncrip − HNRPQ_MOUSE
Q7TMK9
1
0
−1
0
50
100
Distance in Percent

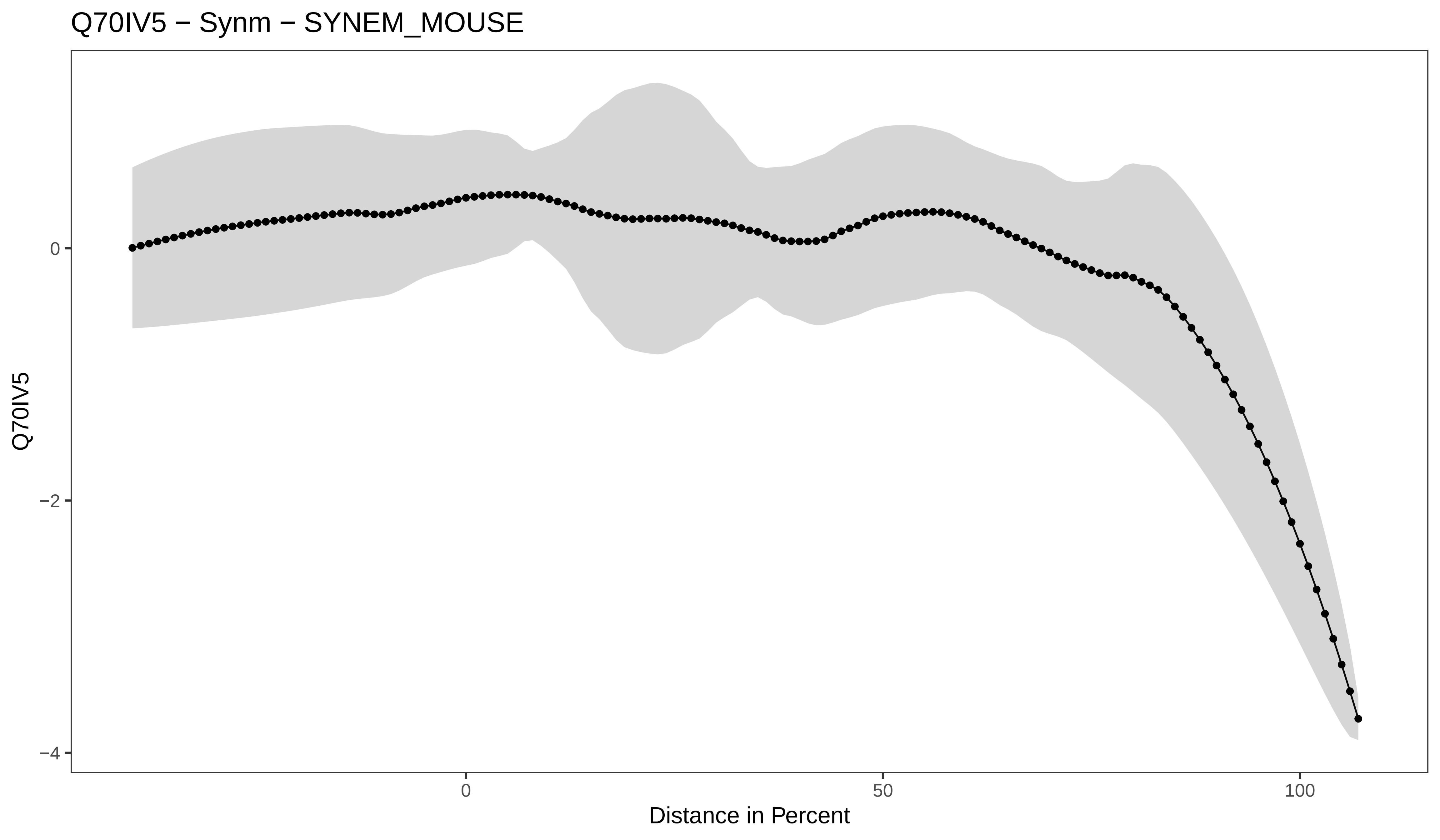

Q70IV5 − Synm − SYNEM_MOUSE
Q70IV5
0
−2
−4
0
50
100
Distance in Percent

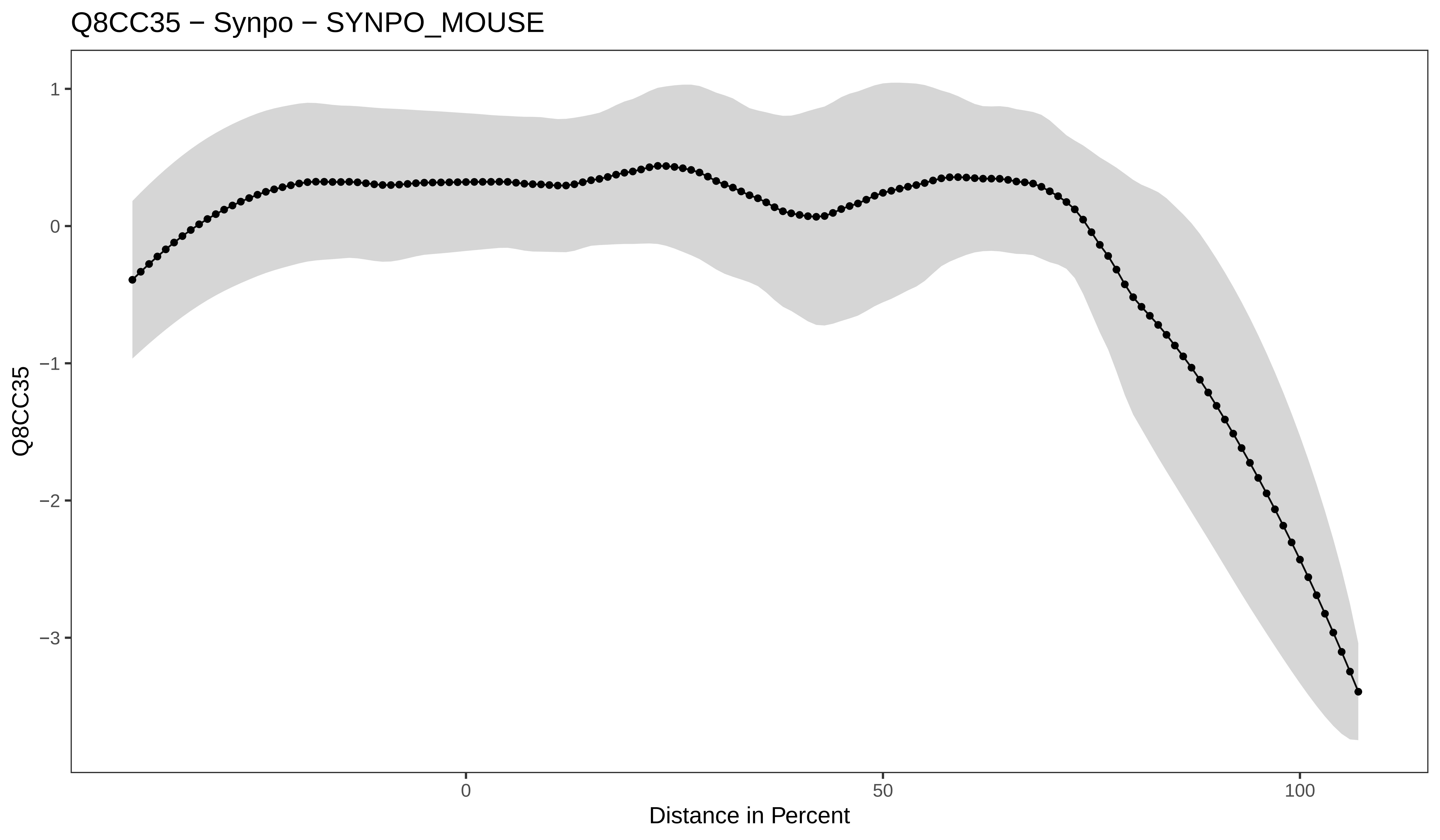

Q8CC35 − Synpo − SYNPO_MOUSE
Q8CC35
1
0
−1
−2
−3
0
50
100
Distance in Percent

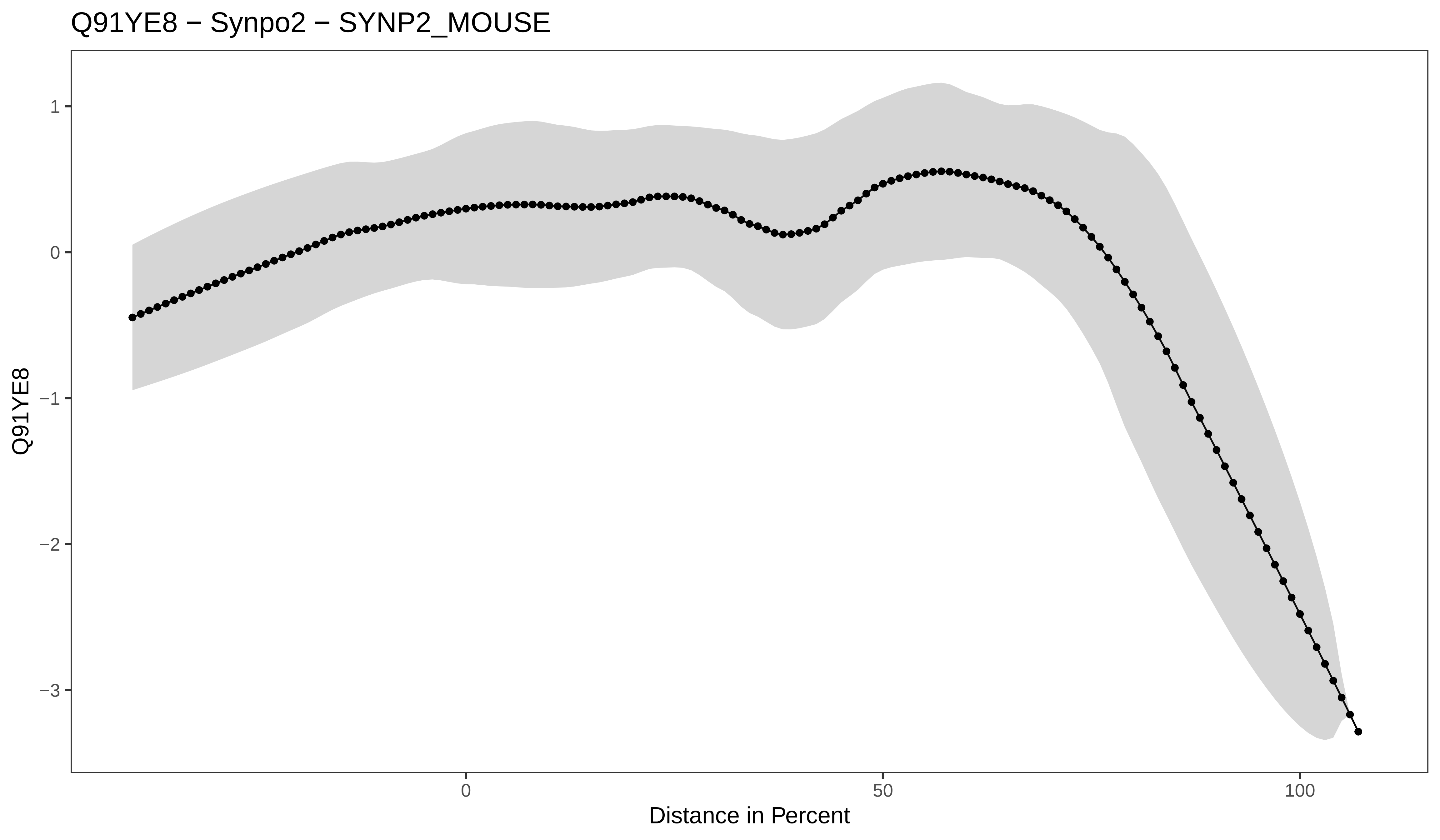

Q91YE8 − Synpo2 − SYNP2_MOUSE
1
0
−1
−2
−3
Q91YE8
0
50
100
Distance in Percent

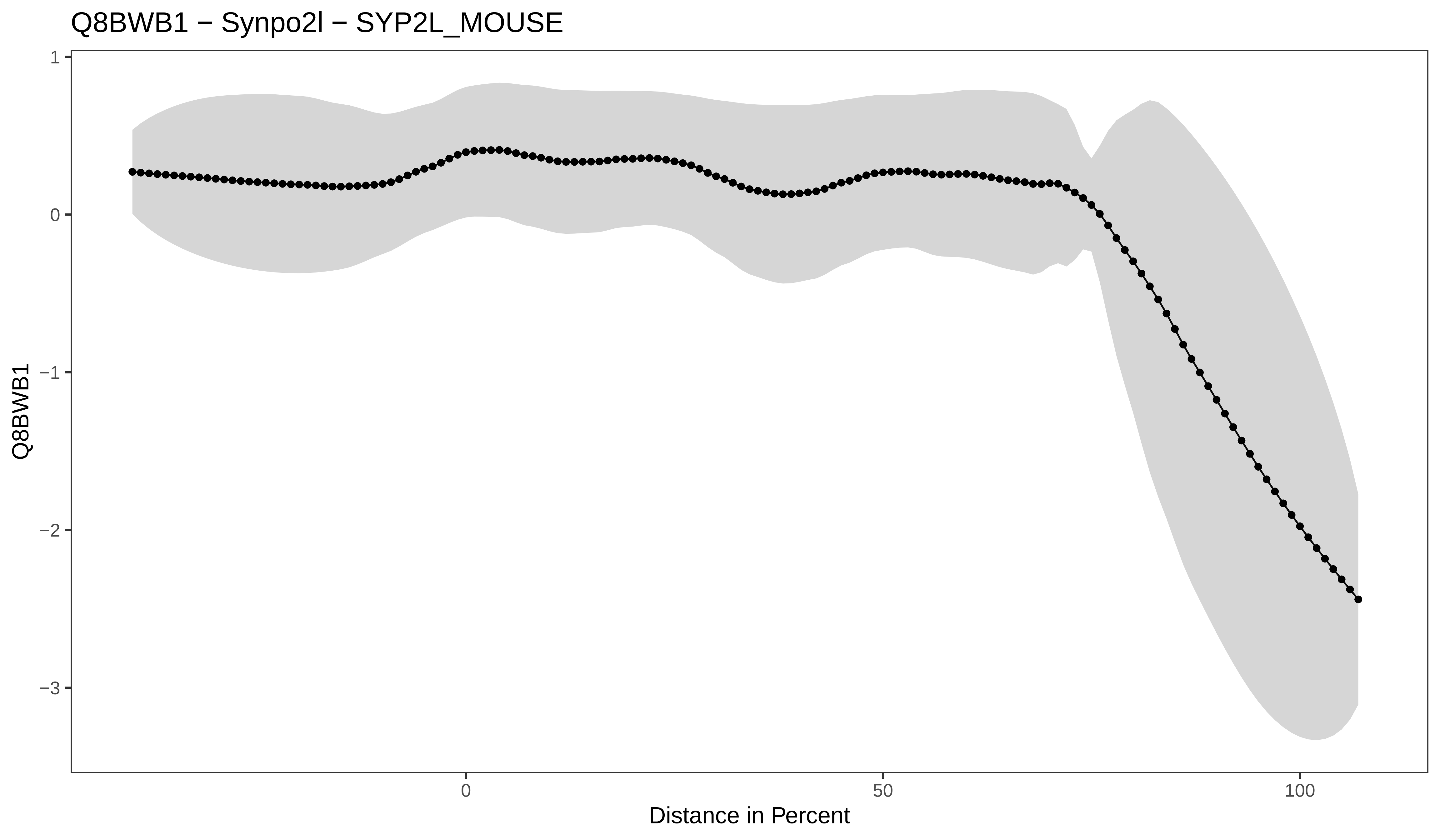
Q8BWB1 − Synpo2l − SYP2L_MOUSE
Q8BWB1
1
0
−1
−2
−3
0
50
100
Distance in Percent

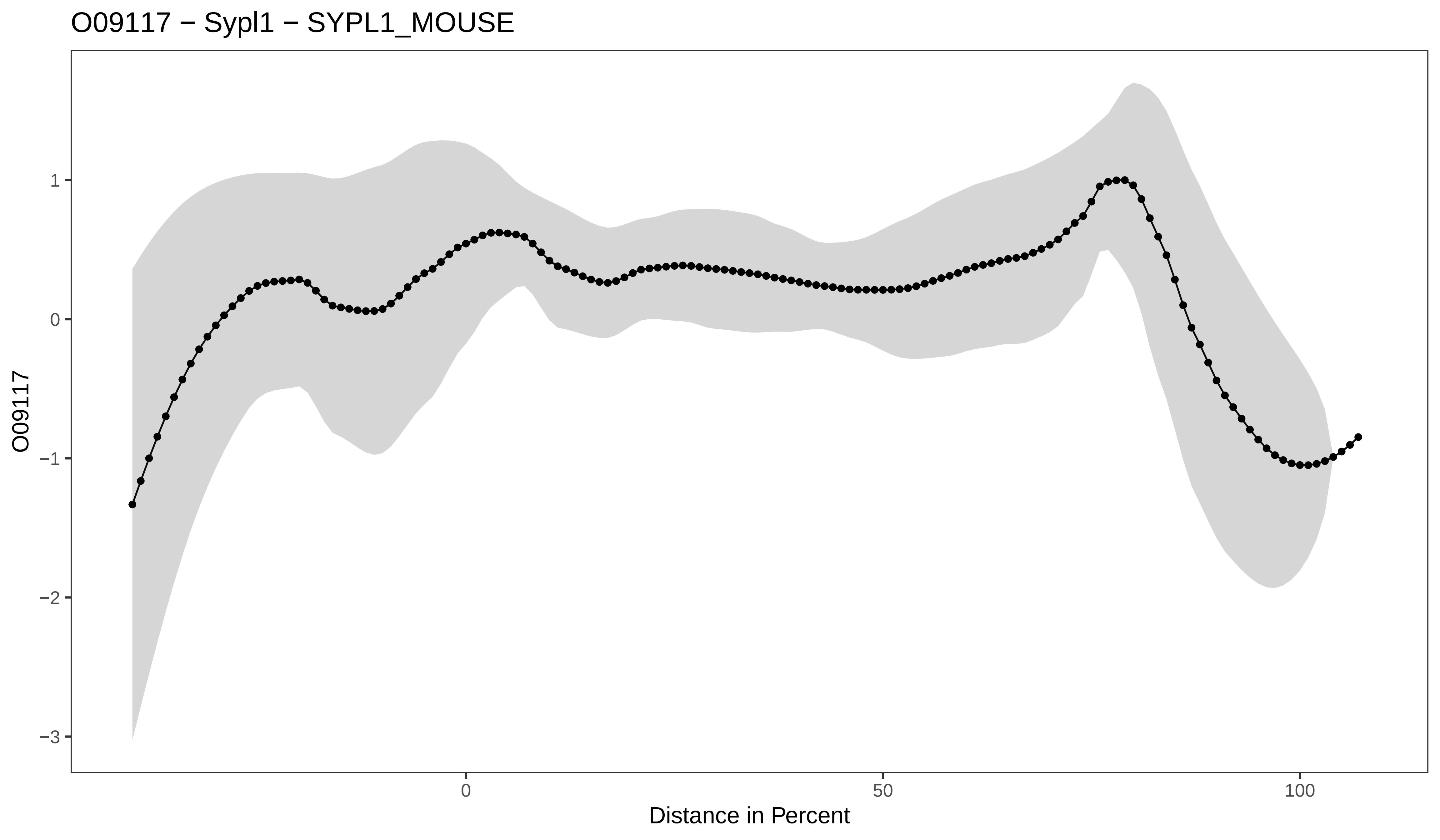
O09117 − Sypl1 − SYPL1_MOUSE
O09117
Distance in Percent
1
0
−1
−2
−3
0
50
100

O89104 − Sypl2 − SYPL2_MOUSE

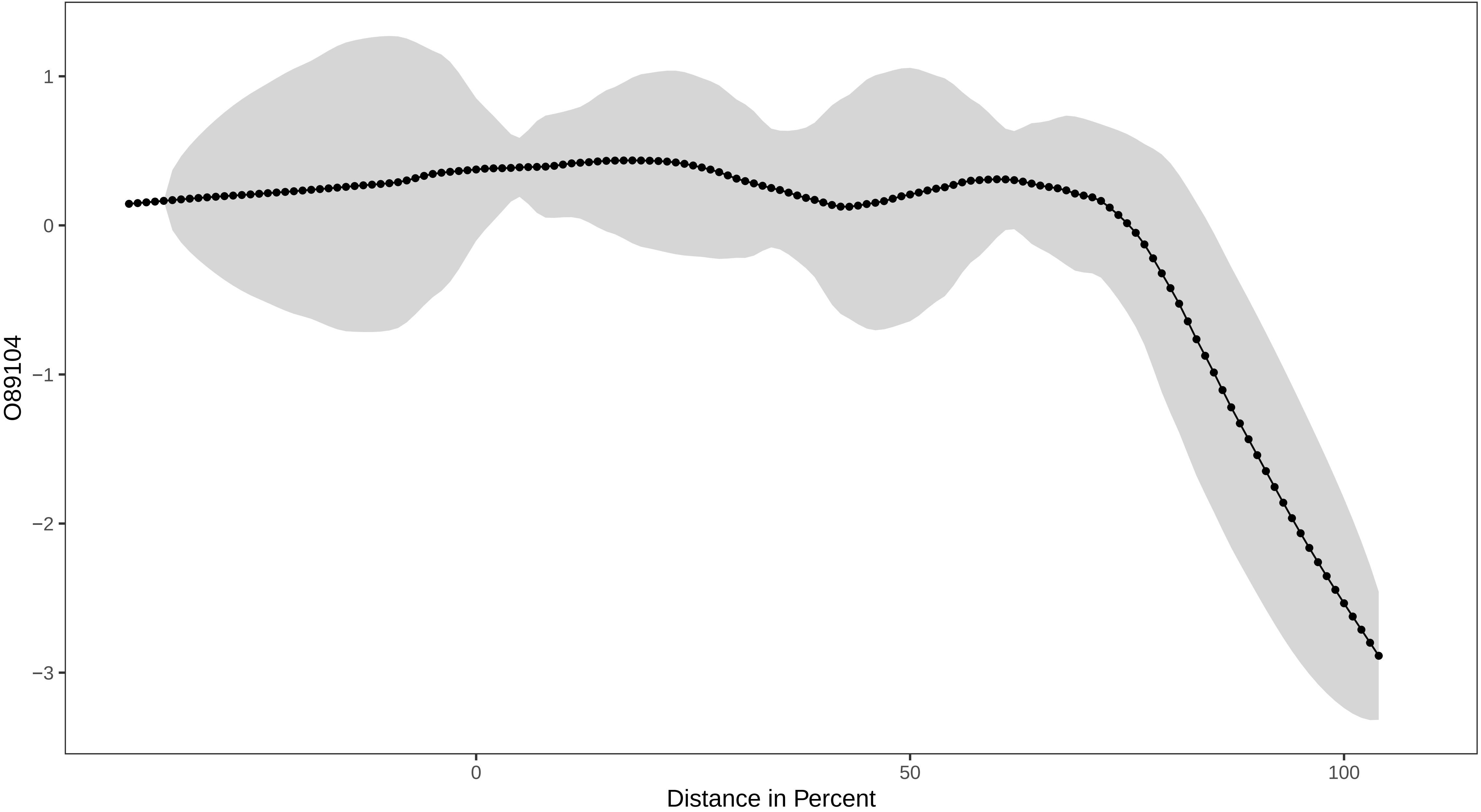

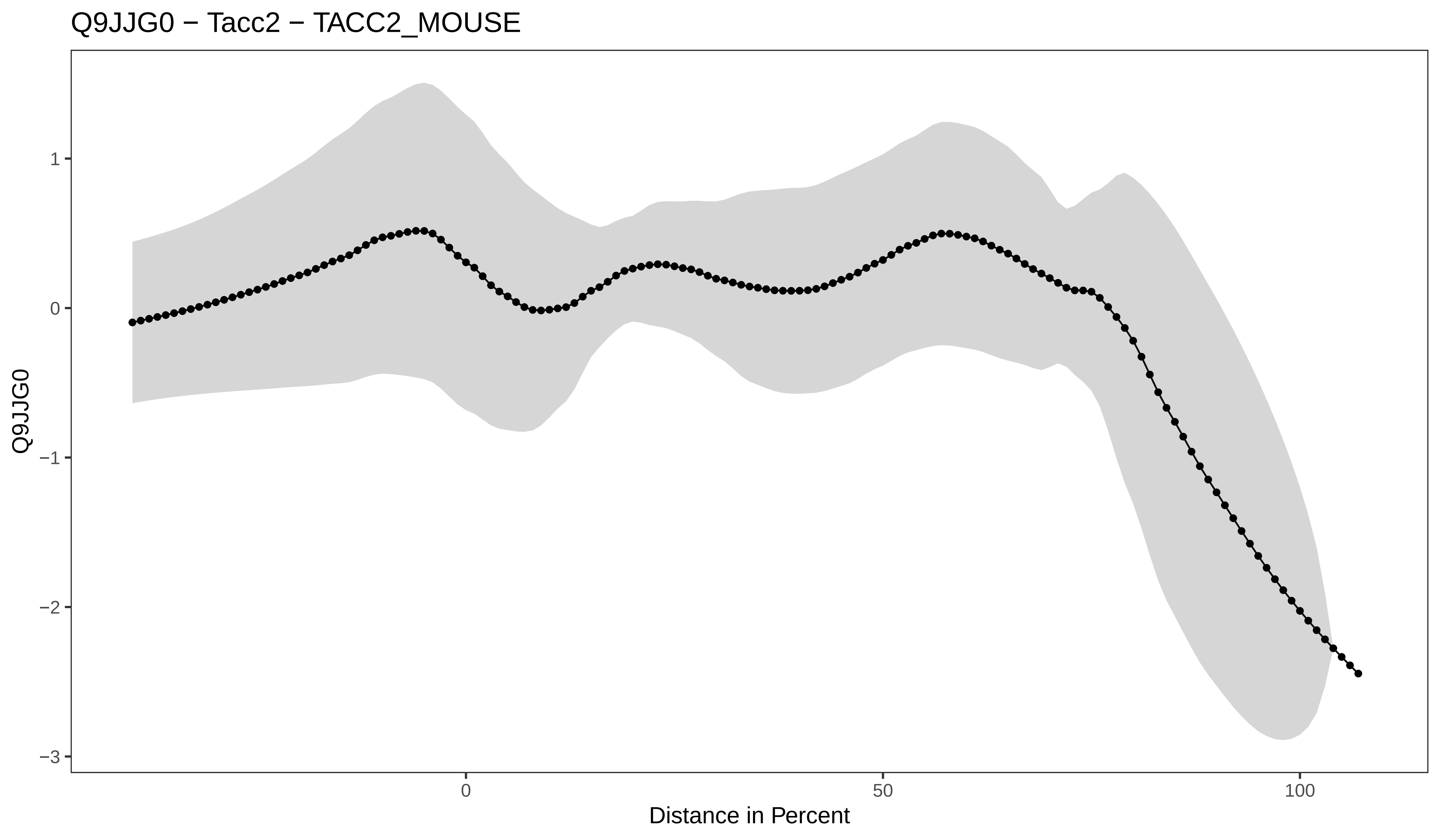
Q9JJG0 − Tacc2 − TACC2_MOUSE
Q9JJG0
1
0
−1
−2
−3
0
50
100
Distance in Percent

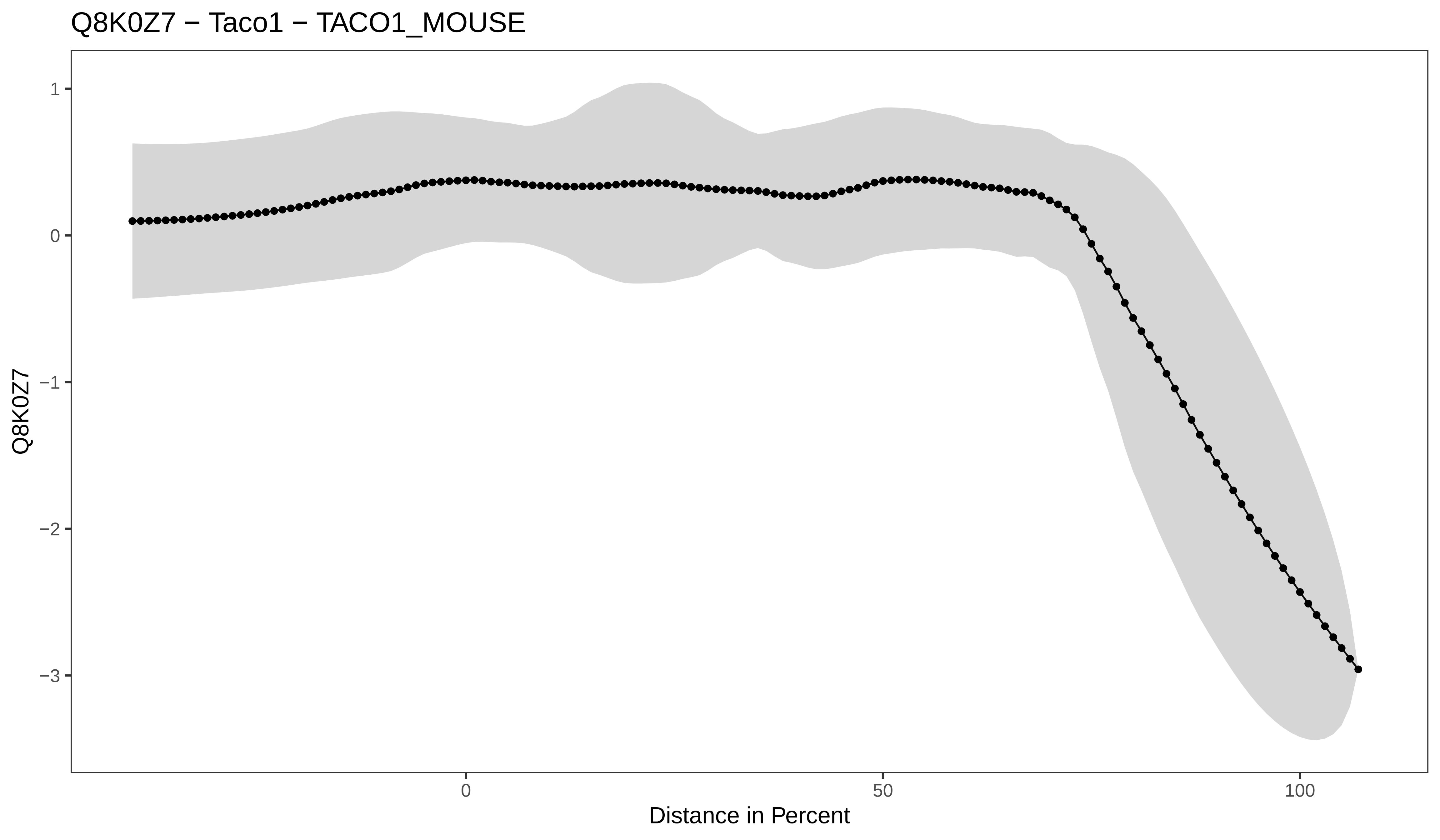

Q8K0Z7 − Taco1 − TACO1_MOUSE
1
0
−1
−2
−3
Q8K0Z7
0
50
100
Distance in Percent

P37804 − Tagln − TAGL_MOUSE

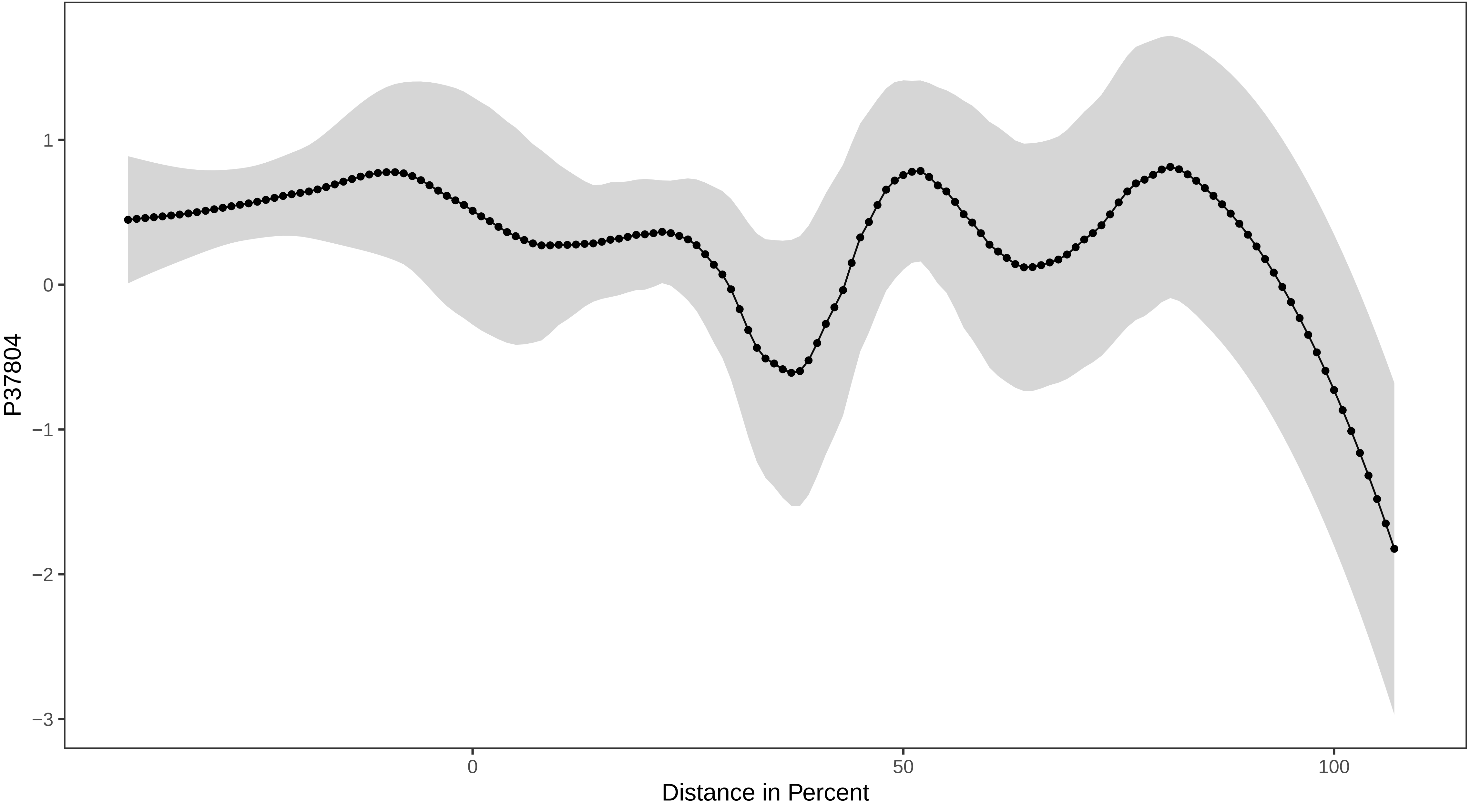

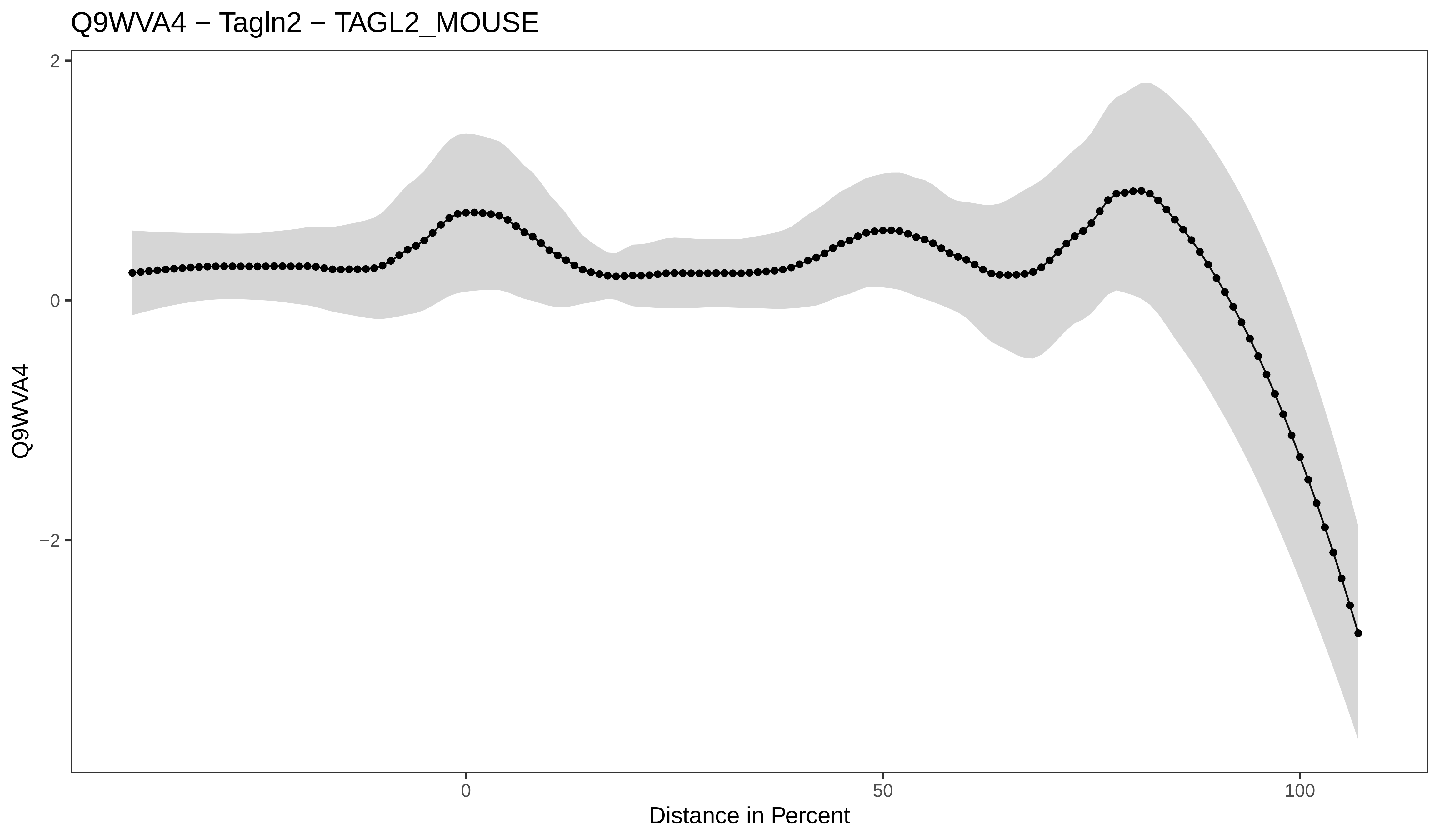
Q9WVA4 − Tagln2 − TAGL2_MOUSE
2
0
−2
Q9WVA4
0
50
100
Distance in Percent

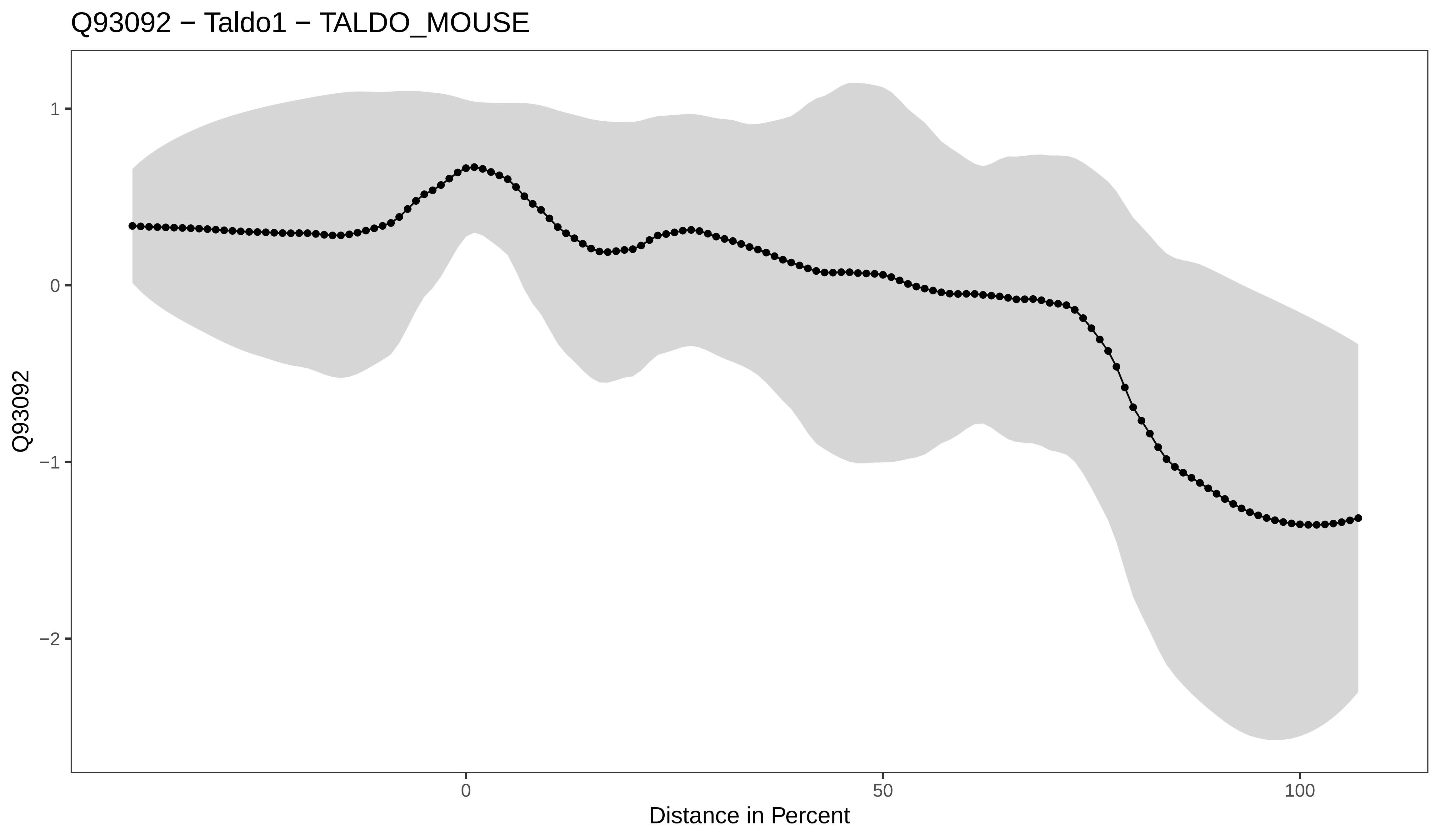

Q93092 − Taldo1 − TALDO_MOUSE
Q93092
1
0
−1
−2
0
50
100
Distance in Percent

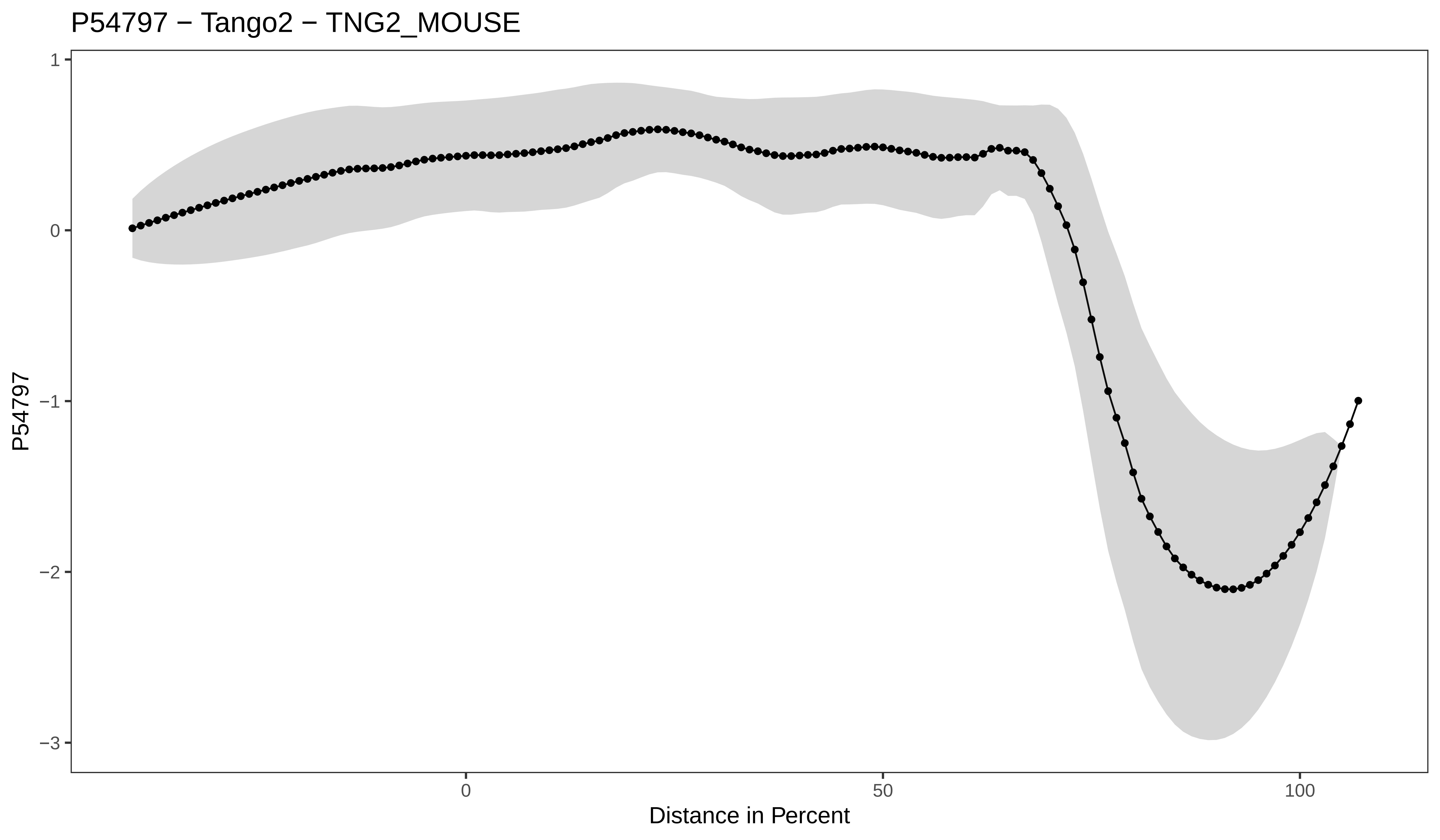

P54797 − Tango2 − TNG2_MOUSE
1
0
−1
−2
−3
P54797
0
50
100
Distance in Percent

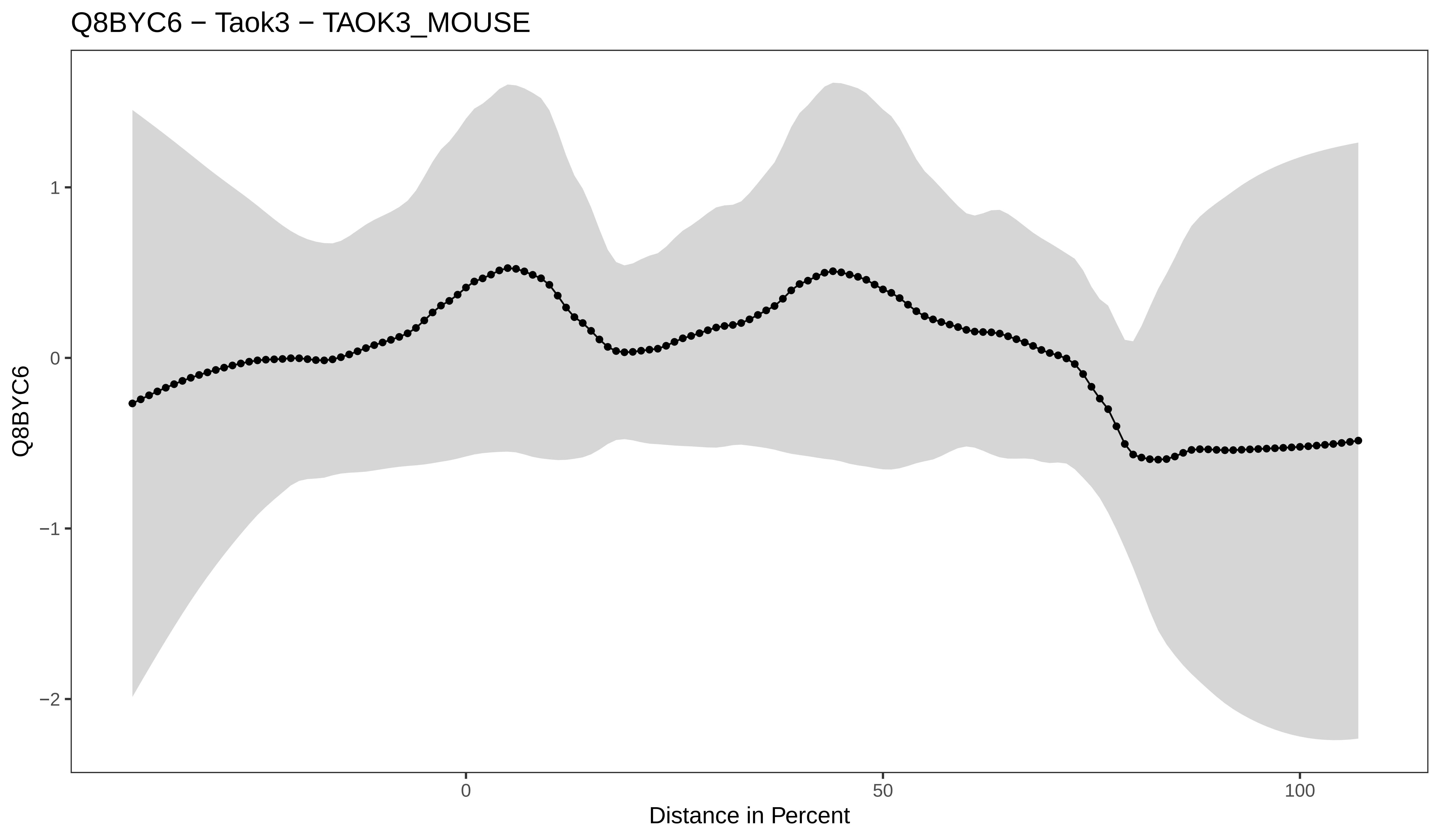
Q8BYC6 − Taok3 − TAOK3_MOUSE
Q8BYC6
Distance in Percent
1
0
−1
−2
0
50
100

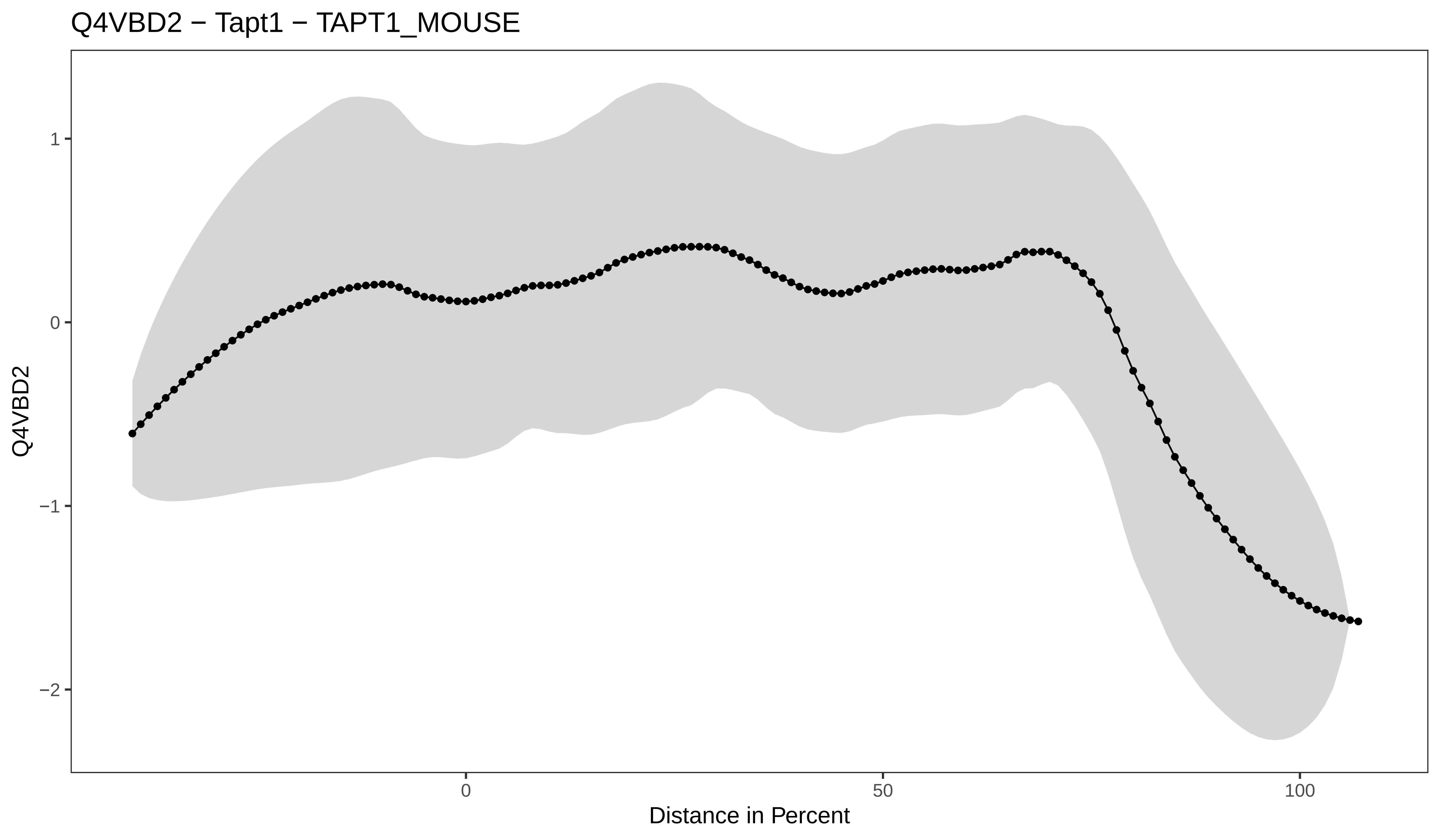

Q4VBD2 − Tapt1 − TAPT1_MOUSE
Q4VBD2
1
0
−1
−2
0
50
100
Distance in Percent

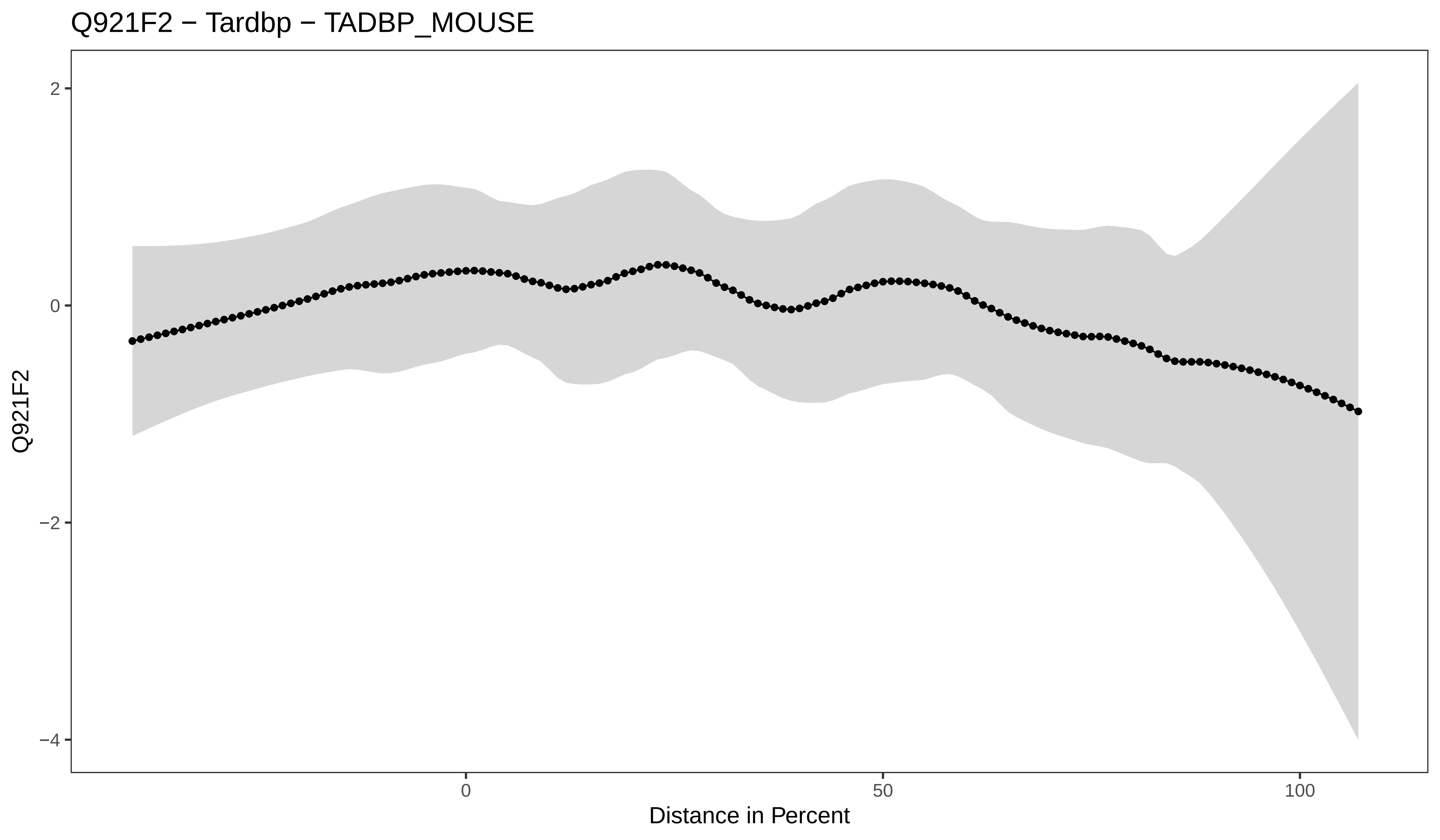

Q921F2 − Tardbp − TADBP_MOUSE
2
0
−2
−4
Q921F2
0
50
100
Distance in Percent

Q9D0R2 − Tars − SYTC_MOUSE

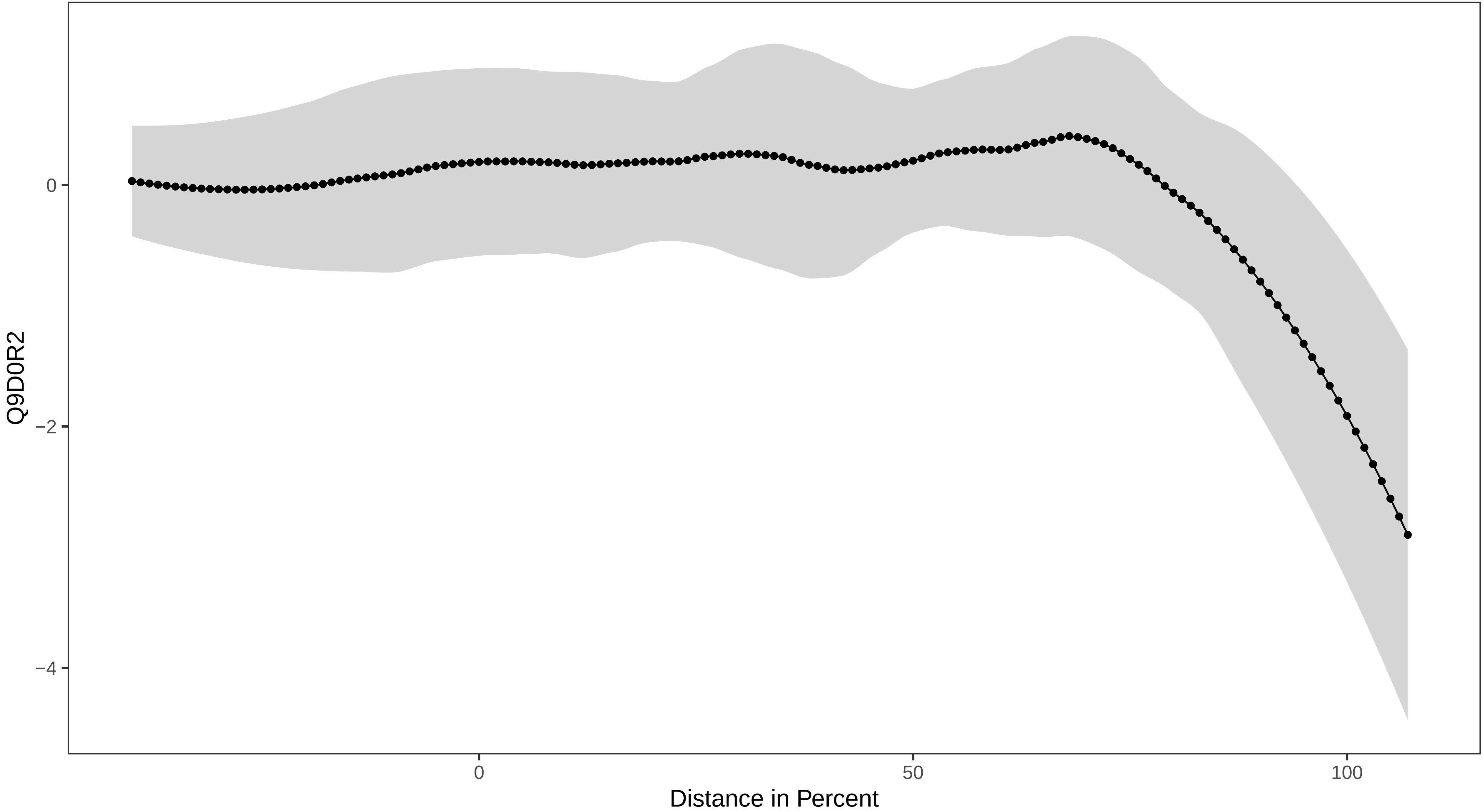

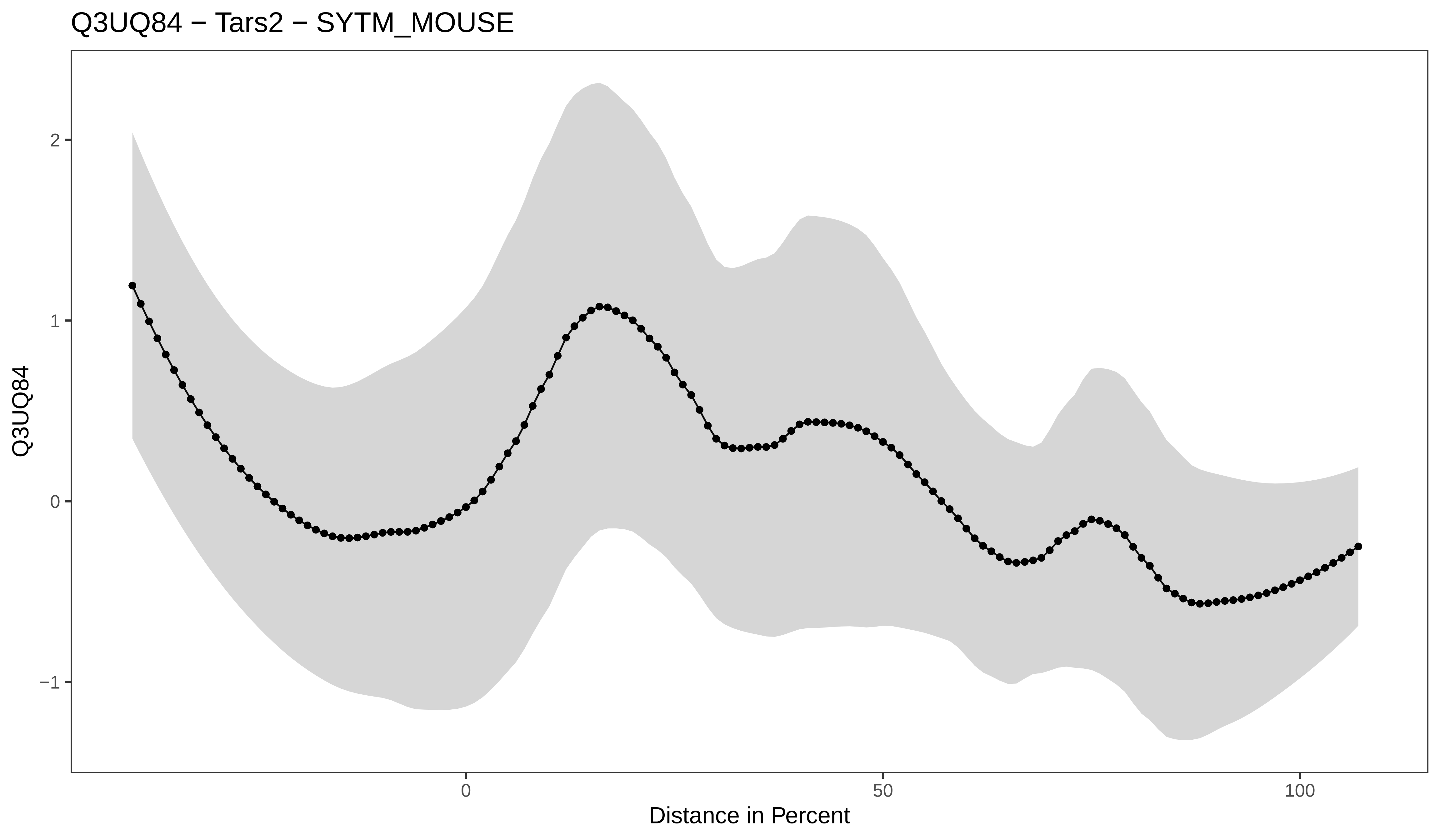

Q3UQ84 − Tars2 − SYTM_MOUSE
Q3UQ84
Distance in Percent
2
1
0
−1
0
50
100

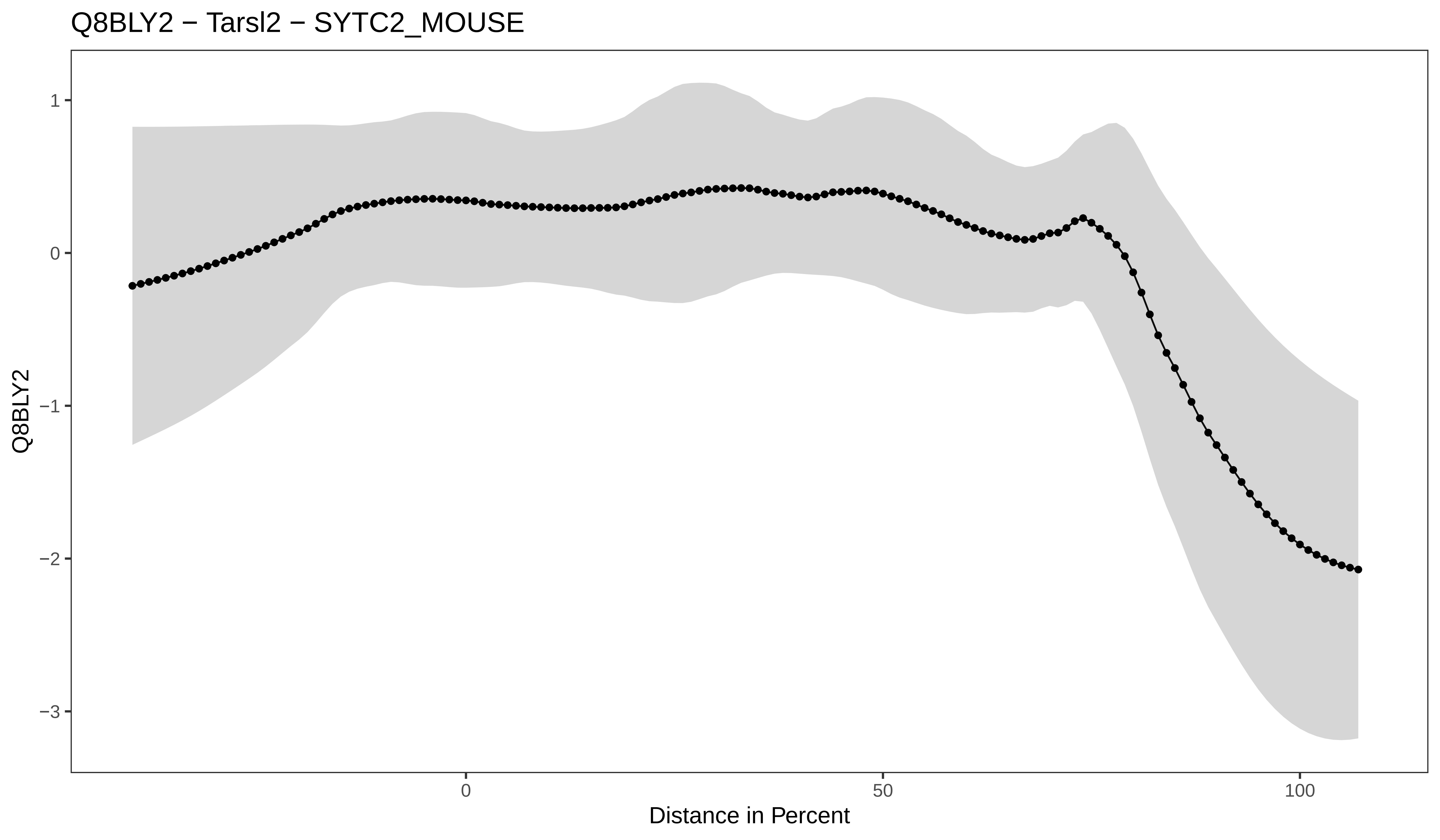
Q8BLY2 − Tarsl2 − SYTC2_MOUSE
Q8BLY2
1
0
−1
−2
−3
0
50
100
Distance in Percent

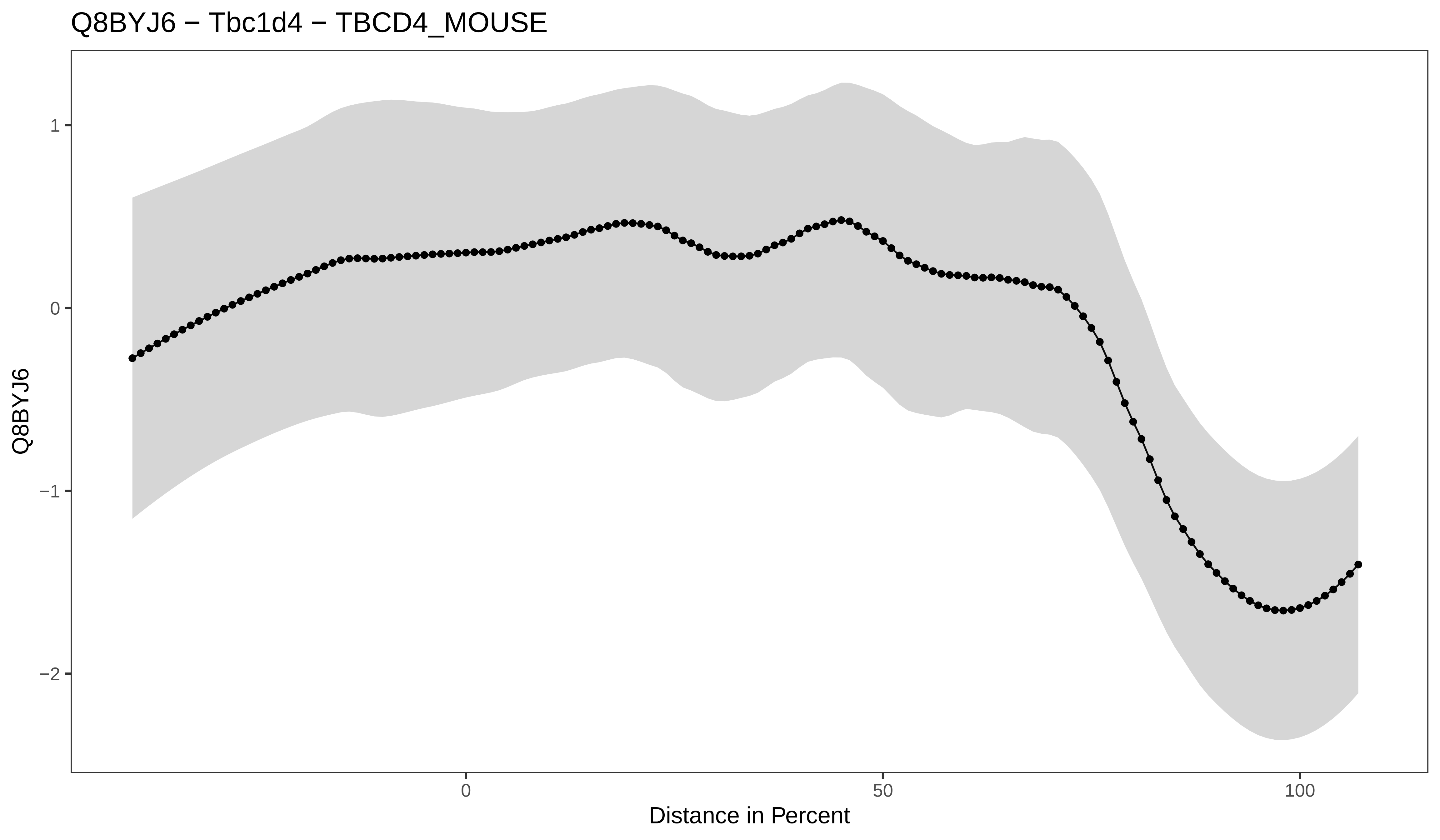
Q8BYJ6 − Tbc1d4 − TBCD4_MOUSE
Q8BYJ6
1
0
−1
−2
0
50
100
Distance in Percent

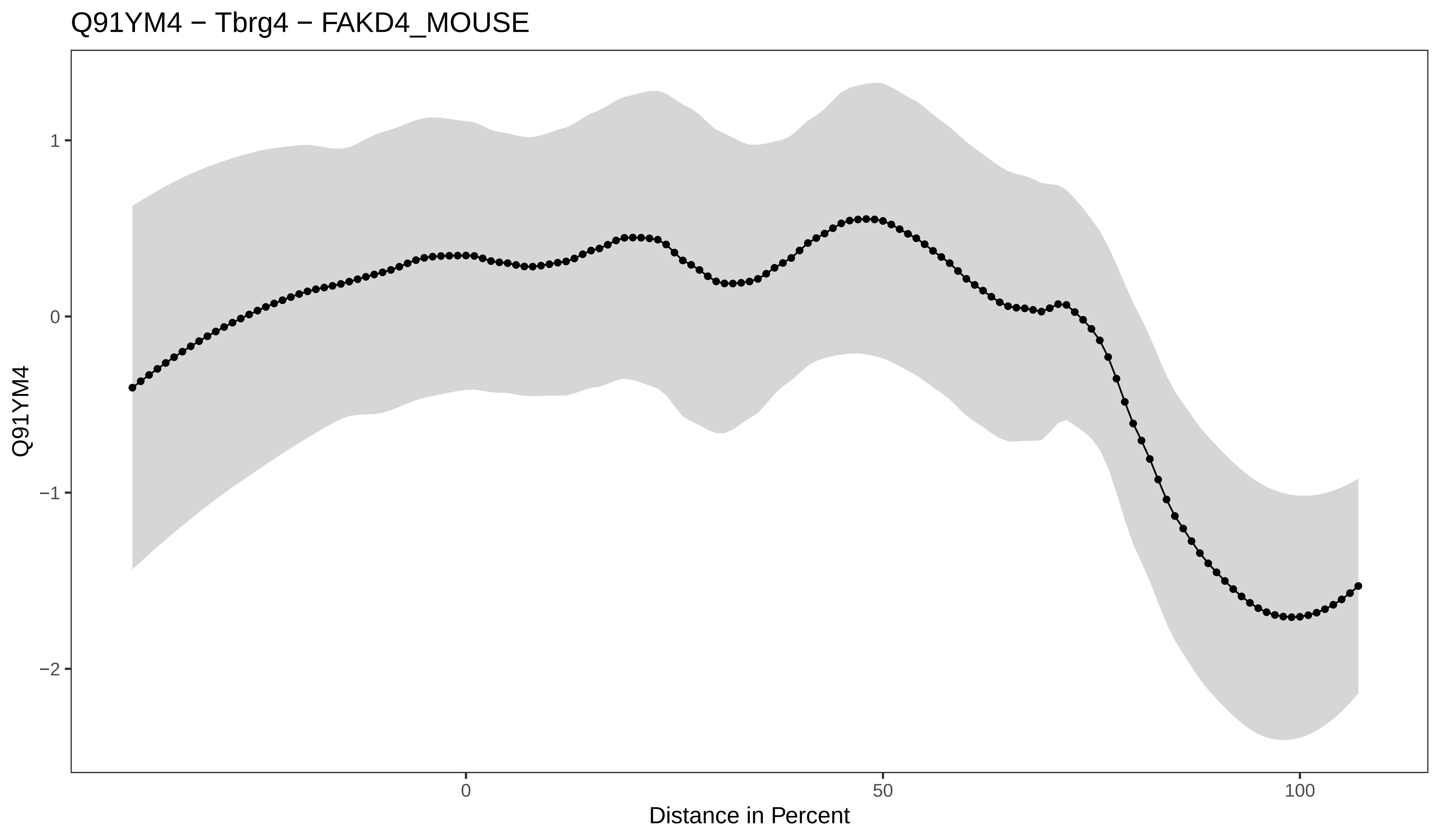

Q91YM4 − Tbrg4 − FAKD4_MOUSE
1
0
−1
−2
Q91YM4
0
50
100
Distance in Percent

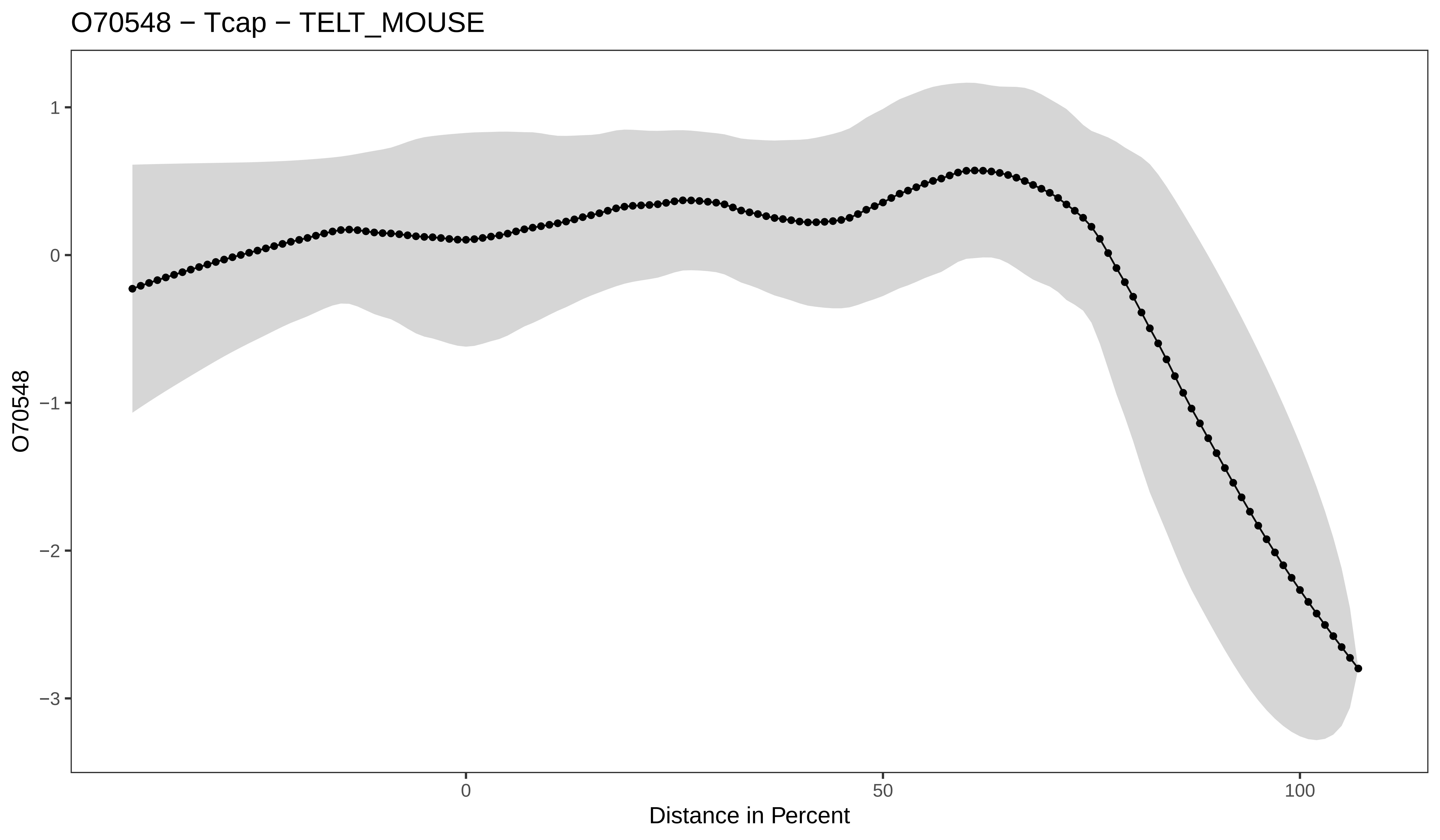

O70548 − Tcap − TELT_MOUSE
1
0
−1
−2
−3
O70548
0
50
100
Distance in Percent

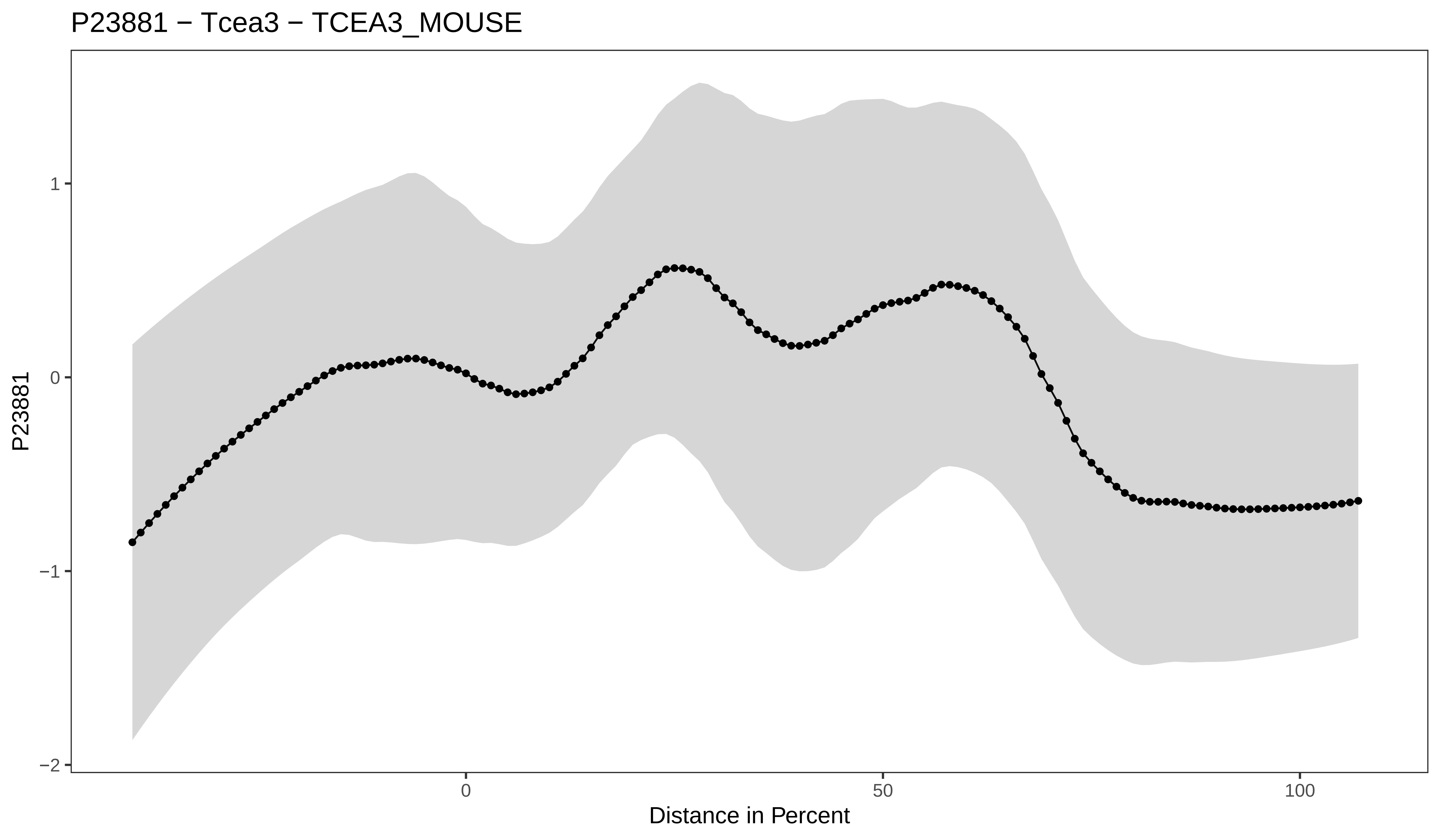
P23881 − Tcea3 − TCEA3_MOUSE
P23881
1
0
−1
−2
0
50
100
Distance in Percent

P11983 − Tcp1 − TCPA_MOUSE

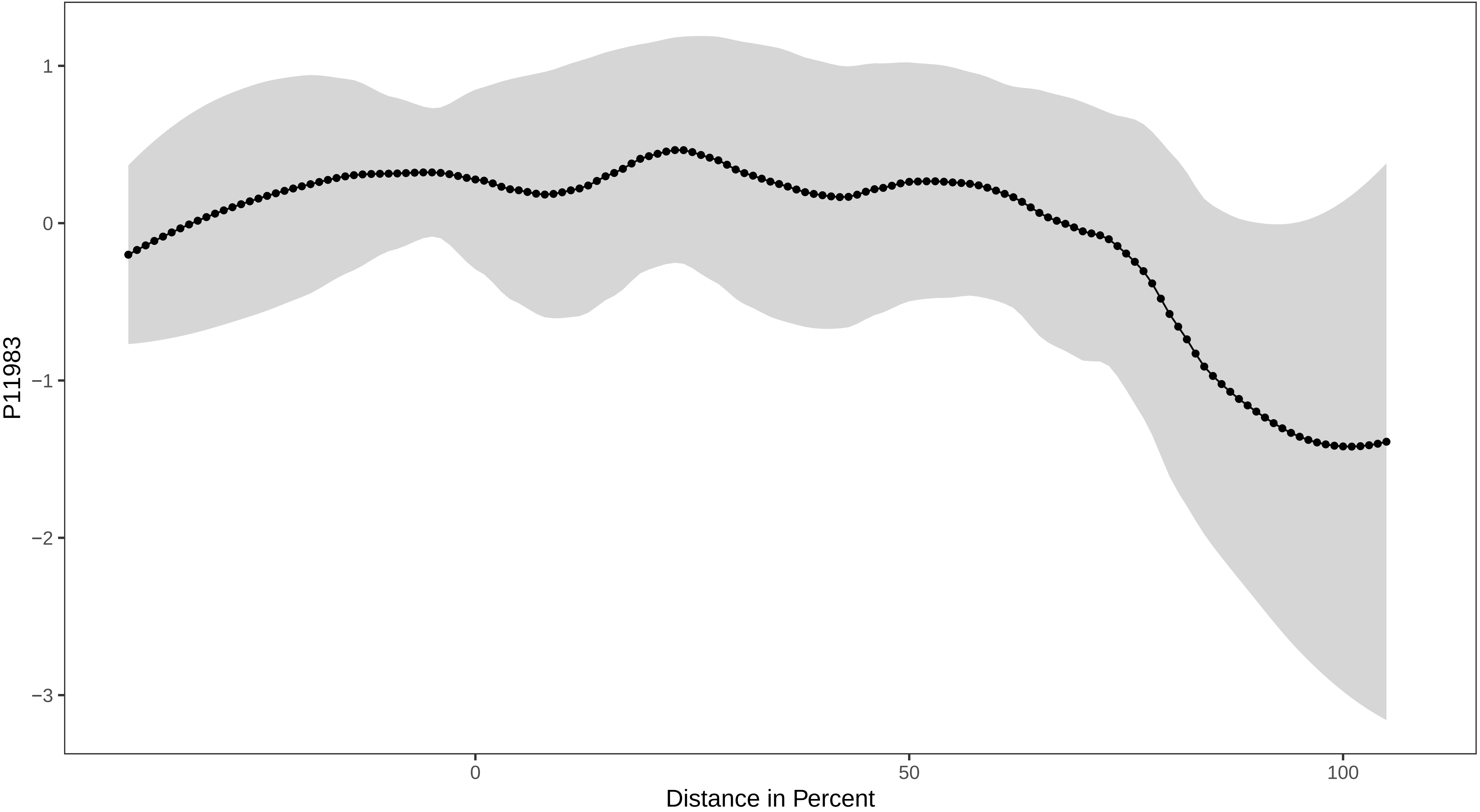

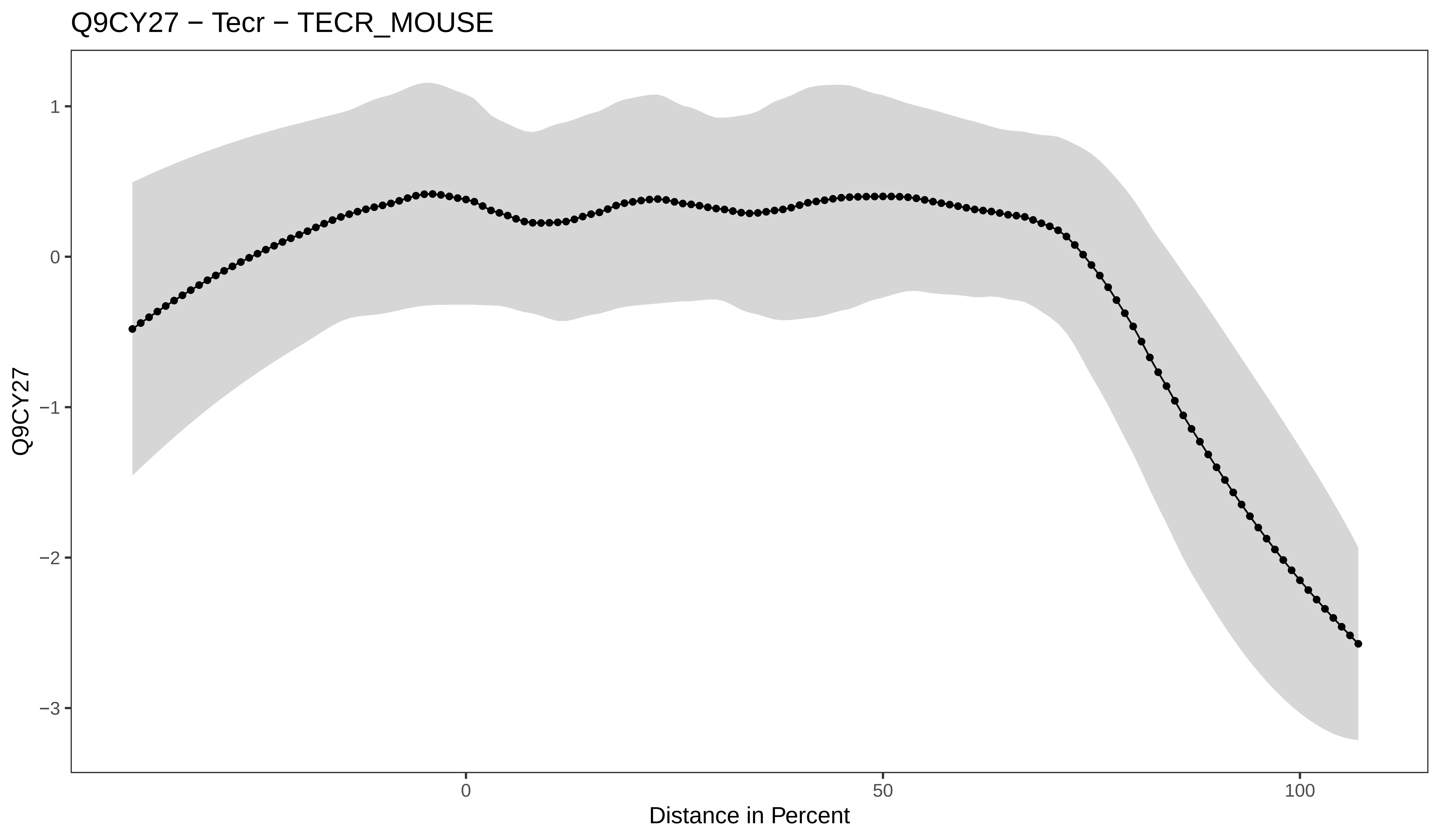

Q9CY27 − Tecr − TECR_MOUSE
Q9CY27
1
0
−1
−2
−3
0
50
100
Distance in Percent

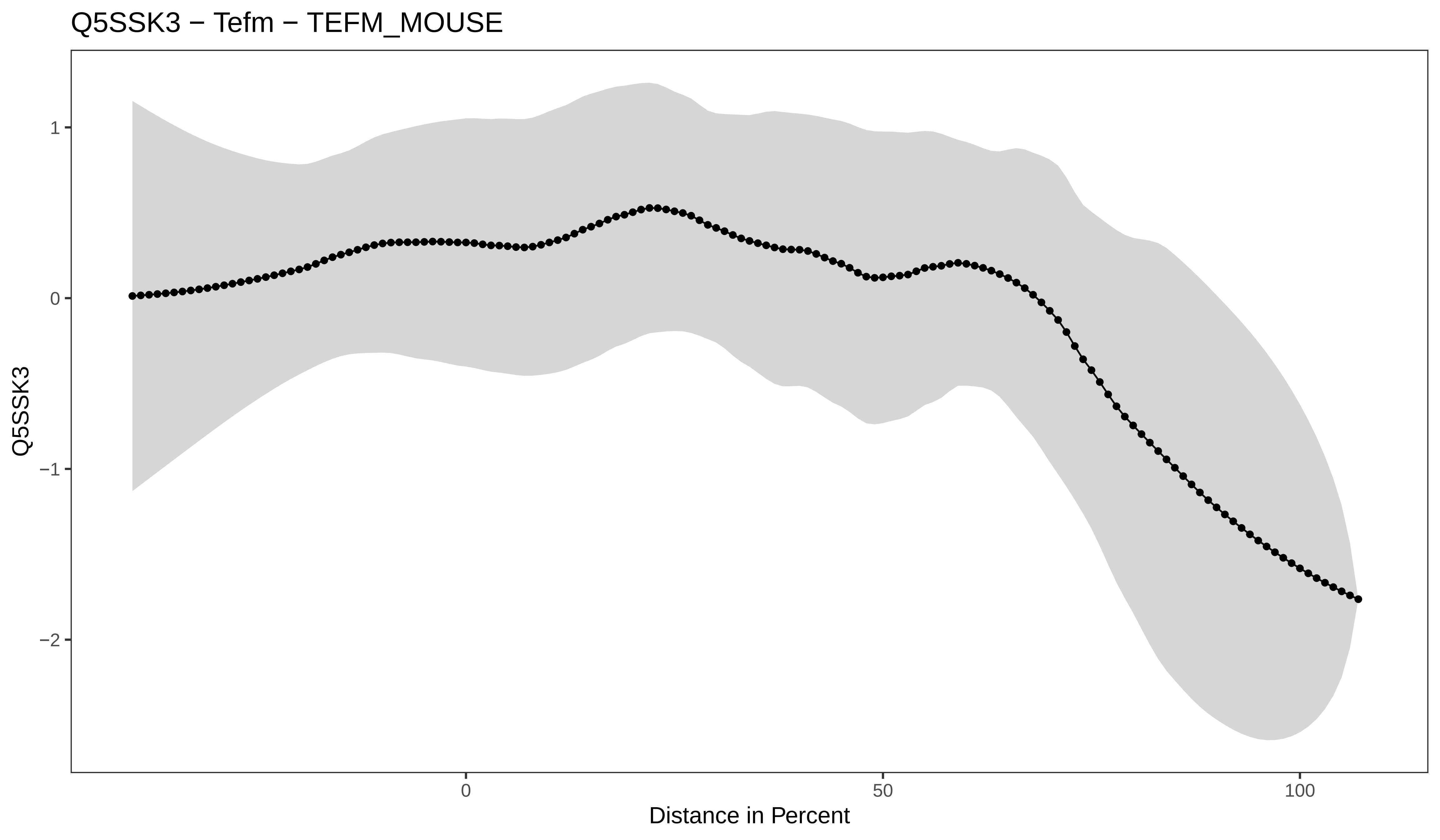
Q5SSK3 − Tefm − TEFM_MOUSE
1
0
−1
−2
Q5SSK3
0
50
100
Distance in Percent

Q921I1 − Tf − TRFE_MOUSE

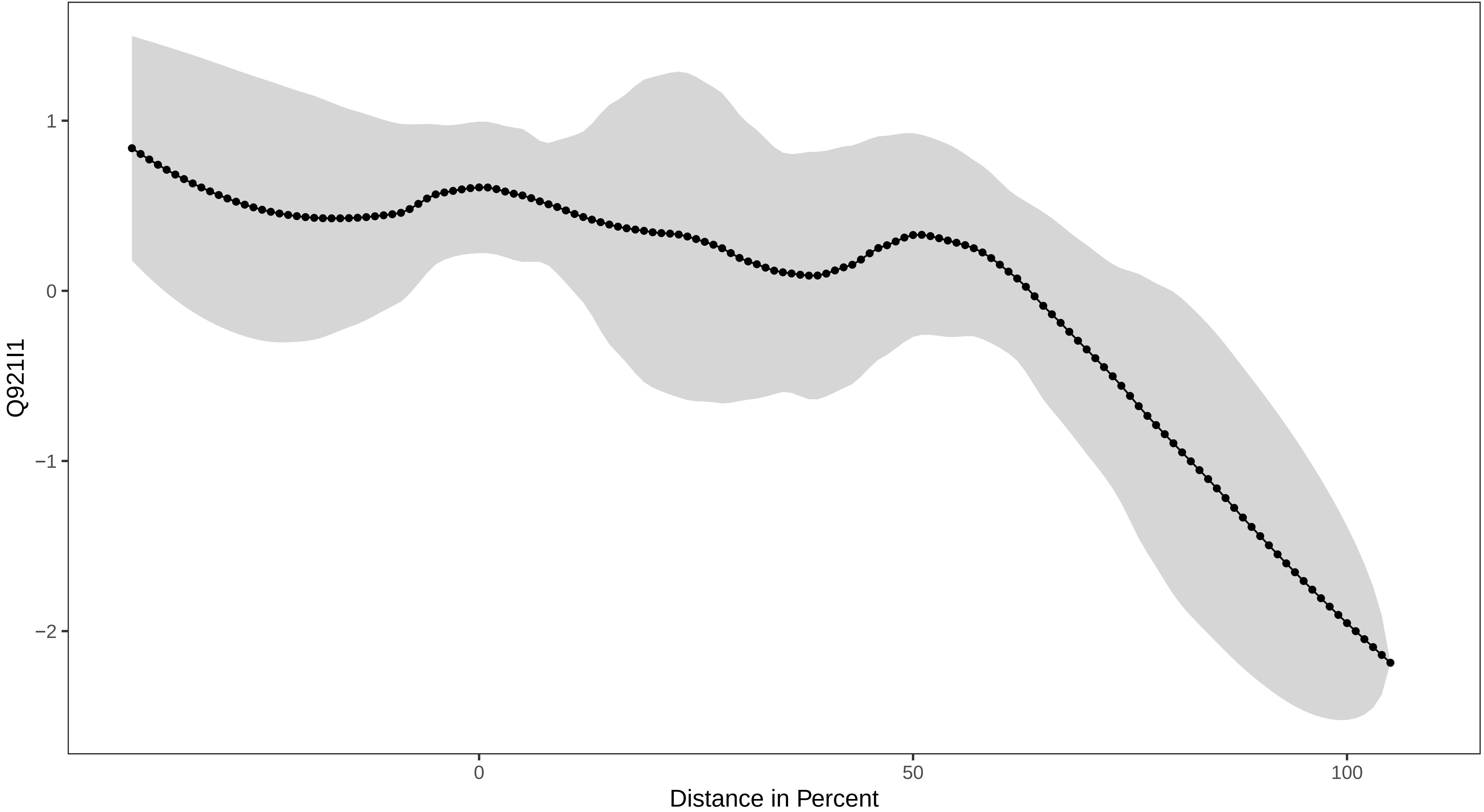

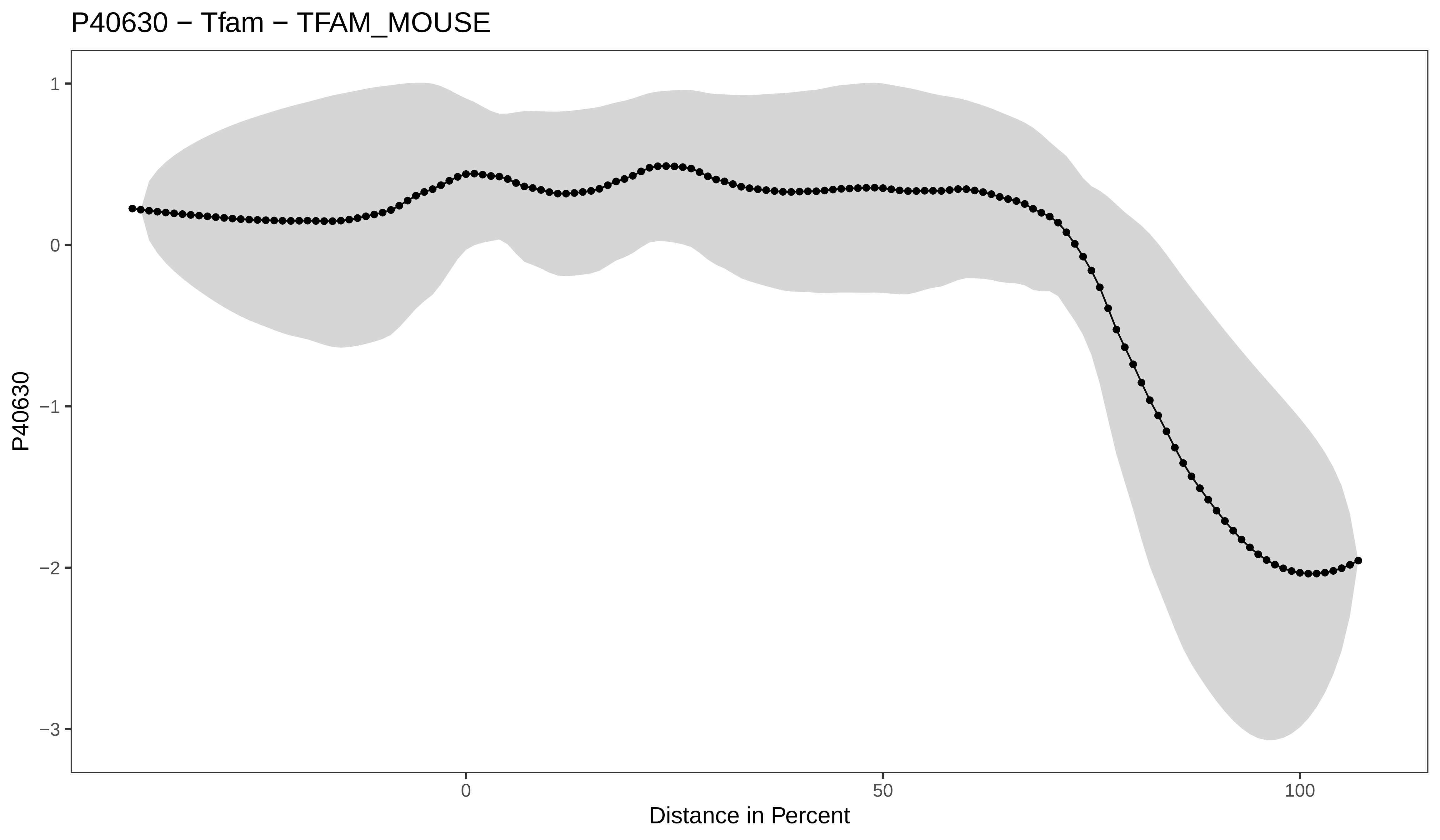
P40630 − Tfam − TFAM_MOUSE
1
0
−1
−2
−3
P40630
0
50
100
Distance in Percent

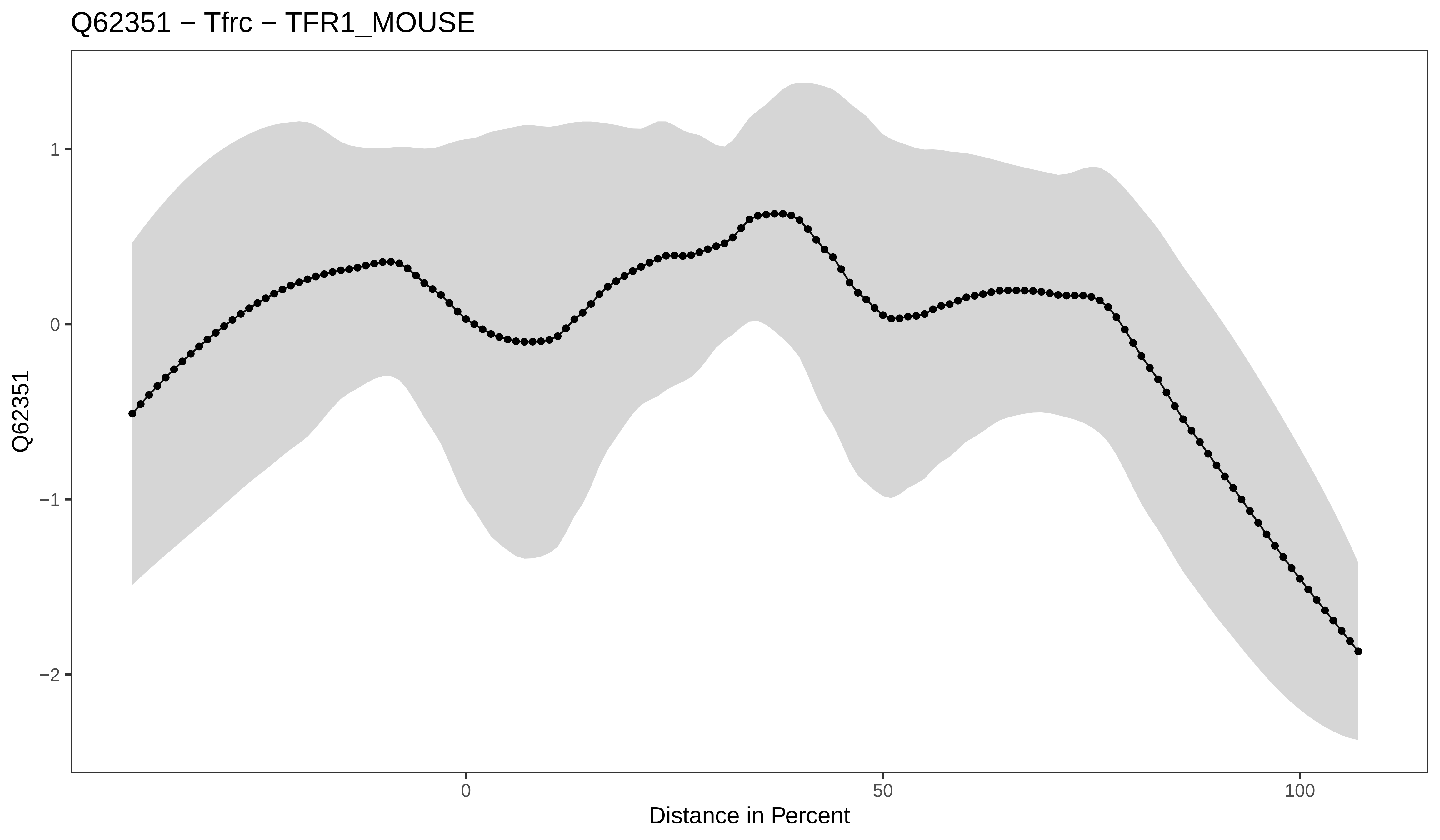
Q62351 − Tfrc − TFR1_MOUSE
Q62351
1
0
−1
−2
0
50
100
Distance in Percent

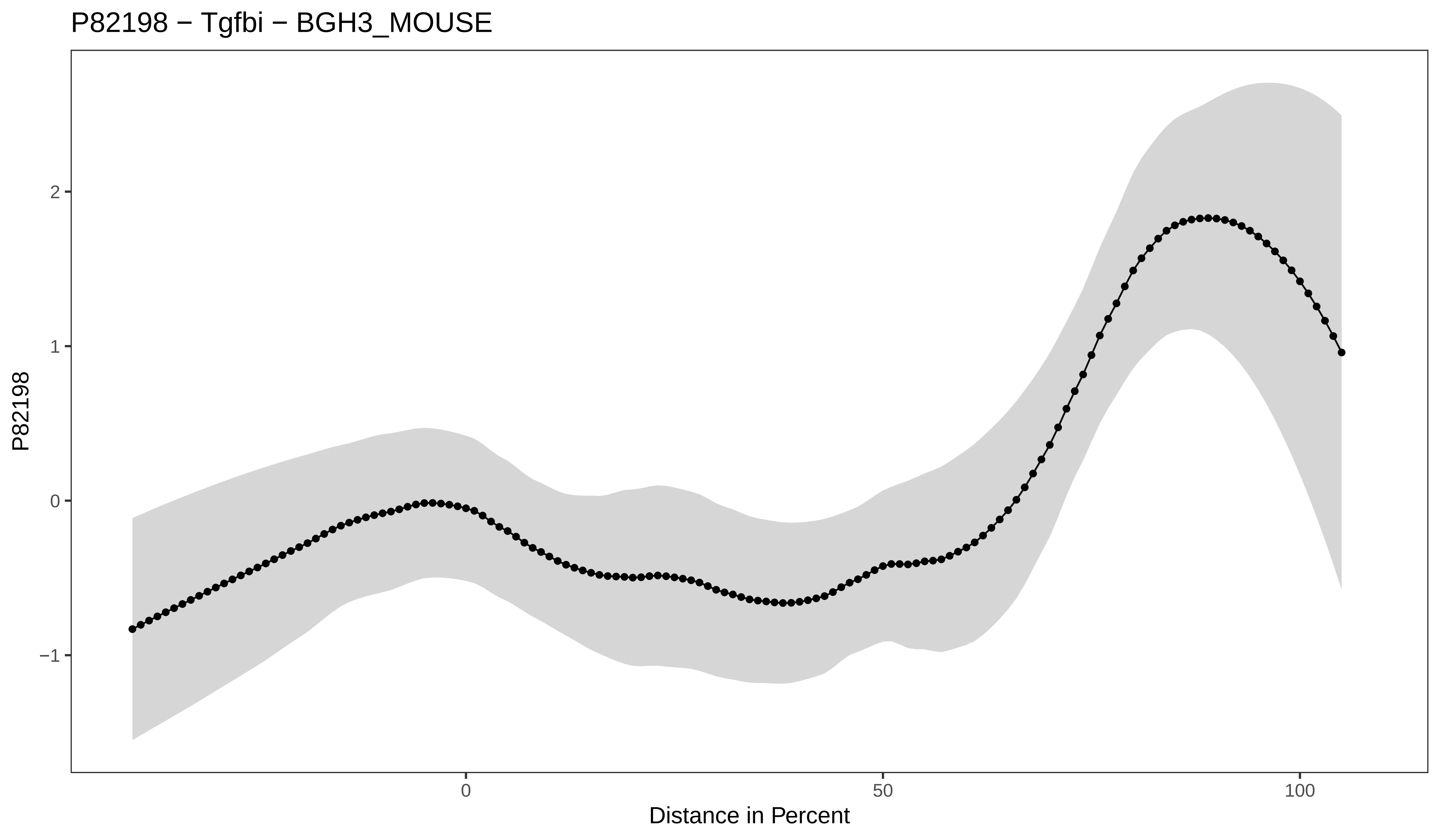
P82198 − Tgfbi − BGH3_MOUSE
P82198
Distance in Percent
2
1
0
−1
0
50
100

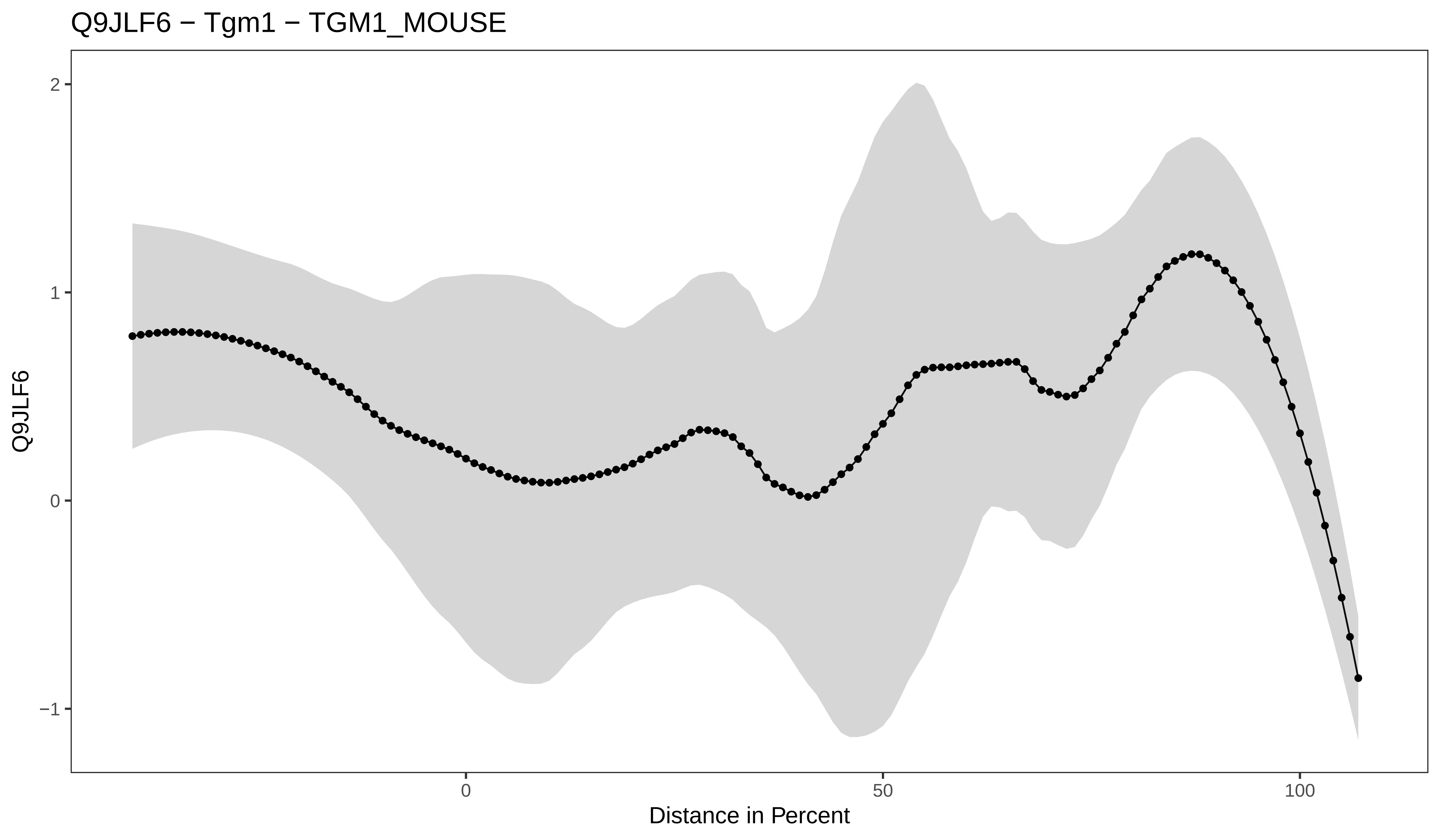
Q9JLF6 − Tgm1 − TGM1_MOUSE
Q9JLF6
Distance in Percent
2
1
0
−1
0
50
100

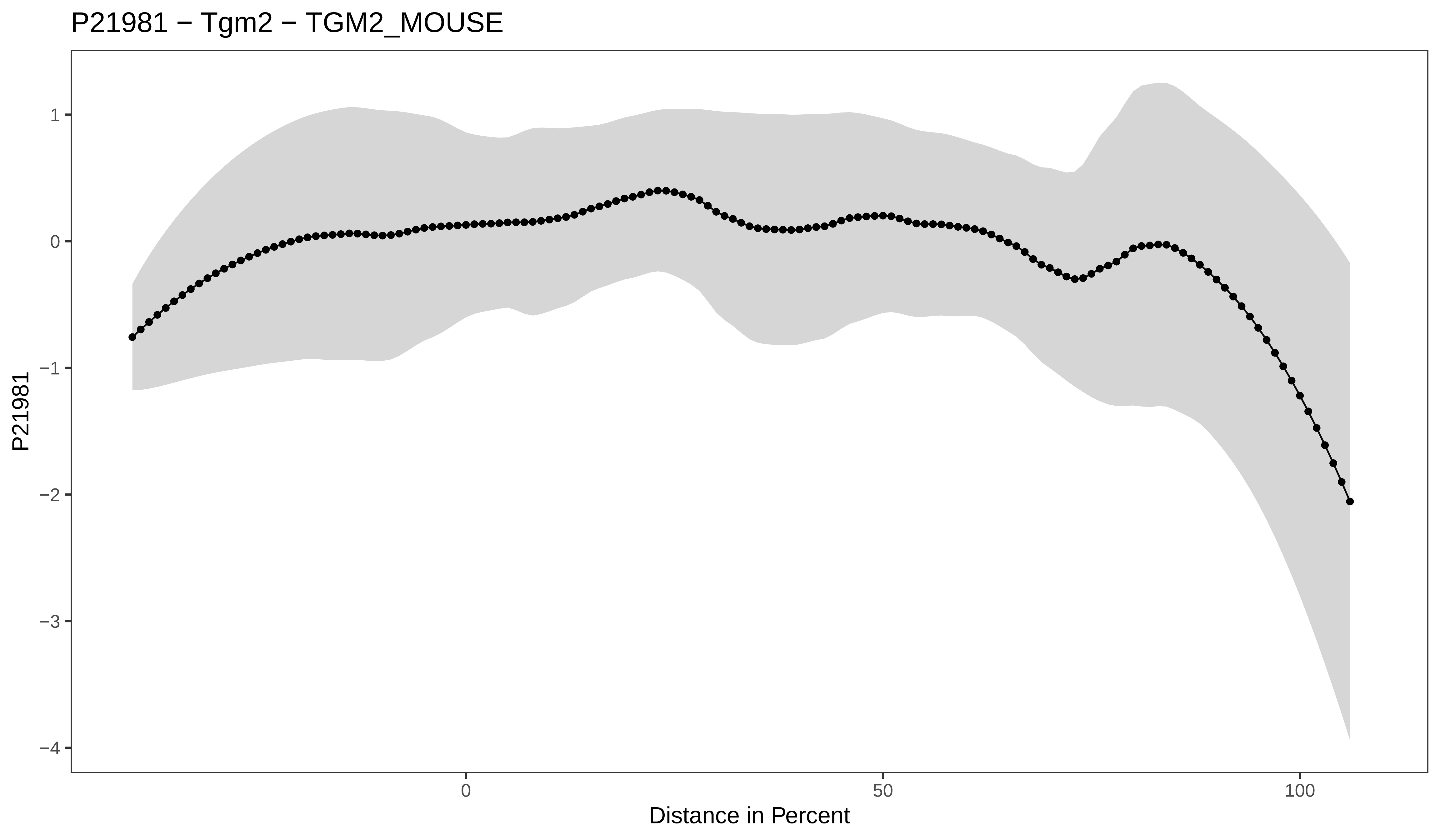

P21981 − Tgm2 − TGM2_MOUSE
1
0
−1
−2
−3
−4
P21981
0
50
100
Distance in Percent

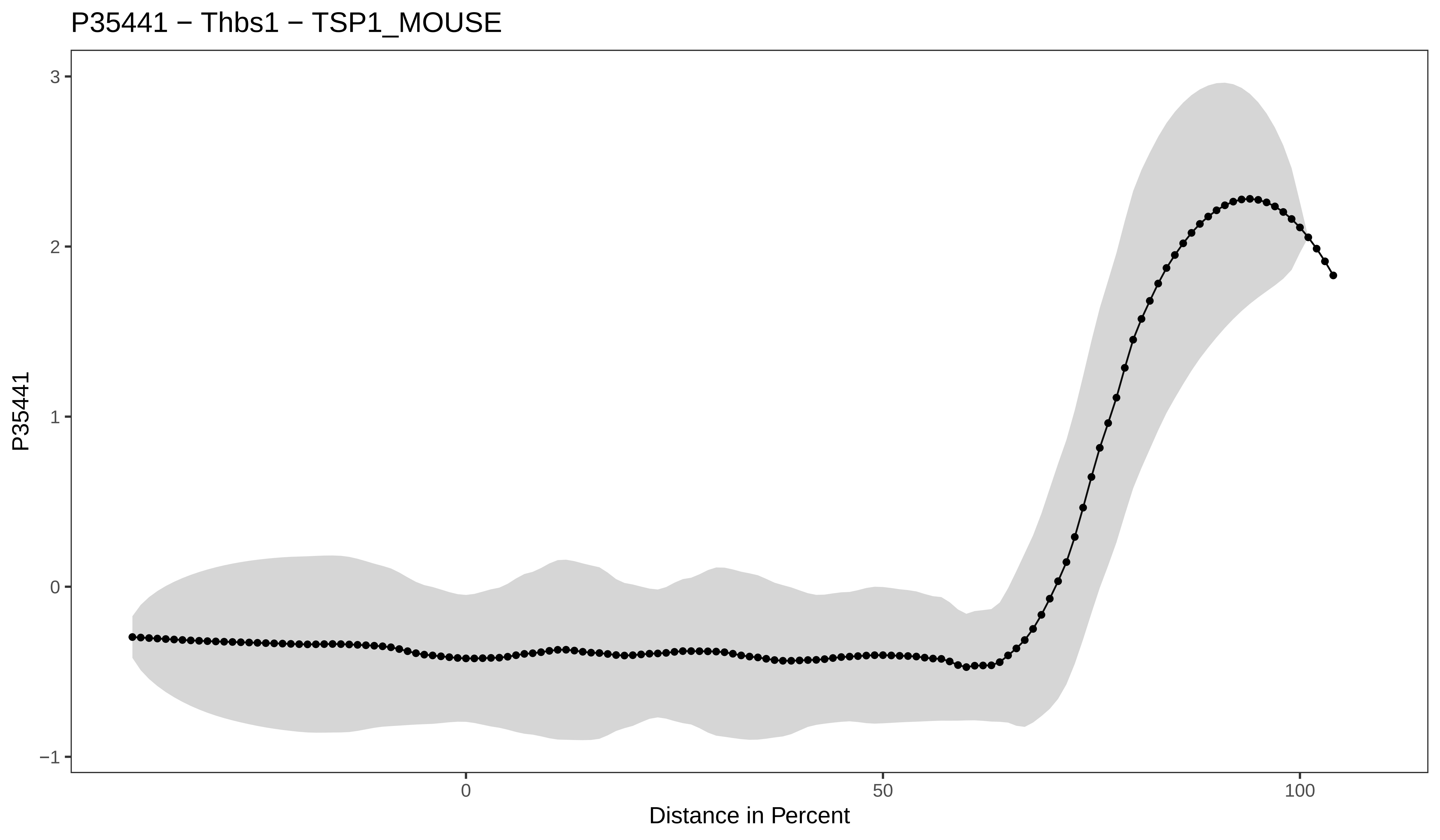

P35441 − Thbs1 − TSP1_MOUSE
P35441
Distance in Percent
3
2
1
0
−1
0
50
100

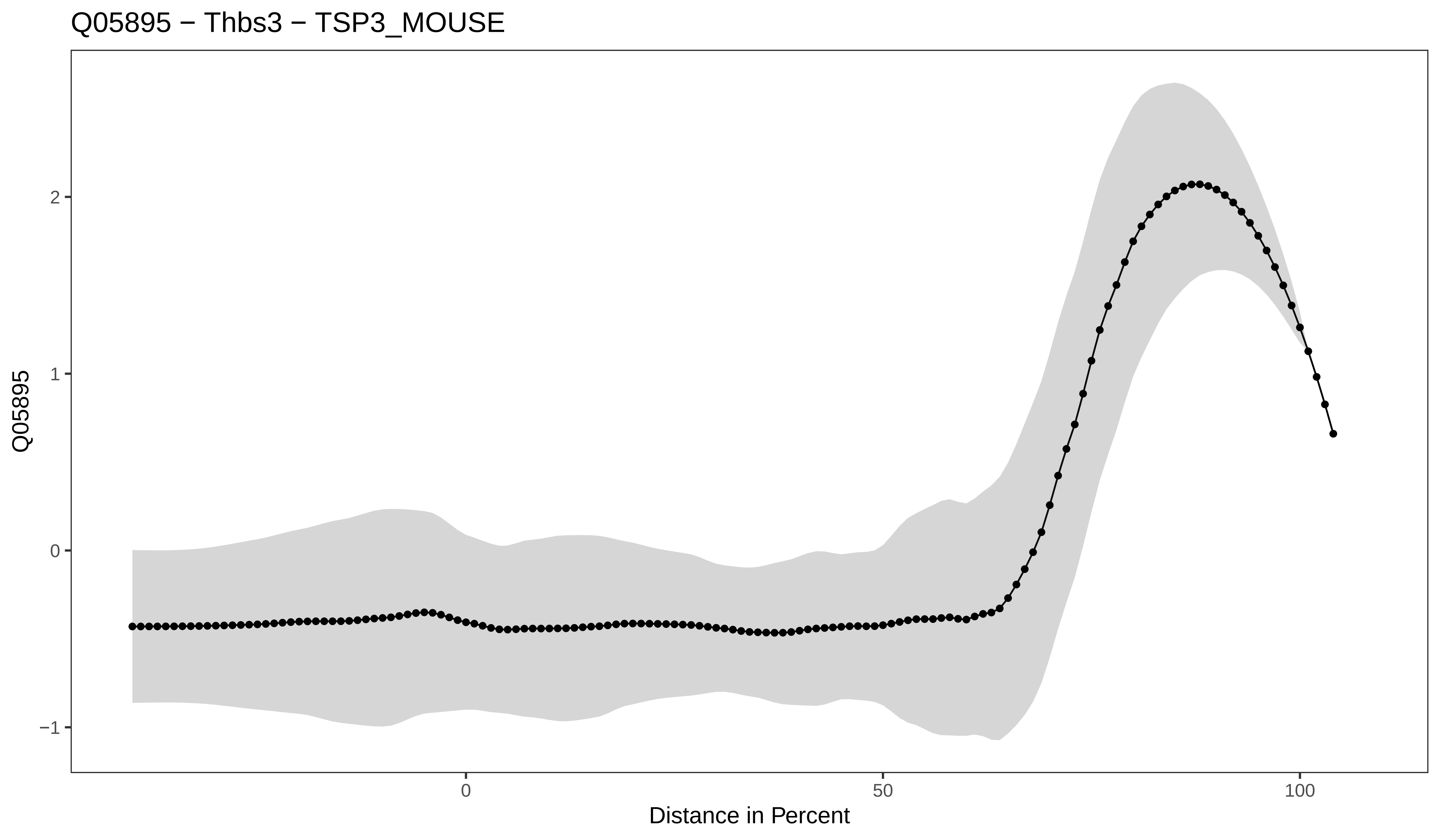

Q05895 − Thbs3 − TSP3_MOUSE
Q05895
Distance in Percent
2
1
0
−1
0
50
100

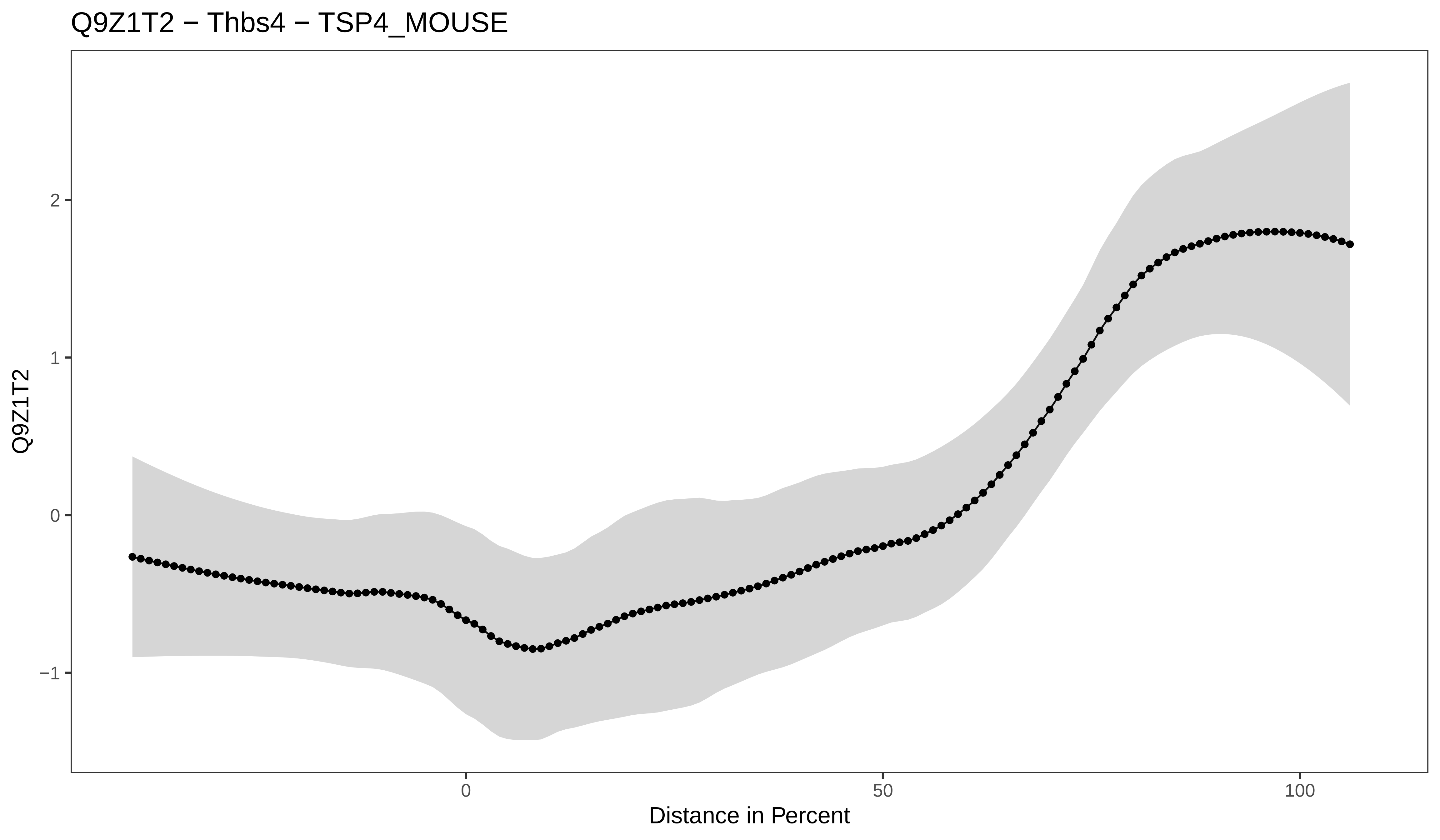

Q9Z1T2 − Thbs4 − TSP4_MOUSE
Q9Z1T2
2
1
0
−1
0
50
100
Distance in Percent

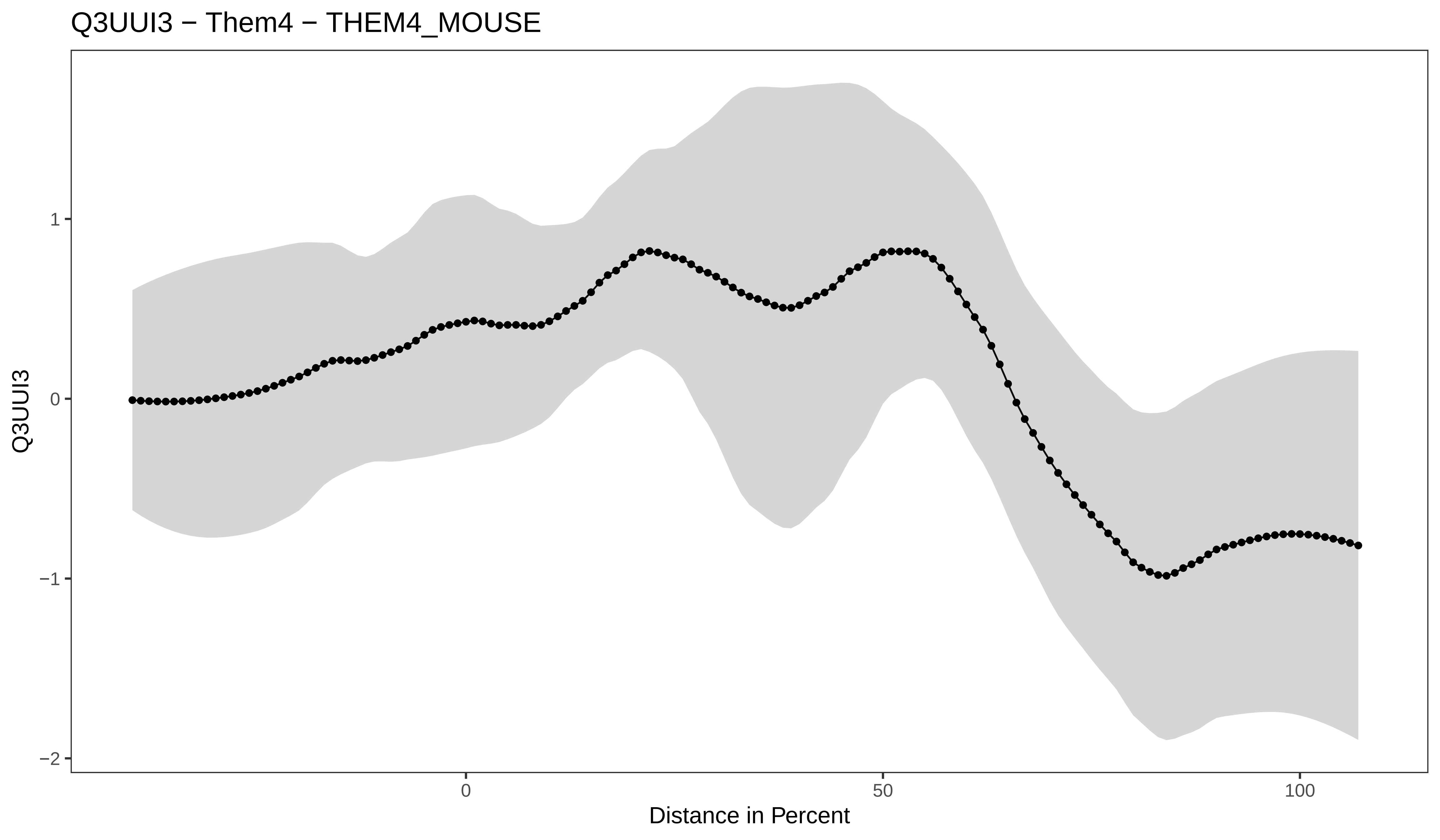

Q3UUI3 − Them4 − THEM4_MOUSE
Q3UUI3
Distance in Percent
1
0
−1
−2
0
50
100

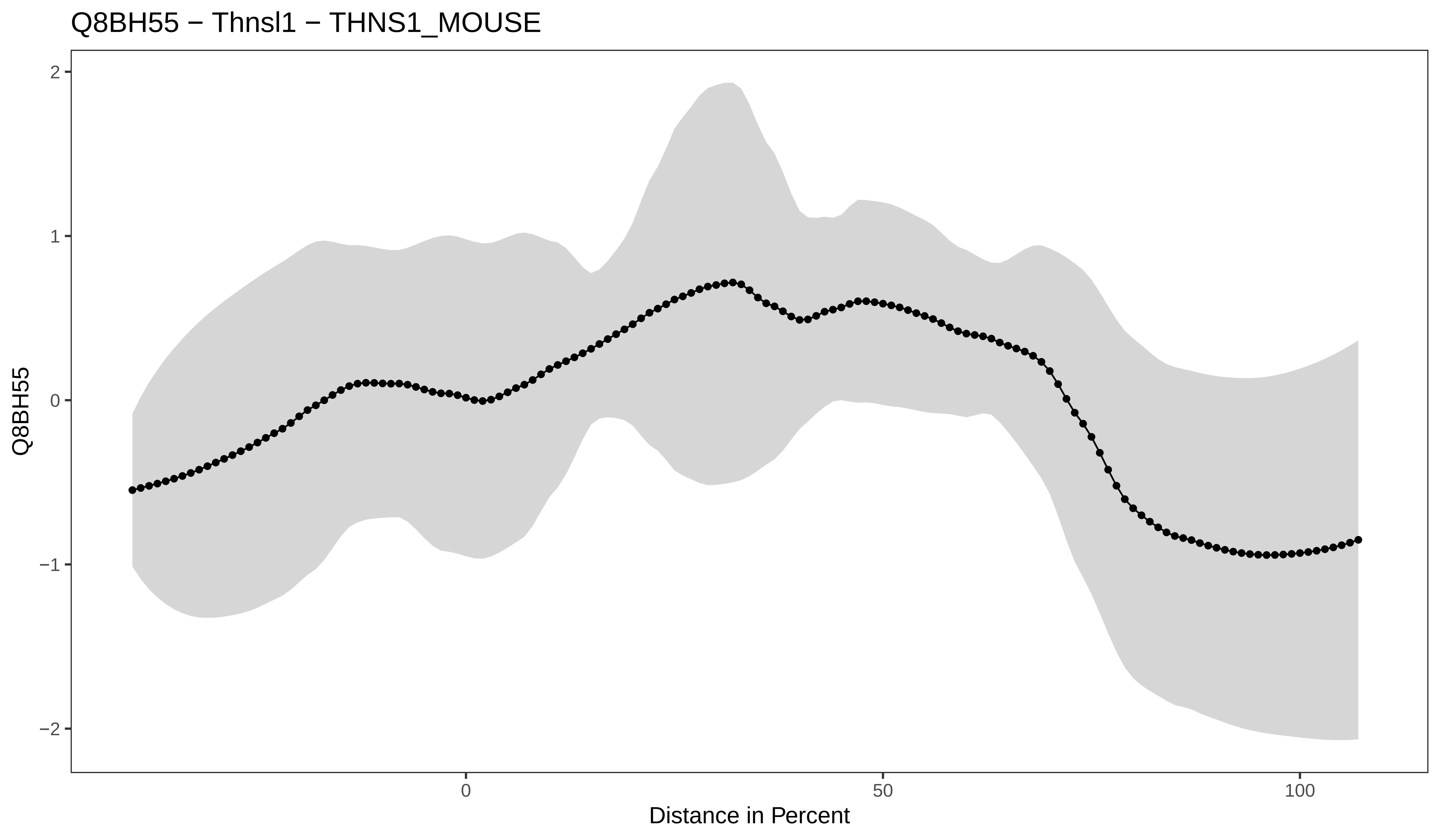

Q8BH55 − Thnsl1 − THNS1_MOUSE
Q8BH55
2
1
0
−1
−2
0
50
100
Distance in Percent

Q8C1A5 − Thop1 − THOP1_MOUSE

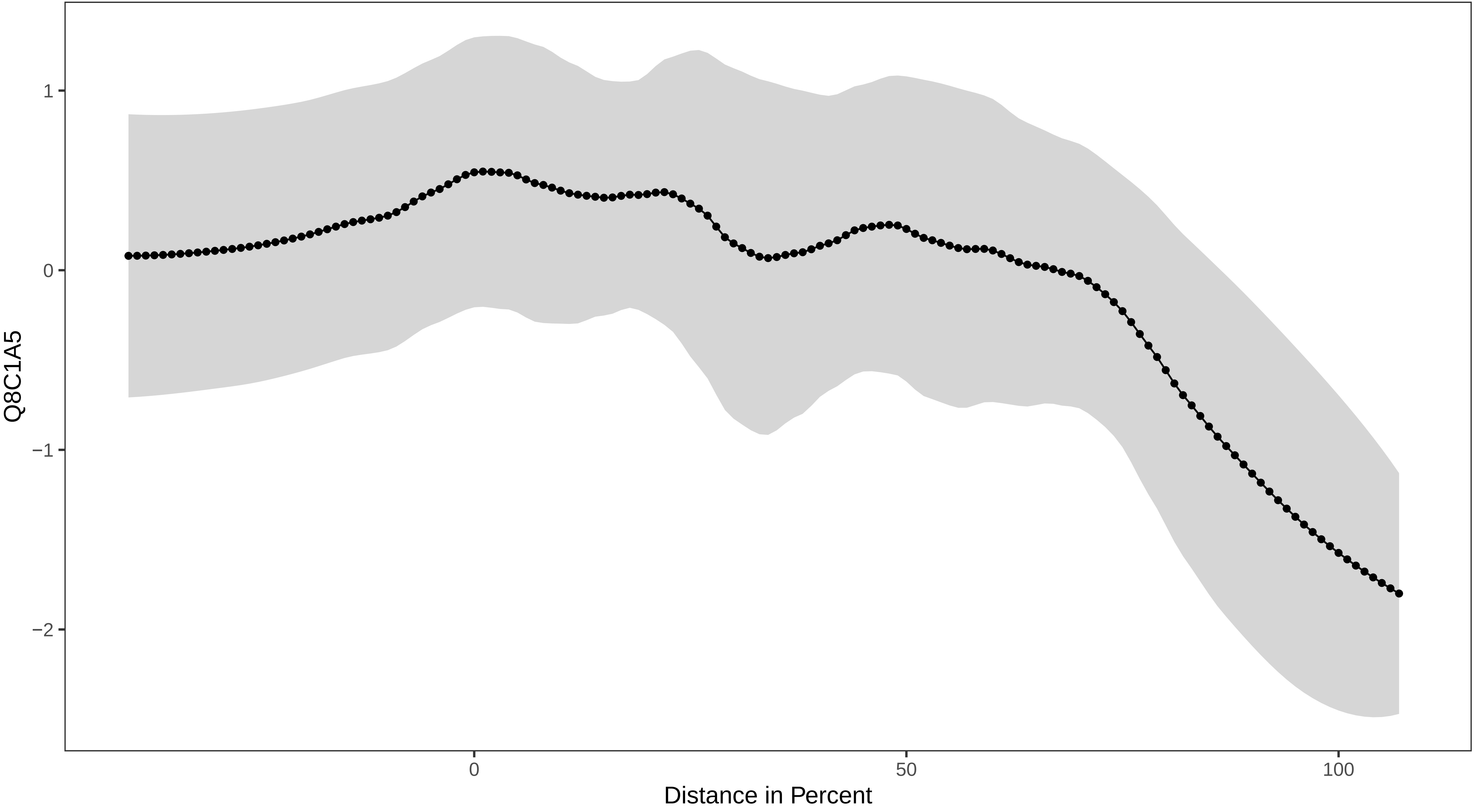

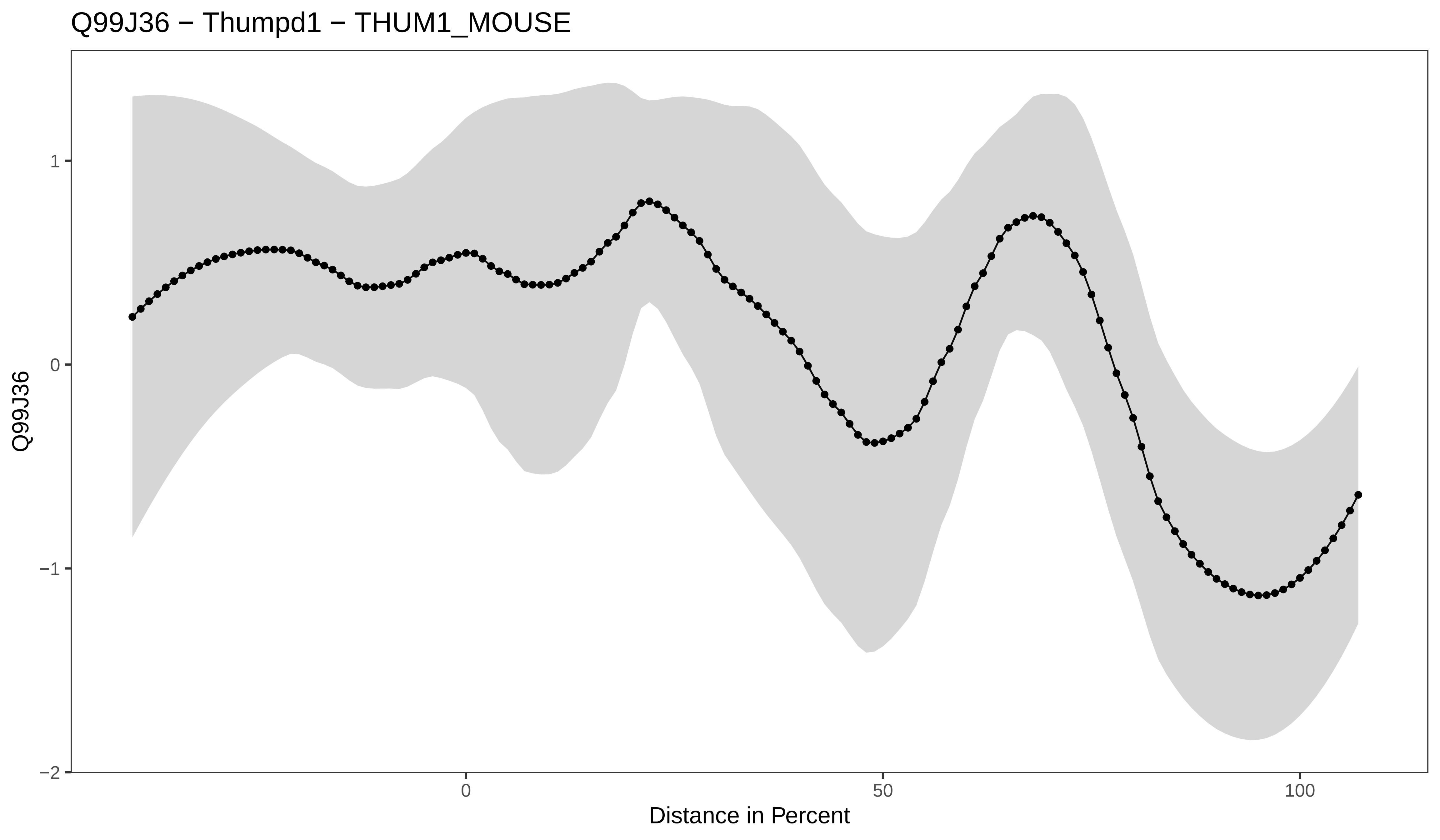

Q99J36 − Thumpd1 − THUM1_MOUSE
Q99J36
1
0
−1
−2
0
50
100
Distance in Percent

P01831 − Thy1 − THY1_MOUSE

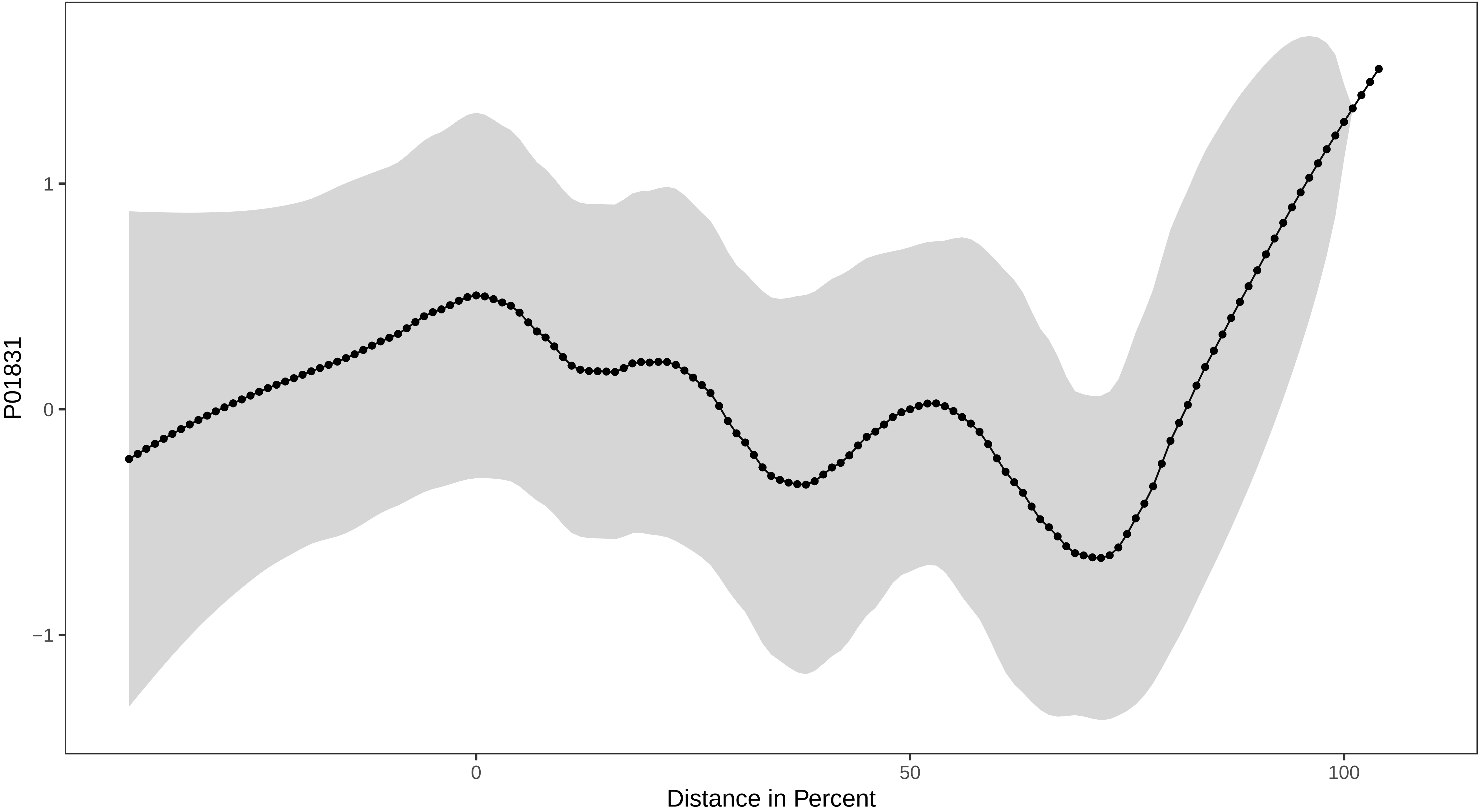

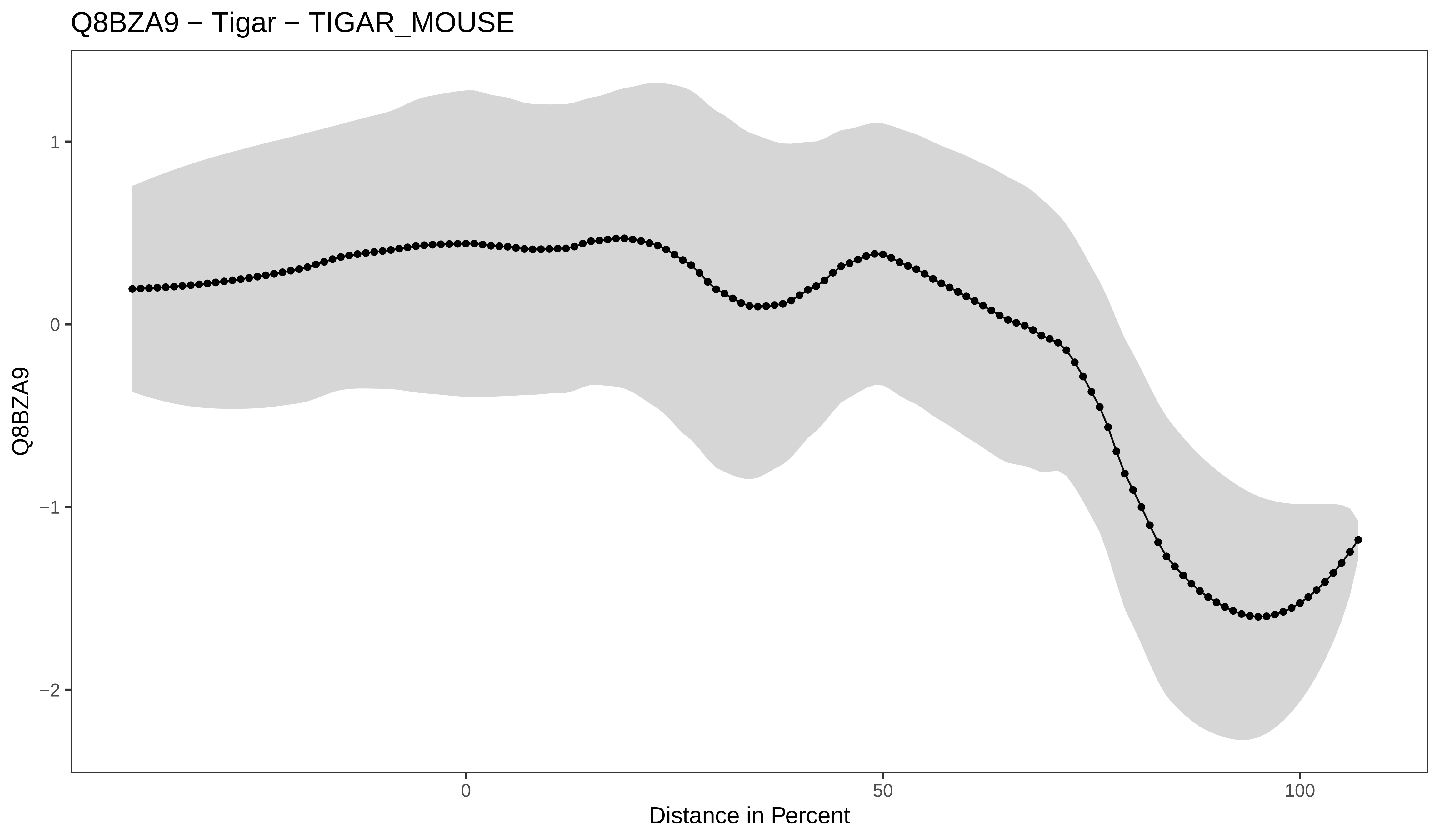

Q8BZA9 − Tigar − TIGAR_MOUSE
Q8BZA9
1
0
−1
−2
0
50
100
Distance in Percent

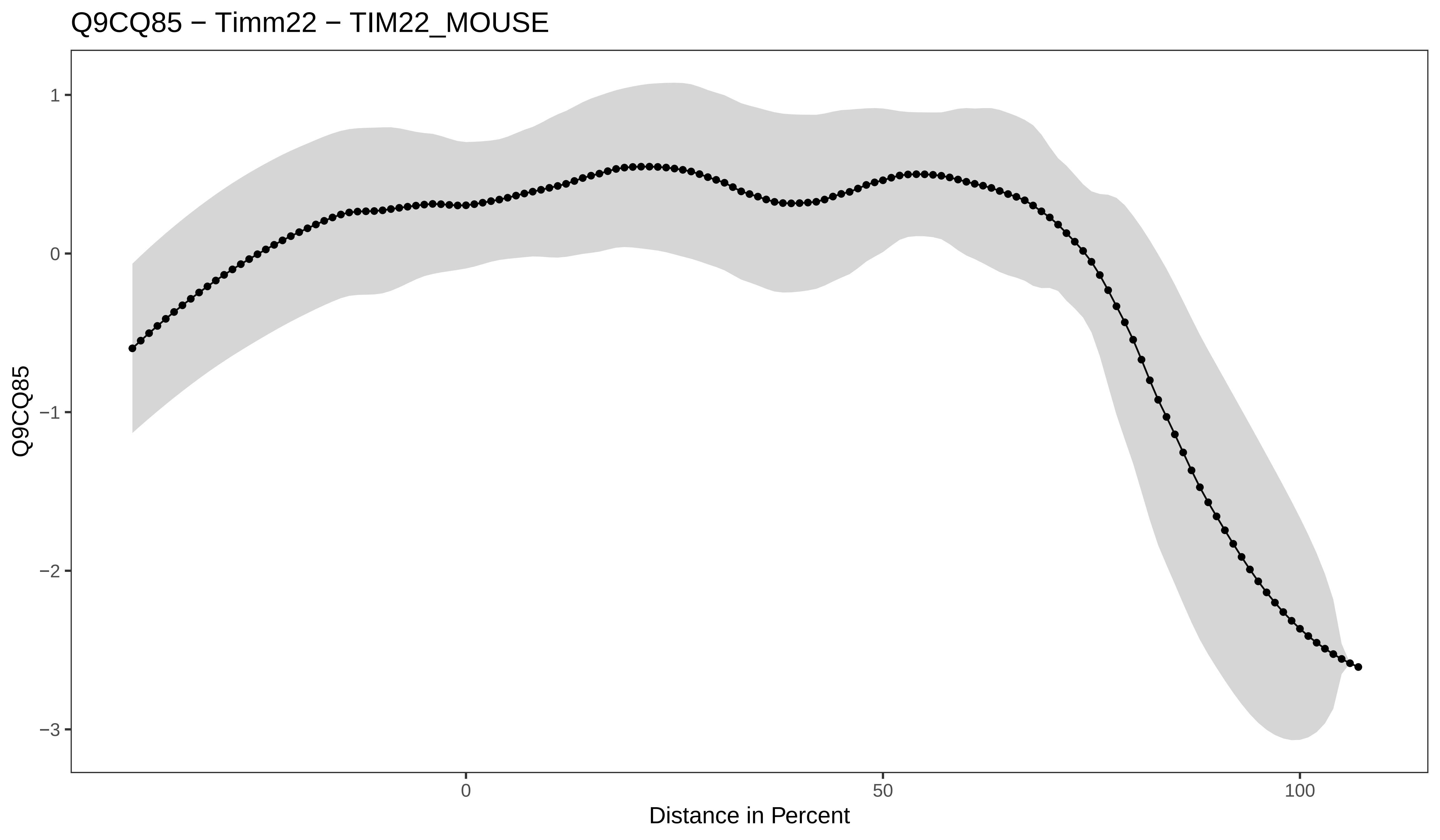

Q9CQ85 − Timm22 − TIM22_MOUSE
Q9CQ85
1
0
−1
−2
−3
0
50
100
Distance in Percent

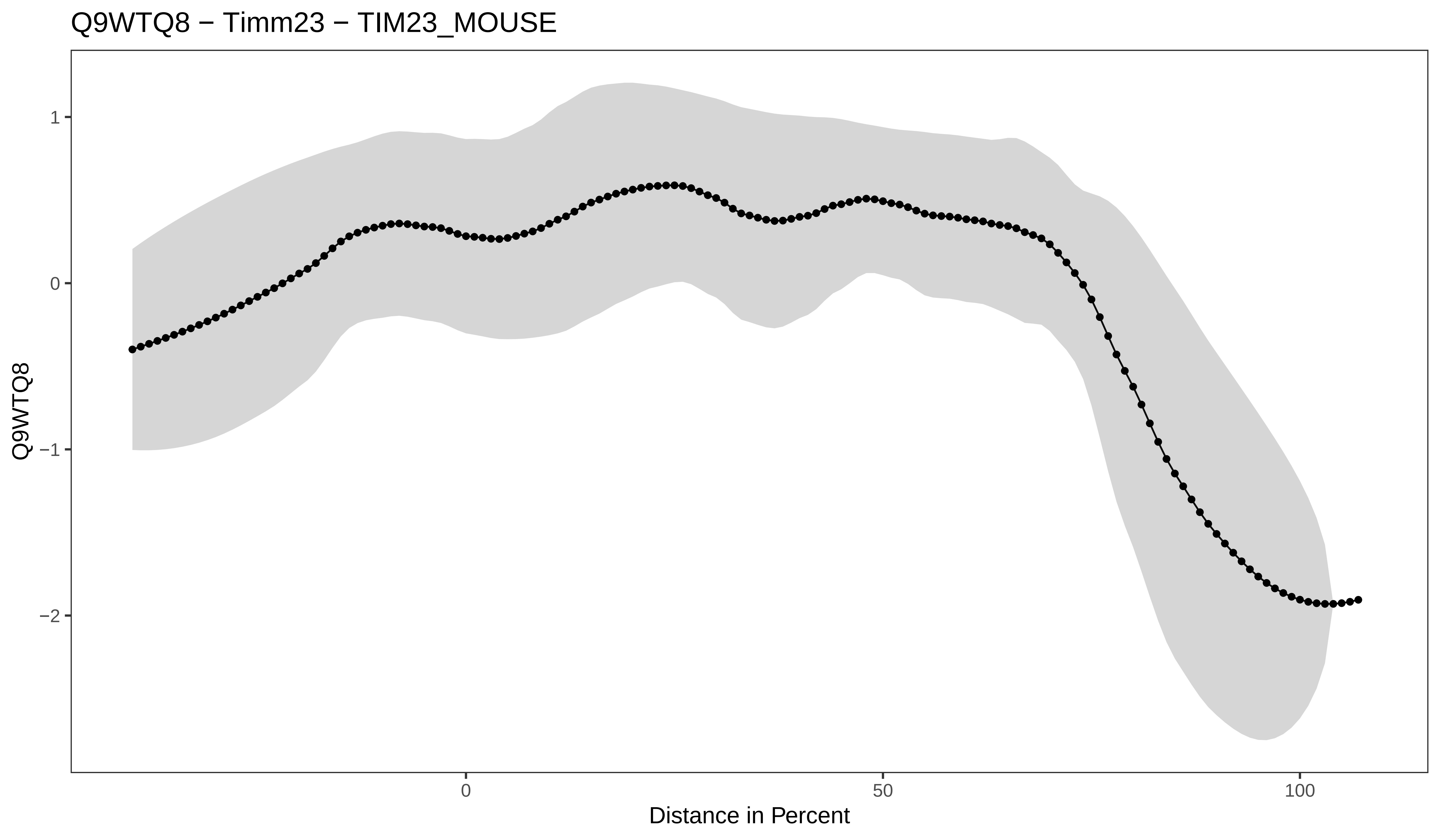

Q9WTQ8 − Timm23 − TIM23_MOUSE
Q9WTQ8
1
0
−1
−2
0
50
100
Distance in Percent

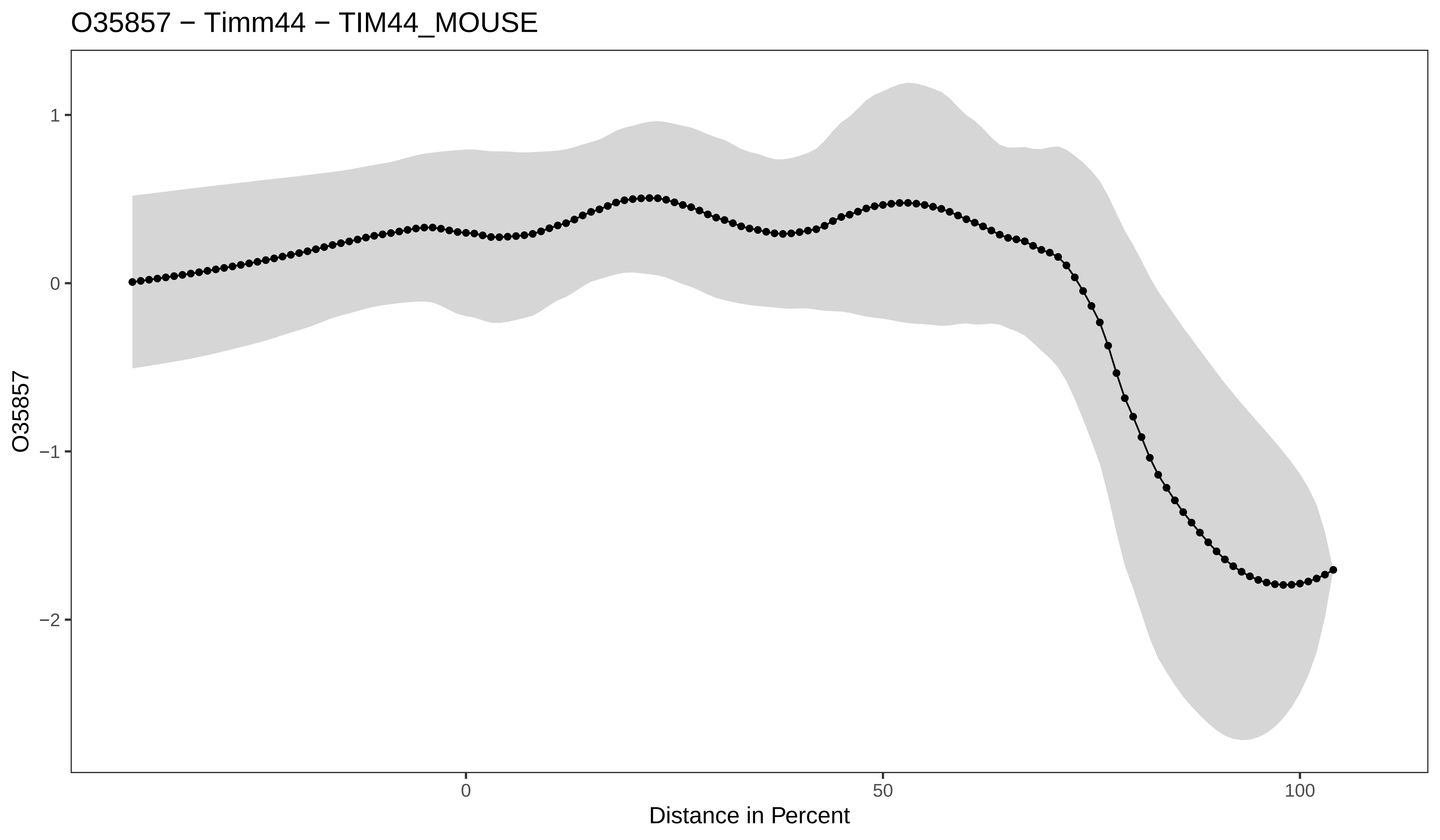
O35857 − Timm44 − TIM44_MOUSE
O35857
1
0
−1
−2
0
50
100
Distance in Percent

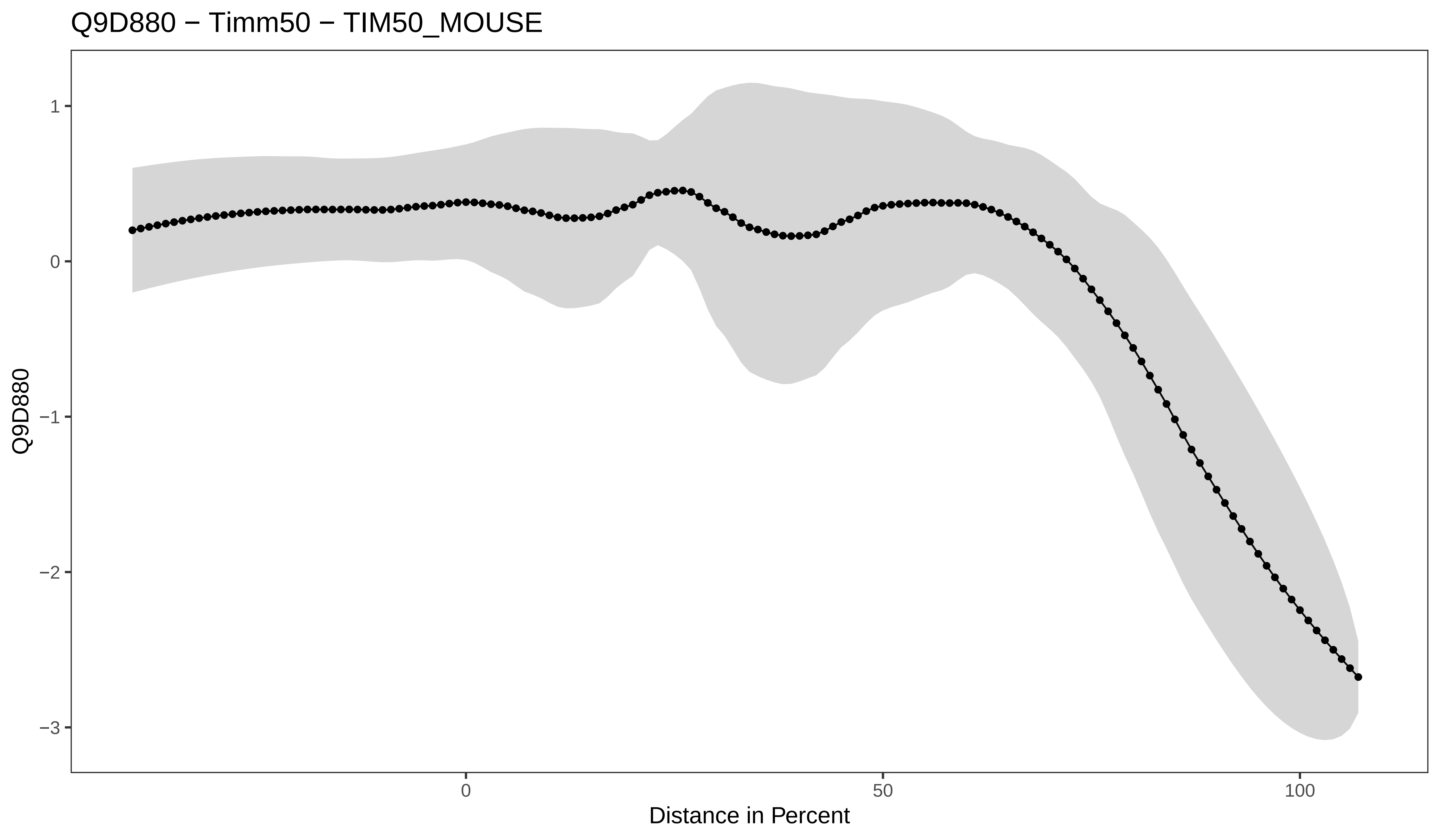

Q9D880 − Timm50 − TIM50_MOUSE
Q9D880
1
0
−1
−2
−3
0
50
100
Distance in Percent

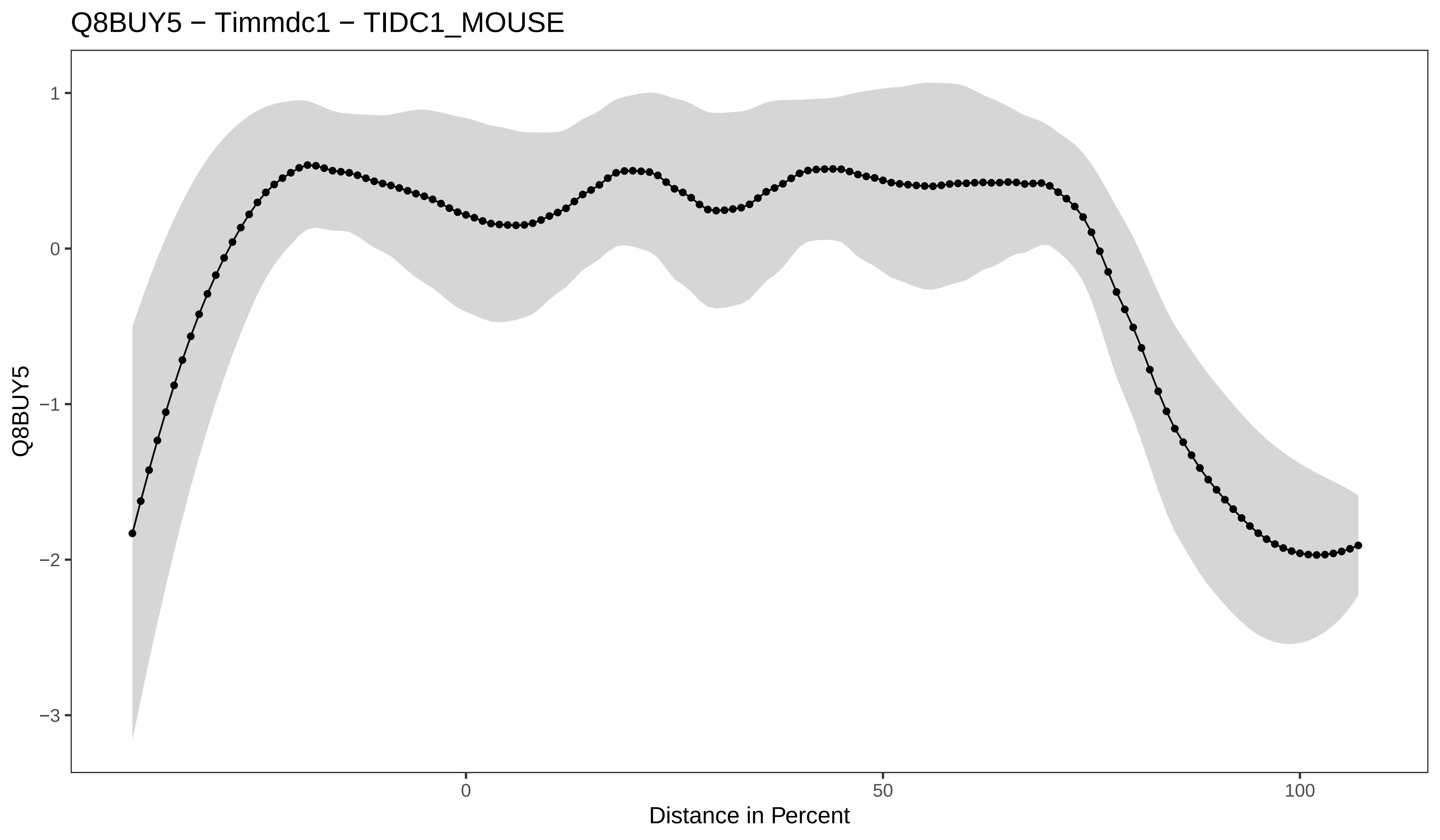
Q8BUY5 − Timmdc1 − TIDC1_MOUSE
1
0
−1
−2
−3
Q8BUY5
0
50
100
Distance in Percent

Q99JR5 − Tinagl1 − TINAL_MOUSE

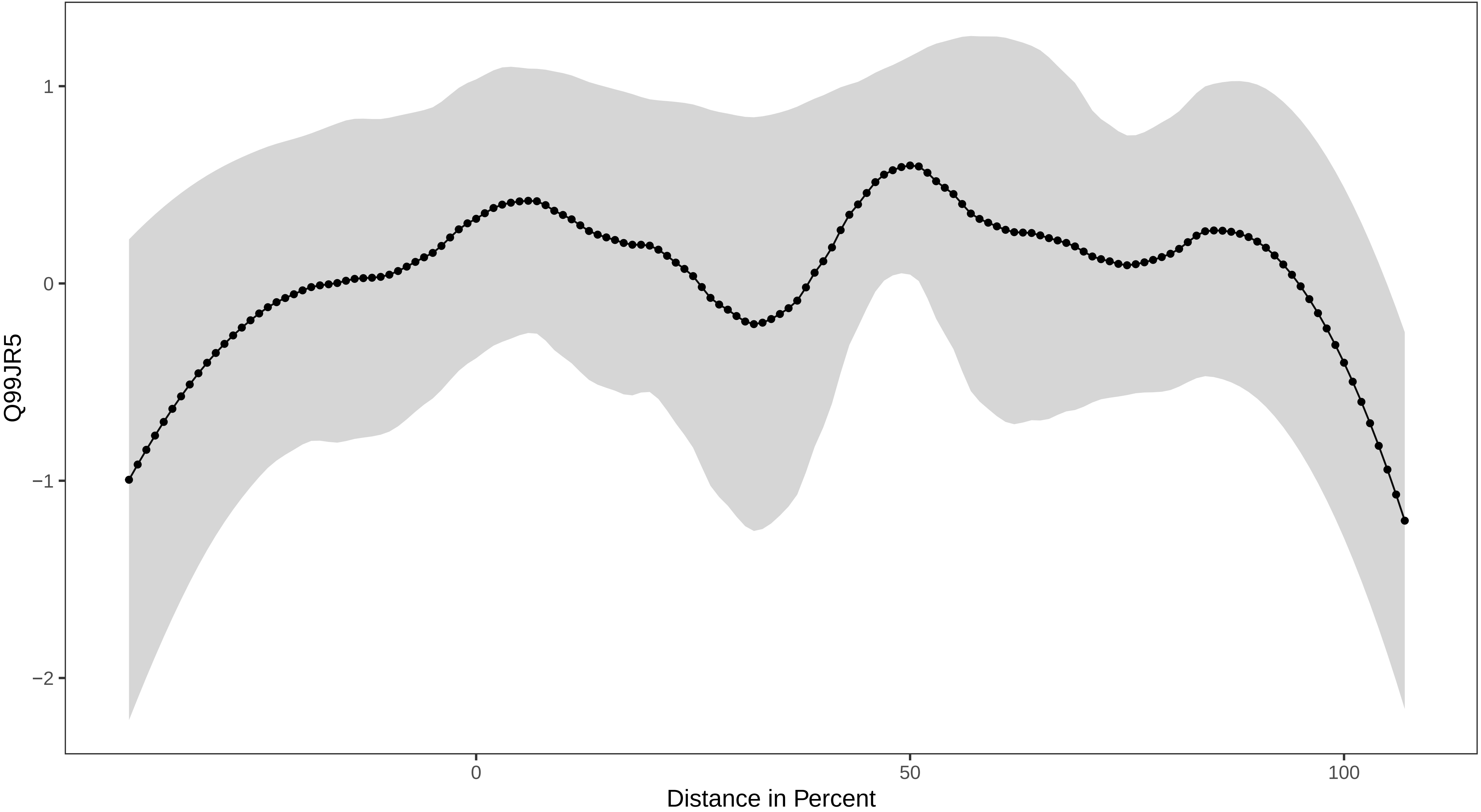

Q8BH58 − Tiprl − TIPRL_MOUSE

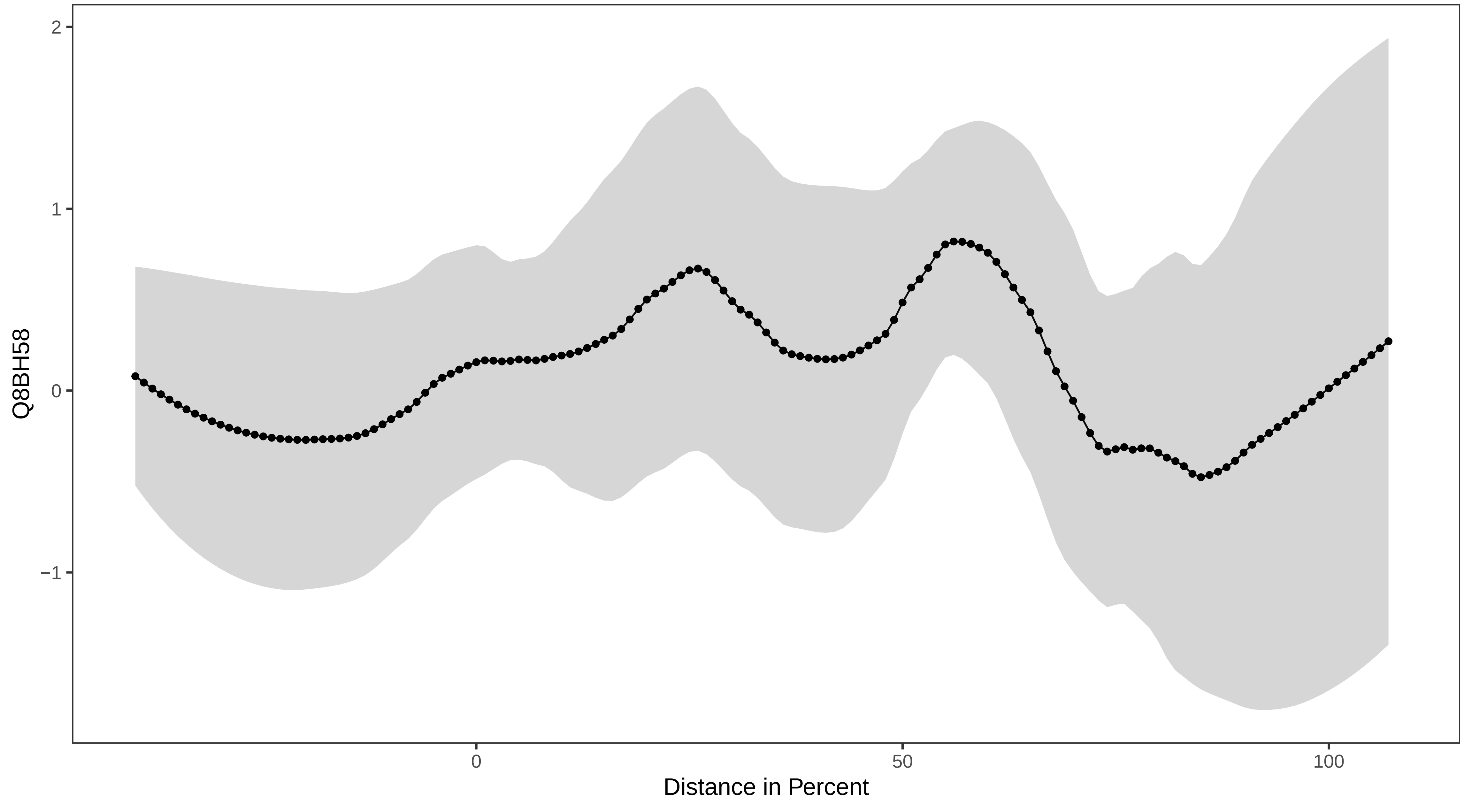

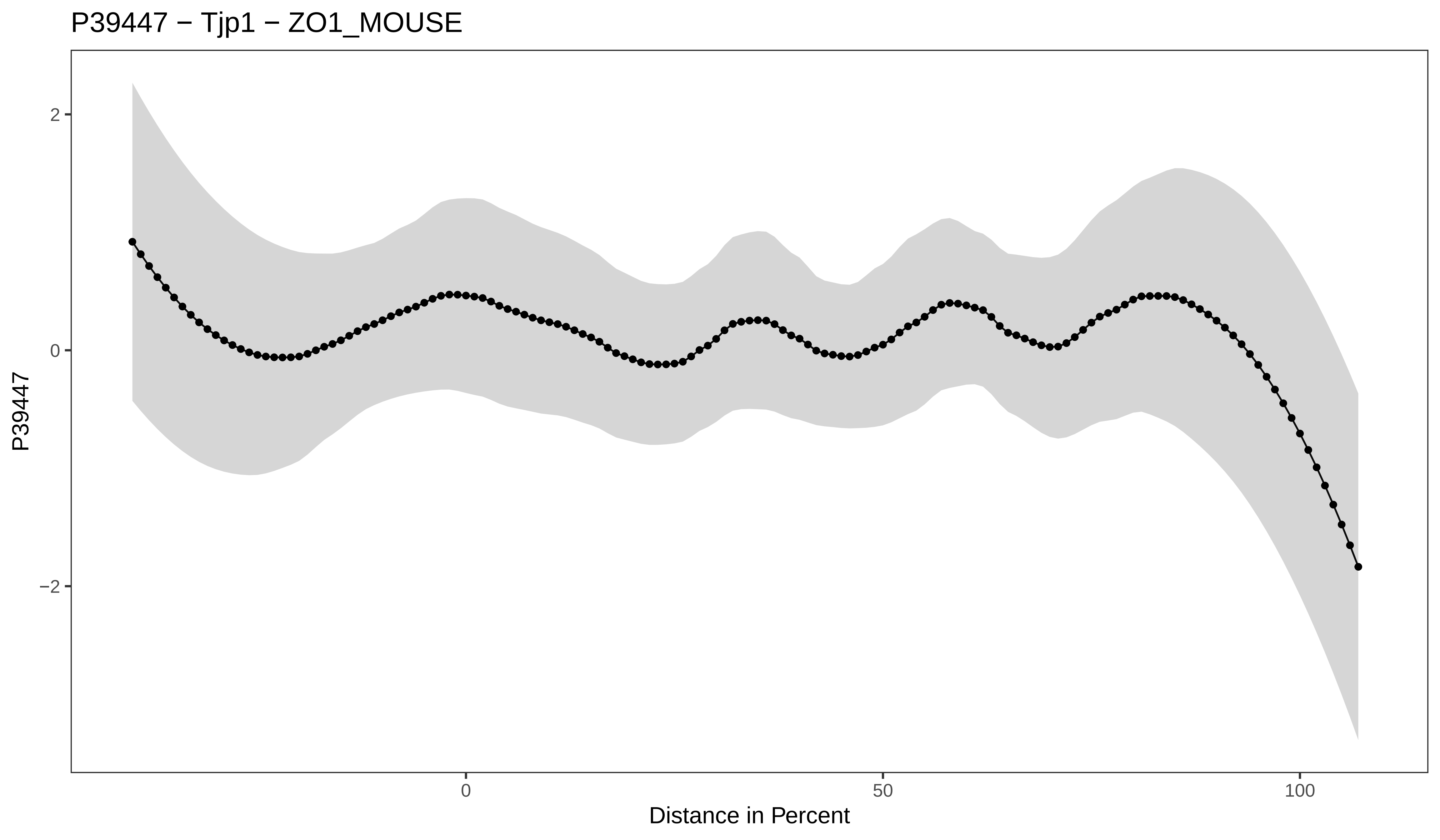

P39447 − Tjp1 − ZO1_MOUSE
2
0
−2
P39447
0
50
100
Distance in Percent

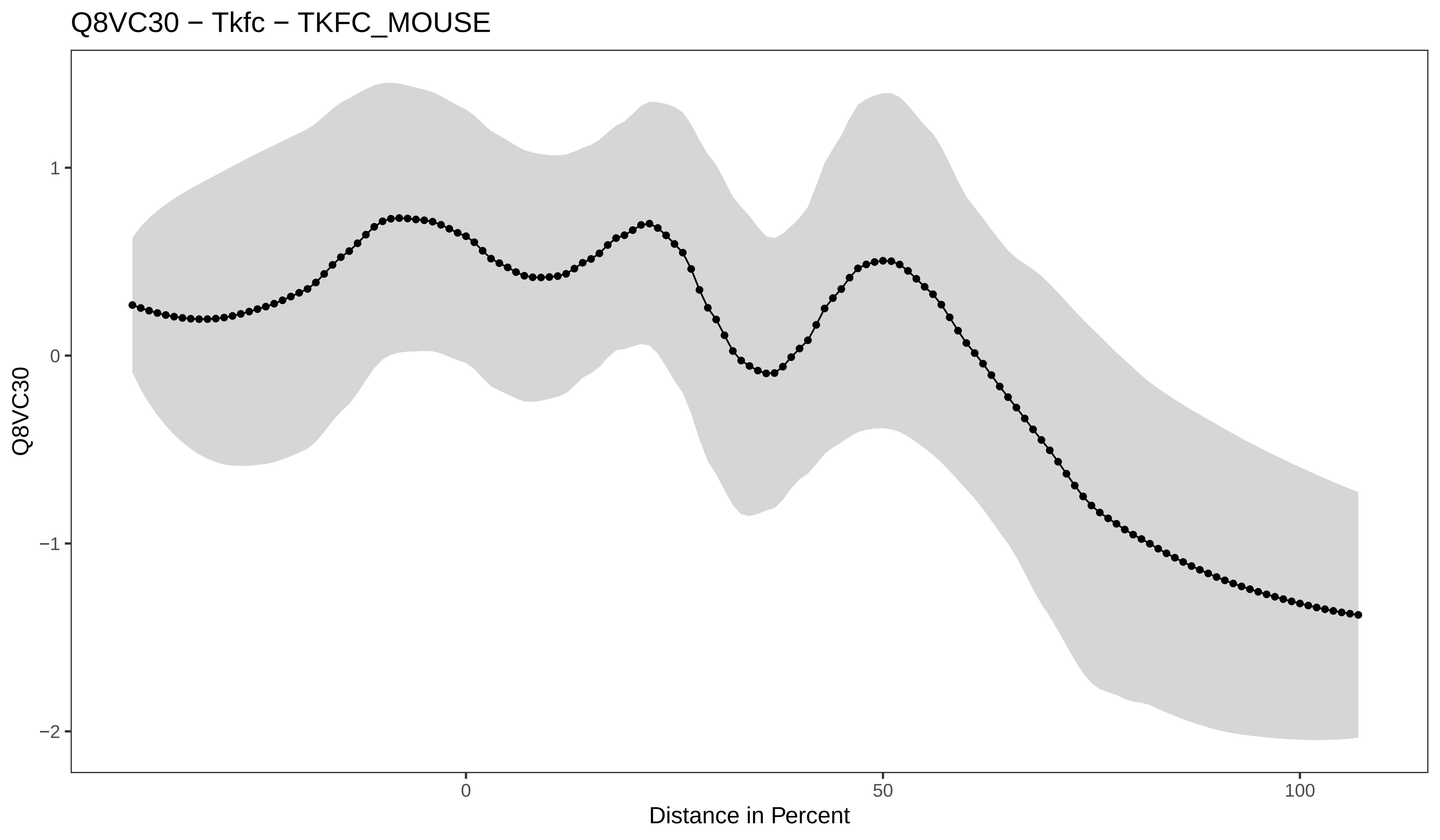

Q8VC30 − Tkfc − TKFC_MOUSE
Q8VC30
1
0
−1
−2
0
50
100
Distance in Percent

P40142 − Tkt − TKT_MOUSE

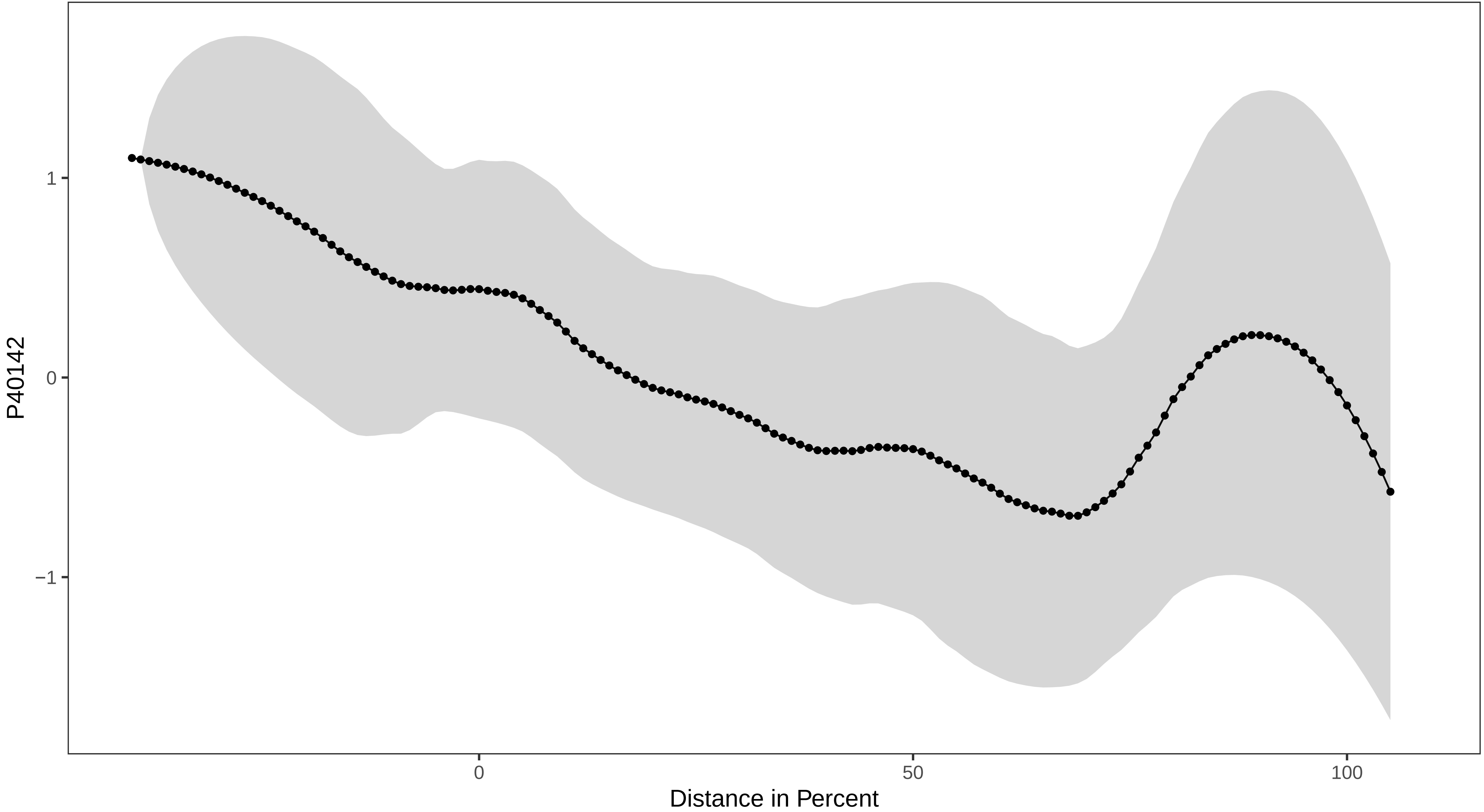

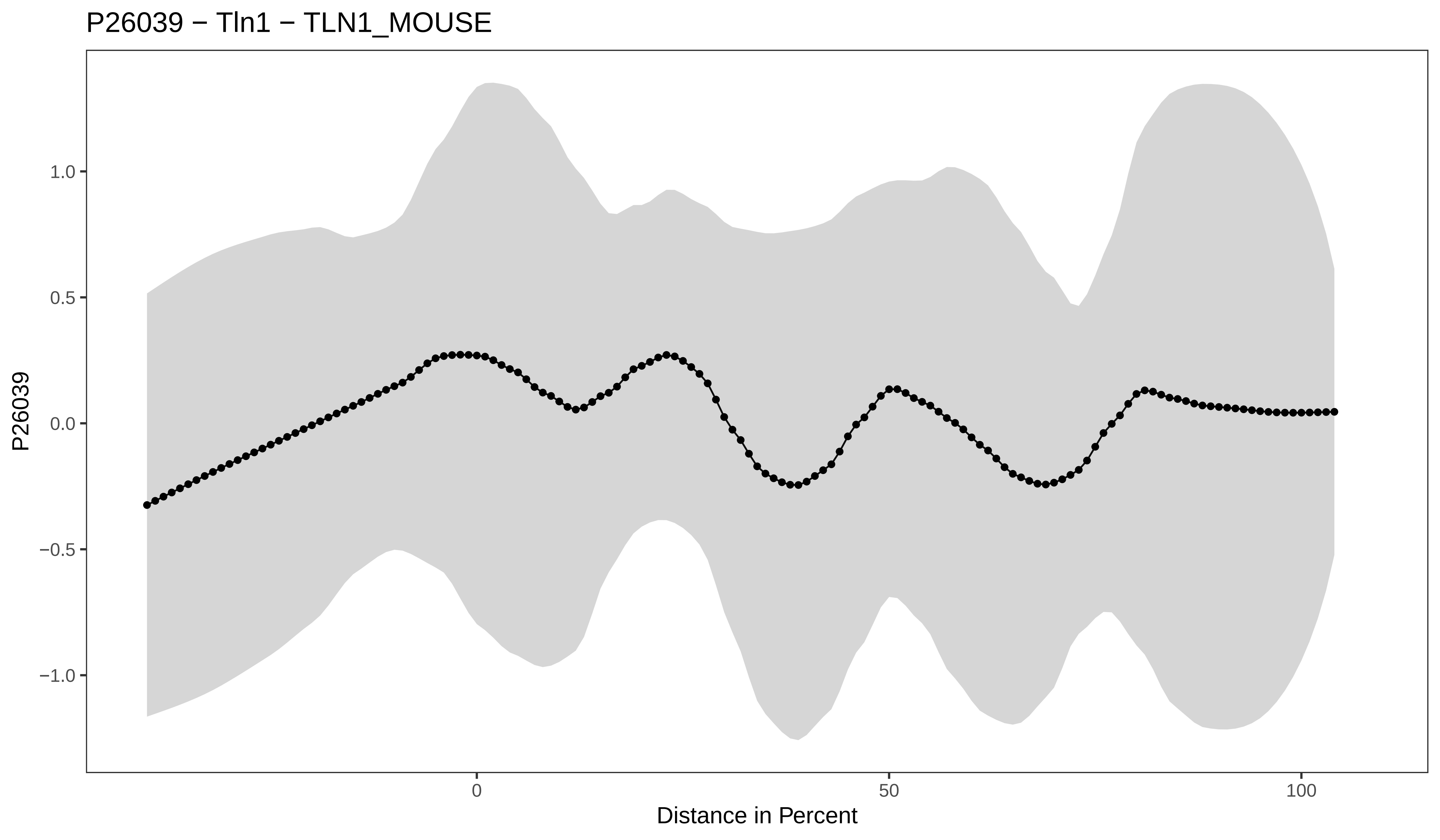

P26039 − Tln1 − TLN1_MOUSE
1.0
0.5
0.0
−0.5
−1.0
P26039
0
50
100
Distance in Percent

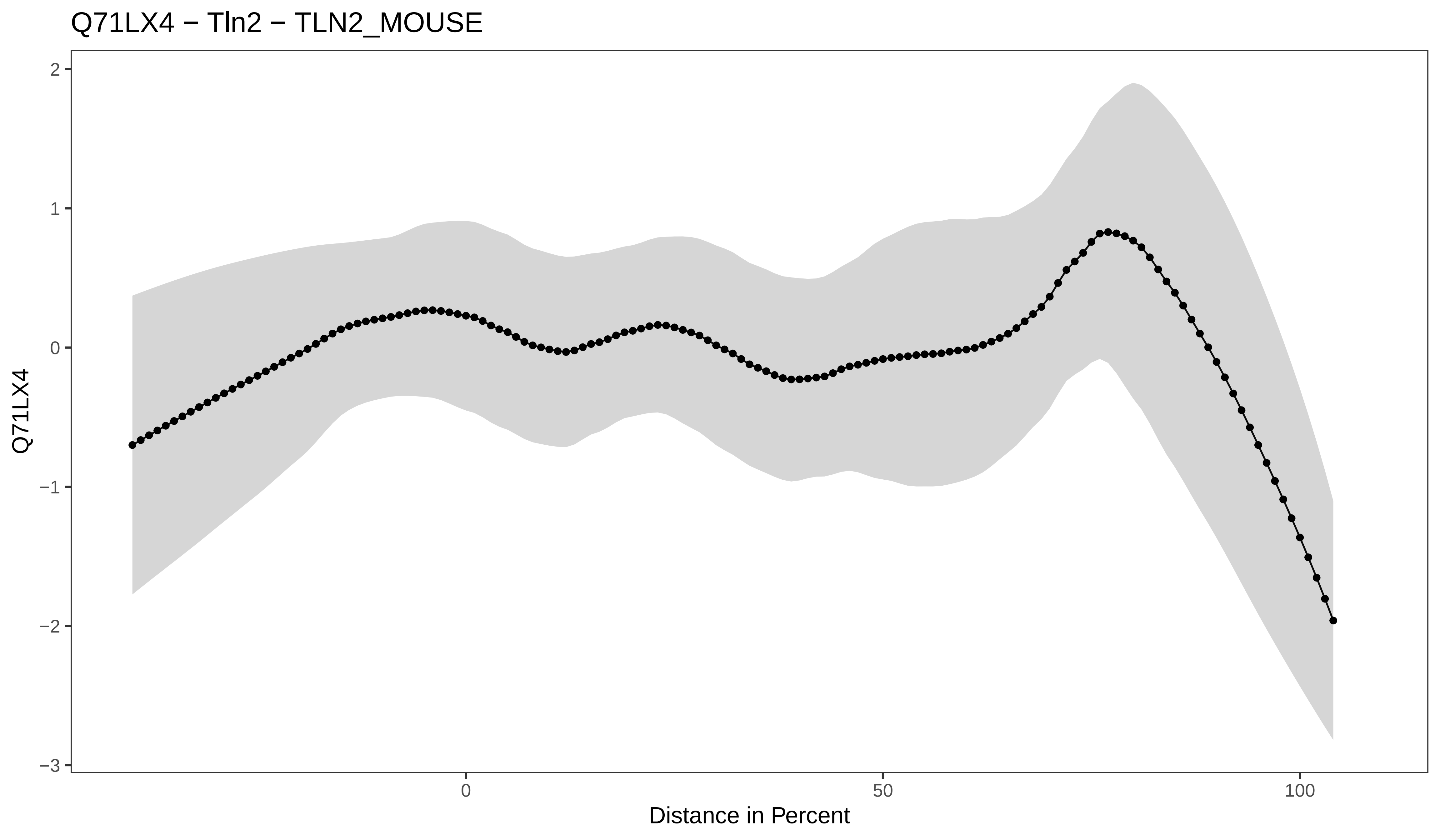
Q71LX4 − Tln2 − TLN2_MOUSE
Q71LX4
2
1
0
−1
−2
−3
0
50
100
Distance in Percent

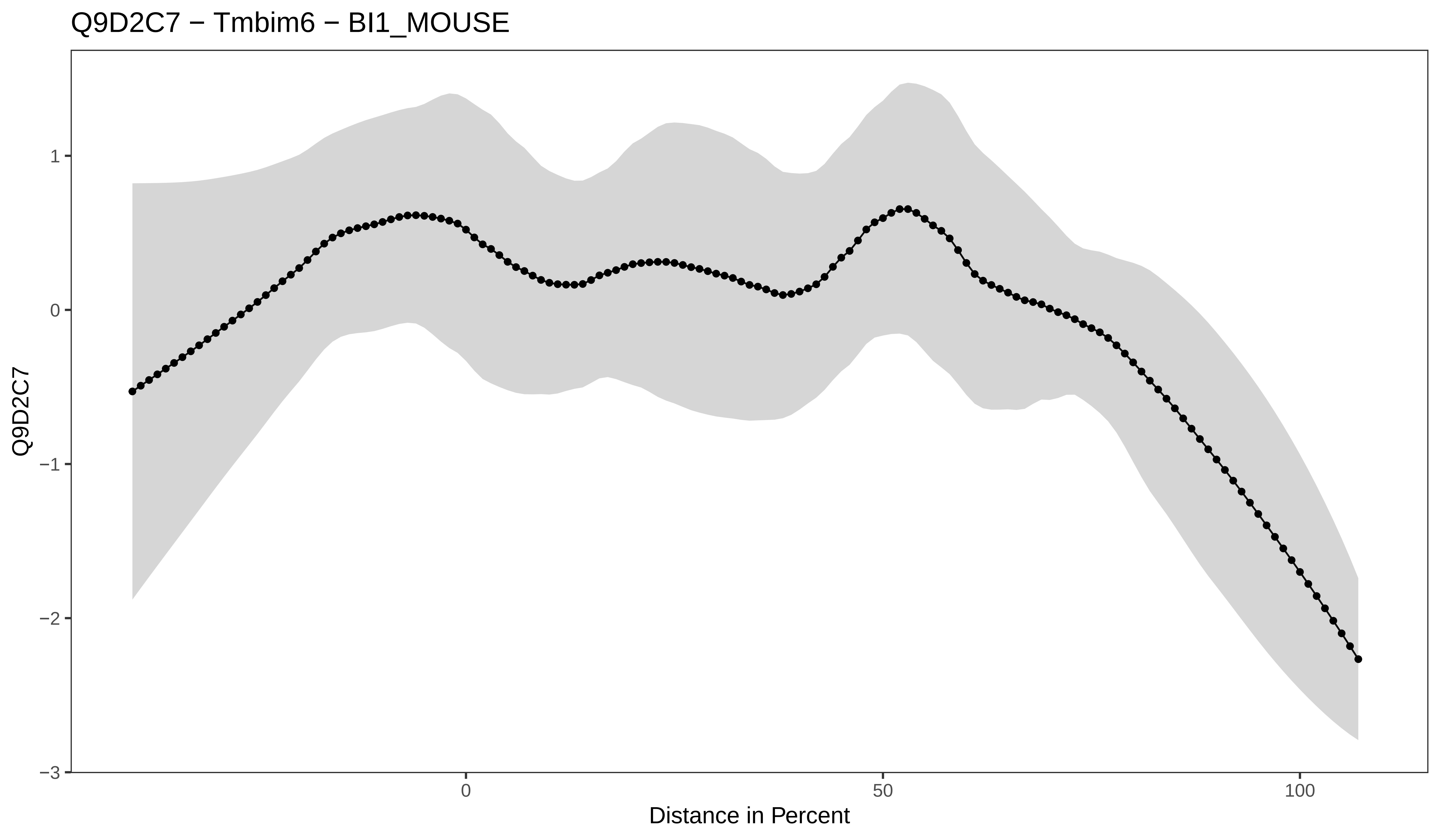

Q9D2C7 − Tmbim6 − BI1_MOUSE
Q9D2C7
1
0
−1
−2
−3
0
50
100
Distance in Percent

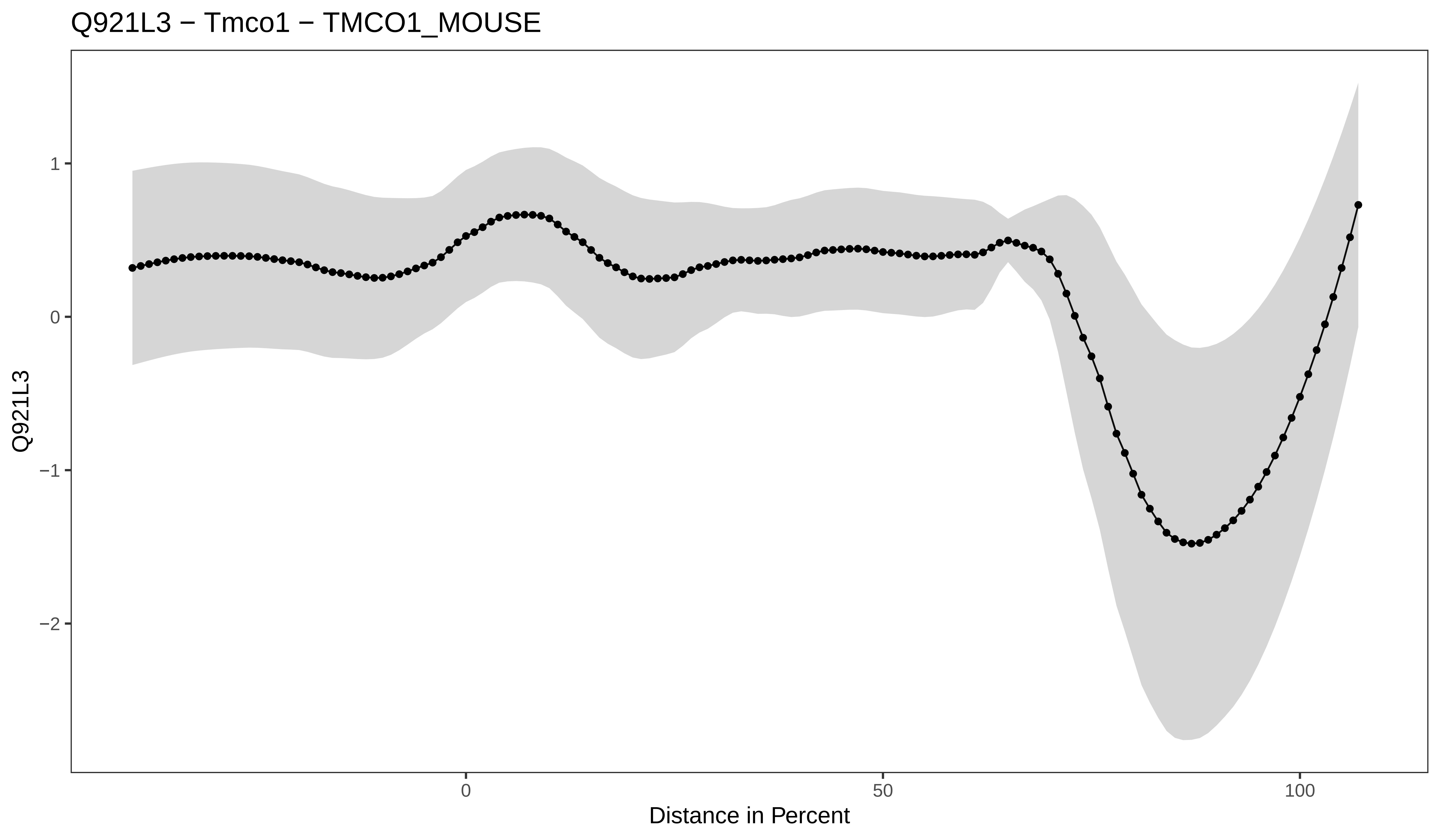
Q921L3 − Tmco1 − TMCO1_MOUSE
Q921L3
1
0
−1
−2
0
50
100
Distance in Percent

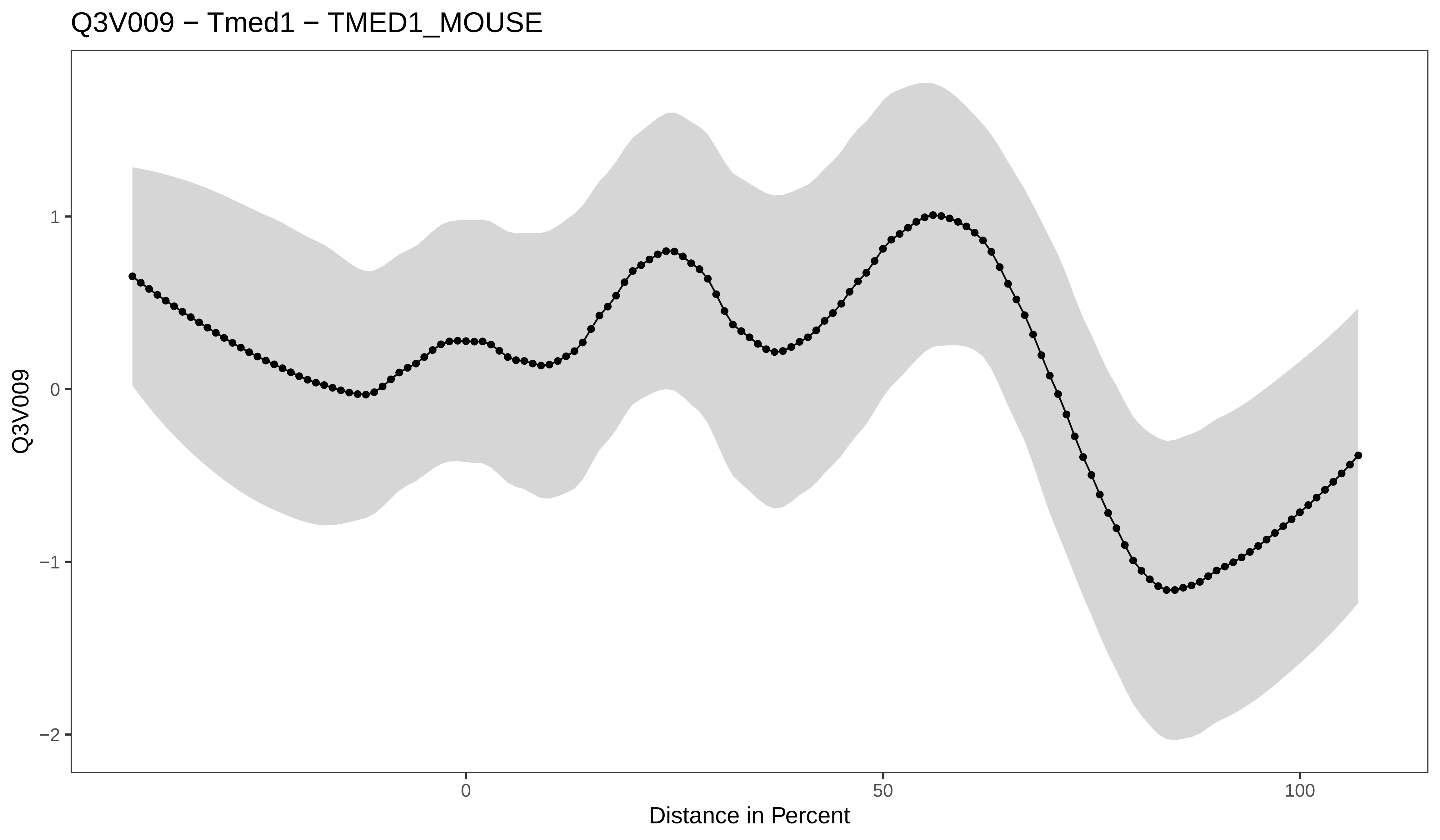

Q3V009 − Tmed1 − TMED1_MOUSE
Q3V009
1
0
−1
−2
0
50
100
Distance in Percent

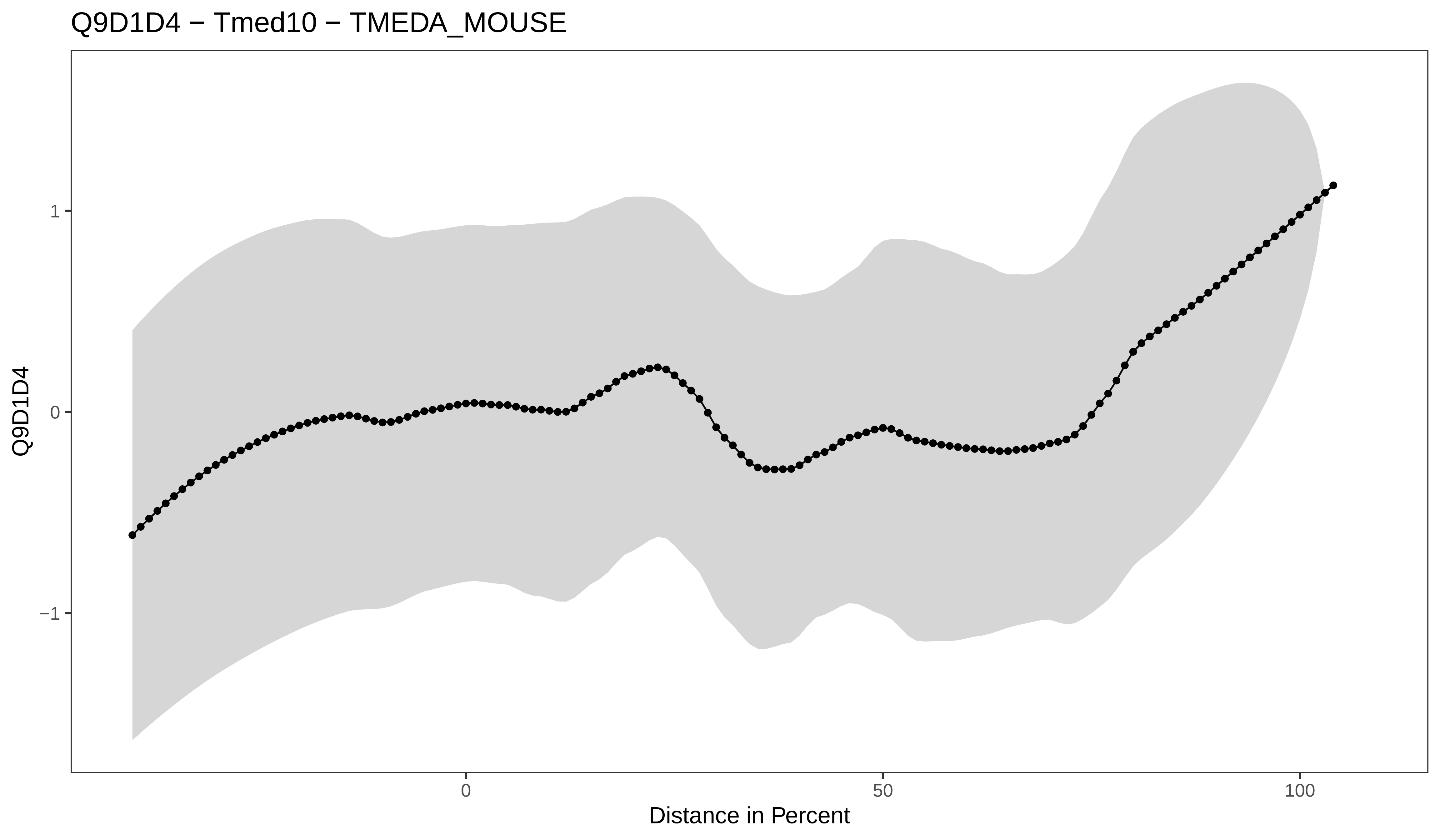

Q9D1D4 − Tmed10 − TMEDA_MOUSE
1
0
−1
Q9D1D4
0
50
100
Distance in Percent

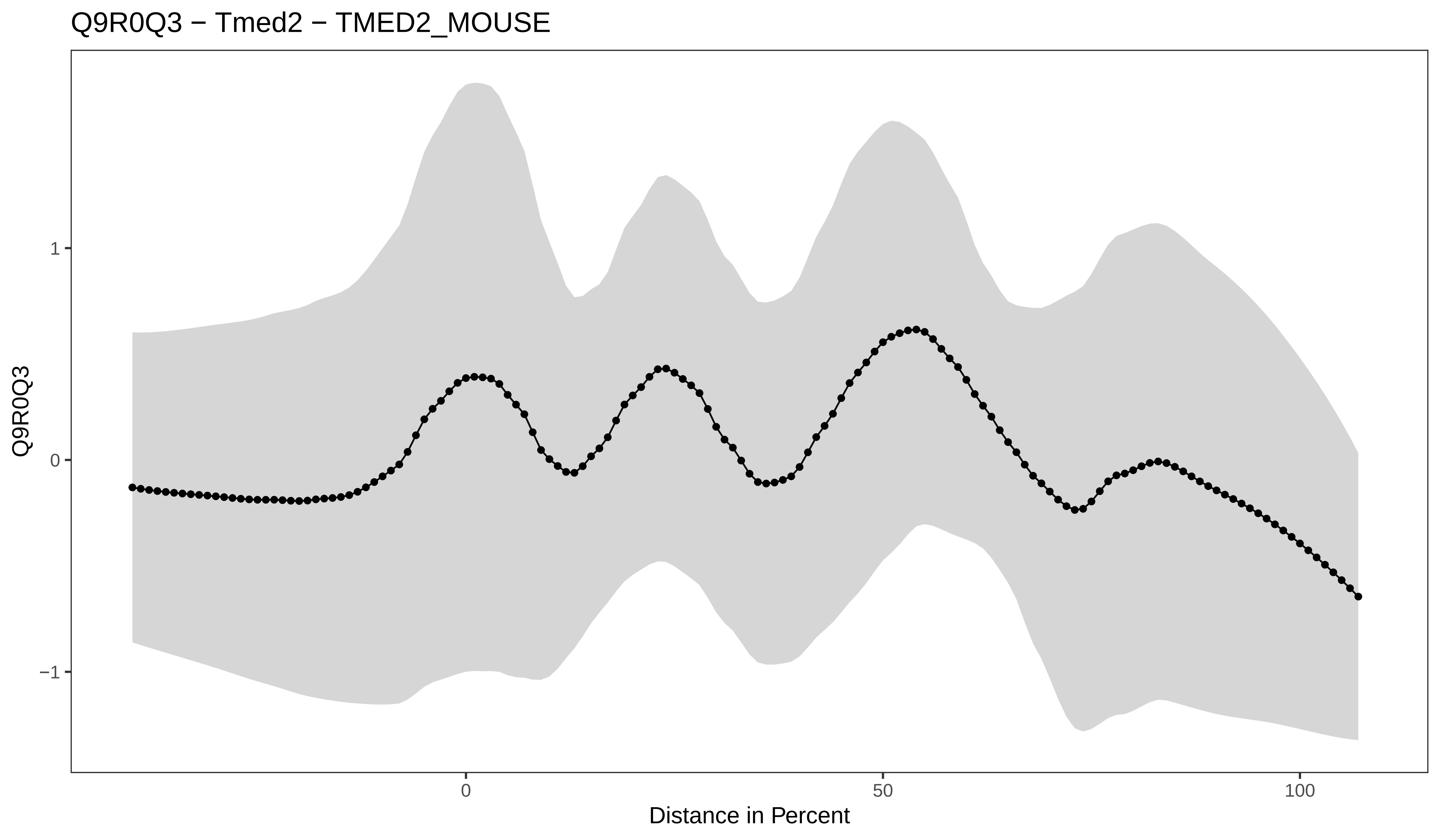

Q9R0Q3 − Tmed2 − TMED2_MOUSE
Q9R0Q3
1
0
−1
0
50
100
Distance in Percent

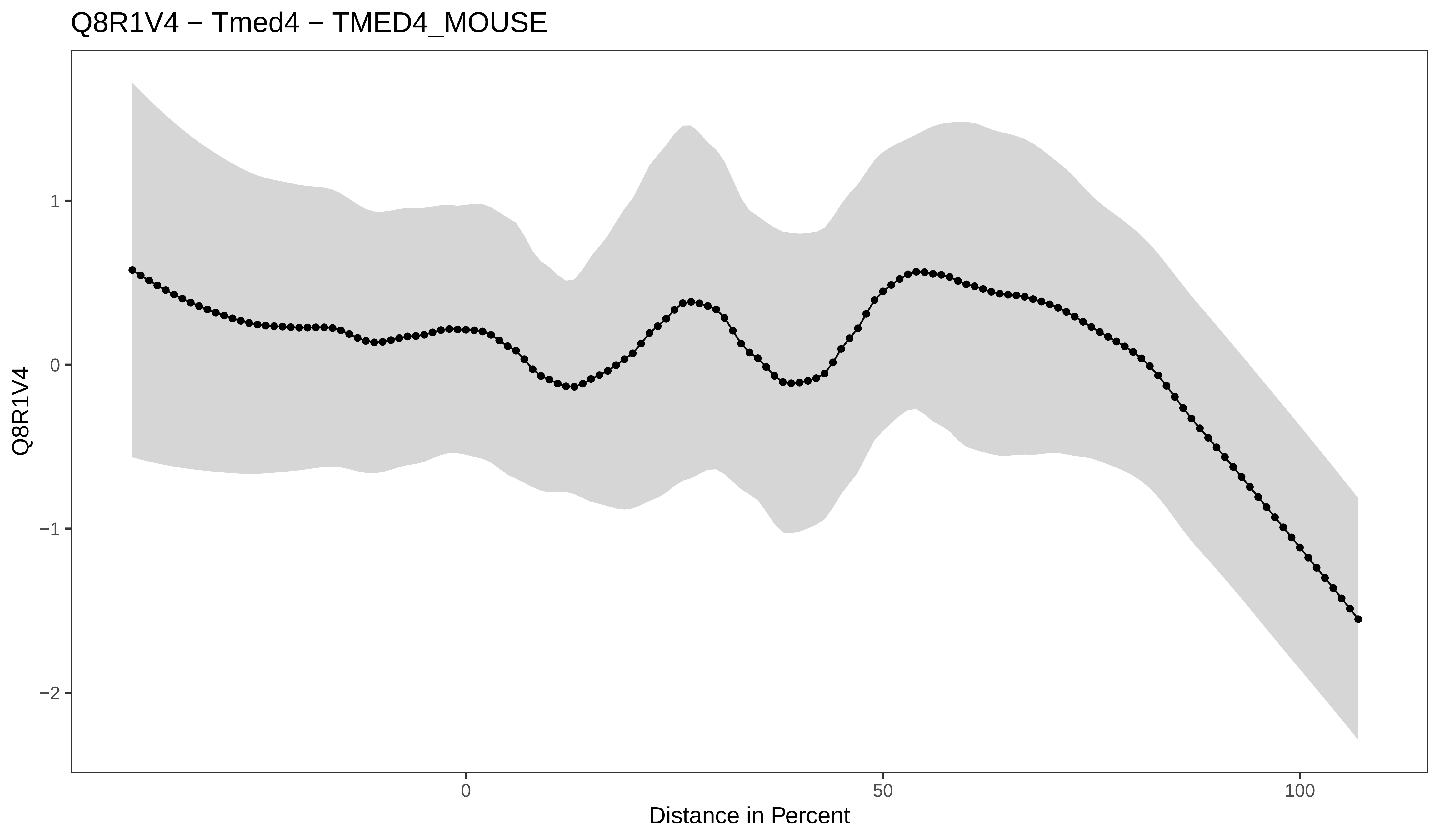

Q8R1V4 − Tmed4 − TMED4_MOUSE
Q8R1V4
1
0
−1
−2
0
50
100
Distance in Percent

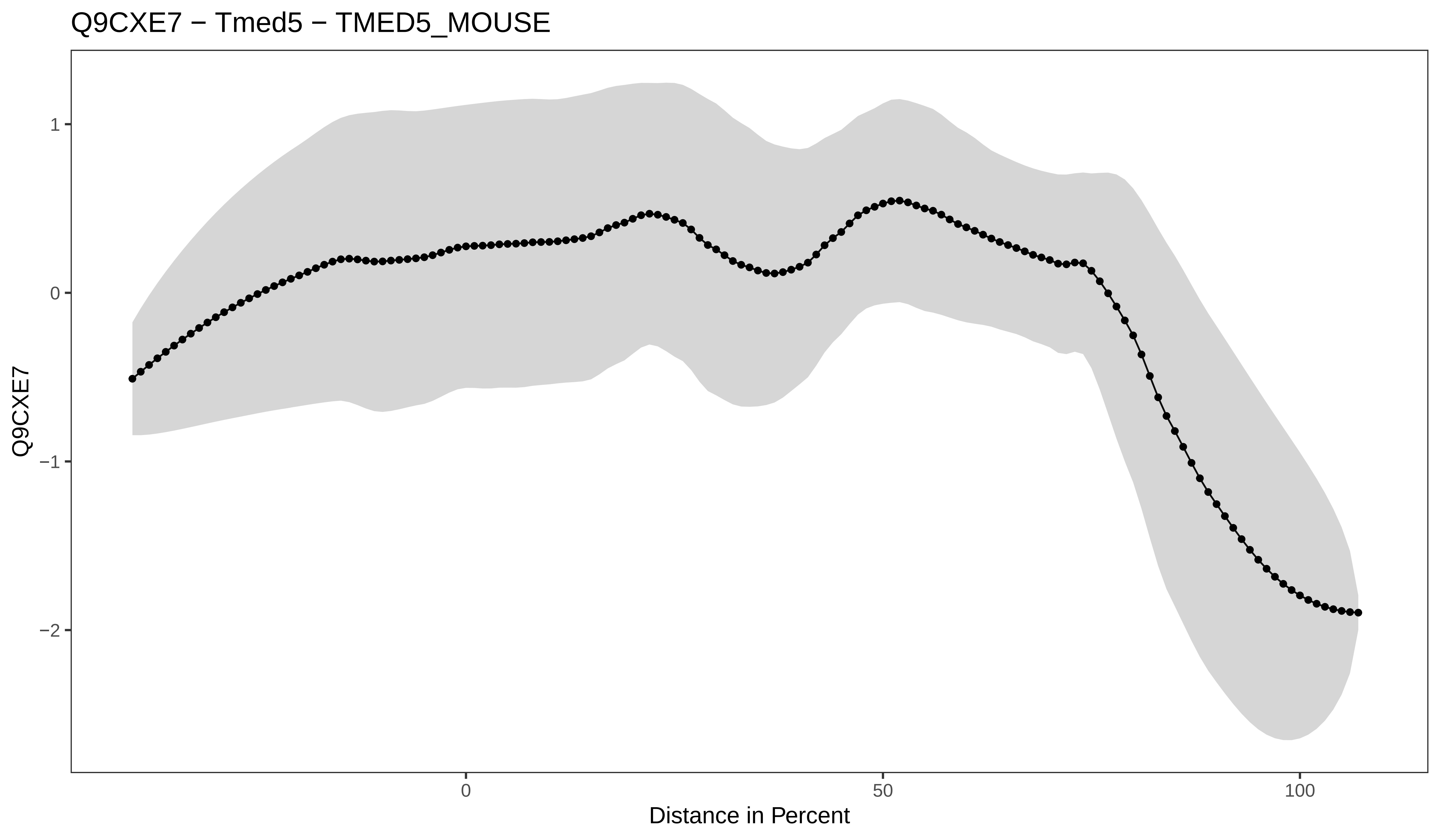
Q9CXE7 − Tmed5 − TMED5_MOUSE
Q9CXE7
Distance in Percent
1
0
−1
−2
0
50
100

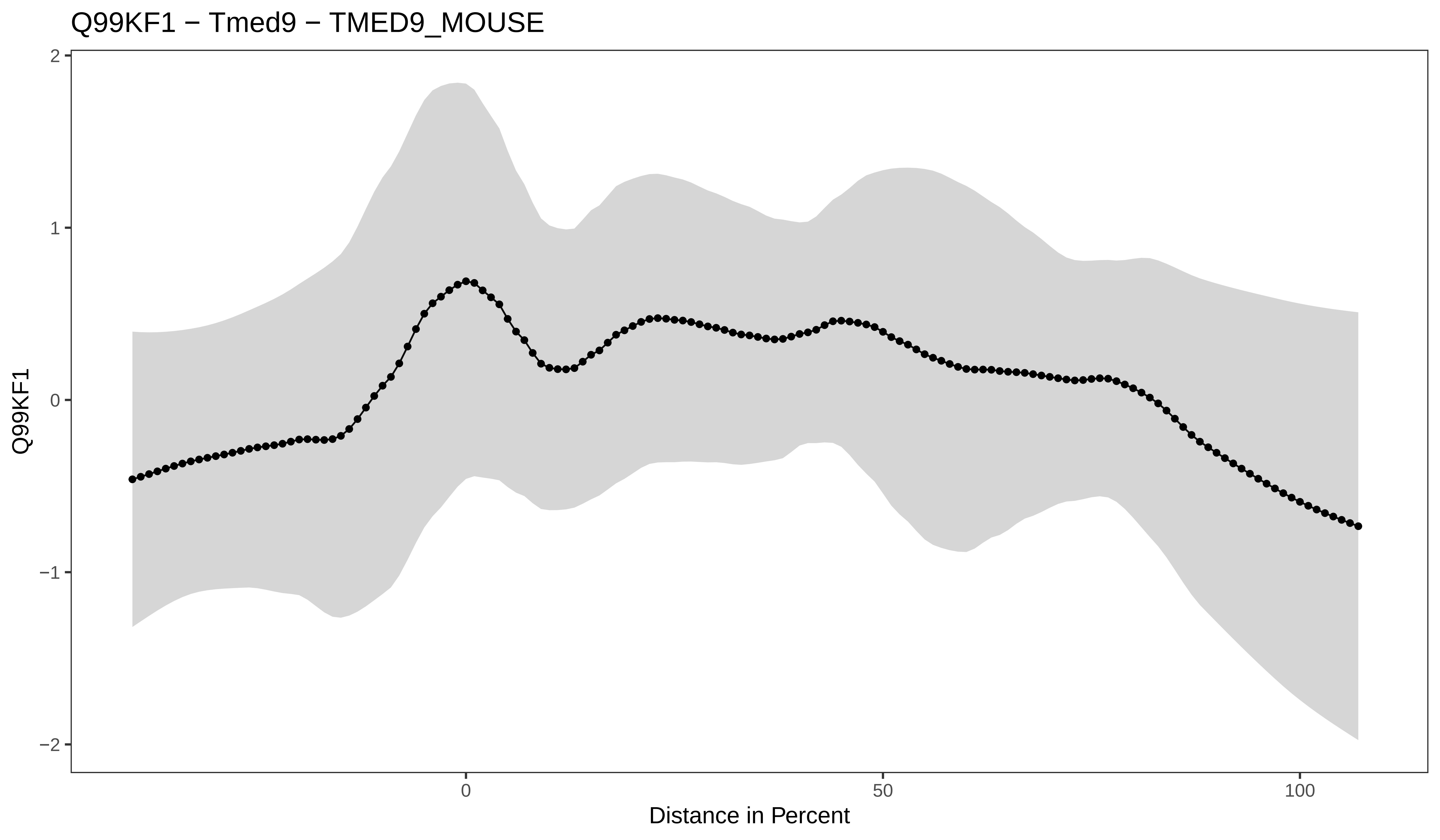

Q99KF1 − Tmed9 − TMED9_MOUSE
Q99KF1
2
1
0
−1
−2
0
50
100
Distance in Percent

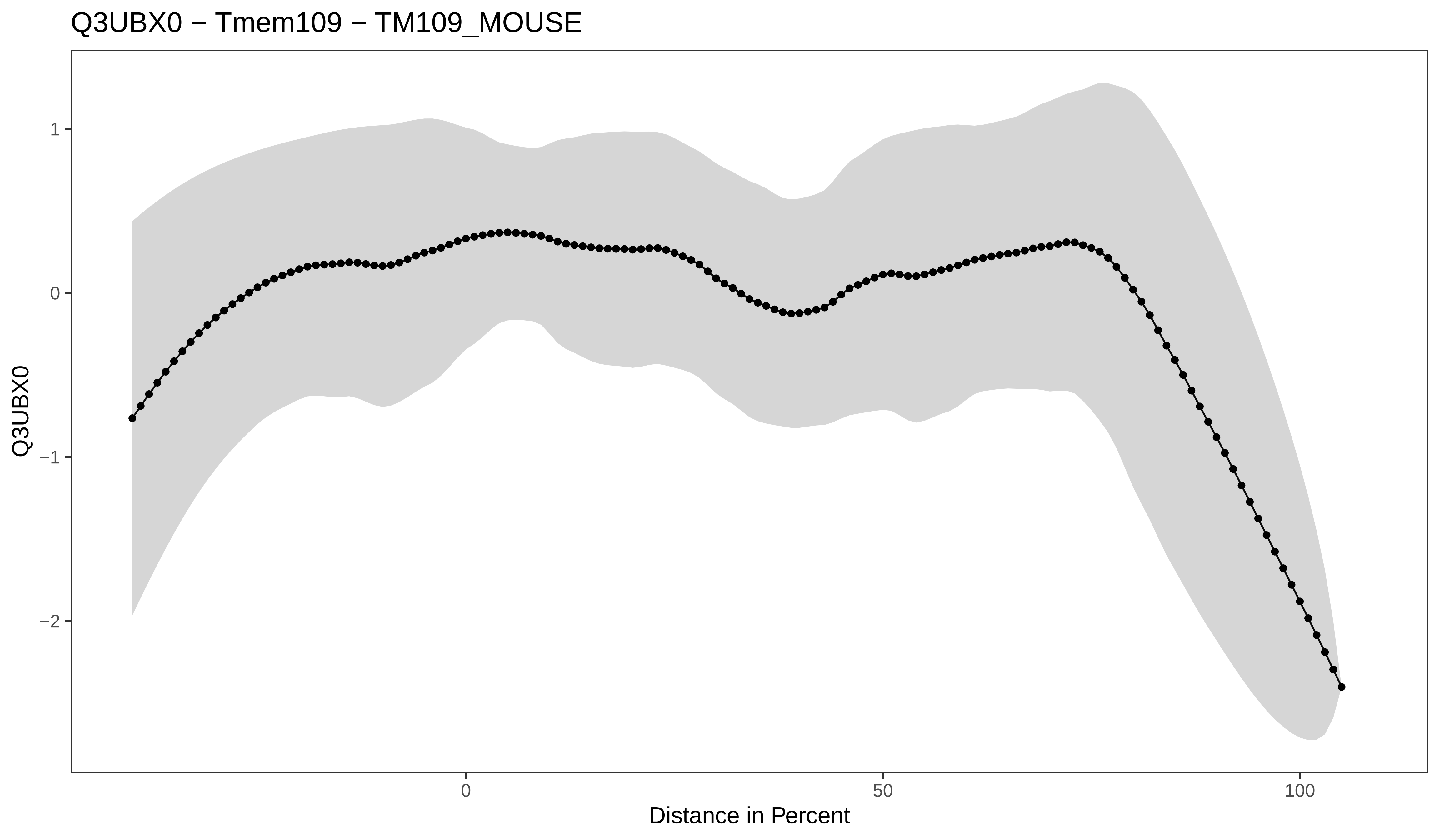

Q3UBX0 − Tmem109 − TM109_MOUSE
Q3UBX0
1
0
−1
−2
0
50
100
Distance in Percent

Q8BK08 − Tmem11 − TMM11_MOUSE

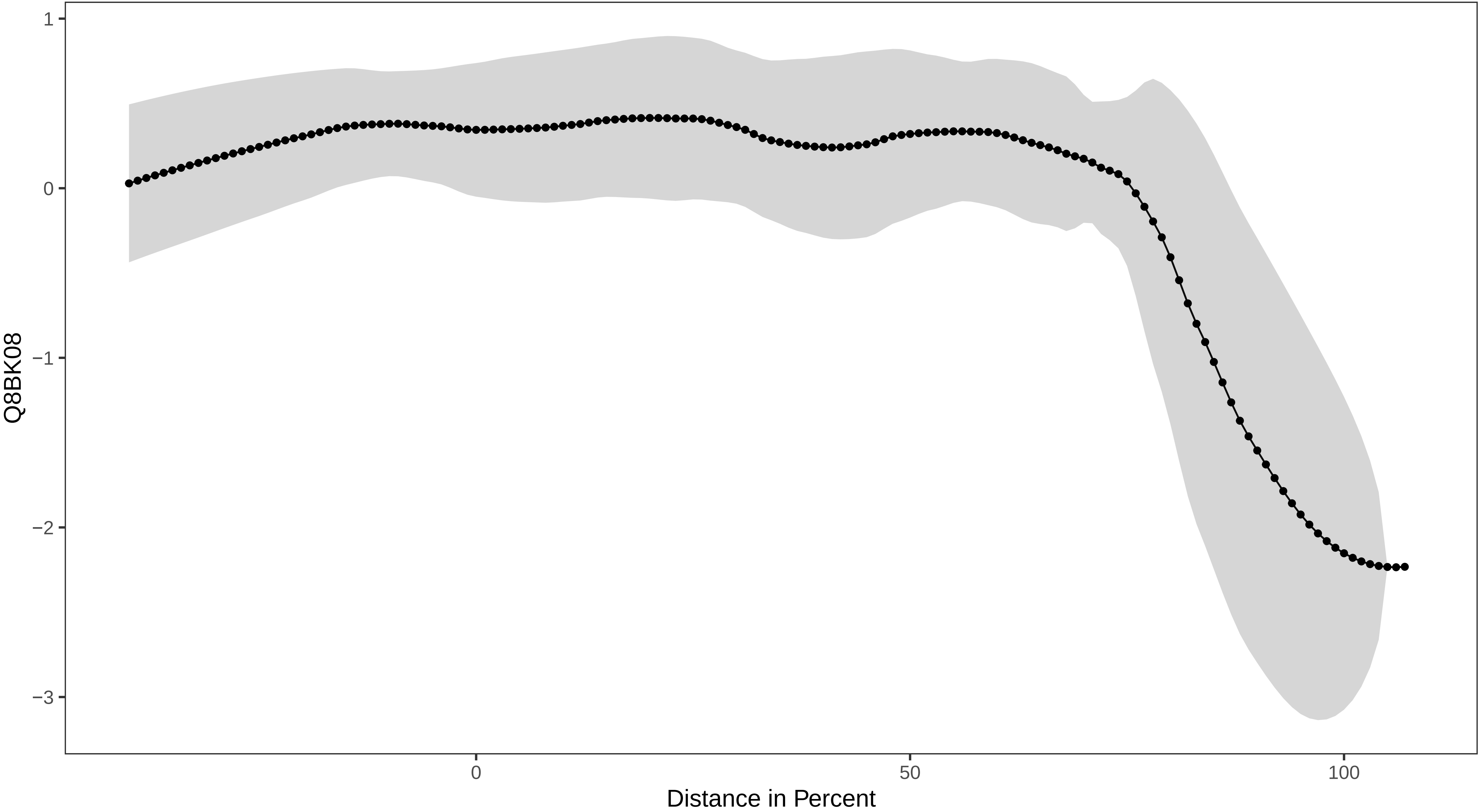

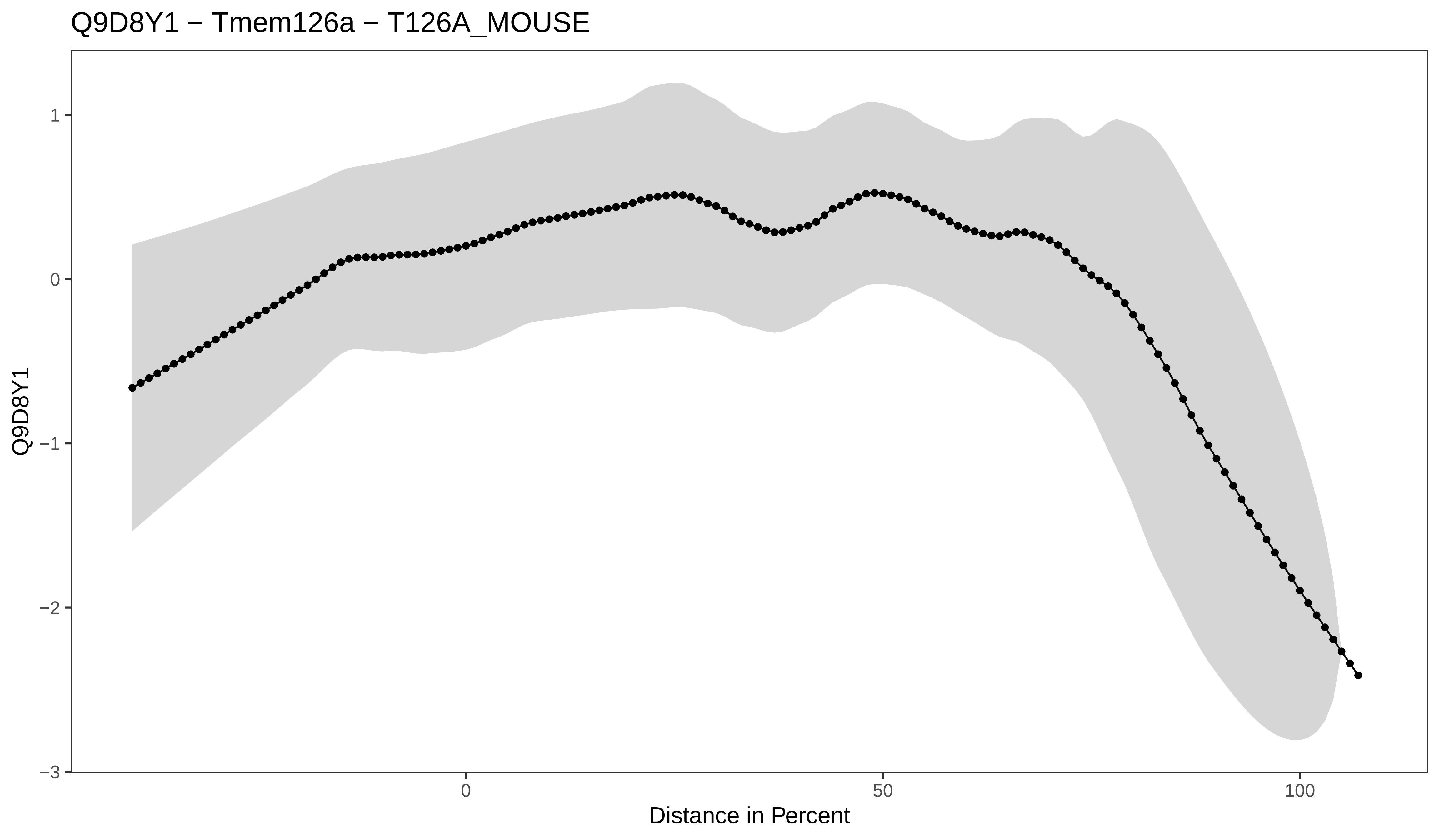

Q9D8Y1 − Tmem126a − T126A_MOUSE
Q9D8Y1
1
0
−1
−2
−3
0
50
100
Distance in Percent

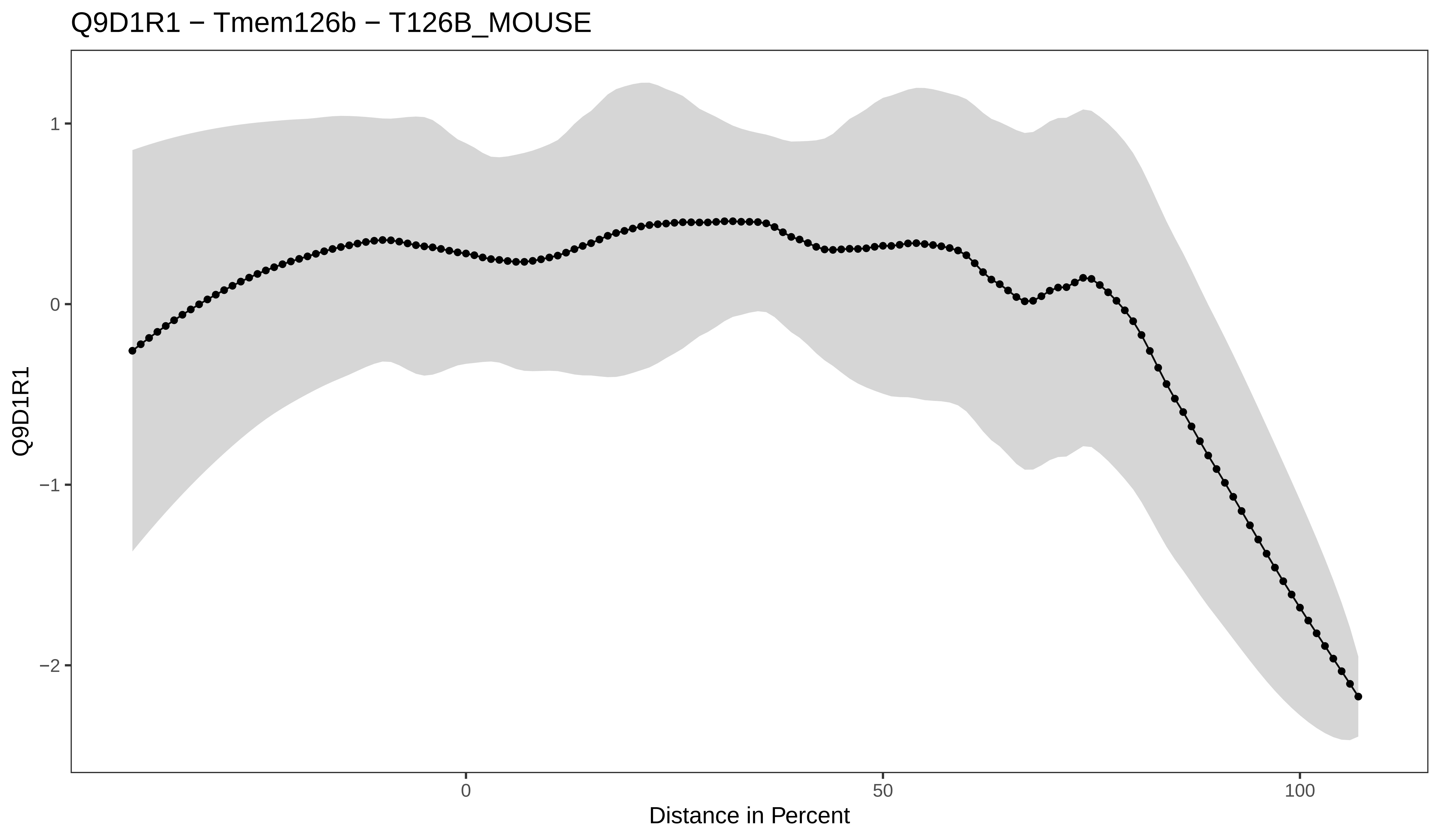

Q9D1R1 − Tmem126b − T126B_MOUSE
Q9D1R1
1
0
−1
−2
0
50
100
Distance in Percent

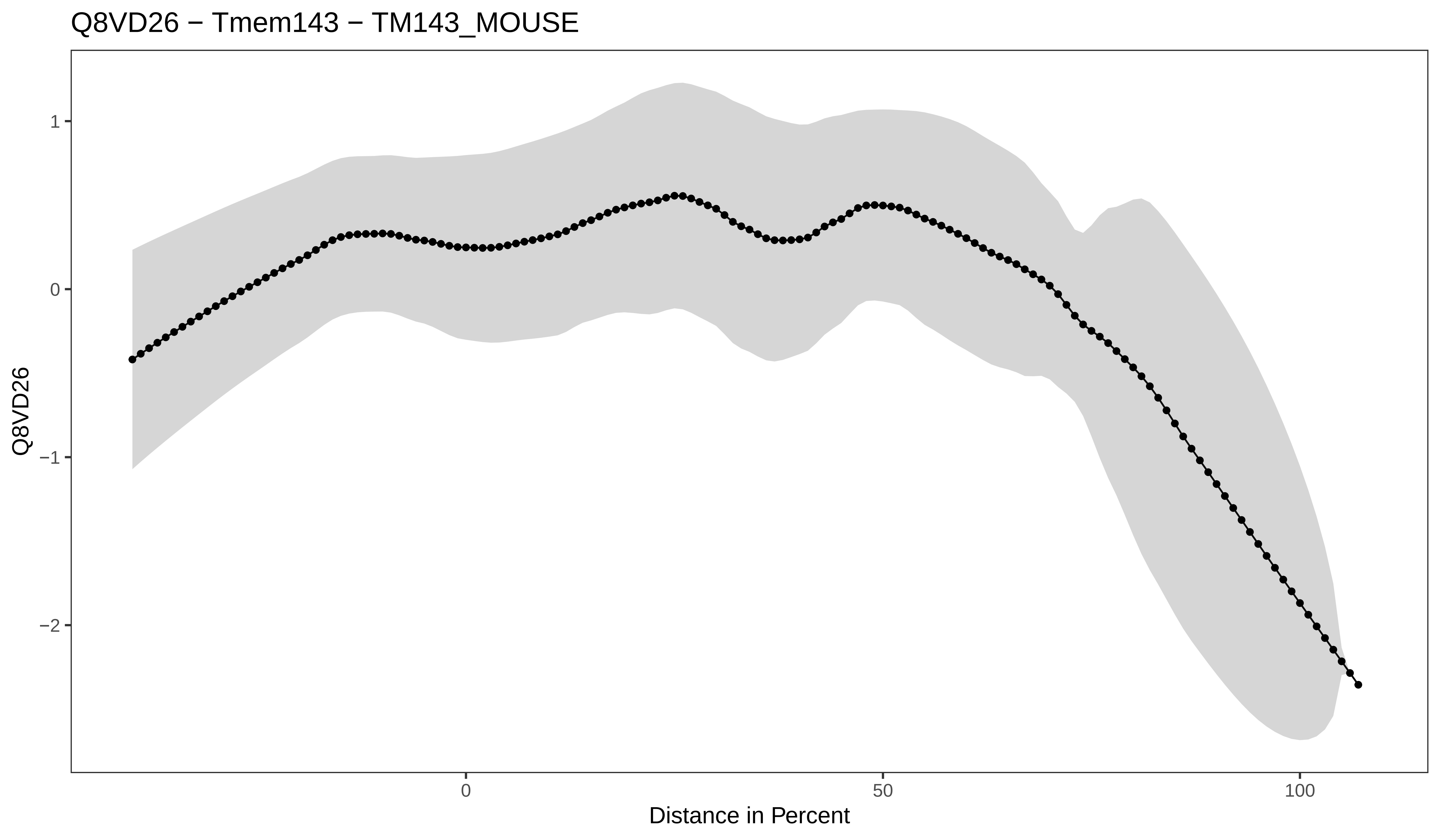

Q8VD26 − Tmem143 − TM143_MOUSE
1
0
−1
−2
Q8VD26
0
50
100
Distance in Percent

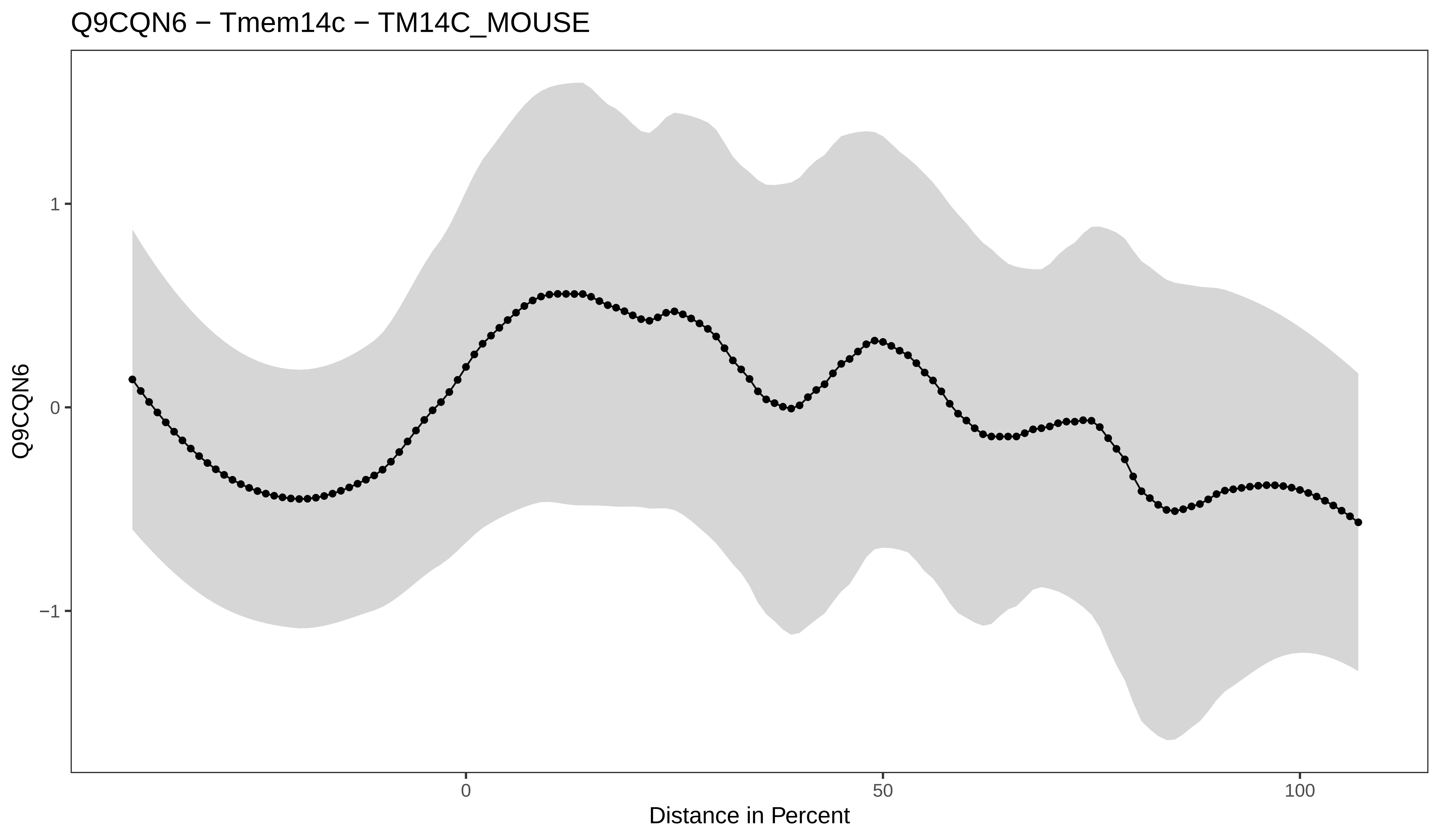

Q9CQN6 − Tmem14c − TM14C_MOUSE
Q9CQN6
1
0
−1
0
50
100
Distance in Percent

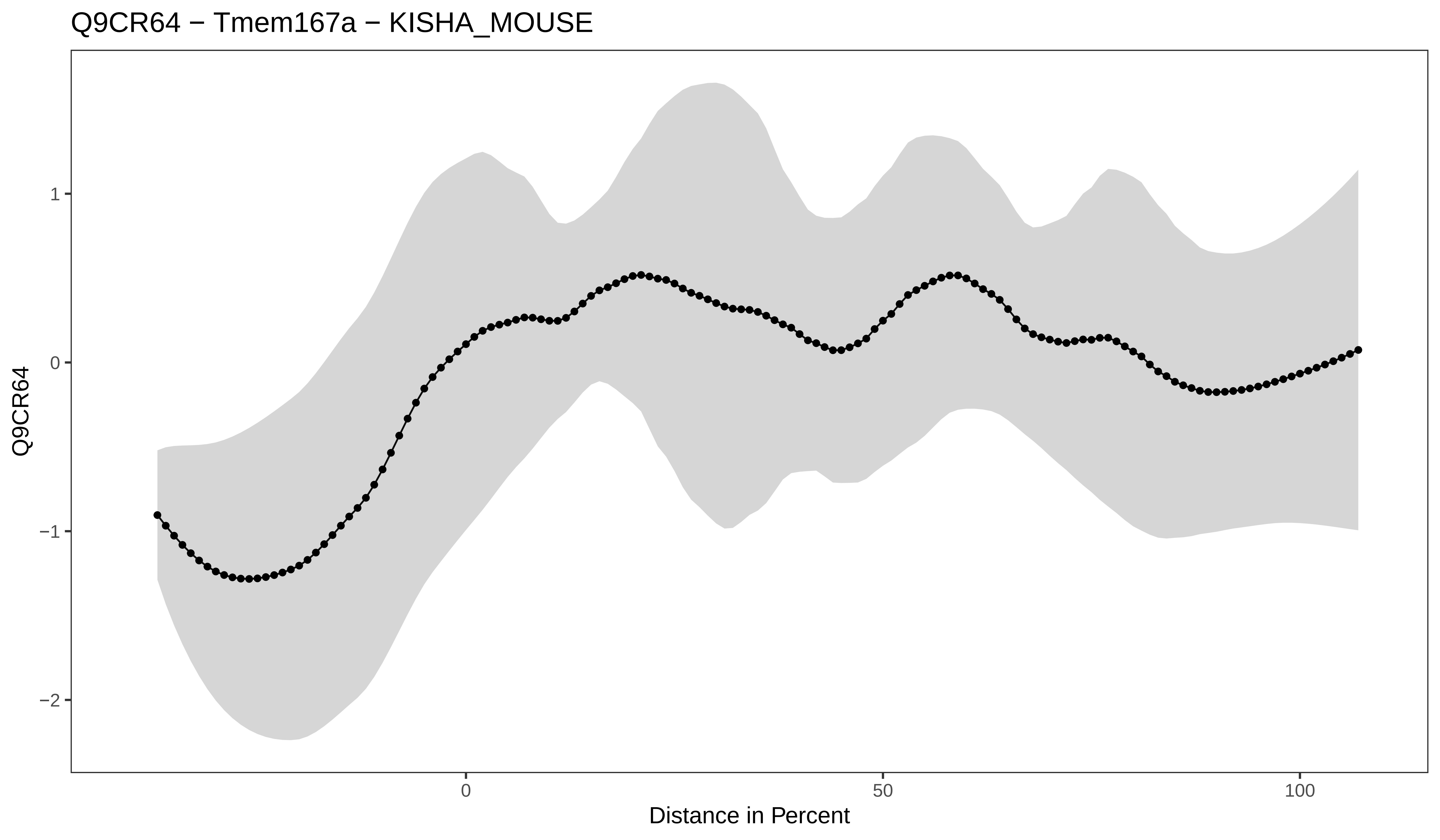

Q9CR64 − Tmem167a − KISHA_MOUSE
Q9CR64
1
0
−1
−2
0
50
100
Distance in Percent

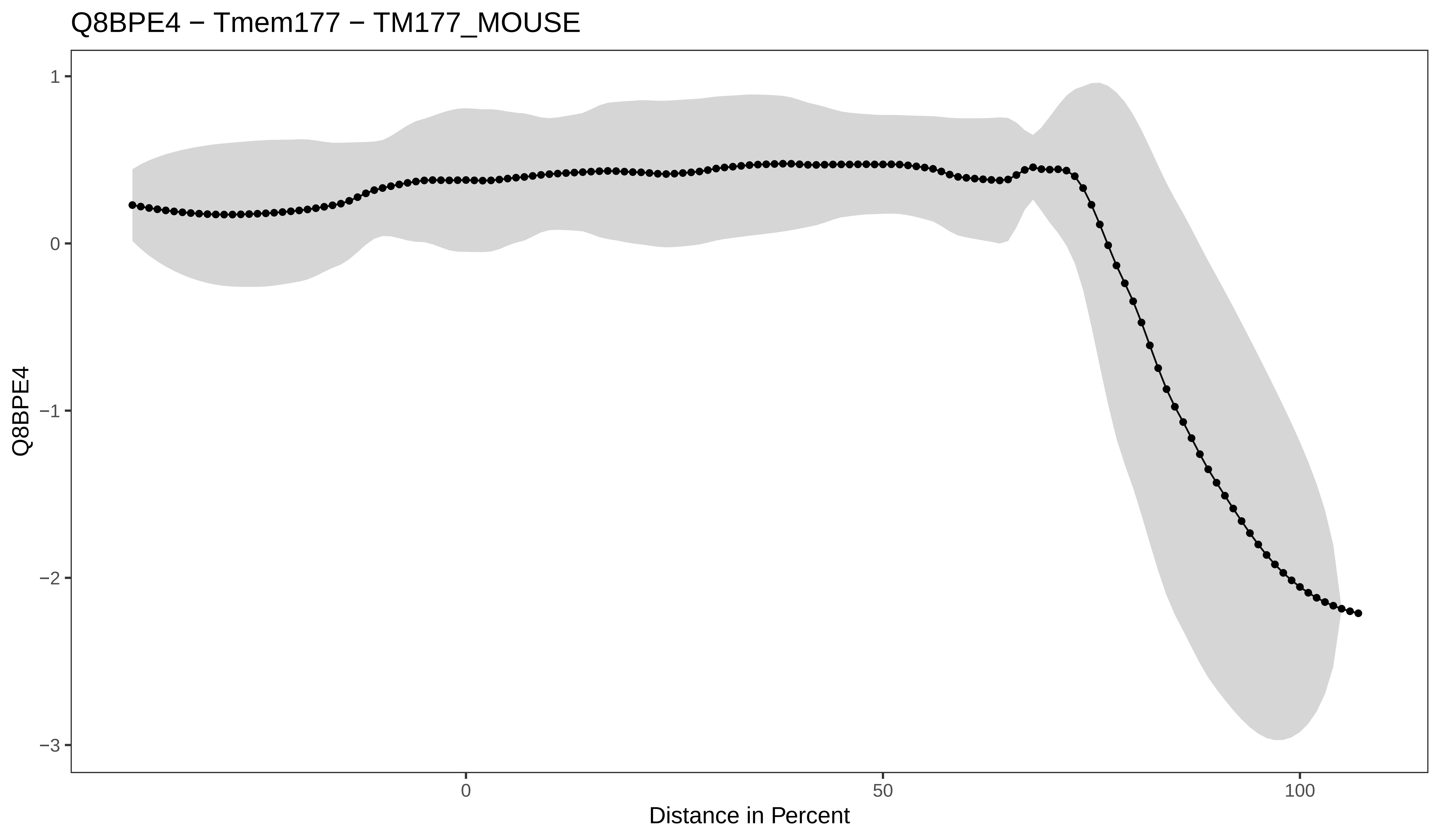

Q8BPE4 − Tmem177 − TM177_MOUSE
1
0
−1
−2
−3
Q8BPE4
0
50
100
Distance in Percent

B2RVY9 − Tmem182 − TM182_MOUSE

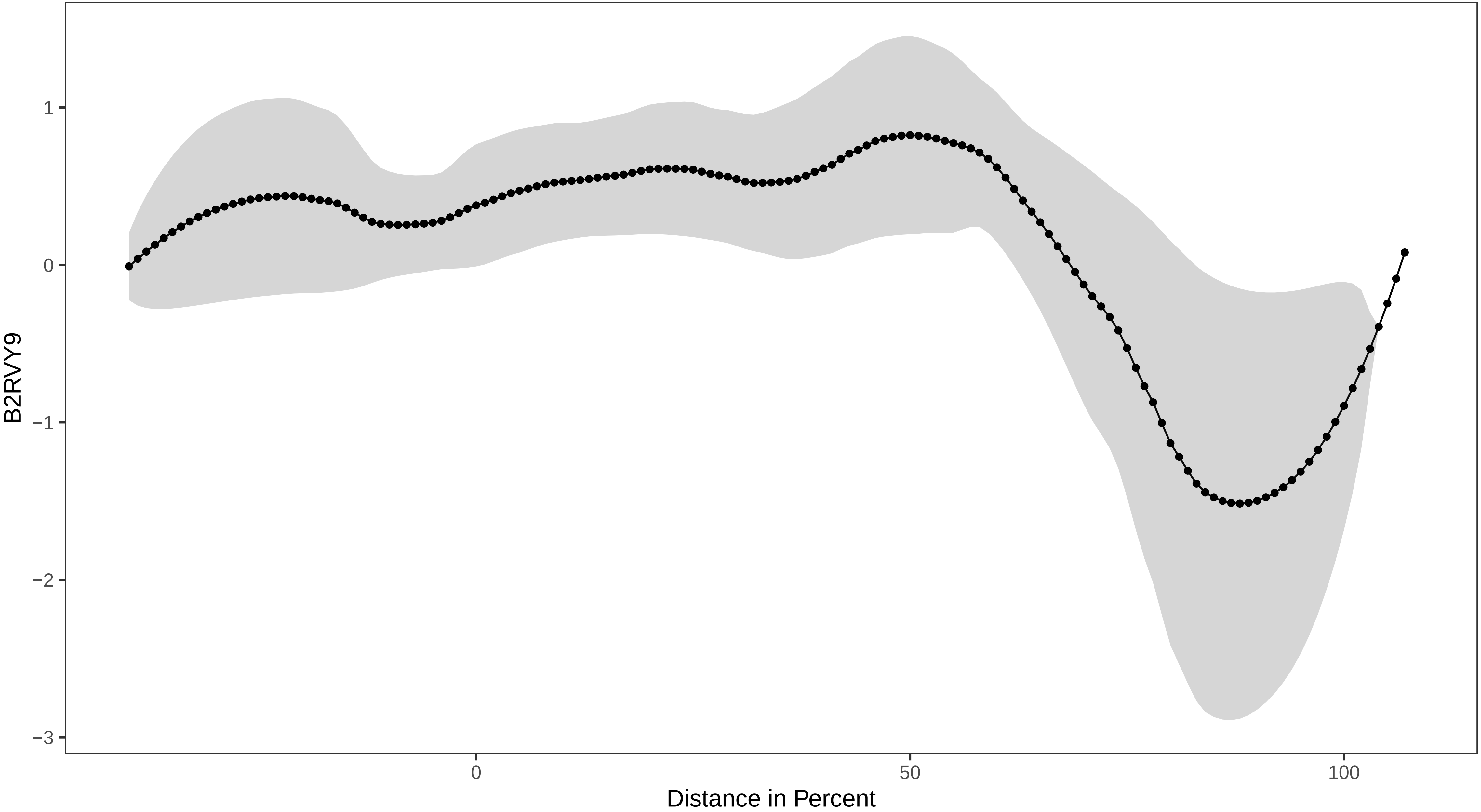

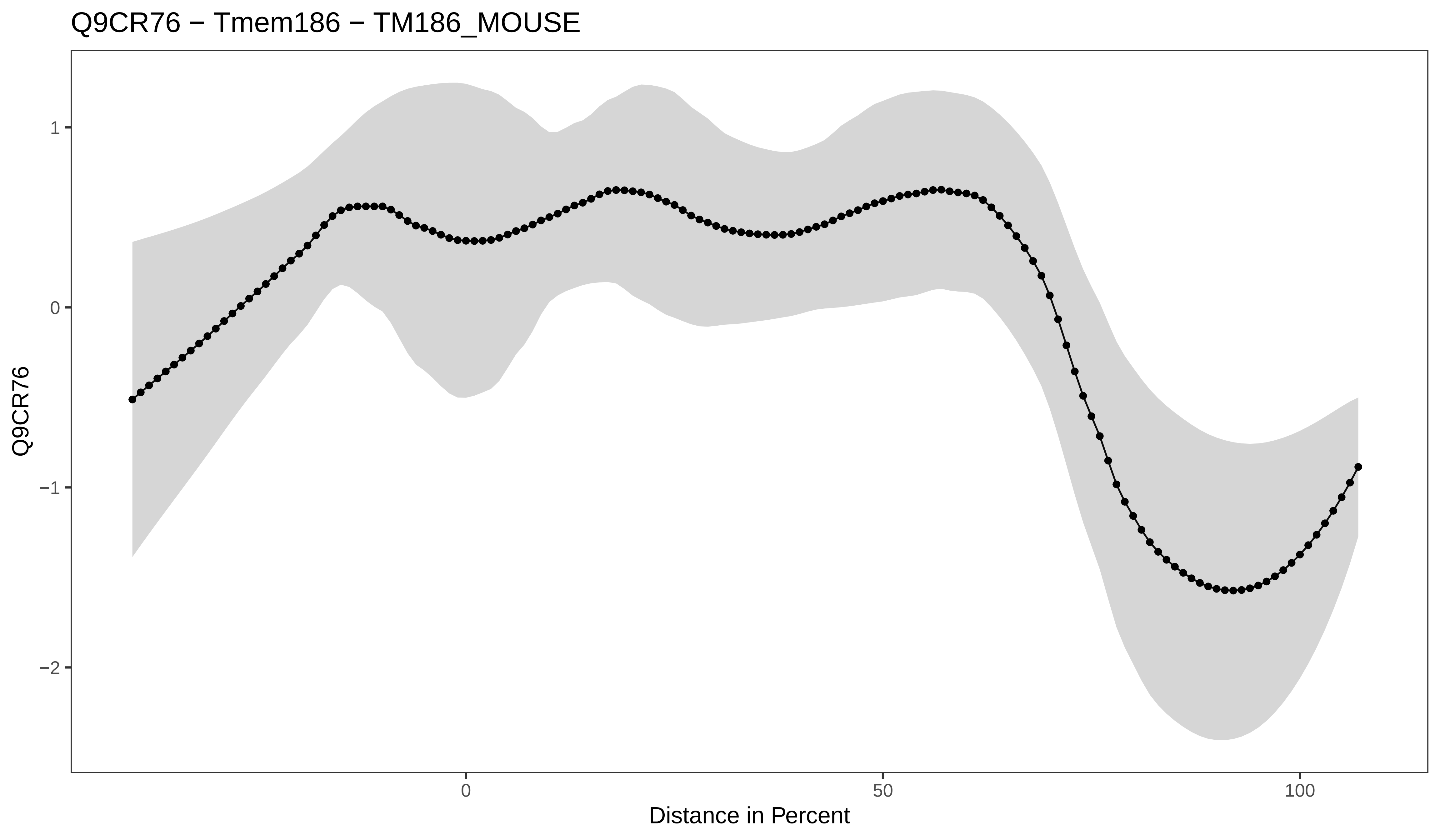
Q9CR76 − Tmem186 − TM186_MOUSE
Q9CR76
Distance in Percent
1
0
−1
−2
0
50
100

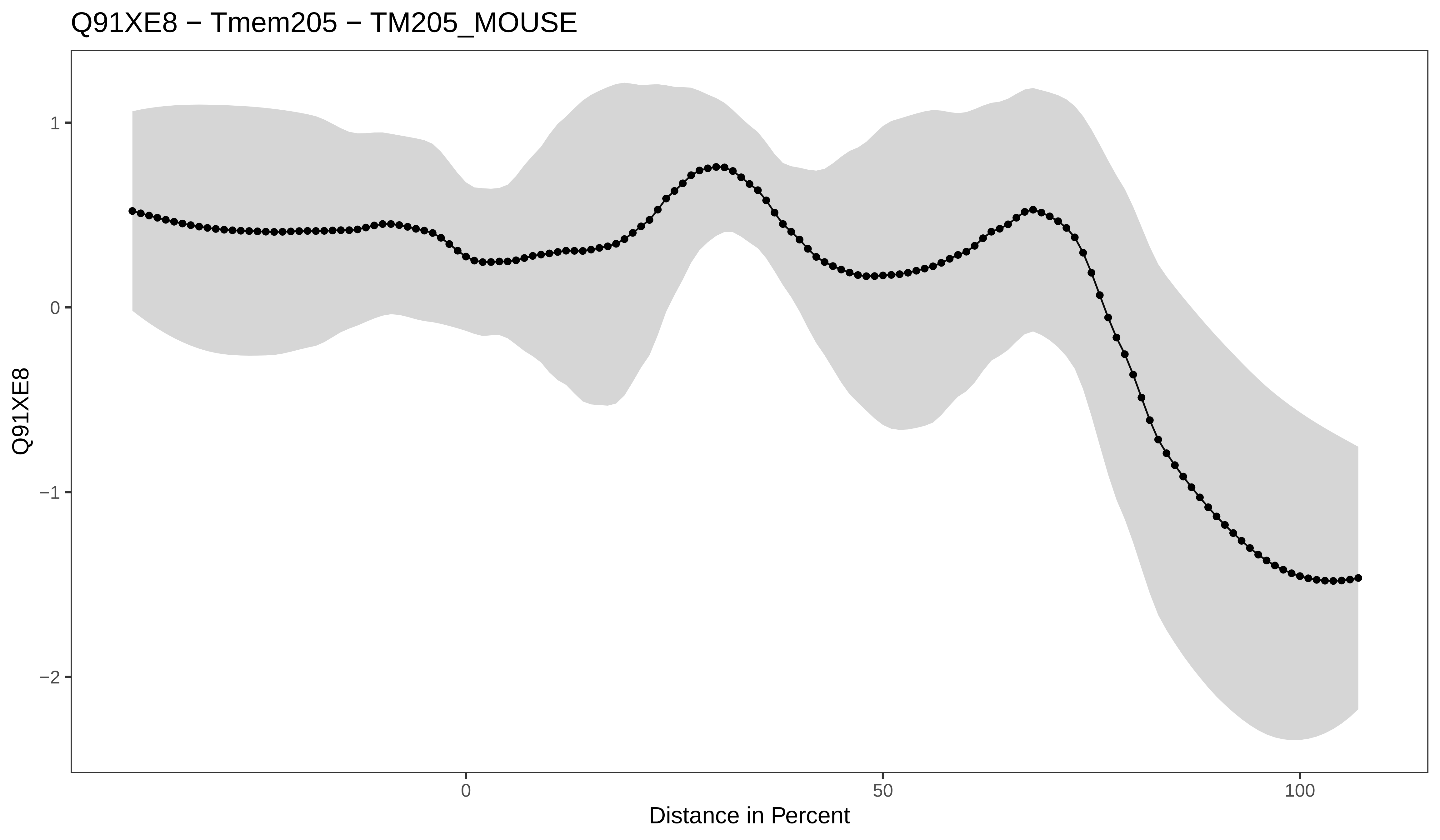

Q91XE8 − Tmem205 − TM205_MOUSE
1
0
−1
−2
Q91XE8
0
50
100
Distance in Percent

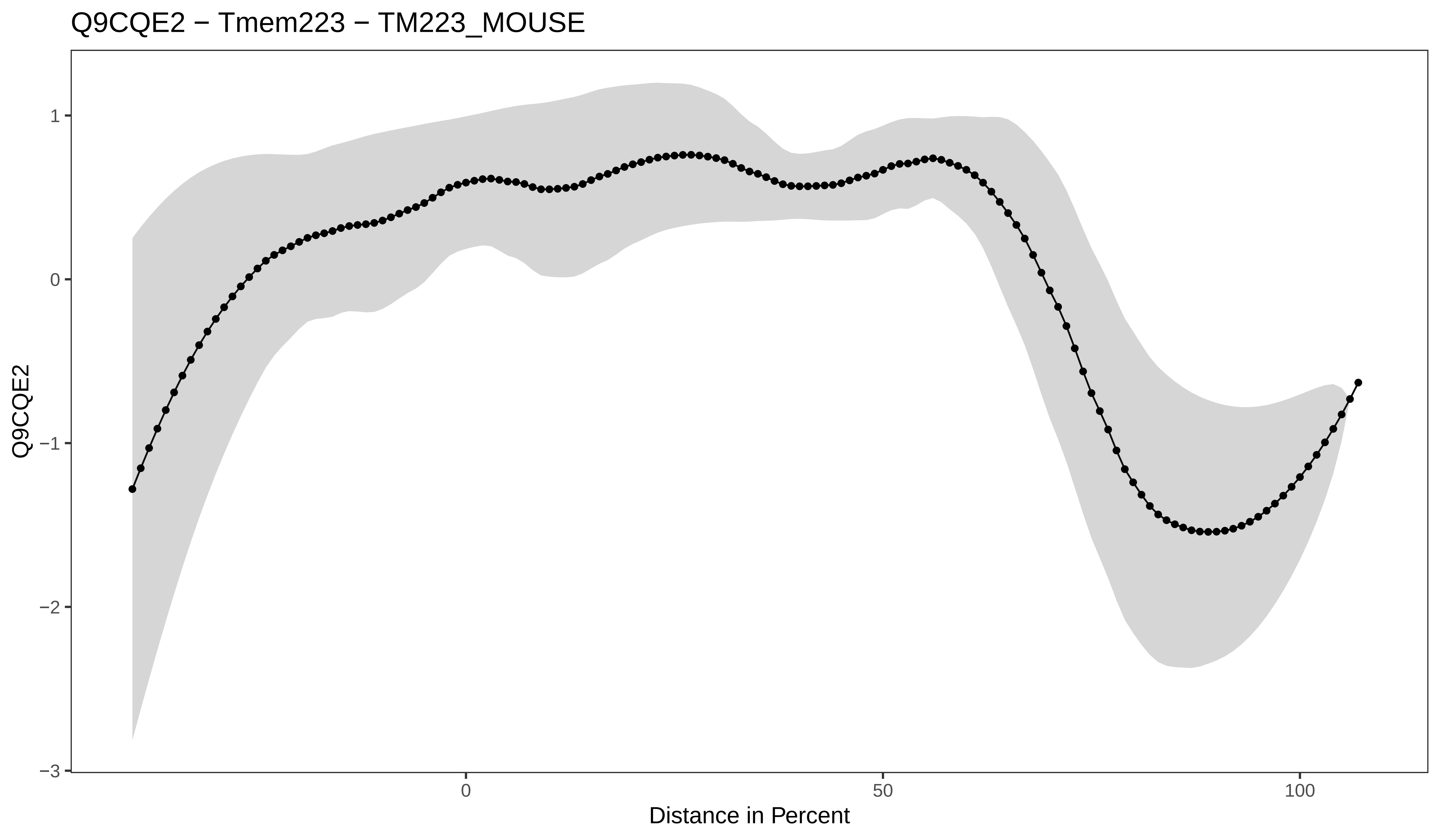
Q9CQE2 − Tmem223 − TM223_MOUSE
1
0
−1
−2
−3
Q9CQE2
0
50
100
Distance in Percent

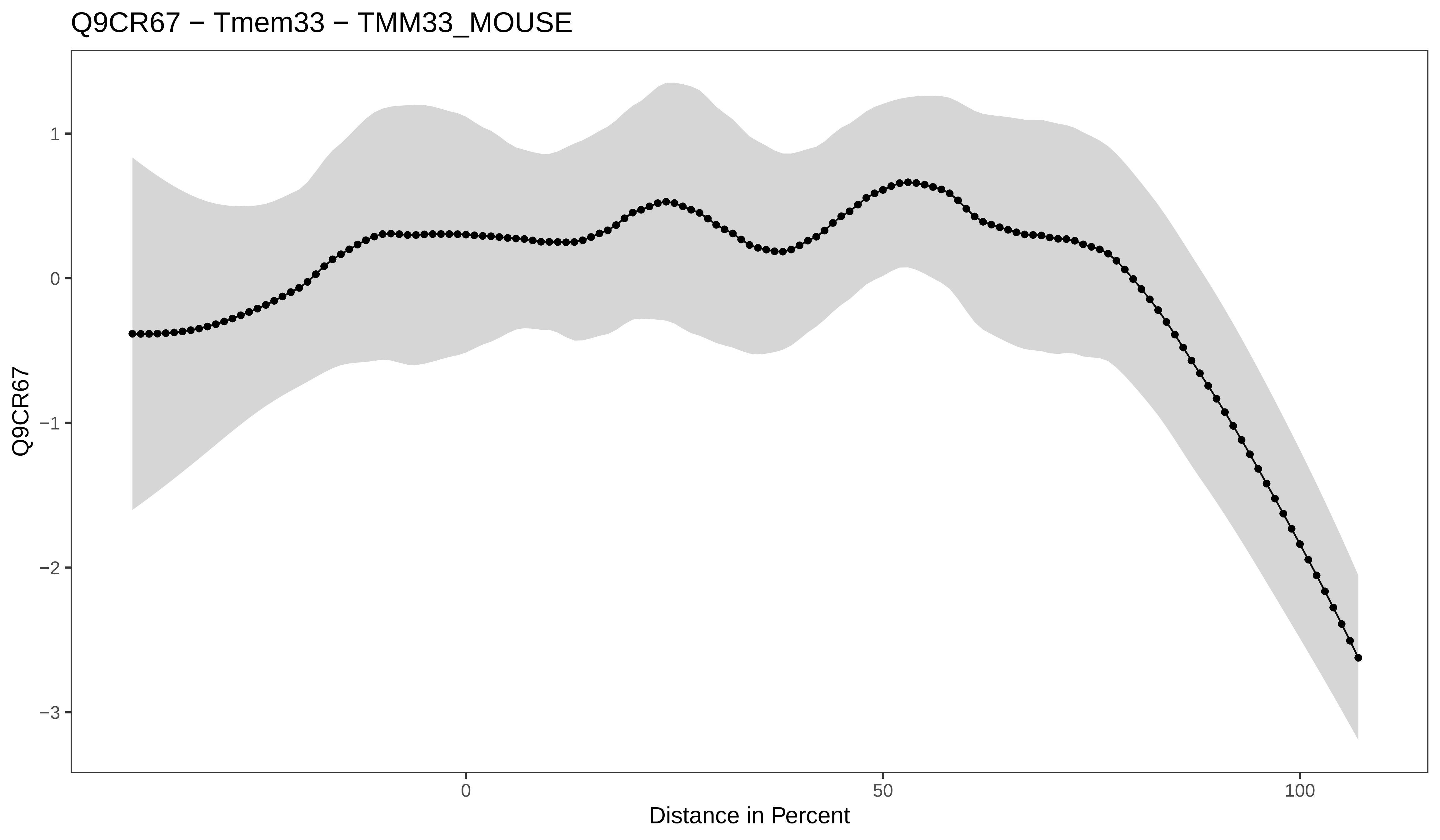
Q9CR67 − Tmem33 − TMM33_MOUSE
Q9CR67
1
0
−1
−2
−3
0
50
100
Distance in Percent

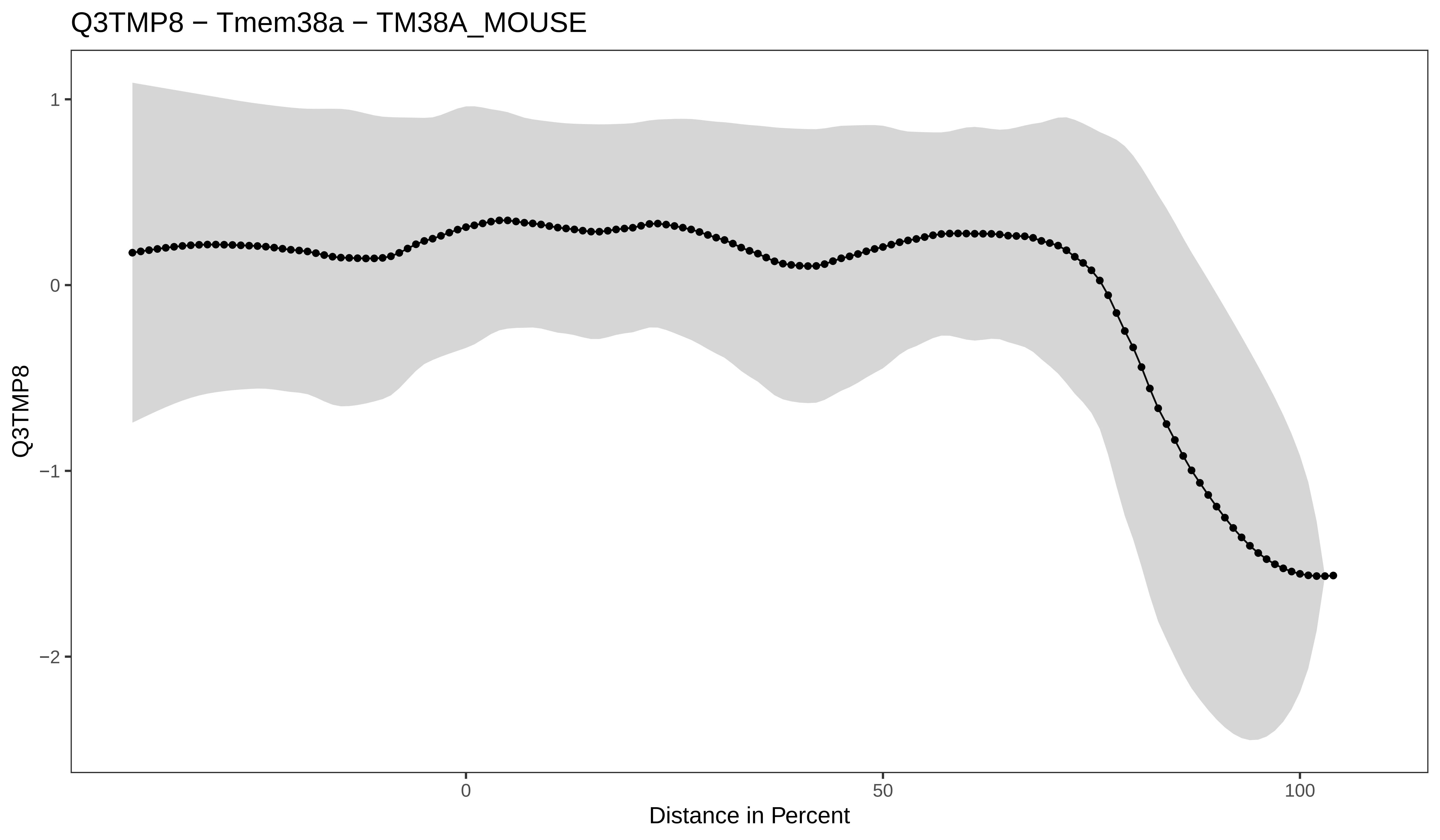

Q3TMP8 − Tmem38a − TM38A_MOUSE
1
0
−1
−2
Q3TMP8
0
50
100
Distance in Percent

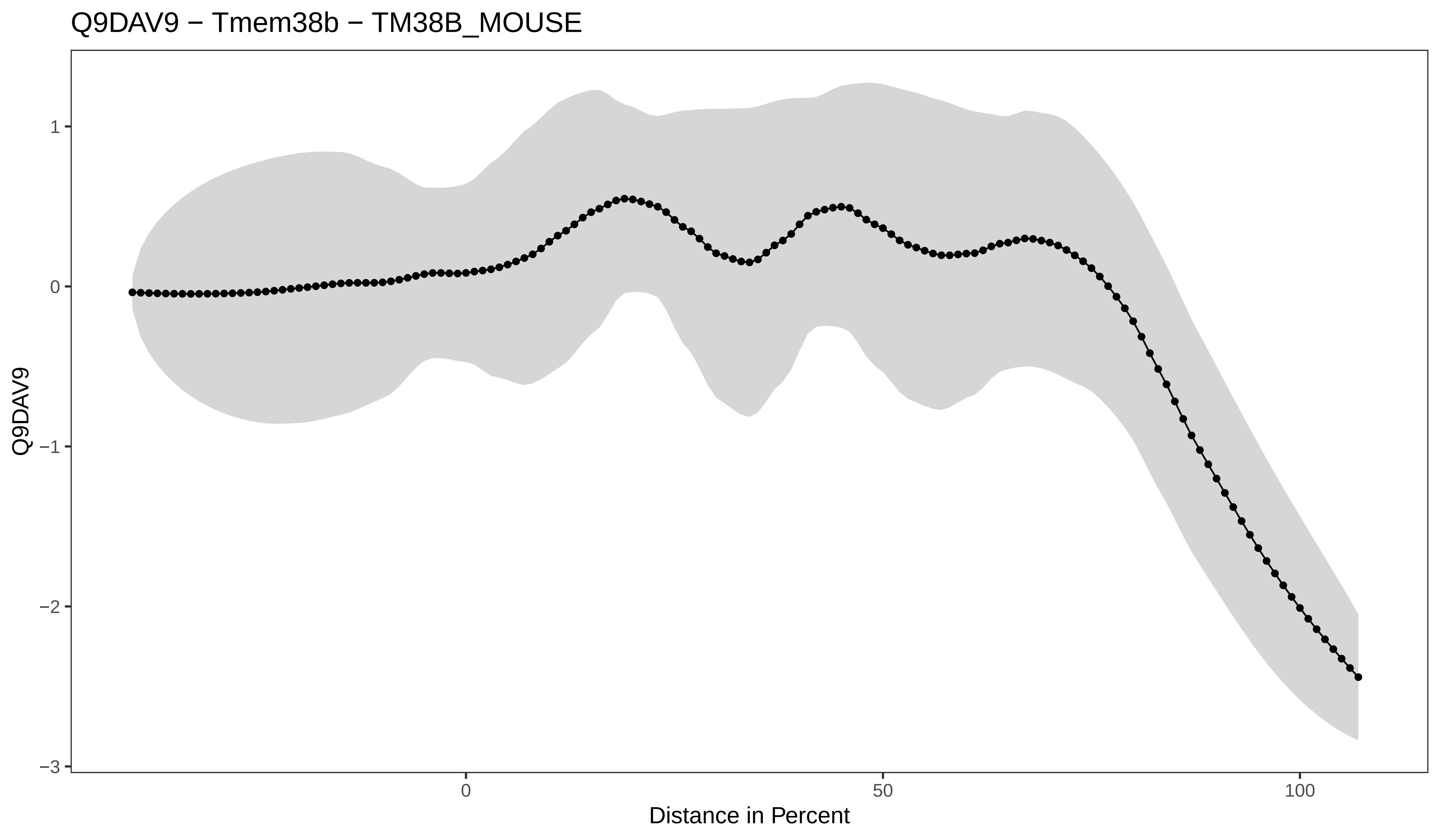

Q9DAV9 − Tmem38b − TM38B_MOUSE
Q9DAV9
1
0
−1
−2
−3
0
50
100
Distance in Percent

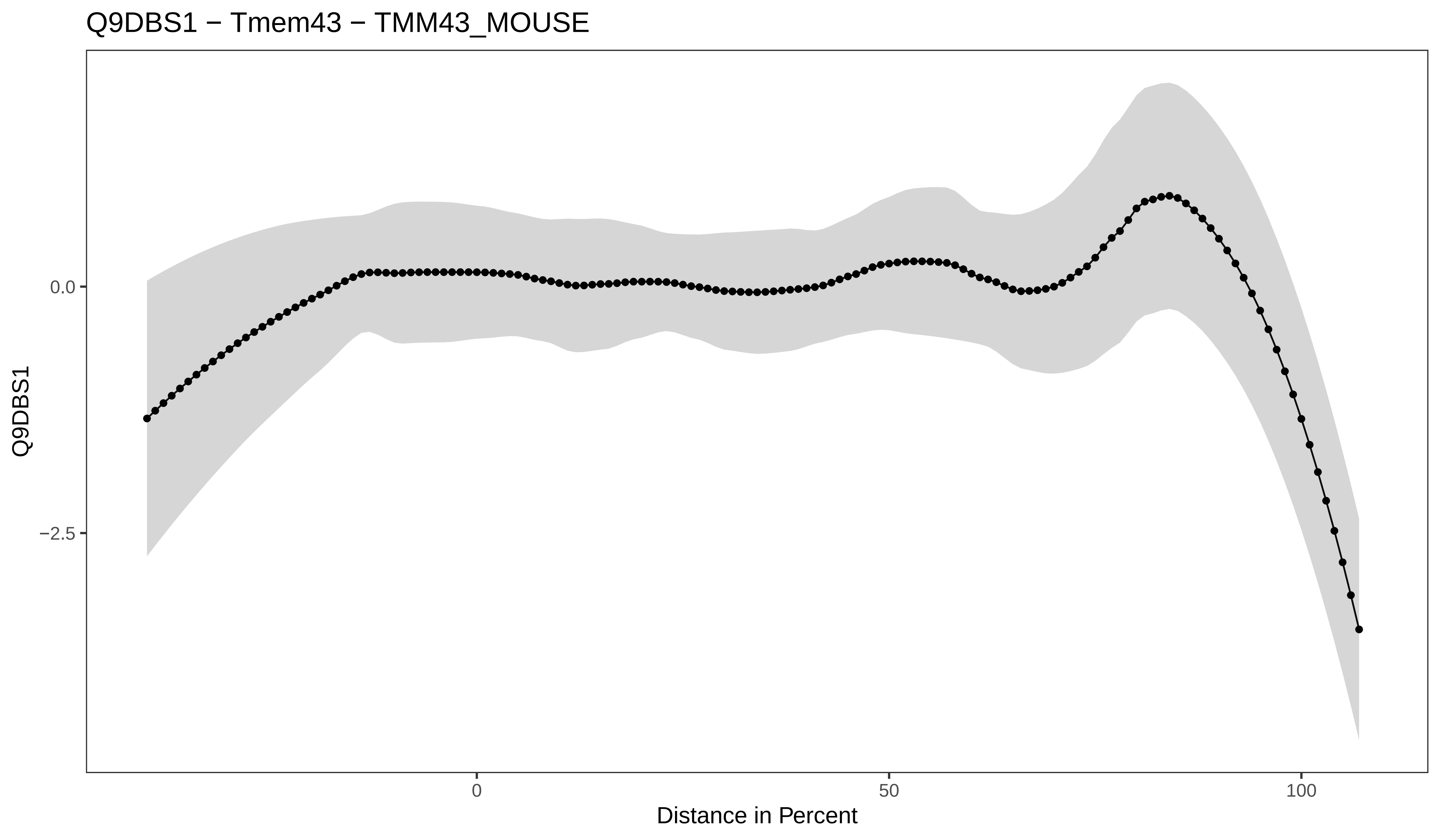

Q9DBS1 − Tmem43 − TMM43_MOUSE
Q9DBS1
0.0
−2.5
0
50
100
Distance in Percent

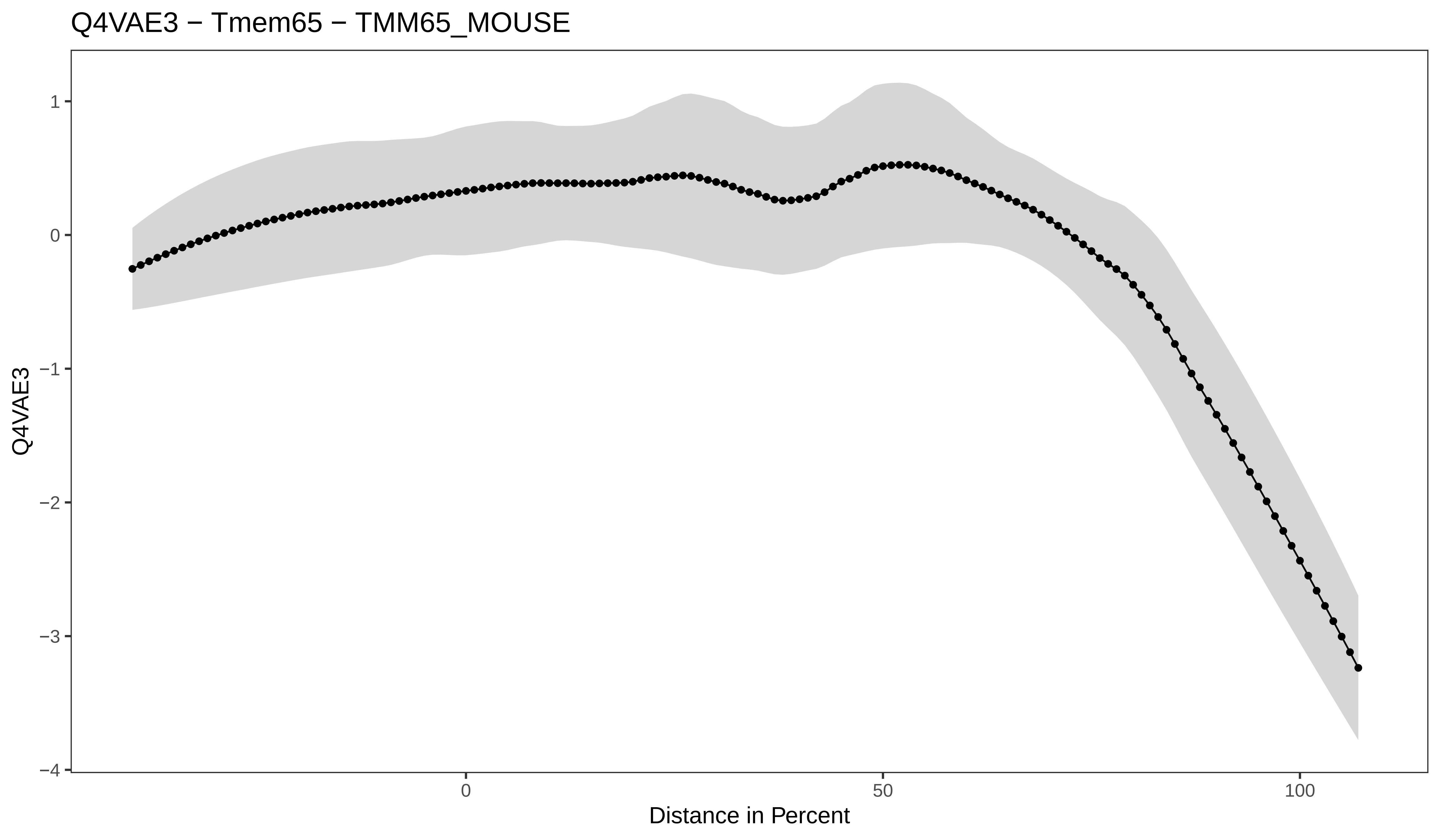

Q4VAE3 − Tmem65 − TMM65_MOUSE
1
0
−1
−2
−3
−4
Q4VAE3
0
50
100
Distance in Percent

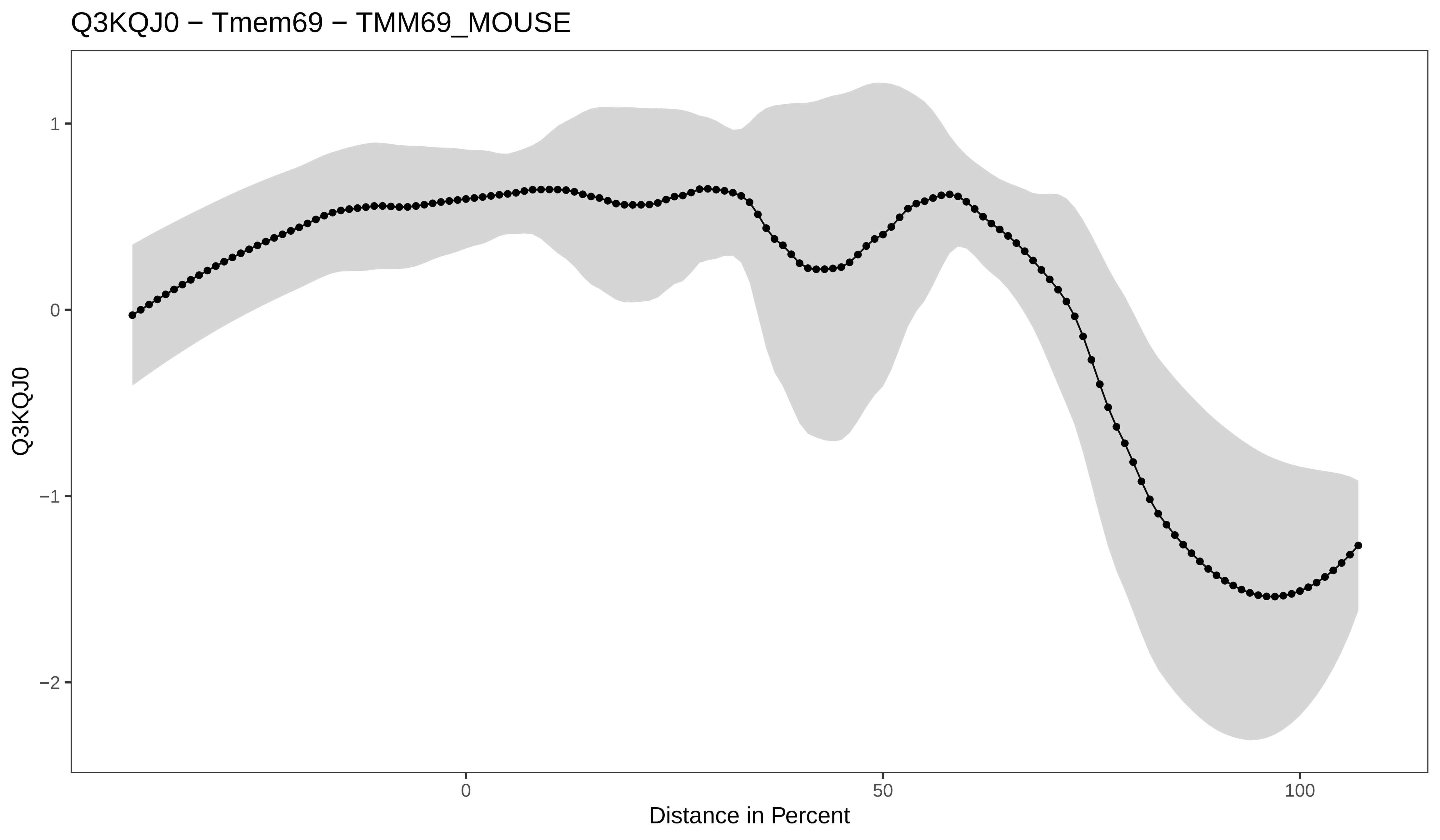
Q3KQJ0 − Tmem69 − TMM69_MOUSE
1
0
−1
−2
Q3KQJ0
0
50
100
Distance in Percent

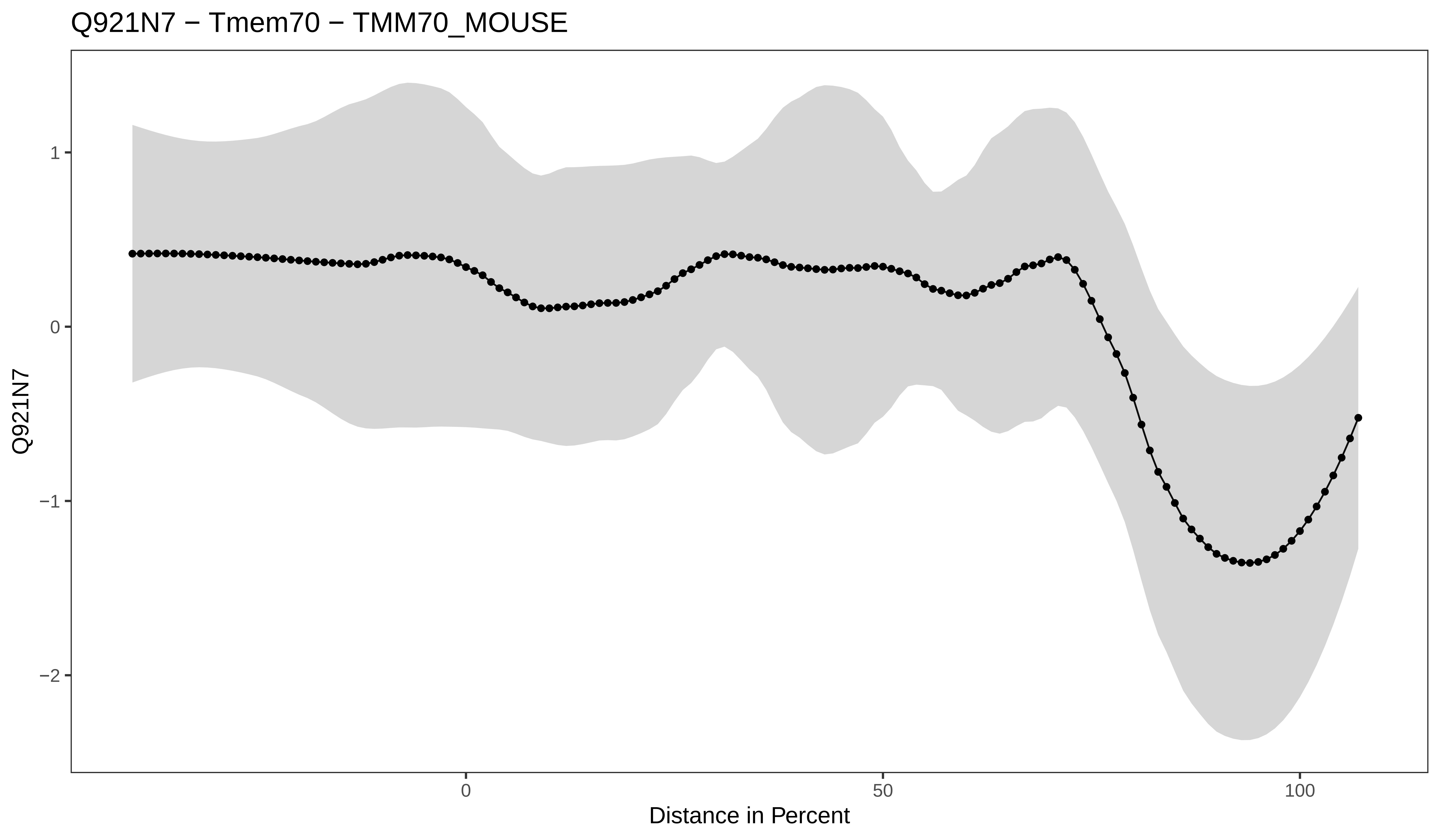

Q921N7 − Tmem70 − TMM70_MOUSE
1
0
−1
−2
Q921N7
0
50
100
Distance in Percent

Q91ZE0 − Tmlhe − TMLH_MOUSE

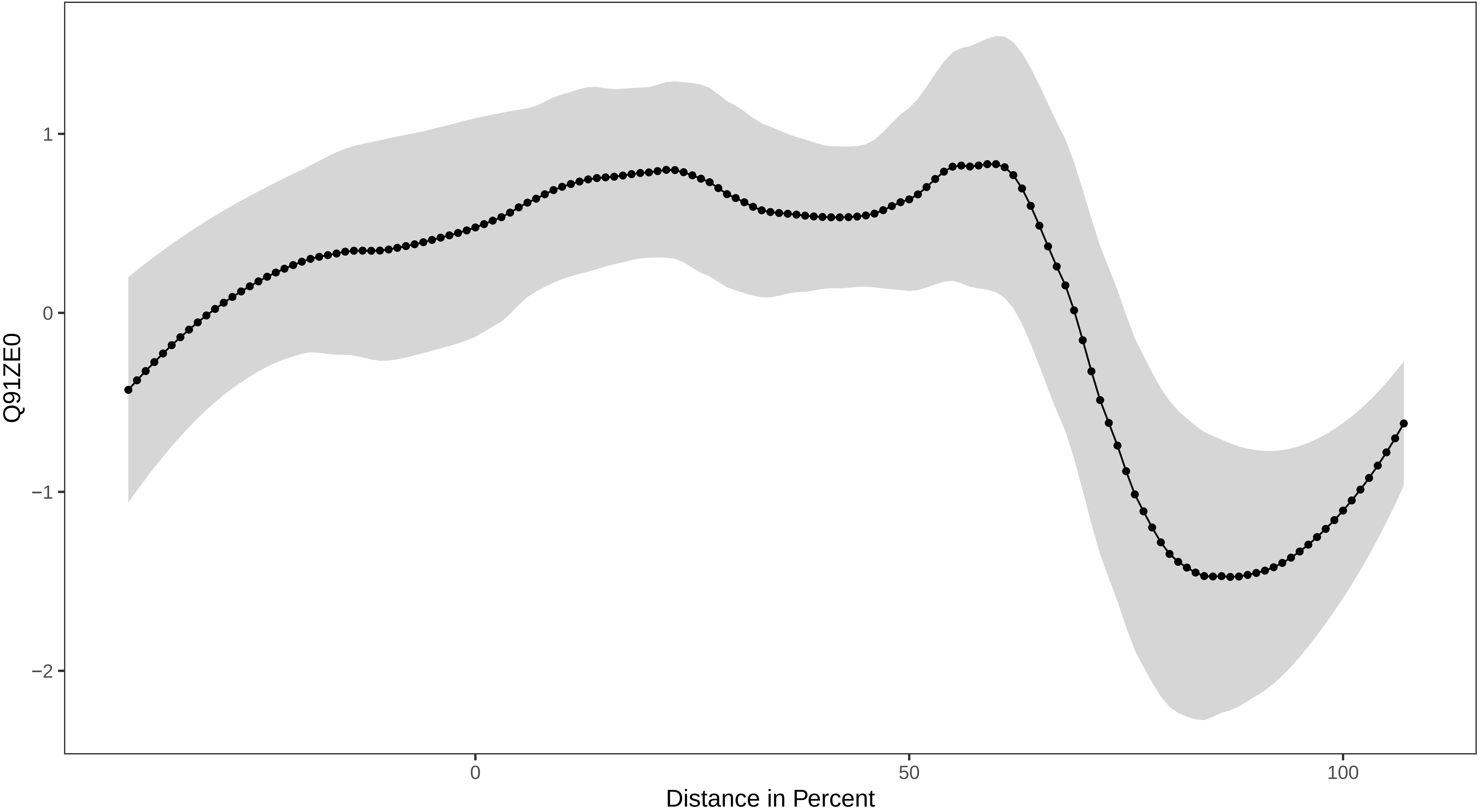

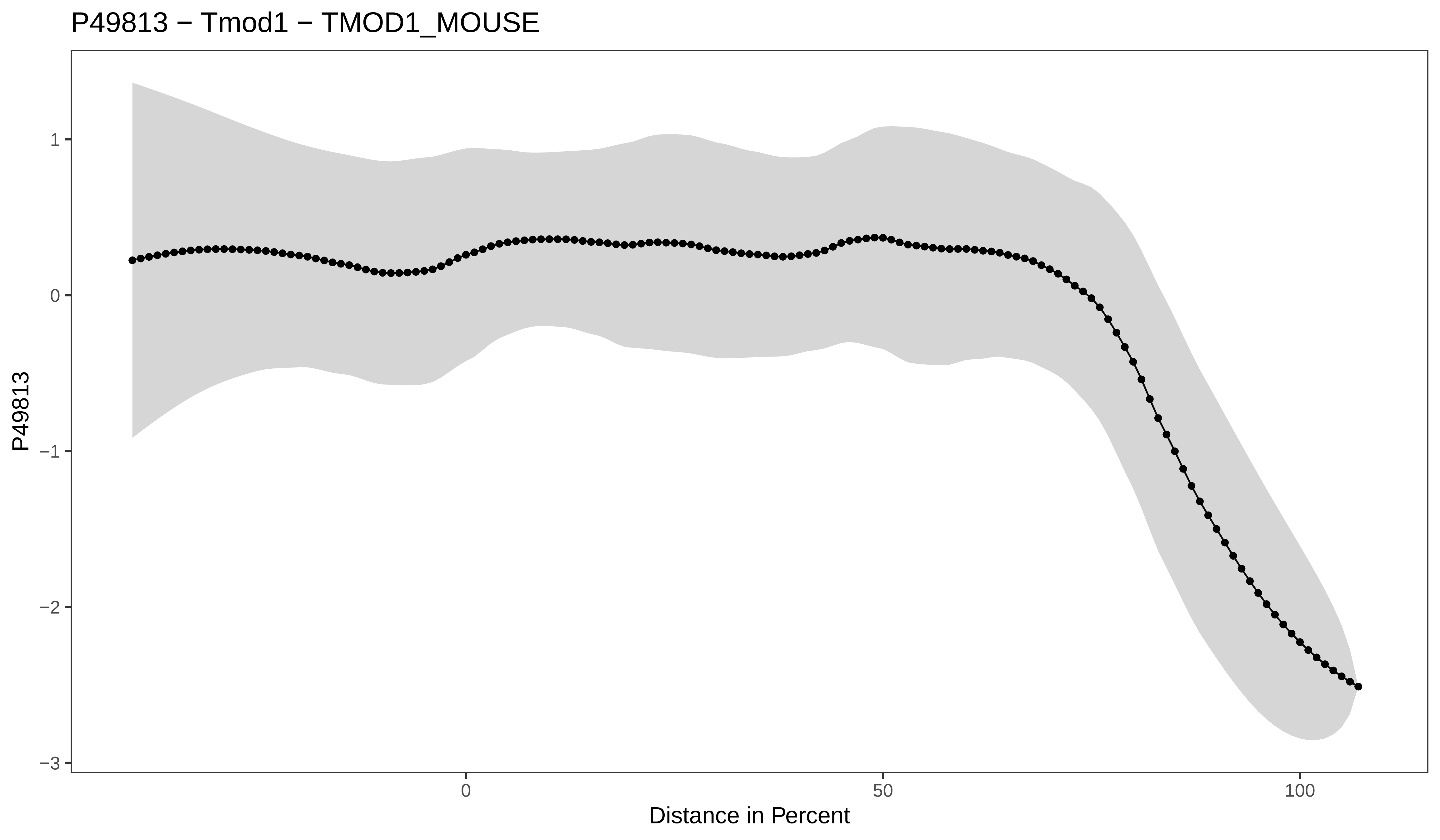

P49813 − Tmod1 − TMOD1_MOUSE
P49813
Distance in Percent
1
0
−1
−2
−3
0
50
100

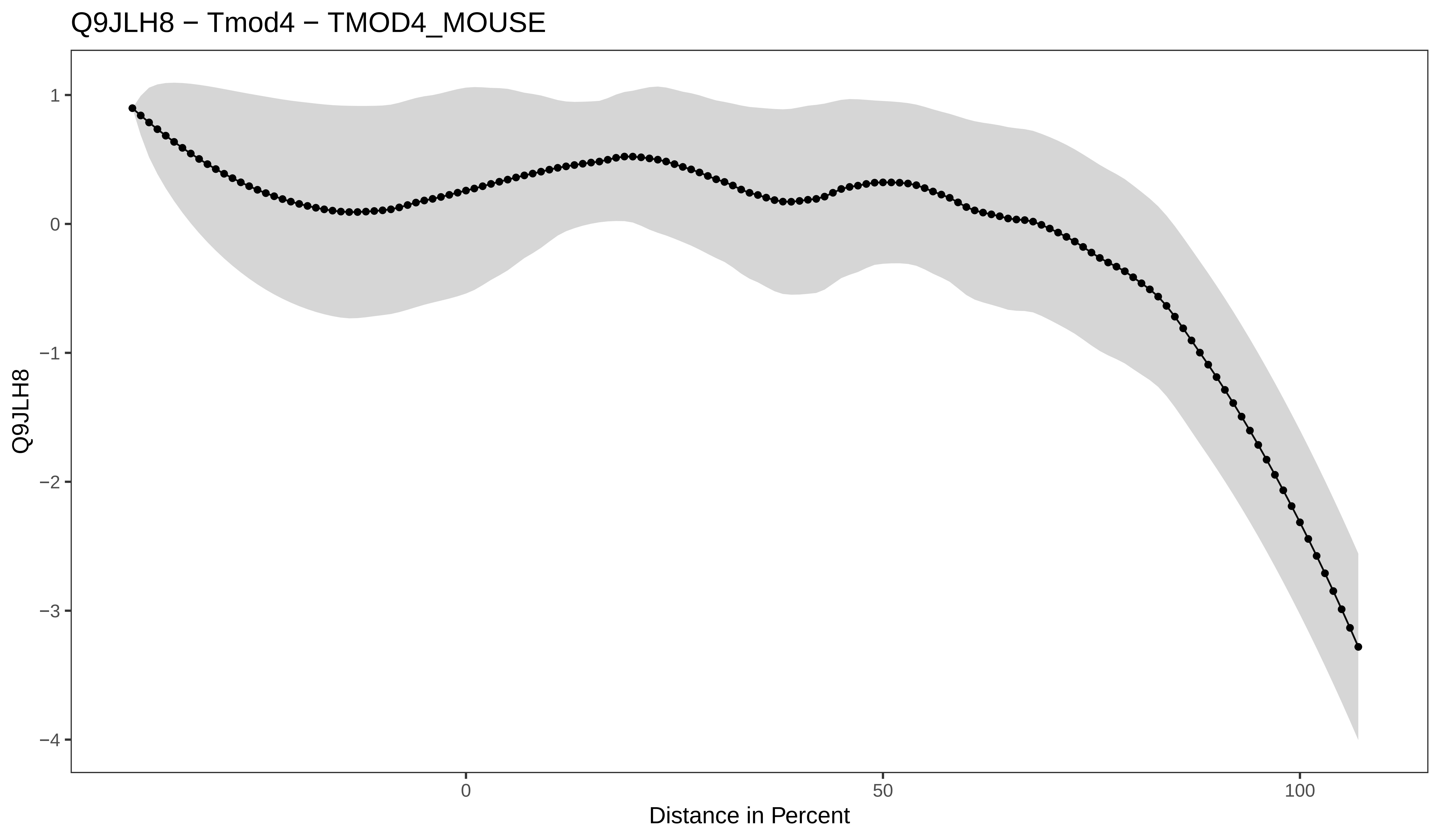

Q9JLH8 − Tmod4 − TMOD4_MOUSE
Q9JLH8
Distance in Percent
1
0
−1
−2
−3
−4
0
50
100

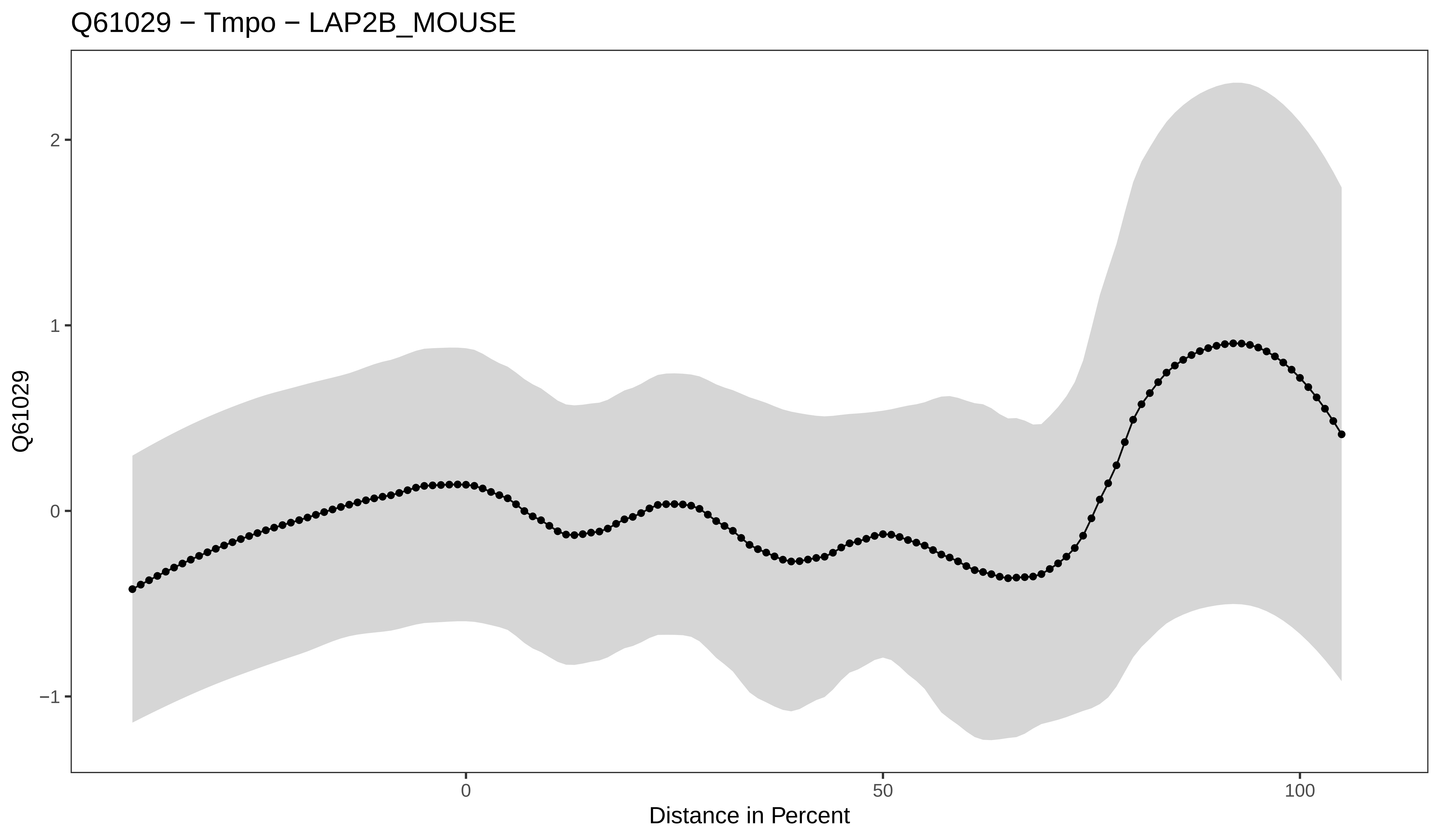

Q61029 − Tmpo − LAP2B_MOUSE
Q61029
2
1
0
−1
0
50
100
Distance in Percent

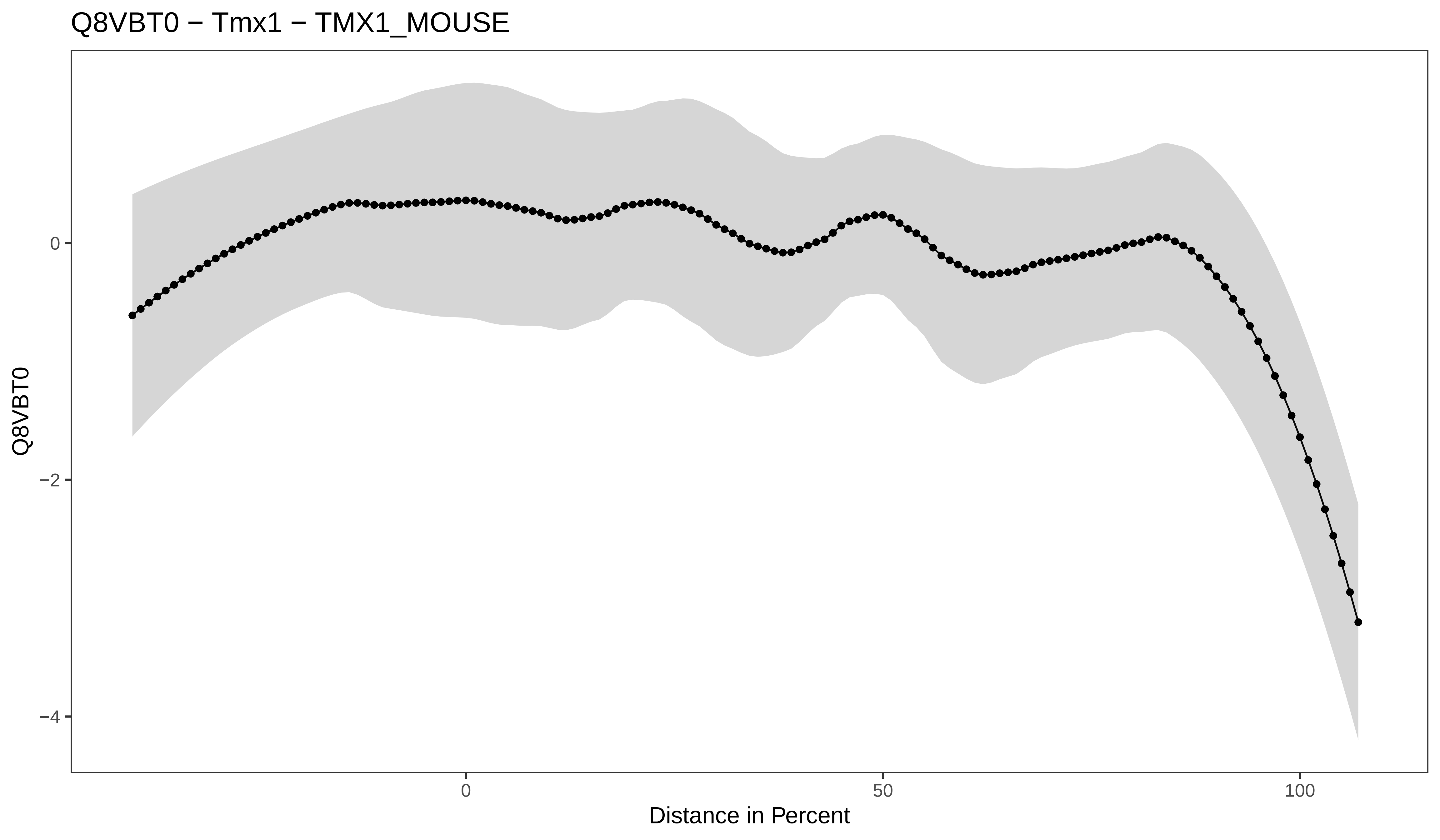

Q8VBT0 − Tmx1 − TMX1_MOUSE
0
−2
−4
Q8VBT0
0
50
100
Distance in Percent

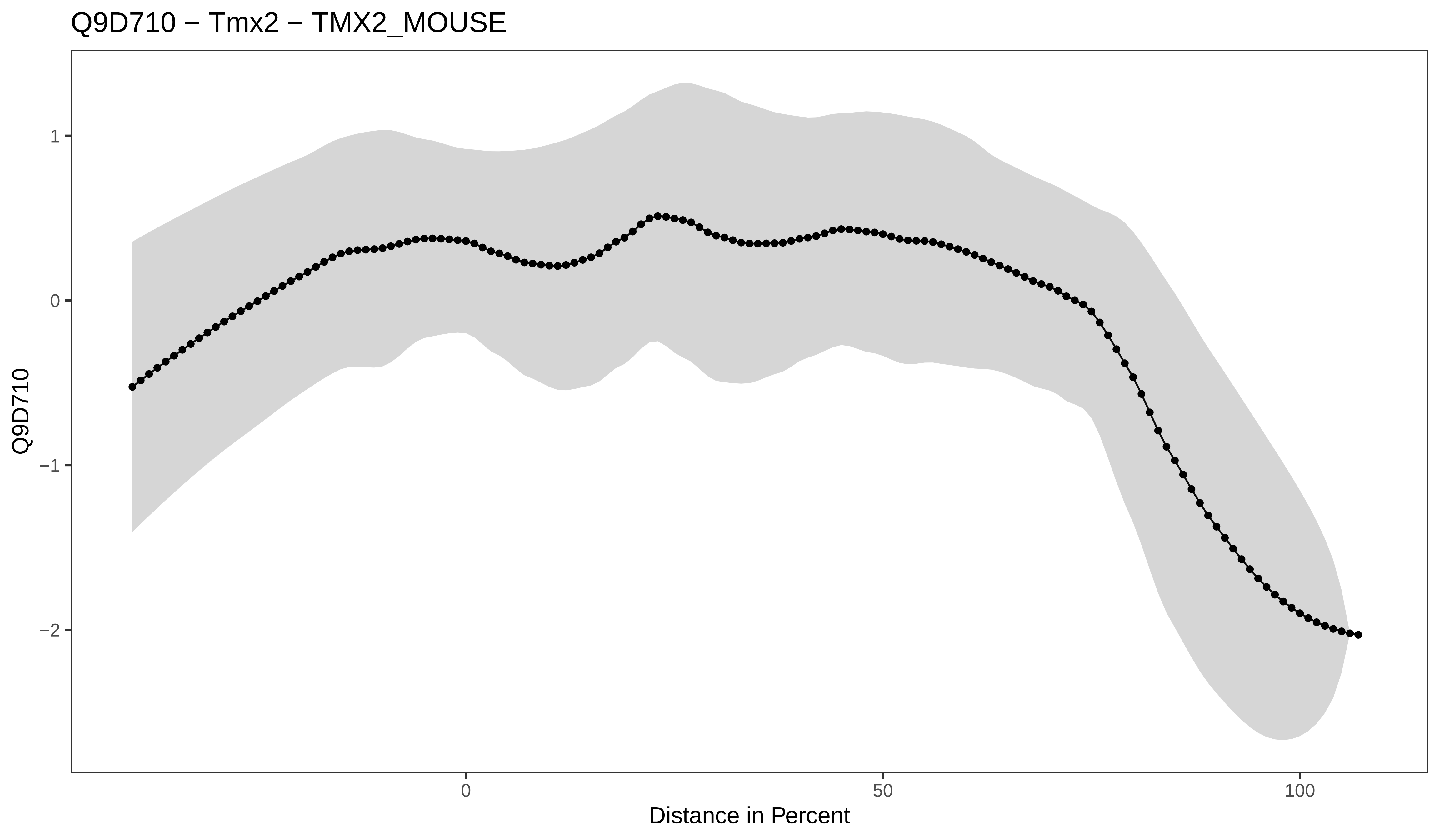

Q9D710 − Tmx2 − TMX2_MOUSE
1
0
−1
−2
Q9D710
0
50
100
Distance in Percent

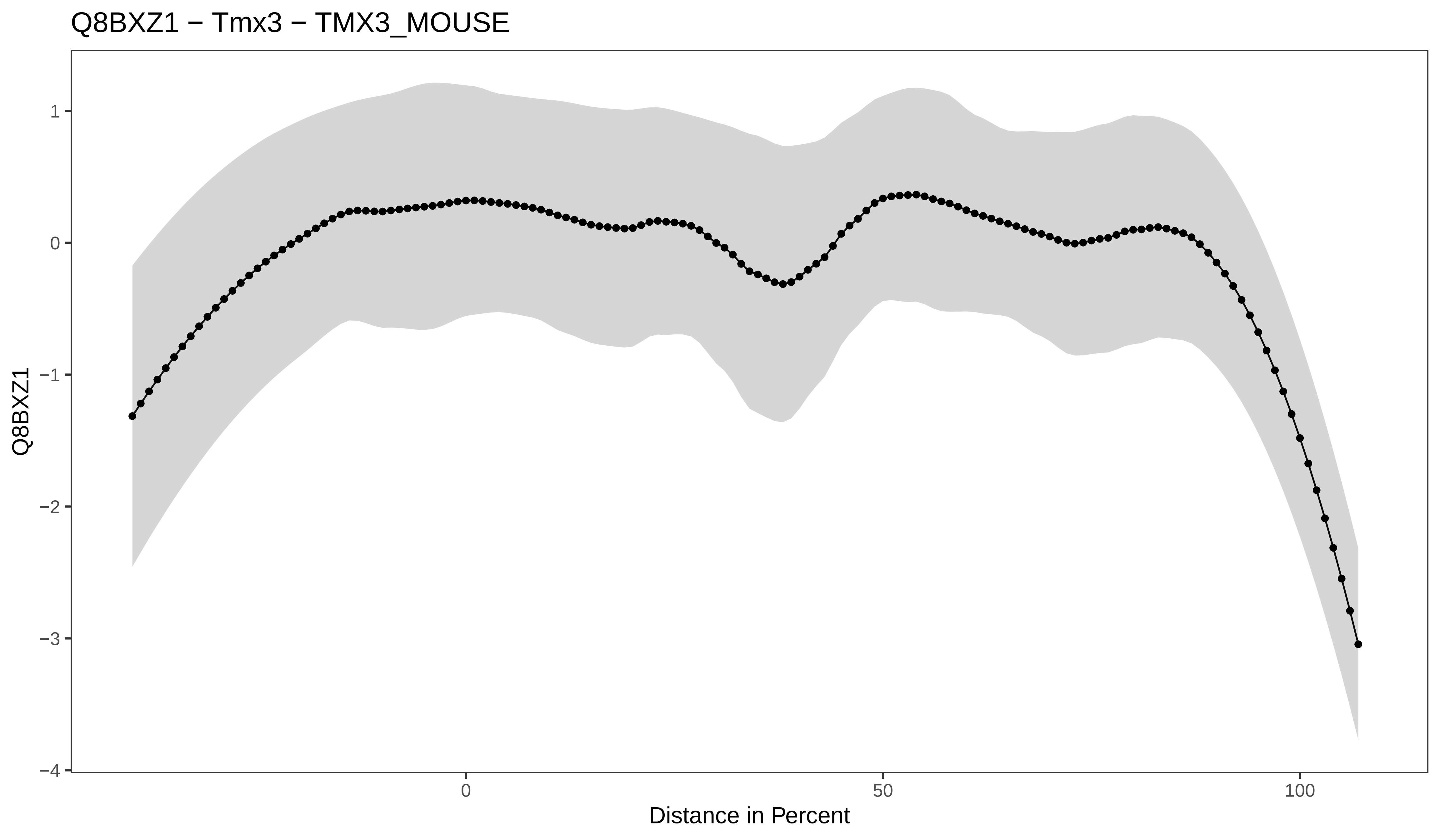
Q8BXZ1 − Tmx3 − TMX3_MOUSE
1
0
−1
−2
−3
−4
Q8BXZ1
0
50
100
Distance in Percent

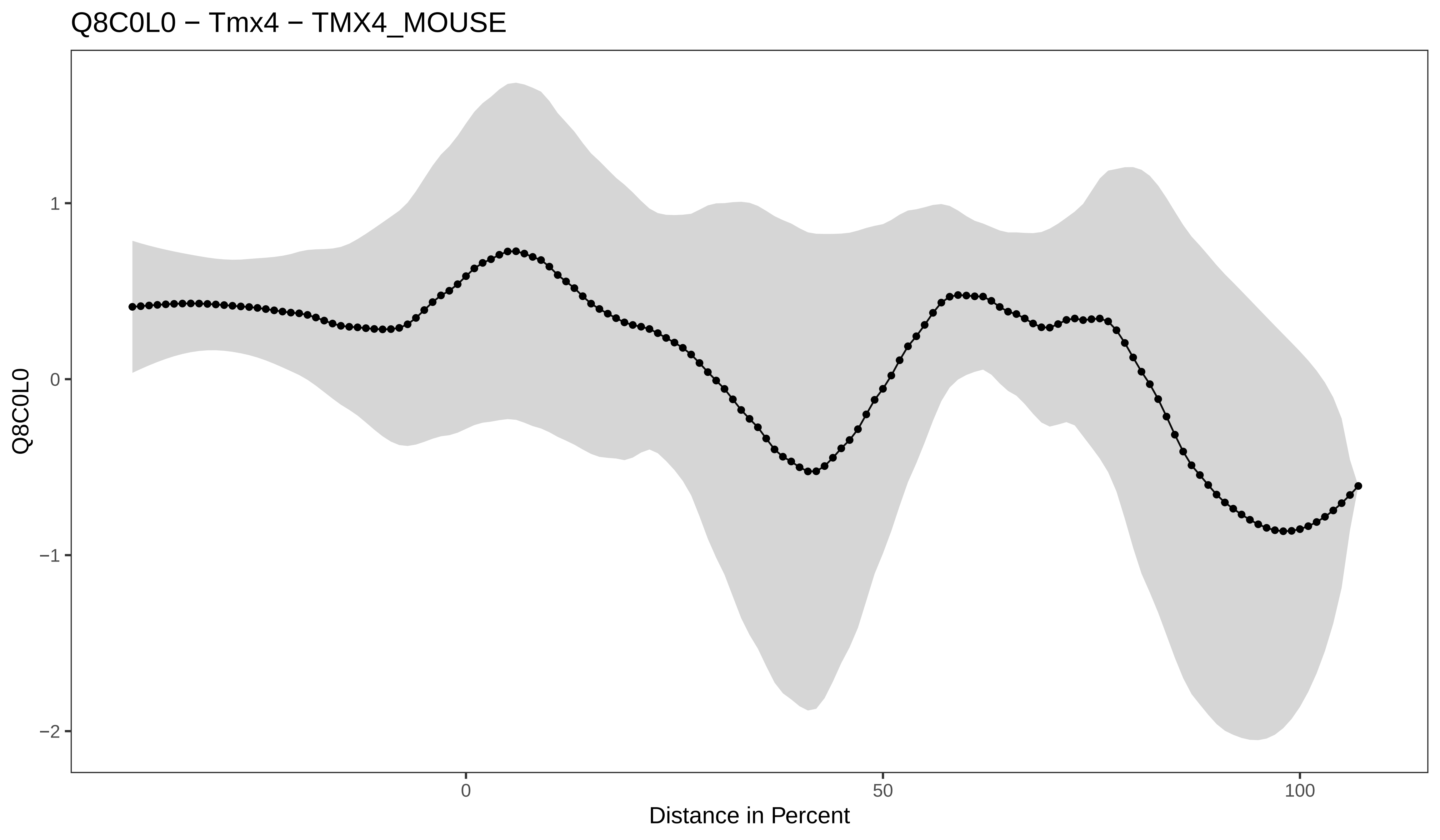

Q8C0L0 − Tmx4 − TMX4_MOUSE
1
0
−1
−2
Q8C0L0
0
50
100
Distance in Percent

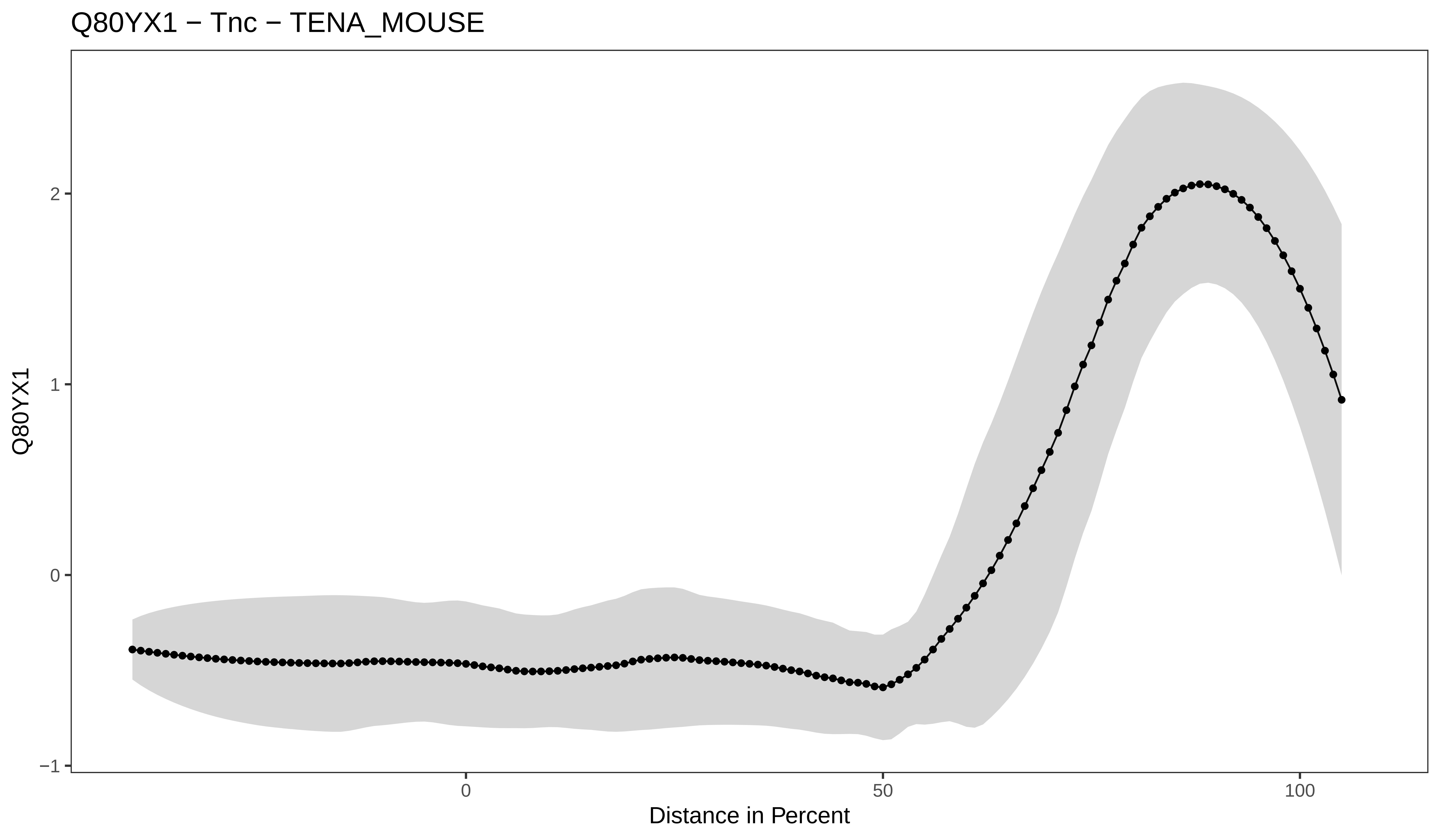

Q80YX1 − Tnc − TENA_MOUSE
Q80YX1
2
1
0
−1
0
50
100
Distance in Percent

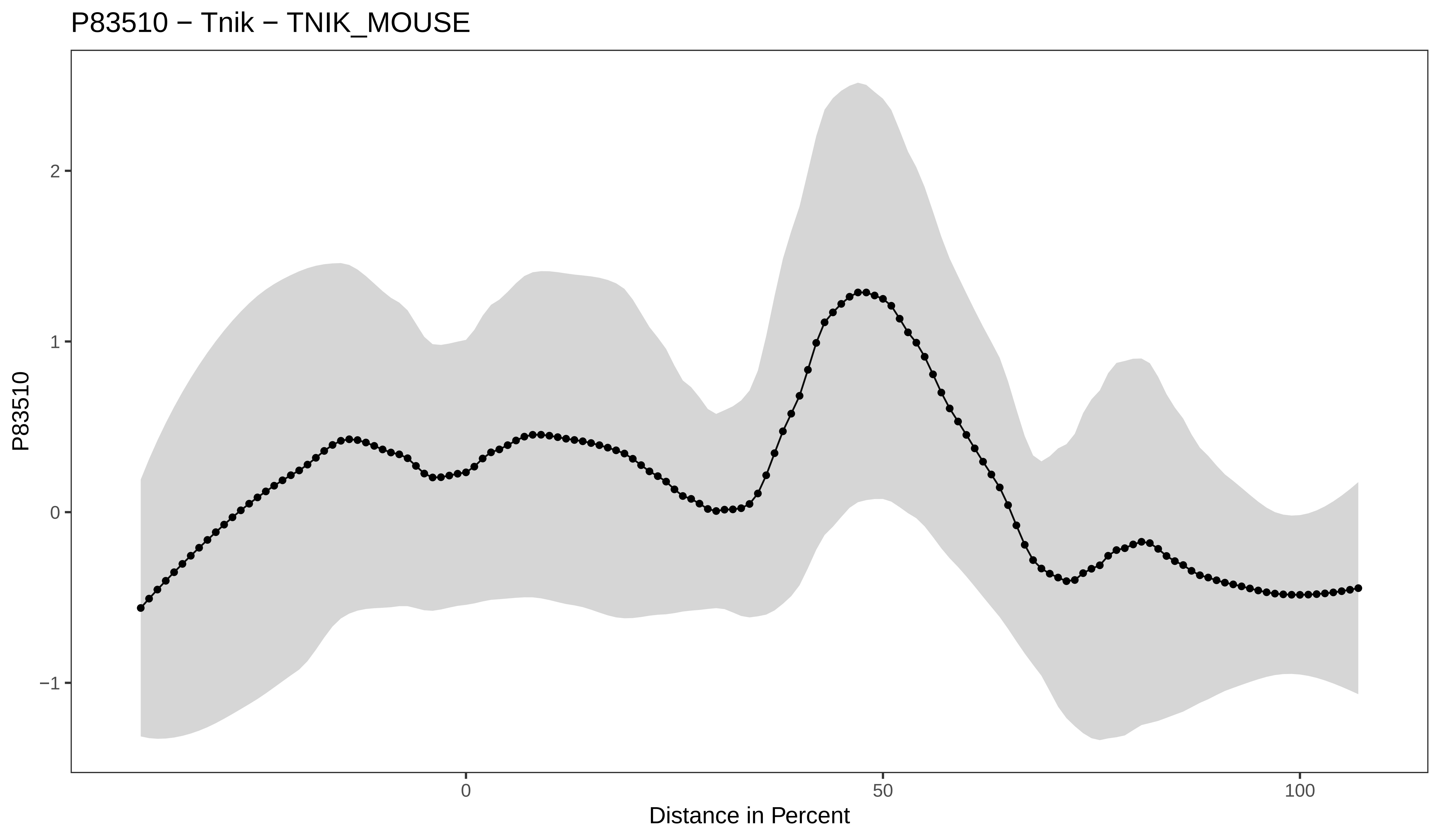

P83510 − Tnik − TNIK_MOUSE
P83510
2
1
0
−1
0
50
100
Distance in Percent

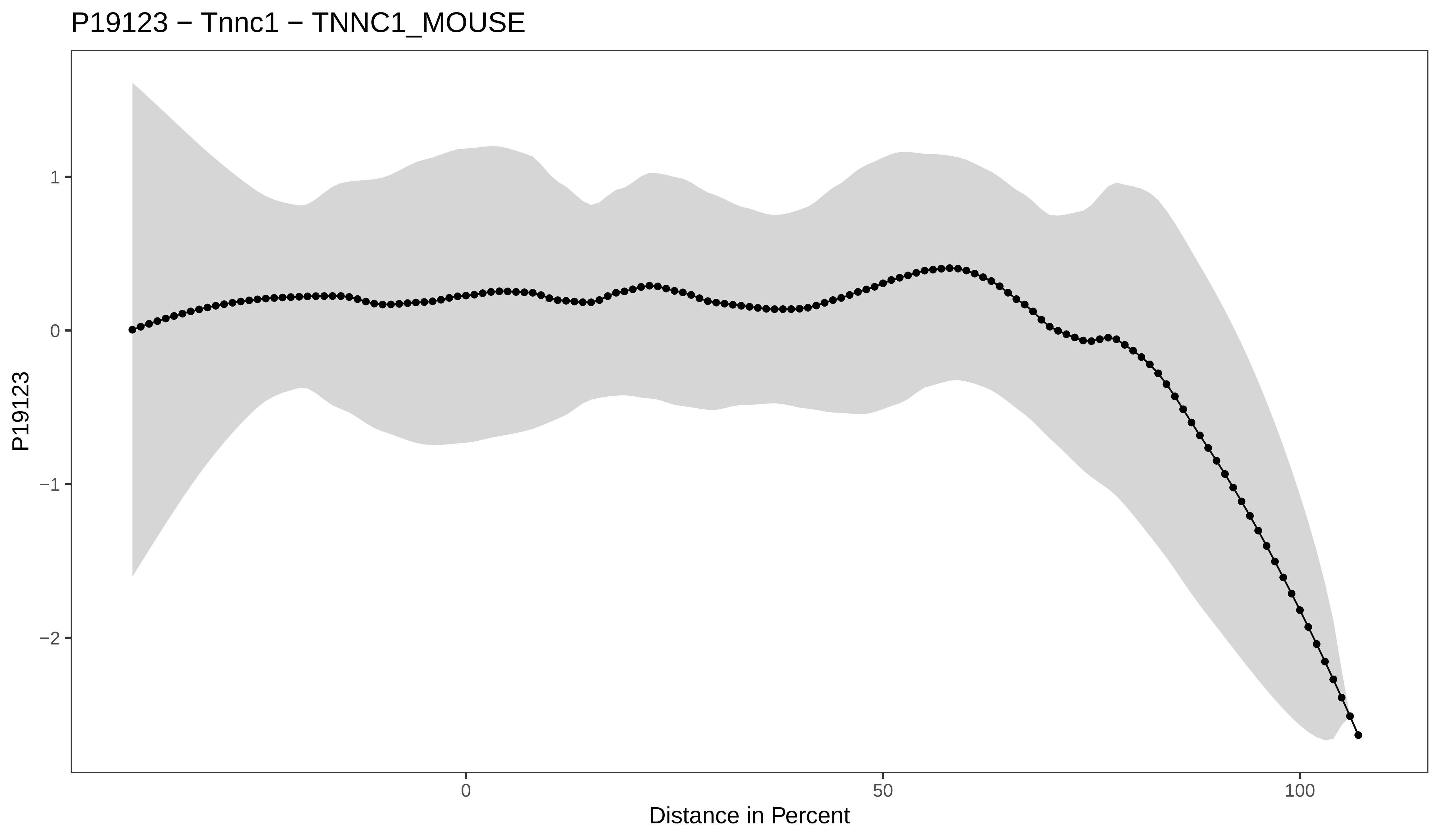

P19123 − Tnnc1 − TNNC1_MOUSE
P19123
1
0
−1
−2
0
50
100
Distance in Percent

P20801 − Tnnc2 − TNNC2_MOUSE

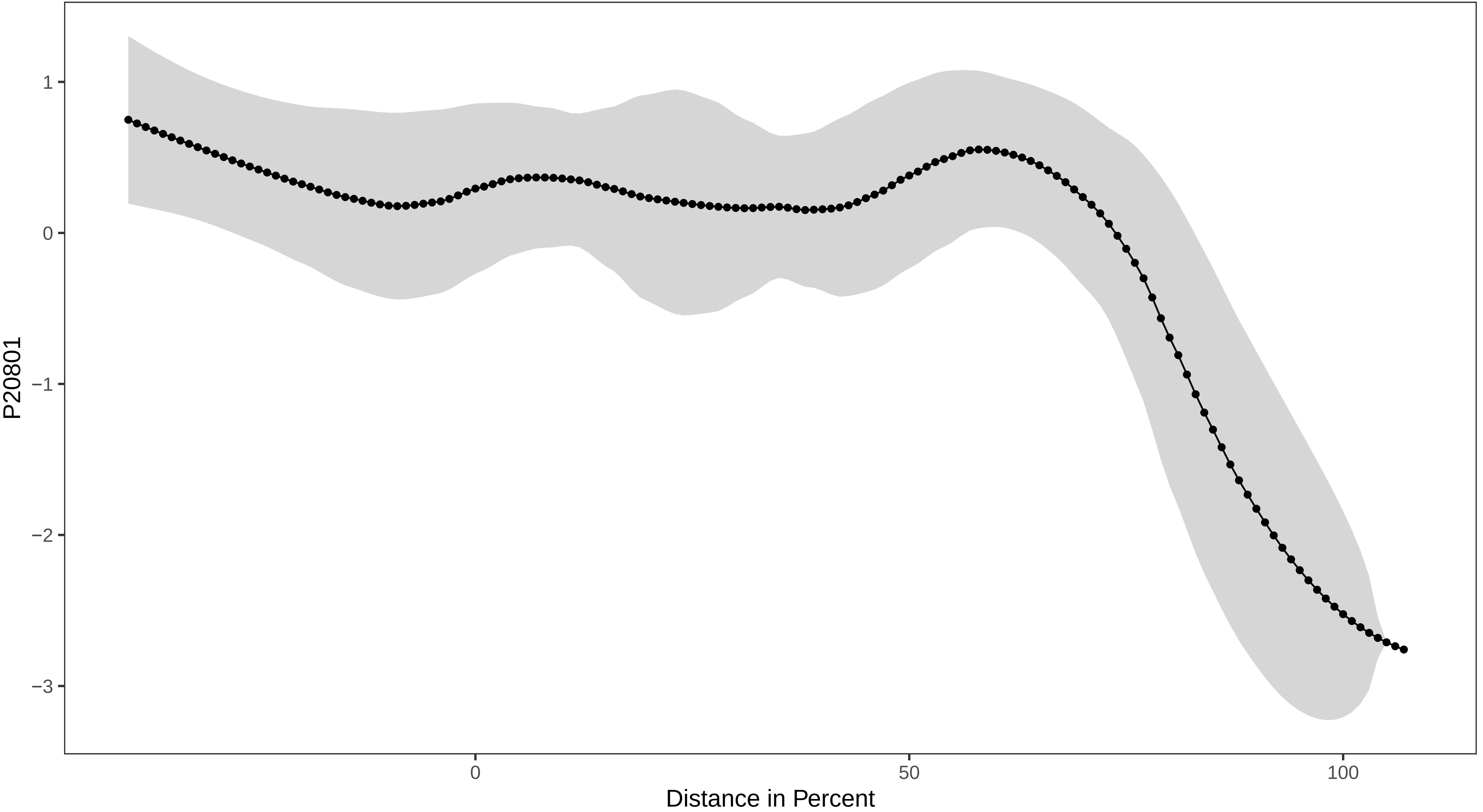

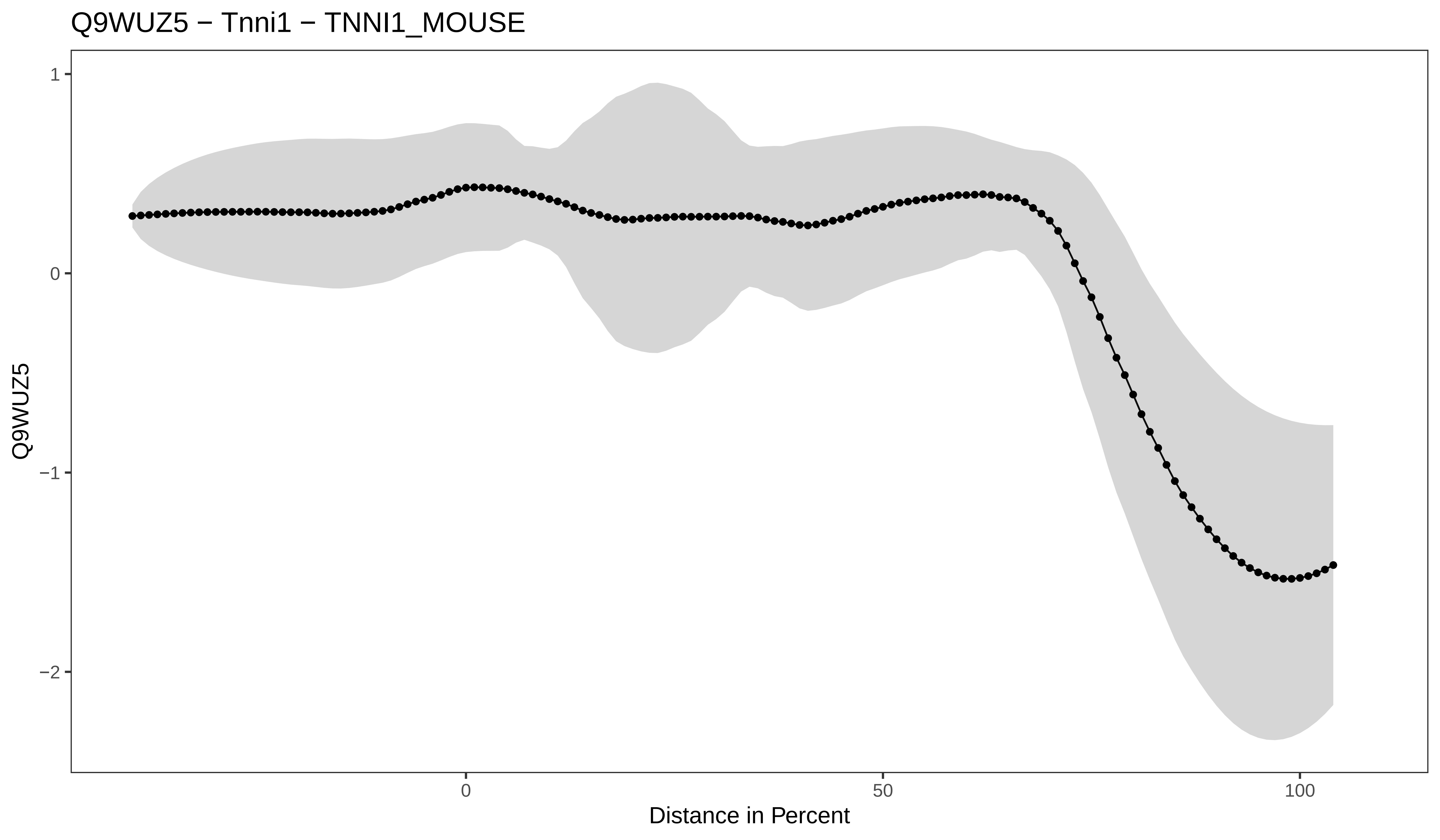
Q9WUZ5 − Tnni1 − TNNI1_MOUSE
1
0
−1
−2
0
50
100
Q9WUZ5
Distance in Percent

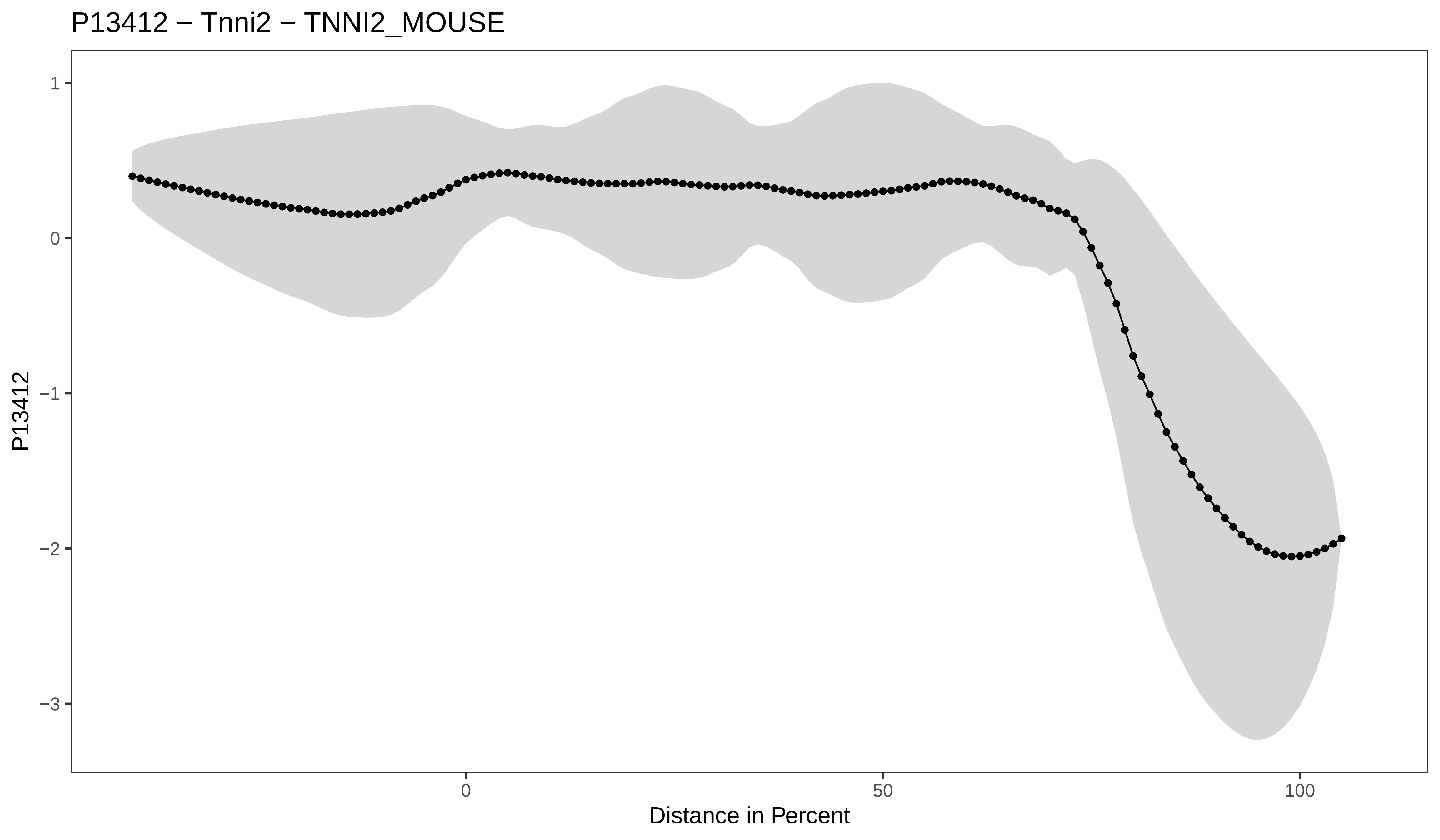
P13412 − Tnni2 − TNNI2_MOUSE
P13412
1
0
−1
−2
−3
0
50
100
Distance in Percent

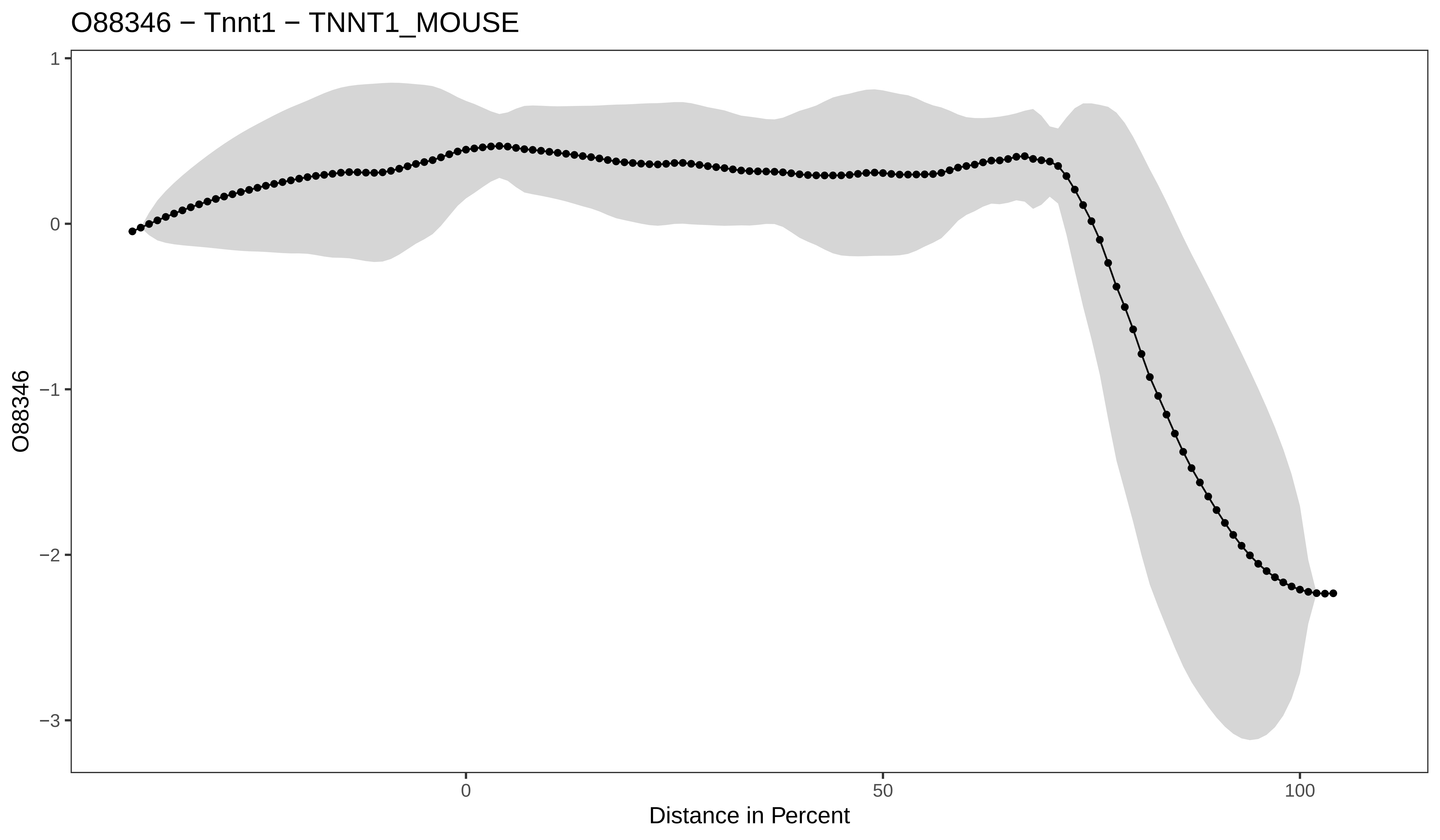
O88346 − Tnnt1 − TNNT1_MOUSE
1
0
−1
−2
−3
O88346
0
50
100
Distance in Percent

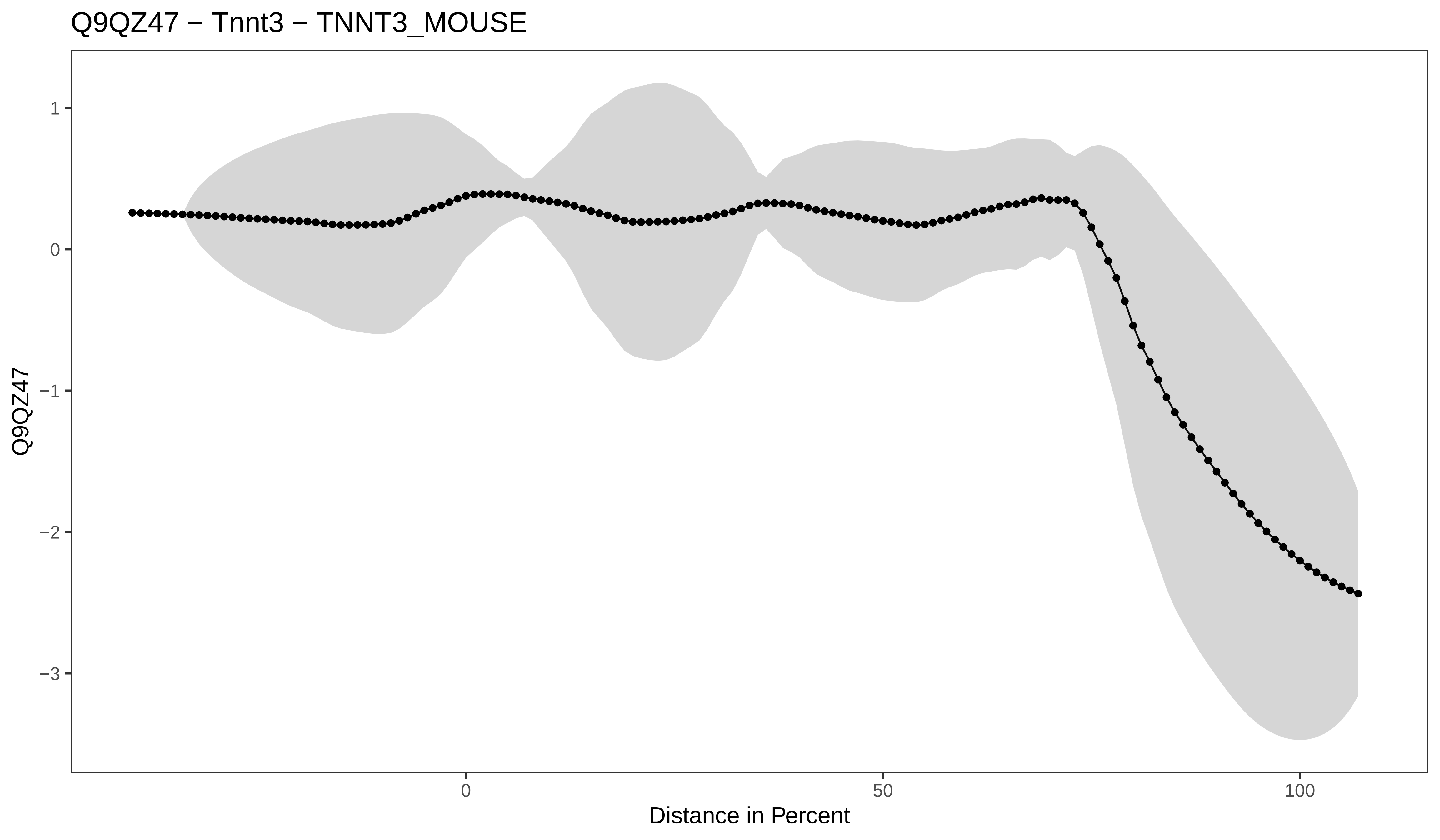

Q9QZ47 − Tnnt3 − TNNT3_MOUSE
1
0
−1
−2
−3
Q9QZ47
0
50
100
Distance in Percent

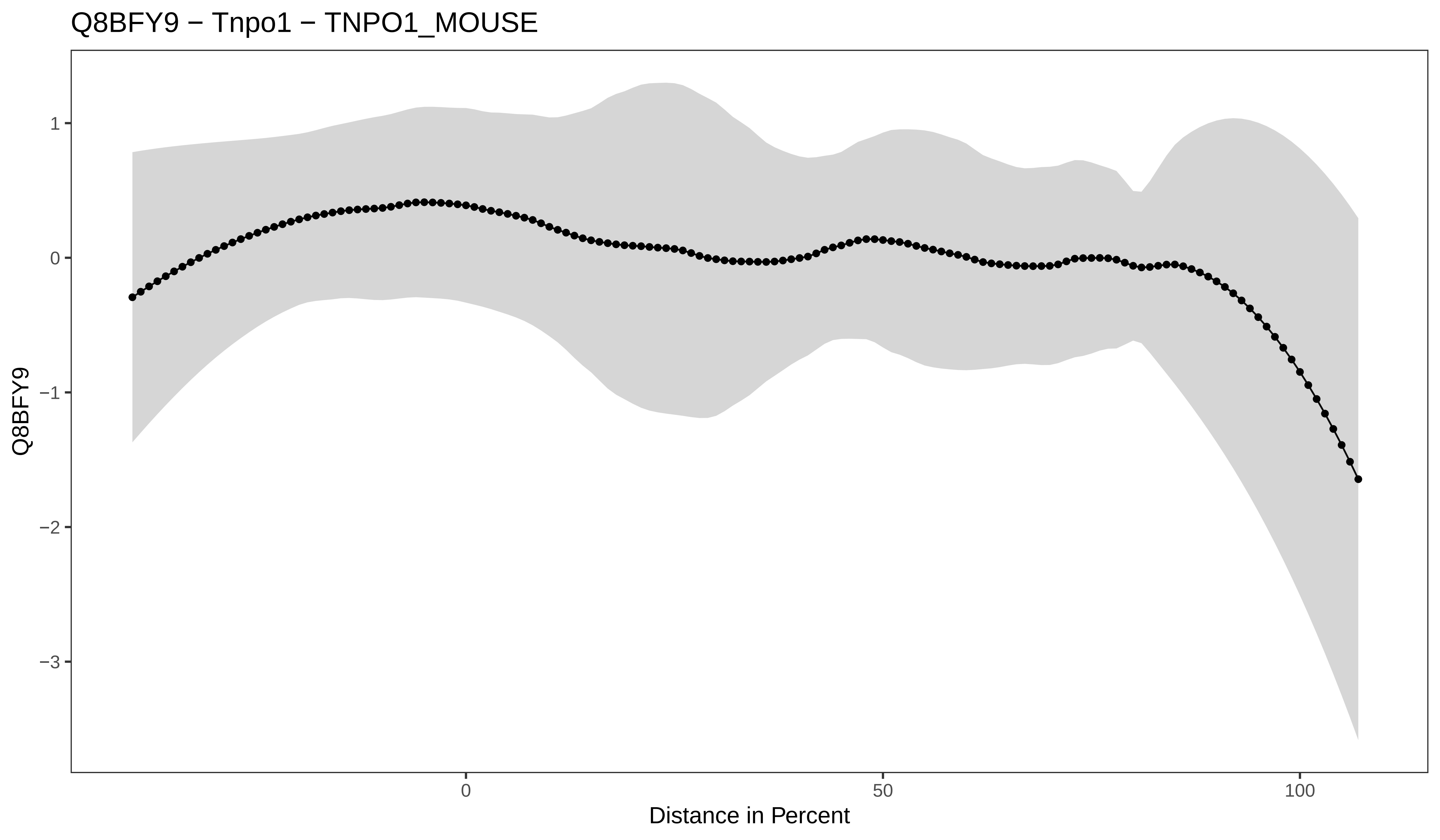
Q8BFY9 − Tnpo1 − TNPO1_MOUSE
Q8BFY9
1
0
−1
−2
−3
0
50
100
Distance in Percent

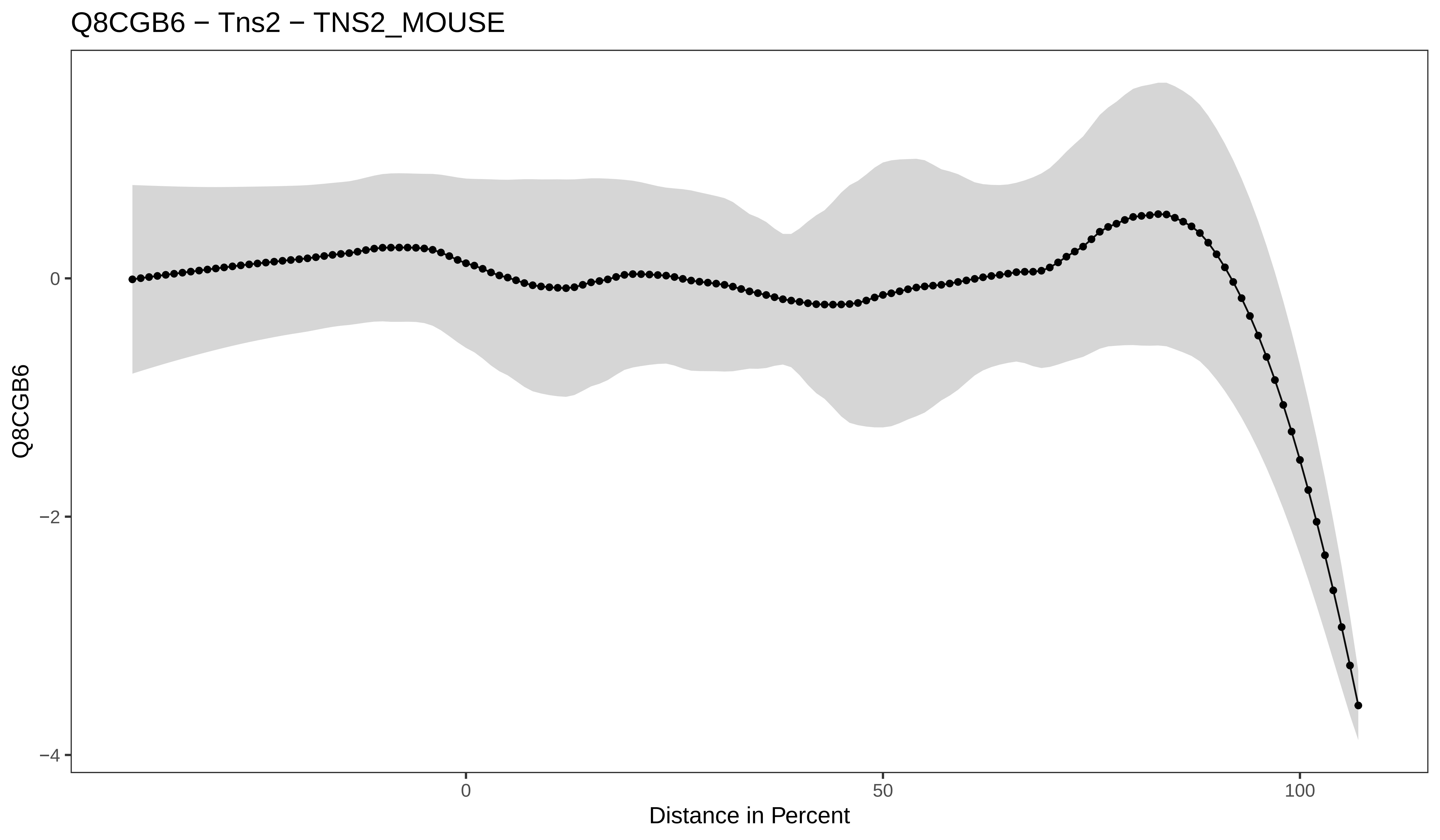

Q8CGB6 − Tns2 − TNS2_MOUSE
Q8CGB6
0
−2
−4
0
50
100
Distance in Percent

O88746 − Tom1 − TOM1_MOUSE

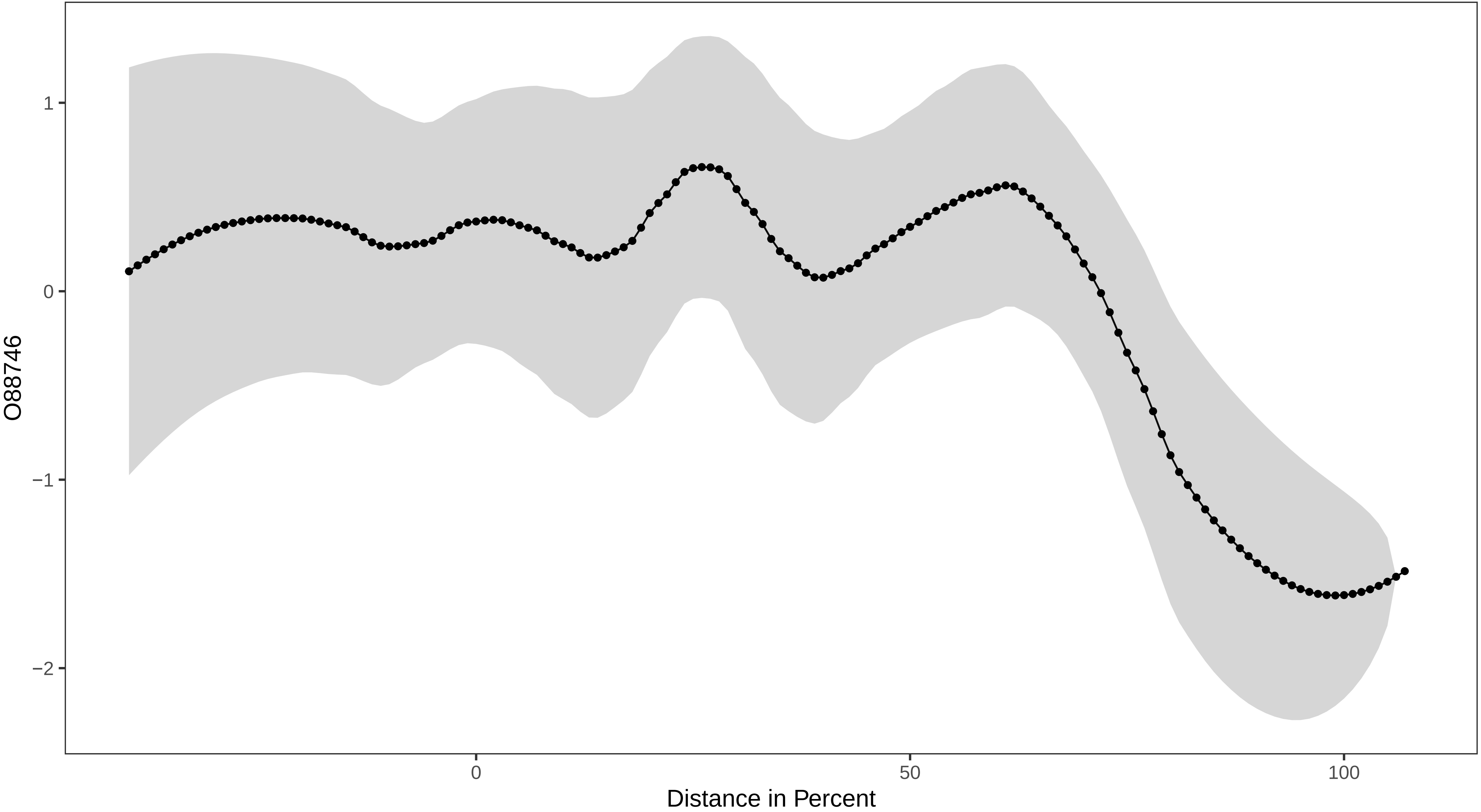

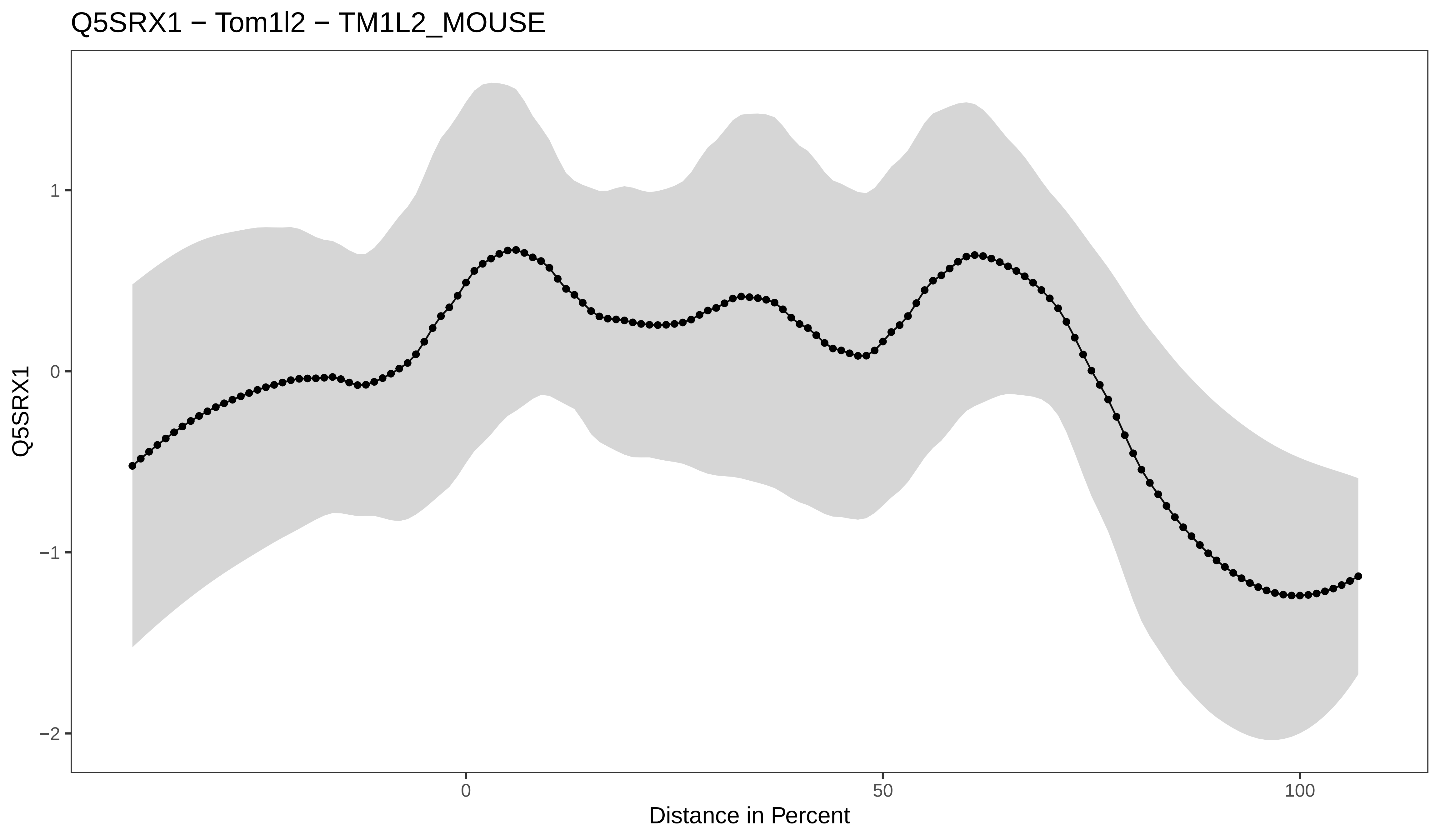

Q5SRX1 − Tom1l2 − TM1L2_MOUSE
Q5SRX1
1
0
−1
−2
0
50
100
Distance in Percent

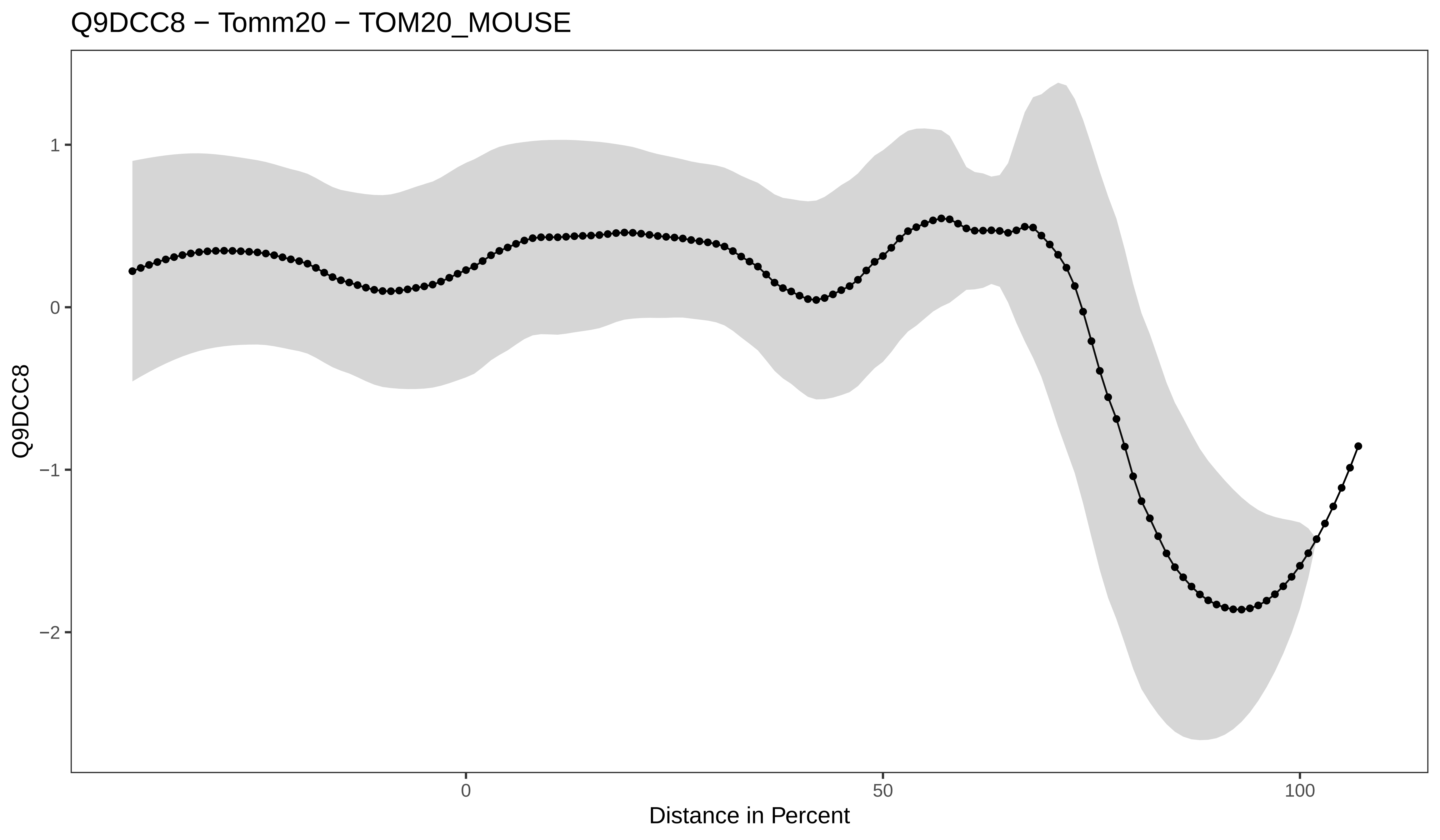

Q9DCC8 − Tomm20 − TOM20_MOUSE
Q9DCC8
1
0
−1
−2
0
50
100
Distance in Percent

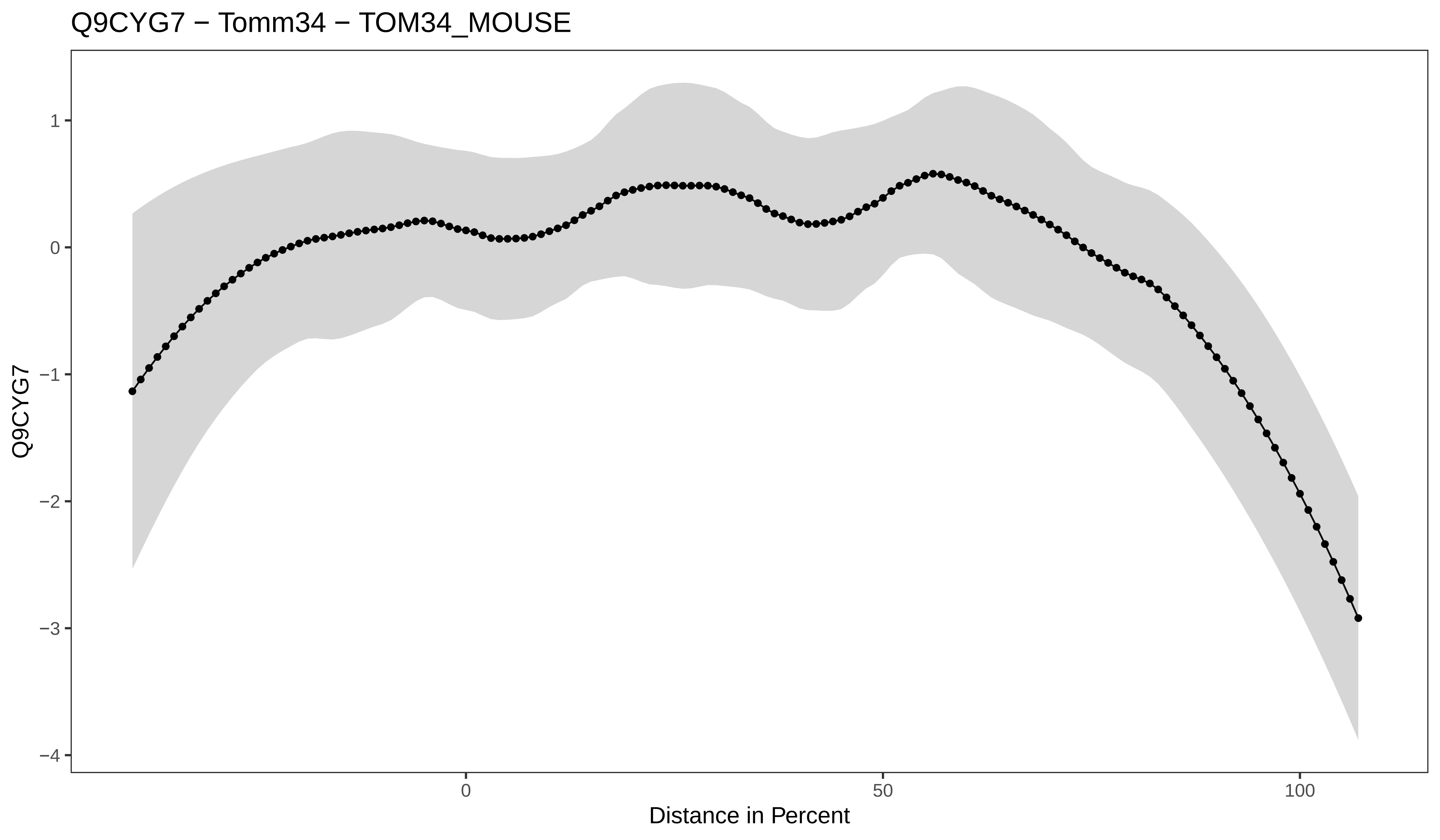
Q9CYG7 − Tomm34 − TOM34_MOUSE
Q9CYG7
Distance in Percent
1
0
−1
−2
−3
−4
0
50
100

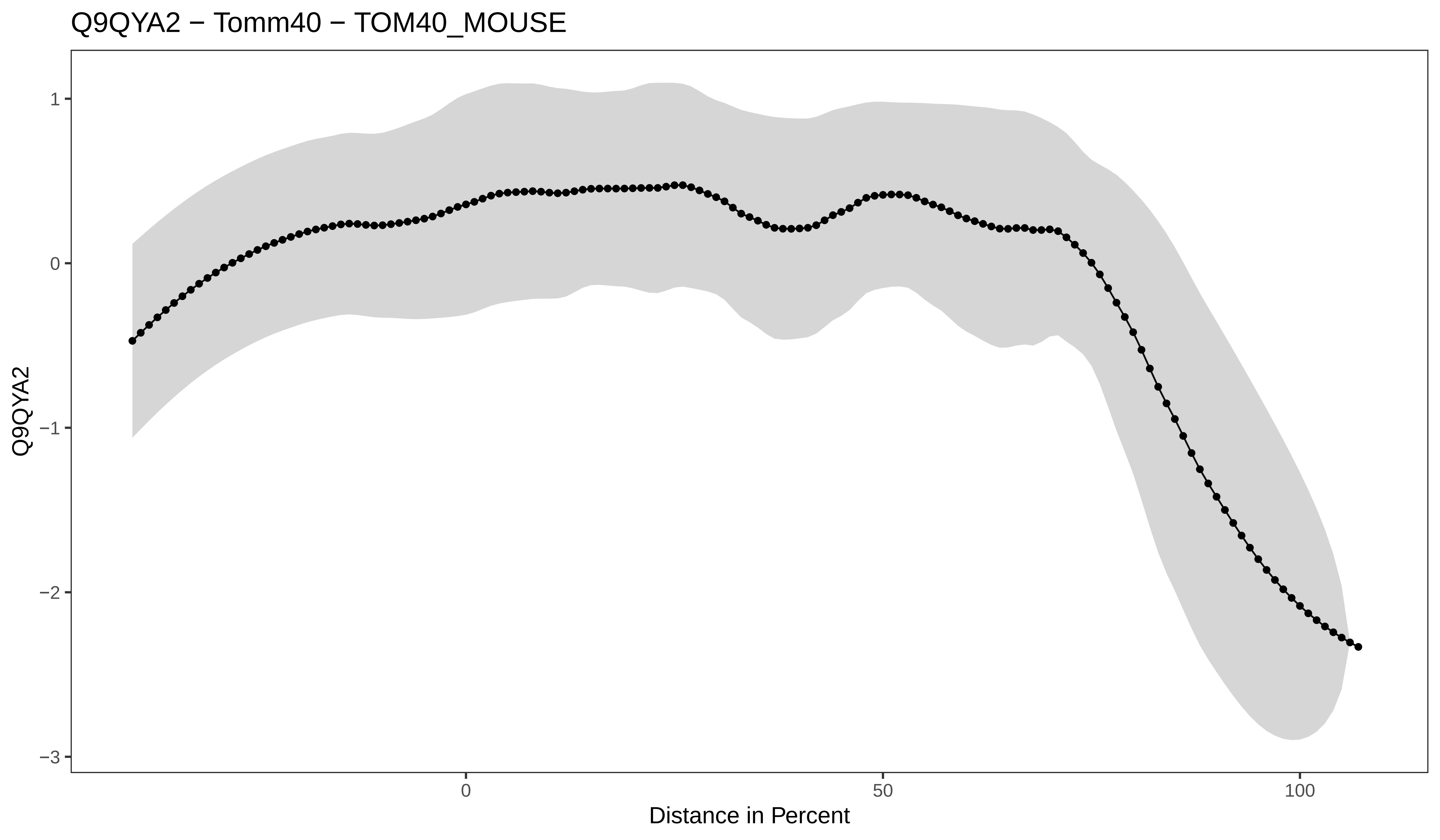

Q9QYA2 − Tomm40 − TOM40_MOUSE
1
0
−1
−2
−3
Q9QYA2
0
50
100
Distance in Percent

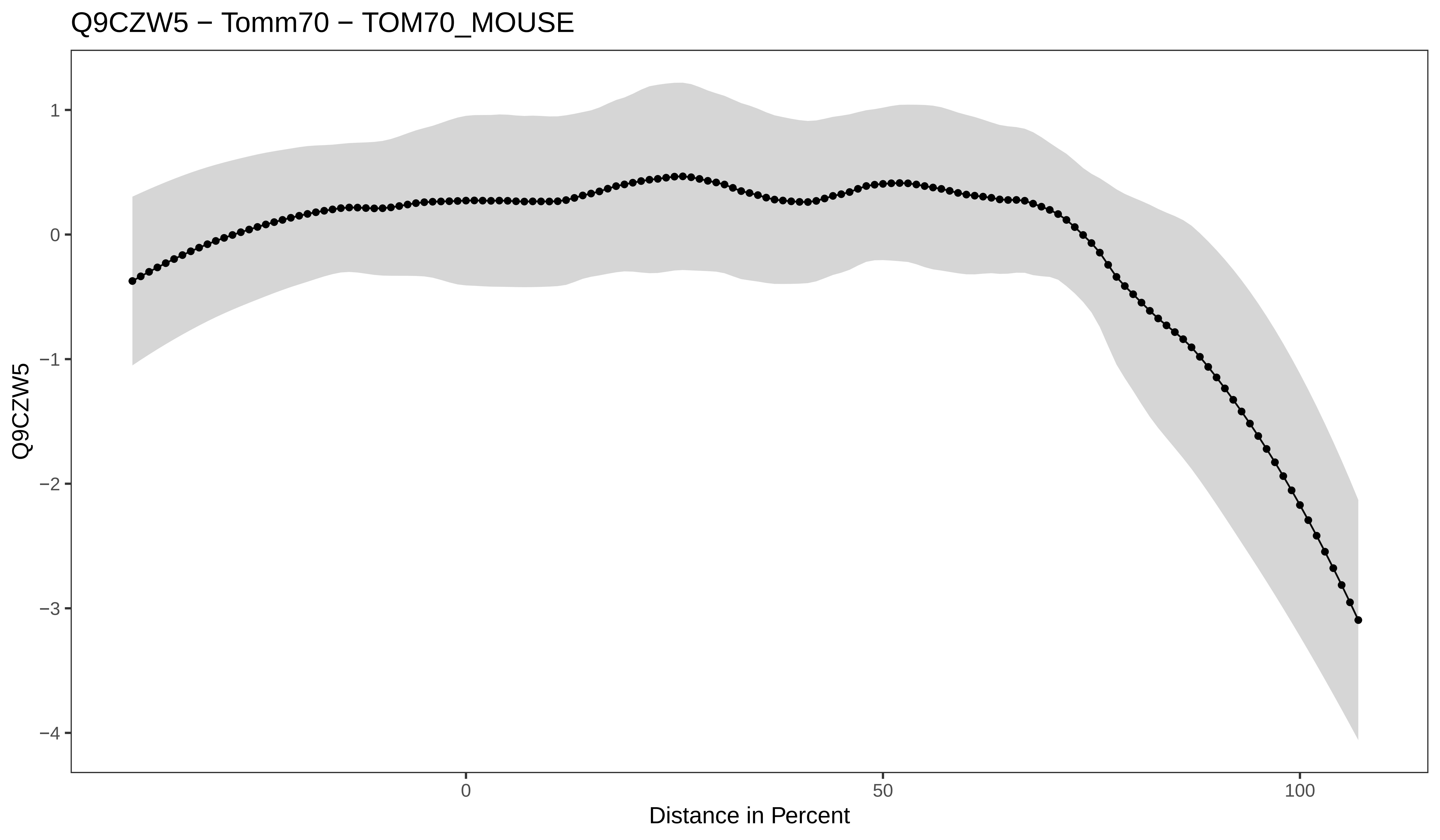
Q9CZW5 − Tomm70 − TOM70_MOUSE
Q9CZW5
Distance in Percent
1
0
−1
−2
−3
−4
0
50
100

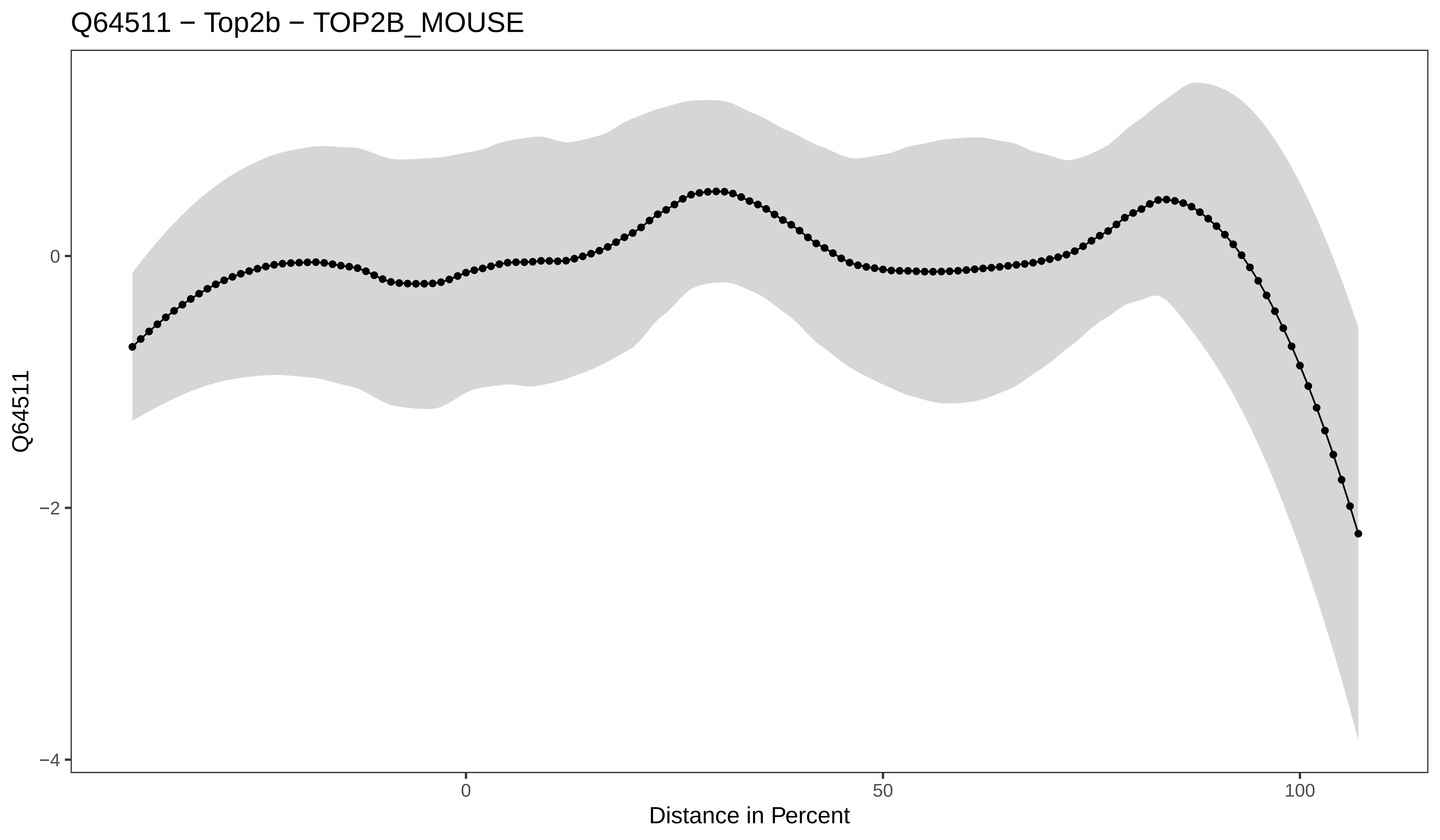
Q64511 − Top2b − TOP2B_MOUSE
Q64511
0
−2
−4
0
50
100
Distance in Percent

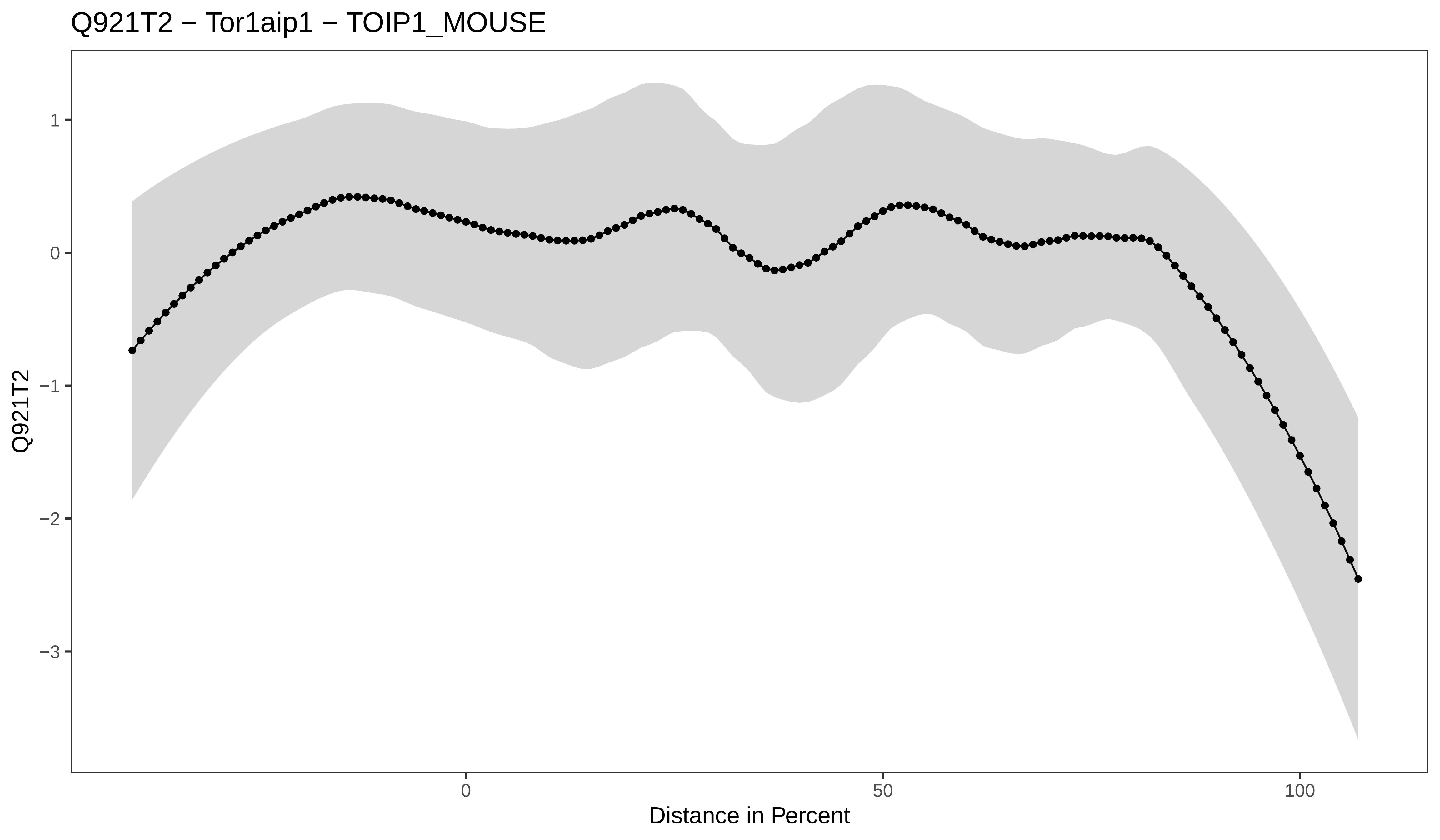

Q921T2 − Tor1aip1 − TOIP1_MOUSE
1
0
−1
−2
−3
Q921T2
0
50
100
Distance in Percent

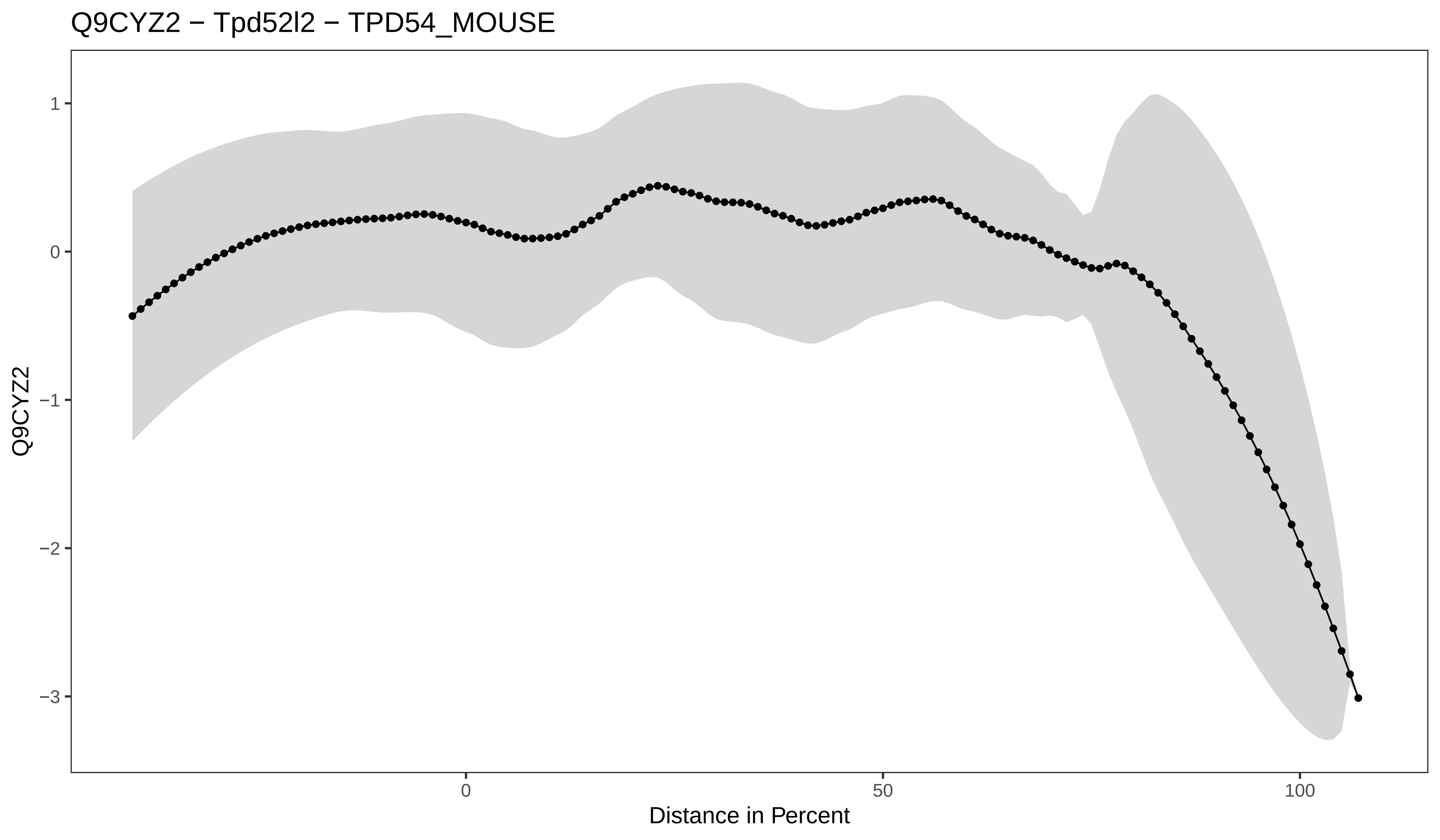
Q9CYZ2 − Tpd52l2 − TPD54_MOUSE
Q9CYZ2
Distance in Percent
1
0
−1
−2
−3
0
50
100

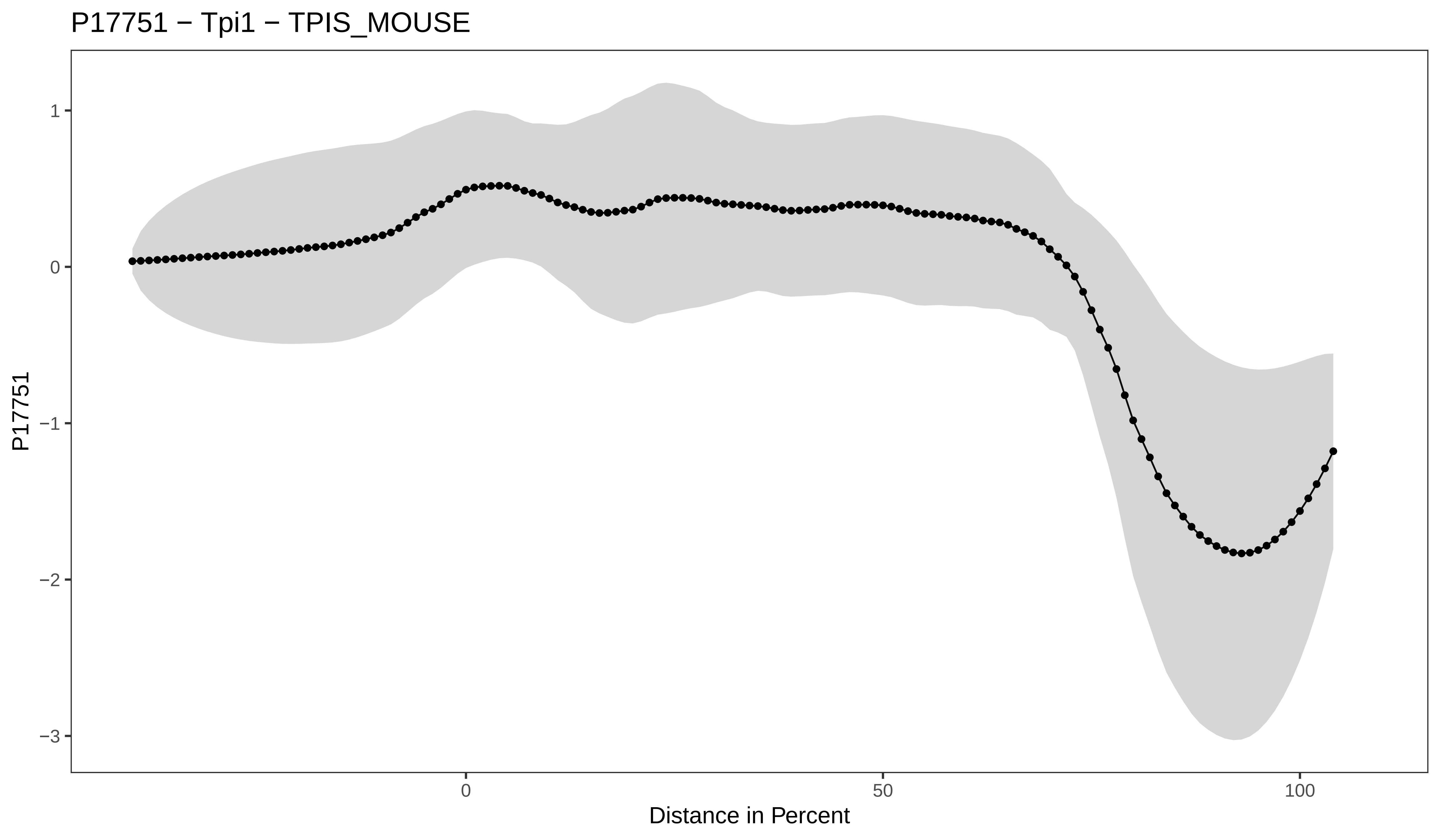

P17751 − Tpi1 − TPIS_MOUSE
P17751
Distance in Percent
1
0
−1
−2
−3
0
50
100

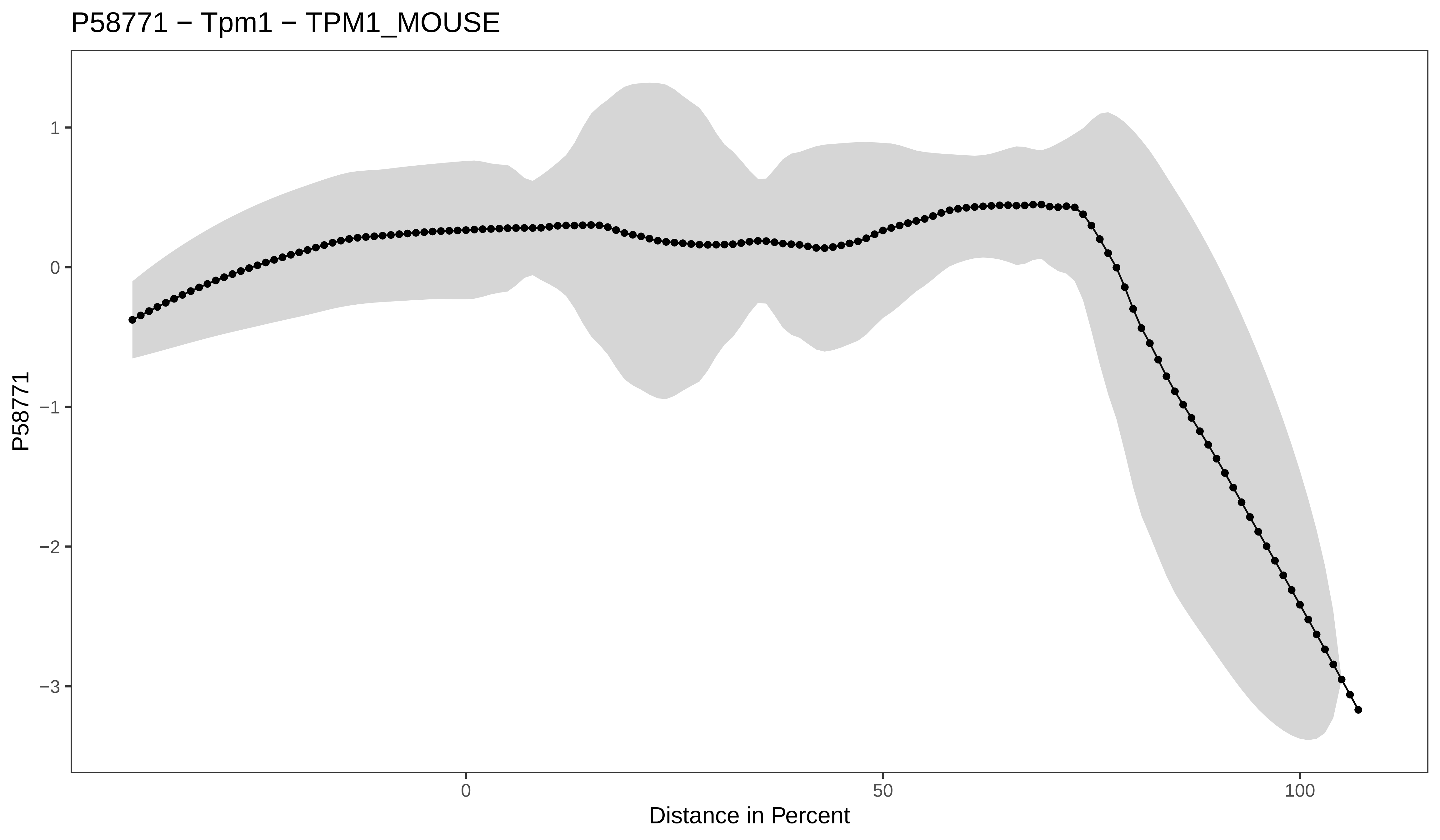

P58771 − Tpm1 − TPM1_MOUSE
1
0
−1
−2
−3
P58771
0
50
100
Distance in Percent

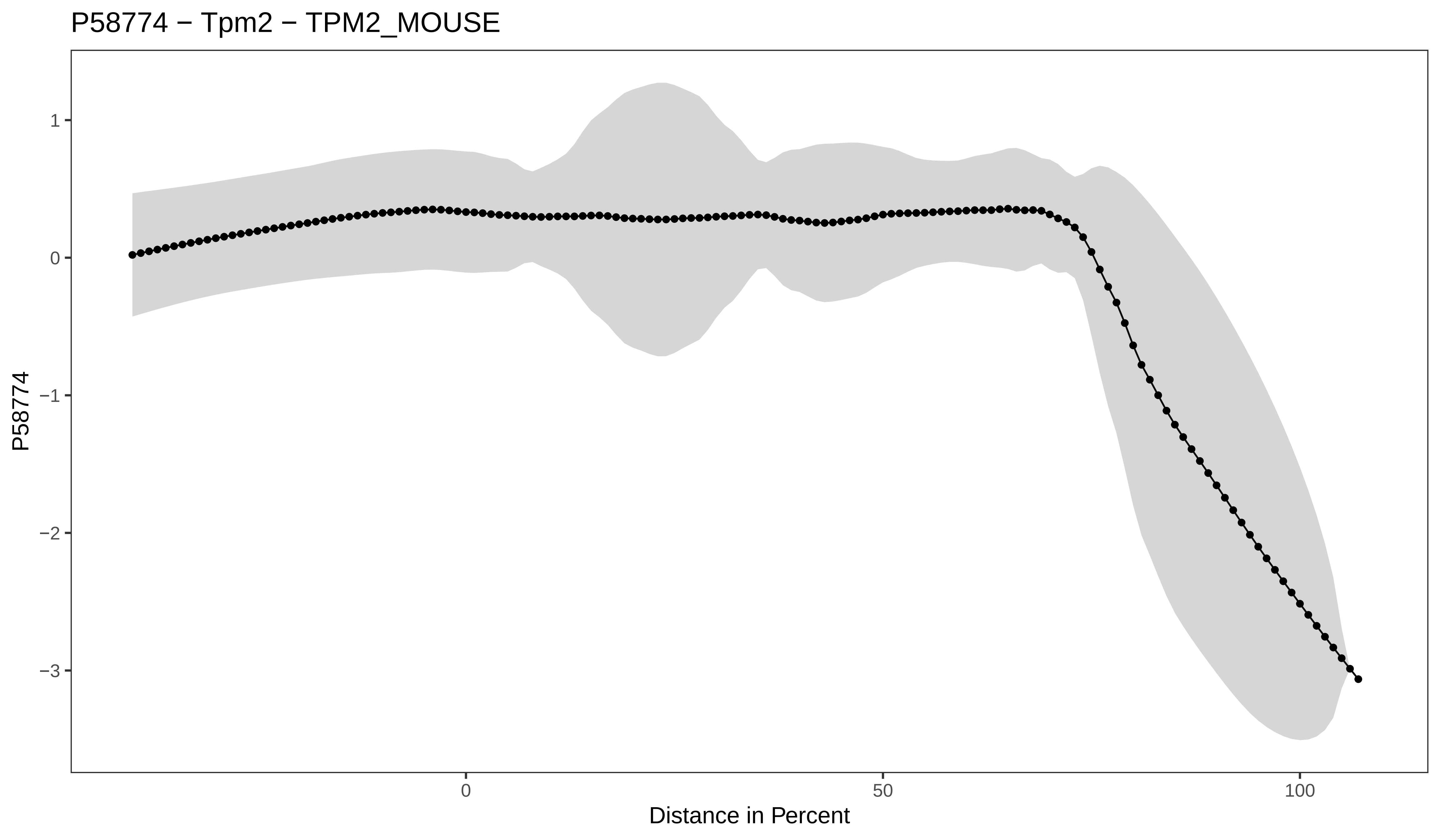

P58774 − Tpm2 − TPM2_MOUSE
1
0
−1
−2
−3
P58774
0
50
100
Distance in Percent

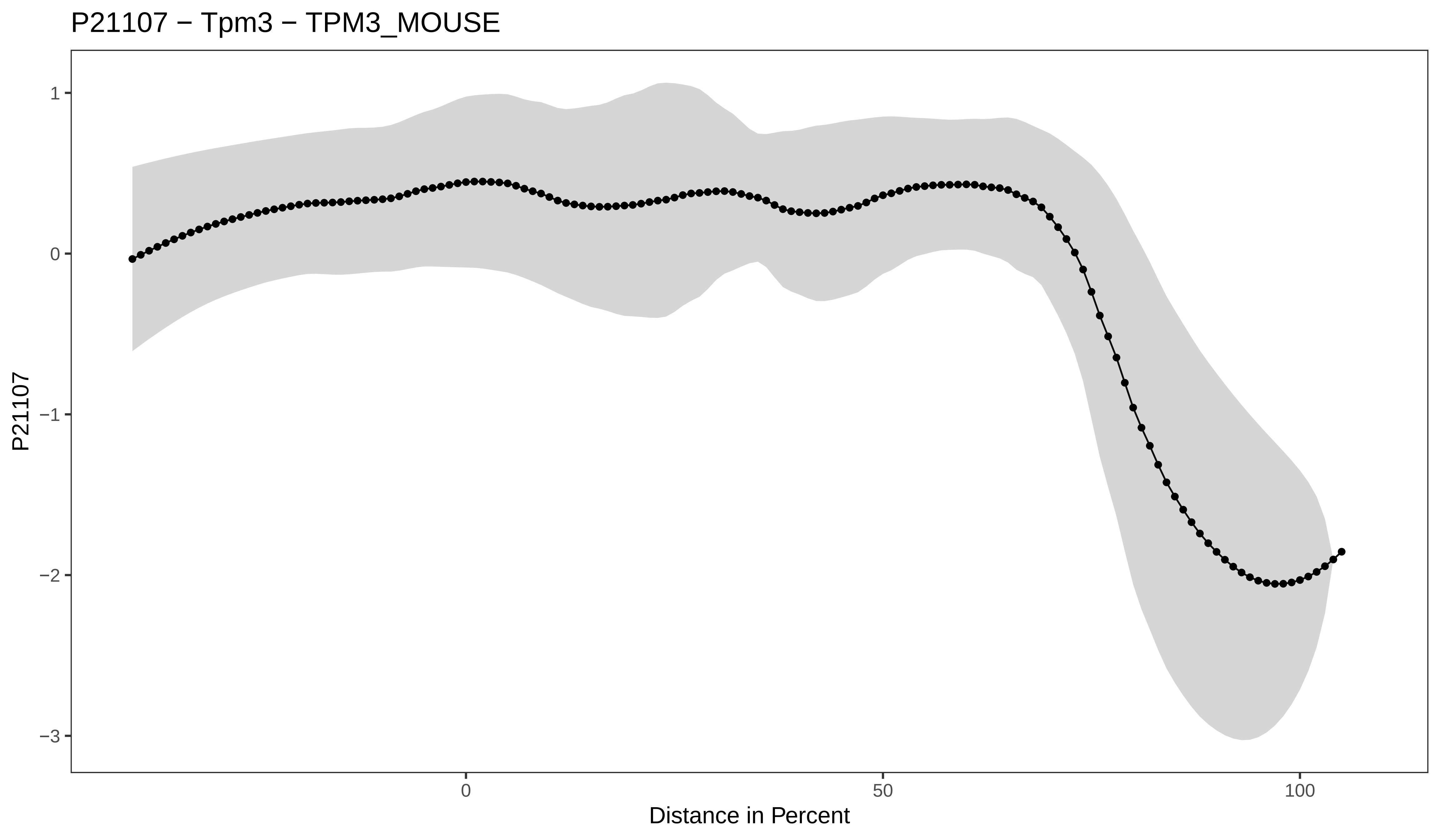

P21107 − Tpm3 − TPM3_MOUSE
1
0
−1
−2
−3
P21107
0
50
100
Distance in Percent

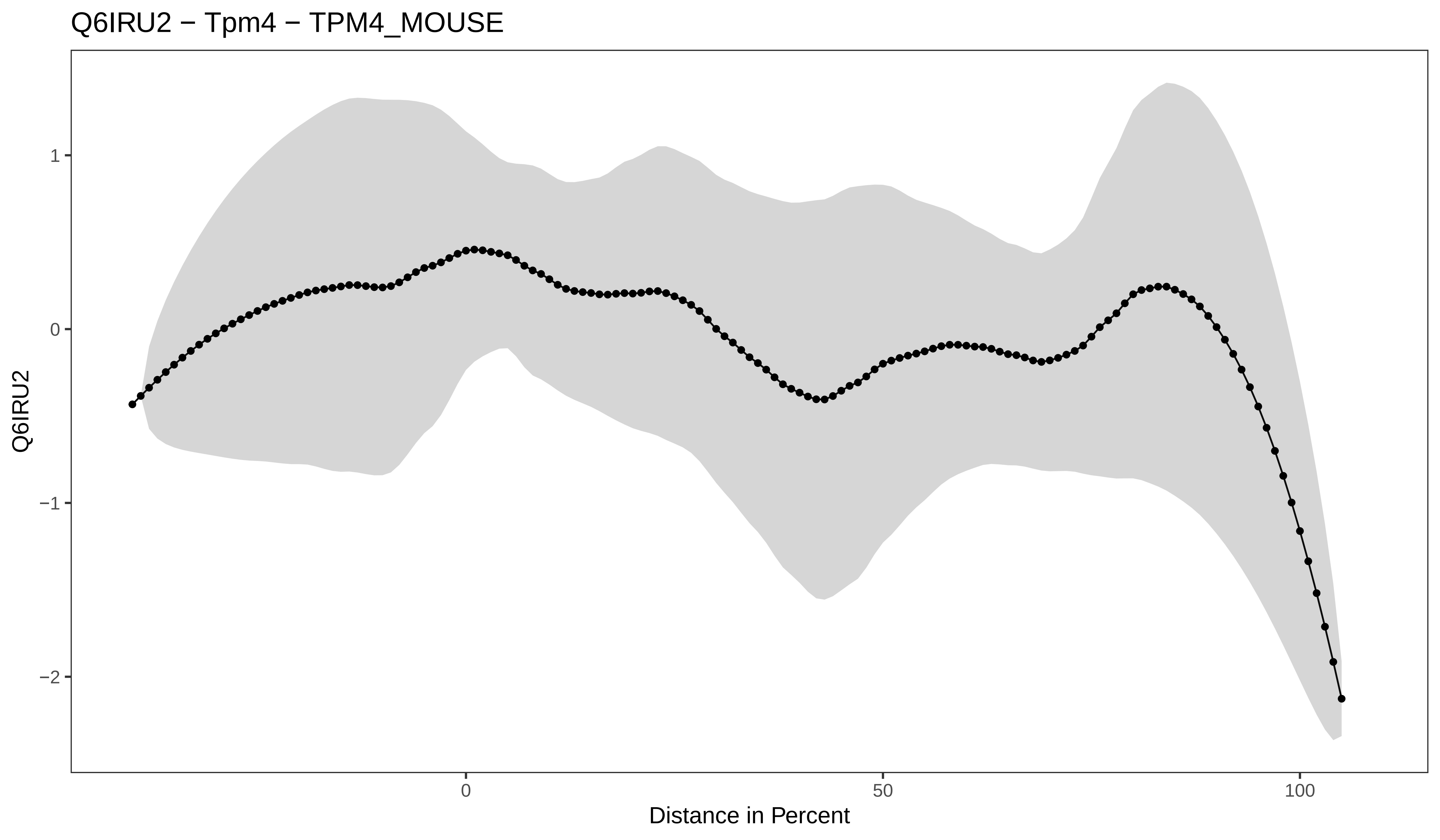

Q6IRU2 − Tpm4 − TPM4_MOUSE
Q6IRU2
1
0
−1
−2
0
50
100
Distance in Percent

Q64514 − Tpp2 − TPP2_MOUSE

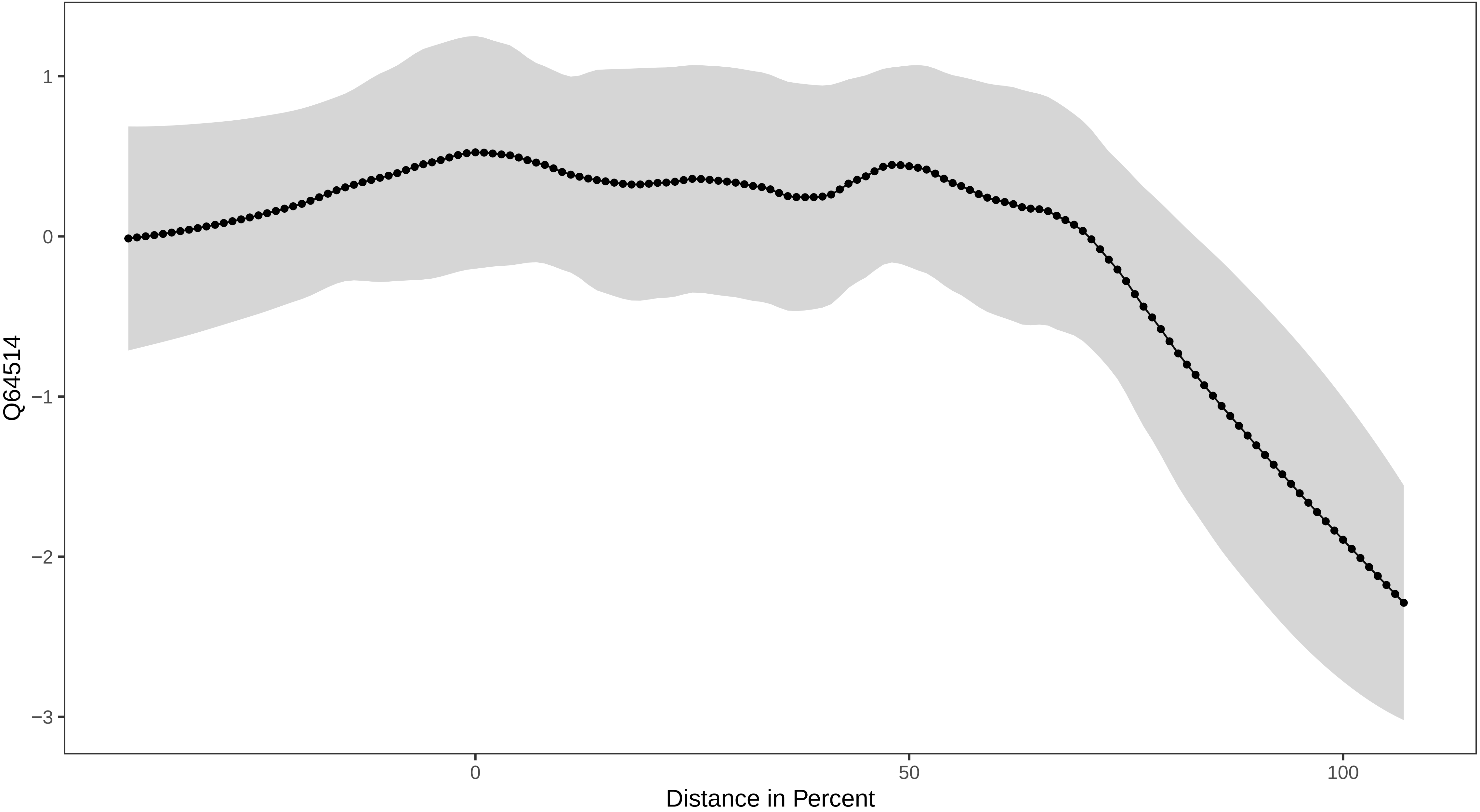

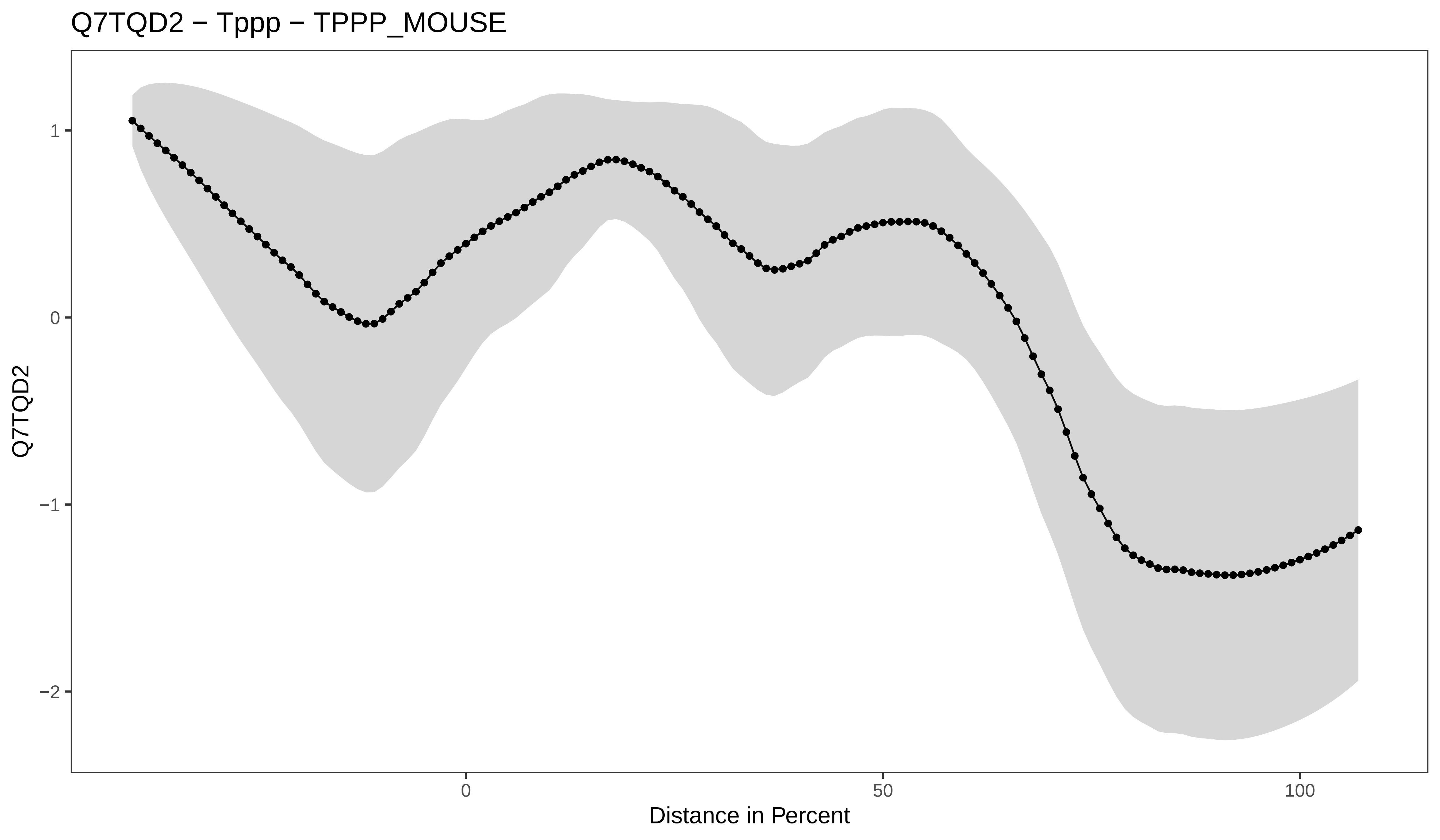
Q7TQD2 − Tppp − TPPP_MOUSE
Q7TQD2
1
0
−1
−2
0
50
100
Distance in Percent

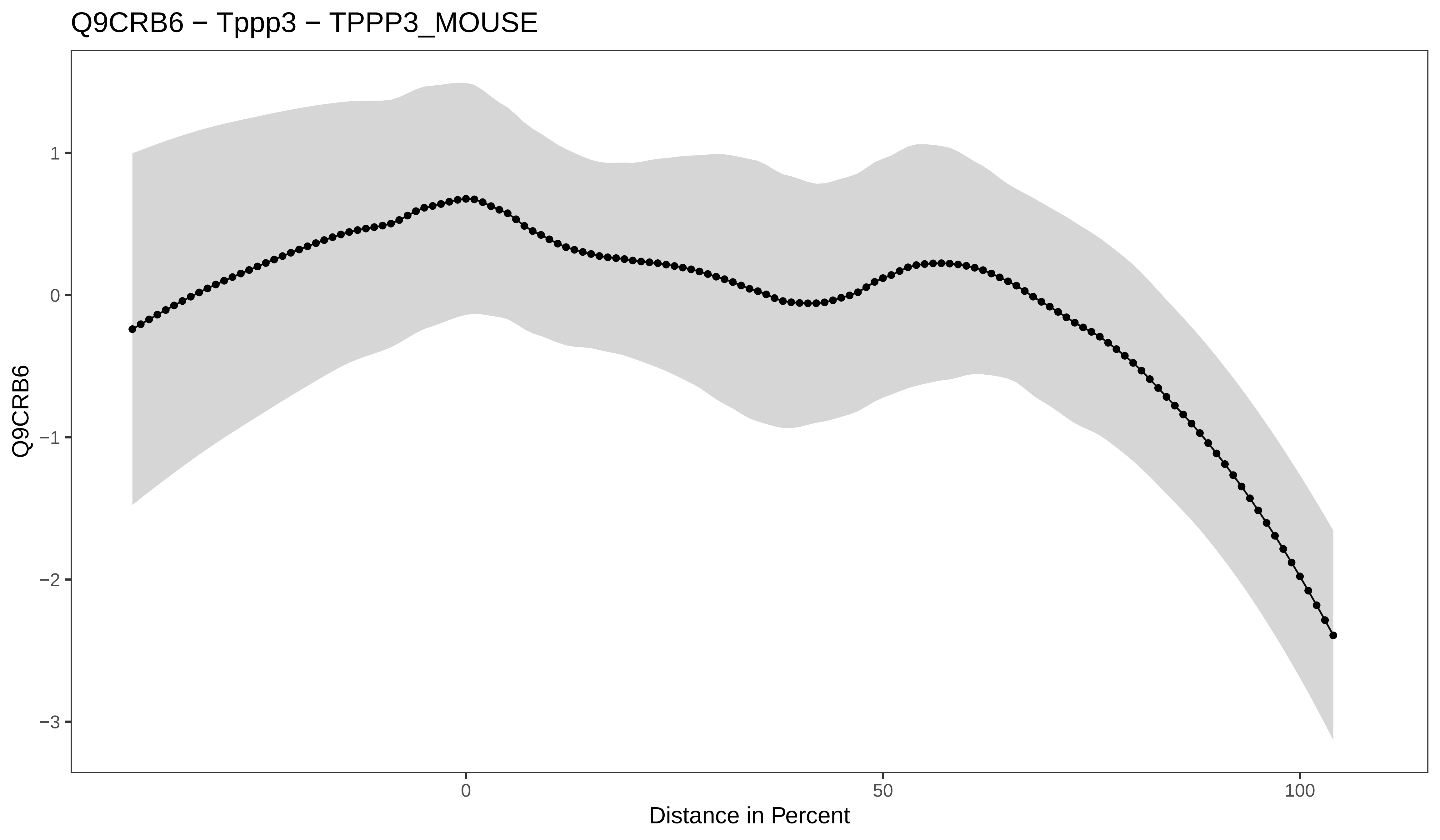
Q9CRB6 − Tppp3 − TPPP3_MOUSE
Q9CRB6
1
0
−1
−2
−3
0
50
100
Distance in Percent

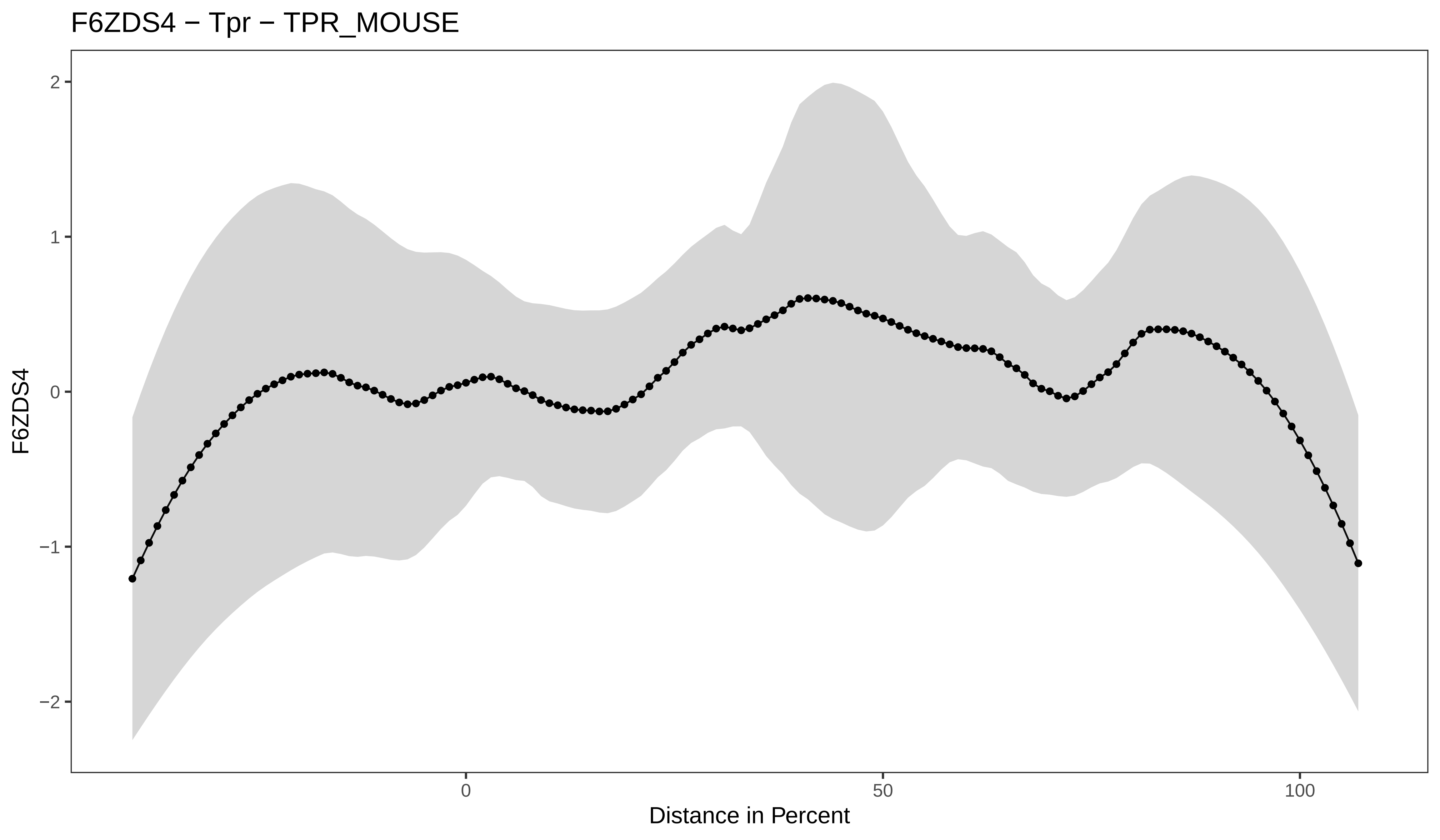
F6ZDS4 − Tpr − TPR_MOUSE
F6ZDS4
Distance in Percent
2
1
0
−1
−2
0
50
100

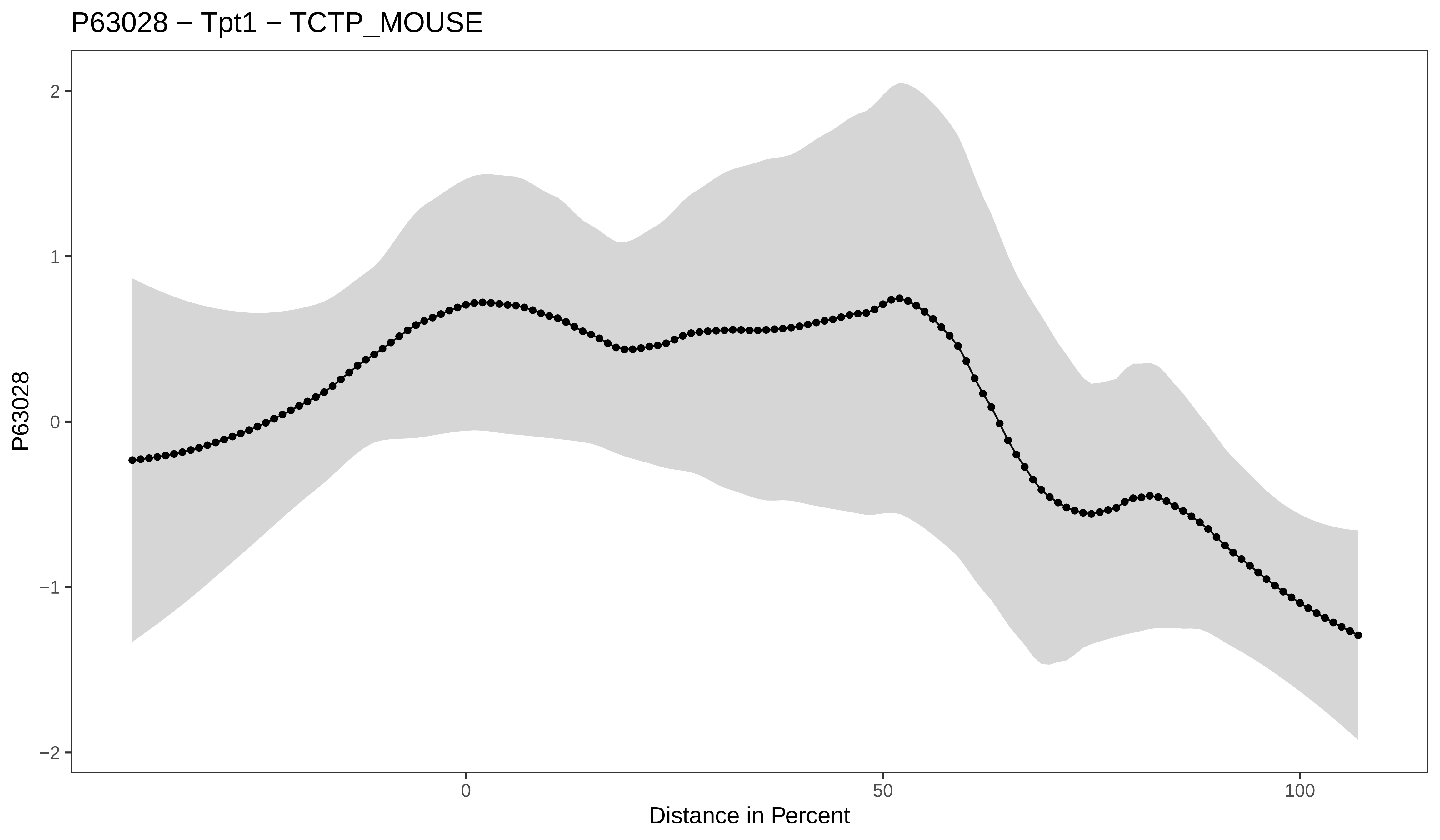
P63028 − Tpt1 − TCTP_MOUSE
P63028
2
1
0
−1
−2
0
50
100
Distance in Percent

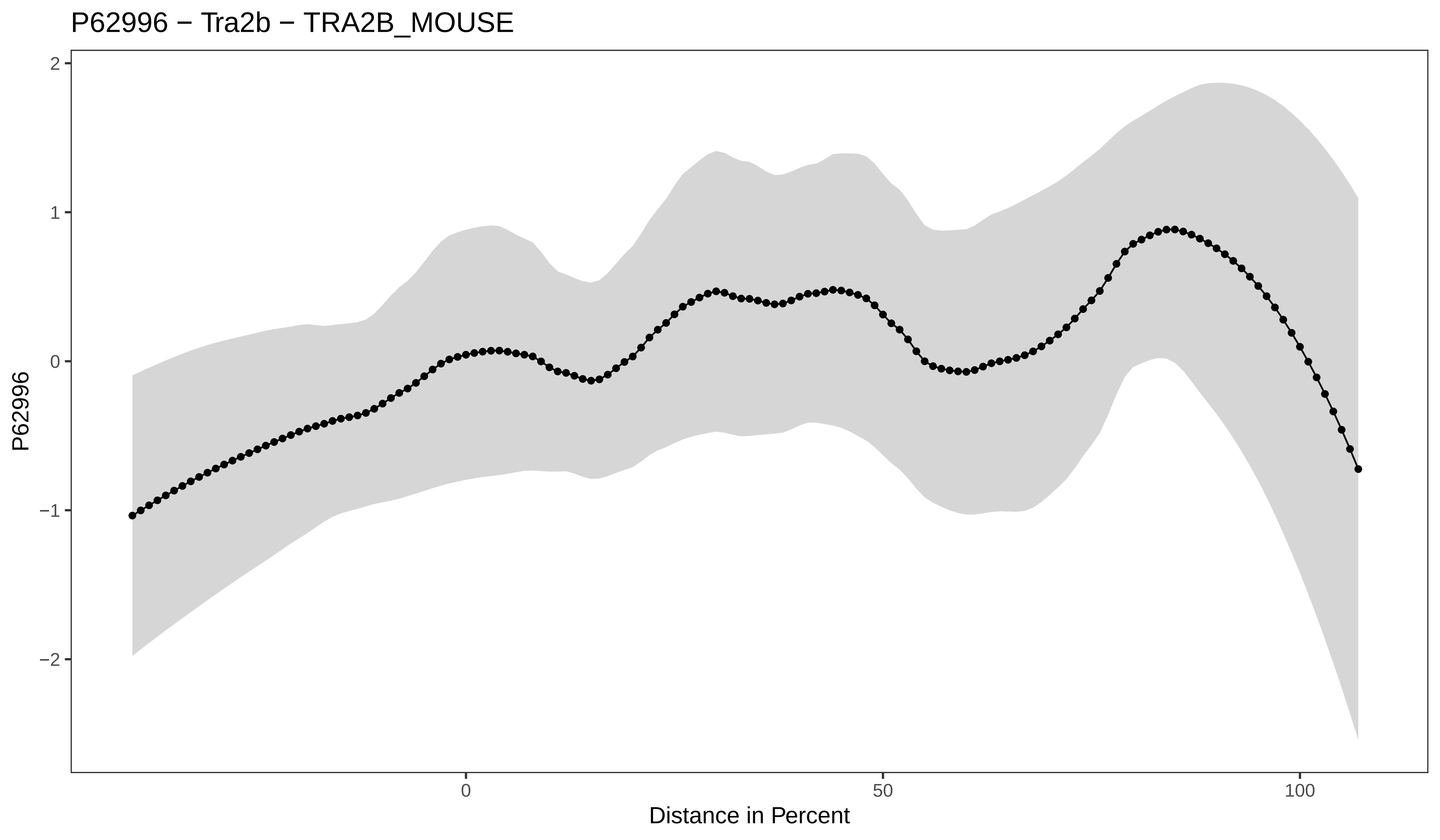

P62996 − Tra2b − TRA2B_MOUSE
P62996
Distance in Percent
2
1
0
−1
−2
0
50
100

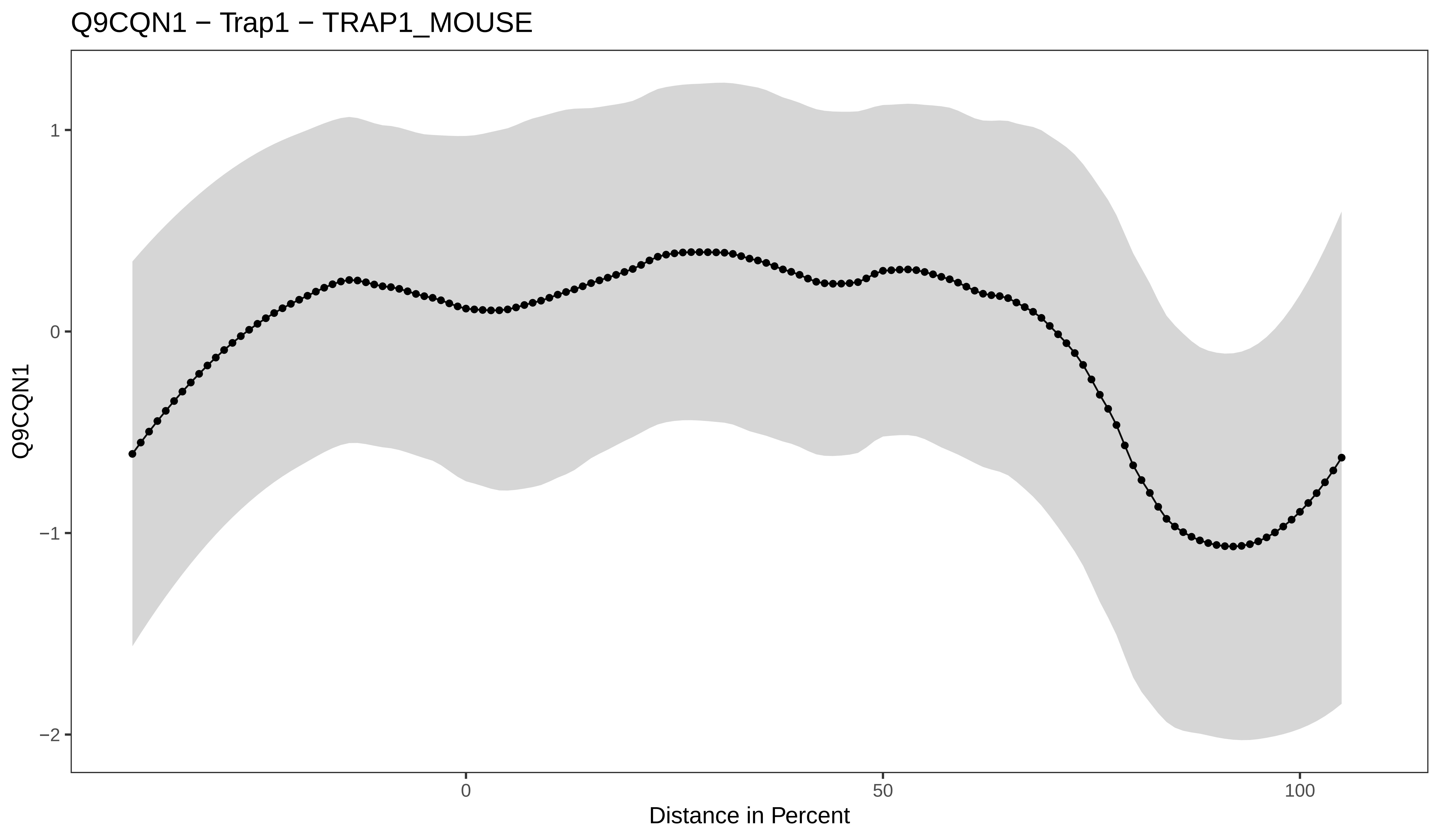
Q9CQN1 − Trap1 − TRAP1_MOUSE
1
0
−1
−2
Q9CQN1
0
50
100
Distance in Percent

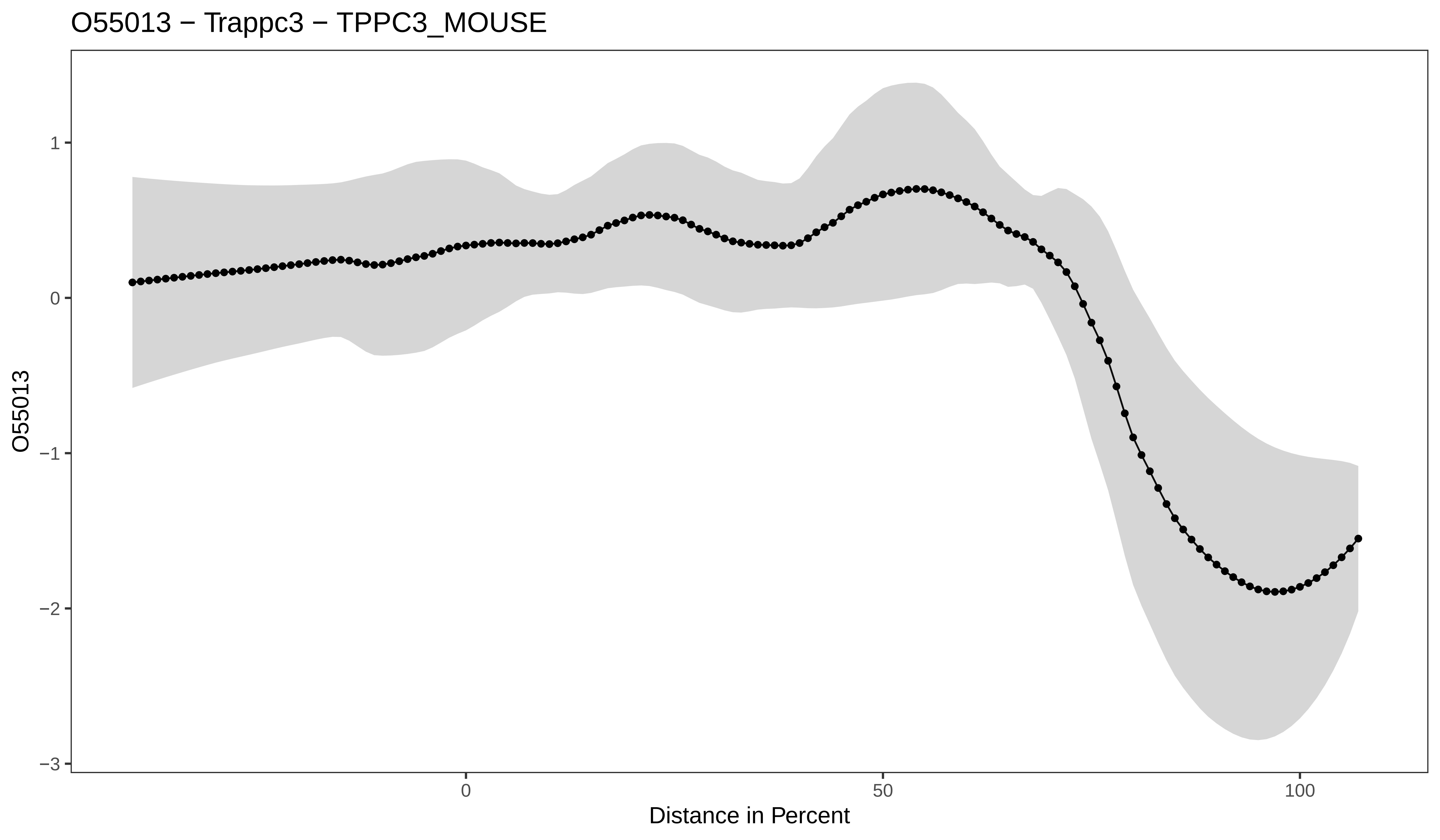

O55013 − Trappc3 − TPPC3_MOUSE
1
0
−1
−2
−3
O55013
0
50
100
Distance in Percent

Q9CQA1 − Trappc5 − TPPC5_MOUSE

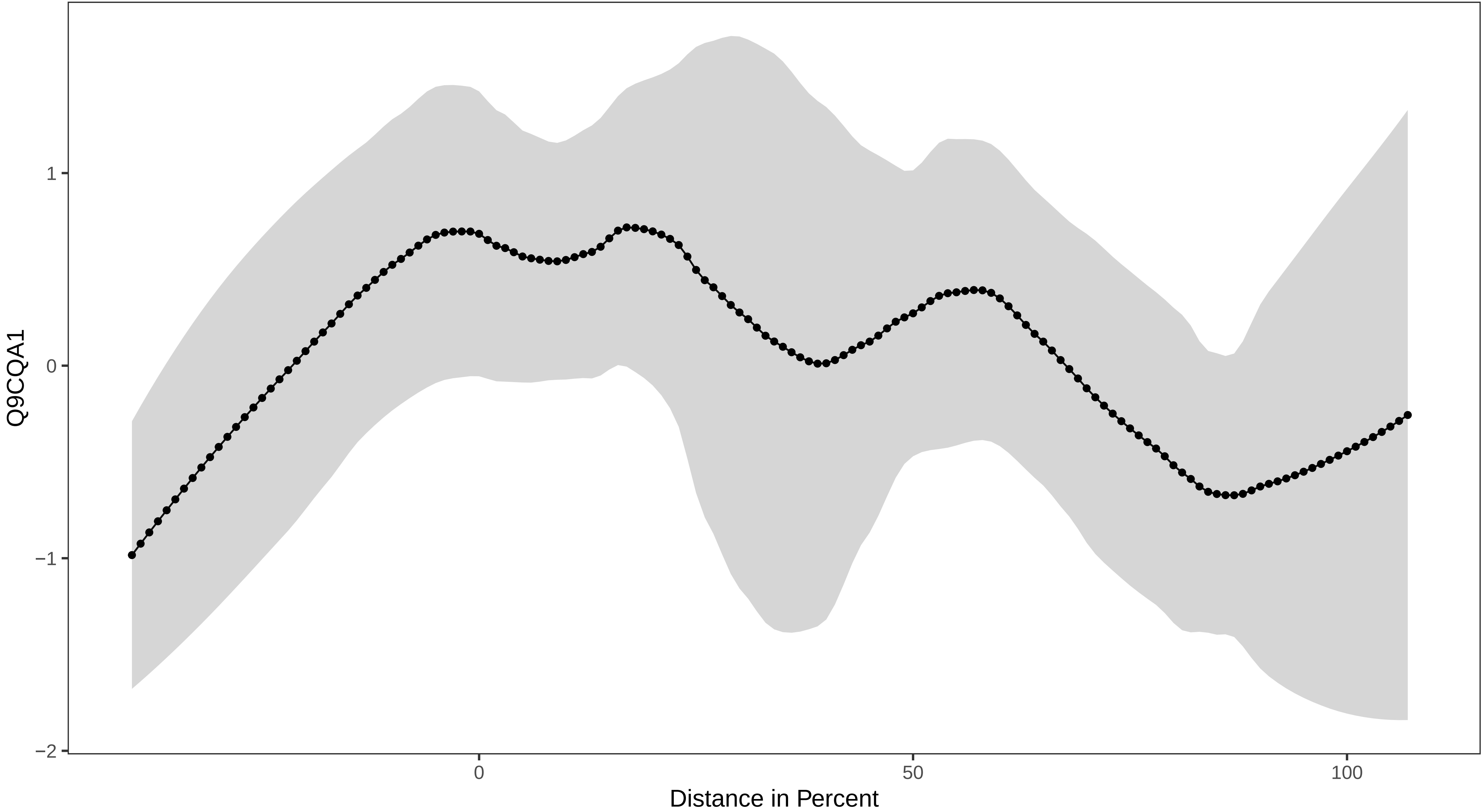

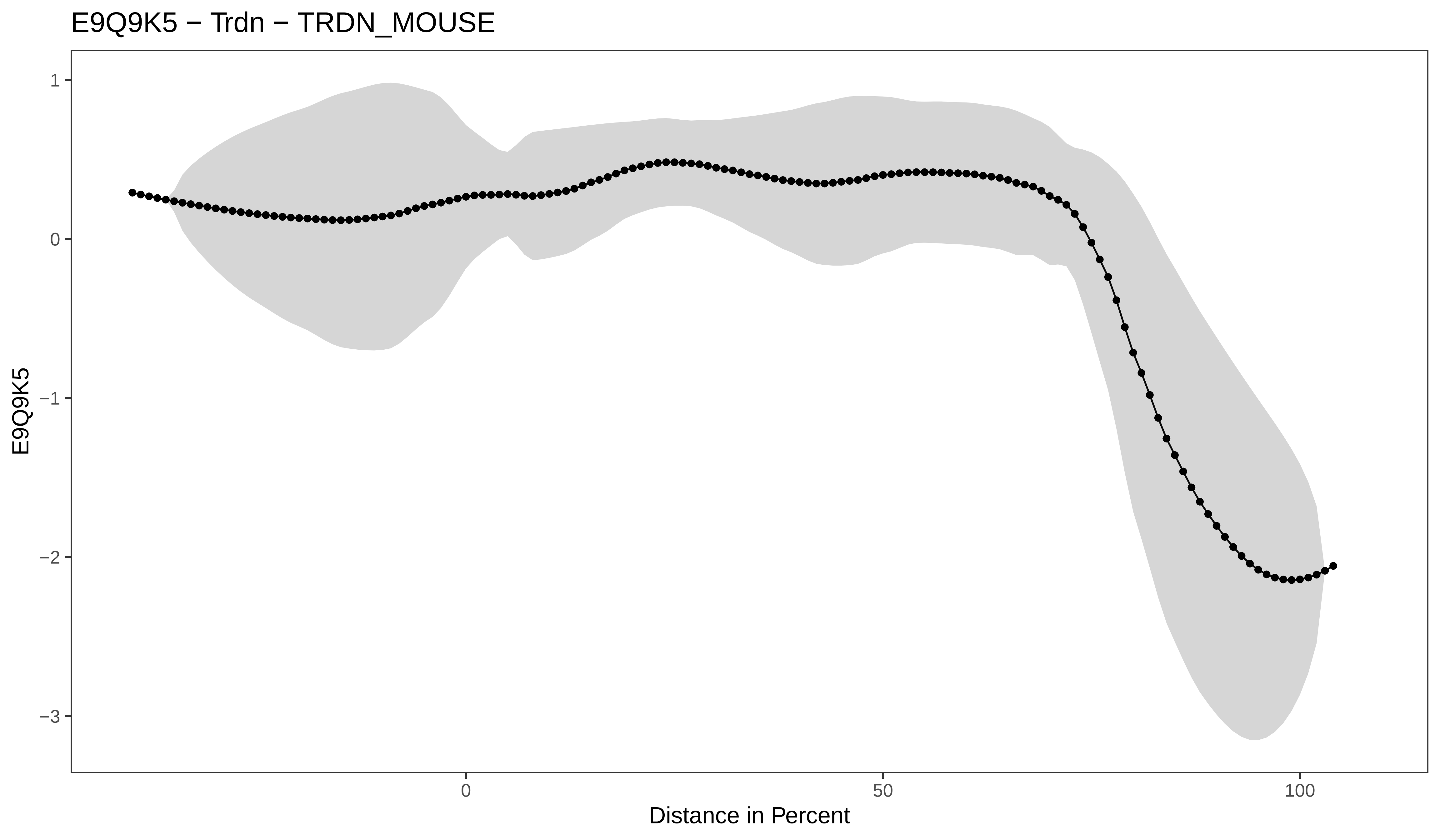

E9Q9K5 − Trdn − TRDN_MOUSE
1
0
−1
−2
−3
E9Q9K5
0
50
100
Distance in Percent

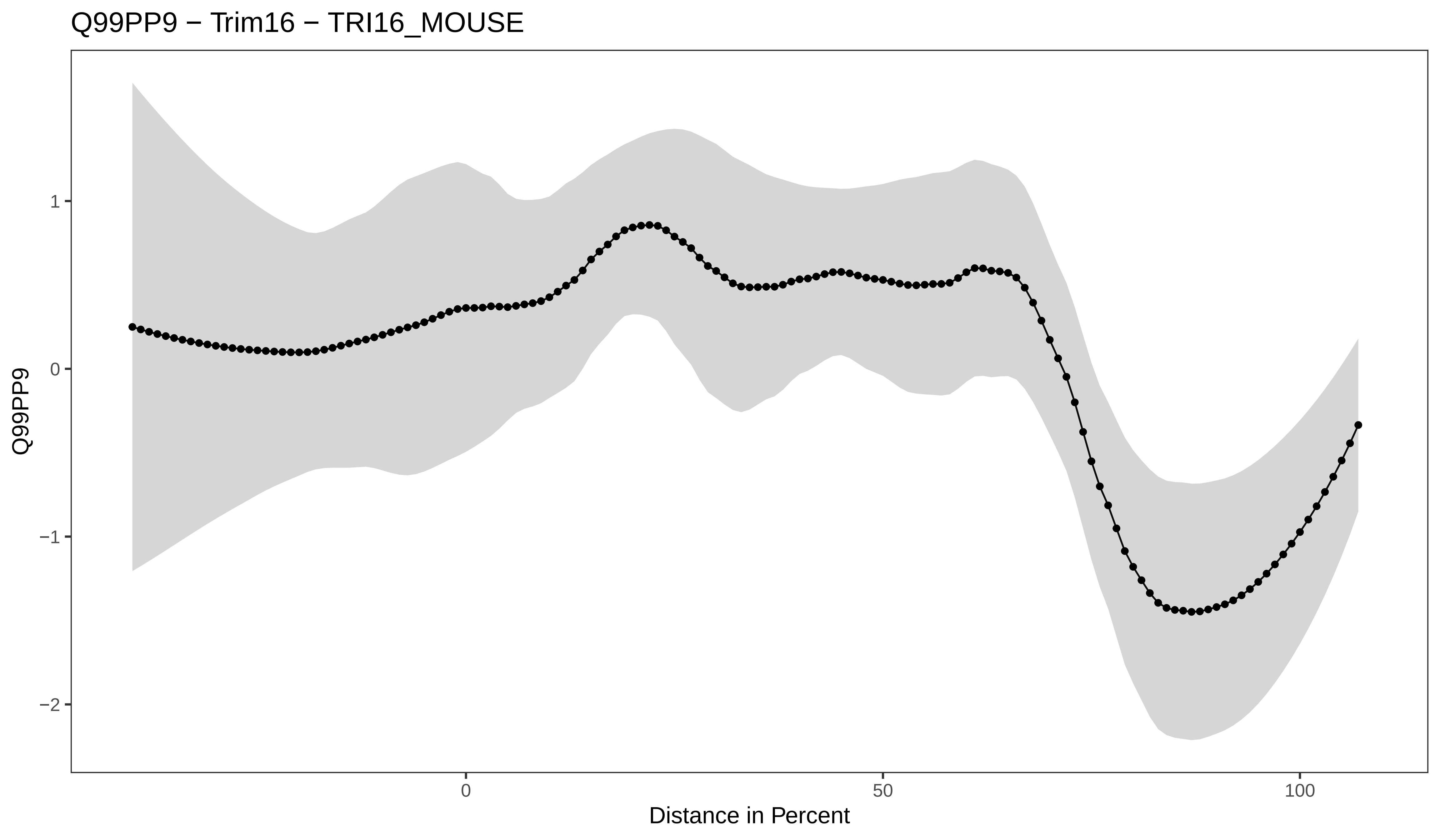
Q99PP9 − Trim16 − TRI16_MOUSE
Q99PP9
1
0
−1
−2
0
50
100
Distance in Percent

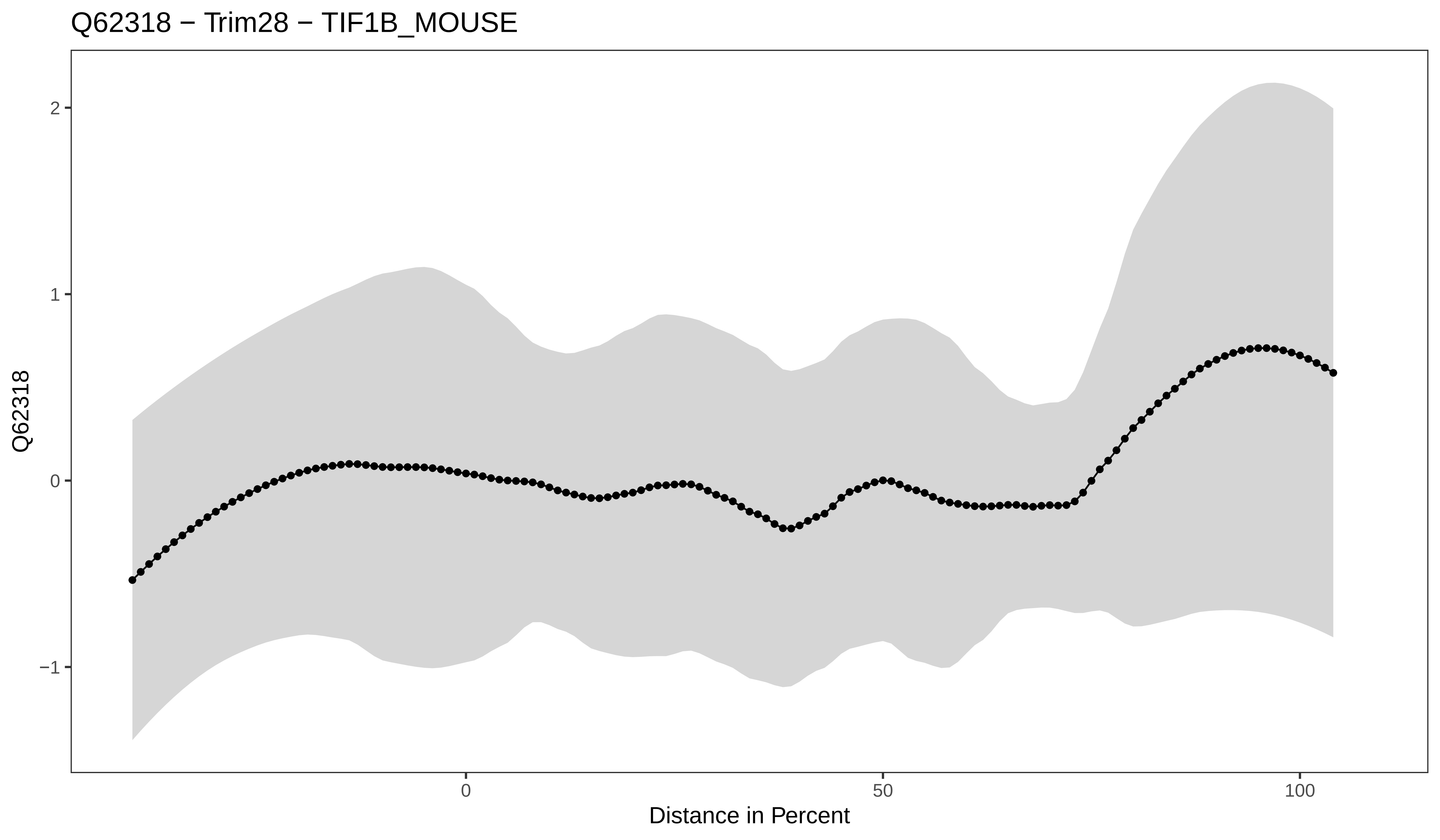
Q62318 − Trim28 − TIF1B_MOUSE
Q62318
2
1
0
−1
0
50
100
Distance in Percent

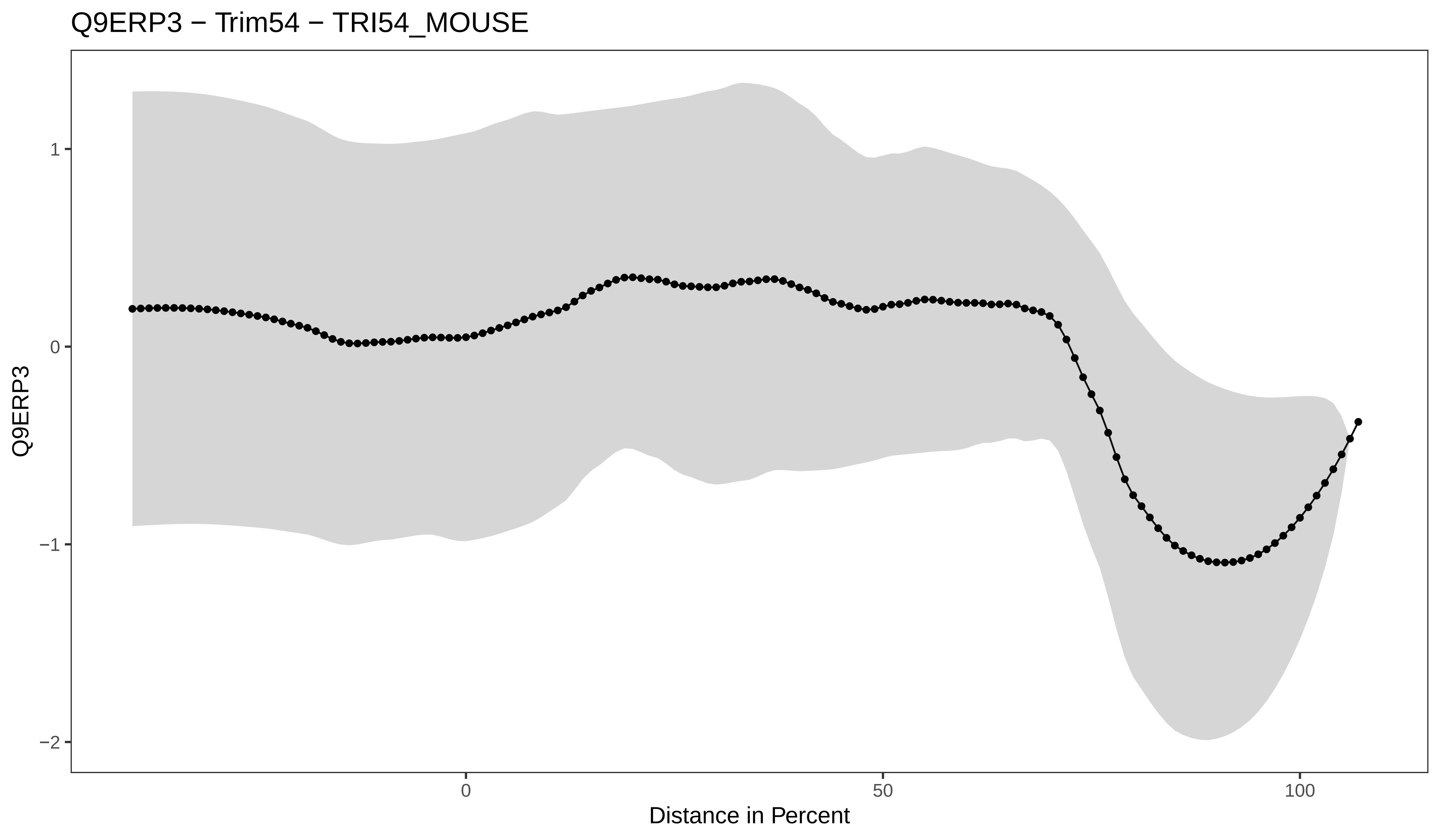
Q9ERP3 − Trim54 − TRI54_MOUSE
Q9ERP3
1
0
−1
−2
0
50
100
Distance in Percent

Q38HM4 − Trim63 − TRI63_MOUSE

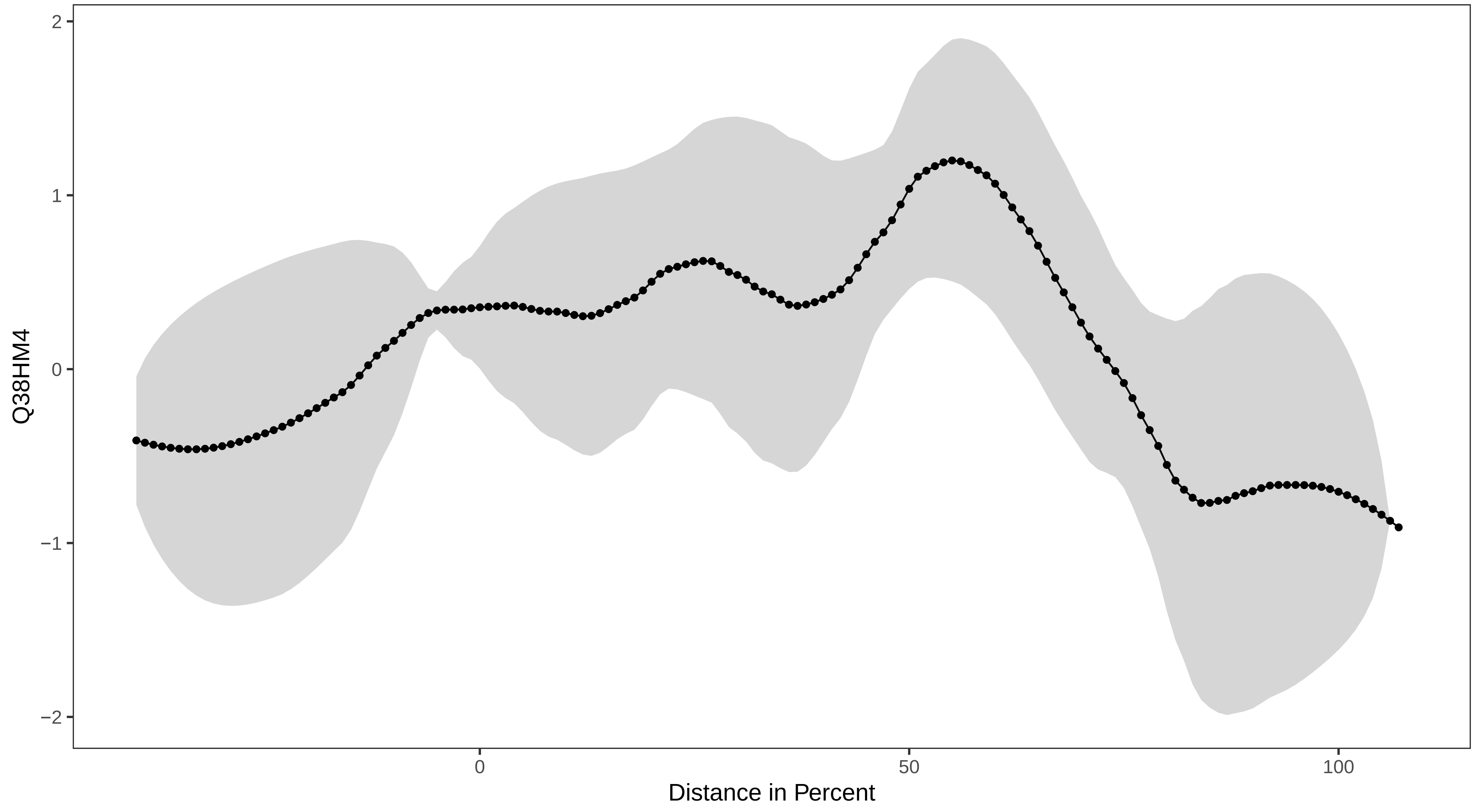

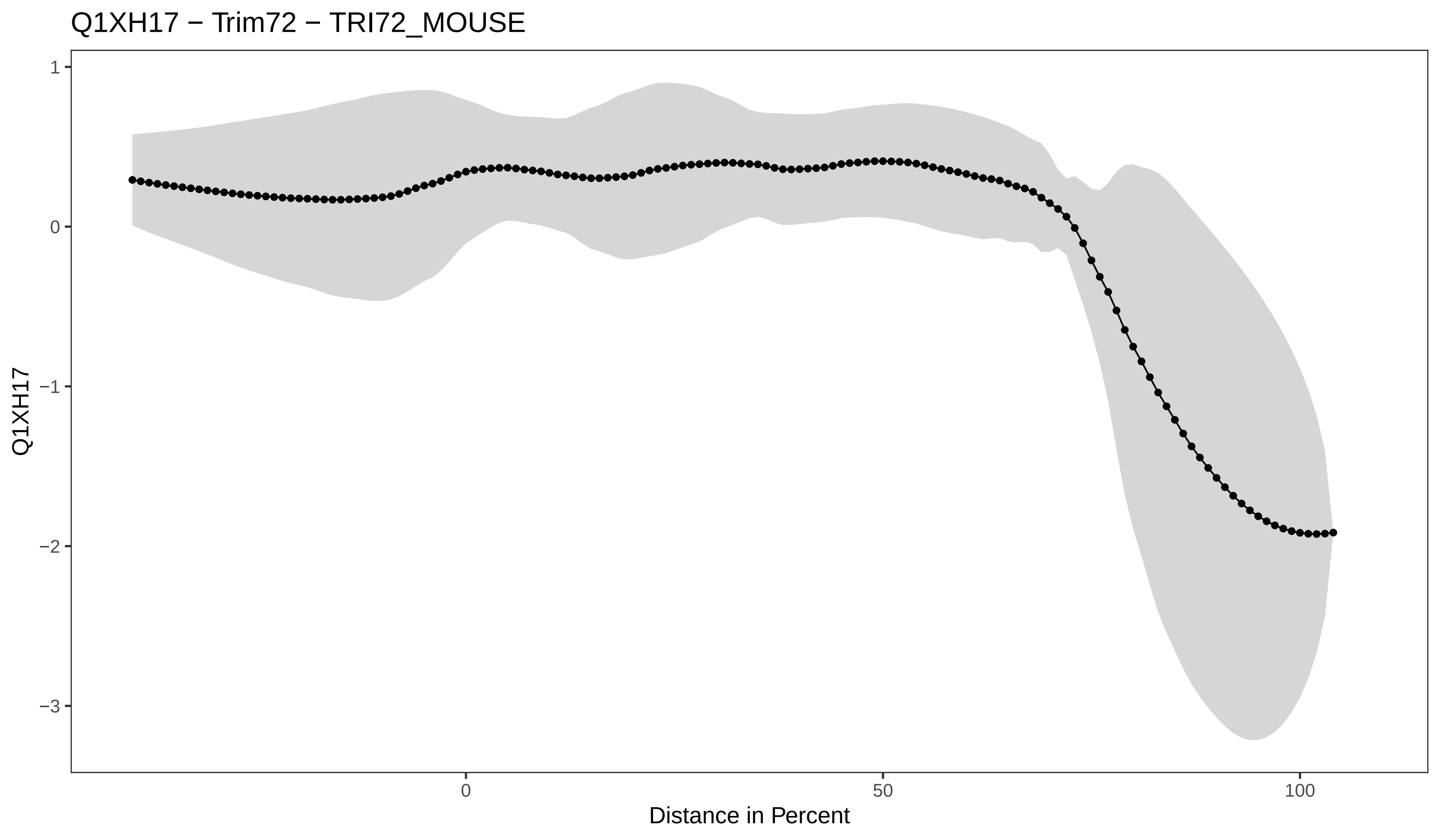

Q1XH17 − Trim72 − TRI72_MOUSE
1
0
−1
−2
−3
Q1XH17
0
50
100
Distance in Percent

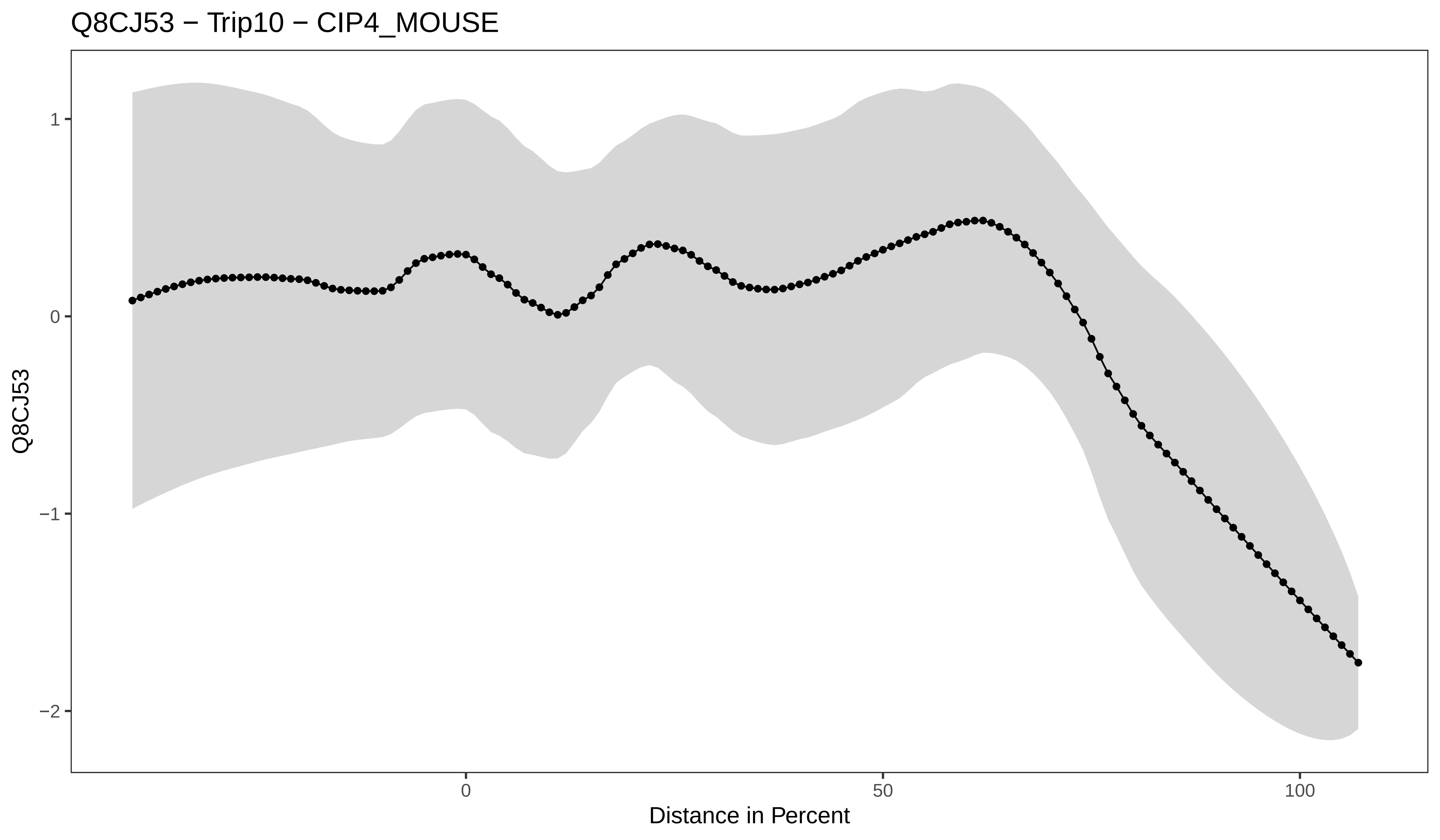

Q8CJ53 − Trip10 − CIP4_MOUSE
Q8CJ53
Distance in Percent
1
0
−1
−2
0
50
100

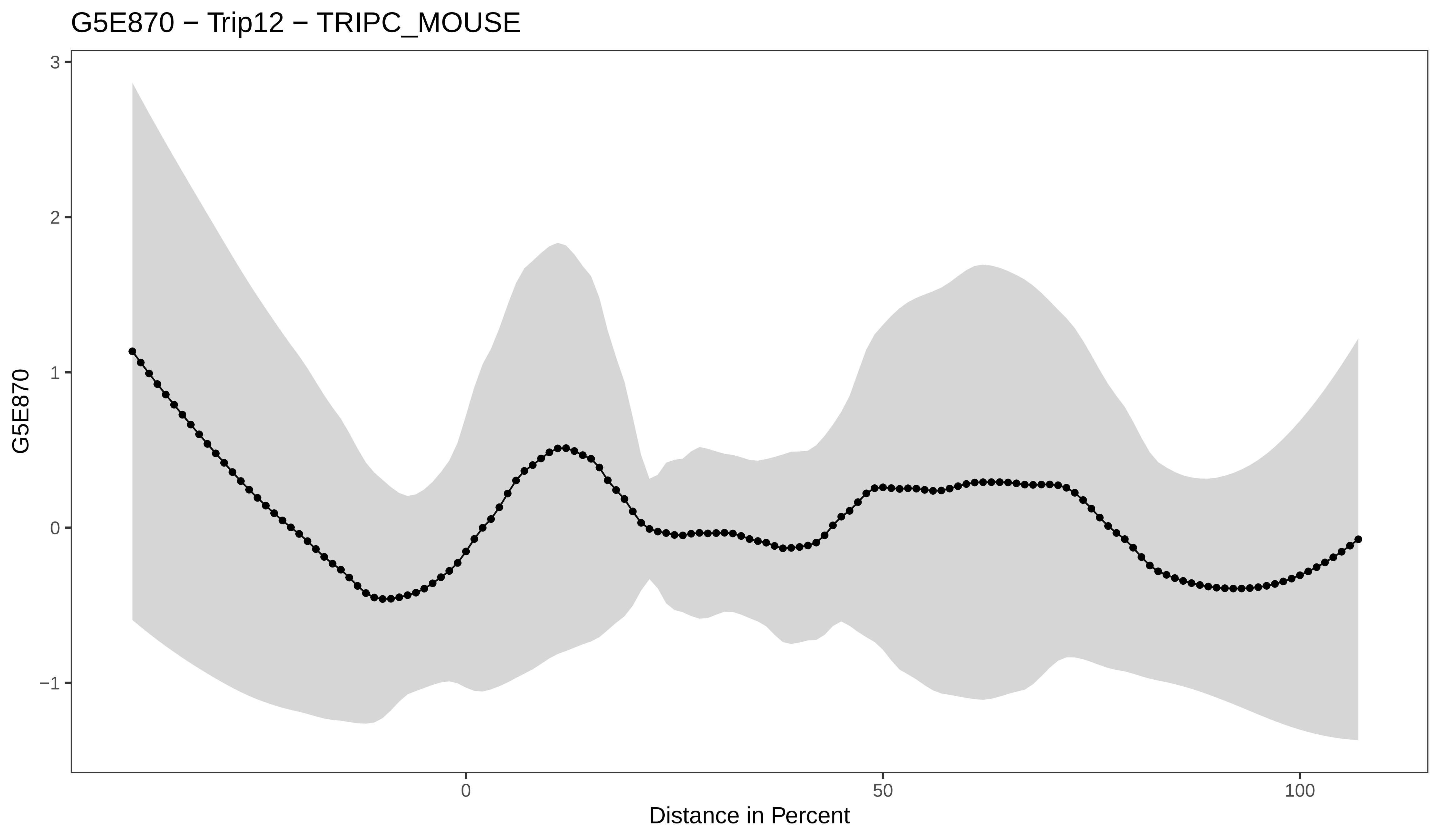
G5E870 − Trip12 − TRIPC_MOUSE
3
2
1
0
−1
G5E870
0
50
100
Distance in Percent

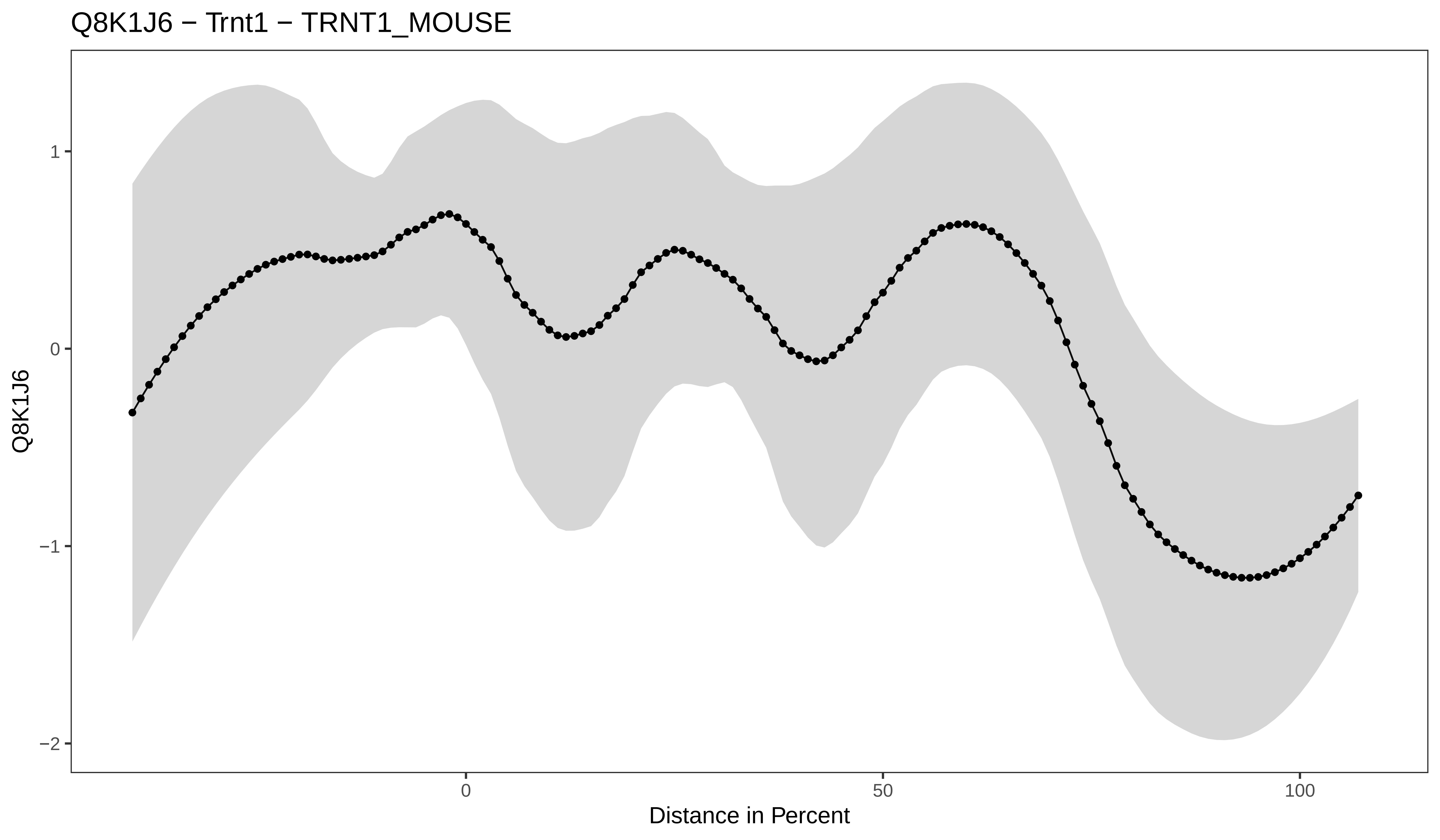

Q8K1J6 − Trnt1 − TRNT1_MOUSE
1
0
−1
−2
Q8K1J6
0
50
100
Distance in Percent

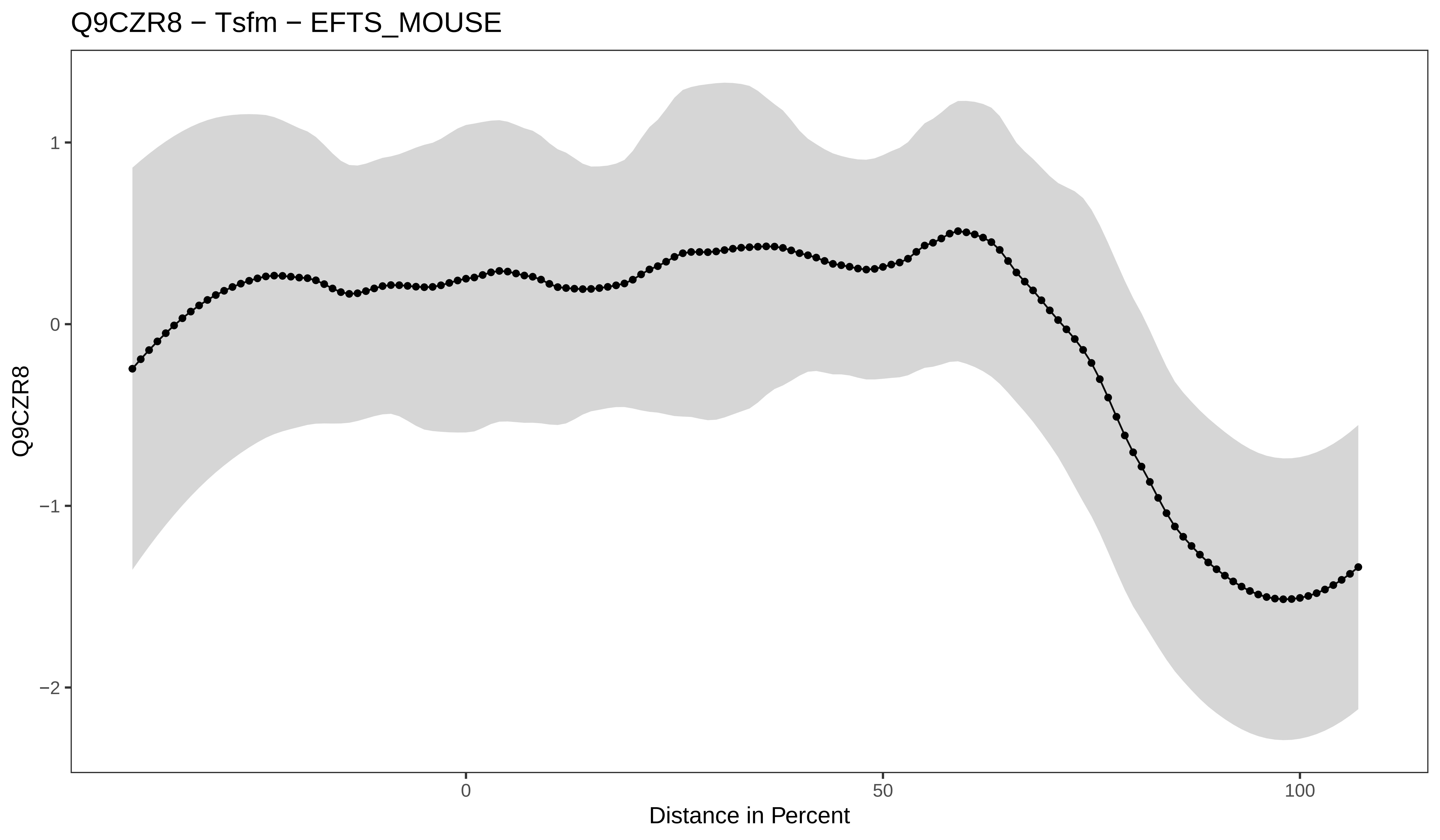

Q9CZR8 − Tsfm − EFTS_MOUSE
1
0
−1
−2
Q9CZR8
0
50
100
Distance in Percent

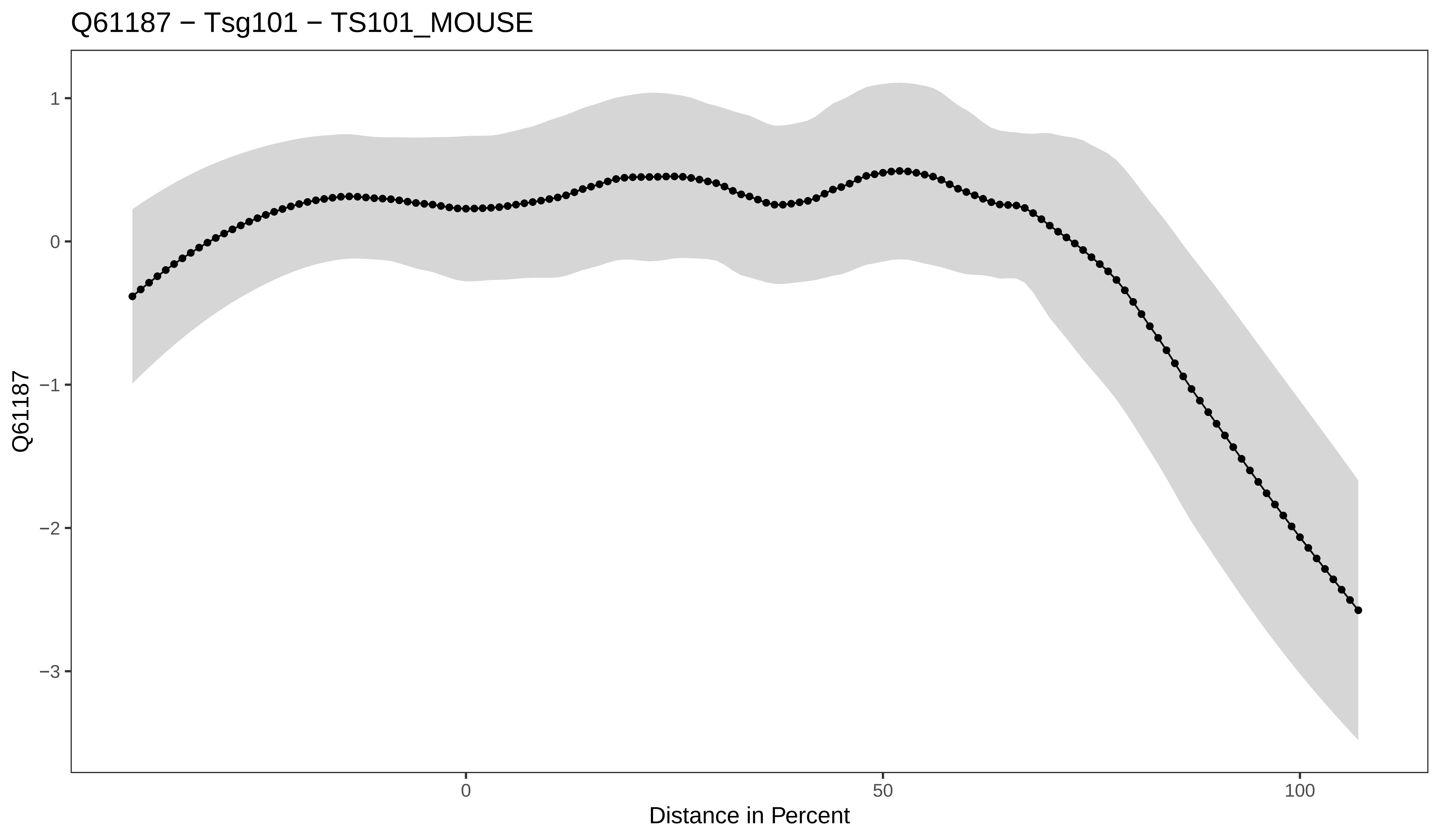

Q61187 − Tsg101 − TS101_MOUSE
1
0
−1
−2
−3
Q61187
0
50
100
Distance in Percent

Q62348 − Tsn − TSN_MOUSE

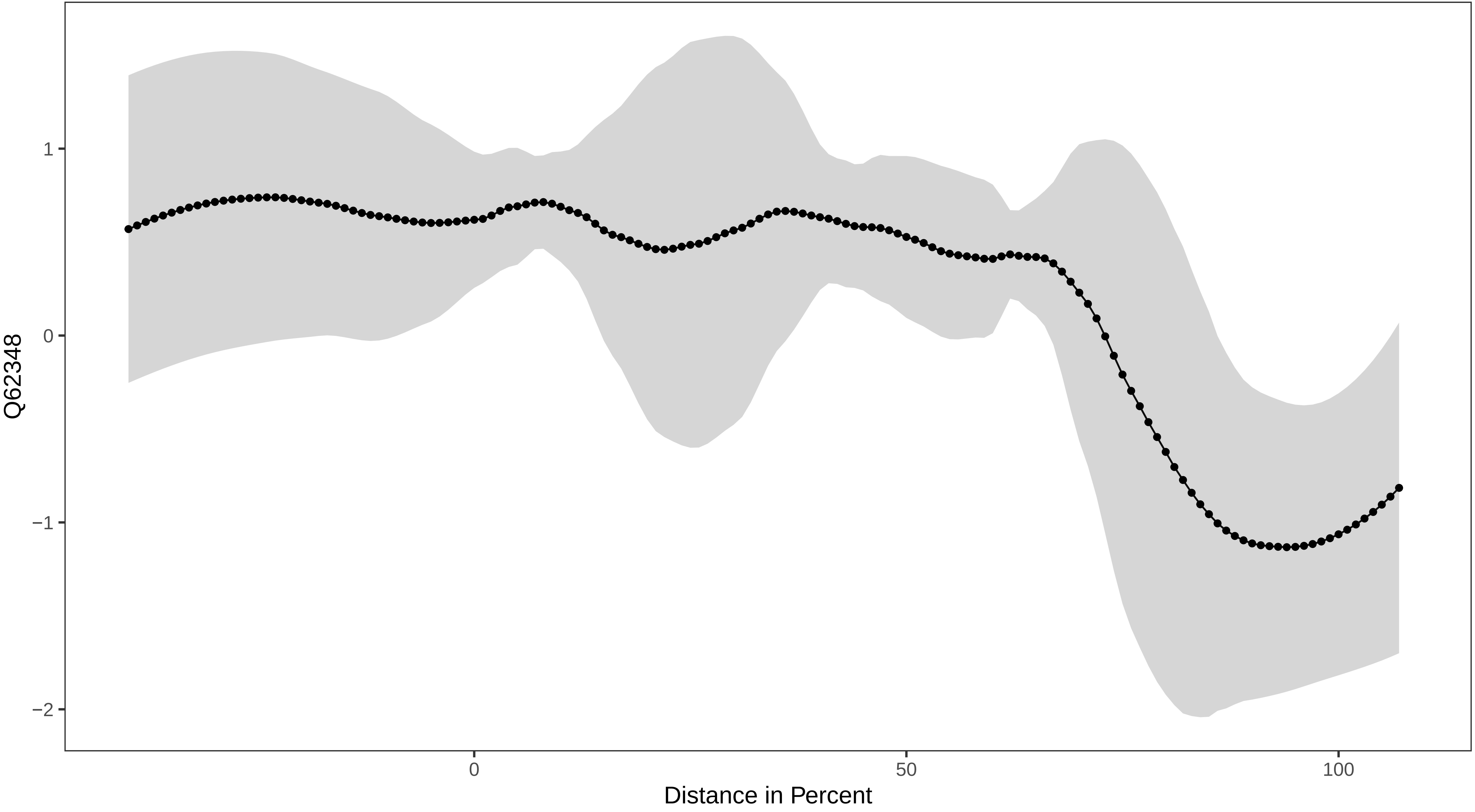

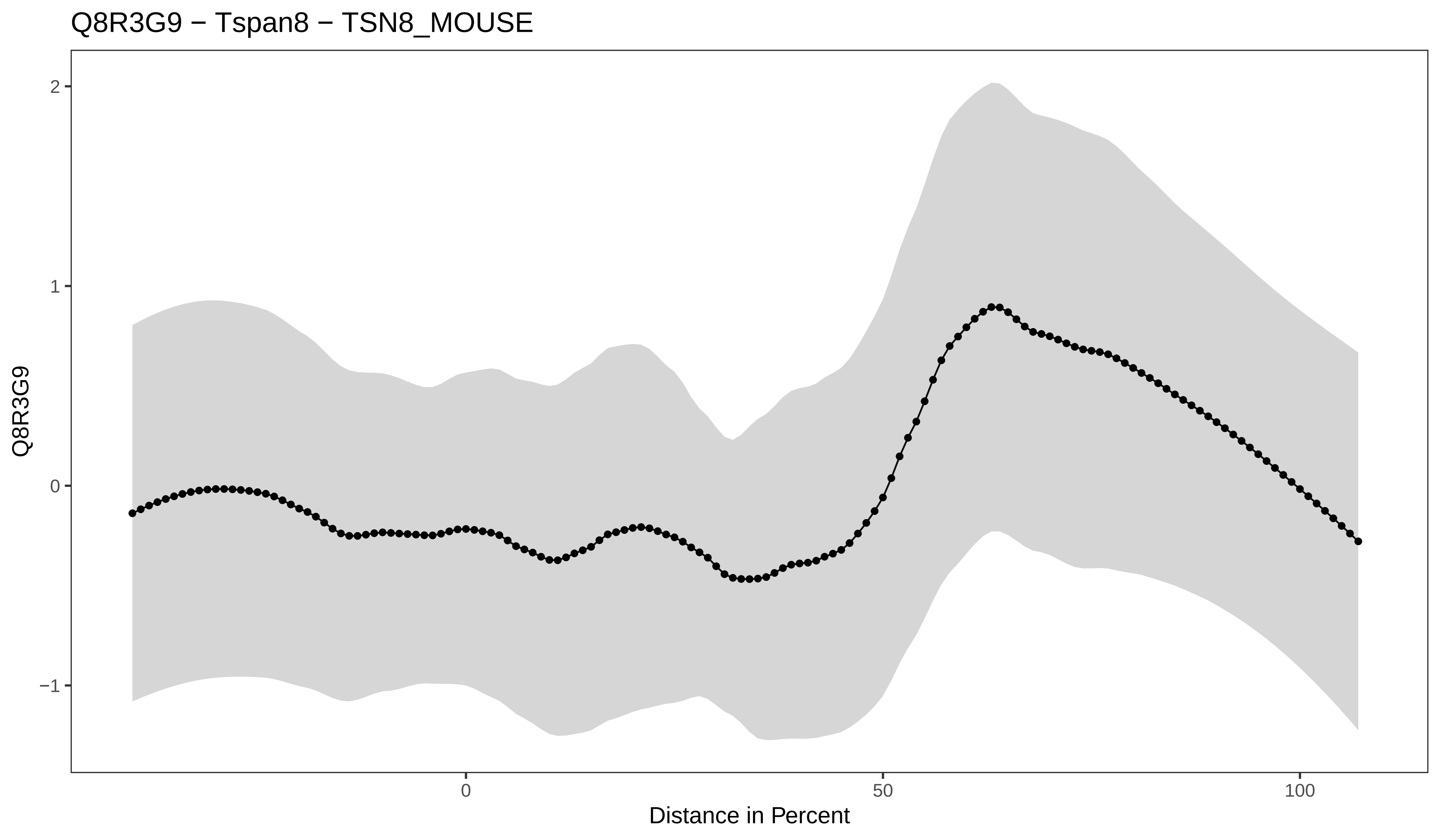
Q8R3G9 − Tspan8 − TSN8_MOUSE
Q8R3G9
Distance in Percent
2
1
0
−1
0
50
100

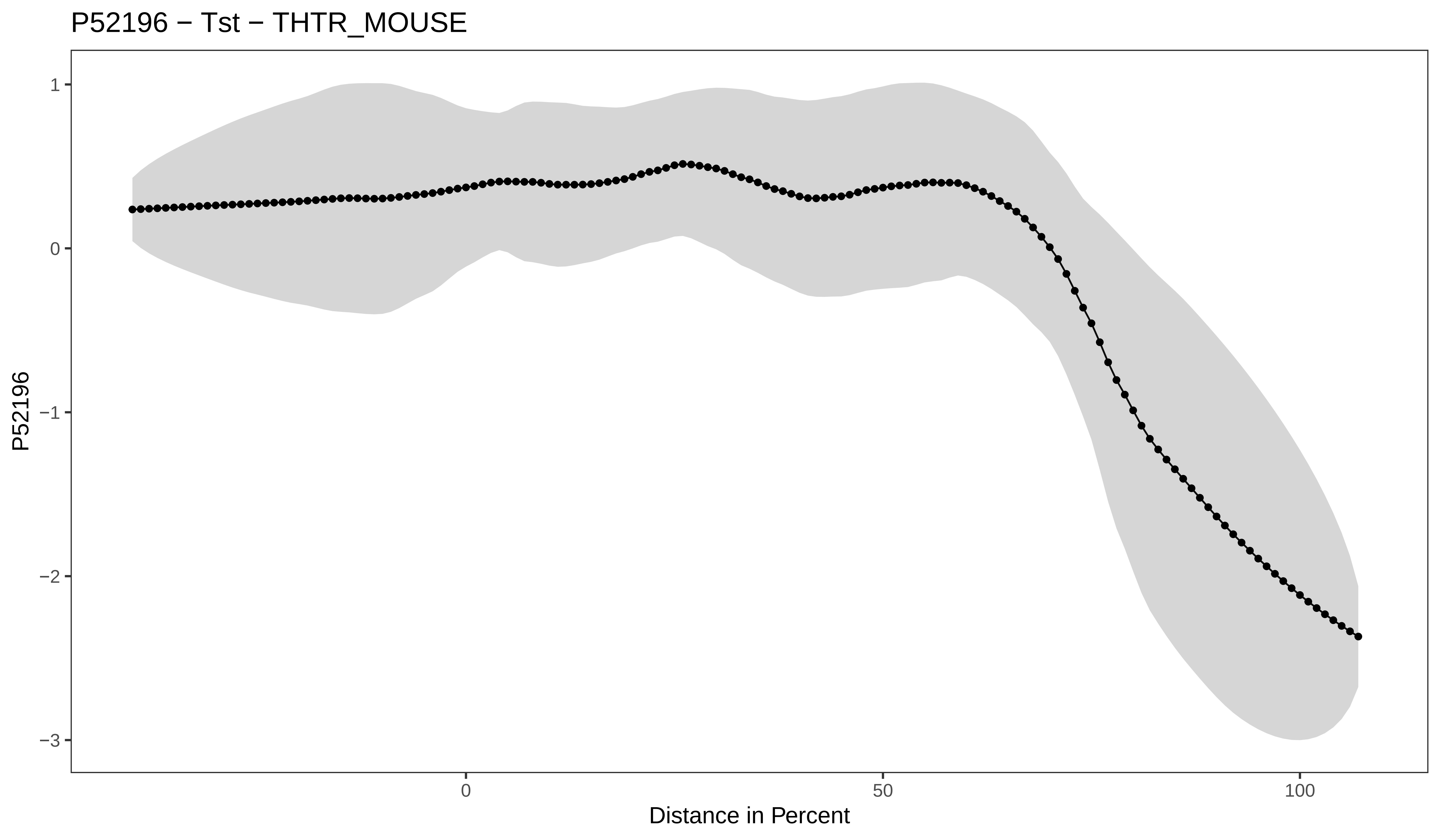

P52196 − Tst − THTR_MOUSE
P52196
1
0
−1
−2
−3
0
50
100
Distance in Percent

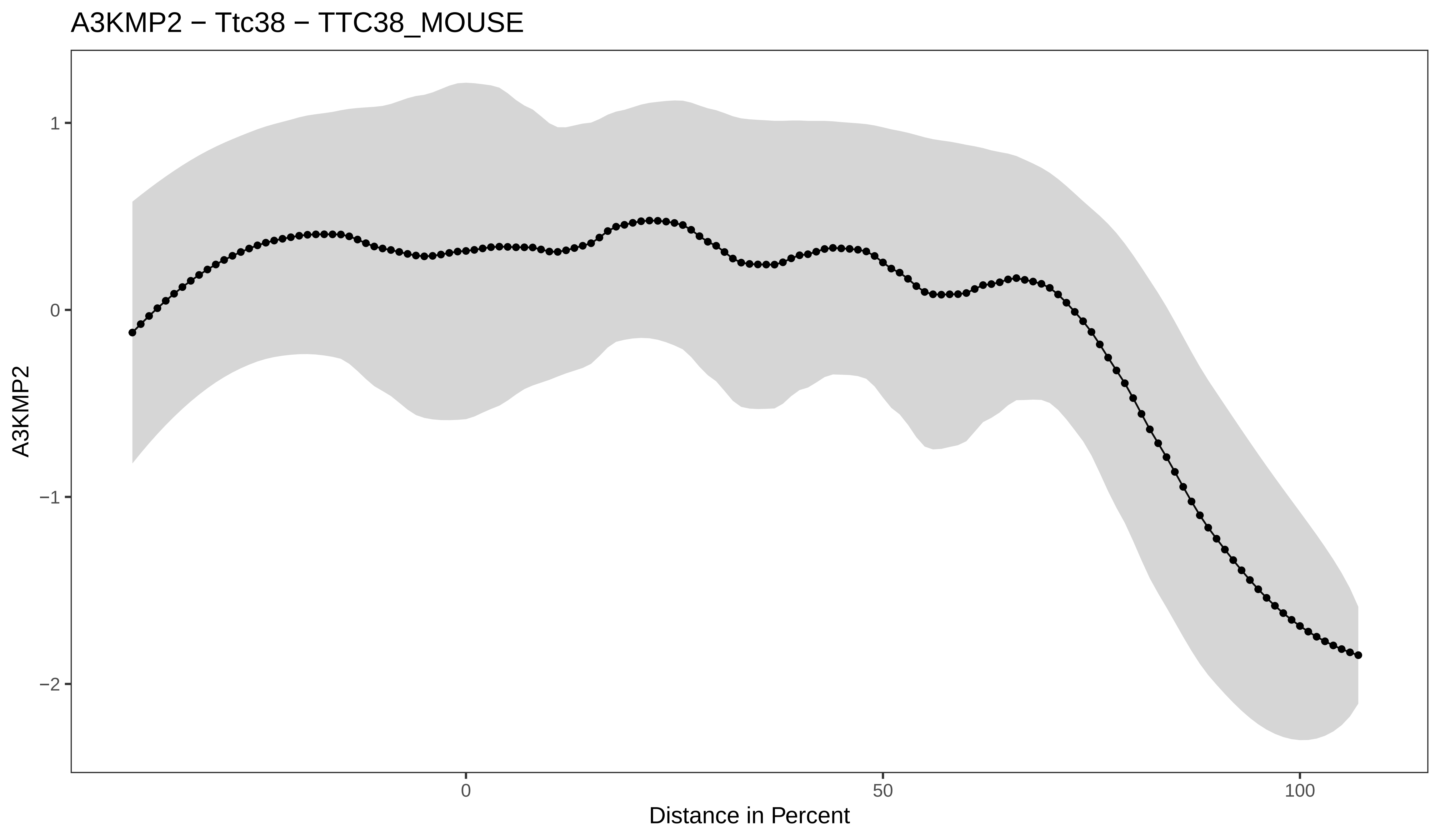

A3KMP2 − Ttc38 − TTC38_MOUSE
A3KMP2
1
0
−1
−2
0
50
100
Distance in Percent

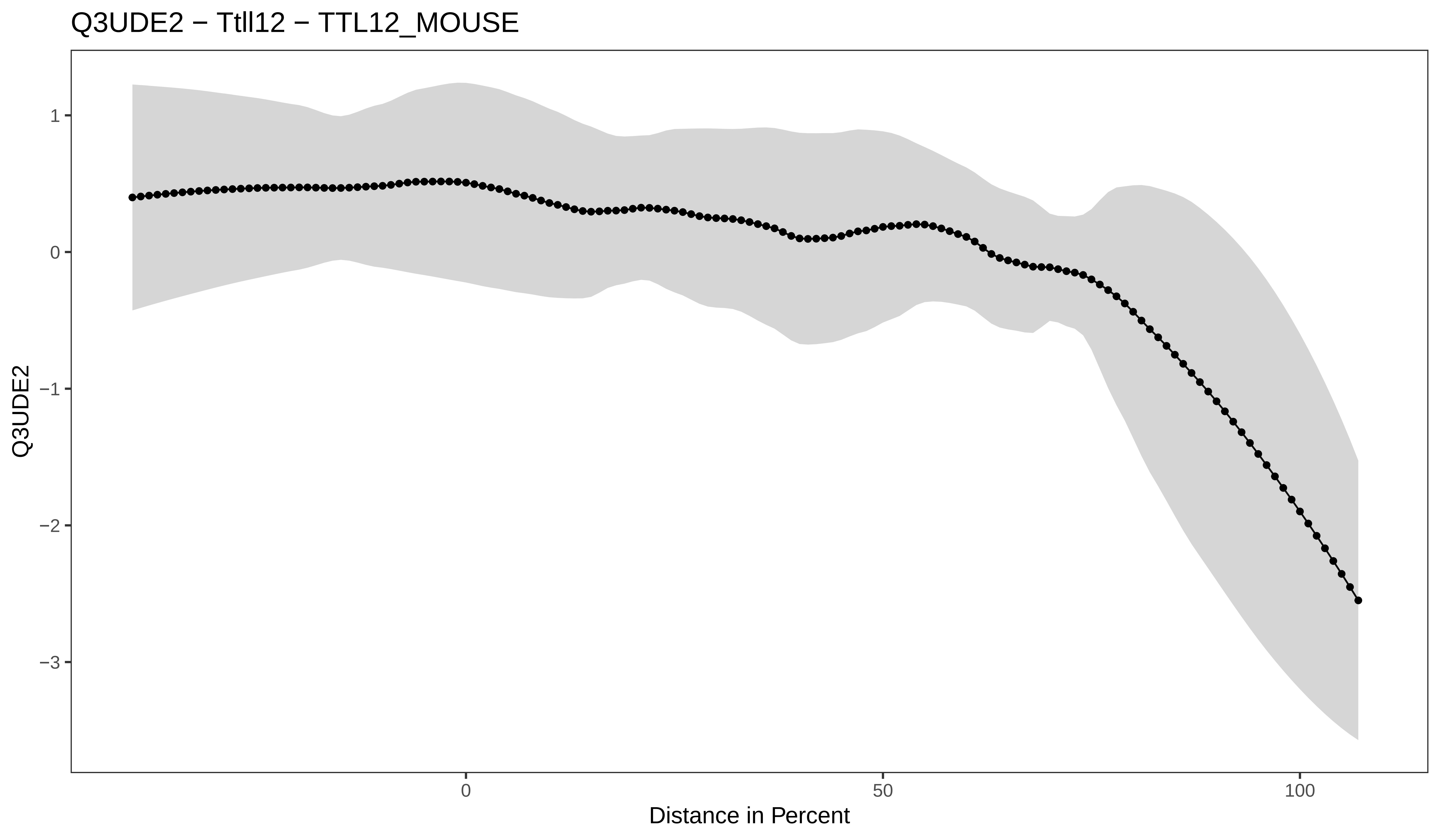
Q3UDE2 − Ttll12 − TTL12_MOUSE
1
0
−1
−2
−3
Q3UDE2
0
50
100
Distance in Percent

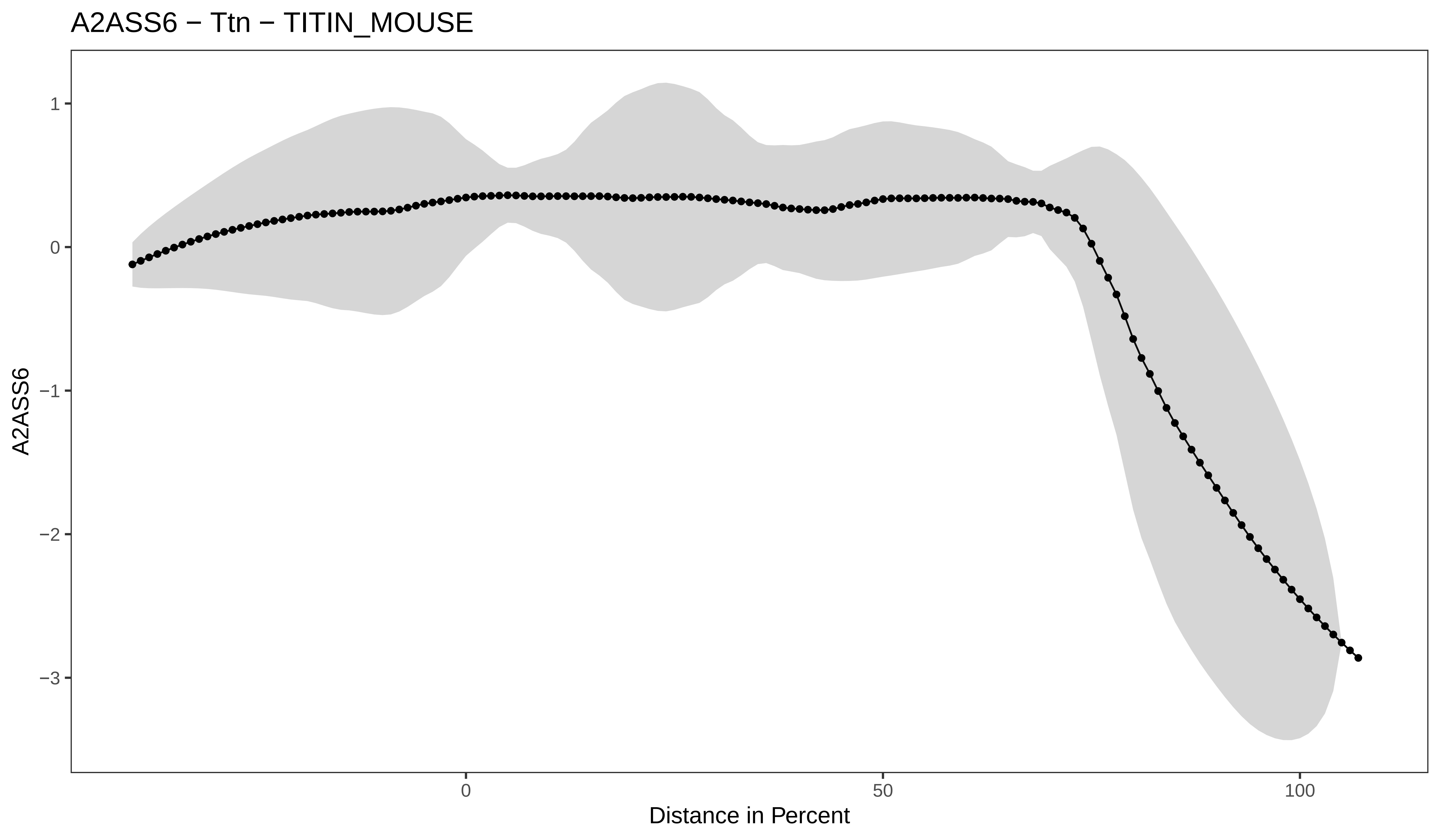
A2ASS6 − Ttn − TITIN_MOUSE
A2ASS6
1
0
−1
−2
−3
0
50
100
Distance in Percent

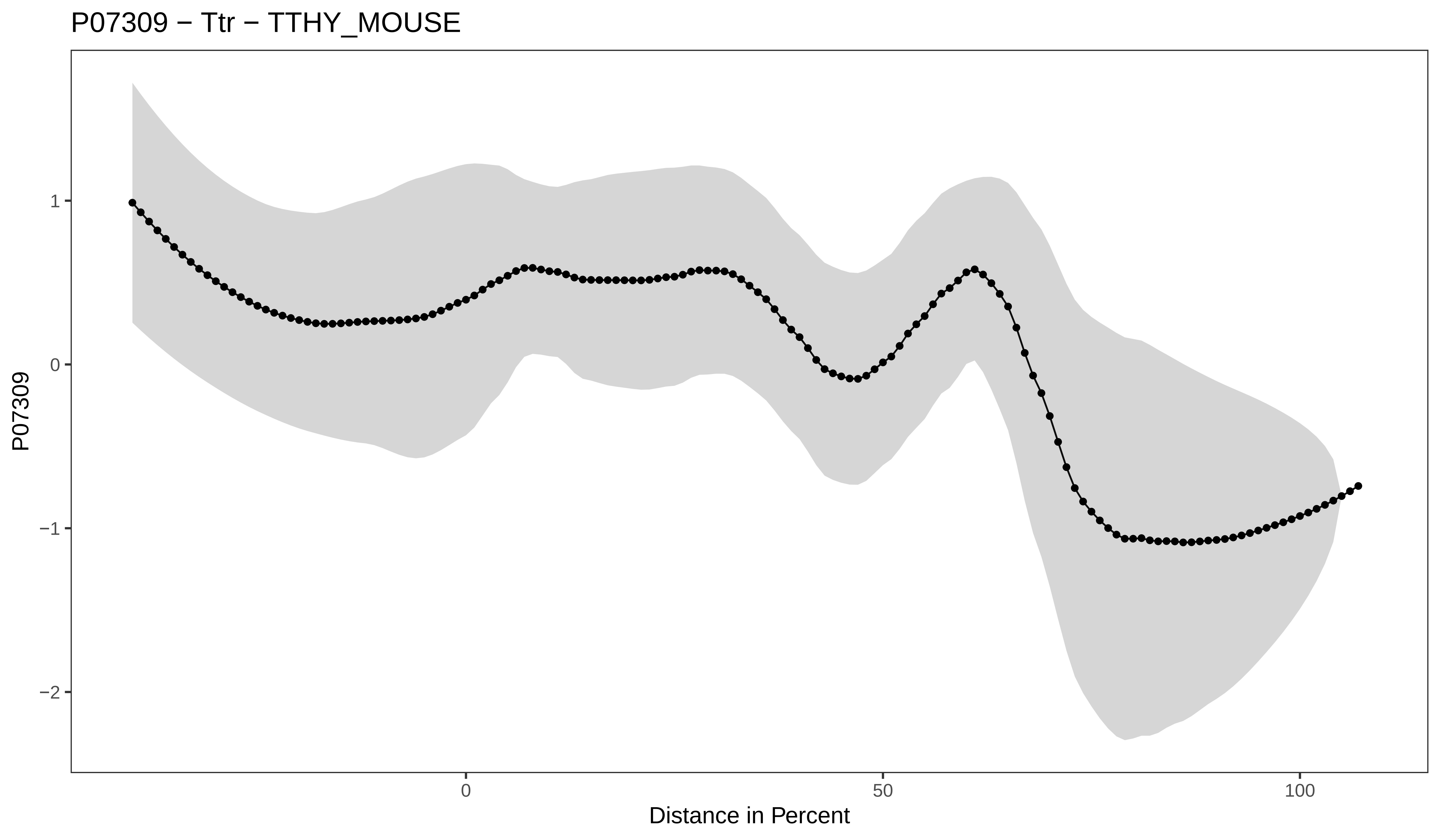

P07309 − Ttr − TTHY_MOUSE
P07309
1
0
−1
−2
0
50
100
Distance in Percent

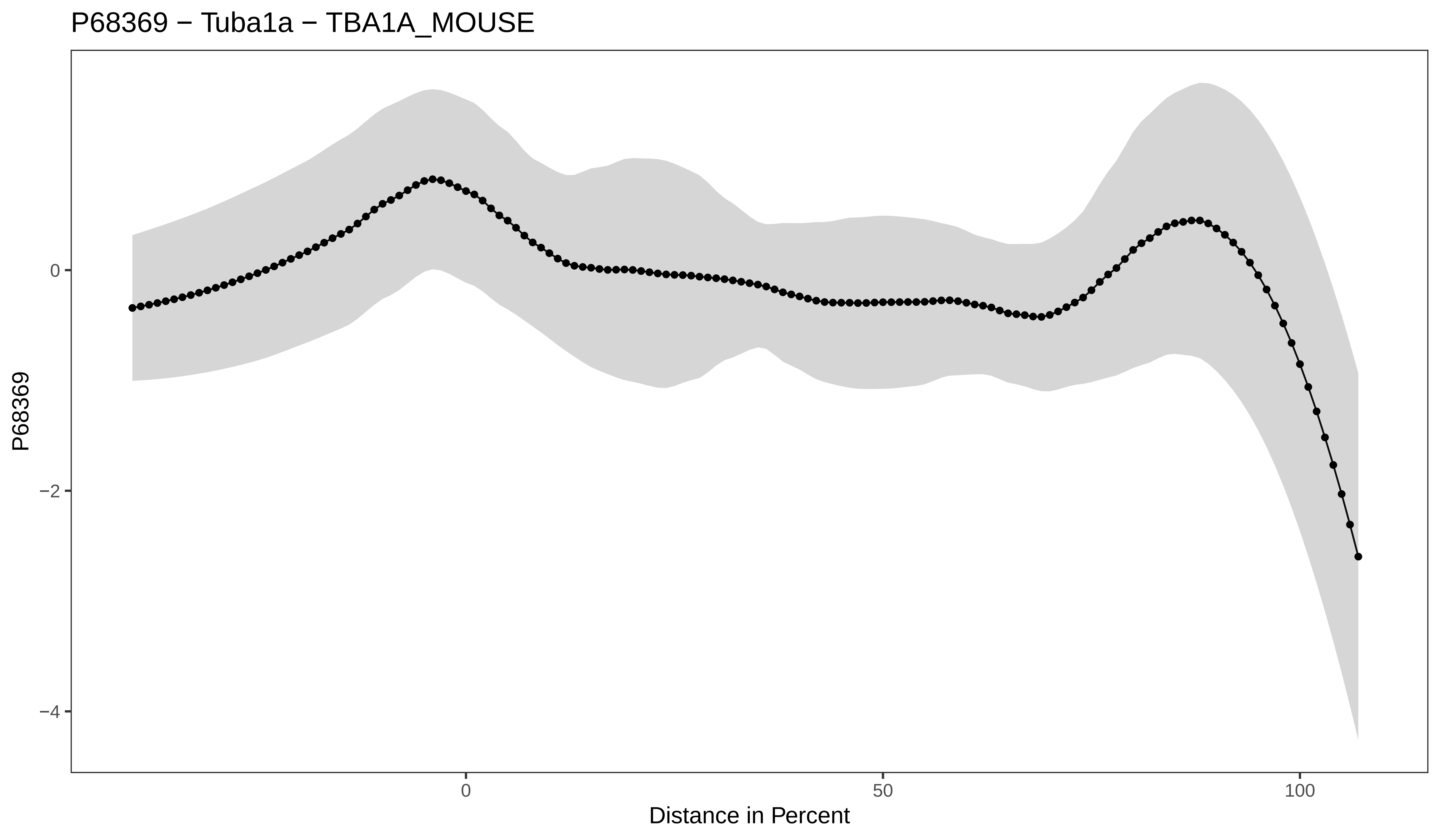
P68369 − Tuba1a − TBA1A_MOUSE
P68369
0
−2
−4
0
50
100
Distance in Percent

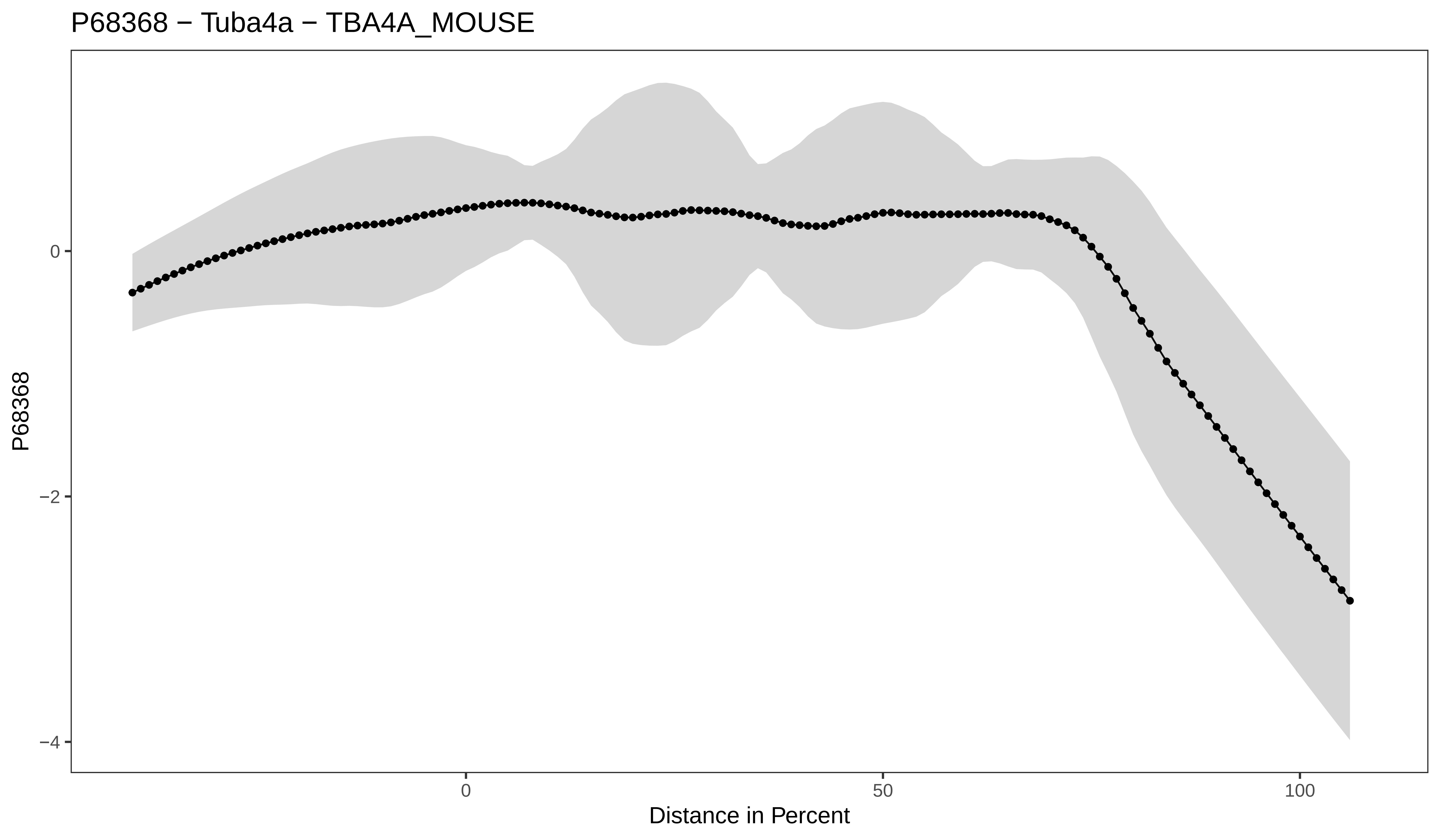

P68368 − Tuba4a − TBA4A_MOUSE
P68368
0
−2
−4
0
50
100
Distance in Percent

Q9JJZ2 − Tuba8 − TBA8_MOUSE

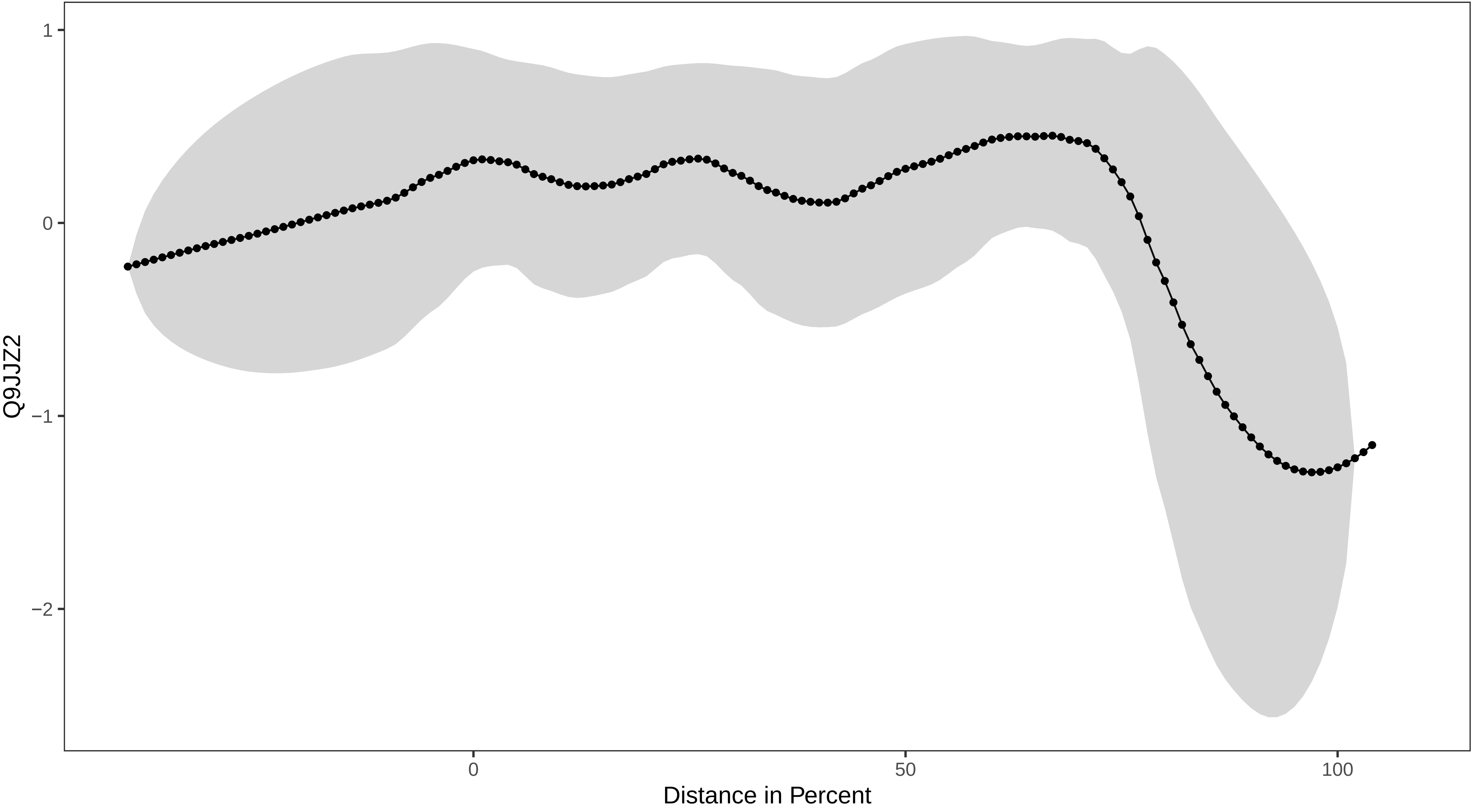

Q7TMM9 − Tubb2a − TBB2A_MOUSE

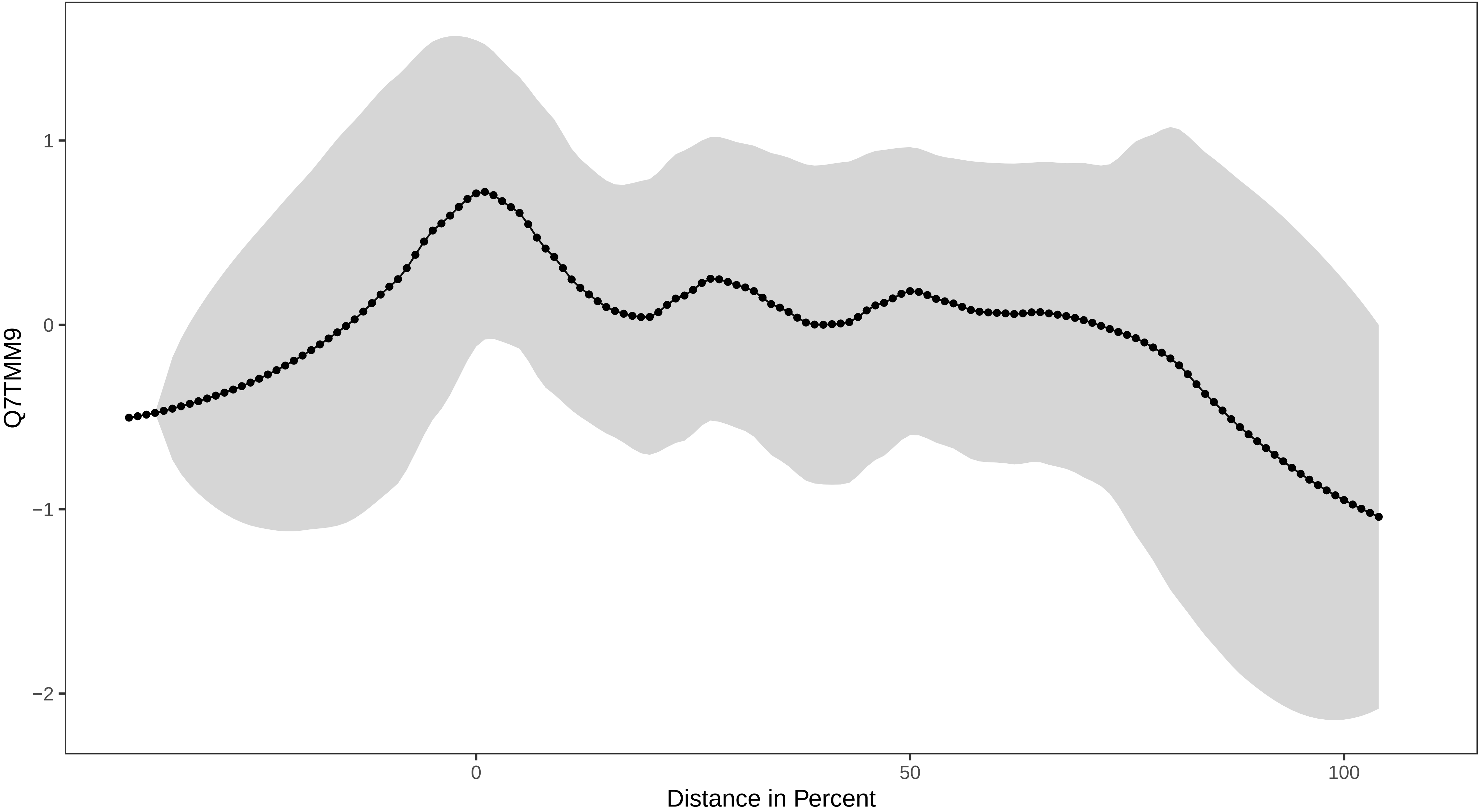

Q9ERD7 − Tubb3 − TBB3_MOUSE

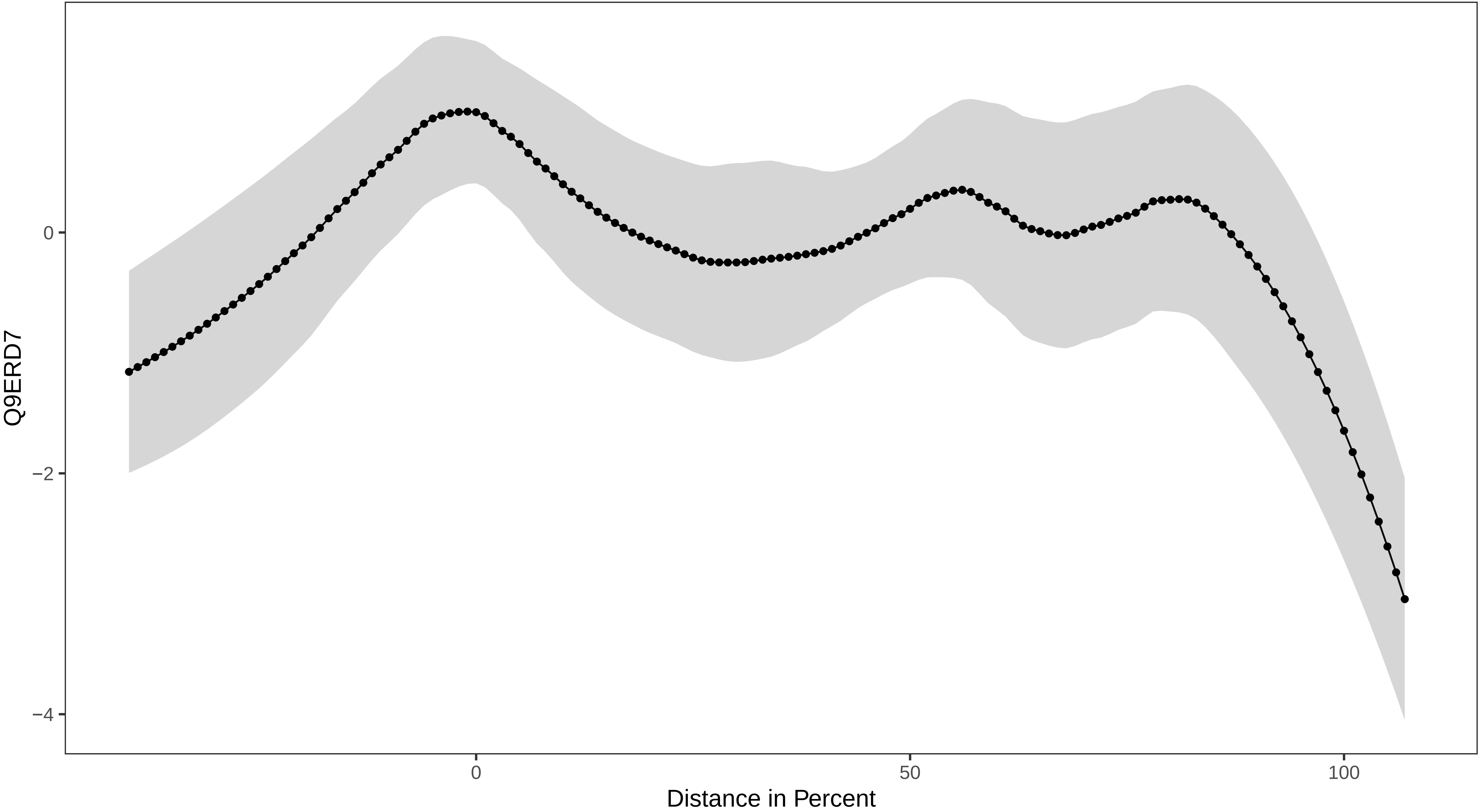

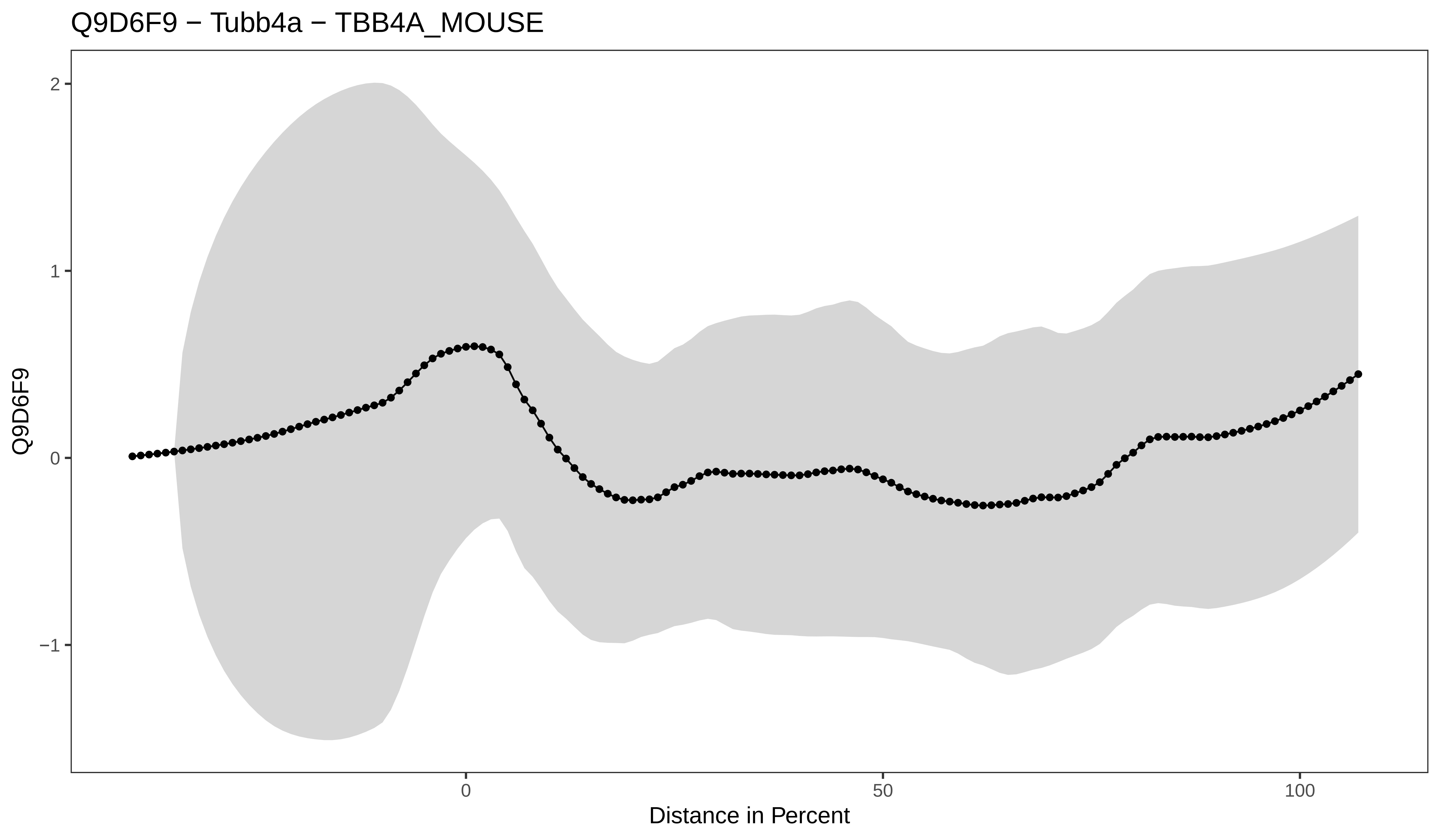

Q9D6F9 − Tubb4a − TBB4A_MOUSE
Q9D6F9
2
1
0
−1
0
50
100
Distance in Percent

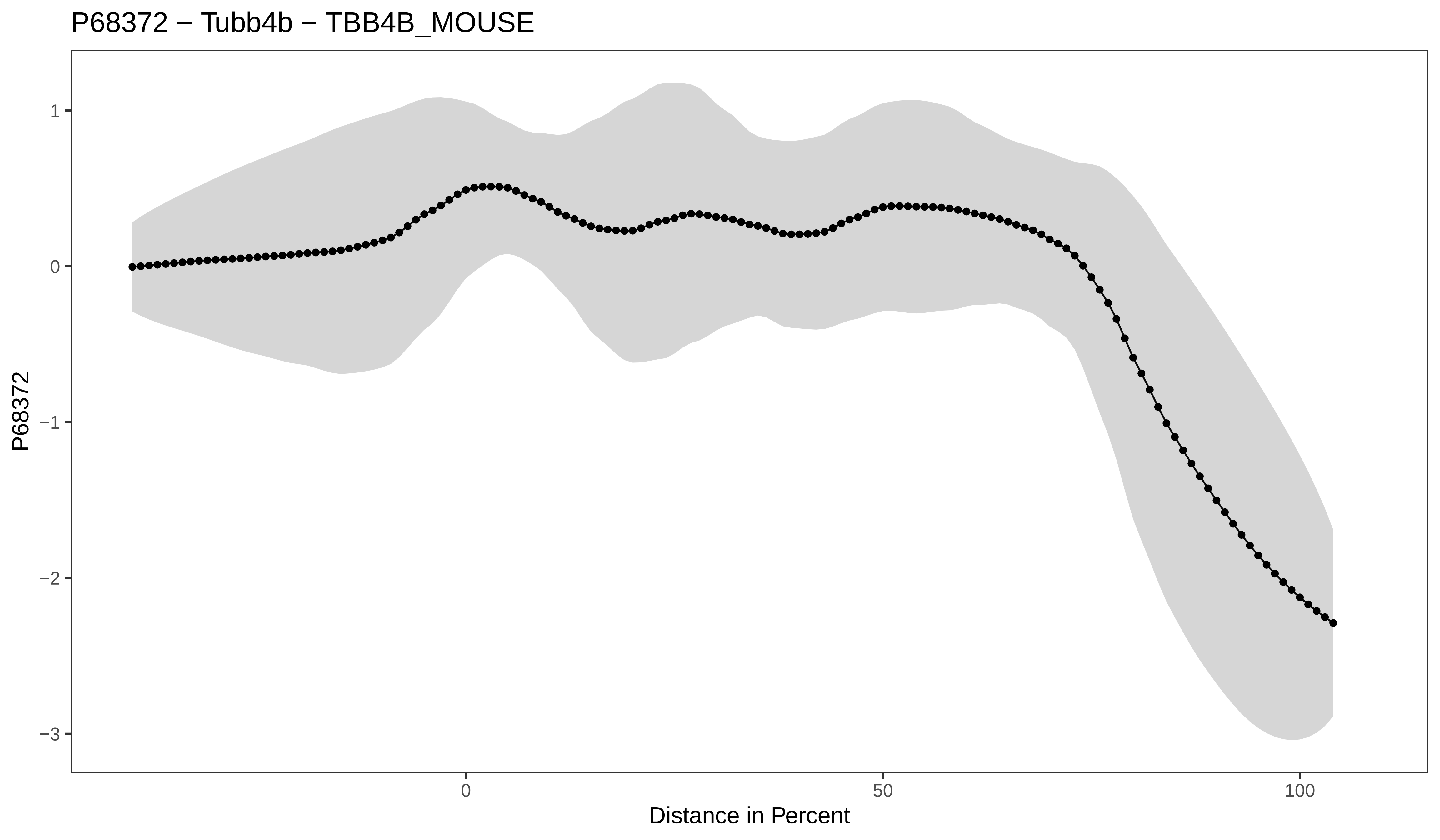

P68372 − Tubb4b − TBB4B_MOUSE
1
0
−1
−2
−3
P68372
0
50
100
Distance in Percent

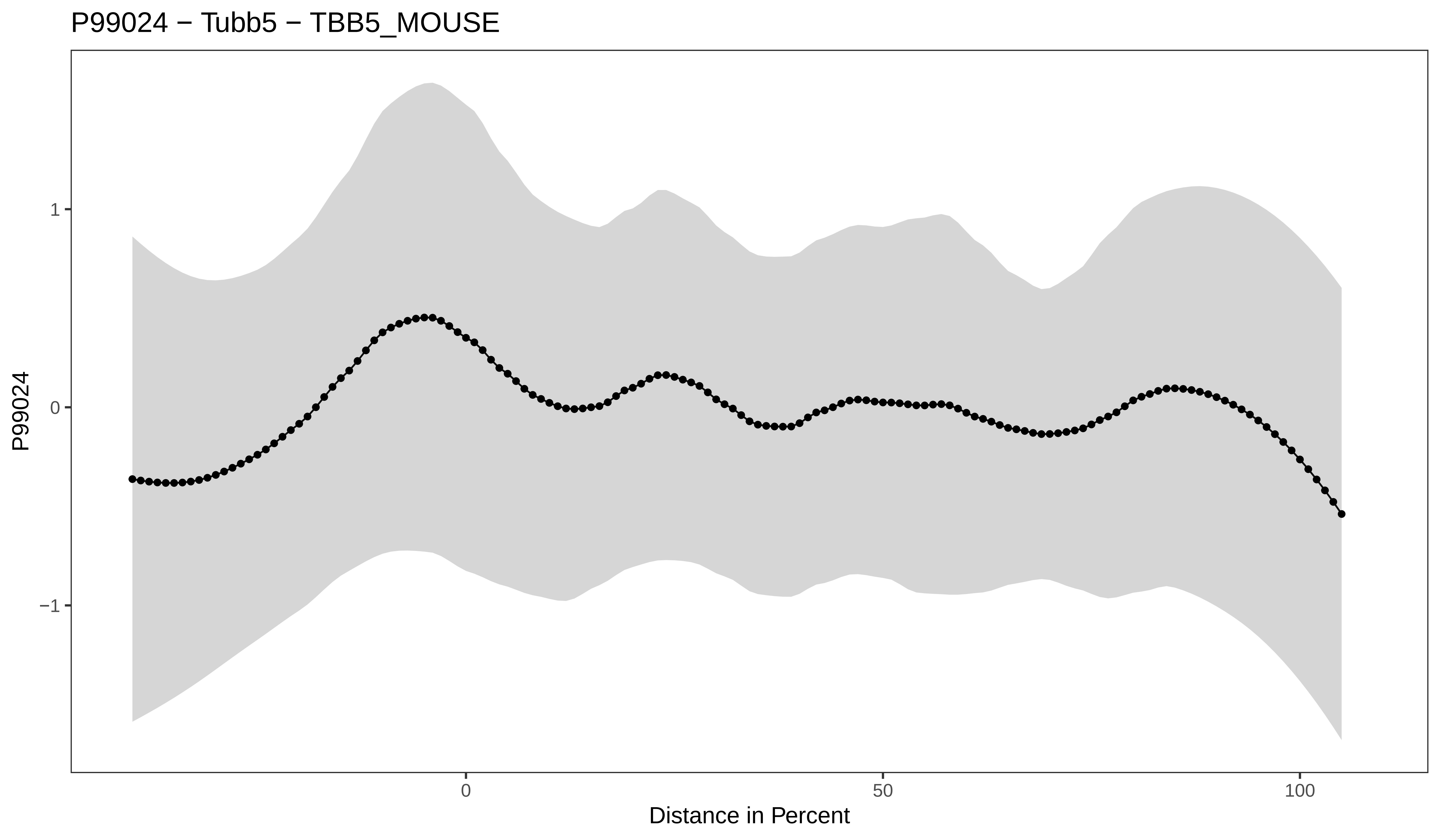

P99024 − Tubb5 − TBB5_MOUSE
P99024
1
0
−1
0
50
100
Distance in Percent

Q922F4 − Tubb6 − TBB6_MOUSE

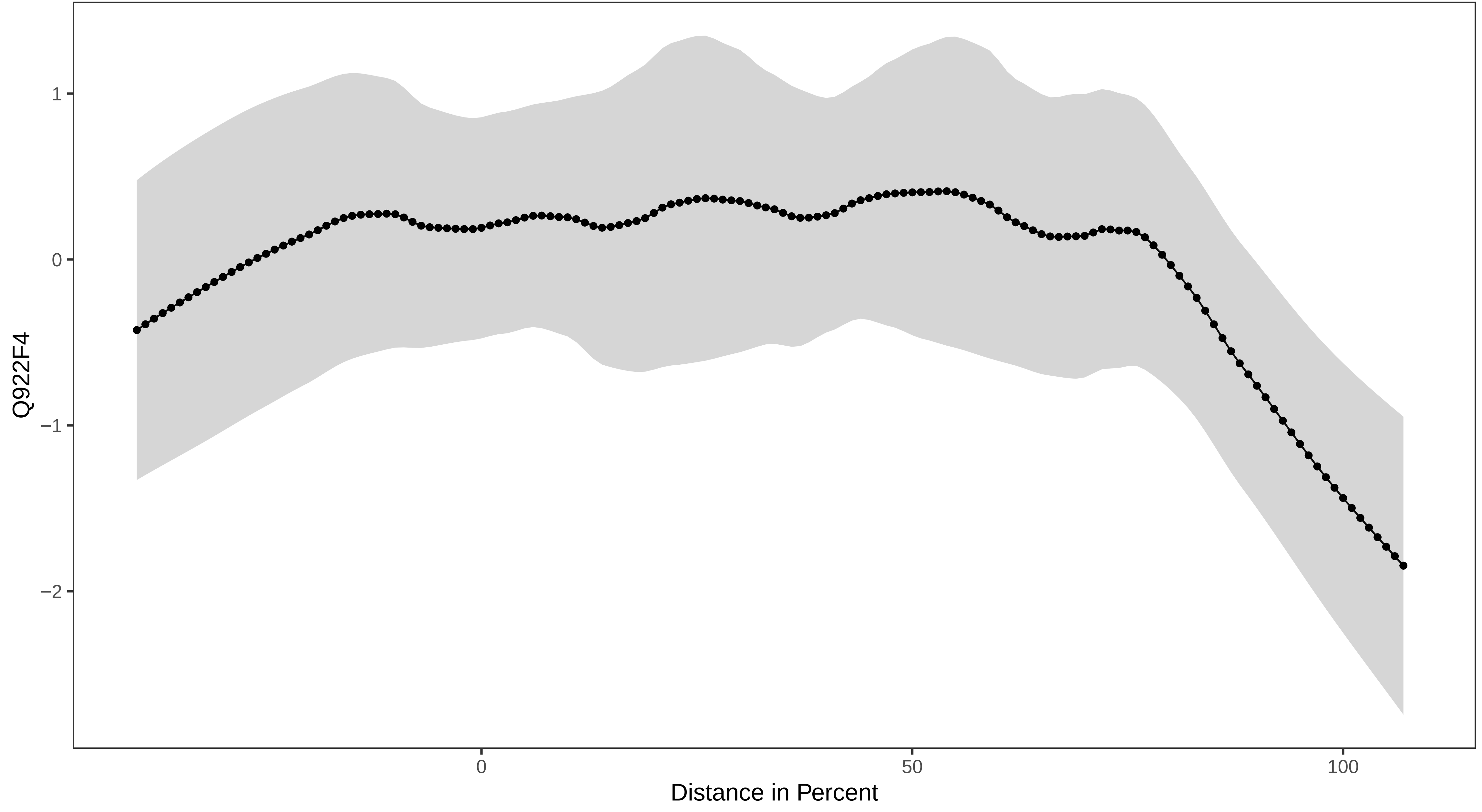

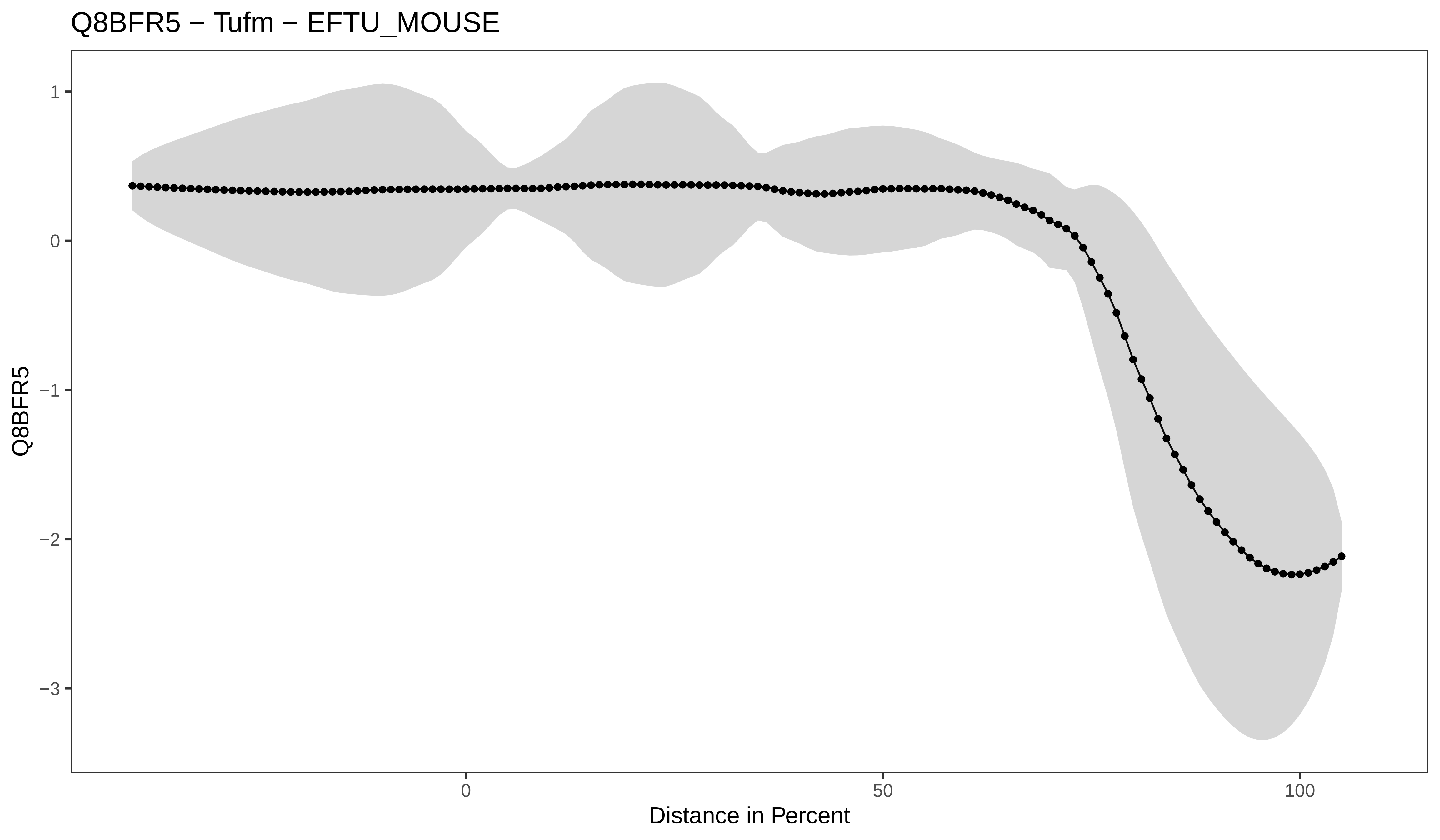
Q8BFR5 − Tufm − EFTU_MOUSE
Q8BFR5
Distance in Percent
1
0
−1
−2
−3
0
50
100

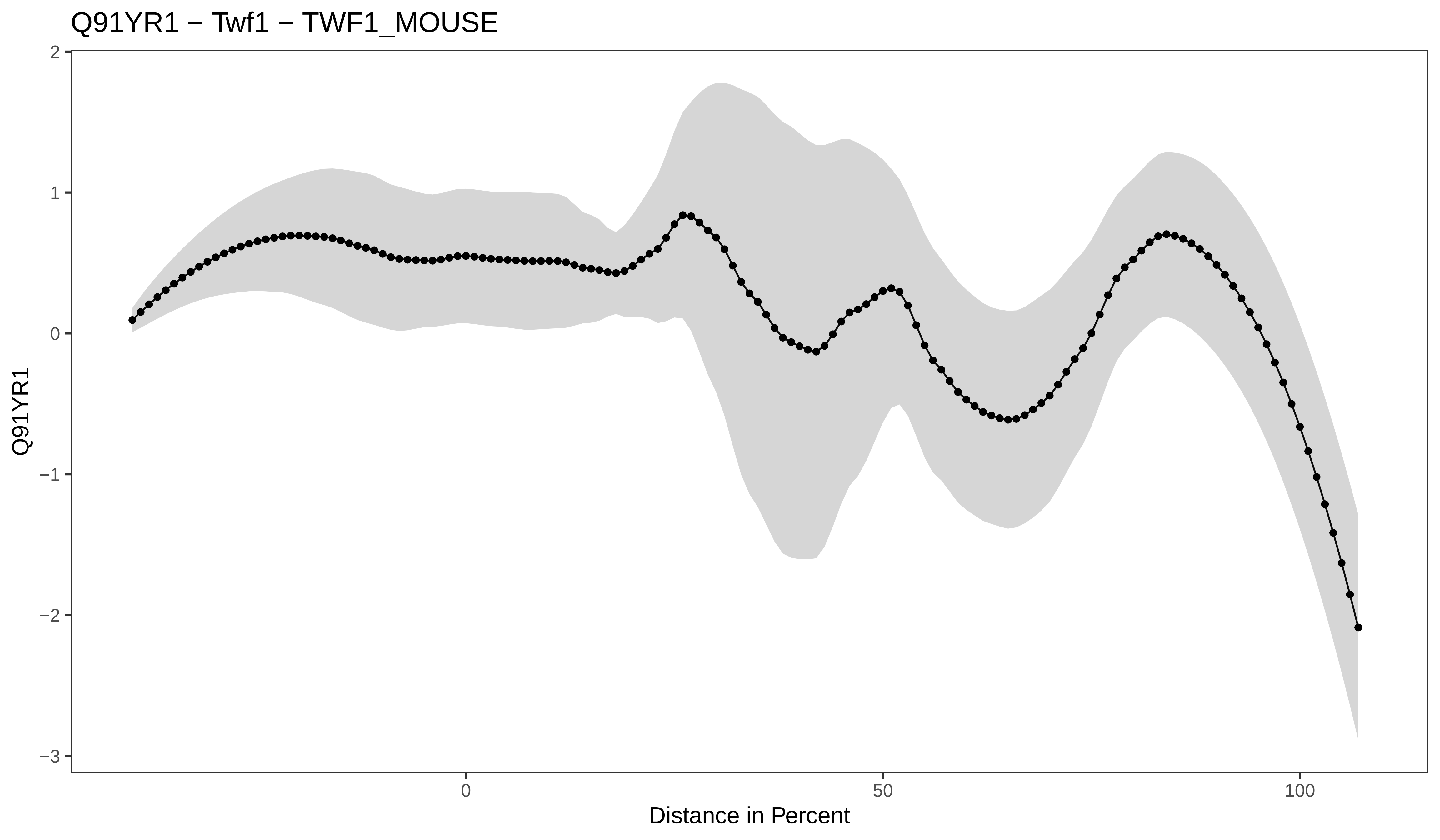

Q91YR1 − Twf1 − TWF1_MOUSE
Q91YR1
Distance in Percent
2
1
0
−1
−2
−3
0
50
100

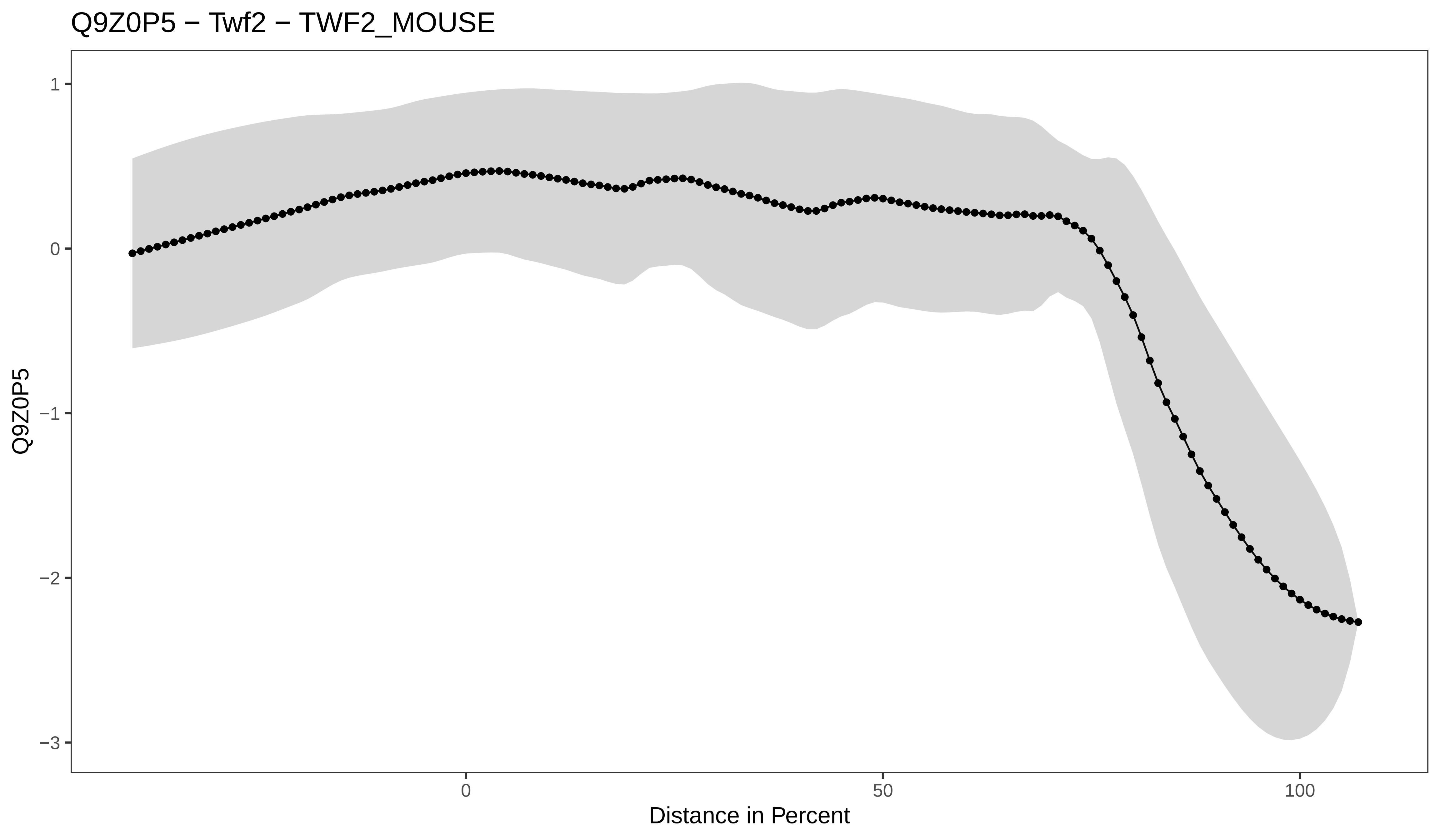

Q9Z0P5 − Twf2 − TWF2_MOUSE
1
0
−1
−2
−3
Q9Z0P5
0
50
100
Distance in Percent

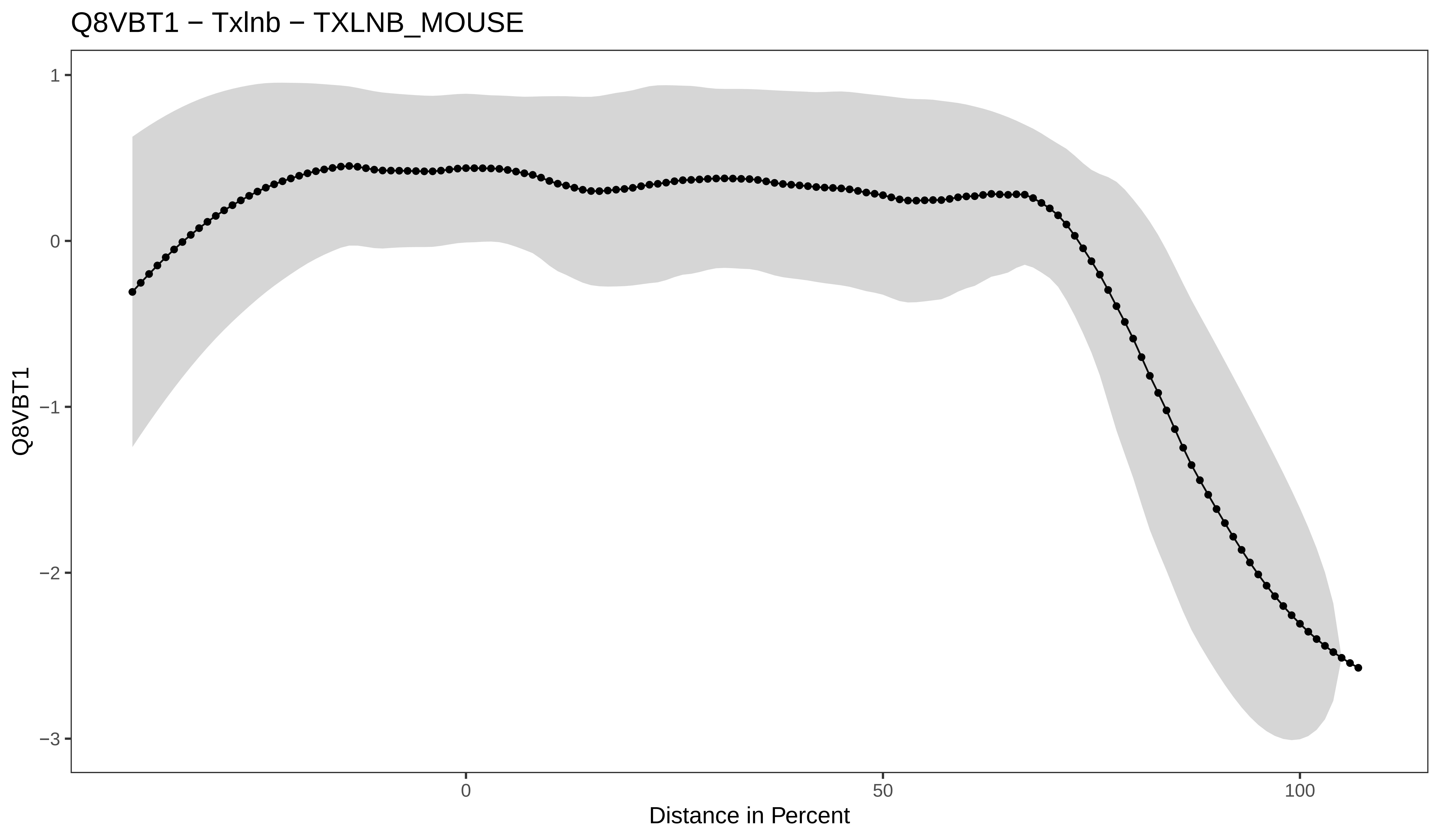
Q8VBT1 − Txlnb − TXLNB_MOUSE
1
0
−1
−2
−3
Q8VBT1
0
50
100
Distance in Percent

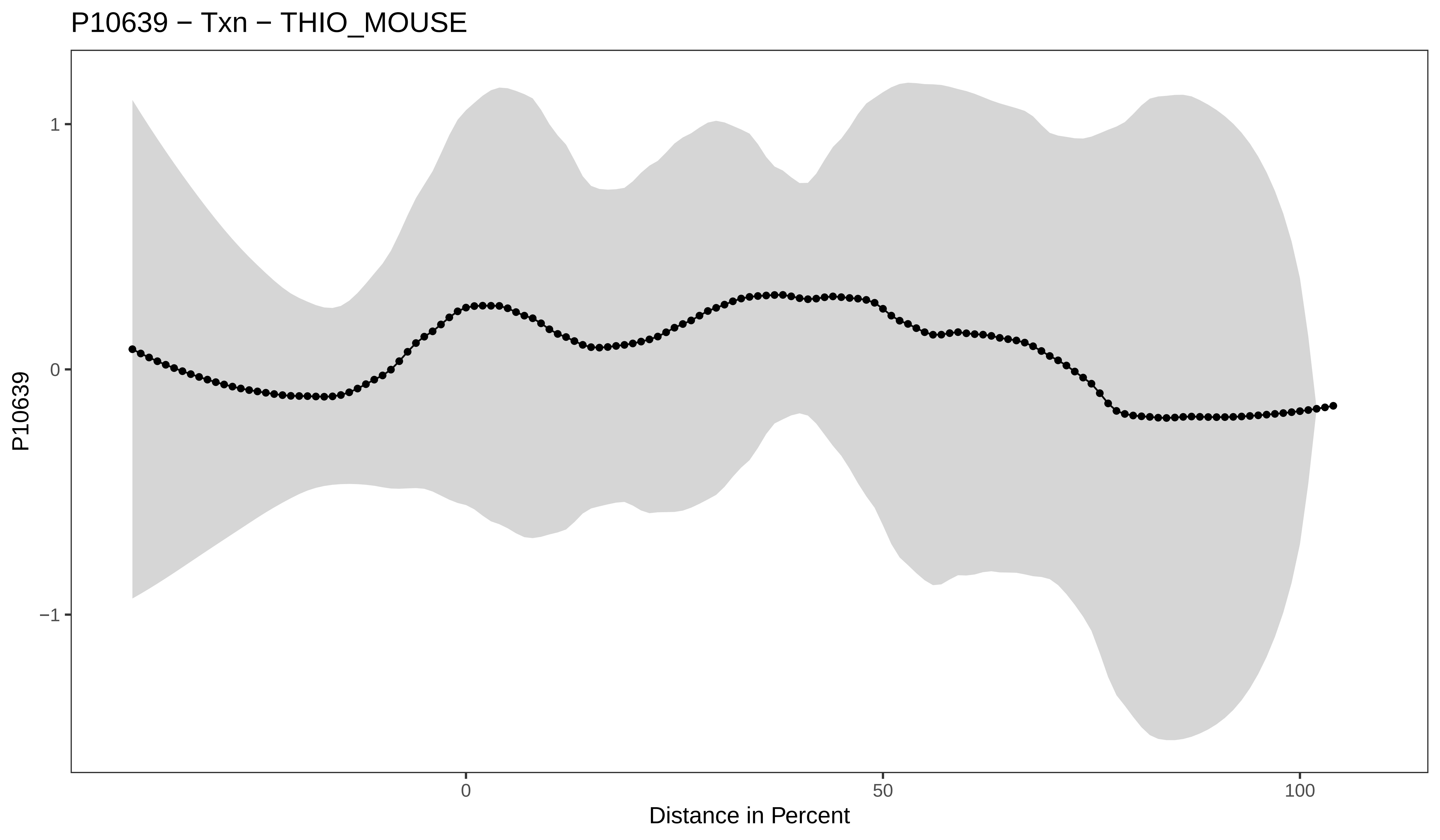
P10639 − Txn − THIO_MOUSE
P10639
1
0
−1
0
50
100
Distance in Percent

Q9CQM5 − Txndc17 − TXD17_MOUSE

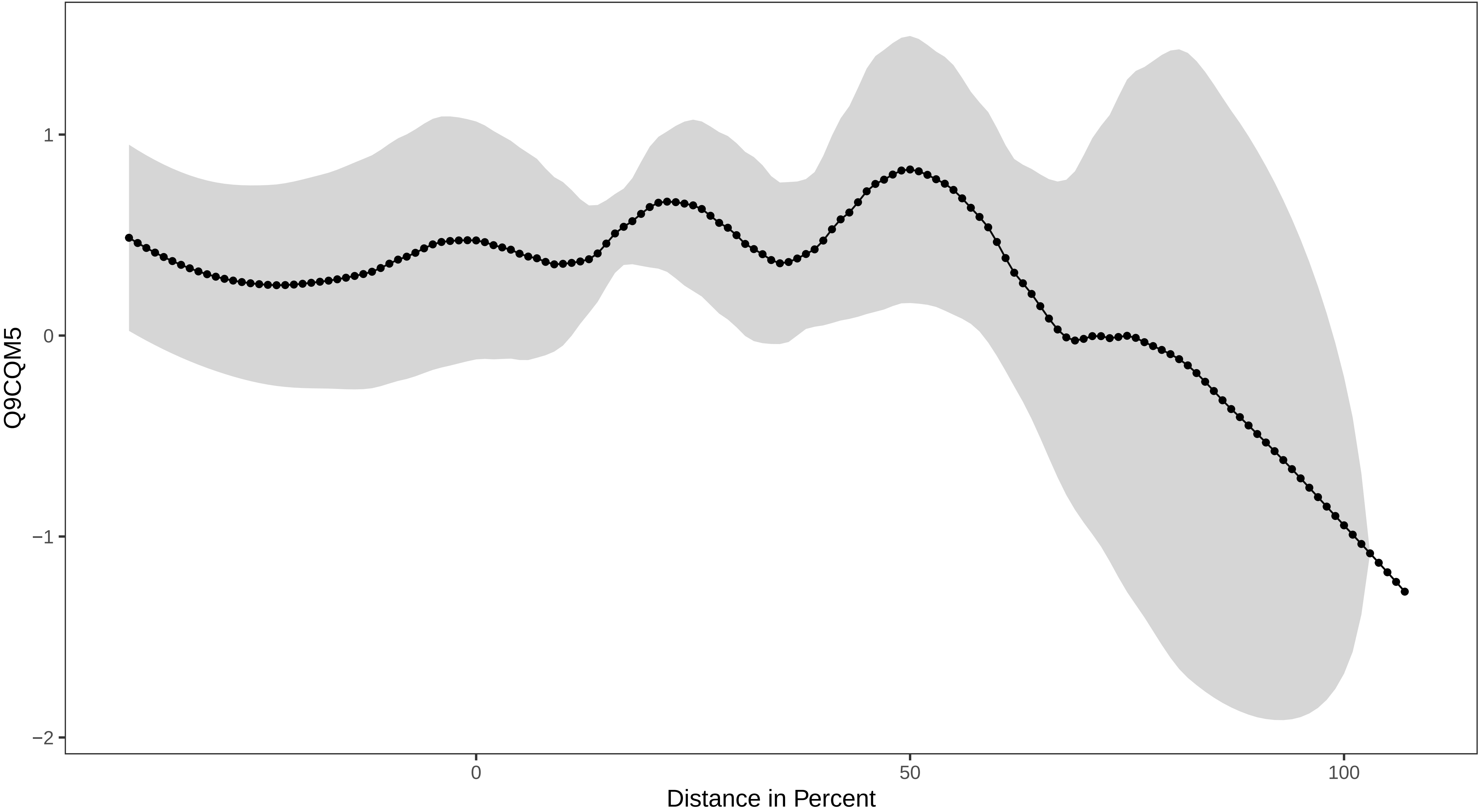

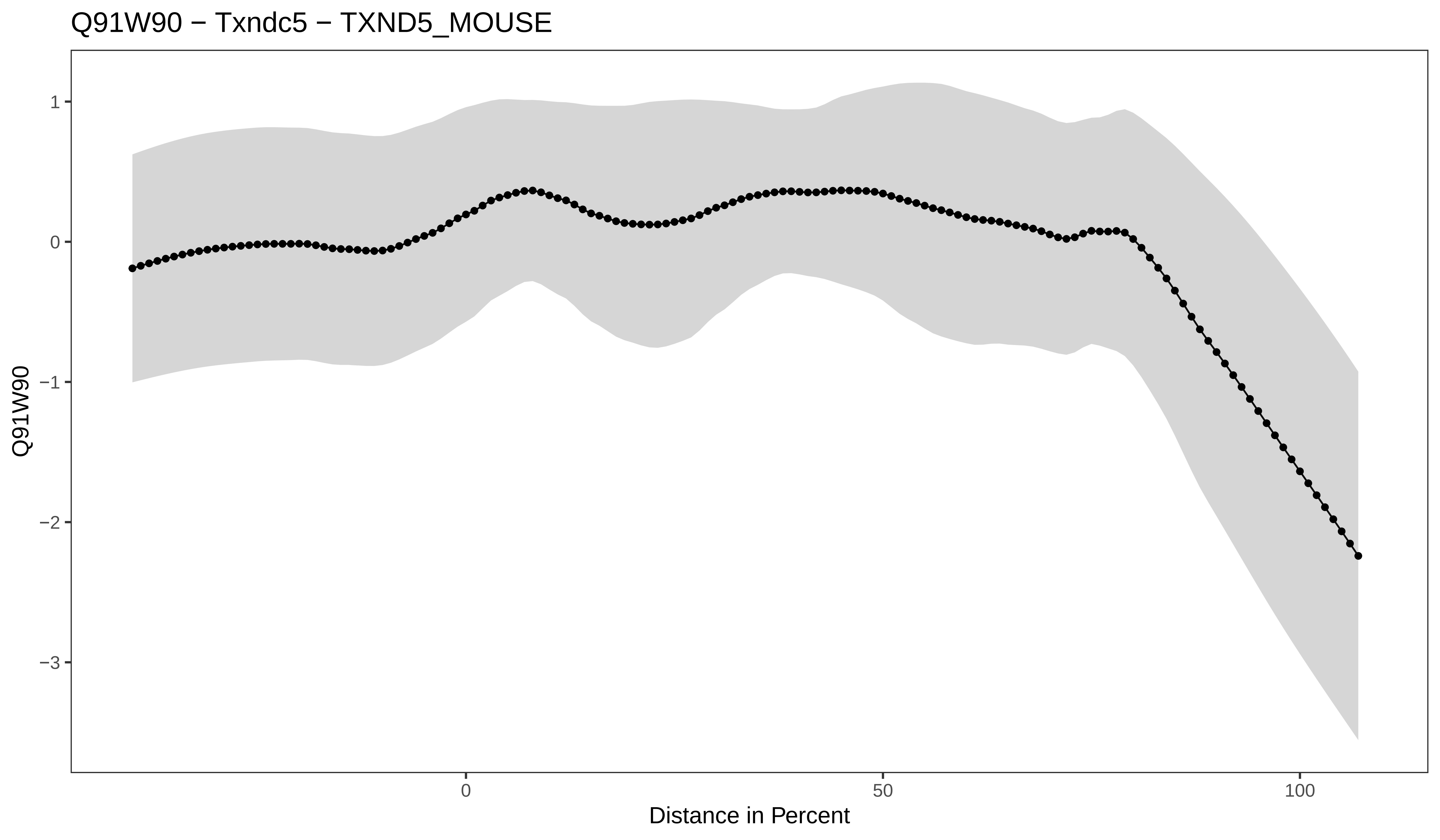

Q91W90 − Txndc5 − TXND5_MOUSE
1
0
−1
−2
−3
Q91W90
0
50
100
Distance in Percent

Q9CQ79 − Txndc9 − TXND9_MOUSE

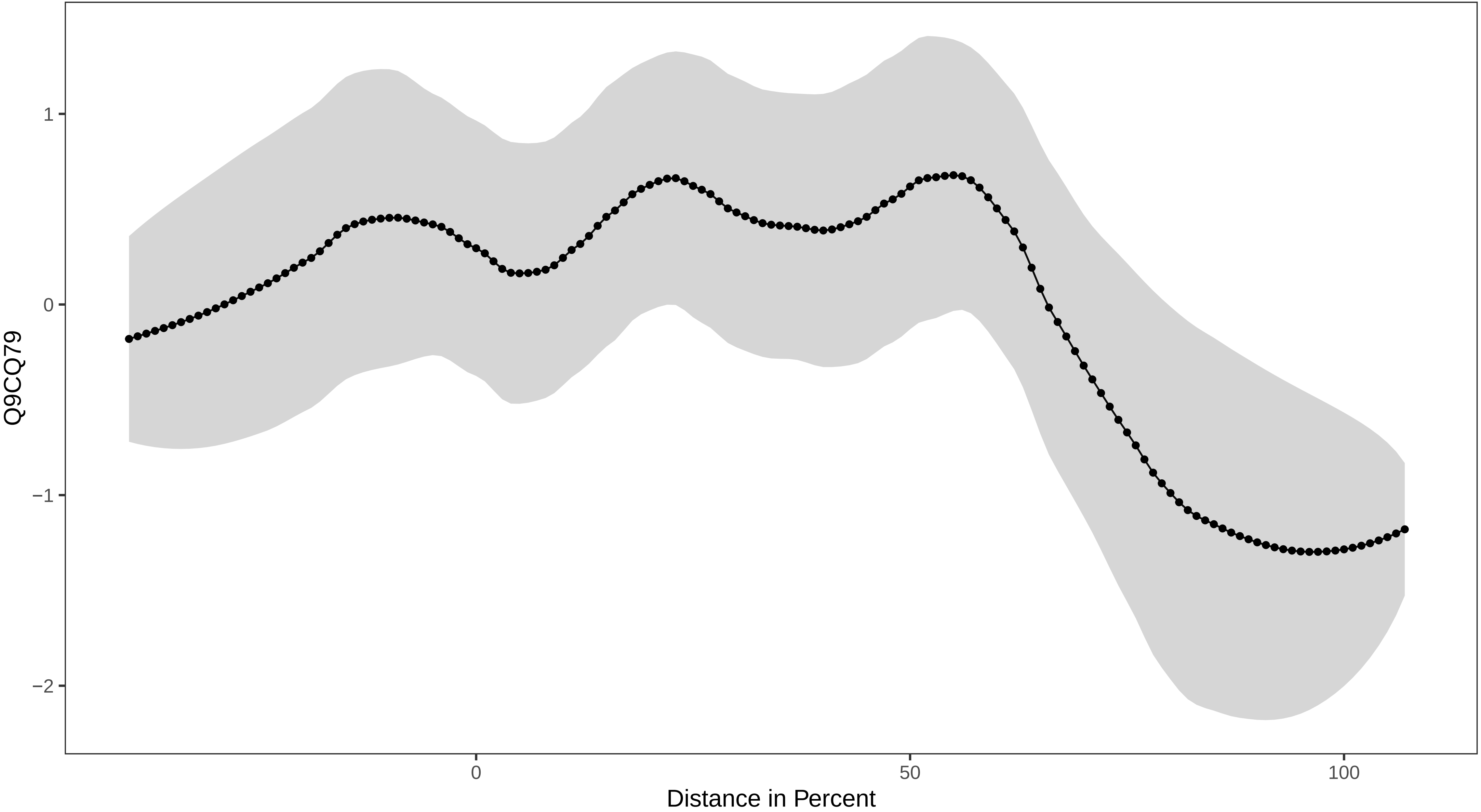

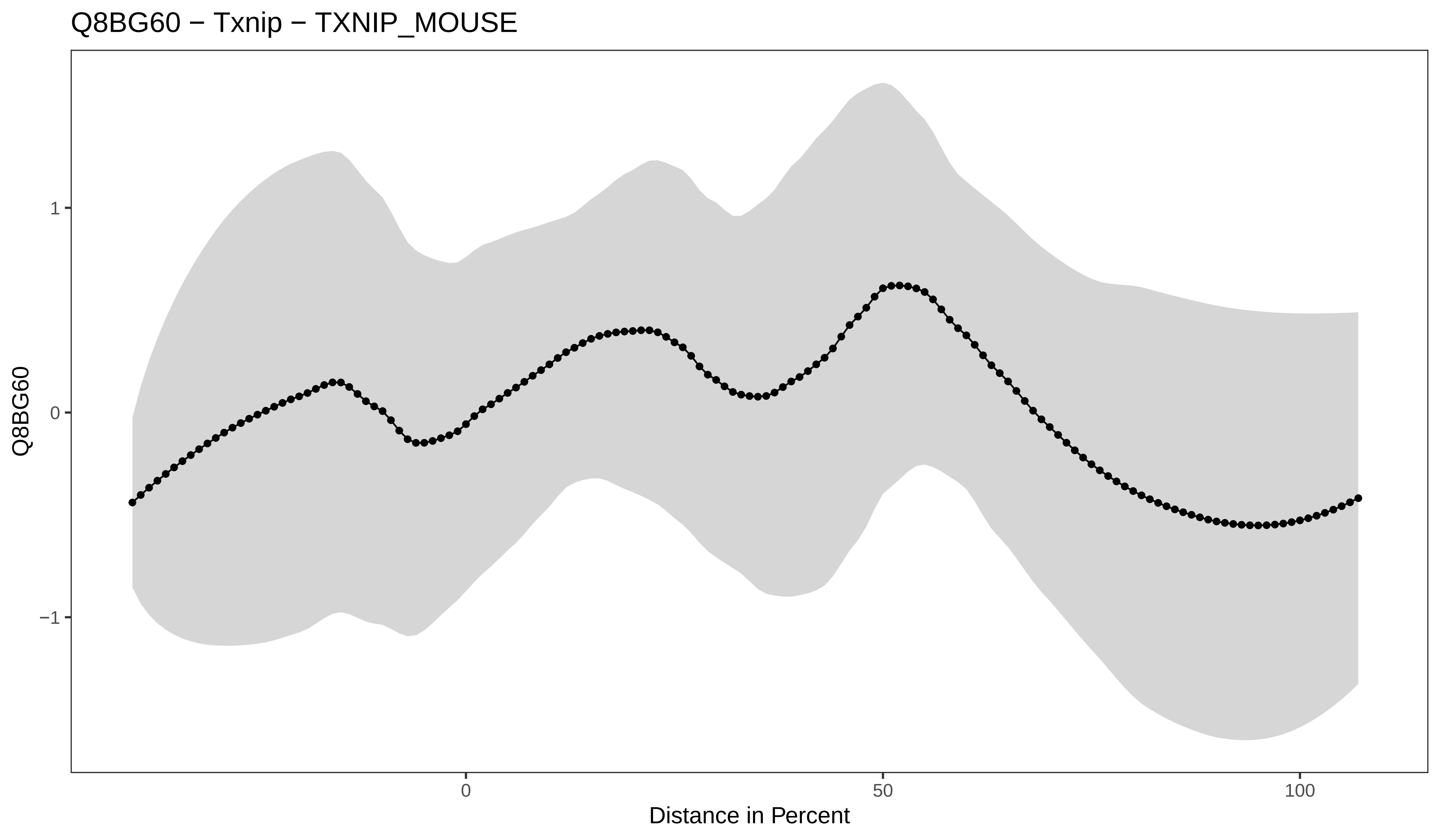
Q8BG60 − Txnip − TXNIP_MOUSE
Q8BG60
1
0
−1
0
50
100
Distance in Percent

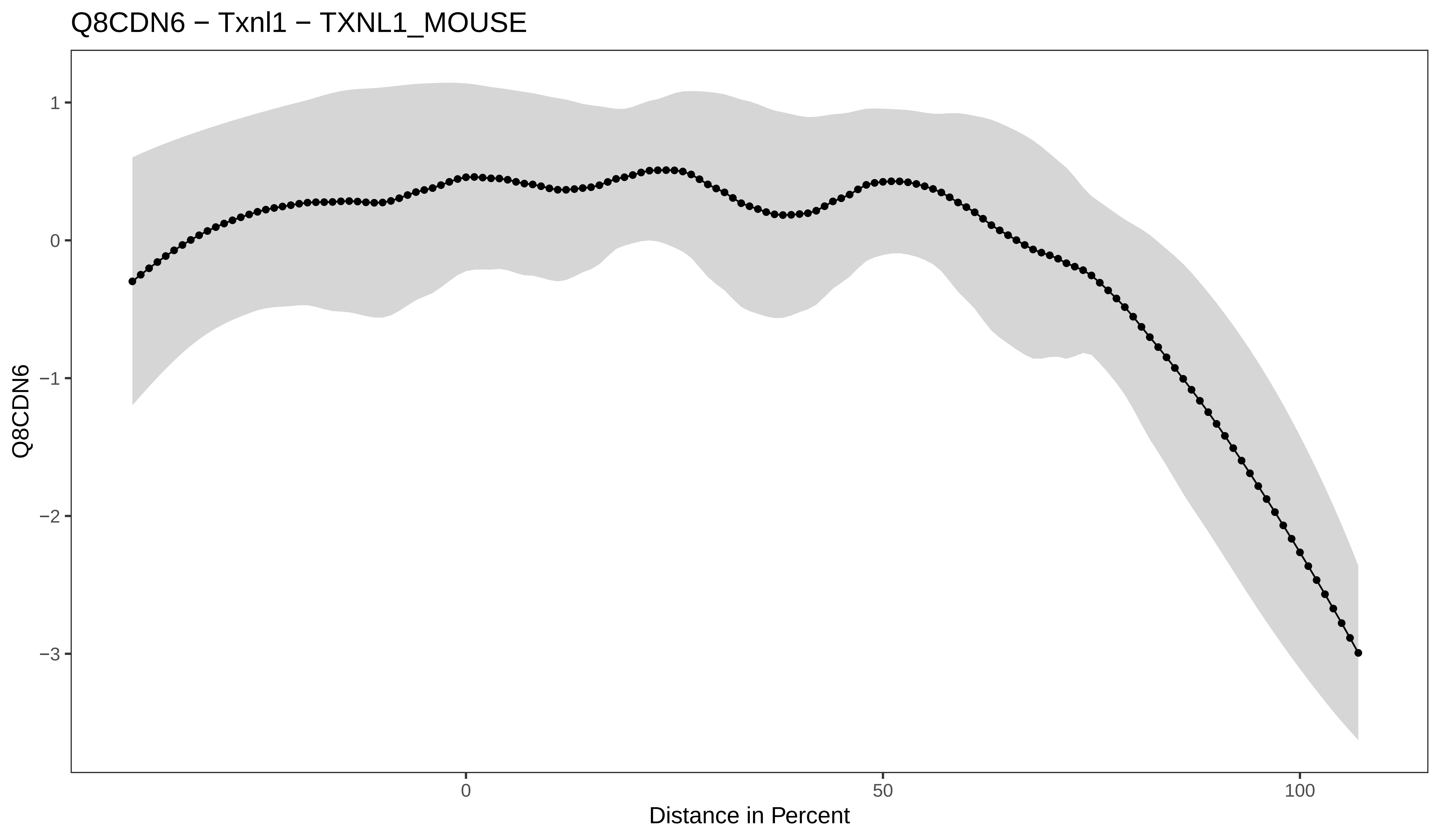

Q8CDN6 − Txnl1 − TXNL1_MOUSE
1
0
−1
−2
−3
Q8CDN6
0
50
100
Distance in Percent

Q9JMH6 − Txnrd1 − TRXR1_MOUSE

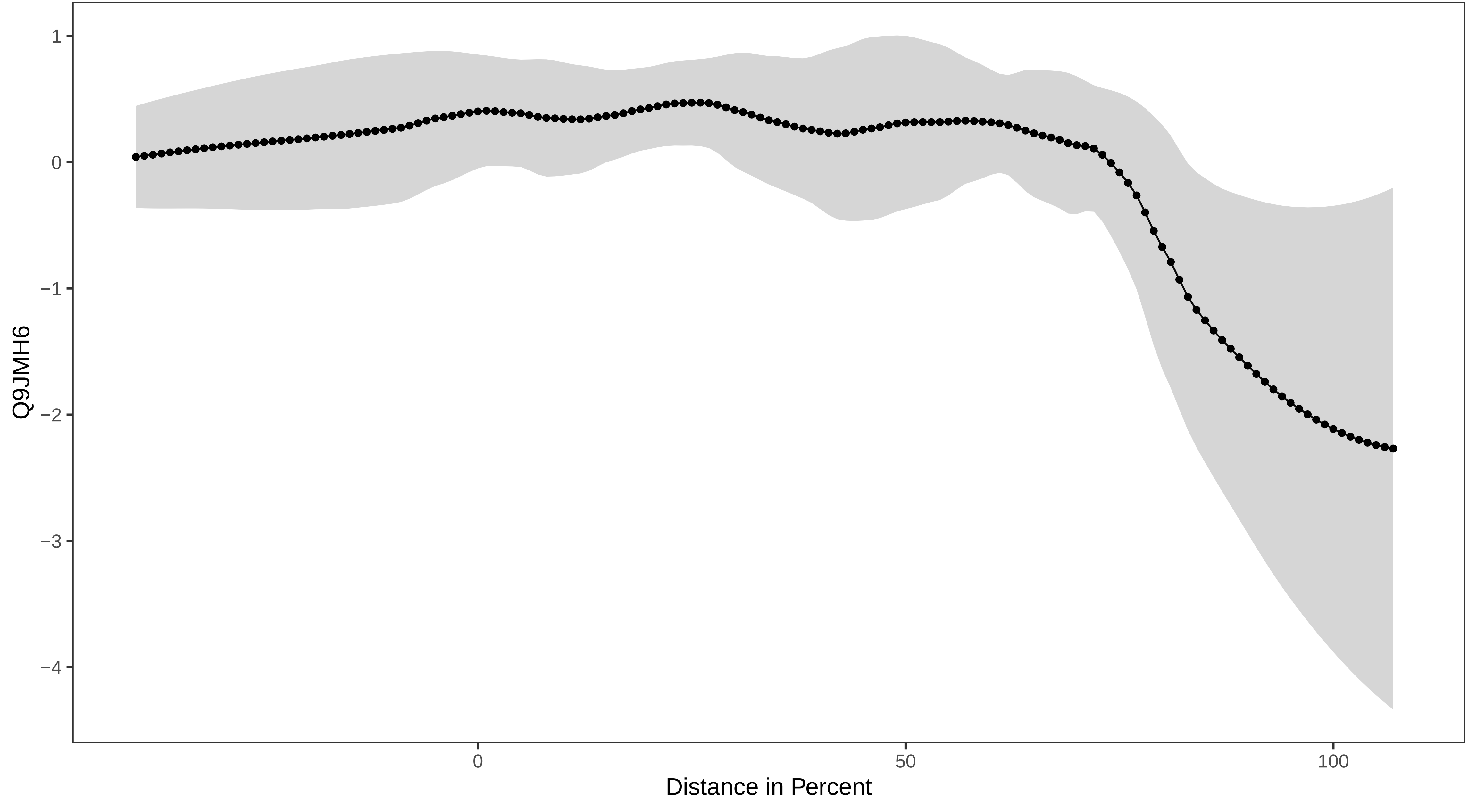

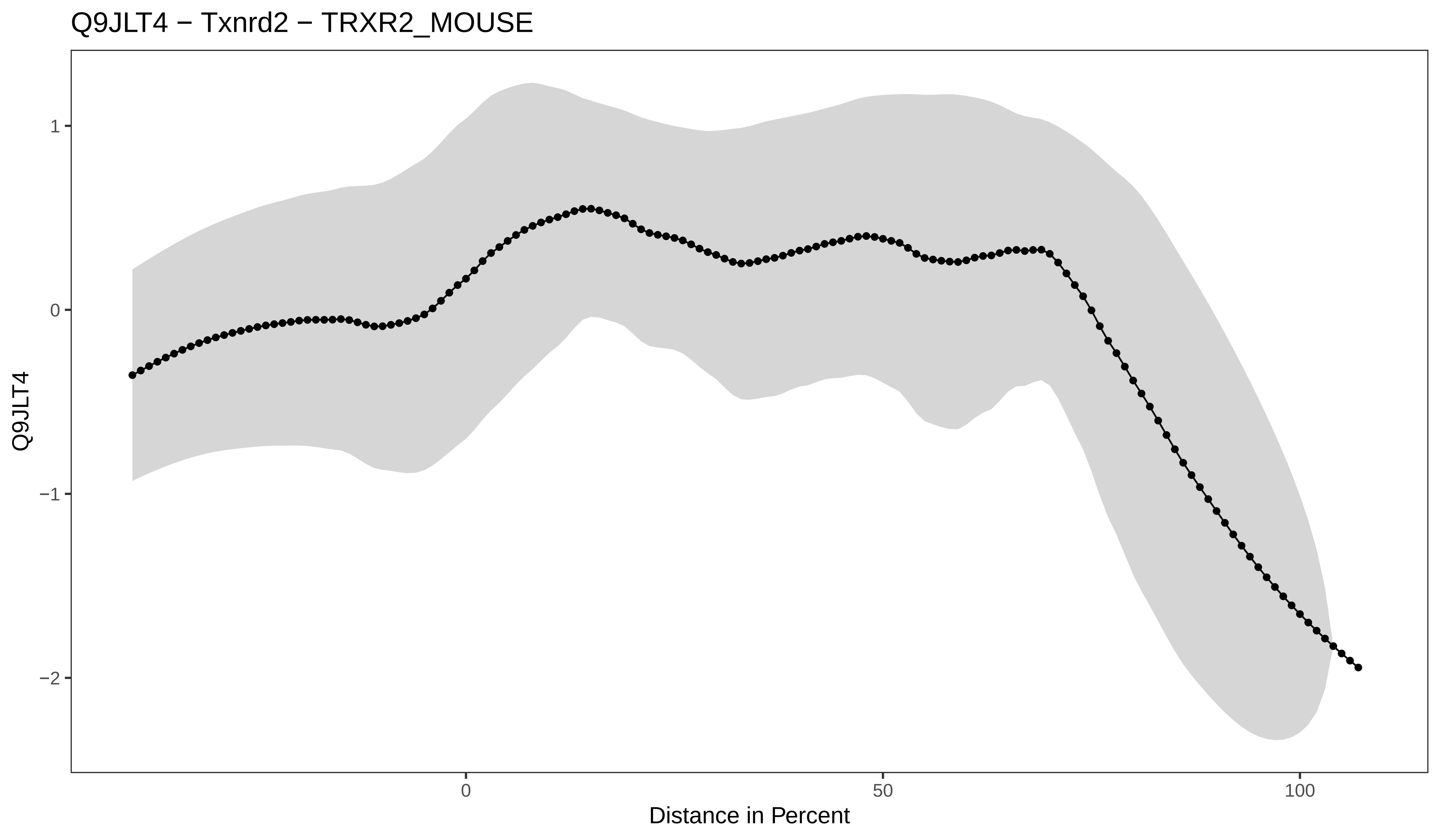

Q9JLT4 − Txnrd2 − TRXR2_MOUSE
Q9JLT4
1
0
−1
−2
0
50
100
Distance in Percent

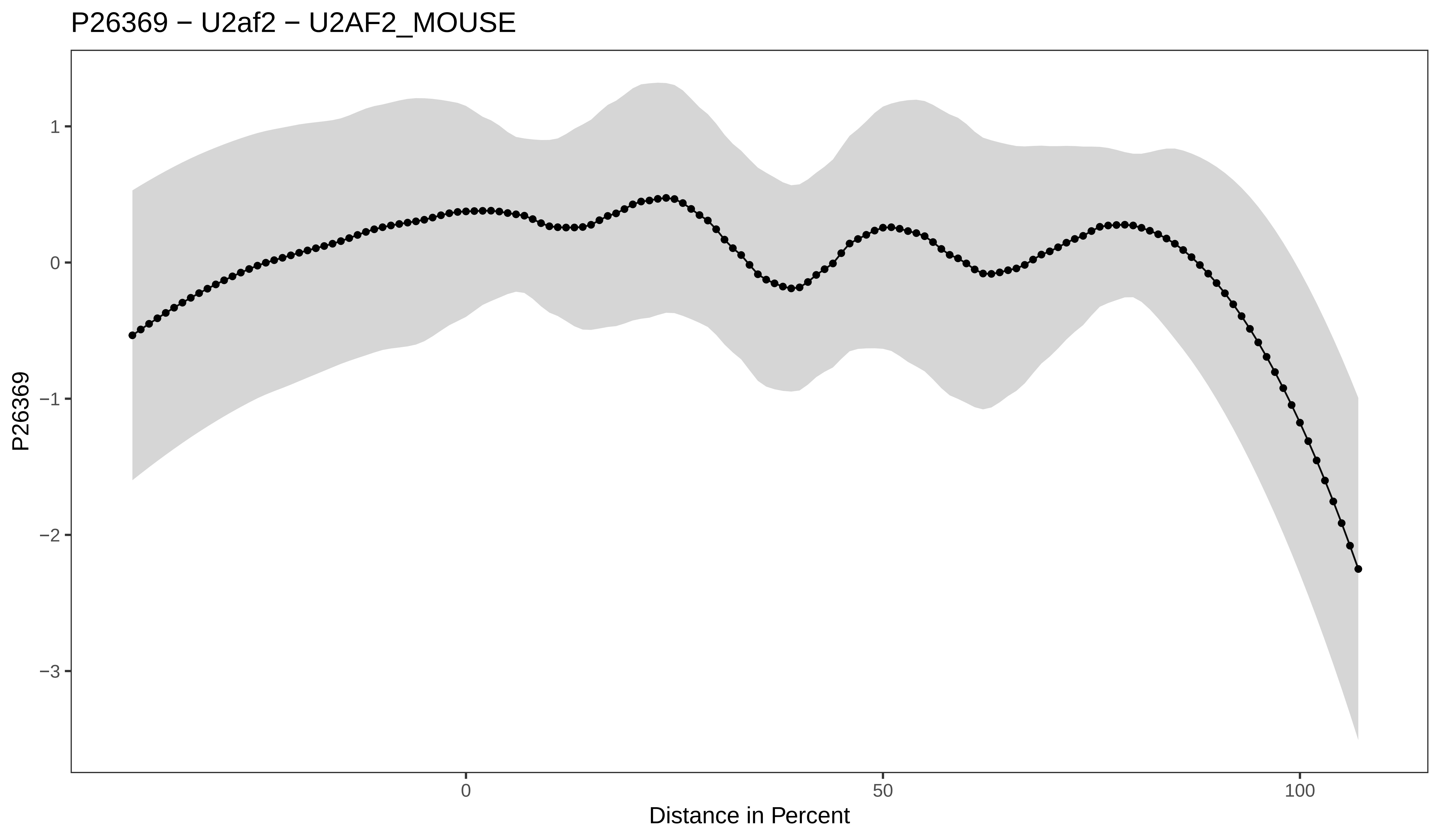
P26369 − U2af2 − U2AF2_MOUSE
1
0
−1
−2
−3
P26369
0
50
100
Distance in Percent

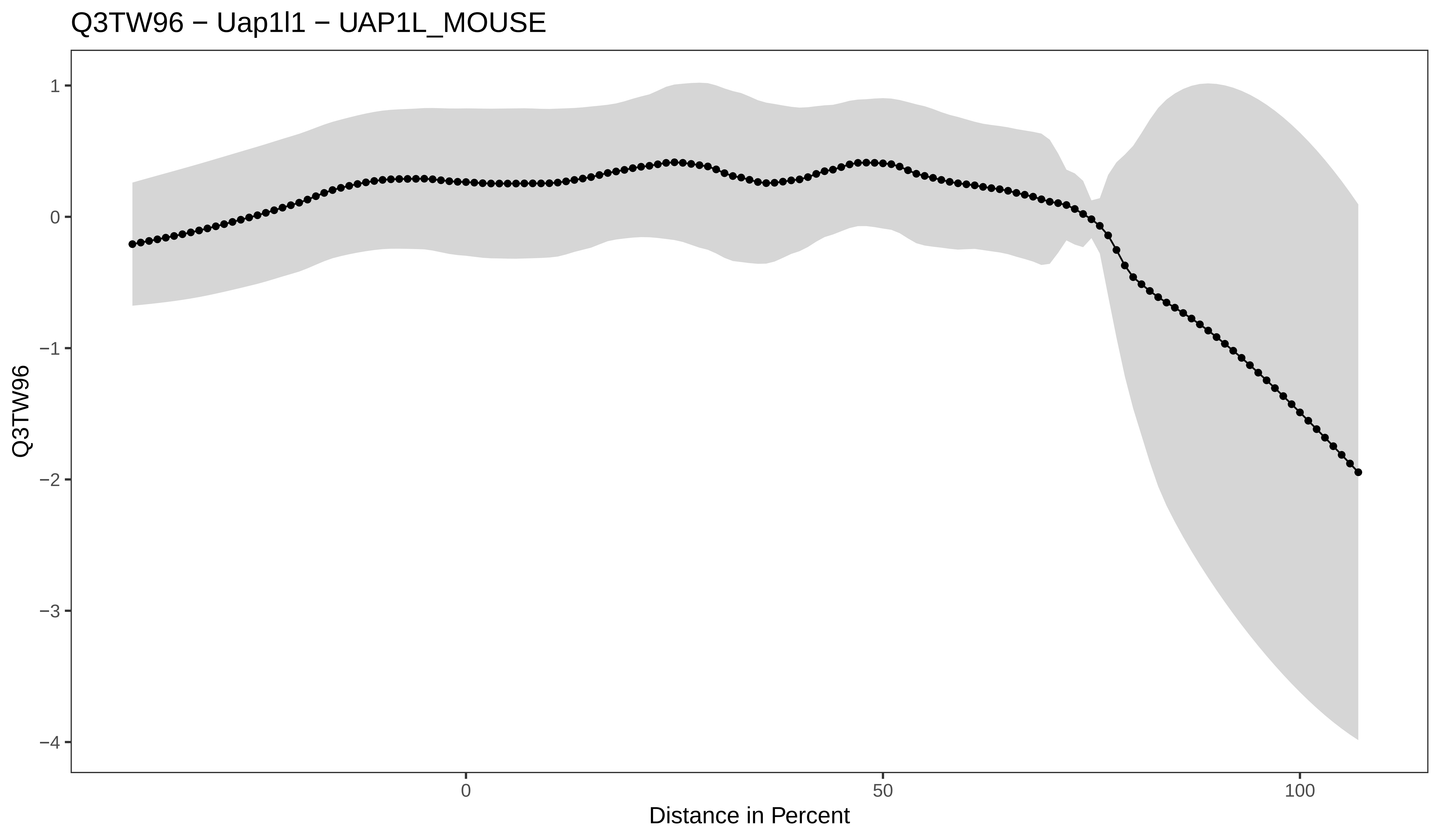
Q3TW96 − Uap1l1 − UAP1L_MOUSE
1
0
−1
−2
−3
−4
Q3TW96
0
50
100
Distance in Percent

Q02053 − Uba1 − UBA1_MOUSE

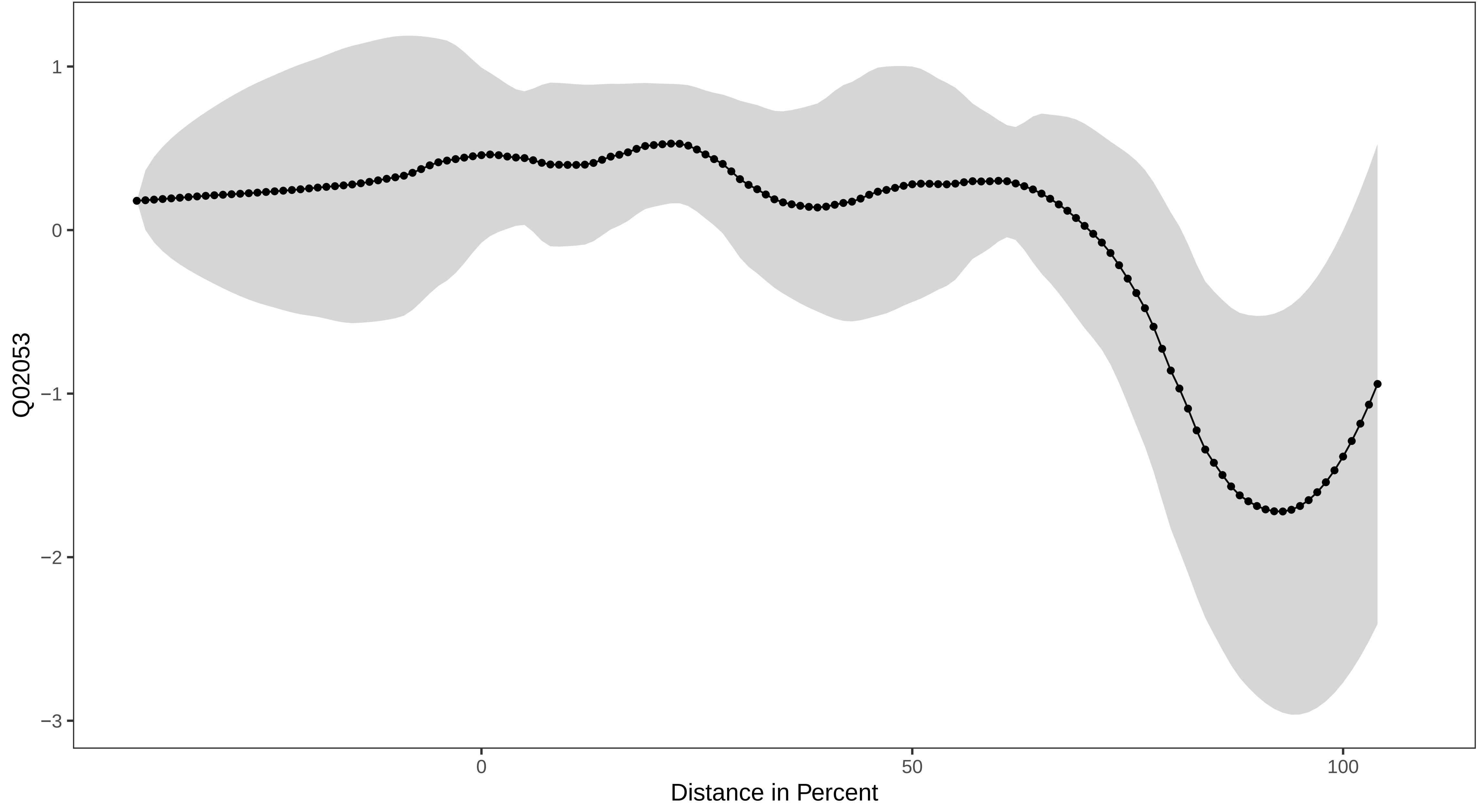

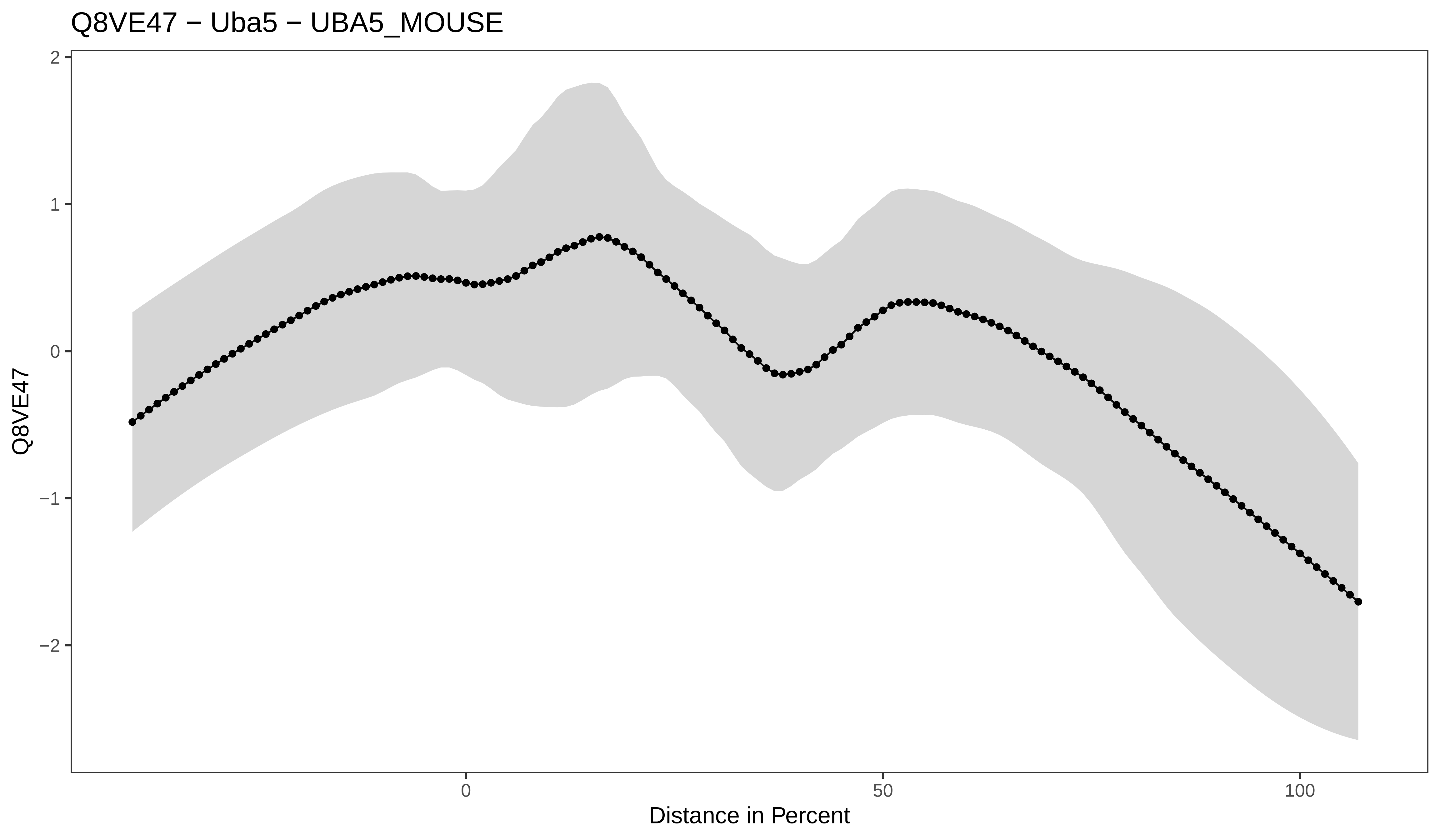

Q8VE47 − Uba5 − UBA5_MOUSE
Q8VE47
Distance in Percent
2
1
0
−1
−2
0
50
100

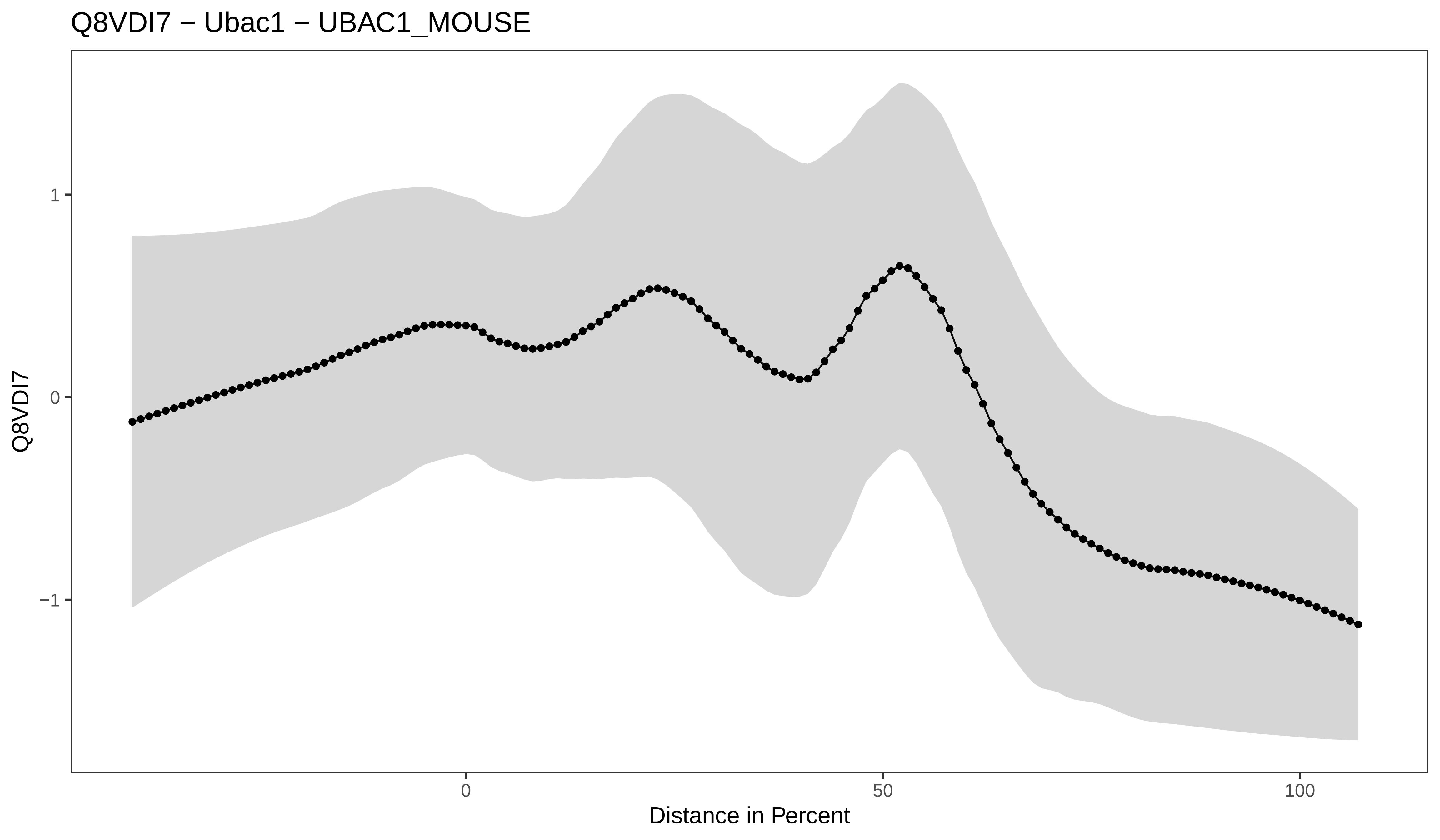

Q8VDI7 − Ubac1 − UBAC1_MOUSE
Q8VDI7
1
0
−1
0
50
100
Distance in Percent

Q80X50 − Ubap2l − UBP2L_MOUSE

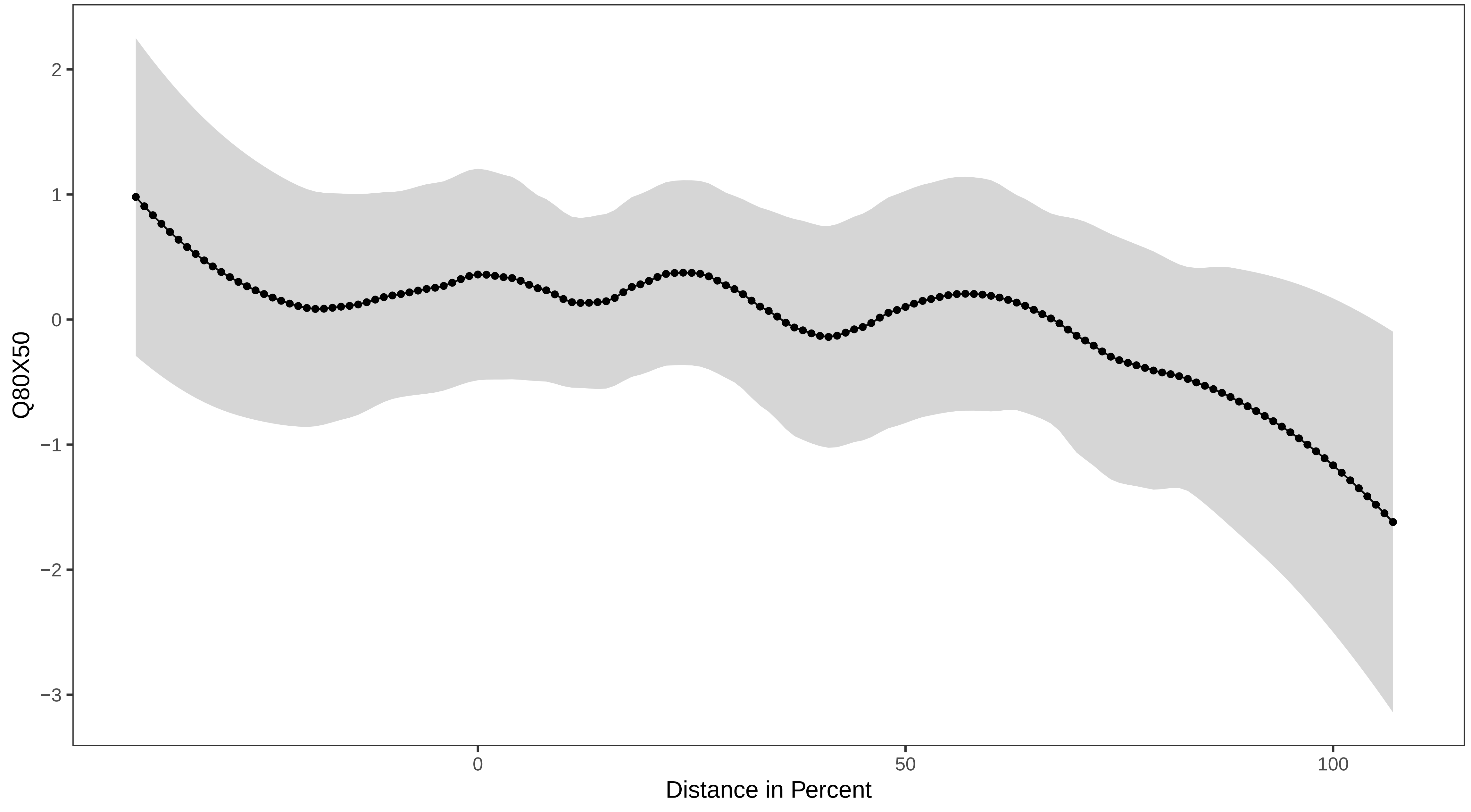

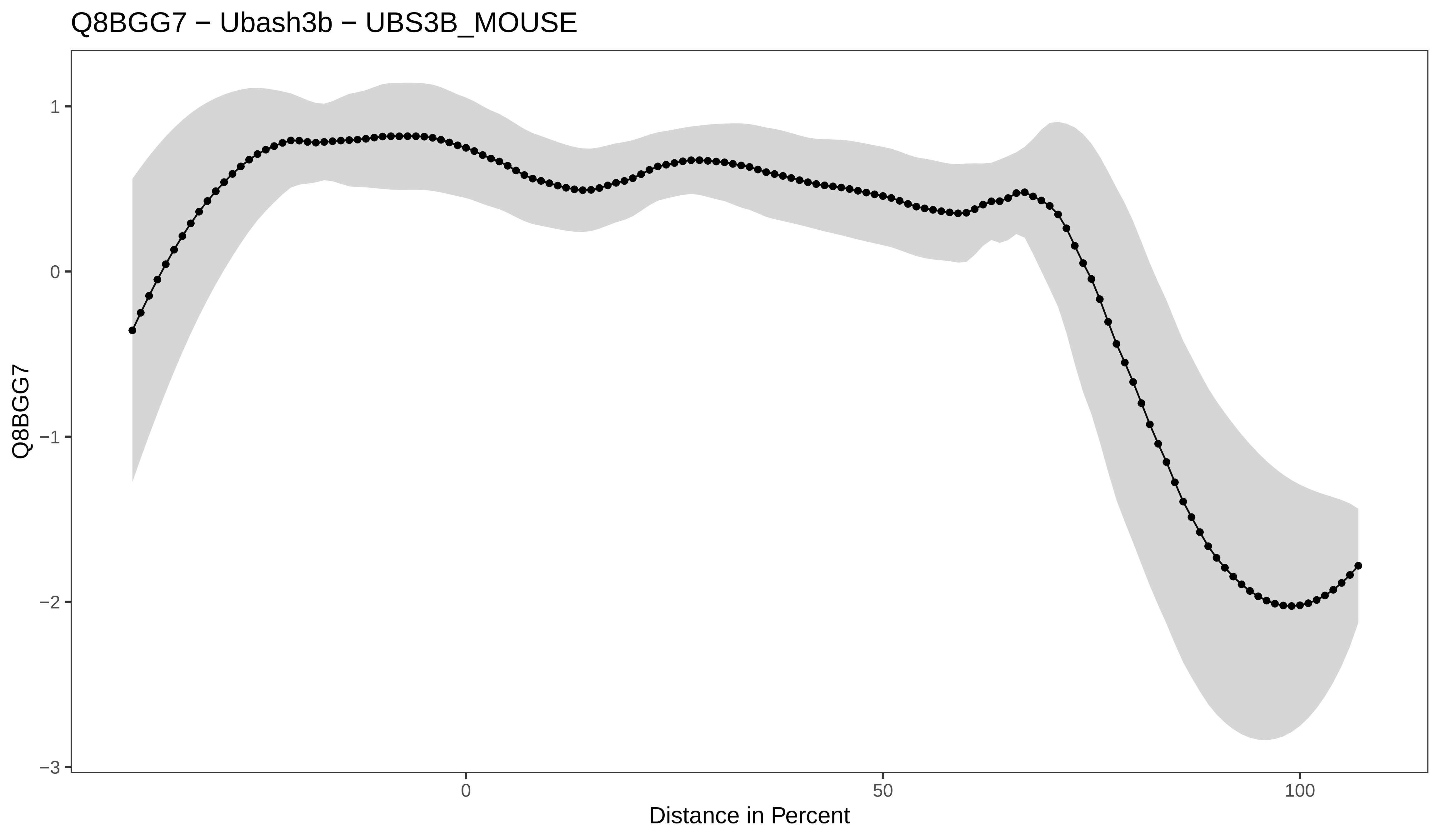

Q8BGG7 − Ubash3b − UBS3B_MOUSE
1
0
−1
−2
−3
Q8BGG7
0
50
100
Distance in Percent

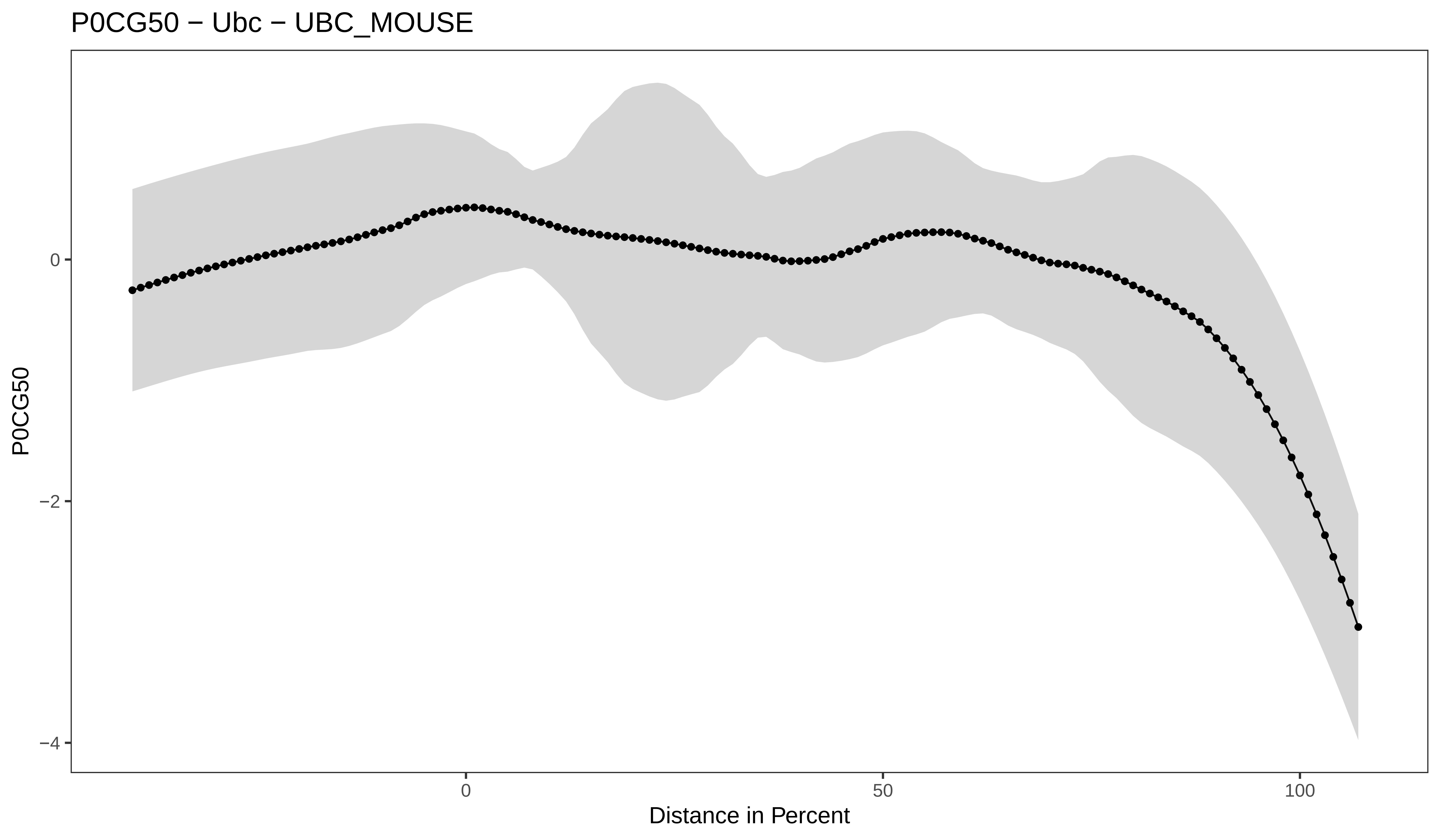
P0CG50 − Ubc − UBC_MOUSE
P0CG50
0
−2
−4
0
50
100
Distance in Percent

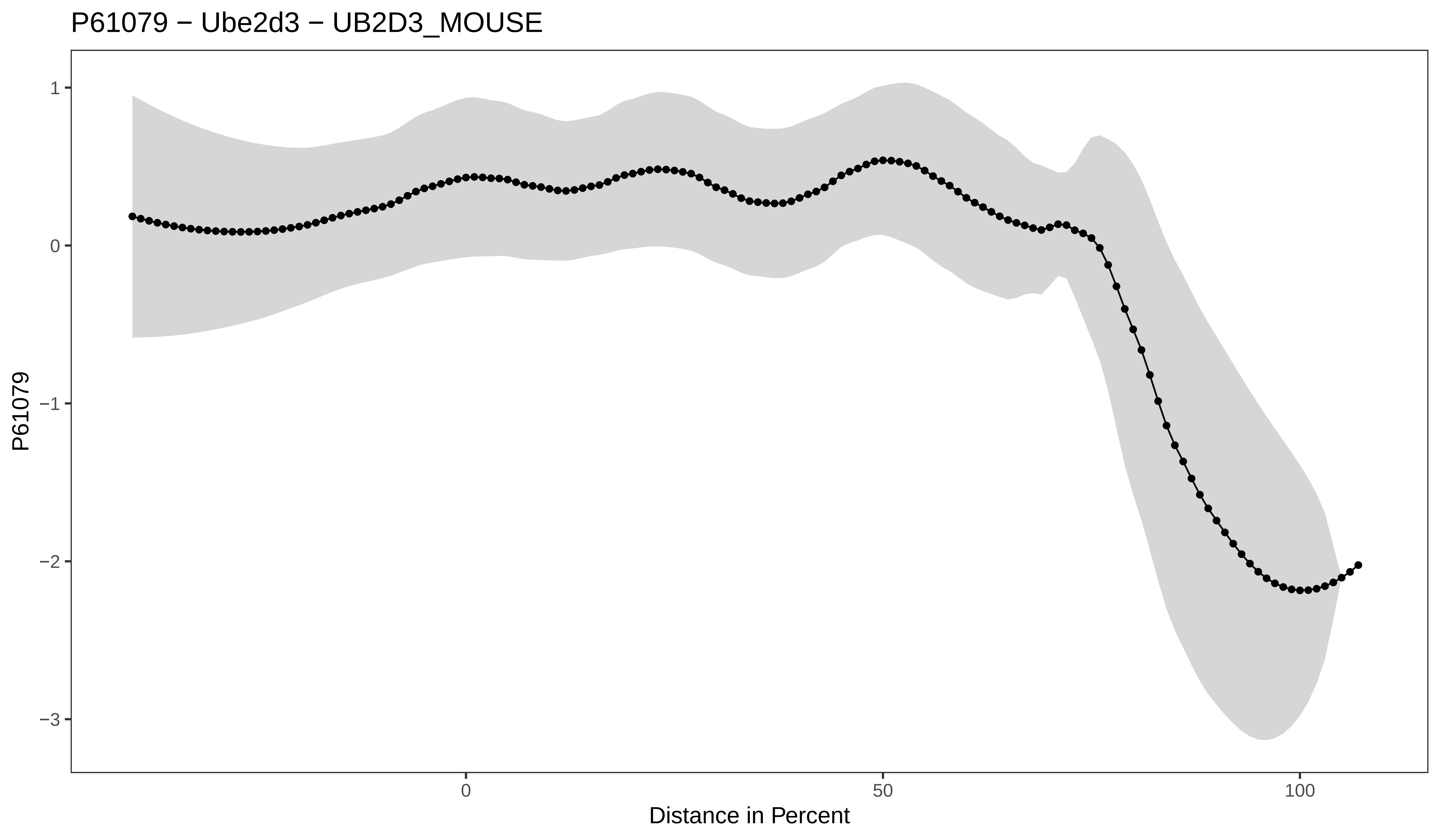
P61079 − Ube2d3 − UB2D3_MOUSE
P61079
1
0
−1
−2
−3
0
50
100
Distance in Percent

P61087 − Ube2k − UBE2K_MOUSE

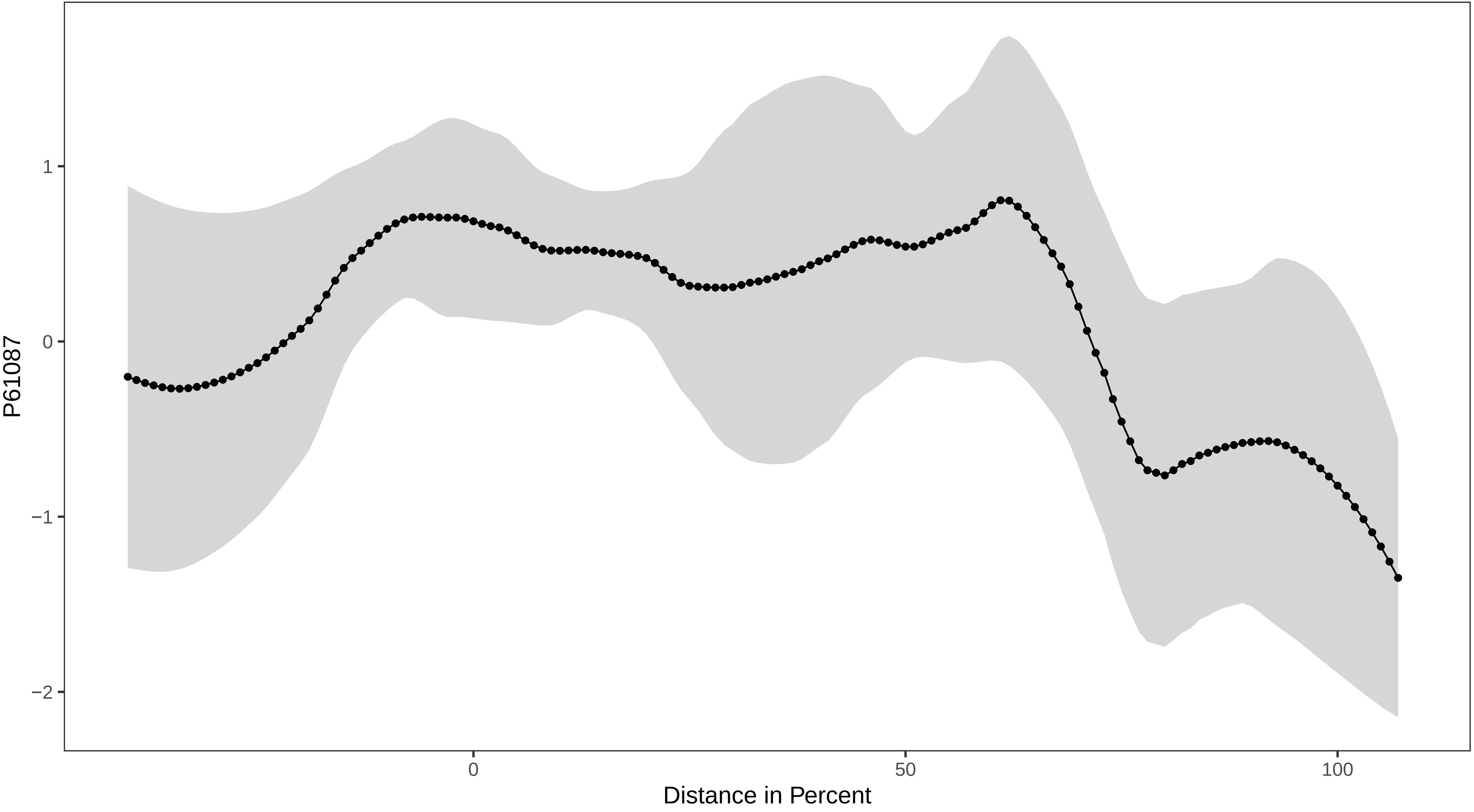

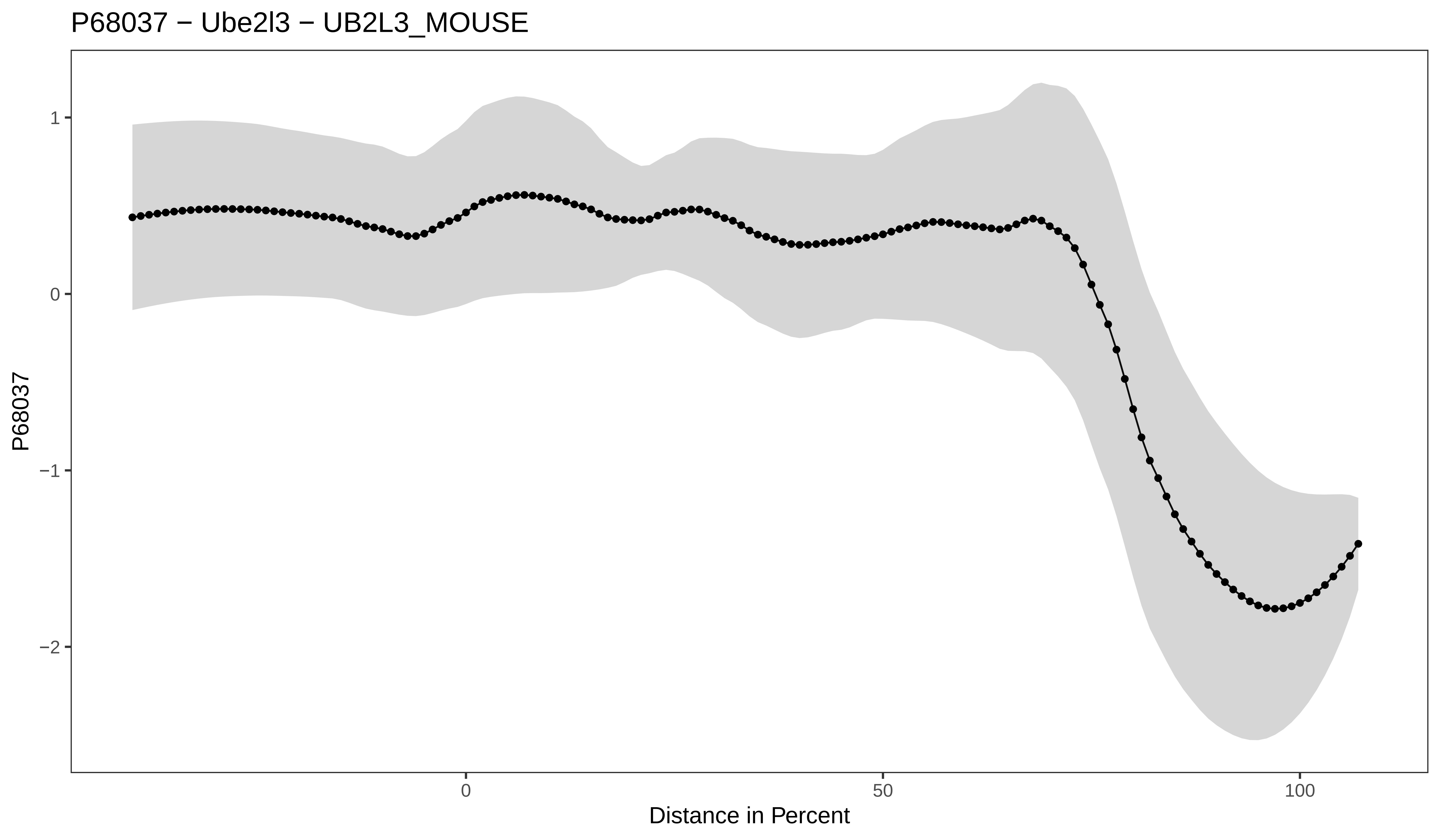
P68037 − Ube2l3 − UB2L3_MOUSE
P68037
1
0
−1
−2
0
50
100
Distance in Percent

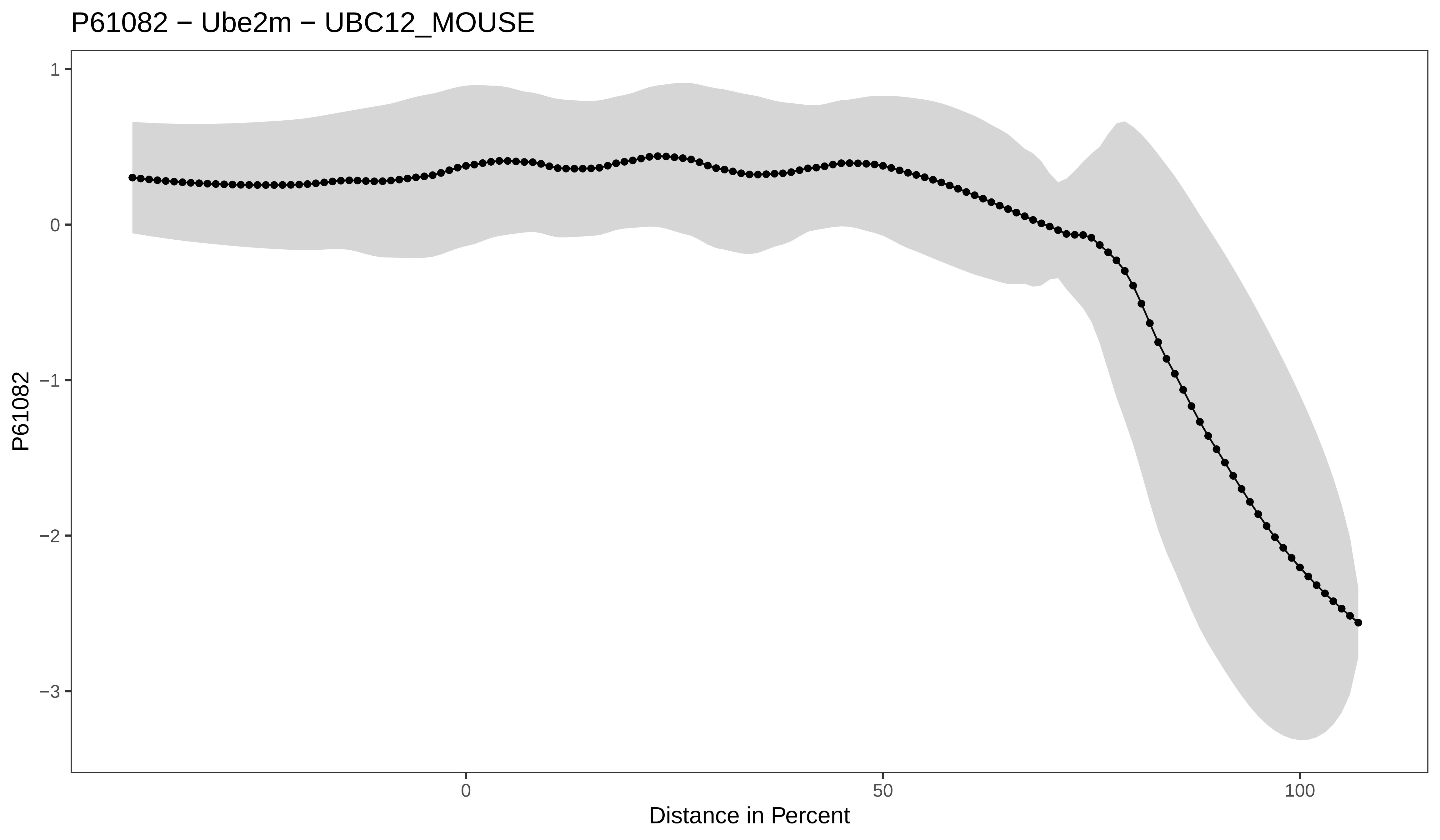

P61082 − Ube2m − UBC12_MOUSE
P61082
Distance in Percent
1
0
−1
−2
−3
0
50
100

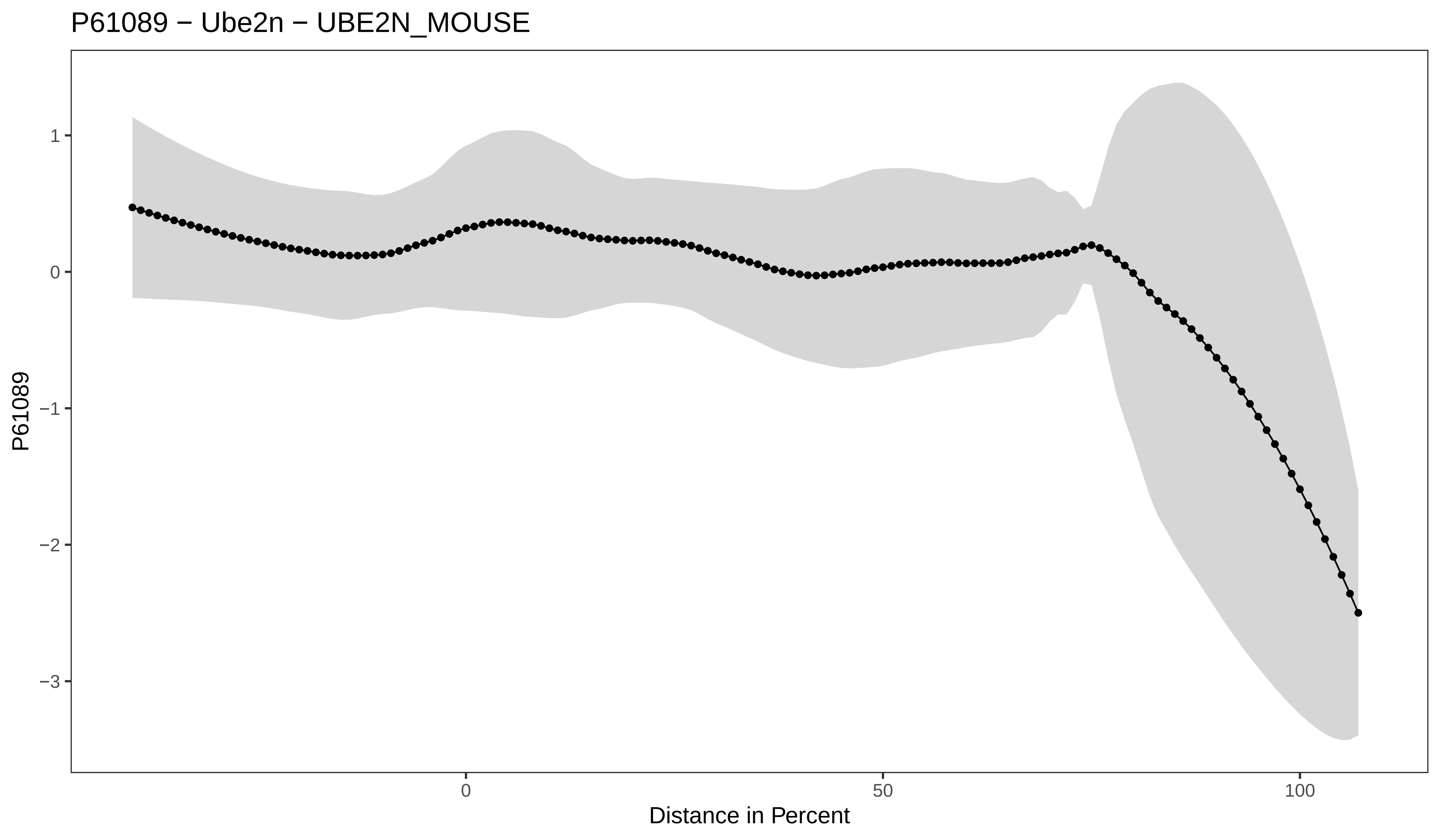
P61089 − Ube2n − UBE2N_MOUSE
P61089
1
0
−1
−2
−3
0
50
100
Distance in Percent

Q6ZPJ3 − Ube2o − UBE2O_MOUSE

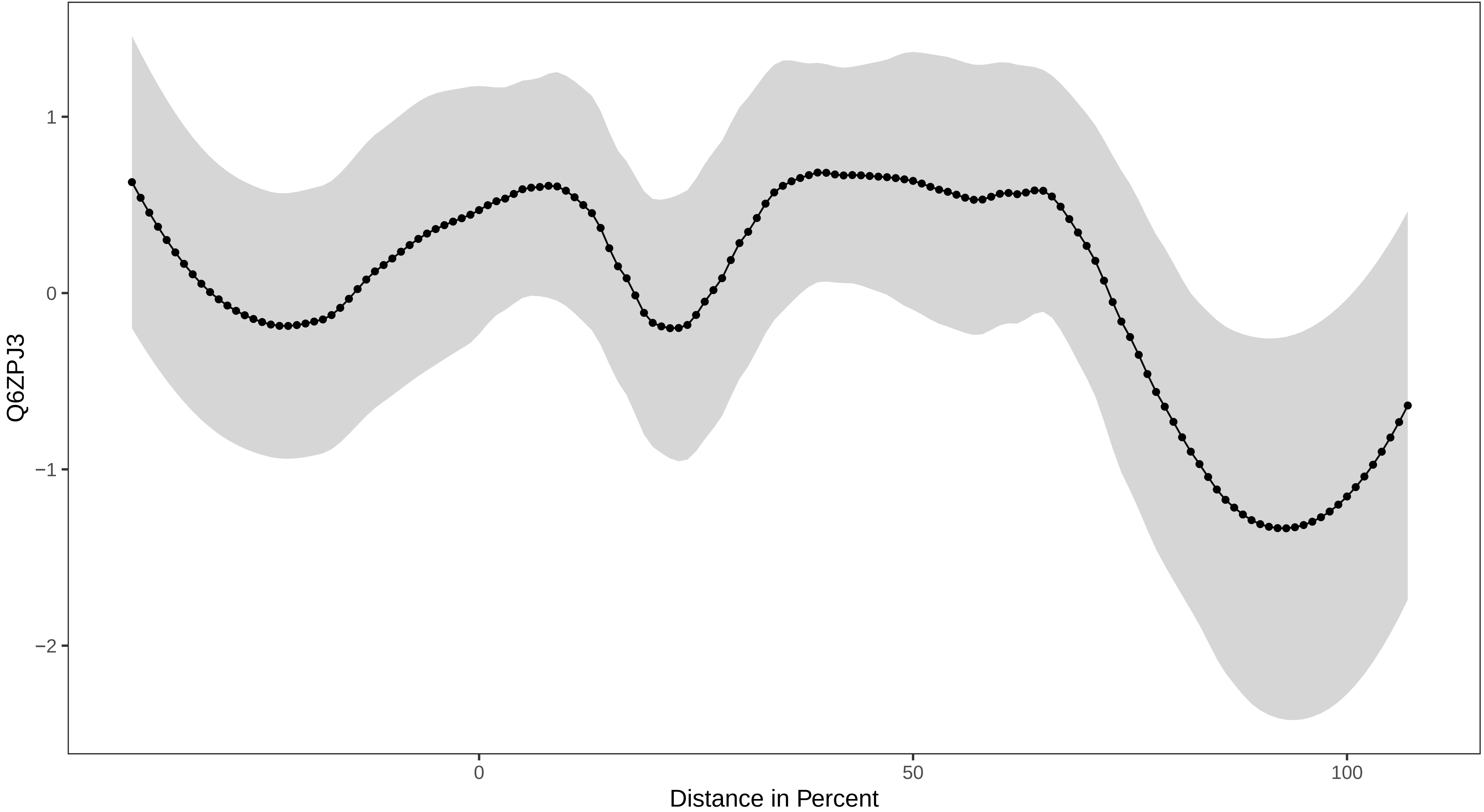

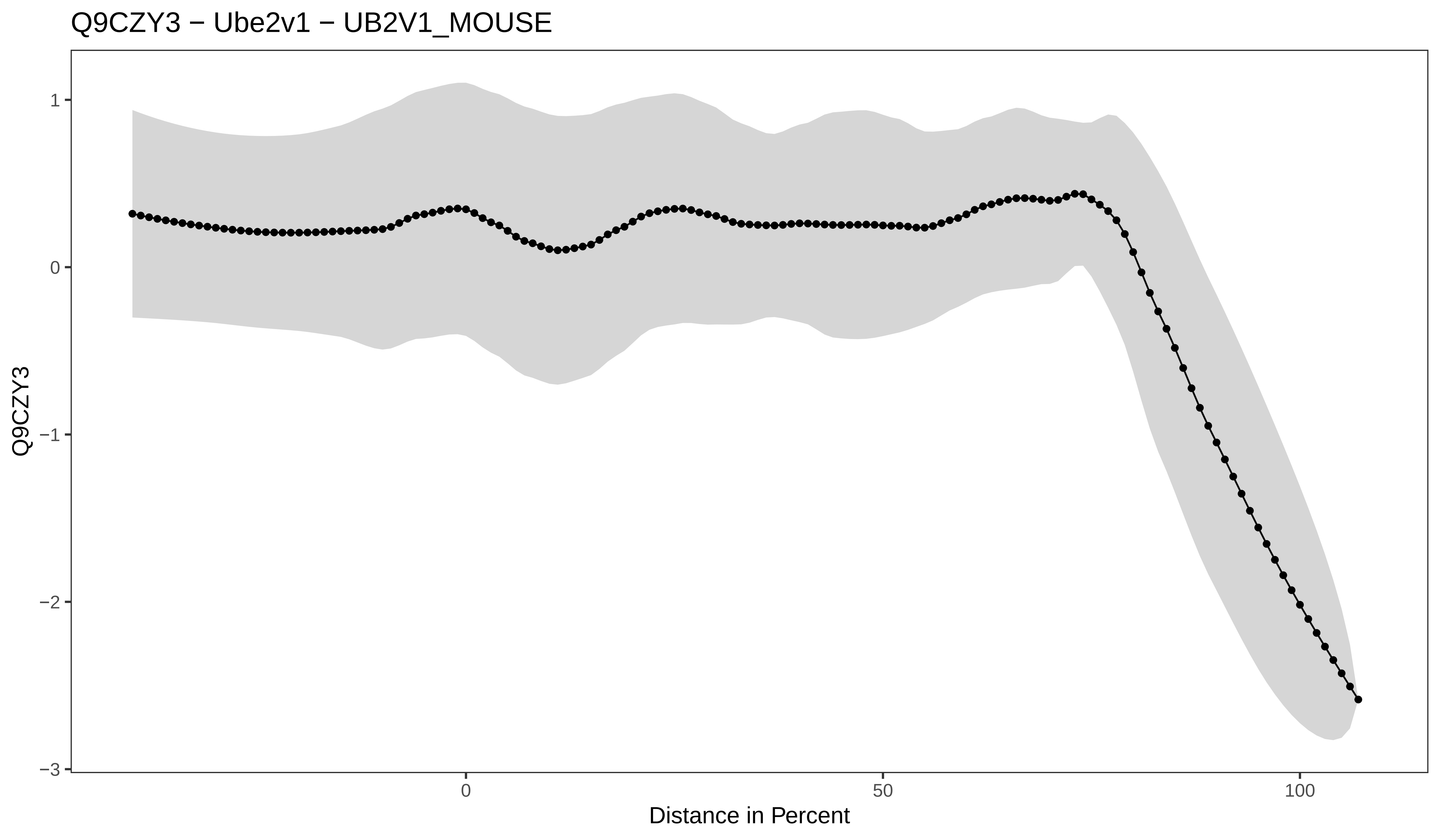

Q9CZY3 − Ube2v1 − UB2V1_MOUSE
Q9CZY3
1
0
−1
−2
−3
0
50
100
Distance in Percent

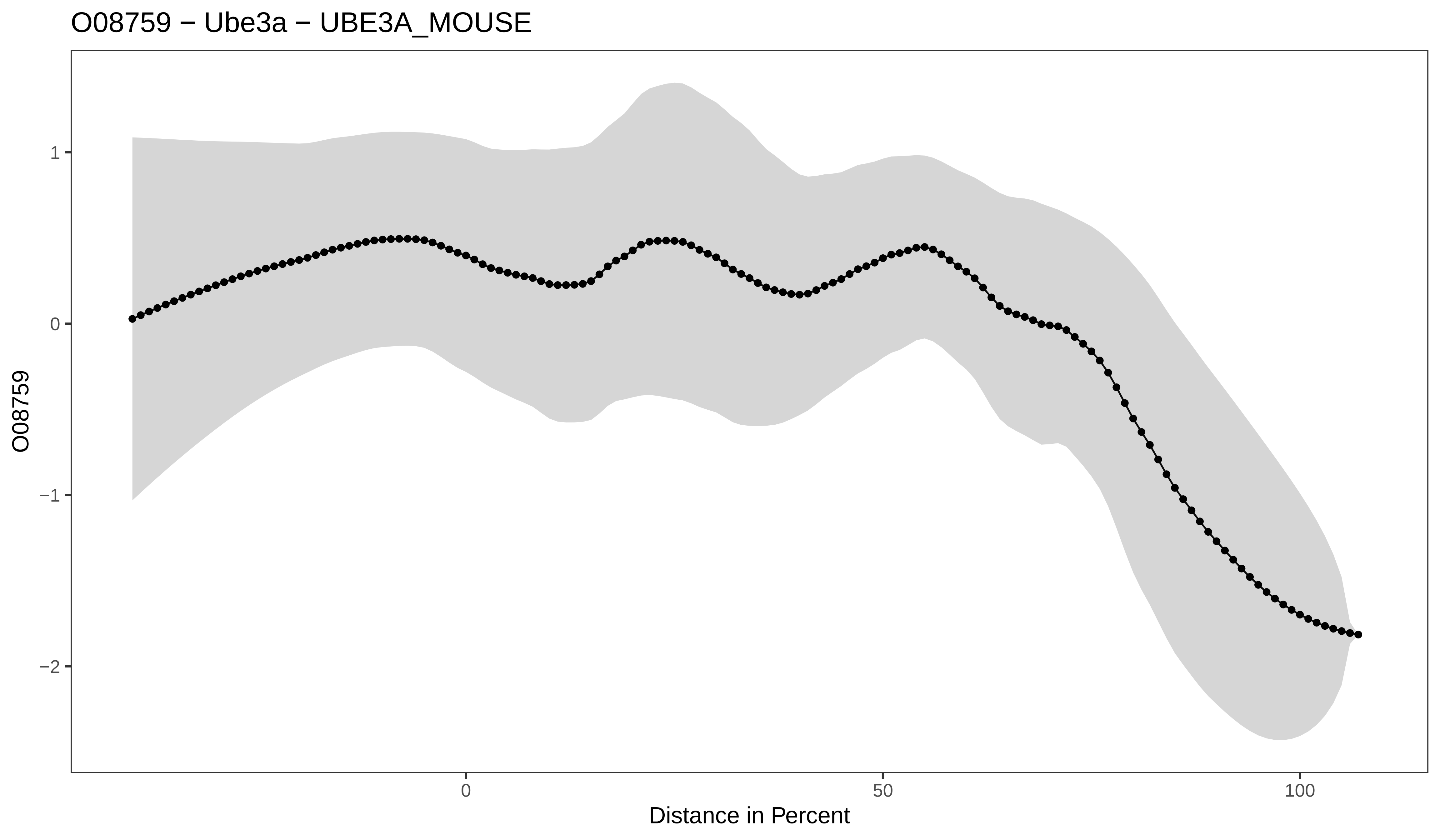
O08759 − Ube3a − UBE3A_MOUSE
O08759
1
0
−1
−2
0
50
100
Distance in Percent

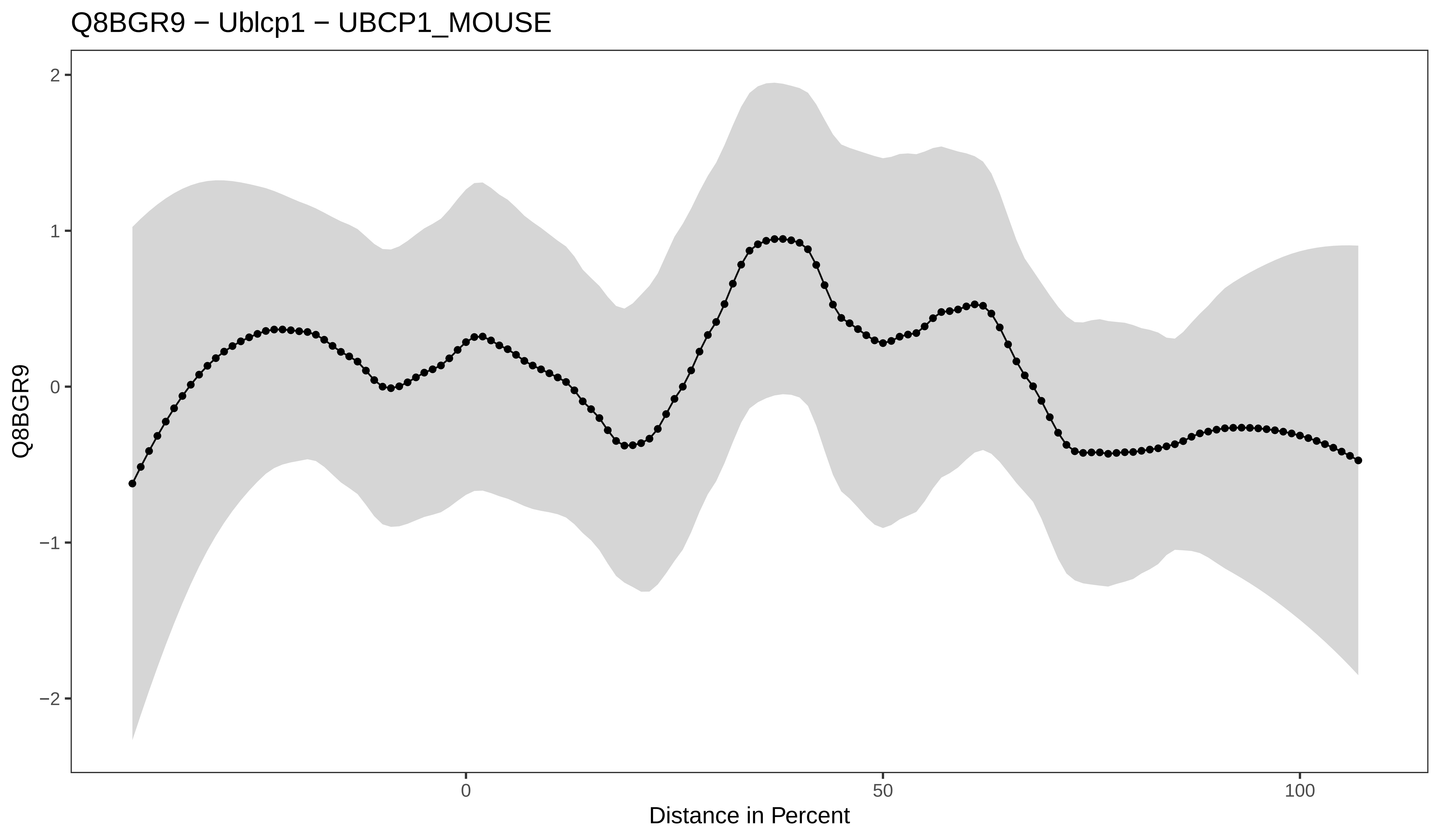
Q8BGR9 − Ublcp1 − UBCP1_MOUSE
Q8BGR9
2
1
0
−1
−2
0
50
100
Distance in Percent

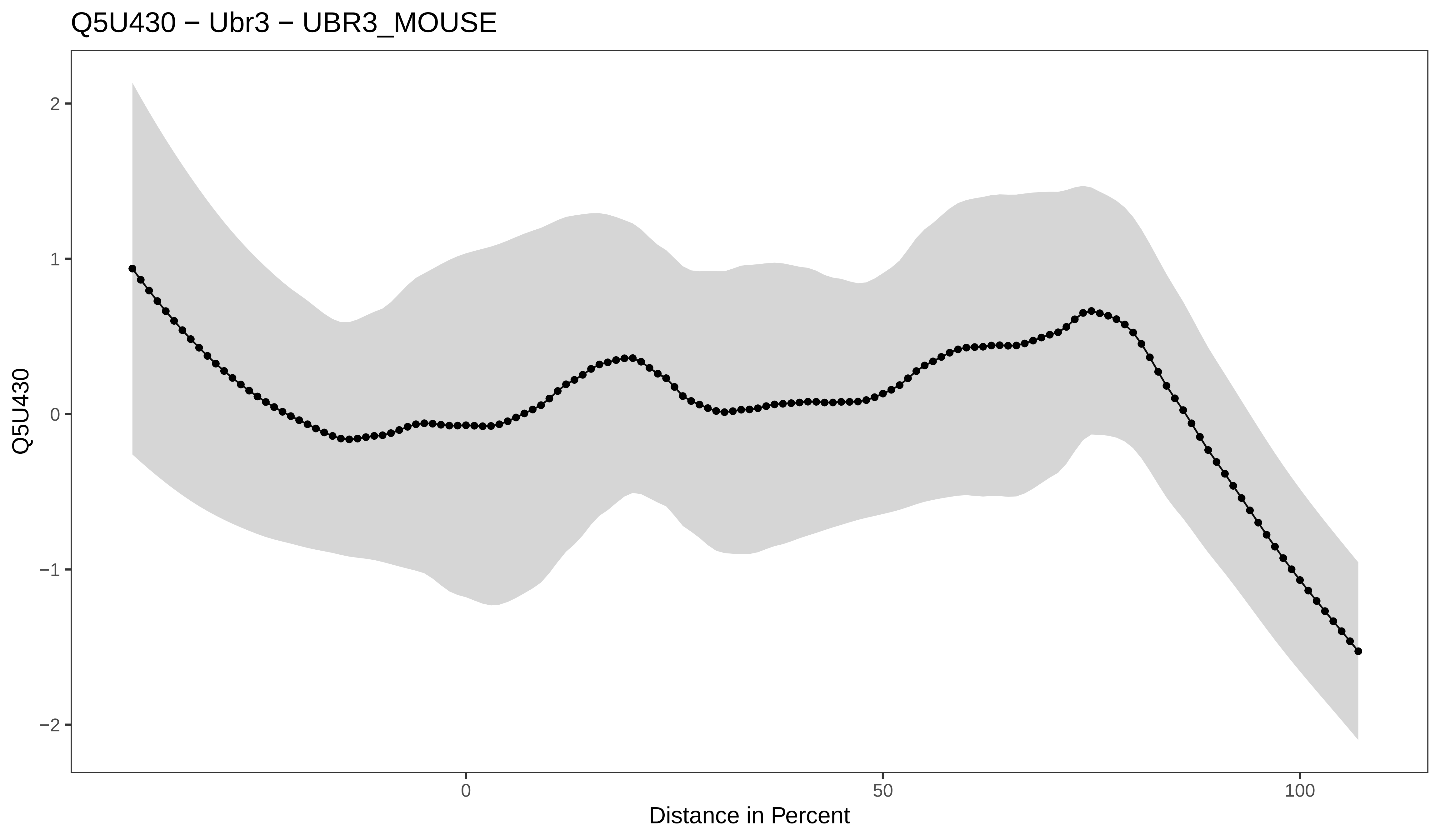
Q5U430 − Ubr3 − UBR3_MOUSE
Q5U430
2
1
0
−1
−2
0
50
100
Distance in Percent

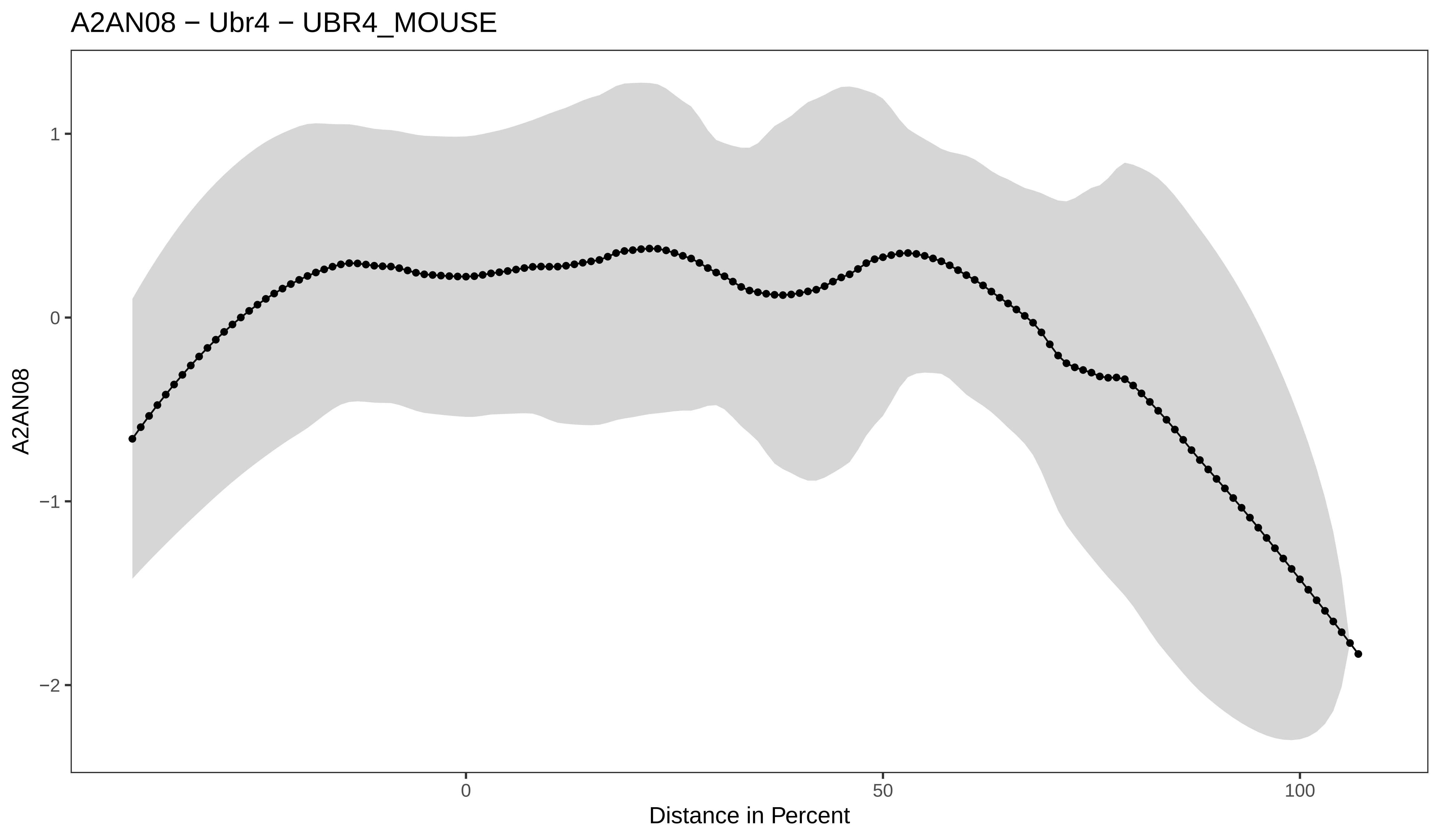

A2AN08 − Ubr4 − UBR4_MOUSE
A2AN08
Distance in Percent
1
0
−1
−2
0
50
100

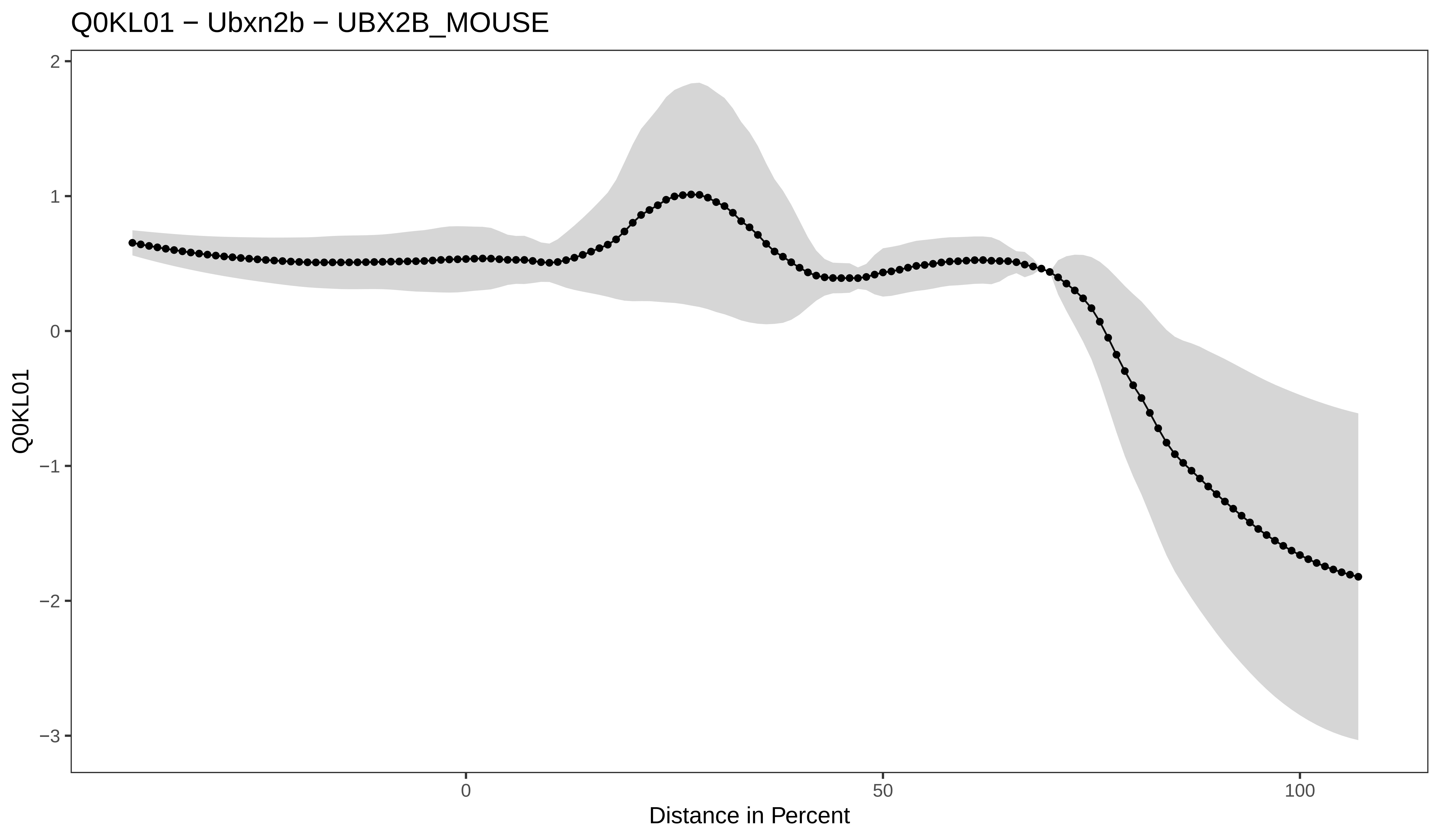

Q0KL01 − Ubxn2b − UBX2B_MOUSE
Q0KL01
Distance in Percent
2
1
0
−1
−2
−3
0
50
100

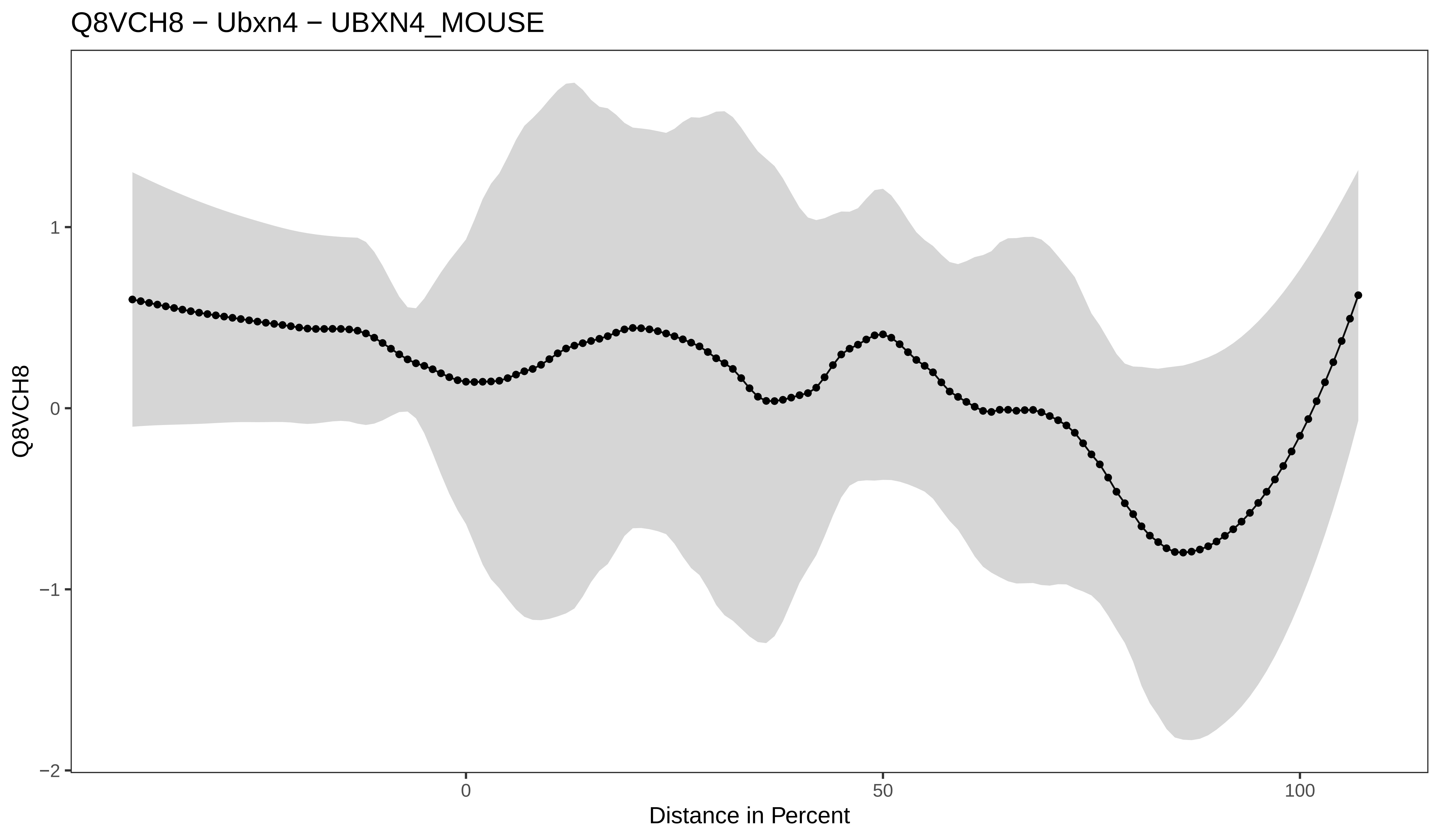

Q8VCH8 − Ubxn4 − UBXN4_MOUSE
Q8VCH8
1
0
−1
−2
0
50
100
Distance in Percent

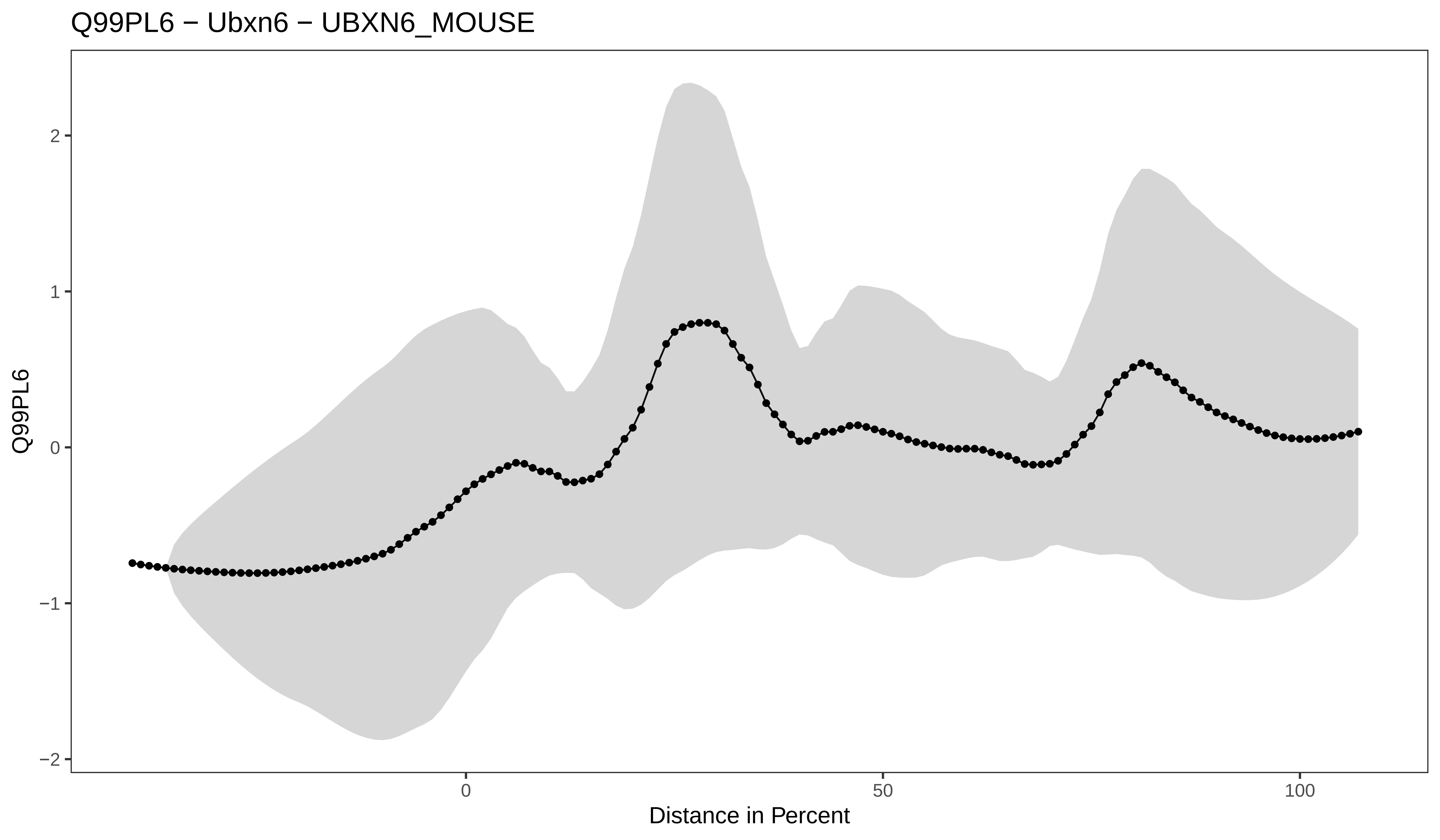

Q99PL6 − Ubxn6 − UBXN6_MOUSE
Q99PL6
2
1
0
−1
−2
0
50
100
Distance in Percent

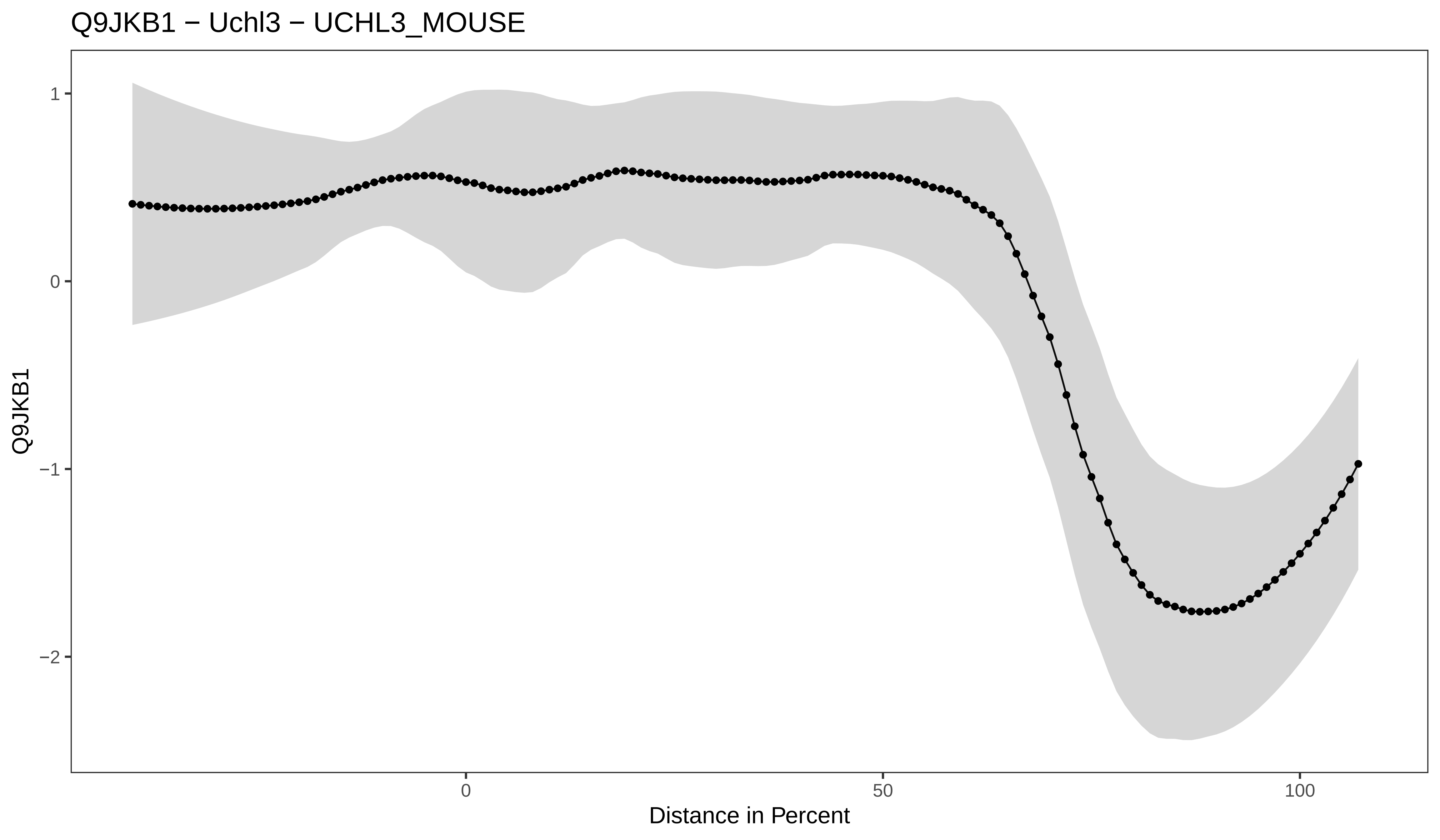
Q9JKB1 − Uchl3 − UCHL3_MOUSE
Q9JKB1
1
0
−1
−2
0
50
100
Distance in Percent

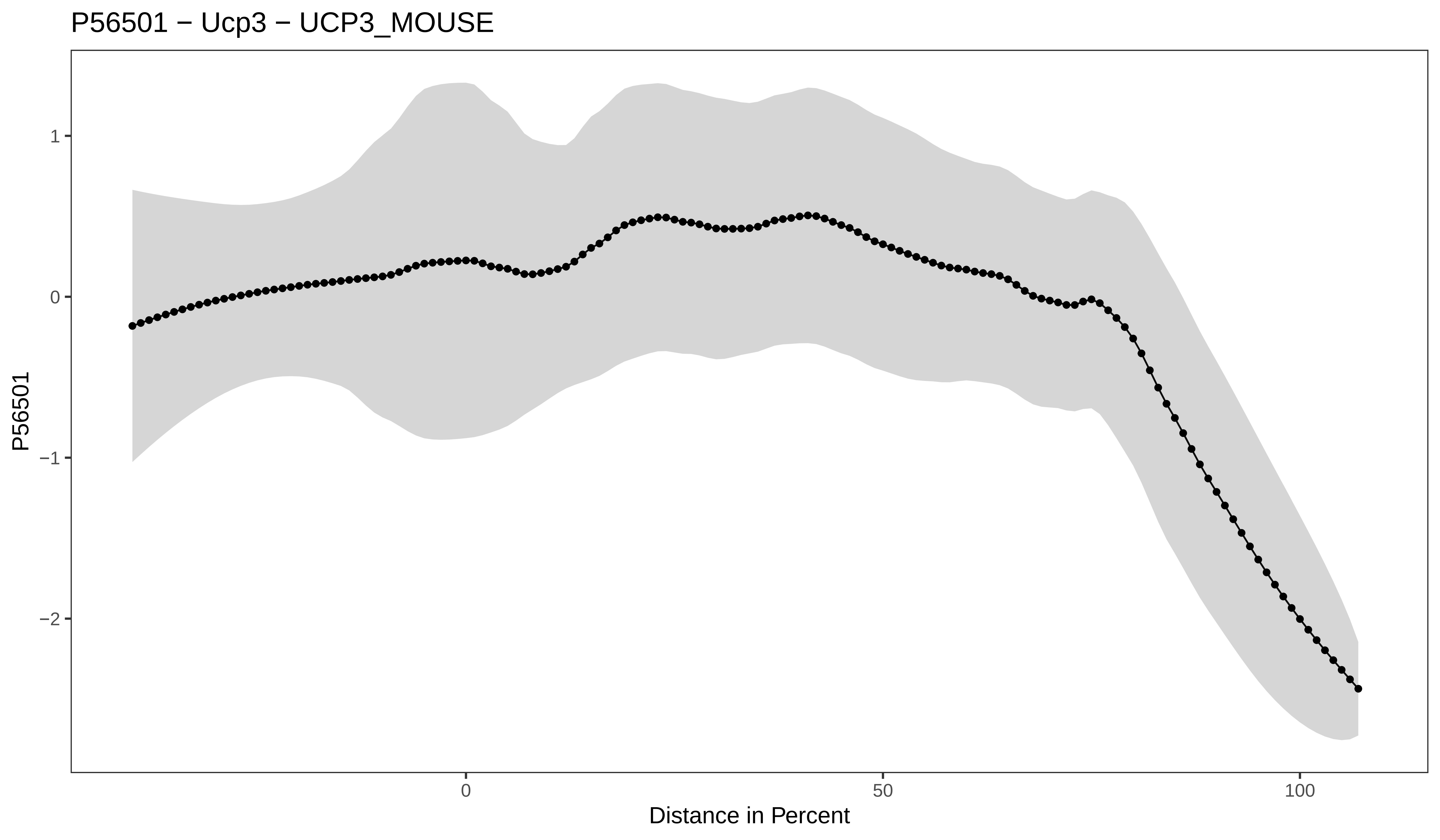

P56501 − Ucp3 − UCP3_MOUSE
1
0
−1
−2
P56501
0
50
100
Distance in Percent

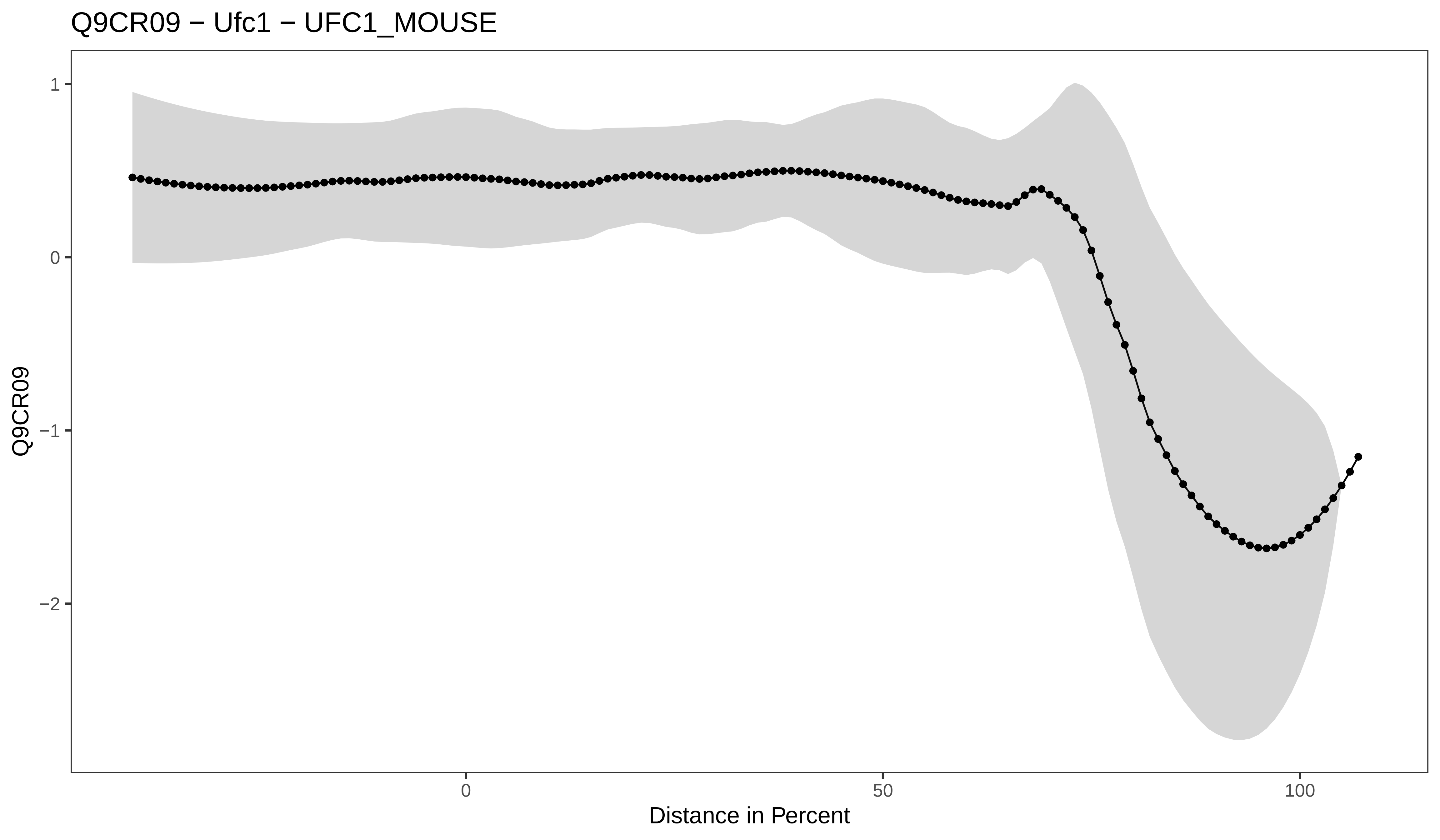

Q9CR09 − Ufc1 − UFC1_MOUSE
1
0
−1
−2
Q9CR09
0
50
100
Distance in Percent

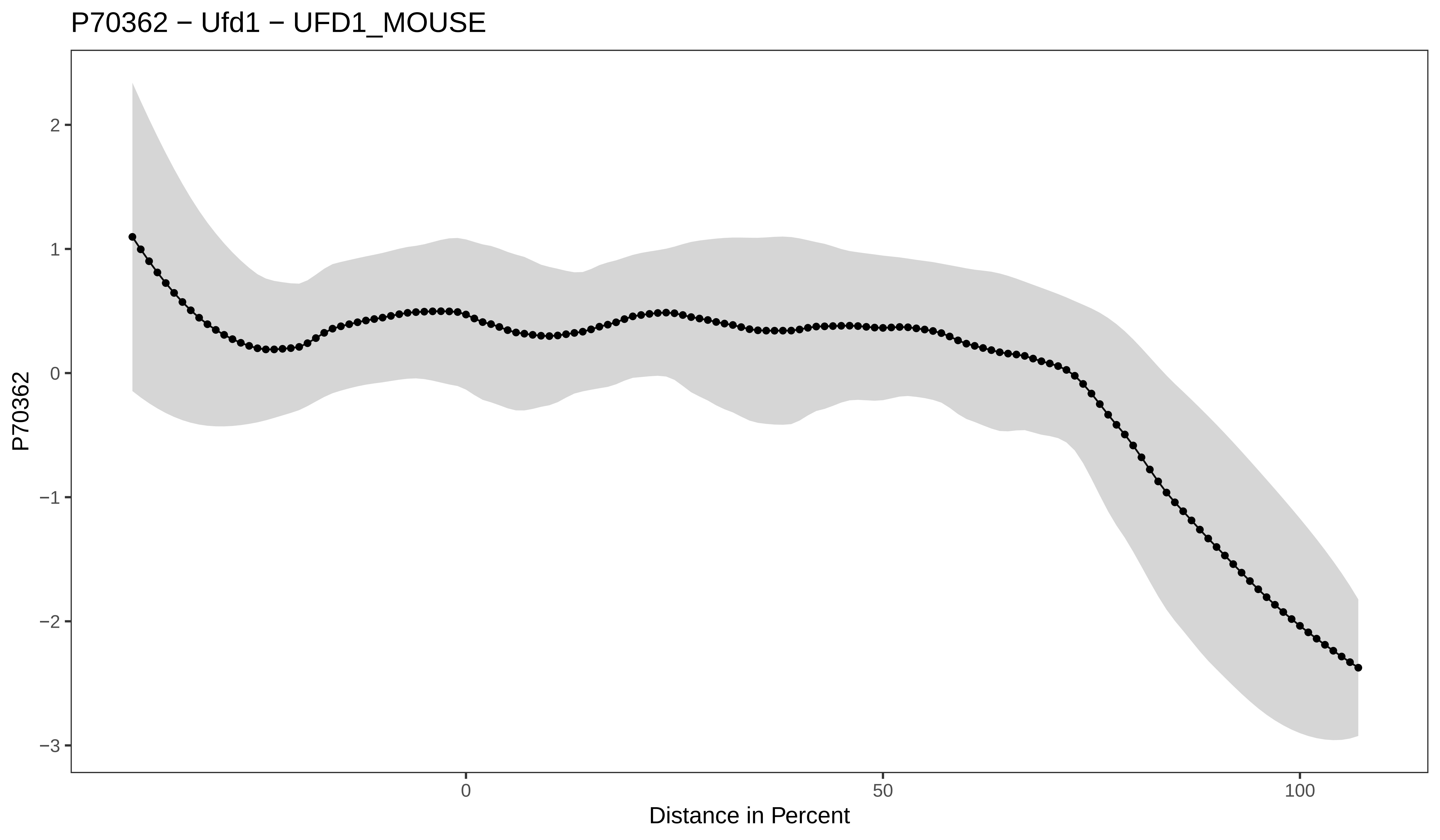

P70362 − Ufd1 − UFD1_MOUSE
P70362
Distance in Percent
2
1
0
−1
−2
−3
0
50
100

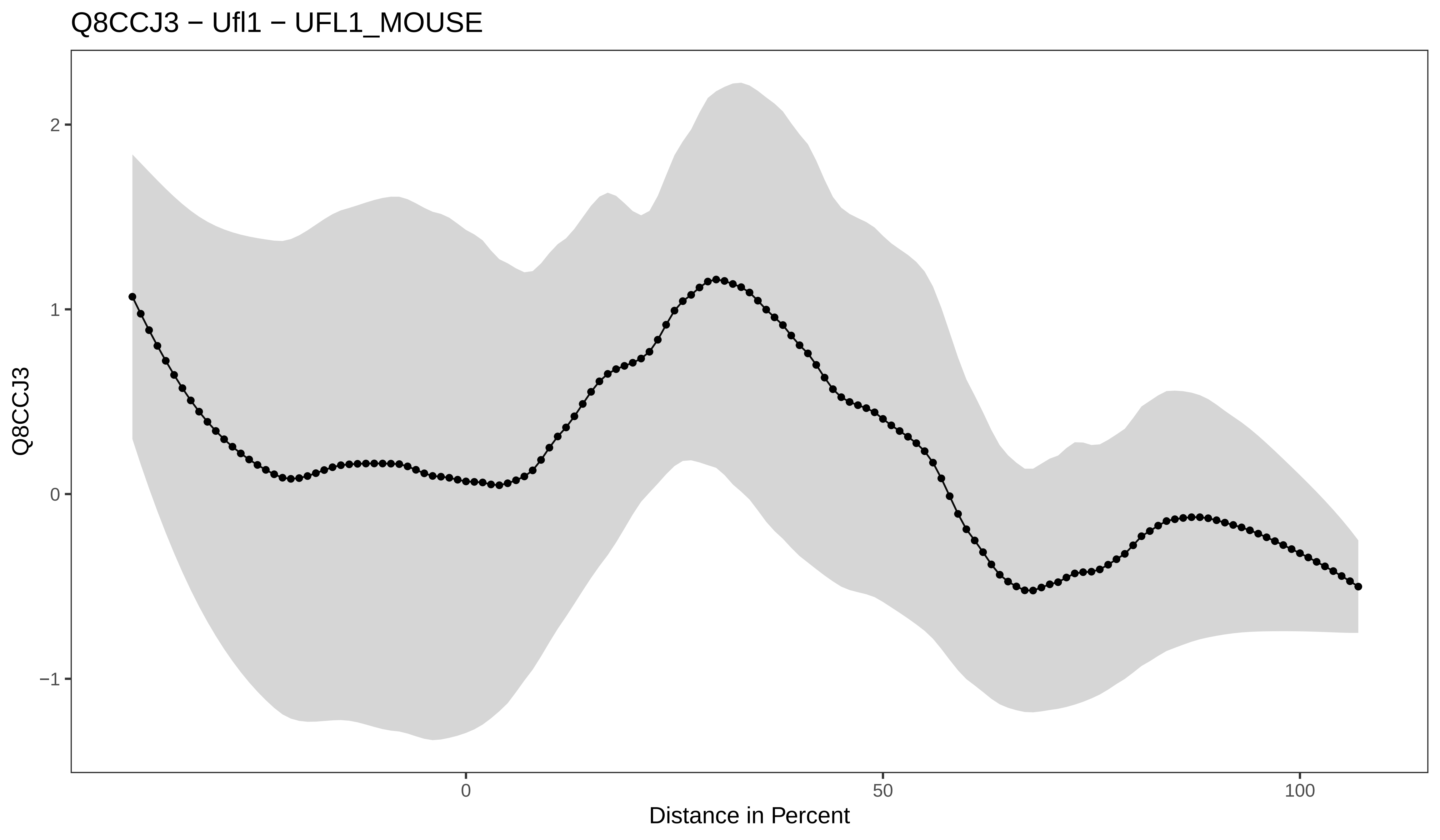

Q8CCJ3 − Ufl1 − UFL1_MOUSE
2
1
0
−1
Q8CCJ3
0
50
100
Distance in Percent

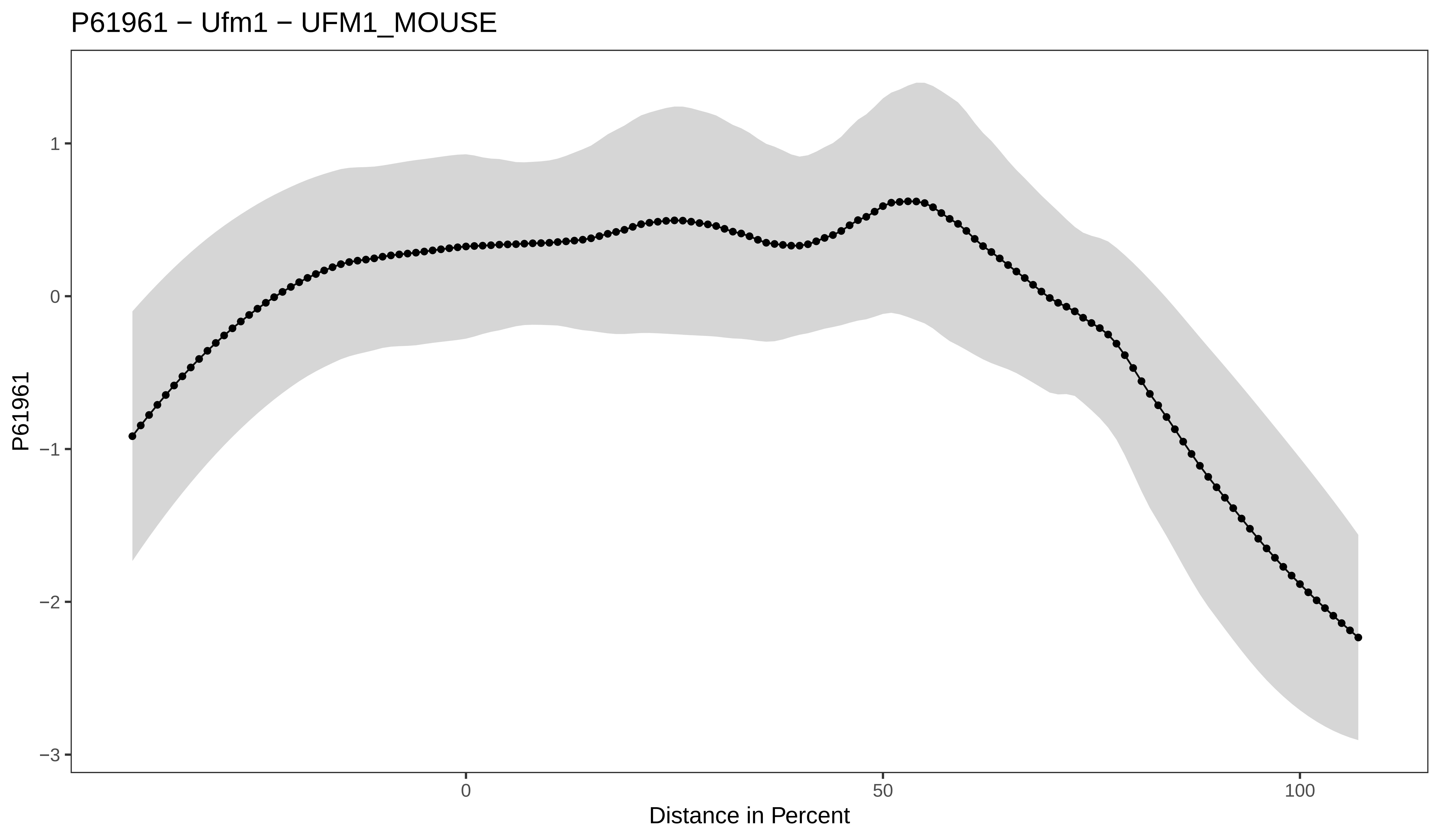
P61961 − Ufm1 − UFM1_MOUSE
P61961
1
0
−1
−2
−3
0
50
100
Distance in Percent

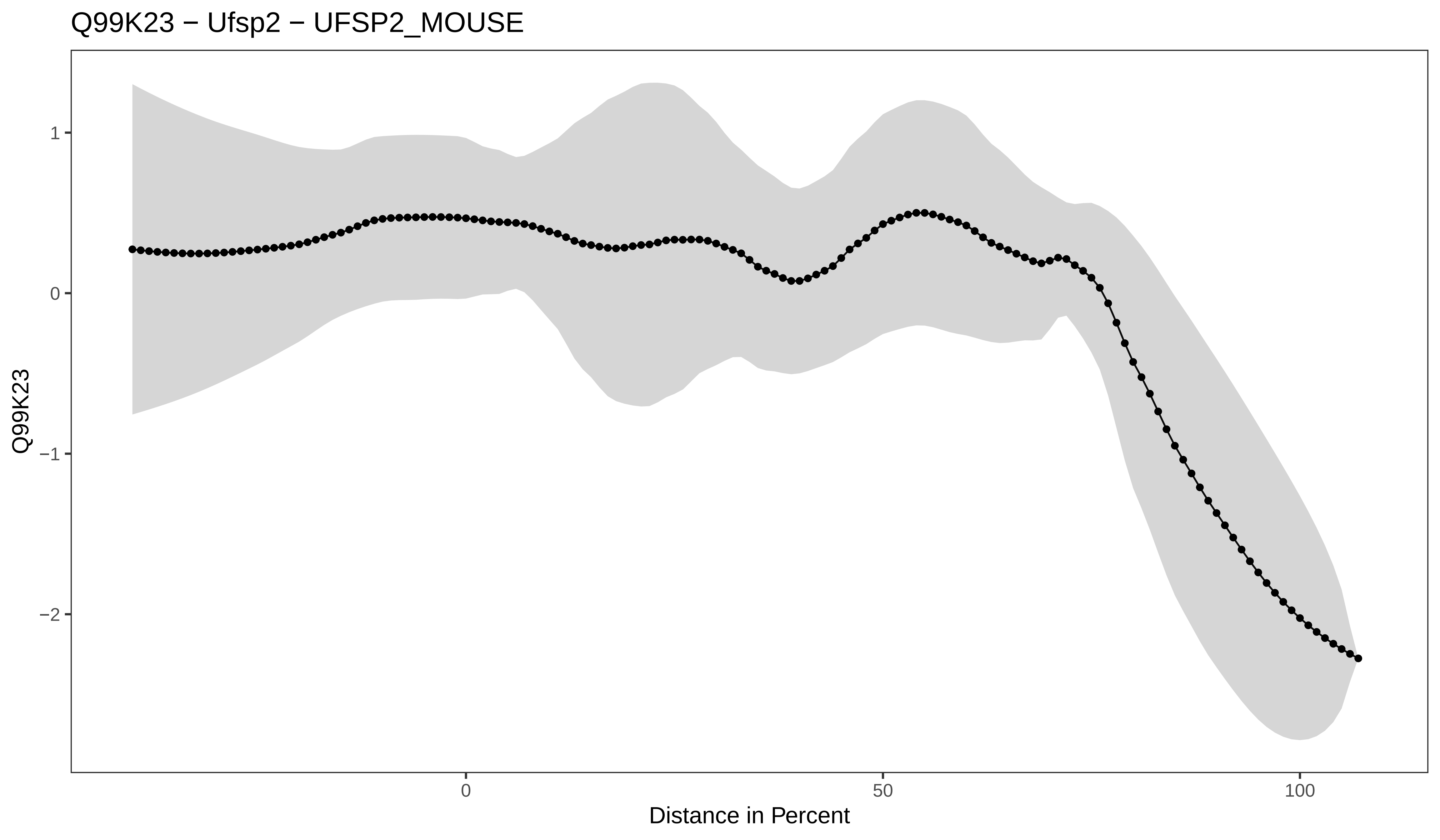
Q99K23 − Ufsp2 − UFSP2_MOUSE
Q99K23
1
0
−1
−2
0
50
100
Distance in Percent

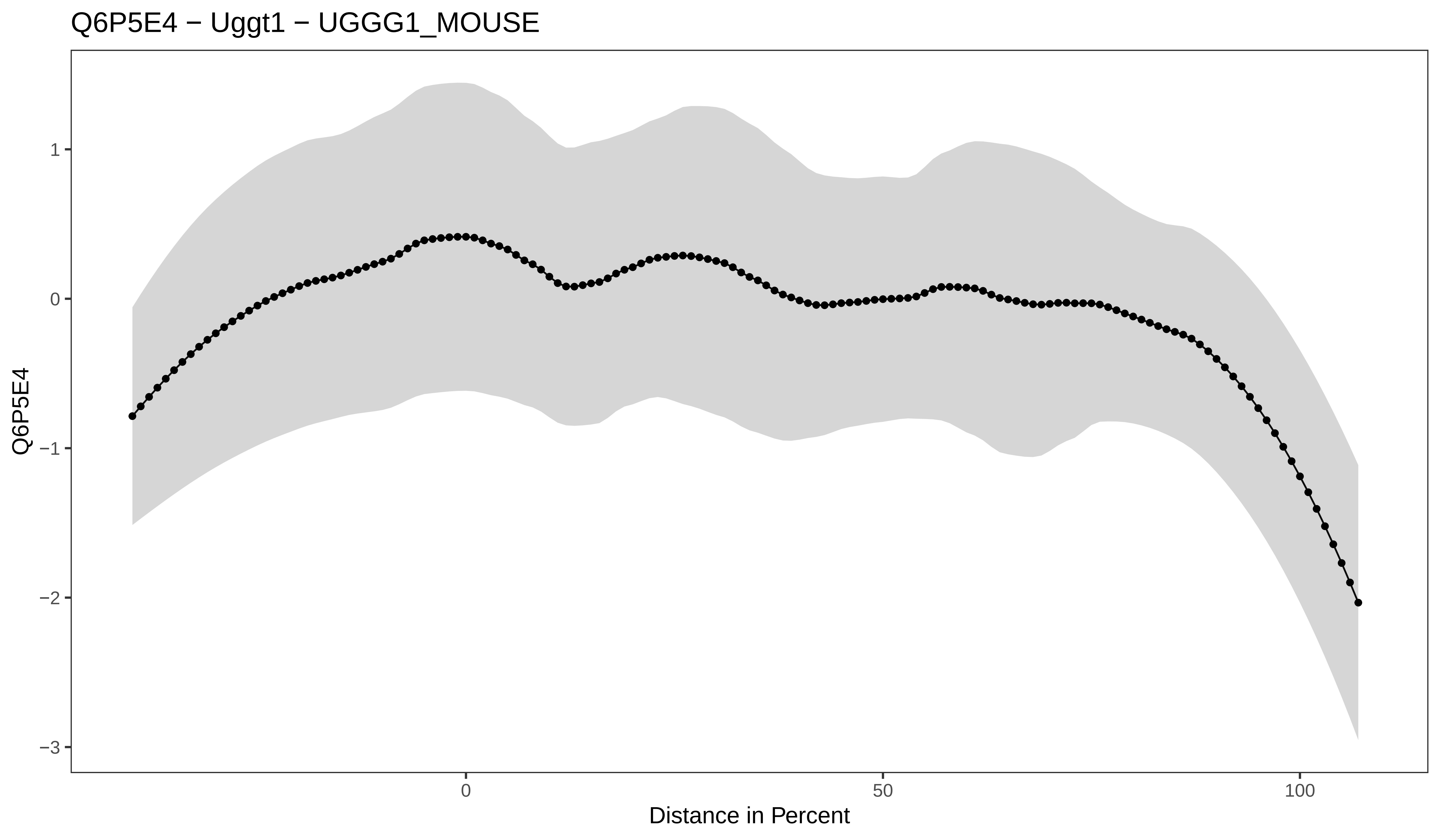

Q6P5E4 − Uggt1 − UGGG1_MOUSE
Q6P5E4
1
0
−1
−2
−3
0
50
100
Distance in Percent

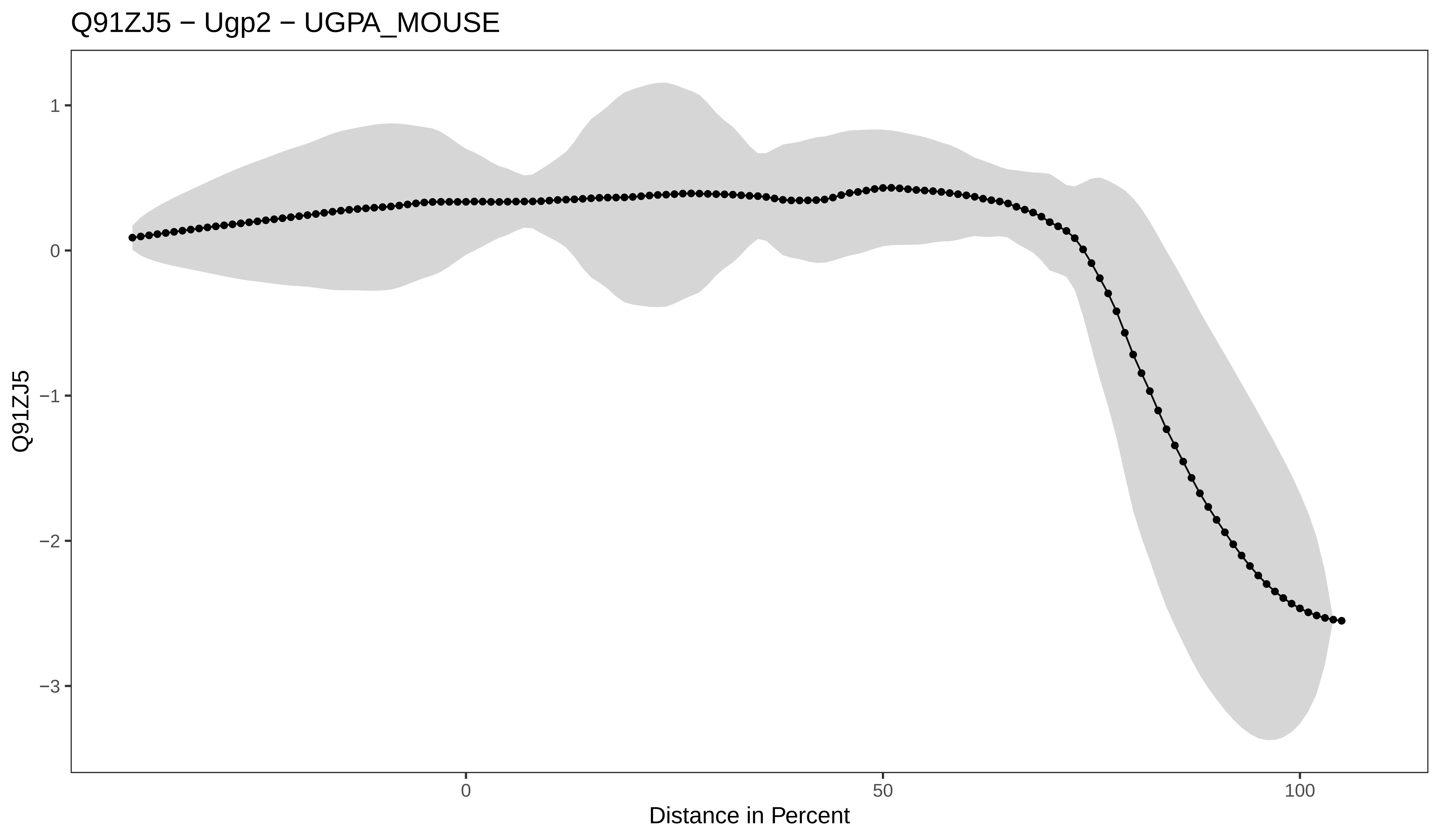

Q91ZJ5 − Ugp2 − UGPA_MOUSE
Q91ZJ5
1
0
−1
−2
−3
0
50
100
Distance in Percent

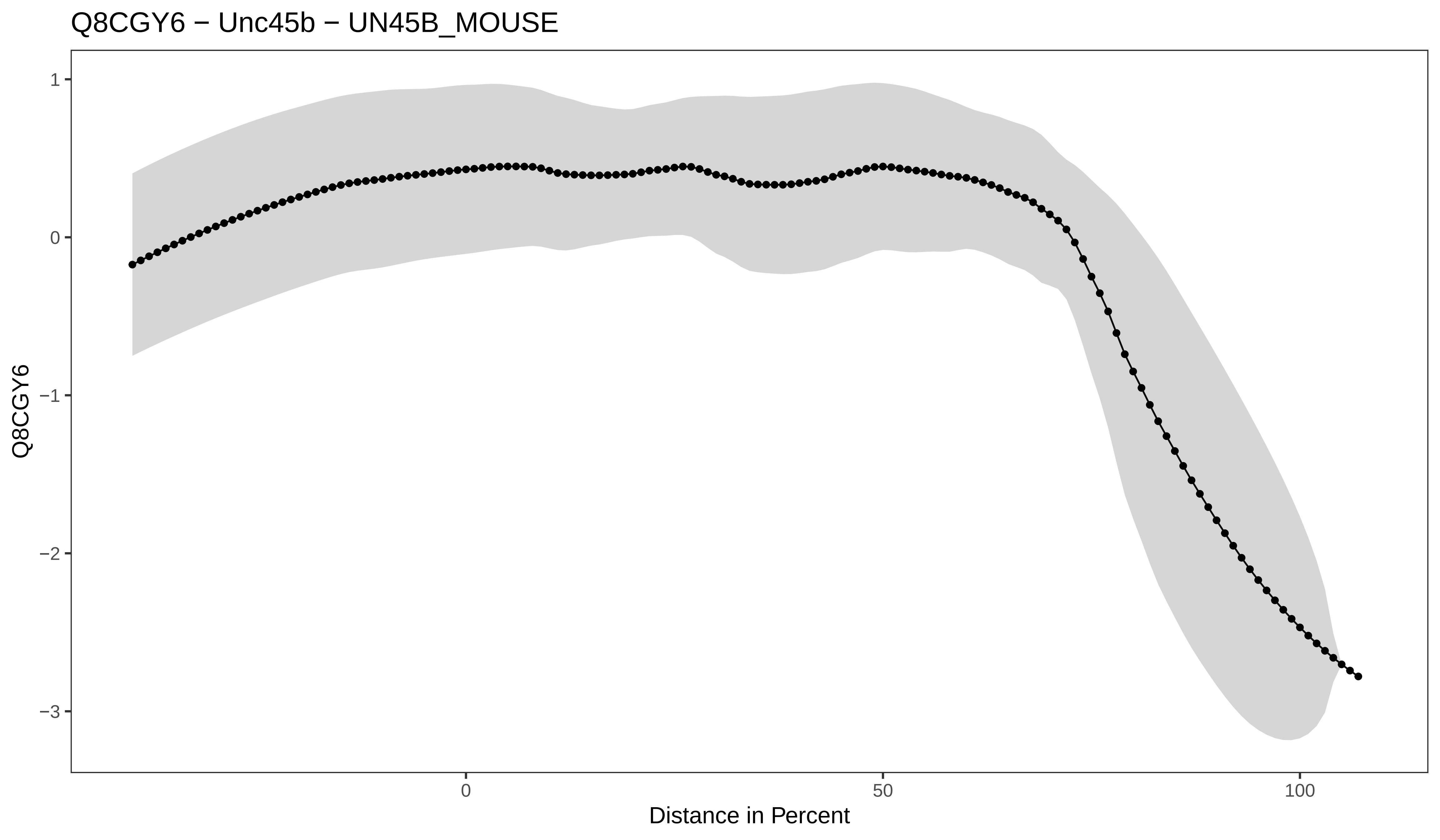

Q8CGY6 − Unc45b − UN45B_MOUSE
1
0
−1
−2
−3
Q8CGY6
0
50
100
Distance in Percent

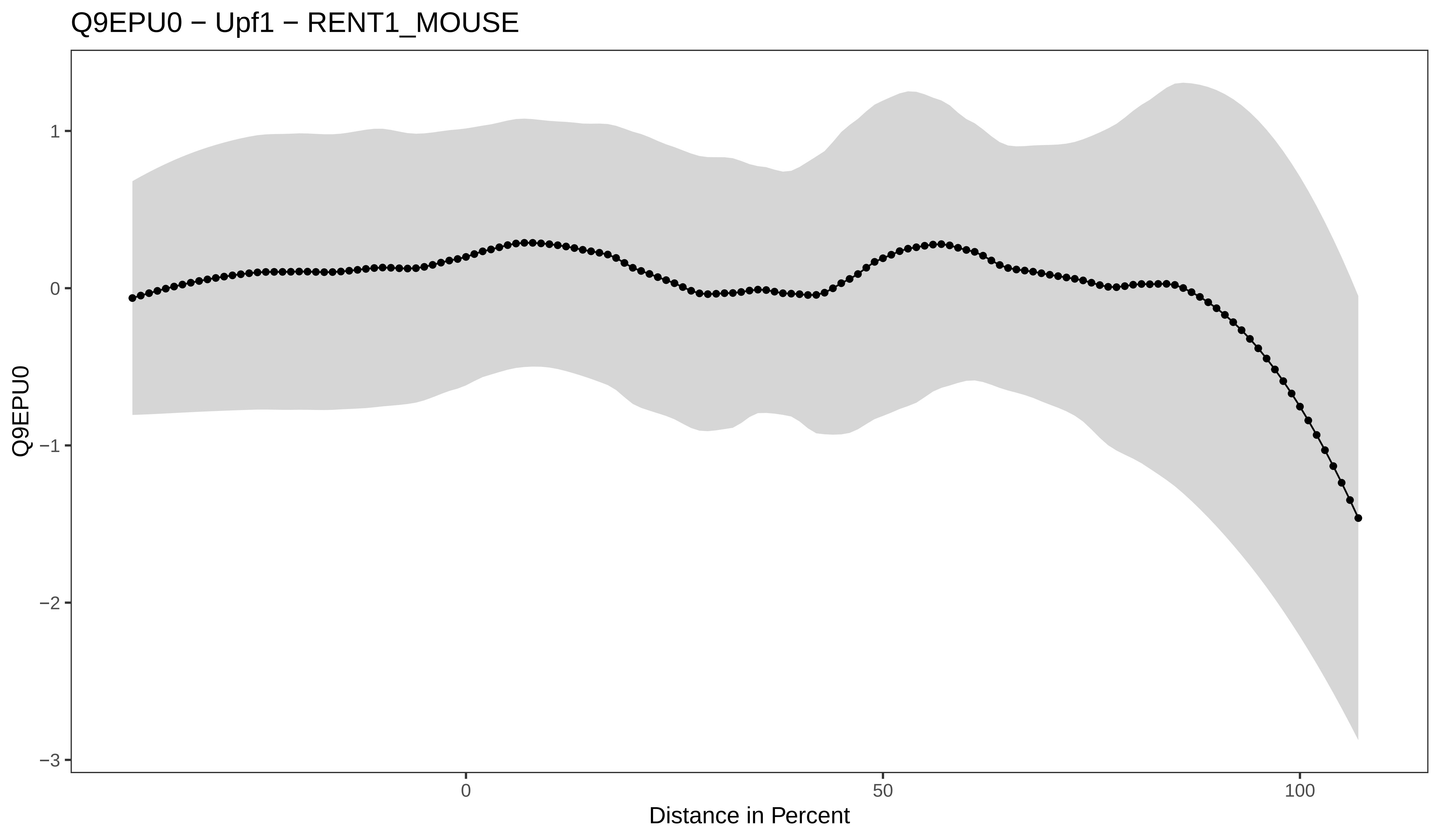

Q9EPU0 − Upf1 − RENT1_MOUSE
Q9EPU0
1
0
−1
−2
−3
0
50
100
Distance in Percent

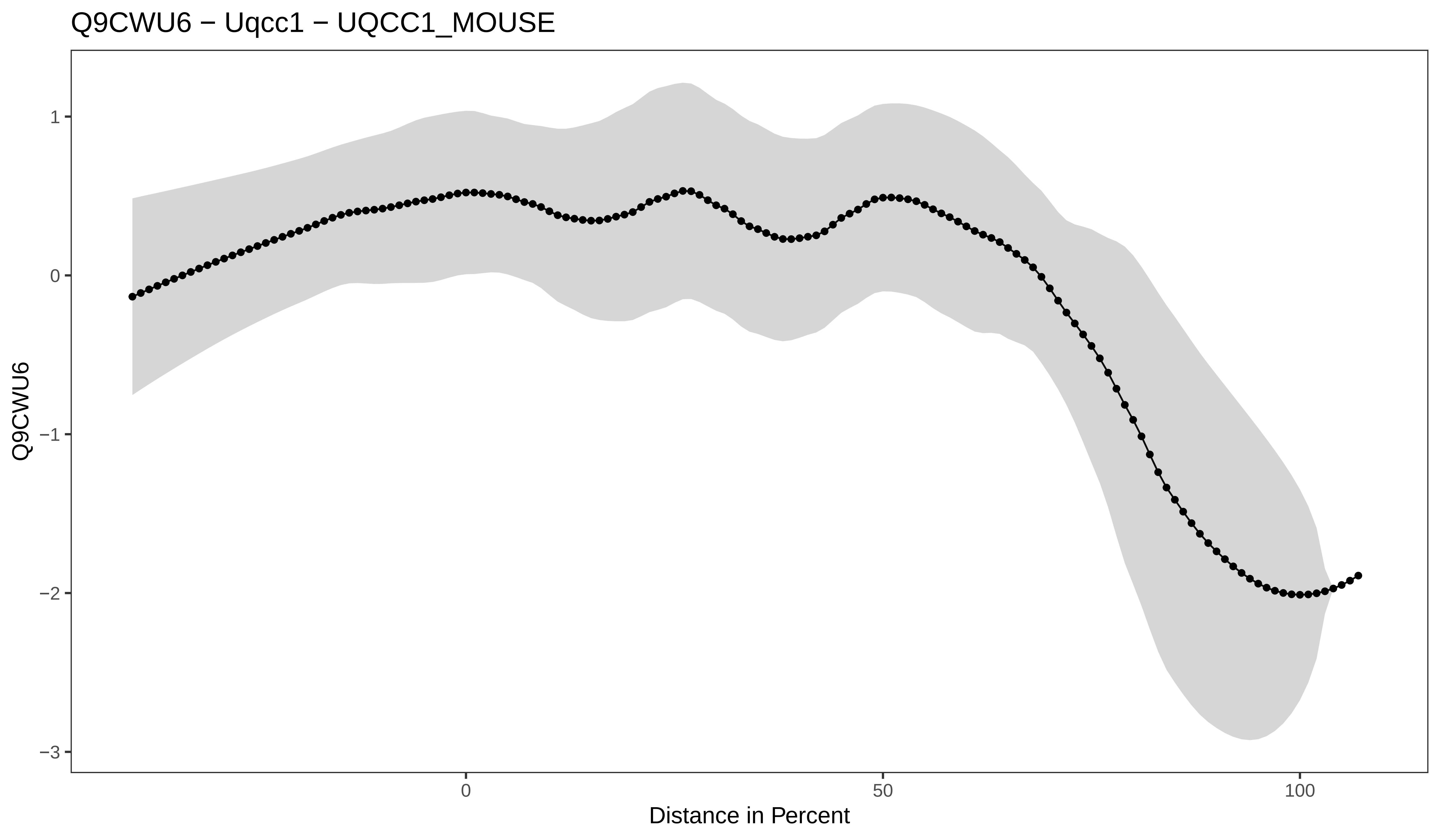

Q9CWU6 − Uqcc1 − UQCC1_MOUSE
1
0
−1
−2
−3
Q9CWU6
0
50
100
Distance in Percent

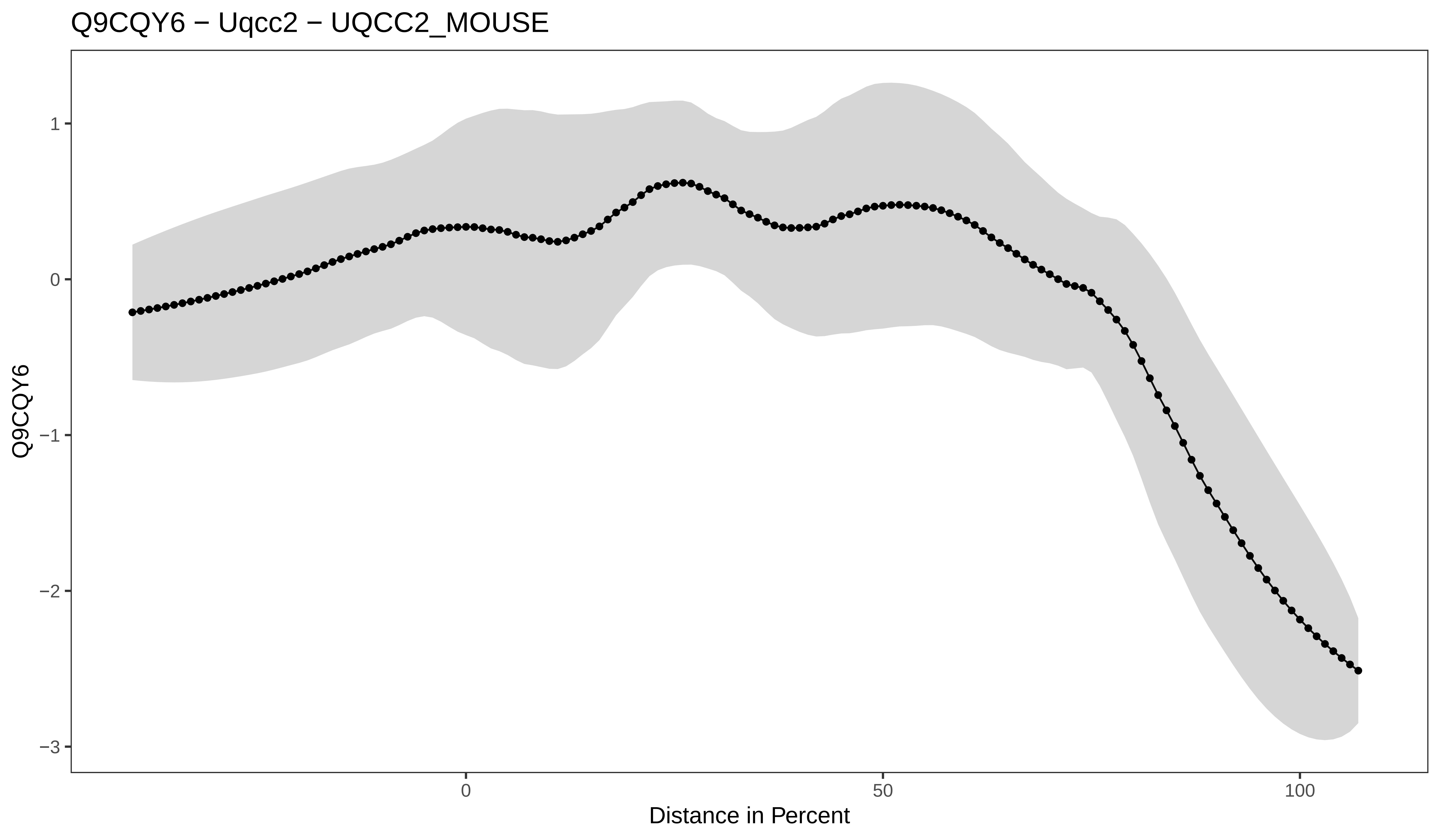

Q9CQY6 − Uqcc2 − UQCC2_MOUSE
Q9CQY6
1
0
−1
−2
−3
0
50
100
Distance in Percent

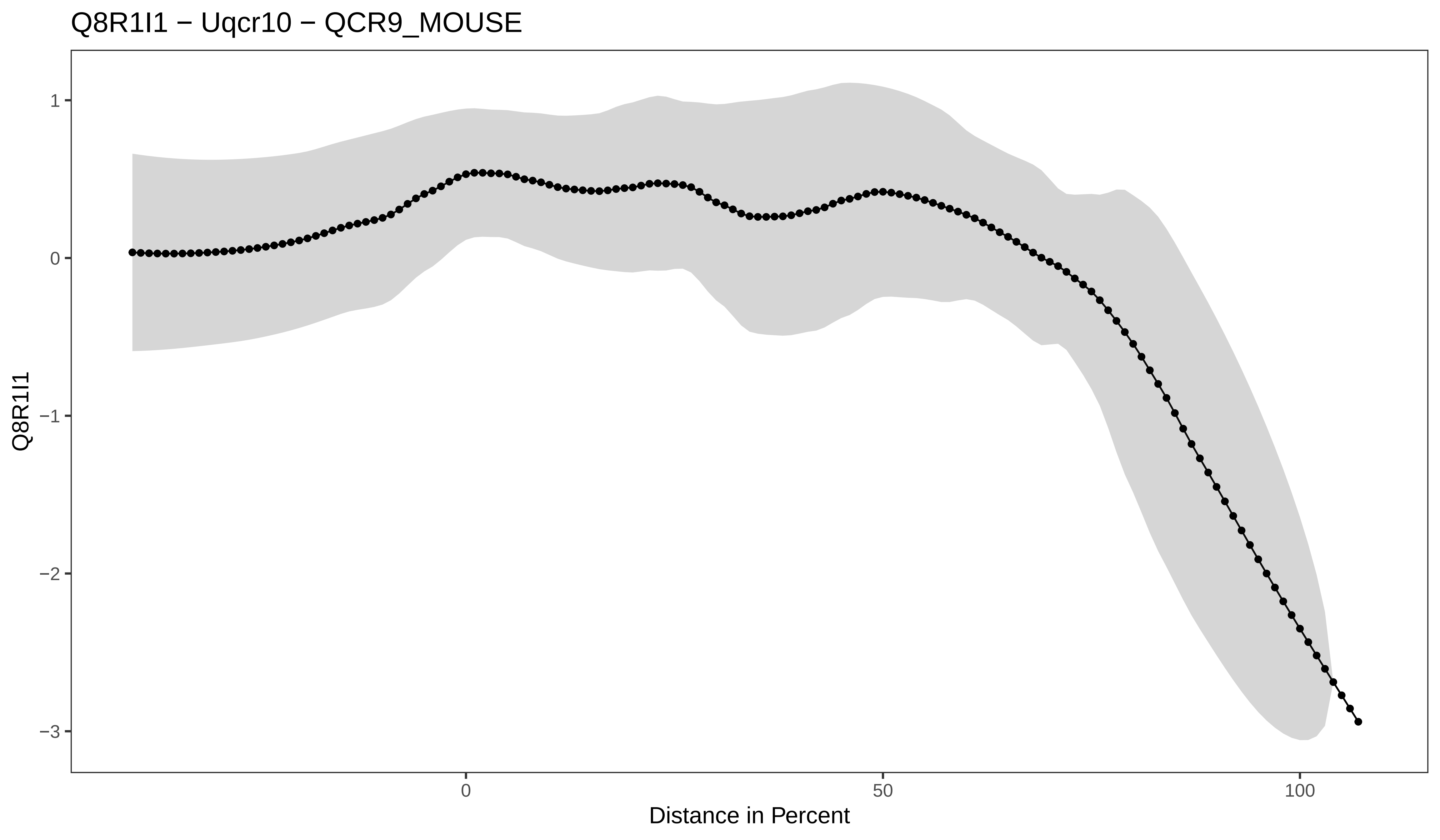

Q8R1I1 − Uqcr10 − QCR9_MOUSE
Q8R1I1
Distance in Percent
1
0
−1
−2
−3
0
50
100

Q9CPX8 − Uqcr11 − QCR10_MOUSE

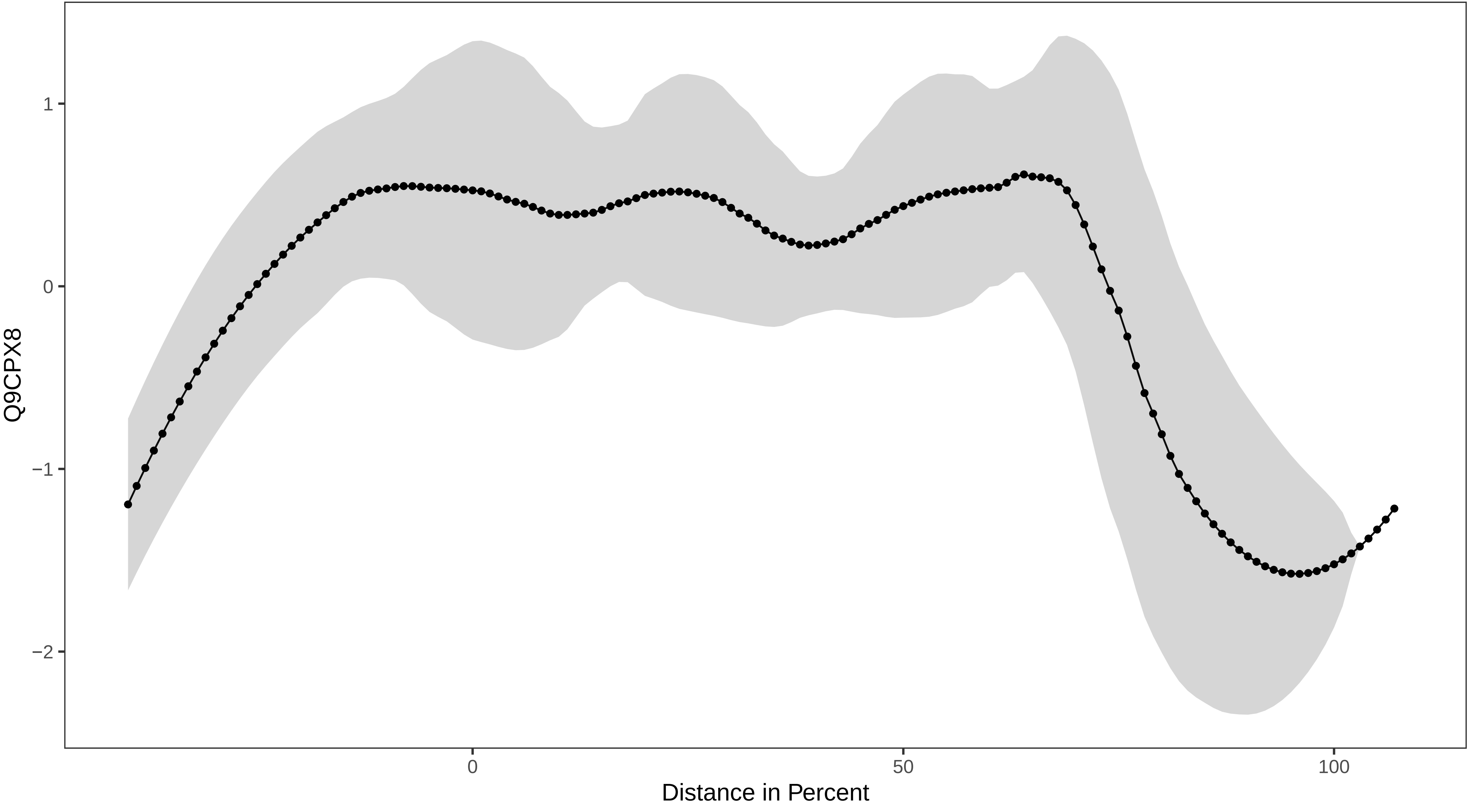

Q9D855 − Uqcrb − QCR7_MOUSE

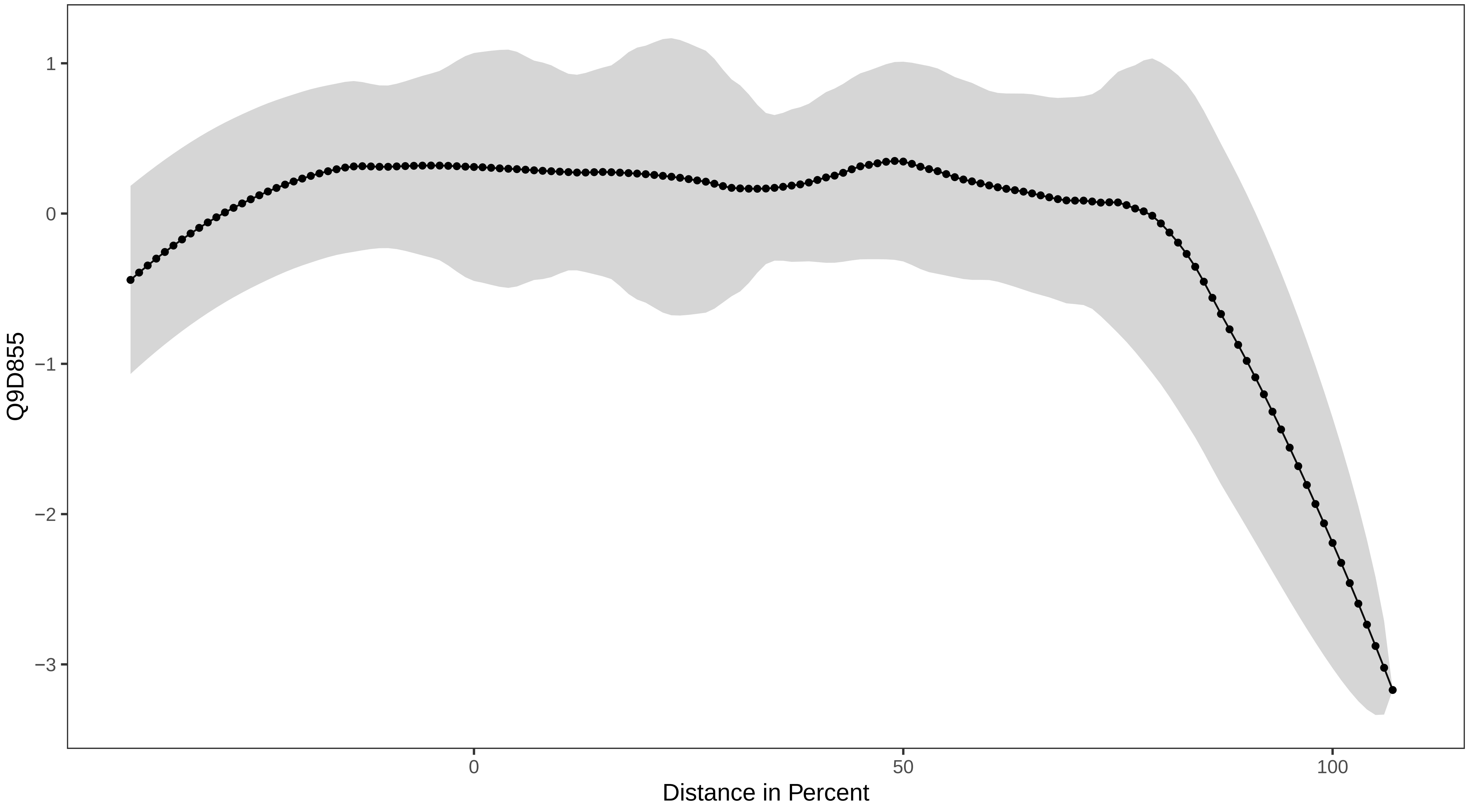

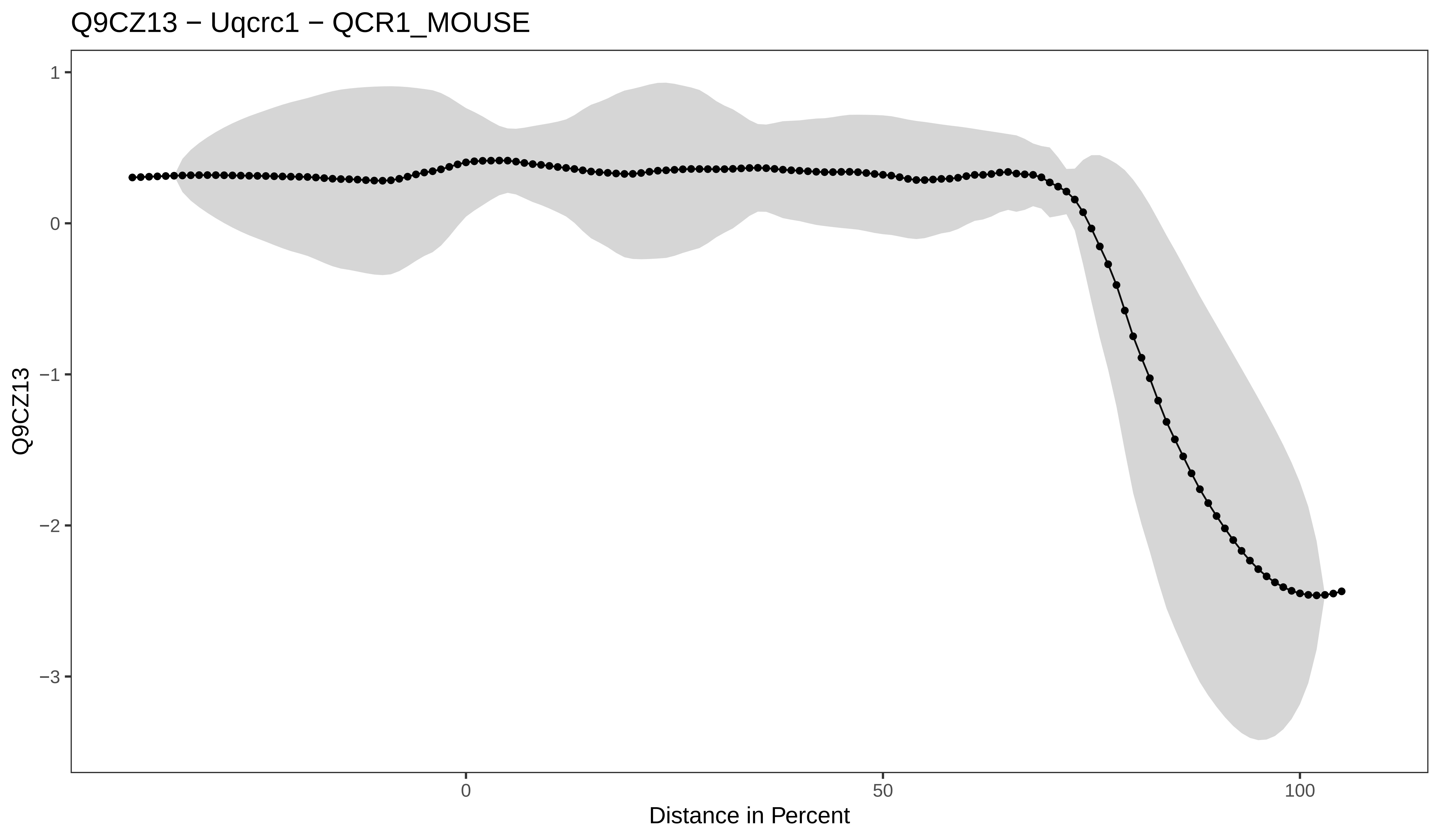

Q9CZ13 − Uqcrc1 − QCR1_MOUSE
1
0
−1
−2
−3
Q9CZ13
0
50
100
Distance in Percent

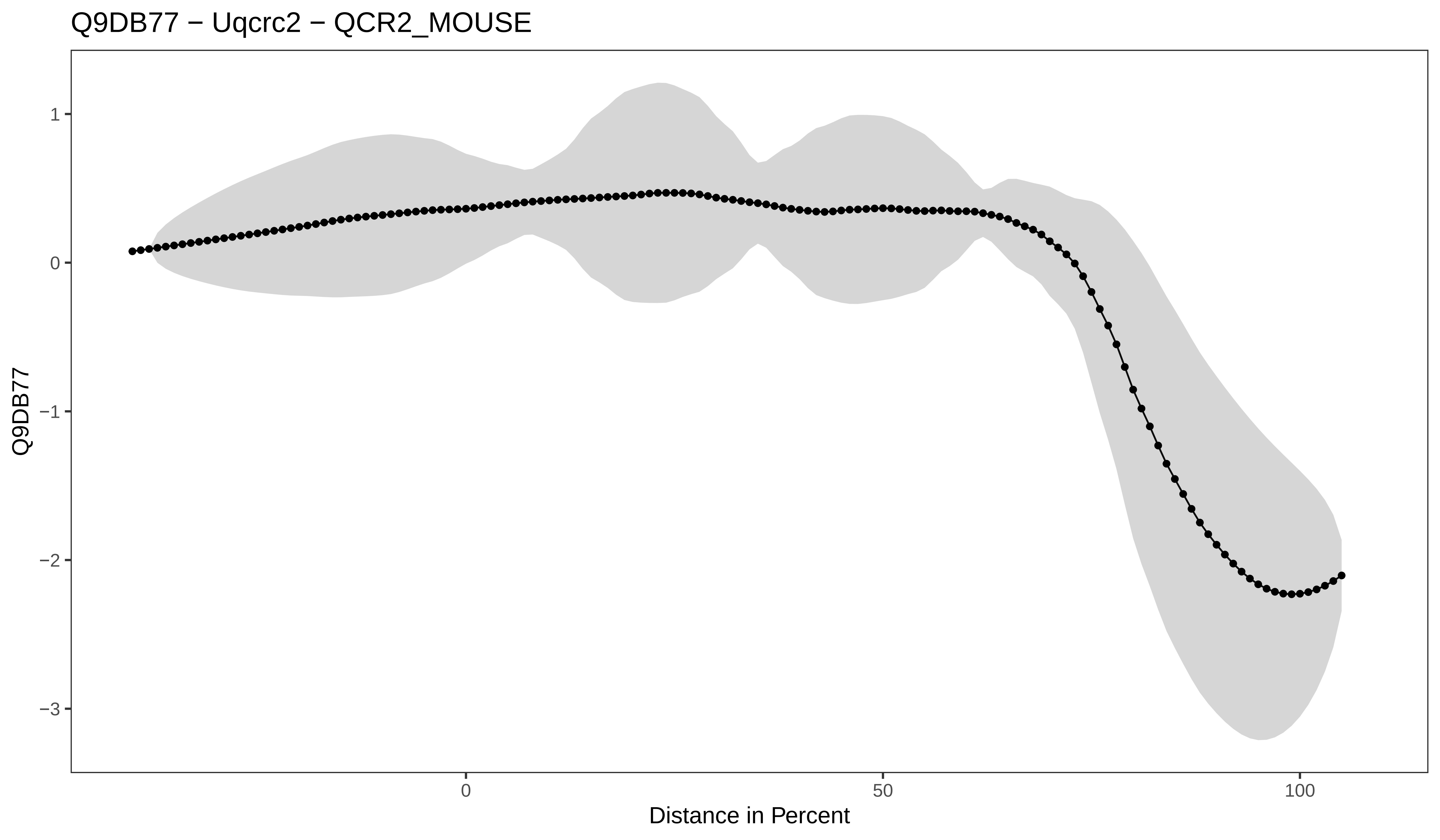

Q9DB77 − Uqcrc2 − QCR2_MOUSE
1
0
−1
−2
−3
Q9DB77
0
50
100
Distance in Percent

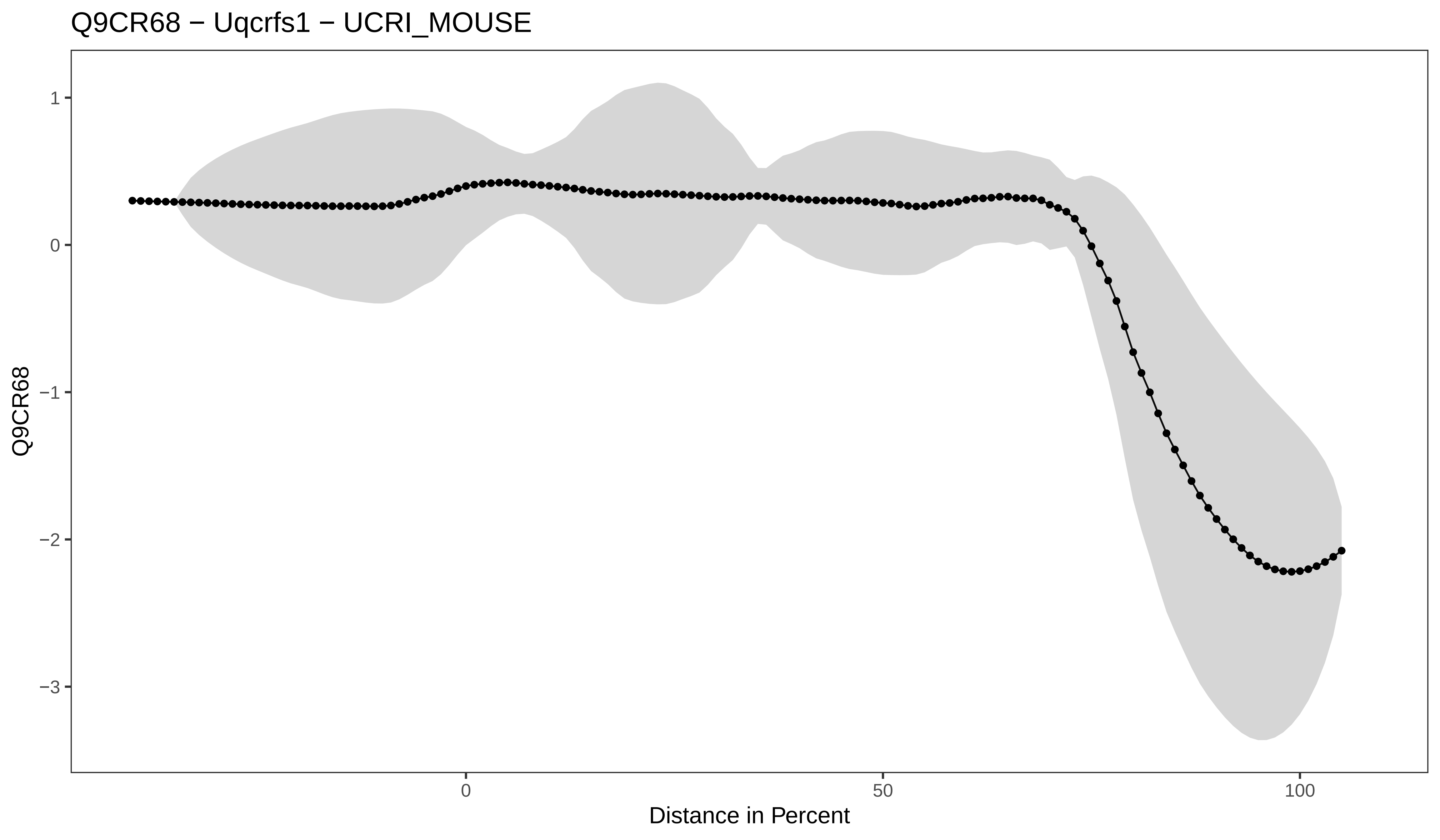
Q9CR68 − Uqcrfs1 − UCRI_MOUSE
Q9CR68
Distance in Percent
1
0
−1
−2
−3
0
50
100

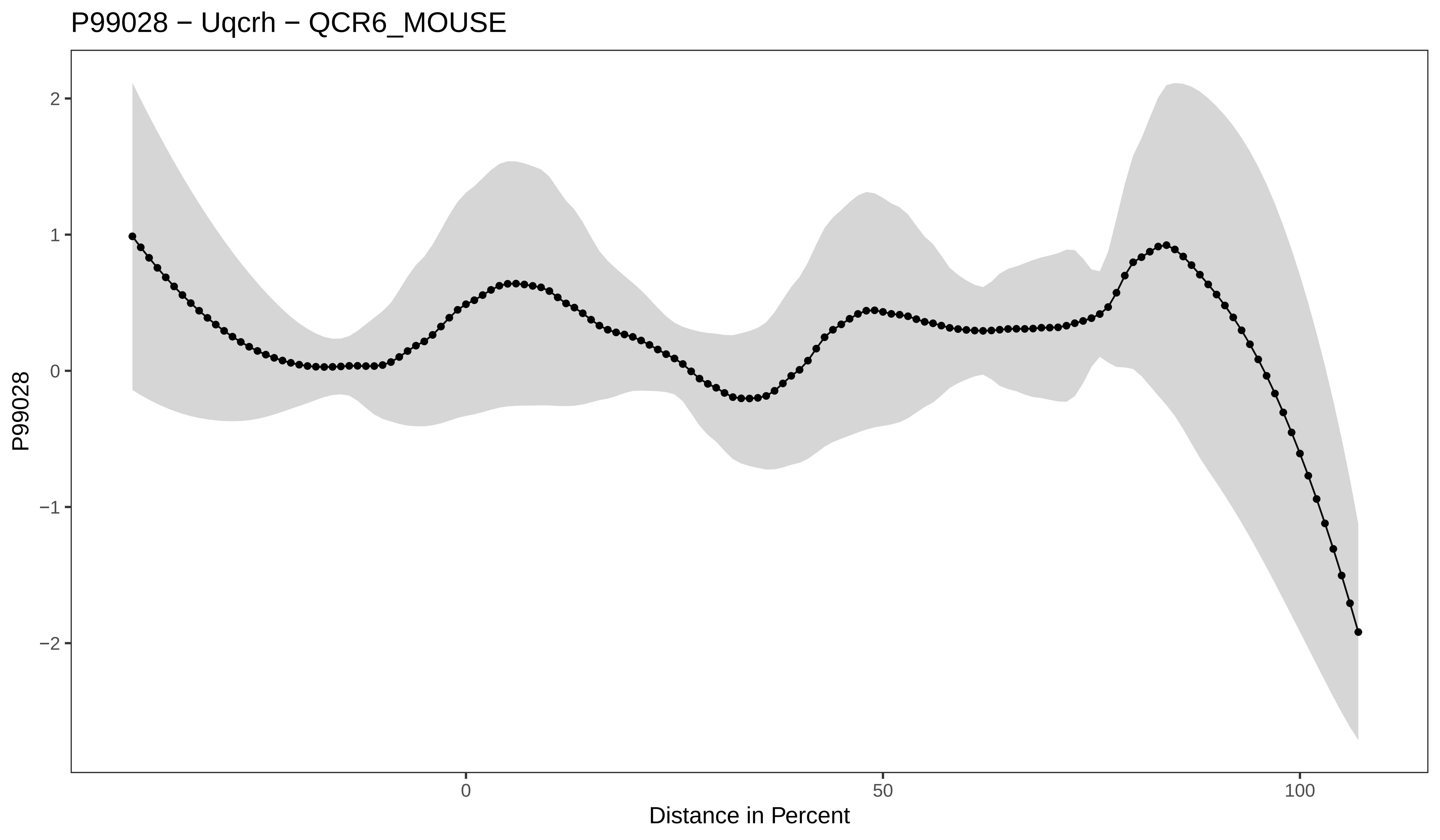

P99028 − Uqcrh − QCR6_MOUSE
2
1
0
−1
−2
P99028
0
50
100
Distance in Percent

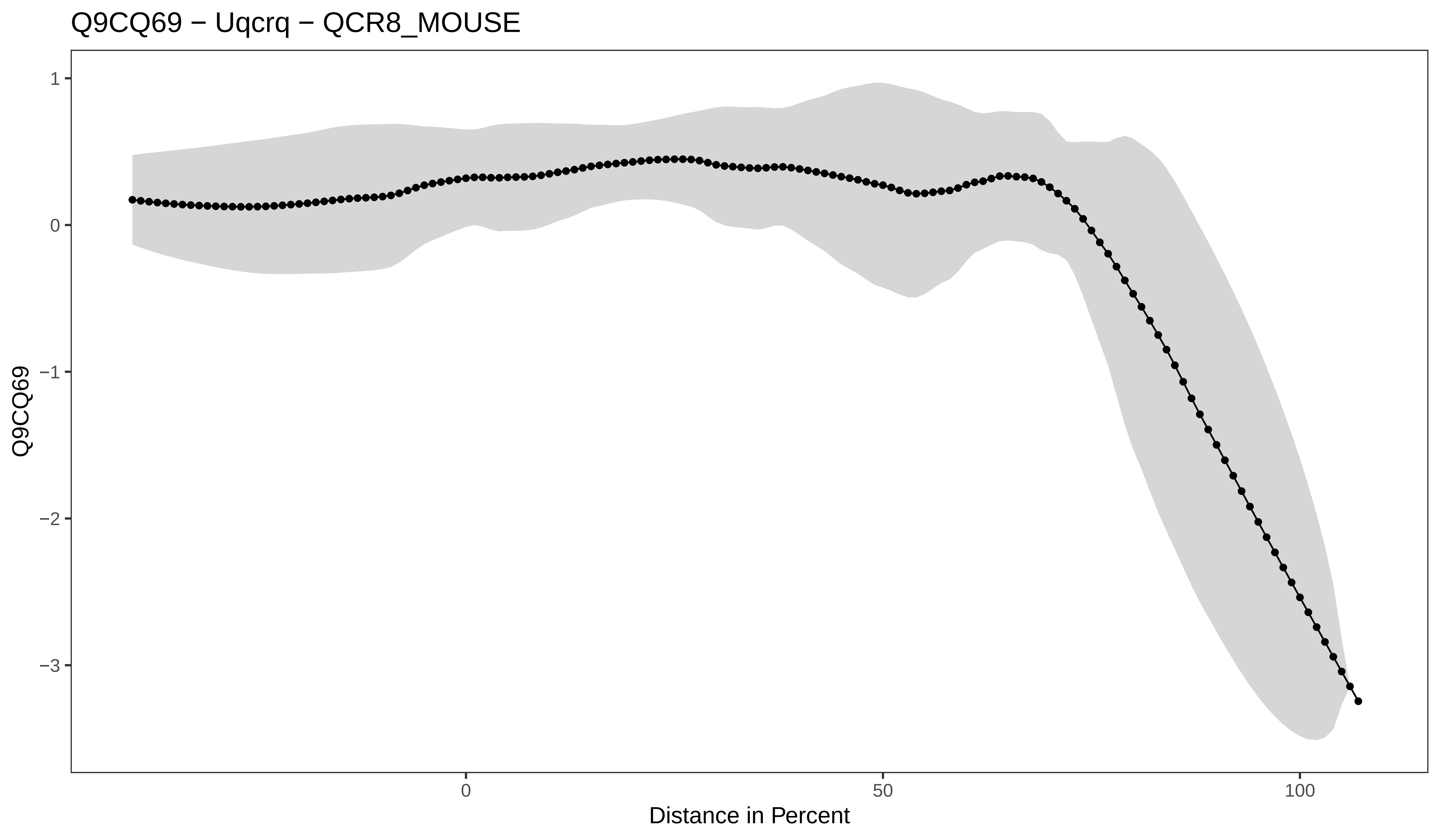

Q9CQ69 − Uqcrq − QCR8_MOUSE
1
0
−1
−2
−3
Q9CQ69
0
50
100
Distance in Percent

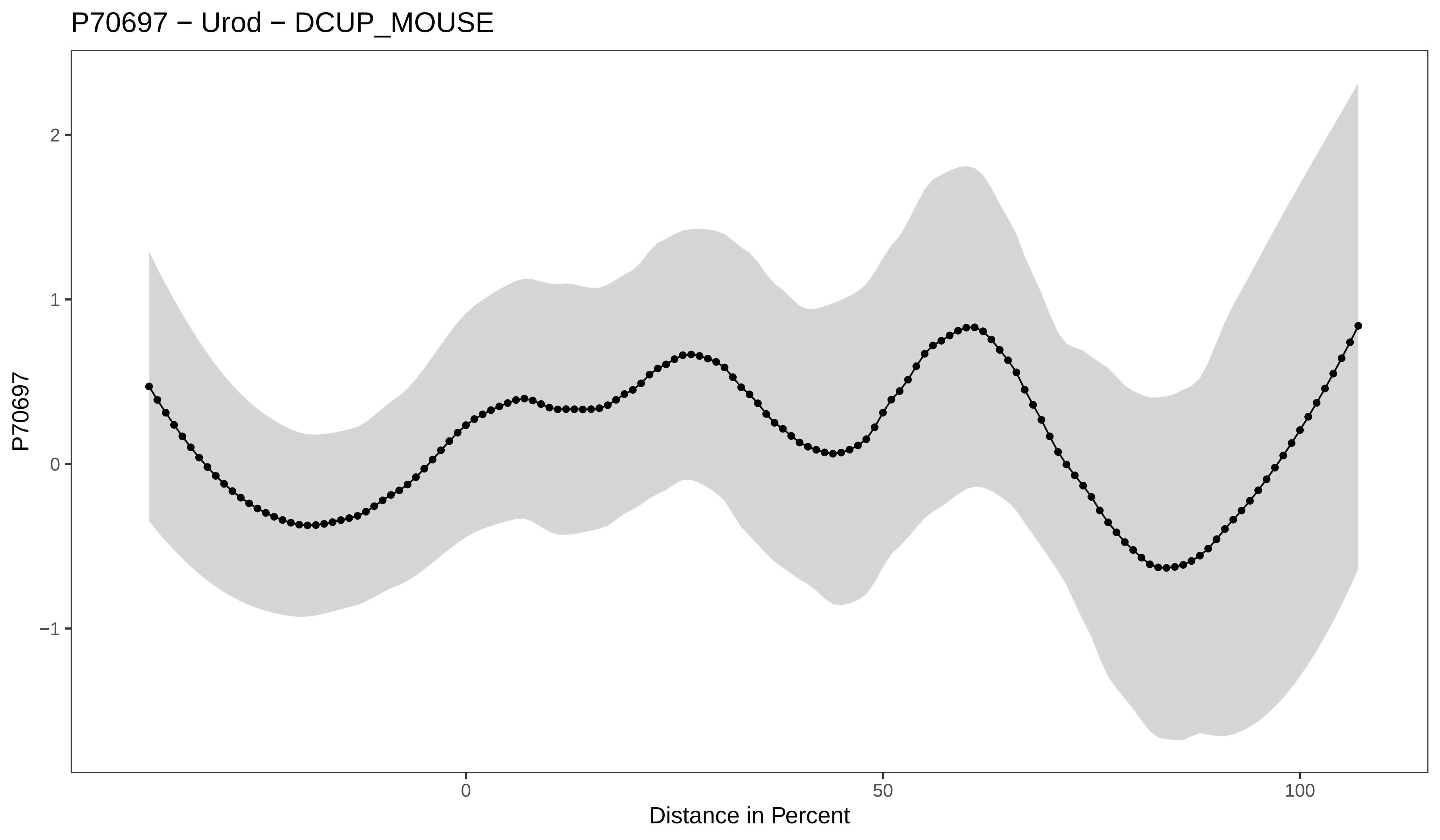
P70697 − Urod − DCUP_MOUSE
P70697
2
1
0
−1
0
50
100
Distance in Percent

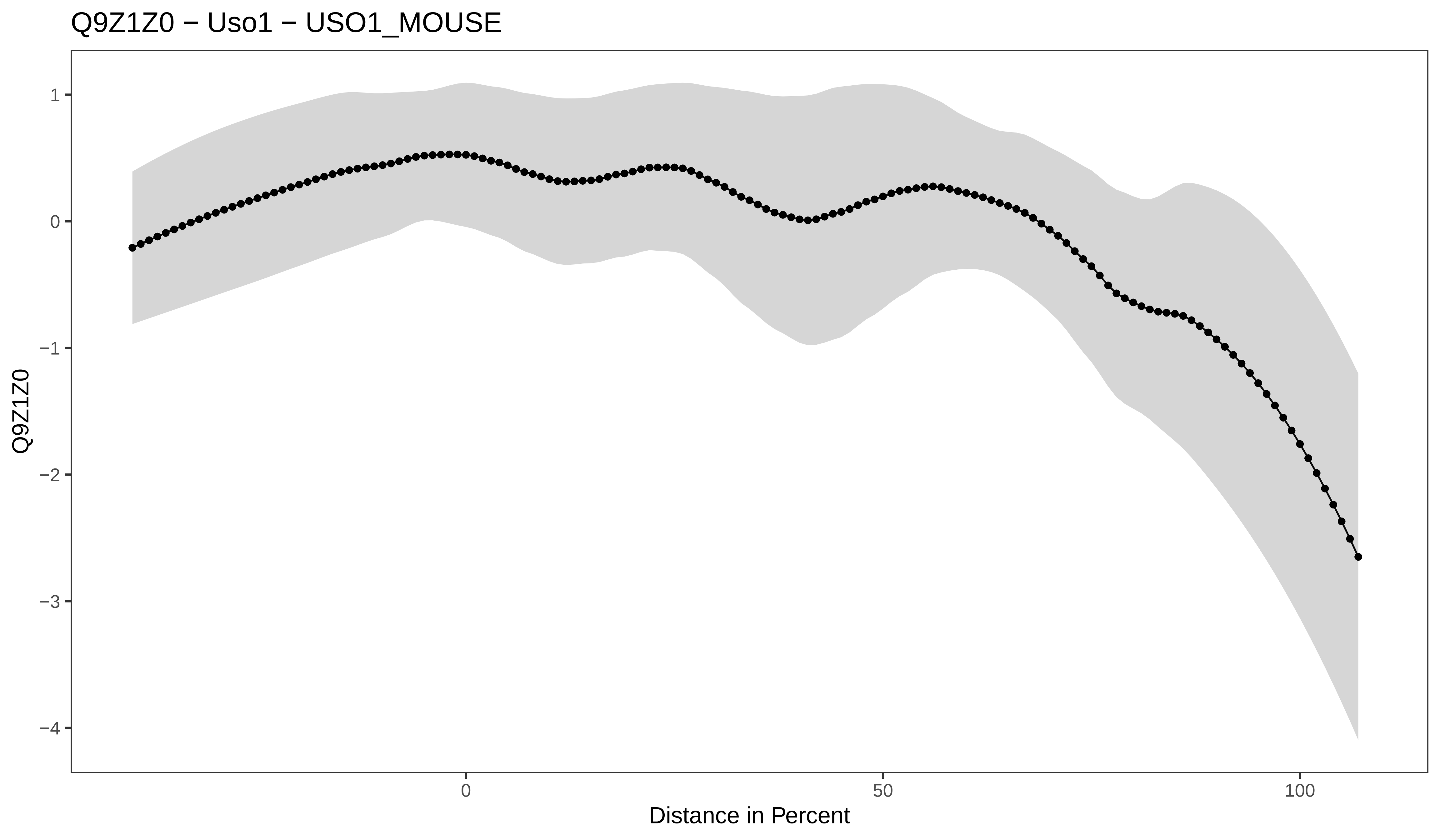

Q9Z1Z0 − Uso1 − USO1_MOUSE
Q9Z1Z0
1
0
−1
−2
−3
−4
0
50
100
Distance in Percent

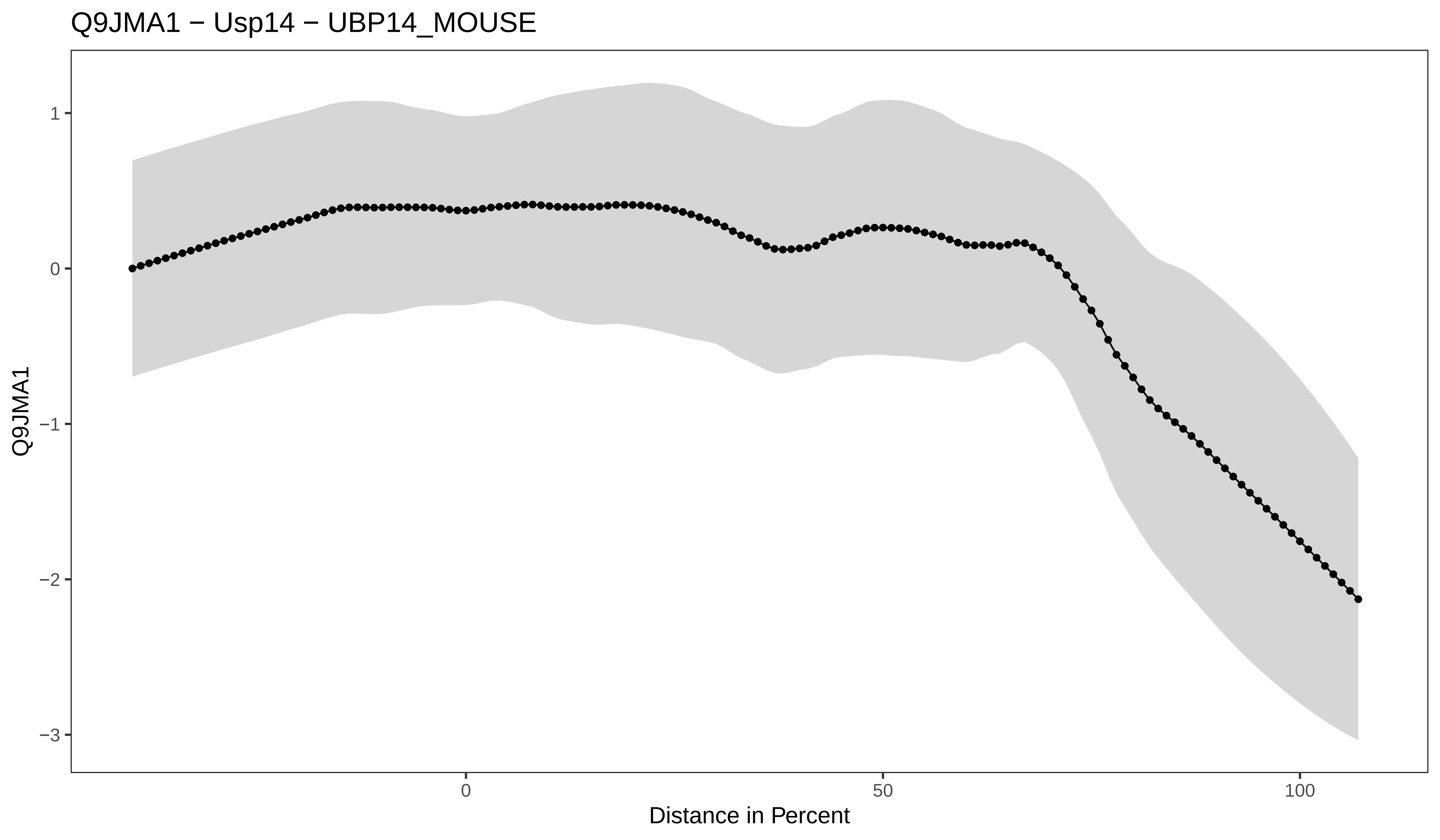
Q9JMA1 − Usp14 − UBP14_MOUSE
Q9JMA1
Distance in Percent
1
0
−1
−2
−3
0
50
100

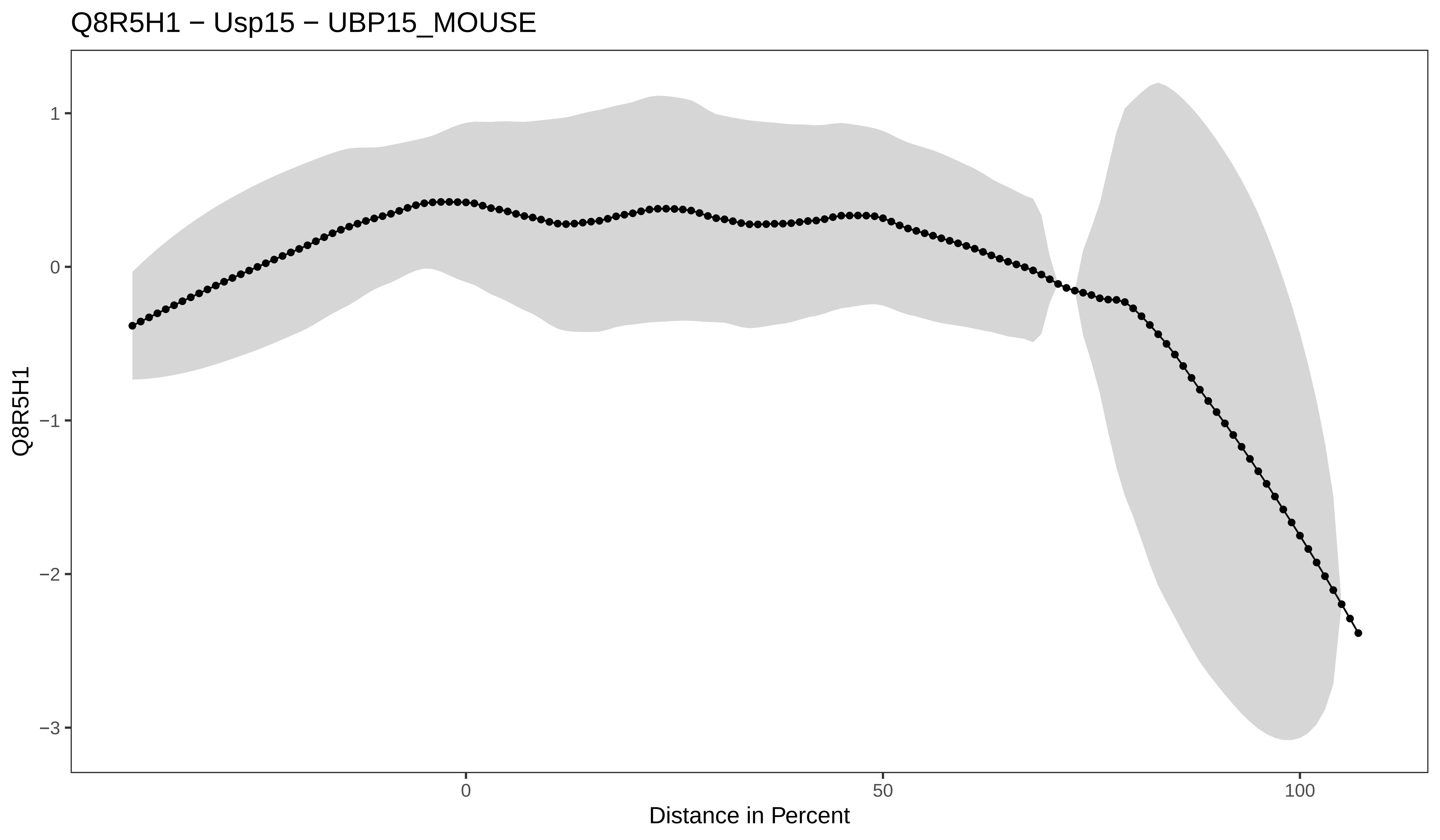
Q8R5H1 − Usp15 − UBP15_MOUSE
1
0
−1
−2
−3
Q8R5H1
0
50
100
Distance in Percent

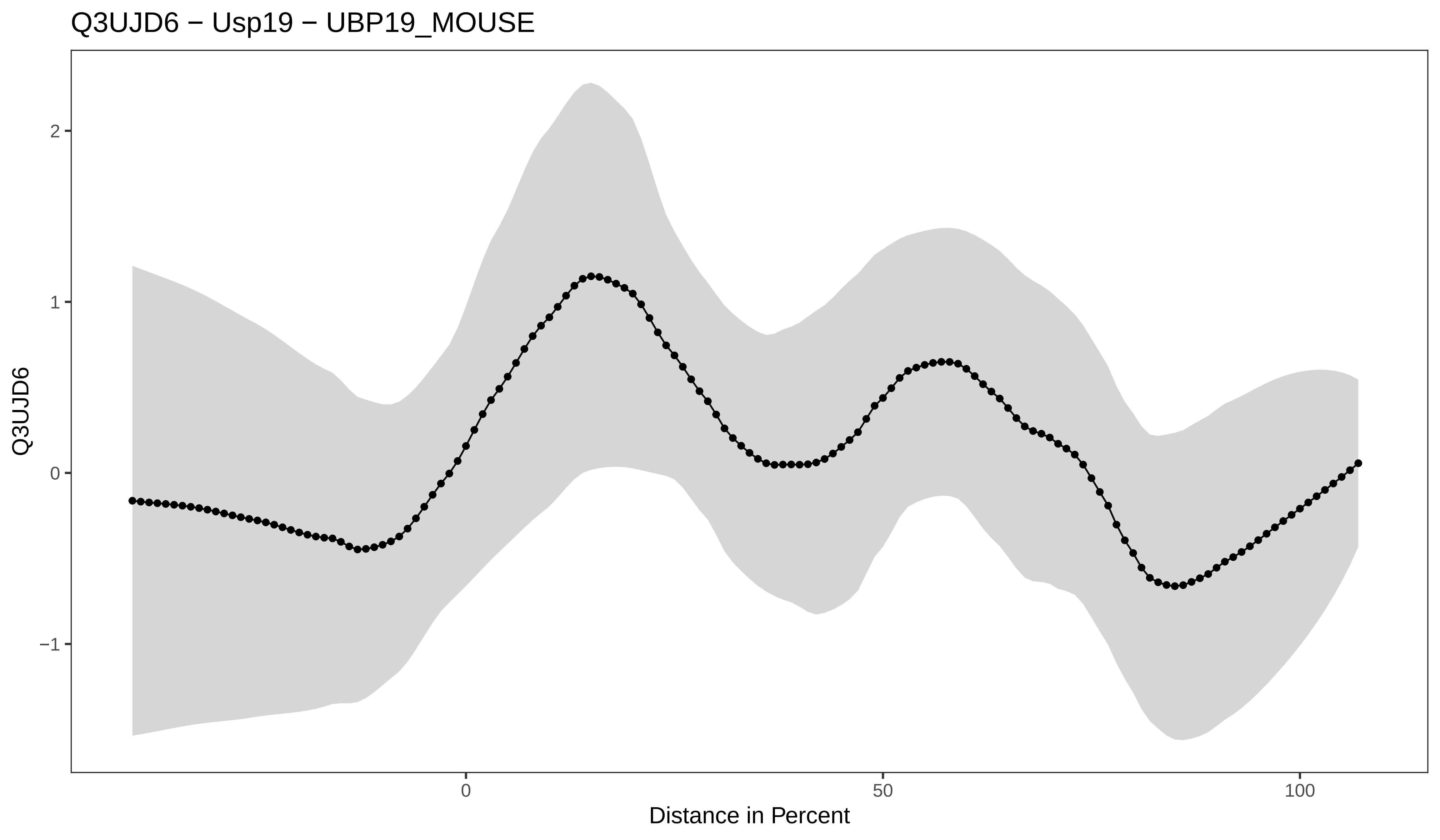

Q3UJD6 − Usp19 − UBP19_MOUSE
Q3UJD6
Distance in Percent
2
1
0
−1
0
50
100

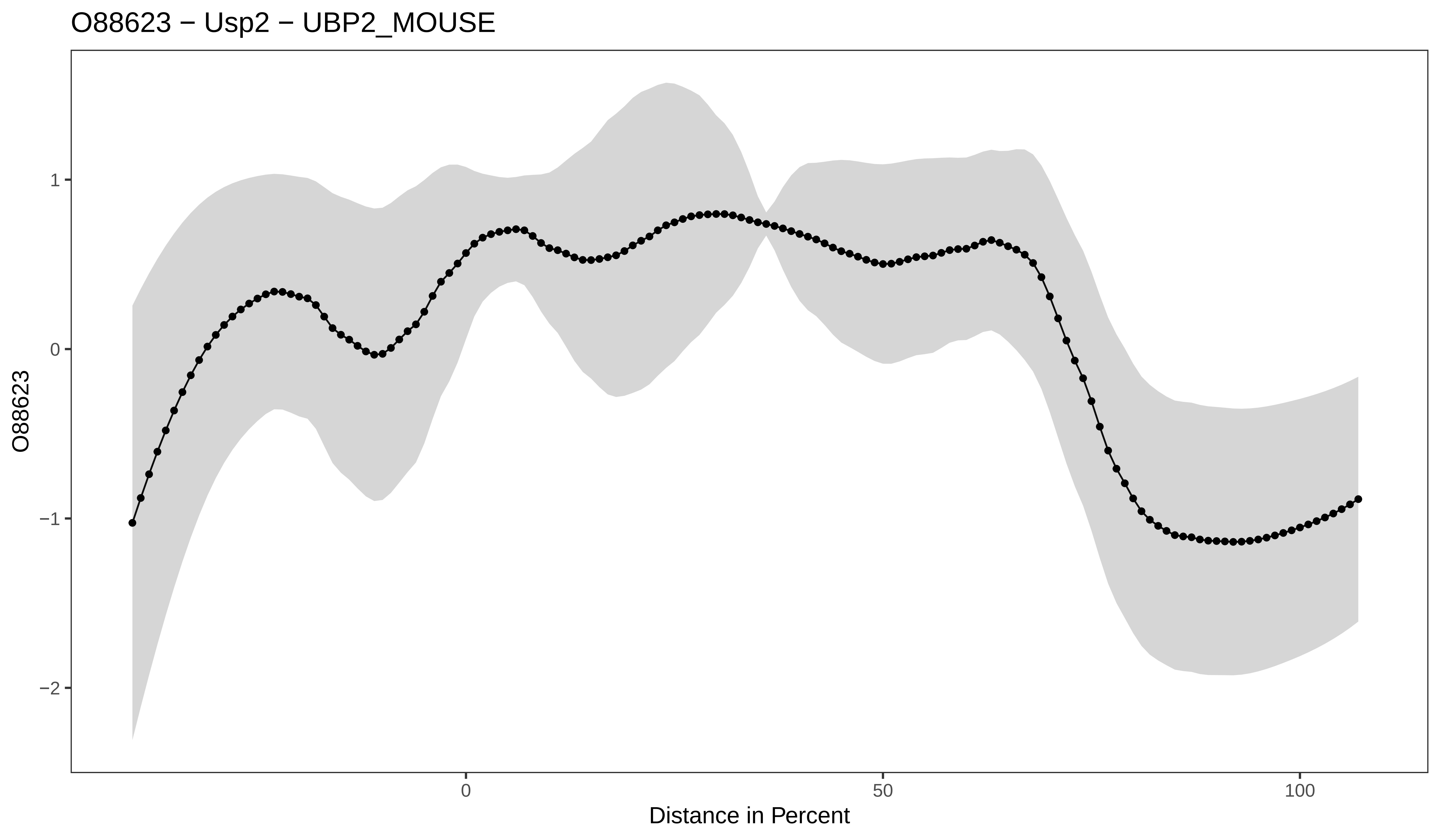

O88623 − Usp2 − UBP2_MOUSE
1
0
−1
−2
O88623
0
50
100
Distance in Percent

B1AY13 − Usp24 − UBP24_MOUSE

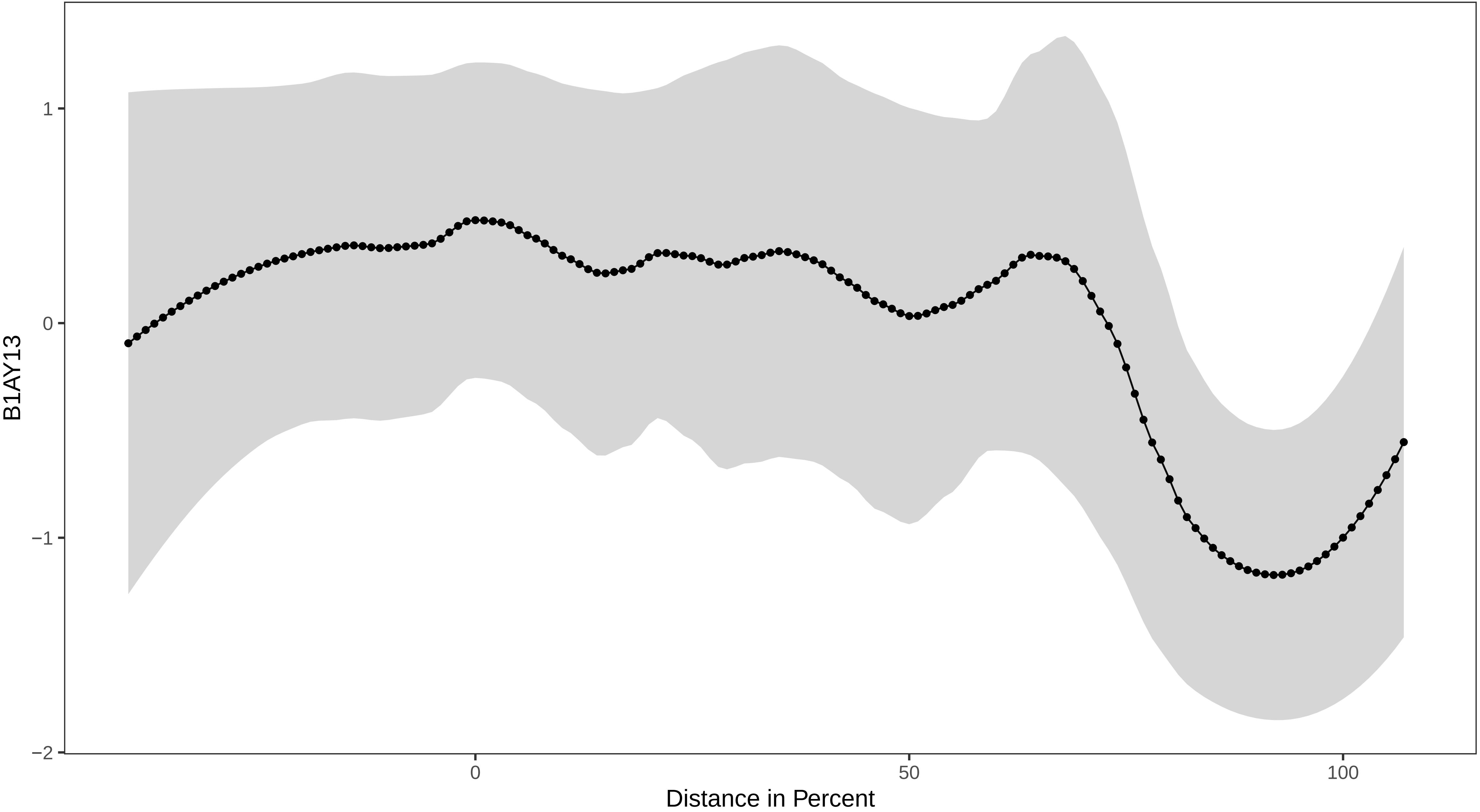

Q3TIX9 − Usp39 − SNUT2_MOUSE

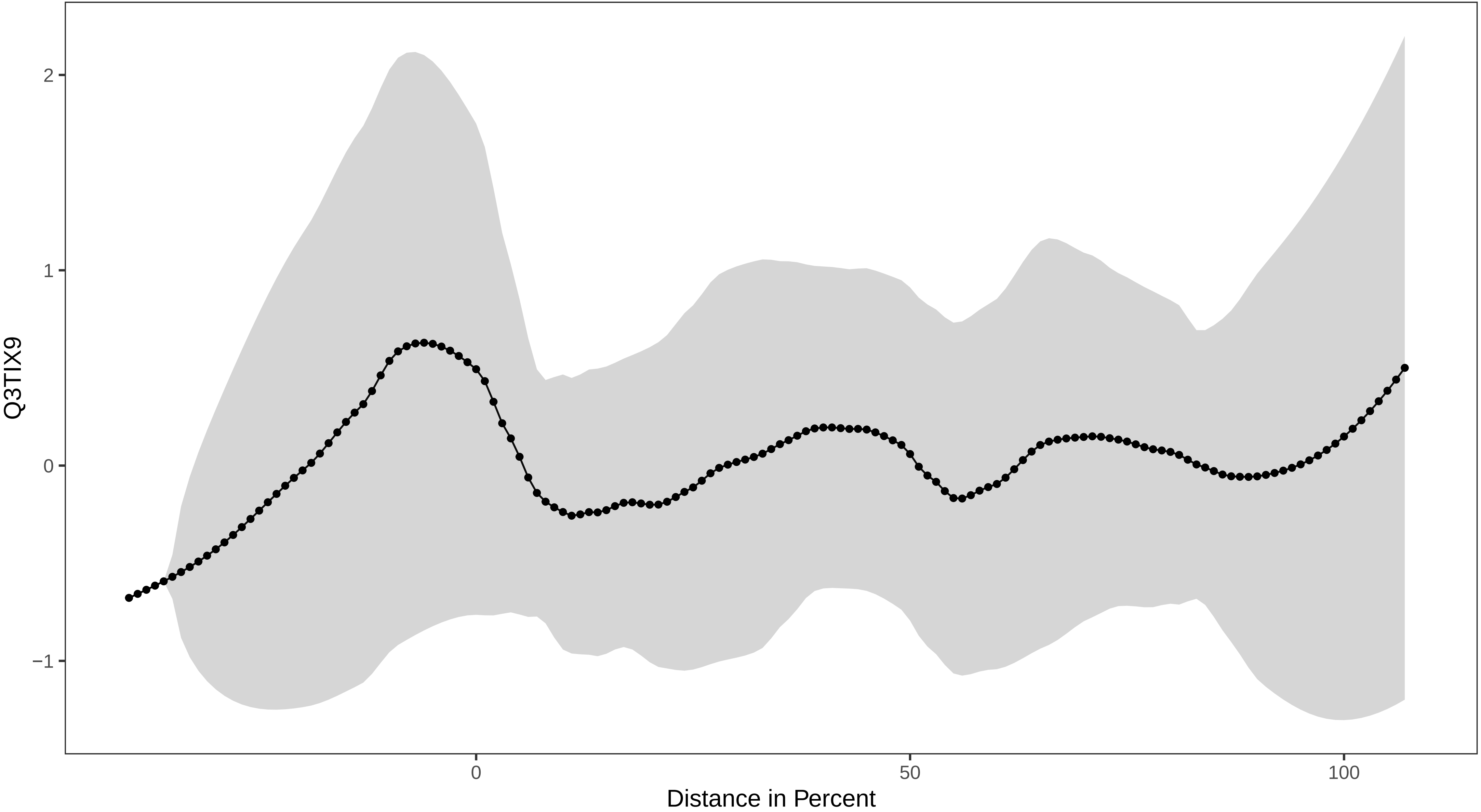

P35123 − Usp4 − UBP4_MOUSE

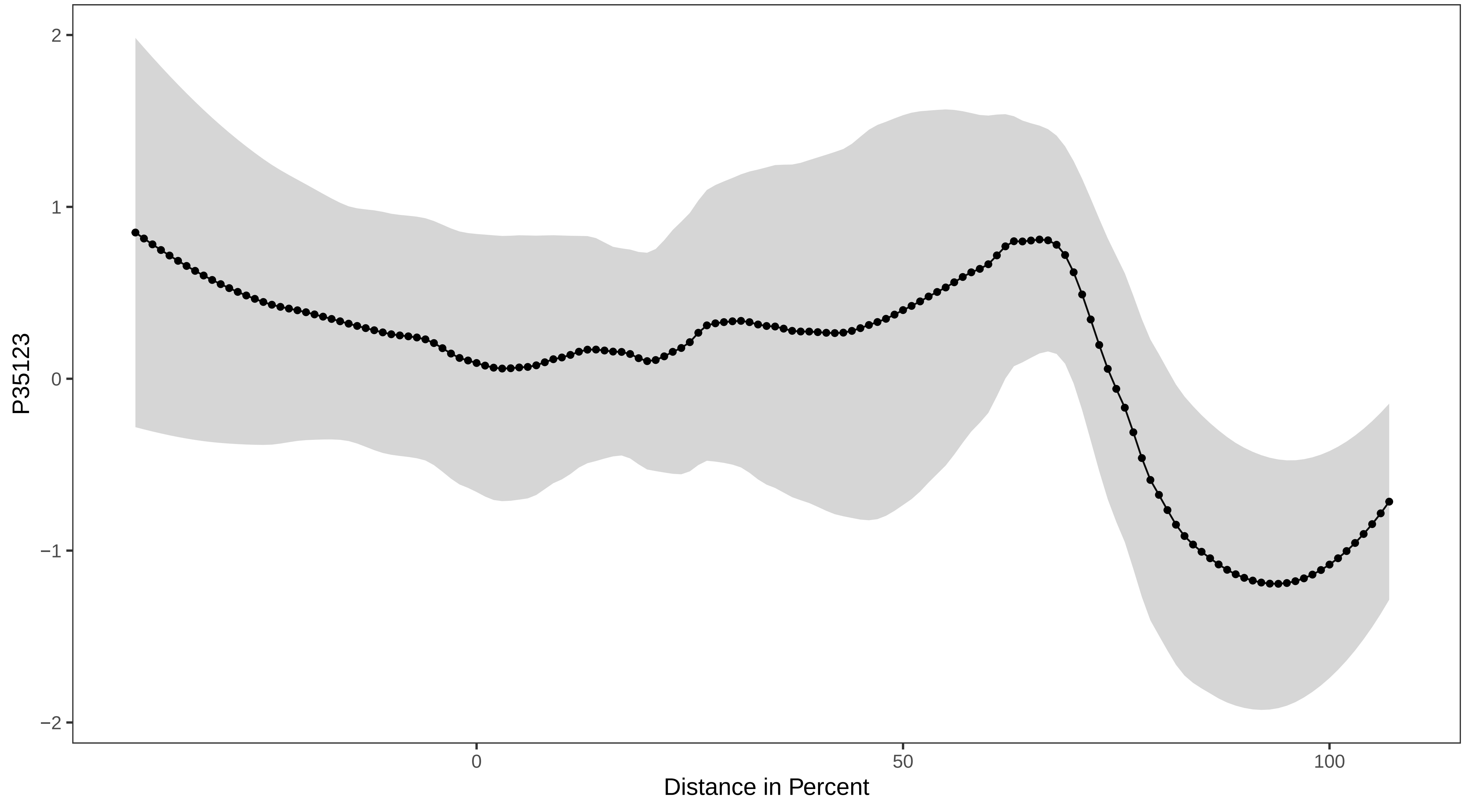

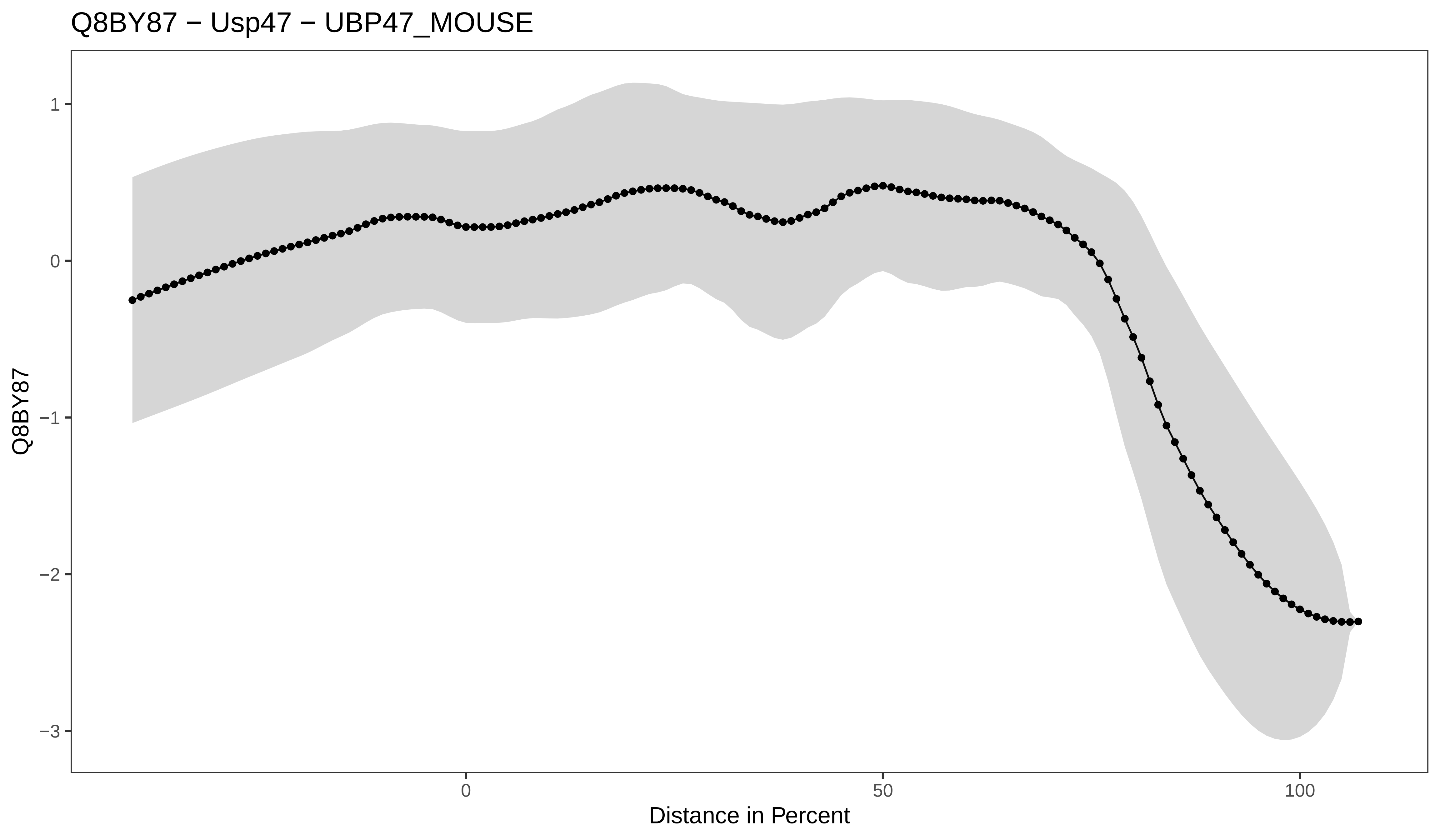

Q8BY87 − Usp47 − UBP47_MOUSE
1
0
−1
−2
−3
Q8BY87
0
50
100
Distance in Percent

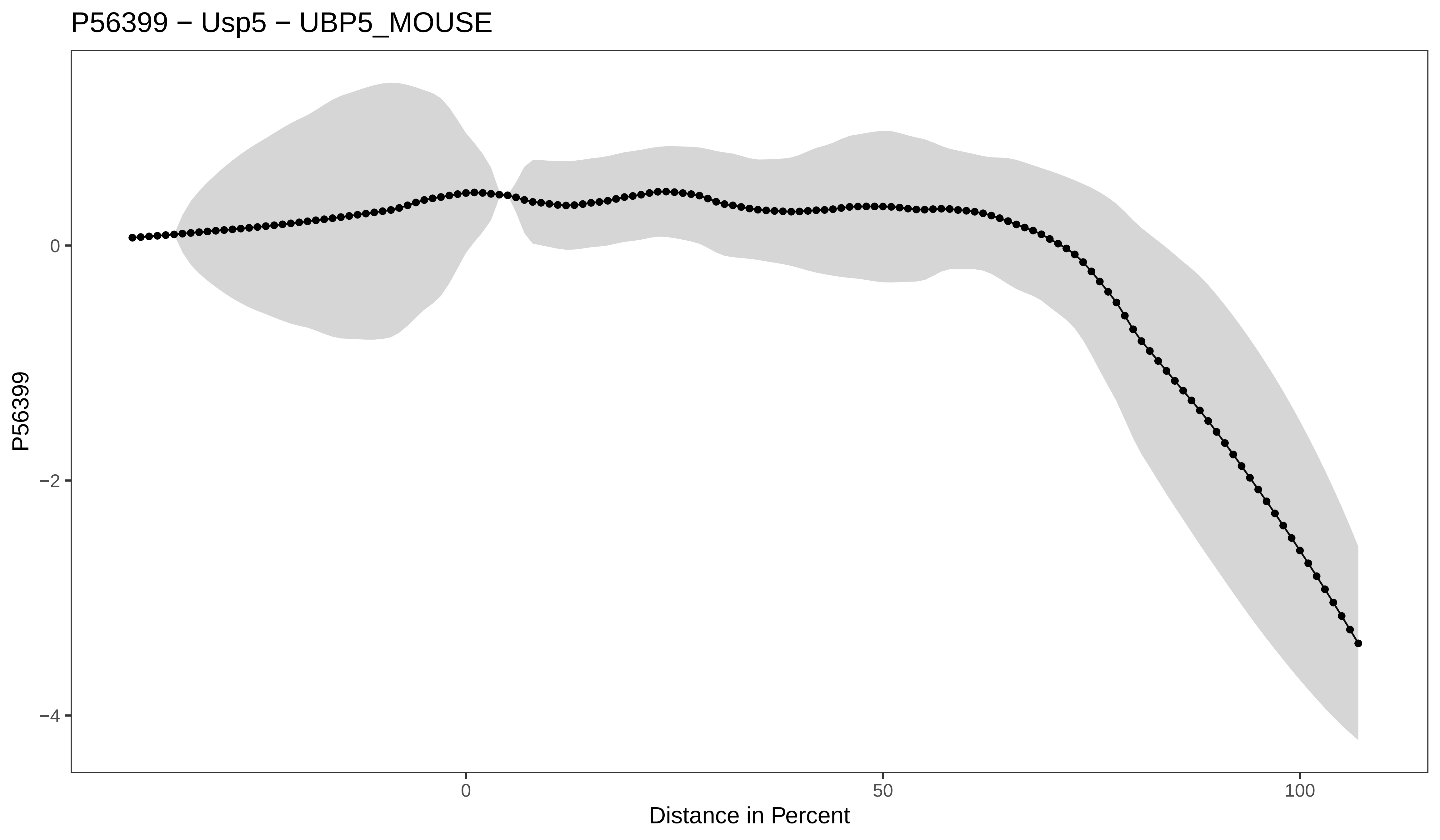

P56399 − Usp5 − UBP5_MOUSE
P56399
0
−2
−4
0
50
100
Distance in Percent

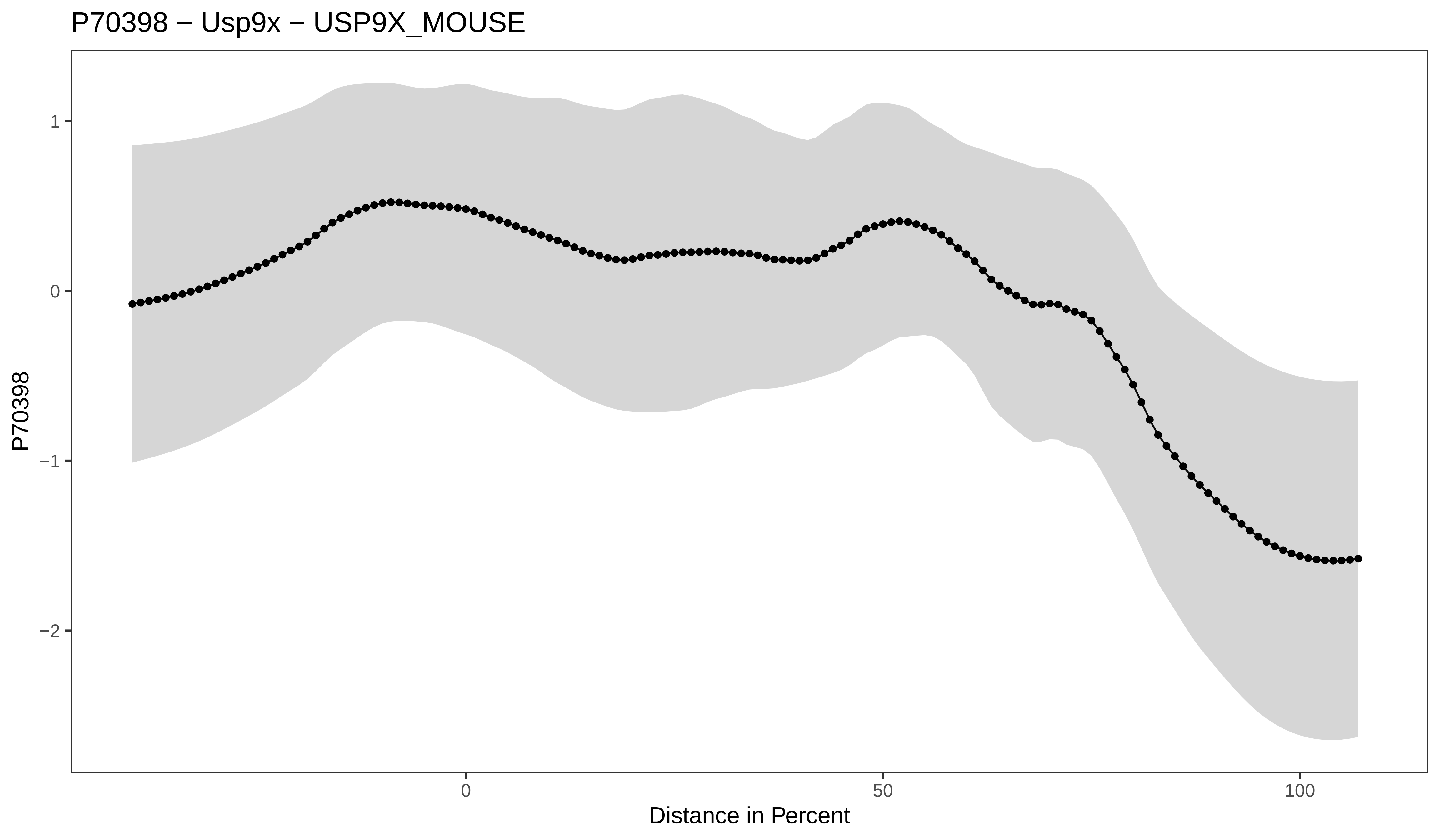
P70398 − Usp9x − USP9X_MOUSE
P70398
1
0
−1
−2
0
50
100
Distance in Percent

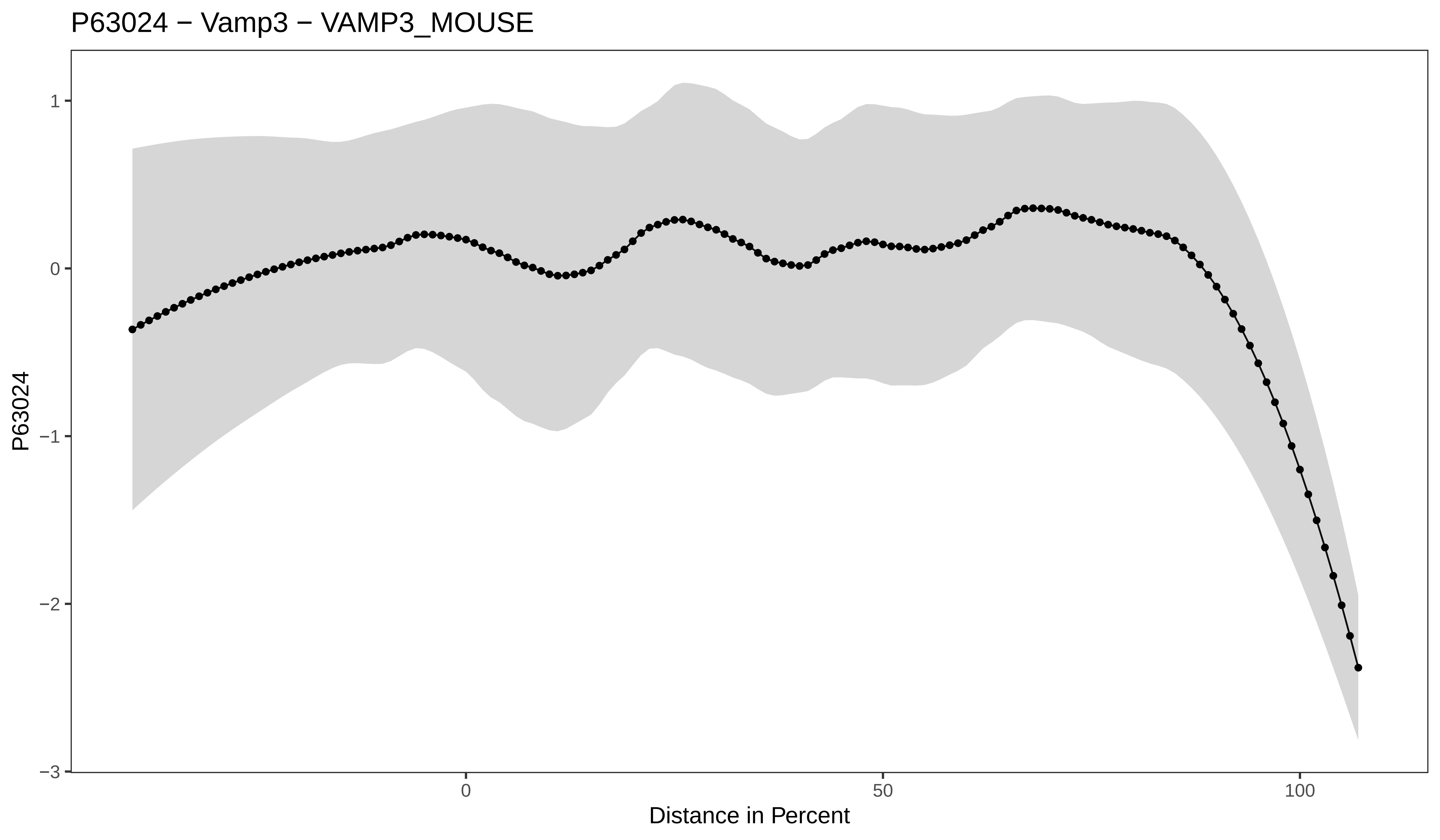

P63024 − Vamp3 − VAMP3_MOUSE
1
0
−1
−2
−3
P63024
0
50
100
Distance in Percent

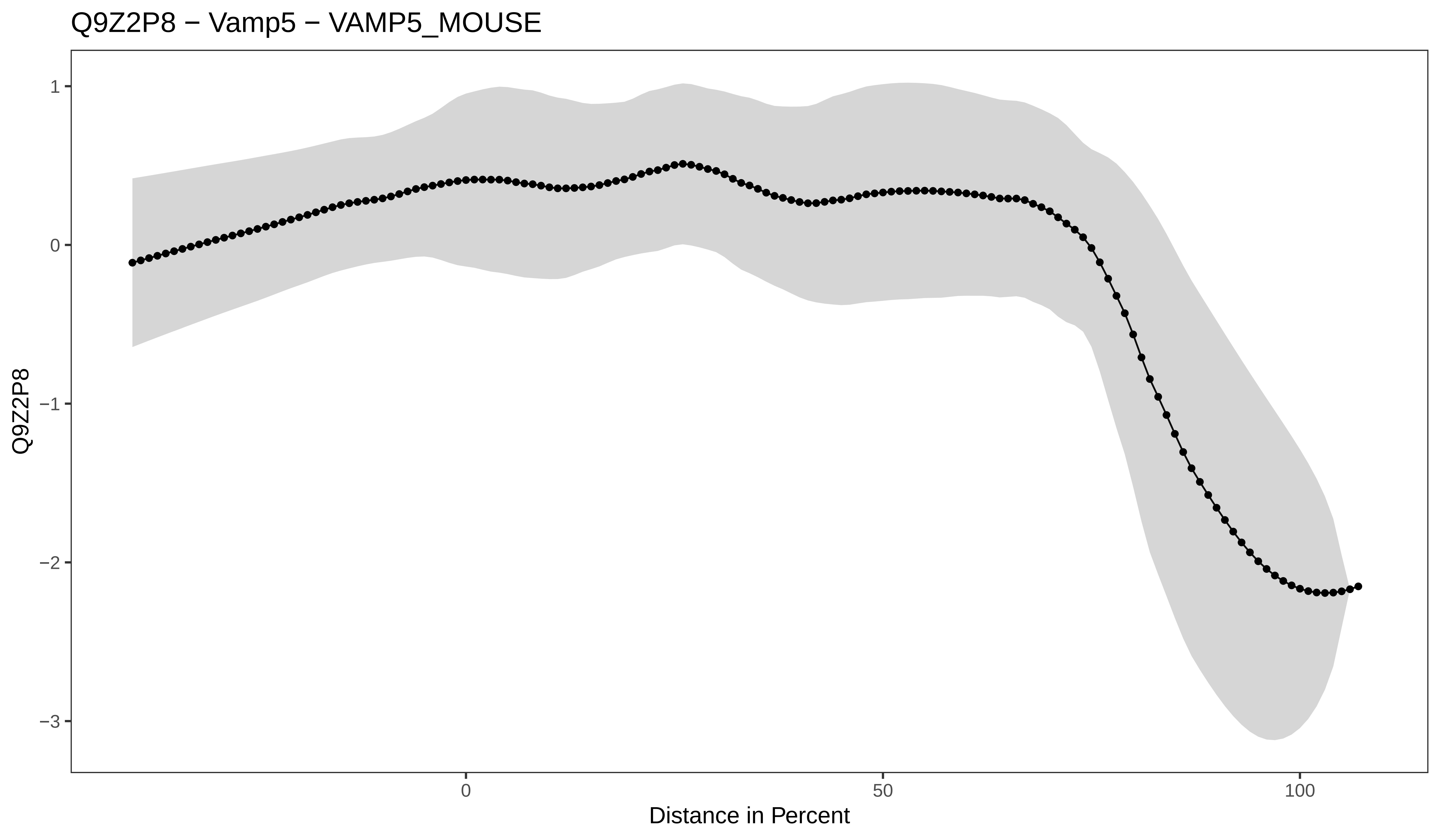

Q9Z2P8 − Vamp5 − VAMP5_MOUSE
1
0
−1
−2
−3
Q9Z2P8
0
50
100
Distance in Percent

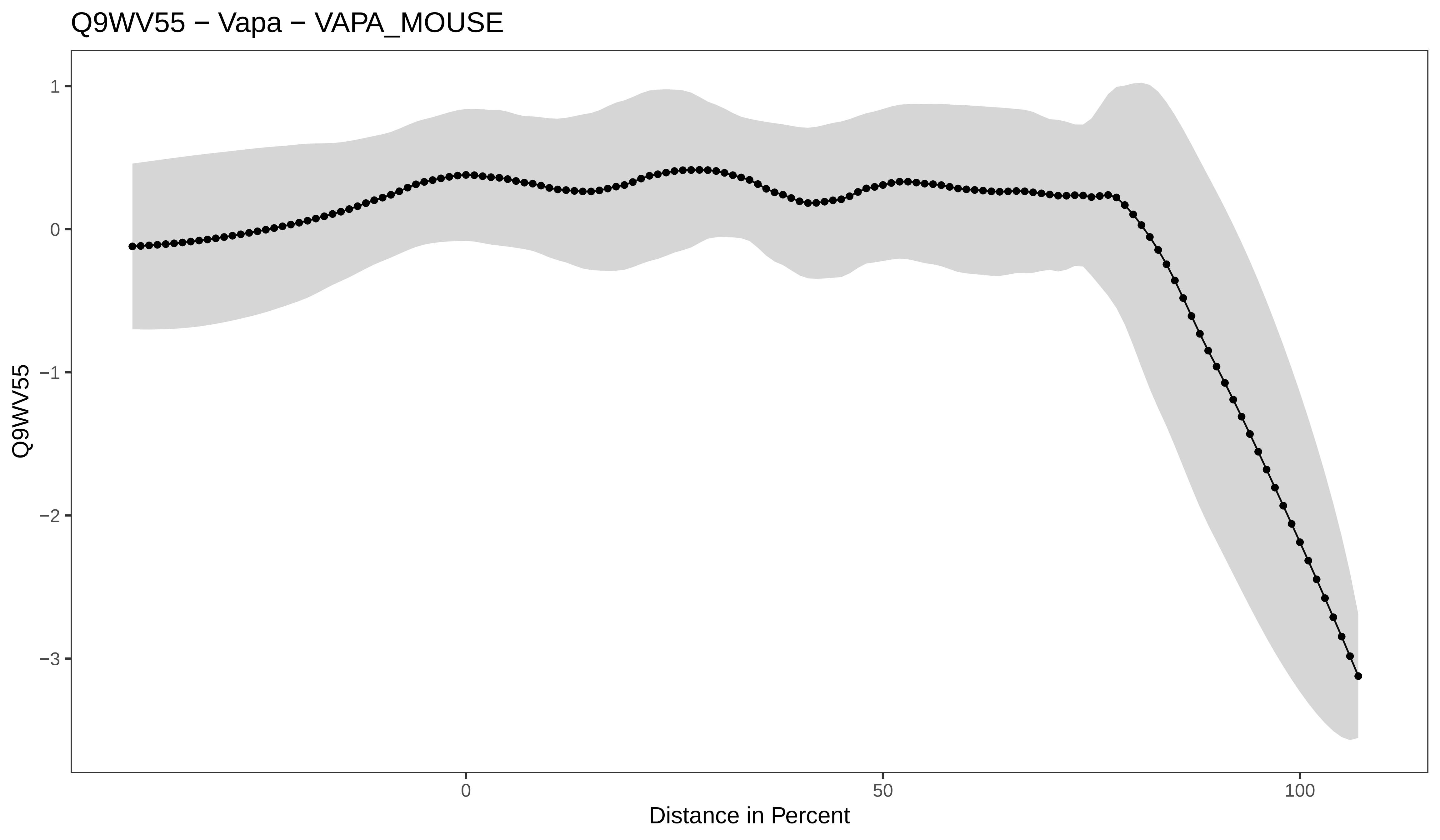

Q9WV55 − Vapa − VAPA_MOUSE
1
0
−1
−2
−3
Q9WV55
0
50
100
Distance in Percent

Q9QY76 − Vapb − VAPB_MOUSE

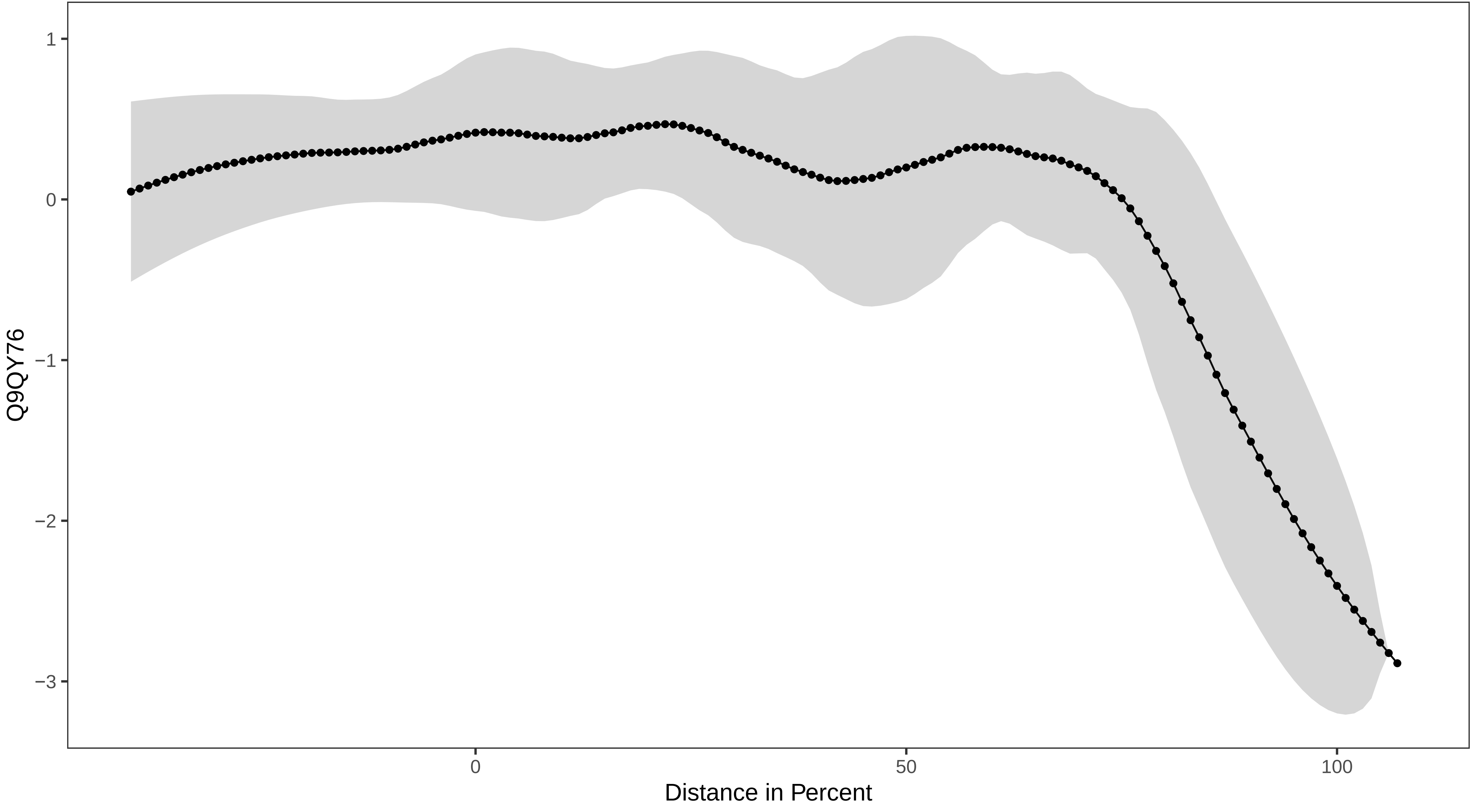

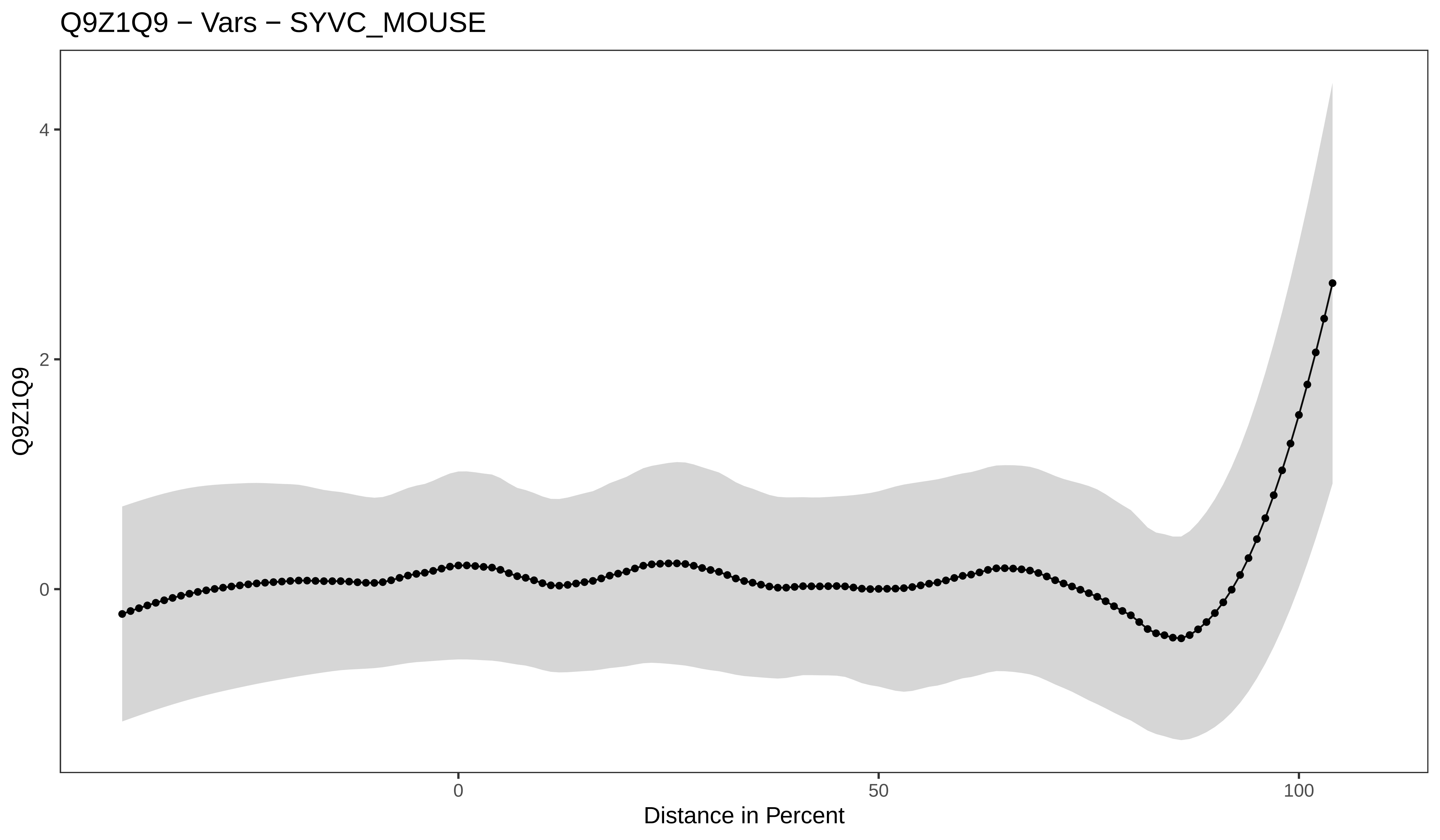

Q9Z1Q9 − Vars − SYVC_MOUSE
Q9Z1Q9
Distance in Percent
0
2
4
0
50
100

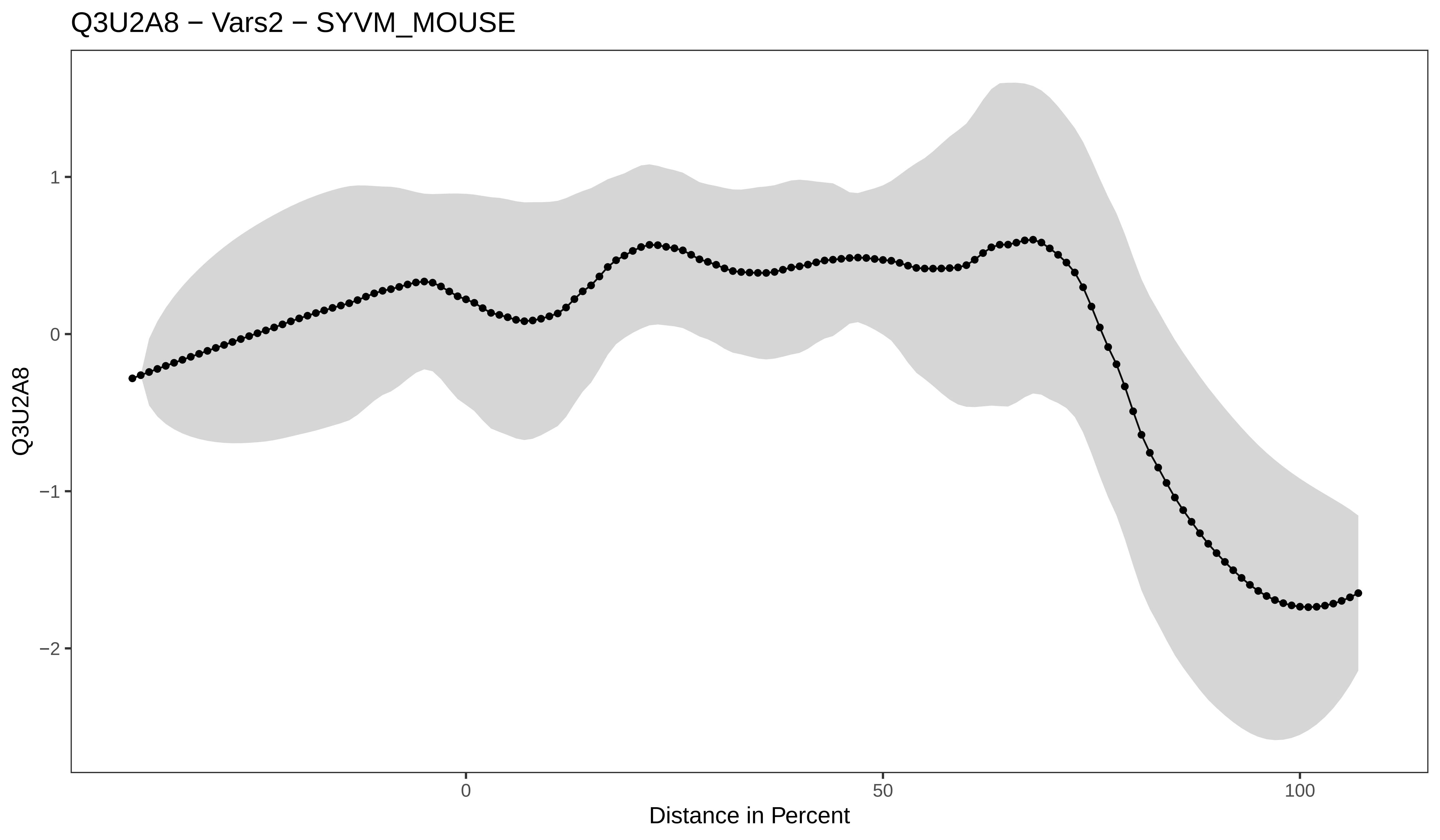
Q3U2A8 − Vars2 − SYVM_MOUSE
1
0
−1
−2
Q3U2A8
0
50
100
Distance in Percent

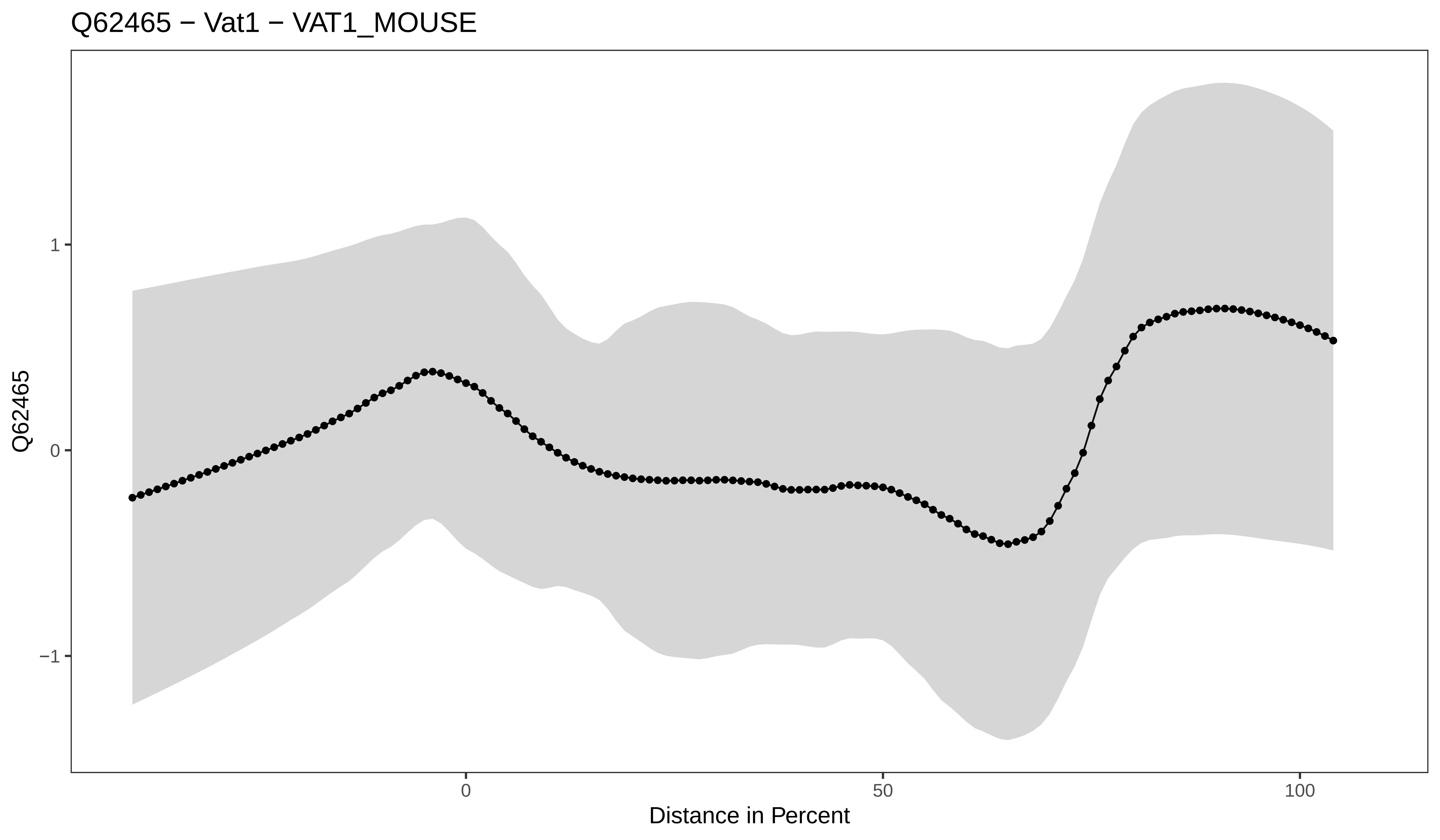

Q62465 − Vat1 − VAT1_MOUSE
Q62465
1
0
−1
0
50
100
Distance in Percent

Q80TB8 − Vat1l − VAT1L_MOUSE

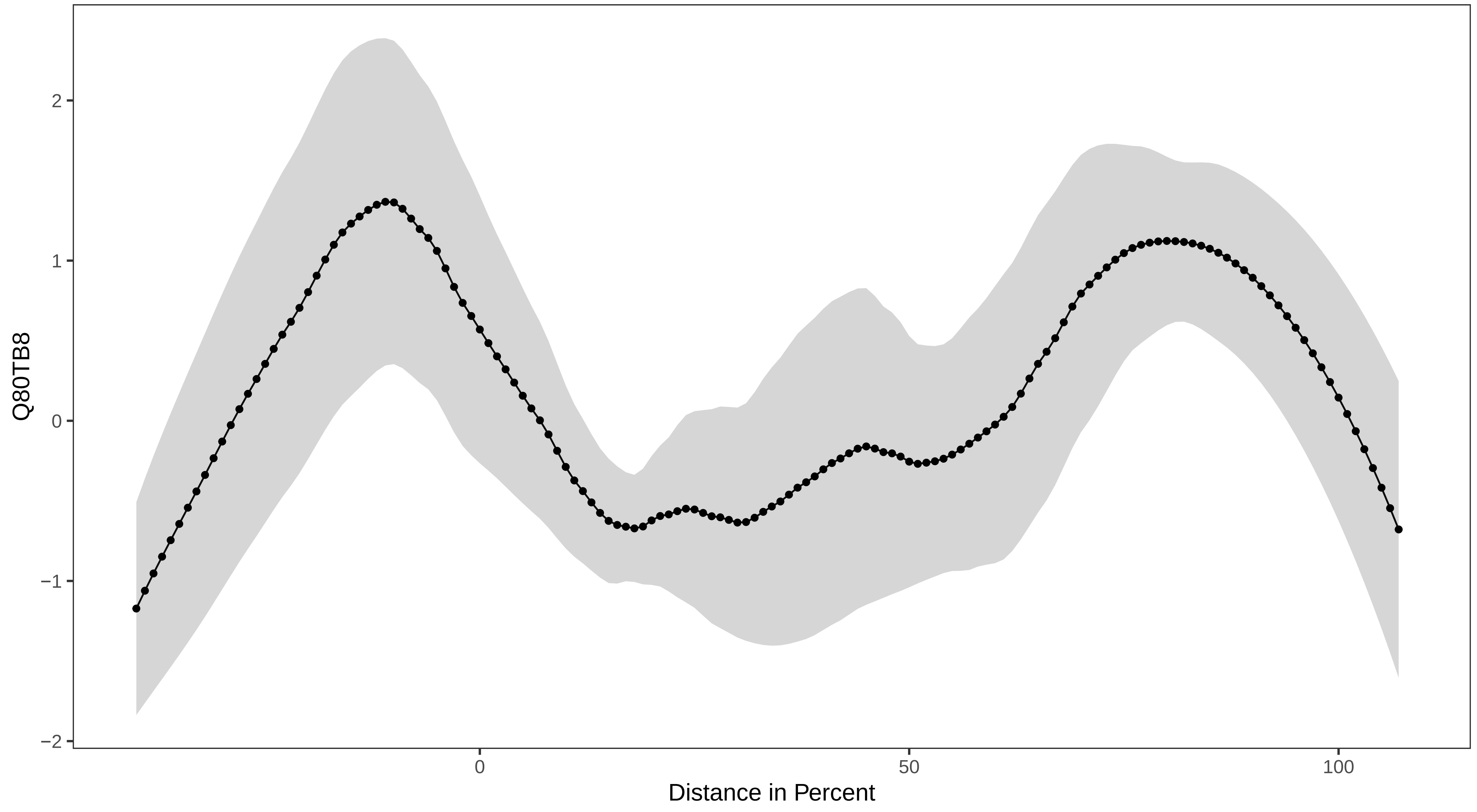

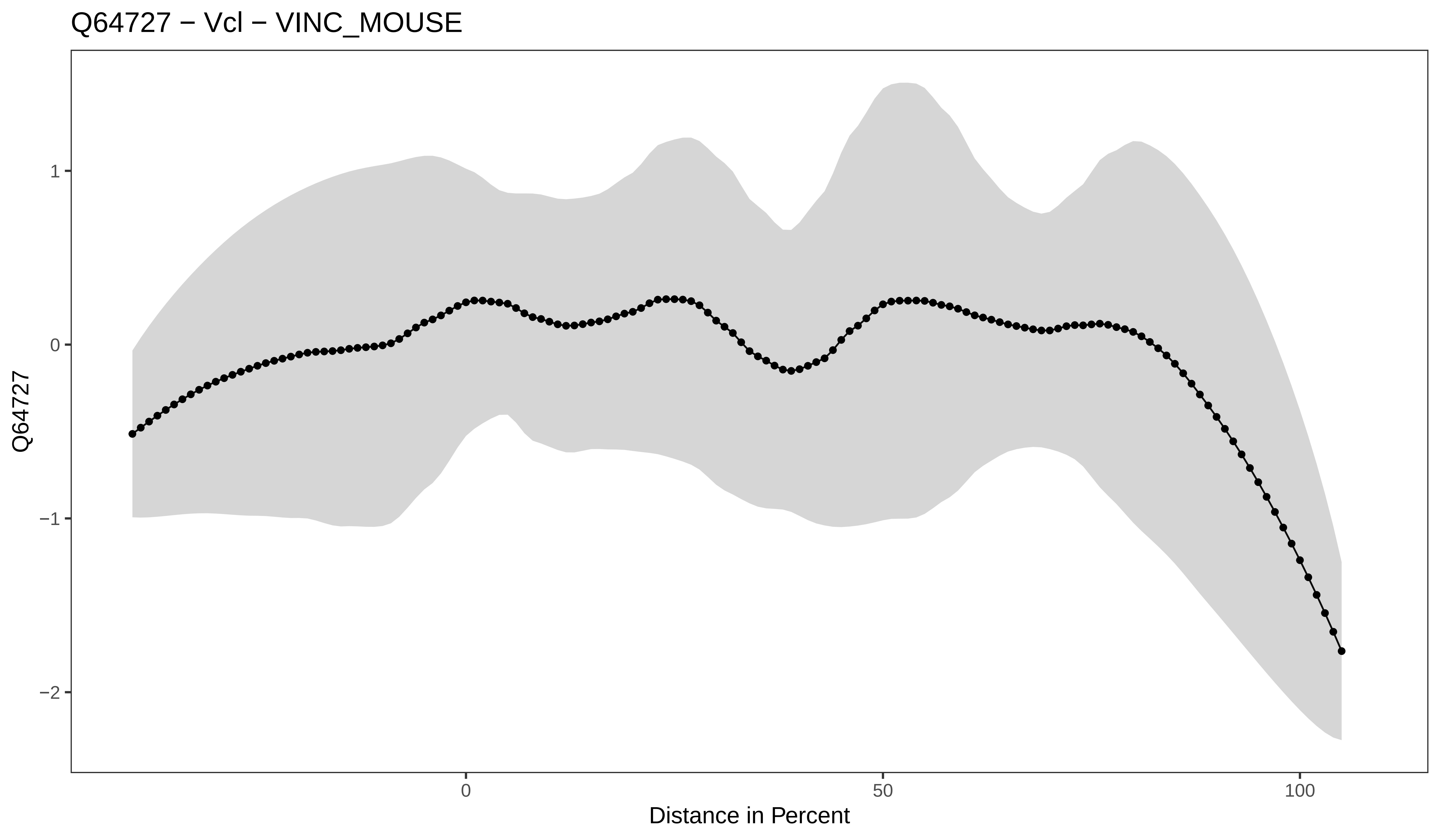

Q64727 − Vcl − VINC_MOUSE
Q64727
1
0
−1
−2
0
50
100
Distance in Percent

Q01853 − Vcp − TERA_MOUSE

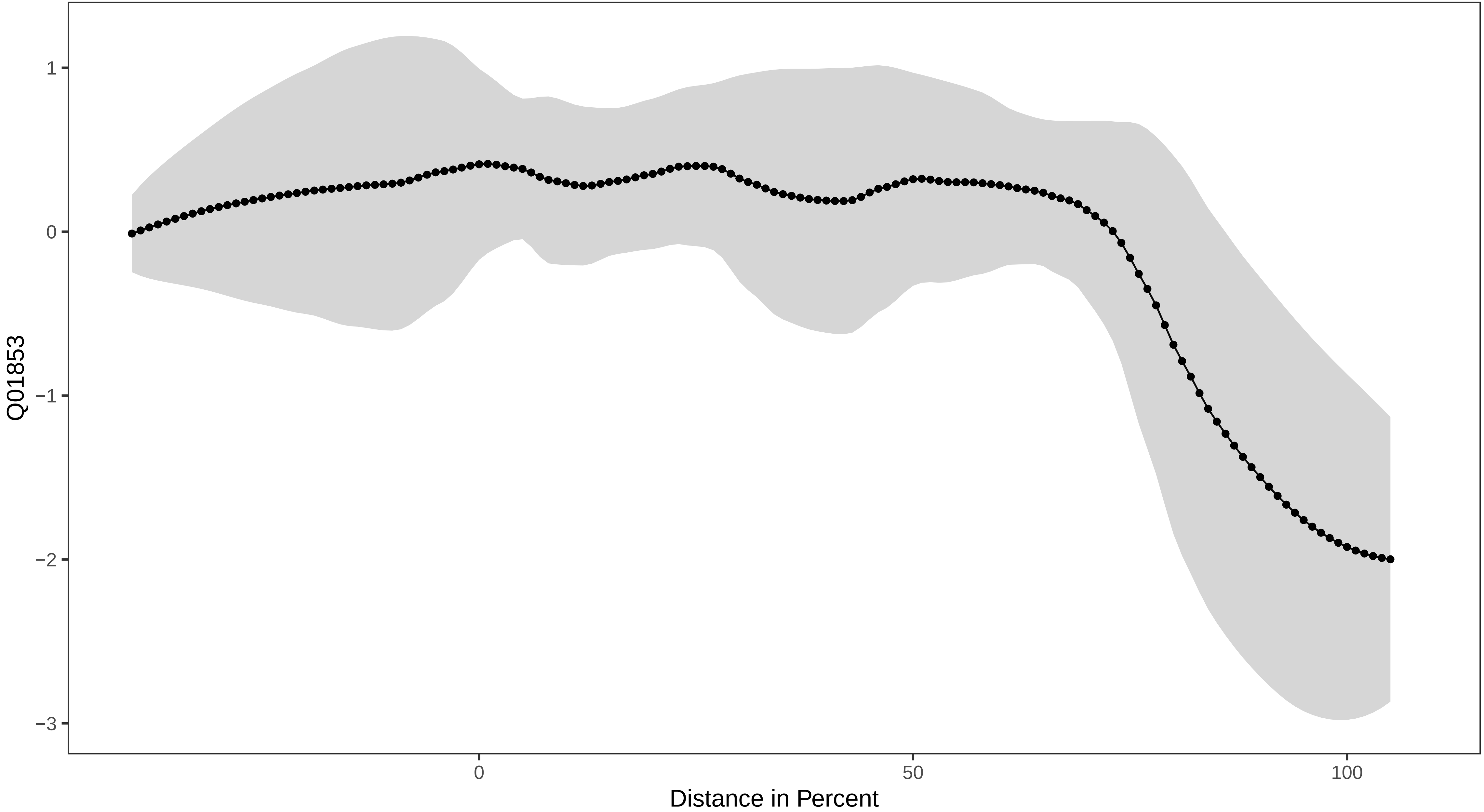

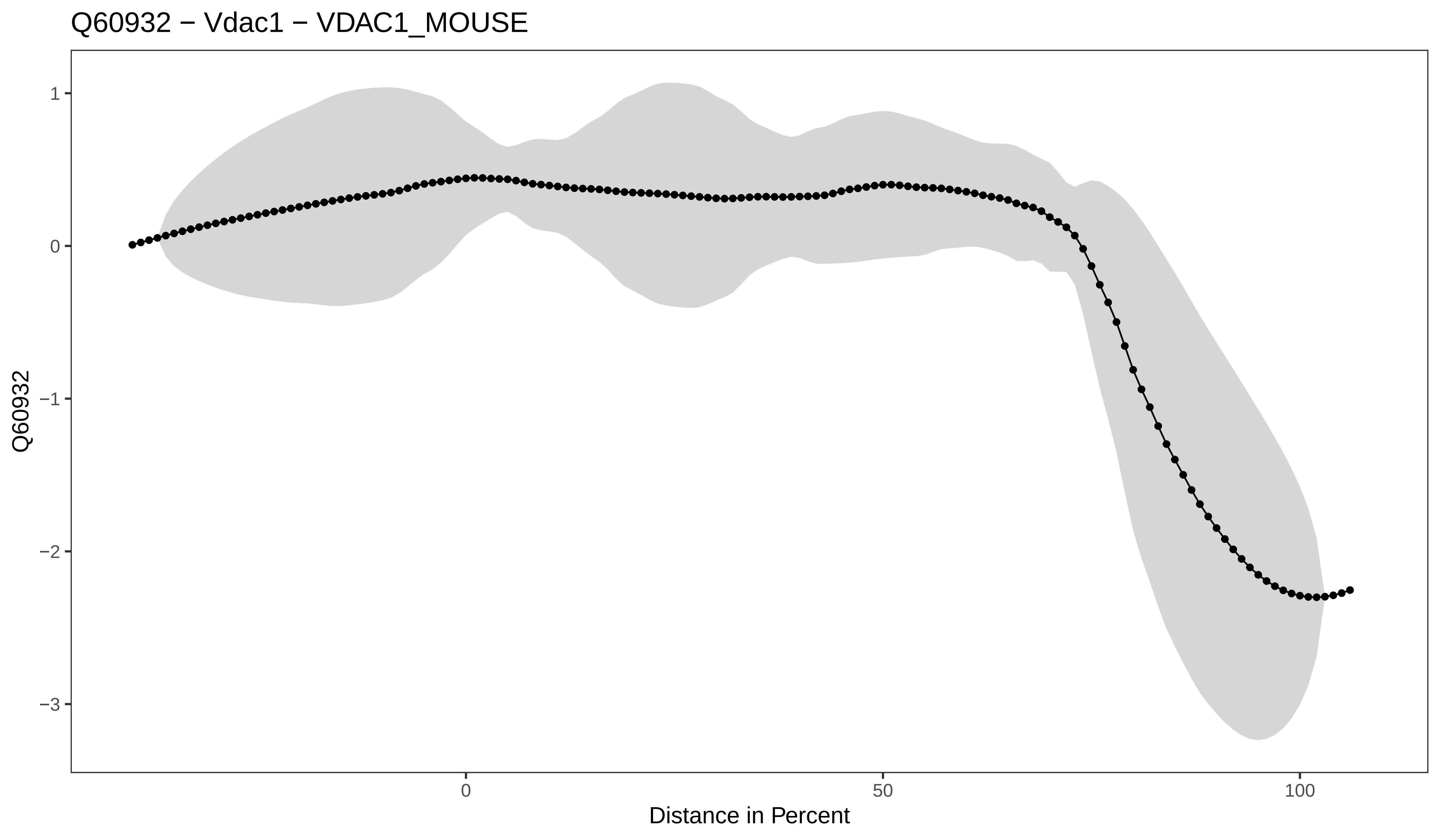

Q60932 − Vdac1 − VDAC1_MOUSE
1
0
−1
−2
−3
Q60932
0
50
100
Distance in Percent

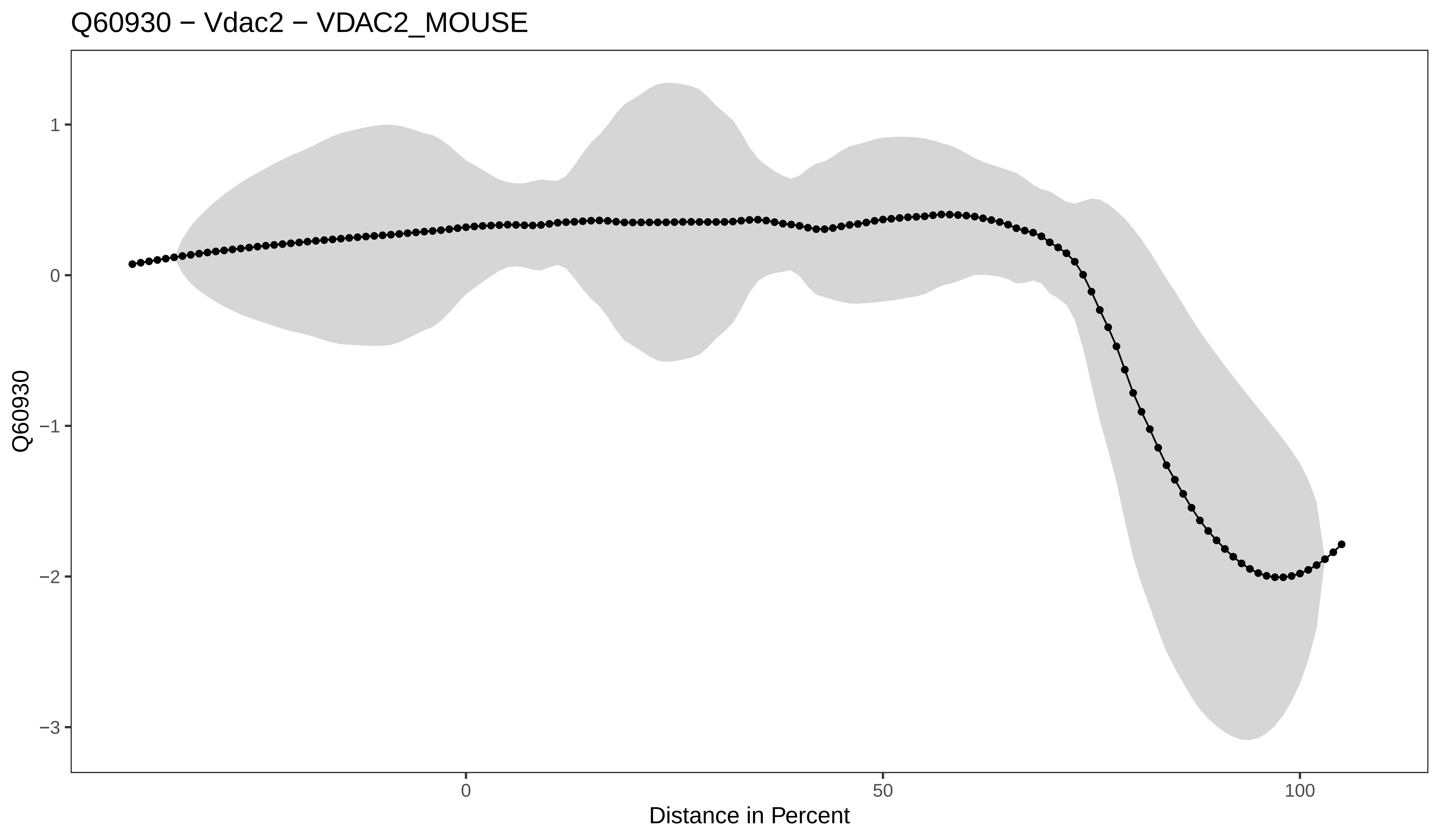
Q60930 − Vdac2 − VDAC2_MOUSE
1
0
−1
−2
−3
Q60930
0
50
100
Distance in Percent

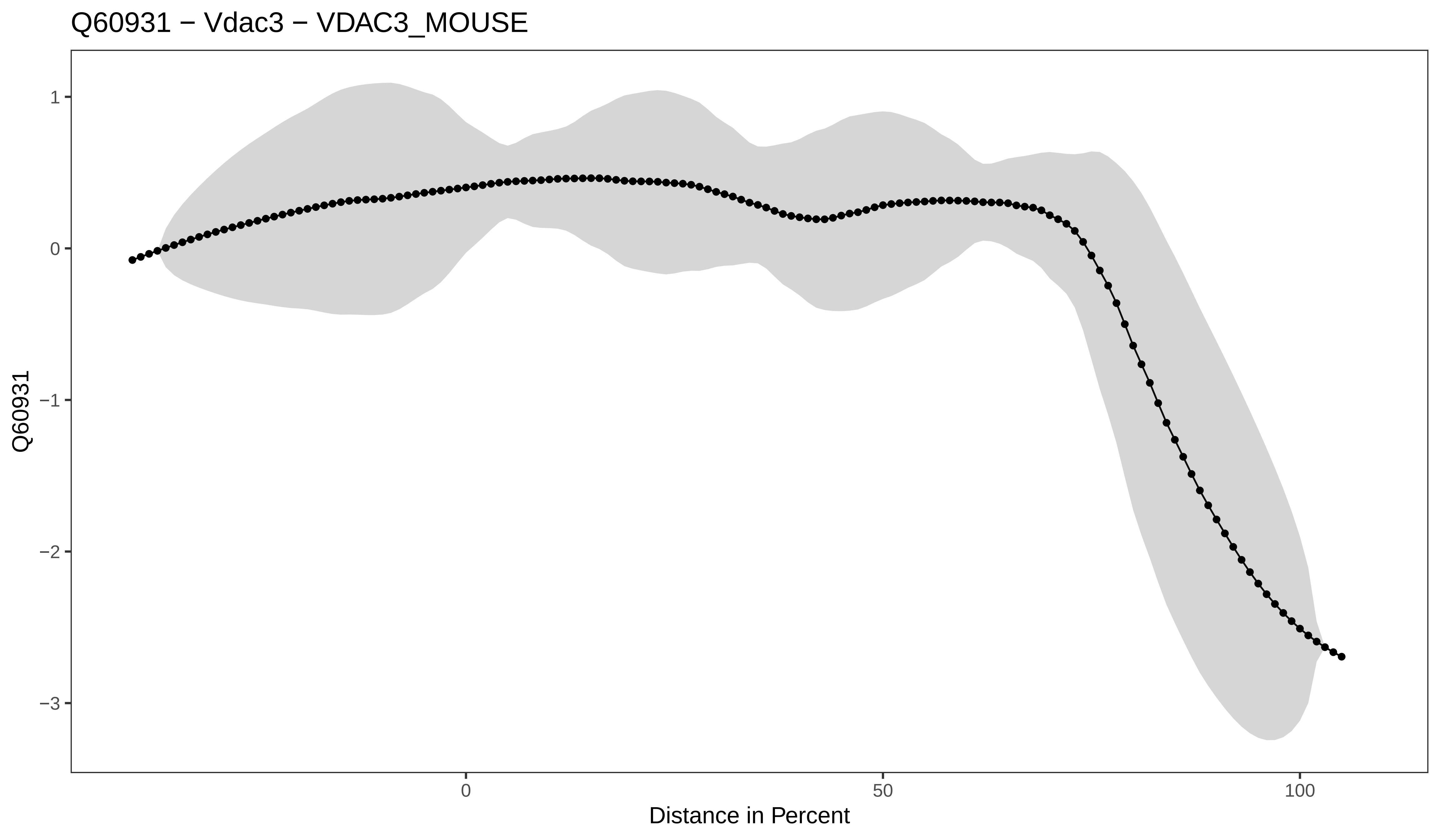
Q60931 − Vdac3 − VDAC3_MOUSE
Q60931
Distance in Percent
1
0
−1
−2
−3
0
50
100

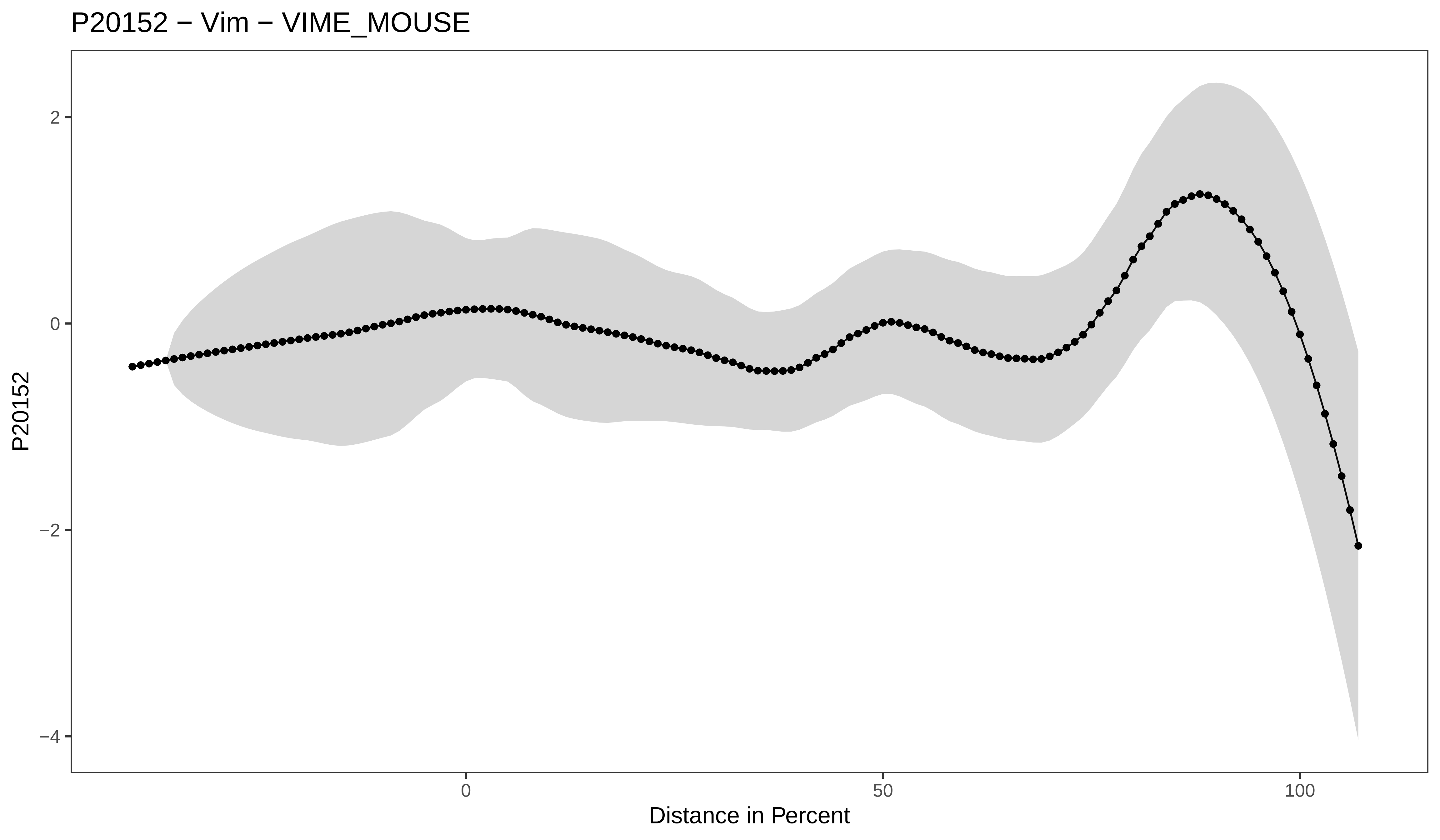

P20152 − Vim − VIME_MOUSE
2
0
−2
−4
P20152
0
50
100
Distance in Percent

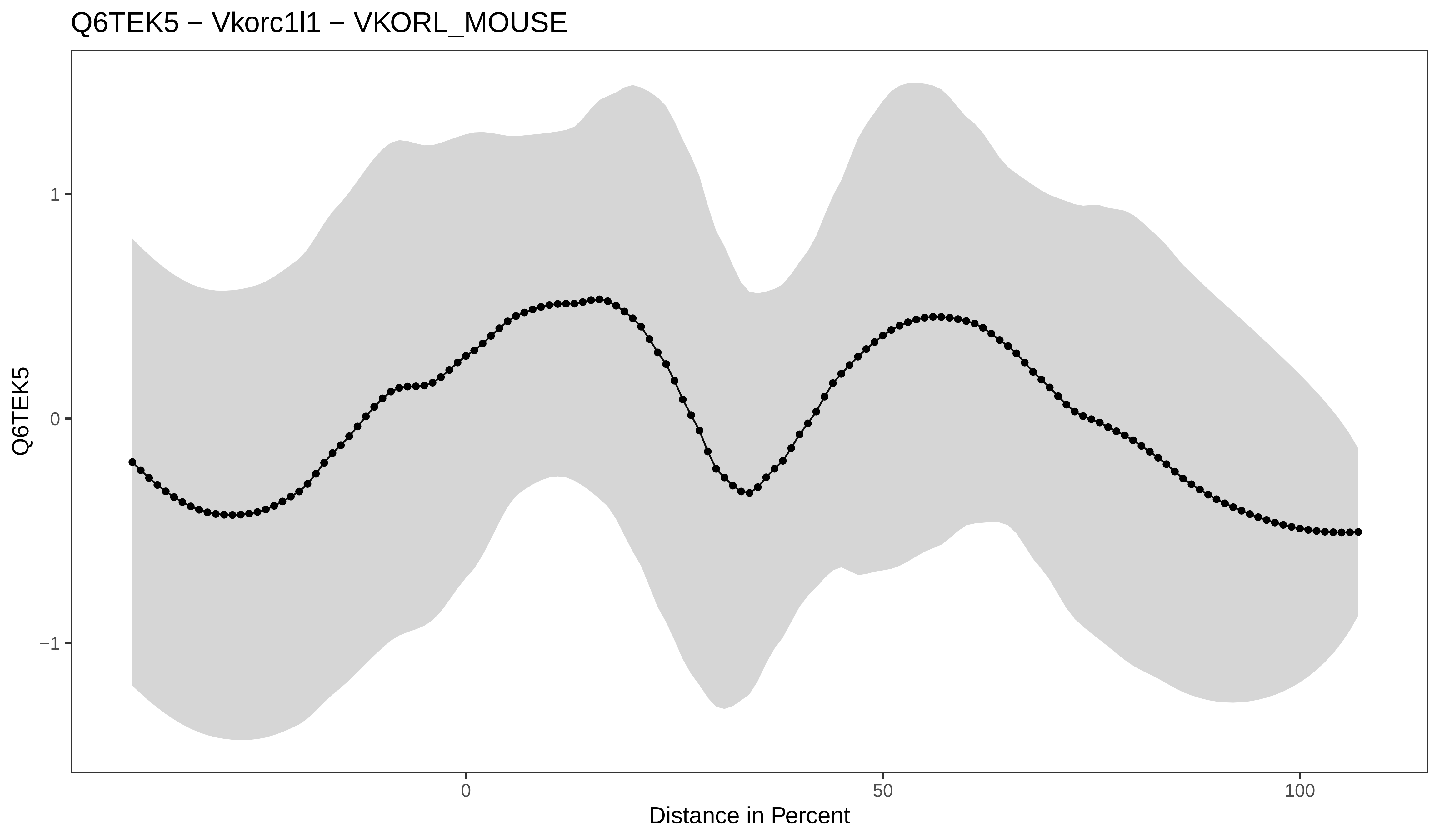
Q6TEK5 − Vkorc1l1 − VKORL_MOUSE
Q6TEK5
1
0
−1
0
50
100
Distance in Percent

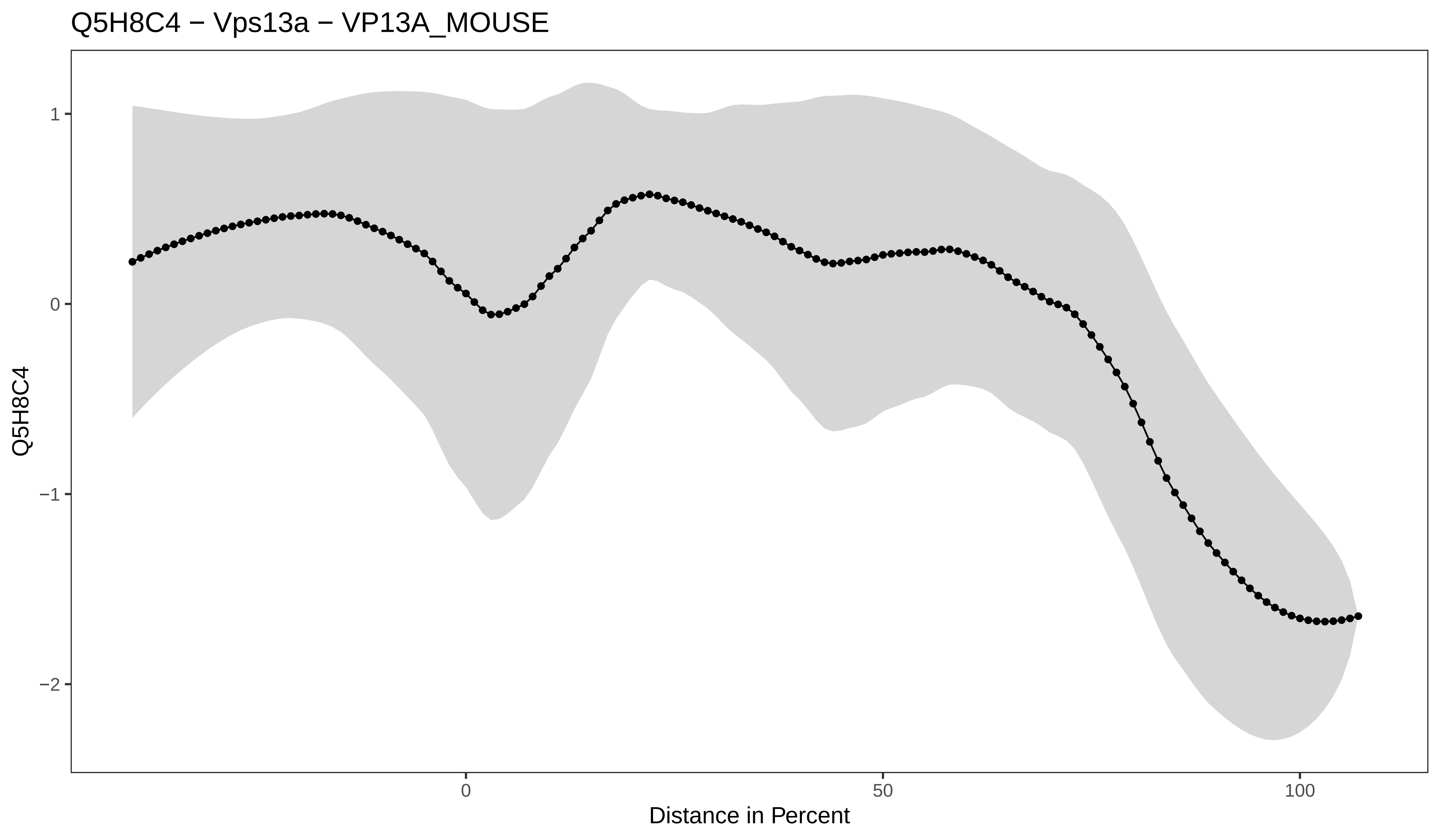
Q5H8C4 − Vps13a − VP13A_MOUSE
Q5H8C4
1
0
−1
−2
0
50
100
Distance in Percent

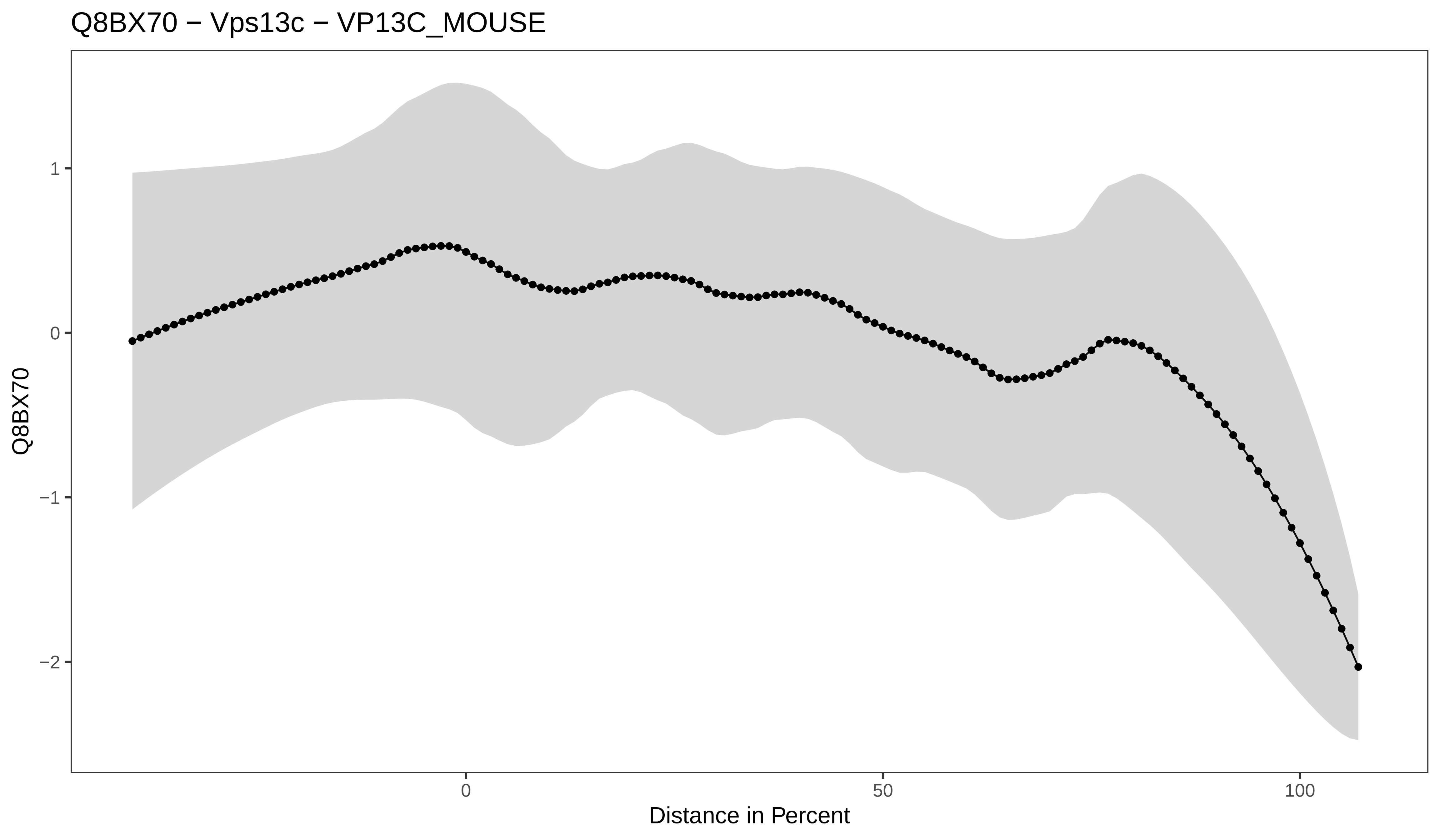
Q8BX70 − Vps13c − VP13C_MOUSE
Q8BX70
1
0
−1
−2
0
50
100
Distance in Percent

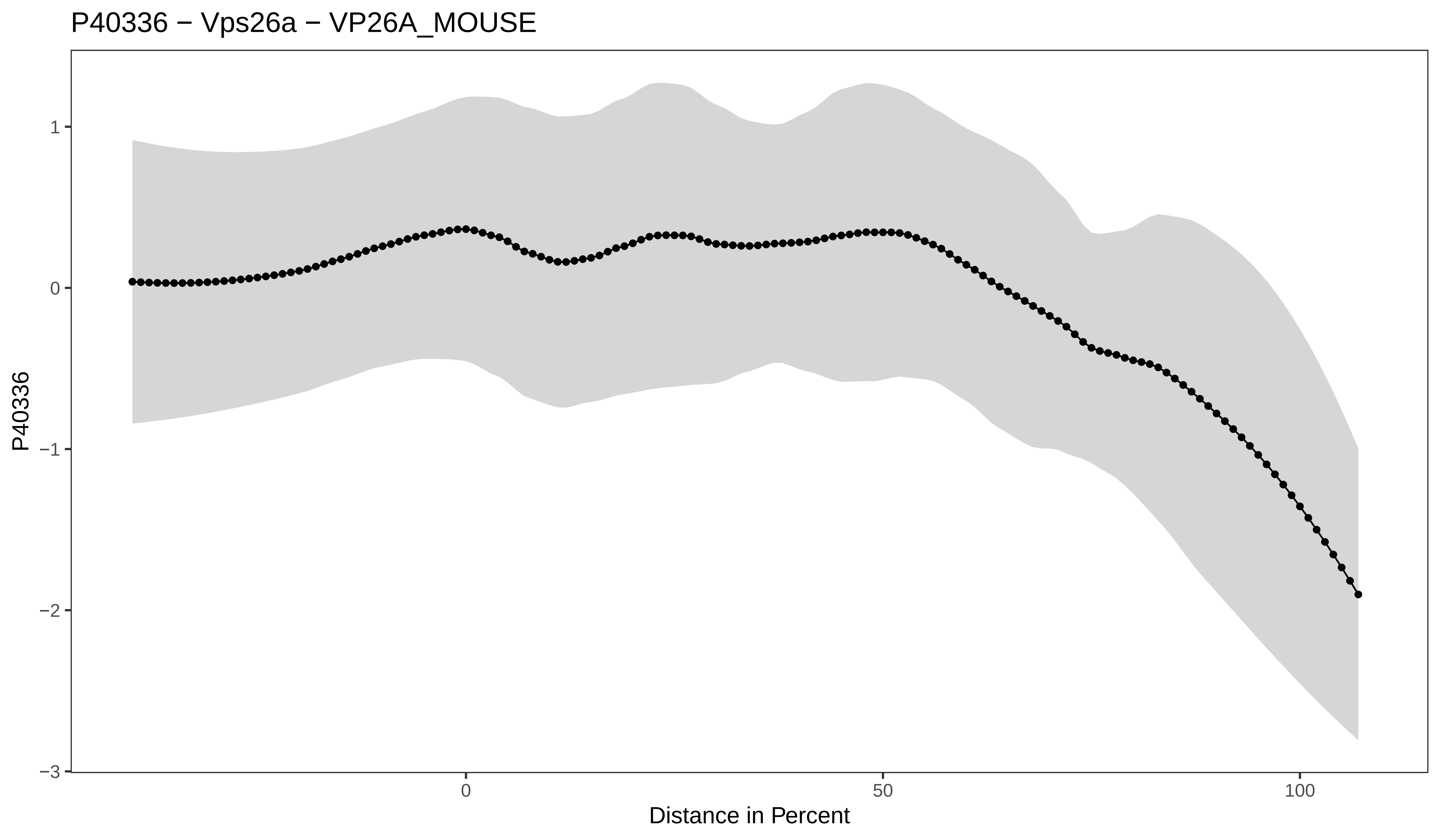

P40336 − Vps26a − VP26A_MOUSE
1
0
−1
−2
−3
P40336
0
50
100
Distance in Percent

Q8C0E2 − Vps26b − VP26B_MOUSE

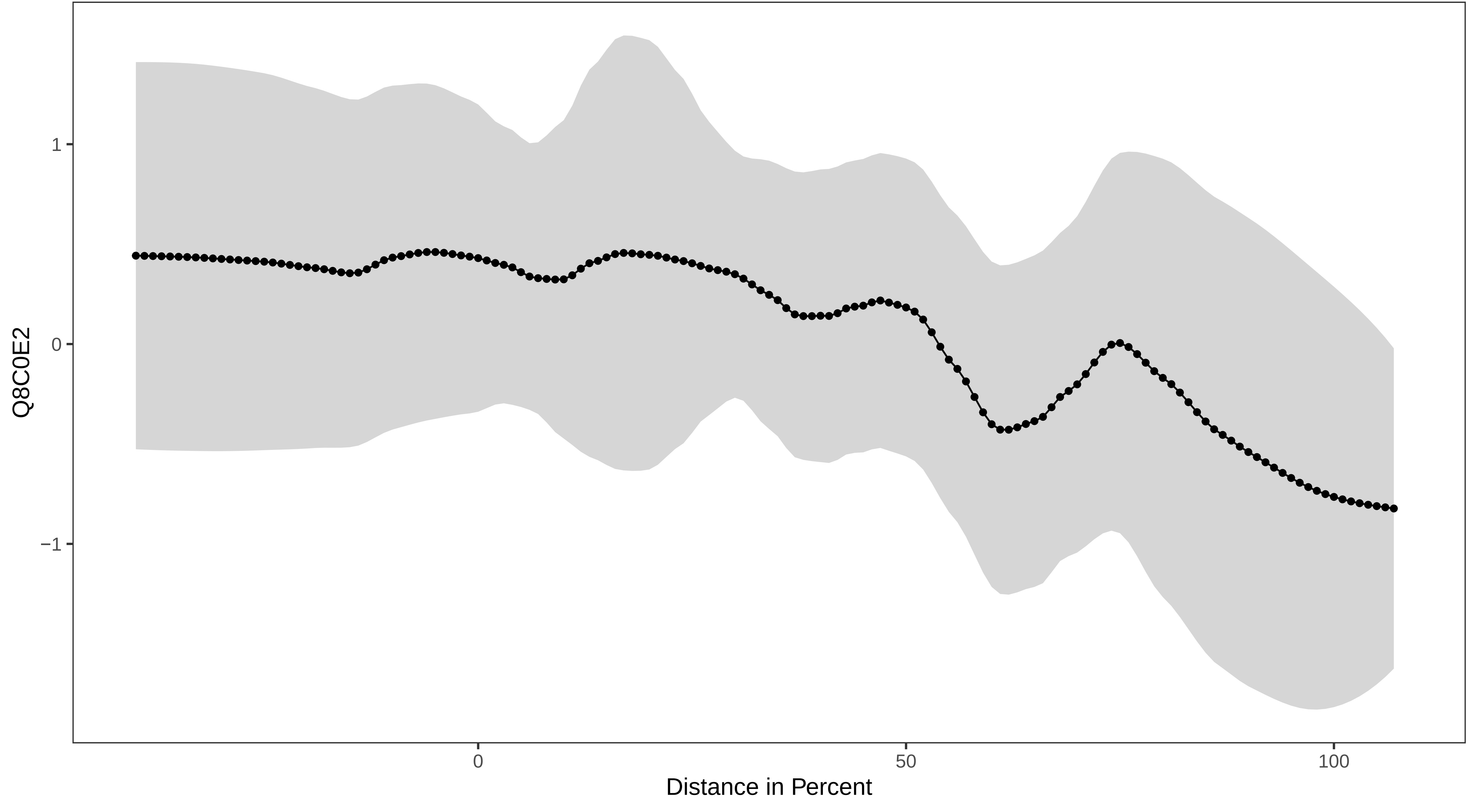

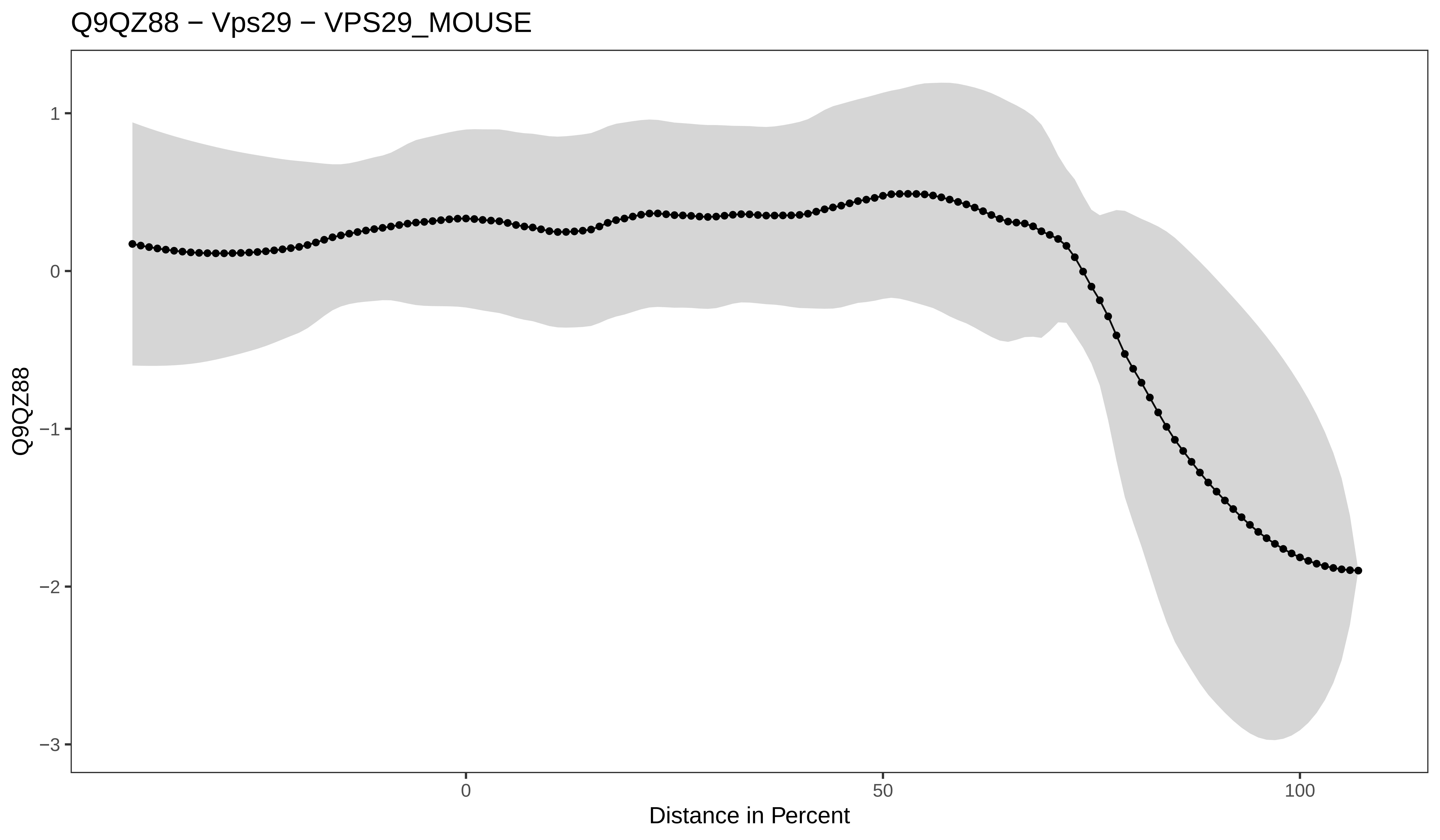

Q9QZ88 − Vps29 − VPS29_MOUSE
1
0
−1
−2
−3
Q9QZ88
0
50
100
Distance in Percent

Q9EQH3 − Vps35 − VPS35_MOUSE

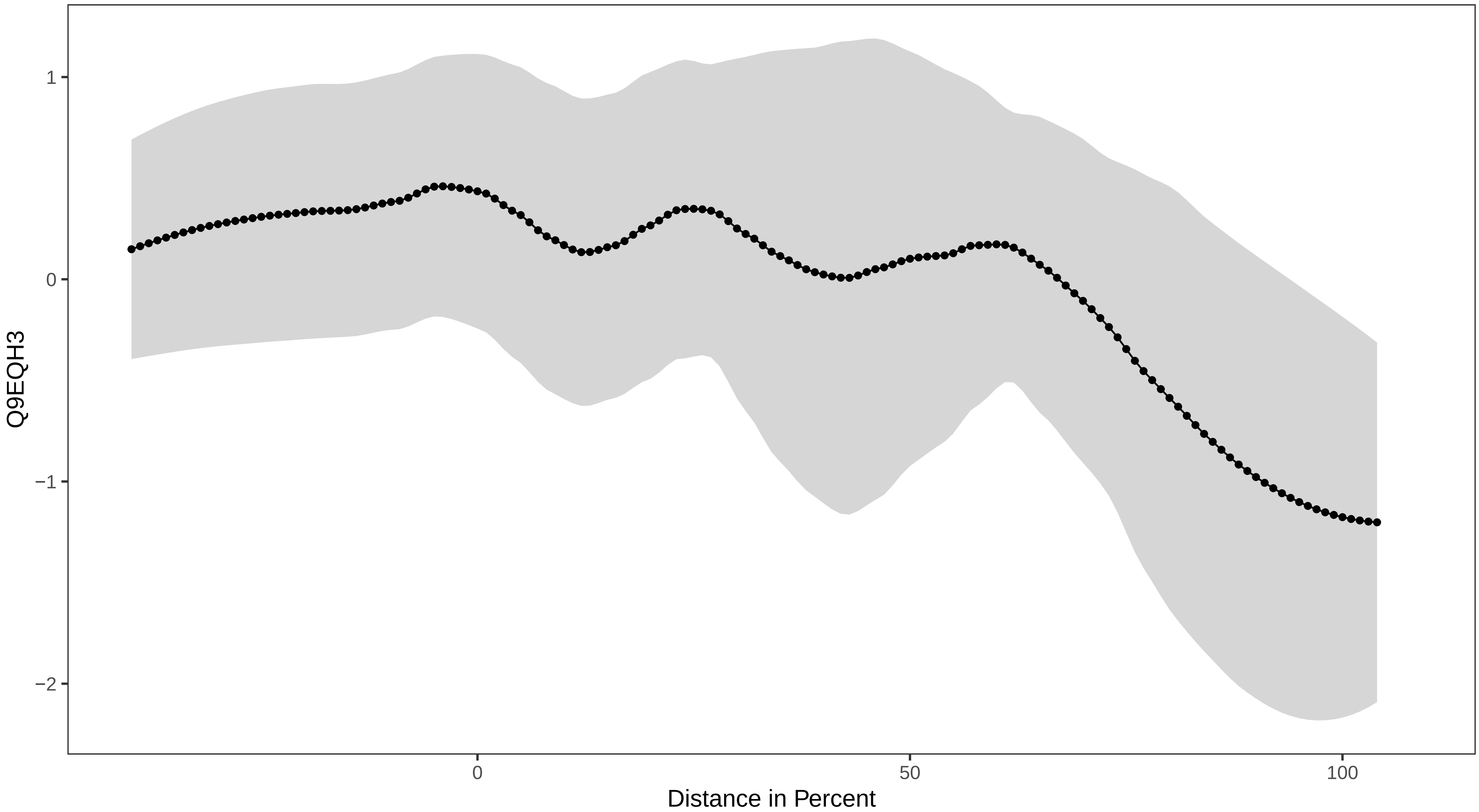

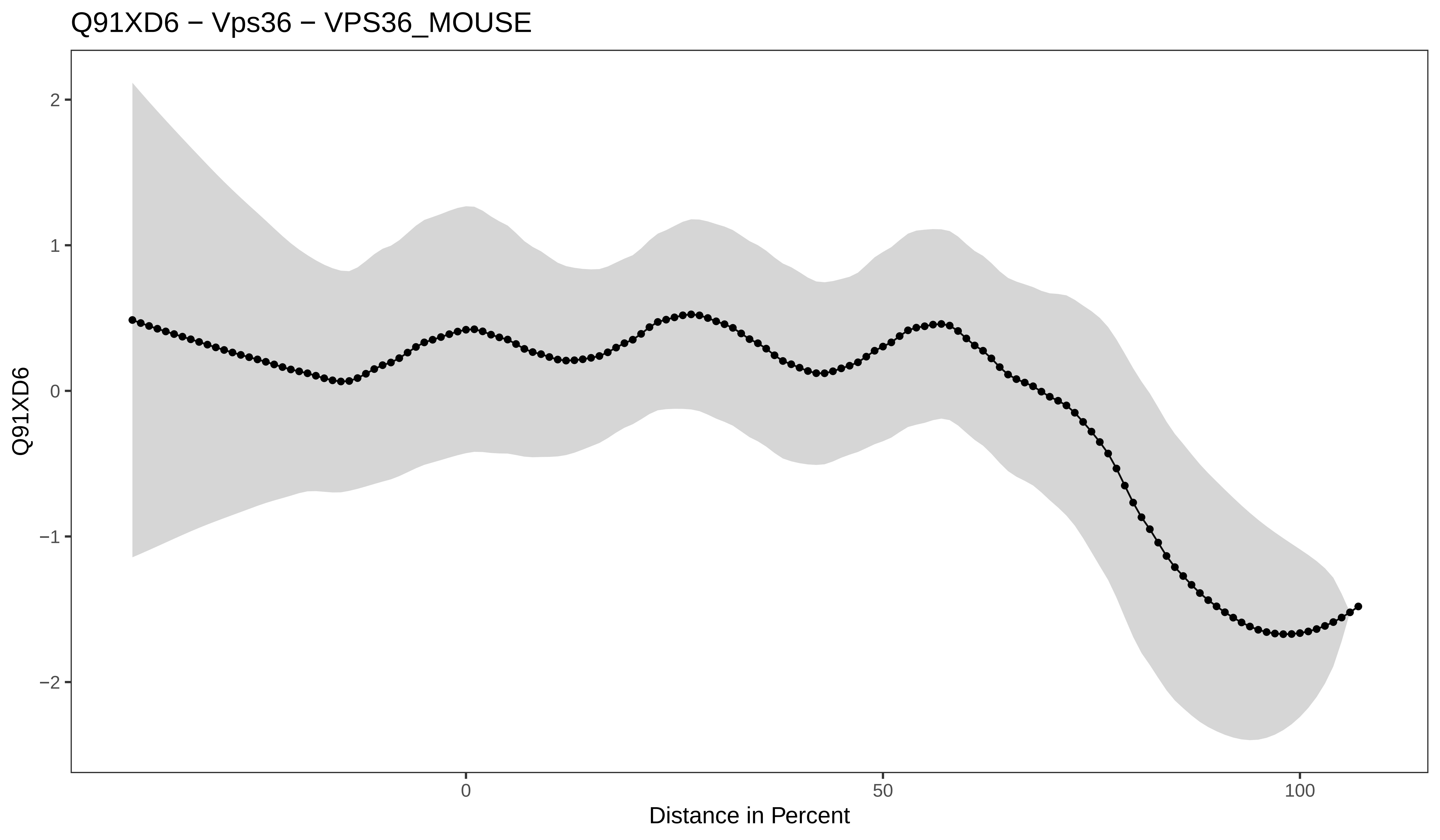

Q91XD6 − Vps36 − VPS36_MOUSE
2
1
0
−1
−2
Q91XD6
0
50
100
Distance in Percent

Q8VEJ9 − Vps4a − VPS4A_MOUSE

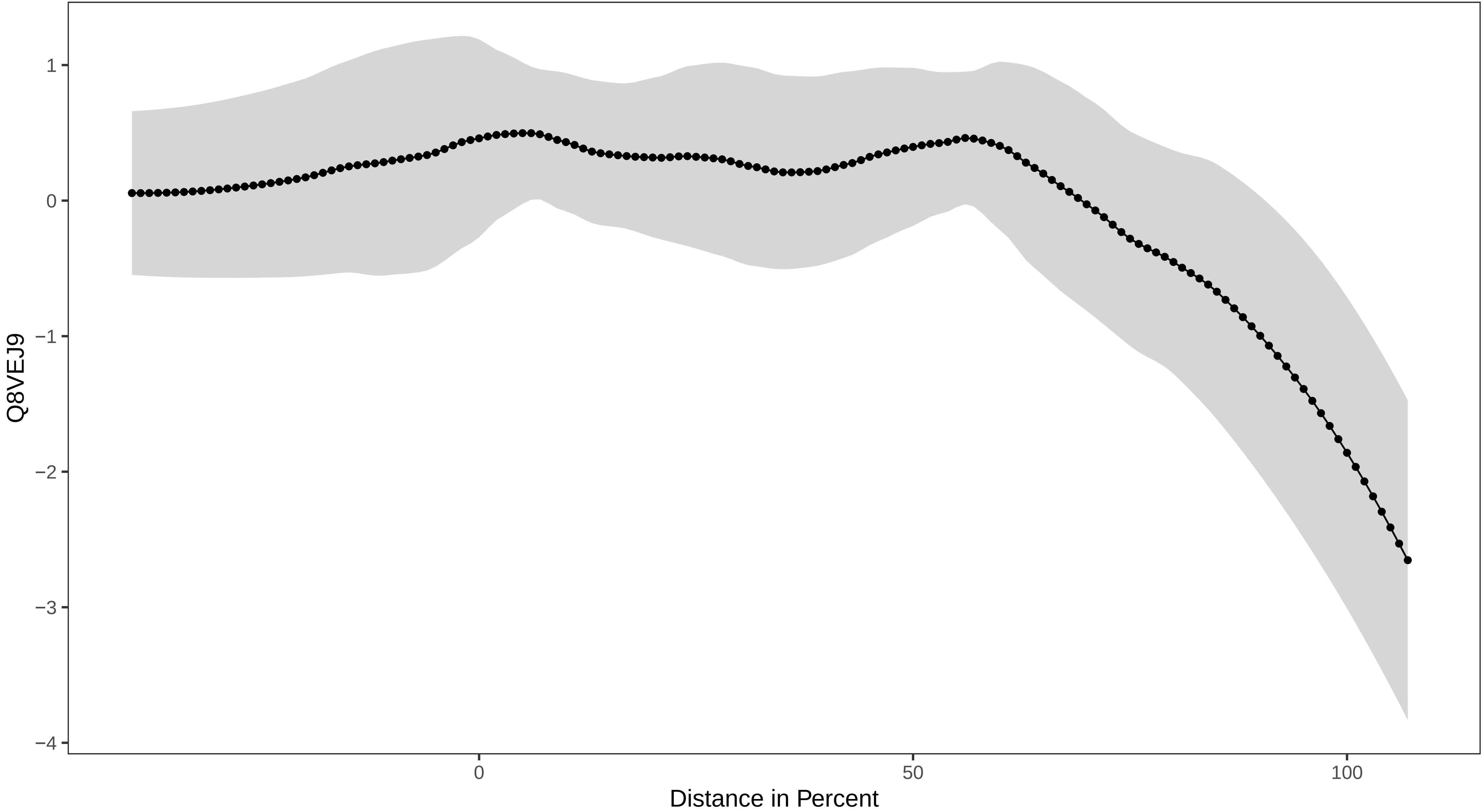

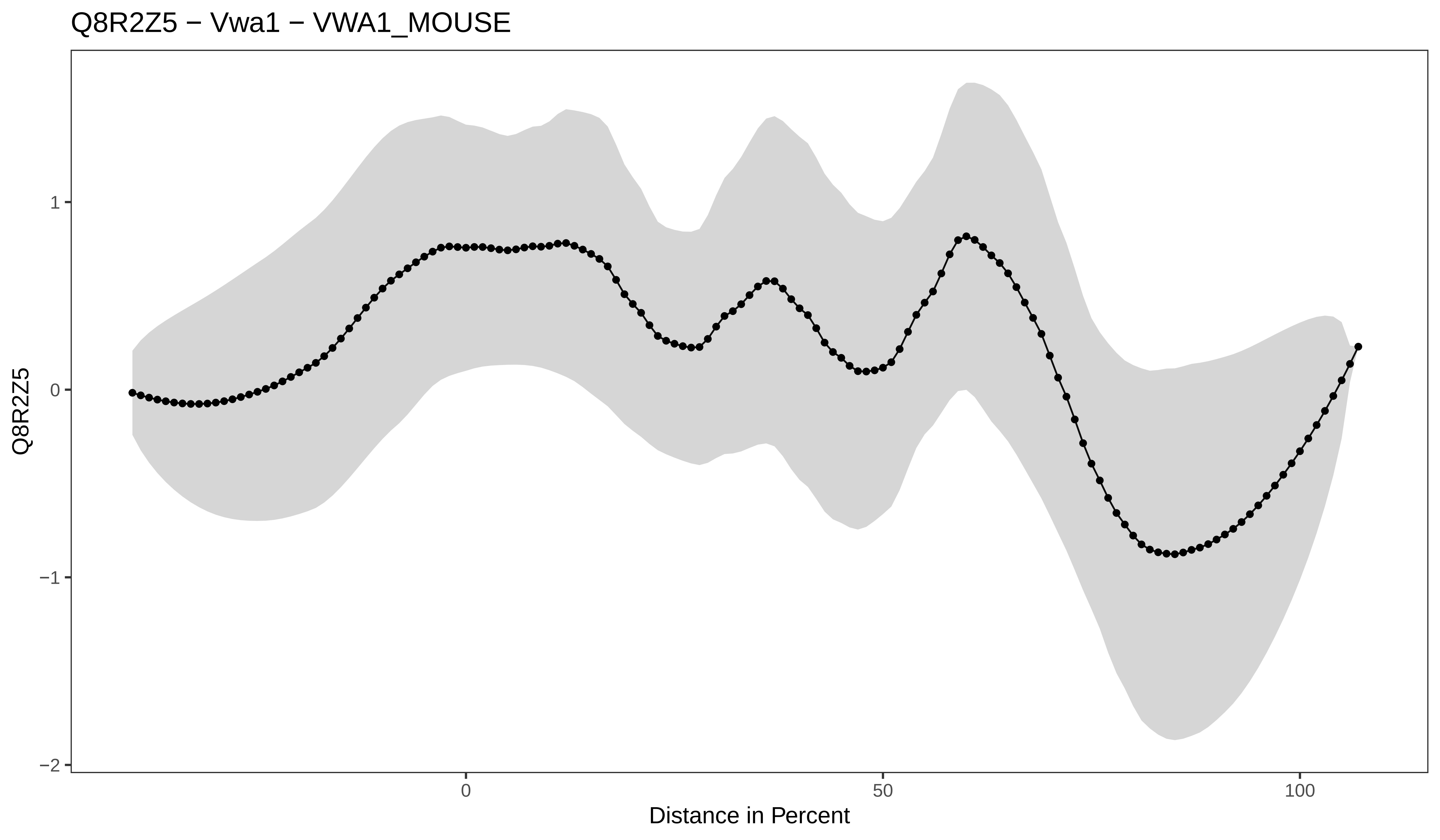
Q8R2Z5 − Vwa1 − VWA1_MOUSE
Q8R2Z5
1
0
−1
−2
0
50
100
Distance in Percent

Q99KC8 − Vwa5a − VMA5A_MOUSE

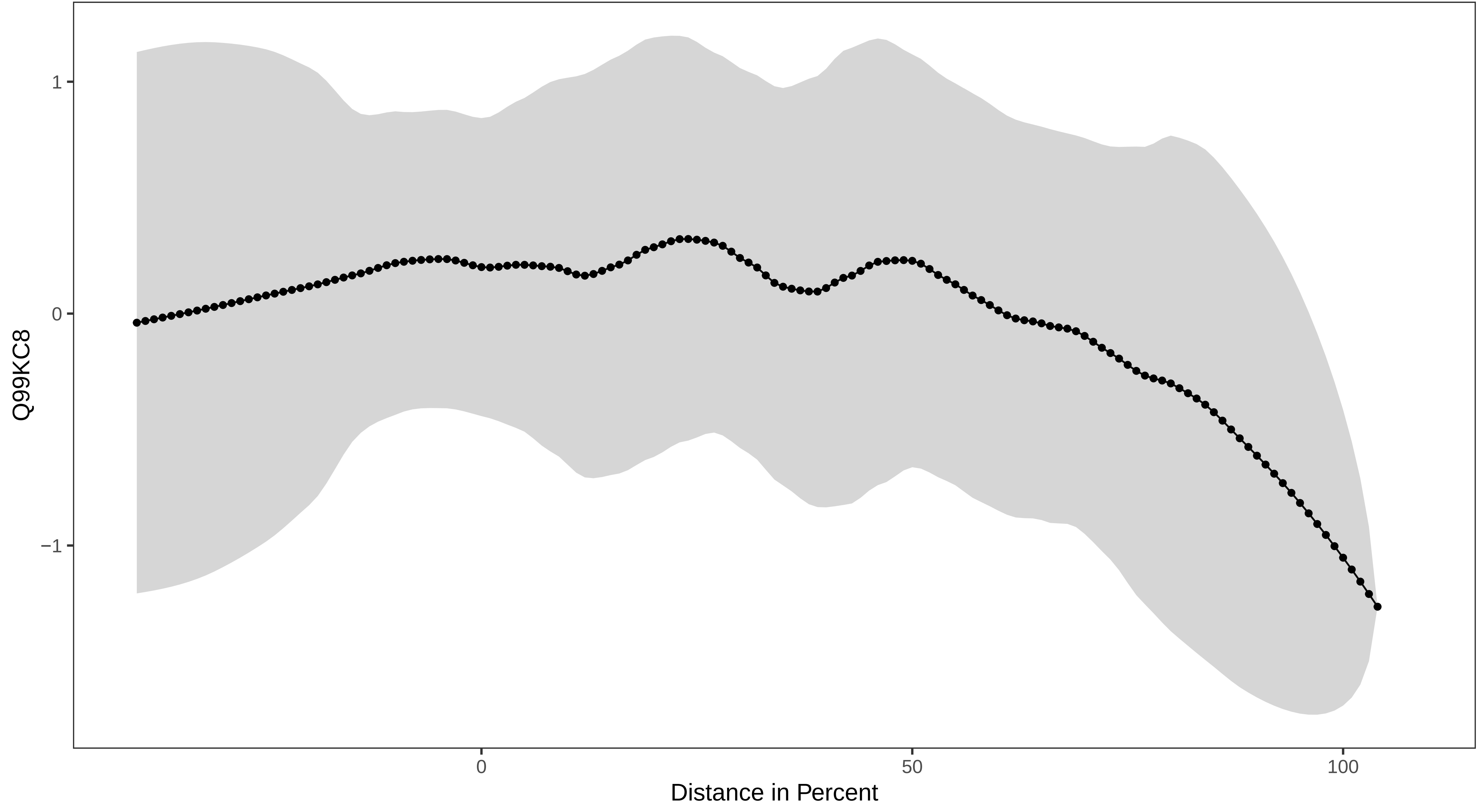

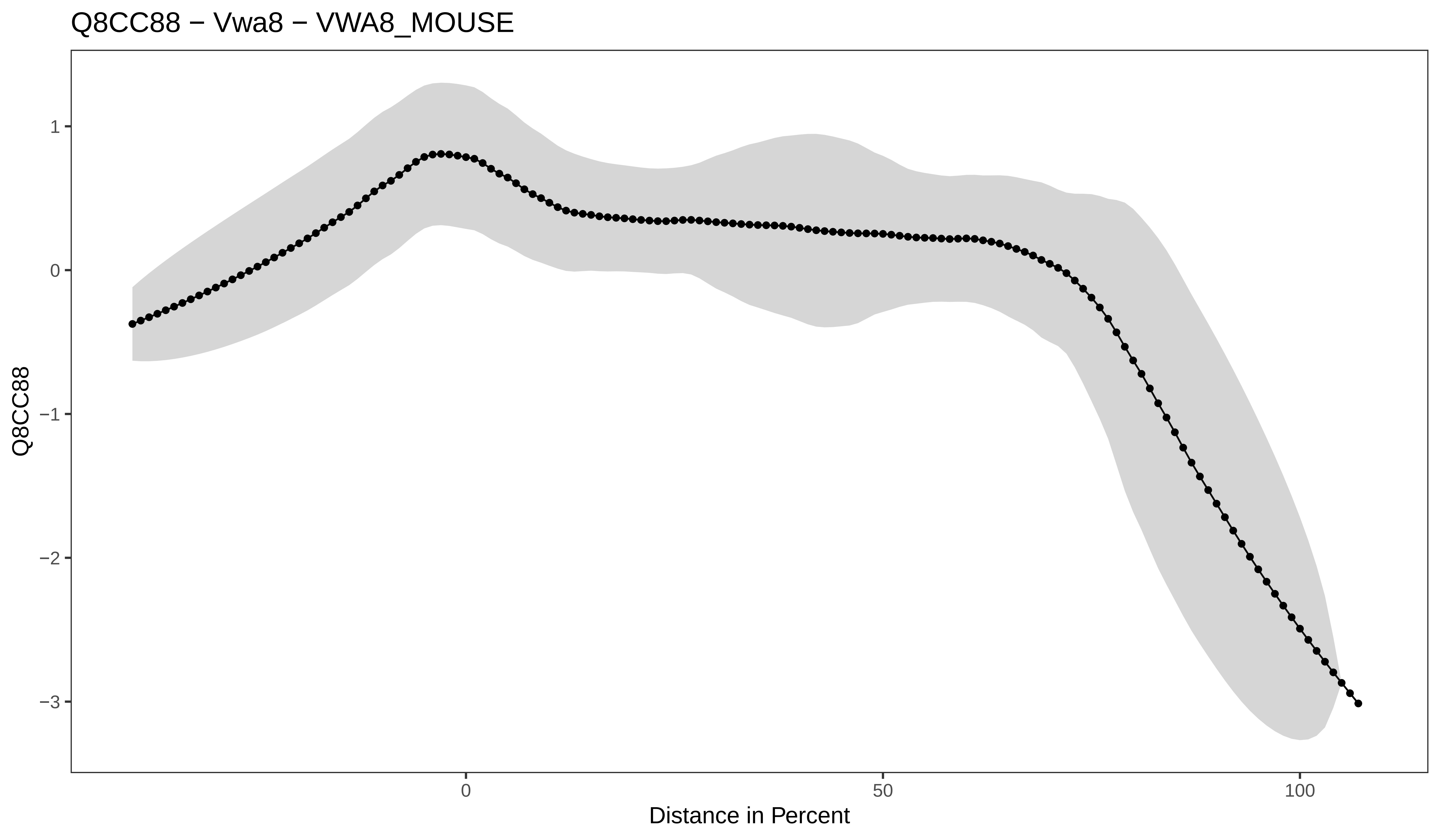
Q8CC88 − Vwa8 − VWA8_MOUSE
1
0
−1
−2
−3
Q8CC88
0
50
100
Distance in Percent

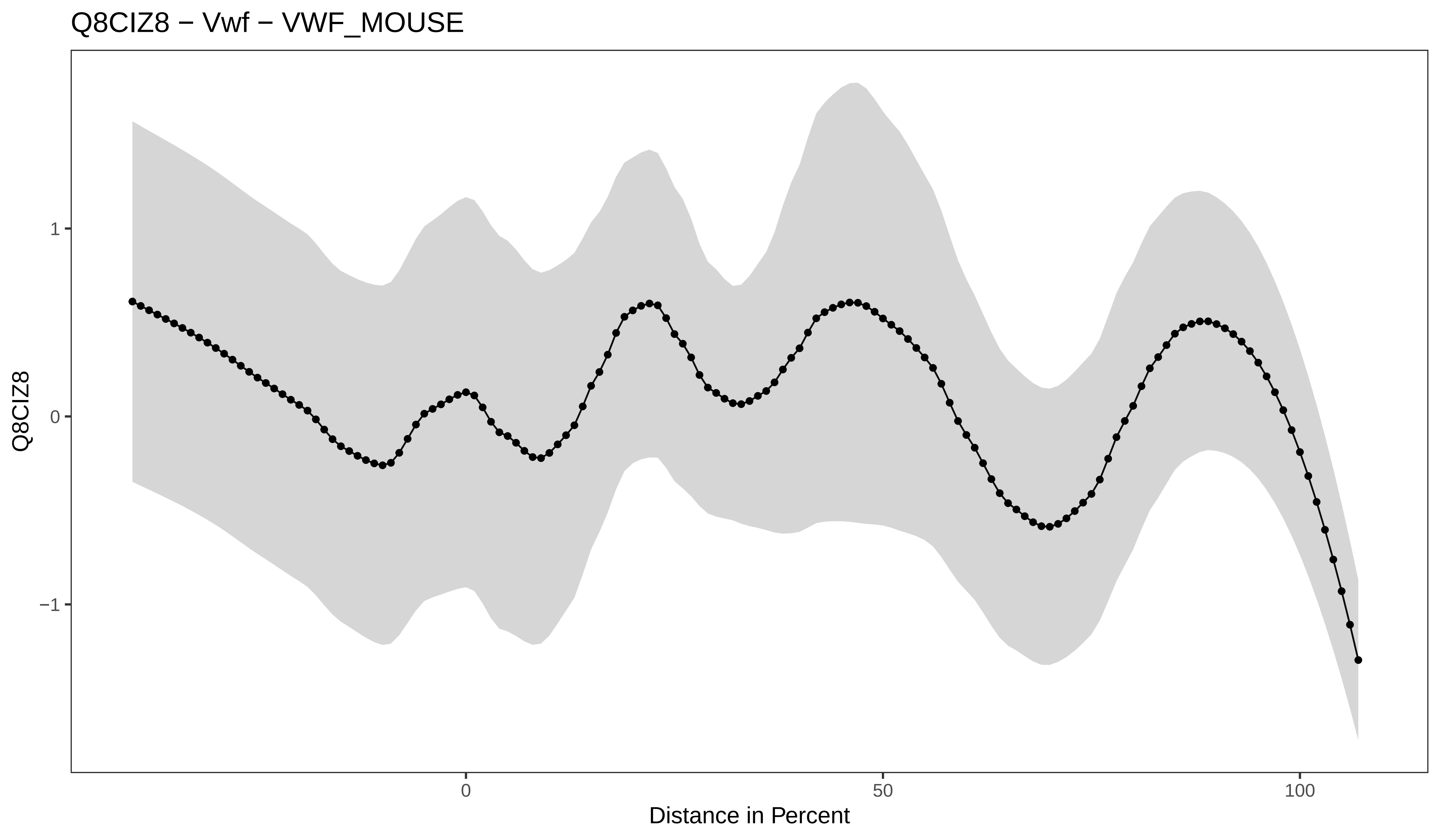

Q8CIZ8 − Vwf − VWF_MOUSE
Q8CIZ8
1
0
−1
0
50
100
Distance in Percent

P32921 − Wars − SYWC_MOUSE

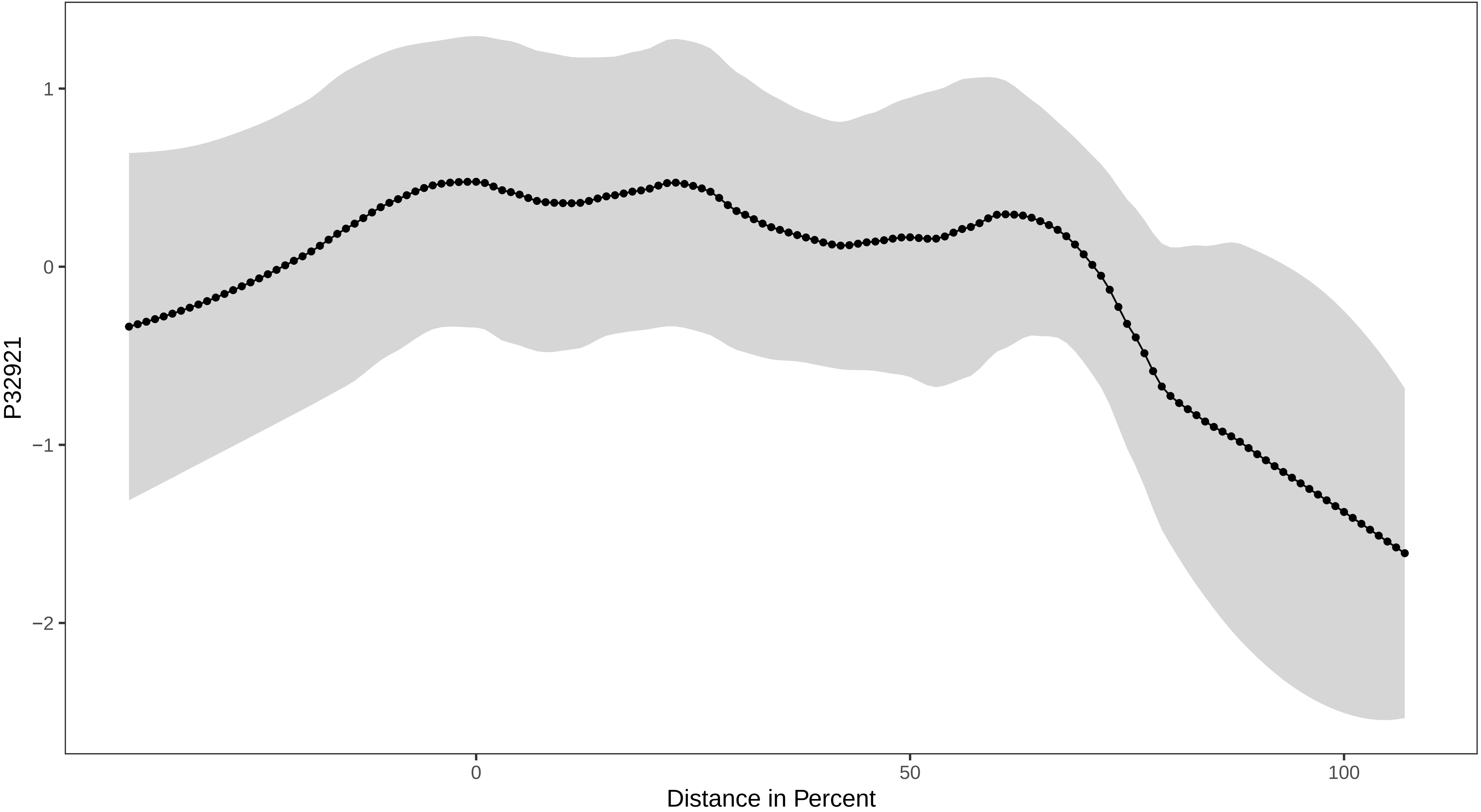

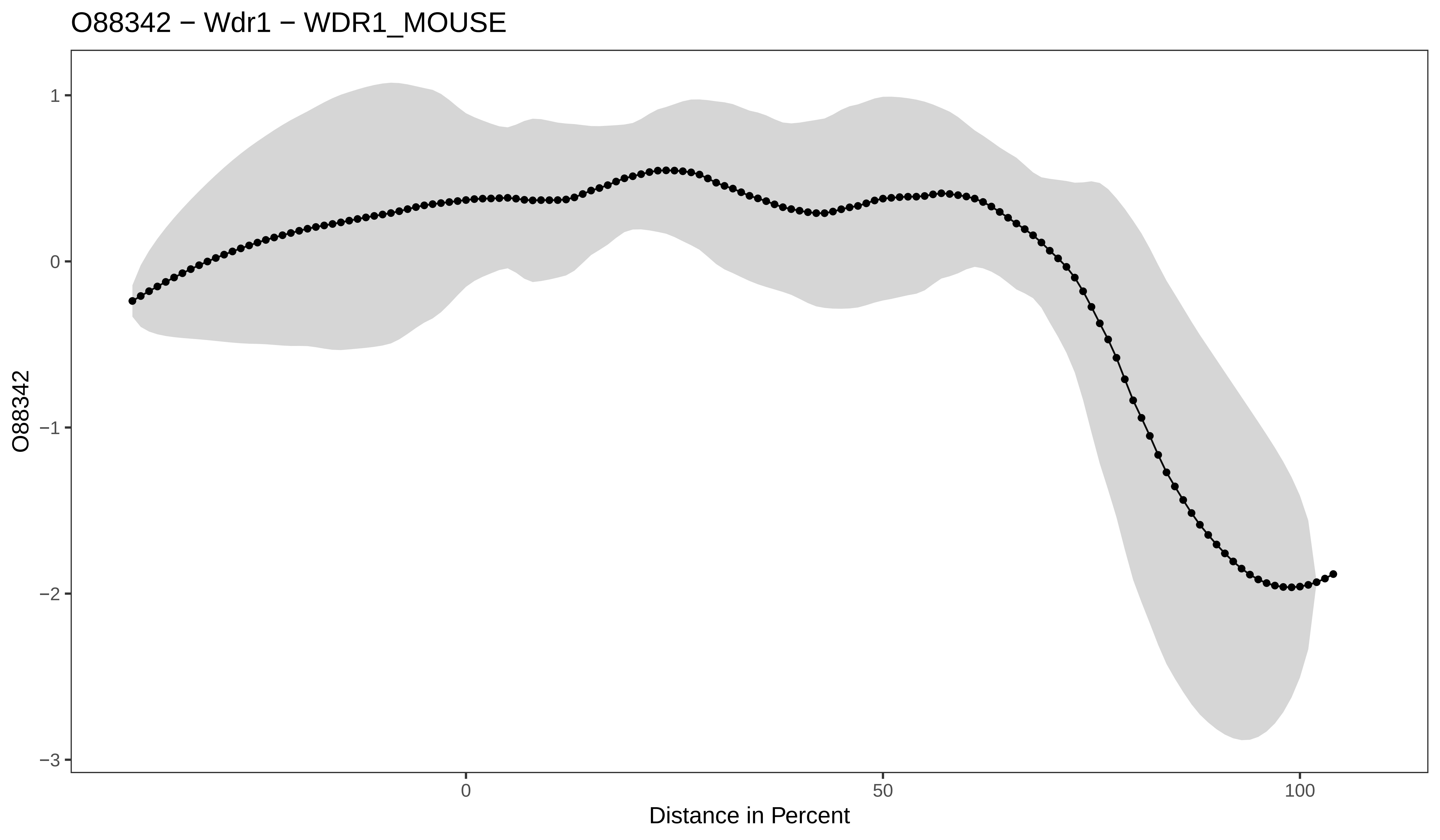

O88342 − Wdr1 − WDR1_MOUSE
1
0
−1
−2
−3
O88342
0
50
100
Distance in Percent

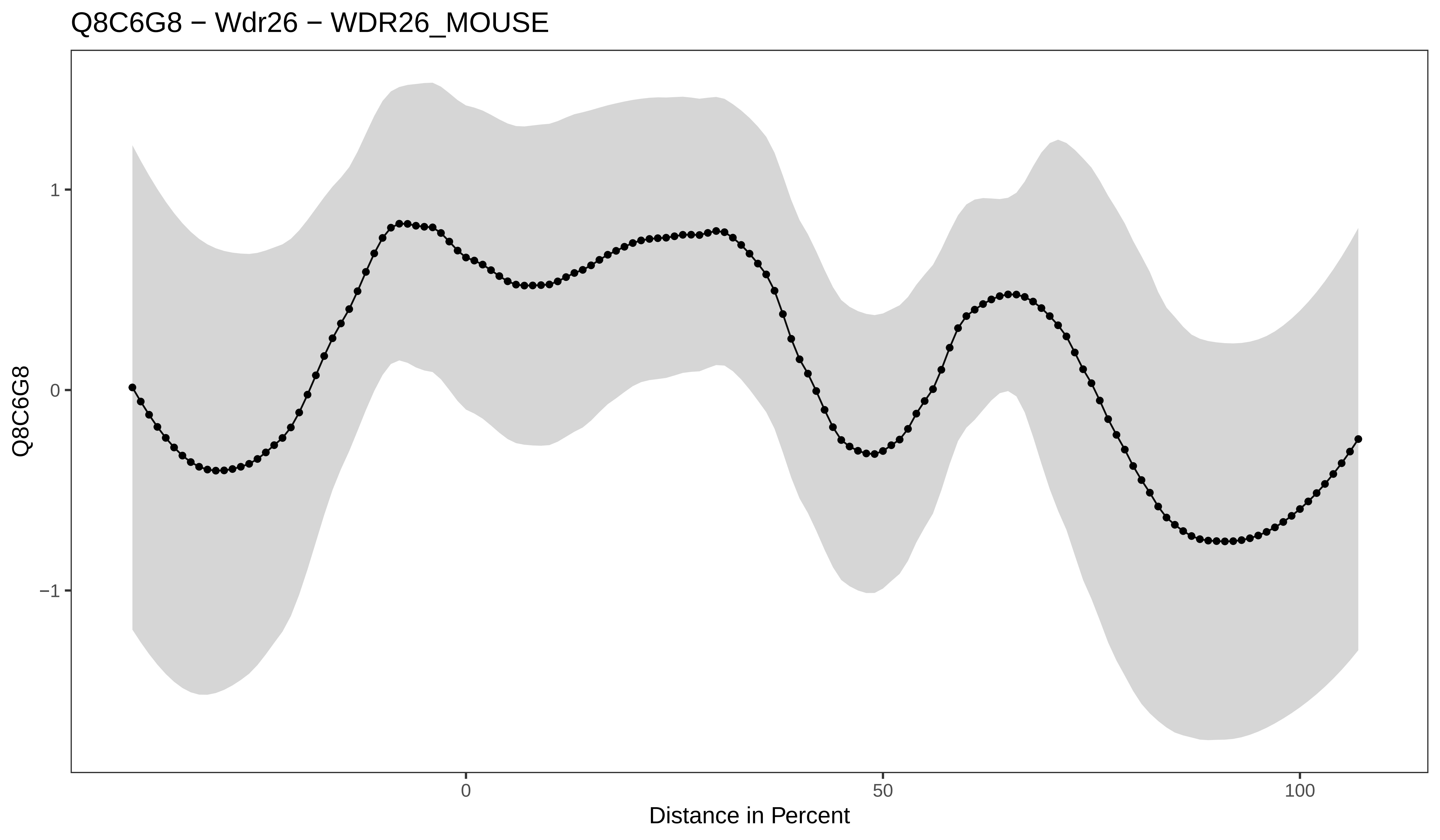

Q8C6G8 − Wdr26 − WDR26_MOUSE
Q8C6G8
1
0
−1
0
50
100
Distance in Percent

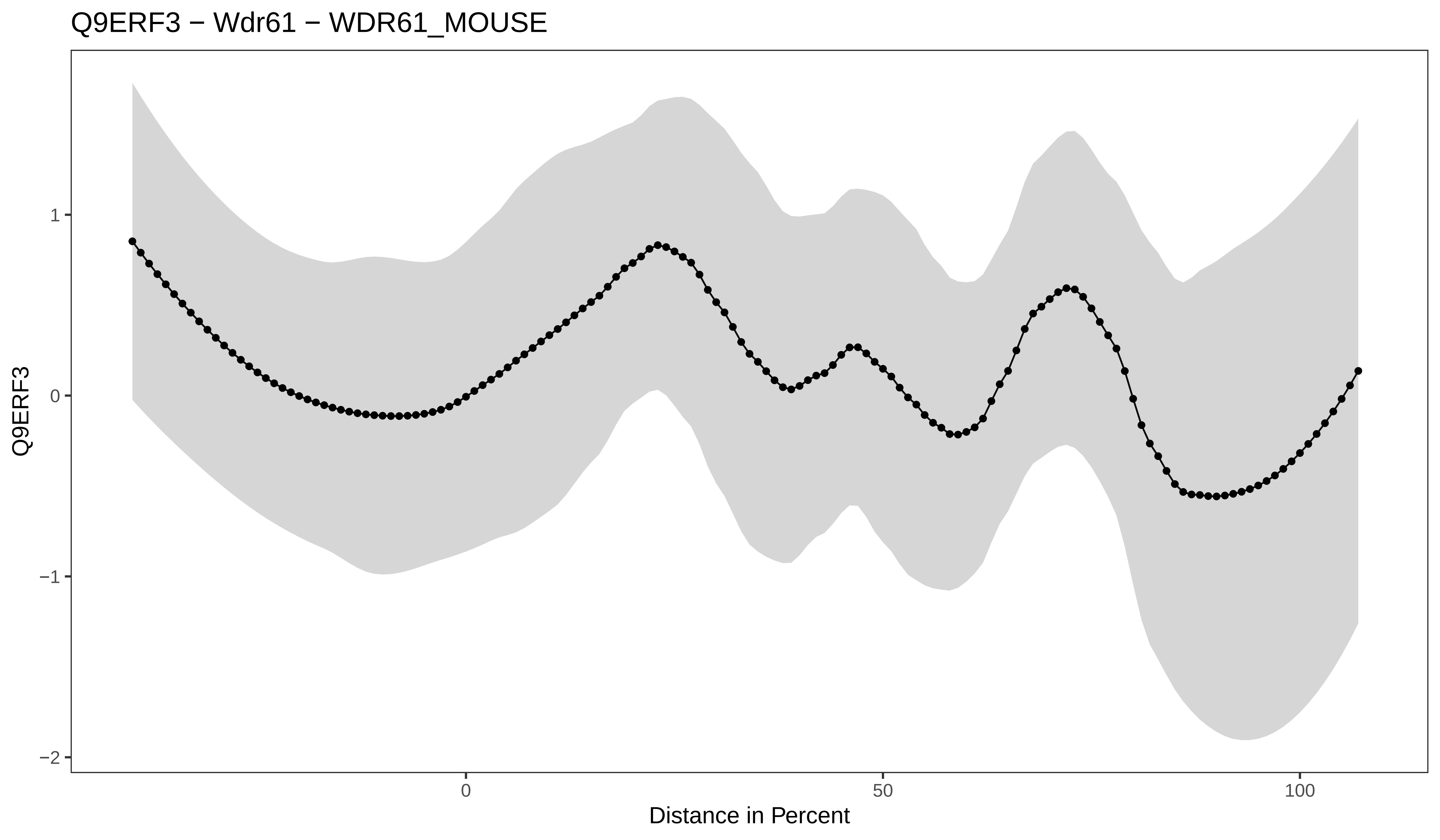

Q9ERF3 − Wdr61 − WDR61_MOUSE
1
0
−1
−2
Q9ERF3
0
50
100
Distance in Percent

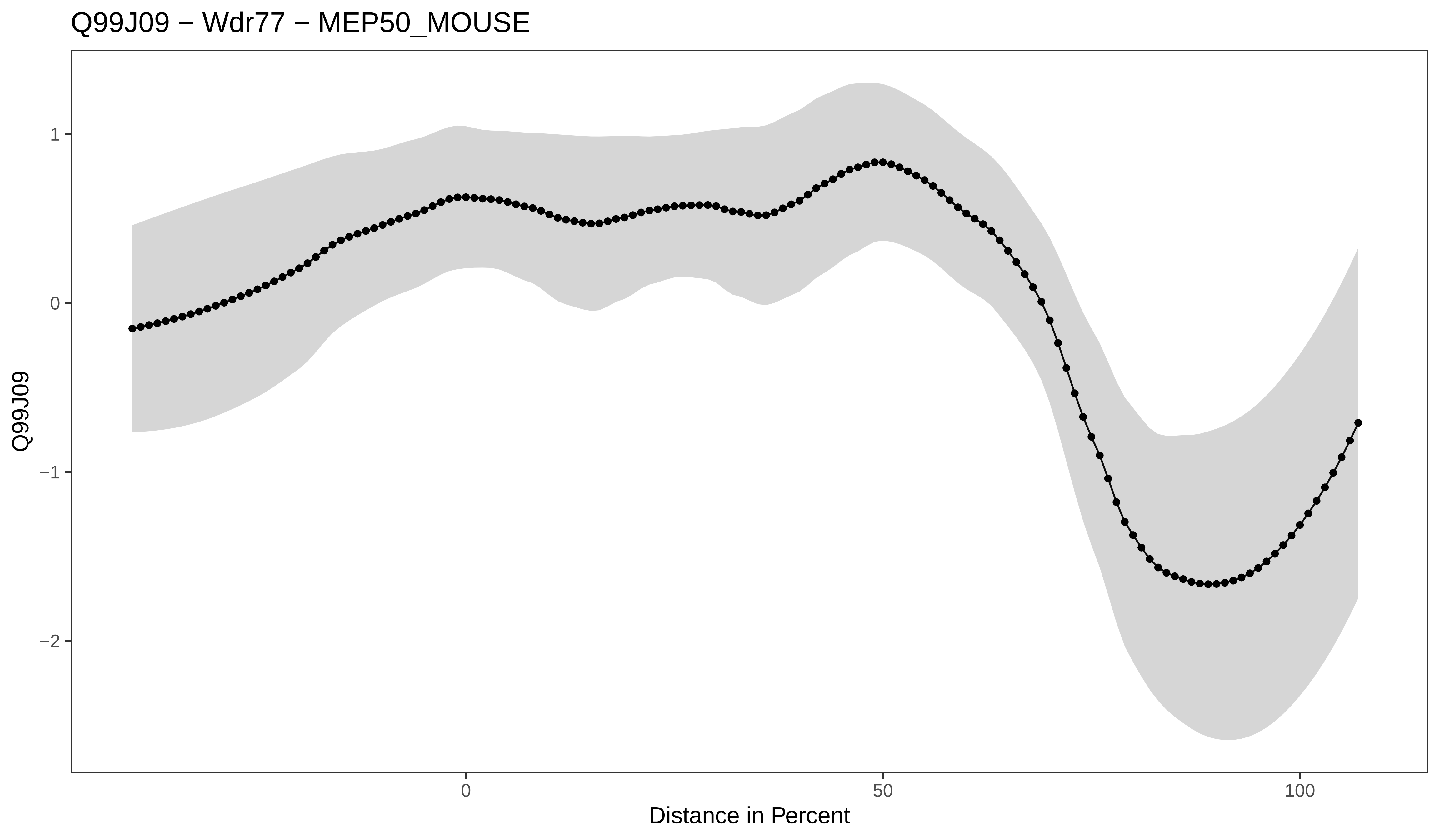

Q99J09 − Wdr77 − MEP50_MOUSE
1
0
−1
−2
Q99J09
0
50
100
Distance in Percent

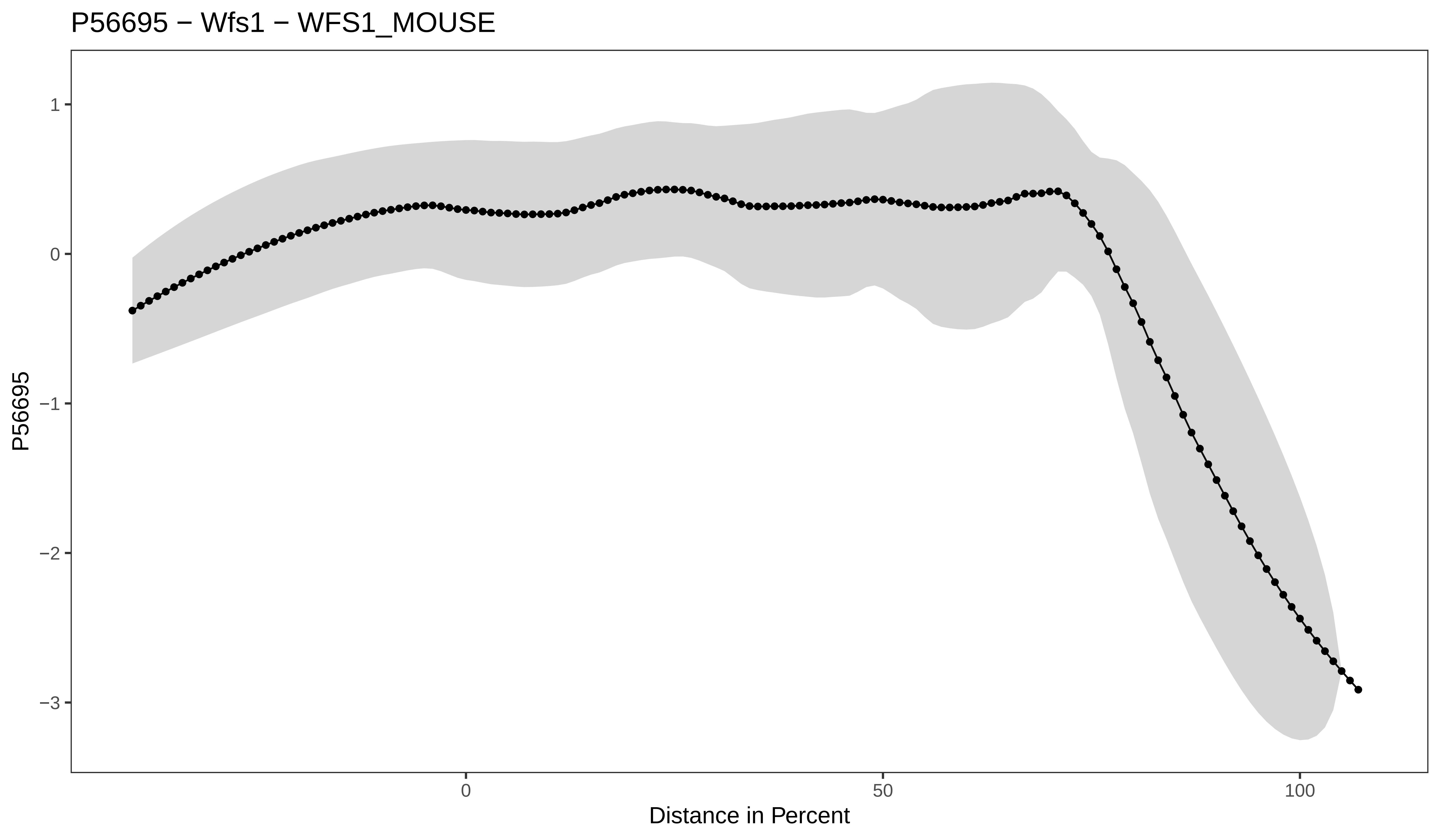

P56695 − Wfs1 − WFS1_MOUSE
1
0
−1
−2
−3
P56695
0
50
100
Distance in Percent

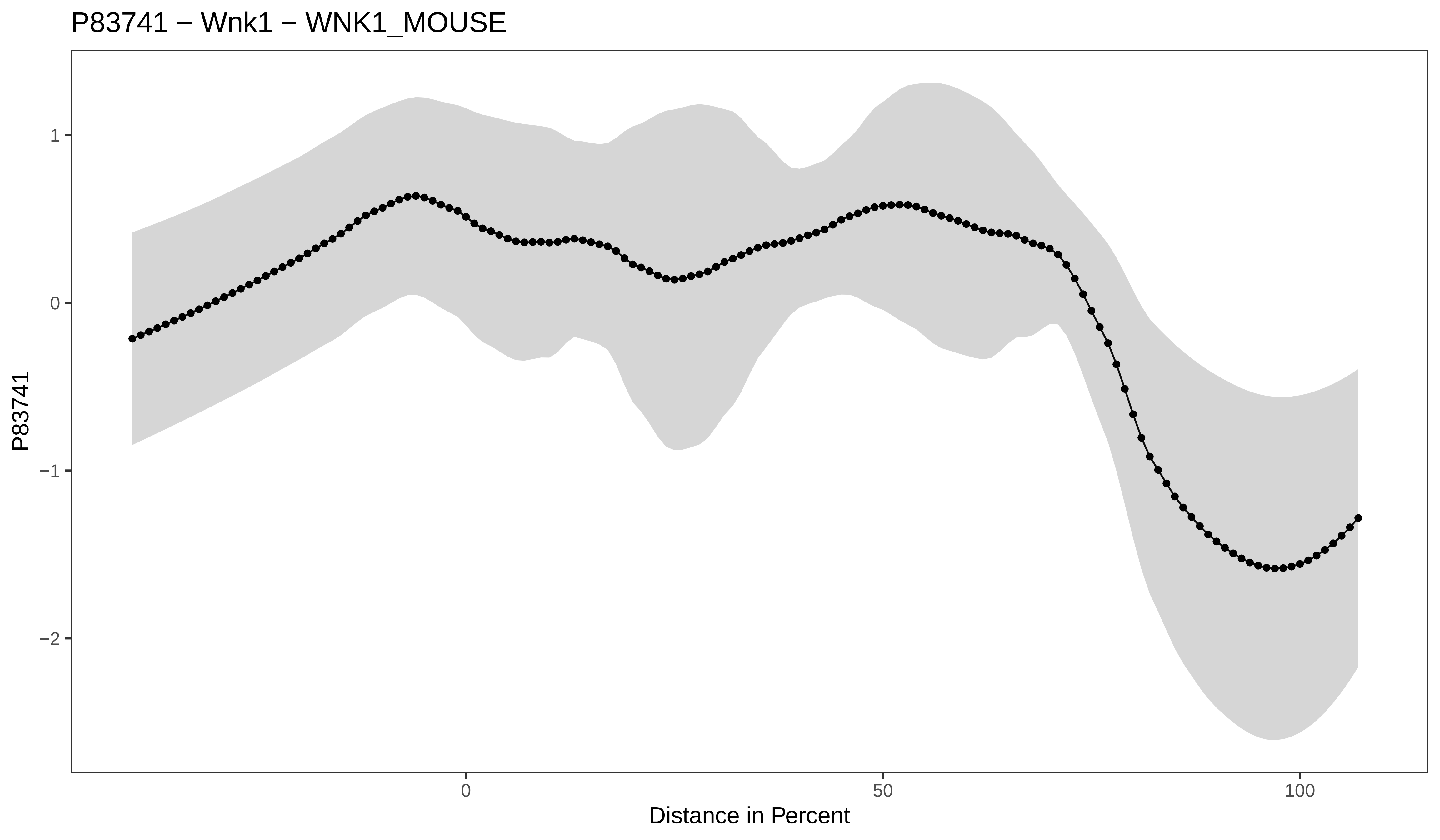

P83741 − Wnk1 − WNK1_MOUSE
P83741
Distance in Percent
1
0
−1
−2
0
50
100

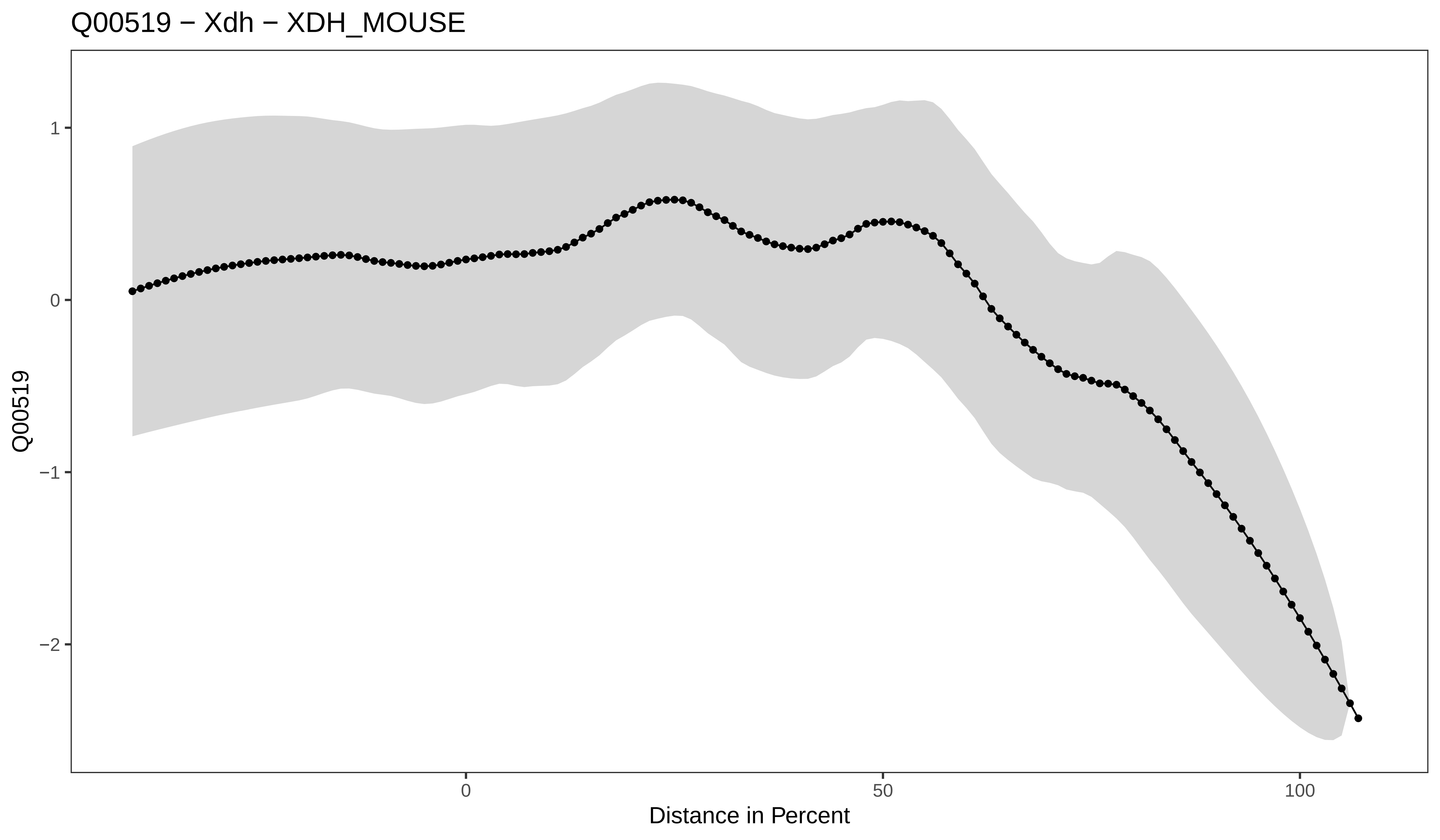

Q00519 − Xdh − XDH_MOUSE
Q00519
1
0
−1
−2
0
50
100
Distance in Percent

O70373 − Xirp1 − XIRP1_MOUSE

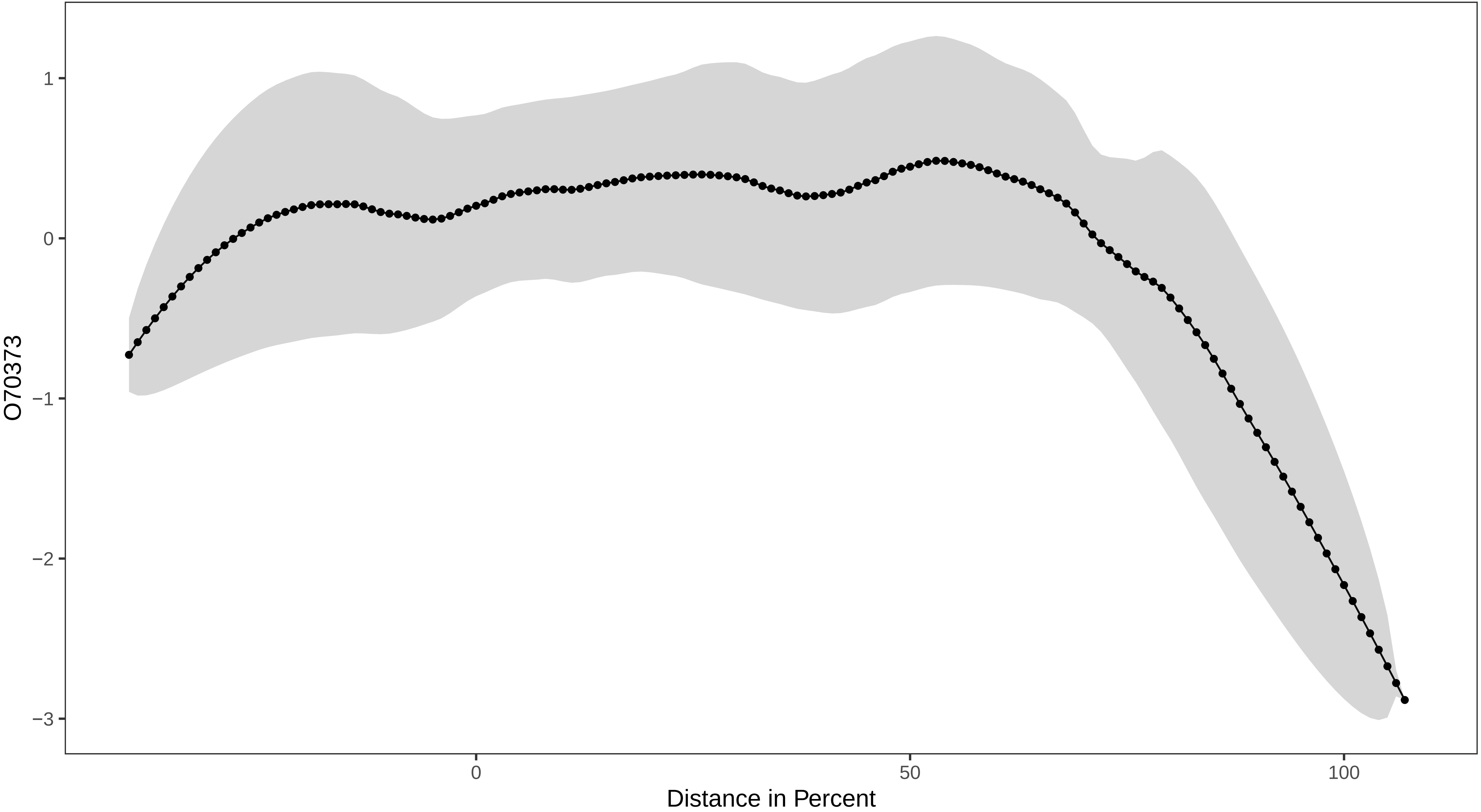

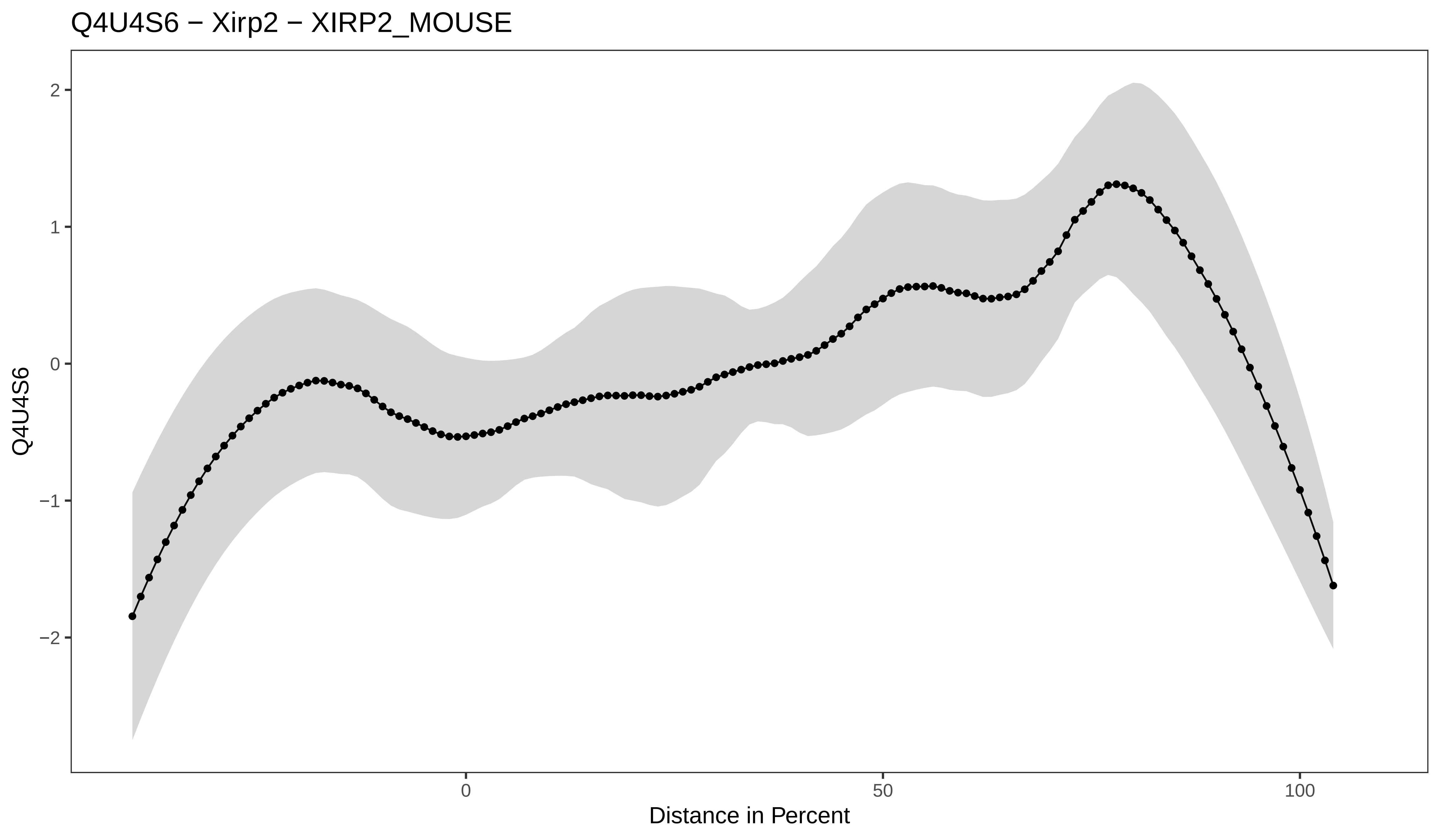

Q4U4S6 − Xirp2 − XIRP2_MOUSE
Q4U4S6
Distance in Percent
2
1
0
−1
−2
0
50
100

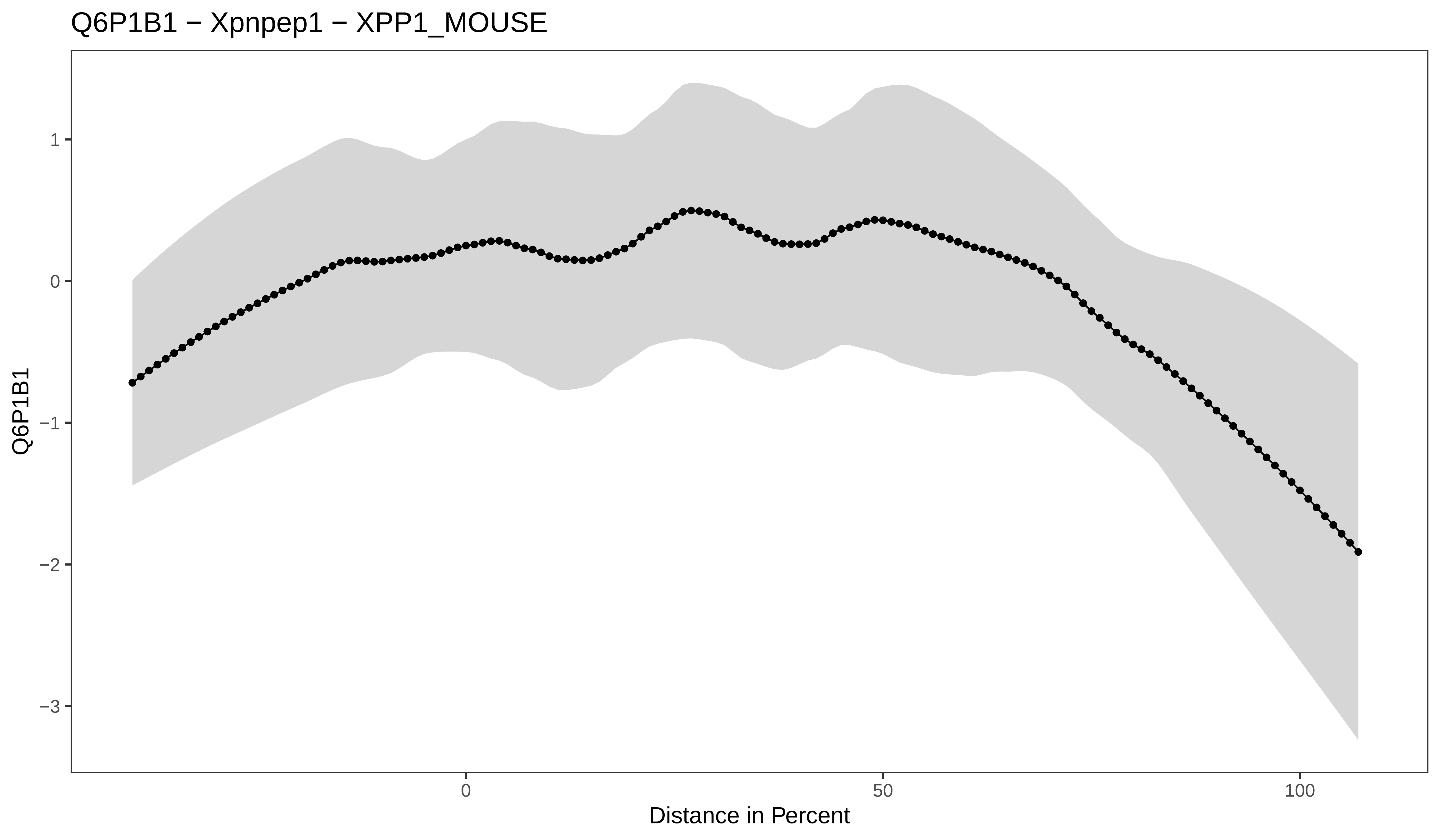

Q6P1B1 − Xpnpep1 − XPP1_MOUSE
1
0
−1
−2
−3
Q6P1B1
0
50
100
Distance in Percent

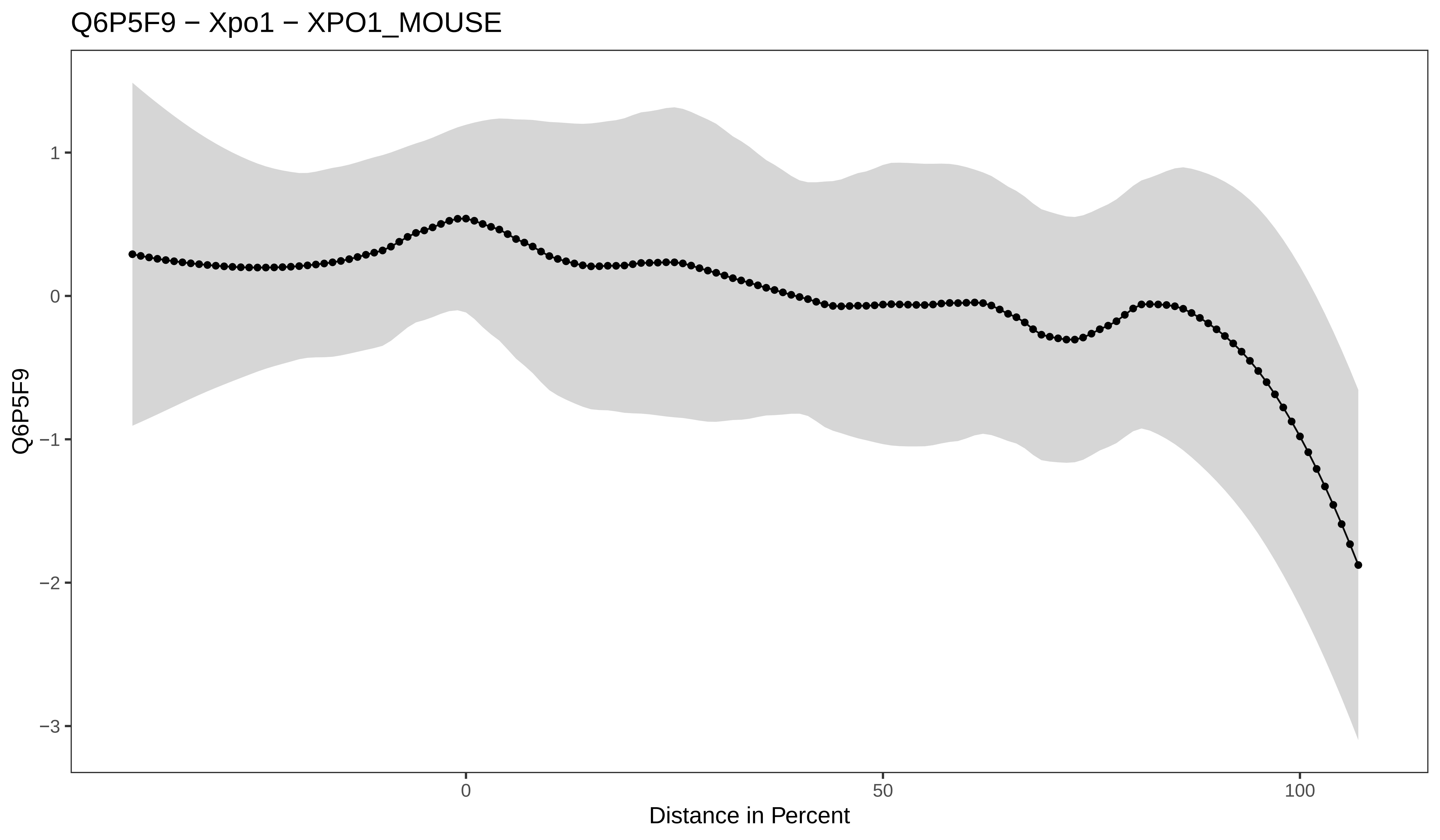
Q6P5F9 − Xpo1 − XPO1_MOUSE
Q6P5F9
1
0
−1
−2
−3
0
50
100
Distance in Percent

Q3U4G3 − Xxylt1 − XXLT1_MOUSE

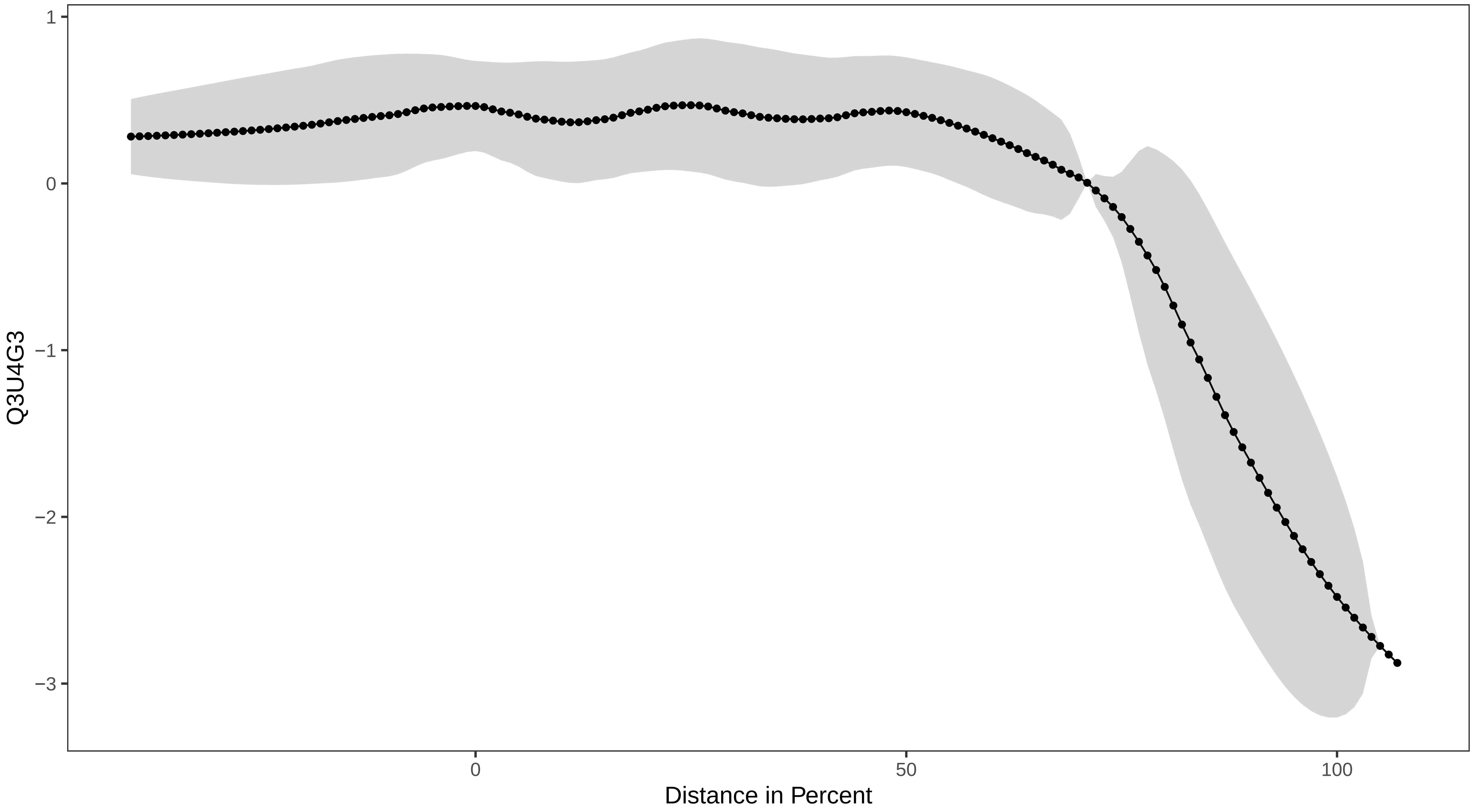

Q91WQ3 − Yars − SYYC_MOUSE

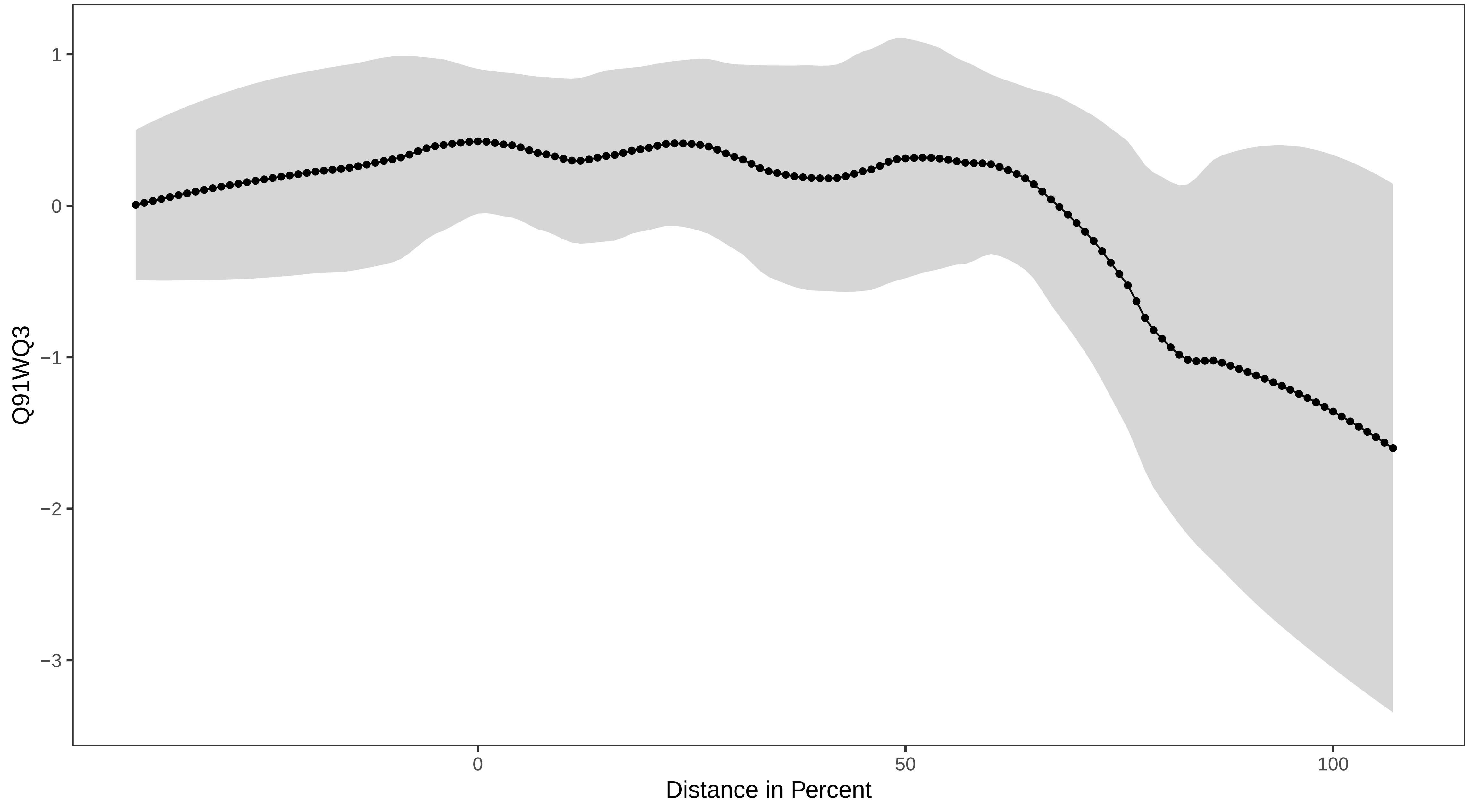

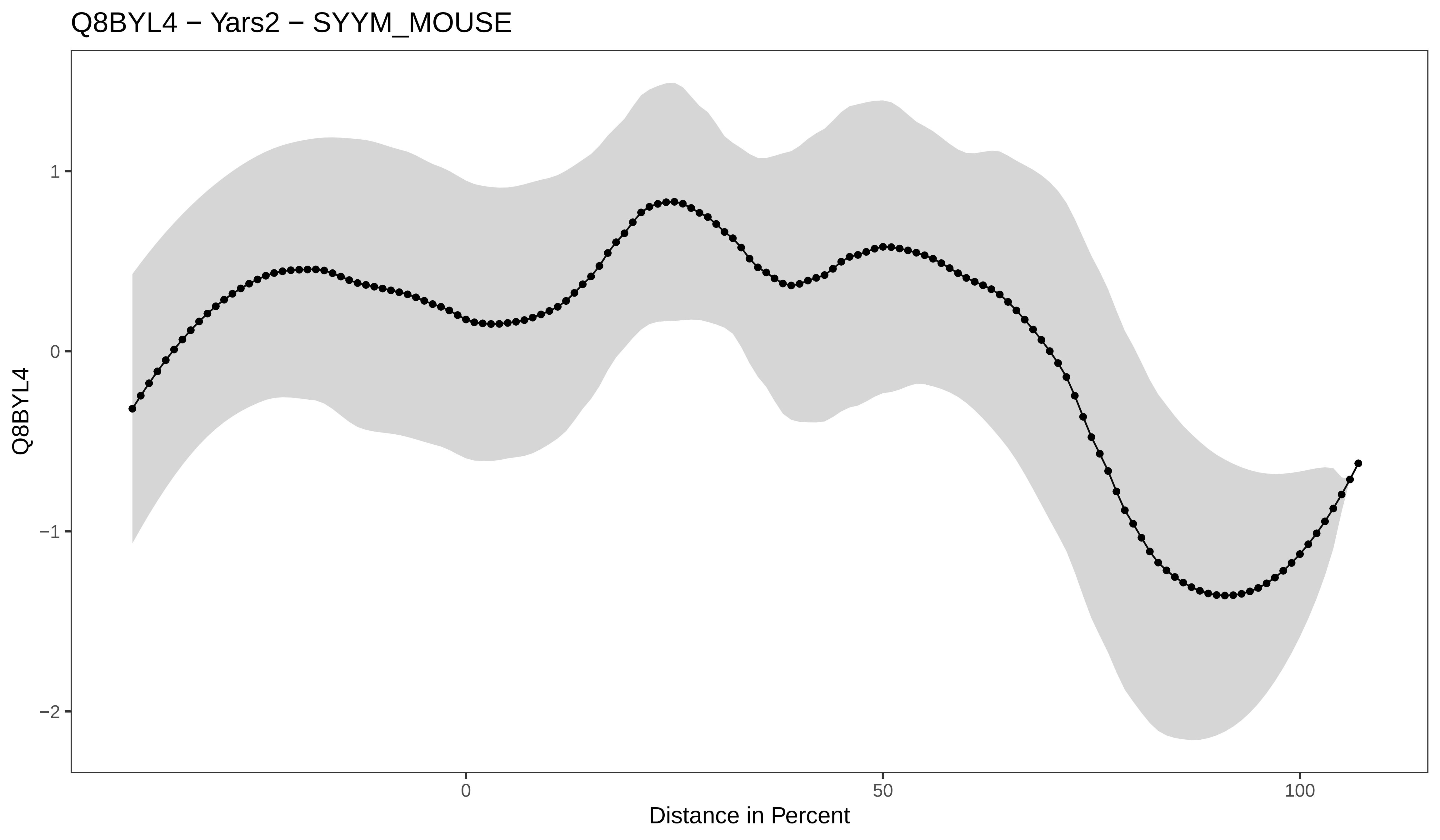
Q8BYL4 − Yars2 − SYYM_MOUSE
Q8BYL4
1
0
−1
−2
0
50
100
Distance in Percent

P62960 − Ybx1 − YBOX1_MOUSE

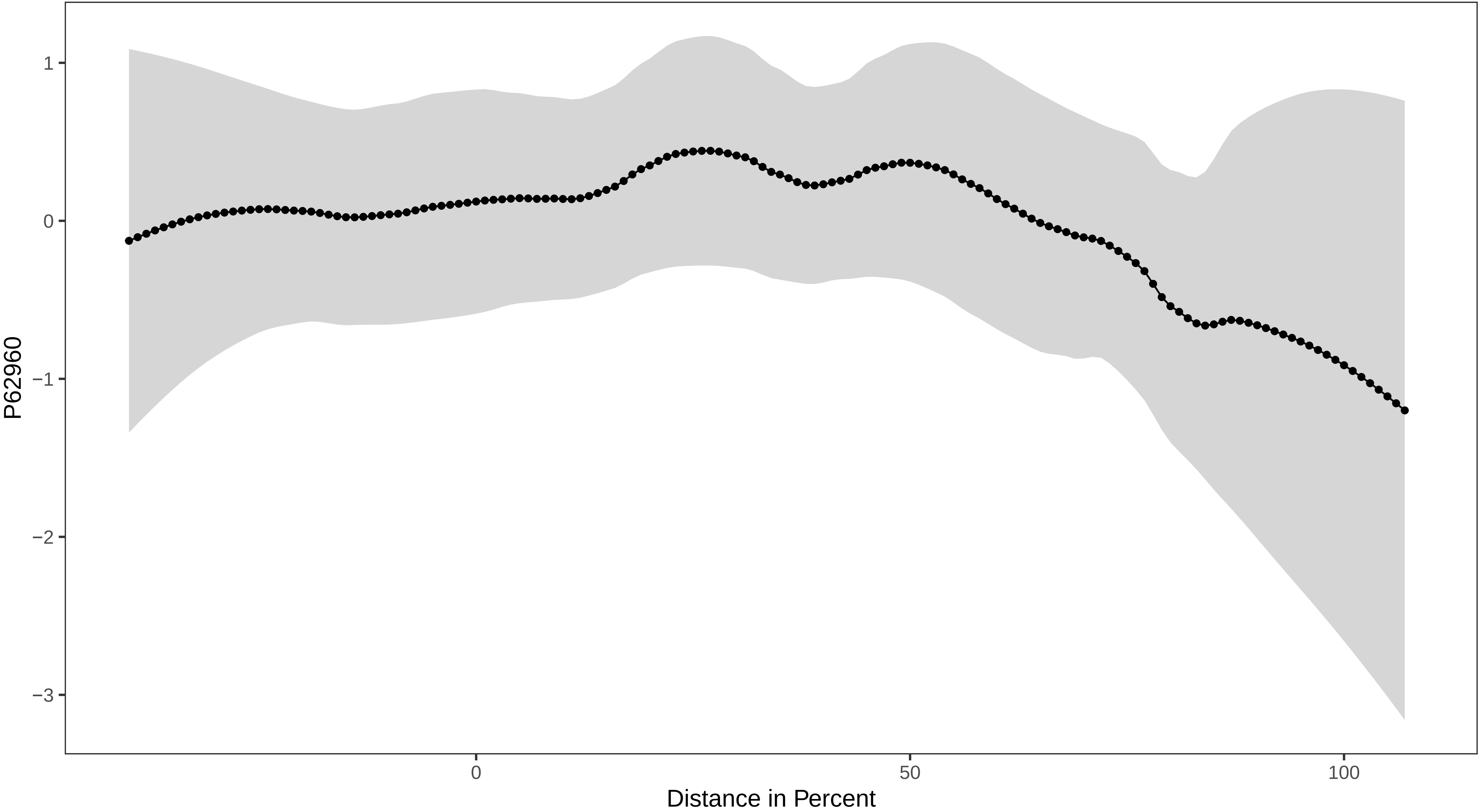

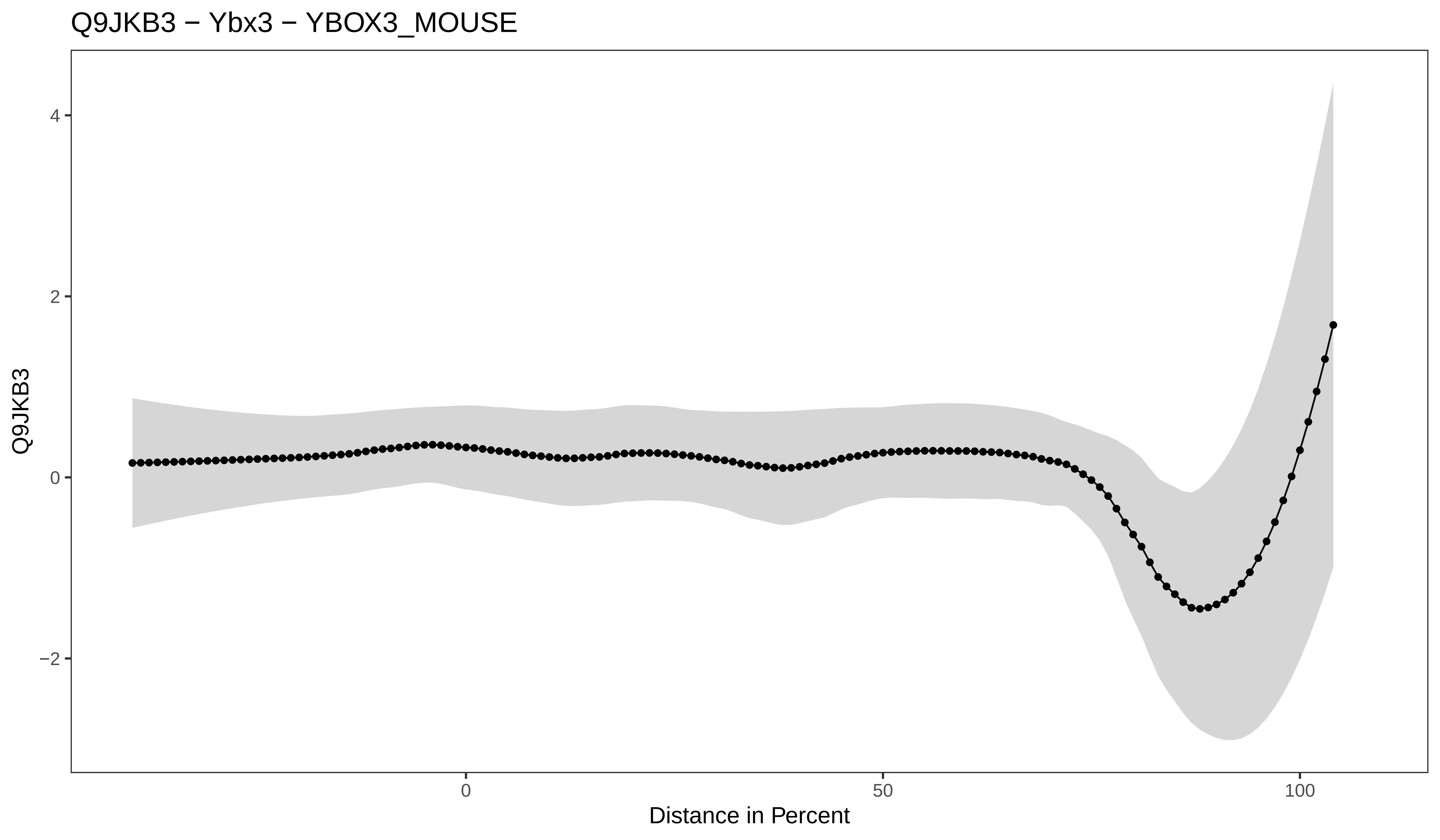

Q9JKB3 − Ybx3 − YBOX3_MOUSE
Q9JKB3
4
2
0
−2
0
50
100
Distance in Percent

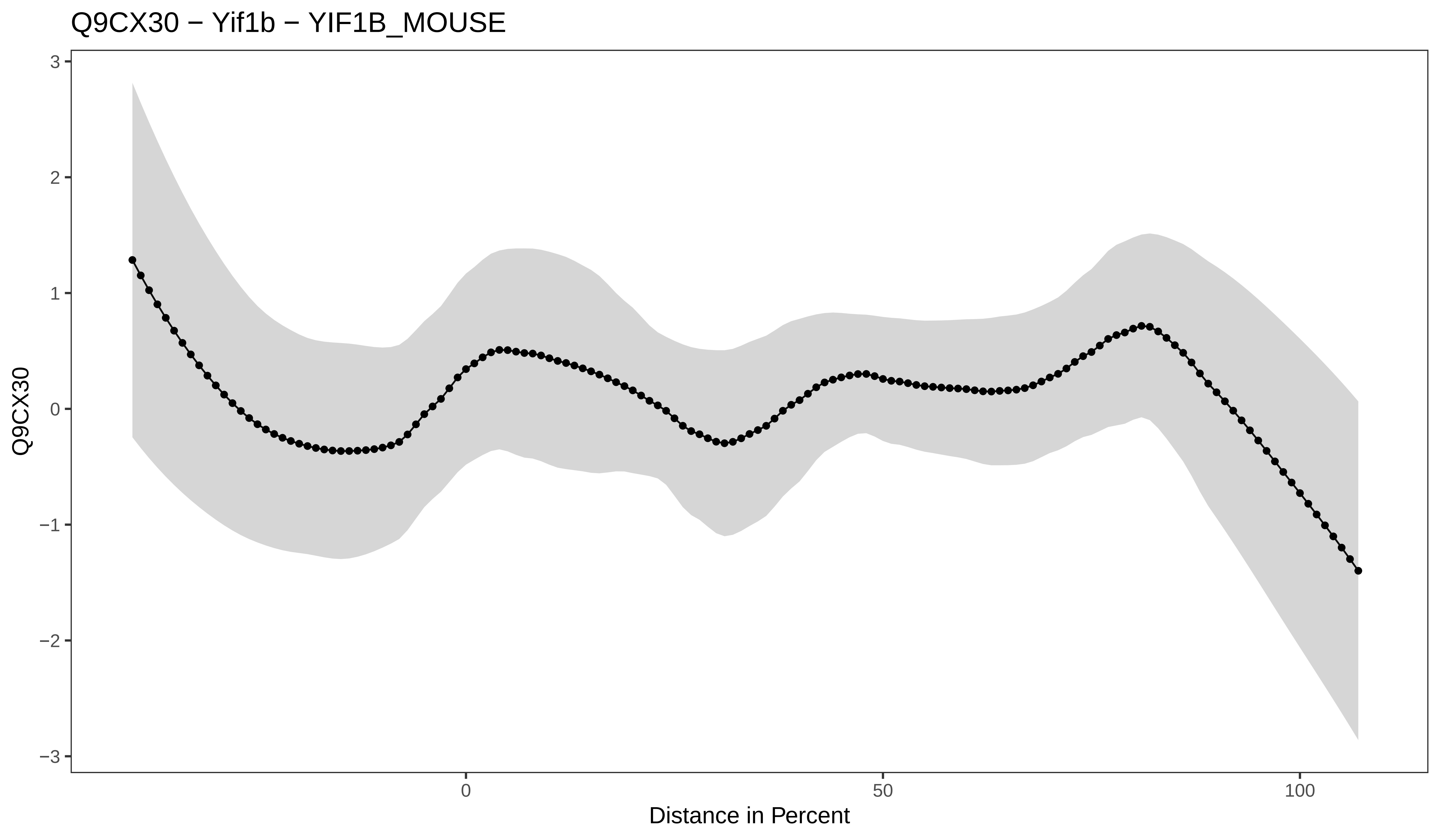

Q9CX30 − Yif1b − YIF1B_MOUSE
Q9CX30
Distance in Percent
3
2
1
0
−1
−2
−3
0
50
100

Q9CQW1 − Ykt6 − YKT6_MOUSE

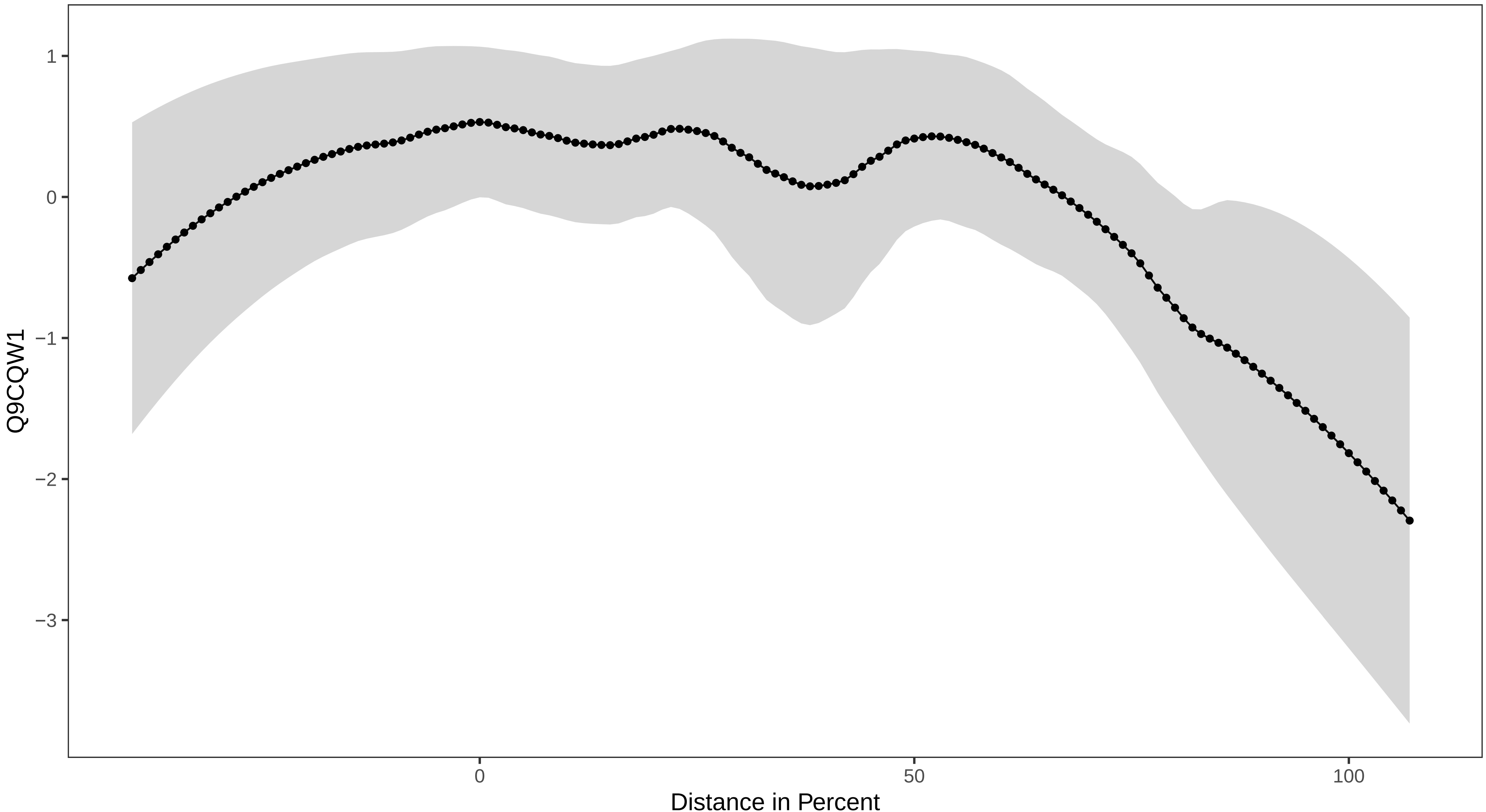

O88967 − Yme1l1 − YMEL1_MOUSE

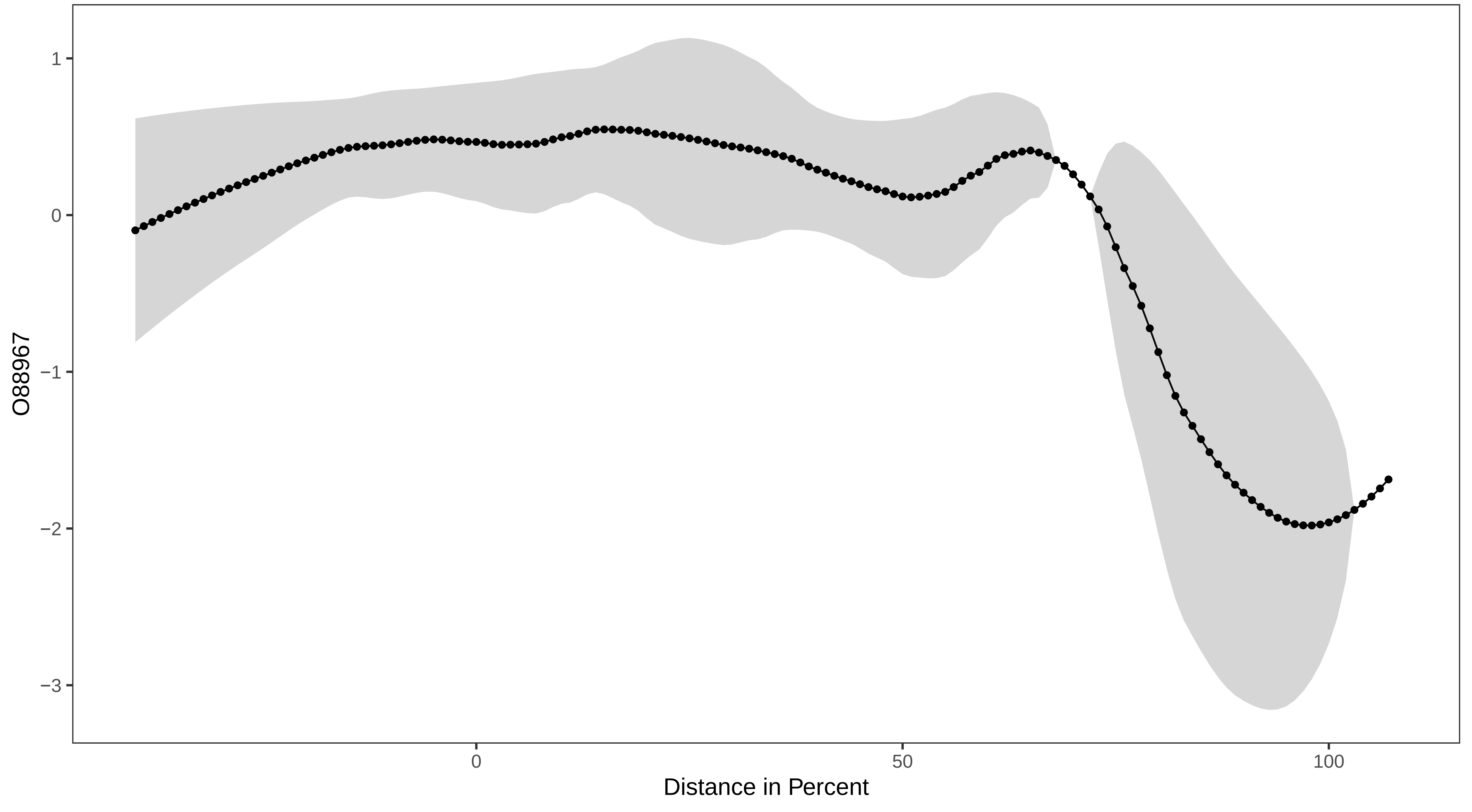

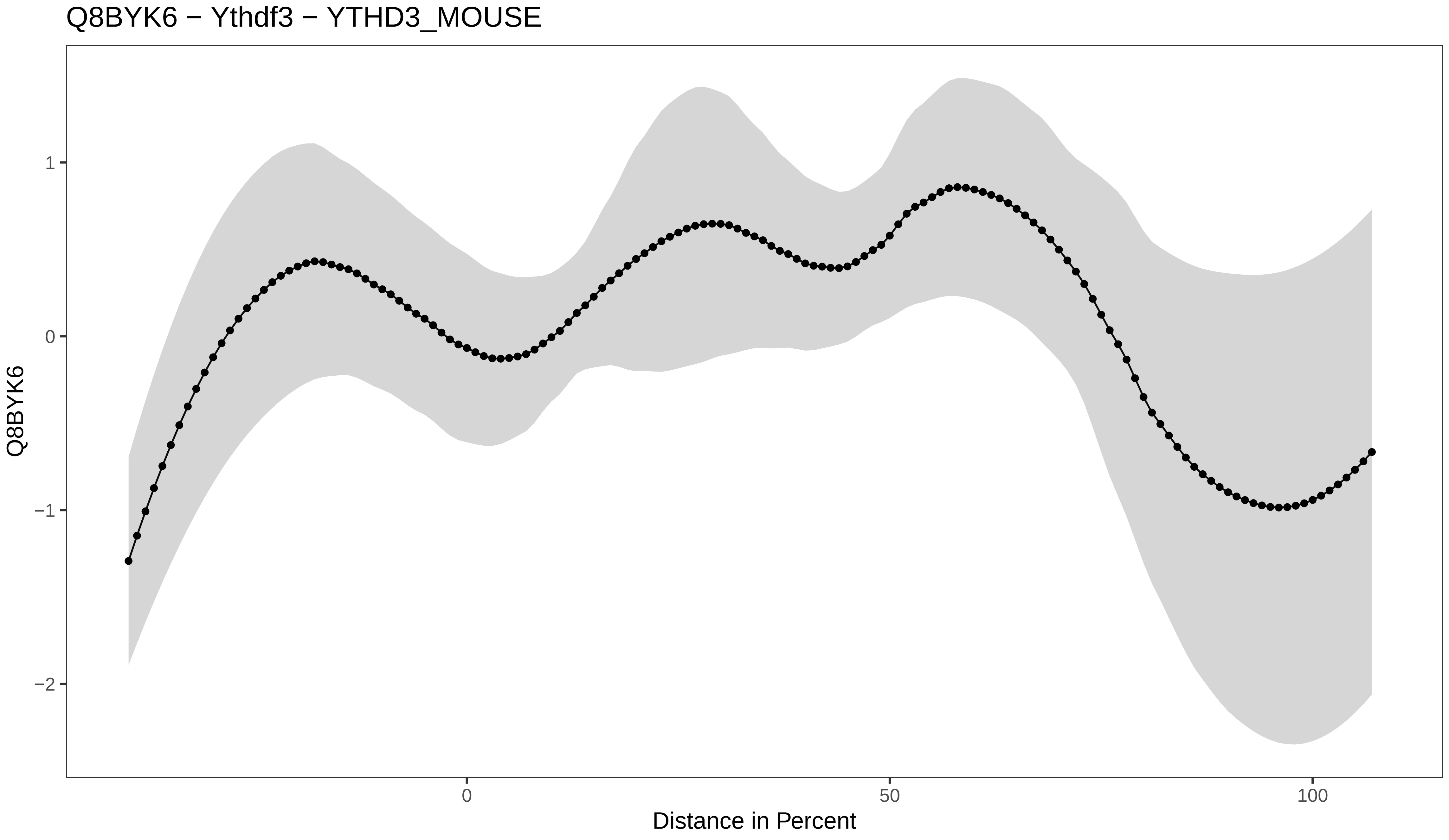
Q8BYK6 − Ythdf3 − YTHD3_MOUSE
Q8BYK6
Distance in Percent
1
0
−1
−2
0
50
100

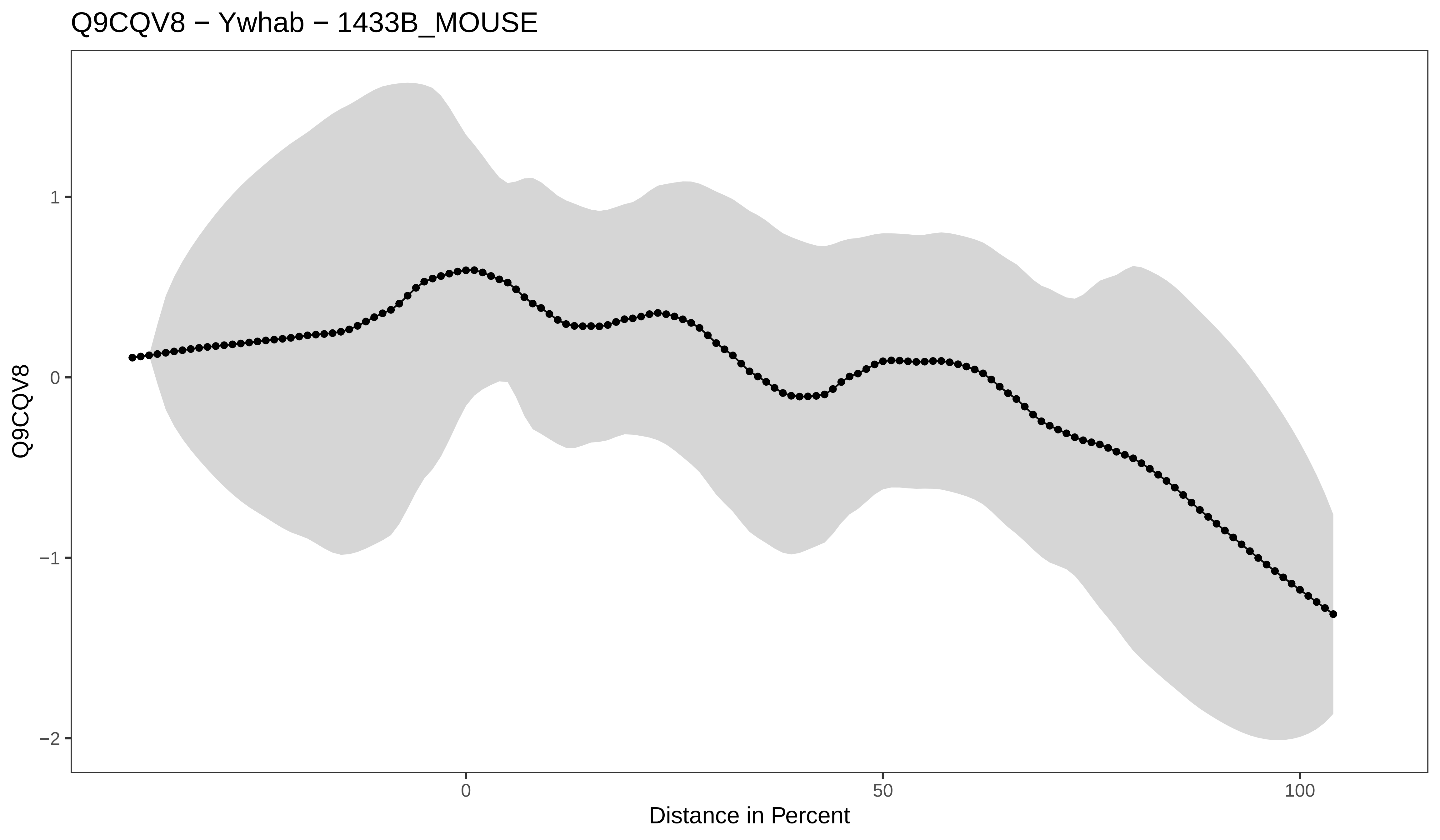

Q9CQV8 − Ywhab − 1433B_MOUSE
Q9CQV8
1
0
−1
−2
0
50
100
Distance in Percent

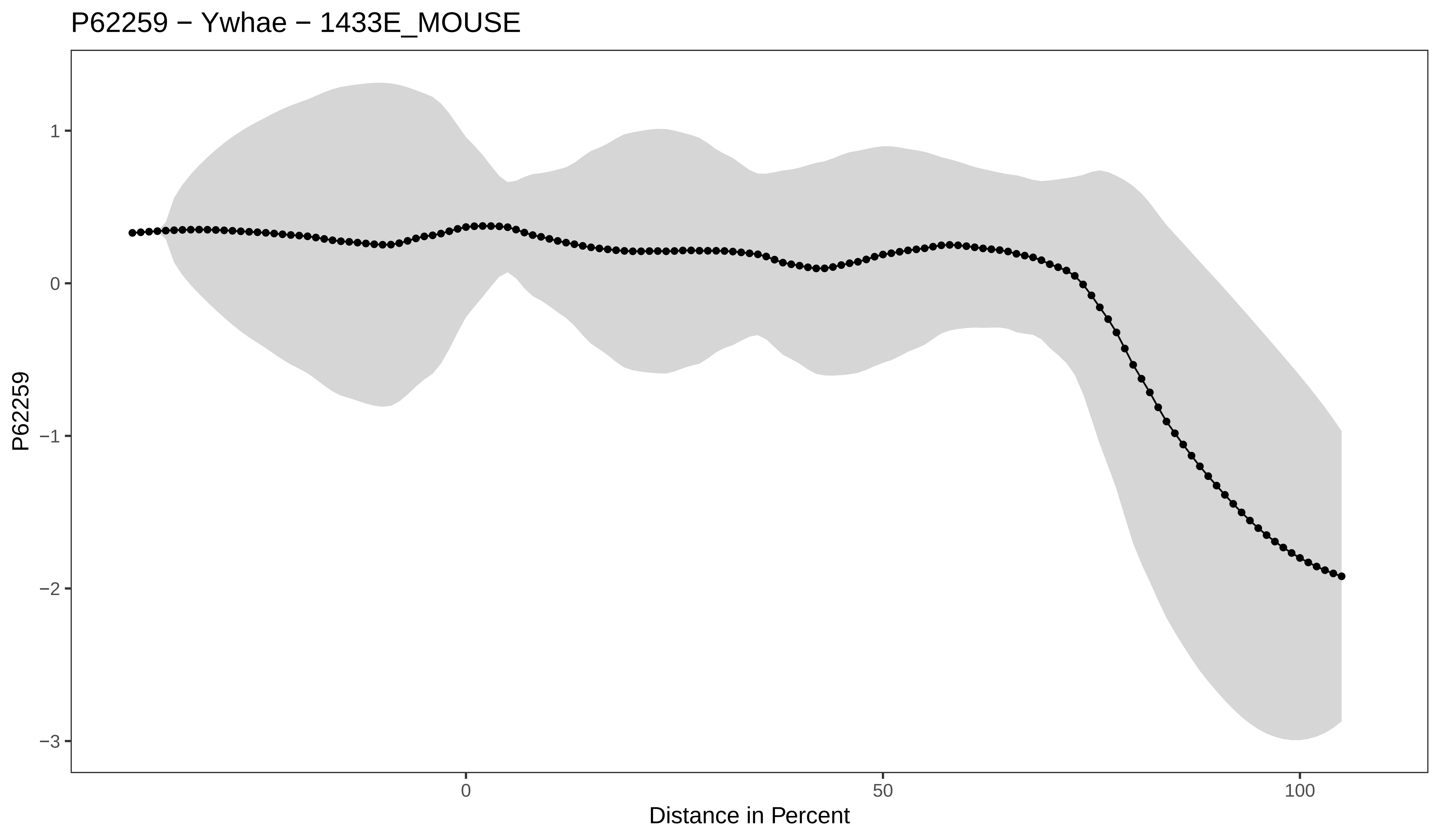
P62259 − Ywhae − 1433E_MOUSE
P62259
Distance in Percent
1
0
−1
−2
−3
0
50
100

P61982 − Ywhag − 1433G_MOUSE

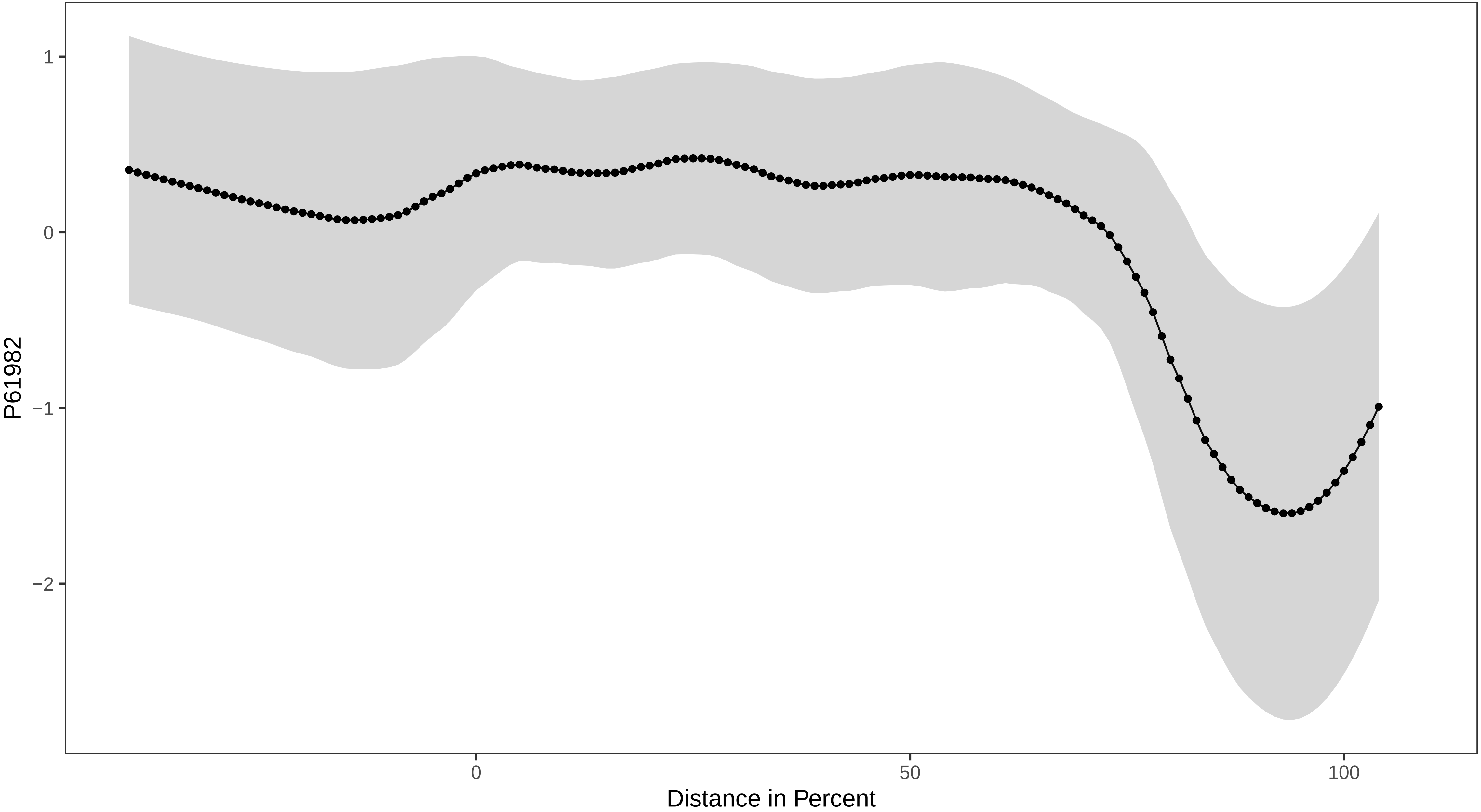

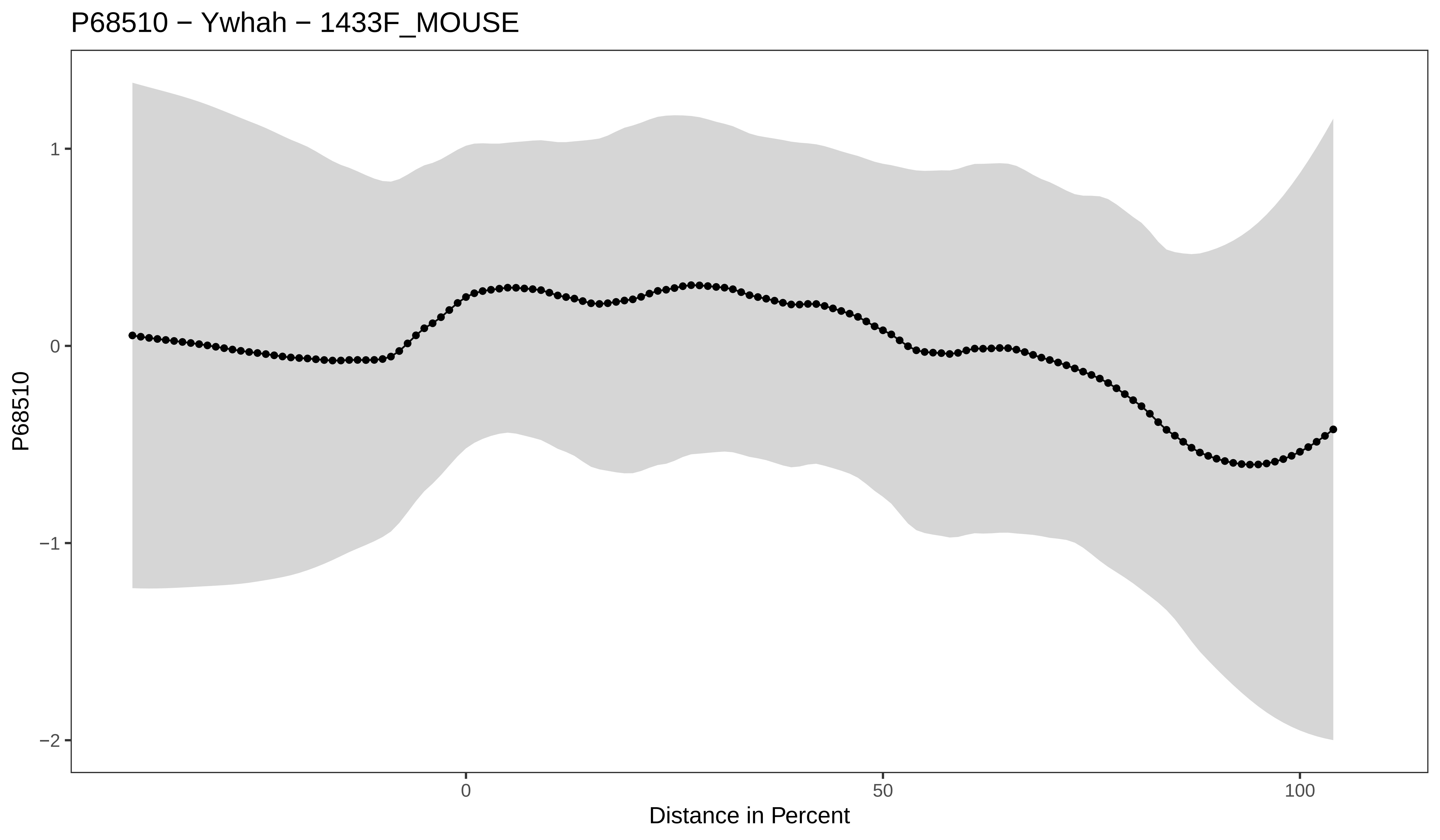
P68510 − Ywhah − 1433F_MOUSE
P68510
1
0
−1
−2
0
50
100
Distance in Percent

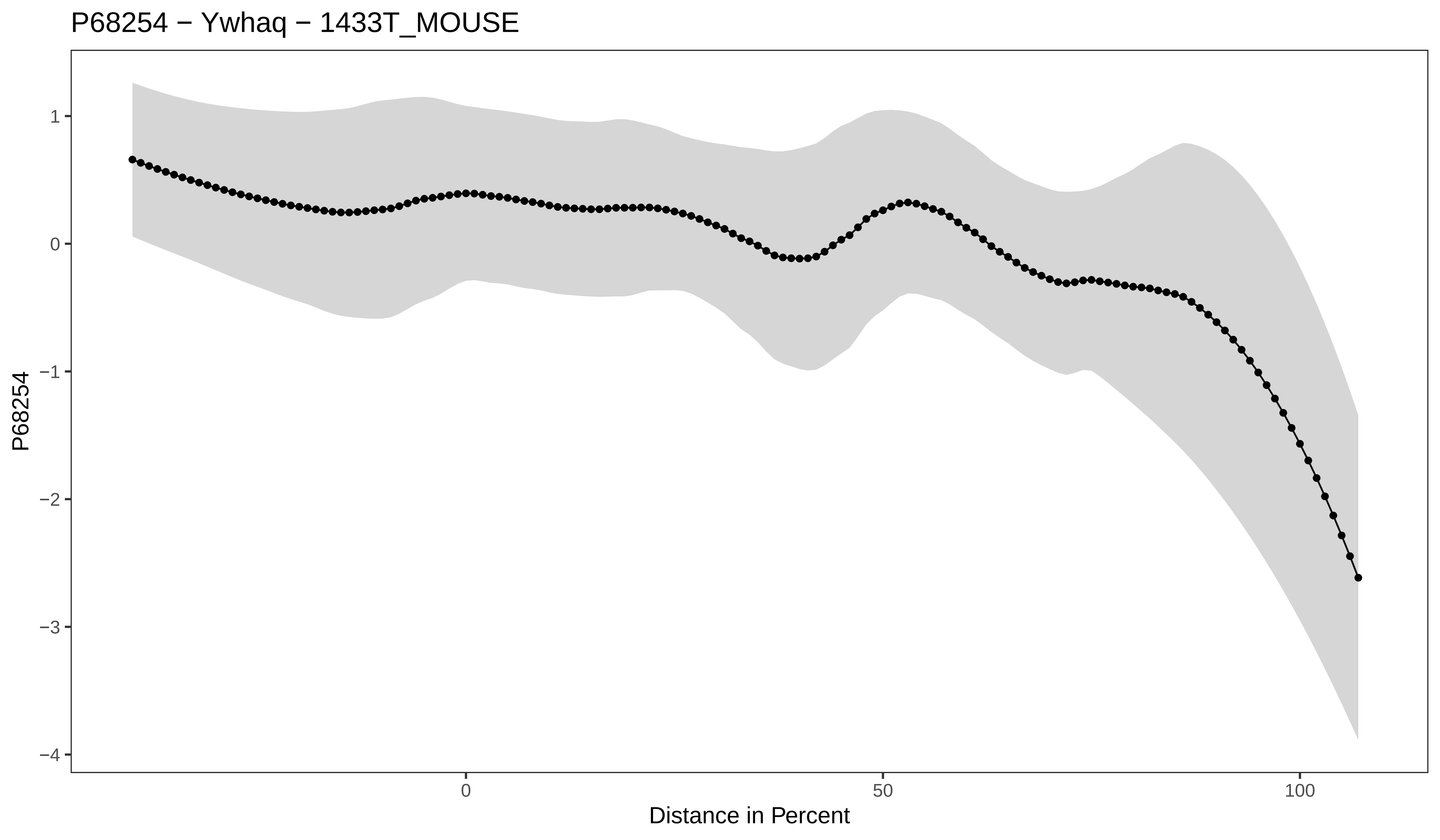

P68254 − Ywhaq − 1433T_MOUSE
P68254
1
0
−1
−2
−3
−4
0
50
100
Distance in Percent

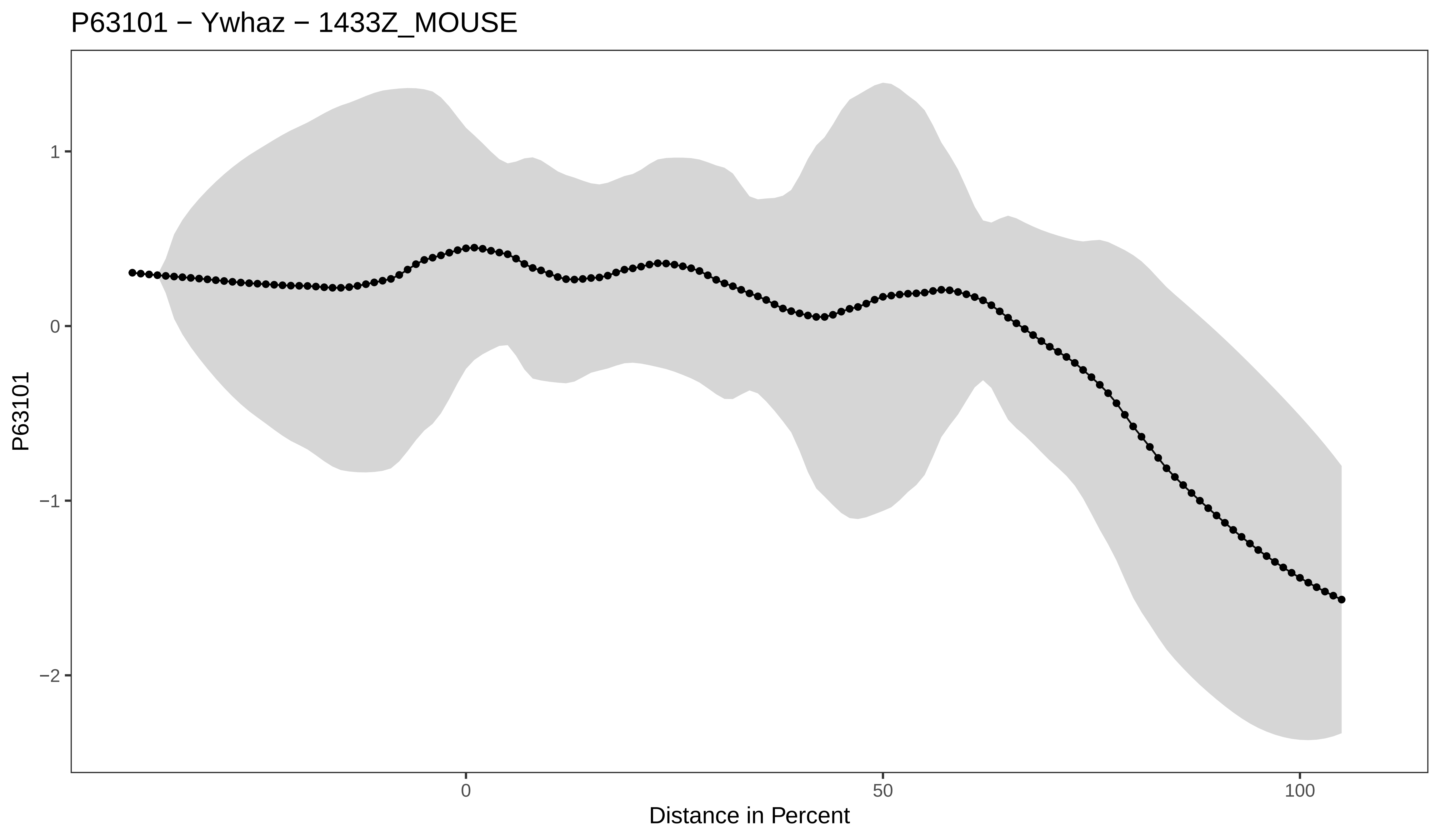
P63101 − Ywhaz − 1433Z_MOUSE
1
0
−1
−2
P63101
0
50
100
Distance in Percent

Q8BGC4 − Zadh2 − PTGR3_MOUSE

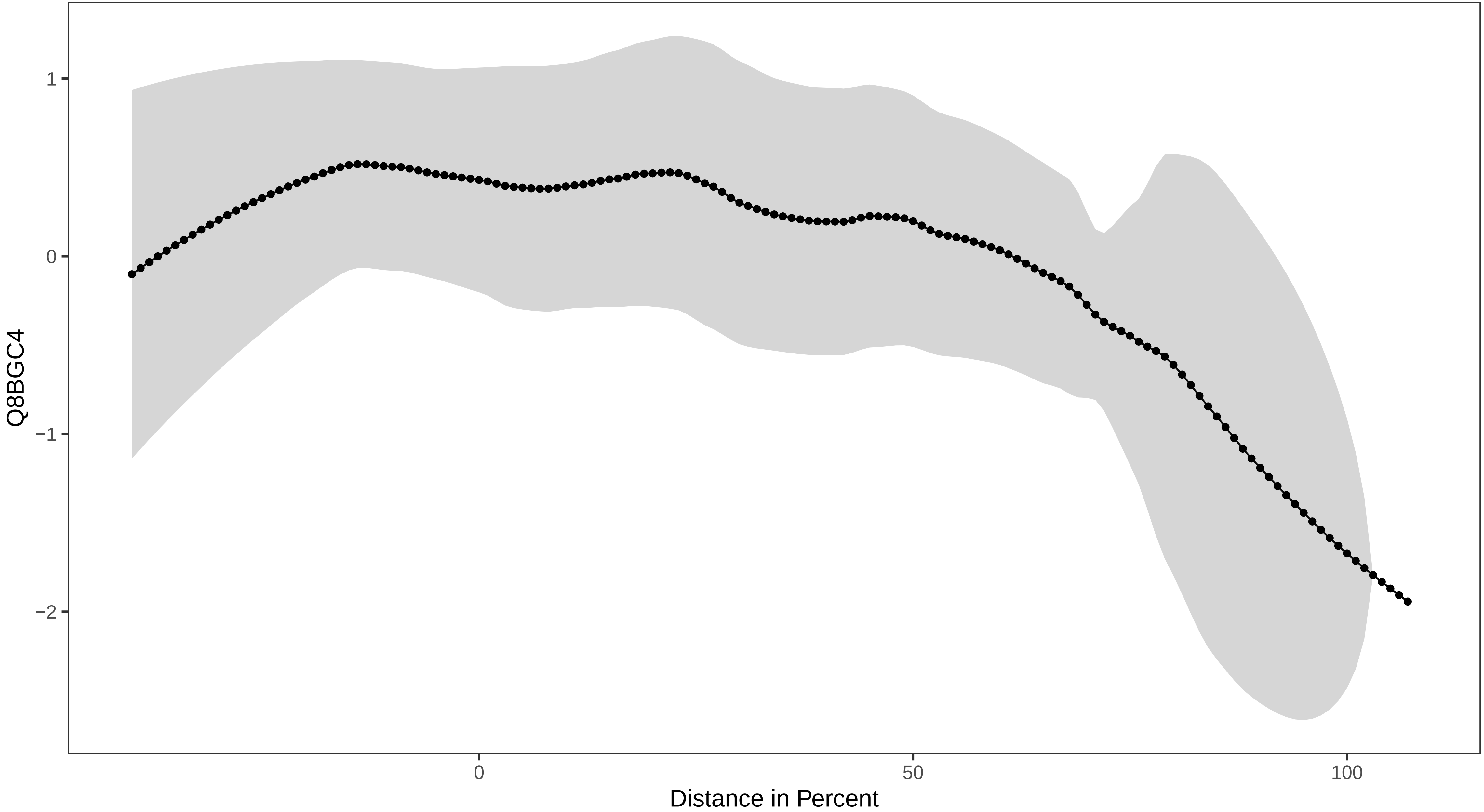

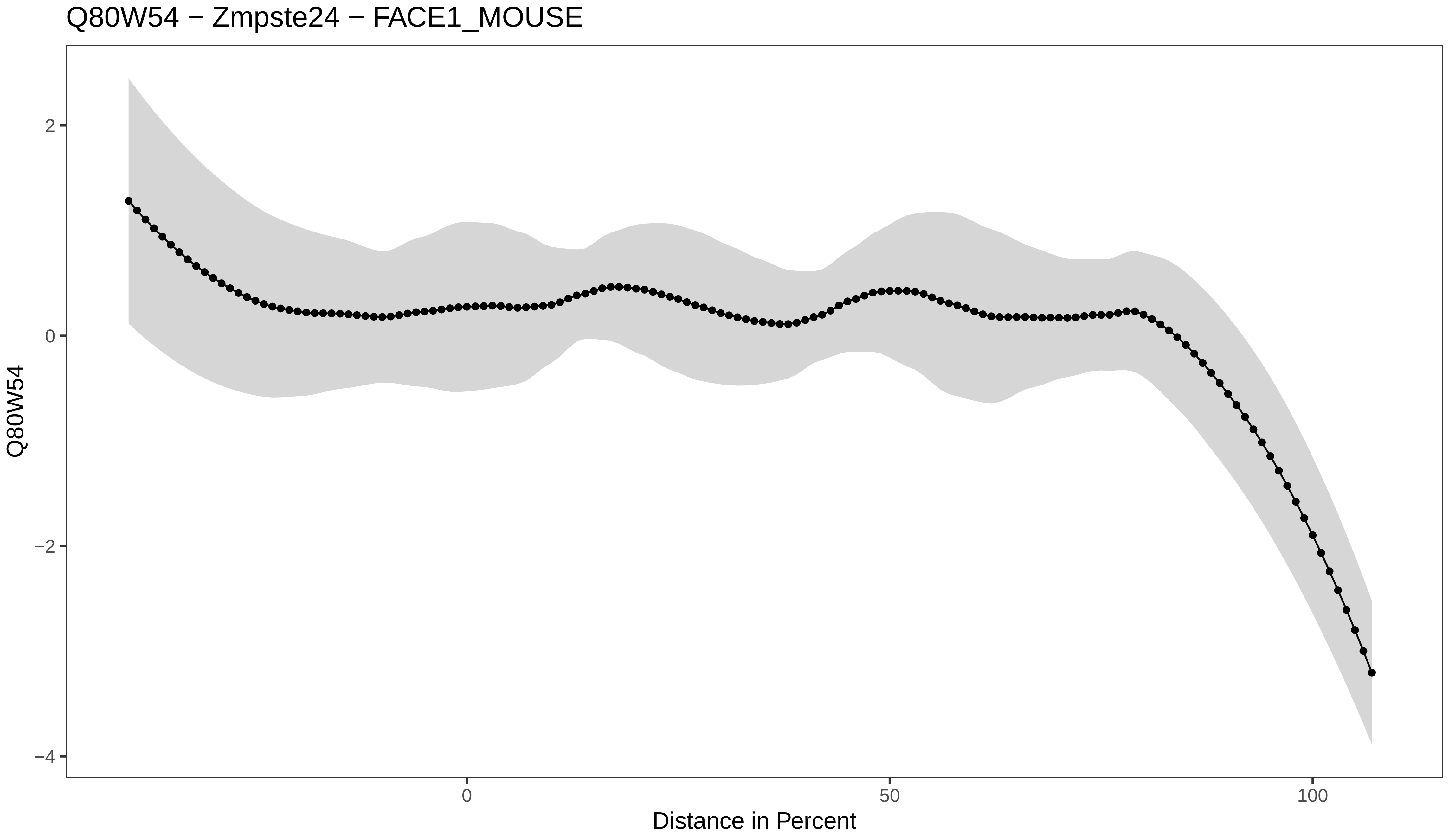

Q80W54 − Zmpste24 − FACE1_MOUSE
Q80W54
2
0
−2
−4
0
50
100
Distance in Percent

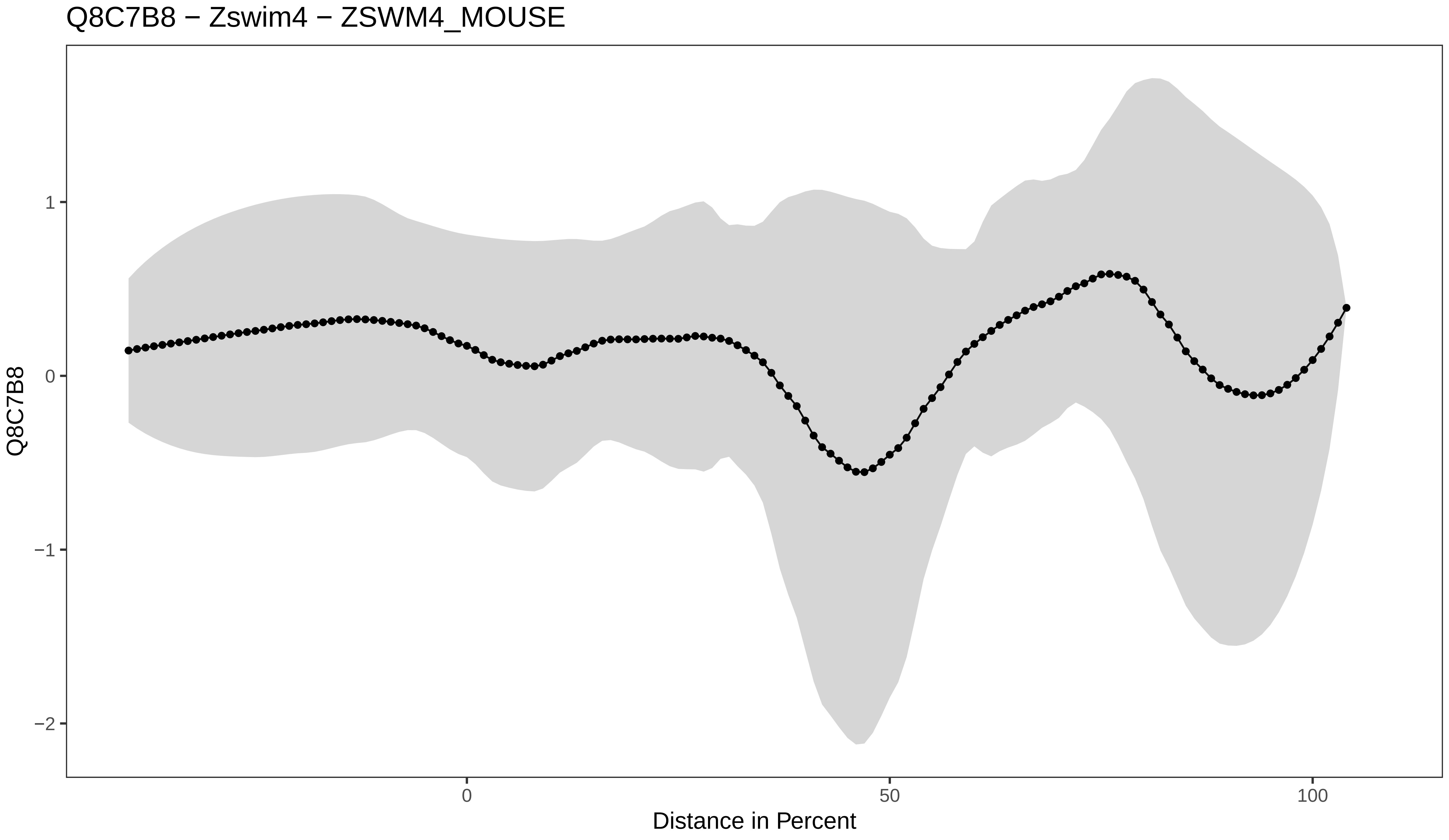

Q8C7B8 − Zswim4 − ZSWM4_MOUSE
Q8C7B8
1
0
−1
−2
0
50
100
Distance in Percent

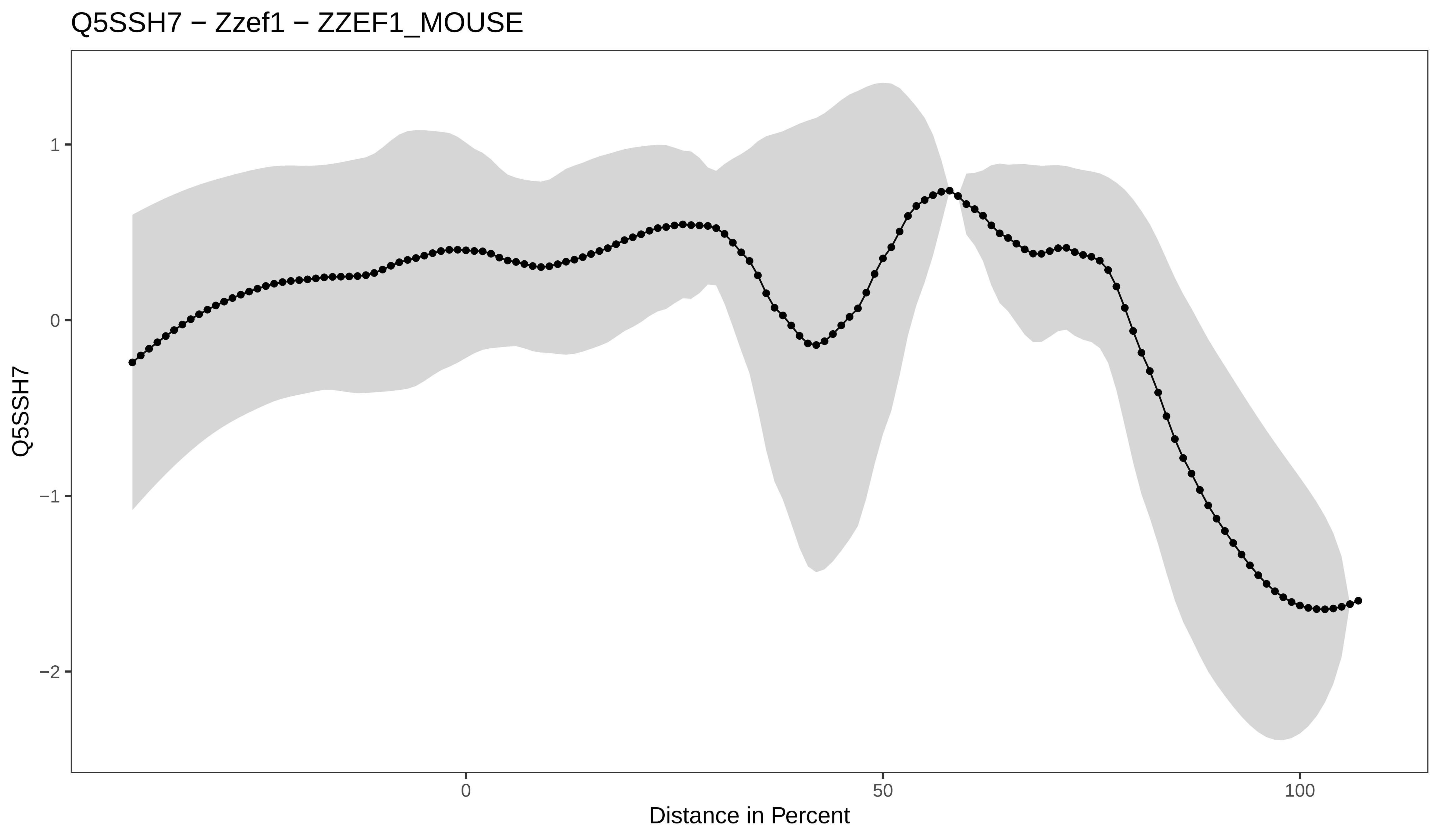

Q5SSH7 − Zzef1 − ZZEF1_MOUSE
Q5SSH7
1
0
−1
−2
0
50
100
Distance in Percent

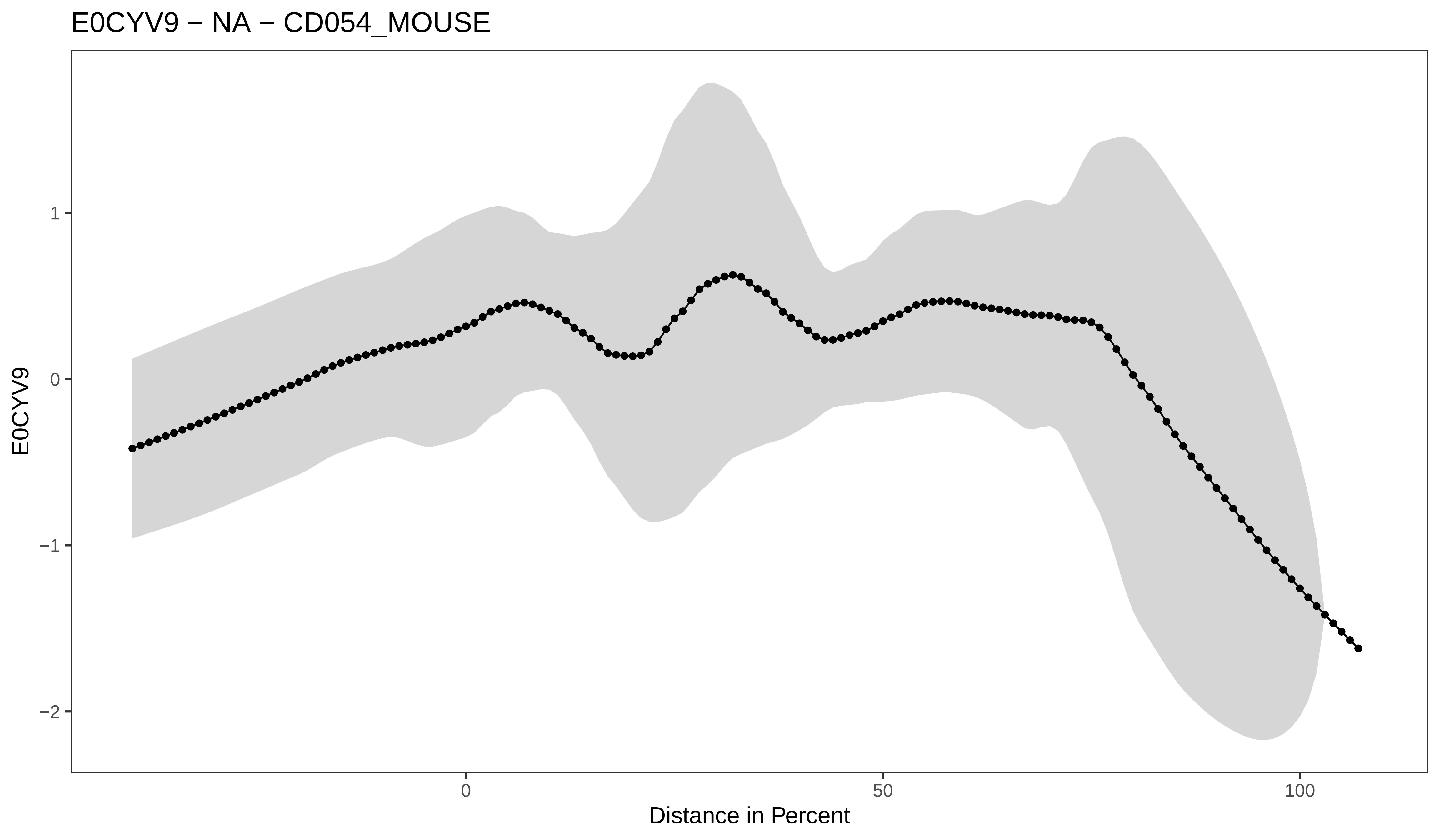

E0CYV9 − NA − CD054_MOUSE
E0CYV9
1
0
−1
−2
0
50
100
Distance in Percent

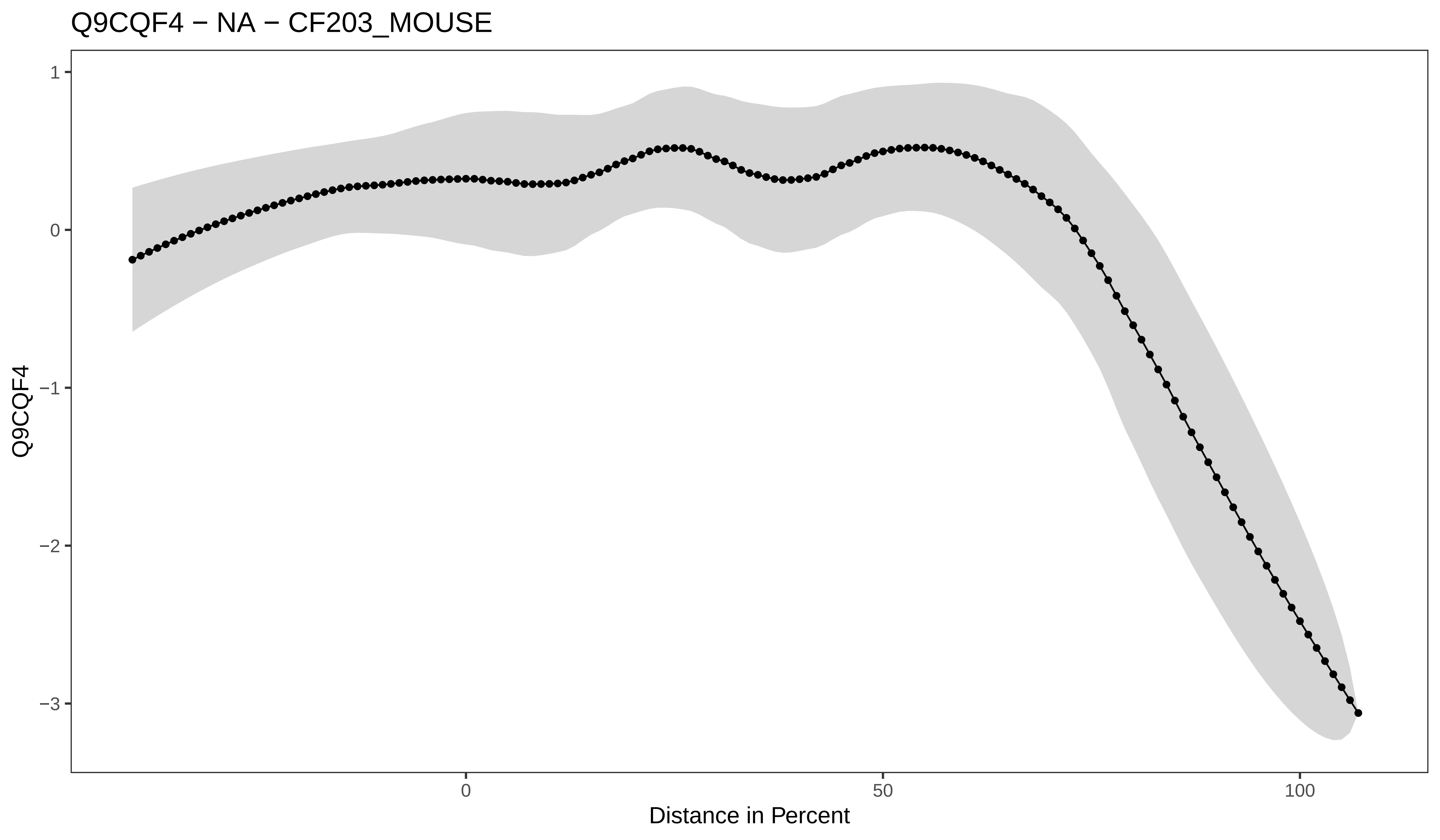
Q9CQF4 − NA − CF203_MOUSE
1
0
−1
−2
−3
Q9CQF4
0
50
100
Distance in Percent

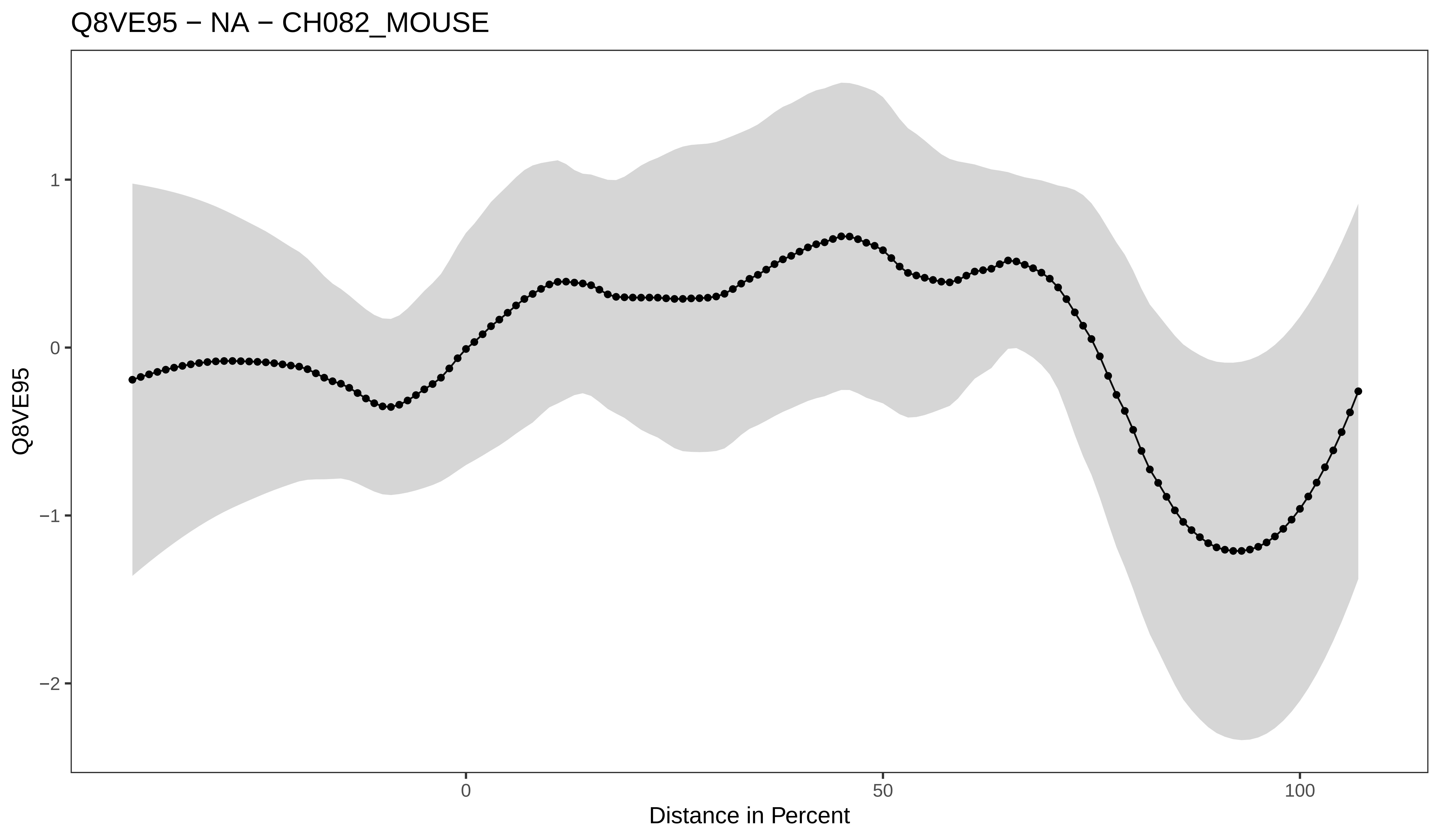

Q8VE95 − NA − CH082_MOUSE
Q8VE95
1
0
−1
−2
0
50
100
Distance in Percent

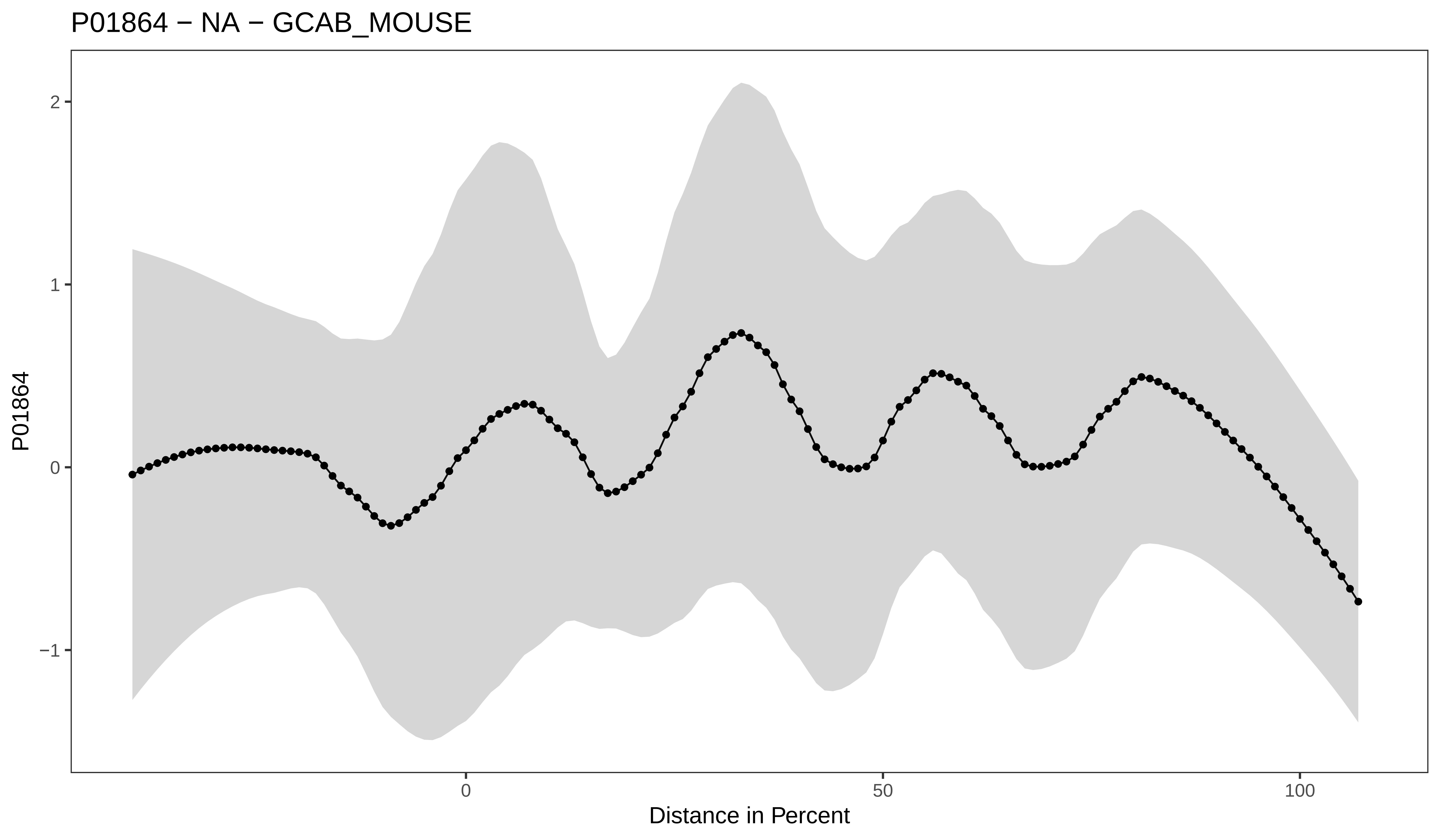

P01864 − NA − GCAB_MOUSE
P01864
Distance in Percent
2
1
0
−1
0
50
100

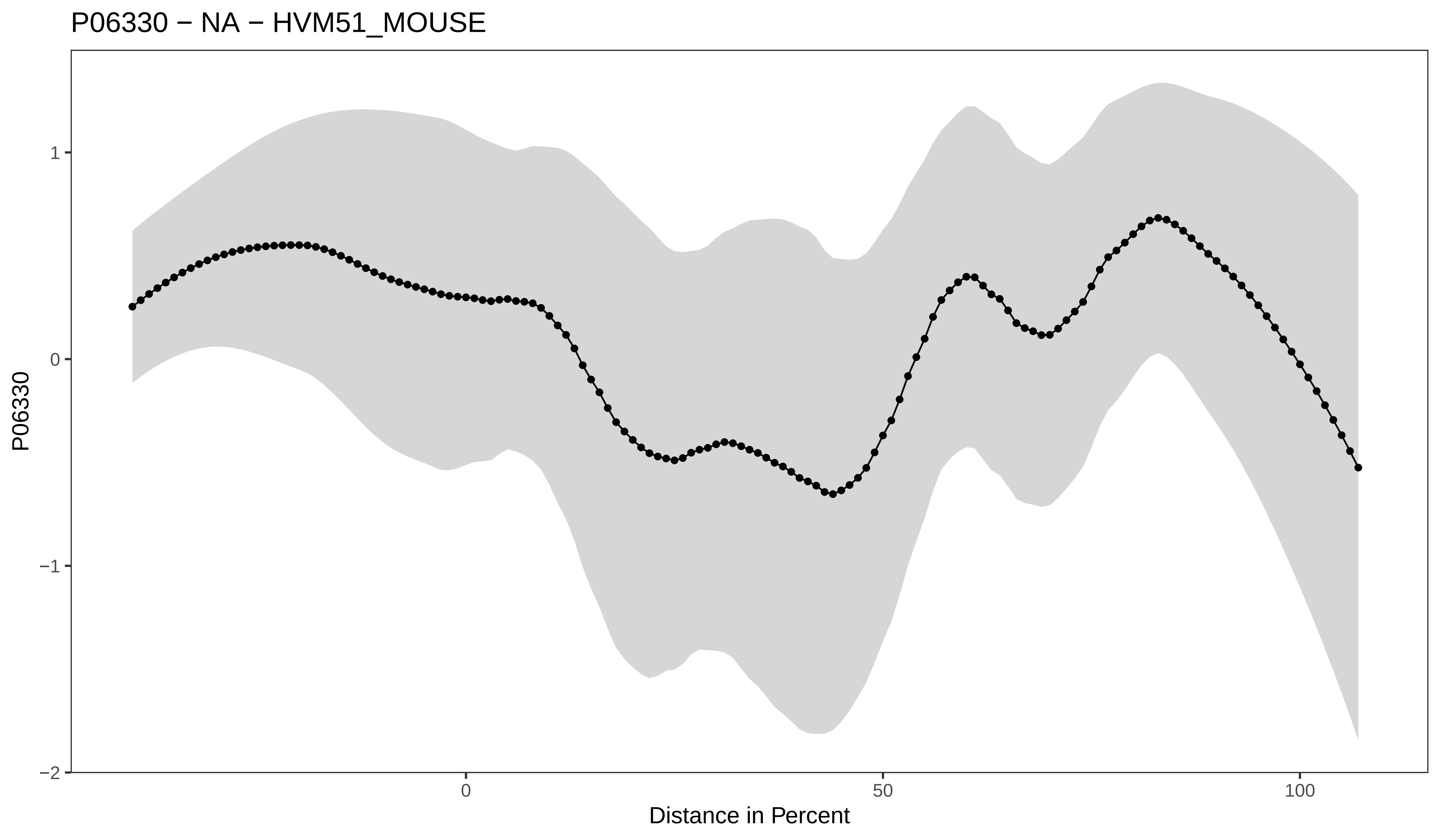
P06330 − NA − HVM51_MOUSE
P06330
1
0
−1
−2
0
50
100
Distance in Percent

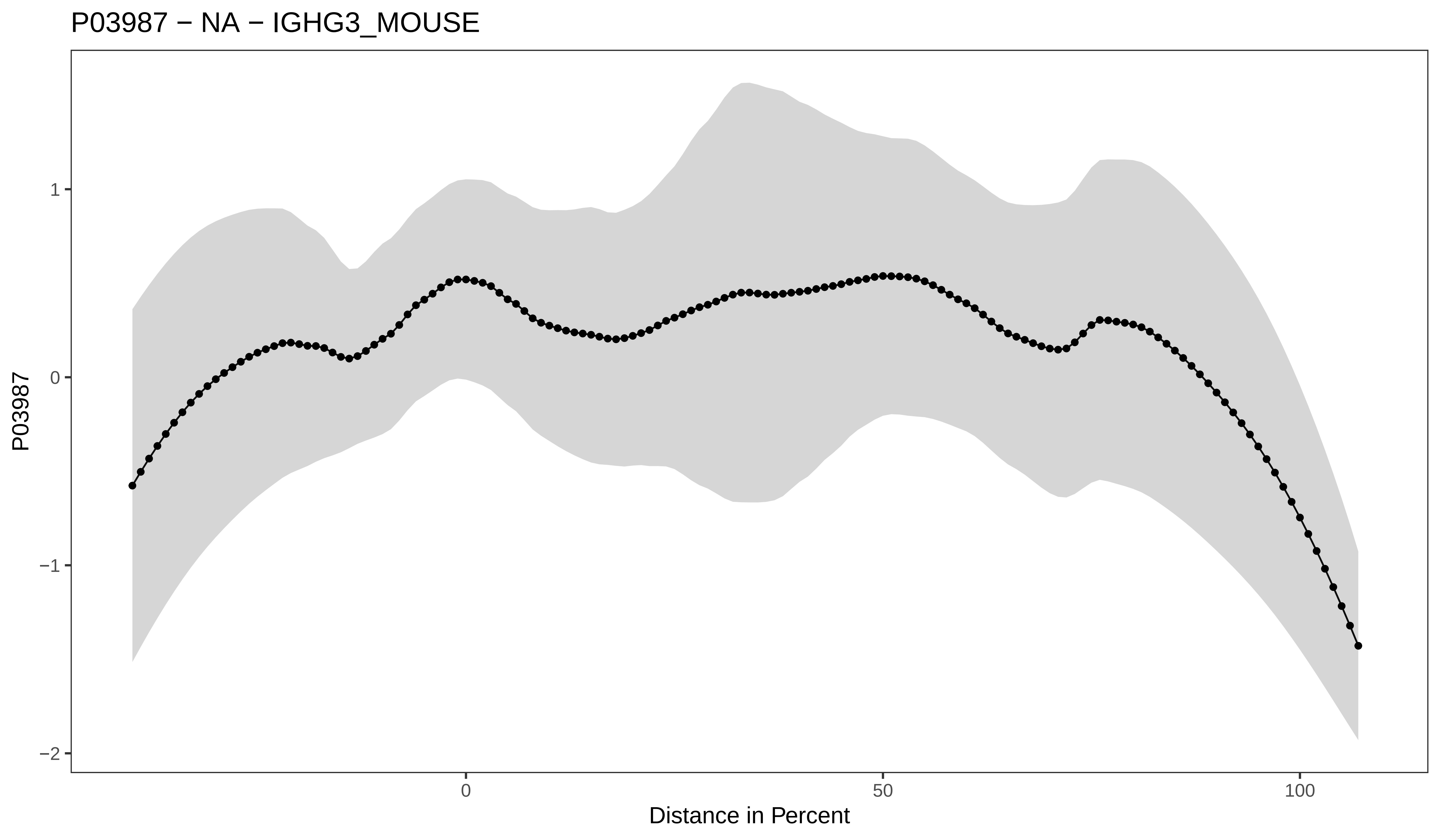
P03987 − NA − IGHG3_MOUSE
P03987
1
0
−1
−2
0
50
100
Distance in Percent
